# High-resolution profile of transcriptomes reveals a role of alternative splicing for modulating response to nitrogen in maize

**DOI:** 10.1186/s12864-020-6769-8

**Published:** 2020-05-11

**Authors:** Yuancong Wang, Jinyan Xu, Min Ge, Lihua Ning, Mengmei Hu, Han Zhao

**Affiliations:** Institute of Crop Germplasm and Biotechnology, Provincial Key Laboratory of Agrobiology, Jiangsu Academy of Agricultural Sciences, Nanjing, 210014 China

**Keywords:** Maize, Alternative splicing, Long-read sequencing, Nitrogen response, *ZmNLP6*

## Abstract

**Background:**

The fluctuation of nitrogen (N) contents profoundly affects the root growth and architecture in maize by altering the expression of thousands of genes. The differentially expressed genes (DEGs) in response to N have been extensively reported. However, information about the effects of N variation on the alternative splicing in genes is limited.

**Results:**

To reveal the effects of N on the transcriptome comprehensively, we studied the N-starved roots of B73 in response to nitrate treatment, using a combination of short-read sequencing (RNA-seq) and long-read sequencing (PacBio-sequencing) techniques. Samples were collected before and 30 min after nitrate supply. RNA-seq analysis revealed that the DEGs in response to N treatment were mainly associated with N metabolism and signal transduction. In addition, we developed a workflow that utilizes the RNA-seq data to improve the quality of long reads, increasing the number of high-quality long reads to about 2.5 times. Using this workflow, we identified thousands of novel isoforms; most of them encoded the known functional domains and were supported by the RNA-seq data. Moreover, we found more than 1000 genes that experienced AS events specifically in the N-treated samples, most of them were not differentially expressed after nitrate supply-these genes mainly related to immunity, molecular modification, and transportation. Notably, we found a transcription factor *ZmNLP6*, a homolog of *AtNLP7*-a well-known regulator for N-response and root growth-generates several isoforms varied in capacities of activating downstream targets specifically after nitrate supply. We found that one of its isoforms has an increased ability to activate downstream genes. Overlaying DEGs and DAP-seq results revealed that many putative targets of ZmNLP6 are involved in regulating N metabolism, suggesting the involvement of ZmNLP6 in the N-response.

**Conclusions:**

Our study shows that many genes, including the transcription factor *ZmNLP6*, are involved in modulating early N-responses in maize through the mechanism of AS rather than altering the transcriptional abundance. Thus, AS plays an important role in maize to adapt N fluctuation.

## Background

As a major worldwide-cultivated crop, maize is not only used for food but also serves as an alternative source for energy production [1]. Nitrogen (N), one of the most important nutrients in the soil, has been extensively used to guarantee the high yield formation of crops [2–4]. Maize plants absorb nitrate from the soil through specific nitrate transporters, such as NRT1.1 [5]. Once taken up by the roots, nitrate is reduced to ammonium through a series of reactions. This process highly depends on two key enzymes, nitrate reductase (NR) and nitrite reductase (NIR) [6, 7].

Plants have evolved complex mechanisms to cope with the variation of N concentrations in the soil. The root system architecture is one of the most important factors that affect N nutrients acquisition efficiency. The lengths of the primary and lateral roots are increased under mild N limitation [8, 9] while decreased due to the delayed development under sever deficient N conditions [10], compared with that of the plants grown under sufficient N conditions. Nitrogen functions not only as a nutrient but also as a signal molecule that coordinates its assimilation with the growth and development of plants [11]. Unveiling the genes in response to N is crucial for understanding the N-regulated network. Using the gene-chip and second-generation sequencing (SGS) technology, several studies have revealed the modifications in the global gene expression by the fluctuation of N availability [12–15]. These N-regulated genes are associated with a wide range of functions, including metabolism, growth, and development. Some of them have promising potential to improve the productions of crops if they are utilized appropriately. For example, *AtCIPK8*, which encodes a protein kinase, was found involved in regulating the low-affinity phase of nitrate response [16]. An N-responsive transcription factor, *OsENOD93–1,* improved the nitrogen use efficiency (NUE) when overexpressed in rice [17]. Besides the protein-coding transcripts, long noncoding RNA (lncRNA) has been demonstrated playing regulatory roles in response to environmental N variation as well [18].

Alternative splicing (AS) is one of the critical regulatory processes in eukaryotes. It greatly contributes to the genomic coding diversity [19–21]. The process of AS substantially enhances the functional complexity while averts increasing the number of genes in the genome. In *Drosophia*, a *DSCAM* gene, which encodes an immunoglobulin superfamily member, has the potential of generating over 38,000 isoforms. This number is more than twice as that of genes in the genome [22]. In humans, more than 90% of genes that harbor multiple exons generate various isoforms through the AS process, indicating that undergoing AS events is universal over intron-containing genes [23]. In addition, a single gene tends to express its splicing isoforms simultaneously, with different transcriptional abundance, though [24], suggesting that different isoforms of an individual gene, in many cases, work coordinately to perform certain functions. For instance, a shorter isoform of *CTCF* in human completes with its canonical isoform for genomic binding and cohesion, thus affects the process of apoptosis by altering the chromatin structure [25].

In addition to the alteration of gene transcriptional abundance, AS adds another layer of modulating the transcriptome to adapt the development stages and variation of the environment [26]. In plants, stresses trigger thousands of genes to experience significantly differential alternative splicing (DAS). Notably, studies showed that only a small fraction of DAS genes, identified under stress conditions, are also differentially expressed genes (DEGs) detected under the same treatment [27, 28], suggesting that AS is independent with gene expression in response to stress. SGS, like RNA-seq, is quite useful in identifying genes that are responded to condition changes by altering the transcriptional abundance (DEGs). However, the short read length of RNA-seq curbs the identification of full-length gene isoforms, for it is challenging to detect the complex AS events precisely [29]. Therefore, using SGS will inevitably ignore a substantial number of genes that respond to environmental changes by altering splicing patterns. Designed by Pacific Biosciences (PacBio), Single-molecule real-time (SMRT) sequencing, which features in long read length, provides a way of overcoming this limitation [29]. A recent study showed that using short reads only captured some one-fifth of splicing isoforms that are identified by SMRT sequencing [30]. However, the SMRT-sequencing flaws in higher error rate and lower throughput, which bottlenecks the accurate quantification of full-length gene isoforms [31]. Luckily, these disadvantages are not a case in the SGS. Thus, a strategy of hybrid sequencing that integrates SGS and SMRT-sequencing overcomes the weaknesses of every single technology alone [29].

The fast progress of sequencing technology allows researchers to study global N-regulatory networks through genomic to agronomic traits. However, limited information is available on the global profile of AS patterns in response to N in maize. In this study, we performed high-resolution transcriptome analyses on the N-treated and untreated samples, using a combination of RNA-seq and SMRT sequencing. We found differentially expressed genes (DEGs) were mainly associated with N metabolism and phytohormones. We used RNA-seq data to correct the long reads and resulted in more than two times of high-confidence reads than that acquired by using long-read sequencing alone. Besides differentially expressed genes (DEGs), we found that N treatment increased about 2000 AS events in the root tissues. Nearly 1000 non-DEGs that experienced AS events in the treated samples specifically were identified; these genes were mainly involved in the processes related to the immunity, molecular modification, and transportation. Furthermore, included in these genes, a transcription factor, *ZmNLP6*, which is a homolog of *AtNLP7*, a master regulator for N-response in Arabidopsis [32–35], generates several splicing isoforms after N treatment specifically. One of its alternative isoforms has a stronger activity of activating downstream targets. Overlapping DAP-seq and RNA-seq results support that ZmNLP6 is involved in modulating early N response and root architecture in maize. Our study shows that AS plays an important role in early N-responses in maize.

## Results

### Experimental system for sample collection

**Fig. 1:**
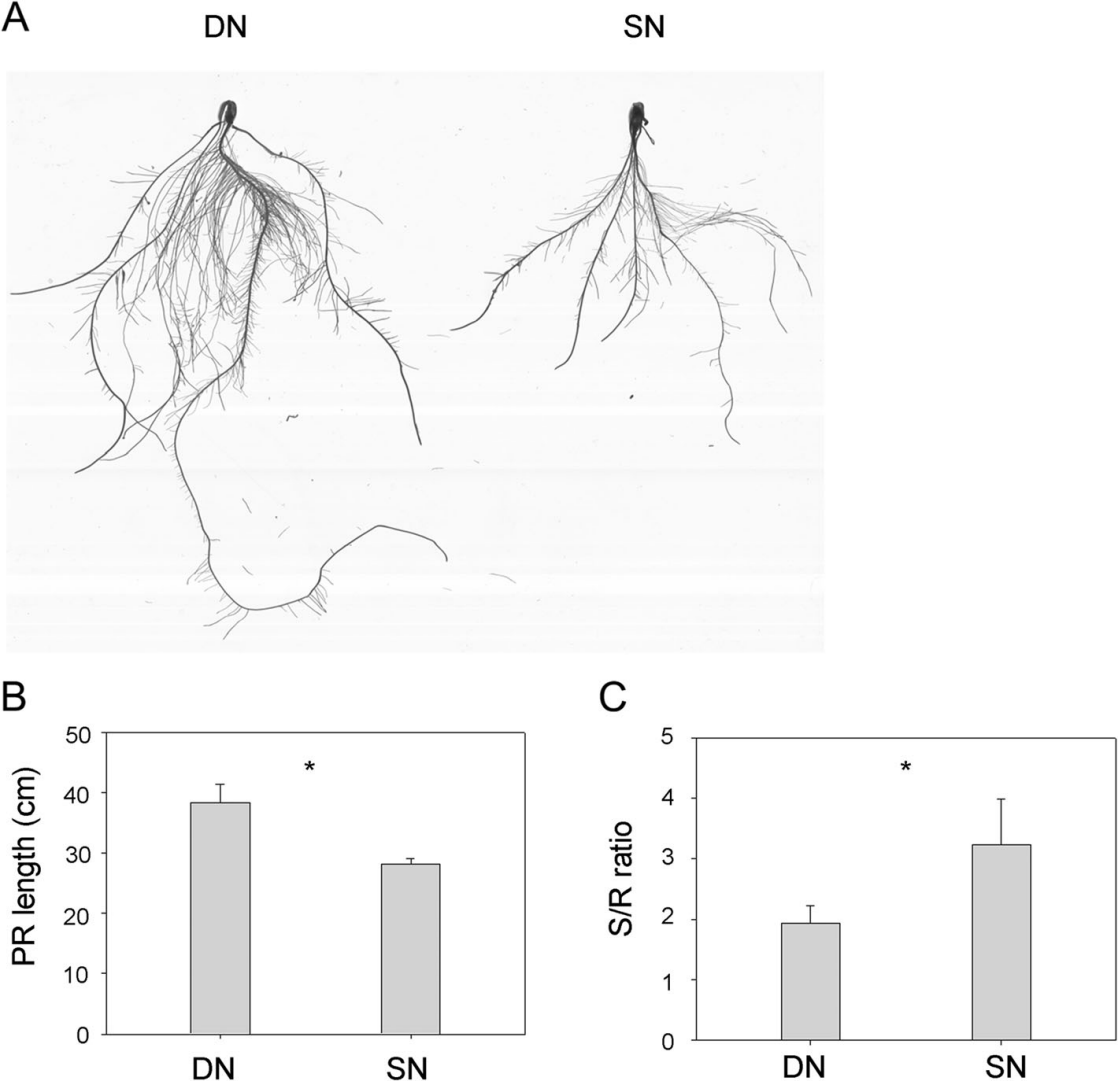
The phenotype of root tissues grown under deficient (DN) and sufficient nitrogen (SN) conditions. **a** The scanned images of two-week-old roots grown under DN and SN conditions, respectively. **b** The primary root lengths of two-week-old seedlings grown under DN and SN conditions, respectively. **c** The ratio of shoot biomass to root biomass (S/R) for plants grown under DN and SN conditions, respectively. The data are expressed as mean ± standard deviation of three separate tests (*n* = 3); “*” represents *p-*values ≤0.05 by student’s *t*-test

We utilized the visible morphological change of root tissues as a way to determine if the seedlings were under nitrogen (N) starvation. Germinated seeds of B73 were cultured using the hydroponic medium with the supply of sufficient N and limited N, respectively (see methods). After 2 weeks, we found that the plants grown under deficient N (DN) conditions developed longer primary root length, compared with that grown under sufficient N (SN) conditions (38.33 cm ± 3.03 vs. 28.13 cm ± 1.0, *p*-value < 0.05, Fig. 1a and b). We next investigated the shoot biomass to root biomass (S/R) ratios, which is an important marker for nutrient starvation [2]. Compared with plants grown under SN conditions, the S/R ratios of plants grown under DN conditions was significantly decreased (3.24 ± 0.75 vs. 1.93 ± 0.30, *P*-value < 0.05, Fig. 1c). These results indicated that the seedlings were suffering the N starvation after 2 weeks of growth under DN conditions.

**Fig. 2:**
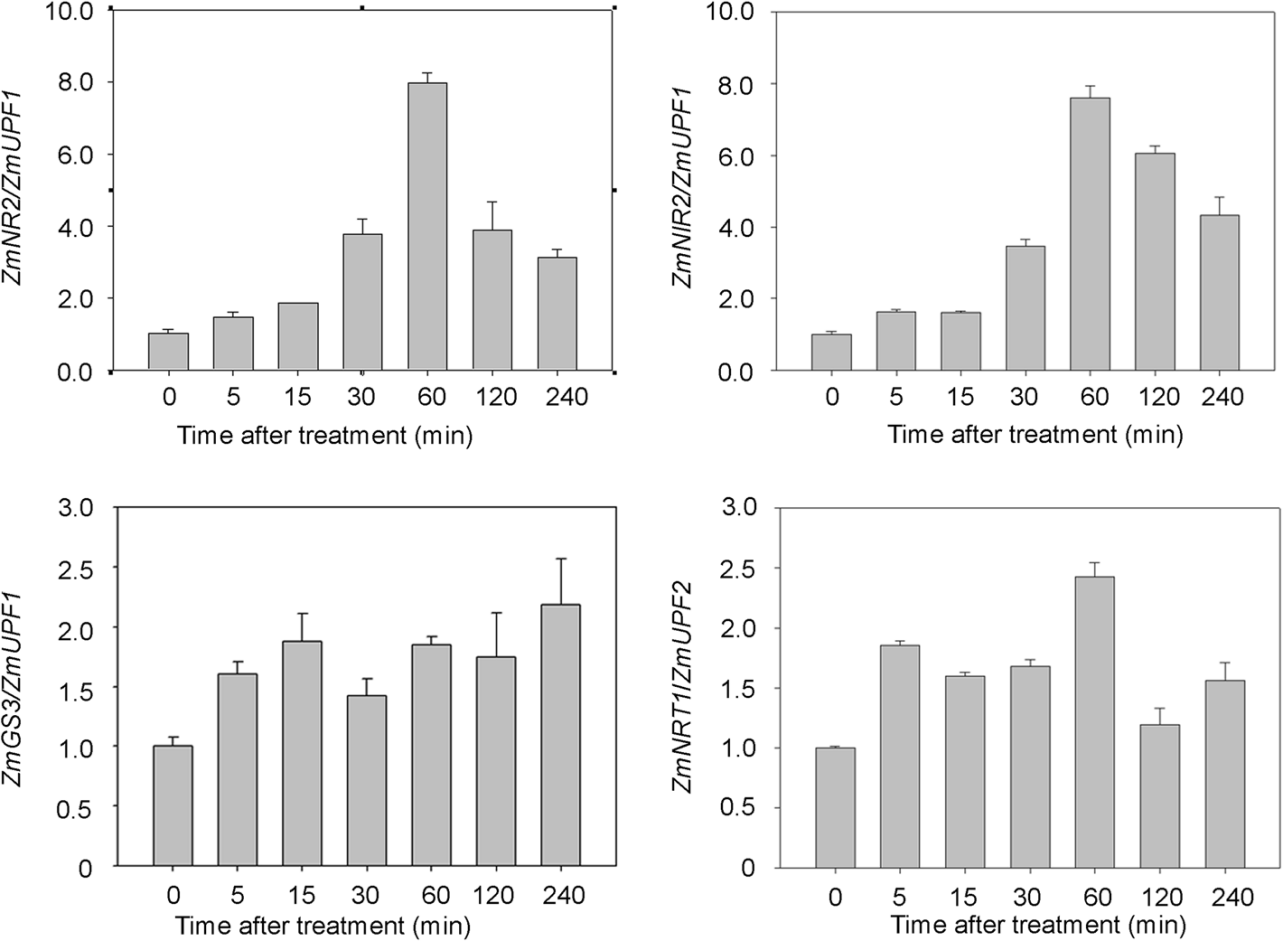
The expression of genes involved in nitrogen (N) uptake and assimilation in response to N. Plants were grown under deficient N conditions for 2 weeks. Expression of *ZmNR2*, *ZmNIR2*, *GS3*, and *ZmNRT1* at a series of time points after nitrate treatment was measured by qRT-PCR. The data are expressed as mean ± standard deviation of three separate tests (*n* = 3)

We further determined how quickly the N-starved roots in response to N by investigating the expression of genes encoding key enzymes involved in N assimilation pathway after nitrate supply at a series of time points. These genes were selected based on the annotation provided on the website of maize genome database (www.maizegdb.org), including *NITRATE REDUCTASE2* (*ZmNR2*, Zm00001d018206), *NITRITE REDUCTASE2* (*ZmNIR2*, Zm00001d052164/Zm00001d052165), *GLUTAMINE SYNTHETASE3* (*ZmGS3*, Zm00001d017958), and *NITRATE TRANSPORTER1* (*ZmNRT1*, Zm00001d054060). Total RNA was extracted from the root tissues of N-starved plants supplied with nitrate at multiple time points (0 min, 5 min, 15 min, 30 min, 60 min, 120 min, 240 min). qPCR showed that the expression of all four genes was significantly up-regulated (about 2–8 times in comparison with 0 min) between 30 and 60 min after the nitrate supply (Fig. 2). These results suggested the N-starved roots of maize seedlings could quickly respond to N (within 30 min) at the transcriptional level.

### RNA-seq identifies early-response genes to nitrate supply in the roots of N-starved plants

To gain a global view of the transcriptome in response to nitrate supply at the transcriptional level, we performed RNA-seq analysis. Total RNA was extracted from the N-starved root tissues of two-week-old seedlings (untreated sample) and that treated with nitrate at 30 min (treated sample), as we showed that the expression of key genes involved in N assimilation respond to N within 30 min (Fig. 2). Libraries for RNA-seq were constructed according to the standard protocol, sequenced on the Illumina HiSeq2500 platform with the pair-ended method (150 bp × 2). We conducted the high-throughput sequencing on three replicates for untreated and treated samples, respectively. Approximately 17–22 million fragments for each sample were processed. The reads that were mapped to cDNA sequences derived from the maize assembly v4 (about 75–80% mapping rate for each sample) were used for further analysis (Supplemental Table S1).

**Fig. 3:**
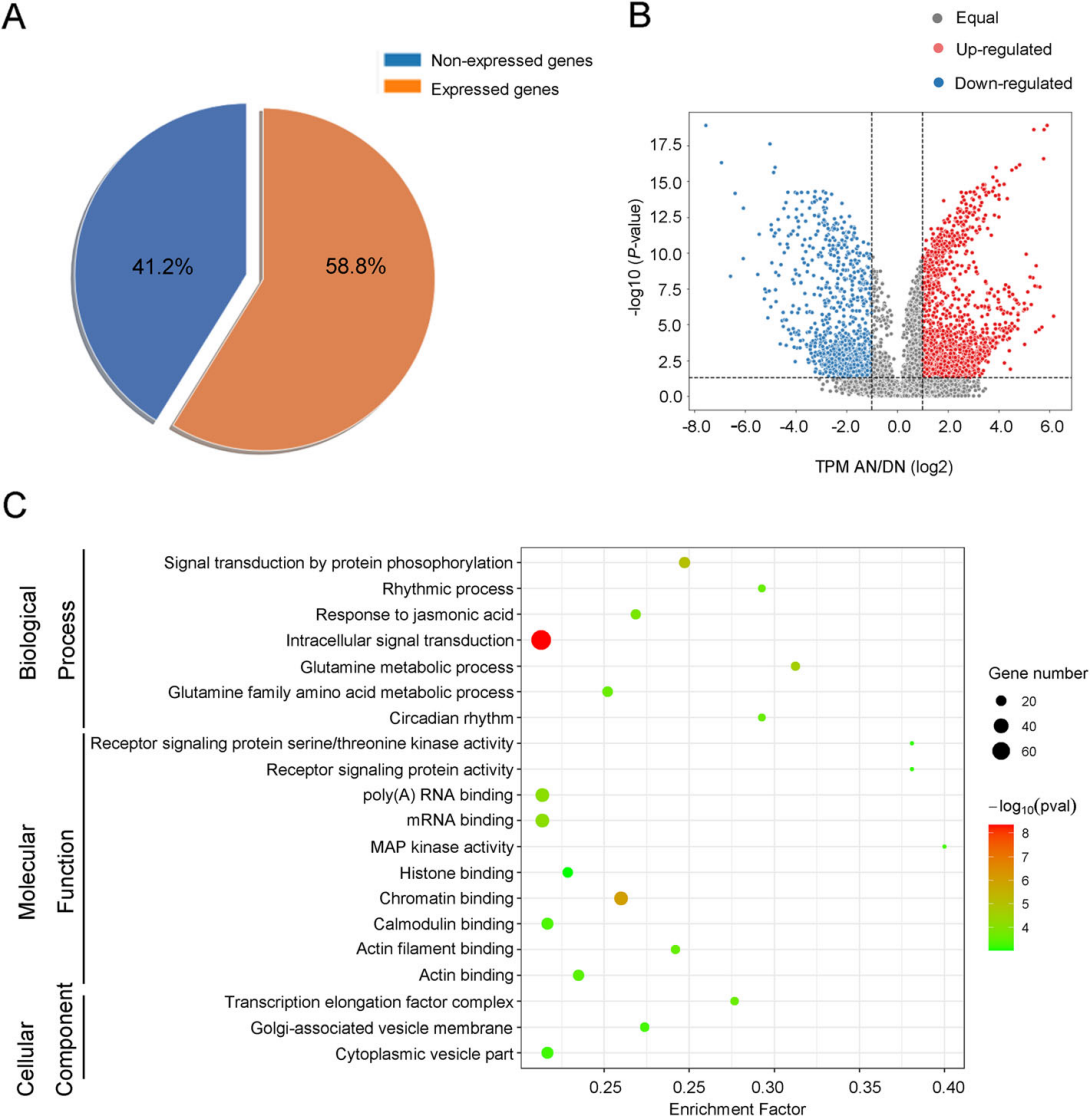
Transcriptome profiling of two-week-old root tissues. RNA was extracted from N-starved roots and that after 30 min of nitrate supply. **a** The ratio of expressed genes in the root tissues of two-week-old seedlings. **b** The volcano plot of log2 fold changes of gene transcriptional abundance. The red and green dots indicate that both more than two fold-changes (x-axis) as well as high statistical significance (−lg of *P*-value, y-axis). **c** Top 20 enriched GO terms of the functionally annotated genes that were responsive to nitrate supply in N-starved plants

We first identified the expressed genes in both untreated and treated samples. The transcriptional abundance of each transcript was calculated using transcript per million (TPM) mapped reads. We found 48,594 expressed transcripts (count-per-million > 1) ≥ 3), which derived from 23,121 genes (Fig. 3a, Supplemental Table S2), accounting for about 58.8% of total gene models. Differentially expressed genes (DEGs) were identified with the threshold of log_2_ expression ratios being either ≥1 or ≤ − 1 and *p* -Values ≤0.05. Based on this criterion, we found 3311 differentially expressed transcripts, which were generated from 2599 genes, after 30 min of N treatment (Supplemental Table S3, Fig. 3b). We also noticed that except *ZmGS3*, the expression of the other three genes detected above (*ZmNR2*, *ZmNIR1*, *ZmNRT1*) was significantly up-regulated, according to the RNA-seq results (Supplemental Table S3). This result demonstrated that our RNA-seq data is in agreement with the qPCR results.

We subjected the DEGs to Gene Ontology (GO) term enrichment analysis. Using the database in *agriGO* (http://bioinfo.cau.edu.cn/agriGO/), 2289 genes were annotated. Results showed that multiple pathways were enriched, including 69 biological processes, 52 molecular functions, and 18 cellular components (Supplemental Table S4). We visualized the GO terms with the top 20 enrichment factors in Fig. 3c. These GO terms consisted of seven biological processes, ten molecular functions, and three cellular components.

In the most enriched biological processes, we found two of them were mainly involved in N assimilation related pathways, including “glutamine metabolic process” (GO:0006541, *p*-value = 2.5e-5, FDR = 0.006) and “glutamine family amino acid metabolic process” (GO:0009064, *p*-value = 2.5e-4, FDR = 0.032). These two GO terms include 15 common genes, such as Zm00001d043845, which encodes a glutamate synthase, was up-regulated in the treated sample. Another gene Zm00001d011357 encoding a ctp synthase was down-regulated after nitrate supply. We also found two GO terms associated with biological rhythmic processes, including “rhythmic process” (GO:0048511, *P*-value = 2.6e-4, FDR = 0.032) and “circadian rhythm” (GO:0007623, *P*-value = 0.00026, FDR = 0.032), suggesting that nitrate supply affects the expression of genes involved in mediating circadian rhythms. For example, Zm00001d045944 (encodes a cryptochrome protein) and Zm00001d006227 (encodes a xap5 circadian timekeeper-like protein) were up-regulated after N treatment. The rest three biological processes are associated with signal transduction, which are “signal transduction by protein phosphorylation” (GO:0023014, *P*-value = 9e-6, FDR = 0.0032), “intracellular signal transduction” (GO:0035556, *P*-value = 4.6e-9, FDR = 8.8e-6), and “response to jasmonic acid” (GO:0009753, *P*-value = 1.5e-4, FDR = 0.021), supporting the conclusions that N functions as a signaling molecular and that the involvement of the plant hormone in modulating the N-response.

The top 20 enriched GO terms include 10 molecular functions. Seven of them were associated with binding, such as “mRNA binding” (GO:0003729, *P*-value = 7.8e-5, FDR = 0.0049), “histone binding” (GO:0042393, *P*-value = 9.8e-4, FDR = 0.047), and “chromatin binding” (GO:0003682, *P*-value = 1.0e-6, FDR = 9.4e-5), suggesting that N treatment altered the transcriptional abundance of genes involved in modulating molecular binding functions. All the other three molecular functions related to signaling activity, including “receptor signaling protein serine/threonine kinase activity” (GO:0004702, *P*-value = 0.00069, FDR = 0.034), “receptor signaling protein activity” (GO:0005057, *P*-value = 0.00069, FDR = 0.034), and “MAP kinase activity” (GO:0004707, *P*-value = 5.3e-4, FDR = 0.028). Besides, three GO terms were classified as cellular components, including “transcription elongation factor complex” (GO:0008023, *P*-value = 2.4e-4, FDR = 0.027), “Golgi-associated vesicle membrane” (GO:0030660, *P*-value = 5.7e-4, FDR = 0.047), and “cytoplasmic vesicle part” (GO:0044433, *P*-value = 0.00061, FDR = 0.047). These GO terms have close relationship with signal transduction, molecular transport, or nucleic acid metabolism. Together, GO enrichment analysis indicated that nitrate supply affects the expression of genes involved in multiple pathways, supporting the idea that N functions as both a key nutrient material and a signal molecular.

### The workflow for long-read data processing and quality checking for the high-confidence reads

To obtain the global profiling of alternative splicing (AS) events in response to N, we performed long-read sequencing on both treated and untreated samples, respectively. We constructed the full-length cDNA libraries using the RNA extracted from the same samples used for performing RNA-seq. Each library was sequenced in one Single-Molecular, Real-Time (SMRT) cell on the Pac-Bio Sequel platform, yielding 7,851,414 and 9,092,052 subreads in the untreated and treated samples, respectively. More than 90% of these reads range from 325 bp–2482 bp (Supplemental Table S5). We used the *IsoSeq3* pipeline (https://anaconda.org/bioconda/isoseq3) to process the data, obtained 419,458 (untreated sample), and 465,176 (treated sample) circular consensus sequencing reads (CCSs). About three-quarters of them were characterized as full-length CCSs, which were subsequently collapsed into non-redundant full-length non-chimeric CCSs (labeled as FLNC CCSs). Compared with the unique FLNC CCSs, slumps in the number of non-redundant high-quality (HQ) isoforms (defined by the IsoSeq3) were observed (8474 HQ isoforms vs. 28,417 FLNC CCSs in the untreated sample, 8612 isoforms vs. 28,461 FLNC CCSs in the treated sample). Based on these HQ isoforms, some 6000 genes were identified in each sample (6045 genes for untreated sample, 6082 for treated sample). This number accounts for about a quarter of the expressed genes identified by RNA-seq (23121). We next explored the range of expression of genes that are in and not in the set of HQ isoforms in the RNA-seq data (labeled as HQ-set genes and Non-HQ-set genes, respectively). In both treated and untreated samples, the expression range of HQ-set genes was significantly higher than that of Non-HQ-set genes (Mann-Whitney U test, *P*-value < 0.05). In the Untreated samples, for the Non-HQ-set genes, the 25th, 75th quantiles, and medians of transcriptional abundance (log2(TPM + 1)) were 0.80, 3.39, and 1.91, while for the HQ-set genes were 0.96, 4.35, and 2.59, respectively. Similar results were observed in the treated samples, values for Non-HQ-set genes were 1.08, 3.54, 2.12, while for the HQ-set genes were 1.44, 4.92, 3.21, respectively (Supplemental Fig. S1). These results suggested that the information for a considerable amount of genes was ignored because of lower throughput of SMRT-sequencing technology when compares with that of RNA-seq technology.

To increase the quality of full-length isoforms from the long-read sequencing, we developed a workflow integrating the RNA-seq data to improve the quality of the FLNC CCSs. As shown in Supplemental Fig. S2, we utilized the RNA-seq data to correct the long reads and validate the chain of splicing junctions (SJs) in each of the FLNC CCSs. Only the sequences with the complete match of the whole chain of SJs were kept for further analysis. Using this workflow, we greatly increased the number of high-confidence full-length transcript isoforms in comparing with that of HQ isoforms (18,414 isoforms for the untreated sample, 20,297 isoforms for the treated sample).

We employed *SQANTI_qc.py* [36] to investigate the qualities of HQ isoform sequences (HQS), non-redundant FLNC CCSs (FLNC), and validated non-redundant FLNC CCSs that were obtained by using our workflow (FLNC-validated), respectively. Results showed that the set of FLNC-validated kept ~ 80% of genes and ~ 70% of isoforms in the set of FLNC. When compared with the collection of HQS, the number of genes in the set of FLNC-validated increased by 1.6 times, and two times for the number of isoforms (Supplemental Fig. S3A and B). Although the set of FLNC-validated contains fewer isoforms than that of FLNC does, it contains more isoforms in the group labeled as full splice match (FSM), which represents perfect reference matches. In both untreated and treated samples, the most gaps between the numbers of isoforms in the sets of FLNC and FLNC-validated were found in the category labeled as Novel Not in Catalog (NNC). About four-fifth of isoforms (82.4% for the untreated sample, 78.8% for the treated sample) belonging to this category were wiped out after SJ validation using RNA-seq data. For the rest of the categories, FLNC-validated kept the major part of the isoforms in that of FLNC correspondingly. Compared with the sets of FLNC and FLNC-validated, HQS has the least number of isoforms in all groups characterized by *SQANTI.qc* (Figure S3C and D).

We next investigated the splicing junctions (SJs) of transcript isoforms. According to the definition in the *SQANTI*, canonical junctions include GT-AG, GC-AG, and AT-AC, SJs otherwise are considered as non-canonical junctions. Compared with the FLNC collection, around four-fifths of the known canonical SJs were also presented in the RNA-seq results for both untreated (77.6%) and treated (82.3%) samples. For other categories, however, the validation process filtered out a major part of SJs that were kept in the category labeled as FLNC-validated. We noted that the set of FLNC-validated filtered out all the known non-canonical SJs, even that were found in the set of HQS, resulting in the decrease of the ratio of non-canonical SJs (the non-canonical SJs account for around 0.5% in HQS and around 0.1% in FLNC-validated). Most parts of the novel SJs, including novel canonical and novel non-canonical, were discarded after using short-read sequencing data to verify each chain of SJs (Supplemental Fig. S4A and B). Except for known non-canonical, the number of SJs in the set of HQS was the least at the other three kinds of SJs. These results suggested that our workflow could efficiently identify the high-confidence isoforms from the long-read sequencing data.

### Characterization and computational validation of novel transcripts

**Fig. 4:**
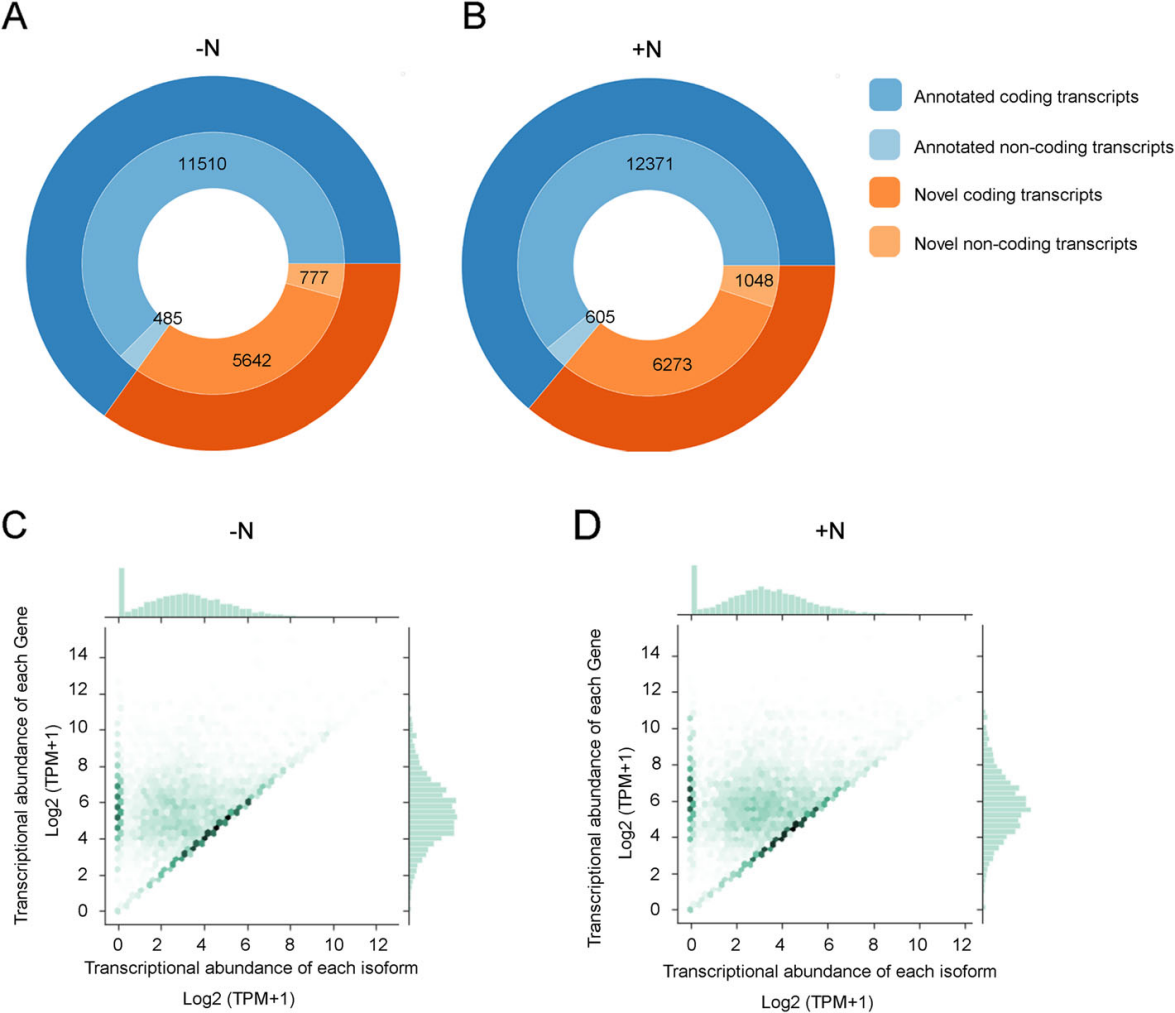
The number and the expression of novel isoforms detected in the N-starved root tissues (−N, untreated sample) and the samples after 30 min nitrate supply (+N, treated sample). **a** The number of annotated and novel transcripts found in untreated samples and treated samples, respectively. **b** The log2 transcriptional abundance of each transcript (x-axis) and its correlated genes (y-axis) calculated using RNA-seq data

In maize, about 45% expressed genes generate various isoforms through AS [37]. For the isoforms in the FLNC-validated category, about one-third of them were classified as novel isoforms (6419 and 7321 in the untreated and treated samples, respectively). The protein-coding potential was calculated using GeneMarkS-T (GMST) algorithm, which is integrated into the *SQANTI.qc*. Results showed that putative protein-coding isoforms account for about 85% of the novel isoforms in total (Fig. 4a and b). To evaluate the extents of authenticity for these potential novel transcripts, we first checked whether the expression of these novel isoforms was supported by RNA-seq data. The transcription abundance of each isoform was quantified by mapping RNA-seq data to the high-confidence cDNA sequence file generated by our workflow. In the untreated sample, the expression of 83.5% of novel isoforms was supported in the RNA-seq (TPM > 1). In the treated sample, the RNA-seq supported novel isoforms account for 73.2% (Fig. 4c and d). In addition, thousands of novel isoforms were highly expressed, for the median TPM are 7.37 in the untreated sample and 8.15 in the treated sample, respectively. Furthermore, the expression of 2425 and 2487 isoforms contribute more than 50% of the transcriptional abundance of their associated genes in the untreated and treated samples, respectively.

**Fig. 5:**
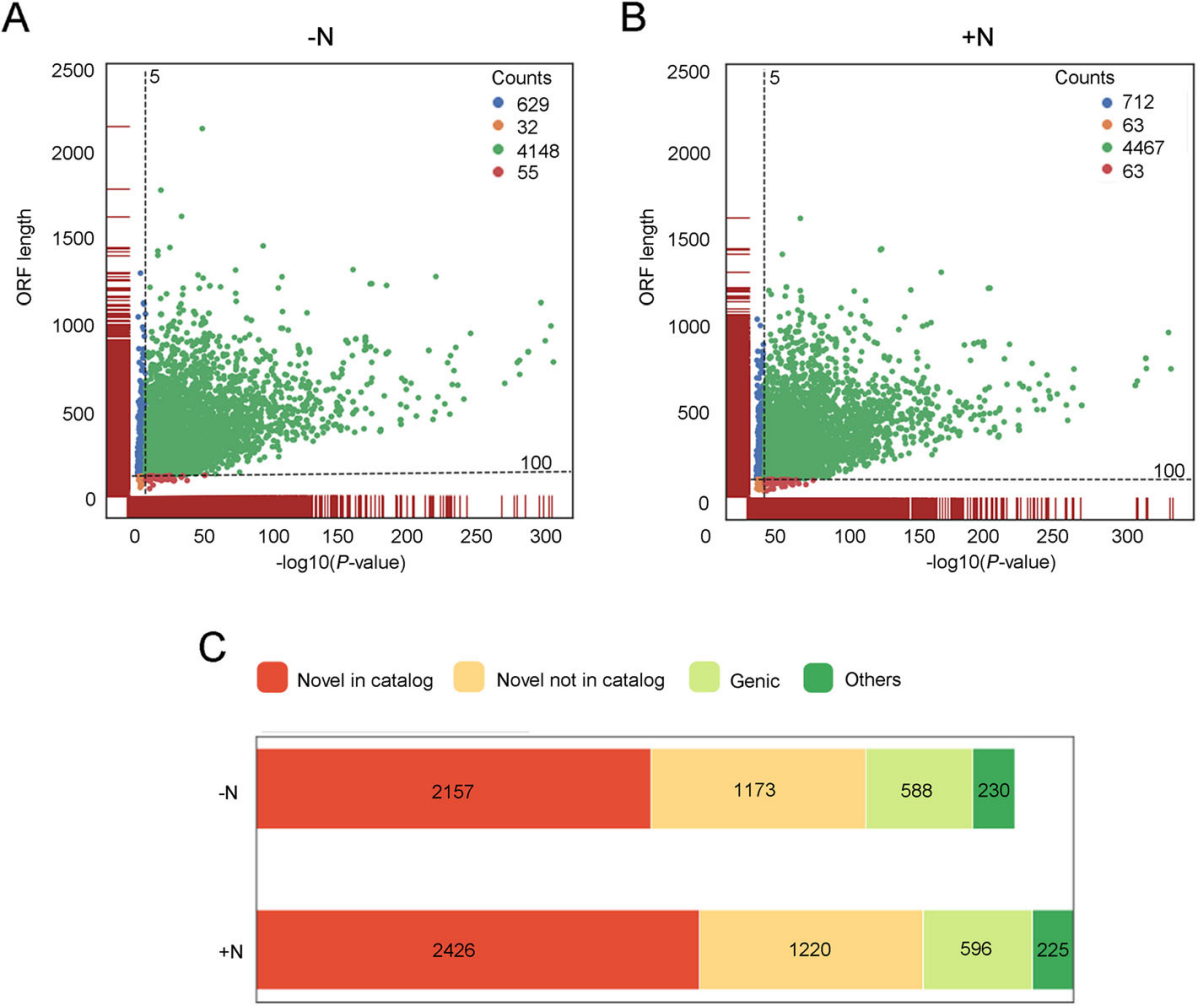
Characterization of novel isoforms and coding potential. **a** and **b** The functional domain analysis and distribution of open reading frame (ORF) lengths in the untreated and treated samples, respectively. The presence of functional domains in the novel isoforms was evaluated using *hmmscan* against the *Pfam* database. **c** The number of novel isoforms (ORF > 100) with functional domains (green dots in A) in each category classified by *SQANTI.qc*

To investigate whether the novel isoforms could function as putative protein-coding genes, we evaluated their capability of coding known protein domains. Their coding potential for of the known proteins domains was assessed using *hmmscan* align the sequences against the *Pfam* database [38]. Using the *P*-value of 10e-5 as a threshold, we found the majority (more than 70%) of novel coding transcripts capable of coding known protein domains. Among these isoforms, 4148 isoforms in the untreated sample, and 4467 isoforms in the treated sample (Fig. 5a and b) harboring ORF longer than 100.

We next investigated the structural categories of these novel protein-coding transcript isoforms. For more than 4000 novel putative protein-coding isoforms identified in each sample, about 50% of them were labeled as Novel in Catalog (NIC), which contains new isoforms with combinations of already annotated SJs or new SJs formed of annotated donors/acceptors (Fig. 5c). Isoforms in this category showed the highest rates of validation in the PCR experiments, among other kinds of categories representing novel transcripts [36]. The second-largest category is Novel Not in Catalog (NNC), which contains the transcripts using novel donors/acceptors. More than one thousand isoforms were included in NNC for each sample, accounting for 28.2 and 27.3% of total novel protein-coding isoforms, respectively. Other than these two categories, less than 600 novel isoforms were classified into the category labeled as Genic, transcripts in this group were overlapped sequences associated with known genes, but contained partial exon and intron (Genic Genomic), or lying completely within the region of the intron. The rest of the isoforms (230 for the untreated sample, 225 for the treated sample) were aligned to the novel transcribed regions. These results suggested that the majority of the novel transcripts were transcribed and have high probabilities of encoding proteins with known functional domains.

We next evaluated the novel non-coding isoforms by looking into the evidence of being functional non-coding RNAs. Based on our analyses, we found about 13.3% of novel transcript isoforms were classified as non-coding transcripts (777 in the untreated sample, 1048 in the treated sample). It is worth mentioning that all the predicted novel non-coding transcripts were longer than 200 bp, which fits the definition of long non-coding RNA (lncRNA). We utilized the *cmscan* option integrated into the infernal tools [39] to identify the novel isoforms that have good matches to the non-coding RNA families in the *Rfam* database [40]. The *Rfam* database is a collection containing multiple sequence alignments and secondary structure profiles that represent non-coding RNA families. As a result, 88 sequences were found had good matches to the *Rfam* non-coding RNA families, resulting in 185 hits (e-value < 0.01, bit scores, and gathering cutoff, Supplemental Fig. S5A).

According to the annotation in the *Rfam* database, more than half (105) of these hits were found belong to small nucleolar RNA families. The second-largest set belonged to microRNA (miR) families, which supposed that are involved in regulating gene expressions. We also found 16 small subunit ribosomal ribonucleic acid (SSU rRNA) family members, which are the smaller parts of the two major RNA components of the ribosome. One particular non-coding novel transcript was found that matched the profile of miR171 identified in the treated sample (ID: PB.6117.1). In the untreated sample, we found an alternative isoform with a shorter 3′ tail (ID: PB.6067.2). Based on our analysis, the mature sequence of predicted miR171 is located in the extended 3′ tail in the treated sample. It is the homolog of *miR171A* (AT3G51375) in the model plant Arabidopsis (Supplemental Fig. S5B). These results suggested that the novel non-coding transcripts possessed longer 3′ tails are involved in response to nitrate supply, either as putative targets or the micro RNA themselves.

### Nitrate treatment decreases the distribution of transcript lengths

As an alternative splicing event often alters the length and coding region of a transcript, we first tested if N treatment had significant effects on the global length distribution of transcripts in the samples. Results showed that nitrate supply significantly changed the length distributions for transcripts (Mann-Whitney U test, *P*-value = 1.74e-79) and coding region sequences (CDS, *P*-value = 5.95e-28). We next evaluated the effects of nitrate supply on these attributes by comparing the 25th, 75th quantiles, and medians between two samples. In all the categories classified, we checked the distributions of isoforms lengths, the number of exons, and coding start/end sites. The ranges of these distributions were generally decreased in the treated sample than that in the untreated sample. For the categories labeled as FSM and ISM, which contain the top two highest-confidence isoforms, the median lengths decreased 8.7 and 18.7%, respectively. For the novel isoforms associated with single known genes, including the categories labeled as NIC, NNC, Genic, and Genic intron, the nitrate supply decreased the median length ranging from 9.1 to 19.9%. The other categories contain the novel isoforms associated with putative novel genes (Genic_intron), antisense known genes (Antisense), and more than one known genes (Fusion). The median length of the isoforms in each of these categories is down-regulated by 19.9, 18.0, and 5.6%, respectively. Similar results were found in the coding regions length stats that the median lengths in the treated sample are about 5.3–23.6% shorter than that in the untreated sample (Supplemental Fig. S6A and B).

We next checked if the nitrate supply affects the distribution of CDS start sites and end sites. Results showed that, at the global level, it significantly advanced the CDS stop sites (Mann-Whitney U test, *P*-value = 2.60e-34) rather than CDS start sites (*P*-value = 0.09). Indeed, compared with the untreated sample, the discrepancies of the medians of CDS start sites in the treated sample were less than 100 bp in most categories, except for that labeled as *Genic intron* (157 bp), which contains the least number of putative protein-coding isoforms (12 in the untreated sample, 28 in the treated sample). For the locations of CDS-end sites, the gaps of different categories between treated and untreated samples ranged from 132 to 257 bp, except the set of Genic intron, which is 533 bp (Supplemental Fig. S6C and D). These results suggested that genes tend to generate shorter isoforms harboring shorter CDS after nitrate supply due to altering the splicing patterns of pre-mRNA in the root tissues.

### Nitrate supply affects AS patterns of thousands of genes

**Fig. 6:**
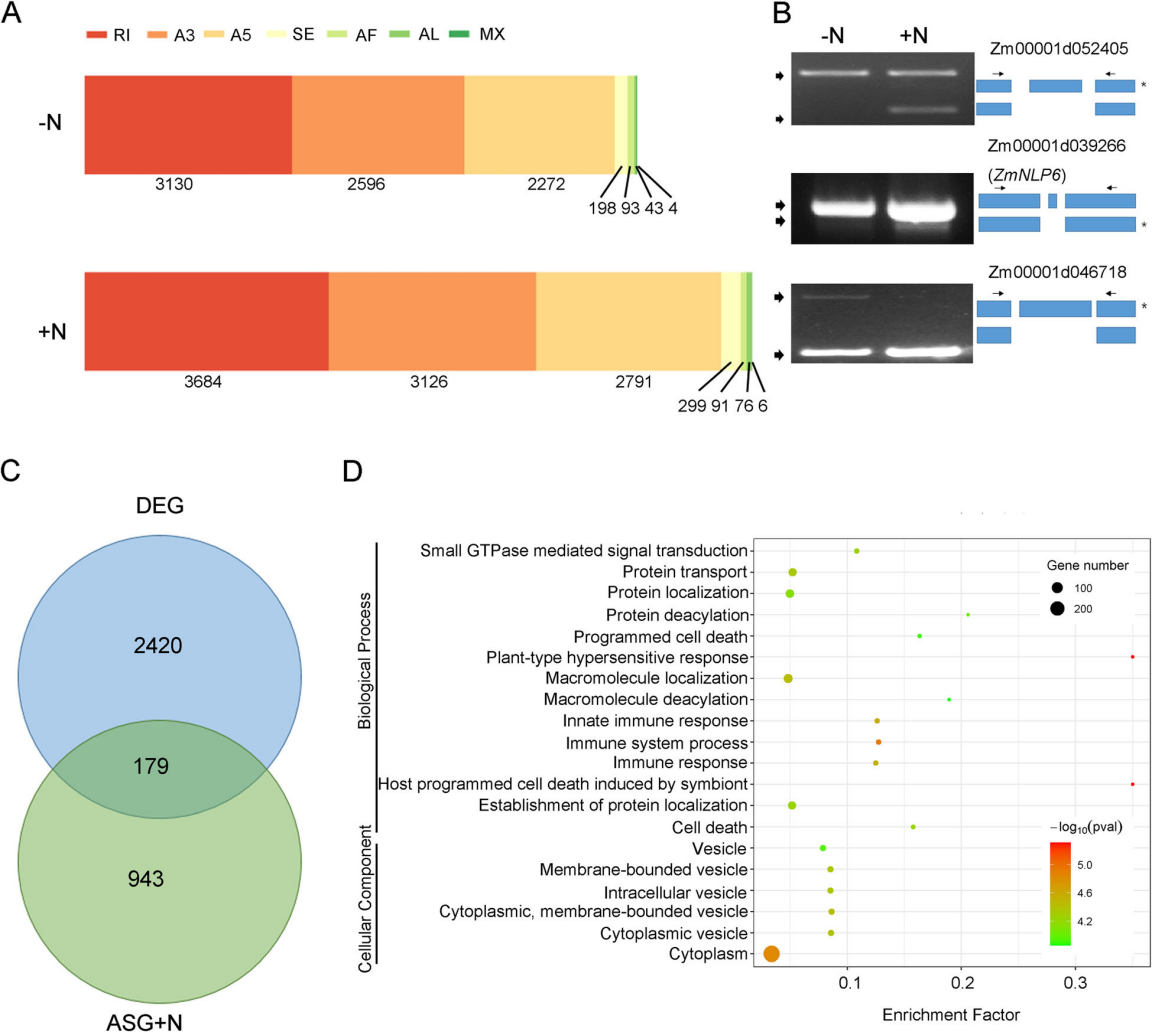
Characterization of AS events affected by nitrate supply. **a** Different types of AS events detected in the samples. The AS events were identified using *SUPPA* software. **b** PCR validation of several AS events, novel isoforms are marked with “*”. **c** Venn diagram of DEGs and genes that specifically experienced AS after nitrate supply (ASG + N). **d** Top 20 enriched pathways in the GO analysis of ASG + N that are not differentially expressed after N treatment

To reveal the detailed effects of N treatment on the AS events, we quantified the number of AS events in the untreated and treated samples, respectively, using *SUPPA* software [41]. In total, we discovered 8336 AS events in the untreated sample. After the nitrate supply, the number of detected AS events was increased by 10,073. *SUPPA* classified the AS events into several groups, including exon skipping (SE), alternative 5′ and 3′ splice sites (A5/A3), mutually exclusive exons (MX), intron retention (RI), and alternative first and last exons (AF/AL). The most abundant type of AS events was RI, which accounts for more than one-third of total events. More than 95% of total events consisted of three types of AS events, including RI, A3, and A5 (Fig. 6a). Using PCR experiment, we validated several AS events in both untreated and treated samples (Fig. 6b).

Based on the AS profiles, we found 1122 genes that specifically experienced AS events after nitrate supply (ASG + N). Most of these genes (943) were not significantly altered the transcriptional abundance after nitrate supply (Fig. 6c). To reveal the functional insights of these genes, we subjected these gene IDs into the GO enrichment analysis, 818 genes were annotated. Results showed 14 biological processes, and 6 cellular components were enriched (Fig. 6d, Supplemental Table S6).

Among the GO terms classified into the set of biological processes, seven of them, including the most significant term, were related to plant immunity. For instance, GO terms like “host programmed cell death induced by symbiont” (GO:0034050, *p*-value = 4.9e-06, FDR = 0.0088), “immune system process” (GO:0002376, *p*-value = 1.2e-5, FDR = 0.014), and “Innate Immune Response” (GO:0045087, *p*-value = 2.7e-5, FDR = 0.021) were enriched in the category of biological process, suggesting that nitrate supply triggers AS events on the genes involved in cell death and immunity response. A homolog (Zm00001d038971) of Necrotic Spotted Lesion 1 (NSL1, AT1G28380), of which dysfunction form confers programmed cell death in Arabidopsis, was undergone AS events typed A5 [42]. A homolog of *RPM1* (Zm00001d021498), which functions as a key regulator in regulating hypersensitive response in Arabidopsis, was undergone A3, A5, and RI to generate different isoforms after nitrate supply [43]. The other enriched biological processes are mainly related to localization, transport, modification and signal transduction, such as “protein localization” (GO:0008104, *p*-value = 7.2e-5, FDR = 0.023), “protein transport” (GO:0015031, *P*-value = 4.7e-5, FDR = 0.021), “protein deacylation” (GO:0035601, *P*-value = 9.1e-5, FDR = 0.027), and “small GTPase mediated signal transduction” (GO:0007264, *P*-value = 5.4e-5, FDR = 0.021). These groups contain genes that encode proteins mainly related to particular cellular components or involved in delivering macromolecules or mediating signal transduction. For example, Zm00001d026312 encodes an ortholog of a transmembrane superfamily member Os06g0650600 in rice, undergoing AS typed A3 after N-treatment. Zm00001d011717 encodes a ras-related protein, which is involved in modulating membrane trafficking, undergoing AS typed A5, A3, and RI.

For the cellular component, the GO terms mainly associated with cytoplasm and vesicle, including “cytoplasm” (GO:0005737, *p*-value = 1.5e-5, FDR = 0.0062), “membrane-bounded vesicle” (GO:0031988, *P*-value = 4.3e-5, FDR = 0.0062), “cytoplasmic vesicle” (GO:0031410, *P*-value =4.1e-5, FDR = 0.0062) and so on (Supplemental Table S6). Genes in these GO terms often related to trafficking and degradation. For instance, Zm00001d018259, which encodes an AUTOPHAGY-RELATED protein (ATG12), was found experiencing A5 and RI after N-treatment. These results suggested that nitrate supply triggers the AS events on genes involved in multiple biological processes that are linked to plant immunity, transportation, and metabolism.

### *ZmNLP6* generated different isoforms in response to nitrate treatment

**Fig. 7:**
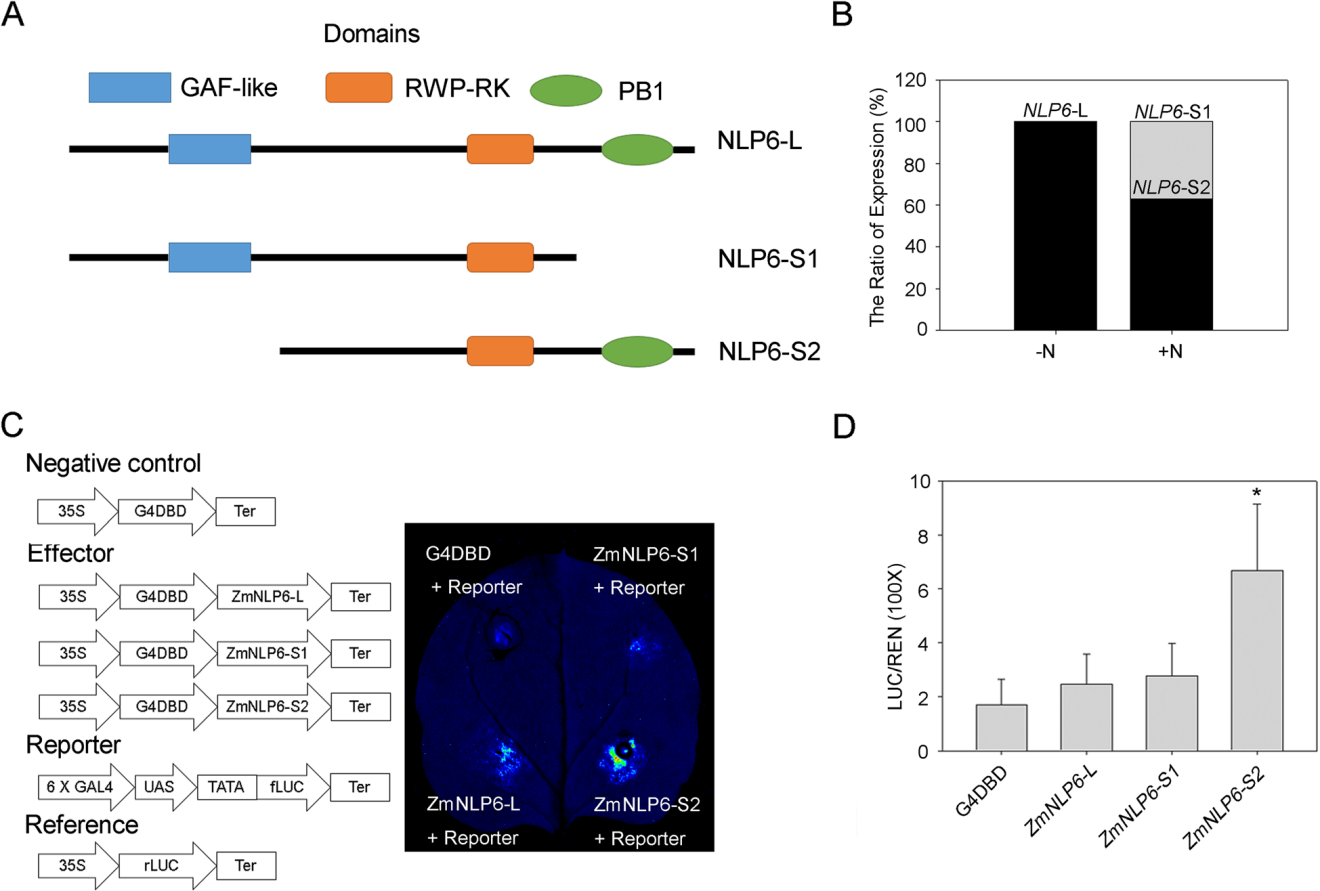
The different *ZmNLP6* isoforms vary in the capacities of activating downstream targets. **a** The diagram of the isoforms of ZmNLP6 identified in this study. **b** The ratio of transcriptional abundance of different *ZmNLP6* isoforms. **c** The diagram of the vectors used in the transient assay. **d** The LUC/REN ratio represents the relative activity of the promoters. The data are expressed as mean ± standard deviation of three separate tests (*n* = 3)

Transcription factors function as key players in regulating N response in maize. Included among the set of ASG + N, we found a transcription factor, *ZmNLP6*, which encodes a NIN-like protein (NLP) family member containing a GAF domain, an RWP-RK domain, and a PB1 domain [44, 45]. *ZmNLP6* was not characterized as a DEG, according to our RNA-seq results. We found one isoform in the untreated sample, and three shorter isoforms in the treated sample were mapped to the *ZmNLP6* gene locus. We subjected the predicted ORFs of these isoforms to the SMART website (http://smart.embl-heidelberg.de/) to find whether these different splicing isoforms contain the domains of ZmNLP6. The longest isoform (*NLP6-L*, PB.3640.1) that was identified in the untreated sample containing all three domains reported in the previous studies. Two shorter isoforms, which specifically found in the treated sample, lack the PB1 domain (*NLP6-S1*, PB.3678.2) and the GAF domain (*NLP6-S2*, PB.3678.1), respectively (Fig. 7a). The other one (PB.3679.1) identified in the treated sample lacks all three domains that were supposed in ZmNLP6. Therefore, we excluded this isoform of *ZmNLP6* in further studies. Combining with the RNA-seq data, we found that the expression of *ZmNLP6-S2* was more than that of *ZmNLP6-S1* in the treated sample, accounting for 56.2% of the total expression of *ZmNLP6* (Fig. 7b).

To identify if these isoforms encode functional proteins, we investigated the ability of each *ZmNLP6* splicing variant for activating downstream genes, using transient expression assay in the tobacco leaves. We combined the sequence encoding binding domain (BD) to the 5′ end of the predicted coding sequences of *NLP6-L*, *NLP6-S1*, and *NLP6-S2*, respectively. The fused sequences were constructed into the p*MDC83* vector, driven by the 35S promoter. These constructs, as well as the vector harboring the *UAS-LUC*, were transformed into *Agrobacterium* (EH105) and injected into tobacco leaves. We found that the injection of *NLP6-S2* combined with the plasmid containing the *UAS-LUC* element resulted in the highest LUC activity (Fig. 7c and d). These results suggested that nitrate supply triggers AS events on *ZmNLP6* to generate different isoforms with various capabilities of activating downstream genes.

### Overlapping DAP-seq and RNA-seq results supports the role of ZmNLP6 as a *trans*-activator

**Fig. 8:**
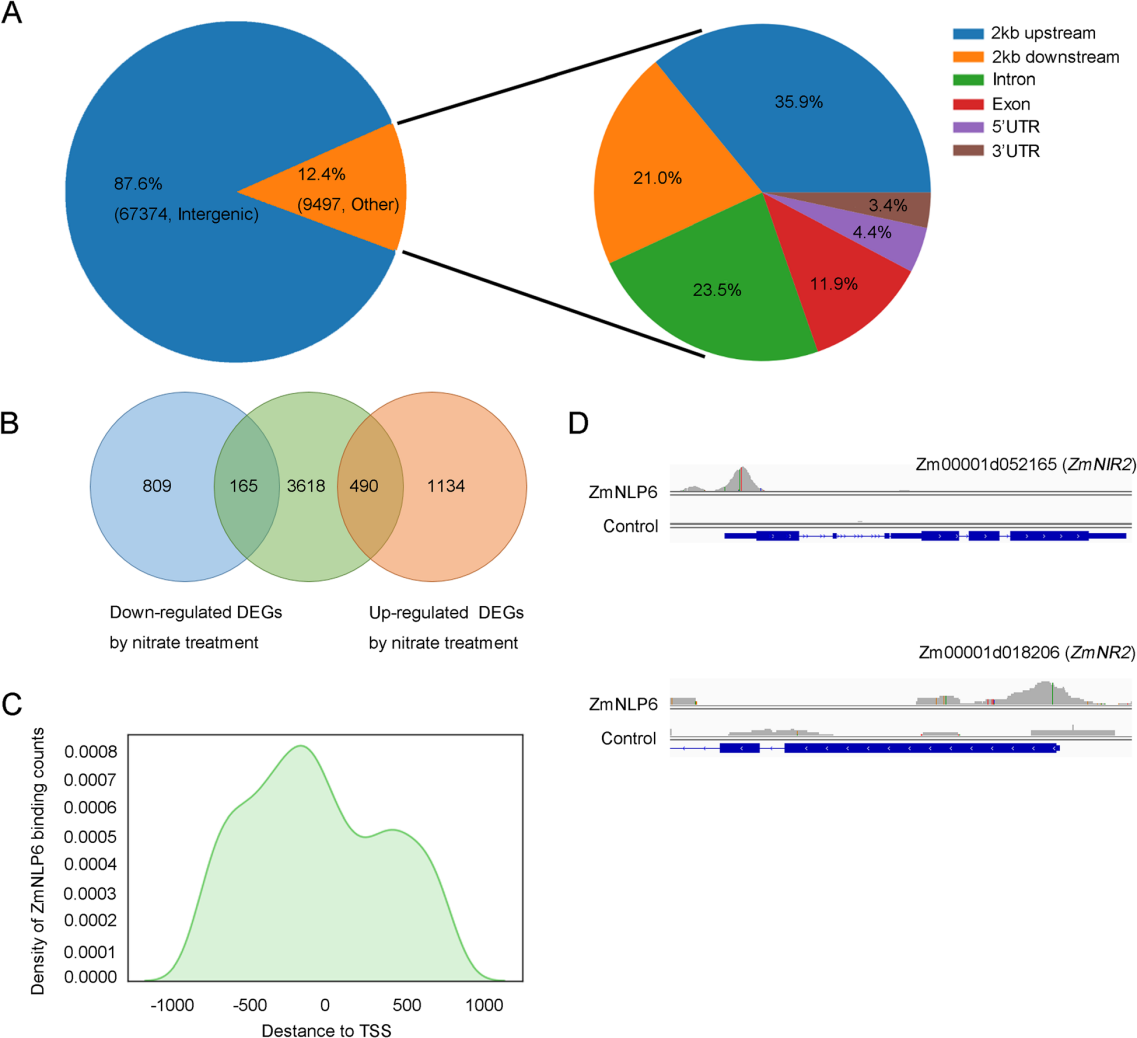
DAP-seq analysis of ZmNLP6. **a** Distribution of ZmNLP6 binding regions in the maize genome. **b** Venn diagram representing a comparison between RNA-Seq and DAP-Seq results. **c** Distribution of ZmNLP6 binding peaks corresponding to the − 1000 to + 1000 -bp region flanking the TSS. **d** ZmNLP6 binding peaks for the *ZmNIR2* and *ZmNR2* are shown in the Integrated Genome Browser

To seek further evidence to support that ZmNLP6 functions as an activator during the early N-response, we performed the DAP-seq experiments. Peaks that are overlapped in two biological replicates were used to identify binding sites. With the cutoff of *q*-value 1e-5 and the genic region defined as 2 kb upstream from the transcription start site (TSS) and downstream of transcription terminate site (TTS), we found 76,871 binding sites. Most of these binding sites (87.6%) were located in the intergenic regions. The remaining peaks (9497) were rested in the areas associated with annotated genes, around one-third (35.9%) within the 2 kb upstream from TSS, 21.0% within 2 kb downstream from TTS, less than 10% laying in the untranslated regions (4.4% for 5’UTR, 3.4% for 3’UTR). The rest peaks were harbored in the coding regions (Fig. 8a).

Combined with the results of RNA-seq, 4273 genes associated with peaks that are not located in the intergenic regions were expressed in the root tissues. Of these genes, 165 were down-regulated, and 490 were up-regulated (Fig. 8b). This distribution was different in comparison with the global distribution of DEGs identified by RNA-seq (*p* < 0.01, χ2 test), suggesting that ZmNLP6 works as a transcription activator. The binding density of ZmNLP6 peaked within the location ranging from − 500 to 0 bp to the TSS (Fig. 8c). Additionally, we found that ZmNLP6 binds to the promoter regions of two key genes in the N-assimilation, which encode ZmNIR2 and ZmNR2 (Fig. 8d). The expression of these two genes was significantly up-regulated after N treatment, suggesting that ZmNLP6 contributes to the up-regulation of these two genes in the transcriptional level. Besides, using GO enrichment analysis, we identified 132 of its putative target genes associated with N metabolism (such as “cellular nitrogen compound metabolic process”-GO:0034641, “regulation of nitrogen compound metabolic process”-GO:0051171, and “organonitrogen compound metabolic process”-GO:1901564, Supplemental Table S7). These putative ZmNLP6 targets contain genes encoding plasma-membrane h + ATPase2 (Zm00001d026490), nitrate reductase (Zm00001d018206), Glutamate Decarboxylase (Zm00001d047981). In addition, several genes that encode predicted transcription factors are also included, such as bHLH family member (Zm00001d023262) MYB family protein (Zm00001d034160), and WRKY22-like protein (Zm00001d044171). These results support that ZmNLP6 functions as a transcription activator and plays a role in regulating the N-response in maize.

## Discussion

In this study, we performed a high-resolution profile of transcriptome in response to N in maize. We developed a workflow that roots from the *STAIR* pipeline [21], integrating the short-read sequencing and long-read sequencing to obtain high-confidence sequences of full-length isoforms. We found 943 non-DEGs that experience AS events in the treated sample specifically. These genes are involved in multiple functions, including immunity, transportation, and metabolism. In particular, we found an NLP family member, *ZmNLP6*, responding to the nitrate supply by changing splicing patterns. Transient expression assay and DAP-seq results suggest that *ZmNLP6* is involved in regulating early N-response in maize.

### Nitrate supply affects the expression of genes related to multiple functions in the N-starved root tissues shortly

N functions as not only a nutrient but also a signal molecule. Shortly (often as early as minutes) after sensing nitrate supply, thousands of genes altered their expression, calling primary nitrate genes. These genes, as well as the N-regulatory network involved in the early N-response, have been well studied in Arabidopsis [11, 46]. Relative less information of nitrate primary genes in maize, however, is documented. Several studies profile the transcriptome in response to nitrate as early as 1 h after N-treatment [47–49] in maize. N-response after this time point was classified as the late N-response, according to the study of Arabidopsis [45]. In this study, we found that the expression of several marker genes, which are involved in N assimilation (*ZmNiR2*, *ZmNR2*, and *GS*), showed a significant increase within 30 min after nitrate supply. These results suggest that N-starved maize seedlings sense N variation swiftly-perhaps only minutes after subjected to supplemental N. A combination of ribosome profiling and RNA-seq suggested that maize plants adjust the responses of transcription and translation independently to drought stress, although moderate correspondence in the fold changes of gene expression and translational levels was found, [50]. Our RNA-seq results revealed that primary nitrate genes in maize are linked to a number of functions, such as functions associated with N assimilation, transportation, binding activity, and phytohormones. A previous proteomic study showed that functions related to amino acid metabolism were found enriched in the proteins that the abundance is affected by hours after N variation [49]. While some GO terms, such as biological processes related to signal transduction, rhythmic process, and phytohormone (jasmonic acid), are not identified in the proteomic analysis, supporting the suggestion that gene expression and the translational level is both moderately correlated and independent in response to the environmental change.

### The combination of short-read and long-read data improves the resolution of annotation on transcriptome

Long-read sequencing greatly improves the chance of finding the novel transcripts and alternative splicing events that are hardly found in short-read sequencing data [37, 51]. In order to decrease the error rates, the major disadvantage of SMRT-sequencing, the latest version of IsoSeq (IsoSeq3) strengthens the criteria for screening high-quality (HQ) isoforms, thus resulting in a fewer number of isoforms, compared with that obtained by prior versions of IsoSeq. This explains, at least partially, the big gaps found between the counts of isoforms in the sets of FLNC CCSs and HQ isoforms, to some extent offsetting the advantage of generating long-reads with lower error rates. In this study, we developed a workflow based on the hybrid-sequencing strategy, moving the number of identified high-confidence full-length isoforms (FLNC-validated) up about 2.2 times. The quality analyses showed that the workflow improves the number of isoforms that are perfectly matched to the annotation of the genome (category labeled as FSM) in comparison with that in the set of HQS. The study shows that all the isoforms randomly picked from this group could be validated using PCR experiments, thus showing the highest rate of validation among all the categories classified by *SQANTI.qc* [36]. Furthermore, the set of FLNC-validated contains even more isoforms classified in the FSM than that FLNC does. This indicates that our workflow not only improves the quality of long reads by correcting their errors but also retains the substantial number of full-length isoforms that are discarded by performing the IsoSeq3 pipeline on the long-read sequencing alone.

Compared with the SJs found in the set of HQS, we found that FLNC-validated contains much more canonical SJs, but less non-canonical SJs. Notably, all known non-canonical SJs are eliminated in the FLNC-validated isoforms, suggesting that RNA-seq does not support most of the non-canonical SJs. We found the ratio of non-canonical SJs account for about 0.1% over the total SJs in maize roots, consistent with the SJs distribution presented in the recent research [30], based on the data derived from maize grown under normal N conditions. Together with our study performed on N-starved roots and N-treated roots, this suggests that the proportions of canonical and non-canonical SJs are relatively stable in response to N variations, compared with AS events affected by nitrate supply.

### Long-read sequencing works better in identifying novel protein-coding isoforms than non-coding RNAs

One tempting feature of using long-read sequencing is that it, in many cases, identifies a substantial number of novel transcript isoforms, according to the corresponding annotation of reference genomes [29, 30, 52]. Generally, there are two types of novel transcripts, one consists of isoforms associated with annotated genes, and the other represents the transcripts locating in the novel transcript regions (NTRs). In the previous study of zebrafish, thousands of novel NTRs were detected due to the rudimentary annotation of the reference genome [51]. In this study, we identified thousands of novel transcripts. The majority of them are associated with known genes, according to the latest version of reference [52], which is also assembled using the long-read sequencing. Similar results were reported in another study made on maize [37], suggesting that the reference annotation is relatively thorough. Compared with the prior version of the maize genome and annotation, a big step of improvement has been made in the current version of the genome. However, the proportions of NTRs and novel isoforms associated with annotated genes changed little, regardless of which version of the maize genome and annotation is used as a reference [30, 51]. This suggests that the status of global RNAs in maize is highly dynamic and sensitive to the environment. Thus, every individual transcriptome test made by the long-read sequencing experiment captures many novel isoforms that are not identified by other experiments.

The majority of the novel isoforms identified in this study are potential protein-coding transcripts. Moreover, a major part of novel protein-coding isoforms was found containing functional domains, using the computational methods based on the annotation of the database. Compared with these coding isoforms and the non-coding transcripts identified in the previous study [18], however, not only the relatively low number of novel non-coding transcripts were identified, but also only the minority of them were found similar to the known functional ones, suggesting that long-read sequencing has its limitation in finding non-coding RNAs (ncRNAs). Since by default, *IsoSeq* pipeline looks for poly-A tails to define full-length transcripts, the low number of ncRNAs found in this study possibly due to the exclusion of very short-read sequences and less likelihood of that ncRNAs are poly (A) tailed [53], suggesting that using current SMRT-sequencing may underestimate the contribution of ncRNA in the global transcriptome.

### Nitrate supply affecting thousands of genes by altering either their expression or splicing patterns

Overall, we found that nitrate supply increased about 2000 AS events. Some 1000 genes were specifically undergone AS events in the treated sample (AS+N genes). However, only a small part of these AS+N genes was also DEGs, consisting with the previous report that most differential AS genes do not alter their expression in response to environmental stresses [27, 28]. Therefore, our results support that AS represents an independent layer of gene regulation in response to the environmental changes. We found that the AS+N genes that are not differentially expressed after N-treatment are involved in some specific functions, such as immunity response, transportation, and localization, suggesting these biological processes are linked to N assimilation and metabolism.

Non-sense mediated decay (NMD), which targets the transcripts with a premature termination codon (PTC), is a quality-control mechanism to prevent unwanted gene products [54]. In this study, we found that the distribution of the transcript lengths is relatively shorter in the treated sample. Moreover, the general distribution of CDS ending sites is shifted ahead in comparison with that in the untreated sample, suggesting that nitrate supply triggers the production of many isoforms harboring PTC. Considering that many AS+N genes are involved in the immunity and autophagy processes, which often function as key factors under stress conditions, it is possible that plants is limiting the production of genes related to stress response after sensing the advent of supplemental nitrate, helping to maximize the assimilation of N-nutrients. For example, ATG8 and ATG12 are key components in the pathway of autophagy, which is crucial for nutrient recycling in maize [55]. In this study, we found that *ATG12* undergone AS specifically in the treated sample, suggesting nitrate affects the splicing pattern of *ATG12*, possible for restricting the production of ATG12 protein by generating “non-productive” RNAs of *ATG12*. This speculation is supported by the observation that *atg12* mutants merely showed severe defects under N starvation, compared with wild-type plants [55]. Collectively, we found that nitrate supply not only affects the transcriptional abundance of many transcripts, it also changes the splicing patterns of thousands of genes.

### ZmNLP6 possibly functions as a trans-activator through AS mechanism

The NIN-like protein family member (NLP), AtNLP7, harboring a DNA-binding domain (RWP-RK) and a PB1 domain, has been proved that functions as a master regulator in regulating the N-response in Arabidopsis [32, 33, 35, 56]. In maize, nine NLP family members were characterized, similar to the case of *AtNLP7*, nitrate supply appears has little effect in changing the transcriptional abundance of *ZmNLP6*, a homolog of *AtNLP7* [44], consisting with our RNA-seq results that *ZmNLP6* is a non-DEG. In our study, however, we found that *ZmNLP6* generated several shorter variants after nitrate supply through alternative splicing. In addition, one of the short isoforms, *ZmNLP6-S2*, which lacks the GAF domain, has an increased capability of activating the expression of downstream *LUC*, suggesting that the GAF domain has a negative role for ZmNLP6 in activating downstream targets.

According to our DAP-seq results, many putative target genes of ZmNLP6 are involved in regulating N-metabolism, including two key enzymes linked to N assimilation named *ZmNR2* and *ZmNIR2.* This result agrees with the finding that ZmNLP6 is able to bind the nitrate-responsive *cis*-element (NRE) presented in the promoter of the *ZmNIR2*, suggesting that ZmNLP6 is a key regulator in the process of assimilating N nutrients. In addition, the overexpression of *ZmNLP6* in Arabidopsis complements the phenotype of *atnlp7–4* [44], restoring the root growth of the mutants, especially under the DN conditions, suggesting functional similarities between *ZmNLP6* and *AtNLP7*. Moreover, the combination of RNA-seq and DAP-seq analyses showed that the number of up-regulated target genes of ZmNLP6 is about three times that of down-regulated ones, supporting the idea that ZmNLP6 functions as a trans-activator. Besides, included in the target genes that are up-regulated after N treatment, more than 100 genes are found directly linked to N metabolism, including a gene related to transport (plasma-membrane h + ATPase2, Zm00001d026490), the enzymes that catalyze early steps of N assimilation (ZmNR2, ZmNIR2), as well as putative transcription factors, suggesting the involvement of ZmNLP6 in the early N-response network. Together, we speculate that ZmNLP6-S2, which generated through AS of *ZmNLP6*, contributes to the activation of these genes after N supply.

## Conclusion

Collectively, our study has identified both DEGs and genes changed splicing patterns in response to nitrate supply, suggesting the role of AS in orchestrating the N-response network in the N-starved root tissues. Our findings could help further uncover the molecular mechanisms underlying the N-response in maize.

## Methods

### Plant materials and growth conditions

The B73 seeds used in this study were obtained from the maize stock center (www.maizegdb.org). The seeds with similar size were prepared were sterilized and germinated on the water-soaked filter paper for 72 h [57]. The germinated seeds were grown in the hydroponic culture of sufficient N (SN) solution and deficient solution (DN) for 2 weeks, respectively. A modified Hoagland nutrient solution [58] was used to culture seedlings, with 15 mM KNO_3_ as SN solution and 0.15 mM KNO_3_ as DN solution. The KCl was used to balance the gap of potassium supply. The root tissues of two-week-old seedlings were harvested for phenotype analysis and subsequent sequencing experiments.

### Quantitative PCR (qPCR) and reverse transcription PCR (RT-PCR)

Total RNA was extracted from root tissues using the SV Total RNA Isolation System kit (Promega, USA). For qPCR, total RNA was collected from samples with different time points after nitrate supply. The DNA was digested using RNase-free DNase I. The Prime ScriptTM RT Reagent kit (Takara, Dalian, China) was used to generate cDNA. The qPCR experiment was performed using a Bio-Rad CFX96 system with SYBR® Premix Ex Taq™ II (Takara, Dalian, China). The gene *ZmUPF1* [59] was used as an internal control. For the RT-PCR experiment, the KOD polymerase (Toyobo) was used on testing AS events in the untreated and treated samples, respectively. The primers used for qPCR and RT-PCR were included in supplemental Table S8.

### RNA-Seq analysis

The library for RNA sequencing was prepared based on Illumina standard instruction (TruSeq Stranded RNA LT Guide). According to the instructions in the Hisequation 2500 user guide, the library DNA was checked for its concentration and size distribution to ensure it met the request of Illumina HiSequation 2500 system before sequencing was performed. Evaluation of reads’ quality was accomplished using *FastQC* [60]. Any reads were removed if they were less than 40 bp using *Btrim* (set the parameter –l = 40) [61], and remaining reads were used for further analysis. cDNA sequence file (downloaded from www.maizegdb.org) derived from Maize B73 genome assembly (V4) [52] was used as a reference. The *Salmon* software (version: 1.1.0) was used for reads mapping to the reference cDNA sequences and calculating the transcript per million (TPM) mapped reads of each transcript using *quasi-mapping* method [62]. The *P*-value of differential expression was calculated in the *R* environment (version 3.6.3, https://www.r-project.org/) using *EdgeR* package [63] (version: 3.28.1), for it has well performance in the identification of DEGs using three biological replicates [64]. *EdgeR* was downloaded from the Bioconductor website (www.bioconductor.org). GO enrichment analysis was performed using *agriGO* (version:2.0) [65].

### The development of the workflow for processing long-read sequencing data

Our workflow for processing the long-read sequencing data are developed based on the *STAIR* pipeline [21]. The original *STAIR* pipeline was used to deal with the similar problem found in long-read sequencing data from rice. It cannot be applied on our data directly due to the upgrade of the *IsoSeq* software and the discrepancies of annotation files between rice and maize. To address this problem, we modified the pipeline in three aspects: first, we wrote a new script that integrates the programs in the *IsoSeq3* for processing the long-read sequencing data to acquire FLNC CCSs (*sub2flnc.py*). Second, we spliced the *STAIR* pipeline into four smaller scripts, enabling us to utilize the softwares freely as long as the results generated by the softwares meet the requirement of each script. Finally, we rewrote most of the scripts and adjusted the parameters in the original pipeline to make the whole workflow can process the sequenced data from maize, the annotation files of high-confidence isoforms obtained using our workflow are listed in supplemental Table S9 and S10, respectively. The codes used in this study is available on the GitHub (https://github.com/www139516/Hybrid-sequencing-workflow).

#### Obtaining the FLNC CCSs and correct the errors

For obtaining the full-length non-chimeric CCSs, we performed the first three steps of the *IsoSeq3* pipeline, including commands of *ccs*, *lima*, and *isoseq3 refine*. The parameters required for these commands were optimized. These three steps were integrated in the pipeline named *sub2flnc.py*. For the error correction of FLNC CCSs, we used the *proovread* software (version: 2.24.1) [66]. For each long-read sequencing dataset, we utilized the corresponding RNA-seq data to correct the long reads, respectively, using default parameters. We also performed the entire *IsoSeq* pipeline (version: 3.0.0) to acquire the high-quality (HQ) isoforms.

#### Mapping the long reads to the reference assembly

The corrected long-reads were mapped to the maize b73 reference assembly (v4) using GMAP (version 2019-01-24) [67]. The mapped reads were sorted using *sort_sam.py*. The sorted isoforms were collapsed into non-redundant isoforms using *collapse_isoform_by_sam.py* (Pacific Bio-sciences) using default parameters. The splicing junctions were detected using STAR software (version: 2.5.3a). According to the STAIR pipeline [21], some parameters were set as follows: outFilterMismatchNmax = 10, alignMatesGapMax = 200,000, alignMatesGapMax = 200,000, chimSegmentMin = 15. The chain of splicing junctions in each non-redundant isoform was validated using *sj_validation.py* to obtain the high-confidence isoforms.

#### Quality checking and AS events calculating

The qualities of obtained long-read sequences were checked using *SQANTI.qc* (36). AS events were calculated by the *SUPPA* pipeline [41] using the GTF files generated by the *SQANTI.qc* with default parameters. These GTF files were obtained based on the high-confidence isoforms obtained by the workflow developed in this study.

### Transient transcriptional activity assay

For the transient expressional assay, the cDNAs of *ZmNLP6*, including full-length CDS (ZmNLP6-L), two shorter isoforms (*ZmNLP6-*S1, *ZmNLP6*-S2) were amplified using P15/P16, P15/P18, P16/P17, respectively. Each amplified DNA fragment was constructed into the vector pMDC83-35S to generate effectors containing different CDSs of *ZmNLP6*. The GAL4/UAS-based system was used to test the transcriptional activities of ZmNLP6-L, ZmNLP6-S1, and ZmNLP6-S2. The firefly luciferase (LUC) gene was placed downstream of 6 × GAL4 binding sites (UAS) to construct the reporter. The control vector harboring *35:: Renilla luciferase* (RLUC) was used as an internal control. All the vectors were transformed into Agrobacterium EH105. The bacterium was infiltrated in the leaves of *N. benthamiana.* The injected plants were well watered and subjected to dark conditions for 24 h. After incubation in the dark, the plants were grown under normal conditions for the next 48 h. The leaves were collected and observed using a low-light cooled CCD imaging apparatus (Tanon 56,200 Multi). The activities of LUC and RLUC were quantified using the Dual Luciferase Reporter Gene Assay Kit (Beyotime, Shanghai, China).

### DNA affinity purification sequencing (DAP-seq)

For DAP-seq, the genomic DNA was isolated from root tissues of two-week-old seedlings (B73) grown under hydroponic culture with sufficient N supply. The genomic library was constructed following the steps in the protocol of Bartlett [68]. The full-length cDNA of *ZmNLP6* was constructed into vector pFN19K, which contains a sequence encoding an HaloTag affinity tag at the 5′ end of the *ZmNLP6.* The Halo fused protein was expressed in the wheat germ system [69]. The Magen Halo-Tag beads was used to collect Halo-ZmNLP6. The collected Halo tagged protein was incubated with the genomic DNA library. The green fluorescent protein (GFP) was used as a control. Two replicates were performed for each sample. The binding DNA was washed and amplified using TruSeq primers. The sequencing was performed on an Illumina HiSeq 2500 platform. The reads were aligned to the maize B73 reference genome (v4). The peaks of DAP-seq were called using MASC2 [70] with the threshold of *q*-value of 0.05.

## Supplementary information

**Additional file 1: Figure S1.** The expression range of genes in different categories in the RNA-seq data. The genes are classified into two categories: genes in the set of high-quality (HQ) isoforms (HQ set genes), and genes are not in the set of HQ isoforms (Non-HQ set genes).

**Additional file 2: Figure S2. T**he workflow for the identification of high-confidence isoforms by combining the data of RNA-seq and PacBio-seq. The circular consensus sequences (CCS) and the full-length non-chimeric CCSs (FLNC CCS) were calculated by *IsoSeq*. The RNA-seq data was used to correct FLNC CCS by software named *Proovread*. The corrected reads were mapped to maize assembly B73 v4 using GMAP with default parameters. The mapped reads were sorted and filtered. The resulted isoforms were collapsed into non-redundant isoforms using *collapse_isoforms_by_sam.py*. The splicing junctions detected in RNA-seq was identified by *STAR*. The non-redundant splicing isoforms were validated by exactly matching each chain of splicing junction with that detected in RNA-seq reads.

**Additional file 3: Figure S3.** The numbers of isoforms and genes identified by different methods. The number of genes (A) and isoforms (B) detected in the sets of high-quality sequences (HQS), FLNC CCS (FLNC), and validated FLNC CCS (FLNC-validated). The number of isoforms in the N-starved root tissues (C) and root tissues after 30 min of nitrate supply (D) classified into different categories by *SQANTI.qc*.

**Additional file 4: Figure S4.** The classification of splicing junctions (SJs) detected in the isoforms detected using *SQANTI.qc*. The profile of SJs detected in the sets of high-quality sequences (HQS), FLNC CCS (FLNC), and validated FLNC CCS (FLNC-validated) in the untreated sample (N-starved root tissues, A) and treated sample (N-starved root tissues with 30 min of nitrate supply, B).

**Additional file 5: Figure S5.** Characterization of putative non-coding novel isoforms. (A) Functional analysis of putative non-coding isoforms. The *cmscan* function in the Infernal tools was used to search for novel non-coding isoforms that match the sequences in *Rfam* database. Each transcript was represented by a single dot. Multiple transcripts can be mapped to the same non-coding RNA family. The points presented in the figure are above a default significance e-value (1e-02). (B) Novel putative miRNA homolog. A novel transcript isoform, containing a sequence that matches the profile of *miR-171* in *Rfam*, was observed in the N-treated sample (+ N). An alternative isoform of this transcript with a shorter 3′ tail is observed in the untreated sample (− N).

**Additional file 6: Figure S6.** The distributions of the isoforms’ features in the untreated sample (−N) and treated sample (+N), respectively. (A) The distribution of the isoform lengths. (B) The distribution of the CDS lengths. (C) The isoform distribution of the CDS start sites. (D) The isoform distribution of the CDS end sites.

**Additional file 7: Table S1.** The information of reads obtained by RNA-seq.

**Additional file 8: Table S2.** Transcriptional abundance (log2 TPM + 1) of genes in RNA-Seq analysis.

**Additional file 9: Table S3.** The fold changes and FDR values based on the RNA-seq analysis.

**Additional file 10: Table S4.** The GO enrichment analysis of DEGs.

**Additional file 11: Table S5.** The distribution of the read lengths generated from Sequel system.

**Additional file 12: Table S6.** GO enrichment analysis of non-DEGs that experienced AS specifally after nitrate supply.

**Additional file 13: Table S7.** The list of ZmNLP6’s putative targets that associated with N metabolism.

**Additional file 14: Table S8.** The primers used in this study.

**Additional file 15: Table S9.** The profile of high-confidence isoforms identified in the untreated sample.

**Additional file 16: Table S10.** The profile of high-confidence isoforms identified in the nitrate-treated sample.

## Data Availability

We have submitted the sequences generated from this study to the NCBI Sequence Read Archive (SRA; https://www.ncbi.nlm.nih.gov/sra) under the access number of PRJNA587226 (https://www.ncbi.nlm.nih.gov/bioproject/PRJNA587226).

## Abbreviations

N: Nitrogen
DN: Deficient nitrogen
SN: Sufficient nitrogen
SGS: Second-generation sequencing
PacBio: Pacific Biosciences
SMRT: Single-molecule real-time
AS: Alternative splicing
DEG: Differentially expressed genes
ASG + N: Genes that are specifically experienced alternative splicing after nitrate supply
NLP: NIN-like protein
NUE: Nitrogen use efficiency
CCS: Circular consensus sequencing reads
FLNC CCSs: Full-length non-chimeric CCSs
SJ: Splicing junction
FSM: Full Splice Match
ISM: Incomplete splice match
NIC: Novel in Catalog
NNC: Novel Not in Catalog
