# Supplementary figures and images for: High-resolution profile of transcriptomes reveals a role of alternative splicing for modulating response to nitrogen in maize
Source: BMC Genomics. 2020 May 11;21:353. doi: 10.1186/s12864-020-6769-8 (PMC7216474; doi:10.1186/s12864-020-6769-8)

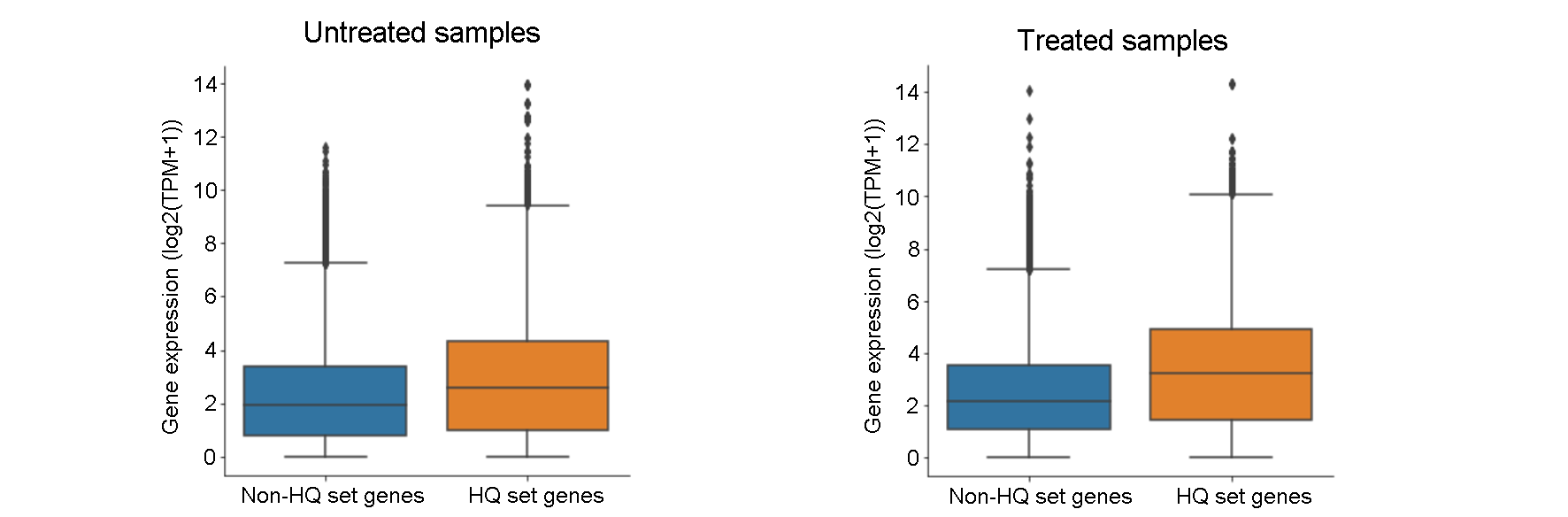

Supplement: Supplementary file 1 — Additional file 1: Figure S1. The expression range of genes in different categories in the RNA-seq data. The genes are classified into two categories: genes in the set of high-quality (HQ) isoforms (HQ set genes), and genes are not in the set of HQ isoforms (Non-HQ set genes). [file 12864_2020_6769_MOESM1_ESM.tif]
