# Supplementary material for: High-resolution profile of transcriptomes reveals a role of alternative splicing for modulating response to nitrogen in maize
Source: BMC Genomics. 2020 May 11;21:353. doi: 10.1186/s12864-020-6769-8 (PMC7216474; doi:10.1186/s12864-020-6769-8)
Supplement: Supplementary file 7 — Additional file 7: Table S1. The information of reads obtained by RNA-seq. [file 12864_2020_6769_MOESM7_ESM.pdf]

Supplemental Table S1. The information of reads obtained by RNA-seq.

Sample	Processed fragments	Mapping rate
Untreated_1	17,500,000	78.68%
Untreated_2	21,500,000	80.99%
Untreated_3	18,500,000	78.98%
Treated_1	22,000,001	75.63%
Treated_2	20,500,000	75.97%
Treated_3	18,000,000	76.19%
