# Supplementary material for: High-resolution profile of transcriptomes reveals a role of alternative splicing for modulating response to nitrogen in maize
Source: BMC Genomics. 2020 May 11;21:353. doi: 10.1186/s12864-020-6769-8 (PMC7216474; doi:10.1186/s12864-020-6769-8)
Supplement: Supplementary file 9 — Additional file 9: Table S3. The fold changes and FDR values based on the RNA-seq analysis. [file 12864_2020_6769_MOESM9_ESM.pdf]

Supplemental table S3: The fold changes and FDR values based on the RNA-seq analysis.

Tracking id	FDR	Fold change	Gene id
Zm00001d011700_T010	0.03504503	1.023512093	Zm00001d011700
Zm00001d039768_T003	0.02141924	-1.448029792	Zm00001d039768
Zm00001d044774_T010	0.01119789	-1.551416901	Zm00001d044774
Zm00001d043849_T001	0.000849431	-2.798773033	Zm00001d043849
Zm00001d000018_T001	0.002555996	2.301780486	Zm00001d000018
Zm00001d000447_T001	0.001203242	-2.016440036	Zm00001d000447
Zm00001d026306_T009	0.04504093	2.464240484	Zm00001d026306
Zm00001d026306_T001	6.64E-07	-2.592734668	Zm00001d026306
Zm00001d010149_T002	0.00037936	-2.407064845	Zm00001d010149
Zm00001d010149_T014	0.001573684	3.346657	Zm00001d010149
Zm00001d034282_T002	1.09E-06	-1.605677916	Zm00001d034282
Zm00001d018562_T006	0.03365733	2.197533677	Zm00001d018562
Zm00001d036780_T001	3.93E-07	5.284079351	Zm00001d036780
Zm00001d009118_T001	0.01730902	-2.306562823	Zm00001d009118
Zm00001d026334_T009	0.000368021	1.408439202	Zm00001d026334
Zm00001d052584_T001	0.004055167	1.886350476	Zm00001d052584
Zm00001d039907_T001	0.01062702	-2.297944203	Zm00001d039907
Zm00001d019115_T024	6.84E-12	1.903984389	Zm00001d019115
Zm00001d031543_T015	0.02346557	1.198804276	Zm00001d031543
Zm00001d040578_T001	0.02029528	-1.182299031	Zm00001d040578
Zm00001d005251_T001	0.02888628	2.506553407	Zm00001d005251
Zm00001d014526_T004	0.01600414	1.301485952	Zm00001d014526
Zm00001d035492_T001	0.02462255	1.337309049	Zm00001d035492
Zm00001d019149_T008	0.04914036	1.399127394	Zm00001d019149
Zm00001d002390_T004	1.96E-07	-1.844699429	Zm00001d002390
Zm00001d005137_T027	0.01306269	1.49562279	Zm00001d005137
Zm00001d005137_T009	0.03174476	1.767458551	Zm00001d005137
Zm00001d020732_T001	0.001261061	-2.07803227	Zm00001d020732
Zm00001d002250_T015	0.001373841	-1.329800398	Zm00001d002250
Zm00001d039165_T029	0.000275609	1.221438113	Zm00001d039165
Zm00001d044600_T010	0.005914915	-2.930891934	Zm00001d044600
Zm00001d028374_T001	0.000309016	2.742594783	Zm00001d028374
Zm00001d028960_T004	0.03427147	2.292524793	Zm00001d028960
Zm00001d028960_T006	0.005922616	2.111916446	Zm00001d028960
Zm00001d028960_T002	0.001230856	2.276507867	Zm00001d028960
Zm00001d019335_T017	3.15E-08	1.129477842	Zm00001d019335
Zm00001d049619_T002	0.003865269	-4.027249618	Zm00001d049619
Zm00001d047566_T015	0.007835597	-2.961466166	Zm00001d047566
Zm00001d016861_T004	4.26E-09	1.678278767	Zm00001d016861
Zm00001d037982_T008	6.11E-09	-1.403761265	Zm00001d037982
Zm00001d015243_T009	0.004668518	-1.613911569	Zm00001d015243
Zm00001d035346_T062	0.04978254	-1.070225562	Zm00001d035346
Zm00001d047412_T002	0.0228443	1.455267521	Zm00001d047412
Zm00001d053554_T001	1.38E-06	-3.563858817	Zm00001d053554
Zm00001d032447_T001	0.000224142	-3.292912291	Zm00001d032447
Zm00001d037246_T008	0.0421692	-1.170428058	Zm00001d037246
Zm00001d006410_T012	0.001315155	-2.363686364	Zm00001d006410
Zm00001d002266_T001	0.000535451	1.056991749	Zm00001d002266

Zm00001d002266_T006	0.001573684	1.743305429	Zm00001d002266
Zm00001d051336_T002	0.001068388	1.850310975	Zm00001d051336
Zm00001d033275_T005	0.000277524	1.357225719	Zm00001d033275
Zm00001d036925_T001	5.03E-10	-4.94713852	Zm00001d036925
Zm00001d027947_T001	3.02E-05	1.478308461	Zm00001d027947
Zm00001d039189_T001	0.02463347	2.104542265	Zm00001d039189
Zm00001d039189_T015	0.01651603	-2.219960774	Zm00001d039189
Zm00001d039189_T007	0.05010859	-1.045729657	Zm00001d039189
Zm00001d039189_T005	1.45E-14	3.25778396	Zm00001d039189
Zm00001d032875_T004	0.0166219	-1.681608551	Zm00001d032875
Zm00001d006593_T012	0.00045129	2.658987719	Zm00001d006593
Zm00001d010572_T015	1.10E-05	2.452467672	Zm00001d010572
Zm00001d010572_T001	1.38E-10	1.280613692	Zm00001d010572
Zm00001d023218_T175	0.003870775	1.933356775	Zm00001d023218
Zm00001d023218_T038	6.39E-07	3.440754511	Zm00001d023218
Zm00001d042512_T007	0.006966048	-2.80246973	Zm00001d042512
Zm00001d028073_T002	0.02454873	1.459599325	Zm00001d028073
Zm00001d049295_T008	0.01468322	2.297144497	Zm00001d049295
Zm00001d038521_T001	2.45E-05	2.666111103	Zm00001d038521
Zm00001d041214_T004	1.53E-10	-1.509899887	Zm00001d041214
Zm00001d041214_T003	0.003956993	2.458329125	Zm00001d041214
Zm00001d013109_T002	0.01199987	2.959018813	Zm00001d013109
Zm00001d013109_T001	0.002924724	1.371735239	Zm00001d013109
Zm00001d006101_T001	0.009848483	3.132120538	Zm00001d006101
Zm00001d003087_T001	0.001248123	1.907166671	Zm00001d003087
Zm00001d003087_T041	0.000929043	-1.894887429	Zm00001d003087
Zm00001d003087_T047	4.97E-05	-3.213051152	Zm00001d003087
Zm00001d047986_T010	0.009760307	2.524696522	Zm00001d047986
Zm00001d047986_T015	6.67E-12	1.952582431	Zm00001d047986
Zm00001d043726_T006	1.15E-10	2.695469325	Zm00001d043726
Zm00001d008289_T001	0.004427436	-2.751058992	Zm00001d008289
Zm00001d010676_T001	0.000319993	3.533234357	Zm00001d010676
Zm00001d014219_T001	0.000869421	-3.227394809	Zm00001d014219
Zm00001d053283_T008	1.16E-08	-1.349110736	Zm00001d053283
Zm00001d034519_T002	0.02672699	-1.910818602	Zm00001d034519
Zm00001d006704_T002	0.005892258	1.383750192	Zm00001d006704
Zm00001d047910_T003	0.000423489	-1.808840241	Zm00001d047910
Zm00001d051102_T001	0.01622968	-1.072813667	Zm00001d051102
Zm00001d050970_T008	0.001171111	-1.776473086	Zm00001d050970
Zm00001d021891_T006	0.03761125	-1.212315399	Zm00001d021891
Zm00001d012953_T001	0.050072	2.028072254	Zm00001d012953
Zm00001d020088_T014	1.91E-08	1.644968242	Zm00001d020088
Zm00001d027483_T001	0.01955821	2.643488416	Zm00001d027483
Zm00001d015856_T021	0.04008909	-2.711344244	Zm00001d015856
Zm00001d028915_T025	4.12E-10	-1.647013502	Zm00001d028915
Zm00001d029180_T021	0.04707027	1.023638672	Zm00001d029180
Zm00001d029180_T007	3.34E-10	1.26150083	Zm00001d029180
Zm00001d028330_T120	0.00037936	1.138808547	Zm00001d028330
Zm00001d034510_T008	0.02815723	1.005260101	Zm00001d034510
Zm00001d044409_T001	0.000104584	-3.179181434	Zm00001d044409
Zm00001d034033_T007	6.67E-10	-2.117048212	Zm00001d034033
Zm00001d048258_T001	1.65E-14	-3.039724848	Zm00001d048258
Zm00001d007050_T001	0.01003933	2.856005468	Zm00001d007050
Zm00001d017487_T006	0.01014068	-1.197014238	Zm00001d017487

Zm00001d026676_T004	0.04693522	-1.235127282	Zm00001d026676
Zm00001d012456_T003	2.21E-06	1.228197396	Zm00001d012456
Zm00001d012456_T004	0.002717702	1.168294848	Zm00001d012456
Zm00001d017983_T001	0.03094646	2.57462095	Zm00001d017983
Zm00001d002618_T001	1.04E-07	4.426980853	Zm00001d002618
Zm00001d021562_T001	0.002632543	3.088775698	Zm00001d021562
Zm00001d018819_T001	2.44E-05	1.184417593	Zm00001d018819
Zm00001d028154_T001	0.000694401	3.408360619	Zm00001d028154
Zm00001d006438_T020	0.02121165	2.200103829	Zm00001d006438
Zm00001d009205_T005	2.35E-13	3.831875931	Zm00001d009205
Zm00001d039670_T008	0.0200189	1.219659748	Zm00001d039670
Zm00001d007307_T009	0.00013693	1.347476943	Zm00001d007307
Zm00001d011429_T003	1.62E-05	2.999090401	Zm00001d011429
Zm00001d036611_T007	0.009851032	1.105730691	Zm00001d036611
Zm00001d028088_T002	0.01573844	1.202655117	Zm00001d028088
Zm00001d048234_T001	0.009062656	-1.752414508	Zm00001d048234
Zm00001d014575_T004	0.02182475	-1.305866692	Zm00001d014575
Zm00001d001857_T001	0.000546337	-2.130620083	Zm00001d001857
Zm00001d052351_T001	0.01535282	2.464147482	Zm00001d052351
Zm00001d005830_T001	0.006170689	-1.303754113	Zm00001d005830
Zm00001d044940_T005	0.00037936	1.791271294	Zm00001d044940
Zm00001d029558_T001	0.0249272	-2.211823279	Zm00001d029558
Zm00001d046226_T001	0.001390205	1.311351671	Zm00001d046226
Zm00001d030131_T005	1.01E-05	3.49268823	Zm00001d030131
Zm00001d030131_T006	8.82E-08	3.825408438	Zm00001d030131
Zm00001d044494_T013	0.02253332	2.064330441	Zm00001d044494
Zm00001d044494_T021	0.01122327	1.963743925	Zm00001d044494
Zm00001d027645_T001	0.04054157	-1.528451868	Zm00001d027645
Zm00001d007835_T004	1.01E-10	-3.030076231	Zm00001d007835
Zm00001d054003_T010	0.005005054	1.033462684	Zm00001d054003
Zm00001d047126_T005	0.02774165	1.046075704	Zm00001d047126
Zm00001d014508_T003	0.01993352	1.088632261	Zm00001d014508
Zm00001d035922_T008	2.14E-11	1.844726888	Zm00001d035922
Zm00001d030164_T120	0.000133025	2.10718035	Zm00001d030164
Zm00001d043469_T004	1.85E-13	2.92898905	Zm00001d043469
Zm00001d043469_T015	0.02994517	1.45409399	Zm00001d043469
Zm00001d043469_T002	0.02454873	2.32949629	Zm00001d043469
Zm00001d035079_T001	5.27E-06	-4.542095038	Zm00001d035079
Zm00001d014977_T007	0.04000108	2.191090002	Zm00001d014977
Zm00001d014977_T028	2.64E-06	2.448845341	Zm00001d014977
Zm00001d042804_T003	0.000580616	2.152124106	Zm00001d042804
Zm00001d042804_T004	0.001400525	1.355128522	Zm00001d042804
Zm00001d042154_T003	0.01140418	-1.530509868	Zm00001d042154
Zm00001d017761_T002	0.00538624	-1.814931769	Zm00001d017761
Zm00001d027743_T004	1.65E-10	1.664807824	Zm00001d027743
Zm00001d038494_T012	0.02925111	1.869618624	Zm00001d038494
Zm00001d038494_T016	0.0257957	-1.697347872	Zm00001d038494
Zm00001d032536_T007	0.01943547	1.080177807	Zm00001d032536
Zm00001d032536_T004	0.01274653	1.598355089	Zm00001d032536
Zm00001d009324_T001	0.009107227	-2.250712388	Zm00001d009324
Zm00001d043274_T008	0.0321622	1.955082845	Zm00001d043274
Zm00001d043274_T002	1.67E-11	-2.041171087	Zm00001d043274
Zm00001d043274_T019	0.000182442	1.729865619	Zm00001d043274
Zm00001d010315_T010	1.33E-12	2.5618716	Zm00001d010315

Zm00001d033451_T001	0.001501543	-1.193711514	Zm00001d033451
Zm00001d016274_T011	0.0252917	1.081216917	Zm00001d016274
Zm00001d016274_T006	0.03174633	2.635951402	Zm00001d016274
Zm00001d041450_T004	3.55E-05	1.903744727	Zm00001d041450
Zm00001d027271_T005	0.0299214	1.651122856	Zm00001d027271
Zm00001d013314_T013	3.54E-07	2.785689389	Zm00001d013314
Zm00001d017660_T003	2.38E-11	1.384685657	Zm00001d017660
Zm00001d041455_T002	0.03182763	-1.166529021	Zm00001d041455
Zm00001d000124_T002	2.41E-09	1.721994134	Zm00001d000124
Zm00001d000124_T005	0.0240988	2.325270509	Zm00001d000124
Zm00001d024998_T146	0.01060455	1.668277571	Zm00001d024998
Zm00001d011564_T001	0.02706902	1.177637638	Zm00001d011564
Zm00001d022491_T009	3.17E-13	-2.212124955	Zm00001d022491
Zm00001d022491_T001	3.38E-12	1.84641542	Zm00001d022491
Zm00001d022491_T008	0.004175771	1.757968251	Zm00001d022491
Zm00001d003855_T007	0.01060017	1.033944152	Zm00001d003855
Zm00001d003855_T002	5.40E-06	1.291835899	Zm00001d003855
Zm00001d011684_T001	0.00405648	1.283241818	Zm00001d011684
Zm00001d011684_T004	3.44E-05	1.403004117	Zm00001d011684
Zm00001d011684_T003	1.51E-10	1.417069953	Zm00001d011684
Zm00001d011684_T002	8.84E-05	3.495842122	Zm00001d011684
Zm00001d041711_T005	1.19E-08	-3.798830172	Zm00001d041711
Zm00001d037970_T018	0.01654858	2.027351721	Zm00001d037970
Zm00001d006762_T003	1.54E-13	2.386060684	Zm00001d006762
Zm00001d011604_T040	1.60E-07	2.267292034	Zm00001d011604
Zm00001d011604_T029	0.02686247	1.178920807	Zm00001d011604
Zm00001d011604_T010	8.01E-10	2.337187303	Zm00001d011604
Zm00001d020195_T001	0.02488927	-2.173293446	Zm00001d020195
Zm00001d024713_T053	0.004573701	1.150569306	Zm00001d024713
Zm00001d024713_T014	4.25E-07	-1.978056347	Zm00001d024713
Zm00001d024713_T013	0.04476943	2.10408453	Zm00001d024713
Zm00001d024713_T042	3.52E-11	1.889376932	Zm00001d024713
Zm00001d038772_T001	0.001609317	-2.761537862	Zm00001d038772
Zm00001d038707_T001	0.01945449	1.938464158	Zm00001d038707
Zm00001d013829_T001	3.69E-05	1.698582396	Zm00001d013829
Zm00001d051838_T002	3.41E-11	-3.460604375	Zm00001d051838
Zm00001d043855_T004	0.00090062	-2.802200261	Zm00001d043855
Zm00001d040694_T001	3.59E-07	1.782310381	Zm00001d040694
Zm00001d034130_T001	0.000132742	3.432909728	Zm00001d034130
Zm00001d014343_T008	8.16E-14	3.031361313	Zm00001d014343
Zm00001d038156_T007	2.84E-13	2.72345286	Zm00001d038156
Zm00001d038156_T008	3.89E-05	2.008359942	Zm00001d038156
Zm00001d011656_T001	2.12E-08	5.490920583	Zm00001d011656
Zm00001d010230_T002	0.000669937	-1.321075044	Zm00001d010230
Zm00001d010230_T004	0.01598815	1.620400726	Zm00001d010230
Zm00001d002159_T001	2.15E-05	-4.278252753	Zm00001d002159
Zm00001d016606_T020	0.04205932	1.915691294	Zm00001d016606
Zm00001d013469_T006	0.006974174	-1.341505476	Zm00001d013469
Zm00001d010375_T001	0.000245961	2.190514082	Zm00001d010375
Zm00001d046454_T004	8.01E-05	1.284324527	Zm00001d046454
Zm00001d000112_T001	0.006586914	3.145500797	Zm00001d000112
Zm00001d000112_T003	0.02626399	1.879746499	Zm00001d000112
Zm00001d000112_T002	0.01102013	1.838017517	Zm00001d000112
Zm00001d000112_T004	0.001556436	3.278460288	Zm00001d000112

Zm00001d043376_T001	1.31E-05	-2.058197371	Zm00001d043376
Zm00001d029868_T004	2.25E-07	1.379672377	Zm00001d029868
Zm00001d018429_T001	0.02561871	-1.82721041	Zm00001d018429
Zm00001d006573_T054	5.78E-13	2.290061498	Zm00001d006573
Zm00001d006573_T001	9.32E-06	2.837328543	Zm00001d006573
Zm00001d051460_T001	0.000937184	2.931896889	Zm00001d051460
Zm00001d009125_T003	1.79E-14	-2.786407147	Zm00001d009125
Zm00001d009125_T002	5.34E-15	3.478423073	Zm00001d009125
Zm00001d009125_T005	0.03767996	3.014990132	Zm00001d009125
Zm00001d024102_T001	0.04707027	1.386516354	Zm00001d024102
Zm00001d002435_T001	0.009744942	2.633962536	Zm00001d002435
Zm00001d031749_T001	0.02824604	-1.681278203	Zm00001d031749
Zm00001d034644_T004	0.004527535	-1.598089619	Zm00001d034644
Zm00001d043998_T006	0.000447457	-2.185084895	Zm00001d043998
Zm00001d052197_T010	0.000708157	1.076770354	Zm00001d052197
Zm00001d018828_T005	1.36E-08	1.191927783	Zm00001d018828
Zm00001d041422_T001	0.01695976	-1.923134766	Zm00001d041422
Zm00001d027472_T002	0.01314356	2.406788821	Zm00001d027472
Zm00001d024468_T002	0.000791633	-2.019044632	Zm00001d024468
Zm00001d045956_T029	0.005659332	-1.008541261	Zm00001d045956
Zm00001d045956_T017	3.15E-13	2.487208404	Zm00001d045956
Zm00001d045956_T001	0.000187249	2.711491084	Zm00001d045956
Zm00001d044232_T001	0.000251832	-1.966504195	Zm00001d044232
Zm00001d002498_T001	0.006844514	-2.043688892	Zm00001d002498
Zm00001d039341_T009	5.07E-16	3.77395362	Zm00001d039341
Zm00001d039341_T133	0.000388444	2.282430363	Zm00001d039341
Zm00001d025128_T011	0.004101034	-2.763132497	Zm00001d025128
Zm00001d051554_T001	0.02685203	1.449838138	Zm00001d051554
Zm00001d051554_T002	0.01554174	2.601000178	Zm00001d051554
Zm00001d019724_T004	0.000425947	1.237502033	Zm00001d019724
Zm00001d019724_T024	0.007188644	1.886412203	Zm00001d019724
Zm00001d033247_T007	0.01306269	-2.29877659	Zm00001d033247
Zm00001d037848_T002	0.02871159	2.348376064	Zm00001d037848
Zm00001d045783_T004	4.37E-09	-1.055596604	Zm00001d045783
Zm00001d047739_T016	0.001556436	1.485810548	Zm00001d047739
Zm00001d047739_T026	0.009347455	1.19244751	Zm00001d047739
Zm00001d047739_T003	6.72E-09	-1.363909309	Zm00001d047739
Zm00001d047739_T043	1.94E-06	-2.961736276	Zm00001d047739
Zm00001d039693_T061	5.35E-11	1.513491143	Zm00001d039693
Zm00001d037743_T001	4.56E-06	-4.440005614	Zm00001d037743
Zm00001d010800_T001	0.001753896	-2.22357178	Zm00001d010800
Zm00001d029297_T003	0.01873517	-1.123259226	Zm00001d029297
Zm00001d047253_T014	3.85E-05	1.172788159	Zm00001d047253
Zm00001d032825_T001	0.001779678	-2.944156942	Zm00001d032825
Zm00001d029028_T001	0.03732086	2.469142289	Zm00001d029028
Zm00001d020046_T001	0.02521944	-2.347296211	Zm00001d020046
Zm00001d036322_T001	0.007360724	-1.532422712	Zm00001d036322
Zm00001d045051_T001	0.0325408	2.427394179	Zm00001d045051
Zm00001d014667_T001	0.02561053	-2.141809968	Zm00001d014667
Zm00001d012156_T051	0.001312528	-1.291491931	Zm00001d012156
Zm00001d012156_T030	4.05E-10	1.021001634	Zm00001d012156
Zm00001d012156_T032	0.003460563	1.737478208	Zm00001d012156
Zm00001d006791_T001	0.000329926	-3.048285051	Zm00001d006791
Zm00001d028472_T007	2.70E-12	2.652338785	Zm00001d028472

Zm00001d003399_T001	0.01753567	2.723362918	Zm00001d003399
Zm00001d033674_T012	5.71E-11	-2.43980121	Zm00001d033674
Zm00001d019472_T005	2.20E-10	-2.01145632	Zm00001d019472
Zm00001d009221_T002	0.02280843	-1.418324346	Zm00001d009221
Zm00001d020881_T002	1.28E-09	1.773152626	Zm00001d020881
Zm00001d020881_T001	0.001343417	-1.664822673	Zm00001d020881
Zm00001d020881_T007	0.008931882	1.436296447	Zm00001d020881
Zm00001d006415_T001	3.12E-11	2.430932091	Zm00001d006415
Zm00001d006415_T002	3.93E-05	-1.259450059	Zm00001d006415
Zm00001d039105_T004	9.76E-09	1.543706374	Zm00001d039105
Zm00001d046581_T012	3.12E-11	2.12653251	Zm00001d046581
Zm00001d025915_T005	6.06E-07	2.10451068	Zm00001d025915
Zm00001d045675_T002	0.00759149	-1.897451332	Zm00001d045675
Zm00001d030373_T007	0.00185145	-2.423036045	Zm00001d030373
Zm00001d023320_T001	0.04913338	1.283150926	Zm00001d023320
Zm00001d015138_T005	8.45E-05	3.293992958	Zm00001d015138
Zm00001d002125_T001	0.001129598	-1.020297462	Zm00001d002125
Zm00001d002125_T016	0.03161488	1.311217767	Zm00001d002125
Zm00001d002125_T006	4.05E-06	2.445135391	Zm00001d002125
Zm00001d043503_T001	0.04072087	-1.07914375	Zm00001d043503
Zm00001d002113_T001	0.04606011	1.055703185	Zm00001d002113
Zm00001d044903_T005	0.00018737	1.795704406	Zm00001d044903
Zm00001d015090_T003	0.01592825	-1.172119079	Zm00001d015090
Zm00001d046468_T003	0.002879391	2.251009527	Zm00001d046468
Zm00001d016705_T001	0.00468038	-2.784221465	Zm00001d016705
Zm00001d051431_T010	0.006338361	2.260871828	Zm00001d051431
Zm00001d051431_T002	2.46E-12	-2.685498287	Zm00001d051431
Zm00001d037732_T004	0.003949247	-1.819493112	Zm00001d037732
Zm00001d014814_T004	0.002725334	-1.128265832	Zm00001d014814
Zm00001d016456_T003	3.38E-08	-1.28276899	Zm00001d016456
Zm00001d021132_T001	0.0140994	-2.841331908	Zm00001d021132
Zm00001d039197_T001	0.03044968	-1.260688931	Zm00001d039197
Zm00001d011619_T006	0.03722719	1.077716383	Zm00001d011619
Zm00001d031882_T001	0.04701349	-1.588816222	Zm00001d031882
Zm00001d030916_T002	3.05E-07	-2.220879145	Zm00001d030916
Zm00001d018904_T001	9.27E-15	3.999817402	Zm00001d018904
Zm00001d018904_T008	0.0394043	-2.053400784	Zm00001d018904
Zm00001d018904_T002	1.86E-11	-2.100257803	Zm00001d018904
Zm00001d014790_T001	0.001629813	-2.840968743	Zm00001d014790
Zm00001d044573_T010	0.03004912	1.879782247	Zm00001d044573
Zm00001d007632_T010	0.0004805	-2.05745583	Zm00001d007632
Zm00001d005808_T006	0.04606011	1.676681313	Zm00001d005808
Zm00001d007089_T048	0.009321786	1.321253495	Zm00001d007089
Zm00001d021915_T002	0.000285564	2.681823421	Zm00001d021915
Zm00001d034383_T001	3.80E-08	-1.409569721	Zm00001d034383
Zm00001d011742_T037	0.007842368	1.836075327	Zm00001d011742
Zm00001d005661_T001	0.02055626	-1.58691574	Zm00001d005661
Zm00001d033670_T003	0.00739911	2.316980673	Zm00001d033670
Zm00001d033670_T001	0.003405378	2.693927973	Zm00001d033670
Zm00001d033670_T004	0.00010633	1.380799811	Zm00001d033670
Zm00001d005849_T001	0.001522709	3.425406576	Zm00001d005849
Zm00001d032598_T023	1.16E-05	-1.481898273	Zm00001d032598
Zm00001d042382_T001	0.02379854	-1.475872818	Zm00001d042382
Zm00001d048311_T003	0.000798901	-1.335156153	Zm00001d048311

Zm00001d048311_T004	3.03E-05	-1.186124448	Zm00001d048311
Zm00001d043775_T018	0.009394971	1.777518245	Zm00001d043775
Zm00001d043775_T015	8.13E-12	-1.716516138	Zm00001d043775
Zm00001d026632_T001	0.01294351	-2.50994903	Zm00001d026632
Zm00001d017895_T007	2.30E-07	-1.823280156	Zm00001d017895
Zm00001d028912_T001	0.000577022	2.113015849	Zm00001d028912
Zm00001d019757_T003	0.03760071	1.394777763	Zm00001d019757
Zm00001d031268_T017	1.38E-10	-1.978477828	Zm00001d031268
Zm00001d002617_T008	0.034458	1.160904844	Zm00001d002617
Zm00001d017590_T001	0.00211038	3.038154466	Zm00001d017590
Zm00001d011816_T002	1.10E-12	-4.972103702	Zm00001d011816
Zm00001d039729_T002	0.004390741	1.247575895	Zm00001d039729
Zm00001d052148_T001	0.01199333	2.944798452	Zm00001d052148
Zm00001d052110_T019	0.01285332	1.237317957	Zm00001d052110
Zm00001d014377_T010	0.001350256	-1.899035752	Zm00001d014377
Zm00001d022084_T001	5.10E-08	-5.170586486	Zm00001d022084
Zm00001d016156_T003	0.001324589	2.044057645	Zm00001d016156
Zm00001d014258_T006	0.01554154	-2.643968103	Zm00001d014258
Zm00001d010743_T001	0.000138621	1.810138455	Zm00001d010743
Zm00001d012641_T001	0.00079548	2.507192651	Zm00001d012641
Zm00001d052118_T001	0.006183364	2.3674722	Zm00001d052118
Zm00001d002889_T007	0.000340227	1.036917797	Zm00001d002889
Zm00001d002889_T019	7.61E-14	-6.067845363	Zm00001d002889
Zm00001d002889_T001	5.60E-05	1.097909989	Zm00001d002889
Zm00001d042066_T005	0.007306547	2.222331134	Zm00001d042066
Zm00001d016410_T008	5.10E-12	-5.444294083	Zm00001d016410
Zm00001d021835_T006	3.55E-05	1.342509947	Zm00001d021835
Zm00001d021835_T005	0.002814932	3.331880815	Zm00001d021835
Zm00001d021835_T003	0.01777235	2.740819775	Zm00001d021835
Zm00001d052751_T044	3.01E-12	1.911132115	Zm00001d052751
Zm00001d039065_T007	3.69E-07	1.045106042	Zm00001d039065
Zm00001d028203_T045	0.01828644	2.187092294	Zm00001d028203
Zm00001d046889_T001	0.001640834	1.490846666	Zm00001d046889
Zm00001d026302_T001	0.03104183	1.448691794	Zm00001d026302
Zm00001d049703_T002	0.04684325	-1.183634855	Zm00001d049703
Zm00001d015468_T001	0.009170631	2.996201808	Zm00001d015468
Zm00001d004624_T001	2.64E-07	3.997097228	Zm00001d004624
Zm00001d027334_T020	2.83E-06	1.435971867	Zm00001d027334
Zm00001d027334_T022	1.61E-12	-1.70735157	Zm00001d027334
Zm00001d027334_T021	1.22E-14	2.5898623	Zm00001d027334
Zm00001d037156_T003	4.07E-05	2.715017599	Zm00001d037156
Zm00001d015212_T006	0.02067213	1.682683922	Zm00001d015212
Zm00001d042084_T059	5.62E-05	2.161196386	Zm00001d042084
Zm00001d033623_T009	2.75E-05	1.713965679	Zm00001d033623
Zm00001d038393_T009	0.00078261	2.267777946	Zm00001d038393
Zm00001d042443_T001	0.000760374	3.628531864	Zm00001d042443
Zm00001d043402_T001	0.0379236	2.196633813	Zm00001d043402
Zm00001d028601_T003	0.009744942	-1.948620274	Zm00001d028601
Zm00001d047536_T007	0.000391823	1.470106788	Zm00001d047536
Zm00001d038776_T001	0.03097032	-1.001014991	Zm00001d038776
Zm00001d002141_T005	0.01728933	-1.789634162	Zm00001d002141
Zm00001d017907_T001	0.04282223	1.950327275	Zm00001d017907
Zm00001d042794_T003	0.00713124	1.094100623	Zm00001d042794
Zm00001d042794_T006	0.000767547	-1.221329773	Zm00001d042794

Zm00001d041105_T007	1.57E-11	1.539820582	Zm00001d041105
Zm00001d041105_T027	0.01056074	1.423376538	Zm00001d041105
Zm00001d041105_T058	6.33E-06	-1.746764657	Zm00001d041105
Zm00001d018443_T002	0.000163855	-3.046816226	Zm00001d018443
Zm00001d034624_T005	0.01325275	2.018342196	Zm00001d034624
Zm00001d034624_T020	2.14E-12	1.33799568	Zm00001d034624
Zm00001d034624_T050	8.23E-06	1.731884801	Zm00001d034624
Zm00001d048096_T014	0.02321164	1.631228327	Zm00001d048096
Zm00001d018303_T011	0.001897768	2.500293147	Zm00001d018303
Zm00001d018303_T001	0.000468585	1.565215436	Zm00001d018303
Zm00001d005127_T021	1.19E-11	-1.793941903	Zm00001d005127
Zm00001d005127_T008	0.00573859	1.324560926	Zm00001d005127
Zm00001d005127_T009	0.000138123	1.958731222	Zm00001d005127
Zm00001d005127_T003	2.30E-14	1.830795656	Zm00001d005127
Zm00001d021160_T001	2.22E-05	5.597374803	Zm00001d021160
Zm00001d053818_T003	0.004513206	2.914057757	Zm00001d053818
Zm00001d053818_T001	0.000733585	1.438676631	Zm00001d053818
Zm00001d052653_T005	0.02558678	2.710261565	Zm00001d052653
Zm00001d052653_T002	0.006418906	2.406924328	Zm00001d052653
Zm00001d052653_T004	0.006919847	3.109701462	Zm00001d052653
Zm00001d045460_T001	2.56E-06	6.165738762	Zm00001d045460
Zm00001d044874_T001	0.000347145	-2.680056628	Zm00001d044874
Zm00001d018289_T014	7.68E-08	1.078620581	Zm00001d018289
Zm00001d005636_T009	7.98E-14	-4.472056085	Zm00001d005636
Zm00001d002020_T002	1.11E-10	-2.654780541	Zm00001d002020
Zm00001d021303_T002	2.68E-05	4.275227472	Zm00001d021303
Zm00001d018965_T001	0.00506932	-2.360609215	Zm00001d018965
Zm00001d011351_T071	0.03585832	-1.62211267	Zm00001d011351
Zm00001d013370_T001	0.005375707	1.97551876	Zm00001d013370
Zm00001d013370_T002	0.000323792	1.925156707	Zm00001d013370
Zm00001d039608_T001	8.58E-05	-2.187093319	Zm00001d039608
Zm00001d039608_T005	0.01111628	1.661004289	Zm00001d039608
Zm00001d039608_T041	0.0060283	1.203025151	Zm00001d039608
Zm00001d013049_T005	0.000500744	1.15636986	Zm00001d013049
Zm00001d014568_T025	0.00146127	1.274849405	Zm00001d014568
Zm00001d052620_T012	0.01532471	-1.815821991	Zm00001d052620
Zm00001d052620_T013	2.71E-10	1.525769984	Zm00001d052620
Zm00001d052620_T001	6.04E-05	-1.914003918	Zm00001d052620
Zm00001d000169_T007	0.0100365	1.909458054	Zm00001d000169
Zm00001d018657_T033	7.17E-14	-2.820551047	Zm00001d018657
Zm00001d018657_T016	0.01606269	2.204856687	Zm00001d018657
Zm00001d046661_T008	1.53E-12	3.236433786	Zm00001d046661
Zm00001d034650_T008	1.32E-11	-2.461294533	Zm00001d034650
Zm00001d034650_T017	2.23E-12	3.254552558	Zm00001d034650
Zm00001d052399_T006	0.02706902	1.946920145	Zm00001d052399
Zm00001d052399_T004	0.01402472	1.354695176	Zm00001d052399
Zm00001d052399_T012	0.000115347	1.426189741	Zm00001d052399
Zm00001d052399_T007	0.01015258	1.046533991	Zm00001d052399
Zm00001d013595_T004	0.04663752	-1.240328946	Zm00001d013595
Zm00001d026691_T003	0.02666365	1.371740371	Zm00001d026691
Zm00001d054049_T001	0.009606413	-2.111638944	Zm00001d054049
Zm00001d006220_T013	0.001117538	-2.096006078	Zm00001d006220
Zm00001d024816_T007	1.69E-11	1.878379235	Zm00001d024816
Zm00001d043701_T001	0.000388444	3.3734276	Zm00001d043701

Zm00001d042864_T004	8.17E-10	2.546490435	Zm00001d042864
Zm00001d051475_T002	0.02888804	-1.708142599	Zm00001d051475
Zm00001d036280_T003	1.44E-11	1.686583911	Zm00001d036280
Zm00001d013193_T002	0.005892258	-2.517051372	Zm00001d013193
Zm00001d045509_T005	0.0191703	-1.26325466	Zm00001d045509
Zm00001d026079_T002	3.06E-12	-3.136375409	Zm00001d026079
Zm00001d021704_T003	0.03030472	-1.927219426	Zm00001d021704
Zm00001d051902_T008	3.15E-13	2.769120601	Zm00001d051902
Zm00001d020396_T003	0.003774627	1.622277074	Zm00001d020396
Zm00001d053736_T001	0.000275609	1.588562011	Zm00001d053736
Zm00001d002128_T003	0.009041647	2.591151034	Zm00001d002128
Zm00001d045534_T002	0.0245	1.622451966	Zm00001d045534
Zm00001d045534_T011	0.01642555	-2.534971492	Zm00001d045534
Zm00001d025551_T005	0.04307801	1.255071687	Zm00001d025551
Zm00001d025817_T045	6.53E-10	-2.309890796	Zm00001d025817
Zm00001d018831_T010	0.009795236	-1.250666616	Zm00001d018831
Zm00001d052731_T001	0.04879383	2.347843849	Zm00001d052731
Zm00001d017555_T008	0.01955655	-3.045724724	Zm00001d017555
Zm00001d053450_T032	0.006224998	1.356790754	Zm00001d053450
Zm00001d039944_T001	3.57E-07	4.78815064	Zm00001d039944
Zm00001d028570_T009	0.006884532	2.497509245	Zm00001d028570
Zm00001d021245_T001	0.0165267	-1.902161008	Zm00001d021245
Zm00001d028017_T001	0.03896328	-1.639816117	Zm00001d028017
Zm00001d007522_T025	2.40E-08	1.085178559	Zm00001d007522
Zm00001d007522_T002	0.01431466	2.611187052	Zm00001d007522
Zm00001d007522_T009	0.02304271	1.841334838	Zm00001d007522
Zm00001d033544_T001	0.0381171	1.175449731	Zm00001d033544
Zm00001d039350_T002	0.04294987	-2.113344369	Zm00001d039350
Zm00001d010719_T004	0.000358318	-1.270384912	Zm00001d010719
Zm00001d010719_T002	0.003224902	1.776355859	Zm00001d010719
Zm00001d018150_T001	0.01478222	-2.621653937	Zm00001d018150
Zm00001d013842_T008	0.009025441	-2.594550496	Zm00001d013842
Zm00001d017568_T005	0.003488553	3.502392605	Zm00001d017568
Zm00001d017568_T023	0.01751306	1.281718688	Zm00001d017568
Zm00001d041632_T009	0.02606139	-1.769684545	Zm00001d041632
Zm00001d023768_T001	0.01119323	-1.988902133	Zm00001d023768
Zm00001d052219_T004	0.02515384	2.253574147	Zm00001d052219
Zm00001d033895_T006	0.02332999	2.145611193	Zm00001d033895
Zm00001d033378_T019	2.77E-14	-2.847254276	Zm00001d033378
Zm00001d025801_T006	0.008473025	-2.412930717	Zm00001d025801
Zm00001d016496_T006	0.03159677	-1.814464403	Zm00001d016496
Zm00001d016496_T014	3.05E-11	2.652457984	Zm00001d016496
Zm00001d012522_T001	0.02370782	2.495828504	Zm00001d012522
Zm00001d044442_T018	1.75E-10	1.366473364	Zm00001d044442
Zm00001d044442_T019	0.02339299	2.259936884	Zm00001d044442
Zm00001d044442_T010	0.000108701	2.922972159	Zm00001d044442
Zm00001d031570_T002	0.03896328	-1.54900891	Zm00001d031570
Zm00001d031570_T003	0.005339206	1.175522623	Zm00001d031570
Zm00001d044546_T001	0.01867124	-2.072758018	Zm00001d044546
Zm00001d016552_T007	0.006217265	-2.105913997	Zm00001d016552
Zm00001d011713_T016	1.08E-08	1.131667414	Zm00001d011713
Zm00001d052530_T001	7.16E-06	4.26941748	Zm00001d052530
Zm00001d016826_T001	0.04419305	2.25281084	Zm00001d016826
Zm00001d027633_T010	0.02590398	1.582056634	Zm00001d027633

Zm00001d005962_T001	0.000959593	3.248543601	Zm00001d005962
Zm00001d038167_T003	0.04949697	1.746843906	Zm00001d038167
Zm00001d014423_T018	0.04090559	-1.305347647	Zm00001d014423
Zm00001d014423_T020	1.25E-05	2.315347432	Zm00001d014423
Zm00001d007631_T001	0.005210231	-3.234875753	Zm00001d007631
Zm00001d003509_T007	0.01424155	2.493180345	Zm00001d003509
Zm00001d003509_T004	0.003072476	2.779214829	Zm00001d003509
Zm00001d002463_T001	0.01075757	-2.749283414	Zm00001d002463
Zm00001d044951_T001	0.04595287	1.04853035	Zm00001d044951
Zm00001d020927_T001	0.001203049	-2.101959189	Zm00001d020927
Zm00001d014954_T005	0.002685015	-2.421881969	Zm00001d014954
Zm00001d014954_T001	0.03238926	-1.234233215	Zm00001d014954
Zm00001d043895_T005	0.002719971	1.483857758	Zm00001d043895
Zm00001d043895_T001	0.00405648	1.21601157	Zm00001d043895
Zm00001d036137_T008	1.81E-10	-3.103591323	Zm00001d036137
Zm00001d036137_T004	0.000999715	1.093895951	Zm00001d036137
Zm00001d027768_T005	4.67E-10	1.759804099	Zm00001d027768
Zm00001d027768_T007	1.52E-08	-1.014402461	Zm00001d027768
Zm00001d036703_T001	0.02653356	-1.977328347	Zm00001d036703
Zm00001d040460_T001	0.04016634	1.998228615	Zm00001d040460
Zm00001d010530_T004	5.33E-12	-2.725467708	Zm00001d010530
Zm00001d047955_T002	0.01506668	2.807905395	Zm00001d047955
Zm00001d017120_T004	9.65E-08	1.986590268	Zm00001d017120
Zm00001d010056_T008	0.000392914	2.566987155	Zm00001d010056
Zm00001d025919_T007	0.01713299	1.690945337	Zm00001d025919
Zm00001d025919_T003	3.31E-07	1.592106678	Zm00001d025919
Zm00001d028752_T001	4.88E-06	4.759916656	Zm00001d028752
Zm00001d047076_T001	0.03728966	-1.828318443	Zm00001d047076
Zm00001d046530_T001	0.03830215	2.564396905	Zm00001d046530
Zm00001d001913_T002	0.01128066	-2.17788154	Zm00001d001913
Zm00001d047911_T014	0.009256586	-2.601914132	Zm00001d047911
Zm00001d012042_T002	0.000565416	-1.706017595	Zm00001d012042
Zm00001d024572_T002	0.04000108	2.554840629	Zm00001d024572
Zm00001d044584_T006	0.004332221	1.625060278	Zm00001d044584
Zm00001d043434_T006	3.01E-12	1.749180202	Zm00001d043434
Zm00001d023774_T001	0.04489514	-1.733502692	Zm00001d023774
Zm00001d038718_T001	0.004610805	2.872130345	Zm00001d038718
Zm00001d038718_T003	1.92E-05	3.880379203	Zm00001d038718
Zm00001d042713_T005	0.005246483	1.254665118	Zm00001d042713
Zm00001d021830_T001	0.01918261	1.568580299	Zm00001d021830
Zm00001d021318_T002	0.000800583	1.002637298	Zm00001d021318
Zm00001d052193_T004	0.006966048	1.578048735	Zm00001d052193
Zm00001d022378_T002	0.02379854	2.38291471	Zm00001d022378
Zm00001d042811_T001	0.02321721	2.537283242	Zm00001d042811
Zm00001d009487_T001	0.004862421	2.992278004	Zm00001d009487
Zm00001d022206_T003	1.52E-06	-1.013828993	Zm00001d022206
Zm00001d028140_T016	1.70E-08	-1.766044868	Zm00001d028140
Zm00001d044443_T001	0.03645088	2.594693096	Zm00001d044443
Zm00001d043158_T001	5.30E-07	-1.018503585	Zm00001d043158
Zm00001d030891_T001	0.01932232	2.847616151	Zm00001d030891
Zm00001d025018_T001	0.03090842	-1.85519427	Zm00001d025018
Zm00001d028838_T001	0.00045105	2.868635824	Zm00001d028838
Zm00001d041186_T005	8.59E-12	2.585111254	Zm00001d041186
Zm00001d053304_T001	5.05E-07	-1.313917538	Zm00001d053304

Zm00001d015025_T001	0.002233729	3.387877732	Zm00001d015025
Zm00001d052923_T001	0.01477296	1.385817273	Zm00001d052923
Zm00001d024567_T015	0.000673934	1.637033256	Zm00001d024567
Zm00001d038642_T001	0.006878684	-1.751513979	Zm00001d038642
Zm00001d028952_T007	1.79E-14	4.016203759	Zm00001d028952
Zm00001d011073_T001	0.01911835	-1.845460086	Zm00001d011073
Zm00001d030863_T001	0.01682926	-1.941343849	Zm00001d030863
Zm00001d018884_T010	0.003054744	2.67379254	Zm00001d018884
Zm00001d015284_T001	0.000238001	-1.299145727	Zm00001d015284
Zm00001d041700_T007	0.02062193	1.125625166	Zm00001d041700
Zm00001d041700_T004	0.04512563	1.311625272	Zm00001d041700
Zm00001d046449_T015	4.98E-17	-6.932454886	Zm00001d046449
Zm00001d036825_T051	1.01E-08	1.007388413	Zm00001d036825
Zm00001d036707_T006	0.01893825	-1.290293299	Zm00001d036707
Zm00001d040748_T034	8.97E-11	1.821489308	Zm00001d040748
Zm00001d040748_T035	7.36E-13	-2.929986623	Zm00001d040748
Zm00001d041083_T003	0.000455444	-1.042321692	Zm00001d041083
Zm00001d041083_T009	0.01122327	1.009742436	Zm00001d041083
Zm00001d018811_T001	0.01548639	1.801230732	Zm00001d018811
Zm00001d005451_T012	9.47E-16	3.932071213	Zm00001d005451
Zm00001d005451_T002	1.00E-11	-1.59344251	Zm00001d005451
Zm00001d005451_T001	0.01175716	2.731821705	Zm00001d005451
Zm00001d016406_T009	0.000197126	1.312791338	Zm00001d016406
Zm00001d005007_T001	0.03648535	-1.777586222	Zm00001d005007
Zm00001d031518_T015	6.72E-09	-1.831864489	Zm00001d031518
Zm00001d025734_T009	0.02178755	1.99461051	Zm00001d025734
Zm00001d025734_T015	0.004247367	2.331555383	Zm00001d025734
Zm00001d029378_T016	0.01637601	-2.307094606	Zm00001d029378
Zm00001d009600_T031	0.003968047	1.083854428	Zm00001d009600
Zm00001d017612_T001	0.02786903	2.550252581	Zm00001d017612
Zm00001d034188_T003	0.000772007	2.781620371	Zm00001d034188
Zm00001d019631_T015	0.000538353	1.427540545	Zm00001d019631
Zm00001d012321_T001	0.003745035	2.780282822	Zm00001d012321
Zm00001d038300_T001	0.04008706	-1.736191264	Zm00001d038300
Zm00001d048546_T013	1.50E-11	1.757681682	Zm00001d048546
Zm00001d014957_T007	1.19E-08	-2.760105114	Zm00001d014957
Zm00001d003679_T001	6.83E-11	-2.623422889	Zm00001d003679
Zm00001d050628_T001	0.01381559	2.151506288	Zm00001d050628
Zm00001d053841_T008	0.03797568	1.705546869	Zm00001d053841
Zm00001d053841_T010	2.35E-09	1.557343119	Zm00001d053841
Zm00001d053841_T005	9.56E-13	-3.084457996	Zm00001d053841
Zm00001d030666_T013	3.55E-11	1.423094876	Zm00001d030666
Zm00001d022000_T051	8.79E-12	-2.531505966	Zm00001d022000
Zm00001d022000_T014	0.005246483	1.588142954	Zm00001d022000
Zm00001d022000_T010	0.000167404	1.86249413	Zm00001d022000
Zm00001d007886_T016	5.49E-06	1.084028288	Zm00001d007886
Zm00001d006948_T001	1.37E-05	1.328363768	Zm00001d006948
Zm00001d000452_T024	0.002528307	1.868155153	Zm00001d000452
Zm00001d019207_T001	0.000378118	3.859369663	Zm00001d019207
Zm00001d003464_T004	0.04903754	-1.20848646	Zm00001d003464
Zm00001d023327_T011	1.63E-15	3.428233101	Zm00001d023327
Zm00001d023327_T003	9.93E-12	1.5636068	Zm00001d023327
Zm00001d017729_T010	0.04406775	2.710775281	Zm00001d017729
Zm00001d017729_T014	0.007309779	1.850950595	Zm00001d017729

Zm00001d025397_T005	1.36E-05	2.686801142	Zm00001d025397
Zm00001d025397_T001	1.01E-07	3.827992651	Zm00001d025397
Zm00001d013865_T005	0.03126852	2.072772174	Zm00001d013865
Zm00001d023316_T002	0.001105106	-2.346419697	Zm00001d023316
Zm00001d002164_T027	0.00090062	-1.081441709	Zm00001d002164
Zm00001d010228_T001	0.000189296	2.04500594	Zm00001d010228
Zm00001d029601_T001	7.14E-05	3.942960225	Zm00001d029601
Zm00001d029601_T002	2.82E-05	4.405385638	Zm00001d029601
Zm00001d029601_T003	0.000156819	3.863659248	Zm00001d029601
Zm00001d005424_T003	0.000806633	1.355760654	Zm00001d005424
Zm00001d049974_T001	1.73E-07	2.422138015	Zm00001d049974
Zm00001d049974_T009	3.53E-12	-1.991970343	Zm00001d049974
Zm00001d028550_T002	1.20E-07	-2.448306398	Zm00001d028550
Zm00001d038762_T009	0.01757082	1.321256861	Zm00001d038762
Zm00001d038762_T018	2.04E-05	1.298336644	Zm00001d038762
Zm00001d038762_T023	0.009908492	1.878323609	Zm00001d038762
Zm00001d038762_T016	0.000241367	2.62547164	Zm00001d038762
Zm00001d015029_T004	2.95E-05	2.088703173	Zm00001d015029
Zm00001d038296_T002	8.79E-08	-3.040788838	Zm00001d038296
Zm00001d032810_T007	0.000259008	3.415018388	Zm00001d032810
Zm00001d032810_T006	0.000263804	2.691992647	Zm00001d032810
Zm00001d032810_T009	4.75E-07	3.073819775	Zm00001d032810
Zm00001d006760_T002	0.02845695	-2.197621578	Zm00001d006760
Zm00001d007369_T021	0.02069359	1.2773044	Zm00001d007369
Zm00001d007369_T018	0.000300769	-1.317711526	Zm00001d007369
Zm00001d031447_T007	1.23E-13	2.469424033	Zm00001d031447
Zm00001d031447_T003	1.26E-10	-1.074621387	Zm00001d031447
Zm00001d049660_T002	0.006314872	2.864019594	Zm00001d049660
Zm00001d013588_T001	0.000134856	-1.158720143	Zm00001d013588
Zm00001d018742_T001	0.03277874	-1.579104889	Zm00001d018742
Zm00001d051795_T001	0.04152779	2.052368786	Zm00001d051795
Zm00001d028261_T012	0.02237672	2.203399016	Zm00001d028261
Zm00001d019217_T003	0.000226353	-1.521165724	Zm00001d019217
Zm00001d024625_T004	0.02729225	-2.156861285	Zm00001d024625
Zm00001d052015_T001	0.005892258	-2.968688039	Zm00001d052015
Zm00001d025892_T001	9.74E-12	2.789880622	Zm00001d025892
Zm00001d051607_T001	2.80E-08	1.167354047	Zm00001d051607
Zm00001d046652_T001	0.0436724	2.474721944	Zm00001d046652
Zm00001d012221_T001	0.000760374	-2.898028485	Zm00001d012221
Zm00001d044551_T017	6.61E-08	-3.643523184	Zm00001d044551
Zm00001d028436_T003	7.66E-14	-2.799042149	Zm00001d028436
Zm00001d028436_T002	4.96E-10	1.779450089	Zm00001d028436
Zm00001d018450_T007	2.73E-06	2.101579953	Zm00001d018450
Zm00001d032449_T002	2.77E-08	-2.673644276	Zm00001d032449
Zm00001d032449_T006	6.01E-15	-3.524838534	Zm00001d032449
Zm00001d032449_T005	0.03485146	1.132064059	Zm00001d032449
Zm00001d032449_T003	1.66E-16	4.518052973	Zm00001d032449
Zm00001d019836_T008	0.02672699	2.102869371	Zm00001d019836
Zm00001d019836_T005	0.04595375	1.923616619	Zm00001d019836
Zm00001d034807_T001	0.01758334	-1.194893958	Zm00001d034807
Zm00001d034807_T005	0.01526785	2.251628175	Zm00001d034807
Zm00001d021207_T001	5.05E-08	3.971652098	Zm00001d021207
Zm00001d027630_T006	2.08E-11	2.218287062	Zm00001d027630
Zm00001d027630_T009	6.85E-14	-2.907199144	Zm00001d027630

Zm00001d028159_T001	0.000388394	3.569764836	Zm00001d028159
Zm00001d053875_T007	1.01E-12	-2.760855171	Zm00001d053875
Zm00001d006059_T001	0.03181655	-1.971445103	Zm00001d006059
Zm00001d009687_T001	0.00113985	-2.79916553	Zm00001d009687
Zm00001d020367_T004	3.41E-11	2.045345814	Zm00001d020367
Zm00001d020367_T003	0.008308776	-1.132321772	Zm00001d020367
Zm00001d048316_T001	0.03725648	2.393415722	Zm00001d048316
Zm00001d030217_T027	0.001178527	-2.653790334	Zm00001d030217
Zm00001d004559_T001	0.00506932	1.15586761	Zm00001d004559
Zm00001d004559_T022	0.03238748	1.194268949	Zm00001d004559
Zm00001d020332_T001	2.97E-05	2.900132865	Zm00001d020332
Zm00001d014967_T003	1.50E-09	-1.435055387	Zm00001d014967
Zm00001d014967_T009	0.003441561	2.41598509	Zm00001d014967
Zm00001d015082_T001	2.08E-12	1.766557196	Zm00001d015082
Zm00001d052111_T033	0.0428905	-1.060901301	Zm00001d052111
Zm00001d018093_T001	0.002196051	1.073812971	Zm00001d018093
Zm00001d045537_T006	0.01967564	1.583594551	Zm00001d045537
Zm00001d018793_T001	0.001943159	3.265720386	Zm00001d018793
Zm00001d012892_T001	1.30E-07	1.916053658	Zm00001d012892
Zm00001d005507_T010	2.44E-07	1.772665693	Zm00001d005507
Zm00001d050247_T001	0.005257717	2.371822462	Zm00001d050247
Zm00001d007324_T004	0.009667038	-2.126513993	Zm00001d007324
Zm00001d005468_T002	0.03639015	-1.983945008	Zm00001d005468
Zm00001d052520_T001	0.04416456	-1.865490417	Zm00001d052520
Zm00001d030935_T021	3.16E-11	1.511923223	Zm00001d030935
Zm00001d035889_T025	0.01814386	1.043327293	Zm00001d035889
Zm00001d035889_T001	0.005324772	2.564780899	Zm00001d035889
Zm00001d034920_T001	0.001261061	-2.255457308	Zm00001d034920
Zm00001d015246_T007	0.000920521	1.272761051	Zm00001d015246
Zm00001d036610_T001	0.00116817	3.070082029	Zm00001d036610
Zm00001d007508_T007	1.87E-07	-1.282579492	Zm00001d007508
Zm00001d020973_T001	4.62E-07	-1.343550212	Zm00001d020973
Zm00001d020429_T001	0.01190273	2.864255917	Zm00001d020429
Zm00001d026040_T001	0.04008706	-1.689721772	Zm00001d026040
Zm00001d051839_T002	0.03715458	1.066613077	Zm00001d051839
Zm00001d025689_T006	4.89E-07	-2.807160814	Zm00001d025689
Zm00001d037346_T030	3.40E-05	1.787913451	Zm00001d037346
Zm00001d037346_T024	0.007365988	2.292464236	Zm00001d037346
Zm00001d037346_T014	3.50E-14	-2.182719357	Zm00001d037346
Zm00001d002366_T007	0.007738702	1.229507016	Zm00001d002366
Zm00001d021653_T001	0.001035991	3.256846864	Zm00001d021653
Zm00001d021653_T002	9.20E-05	2.750771654	Zm00001d021653
Zm00001d029447_T001	0.000188838	-3.387037456	Zm00001d029447
Zm00001d002065_T004	0.0314624	-1.430174049	Zm00001d002065
Zm00001d013046_T004	0.01853475	2.87336693	Zm00001d013046
Zm00001d013046_T005	5.03E-06	1.288494128	Zm00001d013046
Zm00001d033158_T005	0.000117201	-2.012725423	Zm00001d033158
Zm00001d033158_T009	0.0101257	-1.163748829	Zm00001d033158
Zm00001d033158_T011	0.01477598	-1.705535732	Zm00001d033158
Zm00001d042837_T001	0.04727699	-1.51306122	Zm00001d042837
Zm00001d051980_T001	0.000401065	2.52644488	Zm00001d051980
Zm00001d011874_T001	0.008186499	2.766330542	Zm00001d011874
Zm00001d021214_T001	0.01322411	2.34354602	Zm00001d021214
Zm00001d020751_T001	0.01854488	2.354987214	Zm00001d020751

Zm00001d002545_T001	0.05009396	-1.248011109	Zm00001d002545
Zm00001d035089_T005	0.01943884	1.218669935	Zm00001d035089
Zm00001d028619_T003	0.002628689	1.160150882	Zm00001d028619
Zm00001d010213_T005	3.52E-08	2.422196162	Zm00001d010213
Zm00001d037332_T005	1.21E-12	-2.874309476	Zm00001d037332
Zm00001d037332_T001	0.03648535	1.863951618	Zm00001d037332
Zm00001d049706_T028	0.00012109	-1.264398206	Zm00001d049706
Zm00001d049706_T013	1.27E-12	2.226745097	Zm00001d049706
Zm00001d049706_T010	4.64E-11	-1.580857356	Zm00001d049706
Zm00001d006402_T004	2.50E-07	-1.017622583	Zm00001d006402
Zm00001d037772_T014	0.000208615	2.257810127	Zm00001d037772
Zm00001d037772_T013	0.004443898	-1.416584003	Zm00001d037772
Zm00001d009631_T007	0.03956497	1.408093361	Zm00001d009631
Zm00001d009631_T009	0.04270301	1.468608176	Zm00001d009631
Zm00001d044833_T001	0.02224341	2.533074387	Zm00001d044833
Zm00001d044833_T002	3.94E-05	3.791147489	Zm00001d044833
Zm00001d006578_T002	0.003573197	1.144697103	Zm00001d006578
Zm00001d012505_T001	0.001452291	1.999985758	Zm00001d012505
Zm00001d021844_T036	0.000820562	1.208096046	Zm00001d021844
Zm00001d021844_T032	0.000203051	1.875864779	Zm00001d021844
Zm00001d021844_T048	0.005375707	1.052553388	Zm00001d021844
Zm00001d015272_T004	0.01873138	1.918944096	Zm00001d015272
Zm00001d011119_T003	0.0340858	1.859870689	Zm00001d011119
Zm00001d042152_T011	1.28E-09	1.465583155	Zm00001d042152
Zm00001d001978_T133	4.15E-13	-2.476237287	Zm00001d001978
Zm00001d001978_T115	0.009336484	1.052481325	Zm00001d001978
Zm00001d001978_T134	2.27E-12	-2.396876311	Zm00001d001978
Zm00001d001978_T014	4.53E-05	2.521461393	Zm00001d001978
Zm00001d051404_T014	0.000340227	2.204563104	Zm00001d051404
Zm00001d040731_T003	0.007772245	1.540327082	Zm00001d040731
Zm00001d039328_T004	4.38E-07	-3.576811838	Zm00001d039328
Zm00001d029706_T001	0.01858035	-1.530325387	Zm00001d029706
Zm00001d008679_T002	0.000115801	-1.832899593	Zm00001d008679
Zm00001d037962_T010	0.01867386	1.535960235	Zm00001d037962
Zm00001d037962_T017	7.47E-07	1.039823334	Zm00001d037962
Zm00001d048031_T007	2.17E-10	1.75772975	Zm00001d048031
Zm00001d048031_T025	4.27E-05	1.360277485	Zm00001d048031
Zm00001d012291_T001	0.0107304	-1.833993988	Zm00001d012291
Zm00001d029585_T001	4.77E-05	-1.938351857	Zm00001d029585
Zm00001d009749_T009	9.27E-15	3.509975832	Zm00001d009749
Zm00001d015164_T001	0.000191577	3.323229545	Zm00001d015164
Zm00001d005324_T004	4.53E-08	1.380574888	Zm00001d005324
Zm00001d042449_T001	0.03008321	2.327741917	Zm00001d042449
Zm00001d028359_T003	0.008898652	-1.397120774	Zm00001d028359
Zm00001d039919_T002	0.000605558	2.85144348	Zm00001d039919
Zm00001d039563_T013	4.67E-10	1.120445753	Zm00001d039563
Zm00001d039563_T004	0.000349798	1.709034865	Zm00001d039563
Zm00001d011638_T011	1.83E-05	2.231178609	Zm00001d011638
Zm00001d011638_T005	8.12E-10	2.833574482	Zm00001d011638
Zm00001d011638_T008	7.18E-08	1.363060513	Zm00001d011638
Zm00001d011638_T002	1.94E-07	-1.565552449	Zm00001d011638
Zm00001d005936_T001	0.01983709	2.322147906	Zm00001d005936
Zm00001d015406_T001	0.000872907	3.102269405	Zm00001d015406
Zm00001d015406_T003	7.11E-10	-3.522801368	Zm00001d015406

Zm00001d022451_T001	0.01127347	2.911444612	Zm00001d022451
Zm00001d013620_T009	0.0191703	1.602342094	Zm00001d013620
Zm00001d045263_T002	2.60E-08	2.331454135	Zm00001d045263
Zm00001d002644_T023	0.01063015	1.049623473	Zm00001d002644
Zm00001d015289_T001	1.97E-11	1.963093253	Zm00001d015289
Zm00001d027742_T001	0.003152852	-2.985647405	Zm00001d027742
Zm00001d029550_T017	4.34E-08	-2.252352934	Zm00001d029550
Zm00001d048401_T001	0.0228443	-1.108846564	Zm00001d048401
Zm00001d053300_T007	5.10E-12	2.039024499	Zm00001d053300
Zm00001d052888_T009	0.02376368	1.346306069	Zm00001d052888
Zm00001d008727_T001	1.54E-05	-2.650028752	Zm00001d008727
Zm00001d015515_T001	0.005530214	3.141134147	Zm00001d015515
Zm00001d052008_T006	1.98E-05	2.569364583	Zm00001d052008
Zm00001d052008_T004	0.04940148	-1.181215123	Zm00001d052008
Zm00001d033278_T010	7.23E-15	3.043509638	Zm00001d033278
Zm00001d033278_T002	0.000822402	1.823597641	Zm00001d033278
Zm00001d033053_T001	0.00045129	-1.046733644	Zm00001d033053
Zm00001d051542_T071	0.04404459	1.602017559	Zm00001d051542
Zm00001d051542_T076	0.02583579	-1.539833099	Zm00001d051542
Zm00001d048229_T001	0.04443519	-1.57365939	Zm00001d048229
Zm00001d036968_T020	3.58E-12	-1.938212518	Zm00001d036968
Zm00001d036968_T014	1.24E-14	3.237459311	Zm00001d036968
Zm00001d042063_T001	1.00E-05	2.738186634	Zm00001d042063
Zm00001d053262_T037	0.01442294	-2.457944675	Zm00001d053262
Zm00001d053262_T033	0.0180044	1.490402326	Zm00001d053262
Zm00001d037656_T001	0.000128066	-3.237779129	Zm00001d037656
Zm00001d026406_T001	0.005512874	-2.90908532	Zm00001d026406
Zm00001d036535_T001	0.01145971	-2.121521626	Zm00001d036535
Zm00001d034783_T001	0.000105885	3.756461545	Zm00001d034783
Zm00001d017284_T001	0.008296542	2.903468947	Zm00001d017284
Zm00001d025507_T015	0.02622542	1.360492814	Zm00001d025507
Zm00001d028534_T003	3.79E-07	1.556389742	Zm00001d028534
Zm00001d047263_T001	0.009041647	-1.90259916	Zm00001d047263
Zm00001d050917_T001	2.37E-06	1.909155183	Zm00001d050917
Zm00001d017186_T002	0.01054164	2.864930625	Zm00001d017186
Zm00001d053281_T005	0.006622526	-1.811869528	Zm00001d053281
Zm00001d047030_T029	6.66E-13	2.045957631	Zm00001d047030
Zm00001d039411_T003	0.03760071	2.428656968	Zm00001d039411
Zm00001d040466_T002	4.46E-11	-2.175608258	Zm00001d040466
Zm00001d049479_T001	0.004540284	1.760895256	Zm00001d049479
Zm00001d042874_T002	0.004883832	-1.900818516	Zm00001d042874
Zm00001d002158_T001	0.001168745	-1.666886076	Zm00001d002158
Zm00001d014901_T002	0.003015693	-1.004670278	Zm00001d014901
Zm00001d005841_T001	0.01785822	2.17098869	Zm00001d005841
Zm00001d048430_T005	0.0118982	2.249905523	Zm00001d048430
Zm00001d045391_T001	2.59E-10	-6.075121249	Zm00001d045391
Zm00001d044982_T007	0.01045777	1.303315349	Zm00001d044982
Zm00001d044982_T009	0.02586834	2.276816203	Zm00001d044982
Zm00001d008323_T007	3.81E-09	1.761533877	Zm00001d008323
Zm00001d044171_T001	0.009317753	2.6933275	Zm00001d044171
Zm00001d014188_T005	0.000780838	1.543906358	Zm00001d014188
Zm00001d034245_T001	6.25E-10	-2.766566548	Zm00001d034245
Zm00001d021947_T004	0.002445413	1.208078878	Zm00001d021947
Zm00001d021947_T005	3.46E-06	1.937202833	Zm00001d021947

Zm00001d050061_T005	1.04E-08	-1.244950526	Zm00001d050061
Zm00001d010798_T001	0.01481562	2.192282481	Zm00001d010798
Zm00001d027924_T001	0.009891435	-2.297714981	Zm00001d027924
Zm00001d002402_T001	0.03124381	-1.86558718	Zm00001d002402
Zm00001d048268_T001	0.01481829	1.999634499	Zm00001d048268
Zm00001d011379_T004	5.60E-09	1.25777959	Zm00001d011379
Zm00001d028074_T001	1.73E-05	4.267863516	Zm00001d028074
Zm00001d045944_T011	0.00713124	1.261914308	Zm00001d045944
Zm00001d045944_T006	0.03639015	1.554421518	Zm00001d045944
Zm00001d013794_T001	0.01861352	-1.355836301	Zm00001d013794
Zm00001d021279_T001	0.01368227	-1.086966117	Zm00001d021279
Zm00001d028670_T001	0.0252917	-1.635522819	Zm00001d028670
Zm00001d014755_T001	0.009922438	2.844373145	Zm00001d014755
Zm00001d048065_T003	0.0259558	-2.930382356	Zm00001d048065
Zm00001d046055_T001	3.38E-06	-5.10539344	Zm00001d046055
Zm00001d043237_T001	0.04229279	1.203360075	Zm00001d043237
Zm00001d043942_T003	6.78E-10	-4.586912789	Zm00001d043942
Zm00001d040562_T025	0.03332528	1.32898145	Zm00001d040562
Zm00001d040562_T020	0.000687304	3.177306311	Zm00001d040562
Zm00001d002791_T013	0.001627225	1.338844136	Zm00001d002791
Zm00001d042905_T001	0.03495473	1.003859678	Zm00001d042905
Zm00001d035004_T002	0.04075837	1.346864627	Zm00001d035004
Zm00001d035004_T034	3.84E-13	2.29528018	Zm00001d035004
Zm00001d035004_T015	0.000282019	1.689765221	Zm00001d035004
Zm00001d038915_T001	1.93E-07	2.79588415	Zm00001d038915
Zm00001d003119_T002	0.00432903	-1.545327272	Zm00001d003119
Zm00001d003119_T006	4.52E-11	1.80841294	Zm00001d003119
Zm00001d040290_T004	0.0389152	1.047003063	Zm00001d040290
Zm00001d036025_T006	3.94E-05	-2.277205135	Zm00001d036025
Zm00001d008604_T010	0.03778895	1.227506609	Zm00001d008604
Zm00001d052493_T002	0.02845695	-1.98214265	Zm00001d052493
Zm00001d034345_T003	1.05E-06	4.981427138	Zm00001d034345
Zm00001d034345_T002	0.001333162	3.581673259	Zm00001d034345
Zm00001d034345_T001	0.02761546	2.811246911	Zm00001d034345
Zm00001d018217_T009	1.35E-09	1.304692512	Zm00001d018217
Zm00001d018217_T003	0.001034463	2.179366744	Zm00001d018217
Zm00001d043738_T001	4.76E-06	2.349163615	Zm00001d043738
Zm00001d021205_T001	5.61E-09	5.351464905	Zm00001d021205
Zm00001d018482_T005	0.000833983	-1.278049618	Zm00001d018482
Zm00001d011652_T005	0.03774788	1.331278328	Zm00001d011652
Zm00001d022334_T002	0.001508566	1.233518758	Zm00001d022334
Zm00001d020835_T004	0.01320298	1.309201984	Zm00001d020835
Zm00001d020835_T020	0.0389152	-1.253723917	Zm00001d020835
Zm00001d048027_T001	0.001585032	3.028128751	Zm00001d048027
Zm00001d034031_T001	0.03311134	2.707646176	Zm00001d034031
Zm00001d043523_T001	0.006641282	-2.638208058	Zm00001d043523
Zm00001d048870_T001	0.000497198	-2.37645085	Zm00001d048870
Zm00001d011168_T006	1.82E-11	1.617358864	Zm00001d011168
Zm00001d011168_T018	0.003641959	1.711499415	Zm00001d011168
Zm00001d028270_T002	8.36E-07	1.515669761	Zm00001d028270
Zm00001d034492_T019	4.01E-13	3.19084175	Zm00001d034492
Zm00001d002726_T009	2.64E-07	-1.357739578	Zm00001d002726
Zm00001d019897_T003	0.03860996	1.339703883	Zm00001d019897
Zm00001d036122_T001	0.0373054	2.565618557	Zm00001d036122

Zm00001d021946_T001	0.006106844	2.922431949	Zm00001d021946
Zm00001d005012_T015	0.009211255	2.781946858	Zm00001d005012
Zm00001d002658_T014	0.02255438	-1.001612805	Zm00001d002658
Zm00001d013159_T004	1.25E-10	5.08511134	Zm00001d013159
Zm00001d051854_T002	7.85E-05	1.826607651	Zm00001d051854
Zm00001d048236_T013	3.29E-08	1.668836819	Zm00001d048236
Zm00001d043390_T001	0.000468585	3.517381937	Zm00001d043390
Zm00001d018372_T001	0.01537015	2.13979479	Zm00001d018372
Zm00001d005892_T001	5.33E-07	3.443675541	Zm00001d005892
Zm00001d005478_T008	9.41E-05	3.680729806	Zm00001d005478
Zm00001d041750_T006	0.003617779	-3.45254507	Zm00001d041750
Zm00001d023769_T001	5.24E-05	-2.434347628	Zm00001d023769
Zm00001d023769_T002	3.61E-05	1.060791906	Zm00001d023769
Zm00001d034931_T001	0.03776326	1.022743327	Zm00001d034931
Zm00001d045603_T056	0.02680286	1.819606872	Zm00001d045603
Zm00001d038584_T001	0.000475302	2.603423154	Zm00001d038584
Zm00001d014986_T002	0.006958403	1.143818381	Zm00001d014986
Zm00001d039612_T028	1.22E-09	1.122329552	Zm00001d039612
Zm00001d034422_T001	0.03435352	-1.765793691	Zm00001d034422
Zm00001d013477_T010	0.001612382	1.373620161	Zm00001d013477
Zm00001d044254_T006	0.000933882	1.839173258	Zm00001d044254
Zm00001d005151_T001	0.00026702	1.367769978	Zm00001d005151
Zm00001d008176_T008	0.0136329	1.680010076	Zm00001d008176
Zm00001d003108_T005	0.04255884	2.324557823	Zm00001d003108
Zm00001d002388_T002	5.87E-15	-4.314086981	Zm00001d002388
Zm00001d008570_T001	0.01259421	2.713700221	Zm00001d008570
Zm00001d054001_T011	0.03659288	2.184317247	Zm00001d054001
Zm00001d033210_T005	1.24E-19	-7.547061135	Zm00001d033210
Zm00001d045870_T002	0.004838924	1.651463259	Zm00001d045870
Zm00001d001995_T002	0.009270008	1.650282026	Zm00001d001995
Zm00001d036131_T004	0.0109172	-2.551294672	Zm00001d036131
Zm00001d028265_T004	0.02224341	-1.922040344	Zm00001d028265
Zm00001d052578_T001	0.0214763	-1.213984908	Zm00001d052578
Zm00001d049556_T009	1.43E-07	1.066094107	Zm00001d049556
Zm00001d002483_T001	0.000516578	1.07325341	Zm00001d002483
Zm00001d045269_T031	3.33E-13	2.491871022	Zm00001d045269
Zm00001d035207_T001	0.004055167	-1.620916394	Zm00001d035207
Zm00001d042747_T004	4.52E-05	2.223027896	Zm00001d042747
Zm00001d014294_T027	4.68E-05	2.019192835	Zm00001d014294
Zm00001d014294_T008	5.19E-06	2.428448651	Zm00001d014294
Zm00001d014294_T011	0.0441095	2.066664667	Zm00001d014294
Zm00001d014036_T014	3.94E-06	1.706520065	Zm00001d014036
Zm00001d014036_T009	1.15E-06	-2.394022402	Zm00001d014036
Zm00001d017121_T002	0.01823557	-1.561145512	Zm00001d017121
Zm00001d009243_T003	0.02343819	1.731910643	Zm00001d009243
Zm00001d052621_T014	4.35E-05	-4.200226143	Zm00001d052621
Zm00001d053197_T006	0.008258542	2.088150689	Zm00001d053197
Zm00001d041816_T003	0.01441334	1.543378256	Zm00001d041816
Zm00001d018295_T001	9.15E-13	2.410818362	Zm00001d018295
Zm00001d018295_T002	7.12E-13	-2.091724729	Zm00001d018295
Zm00001d046438_T009	0.03327991	1.749945922	Zm00001d046438
Zm00001d011297_T002	0.01727412	-2.122513345	Zm00001d011297
Zm00001d011297_T001	0.003626547	-2.422959169	Zm00001d011297
Zm00001d025141_T003	8.43E-08	2.213767848	Zm00001d025141

Zm00001d025141_T002	2.05E-09	2.700087685	Zm00001d025141
Zm00001d027337_T004	0.001274277	-1.543281561	Zm00001d027337
Zm00001d027337_T012	1.92E-06	1.885040821	Zm00001d027337
Zm00001d038576_T002	0.001560792	1.638151074	Zm00001d038576
Zm00001d011984_T003	0.000233833	-1.375319626	Zm00001d011984
Zm00001d032440_T005	0.04570411	2.284994881	Zm00001d032440
Zm00001d052726_T028	1.26E-08	3.50722665	Zm00001d052726
Zm00001d052726_T004	2.44E-19	5.384092972	Zm00001d052726
Zm00001d052726_T003	1.84E-09	1.146823628	Zm00001d052726
Zm00001d052726_T009	0.000231914	1.265241286	Zm00001d052726
Zm00001d052726_T008	1.36E-07	2.742876444	Zm00001d052726
Zm00001d002161_T001	4.40E-08	-5.050099926	Zm00001d002161
Zm00001d039002_T009	0.000711191	1.580250213	Zm00001d039002
Zm00001d039002_T016	1.67E-11	1.432405999	Zm00001d039002
Zm00001d041504_T004	2.35E-12	-2.370063523	Zm00001d041504
Zm00001d016286_T008	0.01713299	1.264803592	Zm00001d016286
Zm00001d046047_T021	0.000388444	2.072451882	Zm00001d046047
Zm00001d004287_T001	0.0176584	2.203577185	Zm00001d004287
Zm00001d047367_T012	0.04550572	1.140554124	Zm00001d047367
Zm00001d047367_T020	1.60E-07	-1.72320141	Zm00001d047367
Zm00001d027734_T001	0.04241715	2.343630242	Zm00001d027734
Zm00001d023994_T004	0.000129541	-2.849295235	Zm00001d023994
Zm00001d005208_T001	0.000844821	3.547954795	Zm00001d005208
Zm00001d036726_T001	1.15E-06	4.191875243	Zm00001d036726
Zm00001d052666_T009	1.13E-10	1.426348184	Zm00001d052666
Zm00001d040191_T006	5.60E-09	1.032902721	Zm00001d040191
Zm00001d002462_T165	1.20E-09	1.170579938	Zm00001d002462
Zm00001d002462_T087	0.00063647	1.289927538	Zm00001d002462
Zm00001d014491_T001	0.01855432	2.790799055	Zm00001d014491
Zm00001d012821_T002	0.002046598	2.177646174	Zm00001d012821
Zm00001d038865_T012	0.000104571	-2.057645563	Zm00001d038865
Zm00001d038865_T002	0.00080941	-1.369425374	Zm00001d038865
Zm00001d027895_T004	0.001005617	1.423168883	Zm00001d027895
Zm00001d046579_T027	0.007738702	1.064858744	Zm00001d046579
Zm00001d028923_T009	5.53E-12	-3.205847995	Zm00001d028923
Zm00001d042654_T006	2.35E-09	-1.693437634	Zm00001d042654
Zm00001d018158_T001	8.75E-07	3.628329975	Zm00001d018158
Zm00001d005195_T004	2.91E-08	1.254091338	Zm00001d005195
Zm00001d006769_T008	0.000340711	-1.852359448	Zm00001d006769
Zm00001d045042_T015	0.005686021	-2.002243528	Zm00001d045042
Zm00001d033273_T001	0.000294231	1.528418457	Zm00001d033273
Zm00001d005164_T020	0.02700349	1.105964868	Zm00001d005164
Zm00001d005164_T015	0.000260831	2.781069569	Zm00001d005164
Zm00001d005164_T016	1.14E-06	1.560515429	Zm00001d005164
Zm00001d046210_T008	0.004572431	1.364690467	Zm00001d046210
Zm00001d052307_T001	0.02535018	1.614017134	Zm00001d052307
Zm00001d045072_T037	0.01234628	1.121275262	Zm00001d045072
Zm00001d045072_T020	1.54E-13	2.882286981	Zm00001d045072
Zm00001d045072_T044	0.000594653	1.28252956	Zm00001d045072
Zm00001d023732_T005	0.02672227	-1.994111106	Zm00001d023732
Zm00001d019432_T001	1.38E-11	-2.661001265	Zm00001d019432
Zm00001d031933_T007	2.14E-05	1.048930589	Zm00001d031933
Zm00001d022457_T001	0.01573827	-2.092585919	Zm00001d022457
Zm00001d025629_T007	1.23E-12	2.625835145	Zm00001d025629

Zm00001d039522_T006	0.01958328	-2.075806833	Zm00001d039522
Zm00001d010588_T001	3.06E-07	-2.773135992	Zm00001d010588
Zm00001d010588_T003	1.31E-06	-3.37549335	Zm00001d010588
Zm00001d049543_T009	9.76E-09	1.23424952	Zm00001d049543
Zm00001d049543_T013	0.000824048	-1.97642745	Zm00001d049543
Zm00001d049543_T017	4.37E-09	-1.799509992	Zm00001d049543
Zm00001d043703_T004	0.000813969	1.044239431	Zm00001d043703
Zm00001d003420_T001	0.01306162	4.460242966	Zm00001d003420
Zm00001d016679_T001	0.008983932	1.093614902	Zm00001d016679
Zm00001d047786_T001	0.00120544	3.474627229	Zm00001d047786
Zm00001d042828_T001	0.000305188	2.735726403	Zm00001d042828
Zm00001d010640_T001	0.000445504	3.663798781	Zm00001d010640
Zm00001d044579_T001	2.02E-06	-1.170427445	Zm00001d044579
Zm00001d042470_T008	0.001148838	1.200580084	Zm00001d042470
Zm00001d042470_T014	1.61E-06	2.061536602	Zm00001d042470
Zm00001d026289_T003	1.79E-09	-1.331053126	Zm00001d026289
Zm00001d013741_T007	1.52E-12	2.868608542	Zm00001d013741
Zm00001d041670_T001	0.01713299	-1.824050312	Zm00001d041670
Zm00001d017084_T001	0.000919043	2.640909604	Zm00001d017084
Zm00001d050074_T004	0.007540301	1.484127919	Zm00001d050074
Zm00001d050074_T005	2.79E-07	-2.336546168	Zm00001d050074
Zm00001d014658_T005	0.00017617	2.32163668	Zm00001d014658
Zm00001d007422_T006	0.03295965	1.164872227	Zm00001d007422
Zm00001d007422_T012	0.0119098	-3.214563947	Zm00001d007422
Zm00001d034734_T003	1.31E-08	1.279294477	Zm00001d034734
Zm00001d032096_T005	1.59E-07	1.057411171	Zm00001d032096
Zm00001d032120_T001	2.72E-06	3.340482126	Zm00001d032120
Zm00001d017977_T014	0.000521935	-1.609107624	Zm00001d017977
Zm00001d051685_T002	1.05E-06	-1.765226181	Zm00001d051685
Zm00001d018844_T001	0.00012849	3.446335553	Zm00001d018844
Zm00001d017457_T001	0.03372473	2.571877224	Zm00001d017457
Zm00001d027344_T003	3.15E-13	4.006261732	Zm00001d027344
Zm00001d027344_T009	0.002796955	-1.665172084	Zm00001d027344
Zm00001d002119_T009	2.71E-10	1.530393379	Zm00001d002119
Zm00001d006626_T001	0.00268479	3.272179387	Zm00001d006626
Zm00001d037518_T005	0.000379352	1.564357564	Zm00001d037518
Zm00001d017808_T001	6.82E-05	1.730351275	Zm00001d017808
Zm00001d017808_T013	0.04377645	-1.737751264	Zm00001d017808
Zm00001d011473_T002	3.73E-09	1.606532172	Zm00001d011473
Zm00001d036195_T003	1.78E-10	-2.977599098	Zm00001d036195
Zm00001d046187_T001	0.02490188	-1.386280157	Zm00001d046187
Zm00001d009571_T004	1.54E-09	1.816760576	Zm00001d009571
Zm00001d009571_T005	0.03997268	1.571086304	Zm00001d009571
Zm00001d009571_T001	0.03725985	-1.278494419	Zm00001d009571
Zm00001d023658_T002	0.00206067	-2.483592624	Zm00001d023658
Zm00001d043927_T011	2.43E-13	2.06444107	Zm00001d043927
Zm00001d048415_T010	3.64E-10	1.947525216	Zm00001d048415
Zm00001d048415_T012	0.003631449	-1.569935195	Zm00001d048415
Zm00001d034160_T001	0.000303647	3.828217392	Zm00001d034160
Zm00001d011890_T001	0.000241547	-1.7592466	Zm00001d011890
Zm00001d016260_T001	0.02179476	-2.202124895	Zm00001d016260
Zm00001d009726_T003	0.011246	-1.662268733	Zm00001d009726
Zm00001d009309_T001	0.01213194	2.561907488	Zm00001d009309
Zm00001d020727_T005	0.006488149	1.111537412	Zm00001d020727

Zm00001d018413_T017	6.72E-09	2.192631842	Zm00001d018413
Zm00001d045388_T001	0.01390627	-1.719022652	Zm00001d045388
Zm00001d014442_T014	0.04574688	-1.774379591	Zm00001d014442
Zm00001d012797_T007	0.0339815	1.807863986	Zm00001d012797
Zm00001d012797_T001	2.57E-14	-3.750762053	Zm00001d012797
Zm00001d011403_T001	0.02048591	2.033261807	Zm00001d011403
Zm00001d011541_T007	0.004998487	-1.773669633	Zm00001d011541
Zm00001d049049_T004	0.000653854	1.277244719	Zm00001d049049
Zm00001d014726_T012	0.007022294	2.563301273	Zm00001d014726
Zm00001d014726_T005	0.01144665	2.070955928	Zm00001d014726
Zm00001d014726_T008	3.47E-13	-3.984572053	Zm00001d014726
Zm00001d033726_T013	6.40E-13	2.32466808	Zm00001d033726
Zm00001d028339_T004	0.006974629	1.371009551	Zm00001d028339
Zm00001d028339_T007	2.45E-05	-2.157440168	Zm00001d028339
Zm00001d005502_T002	0.0181716	3.195667199	Zm00001d005502
Zm00001d046506_T003	3.65E-08	1.597249314	Zm00001d046506
Zm00001d021893_T002	0.04465781	2.643666995	Zm00001d021893
Zm00001d013831_T015	0.03429718	-1.229825716	Zm00001d013831
Zm00001d000027_T031	0.000238001	1.022408335	Zm00001d000027
Zm00001d037279_T011	0.01374028	2.491567696	Zm00001d037279
Zm00001d037279_T005	0.01503145	2.448476388	Zm00001d037279
Zm00001d038200_T010	0.00436371	2.673325582	Zm00001d038200
Zm00001d012005_T015	5.29E-12	-1.727787353	Zm00001d012005
Zm00001d041382_T001	0.000780838	-3.071343444	Zm00001d041382
Zm00001d006910_T013	1.42E-06	1.451751507	Zm00001d006910
Zm00001d006910_T001	0.001374172	1.382782937	Zm00001d006910
Zm00001d007595_T025	4.26E-09	1.283940374	Zm00001d007595
Zm00001d036118_T001	0.0080401	-2.088950199	Zm00001d036118
Zm00001d045557_T003	0.003289969	3.05404662	Zm00001d045557
Zm00001d045557_T004	0.04298108	2.572943875	Zm00001d045557
Zm00001d045557_T001	0.005053822	3.193339025	Zm00001d045557
Zm00001d052673_T001	0.006878684	3.058653251	Zm00001d052673
Zm00001d039860_T009	0.003818565	2.173250334	Zm00001d039860
Zm00001d045692_T019	0.003015693	1.052486371	Zm00001d045692
Zm00001d045692_T028	0.0169199	1.251629713	Zm00001d045692
Zm00001d037235_T001	0.006220777	1.114822146	Zm00001d037235
Zm00001d002052_T002	0.000273283	-2.348535816	Zm00001d002052
Zm00001d044102_T006	6.83E-07	2.395966581	Zm00001d044102
Zm00001d047761_T006	8.51E-12	2.323238688	Zm00001d047761
Zm00001d047761_T008	7.16E-09	1.319175572	Zm00001d047761
Zm00001d042034_T001	0.02161544	1.768853257	Zm00001d042034
Zm00001d027936_T003	2.55E-05	1.676135289	Zm00001d027936
Zm00001d011233_T018	0.000872228	1.44743646	Zm00001d011233
Zm00001d038791_T001	0.005709369	3.169973062	Zm00001d038791
Zm00001d032724_T001	0.009372412	1.140956306	Zm00001d032724
Zm00001d007121_T057	6.20E-12	1.835297614	Zm00001d007121
Zm00001d017456_T015	7.37E-05	4.066834137	Zm00001d017456
Zm00001d017456_T011	0.002206201	2.171218888	Zm00001d017456
Zm00001d035725_T001	0.01181493	-1.512112192	Zm00001d035725
Zm00001d005969_T008	3.19E-05	1.085411257	Zm00001d005969
Zm00001d021973_T001	0.004978721	3.154190537	Zm00001d021973
Zm00001d021843_T001	0.04606011	-1.755390797	Zm00001d021843
Zm00001d052353_T008	0.003098678	-2.155874524	Zm00001d052353
Zm00001d021806_T027	1.34E-11	1.569578225	Zm00001d021806

Zm00001d021806_T022	0.0421692	1.336238527	Zm00001d021806
Zm00001d031880_T001	0.01214359	2.07677196	Zm00001d031880
Zm00001d013182_T002	0.0312949	-1.838996844	Zm00001d013182
Zm00001d012027_T006	0.000145457	-3.021981723	Zm00001d012027
Zm00001d012027_T014	0.000500744	1.950594528	Zm00001d012027
Zm00001d012027_T001	1.63E-15	4.211800057	Zm00001d012027
Zm00001d034568_T002	1.51E-07	-1.064217907	Zm00001d034568
Zm00001d034568_T006	0.03670383	1.036121247	Zm00001d034568
Zm00001d003958_T017	0.005841413	2.298802582	Zm00001d003958
Zm00001d003958_T013	0.04665552	1.992760625	Zm00001d003958
Zm00001d035044_T010	8.16E-14	2.732431527	Zm00001d035044
Zm00001d035044_T031	3.94E-05	-2.034597327	Zm00001d035044
Zm00001d035044_T024	3.07E-14	-3.349930674	Zm00001d035044
Zm00001d004659_T007	0.006426743	1.62367915	Zm00001d004659
Zm00001d037142_T022	0.002953279	2.820541463	Zm00001d037142
Zm00001d033368_T071	3.07E-14	-4.387735046	Zm00001d033368
Zm00001d033368_T015	3.50E-05	1.00416422	Zm00001d033368
Zm00001d033368_T010	7.11E-12	1.727982811	Zm00001d033368
Zm00001d045109_T003	1.84E-10	1.008389938	Zm00001d045109
Zm00001d015097_T005	1.00E-09	-1.724622779	Zm00001d015097
Zm00001d015097_T004	0.04385265	2.130882473	Zm00001d015097
Zm00001d032319_T017	0.002641253	2.318232169	Zm00001d032319
Zm00001d036409_T001	0.01206564	-2.130642573	Zm00001d036409
Zm00001d012923_T004	0.004527535	-2.302674137	Zm00001d012923
Zm00001d025247_T001	0.01758334	-2.059138668	Zm00001d025247
Zm00001d038270_T001	0.01489828	2.900348875	Zm00001d038270
Zm00001d033909_T001	0.000968584	-3.408745462	Zm00001d033909
Zm00001d044646_T041	3.97E-08	1.029281531	Zm00001d044646
Zm00001d004019_T002	0.01193426	-1.541942717	Zm00001d004019
Zm00001d008791_T005	2.96E-11	-3.734030707	Zm00001d008791
Zm00001d038357_T002	0.006733831	2.71204104	Zm00001d038357
Zm00001d042505_T016	1.29E-05	1.944672247	Zm00001d042505
Zm00001d042505_T001	0.003938874	2.171048828	Zm00001d042505
Zm00001d018276_T003	0.04539599	-1.190300581	Zm00001d018276
Zm00001d004868_T002	0.01193426	-1.29410286	Zm00001d004868
Zm00001d013920_T008	1.13E-10	1.841729357	Zm00001d013920
Zm00001d010790_T028	2.49E-06	1.100120583	Zm00001d010790
Zm00001d027846_T001	0.04570411	-1.690133658	Zm00001d027846
Zm00001d008222_T002	0.0324581	-1.105712995	Zm00001d008222
Zm00001d008222_T001	0.02134347	-1.181212266	Zm00001d008222
Zm00001d033632_T008	0.01578834	1.395179878	Zm00001d033632
Zm00001d048416_T001	0.03463105	2.434905887	Zm00001d048416
Zm00001d041785_T009	0.001916278	-3.328402511	Zm00001d041785
Zm00001d047780_T002	0.02125339	2.024161099	Zm00001d047780
Zm00001d047479_T007	4.27E-05	-1.081670433	Zm00001d047479
Zm00001d032049_T006	0.01362109	-1.929726429	Zm00001d032049
Zm00001d033104_T016	0.007563791	1.649561168	Zm00001d033104
Zm00001d033104_T007	3.06E-05	1.856560847	Zm00001d033104
Zm00001d008788_T004	2.99E-12	2.590213637	Zm00001d008788
Zm00001d002364_T001	0.02171544	2.283684659	Zm00001d002364
Zm00001d024583_T003	0.003503628	1.770459784	Zm00001d024583
Zm00001d046231_T001	1.09E-06	-1.486029255	Zm00001d046231
Zm00001d009186_T001	0.03189684	2.501208341	Zm00001d009186
Zm00001d039789_T001	0.00090062	1.301667247	Zm00001d039789

Zm00001d033788_T003	1.31E-08	-4.068548356	Zm00001d033788
Zm00001d032699_T018	3.30E-09	1.28249172	Zm00001d032699
Zm00001d008548_T001	6.35E-05	-3.192628269	Zm00001d008548
Zm00001d043462_T009	0.02558382	1.192722656	Zm00001d043462
Zm00001d037650_T005	0.03230314	1.770769268	Zm00001d037650
Zm00001d037650_T002	0.04082476	1.816931291	Zm00001d037650
Zm00001d030094_T005	0.000933648	-2.643050291	Zm00001d030094
Zm00001d003187_T002	0.002772078	-1.799284757	Zm00001d003187
Zm00001d003187_T001	9.89E-05	-1.807457095	Zm00001d003187
Zm00001d053427_T011	0.04292993	1.309320058	Zm00001d053427
Zm00001d022388_T012	3.52E-05	1.567492635	Zm00001d022388
Zm00001d022388_T003	0.008251933	2.094079502	Zm00001d022388
Zm00001d022388_T016	1.75E-15	3.447814603	Zm00001d022388
Zm00001d044195_T020	0.001550358	-2.495727588	Zm00001d044195
Zm00001d053580_T005	9.73E-05	-2.362590426	Zm00001d053580
Zm00001d034000_T005	0.03734827	1.15058746	Zm00001d034000
Zm00001d041067_T001	0.01840305	-1.903509585	Zm00001d041067
Zm00001d043277_T008	1.22E-09	1.189341451	Zm00001d043277
Zm00001d006388_T006	0.004411808	-3.493378295	Zm00001d006388
Zm00001d037808_T007	4.40E-11	-4.310393695	Zm00001d037808
Zm00001d019364_T001	0.006499341	-2.667898676	Zm00001d019364
Zm00001d020414_T001	0.0147135	1.227726615	Zm00001d020414
Zm00001d019978_T006	3.65E-08	1.402292028	Zm00001d019978
Zm00001d019978_T010	0.01048202	1.206325889	Zm00001d019978
Zm00001d004664_T001	0.002306494	2.785293718	Zm00001d004664
Zm00001d011425_T001	0.02229829	2.039409545	Zm00001d011425
Zm00001d038921_T001	0.01531128	2.194487011	Zm00001d038921
Zm00001d036337_T001	0.02746855	2.251097814	Zm00001d036337
Zm00001d009160_T001	0.000484721	-2.922530206	Zm00001d009160
Zm00001d000110_T004	0.004536195	1.305428095	Zm00001d000110
Zm00001d022396_T002	0.000678421	1.385840484	Zm00001d022396
Zm00001d013400_T002	0.01049064	-1.042593609	Zm00001d013400
Zm00001d018349_T008	0.04891776	-2.496631179	Zm00001d018349
Zm00001d037693_T002	0.002378541	-1.22035799	Zm00001d037693
Zm00001d034157_T003	0.000329177	-1.013954772	Zm00001d034157
Zm00001d017700_T025	0.000140887	2.261291814	Zm00001d017700
Zm00001d017700_T013	0.01035085	2.184945396	Zm00001d017700
Zm00001d033539_T002	0.05003329	2.325532781	Zm00001d033539
Zm00001d018522_T002	1.16E-12	-2.492442092	Zm00001d018522
Zm00001d030061_T001	0.04667204	1.551931902	Zm00001d030061
Zm00001d017042_T002	0.002593184	1.894673936	Zm00001d017042
Zm00001d017042_T001	0.001005513	-3.395433605	Zm00001d017042
Zm00001d041945_T001	0.01692334	2.311298907	Zm00001d041945
Zm00001d041945_T006	0.0006035	1.685932952	Zm00001d041945
Zm00001d045232_T001	0.005958271	2.139863609	Zm00001d045232
Zm00001d038312_T001	0.009563045	1.782189556	Zm00001d038312
Zm00001d053445_T001	0.001122214	-2.838837486	Zm00001d053445
Zm00001d021646_T014	2.06E-06	2.64975754	Zm00001d021646
Zm00001d008248_T003	0.04241009	2.043314069	Zm00001d008248
Zm00001d008248_T002	0.01477296	2.656045773	Zm00001d008248
Zm00001d027596_T007	0.000136164	2.117276134	Zm00001d027596
Zm00001d023838_T013	0.001992364	-1.864279105	Zm00001d023838
Zm00001d035115_T001	7.46E-14	-3.460140482	Zm00001d035115
Zm00001d025830_T002	9.26E-12	1.704115519	Zm00001d025830

Zm00001d009448_T001	0.000304177	1.80184416	Zm00001d009448
Zm00001d006861_T011	0.03490439	1.104548455	Zm00001d006861
Zm00001d006861_T008	0.000789023	1.237065537	Zm00001d006861
Zm00001d022338_T002	0.00919548	2.207945457	Zm00001d022338
Zm00001d013241_T011	0.001010267	1.579876789	Zm00001d013241
Zm00001d038698_T006	9.96E-05	2.463360595	Zm00001d038698
Zm00001d038698_T008	2.70E-08	1.214475234	Zm00001d038698
Zm00001d045199_T004	8.43E-08	2.571647932	Zm00001d045199
Zm00001d053313_T005	5.19E-10	1.42617595	Zm00001d053313
Zm00001d052167_T001	1.71E-06	4.18398872	Zm00001d052167
Zm00001d027901_T001	1.37E-05	4.425364509	Zm00001d027901
Zm00001d011373_T013	2.23E-11	-2.307986478	Zm00001d011373
Zm00001d022546_T004	0.008135026	2.358401267	Zm00001d022546
Zm00001d022439_T001	0.000275609	2.343080585	Zm00001d022439
Zm00001d024425_T007	0.004726961	1.989400407	Zm00001d024425
Zm00001d037648_T004	0.02793163	1.778124692	Zm00001d037648
Zm00001d041716_T009	0.00602687	-1.819863618	Zm00001d041716
Zm00001d052885_T004	0.02653131	-1.380771348	Zm00001d052885
Zm00001d044506_T002	0.03465657	1.182374825	Zm00001d044506
Zm00001d023304_T004	3.91E-09	1.367180295	Zm00001d023304
Zm00001d049746_T003	6.72E-09	2.261256423	Zm00001d049746
Zm00001d027718_T001	0.000974606	-1.582931745	Zm00001d027718
Zm00001d016402_T024	2.28E-13	-3.089789182	Zm00001d016402
Zm00001d003334_T001	0.01434908	1.072227246	Zm00001d003334
Zm00001d018738_T001	0.000461316	-3.419589997	Zm00001d018738
Zm00001d037975_T048	2.31E-08	1.449345008	Zm00001d037975
Zm00001d041663_T001	0.001606888	-1.715839111	Zm00001d041663
Zm00001d041649_T010	0.02141717	-1.664923444	Zm00001d041649
Zm00001d003485_T001	0.00067674	4.417437932	Zm00001d003485
Zm00001d047854_T006	0.004173226	1.253073389	Zm00001d047854
Zm00001d026126_T001	7.37E-05	3.857681367	Zm00001d026126
Zm00001d028574_T001	5.33E-05	3.074863829	Zm00001d028574
Zm00001d042314_T011	5.34E-08	1.738832764	Zm00001d042314
Zm00001d023311_T007	0.02263356	2.362588447	Zm00001d023311
Zm00001d023311_T029	1.22E-14	-3.265217154	Zm00001d023311
Zm00001d011750_T001	0.01716409	-2.067177373	Zm00001d011750
Zm00001d027899_T001	0.000359435	3.161431461	Zm00001d027899
Zm00001d025031_T003	0.000241971	1.197185466	Zm00001d025031
Zm00001d025031_T004	0.01089689	1.065422896	Zm00001d025031
Zm00001d007470_T001	0.01598815	-1.565897354	Zm00001d007470
Zm00001d027278_T009	4.74E-12	1.413823194	Zm00001d027278
Zm00001d027278_T122	2.23E-08	-2.401851384	Zm00001d027278
Zm00001d003198_T021	1.06E-06	1.216157662	Zm00001d003198
Zm00001d004669_T006	2.80E-08	1.42643585	Zm00001d004669
Zm00001d018439_T008	0.0142963	1.33799075	Zm00001d018439
Zm00001d022445_T001	2.12E-05	-1.26168596	Zm00001d022445
Zm00001d014158_T004	0.01102494	1.51339207	Zm00001d014158
Zm00001d025789_T007	0.005601632	-1.97117956	Zm00001d025789
Zm00001d022502_T009	0.007149287	2.435843148	Zm00001d022502
Zm00001d032855_T005	0.01763469	1.367812353	Zm00001d032855
Zm00001d038436_T001	0.03078523	-2.021394949	Zm00001d038436
Zm00001d042394_T007	0.0373054	-1.661392123	Zm00001d042394
Zm00001d020185_T007	0.004389276	1.069429701	Zm00001d020185
Zm00001d038075_T001	4.03E-05	-2.70749134	Zm00001d038075

Zm00001d037610_T001	0.00904658	-2.261870294	Zm00001d037610
Zm00001d022327_T001	0.0103436	2.808653661	Zm00001d022327
Zm00001d040234_T011	6.53E-10	1.634654015	Zm00001d040234
Zm00001d045459_T001	0.04237526	2.462213649	Zm00001d045459
Zm00001d034776_T008	5.65E-07	-4.985597205	Zm00001d034776
Zm00001d049761_T019	0.0313238	1.041917579	Zm00001d049761
Zm00001d010235_T009	6.53E-10	1.048048846	Zm00001d010235
Zm00001d012661_T001	0.000101475	-1.157747875	Zm00001d012661
Zm00001d052164_T006	7.95E-07	5.111335923	Zm00001d052164
Zm00001d052164_T001	0.009800589	2.381541494	Zm00001d052164
Zm00001d052164_T002	0.001797502	3.061356948	Zm00001d052164
Zm00001d052164_T005	0.002381753	3.328791763	Zm00001d052164
Zm00001d038465_T001	0.03706504	-1.195544795	Zm00001d038465
Zm00001d019591_T001	0.002158908	-2.749952586	Zm00001d019591
Zm00001d010360_T001	0.02685638	-1.625264988	Zm00001d010360
Zm00001d010812_T001	0.001371047	3.164746122	Zm00001d010812
Zm00001d031269_T010	1.58E-09	-4.022499058	Zm00001d031269
Zm00001d008388_T004	0.000270911	-2.545725751	Zm00001d008388
Zm00001d017490_T001	0.02334998	-1.195979532	Zm00001d017490
Zm00001d053575_T007	0.02913084	1.835827441	Zm00001d053575
Zm00001d053575_T005	0.002805118	1.90704686	Zm00001d053575
Zm00001d045202_T005	0.002583044	-1.755338626	Zm00001d045202
Zm00001d006625_T006	0.02207954	1.434316866	Zm00001d006625
Zm00001d005766_T001	8.18E-06	4.520131565	Zm00001d005766
Zm00001d021516_T001	1.14E-07	-2.131998669	Zm00001d021516
Zm00001d039166_T004	0.000134356	-2.4338919	Zm00001d039166
Zm00001d007820_T002	0.01640001	-1.949430359	Zm00001d007820
Zm00001d017865_T006	0.006902401	1.489304958	Zm00001d017865
Zm00001d029613_T001	0.005389852	1.738987709	Zm00001d029613
Zm00001d019670_T010	5.43E-09	1.140931519	Zm00001d019670
Zm00001d019670_T001	0.005458031	2.682306634	Zm00001d019670
Zm00001d021846_T015	0.006622774	1.709770375	Zm00001d021846
Zm00001d052282_T017	2.63E-13	2.065963862	Zm00001d052282
Zm00001d044836_T001	0.03420931	-1.476572643	Zm00001d044836
Zm00001d046039_T024	5.94E-13	1.842029235	Zm00001d046039
Zm00001d046039_T012	4.64E-09	1.156288033	Zm00001d046039
Zm00001d029387_T002	0.004825465	4.224446781	Zm00001d029387
Zm00001d014186_T002	0.03694055	1.291660885	Zm00001d014186
Zm00001d016008_T015	5.60E-09	1.246693539	Zm00001d016008
Zm00001d039006_T008	0.000618797	-2.21137947	Zm00001d039006
Zm00001d014943_T001	0.02255599	1.876753112	Zm00001d014943
Zm00001d036031_T009	0.03467054	3.258630543	Zm00001d036031
Zm00001d030893_T020	0.02575643	1.911315223	Zm00001d030893
Zm00001d030893_T001	0.004213273	1.18435914	Zm00001d030893
Zm00001d030893_T025	6.45E-11	1.805600475	Zm00001d030893
Zm00001d032704_T052	0.007202394	1.184364701	Zm00001d032704
Zm00001d015754_T021	5.68E-10	1.073007372	Zm00001d015754
Zm00001d013151_T001	0.01026064	-1.380220967	Zm00001d013151
Zm00001d049707_T022	1.69E-05	-3.395054519	Zm00001d049707
Zm00001d008906_T008	0.04992561	2.060458863	Zm00001d008906
Zm00001d033109_T008	6.57E-05	-2.397716874	Zm00001d033109
Zm00001d047785_T001	0.006393466	1.242985492	Zm00001d047785
Zm00001d053994_T001	0.01573827	1.039758081	Zm00001d053994
Zm00001d013442_T008	4.90E-06	1.179333491	Zm00001d013442

Zm00001d023521_T001	0.02253332	2.435951665	Zm00001d023521
Zm00001d021297_T016	0.01170181	-2.013354144	Zm00001d021297
Zm00001d047828_T005	0.006079915	-1.727290008	Zm00001d047828
Zm00001d048662_T001	0.007507276	-2.231632461	Zm00001d048662
Zm00001d052886_T003	1.18E-05	2.096070592	Zm00001d052886
Zm00001d052886_T004	0.000339438	2.134287789	Zm00001d052886
Zm00001d052886_T002	0.04127541	1.891177845	Zm00001d052886
Zm00001d027874_T004	0.02091574	-1.582063698	Zm00001d027874
Zm00001d009701_T001	0.000347145	-2.559508282	Zm00001d009701
Zm00001d009474_T001	0.01056074	1.886129662	Zm00001d009474
Zm00001d025011_T001	0.04539508	2.492272802	Zm00001d025011
Zm00001d043407_T004	0.01128066	-2.635888751	Zm00001d043407
Zm00001d002169_T004	0.02873645	1.036968137	Zm00001d002169
Zm00001d021890_T015	0.01149774	1.783068741	Zm00001d021890
Zm00001d021890_T017	6.76E-06	1.38580391	Zm00001d021890
Zm00001d028443_T001	0.0136395	-1.504818538	Zm00001d028443
Zm00001d050606_T035	7.89E-05	3.34232861	Zm00001d050606
Zm00001d014945_T001	0.01195333	-2.23373117	Zm00001d014945
Zm00001d037233_T002	0.007698854	1.105022231	Zm00001d037233
Zm00001d036831_T008	0.003288625	2.908764445	Zm00001d036831
Zm00001d013712_T001	0.004393443	-2.626538044	Zm00001d013712
Zm00001d024494_T001	0.00213664	2.875864588	Zm00001d024494
Zm00001d045022_T001	0.03347087	2.141021208	Zm00001d045022
Zm00001d033557_T006	3.22E-05	-2.866670949	Zm00001d033557
Zm00001d016947_T001	0.000135657	3.099215541	Zm00001d016947
Zm00001d047209_T011	2.92E-07	4.728464004	Zm00001d047209
Zm00001d047209_T020	1.01E-08	3.482334412	Zm00001d047209
Zm00001d047209_T021	1.37E-07	1.240472847	Zm00001d047209
Zm00001d047209_T014	0.000274348	1.694772193	Zm00001d047209
Zm00001d003463_T001	0.00010633	3.663179679	Zm00001d003463
Zm00001d010385_T001	0.01359289	1.775180021	Zm00001d010385
Zm00001d003214_T009	0.000315727	2.04830572	Zm00001d003214
Zm00001d003214_T003	0.000218545	-1.710129684	Zm00001d003214
Zm00001d053515_T006	1.28E-09	1.62970188	Zm00001d053515
Zm00001d006130_T001	0.04346118	2.515809291	Zm00001d006130
Zm00001d005925_T003	0.01193426	1.565127056	Zm00001d005925
Zm00001d005925_T004	0.002291221	2.637080886	Zm00001d005925
Zm00001d047679_T004	0.000584599	1.225588852	Zm00001d047679
Zm00001d053938_T001	0.005570846	2.356869192	Zm00001d053938
Zm00001d026477_T006	0.001736934	2.390387672	Zm00001d026477
Zm00001d002275_T037	9.01E-14	2.480440308	Zm00001d002275
Zm00001d002275_T042	0.00965634	2.830184057	Zm00001d002275
Zm00001d002275_T002	0.01425967	2.249577133	Zm00001d002275
Zm00001d002275_T014	0.003257352	2.67027673	Zm00001d002275
Zm00001d004839_T005	0.01328455	-1.091021877	Zm00001d004839
Zm00001d004839_T003	2.74E-07	-2.716378616	Zm00001d004839
Zm00001d017958_T004	2.73E-05	-1.468872505	Zm00001d017958
Zm00001d038522_T002	0.01535282	2.93645232	Zm00001d038522
Zm00001d003352_T003	0.04099654	-1.142056133	Zm00001d003352
Zm00001d002650_T063	2.20E-05	-3.165503565	Zm00001d002650
Zm00001d025235_T020	1.91E-12	-2.184151342	Zm00001d025235
Zm00001d025235_T007	4.95E-14	3.286712945	Zm00001d025235
Zm00001d017165_T001	1.62E-09	-1.962353639	Zm00001d017165
Zm00001d044510_T031	2.42E-16	-4.876026752	Zm00001d044510

Zm00001d044510_T015	4.25E-05	1.496329253	Zm00001d044510
Zm00001d025081_T001	0.00013693	-3.326711422	Zm00001d025081
Zm00001d025081_T004	3.03E-09	-3.980751774	Zm00001d025081
Zm00001d039518_T012	6.01E-15	-4.02262628	Zm00001d039518
Zm00001d053838_T004	0.000633105	-1.647711967	Zm00001d053838
Zm00001d044101_T001	2.04E-07	-1.352278578	Zm00001d044101
Zm00001d009562_T015	4.38E-13	1.99212445	Zm00001d009562
Zm00001d043439_T011	0.001131893	-1.482252133	Zm00001d043439
Zm00001d048226_T001	0.03591437	2.319730468	Zm00001d048226
Zm00001d042030_T001	0.000228899	-2.405055908	Zm00001d042030
Zm00001d010152_T007	0.01316976	2.701081524	Zm00001d010152
Zm00001d053969_T004	6.92E-09	-2.005022894	Zm00001d053969
Zm00001d036880_T001	0.003956993	-2.228831189	Zm00001d036880
Zm00001d036880_T002	0.01183344	-2.023389356	Zm00001d036880
Zm00001d049459_T002	0.002330117	-2.493036367	Zm00001d049459
Zm00001d008585_T002	0.000934168	1.143971947	Zm00001d008585
Zm00001d018580_T001	0.009927578	1.863060141	Zm00001d018580
Zm00001d048453_T001	0.0136329	2.460133308	Zm00001d048453
Zm00001d053834_T002	0.000288625	-1.412383166	Zm00001d053834
Zm00001d010887_T006	0.03582846	1.130072702	Zm00001d010887
Zm00001d020178_T001	1.76E-14	-2.764669396	Zm00001d020178
Zm00001d014198_T001	0.000776638	-2.557930999	Zm00001d014198
Zm00001d002817_T004	1.70E-08	1.034716748	Zm00001d002817
Zm00001d027447_T007	0.003939115	-2.484055647	Zm00001d027447
Zm00001d049638_T028	0.005533456	-2.278584112	Zm00001d049638
Zm00001d043740_T001	0.003107062	2.991681809	Zm00001d043740
Zm00001d033957_T003	0.0322225	1.003238448	Zm00001d033957
Zm00001d033957_T001	0.004411808	2.744494269	Zm00001d033957
Zm00001d047471_T009	3.71E-05	1.572179146	Zm00001d047471
Zm00001d006169_T001	1.90E-07	3.526922001	Zm00001d006169
Zm00001d036982_T006	0.04351232	1.715511534	Zm00001d036982
Zm00001d011610_T002	2.80E-13	-1.887059997	Zm00001d011610
Zm00001d021668_T005	0.0146133	-1.752472408	Zm00001d021668
Zm00001d011622_T006	1.82E-11	1.886271167	Zm00001d011622
Zm00001d021900_T001	0.04339872	2.240007373	Zm00001d021900
Zm00001d039487_T001	0.0344758	2.449256365	Zm00001d039487
Zm00001d025951_T032	0.000747974	2.252574371	Zm00001d025951
Zm00001d025951_T031	7.59E-05	3.173339124	Zm00001d025951
Zm00001d037001_T002	0.000534889	1.977737255	Zm00001d037001
Zm00001d007785_T001	0.04449306	-1.38626784	Zm00001d007785
Zm00001d042608_T004	0.002685015	1.055568301	Zm00001d042608
Zm00001d017848_T005	5.01E-06	2.413184743	Zm00001d017848
Zm00001d017848_T019	0.002477464	-1.203500614	Zm00001d017848
Zm00001d026263_T007	0.001157211	1.24913429	Zm00001d026263
Zm00001d026263_T005	0.02264751	1.954635753	Zm00001d026263
Zm00001d013984_T012	8.41E-11	2.144712171	Zm00001d013984
Zm00001d004288_T001	0.01472471	2.107666422	Zm00001d004288
Zm00001d036656_T001	0.00290736	2.880813994	Zm00001d036656
Zm00001d036656_T004	0.000239955	2.881649649	Zm00001d036656
Zm00001d012909_T006	0.02718105	-2.931306669	Zm00001d012909
Zm00001d047527_T002	0.01191076	2.28103848	Zm00001d047527
Zm00001d046595_T003	0.000552545	2.236742746	Zm00001d046595
Zm00001d048178_T001	0.02151911	-1.236591251	Zm00001d048178
Zm00001d025360_T001	0.000203969	-3.050343056	Zm00001d025360

Zm00001d047893_T002	0.03011949	-1.005930423	Zm00001d047893
Zm00001d009993_T001	0.002831329	-2.780231974	Zm00001d009993
Zm00001d028786_T003	1.56E-05	-1.469444246	Zm00001d028786
Zm00001d052391_T002	2.45E-11	-4.627923665	Zm00001d052391
Zm00001d053987_T002	6.11E-09	-2.09017203	Zm00001d053987
Zm00001d013039_T001	0.02115378	-2.211763901	Zm00001d013039
Zm00001d004139_T003	2.99E-14	2.539693806	Zm00001d004139
Zm00001d032473_T003	0.04894618	1.123813566	Zm00001d032473
Zm00001d046277_T027	0.001451041	2.179185778	Zm00001d046277
Zm00001d046277_T003	5.15E-07	3.613798291	Zm00001d046277
Zm00001d026629_T001	0.01625687	-1.327103906	Zm00001d026629
Zm00001d017946_T013	9.04E-11	1.31547677	Zm00001d017946
Zm00001d027486_T001	0.00207886	2.191535644	Zm00001d027486
Zm00001d027486_T002	0.000326107	2.841813926	Zm00001d027486
Zm00001d040148_T001	0.006754496	2.16925368	Zm00001d040148
Zm00001d047875_T002	1.27E-08	1.433164868	Zm00001d047875
Zm00001d035470_T001	0.000672201	1.668390668	Zm00001d035470
Zm00001d035470_T012	0.01477598	-1.199425475	Zm00001d035470
Zm00001d035470_T007	2.91E-15	3.358222296	Zm00001d035470
Zm00001d037696_T005	0.002004246	1.122275462	Zm00001d037696
Zm00001d012815_T002	0.004411808	1.382494644	Zm00001d012815
Zm00001d052346_T001	0.002095691	2.734271789	Zm00001d052346
Zm00001d052346_T002	0.02685638	2.254696009	Zm00001d052346
Zm00001d041620_T008	5.31E-10	2.025174992	Zm00001d041620
Zm00001d017793_T003	0.04922677	-1.205344263	Zm00001d017793
Zm00001d047883_T001	0.0200952	2.547328925	Zm00001d047883
Zm00001d007945_T009	0.006314872	1.563878344	Zm00001d007945
Zm00001d037731_T017	0.002572745	1.465770705	Zm00001d037731
Zm00001d028949_T001	0.01356004	2.181220276	Zm00001d028949
Zm00001d028949_T004	0.00095437	1.569224209	Zm00001d028949
Zm00001d028949_T003	0.002075853	2.688250772	Zm00001d028949
Zm00001d020323_T001	0.04515747	2.384264844	Zm00001d020323
Zm00001d042213_T003	7.04E-07	1.489364621	Zm00001d042213
Zm00001d002592_T001	1.34E-05	2.689982045	Zm00001d002592
Zm00001d031992_T001	2.02E-12	-2.727797317	Zm00001d031992
Zm00001d042344_T005	0.02765504	1.305630654	Zm00001d042344
Zm00001d042344_T009	0.000221768	-2.461141568	Zm00001d042344
Zm00001d003616_T007	0.000103032	-4.164100832	Zm00001d003616
Zm00001d003616_T025	0.001748277	-1.919670059	Zm00001d003616
Zm00001d017392_T002	2.48E-12	-2.524064813	Zm00001d017392
Zm00001d008898_T001	0.003015693	-1.752123637	Zm00001d008898
Zm00001d048445_T002	9.12E-09	-1.779708844	Zm00001d048445
Zm00001d026515_T006	0.03493085	1.752232528	Zm00001d026515
Zm00001d026515_T008	0.000142536	-2.6313052	Zm00001d026515
Zm00001d034730_T005	5.15E-15	-3.23548605	Zm00001d034730
Zm00001d010901_T004	0.03752477	-2.298129221	Zm00001d010901
Zm00001d042738_T001	0.03715458	-1.743438207	Zm00001d042738
Zm00001d044593_T008	0.000421721	-1.7576604	Zm00001d044593
Zm00001d034034_T005	0.03974992	1.169961184	Zm00001d034034
Zm00001d018795_T047	0.00017836	1.523646958	Zm00001d018795
Zm00001d044570_T032	0.001309444	1.345673946	Zm00001d044570
Zm00001d044570_T028	3.81E-09	1.313309089	Zm00001d044570
Zm00001d051451_T001	9.73E-05	2.789157301	Zm00001d051451
Zm00001d006866_T001	1.24E-07	-1.566296141	Zm00001d006866

Zm00001d002351_T002	3.84E-05	-2.742714599	Zm00001d002351
Zm00001d002351_T001	0.01261341	-1.371211709	Zm00001d002351
Zm00001d053003_T003	0.01149774	-1.493981603	Zm00001d053003
Zm00001d033931_T006	0.000580616	-1.966542189	Zm00001d033931
Zm00001d012974_T060	0.006958403	-1.847231277	Zm00001d012974
Zm00001d049929_T006	8.91E-05	-1.998479458	Zm00001d049929
Zm00001d033965_T002	0.0372102	1.515122464	Zm00001d033965
Zm00001d017438_T001	0.02309348	-1.702569543	Zm00001d017438
Zm00001d013863_T010	0.002814932	1.813579971	Zm00001d013863
Zm00001d008488_T009	0.009049317	-2.315526243	Zm00001d008488
Zm00001d004564_T005	0.008047309	1.363737419	Zm00001d004564
Zm00001d017872_T006	1.96E-10	2.082402168	Zm00001d017872
Zm00001d000060_T001	0.01229362	1.940451167	Zm00001d000060
Zm00001d026680_T001	0.01022151	-1.124239342	Zm00001d026680
Zm00001d019881_T002	8.37E-05	1.810325571	Zm00001d019881
Zm00001d019881_T003	4.83E-06	2.350934579	Zm00001d019881
Zm00001d011981_T072	1.04E-10	-1.584680238	Zm00001d011981
Zm00001d027293_T008	2.14E-07	1.240680966	Zm00001d027293
Zm00001d030725_T004	0.0252917	3.342441001	Zm00001d030725
Zm00001d030725_T003	0.011246	-1.718912846	Zm00001d030725
Zm00001d051309_T001	0.000148816	3.743389406	Zm00001d051309
Zm00001d002511_T006	0.009606413	-2.545195946	Zm00001d002511
Zm00001d025751_T003	0.01637723	1.220513827	Zm00001d025751
Zm00001d044290_T018	0.01537015	1.842290026	Zm00001d044290
Zm00001d044290_T009	2.05E-11	2.060453491	Zm00001d044290
Zm00001d044303_T009	2.82E-10	1.29982814	Zm00001d044303
Zm00001d044303_T003	0.04407915	1.940605913	Zm00001d044303
Zm00001d028536_T008	0.01477296	1.893488422	Zm00001d028536
Zm00001d030357_T001	2.60E-05	-2.753311	Zm00001d030357
Zm00001d024939_T004	0.008335767	-2.138784386	Zm00001d024939
Zm00001d003533_T002	0.04043864	2.390912826	Zm00001d003533
Zm00001d013202_T001	0.000224843	3.894071654	Zm00001d013202
Zm00001d051656_T001	0.02128297	1.662321857	Zm00001d051656
Zm00001d032815_T015	0.03566678	1.190559616	Zm00001d032815
Zm00001d012155_T016	9.50E-11	1.627224736	Zm00001d012155
Zm00001d012155_T014	0.004365318	1.835359148	Zm00001d012155
Zm00001d012155_T022	0.000197484	1.182041242	Zm00001d012155
Zm00001d012155_T023	6.96E-10	1.398594244	Zm00001d012155
Zm00001d012155_T008	0.000174262	3.452492636	Zm00001d012155
Zm00001d012155_T013	0.001892338	1.450151233	Zm00001d012155
Zm00001d048741_T135	5.17E-07	-1.884444114	Zm00001d048741
Zm00001d048741_T082	1.80E-12	-2.540241138	Zm00001d048741
Zm00001d048741_T008	2.71E-09	1.236094892	Zm00001d048741
Zm00001d014572_T005	0.002765635	-2.544675974	Zm00001d014572
Zm00001d039524_T001	2.35E-08	5.626440521	Zm00001d039524
Zm00001d027665_T007	0.006154785	1.278534713	Zm00001d027665
Zm00001d027665_T014	0.0192532	1.000997647	Zm00001d027665
Zm00001d027285_T001	0.001503204	-1.612178583	Zm00001d027285
Zm00001d020764_T004	0.03578504	2.849771389	Zm00001d020764
Zm00001d042335_T004	0.01808074	1.126368552	Zm00001d042335
Zm00001d042335_T007	8.67E-08	-1.763631407	Zm00001d042335
Zm00001d008333_T006	0.04127541	1.111086457	Zm00001d008333
Zm00001d011426_T002	1.70E-11	-2.056464731	Zm00001d011426
Zm00001d044271_T017	0.001301872	-2.228517034	Zm00001d044271

Zm00001d011655_T001	0.004762117	1.608212047	Zm00001d011655
Zm00001d036763_T001	0.000699734	3.125398696	Zm00001d036763
Zm00001d006533_T024	6.01E-15	3.607288579	Zm00001d006533
Zm00001d027626_T002	0.0043297	1.227119095	Zm00001d027626
Zm00001d027626_T001	0.01901268	-2.408693605	Zm00001d027626
Zm00001d015376_T002	0.001821067	-1.409832878	Zm00001d015376
Zm00001d002000_T009	0.0161855	-1.564710773	Zm00001d002000
Zm00001d008523_T001	1.60E-07	-5.241753202	Zm00001d008523
Zm00001d002126_T001	0.004246457	3.238330133	Zm00001d002126
Zm00001d022041_T002	0.000442113	2.593085868	Zm00001d022041
Zm00001d044096_T005	1.69E-05	1.022530287	Zm00001d044096
Zm00001d044096_T004	0.000113535	2.171978809	Zm00001d044096
Zm00001d017168_T005	0.002048357	-2.72165284	Zm00001d017168
Zm00001d010528_T004	0.02483077	-1.134191326	Zm00001d010528
Zm00001d012701_T021	5.90E-11	-2.438846528	Zm00001d012701
Zm00001d024318_T010	0.02312763	2.083460802	Zm00001d024318
Zm00001d004855_T004	0.0147663	-2.090339161	Zm00001d004855
Zm00001d004855_T005	0.04818964	-1.632877932	Zm00001d004855
Zm00001d005170_T009	0.002306279	1.35198342	Zm00001d005170
Zm00001d005170_T008	0.02706274	1.246716846	Zm00001d005170
Zm00001d032761_T004	0.000259008	-1.385201501	Zm00001d032761
Zm00001d032464_T003	0.02917075	1.305773253	Zm00001d032464
Zm00001d040313_T001	0.00023792	2.051542566	Zm00001d040313
Zm00001d022475_T001	0.001266433	1.462883959	Zm00001d022475
Zm00001d041035_T002	9.93E-11	1.768837041	Zm00001d041035
Zm00001d025240_T009	0.002559064	-2.075003567	Zm00001d025240
Zm00001d048424_T002	0.000248481	-1.087790902	Zm00001d048424
Zm00001d048424_T008	0.03246552	-1.173436974	Zm00001d048424
Zm00001d048424_T006	5.17E-06	-2.946186528	Zm00001d048424
Zm00001d018872_T007	0.006260083	1.234366431	Zm00001d018872
Zm00001d029392_T005	0.02168606	1.032019922	Zm00001d029392
Zm00001d029392_T007	2.67E-05	1.091137502	Zm00001d029392
Zm00001d017096_T025	0.000839604	3.1079675	Zm00001d017096
Zm00001d017096_T017	0.000973999	2.983312888	Zm00001d017096
Zm00001d017096_T021	0.001968686	2.456262594	Zm00001d017096
Zm00001d048272_T011	0.000435301	2.461490856	Zm00001d048272
Zm00001d031676_T006	0.00734409	1.232645147	Zm00001d031676
Zm00001d014344_T036	0.000169193	1.2929539	Zm00001d014344
Zm00001d005051_T001	0.04714113	-1.319372358	Zm00001d005051
Zm00001d019075_T010	0.00914723	-1.690632567	Zm00001d019075
Zm00001d052713_T001	0.03547668	1.069091692	Zm00001d052713
Zm00001d025273_T013	1.61E-12	-3.886770975	Zm00001d025273
Zm00001d019789_T003	0.000952931	2.776274015	Zm00001d019789
Zm00001d047395_T005	0.009667038	1.008296618	Zm00001d047395
Zm00001d037700_T012	0.00238724	-2.778616785	Zm00001d037700
Zm00001d048950_T001	0.04667616	-1.75837955	Zm00001d048950
Zm00001d021180_T004	0.005727571	1.358152418	Zm00001d021180
Zm00001d028825_T003	0.03331115	2.039886273	Zm00001d028825
Zm00001d045185_T001	0.00053586	2.873710731	Zm00001d045185
Zm00001d053890_T001	0.007128408	-1.208848437	Zm00001d053890
Zm00001d044649_T028	0.001632575	1.667371601	Zm00001d044649
Zm00001d042018_T004	0.02546853	-1.940201969	Zm00001d042018
Zm00001d003649_T002	0.02892183	1.658870422	Zm00001d003649
Zm00001d002313_T007	0.003920049	-2.365946404	Zm00001d002313

Zm00001d029490_T016	0.000284972	1.41472309	Zm00001d029490
Zm00001d031847_T021	0.001228936	-2.615663074	Zm00001d031847
Zm00001d031847_T011	0.008554004	3.013108228	Zm00001d031847
Zm00001d049060_T001	0.000241971	5.022721524	Zm00001d049060
Zm00001d013012_T007	0.002602363	-1.229848941	Zm00001d013012
Zm00001d042307_T001	0.02779977	-2.068953451	Zm00001d042307
Zm00001d048099_T002	0.01582338	-1.049140035	Zm00001d048099
Zm00001d048099_T001	0.02631091	-1.903010202	Zm00001d048099
Zm00001d053917_T027	0.02044646	1.076577437	Zm00001d053917
Zm00001d028372_T011	0.02570104	1.60841305	Zm00001d028372
Zm00001d038451_T002	0.000274955	2.51011571	Zm00001d038451
Zm00001d038451_T003	0.005246483	2.478572212	Zm00001d038451
Zm00001d001917_T018	0.003831125	-1.588001319	Zm00001d001917
Zm00001d001917_T021	0.02263356	1.488363037	Zm00001d001917
Zm00001d024463_T008	0.001980112	1.215355982	Zm00001d024463
Zm00001d024463_T004	1.84E-10	-1.13335067	Zm00001d024463
Zm00001d042809_T002	0.0318764	-1.913952606	Zm00001d042809
Zm00001d043766_T008	1.63E-11	1.91373189	Zm00001d043766
Zm00001d018859_T004	0.00396837	-1.629830578	Zm00001d018859
Zm00001d006628_T004	0.002521412	-1.947371756	Zm00001d006628
Zm00001d028572_T006	0.002782443	-1.410693955	Zm00001d028572
Zm00001d033533_T004	0.01095096	1.573775901	Zm00001d033533
Zm00001d041774_T001	0.02081089	-2.289284949	Zm00001d041774
Zm00001d009676_T007	0.003247529	1.449080766	Zm00001d009676
Zm00001d024522_T001	1.99E-08	5.42804365	Zm00001d024522
Zm00001d017911_T001	0.004998382	-2.170410495	Zm00001d017911
Zm00001d040036_T006	0.002628689	1.121770606	Zm00001d040036
Zm00001d040036_T001	0.0141963	-1.11240837	Zm00001d040036
Zm00001d031875_T001	0.001651001	1.073569011	Zm00001d031875
Zm00001d046587_T028	5.75E-09	1.325291004	Zm00001d046587
Zm00001d046587_T026	0.02214	-1.346190929	Zm00001d046587
Zm00001d023262_T003	3.37E-15	3.452003261	Zm00001d023262
Zm00001d008789_T035	1.52E-13	2.603842872	Zm00001d008789
Zm00001d005995_T006	0.001036334	1.185461214	Zm00001d005995
Zm00001d030614_T001	0.005176924	-1.570315022	Zm00001d030614
Zm00001d036961_T008	0.006876689	-1.08806706	Zm00001d036961
Zm00001d034772_T107	0.01225238	-1.430074211	Zm00001d034772
Zm00001d034772_T110	3.91E-09	-3.296439215	Zm00001d034772
Zm00001d029776_T002	1.43E-06	4.342432513	Zm00001d029776
Zm00001d007434_T001	1.88E-05	-2.711650768	Zm00001d007434
Zm00001d007434_T013	0.009549599	-1.500478644	Zm00001d007434
Zm00001d020097_T008	3.28E-10	1.688133335	Zm00001d020097
Zm00001d020097_T006	9.30E-10	-1.186924091	Zm00001d020097
Zm00001d031832_T005	0.004998066	2.101988173	Zm00001d031832
Zm00001d002292_T008	0.009256586	1.802854404	Zm00001d002292
Zm00001d043890_T004	1.00E-05	-2.907986251	Zm00001d043890
Zm00001d010425_T002	0.000433089	-3.956899535	Zm00001d010425
Zm00001d037951_T001	0.01150765	1.60097033	Zm00001d037951
Zm00001d047044_T016	0.002628042	1.461932104	Zm00001d047044
Zm00001d006330_T001	0.03649979	2.493523867	Zm00001d006330
Zm00001d018257_T006	0.01806011	1.651195841	Zm00001d018257
Zm00001d037481_T006	0.003579954	-3.064395783	Zm00001d037481
Zm00001d052149_T001	0.0361763	-1.398766248	Zm00001d052149
Zm00001d052149_T047	1.57E-11	1.665213828	Zm00001d052149

Zm00001d052149_T041	2.31E-05	1.489650598	Zm00001d052149
Zm00001d032989_T005	0.01591506	-1.356753885	Zm00001d032989
Zm00001d051870_T001	0.007975205	-1.919810691	Zm00001d051870
Zm00001d019497_T024	0.01462387	2.05252556	Zm00001d019497
Zm00001d019497_T042	5.25E-12	-4.235205148	Zm00001d019497
Zm00001d019497_T041	0.000992527	-2.50359121	Zm00001d019497
Zm00001d017754_T019	2.86E-15	3.804983114	Zm00001d017754
Zm00001d017754_T021	0.01774747	1.09360577	Zm00001d017754
Zm00001d017754_T039	0.02202181	1.34682298	Zm00001d017754
Zm00001d020036_T001	0.01190599	1.341760256	Zm00001d020036
Zm00001d030106_T107	0.03449392	-1.723737918	Zm00001d030106
Zm00001d024703_T011	0.01161943	-1.558999494	Zm00001d024703
Zm00001d022040_T003	1.53E-12	-1.813647561	Zm00001d022040
Zm00001d022040_T002	0.02081208	2.282091743	Zm00001d022040
Zm00001d007081_T012	3.81E-09	1.344120127	Zm00001d007081
Zm00001d007810_T002	0.005912899	-1.351582573	Zm00001d007810
Zm00001d022335_T005	2.00E-09	-1.54588862	Zm00001d022335
Zm00001d028896_T015	0.004986224	-2.202428248	Zm00001d028896
Zm00001d044266_T001	0.02355239	2.629693217	Zm00001d044266
Zm00001d036395_T013	0.002228826	2.328833557	Zm00001d036395
Zm00001d044447_T007	0.04707027	1.007076821	Zm00001d044447
Zm00001d012816_T003	0.00095619	-1.279221178	Zm00001d012816
Zm00001d018191_T001	0.000251052	2.509806067	Zm00001d018191
Zm00001d021321_T001	0.000370758	3.25363759	Zm00001d021321
Zm00001d043700_T003	2.66E-10	-3.721089248	Zm00001d043700
Zm00001d027487_T019	0.005340142	1.353382147	Zm00001d027487
Zm00001d042887_T001	0.03254374	2.538554011	Zm00001d042887
Zm00001d036784_T001	0.03449192	2.061636879	Zm00001d036784
Zm00001d012956_T001	0.03997268	2.448730691	Zm00001d012956
Zm00001d046255_T027	1.25E-06	1.005484551	Zm00001d046255
Zm00001d013268_T009	0.01617441	1.663019287	Zm00001d013268
Zm00001d047385_T009	0.03706504	1.122837735	Zm00001d047385
Zm00001d011908_T003	1.25E-07	-2.538861793	Zm00001d011908
Zm00001d009927_T009	0.002951811	2.781397095	Zm00001d009927
Zm00001d009927_T008	6.39E-07	3.171799516	Zm00001d009927
Zm00001d009927_T005	0.000191577	2.63883663	Zm00001d009927
Zm00001d025754_T001	0.01455282	-1.82719349	Zm00001d025754
Zm00001d027307_T040	0.003495296	1.864295863	Zm00001d027307
Zm00001d048101_T003	0.03676439	-1.755119565	Zm00001d048101
Zm00001d052943_T009	4.38E-10	1.444275326	Zm00001d052943
Zm00001d021196_T005	0.02255438	-1.312760531	Zm00001d021196
Zm00001d004118_T003	0.002129765	2.644295415	Zm00001d004118
Zm00001d028537_T001	0.0462395	-1.350276367	Zm00001d028537
Zm00001d037165_T001	0.02334998	1.950570245	Zm00001d037165
Zm00001d016856_T011	0.000157029	-1.038611297	Zm00001d016856
Zm00001d009017_T003	0.01476527	-1.062000688	Zm00001d009017
Zm00001d043598_T009	0.009054511	1.090227607	Zm00001d043598
Zm00001d043598_T005	2.44E-19	5.787983666	Zm00001d043598
Zm00001d043598_T002	0.009407985	2.825738877	Zm00001d043598
Zm00001d038728_T007	0.003943145	1.24572272	Zm00001d038728
Zm00001d038728_T003	0.002860823	3.271828335	Zm00001d038728
Zm00001d048927_T001	0.03648535	1.112282515	Zm00001d048927
Zm00001d047881_T005	5.11E-05	-1.224660686	Zm00001d047881
Zm00001d039417_T053	4.77E-10	1.296355818	Zm00001d039417

Zm00001d039474_T003	0.01942718	1.329653144	Zm00001d039474
Zm00001d029872_T006	0.000137405	2.789614695	Zm00001d029872
Zm00001d005590_T001	0.006919847	-1.29415339	Zm00001d005590
Zm00001d034770_T003	4.68E-05	2.956766057	Zm00001d034770
Zm00001d034770_T009	0.009100825	2.185530694	Zm00001d034770
Zm00001d043900_T008	0.000858087	-1.113384497	Zm00001d043900
Zm00001d043900_T006	7.79E-11	1.580079165	Zm00001d043900
Zm00001d047848_T002	0.05001771	-1.462013909	Zm00001d047848
Zm00001d042793_T011	3.73E-09	-1.018974368	Zm00001d042793
Zm00001d042793_T005	1.53E-10	1.337825335	Zm00001d042793
Zm00001d029336_T008	0.03277874	2.121115822	Zm00001d029336
Zm00001d021992_T001	0.01122327	2.442714485	Zm00001d021992
Zm00001d012199_T010	0.02539639	-2.393341812	Zm00001d012199
Zm00001d011357_T004	0.001372749	-2.295764868	Zm00001d011357
Zm00001d007500_T051	7.51E-06	-2.751924427	Zm00001d007500
Zm00001d034018_T002	0.01474121	1.167081308	Zm00001d034018
Zm00001d034018_T010	8.86E-11	1.811555395	Zm00001d034018
Zm00001d002663_T009	3.20E-11	-2.538267382	Zm00001d002663
Zm00001d020958_T002	3.69E-05	-3.214155645	Zm00001d020958
Zm00001d052001_T014	0.005368589	-1.571672085	Zm00001d052001
Zm00001d016857_T001	0.04103975	-1.71164179	Zm00001d016857
Zm00001d002374_T001	0.03198968	-2.185936978	Zm00001d002374
Zm00001d024633_T003	0.002613826	3.041965083	Zm00001d024633
Zm00001d030960_T033	0.03848759	-1.465528382	Zm00001d030960
Zm00001d003068_T001	0.01128472	-1.331491095	Zm00001d003068
Zm00001d044395_T007	0.000294231	2.44816088	Zm00001d044395
Zm00001d025727_T001	0.000196681	-3.230916821	Zm00001d025727
Zm00001d020402_T002	0.01306269	-2.219289668	Zm00001d020402
Zm00001d025167_T005	0.03925128	1.612651597	Zm00001d025167
Zm00001d006789_T013	0.001282657	1.255438929	Zm00001d006789
Zm00001d006789_T019	0.00107198	2.603534719	Zm00001d006789
Zm00001d033375_T001	0.007072787	1.448244169	Zm00001d033375
Zm00001d033375_T015	0.000100728	1.150838287	Zm00001d033375
Zm00001d048501_T001	0.000319487	1.387695798	Zm00001d048501
Zm00001d029976_T008	0.004890922	2.451047354	Zm00001d029976
Zm00001d029976_T009	7.36E-05	2.332917836	Zm00001d029976
Zm00001d029976_T003	0.00207132	1.000926113	Zm00001d029976
Zm00001d029976_T010	0.005900643	1.338129181	Zm00001d029976
Zm00001d029976_T007	0.002796955	1.480316719	Zm00001d029976
Zm00001d048877_T004	7.02E-15	-3.953429612	Zm00001d048877
Zm00001d025567_T001	0.04535247	-1.716759215	Zm00001d025567
Zm00001d017817_T001	0.01048202	-2.183783776	Zm00001d017817
Zm00001d023734_T005	0.005618118	-2.737465065	Zm00001d023734
Zm00001d008189_T005	2.62E-17	5.772725694	Zm00001d008189
Zm00001d012085_T004	1.65E-12	2.887005377	Zm00001d012085
Zm00001d053079_T001	0.000837816	2.473215784	Zm00001d053079
Zm00001d037905_T004	1.82E-07	-2.757247296	Zm00001d037905
Zm00001d039160_T025	6.53E-06	2.967305478	Zm00001d039160
Zm00001d039160_T021	2.05E-05	2.009691285	Zm00001d039160
Zm00001d013371_T009	7.79E-11	2.740931427	Zm00001d013371
Zm00001d036919_T016	0.02676713	-1.879944309	Zm00001d036919
Zm00001d043286_T011	0.000815476	-1.984667924	Zm00001d043286
Zm00001d034433_T001	0.01686346	-2.490030655	Zm00001d034433
Zm00001d036215_T001	0.050072	1.114943693	Zm00001d036215

Zm00001d013857_T005	0.000294231	1.347393426	Zm00001d013857
Zm00001d029839_T007	0.000463721	-1.279409689	Zm00001d029839
Zm00001d029839_T003	6.81E-10	-2.12024455	Zm00001d029839
Zm00001d033154_T001	0.01873517	-1.762723686	Zm00001d033154
Zm00001d017919_T001	0.003224906	-1.151555043	Zm00001d017919
Zm00001d009683_T005	4.14E-09	-1.405377469	Zm00001d009683
Zm00001d030026_T032	1.06E-11	1.377428605	Zm00001d030026
Zm00001d030026_T039	4.38E-10	1.113717958	Zm00001d030026
Zm00001d030026_T004	7.87E-07	1.86147485	Zm00001d030026
Zm00001d030026_T043	2.05E-09	-1.011439322	Zm00001d030026
Zm00001d030026_T042	0.001532887	1.291073869	Zm00001d030026
Zm00001d029443_T006	2.79E-09	3.16029675	Zm00001d029443
Zm00001d047290_T002	0.000247069	3.422142821	Zm00001d047290
Zm00001d039310_T001	0.005921748	-1.778234569	Zm00001d039310
Zm00001d019191_T007	1.20E-07	-1.400551194	Zm00001d019191
Zm00001d018976_T062	0.00919548	-1.71838908	Zm00001d018976
Zm00001d018976_T055	1.65E-14	2.956206531	Zm00001d018976
Zm00001d017720_T009	0.004806727	1.326797249	Zm00001d017720
Zm00001d007175_T001	0.006155769	2.580935866	Zm00001d007175
Zm00001d036301_T007	5.43E-11	1.111701261	Zm00001d036301
Zm00001d032377_T007	0.000818754	1.578438281	Zm00001d032377
Zm00001d046688_T002	0.03495473	-2.079381851	Zm00001d046688
Zm00001d025590_T011	9.56E-13	-2.348847362	Zm00001d025590
Zm00001d012525_T003	0.03867058	1.884157255	Zm00001d012525
Zm00001d049641_T007	4.66E-13	-4.700278702	Zm00001d049641
Zm00001d013947_T001	5.20E-08	5.144506081	Zm00001d013947
Zm00001d027448_T008	0.02242615	1.337861771	Zm00001d027448
Zm00001d021126_T005	0.002685015	2.237260381	Zm00001d021126
Zm00001d047896_T004	0.04663752	2.013279483	Zm00001d047896
Zm00001d011930_T001	0.001750147	2.758551665	Zm00001d011930
Zm00001d038908_T006	0.002146068	2.797063671	Zm00001d038908
Zm00001d047993_T002	1.96E-06	3.561940566	Zm00001d047993
Zm00001d047993_T001	1.90E-05	4.402660091	Zm00001d047993
Zm00001d040004_T001	0.004509663	-2.377871287	Zm00001d040004
Zm00001d005775_T003	0.03619664	1.66804154	Zm00001d005775
Zm00001d011903_T001	0.02300732	-1.634105436	Zm00001d011903
Zm00001d037313_T015	8.55E-11	1.380836939	Zm00001d037313
Zm00001d006433_T014	6.56E-12	-4.288403225	Zm00001d006433
Zm00001d011737_T001	0.001685107	-1.075778688	Zm00001d011737
Zm00001d012751_T021	2.74E-14	-2.856209639	Zm00001d012751
Zm00001d012751_T019	1.75E-15	3.384418472	Zm00001d012751
Zm00001d044478_T015	0.001345215	3.278683119	Zm00001d044478
Zm00001d044478_T059	0.04441707	-1.576697662	Zm00001d044478
Zm00001d044478_T061	2.15E-14	-3.194177768	Zm00001d044478
Zm00001d020227_T024	0.000139328	1.979149911	Zm00001d020227
Zm00001d020227_T005	1.46E-09	1.337366053	Zm00001d020227
Zm00001d020227_T034	6.85E-14	3.048279298	Zm00001d020227
Zm00001d045370_T001	0.001410393	-3.007504084	Zm00001d045370
Zm00001d041954_T002	4.62E-07	1.271878875	Zm00001d041954
Zm00001d041954_T001	2.53E-06	-2.106939002	Zm00001d041954
Zm00001d013743_T001	0.02454213	-2.271332062	Zm00001d013743
Zm00001d028558_T005	0.000823508	1.100525674	Zm00001d028558
Zm00001d017036_T005	0.0419536	-1.666462967	Zm00001d017036
Zm00001d017036_T004	3.61E-05	3.072976408	Zm00001d017036

Zm00001d011654_T001	0.001570084	3.117998113	Zm00001d011654
Zm00001d032362_T003	0.03353728	2.025937395	Zm00001d032362
Zm00001d004979_T003	0.03045399	1.156222922	Zm00001d004979
Zm00001d022258_T005	2.24E-13	-3.759860034	Zm00001d022258
Zm00001d026301_T002	1.11E-11	2.164264922	Zm00001d026301
Zm00001d026301_T004	5.10E-11	-1.605910997	Zm00001d026301
Zm00001d033616_T004	2.82E-12	2.158789941	Zm00001d033616
Zm00001d017780_T004	0.001591827	2.206673346	Zm00001d017780
Zm00001d017780_T002	0.002825	3.226006847	Zm00001d017780
Zm00001d048521_T003	0.01102023	1.094578956	Zm00001d048521
Zm00001d042437_T001	0.02879806	2.626731082	Zm00001d042437
Zm00001d046221_T014	0.01472471	1.730671277	Zm00001d046221
Zm00001d037868_T001	0.000475705	2.432534481	Zm00001d037868
Zm00001d000228_T042	0.003658349	-3.288127237	Zm00001d000228
Zm00001d000228_T040	4.75E-12	2.167081783	Zm00001d000228
Zm00001d012748_T001	0.007698854	1.41600008	Zm00001d012748
Zm00001d012748_T003	1.86E-15	4.026054559	Zm00001d012748
Zm00001d021442_T011	0.03180938	1.067051024	Zm00001d021442
Zm00001d021442_T015	0.04208336	2.457210474	Zm00001d021442
Zm00001d021442_T003	3.77E-06	-2.680146018	Zm00001d021442
Zm00001d038448_T003	0.01084676	2.843652882	Zm00001d038448
Zm00001d038448_T002	6.98E-05	4.010845945	Zm00001d038448
Zm00001d038997_T001	0.000227529	-4.614551951	Zm00001d038997
Zm00001d019507_T010	0.000479665	1.01117963	Zm00001d019507
Zm00001d019507_T032	1.09E-16	4.689461216	Zm00001d019507
Zm00001d031232_T056	8.17E-11	1.544408207	Zm00001d031232
Zm00001d031232_T022	0.001684569	1.046224887	Zm00001d031232
Zm00001d037807_T004	0.04798446	2.3681619	Zm00001d037807
Zm00001d044971_T014	0.01328455	-1.144585188	Zm00001d044971
Zm00001d002132_T001	0.003908299	-2.538556714	Zm00001d002132
Zm00001d031796_T001	4.98E-09	5.238569949	Zm00001d031796
Zm00001d040737_T001	0.001627225	2.100746013	Zm00001d040737
Zm00001d002272_T003	0.0372102	2.538063383	Zm00001d002272
Zm00001d002121_T001	0.006513324	-1.817438133	Zm00001d002121
Zm00001d034777_T008	0.002951811	1.39308626	Zm00001d034777
Zm00001d046927_T001	0.006990354	-2.509833082	Zm00001d046927
Zm00001d018206_T001	0.000425577	3.854302506	Zm00001d018206
Zm00001d032703_T013	0.001297994	2.038892738	Zm00001d032703
Zm00001d008178_T028	0.02165489	-1.787687113	Zm00001d008178
Zm00001d008178_T012	0.01215579	3.050632203	Zm00001d008178
Zm00001d043418_T003	0.01325275	1.091674473	Zm00001d043418
Zm00001d048294_T001	0.001131005	3.615368426	Zm00001d048294
Zm00001d013301_T004	0.007772415	2.067071869	Zm00001d013301
Zm00001d042670_T004	9.63E-11	1.468390403	Zm00001d042670
Zm00001d042670_T041	0.007698854	-1.106150607	Zm00001d042670
Zm00001d048220_T001	6.88E-08	4.526765037	Zm00001d048220
Zm00001d029133_T005	0.001494715	-1.067539578	Zm00001d029133
Zm00001d032475_T001	0.000190425	3.95509365	Zm00001d032475
Zm00001d025323_T011	2.14E-08	1.534687517	Zm00001d025323
Zm00001d025323_T010	0.0164348	2.094388149	Zm00001d025323
Zm00001d025323_T004	0.04031405	2.546870811	Zm00001d025323
Zm00001d025323_T001	0.00012109	1.459667231	Zm00001d025323
Zm00001d025323_T012	0.000326064	2.000572513	Zm00001d025323
Zm00001d024710_T011	0.0146133	1.091888311	Zm00001d024710

Zm00001d046961_T001	0.02135948	-2.286799819	Zm00001d046961
Zm00001d042540_T015	0.00601412	-2.28221241	Zm00001d042540
Zm00001d042540_T007	0.03444594	-1.604048527	Zm00001d042540
Zm00001d037832_T010	0.002644703	1.222030469	Zm00001d037832
Zm00001d037225_T001	0.01033728	1.744233207	Zm00001d037225
Zm00001d007763_T001	0.04091628	-1.181466637	Zm00001d007763
Zm00001d047632_T002	0.003774627	1.450429496	Zm00001d047632
Zm00001d011309_T027	0.01328455	1.00473281	Zm00001d011309
Zm00001d023326_T013	3.69E-15	3.414686124	Zm00001d023326
Zm00001d031891_T021	0.02936899	-2.033579293	Zm00001d031891
Zm00001d022527_T018	8.84E-12	1.735101562	Zm00001d022527
Zm00001d048092_T010	0.004479976	-1.838046232	Zm00001d048092
Zm00001d040660_T001	0.003102858	2.568574293	Zm00001d040660
Zm00001d040660_T002	0.0086703	2.321376316	Zm00001d040660
Zm00001d020188_T002	0.000619347	-2.564594974	Zm00001d020188
Zm00001d043075_T010	8.51E-12	2.195957309	Zm00001d043075
Zm00001d043075_T028	0.001363155	-2.22852697	Zm00001d043075
Zm00001d043075_T005	0.00238057	1.408858915	Zm00001d043075
Zm00001d032339_T001	0.00113879	2.713988963	Zm00001d032339
Zm00001d034834_T001	9.78E-06	2.113429099	Zm00001d034834
Zm00001d034834_T010	0.03107792	-1.985380574	Zm00001d034834
Zm00001d038397_T005	0.000175579	-2.245597383	Zm00001d038397
Zm00001d020965_T008	0.000197484	1.11206652	Zm00001d020965
Zm00001d030682_T001	0.01080912	2.329098094	Zm00001d030682
Zm00001d052165_T001	0.000110548	4.161401272	Zm00001d052165
Zm00001d007280_T001	0.03674033	-2.003709513	Zm00001d007280
Zm00001d044823_T011	0.01856152	1.985651936	Zm00001d044823
Zm00001d034787_T001	2.11E-05	4.529328486	Zm00001d034787
Zm00001d035874_T001	4.59E-05	-3.984727719	Zm00001d035874
Zm00001d000191_T012	0.006370542	1.052551301	Zm00001d000191
Zm00001d036410_T004	7.66E-09	-2.063583313	Zm00001d036410
Zm00001d053082_T001	1.70E-11	2.681752321	Zm00001d053082
Zm00001d048155_T002	4.96E-10	-3.669128074	Zm00001d048155
Zm00001d022205_T005	9.11E-05	2.503750745	Zm00001d022205
Zm00001d000179_T001	0.000403041	3.431764379	Zm00001d000179
Zm00001d040960_T006	0.008916495	-1.620266724	Zm00001d040960
Zm00001d027625_T001	0.01189263	1.999419336	Zm00001d027625
Zm00001d032423_T003	2.37E-09	-4.790667212	Zm00001d032423
Zm00001d032423_T001	0.00224098	-2.663489454	Zm00001d032423
Zm00001d032423_T005	0.02175173	-2.268530182	Zm00001d032423
Zm00001d047762_T003	0.02339581	-1.662457784	Zm00001d047762
Zm00001d047762_T009	0.003185403	2.499180147	Zm00001d047762
Zm00001d042944_T001	0.000455444	-2.133019312	Zm00001d042944
Zm00001d036447_T005	0.006217153	-1.261055512	Zm00001d036447
Zm00001d054016_T002	1.81E-10	1.792411154	Zm00001d054016
Zm00001d017334_T001	0.007616066	2.158834228	Zm00001d017334
Zm00001d005473_T008	0.03110152	1.694821549	Zm00001d005473
Zm00001d026253_T004	1.16E-08	1.109853097	Zm00001d026253
Zm00001d011451_T001	0.00087074	2.990161747	Zm00001d011451
Zm00001d051565_T011	0.006892549	1.005843576	Zm00001d051565
Zm00001d018477_T010	7.18E-12	-2.02210976	Zm00001d018477
Zm00001d018477_T011	0.008110248	2.185937921	Zm00001d018477
Zm00001d052054_T001	0.02638727	-1.494829741	Zm00001d052054
Zm00001d006470_T002	0.000929456	-1.049035363	Zm00001d006470

Zm00001d006034_T001	0.03992146	-2.108437483	Zm00001d006034
Zm00001d003277_T001	1.62E-09	1.668394327	Zm00001d003277
Zm00001d021404_T008	4.06E-11	-1.992582222	Zm00001d021404
Zm00001d043807_T005	0.002259866	-1.732132388	Zm00001d043807
Zm00001d024458_T001	0.03126852	2.517581326	Zm00001d024458
Zm00001d007424_T003	2.50E-07	-1.235610784	Zm00001d007424
Zm00001d038003_T001	0.01286019	-2.492126783	Zm00001d038003
Zm00001d018677_T001	6.91E-05	-2.864634073	Zm00001d018677
Zm00001d018113_T011	0.02764265	1.781327689	Zm00001d018113
Zm00001d026010_T009	6.27E-13	2.637976029	Zm00001d026010
Zm00001d026010_T012	1.06E-16	-4.816861321	Zm00001d026010
Zm00001d035551_T008	0.04606011	-1.184906023	Zm00001d035551
Zm00001d041718_T005	0.02518239	1.008192703	Zm00001d041718
Zm00001d035526_T002	0.01206564	1.60514593	Zm00001d035526
Zm00001d012620_T018	0.009821311	1.133825634	Zm00001d012620
Zm00001d035113_T011	5.37E-12	2.594978441	Zm00001d035113
Zm00001d031957_T001	0.003322704	-1.436741045	Zm00001d031957
Zm00001d037870_T007	2.80E-08	1.406054535	Zm00001d037870
Zm00001d050925_T003	0.000773216	1.262315687	Zm00001d050925
Zm00001d011427_T014	0.000484721	1.456136142	Zm00001d011427
Zm00001d011427_T054	2.44E-10	-1.099528069	Zm00001d011427
Zm00001d021574_T005	0.02012514	1.171171938	Zm00001d021574
Zm00001d034522_T001	2.95E-09	2.087664566	Zm00001d034522
Zm00001d013470_T009	0.04919818	2.004214938	Zm00001d013470
Zm00001d013470_T001	5.42E-10	-2.815127062	Zm00001d013470
Zm00001d052200_T002	0.01141862	-1.092649688	Zm00001d052200
Zm00001d041823_T010	4.62E-07	-1.334636124	Zm00001d041823
Zm00001d014025_T008	0.04527883	1.504024706	Zm00001d014025
Zm00001d014993_T004	1.41E-06	-4.499061238	Zm00001d014993
Zm00001d033754_T001	1.02E-05	1.626537169	Zm00001d033754
Zm00001d019542_T007	0.000196681	1.055884933	Zm00001d019542
Zm00001d004443_T001	0.00709523	2.697461648	Zm00001d004443
Zm00001d043905_T002	0.007316201	-1.636606132	Zm00001d043905
Zm00001d020503_T001	1.97E-05	4.749834404	Zm00001d020503
Zm00001d008390_T006	0.01482443	1.521762681	Zm00001d008390
Zm00001d008390_T009	0.01153328	-1.494875302	Zm00001d008390
Zm00001d032287_T001	1.88E-05	3.540293731	Zm00001d032287
Zm00001d037695_T012	0.03698597	1.17061986	Zm00001d037695
Zm00001d047472_T007	3.29E-05	-3.453887689	Zm00001d047472
Zm00001d045804_T013	1.68E-12	2.970327147	Zm00001d045804
Zm00001d037973_T001	0.005816556	-1.385765421	Zm00001d037973
Zm00001d041900_T006	0.02511518	-1.251160851	Zm00001d041900
Zm00001d027928_T001	0.004041229	2.959059574	Zm00001d027928
Zm00001d047839_T003	4.64E-09	1.237918614	Zm00001d047839
Zm00001d012101_T007	1.90E-08	-2.808203474	Zm00001d012101
Zm00001d012101_T003	0.04606011	1.320092622	Zm00001d012101
Zm00001d028227_T007	0.000196768	-1.289989912	Zm00001d028227
Zm00001d023413_T001	0.001405609	1.45015102	Zm00001d023413
Zm00001d001963_T001	0.000669937	3.562007022	Zm00001d001963
Zm00001d043809_T002	2.93E-06	1.529470493	Zm00001d043809
Zm00001d044641_T009	0.005716143	-1.801491048	Zm00001d044641
Zm00001d049744_T001	3.38E-08	1.251747134	Zm00001d049744
Zm00001d011854_T031	0.000585379	2.779291373	Zm00001d011854
Zm00001d051308_T020	0.01004572	1.040571277	Zm00001d051308

Zm00001d051308_T010	1.04E-06	1.959218764	Zm00001d051308
Zm00001d037099_T012	4.59E-06	2.848417172	Zm00001d037099
Zm00001d047951_T003	0.02219849	1.952772412	Zm00001d047951
Zm00001d032540_T003	0.0140415	1.09125113	Zm00001d032540
Zm00001d029055_T010	7.20E-05	1.561789012	Zm00001d029055
Zm00001d039521_T004	0.01670316	-1.076195978	Zm00001d039521
Zm00001d020915_T002	0.04558476	2.405878357	Zm00001d020915
Zm00001d007606_T001	0.02008212	-1.857333273	Zm00001d007606
Zm00001d046805_T001	0.01640001	2.476653518	Zm00001d046805
Zm00001d052348_T001	0.004352965	1.937682656	Zm00001d052348
Zm00001d035233_T001	0.04322802	-1.793694771	Zm00001d035233
Zm00001d006623_T001	0.02252111	-2.170239248	Zm00001d006623
Zm00001d012761_T005	0.005053822	1.247769959	Zm00001d012761
Zm00001d012761_T007	1.52E-08	-1.322285344	Zm00001d012761
Zm00001d031075_T001	0.03577387	-1.77712344	Zm00001d031075
Zm00001d034616_T002	3.91E-07	1.752753463	Zm00001d034616
Zm00001d013317_T002	0.0496363	2.185527811	Zm00001d013317
Zm00001d035964_T005	2.27E-05	-4.285863946	Zm00001d035964
Zm00001d025557_T001	7.50E-09	-4.126900203	Zm00001d025557
Zm00001d001898_T011	0.004573701	1.199960446	Zm00001d001898
Zm00001d006917_T002	7.65E-10	-2.634337843	Zm00001d006917
Zm00001d015715_T002	0.001236951	-2.640213602	Zm00001d015715
Zm00001d015715_T001	0.00324374	-2.087991308	Zm00001d015715
Zm00001d039043_T006	0.000820562	-1.083885644	Zm00001d039043
Zm00001d022169_T001	0.04054157	1.997399483	Zm00001d022169
Zm00001d006844_T002	0.02151911	-1.385680349	Zm00001d006844
Zm00001d002006_T011	0.01234228	1.417453876	Zm00001d002006
Zm00001d002006_T013	5.11E-06	1.120078339	Zm00001d002006
Zm00001d002006_T016	1.65E-14	4.33867938	Zm00001d002006
Zm00001d037950_T017	8.77E-05	1.809616828	Zm00001d037950
Zm00001d043442_T001	0.001645855	3.457470443	Zm00001d043442
Zm00001d052935_T001	0.04663752	-1.666214006	Zm00001d052935
Zm00001d011258_T002	2.14E-07	1.198259164	Zm00001d011258
Zm00001d011258_T007	0.03157688	1.282283484	Zm00001d011258
Zm00001d017868_T004	0.001679745	1.799352116	Zm00001d017868
Zm00001d017868_T009	9.55E-05	2.004843341	Zm00001d017868
Zm00001d012653_T001	0.02305487	1.233127024	Zm00001d012653
Zm00001d006758_T006	0.005709369	1.955434626	Zm00001d006758
Zm00001d032187_T001	0.000186139	3.91458543	Zm00001d032187
Zm00001d038830_T005	0.006621309	1.585743413	Zm00001d038830
Zm00001d010614_T009	0.000165295	-1.836349669	Zm00001d010614
Zm00001d003495_T009	0.000116863	1.341963806	Zm00001d003495
Zm00001d047676_T005	0.002095691	2.479904118	Zm00001d047676
Zm00001d047548_T001	0.02671151	-2.141207592	Zm00001d047548
Zm00001d028808_T001	0.000628766	3.390090356	Zm00001d028808
Zm00001d014900_T040	0.002528307	1.932788005	Zm00001d014900
Zm00001d032472_T004	3.85E-12	3.143874364	Zm00001d032472
Zm00001d014617_T001	0.000760374	-2.270329831	Zm00001d014617
Zm00001d024755_T009	0.03355314	1.685573263	Zm00001d024755
Zm00001d024755_T003	5.11E-07	3.324214892	Zm00001d024755
Zm00001d024755_T007	0.02673031	1.574014956	Zm00001d024755
Zm00001d024755_T004	0.003590947	1.545357274	Zm00001d024755
Zm00001d010021_T007	1.31E-08	-1.255076171	Zm00001d010021
Zm00001d010021_T002	5.28E-09	1.603082865	Zm00001d010021

Zm00001d017505_T001	7.60E-05	1.057133242	Zm00001d017505
Zm00001d017505_T009	0.001647574	2.636351671	Zm00001d017505
Zm00001d017505_T006	3.13E-07	1.259991559	Zm00001d017505
Zm00001d004396_T016	0.000306523	2.697358866	Zm00001d004396
Zm00001d041569_T009	0.006076389	-2.119654218	Zm00001d041569
Zm00001d037867_T002	0.004389276	1.20210402	Zm00001d037867
Zm00001d037867_T007	0.001280491	2.15281067	Zm00001d037867
Zm00001d037867_T026	0.003000573	1.759950593	Zm00001d037867
Zm00001d046767_T005	0.003343235	-1.878237561	Zm00001d046767
Zm00001d031833_T001	0.02561293	2.55889543	Zm00001d031833
Zm00001d032190_T010	0.002796955	-1.754717514	Zm00001d032190
Zm00001d036448_T001	3.88E-10	-1.516421568	Zm00001d036448
Zm00001d042146_T004	3.15E-05	1.950849503	Zm00001d042146
Zm00001d042146_T001	6.19E-06	1.223173468	Zm00001d042146
Zm00001d028569_T004	1.12E-08	1.592107984	Zm00001d028569
Zm00001d028569_T003	3.15E-08	-1.240806935	Zm00001d028569
Zm00001d036003_T001	3.36E-08	4.220881606	Zm00001d036003
Zm00001d021677_T001	0.01606913	2.179295942	Zm00001d021677
Zm00001d043392_T016	1.65E-09	-2.036705007	Zm00001d043392
Zm00001d043392_T002	0.02334998	1.014283418	Zm00001d043392
Zm00001d043392_T004	0.0169289	1.709750403	Zm00001d043392
Zm00001d000357_T010	0.000477176	1.144770998	Zm00001d000357
Zm00001d000357_T009	0.000512822	1.07175116	Zm00001d000357
Zm00001d000357_T003	0.02147519	2.385530855	Zm00001d000357
Zm00001d045953_T016	0.03949883	1.003117868	Zm00001d045953
Zm00001d029707_T001	0.01144665	-1.781760737	Zm00001d029707
Zm00001d037961_T008	0.002863375	1.51489413	Zm00001d037961
Zm00001d029164_T001	0.0161978	-1.298112943	Zm00001d029164
Zm00001d001993_T001	0.01910192	1.70081013	Zm00001d001993
Zm00001d004898_T001	0.01220849	-1.574277897	Zm00001d004898
Zm00001d028084_T005	1.40E-10	1.439941479	Zm00001d028084
Zm00001d023576_T009	0.000234904	2.29022225	Zm00001d023576
Zm00001d048214_T001	0.04476943	-1.736049529	Zm00001d048214
Zm00001d007300_T011	0.006621309	-1.12242355	Zm00001d007300
Zm00001d002359_T007	0.01118874	-1.418339122	Zm00001d002359
Zm00001d002359_T008	1.10E-13	-2.874040076	Zm00001d002359
Zm00001d048541_T007	3.07E-05	1.528818458	Zm00001d048541
Zm00001d028671_T015	0.001874253	1.703272703	Zm00001d028671
Zm00001d028671_T018	0.01028758	1.307297841	Zm00001d028671
Zm00001d036351_T218	0.003093763	-2.188095524	Zm00001d036351
Zm00001d036351_T009	0.009186909	1.385735505	Zm00001d036351
Zm00001d033455_T001	0.0483012	-1.810069018	Zm00001d033455
Zm00001d026585_T004	0.006779326	1.19628421	Zm00001d026585
Zm00001d032643_T001	0.03529405	-1.967304317	Zm00001d032643
Zm00001d008288_T001	0.000281578	-4.38046484	Zm00001d008288
Zm00001d006607_T055	0.000146005	1.232079836	Zm00001d006607
Zm00001d026706_T011	0.04971279	2.858852846	Zm00001d026706
Zm00001d025012_T020	0.01901268	2.216991076	Zm00001d025012
Zm00001d030678_T003	5.63E-05	-1.498999157	Zm00001d030678
Zm00001d052527_T001	0.01956599	-2.041490924	Zm00001d052527
Zm00001d010710_T008	0.007525957	1.528709044	Zm00001d010710
Zm00001d031344_T001	0.000339742	-3.245116962	Zm00001d031344
Zm00001d031344_T002	9.83E-11	-4.459464502	Zm00001d031344
Zm00001d003981_T001	0.002666247	3.243776826	Zm00001d003981

Zm00001d015435_T020	0.01594071	1.118808899	Zm00001d015435
Zm00001d011158_T012	8.04E-11	1.66519565	Zm00001d011158
Zm00001d005323_T009	0.01624246	1.518283056	Zm00001d005323
Zm00001d013834_T001	0.003542199	3.255129613	Zm00001d013834
Zm00001d031316_T006	0.002796955	-1.663550703	Zm00001d031316
Zm00001d048535_T026	6.67E-10	1.098062406	Zm00001d048535
Zm00001d031660_T002	1.00E-13	3.213266961	Zm00001d031660
Zm00001d048119_T040	0.006510461	-1.957796796	Zm00001d048119
Zm00001d024300_T008	0.001782387	-1.450685587	Zm00001d024300
Zm00001d053578_T001	0.01500842	1.218959898	Zm00001d053578
Zm00001d024225_T032	1.51E-11	1.463464012	Zm00001d024225
Zm00001d041550_T004	0.01048202	-2.145699782	Zm00001d041550
Zm00001d024889_T004	0.000124973	-2.039673065	Zm00001d024889
Zm00001d027539_T002	2.48E-12	-4.965276973	Zm00001d027539
Zm00001d037941_T001	3.50E-05	-3.549849055	Zm00001d037941
Zm00001d037941_T002	7.80E-08	-3.273246105	Zm00001d037941
Zm00001d032030_T001	0.005641901	-2.620562814	Zm00001d032030
Zm00001d012770_T003	0.004479976	-2.434537662	Zm00001d012770
Zm00001d037243_T001	0.01423233	-2.202752002	Zm00001d037243
Zm00001d049187_T001	0.04827422	2.548560527	Zm00001d049187
Zm00001d033240_T003	0.000866567	-2.588616143	Zm00001d033240
Zm00001d033797_T002	8.45E-05	3.578170087	Zm00001d033797
Zm00001d033797_T001	0.007367894	1.724201335	Zm00001d033797
Zm00001d048623_T001	0.006756568	-2.306284688	Zm00001d048623
Zm00001d048623_T002	0.04327008	-1.791577868	Zm00001d048623
Zm00001d002482_T001	0.009394971	1.739123354	Zm00001d002482
Zm00001d027427_T002	0.03117416	-1.79375409	Zm00001d027427
Zm00001d007264_T001	0.03285779	-1.672730483	Zm00001d007264
Zm00001d038675_T120	0.000249454	1.420056167	Zm00001d038675
Zm00001d038675_T187	0.008747332	-1.114717977	Zm00001d038675
Zm00001d038675_T544	2.50E-11	-2.059264445	Zm00001d038675
Zm00001d038675_T201	0.02108144	1.774945124	Zm00001d038675
Zm00001d038675_T182	7.88E-13	1.873867921	Zm00001d038675
Zm00001d038675_T589	0.0422188	-1.259861932	Zm00001d038675
Zm00001d038675_T034	0.0294937	1.792719916	Zm00001d038675
Zm00001d038675_T197	1.53E-10	1.165428411	Zm00001d038675
Zm00001d038675_T056	1.79E-09	1.075638357	Zm00001d038675
Zm00001d038675_T065	0.006217153	1.666286691	Zm00001d038675
Zm00001d032830_T001	0.00309982	-1.601134122	Zm00001d032830
Zm00001d032830_T009	0.05001771	2.031414547	Zm00001d032830
Zm00001d017243_T001	2.92E-06	2.461608986	Zm00001d017243
Zm00001d005736_T036	1.42E-08	-2.361880022	Zm00001d005736
Zm00001d017913_T001	0.001760922	-2.394048899	Zm00001d017913
Zm00001d036161_T018	3.57E-10	1.642868384	Zm00001d036161
Zm00001d038870_T001	0.02815853	-1.889852092	Zm00001d038870
Zm00001d047944_T001	0.02424821	2.278011857	Zm00001d047944
Zm00001d026265_T002	0.000150852	-2.427326235	Zm00001d026265
Zm00001d052937_T001	0.01232703	-1.901837486	Zm00001d052937
Zm00001d053155_T016	6.27E-13	2.701825138	Zm00001d053155
Zm00001d018929_T010	3.27E-08	1.266830101	Zm00001d018929
Zm00001d012900_T001	0.04721739	2.169580629	Zm00001d012900
Zm00001d051419_T003	0.02021864	-2.815788641	Zm00001d051419
Zm00001d003419_T001	0.005206629	3.146493492	Zm00001d003419
Zm00001d054076_T011	0.0299214	1.559382839	Zm00001d054076

Zm00001d054076_T040	0.04000108	-1.235409275	Zm00001d054076
Zm00001d014253_T001	7.15E-07	4.573160869	Zm00001d014253
Zm00001d004099_T006	1.39E-09	1.93582664	Zm00001d004099
Zm00001d002564_T001	0.001416617	1.754354398	Zm00001d002564
Zm00001d042214_T001	0.01506602	2.660829122	Zm00001d042214
Zm00001d022160_T002	2.48E-11	-4.344683695	Zm00001d022160
Zm00001d040827_T001	0.001423487	-1.576133117	Zm00001d040827
Zm00001d022490_T003	0.01911835	1.454552181	Zm00001d022490
Zm00001d022621_T019	0.005500518	-2.608759239	Zm00001d022621
Zm00001d028907_T014	7.83E-05	-1.523353964	Zm00001d028907
Zm00001d013542_T005	0.01442294	-1.289931744	Zm00001d013542
Zm00001d008826_T009	0.01067914	-2.199485473	Zm00001d008826
Zm00001d008826_T015	1.24E-19	5.910344573	Zm00001d008826
Zm00001d008826_T014	0.000149326	2.632281777	Zm00001d008826
Zm00001d008826_T041	0.000142536	2.002638609	Zm00001d008826
Zm00001d008826_T022	5.25E-06	2.582279583	Zm00001d008826
Zm00001d009811_T005	3.64E-10	-1.820950172	Zm00001d009811
Zm00001d045184_T001	0.004070328	2.808367297	Zm00001d045184
Zm00001d033683_T002	0.001075711	-2.898998394	Zm00001d033683
Zm00001d017887_T001	0.000948121	2.222249511	Zm00001d017887
Zm00001d026291_T013	5.80E-05	1.960377463	Zm00001d026291
Zm00001d037214_T002	0.00238057	2.52344656	Zm00001d037214
Zm00001d037214_T006	0.005033695	2.090514266	Zm00001d037214
Zm00001d021410_T001	0.01989623	2.75364622	Zm00001d021410
Zm00001d028404_T001	0.02545254	-2.516168559	Zm00001d028404
Zm00001d050726_T001	0.02555124	1.190876882	Zm00001d050726
Zm00001d027299_T015	0.04573669	-1.273637816	Zm00001d027299
Zm00001d027299_T010	6.54E-15	2.714448638	Zm00001d027299
Zm00001d044008_T001	7.74E-12	1.620733799	Zm00001d044008
Zm00001d049038_T015	1.16E-11	2.942039882	Zm00001d049038
Zm00001d038447_T001	0.000368451	3.724451572	Zm00001d038447
Zm00001d000012_T001	0.003148726	1.487517707	Zm00001d000012
Zm00001d028093_T003	0.000476965	2.145586514	Zm00001d028093
Zm00001d002999_T001	0.000105547	3.51827414	Zm00001d002999
Zm00001d048404_T020	3.69E-12	-1.914016962	Zm00001d048404
Zm00001d048404_T013	6.73E-11	1.567546463	Zm00001d048404
Zm00001d048404_T011	3.55E-12	1.961619704	Zm00001d048404
Zm00001d042239_T001	0.009062656	2.419777145	Zm00001d042239
Zm00001d002736_T001	0.03699476	2.385362365	Zm00001d002736
Zm00001d045550_T001	0.00078261	-1.064842847	Zm00001d045550
Zm00001d045104_T015	2.46E-12	-4.665108872	Zm00001d045104
Zm00001d045104_T014	7.02E-15	-6.395111902	Zm00001d045104
Zm00001d045104_T018	0.02500632	-1.774962259	Zm00001d045104
Zm00001d002357_T001	0.02395949	-1.061910864	Zm00001d002357
Zm00001d006868_T006	0.000528461	2.802169727	Zm00001d006868
Zm00001d037546_T009	0.01213416	2.766099143	Zm00001d037546
Zm00001d037546_T011	8.84E-06	1.308437797	Zm00001d037546
Zm00001d031769_T001	0.0012653	3.286812816	Zm00001d031769
Zm00001d043837_T001	0.02572119	2.589308904	Zm00001d043837
Zm00001d004159_T002	0.003655875	1.181969631	Zm00001d004159
Zm00001d009908_T010	4.28E-07	-1.79117218	Zm00001d009908
Zm00001d028244_T007	0.04491806	2.023246512	Zm00001d028244
Zm00001d002565_T001	0.01209044	2.558557514	Zm00001d002565
Zm00001d012624_T028	1.53E-05	2.281529826	Zm00001d012624

Zm00001d011091_T011	3.22E-10	1.300277037	Zm00001d011091
Zm00001d011091_T036	0.01642345	2.530283861	Zm00001d011091
Zm00001d048461_T004	0.01225238	-2.447597016	Zm00001d048461
Zm00001d044186_T305	0.02906074	-2.342570513	Zm00001d044186
Zm00001d044186_T073	4.86E-11	2.036433216	Zm00001d044186
Zm00001d044186_T304	0.000125942	-1.919525905	Zm00001d044186
Zm00001d044186_T103	0.02593869	1.403446022	Zm00001d044186
Zm00001d013004_T001	0.01035085	1.928980032	Zm00001d013004
Zm00001d015504_T020	0.02345171	-1.912223434	Zm00001d015504
Zm00001d043455_T001	0.03277874	-2.305025262	Zm00001d043455
Zm00001d042132_T001	0.04591214	1.87674106	Zm00001d042132
Zm00001d014413_T013	0.0217161	1.238414327	Zm00001d014413
Zm00001d017592_T001	1.74E-07	3.982988002	Zm00001d017592
Zm00001d003109_T004	2.87E-09	1.862817421	Zm00001d003109
Zm00001d034962_T002	0.000277524	1.000487913	Zm00001d034962
Zm00001d021785_T013	6.63E-11	1.586557234	Zm00001d021785
Zm00001d032115_T001	0.04665552	-1.996081498	Zm00001d032115
Zm00001d047981_T002	1.74E-07	4.63388088	Zm00001d047981
Zm00001d047981_T001	8.31E-07	5.002521917	Zm00001d047981
Zm00001d012211_T006	0.01633919	3.398641342	Zm00001d012211
Zm00001d012211_T009	0.000755736	-2.478468202	Zm00001d012211
Zm00001d052336_T002	0.01524559	-1.148730545	Zm00001d052336
Zm00001d024371_T001	0.0003273	-3.559694547	Zm00001d024371
Zm00001d003655_T014	0.01281526	1.855996209	Zm00001d003655
Zm00001d045298_T002	0.01630111	-1.731980199	Zm00001d045298
Zm00001d041443_T028	2.25E-10	-2.317170205	Zm00001d041443
Zm00001d028262_T083	4.30E-05	-1.657296816	Zm00001d028262
Zm00001d028262_T090	5.01E-05	-1.42314829	Zm00001d028262
Zm00001d030266_T014	5.57E-15	2.876283316	Zm00001d030266
Zm00001d013211_T007	0.01041492	1.305227008	Zm00001d013211
Zm00001d013211_T008	0.0217161	1.063100957	Zm00001d013211
Zm00001d039214_T013	0.003132519	-1.875227017	Zm00001d039214
Zm00001d045889_T001	0.00734409	-1.573760984	Zm00001d045889
Zm00001d009795_T001	4.79E-07	-1.048370665	Zm00001d009795
Zm00001d009795_T006	2.34E-06	1.919530822	Zm00001d009795
Zm00001d011336_T005	0.01528249	-2.63956534	Zm00001d011336
Zm00001d051062_T003	0.03278334	-1.887265763	Zm00001d051062
Zm00001d029375_T017	0.00013693	2.042777371	Zm00001d029375
Zm00001d029375_T001	0.02027865	1.555279426	Zm00001d029375
Zm00001d009761_T010	6.00E-13	-2.861543491	Zm00001d009761
Zm00001d036343_T016	1.33E-10	1.556704728	Zm00001d036343
Zm00001d036343_T008	0.008239117	1.358975373	Zm00001d036343
Zm00001d036343_T019	3.39E-07	-1.318584015	Zm00001d036343
Zm00001d023400_T002	2.13E-05	3.359998647	Zm00001d023400
Zm00001d021192_T002	4.37E-06	-2.822282188	Zm00001d021192
Zm00001d009659_T001	0.008403401	1.831876527	Zm00001d009659
Zm00001d035878_T004	0.005667014	-2.145197197	Zm00001d035878
Zm00001d049409_T005	3.45E-05	3.380217808	Zm00001d049409
Zm00001d049409_T006	1.71E-05	1.678719414	Zm00001d049409
Zm00001d049409_T011	6.21E-08	3.568191515	Zm00001d049409
Zm00001d049409_T009	7.14E-07	3.956316325	Zm00001d049409
Zm00001d011563_T012	0.000476965	1.166743705	Zm00001d011563
Zm00001d017422_T001	0.004200076	2.379919667	Zm00001d017422
Zm00001d003081_T004	0.01316589	-3.201242236	Zm00001d003081

Zm00001d014858_T002	1.39E-10	-3.109529136	Zm00001d014858
Zm00001d018274_T001	0.01614434	1.695680633	Zm00001d018274
Zm00001d008295_T003	3.15E-08	1.085738813	Zm00001d008295
Zm00001d029206_T007	0.02606656	-1.300891703	Zm00001d029206
Zm00001d044138_T001	1.33E-05	4.62414038	Zm00001d044138
Zm00001d044138_T002	4.62E-05	3.03232308	Zm00001d044138
Zm00001d036462_T068	0.04346091	-1.315653995	Zm00001d036462
Zm00001d035616_T004	0.0355567	2.393480669	Zm00001d035616
Zm00001d031943_T005	0.01789897	1.708048296	Zm00001d031943
Zm00001d005779_T169	1.06E-09	-1.142477575	Zm00001d005779
Zm00001d005779_T197	0.001050107	-2.080518029	Zm00001d005779
Zm00001d005779_T106	0.005542545	1.13577774	Zm00001d005779
Zm00001d005779_T006	0.002048092	1.159403587	Zm00001d005779
Zm00001d005779_T171	2.35E-12	2.762219012	Zm00001d005779
Zm00001d005779_T141	1.56E-13	-2.843420939	Zm00001d005779
Zm00001d005779_T002	0.0347692	1.266899415	Zm00001d005779
Zm00001d005779_T174	0.01450116	1.207976183	Zm00001d005779
Zm00001d028267_T005	0.004325522	-2.710852044	Zm00001d028267
Zm00001d017292_T003	2.82E-05	-3.893277614	Zm00001d017292
Zm00001d036609_T009	0.004708579	-1.02446317	Zm00001d036609
Zm00001d036609_T001	4.58E-10	2.164071663	Zm00001d036609
Zm00001d036609_T021	0.000607124	1.259216375	Zm00001d036609
Zm00001d036609_T004	9.04E-11	-1.944382447	Zm00001d036609
Zm00001d048510_T010	2.08E-12	1.697822005	Zm00001d048510
Zm00001d031146_T001	0.03449192	-1.80247473	Zm00001d031146
Zm00001d011428_T005	0.000662792	2.16826459	Zm00001d011428
Zm00001d011428_T004	0.000331982	3.746729169	Zm00001d011428
Zm00001d050689_T001	6.02E-05	3.28134948	Zm00001d050689
Zm00001d026296_T019	0.02420233	-1.170227277	Zm00001d026296
Zm00001d046561_T007	0.0312518	-1.416733895	Zm00001d046561
Zm00001d012881_T001	0.02005525	-1.684349541	Zm00001d012881
Zm00001d023265_T011	5.02E-15	3.757436214	Zm00001d023265
Zm00001d052992_T003	0.01349183	1.665823732	Zm00001d052992
Zm00001d028818_T015	1.35E-05	1.746669346	Zm00001d028818
Zm00001d051796_T004	0.02413379	1.199466538	Zm00001d051796
Zm00001d039464_T001	9.34E-06	3.856398244	Zm00001d039464
Zm00001d029602_T001	0.000108813	4.35241454	Zm00001d029602
Zm00001d002035_T002	0.005677468	-1.924452708	Zm00001d002035
Zm00001d010322_T002	0.001560838	-3.247974157	Zm00001d010322
Zm00001d034387_T005	0.0102476	2.475643789	Zm00001d034387
Zm00001d049734_T001	0.006284918	1.53097879	Zm00001d049734
Zm00001d037557_T020	2.75E-05	1.397143953	Zm00001d037557
Zm00001d043649_T047	2.74E-14	-3.264162381	Zm00001d043649
Zm00001d043649_T056	9.04E-11	2.33003121	Zm00001d043649
Zm00001d048171_T006	0.00040965	1.098951509	Zm00001d048171
Zm00001d048171_T007	0.009489059	1.709748976	Zm00001d048171
Zm00001d029426_T011	0.04606011	-1.496483808	Zm00001d029426
Zm00001d050372_T005	0.02566567	1.006635624	Zm00001d050372
Zm00001d011192_T002	0.000653854	-1.602756335	Zm00001d011192
Zm00001d050099_T001	0.02871159	2.755087646	Zm00001d050099
Zm00001d020602_T045	0.005173865	1.404751923	Zm00001d020602
Zm00001d020602_T037	3.33E-11	1.567411784	Zm00001d020602
Zm00001d044175_T006	0.03726142	2.259392259	Zm00001d044175
Zm00001d021639_T001	0.001411385	1.44010175	Zm00001d021639

Zm00001d042337_T003	0.001567468	-1.280081448	Zm00001d042337
Zm00001d006098_T004	6.75E-05	-1.432752616	Zm00001d006098
Zm00001d044769_T001	3.24E-05	5.45782519	Zm00001d044769
Zm00001d034941_T003	0.004469169	2.351895813	Zm00001d034941
Zm00001d035679_T019	1.35E-10	-2.613249684	Zm00001d035679
Zm00001d001856_T004	0.02276246	-1.265153412	Zm00001d001856
Zm00001d011208_T002	0.025894	-1.767567678	Zm00001d011208
Zm00001d011208_T001	0.03053044	-1.926652693	Zm00001d011208
Zm00001d007769_T002	4.50E-09	1.550801856	Zm00001d007769
Zm00001d036348_T001	0.008267721	-2.058901981	Zm00001d036348
Zm00001d033537_T007	0.04999669	-1.800522116	Zm00001d033537
Zm00001d033246_T005	3.48E-05	-4.048321091	Zm00001d033246
Zm00001d013333_T001	9.02E-05	4.419587667	Zm00001d013333
Zm00001d040263_T010	1.99E-12	3.563573666	Zm00001d040263
Zm00001d040263_T009	2.82E-06	-1.820297135	Zm00001d040263
Zm00001d043309_T015	0.04771357	1.197151117	Zm00001d043309
Zm00001d047105_T008	0.04016634	1.538433607	Zm00001d047105
Zm00001d038225_T003	0.000968173	-1.396206382	Zm00001d038225
Zm00001d025949_T001	0.04071407	1.672334868	Zm00001d025949
Zm00001d045340_T008	0.005342103	-1.239261852	Zm00001d045340
Zm00001d045340_T006	0.007598967	-1.575814177	Zm00001d045340
Zm00001d040311_T007	0.04254089	1.44109485	Zm00001d040311
Zm00001d016401_T001	1.50E-05	5.703747252	Zm00001d016401
Zm00001d042313_T015	0.01035085	1.882483485	Zm00001d042313
Zm00001d042313_T005	3.70E-05	2.937202146	Zm00001d042313
Zm00001d042313_T009	3.45E-07	1.707291554	Zm00001d042313
Zm00001d042313_T011	0.000968173	1.813197124	Zm00001d042313
Zm00001d020702_T001	0.00094777	3.057878851	Zm00001d020702
Zm00001d016139_T001	2.46E-07	-4.780763822	Zm00001d016139
Zm00001d025873_T001	4.72E-06	-4.373029019	Zm00001d025873
Zm00001d000255_T001	2.10E-06	2.781606092	Zm00001d000255
Zm00001d021772_T001	1.25E-06	-3.440455958	Zm00001d021772
Zm00001d038192_T001	0.02312763	-1.93377706	Zm00001d038192
Zm00001d035933_T002	0.04509766	1.231001133	Zm00001d035933
Zm00001d013589_T004	9.63E-11	-2.356424008	Zm00001d013589
Zm00001d017595_T007	3.79E-05	-2.64057586	Zm00001d017595
Zm00001d045784_T002	0.000378118	-4.067425854	Zm00001d045784
Zm00001d052462_T002	3.21E-09	-2.215561329	Zm00001d052462
Zm00001d042979_T005	0.03179747	1.491686729	Zm00001d042979
Zm00001d043667_T004	0.000404364	1.490861633	Zm00001d043667
Zm00001d004215_T003	0.006615702	-1.489198233	Zm00001d004215
Zm00001d024953_T032	3.91E-09	1.286168522	Zm00001d024953
Zm00001d029264_T006	0.001371611	1.122045509	Zm00001d029264
Zm00001d031423_T001	0.03154256	2.205327588	Zm00001d031423
Zm00001d026501_T005	3.28E-05	2.422852931	Zm00001d026501
Zm00001d026501_T006	6.63E-11	1.645095476	Zm00001d026501
Zm00001d053059_T022	0.0177405	-1.24162389	Zm00001d053059
Zm00001d053059_T007	0.007342728	1.066643942	Zm00001d053059
Zm00001d047448_T001	0.002841365	-1.874462484	Zm00001d047448
Zm00001d002089_T043	0.001096538	1.898717817	Zm00001d002089
Zm00001d002089_T003	0.003185403	1.117560633	Zm00001d002089
Zm00001d006227_T002	0.01810291	1.279508864	Zm00001d006227
Zm00001d010755_T003	0.03423702	2.008690388	Zm00001d010755
Zm00001d029527_T005	0.000959593	-2.13004813	Zm00001d029527

Zm00001d053622_T002	6.11E-09	-2.965548586	Zm00001d053622
Zm00001d025914_T001	0.02758849	1.631862822	Zm00001d025914
Zm00001d043071_T003	0.01570661	1.180552966	Zm00001d043071
Zm00001d033649_T003	0.000968173	-2.364766567	Zm00001d033649
Zm00001d000339_T001	0.02344401	2.389168839	Zm00001d000339
Zm00001d053826_T003	8.89E-05	1.201455909	Zm00001d053826
Zm00001d001929_T010	0.007772415	-2.156329892	Zm00001d001929
Zm00001d026439_T094	0.02125534	1.452799247	Zm00001d026439
Zm00001d026439_T072	3.23E-11	1.658350681	Zm00001d026439
Zm00001d026439_T092	5.72E-12	2.68462884	Zm00001d026439
Zm00001d013405_T001	0.03427147	1.819059531	Zm00001d013405
Zm00001d002967_T008	0.02483077	1.174751694	Zm00001d002967
Zm00001d002967_T002	0.005914915	1.27507599	Zm00001d002967
Zm00001d036981_T001	2.35E-13	3.480795737	Zm00001d036981
Zm00001d026490_T006	0.00262847	1.606782658	Zm00001d026490
Zm00001d026490_T010	0.000234547	2.918511564	Zm00001d026490
Zm00001d026490_T002	0.00058544	-1.417094742	Zm00001d026490
Zm00001d026490_T018	0.004469169	1.206109649	Zm00001d026490
Zm00001d044387_T001	2.50E-06	2.267913614	Zm00001d044387
Zm00001d039682_T004	0.005716143	1.317493069	Zm00001d039682
Zm00001d020438_T006	0.003288625	1.127430992	Zm00001d020438
Zm00001d009328_T001	0.000646947	3.215450559	Zm00001d009328
Zm00001d045938_T002	3.65E-08	-1.793803294	Zm00001d045938
Zm00001d015174_T011	0.0002867	-1.804543073	Zm00001d015174
Zm00001d032091_T001	0.001501543	3.187914277	Zm00001d032091
Zm00001d043452_T006	1.20E-09	1.09096313	Zm00001d043452
Zm00001d045431_T003	4.37E-09	-6.576125886	Zm00001d045431
Zm00001d005794_T018	0.001891659	2.612042289	Zm00001d005794
Zm00001d006041_T036	0.0191982	-1.484172024	Zm00001d006041
Zm00001d006041_T007	0.0151662	1.186674166	Zm00001d006041
Zm00001d022316_T002	5.03E-12	-4.191746769	Zm00001d022316
Zm00001d013098_T013	0.000308982	2.107555564	Zm00001d013098
Zm00001d013098_T003	0.0161855	2.840409748	Zm00001d013098
Zm00001d013098_T016	0.001096538	1.271058825	Zm00001d013098
Zm00001d013098_T011	2.15E-09	3.11912241	Zm00001d013098
Zm00001d013098_T015	5.12E-15	-2.919816353	Zm00001d013098
Zm00001d013098_T014	0.01947349	1.12559873	Zm00001d013098
Zm00001d018712_T001	3.78E-05	-3.949617932	Zm00001d018712
Zm00001d007698_T005	0.008959327	2.159489729	Zm00001d007698
Zm00001d007698_T032	8.97E-11	1.615234538	Zm00001d007698
Zm00001d013276_T004	1.08E-13	2.506077466	Zm00001d013276
Zm00001d013276_T013	6.54E-15	-2.822441399	Zm00001d013276
Zm00001d042541_T003	0.000173685	-3.014546759	Zm00001d042541
Zm00001d017261_T015	5.67E-06	1.001502514	Zm00001d017261
Zm00001d009940_T001	0.03596366	-2.023002364	Zm00001d009940
Zm00001d051861_T002	3.81E-09	-3.86689068	Zm00001d051861
Zm00001d046554_T005	4.48E-10	3.015082293	Zm00001d046554
Zm00001d036004_T020	1.17E-09	2.232421056	Zm00001d036004
Zm00001d036004_T006	2.77E-05	-1.54380597	Zm00001d036004
Zm00001d051600_T023	0.02706902	1.944307956	Zm00001d051600
Zm00001d051600_T019	8.48E-09	2.266311748	Zm00001d051600
Zm00001d016463_T014	3.69E-08	-4.797664052	Zm00001d016463
Zm00001d038362_T003	0.004325883	-2.773629402	Zm00001d038362
Zm00001d002770_T017	1.65E-09	1.539092251	Zm00001d002770

Zm00001d048389_T001	0.007530118	2.860513599	Zm00001d048389
Zm00001d032322_T002	4.12E-10	-2.318446192	Zm00001d032322
Zm00001d008511_T137	9.62E-07	1.267968529	Zm00001d008511
Zm00001d008511_T136	7.11E-10	-1.773439587	Zm00001d008511
Zm00001d041514_T010	0.000373808	-1.000304185	Zm00001d041514
Zm00001d048308_T003	0.005221963	-2.250795019	Zm00001d048308
Zm00001d022415_T001	0.01824324	2.873563279	Zm00001d022415
Zm00001d018013_T011	0.000270277	-3.020685252	Zm00001d018013
Zm00001d048089_T044	2.06E-08	1.156146831	Zm00001d048089
Zm00001d032691_T001	7.71E-10	-2.337228282	Zm00001d032691
Zm00001d006428_T010	0.004103803	2.12287065	Zm00001d006428
Zm00001d028055_T001	0.005075715	3.085100479	Zm00001d028055
Zm00001d016770_T005	0.04237526	-2.130388308	Zm00001d016770
Zm00001d005593_T001	0.00365365	-1.750961517	Zm00001d005593
Zm00001d007826_T001	2.90E-11	-2.086229779	Zm00001d007826
Zm00001d043613_T012	1.51E-11	2.191796549	Zm00001d043613
Zm00001d043613_T006	0.007481278	-2.300729452	Zm00001d043613
Zm00001d043613_T008	0.009306966	-1.147108812	Zm00001d043613
Zm00001d019595_T002	0.0109172	-1.03194651	Zm00001d019595
Zm00001d011983_T003	6.06E-05	1.779430422	Zm00001d011983
Zm00001d038473_T001	0.02234554	1.481431963	Zm00001d038473
Zm00001d042627_T001	0.002158908	1.832980376	Zm00001d042627
Zm00001d042627_T012	0.03752477	1.822682142	Zm00001d042627
Zm00001d025799_T005	0.0169199	3.03313693	Zm00001d025799
Zm00001d025799_T001	0.000693551	2.642178441	Zm00001d025799
Zm00001d025799_T004	0.000158741	2.298842219	Zm00001d025799
Zm00001d027295_T003	0.005415469	-1.205817418	Zm00001d027295
Zm00001d031851_T010	0.001225472	-2.486350848	Zm00001d031851
Zm00001d046936_T005	0.04949101	-1.10356787	Zm00001d046936
Zm00001d038420_T001	0.02742794	-2.143049756	Zm00001d038420
Zm00001d006217_T004	0.02460353	2.309292311	Zm00001d006217
Zm00001d014368_T001	0.002285849	-1.861067122	Zm00001d014368
Zm00001d044950_T001	0.01058146	2.14211175	Zm00001d044950
Zm00001d014852_T001	5.52E-05	-3.043666224	Zm00001d014852
Zm00001d003291_T007	0.00891843	-2.017923255	Zm00001d003291
Zm00001d048013_T001	5.27E-08	4.404701946	Zm00001d048013
Zm00001d023376_T007	0.002765635	-2.281887426	Zm00001d023376
Zm00001d050259_T002	0.03883914	1.401721487	Zm00001d050259
Zm00001d018560_T002	0.02189243	2.163944372	Zm00001d018560
Zm00001d021553_T077	5.75E-09	-1.684919304	Zm00001d021553
Zm00001d025982_T001	0.02860418	2.615596705	Zm00001d025982
Zm00001d003762_T002	0.01444297	2.411540117	Zm00001d003762
Zm00001d003762_T001	0.000438989	2.530520803	Zm00001d003762
Zm00001d053609_T069	0.0307919	-1.107591721	Zm00001d053609
Zm00001d053609_T031	7.85E-15	3.08368377	Zm00001d053609
Zm00001d041953_T053	1.11E-10	1.400553291	Zm00001d041953
Zm00001d019586_T025	2.29E-06	1.403595255	Zm00001d019586
Zm00001d019586_T002	0.003920049	1.058540263	Zm00001d019586
Zm00001d010716_T005	0.006131928	1.758325284	Zm00001d010716
Zm00001d052152_T001	0.000203756	2.877019983	Zm00001d052152
Zm00001d032567_T009	0.01045777	1.20243192	Zm00001d032567
Zm00001d032567_T013	7.27E-13	2.525410384	Zm00001d032567
Zm00001d027916_T013	0.04193616	1.063479353	Zm00001d027916
Zm00001d016262_T001	0.03520039	-2.180739392	Zm00001d016262

Zm00001d028429_T019	2.70E-08	-3.111444286	Zm00001d028429
Zm00001d053819_T001	1.06E-10	-1.466945968	Zm00001d053819
Zm00001d032428_T003	3.27E-08	1.012121767	Zm00001d032428
Zm00001d019108_T019	7.45E-11	1.233479397	Zm00001d019108
Zm00001d019108_T083	5.21E-12	1.697353653	Zm00001d019108
Zm00001d040594_T001	0.009728585	1.845064543	Zm00001d040594
Zm00001d039173_T007	0.00049958	-2.057331008	Zm00001d039173
Zm00001d009646_T001	0.03610052	-1.231172656	Zm00001d009646
Zm00001d034584_T001	0.008700137	-1.572383562	Zm00001d034584
Zm00001d016640_T018	3.07E-14	3.327093217	Zm00001d016640
Zm00001d016640_T011	0.007149903	2.33543657	Zm00001d016640
Zm00001d000074_T002	0.00593389	2.821061835	Zm00001d000074
Zm00001d016649_T001	0.03976502	2.295644895	Zm00001d016649
Zm00001d053376_T007	6.12E-10	2.013123316	Zm00001d053376
Zm00001d034490_T003	0.04192531	-1.616632536	Zm00001d034490
Zm00001d024049_T013	7.34E-11	1.90297479	Zm00001d024049
Zm00001d043278_T002	0.011246	1.090382139	Zm00001d043278
Zm00001d010534_T009	3.81E-09	-4.675996427	Zm00001d010534
Zm00001d010534_T008	0.000421441	-3.836407583	Zm00001d010534
Zm00001d049558_T023	2.08E-12	-2.478613524	Zm00001d049558
Zm00001d029004_T107	0.002046598	1.340921769	Zm00001d029004
Zm00001d029004_T003	4.19E-11	1.512773803	Zm00001d029004
Zm00001d052416_T001	0.00995501	2.197680266	Zm00001d052416
Zm00001d035901_T001	3.54E-08	-2.241101555	Zm00001d035901
Zm00001d027667_T012	0.04399682	1.913737427	Zm00001d027667
Zm00001d027667_T007	0.008959327	3.129404492	Zm00001d027667
Zm00001d048020_T001	0.02433133	2.322193133	Zm00001d048020
Zm00001d048020_T002	0.003926032	3.181799653	Zm00001d048020
Zm00001d006037_T001	0.001885319	1.713110724	Zm00001d006037
Zm00001d046445_T001	0.00013768	-2.888804356	Zm00001d046445
Zm00001d030554_T010	0.01264374	1.02356414	Zm00001d030554
Zm00001d014129_T008	8.30E-05	3.055356013	Zm00001d014129
Zm00001d014129_T007	0.01600863	2.760328379	Zm00001d014129
Zm00001d041064_T001	4.13E-12	-2.126967882	Zm00001d041064
Zm00001d042830_T001	0.02456118	2.306863513	Zm00001d042830
Zm00001d021810_T031	0.002179361	1.396380561	Zm00001d021810
Zm00001d021810_T041	0.04428578	-1.834668487	Zm00001d021810
Zm00001d021810_T034	4.49E-13	3.135091375	Zm00001d021810
Zm00001d021810_T022	0.006779326	-1.345925594	Zm00001d021810
Zm00001d047540_T003	0.001647574	1.443463246	Zm00001d047540
Zm00001d045392_T001	3.30E-08	-5.219929999	Zm00001d045392
Zm00001d045392_T003	8.28E-11	-3.606345335	Zm00001d045392
Zm00001d045392_T002	8.99E-14	-3.974106984	Zm00001d045392
Zm00001d005276_T012	0.01987076	-3.236979636	Zm00001d005276
Zm00001d033050_T001	1.61E-06	4.761803455	Zm00001d033050
Zm00001d034553_T010	0.01181493	1.847879644	Zm00001d034553
Zm00001d034553_T001	0.02454834	-1.891111882	Zm00001d034553
Zm00001d034553_T002	0.03122475	2.466814449	Zm00001d034553
Zm00001d003894_T004	0.01513042	2.237528875	Zm00001d003894
Zm00001d018328_T003	6.61E-12	-2.784049973	Zm00001d018328
Zm00001d013052_T001	0.02181402	1.753857183	Zm00001d013052
Zm00001d043797_T006	1.01E-08	1.214227436	Zm00001d043797
Zm00001d003793_T003	0.01092525	1.70970309	Zm00001d003793
Zm00001d032597_T001	0.01081734	-1.894478269	Zm00001d032597

Zm00001d016142_T001	1.42E-06	-3.35686376	Zm00001d016142
Zm00001d029504_T004	1.14E-05	-1.508053495	Zm00001d029504
Zm00001d023741_T008	0.03963874	2.66970099	Zm00001d023741
Zm00001d023741_T021	0.004393869	2.522215595	Zm00001d023741
Zm00001d026362_T003	0.03997268	2.157222691	Zm00001d026362
Zm00001d043504_T016	4.49E-05	1.041649237	Zm00001d043504
Zm00001d013985_T003	5.74E-05	1.37003372	Zm00001d013985
Zm00001d044405_T007	0.002844203	1.422267466	Zm00001d044405
Zm00001d040435_T004	0.02546503	-3.04467682	Zm00001d040435
Zm00001d039648_T001	0.008620609	-2.703134638	Zm00001d039648
Zm00001d052194_T001	0.04441583	-1.457733456	Zm00001d052194
Zm00001d039711_T001	0.005246483	3.110217758	Zm00001d039711
Zm00001d017591_T001	7.68E-08	4.550457438	Zm00001d017591
Zm00001d042416_T003	1.24E-13	2.732700493	Zm00001d042416
Zm00001d042416_T010	0.01214359	1.549440701	Zm00001d042416
Zm00001d042416_T014	0.01756654	1.332866371	Zm00001d042416
Zm00001d042416_T007	4.87E-10	1.54170489	Zm00001d042416
Zm00001d040278_T002	3.28E-10	-3.356418729	Zm00001d040278
Zm00001d013524_T006	0.02561248	1.902843412	Zm00001d013524
Zm00001d024324_T001	0.007698854	2.765151894	Zm00001d024324
Zm00001d035869_T030	0.001765786	-2.243015797	Zm00001d035869
Zm00001d005297_T002	1.19E-08	1.072546511	Zm00001d005297
Zm00001d005297_T012	0.01274653	1.226900725	Zm00001d005297
Zm00001d011618_T001	0.01442608	2.220407987	Zm00001d011618
Zm00001d014383_T001	0.002252959	-1.457295664	Zm00001d014383
Zm00001d035040_T003	4.20E-10	1.633388475	Zm00001d035040
Zm00001d044707_T001	0.04961518	1.755059265	Zm00001d044707
Zm00001d026390_T001	8.15E-06	-3.246889139	Zm00001d026390
Zm00001d011140_T004	0.001249106	1.311099334	Zm00001d011140
Zm00001d053068_T001	0.02166259	-2.642398533	Zm00001d053068
Zm00001d014524_T009	0.001685107	1.001119027	Zm00001d014524
Zm00001d023385_T001	0.02253332	-2.042665514	Zm00001d023385
Zm00001d034205_T001	0.01668756	2.832104403	Zm00001d034205
Zm00001d052840_T020	0.000776638	-1.665135388	Zm00001d052840
Zm00001d004384_T001	0.02081089	-2.117476128	Zm00001d004384
Zm00001d029106_T002	0.005246483	-2.782898612	Zm00001d029106
Zm00001d047690_T003	0.000461795	3.254184544	Zm00001d047690
Zm00001d027932_T001	2.40E-08	4.397744091	Zm00001d027932
Zm00001d036537_T009	3.15E-08	-1.485510087	Zm00001d036537
Zm00001d007955_T001	0.000286815	-3.453329443	Zm00001d007955
Zm00001d003817_T027	0.01943547	-2.109599226	Zm00001d003817
Zm00001d013367_T002	0.03198968	-1.179399045	Zm00001d013367
Zm00001d047663_T001	0.01067552	-1.995417731	Zm00001d047663
Zm00001d021062_T001	2.68E-05	4.476499777	Zm00001d021062
Zm00001d036949_T006	3.03E-09	2.478608645	Zm00001d036949
Zm00001d003865_T001	0.01316589	-2.27046916	Zm00001d003865
Zm00001d021624_T011	2.90E-11	1.510569833	Zm00001d021624
Zm00001d049186_T001	0.03819779	-1.661268255	Zm00001d049186
Zm00001d027511_T001	0.006469929	1.182645516	Zm00001d027511
Zm00001d043950_T001	0.0302166	-1.566643896	Zm00001d043950
Zm00001d006238_T005	0.02995182	1.899362469	Zm00001d006238
Zm00001d016634_T011	0.02770147	2.593553974	Zm00001d016634
Zm00001d016634_T010	1.59E-10	-2.487385093	Zm00001d016634
Zm00001d025111_T022	0.004516829	-1.161401845	Zm00001d025111

Zm00001d028332_T018	6.84E-12	2.420750404	Zm00001d028332
Zm00001d053579_T001	0.001584476	1.954569254	Zm00001d053579
Zm00001d043327_T015	2.95E-09	1.448082571	Zm00001d043327
Zm00001d004509_T001	0.000381868	-1.888977166	Zm00001d004509
Zm00001d037377_T011	6.55E-11	1.872413487	Zm00001d037377
Zm00001d037377_T010	1.01E-10	1.322436134	Zm00001d037377
Zm00001d008573_T001	0.01472471	2.700486944	Zm00001d008573
Zm00001d050218_T001	0.02716411	2.691768706	Zm00001d050218
Zm00001d017923_T001	0.02135948	-2.309963826	Zm00001d017923
Zm00001d039081_T006	0.02420233	-1.871123552	Zm00001d039081
Zm00001d039081_T003	0.01616159	-1.014424558	Zm00001d039081
Zm00001d044555_T017	0.04949101	1.315602882	Zm00001d044555
Zm00001d044555_T002	6.81E-12	2.428944059	Zm00001d044555
Zm00001d006360_T001	0.02013711	-2.101342167	Zm00001d006360
Zm00001d047020_T015	0.03122475	1.546798764	Zm00001d047020
Zm00001d047020_T010	0.01540714	1.017611217	Zm00001d047020
Zm00001d044433_T023	0.01571887	-1.275255608	Zm00001d044433
Zm00001d004881_T001	0.01858893	-2.410535601	Zm00001d004881
Zm00001d045021_T001	0.02307789	-1.808753926	Zm00001d045021
Zm00001d044599_T006	0.04052471	-1.45967688	Zm00001d044599
Zm00001d048612_T001	0.000482044	1.600334535	Zm00001d048612
Zm00001d047481_T004	0.003698888	-1.046717868	Zm00001d047481
Zm00001d023520_T009	9.34E-11	2.060184185	Zm00001d023520
Zm00001d039005_T005	4.13E-12	2.371528205	Zm00001d039005
Zm00001d023796_T040	0.000354328	1.14431249	Zm00001d023796
Zm00001d033389_T001	0.000116308	3.792327483	Zm00001d033389
Zm00001d043625_T004	3.15E-13	-4.296372391	Zm00001d043625
Zm00001d003110_T004	0.03477308	2.041247849	Zm00001d003110
Zm00001d044915_T009	3.27E-08	-1.416388757	Zm00001d044915
Zm00001d039421_T078	0.03950269	-1.26914487	Zm00001d039421
Zm00001d050284_T007	1.09E-06	1.099553134	Zm00001d050284
Zm00001d012161_T002	0.04650707	-1.318642706	Zm00001d012161
Zm00001d032172_T006	0.04288666	-1.15611312	Zm00001d032172
Zm00001d013989_T003	0.004945925	2.524253859	Zm00001d013989
Zm00001d013989_T029	0.000114717	1.107083639	Zm00001d013989
Zm00001d013989_T034	5.81E-05	-2.196823886	Zm00001d013989
Zm00001d043221_T001	0.0272103	1.90889645	Zm00001d043221
Zm00001d030304_T002	0.000983636	3.200025914	Zm00001d030304
Zm00001d038146_T009	0.000134356	2.920331655	Zm00001d038146
Zm00001d038146_T008	0.01286115	2.783934035	Zm00001d038146
Zm00001d002330_T018	1.16E-10	-1.89140988	Zm00001d002330
Zm00001d002330_T016	8.43E-08	-1.045786069	Zm00001d002330
Zm00001d050526_T015	0.000245188	2.216945867	Zm00001d050526
Zm00001d014422_T007	0.01026997	2.078182392	Zm00001d014422
Zm00001d014422_T025	0.001497347	2.86705304	Zm00001d014422
Zm00001d014422_T015	0.0429264	1.8689086	Zm00001d014422
Zm00001d046596_T001	0.009245366	-2.538325952	Zm00001d046596
Zm00001d036542_T001	3.17E-08	-2.985240139	Zm00001d036542
Zm00001d033817_T002	0.04088815	1.81623267	Zm00001d033817
Zm00001d033817_T011	0.04443519	1.724696018	Zm00001d033817
Zm00001d018060_T010	0.01554869	1.421620481	Zm00001d018060
Zm00001d018060_T003	0.02456964	2.376662393	Zm00001d018060
Zm00001d020340_T001	1.69E-06	-1.189069195	Zm00001d020340
Zm00001d040864_T007	0.01396436	1.952880453	Zm00001d040864

Zm00001d038829_T004	0.02012514	1.289358004	Zm00001d038829
Zm00001d037872_T003	8.61E-08	2.843042898	Zm00001d037872
Zm00001d045112_T001	0.006233737	2.191655419	Zm00001d045112
Zm00001d045112_T002	0.0169289	1.054689879	Zm00001d045112
Zm00001d022435_T001	0.000220934	3.099099712	Zm00001d022435
Zm00001d033347_T001	0.000323792	3.738860608	Zm00001d033347
Zm00001d018423_T004	0.003187703	2.742116704	Zm00001d018423
Zm00001d027558_T014	0.01624246	1.261008575	Zm00001d027558
Zm00001d000133_T011	0.001068952	2.871324084	Zm00001d000133
Zm00001d012333_T001	0.00014579	-1.88811063	Zm00001d012333
Zm00001d022286_T002	0.000430339	-2.017881346	Zm00001d022286
Zm00001d037408_T019	0.0116306	1.056764609	Zm00001d037408
Zm00001d015497_T012	0.001917423	1.244488911	Zm00001d015497
Zm00001d052453_T007	0.006179415	1.631573253	Zm00001d052453
Zm00001d014412_T001	0.001992364	3.292644474	Zm00001d014412
Zm00001d032615_T009	4.89E-07	-1.008574412	Zm00001d032615
Zm00001d026111_T087	0.001233524	-1.01942129	Zm00001d026111
Zm00001d054060_T001	0.007943133	3.081175125	Zm00001d054060
Zm00001d031927_T023	0.002960897	-2.144948177	Zm00001d031927
Zm00001d001883_T006	3.12E-11	-2.359537584	Zm00001d001883
Zm00001d043296_T003	0.009394971	-1.910159023	Zm00001d043296
Zm00001d043296_T001	0.02135292	2.445420602	Zm00001d043296
Zm00001d051964_T009	4.38E-10	1.373012486	Zm00001d051964
Zm00001d037507_T023	2.49E-08	-2.813558273	Zm00001d037507
Zm00001d018704_T001	0.000121509	-3.727588207	Zm00001d018704
Zm00001d015314_T011	7.35E-05	1.385677055	Zm00001d015314
Zm00001d007379_T001	0.005339206	-2.635598818	Zm00001d007379
Zm00001d024489_T001	0.04452364	-1.220652725	Zm00001d024489
Zm00001d032937_T001	0.003445404	-1.630233842	Zm00001d032937
Zm00001d012069_T001	0.03953689	2.225032359	Zm00001d012069
Zm00001d036361_T013	0.03525539	-1.165880622	Zm00001d036361
Zm00001d047506_T008	0.01446329	1.135670793	Zm00001d047506
Zm00001d029529_T001	0.003315541	3.298555722	Zm00001d029529
Zm00001d039120_T001	0.005992554	1.93907329	Zm00001d039120
Zm00001d014968_T009	0.04693522	1.118713613	Zm00001d014968
Zm00001d047378_T001	0.001427707	-2.14133414	Zm00001d047378
Zm00001d042641_T013	0.002602753	2.464669881	Zm00001d042641
Zm00001d042641_T005	6.18E-13	-3.61202615	Zm00001d042641
Zm00001d028075_T022	3.71E-10	1.922056228	Zm00001d028075
Zm00001d025855_T040	9.19E-11	1.503661272	Zm00001d025855
Zm00001d007962_T001	0.01650093	2.777328231	Zm00001d007962
Zm00001d020204_T002	0.02296522	1.323027632	Zm00001d020204
Zm00001d014193_T001	0.002796955	-1.501165971	Zm00001d014193
Zm00001d038071_T002	5.59E-08	2.197769302	Zm00001d038071
Zm00001d032956_T014	0.0330477	2.933165969	Zm00001d032956
Zm00001d017308_T056	0.001763861	2.417314573	Zm00001d017308
Zm00001d016836_T002	0.001122518	1.529586622	Zm00001d016836
Zm00001d016836_T003	6.39E-08	-1.116649194	Zm00001d016836
Zm00001d049457_T006	1.56E-10	-1.316247613	Zm00001d049457
Zm00001d049457_T025	2.08E-12	1.605189913	Zm00001d049457
Zm00001d052851_T022	3.23E-06	-1.714422692	Zm00001d052851
Zm00001d014781_T001	0.01743997	-2.21635623	Zm00001d014781
Zm00001d038925_T003	0.001329304	2.84381221	Zm00001d038925
Zm00001d038925_T001	6.23E-07	4.069207295	Zm00001d038925

Zm00001d030166_T012	0.002013217	1.887670539	Zm00001d030166
Zm00001d041055_T001	0.04237526	1.38259495	Zm00001d041055
Zm00001d017935_T001	0.02840871	-2.178853887	Zm00001d017935
Zm00001d020504_T001	0.004107355	3.576382178	Zm00001d020504
Zm00001d016922_T008	4.35E-05	-1.607752778	Zm00001d016922
Zm00001d005090_T023	2.17E-12	1.735420322	Zm00001d005090
Zm00001d005090_T017	1.11E-06	1.148992872	Zm00001d005090
Zm00001d034642_T001	1.93E-05	4.043567302	Zm00001d034642
Zm00001d053016_T001	0.000664899	-2.618145478	Zm00001d053016
Zm00001d027731_T002	0.04060048	-1.804401004	Zm00001d027731
Zm00001d014512_T001	0.01545054	1.247043474	Zm00001d014512
Zm00001d044703_T001	0.006217265	1.390331023	Zm00001d044703
Zm00001d018497_T007	9.23E-08	1.217857642	Zm00001d018497
Zm00001d039865_T003	0.003567712	-2.536151167	Zm00001d039865
Zm00001d027480_T003	0.0002223	-2.200932665	Zm00001d027480
Zm00001d044327_T002	0.000929043	3.451517333	Zm00001d044327
Zm00001d044327_T001	8.15E-06	1.437359541	Zm00001d044327
Zm00001d044327_T003	0.001657253	3.076294794	Zm00001d044327
Zm00001d026262_T001	0.01932412	-2.133162639	Zm00001d026262
Zm00001d011096_T011	0.0101257	1.985592411	Zm00001d011096
Zm00001d036608_T016	0.00309982	-2.44130688	Zm00001d036608
Zm00001d039893_T002	0.02177405	2.647677642	Zm00001d039893
Zm00001d012527_T001	0.02787271	2.564197151	Zm00001d012527
Zm00001d026137_T002	0.01534331	-1.961135997	Zm00001d026137
Zm00001d042673_T001	0.00692208	3.053923645	Zm00001d042673
Zm00001d024735_T001	0.03178132	-1.63419575	Zm00001d024735
Zm00001d048575_T016	0.0217161	2.252560794	Zm00001d048575
Zm00001d048575_T018	0.02978293	1.425679194	Zm00001d048575
Zm00001d005303_T002	0.001189626	-2.658486467	Zm00001d005303
Zm00001d018786_T003	0.00713124	1.166905465	Zm00001d018786
Zm00001d018786_T002	0.01192573	2.649169522	Zm00001d018786
Zm00001d006030_T004	1.30E-07	1.102697115	Zm00001d006030
Zm00001d025739_T026	1.11E-10	-1.915224353	Zm00001d025739
Zm00001d025739_T031	0.02426701	2.199707381	Zm00001d025739
Zm00001d021700_T001	0.0462844	-1.831855381	Zm00001d021700
Zm00001d052208_T001	0.01391558	-1.337060061	Zm00001d052208
Zm00001d014669_T009	2.45E-18	-5.020039584	Zm00001d014669
Zm00001d053672_T007	0.004986224	2.526086825	Zm00001d053672
Zm00001d053672_T002	0.01014068	1.126993399	Zm00001d053672
Zm00001d037769_T001	0.01232763	-1.707719382	Zm00001d037769
Zm00001d023795_T006	0.000208118	2.196658104	Zm00001d023795
Zm00001d023795_T005	2.07E-14	2.637783451	Zm00001d023795
Zm00001d023795_T003	0.01073087	-1.294326145	Zm00001d023795
Zm00001d023795_T030	1.09E-16	3.895822938	Zm00001d023795
Zm00001d053119_T006	0.007967323	-1.186544699	Zm00001d053119
Zm00001d007765_T001	0.009139704	-2.25648869	Zm00001d007765
Zm00001d014804_T001	0.003617779	3.159899897	Zm00001d014804
Zm00001d028560_T034	9.93E-11	2.95330377	Zm00001d028560
Zm00001d028560_T011	0.003015693	2.062448057	Zm00001d028560
Zm00001d038780_T001	8.08E-10	5.479005914	Zm00001d038780
Zm00001d015665_T004	6.12E-08	1.253136259	Zm00001d015665
Zm00001d030239_T012	5.23E-05	1.751092218	Zm00001d030239
Zm00001d032458_T001	3.29E-05	2.583371968	Zm00001d032458
Zm00001d022481_T024	4.20E-10	1.775835759	Zm00001d022481

Zm00001d022481_T019	1.72E-05	1.054828566	Zm00001d022481
Zm00001d022042_T002	5.40E-05	-2.843770353	Zm00001d022042
Zm00001d022042_T006	0.000140887	-1.250985242	Zm00001d022042
Zm00001d032659_T006	0.02676713	1.297403706	Zm00001d032659
Zm00001d026657_T001	0.001106817	2.681490317	Zm00001d026657
Zm00001d021773_T001	0.000718937	-1.887641271	Zm00001d021773
Zm00001d006507_T003	0.03337908	1.616311944	Zm00001d006507
Zm00001d034673_T001	0.03238748	-1.815589689	Zm00001d034673
Zm00001d016136_T001	0.03059728	-1.948366925	Zm00001d016136
Zm00001d038819_T012	2.34E-10	1.10570903	Zm00001d038819
Zm00001d042943_T004	0.001274277	-3.125803995	Zm00001d042943
Zm00001d019547_T005	0.02223345	1.299825324	Zm00001d019547
Zm00001d038016_T001	0.02530813	-2.133716251	Zm00001d038016
Zm00001d021000_T046	2.76E-06	-2.779292067	Zm00001d021000
Zm00001d021000_T022	5.57E-15	3.030206149	Zm00001d021000
Zm00001d021000_T019	0.0168613	1.271650746	Zm00001d021000
Zm00001d021000_T009	4.18E-05	1.865027996	Zm00001d021000
Zm00001d021000_T060	0.005144896	1.363369615	Zm00001d021000
Zm00001d042185_T013	0.01306269	3.200845811	Zm00001d042185
Zm00001d042185_T010	6.90E-05	-2.131896774	Zm00001d042185
Zm00001d053286_T002	2.43E-13	-3.264809317	Zm00001d053286
Zm00001d003707_T001	0.02739288	-1.230578282	Zm00001d003707
Zm00001d029422_T002	0.04870749	1.158579573	Zm00001d029422
Zm00001d029422_T023	0.002718262	1.050098198	Zm00001d029422
Zm00001d044614_T032	2.86E-15	3.159951849	Zm00001d044614
Zm00001d053173_T002	7.04E-07	-2.500269017	Zm00001d053173
Zm00001d042724_T006	0.0357509	1.969846759	Zm00001d042724
Zm00001d013862_T002	0.005374177	-2.026936239	Zm00001d013862
Zm00001d042869_T004	0.000191103	1.338329753	Zm00001d042869
Zm00001d042869_T002	0.000166336	2.089236879	Zm00001d042869
Zm00001d025015_T004	0.000589094	2.927684601	Zm00001d025015
Zm00001d025015_T002	0.006295126	2.81183472	Zm00001d025015
Zm00001d025015_T001	0.004711399	3.214814661	Zm00001d025015
Zm00001d025015_T003	1.96E-07	4.688521825	Zm00001d025015
Zm00001d032811_T001	0.04996583	-1.042425769	Zm00001d032811
Zm00001d005831_T001	0.000627005	-1.841038413	Zm00001d005831
Zm00001d020622_T001	0.00026557	2.649782187	Zm00001d020622
Zm00001d048347_T001	0.04419305	-1.156597843	Zm00001d048347
Zm00001d040312_T001	3.97E-10	1.586211191	Zm00001d040312
Zm00001d029324_T010	5.91E-12	3.708496176	Zm00001d029324
Zm00001d029324_T006	6.25E-13	-3.911510783	Zm00001d029324
Zm00001d031807_T007	3.74E-05	3.538587345	Zm00001d031807
Zm00001d031807_T005	0.000102984	3.484505568	Zm00001d031807
Zm00001d031807_T006	0.00150981	1.967111916	Zm00001d031807
Zm00001d031807_T002	3.70E-05	2.348936056	Zm00001d031807
Zm00001d053412_T001	0.01175516	-1.545081779	Zm00001d053412
Zm00001d053412_T002	1.24E-08	1.622107414	Zm00001d053412
Zm00001d030133_T001	0.003167632	1.098214083	Zm00001d030133
Zm00001d030133_T005	2.41E-09	1.441741103	Zm00001d030133
Zm00001d021875_T001	0.02680286	1.712355829	Zm00001d021875
Zm00001d019453_T001	0.00080775	-2.194594986	Zm00001d019453
Zm00001d021181_T004	0.002266636	2.455060716	Zm00001d021181
Zm00001d021181_T003	0.01110241	1.019796529	Zm00001d021181
Zm00001d022405_T051	0.00952161	1.298570217	Zm00001d022405

Zm00001d022405_T041	0.01458755	1.656644544	Zm00001d022405
Zm00001d026412_T001	0.003580178	-2.437624618	Zm00001d026412
Zm00001d018868_T001	0.0324581	2.491823035	Zm00001d018868
Zm00001d032708_T013	1.79E-09	-1.370094576	Zm00001d032708
Zm00001d032708_T007	1.16E-10	1.811369685	Zm00001d032708
Zm00001d019587_T011	6.32E-09	-2.333542977	Zm00001d019587
Zm00001d005108_T001	0.00011357	3.053411394	Zm00001d005108
Zm00001d043153_T002	0.01282754	-1.839948654	Zm00001d043153
Zm00001d009083_T001	0.008815229	-2.389249821	Zm00001d009083
Zm00001d041582_T005	6.96E-12	2.053839764	Zm00001d041582
Zm00001d028966_T001	0.000254437	1.072040463	Zm00001d028966
Zm00001d028966_T007	0.001899796	2.863330809	Zm00001d028966
Zm00001d028690_T007	0.001433828	2.077649212	Zm00001d028690
Zm00001d007407_T042	3.23E-11	2.284669061	Zm00001d007407
Zm00001d012643_T004	2.96E-11	-2.681669938	Zm00001d012643
Zm00001d004630_T001	0.0295443	1.999824864	Zm00001d004630
Zm00001d043742_T001	3.38E-05	4.25528074	Zm00001d043742
Zm00001d013391_T017	0.03748728	2.069328264	Zm00001d013391
Zm00001d031422_T009	0.002719971	2.130593348	Zm00001d031422
Zm00001d003401_T002	4.02E-08	-1.452039601	Zm00001d003401
Zm00001d021522_T010	0.04486856	-1.228323676	Zm00001d021522
Zm00001d021522_T001	0.003337531	2.392204282	Zm00001d021522
Zm00001d049125_T023	0.03648535	-1.43346046	Zm00001d049125
Zm00001d049125_T005	5.29E-08	2.985745981	Zm00001d049125
Zm00001d027854_T002	0.04241983	-1.400129258	Zm00001d027854
Zm00001d005573_T002	0.03255668	-1.658370292	Zm00001d005573
Zm00001d042028_T008	4.87E-05	1.786944651	Zm00001d042028
Zm00001d053675_T002	1.09E-06	-1.188579756	Zm00001d053675
Zm00001d033297_T011	0.009370386	2.266877438	Zm00001d033297
Zm00001d033297_T001	0.02765694	1.368653682	Zm00001d033297
Zm00001d033297_T006	0.00012849	1.183388055	Zm00001d033297
Zm00001d020685_T011	0.02244514	2.153713445	Zm00001d020685
Zm00001d020685_T004	0.008894986	1.800399145	Zm00001d020685
Zm00001d031289_T003	1.83E-06	1.800293505	Zm00001d031289
Zm00001d044246_T004	0.000921162	-2.174009683	Zm00001d044246
Zm00001d009187_T001	0.03447641	2.457787137	Zm00001d009187
Zm00001d042593_T001	9.88E-06	4.040552811	Zm00001d042593
Zm00001d048409_T006	3.51E-09	1.593599849	Zm00001d048409
Zm00001d018111_T006	5.63E-07	-2.259335802	Zm00001d018111
Zm00001d018111_T005	1.69E-07	2.716866271	Zm00001d018111
Zm00001d018111_T001	0.0394043	1.138256999	Zm00001d018111
Zm00001d036785_T003	0.008259017	1.917996505	Zm00001d036785
Zm00001d045046_T002	6.18E-08	3.638838916	Zm00001d045046
Zm00001d045046_T001	0.00247968	3.360087724	Zm00001d045046
Zm00001d036334_T012	6.11E-09	-1.000709405	Zm00001d036334
Zm00001d036334_T008	0.000636652	-2.03379429	Zm00001d036334
Zm00001d036334_T023	1.86E-11	2.494074763	Zm00001d036334
Zm00001d035695_T005	0.000839403	1.672699967	Zm00001d035695
Zm00001d049379_T016	5.42E-10	-1.581386966	Zm00001d049379
Zm00001d049379_T013	0.04052471	1.032114539	Zm00001d049379
Zm00001d018046_T021	0.04315095	-1.093056173	Zm00001d018046
Zm00001d033049_T001	0.009025441	2.418842051	Zm00001d033049
Zm00001d014895_T002	0.003561752	1.16409673	Zm00001d014895
Zm00001d016675_T016	1.04E-10	1.197555716	Zm00001d016675

Zm00001d042718_T007	0.000626649	2.110637061	Zm00001d042718
Zm00001d009771_T005	9.26E-12	2.442315256	Zm00001d009771
Zm00001d038984_T001	0.02320887	-1.701466605	Zm00001d038984
Zm00001d046494_T001	0.01025653	2.459687953	Zm00001d046494
Zm00001d030518_T029	0.02342941	1.438817011	Zm00001d030518
Zm00001d034757_T011	0.04461803	-1.702106221	Zm00001d034757
Zm00001d014887_T003	3.01E-12	-4.107834761	Zm00001d014887
Zm00001d029667_T001	0.01854344	2.75920676	Zm00001d029667
Zm00001d027340_T010	1.89E-09	-2.393196123	Zm00001d027340
Zm00001d037877_T012	7.65E-05	-1.300119154	Zm00001d037877
Zm00001d037877_T010	0.009025301	-1.672966058	Zm00001d037877
Zm00001d013267_T045	1.58E-09	-2.141883668	Zm00001d013267
Zm00001d013267_T013	0.03877621	1.79603324	Zm00001d013267
Zm00001d047079_T001	0.02334998	1.789685688	Zm00001d047079
Zm00001d047079_T003	0.005006452	-1.53932535	Zm00001d047079
Zm00001d047079_T007	2.49E-08	1.001733201	Zm00001d047079
Zm00001d037019_T001	0.04891776	-1.982724868	Zm00001d037019
Zm00001d036737_T001	0.04509263	2.707057794	Zm00001d036737
Zm00001d016719_T001	2.34E-05	4.103640905	Zm00001d016719
Zm00001d004125_T167	0.000558786	2.469947562	Zm00001d004125
Zm00001d004125_T111	0.03415667	-1.917297563	Zm00001d004125
Zm00001d004125_T174	1.82E-11	-1.33291471	Zm00001d004125
Zm00001d004125_T098	0.01063379	1.844411907	Zm00001d004125
Zm00001d004125_T183	5.06E-14	2.894848227	Zm00001d004125
Zm00001d004125_T036	8.17E-14	2.395246192	Zm00001d004125
Zm00001d032198_T009	0.02062193	-1.998775305	Zm00001d032198
Zm00001d014114_T002	3.22E-10	-1.665219369	Zm00001d014114
Zm00001d039526_T006	0.0454728	1.596786503	Zm00001d039526
Zm00001d042116_T001	0.001651493	3.164744812	Zm00001d042116
Zm00001d034810_T005	9.65E-08	1.179490767	Zm00001d034810
Zm00001d003504_T006	0.000444844	3.262548625	Zm00001d003504
Zm00001d003228_T001	0.04725213	2.370830896	Zm00001d003228
Zm00001d002433_T004	0.006165798	-2.988855988	Zm00001d002433
Zm00001d022436_T007	0.004276488	1.133661975	Zm00001d022436
Zm00001d038808_T015	0.01817166	-1.560973569	Zm00001d038808
Zm00001d038808_T013	0.000294248	-1.519202567	Zm00001d038808
Zm00001d010227_T002	6.57E-05	-1.005166155	Zm00001d010227
Zm00001d020614_T001	0.000189901	3.672222162	Zm00001d020614
Zm00001d020614_T003	1.24E-06	2.478304835	Zm00001d020614
Zm00001d053997_T005	2.50E-11	1.57678616	Zm00001d053997
Zm00001d033984_T006	0.000542957	1.254446415	Zm00001d033984
Zm00001d033984_T007	0.001032461	2.229326094	Zm00001d033984
Zm00001d042975_T002	2.67E-05	1.123227473	Zm00001d042975
Zm00001d042975_T006	0.000149144	1.868856603	Zm00001d042975
Zm00001d016417_T001	0.0367711	-1.243647066	Zm00001d016417
Zm00001d048689_T064	2.97E-12	3.106261429	Zm00001d048689
Zm00001d035609_T001	8.52E-07	5.022307508	Zm00001d035609
Zm00001d005783_T002	0.00707939	2.21524833	Zm00001d005783
Zm00001d013547_T004	0.006217153	-2.916799407	Zm00001d013547
Zm00001d020201_T003	2.16E-07	-2.870299976	Zm00001d020201
Zm00001d022343_T009	0.001852903	1.274953074	Zm00001d022343
Zm00001d053813_T023	0.006647656	2.345252161	Zm00001d053813
Zm00001d053813_T005	0.03022008	1.694621307	Zm00001d053813
Zm00001d010678_T005	0.001624144	1.067735948	Zm00001d010678

Zm00001d023640_T010	0.01668756	1.875866004	Zm00001d023640
Zm00001d023640_T007	6.01E-15	-3.778791739	Zm00001d023640
Zm00001d010012_T001	0.04092962	-1.126540393	Zm00001d010012
Zm00001d041511_T005	0.003609401	1.989956542	Zm00001d041511
Zm00001d030888_T002	0.02669321	-1.853191349	Zm00001d030888
Zm00001d017597_T001	0.006224998	1.637331956	Zm00001d017597
Zm00001d029530_T001	0.009175406	-1.873786165	Zm00001d029530
Zm00001d027259_T001	0.02127052	-1.238756839	Zm00001d027259
Zm00001d022461_T001	7.96E-07	-3.871460104	Zm00001d022461
Zm00001d049061_T001	0.005375707	2.575140803	Zm00001d049061
Zm00001d038587_T010	0.000956301	1.376771722	Zm00001d038587
Zm00001d038587_T032	5.34E-08	1.395783957	Zm00001d038587
Zm00001d038587_T013	0.000110374	1.062677613	Zm00001d038587
Zm00001d028474_T019	5.99E-05	2.111836136	Zm00001d028474
Zm00001d042665_T001	0.03024113	2.426386753	Zm00001d042665
Zm00001d028034_T036	0.04663752	-1.539217821	Zm00001d028034
Zm00001d040477_T006	2.21E-07	-2.700903033	Zm00001d040477
Zm00001d050238_T010	0.006644508	-1.437989588	Zm00001d050238
Zm00001d012212_T001	0.03735116	2.001741227	Zm00001d012212
Zm00001d047897_T002	3.47E-05	2.969950584	Zm00001d047897
Zm00001d005627_T005	0.000923222	-1.321961599	Zm00001d005627
Zm00001d048343_T003	1.92E-06	-2.692361939	Zm00001d048343
Zm00001d002715_T007	0.000758895	1.699117669	Zm00001d002715
Zm00001d002715_T001	0.004520213	1.179029977	Zm00001d002715
Zm00001d043238_T004	0.0365566	2.501460742	Zm00001d043238
Zm00001d010556_T002	0.00265608	1.398419873	Zm00001d010556
Zm00001d041092_T007	0.005206629	1.795731612	Zm00001d041092
Zm00001d021208_T001	3.24E-07	5.006220047	Zm00001d021208
Zm00001d029887_T001	0.003835735	-2.660703256	Zm00001d029887
Zm00001d018737_T001	1.87E-05	-4.105132729	Zm00001d018737
Zm00001d006711_T002	0.00090062	2.987380459	Zm00001d006711
Zm00001d052545_T001	0.02355239	-2.172984927	Zm00001d052545
Zm00001d011130_T003	0.01146315	-1.578302706	Zm00001d011130
Zm00001d003713_T001	0.03045399	1.168104938	Zm00001d003713
Zm00001d052308_T069	0.002379492	2.544328654	Zm00001d052308
Zm00001d052387_T001	0.01491102	-1.879291723	Zm00001d052387
Zm00001d032999_T001	0.02961878	-1.793259381	Zm00001d032999
Zm00001d053935_T047	0.002351675	1.827820046	Zm00001d053935
Zm00001d053935_T015	0.02506163	-1.94090879	Zm00001d053935
Zm00001d053935_T020	0.003578926	1.023461585	Zm00001d053935
Zm00001d038396_T004	0.001092672	1.174749943	Zm00001d038396
Zm00001d025100_T001	0.000698094	-1.879588605	Zm00001d025100
Zm00001d039611_T002	0.03403373	-1.178779143	Zm00001d039611
Zm00001d034721_T001	0.04894618	1.774957044	Zm00001d034721
Zm00001d021816_T001	0.000130794	3.770951681	Zm00001d021816
Zm00001d035542_T002	0.03077473	2.537518558	Zm00001d035542
Zm00001d035542_T001	1.08E-09	3.100540205	Zm00001d035542
Zm00001d020943_T002	1.75E-09	-2.521625186	Zm00001d020943
Zm00001d030051_T002	8.03E-15	-2.589691883	Zm00001d030051
Zm00001d017787_T002	1.16E-11	-2.823176754	Zm00001d017787
Zm00001d046420_T003	1.31E-08	-1.431703697	Zm00001d046420
Zm00001d018161_T002	3.79E-05	4.324818732	Zm00001d018161
Zm00001d018161_T001	0.000110936	4.16413809	Zm00001d018161
Zm00001d008763_T027	6.14E-10	-1.842824161	Zm00001d008763

Zm00001d051690_T006	1.57E-08	1.45006679	Zm00001d051690
Zm00001d029738_T001	0.002899964	3.10224006	Zm00001d029738
Zm00001d041453_T001	0.02309348	2.052213401	Zm00001d041453
Zm00001d010866_T005	0.004390741	1.014883056	Zm00001d010866
Zm00001d010751_T001	0.000992792	-3.033979296	Zm00001d010751
Zm00001d048263_T001	2.02E-06	4.760257997	Zm00001d048263
Zm00001d023303_T007	3.21E-09	1.218041118	Zm00001d023303
Zm00001d038368_T002	0.003297593	-1.693928695	Zm00001d038368
Zm00001d005241_T004	0.00965634	1.72770061	Zm00001d005241
Zm00001d038374_T004	9.92E-05	-3.014655676	Zm00001d038374
Zm00001d021861_T003	0.004516855	1.349156698	Zm00001d021861
Zm00001d047977_T002	0.001169412	-2.293858153	Zm00001d047977
Zm00001d053251_T041	4.04E-06	-1.37119431	Zm00001d053251
Zm00001d027803_T010	4.64E-11	-3.319252935	Zm00001d027803
Zm00001d028497_T001	7.23E-06	1.621520317	Zm00001d028497
Zm00001d009985_T001	2.01E-05	4.150949193	Zm00001d009985
Zm00001d014297_T010	0.01725995	1.96816162	Zm00001d014297
Zm00001d043044_T001	0.01979855	2.35947724	Zm00001d043044
Zm00001d017809_T005	0.02620309	1.688882623	Zm00001d017809
Zm00001d021839_T001	0.04926744	2.510498584	Zm00001d021839
Zm00001d002149_T007	2.66E-11	2.619269133	Zm00001d002149
Zm00001d002149_T005	2.38E-11	-1.137649329	Zm00001d002149
Zm00001d002149_T004	2.71E-09	-1.287614545	Zm00001d002149
Zm00001d039769_T009	0.03222907	2.561719455	Zm00001d039769
Zm00001d039769_T002	0.01318742	1.364468778	Zm00001d039769
Zm00001d033827_T006	3.55E-11	-1.558615933	Zm00001d033827
Zm00001d010720_T008	0.002235212	1.330397589	Zm00001d010720
Zm00001d010720_T005	6.01E-13	3.401218833	Zm00001d010720
Zm00001d037354_T003	0.000653632	1.352625688	Zm00001d037354
Zm00001d021647_T003	0.005868241	1.968563766	Zm00001d021647
Zm00001d014494_T001	1.34E-05	-4.495290415	Zm00001d014494
Zm00001d030659_T006	0.0002213	1.726208929	Zm00001d030659
Zm00001d007943_T009	0.002048092	1.626749235	Zm00001d007943
Zm00001d029046_T015	0.002315768	-1.634053636	Zm00001d029046
Zm00001d029046_T012	2.38E-11	1.880230202	Zm00001d029046
Zm00001d002615_T001	1.22E-09	-2.639227311	Zm00001d002615
Zm00001d031909_T003	3.20E-05	-3.466649355	Zm00001d031909
Zm00001d033364_T074	8.28E-11	1.358010638	Zm00001d033364
Zm00001d033364_T098	0.01492646	-1.558995549	Zm00001d033364
Zm00001d018481_T059	0.006654326	1.889033382	Zm00001d018481
Zm00001d016326_T007	2.20E-10	1.528509969	Zm00001d016326
Zm00001d043502_T003	0.004536195	1.267895507	Zm00001d043502
Zm00001d031657_T003	0.000476965	-2.305778578	Zm00001d031657
Zm00001d023242_T001	0.02249061	-2.172290654	Zm00001d023242
Zm00001d024865_T001	0.01594071	1.927968811	Zm00001d024865
Zm00001d019926_T005	0.000134356	-3.819154076	Zm00001d019926
Zm00001d019926_T001	0.01588722	-1.519135674	Zm00001d019926
Zm00001d037435_T001	0.02010279	2.714225955	Zm00001d037435
Zm00001d028269_T004	0.01817166	1.505850591	Zm00001d028269
Zm00001d008306_T002	0.01849552	1.000534529	Zm00001d008306
Zm00001d008306_T006	0.02919597	1.591654711	Zm00001d008306
Zm00001d014159_T029	0.04422979	1.780360413	Zm00001d014159
Zm00001d014159_T004	5.87E-15	2.525033705	Zm00001d014159
Zm00001d014159_T020	2.17E-10	1.010648408	Zm00001d014159

Zm00001d000001_T001	0.0297118	-2.241570177	Zm00001d000001
Zm00001d042898_T001	0.0398238	1.709640546	Zm00001d042898
Zm00001d008640_T005	0.00184107	-1.504685373	Zm00001d008640
Zm00001d052295_T001	0.001129598	1.214269416	Zm00001d052295
Zm00001d048876_T018	0.000922375	1.168810397	Zm00001d048876
Zm00001d024745_T001	0.0464256	2.468183773	Zm00001d024745
Zm00001d021728_T026	4.43E-05	2.27312336	Zm00001d021728
Zm00001d021728_T054	7.79E-11	2.08575199	Zm00001d021728
Zm00001d045390_T002	3.30E-09	-5.500899139	Zm00001d045390
Zm00001d046939_T004	0.000626649	1.767956559	Zm00001d046939
Zm00001d046939_T002	0.04761544	2.468860256	Zm00001d046939
Zm00001d034364_T003	0.01286819	1.141448779	Zm00001d034364
Zm00001d004771_T002	0.0117398	-1.380083866	Zm00001d004771
Zm00001d053843_T001	5.00E-05	-3.888582559	Zm00001d053843
Zm00001d018226_T011	0.003232376	-1.514171023	Zm00001d018226
Zm00001d048390_T001	0.01786886	-1.432575449	Zm00001d048390
Zm00001d010324_T004	0.000333288	1.139504136	Zm00001d010324
Zm00001d005231_T006	7.01E-05	-1.448284347	Zm00001d005231
Zm00001d008294_T004	1.75E-07	1.137482435	Zm00001d008294
Zm00001d001901_T003	0.002796955	-2.704349343	Zm00001d001901
Zm00001d045192_T016	6.40E-06	-2.288898687	Zm00001d045192
Zm00001d045192_T022	0.02546853	2.001915438	Zm00001d045192
Zm00001d032532_T001	2.68E-05	4.190672095	Zm00001d032532
Zm00001d037448_T015	0.03175747	-2.16626705	Zm00001d037448
Zm00001d038432_T007	1.63E-13	2.836731344	Zm00001d038432
Zm00001d038432_T006	0.001574318	2.149293827	Zm00001d038432
Zm00001d038432_T005	0.03797568	1.475395491	Zm00001d038432
Zm00001d013075_T003	0.01763173	-1.034945215	Zm00001d013075
Zm00001d017707_T005	0.02195526	-1.766614292	Zm00001d017707
Zm00001d047240_T002	0.02815838	2.656178099	Zm00001d047240
Zm00001d000080_T005	0.003015693	-2.278667279	Zm00001d000080
Zm00001d029438_T001	0.02165739	-2.380252517	Zm00001d029438
Zm00001d046509_T004	0.000166376	-2.173122957	Zm00001d046509
Zm00001d028651_T001	0.00947218	-2.406191127	Zm00001d028651
Zm00001d001895_T008	0.000545893	-3.432866238	Zm00001d001895
Zm00001d022132_T008	0.01699508	1.035076625	Zm00001d022132
Zm00001d022132_T001	2.84E-05	1.035084977	Zm00001d022132
Zm00001d022132_T009	2.95E-06	2.545111279	Zm00001d022132
Zm00001d022132_T011	3.77E-07	1.104601725	Zm00001d022132
Zm00001d022132_T014	0.04017497	2.052976383	Zm00001d022132
Zm00001d034073_T001	0.01458755	2.463400214	Zm00001d034073
Zm00001d048785_T002	0.01436987	-1.832975407	Zm00001d048785
Zm00001d048785_T001	0.03712122	-1.648878577	Zm00001d048785
Zm00001d048785_T004	0.01557736	-1.764317782	Zm00001d048785
Zm00001d040651_T001	0.005075492	-2.453801949	Zm00001d040651
Zm00001d040651_T002	0.008985864	-2.434297587	Zm00001d040651
Zm00001d043356_T006	0.04491439	1.39787673	Zm00001d043356
Zm00001d043356_T007	0.01912254	1.691745146	Zm00001d043356
Zm00001d053633_T003	0.001695442	-1.587164452	Zm00001d053633
Zm00001d047574_T002	6.42E-12	2.253011165	Zm00001d047574
Zm00001d022249_T001	0.000306739	-3.41588821	Zm00001d022249
Zm00001d031127_T001	0.007136392	-2.332340488	Zm00001d031127
Zm00001d031127_T002	5.48E-12	-3.930807307	Zm00001d031127
Zm00001d048307_T001	0.000275609	-2.054238164	Zm00001d048307

Zm00001d023797_T003	0.01881593	1.697041236	Zm00001d023797
Zm00001d009727_T009	0.006436882	-1.527068446	Zm00001d009727
Zm00001d051223_T001	0.01509671	-2.007352514	Zm00001d051223
Zm00001d001834_T029	0.02498409	2.850336093	Zm00001d001834
Zm00001d025024_T004	1.24E-13	2.468758865	Zm00001d025024
Zm00001d006911_T001	8.76E-07	4.096603289	Zm00001d006911
Zm00001d008329_T001	0.000713459	3.561620585	Zm00001d008329
Zm00001d051473_T001	0.003084239	-2.761937282	Zm00001d051473
Zm00001d037119_T001	0.002636913	1.596018422	Zm00001d037119
Zm00001d040831_T009	4.37E-09	1.218757628	Zm00001d040831
Zm00001d043805_T007	0.000627005	1.663786986	Zm00001d043805
Zm00001d043805_T002	0.000370571	1.92420745	Zm00001d043805
Zm00001d033846_T001	0.004684965	-2.566585579	Zm00001d033846
Zm00001d018792_T001	0.004041229	2.019297443	Zm00001d018792
Zm00001d018792_T004	7.91E-11	-1.756023274	Zm00001d018792
Zm00001d006350_T006	0.01324222	1.409429341	Zm00001d006350
Zm00001d006350_T008	8.82E-06	-1.865586776	Zm00001d006350
Zm00001d015100_T002	0.02294915	2.621198056	Zm00001d015100
Zm00001d015100_T001	0.009579325	2.990067978	Zm00001d015100
Zm00001d017992_T001	0.02362056	2.454897179	Zm00001d017992
Zm00001d031536_T001	0.04601184	2.154355009	Zm00001d031536
Zm00001d051644_T017	6.59E-13	2.365524164	Zm00001d051644
Zm00001d003048_T001	0.003641959	2.223856917	Zm00001d003048
Zm00001d023706_T014	0.04000108	-1.647203166	Zm00001d023706
Zm00001d009103_T001	0.0039243	-2.693785044	Zm00001d009103
Zm00001d010136_T001	1.69E-06	4.472270429	Zm00001d010136
Zm00001d043653_T015	0.003128451	1.265851399	Zm00001d043653
Zm00001d025232_T006	0.01886424	-1.627356016	Zm00001d025232
Zm00001d045097_T002	1.11E-10	-1.202944107	Zm00001d045097
Zm00001d045097_T001	8.63E-05	1.807785314	Zm00001d045097
Zm00001d029711_T001	0.000712678	2.824322323	Zm00001d029711
Zm00001d044688_T001	0.00071555	3.385612717	Zm00001d044688
Zm00001d046089_T001	0.02827671	-1.934030224	Zm00001d046089
Zm00001d001848_T014	4.75E-12	1.417395906	Zm00001d001848
Zm00001d001848_T024	5.87E-06	1.057284782	Zm00001d001848
Zm00001d001848_T008	0.02206058	2.270820573	Zm00001d001848
Zm00001d001848_T003	4.75E-07	1.579342103	Zm00001d001848
Zm00001d014112_T017	2.70E-08	-2.364542614	Zm00001d014112
Zm00001d035844_T019	7.82E-10	1.211055095	Zm00001d035844
Zm00001d053092_T019	3.91E-09	1.492327875	Zm00001d053092
Zm00001d043829_T001	0.000299493	1.116506205	Zm00001d043829
Zm00001d022518_T001	9.31E-05	-1.542224199	Zm00001d022518
Zm00001d014701_T004	0.000386695	2.639747823	Zm00001d014701
Zm00001d014701_T006	0.01955777	2.383103373	Zm00001d014701
Zm00001d034964_T015	2.68E-05	1.80894957	Zm00001d034964
Zm00001d052233_T003	6.65E-05	1.058192278	Zm00001d052233
Zm00001d048655_T005	2.82E-13	-3.103238198	Zm00001d048655
Zm00001d048655_T008	4.15E-13	3.415594607	Zm00001d048655
Zm00001d048655_T002	7.68E-06	-1.25427509	Zm00001d048655
Zm00001d028260_T003	1.51E-05	-2.397618664	Zm00001d028260
Zm00001d002623_T001	0.001488179	3.198498903	Zm00001d002623
Zm00001d048095_T017	0.000506808	-1.493657574	Zm00001d048095
Zm00001d048095_T005	0.005799023	1.710515584	Zm00001d048095
Zm00001d043845_T013	0.000840076	1.557222385	Zm00001d043845

Zm00001d043845_T076	3.93E-14	2.710824064	Zm00001d043845
Zm00001d002640_T050	3.27E-08	-2.192105276	Zm00001d002640
Zm00001d002640_T025	0.0020163	2.078691533	Zm00001d002640
Zm00001d048734_T002	8.84E-12	-1.98676188	Zm00001d048734
Zm00001d026485_T008	2.29E-09	1.225155102	Zm00001d026485
Zm00001d012100_T039	6.90E-12	-4.861525576	Zm00001d012100
Zm00001d038348_T005	0.008219785	2.142607361	Zm00001d038348
Zm00001d038348_T003	0.02456964	2.02847372	Zm00001d038348
Zm00001d038348_T004	0.04346091	2.007510469	Zm00001d038348
Zm00001d027678_T001	0.03950269	-1.893486268	Zm00001d027678
Zm00001d020140_T023	0.03059454	1.043001388	Zm00001d020140
Zm00001d034940_T053	0.04760415	1.794213537	Zm00001d034940
Zm00001d038326_T007	1.20E-11	-3.649392171	Zm00001d038326
Zm00001d037045_T020	2.73E-11	1.758685419	Zm00001d037045
Zm00001d015366_T002	0.004998066	-1.055787961	Zm00001d015366
Zm00001d005100_T081	1.56E-13	3.041482067	Zm00001d005100
Zm00001d005100_T074	0.004936444	-1.435851195	Zm00001d005100
Zm00001d005100_T020	0.005246483	1.138726572	Zm00001d005100
Zm00001d035570_T026	4.89E-07	-2.840026669	Zm00001d035570
Zm00001d022078_T001	0.009947905	2.631407818	Zm00001d022078
Zm00001d011901_T003	0.03978199	-1.376696081	Zm00001d011901
Zm00001d026591_T001	0.000593003	-1.873756608	Zm00001d026591
Zm00001d049269_T001	0.02098232	1.615605525	Zm00001d049269
Zm00001d053348_T002	0.04489514	1.058644525	Zm00001d053348
Zm00001d021609_T047	0.04063195	1.438957141	Zm00001d021609
Zm00001d011066_T005	0.01855432	2.748243064	Zm00001d011066
Zm00001d011066_T002	0.001935551	2.956398855	Zm00001d011066
Zm00001d011066_T001	0.000170611	3.468053723	Zm00001d011066
Zm00001d050893_T001	0.001189626	-1.624635798	Zm00001d050893
Zm00001d027907_T003	0.00919548	1.619472583	Zm00001d027907
Zm00001d038475_T004	1.37E-11	1.140418559	Zm00001d038475
Zm00001d018603_T004	0.01949981	2.085108525	Zm00001d018603
Zm00001d045635_T060	1.78E-10	1.192166879	Zm00001d045635
Zm00001d015670_T001	0.002451556	-2.578568577	Zm00001d015670
Zm00001d033543_T002	0.000577022	3.2863929	Zm00001d033543
Zm00001d033543_T001	0.006224998	2.791524774	Zm00001d033543
Zm00001d028207_T012	0.02342941	-2.069967448	Zm00001d028207
Zm00001d044358_T001	0.004998066	1.043259367	Zm00001d044358
Zm00001d006621_T005	0.000590857	1.125159181	Zm00001d006621
Zm00001d006621_T003	0.01193426	1.028834913	Zm00001d006621
Zm00001d006621_T004	0.00071786	2.723033984	Zm00001d006621
Zm00001d053566_T002	0.01952936	2.832851906	Zm00001d053566
Zm00001d020069_T002	0.02249061	2.560394484	Zm00001d020069
Zm00001d011041_T001	0.02001912	-1.725099749	Zm00001d011041
Zm00001d052803_T010	0.00281519	-3.517863891	Zm00001d052803
Zm00001d012720_T001	0.04516267	-2.148744714	Zm00001d012720
Zm00001d015279_T006	0.02955793	-1.887012506	Zm00001d015279
Zm00001d032854_T001	0.000463721	-1.902321448	Zm00001d032854
Zm00001d027317_T019	0.000279876	1.106353148	Zm00001d027317
Zm00001d047074_T018	0.001838611	1.113945445	Zm00001d047074
Zm00001d047074_T004	9.24E-05	1.019836963	Zm00001d047074
Zm00001d047074_T012	0.04949171	1.354023151	Zm00001d047074
Zm00001d052697_T009	5.27E-11	-2.539221952	Zm00001d052697
Zm00001d024807_T006	0.009284176	-1.694633802	Zm00001d024807

Zm00001d024807_T011	0.03956497	1.060681849	Zm00001d024807
Zm00001d009337_T036	2.28E-13	2.982944989	Zm00001d009337
Zm00001d047568_T001	0.005130942	2.756663559	Zm00001d047568
Zm00001d043239_T007	0.01175716	1.240029841	Zm00001d043239
Zm00001d043239_T003	0.001040673	2.098995047	Zm00001d043239
Zm00001d043062_T001	0.000546269	2.294778833	Zm00001d043062
Zm00001d003653_T001	0.000546337	-2.009689408	Zm00001d003653
Zm00001d022108_T007	0.002999506	1.012708642	Zm00001d022108
Zm00001d003601_T009	4.41E-07	2.157486309	Zm00001d003601
Zm00001d003601_T007	3.00E-05	1.272250451	Zm00001d003601
Zm00001d003601_T006	0.001290899	1.022637295	Zm00001d003601
Zm00001d037781_T005	9.02E-07	-2.112959064	Zm00001d037781
Zm00001d002695_T014	0.0006054	-2.283868253	Zm00001d002695
Zm00001d002695_T001	0.001167338	3.253367549	Zm00001d002695
Zm00001d030230_T017	4.59E-11	-2.197907402	Zm00001d030230
Zm00001d043463_T001	0.000327806	1.769433648	Zm00001d043463
Zm00001d014101_T001	0.006436882	-1.918228765	Zm00001d014101
Zm00001d027708_T001	0.03847324	2.240077937	Zm00001d027708
Zm00001d027708_T002	0.01656224	2.277604446	Zm00001d027708
Zm00001d023479_T005	7.55E-06	-2.128376613	Zm00001d023479
Zm00001d023479_T009	9.04E-11	1.783642571	Zm00001d023479
Zm00001d047723_T003	3.26E-05	1.162027507	Zm00001d047723
Zm00001d032974_T007	0.002378541	-2.293911328	Zm00001d032974
Zm00001d041570_T001	0.00450406	2.021613554	Zm00001d041570
Zm00001d044281_T030	0.0302166	1.200783087	Zm00001d044281
Zm00001d049204_T001	0.006116759	-1.699352013	Zm00001d049204
Zm00001d025398_T004	0.01893825	1.164340662	Zm00001d025398
Zm00001d029688_T006	2.10E-09	-3.549549587	Zm00001d029688
Zm00001d029688_T005	1.17E-09	2.810764423	Zm00001d029688
Zm00001d039051_T002	0.001656132	-1.154996115	Zm00001d039051
Zm00001d039051_T001	7.47E-10	2.378450234	Zm00001d039051
Zm00001d042826_T001	0.009424738	-2.27098674	Zm00001d042826
Zm00001d013680_T009	0.02507626	-1.4503422	Zm00001d013680
Zm00001d013680_T004	0.01644238	1.819615473	Zm00001d013680
Zm00001d006296_T012	0.001917423	2.222441845	Zm00001d006296
Zm00001d006296_T020	2.70E-05	1.577593574	Zm00001d006296
Zm00001d009312_T031	0.02643233	1.761279379	Zm00001d009312
Zm00001d012196_T006	4.42E-05	-1.30595663	Zm00001d012196
Zm00001d016720_T003	0.02546853	1.485775204	Zm00001d016720
Zm00001d045313_T001	0.003398109	2.784499971	Zm00001d045313
Zm00001d005558_T002	4.07E-12	-1.692087517	Zm00001d005558
Zm00001d005558_T004	0.02203454	-2.021747034	Zm00001d005558
Zm00001d035552_T002	2.87E-05	-1.710682386	Zm00001d035552
Zm00001d046664_T001	0.01585805	2.310095635	Zm00001d046664
Zm00001d038291_T001	0.005789232	2.601434559	Zm00001d038291
Zm00001d029506_T001	1.37E-06	-4.161217288	Zm00001d029506
Zm00001d018103_T001	0.01184787	2.040858122	Zm00001d018103
Zm00001d002875_T021	4.14E-09	-1.84891142	Zm00001d002875
Zm00001d002875_T014	0.04301396	1.269162794	Zm00001d002875
Zm00001d030110_T002	1.02E-14	3.872398218	Zm00001d030110
Zm00001d030110_T029	7.68E-06	1.424236819	Zm00001d030110
Zm00001d030110_T018	0.01652695	-1.87683376	Zm00001d030110
Zm00001d016408_T001	0.006521085	-2.851657539	Zm00001d016408
Zm00001d052243_T001	0.002844203	2.297331338	Zm00001d052243

Zm00001d042762_T001	0.00965634	1.074925556	Zm00001d042762
Zm00001d032961_T004	0.006911083	1.018880438	Zm00001d032961
Zm00001d032961_T012	0.000602129	1.080997462	Zm00001d032961
Zm00001d031259_T003	0.00721882	-1.510404015	Zm00001d031259
Zm00001d034855_T003	0.02760195	-2.241297342	Zm00001d034855
Zm00001d026679_T001	0.02746637	-2.067660591	Zm00001d026679
Zm00001d034277_T001	0.000579806	-2.280946453	Zm00001d034277
Zm00001d004706_T003	0.005033692	2.683068842	Zm00001d004706
Zm00001d022584_T075	0.01865129	1.220933141	Zm00001d022584
Zm00001d023269_T001	0.01346132	-2.494601027	Zm00001d023269
Zm00001d012642_T001	5.03E-07	2.67988066	Zm00001d012642
Zm00001d053746_T001	7.90E-05	3.88832338	Zm00001d053746
Zm00001d025673_T016	1.16E-08	-1.491159946	Zm00001d025673
Zm00001d037215_T005	7.71E-11	2.105020888	Zm00001d037215
Zm00001d037215_T002	1.29E-11	-2.179764302	Zm00001d037215
Zm00001d021770_T019	0.002468871	-1.987304016	Zm00001d021770
Zm00001d021770_T061	1.53E-10	1.21647099	Zm00001d021770
Zm00001d021770_T033	2.26E-11	1.86931381	Zm00001d021770
Zm00001d021770_T002	0.00033877	-2.942415295	Zm00001d021770
Zm00001d052855_T003	0.01493941	-2.007418532	Zm00001d052855
Zm00001d017298_T006	8.27E-05	-2.910069882	Zm00001d017298
Zm00001d017298_T005	0.02371103	2.036716722	Zm00001d017298
Zm00001d001828_T002	0.01042545	2.013458842	Zm00001d001828
Zm00001d040422_T016	0.002484774	1.18919742	Zm00001d040422
Zm00001d048152_T001	7.95E-07	1.067273212	Zm00001d048152
Zm00001d030894_T005	4.94E-08	-2.083826	Zm00001d030894
Zm00001d023859_T001	0.01889758	-1.932668885	Zm00001d023859
Zm00001d052475_T003	0.01229362	-2.183580205	Zm00001d052475
Zm00001d043519_T001	3.30E-08	3.709317301	Zm00001d043519
Zm00001d028325_T003	0.03771189	-1.60674736	Zm00001d028325
Zm00001d028579_T011	0.04634217	-1.044880055	Zm00001d028579
Zm00001d028579_T031	3.88E-06	-1.650730972	Zm00001d028579
Zm00001d028579_T007	0.01945734	1.065288726	Zm00001d028579
Zm00001d021452_T014	0.000121202	2.067409466	Zm00001d021452
Zm00001d009724_T013	0.04052835	-1.881193811	Zm00001d009724
Zm00001d020638_T002	4.62E-07	1.064636796	Zm00001d020638
Zm00001d036359_T001	0.000224456	-1.865003541	Zm00001d036359
Zm00001d036716_T001	0.02834701	1.142716069	Zm00001d036716
Zm00001d041948_T007	0.01622825	-1.178037896	Zm00001d041948
Zm00001d043431_T009	2.91E-08	-1.07852604	Zm00001d043431
Zm00001d044338_T011	0.002873666	-1.59655366	Zm00001d044338
Zm00001d038190_T014	0.03378891	-1.942440673	Zm00001d038190
Zm00001d006759_T013	8.37E-13	-2.414317272	Zm00001d006759
Zm00001d011835_T005	9.03E-06	1.710620029	Zm00001d011835
Zm00001d037536_T023	0.008724765	1.970645851	Zm00001d037536
Zm00001d051754_T010	1.59E-07	-2.10108652	Zm00001d051754
Zm00001d036565_T019	1.02E-05	-2.119113926	Zm00001d036565
Zm00001d045583_T016	0.03546251	-1.586360011	Zm00001d045583
Zm00001d021741_T029	1.32E-07	1.505700395	Zm00001d021741
Zm00001d021741_T085	6.25E-07	3.600317596	Zm00001d021741
Zm00001d007383_T018	1.16E-11	2.362128426	Zm00001d007383
Zm00001d007560_T003	0.0277091	2.241335892	Zm00001d007560
Zm00001d007560_T001	0.002446646	3.174133495	Zm00001d007560
Zm00001d007560_T004	0.0409121	2.307685784	Zm00001d007560

Zm00001d007560_T006	0.04346091	2.206941335	Zm00001d007560
Zm00001d048551_T001	0.001350684	-2.109693077	Zm00001d048551
Zm00001d039697_T001	0.002528307	-1.264521939	Zm00001d039697
Zm00001d044713_T004	0.000127969	-1.69683759	Zm00001d044713
Zm00001d030526_T004	0.00247824	2.554361668	Zm00001d030526
Zm00001d040501_T001	0.000186139	-2.829706819	Zm00001d040501
Zm00001d033714_T001	0.01534331	2.718225825	Zm00001d033714
Zm00001d007549_T002	0.0351872	2.391592715	Zm00001d007549
Zm00001d007549_T003	0.00744532	2.797403446	Zm00001d007549
Zm00001d025963_T001	0.004224988	2.585497029	Zm00001d025963
Zm00001d034701_T016	0.0063288	1.333130903	Zm00001d034701
Zm00001d039589_T015	0.002007262	1.151138073	Zm00001d039589
Zm00001d039589_T179	0.000420611	-2.76133829	Zm00001d039589
Zm00001d039589_T009	1.49E-06	-3.924315113	Zm00001d039589
Zm00001d039589_T035	9.30E-10	1.269752933	Zm00001d039589
Zm00001d039589_T025	0.00355131	1.865214703	Zm00001d039589
Zm00001d044222_T013	4.87E-10	1.556230634	Zm00001d044222
Zm00001d010662_T001	0.000502863	-2.509702496	Zm00001d010662
Zm00001d006446_T001	0.02648586	1.1617695	Zm00001d006446
Zm00001d018619_T001	0.006512038	-1.215583559	Zm00001d018619
Zm00001d053559_T014	3.02E-10	1.305092171	Zm00001d053559
Zm00001d012972_T004	3.11E-07	-2.841414905	Zm00001d012972
Zm00001d026511_T001	0.005393062	1.334234169	Zm00001d026511
Zm00001d026511_T057	6.44E-08	1.843056617	Zm00001d026511
Zm00001d015248_T008	4.35E-05	1.303710081	Zm00001d015248
Zm00001d013612_T002	5.00E-13	-2.894530422	Zm00001d013612
Zm00001d023892_T005	0.000249454	-3.578281176	Zm00001d023892
Zm00001d031939_T003	5.85E-08	-2.62961329	Zm00001d031939
Zm00001d031996_T003	0.00024674	-1.50058024	Zm00001d031996
Zm00001d010961_T001	0.01984524	2.569493439	Zm00001d010961
Zm00001d047283_T009	0.004848206	1.227637631	Zm00001d047283
Zm00001d030915_T001	0.000927301	-2.733987816	Zm00001d030915
Zm00001d012160_T006	0.00965634	-1.983672693	Zm00001d012160
Zm00001d031586_T001	0.01817166	-2.422309351	Zm00001d031586
Zm00001d008312_T002	0.006939942	-2.427849078	Zm00001d008312
Zm00001d005951_T001	0.000347145	3.74660891	Zm00001d005951
Zm00001d005951_T002	6.60E-05	4.204221659	Zm00001d005951
Zm00001d018770_T014	0.01756967	-1.502455526	Zm00001d018770
Zm00001d009376_T001	6.60E-05	1.606528876	Zm00001d009376
Zm00001d014425_T010	0.004509663	1.110426754	Zm00001d014425
Zm00001d026097_T006	0.007572169	-1.841618408	Zm00001d026097
Zm00001d044026_T015	1.94E-12	-1.685340458	Zm00001d044026
Zm00001d044026_T032	1.96E-14	2.999797555	Zm00001d044026
Zm00001d013427_T004	4.86E-11	-2.919074516	Zm00001d013427
Zm00001d008756_T001	0.007311546	3.026500719	Zm00001d008756
Zm00001d039730_T005	0.01225238	-1.928520928	Zm00001d039730
Zm00001d051009_T002	0.0322225	-1.378997569	Zm00001d051009
Zm00001d048515_T002	0.006843194	-1.556309497	Zm00001d048515
Zm00001d019824_T002	0.0109172	-1.832001598	Zm00001d019824
Zm00001d021999_T001	9.93E-12	-3.42999159	Zm00001d021999
Zm00001d022417_T009	0.001199578	1.433539202	Zm00001d022417
Zm00001d022417_T002	0.01052423	1.70722021	Zm00001d022417
Zm00001d044625_T005	0.002333223	-1.002541021	Zm00001d044625
Zm00001d048310_T004	0.03531717	2.486347876	Zm00001d048310

Zm00001d048310_T001	0.02135292	1.072584437	Zm00001d048310
Zm00001d048310_T010	7.87E-05	2.893103439	Zm00001d048310
Zm00001d048310_T009	0.02496468	3.06192436	Zm00001d048310
Zm00001d021119_T008	0.000102984	3.395055053	Zm00001d021119
Zm00001d021119_T007	0.001992364	2.290356477	Zm00001d021119
Zm00001d021119_T010	0.000457603	1.688090682	Zm00001d021119
Zm00001d021119_T009	1.66E-08	4.273793288	Zm00001d021119
Zm00001d021119_T005	6.39E-08	1.404662036	Zm00001d021119
Zm00001d031332_T002	2.96E-11	-2.317743464	Zm00001d031332
Zm00001d027900_T001	0.004624883	2.523797579	Zm00001d027900
Zm00001d010413_T005	0.04604004	2.419335298	Zm00001d010413
Zm00001d010413_T014	7.03E-17	4.825903125	Zm00001d010413
Zm00001d010413_T002	4.75E-07	-4.109805426	Zm00001d010413
Zm00001d012313_T001	0.01325275	1.1757858	Zm00001d012313
Zm00001d007757_T008	4.21E-12	-4.810752258	Zm00001d007757
Zm00001d040571_T019	1.91E-08	1.271846005	Zm00001d040571
Zm00001d000166_T005	3.41E-11	1.953736522	Zm00001d000166
Zm00001d012446_T008	0.04008706	1.921608412	Zm00001d012446
Zm00001d021950_T008	1.31E-08	-1.189439256	Zm00001d021950
Zm00001d021950_T007	0.00167584	1.88817826	Zm00001d021950
Zm00001d012350_T001	0.007572931	-1.386367803	Zm00001d012350
Zm00001d011548_T001	0.03860996	-1.839988445	Zm00001d011548
Zm00001d007139_T007	1.06E-12	2.608964818	Zm00001d007139
Zm00001d045956_T028	0.9979568	0.357246827	Zm00001d045956
Zm00001d045956_T017	3.15E-13	2.487208404	Zm00001d045956
Zm00001d045956_T001	0.000187249	2.711491084	Zm00001d045956
Zm00001d045956_T008	1	0.199119865	Zm00001d045956
Zm00001d023749_T005	0.02072523	0.471014175	Zm00001d023749
Zm00001d023749_T006	0.9624437	0.091991675	Zm00001d023749
Zm00001d023749_T001	0.5693278	-0.438607922	Zm00001d023749
Zm00001d023749_T008	0.9804228	0.33860536	Zm00001d023749
Zm00001d023749_T007	0.3861385	2.372372743	Zm00001d023749
Zm00001d023749_T010	0.493096	0.390284796	Zm00001d023749
Zm00001d019983_T001	0.9380783	0.110521784	Zm00001d019983
Zm00001d045304_T001	0.9849414	0.18181481	Zm00001d045304
Zm00001d016802_T002	0.9954444	0.213988407	Zm00001d016802
Zm00001d048802_T007	0.04092962	0.746598885	Zm00001d048802
Zm00001d048802_T005	0.9672819	0.219912027	Zm00001d048802
Zm00001d048802_T004	0.4354896	1.284186692	Zm00001d048802
Zm00001d048802_T003	0.8564933	0.27541129	Zm00001d048802
Zm00001d025048_T003	0.9893324	0.491276053	Zm00001d025048
Zm00001d015196_T007	0.9832612	0.286082154	Zm00001d015196
Zm00001d015196_T001	0.9930712	0.397622841	Zm00001d015196
Zm00001d029033_T002	0.3366363	1.375623008	Zm00001d029033
Zm00001d029033_T004	0.8854695	0.632611929	Zm00001d029033
Zm00001d029033_T001	0.9399427	0.045939754	Zm00001d029033
Zm00001d044232_T004	0.9804228	0.656045292	Zm00001d044232
Zm00001d044232_T002	0.0652199	0.628243166	Zm00001d044232
Zm00001d044232_T005	0.8660897	0.575085168	Zm00001d044232
Zm00001d044232_T001	0.000251832	-1.966504195	Zm00001d044232
Zm00001d048848_T001	0.908501	-0.357425352	Zm00001d048848
Zm00001d023434_T001	0.718063	-0.084272174	Zm00001d023434
Zm00001d023434_T002	0.997764	0.348572163	Zm00001d023434
Zm00001d032265_T001	0.4229235	1.163451971	Zm00001d032265

Zm00001d048759_T001	0.6406325	-0.388261146	Zm00001d048759
Zm00001d002498_T001	0.006844514	-2.043688892	Zm00001d002498
Zm00001d045119_T001	0.1100612	1.797251295	Zm00001d045119
Zm00001d030338_T001	0.9070578	0.587165454	Zm00001d030338
Zm00001d002345_T001	0.969231	0.512107919	Zm00001d002345
Zm00001d017645_T001	0.9269932	0.605205429	Zm00001d017645
Zm00001d054069_T001	0.7428299	-0.305397826	Zm00001d054069
Zm00001d004496_T001	0.2153703	-1.501664814	Zm00001d004496
Zm00001d052212_T001	0.974492	0.394634631	Zm00001d052212
Zm00001d042476_T002	0.965836	0.596502173	Zm00001d042476
Zm00001d042476_T005	1	0.060406294	Zm00001d042476
Zm00001d038361_T003	0.9804228	0.292862688	Zm00001d038361
Zm00001d038361_T005	0.871546	0.500170666	Zm00001d038361
Zm00001d038361_T002	0.9673473	0.302836758	Zm00001d038361
Zm00001d038361_T006	0.8092097	0.968660913	Zm00001d038361
Zm00001d038361_T001	0.8307555	0.628863001	Zm00001d038361
Zm00001d038361_T008	0.9050366	-0.137639893	Zm00001d038361
Zm00001d014723_T005	0.745718	-0.091007392	Zm00001d014723
Zm00001d012995_T001	0.9134699	-0.15111955	Zm00001d012995
Zm00001d012898_T001	0.8412913	-0.150362282	Zm00001d012898
Zm00001d021709_T001	0.6552456	-0.517849769	Zm00001d021709
Zm00001d051186_T001	0.9673094	0.359293374	Zm00001d051186
Zm00001d033595_T001	0.7646466	0.703128126	Zm00001d033595
Zm00001d043831_T002	0.6053962	0.664146671	Zm00001d043831
Zm00001d043831_T001	0.8786868	-0.064456987	Zm00001d043831
Zm00001d014085_T001	0.9689939	0.631230841	Zm00001d014085
Zm00001d036972_T001	0.5473748	-0.520248651	Zm00001d036972
Zm00001d018703_T001	0.7434928	-0.202197461	Zm00001d018703
Zm00001d018703_T002	0.6524172	-0.462544913	Zm00001d018703
Zm00001d038766_T001	0.7378574	0.601086221	Zm00001d038766
Zm00001d029257_T001	0.8761568	-0.662467029	Zm00001d029257
Zm00001d048218_T001	0.8955831	0.806920379	Zm00001d048218
Zm00001d054038_T001	1	0.309465845	Zm00001d054038
Zm00001d054038_T004	0.1168289	-1.728815056	Zm00001d054038
Zm00001d054038_T002	0.9822138	0.429292899	Zm00001d054038
Zm00001d005256_T001	1	0.229902398	Zm00001d005256
Zm00001d039427_T002	0.9791729	0.572548778	Zm00001d039427
Zm00001d045505_T001	0.9048001	0.761256627	Zm00001d045505
Zm00001d045505_T006	0.9550193	0.5510247	Zm00001d045505
Zm00001d045505_T005	0.2094306	1.051292584	Zm00001d045505
Zm00001d045505_T004	1	0.277196445	Zm00001d045505
Zm00001d045505_T007	0.6012987	-0.768900868	Zm00001d045505
Zm00001d017823_T002	0.9561521	0.070321392	Zm00001d017823
Zm00001d017823_T003	0.3373089	-0.831900447	Zm00001d017823
Zm00001d007038_T004	0.9907928	0.219944893	Zm00001d007038
Zm00001d007038_T003	0.9702242	0.091140173	Zm00001d007038
Zm00001d042578_T001	0.9019013	0.631804264	Zm00001d042578
Zm00001d013597_T003	0.830404	-0.224178206	Zm00001d013597
Zm00001d013597_T002	0.9870394	0.162332505	Zm00001d013597
Zm00001d029044_T006	0.2391684	0.725128367	Zm00001d029044
Zm00001d029044_T004	0.7602652	0.436376031	Zm00001d029044
Zm00001d029044_T002	0.8062829	1.004659135	Zm00001d029044
Zm00001d029044_T007	0.9077762	-0.015987991	Zm00001d029044
Zm00001d029044_T001	0.1826126	-0.264903073	Zm00001d029044

Zm00001d044947_T001	0.7904511	-0.300486016	Zm00001d044947
Zm00001d042363_T005	0.9953345	0.436184952	Zm00001d042363
Zm00001d042363_T013	0.9936409	0.186126611	Zm00001d042363
Zm00001d039341_T001	0.9404125	0.606038109	Zm00001d039341
Zm00001d039341_T009	5.07E-16	3.77395362	Zm00001d039341
Zm00001d039341_T006	0.1481305	0.620526474	Zm00001d039341
Zm00001d039341_T025	0.718063	-0.165045159	Zm00001d039341
Zm00001d039341_T133	0.000388444	2.282430363	Zm00001d039341
Zm00001d039341_T007	0.501854	1.175711883	Zm00001d039341
Zm00001d039341_T012	0.7505997	-0.127065295	Zm00001d039341
Zm00001d039341_T080	0.186917	-0.475369037	Zm00001d039341
Zm00001d039341_T008	0.4022327	-0.091213283	Zm00001d039341
Zm00001d039341_T011	0.8310488	-0.12673032	Zm00001d039341
Zm00001d047750_T001	0.9696449	0.557724733	Zm00001d047750
Zm00001d027894_T001	0.5537557	-0.754962617	Zm00001d027894
Zm00001d006535_T001	0.635589	-0.385839889	Zm00001d006535
Zm00001d018014_T001	0.5983973	-0.232976881	Zm00001d018014
Zm00001d018014_T012	0.9374853	-0.034554034	Zm00001d018014
Zm00001d018014_T002	0.9939021	0.026969304	Zm00001d018014
Zm00001d020950_T001	0.9804228	-0.130629715	Zm00001d020950
Zm00001d025128_T012	0.3139869	1.52985467	Zm00001d025128
Zm00001d025128_T011	0.004101034	-2.763132497	Zm00001d025128
Zm00001d025128_T001	0.9701986	0.389999255	Zm00001d025128
Zm00001d025128_T013	0.1417015	0.744289428	Zm00001d025128
Zm00001d025128_T009	0.90276	0.355091666	Zm00001d025128
Zm00001d025128_T007	1	0.311155261	Zm00001d025128
Zm00001d025128_T008	0.217009	1.361680112	Zm00001d025128
Zm00001d025128_T005	0.9706293	0.234041987	Zm00001d025128
Zm00001d040028_T001	0.6114629	-0.861635943	Zm00001d040028
Zm00001d011746_T003	0.7003081	0.899344305	Zm00001d011746
Zm00001d011746_T002	0.2191815	1.142791633	Zm00001d011746
Zm00001d039157_T007	0.9562711	0.302411024	Zm00001d039157
Zm00001d039157_T002	0.954492	0.078155914	Zm00001d039157
Zm00001d039157_T004	0.8513643	0.273216569	Zm00001d039157
Zm00001d039157_T003	0.4860327	0.66110174	Zm00001d039157
Zm00001d039157_T001	0.9979568	0.319740599	Zm00001d039157
Zm00001d004700_T001	0.8586983	-0.397000743	Zm00001d004700
Zm00001d015962_T001	0.9805897	0.580088629	Zm00001d015962
Zm00001d021754_T011	0.9207666	-0.00977421	Zm00001d021754
Zm00001d021754_T002	0.997764	0.466630418	Zm00001d021754
Zm00001d017177_T005	0.9037807	-0.159130674	Zm00001d017177
Zm00001d017177_T001	0.1382975	-0.730184009	Zm00001d017177
Zm00001d017177_T003	0.9804228	0.164360424	Zm00001d017177
Zm00001d017177_T002	0.07620979	-2.433148968	Zm00001d017177
Zm00001d017177_T007	0.7410689	-0.335915883	Zm00001d017177
Zm00001d017177_T010	0.3847344	1.214306652	Zm00001d017177
Zm00001d017177_T004	0.9109241	0.338855972	Zm00001d017177
Zm00001d017177_T008	0.603064	-0.318683484	Zm00001d017177
Zm00001d044845_T010	0.9139882	0.121754259	Zm00001d044845
Zm00001d044845_T001	0.000405099	0.603122408	Zm00001d044845
Zm00001d044845_T003	0.1476103	1.623707665	Zm00001d044845
Zm00001d044845_T009	0.2398048	2.029961587	Zm00001d044845
Zm00001d006768_T001	0.7602894	0.564291092	Zm00001d006768
Zm00001d025948_T001	0.9177099	0.404663074	Zm00001d025948

Zm00001d011900_T002	0.957148	0.555411832	Zm00001d011900
Zm00001d011900_T001	0.8605736	-0.106499373	Zm00001d011900
Zm00001d018534_T001	0.5718169	-0.629673735	Zm00001d018534
Zm00001d030773_T001	0.9419336	0.057311786	Zm00001d030773
Zm00001d024768_T001	0.749631	-0.276245543	Zm00001d024768
Zm00001d022414_T001	0.9946911	0.474816211	Zm00001d022414
Zm00001d019139_T004	0.6518281	-0.624285609	Zm00001d019139
Zm00001d019139_T005	0.4146736	1.48752349	Zm00001d019139
Zm00001d019139_T008	0.8001345	0.487017899	Zm00001d019139
Zm00001d019139_T006	0.7849294	1.101091469	Zm00001d019139
Zm00001d019139_T003	0.8132343	1.026854169	Zm00001d019139
Zm00001d019139_T001	0.7490886	0.281721493	Zm00001d019139
Zm00001d037158_T001	0.9224387	-0.261090632	Zm00001d037158
Zm00001d024891_T001	0.6658123	1.219997364	Zm00001d024891
Zm00001d007215_T003	0.2005815	2.537294798	Zm00001d007215
Zm00001d007215_T002	1	0.377989015	Zm00001d007215
Zm00001d007215_T001	0.1124224	-1.098177461	Zm00001d007215
Zm00001d046838_T005	0.5698311	-0.262076315	Zm00001d046838
Zm00001d046838_T001	0.991917	0.40721077	Zm00001d046838
Zm00001d017709_T001	0.8033798	0.913496456	Zm00001d017709
Zm00001d051554_T001	0.02685203	1.449838138	Zm00001d051554
Zm00001d051554_T002	0.01554174	2.601000178	Zm00001d051554
Zm00001d039135_T001	0.05822061	-1.990806472	Zm00001d039135
Zm00001d026311_T004	0.2035902	1.771428715	Zm00001d026311
Zm00001d026311_T003	0.431704	0.93327645	Zm00001d026311
Zm00001d026311_T002	0.7479226	1.106469293	Zm00001d026311
Zm00001d000203_T001	1	0.200100286	Zm00001d000203
Zm00001d019724_T058	0.9995663	0.183179906	Zm00001d019724
Zm00001d019724_T004	0.000425947	1.237502033	Zm00001d019724
Zm00001d019724_T011	0.9909153	0.151136793	Zm00001d019724
Zm00001d019724_T041	0.6144495	-0.364973781	Zm00001d019724
Zm00001d019724_T068	0.4783289	-1.065516047	Zm00001d019724
Zm00001d019724_T047	0.03059728	0.70399773	Zm00001d019724
Zm00001d019724_T044	0.8170235	0.312962693	Zm00001d019724
Zm00001d019724_T052	0.2690004	0.561243837	Zm00001d019724
Zm00001d019724_T024	0.007188644	1.886412203	Zm00001d019724
Zm00001d019724_T039	0.844285	0.712102336	Zm00001d019724
Zm00001d019724_T057	0.8348638	-0.188253635	Zm00001d019724
Zm00001d019724_T027	0.6217057	0.659656008	Zm00001d019724
Zm00001d019724_T049	0.09844043	0.740077315	Zm00001d019724
Zm00001d019724_T053	0.107968	0.480410911	Zm00001d019724
Zm00001d019724_T001	0.1732987	0.443651981	Zm00001d019724
Zm00001d019724_T025	0.6824162	0.305354333	Zm00001d019724
Zm00001d019724_T037	0.333158	-0.214214642	Zm00001d019724
Zm00001d014761_T004	0.7626242	-0.258214402	Zm00001d014761
Zm00001d014761_T002	0.9562711	0.347122176	Zm00001d014761
Zm00001d014761_T001	0.7845423	1.013148261	Zm00001d014761
Zm00001d014761_T006	0.4614239	0.751116447	Zm00001d014761
Zm00001d035090_T001	1	0.361596088	Zm00001d035090
Zm00001d035090_T004	0.974657	0.061962164	Zm00001d035090
Zm00001d035090_T003	0.3358965	-1.020106491	Zm00001d035090
Zm00001d011668_T002	1	0.278362441	Zm00001d011668
Zm00001d036431_T001	0.30546	-1.044406677	Zm00001d036431
Zm00001d038118_T003	0.9517262	0.026284033	Zm00001d038118

Zm00001d038118_T001	0.9887584	0.173435331	Zm00001d038118
Zm00001d022037_T001	0.9999526	0.135372107	Zm00001d022037
Zm00001d045007_T001	1	0.336795532	Zm00001d045007
Zm00001d052064_T001	1	0.336548319	Zm00001d052064
Zm00001d044594_T001	0.9046076	0.769061275	Zm00001d044594
Zm00001d025281_T001	0.894937	0.74510692	Zm00001d025281
Zm00001d026322_T001	1	0.304886463	Zm00001d026322
Zm00001d026322_T003	0.8007432	0.999991917	Zm00001d026322
Zm00001d024708_T003	0.1581307	-1.452304724	Zm00001d024708
Zm00001d024708_T001	0.9172003	0.768193277	Zm00001d024708
Zm00001d024708_T002	0.3694523	-0.387751023	Zm00001d024708
Zm00001d026261_T008	0.1271501	1.265480267	Zm00001d026261
Zm00001d026261_T001	0.9374853	0.247846471	Zm00001d026261
Zm00001d026261_T006	0.8031436	0.774957998	Zm00001d026261
Zm00001d033446_T001	0.9508107	0.064529556	Zm00001d033446
Zm00001d008316_T001	0.9856605	0.1141365	Zm00001d008316
Zm00001d043694_T002	0.9695609	0.537826866	Zm00001d043694
Zm00001d043694_T003	0.9469426	0.507455416	Zm00001d043694
Zm00001d043694_T001	0.447037	-0.415322322	Zm00001d043694
Zm00001d015319_T001	0.4052428	-0.757138995	Zm00001d015319
Zm00001d011992_T001	0.7746208	-0.481968861	Zm00001d011992
Zm00001d033247_T003	0.6520542	0.092275226	Zm00001d033247
Zm00001d033247_T001	0.9911485	0.468392631	Zm00001d033247
Zm00001d033247_T007	0.01306269	-2.29877659	Zm00001d033247
Zm00001d033247_T006	0.967161	0.534879655	Zm00001d033247
Zm00001d033247_T002	0.8643636	0.395199724	Zm00001d033247
Zm00001d004993_T006	0.2611065	0.91842338	Zm00001d004993
Zm00001d004993_T003	0.8865624	0.744410969	Zm00001d004993
Zm00001d010061_T002	0.2325006	-1.039402423	Zm00001d010061
Zm00001d010061_T001	0.5410776	-0.782039711	Zm00001d010061
Zm00001d012794_T009	0.9732421	0.022666727	Zm00001d012794
Zm00001d012794_T004	0.8162418	0.412411824	Zm00001d012794
Zm00001d012794_T006	0.5234084	-0.2451375	Zm00001d012794
Zm00001d012794_T010	0.9493302	-0.05700758	Zm00001d012794
Zm00001d012794_T013	0.9252088	0.239599135	Zm00001d012794
Zm00001d012794_T002	0.6445434	-0.367328227	Zm00001d012794
Zm00001d012794_T005	0.2198629	-0.746567773	Zm00001d012794
Zm00001d039564_T013	0.9184323	0.528666363	Zm00001d039564
Zm00001d039564_T005	0.8281771	0.821067315	Zm00001d039564
Zm00001d039564_T019	0.05169209	2.312751908	Zm00001d039564
Zm00001d013709_T004	0.9203655	0.382873712	Zm00001d013709
Zm00001d013709_T001	0.4825899	1.63235058	Zm00001d013709
Zm00001d013709_T003	0.4245857	1.504809572	Zm00001d013709
Zm00001d039296_T006	0.4889069	0.785348244	Zm00001d039296
Zm00001d039296_T003	0.7151095	-0.228002053	Zm00001d039296
Zm00001d039296_T005	0.9429553	0.039897817	Zm00001d039296
Zm00001d039296_T007	0.9689939	0.547096506	Zm00001d039296
Zm00001d006921_T004	0.9031129	-0.088400441	Zm00001d006921
Zm00001d047644_T001	0.8027057	0.947336455	Zm00001d047644
Zm00001d021023_T001	0.8991555	0.729787709	Zm00001d021023
Zm00001d021023_T004	0.06930627	-1.159893903	Zm00001d021023
Zm00001d042553_T001	0.7972183	0.592143509	Zm00001d042553
Zm00001d009823_T001	0.9046076	-0.268998837	Zm00001d009823
Zm00001d022585_T002	0.8145063	-0.293189314	Zm00001d022585

Zm00001d022585_T001	0.8616387	-0.196479307	Zm00001d022585
Zm00001d037848_T005	0.1562235	0.8668338	Zm00001d037848
Zm00001d037848_T002	0.02871159	2.348376064	Zm00001d037848
Zm00001d032295_T001	0.9740325	-0.169446933	Zm00001d032295
Zm00001d043070_T001	0.9781672	0.268319558	Zm00001d043070
Zm00001d045783_T049	0.00071555	0.558802124	Zm00001d045783
Zm00001d045783_T014	0.1129675	1.56461889	Zm00001d045783
Zm00001d045783_T012	0.1052631	0.367546063	Zm00001d045783
Zm00001d045783_T056	0.974657	0.615446587	Zm00001d045783
Zm00001d045783_T046	0.3579474	0.448406086	Zm00001d045783
Zm00001d045783_T013	0.2452899	0.739795368	Zm00001d045783
Zm00001d045783_T047	0.7109402	-0.438312201	Zm00001d045783
Zm00001d045783_T004	4.37E-09	-1.055596604	Zm00001d045783
Zm00001d053643_T002	0.9910452	0.218273761	Zm00001d053643
Zm00001d053643_T003	0.9498685	0.379515477	Zm00001d053643
Zm00001d051632_T001	0.9766874	0.295764581	Zm00001d051632
Zm00001d045154_T001	0.9944247	0.337541534	Zm00001d045154
Zm00001d045154_T003	0.8730771	-0.030327599	Zm00001d045154
Zm00001d021240_T001	0.6292709	1.223712535	Zm00001d021240
Zm00001d017049_T002	0.9782953	0.037136306	Zm00001d017049
Zm00001d017049_T004	0.9957676	0.325223337	Zm00001d017049
Zm00001d017049_T001	0.8744797	-0.12829261	Zm00001d017049
Zm00001d034675_T002	0.9493302	0.657811036	Zm00001d034675
Zm00001d034675_T003	0.8684295	-0.184271565	Zm00001d034675
Zm00001d053432_T001	0.09813161	-0.891470866	Zm00001d053432
Zm00001d040130_T001	0.9541082	0.508653213	Zm00001d040130
Zm00001d000120_T002	0.8816665	-0.484885808	Zm00001d000120
Zm00001d013643_T001	1	0.018805942	Zm00001d013643
Zm00001d052478_T001	0.005306594	0.813563593	Zm00001d052478
Zm00001d052461_T001	0.9557384	-0.003932943	Zm00001d052461
Zm00001d049668_T001	0.6185306	-0.712904507	Zm00001d049668
Zm00001d018057_T001	0.9870394	0.368988423	Zm00001d018057
Zm00001d018057_T002	0.889283	0.475423789	Zm00001d018057
Zm00001d017469_T001	1	0.274156517	Zm00001d017469
Zm00001d003124_T002	1	0.315555152	Zm00001d003124
Zm00001d014199_T002	0.9771057	0.229620973	Zm00001d014199
Zm00001d014199_T004	1	0.186336901	Zm00001d014199
Zm00001d014199_T001	0.7648638	0.982639579	Zm00001d014199
Zm00001d009415_T001	0.8179463	-0.211077228	Zm00001d009415
Zm00001d012476_T001	0.9863483	0.397588774	Zm00001d012476
Zm00001d016543_T002	0.897685	0.30664567	Zm00001d016543
Zm00001d016543_T004	0.7808852	0.416649106	Zm00001d016543
Zm00001d016543_T001	0.943607	-0.042466307	Zm00001d016543
Zm00001d051193_T003	0.9988864	0.449070587	Zm00001d051193
Zm00001d051193_T004	0.2853231	1.448465011	Zm00001d051193
Zm00001d045564_T001	1	0.326019452	Zm00001d045564
Zm00001d015140_T001	0.1155487	-1.396028831	Zm00001d015140
Zm00001d005624_T001	0.9907928	0.227266009	Zm00001d005624
Zm00001d011401_T003	0.9073468	0.543690455	Zm00001d011401
Zm00001d011401_T001	0.9845237	0.207062243	Zm00001d011401
Zm00001d025814_T001	0.6957026	-0.465732474	Zm00001d025814
Zm00001d045031_T001	0.8892107	-0.278034778	Zm00001d045031
Zm00001d047739_T007	0.7970469	0.824327735	Zm00001d047739
Zm00001d047739_T016	0.001556436	1.485810548	Zm00001d047739

Zm00001d047739_T042	0.8995312	0.287530054	Zm00001d047739
Zm00001d047739_T039	0.9988864	0.219699635	Zm00001d047739
Zm00001d047739_T026	0.009347455	1.19244751	Zm00001d047739
Zm00001d047739_T003	6.72E-09	-1.363909309	Zm00001d047739
Zm00001d047739_T025	0.9928864	0.129352258	Zm00001d047739
Zm00001d047739_T009	0.2542693	0.670119875	Zm00001d047739
Zm00001d047739_T002	0.8591203	0.581853083	Zm00001d047739
Zm00001d047739_T043	1.94E-06	-2.961736276	Zm00001d047739
Zm00001d047739_T008	0.7131979	0.454542812	Zm00001d047739
Zm00001d047739_T001	0.1899222	1.077703482	Zm00001d047739
Zm00001d047739_T014	0.07936371	0.957734262	Zm00001d047739
Zm00001d003784_T001	0.07818164	2.015088914	Zm00001d003784
Zm00001d041913_T001	0.9887584	0.139582246	Zm00001d041913
Zm00001d052411_T002	0.9979568	0.312538479	Zm00001d052411
Zm00001d034809_T032	0.9756546	0.174848877	Zm00001d034809
Zm00001d034809_T021	0.5320876	-0.487295637	Zm00001d034809
Zm00001d034809_T026	0.814477	0.590193416	Zm00001d034809
Zm00001d034809_T002	0.9781672	0.450084653	Zm00001d034809
Zm00001d046882_T002	0.09495891	-1.314467941	Zm00001d046882
Zm00001d046882_T001	0.4116763	1.212748902	Zm00001d046882
Zm00001d047662_T011	0.7374518	0.703063369	Zm00001d047662
Zm00001d043387_T002	0.8751525	0.823544796	Zm00001d043387
Zm00001d012755_T016	0.03437276	0.369875745	Zm00001d012755
Zm00001d012755_T054	0.1066148	0.602153014	Zm00001d012755
Zm00001d012755_T002	0.7175572	0.312534041	Zm00001d012755
Zm00001d012755_T004	0.6325449	0.524555339	Zm00001d012755
Zm00001d012755_T056	0.9979568	0.349160802	Zm00001d012755
Zm00001d023596_T001	0.190471	1.466280057	Zm00001d023596
Zm00001d039693_T082	0.8922572	-0.038806783	Zm00001d039693
Zm00001d039693_T054	0.02877072	0.464122293	Zm00001d039693
Zm00001d039693_T043	0.7568663	0.185868747	Zm00001d039693
Zm00001d039693_T061	5.35E-11	1.513491143	Zm00001d039693
Zm00001d039693_T044	0.6075235	0.471683763	Zm00001d039693
Zm00001d039693_T005	0.6398988	0.164109542	Zm00001d039693
Zm00001d037743_T001	4.56E-06	-4.440005614	Zm00001d037743
Zm00001d048481_T001	1	0.374897069	Zm00001d048481
Zm00001d051906_T002	0.9979568	0.149651801	Zm00001d051906
Zm00001d030523_T001	0.9817074	0.365144729	Zm00001d030523
Zm00001d007530_T001	1	0.097299781	Zm00001d007530
Zm00001d034702_T002	0.9361945	0.296731678	Zm00001d034702
Zm00001d034702_T001	0.008942526	-0.789127243	Zm00001d034702
Zm00001d018214_T001	0.9956349	0.452697409	Zm00001d018214
Zm00001d021845_T001	1	0.1855883	Zm00001d021845
Zm00001d001866_T003	0.5717645	0.377165095	Zm00001d001866
Zm00001d001866_T010	0.9097583	0.626852544	Zm00001d001866
Zm00001d001866_T005	0.8706608	0.774226515	Zm00001d001866
Zm00001d042973_T004	0.9745314	0.653071347	Zm00001d042973
Zm00001d042973_T008	0.8593759	0.543002026	Zm00001d042973
Zm00001d042973_T005	0.9573408	0.10311406	Zm00001d042973
Zm00001d042973_T006	0.9845237	0.427480283	Zm00001d042973
Zm00001d042973_T002	0.2072635	1.42098324	Zm00001d042973
Zm00001d053897_T001	0.7799713	0.766410892	Zm00001d053897
Zm00001d053897_T002	0.9334932	0.392182344	Zm00001d053897
Zm00001d033575_T001	0.7294689	1.0302889	Zm00001d033575

Zm00001d033575_T002	0.7891719	0.822424384	Zm00001d033575
Zm00001d005072_T002	0.9061819	0.582741668	Zm00001d005072
Zm00001d005072_T001	0.1160584	-1.915922581	Zm00001d005072
Zm00001d005072_T003	0.7493723	-0.074307468	Zm00001d005072
Zm00001d051403_T003	0.101456	-1.390838079	Zm00001d051403
Zm00001d051403_T001	1	0.379651433	Zm00001d051403
Zm00001d038960_T001	0.5348533	-0.74054074	Zm00001d038960
Zm00001d025062_T001	0.9166356	-0.410297153	Zm00001d025062
Zm00001d021775_T001	0.9833513	0.482565153	Zm00001d021775
Zm00001d014764_T002	0.2719671	0.944430316	Zm00001d014764
Zm00001d014764_T005	0.7801274	0.941282882	Zm00001d014764
Zm00001d054070_T013	0.1056811	1.497232708	Zm00001d054070
Zm00001d054070_T014	0.8035131	-0.058774661	Zm00001d054070
Zm00001d054070_T007	0.03715458	0.615396394	Zm00001d054070
Zm00001d054070_T003	0.8236027	0.218830149	Zm00001d054070
Zm00001d054070_T018	0.2047087	-1.366741817	Zm00001d054070
Zm00001d054070_T002	0.9673473	0.412783167	Zm00001d054070
Zm00001d054070_T005	0.3198266	1.300441707	Zm00001d054070
Zm00001d054070_T015	1	0.34105618	Zm00001d054070
Zm00001d006244_T001	0.8660897	-0.222141488	Zm00001d006244
Zm00001d016797_T006	0.8564479	0.4465093	Zm00001d016797
Zm00001d016797_T002	0.9681793	0.641001716	Zm00001d016797
Zm00001d016797_T009	0.8135961	0.498924733	Zm00001d016797
Zm00001d024872_T001	0.976488	0.043391047	Zm00001d024872
Zm00001d011958_T001	0.9981361	0.282388523	Zm00001d011958
Zm00001d017459_T004	0.9918384	0.077740173	Zm00001d017459
Zm00001d017459_T001	0.6556118	0.414800998	Zm00001d017459
Zm00001d017459_T002	0.9690782	0.015378459	Zm00001d017459
Zm00001d012284_T001	0.9795655	0.159961802	Zm00001d012284
Zm00001d006943_T001	1	0.154337523	Zm00001d006943
Zm00001d010800_T001	0.001753896	-2.22357178	Zm00001d010800
Zm00001d045846_T001	0.9986486	-0.124446295	Zm00001d045846
Zm00001d021664_T001	0.7511561	0.882154791	Zm00001d021664
Zm00001d047701_T002	0.8703457	0.876117346	Zm00001d047701
Zm00001d039287_T002	1	0.423537805	Zm00001d039287
Zm00001d025338_T001	0.6582322	1.011794296	Zm00001d025338
Zm00001d033477_T003	0.4441507	0.43772767	Zm00001d033477
Zm00001d033477_T002	1	0.227532733	Zm00001d033477
Zm00001d050017_T002	1	0.068313728	Zm00001d050017
Zm00001d050017_T003	0.534033	-0.30079611	Zm00001d050017
Zm00001d019944_T003	0.4682753	-0.725924838	Zm00001d019944
Zm00001d019944_T001	0.9988864	0.306363436	Zm00001d019944
Zm00001d019944_T002	0.9971866	-0.020628656	Zm00001d019944
Zm00001d019944_T004	0.3151066	-0.623214776	Zm00001d019944
Zm00001d015423_T001	0.2916362	0.71114026	Zm00001d015423
Zm00001d015423_T003	0.9709887	0.279700231	Zm00001d015423
Zm00001d015423_T006	0.9380783	-0.092085135	Zm00001d015423
Zm00001d033002_T001	0.9410648	0.723969968	Zm00001d033002
Zm00001d053393_T002	0.9718723	0.515781272	Zm00001d053393
Zm00001d053393_T001	0.5308542	0.844287236	Zm00001d053393
Zm00001d029297_T001	0.5653983	1.302838058	Zm00001d029297
Zm00001d029297_T003	0.01873517	-1.123259226	Zm00001d029297
Zm00001d029297_T002	0.08495247	-0.556946569	Zm00001d029297
Zm00001d022215_T001	0.4324221	-0.781224368	Zm00001d022215

Zm00001d034628_T001	0.8175659	-0.583930645	Zm00001d034628
Zm00001d011841_T001	0.6652036	-0.323499915	Zm00001d011841
Zm00001d012229_T001	0.6841106	1.180534906	Zm00001d012229
Zm00001d034514_T001	0.899516	0.4115515	Zm00001d034514
Zm00001d031272_T001	0.7561907	-0.404715186	Zm00001d031272
Zm00001d006226_T001	0.9822138	0.386424772	Zm00001d006226
Zm00001d006226_T002	0.9260032	0.093110193	Zm00001d006226
Zm00001d047106_T001	0.06399284	-1.280356622	Zm00001d047106
Zm00001d013101_T003	0.9988954	0.293210388	Zm00001d013101
Zm00001d013101_T005	0.1580245	-1.328368304	Zm00001d013101
Zm00001d013101_T001	0.9797082	0.158348902	Zm00001d013101
Zm00001d013101_T002	0.9942835	0.075095982	Zm00001d013101
Zm00001d027988_T001	0.1417015	2.073690322	Zm00001d027988
Zm00001d006903_T001	0.8030099	0.769785582	Zm00001d006903
Zm00001d053060_T001	0.6141487	-0.425300663	Zm00001d053060
Zm00001d033223_T004	0.9303773	-0.034313162	Zm00001d033223
Zm00001d033223_T003	0.9797082	0.01558176	Zm00001d033223
Zm00001d023677_T001	0.5257664	-0.680558369	Zm00001d023677
Zm00001d053698_T002	0.9846714	0.525288007	Zm00001d053698
Zm00001d053698_T001	0.7055225	-0.271786565	Zm00001d053698
Zm00001d053698_T004	0.9493302	0.374254364	Zm00001d053698
Zm00001d042024_T001	0.8863085	-0.063128259	Zm00001d042024
Zm00001d004667_T042	0.8896281	-0.021526877	Zm00001d004667
Zm00001d004667_T006	0.9826647	0.25735585	Zm00001d004667
Zm00001d004667_T001	0.8959807	0.792467148	Zm00001d004667
Zm00001d004667_T008	0.8448074	0.223760781	Zm00001d004667
Zm00001d030834_T001	0.8936314	0.582436747	Zm00001d030834
Zm00001d030231_T002	0.579353	-0.328999522	Zm00001d030231
Zm00001d030231_T001	0.9797082	0.465175782	Zm00001d030231
Zm00001d041604_T001	0.9218815	-0.539814042	Zm00001d041604
Zm00001d048129_T002	0.9508258	0.069113209	Zm00001d048129
Zm00001d048129_T004	1	0.440257013	Zm00001d048129
Zm00001d048129_T005	0.6302619	-0.510524115	Zm00001d048129
Zm00001d051856_T005	0.5821194	-0.682683771	Zm00001d051856
Zm00001d051856_T003	0.940651	0.590816325	Zm00001d051856
Zm00001d051856_T010	0.8564933	0.280930341	Zm00001d051856
Zm00001d051856_T007	0.3538957	-0.891971309	Zm00001d051856
Zm00001d051856_T009	0.91126	0.300025918	Zm00001d051856
Zm00001d051856_T002	0.818266	-0.223322267	Zm00001d051856
Zm00001d020183_T009	0.946365	0.140322456	Zm00001d020183
Zm00001d020183_T008	0.3369227	0.845309466	Zm00001d020183
Zm00001d020183_T001	0.9525324	0.672203302	Zm00001d020183
Zm00001d020183_T010	0.1116614	1.693286809	Zm00001d020183
Zm00001d020183_T011	0.8321156	-0.117310754	Zm00001d020183
Zm00001d047253_T002	0.9550193	0.739421717	Zm00001d047253
Zm00001d047253_T014	3.85E-05	1.172788159	Zm00001d047253
Zm00001d047253_T001	0.08698389	-1.024957501	Zm00001d047253
Zm00001d047253_T013	0.5622311	1.103489218	Zm00001d047253
Zm00001d047253_T011	0.2154325	-0.210355239	Zm00001d047253
Zm00001d047253_T004	0.8865624	-0.22492343	Zm00001d047253
Zm00001d038290_T005	0.9979568	0.394031268	Zm00001d038290
Zm00001d038290_T004	0.8546941	0.870595847	Zm00001d038290
Zm00001d053442_T001	0.8935921	-0.188271055	Zm00001d053442
Zm00001d028866_T001	0.9165176	0.036394441	Zm00001d028866

Zm00001d028866_T002	0.1006909	2.014838946	Zm00001d028866
Zm00001d032825_T001	0.001779678	-2.944156942	Zm00001d032825
Zm00001d033279_T001	0.6185122	-0.638484518	Zm00001d033279
Zm00001d029829_T001	0.9265089	0.00141905	Zm00001d029829
Zm00001d018345_T018	0.9760472	0.082761058	Zm00001d018345
Zm00001d018345_T015	0.1701094	-0.965373878	Zm00001d018345
Zm00001d018345_T001	0.8021989	0.396645979	Zm00001d018345
Zm00001d018345_T010	0.2153703	1.059234128	Zm00001d018345
Zm00001d029028_T001	0.03732086	2.469142289	Zm00001d029028
Zm00001d031593_T001	0.8676122	0.849745675	Zm00001d031593
Zm00001d031593_T004	0.9326482	0.264714222	Zm00001d031593
Zm00001d031593_T005	0.2805818	1.227136611	Zm00001d031593
Zm00001d031593_T003	0.9529184	0.070673501	Zm00001d031593
Zm00001d031593_T002	0.6086477	-0.304960719	Zm00001d031593
Zm00001d004372_T004	0.9298013	0.772170143	Zm00001d004372
Zm00001d004372_T002	0.6970851	1.196847786	Zm00001d004372
Zm00001d027318_T001	0.8716622	-0.405585233	Zm00001d027318
Zm00001d024311_T008	0.9909466	0.164391946	Zm00001d024311
Zm00001d024311_T009	0.2179654	1.514792655	Zm00001d024311
Zm00001d036563_T001	0.7414776	-0.299029764	Zm00001d036563
Zm00001d038222_T002	0.425485	1.223768288	Zm00001d038222
Zm00001d038222_T003	0.7313695	1.023290443	Zm00001d038222
Zm00001d006597_T025	0.08157375	1.016834608	Zm00001d006597
Zm00001d006597_T046	0.1442732	-1.611321086	Zm00001d006597
Zm00001d006597_T028	0.9614723	0.600527889	Zm00001d006597
Zm00001d006597_T044	0.9979568	0.295935968	Zm00001d006597
Zm00001d006597_T041	0.459724	1.008573862	Zm00001d006597
Zm00001d044605_T001	0.9914912	0.148913566	Zm00001d044605
Zm00001d011850_T001	0.9609153	-0.027124973	Zm00001d011850
Zm00001d011850_T002	1	0.28515354	Zm00001d011850
Zm00001d043782_T001	0.1330007	2.034665174	Zm00001d043782
Zm00001d011330_T001	1	0.222497072	Zm00001d011330
Zm00001d002968_T001	1	0.18266227	Zm00001d002968
Zm00001d009182_T010	0.728046	1.315320904	Zm00001d009182
Zm00001d009182_T006	0.8583111	-0.224559519	Zm00001d009182
Zm00001d009182_T008	0.2860307	1.106016819	Zm00001d009182
Zm00001d009182_T002	0.8974296	0.894045698	Zm00001d009182
Zm00001d013156_T001	0.9907928	0.382423547	Zm00001d013156
Zm00001d021470_T001	0.9953345	0.177463873	Zm00001d021470
Zm00001d020046_T011	0.7925341	0.285228685	Zm00001d020046
Zm00001d020046_T001	0.02521944	-2.347296211	Zm00001d020046
Zm00001d020046_T007	0.6502105	0.324132785	Zm00001d020046
Zm00001d020046_T010	0.9914036	0.168450823	Zm00001d020046
Zm00001d020046_T009	0.9888172	0.46090071	Zm00001d020046
Zm00001d011125_T001	0.9814175	0.458704236	Zm00001d011125
Zm00001d017806_T002	0.8755291	0.852301818	Zm00001d017806
Zm00001d053623_T001	0.9139882	0.797645022	Zm00001d053623
Zm00001d009971_T001	1	0.278076539	Zm00001d009971
Zm00001d002042_T001	0.1488504	-0.827129011	Zm00001d002042
Zm00001d020315_T001	0.7985128	-0.264005955	Zm00001d020315
Zm00001d040343_T003	0.8021989	-0.149358022	Zm00001d040343
Zm00001d040343_T001	0.968034	0.202551288	Zm00001d040343
Zm00001d040343_T002	0.9664406	0.24554164	Zm00001d040343
Zm00001d040340_T001	0.9954426	0.206931877	Zm00001d040340

Zm00001d021537_T001	0.9173494	-0.140358418	Zm00001d021537
Zm00001d021537_T002	0.9419336	0.030088141	Zm00001d021537
Zm00001d012846_T004	0.1126963	-0.961400163	Zm00001d012846
Zm00001d012846_T001	0.9562304	0.101079456	Zm00001d012846
Zm00001d012846_T002	0.9902733	0.078870884	Zm00001d012846
Zm00001d053293_T001	0.7874734	-0.439973122	Zm00001d053293
Zm00001d015769_T001	0.9493302	0.359418205	Zm00001d015769
Zm00001d005910_T001	0.8192173	0.583452948	Zm00001d005910
Zm00001d009032_T001	0.571282	-0.58875909	Zm00001d009032
Zm00001d048040_T001	0.9726297	0.145189753	Zm00001d048040
Zm00001d019718_T001	0.9781672	0.424641736	Zm00001d019718
Zm00001d006051_T001	0.8719662	-0.188065161	Zm00001d006051
Zm00001d011132_T001	0.9327494	-0.333425931	Zm00001d011132
Zm00001d036322_T002	0.5627844	1.012273646	Zm00001d036322
Zm00001d036322_T001	0.007360724	-1.532422712	Zm00001d036322
Zm00001d005594_T001	0.8884927	-0.087871712	Zm00001d005594
Zm00001d049685_T005	0.9579301	0.114908327	Zm00001d049685
Zm00001d049685_T002	0.9483904	-0.212275297	Zm00001d049685
Zm00001d049685_T004	0.9573272	0.115811115	Zm00001d049685
Zm00001d049685_T003	0.9664406	0.027027914	Zm00001d049685
Zm00001d037612_T002	0.955464	0.250191608	Zm00001d037612
Zm00001d037612_T001	0.9296249	-0.027545678	Zm00001d037612
Zm00001d002787_T001	0.5097071	1.093253149	Zm00001d002787
Zm00001d024521_T001	0.954492	0.518603873	Zm00001d024521
Zm00001d044399_T001	0.8102147	0.542989937	Zm00001d044399
Zm00001d049805_T001	0.997449	0.294261343	Zm00001d049805
Zm00001d040160_T001	0.5697105	-0.538376758	Zm00001d040160
Zm00001d044251_T001	0.506885	0.677891308	Zm00001d044251
Zm00001d044251_T003	0.9760472	0.311091459	Zm00001d044251
Zm00001d044251_T004	0.9902022	0.21280301	Zm00001d044251
Zm00001d044251_T005	0.7792382	-0.476344091	Zm00001d044251
Zm00001d000439_T001	0.2094239	-0.574415781	Zm00001d000439
Zm00001d000439_T005	0.8214774	-0.251081131	Zm00001d000439
Zm00001d000439_T002	0.9692063	0.479690075	Zm00001d000439
Zm00001d006613_T004	0.8028573	0.706921046	Zm00001d006613
Zm00001d006613_T005	0.6530894	-0.463487299	Zm00001d006613
Zm00001d006613_T001	0.9942835	0.50175657	Zm00001d006613
Zm00001d006613_T003	0.2774057	0.472994607	Zm00001d006613
Zm00001d013497_T001	0.9881407	0.280358541	Zm00001d013497
Zm00001d010956_T001	0.5843839	1.342463544	Zm00001d010956
Zm00001d003185_T001	0.9740325	0.169157212	Zm00001d003185
Zm00001d049793_T001	0.9972036	0.232273988	Zm00001d049793
Zm00001d035558_T001	0.002725334	-0.94853639	Zm00001d035558
Zm00001d028922_T001	0.9429712	-0.239596744	Zm00001d028922
Zm00001d050470_T002	0.9984041	0.241409107	Zm00001d050470
Zm00001d031979_T001	1	-0.099044191	Zm00001d031979
Zm00001d003025_T001	0.9104115	-0.057149274	Zm00001d003025
Zm00001d043256_T001	0.9950761	0.34500129	Zm00001d043256
Zm00001d002696_T001	0.998484	0.308391529	Zm00001d002696
Zm00001d020536_T001	0.6354704	-0.46302887	Zm00001d020536
Zm00001d042062_T001	0.9176451	0.485928978	Zm00001d042062
Zm00001d035751_T006	0.8274801	-0.650991832	Zm00001d035751
Zm00001d035751_T002	1	0.23062259	Zm00001d035751
Zm00001d040117_T001	0.9219195	0.693988717	Zm00001d040117

Zm00001d040117_T004	0.7802354	-0.40900419	Zm00001d040117
Zm00001d040117_T002	0.2916362	-1.112937997	Zm00001d040117
Zm00001d040117_T003	0.9836566	0.431267705	Zm00001d040117
Zm00001d031540_T002	0.9804228	0.454962906	Zm00001d031540
Zm00001d031540_T001	0.9962521	0.251864224	Zm00001d031540
Zm00001d047438_T001	0.4744861	1.22787765	Zm00001d047438
Zm00001d025193_T005	0.1501728	-1.225181854	Zm00001d025193
Zm00001d025193_T001	1	0.387086473	Zm00001d025193
Zm00001d048582_T001	0.1614401	-0.669319013	Zm00001d048582
Zm00001d053399_T001	1	0.360418729	Zm00001d053399
Zm00001d053399_T004	0.8028573	0.794433453	Zm00001d053399
Zm00001d037032_T001	0.4292753	-0.593822383	Zm00001d037032
Zm00001d050484_T002	0.9907928	0.187763903	Zm00001d050484
Zm00001d050484_T015	0.4438025	1.008366356	Zm00001d050484
Zm00001d025519_T003	0.6878307	0.800059999	Zm00001d025519
Zm00001d010446_T001	0.7248132	-0.243915703	Zm00001d010446
Zm00001d022139_T001	0.06348214	2.397348723	Zm00001d022139
Zm00001d025475_T001	0.8641671	-0.131751883	Zm00001d025475
Zm00001d051704_T003	0.5695353	0.916429163	Zm00001d051704
Zm00001d051704_T002	0.9775363	0.414959826	Zm00001d051704
Zm00001d048149_T002	0.9690925	0.094289147	Zm00001d048149
Zm00001d012965_T001	0.8720862	-0.26864719	Zm00001d012965
Zm00001d032132_T001	0.9180662	1.190089676	Zm00001d032132
Zm00001d013309_T001	0.877932	0.054196553	Zm00001d013309
Zm00001d045051_T001	0.0325408	2.427394179	Zm00001d045051
Zm00001d008354_T002	1.23E-05	-0.627414799	Zm00001d008354
Zm00001d008354_T006	0.9902022	0.265727141	Zm00001d008354
Zm00001d008354_T001	0.07256374	1.103741129	Zm00001d008354
Zm00001d008354_T005	0.9239172	-0.024878414	Zm00001d008354
Zm00001d008354_T003	0.3550006	0.455040452	Zm00001d008354
Zm00001d014667_T001	0.02561053	-2.141809968	Zm00001d014667
Zm00001d012156_T045	0.2541496	-0.260084977	Zm00001d012156
Zm00001d012156_T051	0.001312528	-1.291491931	Zm00001d012156
Zm00001d012156_T030	4.05E-10	1.021001634	Zm00001d012156
Zm00001d012156_T019	0.4822679	-0.861804395	Zm00001d012156
Zm00001d012156_T018	0.8030099	-0.130651443	Zm00001d012156
Zm00001d012156_T028	0.05880109	0.279510047	Zm00001d012156
Zm00001d012156_T053	0.6882816	-0.409928148	Zm00001d012156
Zm00001d012156_T013	0.3064965	1.508633779	Zm00001d012156
Zm00001d012156_T020	0.8009614	0.715053982	Zm00001d012156
Zm00001d012156_T035	0.000118746	0.736966839	Zm00001d012156
Zm00001d012156_T032	0.003460563	1.737478208	Zm00001d012156
Zm00001d012934_T001	0.4647287	1.485900573	Zm00001d012934
Zm00001d006791_T001	0.000329926	-3.048285051	Zm00001d006791
Zm00001d028472_T002	0.8497683	0.91093419	Zm00001d028472
Zm00001d028472_T006	0.666346	0.685980957	Zm00001d028472
Zm00001d028472_T007	2.70E-12	2.652338785	Zm00001d028472
Zm00001d028472_T004	0.9476864	0.65793479	Zm00001d028472
Zm00001d028472_T001	0.1433069	-0.856176341	Zm00001d028472
Zm00001d003399_T001	0.01753567	2.723362918	Zm00001d003399
Zm00001d029408_T001	0.9083657	0.713327423	Zm00001d029408
Zm00001d034655_T001	0.7255406	1.142465358	Zm00001d034655
Zm00001d014848_T001	0.9720324	0.217076197	Zm00001d014848
Zm00001d017753_T001	0.9988864	0.415914986	Zm00001d017753

Zm00001d017753_T003	1	0.371464308	Zm00001d017753
Zm00001d041767_T004	0.7731926	1.623550345	Zm00001d041767
Zm00001d041767_T001	0.4442914	-0.795081628	Zm00001d041767
Zm00001d041767_T005	0.2325006	0.307245776	Zm00001d041767
Zm00001d041767_T003	0.9606343	0.045453301	Zm00001d041767
Zm00001d018365_T006	0.7169281	-0.067140221	Zm00001d018365
Zm00001d018365_T007	0.3870314	1.207306319	Zm00001d018365
Zm00001d018365_T004	0.4919699	0.04335652	Zm00001d018365
Zm00001d018365_T002	0.9561521	0.25769122	Zm00001d018365
Zm00001d018365_T001	0.9745433	0.052833001	Zm00001d018365
Zm00001d016697_T001	1	-0.033591179	Zm00001d016697
Zm00001d033530_T001	0.9571192	-0.362371034	Zm00001d033530
Zm00001d039790_T001	0.8353463	-0.232655447	Zm00001d039790
Zm00001d033674_T012	5.71E-11	-2.43980121	Zm00001d033674
Zm00001d033674_T002	0.9469426	0.127713754	Zm00001d033674
Zm00001d033674_T009	0.3296668	1.653280548	Zm00001d033674
Zm00001d033674_T005	0.9822138	0.097196314	Zm00001d033674
Zm00001d002811_T002	0.3812438	-0.827977857	Zm00001d002811
Zm00001d002811_T001	0.2042008	-0.886853837	Zm00001d002811
Zm00001d035184_T001	0.8828298	-0.080987986	Zm00001d035184
Zm00001d006194_T001	0.9380783	-0.238707973	Zm00001d006194
Zm00001d019472_T007	1	0.444505019	Zm00001d019472
Zm00001d019472_T006	0.1439251	1.256338909	Zm00001d019472
Zm00001d019472_T001	0.5864438	0.493610839	Zm00001d019472
Zm00001d019472_T003	0.06865764	2.187146078	Zm00001d019472
Zm00001d019472_T002	1	0.405735254	Zm00001d019472
Zm00001d019472_T010	0.09013732	1.874379098	Zm00001d019472
Zm00001d019472_T005	2.20E-10	-2.01145632	Zm00001d019472
Zm00001d019472_T013	0.6325529	1.068141653	Zm00001d019472
Zm00001d014467_T002	0.1184028	2.050675215	Zm00001d014467
Zm00001d014467_T001	0.9804228	0.202975636	Zm00001d014467
Zm00001d053751_T001	0.9441446	0.541855304	Zm00001d053751
Zm00001d027711_T003	0.965836	0.608359011	Zm00001d027711
Zm00001d051320_T007	0.389931	1.036276502	Zm00001d051320
Zm00001d051320_T004	0.9664019	0.090365927	Zm00001d051320
Zm00001d051320_T002	0.5327463	0.308713343	Zm00001d051320
Zm00001d049714_T002	0.9067693	-0.008935268	Zm00001d049714
Zm00001d049714_T001	0.2860325	0.831866621	Zm00001d049714
Zm00001d029648_T001	0.4422995	-0.268235239	Zm00001d029648
Zm00001d018807_T001	0.9469426	0.648104035	Zm00001d018807
Zm00001d006159_T002	1	-0.058387028	Zm00001d006159
Zm00001d006159_T001	0.8172581	-0.560965958	Zm00001d006159
Zm00001d032385_T001	0.8448074	-0.129457485	Zm00001d032385
Zm00001d039816_T002	0.9596377	0.628263801	Zm00001d039816
Zm00001d039816_T001	0.923684	0.691368734	Zm00001d039816
Zm00001d024540_T001	0.9766874	0.182652536	Zm00001d024540
Zm00001d033468_T001	1	0.064021163	Zm00001d033468
Zm00001d009221_T002	0.02280843	-1.418324346	Zm00001d009221
Zm00001d009221_T003	0.5100873	1.062572929	Zm00001d009221
Zm00001d009221_T001	0.9567316	0.67769122	Zm00001d009221
Zm00001d016493_T001	0.7907819	-0.380294898	Zm00001d016493
Zm00001d046005_T001	0.8816665	0.919049753	Zm00001d046005
Zm00001d003422_T001	0.956479	0.383220789	Zm00001d003422
Zm00001d023530_T001	0.8693951	0.384061535	Zm00001d023530

Zm00001d023530_T013	0.9979568	0.052849862	Zm00001d023530
Zm00001d023530_T003	0.2202602	0.359789586	Zm00001d023530
Zm00001d020881_T003	0.1142785	1.857499419	Zm00001d020881
Zm00001d020881_T002	1.28E-09	1.773152626	Zm00001d020881
Zm00001d020881_T006	0.1624781	1.975448984	Zm00001d020881
Zm00001d020881_T001	0.001343417	-1.664822673	Zm00001d020881
Zm00001d020881_T007	0.008931882	1.436296447	Zm00001d020881
Zm00001d048627_T002	0.9902022	0.417325383	Zm00001d048627
Zm00001d048627_T001	0.9673473	0.618825395	Zm00001d048627
Zm00001d020900_T003	1	0.160750289	Zm00001d020900
Zm00001d015581_T001	1	0.369792394	Zm00001d015581
Zm00001d025720_T005	0.8922572	0.463193859	Zm00001d025720
Zm00001d025720_T002	0.9887584	0.226030085	Zm00001d025720
Zm00001d012633_T001	0.9164622	0.809921092	Zm00001d012633
Zm00001d006415_T005	1	0.27323314	Zm00001d006415
Zm00001d006415_T008	1.36E-08	0.799143785	Zm00001d006415
Zm00001d006415_T001	3.12E-11	2.430932091	Zm00001d006415
Zm00001d006415_T004	0.9216521	0.25864145	Zm00001d006415
Zm00001d006415_T018	0.9138633	-0.031437378	Zm00001d006415
Zm00001d006415_T002	3.93E-05	-1.259450059	Zm00001d006415
Zm00001d006415_T006	0.923684	0.32185128	Zm00001d006415
Zm00001d006415_T016	1.16E-06	0.62893693	Zm00001d006415
Zm00001d025957_T001	0.2829145	-1.130208042	Zm00001d025957
Zm00001d027354_T002	0.9089465	-0.006110063	Zm00001d027354
Zm00001d027354_T001	0.9410688	0.062217992	Zm00001d027354
Zm00001d039105_T004	9.76E-09	1.543706374	Zm00001d039105
Zm00001d039105_T003	0.3114566	0.935272074	Zm00001d039105
Zm00001d039105_T001	0.7626242	0.978940585	Zm00001d039105
Zm00001d039105_T005	0.9369505	-0.027731663	Zm00001d039105
Zm00001d028010_T001	0.9895518	0.495080923	Zm00001d028010
Zm00001d053739_T001	0.9681793	0.568835634	Zm00001d053739
Zm00001d016363_T001	0.5618399	-0.972016138	Zm00001d016363
Zm00001d016363_T002	0.8380433	-0.364634922	Zm00001d016363
Zm00001d045206_T003	0.5830307	1.400453165	Zm00001d045206
Zm00001d045206_T001	1	0.128180842	Zm00001d045206
Zm00001d045206_T005	0.7578713	0.895961749	Zm00001d045206
Zm00001d045206_T002	0.3632201	1.544652724	Zm00001d045206
Zm00001d046581_T022	0.9771927	0.015612925	Zm00001d046581
Zm00001d046581_T009	0.8790306	-0.073390105	Zm00001d046581
Zm00001d046581_T010	0.9881957	0.152805661	Zm00001d046581
Zm00001d046581_T001	0.3987447	0.87873294	Zm00001d046581
Zm00001d046581_T005	0.6330708	0.336117066	Zm00001d046581
Zm00001d046581_T012	3.12E-11	2.12653251	Zm00001d046581
Zm00001d046581_T003	0.8227647	0.893496265	Zm00001d046581
Zm00001d031329_T004	0.2930734	-0.478490592	Zm00001d031329
Zm00001d031329_T005	0.6339643	-0.470505502	Zm00001d031329
Zm00001d012717_T004	0.1776326	0.760636642	Zm00001d012717
Zm00001d023922_T005	0.1089785	1.698606812	Zm00001d023922
Zm00001d023922_T006	0.8569937	-0.051965026	Zm00001d023922
Zm00001d023922_T004	0.5081812	0.582615189	Zm00001d023922
Zm00001d023922_T001	0.9988864	0.43422667	Zm00001d023922
Zm00001d014806_T001	0.5408146	0.831767261	Zm00001d014806
Zm00001d045864_T005	0.9766874	0.459083356	Zm00001d045864
Zm00001d045864_T002	0.8545272	0.900544515	Zm00001d045864

Zm00001d045864_T006	0.507122	-1.04813925	Zm00001d045864
Zm00001d034175_T001	0.1590045	-1.490235314	Zm00001d034175
Zm00001d043620_T001	0.9971866	0.308248245	Zm00001d043620
Zm00001d016379_T001	0.05664664	2.127269072	Zm00001d016379
Zm00001d050714_T001	0.7869719	0.518500887	Zm00001d050714
Zm00001d018935_T003	0.8597186	0.670284661	Zm00001d018935
Zm00001d018935_T001	0.03188332	0.628727509	Zm00001d018935
Zm00001d025915_T006	1	0.308737162	Zm00001d025915
Zm00001d025915_T002	0.2204954	-0.82710456	Zm00001d025915
Zm00001d025915_T004	0.01325275	-0.646867065	Zm00001d025915
Zm00001d025915_T007	0.9853731	0.349806927	Zm00001d025915
Zm00001d025915_T005	6.06E-07	2.10451068	Zm00001d025915
Zm00001d049148_T001	0.7888267	-0.22720115	Zm00001d049148
Zm00001d044828_T001	0.9217388	0.715210826	Zm00001d044828
Zm00001d045675_T001	0.4703859	-0.260554811	Zm00001d045675
Zm00001d045675_T002	0.00759149	-1.897451332	Zm00001d045675
Zm00001d045675_T003	0.8898843	0.947589553	Zm00001d045675
Zm00001d045675_T004	0.7571802	1.016940462	Zm00001d045675
Zm00001d037780_T001	0.973825	0.106672567	Zm00001d037780
Zm00001d006527_T001	0.9224387	0.007942036	Zm00001d006527
Zm00001d001987_T003	0.956479	0.046270424	Zm00001d001987
Zm00001d053572_T012	0.7788277	-0.177092552	Zm00001d053572
Zm00001d053572_T007	0.04453576	0.616944219	Zm00001d053572
Zm00001d053572_T004	0.9213038	1.280275574	Zm00001d053572
Zm00001d053572_T009	0.7990254	0.607089042	Zm00001d053572
Zm00001d053572_T010	0.8922384	-0.001121612	Zm00001d053572
Zm00001d053572_T008	0.9719418	0.199227275	Zm00001d053572
Zm00001d035322_T001	0.3583641	-1.010656976	Zm00001d035322
Zm00001d028963_T001	0.9791729	-0.157597481	Zm00001d028963
Zm00001d009719_T002	0.9689939	0.572690282	Zm00001d009719
Zm00001d009719_T003	0.9689939	0.507953095	Zm00001d009719
Zm00001d009719_T001	0.8917973	-0.149262275	Zm00001d009719
Zm00001d052225_T001	0.956184	-0.040897454	Zm00001d052225
Zm00001d007477_T001	0.711844	0.907825001	Zm00001d007477
Zm00001d025674_T001	0.5656355	-0.440634792	Zm00001d025674
Zm00001d034823_T002	0.9767085	0.340715872	Zm00001d034823
Zm00001d030373_T006	0.9845789	0.58138721	Zm00001d030373
Zm00001d030373_T004	0.983226	0.17341099	Zm00001d030373
Zm00001d030373_T007	0.00185145	-2.423036045	Zm00001d030373
Zm00001d030373_T003	0.9872831	0.516161988	Zm00001d030373
Zm00001d030373_T005	0.06071496	-2.060289934	Zm00001d030373
Zm00001d040535_T012	0.8488494	0.188954846	Zm00001d040535
Zm00001d040535_T009	0.1297822	0.450077475	Zm00001d040535
Zm00001d040535_T011	0.6587378	0.31130652	Zm00001d040535
Zm00001d040535_T002	0.8522942	-0.144586619	Zm00001d040535
Zm00001d040535_T004	0.961473	0.107383121	Zm00001d040535
Zm00001d026047_T002	0.9361655	0.699678775	Zm00001d026047
Zm00001d026047_T001	0.4669227	-1.231833867	Zm00001d026047
Zm00001d023320_T004	0.908501	0.72354941	Zm00001d023320
Zm00001d023320_T001	0.04913338	1.283150926	Zm00001d023320
Zm00001d023320_T005	0.919888	0.663054602	Zm00001d023320
Zm00001d015861_T002	0.8987811	-0.107216944	Zm00001d015861
Zm00001d015861_T004	1	0.376781414	Zm00001d015861
Zm00001d015861_T003	0.6313255	-0.28103756	Zm00001d015861

Zm00001d015861_T001	0.9791729	0.126345056	Zm00001d015861
Zm00001d012304_T001	0.07267369	-1.484371918	Zm00001d012304
Zm00001d009495_T001	0.8093303	-0.390176446	Zm00001d009495
Zm00001d018036_T002	0.9812314	0.51236938	Zm00001d018036
Zm00001d018036_T003	0.9216521	-0.063509782	Zm00001d018036
Zm00001d018036_T001	0.8747821	0.371601036	Zm00001d018036
Zm00001d021821_T002	0.8513569	-0.090062114	Zm00001d021821
Zm00001d021821_T001	0.9897446	0.477551787	Zm00001d021821
Zm00001d048687_T002	0.967161	0.563834775	Zm00001d048687
Zm00001d047441_T001	0.9654804	0.444769685	Zm00001d047441
Zm00001d008816_T001	0.5576332	1.412786642	Zm00001d008816
Zm00001d007975_T001	0.8609127	-0.088556979	Zm00001d007975
Zm00001d004460_T006	0.9377241	0.012398244	Zm00001d004460
Zm00001d004460_T009	0.8834929	0.006380948	Zm00001d004460
Zm00001d019852_T004	0.4661639	0.38013646	Zm00001d019852
Zm00001d019852_T005	0.9836566	0.43002574	Zm00001d019852
Zm00001d013886_T001	0.9514141	0.48229817	Zm00001d013886
Zm00001d013886_T002	0.3389411	1.678615428	Zm00001d013886
Zm00001d005230_T001	0.909729	-0.611296553	Zm00001d005230
Zm00001d015791_T001	0.3692327	-0.897924298	Zm00001d015791
Zm00001d039193_T001	0.4093561	-0.204042483	Zm00001d039193
Zm00001d015138_T002	0.06987094	2.152963616	Zm00001d015138
Zm00001d015138_T004	0.6891365	1.007845253	Zm00001d015138
Zm00001d015138_T001	0.9257893	0.837793611	Zm00001d015138
Zm00001d015138_T005	8.45E-05	3.293992958	Zm00001d015138
Zm00001d002125_T013	0.1781613	-0.296756281	Zm00001d002125
Zm00001d002125_T001	0.001129598	-1.020297462	Zm00001d002125
Zm00001d002125_T016	0.03161488	1.311217767	Zm00001d002125
Zm00001d002125_T006	4.05E-06	2.445135391	Zm00001d002125
Zm00001d002125_T018	0.769035	-0.289533816	Zm00001d002125
Zm00001d002125_T024	0.3242782	0.598017516	Zm00001d002125
Zm00001d019306_T001	0.9984041	0.313697612	Zm00001d019306
Zm00001d019306_T002	0.4181514	-0.326488959	Zm00001d019306
Zm00001d044278_T005	1	0.066882515	Zm00001d044278
Zm00001d044278_T003	0.9450101	0.042323009	Zm00001d044278
Zm00001d044278_T001	0.8449395	-0.21476454	Zm00001d044278
Zm00001d003021_T002	0.7492043	-0.416493322	Zm00001d003021
Zm00001d003021_T004	1	0.613590004	Zm00001d003021
Zm00001d021968_T001	0.9979568	0.304298415	Zm00001d021968
Zm00001d034369_T001	0.2642794	-1.188667405	Zm00001d034369
Zm00001d011144_T001	0.957148	0.400737045	Zm00001d011144
Zm00001d011144_T002	0.3940016	1.570919434	Zm00001d011144
Zm00001d022048_T005	0.9881407	0.216810559	Zm00001d022048
Zm00001d022048_T001	0.954492	0.614297075	Zm00001d022048
Zm00001d010902_T002	0.820661	-0.220185676	Zm00001d010902
Zm00001d010902_T004	0.9907739	0.36125938	Zm00001d010902
Zm00001d010902_T003	0.8904146	0.818335877	Zm00001d010902
Zm00001d010902_T001	0.4286831	0.826887263	Zm00001d010902
Zm00001d010902_T007	0.9224378	0.667518461	Zm00001d010902
Zm00001d012875_T001	0.9523023	0.134005947	Zm00001d012875
Zm00001d043503_T001	0.04072087	-1.07914375	Zm00001d043503
Zm00001d024059_T002	0.9975882	0.434764714	Zm00001d024059
Zm00001d011188_T001	0.9419336	0.012404836	Zm00001d011188
Zm00001d002113_T015	0.6936253	-0.004485059	Zm00001d002113

Zm00001d002113_T026	0.8716622	-0.094380729	Zm00001d002113
Zm00001d002113_T011	0.6215505	0.434391548	Zm00001d002113
Zm00001d002113_T025	0.4146004	-0.147121908	Zm00001d002113
Zm00001d002113_T019	2.04E-07	0.539522025	Zm00001d002113
Zm00001d002113_T024	0.836612	0.552094619	Zm00001d002113
Zm00001d002113_T001	0.04606011	1.055703185	Zm00001d002113
Zm00001d009765_T009	0.09302212	-0.97884063	Zm00001d009765
Zm00001d009765_T008	0.7289978	0.022513633	Zm00001d009765
Zm00001d041073_T002	0.8187452	0.339580411	Zm00001d041073
Zm00001d041073_T001	0.9609806	0.633479651	Zm00001d041073
Zm00001d010625_T002	0.658695	1.117110416	Zm00001d010625
Zm00001d010625_T003	0.4044152	-0.553703189	Zm00001d010625
Zm00001d036403_T001	0.6863856	0.40856604	Zm00001d036403
Zm00001d036403_T002	0.9921225	0.21571425	Zm00001d036403
Zm00001d044903_T009	0.9561521	0.137743189	Zm00001d044903
Zm00001d044903_T005	0.00018737	1.795704406	Zm00001d044903
Zm00001d044903_T001	0.9682673	0.169876185	Zm00001d044903
Zm00001d032533_T001	0.9835181	-0.722697144	Zm00001d032533
Zm00001d007507_T006	1	0.087611644	Zm00001d007507
Zm00001d007507_T003	0.02669303	-0.84160919	Zm00001d007507
Zm00001d007507_T005	0.8095705	-0.261237286	Zm00001d007507
Zm00001d007507_T007	0.9747606	0.146985264	Zm00001d007507
Zm00001d007507_T001	0.1113752	1.128122038	Zm00001d007507
Zm00001d015090_T001	0.9355135	0.707099107	Zm00001d015090
Zm00001d015090_T005	0.9165176	-0.386915323	Zm00001d015090
Zm00001d015090_T002	0.998484	-0.13636486	Zm00001d015090
Zm00001d015090_T003	0.01592825	-1.172119079	Zm00001d015090
Zm00001d052415_T001	0.5550339	1.198282133	Zm00001d052415
Zm00001d005679_T001	0.8412913	-0.017232102	Zm00001d005679
Zm00001d046468_T005	0.06313309	1.332615834	Zm00001d046468
Zm00001d046468_T003	0.002879391	2.251009527	Zm00001d046468
Zm00001d046468_T004	0.3973152	0.748151091	Zm00001d046468
Zm00001d046468_T002	0.2225121	-0.897133956	Zm00001d046468
Zm00001d038065_T004	0.957148	0.029156587	Zm00001d038065
Zm00001d038065_T001	0.9839804	0.410910876	Zm00001d038065
Zm00001d038065_T003	0.9791729	0.466525998	Zm00001d038065
Zm00001d038065_T002	0.8702994	-0.095692098	Zm00001d038065
Zm00001d016636_T001	0.8940288	-0.649769263	Zm00001d016636
Zm00001d054057_T001	0.05984445	2.457913262	Zm00001d054057
Zm00001d016705_T002	0.07581914	-1.742201059	Zm00001d016705
Zm00001d016705_T001	0.00468038	-2.784221465	Zm00001d016705
Zm00001d038036_T001	0.8896831	-0.329639101	Zm00001d038036
Zm00001d035817_T001	0.7718694	1.137584816	Zm00001d035817
Zm00001d044515_T001	0.9894615	0.216266977	Zm00001d044515
Zm00001d012824_T001	0.9732421	-0.028932819	Zm00001d012824
Zm00001d045397_T001	0.9348844	0.081299606	Zm00001d045397
Zm00001d031985_T001	0.1974301	-1.436459494	Zm00001d031985
Zm00001d026197_T001	0.1927347	-0.834274352	Zm00001d026197
Zm00001d038543_T004	0.8158142	0.591552608	Zm00001d038543
Zm00001d038543_T001	0.9429712	-0.129387865	Zm00001d038543
Zm00001d051431_T006	0.9701986	0.012256884	Zm00001d051431
Zm00001d051431_T005	0.1670357	1.848076172	Zm00001d051431
Zm00001d051431_T010	0.006338361	2.260871828	Zm00001d051431
Zm00001d051431_T008	0.85068	0.725674463	Zm00001d051431

Zm00001d051431_T002	2.46E-12	-2.685498287	Zm00001d051431
Zm00001d018128_T001	0.9967276	0.298510198	Zm00001d018128
Zm00001d009759_T001	0.9213556	0.701326659	Zm00001d009759
Zm00001d037732_T002	0.9252088	-0.147521686	Zm00001d037732
Zm00001d037732_T004	0.003949247	-1.819493112	Zm00001d037732
Zm00001d037732_T003	0.7712474	-0.115209905	Zm00001d037732
Zm00001d043609_T001	1	0.331809853	Zm00001d043609
Zm00001d043609_T004	0.9404125	0.680890313	Zm00001d043609
Zm00001d014814_T002	0.7214524	-0.411828584	Zm00001d014814
Zm00001d014814_T004	0.002725334	-1.128265832	Zm00001d014814
Zm00001d016743_T001	0.3125023	-0.979592694	Zm00001d016743
Zm00001d042827_T003	0.941922	0.67865759	Zm00001d042827
Zm00001d042827_T001	0.8412913	0.759226898	Zm00001d042827
Zm00001d016456_T007	0.4952796	0.518579798	Zm00001d016456
Zm00001d016456_T001	0.1096824	1.358268588	Zm00001d016456
Zm00001d016456_T003	3.38E-08	-1.28276899	Zm00001d016456
Zm00001d005441_T001	0.8575285	-0.081084477	Zm00001d005441
Zm00001d004481_T001	0.946422	0.408523475	Zm00001d004481
Zm00001d003179_T001	0.9860836	0.128929115	Zm00001d003179
Zm00001d011068_T001	0.9966571	0.336505654	Zm00001d011068
Zm00001d021132_T002	0.3360774	1.032076127	Zm00001d021132
Zm00001d021132_T005	0.8251448	0.926914559	Zm00001d021132
Zm00001d021132_T001	0.0140994	-2.841331908	Zm00001d021132
Zm00001d021132_T004	0.8964117	0.759141054	Zm00001d021132
Zm00001d048299_T002	0.9979568	0.34503414	Zm00001d048299
Zm00001d007767_T001	0.9870394	0.190414719	Zm00001d007767
Zm00001d003426_T002	0.8581679	-0.152192294	Zm00001d003426
Zm00001d003426_T003	0.9003815	0.470781641	Zm00001d003426
Zm00001d039197_T001	0.03044968	-1.260688931	Zm00001d039197
Zm00001d015992_T006	0.8809462	0.836242985	Zm00001d015992
Zm00001d015992_T003	0.9856033	-0.067577802	Zm00001d015992
Zm00001d015992_T001	0.6661718	0.433765643	Zm00001d015992
Zm00001d015992_T002	0.525543	1.405694557	Zm00001d015992
Zm00001d027442_T001	0.8300193	-0.266987388	Zm00001d027442
Zm00001d000382_T001	0.7137782	0.584513297	Zm00001d000382
Zm00001d020837_T001	0.1206554	1.8358676	Zm00001d020837
Zm00001d028707_T003	0.9690782	0.421465927	Zm00001d028707
Zm00001d028707_T002	0.9469426	-0.004094545	Zm00001d028707
Zm00001d028707_T001	1	0.060523157	Zm00001d028707
Zm00001d009508_T001	0.9979568	0.39252943	Zm00001d009508
Zm00001d038755_T001	0.9130008	-0.5270856	Zm00001d038755
Zm00001d021507_T002	1	0.35870847	Zm00001d021507
Zm00001d004222_T001	0.9073468	0.55930391	Zm00001d004222
Zm00001d054105_T001	0.8987811	0.411009208	Zm00001d054105
Zm00001d054105_T007	0.3288311	1.446437675	Zm00001d054105
Zm00001d054105_T009	0.1012839	-1.448394068	Zm00001d054105
Zm00001d054105_T003	0.7251353	0.785272812	Zm00001d054105
Zm00001d054105_T006	0.9308557	-0.293539314	Zm00001d054105
Zm00001d046602_T001	0.9740325	0.137522892	Zm00001d046602
Zm00001d010214_T003	0.2146312	1.706661731	Zm00001d010214
Zm00001d010214_T002	0.5208223	1.357660054	Zm00001d010214
Zm00001d039824_T001	0.852305	0.37364011	Zm00001d039824
Zm00001d035588_T001	0.9486412	0.012286356	Zm00001d035588
Zm00001d052068_T001	0.5259667	-0.831334823	Zm00001d052068

Zm00001d021697_T001	0.8489797	-0.133312827	Zm00001d021697
Zm00001d011619_T004	0.2570953	0.962491109	Zm00001d011619
Zm00001d011619_T006	0.03722719	1.077716383	Zm00001d011619
Zm00001d011619_T003	0.9792387	0.178331363	Zm00001d011619
Zm00001d011619_T007	0.2346697	1.344163087	Zm00001d011619
Zm00001d019895_T001	0.4880775	0.898376722	Zm00001d019895
Zm00001d017925_T004	0.5806888	1.316640857	Zm00001d017925
Zm00001d031882_T001	0.04701349	-1.588816222	Zm00001d031882
Zm00001d043565_T001	1	-0.222669512	Zm00001d043565
Zm00001d021224_T001	0.8619072	-0.1392109	Zm00001d021224
Zm00001d026321_T001	0.9537113	0.499805586	Zm00001d026321
Zm00001d018374_T001	0.9791066	0.138562528	Zm00001d018374
Zm00001d017454_T001	0.6511883	-0.572375248	Zm00001d017454
Zm00001d030916_T002	3.05E-07	-2.220879145	Zm00001d030916
Zm00001d030916_T001	0.9946911	0.503584153	Zm00001d030916
Zm00001d031995_T002	0.9173494	-0.182071949	Zm00001d031995
Zm00001d026460_T001	0.3944431	1.644628396	Zm00001d026460
Zm00001d001902_T001	0.986738	0.33289998	Zm00001d001902
Zm00001d029597_T001	0.8863456	-0.182410509	Zm00001d029597
Zm00001d029597_T002	0.9969123	0.328862897	Zm00001d029597
Zm00001d004545_T001	0.9612081	0.585730615	Zm00001d004545
Zm00001d040276_T001	0.9799748	0.171529155	Zm00001d040276
Zm00001d004414_T001	0.926133	-0.145672348	Zm00001d004414
Zm00001d051674_T016	0.749176	0.273073339	Zm00001d051674
Zm00001d051674_T002	0.08778843	0.804560611	Zm00001d051674
Zm00001d051674_T007	0.09453749	0.645789622	Zm00001d051674
Zm00001d051674_T003	0.9345575	0.143212497	Zm00001d051674
Zm00001d051674_T017	0.9950761	0.118420299	Zm00001d051674
Zm00001d043171_T001	0.9747695	0.193454052	Zm00001d043171
Zm00001d018904_T005	0.8786965	0.904450259	Zm00001d018904
Zm00001d018904_T001	9.27E-15	3.999817402	Zm00001d018904
Zm00001d018904_T003	1.09E-06	0.760309218	Zm00001d018904
Zm00001d018904_T008	0.0394043	-2.053400784	Zm00001d018904
Zm00001d018904_T002	1.86E-11	-2.100257803	Zm00001d018904
Zm00001d014790_T001	0.001629813	-2.840968743	Zm00001d014790
Zm00001d044573_T068	0.9422098	0.056207661	Zm00001d044573
Zm00001d044573_T003	0.876638	0.547525143	Zm00001d044573
Zm00001d044573_T010	0.03004912	1.879782247	Zm00001d044573
Zm00001d044573_T007	0.9907928	-0.050977695	Zm00001d044573
Zm00001d044573_T001	0.2450103	-0.322735421	Zm00001d044573
Zm00001d044573_T070	0.8816374	-0.23503157	Zm00001d044573
Zm00001d053046_T001	0.3492462	1.234228378	Zm00001d053046
Zm00001d036396_T003	0.8945288	-0.05021773	Zm00001d036396
Zm00001d036396_T001	0.9469426	-0.013321424	Zm00001d036396
Zm00001d049351_T001	0.9543237	0.402624113	Zm00001d049351
Zm00001d049351_T002	0.9817074	0.088161113	Zm00001d049351
Zm00001d016765_T001	0.8755938	0.849747234	Zm00001d016765
Zm00001d035561_T001	0.9887889	0.114439404	Zm00001d035561
Zm00001d032356_T001	0.7501	-0.225174584	Zm00001d032356
Zm00001d014774_T001	0.9380783	0.052398004	Zm00001d014774
Zm00001d007632_T006	0.5273755	-0.145764533	Zm00001d007632
Zm00001d007632_T001	0.429391	-0.151934165	Zm00001d007632
Zm00001d007632_T012	0.8571283	0.366411413	Zm00001d007632
Zm00001d007632_T007	7.16E-06	0.395871349	Zm00001d007632

Zm00001d007632_T014	0.8607922	0.611593959	Zm00001d007632
Zm00001d007632_T011	0.9513407	0.429478978	Zm00001d007632
Zm00001d007632_T010	0.0004805	-2.05745583	Zm00001d007632
Zm00001d007632_T002	0.4164012	1.104072028	Zm00001d007632
Zm00001d002537_T001	0.9573434	0.076117361	Zm00001d002537
Zm00001d017878_T001	0.9493302	0.022635487	Zm00001d017878
Zm00001d005808_T003	0.9596377	0.159150423	Zm00001d005808
Zm00001d005808_T002	0.9053391	0.713088852	Zm00001d005808
Zm00001d005808_T010	0.9053391	0.727926061	Zm00001d005808
Zm00001d005808_T006	0.04606011	1.676681313	Zm00001d005808
Zm00001d005808_T001	0.5440777	1.21403753	Zm00001d005808
Zm00001d012918_T003	0.997449	0.232747822	Zm00001d012918
Zm00001d017139_T001	0.2509097	0.936068977	Zm00001d017139
Zm00001d017139_T002	0.6507629	1.279342	Zm00001d017139
Zm00001d029934_T002	0.9218815	0.587909824	Zm00001d029934
Zm00001d015208_T003	0.9412101	0.130911478	Zm00001d015208
Zm00001d015208_T002	0.05464768	0.475233342	Zm00001d015208
Zm00001d045393_T003	0.9100328	0.365774281	Zm00001d045393
Zm00001d029137_T003	0.6377926	-0.310022383	Zm00001d029137
Zm00001d029137_T002	0.2562638	1.016765712	Zm00001d029137
Zm00001d029137_T001	0.8637863	-0.029102285	Zm00001d029137
Zm00001d025964_T001	0.9942835	0.139330992	Zm00001d025964
Zm00001d002184_T002	0.9853786	0.187257613	Zm00001d002184
Zm00001d002184_T001	1	0.240999255	Zm00001d002184
Zm00001d022315_T001	1	0.21182541	Zm00001d022315
Zm00001d022110_T001	0.7865309	-0.403165086	Zm00001d022110
Zm00001d022110_T004	0.5057074	-0.113521409	Zm00001d022110
Zm00001d028232_T007	0.9361945	0.327792019	Zm00001d028232
Zm00001d028232_T004	0.9627389	-0.176809036	Zm00001d028232
Zm00001d007089_T010	0.7778639	-0.250752937	Zm00001d007089
Zm00001d007089_T048	0.009321786	1.321253495	Zm00001d007089
Zm00001d007089_T002	0.2526979	1.191332809	Zm00001d007089
Zm00001d007089_T044	0.9907928	0.142313106	Zm00001d007089
Zm00001d031445_T001	0.937612	0.042014003	Zm00001d031445
Zm00001d007435_T010	0.6165669	-0.898725865	Zm00001d007435
Zm00001d007435_T001	0.9664406	0.173692495	Zm00001d007435
Zm00001d011487_T001	1	0.387553646	Zm00001d011487
Zm00001d047194_T001	0.9565533	0.56305787	Zm00001d047194
Zm00001d049174_T001	0.9659036	0.082326373	Zm00001d049174
Zm00001d029251_T001	1	0.140437641	Zm00001d029251
Zm00001d041959_T001	0.9416512	-0.075661377	Zm00001d041959
Zm00001d045497_T002	0.9918302	0.17875284	Zm00001d045497
Zm00001d021632_T001	0.3783227	1.511032979	Zm00001d021632
Zm00001d020781_T001	1	0.316839391	Zm00001d020781
Zm00001d006931_T002	0.9701986	0.452542715	Zm00001d006931
Zm00001d006931_T001	0.9182861	0.245980084	Zm00001d006931
Zm00001d013090_T002	0.449035	0.581210629	Zm00001d013090
Zm00001d012530_T001	0.565935	-1.183621707	Zm00001d012530
Zm00001d010341_T001	0.9862654	0.446945389	Zm00001d010341
Zm00001d010341_T003	0.6038555	1.09164622	Zm00001d010341
Zm00001d010341_T004	0.8815352	-0.23349032	Zm00001d010341
Zm00001d010341_T014	0.935477	0.371825641	Zm00001d010341
Zm00001d033422_T007	0.01213295	0.930049652	Zm00001d033422
Zm00001d033422_T001	0.9205739	-0.008017161	Zm00001d033422

Zm00001d033422_T005	0.5123429	0.670830951	Zm00001d033422
Zm00001d033422_T004	0.9201237	0.428292242	Zm00001d033422
Zm00001d033422_T006	0.9687311	0.035519028	Zm00001d033422
Zm00001d030551_T001	0.5725302	1.363352335	Zm00001d030551
Zm00001d022322_T001	0.9422537	0.090173589	Zm00001d022322
Zm00001d011766_T001	0.9722469	-0.048805471	Zm00001d011766
Zm00001d052040_T001	0.8001345	1.218830374	Zm00001d052040
Zm00001d007080_T004	0.26606	1.111184753	Zm00001d007080
Zm00001d007080_T002	0.9530931	0.458571939	Zm00001d007080
Zm00001d007080_T001	0.9778436	0.488137109	Zm00001d007080
Zm00001d031159_T002	0.9176653	-0.000415507	Zm00001d031159
Zm00001d038585_T001	0.8706615	0.393565761	Zm00001d038585
Zm00001d048132_T001	0.6524775	-0.861854294	Zm00001d048132
Zm00001d037577_T001	1	0.181295746	Zm00001d037577
Zm00001d005950_T001	0.4821802	1.228457488	Zm00001d005950
Zm00001d025716_T001	0.5140052	-0.526312128	Zm00001d025716
Zm00001d021915_T002	0.000285564	2.681823421	Zm00001d021915
Zm00001d021915_T003	0.2029135	1.787208334	Zm00001d021915
Zm00001d021915_T001	0.4505897	1.482861221	Zm00001d021915
Zm00001d014358_T002	0.9894615	0.495085116	Zm00001d014358
Zm00001d014358_T008	0.7109402	-0.192726296	Zm00001d014358
Zm00001d034383_T001	3.80E-08	-1.409569721	Zm00001d034383
Zm00001d034383_T003	0.8455816	0.77485741	Zm00001d034383
Zm00001d011742_T010	0.8413819	0.171427389	Zm00001d011742
Zm00001d011742_T049	0.2461415	0.235634296	Zm00001d011742
Zm00001d011742_T013	0.8607922	0.084514444	Zm00001d011742
Zm00001d011742_T016	0.000169066	0.705416461	Zm00001d011742
Zm00001d011742_T037	0.007842368	1.836075327	Zm00001d011742
Zm00001d011742_T008	0.9613831	0.420028415	Zm00001d011742
Zm00001d046533_T002	0.9627389	0.162718849	Zm00001d046533
Zm00001d046533_T005	0.1529848	1.104902982	Zm00001d046533
Zm00001d046533_T007	0.9991964	0.030815209	Zm00001d046533
Zm00001d046533_T008	0.03789359	0.52774799	Zm00001d046533
Zm00001d023446_T001	0.997449	0.285887371	Zm00001d023446
Zm00001d002418_T042	0.1566411	0.374461024	Zm00001d002418
Zm00001d002418_T002	2.00E-10	-0.957094702	Zm00001d002418
Zm00001d002418_T044	0.004667046	0.867935192	Zm00001d002418
Zm00001d002418_T025	0.7997379	0.42053808	Zm00001d002418
Zm00001d002418_T051	0.8753765	0.228605722	Zm00001d002418
Zm00001d002418_T045	1	0.034342246	Zm00001d002418
Zm00001d002418_T030	0.2672722	1.288427319	Zm00001d002418
Zm00001d002418_T033	0.5408189	-0.539548136	Zm00001d002418
Zm00001d002418_T001	0.06106929	2.103683161	Zm00001d002418
Zm00001d002418_T026	0.7569064	0.37260709	Zm00001d002418
Zm00001d028565_T003	0.9363424	0.70242055	Zm00001d028565
Zm00001d028565_T002	0.9243706	0.71408039	Zm00001d028565
Zm00001d012957_T002	0.8723809	0.718966813	Zm00001d012957
Zm00001d034338_T001	0.8704456	0.745996758	Zm00001d034338
Zm00001d023220_T001	0.9113566	0.756838758	Zm00001d023220
Zm00001d011225_T002	0.9868821	0.315061817	Zm00001d011225
Zm00001d011225_T003	0.8836975	-0.29538679	Zm00001d011225
Zm00001d011225_T001	0.9977752	0.318096669	Zm00001d011225
Zm00001d005661_T001	0.02055626	-1.58691574	Zm00001d005661
Zm00001d016855_T001	0.6952911	1.256011523	Zm00001d016855

Zm00001d016855_T009	0.9702242	0.158243269	Zm00001d016855
Zm00001d016855_T006	0.07843254	-0.665237769	Zm00001d016855
Zm00001d016855_T007	0.4023166	-0.752372204	Zm00001d016855
Zm00001d016855_T010	0.9053391	0.586276321	Zm00001d016855
Zm00001d016855_T005	0.1349947	0.513342633	Zm00001d016855
Zm00001d038975_T001	0.6914755	-0.595485073	Zm00001d038975
Zm00001d033670_T003	0.00739911	2.316980673	Zm00001d033670
Zm00001d033670_T001	0.003405378	2.693927973	Zm00001d033670
Zm00001d033670_T004	0.00010633	1.380799811	Zm00001d033670
Zm00001d033989_T001	1	0.417754064	Zm00001d033989
Zm00001d005849_T001	0.001522709	3.425406576	Zm00001d005849
Zm00001d029810_T001	0.9979393	0.291932737	Zm00001d029810
Zm00001d028218_T008	0.6901194	0.401979534	Zm00001d028218
Zm00001d028218_T001	0.4812187	1.446086978	Zm00001d028218
Zm00001d028218_T013	1	0.310302681	Zm00001d028218
Zm00001d028218_T009	0.9160053	-0.065678551	Zm00001d028218
Zm00001d028218_T007	0.6750928	-0.407085616	Zm00001d028218
Zm00001d015656_T002	0.9744451	-0.08791209	Zm00001d015656
Zm00001d015656_T001	0.9514141	0.578525663	Zm00001d015656
Zm00001d032598_T023	1.16E-05	-1.481898273	Zm00001d032598
Zm00001d032598_T027	0.3301595	-0.248506659	Zm00001d032598
Zm00001d032598_T014	0.2037316	1.56736066	Zm00001d032598
Zm00001d032598_T012	0.5713699	0.377698959	Zm00001d032598
Zm00001d032598_T003	0.4422995	0.301805551	Zm00001d032598
Zm00001d032598_T024	0.2570953	0.772519079	Zm00001d032598
Zm00001d032598_T019	0.000439089	0.642832184	Zm00001d032598
Zm00001d043798_T001	0.8077635	-0.496574625	Zm00001d043798
Zm00001d012983_T001	0.962254	0.599949817	Zm00001d012983
Zm00001d011573_T001	0.2916362	1.388405811	Zm00001d011573
Zm00001d003224_T005	0.8206098	-0.127340014	Zm00001d003224
Zm00001d003224_T002	0.7681822	0.747065994	Zm00001d003224
Zm00001d042367_T002	0.5988928	-0.530770545	Zm00001d042367
Zm00001d019222_T001	0.3497147	-1.266173646	Zm00001d019222
Zm00001d021371_T001	0.9763597	0.566260304	Zm00001d021371
Zm00001d007294_T001	0.9919298	0.210425668	Zm00001d007294
Zm00001d007294_T002	0.9740325	0.470145038	Zm00001d007294
Zm00001d053878_T001	0.9210327	0.483485092	Zm00001d053878
Zm00001d012380_T002	0.9628432	0.600836796	Zm00001d012380
Zm00001d012380_T004	0.1752002	0.915408383	Zm00001d012380
Zm00001d012380_T001	0.4527616	0.976180323	Zm00001d012380
Zm00001d012380_T006	0.827438	0.577899791	Zm00001d012380
Zm00001d031948_T008	0.9160053	0.470820283	Zm00001d031948
Zm00001d031948_T001	0.9796552	0.355458208	Zm00001d031948
Zm00001d025916_T001	0.07537729	-1.674631074	Zm00001d025916
Zm00001d046910_T001	0.7559329	0.583578258	Zm00001d046910
Zm00001d018246_T016	0.3247936	0.420711745	Zm00001d018246
Zm00001d018246_T005	0.5243524	0.958275659	Zm00001d018246
Zm00001d018246_T001	0.6063001	1.09086449	Zm00001d018246
Zm00001d018246_T018	0.3894524	-0.956687214	Zm00001d018246
Zm00001d018246_T017	0.8533785	-0.660733596	Zm00001d018246
Zm00001d018246_T006	0.9960479	0.434046505	Zm00001d018246
Zm00001d005182_T001	0.9567316	0.542883271	Zm00001d005182
Zm00001d051499_T001	0.9804433	-0.123677198	Zm00001d051499
Zm00001d045107_T001	0.139783	-1.171568927	Zm00001d045107

Zm00001d053215_T001	0.8720862	-0.130700432	Zm00001d053215
Zm00001d053215_T002	0.8862653	-0.560029764	Zm00001d053215
Zm00001d030617_T001	0.9849234	0.088990568	Zm00001d030617
Zm00001d024128_T001	0.9706293	0.031642105	Zm00001d024128
Zm00001d038855_T002	0.8286193	-0.225263808	Zm00001d038855
Zm00001d002501_T001	0.9946911	0.024939654	Zm00001d002501
Zm00001d020909_T001	0.8537544	-0.195047437	Zm00001d020909
Zm00001d053965_T001	0.9745777	0.007885261	Zm00001d053965
Zm00001d047873_T003	0.05015969	1.212775206	Zm00001d047873
Zm00001d047873_T020	0.2190437	-0.598410916	Zm00001d047873
Zm00001d047873_T019	0.9529184	0.111142287	Zm00001d047873
Zm00001d014603_T001	0.852831	-0.234592536	Zm00001d014603
Zm00001d040851_T001	0.4881794	-0.614223949	Zm00001d040851
Zm00001d005586_T001	0.9895395	0.453361565	Zm00001d005586
Zm00001d005279_T001	0.1527884	-1.674053838	Zm00001d005279
Zm00001d012430_T001	0.08974985	1.393529878	Zm00001d012430
Zm00001d044119_T001	0.7712604	0.706423204	Zm00001d044119
Zm00001d042464_T002	1	0.374147885	Zm00001d042464
Zm00001d042382_T001	0.02379854	-1.475872818	Zm00001d042382
Zm00001d026454_T001	0.7300045	-0.217710634	Zm00001d026454
Zm00001d034604_T001	0.9914912	0.400564361	Zm00001d034604
Zm00001d010304_T001	0.923446	0.760261408	Zm00001d010304
Zm00001d048311_T003	0.000798901	-1.335156153	Zm00001d048311
Zm00001d048311_T004	3.03E-05	-1.186124448	Zm00001d048311
Zm00001d033860_T001	0.9040442	-0.115591959	Zm00001d033860
Zm00001d043775_T016	0.285771	1.19389128	Zm00001d043775
Zm00001d043775_T003	0.586985	1.203593704	Zm00001d043775
Zm00001d043775_T012	1.59E-07	0.5817242	Zm00001d043775
Zm00001d043775_T018	0.009394971	1.777518245	Zm00001d043775
Zm00001d043775_T015	8.13E-12	-1.716516138	Zm00001d043775
Zm00001d043775_T008	0.8197974	0.23500467	Zm00001d043775
Zm00001d036356_T004	0.8492405	-0.030446154	Zm00001d036356
Zm00001d036356_T003	0.9666757	0.639902233	Zm00001d036356
Zm00001d036356_T005	0.3363071	-0.717497319	Zm00001d036356
Zm00001d013138_T001	0.8593759	-0.524805236	Zm00001d013138
Zm00001d050811_T002	0.7788277	-0.380650979	Zm00001d050811
Zm00001d053076_T001	0.8825173	0.812459271	Zm00001d053076
Zm00001d031014_T001	0.9851204	0.254853993	Zm00001d031014
Zm00001d031014_T002	0.9421369	0.1523155	Zm00001d031014
Zm00001d007401_T002	0.2642794	-0.147019064	Zm00001d007401
Zm00001d032515_T001	0.3548936	1.279380996	Zm00001d032515
Zm00001d012690_T005	0.8437109	0.570150836	Zm00001d012690
Zm00001d012690_T001	0.84216	-0.305065387	Zm00001d012690
Zm00001d012690_T006	0.9612081	0.3605666	Zm00001d012690
Zm00001d031072_T003	0.7680039	0.525451702	Zm00001d031072
Zm00001d031072_T004	0.2566009	0.478828146	Zm00001d031072
Zm00001d031072_T001	0.993158	0.266454095	Zm00001d031072
Zm00001d018146_T004	0.8389117	0.700658754	Zm00001d018146
Zm00001d018146_T002	0.9945118	0.002889379	Zm00001d018146
Zm00001d018146_T007	0.9765009	0.440780784	Zm00001d018146
Zm00001d018146_T003	0.8835073	0.787615097	Zm00001d018146
Zm00001d018146_T001	0.8107637	0.57041201	Zm00001d018146
Zm00001d022308_T005	1	0.081937927	Zm00001d022308
Zm00001d022308_T003	0.9791729	-0.227793667	Zm00001d022308

Zm00001d048834_T002	0.8053436	-0.248887548	Zm00001d048834
Zm00001d026632_T001	0.01294351	-2.50994903	Zm00001d026632
Zm00001d016301_T001	0.7019361	0.568678904	Zm00001d016301
Zm00001d031651_T001	0.3105729	-1.424038599	Zm00001d031651
Zm00001d031651_T002	0.705921	-0.532500247	Zm00001d031651
Zm00001d017895_T007	2.30E-07	-1.823280156	Zm00001d017895
Zm00001d017895_T006	0.8959807	-0.059611026	Zm00001d017895
Zm00001d017895_T001	0.2764869	1.57669462	Zm00001d017895
Zm00001d030126_T003	0.3016835	-0.885675774	Zm00001d030126
Zm00001d030126_T002	0.7235285	1.453604835	Zm00001d030126
Zm00001d030126_T004	0.06759318	-0.621037416	Zm00001d030126
Zm00001d030126_T015	0.9244973	0.988663384	Zm00001d030126
Zm00001d030126_T008	0.7379612	1.469808873	Zm00001d030126
Zm00001d030126_T005	0.2246004	-0.057295735	Zm00001d030126
Zm00001d030126_T007	0.9968176	0.881136826	Zm00001d030126
Zm00001d044724_T001	0.9121108	0.786836365	Zm00001d044724
Zm00001d044916_T003	1	0.248984861	Zm00001d044916
Zm00001d044916_T006	0.9231466	0.699971381	Zm00001d044916
Zm00001d044916_T007	0.957768	-0.46092035	Zm00001d044916
Zm00001d044916_T005	0.06979879	0.702853123	Zm00001d044916
Zm00001d042722_T008	1	0.139985492	Zm00001d042722
Zm00001d042722_T010	0.8486887	0.397116896	Zm00001d042722
Zm00001d042722_T006	0.9257238	-0.01667515	Zm00001d042722
Zm00001d042722_T007	0.2038697	-0.610623452	Zm00001d042722
Zm00001d042722_T003	0.909729	0.081931269	Zm00001d042722
Zm00001d050712_T001	0.4825899	-0.747528773	Zm00001d050712
Zm00001d025285_T001	0.5980883	-0.331964437	Zm00001d025285
Zm00001d011696_T001	0.9554972	0.666415557	Zm00001d011696
Zm00001d011696_T003	0.9024215	0.825318639	Zm00001d011696
Zm00001d029920_T001	0.3392886	1.618288337	Zm00001d029920
Zm00001d013746_T001	0.1133554	1.514678492	Zm00001d013746
Zm00001d044866_T001	0.9510976	-0.004994423	Zm00001d044866
Zm00001d011348_T001	0.7908437	-0.277763181	Zm00001d011348
Zm00001d004190_T002	0.9778436	0.120510037	Zm00001d004190
Zm00001d004190_T001	0.9673473	0.174679101	Zm00001d004190
Zm00001d003895_T002	0.9768468	0.401382904	Zm00001d003895
Zm00001d035046_T001	0.9899643	0.514402854	Zm00001d035046
Zm00001d038955_T002	0.7564184	1.070122408	Zm00001d038955
Zm00001d038955_T001	0.9968176	0.345183591	Zm00001d038955
Zm00001d038027_T001	0.9747606	0.118070383	Zm00001d038027
Zm00001d038027_T002	0.4786492	-1.043564828	Zm00001d038027
Zm00001d007331_T001	0.2927421	-1.036524388	Zm00001d007331
Zm00001d004917_T001	0.3655498	-0.822834888	Zm00001d004917
Zm00001d001859_T003	0.9310389	0.777712776	Zm00001d001859
Zm00001d049420_T001	0.5196715	1.059918382	Zm00001d049420
Zm00001d044508_T001	1	0.194446478	Zm00001d044508
Zm00001d028912_T001	0.000577022	2.113015849	Zm00001d028912
Zm00001d044564_T002	0.9838213	0.215841424	Zm00001d044564
Zm00001d019757_T002	0.9887584	0.146982401	Zm00001d019757
Zm00001d019757_T006	0.9693993	0.035514424	Zm00001d019757
Zm00001d019757_T003	0.03760071	1.394777763	Zm00001d019757
Zm00001d005899_T003	0.7187138	0.739062287	Zm00001d005899
Zm00001d005899_T001	0.9720425	0.176695505	Zm00001d005899
Zm00001d005899_T002	0.9907928	0.110726358	Zm00001d005899

Zm00001d028211_T002	0.8989636	0.920376529	Zm00001d028211
Zm00001d048014_T001	0.9410648	0.471912509	Zm00001d048014
Zm00001d036451_T001	0.9184323	-0.121673874	Zm00001d036451
Zm00001d036451_T002	0.9689939	0.489979562	Zm00001d036451
Zm00001d028054_T001	0.3066979	1.759636393	Zm00001d028054
Zm00001d028054_T003	0.4035077	0.974496436	Zm00001d028054
Zm00001d028054_T002	0.06805338	0.937103152	Zm00001d028054
Zm00001d002945_T013	0.9812314	0.389094132	Zm00001d002945
Zm00001d002945_T002	0.9293246	0.309333836	Zm00001d002945
Zm00001d002945_T008	0.4237296	-0.268559971	Zm00001d002945
Zm00001d002945_T001	0.9955906	0.414751023	Zm00001d002945
Zm00001d002945_T003	0.5092251	0.356013464	Zm00001d002945
Zm00001d002945_T015	0.8412913	-0.4850018	Zm00001d002945
Zm00001d034552_T001	0.8929316	0.727384326	Zm00001d034552
Zm00001d041833_T007	0.772948	-0.397025263	Zm00001d041833
Zm00001d041833_T013	0.9782953	0.142204219	Zm00001d041833
Zm00001d041833_T018	0.6275807	-0.044009754	Zm00001d041833
Zm00001d041833_T002	0.9717213	0.286610793	Zm00001d041833
Zm00001d041833_T021	0.2164248	-1.719292377	Zm00001d041833
Zm00001d010828_T001	0.9019013	0.431090236	Zm00001d010828
Zm00001d020492_T001	0.4636149	-0.50532364	Zm00001d020492
Zm00001d046723_T001	0.5640102	-0.655003998	Zm00001d046723
Zm00001d044255_T001	0.6646949	-0.498424643	Zm00001d044255
Zm00001d018380_T001	0.7251353	-0.348009601	Zm00001d018380
Zm00001d012513_T001	0.9984041	0.05902951	Zm00001d012513
Zm00001d044394_T004	0.8883561	0.354761133	Zm00001d044394
Zm00001d044394_T005	0.9984041	0.360595363	Zm00001d044394
Zm00001d044394_T001	0.9073468	-0.012947535	Zm00001d044394
Zm00001d044394_T008	0.7594555	0.757315472	Zm00001d044394
Zm00001d044631_T001	0.9956349	0.148820348	Zm00001d044631
Zm00001d010736_T001	0.08416207	-1.547094372	Zm00001d010736
Zm00001d046890_T001	0.872758	-0.220326031	Zm00001d046890
Zm00001d032687_T001	0.9889811	-0.094965846	Zm00001d032687
Zm00001d006825_T001	0.5236638	0.839303177	Zm00001d006825
Zm00001d006825_T003	0.8018398	0.946418401	Zm00001d006825
Zm00001d031268_T003	0.6588175	0.222193169	Zm00001d031268
Zm00001d031268_T010	0.6324192	1.082638766	Zm00001d031268
Zm00001d031268_T016	1	0.358403817	Zm00001d031268
Zm00001d031268_T017	1.38E-10	-1.978477828	Zm00001d031268
Zm00001d050908_T001	0.9703357	1.247523766	Zm00001d050908
Zm00001d050095_T001	1	0.346963625	Zm00001d050095
Zm00001d032852_T003	0.9730613	0.329857554	Zm00001d032852
Zm00001d032852_T001	0.8643636	0.738374606	Zm00001d032852
Zm00001d032852_T002	1	0.204258174	Zm00001d032852
Zm00001d042965_T003	0.9870394	0.240578796	Zm00001d042965
Zm00001d042965_T001	1	0.128496457	Zm00001d042965
Zm00001d002617_T048	0.4417805	-0.695676997	Zm00001d002617
Zm00001d002617_T028	0.6725291	1.076666219	Zm00001d002617
Zm00001d002617_T047	0.3125186	1.587435869	Zm00001d002617
Zm00001d002617_T049	1	0.190982269	Zm00001d002617
Zm00001d002617_T008	0.034458	1.160904844	Zm00001d002617
Zm00001d002617_T001	0.01264374	0.409690747	Zm00001d002617
Zm00001d037703_T001	0.9975882	0.29867578	Zm00001d037703
Zm00001d037771_T001	0.97359	0.19533806	Zm00001d037771

Zm00001d044100_T001	0.8412913	0.5354363	Zm00001d044100
Zm00001d043517_T001	0.6507629	1.297652398	Zm00001d043517
Zm00001d043517_T004	0.9972784	-0.076228164	Zm00001d043517
Zm00001d051421_T005	1	0.31596792	Zm00001d051421
Zm00001d051421_T004	0.9886764	0.101686475	Zm00001d051421
Zm00001d045133_T004	0.9689939	0.884577303	Zm00001d045133
Zm00001d045133_T001	0.9902733	0.333764626	Zm00001d045133
Zm00001d053175_T003	0.9781672	0.040236398	Zm00001d053175
Zm00001d031941_T001	0.9550193	-0.046300376	Zm00001d031941
Zm00001d049332_T001	0.9740325	0.43124549	Zm00001d049332
Zm00001d040456_T002	0.9609806	0.601953882	Zm00001d040456
Zm00001d040456_T001	0.02629931	0.533624502	Zm00001d040456
Zm00001d017590_T001	0.00211038	3.038154466	Zm00001d017590
Zm00001d048086_T001	0.9979568	0.347958554	Zm00001d048086
Zm00001d053354_T002	0.9272378	0.068927199	Zm00001d053354
Zm00001d053354_T004	0.08470891	-1.135185378	Zm00001d053354
Zm00001d053354_T001	0.9184323	0.051175425	Zm00001d053354
Zm00001d019679_T001	0.7946251	-0.403898443	Zm00001d019679
Zm00001d010135_T001	0.956479	0.134091692	Zm00001d010135
Zm00001d010135_T002	0.3114566	0.435287016	Zm00001d010135
Zm00001d010135_T005	0.4233271	0.951723483	Zm00001d010135
Zm00001d010135_T003	0.01481508	0.763668164	Zm00001d010135
Zm00001d031963_T002	0.922516	-0.319994494	Zm00001d031963
Zm00001d031963_T001	0.9410845	0.614314117	Zm00001d031963
Zm00001d031963_T004	0.7806631	-0.384326901	Zm00001d031963
Zm00001d010143_T002	0.8993058	-0.246900221	Zm00001d010143
Zm00001d026627_T001	0.9591873	0.509329748	Zm00001d026627
Zm00001d009556_T001	0.9647033	0.586242663	Zm00001d009556
Zm00001d015148_T001	0.8660897	-0.216331095	Zm00001d015148
Zm00001d015148_T002	0.7078283	-0.351349086	Zm00001d015148
Zm00001d053857_T008	0.9828518	0.502602616	Zm00001d053857
Zm00001d053857_T002	0.7612114	-0.280701723	Zm00001d053857
Zm00001d053857_T006	0.3288556	-1.042621522	Zm00001d053857
Zm00001d053857_T004	1	0.253885915	Zm00001d053857
Zm00001d011816_T001	0.8012203	-0.527918098	Zm00001d011816
Zm00001d011816_T002	1.10E-12	-4.972103702	Zm00001d011816
Zm00001d038930_T001	0.5975461	-0.630012699	Zm00001d038930
Zm00001d010619_T002	0.2982359	0.809656827	Zm00001d010619
Zm00001d010619_T003	0.9311783	0.025862813	Zm00001d010619
Zm00001d010619_T001	0.7042561	0.749345367	Zm00001d010619
Zm00001d031209_T001	0.9666757	0.157087435	Zm00001d031209
Zm00001d030690_T001	0.9242247	-0.098721608	Zm00001d030690
Zm00001d008578_T001	0.4694623	-0.701740049	Zm00001d008578
Zm00001d003448_T002	0.9782953	0.074295946	Zm00001d003448
Zm00001d003448_T001	0.8561115	0.151758762	Zm00001d003448
Zm00001d016031_T001	0.5822407	-0.573411251	Zm00001d016031
Zm00001d018238_T001	0.5509552	-0.590184773	Zm00001d018238
Zm00001d013667_T001	0.9771927	0.194605266	Zm00001d013667
Zm00001d049750_T002	1	0.137188344	Zm00001d049750
Zm00001d024322_T001	0.7505397	-0.695593121	Zm00001d024322
Zm00001d018718_T001	0.6595505	-0.476820375	Zm00001d018718
Zm00001d002198_T002	0.1010856	1.338558972	Zm00001d002198
Zm00001d007548_T001	0.694341	0.796786118	Zm00001d007548
Zm00001d022269_T001	0.9469426	0.034012142	Zm00001d022269

Zm00001d035140_T002	0.4287514	1.458886797	Zm00001d035140
Zm00001d035140_T003	0.9224443	-0.171750809	Zm00001d035140
Zm00001d035140_T001	0.8993062	0.739236193	Zm00001d035140
Zm00001d035140_T004	0.9884825	0.184751736	Zm00001d035140
Zm00001d039729_T002	0.004390741	1.247575895	Zm00001d039729
Zm00001d039729_T016	0.9804228	0.225852636	Zm00001d039729
Zm00001d039729_T021	0.5572941	0.340431921	Zm00001d039729
Zm00001d039729_T013	0.7708328	0.485356771	Zm00001d039729
Zm00001d039729_T005	0.996005	0.178555268	Zm00001d039729
Zm00001d039729_T010	0.9706887	0.069434721	Zm00001d039729
Zm00001d039729_T001	0.6525727	-0.481005009	Zm00001d039729
Zm00001d002572_T005	0.9192339	-0.320961825	Zm00001d002572
Zm00001d015124_T001	0.6818546	0.460078418	Zm00001d015124
Zm00001d031179_T001	0.9869265	0.388403391	Zm00001d031179
Zm00001d038525_T002	0.9825858	0.552745745	Zm00001d038525
Zm00001d038525_T008	0.2062598	0.454573328	Zm00001d038525
Zm00001d052148_T001	0.01199333	2.944798452	Zm00001d052148
Zm00001d025300_T005	0.725373	0.4222127	Zm00001d025300
Zm00001d025300_T003	0.9822138	0.531988753	Zm00001d025300
Zm00001d025300_T004	0.9139991	-0.021088782	Zm00001d025300
Zm00001d025300_T002	0.9399427	0.019424623	Zm00001d025300
Zm00001d034844_T001	0.9969363	0.180789947	Zm00001d034844
Zm00001d047216_T002	0.6625969	1.247663921	Zm00001d047216
Zm00001d038018_T001	0.8581679	-0.240924609	Zm00001d038018
Zm00001d013528_T001	0.8891117	-0.086873571	Zm00001d013528
Zm00001d017524_T001	0.9999526	0.13474975	Zm00001d017524
Zm00001d048391_T001	0.1094786	-1.344751867	Zm00001d048391
Zm00001d023874_T001	0.9173494	-0.045341181	Zm00001d023874
Zm00001d017811_T001	0.9468268	0.027236042	Zm00001d017811
Zm00001d052110_T019	0.01285332	1.237317957	Zm00001d052110
Zm00001d052110_T007	0.9765009	0.423184379	Zm00001d052110
Zm00001d052110_T029	0.1811895	0.389996897	Zm00001d052110
Zm00001d052110_T020	0.8817666	-0.38090194	Zm00001d052110
Zm00001d019047_T001	0.4976511	-0.347818377	Zm00001d019047
Zm00001d051884_T005	0.9264186	0.063863043	Zm00001d051884
Zm00001d051884_T003	0.9173494	0.050158681	Zm00001d051884
Zm00001d051884_T001	0.9352862	0.41970039	Zm00001d051884
Zm00001d051884_T008	0.9936409	0.225461303	Zm00001d051884
Zm00001d051884_T007	0.4407619	0.350424899	Zm00001d051884
Zm00001d033559_T001	1	-0.019916709	Zm00001d033559
Zm00001d013162_T001	0.4500779	-0.429491003	Zm00001d013162
Zm00001d042918_T001	0.9016471	-0.078631776	Zm00001d042918
Zm00001d045181_T006	0.4825899	-0.595790987	Zm00001d045181
Zm00001d045181_T001	0.7251353	0.778061649	Zm00001d045181
Zm00001d045181_T005	0.3655498	0.609959815	Zm00001d045181
Zm00001d045181_T002	0.9816952	0.499279573	Zm00001d045181
Zm00001d042284_T003	0.9930712	0.310325668	Zm00001d042284
Zm00001d042284_T001	0.6876043	-0.278161762	Zm00001d042284
Zm00001d042284_T002	0.9369505	0.647405445	Zm00001d042284
Zm00001d015952_T001	0.9993038	0.396047153	Zm00001d015952
Zm00001d026669_T002	1	0.378278851	Zm00001d026669
Zm00001d014377_T001	0.1054943	1.801854064	Zm00001d014377
Zm00001d014377_T020	0.9513407	-0.006565119	Zm00001d014377
Zm00001d014377_T004	0.09813379	1.46263544	Zm00001d014377

Zm00001d014377_T010	0.001350256	-1.899035752	Zm00001d014377
Zm00001d014377_T008	0.8158142	1.394861412	Zm00001d014377
Zm00001d014377_T013	0.08348055	-0.75259852	Zm00001d014377
Zm00001d014377_T018	0.562828	-0.562055768	Zm00001d014377
Zm00001d018669_T001	1	0.391956349	Zm00001d018669
Zm00001d018669_T006	0.9983139	0.177426817	Zm00001d018669
Zm00001d003607_T001	0.5117928	-0.559487579	Zm00001d003607
Zm00001d003607_T002	0.6742084	0.450405885	Zm00001d003607
Zm00001d049222_T001	1	0.342685541	Zm00001d049222
Zm00001d049222_T002	0.9863681	0.197186049	Zm00001d049222
Zm00001d039615_T001	0.9468268	0.645147907	Zm00001d039615
Zm00001d017721_T001	0.7821468	0.974557199	Zm00001d017721
Zm00001d017721_T003	0.3118193	0.259171701	Zm00001d017721
Zm00001d017721_T004	0.02593757	0.431542786	Zm00001d017721
Zm00001d004143_T001	0.9050121	-0.120060977	Zm00001d004143
Zm00001d047077_T001	0.5713939	-0.308394006	Zm00001d047077
Zm00001d013265_T020	0.8784569	-0.001706049	Zm00001d013265
Zm00001d013265_T011	0.9692109	0.615861593	Zm00001d013265
Zm00001d013265_T001	0.9865038	0.515136302	Zm00001d013265
Zm00001d042500_T002	0.5411872	0.704118188	Zm00001d042500
Zm00001d013596_T001	0.1876594	-1.57833335	Zm00001d013596
Zm00001d032566_T003	0.2902636	0.811557652	Zm00001d032566
Zm00001d032566_T004	0.9050121	0.394292242	Zm00001d032566
Zm00001d032566_T002	1	0.423723894	Zm00001d032566
Zm00001d050216_T001	0.9050121	0.046015746	Zm00001d050216
Zm00001d050216_T006	1	0.264582063	Zm00001d050216
Zm00001d050216_T002	0.8884927	0.487956759	Zm00001d050216
Zm00001d050216_T005	0.9881957	0.156596615	Zm00001d050216
Zm00001d050216_T003	0.9448515	0.631185075	Zm00001d050216
Zm00001d017846_T008	0.8435808	0.969069856	Zm00001d017846
Zm00001d017846_T001	0.577239	0.376084235	Zm00001d017846
Zm00001d017846_T014	0.9907928	-0.015824374	Zm00001d017846
Zm00001d017846_T002	0.08848785	0.755543604	Zm00001d017846
Zm00001d031487_T012	0.9833133	0.53990735	Zm00001d031487
Zm00001d031487_T005	0.0738481	-1.276198949	Zm00001d031487
Zm00001d031487_T003	0.174228	1.962037958	Zm00001d031487
Zm00001d031487_T011	0.9825858	0.245731144	Zm00001d031487
Zm00001d022084_T001	5.10E-08	-5.170586486	Zm00001d022084
Zm00001d012520_T001	0.7538479	0.753281163	Zm00001d012520
Zm00001d050434_T002	0.9489142	0.466945398	Zm00001d050434
Zm00001d016156_T003	0.001324589	2.044057645	Zm00001d016156
Zm00001d025271_T001	0.9979568	0.35888852	Zm00001d025271
Zm00001d015088_T002	0.9909153	0.298722503	Zm00001d015088
Zm00001d053776_T004	0.9947747	0.492954511	Zm00001d053776
Zm00001d053776_T016	0.667889	-0.603855785	Zm00001d053776
Zm00001d053776_T015	0.9561521	0.608285295	Zm00001d053776
Zm00001d002341_T001	1	0.215440893	Zm00001d002341
Zm00001d014258_T001	0.8486461	-0.171077514	Zm00001d014258
Zm00001d014258_T004	0.1517793	-1.215913416	Zm00001d014258
Zm00001d014258_T002	0.4146004	-0.150407294	Zm00001d014258
Zm00001d014258_T006	0.01554154	-2.643968103	Zm00001d014258
Zm00001d014258_T003	0.3706063	-0.527087574	Zm00001d014258
Zm00001d014258_T007	0.3358121	-0.865768067	Zm00001d014258
Zm00001d052783_T001	0.8865624	-0.386675818	Zm00001d052783

Zm00001d003668_T001	0.9797082	0.588461377	Zm00001d003668
Zm00001d031236_T012	0.972985	0.014092687	Zm00001d031236
Zm00001d034773_T008	0.2329697	-0.771226161	Zm00001d034773
Zm00001d034773_T001	0.1079908	-0.208967431	Zm00001d034773
Zm00001d034773_T002	0.9275747	0.070218036	Zm00001d034773
Zm00001d024926_T001	0.9834601	0.56744544	Zm00001d024926
Zm00001d010743_T001	0.000138621	1.810138455	Zm00001d010743
Zm00001d022274_T001	0.9843847	0.204745821	Zm00001d022274
Zm00001d040985_T002	1	0.175765172	Zm00001d040985
Zm00001d039456_T001	0.9646442	0.151226641	Zm00001d039456
Zm00001d042761_T006	0.2510212	-1.779655321	Zm00001d042761
Zm00001d042761_T001	0.4386149	-0.42508414	Zm00001d042761
Zm00001d042761_T004	0.9690925	0.335264468	Zm00001d042761
Zm00001d042761_T002	0.9693993	0.571096222	Zm00001d042761
Zm00001d006505_T002	0.7353854	0.836697639	Zm00001d006505
Zm00001d042796_T001	0.7407179	-0.506426469	Zm00001d042796
Zm00001d046735_T001	0.7945048	0.514500519	Zm00001d046735
Zm00001d027296_T003	0.7322486	-0.253974708	Zm00001d027296
Zm00001d027296_T001	0.7063456	-0.221753015	Zm00001d027296
Zm00001d027392_T049	0.03198968	-0.560599033	Zm00001d027392
Zm00001d027392_T069	0.05103414	1.008083711	Zm00001d027392
Zm00001d027392_T071	0.9224443	0.06258718	Zm00001d027392
Zm00001d027392_T060	0.3294905	1.158950807	Zm00001d027392
Zm00001d027392_T015	0.8922975	0.322401671	Zm00001d027392
Zm00001d027392_T065	0.921164	0.224760891	Zm00001d027392
Zm00001d045632_T002	1	0.374210037	Zm00001d045632
Zm00001d045632_T005	0.6910721	-0.492367905	Zm00001d045632
Zm00001d045632_T004	0.1246229	-0.313760904	Zm00001d045632
Zm00001d047396_T002	0.9970835	0.249027384	Zm00001d047396
Zm00001d047396_T001	0.9907928	0.096000704	Zm00001d047396
Zm00001d035637_T001	0.9894615	1.956909248	Zm00001d035637
Zm00001d027869_T006	0.9979568	0.113102593	Zm00001d027869
Zm00001d027869_T003	0.6768588	-0.720426305	Zm00001d027869
Zm00001d027869_T007	0.3694368	0.72093717	Zm00001d027869
Zm00001d027869_T005	0.9956119	0.121347488	Zm00001d027869
Zm00001d027869_T001	0.9782953	0.548291147	Zm00001d027869
Zm00001d027763_T001	0.2916362	1.711735383	Zm00001d027763
Zm00001d039174_T001	0.2212161	0.930983606	Zm00001d039174
Zm00001d003878_T001	0.9219195	-0.03378604	Zm00001d003878
Zm00001d003878_T002	0.9945076	0.450786158	Zm00001d003878
Zm00001d012641_T001	0.00079548	2.507192651	Zm00001d012641
Zm00001d052118_T001	0.006183364	2.3674722	Zm00001d052118
Zm00001d026370_T001	0.9791729	0.183370057	Zm00001d026370
Zm00001d026370_T003	0.8706608	0.357893856	Zm00001d026370
Zm00001d022209_T001	0.975323	0.180872916	Zm00001d022209
Zm00001d002889_T007	0.000340227	1.036917797	Zm00001d002889
Zm00001d002889_T005	0.5806888	0.78109347	Zm00001d002889
Zm00001d002889_T019	7.61E-14	-6.067845363	Zm00001d002889
Zm00001d002889_T006	0.8998563	0.847528508	Zm00001d002889
Zm00001d002889_T001	5.60E-05	1.097909989	Zm00001d002889
Zm00001d002889_T003	1	0.281967203	Zm00001d002889
Zm00001d002889_T017	1	0.140146582	Zm00001d002889
Zm00001d002889_T004	0.8919896	0.752348107	Zm00001d002889
Zm00001d030968_T003	0.9313866	0.403541394	Zm00001d030968

Zm00001d030968_T006	0.9975882	0.342723031	Zm00001d030968
Zm00001d030968_T010	0.1286885	-1.407136774	Zm00001d030968
Zm00001d030968_T001	0.7727013	0.426742599	Zm00001d030968
Zm00001d030968_T004	0.4380979	0.37462138	Zm00001d030968
Zm00001d032939_T001	0.6030192	-0.487712808	Zm00001d032939
Zm00001d027361_T001	0.1614894	-1.322410693	Zm00001d027361
Zm00001d015274_T005	0.9269932	-0.244380193	Zm00001d015274
Zm00001d015274_T001	0.950155	0.57533533	Zm00001d015274
Zm00001d015274_T002	0.8591203	-0.093606557	Zm00001d015274
Zm00001d042066_T042	0.9361945	0.042934558	Zm00001d042066
Zm00001d042066_T028	0.9127665	0.007246494	Zm00001d042066
Zm00001d042066_T044	0.98291	0.080790391	Zm00001d042066
Zm00001d042066_T023	0.6002177	0.42937627	Zm00001d042066
Zm00001d042066_T030	0.2138161	0.643495237	Zm00001d042066
Zm00001d042066_T002	0.2293814	-0.315572884	Zm00001d042066
Zm00001d042066_T005	0.007306547	2.222331134	Zm00001d042066
Zm00001d042066_T009	0.9160053	0.734812844	Zm00001d042066
Zm00001d022386_T001	0.9422098	0.055109684	Zm00001d022386
Zm00001d006102_T001	0.8031436	1.016933482	Zm00001d006102
Zm00001d006102_T003	0.9563232	0.086291211	Zm00001d006102
Zm00001d006102_T004	0.2604053	0.452655754	Zm00001d006102
Zm00001d006102_T005	0.5377194	0.853430516	Zm00001d006102
Zm00001d025681_T001	0.9935164	0.483831623	Zm00001d025681
Zm00001d008424_T001	0.9706293	0.572212233	Zm00001d008424
Zm00001d017030_T001	0.9965998	0.334315722	Zm00001d017030
Zm00001d003164_T001	0.9272378	-0.154630696	Zm00001d003164
Zm00001d029371_T001	0.1328218	2.127645236	Zm00001d029371
Zm00001d014292_T001	0.9478397	0.477038177	Zm00001d014292
Zm00001d042567_T004	0.6318256	0.600084626	Zm00001d042567
Zm00001d042567_T001	0.9938825	0.18139712	Zm00001d042567
Zm00001d042567_T006	0.9410845	-0.047520744	Zm00001d042567
Zm00001d042567_T003	0.894759	-0.11432103	Zm00001d042567
Zm00001d009085_T001	0.9936409	0.330641527	Zm00001d009085
Zm00001d004090_T002	0.9987435	-0.005989821	Zm00001d004090
Zm00001d004090_T005	0.9161657	-0.007876543	Zm00001d004090
Zm00001d004090_T007	0.8665954	-0.268804469	Zm00001d004090
Zm00001d010478_T001	0.9469426	0.657118117	Zm00001d010478
Zm00001d018179_T005	0.9766874	0.38475494	Zm00001d018179
Zm00001d018179_T019	0.9106579	-0.391094548	Zm00001d018179
Zm00001d018179_T003	0.8751525	-0.14358421	Zm00001d018179
Zm00001d018179_T002	0.01111605	0.685989676	Zm00001d018179
Zm00001d018179_T001	0.7984409	0.310266324	Zm00001d018179
Zm00001d016410_T006	0.9200919	-0.060704754	Zm00001d016410
Zm00001d016410_T001	0.07440596	-1.81256288	Zm00001d016410
Zm00001d016410_T002	0.9988095	0.298973496	Zm00001d016410
Zm00001d016410_T008	5.10E-12	-5.444294083	Zm00001d016410
Zm00001d016410_T003	0.9863483	0.097272609	Zm00001d016410
Zm00001d035766_T001	0.5780648	1.164665187	Zm00001d035766
Zm00001d043634_T001	0.6736705	1.192113893	Zm00001d043634
Zm00001d005751_T002	0.9668789	0.086571583	Zm00001d005751
Zm00001d021877_T008	0.6946848	0.406589657	Zm00001d021877
Zm00001d021877_T001	0.941922	0.320963206	Zm00001d021877
Zm00001d021877_T002	0.8660897	0.592585757	Zm00001d021877
Zm00001d013003_T001	0.07287871	2.278501947	Zm00001d013003

Zm00001d021835_T004	0.1011991	0.800268648	Zm00001d021835
Zm00001d021835_T006	3.55E-05	1.342509947	Zm00001d021835
Zm00001d021835_T005	0.002814932	3.331880815	Zm00001d021835
Zm00001d021835_T003	0.01777235	2.740819775	Zm00001d021835
Zm00001d021835_T007	0.4609193	0.844747797	Zm00001d021835
Zm00001d021835_T001	0.6231219	0.162521157	Zm00001d021835
Zm00001d006731_T003	0.2525806	-0.580060984	Zm00001d006731
Zm00001d006731_T006	0.9805029	0.162993054	Zm00001d006731
Zm00001d006731_T005	0.7533768	1.155121614	Zm00001d006731
Zm00001d006731_T004	0.3163699	0.974571619	Zm00001d006731
Zm00001d035578_T001	0.9839367	0.547188868	Zm00001d035578
Zm00001d017379_T001	0.6082229	-0.866629683	Zm00001d017379
Zm00001d016648_T001	0.6932474	0.781198616	Zm00001d016648
Zm00001d048418_T001	0.2022097	-1.198886042	Zm00001d048418
Zm00001d044457_T001	0.8030099	-0.147605966	Zm00001d044457
Zm00001d020804_T001	0.5348533	-0.63293289	Zm00001d020804
Zm00001d039636_T001	0.910614	0.022827273	Zm00001d039636
Zm00001d008238_T001	0.9463326	-0.01586964	Zm00001d008238
Zm00001d013627_T001	0.8767824	0.916758466	Zm00001d013627
Zm00001d031002_T004	0.8585804	0.124017735	Zm00001d031002
Zm00001d031002_T003	0.908501	-0.075177442	Zm00001d031002
Zm00001d031002_T005	0.7714187	-0.449637756	Zm00001d031002
Zm00001d005864_T001	0.9872831	-0.187879909	Zm00001d005864
Zm00001d010287_T002	0.9730613	0.141975249	Zm00001d010287
Zm00001d050401_T002	0.7293985	-0.330908268	Zm00001d050401
Zm00001d050401_T004	0.9211172	0.359132415	Zm00001d050401
Zm00001d050401_T005	0.9804228	0.130419009	Zm00001d050401
Zm00001d050401_T001	0.9690782	0.600559673	Zm00001d050401
Zm00001d051244_T001	0.4471095	1.444375603	Zm00001d051244
Zm00001d051244_T002	0.9740325	0.389004433	Zm00001d051244
Zm00001d027844_T001	0.7682041	-0.487234208	Zm00001d027844
Zm00001d008844_T001	0.9673473	-0.066349639	Zm00001d008844
Zm00001d052751_T091	0.7943703	-0.195025526	Zm00001d052751
Zm00001d052751_T087	1	0.070697909	Zm00001d052751
Zm00001d052751_T007	0.9982897	-0.003670087	Zm00001d052751
Zm00001d052751_T081	0.5879461	0.718639417	Zm00001d052751
Zm00001d052751_T044	3.01E-12	1.911132115	Zm00001d052751
Zm00001d052751_T045	0.698243	-0.025801687	Zm00001d052751
Zm00001d052751_T083	0.5537579	0.583674474	Zm00001d052751
Zm00001d052751_T019	1.27E-08	0.861956296	Zm00001d052751
Zm00001d032293_T001	1	0.375345978	Zm00001d032293
Zm00001d039065_T007	3.69E-07	1.045106042	Zm00001d039065
Zm00001d039065_T008	0.9160053	0.171346033	Zm00001d039065
Zm00001d039065_T004	0.9530971	0.106405597	Zm00001d039065
Zm00001d039065_T005	0.3838702	0.895466715	Zm00001d039065
Zm00001d033706_T001	0.744734	-0.499478772	Zm00001d033706
Zm00001d028203_T031	0.05168277	0.926494798	Zm00001d028203
Zm00001d028203_T045	0.01828644	2.187092294	Zm00001d028203
Zm00001d028203_T017	0.6043003	0.623416325	Zm00001d028203
Zm00001d028203_T051	0.1805724	-0.971055706	Zm00001d028203
Zm00001d003019_T004	0.7412991	0.602201385	Zm00001d003019
Zm00001d007806_T002	0.9386141	0.05893939	Zm00001d007806
Zm00001d007806_T008	0.3486589	0.312434694	Zm00001d007806
Zm00001d007806_T001	0.9979568	0.385982025	Zm00001d007806

Zm00001d007806_T006	0.9618682	0.612819331	Zm00001d007806
Zm00001d024094_T003	0.5713703	-0.124115262	Zm00001d024094
Zm00001d024094_T008	0.6284759	0.727208175	Zm00001d024094
Zm00001d029539_T001	0.9926496	0.190600702	Zm00001d029539
Zm00001d029539_T002	0.8913084	0.761695077	Zm00001d029539
Zm00001d029539_T003	0.7051648	1.081169441	Zm00001d029539
Zm00001d036715_T001	0.9914912	0.510583766	Zm00001d036715
Zm00001d001789_T002	0.9563232	0.589619316	Zm00001d001789
Zm00001d001789_T001	0.9326774	-0.040405365	Zm00001d001789
Zm00001d001789_T003	0.7951378	0.766289611	Zm00001d001789
Zm00001d026544_T005	0.9888172	0.040486112	Zm00001d026544
Zm00001d044497_T006	0.8213461	-0.178108082	Zm00001d044497
Zm00001d044497_T004	0.5988362	1.216355618	Zm00001d044497
Zm00001d044497_T001	0.9514141	-0.082840428	Zm00001d044497
Zm00001d044497_T007	0.7312651	-0.416662412	Zm00001d044497
Zm00001d043881_T001	0.9832092	0.033300512	Zm00001d043881
Zm00001d048614_T002	0.803912	-0.143215022	Zm00001d048614
Zm00001d048614_T001	0.9870394	0.196024143	Zm00001d048614
Zm00001d053901_T002	0.9833565	0.494902715	Zm00001d053901
Zm00001d025437_T002	0.5720588	1.030509413	Zm00001d025437
Zm00001d025437_T001	0.5793913	1.136299494	Zm00001d025437
Zm00001d042503_T001	0.9718723	0.201796713	Zm00001d042503
Zm00001d016225_T001	0.9311783	0.736552582	Zm00001d016225
Zm00001d026703_T001	0.7311884	-0.441040422	Zm00001d026703
Zm00001d026703_T003	0.9257238	-0.135131344	Zm00001d026703
Zm00001d026703_T004	0.9965998	0.130101513	Zm00001d026703
Zm00001d026703_T005	0.8114042	-0.526919422	Zm00001d026703
Zm00001d013439_T007	0.8097414	-0.148612974	Zm00001d013439
Zm00001d013439_T004	0.9727583	0.614778971	Zm00001d013439
Zm00001d013439_T003	0.9099617	0.089213015	Zm00001d013439
Zm00001d013439_T014	0.2496307	-1.255549451	Zm00001d013439
Zm00001d013439_T013	0.745469	0.405769853	Zm00001d013439
Zm00001d013439_T006	0.795829	0.369882401	Zm00001d013439
Zm00001d004837_T001	0.4752918	-0.764741089	Zm00001d004837
Zm00001d004837_T002	0.4362603	-0.771665397	Zm00001d004837
Zm00001d029226_T003	0.1334032	0.773397654	Zm00001d029226
Zm00001d001808_T001	1	0.287553394	Zm00001d001808
Zm00001d001808_T004	0.9950761	0.301787767	Zm00001d001808
Zm00001d010183_T001	0.2860325	-0.962166178	Zm00001d010183
Zm00001d046207_T001	0.9902022	0.263899031	Zm00001d046207
Zm00001d014030_T001	1	0.287646404	Zm00001d014030
Zm00001d004310_T001	0.4890752	-1.645115532	Zm00001d004310
Zm00001d014748_T001	0.9384219	-0.005605219	Zm00001d014748
Zm00001d021702_T001	0.9946911	0.295083017	Zm00001d021702
Zm00001d016973_T001	0.3947604	1.117104519	Zm00001d016973
Zm00001d051060_T004	0.9766874	0.140729231	Zm00001d051060
Zm00001d004768_T002	0.9933044	0.300386408	Zm00001d004768
Zm00001d004768_T004	0.9867505	-0.20040393	Zm00001d004768
Zm00001d004768_T003	0.9984041	0.14274175	Zm00001d004768
Zm00001d004768_T001	0.9881407	0.513554284	Zm00001d004768
Zm00001d015893_T004	0.9878022	0.130660742	Zm00001d015893
Zm00001d015893_T001	0.9791729	0.273029226	Zm00001d015893
Zm00001d046889_T001	0.001640834	1.490846666	Zm00001d046889
Zm00001d046889_T003	0.9954444	0.46718395	Zm00001d046889

Zm00001d046889_T006	0.8412913	0.153790268	Zm00001d046889
Zm00001d046889_T005	0.9380783	-0.069952231	Zm00001d046889
Zm00001d046889_T007	0.09191856	1.442600246	Zm00001d046889
Zm00001d010104_T001	0.6453183	-0.620699937	Zm00001d010104
Zm00001d002302_T004	0.9756546	0.1998177	Zm00001d002302
Zm00001d002302_T008	1	0.313132218	Zm00001d002302
Zm00001d002302_T001	0.8050884	0.741069712	Zm00001d002302
Zm00001d002302_T013	1	0.113783674	Zm00001d002302
Zm00001d002302_T006	0.9086619	0.00036211	Zm00001d002302
Zm00001d002302_T009	0.7209242	-0.213296679	Zm00001d002302
Zm00001d002302_T003	0.4723494	-0.175887983	Zm00001d002302
Zm00001d035540_T002	0.9974734	0.332694391	Zm00001d035540
Zm00001d028397_T008	0.8218874	1.117193814	Zm00001d028397
Zm00001d028397_T005	0.7697196	0.649623758	Zm00001d028397
Zm00001d028397_T001	0.9988864	0.455429415	Zm00001d028397
Zm00001d028397_T003	0.3725903	0.635851652	Zm00001d028397
Zm00001d031816_T001	0.9519258	0.423728071	Zm00001d031816
Zm00001d041947_T001	0.7289978	0.938158795	Zm00001d041947
Zm00001d025798_T013	0.6441703	-0.461434533	Zm00001d025798
Zm00001d025798_T014	0.9822138	0.119439711	Zm00001d025798
Zm00001d025798_T012	0.9994624	0.29985384	Zm00001d025798
Zm00001d025798_T005	0.995201	0.141436867	Zm00001d025798
Zm00001d035020_T001	0.9451281	0.250463078	Zm00001d035020
Zm00001d040314_T003	0.4771864	-1.01572435	Zm00001d040314
Zm00001d040314_T001	0.9583056	0.083556859	Zm00001d040314
Zm00001d026302_T003	0.9734722	0.513909367	Zm00001d026302
Zm00001d026302_T001	0.03104183	1.448691794	Zm00001d026302
Zm00001d002163_T003	0.8518279	0.865191329	Zm00001d002163
Zm00001d002163_T005	0.8774597	0.529542999	Zm00001d002163
Zm00001d002163_T004	0.8267985	-0.429341139	Zm00001d002163
Zm00001d049703_T002	0.04684325	-1.183634855	Zm00001d049703
Zm00001d049703_T001	0.9533693	0.028043358	Zm00001d049703
Zm00001d027679_T001	0.5061365	-0.648490255	Zm00001d027679
Zm00001d003262_T002	0.2525169	1.76325285	Zm00001d003262
Zm00001d003262_T005	0.7932535	0.843229202	Zm00001d003262
Zm00001d003262_T004	0.08362855	1.90964544	Zm00001d003262
Zm00001d003262_T003	0.5299898	-1.171005628	Zm00001d003262
Zm00001d005692_T001	0.8591203	-0.195744502	Zm00001d005692
Zm00001d046937_T001	0.221483	-1.140167927	Zm00001d046937
Zm00001d037305_T001	0.6106457	0.885244338	Zm00001d037305
Zm00001d003106_T001	0.9441469	0.118739519	Zm00001d003106
Zm00001d003106_T003	0.602511	1.284603092	Zm00001d003106
Zm00001d003106_T002	0.8958882	-0.011782121	Zm00001d003106
Zm00001d018297_T001	0.9979568	0.25503442	Zm00001d018297
Zm00001d018297_T003	0.7440932	1.003622307	Zm00001d018297
Zm00001d002536_T001	0.9867452	0.334464802	Zm00001d002536
Zm00001d015468_T001	0.009170631	2.996201808	Zm00001d015468
Zm00001d047766_T001	0.8376343	-0.340643205	Zm00001d047766
Zm00001d027976_T002	0.9832092	0.511444714	Zm00001d027976
Zm00001d027976_T003	0.3409531	1.200644959	Zm00001d027976
Zm00001d004624_T001	2.64E-07	3.997097228	Zm00001d004624
Zm00001d017871_T001	0.9749728	0.388540364	Zm00001d017871
Zm00001d017871_T005	0.5234084	-0.812889922	Zm00001d017871
Zm00001d051866_T013	1	0.273295709	Zm00001d051866

Zm00001d051866_T004	0.1279806	1.13909072	Zm00001d051866
Zm00001d051866_T016	0.4449888	-0.192558856	Zm00001d051866
Zm00001d051866_T006	0.695703	-0.193578276	Zm00001d051866
Zm00001d047382_T002	0.977606	0.142989557	Zm00001d047382
Zm00001d047382_T001	0.954984	0.638519241	Zm00001d047382
Zm00001d049991_T001	0.9513407	0.031307036	Zm00001d049991
Zm00001d020620_T001	0.9232281	0.517011855	Zm00001d020620
Zm00001d027334_T028	0.8864089	-0.037566134	Zm00001d027334
Zm00001d027334_T020	2.83E-06	1.435971867	Zm00001d027334
Zm00001d027334_T001	0.4396083	1.019942511	Zm00001d027334
Zm00001d027334_T022	1.61E-12	-1.70735157	Zm00001d027334
Zm00001d027334_T021	1.22E-14	2.5898623	Zm00001d027334
Zm00001d052781_T005	0.9083745	0.006855143	Zm00001d052781
Zm00001d052781_T002	0.758035	-0.091859348	Zm00001d052781
Zm00001d052781_T010	0.8483996	0.010770531	Zm00001d052781
Zm00001d037156_T019	0.5390482	0.418821425	Zm00001d037156
Zm00001d037156_T003	4.07E-05	2.715017599	Zm00001d037156
Zm00001d037156_T065	0.285936	0.404588211	Zm00001d037156
Zm00001d037156_T041	0.8183889	0.161335559	Zm00001d037156
Zm00001d037156_T012	0.9813293	0.260106797	Zm00001d037156
Zm00001d037156_T074	0.000477176	0.418604891	Zm00001d037156
Zm00001d037156_T056	0.2760977	-1.132668926	Zm00001d037156
Zm00001d037156_T085	0.4150157	1.037992181	Zm00001d037156
Zm00001d037156_T030	0.8694217	0.16043205	Zm00001d037156
Zm00001d037156_T024	0.1981174	-0.767469083	Zm00001d037156
Zm00001d038082_T008	1	0.173667735	Zm00001d038082
Zm00001d038082_T007	0.9782953	-0.030360075	Zm00001d038082
Zm00001d038082_T001	0.9756546	0.563754172	Zm00001d038082
Zm00001d038082_T003	0.6361249	0.569922613	Zm00001d038082
Zm00001d038082_T006	0.6602891	-0.084836736	Zm00001d038082
Zm00001d038082_T005	0.7061489	0.310689613	Zm00001d038082
Zm00001d015212_T006	0.02067213	1.682683922	Zm00001d015212
Zm00001d015212_T001	0.9481577	0.088403665	Zm00001d015212
Zm00001d015212_T005	0.05292041	-1.308953242	Zm00001d015212
Zm00001d015212_T004	0.9880075	0.567304873	Zm00001d015212
Zm00001d011298_T004	0.9745948	0.211226866	Zm00001d011298
Zm00001d011298_T001	0.909729	0.60973207	Zm00001d011298
Zm00001d038647_T001	0.6357008	-0.803340605	Zm00001d038647
Zm00001d042084_T052	1	0.874941193	Zm00001d042084
Zm00001d042084_T047	0.9694613	0.063438168	Zm00001d042084
Zm00001d042084_T011	0.3306925	1.54938098	Zm00001d042084
Zm00001d042084_T035	0.2268865	-1.174778988	Zm00001d042084
Zm00001d042084_T059	5.62E-05	2.161196386	Zm00001d042084
Zm00001d042084_T037	0.5258767	1.597231728	Zm00001d042084
Zm00001d042084_T020	1	0.545564298	Zm00001d042084
Zm00001d042084_T076	0.9884437	0.219883494	Zm00001d042084
Zm00001d042084_T081	0.08052306	1.192500539	Zm00001d042084
Zm00001d042084_T001	0.005501992	-0.811161689	Zm00001d042084
Zm00001d002603_T001	0.9817074	0.240647854	Zm00001d002603
Zm00001d018508_T001	0.9222107	0.305946029	Zm00001d018508
Zm00001d010336_T001	0.9931431	0.053216197	Zm00001d010336
Zm00001d010336_T002	0.9501111	0.110608949	Zm00001d010336
Zm00001d028167_T001	0.4641815	1.512007549	Zm00001d028167
Zm00001d026584_T003	0.858862	0.724381919	Zm00001d026584

Zm00001d026584_T004	0.9832092	0.484797065	Zm00001d026584
Zm00001d026584_T001	0.9484361	0.424518146	Zm00001d026584
Zm00001d026584_T005	0.9965998	0.160052997	Zm00001d026584
Zm00001d013426_T002	0.899177	0.838559888	Zm00001d013426
Zm00001d033623_T004	0.9344122	0.693235021	Zm00001d033623
Zm00001d033623_T002	0.998762	0.400714271	Zm00001d033623
Zm00001d033623_T015	0.6134185	-0.824946788	Zm00001d033623
Zm00001d033623_T010	0.9213679	0.826006694	Zm00001d033623
Zm00001d033623_T016	0.6946859	1.238043643	Zm00001d033623
Zm00001d033623_T009	2.75E-05	1.713965679	Zm00001d033623
Zm00001d027852_T001	0.737932	-0.307780584	Zm00001d027852
Zm00001d049510_T001	0.9454966	0.147121866	Zm00001d049510
Zm00001d049510_T002	0.9895224	0.534981331	Zm00001d049510
Zm00001d049510_T004	0.6324339	-0.468453834	Zm00001d049510
Zm00001d031454_T001	0.5582612	1.353574982	Zm00001d031454
Zm00001d029099_T001	0.9719113	0.236477999	Zm00001d029099
Zm00001d004848_T001	0.737932	1.008061467	Zm00001d004848
Zm00001d038393_T009	0.00078261	2.267777946	Zm00001d038393
Zm00001d038393_T013	0.6318256	-0.377824437	Zm00001d038393
Zm00001d038393_T004	0.2546213	0.562432845	Zm00001d038393
Zm00001d042443_T001	0.000760374	3.628531864	Zm00001d042443
Zm00001d025704_T001	1	0.228478886	Zm00001d025704
Zm00001d043402_T001	0.0379236	2.196633813	Zm00001d043402
Zm00001d018901_T002	0.8825173	-0.130008365	Zm00001d018901
Zm00001d010574_T001	0.8512609	-0.522231935	Zm00001d010574
Zm00001d007395_T001	0.9573272	0.121598741	Zm00001d007395
Zm00001d034457_T003	0.9528476	0.035253826	Zm00001d034457
Zm00001d034457_T004	0.9971866	0.211172265	Zm00001d034457
Zm00001d034457_T001	0.9514141	0.613932513	Zm00001d034457
Zm00001d034457_T002	0.9252088	-0.0238025	Zm00001d034457
Zm00001d024804_T003	0.1275272	0.776165842	Zm00001d024804
Zm00001d024804_T002	0.9974734	0.320155901	Zm00001d024804
Zm00001d051329_T003	0.8726017	0.593235018	Zm00001d051329
Zm00001d034565_T001	0.2802526	-0.836254873	Zm00001d034565
Zm00001d034565_T004	0.3781355	1.436112354	Zm00001d034565
Zm00001d013658_T001	0.9451369	0.089279786	Zm00001d013658
Zm00001d033795_T010	0.63477	0.541589763	Zm00001d033795
Zm00001d033795_T003	1	0.076521173	Zm00001d033795
Zm00001d033795_T006	0.9422098	0.106947804	Zm00001d033795
Zm00001d033795_T005	1.42E-06	0.699473904	Zm00001d033795
Zm00001d033795_T012	0.8830711	0.256997868	Zm00001d033795
Zm00001d033795_T008	0.2282332	1.343835998	Zm00001d033795
Zm00001d007269_T001	0.7641977	0.833072512	Zm00001d007269
Zm00001d028987_T004	0.852305	-0.103541809	Zm00001d028987
Zm00001d028987_T002	0.4141096	1.539765692	Zm00001d028987
Zm00001d016558_T001	0.9355135	0.610329607	Zm00001d016558
Zm00001d043986_T008	0.4821692	0.314615407	Zm00001d043986
Zm00001d043986_T004	0.9914912	0.311640739	Zm00001d043986
Zm00001d043986_T010	0.5549472	-0.838283983	Zm00001d043986
Zm00001d043986_T006	0.101892	0.284797253	Zm00001d043986
Zm00001d041216_T001	0.8740066	-0.360634795	Zm00001d041216
Zm00001d028601_T002	0.5718096	1.341641857	Zm00001d028601
Zm00001d028601_T003	0.009744942	-1.948620274	Zm00001d028601
Zm00001d028601_T001	0.4719488	0.995589767	Zm00001d028601

Zm00001d037644_T001	0.2273681	-1.262879336	Zm00001d037644
Zm00001d022234_T001	0.9404125	-0.043499191	Zm00001d022234
Zm00001d040721_T002	0.9894615	0.289794046	Zm00001d040721
Zm00001d047536_T007	0.000391823	1.470106788	Zm00001d047536
Zm00001d047536_T001	0.6891365	0.210595396	Zm00001d047536
Zm00001d047536_T016	4.38E-07	0.556958288	Zm00001d047536
Zm00001d047536_T058	1	0.44438165	Zm00001d047536
Zm00001d047536_T035	0.811516	0.709749628	Zm00001d047536
Zm00001d041595_T001	0.9956349	0.195250435	Zm00001d041595
Zm00001d007889_T009	0.453061	0.702397239	Zm00001d007889
Zm00001d007889_T002	0.389058	1.893847777	Zm00001d007889
Zm00001d007889_T001	0.9380783	0.312985337	Zm00001d007889
Zm00001d007889_T007	0.3391655	-1.200056739	Zm00001d007889
Zm00001d052074_T008	0.7939944	1.072691395	Zm00001d052074
Zm00001d052074_T009	0.3027291	2.064408904	Zm00001d052074
Zm00001d052074_T011	0.3540453	0.607586243	Zm00001d052074
Zm00001d052074_T001	0.6644959	0.397213656	Zm00001d052074
Zm00001d052074_T004	0.9974734	0.243555677	Zm00001d052074
Zm00001d052074_T010	0.9870394	0.475291127	Zm00001d052074
Zm00001d052074_T007	0.9887889	0.190849487	Zm00001d052074
Zm00001d052074_T005	0.9160053	0.225339942	Zm00001d052074
Zm00001d047439_T001	0.9890668	0.49710602	Zm00001d047439
Zm00001d016980_T001	0.8516212	0.668183511	Zm00001d016980
Zm00001d022590_T017	0.317329	0.196519354	Zm00001d022590
Zm00001d022590_T020	0.215298	-0.449069624	Zm00001d022590
Zm00001d022590_T013	0.9902733	0.235931567	Zm00001d022590
Zm00001d022590_T012	0.007378051	0.43818312	Zm00001d022590
Zm00001d033951_T001	1	0.020284458	Zm00001d033951
Zm00001d017363_T001	0.9237473	-0.021795629	Zm00001d017363
Zm00001d037792_T001	0.6275807	-0.375970611	Zm00001d037792
Zm00001d012607_T001	0.9529184	0.490740068	Zm00001d012607
Zm00001d006630_T001	0.9242611	-0.046432866	Zm00001d006630
Zm00001d028464_T043	0.5732194	-1.146085498	Zm00001d028464
Zm00001d028464_T037	0.9833133	0.518478036	Zm00001d028464
Zm00001d028464_T035	0.5490212	0.587357983	Zm00001d028464
Zm00001d028464_T028	0.9835181	0.273792838	Zm00001d028464
Zm00001d028464_T026	0.4106274	0.434220762	Zm00001d028464
Zm00001d043267_T002	0.9441446	-0.13926028	Zm00001d043267
Zm00001d043267_T001	0.1577885	1.944653194	Zm00001d043267
Zm00001d007687_T001	0.9800692	0.103334117	Zm00001d007687
Zm00001d038359_T001	0.9822138	0.447804744	Zm00001d038359
Zm00001d038359_T006	0.9130008	0.165141974	Zm00001d038359
Zm00001d038359_T007	0.7568663	-0.793530827	Zm00001d038359
Zm00001d038359_T003	0.718063	0.07353442	Zm00001d038359
Zm00001d018200_T001	0.8585804	0.805582629	Zm00001d018200
Zm00001d006610_T001	1	0.282526028	Zm00001d006610
Zm00001d035050_T003	0.4403985	0.760762728	Zm00001d035050
Zm00001d035050_T005	0.9781672	0.174691814	Zm00001d035050
Zm00001d035050_T001	0.9975532	0.335225231	Zm00001d035050
Zm00001d035050_T010	0.4106274	0.481809271	Zm00001d035050
Zm00001d035050_T007	0.7682041	1.067948671	Zm00001d035050
Zm00001d028191_T001	0.9570268	0.547599573	Zm00001d028191
Zm00001d028191_T004	0.7174592	0.534958372	Zm00001d028191
Zm00001d028191_T003	0.9953345	0.318430379	Zm00001d028191

Zm00001d028191_T002	0.9365362	0.527567706	Zm00001d028191
Zm00001d038776_T001	0.03097032	-1.001014991	Zm00001d038776
Zm00001d038776_T002	0.9221549	0.845949648	Zm00001d038776
Zm00001d038776_T003	0.9702242	0.159299751	Zm00001d038776
Zm00001d029194_T001	0.9979568	0.204846476	Zm00001d029194
Zm00001d046742_T001	1	0.381738858	Zm00001d046742
Zm00001d046742_T004	0.4096848	-0.572289408	Zm00001d046742
Zm00001d046742_T002	0.9173494	-0.000580361	Zm00001d046742
Zm00001d043619_T001	0.997764	0.322335981	Zm00001d043619
Zm00001d007838_T001	0.6493892	0.699220728	Zm00001d007838
Zm00001d007070_T001	0.8722055	-0.086024698	Zm00001d007070
Zm00001d052229_T001	0.8913084	-0.308664027	Zm00001d052229
Zm00001d034083_T001	0.9189175	-0.270733416	Zm00001d034083
Zm00001d027748_T001	0.4492632	-0.400715951	Zm00001d027748
Zm00001d021659_T005	0.07060489	2.424690519	Zm00001d021659
Zm00001d021659_T011	2.14E-07	0.627265897	Zm00001d021659
Zm00001d021659_T015	0.9493302	1.213100985	Zm00001d021659
Zm00001d021659_T013	0.4890341	2.144969946	Zm00001d021659
Zm00001d016294_T001	0.2217688	-1.308266467	Zm00001d016294
Zm00001d045277_T002	0.9804228	-0.073071493	Zm00001d045277
Zm00001d005764_T005	0.7560147	0.797866539	Zm00001d005764
Zm00001d005764_T001	0.424441	1.373851794	Zm00001d005764
Zm00001d051898_T001	0.9123976	0.71591692	Zm00001d051898
Zm00001d035439_T002	0.4977061	-0.330145814	Zm00001d035439
Zm00001d035439_T001	0.7187138	-0.390470348	Zm00001d035439
Zm00001d049265_T001	0.9543425	0.080714409	Zm00001d049265
Zm00001d008753_T001	0.6450309	-0.652505998	Zm00001d008753
Zm00001d009741_T001	0.9486412	0.037089712	Zm00001d009741
Zm00001d047917_T001	0.9693993	0.576378094	Zm00001d047917
Zm00001d026447_T001	0.5307668	-1.228269253	Zm00001d026447
Zm00001d008968_T001	0.2146351	-0.995289128	Zm00001d008968
Zm00001d037265_T001	0.8350279	0.245906063	Zm00001d037265
Zm00001d037265_T007	0.9974734	0.201632327	Zm00001d037265
Zm00001d037265_T006	0.3015167	-0.17296727	Zm00001d037265
Zm00001d037265_T004	0.9469426	0.466146281	Zm00001d037265
Zm00001d037265_T005	0.7810295	1.080117912	Zm00001d037265
Zm00001d037261_T001	0.9950006	0.330558975	Zm00001d037261
Zm00001d013700_T001	0.9894615	-0.076385616	Zm00001d013700
Zm00001d002141_T005	0.01728933	-1.789634162	Zm00001d002141
Zm00001d002141_T003	0.9722469	0.155070445	Zm00001d002141
Zm00001d002141_T021	0.6481441	-0.415099534	Zm00001d002141
Zm00001d002141_T022	0.7421276	-0.309304072	Zm00001d002141
Zm00001d002141_T001	0.9871621	0.087179563	Zm00001d002141
Zm00001d002141_T004	0.8495532	0.488793807	Zm00001d002141
Zm00001d002141_T008	0.3289004	0.909948884	Zm00001d002141
Zm00001d002141_T012	0.8503172	0.588317876	Zm00001d002141
Zm00001d002141_T010	0.6504599	-0.575229541	Zm00001d002141
Zm00001d017907_T001	0.04282223	1.950327275	Zm00001d017907
Zm00001d040880_T001	0.8537544	0.856178521	Zm00001d040880
Zm00001d008311_T003	0.7923921	0.499750904	Zm00001d008311
Zm00001d008311_T001	0.971204	0.363129757	Zm00001d008311
Zm00001d042794_T005	1	0.451492413	Zm00001d042794
Zm00001d042794_T010	0.2368548	-0.613599538	Zm00001d042794
Zm00001d042794_T001	0.4227896	-0.637478697	Zm00001d042794

Zm00001d042794_T004	0.3030629	1.335028157	Zm00001d042794
Zm00001d042794_T003	0.00713124	1.094100623	Zm00001d042794
Zm00001d042794_T006	0.000767547	-1.221329773	Zm00001d042794
Zm00001d020664_T027	0.7545745	0.399417955	Zm00001d020664
Zm00001d020664_T023	0.515033	-0.042772016	Zm00001d020664
Zm00001d020664_T012	0.8435808	0.356361132	Zm00001d020664
Zm00001d020664_T022	0.01230683	-0.250649779	Zm00001d020664
Zm00001d020664_T002	0.9469426	0.220499616	Zm00001d020664
Zm00001d020664_T009	1	0.370908605	Zm00001d020664
Zm00001d025050_T002	0.9312703	0.816117052	Zm00001d025050
Zm00001d025050_T004	0.5784042	1.360991291	Zm00001d025050
Zm00001d025050_T001	0.9754972	0.240941769	Zm00001d025050
Zm00001d025050_T006	0.9370449	0.286114753	Zm00001d025050
Zm00001d025050_T005	1	0.80124005	Zm00001d025050
Zm00001d021282_T003	0.9021539	-0.338533542	Zm00001d021282
Zm00001d021282_T001	0.908947	-0.310702204	Zm00001d021282
Zm00001d041105_T007	1.57E-11	1.539820582	Zm00001d041105
Zm00001d041105_T027	0.01056074	1.423376538	Zm00001d041105
Zm00001d041105_T047	0.1196915	2.034999329	Zm00001d041105
Zm00001d041105_T017	0.9455514	0.173621569	Zm00001d041105
Zm00001d041105_T066	0.9196877	0.026288165	Zm00001d041105
Zm00001d041105_T058	6.33E-06	-1.746764657	Zm00001d041105
Zm00001d041105_T033	0.101456	0.714416969	Zm00001d041105
Zm00001d041105_T005	0.4386149	1.007840345	Zm00001d041105
Zm00001d045833_T001	0.8958882	-0.334383991	Zm00001d045833
Zm00001d018443_T002	0.000163855	-3.046816226	Zm00001d018443
Zm00001d018443_T001	0.8888255	0.899722163	Zm00001d018443
Zm00001d006132_T001	0.9745948	0.446767178	Zm00001d006132
Zm00001d024464_T002	0.8822992	-0.220956716	Zm00001d024464
Zm00001d049764_T001	1	0.430590801	Zm00001d049764
Zm00001d015525_T001	0.9835181	-0.008944168	Zm00001d015525
Zm00001d038330_T001	1	0.375105793	Zm00001d038330
Zm00001d029855_T001	0.8019448	-0.23046207	Zm00001d029855
Zm00001d038480_T001	0.8079397	0.93105257	Zm00001d038480
Zm00001d034624_T015	0.2861677	0.276372367	Zm00001d034624
Zm00001d034624_T012	0.3852872	1.301291155	Zm00001d034624
Zm00001d034624_T032	0.06751384	0.520640177	Zm00001d034624
Zm00001d034624_T038	0.07256374	1.279384752	Zm00001d034624
Zm00001d034624_T005	0.01325275	2.018342196	Zm00001d034624
Zm00001d034624_T020	2.14E-12	1.33799568	Zm00001d034624
Zm00001d034624_T050	8.23E-06	1.731884801	Zm00001d034624
Zm00001d034624_T006	0.5950499	-0.449205669	Zm00001d034624
Zm00001d034624_T009	0.06076514	0.826074686	Zm00001d034624
Zm00001d034624_T052	0.2525806	-0.460426673	Zm00001d034624
Zm00001d052636_T001	0.9988864	0.224410403	Zm00001d052636
Zm00001d004812_T006	0.4701937	0.676277097	Zm00001d004812
Zm00001d004812_T002	0.9377241	0.102875405	Zm00001d004812
Zm00001d004812_T005	0.9693993	0.168654377	Zm00001d004812
Zm00001d004812_T008	0.6936253	-0.319107484	Zm00001d004812
Zm00001d019280_T001	0.2735451	-1.244017271	Zm00001d019280
Zm00001d019280_T002	0.2510212	-1.17039174	Zm00001d019280
Zm00001d008559_T002	0.9043641	-0.088754219	Zm00001d008559
Zm00001d031712_T020	0.4604152	-1.133403976	Zm00001d031712
Zm00001d031712_T001	0.02134347	-0.593735444	Zm00001d031712

Zm00001d031712_T003	0.8099578	0.992389529	Zm00001d031712
Zm00001d031712_T005	0.9912886	0.181750321	Zm00001d031712
Zm00001d031712_T006	0.8678944	1.428130114	Zm00001d031712
Zm00001d031712_T014	0.5226927	-0.406591161	Zm00001d031712
Zm00001d031712_T018	0.9822138	0.203399159	Zm00001d031712
Zm00001d040110_T001	0.7654384	-0.797157985	Zm00001d040110
Zm00001d052952_T001	0.5774828	-0.841747764	Zm00001d052952
Zm00001d034638_T001	0.6818546	-0.209509926	Zm00001d034638
Zm00001d034638_T003	0.961399	0.085094115	Zm00001d034638
Zm00001d000170_T001	0.4442914	-0.613639645	Zm00001d000170
Zm00001d038104_T009	0.7100078	1.140796035	Zm00001d038104
Zm00001d038104_T032	0.5204602	-0.586531052	Zm00001d038104
Zm00001d038104_T031	0.4972742	0.402005055	Zm00001d038104
Zm00001d038104_T027	0.05487843	0.838475685	Zm00001d038104
Zm00001d038104_T021	6.67E-08	0.879624769	Zm00001d038104
Zm00001d011520_T001	1	0.153280143	Zm00001d011520
Zm00001d012671_T001	0.9275987	-0.018664408	Zm00001d012671
Zm00001d012671_T002	0.9377241	-0.08800683	Zm00001d012671
Zm00001d028973_T001	0.9872831	-0.010165541	Zm00001d028973
Zm00001d049141_T002	0.07429845	-0.931309199	Zm00001d049141
Zm00001d031790_T002	0.9001906	-0.021068516	Zm00001d031790
Zm00001d031790_T001	0.9173494	-0.068240824	Zm00001d031790
Zm00001d041787_T001	0.7520438	0.807584598	Zm00001d041787
Zm00001d024876_T001	0.9970835	0.197959195	Zm00001d024876
Zm00001d044552_T002	0.7888267	0.851555009	Zm00001d044552
Zm00001d044552_T004	0.9269932	0.161211563	Zm00001d044552
Zm00001d044552_T001	0.925875	0.257771313	Zm00001d044552
Zm00001d044552_T006	0.7490886	-0.083272319	Zm00001d044552
Zm00001d044552_T011	0.9902928	0.462952643	Zm00001d044552
Zm00001d027517_T003	0.9364913	-0.383115718	Zm00001d027517
Zm00001d027517_T002	0.5440777	-0.391846935	Zm00001d027517
Zm00001d027517_T001	0.9718723	0.509225447	Zm00001d027517
Zm00001d025103_T001	0.9781672	0.239909941	Zm00001d025103
Zm00001d048096_T014	0.02321164	1.631228327	Zm00001d048096
Zm00001d048096_T019	0.5384294	0.219935211	Zm00001d048096
Zm00001d048096_T013	0.8543794	-0.093214036	Zm00001d048096
Zm00001d048096_T009	1.14E-09	0.938044288	Zm00001d048096
Zm00001d048096_T001	0.8032834	0.596184516	Zm00001d048096
Zm00001d024831_T001	0.9611506	0.090050623	Zm00001d024831
Zm00001d016621_T001	0.9020229	-0.11927732	Zm00001d016621
Zm00001d004078_T001	0.9016285	-0.105723317	Zm00001d004078
Zm00001d045774_T001	0.9486412	0.486771581	Zm00001d045774
Zm00001d020793_T001	0.770492	-0.702665615	Zm00001d020793
Zm00001d034937_T001	0.9173494	-0.213535424	Zm00001d034937
Zm00001d018105_T001	0.1486629	1.734745283	Zm00001d018105
Zm00001d018647_T001	0.9987435	0.164395191	Zm00001d018647
Zm00001d017323_T001	0.4280489	1.333457153	Zm00001d017323
Zm00001d028311_T001	0.9037324	-0.048206259	Zm00001d028311
Zm00001d027875_T001	1	0.193586259	Zm00001d027875
Zm00001d012469_T001	0.8767824	-0.001389898	Zm00001d012469
Zm00001d014909_T004	1	0.416197071	Zm00001d014909
Zm00001d014909_T007	0.5864438	0.324105869	Zm00001d014909
Zm00001d014909_T006	0.8716622	0.383338801	Zm00001d014909
Zm00001d041088_T002	0.7585131	1.368337775	Zm00001d041088

Zm00001d021261_T005	0.909739	0.523669941	Zm00001d021261
Zm00001d021261_T003	0.7735802	0.762359474	Zm00001d021261
Zm00001d048947_T001	0.1611849	-1.470175515	Zm00001d048947
Zm00001d018303_T011	0.001897768	2.500293147	Zm00001d018303
Zm00001d018303_T013	0.9367478	0.029741842	Zm00001d018303
Zm00001d018303_T001	0.000468585	1.565215436	Zm00001d018303
Zm00001d018303_T006	0.4055182	1.341786748	Zm00001d018303
Zm00001d018303_T002	0.8701267	0.697102453	Zm00001d018303
Zm00001d018303_T008	0.3469239	0.260153993	Zm00001d018303
Zm00001d043093_T099	0.9532226	0.283121949	Zm00001d043093
Zm00001d043093_T020	0.01831185	0.373541475	Zm00001d043093
Zm00001d043093_T077	0.01380097	0.519750355	Zm00001d043093
Zm00001d043093_T102	0.6310045	-0.876866392	Zm00001d043093
Zm00001d043093_T040	0.07111099	0.368869022	Zm00001d043093
Zm00001d022403_T001	0.7669786	-0.420318823	Zm00001d022403
Zm00001d034836_T001	0.5004602	-0.755432975	Zm00001d034836
Zm00001d005127_T024	0.2970503	1.556521456	Zm00001d005127
Zm00001d005127_T001	0.7228647	0.229521874	Zm00001d005127
Zm00001d005127_T016	0.9756546	0.127792813	Zm00001d005127
Zm00001d005127_T026	0.8545272	-0.374672078	Zm00001d005127
Zm00001d005127_T021	1.19E-11	-1.793941903	Zm00001d005127
Zm00001d005127_T008	0.00573859	1.324560926	Zm00001d005127
Zm00001d005127_T018	0.9264186	0.110966462	Zm00001d005127
Zm00001d005127_T009	0.000138123	1.958731222	Zm00001d005127
Zm00001d005127_T023	0.9579758	0.511816481	Zm00001d005127
Zm00001d005127_T003	2.30E-14	1.830795656	Zm00001d005127
Zm00001d027556_T001	0.9942091	0.267279806	Zm00001d027556
Zm00001d027425_T001	0.9529184	0.599595251	Zm00001d027425
Zm00001d027425_T002	0.9380783	0.669873209	Zm00001d027425
Zm00001d052107_T005	0.5472547	-0.253115252	Zm00001d052107
Zm00001d052107_T006	0.9131638	0.180598568	Zm00001d052107
Zm00001d052107_T003	0.946422	0.097748534	Zm00001d052107
Zm00001d031801_T001	0.6745129	-0.601253026	Zm00001d031801
Zm00001d029846_T001	0.956184	0.539754797	Zm00001d029846
Zm00001d021160_T001	2.22E-05	5.597374803	Zm00001d021160
Zm00001d020946_T002	0.9781672	0.180836889	Zm00001d020946
Zm00001d020946_T006	1	0.142692266	Zm00001d020946
Zm00001d020946_T001	0.5471937	0.675345372	Zm00001d020946
Zm00001d020946_T007	1	0.341079424	Zm00001d020946
Zm00001d020946_T004	0.9466261	0.122034699	Zm00001d020946
Zm00001d029806_T001	0.8972022	-0.192488597	Zm00001d029806
Zm00001d013393_T001	0.9988954	0.225043514	Zm00001d013393
Zm00001d042056_T001	0.06402929	2.145852215	Zm00001d042056
Zm00001d017979_T004	0.9840043	0.239688868	Zm00001d017979
Zm00001d017979_T002	0.2205171	1.889329222	Zm00001d017979
Zm00001d015521_T001	0.9894727	0.549813192	Zm00001d015521
Zm00001d003112_T005	0.9173494	0.357976792	Zm00001d003112
Zm00001d003112_T015	0.4442914	-0.642339323	Zm00001d003112
Zm00001d003112_T016	0.9833133	0.161696934	Zm00001d003112
Zm00001d003112_T018	0.4487097	0.244117405	Zm00001d003112
Zm00001d003112_T012	0.9734722	0.03392194	Zm00001d003112
Zm00001d003112_T010	0.9468268	0.5116257	Zm00001d003112
Zm00001d000349_T001	0.9689939	0.331706775	Zm00001d000349
Zm00001d046656_T005	0.5843839	-1.00227287	Zm00001d046656

Zm00001d046656_T001	0.9918315	0.223649053	Zm00001d046656
Zm00001d028699_T059	0.9840043	0.160842526	Zm00001d028699
Zm00001d028699_T009	0.07992954	1.859126479	Zm00001d028699
Zm00001d028699_T018	0.3793662	0.352385178	Zm00001d028699
Zm00001d028699_T006	0.001345107	0.960444917	Zm00001d028699
Zm00001d028699_T055	0.8836195	-0.028160227	Zm00001d028699
Zm00001d028699_T056	0.1685404	0.350204424	Zm00001d028699
Zm00001d028699_T052	0.5913892	-0.419208438	Zm00001d028699
Zm00001d028699_T007	0.9723861	0.272178519	Zm00001d028699
Zm00001d028699_T017	0.7912425	0.362360805	Zm00001d028699
Zm00001d028699_T049	0.9378699	0.330849119	Zm00001d028699
Zm00001d028699_T061	0.776742	0.446349105	Zm00001d028699
Zm00001d010281_T001	0.9628432	0.512467524	Zm00001d010281
Zm00001d053818_T003	0.004513206	2.914057757	Zm00001d053818
Zm00001d053818_T002	0.0563461	2.100174899	Zm00001d053818
Zm00001d053818_T001	0.000733585	1.438676631	Zm00001d053818
Zm00001d053818_T004	0.06908877	2.138923652	Zm00001d053818
Zm00001d022185_T010	0.04346091	0.801152932	Zm00001d022185
Zm00001d022185_T004	0.2419534	-0.531405962	Zm00001d022185
Zm00001d022185_T007	1.76E-05	0.872982629	Zm00001d022185
Zm00001d022185_T008	0.3541203	1.699533268	Zm00001d022185
Zm00001d022185_T009	0.5273361	-0.531225257	Zm00001d022185
Zm00001d045571_T001	0.7452824	-0.271815406	Zm00001d045571
Zm00001d052653_T005	0.02558678	2.710261565	Zm00001d052653
Zm00001d052653_T006	0.461503	1.226933318	Zm00001d052653
Zm00001d052653_T002	0.006418906	2.406924328	Zm00001d052653
Zm00001d052653_T004	0.006919847	3.109701462	Zm00001d052653
Zm00001d007554_T001	0.9962521	0.340218685	Zm00001d007554
Zm00001d018069_T001	0.7584314	-0.733465355	Zm00001d018069
Zm00001d045460_T001	2.56E-06	6.165738762	Zm00001d045460
Zm00001d044874_T001	0.000347145	-2.680056628	Zm00001d044874
Zm00001d034423_T001	1	0.364714945	Zm00001d034423
Zm00001d029745_T005	1	0.070117931	Zm00001d029745
Zm00001d024141_T001	0.9449241	0.752323044	Zm00001d024141
Zm00001d013032_T001	0.2917536	1.607781658	Zm00001d013032
Zm00001d039460_T002	0.6989997	-0.318758125	Zm00001d039460
Zm00001d039460_T001	0.9907928	0.174642337	Zm00001d039460
Zm00001d029234_T008	0.3035511	1.032680753	Zm00001d029234
Zm00001d029234_T002	1	0.087271176	Zm00001d029234
Zm00001d029234_T007	0.3941547	0.402315742	Zm00001d029234
Zm00001d029234_T003	0.6683938	1.072606203	Zm00001d029234
Zm00001d029234_T010	0.07256374	0.858469579	Zm00001d029234
Zm00001d042646_T001	0.9687311	0.458408025	Zm00001d042646
Zm00001d053117_T001	0.2982359	-0.913004017	Zm00001d053117
Zm00001d052911_T005	0.9804433	0.212948748	Zm00001d052911
Zm00001d052911_T003	0.9907739	0.302236593	Zm00001d052911
Zm00001d052911_T001	0.8377512	-0.150408941	Zm00001d052911
Zm00001d052153_T003	0.794911	0.445468839	Zm00001d052153
Zm00001d052153_T001	0.9573272	0.089488684	Zm00001d052153
Zm00001d023655_T001	0.8747821	0.830441444	Zm00001d023655
Zm00001d039104_T001	0.8656506	-0.072263275	Zm00001d039104
Zm00001d045282_T001	0.9907928	0.196231212	Zm00001d045282
Zm00001d023669_T001	0.3617595	-0.883392746	Zm00001d023669
Zm00001d035820_T002	0.9804228	0.504520566	Zm00001d035820

Zm00001d023261_T001	0.5571558	-0.668429167	Zm00001d023261
Zm00001d021788_T001	0.8650248	-0.124712908	Zm00001d021788
Zm00001d018289_T001	0.9687311	0.211495128	Zm00001d018289
Zm00001d018289_T014	7.68E-08	1.078620581	Zm00001d018289
Zm00001d018289_T011	0.9886764	0.063667168	Zm00001d018289
Zm00001d018289_T027	0.2030603	-0.807798524	Zm00001d018289
Zm00001d018289_T018	0.008737931	0.839954275	Zm00001d018289
Zm00001d018289_T026	0.6750928	1.043551014	Zm00001d018289
Zm00001d005636_T001	0.9902022	0.312457572	Zm00001d005636
Zm00001d005636_T009	7.98E-14	-4.472056085	Zm00001d005636
Zm00001d005636_T003	0.9941267	0.654530199	Zm00001d005636
Zm00001d020471_T001	0.9986486	0.449627092	Zm00001d020471
Zm00001d020471_T003	0.9524127	0.508183279	Zm00001d020471
Zm00001d020471_T002	0.6324875	1.15769733	Zm00001d020471
Zm00001d011565_T005	0.9610587	-0.001219234	Zm00001d011565
Zm00001d011565_T002	0.9898682	0.284067558	Zm00001d011565
Zm00001d011565_T001	0.9690782	0.446261774	Zm00001d011565
Zm00001d011565_T003	0.8664286	-0.105967503	Zm00001d011565
Zm00001d011565_T009	0.8958197	0.698422412	Zm00001d011565
Zm00001d011565_T006	0.2332303	-0.967186691	Zm00001d011565
Zm00001d011565_T010	0.9782953	0.114014265	Zm00001d011565
Zm00001d011565_T008	0.5473748	0.958246621	Zm00001d011565
Zm00001d011565_T004	0.9173494	0.75877353	Zm00001d011565
Zm00001d050258_T004	0.8412913	1.213590626	Zm00001d050258
Zm00001d050258_T001	0.5613757	-0.285238992	Zm00001d050258
Zm00001d050258_T003	0.9604475	0.080141399	Zm00001d050258
Zm00001d011869_T001	0.9766874	0.304276109	Zm00001d011869
Zm00001d011350_T001	0.6403525	0.832570587	Zm00001d011350
Zm00001d017719_T001	0.4701937	-1.08928103	Zm00001d017719
Zm00001d004554_T002	0.9043641	-0.119957295	Zm00001d004554
Zm00001d032837_T001	0.9580985	0.025883616	Zm00001d032837
Zm00001d009260_T004	0.4146004	-1.417939397	Zm00001d009260
Zm00001d009260_T002	0.9618192	-0.040639154	Zm00001d009260
Zm00001d034677_T001	0.8825173	-0.364111062	Zm00001d034677
Zm00001d034150_T001	0.8273397	-0.18060822	Zm00001d034150
Zm00001d026505_T001	0.7304045	-0.55730159	Zm00001d026505
Zm00001d040678_T010	0.9416089	0.033086364	Zm00001d040678
Zm00001d040678_T001	0.3596799	1.053521028	Zm00001d040678
Zm00001d040678_T014	0.5196622	-0.615199908	Zm00001d040678
Zm00001d040678_T013	0.03182725	0.335154828	Zm00001d040678
Zm00001d040678_T004	0.5184576	0.90438822	Zm00001d040678
Zm00001d040678_T003	0.8767824	-0.059695631	Zm00001d040678
Zm00001d009820_T001	0.1177057	-1.881438768	Zm00001d009820
Zm00001d033645_T001	0.8957497	0.850853522	Zm00001d033645
Zm00001d009684_T001	0.3288952	1.43705988	Zm00001d009684
Zm00001d032591_T003	0.9619942	0.678739246	Zm00001d032591
Zm00001d032591_T001	0.8031436	0.288360324	Zm00001d032591
Zm00001d031670_T002	0.7727043	0.751874494	Zm00001d031670
Zm00001d031670_T001	0.9336646	0.81432878	Zm00001d031670
Zm00001d022313_T001	0.9398057	0.046767883	Zm00001d022313
Zm00001d038380_T005	0.2805818	-2.275881901	Zm00001d038380
Zm00001d038380_T001	0.9210327	0.717153127	Zm00001d038380
Zm00001d038380_T003	0.139783	-1.546168664	Zm00001d038380
Zm00001d038380_T006	0.4261766	-0.504125042	Zm00001d038380

Zm00001d028771_T011	0.8622539	-0.162188474	Zm00001d028771
Zm00001d028771_T002	0.8637863	-0.03632217	Zm00001d028771
Zm00001d053055_T001	0.9687311	0.155613042	Zm00001d053055
Zm00001d033190_T006	0.8252955	-0.502734609	Zm00001d033190
Zm00001d048727_T001	0.9979568	-0.055837372	Zm00001d048727
Zm00001d034533_T001	0.5875107	-0.476580798	Zm00001d034533
Zm00001d034533_T003	0.9892921	0.118049152	Zm00001d034533
Zm00001d034590_T002	0.9563232	0.185383612	Zm00001d034590
Zm00001d034590_T009	0.4080269	-0.381168336	Zm00001d034590
Zm00001d034590_T007	0.9529184	-0.022664607	Zm00001d034590
Zm00001d034590_T012	1	0.698021589	Zm00001d034590
Zm00001d024738_T001	0.8328444	-0.343812131	Zm00001d024738
Zm00001d040393_T001	0.9931431	0.280215103	Zm00001d040393
Zm00001d052690_T001	0.8050292	-0.203384222	Zm00001d052690
Zm00001d019775_T001	0.9468268	-0.020947014	Zm00001d019775
Zm00001d029365_T001	0.5864438	1.071337399	Zm00001d029365
Zm00001d029365_T002	0.8866937	-0.207221952	Zm00001d029365
Zm00001d042005_T003	0.8865624	-0.149322689	Zm00001d042005
Zm00001d012241_T001	0.5451508	-0.792429509	Zm00001d012241
Zm00001d028439_T001	0.9377241	0.059689601	Zm00001d028439
Zm00001d002020_T005	1	-0.326639129	Zm00001d002020
Zm00001d002020_T004	0.8565397	-0.17359164	Zm00001d002020
Zm00001d002020_T001	0.3584875	-0.453195805	Zm00001d002020
Zm00001d002020_T002	1.11E-10	-2.654780541	Zm00001d002020
Zm00001d000123_T001	0.07730424	0.833666546	Zm00001d000123
Zm00001d000123_T003	0.9070578	0.665334689	Zm00001d000123
Zm00001d033786_T001	0.1950386	1.917689671	Zm00001d033786
Zm00001d033786_T004	0.9950703	0.356317567	Zm00001d033786
Zm00001d048787_T001	0.6971821	0.797477268	Zm00001d048787
Zm00001d051659_T001	0.7339388	-0.880121654	Zm00001d051659
Zm00001d032945_T001	0.7021418	-0.408963859	Zm00001d032945
Zm00001d051669_T003	0.5582612	-0.21470581	Zm00001d051669
Zm00001d040920_T001	0.8985024	0.760026835	Zm00001d040920
Zm00001d030639_T002	0.9344122	0.039240708	Zm00001d030639
Zm00001d030639_T003	0.08864449	-0.993532262	Zm00001d030639
Zm00001d016835_T018	0.2848861	0.691778231	Zm00001d016835
Zm00001d016835_T006	0.9863483	0.283537031	Zm00001d016835
Zm00001d052380_T001	0.8193651	-0.158938843	Zm00001d052380
Zm00001d023979_T002	0.3877192	1.340472852	Zm00001d023979
Zm00001d023979_T004	0.909186	0.5272418	Zm00001d023979
Zm00001d021303_T002	2.68E-05	4.275227472	Zm00001d021303
Zm00001d018965_T001	0.00506932	-2.360609215	Zm00001d018965
Zm00001d034561_T001	0.9269932	-0.277281129	Zm00001d034561
Zm00001d034561_T002	0.8987811	-0.435220771	Zm00001d034561
Zm00001d029025_T001	0.8239342	0.416197245	Zm00001d029025
Zm00001d027325_T003	0.8974665	0.576160804	Zm00001d027325
Zm00001d027325_T001	0.9630274	0.078409669	Zm00001d027325
Zm00001d018494_T001	0.9322451	0.582202801	Zm00001d018494
Zm00001d018494_T002	0.9112075	-0.136747855	Zm00001d018494
Zm00001d018494_T003	0.7289978	-0.647266475	Zm00001d018494
Zm00001d032694_T001	0.07146096	2.025663244	Zm00001d032694
Zm00001d050967_T001	0.9219195	0.451937517	Zm00001d050967
Zm00001d011351_T070	0.03857281	0.768556423	Zm00001d011351
Zm00001d011351_T067	0.989787	0.307126819	Zm00001d011351

Zm00001d011351_T031	0.7792228	-0.155169273	Zm00001d011351
Zm00001d011351_T008	0.7761352	-0.250419537	Zm00001d011351
Zm00001d011351_T036	0.04916801	0.517879058	Zm00001d011351
Zm00001d011351_T055	0.4955205	0.477260339	Zm00001d011351
Zm00001d011351_T003	0.6033538	0.943595343	Zm00001d011351
Zm00001d011351_T035	0.8951059	0.301331359	Zm00001d011351
Zm00001d011351_T007	0.3757326	0.536063272	Zm00001d011351
Zm00001d011351_T071	0.03585832	-1.62211267	Zm00001d011351
Zm00001d011351_T029	0.9928593	0.038574272	Zm00001d011351
Zm00001d036251_T001	0.9514141	0.298007106	Zm00001d036251
Zm00001d032430_T001	0.5717924	0.573160626	Zm00001d032430
Zm00001d032430_T002	0.9781672	0.108125906	Zm00001d032430
Zm00001d041489_T005	0.9037324	0.397445199	Zm00001d041489
Zm00001d041489_T002	0.9173494	0.043154666	Zm00001d041489
Zm00001d041489_T008	0.07220675	0.594102689	Zm00001d041489
Zm00001d006763_T001	0.8001654	-0.290712581	Zm00001d006763
Zm00001d006763_T002	0.6519999	1.113218921	Zm00001d006763
Zm00001d015037_T002	1	0.302407706	Zm00001d015037
Zm00001d015037_T003	0.4263097	-0.488689848	Zm00001d015037
Zm00001d015037_T004	1	0.297639641	Zm00001d015037
Zm00001d010759_T001	0.973825	0.078586056	Zm00001d010759
Zm00001d052323_T001	0.971204	0.191798063	Zm00001d052323
Zm00001d044513_T008	0.4187707	0.352646842	Zm00001d044513
Zm00001d044513_T011	0.9507337	0.053247566	Zm00001d044513
Zm00001d014976_T001	0.1322796	-1.147021203	Zm00001d014976
Zm00001d013410_T001	0.0797978	1.910144272	Zm00001d013410
Zm00001d013410_T002	0.9666757	0.200935103	Zm00001d013410
Zm00001d013410_T004	0.7287219	1.7918838	Zm00001d013410
Zm00001d000436_T004	0.8928585	0.639177447	Zm00001d000436
Zm00001d000436_T001	0.8014974	0.995571565	Zm00001d000436
Zm00001d000436_T002	0.1082702	-1.336022088	Zm00001d000436
Zm00001d046700_T003	0.6783686	-0.448763498	Zm00001d046700
Zm00001d029647_T001	0.9090332	0.498953548	Zm00001d029647
Zm00001d042023_T001	0.5570384	-0.729097401	Zm00001d042023
Zm00001d042023_T002	0.9573272	1.111592031	Zm00001d042023
Zm00001d031094_T001	0.5140052	-0.789838946	Zm00001d031094
Zm00001d021634_T001	0.745469	-0.80790713	Zm00001d021634
Zm00001d018403_T003	0.974657	-0.43444065	Zm00001d018403
Zm00001d018403_T004	0.9688529	-0.041202956	Zm00001d018403
Zm00001d018403_T005	0.5269468	0.465432674	Zm00001d018403
Zm00001d013370_T001	0.005375707	1.97551876	Zm00001d013370
Zm00001d013370_T003	0.007443683	0.99865179	Zm00001d013370
Zm00001d013370_T002	0.000323792	1.925156707	Zm00001d013370
Zm00001d014382_T005	0.3097887	1.474515196	Zm00001d014382
Zm00001d014382_T001	0.9596377	0.139823544	Zm00001d014382
Zm00001d014382_T004	0.9237617	0.425835578	Zm00001d014382
Zm00001d036568_T006	0.906046	0.063781573	Zm00001d036568
Zm00001d036568_T013	0.9895224	0.242343444	Zm00001d036568
Zm00001d036568_T003	0.9524127	-0.17391079	Zm00001d036568
Zm00001d036568_T002	0.9196877	-0.27595888	Zm00001d036568
Zm00001d048134_T001	0.9429712	0.580514002	Zm00001d048134
Zm00001d028410_T001	0.0161377	-0.884479949	Zm00001d028410
Zm00001d052874_T001	0.9945118	-0.176448523	Zm00001d052874
Zm00001d027512_T002	0.7778639	-0.364552383	Zm00001d027512

Zm00001d036216_T001	0.4976887	1.231167321	Zm00001d036216
Zm00001d042305_T001	0.9027598	-0.252433486	Zm00001d042305
Zm00001d021545_T006	0.04206673	-0.759578957	Zm00001d021545
Zm00001d021545_T001	0.8987811	0.530038818	Zm00001d021545
Zm00001d020617_T001	0.6554074	-0.380873211	Zm00001d020617
Zm00001d039608_T001	8.58E-05	-2.187093319	Zm00001d039608
Zm00001d039608_T005	0.01111628	1.661004289	Zm00001d039608
Zm00001d039608_T041	0.0060283	1.203025151	Zm00001d039608
Zm00001d039608_T045	0.5719089	-0.635231255	Zm00001d039608
Zm00001d039608_T014	0.9968176	0.194000553	Zm00001d039608
Zm00001d039608_T022	0.874819	0.552657412	Zm00001d039608
Zm00001d051396_T007	0.9326774	0.128074566	Zm00001d051396
Zm00001d051396_T016	0.6171076	0.71733906	Zm00001d051396
Zm00001d051396_T021	0.03944307	0.479885432	Zm00001d051396
Zm00001d049628_T001	0.9046438	-0.0412269	Zm00001d049628
Zm00001d049628_T002	0.9468268	0.332477845	Zm00001d049628
Zm00001d002085_T001	0.9180865	0.792891141	Zm00001d002085
Zm00001d048513_T001	0.9965998	0.168311058	Zm00001d048513
Zm00001d024528_T001	0.7852613	0.643034915	Zm00001d024528
Zm00001d032688_T001	0.455428	-0.797029687	Zm00001d032688
Zm00001d041960_T001	0.5576423	-0.966782454	Zm00001d041960
Zm00001d030833_T001	1	0.176392919	Zm00001d030833
Zm00001d040147_T003	0.9968176	0.01872741	Zm00001d040147
Zm00001d040147_T001	0.9979568	0.17564161	Zm00001d040147
Zm00001d046112_T002	0.8864089	-0.124210936	Zm00001d046112
Zm00001d046112_T003	0.909602	-0.066916455	Zm00001d046112
Zm00001d021490_T001	0.355126	1.510623084	Zm00001d021490
Zm00001d021490_T004	0.957148	0.327137238	Zm00001d021490
Zm00001d013049_T002	0.4422995	1.386633253	Zm00001d013049
Zm00001d013049_T006	0.1741809	0.962894369	Zm00001d013049
Zm00001d013049_T005	0.000500744	1.15636986	Zm00001d013049
Zm00001d014568_T025	0.00146127	1.274849405	Zm00001d014568
Zm00001d014568_T007	0.6717227	0.370000553	Zm00001d014568
Zm00001d014568_T037	0.8141108	-0.173498026	Zm00001d014568
Zm00001d014568_T028	0.04179364	0.47420759	Zm00001d014568
Zm00001d014568_T035	0.9881407	0.115485646	Zm00001d014568
Zm00001d006822_T001	0.9651683	0.100198115	Zm00001d006822
Zm00001d029889_T034	0.9404125	0.735114262	Zm00001d029889
Zm00001d029889_T003	0.9824348	0.553131964	Zm00001d029889
Zm00001d029889_T032	0.3649839	1.234267515	Zm00001d029889
Zm00001d029889_T038	0.9257893	0.416137257	Zm00001d029889
Zm00001d036152_T001	0.811177	0.596894597	Zm00001d036152
Zm00001d053641_T002	0.9249846	-0.278492996	Zm00001d053641
Zm00001d053641_T003	0.9979568	0.131759713	Zm00001d053641
Zm00001d053641_T001	0.3403653	-0.632901016	Zm00001d053641
Zm00001d028352_T001	0.9455821	0.300902309	Zm00001d028352
Zm00001d028352_T003	0.2007953	0.46788088	Zm00001d028352
Zm00001d028352_T007	0.2491267	-1.224011915	Zm00001d028352
Zm00001d028352_T002	0.983226	0.397635658	Zm00001d028352
Zm00001d018977_T002	0.01008423	0.75869308	Zm00001d018977
Zm00001d018977_T021	0.9860836	0.05438806	Zm00001d018977
Zm00001d018977_T091	0.4092696	-0.722726966	Zm00001d018977
Zm00001d028616_T001	0.6118682	-0.495047691	Zm00001d028616
Zm00001d014876_T001	0.3312671	1.638666807	Zm00001d014876

Zm00001d014876_T045	0.7109402	-0.256927089	Zm00001d014876
Zm00001d014876_T015	0.8581699	1.412904593	Zm00001d014876
Zm00001d014876_T003	0.9887584	0.346562937	Zm00001d014876
Zm00001d014876_T037	0.4546327	1.389168511	Zm00001d014876
Zm00001d014876_T044	0.4261766	-0.699421819	Zm00001d014876
Zm00001d006167_T004	0.9181741	0.3321714	Zm00001d006167
Zm00001d006167_T003	0.9870394	0.441957333	Zm00001d006167
Zm00001d006167_T001	0.4601888	-0.481609624	Zm00001d006167
Zm00001d034714_T001	1	0.450084656	Zm00001d034714
Zm00001d052620_T011	0.3495559	0.284459734	Zm00001d052620
Zm00001d052620_T005	0.9944696	0.295571263	Zm00001d052620
Zm00001d052620_T016	0.2095745	1.397866268	Zm00001d052620
Zm00001d052620_T015	0.9979568	1.10894109	Zm00001d052620
Zm00001d052620_T012	0.01532471	-1.815821991	Zm00001d052620
Zm00001d052620_T007	0.9930712	0.428563541	Zm00001d052620
Zm00001d052620_T013	2.71E-10	1.525769984	Zm00001d052620
Zm00001d052620_T001	6.04E-05	-1.914003918	Zm00001d052620
Zm00001d052620_T003	0.07420193	-0.764140734	Zm00001d052620
Zm00001d053822_T002	0.9979568	0.276186304	Zm00001d053822
Zm00001d022197_T001	0.8677544	-0.569995717	Zm00001d022197
Zm00001d042642_T001	0.5186912	0.864653573	Zm00001d042642
Zm00001d000169_T002	0.01225238	0.372331675	Zm00001d000169
Zm00001d000169_T007	0.0100365	1.909458054	Zm00001d000169
Zm00001d000169_T029	3.52E-08	0.844120828	Zm00001d000169
Zm00001d000169_T016	0.535136	1.309739612	Zm00001d000169
Zm00001d000169_T032	0.4889069	-0.64358791	Zm00001d000169
Zm00001d000169_T014	0.8513643	0.161104802	Zm00001d000169
Zm00001d000169_T009	0.9740325	0.165540778	Zm00001d000169
Zm00001d000169_T027	0.897685	-0.20652553	Zm00001d000169
Zm00001d037489_T002	0.9596377	-0.172624163	Zm00001d037489
Zm00001d037489_T005	0.1640339	-1.108891837	Zm00001d037489
Zm00001d037489_T004	0.2846089	-1.459718559	Zm00001d037489
Zm00001d028471_T002	0.9538039	0.065082783	Zm00001d028471
Zm00001d053613_T005	0.5743822	0.944789471	Zm00001d053613
Zm00001d053613_T006	0.7059983	0.84462531	Zm00001d053613
Zm00001d053613_T002	0.9858855	0.199363169	Zm00001d053613
Zm00001d053613_T001	0.9969123	0.240520119	Zm00001d053613
Zm00001d053613_T003	1	0.149945903	Zm00001d053613
Zm00001d018657_T004	0.3173707	1.340324787	Zm00001d018657
Zm00001d018657_T006	0.9268445	0.232146995	Zm00001d018657
Zm00001d018657_T001	0.7606712	0.028491282	Zm00001d018657
Zm00001d018657_T033	7.17E-14	-2.820551047	Zm00001d018657
Zm00001d018657_T016	0.01606269	2.204856687	Zm00001d018657
Zm00001d006154_T001	0.9537113	0.042232921	Zm00001d006154
Zm00001d037446_T001	0.9609806	0.136332384	Zm00001d037446
Zm00001d037446_T004	0.718063	-0.333914279	Zm00001d037446
Zm00001d037446_T007	0.9256115	0.304008375	Zm00001d037446
Zm00001d000194_T001	0.8558921	0.637795156	Zm00001d000194
Zm00001d034669_T001	0.9806244	0.153661845	Zm00001d034669
Zm00001d048836_T001	0.6470421	-0.454534444	Zm00001d048836
Zm00001d008468_T001	0.5908824	-0.454063766	Zm00001d008468
Zm00001d038341_T001	0.7289978	0.488685624	Zm00001d038341
Zm00001d045808_T065	0.2212562	-0.289031349	Zm00001d045808
Zm00001d045808_T037	7.69E-05	0.680898386	Zm00001d045808

Zm00001d045808_T025	0.7952435	-0.255260618	Zm00001d045808
Zm00001d045808_T031	0.9907928	0.016003217	Zm00001d045808
Zm00001d045808_T066	1	0.245379911	Zm00001d045808
Zm00001d045808_T001	0.9017638	0.660885177	Zm00001d045808
Zm00001d045808_T008	0.04523809	0.909699497	Zm00001d045808
Zm00001d021087_T003	0.8693754	0.838270827	Zm00001d021087
Zm00001d029038_T001	0.6445434	0.960181052	Zm00001d029038
Zm00001d043984_T001	0.9031129	-0.218360169	Zm00001d043984
Zm00001d046044_T001	0.9727064	0.249978249	Zm00001d046044
Zm00001d013073_T002	0.1922848	-1.106142477	Zm00001d013073
Zm00001d013073_T001	0.9669771	0.529400204	Zm00001d013073
Zm00001d028929_T001	0.9627389	-0.004159835	Zm00001d028929
Zm00001d049387_T001	0.9195989	-0.45001145	Zm00001d049387
Zm00001d007357_T003	0.6552247	-0.444437122	Zm00001d007357
Zm00001d007357_T001	0.7646718	-0.399446488	Zm00001d007357
Zm00001d007357_T005	0.5347419	1.233751826	Zm00001d007357
Zm00001d053620_T008	0.4146004	0.712517037	Zm00001d053620
Zm00001d053620_T042	0.8883116	0.297654748	Zm00001d053620
Zm00001d053620_T005	0.6044465	-0.115929006	Zm00001d053620
Zm00001d053620_T006	0.9514141	0.12353627	Zm00001d053620
Zm00001d053620_T026	0.07537729	0.545423798	Zm00001d053620
Zm00001d053620_T024	0.8333362	0.71740122	Zm00001d053620
Zm00001d053620_T031	0.000205957	0.695930006	Zm00001d053620
Zm00001d053620_T039	0.003908299	0.847839032	Zm00001d053620
Zm00001d053620_T016	0.4702431	1.573619608	Zm00001d053620
Zm00001d028887_T001	0.9962972	0.471361151	Zm00001d028887
Zm00001d012086_T004	0.9037002	0.847565508	Zm00001d012086
Zm00001d012086_T011	0.3838671	-0.558975815	Zm00001d012086
Zm00001d012086_T010	0.7561907	0.364611682	Zm00001d012086
Zm00001d012086_T002	0.7983447	-0.163252732	Zm00001d012086
Zm00001d012086_T012	0.3296668	0.62208722	Zm00001d012086
Zm00001d010834_T002	1	0.319159546	Zm00001d010834
Zm00001d010834_T003	0.9469426	0.232038937	Zm00001d010834
Zm00001d007770_T001	0.8578953	-0.127777209	Zm00001d007770
Zm00001d020325_T001	0.9721766	0.636398632	Zm00001d020325
Zm00001d017968_T001	0.9371934	-0.154237458	Zm00001d017968
Zm00001d036300_T001	0.9825388	0.473720268	Zm00001d036300
Zm00001d040188_T001	0.6673107	-0.452633855	Zm00001d040188
Zm00001d045044_T001	0.9135335	-0.016528915	Zm00001d045044
Zm00001d010269_T001	0.822299	0.906199728	Zm00001d010269
Zm00001d000086_T001	0.919888	-0.104518125	Zm00001d000086
Zm00001d000113_T001	0.9579758	-0.264206952	Zm00001d000113
Zm00001d002058_T001	0.9507337	0.063504756	Zm00001d002058
Zm00001d042954_T001	0.7052177	-0.35155685	Zm00001d042954
Zm00001d005933_T001	0.06223747	1.493165914	Zm00001d005933
Zm00001d010040_T001	0.9693993	-0.265587718	Zm00001d010040
Zm00001d016692_T001	1	-0.096006847	Zm00001d016692
Zm00001d034627_T001	0.9804228	0.242501196	Zm00001d034627
Zm00001d034627_T003	0.8863456	-0.038017516	Zm00001d034627
Zm00001d049155_T014	0.4530974	1.104892879	Zm00001d049155
Zm00001d049155_T020	0.3592677	1.181065747	Zm00001d049155
Zm00001d049155_T002	0.7565187	-0.070817697	Zm00001d049155
Zm00001d049155_T005	0.5535826	0.453156558	Zm00001d049155
Zm00001d049155_T018	0.03831914	0.532744864	Zm00001d049155

Zm00001d049155_T030	0.9971866	0.002242664	Zm00001d049155
Zm00001d049155_T024	0.8262962	0.241483781	Zm00001d049155
Zm00001d049155_T025	0.9324801	0.77317665	Zm00001d049155
Zm00001d029129_T002	0.716106	0.645733714	Zm00001d029129
Zm00001d029129_T003	0.9760472	0.128930352	Zm00001d029129
Zm00001d029129_T006	0.8645432	0.644851424	Zm00001d029129
Zm00001d029129_T001	0.5711067	0.40166055	Zm00001d029129
Zm00001d031008_T002	0.7688599	0.20969617	Zm00001d031008
Zm00001d031008_T001	0.8379797	-0.213379146	Zm00001d031008
Zm00001d030220_T001	0.7688599	-0.333764736	Zm00001d030220
Zm00001d047719_T001	0.9037807	-0.04436508	Zm00001d047719
Zm00001d032884_T001	0.9363424	0.590531359	Zm00001d032884
Zm00001d044434_T001	0.6086477	1.335060734	Zm00001d044434
Zm00001d034892_T001	0.8825058	-0.038686132	Zm00001d034892
Zm00001d012937_T001	0.880737	-0.446877917	Zm00001d012937
Zm00001d041601_T001	0.8645432	0.787215391	Zm00001d041601
Zm00001d041601_T002	0.2974375	-1.394266636	Zm00001d041601
Zm00001d038229_T002	0.9778436	0.248557352	Zm00001d038229
Zm00001d038229_T001	0.9468268	-0.137500569	Zm00001d038229
Zm00001d018187_T001	0.9894615	0.487777727	Zm00001d018187
Zm00001d044937_T001	0.9171732	-0.057418918	Zm00001d044937
Zm00001d029754_T001	0.9355135	0.079859336	Zm00001d029754
Zm00001d029754_T004	0.9027598	0.662389676	Zm00001d029754
Zm00001d026017_T001	0.2416802	-0.932413446	Zm00001d026017
Zm00001d002476_T001	0.3861376	-0.796835518	Zm00001d002476
Zm00001d039266_T005	0.02942365	0.94793914	Zm00001d039266
Zm00001d039266_T002	0.4926865	-0.451908283	Zm00001d039266
Zm00001d039266_T001	0.6824162	0.697002748	Zm00001d039266
Zm00001d039266_T003	1	0.286362391	Zm00001d039266
Zm00001d039266_T004	0.7989126	-0.082574588	Zm00001d039266
Zm00001d039266_T015	0.9870394	0.194399822	Zm00001d039266
Zm00001d045837_T002	0.9242136	0.662432053	Zm00001d045837
Zm00001d045837_T001	0.940778	-0.442874637	Zm00001d045837
Zm00001d032950_T003	0.9395327	0.783893771	Zm00001d032950
Zm00001d032950_T002	0.9781672	0.542841085	Zm00001d032950
Zm00001d043616_T001	0.9237037	-0.05440844	Zm00001d043616
Zm00001d038839_T002	0.9486412	-0.467651016	Zm00001d038839
Zm00001d019311_T001	0.8950139	0.777715912	Zm00001d019311
Zm00001d032078_T001	0.7199158	0.966200758	Zm00001d032078
Zm00001d034494_T001	0.1543317	1.999020176	Zm00001d034494
Zm00001d039787_T001	0.5719089	1.289141652	Zm00001d039787
Zm00001d039787_T008	0.574542	-0.429330813	Zm00001d039787
Zm00001d039787_T006	0.2982377	1.450250233	Zm00001d039787
Zm00001d039787_T007	0.07567414	0.565350845	Zm00001d039787
Zm00001d039787_T002	0.7159087	0.358359005	Zm00001d039787
Zm00001d027850_T003	1	0.217290464	Zm00001d027850
Zm00001d046661_T008	1.53E-12	3.236433786	Zm00001d046661
Zm00001d046661_T005	0.6445434	1.16328499	Zm00001d046661
Zm00001d046661_T002	0.7475575	-0.298910157	Zm00001d046661
Zm00001d046661_T007	0.9909466	0.47380776	Zm00001d046661
Zm00001d046661_T003	0.5537579	-0.614419023	Zm00001d046661
Zm00001d013077_T003	0.4062887	-0.422909556	Zm00001d013077
Zm00001d048505_T002	0.7912425	-0.220365712	Zm00001d048505
Zm00001d023210_T001	0.007667184	-0.972502096	Zm00001d023210

Zm00001d045577_T002	0.9991964	0.182003833	Zm00001d045577
Zm00001d034650_T008	1.32E-11	-2.461294533	Zm00001d034650
Zm00001d034650_T017	2.23E-12	3.254552558	Zm00001d034650
Zm00001d034650_T016	1.66E-07	0.575167114	Zm00001d034650
Zm00001d034650_T007	0.9206245	0.308380471	Zm00001d034650
Zm00001d034650_T004	0.9747695	0.576061427	Zm00001d034650
Zm00001d028490_T002	0.4845703	1.426418231	Zm00001d028490
Zm00001d028490_T005	0.6916675	0.359918959	Zm00001d028490
Zm00001d028490_T008	0.4416749	-1.469865871	Zm00001d028490
Zm00001d028490_T011	0.9851204	0.148648251	Zm00001d028490
Zm00001d052399_T006	0.02706902	1.946920145	Zm00001d052399
Zm00001d052399_T009	1	0.308534142	Zm00001d052399
Zm00001d052399_T003	0.4873881	1.336122522	Zm00001d052399
Zm00001d052399_T018	0.8924469	1.397194442	Zm00001d052399
Zm00001d052399_T004	0.01402472	1.354695176	Zm00001d052399
Zm00001d052399_T017	0.9979568	0.114539219	Zm00001d052399
Zm00001d052399_T012	0.000115347	1.426189741	Zm00001d052399
Zm00001d052399_T008	0.4937049	-0.752248729	Zm00001d052399
Zm00001d052399_T007	0.01015258	1.046533991	Zm00001d052399
Zm00001d052399_T001	0.5398956	1.415504847	Zm00001d052399
Zm00001d052399_T010	0.1339387	0.552037289	Zm00001d052399
Zm00001d004616_T009	0.9832092	0.094783385	Zm00001d004616
Zm00001d004616_T001	0.9410688	-0.00132241	Zm00001d004616
Zm00001d004616_T011	0.7626242	0.558828648	Zm00001d004616
Zm00001d004616_T004	0.9718605	0.560943997	Zm00001d004616
Zm00001d004616_T014	0.2073293	0.309821686	Zm00001d004616
Zm00001d004616_T010	0.3823269	0.767563047	Zm00001d004616
Zm00001d004616_T007	0.5127506	0.936405884	Zm00001d004616
Zm00001d004616_T012	0.3795013	-0.111680446	Zm00001d004616
Zm00001d004616_T015	0.9979568	0.059771332	Zm00001d004616
Zm00001d004616_T008	0.9493302	0.032547978	Zm00001d004616
Zm00001d004616_T002	0.9914912	0.442536598	Zm00001d004616
Zm00001d034373_T001	0.7437035	1.15056551	Zm00001d034373
Zm00001d039712_T003	0.9404125	-0.195875184	Zm00001d039712
Zm00001d039712_T004	0.971204	0.805231623	Zm00001d039712
Zm00001d039712_T002	0.5440777	1.087918299	Zm00001d039712
Zm00001d039712_T001	0.9950761	0.562030149	Zm00001d039712
Zm00001d039712_T005	0.9860676	0.257091428	Zm00001d039712
Zm00001d039311_T002	0.3497147	-0.8831439	Zm00001d039311
Zm00001d039311_T001	0.9016285	-0.045239559	Zm00001d039311
Zm00001d034085_T001	0.0733255	-0.731666013	Zm00001d034085
Zm00001d034085_T003	0.4964992	1.149142638	Zm00001d034085
Zm00001d013595_T001	0.9817074	0.512029165	Zm00001d013595
Zm00001d013595_T003	0.4380979	-0.873316194	Zm00001d013595
Zm00001d013595_T004	0.04663752	-1.240328946	Zm00001d013595
Zm00001d033211_T002	0.9821324	0.520681889	Zm00001d033211
Zm00001d038366_T001	0.5111755	1.45840535	Zm00001d038366
Zm00001d013383_T001	0.9690925	0.148171103	Zm00001d013383
Zm00001d026691_T009	0.1204448	-0.483258101	Zm00001d026691
Zm00001d026691_T007	0.04874817	0.748467596	Zm00001d026691
Zm00001d026691_T019	0.6129944	-0.482724831	Zm00001d026691
Zm00001d026691_T001	0.001468961	-0.665177049	Zm00001d026691
Zm00001d026691_T003	0.02666365	1.371740371	Zm00001d026691
Zm00001d026691_T006	0.1381169	1.207519857	Zm00001d026691

Zm00001d054049_T001	0.009606413	-2.111638944	Zm00001d054049
Zm00001d054049_T002	0.3388363	1.686629277	Zm00001d054049
Zm00001d008587_T001	0.4046727	1.337472432	Zm00001d008587
Zm00001d006220_T013	0.001117538	-2.096006078	Zm00001d006220
Zm00001d006220_T006	0.8439431	-0.220750028	Zm00001d006220
Zm00001d006220_T007	0.9717919	0.465236803	Zm00001d006220
Zm00001d009939_T001	0.5097225	1.21262944	Zm00001d009939
Zm00001d046348_T008	0.2295689	1.291008017	Zm00001d046348
Zm00001d037208_T002	0.7275347	0.682216351	Zm00001d037208
Zm00001d013656_T001	1	-0.102768116	Zm00001d013656
Zm00001d029248_T002	0.8226641	-0.223851732	Zm00001d029248
Zm00001d002950_T003	0.6086477	0.847921559	Zm00001d002950
Zm00001d002950_T012	0.03437276	-0.291525419	Zm00001d002950
Zm00001d002950_T016	0.3244552	-0.958360202	Zm00001d002950
Zm00001d053396_T001	0.7042561	-0.41869068	Zm00001d053396
Zm00001d009222_T002	0.9979568	0.359106555	Zm00001d009222
Zm00001d009222_T001	0.9845237	0.163626836	Zm00001d009222
Zm00001d016542_T001	1	0.259339615	Zm00001d016542
Zm00001d005629_T001	0.6552456	1.115494096	Zm00001d005629
Zm00001d039694_T001	0.4002383	-1.179692676	Zm00001d039694
Zm00001d042807_T003	0.7292566	1.049066072	Zm00001d042807
Zm00001d042807_T001	0.7489533	-0.320590009	Zm00001d042807
Zm00001d012128_T003	0.6053962	1.322169329	Zm00001d012128
Zm00001d012128_T005	0.5408683	-0.57126104	Zm00001d012128
Zm00001d020209_T001	0.8853366	0.633623473	Zm00001d020209
Zm00001d020209_T005	0.1893875	-1.350594694	Zm00001d020209
Zm00001d020209_T003	0.5179617	-0.363043278	Zm00001d020209
Zm00001d020209_T002	0.8863643	0.796289207	Zm00001d020209
Zm00001d024816_T025	0.9447869	-0.050319792	Zm00001d024816
Zm00001d024816_T002	0.5793913	0.551399579	Zm00001d024816
Zm00001d024816_T001	0.9749249	0.234010362	Zm00001d024816
Zm00001d024816_T009	0.8748755	-0.264660898	Zm00001d024816
Zm00001d024816_T007	1.69E-11	1.878379235	Zm00001d024816
Zm00001d021417_T001	0.9531608	-0.282007409	Zm00001d021417
Zm00001d021777_T002	0.6258249	-0.389399439	Zm00001d021777
Zm00001d040554_T001	0.9475565	0.455237459	Zm00001d040554
Zm00001d026576_T001	0.7251353	-0.43741527	Zm00001d026576
Zm00001d043701_T001	0.000388444	3.3734276	Zm00001d043701
Zm00001d008357_T004	0.7770297	2.004918401	Zm00001d008357
Zm00001d008357_T001	0.9928864	0.402178914	Zm00001d008357
Zm00001d008357_T008	0.8688416	-0.300265117	Zm00001d008357
Zm00001d008357_T010	0.5563797	0.73048733	Zm00001d008357
Zm00001d010466_T001	0.910614	0.780236474	Zm00001d010466
Zm00001d027884_T005	0.9664736	0.154548492	Zm00001d027884
Zm00001d027884_T003	0.9329122	0.755161835	Zm00001d027884
Zm00001d015945_T001	1	0.249070711	Zm00001d015945
Zm00001d051014_T004	0.8452909	0.930992364	Zm00001d051014
Zm00001d051014_T008	0.02488927	0.360752111	Zm00001d051014
Zm00001d051014_T005	0.9887889	0.393870877	Zm00001d051014
Zm00001d012067_T004	0.7825891	0.822268461	Zm00001d012067
Zm00001d012067_T003	0.9887584	0.301588531	Zm00001d012067
Zm00001d037714_T001	0.9909466	-0.002096065	Zm00001d037714
Zm00001d025539_T014	0.1214442	-1.778491437	Zm00001d025539
Zm00001d025539_T006	0.9498304	-0.273344418	Zm00001d025539

Zm00001d025539_T001	0.9843194	-0.032008147	Zm00001d025539
Zm00001d025539_T013	0.8695779	0.787434102	Zm00001d025539
Zm00001d025539_T004	1	0.259407841	Zm00001d025539
Zm00001d025539_T005	0.9785005	0.433789205	Zm00001d025539
Zm00001d025539_T002	0.8767824	0.533651352	Zm00001d025539
Zm00001d025539_T003	0.7602652	0.549754791	Zm00001d025539
Zm00001d021204_T002	0.9312703	0.549563542	Zm00001d021204
Zm00001d014458_T001	0.2619882	1.629486827	Zm00001d014458
Zm00001d014458_T003	0.9037002	-0.04403836	Zm00001d014458
Zm00001d010212_T002	0.9955906	0.133630072	Zm00001d010212
Zm00001d035717_T001	0.9817074	0.52279786	Zm00001d035717
Zm00001d010632_T001	0.6767305	-0.911446695	Zm00001d010632
Zm00001d010632_T002	0.6517582	-0.936490359	Zm00001d010632
Zm00001d041856_T001	0.9487214	-0.41250711	Zm00001d041856
Zm00001d020292_T001	0.8056124	-0.194273527	Zm00001d020292
Zm00001d038708_T001	0.1048346	1.913094009	Zm00001d038708
Zm00001d042864_T006	0.5796852	-1.565123613	Zm00001d042864
Zm00001d042864_T001	0.06970238	1.869882058	Zm00001d042864
Zm00001d042864_T004	8.17E-10	2.546490435	Zm00001d042864
Zm00001d042864_T005	0.988575	0.173728105	Zm00001d042864
Zm00001d042864_T003	0.9965998	0.273222058	Zm00001d042864
Zm00001d042864_T002	0.9458846	0.625171509	Zm00001d042864
Zm00001d043651_T001	0.9832092	0.102563108	Zm00001d043651
Zm00001d043651_T002	0.8660897	0.836655859	Zm00001d043651
Zm00001d051475_T002	0.02888804	-1.708142599	Zm00001d051475
Zm00001d031323_T001	0.9881407	0.222518734	Zm00001d031323
Zm00001d036280_T002	0.8322028	-0.122578023	Zm00001d036280
Zm00001d036280_T003	1.44E-11	1.686583911	Zm00001d036280
Zm00001d004895_T001	0.9075969	-0.244116592	Zm00001d004895
Zm00001d052485_T011	0.8916793	0.07713196	Zm00001d052485
Zm00001d052485_T001	0.3221433	1.673693293	Zm00001d052485
Zm00001d052485_T002	0.7176505	-0.34496522	Zm00001d052485
Zm00001d052485_T015	0.411963	0.769995849	Zm00001d052485
Zm00001d019698_T001	0.9290639	0.67138814	Zm00001d019698
Zm00001d045788_T001	0.7808781	-0.256754428	Zm00001d045788
Zm00001d052058_T001	0.4403985	-0.978907274	Zm00001d052058
Zm00001d011087_T002	0.997449	0.300712521	Zm00001d011087
Zm00001d011087_T003	1	0.134171159	Zm00001d011087
Zm00001d011087_T004	0.935477	0.038656678	Zm00001d011087
Zm00001d053541_T002	0.9840918	0.499951578	Zm00001d053541
Zm00001d044923_T001	0.9210235	-0.797059689	Zm00001d044923
Zm00001d013193_T002	0.005892258	-2.517051372	Zm00001d013193
Zm00001d046440_T001	0.9693993	0.121319163	Zm00001d046440
Zm00001d026149_T001	0.4553805	0.404030123	Zm00001d026149
Zm00001d043696_T006	0.02830027	0.682764256	Zm00001d043696
Zm00001d043696_T001	0.9846714	0.463299274	Zm00001d043696
Zm00001d043696_T007	0.7452824	-0.085271339	Zm00001d043696
Zm00001d043696_T004	0.9909466	0.769542706	Zm00001d043696
Zm00001d040597_T001	0.9749966	0.104377901	Zm00001d040597
Zm00001d048669_T001	0.4974605	-0.560389343	Zm00001d048669
Zm00001d048669_T002	0.07676088	-1.230423856	Zm00001d048669
Zm00001d045509_T005	0.0191703	-1.26325466	Zm00001d045509
Zm00001d045509_T007	0.5698311	-0.771398948	Zm00001d045509
Zm00001d045509_T004	0.9994624	0.198742781	Zm00001d045509

Zm00001d045509_T006	0.9824516	0.463075633	Zm00001d045509
Zm00001d045509_T002	0.9262099	1.138186737	Zm00001d045509
Zm00001d047174_T001	0.9782953	0.223445425	Zm00001d047174
Zm00001d007327_T001	0.9703357	-0.07353838	Zm00001d007327
Zm00001d008737_T001	0.9781672	-0.067253539	Zm00001d008737
Zm00001d037251_T001	0.9745948	-0.04931125	Zm00001d037251
Zm00001d027355_T001	0.9791729	0.488581714	Zm00001d027355
Zm00001d026079_T002	3.06E-12	-3.136375409	Zm00001d026079
Zm00001d026079_T001	0.9344122	0.768332395	Zm00001d026079
Zm00001d021704_T001	0.9884825	0.111952362	Zm00001d021704
Zm00001d021704_T003	0.03030472	-1.927219426	Zm00001d021704
Zm00001d021704_T002	1	0.150026615	Zm00001d021704
Zm00001d051902_T004	0.7079838	1.14311669	Zm00001d051902
Zm00001d051902_T008	3.15E-13	2.769120601	Zm00001d051902
Zm00001d051902_T002	0.941809	0.077471946	Zm00001d051902
Zm00001d028187_T003	0.107926	0.972906083	Zm00001d028187
Zm00001d028187_T001	0.9804433	0.563673418	Zm00001d028187
Zm00001d028187_T004	0.1592738	0.628494071	Zm00001d028187
Zm00001d024421_T001	0.9326774	0.563760855	Zm00001d024421
Zm00001d024421_T002	0.9846714	0.287481424	Zm00001d024421
Zm00001d018017_T001	0.7262357	0.944408197	Zm00001d018017
Zm00001d007442_T001	0.9690782	0.123071292	Zm00001d007442
Zm00001d035999_T002	0.9765009	0.18368568	Zm00001d035999
Zm00001d044683_T001	0.9446379	-0.126305509	Zm00001d044683
Zm00001d037315_T011	0.9780511	0.015623769	Zm00001d037315
Zm00001d037315_T019	0.3497147	0.525236345	Zm00001d037315
Zm00001d037315_T020	0.75426	0.843271265	Zm00001d037315
Zm00001d037315_T009	0.5954619	-0.118736161	Zm00001d037315
Zm00001d037315_T004	0.6645568	0.564521572	Zm00001d037315
Zm00001d037315_T015	0.8883561	1.290193071	Zm00001d037315
Zm00001d037315_T001	0.1677044	0.806416876	Zm00001d037315
Zm00001d048613_T001	0.8757823	0.626966233	Zm00001d048613
Zm00001d046913_T001	0.7265805	-0.316234638	Zm00001d046913
Zm00001d034641_T001	0.2589628	1.458941987	Zm00001d034641
Zm00001d022212_T002	0.3010905	-0.722437245	Zm00001d022212
Zm00001d022212_T001	0.9582899	0.044266079	Zm00001d022212
Zm00001d020396_T003	0.003774627	1.622277074	Zm00001d020396
Zm00001d020396_T004	0.3497147	1.746905487	Zm00001d020396
Zm00001d014192_T001	0.9919298	0.222286269	Zm00001d014192
Zm00001d002396_T021	0.3935846	-0.469127886	Zm00001d002396
Zm00001d002396_T012	0.2836969	0.955079893	Zm00001d002396
Zm00001d002396_T005	0.01662821	-0.497229411	Zm00001d002396
Zm00001d002396_T027	0.2566009	-0.703835982	Zm00001d002396
Zm00001d036654_T001	0.9690782	0.089797854	Zm00001d036654
Zm00001d036654_T002	0.8357582	0.925019843	Zm00001d036654
Zm00001d036654_T003	0.9679145	0.324005424	Zm00001d036654
Zm00001d045149_T001	0.7169281	-0.290073762	Zm00001d045149
Zm00001d011179_T001	0.9563232	-0.022691558	Zm00001d011179
Zm00001d008870_T006	1	0.215883571	Zm00001d008870
Zm00001d008870_T001	0.4516145	-1.574358818	Zm00001d008870
Zm00001d008870_T007	0.9127665	0.695533354	Zm00001d008870
Zm00001d008870_T004	0.497443	0.553690616	Zm00001d008870
Zm00001d021572_T001	0.7167212	0.426027836	Zm00001d021572
Zm00001d021036_T001	0.9852645	0.463344427	Zm00001d021036

Zm00001d044090_T004	0.4359356	0.61245327	Zm00001d044090
Zm00001d044090_T002	0.9827298	0.382334176	Zm00001d044090
Zm00001d044090_T001	0.9860836	0.196512337	Zm00001d044090
Zm00001d009022_T001	0.2496307	-0.705424625	Zm00001d009022
Zm00001d053736_T001	0.000275609	1.588562011	Zm00001d053736
Zm00001d053026_T001	0.9791729	0.040933535	Zm00001d053026
Zm00001d045383_T013	0.09013732	-0.313951463	Zm00001d045383
Zm00001d045383_T012	0.6625969	0.354619218	Zm00001d045383
Zm00001d045383_T005	0.7235553	0.360328379	Zm00001d045383
Zm00001d045383_T004	1	0.349862492	Zm00001d045383
Zm00001d051484_T001	0.1606259	-1.121420624	Zm00001d051484
Zm00001d016928_T001	0.6608757	0.669829987	Zm00001d016928
Zm00001d016928_T005	0.8836975	0.911183713	Zm00001d016928
Zm00001d016928_T008	0.6963657	-0.365922191	Zm00001d016928
Zm00001d016928_T010	0.8031436	1.020297868	Zm00001d016928
Zm00001d008256_T002	0.9792387	0.54898884	Zm00001d008256
Zm00001d008256_T005	0.2809344	-0.54216022	Zm00001d008256
Zm00001d051166_T001	0.3481719	1.550863686	Zm00001d051166
Zm00001d008308_T001	0.7565187	0.878153219	Zm00001d008308
Zm00001d042269_T001	0.9351594	0.058241295	Zm00001d042269
Zm00001d025210_T001	0.4578502	0.424399095	Zm00001d025210
Zm00001d025210_T002	0.9745777	0.215057585	Zm00001d025210
Zm00001d019404_T001	1	0.300906062	Zm00001d019404
Zm00001d033552_T004	0.1947403	-0.372666622	Zm00001d033552
Zm00001d045500_T001	0.2980414	-0.990360312	Zm00001d045500
Zm00001d045500_T002	0.9508258	-0.160053784	Zm00001d045500
Zm00001d052651_T001	0.1230305	-1.438267098	Zm00001d052651
Zm00001d020953_T001	0.2032568	-1.096734489	Zm00001d020953
Zm00001d002939_T001	0.7454538	-0.2648305	Zm00001d002939
Zm00001d002939_T002	0.6165407	-0.429305044	Zm00001d002939
Zm00001d027995_T001	0.9975882	0.389046765	Zm00001d027995
Zm00001d022096_T001	0.9887889	0.297226244	Zm00001d022096
Zm00001d052775_T001	0.8267985	0.876450546	Zm00001d052775
Zm00001d002128_T005	0.8988765	0.762897173	Zm00001d002128
Zm00001d002128_T004	0.9618235	-0.044648486	Zm00001d002128
Zm00001d002128_T001	0.8585804	0.827572705	Zm00001d002128
Zm00001d002128_T002	0.7856839	0.979851902	Zm00001d002128
Zm00001d002128_T003	0.009041647	2.591151034	Zm00001d002128
Zm00001d041191_T001	0.9404125	-0.142594038	Zm00001d041191
Zm00001d046745_T001	0.5566459	1.130200995	Zm00001d046745
Zm00001d020401_T001	0.9781672	-0.084308719	Zm00001d020401
Zm00001d040315_T001	0.9356767	-0.209030379	Zm00001d040315
Zm00001d051833_T001	0.9851204	0.216562403	Zm00001d051833
Zm00001d028457_T001	0.8020576	0.652173717	Zm00001d028457
Zm00001d045534_T001	0.1945933	0.502021152	Zm00001d045534
Zm00001d045534_T002	0.0245	1.622451966	Zm00001d045534
Zm00001d045534_T008	0.1912529	0.590232091	Zm00001d045534
Zm00001d045534_T011	0.01642555	-2.534971492	Zm00001d045534
Zm00001d045534_T004	0.449035	1.312549389	Zm00001d045534
Zm00001d045534_T010	0.8616666	1.002419495	Zm00001d045534
Zm00001d014960_T001	0.6099833	1.191128512	Zm00001d014960
Zm00001d026131_T001	1	0.298295774	Zm00001d026131
Zm00001d029432_T003	0.9954426	0.289589508	Zm00001d029432
Zm00001d043305_T001	0.8486461	0.916138894	Zm00001d043305

Zm00001d043305_T004	0.9624437	-0.074921732	Zm00001d043305
Zm00001d043305_T003	0.3953947	0.409078498	Zm00001d043305
Zm00001d043305_T009	0.139783	1.40444174	Zm00001d043305
Zm00001d043305_T008	0.2468051	0.58942322	Zm00001d043305
Zm00001d007058_T004	0.8950139	0.280131893	Zm00001d007058
Zm00001d007058_T007	1	0.03351769	Zm00001d007058
Zm00001d007058_T014	0.9224378	0.296532363	Zm00001d007058
Zm00001d007058_T012	0.9169169	0.482868202	Zm00001d007058
Zm00001d007058_T001	0.9585454	0.587967211	Zm00001d007058
Zm00001d013611_T001	0.6816752	1.115279609	Zm00001d013611
Zm00001d047908_T001	0.8513643	0.949357294	Zm00001d047908
Zm00001d047908_T002	0.7194959	0.984170645	Zm00001d047908
Zm00001d018633_T001	0.5137795	0.704305567	Zm00001d018633
Zm00001d037275_T001	0.8517246	-0.602646445	Zm00001d037275
Zm00001d018979_T001	0.774379	-0.611819341	Zm00001d018979
Zm00001d026562_T001	0.8643636	-0.309234997	Zm00001d026562
Zm00001d006361_T001	0.8498341	-0.466925459	Zm00001d006361
Zm00001d020695_T001	0.9390501	-0.004222356	Zm00001d020695
Zm00001d013742_T001	0.9213752	0.638508693	Zm00001d013742
Zm00001d021902_T002	0.9182563	0.762102885	Zm00001d021902
Zm00001d021902_T003	0.6439227	-0.584544198	Zm00001d021902
Zm00001d014809_T002	0.8561115	-0.145376029	Zm00001d014809
Zm00001d014809_T004	0.517208	0.517687259	Zm00001d014809
Zm00001d011624_T002	0.2699928	-0.919131944	Zm00001d011624
Zm00001d011624_T001	1	0.332389523	Zm00001d011624
Zm00001d033741_T001	0.2491267	0.935546198	Zm00001d033741
Zm00001d031060_T001	0.974657	0.136120898	Zm00001d031060
Zm00001d015722_T001	0.5699081	1.004020502	Zm00001d015722
Zm00001d017145_T003	0.0372102	-0.79029796	Zm00001d017145
Zm00001d017145_T002	0.9201341	0.712288233	Zm00001d017145
Zm00001d022281_T001	0.90276	-0.029009737	Zm00001d022281
Zm00001d019642_T002	0.3217566	-0.465206341	Zm00001d019642
Zm00001d019642_T001	0.8720321	0.329526962	Zm00001d019642
Zm00001d019642_T006	0.9979568	0.10128994	Zm00001d019642
Zm00001d025551_T003	0.8961192	-0.070419732	Zm00001d025551
Zm00001d025551_T001	0.6325529	0.797866037	Zm00001d025551
Zm00001d025551_T005	0.04307801	1.255071687	Zm00001d025551
Zm00001d012648_T001	0.6086477	1.224720065	Zm00001d012648
Zm00001d019700_T017	0.04074756	-0.987686315	Zm00001d019700
Zm00001d019700_T004	0.9740325	0.186474471	Zm00001d019700
Zm00001d019700_T001	0.477074	1.333312118	Zm00001d019700
Zm00001d031587_T002	0.6783244	0.442540674	Zm00001d031587
Zm00001d031587_T001	0.8717403	0.554759929	Zm00001d031587
Zm00001d002521_T001	0.941809	1.277184489	Zm00001d002521
Zm00001d004409_T001	0.9843321	-0.0629673	Zm00001d004409
Zm00001d039733_T001	0.7591435	-0.896751834	Zm00001d039733
Zm00001d047050_T001	0.8922781	-0.093670521	Zm00001d047050
Zm00001d014145_T001	0.9354647	0.056680492	Zm00001d014145
Zm00001d025817_T041	0.9918302	0.036058495	Zm00001d025817
Zm00001d025817_T008	0.684319	-0.609069877	Zm00001d025817
Zm00001d025817_T028	0.7990254	0.211101048	Zm00001d025817
Zm00001d025817_T025	3.55E-06	0.650876536	Zm00001d025817
Zm00001d025817_T001	0.2140375	1.510486247	Zm00001d025817
Zm00001d025817_T045	6.53E-10	-2.309890796	Zm00001d025817

Zm00001d025817_T038	0.926928	0.662088618	Zm00001d025817
Zm00001d053928_T001	0.9975882	0.261327592	Zm00001d053928
Zm00001d042639_T006	0.8517246	0.872153803	Zm00001d042639
Zm00001d042639_T004	0.05103414	-0.550552821	Zm00001d042639
Zm00001d042639_T001	0.3163102	-0.859313693	Zm00001d042639
Zm00001d018831_T010	0.009795236	-1.250666616	Zm00001d018831
Zm00001d018831_T007	0.6430278	1.109094534	Zm00001d018831
Zm00001d018831_T004	0.7807489	-0.216657938	Zm00001d018831
Zm00001d018831_T003	0.9709887	-0.106450858	Zm00001d018831
Zm00001d018831_T002	0.9951335	0.540330211	Zm00001d018831
Zm00001d005431_T001	0.8494782	0.880671925	Zm00001d005431
Zm00001d005431_T005	0.9618822	0.264668079	Zm00001d005431
Zm00001d005431_T007	0.9514628	0.500784888	Zm00001d005431
Zm00001d047895_T001	0.9785005	0.21208985	Zm00001d047895
Zm00001d045757_T001	0.8976475	-0.031007994	Zm00001d045757
Zm00001d039904_T001	0.6015602	-0.337209481	Zm00001d039904
Zm00001d039904_T011	0.9274507	-0.030776679	Zm00001d039904
Zm00001d039904_T006	0.9965274	0.467832365	Zm00001d039904
Zm00001d039904_T004	0.4570433	0.883492589	Zm00001d039904
Zm00001d049533_T001	0.8412913	0.353230422	Zm00001d049533
Zm00001d018412_T001	0.9962521	-0.680351637	Zm00001d018412
Zm00001d027699_T001	0.8825058	-0.280210492	Zm00001d027699
Zm00001d027699_T002	0.852305	-0.039978101	Zm00001d027699
Zm00001d037240_T005	1	0.195003707	Zm00001d037240
Zm00001d037240_T003	0.8631191	-0.051327247	Zm00001d037240
Zm00001d037240_T006	1	0.030139193	Zm00001d037240
Zm00001d032316_T001	0.997764	0.311051624	Zm00001d032316
Zm00001d052731_T001	0.04879383	2.347843849	Zm00001d052731
Zm00001d034615_T001	0.9907928	0.302475857	Zm00001d034615
Zm00001d017555_T002	0.9945076	0.29393019	Zm00001d017555
Zm00001d017555_T008	0.01955655	-3.045724724	Zm00001d017555
Zm00001d021980_T001	1	0.165744365	Zm00001d021980
Zm00001d023799_T001	0.1904181	1.143140082	Zm00001d023799
Zm00001d053450_T062	0.1614401	0.468628426	Zm00001d053450
Zm00001d053450_T032	0.006224998	1.356790754	Zm00001d053450
Zm00001d053450_T085	0.7852993	-0.445282169	Zm00001d053450
Zm00001d053450_T065	1	-0.029322702	Zm00001d053450
Zm00001d053450_T029	0.6115361	0.18177838	Zm00001d053450
Zm00001d053450_T089	0.9177108	-0.054094708	Zm00001d053450
Zm00001d039944_T001	3.57E-07	4.78815064	Zm00001d039944
Zm00001d029906_T001	0.909351	-0.109954706	Zm00001d029906
Zm00001d036063_T001	0.9104115	0.380067219	Zm00001d036063
Zm00001d042536_T001	0.8027057	1.0117509	Zm00001d042536
Zm00001d048686_T001	0.9804228	-0.075529787	Zm00001d048686
Zm00001d017353_T001	0.9979568	0.348302833	Zm00001d017353
Zm00001d028570_T006	0.972985	0.170738878	Zm00001d028570
Zm00001d028570_T007	0.2736137	1.622791567	Zm00001d028570
Zm00001d028570_T002	0.5166741	-0.095035857	Zm00001d028570
Zm00001d028570_T010	0.989787	0.097733412	Zm00001d028570
Zm00001d028570_T001	0.517789	0.361670498	Zm00001d028570
Zm00001d028570_T005	0.8956671	-0.032894986	Zm00001d028570
Zm00001d028570_T009	0.006884532	2.497509245	Zm00001d028570
Zm00001d016512_T001	0.9008366	0.820582701	Zm00001d016512
Zm00001d016512_T002	0.9953345	0.149531901	Zm00001d016512

Zm00001d005871_T002	0.44606	-0.53259463	Zm00001d005871
Zm00001d037291_T003	0.8393168	0.953576611	Zm00001d037291
Zm00001d037291_T001	0.9173494	0.795869421	Zm00001d037291
Zm00001d051756_T008	0.3996069	-0.501527503	Zm00001d051756
Zm00001d051756_T004	0.163796	1.612476408	Zm00001d051756
Zm00001d019985_T001	0.749176	-0.241572388	Zm00001d019985
Zm00001d048045_T002	0.9296249	-0.331569915	Zm00001d048045
Zm00001d048045_T001	1	0.213774308	Zm00001d048045
Zm00001d021245_T001	0.0165267	-1.902161008	Zm00001d021245
Zm00001d005851_T001	0.8955831	0.430022322	Zm00001d005851
Zm00001d010275_T001	0.9979568	0.027948035	Zm00001d010275
Zm00001d042088_T001	0.5570384	-0.487301505	Zm00001d042088
Zm00001d008520_T001	0.07581914	0.620301608	Zm00001d008520
Zm00001d008520_T005	0.9968176	0.361704099	Zm00001d008520
Zm00001d008520_T009	0.9058125	-0.068525494	Zm00001d008520
Zm00001d008520_T002	0.7309251	0.914242712	Zm00001d008520
Zm00001d050884_T005	0.9983825	0.257780657	Zm00001d050884
Zm00001d050884_T001	0.990751	0.390752045	Zm00001d050884
Zm00001d028017_T001	0.03896328	-1.639816117	Zm00001d028017
Zm00001d007522_T026	0.4355767	1.55838115	Zm00001d007522
Zm00001d007522_T025	2.40E-08	1.085178559	Zm00001d007522
Zm00001d007522_T002	0.01431466	2.611187052	Zm00001d007522
Zm00001d007522_T009	0.02304271	1.841334838	Zm00001d007522
Zm00001d007522_T021	0.07943446	1.192035934	Zm00001d007522
Zm00001d007522_T028	0.8677531	0.112096141	Zm00001d007522
Zm00001d003848_T003	0.1501558	1.93377604	Zm00001d003848
Zm00001d003848_T001	0.4034828	1.548549564	Zm00001d003848
Zm00001d050440_T002	0.9717213	0.073726659	Zm00001d050440
Zm00001d050440_T003	0.9278539	0.574443076	Zm00001d050440
Zm00001d050440_T001	0.6725985	-0.479621342	Zm00001d050440
Zm00001d003669_T001	0.4919207	1.399904498	Zm00001d003669
Zm00001d047843_T001	0.9907928	0.245170531	Zm00001d047843
Zm00001d047843_T002	1	0.394530126	Zm00001d047843
Zm00001d038878_T001	0.9669766	0.501836915	Zm00001d038878
Zm00001d010443_T001	0.9741154	0.132482831	Zm00001d010443
Zm00001d031129_T001	0.973825	0.159496197	Zm00001d031129
Zm00001d007044_T001	0.8746701	0.842213172	Zm00001d007044
Zm00001d001864_T004	0.2591129	-0.447149729	Zm00001d001864
Zm00001d001864_T003	0.5466134	0.563985184	Zm00001d001864
Zm00001d001864_T005	0.9048001	-0.156467724	Zm00001d001864
Zm00001d051671_T024	0.9252088	-0.137446703	Zm00001d051671
Zm00001d051671_T028	0.7253704	0.722942363	Zm00001d051671
Zm00001d051671_T016	0.9130008	-0.141076006	Zm00001d051671
Zm00001d051671_T025	0.9067774	1.062022209	Zm00001d051671
Zm00001d051671_T044	0.1447705	0.62977157	Zm00001d051671
Zm00001d051671_T042	0.004033773	0.984286405	Zm00001d051671
Zm00001d051671_T010	0.8427421	0.94636436	Zm00001d051671
Zm00001d034886_T001	0.954492	0.131296924	Zm00001d034886
Zm00001d038803_T001	0.9846446	0.178586421	Zm00001d038803
Zm00001d033544_T002	0.2966369	1.019184297	Zm00001d033544
Zm00001d033544_T001	0.0381171	1.175449731	Zm00001d033544
Zm00001d046404_T001	0.2693993	-1.109480167	Zm00001d046404
Zm00001d016714_T001	0.4729109	1.337758426	Zm00001d016714
Zm00001d016714_T002	0.7682041	0.938473384	Zm00001d016714

Zm00001d016714_T003	0.3500337	1.547358087	Zm00001d016714
Zm00001d019873_T001	0.9187556	-0.341462794	Zm00001d019873
Zm00001d052865_T001	0.8031754	-0.340868133	Zm00001d052865
Zm00001d015612_T001	1	0.127403644	Zm00001d015612
Zm00001d030675_T002	0.9872831	0.513939172	Zm00001d030675
Zm00001d038023_T001	0.7351951	1.093317409	Zm00001d038023
Zm00001d042767_T001	0.497443	1.394092665	Zm00001d042767
Zm00001d036735_T002	0.715186	0.773192488	Zm00001d036735
Zm00001d039350_T001	0.9539869	0.028690565	Zm00001d039350
Zm00001d039350_T002	0.04294987	-2.113344369	Zm00001d039350
Zm00001d010719_T004	0.000358318	-1.270384912	Zm00001d010719
Zm00001d010719_T002	0.003224902	1.776355859	Zm00001d010719
Zm00001d010719_T005	0.8508017	-0.194733043	Zm00001d010719
Zm00001d010719_T007	3.61E-05	0.277298941	Zm00001d010719
Zm00001d010719_T008	0.6568508	0.540643841	Zm00001d010719
Zm00001d010719_T006	0.6409895	-0.468811236	Zm00001d010719
Zm00001d052905_T002	0.9246704	0.081757665	Zm00001d052905
Zm00001d011228_T006	0.1219709	1.851538133	Zm00001d011228
Zm00001d011228_T002	0.164302	1.884831273	Zm00001d011228
Zm00001d003067_T001	0.8535962	0.606633362	Zm00001d003067
Zm00001d044464_T002	0.7881315	-0.851609247	Zm00001d044464
Zm00001d044464_T001	0.8520487	-0.304312064	Zm00001d044464
Zm00001d007200_T004	0.971204	0.344708898	Zm00001d007200
Zm00001d007200_T009	0.2572096	-0.334315979	Zm00001d007200
Zm00001d032467_T001	0.9988271	0.291751376	Zm00001d032467
Zm00001d032155_T001	0.9451281	0.610735515	Zm00001d032155
Zm00001d012467_T001	0.2870002	-0.503677528	Zm00001d012467
Zm00001d006343_T001	0.98291	0.325557861	Zm00001d006343
Zm00001d039547_T015	0.9806244	0.202308606	Zm00001d039547
Zm00001d039547_T012	0.897685	-0.00908488	Zm00001d039547
Zm00001d039547_T002	0.9388219	0.141097072	Zm00001d039547
Zm00001d039547_T008	0.9347957	-0.255004812	Zm00001d039547
Zm00001d047478_T001	0.9991964	0.05816143	Zm00001d047478
Zm00001d018150_T001	0.01478222	-2.621653937	Zm00001d018150
Zm00001d033902_T001	0.2267046	-1.233492602	Zm00001d033902
Zm00001d028273_T001	0.9269932	0.646931834	Zm00001d028273
Zm00001d006430_T005	0.8412913	0.473219779	Zm00001d006430
Zm00001d006430_T004	0.5549472	0.566976203	Zm00001d006430
Zm00001d006430_T007	0.3014141	0.580838959	Zm00001d006430
Zm00001d006430_T002	0.9207666	-0.065010734	Zm00001d006430
Zm00001d006430_T001	0.9535734	0.523199379	Zm00001d006430
Zm00001d006566_T002	0.760208	-0.553912264	Zm00001d006566
Zm00001d014492_T001	0.9393171	0.186116802	Zm00001d014492
Zm00001d007610_T002	0.9172003	-0.087586454	Zm00001d007610
Zm00001d007610_T003	0.7743281	0.964881232	Zm00001d007610
Zm00001d001953_T003	1	0.551376139	Zm00001d001953
Zm00001d001953_T001	0.9804228	0.283380601	Zm00001d001953
Zm00001d039446_T001	0.9666757	0.158758452	Zm00001d039446
Zm00001d050921_T001	0.8952297	-0.068475023	Zm00001d050921
Zm00001d050921_T002	0.8054423	-0.383966712	Zm00001d050921
Zm00001d050921_T003	0.6646236	-0.572323031	Zm00001d050921
Zm00001d038405_T001	0.6014554	0.913511598	Zm00001d038405
Zm00001d024765_T001	0.9788505	0.178312677	Zm00001d024765
Zm00001d043590_T001	0.8594358	-0.267918008	Zm00001d043590

Zm00001d012931_T001	0.9974734	0.426576084	Zm00001d012931
Zm00001d027983_T001	0.626109	0.954127625	Zm00001d027983
Zm00001d047307_T001	0.1334032	0.828931512	Zm00001d047307
Zm00001d043185_T001	0.8021989	-0.281314237	Zm00001d043185
Zm00001d013042_T001	0.1878813	-1.408827098	Zm00001d013042
Zm00001d016131_T001	0.9817074	0.320669341	Zm00001d016131
Zm00001d016131_T005	1	0.083513992	Zm00001d016131
Zm00001d016131_T003	0.9914912	0.487379968	Zm00001d016131
Zm00001d016131_T006	0.9257893	0.692555096	Zm00001d016131
Zm00001d030032_T001	0.1535144	-1.290668507	Zm00001d030032
Zm00001d030032_T002	0.9149322	-0.055437401	Zm00001d030032
Zm00001d039594_T001	0.8987811	-0.034290826	Zm00001d039594
Zm00001d002673_T001	0.5597598	-0.559299884	Zm00001d002673
Zm00001d040035_T001	0.3504849	-0.824188943	Zm00001d040035
Zm00001d024155_T001	0.9563232	0.460381352	Zm00001d024155
Zm00001d013842_T008	0.009025441	-2.594550496	Zm00001d013842
Zm00001d013842_T007	0.774379	-0.255077796	Zm00001d013842
Zm00001d013842_T003	0.9685163	0.635783461	Zm00001d013842
Zm00001d043325_T001	0.9689939	0.316442145	Zm00001d043325
Zm00001d017834_T001	0.9868196	0.34035598	Zm00001d017834
Zm00001d029757_T001	0.9585454	-0.060617369	Zm00001d029757
Zm00001d017568_T004	0.9719113	-0.646879626	Zm00001d017568
Zm00001d017568_T005	0.003488553	3.502392605	Zm00001d017568
Zm00001d017568_T015	0.7561907	0.38945107	Zm00001d017568
Zm00001d017568_T008	0.6511883	-0.068907201	Zm00001d017568
Zm00001d017568_T020	0.9979568	-0.015695453	Zm00001d017568
Zm00001d017568_T001	0.07457948	-0.810133558	Zm00001d017568
Zm00001d017568_T023	0.01751306	1.281718688	Zm00001d017568
Zm00001d006440_T002	0.9911485	0.246260205	Zm00001d006440
Zm00001d006440_T003	0.7285679	0.620894676	Zm00001d006440
Zm00001d038553_T001	0.7142338	-0.360800347	Zm00001d038553
Zm00001d041632_T005	0.8197432	0.997335375	Zm00001d041632
Zm00001d041632_T002	0.3870314	1.145992229	Zm00001d041632
Zm00001d041632_T009	0.02606139	-1.769684545	Zm00001d041632
Zm00001d047269_T001	0.9889811	0.051756123	Zm00001d047269
Zm00001d010229_T001	0.7389826	-0.323763	Zm00001d010229
Zm00001d021344_T003	0.8213533	0.849171709	Zm00001d021344
Zm00001d021344_T004	0.208031	0.50571405	Zm00001d021344
Zm00001d021344_T006	0.67455	-0.296250058	Zm00001d021344
Zm00001d021344_T005	0.4179542	-0.39346025	Zm00001d021344
Zm00001d021344_T002	0.9900377	0.517438314	Zm00001d021344
Zm00001d034996_T002	0.9334932	0.069665248	Zm00001d034996
Zm00001d034996_T001	0.01769584	0.763907489	Zm00001d034996
Zm00001d034996_T004	0.9100328	0.241795509	Zm00001d034996
Zm00001d034996_T013	0.146054	0.690998099	Zm00001d034996
Zm00001d034996_T006	0.9513407	0.659615618	Zm00001d034996
Zm00001d039138_T016	0.2974375	1.735543434	Zm00001d039138
Zm00001d039138_T009	0.9916733	0.52231183	Zm00001d039138
Zm00001d039138_T002	0.2266716	1.379785599	Zm00001d039138
Zm00001d039138_T008	0.4604152	-0.699280044	Zm00001d039138
Zm00001d036727_T001	0.8439792	0.931036801	Zm00001d036727
Zm00001d011676_T001	0.9361945	0.746290877	Zm00001d011676
Zm00001d052591_T001	0.8285332	0.886380419	Zm00001d052591
Zm00001d029548_T015	0.7561027	0.378889746	Zm00001d029548

Zm00001d029548_T008	1	0.174229308	Zm00001d029548
Zm00001d029548_T009	0.8788	-0.148403593	Zm00001d029548
Zm00001d029548_T001	0.9508314	0.064298971	Zm00001d029548
Zm00001d029548_T006	0.9326774	0.535567463	Zm00001d029548
Zm00001d029548_T012	0.9804228	0.104969762	Zm00001d029548
Zm00001d053453_T001	0.9419336	0.754037601	Zm00001d053453
Zm00001d053453_T019	0.9930712	0.08150433	Zm00001d053453
Zm00001d053453_T002	0.7518152	-0.230369221	Zm00001d053453
Zm00001d053453_T018	0.8031436	0.974721191	Zm00001d053453
Zm00001d053453_T015	0.9573435	0.235570608	Zm00001d053453
Zm00001d051015_T001	0.6118255	1.103102651	Zm00001d051015
Zm00001d038562_T001	0.971204	0.455455456	Zm00001d038562
Zm00001d023768_T001	0.01119323	-1.988902133	Zm00001d023768
Zm00001d052219_T004	0.02515384	2.253574147	Zm00001d052219
Zm00001d052219_T001	0.9895224	0.482760451	Zm00001d052219
Zm00001d029272_T015	0.7852993	-0.390664251	Zm00001d029272
Zm00001d029272_T005	0.9932384	0.204779305	Zm00001d029272
Zm00001d029272_T013	0.1844259	-1.605422733	Zm00001d029272
Zm00001d029272_T002	0.7194777	-0.10013753	Zm00001d029272
Zm00001d029272_T003	0.7439782	1.055612467	Zm00001d029272
Zm00001d041331_T001	0.5112002	-0.537653147	Zm00001d041331
Zm00001d041331_T003	0.4782426	-1.013748365	Zm00001d041331
Zm00001d047859_T002	0.6176648	-0.23430083	Zm00001d047859
Zm00001d047859_T001	0.6552247	-0.457965886	Zm00001d047859
Zm00001d048988_T034	0.08679718	-0.742580894	Zm00001d048988
Zm00001d048988_T033	0.9342381	0.047558833	Zm00001d048988
Zm00001d048988_T007	0.9073468	0.709852667	Zm00001d048988
Zm00001d048988_T016	0.03182725	0.263904344	Zm00001d048988
Zm00001d048988_T015	0.5719089	-0.144921317	Zm00001d048988
Zm00001d008940_T003	0.8966978	0.516385222	Zm00001d008940
Zm00001d008940_T002	0.8591388	0.936570626	Zm00001d008940
Zm00001d008940_T004	0.989787	0.194432764	Zm00001d008940
Zm00001d036904_T001	0.02542031	-0.616037555	Zm00001d036904
Zm00001d036904_T002	0.9224179	0.012416871	Zm00001d036904
Zm00001d036904_T003	0.8746701	0.06396555	Zm00001d036904
Zm00001d042653_T003	0.7354246	-0.413345389	Zm00001d042653
Zm00001d042653_T004	0.6223043	-0.711303483	Zm00001d042653
Zm00001d042653_T002	0.9219195	-0.067358289	Zm00001d042653
Zm00001d042653_T001	0.4497863	-0.647545921	Zm00001d042653
Zm00001d051635_T001	0.9959972	0.58583188	Zm00001d051635
Zm00001d002373_T013	0.9272378	0.223231396	Zm00001d002373
Zm00001d002373_T005	0.8993058	-0.058430009	Zm00001d002373
Zm00001d002373_T015	0.9869265	0.873425287	Zm00001d002373
Zm00001d027861_T001	0.9979568	0.356458586	Zm00001d027861
Zm00001d012447_T006	0.3207142	0.657011897	Zm00001d012447
Zm00001d012447_T001	0.6658123	0.709884871	Zm00001d012447
Zm00001d012447_T003	0.9416622	-0.129492092	Zm00001d012447
Zm00001d012447_T005	0.4246306	1.066664094	Zm00001d012447
Zm00001d012447_T002	0.2059265	0.985579668	Zm00001d012447
Zm00001d039362_T002	0.9050121	0.000224093	Zm00001d039362
Zm00001d038684_T005	0.99543	0.140117117	Zm00001d038684
Zm00001d038684_T003	0.9763597	0.164105765	Zm00001d038684
Zm00001d017877_T001	1	0.428057079	Zm00001d017877
Zm00001d045948_T003	0.8984709	0.484507735	Zm00001d045948

Zm00001d045948_T002	0.6386265	0.856708914	Zm00001d045948
Zm00001d045948_T006	0.897371	-0.112205552	Zm00001d045948
Zm00001d020630_T001	0.9781672	-0.202476556	Zm00001d020630
Zm00001d036008_T001	1	0.383077215	Zm00001d036008
Zm00001d023715_T024	0.8660897	-0.138034194	Zm00001d023715
Zm00001d023715_T009	0.972985	0.147865471	Zm00001d023715
Zm00001d023715_T003	0.577239	0.36503515	Zm00001d023715
Zm00001d023715_T023	0.8224736	0.259465265	Zm00001d023715
Zm00001d023715_T004	0.3910481	-0.49425873	Zm00001d023715
Zm00001d023715_T017	0.9222337	0.466471142	Zm00001d023715
Zm00001d041374_T001	0.9533693	0.014179455	Zm00001d041374
Zm00001d046305_T001	0.7485836	-0.310330514	Zm00001d046305
Zm00001d022434_T001	0.7395627	1.102453262	Zm00001d022434
Zm00001d042245_T001	0.6696627	1.170357995	Zm00001d042245
Zm00001d038064_T001	0.6749969	1.127340208	Zm00001d038064
Zm00001d025362_T002	0.9689939	0.437124065	Zm00001d025362
Zm00001d025362_T001	0.550715	-0.405567864	Zm00001d025362
Zm00001d037533_T001	0.4137215	-1.040464481	Zm00001d037533
Zm00001d051859_T001	0.9107561	-0.42508942	Zm00001d051859
Zm00001d003153_T007	0.4146004	-0.522931146	Zm00001d003153
Zm00001d003153_T008	0.8453901	0.775988895	Zm00001d003153
Zm00001d003153_T001	0.5327463	0.126185268	Zm00001d003153
Zm00001d003153_T010	0.08520203	-0.74691962	Zm00001d003153
Zm00001d033895_T002	0.1740006	1.815160911	Zm00001d033895
Zm00001d033895_T010	0.1003679	1.101087348	Zm00001d033895
Zm00001d033895_T006	0.02332999	2.145611193	Zm00001d033895
Zm00001d033895_T008	0.8179463	0.896609799	Zm00001d033895
Zm00001d033895_T009	0.9608605	0.124165996	Zm00001d033895
Zm00001d048732_T003	0.8751967	0.575946077	Zm00001d048732
Zm00001d048732_T001	0.9487214	0.445555702	Zm00001d048732
Zm00001d048732_T002	0.3674032	0.358433954	Zm00001d048732
Zm00001d048732_T004	0.05876722	-0.832220867	Zm00001d048732
Zm00001d048732_T005	0.9807381	0.551633832	Zm00001d048732
Zm00001d036285_T001	0.9968176	0.285566263	Zm00001d036285
Zm00001d022323_T001	0.5718169	-0.475053026	Zm00001d022323
Zm00001d011825_T001	0.8883561	-0.398455685	Zm00001d011825
Zm00001d034206_T001	0.9945118	0.382984146	Zm00001d034206
Zm00001d015102_T001	0.9944247	0.223804782	Zm00001d015102
Zm00001d011049_T001	0.9173494	-0.274025926	Zm00001d011049
Zm00001d033378_T007	0.4230609	0.524025006	Zm00001d033378
Zm00001d033378_T010	0.000409011	0.561187142	Zm00001d033378
Zm00001d033378_T018	0.1007136	1.747031747	Zm00001d033378
Zm00001d033378_T006	0.5340174	0.454437577	Zm00001d033378
Zm00001d033378_T012	0.6952911	0.632669034	Zm00001d033378
Zm00001d033378_T019	2.77E-14	-2.847254276	Zm00001d033378
Zm00001d033378_T022	0.4786492	-0.772853829	Zm00001d033378
Zm00001d033378_T004	0.8515562	0.978472024	Zm00001d033378
Zm00001d033378_T016	0.4819201	1.936551603	Zm00001d033378
Zm00001d033378_T020	0.4277454	0.934968975	Zm00001d033378
Zm00001d010587_T001	0.9186216	0.593416081	Zm00001d010587
Zm00001d020354_T001	0.8872096	-1.139467644	Zm00001d020354
Zm00001d052540_T001	0.9027603	0.534741133	Zm00001d052540
Zm00001d044168_T001	0.965919	-0.027791766	Zm00001d044168
Zm00001d035029_T002	0.9979568	0.762000153	Zm00001d035029

Zm00001d035029_T008	0.2546519	-1.355684259	Zm00001d035029
Zm00001d035029_T007	0.6106611	0.834563614	Zm00001d035029
Zm00001d035029_T001	0.8934074	0.471702753	Zm00001d035029
Zm00001d035029_T009	0.9539869	0.275627514	Zm00001d035029
Zm00001d035029_T005	0.9334932	0.01809854	Zm00001d035029
Zm00001d035029_T010	0.2463927	-1.146740108	Zm00001d035029
Zm00001d033421_T001	0.7010603	-0.540759956	Zm00001d033421
Zm00001d054044_T001	0.1260325	-1.254319418	Zm00001d054044
Zm00001d019220_T001	0.8993062	-0.080508767	Zm00001d019220
Zm00001d014043_T001	0.5913892	1.21121731	Zm00001d014043
Zm00001d045347_T001	0.2567004	-0.77986358	Zm00001d045347
Zm00001d012820_T001	0.7379612	-0.615673815	Zm00001d012820
Zm00001d038696_T001	0.4029716	-1.328649863	Zm00001d038696
Zm00001d025801_T007	0.8613703	2.312691646	Zm00001d025801
Zm00001d025801_T001	0.9969123	0.355923241	Zm00001d025801
Zm00001d025801_T006	0.008473025	-2.412930717	Zm00001d025801
Zm00001d025801_T005	0.03668304	-0.601733147	Zm00001d025801
Zm00001d037852_T008	0.9586437	0.267896934	Zm00001d037852
Zm00001d037852_T005	0.1870898	1.054198215	Zm00001d037852
Zm00001d037852_T017	0.03728966	0.836146036	Zm00001d037852
Zm00001d037852_T016	0.9998278	0.315156401	Zm00001d037852
Zm00001d048007_T003	0.8686467	-0.171362291	Zm00001d048007
Zm00001d048007_T002	0.9429712	0.149539402	Zm00001d048007
Zm00001d053322_T001	0.278442	-0.902521634	Zm00001d053322
Zm00001d020936_T001	0.7175167	1.088002765	Zm00001d020936
Zm00001d020936_T002	0.139783	-0.916037141	Zm00001d020936
Zm00001d036415_T001	0.9999076	0.226214776	Zm00001d036415
Zm00001d019078_T001	0.9781672	0.494739962	Zm00001d019078
Zm00001d019078_T002	0.9419336	0.038813487	Zm00001d019078
Zm00001d013982_T002	0.9979568	0.12776056	Zm00001d013982
Zm00001d028197_T001	0.9487214	-0.010267241	Zm00001d028197
Zm00001d008545_T001	0.8728996	-0.466965058	Zm00001d008545
Zm00001d011434_T001	0.2398048	1.040990235	Zm00001d011434
Zm00001d028593_T004	0.9906533	0.82507874	Zm00001d028593
Zm00001d028593_T003	0.9745948	0.167959681	Zm00001d028593
Zm00001d038690_T001	0.9374853	0.659685269	Zm00001d038690
Zm00001d046538_T005	0.2147503	-1.203288833	Zm00001d046538
Zm00001d046538_T004	0.8743763	0.845397431	Zm00001d046538
Zm00001d046538_T002	0.8383745	-0.11468537	Zm00001d046538
Zm00001d046538_T001	0.3548936	0.7074515	Zm00001d046538
Zm00001d010974_T029	0.2929405	0.958759988	Zm00001d010974
Zm00001d010974_T016	0.9296811	0.701324454	Zm00001d010974
Zm00001d010974_T014	0.005040824	-0.497085698	Zm00001d010974
Zm00001d010974_T075	0.01481508	0.555283049	Zm00001d010974
Zm00001d010974_T063	0.9223663	-0.059419757	Zm00001d010974
Zm00001d010974_T017	0.8929316	-0.009907264	Zm00001d010974
Zm00001d010974_T032	0.5582612	0.266293833	Zm00001d010974
Zm00001d010974_T005	0.1410912	2.501697216	Zm00001d010974
Zm00001d010974_T024	0.6316708	-0.516156191	Zm00001d010974
Zm00001d010974_T001	0.03648535	0.566123991	Zm00001d010974
Zm00001d010974_T065	0.8125642	0.591318848	Zm00001d010974
Zm00001d010974_T037	8.50E-09	0.968235688	Zm00001d010974
Zm00001d010974_T045	0.5473404	0.619213039	Zm00001d010974
Zm00001d042935_T001	0.2274738	-1.676376906	Zm00001d042935

Zm00001d045972_T001	0.1470238	1.698251855	Zm00001d045972
Zm00001d035514_T016	0.7810295	-0.173880412	Zm00001d035514
Zm00001d035514_T005	0.8414741	-0.033119964	Zm00001d035514
Zm00001d035514_T012	0.9744451	-0.292043163	Zm00001d035514
Zm00001d035514_T007	0.7020458	1.051249292	Zm00001d035514
Zm00001d035514_T014	0.5086617	-0.767855753	Zm00001d035514
Zm00001d001944_T001	0.7418111	-0.299061342	Zm00001d001944
Zm00001d021342_T001	0.3247177	1.377899822	Zm00001d021342
Zm00001d021342_T021	0.6891365	0.625490603	Zm00001d021342
Zm00001d021342_T002	0.7989126	0.283565752	Zm00001d021342
Zm00001d021342_T009	0.2009937	1.365284556	Zm00001d021342
Zm00001d027714_T001	0.9907928	0.202970698	Zm00001d027714
Zm00001d008613_T001	0.8291674	-0.219344864	Zm00001d008613
Zm00001d042792_T001	0.9981361	0.278369408	Zm00001d042792
Zm00001d042792_T003	0.9717919	0.154749207	Zm00001d042792
Zm00001d042792_T002	0.7624517	0.808226154	Zm00001d042792
Zm00001d042792_T004	0.8412913	0.127667039	Zm00001d042792
Zm00001d037900_T001	1	0.350973039	Zm00001d037900
Zm00001d034069_T001	0.6450309	-0.413020189	Zm00001d034069
Zm00001d044427_T001	0.7907819	0.992638267	Zm00001d044427
Zm00001d021225_T004	1	0.237619487	Zm00001d021225
Zm00001d021225_T001	0.926928	-0.191366306	Zm00001d021225
Zm00001d021225_T002	0.9073468	-0.157769262	Zm00001d021225
Zm00001d053887_T001	0.9312703	-0.137169832	Zm00001d053887
Zm00001d038672_T003	0.2771512	0.822430601	Zm00001d038672
Zm00001d038672_T001	0.9979568	0.432355176	Zm00001d038672
Zm00001d038672_T002	0.1584752	2.209126715	Zm00001d038672
Zm00001d038672_T004	0.8032834	0.638536755	Zm00001d038672
Zm00001d038672_T006	0.9956349	0.338021517	Zm00001d038672
Zm00001d029635_T001	0.1805724	-0.732235342	Zm00001d029635
Zm00001d012895_T005	0.9845789	0.285993149	Zm00001d012895
Zm00001d011687_T001	0.9217388	0.662594338	Zm00001d011687
Zm00001d029031_T002	1	0.379406873	Zm00001d029031
Zm00001d029031_T004	0.3400608	0.616236321	Zm00001d029031
Zm00001d029031_T003	0.3437937	0.786210836	Zm00001d029031
Zm00001d020984_T001	0.9689939	0.134585406	Zm00001d020984
Zm00001d038508_T001	0.9942767	0.295402715	Zm00001d038508
Zm00001d032326_T006	0.05021313	0.434626005	Zm00001d032326
Zm00001d032326_T004	0.8819727	-0.022963101	Zm00001d032326
Zm00001d032326_T003	0.9419336	0.044801911	Zm00001d032326
Zm00001d032326_T005	0.9416089	0.002498944	Zm00001d032326
Zm00001d032326_T002	0.5054762	-0.340462375	Zm00001d032326
Zm00001d020011_T001	0.9986486	0.255513462	Zm00001d020011
Zm00001d052344_T003	0.8263348	-0.135953429	Zm00001d052344
Zm00001d052344_T005	0.215622	1.230624318	Zm00001d052344
Zm00001d052344_T004	0.996299	0.312549147	Zm00001d052344
Zm00001d015639_T001	0.7063456	-1.020070879	Zm00001d015639
Zm00001d027896_T001	0.9881407	0.15214376	Zm00001d027896
Zm00001d049625_T002	0.9845789	0.183900917	Zm00001d049625
Zm00001d022551_T001	0.9514141	0.105013711	Zm00001d022551
Zm00001d022551_T003	0.1976354	-0.592404661	Zm00001d022551
Zm00001d025891_T001	0.816571	-0.586279322	Zm00001d025891
Zm00001d016496_T010	1	0.69494488	Zm00001d016496
Zm00001d016496_T013	0.9887584	0.233300695	Zm00001d016496

Zm00001d016496_T005	0.9740325	0.653094944	Zm00001d016496
Zm00001d016496_T006	0.03159677	-1.814464403	Zm00001d016496
Zm00001d016496_T001	1	0.155697369	Zm00001d016496
Zm00001d016496_T014	3.05E-11	2.652457984	Zm00001d016496
Zm00001d027484_T003	1	0.389263059	Zm00001d027484
Zm00001d027484_T006	0.8380736	0.376064399	Zm00001d027484
Zm00001d027484_T005	0.2443389	0.839311487	Zm00001d027484
Zm00001d038866_T003	0.9867452	0.766199319	Zm00001d038866
Zm00001d038866_T005	0.6314583	1.155190662	Zm00001d038866
Zm00001d038866_T002	0.9751997	0.183389938	Zm00001d038866
Zm00001d038866_T004	0.9429712	-0.219489388	Zm00001d038866
Zm00001d008405_T001	1	0.118415133	Zm00001d008405
Zm00001d011790_T001	0.743337	-0.309677831	Zm00001d011790
Zm00001d011790_T002	1	0.204915185	Zm00001d011790
Zm00001d038530_T006	0.8678783	-0.0264854	Zm00001d038530
Zm00001d038530_T011	0.9828518	0.236061686	Zm00001d038530
Zm00001d038530_T010	0.634183	1.091508024	Zm00001d038530
Zm00001d038530_T009	1	0.158555457	Zm00001d038530
Zm00001d038530_T005	0.4574813	0.353952008	Zm00001d038530
Zm00001d038530_T008	0.6839661	0.407303077	Zm00001d038530
Zm00001d038530_T007	0.08229212	1.017819968	Zm00001d038530
Zm00001d032510_T001	0.4859645	-0.518706368	Zm00001d032510
Zm00001d047760_T001	0.9508107	0.077784083	Zm00001d047760
Zm00001d012522_T001	0.02370782	2.495828504	Zm00001d012522
Zm00001d045559_T001	1	0.312359959	Zm00001d045559
Zm00001d011370_T001	0.9986486	0.386776609	Zm00001d011370
Zm00001d003227_T001	0.885586	-0.051845902	Zm00001d003227
Zm00001d003227_T006	0.8041769	0.390544514	Zm00001d003227
Zm00001d016916_T003	0.9528476	0.090933289	Zm00001d016916
Zm00001d016916_T004	0.9303773	0.632691923	Zm00001d016916
Zm00001d016916_T002	0.3655498	0.926475569	Zm00001d016916
Zm00001d028100_T006	0.9941715	0.271630605	Zm00001d028100
Zm00001d028100_T004	0.5775553	0.57985275	Zm00001d028100
Zm00001d028100_T005	0.710892	0.728772507	Zm00001d028100
Zm00001d028100_T007	0.8533785	0.378026137	Zm00001d028100
Zm00001d028100_T001	0.9817074	0.159163003	Zm00001d028100
Zm00001d028100_T003	0.7858094	-0.186991905	Zm00001d028100
Zm00001d002625_T003	0.666346	1.177055548	Zm00001d002625
Zm00001d009292_T001	0.9326482	-0.27112282	Zm00001d009292
Zm00001d020639_T002	0.9377241	0.098006879	Zm00001d020639
Zm00001d020639_T003	4.94E-08	-0.90559045	Zm00001d020639
Zm00001d020639_T005	0.8987811	0.788615074	Zm00001d020639
Zm00001d044442_T021	7.60E-05	0.871317996	Zm00001d044442
Zm00001d044442_T007	0.3631355	1.439665496	Zm00001d044442
Zm00001d044442_T015	0.002446646	0.563423184	Zm00001d044442
Zm00001d044442_T018	1.75E-10	1.366473364	Zm00001d044442
Zm00001d044442_T020	0.00111428	0.792865887	Zm00001d044442
Zm00001d044442_T019	0.02339299	2.259936884	Zm00001d044442
Zm00001d044442_T010	0.000108701	2.922972159	Zm00001d044442
Zm00001d031570_T001	0.9706887	0.594045834	Zm00001d031570
Zm00001d031570_T002	0.03896328	-1.54900891	Zm00001d031570
Zm00001d031570_T003	0.005339206	1.175522623	Zm00001d031570
Zm00001d024212_T001	0.6062783	-0.719692179	Zm00001d024212
Zm00001d024212_T007	0.7640707	1.091193625	Zm00001d024212

Zm00001d024212_T014	0.9931009	0.044836901	Zm00001d024212
Zm00001d024212_T015	0.2800303	-0.445331806	Zm00001d024212
Zm00001d024212_T002	0.852305	0.678079682	Zm00001d024212
Zm00001d044546_T001	0.01867124	-2.072758018	Zm00001d044546
Zm00001d039920_T001	0.9213556	0.033553776	Zm00001d039920
Zm00001d026348_T002	0.9673094	0.381242312	Zm00001d026348
Zm00001d026348_T003	0.920283	0.54118897	Zm00001d026348
Zm00001d026348_T001	0.4245499	-1.127537398	Zm00001d026348
Zm00001d028089_T001	0.761594	0.59435683	Zm00001d028089
Zm00001d042362_T001	0.6494559	-0.204037767	Zm00001d042362
Zm00001d018506_T002	0.9701986	0.104651497	Zm00001d018506
Zm00001d018506_T001	0.1078102	-0.743714292	Zm00001d018506
Zm00001d029051_T001	0.6318807	1.13957818	Zm00001d029051
Zm00001d016552_T004	0.9993872	0.152117357	Zm00001d016552
Zm00001d016552_T005	0.7788277	0.781700994	Zm00001d016552
Zm00001d016552_T002	0.9673473	0.13171617	Zm00001d016552
Zm00001d016552_T008	0.8762906	0.816425892	Zm00001d016552
Zm00001d016552_T007	0.006217265	-2.105913997	Zm00001d016552
Zm00001d016552_T001	0.9693993	0.073204265	Zm00001d016552
Zm00001d016552_T003	0.3562694	1.466219784	Zm00001d016552
Zm00001d011713_T005	0.9860836	0.302861346	Zm00001d011713
Zm00001d011713_T008	0.09187252	0.868260382	Zm00001d011713
Zm00001d011713_T010	0.03972663	0.293154029	Zm00001d011713
Zm00001d011713_T016	1.08E-08	1.131667414	Zm00001d011713
Zm00001d011713_T001	0.1098002	1.2560234	Zm00001d011713
Zm00001d011713_T009	0.5906408	0.526862227	Zm00001d011713
Zm00001d044315_T001	0.5027472	-0.203440743	Zm00001d044315
Zm00001d044315_T005	0.7134188	-0.157483205	Zm00001d044315
Zm00001d044315_T010	0.2061804	-0.803008459	Zm00001d044315
Zm00001d044315_T013	0.9771057	0.55507649	Zm00001d044315
Zm00001d044315_T009	0.05053288	-0.308253136	Zm00001d044315
Zm00001d044315_T002	0.9487214	0.099327384	Zm00001d044315
Zm00001d003381_T001	0.4550144	-0.780352327	Zm00001d003381
Zm00001d009572_T001	0.9740325	0.455887304	Zm00001d009572
Zm00001d005642_T003	0.6331067	-1.025322988	Zm00001d005642
Zm00001d005642_T001	0.7488344	-0.808007894	Zm00001d005642
Zm00001d052530_T001	7.16E-06	4.26941748	Zm00001d052530
Zm00001d043125_T001	0.6165106	-0.661500005	Zm00001d043125
Zm00001d016826_T001	0.04419305	2.25281084	Zm00001d016826
Zm00001d048190_T001	0.8720862	-0.199040691	Zm00001d048190
Zm00001d048190_T004	0.9856605	0.23078031	Zm00001d048190
Zm00001d048190_T002	0.7712604	-0.340780591	Zm00001d048190
Zm00001d042427_T001	0.9535734	0.090279225	Zm00001d042427
Zm00001d006255_T001	0.8325302	-0.163250346	Zm00001d006255
Zm00001d030812_T001	0.9281046	-0.187921959	Zm00001d030812
Zm00001d027633_T010	0.02590398	1.582056634	Zm00001d027633
Zm00001d027633_T007	0.6500231	0.362822538	Zm00001d027633
Zm00001d027633_T011	0.4778277	1.076335199	Zm00001d027633
Zm00001d051528_T004	0.6717868	-0.400964649	Zm00001d051528
Zm00001d051528_T005	0.9210513	0.818238272	Zm00001d051528
Zm00001d051528_T002	0.8006031	0.410807001	Zm00001d051528
Zm00001d051528_T009	0.7501	1.63763841	Zm00001d051528
Zm00001d025566_T001	0.7292566	0.719454792	Zm00001d025566
Zm00001d005962_T001	0.000959593	3.248543601	Zm00001d005962

Zm00001d013057_T001	0.3360989	1.517904571	Zm00001d013057
Zm00001d040757_T002	0.9930712	0.313337952	Zm00001d040757
Zm00001d024778_T001	0.8977775	2.221695099	Zm00001d024778
Zm00001d011821_T004	0.7273213	0.87196818	Zm00001d011821
Zm00001d011821_T009	0.934595	0.232736571	Zm00001d011821
Zm00001d011821_T008	0.7771984	0.504386136	Zm00001d011821
Zm00001d011821_T001	0.9945592	0.516729866	Zm00001d011821
Zm00001d011821_T011	0.9024215	-0.086660849	Zm00001d011821
Zm00001d011821_T002	0.3811358	0.839887156	Zm00001d011821
Zm00001d013429_T001	0.9139882	-0.029244318	Zm00001d013429
Zm00001d018616_T001	0.3815032	-0.442973184	Zm00001d018616
Zm00001d018616_T004	0.9833565	0.163637318	Zm00001d018616
Zm00001d011830_T004	0.9404125	0.500104064	Zm00001d011830
Zm00001d011830_T005	0.4254374	0.634663589	Zm00001d011830
Zm00001d035775_T001	0.07396433	2.30116173	Zm00001d035775
Zm00001d010305_T002	0.5552199	0.507005243	Zm00001d010305
Zm00001d010305_T001	0.09143854	-1.356546308	Zm00001d010305
Zm00001d010305_T004	0.8825173	0.818398308	Zm00001d010305
Zm00001d038167_T001	0.5864438	1.304646358	Zm00001d038167
Zm00001d038167_T003	0.04949697	1.746843906	Zm00001d038167
Zm00001d023379_T001	0.9975882	0.36833933	Zm00001d023379
Zm00001d049376_T001	1	0.428498369	Zm00001d049376
Zm00001d038151_T001	0.5714061	-0.449547476	Zm00001d038151
Zm00001d035598_T001	0.9344122	0.737008248	Zm00001d035598
Zm00001d028426_T001	0.8092097	-0.579405057	Zm00001d028426
Zm00001d036651_T004	0.5437196	0.884471175	Zm00001d036651
Zm00001d036651_T005	1	0.228647446	Zm00001d036651
Zm00001d020901_T001	1	0.339494595	Zm00001d020901
Zm00001d039102_T001	1	0.293387669	Zm00001d039102
Zm00001d014423_T008	0.9749249	1.288688036	Zm00001d014423
Zm00001d014423_T005	0.7511561	1.323902974	Zm00001d014423
Zm00001d014423_T023	0.9278769	0.795837081	Zm00001d014423
Zm00001d014423_T018	0.04090559	-1.305347647	Zm00001d014423
Zm00001d014423_T003	0.04784289	-0.47665482	Zm00001d014423
Zm00001d014423_T013	0.8068391	0.739155102	Zm00001d014423
Zm00001d014423_T022	0.8322305	-0.209241856	Zm00001d014423
Zm00001d014423_T019	0.07155595	-1.650435774	Zm00001d014423
Zm00001d014423_T020	1.25E-05	2.315347432	Zm00001d014423
Zm00001d014423_T002	1	0.107659461	Zm00001d014423
Zm00001d014423_T009	0.9986486	0.198474467	Zm00001d014423
Zm00001d014423_T021	0.5695353	2.051227006	Zm00001d014423
Zm00001d014423_T001	0.93919	0.022936558	Zm00001d014423
Zm00001d014423_T015	0.1113752	-0.941664718	Zm00001d014423
Zm00001d007631_T001	0.005210231	-3.234875753	Zm00001d007631
Zm00001d005081_T001	0.9907928	-0.07851135	Zm00001d005081
Zm00001d041625_T005	0.9252088	-0.032189505	Zm00001d041625
Zm00001d041625_T004	0.6134001	-0.13729706	Zm00001d041625
Zm00001d041625_T006	0.8232235	0.308206312	Zm00001d041625
Zm00001d020436_T001	0.9911688	0.193480186	Zm00001d020436
Zm00001d034133_T001	0.7011505	-0.281889134	Zm00001d034133
Zm00001d044260_T004	0.9817074	0.331598133	Zm00001d044260
Zm00001d044260_T001	0.519412	-0.392169339	Zm00001d044260
Zm00001d044260_T002	0.865651	-0.060708928	Zm00001d044260
Zm00001d025663_T004	0.9782953	0.408851307	Zm00001d025663

Zm00001d025663_T002	0.6470421	-0.436663933	Zm00001d025663
Zm00001d025663_T001	0.8412913	0.674709347	Zm00001d025663
Zm00001d029672_T003	0.9636929	0.065589628	Zm00001d029672
Zm00001d003754_T001	0.02506163	-0.570053567	Zm00001d003754
Zm00001d033529_T001	0.9468268	0.042475882	Zm00001d033529
Zm00001d013249_T001	0.9519347	0.608662695	Zm00001d013249
Zm00001d046586_T001	0.7287219	1.055460148	Zm00001d046586
Zm00001d004089_T001	0.9091175	-0.177172596	Zm00001d004089
Zm00001d009780_T005	0.9765009	0.449484655	Zm00001d009780
Zm00001d009780_T006	0.9468268	-0.209607624	Zm00001d009780
Zm00001d009780_T001	0.774518	0.651564182	Zm00001d009780
Zm00001d034665_T001	0.9804228	0.432803153	Zm00001d034665
Zm00001d043430_T001	0.6182353	-0.813764476	Zm00001d043430
Zm00001d034037_T001	0.9745777	0.118614072	Zm00001d034037
Zm00001d003509_T009	0.5030977	1.349274443	Zm00001d003509
Zm00001d003509_T001	0.4059118	1.572041095	Zm00001d003509
Zm00001d003509_T007	0.01424155	2.493180345	Zm00001d003509
Zm00001d003509_T004	0.003072476	2.779214829	Zm00001d003509
Zm00001d049821_T001	0.9924986	0.305978546	Zm00001d049821
Zm00001d052999_T001	0.8279885	-0.183278479	Zm00001d052999
Zm00001d046803_T001	0.90276	-0.307898381	Zm00001d046803
Zm00001d020133_T001	0.820661	-0.730592285	Zm00001d020133
Zm00001d028669_T010	0.3852872	0.777049169	Zm00001d028669
Zm00001d028669_T020	0.9856605	0.305559249	Zm00001d028669
Zm00001d028669_T006	0.91126	0.141912615	Zm00001d028669
Zm00001d026618_T002	0.7375754	0.64509682	Zm00001d026618
Zm00001d026618_T001	0.2502162	0.9634933	Zm00001d026618
Zm00001d053816_T003	0.9902022	0.160467007	Zm00001d053816
Zm00001d053816_T008	0.6321049	-0.301854801	Zm00001d053816
Zm00001d053816_T010	0.961399	0.080001878	Zm00001d053816
Zm00001d053816_T009	0.6183146	-0.646897226	Zm00001d053816
Zm00001d053816_T006	0.7868558	-0.199444582	Zm00001d053816
Zm00001d053816_T007	0.6967776	-0.444288383	Zm00001d053816
Zm00001d053816_T011	0.7885216	0.529198021	Zm00001d053816
Zm00001d053816_T005	0.003885444	0.952928685	Zm00001d053816
Zm00001d053816_T013	0.8628261	-0.055501238	Zm00001d053816
Zm00001d054014_T001	0.8854695	-0.104175142	Zm00001d054014
Zm00001d025861_T001	0.9979568	0.235048279	Zm00001d025861
Zm00001d009774_T001	0.9975532	0.404452228	Zm00001d009774
Zm00001d021421_T015	0.9988063	1.820065194	Zm00001d021421
Zm00001d021421_T002	0.9887584	0.298365065	Zm00001d021421
Zm00001d021421_T010	0.9741154	0.567293394	Zm00001d021421
Zm00001d021421_T016	0.9693993	1.145076304	Zm00001d021421
Zm00001d021421_T020	0.9836566	1.307638308	Zm00001d021421
Zm00001d021421_T018	0.8677544	0.333850451	Zm00001d021421
Zm00001d037733_T001	0.9872831	0.181404617	Zm00001d037733
Zm00001d014261_T066	0.2067536	0.797109288	Zm00001d014261
Zm00001d014261_T056	0.8053436	-0.326802021	Zm00001d014261
Zm00001d014261_T049	0.000234904	0.846889712	Zm00001d014261
Zm00001d014261_T048	0.4292753	-0.150024673	Zm00001d014261
Zm00001d014261_T058	0.6523514	-0.085371721	Zm00001d014261
Zm00001d014261_T059	0.6235959	0.604324605	Zm00001d014261
Zm00001d014261_T035	0.6107148	-0.22393533	Zm00001d014261
Zm00001d014261_T047	0.9550193	0.21962446	Zm00001d014261

Zm00001d002463_T001	0.01075757	-2.749283414	Zm00001d002463
Zm00001d035610_T001	0.7155054	-0.212368663	Zm00001d035610
Zm00001d041597_T001	0.8549534	-0.23721952	Zm00001d041597
Zm00001d020706_T001	0.9664406	0.121252457	Zm00001d020706
Zm00001d044669_T001	0.6511883	-0.405865743	Zm00001d044669
Zm00001d033155_T002	0.7565187	-0.261434764	Zm00001d033155
Zm00001d033155_T001	0.7860927	0.982843609	Zm00001d033155
Zm00001d017575_T001	1	0.274942481	Zm00001d017575
Zm00001d044951_T001	0.04595287	1.04853035	Zm00001d044951
Zm00001d043803_T002	0.9897942	0.076931621	Zm00001d043803
Zm00001d048526_T001	0.8638849	-0.45708311	Zm00001d048526
Zm00001d032099_T004	0.9979568	0.381937982	Zm00001d032099
Zm00001d037342_T001	0.6926364	-0.559256857	Zm00001d037342
Zm00001d045435_T011	0.5122191	-0.826434148	Zm00001d045435
Zm00001d045435_T005	0.9914064	0.120495959	Zm00001d045435
Zm00001d045435_T001	0.9847267	0.492416252	Zm00001d045435
Zm00001d045435_T010	0.01572311	0.741141403	Zm00001d045435
Zm00001d041608_T001	0.3176511	1.652926122	Zm00001d041608
Zm00001d020927_T001	0.001203049	-2.101959189	Zm00001d020927
Zm00001d033412_T001	0.8593984	-0.208799075	Zm00001d033412
Zm00001d042661_T001	0.9970835	0.327333832	Zm00001d042661
Zm00001d042661_T002	0.8557307	0.198722538	Zm00001d042661
Zm00001d053985_T001	0.9562711	0.053991513	Zm00001d053985
Zm00001d046158_T001	0.2153746	-1.973750066	Zm00001d046158
Zm00001d010037_T001	0.9502794	0.438427933	Zm00001d010037
Zm00001d047705_T001	0.07256374	-2.154924699	Zm00001d047705
Zm00001d048180_T001	0.9361945	0.051525313	Zm00001d048180
Zm00001d051140_T001	0.7921111	-0.326402773	Zm00001d051140
Zm00001d014954_T006	1	-0.006282557	Zm00001d014954
Zm00001d014954_T003	0.9515686	0.042922239	Zm00001d014954
Zm00001d014954_T005	0.002685015	-2.421881969	Zm00001d014954
Zm00001d014954_T001	0.03238926	-1.234233215	Zm00001d014954
Zm00001d014954_T004	0.6465256	-0.636451215	Zm00001d014954
Zm00001d027478_T001	0.926825	-0.497058605	Zm00001d027478
Zm00001d053228_T002	0.9469426	-0.240771137	Zm00001d053228
Zm00001d053228_T001	0.8585804	-0.245863817	Zm00001d053228
Zm00001d038739_T003	0.8018393	0.817743483	Zm00001d038739
Zm00001d038739_T001	0.09363969	2.273114964	Zm00001d038739
Zm00001d042039_T001	0.8805297	0.416508424	Zm00001d042039
Zm00001d038963_T001	0.8987811	-0.049799824	Zm00001d038963
Zm00001d009595_T001	0.7626242	0.564827648	Zm00001d009595
Zm00001d021467_T005	0.9303773	-0.021276947	Zm00001d021467
Zm00001d021467_T003	0.9607878	0.352464862	Zm00001d021467
Zm00001d021467_T007	0.8412913	-0.49192319	Zm00001d021467
Zm00001d021467_T004	0.9580985	1.067186148	Zm00001d021467
Zm00001d021467_T002	0.9673094	0.753162745	Zm00001d021467
Zm00001d013127_T003	0.4801967	1.254299446	Zm00001d013127
Zm00001d043895_T005	0.002719971	1.483857758	Zm00001d043895
Zm00001d043895_T001	0.00405648	1.21601157	Zm00001d043895
Zm00001d043895_T004	0.8494782	0.265237078	Zm00001d043895
Zm00001d043895_T002	0.9885392	0.466541023	Zm00001d043895
Zm00001d001908_T002	0.9747606	0.409712258	Zm00001d001908
Zm00001d001908_T001	0.9849315	0.448215857	Zm00001d001908
Zm00001d030661_T001	0.9404125	0.032366447	Zm00001d030661

Zm00001d002706_T003	0.8001345	0.557018774	Zm00001d002706
Zm00001d036137_T005	0.941809	0.712046341	Zm00001d036137
Zm00001d036137_T008	1.81E-10	-3.103591323	Zm00001d036137
Zm00001d036137_T004	0.000999715	1.093895951	Zm00001d036137
Zm00001d036137_T003	0.9303773	0.026732579	Zm00001d036137
Zm00001d031799_T001	0.9833565	0.136348058	Zm00001d031799
Zm00001d003866_T001	0.9977752	0.314306348	Zm00001d003866
Zm00001d017621_T001	0.6688463	-0.640662789	Zm00001d017621
Zm00001d045191_T001	0.7634101	1.020688875	Zm00001d045191
Zm00001d003884_T001	0.3200182	-0.851690137	Zm00001d003884
Zm00001d025726_T001	0.9959972	0.464055984	Zm00001d025726
Zm00001d017209_T001	0.8986911	0.556743202	Zm00001d017209
Zm00001d050051_T002	0.9732421	-0.205938246	Zm00001d050051
Zm00001d050051_T001	0.9570177	0.551226711	Zm00001d050051
Zm00001d047869_T009	0.9706887	0.417745617	Zm00001d047869
Zm00001d047869_T007	0.1611689	0.922027931	Zm00001d047869
Zm00001d047869_T003	0.7986776	0.078427723	Zm00001d047869
Zm00001d047869_T005	0.9712373	0.173113261	Zm00001d047869
Zm00001d047869_T016	0.05522322	-0.415615036	Zm00001d047869
Zm00001d047869_T010	0.8874251	0.557559137	Zm00001d047869
Zm00001d015990_T001	0.8440444	0.58103877	Zm00001d015990
Zm00001d012415_T001	0.08672315	2.112465623	Zm00001d012415
Zm00001d044068_T001	0.9860836	0.436963707	Zm00001d044068
Zm00001d027768_T002	0.2225404	0.31903377	Zm00001d027768
Zm00001d027768_T005	4.67E-10	1.759804099	Zm00001d027768
Zm00001d027768_T014	0.6767305	0.33827993	Zm00001d027768
Zm00001d027768_T012	0.7609047	0.32822629	Zm00001d027768
Zm00001d027768_T007	1.52E-08	-1.014402461	Zm00001d027768
Zm00001d027768_T016	0.5757967	0.874508831	Zm00001d027768
Zm00001d027768_T023	0.9269932	0.631289898	Zm00001d027768
Zm00001d015657_T001	0.9444056	-0.283082952	Zm00001d015657
Zm00001d042799_T001	0.8747821	-0.28241782	Zm00001d042799
Zm00001d037188_T001	0.7425196	0.877912703	Zm00001d037188
Zm00001d051149_T001	0.79251	0.639498477	Zm00001d051149
Zm00001d008396_T001	0.7682041	-0.366211166	Zm00001d008396
Zm00001d011991_T001	0.9550193	0.501502573	Zm00001d011991
Zm00001d052452_T001	0.7101323	-0.62947238	Zm00001d052452
Zm00001d041707_T002	0.9959972	0.402142107	Zm00001d041707
Zm00001d041707_T003	0.9932666	0.009381158	Zm00001d041707
Zm00001d037666_T001	0.9951827	0.321211692	Zm00001d037666
Zm00001d036703_T001	0.02653356	-1.977328347	Zm00001d036703
Zm00001d047404_T001	0.9979568	0.339113423	Zm00001d047404
Zm00001d044142_T001	0.956184	0.118182827	Zm00001d044142
Zm00001d029143_T005	0.5325504	-0.617772082	Zm00001d029143
Zm00001d029143_T003	0.3443331	1.28849535	Zm00001d029143
Zm00001d029143_T001	0.9101584	0.324303779	Zm00001d029143
Zm00001d032222_T001	0.971204	0.483780223	Zm00001d032222
Zm00001d032222_T002	0.7167212	-0.855625278	Zm00001d032222
Zm00001d044287_T002	0.9311783	0.031397072	Zm00001d044287
Zm00001d044287_T001	0.8661802	-0.288287971	Zm00001d044287
Zm00001d040460_T001	0.04016634	1.998228615	Zm00001d040460
Zm00001d035756_T001	0.7912425	0.898517464	Zm00001d035756
Zm00001d000038_T001	0.9109241	-0.027037552	Zm00001d000038
Zm00001d039758_T006	0.997449	0.464536322	Zm00001d039758

Zm00001d039758_T002	0.04570411	0.856201814	Zm00001d039758
Zm00001d032606_T003	0.09245531	0.920885235	Zm00001d032606
Zm00001d032606_T001	0.9311783	0.281852837	Zm00001d032606
Zm00001d032606_T002	0.9082406	0.58684787	Zm00001d032606
Zm00001d052738_T001	0.9846714	0.401568638	Zm00001d052738
Zm00001d051441_T001	1	0.372541902	Zm00001d051441
Zm00001d000087_T002	0.9835413	0.272838051	Zm00001d000087
Zm00001d019934_T001	0.9821324	0.193934631	Zm00001d019934
Zm00001d005989_T001	0.2690004	-0.066295834	Zm00001d005989
Zm00001d051872_T001	0.957148	0.587587282	Zm00001d051872
Zm00001d014769_T001	0.8321156	-0.099215191	Zm00001d014769
Zm00001d014769_T002	0.8806256	-0.105855464	Zm00001d014769
Zm00001d007572_T001	0.7056926	-0.194501907	Zm00001d007572
Zm00001d007118_T003	0.9970835	-0.066092948	Zm00001d007118
Zm00001d007118_T004	0.9404125	0.36673441	Zm00001d007118
Zm00001d007118_T001	0.9979568	1.527732448	Zm00001d007118
Zm00001d007118_T005	0.9835181	0.047125918	Zm00001d007118
Zm00001d016845_T001	0.9396569	-0.074096564	Zm00001d016845
Zm00001d007858_T001	0.9673094	0.11909304	Zm00001d007858
Zm00001d052385_T001	0.9722469	0.188533998	Zm00001d052385
Zm00001d052407_T001	0.7805254	-0.230228482	Zm00001d052407
Zm00001d052407_T002	1	0.370541875	Zm00001d052407
Zm00001d037904_T001	1	0.112469108	Zm00001d037904
Zm00001d016570_T001	0.9708541	0.396662445	Zm00001d016570
Zm00001d037180_T001	0.7585131	0.860116409	Zm00001d037180
Zm00001d013561_T001	0.481901	1.209402684	Zm00001d013561
Zm00001d028733_T002	0.9512833	0.10634051	Zm00001d028733
Zm00001d028733_T001	0.8896831	0.820595793	Zm00001d028733
Zm00001d028733_T003	0.01062233	-0.65579546	Zm00001d028733
Zm00001d009123_T001	0.9967276	0.268434923	Zm00001d009123
Zm00001d022624_T001	0.7863329	-0.141643246	Zm00001d022624
Zm00001d039072_T013	0.8515562	0.559912502	Zm00001d039072
Zm00001d039072_T001	0.9139882	0.651045527	Zm00001d039072
Zm00001d039072_T017	0.2002595	0.547561929	Zm00001d039072
Zm00001d039072_T015	0.01328455	0.538237085	Zm00001d039072
Zm00001d025132_T001	0.9689939	0.190255527	Zm00001d025132
Zm00001d010530_T005	0.7120216	0.697733526	Zm00001d010530
Zm00001d010530_T002	0.9791729	0.578164366	Zm00001d010530
Zm00001d010530_T004	5.33E-12	-2.725467708	Zm00001d010530
Zm00001d047955_T002	0.01506668	2.807905395	Zm00001d047955
Zm00001d047955_T001	0.6002265	1.317221966	Zm00001d047955
Zm00001d018133_T018	0.9779851	0.233649845	Zm00001d018133
Zm00001d018133_T002	0.2015088	1.506800712	Zm00001d018133
Zm00001d020176_T001	0.9745777	0.136257057	Zm00001d020176
Zm00001d038117_T001	0.9740325	-0.244095971	Zm00001d038117
Zm00001d014035_T001	0.923446	0.357464181	Zm00001d014035
Zm00001d002669_T001	0.09082919	1.316097027	Zm00001d002669
Zm00001d002669_T002	0.1742488	1.36134657	Zm00001d002669
Zm00001d045845_T001	0.9430459	-0.003009945	Zm00001d045845
Zm00001d027805_T004	0.9740325	0.463273245	Zm00001d027805
Zm00001d027805_T005	0.9201237	0.394979864	Zm00001d027805
Zm00001d048147_T006	0.5149153	-0.818789002	Zm00001d048147
Zm00001d048147_T001	0.9693993	0.268026414	Zm00001d048147
Zm00001d048147_T003	1	0.266950668	Zm00001d048147

Zm00001d030587_T001	0.9730613	-0.390327395	Zm00001d030587
Zm00001d051337_T002	0.9845237	0.156060433	Zm00001d051337
Zm00001d051337_T001	0.8922572	0.346367381	Zm00001d051337
Zm00001d028170_T001	0.9766874	0.113227626	Zm00001d028170
Zm00001d008859_T001	0.9845237	0.420609595	Zm00001d008859
Zm00001d009480_T001	0.440598	-0.84914718	Zm00001d009480
Zm00001d022537_T001	0.7406865	0.623230709	Zm00001d022537
Zm00001d017120_T003	0.6200215	-0.618501316	Zm00001d017120
Zm00001d017120_T001	0.9252088	0.020643881	Zm00001d017120
Zm00001d017120_T004	9.65E-08	1.986590268	Zm00001d017120
Zm00001d017920_T001	0.9988954	0.449141173	Zm00001d017920
Zm00001d016531_T001	0.9338972	0.063329369	Zm00001d016531
Zm00001d004877_T001	0.6839661	0.369996104	Zm00001d004877
Zm00001d007718_T002	0.6818546	-0.565423987	Zm00001d007718
Zm00001d007718_T005	0.220834	-0.606756376	Zm00001d007718
Zm00001d015599_T001	0.9791729	0.481626346	Zm00001d015599
Zm00001d027305_T001	0.1176287	-0.656944584	Zm00001d027305
Zm00001d027305_T002	0.9689939	0.62107189	Zm00001d027305
Zm00001d053372_T001	0.9979568	0.447601366	Zm00001d053372
Zm00001d012604_T001	0.7613827	0.859539627	Zm00001d012604
Zm00001d044717_T001	0.5988928	1.260461982	Zm00001d044717
Zm00001d008782_T001	0.2164077	-0.806052443	Zm00001d008782
Zm00001d010056_T021	0.9969123	0.752518738	Zm00001d010056
Zm00001d010056_T024	0.1257477	-2.38142983	Zm00001d010056
Zm00001d010056_T008	0.000392914	2.566987155	Zm00001d010056
Zm00001d010056_T025	0.7109402	-0.548804591	Zm00001d010056
Zm00001d010056_T017	0.9833513	0.404089205	Zm00001d010056
Zm00001d010056_T023	0.9073468	0.254788636	Zm00001d010056
Zm00001d010056_T004	0.9833513	0.551904343	Zm00001d010056
Zm00001d012922_T003	0.8241665	-0.136051047	Zm00001d012922
Zm00001d029455_T001	0.9416622	-0.004489124	Zm00001d029455
Zm00001d025919_T007	0.01713299	1.690945337	Zm00001d025919
Zm00001d025919_T004	0.5131665	0.755183077	Zm00001d025919
Zm00001d025919_T011	0.4441507	0.992071131	Zm00001d025919
Zm00001d025919_T008	0.7619918	0.575186336	Zm00001d025919
Zm00001d025919_T003	3.31E-07	1.592106678	Zm00001d025919
Zm00001d025919_T002	0.2614736	1.57684158	Zm00001d025919
Zm00001d025919_T009	0.1316667	1.632175504	Zm00001d025919
Zm00001d039297_T001	0.5490561	-0.785239543	Zm00001d039297
Zm00001d045053_T001	0.7546971	0.824326557	Zm00001d045053
Zm00001d029699_T002	0.416815	1.397484853	Zm00001d029699
Zm00001d022120_T001	1	0.26096769	Zm00001d022120
Zm00001d004992_T002	0.972515	0.128283304	Zm00001d004992
Zm00001d004992_T001	0.9894615	0.081724285	Zm00001d004992
Zm00001d016767_T001	0.8869562	-0.286346829	Zm00001d016767
Zm00001d040689_T002	0.9804433	0.517800617	Zm00001d040689
Zm00001d040689_T006	0.7458694	-0.397887839	Zm00001d040689
Zm00001d040689_T005	1	0.112240906	Zm00001d040689
Zm00001d014039_T001	0.8337863	-0.216594058	Zm00001d014039
Zm00001d014039_T002	0.8995312	0.768921734	Zm00001d014039
Zm00001d050319_T001	0.9682673	0.082024448	Zm00001d050319
Zm00001d028752_T001	4.88E-06	4.759916656	Zm00001d028752
Zm00001d002256_T014	0.8720321	-0.011080037	Zm00001d002256
Zm00001d002256_T008	0.03963435	0.716779442	Zm00001d002256

Zm00001d002256_T012	0.6250445	-0.153647787	Zm00001d002256
Zm00001d002639_T001	0.983226	0.207272378	Zm00001d002639
Zm00001d048471_T001	0.9870394	0.243688605	Zm00001d048471
Zm00001d030732_T001	0.9311783	0.070580646	Zm00001d030732
Zm00001d047076_T001	0.03728966	-1.828318443	Zm00001d047076
Zm00001d047076_T003	0.7369802	1.092532111	Zm00001d047076
Zm00001d013037_T002	0.4661639	-0.861825633	Zm00001d013037
Zm00001d013037_T001	0.2004159	-1.085619553	Zm00001d013037
Zm00001d041536_T001	0.7820614	-0.233400667	Zm00001d041536
Zm00001d026213_T004	0.07730424	0.592726045	Zm00001d026213
Zm00001d026213_T006	0.8677531	-0.092119441	Zm00001d026213
Zm00001d026213_T003	0.7262247	1.019634721	Zm00001d026213
Zm00001d026213_T001	0.4146004	-0.215587717	Zm00001d026213
Zm00001d026213_T005	0.7900674	-0.24984513	Zm00001d026213
Zm00001d006705_T001	0.7499604	0.852322294	Zm00001d006705
Zm00001d005648_T002	0.7664596	-0.32390582	Zm00001d005648
Zm00001d046530_T001	0.03830215	2.564396905	Zm00001d046530
Zm00001d016760_T001	0.9932384	-0.071199665	Zm00001d016760
Zm00001d003658_T001	0.9583056	0.018353508	Zm00001d003658
Zm00001d001913_T001	0.9810094	0.206233733	Zm00001d001913
Zm00001d001913_T003	0.3376079	-0.124389889	Zm00001d001913
Zm00001d001913_T005	0.7078717	-0.973311569	Zm00001d001913
Zm00001d001913_T004	0.9463296	0.049026903	Zm00001d001913
Zm00001d001913_T002	0.01128066	-2.17788154	Zm00001d001913
Zm00001d049683_T001	0.8158416	1.026441726	Zm00001d049683
Zm00001d053284_T001	0.9429712	-0.084716427	Zm00001d053284
Zm00001d053284_T003	0.1858922	0.687214849	Zm00001d053284
Zm00001d053284_T004	0.268975	-0.65057761	Zm00001d053284
Zm00001d053284_T005	0.4179456	1.068546427	Zm00001d053284
Zm00001d017822_T001	0.9776209	0.735454017	Zm00001d017822
Zm00001d017900_T001	0.9979568	0.158617313	Zm00001d017900
Zm00001d023465_T002	0.9756546	0.167727683	Zm00001d023465
Zm00001d023465_T001	0.9272378	-0.074067234	Zm00001d023465
Zm00001d036108_T003	0.996239	0.203999896	Zm00001d036108
Zm00001d026366_T002	0.9975882	0.418656296	Zm00001d026366
Zm00001d023615_T001	0.8545272	-0.202335702	Zm00001d023615
Zm00001d025425_T001	0.9367382	-0.324101841	Zm00001d025425
Zm00001d031580_T001	0.3899945	1.526676068	Zm00001d031580
Zm00001d031580_T002	0.8079251	0.805677645	Zm00001d031580
Zm00001d036971_T012	0.9706293	0.101086283	Zm00001d036971
Zm00001d036971_T011	0.850843	0.661467037	Zm00001d036971
Zm00001d036971_T024	0.1331007	1.980269442	Zm00001d036971
Zm00001d036971_T003	0.8660064	0.505220511	Zm00001d036971
Zm00001d036971_T045	0.4196849	-0.780166376	Zm00001d036971
Zm00001d036971_T042	0.9701986	0.064490681	Zm00001d036971
Zm00001d036971_T044	0.9918302	0.161729324	Zm00001d036971
Zm00001d036971_T025	0.9541864	0.127679409	Zm00001d036971
Zm00001d013166_T001	0.9773181	0.565381575	Zm00001d013166
Zm00001d033419_T001	0.5426998	-0.706917316	Zm00001d033419
Zm00001d043224_T001	1	0.109835523	Zm00001d043224
Zm00001d021269_T015	0.6231517	-0.371772138	Zm00001d021269
Zm00001d021269_T018	0.8665839	-0.071249787	Zm00001d021269
Zm00001d025144_T003	0.941922	0.075846839	Zm00001d025144
Zm00001d025144_T001	0.7258102	1.063104229	Zm00001d025144

Zm00001d047911_T007	1	0.071507388	Zm00001d047911
Zm00001d047911_T010	0.3431817	1.593745776	Zm00001d047911
Zm00001d047911_T005	1	0.350600811	Zm00001d047911
Zm00001d047911_T004	0.6050512	-0.463908617	Zm00001d047911
Zm00001d047911_T009	0.1616209	-0.575050355	Zm00001d047911
Zm00001d047911_T006	0.7404798	0.667597442	Zm00001d047911
Zm00001d047911_T001	0.7697196	0.72817563	Zm00001d047911
Zm00001d047911_T014	0.009256586	-2.601914132	Zm00001d047911
Zm00001d025667_T001	0.9951827	0.409210911	Zm00001d025667
Zm00001d012042_T002	0.000565416	-1.706017595	Zm00001d012042
Zm00001d012042_T001	0.9779656	-0.1649651	Zm00001d012042
Zm00001d024572_T002	0.04000108	2.554840629	Zm00001d024572
Zm00001d044584_T019	0.5533841	-1.180428287	Zm00001d044584
Zm00001d044584_T003	0.9693993	0.166073463	Zm00001d044584
Zm00001d044584_T005	0.7016731	0.129799389	Zm00001d044584
Zm00001d044584_T013	0.9824516	-0.318887769	Zm00001d044584
Zm00001d044584_T006	0.004332221	1.625060278	Zm00001d044584
Zm00001d044584_T011	0.2575978	0.531485255	Zm00001d044584
Zm00001d044584_T020	0.1260325	1.429833763	Zm00001d044584
Zm00001d044224_T001	0.9804228	0.389899943	Zm00001d044224
Zm00001d022593_T001	0.4009979	1.406539726	Zm00001d022593
Zm00001d031033_T001	0.4274324	-0.606969734	Zm00001d031033
Zm00001d048908_T001	0.9278769	0.159578714	Zm00001d048908
Zm00001d048908_T002	0.5680339	1.329010913	Zm00001d048908
Zm00001d008617_T006	0.3355076	-0.339021394	Zm00001d008617
Zm00001d008617_T005	0.07515851	1.413173145	Zm00001d008617
Zm00001d008617_T001	0.9887889	0.297121131	Zm00001d008617
Zm00001d006644_T002	0.9999183	0.376344965	Zm00001d006644
Zm00001d017559_T002	0.6593598	-0.447810156	Zm00001d017559
Zm00001d043434_T037	0.7626242	1.084214874	Zm00001d043434
Zm00001d043434_T035	0.05224483	1.082256139	Zm00001d043434
Zm00001d043434_T005	0.07482195	-1.200561404	Zm00001d043434
Zm00001d043434_T006	3.01E-12	1.749180202	Zm00001d043434
Zm00001d043434_T036	3.65E-05	0.905407129	Zm00001d043434
Zm00001d043434_T003	0.8565397	-0.153142236	Zm00001d043434
Zm00001d042193_T006	1	0.275167065	Zm00001d042193
Zm00001d042193_T003	0.7333	-0.355065194	Zm00001d042193
Zm00001d042193_T008	0.8913084	0.318831423	Zm00001d042193
Zm00001d042193_T007	0.4431901	-0.669745866	Zm00001d042193
Zm00001d042193_T002	0.9119921	-0.145662358	Zm00001d042193
Zm00001d054089_T001	0.9942835	0.339511969	Zm00001d054089
Zm00001d025542_T002	0.9689939	0.450090978	Zm00001d025542
Zm00001d011745_T002	0.9273903	0.542496393	Zm00001d011745
Zm00001d011745_T004	0.06327499	0.634164392	Zm00001d011745
Zm00001d048273_T001	0.2697012	-1.187899795	Zm00001d048273
Zm00001d046603_T001	0.8616387	-0.139820359	Zm00001d046603
Zm00001d006594_T001	0.6967776	-0.822766016	Zm00001d006594
Zm00001d008233_T002	0.9956349	0.295204051	Zm00001d008233
Zm00001d008233_T005	0.9145885	0.369221949	Zm00001d008233
Zm00001d052748_T001	0.9740325	0.56848653	Zm00001d052748
Zm00001d026589_T001	0.8494782	-0.382023425	Zm00001d026589
Zm00001d028702_T001	0.9720956	0.096939745	Zm00001d028702
Zm00001d000094_T002	0.8256776	-0.165169319	Zm00001d000094
Zm00001d040162_T004	0.9988271	0.440360535	Zm00001d040162

Zm00001d040162_T006	1	0.32022267	Zm00001d040162
Zm00001d040162_T010	0.08045219	-0.776275827	Zm00001d040162
Zm00001d040162_T002	0.9469426	0.100463704	Zm00001d040162
Zm00001d040162_T008	0.02483077	0.745786865	Zm00001d040162
Zm00001d040162_T003	0.1219709	1.434696725	Zm00001d040162
Zm00001d019040_T006	1	0.08934123	Zm00001d019040
Zm00001d019040_T013	0.7992064	-0.212043679	Zm00001d019040
Zm00001d019040_T012	0.9257893	0.499384526	Zm00001d019040
Zm00001d019040_T004	0.9689939	0.154082707	Zm00001d019040
Zm00001d019040_T005	0.9881407	0.037857624	Zm00001d019040
Zm00001d019040_T007	0.8587779	0.88356455	Zm00001d019040
Zm00001d005724_T001	0.9930712	-0.11260972	Zm00001d005724
Zm00001d020609_T003	0.5853819	1.051653219	Zm00001d020609
Zm00001d020609_T001	0.4753078	1.429215357	Zm00001d020609
Zm00001d020609_T004	0.9343956	0.749213736	Zm00001d020609
Zm00001d016577_T001	0.06674829	-0.730453733	Zm00001d016577
Zm00001d016577_T005	0.8158142	-0.240986654	Zm00001d016577
Zm00001d016577_T002	0.8031436	-0.25188667	Zm00001d016577
Zm00001d023664_T001	0.2154325	1.800713085	Zm00001d023664
Zm00001d051323_T003	1	0.131368068	Zm00001d051323
Zm00001d051323_T001	0.8103289	-0.184420834	Zm00001d051323
Zm00001d002072_T001	0.5718096	1.0993313	Zm00001d002072
Zm00001d012666_T003	0.7011505	1.02384861	Zm00001d012666
Zm00001d012666_T001	0.8919874	0.33153229	Zm00001d012666
Zm00001d049830_T002	0.8414741	-0.425972731	Zm00001d049830
Zm00001d015585_T001	0.9559227	0.010242719	Zm00001d015585
Zm00001d006659_T001	0.7923921	0.837504826	Zm00001d006659
Zm00001d032420_T001	0.9419336	-0.004305686	Zm00001d032420
Zm00001d049698_T001	0.935477	0.735885844	Zm00001d049698
Zm00001d043117_T002	0.9664994	0.153063303	Zm00001d043117
Zm00001d005909_T001	0.9973528	0.279312853	Zm00001d005909
Zm00001d009549_T001	0.9912886	0.233441746	Zm00001d009549
Zm00001d053195_T001	0.8908955	-0.093057719	Zm00001d053195
Zm00001d050436_T001	0.972861	0.069588546	Zm00001d050436
Zm00001d025865_T002	0.4407493	0.817456075	Zm00001d025865
Zm00001d025865_T001	0.941922	0.704056249	Zm00001d025865
Zm00001d038688_T001	0.7739226	0.824705809	Zm00001d038688
Zm00001d038688_T003	0.5650342	-1.088855138	Zm00001d038688
Zm00001d051951_T005	0.8031436	0.667788861	Zm00001d051951
Zm00001d051951_T002	0.966464	0.651749614	Zm00001d051951
Zm00001d051951_T006	0.4146004	-1.114469455	Zm00001d051951
Zm00001d051951_T004	1	0.307995746	Zm00001d051951
Zm00001d051951_T010	0.9531886	0.070524626	Zm00001d051951
Zm00001d051951_T009	0.5384294	0.692011797	Zm00001d051951
Zm00001d048533_T001	0.6099833	1.282500246	Zm00001d048533
Zm00001d052314_T004	0.826773	0.349031114	Zm00001d052314
Zm00001d052314_T003	0.9002196	0.619785097	Zm00001d052314
Zm00001d052314_T034	0.5459845	1.680729125	Zm00001d052314
Zm00001d052314_T007	0.2935147	0.971574226	Zm00001d052314
Zm00001d052314_T011	0.5623656	1.129797265	Zm00001d052314
Zm00001d023774_T001	0.04489514	-1.733502692	Zm00001d023774
Zm00001d028204_T004	0.9747606	0.072816495	Zm00001d028204
Zm00001d000305_T003	0.9830387	0.558391441	Zm00001d000305
Zm00001d000305_T002	0.926928	0.131817518	Zm00001d000305

Zm00001d038718_T001	0.004610805	2.872130345	Zm00001d038718
Zm00001d038718_T003	1.92E-05	3.880379203	Zm00001d038718
Zm00001d037427_T001	0.8902687	-0.002806009	Zm00001d037427
Zm00001d037427_T003	0.772948	-0.137380611	Zm00001d037427
Zm00001d037427_T005	0.8537544	-0.07413092	Zm00001d037427
Zm00001d037427_T004	0.9567316	0.096368926	Zm00001d037427
Zm00001d037427_T002	0.9979568	0.354507448	Zm00001d037427
Zm00001d024908_T010	0.01538637	0.636158725	Zm00001d024908
Zm00001d024908_T008	0.8585804	0.829734257	Zm00001d024908
Zm00001d024908_T004	0.4721293	-0.944139958	Zm00001d024908
Zm00001d024908_T009	0.9902022	-0.097396429	Zm00001d024908
Zm00001d024908_T011	1	0.078449918	Zm00001d024908
Zm00001d024908_T001	0.9941329	0.316135532	Zm00001d024908
Zm00001d037192_T001	0.9618192	0.024308343	Zm00001d037192
Zm00001d012929_T002	0.954492	0.102507068	Zm00001d012929
Zm00001d012929_T004	0.4070866	-0.394057588	Zm00001d012929
Zm00001d020516_T002	0.1709638	0.744080125	Zm00001d020516
Zm00001d051458_T007	0.9429712	0.080094647	Zm00001d051458
Zm00001d051458_T006	0.8677544	-0.136828918	Zm00001d051458
Zm00001d051458_T005	0.4063448	0.319696389	Zm00001d051458
Zm00001d051458_T001	0.9352321	0.529729195	Zm00001d051458
Zm00001d023905_T001	0.8342838	0.968167388	Zm00001d023905
Zm00001d050488_T001	0.522218	1.122942966	Zm00001d050488
Zm00001d042713_T008	0.3899945	-1.052766411	Zm00001d042713
Zm00001d042713_T005	0.005246483	1.254665118	Zm00001d042713
Zm00001d042713_T006	0.9580876	0.407007321	Zm00001d042713
Zm00001d042713_T002	0.5034905	0.455731103	Zm00001d042713
Zm00001d042713_T007	0.9886764	-0.210211101	Zm00001d042713
Zm00001d035246_T001	0.8549534	-0.173971075	Zm00001d035246
Zm00001d043338_T001	0.8751573	-0.333439108	Zm00001d043338
Zm00001d034760_T001	0.955383	-0.437703715	Zm00001d034760
Zm00001d019593_T001	0.7594555	-0.512544296	Zm00001d019593
Zm00001d028692_T005	0.2329183	1.798131923	Zm00001d028692
Zm00001d052680_T001	0.9979393	0.088041476	Zm00001d052680
Zm00001d052680_T007	0.9246704	-0.189166472	Zm00001d052680
Zm00001d052680_T008	0.8843193	-0.376557192	Zm00001d052680
Zm00001d024543_T002	0.1623075	1.55633787	Zm00001d024543
Zm00001d024543_T003	0.1339192	0.913697803	Zm00001d024543
Zm00001d024543_T001	0.1873585	1.755631057	Zm00001d024543
Zm00001d026597_T001	0.9627438	0.38092876	Zm00001d026597
Zm00001d052889_T001	0.9160053	0.687213291	Zm00001d052889
Zm00001d009196_T001	0.9946911	0.108266316	Zm00001d009196
Zm00001d033912_T002	1	0.32366345	Zm00001d033912
Zm00001d033912_T001	0.968737	0.477291381	Zm00001d033912
Zm00001d044335_T001	0.9220919	-0.315479921	Zm00001d044335
Zm00001d043060_T001	0.4003602	1.164965071	Zm00001d043060
Zm00001d045905_T001	0.9210513	-0.435063112	Zm00001d045905
Zm00001d052189_T001	0.6721904	-0.342220714	Zm00001d052189
Zm00001d048414_T002	0.9380783	-0.174908503	Zm00001d048414
Zm00001d018857_T001	0.1266122	-1.601538301	Zm00001d018857
Zm00001d039375_T001	0.645777	-1.041333062	Zm00001d039375
Zm00001d038577_T001	0.9740325	0.512264305	Zm00001d038577
Zm00001d038577_T002	0.9518042	0.41865926	Zm00001d038577
Zm00001d029100_T001	0.987516	0.361321746	Zm00001d029100

Zm00001d013725_T001	0.9894615	0.292386429	Zm00001d013725
Zm00001d046823_T001	0.9969123	0.18461762	Zm00001d046823
Zm00001d034588_T001	0.7743716	-0.574232318	Zm00001d034588
Zm00001d050851_T001	0.9182861	0.042693146	Zm00001d050851
Zm00001d050851_T007	0.5138741	1.154023676	Zm00001d050851
Zm00001d050851_T005	0.9804433	0.392345082	Zm00001d050851
Zm00001d020721_T001	0.6962193	-0.306521198	Zm00001d020721
Zm00001d006879_T001	0.9384219	-0.351361599	Zm00001d006879
Zm00001d025206_T001	0.9689939	0.183127303	Zm00001d025206
Zm00001d021830_T003	0.1410912	-0.686011086	Zm00001d021830
Zm00001d021830_T001	0.01918261	1.568580299	Zm00001d021830
Zm00001d021830_T002	0.9855106	0.186874801	Zm00001d021830
Zm00001d025670_T001	0.2214361	-0.507111068	Zm00001d025670
Zm00001d021318_T005	0.8405128	-0.216517435	Zm00001d021318
Zm00001d021318_T004	0.5956992	-0.365384919	Zm00001d021318
Zm00001d021318_T006	1	0.425589988	Zm00001d021318
Zm00001d021318_T007	0.933055	0.658251181	Zm00001d021318
Zm00001d021318_T002	0.000800583	1.002637298	Zm00001d021318
Zm00001d024179_T001	0.9673473	-0.11264556	Zm00001d024179
Zm00001d030282_T015	0.06372667	0.622849117	Zm00001d030282
Zm00001d030282_T035	0.6275807	0.342751418	Zm00001d030282
Zm00001d030282_T008	0.9171372	0.267373586	Zm00001d030282
Zm00001d033620_T001	0.9265089	-0.181946139	Zm00001d033620
Zm00001d021299_T002	0.9676336	0.189150104	Zm00001d021299
Zm00001d021299_T001	0.9748076	0.237025277	Zm00001d021299
Zm00001d039754_T001	0.811032	0.463699203	Zm00001d039754
Zm00001d052193_T007	0.7146325	0.193436498	Zm00001d052193
Zm00001d052193_T014	0.8765337	-0.110555491	Zm00001d052193
Zm00001d052193_T004	0.006966048	1.578048735	Zm00001d052193
Zm00001d052193_T005	0.9740325	0.110011341	Zm00001d052193
Zm00001d022378_T120	0.8851769	0.826084999	Zm00001d022378
Zm00001d022378_T127	0.4557436	0.686507845	Zm00001d022378
Zm00001d022378_T044	1	-0.088736398	Zm00001d022378
Zm00001d022378_T064	0.8031436	0.237745517	Zm00001d022378
Zm00001d022378_T130	0.2917536	0.991497332	Zm00001d022378
Zm00001d022378_T124	0.008267721	0.596663914	Zm00001d022378
Zm00001d022378_T002	0.02379854	2.38291471	Zm00001d022378
Zm00001d022378_T065	2.93E-06	0.53006542	Zm00001d022378
Zm00001d022378_T131	0.8585804	-0.002949242	Zm00001d022378
Zm00001d022378_T132	0.9701986	-0.181230642	Zm00001d022378
Zm00001d022378_T128	0.8752115	-0.048888425	Zm00001d022378
Zm00001d039776_T001	0.9015226	0.454705645	Zm00001d039776
Zm00001d011100_T002	0.9469426	0.049065637	Zm00001d011100
Zm00001d019400_T001	0.4359356	1.672582566	Zm00001d019400
Zm00001d033194_T001	0.6315881	-0.469130888	Zm00001d033194
Zm00001d040292_T001	0.822299	-0.519149707	Zm00001d040292
Zm00001d039407_T002	0.2721567	1.810084617	Zm00001d039407
Zm00001d039407_T003	0.9557384	0.631086746	Zm00001d039407
Zm00001d032434_T001	0.684769	-0.598147119	Zm00001d032434
Zm00001d007229_T001	0.7296232	-0.156411641	Zm00001d007229
Zm00001d007229_T002	0.9689939	0.188074554	Zm00001d007229
Zm00001d006667_T001	0.9822138	0.521233	Zm00001d006667
Zm00001d006667_T005	0.7952435	-0.419613703	Zm00001d006667
Zm00001d006368_T003	1	0.283763722	Zm00001d006368

Zm00001d031434_T001	0.113956	-1.443755252	Zm00001d031434
Zm00001d027571_T003	0.8557307	0.94736321	Zm00001d027571
Zm00001d027571_T006	0.7989126	0.448576384	Zm00001d027571
Zm00001d027571_T004	0.2518892	-0.414062914	Zm00001d027571
Zm00001d019703_T001	0.9992454	0.338551466	Zm00001d019703
Zm00001d031220_T001	0.5715414	-0.960977454	Zm00001d031220
Zm00001d013551_T001	0.8412913	-0.558192129	Zm00001d013551
Zm00001d053648_T008	1	0.221156438	Zm00001d053648
Zm00001d053648_T002	0.9817074	0.15394991	Zm00001d053648
Zm00001d053648_T007	0.3331634	0.512770924	Zm00001d053648
Zm00001d020569_T001	0.8494747	-0.191294676	Zm00001d020569
Zm00001d042811_T001	0.02321721	2.537283242	Zm00001d042811
Zm00001d043548_T001	0.8859511	-0.083387711	Zm00001d043548
Zm00001d042763_T001	0.9363242	0.298112283	Zm00001d042763
Zm00001d047388_T003	0.6993528	0.527781995	Zm00001d047388
Zm00001d047388_T002	0.9902022	0.284912152	Zm00001d047388
Zm00001d052306_T003	0.9720425	0.639821024	Zm00001d052306
Zm00001d024709_T004	0.9441469	0.029092533	Zm00001d024709
Zm00001d024709_T001	1	0.117528949	Zm00001d024709
Zm00001d025846_T001	0.8267985	-0.192740028	Zm00001d025846
Zm00001d025846_T002	0.9419336	4.67E-05	Zm00001d025846
Zm00001d040238_T005	0.9694074	0.023701721	Zm00001d040238
Zm00001d040238_T001	0.9931431	0.263663352	Zm00001d040238
Zm00001d040238_T004	0.9724404	0.074176162	Zm00001d040238
Zm00001d002940_T003	0.7889719	-0.08869073	Zm00001d002940
Zm00001d002940_T001	7.68E-08	0.803348195	Zm00001d002940
Zm00001d040125_T001	0.6182279	-0.353665252	Zm00001d040125
Zm00001d048772_T001	0.9975882	0.143663959	Zm00001d048772
Zm00001d048224_T001	0.8080468	-0.578989814	Zm00001d048224
Zm00001d023652_T002	0.9693993	0.121353783	Zm00001d023652
Zm00001d005503_T001	0.7757188	-0.587256871	Zm00001d005503
Zm00001d007830_T001	0.9192385	0.668316028	Zm00001d007830
Zm00001d053778_T001	0.850838	0.921946691	Zm00001d053778
Zm00001d031706_T001	0.9659036	0.650823848	Zm00001d031706
Zm00001d049595_T001	0.9916087	0.354084362	Zm00001d049595
Zm00001d037783_T001	0.5418433	-0.452222441	Zm00001d037783
Zm00001d028370_T001	0.9781672	0.261535454	Zm00001d028370
Zm00001d034833_T001	0.741153	-0.267908488	Zm00001d034833
Zm00001d034833_T004	0.9369006	0.677941667	Zm00001d034833
Zm00001d034833_T003	0.4977317	-0.789283197	Zm00001d034833
Zm00001d034833_T002	0.6852171	0.430144425	Zm00001d034833
Zm00001d024600_T002	0.7020458	-0.005073763	Zm00001d024600
Zm00001d024600_T003	0.1510538	0.596202618	Zm00001d024600
Zm00001d049450_T005	0.7294039	0.777388217	Zm00001d049450
Zm00001d049450_T002	0.2546213	-0.431845777	Zm00001d049450
Zm00001d049450_T008	0.7895843	0.351843411	Zm00001d049450
Zm00001d037797_T001	0.7775347	0.723549184	Zm00001d037797
Zm00001d037797_T003	0.9321001	0.027455307	Zm00001d037797
Zm00001d006894_T001	0.9563257	0.051980668	Zm00001d006894
Zm00001d029281_T005	0.912978	0.007048135	Zm00001d029281
Zm00001d029281_T001	0.9543419	0.955561511	Zm00001d029281
Zm00001d029281_T004	0.6801401	-0.241766736	Zm00001d029281
Zm00001d029281_T007	1	0.294805448	Zm00001d029281
Zm00001d049360_T002	0.3722179	0.912916568	Zm00001d049360

Zm00001d049360_T001	0.9232446	0.185391927	Zm00001d049360
Zm00001d049360_T003	0.9983139	0.40945496	Zm00001d049360
Zm00001d049360_T005	0.9766874	1.254204992	Zm00001d049360
Zm00001d031717_T001	0.4723494	-0.913857114	Zm00001d031717
Zm00001d031619_T002	0.7594555	0.72613557	Zm00001d031619
Zm00001d031619_T001	0.4187009	1.509031843	Zm00001d031619
Zm00001d034752_T007	0.7651954	-0.208407597	Zm00001d034752
Zm00001d009487_T001	0.004862421	2.992278004	Zm00001d009487
Zm00001d052425_T015	0.518947	0.467903565	Zm00001d052425
Zm00001d052425_T008	0.4842372	1.590172416	Zm00001d052425
Zm00001d052425_T010	0.3183941	0.449574923	Zm00001d052425
Zm00001d052756_T002	0.8125642	-0.342170114	Zm00001d052756
Zm00001d052756_T003	0.7489032	-0.386090514	Zm00001d052756
Zm00001d052756_T001	0.9979568	0.379554696	Zm00001d052756
Zm00001d043446_T001	0.9451369	-0.011340507	Zm00001d043446
Zm00001d045136_T001	0.9673473	0.188288322	Zm00001d045136
Zm00001d042993_T001	0.4537759	1.556026981	Zm00001d042993
Zm00001d052605_T001	0.5585191	-0.431821888	Zm00001d052605
Zm00001d053006_T001	0.7193211	-0.185640074	Zm00001d053006
Zm00001d034875_T001	0.6110258	1.201100125	Zm00001d034875
Zm00001d048085_T003	0.4723494	-0.854682687	Zm00001d048085
Zm00001d048085_T002	0.7978964	-0.279110782	Zm00001d048085
Zm00001d048085_T001	0.707391	-0.360722932	Zm00001d048085
Zm00001d022206_T005	0.9689939	0.057195451	Zm00001d022206
Zm00001d022206_T003	1.52E-06	-1.013828993	Zm00001d022206
Zm00001d015758_T001	0.7623518	0.311080648	Zm00001d015758
Zm00001d015758_T002	0.954492	0.326622049	Zm00001d015758
Zm00001d028140_T001	0.8495532	0.931224136	Zm00001d028140
Zm00001d028140_T009	0.9334932	0.237668253	Zm00001d028140
Zm00001d028140_T016	1.70E-08	-1.766044868	Zm00001d028140
Zm00001d028140_T007	0.7626242	0.935380473	Zm00001d028140
Zm00001d028140_T002	1.42E-06	0.650741889	Zm00001d028140
Zm00001d028140_T014	0.5137998	0.835234906	Zm00001d028140
Zm00001d028140_T005	0.6394238	1.159585772	Zm00001d028140
Zm00001d028140_T008	0.9422823	0.483133137	Zm00001d028140
Zm00001d028140_T015	0.4586954	0.807571698	Zm00001d028140
Zm00001d015609_T021	0.6386265	0.845594524	Zm00001d015609
Zm00001d015609_T009	0.3921441	-0.90056445	Zm00001d015609
Zm00001d015609_T025	0.05999172	1.707715826	Zm00001d015609
Zm00001d025584_T001	0.9276061	0.653976733	Zm00001d025584
Zm00001d044443_T001	0.03645088	2.594693096	Zm00001d044443
Zm00001d013432_T001	0.9487214	0.596973143	Zm00001d013432
Zm00001d050174_T001	0.9561521	0.648846293	Zm00001d050174
Zm00001d048346_T001	0.7137316	-0.389366446	Zm00001d048346
Zm00001d014820_T001	0.9285812	-0.004286352	Zm00001d014820
Zm00001d040720_T001	0.9936409	0.333424522	Zm00001d040720
Zm00001d009731_T003	0.2780484	0.458574416	Zm00001d009731
Zm00001d009731_T002	0.5715685	-0.319080794	Zm00001d009731
Zm00001d040111_T001	0.9824348	0.277962072	Zm00001d040111
Zm00001d006020_T001	0.5158356	0.884882074	Zm00001d006020
Zm00001d039345_T001	0.1059111	-0.63231692	Zm00001d039345
Zm00001d008173_T001	0.8825173	-0.152663026	Zm00001d008173
Zm00001d020412_T001	0.3547256	-0.744139355	Zm00001d020412
Zm00001d013153_T001	0.3865855	-0.768585648	Zm00001d013153

Zm00001d032044_T005	0.3547256	0.952827331	Zm00001d032044
Zm00001d032044_T003	0.9216521	0.026709317	Zm00001d032044
Zm00001d032044_T004	0.3166233	1.740021831	Zm00001d032044
Zm00001d045043_T005	0.9469426	0.586374046	Zm00001d045043
Zm00001d045043_T001	0.8660897	0.054609419	Zm00001d045043
Zm00001d045043_T006	0.6313255	0.521891631	Zm00001d045043
Zm00001d045043_T004	0.724133	1.077688538	Zm00001d045043
Zm00001d011770_T001	0.8643636	0.905570994	Zm00001d011770
Zm00001d027532_T002	0.4233885	-0.589200868	Zm00001d027532
Zm00001d027532_T001	0.4380979	-0.122806312	Zm00001d027532
Zm00001d043815_T005	0.9334932	0.009221598	Zm00001d043815
Zm00001d043815_T006	0.9100328	-0.129205184	Zm00001d043815
Zm00001d030665_T001	0.9154179	-0.356379481	Zm00001d030665
Zm00001d043158_T002	1	0.610652429	Zm00001d043158
Zm00001d043158_T003	0.5269468	-0.687971112	Zm00001d043158
Zm00001d043158_T001	5.30E-07	-1.018503585	Zm00001d043158
Zm00001d039534_T006	0.8031436	0.422632317	Zm00001d039534
Zm00001d039534_T001	0.9429712	0.244760776	Zm00001d039534
Zm00001d039534_T008	0.7410676	-0.29280873	Zm00001d039534
Zm00001d007328_T005	0.4531805	-0.653762516	Zm00001d007328
Zm00001d007328_T004	0.9441469	0.541277012	Zm00001d007328
Zm00001d007328_T001	0.264061	0.82130342	Zm00001d007328
Zm00001d027982_T002	0.9266346	0.052407661	Zm00001d027982
Zm00001d027982_T003	0.5750164	0.622902951	Zm00001d027982
Zm00001d027982_T006	0.7565187	0.308553777	Zm00001d027982
Zm00001d027982_T001	0.06064403	-1.046059365	Zm00001d027982
Zm00001d008427_T001	0.7901736	0.407869121	Zm00001d008427
Zm00001d008772_T002	0.294216	0.745485087	Zm00001d008772
Zm00001d008772_T003	0.8686467	0.225330242	Zm00001d008772
Zm00001d051535_T004	0.7509844	-0.087238431	Zm00001d051535
Zm00001d051535_T002	0.9860492	0.207596752	Zm00001d051535
Zm00001d018498_T002	0.9846714	0.230432724	Zm00001d018498
Zm00001d018498_T004	0.9954444	0.244501171	Zm00001d018498
Zm00001d021363_T001	0.9929973	0.252011698	Zm00001d021363
Zm00001d047183_T001	0.01092607	-0.641920618	Zm00001d047183
Zm00001d047183_T007	0.3411233	1.093273273	Zm00001d047183
Zm00001d047183_T013	0.9692109	0.602935247	Zm00001d047183
Zm00001d031473_T001	0.960704	0.125657383	Zm00001d031473
Zm00001d041789_T003	0.941922	0.030040007	Zm00001d041789
Zm00001d041789_T001	0.9979877	0.307565187	Zm00001d041789
Zm00001d041789_T002	0.6632799	0.710360819	Zm00001d041789
Zm00001d034410_T001	0.9706887	0.128198407	Zm00001d034410
Zm00001d032396_T002	0.8685001	-0.144832868	Zm00001d032396
Zm00001d025576_T001	0.1349947	-1.673995547	Zm00001d025576
Zm00001d016159_T001	0.5023431	-1.01464037	Zm00001d016159
Zm00001d021365_T014	0.9740325	0.105433511	Zm00001d021365
Zm00001d021365_T001	0.3836885	0.611393902	Zm00001d021365
Zm00001d014666_T001	0.6976864	-0.398909859	Zm00001d014666
Zm00001d030891_T001	0.01932232	2.847616151	Zm00001d030891
Zm00001d029196_T001	0.9956688	0.360347174	Zm00001d029196
Zm00001d029196_T002	0.1799314	1.794566916	Zm00001d029196
Zm00001d025018_T001	0.03090842	-1.85519427	Zm00001d025018
Zm00001d010740_T001	0.2136911	-1.68060076	Zm00001d010740
Zm00001d015194_T001	0.9556141	0.080262121	Zm00001d015194

Zm00001d034358_T002	0.9994997	0.356632159	Zm00001d034358
Zm00001d034358_T001	0.9804228	0.142336415	Zm00001d034358
Zm00001d008849_T001	1	0.313197137	Zm00001d008849
Zm00001d006562_T001	0.8937896	-0.152649288	Zm00001d006562
Zm00001d018384_T001	0.3783227	-0.645387346	Zm00001d018384
Zm00001d018384_T004	0.5864438	1.116924877	Zm00001d018384
Zm00001d020064_T001	0.6993528	0.873074478	Zm00001d020064
Zm00001d020064_T002	0.9766874	0.250728682	Zm00001d020064
Zm00001d041782_T001	0.2338394	-1.040335894	Zm00001d041782
Zm00001d004451_T001	0.7114961	-0.56254379	Zm00001d004451
Zm00001d008602_T002	0.4842546	-0.688835138	Zm00001d008602
Zm00001d008602_T001	0.8377512	-0.095016213	Zm00001d008602
Zm00001d008602_T003	0.8987811	-0.047641134	Zm00001d008602
Zm00001d014553_T001	0.9947747	0.319449025	Zm00001d014553
Zm00001d024376_T001	0.7185621	0.882479866	Zm00001d024376
Zm00001d022181_T001	0.3224744	0.313389708	Zm00001d022181
Zm00001d022181_T010	0.1317675	0.784867469	Zm00001d022181
Zm00001d022181_T004	0.01911784	-0.572313249	Zm00001d022181
Zm00001d022181_T006	0.2829145	0.748072215	Zm00001d022181
Zm00001d022181_T013	0.12478	0.567464152	Zm00001d022181
Zm00001d022181_T008	0.9469426	0.34969182	Zm00001d022181
Zm00001d022181_T005	0.909186	1.140201121	Zm00001d022181
Zm00001d022257_T001	0.8865624	-0.153715982	Zm00001d022257
Zm00001d027254_T001	0.9113667	-0.00034729	Zm00001d027254
Zm00001d014988_T001	0.9374853	0.054380412	Zm00001d014988
Zm00001d047015_T059	0.9664736	0.529600504	Zm00001d047015
Zm00001d047015_T022	0.002381753	0.688287441	Zm00001d047015
Zm00001d047015_T041	0.001837801	0.707921285	Zm00001d047015
Zm00001d047015_T002	0.2935147	0.292083194	Zm00001d047015
Zm00001d051039_T005	0.9380783	0.495732312	Zm00001d051039
Zm00001d051039_T007	0.7727043	-0.080304843	Zm00001d051039
Zm00001d051039_T004	0.700482	0.445792357	Zm00001d051039
Zm00001d051039_T010	0.9690782	0.447847589	Zm00001d051039
Zm00001d051039_T002	0.2982377	-0.178554574	Zm00001d051039
Zm00001d051039_T003	0.9355135	-0.017900843	Zm00001d051039
Zm00001d009695_T001	0.7232651	-0.450094045	Zm00001d009695
Zm00001d029519_T001	0.9979393	0.396172997	Zm00001d029519
Zm00001d032582_T006	1	0.113225935	Zm00001d032582
Zm00001d032582_T003	0.1680969	0.726780258	Zm00001d032582
Zm00001d032582_T007	0.4954022	0.830730535	Zm00001d032582
Zm00001d028606_T002	0.9514141	0.697707322	Zm00001d028606
Zm00001d028606_T004	0.3795013	0.780857946	Zm00001d028606
Zm00001d039568_T002	0.878248	-0.137959131	Zm00001d039568
Zm00001d010936_T001	0.1652317	0.500045573	Zm00001d010936
Zm00001d028838_T001	0.00045105	2.868635824	Zm00001d028838
Zm00001d018359_T001	0.6375369	-0.46210491	Zm00001d018359
Zm00001d018359_T003	0.7881977	-0.332886729	Zm00001d018359
Zm00001d018359_T002	0.9703357	0.614982464	Zm00001d018359
Zm00001d036952_T001	1	0.128601508	Zm00001d036952
Zm00001d042442_T002	0.5282092	0.356103787	Zm00001d042442
Zm00001d042442_T004	0.8716622	0.832391218	Zm00001d042442
Zm00001d006685_T001	0.9919298	0.358619565	Zm00001d006685
Zm00001d011183_T001	0.9219195	0.645100399	Zm00001d011183
Zm00001d035228_T001	0.9973914	0.31864525	Zm00001d035228

Zm00001d034932_T001	0.9681793	0.101354797	Zm00001d034932
Zm00001d030910_T002	0.9543237	-0.048403648	Zm00001d030910
Zm00001d030910_T001	0.3861376	-0.530236676	Zm00001d030910
Zm00001d045812_T001	0.8681121	-0.48076204	Zm00001d045812
Zm00001d019354_T006	0.03127381	0.857248634	Zm00001d019354
Zm00001d019354_T001	0.9942835	0.155585784	Zm00001d019354
Zm00001d045402_T001	0.5715685	-0.222892526	Zm00001d045402
Zm00001d050608_T001	0.9244973	-0.047758187	Zm00001d050608
Zm00001d013025_T002	0.7167212	-0.443858007	Zm00001d013025
Zm00001d031120_T003	0.9979568	0.497943459	Zm00001d031120
Zm00001d031120_T004	0.7853478	0.51705762	Zm00001d031120
Zm00001d041186_T006	0.3541203	1.222068986	Zm00001d041186
Zm00001d041186_T001	0.9698611	0.677551046	Zm00001d041186
Zm00001d041186_T005	8.59E-12	2.585111254	Zm00001d041186
Zm00001d041186_T003	0.9235467	0.008732434	Zm00001d041186
Zm00001d041186_T009	0.953745	0.089997259	Zm00001d041186
Zm00001d013008_T001	0.9846714	-0.075779676	Zm00001d013008
Zm00001d035947_T003	1	0.193832142	Zm00001d035947
Zm00001d035947_T001	0.9907928	0.22951427	Zm00001d035947
Zm00001d017170_T002	0.323944	-0.316545647	Zm00001d017170
Zm00001d038859_T002	0.9173494	0.673079095	Zm00001d038859
Zm00001d038859_T010	0.6838014	-0.695832254	Zm00001d038859
Zm00001d042801_T001	0.3212723	-0.847804017	Zm00001d042801
Zm00001d017642_T002	0.2385656	-0.816979486	Zm00001d017642
Zm00001d039001_T002	0.1176287	-1.762575374	Zm00001d039001
Zm00001d024890_T001	0.9765009	0.037567729	Zm00001d024890
Zm00001d033495_T001	0.8580945	0.35469572	Zm00001d033495
Zm00001d027403_T002	0.6398988	-0.22098599	Zm00001d027403
Zm00001d027403_T001	0.6748421	-0.403228214	Zm00001d027403
Zm00001d053304_T001	5.05E-07	-1.313917538	Zm00001d053304
Zm00001d010339_T001	0.8031028	-0.422620692	Zm00001d010339
Zm00001d024492_T001	1	0.220082676	Zm00001d024492
Zm00001d003670_T003	0.2403914	1.462317265	Zm00001d003670
Zm00001d024187_T001	0.7481738	1.158482033	Zm00001d024187
Zm00001d032649_T001	0.954492	-0.022707984	Zm00001d032649
Zm00001d004526_T001	0.9760472	0.60305589	Zm00001d004526
Zm00001d020110_T001	0.8252955	-0.342824672	Zm00001d020110
Zm00001d037429_T001	0.9902733	0.462628761	Zm00001d037429
Zm00001d008795_T001	0.9419336	0.469795968	Zm00001d008795
Zm00001d046396_T001	0.9865038	0.495088405	Zm00001d046396
Zm00001d044202_T001	0.7216201	1.025852877	Zm00001d044202
Zm00001d021435_T001	0.4838289	-0.866954337	Zm00001d021435
Zm00001d029254_T001	0.9673094	0.084392253	Zm00001d029254
Zm00001d051291_T006	0.7187138	0.605959349	Zm00001d051291
Zm00001d051291_T005	0.1350855	1.180326872	Zm00001d051291
Zm00001d051291_T002	0.9706887	0.209703845	Zm00001d051291
Zm00001d007446_T002	0.9782953	0.156189854	Zm00001d007446
Zm00001d007446_T001	0.7658741	-0.647242377	Zm00001d007446
Zm00001d007446_T005	0.8336703	-0.164979114	Zm00001d007446
Zm00001d019672_T001	0.9447869	-0.305104761	Zm00001d019672
Zm00001d019672_T002	0.9361945	0.399457224	Zm00001d019672
Zm00001d015025_T001	0.002233729	3.387877732	Zm00001d015025
Zm00001d054005_T007	1	0.386041044	Zm00001d054005
Zm00001d054005_T001	0.9765009	-0.010016857	Zm00001d054005

Zm00001d017264_T001	0.6818546	-0.201635546	Zm00001d017264
Zm00001d044042_T001	0.9563257	0.071132575	Zm00001d044042
Zm00001d026480_T001	0.1272455	-1.285834967	Zm00001d026480
Zm00001d039762_T002	0.08378101	-1.527370037	Zm00001d039762
Zm00001d052923_T001	0.01477296	1.385817273	Zm00001d052923
Zm00001d016002_T001	1	0.254452699	Zm00001d016002
Zm00001d016002_T002	0.3497147	-0.498435112	Zm00001d016002
Zm00001d016002_T003	0.8492658	0.575789585	Zm00001d016002
Zm00001d046629_T001	1	0.147759708	Zm00001d046629
Zm00001d020828_T001	0.9130008	0.772393375	Zm00001d020828
Zm00001d038376_T002	0.7852993	-0.213716497	Zm00001d038376
Zm00001d038376_T001	0.9945592	-0.000695722	Zm00001d038376
Zm00001d008322_T004	0.9419336	-0.056055311	Zm00001d008322
Zm00001d008322_T008	0.8001345	0.228794043	Zm00001d008322
Zm00001d008322_T005	1	0.179600942	Zm00001d008322
Zm00001d008322_T003	0.9216521	0.025183138	Zm00001d008322
Zm00001d008322_T006	0.000358577	0.999253765	Zm00001d008322
Zm00001d024567_T011	0.7915647	0.942459989	Zm00001d024567
Zm00001d024567_T015	0.000673934	1.637033256	Zm00001d024567
Zm00001d024567_T005	0.8982893	0.600646721	Zm00001d024567
Zm00001d024567_T006	0.9967513	0.101308609	Zm00001d024567
Zm00001d013708_T001	0.2208276	-1.715833558	Zm00001d013708
Zm00001d013708_T002	1	0.109295637	Zm00001d013708
Zm00001d006588_T001	0.9821324	0.131557232	Zm00001d006588
Zm00001d040248_T001	0.3181489	-0.33552211	Zm00001d040248
Zm00001d006714_T001	0.9499689	-0.074792993	Zm00001d006714
Zm00001d044103_T001	0.909729	-0.056680693	Zm00001d044103
Zm00001d044103_T003	0.993158	0.262161163	Zm00001d044103
Zm00001d029389_T001	0.9745433	0.114732616	Zm00001d029389
Zm00001d021962_T001	0.9739189	0.152901446	Zm00001d021962
Zm00001d053605_T001	0.5718096	-0.657843308	Zm00001d053605
Zm00001d013336_T006	0.9738836	0.208050306	Zm00001d013336
Zm00001d013336_T001	0.9828518	0.263232877	Zm00001d013336
Zm00001d048219_T001	0.9016285	0.566418968	Zm00001d048219
Zm00001d038642_T001	0.006878684	-1.751513979	Zm00001d038642
Zm00001d043194_T002	0.8569937	-0.420986925	Zm00001d043194
Zm00001d024990_T001	0.9535734	0.12555533	Zm00001d024990
Zm00001d049730_T002	0.6625969	1.151316279	Zm00001d049730
Zm00001d049730_T003	0.9718747	0.283789776	Zm00001d049730
Zm00001d049730_T001	0.903659	0.612797891	Zm00001d049730
Zm00001d049730_T004	0.2987723	-0.785698996	Zm00001d049730
Zm00001d028952_T002	0.9833133	0.584095647	Zm00001d028952
Zm00001d028952_T003	0.00282118	0.79486967	Zm00001d028952
Zm00001d028952_T006	0.7446163	-0.591796859	Zm00001d028952
Zm00001d028952_T005	0.4063448	0.318770094	Zm00001d028952
Zm00001d028952_T007	1.79E-14	4.016203759	Zm00001d028952
Zm00001d028952_T004	0.9493302	0.2805379	Zm00001d028952
Zm00001d011073_T001	0.01911835	-1.845460086	Zm00001d011073
Zm00001d002446_T001	0.9172003	0.007719862	Zm00001d002446
Zm00001d030863_T006	0.9954426	0.445118795	Zm00001d030863
Zm00001d030863_T005	0.8533785	0.768635208	Zm00001d030863
Zm00001d030863_T001	0.01682926	-1.941343849	Zm00001d030863
Zm00001d030863_T004	2.53E-06	-0.589699719	Zm00001d030863
Zm00001d018884_T003	0.8424588	0.829541045	Zm00001d018884

Zm00001d018884_T013	0.6275807	1.043558715	Zm00001d018884
Zm00001d018884_T008	0.3405091	0.951386333	Zm00001d018884
Zm00001d018884_T021	0.8883561	0.418316389	Zm00001d018884
Zm00001d018884_T004	0.9666757	0.714622982	Zm00001d018884
Zm00001d018884_T002	0.01628935	-0.836441378	Zm00001d018884
Zm00001d018884_T011	0.979187	0.034414613	Zm00001d018884
Zm00001d018884_T010	0.003054744	2.67379254	Zm00001d018884
Zm00001d020941_T001	0.9855117	0.12949454	Zm00001d020941
Zm00001d045169_T001	0.8168128	-0.22155064	Zm00001d045169
Zm00001d024325_T001	0.9529184	0.388349722	Zm00001d024325
Zm00001d024325_T002	0.9749249	0.204853682	Zm00001d024325
Zm00001d031747_T001	0.9253672	0.029966792	Zm00001d031747
Zm00001d031747_T002	0.5006858	-0.572200441	Zm00001d031747
Zm00001d034090_T001	0.7526664	0.94602161	Zm00001d034090
Zm00001d018990_T001	0.684563	-0.465333007	Zm00001d018990
Zm00001d032710_T001	0.8961437	0.710397768	Zm00001d032710
Zm00001d032710_T003	0.4849772	1.25027772	Zm00001d032710
Zm00001d025008_T001	0.9533693	0.109068903	Zm00001d025008
Zm00001d025460_T002	0.05243014	-0.908550322	Zm00001d025460
Zm00001d025460_T001	0.9180865	0.730165229	Zm00001d025460
Zm00001d015284_T001	0.000238001	-1.299145727	Zm00001d015284
Zm00001d014745_T001	0.9760472	0.166931934	Zm00001d014745
Zm00001d046178_T001	0.9855106	0.238996166	Zm00001d046178
Zm00001d047990_T002	0.973825	0.169024794	Zm00001d047990
Zm00001d042931_T001	0.9173494	-0.054956893	Zm00001d042931
Zm00001d020874_T001	0.907301	-0.164964861	Zm00001d020874
Zm00001d042523_T008	0.8611836	0.135963758	Zm00001d042523
Zm00001d042523_T042	0.8142464	-0.197965793	Zm00001d042523
Zm00001d042523_T018	0.01163179	0.578930037	Zm00001d042523
Zm00001d042523_T019	0.9050121	0.08994881	Zm00001d042523
Zm00001d042523_T030	0.0273589	0.862319683	Zm00001d042523
Zm00001d042523_T001	0.8961518	0.468146179	Zm00001d042523
Zm00001d049152_T001	0.557673	1.201922165	Zm00001d049152
Zm00001d009247_T001	0.5577356	-1.459419926	Zm00001d009247
Zm00001d031659_T001	1	0.385981731	Zm00001d031659
Zm00001d024661_T001	0.9846714	0.445165148	Zm00001d024661
Zm00001d026271_T001	0.2304095	-1.585987042	Zm00001d026271
Zm00001d042862_T001	0.9979393	-0.195636848	Zm00001d042862
Zm00001d049913_T001	0.9951827	0.478771562	Zm00001d049913
Zm00001d006947_T001	0.4647287	0.794293097	Zm00001d006947
Zm00001d004843_T003	0.7889615	-0.435318397	Zm00001d004843
Zm00001d003812_T002	0.956184	0.162574313	Zm00001d003812
Zm00001d048959_T001	0.9999526	0.368194969	Zm00001d048959
Zm00001d048959_T002	0.9596377	-0.041173579	Zm00001d048959
Zm00001d051069_T001	0.9859755	0.200466779	Zm00001d051069
Zm00001d041175_T006	0.8786965	0.630107562	Zm00001d041175
Zm00001d041175_T005	0.8888255	-0.122854174	Zm00001d041175
Zm00001d023910_T001	0.9682673	0.522789589	Zm00001d023910
Zm00001d025645_T001	0.1272455	-1.969829742	Zm00001d025645
Zm00001d010782_T009	0.1538332	1.712962491	Zm00001d010782
Zm00001d042676_T001	0.909729	0.808577317	Zm00001d042676
Zm00001d042676_T002	0.9337571	0.502619653	Zm00001d042676
Zm00001d053434_T001	0.1447705	-1.318490104	Zm00001d053434
Zm00001d041700_T006	0.616338	0.548370852	Zm00001d041700

Zm00001d041700_T005	0.7122217	0.262523808	Zm00001d041700
Zm00001d041700_T007	0.02062193	1.125625166	Zm00001d041700
Zm00001d041700_T004	0.04512563	1.311625272	Zm00001d041700
Zm00001d041700_T001	0.8883561	0.094413387	Zm00001d041700
Zm00001d015290_T001	0.9498304	0.701971292	Zm00001d015290
Zm00001d039305_T008	0.7923921	-0.142611362	Zm00001d039305
Zm00001d039305_T001	0.9722469	0.196011627	Zm00001d039305
Zm00001d039305_T003	1	0.005959045	Zm00001d039305
Zm00001d039305_T007	0.5189688	-0.787838578	Zm00001d039305
Zm00001d039211_T002	0.9868533	0.113829924	Zm00001d039211
Zm00001d039211_T003	0.8783081	-0.09964165	Zm00001d039211
Zm00001d039211_T005	0.9926496	0.299855534	Zm00001d039211
Zm00001d050716_T003	0.1530603	1.421931146	Zm00001d050716
Zm00001d050716_T005	0.6814077	-0.267254632	Zm00001d050716
Zm00001d050716_T001	0.9399441	0.53658344	Zm00001d050716
Zm00001d050716_T010	0.9912886	0.545345492	Zm00001d050716
Zm00001d045578_T001	0.8020385	-0.479651615	Zm00001d045578
Zm00001d031668_T001	0.9249382	-0.03804125	Zm00001d031668
Zm00001d032338_T002	0.9944857	0.362023189	Zm00001d032338
Zm00001d032338_T001	0.9852645	0.52305372	Zm00001d032338
Zm00001d046865_T002	0.2015088	-0.425747452	Zm00001d046865
Zm00001d007503_T001	0.6768588	1.152372264	Zm00001d007503
Zm00001d005598_T001	0.9046076	0.46433251	Zm00001d005598
Zm00001d028214_T001	0.7406613	1.115622998	Zm00001d028214
Zm00001d026026_T001	0.9914036	0.14751223	Zm00001d026026
Zm00001d008410_T001	0.5236656	-0.863596901	Zm00001d008410
Zm00001d046449_T003	0.02234554	-0.459424798	Zm00001d046449
Zm00001d046449_T004	0.9892558	0.06704108	Zm00001d046449
Zm00001d046449_T005	0.9677364	0.204019999	Zm00001d046449
Zm00001d046449_T015	4.98E-17	-6.932454886	Zm00001d046449
Zm00001d037828_T001	0.7721703	-0.42852143	Zm00001d037828
Zm00001d038972_T002	0.2093531	0.604035016	Zm00001d038972
Zm00001d038972_T025	0.1279806	0.600984183	Zm00001d038972
Zm00001d038972_T015	0.6989821	-0.331120198	Zm00001d038972
Zm00001d038972_T010	0.2142346	2.045725954	Zm00001d038972
Zm00001d038972_T004	0.6580146	0.651688111	Zm00001d038972
Zm00001d038972_T007	0.8513643	-0.115828958	Zm00001d038972
Zm00001d038972_T001	0.9596377	0.149167072	Zm00001d038972
Zm00001d012917_T001	0.509262	-0.625068941	Zm00001d012917
Zm00001d020538_T001	0.8585804	0.587072272	Zm00001d020538
Zm00001d046363_T001	1	0.204320715	Zm00001d046363
Zm00001d032350_T007	0.9468941	0.695789319	Zm00001d032350
Zm00001d032350_T002	0.7289978	0.553795564	Zm00001d032350
Zm00001d032350_T001	0.9975882	0.445746358	Zm00001d032350
Zm00001d032350_T006	0.937572	0.752482357	Zm00001d032350
Zm00001d032350_T004	0.9693993	0.070034295	Zm00001d032350
Zm00001d028022_T003	0.6501259	1.063097762	Zm00001d028022
Zm00001d028022_T001	0.6275807	0.175355131	Zm00001d028022
Zm00001d049193_T001	0.997764	-0.103887824	Zm00001d049193
Zm00001d020675_T001	0.3128178	1.453784663	Zm00001d020675
Zm00001d029910_T002	0.9771927	0.878174705	Zm00001d029910
Zm00001d029910_T001	0.9690925	0.826833034	Zm00001d029910
Zm00001d044412_T002	0.9936409	0.439410139	Zm00001d044412
Zm00001d016342_T001	0.6655267	-0.582236868	Zm00001d016342

Zm00001d010822_T001	0.9821324	0.377807444	Zm00001d010822
Zm00001d031971_T001	0.866232	0.745667056	Zm00001d031971
Zm00001d031971_T002	0.8113478	0.860834378	Zm00001d031971
Zm00001d044814_T010	0.6284759	0.539203501	Zm00001d044814
Zm00001d044814_T009	0.2038697	1.224413035	Zm00001d044814
Zm00001d044814_T001	0.6182279	1.338944338	Zm00001d044814
Zm00001d044814_T017	0.9311783	0.775242002	Zm00001d044814
Zm00001d044814_T018	0.6934931	0.861731965	Zm00001d044814
Zm00001d044814_T013	0.8881183	0.562061685	Zm00001d044814
Zm00001d033088_T001	0.9073468	0.358037538	Zm00001d033088
Zm00001d051405_T001	0.9959972	0.198847428	Zm00001d051405
Zm00001d012078_T001	0.9416622	-0.109494213	Zm00001d012078
Zm00001d031988_T002	0.4629696	0.642858701	Zm00001d031988
Zm00001d031988_T001	0.9975882	0.361018305	Zm00001d031988
Zm00001d053172_T001	0.9486412	-0.071993901	Zm00001d053172
Zm00001d053172_T002	0.9956119	0.227880927	Zm00001d053172
Zm00001d036825_T007	0.8350279	-0.139674159	Zm00001d036825
Zm00001d036825_T051	1.01E-08	1.007388413	Zm00001d036825
Zm00001d036825_T023	0.6572222	0.80837573	Zm00001d036825
Zm00001d036825_T033	0.9782953	0.115121834	Zm00001d036825
Zm00001d036825_T028	0.1102471	0.793321918	Zm00001d036825
Zm00001d036825_T013	0.04732668	0.770090414	Zm00001d036825
Zm00001d036825_T052	6.56E-06	0.760038951	Zm00001d036825
Zm00001d036825_T002	0.9954426	0.065776819	Zm00001d036825
Zm00001d036825_T065	0.4958139	0.642922131	Zm00001d036825
Zm00001d036825_T055	1	0.150710628	Zm00001d036825
Zm00001d036825_T016	0.9892609	0.241771235	Zm00001d036825
Zm00001d036825_T073	0.222474	-0.868478593	Zm00001d036825
Zm00001d036825_T010	0.9173494	0.138651346	Zm00001d036825
Zm00001d036825_T026	0.497443	0.267415118	Zm00001d036825
Zm00001d036707_T001	0.4422995	1.387032827	Zm00001d036707
Zm00001d036707_T006	0.01893825	-1.290293299	Zm00001d036707
Zm00001d030343_T001	0.9979568	0.401739365	Zm00001d030343
Zm00001d009599_T001	0.5913892	0.705856421	Zm00001d009599
Zm00001d016491_T001	0.7485644	-0.461084519	Zm00001d016491
Zm00001d033186_T001	0.9281046	-0.307042673	Zm00001d033186
Zm00001d027794_T001	0.8581679	-0.522111652	Zm00001d027794
Zm00001d027794_T002	0.9252088	-0.297754932	Zm00001d027794
Zm00001d018324_T001	0.4052048	-1.02728499	Zm00001d018324
Zm00001d029988_T001	0.9469426	0.056008454	Zm00001d029988
Zm00001d005997_T001	0.3706063	-0.758182346	Zm00001d005997
Zm00001d005997_T002	0.819501	0.985143616	Zm00001d005997
Zm00001d019856_T007	0.2619882	1.315232091	Zm00001d019856
Zm00001d019856_T010	0.08451169	0.484874566	Zm00001d019856
Zm00001d019856_T009	0.8686467	0.171948357	Zm00001d019856
Zm00001d019856_T003	0.7506084	-0.220355348	Zm00001d019856
Zm00001d026463_T001	0.954492	0.077825253	Zm00001d026463
Zm00001d037659_T001	0.8906466	0.433482449	Zm00001d037659
Zm00001d003212_T001	0.8625537	0.695911594	Zm00001d003212
Zm00001d003212_T003	0.644271	1.100649871	Zm00001d003212
Zm00001d012749_T001	0.556323	1.278402448	Zm00001d012749
Zm00001d046200_T002	0.05256956	-0.50244202	Zm00001d046200
Zm00001d046200_T005	0.9024791	-0.12015489	Zm00001d046200
Zm00001d046200_T004	1	0.1144341	Zm00001d046200

Zm00001d028096_T031	0.6086477	0.760939972	Zm00001d028096
Zm00001d028096_T004	0.9173494	-0.125791332	Zm00001d028096
Zm00001d040748_T039	0.3202116	0.968621349	Zm00001d040748
Zm00001d040748_T048	0.7603304	1.237616901	Zm00001d040748
Zm00001d040748_T063	0.2090094	0.603526406	Zm00001d040748
Zm00001d040748_T037	0.5495068	0.313846772	Zm00001d040748
Zm00001d040748_T034	8.97E-11	1.821489308	Zm00001d040748
Zm00001d040748_T021	0.5196715	0.394038798	Zm00001d040748
Zm00001d040748_T087	0.4801008	1.05581097	Zm00001d040748
Zm00001d040748_T035	7.36E-13	-2.929986623	Zm00001d040748
Zm00001d041518_T001	0.8832328	0.675151483	Zm00001d041518
Zm00001d039498_T002	0.9897446	0.43110109	Zm00001d039498
Zm00001d039498_T003	0.6556118	0.472667996	Zm00001d039498
Zm00001d053548_T003	0.907301	-0.037150016	Zm00001d053548
Zm00001d053548_T004	0.7138265	1.066989525	Zm00001d053548
Zm00001d039316_T001	0.9954444	0.125052907	Zm00001d039316
Zm00001d039316_T004	0.9846446	0.178228668	Zm00001d039316
Zm00001d026066_T001	0.9173494	-0.042752048	Zm00001d026066
Zm00001d034064_T001	0.972985	0.23054147	Zm00001d034064
Zm00001d051840_T004	0.3070924	-1.535414823	Zm00001d051840
Zm00001d051840_T001	0.3195751	1.664566602	Zm00001d051840
Zm00001d051840_T003	0.3693044	-1.162366503	Zm00001d051840
Zm00001d013271_T001	0.9942835	0.261701216	Zm00001d013271
Zm00001d013935_T008	0.2731306	-0.824517215	Zm00001d013935
Zm00001d013935_T004	0.4583711	0.599212011	Zm00001d013935
Zm00001d013935_T003	0.9410845	0.633478362	Zm00001d013935
Zm00001d013935_T009	0.9214856	0.384275683	Zm00001d013935
Zm00001d013935_T006	0.6724646	1.18515311	Zm00001d013935
Zm00001d038998_T001	0.918657	0.602728117	Zm00001d038998
Zm00001d038998_T003	0.9224378	0.658011933	Zm00001d038998
Zm00001d045204_T001	0.8667498	-0.288786236	Zm00001d045204
Zm00001d041083_T016	0.8580945	0.257906523	Zm00001d041083
Zm00001d041083_T003	0.000455444	-1.042321692	Zm00001d041083
Zm00001d041083_T005	0.8113478	-0.188146052	Zm00001d041083
Zm00001d041083_T004	0.02963837	0.865508945	Zm00001d041083
Zm00001d041083_T014	0.6468965	0.519033045	Zm00001d041083
Zm00001d041083_T006	0.03715458	0.535437422	Zm00001d041083
Zm00001d041083_T017	0.342013	0.530064224	Zm00001d041083
Zm00001d041083_T010	0.000368021	0.391444904	Zm00001d041083
Zm00001d041083_T009	0.01122327	1.009742436	Zm00001d041083
Zm00001d041083_T008	0.2754622	0.44182999	Zm00001d041083
Zm00001d041083_T030	0.9645314	0.04195435	Zm00001d041083
Zm00001d041083_T015	0.8647028	0.392303246	Zm00001d041083
Zm00001d041083_T029	0.9440782	-0.049059099	Zm00001d041083
Zm00001d036404_T003	0.9003815	0.197731648	Zm00001d036404
Zm00001d022582_T002	0.8791564	0.895939547	Zm00001d022582
Zm00001d024083_T001	0.9979568	0.245292996	Zm00001d024083
Zm00001d038977_T001	0.6472815	-0.919955305	Zm00001d038977
Zm00001d021752_T001	0.5567925	1.361595192	Zm00001d021752
Zm00001d001811_T001	0.9446379	-0.026312488	Zm00001d001811
Zm00001d050489_T001	0.9554387	0.599968354	Zm00001d050489
Zm00001d002066_T001	0.9722469	-0.004695635	Zm00001d002066
Zm00001d023239_T001	0.9612591	0.151612618	Zm00001d023239
Zm00001d013797_T003	0.9451369	0.43071491	Zm00001d013797

Zm00001d013797_T001	0.7289978	-0.277865776	Zm00001d013797
Zm00001d013797_T006	0.8821167	0.516011626	Zm00001d013797
Zm00001d013797_T007	0.5963791	1.143806093	Zm00001d013797
Zm00001d013797_T002	0.6250123	0.405639502	Zm00001d013797
Zm00001d013797_T005	0.7831859	0.577827779	Zm00001d013797
Zm00001d033925_T001	0.8624945	-0.181529095	Zm00001d033925
Zm00001d043920_T001	0.7080786	0.661597246	Zm00001d043920
Zm00001d044734_T001	0.05998958	1.228285032	Zm00001d044734
Zm00001d018811_T001	0.01548639	1.801230732	Zm00001d018811
Zm00001d005451_T070	0.8533785	0.996257107	Zm00001d005451
Zm00001d005451_T012	9.47E-16	3.932071213	Zm00001d005451
Zm00001d005451_T002	1.00E-11	-1.59344251	Zm00001d005451
Zm00001d005451_T001	0.01175716	2.731821705	Zm00001d005451
Zm00001d007940_T002	0.8414741	-0.496465902	Zm00001d007940
Zm00001d043508_T002	0.9912886	0.193319736	Zm00001d043508
Zm00001d043508_T001	0.9337571	-0.043444259	Zm00001d043508
Zm00001d009627_T002	0.7751076	0.5603695	Zm00001d009627
Zm00001d009627_T001	0.9173494	-0.033415601	Zm00001d009627
Zm00001d012564_T001	0.900661	-0.087717282	Zm00001d012564
Zm00001d029333_T012	0.8001345	-0.147462205	Zm00001d029333
Zm00001d029333_T009	0.3202116	0.740458905	Zm00001d029333
Zm00001d029333_T014	0.8720862	-0.093303799	Zm00001d029333
Zm00001d029333_T008	0.6129837	0.835980381	Zm00001d029333
Zm00001d028107_T001	0.962254	0.018969987	Zm00001d028107
Zm00001d038995_T001	0.9979568	-0.277855309	Zm00001d038995
Zm00001d038995_T002	0.9596377	-0.04065903	Zm00001d038995
Zm00001d018529_T001	0.9293689	-0.001569068	Zm00001d018529
Zm00001d031860_T005	0.6577149	-0.277907128	Zm00001d031860
Zm00001d031860_T004	0.9312703	-0.01437877	Zm00001d031860
Zm00001d025508_T001	0.6782305	0.625419825	Zm00001d025508
Zm00001d002496_T001	0.889992	0.700470425	Zm00001d002496
Zm00001d028994_T005	0.06265963	-0.772728023	Zm00001d028994
Zm00001d028994_T010	0.4184832	0.5016959	Zm00001d028994
Zm00001d028994_T009	0.8307555	1.265872477	Zm00001d028994
Zm00001d038185_T001	0.8371282	-0.199431639	Zm00001d038185
Zm00001d011534_T002	0.6411729	-0.80857335	Zm00001d011534
Zm00001d011534_T003	0.7751061	-0.522648919	Zm00001d011534
Zm00001d029048_T001	1	0.360809124	Zm00001d029048
Zm00001d002241_T001	0.9872831	0.440502579	Zm00001d002241
Zm00001d017651_T001	0.9673473	0.572656282	Zm00001d017651
Zm00001d032517_T002	0.962254	0.477283307	Zm00001d032517
Zm00001d032517_T001	0.8113478	0.495299996	Zm00001d032517
Zm00001d002154_T002	0.965836	0.016654124	Zm00001d002154
Zm00001d002154_T006	0.9037324	-0.048427482	Zm00001d002154
Zm00001d002154_T005	0.9817074	0.338199084	Zm00001d002154
Zm00001d002154_T008	0.5204602	-0.456130114	Zm00001d002154
Zm00001d044401_T001	0.9779851	0.279543399	Zm00001d044401
Zm00001d052719_T002	0.7136625	-0.480739767	Zm00001d052719
Zm00001d010434_T001	0.5050116	-1.130762811	Zm00001d010434
Zm00001d007295_T003	0.3082157	0.596265267	Zm00001d007295
Zm00001d007295_T002	0.204983	1.484289131	Zm00001d007295
Zm00001d007295_T001	0.6958637	-0.377089256	Zm00001d007295
Zm00001d040203_T008	0.000169449	0.816452712	Zm00001d040203
Zm00001d040203_T001	0.910709	0.738041738	Zm00001d040203

Zm00001d021138_T003	0.04092962	-0.508593297	Zm00001d021138
Zm00001d021138_T005	0.5079763	0.519389439	Zm00001d021138
Zm00001d021138_T004	0.9422098	0.679790816	Zm00001d021138
Zm00001d014947_T001	0.5053493	-0.637740127	Zm00001d014947
Zm00001d014180_T006	0.050072	-0.254218467	Zm00001d014180
Zm00001d014180_T014	0.7682041	-0.18258218	Zm00001d014180
Zm00001d047849_T001	0.9706293	0.1443764	Zm00001d047849
Zm00001d049449_T003	0.7626242	1.116831495	Zm00001d049449
Zm00001d049449_T005	0.8455957	1.148763696	Zm00001d049449
Zm00001d049449_T007	0.03525539	-0.693265506	Zm00001d049449
Zm00001d016655_T001	0.1311444	-1.411652825	Zm00001d016655
Zm00001d002748_T001	0.963405	0.158872006	Zm00001d002748
Zm00001d048609_T001	0.6166973	-0.626056597	Zm00001d048609
Zm00001d016406_T015	0.1941301	-0.772854599	Zm00001d016406
Zm00001d016406_T001	0.3105729	0.731699866	Zm00001d016406
Zm00001d016406_T009	0.000197126	1.312791338	Zm00001d016406
Zm00001d016406_T013	0.7438361	0.586763417	Zm00001d016406
Zm00001d016406_T017	0.6466858	-0.099822178	Zm00001d016406
Zm00001d016406_T016	0.7336597	0.235246854	Zm00001d016406
Zm00001d016406_T004	0.9740325	0.233713221	Zm00001d016406
Zm00001d053963_T002	0.7483274	-0.454397439	Zm00001d053963
Zm00001d053963_T003	0.9664406	-0.033687113	Zm00001d053963
Zm00001d053963_T001	0.9340079	0.008989306	Zm00001d053963
Zm00001d043595_T005	0.1447705	0.597333013	Zm00001d043595
Zm00001d043595_T004	0.9184323	0.771545543	Zm00001d043595
Zm00001d013225_T005	0.470047	0.799533208	Zm00001d013225
Zm00001d013225_T003	0.1439251	0.894112446	Zm00001d013225
Zm00001d013225_T001	0.9416622	-0.01102527	Zm00001d013225
Zm00001d006502_T001	0.9194411	-0.596532189	Zm00001d006502
Zm00001d000008_T001	0.768152	0.765594523	Zm00001d000008
Zm00001d043520_T001	0.2489636	-0.952381942	Zm00001d043520
Zm00001d030301_T003	0.9907928	0.060794431	Zm00001d030301
Zm00001d030301_T002	0.6201746	0.747413744	Zm00001d030301
Zm00001d007531_T001	0.987516	0.285668752	Zm00001d007531
Zm00001d024001_T001	0.7946251	-0.260591188	Zm00001d024001
Zm00001d043170_T001	0.9441469	-0.338910204	Zm00001d043170
Zm00001d052198_T009	0.8537544	-0.090628047	Zm00001d052198
Zm00001d052198_T001	0.7451122	0.96641173	Zm00001d052198
Zm00001d052198_T003	0.8846418	-0.161881192	Zm00001d052198
Zm00001d052198_T008	0.6466858	0.604130031	Zm00001d052198
Zm00001d045174_T001	0.5842609	-0.991929586	Zm00001d045174
Zm00001d020117_T002	0.01306269	0.970091232	Zm00001d020117
Zm00001d020117_T001	0.2329183	1.171897619	Zm00001d020117
Zm00001d028868_T001	0.9201237	0.031837064	Zm00001d028868
Zm00001d028868_T002	0.843747	0.499330005	Zm00001d028868
Zm00001d020374_T001	0.001211642	0.432686273	Zm00001d020374
Zm00001d020374_T002	1	0.279436225	Zm00001d020374
Zm00001d014685_T009	0.5853988	1.172097197	Zm00001d014685
Zm00001d014685_T005	0.1826126	0.272082139	Zm00001d014685
Zm00001d014685_T002	0.91126	0.16313015	Zm00001d014685
Zm00001d005007_T001	0.03648535	-1.777586222	Zm00001d005007
Zm00001d032139_T002	0.8219372	-0.470955394	Zm00001d032139
Zm00001d032139_T005	0.9242611	0.263398183	Zm00001d032139
Zm00001d037258_T002	0.9184323	-0.013989558	Zm00001d037258

Zm00001d037258_T001	0.5316541	-0.510587317	Zm00001d037258
Zm00001d012884_T001	0.941922	0.055458097	Zm00001d012884
Zm00001d012884_T007	0.2326127	0.775882564	Zm00001d012884
Zm00001d003037_T001	0.3861376	1.242723351	Zm00001d003037
Zm00001d003445_T001	0.8376343	0.496333279	Zm00001d003445
Zm00001d003445_T002	0.3020017	1.235668646	Zm00001d003445
Zm00001d018545_T001	0.9894218	0.267556396	Zm00001d018545
Zm00001d018545_T003	0.289909	-1.149619704	Zm00001d018545
Zm00001d020392_T002	0.9147333	-0.067254033	Zm00001d020392
Zm00001d010116_T001	0.9771927	0.136168666	Zm00001d010116
Zm00001d004960_T004	1	0.261896386	Zm00001d004960
Zm00001d004960_T001	0.8993062	-0.030276209	Zm00001d004960
Zm00001d017213_T001	0.9513815	0.091963112	Zm00001d017213
Zm00001d029410_T001	0.9701986	0.564055857	Zm00001d029410
Zm00001d029410_T002	1	0.210201996	Zm00001d029410
Zm00001d007339_T002	0.8894168	-0.041329932	Zm00001d007339
Zm00001d007339_T001	0.7603034	-0.356167192	Zm00001d007339
Zm00001d003202_T001	0.5890036	0.867150164	Zm00001d003202
Zm00001d035039_T001	0.4029638	1.625209845	Zm00001d035039
Zm00001d045479_T001	0.7328249	1.122373744	Zm00001d045479
Zm00001d043317_T001	0.7091158	-0.474174909	Zm00001d043317
Zm00001d033869_T001	0.9825858	0.285245858	Zm00001d033869
Zm00001d036771_T001	0.5605484	0.581679402	Zm00001d036771
Zm00001d033366_T002	0.9543237	0.102361686	Zm00001d033366
Zm00001d038795_T001	0.9851204	1.470141142	Zm00001d038795
Zm00001d009652_T002	0.2910059	1.657987356	Zm00001d009652
Zm00001d009652_T006	0.6053962	-1.024341526	Zm00001d009652
Zm00001d009652_T004	0.6730524	-0.816826777	Zm00001d009652
Zm00001d009652_T005	0.4233271	-0.498124792	Zm00001d009652
Zm00001d051889_T002	0.9892044	0.250004883	Zm00001d051889
Zm00001d051889_T001	0.629494	-0.226999406	Zm00001d051889
Zm00001d021017_T001	0.4263859	-1.139200044	Zm00001d021017
Zm00001d018842_T001	0.9760472	0.567771618	Zm00001d018842
Zm00001d052162_T001	0.4114435	-1.03490564	Zm00001d052162
Zm00001d031518_T015	6.72E-09	-1.831864489	Zm00001d031518
Zm00001d031518_T006	0.3763648	1.268672578	Zm00001d031518
Zm00001d031518_T012	0.399447	0.703125744	Zm00001d031518
Zm00001d038161_T001	0.5715414	-0.380364931	Zm00001d038161
Zm00001d018377_T003	0.3777434	0.79279588	Zm00001d018377
Zm00001d018377_T010	0.9832612	0.281503963	Zm00001d018377
Zm00001d018377_T002	1	0.392948385	Zm00001d018377
Zm00001d042706_T002	0.4825809	0.495436203	Zm00001d042706
Zm00001d038885_T001	1	0.393768369	Zm00001d038885
Zm00001d038885_T002	0.9265089	0.166759061	Zm00001d038885
Zm00001d048682_T001	0.7807489	-0.14479359	Zm00001d048682
Zm00001d045606_T004	0.9768996	0.15420217	Zm00001d045606
Zm00001d025734_T017	0.827438	0.538256909	Zm00001d025734
Zm00001d025734_T008	0.995534	0.409178559	Zm00001d025734
Zm00001d025734_T009	0.02178755	1.99461051	Zm00001d025734
Zm00001d025734_T003	0.9486412	0.083149102	Zm00001d025734
Zm00001d025734_T002	1	0.288773037	Zm00001d025734
Zm00001d025734_T015	0.004247367	2.331555383	Zm00001d025734
Zm00001d025734_T014	0.220755	-0.745139088	Zm00001d025734
Zm00001d042142_T001	0.9303773	0.525489453	Zm00001d042142

Zm00001d029378_T003	0.9659036	0.529692477	Zm00001d029378
Zm00001d029378_T007	0.6417266	0.256350844	Zm00001d029378
Zm00001d029378_T002	0.9413175	0.105673972	Zm00001d029378
Zm00001d029378_T005	0.963405	0.022336987	Zm00001d029378
Zm00001d029378_T001	9.65E-08	-0.912973111	Zm00001d029378
Zm00001d029378_T010	0.3183965	0.781856964	Zm00001d029378
Zm00001d029378_T004	0.7697196	0.225188849	Zm00001d029378
Zm00001d029378_T016	0.01637601	-2.307094606	Zm00001d029378
Zm00001d048574_T003	0.5134498	-1.23136508	Zm00001d048574
Zm00001d048574_T002	1	0.408945911	Zm00001d048574
Zm00001d053979_T001	0.5966057	-0.864953982	Zm00001d053979
Zm00001d051005_T001	1	0.25438548	Zm00001d051005
Zm00001d014863_T001	0.8678673	-0.095044804	Zm00001d014863
Zm00001d014863_T002	0.9873876	0.176103084	Zm00001d014863
Zm00001d009600_T003	0.5507795	0.457950868	Zm00001d009600
Zm00001d009600_T035	0.6137117	0.584443672	Zm00001d009600
Zm00001d009600_T031	0.003968047	1.083854428	Zm00001d009600
Zm00001d009600_T017	0.8705753	-0.183050077	Zm00001d009600
Zm00001d009600_T011	0.2292281	-0.751009905	Zm00001d009600
Zm00001d009600_T036	0.9300784	0.089875342	Zm00001d009600
Zm00001d009600_T024	0.8050292	0.527244738	Zm00001d009600
Zm00001d017397_T001	0.2314949	-1.245455398	Zm00001d017397
Zm00001d027859_T001	0.7253613	-0.436594582	Zm00001d027859
Zm00001d010880_T003	0.2666056	0.933983667	Zm00001d010880
Zm00001d010880_T002	0.9326774	0.038981285	Zm00001d010880
Zm00001d001860_T001	0.640854	0.834550337	Zm00001d001860
Zm00001d029052_T001	0.6620718	0.843270531	Zm00001d029052
Zm00001d043091_T003	0.6053962	1.340624815	Zm00001d043091
Zm00001d043091_T005	0.4832366	0.697359173	Zm00001d043091
Zm00001d043091_T001	0.4461521	0.839704978	Zm00001d043091
Zm00001d043091_T004	0.4825899	1.098691584	Zm00001d043091
Zm00001d016704_T001	1	0.325585897	Zm00001d016704
Zm00001d045482_T010	0.9454034	0.068619638	Zm00001d045482
Zm00001d045482_T014	0.6260085	0.725041634	Zm00001d045482
Zm00001d045482_T001	0.955464	0.711536342	Zm00001d045482
Zm00001d045482_T013	0.8795714	-0.17538101	Zm00001d045482
Zm00001d045482_T009	0.9785005	0.105570894	Zm00001d045482
Zm00001d045482_T012	0.9708927	0.603358225	Zm00001d045482
Zm00001d014065_T001	0.9467328	-0.027659802	Zm00001d014065
Zm00001d034145_T001	0.5890036	1.029294481	Zm00001d034145
Zm00001d018183_T001	0.9703357	0.607449704	Zm00001d018183
Zm00001d034915_T005	0.9503115	0.018400493	Zm00001d034915
Zm00001d034915_T004	0.8656238	0.477099414	Zm00001d034915
Zm00001d034915_T002	0.9091175	-0.024213214	Zm00001d034915
Zm00001d041850_T001	0.7437035	-0.294095907	Zm00001d041850
Zm00001d017612_T001	0.02786903	2.550252581	Zm00001d017612
Zm00001d034188_T001	0.9048001	0.730106809	Zm00001d034188
Zm00001d034188_T003	0.000772007	2.781620371	Zm00001d034188
Zm00001d034188_T002	0.7158349	1.234834762	Zm00001d034188
Zm00001d049054_T002	0.910604	0.633257748	Zm00001d049054
Zm00001d050988_T001	0.7631455	-0.46269078	Zm00001d050988
Zm00001d018082_T001	0.9889197	0.218869368	Zm00001d018082
Zm00001d019631_T004	0.9956046	0.481019081	Zm00001d019631
Zm00001d019631_T010	0.9846714	0.568162579	Zm00001d019631

Zm00001d019631_T006	0.9037807	-0.092647972	Zm00001d019631
Zm00001d019631_T015	0.000538353	1.427540545	Zm00001d019631
Zm00001d012321_T001	0.003745035	2.780282822	Zm00001d012321
Zm00001d027463_T001	0.9361945	0.445966488	Zm00001d027463
Zm00001d008177_T001	0.9847136	0.509793086	Zm00001d008177
Zm00001d037899_T001	1	0.22947515	Zm00001d037899
Zm00001d038300_T002	0.2153519	-1.117844461	Zm00001d038300
Zm00001d038300_T001	0.04008706	-1.736191264	Zm00001d038300
Zm00001d043997_T007	0.9821324	0.49861512	Zm00001d043997
Zm00001d043997_T010	0.07677929	-1.506600973	Zm00001d043997
Zm00001d043997_T002	0.9031129	0.636179757	Zm00001d043997
Zm00001d043997_T008	0.9429712	-0.012454678	Zm00001d043997
Zm00001d043997_T004	0.9914064	0.125494442	Zm00001d043997
Zm00001d040222_T001	0.7312651	0.335744798	Zm00001d040222
Zm00001d034366_T004	0.9907928	0.576187491	Zm00001d034366
Zm00001d034366_T005	0.007750285	0.845203616	Zm00001d034366
Zm00001d034366_T017	0.8533785	-0.108890627	Zm00001d034366
Zm00001d034366_T010	0.1297822	1.04155844	Zm00001d034366
Zm00001d034366_T008	0.8158416	0.040243628	Zm00001d034366
Zm00001d002836_T004	0.9563232	0.43203717	Zm00001d002836
Zm00001d002836_T006	1	0.28318117	Zm00001d002836
Zm00001d039685_T001	0.9792387	0.228457229	Zm00001d039685
Zm00001d048546_T004	0.000599858	0.902065668	Zm00001d048546
Zm00001d048546_T016	0.9377241	0.063658311	Zm00001d048546
Zm00001d048546_T001	0.4988467	-0.048379118	Zm00001d048546
Zm00001d048546_T005	0.5448104	0.249061229	Zm00001d048546
Zm00001d048546_T013	1.50E-11	1.757681682	Zm00001d048546
Zm00001d048546_T033	0.5254702	-0.578014063	Zm00001d048546
Zm00001d048546_T011	0.1420257	0.408444265	Zm00001d048546
Zm00001d048546_T014	0.9501111	0.184369004	Zm00001d048546
Zm00001d012091_T001	0.8650248	0.846861356	Zm00001d012091
Zm00001d015662_T001	0.8099107	-0.522828166	Zm00001d015662
Zm00001d014957_T012	0.9609506	0.402277618	Zm00001d014957
Zm00001d014957_T007	1.19E-08	-2.760105114	Zm00001d014957
Zm00001d014957_T001	0.9975882	0.323813082	Zm00001d014957
Zm00001d014957_T005	0.8681121	-0.657249851	Zm00001d014957
Zm00001d019344_T001	0.9061819	-0.411977714	Zm00001d019344
Zm00001d019344_T002	0.8155407	-0.375022974	Zm00001d019344
Zm00001d046135_T001	0.954492	0.083562972	Zm00001d046135
Zm00001d046135_T002	0.5637372	1.087579497	Zm00001d046135
Zm00001d003679_T004	1	0.063797973	Zm00001d003679
Zm00001d003679_T006	0.2157717	1.605938099	Zm00001d003679
Zm00001d003679_T001	6.83E-11	-2.623422889	Zm00001d003679
Zm00001d017419_T001	0.7998707	0.930857047	Zm00001d017419
Zm00001d034030_T001	0.07883407	0.743267509	Zm00001d034030
Zm00001d034030_T014	0.4060281	-0.585396005	Zm00001d034030
Zm00001d047005_T001	0.4808446	-0.996075989	Zm00001d047005
Zm00001d045118_T001	0.3411233	-1.234817275	Zm00001d045118
Zm00001d036796_T002	0.9135067	-0.070509762	Zm00001d036796
Zm00001d036796_T003	0.9030616	0.58234578	Zm00001d036796
Zm00001d036796_T004	0.9173494	-0.025986979	Zm00001d036796
Zm00001d032530_T001	0.9719113	0.15883842	Zm00001d032530
Zm00001d022360_T002	0.8287709	-0.171558605	Zm00001d022360
Zm00001d022360_T004	0.7435861	0.850209807	Zm00001d022360

Zm00001d022360_T003	0.3161129	-0.5966226	Zm00001d022360
Zm00001d047424_T001	0.7971012	-0.241118605	Zm00001d047424
Zm00001d043200_T001	0.5418278	0.88416337	Zm00001d043200
Zm00001d006097_T001	0.4373102	-0.734276007	Zm00001d006097
Zm00001d050628_T001	0.01381559	2.151506288	Zm00001d050628
Zm00001d023694_T006	0.2368548	-0.790690826	Zm00001d023694
Zm00001d023694_T001	1	0.367778979	Zm00001d023694
Zm00001d023694_T007	0.9659036	0.482899405	Zm00001d023694
Zm00001d023694_T005	0.8050292	0.662333314	Zm00001d023694
Zm00001d013208_T001	0.06978913	2.058551431	Zm00001d013208
Zm00001d053841_T008	0.03797568	1.705546869	Zm00001d053841
Zm00001d053841_T012	0.8710199	0.671703116	Zm00001d053841
Zm00001d053841_T011	1	0.103075784	Zm00001d053841
Zm00001d053841_T001	0.8209981	0.849721226	Zm00001d053841
Zm00001d053841_T014	0.8573221	1.022148745	Zm00001d053841
Zm00001d053841_T009	0.1476397	-0.62526085	Zm00001d053841
Zm00001d053841_T010	2.35E-09	1.557343119	Zm00001d053841
Zm00001d053841_T005	9.56E-13	-3.084457996	Zm00001d053841
Zm00001d044704_T018	0.9380783	0.312971806	Zm00001d044704
Zm00001d044704_T005	0.9402255	0.008907203	Zm00001d044704
Zm00001d044704_T019	0.8993062	-0.102717472	Zm00001d044704
Zm00001d044704_T012	0.1512123	0.44861273	Zm00001d044704
Zm00001d044704_T002	0.927255	0.22315638	Zm00001d044704
Zm00001d044704_T014	0.6826913	0.261463743	Zm00001d044704
Zm00001d013503_T004	0.9207666	-0.069393535	Zm00001d013503
Zm00001d013503_T003	0.9297869	0.038785552	Zm00001d013503
Zm00001d013503_T001	0.9596377	0.145630187	Zm00001d013503
Zm00001d004340_T002	0.9791729	0.451927974	Zm00001d004340
Zm00001d029361_T001	0.8043459	-0.657370455	Zm00001d029361
Zm00001d030666_T027	0.1769716	0.232982071	Zm00001d030666
Zm00001d030666_T013	3.55E-11	1.423094876	Zm00001d030666
Zm00001d030666_T002	1.35E-09	0.816965495	Zm00001d030666
Zm00001d030666_T130	0.9550193	0.16656827	Zm00001d030666
Zm00001d030666_T128	0.9596377	0.52766834	Zm00001d030666
Zm00001d030666_T006	0.1453031	0.540921717	Zm00001d030666
Zm00001d030666_T110	0.07431695	-0.732001359	Zm00001d030666
Zm00001d030666_T048	0.1178563	-0.699486985	Zm00001d030666
Zm00001d030666_T004	0.3799352	0.443626617	Zm00001d030666
Zm00001d016029_T001	0.9822138	0.126292828	Zm00001d016029
Zm00001d016029_T002	0.9184323	0.385157389	Zm00001d016029
Zm00001d024396_T001	0.09603243	0.509145744	Zm00001d024396
Zm00001d026182_T001	0.9945076	0.111874335	Zm00001d026182
Zm00001d019123_T004	0.3896536	0.941192731	Zm00001d019123
Zm00001d019123_T001	0.8883002	0.787243837	Zm00001d019123
Zm00001d019123_T003	0.9207666	0.386648228	Zm00001d019123
Zm00001d014906_T007	0.9795655	0.185696259	Zm00001d014906
Zm00001d014906_T005	0.9171885	0.816708809	Zm00001d014906
Zm00001d014906_T002	0.03180481	0.838809968	Zm00001d014906
Zm00001d014906_T004	1	0.159435408	Zm00001d014906
Zm00001d043681_T001	1	0.25636451	Zm00001d043681
Zm00001d023792_T016	0.9356767	-0.019504428	Zm00001d023792
Zm00001d023792_T009	0.530398	-0.373570451	Zm00001d023792
Zm00001d023792_T001	0.06021621	1.036096222	Zm00001d023792
Zm00001d023792_T014	0.1447705	0.591005714	Zm00001d023792

Zm00001d023792_T008	0.9091907	0.491329343	Zm00001d023792
Zm00001d023792_T012	0.4237296	0.27152111	Zm00001d023792
Zm00001d040109_T001	0.2913798	-1.078848266	Zm00001d040109
Zm00001d024919_T001	0.9860836	0.148504306	Zm00001d024919
Zm00001d036386_T001	1	0.352614979	Zm00001d036386
Zm00001d003103_T001	0.8443172	-0.199610923	Zm00001d003103
Zm00001d036293_T003	0.5421999	-0.651713872	Zm00001d036293
Zm00001d036293_T001	0.9918302	0.211268984	Zm00001d036293
Zm00001d036293_T002	0.9673473	-0.005528287	Zm00001d036293
Zm00001d003549_T001	0.9887889	0.130591756	Zm00001d003549
Zm00001d007822_T001	0.8300901	-0.556570137	Zm00001d007822
Zm00001d007075_T011	1.65E-09	0.941164352	Zm00001d007075
Zm00001d007075_T075	0.000305096	0.580309143	Zm00001d007075
Zm00001d007075_T073	0.9988864	0.232172974	Zm00001d007075
Zm00001d007075_T065	0.5016566	1.25835792	Zm00001d007075
Zm00001d007075_T080	0.971204	0.221518311	Zm00001d007075
Zm00001d007075_T064	1.33E-06	-0.373860792	Zm00001d007075
Zm00001d007075_T008	0.3387965	1.499108545	Zm00001d007075
Zm00001d002610_T001	0.9543425	0.389258306	Zm00001d002610
Zm00001d013885_T001	0.9868533	0.399222716	Zm00001d013885
Zm00001d046260_T001	0.3221433	-0.615738027	Zm00001d046260
Zm00001d044970_T001	0.9337571	0.545067374	Zm00001d044970
Zm00001d022000_T049	0.000553383	0.734019485	Zm00001d022000
Zm00001d022000_T026	0.7619918	0.354359729	Zm00001d022000
Zm00001d022000_T052	0.277436	-0.863059076	Zm00001d022000
Zm00001d022000_T012	0.9781672	0.089343251	Zm00001d022000
Zm00001d022000_T056	0.5810332	-0.13141327	Zm00001d022000
Zm00001d022000_T020	0.6974118	0.788730401	Zm00001d022000
Zm00001d022000_T053	0.9668789	0.525738094	Zm00001d022000
Zm00001d022000_T003	0.6968709	0.471038551	Zm00001d022000
Zm00001d022000_T046	0.1787789	-0.432488001	Zm00001d022000
Zm00001d022000_T009	0.6783996	0.953999247	Zm00001d022000
Zm00001d022000_T051	8.79E-12	-2.531505966	Zm00001d022000
Zm00001d022000_T030	0.08012154	0.340624457	Zm00001d022000
Zm00001d022000_T022	0.7680039	0.634813286	Zm00001d022000
Zm00001d022000_T029	1	0.241152667	Zm00001d022000
Zm00001d022000_T058	0.8660897	-0.088168866	Zm00001d022000
Zm00001d022000_T057	0.7982818	0.650374971	Zm00001d022000
Zm00001d022000_T011	0.8896281	-0.046873479	Zm00001d022000
Zm00001d022000_T047	0.7353854	-0.15388058	Zm00001d022000
Zm00001d022000_T059	0.1455866	-1.618648323	Zm00001d022000
Zm00001d022000_T014	0.005246483	1.588142954	Zm00001d022000
Zm00001d022000_T040	0.4418316	0.584700809	Zm00001d022000
Zm00001d022000_T004	0.05931006	-0.767978455	Zm00001d022000
Zm00001d022000_T033	0.5622303	0.403743591	Zm00001d022000
Zm00001d022000_T010	0.000167404	1.86249413	Zm00001d022000
Zm00001d022000_T054	0.8357582	0.194483199	Zm00001d022000
Zm00001d049416_T001	0.9791729	0.237972042	Zm00001d049416
Zm00001d049416_T003	0.9840043	0.24443391	Zm00001d049416
Zm00001d019990_T001	0.8993062	-0.091317104	Zm00001d019990
Zm00001d023811_T001	0.477159	-0.791516108	Zm00001d023811
Zm00001d047516_T001	0.8861003	0.759347916	Zm00001d047516
Zm00001d036338_T001	0.1348894	2.006633283	Zm00001d036338
Zm00001d006582_T002	0.6086477	1.139653713	Zm00001d006582

Zm00001d045194_T001	0.8219372	-0.649090077	Zm00001d045194
Zm00001d010243_T001	0.9624987	0.630336191	Zm00001d010243
Zm00001d010243_T005	0.3319631	1.725328548	Zm00001d010243
Zm00001d018565_T001	0.9138633	0.837288915	Zm00001d018565
Zm00001d013099_T001	0.9513815	-0.083312	Zm00001d013099
Zm00001d046465_T002	0.907301	-0.594978353	Zm00001d046465
Zm00001d046465_T001	0.799595	-0.465967393	Zm00001d046465
Zm00001d051314_T001	0.5521461	1.087747698	Zm00001d051314
Zm00001d032146_T001	0.7831859	-0.337508615	Zm00001d032146
Zm00001d027399_T001	0.9404125	-0.277656396	Zm00001d027399
Zm00001d054110_T001	0.1019144	0.460712251	Zm00001d054110
Zm00001d054110_T007	0.001159928	0.540899932	Zm00001d054110
Zm00001d054110_T003	0.4367675	-0.244495276	Zm00001d054110
Zm00001d035067_T001	0.8737073	-0.170213169	Zm00001d035067
Zm00001d038733_T001	0.8842271	-0.073453889	Zm00001d038733
Zm00001d044302_T001	0.6845507	-0.350647852	Zm00001d044302
Zm00001d010618_T004	0.214971	-0.836762948	Zm00001d010618
Zm00001d010618_T002	0.9514141	-0.178030776	Zm00001d010618
Zm00001d010618_T001	0.9981278	0.345741883	Zm00001d010618
Zm00001d012205_T001	0.9870394	0.362344498	Zm00001d012205
Zm00001d007886_T006	0.3746932	-0.814539479	Zm00001d007886
Zm00001d007886_T016	5.49E-06	1.084028288	Zm00001d007886
Zm00001d007886_T015	0.1317222	1.245721644	Zm00001d007886
Zm00001d006948_T001	1.37E-05	1.328363768	Zm00001d006948
Zm00001d006473_T001	0.9690925	0.145144615	Zm00001d006473
Zm00001d011256_T001	0.6086477	0.644812213	Zm00001d011256
Zm00001d052624_T004	0.9845789	0.348367797	Zm00001d052624
Zm00001d052624_T002	0.9296811	0.055639935	Zm00001d052624
Zm00001d052624_T007	0.9223663	-0.058515945	Zm00001d052624
Zm00001d052624_T003	0.9984041	0.178983307	Zm00001d052624
Zm00001d023817_T001	0.9701986	0.557999916	Zm00001d023817
Zm00001d023817_T004	0.8492405	-0.150638351	Zm00001d023817
Zm00001d023817_T002	0.757578	0.464000726	Zm00001d023817
Zm00001d010023_T001	0.8099578	0.870227699	Zm00001d010023
Zm00001d010023_T008	0.5307031	0.739158458	Zm00001d010023
Zm00001d010023_T010	0.2545977	0.255540471	Zm00001d010023
Zm00001d010023_T016	0.3015167	-1.106469824	Zm00001d010023
Zm00001d010023_T013	0.5038499	0.760920192	Zm00001d010023
Zm00001d004978_T001	0.926133	0.424435887	Zm00001d004978
Zm00001d050103_T004	0.9660058	0.59199643	Zm00001d050103
Zm00001d050103_T007	0.8904578	0.562748505	Zm00001d050103
Zm00001d050103_T005	0.8883561	0.319668141	Zm00001d050103
Zm00001d050103_T002	0.997764	0.474829148	Zm00001d050103
Zm00001d032791_T001	0.7008573	-0.395974644	Zm00001d032791
Zm00001d032981_T004	0.9867452	0.081300821	Zm00001d032981
Zm00001d032981_T003	0.9822138	0.429416171	Zm00001d032981
Zm00001d003646_T003	0.850642	-0.131428015	Zm00001d003646
Zm00001d003646_T001	0.726958	-0.246683806	Zm00001d003646
Zm00001d039354_T001	0.4219823	-1.200217838	Zm00001d039354
Zm00001d016164_T007	0.8555166	-0.290822423	Zm00001d016164
Zm00001d016164_T001	0.8031436	-0.321096719	Zm00001d016164
Zm00001d016164_T006	0.9902022	0.337702507	Zm00001d016164
Zm00001d041958_T001	0.8751525	-0.096175597	Zm00001d041958
Zm00001d050694_T001	0.9657321	0.433540708	Zm00001d050694

Zm00001d019184_T002	0.6844831	-0.336014071	Zm00001d019184
Zm00001d018870_T001	0.9429712	0.074422025	Zm00001d018870
Zm00001d014161_T001	0.7458694	-0.439898658	Zm00001d014161
Zm00001d000452_T019	0.006621309	0.555031725	Zm00001d000452
Zm00001d000452_T027	0.3376532	1.134417282	Zm00001d000452
Zm00001d000452_T007	0.8529547	-0.09586395	Zm00001d000452
Zm00001d000452_T010	0.7712474	-0.1225664	Zm00001d000452
Zm00001d000452_T001	0.8922572	0.522327103	Zm00001d000452
Zm00001d000452_T012	0.000194122	0.839513758	Zm00001d000452
Zm00001d000452_T024	0.002528307	1.868155153	Zm00001d000452
Zm00001d000452_T009	0.4848011	0.559297348	Zm00001d000452
Zm00001d007255_T002	0.1054943	-0.604379366	Zm00001d007255
Zm00001d007255_T001	0.8267985	-0.163547104	Zm00001d007255
Zm00001d027580_T001	0.9361702	-0.007587756	Zm00001d027580
Zm00001d007529_T001	0.3212177	1.345430526	Zm00001d007529
Zm00001d047191_T001	0.748607	-0.27943179	Zm00001d047191
Zm00001d047191_T004	0.5014025	1.336126632	Zm00001d047191
Zm00001d047191_T008	0.07040175	-0.480062896	Zm00001d047191
Zm00001d019207_T001	0.000378118	3.859369663	Zm00001d019207
Zm00001d016198_T001	0.2143036	0.616974124	Zm00001d016198
Zm00001d016198_T003	0.5292925	-0.630899295	Zm00001d016198
Zm00001d016198_T006	0.000158043	0.763490564	Zm00001d016198
Zm00001d012993_T001	0.6041001	-0.553820525	Zm00001d012993
Zm00001d047266_T001	0.9596377	0.053632858	Zm00001d047266
Zm00001d034706_T001	0.4943505	-0.581812037	Zm00001d034706
Zm00001d045048_T001	0.1416275	-0.638038372	Zm00001d045048
Zm00001d033312_T003	0.745718	-0.536505884	Zm00001d033312
Zm00001d033312_T002	0.3544526	-1.250637152	Zm00001d033312
Zm00001d033312_T004	0.6038555	-0.980669026	Zm00001d033312
Zm00001d002733_T001	0.9361702	-0.280668594	Zm00001d002733
Zm00001d007764_T001	0.9239172	0.699734626	Zm00001d007764
Zm00001d035308_T001	0.8336035	-0.261061761	Zm00001d035308
Zm00001d046422_T001	1	0.425628537	Zm00001d046422
Zm00001d044898_T001	0.8964117	0.546711599	Zm00001d044898
Zm00001d011902_T001	0.6416932	-0.985849635	Zm00001d011902
Zm00001d038792_T001	0.9988864	0.43950073	Zm00001d038792
Zm00001d018699_T001	0.6007475	-0.852177682	Zm00001d018699
Zm00001d013599_T005	0.9563232	0.556260142	Zm00001d013599
Zm00001d013599_T011	0.9690925	0.169112216	Zm00001d013599
Zm00001d013599_T001	0.5411248	-0.264357888	Zm00001d013599
Zm00001d013599_T016	0.8747821	0.777948868	Zm00001d013599
Zm00001d047767_T004	1	0.176877877	Zm00001d047767
Zm00001d047767_T003	1	0.096182837	Zm00001d047767
Zm00001d047767_T001	0.950155	-0.092632748	Zm00001d047767
Zm00001d026005_T001	0.8665954	-0.165760757	Zm00001d026005
Zm00001d026307_T001	0.9050366	-0.194628268	Zm00001d026307
Zm00001d007486_T001	0.9417709	0.026531439	Zm00001d007486
Zm00001d015927_T001	0.9966965	0.435460429	Zm00001d015927
Zm00001d031231_T001	0.2332303	-0.877324176	Zm00001d031231
Zm00001d031231_T003	1	0.349485482	Zm00001d031231
Zm00001d031231_T017	0.659289	0.651149497	Zm00001d031231
Zm00001d031231_T010	0.894759	-0.004167424	Zm00001d031231
Zm00001d031231_T024	1	0.120319464	Zm00001d031231
Zm00001d031231_T008	0.5935355	1.062184479	Zm00001d031231

Zm00001d005542_T007	0.866232	-0.572640578	Zm00001d005542
Zm00001d005542_T002	0.9845237	0.230172264	Zm00001d005542
Zm00001d051527_T001	0.906282	0.483503224	Zm00001d051527
Zm00001d051527_T002	0.9524127	0.441606183	Zm00001d051527
Zm00001d036181_T001	0.9217388	0.647199401	Zm00001d036181
Zm00001d015151_T020	0.5292925	-0.13350603	Zm00001d015151
Zm00001d015151_T007	0.9100328	0.011940142	Zm00001d015151
Zm00001d015151_T005	0.8347734	0.535303606	Zm00001d015151
Zm00001d015151_T018	0.7253704	0.248511681	Zm00001d015151
Zm00001d021376_T005	0.358229	0.621542191	Zm00001d021376
Zm00001d021376_T003	0.909729	0.488028326	Zm00001d021376
Zm00001d012559_T005	0.5525447	1.296061878	Zm00001d012559
Zm00001d012559_T002	0.997764	0.348592719	Zm00001d012559
Zm00001d012559_T009	0.9902733	0.220558091	Zm00001d012559
Zm00001d012559_T008	0.8701267	0.258156678	Zm00001d012559
Zm00001d046370_T001	1	0.138421968	Zm00001d046370
Zm00001d022420_T001	0.7479767	0.943140381	Zm00001d022420
Zm00001d017118_T001	0.7259507	-0.446648207	Zm00001d017118
Zm00001d052288_T001	0.6087467	0.934578239	Zm00001d052288
Zm00001d018367_T001	0.3767085	-0.833449462	Zm00001d018367
Zm00001d003464_T002	0.9291214	0.683937621	Zm00001d003464
Zm00001d003464_T003	0.7020458	-0.372686392	Zm00001d003464
Zm00001d003464_T004	0.04903754	-1.20848646	Zm00001d003464
Zm00001d009873_T001	0.4121484	1.187447472	Zm00001d009873
Zm00001d009873_T002	0.5856273	0.832198523	Zm00001d009873
Zm00001d008477_T001	0.8031436	0.947981544	Zm00001d008477
Zm00001d008477_T014	0.9249382	0.282292468	Zm00001d008477
Zm00001d008477_T009	0.2449234	-1.068657527	Zm00001d008477
Zm00001d026427_T001	1	0.095673215	Zm00001d026427
Zm00001d031191_T001	0.995534	-0.122305927	Zm00001d031191
Zm00001d010927_T001	1	0.412276233	Zm00001d010927
Zm00001d001882_T005	0.4029716	-0.496884579	Zm00001d001882
Zm00001d001882_T004	0.8341208	-0.552058993	Zm00001d001882
Zm00001d003309_T002	0.9195989	0.055066199	Zm00001d003309
Zm00001d003309_T003	0.6004941	-0.414495371	Zm00001d003309
Zm00001d003309_T001	0.776742	-0.130053095	Zm00001d003309
Zm00001d004753_T001	0.9975882	0.276648967	Zm00001d004753
Zm00001d004196_T004	0.8001345	0.23177247	Zm00001d004196
Zm00001d004196_T005	1	0.054491218	Zm00001d004196
Zm00001d004196_T002	0.5138329	0.678803713	Zm00001d004196
Zm00001d012782_T001	0.1967676	-0.957398237	Zm00001d012782
Zm00001d023895_T002	0.9730613	0.090484146	Zm00001d023895
Zm00001d005395_T005	0.6761587	1.551484744	Zm00001d005395
Zm00001d005395_T007	0.2369807	0.722313284	Zm00001d005395
Zm00001d005395_T008	0.9673094	0.098321687	Zm00001d005395
Zm00001d005395_T001	0.03525539	-0.628553215	Zm00001d005395
Zm00001d053425_T001	0.9573272	0.653675453	Zm00001d053425
Zm00001d021557_T001	0.9237037	-0.01787662	Zm00001d021557
Zm00001d031962_T001	0.5717924	-0.546450116	Zm00001d031962
Zm00001d008273_T001	0.3015167	-0.437058291	Zm00001d008273
Zm00001d041070_T001	0.9812314	0.51732023	Zm00001d041070
Zm00001d040839_T001	0.6278106	-0.557716413	Zm00001d040839
Zm00001d023327_T001	0.2820123	-0.866953896	Zm00001d023327
Zm00001d023327_T011	1.63E-15	3.428233101	Zm00001d023327

Zm00001d023327_T008	0.02477549	-0.660352585	Zm00001d023327
Zm00001d023327_T003	9.93E-12	1.5636068	Zm00001d023327
Zm00001d023327_T010	0.9945592	0.007806802	Zm00001d023327
Zm00001d023327_T002	0.6082229	0.799326226	Zm00001d023327
Zm00001d003950_T003	0.9542458	0.413211693	Zm00001d003950
Zm00001d003950_T002	0.9075976	0.672208061	Zm00001d003950
Zm00001d003950_T001	0.9303773	0.085204719	Zm00001d003950
Zm00001d031133_T001	0.9334932	0.365964393	Zm00001d031133
Zm00001d037300_T001	0.5579636	-0.890436277	Zm00001d037300
Zm00001d017729_T013	0.3199106	-0.501921566	Zm00001d017729
Zm00001d017729_T002	0.1048346	0.771172271	Zm00001d017729
Zm00001d017729_T029	0.3823206	0.703680083	Zm00001d017729
Zm00001d017729_T008	0.5756796	1.110594633	Zm00001d017729
Zm00001d017729_T010	0.04406775	2.710775281	Zm00001d017729
Zm00001d017729_T028	0.4665724	0.47288965	Zm00001d017729
Zm00001d017729_T042	0.9760516	1.189905322	Zm00001d017729
Zm00001d017729_T014	0.007309779	1.850950595	Zm00001d017729
Zm00001d017729_T007	0.158266	1.208942343	Zm00001d017729
Zm00001d017729_T017	0.3790296	1.871461702	Zm00001d017729
Zm00001d017729_T001	0.2431378	1.199628694	Zm00001d017729
Zm00001d030934_T004	0.9942091	0.080624075	Zm00001d030934
Zm00001d030934_T001	0.6648793	-0.509312505	Zm00001d030934
Zm00001d030934_T003	0.9219195	0.424462598	Zm00001d030934
Zm00001d025397_T005	1.36E-05	2.686801142	Zm00001d025397
Zm00001d025397_T001	1.01E-07	3.827992651	Zm00001d025397
Zm00001d025397_T006	0.8522942	0.883472665	Zm00001d025397
Zm00001d025397_T002	0.9361945	0.729184762	Zm00001d025397
Zm00001d040665_T002	0.07730424	0.65027722	Zm00001d040665
Zm00001d040665_T007	0.9979568	0.44138116	Zm00001d040665
Zm00001d030579_T001	1	0.376906323	Zm00001d030579
Zm00001d045495_T001	0.4380979	-0.772765003	Zm00001d045495
Zm00001d021351_T001	0.6284759	0.482852188	Zm00001d021351
Zm00001d021351_T002	1	0.277283654	Zm00001d021351
Zm00001d021351_T004	0.9833133	0.217218222	Zm00001d021351
Zm00001d013865_T003	0.63821	-0.508625632	Zm00001d013865
Zm00001d013865_T001	0.9526971	0.091329994	Zm00001d013865
Zm00001d013865_T005	0.03126852	2.072772174	Zm00001d013865
Zm00001d000017_T001	0.0520257	1.729534345	Zm00001d000017
Zm00001d026670_T001	0.98291	0.10699929	Zm00001d026670
Zm00001d022017_T001	0.1659623	1.664509444	Zm00001d022017
Zm00001d016996_T001	0.8517246	-0.212933246	Zm00001d016996
Zm00001d040455_T001	0.8825173	-0.072999088	Zm00001d040455
Zm00001d038709_T004	0.8365671	1.107480201	Zm00001d038709
Zm00001d038709_T003	1	0.242968374	Zm00001d038709
Zm00001d038709_T005	0.9563257	-0.252503412	Zm00001d038709
Zm00001d038709_T002	0.5162062	-0.132043295	Zm00001d038709
Zm00001d042087_T001	0.9982897	0.265556237	Zm00001d042087
Zm00001d029721_T002	0.5023988	-0.422795718	Zm00001d029721
Zm00001d023316_T004	0.2332303	1.098098932	Zm00001d023316
Zm00001d023316_T002	0.001105106	-2.346419697	Zm00001d023316
Zm00001d023316_T001	0.9539869	0.330189413	Zm00001d023316
Zm00001d023316_T003	0.9872831	0.209208874	Zm00001d023316
Zm00001d004055_T005	1	0.261221634	Zm00001d004055
Zm00001d004055_T002	0.657881	-0.270785168	Zm00001d004055

Zm00001d004055_T003	0.9326774	0.055289594	Zm00001d004055
Zm00001d031431_T001	0.8672467	-0.479441697	Zm00001d031431
Zm00001d014599_T001	0.9215988	-0.099982263	Zm00001d014599
Zm00001d037079_T001	1	0.275335431	Zm00001d037079
Zm00001d035157_T002	0.6724442	1.272912131	Zm00001d035157
Zm00001d035157_T001	0.9975882	0.513666192	Zm00001d035157
Zm00001d053759_T001	0.8789841	-0.409843091	Zm00001d053759
Zm00001d001855_T002	0.947501	0.093349621	Zm00001d001855
Zm00001d026489_T001	0.9732421	0.073689266	Zm00001d026489
Zm00001d037236_T001	0.9886764	0.220680256	Zm00001d037236
Zm00001d052075_T001	0.768152	-0.758999821	Zm00001d052075
Zm00001d052075_T003	0.09262102	-1.523346078	Zm00001d052075
Zm00001d052075_T002	0.9979568	0.227064935	Zm00001d052075
Zm00001d018097_T001	0.8641671	-0.27116485	Zm00001d018097
Zm00001d002164_T018	0.02386597	-0.411553031	Zm00001d002164
Zm00001d002164_T020	1	0.131573305	Zm00001d002164
Zm00001d002164_T017	2.95E-07	0.620807872	Zm00001d002164
Zm00001d002164_T021	0.623486	-0.145960767	Zm00001d002164
Zm00001d002164_T016	0.8239342	0.672720914	Zm00001d002164
Zm00001d002164_T008	0.947501	0.564705976	Zm00001d002164
Zm00001d002164_T011	0.2922198	1.157187183	Zm00001d002164
Zm00001d002164_T006	0.9832092	0.549630081	Zm00001d002164
Zm00001d002164_T013	0.9078934	-0.058972581	Zm00001d002164
Zm00001d002164_T027	0.00090062	-1.081441709	Zm00001d002164
Zm00001d002828_T001	0.6480001	0.897069386	Zm00001d002828
Zm00001d047970_T001	0.3423638	-0.817241602	Zm00001d047970
Zm00001d052500_T001	0.7400422	-0.5733631	Zm00001d052500
Zm00001d048476_T001	0.9664406	-0.016945565	Zm00001d048476
Zm00001d008901_T001	0.8872096	-0.136060501	Zm00001d008901
Zm00001d031005_T009	0.8050292	-0.149023029	Zm00001d031005
Zm00001d031005_T003	0.3551811	1.22173877	Zm00001d031005
Zm00001d031005_T004	1.24E-08	0.837713736	Zm00001d031005
Zm00001d024934_T001	0.4815614	1.32616979	Zm00001d024934
Zm00001d049541_T001	0.9469426	0.677175629	Zm00001d049541
Zm00001d048868_T002	0.9962521	0.335026427	Zm00001d048868
Zm00001d048868_T001	0.9763286	0.078738766	Zm00001d048868
Zm00001d048868_T004	0.935477	0.586920142	Zm00001d048868
Zm00001d031290_T001	0.6667451	1.268411617	Zm00001d031290
Zm00001d031058_T007	0.3230455	0.871607911	Zm00001d031058
Zm00001d031058_T006	0.9043641	0.91594549	Zm00001d031058
Zm00001d031058_T010	1	0.28358464	Zm00001d031058
Zm00001d031058_T005	0.215048	1.301112734	Zm00001d031058
Zm00001d031058_T001	0.7020458	-0.110596913	Zm00001d031058
Zm00001d002936_T010	0.2154325	1.779224724	Zm00001d002936
Zm00001d002936_T038	0.5455802	-0.181817874	Zm00001d002936
Zm00001d002936_T041	0.8713206	-0.331911067	Zm00001d002936
Zm00001d002936_T042	0.9515686	-0.152999269	Zm00001d002936
Zm00001d002936_T043	0.8957792	-0.101765152	Zm00001d002936
Zm00001d002936_T003	0.9817074	0.122751423	Zm00001d002936
Zm00001d042843_T001	0.8488494	-0.222453485	Zm00001d042843
Zm00001d010228_T001	0.000189296	2.04500594	Zm00001d010228
Zm00001d033993_T014	0.008106114	0.498541639	Zm00001d033993
Zm00001d033993_T006	0.9719113	0.556616875	Zm00001d033993
Zm00001d029601_T001	7.14E-05	3.942960225	Zm00001d029601

Zm00001d029601_T002	2.82E-05	4.405385638	Zm00001d029601
Zm00001d029601_T003	0.000156819	3.863659248	Zm00001d029601
Zm00001d048702_T001	0.9596377	0.106918556	Zm00001d048702
Zm00001d048702_T002	0.9914912	0.311892286	Zm00001d048702
Zm00001d042266_T001	0.7128893	-0.354959325	Zm00001d042266
Zm00001d034991_T001	0.9843847	0.349833755	Zm00001d034991
Zm00001d032546_T001	0.2140452	1.94380368	Zm00001d032546
Zm00001d034424_T001	0.8376132	0.260251283	Zm00001d034424
Zm00001d034424_T003	0.6652036	0.981909305	Zm00001d034424
Zm00001d048417_T001	0.9261039	0.628015075	Zm00001d048417
Zm00001d005424_T006	0.8027057	0.703818557	Zm00001d005424
Zm00001d005424_T005	0.989787	0.257045864	Zm00001d005424
Zm00001d005424_T003	0.000806633	1.355760654	Zm00001d005424
Zm00001d033205_T001	0.2803683	-0.719826189	Zm00001d033205
Zm00001d049974_T019	0.4819918	0.694667308	Zm00001d049974
Zm00001d049974_T043	0.644595	-0.738411645	Zm00001d049974
Zm00001d049974_T026	0.7766169	-0.100864068	Zm00001d049974
Zm00001d049974_T042	0.9460688	0.357111017	Zm00001d049974
Zm00001d049974_T001	1.73E-07	2.422138015	Zm00001d049974
Zm00001d049974_T007	0.2349161	1.146621745	Zm00001d049974
Zm00001d049974_T009	3.53E-12	-1.991970343	Zm00001d049974
Zm00001d048487_T004	0.9968176	0.293645291	Zm00001d048487
Zm00001d048487_T002	0.3849533	-0.144085737	Zm00001d048487
Zm00001d048487_T003	0.666332	0.478596067	Zm00001d048487
Zm00001d037112_T001	0.9029876	-0.043183173	Zm00001d037112
Zm00001d017420_T002	0.6266648	1.036594374	Zm00001d017420
Zm00001d017420_T001	0.1611849	1.026703262	Zm00001d017420
Zm00001d029627_T001	0.5814472	0.524708681	Zm00001d029627
Zm00001d029627_T002	0.974657	0.048058248	Zm00001d029627
Zm00001d028550_T001	0.9699723	0.137035661	Zm00001d028550
Zm00001d028550_T003	0.1501728	-0.8966344	Zm00001d028550
Zm00001d028550_T002	1.20E-07	-2.448306398	Zm00001d028550
Zm00001d036283_T002	0.9902022	0.279674817	Zm00001d036283
Zm00001d036283_T003	0.4113115	-0.75284877	Zm00001d036283
Zm00001d036283_T001	0.9781672	0.019074178	Zm00001d036283
Zm00001d038762_T009	0.01757082	1.321256861	Zm00001d038762
Zm00001d038762_T018	2.04E-05	1.298336644	Zm00001d038762
Zm00001d038762_T023	0.009908492	1.878323609	Zm00001d038762
Zm00001d038762_T001	0.8641957	0.703537091	Zm00001d038762
Zm00001d038762_T022	0.8412913	0.968449838	Zm00001d038762
Zm00001d038762_T016	0.000241367	2.62547164	Zm00001d038762
Zm00001d015029_T004	2.95E-05	2.088703173	Zm00001d015029
Zm00001d015029_T002	1	0.362158684	Zm00001d015029
Zm00001d028086_T001	0.8292199	-0.104976017	Zm00001d028086
Zm00001d013873_T001	0.9823248	0.207628802	Zm00001d013873
Zm00001d028728_T009	0.4647287	-0.35638213	Zm00001d028728
Zm00001d028728_T004	0.7425238	0.598703706	Zm00001d028728
Zm00001d028728_T007	0.9924805	0.265738405	Zm00001d028728
Zm00001d028728_T005	0.1241083	-0.887419181	Zm00001d028728
Zm00001d046696_T001	0.5423554	-0.919307353	Zm00001d046696
Zm00001d039066_T002	0.9701986	0.498652667	Zm00001d039066
Zm00001d001832_T001	0.401517	1.154020493	Zm00001d001832
Zm00001d038296_T002	8.79E-08	-3.040788838	Zm00001d038296
Zm00001d038296_T001	0.1025055	-1.350411956	Zm00001d038296

Zm00001d036718_T001	0.585748	0.886489885	Zm00001d036718
Zm00001d036718_T002	0.9907928	0.223144407	Zm00001d036718
Zm00001d003002_T001	0.9968176	0.228894437	Zm00001d003002
Zm00001d034885_T002	1	0.400914022	Zm00001d034885
Zm00001d032810_T003	0.5479918	1.096947604	Zm00001d032810
Zm00001d032810_T013	0.7923921	0.380434519	Zm00001d032810
Zm00001d032810_T007	0.000259008	3.415018388	Zm00001d032810
Zm00001d032810_T014	0.6190151	1.014565659	Zm00001d032810
Zm00001d032810_T004	0.1415154	1.860683414	Zm00001d032810
Zm00001d032810_T010	0.652668	1.073851861	Zm00001d032810
Zm00001d032810_T006	0.000263804	2.691992647	Zm00001d032810
Zm00001d032810_T009	4.75E-07	3.073819775	Zm00001d032810
Zm00001d032810_T002	0.5538176	1.378650749	Zm00001d032810
Zm00001d032810_T005	1	0.99837399	Zm00001d032810
Zm00001d032810_T011	0.8785063	1.189383662	Zm00001d032810
Zm00001d032810_T008	0.5368684	1.448456858	Zm00001d032810
Zm00001d028396_T003	1	0.345386895	Zm00001d028396
Zm00001d045566_T001	0.923446	0.052670239	Zm00001d045566
Zm00001d044267_T003	0.9925955	0.142584316	Zm00001d044267
Zm00001d015052_T001	0.8089972	0.514558537	Zm00001d015052
Zm00001d023721_T001	0.923446	0.03557812	Zm00001d023721
Zm00001d053518_T001	0.4125465	1.304762993	Zm00001d053518
Zm00001d003851_T003	0.7270513	0.975584066	Zm00001d003851
Zm00001d003851_T002	0.8424588	0.952049636	Zm00001d003851
Zm00001d003851_T004	0.9279367	0.331592503	Zm00001d003851
Zm00001d003851_T001	0.8636447	0.695047204	Zm00001d003851
Zm00001d003492_T001	0.9291214	0.709723777	Zm00001d003492
Zm00001d051247_T002	0.8660897	0.694265427	Zm00001d051247
Zm00001d051247_T001	0.9813293	0.155072333	Zm00001d051247
Zm00001d047303_T001	0.8701267	-0.460492945	Zm00001d047303
Zm00001d031308_T001	0.9690925	-0.469681832	Zm00001d031308
Zm00001d007352_T001	0.9702242	-0.138868714	Zm00001d007352
Zm00001d029020_T002	0.9979568	0.457335286	Zm00001d029020
Zm00001d029020_T004	0.1195596	1.570442217	Zm00001d029020
Zm00001d029020_T005	0.05050405	-1.048620986	Zm00001d029020
Zm00001d028184_T001	0.9741926	0.046932579	Zm00001d028184
Zm00001d028184_T002	0.9180865	-0.258071657	Zm00001d028184
Zm00001d006760_T001	0.9529184	0.506657914	Zm00001d006760
Zm00001d006760_T002	0.02845695	-2.197621578	Zm00001d006760
Zm00001d037096_T008	1	0.014217175	Zm00001d037096
Zm00001d037096_T007	1	0.115598418	Zm00001d037096
Zm00001d037096_T001	0.9843639	0.175438015	Zm00001d037096
Zm00001d013750_T001	0.9279367	-0.320801115	Zm00001d013750
Zm00001d007011_T001	0.8884142	1.527614212	Zm00001d007011
Zm00001d048890_T002	0.5572941	-0.377365881	Zm00001d048890
Zm00001d048890_T003	0.9947747	0.275927373	Zm00001d048890
Zm00001d048890_T004	1	0.265613158	Zm00001d048890
Zm00001d038170_T002	0.9753621	0.473167435	Zm00001d038170
Zm00001d038170_T003	0.8721631	-0.166705957	Zm00001d038170
Zm00001d014026_T001	0.9368844	0.049588374	Zm00001d014026
Zm00001d007369_T013	0.5635628	1.049927483	Zm00001d007369
Zm00001d007369_T020	0.8522726	-0.047649741	Zm00001d007369
Zm00001d007369_T021	0.02069359	1.2773044	Zm00001d007369
Zm00001d007369_T018	0.000300769	-1.317711526	Zm00001d007369

Zm00001d007369_T010	0.04476943	-0.82652147	Zm00001d007369
Zm00001d007369_T006	1.84E-07	0.636424743	Zm00001d007369
Zm00001d007369_T009	0.8840168	0.322457121	Zm00001d007369
Zm00001d007369_T004	0.288999	-0.230648834	Zm00001d007369
Zm00001d007369_T019	0.5864438	-0.44612041	Zm00001d007369
Zm00001d007369_T014	0.9422098	0.09011758	Zm00001d007369
Zm00001d044647_T003	1	0.15282023	Zm00001d044647
Zm00001d031447_T004	0.7751076	-0.225741657	Zm00001d031447
Zm00001d031447_T012	0.4178262	0.474616437	Zm00001d031447
Zm00001d031447_T001	0.7011253	0.997165587	Zm00001d031447
Zm00001d031447_T014	0.2840735	-0.646095379	Zm00001d031447
Zm00001d031447_T007	1.23E-13	2.469424033	Zm00001d031447
Zm00001d031447_T003	1.26E-10	-1.074621387	Zm00001d031447
Zm00001d029010_T006	0.9945118	0.124347212	Zm00001d029010
Zm00001d029010_T001	0.6357777	-0.116292128	Zm00001d029010
Zm00001d029010_T005	1	0.208268679	Zm00001d029010
Zm00001d029010_T002	0.7667736	0.833085255	Zm00001d029010
Zm00001d039256_T001	0.1164957	-1.616858873	Zm00001d039256
Zm00001d049660_T002	0.006314872	2.864019594	Zm00001d049660
Zm00001d049660_T001	0.09184437	1.650729718	Zm00001d049660
Zm00001d030153_T001	0.158266	-1.715742385	Zm00001d030153
Zm00001d002079_T001	0.9979568	0.027244632	Zm00001d002079
Zm00001d018530_T001	0.8503172	-0.468330184	Zm00001d018530
Zm00001d013588_T001	0.000134856	-1.158720143	Zm00001d013588
Zm00001d047573_T001	0.145781	-1.313416367	Zm00001d047573
Zm00001d013546_T006	0.9918384	0.130356015	Zm00001d013546
Zm00001d013546_T002	1	0.292218325	Zm00001d013546
Zm00001d013546_T004	0.9786754	0.029208389	Zm00001d013546
Zm00001d002524_T001	0.6340221	0.771788616	Zm00001d002524
Zm00001d007688_T002	0.8780771	0.188538795	Zm00001d007688
Zm00001d007688_T001	0.9596377	0.496321975	Zm00001d007688
Zm00001d008871_T001	0.8647028	-0.178406533	Zm00001d008871
Zm00001d031694_T001	0.9913436	-0.227818091	Zm00001d031694
Zm00001d031694_T006	0.6503853	0.340465722	Zm00001d031694
Zm00001d031694_T005	0.8412913	0.827290098	Zm00001d031694
Zm00001d031694_T002	0.9203655	0.608315711	Zm00001d031694
Zm00001d031694_T009	0.8412913	1.06635994	Zm00001d031694
Zm00001d031694_T015	0.9843847	1.007210947	Zm00001d031694
Zm00001d002012_T004	1	0.447004148	Zm00001d002012
Zm00001d002012_T005	0.3642154	-1.313116384	Zm00001d002012
Zm00001d002012_T001	0.8695779	-0.074942307	Zm00001d002012
Zm00001d044854_T001	0.961399	0.027196828	Zm00001d044854
Zm00001d044854_T003	0.9524127	0.607186448	Zm00001d044854
Zm00001d039974_T001	0.7296512	-0.888174908	Zm00001d039974
Zm00001d000243_T001	0.9893214	0.078626902	Zm00001d000243
Zm00001d013943_T001	0.9988954	0.018683675	Zm00001d013943
Zm00001d027719_T001	0.9285797	0.614213712	Zm00001d027719
Zm00001d039349_T002	0.9868533	0.301512825	Zm00001d039349
Zm00001d046788_T001	0.9747606	0.190727283	Zm00001d046788
Zm00001d009960_T001	0.4421408	1.494885116	Zm00001d009960
Zm00001d038944_T003	1	0.359555605	Zm00001d038944
Zm00001d038944_T002	0.946408	0.116801815	Zm00001d038944
Zm00001d038944_T004	0.8984125	0.800952282	Zm00001d038944
Zm00001d038944_T009	0.2903147	-0.985014035	Zm00001d038944

Zm00001d018742_T001	0.03277874	-1.579104889	Zm00001d018742
Zm00001d018742_T005	0.001529479	-0.953925704	Zm00001d018742
Zm00001d032498_T001	0.827438	-0.711809876	Zm00001d032498
Zm00001d051795_T001	0.04152779	2.052368786	Zm00001d051795
Zm00001d028679_T002	0.9907928	0.328182099	Zm00001d028679
Zm00001d028679_T001	0.9966072	0.260529366	Zm00001d028679
Zm00001d009936_T001	0.8881012	-0.211279486	Zm00001d009936
Zm00001d010512_T001	0.9099617	0.009537796	Zm00001d010512
Zm00001d017956_T001	0.7697196	-0.379584538	Zm00001d017956
Zm00001d035765_T001	0.7658902	-0.355882253	Zm00001d035765
Zm00001d035765_T002	0.6503092	-0.643484702	Zm00001d035765
Zm00001d020445_T001	0.9994451	0.170261391	Zm00001d020445
Zm00001d049173_T001	0.1664354	-1.052586011	Zm00001d049173
Zm00001d029499_T001	0.8412913	-0.202599475	Zm00001d029499
Zm00001d029499_T002	0.9782953	0.19830972	Zm00001d029499
Zm00001d022152_T001	0.9979568	0.364783338	Zm00001d022152
Zm00001d002111_T001	0.9855117	-0.065516958	Zm00001d002111
Zm00001d028261_T001	1	-0.251541288	Zm00001d028261
Zm00001d028261_T013	0.749176	0.248433269	Zm00001d028261
Zm00001d028261_T010	0.5717645	-0.198115713	Zm00001d028261
Zm00001d028261_T012	0.02237672	2.203399016	Zm00001d028261
Zm00001d028261_T007	0.8637863	-0.147580406	Zm00001d028261
Zm00001d045385_T001	0.3074488	-1.215646975	Zm00001d045385
Zm00001d030940_T001	0.1840497	-0.609774709	Zm00001d030940
Zm00001d023342_T001	0.6812088	0.959172958	Zm00001d023342
Zm00001d024813_T002	0.9804228	0.3414448	Zm00001d024813
Zm00001d019217_T003	0.000226353	-1.521165724	Zm00001d019217
Zm00001d019217_T002	0.954492	0.119990594	Zm00001d019217
Zm00001d019217_T005	0.9851204	0.164912326	Zm00001d019217
Zm00001d019217_T004	0.5857886	1.271059195	Zm00001d019217
Zm00001d015985_T006	0.670825	0.396933903	Zm00001d015985
Zm00001d015985_T005	0.5658009	-0.519780569	Zm00001d015985
Zm00001d039465_T001	1	0.431745335	Zm00001d039465
Zm00001d005399_T001	0.9237617	-0.061884269	Zm00001d005399
Zm00001d024625_T002	0.9706887	0.594015541	Zm00001d024625
Zm00001d024625_T004	0.02729225	-2.156861285	Zm00001d024625
Zm00001d050616_T003	0.4555994	1.252858805	Zm00001d050616
Zm00001d050616_T001	0.9730206	0.177434614	Zm00001d050616
Zm00001d050616_T002	0.04667204	-0.573661103	Zm00001d050616
Zm00001d037052_T003	0.9429712	0.350941768	Zm00001d037052
Zm00001d037052_T002	1	0.063953539	Zm00001d037052
Zm00001d046681_T001	0.9695018	0.340437877	Zm00001d046681
Zm00001d039931_T005	0.9257893	0.657036767	Zm00001d039931
Zm00001d039931_T001	1	0.169871646	Zm00001d039931
Zm00001d039931_T003	1	0.290723801	Zm00001d039931
Zm00001d052015_T001	0.005892258	-2.968688039	Zm00001d052015
Zm00001d001772_T001	0.7324169	-0.807438355	Zm00001d001772
Zm00001d028554_T001	0.9766874	0.889177909	Zm00001d028554
Zm00001d028554_T002	0.7590372	-0.256256621	Zm00001d028554
Zm00001d028554_T003	0.1273693	-1.159446593	Zm00001d028554
Zm00001d004469_T001	0.9874456	0.072440465	Zm00001d004469
Zm00001d017883_T001	1	0.502793398	Zm00001d017883
Zm00001d017883_T031	0.9766874	0.273641237	Zm00001d017883
Zm00001d034610_T003	0.9791729	0.3323023	Zm00001d034610

Zm00001d034610_T002	0.9999526	0.426543853	Zm00001d034610
Zm00001d043067_T025	1	0.338828688	Zm00001d043067
Zm00001d043067_T002	1	0.240754976	Zm00001d043067
Zm00001d043067_T011	0.4096848	1.640911202	Zm00001d043067
Zm00001d049743_T001	0.9201237	0.046986496	Zm00001d049743
Zm00001d012034_T002	0.7063818	0.803534555	Zm00001d012034
Zm00001d012034_T003	0.9689939	0.3175777	Zm00001d012034
Zm00001d012034_T004	0.9683589	0.371170166	Zm00001d012034
Zm00001d022065_T001	0.5849395	-0.575174233	Zm00001d022065
Zm00001d024412_T002	0.1446389	0.469689755	Zm00001d024412
Zm00001d024412_T005	0.8412913	0.535444428	Zm00001d024412
Zm00001d040008_T010	0.09528713	1.377550253	Zm00001d040008
Zm00001d040008_T002	0.4527616	1.239873458	Zm00001d040008
Zm00001d040008_T006	0.02854976	0.464976179	Zm00001d040008
Zm00001d040008_T009	0.2236565	-0.587989899	Zm00001d040008
Zm00001d040008_T004	0.8630483	1.10437807	Zm00001d040008
Zm00001d040008_T008	0.9580985	0.246087419	Zm00001d040008
Zm00001d020502_T001	0.9237617	-0.066183395	Zm00001d020502
Zm00001d010033_T003	0.9370449	7.84E-05	Zm00001d010033
Zm00001d010033_T001	0.9180662	0.787399843	Zm00001d010033
Zm00001d003346_T001	0.3777631	0.197820786	Zm00001d003346
Zm00001d028903_T001	0.9335676	0.719010141	Zm00001d028903
Zm00001d038874_T002	0.9706293	0.125874046	Zm00001d038874
Zm00001d030561_T009	1	0.381108319	Zm00001d030561
Zm00001d030561_T008	0.9048001	0.562759415	Zm00001d030561
Zm00001d030561_T003	0.9909466	0.305955607	Zm00001d030561
Zm00001d030561_T006	0.9205739	0.142994392	Zm00001d030561
Zm00001d003664_T002	0.9936409	-0.093813307	Zm00001d003664
Zm00001d003664_T001	0.7702266	1.055300841	Zm00001d003664
Zm00001d025892_T012	0.9543237	-0.227871915	Zm00001d025892
Zm00001d025892_T005	0.211796	1.37042272	Zm00001d025892
Zm00001d025892_T001	9.74E-12	2.789880622	Zm00001d025892
Zm00001d025892_T016	0.9804228	0.246506453	Zm00001d025892
Zm00001d025892_T002	0.9419336	0.685927995	Zm00001d025892
Zm00001d025892_T018	0.9689939	0.64396145	Zm00001d025892
Zm00001d025892_T008	0.5537579	1.019300433	Zm00001d025892
Zm00001d025892_T017	0.4576971	1.766813372	Zm00001d025892
Zm00001d008393_T003	0.9987435	0.263624152	Zm00001d008393
Zm00001d008393_T002	0.971204	0.067351395	Zm00001d008393
Zm00001d050334_T001	0.9950703	0.205251409	Zm00001d050334
Zm00001d012611_T001	0.9831532	0.115769145	Zm00001d012611
Zm00001d016486_T001	0.06190135	1.487821281	Zm00001d016486
Zm00001d016486_T011	0.2341066	-1.393386157	Zm00001d016486
Zm00001d016486_T006	0.8830977	0.279638435	Zm00001d016486
Zm00001d016486_T004	0.9781672	0.131204148	Zm00001d016486
Zm00001d016486_T005	0.8030099	0.706259656	Zm00001d016486
Zm00001d016486_T003	0.6673479	0.620944887	Zm00001d016486
Zm00001d024819_T004	0.1003013	0.72663005	Zm00001d024819
Zm00001d024819_T007	0.923446	0.565758153	Zm00001d024819
Zm00001d024819_T003	0.7761661	-0.051768869	Zm00001d024819
Zm00001d012521_T001	0.01955821	0.797493014	Zm00001d012521
Zm00001d028689_T001	0.8908955	0.896637502	Zm00001d028689
Zm00001d045511_T001	0.7921111	-0.352694772	Zm00001d045511
Zm00001d009416_T001	0.708674	-0.709818665	Zm00001d009416

Zm00001d038268_T001	1	0.382618459	Zm00001d038268
Zm00001d017468_T001	0.7838436	-0.313875939	Zm00001d017468
Zm00001d019481_T002	0.4701937	-1.060405148	Zm00001d019481
Zm00001d019481_T001	0.9517318	-0.261260313	Zm00001d019481
Zm00001d016684_T030	0.9706887	0.095065878	Zm00001d016684
Zm00001d016684_T001	0.6140391	-0.714586259	Zm00001d016684
Zm00001d028827_T004	1	0.349363866	Zm00001d028827
Zm00001d028827_T002	0.6134185	1.108722841	Zm00001d028827
Zm00001d007313_T001	0.9881407	0.36794147	Zm00001d007313
Zm00001d021711_T001	0.2774208	-1.31032184	Zm00001d021711
Zm00001d022164_T002	0.9334932	-0.576609769	Zm00001d022164
Zm00001d022164_T001	0.9882261	-0.162285088	Zm00001d022164
Zm00001d040155_T005	0.9766874	0.331723762	Zm00001d040155
Zm00001d040155_T003	1	0.344756144	Zm00001d040155
Zm00001d051607_T007	0.9804228	0.179523291	Zm00001d051607
Zm00001d051607_T004	0.4825966	0.645160126	Zm00001d051607
Zm00001d051607_T001	2.80E-08	1.167354047	Zm00001d051607
Zm00001d050741_T008	0.9686323	0.082753147	Zm00001d050741
Zm00001d050741_T005	0.1170004	1.34434672	Zm00001d050741
Zm00001d050741_T007	0.9596377	0.178952933	Zm00001d050741
Zm00001d050741_T004	0.9193633	-0.044908149	Zm00001d050741
Zm00001d050741_T006	0.7246447	-0.23573687	Zm00001d050741
Zm00001d050741_T003	0.9878764	0.474657287	Zm00001d050741
Zm00001d046652_T001	0.0436724	2.474721944	Zm00001d046652
Zm00001d010159_T003	0.7008849	-0.43411811	Zm00001d010159
Zm00001d010159_T002	0.7820614	1.072033841	Zm00001d010159
Zm00001d023499_T002	0.9791066	0.489048455	Zm00001d023499
Zm00001d023499_T001	0.9714345	0.147374201	Zm00001d023499
Zm00001d005484_T001	0.9429712	0.158273081	Zm00001d005484
Zm00001d038514_T001	0.7427189	-0.784450019	Zm00001d038514
Zm00001d001767_T001	0.3174539	-0.898856352	Zm00001d001767
Zm00001d007605_T012	2.18E-06	0.833980412	Zm00001d007605
Zm00001d021403_T001	0.9979568	0.34357254	Zm00001d021403
Zm00001d028482_T001	0.6280338	-0.478705492	Zm00001d028482
Zm00001d031266_T001	0.8105481	-0.444017221	Zm00001d031266
Zm00001d012221_T001	0.000760374	-2.898028485	Zm00001d012221
Zm00001d025862_T002	0.7998435	0.953318124	Zm00001d025862
Zm00001d025862_T001	0.9352321	0.309308271	Zm00001d025862
Zm00001d012119_T002	0.9805897	0.05183955	Zm00001d012119
Zm00001d012119_T001	1	0.25228874	Zm00001d012119
Zm00001d052543_T004	0.6009127	0.573291039	Zm00001d052543
Zm00001d052543_T003	0.8964117	0.733098956	Zm00001d052543
Zm00001d052543_T001	0.9872831	0.534617049	Zm00001d052543
Zm00001d030533_T001	0.8134743	-0.119740598	Zm00001d030533
Zm00001d030533_T003	0.9627389	0.156234201	Zm00001d030533
Zm00001d030533_T004	0.6957026	0.517064334	Zm00001d030533
Zm00001d030751_T001	0.000110936	-0.637970878	Zm00001d030751
Zm00001d003434_T001	0.7408387	-0.37380221	Zm00001d003434
Zm00001d053704_T001	0.909186	-0.169203513	Zm00001d053704
Zm00001d020725_T001	0.3183965	-0.910652851	Zm00001d020725
Zm00001d044551_T011	0.4270217	0.270756022	Zm00001d044551
Zm00001d044551_T017	6.61E-08	-3.643523184	Zm00001d044551
Zm00001d044551_T004	0.2369807	1.016542452	Zm00001d044551
Zm00001d044551_T009	0.9817074	0.453515052	Zm00001d044551

Zm00001d044551_T003	0.8993062	0.797918609	Zm00001d044551
Zm00001d044551_T013	0.9352862	0.528889685	Zm00001d044551
Zm00001d045973_T003	0.9981278	0.226600124	Zm00001d045973
Zm00001d034550_T001	0.8126103	-0.283536956	Zm00001d034550
Zm00001d020038_T001	0.9514141	0.667845716	Zm00001d020038
Zm00001d020038_T003	0.9160053	0.002464114	Zm00001d020038
Zm00001d020038_T002	0.9781672	0.30204965	Zm00001d020038
Zm00001d020038_T005	0.3876593	1.34438256	Zm00001d020038
Zm00001d027555_T001	0.9936756	0.128023688	Zm00001d027555
Zm00001d026171_T001	0.9442514	-0.085367847	Zm00001d026171
Zm00001d013521_T001	0.6062927	0.759824288	Zm00001d013521
Zm00001d029886_T001	0.4032014	1.468984809	Zm00001d029886
Zm00001d018613_T006	0.9887889	0.473475927	Zm00001d018613
Zm00001d018613_T009	0.6220826	-0.878701604	Zm00001d018613
Zm00001d018613_T015	0.7009673	-0.696783606	Zm00001d018613
Zm00001d018613_T011	0.9766874	0.321237613	Zm00001d018613
Zm00001d002423_T001	0.9975882	0.23234173	Zm00001d002423
Zm00001d018891_T004	0.8643636	0.121004285	Zm00001d018891
Zm00001d018891_T001	0.8503112	0.388980019	Zm00001d018891
Zm00001d001899_T001	0.745806	-0.698138216	Zm00001d001899
Zm00001d002452_T001	0.9523689	0.092145471	Zm00001d002452
Zm00001d039477_T001	0.9503115	0.071342305	Zm00001d039477
Zm00001d018490_T007	0.9771927	0.377993825	Zm00001d018490
Zm00001d018490_T004	0.9257893	0.663207165	Zm00001d018490
Zm00001d018490_T002	0.923446	0.209799555	Zm00001d018490
Zm00001d033870_T004	0.909729	-0.039639315	Zm00001d033870
Zm00001d033870_T003	0.9262099	0.676945976	Zm00001d033870
Zm00001d049848_T001	0.9664406	-0.399138045	Zm00001d049848
Zm00001d011975_T007	0.9291214	-0.01584442	Zm00001d011975
Zm00001d011975_T008	0.7493723	0.212772283	Zm00001d011975
Zm00001d011975_T002	0.8498341	0.720587765	Zm00001d011975
Zm00001d039714_T001	0.9804281	0.446016123	Zm00001d039714
Zm00001d042166_T007	0.9825858	0.050719611	Zm00001d042166
Zm00001d046714_T003	1	0.363393235	Zm00001d046714
Zm00001d046714_T004	0.9372575	0.008808591	Zm00001d046714
Zm00001d046714_T001	0.983226	0.205790018	Zm00001d046714
Zm00001d007381_T001	0.7194959	-0.352684165	Zm00001d007381
Zm00001d028484_T002	0.9377241	0.008162216	Zm00001d028484
Zm00001d028436_T003	7.66E-14	-2.799042149	Zm00001d028436
Zm00001d028436_T001	0.8891117	0.925606653	Zm00001d028436
Zm00001d028436_T002	4.96E-10	1.779450089	Zm00001d028436
Zm00001d043581_T001	0.9673473	0.189789289	Zm00001d043581
Zm00001d028664_T001	0.7949312	0.572018079	Zm00001d028664
Zm00001d018450_T006	0.5718169	0.266334364	Zm00001d018450
Zm00001d018450_T004	0.190237	1.02905673	Zm00001d018450
Zm00001d018450_T015	0.2322337	-0.685704539	Zm00001d018450
Zm00001d018450_T007	2.73E-06	2.101579953	Zm00001d018450
Zm00001d018450_T001	0.9190176	0.036275963	Zm00001d018450
Zm00001d042494_T004	0.733097	-0.091436066	Zm00001d042494
Zm00001d042494_T017	0.9825058	0.59275424	Zm00001d042494
Zm00001d042494_T015	0.5831618	0.252386195	Zm00001d042494
Zm00001d042494_T005	0.5092251	-0.229264262	Zm00001d042494
Zm00001d042494_T013	0.000221303	0.965468424	Zm00001d042494
Zm00001d042494_T001	0.8786965	0.455634679	Zm00001d042494

Zm00001d042494_T024	0.2285677	0.917690323	Zm00001d042494
Zm00001d020692_T002	0.2412176	1.039510846	Zm00001d020692
Zm00001d020692_T001	0.2400462	1.638045307	Zm00001d020692
Zm00001d002980_T003	0.9791729	0.200938824	Zm00001d002980
Zm00001d003709_T001	0.4304365	-1.381347862	Zm00001d003709
Zm00001d050913_T001	0.9850313	0.208860836	Zm00001d050913
Zm00001d012238_T001	0.6134185	1.299291351	Zm00001d012238
Zm00001d012238_T017	0.07958438	0.90540202	Zm00001d012238
Zm00001d012238_T018	0.7888267	0.914784603	Zm00001d012238
Zm00001d012238_T009	0.4475935	1.639791589	Zm00001d012238
Zm00001d012238_T014	0.2087765	0.401775023	Zm00001d012238
Zm00001d012238_T011	0.936261	1.414586132	Zm00001d012238
Zm00001d026532_T008	0.9846714	0.53050412	Zm00001d026532
Zm00001d026532_T012	0.7945048	0.325063827	Zm00001d026532
Zm00001d026532_T015	0.4548651	-1.012858825	Zm00001d026532
Zm00001d026532_T013	0.9817074	0.044013901	Zm00001d026532
Zm00001d045015_T001	0.8638849	0.618910484	Zm00001d045015
Zm00001d039200_T001	0.9356767	0.038006808	Zm00001d039200
Zm00001d001903_T006	0.9988864	1.213948213	Zm00001d001903
Zm00001d001903_T001	0.996299	0.350551582	Zm00001d001903
Zm00001d001903_T005	0.9364913	0.025970181	Zm00001d001903
Zm00001d013607_T005	0.6907306	-0.462927732	Zm00001d013607
Zm00001d013607_T001	0.7292793	1.120585252	Zm00001d013607
Zm00001d013607_T006	0.2189485	-0.98857424	Zm00001d013607
Zm00001d032449_T008	0.001410321	0.915923266	Zm00001d032449
Zm00001d032449_T002	2.77E-08	-2.673644276	Zm00001d032449
Zm00001d032449_T006	6.01E-15	-3.524838534	Zm00001d032449
Zm00001d032449_T005	0.03485146	1.132064059	Zm00001d032449
Zm00001d032449_T003	1.66E-16	4.518052973	Zm00001d032449
Zm00001d032449_T010	0.9942835	0.04335188	Zm00001d032449
Zm00001d021974_T002	0.9817074	0.210053276	Zm00001d021974
Zm00001d031457_T009	0.1177057	1.438063423	Zm00001d031457
Zm00001d031457_T004	1.92E-05	0.729169977	Zm00001d031457
Zm00001d031457_T002	0.2091699	-0.241132458	Zm00001d031457
Zm00001d043179_T001	0.9033096	-0.033917773	Zm00001d043179
Zm00001d029657_T001	1	0.378872686	Zm00001d029657
Zm00001d029657_T002	0.8580945	0.291258973	Zm00001d029657
Zm00001d037682_T001	0.4825966	-0.467531626	Zm00001d037682
Zm00001d013425_T001	0.8819727	-0.115860505	Zm00001d013425
Zm00001d019836_T008	0.02672699	2.102869371	Zm00001d019836
Zm00001d019836_T010	0.9870394	-0.016742318	Zm00001d019836
Zm00001d019836_T001	0.9419336	0.332979884	Zm00001d019836
Zm00001d019836_T005	0.04595375	1.923616619	Zm00001d019836
Zm00001d019836_T009	0.8581713	0.651360999	Zm00001d019836
Zm00001d019836_T006	0.9804228	-0.290404161	Zm00001d019836
Zm00001d036084_T004	1	0.17105742	Zm00001d036084
Zm00001d036084_T006	0.8412913	0.506353848	Zm00001d036084
Zm00001d036084_T003	0.7242054	0.227291951	Zm00001d036084
Zm00001d015737_T002	0.7638108	-0.515278002	Zm00001d015737
Zm00001d019688_T001	0.7011505	-0.882059732	Zm00001d019688
Zm00001d028337_T003	1	0.33426618	Zm00001d028337
Zm00001d047034_T003	1	0.257615159	Zm00001d047034
Zm00001d047034_T001	0.7325823	1.051061148	Zm00001d047034
Zm00001d034807_T002	0.1028434	-0.486285067	Zm00001d034807

Zm00001d034807_T004	0.8132343	-0.294304966	Zm00001d034807
Zm00001d034807_T007	0.3898578	1.107057652	Zm00001d034807
Zm00001d034807_T003	0.3003804	-1.231693553	Zm00001d034807
Zm00001d034807_T001	0.01758334	-1.194893958	Zm00001d034807
Zm00001d034807_T006	0.6082229	1.605147142	Zm00001d034807
Zm00001d034807_T005	0.01526785	2.251628175	Zm00001d034807
Zm00001d053636_T002	0.9563257	0.023301297	Zm00001d053636
Zm00001d053636_T001	0.7079447	-0.501125777	Zm00001d053636
Zm00001d035605_T001	0.4881794	-0.763438121	Zm00001d035605
Zm00001d016442_T007	0.9668789	0.536938458	Zm00001d016442
Zm00001d016442_T009	0.477074	0.545469663	Zm00001d016442
Zm00001d016442_T004	0.6217247	-0.1104716	Zm00001d016442
Zm00001d016442_T008	0.8412913	0.428135398	Zm00001d016442
Zm00001d042786_T001	0.9714345	0.072494124	Zm00001d042786
Zm00001d039675_T001	0.9930712	0.459034663	Zm00001d039675
Zm00001d039675_T003	0.1090245	0.993557249	Zm00001d039675
Zm00001d031581_T003	0.9309468	-0.089414513	Zm00001d031581
Zm00001d031581_T004	0.9979568	0.230592655	Zm00001d031581
Zm00001d031581_T007	0.6062927	1.248960694	Zm00001d031581
Zm00001d031581_T001	0.940651	0.405447119	Zm00001d031581
Zm00001d029286_T001	0.9037002	-0.027631918	Zm00001d029286
Zm00001d000135_T001	0.9872831	0.26250558	Zm00001d000135
Zm00001d021207_T001	5.05E-08	3.971652098	Zm00001d021207
Zm00001d051338_T001	0.7746801	0.720088686	Zm00001d051338
Zm00001d051338_T002	0.2546519	1.511741475	Zm00001d051338
Zm00001d017384_T016	0.923446	0.447555774	Zm00001d017384
Zm00001d017384_T008	0.835901	0.47670306	Zm00001d017384
Zm00001d017384_T011	0.8518279	1.012938714	Zm00001d017384
Zm00001d010309_T012	0.8987811	-0.125934568	Zm00001d010309
Zm00001d010309_T007	0.02346557	0.414905679	Zm00001d010309
Zm00001d010309_T004	0.6771289	1.044162979	Zm00001d010309
Zm00001d010309_T009	0.06720617	0.592117271	Zm00001d010309
Zm00001d010309_T011	0.7194959	0.390538179	Zm00001d010309
Zm00001d010309_T008	0.214251	0.490209853	Zm00001d010309
Zm00001d019372_T001	0.9765009	0.055616901	Zm00001d019372
Zm00001d014983_T001	0.5289854	1.413230628	Zm00001d014983
Zm00001d038439_T001	0.215298	0.910128256	Zm00001d038439
Zm00001d032505_T002	0.9442599	0.616034657	Zm00001d032505
Zm00001d032505_T001	0.6575309	-0.365226563	Zm00001d032505
Zm00001d025827_T002	0.952325	0.484548402	Zm00001d025827
Zm00001d037674_T001	0.9778436	0.075587634	Zm00001d037674
Zm00001d043477_T002	1	0.446280844	Zm00001d043477
Zm00001d043477_T001	0.2802526	1.882410796	Zm00001d043477
Zm00001d034107_T001	0.5849395	-0.428955939	Zm00001d034107
Zm00001d006106_T001	0.07332769	2.02927478	Zm00001d006106
Zm00001d036428_T002	0.9302721	0.717414011	Zm00001d036428
Zm00001d003613_T002	0.9559227	0.104420853	Zm00001d003613
Zm00001d018957_T001	0.6470421	-0.493236797	Zm00001d018957
Zm00001d052270_T008	0.6952911	-0.469430152	Zm00001d052270
Zm00001d052270_T005	0.9979568	0.195108698	Zm00001d052270
Zm00001d052270_T007	0.9224443	-0.043265469	Zm00001d052270
Zm00001d052270_T010	0.3360774	0.684052351	Zm00001d052270
Zm00001d018967_T001	0.8836195	-0.13135545	Zm00001d018967
Zm00001d021120_T001	0.9693993	0.539036824	Zm00001d021120

Zm00001d001937_T001	0.7351951	-0.734550944	Zm00001d001937
Zm00001d001937_T002	0.9918302	0.260110737	Zm00001d001937
Zm00001d001937_T005	0.584304	-1.121506067	Zm00001d001937
Zm00001d001937_T004	1	0.204571722	Zm00001d001937
Zm00001d018350_T001	0.919888	-0.073210602	Zm00001d018350
Zm00001d021511_T001	0.8097414	-0.373935633	Zm00001d021511
Zm00001d044535_T001	0.8922781	0.790940683	Zm00001d044535
Zm00001d002607_T001	0.1332405	-1.316381715	Zm00001d002607
Zm00001d018478_T001	0.8647028	-0.514038456	Zm00001d018478
Zm00001d002641_T001	0.8663602	0.695187554	Zm00001d002641
Zm00001d008588_T001	0.3858297	1.486687796	Zm00001d008588
Zm00001d042880_T003	0.897685	-0.153481442	Zm00001d042880
Zm00001d018911_T001	0.8203096	-0.174832995	Zm00001d018911
Zm00001d030002_T002	0.8705378	-0.142263857	Zm00001d030002
Zm00001d023538_T006	0.9335833	0.209868666	Zm00001d023538
Zm00001d023538_T004	0.2138161	0.69462693	Zm00001d023538
Zm00001d023538_T002	0.8558921	0.913744649	Zm00001d023538
Zm00001d039512_T008	0.2626567	0.863154291	Zm00001d039512
Zm00001d039512_T014	0.4508973	-0.648475016	Zm00001d039512
Zm00001d039512_T006	0.6947003	-0.389914984	Zm00001d039512
Zm00001d039512_T005	0.8063892	0.397137693	Zm00001d039512
Zm00001d039512_T013	0.9356767	0.527267443	Zm00001d039512
Zm00001d044987_T007	1	0.09807652	Zm00001d044987
Zm00001d044987_T006	0.9326482	0.08578388	Zm00001d044987
Zm00001d027630_T006	2.08E-11	2.218287062	Zm00001d027630
Zm00001d027630_T001	0.9666399	0.191974523	Zm00001d027630
Zm00001d027630_T003	0.9498304	0.719331648	Zm00001d027630
Zm00001d027630_T009	6.85E-14	-2.907199144	Zm00001d027630
Zm00001d027630_T002	0.8613703	0.71661739	Zm00001d027630
Zm00001d027630_T008	0.4579981	2.013850695	Zm00001d027630
Zm00001d028159_T001	0.000388394	3.569764836	Zm00001d028159
Zm00001d028875_T001	0.6418619	0.498398363	Zm00001d028875
Zm00001d027236_T001	1	0.137833995	Zm00001d027236
Zm00001d052043_T003	0.8187452	0.392242732	Zm00001d052043
Zm00001d052043_T014	0.4574952	0.523320776	Zm00001d052043
Zm00001d003319_T001	7.47E-07	0.409743815	Zm00001d003319
Zm00001d003319_T002	0.8192173	0.23636675	Zm00001d003319
Zm00001d003319_T003	0.04178595	-0.304500149	Zm00001d003319
Zm00001d042183_T002	0.9049007	0.654554466	Zm00001d042183
Zm00001d042183_T001	0.9224443	-0.003840419	Zm00001d042183
Zm00001d042183_T003	0.8591616	0.440259585	Zm00001d042183
Zm00001d044391_T002	0.9519347	-0.200012642	Zm00001d044391
Zm00001d044391_T003	0.9050121	0.501265827	Zm00001d044391
Zm00001d050960_T001	0.7297076	-0.167430136	Zm00001d050960
Zm00001d021387_T001	0.9781672	0.389167172	Zm00001d021387
Zm00001d012969_T002	0.9760472	0.104343257	Zm00001d012969
Zm00001d012969_T008	0.8566319	0.698361325	Zm00001d012969
Zm00001d012969_T011	0.9788508	0.182385024	Zm00001d012969
Zm00001d012969_T009	1	0.09840419	Zm00001d012969
Zm00001d052053_T004	0.4665527	1.311513036	Zm00001d052053
Zm00001d052053_T001	0.4890076	1.17587478	Zm00001d052053
Zm00001d052053_T003	0.9633184	0.63141371	Zm00001d052053
Zm00001d036720_T001	0.4570203	1.03096367	Zm00001d036720
Zm00001d049377_T009	0.2642794	-0.341450927	Zm00001d049377

Zm00001d049377_T014	0.565911	0.68794313	Zm00001d049377
Zm00001d053875_T001	0.4425107	1.545214632	Zm00001d053875
Zm00001d053875_T003	0.08809057	1.497225733	Zm00001d053875
Zm00001d053875_T007	1.01E-12	-2.760855171	Zm00001d053875
Zm00001d038933_T001	0.7273676	-0.497382931	Zm00001d038933
Zm00001d048066_T001	0.9022629	-0.085544791	Zm00001d048066
Zm00001d013626_T001	0.9326774	-0.200177733	Zm00001d013626
Zm00001d013626_T003	0.7610869	0.462190834	Zm00001d013626
Zm00001d006059_T001	0.03181655	-1.971445103	Zm00001d006059
Zm00001d025705_T006	0.8625537	0.572223433	Zm00001d025705
Zm00001d025705_T005	0.9895224	0.103381682	Zm00001d025705
Zm00001d025705_T009	0.9939021	0.336854887	Zm00001d025705
Zm00001d025705_T010	0.8142464	-0.218370637	Zm00001d025705
Zm00001d025705_T013	0.7383611	0.269906834	Zm00001d025705
Zm00001d000403_T001	0.9550374	0.129874705	Zm00001d000403
Zm00001d010575_T001	0.7169281	-0.27891133	Zm00001d010575
Zm00001d035965_T004	0.9833565	0.039931161	Zm00001d035965
Zm00001d035965_T001	0.4096933	-0.686588336	Zm00001d035965
Zm00001d035965_T002	0.7351717	-0.368066772	Zm00001d035965
Zm00001d027302_T004	0.8715993	-0.106694956	Zm00001d027302
Zm00001d027302_T001	0.6824162	-0.092907223	Zm00001d027302
Zm00001d002585_T006	0.9753213	0.571305279	Zm00001d002585
Zm00001d002585_T009	0.7868558	0.307216896	Zm00001d002585
Zm00001d002585_T008	0.3176511	-1.172057516	Zm00001d002585
Zm00001d032402_T001	0.9213679	0.498387382	Zm00001d032402
Zm00001d006918_T001	0.9003815	-0.071823467	Zm00001d006918
Zm00001d032661_T001	0.8746701	-0.122352097	Zm00001d032661
Zm00001d031088_T001	0.9747606	0.619497127	Zm00001d031088
Zm00001d009687_T001	0.00113985	-2.79916553	Zm00001d009687
Zm00001d019576_T001	0.9817074	0.067805157	Zm00001d019576
Zm00001d026280_T001	0.9954444	0.446654736	Zm00001d026280
Zm00001d000293_T001	0.1343369	1.34059093	Zm00001d000293
Zm00001d000293_T002	0.854295	-0.089775421	Zm00001d000293
Zm00001d029814_T001	0.9706293	0.510079493	Zm00001d029814
Zm00001d047537_T003	0.8051963	-0.015599479	Zm00001d047537
Zm00001d047537_T004	0.4396083	1.182640707	Zm00001d047537
Zm00001d004354_T001	0.7594555	0.834276134	Zm00001d004354
Zm00001d004354_T002	0.9828128	0.353252525	Zm00001d004354
Zm00001d020367_T004	3.41E-11	2.045345814	Zm00001d020367
Zm00001d020367_T001	0.925875	0.331472279	Zm00001d020367
Zm00001d020367_T003	0.008308776	-1.132321772	Zm00001d020367
Zm00001d023953_T001	0.7322202	-0.459110904	Zm00001d023953
Zm00001d007090_T002	0.9354647	0.049316887	Zm00001d007090
Zm00001d007090_T001	1	0.050597891	Zm00001d007090
Zm00001d007090_T003	0.7109402	0.556351556	Zm00001d007090
Zm00001d018418_T001	0.9824348	0.162415012	Zm00001d018418
Zm00001d041679_T001	0.9410688	0.0304987	Zm00001d041679
Zm00001d041679_T006	0.9664736	0.21641711	Zm00001d041679
Zm00001d041679_T004	0.9648856	0.226860369	Zm00001d041679
Zm00001d039841_T001	0.7458694	-0.52010535	Zm00001d039841
Zm00001d022134_T001	0.6478223	-0.588610069	Zm00001d022134
Zm00001d022134_T003	0.6260085	-0.876269044	Zm00001d022134
Zm00001d022134_T002	0.9543237	-0.167164313	Zm00001d022134
Zm00001d013342_T001	0.9931431	0.328116135	Zm00001d013342

Zm00001d035053_T003	0.6154832	0.857193272	Zm00001d035053
Zm00001d035053_T001	0.7735802	0.631646616	Zm00001d035053
Zm00001d024867_T001	0.3133174	1.429940961	Zm00001d024867
Zm00001d045326_T003	0.6144625	1.024479482	Zm00001d045326
Zm00001d045326_T001	0.8127863	1.02064864	Zm00001d045326
Zm00001d045721_T001	0.5109956	-0.592033382	Zm00001d045721
Zm00001d033303_T004	0.5693278	1.327544258	Zm00001d033303
Zm00001d033303_T006	0.4553805	0.430576715	Zm00001d033303
Zm00001d033303_T008	0.9988864	0.356631118	Zm00001d033303
Zm00001d044934_T001	0.9884825	0.454351058	Zm00001d044934
Zm00001d020572_T002	0.9872831	0.060820395	Zm00001d020572
Zm00001d048131_T001	0.06731914	2.236717911	Zm00001d048131
Zm00001d043929_T001	0.941922	-0.199762169	Zm00001d043929
Zm00001d026269_T001	1	0.284336966	Zm00001d026269
Zm00001d028783_T001	1	0.138786985	Zm00001d028783
Zm00001d018501_T001	0.6673107	-0.619804431	Zm00001d018501
Zm00001d030851_T001	0.6182122	-0.401680181	Zm00001d030851
Zm00001d053798_T004	1	0.341465144	Zm00001d053798
Zm00001d053798_T001	0.6951531	-0.393282591	Zm00001d053798
Zm00001d021469_T005	0.2891367	-0.487033468	Zm00001d021469
Zm00001d021469_T008	0.06158678	0.412021188	Zm00001d021469
Zm00001d021469_T002	0.001006703	0.896031343	Zm00001d021469
Zm00001d021469_T001	0.7853478	-0.001950053	Zm00001d021469
Zm00001d035084_T001	0.2923172	-0.933109943	Zm00001d035084
Zm00001d009435_T001	0.6470421	0.786030309	Zm00001d009435
Zm00001d033642_T004	0.457673	-1.07849935	Zm00001d033642
Zm00001d033642_T001	0.9100716	0.828029179	Zm00001d033642
Zm00001d033642_T003	0.9160053	-0.0302598	Zm00001d033642
Zm00001d009445_T002	0.007518018	-0.566796727	Zm00001d009445
Zm00001d009445_T001	0.9832092	0.20275201	Zm00001d009445
Zm00001d037515_T001	0.1311444	-1.796400398	Zm00001d037515
Zm00001d045836_T001	0.9979568	0.336909244	Zm00001d045836
Zm00001d048316_T001	0.03725648	2.393415722	Zm00001d048316
Zm00001d018468_T002	0.9988864	0.185343743	Zm00001d018468
Zm00001d018468_T003	0.9539779	0.070197709	Zm00001d018468
Zm00001d018468_T001	0.7773592	-0.173439669	Zm00001d018468
Zm00001d053975_T007	0.366346	-0.858948024	Zm00001d053975
Zm00001d053975_T004	0.008230499	0.97566473	Zm00001d053975
Zm00001d053975_T002	0.9968176	0.315013987	Zm00001d053975
Zm00001d053975_T003	0.1259396	-0.666296152	Zm00001d053975
Zm00001d053975_T006	0.9048001	0.360054486	Zm00001d053975
Zm00001d030217_T012	0.5273755	-0.321465998	Zm00001d030217
Zm00001d030217_T027	0.001178527	-2.653790334	Zm00001d030217
Zm00001d030217_T001	0.8186827	0.702939241	Zm00001d030217
Zm00001d030217_T021	0.5936067	-0.443318462	Zm00001d030217
Zm00001d030217_T009	0.491591	-0.283057473	Zm00001d030217
Zm00001d030217_T029	0.1008288	-1.22257229	Zm00001d030217
Zm00001d030217_T017	0.09591861	3.016651017	Zm00001d030217
Zm00001d030217_T004	0.9852645	0.687279374	Zm00001d030217
Zm00001d025746_T001	0.9719113	0.183727142	Zm00001d025746
Zm00001d014405_T001	0.9867452	0.196824002	Zm00001d014405
Zm00001d032683_T001	0.9673473	0.174234773	Zm00001d032683
Zm00001d004559_T001	0.00506932	1.15586761	Zm00001d004559
Zm00001d004559_T027	9.62E-07	0.612365551	Zm00001d004559

Zm00001d004559_T028	0.186917	0.821259655	Zm00001d004559
Zm00001d004559_T022	0.03238748	1.194268949	Zm00001d004559
Zm00001d004559_T008	0.4698441	1.514816325	Zm00001d004559
Zm00001d048690_T001	0.2198998	-0.797542157	Zm00001d048690
Zm00001d047757_T010	0.4860327	-0.275240768	Zm00001d047757
Zm00001d047757_T001	0.9944178	0.265279805	Zm00001d047757
Zm00001d047757_T004	0.8700564	-0.073137803	Zm00001d047757
Zm00001d047757_T008	0.9621967	0.433037097	Zm00001d047757
Zm00001d047757_T014	0.3487622	-1.348789834	Zm00001d047757
Zm00001d047757_T009	0.8825173	0.829133321	Zm00001d047757
Zm00001d035253_T001	0.8575285	0.587497652	Zm00001d035253
Zm00001d007862_T001	0.9195778	0.045711839	Zm00001d007862
Zm00001d005986_T001	0.9860492	0.473417537	Zm00001d005986
Zm00001d049156_T002	0.7697196	-0.601885912	Zm00001d049156
Zm00001d049156_T003	0.3543475	-1.311168007	Zm00001d049156
Zm00001d049156_T001	0.139268	-1.703528383	Zm00001d049156
Zm00001d003930_T001	0.5294537	-0.866705832	Zm00001d003930
Zm00001d016529_T002	1	0.185616033	Zm00001d016529
Zm00001d048799_T001	0.8747821	-0.173546096	Zm00001d048799
Zm00001d006315_T001	0.9909466	0.245285966	Zm00001d006315
Zm00001d004309_T001	0.9950006	1.523217935	Zm00001d004309
Zm00001d001880_T001	0.7807489	-0.133636369	Zm00001d001880
Zm00001d001880_T002	0.8413408	-0.496303278	Zm00001d001880
Zm00001d001880_T003	0.745806	-0.497599517	Zm00001d001880
Zm00001d020332_T001	2.97E-05	2.900132865	Zm00001d020332
Zm00001d014967_T003	1.50E-09	-1.435055387	Zm00001d014967
Zm00001d014967_T009	0.003441561	2.41598509	Zm00001d014967
Zm00001d014967_T010	0.9942835	0.120126153	Zm00001d014967
Zm00001d014967_T008	0.9701341	0.504980774	Zm00001d014967
Zm00001d014967_T001	0.9945592	0.430747164	Zm00001d014967
Zm00001d014967_T039	0.7790464	-0.285412123	Zm00001d014967
Zm00001d007573_T001	0.7289978	-0.387332351	Zm00001d007573
Zm00001d009564_T001	0.9791729	0.538734048	Zm00001d009564
Zm00001d048454_T005	0.9210327	0.275207687	Zm00001d048454
Zm00001d018230_T001	0.9326879	0.706720178	Zm00001d018230
Zm00001d002744_T001	0.7389125	-0.788512922	Zm00001d002744
Zm00001d014692_T001	0.9914036	0.073674344	Zm00001d014692
Zm00001d035527_T001	0.9739189	0.5524893	Zm00001d035527
Zm00001d052492_T001	0.3563242	-0.77771932	Zm00001d052492
Zm00001d032669_T001	0.9559189	0.158100339	Zm00001d032669
Zm00001d021633_T007	0.8001345	-0.22266589	Zm00001d021633
Zm00001d021633_T003	0.9722469	-0.03417486	Zm00001d021633
Zm00001d021633_T006	0.9845237	0.101676359	Zm00001d021633
Zm00001d021633_T012	0.5199848	-0.334014769	Zm00001d021633
Zm00001d021633_T005	0.9957373	0.060912274	Zm00001d021633
Zm00001d021633_T010	0.9894615	0.158233406	Zm00001d021633
Zm00001d021633_T009	0.135934	1.01936853	Zm00001d021633
Zm00001d021633_T011	0.9792387	0.355802058	Zm00001d021633
Zm00001d015082_T010	0.06745171	0.716053044	Zm00001d015082
Zm00001d015082_T001	2.08E-12	1.766557196	Zm00001d015082
Zm00001d015082_T015	0.000496965	0.586590704	Zm00001d015082
Zm00001d015082_T036	0.9320554	0.710296279	Zm00001d015082
Zm00001d015082_T014	0.01442294	0.59736717	Zm00001d015082
Zm00001d015082_T037	0.5552199	0.593559066	Zm00001d015082

Zm00001d015082_T026	0.1425146	0.935739541	Zm00001d015082
Zm00001d012674_T004	0.9740325	0.558809231	Zm00001d012674
Zm00001d012674_T011	0.9384219	0.603712173	Zm00001d012674
Zm00001d012674_T013	0.8412913	-0.573759251	Zm00001d012674
Zm00001d012674_T008	0.9835181	0.317256133	Zm00001d012674
Zm00001d012674_T006	0.8128711	0.838262053	Zm00001d012674
Zm00001d012674_T009	0.9967276	-0.070522155	Zm00001d012674
Zm00001d012674_T007	0.6970851	0.899979656	Zm00001d012674
Zm00001d004086_T001	0.9782953	0.509288086	Zm00001d004086
Zm00001d035234_T001	0.8445154	0.667437088	Zm00001d035234
Zm00001d006864_T003	0.1585484	-0.210772935	Zm00001d006864
Zm00001d006864_T043	0.3497147	-0.581874994	Zm00001d006864
Zm00001d006864_T040	0.6086477	0.144026593	Zm00001d006864
Zm00001d006864_T021	0.6129837	1.149990108	Zm00001d006864
Zm00001d038384_T001	1	-0.169681015	Zm00001d038384
Zm00001d006070_T005	0.8585804	0.376233745	Zm00001d006070
Zm00001d006070_T002	0.9969123	0.384278075	Zm00001d006070
Zm00001d006070_T008	0.9135335	-0.882763284	Zm00001d006070
Zm00001d006070_T003	0.90591	0.502654937	Zm00001d006070
Zm00001d006070_T004	0.8997501	0.663802186	Zm00001d006070
Zm00001d012404_T003	0.8908955	0.771290564	Zm00001d012404
Zm00001d029630_T001	0.2252392	-1.808188255	Zm00001d029630
Zm00001d052941_T001	0.8478451	-0.157628741	Zm00001d052941
Zm00001d031754_T001	0.8412913	0.565061631	Zm00001d031754
Zm00001d012464_T002	0.8281834	0.638433132	Zm00001d012464
Zm00001d012464_T003	0.7921111	0.380783097	Zm00001d012464
Zm00001d003904_T003	0.900979	-0.019751415	Zm00001d003904
Zm00001d003904_T007	0.903659	-0.27987122	Zm00001d003904
Zm00001d003904_T001	0.8064667	-0.316305238	Zm00001d003904
Zm00001d003904_T004	0.8951059	0.60313197	Zm00001d003904
Zm00001d012482_T001	0.06899302	0.958062105	Zm00001d012482
Zm00001d025379_T001	0.9871621	0.184898725	Zm00001d025379
Zm00001d029331_T006	0.5570384	-0.9942539	Zm00001d029331
Zm00001d029331_T003	0.98291	0.291849897	Zm00001d029331
Zm00001d029331_T001	0.9706887	0.585645198	Zm00001d029331
Zm00001d048463_T004	0.9763608	0.586379241	Zm00001d048463
Zm00001d048463_T005	0.8197974	0.3753051	Zm00001d048463
Zm00001d048463_T006	0.9956119	0.135085505	Zm00001d048463
Zm00001d028452_T001	0.8197974	-0.113889713	Zm00001d028452
Zm00001d031954_T005	0.957148	-0.088524918	Zm00001d031954
Zm00001d003066_T002	0.8660897	-0.127138905	Zm00001d003066
Zm00001d003066_T004	0.6019353	-0.595925665	Zm00001d003066
Zm00001d033370_T002	0.9791729	0.451242731	Zm00001d033370
Zm00001d033370_T007	0.9469426	-0.158279286	Zm00001d033370
Zm00001d033370_T006	0.9850313	0.250499667	Zm00001d033370
Zm00001d052059_T001	0.5103529	-0.642574087	Zm00001d052059
Zm00001d053563_T004	0.9956119	0.431138451	Zm00001d053563
Zm00001d053563_T003	0.9210327	0.374704672	Zm00001d053563
Zm00001d053563_T002	0.9833568	0.498030588	Zm00001d053563
Zm00001d037495_T002	1	0.321920447	Zm00001d037495
Zm00001d014735_T001	0.9745777	0.207033479	Zm00001d014735
Zm00001d041993_T001	0.015044	-0.818046413	Zm00001d041993
Zm00001d041993_T003	0.9936589	0.007025555	Zm00001d041993
Zm00001d045121_T001	0.7785869	-0.48159743	Zm00001d045121

Zm00001d025325_T003	0.3497147	-0.740080077	Zm00001d025325
Zm00001d025325_T002	0.3082157	-0.961325742	Zm00001d025325
Zm00001d020242_T008	0.9988864	0.087476741	Zm00001d020242
Zm00001d020242_T004	0.9663913	0.565803519	Zm00001d020242
Zm00001d020242_T001	0.9869996	0.230944196	Zm00001d020242
Zm00001d052404_T003	1	0.33185559	Zm00001d052404
Zm00001d052404_T002	0.9740325	0.120003002	Zm00001d052404
Zm00001d052404_T004	0.9037324	0.468181556	Zm00001d052404
Zm00001d052404_T005	0.9419336	0.405758291	Zm00001d052404
Zm00001d009847_T001	0.8834406	-0.434702176	Zm00001d009847
Zm00001d009594_T001	0.9410845	0.051924758	Zm00001d009594
Zm00001d009594_T004	0.9886745	0.285903115	Zm00001d009594
Zm00001d009594_T003	0.6787725	-0.067364871	Zm00001d009594
Zm00001d003276_T001	0.8740066	0.539607966	Zm00001d003276
Zm00001d052111_T034	0.5864438	0.79853757	Zm00001d052111
Zm00001d052111_T025	0.3818599	0.255181246	Zm00001d052111
Zm00001d052111_T020	0.002796955	0.367720601	Zm00001d052111
Zm00001d052111_T008	0.1451373	1.191858479	Zm00001d052111
Zm00001d052111_T032	0.5336382	1.510108604	Zm00001d052111
Zm00001d052111_T033	0.0428905	-1.060901301	Zm00001d052111
Zm00001d052111_T014	0.2912906	0.468227336	Zm00001d052111
Zm00001d052111_T005	0.9693993	0.638691882	Zm00001d052111
Zm00001d008856_T004	0.9706887	0.18058755	Zm00001d008856
Zm00001d008856_T008	0.556323	-0.618137093	Zm00001d008856
Zm00001d008856_T005	0.605961	-0.371140712	Zm00001d008856
Zm00001d008856_T001	0.7146325	-0.432722725	Zm00001d008856
Zm00001d016851_T001	0.9588234	0.05645318	Zm00001d016851
Zm00001d034036_T007	0.4824723	0.963351327	Zm00001d034036
Zm00001d034036_T005	0.9469426	0.289570591	Zm00001d034036
Zm00001d034036_T004	0.3836885	1.400776904	Zm00001d034036
Zm00001d033405_T003	0.8807673	-0.082856549	Zm00001d033405
Zm00001d050245_T001	0.9129118	-0.089678706	Zm00001d050245
Zm00001d018093_T001	0.002196051	1.073812971	Zm00001d018093
Zm00001d038318_T007	0.8896102	0.762204216	Zm00001d038318
Zm00001d038318_T008	0.9138633	0.732734878	Zm00001d038318
Zm00001d038318_T011	0.3059054	-1.130802325	Zm00001d038318
Zm00001d000168_T001	0.9221549	0.025992671	Zm00001d000168
Zm00001d009134_T001	0.953745	0.018999343	Zm00001d009134
Zm00001d011364_T001	1	0.283833126	Zm00001d011364
Zm00001d045537_T018	0.9187004	0.443257014	Zm00001d045537
Zm00001d045537_T010	0.2546213	1.528570116	Zm00001d045537
Zm00001d045537_T019	0.5902617	1.08986086	Zm00001d045537
Zm00001d045537_T011	0.203535	1.651817492	Zm00001d045537
Zm00001d045537_T021	0.8890931	0.262700695	Zm00001d045537
Zm00001d045537_T003	0.7907819	0.714243369	Zm00001d045537
Zm00001d045537_T006	0.01967564	1.583594551	Zm00001d045537
Zm00001d018793_T001	0.001943159	3.265720386	Zm00001d018793
Zm00001d014883_T001	0.7074815	-0.700703046	Zm00001d014883
Zm00001d014716_T002	0.6843488	-0.335060713	Zm00001d014716
Zm00001d016823_T001	0.2153703	1.239226917	Zm00001d016823
Zm00001d049286_T001	0.000833983	-0.499161266	Zm00001d049286
Zm00001d049286_T003	0.546505	-0.377200245	Zm00001d049286
Zm00001d049286_T002	1	0.255674265	Zm00001d049286
Zm00001d012892_T002	0.4250221	-0.516003156	Zm00001d012892

Zm00001d012892_T005	0.9562304	0.686011573	Zm00001d012892
Zm00001d012892_T006	0.6812088	-0.33087366	Zm00001d012892
Zm00001d012892_T001	1.30E-07	1.916053658	Zm00001d012892
Zm00001d046263_T001	0.9822138	0.236227193	Zm00001d046263
Zm00001d046263_T002	0.2913804	0.459929167	Zm00001d046263
Zm00001d046263_T004	0.3284493	1.042993257	Zm00001d046263
Zm00001d005507_T002	0.556738	0.803493076	Zm00001d005507
Zm00001d005507_T013	0.1565832	-0.904732736	Zm00001d005507
Zm00001d005507_T010	2.44E-07	1.772665693	Zm00001d005507
Zm00001d005507_T005	0.536983	1.18972239	Zm00001d005507
Zm00001d020087_T001	0.5864438	0.805240808	Zm00001d020087
Zm00001d012655_T002	0.9912983	0.428312304	Zm00001d012655
Zm00001d006295_T009	0.4386149	0.391968726	Zm00001d006295
Zm00001d006295_T005	0.9303773	0.483530084	Zm00001d006295
Zm00001d006295_T006	0.9862402	0.448705195	Zm00001d006295
Zm00001d006295_T011	0.8264047	0.389111585	Zm00001d006295
Zm00001d021822_T005	0.1920144	0.937918486	Zm00001d021822
Zm00001d021822_T002	0.9014274	-0.501809944	Zm00001d021822
Zm00001d000066_T001	0.8125495	-0.528663629	Zm00001d000066
Zm00001d050247_T001	0.005257717	2.371822462	Zm00001d050247
Zm00001d018822_T001	0.7249455	-0.530832906	Zm00001d018822
Zm00001d007324_T004	0.009667038	-2.126513993	Zm00001d007324
Zm00001d007324_T005	0.9949037	0.366026725	Zm00001d007324
Zm00001d025869_T001	0.3212723	0.737792392	Zm00001d025869
Zm00001d025869_T003	0.4900864	1.365642921	Zm00001d025869
Zm00001d025869_T002	0.1989366	1.574687603	Zm00001d025869
Zm00001d025869_T004	0.5437049	0.856953954	Zm00001d025869
Zm00001d025869_T005	0.3949594	0.889984801	Zm00001d025869
Zm00001d013461_T001	0.2997051	-1.026922932	Zm00001d013461
Zm00001d006002_T001	0.945968	-0.235583003	Zm00001d006002
Zm00001d005468_T001	0.9534335	0.547226454	Zm00001d005468
Zm00001d005468_T002	0.03639015	-1.983945008	Zm00001d005468
Zm00001d013729_T004	1	0.152047382	Zm00001d013729
Zm00001d013729_T002	0.9909153	0.252281626	Zm00001d013729
Zm00001d009494_T001	0.1616926	-0.806065316	Zm00001d009494
Zm00001d052520_T001	0.04416456	-1.865490417	Zm00001d052520
Zm00001d002674_T001	0.9835181	0.126536656	Zm00001d002674
Zm00001d019562_T001	0.9134699	-0.470224509	Zm00001d019562
Zm00001d019562_T002	0.872254	-0.02434219	Zm00001d019562
Zm00001d005059_T002	0.9621967	0.165900274	Zm00001d005059
Zm00001d005059_T004	0.7368534	-0.098376619	Zm00001d005059
Zm00001d034380_T001	1	0.382669766	Zm00001d034380
Zm00001d037452_T001	0.9702493	-0.022263935	Zm00001d037452
Zm00001d037452_T003	0.8991659	0.566793648	Zm00001d037452
Zm00001d028778_T002	0.7481233	-0.308490886	Zm00001d028778
Zm00001d003283_T001	0.9326774	0.674161595	Zm00001d003283
Zm00001d012171_T001	0.9965998	0.014650057	Zm00001d012171
Zm00001d014994_T001	0.9979568	0.393570421	Zm00001d014994
Zm00001d005016_T003	0.9370633	-0.006154263	Zm00001d005016
Zm00001d005016_T007	0.5640366	1.220497574	Zm00001d005016
Zm00001d005016_T004	0.437589	1.049584513	Zm00001d005016
Zm00001d014894_T001	0.9410845	-0.134531986	Zm00001d014894
Zm00001d016471_T001	0.360669	-0.814191283	Zm00001d016471
Zm00001d034253_T001	0.8428212	-0.08398714	Zm00001d034253

Zm00001d043137_T006	0.6589089	1.726777398	Zm00001d043137
Zm00001d043137_T001	0.852305	-0.532268019	Zm00001d043137
Zm00001d043137_T005	0.972985	0.236101534	Zm00001d043137
Zm00001d030935_T019	0.2587265	0.255524007	Zm00001d030935
Zm00001d030935_T021	3.16E-11	1.511923223	Zm00001d030935
Zm00001d030935_T002	0.8412913	0.098239889	Zm00001d030935
Zm00001d030935_T007	0.09551638	0.499507426	Zm00001d030935
Zm00001d030935_T001	0.01614531	0.539424026	Zm00001d030935
Zm00001d030935_T023	0.9141981	0.043159717	Zm00001d030935
Zm00001d030935_T029	0.3495559	-0.888375752	Zm00001d030935
Zm00001d002896_T001	0.7345621	-0.337660046	Zm00001d002896
Zm00001d029232_T001	0.9206245	0.483981212	Zm00001d029232
Zm00001d035889_T005	0.98291	0.575687641	Zm00001d035889
Zm00001d035889_T021	0.5332769	0.469918238	Zm00001d035889
Zm00001d035889_T025	0.01814386	1.043327293	Zm00001d035889
Zm00001d035889_T036	0.4395938	0.333394021	Zm00001d035889
Zm00001d035889_T022	0.5328547	0.222416539	Zm00001d035889
Zm00001d035889_T001	0.005324772	2.564780899	Zm00001d035889
Zm00001d026252_T001	0.9429978	0.001920231	Zm00001d026252
Zm00001d000122_T005	0.337622	0.473490437	Zm00001d000122
Zm00001d008408_T001	0.8827359	0.676806623	Zm00001d008408
Zm00001d048201_T001	0.8643636	0.20666273	Zm00001d048201
Zm00001d048201_T012	1	0.455232683	Zm00001d048201
Zm00001d048201_T009	0.9880075	0.151850262	Zm00001d048201
Zm00001d012265_T001	0.9187559	0.627884833	Zm00001d012265
Zm00001d017536_T001	0.5883436	1.289969002	Zm00001d017536
Zm00001d007436_T002	0.9765009	0.120036081	Zm00001d007436
Zm00001d007436_T004	0.6538483	0.922257187	Zm00001d007436
Zm00001d007436_T006	0.9216521	0.445990891	Zm00001d007436
Zm00001d007436_T003	0.9797082	0.087569437	Zm00001d007436
Zm00001d018962_T001	0.1238066	-1.282919344	Zm00001d018962
Zm00001d034920_T001	0.001261061	-2.255457308	Zm00001d034920
Zm00001d017103_T001	0.3706063	-0.445806197	Zm00001d017103
Zm00001d017103_T005	0.9749152	0.061383936	Zm00001d017103
Zm00001d035957_T001	0.9913455	0.454879487	Zm00001d035957
Zm00001d030328_T001	0.5613757	-0.29755101	Zm00001d030328
Zm00001d032921_T002	0.9915818	0.40571004	Zm00001d032921
Zm00001d001846_T001	0.9975882	0.49342555	Zm00001d001846
Zm00001d044038_T001	0.919127	0.748205597	Zm00001d044038
Zm00001d033322_T001	0.8656506	0.777685078	Zm00001d033322
Zm00001d017608_T001	0.9766874	0.371586946	Zm00001d017608
Zm00001d033505_T001	0.9825858	0.494002812	Zm00001d033505
Zm00001d021621_T022	0.6538483	-0.476528545	Zm00001d021621
Zm00001d021621_T010	0.2905294	1.412225392	Zm00001d021621
Zm00001d021621_T016	0.8720321	0.329528083	Zm00001d021621
Zm00001d041725_T001	0.9103625	0.502902732	Zm00001d041725
Zm00001d038189_T004	0.8027057	0.553365774	Zm00001d038189
Zm00001d038189_T001	0.05225416	-0.514747763	Zm00001d038189
Zm00001d038189_T014	0.9180865	0.030580593	Zm00001d038189
Zm00001d007160_T001	0.6086477	-0.533399566	Zm00001d007160
Zm00001d015921_T001	0.1738057	-1.282162439	Zm00001d015921
Zm00001d044690_T001	0.8031436	0.692104566	Zm00001d044690
Zm00001d005970_T001	0.198071	1.452999664	Zm00001d005970
Zm00001d021936_T001	0.9908137	0.310158789	Zm00001d021936

Zm00001d027937_T014	0.675716	-0.213829195	Zm00001d027937
Zm00001d011562_T004	0.9160053	-0.066455607	Zm00001d011562
Zm00001d011562_T001	1	0.194766781	Zm00001d011562
Zm00001d011562_T002	0.9689939	0.104041256	Zm00001d011562
Zm00001d020053_T001	0.7720277	-0.749680017	Zm00001d020053
Zm00001d020053_T003	0.4100322	1.238729497	Zm00001d020053
Zm00001d020053_T002	0.4471095	-0.689937989	Zm00001d020053
Zm00001d015246_T004	0.6579839	-0.081790053	Zm00001d015246
Zm00001d015246_T015	0.9771057	0.198770671	Zm00001d015246
Zm00001d015246_T009	0.9785005	-0.00345752	Zm00001d015246
Zm00001d015246_T002	0.2526036	0.950002863	Zm00001d015246
Zm00001d015246_T007	0.000920521	1.272761051	Zm00001d015246
Zm00001d015246_T013	0.3020148	-0.572341366	Zm00001d015246
Zm00001d015246_T012	0.7114361	0.339838345	Zm00001d015246
Zm00001d053682_T002	0.6597828	-0.834878285	Zm00001d053682
Zm00001d053682_T001	0.9954426	0.24547252	Zm00001d053682
Zm00001d040627_T001	0.9843639	0.299101447	Zm00001d040627
Zm00001d044220_T009	0.1527884	0.224617126	Zm00001d044220
Zm00001d044220_T006	0.9791674	0.183859042	Zm00001d044220
Zm00001d005156_T005	0.4881794	0.230660359	Zm00001d005156
Zm00001d005156_T002	0.9946646	0.28746369	Zm00001d005156
Zm00001d003889_T001	0.9361945	-0.022194385	Zm00001d003889
Zm00001d003889_T002	0.5369562	-1.087193996	Zm00001d003889
Zm00001d025075_T002	0.9001702	0.326736552	Zm00001d025075
Zm00001d025075_T004	0.9134699	0.409200125	Zm00001d025075
Zm00001d025075_T003	0.9578348	0.041569467	Zm00001d025075
Zm00001d025075_T001	0.7614965	-0.143063291	Zm00001d025075
Zm00001d017268_T001	0.8061103	0.771062417	Zm00001d017268
Zm00001d017390_T001	0.9821463	0.440923212	Zm00001d017390
Zm00001d048312_T001	0.7904511	0.790732671	Zm00001d048312
Zm00001d013089_T001	0.4698008	-0.769299564	Zm00001d013089
Zm00001d013089_T002	0.799639	0.7343693	Zm00001d013089
Zm00001d003757_T001	0.7412102	-0.667955964	Zm00001d003757
Zm00001d050068_T001	0.8530103	-0.443758113	Zm00001d050068
Zm00001d027835_T001	0.1020373	1.493511909	Zm00001d027835
Zm00001d012546_T003	0.971204	0.523859645	Zm00001d012546
Zm00001d026513_T001	0.8099578	0.6976092	Zm00001d026513
Zm00001d045763_T002	0.5730988	1.318391426	Zm00001d045763
Zm00001d012836_T001	0.1906729	-1.251678658	Zm00001d012836
Zm00001d029009_T002	0.9609806	0.568083542	Zm00001d029009
Zm00001d029009_T004	0.3389645	0.444675412	Zm00001d029009
Zm00001d009879_T002	1	0.288878176	Zm00001d009879
Zm00001d036610_T001	0.00116817	3.070082029	Zm00001d036610
Zm00001d053881_T001	0.9817074	0.606525453	Zm00001d053881
Zm00001d014058_T006	0.8508148	-0.111075278	Zm00001d014058
Zm00001d014058_T007	0.9979568	0.942476383	Zm00001d014058
Zm00001d014058_T008	0.1608582	-0.585059913	Zm00001d014058
Zm00001d014058_T001	0.4902998	0.00911988	Zm00001d014058
Zm00001d014058_T005	0.6680426	-0.404031958	Zm00001d014058
Zm00001d036315_T001	0.6470421	0.86210029	Zm00001d036315
Zm00001d047868_T001	0.9192642	-0.017883822	Zm00001d047868
Zm00001d002798_T001	0.08857859	-2.129720039	Zm00001d002798
Zm00001d029543_T001	0.5415683	-1.013718649	Zm00001d029543
Zm00001d007508_T009	0.4823171	-1.139898993	Zm00001d007508

Zm00001d007508_T008	0.4029381	-0.210227819	Zm00001d007508
Zm00001d007508_T007	1.87E-07	-1.282579492	Zm00001d007508
Zm00001d007508_T006	0.9216521	1.618899634	Zm00001d007508
Zm00001d017199_T001	0.805584	0.735258758	Zm00001d017199
Zm00001d038004_T001	0.98291	-0.066862576	Zm00001d038004
Zm00001d015714_T006	0.9781672	0.180225485	Zm00001d015714
Zm00001d015714_T002	0.7472497	1.596608369	Zm00001d015714
Zm00001d051251_T003	0.9954426	0.338551502	Zm00001d051251
Zm00001d031937_T002	0.9190522	-0.194570924	Zm00001d031937
Zm00001d012426_T001	0.6605884	0.922614217	Zm00001d012426
Zm00001d020973_T003	0.8412913	-0.174863174	Zm00001d020973
Zm00001d020973_T004	0.6632799	-0.127783008	Zm00001d020973
Zm00001d020973_T001	4.62E-07	-1.343550212	Zm00001d020973
Zm00001d033062_T001	0.1439251	-0.809332875	Zm00001d033062
Zm00001d035760_T001	0.9206245	-0.202131516	Zm00001d035760
Zm00001d008597_T004	0.9384219	0.048749332	Zm00001d008597
Zm00001d020429_T001	0.01190273	2.864255917	Zm00001d020429
Zm00001d038840_T001	0.945968	0.016336352	Zm00001d038840
Zm00001d010824_T001	0.9942835	0.294916222	Zm00001d010824
Zm00001d022275_T003	0.3836954	-1.1705444	Zm00001d022275
Zm00001d022275_T001	0.956053	0.602114162	Zm00001d022275
Zm00001d000419_T001	0.8519292	0.6698669	Zm00001d000419
Zm00001d024058_T001	0.9962521	0.33846889	Zm00001d024058
Zm00001d016744_T001	0.9765009	0.127782332	Zm00001d016744
Zm00001d026040_T001	0.04008706	-1.689721772	Zm00001d026040
Zm00001d051568_T001	0.8430106	-0.131309822	Zm00001d051568
Zm00001d002252_T001	0.9441469	0.695154011	Zm00001d002252
Zm00001d001781_T001	0.8998563	-0.040484193	Zm00001d001781
Zm00001d014897_T001	0.9918302	0.506215472	Zm00001d014897
Zm00001d014897_T003	0.2280926	-1.342837229	Zm00001d014897
Zm00001d035772_T006	0.1142947	0.693781375	Zm00001d035772
Zm00001d035772_T005	0.1440511	-0.394437851	Zm00001d035772
Zm00001d035772_T001	0.6026247	0.043644042	Zm00001d035772
Zm00001d035772_T004	0.9107993	0.836254656	Zm00001d035772
Zm00001d035772_T007	1	0.201639313	Zm00001d035772
Zm00001d035772_T002	0.9535734	0.646792241	Zm00001d035772
Zm00001d035772_T003	0.7449399	-0.61383011	Zm00001d035772
Zm00001d027394_T001	0.7136625	-0.971348455	Zm00001d027394
Zm00001d039452_T003	0.9818992	0.251418902	Zm00001d039452
Zm00001d039452_T001	0.9627389	0.173718554	Zm00001d039452
Zm00001d039452_T002	1	0.410504968	Zm00001d039452
Zm00001d015200_T002	0.9802412	0.154775789	Zm00001d015200
Zm00001d042583_T001	0.9974734	0.224712189	Zm00001d042583
Zm00001d022347_T001	0.9469426	0.06655862	Zm00001d022347
Zm00001d011970_T001	0.9889811	0.274773683	Zm00001d011970
Zm00001d028102_T001	0.9693045	0.10531308	Zm00001d028102
Zm00001d047492_T001	0.1459499	-1.377979397	Zm00001d047492
Zm00001d013440_T001	0.8318191	-0.31690314	Zm00001d013440
Zm00001d051480_T001	0.6779665	-0.356487247	Zm00001d051480
Zm00001d051839_T003	0.9984041	0.033400018	Zm00001d051839
Zm00001d051839_T009	0.3182957	0.433654805	Zm00001d051839
Zm00001d051839_T015	0.9375274	0.849351879	Zm00001d051839
Zm00001d051839_T002	0.03715458	1.066613077	Zm00001d051839
Zm00001d051839_T006	0.8383745	0.41638098	Zm00001d051839

Zm00001d036573_T004	0.1277345	1.306317877	Zm00001d036573
Zm00001d036573_T002	0.9233022	0.000316279	Zm00001d036573
Zm00001d036573_T005	0.5718169	0.515937194	Zm00001d036573
Zm00001d036573_T001	0.930095	-0.038335701	Zm00001d036573
Zm00001d010514_T001	0.9946911	0.269471938	Zm00001d010514
Zm00001d037871_T005	1	0.336834803	Zm00001d037871
Zm00001d037871_T006	0.974657	0.057056758	Zm00001d037871
Zm00001d005358_T001	0.9493302	0.129458803	Zm00001d005358
Zm00001d012708_T004	0.9942091	0.172600847	Zm00001d012708
Zm00001d012708_T003	0.9237921	0.566820168	Zm00001d012708
Zm00001d027595_T001	0.7613681	-0.436599884	Zm00001d027595
Zm00001d049442_T013	0.8044932	0.809571429	Zm00001d049442
Zm00001d049442_T010	0.8819727	0.455095292	Zm00001d049442
Zm00001d049442_T005	0.9947387	0.277562051	Zm00001d049442
Zm00001d037816_T002	0.1134387	-0.859603406	Zm00001d037816
Zm00001d043207_T002	0.957265	0.023942883	Zm00001d043207
Zm00001d043207_T003	0.7305041	-0.339885409	Zm00001d043207
Zm00001d024612_T013	0.8486887	-0.073246383	Zm00001d024612
Zm00001d024612_T019	0.755035	0.590760521	Zm00001d024612
Zm00001d024612_T014	0.7055225	0.417831973	Zm00001d024612
Zm00001d024612_T038	0.6578675	0.430285383	Zm00001d024612
Zm00001d024612_T047	0.9975882	0.326119309	Zm00001d024612
Zm00001d024612_T066	0.6689911	0.440080637	Zm00001d024612
Zm00001d024612_T041	0.8958882	0.213920111	Zm00001d024612
Zm00001d024612_T039	0.107968	0.58714857	Zm00001d024612
Zm00001d024612_T023	0.9822138	0.322954706	Zm00001d024612
Zm00001d024612_T004	0.9293689	0.137403551	Zm00001d024612
Zm00001d024612_T031	0.991004	0.180422444	Zm00001d024612
Zm00001d024612_T053	0.9257893	0.329527511	Zm00001d024612
Zm00001d024612_T020	0.3537794	0.632921311	Zm00001d024612
Zm00001d024612_T067	0.02875644	-0.797857883	Zm00001d024612
Zm00001d024612_T015	0.9074725	0.635661049	Zm00001d024612
Zm00001d024612_T069	0.1798861	-1.397561862	Zm00001d024612
Zm00001d024612_T018	0.1966199	0.394243685	Zm00001d024612
Zm00001d024612_T007	0.8906466	-0.118232712	Zm00001d024612
Zm00001d024612_T029	0.7146325	0.520085705	Zm00001d024612
Zm00001d048194_T002	0.9745084	0.078302791	Zm00001d048194
Zm00001d018719_T001	0.9822138	0.247465429	Zm00001d018719
Zm00001d025689_T006	4.89E-07	-2.807160814	Zm00001d025689
Zm00001d025689_T001	0.9928593	0.196710746	Zm00001d025689
Zm00001d025689_T003	0.9690782	0.584764653	Zm00001d025689
Zm00001d051502_T004	0.9037324	0.787812741	Zm00001d051502
Zm00001d051502_T006	0.3579474	0.992607032	Zm00001d051502
Zm00001d051502_T001	0.8409438	0.777703799	Zm00001d051502
Zm00001d051502_T005	0.007924806	0.816629768	Zm00001d051502
Zm00001d032275_T001	0.9356767	-0.043324237	Zm00001d032275
Zm00001d015127_T001	0.556323	-0.887495513	Zm00001d015127
Zm00001d037346_T016	0.1358919	1.4687326	Zm00001d037346
Zm00001d037346_T030	3.40E-05	1.787913451	Zm00001d037346
Zm00001d037346_T043	0.5260431	1.110257234	Zm00001d037346
Zm00001d037346_T017	0.342013	0.881741099	Zm00001d037346
Zm00001d037346_T024	0.007365988	2.292464236	Zm00001d037346
Zm00001d037346_T041	0.3301595	0.693902278	Zm00001d037346
Zm00001d037346_T009	0.4715806	0.582352494	Zm00001d037346

Zm00001d037346_T047	0.7931524	-0.232898449	Zm00001d037346
Zm00001d037346_T045	0.09841706	2.115078469	Zm00001d037346
Zm00001d037346_T003	0.8726127	0.26676952	Zm00001d037346
Zm00001d037346_T014	3.50E-14	-2.182719357	Zm00001d037346
Zm00001d037346_T040	1	0.077076966	Zm00001d037346
Zm00001d037346_T028	1	0.140894257	Zm00001d037346
Zm00001d037346_T039	0.07387502	0.928165213	Zm00001d037346
Zm00001d011845_T001	0.9470409	-0.14543346	Zm00001d011845
Zm00001d002366_T007	0.007738702	1.229507016	Zm00001d002366
Zm00001d002366_T004	0.9498304	0.034264894	Zm00001d002366
Zm00001d002366_T003	0.8188746	0.833727204	Zm00001d002366
Zm00001d002366_T008	0.8342838	0.247467977	Zm00001d002366
Zm00001d002366_T006	0.1842201	-0.565691175	Zm00001d002366
Zm00001d014471_T001	0.9429712	0.333696738	Zm00001d014471
Zm00001d010162_T001	0.998762	0.389879567	Zm00001d010162
Zm00001d006930_T001	0.8117344	-0.609319744	Zm00001d006930
Zm00001d021653_T001	0.001035991	3.256846864	Zm00001d021653
Zm00001d021653_T002	9.20E-05	2.750771654	Zm00001d021653
Zm00001d024333_T001	0.9768802	0.360477936	Zm00001d024333
Zm00001d024333_T003	0.9733967	0.302242401	Zm00001d024333
Zm00001d051174_T003	0.9894615	0.115588103	Zm00001d051174
Zm00001d051174_T002	0.9984041	0.411274278	Zm00001d051174
Zm00001d021567_T001	0.7900674	1.052703176	Zm00001d021567
Zm00001d023620_T001	0.4701937	-0.935158467	Zm00001d023620
Zm00001d051424_T001	0.9532189	0.525299069	Zm00001d051424
Zm00001d016866_T002	0.796163	0.995641311	Zm00001d016866
Zm00001d016866_T001	0.9213679	0.564411513	Zm00001d016866
Zm00001d039899_T001	0.9659036	-0.011669534	Zm00001d039899
Zm00001d005756_T014	0.8704891	-0.187541125	Zm00001d005756
Zm00001d005756_T003	0.655817	0.85196785	Zm00001d005756
Zm00001d005756_T001	0.1440511	0.47405865	Zm00001d005756
Zm00001d004123_T003	0.9160053	0.654844262	Zm00001d004123
Zm00001d004123_T001	0.9720425	0.521905362	Zm00001d004123
Zm00001d030347_T001	0.9778436	0.491192124	Zm00001d030347
Zm00001d029447_T001	0.000188838	-3.387037456	Zm00001d029447
Zm00001d035047_T002	0.706283	-0.302887379	Zm00001d035047
Zm00001d035047_T001	0.6984773	-0.279736705	Zm00001d035047
Zm00001d035047_T003	0.3392838	-0.33226891	Zm00001d035047
Zm00001d002065_T001	0.8747821	-0.050791433	Zm00001d002065
Zm00001d002065_T006	1	0.400610058	Zm00001d002065
Zm00001d002065_T004	0.0314624	-1.430174049	Zm00001d002065
Zm00001d002065_T012	0.5472547	-0.868924337	Zm00001d002065
Zm00001d002065_T008	0.9730261	0.115654738	Zm00001d002065
Zm00001d026239_T007	0.8557307	0.854379392	Zm00001d026239
Zm00001d026239_T008	0.8788699	-0.087814309	Zm00001d026239
Zm00001d026239_T001	0.622153	-0.333303947	Zm00001d026239
Zm00001d029223_T003	1	0.134223103	Zm00001d029223
Zm00001d029223_T001	0.1257966	1.562603435	Zm00001d029223
Zm00001d029223_T015	0.8819727	0.860211557	Zm00001d029223
Zm00001d005099_T003	0.9954444	0.39825858	Zm00001d005099
Zm00001d005099_T001	0.8129337	0.265155294	Zm00001d005099
Zm00001d013046_T001	0.1944318	2.058976443	Zm00001d013046
Zm00001d013046_T004	0.01853475	2.87336693	Zm00001d013046
Zm00001d013046_T005	5.03E-06	1.288494128	Zm00001d013046

Zm00001d013046_T006	0.6873614	1.167428854	Zm00001d013046
Zm00001d033551_T047	0.9408399	0.165037849	Zm00001d033551
Zm00001d033551_T033	0.1159359	0.598366098	Zm00001d033551
Zm00001d043971_T001	0.9924805	0.101215655	Zm00001d043971
Zm00001d051380_T001	0.5196715	-0.758467906	Zm00001d051380
Zm00001d048588_T001	0.7680039	0.724401296	Zm00001d048588
Zm00001d034166_T023	0.9843847	0.169086787	Zm00001d034166
Zm00001d034166_T007	0.9887435	0.275396972	Zm00001d034166
Zm00001d034166_T008	0.9539896	0.037919805	Zm00001d034166
Zm00001d051592_T001	0.945968	0.416132508	Zm00001d051592
Zm00001d033589_T001	0.7771984	-0.641908426	Zm00001d033589
Zm00001d002596_T001	0.2090746	-1.125804753	Zm00001d002596
Zm00001d036789_T003	0.7292793	0.956143063	Zm00001d036789
Zm00001d036789_T005	1	0.072778085	Zm00001d036789
Zm00001d022569_T001	0.9845237	-0.238100885	Zm00001d022569
Zm00001d005392_T002	0.2855784	-0.968967559	Zm00001d005392
Zm00001d005392_T001	0.2056602	-0.381878878	Zm00001d005392
Zm00001d047583_T002	0.859708	0.77422943	Zm00001d047583
Zm00001d047583_T003	0.5509552	0.560212	Zm00001d047583
Zm00001d047583_T001	0.8486461	-0.13909728	Zm00001d047583
Zm00001d033158_T001	0.3224744	-0.249721792	Zm00001d033158
Zm00001d033158_T004	0.967161	0.453837828	Zm00001d033158
Zm00001d033158_T005	0.000117201	-2.012725423	Zm00001d033158
Zm00001d033158_T009	0.0101257	-1.163748829	Zm00001d033158
Zm00001d033158_T006	0.1779352	-0.635397568	Zm00001d033158
Zm00001d033158_T007	0.9817074	0.119684321	Zm00001d033158
Zm00001d033158_T011	0.01477598	-1.705535732	Zm00001d033158
Zm00001d000035_T001	0.9782953	0.142420645	Zm00001d000035
Zm00001d040052_T001	1	0.155454769	Zm00001d040052
Zm00001d007067_T002	0.8107845	-0.289401872	Zm00001d007067
Zm00001d007067_T003	0.9040872	0.768604202	Zm00001d007067
Zm00001d007067_T001	0.9706887	0.145308653	Zm00001d007067
Zm00001d045078_T001	0.5572941	-0.536781759	Zm00001d045078
Zm00001d001962_T001	0.1293734	0.300224291	Zm00001d001962
Zm00001d001962_T014	0.9863483	0.562108086	Zm00001d001962
Zm00001d001962_T013	0.07948208	0.528227731	Zm00001d001962
Zm00001d001962_T020	0.1424006	0.427773121	Zm00001d001962
Zm00001d001962_T017	0.8660897	0.926662819	Zm00001d001962
Zm00001d001962_T031	0.5864438	-0.591720572	Zm00001d001962
Zm00001d042837_T001	0.04727699	-1.51306122	Zm00001d042837
Zm00001d038920_T001	0.6767305	-0.458815784	Zm00001d038920
Zm00001d007241_T001	0.8591203	0.886735998	Zm00001d007241
Zm00001d007241_T003	0.2053901	1.40793229	Zm00001d007241
Zm00001d021031_T002	0.2568783	-0.906592543	Zm00001d021031
Zm00001d021031_T001	0.9410845	0.593580559	Zm00001d021031
Zm00001d021031_T006	0.9682673	0.099135889	Zm00001d021031
Zm00001d021031_T003	0.816571	0.723947946	Zm00001d021031
Zm00001d029203_T002	0.6023287	-0.568180076	Zm00001d029203
Zm00001d029203_T001	0.3966246	-0.839917801	Zm00001d029203
Zm00001d047876_T001	0.8591203	-0.234410965	Zm00001d047876
Zm00001d047876_T003	1	0.218615872	Zm00001d047876
Zm00001d053358_T001	0.8543977	-0.240646349	Zm00001d053358
Zm00001d051865_T001	0.1535144	-1.070053964	Zm00001d051865
Zm00001d006670_T002	0.8884927	0.697186499	Zm00001d006670

Zm00001d015956_T001	0.8880558	-0.13633444	Zm00001d015956
Zm00001d015956_T003	1	0.160168333	Zm00001d015956
Zm00001d018554_T001	0.9987435	0.188945807	Zm00001d018554
Zm00001d050382_T005	0.5748211	-0.310705564	Zm00001d050382
Zm00001d050382_T010	0.9781672	0.156368864	Zm00001d050382
Zm00001d050382_T007	0.956184	0.19941839	Zm00001d050382
Zm00001d050382_T001	0.8862653	0.088621417	Zm00001d050382
Zm00001d043990_T001	0.8267985	-0.468607005	Zm00001d043990
Zm00001d007111_T001	1	0.396266057	Zm00001d007111
Zm00001d003725_T001	0.7010603	-0.440031358	Zm00001d003725
Zm00001d043516_T001	0.9834601	0.239515525	Zm00001d043516
Zm00001d020644_T001	0.96672	-0.02906152	Zm00001d020644
Zm00001d051980_T001	0.000401065	2.52644488	Zm00001d051980
Zm00001d051980_T004	0.8389117	0.102814151	Zm00001d051980
Zm00001d051980_T010	0.9693222	0.686952273	Zm00001d051980
Zm00001d051980_T006	0.0877477	1.839809875	Zm00001d051980
Zm00001d051980_T003	0.6023541	0.272734004	Zm00001d051980
Zm00001d042461_T001	0.1607286	2.014228346	Zm00001d042461
Zm00001d011874_T001	0.008186499	2.766330542	Zm00001d011874
Zm00001d014565_T001	0.8555271	-0.331027507	Zm00001d014565
Zm00001d021214_T001	0.01322411	2.34354602	Zm00001d021214
Zm00001d011165_T002	0.9272378	0.006913505	Zm00001d011165
Zm00001d048017_T006	0.8757823	0.81447534	Zm00001d048017
Zm00001d048017_T004	0.908501	0.078534381	Zm00001d048017
Zm00001d050810_T001	0.9501111	0.016308357	Zm00001d050810
Zm00001d012818_T001	0.9860836	0.128491972	Zm00001d012818
Zm00001d036080_T002	0.9804228	-0.127437167	Zm00001d036080
Zm00001d036080_T001	0.9596377	-0.342006504	Zm00001d036080
Zm00001d015101_T001	0.8819727	-0.072761367	Zm00001d015101
Zm00001d015101_T002	0.9582981	0.319022332	Zm00001d015101
Zm00001d032357_T001	0.9812314	0.206227814	Zm00001d032357
Zm00001d020751_T001	0.01854488	2.354987214	Zm00001d020751
Zm00001d034054_T001	0.7305041	0.276844026	Zm00001d034054
Zm00001d034054_T008	0.571366	-0.771090149	Zm00001d034054
Zm00001d034054_T006	0.941922	0.524820613	Zm00001d034054
Zm00001d002545_T004	0.4099581	-0.49841874	Zm00001d002545
Zm00001d002545_T001	0.05009396	-1.248011109	Zm00001d002545
Zm00001d002545_T002	0.6220253	-0.341341348	Zm00001d002545
Zm00001d031560_T002	0.9149322	-0.187647915	Zm00001d031560
Zm00001d031560_T003	0.6466858	-1.024318613	Zm00001d031560
Zm00001d011632_T001	1	0.305724205	Zm00001d011632
Zm00001d011632_T002	0.6456233	1.023578202	Zm00001d011632
Zm00001d011632_T003	0.7631862	-0.484512044	Zm00001d011632
Zm00001d034521_T001	0.9906533	0.135657191	Zm00001d034521
Zm00001d035089_T003	0.000326064	0.768003616	Zm00001d035089
Zm00001d035089_T005	0.01943884	1.218669935	Zm00001d035089
Zm00001d050658_T001	0.9880075	0.11432156	Zm00001d050658
Zm00001d051645_T001	0.5292611	-0.783899923	Zm00001d051645
Zm00001d043785_T002	0.9936409	0.27344927	Zm00001d043785
Zm00001d043785_T003	0.8264567	0.537843648	Zm00001d043785
Zm00001d043785_T006	0.4396896	0.952792146	Zm00001d043785
Zm00001d028631_T002	0.9773578	0.178323324	Zm00001d028631
Zm00001d033858_T001	0.6629026	0.693281662	Zm00001d033858
Zm00001d033858_T002	1	0.292929209	Zm00001d033858

Zm00001d027485_T001	0.586618	-0.715853991	Zm00001d027485
Zm00001d028226_T001	0.4289876	-0.877594915	Zm00001d028226
Zm00001d011576_T001	0.8681121	0.233043322	Zm00001d011576
Zm00001d048385_T001	0.9322451	-0.038424629	Zm00001d048385
Zm00001d048385_T002	0.8990393	0.504850483	Zm00001d048385
Zm00001d023807_T002	0.9907928	0.326045275	Zm00001d023807
Zm00001d012448_T001	0.9689939	0.119270943	Zm00001d012448
Zm00001d028619_T003	0.002628689	1.160150882	Zm00001d028619
Zm00001d028619_T017	0.9825858	0.456617578	Zm00001d028619
Zm00001d028619_T015	0.7187138	0.958393466	Zm00001d028619
Zm00001d028619_T004	0.0483178	0.962094011	Zm00001d028619
Zm00001d028619_T001	0.9833565	0.473008769	Zm00001d028619
Zm00001d028619_T013	0.3342888	0.516023968	Zm00001d028619
Zm00001d028619_T002	0.5537579	1.20556492	Zm00001d028619
Zm00001d043904_T001	0.8746701	-0.257933401	Zm00001d043904
Zm00001d014204_T001	0.8057128	-0.251101591	Zm00001d014204
Zm00001d033541_T002	1	0.059245888	Zm00001d033541
Zm00001d016235_T001	0.5387165	1.038519745	Zm00001d016235
Zm00001d023579_T001	0.873307	0.877893779	Zm00001d023579
Zm00001d023579_T002	0.9747606	0.163656095	Zm00001d023579
Zm00001d053109_T001	0.522218	-0.180538838	Zm00001d053109
Zm00001d022229_T001	1	0.153995487	Zm00001d022229
Zm00001d002199_T002	0.7431788	0.586635296	Zm00001d002199
Zm00001d024424_T001	0.8891117	0.336857548	Zm00001d024424
Zm00001d025907_T001	0.9187493	-0.01628578	Zm00001d025907
Zm00001d053162_T003	0.9781672	0.521281681	Zm00001d053162
Zm00001d010213_T003	0.8909361	0.68146684	Zm00001d010213
Zm00001d010213_T002	0.8791987	0.706714995	Zm00001d010213
Zm00001d010213_T004	0.2719671	-1.969959387	Zm00001d010213
Zm00001d010213_T005	3.52E-08	2.422196162	Zm00001d010213
Zm00001d010213_T001	0.9361945	0.633845079	Zm00001d010213
Zm00001d054093_T006	0.8668602	-0.599221527	Zm00001d054093
Zm00001d022307_T002	0.7224803	-0.663815874	Zm00001d022307
Zm00001d049364_T001	0.8557049	0.831454042	Zm00001d049364
Zm00001d028590_T001	0.8660897	-0.454682376	Zm00001d028590
Zm00001d048281_T001	0.1379797	1.299188823	Zm00001d048281
Zm00001d006064_T001	0.850642	-0.528003234	Zm00001d006064
Zm00001d012982_T001	1	0.188750289	Zm00001d012982
Zm00001d047842_T001	0.3941988	1.323608081	Zm00001d047842
Zm00001d047842_T006	0.08392805	-2.087320778	Zm00001d047842
Zm00001d047842_T007	0.2445116	1.27067172	Zm00001d047842
Zm00001d047842_T004	0.8883561	0.782196979	Zm00001d047842
Zm00001d033334_T001	0.9224443	0.760714738	Zm00001d033334
Zm00001d033334_T008	0.7195936	0.331877277	Zm00001d033334
Zm00001d033334_T010	0.9712373	0.264180244	Zm00001d033334
Zm00001d006364_T001	0.113946	-0.736692251	Zm00001d006364
Zm00001d043871_T001	0.7565187	-0.133875841	Zm00001d043871
Zm00001d046060_T005	0.971204	0.356922185	Zm00001d046060
Zm00001d046060_T001	0.8830711	0.752164531	Zm00001d046060
Zm00001d005203_T001	0.6325449	-0.789206138	Zm00001d005203
Zm00001d051875_T001	0.9326774	0.087105322	Zm00001d051875
Zm00001d035014_T001	0.9201237	-0.099941576	Zm00001d035014
Zm00001d032902_T001	0.7105308	-0.389359769	Zm00001d032902
Zm00001d017791_T003	0.6450292	1.138206191	Zm00001d017791

Zm00001d017791_T001	0.8667498	1.032888378	Zm00001d017791
Zm00001d046033_T001	0.9693382	0.530261682	Zm00001d046033
Zm00001d011762_T001	1	0.22598861	Zm00001d011762
Zm00001d035012_T001	0.9948424	0.234772905	Zm00001d035012
Zm00001d045108_T001	0.9334932	-0.121352673	Zm00001d045108
Zm00001d032565_T001	0.8109283	-0.300148018	Zm00001d032565
Zm00001d030777_T002	0.6231523	0.782562009	Zm00001d030777
Zm00001d045589_T001	0.2040851	-1.044573609	Zm00001d045589
Zm00001d053968_T004	0.6894164	0.73656512	Zm00001d053968
Zm00001d053968_T001	0.000939984	-0.768245244	Zm00001d053968
Zm00001d053968_T007	0.4163478	1.40177947	Zm00001d053968
Zm00001d053968_T006	0.9395327	-0.056117411	Zm00001d053968
Zm00001d031267_T001	0.9817074	0.483789878	Zm00001d031267
Zm00001d037332_T003	0.001767431	0.470159559	Zm00001d037332
Zm00001d037332_T007	0.9509252	0.253363399	Zm00001d037332
Zm00001d037332_T005	1.21E-12	-2.874309476	Zm00001d037332
Zm00001d037332_T008	0.4527189	1.472685557	Zm00001d037332
Zm00001d037332_T001	0.03648535	1.863951618	Zm00001d037332
Zm00001d037332_T004	0.967161	0.043631224	Zm00001d037332
Zm00001d049706_T018	0.7830446	-0.096502945	Zm00001d049706
Zm00001d049706_T003	0.04999669	0.669371081	Zm00001d049706
Zm00001d049706_T028	0.00012109	-1.264398206	Zm00001d049706
Zm00001d049706_T013	1.27E-12	2.226745097	Zm00001d049706
Zm00001d049706_T010	4.64E-11	-1.580857356	Zm00001d049706
Zm00001d049706_T001	0.7169281	-0.061794815	Zm00001d049706
Zm00001d049706_T009	0.7753093	-0.233383351	Zm00001d049706
Zm00001d019641_T001	0.8968577	0.268530556	Zm00001d019641
Zm00001d019641_T002	0.2131685	-0.78455664	Zm00001d019641
Zm00001d040986_T001	0.9468268	-0.218339847	Zm00001d040986
Zm00001d037109_T002	0.9535734	0.699435015	Zm00001d037109
Zm00001d037109_T005	0.146054	0.701634151	Zm00001d037109
Zm00001d037109_T001	0.7442388	0.861719613	Zm00001d037109
Zm00001d037109_T006	0.8896281	0.891278303	Zm00001d037109
Zm00001d037109_T007	0.8720862	-0.017711044	Zm00001d037109
Zm00001d009409_T004	0.9979568	0.346652907	Zm00001d009409
Zm00001d048565_T001	0.7582314	-0.430809123	Zm00001d048565
Zm00001d006402_T003	0.7933933	0.526801665	Zm00001d006402
Zm00001d006402_T004	2.50E-07	-1.017622583	Zm00001d006402
Zm00001d006402_T002	0.745718	0.618007921	Zm00001d006402
Zm00001d034502_T002	0.7925341	-0.067432245	Zm00001d034502
Zm00001d034502_T003	0.6648793	0.99295858	Zm00001d034502
Zm00001d034502_T001	0.06712735	-1.063512806	Zm00001d034502
Zm00001d007347_T001	0.3003804	-1.121362344	Zm00001d007347
Zm00001d037772_T002	0.2645158	0.586641593	Zm00001d037772
Zm00001d037772_T001	0.9311783	0.873132745	Zm00001d037772
Zm00001d037772_T003	0.103478	0.528533914	Zm00001d037772
Zm00001d037772_T014	0.000208615	2.257810127	Zm00001d037772
Zm00001d037772_T013	0.004443898	-1.416584003	Zm00001d037772
Zm00001d004176_T001	0.965347	0.151055801	Zm00001d004176
Zm00001d011828_T003	0.8987811	-0.038184024	Zm00001d011828
Zm00001d011828_T002	0.24481	-0.562452364	Zm00001d011828
Zm00001d036529_T001	0.923446	-0.168542298	Zm00001d036529
Zm00001d005881_T011	0.7428299	0.860887024	Zm00001d005881
Zm00001d033749_T001	0.9968176	0.355438357	Zm00001d033749

Zm00001d019225_T001	0.5698311	-0.540441707	Zm00001d019225
Zm00001d029064_T001	1	0.375718489	Zm00001d029064
Zm00001d053210_T001	0.8746701	0.484316265	Zm00001d053210
Zm00001d008531_T005	0.9468268	0.336583419	Zm00001d008531
Zm00001d008531_T007	0.1778349	1.457661786	Zm00001d008531
Zm00001d008531_T006	0.7842749	0.624822158	Zm00001d008531
Zm00001d008531_T001	0.9446379	0.110469598	Zm00001d008531
Zm00001d008531_T004	0.708953	0.286145021	Zm00001d008531
Zm00001d017874_T001	0.4012088	1.509934476	Zm00001d017874
Zm00001d017998_T001	0.4310439	-0.890003513	Zm00001d017998
Zm00001d009631_T007	0.03956497	1.408093361	Zm00001d009631
Zm00001d009631_T001	0.5088062	1.299549598	Zm00001d009631
Zm00001d009631_T008	0.07457948	2.016297441	Zm00001d009631
Zm00001d009631_T009	0.04270301	1.468608176	Zm00001d009631
Zm00001d037859_T002	0.9580867	-0.025887383	Zm00001d037859
Zm00001d020825_T001	0.9544587	0.047056492	Zm00001d020825
Zm00001d044833_T001	0.02224341	2.533074387	Zm00001d044833
Zm00001d044833_T002	3.94E-05	3.791147489	Zm00001d044833
Zm00001d041696_T001	0.9228108	0.59857957	Zm00001d041696
Zm00001d047658_T001	0.8107845	0.928718716	Zm00001d047658
Zm00001d006578_T003	0.1293734	2.141146759	Zm00001d006578
Zm00001d006578_T004	0.3140033	1.560535093	Zm00001d006578
Zm00001d006578_T001	0.7120282	1.144850754	Zm00001d006578
Zm00001d006578_T002	0.003573197	1.144697103	Zm00001d006578
Zm00001d021607_T001	1	0.371220581	Zm00001d021607
Zm00001d039032_T001	0.7300888	0.635812927	Zm00001d039032
Zm00001d026345_T003	0.7020458	-0.656629672	Zm00001d026345
Zm00001d026345_T002	0.9818452	0.406291248	Zm00001d026345
Zm00001d034350_T003	0.7866898	-0.028393974	Zm00001d034350
Zm00001d034350_T010	0.8031436	0.971561125	Zm00001d034350
Zm00001d034350_T002	0.8908501	0.053664648	Zm00001d034350
Zm00001d034350_T009	0.6689911	0.962166819	Zm00001d034350
Zm00001d047355_T001	0.9222107	0.63142851	Zm00001d047355
Zm00001d053882_T001	0.9171732	0.782517455	Zm00001d053882
Zm00001d012505_T001	0.001452291	1.999985758	Zm00001d012505
Zm00001d013020_T001	0.8256776	0.708422146	Zm00001d013020
Zm00001d012812_T001	0.962254	0.129271628	Zm00001d012812
Zm00001d016130_T001	0.1669576	2.043045479	Zm00001d016130
Zm00001d016130_T002	0.2536137	1.822854146	Zm00001d016130
Zm00001d022159_T001	0.957768	0.056980168	Zm00001d022159
Zm00001d022159_T002	0.991538	0.25197066	Zm00001d022159
Zm00001d022391_T001	0.8958882	0.67183382	Zm00001d022391
Zm00001d022391_T002	6.56E-06	0.671124373	Zm00001d022391
Zm00001d013444_T004	0.4552715	0.30477361	Zm00001d013444
Zm00001d013444_T002	0.8817916	0.87822954	Zm00001d013444
Zm00001d050604_T011	0.07443173	1.818556048	Zm00001d050604
Zm00001d050604_T009	0.0982823	0.993285317	Zm00001d050604
Zm00001d050604_T015	0.4895863	-0.778492098	Zm00001d050604
Zm00001d050604_T002	0.91126	0.242258753	Zm00001d050604
Zm00001d050604_T006	0.9701986	-0.040118758	Zm00001d050604
Zm00001d050604_T010	0.749176	0.351717127	Zm00001d050604
Zm00001d016894_T002	0.5080934	1.306486116	Zm00001d016894
Zm00001d007233_T001	1	0.250962251	Zm00001d007233
Zm00001d047184_T002	0.8958882	-0.150371672	Zm00001d047184

Zm00001d047184_T001	0.705921	-0.26067559	Zm00001d047184
Zm00001d037098_T005	0.6134185	-0.829436152	Zm00001d037098
Zm00001d037098_T001	0.447037	1.274218157	Zm00001d037098
Zm00001d037098_T004	0.3893584	0.939514265	Zm00001d037098
Zm00001d037098_T006	0.1003679	1.106541839	Zm00001d037098
Zm00001d031597_T001	1	0.01400502	Zm00001d031597
Zm00001d026312_T001	0.8535962	-0.287710004	Zm00001d026312
Zm00001d026312_T004	0.9719113	0.403334394	Zm00001d026312
Zm00001d026312_T005	0.9519347	0.004024106	Zm00001d026312
Zm00001d026312_T002	0.9886837	0.234347909	Zm00001d026312
Zm00001d026312_T003	1	0.188862181	Zm00001d026312
Zm00001d026312_T007	0.8224042	0.755242095	Zm00001d026312
Zm00001d020972_T005	0.06348214	0.601867515	Zm00001d020972
Zm00001d020972_T003	0.4925065	1.366426815	Zm00001d020972
Zm00001d020972_T009	0.9121108	0.791354165	Zm00001d020972
Zm00001d021844_T026	1.04E-05	0.842534246	Zm00001d021844
Zm00001d021844_T021	0.6936253	0.917214295	Zm00001d021844
Zm00001d021844_T049	0.2572389	-0.861725611	Zm00001d021844
Zm00001d021844_T009	0.003015693	0.65002806	Zm00001d021844
Zm00001d021844_T027	0.01652636	0.512808711	Zm00001d021844
Zm00001d021844_T036	0.000820562	1.208096046	Zm00001d021844
Zm00001d021844_T071	0.536983	-0.556885484	Zm00001d021844
Zm00001d021844_T030	0.7333	-0.3412259	Zm00001d021844
Zm00001d021844_T077	0.1177128	-1.540883198	Zm00001d021844
Zm00001d021844_T024	0.2698646	0.883883337	Zm00001d021844
Zm00001d021844_T061	0.01181493	0.548601485	Zm00001d021844
Zm00001d021844_T054	0.9973181	0.307759032	Zm00001d021844
Zm00001d021844_T032	0.000203051	1.875864779	Zm00001d021844
Zm00001d021844_T057	0.9975882	0.04118451	Zm00001d021844
Zm00001d021844_T075	0.9782953	0.041497027	Zm00001d021844
Zm00001d021844_T025	0.8564933	-0.160275034	Zm00001d021844
Zm00001d021844_T058	0.01475604	0.990086009	Zm00001d021844
Zm00001d021844_T044	0.1950386	0.317083799	Zm00001d021844
Zm00001d021844_T048	0.005375707	1.052553388	Zm00001d021844
Zm00001d025294_T001	0.8075512	-0.307957589	Zm00001d025294
Zm00001d033635_T002	0.9016285	-0.075483693	Zm00001d033635
Zm00001d017607_T001	0.7952435	-0.079372592	Zm00001d017607
Zm00001d014280_T001	0.9766874	0.615766279	Zm00001d014280
Zm00001d014280_T003	0.3713446	0.356994555	Zm00001d014280
Zm00001d007039_T001	0.8575285	-0.123632148	Zm00001d007039
Zm00001d023294_T001	0.4283203	-0.686463919	Zm00001d023294
Zm00001d010204_T001	1	0.147202206	Zm00001d010204
Zm00001d006184_T001	0.8139802	0.867792729	Zm00001d006184
Zm00001d043159_T001	0.8489797	0.883714523	Zm00001d043159
Zm00001d050831_T001	0.9160957	0.605352946	Zm00001d050831
Zm00001d016492_T001	1	-0.21235935	Zm00001d016492
Zm00001d048336_T005	0.7454635	0.264210641	Zm00001d048336
Zm00001d048336_T004	0.9514141	0.057287665	Zm00001d048336
Zm00001d039653_T003	0.9524127	0.016260282	Zm00001d039653
Zm00001d039653_T009	0.9660311	0.112235457	Zm00001d039653
Zm00001d039653_T005	0.4439665	-0.064182009	Zm00001d039653
Zm00001d039653_T002	1	0.287120616	Zm00001d039653
Zm00001d039653_T015	0.02643233	0.925124046	Zm00001d039653
Zm00001d039653_T007	0.6672656	-0.104012113	Zm00001d039653

Zm00001d008299_T002	0.9315495	-0.021869512	Zm00001d008299
Zm00001d021418_T001	0.9745948	-0.184844254	Zm00001d021418
Zm00001d019219_T001	0.9096001	-0.319648274	Zm00001d019219
Zm00001d019219_T003	0.07180163	-2.010837613	Zm00001d019219
Zm00001d015272_T021	0.5368112	0.968394062	Zm00001d015272
Zm00001d015272_T011	0.9979568	0.027318036	Zm00001d015272
Zm00001d015272_T024	0.9122528	0.00837912	Zm00001d015272
Zm00001d015272_T013	0.9159727	0.399224157	Zm00001d015272
Zm00001d015272_T012	0.4879596	0.858606441	Zm00001d015272
Zm00001d015272_T023	0.9825858	0.203862229	Zm00001d015272
Zm00001d015272_T008	0.000329177	0.685358073	Zm00001d015272
Zm00001d015272_T004	0.01873138	1.918944096	Zm00001d015272
Zm00001d032644_T002	0.8376861	0.463621041	Zm00001d032644
Zm00001d048542_T001	0.9706887	0.117453701	Zm00001d048542
Zm00001d047906_T001	0.9986486	0.132539091	Zm00001d047906
Zm00001d047906_T006	0.9910452	0.048871873	Zm00001d047906
Zm00001d047906_T009	0.2133145	1.473299418	Zm00001d047906
Zm00001d047906_T012	0.6248209	1.084871748	Zm00001d047906
Zm00001d047906_T010	0.3181489	0.553167872	Zm00001d047906
Zm00001d047906_T011	0.944802	-0.086738929	Zm00001d047906
Zm00001d047906_T007	0.7789533	0.562711266	Zm00001d047906
Zm00001d047906_T008	0.9627389	-0.021784023	Zm00001d047906
Zm00001d047906_T013	0.9561521	-0.262217115	Zm00001d047906
Zm00001d033979_T001	0.9400285	-0.152067138	Zm00001d033979
Zm00001d048159_T001	0.7220539	-0.469971179	Zm00001d048159
Zm00001d012752_T008	0.9050121	0.466349563	Zm00001d012752
Zm00001d023575_T001	0.9984041	0.202953273	Zm00001d023575
Zm00001d011119_T008	0.9833565	0.235904146	Zm00001d011119
Zm00001d011119_T016	0.6217057	0.469217899	Zm00001d011119
Zm00001d011119_T017	0.7599359	0.332798774	Zm00001d011119
Zm00001d011119_T019	0.9369505	0.653718937	Zm00001d011119
Zm00001d011119_T023	0.9766874	0.049517228	Zm00001d011119
Zm00001d011119_T013	0.9469426	0.062800563	Zm00001d011119
Zm00001d011119_T022	0.2078632	1.134797891	Zm00001d011119
Zm00001d011119_T011	0.4422995	0.889488008	Zm00001d011119
Zm00001d011119_T018	0.9851204	1.123841102	Zm00001d011119
Zm00001d011119_T015	0.9139882	-0.088563646	Zm00001d011119
Zm00001d011119_T003	0.0340858	1.859870689	Zm00001d011119
Zm00001d025296_T002	0.7194918	-0.484986848	Zm00001d025296
Zm00001d025296_T001	0.5328547	-0.723604907	Zm00001d025296
Zm00001d042152_T002	0.8529214	-0.130106618	Zm00001d042152
Zm00001d042152_T015	0.7885216	-0.113593265	Zm00001d042152
Zm00001d042152_T011	1.28E-09	1.465583155	Zm00001d042152
Zm00001d042152_T012	0.8857953	0.571981564	Zm00001d042152
Zm00001d042152_T006	1	0.102852394	Zm00001d042152
Zm00001d042152_T008	0.8665954	-0.08153438	Zm00001d042152
Zm00001d042152_T005	0.5902617	1.276537814	Zm00001d042152
Zm00001d001978_T090	1.57E-08	0.784705519	Zm00001d001978
Zm00001d001978_T133	4.15E-13	-2.476237287	Zm00001d001978
Zm00001d001978_T115	0.009336484	1.052481325	Zm00001d001978
Zm00001d001978_T137	0.9817074	0.074554976	Zm00001d001978
Zm00001d001978_T134	2.27E-12	-2.396876311	Zm00001d001978
Zm00001d001978_T121	0.05539594	2.205803181	Zm00001d001978
Zm00001d001978_T014	4.53E-05	2.521461393	Zm00001d001978

Zm00001d001978_T131	0.02653131	0.67192682	Zm00001d001978
Zm00001d001978_T001	0.1017596	1.49667778	Zm00001d001978
Zm00001d001978_T122	0.3668531	1.046658901	Zm00001d001978
Zm00001d001978_T009	0.04824454	0.443413585	Zm00001d001978
Zm00001d047275_T003	0.5220513	0.640571077	Zm00001d047275
Zm00001d031217_T002	0.9380783	0.746748599	Zm00001d031217
Zm00001d031217_T005	0.3636519	0.395392526	Zm00001d031217
Zm00001d042899_T001	0.4393923	1.46852379	Zm00001d042899
Zm00001d042899_T004	0.8839371	0.666550446	Zm00001d042899
Zm00001d042899_T002	0.8583496	0.899308496	Zm00001d042899
Zm00001d042899_T003	0.6391307	0.580877103	Zm00001d042899
Zm00001d027800_T001	0.7389826	0.701672482	Zm00001d027800
Zm00001d045417_T003	0.9311027	0.413886415	Zm00001d045417
Zm00001d045417_T001	0.9358417	0.075789767	Zm00001d045417
Zm00001d045417_T002	0.3706063	1.16297393	Zm00001d045417
Zm00001d036091_T001	0.7516958	-0.452150194	Zm00001d036091
Zm00001d038012_T001	0.9569719	0.576467908	Zm00001d038012
Zm00001d051404_T015	0.6619288	-0.363915972	Zm00001d051404
Zm00001d051404_T001	0.6347225	-0.354840045	Zm00001d051404
Zm00001d051404_T014	0.000340227	2.204563104	Zm00001d051404
Zm00001d006243_T025	0.5909717	0.899484294	Zm00001d006243
Zm00001d006243_T016	0.9045566	0.311430638	Zm00001d006243
Zm00001d006243_T002	1.52E-06	0.754647058	Zm00001d006243
Zm00001d013122_T003	0.9048001	0.227823148	Zm00001d013122
Zm00001d013122_T012	0.6280761	1.049714218	Zm00001d013122
Zm00001d013122_T015	0.9717919	0.140931818	Zm00001d013122
Zm00001d013122_T014	1	0.105572307	Zm00001d013122
Zm00001d002051_T001	0.9942835	0.329626963	Zm00001d002051
Zm00001d042972_T001	0.5936067	1.116594695	Zm00001d042972
Zm00001d042972_T007	0.3928501	-0.562137387	Zm00001d042972
Zm00001d042972_T004	0.8376343	0.352426591	Zm00001d042972
Zm00001d042972_T002	0.920208	0.028784828	Zm00001d042972
Zm00001d003525_T003	0.9279367	0.25679208	Zm00001d003525
Zm00001d031894_T003	0.9706293	0.050233842	Zm00001d031894
Zm00001d031894_T006	0.9869265	0.202489295	Zm00001d031894
Zm00001d042141_T001	0.5759563	1.220497569	Zm00001d042141
Zm00001d028597_T002	0.9073468	-0.005959764	Zm00001d028597
Zm00001d028597_T007	0.4302501	0.532332425	Zm00001d028597
Zm00001d012595_T001	0.9486412	0.466392859	Zm00001d012595
Zm00001d010583_T001	0.9730613	0.086437241	Zm00001d010583
Zm00001d040118_T001	0.6954735	-0.866312198	Zm00001d040118
Zm00001d025339_T006	0.9326774	0.61823923	Zm00001d025339
Zm00001d025339_T004	0.8276231	-0.134839597	Zm00001d025339
Zm00001d025339_T002	1	-0.167873701	Zm00001d025339
Zm00001d025339_T001	0.9673094	0.309653314	Zm00001d025339
Zm00001d025339_T003	0.9933044	-0.257923924	Zm00001d025339
Zm00001d030998_T015	0.6967776	0.809284526	Zm00001d030998
Zm00001d030998_T020	0.2566009	-0.400892951	Zm00001d030998
Zm00001d030998_T019	0.9747714	0.169906922	Zm00001d030998
Zm00001d030998_T016	0.3605911	0.985021411	Zm00001d030998
Zm00001d037616_T001	0.9024791	0.690647035	Zm00001d037616
Zm00001d040731_T002	0.9171653	-0.022235208	Zm00001d040731
Zm00001d040731_T004	0.8987811	0.698998981	Zm00001d040731
Zm00001d040731_T003	0.007772245	1.540327082	Zm00001d040731

Zm00001d012052_T001	0.6450309	-0.786427856	Zm00001d012052
Zm00001d005257_T001	0.9963508	-0.014761375	Zm00001d005257
Zm00001d005257_T002	0.6325449	0.947882717	Zm00001d005257
Zm00001d005257_T006	0.3936871	1.284354518	Zm00001d005257
Zm00001d038336_T001	0.9429712	0.494947666	Zm00001d038336
Zm00001d032739_T007	0.2860629	-0.614313371	Zm00001d032739
Zm00001d032739_T004	0.8031436	-0.129062097	Zm00001d032739
Zm00001d032739_T002	0.6570312	-0.242268867	Zm00001d032739
Zm00001d032739_T005	0.9988864	0.156315976	Zm00001d032739
Zm00001d032739_T006	0.3492462	-0.645762771	Zm00001d032739
Zm00001d032739_T001	0.8720862	0.177568017	Zm00001d032739
Zm00001d032739_T009	0.9988954	0.363062329	Zm00001d032739
Zm00001d051561_T001	0.960704	0.549023924	Zm00001d051561
Zm00001d003390_T001	0.8595871	-0.301706267	Zm00001d003390
Zm00001d030748_T001	0.8486887	0.967979009	Zm00001d030748
Zm00001d010493_T001	0.9037002	-0.248793045	Zm00001d010493
Zm00001d039328_T001	0.3323541	-0.399142438	Zm00001d039328
Zm00001d039328_T002	0.2183827	-0.868858965	Zm00001d039328
Zm00001d039328_T004	4.38E-07	-3.576811838	Zm00001d039328
Zm00001d039328_T006	0.05243369	-1.503842691	Zm00001d039328
Zm00001d027319_T006	0.8974665	0.617105096	Zm00001d027319
Zm00001d027319_T001	0.9683047	0.495815345	Zm00001d027319
Zm00001d029706_T001	0.01858035	-1.530325387	Zm00001d029706
Zm00001d005030_T005	0.3385848	1.043847511	Zm00001d005030
Zm00001d005030_T006	0.6890838	-0.691498103	Zm00001d005030
Zm00001d005030_T001	0.9979568	0.18597751	Zm00001d005030
Zm00001d005030_T008	0.4146004	0.450800137	Zm00001d005030
Zm00001d005030_T004	0.5134498	-0.305326234	Zm00001d005030
Zm00001d016100_T001	0.9906533	0.178153632	Zm00001d016100
Zm00001d013644_T002	0.9751899	0.081332122	Zm00001d013644
Zm00001d013644_T004	0.9019262	0.895105955	Zm00001d013644
Zm00001d013644_T003	0.5756796	-0.074576026	Zm00001d013644
Zm00001d011643_T001	0.9846714	0.390835257	Zm00001d011643
Zm00001d008679_T008	0.942537	1.462853387	Zm00001d008679
Zm00001d008679_T002	0.000115801	-1.832899593	Zm00001d008679
Zm00001d008679_T009	0.9851204	0.107545419	Zm00001d008679
Zm00001d008679_T005	0.9945118	0.315880529	Zm00001d008679
Zm00001d008679_T010	0.05933188	1.854057801	Zm00001d008679
Zm00001d048777_T014	0.1252127	0.411996376	Zm00001d048777
Zm00001d048777_T006	0.9303773	0.084044396	Zm00001d048777
Zm00001d048777_T017	0.6543848	1.065541534	Zm00001d048777
Zm00001d048777_T026	0.7179988	-0.446990129	Zm00001d048777
Zm00001d048777_T018	0.9853158	0.256590905	Zm00001d048777
Zm00001d048777_T013	0.1051593	-0.742930728	Zm00001d048777
Zm00001d048777_T008	0.7400422	1.242849215	Zm00001d048777
Zm00001d024510_T002	0.9894615	0.397304768	Zm00001d024510
Zm00001d024510_T004	0.8495463	0.652796233	Zm00001d024510
Zm00001d024510_T001	0.5730137	-0.771092783	Zm00001d024510
Zm00001d044656_T001	0.9846714	0.351561721	Zm00001d044656
Zm00001d037962_T012	0.4538623	-2.641298539	Zm00001d037962
Zm00001d037962_T011	0.5682962	2.596471717	Zm00001d037962
Zm00001d037962_T010	0.01867386	1.535960235	Zm00001d037962
Zm00001d037962_T017	7.47E-07	1.039823334	Zm00001d037962
Zm00001d037962_T008	0.3105424	-1.171171118	Zm00001d037962

Zm00001d037962_T013	0.07920356	-1.560173066	Zm00001d037962
Zm00001d045560_T001	0.9937224	0.316457713	Zm00001d045560
Zm00001d042395_T001	0.9993872	0.123421104	Zm00001d042395
Zm00001d044481_T002	0.8760894	-0.070210479	Zm00001d044481
Zm00001d044481_T006	0.6379439	1.201071091	Zm00001d044481
Zm00001d044481_T005	0.7831859	0.297010857	Zm00001d044481
Zm00001d044481_T004	0.8987811	-0.03715611	Zm00001d044481
Zm00001d018182_T001	1	0.388628137	Zm00001d018182
Zm00001d011365_T001	0.5539906	-0.444469716	Zm00001d011365
Zm00001d010679_T001	0.9139882	-0.068369669	Zm00001d010679
Zm00001d018698_T001	0.8392925	0.791089506	Zm00001d018698
Zm00001d048031_T024	0.8037772	0.746316101	Zm00001d048031
Zm00001d048031_T009	0.5446804	1.436280848	Zm00001d048031
Zm00001d048031_T040	0.9745777	0.258929179	Zm00001d048031
Zm00001d048031_T007	2.17E-10	1.75772975	Zm00001d048031
Zm00001d048031_T048	0.3273212	0.366050265	Zm00001d048031
Zm00001d048031_T018	0.3288849	0.695109596	Zm00001d048031
Zm00001d048031_T082	1.33E-06	0.984327669	Zm00001d048031
Zm00001d048031_T020	0.7552081	0.934053557	Zm00001d048031
Zm00001d048031_T065	0.750706	0.401749519	Zm00001d048031
Zm00001d048031_T025	4.27E-05	1.360277485	Zm00001d048031
Zm00001d048031_T066	1	0.047432712	Zm00001d048031
Zm00001d048031_T015	0.3564059	0.67012375	Zm00001d048031
Zm00001d008693_T003	1	-0.371632451	Zm00001d008693
Zm00001d008693_T002	0.954492	0.575479897	Zm00001d008693
Zm00001d012856_T001	0.8746701	-0.051656877	Zm00001d012856
Zm00001d012856_T003	0.8252955	0.2624059	Zm00001d012856
Zm00001d029357_T001	0.9469426	0.276643223	Zm00001d029357
Zm00001d043414_T001	0.9788508	0.203702479	Zm00001d043414
Zm00001d043414_T003	0.7831859	0.252815481	Zm00001d043414
Zm00001d012291_T001	0.0107304	-1.833993988	Zm00001d012291
Zm00001d023305_T001	0.9604475	-0.167643854	Zm00001d023305
Zm00001d009411_T002	0.9979568	0.24709157	Zm00001d009411
Zm00001d050121_T001	0.9501111	-0.071713027	Zm00001d050121
Zm00001d029585_T001	4.77E-05	-1.938351857	Zm00001d029585
Zm00001d009749_T009	9.27E-15	3.509975832	Zm00001d009749
Zm00001d009749_T021	0.9203655	0.265245298	Zm00001d009749
Zm00001d009749_T012	0.9514141	0.033207728	Zm00001d009749
Zm00001d009749_T017	0.5307031	-0.202753684	Zm00001d009749
Zm00001d009749_T024	0.8889484	0.198151752	Zm00001d009749
Zm00001d009749_T005	0.9948667	1.331779111	Zm00001d009749
Zm00001d009749_T001	0.04663752	-0.6297927	Zm00001d009749
Zm00001d009749_T023	0.8884142	-0.469123298	Zm00001d009749
Zm00001d009749_T006	0.01458344	-0.914472044	Zm00001d009749
Zm00001d026689_T007	0.8859449	-0.110373454	Zm00001d026689
Zm00001d026689_T006	0.8637863	-0.025444658	Zm00001d026689
Zm00001d015164_T001	0.000191577	3.323229545	Zm00001d015164
Zm00001d030213_T001	0.8776306	0.795147347	Zm00001d030213
Zm00001d006640_T001	0.9884825	0.514350083	Zm00001d006640
Zm00001d006640_T002	0.9657993	0.400494111	Zm00001d006640
Zm00001d006476_T005	0.6818546	-0.132705096	Zm00001d006476
Zm00001d050838_T001	0.8648969	-0.141167374	Zm00001d050838
Zm00001d050838_T002	0.08984708	-1.652179028	Zm00001d050838
Zm00001d006886_T002	0.6644959	-0.362879058	Zm00001d006886

Zm00001d006886_T003	0.1134387	-0.545149858	Zm00001d006886
Zm00001d011446_T001	0.584722	-0.475303084	Zm00001d011446
Zm00001d012102_T004	0.9709778	0.354715255	Zm00001d012102
Zm00001d012102_T005	0.9944857	0.106084404	Zm00001d012102
Zm00001d012102_T001	0.8819727	0.699506269	Zm00001d012102
Zm00001d005324_T002	0.883637	-0.064167367	Zm00001d005324
Zm00001d005324_T003	0.9323429	0.023085978	Zm00001d005324
Zm00001d005324_T004	4.53E-08	1.380574888	Zm00001d005324
Zm00001d027585_T001	0.6974118	-0.455439294	Zm00001d027585
Zm00001d030947_T002	0.9805897	0.162819925	Zm00001d030947
Zm00001d030947_T004	0.9867452	0.248290732	Zm00001d030947
Zm00001d042453_T002	0.9719113	0.176085315	Zm00001d042453
Zm00001d042453_T001	1	0.343474339	Zm00001d042453
Zm00001d043943_T002	0.9695609	0.137705422	Zm00001d043943
Zm00001d043943_T003	0.6239087	-0.24184895	Zm00001d043943
Zm00001d031013_T008	0.9469426	0.07962605	Zm00001d031013
Zm00001d031013_T005	0.7155748	0.709409883	Zm00001d031013
Zm00001d031013_T007	0.1921001	0.742309578	Zm00001d031013
Zm00001d031013_T006	0.6568508	0.727320438	Zm00001d031013
Zm00001d031013_T002	0.5822629	0.988658175	Zm00001d031013
Zm00001d037267_T003	0.8580945	-0.099293824	Zm00001d037267
Zm00001d037267_T002	0.9911485	0.053363604	Zm00001d037267
Zm00001d037267_T001	0.930095	-0.089783794	Zm00001d037267
Zm00001d045213_T004	0.57755	-0.557391779	Zm00001d045213
Zm00001d045213_T002	0.9968176	0.386819405	Zm00001d045213
Zm00001d005027_T002	0.9396782	0.664274681	Zm00001d005027
Zm00001d005027_T001	0.9988864	0.332483676	Zm00001d005027
Zm00001d005027_T004	0.9210235	0.446008577	Zm00001d005027
Zm00001d036626_T001	0.997764	-0.030198882	Zm00001d036626
Zm00001d033967_T001	0.5541374	0.956222788	Zm00001d033967
Zm00001d005271_T001	1	0.04181017	Zm00001d005271
Zm00001d005271_T002	0.9469426	0.667141991	Zm00001d005271
Zm00001d033594_T001	1	0.266581367	Zm00001d033594
Zm00001d028189_T001	0.9790102	0.208047	Zm00001d028189
Zm00001d028189_T003	0.9524127	0.283343494	Zm00001d028189
Zm00001d042449_T001	0.03008321	2.327741917	Zm00001d042449
Zm00001d039095_T001	0.836612	-0.133650506	Zm00001d039095
Zm00001d031490_T006	0.0318764	0.994672017	Zm00001d031490
Zm00001d031490_T004	0.5021575	0.938691794	Zm00001d031490
Zm00001d031490_T003	0.989392	0.275859845	Zm00001d031490
Zm00001d028359_T003	0.008898652	-1.397120774	Zm00001d028359
Zm00001d028359_T001	0.9848773	0.572987572	Zm00001d028359
Zm00001d020606_T002	0.9982897	0.108794026	Zm00001d020606
Zm00001d018471_T001	0.5875794	-0.773943462	Zm00001d018471
Zm00001d018471_T003	0.9239352	-0.064388861	Zm00001d018471
Zm00001d005396_T001	0.1884672	-2.110765824	Zm00001d005396
Zm00001d005396_T002	0.9338972	0.55635522	Zm00001d005396
Zm00001d046434_T006	0.9740325	0.523582738	Zm00001d046434
Zm00001d046434_T001	0.9596377	0.027889744	Zm00001d046434
Zm00001d046434_T002	0.9860836	0.475758693	Zm00001d046434
Zm00001d015851_T009	0.7313702	0.623991841	Zm00001d015851
Zm00001d015851_T010	0.3928501	-0.641182165	Zm00001d015851
Zm00001d015851_T003	0.9073468	0.816846906	Zm00001d015851
Zm00001d038746_T001	0.9265089	-0.054233417	Zm00001d038746

Zm00001d042910_T005	0.9919298	0.522832733	Zm00001d042910
Zm00001d042910_T008	0.9221126	0.459320608	Zm00001d042910
Zm00001d042910_T010	0.3286508	-0.365253101	Zm00001d042910
Zm00001d012320_T001	0.9343437	0.472902039	Zm00001d012320
Zm00001d042695_T001	0.98291	0.522158173	Zm00001d042695
Zm00001d042695_T009	0.09762222	1.544566486	Zm00001d042695
Zm00001d006884_T003	0.6724646	0.253651997	Zm00001d006884
Zm00001d006884_T004	0.4891923	0.78555307	Zm00001d006884
Zm00001d006884_T002	0.9968176	0.440141939	Zm00001d006884
Zm00001d051068_T001	0.3071359	0.786306382	Zm00001d051068
Zm00001d008500_T001	0.6839097	-0.775939996	Zm00001d008500
Zm00001d039919_T001	0.3171013	1.494117057	Zm00001d039919
Zm00001d039919_T002	0.000605558	2.85144348	Zm00001d039919
Zm00001d020067_T001	0.5853988	-0.465442245	Zm00001d020067
Zm00001d039925_T001	0.8600981	-0.437554003	Zm00001d039925
Zm00001d030688_T006	0.8001345	-0.979749002	Zm00001d030688
Zm00001d030688_T002	0.8600981	-0.251653243	Zm00001d030688
Zm00001d047293_T001	0.9817074	0.123906059	Zm00001d047293
Zm00001d028417_T005	0.9123976	0.42423485	Zm00001d028417
Zm00001d028417_T002	0.3648281	1.59684522	Zm00001d028417
Zm00001d028417_T001	0.9090332	0.847533213	Zm00001d028417
Zm00001d016378_T002	0.4635996	0.569799306	Zm00001d016378
Zm00001d016378_T004	0.9020229	0.151235933	Zm00001d016378
Zm00001d016378_T005	0.9529184	-0.002481556	Zm00001d016378
Zm00001d016378_T003	0.9469426	0.745100408	Zm00001d016378
Zm00001d025307_T001	0.2002595	1.70999914	Zm00001d025307
Zm00001d025307_T002	0.004954152	0.517613219	Zm00001d025307
Zm00001d028711_T003	0.6450309	1.019588572	Zm00001d028711
Zm00001d047361_T001	0.8377512	0.336398026	Zm00001d047361
Zm00001d047361_T004	0.9706887	0.088819505	Zm00001d047361
Zm00001d047361_T002	0.2385188	-0.346345623	Zm00001d047361
Zm00001d031104_T005	0.9947747	0.212206772	Zm00001d031104
Zm00001d002283_T004	0.5713939	0.663532623	Zm00001d002283
Zm00001d002283_T002	0.9216521	0.743145683	Zm00001d002283
Zm00001d002283_T001	0.961399	0.094521229	Zm00001d002283
Zm00001d024292_T001	0.9706887	0.502547466	Zm00001d024292
Zm00001d024292_T002	0.7339517	-0.288239246	Zm00001d024292
Zm00001d033029_T001	0.5652791	0.966584809	Zm00001d033029
Zm00001d026487_T002	0.7009673	-0.345915854	Zm00001d026487
Zm00001d026487_T001	0.8505918	0.020978079	Zm00001d026487
Zm00001d052509_T001	0.6354704	-0.479764033	Zm00001d052509
Zm00001d039563_T013	4.67E-10	1.120445753	Zm00001d039563
Zm00001d039563_T004	0.000349798	1.709034865	Zm00001d039563
Zm00001d039563_T039	0.008403401	0.515562293	Zm00001d039563
Zm00001d045520_T001	0.9968176	0.290423001	Zm00001d045520
Zm00001d034440_T001	0.5079763	-0.65181499	Zm00001d034440
Zm00001d052186_T001	0.9514141	0.103824433	Zm00001d052186
Zm00001d002439_T001	0.9986486	0.158041853	Zm00001d002439
Zm00001d016285_T001	0.4163319	-0.661813266	Zm00001d016285
Zm00001d038127_T001	0.98291	0.512506047	Zm00001d038127
Zm00001d020417_T001	0.5275577	-0.71786792	Zm00001d020417
Zm00001d042845_T001	0.9815875	0.163148286	Zm00001d042845
Zm00001d047220_T003	0.659943	-0.409778093	Zm00001d047220
Zm00001d047220_T001	0.9868821	0.448906851	Zm00001d047220

Zm00001d038388_T001	0.6587378	-0.472749813	Zm00001d038388
Zm00001d003729_T001	0.9921225	-0.061696884	Zm00001d003729
Zm00001d034948_T003	0.9510902	0.55917071	Zm00001d034948
Zm00001d006536_T001	0.2196702	-0.995976385	Zm00001d006536
Zm00001d017158_T001	0.9246704	0.019815604	Zm00001d017158
Zm00001d010793_T001	0.8747821	-0.162602254	Zm00001d010793
Zm00001d010793_T005	0.9656136	-0.049645253	Zm00001d010793
Zm00001d031777_T001	0.6504599	1.24183417	Zm00001d031777
Zm00001d019004_T002	0.1763656	-0.771625452	Zm00001d019004
Zm00001d033906_T001	1	0.34716232	Zm00001d033906
Zm00001d011638_T012	0.1301507	0.630163439	Zm00001d011638
Zm00001d011638_T004	0.05328006	2.385083263	Zm00001d011638
Zm00001d011638_T011	1.83E-05	2.231178609	Zm00001d011638
Zm00001d011638_T005	8.12E-10	2.833574482	Zm00001d011638
Zm00001d011638_T010	0.1545218	0.860491229	Zm00001d011638
Zm00001d011638_T008	7.18E-08	1.363060513	Zm00001d011638
Zm00001d011638_T002	1.94E-07	-1.565552449	Zm00001d011638
Zm00001d003510_T001	0.5437812	-0.522724981	Zm00001d003510
Zm00001d003013_T001	0.9825858	0.327751673	Zm00001d003013
Zm00001d013209_T001	0.6375985	0.956173943	Zm00001d013209
Zm00001d005936_T001	0.01983709	2.322147906	Zm00001d005936
Zm00001d006409_T002	0.9975882	0.211104661	Zm00001d006409
Zm00001d006409_T001	0.9219195	-0.014748622	Zm00001d006409
Zm00001d006409_T004	0.8031436	0.432151412	Zm00001d006409
Zm00001d009374_T001	0.983226	0.363361538	Zm00001d009374
Zm00001d052971_T004	1	0.394696784	Zm00001d052971
Zm00001d052971_T005	0.2439418	-0.649763921	Zm00001d052971
Zm00001d052971_T001	0.8950139	-0.006606258	Zm00001d052971
Zm00001d052971_T003	0.8557307	-0.115614836	Zm00001d052971
Zm00001d012634_T008	0.0581026	0.939994944	Zm00001d012634
Zm00001d012634_T001	0.670825	1.286912533	Zm00001d012634
Zm00001d022106_T001	0.5711435	-0.56271669	Zm00001d022106
Zm00001d042768_T001	0.9264969	0.487440778	Zm00001d042768
Zm00001d045030_T001	0.9902022	-0.192372677	Zm00001d045030
Zm00001d017789_T001	0.1371094	-1.630927739	Zm00001d017789
Zm00001d016616_T002	0.9246704	-0.040893025	Zm00001d016616
Zm00001d025082_T002	0.5705692	-0.510247669	Zm00001d025082
Zm00001d021264_T001	0.9575224	0.345606565	Zm00001d021264
Zm00001d010832_T001	1	0.027250366	Zm00001d010832
Zm00001d052797_T003	0.9984041	0.353678807	Zm00001d052797
Zm00001d052797_T002	0.9817074	0.356590828	Zm00001d052797
Zm00001d052797_T001	0.4418602	0.490136745	Zm00001d052797
Zm00001d052797_T005	0.1365783	-1.947245167	Zm00001d052797
Zm00001d016815_T001	0.8834929	-0.047239428	Zm00001d016815
Zm00001d019915_T001	0.9486412	-0.081750743	Zm00001d019915
Zm00001d050011_T004	0.666449	0.936015569	Zm00001d050011
Zm00001d050011_T003	0.4784037	1.301606799	Zm00001d050011
Zm00001d050011_T005	0.2139058	1.504058524	Zm00001d050011
Zm00001d050011_T001	0.8648059	0.843874209	Zm00001d050011
Zm00001d022607_T001	1	0.241901303	Zm00001d022607
Zm00001d032386_T003	0.9486412	0.130252969	Zm00001d032386
Zm00001d032386_T004	0.4332539	-0.265280927	Zm00001d032386
Zm00001d032386_T001	0.09972704	-1.225195389	Zm00001d032386
Zm00001d008562_T001	0.2246498	-1.465459771	Zm00001d008562

Zm00001d038540_T001	0.9877195	0.444100743	Zm00001d038540
Zm00001d047116_T001	0.09013732	-1.688596421	Zm00001d047116
Zm00001d015406_T001	0.000872907	3.102269405	Zm00001d015406
Zm00001d015406_T003	7.11E-10	-3.522801368	Zm00001d015406
Zm00001d027676_T001	0.8816665	0.77237314	Zm00001d027676
Zm00001d022451_T001	0.01127347	2.911444612	Zm00001d022451
Zm00001d013620_T025	0.9343437	0.082779875	Zm00001d013620
Zm00001d013620_T032	0.5318472	-0.267501632	Zm00001d013620
Zm00001d013620_T012	0.9804228	0.042595319	Zm00001d013620
Zm00001d013620_T029	0.9216521	0.181283729	Zm00001d013620
Zm00001d013620_T006	0.9580876	0.051159934	Zm00001d013620
Zm00001d013620_T009	0.0191703	1.602342094	Zm00001d013620
Zm00001d013620_T002	0.8435808	0.032388846	Zm00001d013620
Zm00001d013620_T001	0.7302394	1.290594349	Zm00001d013620
Zm00001d033456_T001	0.2604053	1.72837692	Zm00001d033456
Zm00001d028768_T001	0.910614	0.333002156	Zm00001d028768
Zm00001d032131_T001	0.9475709	0.010169082	Zm00001d032131
Zm00001d028114_T001	0.6946848	-0.275267253	Zm00001d028114
Zm00001d007076_T001	0.1131556	-1.412882552	Zm00001d007076
Zm00001d045263_T001	0.879484	-0.219893198	Zm00001d045263
Zm00001d045263_T002	2.60E-08	2.331454135	Zm00001d045263
Zm00001d045263_T003	0.923446	0.603499307	Zm00001d045263
Zm00001d002644_T018	0.9043641	0.400591955	Zm00001d002644
Zm00001d002644_T052	0.650961	-0.239671278	Zm00001d002644
Zm00001d002644_T023	0.01063015	1.049623473	Zm00001d002644
Zm00001d002644_T055	0.9954444	-0.069962072	Zm00001d002644
Zm00001d029649_T002	0.9588604	0.134937797	Zm00001d029649
Zm00001d023791_T002	1	0.354455882	Zm00001d023791
Zm00001d034689_T003	0.9016285	-0.037464601	Zm00001d034689
Zm00001d002387_T003	0.8411786	-0.481499716	Zm00001d002387
Zm00001d050705_T001	0.850642	-0.411717118	Zm00001d050705
Zm00001d033472_T002	0.7869719	0.522755157	Zm00001d033472
Zm00001d033472_T001	0.9468268	0.053617418	Zm00001d033472
Zm00001d043052_T004	0.760208	1.034190709	Zm00001d043052
Zm00001d043052_T009	0.1235256	2.047970487	Zm00001d043052
Zm00001d043052_T008	0.9791729	0.227407439	Zm00001d043052
Zm00001d043052_T003	0.240144	0.905515973	Zm00001d043052
Zm00001d043052_T001	0.6273738	-0.518404381	Zm00001d043052
Zm00001d053369_T001	0.2038697	-1.148376483	Zm00001d053369
Zm00001d008581_T001	1	0.414962619	Zm00001d008581
Zm00001d045728_T003	0.684563	-0.079778425	Zm00001d045728
Zm00001d045728_T008	0.8329158	-0.119777158	Zm00001d045728
Zm00001d045728_T004	0.9912564	0.513149406	Zm00001d045728
Zm00001d045728_T005	1	0.185397765	Zm00001d045728
Zm00001d012263_T003	0.873307	0.738799196	Zm00001d012263
Zm00001d012263_T006	0.9817074	-0.083436422	Zm00001d012263
Zm00001d012263_T005	0.1353026	0.410916195	Zm00001d012263
Zm00001d038668_T001	0.8660897	-0.352774224	Zm00001d038668
Zm00001d017863_T006	0.9770343	0.091038659	Zm00001d017863
Zm00001d017863_T004	0.7744288	-0.354950309	Zm00001d017863
Zm00001d051313_T001	0.9804228	0.124764271	Zm00001d051313
Zm00001d028304_T001	0.4359675	-0.788696833	Zm00001d028304
Zm00001d040888_T002	1	0.030644341	Zm00001d040888
Zm00001d040888_T001	0.9933725	0.364933627	Zm00001d040888

Zm00001d040888_T004	0.7632146	0.444641479	Zm00001d040888
Zm00001d038205_T002	0.9760472	0.539002422	Zm00001d038205
Zm00001d038205_T003	0.2140375	0.821121844	Zm00001d038205
Zm00001d015289_T001	1.97E-11	1.963093253	Zm00001d015289
Zm00001d015289_T002	0.7440932	1.087784917	Zm00001d015289
Zm00001d015289_T009	0.9666757	0.155037546	Zm00001d015289
Zm00001d015289_T007	0.05168277	1.940830649	Zm00001d015289
Zm00001d015289_T004	0.6716447	0.22708258	Zm00001d015289
Zm00001d023331_T001	1	0.172771052	Zm00001d023331
Zm00001d028691_T002	0.9884825	0.249518012	Zm00001d028691
Zm00001d022193_T002	0.2982377	0.806389429	Zm00001d022193
Zm00001d029703_T001	0.3842638	-0.82118289	Zm00001d029703
Zm00001d039280_T012	0.3085371	-1.180941464	Zm00001d039280
Zm00001d039280_T005	0.4925065	-0.268148038	Zm00001d039280
Zm00001d039280_T010	0.7746801	-0.142207179	Zm00001d039280
Zm00001d039280_T008	0.7773575	-0.210893627	Zm00001d039280
Zm00001d039280_T009	0.6593598	0.848631618	Zm00001d039280
Zm00001d039280_T007	0.9252028	0.555175227	Zm00001d039280
Zm00001d039280_T004	0.9868533	0.227447395	Zm00001d039280
Zm00001d039280_T001	0.9693993	0.563211926	Zm00001d039280
Zm00001d039280_T006	0.9089465	0.491362956	Zm00001d039280
Zm00001d045050_T001	0.9730613	0.532081917	Zm00001d045050
Zm00001d045050_T002	0.974657	0.560549985	Zm00001d045050
Zm00001d024839_T001	0.9885729	0.386748475	Zm00001d024839
Zm00001d035367_T001	0.7289978	-0.499508161	Zm00001d035367
Zm00001d035750_T001	0.9223663	0.522065434	Zm00001d035750
Zm00001d040235_T005	0.9565533	0.625367968	Zm00001d040235
Zm00001d040235_T001	0.8513643	0.00257788	Zm00001d040235
Zm00001d040235_T002	0.8861268	-0.517656235	Zm00001d040235
Zm00001d040235_T003	0.6781079	0.648260901	Zm00001d040235
Zm00001d032442_T003	0.9442138	-0.311932019	Zm00001d032442
Zm00001d032442_T007	0.8587779	-0.528606951	Zm00001d032442
Zm00001d032442_T008	0.9139882	-0.290942534	Zm00001d032442
Zm00001d032442_T005	0.3397699	2.181946114	Zm00001d032442
Zm00001d030138_T001	0.9984041	-0.235127323	Zm00001d030138
Zm00001d028285_T001	0.842741	0.910524972	Zm00001d028285
Zm00001d028285_T002	0.8476927	0.002838827	Zm00001d028285
Zm00001d027742_T001	0.003152852	-2.985647405	Zm00001d027742
Zm00001d032266_T004	0.9534335	0.25400132	Zm00001d032266
Zm00001d032266_T001	0.966464	-0.090212383	Zm00001d032266
Zm00001d029550_T008	0.9732421	0.393400528	Zm00001d029550
Zm00001d029550_T002	0.390328	0.591688896	Zm00001d029550
Zm00001d029550_T004	0.8032834	0.099267305	Zm00001d029550
Zm00001d029550_T005	0.9809771	0.137325764	Zm00001d029550
Zm00001d029550_T017	4.34E-08	-2.252352934	Zm00001d029550
Zm00001d029550_T007	0.9293689	0.348998066	Zm00001d029550
Zm00001d029550_T016	0.836612	-0.162756836	Zm00001d029550
Zm00001d029550_T006	0.004986224	0.663862458	Zm00001d029550
Zm00001d012600_T002	0.7056782	-0.237487177	Zm00001d012600
Zm00001d023477_T001	0.36742	-0.852181643	Zm00001d023477
Zm00001d041221_T001	1	0.232212598	Zm00001d041221
Zm00001d010198_T001	1	0.389769634	Zm00001d010198
Zm00001d006608_T001	0.9979568	0.211955563	Zm00001d006608
Zm00001d022598_T001	0.8625537	-0.093782619	Zm00001d022598

Zm00001d034414_T002	0.717473	0.884704015	Zm00001d034414
Zm00001d048401_T001	0.0228443	-1.108846564	Zm00001d048401
Zm00001d010693_T001	0.8989951	0.582193772	Zm00001d010693
Zm00001d014349_T001	0.2094239	-1.429897591	Zm00001d014349
Zm00001d053300_T005	0.8746701	0.014364214	Zm00001d053300
Zm00001d053300_T003	0.3975197	-0.681152598	Zm00001d053300
Zm00001d053300_T001	0.9571192	0.506908561	Zm00001d053300
Zm00001d053300_T007	5.10E-12	2.039024499	Zm00001d053300
Zm00001d035187_T001	0.9907928	0.103345007	Zm00001d035187
Zm00001d035187_T002	0.9344122	0.359441951	Zm00001d035187
Zm00001d014958_T001	0.9519347	0.066817921	Zm00001d014958
Zm00001d033046_T002	0.9673473	0.144297333	Zm00001d033046
Zm00001d033046_T001	0.9073468	0.330445318	Zm00001d033046
Zm00001d018649_T001	0.9933044	0.223860248	Zm00001d018649
Zm00001d018649_T002	0.8830977	-0.158402081	Zm00001d018649
Zm00001d039532_T001	0.9722469	0.178689673	Zm00001d039532
Zm00001d009339_T001	0.9668789	0.502989088	Zm00001d009339
Zm00001d009339_T003	0.7412102	-0.764122902	Zm00001d009339
Zm00001d012659_T006	0.9292973	0.249608594	Zm00001d012659
Zm00001d012659_T008	0.8612451	0.558754917	Zm00001d012659
Zm00001d012659_T007	0.9501111	0.043678754	Zm00001d012659
Zm00001d012659_T005	0.5857168	1.096466324	Zm00001d012659
Zm00001d026586_T003	0.8591203	0.901192558	Zm00001d026586
Zm00001d026586_T002	0.9380783	0.411312234	Zm00001d026586
Zm00001d026586_T004	0.9693993	0.593144	Zm00001d026586
Zm00001d021324_T001	0.7536272	0.560347944	Zm00001d021324
Zm00001d018309_T015	0.6970851	0.395974857	Zm00001d018309
Zm00001d018309_T003	0.9673473	0.616540048	Zm00001d018309
Zm00001d040659_T001	1	0.156334701	Zm00001d040659
Zm00001d050283_T002	0.9706293	0.238328856	Zm00001d050283
Zm00001d010445_T001	0.6354704	-0.588651344	Zm00001d010445
Zm00001d010445_T002	0.9635696	0.123677524	Zm00001d010445
Zm00001d003796_T003	0.9833513	-0.235795312	Zm00001d003796
Zm00001d053650_T001	0.6761587	-0.497865539	Zm00001d053650
Zm00001d009476_T001	0.6260085	-0.618917002	Zm00001d009476
Zm00001d009476_T002	0.9419336	-0.047975511	Zm00001d009476
Zm00001d052888_T009	0.02376368	1.346306069	Zm00001d052888
Zm00001d052888_T003	0.9918929	0.499605114	Zm00001d052888
Zm00001d052888_T020	0.9988864	0.025481181	Zm00001d052888
Zm00001d016306_T001	0.2419153	-1.02917838	Zm00001d016306
Zm00001d031804_T001	0.4823693	-0.837913612	Zm00001d031804
Zm00001d040344_T001	0.9967276	0.498899981	Zm00001d040344
Zm00001d008727_T004	0.6812088	1.445965047	Zm00001d008727
Zm00001d008727_T003	0.9753621	0.14186343	Zm00001d008727
Zm00001d008727_T001	1.54E-05	-2.650028752	Zm00001d008727
Zm00001d008727_T002	0.7641977	-0.135479224	Zm00001d008727
Zm00001d008727_T006	0.6026209	-0.0428772	Zm00001d008727
Zm00001d050219_T017	0.8112903	-0.191002421	Zm00001d050219
Zm00001d050219_T006	0.2799099	0.913522745	Zm00001d050219
Zm00001d050219_T018	0.9749249	0.242164588	Zm00001d050219
Zm00001d050219_T009	0.6172111	1.092341116	Zm00001d050219
Zm00001d018409_T004	0.9822138	0.556732335	Zm00001d018409
Zm00001d007261_T001	0.889992	-0.151697194	Zm00001d007261
Zm00001d047470_T002	0.7481233	-0.32932272	Zm00001d047470

Zm00001d047470_T003	0.8158142	0.784111756	Zm00001d047470
Zm00001d047470_T001	0.993158	0.139460127	Zm00001d047470
Zm00001d047470_T004	0.08470891	-1.296698652	Zm00001d047470
Zm00001d047149_T001	0.9701986	0.114782927	Zm00001d047149
Zm00001d008462_T001	0.4191028	-0.125177943	Zm00001d008462
Zm00001d008462_T002	0.4182188	-0.713753924	Zm00001d008462
Zm00001d028755_T002	0.923446	-0.097762464	Zm00001d028755
Zm00001d028755_T003	0.07608185	1.804231237	Zm00001d028755
Zm00001d020511_T003	0.9979568	0.243468521	Zm00001d020511
Zm00001d038093_T002	0.3757326	0.532585804	Zm00001d038093
Zm00001d038093_T007	0.9846714	0.25993961	Zm00001d038093
Zm00001d046997_T001	0.6504599	0.988184549	Zm00001d046997
Zm00001d052354_T006	0.6906858	0.356630994	Zm00001d052354
Zm00001d052354_T001	0.2375452	-1.587960774	Zm00001d052354
Zm00001d052354_T005	0.962254	0.617302108	Zm00001d052354
Zm00001d047938_T001	0.4845681	1.407974584	Zm00001d047938
Zm00001d014278_T001	0.9673473	0.088042319	Zm00001d014278
Zm00001d035007_T001	0.9062902	0.774151178	Zm00001d035007
Zm00001d048163_T001	0.8924209	-0.435449261	Zm00001d048163
Zm00001d014486_T002	0.9171732	-0.063416561	Zm00001d014486
Zm00001d043019_T001	0.8158416	0.958636542	Zm00001d043019
Zm00001d043019_T005	0.9562304	1.290968308	Zm00001d043019
Zm00001d043019_T002	0.7169281	1.213307158	Zm00001d043019
Zm00001d018806_T001	0.9582109	0.548026389	Zm00001d018806
Zm00001d015515_T001	0.005530214	3.141134147	Zm00001d015515
Zm00001d043333_T001	0.8230063	-0.235206661	Zm00001d043333
Zm00001d052008_T005	0.2009937	0.844197455	Zm00001d052008
Zm00001d052008_T001	0.6966097	1.187887259	Zm00001d052008
Zm00001d052008_T006	1.98E-05	2.569364583	Zm00001d052008
Zm00001d052008_T004	0.04940148	-1.181215123	Zm00001d052008
Zm00001d033278_T014	0.09369613	0.425226725	Zm00001d033278
Zm00001d033278_T010	7.23E-15	3.043509638	Zm00001d033278
Zm00001d033278_T001	0.006436882	0.287497494	Zm00001d033278
Zm00001d033278_T011	0.1349403	2.059607932	Zm00001d033278
Zm00001d033278_T007	0.9263167	0.072105122	Zm00001d033278
Zm00001d033278_T012	0.5010043	0.142647217	Zm00001d033278
Zm00001d033278_T016	0.8021989	0.270818776	Zm00001d033278
Zm00001d033278_T002	0.000822402	1.823597641	Zm00001d033278
Zm00001d033053_T001	0.00045129	-1.046733644	Zm00001d033053
Zm00001d012718_T001	0.896517	-0.068587937	Zm00001d012718
Zm00001d050310_T001	0.8641671	-0.149818693	Zm00001d050310
Zm00001d030184_T002	0.9785379	0.501887058	Zm00001d030184
Zm00001d022102_T001	0.9469426	-0.030427104	Zm00001d022102
Zm00001d003184_T001	0.0667426	-1.419467566	Zm00001d003184
Zm00001d043443_T001	0.6868477	-0.397852241	Zm00001d043443
Zm00001d017770_T002	0.9666757	-0.018338266	Zm00001d017770
Zm00001d032874_T004	0.3181489	0.280981101	Zm00001d032874
Zm00001d051542_T046	0.003370189	-0.757797402	Zm00001d051542
Zm00001d051542_T050	0.8883561	0.157478044	Zm00001d051542
Zm00001d051542_T043	0.9503115	0.200120819	Zm00001d051542
Zm00001d051542_T071	0.04404459	1.602017559	Zm00001d051542
Zm00001d051542_T076	0.02583579	-1.539833099	Zm00001d051542
Zm00001d048229_T001	0.04443519	-1.57365939	Zm00001d048229
Zm00001d014582_T001	0.5859375	-0.516430429	Zm00001d014582

Zm00001d020233_T001	1	0.362681497	Zm00001d020233
Zm00001d041476_T001	0.8638849	0.69320663	Zm00001d041476
Zm00001d012667_T018	0.9173494	0.263767555	Zm00001d012667
Zm00001d012667_T006	0.9050121	-0.084962179	Zm00001d012667
Zm00001d012667_T029	0.7989126	0.935575684	Zm00001d012667
Zm00001d012667_T024	0.9290834	-0.165651848	Zm00001d012667
Zm00001d012667_T005	0.1851557	0.58918262	Zm00001d012667
Zm00001d012667_T031	0.9186216	0.366321503	Zm00001d012667
Zm00001d012667_T012	0.3642654	0.723493473	Zm00001d012667
Zm00001d012667_T009	0.000960117	0.671038558	Zm00001d012667
Zm00001d012667_T001	0.9817074	0.366875814	Zm00001d012667
Zm00001d012667_T004	0.9584416	0.60591379	Zm00001d012667
Zm00001d012667_T030	0.06970238	-2.278898961	Zm00001d012667
Zm00001d050134_T001	0.8860576	0.779467707	Zm00001d050134
Zm00001d035475_T001	0.6313899	-0.214683865	Zm00001d035475
Zm00001d035475_T002	0.2356674	-0.998552874	Zm00001d035475
Zm00001d030372_T001	0.5537557	-0.575009672	Zm00001d030372
Zm00001d004231_T001	1	0.031541066	Zm00001d004231
Zm00001d002941_T001	0.9843847	0.266754458	Zm00001d002941
Zm00001d002941_T002	0.8718957	-0.095036484	Zm00001d002941
Zm00001d036968_T017	0.3082157	-0.346569404	Zm00001d036968
Zm00001d036968_T011	0.9215988	0.010725989	Zm00001d036968
Zm00001d036968_T019	0.4146004	0.434954724	Zm00001d036968
Zm00001d036968_T010	0.4240522	1.3517683	Zm00001d036968
Zm00001d036968_T020	3.58E-12	-1.938212518	Zm00001d036968
Zm00001d036968_T014	1.24E-14	3.237459311	Zm00001d036968
Zm00001d016000_T009	0.08289152	0.842947994	Zm00001d016000
Zm00001d016000_T002	0.4319576	-0.395982286	Zm00001d016000
Zm00001d016000_T010	0.7485836	-0.300206508	Zm00001d016000
Zm00001d016000_T003	0.9361945	0.541531399	Zm00001d016000
Zm00001d016000_T008	0.6649899	-0.468976979	Zm00001d016000
Zm00001d016000_T004	0.1563286	1.70883655	Zm00001d016000
Zm00001d016000_T001	0.9196877	0.454916171	Zm00001d016000
Zm00001d003142_T001	0.1137443	-0.780629828	Zm00001d003142
Zm00001d030062_T003	0.9751728	0.285514617	Zm00001d030062
Zm00001d008715_T001	0.8581713	-0.07894626	Zm00001d008715
Zm00001d050300_T002	0.9745948	0.038521841	Zm00001d050300
Zm00001d050300_T001	0.7128156	-0.573277246	Zm00001d050300
Zm00001d042063_T001	1.00E-05	2.738186634	Zm00001d042063
Zm00001d008764_T001	0.6745773	-0.228704161	Zm00001d008764
Zm00001d027446_T001	1	0.368366025	Zm00001d027446
Zm00001d008196_T001	0.06553736	-1.900183291	Zm00001d008196
Zm00001d018725_T001	0.5117107	-0.687927805	Zm00001d018725
Zm00001d045646_T001	0.9869996	0.193381416	Zm00001d045646
Zm00001d019065_T001	0.7565187	0.615699721	Zm00001d019065
Zm00001d020961_T001	0.3465241	1.615569206	Zm00001d020961
Zm00001d020961_T002	0.3857707	0.768734581	Zm00001d020961
Zm00001d034729_T002	0.4660706	-0.665309972	Zm00001d034729
Zm00001d034729_T001	0.6967776	-0.166343298	Zm00001d034729
Zm00001d023919_T001	0.910709	0.021328987	Zm00001d023919
Zm00001d009161_T002	0.7761227	-0.218337933	Zm00001d009161
Zm00001d016105_T001	0.7153452	-0.421903299	Zm00001d016105
Zm00001d053713_T001	0.9368844	0.355732437	Zm00001d053713
Zm00001d053262_T031	0.3961965	0.177420547	Zm00001d053262

Zm00001d053262_T030	0.6120091	1.050078807	Zm00001d053262
Zm00001d053262_T016	3.57E-10	0.936515453	Zm00001d053262
Zm00001d053262_T037	0.01442294	-2.457944675	Zm00001d053262
Zm00001d053262_T033	0.0180044	1.490402326	Zm00001d053262
Zm00001d053262_T036	0.7987321	-0.121941658	Zm00001d053262
Zm00001d053262_T014	0.1757415	-0.183724062	Zm00001d053262
Zm00001d009004_T011	0.447037	-0.58533856	Zm00001d009004
Zm00001d009004_T001	0.6138171	1.052616521	Zm00001d009004
Zm00001d020688_T001	0.8964117	-0.085678689	Zm00001d020688
Zm00001d037656_T001	0.000128066	-3.237779129	Zm00001d037656
Zm00001d017107_T001	0.5795936	-0.762197853	Zm00001d017107
Zm00001d024605_T001	0.9355135	0.662112574	Zm00001d024605
Zm00001d027576_T003	0.684319	-0.199110827	Zm00001d027576
Zm00001d027576_T001	0.4811797	-0.654088989	Zm00001d027576
Zm00001d027576_T005	0.7239171	-0.507076732	Zm00001d027576
Zm00001d027576_T002	0.9682673	0.049757822	Zm00001d027576
Zm00001d027576_T004	0.8107845	-0.147888103	Zm00001d027576
Zm00001d049753_T003	0.9870394	0.135930159	Zm00001d049753
Zm00001d049753_T002	0.9090332	-0.104805194	Zm00001d049753
Zm00001d018869_T001	0.7648638	0.964412914	Zm00001d018869
Zm00001d004956_T002	0.970085	0.177389698	Zm00001d004956
Zm00001d004956_T003	0.9968023	0.227128991	Zm00001d004956
Zm00001d004956_T001	0.9219195	0.036704733	Zm00001d004956
Zm00001d011785_T001	0.7619918	-0.814112118	Zm00001d011785
Zm00001d020184_T001	0.506927	-0.996806963	Zm00001d020184
Zm00001d026406_T001	0.005512874	-2.90908532	Zm00001d026406
Zm00001d006682_T002	0.3191802	1.398190892	Zm00001d006682
Zm00001d006682_T001	0.840693	0.901788142	Zm00001d006682
Zm00001d049188_T002	0.680648	0.805305262	Zm00001d049188
Zm00001d049188_T007	0.9404125	0.19963741	Zm00001d049188
Zm00001d049188_T005	0.9446484	0.102161793	Zm00001d049188
Zm00001d017677_T003	0.5023988	0.844757448	Zm00001d017677
Zm00001d017677_T002	0.9907928	0.211754	Zm00001d017677
Zm00001d036535_T001	0.01145971	-2.121521626	Zm00001d036535
Zm00001d034783_T001	0.000105885	3.756461545	Zm00001d034783
Zm00001d017284_T001	0.008296542	2.903468947	Zm00001d017284
Zm00001d025037_T001	0.4763999	-0.501807224	Zm00001d025037
Zm00001d030171_T001	0.967161	0.175549658	Zm00001d030171
Zm00001d049581_T001	0.8951107	-0.12801533	Zm00001d049581
Zm00001d047634_T002	0.9851204	0.1277602	Zm00001d047634
Zm00001d047634_T001	0.9556568	-0.116905412	Zm00001d047634
Zm00001d024999_T002	0.8031436	-0.422102434	Zm00001d024999
Zm00001d009424_T013	0.5632829	-0.466764008	Zm00001d009424
Zm00001d009424_T010	0.1166311	1.761462907	Zm00001d009424
Zm00001d009424_T011	0.139268	0.856914519	Zm00001d009424
Zm00001d025507_T017	0.9666757	0.420281259	Zm00001d025507
Zm00001d025507_T020	0.7233733	0.248982955	Zm00001d025507
Zm00001d025507_T008	0.7559329	-0.346269314	Zm00001d025507
Zm00001d025507_T019	0.8993062	-0.133110747	Zm00001d025507
Zm00001d025507_T014	0.5813702	-0.88648786	Zm00001d025507
Zm00001d025507_T015	0.02622542	1.360492814	Zm00001d025507
Zm00001d025507_T004	0.8660897	-0.02002972	Zm00001d025507
Zm00001d025507_T013	0.8231712	0.291131199	Zm00001d025507
Zm00001d025507_T018	0.8412913	0.450076254	Zm00001d025507

Zm00001d045472_T002	1	0.288985676	Zm00001d045472
Zm00001d045472_T001	0.8913467	-0.15742923	Zm00001d045472
Zm00001d047603_T005	1	0.153790075	Zm00001d047603
Zm00001d047603_T003	0.8643636	-0.44951366	Zm00001d047603
Zm00001d047603_T001	0.9984041	0.291032894	Zm00001d047603
Zm00001d042093_T002	0.9845237	0.145692888	Zm00001d042093
Zm00001d042093_T001	0.9107561	-0.110038693	Zm00001d042093
Zm00001d012832_T001	0.9418033	-0.150878689	Zm00001d012832
Zm00001d012832_T004	0.4426218	-1.035293845	Zm00001d012832
Zm00001d049886_T001	0.9881407	0.18454637	Zm00001d049886
Zm00001d047252_T001	0.4698008	1.098594903	Zm00001d047252
Zm00001d028534_T005	0.1225923	1.789802587	Zm00001d028534
Zm00001d028534_T006	0.001554726	0.889559734	Zm00001d028534
Zm00001d028534_T003	3.79E-07	1.556389742	Zm00001d028534
Zm00001d028534_T001	0.9311783	-0.358511549	Zm00001d028534
Zm00001d047263_T001	0.009041647	-1.90259916	Zm00001d047263
Zm00001d038752_T001	0.03196455	0.964426386	Zm00001d038752
Zm00001d038752_T005	0.7990254	0.250366773	Zm00001d038752
Zm00001d038752_T006	0.9024215	-0.039781029	Zm00001d038752
Zm00001d012787_T002	0.8499233	0.548938059	Zm00001d012787
Zm00001d015884_T001	0.9517262	0.565586029	Zm00001d015884
Zm00001d015485_T001	0.982665	0.097270543	Zm00001d015485
Zm00001d050917_T003	0.7680039	1.592208351	Zm00001d050917
Zm00001d050917_T002	0.4753371	1.566075001	Zm00001d050917
Zm00001d050917_T001	2.37E-06	1.909155183	Zm00001d050917
Zm00001d027728_T001	0.9979568	0.463182596	Zm00001d027728
Zm00001d052996_T001	0.2908892	1.057539932	Zm00001d052996
Zm00001d052996_T002	0.1867714	1.021900441	Zm00001d052996
Zm00001d049657_T001	0.2981136	-1.443259143	Zm00001d049657
Zm00001d017837_T001	0.8695779	0.748512027	Zm00001d017837
Zm00001d048634_T001	0.9567316	0.539958987	Zm00001d048634
Zm00001d017360_T004	0.1191683	2.223275432	Zm00001d017360
Zm00001d017360_T006	0.9293689	-0.015359808	Zm00001d017360
Zm00001d017360_T016	0.1210355	-1.442150887	Zm00001d017360
Zm00001d017360_T009	0.9846714	-0.003979267	Zm00001d017360
Zm00001d017360_T008	0.4087458	0.513522344	Zm00001d017360
Zm00001d017360_T003	0.09112037	0.723400129	Zm00001d017360
Zm00001d027955_T001	0.8616387	-0.343250467	Zm00001d027955
Zm00001d022425_T002	0.897685	0.817959478	Zm00001d022425
Zm00001d022425_T009	0.9657993	0.299201051	Zm00001d022425
Zm00001d022425_T001	0.5411491	-0.586437022	Zm00001d022425
Zm00001d014097_T001	1	0.372159693	Zm00001d014097
Zm00001d037736_T001	0.8982893	-0.416028285	Zm00001d037736
Zm00001d014062_T001	0.9975532	0.248703892	Zm00001d014062
Zm00001d052375_T002	1	0.009767953	Zm00001d052375
Zm00001d052375_T001	0.8293093	-0.651439814	Zm00001d052375
Zm00001d034916_T001	0.2747861	-1.035012215	Zm00001d034916
Zm00001d010366_T001	0.9860836	0.106555985	Zm00001d010366
Zm00001d017186_T002	0.01054164	2.864930625	Zm00001d017186
Zm00001d017186_T003	0.338859	1.749091119	Zm00001d017186
Zm00001d017186_T001	0.9359529	0.948792422	Zm00001d017186
Zm00001d006142_T003	0.07773638	1.492483071	Zm00001d006142
Zm00001d006142_T006	0.1460911	-1.012535473	Zm00001d006142
Zm00001d006142_T002	0.3870314	1.749858337	Zm00001d006142

Zm00001d006142_T011	0.8309214	-0.188674624	Zm00001d006142
Zm00001d025573_T001	0.0404945	-0.673599261	Zm00001d025573
Zm00001d006342_T002	0.4817874	-0.378330574	Zm00001d006342
Zm00001d006342_T003	0.9160053	-0.056042135	Zm00001d006342
Zm00001d032555_T011	0.5730988	0.847777111	Zm00001d032555
Zm00001d032555_T008	0.9979568	0.790076429	Zm00001d032555
Zm00001d032555_T005	0.2653595	-0.310065108	Zm00001d032555
Zm00001d032555_T014	0.3764439	-0.83098425	Zm00001d032555
Zm00001d032555_T010	0.9988954	0.27352481	Zm00001d032555
Zm00001d032555_T002	0.1211086	1.752742365	Zm00001d032555
Zm00001d008890_T001	0.8974665	0.58663307	Zm00001d008890
Zm00001d049922_T002	0.9422098	0.45796521	Zm00001d049922
Zm00001d041702_T001	0.9518042	0.054907556	Zm00001d041702
Zm00001d021617_T001	0.9962521	0.430109998	Zm00001d021617
Zm00001d021617_T002	0.8107637	0.344254851	Zm00001d021617
Zm00001d053281_T008	0.1847748	-1.064605669	Zm00001d053281
Zm00001d053281_T005	0.006622526	-1.811869528	Zm00001d053281
Zm00001d053281_T007	0.923446	0.008251599	Zm00001d053281
Zm00001d053281_T006	0.7408678	-0.208489395	Zm00001d053281
Zm00001d053281_T001	0.9582109	1.609178405	Zm00001d053281
Zm00001d051740_T006	0.443366	0.619061224	Zm00001d051740
Zm00001d051740_T007	0.5549472	0.338611695	Zm00001d051740
Zm00001d051740_T002	0.9519347	0.095747341	Zm00001d051740
Zm00001d004468_T003	0.987314	0.114808884	Zm00001d004468
Zm00001d029212_T010	0.066948	0.666444185	Zm00001d029212
Zm00001d029212_T009	0.9791729	1.303771292	Zm00001d029212
Zm00001d029212_T004	0.6142635	1.399094788	Zm00001d029212
Zm00001d040644_T001	0.8819727	-0.128874288	Zm00001d040644
Zm00001d006752_T001	0.6074342	-0.446673993	Zm00001d006752
Zm00001d006752_T002	0.7285679	0.712712272	Zm00001d006752
Zm00001d035720_T001	0.8457647	-0.205754172	Zm00001d035720
Zm00001d035720_T002	1	0.41467033	Zm00001d035720
Zm00001d039046_T003	0.587212	-0.173992931	Zm00001d039046
Zm00001d039046_T006	0.09458322	1.030744699	Zm00001d039046
Zm00001d039046_T001	0.07663943	-0.695990776	Zm00001d039046
Zm00001d039046_T024	0.3314652	-1.066654611	Zm00001d039046
Zm00001d039046_T011	0.8213533	0.906008542	Zm00001d039046
Zm00001d048857_T004	0.9334729	0.42297192	Zm00001d048857
Zm00001d048857_T003	0.2450578	1.221736391	Zm00001d048857
Zm00001d048857_T006	0.8835073	0.844291101	Zm00001d048857
Zm00001d048857_T002	0.3144119	-0.321961684	Zm00001d048857
Zm00001d026475_T001	1	0.317778569	Zm00001d026475
Zm00001d023737_T001	0.9072584	-0.283018983	Zm00001d023737
Zm00001d047030_T029	6.66E-13	2.045957631	Zm00001d047030
Zm00001d047030_T001	0.941809	0.858616396	Zm00001d047030
Zm00001d047030_T074	0.9668789	0.200863266	Zm00001d047030
Zm00001d047030_T011	0.4698441	1.385821919	Zm00001d047030
Zm00001d047030_T085	0.8858223	-0.214095907	Zm00001d047030
Zm00001d047030_T047	0.2358353	0.467339225	Zm00001d047030
Zm00001d047030_T023	0.002016075	0.888797142	Zm00001d047030
Zm00001d047030_T018	0.3428643	-0.81302564	Zm00001d047030
Zm00001d029628_T001	0.2575624	-1.500284085	Zm00001d029628
Zm00001d039411_T009	0.9543237	0.169129724	Zm00001d039411
Zm00001d039411_T002	0.2208276	-1.498022135	Zm00001d039411

Zm00001d039411_T001	0.9573434	0.401899326	Zm00001d039411
Zm00001d039411_T003	0.03760071	2.428656968	Zm00001d039411
Zm00001d026675_T004	0.7085474	-0.324467183	Zm00001d026675
Zm00001d026675_T002	0.9659371	0.135772368	Zm00001d026675
Zm00001d029783_T003	0.7943953	0.982270506	Zm00001d029783
Zm00001d029783_T004	0.9709887	0.394163378	Zm00001d029783
Zm00001d029783_T001	0.2774216	-0.94519686	Zm00001d029783
Zm00001d006725_T001	0.9216521	0.715500336	Zm00001d006725
Zm00001d025922_T001	0.5946447	1.199284692	Zm00001d025922
Zm00001d016364_T001	0.2669525	-1.044357026	Zm00001d016364
Zm00001d016364_T003	0.2004267	-0.680613975	Zm00001d016364
Zm00001d016084_T004	0.1229731	0.6084399	Zm00001d016084
Zm00001d016084_T005	0.7759478	-0.138137784	Zm00001d016084
Zm00001d016084_T002	1	0.392764427	Zm00001d016084
Zm00001d040466_T001	0.9702242	0.643411203	Zm00001d040466
Zm00001d040466_T002	4.46E-11	-2.175608258	Zm00001d040466
Zm00001d027499_T001	0.8605736	0.412990114	Zm00001d027499
Zm00001d049479_T002	0.9766874	0.220692263	Zm00001d049479
Zm00001d049479_T004	0.4523558	0.921627512	Zm00001d049479
Zm00001d049479_T001	0.004540284	1.760895256	Zm00001d049479
Zm00001d019185_T005	0.5570384	0.368916047	Zm00001d019185
Zm00001d030361_T001	0.4565762	-0.745735787	Zm00001d030361
Zm00001d029118_T001	0.9979393	0.294882029	Zm00001d029118
Zm00001d029118_T003	0.4096848	0.757464261	Zm00001d029118
Zm00001d049738_T001	0.865651	-0.283033175	Zm00001d049738
Zm00001d005298_T001	0.954492	0.133482908	Zm00001d005298
Zm00001d021390_T004	0.8896281	-0.072853788	Zm00001d021390
Zm00001d021390_T005	0.909729	0.290045503	Zm00001d021390
Zm00001d021390_T001	1	0.352457704	Zm00001d021390
Zm00001d021390_T003	0.5813054	0.667273175	Zm00001d021390
Zm00001d000042_T002	0.8498341	0.790850486	Zm00001d000042
Zm00001d000042_T001	0.8894168	-0.015134627	Zm00001d000042
Zm00001d039637_T001	0.9237921	0.004712138	Zm00001d039637
Zm00001d011570_T004	0.9912983	0.173872313	Zm00001d011570
Zm00001d011570_T001	0.9867452	0.31962217	Zm00001d011570
Zm00001d011570_T006	0.3909958	1.248286913	Zm00001d011570
Zm00001d016878_T001	0.9404125	0.015555048	Zm00001d016878
Zm00001d045894_T005	0.9842144	0.782745818	Zm00001d045894
Zm00001d045894_T001	0.9743741	0.254806636	Zm00001d045894
Zm00001d045894_T004	0.8935288	-0.341359218	Zm00001d045894
Zm00001d045894_T002	0.7029928	1.074899541	Zm00001d045894
Zm00001d045894_T003	0.9037807	0.511121347	Zm00001d045894
Zm00001d046460_T001	0.8859016	-0.132909722	Zm00001d046460
Zm00001d042874_T002	0.004883832	-1.900818516	Zm00001d042874
Zm00001d042874_T005	0.9985916	0.433921542	Zm00001d042874
Zm00001d042874_T004	0.9807381	1.359657445	Zm00001d042874
Zm00001d043449_T001	0.9693993	0.080544423	Zm00001d043449
Zm00001d006256_T001	0.449035	-1.092173387	Zm00001d006256
Zm00001d051334_T001	0.6207301	1.171760197	Zm00001d051334
Zm00001d045231_T002	0.3461501	0.974719819	Zm00001d045231
Zm00001d050139_T006	0.7588107	-0.198056787	Zm00001d050139
Zm00001d050139_T004	0.8224042	0.609968913	Zm00001d050139
Zm00001d050139_T007	0.9903483	0.284886733	Zm00001d050139
Zm00001d024393_T001	0.9877195	0.472096837	Zm00001d024393

Zm00001d024393_T003	0.9975882	0.413162395	Zm00001d024393
Zm00001d024393_T002	1	0.155628154	Zm00001d024393
Zm00001d027683_T001	0.9965998	0.25305348	Zm00001d027683
Zm00001d002158_T001	0.001168745	-1.666886076	Zm00001d002158
Zm00001d014901_T001	0.9778436	0.597090791	Zm00001d014901
Zm00001d014901_T006	0.9380783	0.556577295	Zm00001d014901
Zm00001d014901_T002	0.003015693	-1.004670278	Zm00001d014901
Zm00001d052795_T001	0.5547266	1.228314396	Zm00001d052795
Zm00001d005841_T001	0.01785822	2.17098869	Zm00001d005841
Zm00001d015060_T001	0.8958882	0.635536335	Zm00001d015060
Zm00001d015060_T002	0.9890668	0.380044228	Zm00001d015060
Zm00001d024347_T001	0.9566139	-0.071924938	Zm00001d024347
Zm00001d037574_T001	0.6354554	-0.753411061	Zm00001d037574
Zm00001d031505_T001	0.9605953	0.103493978	Zm00001d031505
Zm00001d031505_T002	0.8695779	0.534968652	Zm00001d031505
Zm00001d045128_T001	0.8784177	0.850643131	Zm00001d045128
Zm00001d033317_T001	0.962808	-0.155815936	Zm00001d033317
Zm00001d048430_T014	0.002096923	0.957711089	Zm00001d048430
Zm00001d048430_T001	2.02E-06	0.641320363	Zm00001d048430
Zm00001d048430_T002	0.7187138	0.850752581	Zm00001d048430
Zm00001d048430_T016	0.9886764	0.285153279	Zm00001d048430
Zm00001d048430_T005	0.0118982	2.249905523	Zm00001d048430
Zm00001d012492_T001	0.596228	-0.287249634	Zm00001d012492
Zm00001d043422_T001	0.2630185	-1.569953637	Zm00001d043422
Zm00001d022602_T001	0.9781672	-0.000287169	Zm00001d022602
Zm00001d045391_T001	2.59E-10	-6.075121249	Zm00001d045391
Zm00001d044982_T007	0.01045777	1.303315349	Zm00001d044982
Zm00001d044982_T015	0.1155712	1.589180514	Zm00001d044982
Zm00001d044982_T011	0.2454012	0.630107135	Zm00001d044982
Zm00001d044982_T016	0.06421459	-1.478173381	Zm00001d044982
Zm00001d044982_T010	0.1386783	1.653438536	Zm00001d044982
Zm00001d044982_T009	0.02586834	2.276816203	Zm00001d044982
Zm00001d042333_T001	1	0.382041897	Zm00001d042333
Zm00001d009667_T001	1	0.346986664	Zm00001d009667
Zm00001d023282_T001	0.7978964	-0.299438182	Zm00001d023282
Zm00001d028366_T001	0.3173354	-0.94375019	Zm00001d028366
Zm00001d034800_T001	0.1106445	1.099409364	Zm00001d034800
Zm00001d008731_T005	0.2840735	0.782830375	Zm00001d008731
Zm00001d008731_T003	0.8498341	-0.341511813	Zm00001d008731
Zm00001d008731_T006	0.9962521	0.0621224	Zm00001d008731
Zm00001d028725_T001	0.3764439	1.628951709	Zm00001d028725
Zm00001d007125_T001	0.9791729	0.033599987	Zm00001d007125
Zm00001d034404_T001	0.9177108	-0.254166872	Zm00001d034404
Zm00001d035425_T001	0.05406662	1.73842436	Zm00001d035425
Zm00001d008323_T007	3.81E-09	1.761533877	Zm00001d008323
Zm00001d008323_T008	0.3894524	0.762332359	Zm00001d008323
Zm00001d008323_T004	0.9619176	0.133299389	Zm00001d008323
Zm00001d008323_T011	0.1094786	1.009768458	Zm00001d008323
Zm00001d008323_T017	0.1456397	0.72389511	Zm00001d008323
Zm00001d049046_T002	0.8741963	0.783091899	Zm00001d049046
Zm00001d009849_T005	0.9903234	0.159026081	Zm00001d009849
Zm00001d009849_T001	0.9732421	0.557615157	Zm00001d009849
Zm00001d010924_T001	0.4929873	-0.70151054	Zm00001d010924
Zm00001d005833_T003	0.2637611	-0.894118506	Zm00001d005833

Zm00001d005833_T004	0.9463296	0.077790856	Zm00001d005833
Zm00001d005833_T001	0.9356767	0.444572284	Zm00001d005833
Zm00001d044171_T001	0.009317753	2.6933275	Zm00001d044171
Zm00001d007105_T002	0.6934931	-0.417705231	Zm00001d007105
Zm00001d018789_T001	0.9244098	0.744571719	Zm00001d018789
Zm00001d003050_T001	0.9827917	0.354748174	Zm00001d003050
Zm00001d043834_T002	0.9988063	0.375915428	Zm00001d043834
Zm00001d032188_T001	0.9237921	0.74480479	Zm00001d032188
Zm00001d032188_T002	0.7010603	1.140363297	Zm00001d032188
Zm00001d017403_T001	0.7973501	0.968598422	Zm00001d017403
Zm00001d010814_T001	0.5857921	1.34903249	Zm00001d010814
Zm00001d032707_T001	0.8987811	0.579130171	Zm00001d032707
Zm00001d036245_T009	0.2922198	-0.713957332	Zm00001d036245
Zm00001d036245_T005	0.9725269	0.591065161	Zm00001d036245
Zm00001d036245_T001	1	0.267241238	Zm00001d036245
Zm00001d023923_T001	1	0.407816525	Zm00001d023923
Zm00001d052614_T001	0.6705583	-0.560147021	Zm00001d052614
Zm00001d053177_T001	0.8899623	-0.086839259	Zm00001d053177
Zm00001d053177_T002	0.7497503	0.353410681	Zm00001d053177
Zm00001d030796_T007	0.9770343	0.37722169	Zm00001d030796
Zm00001d030796_T003	0.8031754	-0.132049175	Zm00001d030796
Zm00001d030796_T006	0.8095986	-0.309656524	Zm00001d030796
Zm00001d015450_T002	0.964982	0.178800609	Zm00001d015450
Zm00001d028807_T001	0.4167857	-1.35379952	Zm00001d028807
Zm00001d028807_T002	0.6357777	-0.652570062	Zm00001d028807
Zm00001d029560_T001	0.3222139	-0.677140094	Zm00001d029560
Zm00001d014188_T005	0.000780838	1.543906358	Zm00001d014188
Zm00001d014188_T034	0.9888172	0.079623515	Zm00001d014188
Zm00001d014188_T033	0.9831949	0.016267375	Zm00001d014188
Zm00001d014188_T035	0.7429199	-0.227016891	Zm00001d014188
Zm00001d025793_T004	0.8224736	0.198004764	Zm00001d025793
Zm00001d025793_T002	0.9979393	0.333405732	Zm00001d025793
Zm00001d032911_T001	0.6206868	-0.569283798	Zm00001d032911
Zm00001d043386_T001	0.3497147	0.975809734	Zm00001d043386
Zm00001d020851_T002	0.5189688	-0.447343517	Zm00001d020851
Zm00001d020851_T001	0.5698311	0.615748187	Zm00001d020851
Zm00001d048422_T001	0.9747606	0.113657468	Zm00001d048422
Zm00001d003256_T001	0.9963927	0.482451814	Zm00001d003256
Zm00001d031539_T003	0.8252955	-0.314456501	Zm00001d031539
Zm00001d031539_T001	0.6970851	-0.431119328	Zm00001d031539
Zm00001d013243_T002	0.5613757	-0.461066729	Zm00001d013243
Zm00001d010171_T001	0.164302	1.030710118	Zm00001d010171
Zm00001d010171_T003	0.2546213	1.495813668	Zm00001d010171
Zm00001d010171_T002	0.9173494	-0.150037349	Zm00001d010171
Zm00001d003555_T002	0.9210235	0.402242296	Zm00001d003555
Zm00001d045315_T002	0.9821324	0.160973626	Zm00001d045315
Zm00001d045315_T004	0.9543425	1.105713017	Zm00001d045315
Zm00001d037239_T001	0.971204	0.160086026	Zm00001d037239
Zm00001d040733_T001	0.6812088	-0.402807699	Zm00001d040733
Zm00001d040733_T002	0.8665954	0.592649189	Zm00001d040733
Zm00001d039854_T001	0.9984041	0.293986076	Zm00001d039854
Zm00001d034245_T003	0.7430942	0.673990173	Zm00001d034245
Zm00001d034245_T009	0.6620718	1.191714458	Zm00001d034245
Zm00001d034245_T001	6.25E-10	-2.766566548	Zm00001d034245

Zm00001d034245_T014	0.6735594	0.598174498	Zm00001d034245
Zm00001d045607_T001	0.8027057	-0.491898385	Zm00001d045607
Zm00001d007881_T004	1	0.68220677	Zm00001d007881
Zm00001d007881_T010	0.8545986	-0.963941203	Zm00001d007881
Zm00001d007881_T005	0.9728856	0.103355791	Zm00001d007881
Zm00001d021947_T004	0.002445413	1.208078878	Zm00001d021947
Zm00001d021947_T005	3.46E-06	1.937202833	Zm00001d021947
Zm00001d021947_T002	0.7697196	1.095619187	Zm00001d021947
Zm00001d006658_T003	0.9986486	0.356373166	Zm00001d006658
Zm00001d006658_T005	0.1874475	0.80543166	Zm00001d006658
Zm00001d006658_T007	0.6201746	0.265489764	Zm00001d006658
Zm00001d006658_T002	0.2295978	0.696468068	Zm00001d006658
Zm00001d005301_T015	0.8139802	0.244942568	Zm00001d005301
Zm00001d005301_T016	0.830404	0.47836505	Zm00001d005301
Zm00001d005301_T012	0.5361006	0.205294386	Zm00001d005301
Zm00001d040834_T001	0.8139802	-0.627739461	Zm00001d040834
Zm00001d037356_T001	0.8746701	-0.054332128	Zm00001d037356
Zm00001d017504_T001	0.9983524	0.125873786	Zm00001d017504
Zm00001d050061_T005	1.04E-08	-1.244950526	Zm00001d050061
Zm00001d050061_T006	0.9690925	0.197826158	Zm00001d050061
Zm00001d050061_T002	0.9166356	0.575126518	Zm00001d050061
Zm00001d026284_T001	0.9975882	-0.020042257	Zm00001d026284
Zm00001d049435_T001	0.09183753	1.496051316	Zm00001d049435
Zm00001d027642_T001	0.9851204	0.134952816	Zm00001d027642
Zm00001d028408_T001	0.2309798	1.00405216	Zm00001d028408
Zm00001d017830_T011	0.5395632	1.367592151	Zm00001d017830
Zm00001d017830_T012	0.8940288	1.223032813	Zm00001d017830
Zm00001d017830_T001	0.091426	0.004389445	Zm00001d017830
Zm00001d017830_T003	0.9706887	0.0205001	Zm00001d017830
Zm00001d017830_T014	0.2856065	0.729070463	Zm00001d017830
Zm00001d017830_T009	0.5622303	1.157262135	Zm00001d017830
Zm00001d030421_T001	0.9688529	0.548932267	Zm00001d030421
Zm00001d037553_T002	0.08686142	-0.675382551	Zm00001d037553
Zm00001d037553_T003	0.9781672	0.172112643	Zm00001d037553
Zm00001d037553_T001	0.9224443	0.203504006	Zm00001d037553
Zm00001d031700_T003	0.7842749	-0.126322101	Zm00001d031700
Zm00001d031700_T002	0.9709887	0.622483674	Zm00001d031700
Zm00001d031700_T004	0.9902733	0.142063431	Zm00001d031700
Zm00001d023235_T001	0.6480001	0.851548685	Zm00001d023235
Zm00001d023235_T002	0.6436915	-0.522905509	Zm00001d023235
Zm00001d017415_T001	0.8885217	-0.347673244	Zm00001d017415
Zm00001d038831_T001	0.8439431	-0.13917923	Zm00001d038831
Zm00001d024751_T001	0.5626026	1.314532596	Zm00001d024751
Zm00001d017237_T003	0.9747695	0.053919804	Zm00001d017237
Zm00001d041710_T004	0.9296811	0.708751538	Zm00001d041710
Zm00001d041710_T006	0.9168913	0.440802974	Zm00001d041710
Zm00001d041710_T007	1	0.068794998	Zm00001d041710
Zm00001d041710_T005	0.5277554	-0.227885947	Zm00001d041710
Zm00001d041710_T002	0.9766874	0.603981639	Zm00001d041710
Zm00001d039535_T001	0.9779656	0.577664838	Zm00001d039535
Zm00001d049073_T001	0.8412913	0.54695362	Zm00001d049073
Zm00001d010798_T001	0.01481562	2.192282481	Zm00001d010798
Zm00001d032213_T001	0.06982753	1.988854542	Zm00001d032213
Zm00001d027924_T001	0.009891435	-2.297714981	Zm00001d027924

Zm00001d002402_T002	0.652668	-0.409407992	Zm00001d002402
Zm00001d002402_T001	0.03124381	-1.86558718	Zm00001d002402
Zm00001d022021_T001	0.8886159	-0.02149589	Zm00001d022021
Zm00001d002890_T001	0.7100246	-0.517109219	Zm00001d002890
Zm00001d019173_T001	0.9944178	0.516354424	Zm00001d019173
Zm00001d005001_T003	0.8553049	0.408517426	Zm00001d005001
Zm00001d005001_T001	0.9950703	0.326509781	Zm00001d005001
Zm00001d005001_T002	0.9139882	0.712927894	Zm00001d005001
Zm00001d005654_T001	0.9184323	0.23696306	Zm00001d005654
Zm00001d032539_T001	0.8612451	0.921808411	Zm00001d032539
Zm00001d044132_T001	0.2344999	0.758605073	Zm00001d044132
Zm00001d044132_T002	0.5380217	0.897682669	Zm00001d044132
Zm00001d044801_T001	0.5715832	0.79268475	Zm00001d044801
Zm00001d009825_T001	0.7528247	0.832441593	Zm00001d009825
Zm00001d036524_T002	0.8023277	-0.13974804	Zm00001d036524
Zm00001d036524_T001	0.9702644	0.614633081	Zm00001d036524
Zm00001d039483_T001	0.9745777	0.176121716	Zm00001d039483
Zm00001d000432_T001	0.2575978	-1.009658216	Zm00001d000432
Zm00001d000432_T003	0.9370449	0.778960195	Zm00001d000432
Zm00001d009288_T012	0.757578	0.715466733	Zm00001d009288
Zm00001d009288_T016	0.3541203	-0.940433361	Zm00001d009288
Zm00001d009288_T003	0.8896102	0.909284088	Zm00001d009288
Zm00001d009288_T006	0.8412913	0.299501421	Zm00001d009288
Zm00001d033388_T002	0.9711278	0.625754821	Zm00001d033388
Zm00001d033388_T003	0.9791729	0.246373314	Zm00001d033388
Zm00001d052216_T001	0.9855117	-0.11497697	Zm00001d052216
Zm00001d007208_T001	0.5698311	1.239652382	Zm00001d007208
Zm00001d040076_T016	0.4178252	0.61215052	Zm00001d040076
Zm00001d040076_T001	0.909186	0.018624696	Zm00001d040076
Zm00001d040076_T011	0.5853819	0.620944407	Zm00001d040076
Zm00001d040076_T002	0.4282459	0.692310469	Zm00001d040076
Zm00001d040076_T010	0.03710512	0.685171438	Zm00001d040076
Zm00001d040076_T008	0.9950703	0.403930674	Zm00001d040076
Zm00001d049220_T001	0.3695075	1.512074584	Zm00001d049220
Zm00001d003840_T001	0.3190763	-1.063493864	Zm00001d003840
Zm00001d048268_T001	0.01481829	1.999634499	Zm00001d048268
Zm00001d033172_T003	0.7997379	0.631204793	Zm00001d033172
Zm00001d033172_T005	0.990751	0.260334532	Zm00001d033172
Zm00001d033172_T009	0.5199848	0.865469066	Zm00001d033172
Zm00001d033172_T007	0.2879626	-0.472514374	Zm00001d033172
Zm00001d011379_T016	0.85068	0.791276221	Zm00001d011379
Zm00001d011379_T023	0.1159818	0.586939787	Zm00001d011379
Zm00001d011379_T004	5.60E-09	1.25777959	Zm00001d011379
Zm00001d011379_T005	0.8926364	0.058619316	Zm00001d011379
Zm00001d011379_T007	0.6467502	-0.190796109	Zm00001d011379
Zm00001d035612_T001	1	0.22257788	Zm00001d035612
Zm00001d025905_T003	0.9942835	-0.082976743	Zm00001d025905
Zm00001d025905_T004	0.241511	0.996456054	Zm00001d025905
Zm00001d028074_T001	1.73E-05	4.267863516	Zm00001d028074
Zm00001d035649_T001	0.8645719	-0.232764839	Zm00001d035649
Zm00001d053665_T001	0.8687089	0.844016612	Zm00001d053665
Zm00001d053665_T002	0.9863483	0.400174507	Zm00001d053665
Zm00001d012175_T001	0.9954426	0.267090137	Zm00001d012175
Zm00001d012175_T003	0.996005	0.348427364	Zm00001d012175

Zm00001d012175_T004	0.9832092	0.480734488	Zm00001d012175
Zm00001d012175_T002	0.9302759	0.178411946	Zm00001d012175
Zm00001d016902_T004	0.9687311	-0.048669145	Zm00001d016902
Zm00001d016902_T003	0.8825173	0.885741859	Zm00001d016902
Zm00001d016902_T002	0.9554458	0.267404245	Zm00001d016902
Zm00001d030340_T039	0.1526397	0.235599608	Zm00001d030340
Zm00001d030340_T060	0.7235553	0.46113437	Zm00001d030340
Zm00001d030340_T024	0.8787604	-0.048338335	Zm00001d030340
Zm00001d030340_T003	0.8135961	0.662001261	Zm00001d030340
Zm00001d030340_T019	0.2708531	0.461031157	Zm00001d030340
Zm00001d044618_T001	0.5341861	-0.393197467	Zm00001d044618
Zm00001d047960_T002	1	0.401191589	Zm00001d047960
Zm00001d047960_T004	0.308051	1.089487492	Zm00001d047960
Zm00001d047960_T001	0.4642969	-0.379294971	Zm00001d047960
Zm00001d028514_T001	0.5147437	1.303346553	Zm00001d028514
Zm00001d028514_T002	0.03449192	0.75080876	Zm00001d028514
Zm00001d052845_T002	0.9593283	0.154644858	Zm00001d052845
Zm00001d033574_T001	0.9007927	-0.537345982	Zm00001d033574
Zm00001d045944_T008	0.4351411	0.725022185	Zm00001d045944
Zm00001d045944_T007	0.2355554	1.703364702	Zm00001d045944
Zm00001d045944_T005	0.2738479	0.679567645	Zm00001d045944
Zm00001d045944_T011	0.00713124	1.261914308	Zm00001d045944
Zm00001d045944_T006	0.03639015	1.554421518	Zm00001d045944
Zm00001d045944_T002	0.3689534	-1.215949717	Zm00001d045944
Zm00001d045747_T005	0.416342	-0.858793313	Zm00001d045747
Zm00001d045747_T004	0.7585131	0.878183174	Zm00001d045747
Zm00001d046766_T003	0.03060013	-0.738555089	Zm00001d046766
Zm00001d046766_T001	0.4860327	0.574512733	Zm00001d046766
Zm00001d046766_T005	0.8863456	-0.147162878	Zm00001d046766
Zm00001d016992_T001	0.6343135	2.484283737	Zm00001d016992
Zm00001d052813_T001	0.1816976	-0.494980821	Zm00001d052813
Zm00001d049359_T001	0.9979568	0.286618573	Zm00001d049359
Zm00001d001945_T017	0.5936419	0.231852184	Zm00001d001945
Zm00001d042117_T002	0.8183889	-0.157338226	Zm00001d042117
Zm00001d042117_T004	0.5844177	-0.116107347	Zm00001d042117
Zm00001d042117_T001	0.5478438	-0.979253735	Zm00001d042117
Zm00001d012536_T006	0.9367198	0.472193724	Zm00001d012536
Zm00001d012536_T002	1	0.375076305	Zm00001d012536
Zm00001d012536_T009	0.9390517	0.371006106	Zm00001d012536
Zm00001d028238_T001	0.7991382	0.847183844	Zm00001d028238
Zm00001d028238_T009	0.8031436	-0.260846772	Zm00001d028238
Zm00001d028238_T007	0.577886	0.468177046	Zm00001d028238
Zm00001d028238_T012	0.2879626	0.279294654	Zm00001d028238
Zm00001d020554_T001	0.550715	1.164270899	Zm00001d020554
Zm00001d012396_T001	0.7531776	0.372677972	Zm00001d012396
Zm00001d012396_T005	0.7909041	0.346066102	Zm00001d012396
Zm00001d012396_T006	0.8545272	0.305781341	Zm00001d012396
Zm00001d012396_T007	0.377256	2.164089056	Zm00001d012396
Zm00001d002761_T001	0.5208223	-0.570386125	Zm00001d002761
Zm00001d040857_T001	0.9100328	-0.030931387	Zm00001d040857
Zm00001d011558_T001	0.8102147	-0.643400174	Zm00001d011558
Zm00001d010416_T002	0.9979568	0.331496042	Zm00001d010416
Zm00001d005402_T003	0.8158416	0.445844676	Zm00001d005402
Zm00001d005402_T005	1	0.157444685	Zm00001d005402

Zm00001d005402_T008	0.4179542	-1.150450487	Zm00001d005402
Zm00001d005402_T007	0.7097086	0.387152385	Zm00001d005402
Zm00001d005402_T004	0.8660897	0.294657095	Zm00001d005402
Zm00001d033383_T001	0.9950703	0.331502389	Zm00001d033383
Zm00001d033383_T002	0.5597598	-0.312808407	Zm00001d033383
Zm00001d034059_T005	0.9171732	-0.128739713	Zm00001d034059
Zm00001d028999_T001	0.941809	0.056704944	Zm00001d028999
Zm00001d044074_T001	1	0.232183188	Zm00001d044074
Zm00001d013794_T001	0.01861352	-1.355836301	Zm00001d013794
Zm00001d040766_T001	0.9979568	0.306191423	Zm00001d040766
Zm00001d029214_T001	0.9894615	0.41064028	Zm00001d029214
Zm00001d029214_T006	0.3913208	0.457925843	Zm00001d029214
Zm00001d029214_T005	0.7831859	0.448754573	Zm00001d029214
Zm00001d044500_T001	1	0.36192868	Zm00001d044500
Zm00001d044500_T002	0.9701986	0.328195282	Zm00001d044500
Zm00001d006195_T001	0.8882695	-0.142363983	Zm00001d006195
Zm00001d012438_T001	0.9654721	0.508664326	Zm00001d012438
Zm00001d021279_T001	0.01368227	-1.086966117	Zm00001d021279
Zm00001d026381_T003	0.9887584	0.456808687	Zm00001d026381
Zm00001d026381_T002	0.9868533	0.192290897	Zm00001d026381
Zm00001d031969_T001	0.9770343	0.106743077	Zm00001d031969
Zm00001d031969_T002	0.8354517	-0.370649773	Zm00001d031969
Zm00001d000150_T001	0.9743605	0.553368132	Zm00001d000150
Zm00001d028670_T001	0.0252917	-1.635522819	Zm00001d028670
Zm00001d048111_T001	0.7074815	0.99015146	Zm00001d048111
Zm00001d050612_T008	0.9825858	0.148439314	Zm00001d050612
Zm00001d050612_T001	0.2679983	0.842843682	Zm00001d050612
Zm00001d027283_T001	0.522218	-0.892166488	Zm00001d027283
Zm00001d020358_T001	0.6412909	-0.473561268	Zm00001d020358
Zm00001d022614_T002	0.9832092	0.20140706	Zm00001d022614
Zm00001d022614_T001	0.9563232	0.073728649	Zm00001d022614
Zm00001d014755_T001	0.009922438	2.844373145	Zm00001d014755
Zm00001d017166_T002	0.8625537	0.620070297	Zm00001d017166
Zm00001d017166_T001	0.9139882	0.734532847	Zm00001d017166
Zm00001d032035_T001	0.6674901	1.248310621	Zm00001d032035
Zm00001d025513_T001	1	0.364939836	Zm00001d025513
Zm00001d039499_T002	0.8031436	1.038905275	Zm00001d039499
Zm00001d034740_T001	0.9233022	-0.053157366	Zm00001d034740
Zm00001d034740_T002	0.7430929	-0.235426304	Zm00001d034740
Zm00001d048065_T002	0.7759478	1.049066977	Zm00001d048065
Zm00001d048065_T001	0.9719113	0.432835163	Zm00001d048065
Zm00001d048065_T003	0.0259558	-2.930382356	Zm00001d048065
Zm00001d008622_T002	0.9814486	0.466457309	Zm00001d008622
Zm00001d008622_T001	0.8964117	0.034176468	Zm00001d008622
Zm00001d046055_T001	3.38E-06	-5.10539344	Zm00001d046055
Zm00001d014124_T004	0.09324703	2.248629904	Zm00001d014124
Zm00001d014124_T003	0.9173494	1.797294029	Zm00001d014124
Zm00001d014124_T001	0.000813969	-0.998238917	Zm00001d014124
Zm00001d014124_T006	0.3437881	0.969081699	Zm00001d014124
Zm00001d014674_T005	0.7262357	0.576544471	Zm00001d014674
Zm00001d014674_T001	0.9378338	0.657962732	Zm00001d014674
Zm00001d014674_T004	0.4860327	0.37573885	Zm00001d014674
Zm00001d020018_T001	0.1504172	0.753696817	Zm00001d020018
Zm00001d013026_T003	0.9995806	0.263729151	Zm00001d013026

Zm00001d013026_T002	0.9887584	0.341392004	Zm00001d013026
Zm00001d013026_T001	0.4723494	0.667420986	Zm00001d013026
Zm00001d013026_T004	0.7820223	-0.169131309	Zm00001d013026
Zm00001d005144_T006	0.9825858	0.084603671	Zm00001d005144
Zm00001d005144_T010	0.9647033	0.071823837	Zm00001d005144
Zm00001d005144_T001	0.9896486	0.447177239	Zm00001d005144
Zm00001d005144_T012	0.7945048	-0.213745522	Zm00001d005144
Zm00001d005144_T005	0.4437212	1.213408472	Zm00001d005144
Zm00001d026248_T001	0.9023635	0.723338177	Zm00001d026248
Zm00001d048717_T003	0.9031129	0.471337929	Zm00001d048717
Zm00001d002847_T001	0.2790987	1.186254923	Zm00001d002847
Zm00001d053100_T004	0.7664596	0.053388349	Zm00001d053100
Zm00001d053100_T005	0.541024	-0.69200318	Zm00001d053100
Zm00001d053100_T003	0.9468268	0.568589532	Zm00001d053100
Zm00001d053100_T001	0.6580146	0.625272533	Zm00001d053100
Zm00001d052234_T001	0.956479	-0.132198787	Zm00001d052234
Zm00001d044700_T001	0.8647028	0.63474309	Zm00001d044700
Zm00001d038903_T002	0.8859449	0.645832042	Zm00001d038903
Zm00001d038903_T001	0.8689882	0.667409266	Zm00001d038903
Zm00001d024652_T001	0.4537759	1.177627396	Zm00001d024652
Zm00001d051429_T001	0.4250774	1.322411178	Zm00001d051429
Zm00001d047801_T002	0.6632816	-0.092067717	Zm00001d047801
Zm00001d047801_T001	0.9763286	0.496568977	Zm00001d047801
Zm00001d044839_T001	0.1344871	-1.021716424	Zm00001d044839
Zm00001d032807_T001	0.8348638	-0.215025106	Zm00001d032807
Zm00001d032807_T002	0.7109402	-0.008037255	Zm00001d032807
Zm00001d051995_T001	0.9031129	-0.110447485	Zm00001d051995
Zm00001d049163_T003	0.9621967	0.066313504	Zm00001d049163
Zm00001d049163_T006	0.9877195	0.185253004	Zm00001d049163
Zm00001d049163_T001	0.9681793	0.139596923	Zm00001d049163
Zm00001d014770_T001	0.8819999	-0.156009841	Zm00001d014770
Zm00001d023987_T007	0.356795	-1.165511935	Zm00001d023987
Zm00001d023987_T008	0.6891365	0.211417888	Zm00001d023987
Zm00001d023987_T002	0.9778436	0.223514969	Zm00001d023987
Zm00001d023987_T004	0.8906466	0.321335018	Zm00001d023987
Zm00001d023987_T006	0.2648796	-0.852417626	Zm00001d023987
Zm00001d032160_T001	1	0.324753709	Zm00001d032160
Zm00001d006775_T001	0.9957676	0.121745901	Zm00001d006775
Zm00001d044729_T001	1	0.300242716	Zm00001d044729
Zm00001d043580_T001	0.8946287	-0.059094779	Zm00001d043580
Zm00001d043237_T001	0.04229279	1.203360075	Zm00001d043237
Zm00001d024004_T001	0.9781672	0.211370685	Zm00001d024004
Zm00001d011811_T002	0.9469426	0.002789917	Zm00001d011811
Zm00001d044451_T001	1	0.414936674	Zm00001d044451
Zm00001d044451_T002	0.875998	0.692673438	Zm00001d044451
Zm00001d043942_T002	0.6578675	-0.654458804	Zm00001d043942
Zm00001d043942_T003	6.78E-10	-4.586912789	Zm00001d043942
Zm00001d051359_T001	0.7357641	-0.458558819	Zm00001d051359
Zm00001d017952_T002	0.338859	1.614585795	Zm00001d017952
Zm00001d014179_T001	0.8731348	-0.07785035	Zm00001d014179
Zm00001d015130_T001	0.9666249	0.127409785	Zm00001d015130
Zm00001d040562_T007	0.12948	0.585282702	Zm00001d040562
Zm00001d040562_T006	0.983226	0.530460909	Zm00001d040562
Zm00001d040562_T025	0.03332528	1.32898145	Zm00001d040562

Zm00001d040562_T017	0.2153746	1.449576037	Zm00001d040562
Zm00001d040562_T009	0.009041647	0.758164883	Zm00001d040562
Zm00001d040562_T005	0.8092097	0.216392034	Zm00001d040562
Zm00001d040562_T020	0.000687304	3.177306311	Zm00001d040562
Zm00001d040562_T019	0.1066548	1.145645044	Zm00001d040562
Zm00001d040562_T013	0.586618	1.354198309	Zm00001d040562
Zm00001d002791_T011	0.8647916	0.597599743	Zm00001d002791
Zm00001d002791_T007	0.5256861	-0.774669807	Zm00001d002791
Zm00001d002791_T009	1	0.077473637	Zm00001d002791
Zm00001d002791_T006	0.9221549	-0.036732756	Zm00001d002791
Zm00001d002791_T013	0.001627225	1.338844136	Zm00001d002791
Zm00001d002791_T001	0.9422098	0.523570661	Zm00001d002791
Zm00001d049361_T002	0.9657314	0.165743094	Zm00001d049361
Zm00001d049361_T003	0.9090332	0.941513143	Zm00001d049361
Zm00001d051806_T001	0.8032834	-0.225159505	Zm00001d051806
Zm00001d051806_T002	0.8056055	0.890536721	Zm00001d051806
Zm00001d005082_T001	0.3685079	-0.492806271	Zm00001d005082
Zm00001d041855_T003	0.9806983	0.472518028	Zm00001d041855
Zm00001d041855_T006	0.1357514	1.501581863	Zm00001d041855
Zm00001d041855_T001	0.9458586	-0.013693663	Zm00001d041855
Zm00001d041855_T005	0.9856605	0.229459337	Zm00001d041855
Zm00001d045305_T001	0.6921285	-0.451390155	Zm00001d045305
Zm00001d047449_T032	0.8980665	0.413081576	Zm00001d047449
Zm00001d047449_T094	0.6963657	-0.301536296	Zm00001d047449
Zm00001d047449_T007	1	0.540368564	Zm00001d047449
Zm00001d051881_T001	0.9262099	0.005056716	Zm00001d051881
Zm00001d013416_T001	0.3699845	-0.423210271	Zm00001d013416
Zm00001d000093_T001	0.4437212	0.729365335	Zm00001d000093
Zm00001d012251_T003	0.7831859	-0.169067912	Zm00001d012251
Zm00001d016477_T001	0.9573272	0.616569369	Zm00001d016477
Zm00001d047089_T001	0.9627389	0.010517215	Zm00001d047089
Zm00001d025528_T002	0.03495473	0.694822303	Zm00001d025528
Zm00001d025528_T001	0.2917536	-1.251420572	Zm00001d025528
Zm00001d025528_T003	0.07900722	-0.92755304	Zm00001d025528
Zm00001d039590_T004	0.9719113	0.23890016	Zm00001d039590
Zm00001d039590_T001	0.9791729	0.401089901	Zm00001d039590
Zm00001d005816_T001	0.67913	1.122468963	Zm00001d005816
Zm00001d043578_T014	0.9791729	0.136320447	Zm00001d043578
Zm00001d043578_T013	0.4988369	0.251294603	Zm00001d043578
Zm00001d042905_T001	0.03495473	1.003859678	Zm00001d042905
Zm00001d043105_T001	0.1429392	1.449894886	Zm00001d043105
Zm00001d011797_T002	0.4332539	-0.819997493	Zm00001d011797
Zm00001d037946_T001	0.4698008	1.361856273	Zm00001d037946
Zm00001d021689_T001	0.74819	-0.188193071	Zm00001d021689
Zm00001d031201_T001	0.9914064	0.319015513	Zm00001d031201
Zm00001d045625_T001	0.9950703	0.215057686	Zm00001d045625
Zm00001d032334_T002	0.4167857	1.278062173	Zm00001d032334
Zm00001d043734_T001	1	0.345937917	Zm00001d043734
Zm00001d043860_T009	0.9979568	0.324193303	Zm00001d043860
Zm00001d043860_T004	0.5514634	1.044327258	Zm00001d043860
Zm00001d043860_T005	0.5507795	0.571431579	Zm00001d043860
Zm00001d036750_T001	0.5742833	1.288675074	Zm00001d036750
Zm00001d050781_T002	0.9692063	0.585803572	Zm00001d050781
Zm00001d010292_T001	0.74303	0.970684882	Zm00001d010292

Zm00001d035037_T001	0.9429712	1.206442087	Zm00001d035037
Zm00001d035037_T008	0.9736468	0.110248999	Zm00001d035037
Zm00001d035037_T003	0.9984041	0.34714968	Zm00001d035037
Zm00001d035037_T006	0.9510018	-0.215011233	Zm00001d035037
Zm00001d020986_T002	0.9050121	-0.037354404	Zm00001d020986
Zm00001d029105_T001	0.9891237	0.261570768	Zm00001d029105
Zm00001d005168_T006	0.7020458	-0.200467983	Zm00001d005168
Zm00001d005168_T003	0.2080144	1.387789827	Zm00001d005168
Zm00001d018393_T001	0.910614	-0.42855934	Zm00001d018393
Zm00001d003775_T001	0.9722469	-0.244809569	Zm00001d003775
Zm00001d022541_T003	0.9138633	-0.083535316	Zm00001d022541
Zm00001d022541_T004	0.9673473	0.443951076	Zm00001d022541
Zm00001d022541_T002	0.8836975	-0.078028581	Zm00001d022541
Zm00001d013573_T001	0.6453183	-0.470616184	Zm00001d013573
Zm00001d037018_T001	0.8807115	-0.526559689	Zm00001d037018
Zm00001d048492_T002	0.6827367	-0.694160889	Zm00001d048492
Zm00001d048492_T003	0.9888172	0.146269517	Zm00001d048492
Zm00001d050642_T001	0.5912583	1.042507753	Zm00001d050642
Zm00001d049831_T005	0.09384546	-1.495168031	Zm00001d049831
Zm00001d049831_T002	0.9676125	0.131162021	Zm00001d049831
Zm00001d049428_T001	0.3497147	1.378865821	Zm00001d049428
Zm00001d012425_T003	0.9980559	-0.021491723	Zm00001d012425
Zm00001d012425_T002	0.929519	-0.270085644	Zm00001d012425
Zm00001d025402_T006	0.9748076	0.518040889	Zm00001d025402
Zm00001d025402_T002	0.9771927	0.489994647	Zm00001d025402
Zm00001d014944_T002	0.5448225	-0.663322563	Zm00001d014944
Zm00001d014944_T001	0.06031978	-1.479970696	Zm00001d014944
Zm00001d028951_T001	0.9832612	0.132726621	Zm00001d028951
Zm00001d022560_T002	0.6929566	-0.281542715	Zm00001d022560
Zm00001d022560_T001	0.4508973	0.684105843	Zm00001d022560
Zm00001d022560_T003	0.5868947	0.438363385	Zm00001d022560
Zm00001d052026_T001	0.9237617	0.449519435	Zm00001d052026
Zm00001d037803_T002	0.923446	0.586231119	Zm00001d037803
Zm00001d037803_T001	0.4226019	0.358813762	Zm00001d037803
Zm00001d035004_T002	0.04075837	1.346864627	Zm00001d035004
Zm00001d035004_T039	0.7301819	1.053249824	Zm00001d035004
Zm00001d035004_T034	3.84E-13	2.29528018	Zm00001d035004
Zm00001d035004_T015	0.000282019	1.689765221	Zm00001d035004
Zm00001d035004_T026	0.05110875	0.34331225	Zm00001d035004
Zm00001d026352_T001	0.8412913	-0.587700119	Zm00001d026352
Zm00001d009628_T001	0.9845237	-0.085455691	Zm00001d009628
Zm00001d050872_T001	0.1557051	2.051744383	Zm00001d050872
Zm00001d004126_T001	0.9237617	0.606345203	Zm00001d004126
Zm00001d010703_T007	0.5795936	1.331660262	Zm00001d010703
Zm00001d010703_T009	0.9571192	0.170513034	Zm00001d010703
Zm00001d010703_T010	0.8498341	-0.205822494	Zm00001d010703
Zm00001d010703_T005	0.966464	0.315859517	Zm00001d010703
Zm00001d038729_T001	0.08778843	-1.607534578	Zm00001d038729
Zm00001d033112_T001	0.6364634	0.96556605	Zm00001d033112
Zm00001d033112_T006	0.4321944	-0.963454899	Zm00001d033112
Zm00001d024756_T005	0.9356767	-0.017751102	Zm00001d024756
Zm00001d038915_T001	1.93E-07	2.79588415	Zm00001d038915
Zm00001d017833_T001	0.9979568	0.41576018	Zm00001d017833
Zm00001d017833_T002	0.2953667	-0.717286608	Zm00001d017833

Zm00001d038552_T001	0.8247952	0.82153923	Zm00001d038552
Zm00001d008623_T001	1	0.278290069	Zm00001d008623
Zm00001d013038_T001	0.5441931	1.042034026	Zm00001d013038
Zm00001d017876_T007	0.9718747	0.060732051	Zm00001d017876
Zm00001d017876_T010	0.09126143	1.240845999	Zm00001d017876
Zm00001d017876_T012	0.2285031	0.733926903	Zm00001d017876
Zm00001d017876_T011	0.6700298	0.275336302	Zm00001d017876
Zm00001d017876_T006	0.7446163	0.805450406	Zm00001d017876
Zm00001d017876_T009	0.9907928	0.261629679	Zm00001d017876
Zm00001d017876_T018	0.3110193	1.602557874	Zm00001d017876
Zm00001d021699_T001	0.9213556	-0.035144205	Zm00001d021699
Zm00001d003119_T002	0.00432903	-1.545327272	Zm00001d003119
Zm00001d003119_T001	0.608209	1.14793703	Zm00001d003119
Zm00001d003119_T006	4.52E-11	1.80841294	Zm00001d003119
Zm00001d003446_T003	0.9543237	0.12527485	Zm00001d003446
Zm00001d003446_T002	0.4045966	-0.763778411	Zm00001d003446
Zm00001d013554_T001	0.8537544	-0.158425623	Zm00001d013554
Zm00001d046313_T003	0.9814486	-0.288503527	Zm00001d046313
Zm00001d046313_T001	0.9970835	0.194421287	Zm00001d046313
Zm00001d034630_T001	1	0.406931331	Zm00001d034630
Zm00001d028649_T002	0.7214524	1.123608488	Zm00001d028649
Zm00001d028649_T001	0.1291359	1.829488068	Zm00001d028649
Zm00001d034270_T001	0.9942835	-0.048458809	Zm00001d034270
Zm00001d034270_T002	0.9726297	0.198781891	Zm00001d034270
Zm00001d040290_T003	0.8894154	0.858770897	Zm00001d040290
Zm00001d040290_T005	1	-0.037843061	Zm00001d040290
Zm00001d040290_T004	0.0389152	1.047003063	Zm00001d040290
Zm00001d013185_T001	0.1651526	1.978308165	Zm00001d013185
Zm00001d006516_T001	0.9689939	0.105246468	Zm00001d006516
Zm00001d017522_T011	0.9779851	0.182746028	Zm00001d017522
Zm00001d017522_T007	0.5606519	0.90277984	Zm00001d017522
Zm00001d017522_T018	0.8918362	0.887662628	Zm00001d017522
Zm00001d017522_T016	0.9604475	0.370652465	Zm00001d017522
Zm00001d017522_T020	0.3700837	0.554097391	Zm00001d017522
Zm00001d017522_T023	0.7914396	-0.596001476	Zm00001d017522
Zm00001d017522_T012	0.1097282	2.117222618	Zm00001d017522
Zm00001d017522_T009	0.2917536	0.699973169	Zm00001d017522
Zm00001d011276_T001	0.306157	1.598382718	Zm00001d011276
Zm00001d053864_T003	0.9375274	-0.073385203	Zm00001d053864
Zm00001d028623_T001	0.9719791	0.217421214	Zm00001d028623
Zm00001d028623_T002	0.2727417	0.491040307	Zm00001d028623
Zm00001d041734_T001	0.9766874	0.194522879	Zm00001d041734
Zm00001d013962_T002	0.9979568	0.045080864	Zm00001d013962
Zm00001d013962_T004	0.9907928	1.047824201	Zm00001d013962
Zm00001d013962_T001	0.02195903	-0.248309554	Zm00001d013962
Zm00001d036025_T005	0.9832092	0.455391688	Zm00001d036025
Zm00001d036025_T001	0.9894615	0.472184928	Zm00001d036025
Zm00001d036025_T006	3.94E-05	-2.277205135	Zm00001d036025
Zm00001d036025_T003	0.2396674	-0.620462642	Zm00001d036025
Zm00001d036025_T007	0.9845237	0.110103033	Zm00001d036025
Zm00001d008941_T008	0.9979568	0.22636441	Zm00001d008941
Zm00001d008941_T004	0.9550193	0.177167287	Zm00001d008941
Zm00001d012420_T004	0.8050292	-0.593641009	Zm00001d012420
Zm00001d012420_T002	0.3720939	-0.474781386	Zm00001d012420

Zm00001d012420_T003	0.8987811	0.675018662	Zm00001d012420
Zm00001d043420_T001	0.6432857	-0.449168317	Zm00001d043420
Zm00001d022059_T001	0.9975882	0.215364837	Zm00001d022059
Zm00001d027298_T001	0.6269582	-0.445313133	Zm00001d027298
Zm00001d033976_T001	0.8677544	-0.190788782	Zm00001d033976
Zm00001d033976_T004	0.2001709	-0.352436174	Zm00001d033976
Zm00001d028603_T005	0.6921285	-0.354588362	Zm00001d028603
Zm00001d028603_T001	0.9702242	0.222969514	Zm00001d028603
Zm00001d028603_T006	0.9278769	0.69205308	Zm00001d028603
Zm00001d004790_T006	0.6929566	1.038432279	Zm00001d004790
Zm00001d004790_T002	0.9740325	0.238835114	Zm00001d004790
Zm00001d008604_T011	0.9870394	0.271966528	Zm00001d008604
Zm00001d008604_T003	0.1139375	1.915156285	Zm00001d008604
Zm00001d008604_T007	0.1932887	1.10610702	Zm00001d008604
Zm00001d008604_T005	0.9396569	0.293918132	Zm00001d008604
Zm00001d008604_T001	0.4008348	1.501970791	Zm00001d008604
Zm00001d008604_T004	0.908501	0.694331046	Zm00001d008604
Zm00001d008604_T010	0.03778895	1.227506609	Zm00001d008604
Zm00001d012532_T001	0.9945118	0.340198921	Zm00001d012532
Zm00001d002382_T002	0.7885216	0.956166251	Zm00001d002382
Zm00001d002382_T001	0.77179	0.878204413	Zm00001d002382
Zm00001d015131_T002	0.8537544	0.759184657	Zm00001d015131
Zm00001d015131_T005	0.965836	0.149528804	Zm00001d015131
Zm00001d015131_T001	0.9954649	0.313453962	Zm00001d015131
Zm00001d043842_T008	0.9264186	0.942990202	Zm00001d043842
Zm00001d043842_T007	0.9369505	0.428243058	Zm00001d043842
Zm00001d043842_T004	0.7296512	0.171514149	Zm00001d043842
Zm00001d043842_T013	0.7481801	-0.358721743	Zm00001d043842
Zm00001d043842_T003	0.6395973	1.204765168	Zm00001d043842
Zm00001d023264_T004	0.5451508	-0.501947248	Zm00001d023264
Zm00001d023264_T002	1	0.365635643	Zm00001d023264
Zm00001d023264_T007	0.5509641	-0.695706169	Zm00001d023264
Zm00001d033420_T001	0.6014554	-0.642547357	Zm00001d033420
Zm00001d034163_T001	0.1538809	-1.230808792	Zm00001d034163
Zm00001d052488_T002	0.891579	-0.015237079	Zm00001d052488
Zm00001d018984_T001	0.9369505	-0.210559263	Zm00001d018984
Zm00001d015213_T001	0.3919095	-0.802287346	Zm00001d015213
Zm00001d036370_T001	0.2015088	-1.321290359	Zm00001d036370
Zm00001d037786_T001	0.7619994	0.727570368	Zm00001d037786
Zm00001d011555_T001	1	0.041060497	Zm00001d011555
Zm00001d017767_T002	0.8031436	0.912839399	Zm00001d017767
Zm00001d017767_T003	0.7712604	0.602193794	Zm00001d017767
Zm00001d044880_T001	0.9627389	-0.216390655	Zm00001d044880
Zm00001d004810_T002	0.9332923	0.010303661	Zm00001d004810
Zm00001d004810_T001	0.8129337	-0.134032609	Zm00001d004810
Zm00001d000011_T007	0.4146004	-0.226630431	Zm00001d000011
Zm00001d000011_T001	0.6891365	-0.276541784	Zm00001d000011
Zm00001d000011_T024	0.6395973	0.718289917	Zm00001d000011
Zm00001d000011_T002	0.9469426	0.472606209	Zm00001d000011
Zm00001d000011_T019	0.9979568	0.099190871	Zm00001d000011
Zm00001d000011_T022	0.8320654	0.372946796	Zm00001d000011
Zm00001d013116_T001	0.6733323	-0.116018428	Zm00001d013116
Zm00001d013116_T004	0.9984041	-0.287369753	Zm00001d013116
Zm00001d045462_T001	0.9860836	0.356166558	Zm00001d045462

Zm00001d045462_T006	0.7193211	0.46590692	Zm00001d045462
Zm00001d052146_T001	0.9846871	0.079276266	Zm00001d052146
Zm00001d051422_T001	0.906282	0.041396854	Zm00001d051422
Zm00001d003496_T001	0.9193633	0.518120633	Zm00001d003496
Zm00001d038022_T001	0.9867657	0.274164291	Zm00001d038022
Zm00001d052493_T001	0.1336234	-1.239775761	Zm00001d052493
Zm00001d052493_T003	0.1373956	-1.472726262	Zm00001d052493
Zm00001d052493_T002	0.02845695	-1.98214265	Zm00001d052493
Zm00001d048372_T002	0.9954444	0.333011917	Zm00001d048372
Zm00001d044059_T001	0.9833133	0.511100601	Zm00001d044059
Zm00001d041416_T003	0.9061819	-0.066061919	Zm00001d041416
Zm00001d041416_T002	0.6869898	-0.824865363	Zm00001d041416
Zm00001d041416_T004	0.9466261	-0.007938317	Zm00001d041416
Zm00001d041416_T001	0.7194777	-0.363262455	Zm00001d041416
Zm00001d019279_T001	0.9161657	-0.095302795	Zm00001d019279
Zm00001d040320_T004	0.02834793	0.919455901	Zm00001d040320
Zm00001d040320_T002	0.9666757	0.461135983	Zm00001d040320
Zm00001d052749_T001	0.835901	-0.416357008	Zm00001d052749
Zm00001d038123_T002	0.98291	0.531283462	Zm00001d038123
Zm00001d038123_T004	0.7631455	0.814850803	Zm00001d038123
Zm00001d043095_T001	0.9909153	0.003214496	Zm00001d043095
Zm00001d043095_T002	0.8941695	0.013627128	Zm00001d043095
Zm00001d016132_T001	0.8656506	0.656177543	Zm00001d016132
Zm00001d045421_T001	0.9804228	0.435269848	Zm00001d045421
Zm00001d028810_T001	0.9466213	0.076387617	Zm00001d028810
Zm00001d034345_T003	1.05E-06	4.981427138	Zm00001d034345
Zm00001d034345_T002	0.001333162	3.581673259	Zm00001d034345
Zm00001d034345_T001	0.02761546	2.811246911	Zm00001d034345
Zm00001d053309_T001	0.1516716	1.003903572	Zm00001d053309
Zm00001d017643_T001	0.8445671	0.823661717	Zm00001d017643
Zm00001d025750_T019	0.929138	0.287573026	Zm00001d025750
Zm00001d025750_T011	0.9470137	0.693250319	Zm00001d025750
Zm00001d025750_T012	0.7810295	-0.084130749	Zm00001d025750
Zm00001d025750_T024	0.399447	-0.669119056	Zm00001d025750
Zm00001d041740_T001	0.9732421	0.583675667	Zm00001d041740
Zm00001d015613_T001	0.9765009	0.112021368	Zm00001d015613
Zm00001d015613_T002	0.8987811	-0.15986542	Zm00001d015613
Zm00001d053685_T005	0.9311405	1.245732023	Zm00001d053685
Zm00001d053685_T007	0.4758724	0.02380843	Zm00001d053685
Zm00001d042766_T001	0.4579875	-0.959932918	Zm00001d042766
Zm00001d029919_T002	0.995534	0.3815141	Zm00001d029919
Zm00001d007433_T001	0.101892	-0.589026393	Zm00001d007433
Zm00001d048737_T001	0.5731688	-0.494933283	Zm00001d048737
Zm00001d022364_T001	0.586618	-0.952366975	Zm00001d022364
Zm00001d022364_T003	0.8840817	0.877587076	Zm00001d022364
Zm00001d025710_T002	0.8929316	-0.038676421	Zm00001d025710
Zm00001d038845_T004	0.000190973	0.766262911	Zm00001d038845
Zm00001d038845_T007	0.9720425	0.31985676	Zm00001d038845
Zm00001d038845_T011	7.47E-07	-0.815574074	Zm00001d038845
Zm00001d038845_T006	0.6182353	-0.022461876	Zm00001d038845
Zm00001d038845_T012	0.9909153	0.109987027	Zm00001d038845
Zm00001d043358_T001	0.8865624	-0.061368993	Zm00001d043358
Zm00001d012887_T001	0.8272327	0.936835226	Zm00001d012887
Zm00001d012887_T002	0.8863456	0.657519208	Zm00001d012887

Zm00001d040743_T001	0.9971824	0.279310147	Zm00001d040743
Zm00001d016850_T002	0.9333354	-0.448213197	Zm00001d016850
Zm00001d010975_T005	0.4282459	0.672443458	Zm00001d010975
Zm00001d010975_T004	0.8050292	0.853143027	Zm00001d010975
Zm00001d010975_T006	0.9402253	0.032610971	Zm00001d010975
Zm00001d010975_T003	0.9902022	0.269349692	Zm00001d010975
Zm00001d010975_T001	0.8152824	0.996744157	Zm00001d010975
Zm00001d052083_T019	0.002206201	0.766249876	Zm00001d052083
Zm00001d052083_T006	0.5467906	-0.270873309	Zm00001d052083
Zm00001d024213_T001	0.1699706	-1.32382354	Zm00001d024213
Zm00001d041911_T033	0.9229401	0.42737321	Zm00001d041911
Zm00001d041911_T027	0.3004476	0.983332069	Zm00001d041911
Zm00001d041911_T026	0.2612921	-0.767671726	Zm00001d041911
Zm00001d041911_T011	0.9429712	0.673875023	Zm00001d041911
Zm00001d041911_T030	0.9902733	0.091883042	Zm00001d041911
Zm00001d041911_T025	0.923446	0.335852569	Zm00001d041911
Zm00001d048893_T001	1	0.370002125	Zm00001d048893
Zm00001d034279_T001	0.8885778	-0.252387882	Zm00001d034279
Zm00001d018217_T004	0.9354647	0.634559404	Zm00001d018217
Zm00001d018217_T009	1.35E-09	1.304692512	Zm00001d018217
Zm00001d018217_T002	0.7114361	0.89949568	Zm00001d018217
Zm00001d018217_T003	0.001034463	2.179366744	Zm00001d018217
Zm00001d030967_T005	0.462123	-1.033528712	Zm00001d030967
Zm00001d030967_T002	0.7575362	1.074160287	Zm00001d030967
Zm00001d030967_T006	0.9872831	0.249948568	Zm00001d030967
Zm00001d030967_T007	0.6610974	-0.199108394	Zm00001d030967
Zm00001d030967_T010	0.7419821	-0.295923207	Zm00001d030967
Zm00001d020963_T002	0.05280356	-1.453147557	Zm00001d020963
Zm00001d020963_T001	1	0.422022757	Zm00001d020963
Zm00001d012736_T001	0.3129507	-1.375869858	Zm00001d012736
Zm00001d000041_T001	0.9792387	0.000184125	Zm00001d000041
Zm00001d018625_T021	0.9196677	0.339418761	Zm00001d018625
Zm00001d029170_T002	0.8421761	-0.137798947	Zm00001d029170
Zm00001d029170_T005	0.9232446	0.401079595	Zm00001d029170
Zm00001d029170_T003	0.9693993	0.287047095	Zm00001d029170
Zm00001d019304_T001	0.7552081	-0.781252007	Zm00001d019304
Zm00001d051890_T001	0.9037324	0.576291799	Zm00001d051890
Zm00001d010711_T002	0.866781	-0.228621821	Zm00001d010711
Zm00001d010711_T001	0.1170004	1.70537251	Zm00001d010711
Zm00001d053259_T004	0.9469426	0.008601913	Zm00001d053259
Zm00001d053259_T001	0.1296685	0.879977911	Zm00001d053259
Zm00001d053259_T003	0.9839988	0.521425417	Zm00001d053259
Zm00001d021956_T006	0.5220513	0.955389664	Zm00001d021956
Zm00001d021956_T007	0.4776988	0.60030555	Zm00001d021956
Zm00001d021956_T002	0.9746071	0.191071413	Zm00001d021956
Zm00001d021956_T008	0.2387791	0.615349823	Zm00001d021956
Zm00001d011817_T002	0.9513407	0.355104843	Zm00001d011817
Zm00001d011817_T001	0.3953947	-0.266229403	Zm00001d011817
Zm00001d011817_T003	0.9907928	0.201870327	Zm00001d011817
Zm00001d030656_T001	0.9563232	0.68932783	Zm00001d030656
Zm00001d046093_T001	0.9992342	0.361491675	Zm00001d046093
Zm00001d033572_T004	0.9127665	-0.023370219	Zm00001d033572
Zm00001d033572_T001	0.9706293	-0.131618044	Zm00001d033572
Zm00001d033572_T006	0.5844177	0.537504859	Zm00001d033572

Zm00001d029217_T001	1	0.014890149	Zm00001d029217
Zm00001d007151_T011	0.3551811	0.31790947	Zm00001d007151
Zm00001d007151_T009	0.9770343	0.356607024	Zm00001d007151
Zm00001d007151_T004	0.3603685	-0.270718378	Zm00001d007151
Zm00001d027962_T001	0.9563232	0.655223525	Zm00001d027962
Zm00001d004919_T001	0.3919095	-0.734448842	Zm00001d004919
Zm00001d012269_T004	0.6023446	1.087190891	Zm00001d012269
Zm00001d012269_T002	0.9171166	0.609238317	Zm00001d012269
Zm00001d012269_T003	0.139268	-0.966200058	Zm00001d012269
Zm00001d012269_T006	0.6546079	1.246067683	Zm00001d012269
Zm00001d023635_T001	0.9904282	0.24776219	Zm00001d023635
Zm00001d005064_T002	0.8730771	-0.134976435	Zm00001d005064
Zm00001d035962_T001	0.1947403	1.873656357	Zm00001d035962
Zm00001d028210_T001	0.9654804	0.15870443	Zm00001d028210
Zm00001d007514_T001	0.6291038	-0.814333838	Zm00001d007514
Zm00001d033363_T001	0.6952911	-0.772971617	Zm00001d033363
Zm00001d044415_T002	0.7942492	-0.805134541	Zm00001d044415
Zm00001d044415_T001	0.8479713	0.694657344	Zm00001d044415
Zm00001d003226_T001	0.6074067	0.75906582	Zm00001d003226
Zm00001d043738_T001	4.76E-06	2.349163615	Zm00001d043738
Zm00001d019259_T001	0.9293689	0.011885463	Zm00001d019259
Zm00001d024088_T001	0.989787	0.297427601	Zm00001d024088
Zm00001d003534_T001	0.221452	1.015139433	Zm00001d003534
Zm00001d044130_T002	0.8730771	-0.497518888	Zm00001d044130
Zm00001d029684_T001	0.4827226	1.333200797	Zm00001d029684
Zm00001d028957_T001	0.6503853	-0.620357082	Zm00001d028957
Zm00001d045350_T002	0.9932384	0.066665842	Zm00001d045350
Zm00001d045350_T001	0.9722469	0.231849772	Zm00001d045350
Zm00001d045350_T003	0.3655498	1.098758294	Zm00001d045350
Zm00001d000126_T001	0.8751525	-0.231212399	Zm00001d000126
Zm00001d035143_T001	0.8757833	0.287469443	Zm00001d035143
Zm00001d042041_T001	0.108483	1.573318847	Zm00001d042041
Zm00001d012844_T005	0.1286688	-1.289956256	Zm00001d012844
Zm00001d012844_T002	1	0.412482357	Zm00001d012844
Zm00001d021205_T001	5.61E-09	5.351464905	Zm00001d021205
Zm00001d021420_T001	0.9361945	-0.006572306	Zm00001d021420
Zm00001d021420_T002	0.8686467	-0.191189753	Zm00001d021420
Zm00001d021420_T003	0.9791729	0.459981946	Zm00001d021420
Zm00001d047306_T005	0.9735632	0.562307337	Zm00001d047306
Zm00001d047306_T012	0.9252088	-0.060101724	Zm00001d047306
Zm00001d013022_T001	0.9845237	0.397324654	Zm00001d013022
Zm00001d006746_T001	0.8307555	0.593395409	Zm00001d006746
Zm00001d018482_T005	0.000833983	-1.278049618	Zm00001d018482
Zm00001d018482_T004	0.8495346	0.03363732	Zm00001d018482
Zm00001d018482_T001	0.8828298	0.893334776	Zm00001d018482
Zm00001d018482_T003	0.6453567	0.314884965	Zm00001d018482
Zm00001d005372_T003	0.8929316	-0.233849503	Zm00001d005372
Zm00001d005372_T002	0.817505	1.033608469	Zm00001d005372
Zm00001d005372_T016	0.9872831	0.128644021	Zm00001d005372
Zm00001d005372_T009	0.701515	0.530452247	Zm00001d005372
Zm00001d005372_T006	0.9776645	0.237439845	Zm00001d005372
Zm00001d009660_T001	0.366346	-0.876172635	Zm00001d009660
Zm00001d052445_T020	0.6278434	0.354642161	Zm00001d052445
Zm00001d052445_T001	0.2365219	0.779016936	Zm00001d052445

Zm00001d052445_T012	4.09E-06	0.762376455	Zm00001d052445
Zm00001d052445_T008	1	0.426521187	Zm00001d052445
Zm00001d004438_T001	1	0.167219666	Zm00001d004438
Zm00001d011304_T001	0.5451899	-0.364991769	Zm00001d011304
Zm00001d025098_T007	0.6841106	0.346038519	Zm00001d025098
Zm00001d025098_T003	0.5831618	0.876657196	Zm00001d025098
Zm00001d025098_T008	0.8412913	0.433716639	Zm00001d025098
Zm00001d025098_T005	1	0.352080921	Zm00001d025098
Zm00001d042720_T006	0.05203429	0.76930474	Zm00001d042720
Zm00001d042720_T007	0.6794962	0.474518239	Zm00001d042720
Zm00001d042720_T001	0.7137316	0.461925408	Zm00001d042720
Zm00001d042720_T002	0.08311592	-1.70777056	Zm00001d042720
Zm00001d042720_T005	0.8112903	0.889989783	Zm00001d042720
Zm00001d034995_T001	0.3016085	1.414389354	Zm00001d034995
Zm00001d047201_T002	0.3382827	1.160992849	Zm00001d047201
Zm00001d047201_T003	0.956479	-0.037608973	Zm00001d047201
Zm00001d013953_T001	0.827438	0.720713127	Zm00001d013953
Zm00001d011652_T007	0.7298446	-0.300785886	Zm00001d011652
Zm00001d011652_T010	0.5591127	0.533596154	Zm00001d011652
Zm00001d011652_T011	0.9884825	0.386642381	Zm00001d011652
Zm00001d011652_T003	0.362479	0.616633816	Zm00001d011652
Zm00001d011652_T012	0.811177	0.208050786	Zm00001d011652
Zm00001d011652_T005	0.03774788	1.331278328	Zm00001d011652
Zm00001d037759_T001	0.918657	0.493782976	Zm00001d037759
Zm00001d000226_T009	0.7122217	0.658192681	Zm00001d000226
Zm00001d000226_T001	1	0.281031461	Zm00001d000226
Zm00001d027588_T003	0.5377194	0.672336311	Zm00001d027588
Zm00001d027588_T001	0.9558093	0.093431977	Zm00001d027588
Zm00001d027588_T002	0.9218815	0.638300503	Zm00001d027588
Zm00001d013010_T005	0.8929316	-0.06040933	Zm00001d013010
Zm00001d013010_T003	0.4219823	-0.353718889	Zm00001d013010
Zm00001d013010_T001	0.8987811	-0.261536063	Zm00001d013010
Zm00001d037818_T001	0.003423432	-0.488555384	Zm00001d037818
Zm00001d037818_T002	0.909186	-0.016458251	Zm00001d037818
Zm00001d009365_T002	0.8599886	0.465676865	Zm00001d009365
Zm00001d009365_T001	0.9792387	0.499303622	Zm00001d009365
Zm00001d031533_T001	0.8660897	-0.107405673	Zm00001d031533
Zm00001d020461_T001	1	0.442870992	Zm00001d020461
Zm00001d011227_T001	0.9701986	0.342665173	Zm00001d011227
Zm00001d033704_T008	0.5887817	1.213968036	Zm00001d033704
Zm00001d033704_T007	0.8840817	-0.06332894	Zm00001d033704
Zm00001d033704_T001	0.9732421	0.357779492	Zm00001d033704
Zm00001d002420_T001	0.8929316	0.59029572	Zm00001d002420
Zm00001d003807_T010	0.768921	0.69774922	Zm00001d003807
Zm00001d003807_T001	0.5344384	-0.532204905	Zm00001d003807
Zm00001d003807_T004	0.9673094	0.418448029	Zm00001d003807
Zm00001d040780_T005	0.5196715	-0.600403541	Zm00001d040780
Zm00001d040780_T002	0.9475135	0.045751148	Zm00001d040780
Zm00001d040780_T003	0.9782953	0.16639538	Zm00001d040780
Zm00001d040780_T004	1	0.305043881	Zm00001d040780
Zm00001d047245_T001	1	0.358147596	Zm00001d047245
Zm00001d043914_T001	1	0.370542573	Zm00001d043914
Zm00001d033294_T007	0.1785712	-0.580991314	Zm00001d033294
Zm00001d033294_T008	0.7912425	0.573979504	Zm00001d033294

Zm00001d033294_T003	0.9468268	0.059319182	Zm00001d033294
Zm00001d033294_T005	0.9997235	0.109313372	Zm00001d033294
Zm00001d031427_T001	0.4508973	-0.405043368	Zm00001d031427
Zm00001d031611_T002	0.2808487	-1.407899228	Zm00001d031611
Zm00001d031611_T001	0.7807489	-0.470202261	Zm00001d031611
Zm00001d022334_T002	0.001508566	1.233518758	Zm00001d022334
Zm00001d022334_T001	0.2981555	1.643019277	Zm00001d022334
Zm00001d003897_T001	0.7273213	0.915328531	Zm00001d003897
Zm00001d018552_T001	0.9083745	-0.106645703	Zm00001d018552
Zm00001d036278_T003	1	0.004089937	Zm00001d036278
Zm00001d020902_T002	0.9819415	0.514706444	Zm00001d020902
Zm00001d032079_T001	0.5127506	1.360103218	Zm00001d032079
Zm00001d020835_T012	1	0.191276218	Zm00001d020835
Zm00001d020835_T027	0.6621234	-0.075813703	Zm00001d020835
Zm00001d020835_T004	0.01320298	1.309201984	Zm00001d020835
Zm00001d020835_T020	0.0389152	-1.253723917	Zm00001d020835
Zm00001d020835_T011	0.006598714	0.561250322	Zm00001d020835
Zm00001d020835_T003	0.7432468	-0.381185917	Zm00001d020835
Zm00001d020835_T014	0.8367787	-0.071209338	Zm00001d020835
Zm00001d007035_T001	0.5698127	-0.858885844	Zm00001d007035
Zm00001d016898_T001	0.3903214	-0.606269054	Zm00001d016898
Zm00001d022573_T001	0.9880075	0.251982102	Zm00001d022573
Zm00001d048027_T001	0.001585032	3.028128751	Zm00001d048027
Zm00001d034031_T001	0.03311134	2.707646176	Zm00001d034031
Zm00001d047600_T004	0.7063818	1.51757321	Zm00001d047600
Zm00001d047600_T003	0.8513643	0.981877274	Zm00001d047600
Zm00001d044276_T001	0.3823269	-0.518103524	Zm00001d044276
Zm00001d043523_T001	0.006641282	-2.638208058	Zm00001d043523
Zm00001d022394_T001	0.9252088	0.187265059	Zm00001d022394
Zm00001d022394_T002	0.2417871	-0.407842125	Zm00001d022394
Zm00001d012785_T001	0.9186216	-0.041081538	Zm00001d012785
Zm00001d024432_T001	0.9139882	0.707363632	Zm00001d024432
Zm00001d018003_T001	1	-0.079960531	Zm00001d018003
Zm00001d027769_T003	0.3985671	-0.373449279	Zm00001d027769
Zm00001d027769_T002	0.9944178	0.443146402	Zm00001d027769
Zm00001d035599_T001	0.9745777	0.552932847	Zm00001d035599
Zm00001d020442_T001	0.9293689	-0.417297862	Zm00001d020442
Zm00001d053817_T001	0.9804228	0.384731926	Zm00001d053817
Zm00001d048870_T001	0.000497198	-2.37645085	Zm00001d048870
Zm00001d002772_T001	0.9360579	0.395481687	Zm00001d002772
Zm00001d033156_T001	0.4052428	-1.125007157	Zm00001d033156
Zm00001d037636_T008	1	0.469322959	Zm00001d037636
Zm00001d037636_T006	0.452973	2.525252409	Zm00001d037636
Zm00001d037636_T001	0.8039029	0.617685659	Zm00001d037636
Zm00001d037636_T005	0.06165335	2.473372345	Zm00001d037636
Zm00001d011149_T001	0.9690925	0.016716862	Zm00001d011149
Zm00001d011149_T002	0.8050292	-0.549721525	Zm00001d011149
Zm00001d038136_T004	0.8127863	0.556092803	Zm00001d038136
Zm00001d038136_T002	0.9970835	0.389749086	Zm00001d038136
Zm00001d011168_T010	0.5591883	0.443217214	Zm00001d011168
Zm00001d011168_T006	1.82E-11	1.617358864	Zm00001d011168
Zm00001d011168_T011	0.8643636	0.433669138	Zm00001d011168
Zm00001d011168_T017	0.9543237	0.226794285	Zm00001d011168
Zm00001d011168_T007	0.9998515	0.2984683	Zm00001d011168

Zm00001d011168_T002	0.00162061	0.759937024	Zm00001d011168
Zm00001d011168_T008	0.425485	-0.341186457	Zm00001d011168
Zm00001d011168_T005	0.3486589	0.898302158	Zm00001d011168
Zm00001d011168_T018	0.003641959	1.711499415	Zm00001d011168
Zm00001d011168_T016	0.9762449	-0.422862265	Zm00001d011168
Zm00001d025777_T007	0.1562235	1.125497641	Zm00001d025777
Zm00001d025777_T009	0.9326482	0.76006179	Zm00001d025777
Zm00001d025777_T004	0.7765963	0.98422354	Zm00001d025777
Zm00001d025966_T001	0.986738	0.408039036	Zm00001d025966
Zm00001d039103_T001	0.9037324	0.579026324	Zm00001d039103
Zm00001d025133_T001	1	0.219335531	Zm00001d025133
Zm00001d040953_T001	0.8554995	0.771605384	Zm00001d040953
Zm00001d028270_T002	8.36E-07	1.515669761	Zm00001d028270
Zm00001d036650_T001	1	-0.053397675	Zm00001d036650
Zm00001d042279_T001	0.9446379	0.088662803	Zm00001d042279
Zm00001d034492_T024	0.9887889	0.970759274	Zm00001d034492
Zm00001d034492_T007	0.3497147	0.586190494	Zm00001d034492
Zm00001d034492_T006	0.7797345	-0.054493174	Zm00001d034492
Zm00001d034492_T019	4.01E-13	3.19084175	Zm00001d034492
Zm00001d033300_T001	0.522218	0.906416687	Zm00001d033300
Zm00001d053930_T003	1	0.294269553	Zm00001d053930
Zm00001d053930_T001	0.8512136	-0.185554385	Zm00001d053930
Zm00001d053930_T002	0.9356767	0.006035142	Zm00001d053930
Zm00001d015421_T001	0.9979568	0.330423989	Zm00001d015421
Zm00001d015421_T002	0.9563232	0.617053996	Zm00001d015421
Zm00001d009618_T001	0.9910452	0.18807296	Zm00001d009618
Zm00001d041179_T006	0.9252088	0.574479519	Zm00001d041179
Zm00001d021591_T001	0.9257238	0.605064066	Zm00001d021591
Zm00001d053316_T003	1	0.353763298	Zm00001d053316
Zm00001d053316_T012	1	0.326995991	Zm00001d053316
Zm00001d053316_T008	0.757295	0.395917213	Zm00001d053316
Zm00001d053316_T001	1	0.206795434	Zm00001d053316
Zm00001d053316_T011	0.7778639	0.543064707	Zm00001d053316
Zm00001d053316_T007	0.468282	-0.562443493	Zm00001d053316
Zm00001d053316_T006	0.313256	0.842558538	Zm00001d053316
Zm00001d045547_T001	0.9298144	-0.073179818	Zm00001d045547
Zm00001d016876_T001	0.8111431	-0.325980119	Zm00001d016876
Zm00001d004193_T004	0.05702114	0.330294443	Zm00001d004193
Zm00001d004193_T001	0.9811162	0.139238374	Zm00001d004193
Zm00001d004193_T003	0.8863456	-0.070945267	Zm00001d004193
Zm00001d004193_T005	0.9969123	0.066554442	Zm00001d004193
Zm00001d004033_T001	1	0.386459364	Zm00001d004033
Zm00001d002868_T001	0.8749298	-0.584668985	Zm00001d002868
Zm00001d002726_T008	0.5622303	1.218825238	Zm00001d002726
Zm00001d002726_T001	0.05061282	1.124250561	Zm00001d002726
Zm00001d002726_T009	2.64E-07	-1.357739578	Zm00001d002726
Zm00001d019897_T003	0.03860996	1.339703883	Zm00001d019897
Zm00001d019897_T001	0.1181191	0.83484275	Zm00001d019897
Zm00001d026621_T006	0.1115767	-0.46562683	Zm00001d026621
Zm00001d026621_T019	0.5766968	0.544678309	Zm00001d026621
Zm00001d026621_T016	2.79E-07	0.8562325	Zm00001d026621
Zm00001d026621_T009	0.9979568	0.129496126	Zm00001d026621
Zm00001d026621_T018	0.02555124	0.898711089	Zm00001d026621
Zm00001d026621_T007	0.9132406	0.748791076	Zm00001d026621

Zm00001d024712_T002	0.9432413	0.060921015	Zm00001d024712
Zm00001d024712_T001	0.9596377	-0.025853468	Zm00001d024712
Zm00001d049079_T001	0.7077089	-0.462248539	Zm00001d049079
Zm00001d007254_T001	0.8591203	-0.079735966	Zm00001d007254
Zm00001d002319_T001	0.8349995	0.678907581	Zm00001d002319
Zm00001d021641_T018	0.09744749	-0.464470716	Zm00001d021641
Zm00001d021641_T016	0.9224378	0.381129316	Zm00001d021641
Zm00001d021641_T002	0.9222574	0.103773873	Zm00001d021641
Zm00001d021641_T008	0.9479007	0.229142126	Zm00001d021641
Zm00001d021641_T004	0.9264186	0.547218095	Zm00001d021641
Zm00001d045483_T004	0.5988928	0.980795548	Zm00001d045483
Zm00001d045483_T001	0.9727334	0.584923724	Zm00001d045483
Zm00001d045483_T003	0.3934729	0.557902442	Zm00001d045483
Zm00001d048573_T001	0.9771057	0.074699616	Zm00001d048573
Zm00001d048606_T001	0.690706	1.124873507	Zm00001d048606
Zm00001d024963_T001	0.9895224	0.194932415	Zm00001d024963
Zm00001d024963_T002	0.1629598	-0.262403751	Zm00001d024963
Zm00001d009028_T002	0.976488	0.154499224	Zm00001d009028
Zm00001d013997_T002	0.2146351	-1.073441435	Zm00001d013997
Zm00001d036598_T001	0.8513643	-0.127980012	Zm00001d036598
Zm00001d036598_T008	0.09679593	1.0393468	Zm00001d036598
Zm00001d036598_T004	0.8001345	0.905490108	Zm00001d036598
Zm00001d032906_T001	0.2509097	-0.85518692	Zm00001d032906
Zm00001d036122_T001	0.0373054	2.565618557	Zm00001d036122
Zm00001d028235_T001	0.4104756	-0.555881509	Zm00001d028235
Zm00001d022144_T002	0.9235467	0.725831412	Zm00001d022144
Zm00001d049881_T001	0.9914326	0.32916047	Zm00001d049881
Zm00001d014879_T004	0.9894615	0.568346736	Zm00001d014879
Zm00001d021730_T001	0.8837967	-0.407502122	Zm00001d021730
Zm00001d021946_T001	0.006106844	2.922431949	Zm00001d021946
Zm00001d021927_T001	0.880737	-0.138801085	Zm00001d021927
Zm00001d009907_T001	0.8201926	-0.323807321	Zm00001d009907
Zm00001d020976_T001	0.5572653	-0.340930102	Zm00001d020976
Zm00001d005012_T004	0.9396809	0.49074406	Zm00001d005012
Zm00001d005012_T014	0.7737238	0.73699597	Zm00001d005012
Zm00001d005012_T013	0.4422995	-0.509217775	Zm00001d005012
Zm00001d005012_T015	0.009211255	2.781946858	Zm00001d005012
Zm00001d005012_T006	0.9203655	0.147137161	Zm00001d005012
Zm00001d005012_T002	0.9749249	0.101897874	Zm00001d005012
Zm00001d002658_T015	0.07730424	0.776864594	Zm00001d002658
Zm00001d002658_T007	0.614631	0.262219343	Zm00001d002658
Zm00001d002658_T002	0.566256	0.862243645	Zm00001d002658
Zm00001d002658_T014	0.02255438	-1.001612805	Zm00001d002658
Zm00001d002658_T016	0.1596683	1.408061724	Zm00001d002658
Zm00001d037725_T001	0.96672	0.148975992	Zm00001d037725
Zm00001d006458_T001	0.8881012	1.017714593	Zm00001d006458
Zm00001d036704_T002	0.01540208	0.921908971	Zm00001d036704
Zm00001d036704_T001	0.8884142	0.797415014	Zm00001d036704
Zm00001d018019_T001	0.9300784	0.429193346	Zm00001d018019
Zm00001d013159_T003	0.98291	0.182009889	Zm00001d013159
Zm00001d013159_T001	0.8321156	-0.192844946	Zm00001d013159
Zm00001d013159_T004	1.25E-10	5.08511134	Zm00001d013159
Zm00001d012515_T002	0.8746701	-0.021011618	Zm00001d012515
Zm00001d012515_T001	0.5322043	0.056643104	Zm00001d012515

Zm00001d045503_T001	1	0.046893429	Zm00001d045503
Zm00001d039372_T003	0.85068	-0.407471207	Zm00001d039372
Zm00001d039372_T001	0.844285	-0.329735858	Zm00001d039372
Zm00001d032849_T002	0.06650348	-1.485903401	Zm00001d032849
Zm00001d032849_T001	0.06978913	-1.724747034	Zm00001d032849
Zm00001d006118_T001	0.9797082	0.32599733	Zm00001d006118
Zm00001d006118_T002	1	0.353985999	Zm00001d006118
Zm00001d011613_T001	0.923446	-0.036901594	Zm00001d011613
Zm00001d051854_T002	7.85E-05	1.826607651	Zm00001d051854
Zm00001d051854_T001	0.9285797	0.721093068	Zm00001d051854
Zm00001d051854_T003	0.9257893	0.670555669	Zm00001d051854
Zm00001d014924_T001	0.4274324	-0.38517935	Zm00001d014924
Zm00001d014924_T004	0.9224387	0.703458297	Zm00001d014924
Zm00001d014924_T005	0.698243	-0.113598986	Zm00001d014924
Zm00001d014924_T002	0.8179463	-0.258720358	Zm00001d014924
Zm00001d012158_T001	0.9911485	0.208323293	Zm00001d012158
Zm00001d048236_T005	0.5269554	0.426671985	Zm00001d048236
Zm00001d048236_T008	0.5207004	-0.59136857	Zm00001d048236
Zm00001d048236_T009	0.9673473	0.067483485	Zm00001d048236
Zm00001d048236_T001	0.6284759	-0.268721698	Zm00001d048236
Zm00001d048236_T013	3.29E-08	1.668836819	Zm00001d048236
Zm00001d048236_T015	0.9867591	0.004889005	Zm00001d048236
Zm00001d048997_T001	0.9565144	-0.253802307	Zm00001d048997
Zm00001d006581_T001	0.6919639	-0.471131236	Zm00001d006581
Zm00001d010519_T001	0.9689939	0.126785414	Zm00001d010519
Zm00001d043563_T004	0.9817074	0.570797718	Zm00001d043563
Zm00001d043563_T003	0.9237037	0.635263143	Zm00001d043563
Zm00001d043563_T005	0.8158416	-0.408603011	Zm00001d043563
Zm00001d043563_T002	1	0.413739889	Zm00001d043563
Zm00001d001900_T002	1	0.401625498	Zm00001d001900
Zm00001d001900_T001	1	-0.006311734	Zm00001d001900
Zm00001d001900_T003	0.2306466	1.63433467	Zm00001d001900
Zm00001d021662_T002	0.8418438	-0.124195503	Zm00001d021662
Zm00001d021662_T001	0.8896831	-0.013218399	Zm00001d021662
Zm00001d008495_T001	0.5430102	1.268017152	Zm00001d008495
Zm00001d045519_T003	0.9986486	0.176820287	Zm00001d045519
Zm00001d045519_T005	0.6377926	1.137771957	Zm00001d045519
Zm00001d045519_T001	0.3686542	1.663316529	Zm00001d045519
Zm00001d045519_T004	0.9179118	0.023324907	Zm00001d045519
Zm00001d029847_T001	0.6086477	1.098769859	Zm00001d029847
Zm00001d003773_T006	0.5864542	-0.885053463	Zm00001d003773
Zm00001d003773_T007	0.5718169	0.316617647	Zm00001d003773
Zm00001d003773_T002	0.2222811	1.982935796	Zm00001d003773
Zm00001d003773_T001	0.8825173	0.105472202	Zm00001d003773
Zm00001d003773_T008	0.9469426	0.924435311	Zm00001d003773
Zm00001d040628_T003	0.9786184	0.000969218	Zm00001d040628
Zm00001d040628_T002	0.5878314	-0.935770907	Zm00001d040628
Zm00001d025117_T001	0.8990393	-0.135507173	Zm00001d025117
Zm00001d026360_T001	0.323944	-0.724053072	Zm00001d026360
Zm00001d043390_T001	0.000468585	3.517381937	Zm00001d043390
Zm00001d018372_T001	0.01537015	2.13979479	Zm00001d018372
Zm00001d008293_T001	0.2981772	-0.240618377	Zm00001d008293
Zm00001d008293_T004	0.5447165	0.73645806	Zm00001d008293
Zm00001d008293_T006	0.9745777	0.235507787	Zm00001d008293

Zm00001d008293_T007	0.5988928	-0.438537701	Zm00001d008293
Zm00001d053452_T004	0.9979568	0.304640541	Zm00001d053452
Zm00001d053452_T003	0.9468268	0.674243322	Zm00001d053452
Zm00001d023462_T002	0.9845237	0.422836798	Zm00001d023462
Zm00001d035757_T009	0.1574436	-1.057921142	Zm00001d035757
Zm00001d035757_T002	0.5795647	1.131260301	Zm00001d035757
Zm00001d035757_T006	0.9104115	-0.010682698	Zm00001d035757
Zm00001d035757_T003	0.4072293	-0.370236884	Zm00001d035757
Zm00001d035757_T007	0.9177099	-0.056476028	Zm00001d035757
Zm00001d024770_T001	0.2235678	-0.923936388	Zm00001d024770
Zm00001d042361_T002	1	0.31540781	Zm00001d042361
Zm00001d046810_T002	0.3870314	0.447663328	Zm00001d046810
Zm00001d046810_T005	0.9293689	1.045985656	Zm00001d046810
Zm00001d046810_T003	0.7760798	0.055836343	Zm00001d046810
Zm00001d043314_T001	0.9791729	0.017201359	Zm00001d043314
Zm00001d014378_T001	0.9747606	0.211449068	Zm00001d014378
Zm00001d011769_T001	0.3000263	-0.728056563	Zm00001d011769
Zm00001d021335_T003	0.4629422	-0.777700903	Zm00001d021335
Zm00001d021335_T004	0.9760472	0.075431522	Zm00001d021335
Zm00001d021335_T005	0.7095161	-0.13263115	Zm00001d021335
Zm00001d005892_T001	5.33E-07	3.443675541	Zm00001d005892
Zm00001d038740_T001	0.7449732	-0.697152865	Zm00001d038740
Zm00001d038740_T002	0.6996374	-0.647927367	Zm00001d038740
Zm00001d032410_T001	0.3412344	-1.17845404	Zm00001d032410
Zm00001d025735_T001	0.06021589	1.939877386	Zm00001d025735
Zm00001d012302_T001	0.556323	-0.709613163	Zm00001d012302
Zm00001d046286_T001	0.8321156	0.80309022	Zm00001d046286
Zm00001d017945_T001	0.9894615	0.267762871	Zm00001d017945
Zm00001d029797_T002	0.9689939	-0.25180847	Zm00001d029797
Zm00001d036475_T002	0.1774305	1.716011185	Zm00001d036475
Zm00001d036475_T003	0.9766874	0.622560601	Zm00001d036475
Zm00001d036475_T009	0.9771927	0.603842049	Zm00001d036475
Zm00001d036475_T006	0.2454949	0.650277117	Zm00001d036475
Zm00001d036475_T001	0.1272455	0.704174702	Zm00001d036475
Zm00001d036475_T008	0.05931006	2.262199224	Zm00001d036475
Zm00001d048393_T001	0.4586744	1.334733551	Zm00001d048393
Zm00001d005478_T026	0.666346	1.262234541	Zm00001d005478
Zm00001d005478_T025	0.9682673	0.62401408	Zm00001d005478
Zm00001d005478_T009	0.03577387	0.744629925	Zm00001d005478
Zm00001d005478_T021	0.8296999	0.962968767	Zm00001d005478
Zm00001d005478_T001	0.4850136	-0.886423853	Zm00001d005478
Zm00001d005478_T007	0.9884825	0.899280906	Zm00001d005478
Zm00001d005478_T003	0.001045684	0.905289408	Zm00001d005478
Zm00001d005478_T008	9.41E-05	3.680729806	Zm00001d005478
Zm00001d005478_T005	0.006843194	0.529847423	Zm00001d005478
Zm00001d013144_T001	0.897685	-0.19366758	Zm00001d013144
Zm00001d036077_T003	0.9689939	0.120610975	Zm00001d036077
Zm00001d036077_T001	0.8817666	-0.222395117	Zm00001d036077
Zm00001d036077_T002	0.2891877	-1.115313809	Zm00001d036077
Zm00001d018507_T002	0.9781672	-0.142588352	Zm00001d018507
Zm00001d018507_T001	0.4228909	0.583374203	Zm00001d018507
Zm00001d044459_T001	0.9087929	-0.166723105	Zm00001d044459
Zm00001d044459_T005	0.8537392	1.932923951	Zm00001d044459
Zm00001d044459_T004	0.9970835	0.133509231	Zm00001d044459

Zm00001d044459_T008	0.6589018	-0.897133093	Zm00001d044459
Zm00001d033402_T001	0.8097969	-0.643060183	Zm00001d033402
Zm00001d033402_T002	0.8223151	-0.484884802	Zm00001d033402
Zm00001d038059_T001	1	0.384753078	Zm00001d038059
Zm00001d041698_T001	0.9766291	0.529467739	Zm00001d041698
Zm00001d018208_T001	0.8192173	-0.371520317	Zm00001d018208
Zm00001d041750_T001	0.5352956	-0.554219691	Zm00001d041750
Zm00001d041750_T004	0.8824261	-0.236543793	Zm00001d041750
Zm00001d041750_T002	0.891695	-0.147229469	Zm00001d041750
Zm00001d041750_T008	0.8613703	-0.063964134	Zm00001d041750
Zm00001d041750_T006	0.003617779	-3.45254507	Zm00001d041750
Zm00001d023769_T001	5.24E-05	-2.434347628	Zm00001d023769
Zm00001d023769_T002	3.61E-05	1.060791906	Zm00001d023769
Zm00001d041299_T001	0.9393274	-0.091916336	Zm00001d041299
Zm00001d020163_T001	0.2317185	-0.860973113	Zm00001d020163
Zm00001d004758_T001	0.9047923	-0.292432917	Zm00001d004758
Zm00001d048491_T001	0.9782953	-0.202806144	Zm00001d048491
Zm00001d003857_T001	0.8991659	0.879082995	Zm00001d003857
Zm00001d003857_T002	0.6076098	-0.767273301	Zm00001d003857
Zm00001d034931_T001	0.03776326	1.022743327	Zm00001d034931
Zm00001d034931_T008	0.05061282	-0.663882795	Zm00001d034931
Zm00001d034931_T015	0.4311128	0.348926243	Zm00001d034931
Zm00001d034931_T011	0.8640292	0.408466681	Zm00001d034931
Zm00001d034931_T006	1.27E-08	0.807008184	Zm00001d034931
Zm00001d034931_T034	0.9281	0.153991065	Zm00001d034931
Zm00001d034931_T032	0.9410688	0.736252607	Zm00001d034931
Zm00001d044768_T001	0.9693993	0.062980379	Zm00001d044768
Zm00001d015917_T001	0.8592494	0.766515722	Zm00001d015917
Zm00001d031653_T002	0.5257585	-0.621729338	Zm00001d031653
Zm00001d005004_T001	0.954492	0.166166684	Zm00001d005004
Zm00001d014246_T003	1	0.34089777	Zm00001d014246
Zm00001d014246_T010	0.6411729	-0.782668805	Zm00001d014246
Zm00001d014246_T009	0.8239342	0.35307715	Zm00001d014246
Zm00001d021729_T003	0.478267	1.225127824	Zm00001d021729
Zm00001d021729_T001	1	0.406075876	Zm00001d021729
Zm00001d045954_T004	0.8690525	0.808851211	Zm00001d045954
Zm00001d051628_T002	0.9154044	0.757052504	Zm00001d051628
Zm00001d051628_T001	0.9954444	0.269423472	Zm00001d051628
Zm00001d046616_T001	0.9889197	0.306918697	Zm00001d046616
Zm00001d038528_T001	0.7428299	-0.490059355	Zm00001d038528
Zm00001d033018_T001	0.8548987	-0.206761928	Zm00001d033018
Zm00001d049980_T001	0.8556442	0.888620324	Zm00001d049980
Zm00001d026371_T001	0.8537544	-0.194614919	Zm00001d026371
Zm00001d011486_T003	0.589378	-0.502285234	Zm00001d011486
Zm00001d011486_T001	0.9701986	0.195100265	Zm00001d011486
Zm00001d034129_T002	0.9975882	0.255080101	Zm00001d034129
Zm00001d034129_T001	0.9300784	0.749208162	Zm00001d034129
Zm00001d034336_T005	0.9979568	0.127155441	Zm00001d034336
Zm00001d026022_T001	0.3615897	-0.904992206	Zm00001d026022
Zm00001d026022_T005	0.9895224	0.468381375	Zm00001d026022
Zm00001d019905_T004	0.6724646	-0.117474431	Zm00001d019905
Zm00001d019905_T005	1	0.117940321	Zm00001d019905
Zm00001d009596_T001	0.7978964	-0.152851624	Zm00001d009596
Zm00001d042662_T006	0.4552715	-0.25677437	Zm00001d042662

Zm00001d042662_T005	0.07420193	0.325612328	Zm00001d042662
Zm00001d042662_T001	0.5795936	1.046756162	Zm00001d042662
Zm00001d045603_T009	6.96E-10	0.995965723	Zm00001d045603
Zm00001d045603_T061	0.03182725	0.270181023	Zm00001d045603
Zm00001d045603_T051	0.1228982	0.579143991	Zm00001d045603
Zm00001d045603_T010	0.09881809	0.577457557	Zm00001d045603
Zm00001d045603_T029	0.7907819	0.020227788	Zm00001d045603
Zm00001d045603_T056	0.02680286	1.819606872	Zm00001d045603
Zm00001d045603_T069	0.8412913	-0.119355592	Zm00001d045603
Zm00001d045603_T068	0.1310827	-0.377828767	Zm00001d045603
Zm00001d043378_T001	0.8940288	0.503911333	Zm00001d043378
Zm00001d015379_T001	0.9272378	-0.170384643	Zm00001d015379
Zm00001d038584_T001	0.000475302	2.603423154	Zm00001d038584
Zm00001d014986_T002	0.006958403	1.143818381	Zm00001d014986
Zm00001d014986_T007	0.4422995	-0.2513528	Zm00001d014986
Zm00001d014986_T001	0.205062	0.377184173	Zm00001d014986
Zm00001d014986_T005	0.1592859	-0.994571016	Zm00001d014986
Zm00001d014986_T009	0.5401027	-0.079555694	Zm00001d014986
Zm00001d014986_T004	0.8092097	-0.218636218	Zm00001d014986
Zm00001d014986_T006	0.1221112	1.337710775	Zm00001d014986
Zm00001d027838_T001	0.9469426	0.016949301	Zm00001d027838
Zm00001d016501_T001	0.9570268	0.126755545	Zm00001d016501
Zm00001d017242_T001	0.7369755	-0.726236877	Zm00001d017242
Zm00001d034832_T008	0.8686467	0.202277916	Zm00001d034832
Zm00001d034832_T006	0.4270038	0.664816004	Zm00001d034832
Zm00001d034832_T002	0.9311783	0.019478646	Zm00001d034832
Zm00001d039612_T001	0.1486779	0.982511068	Zm00001d039612
Zm00001d039612_T013	0.01790185	0.926055946	Zm00001d039612
Zm00001d039612_T028	1.22E-09	1.122329552	Zm00001d039612
Zm00001d039612_T033	0.05575778	0.975027404	Zm00001d039612
Zm00001d039612_T025	0.7513339	0.106407452	Zm00001d039612
Zm00001d039612_T009	0.3148382	0.331595919	Zm00001d039612
Zm00001d028160_T001	0.7626242	0.897074441	Zm00001d028160
Zm00001d037080_T001	0.9515686	0.09181248	Zm00001d037080
Zm00001d014272_T002	1	0.157995123	Zm00001d014272
Zm00001d014272_T001	0.9992927	0.08652818	Zm00001d014272
Zm00001d007072_T001	0.9771927	0.476057777	Zm00001d007072
Zm00001d039404_T001	0.7408387	1.005964851	Zm00001d039404
Zm00001d012562_T001	0.9563232	-0.024395413	Zm00001d012562
Zm00001d008397_T001	0.3354744	-1.293570591	Zm00001d008397
Zm00001d011838_T001	0.983226	0.263306273	Zm00001d011838
Zm00001d032600_T001	0.8111431	-0.22076831	Zm00001d032600
Zm00001d034422_T003	0.6696579	-0.81376905	Zm00001d034422
Zm00001d034422_T002	0.8001345	-0.713654067	Zm00001d034422
Zm00001d034422_T001	0.03435352	-1.765793691	Zm00001d034422
Zm00001d043272_T002	0.7134188	1.117056005	Zm00001d043272
Zm00001d019550_T003	0.9766874	0.438272751	Zm00001d019550
Zm00001d019550_T002	0.98291	0.137397858	Zm00001d019550
Zm00001d019550_T001	0.98291	0.232875848	Zm00001d019550
Zm00001d005043_T001	0.9493302	0.430583702	Zm00001d005043
Zm00001d011117_T001	0.9293689	0.016184008	Zm00001d011117
Zm00001d011060_T001	0.8031436	-0.242904134	Zm00001d011060
Zm00001d032031_T002	0.9979568	0.128081275	Zm00001d032031
Zm00001d032031_T001	0.9818381	0.169126572	Zm00001d032031

Zm00001d017072_T001	0.8757823	-0.052463125	Zm00001d017072
Zm00001d017072_T002	0.8605736	-0.017332325	Zm00001d017072
Zm00001d017072_T006	0.995534	0.339802045	Zm00001d017072
Zm00001d017072_T008	1	1.392654895	Zm00001d017072
Zm00001d034838_T002	0.2317185	-0.417838997	Zm00001d034838
Zm00001d030235_T002	0.9302759	0.022016887	Zm00001d030235
Zm00001d030235_T001	0.8537544	0.651461169	Zm00001d030235
Zm00001d006547_T001	0.8694217	-0.440217611	Zm00001d006547
Zm00001d045475_T001	0.4827611	-0.99075678	Zm00001d045475
Zm00001d034867_T001	0.9791729	0.214791357	Zm00001d034867
Zm00001d015527_T002	0.7289978	1.08922108	Zm00001d015527
Zm00001d015527_T004	0.2899461	0.716057212	Zm00001d015527
Zm00001d009738_T001	0.8864089	0.307294078	Zm00001d009738
Zm00001d025575_T002	0.9760472	0.256457437	Zm00001d025575
Zm00001d039575_T001	0.4001127	1.547438454	Zm00001d039575
Zm00001d042833_T001	0.4082324	-0.77502163	Zm00001d042833
Zm00001d035413_T003	0.9936756	0.190502417	Zm00001d035413
Zm00001d035413_T001	0.8881183	0.745307538	Zm00001d035413
Zm00001d035413_T004	0.9766874	0.172823331	Zm00001d035413
Zm00001d013418_T001	0.4490888	-0.697857889	Zm00001d013418
Zm00001d011178_T001	0.9422098	0.591331006	Zm00001d011178
Zm00001d034935_T001	0.8183889	0.140824469	Zm00001d034935
Zm00001d034935_T007	0.9706293	0.232787223	Zm00001d034935
Zm00001d034935_T011	0.3574189	0.785121802	Zm00001d034935
Zm00001d034935_T003	0.01758334	-0.217756022	Zm00001d034935
Zm00001d017090_T001	0.8913084	-0.212035127	Zm00001d017090
Zm00001d025989_T001	0.1813603	-0.737447188	Zm00001d025989
Zm00001d002084_T009	0.9565533	-0.024313682	Zm00001d002084
Zm00001d002084_T004	0.9941715	0.252613898	Zm00001d002084
Zm00001d002084_T011	0.3225746	-0.793795548	Zm00001d002084
Zm00001d013477_T001	0.2826256	1.472878291	Zm00001d013477
Zm00001d013477_T008	0.9768802	0.181262731	Zm00001d013477
Zm00001d013477_T012	0.08463804	1.240237243	Zm00001d013477
Zm00001d013477_T010	0.001612382	1.373620161	Zm00001d013477
Zm00001d009431_T003	0.9846714	0.246333201	Zm00001d009431
Zm00001d009431_T002	0.7578559	0.985679853	Zm00001d009431
Zm00001d052987_T001	1	0.401552219	Zm00001d052987
Zm00001d044254_T007	0.3320841	-1.331465241	Zm00001d044254
Zm00001d044254_T001	0.1614455	1.750344566	Zm00001d044254
Zm00001d044254_T003	0.5192183	-0.88532845	Zm00001d044254
Zm00001d044254_T006	0.000933882	1.839173258	Zm00001d044254
Zm00001d016059_T001	0.909739	-0.338560864	Zm00001d016059
Zm00001d052757_T001	0.9804228	-0.289434176	Zm00001d052757
Zm00001d005151_T001	0.00026702	1.367769978	Zm00001d005151
Zm00001d005151_T004	0.9722469	0.570745973	Zm00001d005151
Zm00001d005151_T006	0.8412913	0.197783083	Zm00001d005151
Zm00001d005151_T007	1	0.533060742	Zm00001d005151
Zm00001d049347_T001	0.9302759	-0.267987249	Zm00001d049347
Zm00001d043249_T001	0.9371934	0.538907535	Zm00001d043249
Zm00001d012249_T001	0.4332539	-0.673873188	Zm00001d012249
Zm00001d008176_T015	0.4657833	1.658997196	Zm00001d008176
Zm00001d008176_T008	0.0136329	1.680010076	Zm00001d008176
Zm00001d008176_T006	0.3720939	1.529662809	Zm00001d008176
Zm00001d051511_T001	0.9666757	0.09616862	Zm00001d051511

Zm00001d032990_T001	0.9811162	0.155236843	Zm00001d032990
Zm00001d046660_T001	0.9747695	0.157650039	Zm00001d046660
Zm00001d043857_T001	1	0.231479495	Zm00001d043857
Zm00001d007231_T002	1	0.151310501	Zm00001d007231
Zm00001d006397_T001	0.342158	0.935590459	Zm00001d006397
Zm00001d019678_T001	0.9791729	0.789049204	Zm00001d019678
Zm00001d022400_T003	0.9619517	0.028711699	Zm00001d022400
Zm00001d022400_T004	0.9884525	0.324543724	Zm00001d022400
Zm00001d022400_T005	0.8605736	-0.215744188	Zm00001d022400
Zm00001d022400_T006	0.5116079	-0.828730215	Zm00001d022400
Zm00001d010795_T001	0.7010603	0.878889143	Zm00001d010795
Zm00001d050645_T003	0.8103289	0.928307191	Zm00001d050645
Zm00001d050645_T002	0.9805897	0.525327888	Zm00001d050645
Zm00001d042133_T018	0.440368	-0.983301942	Zm00001d042133
Zm00001d042133_T004	0.9988864	0.424067309	Zm00001d042133
Zm00001d042133_T001	0.9107561	0.57650391	Zm00001d042133
Zm00001d025382_T001	0.9781672	0.258875799	Zm00001d025382
Zm00001d012423_T001	0.677308	-0.450970214	Zm00001d012423
Zm00001d028566_T001	0.8578122	-0.115725716	Zm00001d028566
Zm00001d028566_T002	0.1362314	2.035096704	Zm00001d028566
Zm00001d003108_T003	0.842741	-0.437227407	Zm00001d003108
Zm00001d003108_T007	0.7581615	0.640594616	Zm00001d003108
Zm00001d003108_T001	1	0.28107299	Zm00001d003108
Zm00001d003108_T004	0.9867657	0.255354073	Zm00001d003108
Zm00001d003108_T005	0.04255884	2.324557823	Zm00001d003108
Zm00001d038682_T001	0.7626242	-0.376027743	Zm00001d038682
Zm00001d010465_T001	0.9825385	0.393587421	Zm00001d010465
Zm00001d049305_T006	0.07199362	-1.7471508	Zm00001d049305
Zm00001d049305_T005	0.598789	-0.288783032	Zm00001d049305
Zm00001d049305_T001	0.9446484	-0.142835148	Zm00001d049305
Zm00001d049305_T003	0.669171	-0.572747357	Zm00001d049305
Zm00001d037343_T001	1	0.22808329	Zm00001d037343
Zm00001d037343_T003	0.9745948	0.492232239	Zm00001d037343
Zm00001d025108_T001	0.7194121	0.80610665	Zm00001d025108
Zm00001d050184_T001	1	0.355176194	Zm00001d050184
Zm00001d050184_T003	0.6727656	1.137301497	Zm00001d050184
Zm00001d050184_T011	0.9868533	0.125719501	Zm00001d050184
Zm00001d050184_T010	0.8689882	-0.120523923	Zm00001d050184
Zm00001d050184_T002	0.1664354	1.281288497	Zm00001d050184
Zm00001d006631_T001	0.9686323	0.542008482	Zm00001d006631
Zm00001d003982_T001	0.8224736	-0.273300624	Zm00001d003982
Zm00001d043599_T001	0.5722588	-0.48899678	Zm00001d043599
Zm00001d024803_T001	0.9791729	0.225418534	Zm00001d024803
Zm00001d029720_T001	0.6705583	-0.956146279	Zm00001d029720
Zm00001d053658_T002	0.9869996	0.488729255	Zm00001d053658
Zm00001d053658_T001	0.9979568	0.425361683	Zm00001d053658
Zm00001d010178_T001	0.7399105	-0.303568657	Zm00001d010178
Zm00001d002388_T007	0.4817122	0.735554064	Zm00001d002388
Zm00001d002388_T003	0.609919	1.32459308	Zm00001d002388
Zm00001d002388_T002	5.87E-15	-4.314086981	Zm00001d002388
Zm00001d017659_T001	0.8624945	-0.499533611	Zm00001d017659
Zm00001d013128_T001	0.9442138	-0.211482431	Zm00001d013128
Zm00001d013128_T005	0.9046076	0.457012654	Zm00001d013128
Zm00001d013128_T004	0.8412913	-0.127895512	Zm00001d013128

Zm00001d017138_T001	0.9447869	0.079900878	Zm00001d017138
Zm00001d052820_T001	0.9469426	0.066369989	Zm00001d052820
Zm00001d048740_T001	0.5507795	-0.551138934	Zm00001d048740
Zm00001d038335_T001	0.9752909	-0.198207145	Zm00001d038335
Zm00001d022111_T002	0.6106534	-0.594461198	Zm00001d022111
Zm00001d022111_T001	0.9533693	0.112342352	Zm00001d022111
Zm00001d008570_T001	0.01259421	2.713700221	Zm00001d008570
Zm00001d019579_T008	0.9877195	0.348542949	Zm00001d019579
Zm00001d019579_T002	0.9979568	0.217819312	Zm00001d019579
Zm00001d019579_T007	0.9902733	0.408039387	Zm00001d019579
Zm00001d005228_T001	0.9232446	-0.042474932	Zm00001d005228
Zm00001d004702_T006	0.957148	0.340442404	Zm00001d004702
Zm00001d004702_T003	0.8170081	1.007786982	Zm00001d004702
Zm00001d004702_T002	0.006762321	-0.93598539	Zm00001d004702
Zm00001d004702_T004	1	0.084399818	Zm00001d004702
Zm00001d012127_T001	0.9979568	0.139338884	Zm00001d012127
Zm00001d054001_T002	0.9947739	0.261756085	Zm00001d054001
Zm00001d054001_T011	0.03659288	2.184317247	Zm00001d054001
Zm00001d054001_T016	0.9681793	0.390499747	Zm00001d054001
Zm00001d054001_T022	0.9747695	-0.035359894	Zm00001d054001
Zm00001d054001_T001	0.0189919	0.796102689	Zm00001d054001
Zm00001d054001_T028	0.09783201	1.155171983	Zm00001d054001
Zm00001d054001_T004	0.05329398	-0.845437651	Zm00001d054001
Zm00001d042441_T003	0.164302	-1.323130944	Zm00001d042441
Zm00001d042441_T001	0.9689939	0.160857616	Zm00001d042441
Zm00001d029704_T001	0.5718169	-0.872088868	Zm00001d029704
Zm00001d002958_T001	0.5613757	1.205297952	Zm00001d002958
Zm00001d036558_T001	0.9402253	-0.011875277	Zm00001d036558
Zm00001d029077_T001	0.9886764	0.065613311	Zm00001d029077
Zm00001d029077_T002	0.6882326	-0.346236278	Zm00001d029077
Zm00001d042373_T002	0.7209242	0.743785244	Zm00001d042373
Zm00001d042373_T001	0.960747	0.674287069	Zm00001d042373
Zm00001d042373_T003	0.9429712	0.400744656	Zm00001d042373
Zm00001d011347_T002	0.9850313	0.220253718	Zm00001d011347
Zm00001d011347_T001	0.7315461	1.133826852	Zm00001d011347
Zm00001d033210_T002	0.909729	-0.023668445	Zm00001d033210
Zm00001d033210_T005	1.24E-19	-7.547061135	Zm00001d033210
Zm00001d033210_T004	0.4909635	-0.377724477	Zm00001d033210
Zm00001d033210_T001	0.9100328	0.211980424	Zm00001d033210
Zm00001d048884_T001	0.9614723	-0.278396896	Zm00001d048884
Zm00001d017678_T001	0.9078287	-0.116827875	Zm00001d017678
Zm00001d016982_T001	0.9693993	0.450164274	Zm00001d016982
Zm00001d024913_T001	0.5217931	-0.78080826	Zm00001d024913
Zm00001d045870_T001	0.9832092	0.320436985	Zm00001d045870
Zm00001d045870_T002	0.004838924	1.651463259	Zm00001d045870
Zm00001d045870_T003	0.8050292	0.988409808	Zm00001d045870
Zm00001d053208_T001	0.9451369	0.037135809	Zm00001d053208
Zm00001d001995_T001	0.07730424	-1.189941672	Zm00001d001995
Zm00001d001995_T002	0.009270008	1.650282026	Zm00001d001995
Zm00001d029121_T004	0.302257	0.311315161	Zm00001d029121
Zm00001d029121_T001	0.325998	-0.9462662	Zm00001d029121
Zm00001d029121_T003	0.341302	0.404870421	Zm00001d029121
Zm00001d029121_T005	0.9970835	0.400596571	Zm00001d029121
Zm00001d029121_T002	0.4254374	0.395896092	Zm00001d029121

Zm00001d034532_T001	0.805584	0.784920032	Zm00001d034532
Zm00001d034532_T002	0.9432413	0.071872857	Zm00001d034532
Zm00001d036131_T001	0.9833565	0.492474334	Zm00001d036131
Zm00001d036131_T004	0.0109172	-2.551294672	Zm00001d036131
Zm00001d028265_T004	0.02224341	-1.922040344	Zm00001d028265
Zm00001d028265_T002	0.1003013	1.959075368	Zm00001d028265
Zm00001d028265_T005	0.754061	1.431096037	Zm00001d028265
Zm00001d028265_T001	1	0.167653404	Zm00001d028265
Zm00001d028265_T007	0.9613188	0.029303755	Zm00001d028265
Zm00001d054010_T001	0.9269932	0.607935838	Zm00001d054010
Zm00001d036532_T001	0.3217566	1.548531379	Zm00001d036532
Zm00001d008380_T001	0.9927847	0.295799342	Zm00001d008380
Zm00001d008380_T002	0.224247	-0.902065512	Zm00001d008380
Zm00001d010442_T001	0.9404125	0.545477268	Zm00001d010442
Zm00001d013326_T001	0.1220945	-1.900930938	Zm00001d013326
Zm00001d014181_T001	0.9860836	0.395350007	Zm00001d014181
Zm00001d052578_T001	0.0214763	-1.213984908	Zm00001d052578
Zm00001d023216_T001	0.6185593	-0.523594556	Zm00001d023216
Zm00001d036201_T001	0.8776881	-0.175917053	Zm00001d036201
Zm00001d046016_T001	0.8705498	0.924562777	Zm00001d046016
Zm00001d031489_T003	0.9979393	0.463800156	Zm00001d031489
Zm00001d031489_T002	0.9561521	0.093078335	Zm00001d031489
Zm00001d031489_T004	0.3564291	1.100367895	Zm00001d031489
Zm00001d049556_T009	1.43E-07	1.066094107	Zm00001d049556
Zm00001d049556_T001	0.9179118	-0.006375122	Zm00001d049556
Zm00001d021817_T001	0.08280766	2.28816295	Zm00001d021817
Zm00001d047272_T001	0.9344122	-0.402616693	Zm00001d047272
Zm00001d051576_T001	0.9303773	0.181855547	Zm00001d051576
Zm00001d043045_T001	0.7518152	-0.643351229	Zm00001d043045
Zm00001d032868_T001	0.08197566	1.004511581	Zm00001d032868
Zm00001d008720_T002	0.9689939	0.077150454	Zm00001d008720
Zm00001d020669_T001	0.9809965	0.269243133	Zm00001d020669
Zm00001d002483_T006	0.1127161	-0.845756415	Zm00001d002483
Zm00001d002483_T004	0.8451052	0.477598206	Zm00001d002483
Zm00001d002483_T001	0.000516578	1.07325341	Zm00001d002483
Zm00001d045269_T014	0.003280308	0.671128471	Zm00001d045269
Zm00001d045269_T031	3.33E-13	2.491871022	Zm00001d045269
Zm00001d045269_T039	0.5001864	0.807838115	Zm00001d045269
Zm00001d045269_T004	0.02555124	0.715897525	Zm00001d045269
Zm00001d045269_T011	0.220759	0.981456026	Zm00001d045269
Zm00001d045269_T012	0.228634	0.601472241	Zm00001d045269
Zm00001d045269_T042	0.9706887	0.590941218	Zm00001d045269
Zm00001d053011_T006	0.2803683	0.398195357	Zm00001d053011
Zm00001d053011_T003	0.9704112	0.500623295	Zm00001d053011
Zm00001d053011_T004	0.005044807	0.95393553	Zm00001d053011
Zm00001d022525_T001	0.8056124	-0.210065788	Zm00001d022525
Zm00001d016551_T001	0.1895778	-0.79234427	Zm00001d016551
Zm00001d016551_T002	0.8099858	-0.201661236	Zm00001d016551
Zm00001d033021_T001	0.4482327	1.2505841	Zm00001d033021
Zm00001d010191_T001	0.997764	0.128560648	Zm00001d010191
Zm00001d023424_T001	0.8916793	-0.118021504	Zm00001d023424
Zm00001d035207_T001	0.004055167	-1.620916394	Zm00001d035207
Zm00001d035207_T002	0.9840918	0.591184378	Zm00001d035207
Zm00001d035207_T003	0.7899426	0.924125012	Zm00001d035207

Zm00001d029039_T001	0.9664393	0.5831042	Zm00001d029039
Zm00001d008815_T002	0.9835181	0.136864123	Zm00001d008815
Zm00001d042747_T004	4.52E-05	2.223027896	Zm00001d042747
Zm00001d042747_T001	0.9868533	0.325280306	Zm00001d042747
Zm00001d042747_T006	0.3649839	0.797117209	Zm00001d042747
Zm00001d042747_T002	1	0.309338638	Zm00001d042747
Zm00001d042747_T008	0.01048202	0.961393431	Zm00001d042747
Zm00001d042747_T005	0.2650686	1.618490472	Zm00001d042747
Zm00001d048841_T001	0.9930712	0.189315046	Zm00001d048841
Zm00001d014294_T007	0.02913084	0.277041714	Zm00001d014294
Zm00001d014294_T027	4.68E-05	2.019192835	Zm00001d014294
Zm00001d014294_T021	0.7942213	1.102146684	Zm00001d014294
Zm00001d014294_T008	5.19E-06	2.428448651	Zm00001d014294
Zm00001d014294_T011	0.0441095	2.066664667	Zm00001d014294
Zm00001d014294_T009	1.06E-07	-0.353360503	Zm00001d014294
Zm00001d008457_T001	0.9355135	0.039040683	Zm00001d008457
Zm00001d014036_T014	3.94E-06	1.706520065	Zm00001d014036
Zm00001d014036_T007	0.6085458	0.828930724	Zm00001d014036
Zm00001d014036_T002	0.8935921	0.047381323	Zm00001d014036
Zm00001d014036_T005	0.6945741	1.202614769	Zm00001d014036
Zm00001d014036_T009	1.15E-06	-2.394022402	Zm00001d014036
Zm00001d014036_T018	0.2004267	0.4291432	Zm00001d014036
Zm00001d014036_T003	0.2982359	0.794166621	Zm00001d014036
Zm00001d041920_T001	0.9149322	0.733460862	Zm00001d041920
Zm00001d017121_T002	0.01823557	-1.561145512	Zm00001d017121
Zm00001d017121_T001	0.4485954	-0.734795585	Zm00001d017121
Zm00001d009243_T001	0.6118682	1.334924165	Zm00001d009243
Zm00001d009243_T002	0.716456	1.1446437	Zm00001d009243
Zm00001d009243_T003	0.02343819	1.731910643	Zm00001d009243
Zm00001d015970_T002	0.9353429	0.240511657	Zm00001d015970
Zm00001d015970_T003	0.9518412	-0.493226272	Zm00001d015970
Zm00001d040768_T001	0.7954824	-0.198871576	Zm00001d040768
Zm00001d012677_T015	0.8704382	0.478667839	Zm00001d012677
Zm00001d012677_T007	0.5849574	0.801347442	Zm00001d012677
Zm00001d012677_T001	0.7545745	0.780867866	Zm00001d012677
Zm00001d012677_T011	0.990751	0.2676066	Zm00001d012677
Zm00001d036678_T001	1	0.339709108	Zm00001d036678
Zm00001d036678_T007	0.01955821	0.37578273	Zm00001d036678
Zm00001d052621_T004	0.9442514	0.176857287	Zm00001d052621
Zm00001d052621_T008	0.9690782	0.315468309	Zm00001d052621
Zm00001d052621_T012	0.1418279	1.069034805	Zm00001d052621
Zm00001d052621_T001	0.9812314	0.382284253	Zm00001d052621
Zm00001d052621_T014	4.35E-05	-4.200226143	Zm00001d052621
Zm00001d052621_T006	0.6826913	0.343628783	Zm00001d052621
Zm00001d052621_T005	0.7881977	-0.219027094	Zm00001d052621
Zm00001d052621_T007	0.9404125	1.082523899	Zm00001d052621
Zm00001d052621_T002	0.3096524	-0.693392536	Zm00001d052621
Zm00001d053197_T015	0.745469	-0.257743259	Zm00001d053197
Zm00001d053197_T006	0.008258542	2.088150689	Zm00001d053197
Zm00001d053197_T002	0.9514141	0.172155442	Zm00001d053197
Zm00001d053197_T014	0.7294689	0.835241319	Zm00001d053197
Zm00001d053197_T011	0.003668704	0.663050119	Zm00001d053197
Zm00001d053197_T004	0.9396569	1.197223211	Zm00001d053197
Zm00001d053197_T018	0.7626242	-0.698295526	Zm00001d053197

Zm00001d053197_T005	0.05875368	-2.132806804	Zm00001d053197
Zm00001d026645_T020	0.973825	-0.004885978	Zm00001d026645
Zm00001d026645_T016	1.23E-05	0.473818799	Zm00001d026645
Zm00001d026645_T004	0.6007866	-0.105584764	Zm00001d026645
Zm00001d026645_T015	0.9945076	0.271135468	Zm00001d026645
Zm00001d026645_T007	0.1619854	1.126763016	Zm00001d026645
Zm00001d014641_T001	0.9090332	-0.00752984	Zm00001d014641
Zm00001d009773_T001	0.3105424	-1.018041514	Zm00001d009773
Zm00001d022225_T001	0.2135821	1.816739379	Zm00001d022225
Zm00001d016816_T002	0.9637995	0.136480057	Zm00001d016816
Zm00001d046728_T001	0.4355187	-0.942176714	Zm00001d046728
Zm00001d020593_T006	0.9979568	0.346999347	Zm00001d020593
Zm00001d020593_T002	0.08342142	1.902102669	Zm00001d020593
Zm00001d052322_T001	0.9583056	0.010962202	Zm00001d052322
Zm00001d041322_T002	0.7897201	-0.553182184	Zm00001d041322
Zm00001d010835_T003	0.4163319	-0.989334068	Zm00001d010835
Zm00001d010835_T005	0.7365938	-0.10362716	Zm00001d010835
Zm00001d010835_T006	0.9832612	0.474860267	Zm00001d010835
Zm00001d051047_T001	0.745469	0.844991182	Zm00001d051047
Zm00001d032670_T021	0.6324192	-0.351498002	Zm00001d032670
Zm00001d032670_T009	0.7020332	-0.252143696	Zm00001d032670
Zm00001d032670_T013	0.9760472	-0.027099702	Zm00001d032670
Zm00001d032670_T011	0.9269932	0.037307933	Zm00001d032670
Zm00001d032670_T001	0.164302	-0.204740211	Zm00001d032670
Zm00001d032670_T020	0.8071043	0.691693114	Zm00001d032670
Zm00001d032670_T003	0.8114815	-0.135039242	Zm00001d032670
Zm00001d032670_T006	0.1329691	0.385285648	Zm00001d032670
Zm00001d034893_T002	0.9867452	0.037136309	Zm00001d034893
Zm00001d034893_T001	0.9683047	0.338127203	Zm00001d034893
Zm00001d034893_T004	0.9740325	0.138430161	Zm00001d034893
Zm00001d031671_T001	0.9702493	0.700212346	Zm00001d031671
Zm00001d022542_T002	0.9273672	0.766254689	Zm00001d022542
Zm00001d022542_T004	0.5408146	1.108032629	Zm00001d022542
Zm00001d022542_T007	0.9563232	-0.013446543	Zm00001d022542
Zm00001d022542_T005	0.9708541	0.569698034	Zm00001d022542
Zm00001d022542_T003	0.997764	0.321704665	Zm00001d022542
Zm00001d022542_T001	0.8934143	0.924025006	Zm00001d022542
Zm00001d022542_T006	0.976488	0.237677135	Zm00001d022542
Zm00001d029753_T003	0.9740325	0.367777173	Zm00001d029753
Zm00001d029753_T002	0.997764	0.371912192	Zm00001d029753
Zm00001d013136_T001	1	0.167947897	Zm00001d013136
Zm00001d008307_T001	0.9109605	0.653244643	Zm00001d008307
Zm00001d041816_T005	0.9419336	0.416385883	Zm00001d041816
Zm00001d041816_T004	0.2004267	-0.523409666	Zm00001d041816
Zm00001d041816_T002	0.3423638	-0.259221624	Zm00001d041816
Zm00001d041816_T003	0.01441334	1.543378256	Zm00001d041816
Zm00001d049665_T001	0.8517335	-0.131717859	Zm00001d049665
Zm00001d013362_T001	0.5157215	1.346039421	Zm00001d013362
Zm00001d034049_T002	0.8907908	0.939715704	Zm00001d034049
Zm00001d034049_T001	1	0.498674516	Zm00001d034049
Zm00001d034049_T003	0.9537113	0.090266543	Zm00001d034049
Zm00001d027756_T002	0.9744451	0.461227078	Zm00001d027756
Zm00001d027756_T001	0.1559427	-0.63015557	Zm00001d027756
Zm00001d052709_T002	0.2518892	0.608308768	Zm00001d052709

Zm00001d052709_T010	0.470047	0.710300963	Zm00001d052709
Zm00001d052709_T013	0.8597186	-0.429848792	Zm00001d052709
Zm00001d052709_T004	0.8097523	1.011449772	Zm00001d052709
Zm00001d052709_T005	0.9799902	0.175804913	Zm00001d052709
Zm00001d052709_T003	0.6123019	0.977324048	Zm00001d052709
Zm00001d052709_T008	0.7318368	0.483988709	Zm00001d052709
Zm00001d041124_T017	0.3173447	0.333499737	Zm00001d041124
Zm00001d041124_T007	0.897685	-0.021707886	Zm00001d041124
Zm00001d041124_T005	0.9933044	0.325239011	Zm00001d041124
Zm00001d041124_T010	0.03760071	0.578633417	Zm00001d041124
Zm00001d014032_T001	0.4163319	-1.387602802	Zm00001d014032
Zm00001d001802_T001	0.7187348	0.511667925	Zm00001d001802
Zm00001d046336_T001	0.03306112	-0.592340643	Zm00001d046336
Zm00001d046336_T002	0.9950703	0.334406436	Zm00001d046336
Zm00001d006808_T002	0.9311783	0.48470663	Zm00001d006808
Zm00001d006808_T001	0.8802656	-0.37453927	Zm00001d006808
Zm00001d006944_T003	1	0.290239743	Zm00001d006944
Zm00001d006944_T002	0.4800309	1.339170008	Zm00001d006944
Zm00001d006944_T004	0.6876043	0.560457737	Zm00001d006944
Zm00001d020273_T001	0.5706512	-0.619073552	Zm00001d020273
Zm00001d048705_T001	0.7445049	-0.278964945	Zm00001d048705
Zm00001d027502_T001	0.3908747	-0.494530154	Zm00001d027502
Zm00001d027502_T002	0.9984041	0.258974175	Zm00001d027502
Zm00001d052776_T001	0.5886552	-1.177964401	Zm00001d052776
Zm00001d018295_T001	9.15E-13	2.410818362	Zm00001d018295
Zm00001d018295_T002	7.12E-13	-2.091724729	Zm00001d018295
Zm00001d018295_T007	0.9988954	0.342797927	Zm00001d018295
Zm00001d032185_T001	0.8854695	-0.099923431	Zm00001d032185
Zm00001d016106_T001	1	0.343616129	Zm00001d016106
Zm00001d016106_T003	0.9441446	0.642239845	Zm00001d016106
Zm00001d050211_T001	0.7213008	0.956733534	Zm00001d050211
Zm00001d028894_T003	0.3469239	0.708949234	Zm00001d028894
Zm00001d028894_T001	0.9971866	0.25605411	Zm00001d028894
Zm00001d037590_T001	0.9894615	0.485000204	Zm00001d037590
Zm00001d037590_T004	0.6509354	-0.881942294	Zm00001d037590
Zm00001d052793_T001	0.9233973	0.775568587	Zm00001d052793
Zm00001d034768_T002	0.9906533	0.250442806	Zm00001d034768
Zm00001d034768_T005	0.9860836	0.820569867	Zm00001d034768
Zm00001d034768_T003	0.9396279	-0.102699883	Zm00001d034768
Zm00001d034768_T001	1	0.378938926	Zm00001d034768
Zm00001d005959_T001	0.6530894	0.935688791	Zm00001d005959
Zm00001d024516_T001	0.9514839	0.693275452	Zm00001d024516
Zm00001d048954_T001	0.9950703	0.298850089	Zm00001d048954
Zm00001d027802_T002	0.6747252	0.771880112	Zm00001d027802
Zm00001d027802_T004	0.780547	0.939182711	Zm00001d027802
Zm00001d027802_T001	0.7294689	1.11997031	Zm00001d027802
Zm00001d027802_T003	0.9745948	0.610048194	Zm00001d027802
Zm00001d023724_T006	0.957148	0.43875376	Zm00001d023724
Zm00001d023724_T015	0.7439474	0.813592308	Zm00001d023724
Zm00001d023724_T012	0.7439474	-0.87032183	Zm00001d023724
Zm00001d023724_T001	0.830619	0.744525293	Zm00001d023724
Zm00001d023724_T007	0.8784569	-0.037859471	Zm00001d023724
Zm00001d023724_T008	0.4860327	0.452009892	Zm00001d023724
Zm00001d023724_T017	1	0.301384822	Zm00001d023724

Zm00001d023724_T011	0.4738391	-1.029909343	Zm00001d023724
Zm00001d023724_T003	0.9384219	0.05057398	Zm00001d023724
Zm00001d006027_T002	0.9975882	0.319681047	Zm00001d006027
Zm00001d029777_T001	0.1286952	1.901753175	Zm00001d029777
Zm00001d035797_T001	0.9262947	0.394738618	Zm00001d035797
Zm00001d028399_T001	0.9766874	0.557934398	Zm00001d028399
Zm00001d021898_T001	0.9524127	0.045425385	Zm00001d021898
Zm00001d020395_T002	0.9706887	0.406441852	Zm00001d020395
Zm00001d046438_T014	0.8835073	-0.156260717	Zm00001d046438
Zm00001d046438_T009	0.03327991	1.749945922	Zm00001d046438
Zm00001d046438_T013	0.5140052	0.712080453	Zm00001d046438
Zm00001d041857_T002	0.7882797	0.83853787	Zm00001d041857
Zm00001d041857_T001	0.9860836	0.478931966	Zm00001d041857
Zm00001d011297_T002	0.01727412	-2.122513345	Zm00001d011297
Zm00001d011297_T001	0.003626547	-2.422959169	Zm00001d011297
Zm00001d028547_T001	0.6394238	-0.601408846	Zm00001d028547
Zm00001d040666_T002	0.9664406	0.104648608	Zm00001d040666
Zm00001d040666_T003	0.973825	0.439003039	Zm00001d040666
Zm00001d035864_T001	0.7309251	0.631694815	Zm00001d035864
Zm00001d040735_T003	0.6227737	1.143006989	Zm00001d040735
Zm00001d040735_T004	0.4730185	1.261916117	Zm00001d040735
Zm00001d025141_T003	8.43E-08	2.213767848	Zm00001d025141
Zm00001d025141_T002	2.05E-09	2.700087685	Zm00001d025141
Zm00001d037479_T001	0.6458823	-1.027878576	Zm00001d037479
Zm00001d042262_T004	0.9770343	0.225241672	Zm00001d042262
Zm00001d027337_T004	0.001274277	-1.543281561	Zm00001d027337
Zm00001d027337_T012	1.92E-06	1.885040821	Zm00001d027337
Zm00001d053749_T001	0.9666757	0.131740783	Zm00001d053749
Zm00001d005315_T001	0.6814077	1.033254915	Zm00001d005315
Zm00001d050897_T002	0.8600403	0.47434636	Zm00001d050897
Zm00001d050897_T001	1	0.300632391	Zm00001d050897
Zm00001d025875_T001	0.9429712	0.105090546	Zm00001d025875
Zm00001d037250_T001	0.7368099	-0.3297659	Zm00001d037250
Zm00001d013431_T001	0.9028534	0.585057928	Zm00001d013431
Zm00001d013431_T003	0.9860836	-0.043839998	Zm00001d013431
Zm00001d015399_T003	0.509262	0.87283258	Zm00001d015399
Zm00001d015399_T005	0.6752822	-0.59329489	Zm00001d015399
Zm00001d015399_T002	0.954492	0.085352687	Zm00001d015399
Zm00001d015399_T004	0.9311783	-0.32643747	Zm00001d015399
Zm00001d017087_T001	0.9822138	0.068302308	Zm00001d017087
Zm00001d003012_T001	0.3870314	1.488093653	Zm00001d003012
Zm00001d018732_T004	0.9979393	0.244015221	Zm00001d018732
Zm00001d018732_T005	0.4341643	-0.311754853	Zm00001d018732
Zm00001d018732_T007	1	0.287317401	Zm00001d018732
Zm00001d018732_T001	0.9730613	0.242637965	Zm00001d018732
Zm00001d018732_T002	0.8842271	-0.066314145	Zm00001d018732
Zm00001d009747_T001	0.9550374	0.563342575	Zm00001d009747
Zm00001d035683_T001	0.9627389	1.767620894	Zm00001d035683
Zm00001d026551_T002	0.9285764	-0.129720767	Zm00001d026551
Zm00001d039415_T001	0.2396674	-0.457428518	Zm00001d039415
Zm00001d052929_T001	1	0.334715744	Zm00001d052929
Zm00001d052929_T004	0.9742379	0.058289499	Zm00001d052929
Zm00001d038097_T004	0.9616398	-0.131371123	Zm00001d038097
Zm00001d038097_T002	0.2756333	0.963598334	Zm00001d038097

Zm00001d035948_T005	0.9817074	-0.127623211	Zm00001d035948
Zm00001d035948_T001	0.1893056	-1.122293291	Zm00001d035948
Zm00001d035948_T004	0.5850461	-0.610716529	Zm00001d035948
Zm00001d035948_T002	0.6444327	-0.123689808	Zm00001d035948
Zm00001d037926_T001	0.9404125	-0.129785216	Zm00001d037926
Zm00001d031759_T001	0.5328025	-0.434427943	Zm00001d031759
Zm00001d013234_T001	0.6952911	0.982125345	Zm00001d013234
Zm00001d052618_T001	0.8688441	-0.138063005	Zm00001d052618
Zm00001d020584_T001	0.4860327	-0.624946359	Zm00001d020584
Zm00001d038546_T005	0.9518412	0.618262456	Zm00001d038546
Zm00001d038546_T002	0.07990611	-0.996857076	Zm00001d038546
Zm00001d026684_T001	0.7146325	-0.379598839	Zm00001d026684
Zm00001d045800_T001	0.3407954	1.387854643	Zm00001d045800
Zm00001d036467_T001	0.8819727	-0.525889457	Zm00001d036467
Zm00001d028181_T001	0.974657	-0.1446181	Zm00001d028181
Zm00001d039967_T001	0.6254218	-0.392550803	Zm00001d039967
Zm00001d028205_T003	0.893908	-0.080056642	Zm00001d028205
Zm00001d044021_T001	0.9121108	0.729560405	Zm00001d044021
Zm00001d030992_T001	0.9926496	0.026736298	Zm00001d030992
Zm00001d013810_T002	1	0.096902465	Zm00001d013810
Zm00001d013810_T001	0.5259724	1.404804431	Zm00001d013810
Zm00001d013810_T003	0.5537579	-0.304617304	Zm00001d013810
Zm00001d012979_T006	0.4625124	-0.986162548	Zm00001d012979
Zm00001d012979_T003	0.9778436	0.064043844	Zm00001d012979
Zm00001d012979_T020	0.9514141	0.291853328	Zm00001d012979
Zm00001d012979_T009	0.1566404	0.452785471	Zm00001d012979
Zm00001d012979_T011	0.9845237	0.014651385	Zm00001d012979
Zm00001d012979_T035	0.07907404	-0.598073958	Zm00001d012979
Zm00001d012979_T004	0.8607922	-0.224606468	Zm00001d012979
Zm00001d012979_T024	0.4395938	-0.238806217	Zm00001d012979
Zm00001d029130_T001	0.9689939	0.494994902	Zm00001d029130
Zm00001d050253_T003	0.9704269	0.167478364	Zm00001d050253
Zm00001d050253_T006	0.3622612	0.228380368	Zm00001d050253
Zm00001d050253_T004	0.9842144	0.451312732	Zm00001d050253
Zm00001d050253_T001	0.8859449	-0.130878297	Zm00001d050253
Zm00001d032561_T001	0.9886764	0.216046049	Zm00001d032561
Zm00001d007441_T001	0.8992717	0.844286437	Zm00001d007441
Zm00001d007441_T002	0.7074815	0.342376715	Zm00001d007441
Zm00001d023762_T001	0.9804228	0.230171294	Zm00001d023762
Zm00001d043228_T001	0.9584416	0.084654406	Zm00001d043228
Zm00001d051901_T001	0.1181155	1.596761354	Zm00001d051901
Zm00001d036050_T001	0.9878764	0.242569064	Zm00001d036050
Zm00001d026235_T001	1	0.17931058	Zm00001d026235
Zm00001d019414_T001	0.09187252	-1.393972478	Zm00001d019414
Zm00001d027402_T002	0.9048001	0.442148784	Zm00001d027402
Zm00001d038576_T001	0.5954619	1.269293308	Zm00001d038576
Zm00001d038576_T002	0.001560792	1.638151074	Zm00001d038576
Zm00001d052185_T001	0.9968176	0.509894931	Zm00001d052185
Zm00001d037290_T002	0.8613703	-0.138623751	Zm00001d037290
Zm00001d038478_T001	0.05781038	2.05393044	Zm00001d038478
Zm00001d029705_T069	0.1514631	-0.661717993	Zm00001d029705
Zm00001d029705_T040	0.9361945	0.650162051	Zm00001d029705
Zm00001d029705_T042	0.3658434	0.774263073	Zm00001d029705
Zm00001d029705_T043	0.9967995	0.13192088	Zm00001d029705

Zm00001d029705_T029	0.5591127	0.788467542	Zm00001d029705
Zm00001d029705_T039	0.9979568	0.108792816	Zm00001d029705
Zm00001d029705_T028	1	0.118182901	Zm00001d029705
Zm00001d029705_T046	0.164302	-0.234995882	Zm00001d029705
Zm00001d029705_T008	0.7211861	0.407366267	Zm00001d029705
Zm00001d029705_T004	0.8717403	0.000480009	Zm00001d029705
Zm00001d053726_T001	0.9801246	0.499753866	Zm00001d053726
Zm00001d005638_T001	0.9232281	0.171573369	Zm00001d005638
Zm00001d005638_T014	0.9850313	0.574911735	Zm00001d005638
Zm00001d006109_T001	0.954492	-0.308215257	Zm00001d006109
Zm00001d004523_T001	0.9493302	-0.527347626	Zm00001d004523
Zm00001d051307_T001	0.8740066	0.909094315	Zm00001d051307
Zm00001d038535_T006	0.4744861	-0.888504946	Zm00001d038535
Zm00001d038535_T003	0.2982359	-0.967097526	Zm00001d038535
Zm00001d038535_T004	0.7818859	-0.688614593	Zm00001d038535
Zm00001d038535_T005	0.9753621	0.112133927	Zm00001d038535
Zm00001d025079_T001	1	0.31092443	Zm00001d025079
Zm00001d043157_T002	0.9860836	0.14306843	Zm00001d043157
Zm00001d017847_T001	0.9983139	0.103182776	Zm00001d017847
Zm00001d017847_T002	0.8376343	-0.298093504	Zm00001d017847
Zm00001d024298_T001	0.5698311	-0.572829221	Zm00001d024298
Zm00001d028826_T001	0.1807531	1.906128052	Zm00001d028826
Zm00001d024034_T001	0.7409461	0.88970985	Zm00001d024034
Zm00001d047124_T001	0.9760472	0.529445285	Zm00001d047124
Zm00001d018475_T001	0.07990611	1.525089545	Zm00001d018475
Zm00001d011984_T004	0.9805897	0.07809719	Zm00001d011984
Zm00001d011984_T001	0.684563	0.9997894	Zm00001d011984
Zm00001d011984_T003	0.000233833	-1.375319626	Zm00001d011984
Zm00001d011334_T003	0.8615634	0.737328663	Zm00001d011334
Zm00001d011334_T002	0.8533785	0.82250949	Zm00001d011334
Zm00001d045336_T001	0.9863483	0.215491263	Zm00001d045336
Zm00001d032440_T005	0.04570411	2.284994881	Zm00001d032440
Zm00001d032440_T004	0.8056124	0.343342944	Zm00001d032440
Zm00001d032440_T006	0.1996314	-1.247301554	Zm00001d032440
Zm00001d032440_T009	0.1574436	0.98559616	Zm00001d032440
Zm00001d052726_T015	0.222943	0.322914939	Zm00001d052726
Zm00001d052726_T045	0.7948449	-0.197842033	Zm00001d052726
Zm00001d052726_T028	1.26E-08	3.50722665	Zm00001d052726
Zm00001d052726_T004	2.44E-19	5.384092972	Zm00001d052726
Zm00001d052726_T007	0.1142433	1.395160992	Zm00001d052726
Zm00001d052726_T003	1.84E-09	1.146823628	Zm00001d052726
Zm00001d052726_T012	3.90E-07	0.644673533	Zm00001d052726
Zm00001d052726_T011	0.8064667	-0.145733371	Zm00001d052726
Zm00001d052726_T009	0.000231914	1.265241286	Zm00001d052726
Zm00001d052726_T016	0.1851529	0.634398122	Zm00001d052726
Zm00001d052726_T024	0.9332923	0.039760288	Zm00001d052726
Zm00001d052726_T029	0.9745777	0.133988331	Zm00001d052726
Zm00001d052726_T018	0.08495247	0.447097063	Zm00001d052726
Zm00001d052726_T032	0.08707621	0.536277879	Zm00001d052726
Zm00001d052726_T008	1.36E-07	2.742876444	Zm00001d052726
Zm00001d052726_T034	0.3735722	0.579221986	Zm00001d052726
Zm00001d052726_T039	0.8862653	0.402311733	Zm00001d052726
Zm00001d052726_T006	0.2619882	1.19156544	Zm00001d052726
Zm00001d052726_T010	0.4310578	1.511385911	Zm00001d052726

Zm00001d052726_T001	0.2035492	0.580807839	Zm00001d052726
Zm00001d052726_T030	0.3148382	0.315180895	Zm00001d052726
Zm00001d003396_T001	1	0.214780574	Zm00001d003396
Zm00001d006499_T002	0.8917344	-0.047075547	Zm00001d006499
Zm00001d030143_T001	0.9979568	0.171903329	Zm00001d030143
Zm00001d020626_T001	0.9693993	0.017967163	Zm00001d020626
Zm00001d001865_T001	0.8685001	0.516521374	Zm00001d001865
Zm00001d035937_T001	0.8922781	-0.054926351	Zm00001d035937
Zm00001d002161_T001	4.40E-08	-5.050099926	Zm00001d002161
Zm00001d038574_T004	0.7511396	-0.327838188	Zm00001d038574
Zm00001d038574_T002	0.9945118	0.115597672	Zm00001d038574
Zm00001d038574_T001	0.873307	-0.175826779	Zm00001d038574
Zm00001d034022_T001	0.9979568	0.161671803	Zm00001d034022
Zm00001d025003_T004	0.9173494	0.001726281	Zm00001d025003
Zm00001d025003_T001	0.5433327	1.212087544	Zm00001d025003
Zm00001d039619_T005	0.9778436	-0.144625337	Zm00001d039619
Zm00001d039619_T001	0.8354517	-0.028134799	Zm00001d039619
Zm00001d039619_T003	0.718063	0.181250924	Zm00001d039619
Zm00001d039619_T009	0.4359356	-1.395062378	Zm00001d039619
Zm00001d015747_T012	0.9449404	0.02652394	Zm00001d015747
Zm00001d015747_T024	0.8050292	0.603069761	Zm00001d015747
Zm00001d015747_T008	0.8192173	0.603235151	Zm00001d015747
Zm00001d015747_T022	0.7146325	0.276198432	Zm00001d015747
Zm00001d009658_T001	0.07034708	1.270825919	Zm00001d009658
Zm00001d037307_T001	0.7672826	1.001477956	Zm00001d037307
Zm00001d044671_T001	1	-0.225077578	Zm00001d044671
Zm00001d010863_T001	0.962254	0.155934837	Zm00001d010863
Zm00001d010863_T002	0.1255172	-1.545003576	Zm00001d010863
Zm00001d045403_T002	0.941922	-0.031336291	Zm00001d045403
Zm00001d012232_T003	0.9969123	0.221773681	Zm00001d012232
Zm00001d012232_T001	0.9878022	0.382775157	Zm00001d012232
Zm00001d012232_T002	0.9317791	-0.093855134	Zm00001d012232
Zm00001d050173_T001	0.9628835	0.033630702	Zm00001d050173
Zm00001d024982_T001	0.5027571	-1.181759296	Zm00001d024982
Zm00001d024982_T002	0.5424378	-0.892232267	Zm00001d024982
Zm00001d007199_T001	0.5756796	1.192431993	Zm00001d007199
Zm00001d007267_T002	0.139268	-1.069673898	Zm00001d007267
Zm00001d050026_T005	0.8863456	0.546782712	Zm00001d050026
Zm00001d050026_T003	0.4490888	0.619934799	Zm00001d050026
Zm00001d050026_T001	0.9845237	0.453360862	Zm00001d050026
Zm00001d012647_T001	0.7494913	0.989198738	Zm00001d012647
Zm00001d012647_T002	0.2123672	1.166416673	Zm00001d012647
Zm00001d039002_T004	1	0.425455195	Zm00001d039002
Zm00001d039002_T021	0.941922	0.06147074	Zm00001d039002
Zm00001d039002_T009	0.000711191	1.580250213	Zm00001d039002
Zm00001d039002_T016	1.67E-11	1.432405999	Zm00001d039002
Zm00001d039002_T027	0.1535144	0.595491492	Zm00001d039002
Zm00001d039002_T012	0.02184252	0.439090457	Zm00001d039002
Zm00001d008239_T001	0.9628812	0.128521337	Zm00001d008239
Zm00001d030672_T001	0.6993528	1.091252628	Zm00001d030672
Zm00001d041504_T004	2.35E-12	-2.370063523	Zm00001d041504
Zm00001d041504_T002	0.9818386	0.652155663	Zm00001d041504
Zm00001d010103_T003	0.9563232	0.596911088	Zm00001d010103
Zm00001d045738_T001	0.9361945	0.290804636	Zm00001d045738

Zm00001d016286_T011	0.5307031	0.683468119	Zm00001d016286
Zm00001d016286_T008	0.01713299	1.264803592	Zm00001d016286
Zm00001d016286_T001	0.5658691	-0.465554706	Zm00001d016286
Zm00001d007932_T001	0.98291	0.200798234	Zm00001d007932
Zm00001d007932_T002	0.7945048	0.436300142	Zm00001d007932
Zm00001d016431_T001	0.5717773	-0.415295256	Zm00001d016431
Zm00001d009918_T001	0.9640775	-0.010866098	Zm00001d009918
Zm00001d046047_T012	0.2181437	0.604185741	Zm00001d046047
Zm00001d046047_T009	0.2679983	0.847315788	Zm00001d046047
Zm00001d046047_T007	0.9101904	-0.018395433	Zm00001d046047
Zm00001d046047_T021	0.000388444	2.072451882	Zm00001d046047
Zm00001d046047_T001	0.9369505	0.338381395	Zm00001d046047
Zm00001d046047_T022	0.9497169	0.300786204	Zm00001d046047
Zm00001d006180_T001	0.8031754	0.763428071	Zm00001d006180
Zm00001d028050_T001	1	0.394481317	Zm00001d028050
Zm00001d041961_T001	0.9732421	0.05250558	Zm00001d041961
Zm00001d053595_T002	0.9847267	0.506043867	Zm00001d053595
Zm00001d053595_T005	0.1663456	0.283475613	Zm00001d053595
Zm00001d053595_T003	0.851347	-0.021613402	Zm00001d053595
Zm00001d053595_T004	0.9580985	0.676862726	Zm00001d053595
Zm00001d036620_T001	1	0.297298524	Zm00001d036620
Zm00001d027652_T001	0.7778727	-0.41094813	Zm00001d027652
Zm00001d010536_T001	0.9468941	-0.148429896	Zm00001d010536
Zm00001d043767_T001	0.9503115	0.004528101	Zm00001d043767
Zm00001d043767_T003	0.9944857	0.161366898	Zm00001d043767
Zm00001d043767_T006	0.957148	0.01117205	Zm00001d043767
Zm00001d043767_T004	0.4119147	-0.508891249	Zm00001d043767
Zm00001d029572_T001	0.9071546	0.449687595	Zm00001d029572
Zm00001d006503_T001	0.1601313	1.992455663	Zm00001d006503
Zm00001d030363_T001	0.822299	-0.495243868	Zm00001d030363
Zm00001d002021_T001	0.8620085	-0.491294371	Zm00001d002021
Zm00001d022622_T007	0.8197974	-0.126083422	Zm00001d022622
Zm00001d022622_T001	0.1213982	3.199584826	Zm00001d022622
Zm00001d022622_T003	0.9666757	1.520707368	Zm00001d022622
Zm00001d022622_T004	0.9779851	0.309368435	Zm00001d022622
Zm00001d047330_T001	0.1106445	1.376114185	Zm00001d047330
Zm00001d004287_T001	0.0176584	2.203577185	Zm00001d004287
Zm00001d006880_T001	0.9979568	0.180421761	Zm00001d006880
Zm00001d012138_T001	0.462123	-0.643275642	Zm00001d012138
Zm00001d047367_T015	0.3402558	1.009427667	Zm00001d047367
Zm00001d047367_T001	0.5513525	1.220194191	Zm00001d047367
Zm00001d047367_T016	0.9791729	0.415528426	Zm00001d047367
Zm00001d047367_T019	0.1323863	-1.173501563	Zm00001d047367
Zm00001d047367_T010	0.003720932	0.68001842	Zm00001d047367
Zm00001d047367_T006	0.8929316	0.770465708	Zm00001d047367
Zm00001d047367_T014	0.852305	0.388399759	Zm00001d047367
Zm00001d047367_T012	0.04550572	1.140554124	Zm00001d047367
Zm00001d047367_T003	0.8720321	1.05738318	Zm00001d047367
Zm00001d047367_T008	0.6976092	0.42373336	Zm00001d047367
Zm00001d047367_T013	0.7613827	0.904408692	Zm00001d047367
Zm00001d047367_T002	0.9498957	0.102454774	Zm00001d047367
Zm00001d047367_T020	1.60E-07	-1.72320141	Zm00001d047367
Zm00001d012413_T001	0.9306419	0.576171899	Zm00001d012413
Zm00001d038381_T001	0.8720862	-0.327057578	Zm00001d038381

Zm00001d053735_T001	0.7682041	0.579575797	Zm00001d053735
Zm00001d053217_T001	0.9514141	0.006859219	Zm00001d053217
Zm00001d029047_T001	0.8664689	0.965130832	Zm00001d029047
Zm00001d005682_T001	0.8743698	0.863890818	Zm00001d005682
Zm00001d051534_T001	0.935477	0.531517939	Zm00001d051534
Zm00001d035171_T001	0.6485803	-0.906921129	Zm00001d035171
Zm00001d044654_T001	0.05519073	-0.478330827	Zm00001d044654
Zm00001d022272_T004	0.1280206	-1.667631824	Zm00001d022272
Zm00001d022272_T003	0.9781672	0.474460598	Zm00001d022272
Zm00001d022272_T001	0.8705378	-0.052467121	Zm00001d022272
Zm00001d022272_T002	0.6506202	1.026070745	Zm00001d022272
Zm00001d044316_T001	0.9203655	0.34384387	Zm00001d044316
Zm00001d048776_T001	0.7809834	-0.265993605	Zm00001d048776
Zm00001d047932_T001	0.9999526	0.220285892	Zm00001d047932
Zm00001d011352_T001	0.9486412	0.122271735	Zm00001d011352
Zm00001d031213_T001	0.3497147	-0.804253076	Zm00001d031213
Zm00001d025617_T009	0.9824348	-0.010355446	Zm00001d025617
Zm00001d025617_T005	0.8056124	0.331951092	Zm00001d025617
Zm00001d025617_T002	0.4025336	0.756090052	Zm00001d025617
Zm00001d025617_T006	0.08984708	-0.227405499	Zm00001d025617
Zm00001d025617_T003	0.9404125	0.924348599	Zm00001d025617
Zm00001d025617_T001	0.8105266	-0.222630616	Zm00001d025617
Zm00001d034797_T001	0.9072584	-0.029753081	Zm00001d034797
Zm00001d036708_T001	0.9863483	-0.232233475	Zm00001d036708
Zm00001d009364_T001	0.9918302	0.448327305	Zm00001d009364
Zm00001d027734_T006	0.8714678	0.833771217	Zm00001d027734
Zm00001d027734_T001	0.04241715	2.343630242	Zm00001d027734
Zm00001d027734_T007	0.9398534	0.114512697	Zm00001d027734
Zm00001d027734_T002	0.9416622	0.07964287	Zm00001d027734
Zm00001d027734_T005	0.7097086	-0.397557978	Zm00001d027734
Zm00001d050043_T006	0.4029716	-0.513198504	Zm00001d050043
Zm00001d050043_T005	1	0.526695876	Zm00001d050043
Zm00001d050043_T004	0.900806	-0.09816096	Zm00001d050043
Zm00001d050043_T002	0.4403985	-0.285783239	Zm00001d050043
Zm00001d039480_T001	0.946422	0.27257396	Zm00001d039480
Zm00001d023994_T004	0.000129541	-2.849295235	Zm00001d023994
Zm00001d023994_T003	0.6528028	-0.378658227	Zm00001d023994
Zm00001d023994_T002	0.8064667	-0.213270093	Zm00001d023994
Zm00001d005208_T001	0.000844821	3.547954795	Zm00001d005208
Zm00001d032806_T001	0.9197676	-0.011830223	Zm00001d032806
Zm00001d014765_T001	0.9419336	-0.010036809	Zm00001d014765
Zm00001d011912_T004	0.2770066	-0.902929657	Zm00001d011912
Zm00001d042772_T002	0.8757546	-0.185148563	Zm00001d042772
Zm00001d042772_T004	0.6781675	-0.577616417	Zm00001d042772
Zm00001d042772_T003	0.5936419	-0.169842786	Zm00001d042772
Zm00001d036726_T001	1.15E-06	4.191875243	Zm00001d036726
Zm00001d025901_T001	0.9050121	-0.070324862	Zm00001d025901
Zm00001d033483_T001	0.9846714	0.502259536	Zm00001d033483
Zm00001d041708_T002	0.07730424	0.897382205	Zm00001d041708
Zm00001d041708_T001	0.9521719	0.091241648	Zm00001d041708
Zm00001d036454_T004	0.6284	-0.440390026	Zm00001d036454
Zm00001d036454_T002	0.9160053	0.225159762	Zm00001d036454
Zm00001d036454_T005	0.416815	-0.754668574	Zm00001d036454
Zm00001d036454_T003	0.5273755	-0.677799414	Zm00001d036454

Zm00001d036454_T001	0.9469426	0.033296094	Zm00001d036454
Zm00001d008369_T006	0.9745948	0.566871366	Zm00001d008369
Zm00001d008369_T008	0.5411872	-0.674441612	Zm00001d008369
Zm00001d008369_T004	0.9233022	-0.028860417	Zm00001d008369
Zm00001d053883_T001	0.9160053	-0.096987242	Zm00001d053883
Zm00001d052933_T001	0.8896102	-0.062499686	Zm00001d052933
Zm00001d042050_T001	0.9365531	0.011763598	Zm00001d042050
Zm00001d033682_T004	0.9824348	0.19937714	Zm00001d033682
Zm00001d033682_T002	1	0.424185518	Zm00001d033682
Zm00001d033855_T003	0.9706887	0.554144796	Zm00001d033855
Zm00001d033855_T002	0.7490886	1.100849609	Zm00001d033855
Zm00001d033855_T004	0.4949263	1.5141482	Zm00001d033855
Zm00001d050008_T001	0.2015088	1.863311118	Zm00001d050008
Zm00001d011283_T005	0.9860836	0.373638042	Zm00001d011283
Zm00001d011283_T002	0.7234221	-0.33935933	Zm00001d011283
Zm00001d011283_T006	0.7980206	0.642691138	Zm00001d011283
Zm00001d043814_T001	0.8041769	0.647158088	Zm00001d043814
Zm00001d043814_T002	1	0.323249167	Zm00001d043814
Zm00001d043814_T003	0.2790987	-1.467393228	Zm00001d043814
Zm00001d052666_T009	1.13E-10	1.426348184	Zm00001d052666
Zm00001d052666_T103	0.9201237	-0.457626451	Zm00001d052666
Zm00001d052666_T100	0.4760609	-0.593815297	Zm00001d052666
Zm00001d052666_T089	0.7458694	-0.043600719	Zm00001d052666
Zm00001d052666_T017	2.04E-07	0.805794304	Zm00001d052666
Zm00001d004733_T001	0.511966	-1.243004708	Zm00001d004733
Zm00001d028890_T014	0.6258249	0.969320975	Zm00001d028890
Zm00001d028890_T008	1	0.111574806	Zm00001d028890
Zm00001d028890_T018	0.7095161	0.987740049	Zm00001d028890
Zm00001d028890_T005	0.1010062	-0.454964656	Zm00001d028890
Zm00001d022321_T010	0.9582899	0.424759944	Zm00001d022321
Zm00001d022321_T012	0.9786754	0.312695457	Zm00001d022321
Zm00001d022321_T006	0.9173494	-0.064738394	Zm00001d022321
Zm00001d022321_T001	0.9825858	0.39247317	Zm00001d022321
Zm00001d021774_T001	0.9867452	0.125999346	Zm00001d021774
Zm00001d003047_T001	0.9654804	0.394747713	Zm00001d003047
Zm00001d013534_T002	0.7227713	-0.486133046	Zm00001d013534
Zm00001d013534_T001	0.9687311	0.05163485	Zm00001d013534
Zm00001d011971_T001	0.9320554	0.016491143	Zm00001d011971
Zm00001d014671_T001	0.9999526	0.42490191	Zm00001d014671
Zm00001d005264_T001	1	0.434512478	Zm00001d005264
Zm00001d017593_T002	0.1826126	1.285945925	Zm00001d017593
Zm00001d017593_T001	0.05692626	1.272363421	Zm00001d017593
Zm00001d019312_T001	0.3221815	-1.013436929	Zm00001d019312
Zm00001d019312_T002	0.4178453	-0.866545256	Zm00001d019312
Zm00001d039634_T001	0.9184323	0.00135974	Zm00001d039634
Zm00001d032668_T001	0.6948891	-0.563521045	Zm00001d032668
Zm00001d030603_T001	0.4028633	-0.693058008	Zm00001d030603
Zm00001d040244_T003	0.9429712	0.366634091	Zm00001d040244
Zm00001d040244_T001	0.9954426	0.145569511	Zm00001d040244
Zm00001d037810_T020	0.7400422	-0.111786786	Zm00001d037810
Zm00001d037810_T005	1	0.02289921	Zm00001d037810
Zm00001d037810_T024	0.9318116	0.080305077	Zm00001d037810
Zm00001d037810_T026	0.5215715	-0.522849034	Zm00001d037810
Zm00001d037810_T016	0.3071359	-0.42797488	Zm00001d037810

Zm00001d037810_T001	0.02212787	-0.824793843	Zm00001d037810
Zm00001d042078_T001	0.923446	-0.304460796	Zm00001d042078
Zm00001d053731_T001	0.96809	-0.158108307	Zm00001d053731
Zm00001d007502_T001	1	0.218346146	Zm00001d007502
Zm00001d036016_T001	0.1428962	1.615187855	Zm00001d036016
Zm00001d031640_T001	0.8031436	-0.290260141	Zm00001d031640
Zm00001d014497_T001	0.9817074	0.236545215	Zm00001d014497
Zm00001d013009_T001	0.9018448	-0.163250709	Zm00001d013009
Zm00001d044037_T001	0.9641165	0.105271704	Zm00001d044037
Zm00001d040191_T006	5.60E-09	1.032902721	Zm00001d040191
Zm00001d040191_T001	0.9779656	0.575102167	Zm00001d040191
Zm00001d040191_T007	6.92E-09	-0.792824001	Zm00001d040191
Zm00001d040191_T008	1	0.196052529	Zm00001d040191
Zm00001d020505_T001	0.5440777	1.246667235	Zm00001d020505
Zm00001d002462_T021	0.5183158	0.704561225	Zm00001d002462
Zm00001d002462_T067	0.2719242	0.439157983	Zm00001d002462
Zm00001d002462_T074	0.6501259	0.686837452	Zm00001d002462
Zm00001d002462_T060	0.8591203	0.136104142	Zm00001d002462
Zm00001d002462_T191	0.3450234	1.790733663	Zm00001d002462
Zm00001d002462_T179	0.2782826	0.663973972	Zm00001d002462
Zm00001d002462_T165	1.20E-09	1.170579938	Zm00001d002462
Zm00001d002462_T011	1	0.030911264	Zm00001d002462
Zm00001d002462_T185	0.8913084	-0.164734489	Zm00001d002462
Zm00001d002462_T181	0.6572222	-0.088110849	Zm00001d002462
Zm00001d002462_T189	0.9363424	0.832112272	Zm00001d002462
Zm00001d002462_T149	0.4342973	0.611818792	Zm00001d002462
Zm00001d002462_T176	0.3237726	1.473072728	Zm00001d002462
Zm00001d002462_T187	0.9867938	-0.032397336	Zm00001d002462
Zm00001d002462_T073	0.5572292	-0.403049038	Zm00001d002462
Zm00001d002462_T079	0.00012109	0.847841782	Zm00001d002462
Zm00001d002462_T171	0.8941695	0.024232104	Zm00001d002462
Zm00001d002462_T116	0.001432304	0.662812999	Zm00001d002462
Zm00001d002462_T001	0.000805013	0.510289587	Zm00001d002462
Zm00001d002462_T190	0.5903112	0.475672553	Zm00001d002462
Zm00001d002462_T087	0.00063647	1.289927538	Zm00001d002462
Zm00001d042372_T002	1	0.067232219	Zm00001d042372
Zm00001d037050_T001	0.6201695	-0.243899226	Zm00001d037050
Zm00001d037050_T002	0.9429712	0.060049794	Zm00001d037050
Zm00001d006755_T001	0.9822138	0.482880182	Zm00001d006755
Zm00001d018282_T001	0.9722469	0.622375085	Zm00001d018282
Zm00001d045978_T005	0.9979568	0.216134743	Zm00001d045978
Zm00001d051474_T001	0.6508204	-0.378962717	Zm00001d051474
Zm00001d014491_T001	0.01855432	2.790799055	Zm00001d014491
Zm00001d012435_T001	0.7531776	-0.281895489	Zm00001d012435
Zm00001d008663_T002	0.9887889	0.472662058	Zm00001d008663
Zm00001d005008_T003	0.04573669	-0.499931383	Zm00001d005008
Zm00001d005008_T004	0.9166818	-0.138040479	Zm00001d005008
Zm00001d029120_T005	0.2801949	-0.808698783	Zm00001d029120
Zm00001d029120_T002	0.05938391	2.040891517	Zm00001d029120
Zm00001d029120_T001	0.09000944	0.963514843	Zm00001d029120
Zm00001d029120_T004	0.7097086	-0.05864286	Zm00001d029120
Zm00001d029120_T014	0.5811896	-0.312006779	Zm00001d029120
Zm00001d029120_T010	0.4341643	1.834787353	Zm00001d029120
Zm00001d029120_T008	0.02845695	0.612972709	Zm00001d029120

Zm00001d052213_T004	0.2982359	-0.572418628	Zm00001d052213
Zm00001d052213_T001	0.9988864	0.342094514	Zm00001d052213
Zm00001d052213_T003	0.5424653	1.089377614	Zm00001d052213
Zm00001d047560_T001	0.1934531	-1.447754802	Zm00001d047560
Zm00001d012821_T007	0.4647287	1.284413935	Zm00001d012821
Zm00001d012821_T002	0.002046598	2.177646174	Zm00001d012821
Zm00001d012821_T009	0.9500229	0.11009787	Zm00001d012821
Zm00001d012821_T003	0.1966199	-0.344590195	Zm00001d012821
Zm00001d012821_T004	0.9475709	0.136924265	Zm00001d012821
Zm00001d012821_T008	0.677308	-0.28531224	Zm00001d012821
Zm00001d012821_T005	0.8819513	0.700143094	Zm00001d012821
Zm00001d038865_T001	0.8513643	-0.456450461	Zm00001d038865
Zm00001d038865_T012	0.000104571	-2.057645563	Zm00001d038865
Zm00001d038865_T002	0.00080941	-1.369425374	Zm00001d038865
Zm00001d038865_T006	0.8863456	-0.391648845	Zm00001d038865
Zm00001d038865_T009	0.8545272	-0.541144723	Zm00001d038865
Zm00001d038865_T013	0.6338908	-1.04289574	Zm00001d038865
Zm00001d009011_T002	0.7901736	-0.186306629	Zm00001d009011
Zm00001d038531_T001	0.4860327	-0.71824675	Zm00001d038531
Zm00001d038531_T004	0.9883036	0.209351814	Zm00001d038531
Zm00001d038531_T003	0.98291	0.481483675	Zm00001d038531
Zm00001d044301_T002	0.8585804	0.572315296	Zm00001d044301
Zm00001d044301_T001	0.4641164	1.509745793	Zm00001d044301
Zm00001d027895_T004	0.001005617	1.423168883	Zm00001d027895
Zm00001d027895_T014	0.197702	1.519249171	Zm00001d027895
Zm00001d027895_T006	0.1671483	0.8276036	Zm00001d027895
Zm00001d027895_T019	0.9722469	0.182492774	Zm00001d027895
Zm00001d027895_T002	0.9979568	0.357850143	Zm00001d027895
Zm00001d027895_T032	0.8559428	-0.156484755	Zm00001d027895
Zm00001d023867_T013	0.6185011	0.483951685	Zm00001d023867
Zm00001d023867_T017	0.1565832	-0.741102424	Zm00001d023867
Zm00001d023867_T003	0.7889601	1.012944211	Zm00001d023867
Zm00001d023867_T001	0.7483641	0.35859499	Zm00001d023867
Zm00001d028596_T001	0.983226	0.452175238	Zm00001d028596
Zm00001d029980_T001	0.8778391	0.216201781	Zm00001d029980
Zm00001d029980_T003	0.9203655	-0.062802427	Zm00001d029980
Zm00001d002956_T003	0.6395973	0.849718599	Zm00001d002956
Zm00001d002956_T004	0.99345	0.210435489	Zm00001d002956
Zm00001d002956_T005	0.4146004	1.96596116	Zm00001d002956
Zm00001d002956_T001	0.7904894	-0.221136169	Zm00001d002956
Zm00001d049192_T002	0.9361945	0.730217933	Zm00001d049192
Zm00001d049192_T001	0.6439227	0.383355991	Zm00001d049192
Zm00001d026273_T003	0.9975882	0.263434192	Zm00001d026273
Zm00001d010910_T002	0.8293703	0.548618774	Zm00001d010910
Zm00001d010910_T001	0.9693993	0.05915622	Zm00001d010910
Zm00001d010910_T004	0.412226	-1.103819805	Zm00001d010910
Zm00001d019479_T002	0.01477598	-0.878618494	Zm00001d019479
Zm00001d019479_T006	0.9988864	0.209184602	Zm00001d019479
Zm00001d007204_T003	0.967161	0.01782993	Zm00001d007204
Zm00001d042102_T001	1	0.118962702	Zm00001d042102
Zm00001d033734_T002	0.9959972	0.5215756	Zm00001d033734
Zm00001d043280_T001	1	0.336779383	Zm00001d043280
Zm00001d043280_T006	0.9993872	0.39616909	Zm00001d043280
Zm00001d043280_T008	0.98291	0.188511318	Zm00001d043280

Zm00001d047793_T001	0.7927022	-0.263108779	Zm00001d047793
Zm00001d049260_T001	0.9988864	0.358669878	Zm00001d049260
Zm00001d025652_T005	0.8142464	0.720834866	Zm00001d025652
Zm00001d025652_T004	0.8591203	-0.179801167	Zm00001d025652
Zm00001d005482_T001	0.9422098	-0.08719025	Zm00001d005482
Zm00001d005482_T002	0.7483641	-0.329492176	Zm00001d005482
Zm00001d019492_T001	0.9071836	-0.077456655	Zm00001d019492
Zm00001d012891_T001	0.9469426	0.01856113	Zm00001d012891
Zm00001d013043_T003	0.3166233	-0.847136484	Zm00001d013043
Zm00001d013043_T001	0.5954619	-0.573104074	Zm00001d013043
Zm00001d013043_T004	0.999573	0.285317445	Zm00001d013043
Zm00001d024082_T001	0.8695779	-0.254678288	Zm00001d024082
Zm00001d048090_T002	0.9311027	0.064949145	Zm00001d048090
Zm00001d021227_T003	0.8617755	0.91571048	Zm00001d021227
Zm00001d021227_T004	0.091622	-1.381442749	Zm00001d021227
Zm00001d021227_T001	0.447037	1.214503511	Zm00001d021227
Zm00001d052239_T001	0.9979568	0.320491589	Zm00001d052239
Zm00001d003457_T001	0.850642	-0.295263248	Zm00001d003457
Zm00001d002751_T001	0.9570268	0.252257385	Zm00001d002751
Zm00001d002751_T004	0.9535734	0.30333668	Zm00001d002751
Zm00001d002751_T002	0.9718747	0.158887122	Zm00001d002751
Zm00001d039546_T001	0.9832612	0.267620054	Zm00001d039546
Zm00001d039546_T003	0.7740061	-0.249814519	Zm00001d039546
Zm00001d039546_T011	0.9851204	-0.007131684	Zm00001d039546
Zm00001d039546_T010	0.6625969	0.886683565	Zm00001d039546
Zm00001d014533_T002	0.9944178	0.434064927	Zm00001d014533
Zm00001d014533_T003	1	0.165558673	Zm00001d014533
Zm00001d014533_T001	0.9596377	0.086862605	Zm00001d014533
Zm00001d011241_T001	0.9298013	0.553756048	Zm00001d011241
Zm00001d014911_T017	0.7051648	0.442365388	Zm00001d014911
Zm00001d014911_T002	0.8219372	-0.106643959	Zm00001d014911
Zm00001d014911_T011	0.6190042	0.856561961	Zm00001d014911
Zm00001d014911_T019	0.9846714	0.063745135	Zm00001d014911
Zm00001d014911_T008	0.06187513	-1.194713886	Zm00001d014911
Zm00001d014911_T015	0.9706293	0.346457904	Zm00001d014911
Zm00001d020426_T001	0.998484	0.309238989	Zm00001d020426
Zm00001d039361_T001	0.8047831	0.923986321	Zm00001d039361
Zm00001d011843_T001	0.9862402	0.385850137	Zm00001d011843
Zm00001d040429_T007	0.3100253	-1.202025888	Zm00001d040429
Zm00001d040429_T012	0.1735732	0.351370057	Zm00001d040429
Zm00001d040429_T003	0.9867452	0.501146716	Zm00001d040429
Zm00001d017288_T001	0.9950761	0.397000312	Zm00001d017288
Zm00001d036905_T004	0.8231712	-0.164169606	Zm00001d036905
Zm00001d036905_T006	0.5954619	-0.002515826	Zm00001d036905
Zm00001d036905_T001	0.2110444	2.266246761	Zm00001d036905
Zm00001d036905_T003	1	0.106636685	Zm00001d036905
Zm00001d015186_T001	0.8955831	0.864615121	Zm00001d015186
Zm00001d027373_T004	0.9717213	0.124652092	Zm00001d027373
Zm00001d014341_T001	0.3009522	-0.835731581	Zm00001d014341
Zm00001d040789_T004	0.9580985	0.043444696	Zm00001d040789
Zm00001d040789_T002	0.9986486	0.430084154	Zm00001d040789
Zm00001d037972_T001	0.8340819	-0.774124226	Zm00001d037972
Zm00001d032263_T002	0.9216521	0.372472834	Zm00001d032263
Zm00001d032263_T001	0.9984041	0.152781441	Zm00001d032263

Zm00001d039110_T001	0.5495068	1.121678884	Zm00001d039110
Zm00001d046579_T029	0.2793723	1.396277693	Zm00001d046579
Zm00001d046579_T020	4.35E-05	0.700488975	Zm00001d046579
Zm00001d046579_T003	0.6231517	0.484291836	Zm00001d046579
Zm00001d046579_T034	0.3975776	-0.764635164	Zm00001d046579
Zm00001d046579_T031	0.4701937	0.364365752	Zm00001d046579
Zm00001d046579_T016	0.1185825	1.719959955	Zm00001d046579
Zm00001d046579_T027	0.007738702	1.064858744	Zm00001d046579
Zm00001d017071_T001	0.8896831	-0.052705255	Zm00001d017071
Zm00001d028923_T002	0.934325	0.728948808	Zm00001d028923
Zm00001d028923_T005	0.6012987	-0.852817443	Zm00001d028923
Zm00001d028923_T004	0.7732332	0.483185651	Zm00001d028923
Zm00001d028923_T008	0.9664736	0.291109497	Zm00001d028923
Zm00001d028923_T009	5.53E-12	-3.205847995	Zm00001d028923
Zm00001d017649_T001	0.852305	0.907744036	Zm00001d017649
Zm00001d017169_T001	0.9565958	-0.305149209	Zm00001d017169
Zm00001d034152_T002	0.8569937	0.957900407	Zm00001d034152
Zm00001d034152_T005	0.09028577	0.929075691	Zm00001d034152
Zm00001d042654_T003	0.9404627	0.593149934	Zm00001d042654
Zm00001d042654_T011	0.07220675	0.804563376	Zm00001d042654
Zm00001d042654_T009	0.9745948	0.153826168	Zm00001d042654
Zm00001d042654_T006	2.35E-09	-1.693437634	Zm00001d042654
Zm00001d026308_T001	0.9686323	0.380382917	Zm00001d026308
Zm00001d035617_T001	0.148906	-2.924487943	Zm00001d035617
Zm00001d031265_T034	0.9127665	-0.038203575	Zm00001d031265
Zm00001d031265_T024	0.9219195	0.003737434	Zm00001d031265
Zm00001d031265_T023	0.9090332	-0.075053827	Zm00001d031265
Zm00001d031265_T028	0.9543237	0.038837757	Zm00001d031265
Zm00001d013967_T019	0.8321156	-0.120511774	Zm00001d013967
Zm00001d013967_T015	0.04830133	0.918849217	Zm00001d013967
Zm00001d021016_T001	1	0.37480305	Zm00001d021016
Zm00001d020989_T011	0.9647033	0.427999816	Zm00001d020989
Zm00001d020989_T014	0.5640102	-0.451661084	Zm00001d020989
Zm00001d020989_T008	0.7212793	0.694528735	Zm00001d020989
Zm00001d025407_T001	0.8958882	-0.261875281	Zm00001d025407
Zm00001d016800_T001	1	0.366179705	Zm00001d016800
Zm00001d034108_T008	0.683785	0.777260051	Zm00001d034108
Zm00001d034108_T005	0.6468352	-0.15959153	Zm00001d034108
Zm00001d034108_T001	1	0.0899368	Zm00001d034108
Zm00001d034108_T006	0.8647028	-0.71160372	Zm00001d034108
Zm00001d034108_T007	0.172777	2.339294818	Zm00001d034108
Zm00001d034108_T009	0.3095834	-0.709210874	Zm00001d034108
Zm00001d007360_T001	0.517208	-0.667538185	Zm00001d007360
Zm00001d018158_T001	8.75E-07	3.628329975	Zm00001d018158
Zm00001d014153_T001	0.5864438	-0.799847354	Zm00001d014153
Zm00001d024016_T001	0.9988864	0.131974004	Zm00001d024016
Zm00001d036086_T001	0.894759	-0.104724703	Zm00001d036086
Zm00001d005350_T011	0.885586	-0.091529887	Zm00001d005350
Zm00001d005350_T006	0.1560295	0.347421981	Zm00001d005350
Zm00001d005350_T001	0.7059883	0.534784321	Zm00001d005350
Zm00001d040446_T001	0.9779851	0.589096491	Zm00001d040446
Zm00001d051588_T001	0.9617681	-0.234663268	Zm00001d051588
Zm00001d052457_T001	0.7315461	1.181788246	Zm00001d052457
Zm00001d052457_T002	0.9910333	0.094368495	Zm00001d052457

Zm00001d050609_T001	0.8537544	0.566119729	Zm00001d050609
Zm00001d015129_T001	0.9334932	0.486262943	Zm00001d015129
Zm00001d005195_T003	0.01234545	-0.606236728	Zm00001d005195
Zm00001d005195_T008	0.1326286	1.079953772	Zm00001d005195
Zm00001d005195_T013	0.7737238	0.088446095	Zm00001d005195
Zm00001d005195_T014	0.7737238	-0.242561403	Zm00001d005195
Zm00001d005195_T007	0.5970131	-0.812269616	Zm00001d005195
Zm00001d005195_T001	0.9878764	0.193107767	Zm00001d005195
Zm00001d005195_T004	2.91E-08	1.254091338	Zm00001d005195
Zm00001d005195_T006	0.8659393	0.248567832	Zm00001d005195
Zm00001d005195_T002	0.05922369	1.071424023	Zm00001d005195
Zm00001d008530_T001	0.8670002	-0.29064523	Zm00001d008530
Zm00001d022432_T003	0.9690925	-0.041406976	Zm00001d022432
Zm00001d022432_T001	0.9201237	0.789370076	Zm00001d022432
Zm00001d022432_T004	0.435442	-0.609193864	Zm00001d022432
Zm00001d022432_T002	0.2271365	-0.796234675	Zm00001d022432
Zm00001d032754_T002	0.9902022	0.212920327	Zm00001d032754
Zm00001d032754_T003	0.9509252	0.317871075	Zm00001d032754
Zm00001d032754_T001	0.4137215	-0.7318298	Zm00001d032754
Zm00001d037943_T001	0.6394238	-0.637006518	Zm00001d037943
Zm00001d006769_T001	0.3614644	1.195240366	Zm00001d006769
Zm00001d006769_T008	0.000340711	-1.852359448	Zm00001d006769
Zm00001d006769_T003	1	0.365690657	Zm00001d006769
Zm00001d006769_T009	0.9442138	0.039673154	Zm00001d006769
Zm00001d017041_T001	0.1698955	-1.112261985	Zm00001d017041
Zm00001d044694_T001	0.9734722	0.42203925	Zm00001d044694
Zm00001d044694_T002	0.9970835	0.4119768	Zm00001d044694
Zm00001d016948_T001	0.07378614	2.774629044	Zm00001d016948
Zm00001d029826_T003	0.9061819	-0.472615115	Zm00001d029826
Zm00001d029826_T006	0.955464	-0.410747738	Zm00001d029826
Zm00001d029826_T002	0.974657	0.086209369	Zm00001d029826
Zm00001d007799_T002	0.8010758	0.476338202	Zm00001d007799
Zm00001d007799_T001	0.8175516	-0.215699731	Zm00001d007799
Zm00001d033504_T001	0.9702457	0.167722622	Zm00001d033504
Zm00001d013641_T001	0.998484	0.127937219	Zm00001d013641
Zm00001d036035_T001	0.6530894	-0.892942818	Zm00001d036035
Zm00001d021189_T001	0.9945118	0.266917457	Zm00001d021189
Zm00001d035747_T021	0.7697196	1.057060709	Zm00001d035747
Zm00001d035747_T009	0.2853231	0.751579292	Zm00001d035747
Zm00001d035747_T013	0.7594555	0.214159851	Zm00001d035747
Zm00001d035747_T096	0.9745777	0.216442217	Zm00001d035747
Zm00001d035747_T027	0.622153	0.295427255	Zm00001d035747
Zm00001d004009_T004	0.8993746	-0.416401682	Zm00001d004009
Zm00001d004009_T007	0.103478	-2.020918333	Zm00001d004009
Zm00001d004009_T005	0.3212723	-1.127775241	Zm00001d004009
Zm00001d004009_T006	0.8929316	-0.063923004	Zm00001d004009
Zm00001d000039_T001	0.6507629	1.020392137	Zm00001d000039
Zm00001d000039_T002	0.1797941	-0.403883965	Zm00001d000039
Zm00001d046590_T001	0.9987435	0.461974284	Zm00001d046590
Zm00001d046590_T003	0.3299169	1.053930743	Zm00001d046590
Zm00001d029978_T003	0.6185011	0.381833448	Zm00001d029978
Zm00001d029978_T004	1	0.085438738	Zm00001d029978
Zm00001d029978_T002	0.9791729	0.155527847	Zm00001d029978
Zm00001d029978_T001	0.9514141	0.074347129	Zm00001d029978

Zm00001d022496_T001	0.9193633	0.744939772	Zm00001d022496
Zm00001d031459_T001	0.9867452	0.127276707	Zm00001d031459
Zm00001d039163_T002	0.9690925	0.583594589	Zm00001d039163
Zm00001d045042_T020	0.235536	-1.108817736	Zm00001d045042
Zm00001d045042_T002	0.03688028	-0.592386708	Zm00001d045042
Zm00001d045042_T007	1	0.701935504	Zm00001d045042
Zm00001d045042_T013	0.9186216	0.402288613	Zm00001d045042
Zm00001d045042_T015	0.005686021	-2.002243528	Zm00001d045042
Zm00001d045042_T010	0.000630292	0.606148327	Zm00001d045042
Zm00001d045042_T005	0.7777722	-0.0568646	Zm00001d045042
Zm00001d045042_T019	0.9719113	1.189816431	Zm00001d045042
Zm00001d019059_T001	0.8529478	-0.289805963	Zm00001d019059
Zm00001d033273_T006	0.8746701	-0.093071955	Zm00001d033273
Zm00001d033273_T001	0.000294231	1.528418457	Zm00001d033273
Zm00001d033273_T010	0.1585484	-1.544958191	Zm00001d033273
Zm00001d033273_T009	0.1692579	1.579921824	Zm00001d033273
Zm00001d010954_T001	0.8489797	-0.27160382	Zm00001d010954
Zm00001d023492_T001	0.5455802	1.006229982	Zm00001d023492
Zm00001d023492_T002	0.9229883	-0.101889273	Zm00001d023492
Zm00001d024767_T005	0.7877461	-0.250117218	Zm00001d024767
Zm00001d024767_T007	0.9950703	1.259126146	Zm00001d024767
Zm00001d024767_T008	0.3952681	-1.15771655	Zm00001d024767
Zm00001d024767_T002	0.8863456	0.947137483	Zm00001d024767
Zm00001d024767_T003	0.9936409	0.31300141	Zm00001d024767
Zm00001d026236_T001	0.9400055	-0.131153524	Zm00001d026236
Zm00001d052718_T001	0.7270031	-0.574178106	Zm00001d052718
Zm00001d034814_T002	0.5587524	0.908342481	Zm00001d034814
Zm00001d034814_T001	0.954492	0.640937977	Zm00001d034814
Zm00001d012339_T001	0.7319413	-0.503320704	Zm00001d012339
Zm00001d005164_T010	0.4052048	0.83780393	Zm00001d005164
Zm00001d005164_T020	0.02700349	1.105964868	Zm00001d005164
Zm00001d005164_T015	0.000260831	2.781069569	Zm00001d005164
Zm00001d005164_T016	1.14E-06	1.560515429	Zm00001d005164
Zm00001d005164_T011	0.04054157	0.456280661	Zm00001d005164
Zm00001d005164_T001	0.6523514	1.261717915	Zm00001d005164
Zm00001d053352_T001	0.9748909	-0.05488783	Zm00001d053352
Zm00001d051427_T008	0.8345927	0.815542816	Zm00001d051427
Zm00001d051427_T004	0.9887584	0.510725628	Zm00001d051427
Zm00001d051427_T006	0.812161	-0.54106527	Zm00001d051427
Zm00001d051427_T003	0.9887584	0.445966832	Zm00001d051427
Zm00001d002919_T001	0.9921225	0.267634364	Zm00001d002919
Zm00001d024406_T002	0.9939021	0.195925087	Zm00001d024406
Zm00001d037729_T001	0.3353271	-0.846849158	Zm00001d037729
Zm00001d005038_T001	0.5709823	-0.630276808	Zm00001d005038
Zm00001d023223_T001	0.5096243	-0.822125437	Zm00001d023223
Zm00001d023223_T003	0.3015167	-0.625504127	Zm00001d023223
Zm00001d032520_T001	0.5269508	1.136421223	Zm00001d032520
Zm00001d032520_T002	0.9903568	0.469201026	Zm00001d032520
Zm00001d024222_T001	0.9923429	0.192371838	Zm00001d024222
Zm00001d002837_T003	0.9177108	0.406479773	Zm00001d002837
Zm00001d002837_T002	0.9954444	0.302817268	Zm00001d002837
Zm00001d016950_T001	0.8210082	0.822839625	Zm00001d016950
Zm00001d016950_T002	0.5311736	1.277830093	Zm00001d016950
Zm00001d050575_T004	0.6214202	-0.223042893	Zm00001d050575

Zm00001d050575_T001	0.9201341	0.766196243	Zm00001d050575
Zm00001d053254_T001	0.9073468	-0.018763958	Zm00001d053254
Zm00001d017618_T001	0.8310488	0.531578197	Zm00001d017618
Zm00001d006016_T001	0.8160256	0.584734871	Zm00001d006016
Zm00001d027291_T001	0.525543	-0.647663746	Zm00001d027291
Zm00001d046210_T003	0.2270984	1.517280772	Zm00001d046210
Zm00001d046210_T006	0.4959955	0.89745242	Zm00001d046210
Zm00001d046210_T013	0.9791066	0.158616226	Zm00001d046210
Zm00001d046210_T009	0.7223966	0.609632796	Zm00001d046210
Zm00001d046210_T012	0.5864438	1.315626357	Zm00001d046210
Zm00001d046210_T002	0.941809	0.595934966	Zm00001d046210
Zm00001d046210_T004	0.4286831	1.231825325	Zm00001d046210
Zm00001d046210_T011	0.03830215	-0.788883884	Zm00001d046210
Zm00001d046210_T008	0.004572431	1.364690467	Zm00001d046210
Zm00001d046210_T005	0.4758019	1.133768519	Zm00001d046210
Zm00001d046210_T010	0.7367876	0.423244853	Zm00001d046210
Zm00001d046210_T014	0.9441446	-0.234451532	Zm00001d046210
Zm00001d030391_T001	0.9845237	0.474905391	Zm00001d030391
Zm00001d038980_T001	0.9975882	0.236647018	Zm00001d038980
Zm00001d014953_T001	0.6197322	0.306820684	Zm00001d014953
Zm00001d014953_T002	1	0.364252785	Zm00001d014953
Zm00001d043617_T001	0.07601567	-0.718079213	Zm00001d043617
Zm00001d037751_T001	0.8489797	0.663276885	Zm00001d037751
Zm00001d032980_T046	0.1339192	-0.424577954	Zm00001d032980
Zm00001d032980_T052	0.9561001	0.092508282	Zm00001d032980
Zm00001d032980_T056	0.923446	0.401321289	Zm00001d032980
Zm00001d032980_T031	0.9971866	0.077316415	Zm00001d032980
Zm00001d032980_T053	0.8886159	-0.097031749	Zm00001d032980
Zm00001d032980_T049	0.4045966	1.357468327	Zm00001d032980
Zm00001d032980_T020	0.8357705	0.668928678	Zm00001d032980
Zm00001d032980_T006	0.9690925	0.606214991	Zm00001d032980
Zm00001d032980_T054	0.5340174	0.313004943	Zm00001d032980
Zm00001d029965_T001	0.9979568	0.364857279	Zm00001d029965
Zm00001d006787_T001	0.9207666	-0.059181731	Zm00001d006787
Zm00001d052401_T001	0.9422098	0.619390015	Zm00001d052401
Zm00001d019890_T001	0.9533988	0.147251678	Zm00001d019890
Zm00001d044753_T001	0.9970835	0.292853971	Zm00001d044753
Zm00001d010731_T002	0.8832328	-0.147621949	Zm00001d010731
Zm00001d010731_T001	0.9887889	0.266982898	Zm00001d010731
Zm00001d012165_T001	0.6059172	-0.931055368	Zm00001d012165
Zm00001d052307_T015	0.8450595	-0.122662716	Zm00001d052307
Zm00001d052307_T001	0.02535018	1.614017134	Zm00001d052307
Zm00001d052307_T002	0.6967776	-0.405446981	Zm00001d052307
Zm00001d045072_T029	0.806084	-0.109588651	Zm00001d045072
Zm00001d045072_T012	0.7700786	-0.271916189	Zm00001d045072
Zm00001d045072_T037	0.01234628	1.121275262	Zm00001d045072
Zm00001d045072_T010	0.2217688	0.482634355	Zm00001d045072
Zm00001d045072_T004	0.7020458	0.256386531	Zm00001d045072
Zm00001d045072_T033	0.5268107	0.791170398	Zm00001d045072
Zm00001d045072_T003	0.9614723	0.224341473	Zm00001d045072
Zm00001d045072_T020	1.54E-13	2.882286981	Zm00001d045072
Zm00001d045072_T035	0.000267083	0.660310967	Zm00001d045072
Zm00001d045072_T028	0.6028549	1.025714199	Zm00001d045072
Zm00001d045072_T021	0.01195333	-0.916817394	Zm00001d045072

Zm00001d045072_T026	0.9953345	0.264148477	Zm00001d045072
Zm00001d045072_T001	0.9469426	0.516924549	Zm00001d045072
Zm00001d045072_T002	0.000266261	0.994828378	Zm00001d045072
Zm00001d045072_T044	0.000594653	1.28252956	Zm00001d045072
Zm00001d051567_T001	0.2276321	-0.735110646	Zm00001d051567
Zm00001d007117_T002	0.745718	-0.238330694	Zm00001d007117
Zm00001d040874_T001	0.7236841	-0.95861419	Zm00001d040874
Zm00001d008641_T001	0.9846714	0.077158223	Zm00001d008641
Zm00001d021480_T001	0.9867452	0.498751899	Zm00001d021480
Zm00001d021480_T003	0.9931431	0.115178964	Zm00001d021480
Zm00001d021480_T004	0.9336646	0.459182497	Zm00001d021480
Zm00001d005450_T005	0.9216521	0.740202666	Zm00001d005450
Zm00001d005450_T011	0.1236971	-1.790606953	Zm00001d005450
Zm00001d005450_T002	0.2834191	1.105878192	Zm00001d005450
Zm00001d029350_T001	0.5830262	0.69369366	Zm00001d029350
Zm00001d009522_T002	0.4988369	1.233622481	Zm00001d009522
Zm00001d009522_T003	0.8707508	-0.200814003	Zm00001d009522
Zm00001d009522_T004	0.8704382	-0.20974346	Zm00001d009522
Zm00001d023732_T002	0.6809549	0.744985428	Zm00001d023732
Zm00001d023732_T005	0.02672227	-1.994111106	Zm00001d023732
Zm00001d023732_T003	0.000508588	-0.994324566	Zm00001d023732
Zm00001d019432_T008	0.07730424	1.243236997	Zm00001d019432
Zm00001d019432_T010	0.221452	0.757534376	Zm00001d019432
Zm00001d019432_T002	0.5421999	1.344627077	Zm00001d019432
Zm00001d019432_T011	0.8993058	0.974933584	Zm00001d019432
Zm00001d019432_T007	0.1259588	0.454408372	Zm00001d019432
Zm00001d019432_T001	1.38E-11	-2.661001265	Zm00001d019432
Zm00001d039343_T001	0.9491569	0.057358942	Zm00001d039343
Zm00001d039343_T002	0.9052534	0.002223574	Zm00001d039343
Zm00001d020214_T001	0.8591991	-0.523674112	Zm00001d020214
Zm00001d031933_T007	2.14E-05	1.048930589	Zm00001d031933
Zm00001d031933_T005	0.9418022	0.093950189	Zm00001d031933
Zm00001d031933_T002	0.07431695	1.171009641	Zm00001d031933
Zm00001d031933_T001	1	0.373625177	Zm00001d031933
Zm00001d031933_T003	0.8677544	0.905214139	Zm00001d031933
Zm00001d031933_T010	0.833905	0.739985391	Zm00001d031933
Zm00001d048364_T001	0.9303773	-0.004671657	Zm00001d048364
Zm00001d048364_T002	0.8936314	0.763162002	Zm00001d048364
Zm00001d032152_T001	0.7215401	1.14068172	Zm00001d032152
Zm00001d017957_T001	0.8585804	0.648064206	Zm00001d017957
Zm00001d017957_T003	0.9768468	0.237246456	Zm00001d017957
Zm00001d017957_T002	0.9945118	0.453184817	Zm00001d017957
Zm00001d029053_T001	2.93E-06	-0.388012826	Zm00001d029053
Zm00001d029053_T010	0.1057642	1.696956823	Zm00001d029053
Zm00001d029053_T011	0.03830215	0.525842928	Zm00001d029053
Zm00001d029053_T003	0.09695879	-0.690903517	Zm00001d029053
Zm00001d029053_T004	0.9988864	0.130922817	Zm00001d029053
Zm00001d014862_T001	0.9692063	0.663839595	Zm00001d014862
Zm00001d014862_T011	0.7682041	1.297739678	Zm00001d014862
Zm00001d025820_T001	0.8767824	-0.024661844	Zm00001d025820
Zm00001d028406_T001	0.2764096	1.751679917	Zm00001d028406
Zm00001d023583_T005	0.7480195	0.678510542	Zm00001d023583
Zm00001d023583_T002	0.6465256	-0.131483853	Zm00001d023583
Zm00001d013487_T001	0.5980883	2.761307266	Zm00001d013487

Zm00001d016827_T002	0.98291	-0.035092542	Zm00001d016827
Zm00001d016827_T003	0.7708874	-0.234101877	Zm00001d016827
Zm00001d016827_T004	0.4531805	1.236137613	Zm00001d016827
Zm00001d022457_T001	0.01573827	-2.092585919	Zm00001d022457
Zm00001d025629_T003	1	0.230365023	Zm00001d025629
Zm00001d025629_T004	0.8693951	-0.018422019	Zm00001d025629
Zm00001d025629_T007	1.23E-12	2.625835145	Zm00001d025629
Zm00001d025629_T001	0.1002615	-0.546634819	Zm00001d025629
Zm00001d023896_T003	0.6846487	-0.4805096	Zm00001d023896
Zm00001d000400_T001	0.3692804	1.371766895	Zm00001d000400
Zm00001d029921_T001	0.579353	1.198386187	Zm00001d029921
Zm00001d027466_T001	0.8557307	0.916019077	Zm00001d027466
Zm00001d007700_T001	0.1608814	-1.37767692	Zm00001d007700
Zm00001d043451_T001	0.9717919	0.157074713	Zm00001d043451
Zm00001d039522_T002	0.3774847	1.286295069	Zm00001d039522
Zm00001d039522_T018	0.5622303	1.480367354	Zm00001d039522
Zm00001d039522_T023	0.148906	0.4758104	Zm00001d039522
Zm00001d039522_T006	0.01958328	-2.075806833	Zm00001d039522
Zm00001d039522_T014	0.3514	1.432892663	Zm00001d039522
Zm00001d039522_T024	0.9724085	0.252681581	Zm00001d039522
Zm00001d042730_T001	0.8125642	-0.429659939	Zm00001d042730
Zm00001d017537_T001	0.3777631	-0.724209186	Zm00001d017537
Zm00001d045175_T001	0.5224775	1.189008925	Zm00001d045175
Zm00001d010588_T001	3.06E-07	-2.773135992	Zm00001d010588
Zm00001d010588_T003	1.31E-06	-3.37549335	Zm00001d010588
Zm00001d026004_T001	0.2646802	-0.783468664	Zm00001d026004
Zm00001d044261_T004	0.3336427	-0.85128535	Zm00001d044261
Zm00001d044261_T002	0.8569937	-0.112396552	Zm00001d044261
Zm00001d044261_T001	0.9404125	0.108610999	Zm00001d044261
Zm00001d034217_T001	0.5831037	-0.533557381	Zm00001d034217
Zm00001d011719_T008	0.3071359	-0.549437494	Zm00001d011719
Zm00001d011719_T007	0.6723947	-0.247049447	Zm00001d011719
Zm00001d011719_T006	0.9867452	0.261618385	Zm00001d011719
Zm00001d011719_T001	0.7419821	-0.088801296	Zm00001d011719
Zm00001d011719_T003	0.2287867	0.52369163	Zm00001d011719
Zm00001d014005_T001	0.9513906	-0.313870349	Zm00001d014005
Zm00001d053416_T001	1	0.285670894	Zm00001d053416
Zm00001d048545_T006	0.7080786	0.562258797	Zm00001d048545
Zm00001d048545_T001	0.955383	0.681375095	Zm00001d048545
Zm00001d048545_T008	0.8325852	-0.136028954	Zm00001d048545
Zm00001d048545_T007	0.6082229	-0.298672989	Zm00001d048545
Zm00001d021558_T001	0.9988063	0.402208763	Zm00001d021558
Zm00001d021558_T003	0.9760472	0.486828506	Zm00001d021558
Zm00001d021558_T002	0.9740325	0.346439625	Zm00001d021558
Zm00001d014417_T002	0.9971866	0.158314287	Zm00001d014417
Zm00001d014417_T003	0.9833513	0.166350496	Zm00001d014417
Zm00001d015473_T002	0.9867452	-0.211169121	Zm00001d015473
Zm00001d015473_T001	1	0.386557866	Zm00001d015473
Zm00001d049543_T001	0.5347419	-0.301893222	Zm00001d049543
Zm00001d049543_T011	0.9845789	0.194401407	Zm00001d049543
Zm00001d049543_T009	9.76E-09	1.23424952	Zm00001d049543
Zm00001d049543_T007	0.9760472	0.02566317	Zm00001d049543
Zm00001d049543_T013	0.000824048	-1.97642745	Zm00001d049543
Zm00001d049543_T016	0.8964117	-0.146433024	Zm00001d049543

Zm00001d049543_T017	4.37E-09	-1.799509992	Zm00001d049543
Zm00001d037738_T001	0.7984948	0.937215988	Zm00001d037738
Zm00001d036917_T009	0.03860996	0.466706671	Zm00001d036917
Zm00001d032285_T001	0.9745084	0.177565248	Zm00001d032285
Zm00001d043703_T008	0.08778843	0.670238325	Zm00001d043703
Zm00001d043703_T011	0.9862794	0.131492559	Zm00001d043703
Zm00001d043703_T003	0.9666757	0.383696913	Zm00001d043703
Zm00001d043703_T007	0.3827309	1.193842027	Zm00001d043703
Zm00001d043703_T004	0.000813969	1.044239431	Zm00001d043703
Zm00001d011945_T002	0.9984041	0.309571933	Zm00001d011945
Zm00001d014436_T013	0.9897446	0.484903405	Zm00001d014436
Zm00001d014436_T010	0.6742084	-0.450840951	Zm00001d014436
Zm00001d014436_T001	0.2796168	-0.727729145	Zm00001d014436
Zm00001d014436_T002	0.9953345	0.272227888	Zm00001d014436
Zm00001d014436_T011	0.9903234	0.073195743	Zm00001d014436
Zm00001d033890_T002	0.9673473	0.131720085	Zm00001d033890
Zm00001d033890_T001	0.9924805	0.32573269	Zm00001d033890
Zm00001d003420_T001	0.01306162	4.460242966	Zm00001d003420
Zm00001d044423_T001	0.8368466	-0.430553448	Zm00001d044423
Zm00001d049375_T001	0.6466858	-0.5083993	Zm00001d049375
Zm00001d016679_T003	0.8581713	-0.082978729	Zm00001d016679
Zm00001d016679_T005	0.3649839	0.636669502	Zm00001d016679
Zm00001d016679_T006	0.8452909	-0.079298685	Zm00001d016679
Zm00001d016679_T008	0.8961518	-0.040833305	Zm00001d016679
Zm00001d016679_T001	0.008983932	1.093614902	Zm00001d016679
Zm00001d047786_T001	0.00120544	3.474627229	Zm00001d047786
Zm00001d037438_T002	0.9918544	0.35835583	Zm00001d037438
Zm00001d037438_T007	0.8641671	0.28835626	Zm00001d037438
Zm00001d037438_T006	1	0.257959947	Zm00001d037438
Zm00001d037438_T001	0.3960368	0.376501522	Zm00001d037438
Zm00001d018830_T001	0.9804228	0.309126338	Zm00001d018830
Zm00001d043242_T001	0.9664736	0.433635917	Zm00001d043242
Zm00001d021707_T001	0.9070578	-0.345496762	Zm00001d021707
Zm00001d033604_T001	0.6976102	0.685120647	Zm00001d033604
Zm00001d007187_T003	0.7628677	-0.249230275	Zm00001d007187
Zm00001d007187_T002	0.8892594	0.807669067	Zm00001d007187
Zm00001d042828_T001	0.000305188	2.735726403	Zm00001d042828
Zm00001d028119_T008	0.2065419	0.395123826	Zm00001d028119
Zm00001d028119_T019	0.04713635	0.750078565	Zm00001d028119
Zm00001d028119_T018	0.1429392	-0.451731779	Zm00001d028119
Zm00001d010640_T001	0.000445504	3.663798781	Zm00001d010640
Zm00001d045144_T001	0.550715	-0.752421454	Zm00001d045144
Zm00001d027991_T003	0.6552247	1.089500631	Zm00001d027991
Zm00001d027991_T001	0.4792685	1.448544879	Zm00001d027991
Zm00001d012015_T001	0.6023541	1.237479447	Zm00001d012015
Zm00001d044579_T001	2.02E-06	-1.170427445	Zm00001d044579
Zm00001d051836_T001	0.90276	-0.140993436	Zm00001d051836
Zm00001d019719_T001	0.5368684	-0.481286765	Zm00001d019719
Zm00001d042470_T004	0.3967188	0.708729618	Zm00001d042470
Zm00001d042470_T008	0.001148838	1.200580084	Zm00001d042470
Zm00001d042470_T012	0.7782328	0.72850753	Zm00001d042470
Zm00001d042470_T014	1.61E-06	2.061536602	Zm00001d042470
Zm00001d042470_T007	0.9944857	0.418730145	Zm00001d042470
Zm00001d042470_T006	0.4730617	0.643756153	Zm00001d042470

Zm00001d009513_T001	0.9524127	-0.312934897	Zm00001d009513
Zm00001d009513_T004	0.8522942	0.73369167	Zm00001d009513
Zm00001d009513_T002	0.8488448	0.612275362	Zm00001d009513
Zm00001d009513_T003	0.9804228	0.090862086	Zm00001d009513
Zm00001d039607_T001	0.9916733	0.204152221	Zm00001d039607
Zm00001d013014_T001	0.6419432	0.490507282	Zm00001d013014
Zm00001d013014_T003	0.7475609	0.982559223	Zm00001d013014
Zm00001d018344_T001	0.3464027	-1.135530983	Zm00001d018344
Zm00001d026289_T001	0.4748531	-0.190953185	Zm00001d026289
Zm00001d026289_T004	0.9514141	0.525622287	Zm00001d026289
Zm00001d026289_T003	1.79E-09	-1.331053126	Zm00001d026289
Zm00001d027536_T005	0.002252959	0.396569947	Zm00001d027536
Zm00001d027536_T003	0.5359966	-0.187876981	Zm00001d027536
Zm00001d027536_T004	0.5718169	-0.173215671	Zm00001d027536
Zm00001d027536_T008	0.9213679	0.679226454	Zm00001d027536
Zm00001d032663_T001	0.6282939	-0.739226393	Zm00001d032663
Zm00001d002257_T001	0.9840043	0.506442132	Zm00001d002257
Zm00001d002257_T005	0.9173494	0.492876946	Zm00001d002257
Zm00001d002599_T001	0.5272498	-0.511689445	Zm00001d002599
Zm00001d002599_T002	0.9507337	0.519962257	Zm00001d002599
Zm00001d048825_T003	0.5570384	-0.810886439	Zm00001d048825
Zm00001d048825_T001	0.7914025	-0.540154471	Zm00001d048825
Zm00001d021930_T001	0.8031436	1.026825626	Zm00001d021930
Zm00001d021930_T005	0.1034438	1.790156304	Zm00001d021930
Zm00001d021930_T002	0.1375053	1.932962153	Zm00001d021930
Zm00001d011422_T001	0.3071359	-0.729154527	Zm00001d011422
Zm00001d039147_T001	0.8267985	1.001508232	Zm00001d039147
Zm00001d013741_T007	1.52E-12	2.868608542	Zm00001d013741
Zm00001d013741_T001	0.9254376	0.020594163	Zm00001d013741
Zm00001d013741_T003	0.9139422	0.068702494	Zm00001d013741
Zm00001d013741_T008	0.9778436	0.076381502	Zm00001d013741
Zm00001d013741_T002	0.5822629	-0.500940031	Zm00001d013741
Zm00001d041670_T001	0.01713299	-1.824050312	Zm00001d041670
Zm00001d002440_T001	1	0.444208398	Zm00001d002440
Zm00001d002440_T003	0.05523257	1.527693034	Zm00001d002440
Zm00001d002440_T004	0.4531805	1.258906034	Zm00001d002440
Zm00001d002440_T002	0.9825858	0.547775748	Zm00001d002440
Zm00001d050715_T002	0.8300901	0.786572182	Zm00001d050715
Zm00001d028363_T001	1	0.403238058	Zm00001d028363
Zm00001d037857_T001	0.776742	0.640877924	Zm00001d037857
Zm00001d047972_T001	0.6839661	-0.433113202	Zm00001d047972
Zm00001d047972_T002	0.8999046	-0.036051466	Zm00001d047972
Zm00001d000444_T001	0.9914912	0.281498635	Zm00001d000444
Zm00001d029066_T021	0.5754586	0.796799383	Zm00001d029066
Zm00001d029066_T017	0.2944197	0.59665167	Zm00001d029066
Zm00001d029066_T010	0.6322656	0.761095255	Zm00001d029066
Zm00001d029066_T004	2.02E-06	0.59063861	Zm00001d029066
Zm00001d041726_T001	0.4959633	-0.672068548	Zm00001d041726
Zm00001d020424_T002	0.7831147	0.874291111	Zm00001d020424
Zm00001d020424_T011	0.3286812	0.435114278	Zm00001d020424
Zm00001d017084_T001	0.000919043	2.640909604	Zm00001d017084
Zm00001d008327_T001	0.4754561	-0.712529519	Zm00001d008327
Zm00001d050074_T004	0.007540301	1.484127919	Zm00001d050074
Zm00001d050074_T007	0.9184323	0.251381849	Zm00001d050074

Zm00001d050074_T002	0.9930712	0.351063145	Zm00001d050074
Zm00001d050074_T005	2.79E-07	-2.336546168	Zm00001d050074
Zm00001d050074_T003	0.9086616	0.42872545	Zm00001d050074
Zm00001d010671_T002	0.9157081	0.709081034	Zm00001d010671
Zm00001d043025_T001	0.1684396	1.840539176	Zm00001d043025
Zm00001d025036_T003	0.1234187	0.791932952	Zm00001d025036
Zm00001d025036_T002	0.224247	-0.654724258	Zm00001d025036
Zm00001d042302_T005	0.6523514	-0.454929602	Zm00001d042302
Zm00001d042302_T001	0.6366096	1.212199733	Zm00001d042302
Zm00001d042302_T004	0.3105424	0.456422756	Zm00001d042302
Zm00001d042302_T006	0.2689005	0.27418525	Zm00001d042302
Zm00001d018943_T002	0.9416622	0.448048256	Zm00001d018943
Zm00001d018943_T001	0.1767827	-0.732850185	Zm00001d018943
Zm00001d038035_T004	0.983226	0.101285254	Zm00001d038035
Zm00001d038035_T002	0.967161	0.119715166	Zm00001d038035
Zm00001d038035_T005	0.8192173	-0.534802241	Zm00001d038035
Zm00001d006472_T003	0.1159519	1.641145578	Zm00001d006472
Zm00001d006472_T030	0.7265805	-0.316712727	Zm00001d006472
Zm00001d006472_T029	0.4090471	1.159637208	Zm00001d006472
Zm00001d042338_T001	0.3889651	0.431160325	Zm00001d042338
Zm00001d042338_T002	0.9563232	0.675990835	Zm00001d042338
Zm00001d039519_T001	0.9817074	0.470072617	Zm00001d039519
Zm00001d016723_T001	0.7808852	-0.311741752	Zm00001d016723
Zm00001d013692_T001	0.9817074	0.250589793	Zm00001d013692
Zm00001d007341_T001	0.5402748	-0.916939056	Zm00001d007341
Zm00001d034137_T001	0.9139882	-0.057875533	Zm00001d034137
Zm00001d002312_T004	0.5440777	0.393301441	Zm00001d002312
Zm00001d020908_T006	0.9872831	0.095353698	Zm00001d020908
Zm00001d020908_T004	1	0.364568054	Zm00001d020908
Zm00001d054017_T006	0.9052981	-0.415750628	Zm00001d054017
Zm00001d054017_T004	0.972985	0.139868844	Zm00001d054017
Zm00001d054017_T007	0.9539896	0.529317598	Zm00001d054017
Zm00001d054017_T005	0.9766874	0.410154131	Zm00001d054017
Zm00001d054017_T002	0.8996347	0.091029049	Zm00001d054017
Zm00001d014658_T005	0.00017617	2.32163668	Zm00001d014658
Zm00001d014658_T001	0.5715685	1.411476104	Zm00001d014658
Zm00001d014658_T002	0.07600847	2.12255784	Zm00001d014658
Zm00001d014658_T003	0.8280723	-0.194179253	Zm00001d014658
Zm00001d031766_T001	0.9942835	0.177670208	Zm00001d031766
Zm00001d026594_T001	0.07528435	-1.62930142	Zm00001d026594
Zm00001d048728_T001	0.9644829	0.517840647	Zm00001d048728
Zm00001d007422_T008	0.7458694	1.10491586	Zm00001d007422
Zm00001d007422_T006	0.03295965	1.164872227	Zm00001d007422
Zm00001d007422_T001	0.1637088	-0.245597543	Zm00001d007422
Zm00001d007422_T012	0.0119098	-3.214563947	Zm00001d007422
Zm00001d007422_T011	0.9884825	0.165206354	Zm00001d007422
Zm00001d007422_T010	0.8376343	1.067561563	Zm00001d007422
Zm00001d007422_T007	0.6646236	-0.180751158	Zm00001d007422
Zm00001d038734_T004	0.9868821	0.226385267	Zm00001d038734
Zm00001d038734_T001	0.822299	-0.227073828	Zm00001d038734
Zm00001d038734_T006	0.9213679	0.039711994	Zm00001d038734
Zm00001d042669_T003	0.6958637	-0.387809532	Zm00001d042669
Zm00001d042669_T006	0.5637372	0.431353217	Zm00001d042669
Zm00001d042669_T005	0.7827148	1.037684595	Zm00001d042669

Zm00001d042669_T001	0.3924189	1.109620093	Zm00001d042669
Zm00001d042669_T004	0.4124502	1.477126507	Zm00001d042669
Zm00001d037337_T001	0.8617755	-0.304502302	Zm00001d037337
Zm00001d048094_T001	0.8363027	-0.606419278	Zm00001d048094
Zm00001d049613_T001	0.9752014	0.145965904	Zm00001d049613
Zm00001d028454_T001	0.8896281	-0.07727485	Zm00001d028454
Zm00001d043902_T001	0.1767173	1.888765554	Zm00001d043902
Zm00001d013219_T001	0.644634	-0.459484021	Zm00001d013219
Zm00001d012407_T004	0.9237037	0.798330086	Zm00001d012407
Zm00001d012407_T006	0.6466858	-0.472535619	Zm00001d012407
Zm00001d012407_T001	0.780135	-0.121617767	Zm00001d012407
Zm00001d012407_T007	0.8922178	-0.533760748	Zm00001d012407
Zm00001d001786_T004	0.9785005	0.240111536	Zm00001d001786
Zm00001d012022_T001	0.08979202	-1.004201155	Zm00001d012022
Zm00001d006604_T001	0.9201237	-0.383511712	Zm00001d006604
Zm00001d048449_T001	0.9979568	0.257953756	Zm00001d048449
Zm00001d035748_T001	0.5238472	1.046992734	Zm00001d035748
Zm00001d038946_T002	0.9363242	0.552141115	Zm00001d038946
Zm00001d028841_T001	0.9529184	-0.033531956	Zm00001d028841
Zm00001d040966_T001	0.9846714	0.108082694	Zm00001d040966
Zm00001d015265_T003	0.4443554	0.858113485	Zm00001d015265
Zm00001d015265_T001	0.9541039	0.145556548	Zm00001d015265
Zm00001d034734_T007	0.3352337	-0.57313989	Zm00001d034734
Zm00001d034734_T004	0.4430193	0.356834598	Zm00001d034734
Zm00001d034734_T003	1.31E-08	1.279294477	Zm00001d034734
Zm00001d034734_T001	0.3128816	0.937143583	Zm00001d034734
Zm00001d036426_T001	0.8785063	-0.165484845	Zm00001d036426
Zm00001d017692_T001	1	0.142902416	Zm00001d017692
Zm00001d035913_T004	0.9944247	-0.013781426	Zm00001d035913
Zm00001d035913_T010	0.7367876	-0.035717146	Zm00001d035913
Zm00001d035913_T007	0.8554968	0.156748019	Zm00001d035913
Zm00001d035913_T003	0.9206245	-0.019833157	Zm00001d035913
Zm00001d032096_T012	0.9748733	0.417602691	Zm00001d032096
Zm00001d032096_T010	0.7682041	0.458885494	Zm00001d032096
Zm00001d032096_T005	1.59E-07	1.057411171	Zm00001d032096
Zm00001d039053_T001	0.7855752	-0.293032847	Zm00001d039053
Zm00001d012992_T002	0.6625969	-0.832860845	Zm00001d012992
Zm00001d023629_T001	0.989392	0.493036613	Zm00001d023629
Zm00001d048222_T001	0.9804433	0.110245136	Zm00001d048222
Zm00001d052317_T002	0.9787989	0.123806907	Zm00001d052317
Zm00001d030638_T002	0.9893214	0.24482181	Zm00001d030638
Zm00001d028129_T002	0.785169	0.99317073	Zm00001d028129
Zm00001d032603_T001	0.8958882	-0.084033568	Zm00001d032603
Zm00001d032603_T002	0.9201237	-0.024171388	Zm00001d032603
Zm00001d047912_T002	0.9224378	-0.033612034	Zm00001d047912
Zm00001d029869_T001	0.4647287	-0.748815671	Zm00001d029869
Zm00001d037004_T001	0.9846714	0.40113309	Zm00001d037004
Zm00001d037004_T004	0.1129566	1.068979849	Zm00001d037004
Zm00001d031221_T001	0.9950081	0.438127419	Zm00001d031221
Zm00001d042179_T001	0.5353057	-0.698794775	Zm00001d042179
Zm00001d035603_T001	0.6092595	-0.480510701	Zm00001d035603
Zm00001d006521_T001	0.9706293	-0.044873537	Zm00001d006521
Zm00001d043056_T006	0.9942091	0.458743845	Zm00001d043056
Zm00001d053207_T001	0.8374775	0.359421345	Zm00001d053207

Zm00001d032120_T001	2.72E-06	3.340482126	Zm00001d032120
Zm00001d044581_T002	0.8102147	0.826943943	Zm00001d044581
Zm00001d044581_T015	0.8580945	-0.075650281	Zm00001d044581
Zm00001d012081_T001	0.5730328	-0.581450938	Zm00001d012081
Zm00001d042192_T001	0.9947739	0.24822494	Zm00001d042192
Zm00001d037163_T002	1	0.126511019	Zm00001d037163
Zm00001d042962_T002	0.8721235	0.851087555	Zm00001d042962
Zm00001d042962_T007	0.9542135	0.423466733	Zm00001d042962
Zm00001d021073_T002	0.9961549	0.252065578	Zm00001d021073
Zm00001d021073_T004	1	0.155144132	Zm00001d021073
Zm00001d011525_T001	0.6185122	1.110213666	Zm00001d011525
Zm00001d011525_T008	0.8288235	-0.131064411	Zm00001d011525
Zm00001d011525_T007	0.9440782	0.109045888	Zm00001d011525
Zm00001d011525_T004	0.5717645	0.449283376	Zm00001d011525
Zm00001d031134_T001	0.8743696	-0.076166323	Zm00001d031134
Zm00001d017977_T002	0.8922781	0.60870324	Zm00001d017977
Zm00001d017977_T014	0.000521935	-1.609107624	Zm00001d017977
Zm00001d017977_T016	0.05869493	0.788399249	Zm00001d017977
Zm00001d017977_T006	0.1539575	0.868833368	Zm00001d017977
Zm00001d017977_T013	0.5343441	1.315956594	Zm00001d017977
Zm00001d040010_T004	0.1272455	0.362268158	Zm00001d040010
Zm00001d040010_T003	0.9979568	0.48853276	Zm00001d040010
Zm00001d027457_T001	0.6661421	-0.786754014	Zm00001d027457
Zm00001d001914_T002	0.9628835	0.105420801	Zm00001d001914
Zm00001d001914_T003	0.9219195	0.716801727	Zm00001d001914
Zm00001d001914_T001	0.9203655	-0.04131537	Zm00001d001914
Zm00001d017840_T001	0.9618822	0.027319471	Zm00001d017840
Zm00001d024597_T001	0.9869265	0.465309348	Zm00001d024597
Zm00001d017435_T001	0.7289978	-0.437072815	Zm00001d017435
Zm00001d027359_T001	0.9069584	-0.036840209	Zm00001d027359
Zm00001d051685_T002	1.05E-06	-1.765226181	Zm00001d051685
Zm00001d051685_T001	0.9706293	0.173368934	Zm00001d051685
Zm00001d021051_T004	0.9979568	0.187301075	Zm00001d021051
Zm00001d021051_T002	0.9791729	0.158601863	Zm00001d021051
Zm00001d021051_T003	0.9945118	0.04922153	Zm00001d021051
Zm00001d021051_T001	0.8545272	0.280918389	Zm00001d021051
Zm00001d053223_T001	0.7636892	-0.375832191	Zm00001d053223
Zm00001d030007_T001	0.7810295	-0.268059608	Zm00001d030007
Zm00001d007868_T001	0.2935147	1.445295275	Zm00001d007868
Zm00001d027525_T001	1	0.201685465	Zm00001d027525
Zm00001d008956_T001	0.9771927	0.316880126	Zm00001d008956
Zm00001d018844_T001	0.00012849	3.446335553	Zm00001d018844
Zm00001d020170_T003	1	0.077315795	Zm00001d020170
Zm00001d020170_T001	0.9912983	0.522821709	Zm00001d020170
Zm00001d044189_T001	1	0.353980652	Zm00001d044189
Zm00001d023271_T002	0.8720862	-0.106421227	Zm00001d023271
Zm00001d007390_T002	0.8952297	-0.519337664	Zm00001d007390
Zm00001d053632_T002	1	0.23385589	Zm00001d053632
Zm00001d053632_T001	0.7128296	-0.507561043	Zm00001d053632
Zm00001d016665_T001	0.9570268	0.019514541	Zm00001d016665
Zm00001d016665_T002	0.9469426	-0.092926458	Zm00001d016665
Zm00001d044922_T001	0.9237921	0.532357182	Zm00001d044922
Zm00001d044922_T002	0.923446	0.446933702	Zm00001d044922
Zm00001d036836_T001	0.9164622	-0.409312462	Zm00001d036836

Zm00001d021802_T003	0.5964536	0.765644308	Zm00001d021802
Zm00001d021802_T002	0.9160053	0.011185709	Zm00001d021802
Zm00001d021802_T004	0.9887889	0.348213336	Zm00001d021802
Zm00001d021802_T005	0.1250366	1.65799379	Zm00001d021802
Zm00001d047241_T005	0.9887584	0.137820546	Zm00001d047241
Zm00001d047241_T003	0.01751662	-0.756164206	Zm00001d047241
Zm00001d047241_T006	0.82705	0.404807801	Zm00001d047241
Zm00001d047241_T004	0.9979393	0.495365845	Zm00001d047241
Zm00001d048451_T001	0.5467906	-0.293105309	Zm00001d048451
Zm00001d032636_T001	0.6558718	-0.503675856	Zm00001d032636
Zm00001d015568_T003	0.897685	0.403713249	Zm00001d015568
Zm00001d015568_T002	0.9303773	-0.175920615	Zm00001d015568
Zm00001d047236_T003	0.9311027	0.420925133	Zm00001d047236
Zm00001d043473_T024	0.01563112	0.691468347	Zm00001d043473
Zm00001d043473_T016	0.3005677	0.281659328	Zm00001d043473
Zm00001d043473_T009	0.9724085	0.160047744	Zm00001d043473
Zm00001d043473_T025	0.9446321	0.443244275	Zm00001d043473
Zm00001d043473_T010	0.0689712	1.126670463	Zm00001d043473
Zm00001d043473_T001	0.7492661	0.434598077	Zm00001d043473
Zm00001d043473_T008	0.9529094	0.300871164	Zm00001d043473
Zm00001d043473_T012	0.6733323	1.203478118	Zm00001d043473
Zm00001d013828_T003	0.4425935	0.875183924	Zm00001d013828
Zm00001d013828_T002	0.9956182	0.26264828	Zm00001d013828
Zm00001d013828_T001	1	0.130194732	Zm00001d013828
Zm00001d033932_T001	0.9930712	0.458881528	Zm00001d033932
Zm00001d033932_T004	0.8730113	0.251995336	Zm00001d033932
Zm00001d022092_T001	0.9753621	0.024544735	Zm00001d022092
Zm00001d030021_T001	0.9481543	-0.270561851	Zm00001d030021
Zm00001d030021_T005	0.3091353	-0.689086109	Zm00001d030021
Zm00001d044621_T001	0.7483274	-0.55847184	Zm00001d044621
Zm00001d053828_T001	0.9429712	-0.088885783	Zm00001d053828
Zm00001d017708_T002	0.9897446	0.16167541	Zm00001d017708
Zm00001d017708_T001	0.9979568	0.036015597	Zm00001d017708
Zm00001d017206_T001	1	0.123955702	Zm00001d017206
Zm00001d050583_T001	0.8357705	0.784107285	Zm00001d050583
Zm00001d049716_T001	0.8720862	-0.131423594	Zm00001d049716
Zm00001d032547_T012	0.8987811	1.210367863	Zm00001d032547
Zm00001d032547_T007	0.1627117	1.256411115	Zm00001d032547
Zm00001d032547_T011	0.2058947	1.261913403	Zm00001d032547
Zm00001d032547_T002	0.5951634	0.011204182	Zm00001d032547
Zm00001d032547_T004	0.9206245	0.753218632	Zm00001d032547
Zm00001d032547_T006	0.3426298	-0.54604992	Zm00001d032547
Zm00001d017457_T001	0.03372473	2.571877224	Zm00001d017457
Zm00001d009426_T006	0.9581709	0.058652843	Zm00001d009426
Zm00001d019752_T001	0.9979568	0.373445854	Zm00001d019752
Zm00001d013468_T001	0.5954619	0.496196429	Zm00001d013468
Zm00001d013468_T003	0.02346557	0.978854896	Zm00001d013468
Zm00001d013468_T002	0.06691707	2.342418065	Zm00001d013468
Zm00001d034618_T002	0.8836195	-0.115296747	Zm00001d034618
Zm00001d031569_T001	0.9690925	0.655727091	Zm00001d031569
Zm00001d012963_T001	0.6967776	-0.271181521	Zm00001d012963
Zm00001d047712_T001	0.889796	-0.069832418	Zm00001d047712
Zm00001d012108_T001	0.8950139	0.285046381	Zm00001d012108
Zm00001d012108_T012	0.9321001	-0.010169398	Zm00001d012108

Zm00001d012108_T011	0.8494782	-0.287884563	Zm00001d012108
Zm00001d012108_T006	0.000405099	0.795765306	Zm00001d012108
Zm00001d012108_T009	0.7063456	0.510187401	Zm00001d012108
Zm00001d012108_T002	1	0.424504616	Zm00001d012108
Zm00001d006798_T012	0.4661639	0.447050058	Zm00001d006798
Zm00001d006798_T002	0.3243413	0.771414534	Zm00001d006798
Zm00001d006798_T003	0.6466858	0.614064637	Zm00001d006798
Zm00001d006798_T001	0.3746502	-0.761974592	Zm00001d006798
Zm00001d006798_T013	0.9768996	0.107799062	Zm00001d006798
Zm00001d006798_T007	0.05891666	0.785086398	Zm00001d006798
Zm00001d006798_T020	0.004269411	0.9083365	Zm00001d006798
Zm00001d005785_T014	0.9981361	0.062667673	Zm00001d005785
Zm00001d005785_T011	0.9851204	0.293400984	Zm00001d005785
Zm00001d005785_T002	0.8987811	0.251894708	Zm00001d005785
Zm00001d005785_T005	0.5707685	-0.213107962	Zm00001d005785
Zm00001d028543_T001	0.9760472	0.227558721	Zm00001d028543
Zm00001d010261_T001	0.8156202	-0.705881342	Zm00001d010261
Zm00001d013100_T001	0.954984	0.127102595	Zm00001d013100
Zm00001d013100_T002	0.8093695	-0.615813657	Zm00001d013100
Zm00001d027344_T005	0.8941489	0.096902892	Zm00001d027344
Zm00001d027344_T006	0.7907819	1.177361291	Zm00001d027344
Zm00001d027344_T001	0.6470421	-0.406394005	Zm00001d027344
Zm00001d027344_T003	3.15E-13	4.006261732	Zm00001d027344
Zm00001d027344_T009	0.002796955	-1.665172084	Zm00001d027344
Zm00001d027344_T008	0.9160053	0.391310282	Zm00001d027344
Zm00001d002450_T002	0.7464064	-0.673527222	Zm00001d002450
Zm00001d045465_T001	0.995534	0.412976016	Zm00001d045465
Zm00001d050265_T002	0.7790464	-0.339625697	Zm00001d050265
Zm00001d050265_T001	0.9840918	0.41989717	Zm00001d050265
Zm00001d018776_T019	0.9975882	0.162602559	Zm00001d018776
Zm00001d018776_T007	0.9249382	0.183016009	Zm00001d018776
Zm00001d018776_T005	0.3484876	0.636086248	Zm00001d018776
Zm00001d018776_T002	0.7527904	-0.105743978	Zm00001d018776
Zm00001d030542_T001	0.2622146	-0.691901466	Zm00001d030542
Zm00001d026547_T001	0.9385406	-0.144239871	Zm00001d026547
Zm00001d013076_T001	0.6983273	-0.422439559	Zm00001d013076
Zm00001d002711_T002	0.7454635	0.59840393	Zm00001d002711
Zm00001d002711_T003	0.9756546	0.16329076	Zm00001d002711
Zm00001d002711_T001	0.9567316	0.496792502	Zm00001d002711
Zm00001d022516_T001	0.6354554	0.943206149	Zm00001d022516
Zm00001d004709_T001	0.9529184	0.580579543	Zm00001d004709
Zm00001d039695_T001	0.7231383	1.158328503	Zm00001d039695
Zm00001d037426_T001	0.8450197	0.557381468	Zm00001d037426
Zm00001d011788_T001	0.5814152	-0.335494777	Zm00001d011788
Zm00001d020636_T003	0.9792387	0.264382927	Zm00001d020636
Zm00001d020636_T001	0.9803901	0.541998775	Zm00001d020636
Zm00001d002119_T020	0.6436915	-0.382916598	Zm00001d002119
Zm00001d002119_T009	2.71E-10	1.530393379	Zm00001d002119
Zm00001d002119_T006	0.3647722	-0.315911698	Zm00001d002119
Zm00001d045667_T001	0.7997379	-0.221273008	Zm00001d045667
Zm00001d034575_T002	0.9971866	0.074515252	Zm00001d034575
Zm00001d034575_T001	1	0.408341446	Zm00001d034575
Zm00001d041539_T001	0.1545218	-1.646700797	Zm00001d041539
Zm00001d041539_T002	0.1396451	-1.709248826	Zm00001d041539

Zm00001d016072_T002	0.9413175	-0.170033165	Zm00001d016072
Zm00001d003643_T003	0.8236316	-0.062821662	Zm00001d003643
Zm00001d003643_T005	0.4312879	1.751350794	Zm00001d003643
Zm00001d003643_T006	0.9369505	0.013566974	Zm00001d003643
Zm00001d028643_T001	0.9104115	0.789473981	Zm00001d028643
Zm00001d036970_T001	0.9967513	0.302802098	Zm00001d036970
Zm00001d006626_T001	0.00268479	3.272179387	Zm00001d006626
Zm00001d053716_T001	0.9128485	-0.085925457	Zm00001d053716
Zm00001d045127_T002	0.9921225	0.433739304	Zm00001d045127
Zm00001d015767_T002	0.9745777	0.025572689	Zm00001d015767
Zm00001d016118_T001	0.4324156	-0.466907622	Zm00001d016118
Zm00001d037518_T003	0.4650567	-0.773357008	Zm00001d037518
Zm00001d037518_T005	0.000379352	1.564357564	Zm00001d037518
Zm00001d037518_T008	0.9170869	-0.132229336	Zm00001d037518
Zm00001d029246_T001	0.3884952	-0.754389934	Zm00001d029246
Zm00001d017808_T009	1	0.260496298	Zm00001d017808
Zm00001d017808_T012	0.9187493	0.205597138	Zm00001d017808
Zm00001d017808_T001	6.82E-05	1.730351275	Zm00001d017808
Zm00001d017808_T008	0.7573198	0.711831506	Zm00001d017808
Zm00001d017808_T013	0.04377645	-1.737751264	Zm00001d017808
Zm00001d017808_T014	0.8486887	0.327405674	Zm00001d017808
Zm00001d016170_T001	0.8996162	-0.275434973	Zm00001d016170
Zm00001d019376_T007	0.6358713	-0.203591873	Zm00001d019376
Zm00001d003349_T001	0.8375108	0.812714925	Zm00001d003349
Zm00001d040941_T001	0.09843617	-1.7922999	Zm00001d040941
Zm00001d040941_T002	0.959308	0.574335664	Zm00001d040941
Zm00001d040941_T006	0.5697105	1.149976391	Zm00001d040941
Zm00001d011473_T002	3.73E-09	1.606532172	Zm00001d011473
Zm00001d011473_T006	0.2801551	1.516335338	Zm00001d011473
Zm00001d028687_T001	0.9371235	0.79132399	Zm00001d028687
Zm00001d044475_T001	0.9892609	0.254708854	Zm00001d044475
Zm00001d031913_T001	0.2546519	-1.106823639	Zm00001d031913
Zm00001d031913_T004	0.8607922	0.914469533	Zm00001d031913
Zm00001d017768_T008	0.112599	1.955001558	Zm00001d017768
Zm00001d017768_T001	0.347955	1.239146991	Zm00001d017768
Zm00001d017768_T013	0.1499043	0.798203809	Zm00001d017768
Zm00001d017768_T003	0.5902617	0.925599685	Zm00001d017768
Zm00001d042096_T001	0.3541203	-1.300075658	Zm00001d042096
Zm00001d036195_T002	0.4309206	1.56729993	Zm00001d036195
Zm00001d036195_T003	1.78E-10	-2.977599098	Zm00001d036195
Zm00001d036195_T001	0.4003602	0.369898363	Zm00001d036195
Zm00001d053649_T003	0.7712256	1.986321817	Zm00001d053649
Zm00001d053649_T002	0.07550872	-1.716402921	Zm00001d053649
Zm00001d046187_T001	0.02490188	-1.386280157	Zm00001d046187
Zm00001d010965_T001	0.9311783	-0.377067689	Zm00001d010965
Zm00001d020381_T011	0.6140391	0.665840391	Zm00001d020381
Zm00001d020381_T004	0.6217057	1.777336428	Zm00001d020381
Zm00001d020381_T010	0.9507998	0.623441916	Zm00001d020381
Zm00001d020381_T001	0.7146978	-0.088381845	Zm00001d020381
Zm00001d020381_T005	0.8483996	-0.189561549	Zm00001d020381
Zm00001d041395_T010	0.8376343	-0.139297757	Zm00001d041395
Zm00001d041395_T002	0.2518892	1.355842395	Zm00001d041395
Zm00001d041395_T003	0.3120537	0.510459573	Zm00001d041395
Zm00001d042045_T001	0.9541864	0.605823902	Zm00001d042045

Zm00001d002370_T001	0.3947262	1.59680398	Zm00001d002370
Zm00001d031000_T001	0.956053	-0.252166912	Zm00001d031000
Zm00001d045220_T001	0.8935288	-0.122835852	Zm00001d045220
Zm00001d042626_T001	0.9466531	0.438172235	Zm00001d042626
Zm00001d007387_T005	0.8641671	0.473119761	Zm00001d007387
Zm00001d007387_T004	0.7866898	0.106658684	Zm00001d007387
Zm00001d032029_T003	0.910614	-0.017212536	Zm00001d032029
Zm00001d009571_T007	0.4416956	1.514651179	Zm00001d009571
Zm00001d009571_T004	1.54E-09	1.816760576	Zm00001d009571
Zm00001d009571_T005	0.03997268	1.571086304	Zm00001d009571
Zm00001d009571_T008	0.6389363	0.617162414	Zm00001d009571
Zm00001d009571_T001	0.03725985	-1.278494419	Zm00001d009571
Zm00001d018512_T001	0.9768802	0.502555474	Zm00001d018512
Zm00001d018512_T002	0.983226	0.227711762	Zm00001d018512
Zm00001d023658_T009	0.8953011	0.424061536	Zm00001d023658
Zm00001d023658_T008	0.8107637	0.271145484	Zm00001d023658
Zm00001d023658_T007	0.08007737	0.847614396	Zm00001d023658
Zm00001d023658_T003	0.9760472	0.520952361	Zm00001d023658
Zm00001d023658_T001	0.3404176	1.819102871	Zm00001d023658
Zm00001d023658_T002	0.00206067	-2.483592624	Zm00001d023658
Zm00001d023658_T004	0.824532	0.946093972	Zm00001d023658
Zm00001d004640_T001	0.9335676	0.677610289	Zm00001d004640
Zm00001d004640_T002	0.9840043	0.209276433	Zm00001d004640
Zm00001d025353_T020	0.9991964	0.091268521	Zm00001d025353
Zm00001d025353_T001	0.9174933	0.675957915	Zm00001d025353
Zm00001d025353_T019	0.3706063	-0.625082565	Zm00001d025353
Zm00001d025353_T010	0.9399441	0.041863763	Zm00001d025353
Zm00001d007130_T001	0.921164	-0.068322897	Zm00001d007130
Zm00001d051516_T001	0.6156484	-0.26896797	Zm00001d051516
Zm00001d024672_T001	0.923446	0.5827492	Zm00001d024672
Zm00001d041497_T003	0.5174561	0.064385512	Zm00001d041497
Zm00001d041497_T008	0.1870898	-1.206839899	Zm00001d041497
Zm00001d041497_T005	0.2339788	1.248878993	Zm00001d041497
Zm00001d041694_T001	0.05789985	0.323943489	Zm00001d041694
Zm00001d043927_T011	2.43E-13	2.06444107	Zm00001d043927
Zm00001d043927_T002	1	0.29261993	Zm00001d043927
Zm00001d043927_T009	0.9416089	0.115874384	Zm00001d043927
Zm00001d043927_T010	0.5974852	0.707425433	Zm00001d043927
Zm00001d040348_T003	0.02332999	0.66285035	Zm00001d040348
Zm00001d040348_T005	0.5570384	0.395120407	Zm00001d040348
Zm00001d040348_T001	0.9508258	0.495058263	Zm00001d040348
Zm00001d013499_T028	0.3748743	0.855094757	Zm00001d013499
Zm00001d013499_T029	0.6480001	0.702590617	Zm00001d013499
Zm00001d013499_T002	0.9828518	0.485823599	Zm00001d013499
Zm00001d013499_T027	0.907301	0.263435179	Zm00001d013499
Zm00001d013499_T025	0.07199362	0.50073257	Zm00001d013499
Zm00001d016521_T001	0.8643636	0.751001524	Zm00001d016521
Zm00001d048415_T009	0.8781102	-0.107155714	Zm00001d048415
Zm00001d048415_T015	0.2146959	0.394003424	Zm00001d048415
Zm00001d048415_T007	0.5715685	-0.256023717	Zm00001d048415
Zm00001d048415_T022	1	0.103585583	Zm00001d048415
Zm00001d048415_T010	3.64E-10	1.947525216	Zm00001d048415
Zm00001d048415_T012	0.003631449	-1.569935195	Zm00001d048415
Zm00001d048415_T016	0.7167212	-0.135687068	Zm00001d048415

Zm00001d048415_T019	0.1530791	1.82495331	Zm00001d048415
Zm00001d048415_T008	0.797205	-0.240702259	Zm00001d048415
Zm00001d048415_T018	0.3314652	0.892381017	Zm00001d048415
Zm00001d034160_T001	0.000303647	3.828217392	Zm00001d034160
Zm00001d035316_T002	0.8541018	0.438577379	Zm00001d035316
Zm00001d041138_T003	0.8881012	-0.11007878	Zm00001d041138
Zm00001d041138_T001	0.571018	0.42076003	Zm00001d041138
Zm00001d041138_T006	0.5560821	1.28802964	Zm00001d041138
Zm00001d041138_T005	0.7011611	-0.886170007	Zm00001d041138
Zm00001d041138_T004	0.980412	0.241762381	Zm00001d041138
Zm00001d039395_T001	0.7840852	-0.362014118	Zm00001d039395
Zm00001d020703_T001	0.6887207	1.182977084	Zm00001d020703
Zm00001d011890_T001	0.000241547	-1.7592466	Zm00001d011890
Zm00001d049476_T024	0.9582109	-0.321575818	Zm00001d049476
Zm00001d049476_T166	0.9232281	-0.492466547	Zm00001d049476
Zm00001d019726_T001	0.1349847	1.694486786	Zm00001d019726
Zm00001d009077_T002	0.995534	0.460856309	Zm00001d009077
Zm00001d009077_T003	0.3495559	0.450039753	Zm00001d009077
Zm00001d009077_T004	0.7626242	0.352198631	Zm00001d009077
Zm00001d036177_T003	0.9515686	0.505637735	Zm00001d036177
Zm00001d012393_T001	0.957148	0.571281033	Zm00001d012393
Zm00001d012393_T002	1	0.21668955	Zm00001d012393
Zm00001d012393_T004	0.1863399	-1.488794808	Zm00001d012393
Zm00001d013683_T002	0.9580876	0.138988874	Zm00001d013683
Zm00001d013683_T009	1	0.131808067	Zm00001d013683
Zm00001d013683_T001	0.7414578	1.077995947	Zm00001d013683
Zm00001d024828_T002	0.9031129	0.274382335	Zm00001d024828
Zm00001d024828_T004	0.7375754	-0.299615436	Zm00001d024828
Zm00001d024828_T001	0.8704456	-0.064299766	Zm00001d024828
Zm00001d024828_T005	0.9599327	0.551678754	Zm00001d024828
Zm00001d038891_T002	0.07466391	2.214195062	Zm00001d038891
Zm00001d038891_T004	0.9887584	0.452947335	Zm00001d038891
Zm00001d049760_T001	0.9836566	0.193454019	Zm00001d049760
Zm00001d017967_T001	0.241511	0.45872621	Zm00001d017967
Zm00001d017967_T003	0.003017622	0.385890527	Zm00001d017967
Zm00001d017967_T006	0.4607854	0.950532903	Zm00001d017967
Zm00001d017967_T002	1	0.529436716	Zm00001d017967
Zm00001d042306_T004	0.8571283	-0.135467811	Zm00001d042306
Zm00001d037191_T001	0.8819727	0.449818361	Zm00001d037191
Zm00001d037191_T002	0.9256716	0.484047339	Zm00001d037191
Zm00001d016260_T001	0.02179476	-2.202124895	Zm00001d016260
Zm00001d053626_T001	0.8650248	-0.119102499	Zm00001d053626
Zm00001d053626_T002	0.9563232	-0.070379471	Zm00001d053626
Zm00001d052723_T004	1.03E-06	-0.293120189	Zm00001d052723
Zm00001d052723_T010	1	0.73735312	Zm00001d052723
Zm00001d052723_T002	0.9100328	0.848671914	Zm00001d052723
Zm00001d052723_T009	0.6798061	0.68618372	Zm00001d052723
Zm00001d037711_T001	0.9792387	0.117119031	Zm00001d037711
Zm00001d009726_T001	0.9867452	0.081570255	Zm00001d009726
Zm00001d009726_T003	0.011246	-1.662268733	Zm00001d009726
Zm00001d012936_T001	0.7262104	-0.616728063	Zm00001d012936
Zm00001d024967_T006	0.2727417	0.813812682	Zm00001d024967
Zm00001d024967_T003	0.8637863	0.824649762	Zm00001d024967
Zm00001d024967_T005	0.7213635	0.298388335	Zm00001d024967

Zm00001d024967_T002	0.9781672	0.263101781	Zm00001d024967
Zm00001d051457_T002	1	0.288657012	Zm00001d051457
Zm00001d051457_T001	0.9359529	0.730945141	Zm00001d051457
Zm00001d019950_T002	0.8537868	-0.475556155	Zm00001d019950
Zm00001d019950_T001	0.8676122	0.684325519	Zm00001d019950
Zm00001d032480_T001	0.9050121	0.77091241	Zm00001d032480
Zm00001d032480_T002	0.900979	0.809109191	Zm00001d032480
Zm00001d009309_T001	0.01213194	2.561907488	Zm00001d009309
Zm00001d013154_T001	0.7474038	1.157616123	Zm00001d013154
Zm00001d031480_T001	0.8628261	-0.179511021	Zm00001d031480
Zm00001d043510_T001	0.3255715	-0.888085334	Zm00001d043510
Zm00001d043510_T002	0.5383648	1.121905258	Zm00001d043510
Zm00001d020646_T001	0.7414776	-0.385709912	Zm00001d020646
Zm00001d020727_T002	0.02276246	0.834615005	Zm00001d020727
Zm00001d020727_T022	0.9778436	-0.0011021	Zm00001d020727
Zm00001d020727_T020	0.8063892	0.388019399	Zm00001d020727
Zm00001d020727_T001	0.2642794	-0.18083932	Zm00001d020727
Zm00001d020727_T005	0.006488149	1.111537412	Zm00001d020727
Zm00001d020727_T016	0.2093531	2.477117012	Zm00001d020727
Zm00001d015730_T001	0.8937061	-0.627358479	Zm00001d015730
Zm00001d044017_T001	0.9840918	0.231868111	Zm00001d044017
Zm00001d047639_T001	0.908501	0.698924907	Zm00001d047639
Zm00001d047639_T005	0.9894615	0.470820507	Zm00001d047639
Zm00001d047639_T002	1	0.459325217	Zm00001d047639
Zm00001d047639_T003	0.4661639	-0.090529419	Zm00001d047639
Zm00001d047639_T004	0.9557384	0.765639596	Zm00001d047639
Zm00001d053610_T004	0.8513643	0.869245567	Zm00001d053610
Zm00001d053610_T005	0.9648856	0.353871346	Zm00001d053610
Zm00001d053610_T003	0.8195759	-0.197093945	Zm00001d053610
Zm00001d010610_T001	0.9825858	0.501574331	Zm00001d010610
Zm00001d052174_T001	0.9902733	0.40487107	Zm00001d052174
Zm00001d032979_T001	0.8300901	0.87249594	Zm00001d032979
Zm00001d032979_T010	0.7273213	-0.494798564	Zm00001d032979
Zm00001d032979_T006	0.2790987	0.868710425	Zm00001d032979
Zm00001d032979_T003	0.5259601	1.397990714	Zm00001d032979
Zm00001d003183_T001	0.2150214	-1.035153834	Zm00001d003183
Zm00001d002962_T001	0.8350279	0.946898692	Zm00001d002962
Zm00001d018413_T012	0.3497147	0.336070594	Zm00001d018413
Zm00001d018413_T017	6.72E-09	2.192631842	Zm00001d018413
Zm00001d018413_T002	0.9486412	0.249502643	Zm00001d018413
Zm00001d018413_T015	0.9998515	0.318039134	Zm00001d018413
Zm00001d018413_T008	0.9856916	0.411968893	Zm00001d018413
Zm00001d018413_T001	0.4471095	0.682837624	Zm00001d018413
Zm00001d029497_T001	0.7601352	1.018908168	Zm00001d029497
Zm00001d003962_T001	0.6993528	0.800743004	Zm00001d003962
Zm00001d053964_T001	0.8896831	-0.190729175	Zm00001d053964
Zm00001d039989_T001	0.9142822	0.806614973	Zm00001d039989
Zm00001d039183_T001	0.9418022	0.675807433	Zm00001d039183
Zm00001d048270_T002	0.5127506	-0.801303968	Zm00001d048270
Zm00001d048270_T001	0.8802656	0.666860596	Zm00001d048270
Zm00001d044095_T001	0.1350887	1.998970255	Zm00001d044095
Zm00001d050735_T001	0.9563257	0.426056858	Zm00001d050735
Zm00001d050735_T003	0.8830711	0.538254198	Zm00001d050735
Zm00001d029710_T001	0.1680022	1.046783226	Zm00001d029710

Zm00001d042315_T001	0.6436915	2.294378677	Zm00001d042315
Zm00001d039703_T001	0.9166512	-0.016707216	Zm00001d039703
Zm00001d022211_T001	0.7109402	0.719752718	Zm00001d022211
Zm00001d042312_T008	0.9619942	0.150527054	Zm00001d042312
Zm00001d042312_T013	0.4044152	1.500897476	Zm00001d042312
Zm00001d042312_T002	0.9563232	0.676471589	Zm00001d042312
Zm00001d042312_T003	0.9863658	0.180633854	Zm00001d042312
Zm00001d012707_T006	0.8660897	0.630019461	Zm00001d012707
Zm00001d012707_T001	0.8760345	0.520439362	Zm00001d012707
Zm00001d034804_T007	1	0.156704087	Zm00001d034804
Zm00001d034804_T001	0.9344122	-0.004001093	Zm00001d034804
Zm00001d006512_T002	0.9605054	0.652716177	Zm00001d006512
Zm00001d006512_T004	1	0.464501221	Zm00001d006512
Zm00001d040202_T002	0.6903979	-0.60867026	Zm00001d040202
Zm00001d040202_T005	0.9894615	0.318259673	Zm00001d040202
Zm00001d029059_T001	0.7594555	-0.196613097	Zm00001d029059
Zm00001d001835_T001	0.945968	-0.008035205	Zm00001d001835
Zm00001d025238_T001	0.9855106	0.504883308	Zm00001d025238
Zm00001d016473_T001	0.9614723	0.231093713	Zm00001d016473
Zm00001d022355_T001	0.8663036	-0.140137892	Zm00001d022355
Zm00001d045388_T001	0.01390627	-1.719022652	Zm00001d045388
Zm00001d043367_T001	0.9206823	-0.027185544	Zm00001d043367
Zm00001d006813_T001	0.2981646	-0.964957381	Zm00001d006813
Zm00001d036318_T001	1	0.342328267	Zm00001d036318
Zm00001d007577_T001	0.9753288	0.176261613	Zm00001d007577
Zm00001d047834_T002	0.9766874	0.053836599	Zm00001d047834
Zm00001d047834_T007	0.958561	0.552421544	Zm00001d047834
Zm00001d047834_T018	0.07443814	-2.04187024	Zm00001d047834
Zm00001d047834_T016	0.9037807	0.558105406	Zm00001d047834
Zm00001d047834_T012	0.9988864	0.327537166	Zm00001d047834
Zm00001d029390_T003	0.4457952	-0.217291207	Zm00001d029390
Zm00001d029390_T007	0.6328923	-0.192830216	Zm00001d029390
Zm00001d029390_T008	0.6486663	-0.315336176	Zm00001d029390
Zm00001d007624_T001	0.4131629	-1.216295435	Zm00001d007624
Zm00001d014442_T009	0.4014977	0.319933722	Zm00001d014442
Zm00001d014442_T014	0.04574688	-1.774379591	Zm00001d014442
Zm00001d014442_T001	0.5081017	0.821995763	Zm00001d014442
Zm00001d014442_T010	0.6768588	0.371968149	Zm00001d014442
Zm00001d014442_T011	0.3042782	1.75001101	Zm00001d014442
Zm00001d014442_T008	0.4609193	1.37625647	Zm00001d014442
Zm00001d011778_T001	0.9269932	-0.042541036	Zm00001d011778
Zm00001d034517_T001	0.9914912	0.269018515	Zm00001d034517
Zm00001d046613_T004	0.4146004	0.346466518	Zm00001d046613
Zm00001d046613_T005	1	0.21994007	Zm00001d046613
Zm00001d046613_T008	0.9390517	0.010117079	Zm00001d046613
Zm00001d020485_T001	0.9906533	0.371563604	Zm00001d020485
Zm00001d029177_T003	0.9936409	0.218147324	Zm00001d029177
Zm00001d029177_T001	0.7642882	-0.280976586	Zm00001d029177
Zm00001d043551_T001	0.9410845	0.441574897	Zm00001d043551
Zm00001d043551_T003	0.9919298	0.437256738	Zm00001d043551
Zm00001d029675_T001	0.866232	0.601736239	Zm00001d029675
Zm00001d008601_T001	0.7889719	-0.72510238	Zm00001d008601
Zm00001d012797_T011	1	0.311779007	Zm00001d012797
Zm00001d012797_T012	0.4831314	0.721816848	Zm00001d012797

Zm00001d012797_T006	0.2693973	-0.435550392	Zm00001d012797
Zm00001d012797_T014	0.9770343	0.009616889	Zm00001d012797
Zm00001d012797_T013	0.4193179	1.672397154	Zm00001d012797
Zm00001d012797_T010	0.3953411	1.134981052	Zm00001d012797
Zm00001d012797_T007	0.0339815	1.807863986	Zm00001d012797
Zm00001d012797_T009	0.2032568	0.369458559	Zm00001d012797
Zm00001d012797_T015	0.02463906	-0.621732402	Zm00001d012797
Zm00001d012797_T001	2.57E-14	-3.750762053	Zm00001d012797
Zm00001d016373_T010	0.9647033	0.335240386	Zm00001d016373
Zm00001d016373_T003	0.9160053	0.831496076	Zm00001d016373
Zm00001d016373_T007	0.8028573	-0.513609149	Zm00001d016373
Zm00001d048354_T001	0.9635231	0.42886021	Zm00001d048354
Zm00001d023560_T002	0.9867452	0.198192277	Zm00001d023560
Zm00001d023560_T001	0.9583056	0.07092718	Zm00001d023560
Zm00001d025047_T001	0.8471986	0.799451956	Zm00001d025047
Zm00001d011403_T001	0.02048591	2.033261807	Zm00001d011403
Zm00001d016453_T001	0.8074585	0.631241088	Zm00001d016453
Zm00001d022489_T002	0.9621967	0.019043368	Zm00001d022489
Zm00001d022489_T001	0.9706887	0.231289618	Zm00001d022489
Zm00001d022489_T003	0.9361945	-0.159581405	Zm00001d022489
Zm00001d038737_T001	0.5753645	-0.748718266	Zm00001d038737
Zm00001d018358_T001	0.438342	-0.611861075	Zm00001d018358
Zm00001d025023_T002	0.9939021	0.464443694	Zm00001d025023
Zm00001d025023_T001	0.8757833	-0.158223571	Zm00001d025023
Zm00001d014989_T001	0.6508204	-0.537900706	Zm00001d014989
Zm00001d052519_T007	0.8434744	-0.181719796	Zm00001d052519
Zm00001d052519_T004	0.7333	0.273717123	Zm00001d052519
Zm00001d009473_T001	0.9311674	0.054774164	Zm00001d009473
Zm00001d006591_T001	0.7934233	0.655889326	Zm00001d006591
Zm00001d017866_T001	0.9519347	0.696434943	Zm00001d017866
Zm00001d017866_T002	1	0.383439586	Zm00001d017866
Zm00001d017866_T003	0.978168	0.608896903	Zm00001d017866
Zm00001d037596_T001	0.9613462	0.045509528	Zm00001d037596
Zm00001d037596_T002	0.9766874	0.557287304	Zm00001d037596
Zm00001d037596_T003	0.7433188	0.887194659	Zm00001d037596
Zm00001d005140_T004	0.7345787	-0.052106927	Zm00001d005140
Zm00001d005140_T002	0.9686323	0.044019358	Zm00001d005140
Zm00001d011541_T002	0.9845237	0.51630151	Zm00001d011541
Zm00001d011541_T009	0.9852645	0.244741995	Zm00001d011541
Zm00001d011541_T007	0.004998487	-1.773669633	Zm00001d011541
Zm00001d011541_T003	0.005044807	-0.604243997	Zm00001d011541
Zm00001d011541_T008	0.1799392	1.901898658	Zm00001d011541
Zm00001d008296_T001	0.1272455	2.009781807	Zm00001d008296
Zm00001d012282_T001	0.5549472	-1.007329405	Zm00001d012282
Zm00001d046083_T001	0.9686323	0.129972128	Zm00001d046083
Zm00001d022180_T007	0.6053145	-0.972559175	Zm00001d022180
Zm00001d022180_T009	0.5815227	-0.694514527	Zm00001d022180
Zm00001d022180_T001	0.8007432	-0.331829268	Zm00001d022180
Zm00001d050768_T003	0.2208276	-1.221014324	Zm00001d050768
Zm00001d051629_T008	0.9426876	0.724798856	Zm00001d051629
Zm00001d051629_T001	0.07645399	-1.650111215	Zm00001d051629
Zm00001d015168_T001	0.8412913	-0.160007055	Zm00001d015168
Zm00001d008708_T001	0.9637995	0.174163683	Zm00001d008708
Zm00001d049049_T004	0.000653854	1.277244719	Zm00001d049049

Zm00001d049049_T003	0.06455868	0.929724288	Zm00001d049049
Zm00001d049049_T002	0.7626242	1.064292656	Zm00001d049049
Zm00001d038512_T001	0.980286	0.396279624	Zm00001d038512
Zm00001d053984_T002	0.9524127	0.102229769	Zm00001d053984
Zm00001d053984_T003	0.9184323	0.528973971	Zm00001d053984
Zm00001d048888_T001	0.9627389	0.046160648	Zm00001d048888
Zm00001d023590_T005	0.9998515	0.765995983	Zm00001d023590
Zm00001d023590_T007	0.2922198	1.070272616	Zm00001d023590
Zm00001d023590_T002	0.9765009	0.253708113	Zm00001d023590
Zm00001d023590_T004	0.4698008	-0.188161912	Zm00001d023590
Zm00001d023590_T006	0.08874285	0.440672002	Zm00001d023590
Zm00001d037059_T027	0.962254	0.091456488	Zm00001d037059
Zm00001d037059_T016	0.432426	0.282808095	Zm00001d037059
Zm00001d037059_T005	0.02533317	0.642999727	Zm00001d037059
Zm00001d037059_T004	0.05703555	0.472242184	Zm00001d037059
Zm00001d037059_T026	0.7518598	-0.237797566	Zm00001d037059
Zm00001d047749_T001	0.8984709	-0.039427743	Zm00001d047749
Zm00001d018636_T002	0.2295978	-1.143952219	Zm00001d018636
Zm00001d018636_T003	0.8342677	-0.153597757	Zm00001d018636
Zm00001d014726_T010	0.4466862	1.560216482	Zm00001d014726
Zm00001d014726_T011	0.6466858	1.317925145	Zm00001d014726
Zm00001d014726_T012	0.007022294	2.563301273	Zm00001d014726
Zm00001d014726_T005	0.01144665	2.070955928	Zm00001d014726
Zm00001d014726_T008	3.47E-13	-3.984572053	Zm00001d014726
Zm00001d032718_T001	0.8384064	-0.201769862	Zm00001d032718
Zm00001d001933_T001	0.6485803	-0.564507911	Zm00001d001933
Zm00001d029762_T001	0.6010203	-0.551927642	Zm00001d029762
Zm00001d029762_T002	0.944166	0.046791636	Zm00001d029762
Zm00001d029762_T004	0.6565447	1.172042935	Zm00001d029762
Zm00001d029762_T008	0.907938	0.869785168	Zm00001d029762
Zm00001d037966_T008	0.9870394	-0.026727921	Zm00001d037966
Zm00001d037966_T005	0.9834601	0.108895476	Zm00001d037966
Zm00001d037966_T002	0.9393171	0.394495072	Zm00001d037966
Zm00001d037966_T004	0.9285812	0.409051452	Zm00001d037966
Zm00001d037966_T006	0.9934917	0.16561444	Zm00001d037966
Zm00001d037966_T001	0.6594369	1.13368405	Zm00001d037966
Zm00001d040268_T012	0.4672873	0.340459339	Zm00001d040268
Zm00001d040268_T008	0.534033	0.853413038	Zm00001d040268
Zm00001d040268_T002	0.9482354	0.05474204	Zm00001d040268
Zm00001d033726_T013	6.40E-13	2.32466808	Zm00001d033726
Zm00001d033726_T036	0.877725	0.832513382	Zm00001d033726
Zm00001d033726_T011	0.8584358	0.570027821	Zm00001d033726
Zm00001d033726_T038	0.7475609	-0.684491928	Zm00001d033726
Zm00001d033726_T007	0.8098845	0.862331979	Zm00001d033726
Zm00001d006127_T002	1	0.331800112	Zm00001d006127
Zm00001d006127_T001	0.5443214	-0.970338732	Zm00001d006127
Zm00001d006127_T003	0.9748076	0.519041893	Zm00001d006127
Zm00001d019643_T009	0.4723494	0.309925605	Zm00001d019643
Zm00001d019643_T001	0.9979568	0.16540147	Zm00001d019643
Zm00001d019643_T002	0.7520438	-0.253852962	Zm00001d019643
Zm00001d028339_T001	0.917446	0.142753607	Zm00001d028339
Zm00001d028339_T023	0.9988864	0.125742575	Zm00001d028339
Zm00001d028339_T004	0.006974629	1.371009551	Zm00001d028339
Zm00001d028339_T018	0.5385297	0.40928933	Zm00001d028339

Zm00001d028339_T007	2.45E-05	-2.157440168	Zm00001d028339
Zm00001d005502_T003	0.891216	-0.061164168	Zm00001d005502
Zm00001d005502_T002	0.0181716	3.195667199	Zm00001d005502
Zm00001d005502_T015	0.3648281	-0.905225229	Zm00001d005502
Zm00001d005502_T018	0.9118093	-0.02805137	Zm00001d005502
Zm00001d005502_T009	1.20E-09	0.947613591	Zm00001d005502
Zm00001d044204_T001	1	0.187836428	Zm00001d044204
Zm00001d046506_T001	1	0.414454257	Zm00001d046506
Zm00001d046506_T003	3.65E-08	1.597249314	Zm00001d046506
Zm00001d046506_T004	0.4786492	0.848464637	Zm00001d046506
Zm00001d037537_T003	0.9856605	0.848571035	Zm00001d037537
Zm00001d037537_T002	0.8099107	0.930709039	Zm00001d037537
Zm00001d003911_T008	0.543223	2.199117958	Zm00001d003911
Zm00001d003911_T001	0.6343135	-0.37909551	Zm00001d003911
Zm00001d003911_T011	0.9745777	0.552614897	Zm00001d003911
Zm00001d003911_T010	0.6695932	-0.325341165	Zm00001d003911
Zm00001d035198_T001	1	0.302177327	Zm00001d035198
Zm00001d032045_T001	0.9956119	0.214362991	Zm00001d032045
Zm00001d041882_T003	0.9490518	-0.332449821	Zm00001d041882
Zm00001d041882_T001	0.8256776	-0.584010369	Zm00001d041882
Zm00001d041882_T002	0.945968	-0.050714128	Zm00001d041882
Zm00001d047737_T001	0.910614	-0.052981875	Zm00001d047737
Zm00001d044691_T001	0.9927201	0.282506394	Zm00001d044691
Zm00001d039626_T003	0.9968176	0.229520127	Zm00001d039626
Zm00001d039626_T001	0.9515686	0.035533155	Zm00001d039626
Zm00001d043299_T001	0.09069548	-1.729824206	Zm00001d043299
Zm00001d021893_T003	0.9730613	0.202959048	Zm00001d021893
Zm00001d021893_T005	0.2690004	0.477322906	Zm00001d021893
Zm00001d021893_T002	0.04465781	2.643666995	Zm00001d021893
Zm00001d021893_T004	0.5795936	-0.745296229	Zm00001d021893
Zm00001d007890_T005	0.9006781	1.026393777	Zm00001d007890
Zm00001d007890_T006	0.09796945	-1.772742418	Zm00001d007890
Zm00001d007890_T003	0.9732421	0.310344636	Zm00001d007890
Zm00001d007890_T001	0.9443426	0.20575144	Zm00001d007890
Zm00001d037613_T004	0.7659004	-0.063865231	Zm00001d037613
Zm00001d037613_T005	0.8625537	0.424307681	Zm00001d037613
Zm00001d037613_T001	0.9765009	0.543534195	Zm00001d037613
Zm00001d006561_T001	0.7406865	-0.721792692	Zm00001d006561
Zm00001d045581_T001	0.9189175	-0.361388562	Zm00001d045581
Zm00001d018243_T002	0.2238491	-1.44232571	Zm00001d018243
Zm00001d018243_T001	0.9176356	-0.048517922	Zm00001d018243
Zm00001d019100_T001	0.9862402	0.206180012	Zm00001d019100
Zm00001d050357_T004	0.9682673	0.146759254	Zm00001d050357
Zm00001d050357_T007	0.9804433	0.092971264	Zm00001d050357
Zm00001d050357_T001	0.8330084	-0.119881079	Zm00001d050357
Zm00001d050357_T006	0.9746187	0.101960966	Zm00001d050357
Zm00001d050357_T005	0.9988762	0.119185974	Zm00001d050357
Zm00001d013831_T012	0.9073468	0.77841504	Zm00001d013831
Zm00001d013831_T013	0.5588216	0.738948371	Zm00001d013831
Zm00001d013831_T002	0.6504599	-0.406758616	Zm00001d013831
Zm00001d013831_T011	0.7175167	1.065432263	Zm00001d013831
Zm00001d013831_T010	0.9771057	0.552718244	Zm00001d013831
Zm00001d013831_T014	0.9356767	-0.082230078	Zm00001d013831
Zm00001d013831_T015	0.03429718	-1.229825716	Zm00001d013831

Zm00001d013831_T006	0.9283222	0.667545726	Zm00001d013831
Zm00001d040898_T001	0.9416622	0.55049614	Zm00001d040898
Zm00001d052010_T002	1	0.027885113	Zm00001d052010
Zm00001d029074_T003	0.9970835	0.407471986	Zm00001d029074
Zm00001d029074_T002	0.9609806	0.037675757	Zm00001d029074
Zm00001d048483_T001	0.9125205	-0.130010819	Zm00001d048483
Zm00001d048483_T002	0.9543425	-0.006124367	Zm00001d048483
Zm00001d048483_T003	0.9429712	-0.092889709	Zm00001d048483
Zm00001d001824_T001	0.9557384	-0.265475237	Zm00001d001824
Zm00001d049708_T001	0.50213	-0.559929641	Zm00001d049708
Zm00001d049708_T008	0.3295805	1.231142953	Zm00001d049708
Zm00001d049708_T006	0.8645432	0.710923792	Zm00001d049708
Zm00001d017076_T001	0.8213809	-0.299820232	Zm00001d017076
Zm00001d005437_T001	0.9791729	0.032161096	Zm00001d005437
Zm00001d013543_T007	0.5962054	-0.368966074	Zm00001d013543
Zm00001d013543_T009	0.9216521	0.022305883	Zm00001d013543
Zm00001d013543_T005	0.0606529	1.141603189	Zm00001d013543
Zm00001d013543_T002	0.5154504	1.036521224	Zm00001d013543
Zm00001d005733_T002	1	0.042105789	Zm00001d005733
Zm00001d005733_T001	0.5825606	-0.776353169	Zm00001d005733
Zm00001d009957_T001	0.9429712	0.048607862	Zm00001d009957
Zm00001d016732_T005	0.3966246	0.690162969	Zm00001d016732
Zm00001d016732_T003	0.7766169	-0.282546355	Zm00001d016732
Zm00001d040051_T039	0.954984	-0.305759622	Zm00001d040051
Zm00001d021026_T001	0.8031436	-0.27955652	Zm00001d021026
Zm00001d002235_T027	0.6033645	0.40096095	Zm00001d002235
Zm00001d002235_T009	0.8603613	-0.110423744	Zm00001d002235
Zm00001d002235_T019	0.5924463	0.772338329	Zm00001d002235
Zm00001d002235_T026	1	0.153293764	Zm00001d002235
Zm00001d002235_T017	0.01806011	0.797353215	Zm00001d002235
Zm00001d002235_T006	0.1316503	0.52651356	Zm00001d002235
Zm00001d002235_T010	0.7613617	0.13049492	Zm00001d002235
Zm00001d002235_T008	0.8050292	0.757001347	Zm00001d002235
Zm00001d035899_T001	0.6329403	-0.518595372	Zm00001d035899
Zm00001d000027_T029	0.0584017	0.687683492	Zm00001d000027
Zm00001d000027_T020	0.9404125	0.153571456	Zm00001d000027
Zm00001d000027_T031	0.000238001	1.022408335	Zm00001d000027
Zm00001d000027_T035	0.85068	-0.60271535	Zm00001d000027
Zm00001d000027_T007	0.9765009	0.25233872	Zm00001d000027
Zm00001d000027_T030	0.7194959	-0.113591953	Zm00001d000027
Zm00001d021823_T002	0.7658741	1.105399509	Zm00001d021823
Zm00001d021823_T004	0.3102519	0.239503594	Zm00001d021823
Zm00001d021823_T003	0.852831	0.292510655	Zm00001d021823
Zm00001d037279_T009	0.002717702	0.7318395	Zm00001d037279
Zm00001d037279_T011	0.01374028	2.491567696	Zm00001d037279
Zm00001d037279_T002	0.8886159	-0.024746538	Zm00001d037279
Zm00001d037279_T005	0.01503145	2.448476388	Zm00001d037279
Zm00001d037279_T013	0.9868554	0.299061805	Zm00001d037279
Zm00001d022631_T002	0.9887584	0.389528924	Zm00001d022631
Zm00001d022631_T001	0.677308	0.248653761	Zm00001d022631
Zm00001d022631_T003	0.9887889	0.296734031	Zm00001d022631
Zm00001d042863_T001	0.954492	0.056995266	Zm00001d042863
Zm00001d053168_T001	0.8272469	0.617634267	Zm00001d053168
Zm00001d008489_T001	0.7946466	0.887759241	Zm00001d008489

Zm00001d018099_T002	0.8829082	0.665114716	Zm00001d018099
Zm00001d018099_T001	0.9311027	-0.01507908	Zm00001d018099
Zm00001d016567_T001	0.9216521	2.009969814	Zm00001d016567
Zm00001d038200_T001	0.745718	-0.064219557	Zm00001d038200
Zm00001d038200_T005	0.8591203	1.307456287	Zm00001d038200
Zm00001d038200_T010	0.00436371	2.673325582	Zm00001d038200
Zm00001d038200_T004	0.05268763	0.855434075	Zm00001d038200
Zm00001d038200_T008	0.9336646	-0.24157691	Zm00001d038200
Zm00001d038200_T003	0.9451369	0.109619149	Zm00001d038200
Zm00001d038200_T009	0.5088062	0.685053648	Zm00001d038200
Zm00001d012005_T024	0.851347	0.896755999	Zm00001d012005
Zm00001d012005_T004	0.5813054	1.337022768	Zm00001d012005
Zm00001d012005_T025	0.6702673	1.050630276	Zm00001d012005
Zm00001d012005_T002	0.9928864	0.10463187	Zm00001d012005
Zm00001d012005_T015	5.29E-12	-1.727787353	Zm00001d012005
Zm00001d041382_T001	0.000780838	-3.071343444	Zm00001d041382
Zm00001d043806_T001	0.9197844	-0.169718498	Zm00001d043806
Zm00001d033641_T001	0.9804228	0.148023747	Zm00001d033641
Zm00001d033641_T003	0.98291	0.413088243	Zm00001d033641
Zm00001d033641_T005	0.897685	-0.054713001	Zm00001d033641
Zm00001d033641_T004	1	0.105439137	Zm00001d033641
Zm00001d033232_T001	0.9771927	0.055870781	Zm00001d033232
Zm00001d041111_T001	0.6772176	1.049640161	Zm00001d041111
Zm00001d041111_T002	0.3973189	1.624506887	Zm00001d041111
Zm00001d028575_T001	0.9931431	0.333049398	Zm00001d028575
Zm00001d013755_T003	0.926133	0.651938168	Zm00001d013755
Zm00001d013755_T002	0.8752644	0.796262786	Zm00001d013755
Zm00001d017821_T002	0.2349161	1.391889375	Zm00001d017821
Zm00001d017821_T001	0.3716692	1.290177263	Zm00001d017821
Zm00001d013335_T001	0.9619176	0.537973264	Zm00001d013335
Zm00001d045009_T001	0.9033985	0.155009565	Zm00001d045009
Zm00001d006910_T009	0.9988864	0.236124648	Zm00001d006910
Zm00001d006910_T004	0.5260431	-0.542583768	Zm00001d006910
Zm00001d006910_T015	0.7443376	-1.502862176	Zm00001d006910
Zm00001d006910_T008	0.1883701	1.314267895	Zm00001d006910
Zm00001d006910_T013	1.42E-06	1.451751507	Zm00001d006910
Zm00001d006910_T001	0.001374172	1.382782937	Zm00001d006910
Zm00001d006910_T010	0.8457647	0.984090494	Zm00001d006910
Zm00001d006910_T005	0.3679441	-0.718694442	Zm00001d006910
Zm00001d006910_T011	0.8807115	0.156170488	Zm00001d006910
Zm00001d049826_T001	0.8010758	-0.554044109	Zm00001d049826
Zm00001d007595_T017	0.9374953	0.009154938	Zm00001d007595
Zm00001d007595_T013	0.8842271	0.337549458	Zm00001d007595
Zm00001d007595_T025	4.26E-09	1.283940374	Zm00001d007595
Zm00001d007595_T012	0.9201237	0.000377037	Zm00001d007595
Zm00001d007595_T014	0.9768802	0.497554354	Zm00001d007595
Zm00001d007595_T027	0.9164622	0.515183685	Zm00001d007595
Zm00001d007595_T022	0.9402253	0.255675179	Zm00001d007595
Zm00001d007595_T010	0.8077635	-0.140960367	Zm00001d007595
Zm00001d043389_T001	0.2175243	1.912290822	Zm00001d043389
Zm00001d043389_T003	0.2756202	1.078636216	Zm00001d043389
Zm00001d034183_T003	0.9455715	0.278657776	Zm00001d034183
Zm00001d034183_T006	0.9657993	0.480206914	Zm00001d034183
Zm00001d034183_T002	1	0.298625211	Zm00001d034183

Zm00001d034183_T004	0.7343055	-0.225799466	Zm00001d034183
Zm00001d024493_T008	0.9903568	0.245998241	Zm00001d024493
Zm00001d024493_T002	0.3113324	1.742624282	Zm00001d024493
Zm00001d024493_T009	0.7957286	-0.359771485	Zm00001d024493
Zm00001d024493_T003	0.1574436	1.281626338	Zm00001d024493
Zm00001d024493_T006	0.9693993	0.153864939	Zm00001d024493
Zm00001d053753_T007	0.6088201	-0.208608322	Zm00001d053753
Zm00001d053753_T001	0.8865278	0.636381282	Zm00001d053753
Zm00001d053753_T003	0.9180662	0.808561855	Zm00001d053753
Zm00001d053753_T013	0.4583128	-0.731046434	Zm00001d053753
Zm00001d053753_T008	0.9177108	-0.025380878	Zm00001d053753
Zm00001d021093_T001	0.4386149	-1.035835109	Zm00001d021093
Zm00001d005532_T001	0.9979568	-0.039990854	Zm00001d005532
Zm00001d005532_T003	0.8026836	0.889163721	Zm00001d005532
Zm00001d005532_T002	0.9850313	0.032676899	Zm00001d005532
Zm00001d042932_T001	0.9749152	0.120890495	Zm00001d042932
Zm00001d037120_T001	0.9545374	0.134133517	Zm00001d037120
Zm00001d042148_T001	0.9210327	0.5988139	Zm00001d042148
Zm00001d019759_T001	0.7759327	0.998142751	Zm00001d019759
Zm00001d042281_T001	0.8257365	-0.188585583	Zm00001d042281
Zm00001d036118_T001	0.0080401	-2.088950199	Zm00001d036118
Zm00001d045557_T003	0.003289969	3.05404662	Zm00001d045557
Zm00001d045557_T004	0.04298108	2.572943875	Zm00001d045557
Zm00001d045557_T001	0.005053822	3.193339025	Zm00001d045557
Zm00001d037449_T002	1	0.226131084	Zm00001d037449
Zm00001d024420_T005	0.9469426	0.016172943	Zm00001d024420
Zm00001d024420_T001	0.909729	-0.011813016	Zm00001d024420
Zm00001d024420_T006	1	0.225871537	Zm00001d024420
Zm00001d006192_T002	0.9859755	0.181122956	Zm00001d006192
Zm00001d006192_T001	0.988575	0.28899685	Zm00001d006192
Zm00001d041444_T002	0.4751894	-0.671203041	Zm00001d041444
Zm00001d039929_T001	0.6700298	0.680021066	Zm00001d039929
Zm00001d028130_T001	0.9213679	-0.071949059	Zm00001d028130
Zm00001d028130_T002	1	0.050427805	Zm00001d028130
Zm00001d033187_T002	0.8219372	0.897032947	Zm00001d033187
Zm00001d033187_T003	0.8950139	-0.15954459	Zm00001d033187
Zm00001d033187_T001	0.8743087	0.897978901	Zm00001d033187
Zm00001d009638_T001	0.9673473	0.185888286	Zm00001d009638
Zm00001d038038_T001	0.9998515	0.12222104	Zm00001d038038
Zm00001d002789_T001	0.8107637	-0.128411235	Zm00001d002789
Zm00001d051967_T001	0.8864089	-0.102780918	Zm00001d051967
Zm00001d002969_T001	0.5209964	-1.024124032	Zm00001d002969
Zm00001d045094_T001	0.9335862	-0.065459092	Zm00001d045094
Zm00001d020526_T001	0.0249272	-0.435355281	Zm00001d020526
Zm00001d052922_T005	0.9614723	0.32538244	Zm00001d052922
Zm00001d052922_T009	0.9225227	0.033102236	Zm00001d052922
Zm00001d052922_T007	0.9945118	0.392121799	Zm00001d052922
Zm00001d052922_T010	0.9281046	-0.073010372	Zm00001d052922
Zm00001d052673_T001	0.006878684	3.058653251	Zm00001d052673
Zm00001d052673_T002	0.9195654	0.628944014	Zm00001d052673
Zm00001d016248_T001	0.9370449	0.072625186	Zm00001d016248
Zm00001d028414_T003	0.9801246	0.379879269	Zm00001d028414
Zm00001d028414_T001	0.9489956	0.175109259	Zm00001d028414
Zm00001d015026_T004	0.9681793	0.556431727	Zm00001d015026

Zm00001d015026_T001	0.988757	0.268929181	Zm00001d015026
Zm00001d015026_T002	0.9805897	0.080490418	Zm00001d015026
Zm00001d026483_T004	0.8894168	-0.077483385	Zm00001d026483
Zm00001d026483_T001	0.7065431	1.204721728	Zm00001d026483
Zm00001d026483_T002	0.883637	0.599539213	Zm00001d026483
Zm00001d042800_T001	0.967161	0.37336934	Zm00001d042800
Zm00001d013177_T004	0.1330891	-0.352765962	Zm00001d013177
Zm00001d039860_T007	0.2616224	-0.911153968	Zm00001d039860
Zm00001d039860_T011	0.6745773	0.6866099	Zm00001d039860
Zm00001d039860_T008	0.9627389	0.69321503	Zm00001d039860
Zm00001d039860_T002	0.8455957	0.724319431	Zm00001d039860
Zm00001d039860_T009	0.003818565	2.173250334	Zm00001d039860
Zm00001d039860_T013	0.7803208	0.357032925	Zm00001d039860
Zm00001d052480_T001	0.1242419	-1.374480861	Zm00001d052480
Zm00001d040310_T001	0.7648702	0.967075009	Zm00001d040310
Zm00001d037609_T003	0.02824001	-0.968851634	Zm00001d037609
Zm00001d037609_T006	0.8680274	0.336297234	Zm00001d037609
Zm00001d037609_T002	0.9664406	0.100954771	Zm00001d037609
Zm00001d037609_T001	0.1565832	-0.722030857	Zm00001d037609
Zm00001d015058_T003	0.910604	-0.320758336	Zm00001d015058
Zm00001d015058_T001	0.9363242	-0.126597244	Zm00001d015058
Zm00001d045692_T019	0.003015693	1.052486371	Zm00001d045692
Zm00001d045692_T028	0.0169199	1.251629713	Zm00001d045692
Zm00001d045692_T029	0.306157	0.227731525	Zm00001d045692
Zm00001d045692_T031	0.9563232	0.150482066	Zm00001d045692
Zm00001d024757_T001	0.5896544	-0.649762218	Zm00001d024757
Zm00001d048698_T006	0.7194121	0.488433389	Zm00001d048698
Zm00001d048698_T004	0.3367371	-0.727334095	Zm00001d048698
Zm00001d048698_T003	0.4698008	-0.665109621	Zm00001d048698
Zm00001d048698_T005	0.8008612	-0.241356376	Zm00001d048698
Zm00001d027971_T001	0.8267006	0.613573101	Zm00001d027971
Zm00001d027971_T003	0.2518892	-1.00435679	Zm00001d027971
Zm00001d027971_T004	0.7889662	-0.803756154	Zm00001d027971
Zm00001d027971_T002	0.8256776	0.953174781	Zm00001d027971
Zm00001d037235_T001	0.006220777	1.114822146	Zm00001d037235
Zm00001d039461_T001	0.4164664	1.366929678	Zm00001d039461
Zm00001d017991_T001	0.2425907	0.848750697	Zm00001d017991
Zm00001d016998_T001	0.9834756	0.182632612	Zm00001d016998
Zm00001d015008_T001	0.9840918	0.289066839	Zm00001d015008
Zm00001d046836_T003	0.9918302	0.242433576	Zm00001d046836
Zm00001d052123_T001	0.8209051	-0.354567225	Zm00001d052123
Zm00001d002052_T003	0.648526	0.929734806	Zm00001d002052
Zm00001d002052_T001	0.1757415	2.019035486	Zm00001d002052
Zm00001d002052_T002	0.000273283	-2.348535816	Zm00001d002052
Zm00001d007445_T001	0.4153808	-0.745272813	Zm00001d007445
Zm00001d040259_T002	0.8819727	-0.097698351	Zm00001d040259
Zm00001d040259_T004	0.9429712	0.051166382	Zm00001d040259
Zm00001d028392_T001	0.923446	-0.159418147	Zm00001d028392
Zm00001d044102_T006	6.83E-07	2.395966581	Zm00001d044102
Zm00001d044102_T001	0.9673473	0.16185473	Zm00001d044102
Zm00001d029534_T001	0.9689939	0.050660941	Zm00001d029534
Zm00001d029534_T002	1	0.376515004	Zm00001d029534
Zm00001d049452_T001	0.9264186	0.506819527	Zm00001d049452
Zm00001d021943_T004	0.8643636	-0.068359451	Zm00001d021943

Zm00001d021943_T003	0.8680274	-0.469454078	Zm00001d021943
Zm00001d007016_T001	0.9791066	0.446111255	Zm00001d007016
Zm00001d007951_T001	0.9617391	0.099672618	Zm00001d007951
Zm00001d026297_T001	0.9419336	0.59498274	Zm00001d026297
Zm00001d052473_T003	0.6076098	-0.613784567	Zm00001d052473
Zm00001d052473_T001	2.53E-06	0.466135406	Zm00001d052473
Zm00001d052473_T002	0.23288	-1.063013075	Zm00001d052473
Zm00001d014882_T006	0.6033645	-0.443975553	Zm00001d014882
Zm00001d014882_T005	0.9994624	0.23358925	Zm00001d014882
Zm00001d029134_T002	0.9237037	-0.066826904	Zm00001d029134
Zm00001d029134_T005	5.11E-05	0.633846578	Zm00001d029134
Zm00001d047761_T005	0.8890998	0.4292286	Zm00001d047761
Zm00001d047761_T009	0.6141894	1.455000359	Zm00001d047761
Zm00001d047761_T001	0.1685404	1.070010232	Zm00001d047761
Zm00001d047761_T014	0.9822138	0.413363087	Zm00001d047761
Zm00001d047761_T015	0.3677159	0.09913166	Zm00001d047761
Zm00001d047761_T012	0.9596377	0.473135546	Zm00001d047761
Zm00001d047761_T006	8.51E-12	2.323238688	Zm00001d047761
Zm00001d047761_T008	7.16E-09	1.319175572	Zm00001d047761
Zm00001d002694_T005	0.9213556	0.718073271	Zm00001d002694
Zm00001d024239_T001	0.9740325	-0.439939858	Zm00001d024239
Zm00001d042034_T001	0.02161544	1.768853257	Zm00001d042034
Zm00001d042034_T004	0.9617391	0.16460534	Zm00001d042034
Zm00001d042034_T003	0.731653	-0.300905141	Zm00001d042034
Zm00001d051001_T004	0.5954619	-0.611510342	Zm00001d051001
Zm00001d051001_T002	0.8021989	-0.255593712	Zm00001d051001
Zm00001d008987_T001	0.9811162	0.482509424	Zm00001d008987
Zm00001d049557_T002	0.8982893	-0.573748369	Zm00001d049557
Zm00001d021883_T001	0.9906533	0.173106652	Zm00001d021883
Zm00001d004248_T001	0.9291337	0.008554748	Zm00001d004248
Zm00001d010388_T001	0.1811895	3.008210869	Zm00001d010388
Zm00001d027936_T003	2.55E-05	1.676135289	Zm00001d027936
Zm00001d027936_T009	0.2342148	-1.069686875	Zm00001d027936
Zm00001d027936_T007	0.9846714	0.143782303	Zm00001d027936
Zm00001d027936_T004	0.8886661	0.000542793	Zm00001d027936
Zm00001d027936_T005	0.51701	1.390527929	Zm00001d027936
Zm00001d045064_T001	0.9768802	0.037552344	Zm00001d045064
Zm00001d049832_T001	1	0.305458292	Zm00001d049832
Zm00001d028230_T001	0.8440444	0.95885631	Zm00001d028230
Zm00001d002860_T001	0.9906533	0.1165239	Zm00001d002860
Zm00001d012170_T002	0.8647831	0.911361435	Zm00001d012170
Zm00001d012170_T001	0.8409106	-0.181558247	Zm00001d012170
Zm00001d036613_T001	1	0.144722939	Zm00001d036613
Zm00001d022031_T005	0.8356083	0.732793772	Zm00001d022031
Zm00001d022031_T001	0.8825173	-0.152629915	Zm00001d022031
Zm00001d022031_T002	0.9909153	0.141166231	Zm00001d022031
Zm00001d020454_T003	0.8031436	1.021565526	Zm00001d020454
Zm00001d020454_T002	0.7187088	1.127225549	Zm00001d020454
Zm00001d020454_T004	0.9914064	0.392849449	Zm00001d020454
Zm00001d024752_T001	0.9126586	0.638581627	Zm00001d024752
Zm00001d028806_T001	0.3704188	1.445203037	Zm00001d028806
Zm00001d053661_T003	0.7213008	-0.548812104	Zm00001d053661
Zm00001d008730_T001	0.8964117	-0.189655819	Zm00001d008730
Zm00001d011233_T050	0.9469426	0.092111875	Zm00001d011233

Zm00001d011233_T026	0.8894168	0.276409175	Zm00001d011233
Zm00001d011233_T019	0.6329403	-0.365563235	Zm00001d011233
Zm00001d011233_T018	0.000872228	1.44743646	Zm00001d011233
Zm00001d011233_T041	0.8842271	0.180290284	Zm00001d011233
Zm00001d011233_T001	0.1936436	0.482197723	Zm00001d011233
Zm00001d011233_T007	0.0691493	1.88882595	Zm00001d011233
Zm00001d011233_T006	0.03130693	-0.389415125	Zm00001d011233
Zm00001d032899_T001	0.9596377	0.087355521	Zm00001d032899
Zm00001d029662_T001	0.8077635	0.987206052	Zm00001d029662
Zm00001d041831_T003	0.9563257	0.21090318	Zm00001d041831
Zm00001d041831_T001	1	0.48739866	Zm00001d041831
Zm00001d041831_T002	0.6146141	1.354991308	Zm00001d041831
Zm00001d038824_T001	0.3966246	-0.829468121	Zm00001d038824
Zm00001d038824_T002	0.8027057	-0.064023823	Zm00001d038824
Zm00001d003841_T002	0.3097848	1.600267845	Zm00001d003841
Zm00001d051111_T018	0.001427798	-0.430696187	Zm00001d051111
Zm00001d034125_T002	0.9702252	0.198678501	Zm00001d034125
Zm00001d034125_T001	0.9668709	0.616403456	Zm00001d034125
Zm00001d050116_T003	0.6737073	-0.794966724	Zm00001d050116
Zm00001d050116_T001	0.6947003	0.811668129	Zm00001d050116
Zm00001d050116_T002	0.6406325	1.091646276	Zm00001d050116
Zm00001d009740_T003	0.7405617	-0.366331617	Zm00001d009740
Zm00001d009740_T002	0.9868533	-1.51E-05	Zm00001d009740
Zm00001d018894_T003	0.586618	-0.266760978	Zm00001d018894
Zm00001d018894_T007	0.9791729	0.121833691	Zm00001d018894
Zm00001d018894_T009	0.741153	-0.484446158	Zm00001d018894
Zm00001d018894_T001	0.6703771	1.469964179	Zm00001d018894
Zm00001d018894_T008	0.7414776	-0.110893572	Zm00001d018894
Zm00001d018902_T001	0.9840043	0.476900943	Zm00001d018902
Zm00001d041323_T012	0.9748076	0.096053367	Zm00001d041323
Zm00001d041323_T003	0.5430222	0.701787463	Zm00001d041323
Zm00001d048202_T002	1	0.128234678	Zm00001d048202
Zm00001d048202_T001	0.9894615	-0.04640024	Zm00001d048202
Zm00001d052112_T003	0.4799121	-0.614436047	Zm00001d052112
Zm00001d052112_T002	0.6394238	-0.437948552	Zm00001d052112
Zm00001d017503_T003	0.2235678	-0.86949637	Zm00001d017503
Zm00001d017503_T004	0.4562375	-0.752245997	Zm00001d017503
Zm00001d009167_T001	0.9687311	0.035995374	Zm00001d009167
Zm00001d040381_T001	0.9669771	0.120923526	Zm00001d040381
Zm00001d017616_T001	0.9921225	0.131126394	Zm00001d017616
Zm00001d039213_T002	0.8031436	-0.4283718	Zm00001d039213
Zm00001d039213_T001	0.8533785	-0.447558457	Zm00001d039213
Zm00001d047115_T004	0.5309312	-1.071498681	Zm00001d047115
Zm00001d018258_T004	0.3212177	0.713263211	Zm00001d018258
Zm00001d018258_T009	0.9913523	0.178041204	Zm00001d018258
Zm00001d018258_T012	0.02449193	0.814591951	Zm00001d018258
Zm00001d018258_T013	0.8786231	0.870216409	Zm00001d018258
Zm00001d018258_T011	0.8809072	0.726818734	Zm00001d018258
Zm00001d018258_T001	0.2604053	0.542276762	Zm00001d018258
Zm00001d029078_T001	0.9055246	-0.129483488	Zm00001d029078
Zm00001d038648_T003	0.9694613	0.606654881	Zm00001d038648
Zm00001d038648_T005	0.5582612	-0.293406319	Zm00001d038648
Zm00001d038648_T001	0.09028577	1.426223017	Zm00001d038648
Zm00001d038648_T004	0.8039029	0.692070277	Zm00001d038648

Zm00001d038648_T002	0.5225862	1.227225568	Zm00001d038648
Zm00001d002416_T002	0.9781672	0.336349224	Zm00001d002416
Zm00001d002416_T001	0.9238183	0.702262434	Zm00001d002416
Zm00001d050365_T001	0.6579417	-0.422850005	Zm00001d050365
Zm00001d050365_T003	0.7160115	-0.268479957	Zm00001d050365
Zm00001d050365_T002	0.2872619	-0.672231237	Zm00001d050365
Zm00001d050365_T004	0.1989189	-1.565988807	Zm00001d050365
Zm00001d023447_T001	1	0.163019566	Zm00001d023447
Zm00001d035235_T001	0.9968176	0.15840885	Zm00001d035235
Zm00001d039537_T053	0.3667933	0.377405523	Zm00001d039537
Zm00001d039537_T068	0.6947028	0.113175649	Zm00001d039537
Zm00001d039537_T069	0.5936419	0.306688127	Zm00001d039537
Zm00001d039537_T048	6.64E-07	0.481236642	Zm00001d039537
Zm00001d039537_T002	0.2526012	-0.783803551	Zm00001d039537
Zm00001d039537_T019	0.1767827	0.338670182	Zm00001d039537
Zm00001d039537_T032	0.8213498	0.755778246	Zm00001d039537
Zm00001d039537_T018	0.4849772	1.19827059	Zm00001d039537
Zm00001d039537_T067	0.002460833	0.423765229	Zm00001d039537
Zm00001d039537_T024	0.7224237	0.53362131	Zm00001d039537
Zm00001d039537_T066	0.9177099	0.170425265	Zm00001d039537
Zm00001d039537_T036	0.2292281	-0.671393803	Zm00001d039537
Zm00001d039537_T039	0.7830318	0.571591299	Zm00001d039537
Zm00001d048437_T002	0.9524127	0.094529195	Zm00001d048437
Zm00001d048437_T001	0.2845836	-1.194047263	Zm00001d048437
Zm00001d048496_T004	0.5934246	-0.425770923	Zm00001d048496
Zm00001d048496_T001	0.8518279	0.921449059	Zm00001d048496
Zm00001d025431_T001	0.7211554	0.600917558	Zm00001d025431
Zm00001d025431_T002	0.8783081	0.906926858	Zm00001d025431
Zm00001d025431_T003	0.30546	1.352031654	Zm00001d025431
Zm00001d044004_T001	0.2536137	-1.297060228	Zm00001d044004
Zm00001d038791_T001	0.005709369	3.169973062	Zm00001d038791
Zm00001d016736_T001	0.9945592	0.212049831	Zm00001d016736
Zm00001d037227_T004	0.8125642	-0.319644218	Zm00001d037227
Zm00001d037227_T008	0.8221926	0.766356666	Zm00001d037227
Zm00001d037227_T002	0.8937133	0.031359556	Zm00001d037227
Zm00001d037227_T007	0.7660283	0.606806385	Zm00001d037227
Zm00001d037227_T005	0.8936884	1.069505526	Zm00001d037227
Zm00001d037227_T003	1	0.328992121	Zm00001d037227
Zm00001d038101_T003	0.9778436	0.239095771	Zm00001d038101
Zm00001d038101_T002	0.8160256	1.04096947	Zm00001d038101
Zm00001d038101_T001	0.9664406	0.448952983	Zm00001d038101
Zm00001d010576_T002	0.9732421	0.068465661	Zm00001d010576
Zm00001d010576_T003	0.6156484	-0.296081344	Zm00001d010576
Zm00001d024470_T002	1.41E-08	0.960590932	Zm00001d024470
Zm00001d024470_T001	0.8064667	-0.121468406	Zm00001d024470
Zm00001d023888_T001	0.7073686	-0.528444765	Zm00001d023888
Zm00001d009591_T002	0.3111055	-0.820749849	Zm00001d009591
Zm00001d009591_T003	0.6318866	1.191939441	Zm00001d009591
Zm00001d037321_T015	0.003015693	0.740620505	Zm00001d037321
Zm00001d037321_T031	0.7235285	1.039145975	Zm00001d037321
Zm00001d037321_T013	0.2247608	0.646965977	Zm00001d037321
Zm00001d037321_T024	0.9982897	0.170832816	Zm00001d037321
Zm00001d037321_T033	0.9797082	0.348770113	Zm00001d037321
Zm00001d037321_T002	0.5943038	0.354709267	Zm00001d037321

Zm00001d032724_T001	0.009372412	1.140956306	Zm00001d032724
Zm00001d016158_T001	0.7454538	-0.712531368	Zm00001d016158
Zm00001d009848_T001	0.7158349	-0.604499459	Zm00001d009848
Zm00001d009848_T002	0.9298424	0.023113354	Zm00001d009848
Zm00001d026672_T001	0.9790556	0.491551789	Zm00001d026672
Zm00001d005928_T002	0.9190176	0.635422596	Zm00001d005928
Zm00001d007121_T052	0.9979568	0.115991822	Zm00001d007121
Zm00001d007121_T057	6.20E-12	1.835297614	Zm00001d007121
Zm00001d007121_T066	0.9805897	0.227770007	Zm00001d007121
Zm00001d007121_T022	0.8410195	0.34728569	Zm00001d007121
Zm00001d007121_T049	0.03970719	-0.611215267	Zm00001d007121
Zm00001d007121_T063	0.9180662	0.037590494	Zm00001d007121
Zm00001d007121_T044	0.08479858	0.820254829	Zm00001d007121
Zm00001d007121_T026	0.265603	0.333715674	Zm00001d007121
Zm00001d007121_T004	0.9921225	0.207690465	Zm00001d007121
Zm00001d007121_T033	0.906282	0.053209667	Zm00001d007121
Zm00001d007121_T065	0.01955821	0.964576154	Zm00001d007121
Zm00001d007121_T001	0.8667498	0.56029861	Zm00001d007121
Zm00001d007121_T046	0.4347579	0.698272436	Zm00001d007121
Zm00001d017456_T009	0.1365583	1.109682542	Zm00001d017456
Zm00001d017456_T015	7.37E-05	4.066834137	Zm00001d017456
Zm00001d017456_T005	0.002685015	0.516771503	Zm00001d017456
Zm00001d017456_T014	0.07431695	-2.065259648	Zm00001d017456
Zm00001d017456_T004	0.9747606	0.472486031	Zm00001d017456
Zm00001d017456_T010	0.8572948	0.725717596	Zm00001d017456
Zm00001d017456_T012	0.5719089	1.275853834	Zm00001d017456
Zm00001d017456_T011	0.002206201	2.171218888	Zm00001d017456
Zm00001d017456_T001	0.9559227	0.295757328	Zm00001d017456
Zm00001d047618_T001	0.90276	0.566249973	Zm00001d047618
Zm00001d035725_T001	0.01181493	-1.512112192	Zm00001d035725
Zm00001d053080_T002	0.8669288	0.856566505	Zm00001d053080
Zm00001d005969_T008	3.19E-05	1.085411257	Zm00001d005969
Zm00001d005969_T012	0.6283395	1.220790896	Zm00001d005969
Zm00001d005969_T003	0.8179463	0.300525868	Zm00001d005969
Zm00001d005969_T009	0.1252127	1.803878899	Zm00001d005969
Zm00001d005969_T013	0.06865764	1.479022107	Zm00001d005969
Zm00001d005969_T005	0.000368021	0.775842715	Zm00001d005969
Zm00001d005969_T004	0.006044335	0.576785341	Zm00001d005969
Zm00001d005969_T015	0.9781672	0.509912251	Zm00001d005969
Zm00001d003575_T001	0.5852266	-0.592279723	Zm00001d003575
Zm00001d030766_T001	0.9850116	0.377653091	Zm00001d030766
Zm00001d015134_T001	0.9500229	-0.000508148	Zm00001d015134
Zm00001d048875_T001	0.923446	0.62246286	Zm00001d048875
Zm00001d029636_T001	0.5892262	1.358013403	Zm00001d029636
Zm00001d045862_T001	0.8816665	0.598744594	Zm00001d045862
Zm00001d038523_T001	0.5488962	-1.067564807	Zm00001d038523
Zm00001d003076_T002	0.1234187	-1.114154286	Zm00001d003076
Zm00001d003076_T001	1	0.339946397	Zm00001d003076
Zm00001d021973_T001	0.004978721	3.154190537	Zm00001d021973
Zm00001d047058_T003	0.6222811	-0.967237267	Zm00001d047058
Zm00001d047058_T001	0.9778436	0.079706036	Zm00001d047058
Zm00001d033475_T001	0.7921111	0.549810477	Zm00001d033475
Zm00001d038623_T002	1	0.341732671	Zm00001d038623
Zm00001d039451_T004	0.8027057	-0.170735966	Zm00001d039451

Zm00001d039451_T007	0.9754972	0.089907384	Zm00001d039451
Zm00001d039451_T006	0.3010905	-0.889835384	Zm00001d039451
Zm00001d049097_T005	0.9706887	0.166354747	Zm00001d049097
Zm00001d049097_T003	0.6059172	-0.599235892	Zm00001d049097
Zm00001d049097_T001	0.9628812	0.067825594	Zm00001d049097
Zm00001d049097_T006	0.7067229	-0.996926251	Zm00001d049097
Zm00001d049097_T007	0.6127499	1.198316059	Zm00001d049097
Zm00001d049097_T002	0.6843488	0.31850604	Zm00001d049097
Zm00001d011204_T001	0.923446	-0.312178157	Zm00001d011204
Zm00001d048856_T005	0.3764439	-1.075673172	Zm00001d048856
Zm00001d048856_T003	0.8883561	-0.082599078	Zm00001d048856
Zm00001d024497_T001	0.8839371	-0.033933821	Zm00001d024497
Zm00001d011413_T001	0.2021231	-0.8138855	Zm00001d011413
Zm00001d036898_T001	0.7419821	-0.815715542	Zm00001d036898
Zm00001d026195_T001	0.8706799	-0.037362126	Zm00001d026195
Zm00001d026195_T005	0.9369505	0.110206482	Zm00001d026195
Zm00001d035876_T007	0.117549	0.797726775	Zm00001d035876
Zm00001d035876_T004	0.1577745	-0.835697207	Zm00001d035876
Zm00001d035876_T002	0.621424	-0.337577002	Zm00001d035876
Zm00001d035876_T009	0.9493302	-0.024935816	Zm00001d035876
Zm00001d043972_T001	0.8637863	-0.305032864	Zm00001d043972
Zm00001d015092_T001	0.9719418	-0.009068513	Zm00001d015092
Zm00001d017927_T001	0.8587779	-0.286934441	Zm00001d017927
Zm00001d021843_T001	0.04606011	-1.755390797	Zm00001d021843
Zm00001d052353_T006	1	0.291280533	Zm00001d052353
Zm00001d052353_T004	0.745718	0.283581553	Zm00001d052353
Zm00001d052353_T008	0.003098678	-2.155874524	Zm00001d052353
Zm00001d052353_T001	0.9563232	0.588054427	Zm00001d052353
Zm00001d040416_T004	0.8107637	0.881012553	Zm00001d040416
Zm00001d040416_T003	0.5717773	-0.85426413	Zm00001d040416
Zm00001d010825_T001	0.9894615	0.496931487	Zm00001d010825
Zm00001d010825_T002	0.9745777	0.068385148	Zm00001d010825
Zm00001d011765_T002	0.897685	-0.11835608	Zm00001d011765
Zm00001d048924_T001	0.8857953	0.891759423	Zm00001d048924
Zm00001d051977_T001	0.9766874	0.642282851	Zm00001d051977
Zm00001d027998_T001	0.3769336	-1.196524454	Zm00001d027998
Zm00001d048233_T001	0.2550154	1.729643647	Zm00001d048233
Zm00001d021806_T028	0.04008706	0.960712172	Zm00001d021806
Zm00001d021806_T016	0.8134945	-0.238163411	Zm00001d021806
Zm00001d021806_T014	0.1205693	-0.484255987	Zm00001d021806
Zm00001d021806_T027	1.34E-11	1.569578225	Zm00001d021806
Zm00001d021806_T022	0.0421692	1.336238527	Zm00001d021806
Zm00001d021806_T032	4.33E-07	0.757715688	Zm00001d021806
Zm00001d021806_T068	0.05955382	0.306459133	Zm00001d021806
Zm00001d021806_T034	0.7952045	1.040455799	Zm00001d021806
Zm00001d021806_T049	0.4270217	0.200658943	Zm00001d021806
Zm00001d024753_T001	0.9380783	0.533864832	Zm00001d024753
Zm00001d012710_T002	0.9825014	0.138022193	Zm00001d012710
Zm00001d011195_T001	0.9669175	0.250983379	Zm00001d011195
Zm00001d011195_T002	0.8027057	0.888961955	Zm00001d011195
Zm00001d025717_T001	0.9855117	0.516416076	Zm00001d025717
Zm00001d027479_T002	0.9791729	0.187967667	Zm00001d027479
Zm00001d002876_T001	0.9865262	0.52135691	Zm00001d002876
Zm00001d009617_T003	0.9486412	0.634575215	Zm00001d009617

Zm00001d009617_T001	0.03517382	-0.512631197	Zm00001d009617
Zm00001d021721_T005	0.6745773	-0.487665101	Zm00001d021721
Zm00001d021721_T003	0.737932	1.099378557	Zm00001d021721
Zm00001d021721_T001	0.8953011	-0.049684762	Zm00001d021721
Zm00001d013732_T001	0.9849234	0.209132396	Zm00001d013732
Zm00001d032181_T002	1	0.196257978	Zm00001d032181
Zm00001d032181_T001	0.8428212	-0.544865334	Zm00001d032181
Zm00001d031471_T001	0.8985561	-0.019787932	Zm00001d031471
Zm00001d027534_T001	0.4298843	-0.680640526	Zm00001d027534
Zm00001d006268_T001	0.986738	0.082606266	Zm00001d006268
Zm00001d050466_T001	0.1512844	-1.400802715	Zm00001d050466
Zm00001d014275_T002	1	0.400358583	Zm00001d014275
Zm00001d014275_T003	0.2443389	0.512671757	Zm00001d014275
Zm00001d039799_T001	0.9160053	0.454525771	Zm00001d039799
Zm00001d039506_T002	0.8951107	0.827101721	Zm00001d039506
Zm00001d006274_T002	0.6594369	0.693230556	Zm00001d006274
Zm00001d006274_T001	0.9561521	0.026505301	Zm00001d006274
Zm00001d006274_T005	0.07966903	-0.636515197	Zm00001d006274
Zm00001d007977_T001	0.4115493	-1.152333553	Zm00001d007977
Zm00001d027680_T002	0.8581713	-0.344910465	Zm00001d027680
Zm00001d043560_T001	0.8581679	-0.132264389	Zm00001d043560
Zm00001d043560_T007	0.995201	0.168032263	Zm00001d043560
Zm00001d043560_T005	0.7945048	0.267145685	Zm00001d043560
Zm00001d052796_T004	0.8955831	-0.176660974	Zm00001d052796
Zm00001d052796_T002	0.5784042	-0.753800534	Zm00001d052796
Zm00001d052796_T001	0.05668191	-1.41891342	Zm00001d052796
Zm00001d042619_T002	0.9902733	0.592436409	Zm00001d042619
Zm00001d042619_T001	0.8684295	0.920722276	Zm00001d042619
Zm00001d042619_T003	0.7626242	-0.037771264	Zm00001d042619
Zm00001d011122_T001	0.9811162	0.142659797	Zm00001d011122
Zm00001d050054_T001	0.9563988	-0.173364436	Zm00001d050054
Zm00001d051501_T001	0.955464	-0.066890896	Zm00001d051501
Zm00001d003756_T001	0.8884142	-0.36459291	Zm00001d003756
Zm00001d029102_T001	0.319674	-1.136485184	Zm00001d029102
Zm00001d010048_T004	0.956053	0.285603838	Zm00001d010048
Zm00001d010048_T003	0.552097	-0.419494282	Zm00001d010048
Zm00001d010048_T001	0.8267985	0.940018696	Zm00001d010048
Zm00001d010048_T002	0.9210235	0.801380054	Zm00001d010048
Zm00001d004775_T006	0.9770343	0.221773651	Zm00001d004775
Zm00001d011461_T001	0.8152824	0.547613657	Zm00001d011461
Zm00001d031880_T001	0.01214359	2.07677196	Zm00001d031880
Zm00001d002032_T045	0.2153703	0.71055983	Zm00001d002032
Zm00001d002032_T026	0.4596739	0.464599107	Zm00001d002032
Zm00001d002032_T044	0.1563559	1.30587854	Zm00001d002032
Zm00001d002032_T024	0.2786734	0.573239807	Zm00001d002032
Zm00001d002032_T022	0.2374233	0.909764383	Zm00001d002032
Zm00001d002032_T013	0.001703929	0.808519113	Zm00001d002032
Zm00001d002032_T002	0.9747606	0.14557033	Zm00001d002032
Zm00001d002032_T033	0.7416612	-0.224613392	Zm00001d002032
Zm00001d002032_T001	0.9090332	-0.049158211	Zm00001d002032
Zm00001d002032_T015	0.4363247	0.90860439	Zm00001d002032
Zm00001d002032_T034	0.005416588	0.545359283	Zm00001d002032
Zm00001d002032_T003	0.9835181	0.189829941	Zm00001d002032
Zm00001d002032_T010	0.0353277	0.894446842	Zm00001d002032

Zm00001d002032_T020	0.9596377	0.097654124	Zm00001d002032
Zm00001d002032_T043	0.04197626	0.360474156	Zm00001d002032
Zm00001d045657_T001	0.1389129	1.850851348	Zm00001d045657
Zm00001d046400_T002	0.8053357	0.568445471	Zm00001d046400
Zm00001d046400_T001	0.8996827	-0.048454386	Zm00001d046400
Zm00001d018421_T001	0.9736468	0.498463958	Zm00001d018421
Zm00001d018421_T006	0.9232446	0.272434762	Zm00001d018421
Zm00001d018421_T003	0.9613188	0.144194812	Zm00001d018421
Zm00001d018421_T002	0.6875139	1.174016317	Zm00001d018421
Zm00001d018421_T005	1	0.514096405	Zm00001d018421
Zm00001d018421_T004	0.8643636	1.316968231	Zm00001d018421
Zm00001d029801_T001	0.9031129	-0.205011029	Zm00001d029801
Zm00001d048142_T001	0.6358713	1.097587014	Zm00001d048142
Zm00001d002897_T001	0.6019545	-0.59529635	Zm00001d002897
Zm00001d013182_T002	0.0312949	-1.838996844	Zm00001d013182
Zm00001d013182_T004	0.8744797	-0.068984939	Zm00001d013182
Zm00001d013182_T003	0.6154071	-0.355481599	Zm00001d013182
Zm00001d012027_T006	0.000145457	-3.021981723	Zm00001d012027
Zm00001d012027_T014	0.000500744	1.950594528	Zm00001d012027
Zm00001d012027_T001	1.63E-15	4.211800057	Zm00001d012027
Zm00001d045098_T001	0.9745777	0.203498621	Zm00001d045098
Zm00001d007296_T001	0.9855106	0.205133408	Zm00001d007296
Zm00001d003890_T001	0.7082091	-0.377255625	Zm00001d003890
Zm00001d028775_T001	0.7017446	-0.497837564	Zm00001d028775
Zm00001d025931_T003	0.9100954	0.729530352	Zm00001d025931
Zm00001d025931_T001	0.9312703	-0.092800238	Zm00001d025931
Zm00001d002377_T002	0.7986776	-0.265526103	Zm00001d002377
Zm00001d018394_T003	0.9160053	0.069484727	Zm00001d018394
Zm00001d018394_T001	0.9993872	0.327900762	Zm00001d018394
Zm00001d046568_T001	0.9892609	0.238375445	Zm00001d046568
Zm00001d046568_T003	0.9974023	0.233880861	Zm00001d046568
Zm00001d018557_T001	0.811176	-0.65168915	Zm00001d018557
Zm00001d042978_T001	0.9013236	0.396432789	Zm00001d042978
Zm00001d037886_T003	0.7548788	-0.431848641	Zm00001d037886
Zm00001d037886_T004	0.9210327	-0.116436175	Zm00001d037886
Zm00001d037886_T002	1	0.184849132	Zm00001d037886
Zm00001d026678_T001	0.6220803	-0.596832881	Zm00001d026678
Zm00001d036024_T003	0.4821209	1.394779963	Zm00001d036024
Zm00001d036024_T001	0.9982897	0.427211625	Zm00001d036024
Zm00001d036024_T005	0.7194959	1.002380854	Zm00001d036024
Zm00001d036024_T004	0.8776881	-0.103306502	Zm00001d036024
Zm00001d009117_T002	0.852305	-0.334197513	Zm00001d009117
Zm00001d022122_T001	0.8874701	-0.070883424	Zm00001d022122
Zm00001d009264_T001	0.6134185	-0.587006665	Zm00001d009264
Zm00001d018382_T020	0.2489636	1.080970012	Zm00001d018382
Zm00001d018382_T033	0.8922781	0.863145436	Zm00001d018382
Zm00001d018382_T001	0.9939021	0.28160051	Zm00001d018382
Zm00001d018382_T040	0.1504172	0.361741762	Zm00001d018382
Zm00001d018382_T019	1.52E-06	0.391991024	Zm00001d018382
Zm00001d018382_T028	0.3102519	-0.152421717	Zm00001d018382
Zm00001d018382_T003	0.7568663	0.820041374	Zm00001d018382
Zm00001d007947_T003	0.9782953	0.269053639	Zm00001d007947
Zm00001d007947_T001	0.8721631	0.927692344	Zm00001d007947
Zm00001d037565_T001	1	0.118630159	Zm00001d037565

Zm00001d037565_T002	0.9887584	0.159418136	Zm00001d037565
Zm00001d034568_T005	0.2397673	1.204743371	Zm00001d034568
Zm00001d034568_T003	5.53E-06	0.920850451	Zm00001d034568
Zm00001d034568_T004	0.9984041	0.14829049	Zm00001d034568
Zm00001d034568_T002	1.51E-07	-1.064217907	Zm00001d034568
Zm00001d034568_T006	0.03670383	1.036121247	Zm00001d034568
Zm00001d034568_T001	0.9138633	-0.004178746	Zm00001d034568
Zm00001d012830_T002	0.8746701	-0.169806705	Zm00001d012830
Zm00001d028895_T001	0.9746429	0.117647489	Zm00001d028895
Zm00001d008229_T001	0.405448	-0.529757796	Zm00001d008229
Zm00001d032162_T001	0.9797082	-0.021906479	Zm00001d032162
Zm00001d044816_T001	0.8860574	0.740589191	Zm00001d044816
Zm00001d034992_T001	0.7968244	-0.576140981	Zm00001d034992
Zm00001d010884_T001	0.6470421	-0.274373965	Zm00001d010884
Zm00001d016969_T001	0.9419336	-0.039319992	Zm00001d016969
Zm00001d020490_T001	0.2642794	-1.007512242	Zm00001d020490
Zm00001d009488_T004	0.4430193	2.348593201	Zm00001d009488
Zm00001d009488_T001	1	0.509444076	Zm00001d009488
Zm00001d009488_T003	0.9381687	0.060373249	Zm00001d009488
Zm00001d009488_T002	0.98291	0.301090955	Zm00001d009488
Zm00001d003958_T017	0.005841413	2.298802582	Zm00001d003958
Zm00001d003958_T011	0.4448541	-0.14196267	Zm00001d003958
Zm00001d003958_T013	0.04665552	1.992760625	Zm00001d003958
Zm00001d003958_T007	0.9703564	0.639781142	Zm00001d003958
Zm00001d052025_T001	0.6159427	-1.003047335	Zm00001d052025
Zm00001d015837_T003	0.9975882	0.098887581	Zm00001d015837
Zm00001d015837_T001	0.8853366	0.795865443	Zm00001d015837
Zm00001d038932_T001	0.6724442	-0.583574202	Zm00001d038932
Zm00001d011551_T001	0.1021675	-0.965769135	Zm00001d011551
Zm00001d035044_T010	8.16E-14	2.732431527	Zm00001d035044
Zm00001d035044_T004	0.7921111	-0.113682592	Zm00001d035044
Zm00001d035044_T001	0.3870314	1.467884748	Zm00001d035044
Zm00001d035044_T023	0.3183965	-0.514689575	Zm00001d035044
Zm00001d035044_T011	0.3428189	-0.430829553	Zm00001d035044
Zm00001d035044_T031	3.94E-05	-2.034597327	Zm00001d035044
Zm00001d035044_T024	3.07E-14	-3.349930674	Zm00001d035044
Zm00001d035044_T002	0.5057074	1.219723402	Zm00001d035044
Zm00001d035044_T003	0.7419821	1.701610545	Zm00001d035044
Zm00001d033159_T001	0.01610476	0.885747018	Zm00001d033159
Zm00001d033159_T002	0.945968	0.056528924	Zm00001d033159
Zm00001d002790_T001	0.9825209	0.21463524	Zm00001d002790
Zm00001d002790_T002	0.7454635	0.850131262	Zm00001d002790
Zm00001d009928_T001	0.3893584	-0.49823918	Zm00001d009928
Zm00001d004659_T001	0.2830018	-0.833837791	Zm00001d004659
Zm00001d004659_T011	0.1421277	0.954992485	Zm00001d004659
Zm00001d004659_T012	0.6625248	0.913981192	Zm00001d004659
Zm00001d004659_T007	0.006426743	1.62367915	Zm00001d004659
Zm00001d004659_T004	0.3000263	0.606662042	Zm00001d004659
Zm00001d002611_T001	0.6001859	0.605276544	Zm00001d002611
Zm00001d002611_T002	0.9897446	0.176605803	Zm00001d002611
Zm00001d017254_T002	0.7103637	-0.569563424	Zm00001d017254
Zm00001d002253_T001	0.6424899	-0.528302485	Zm00001d002253
Zm00001d035753_T001	0.708953	0.937074923	Zm00001d035753
Zm00001d043735_T001	0.7568663	0.740752728	Zm00001d043735

Zm00001d038149_T004	0.274306	0.4857353	Zm00001d038149
Zm00001d038149_T003	1	0.37921891	Zm00001d038149
Zm00001d038149_T005	0.4880775	-0.694018318	Zm00001d038149
Zm00001d012913_T008	0.9189175	0.870815945	Zm00001d012913
Zm00001d012913_T009	0.8288594	-0.026851544	Zm00001d012913
Zm00001d012913_T005	0.4798122	0.288326003	Zm00001d012913
Zm00001d044726_T001	0.9627389	0.564880372	Zm00001d044726
Zm00001d026625_T001	0.6571086	-0.863935156	Zm00001d026625
Zm00001d026625_T002	0.6802613	-0.733155929	Zm00001d026625
Zm00001d043935_T001	0.366346	1.583620865	Zm00001d043935
Zm00001d032257_T001	0.9664406	0.629337603	Zm00001d032257
Zm00001d032257_T003	0.7439474	-0.678216692	Zm00001d032257
Zm00001d049440_T006	0.9429712	-0.107647538	Zm00001d049440
Zm00001d049440_T003	0.8885217	0.680761103	Zm00001d049440
Zm00001d049440_T004	0.4956541	1.038881472	Zm00001d049440
Zm00001d049440_T002	0.2545977	-0.313173665	Zm00001d049440
Zm00001d049440_T005	0.9670588	0.74955084	Zm00001d049440
Zm00001d010655_T001	0.8179463	-0.258605118	Zm00001d010655
Zm00001d012459_T001	0.9416622	-0.030187371	Zm00001d012459
Zm00001d011694_T001	0.8050292	-0.271898452	Zm00001d011694
Zm00001d048362_T001	0.9942835	0.406540234	Zm00001d048362
Zm00001d012378_T001	0.8881012	0.405695317	Zm00001d012378
Zm00001d037142_T012	0.7894872	-0.620912862	Zm00001d037142
Zm00001d037142_T007	0.164302	0.574950694	Zm00001d037142
Zm00001d037142_T002	0.9918302	0.598940905	Zm00001d037142
Zm00001d037142_T022	0.002953279	2.820541463	Zm00001d037142
Zm00001d037142_T001	0.150217	-0.470045375	Zm00001d037142
Zm00001d007257_T001	0.8383011	-0.343558929	Zm00001d007257
Zm00001d018713_T002	0.8883561	-0.064324805	Zm00001d018713
Zm00001d025326_T001	0.8676122	0.3886731	Zm00001d025326
Zm00001d025326_T002	0.7876948	0.975837346	Zm00001d025326
Zm00001d005344_T001	0.7047956	-0.582710055	Zm00001d005344
Zm00001d005344_T002	1	0.19576587	Zm00001d005344
Zm00001d027368_T001	0.3900878	-1.258610961	Zm00001d027368
Zm00001d031674_T004	0.9059564	-0.004720149	Zm00001d031674
Zm00001d045937_T001	0.9537771	0.166019783	Zm00001d045937
Zm00001d021867_T001	0.9724085	0.14508186	Zm00001d021867
Zm00001d004705_T001	0.1542609	0.983099719	Zm00001d004705
Zm00001d040611_T004	0.1750389	0.294269531	Zm00001d040611
Zm00001d040611_T005	0.6460225	-0.120832565	Zm00001d040611
Zm00001d040611_T002	0.6778807	-0.412054494	Zm00001d040611
Zm00001d034407_T001	0.4462128	-1.172156136	Zm00001d034407
Zm00001d044133_T002	0.5618486	0.330842053	Zm00001d044133
Zm00001d044133_T008	1	0.175187622	Zm00001d044133
Zm00001d044133_T011	1	0.460440861	Zm00001d044133
Zm00001d044133_T009	1	0.376752658	Zm00001d044133
Zm00001d044133_T001	0.8385304	-0.069913682	Zm00001d044133
Zm00001d044133_T014	0.4812187	0.496258957	Zm00001d044133
Zm00001d044133_T010	0.9213556	0.040324646	Zm00001d044133
Zm00001d042758_T001	0.3301595	0.984758271	Zm00001d042758
Zm00001d038408_T001	0.7438361	-0.593581038	Zm00001d038408
Zm00001d027721_T001	0.9984041	0.267065868	Zm00001d027721
Zm00001d012612_T001	0.9392687	0.346572042	Zm00001d012612
Zm00001d025337_T001	0.9690782	0.392688772	Zm00001d025337

Zm00001d047597_T001	0.9965728	-0.01665043	Zm00001d047597
Zm00001d033489_T001	0.9567316	0.09843749	Zm00001d033489
Zm00001d015126_T001	0.1006821	-1.389683045	Zm00001d015126
Zm00001d033368_T071	3.07E-14	-4.387735046	Zm00001d033368
Zm00001d033368_T064	0.9907928	0.228166684	Zm00001d033368
Zm00001d033368_T062	0.2238888	1.38240235	Zm00001d033368
Zm00001d033368_T030	0.461503	-0.415143915	Zm00001d033368
Zm00001d033368_T065	1	0.01304567	Zm00001d033368
Zm00001d033368_T006	0.9046076	0.011918494	Zm00001d033368
Zm00001d033368_T015	3.50E-05	1.00416422	Zm00001d033368
Zm00001d033368_T010	7.11E-12	1.727982811	Zm00001d033368
Zm00001d033368_T066	0.9804228	0.286563938	Zm00001d033368
Zm00001d033368_T045	0.09453749	1.818321495	Zm00001d033368
Zm00001d053020_T001	0.1194751	-1.028559791	Zm00001d053020
Zm00001d013273_T003	1	0.343766252	Zm00001d013273
Zm00001d013273_T001	0.9666757	0.218862602	Zm00001d013273
Zm00001d050498_T001	0.8821903	-0.108469423	Zm00001d050498
Zm00001d050498_T002	0.9519258	0.238747223	Zm00001d050498
Zm00001d010500_T001	0.9732421	0.174677247	Zm00001d010500
Zm00001d032570_T001	0.997764	0.403698455	Zm00001d032570
Zm00001d032570_T002	0.9550193	0.393079644	Zm00001d032570
Zm00001d019256_T001	0.4176155	-0.667001032	Zm00001d019256
Zm00001d043861_T001	0.8775735	0.905497614	Zm00001d043861
Zm00001d043932_T001	0.3595196	1.222965647	Zm00001d043932
Zm00001d043506_T005	0.9160053	0.118403435	Zm00001d043506
Zm00001d043506_T004	0.6494559	0.474387794	Zm00001d043506
Zm00001d008690_T001	0.9224387	0.005598581	Zm00001d008690
Zm00001d002797_T001	1	0.053550779	Zm00001d002797
Zm00001d002797_T008	0.7262357	-0.357876353	Zm00001d002797
Zm00001d002797_T003	0.8866361	0.848440523	Zm00001d002797
Zm00001d002797_T005	0.6858852	0.857679032	Zm00001d002797
Zm00001d004467_T001	0.5523216	-0.553002754	Zm00001d004467
Zm00001d042389_T001	1	0.197353461	Zm00001d042389
Zm00001d015658_T002	0.9596377	0.317155928	Zm00001d015658
Zm00001d015658_T001	1	0.172878039	Zm00001d015658
Zm00001d015410_T002	0.5437049	-0.521714652	Zm00001d015410
Zm00001d015410_T001	0.7746801	-0.19647842	Zm00001d015410
Zm00001d015410_T003	0.1701341	-1.461382322	Zm00001d015410
Zm00001d003223_T001	0.9235467	0.38492653	Zm00001d003223
Zm00001d031187_T001	0.9203655	-0.061955079	Zm00001d031187
Zm00001d031187_T002	0.9860836	0.394112082	Zm00001d031187
Zm00001d016853_T001	0.8660897	-0.102203046	Zm00001d016853
Zm00001d039062_T014	0.8819513	-0.109770421	Zm00001d039062
Zm00001d039062_T006	0.06304775	0.512361387	Zm00001d039062
Zm00001d031793_T002	1	0.305257245	Zm00001d031793
Zm00001d031793_T001	0.748607	0.496276253	Zm00001d031793
Zm00001d007156_T001	0.9817074	0.115784903	Zm00001d007156
Zm00001d018709_T002	0.5537557	-0.517611703	Zm00001d018709
Zm00001d012518_T004	0.8353818	-0.09717185	Zm00001d012518
Zm00001d012518_T008	0.9257893	1.757031342	Zm00001d012518
Zm00001d012518_T001	0.02255438	-0.804203141	Zm00001d012518
Zm00001d053211_T003	0.9979568	0.198771182	Zm00001d053211
Zm00001d053211_T002	0.05225416	0.741097633	Zm00001d053211
Zm00001d053211_T008	0.9998515	0.355673472	Zm00001d053211

Zm00001d053211_T005	0.4604035	1.120715618	Zm00001d053211
Zm00001d026426_T005	0.9860836	0.264314056	Zm00001d026426
Zm00001d026426_T001	0.6627547	-0.373896889	Zm00001d026426
Zm00001d012551_T001	0.745806	-0.623897483	Zm00001d012551
Zm00001d045109_T031	0.7267731	-0.323371499	Zm00001d045109
Zm00001d045109_T003	1.84E-10	1.008389938	Zm00001d045109
Zm00001d045109_T037	0.3450699	-1.311031745	Zm00001d045109
Zm00001d045109_T034	1	0.360538552	Zm00001d045109
Zm00001d045109_T010	0.1750262	1.752042772	Zm00001d045109
Zm00001d045109_T008	0.01906927	0.634239325	Zm00001d045109
Zm00001d045109_T022	0.2687567	0.427475917	Zm00001d045109
Zm00001d045109_T036	0.8747821	0.128520177	Zm00001d045109
Zm00001d052794_T001	0.9073212	0.504035224	Zm00001d052794
Zm00001d003471_T001	0.3247936	-0.842959956	Zm00001d003471
Zm00001d034486_T002	0.9645916	0.102152648	Zm00001d034486
Zm00001d000046_T002	0.268756	1.413303252	Zm00001d000046
Zm00001d000046_T001	0.9571192	0.511965309	Zm00001d000046
Zm00001d022251_T002	0.9991964	0.378537456	Zm00001d022251
Zm00001d052785_T001	0.9530658	0.059952358	Zm00001d052785
Zm00001d033650_T001	0.9907928	0.24383102	Zm00001d033650
Zm00001d015097_T003	0.8488494	0.581040352	Zm00001d015097
Zm00001d015097_T001	1	0.319591671	Zm00001d015097
Zm00001d015097_T005	1.00E-09	-1.724622779	Zm00001d015097
Zm00001d015097_T004	0.04385265	2.130882473	Zm00001d015097
Zm00001d013958_T003	0.9949999	0.329035366	Zm00001d013958
Zm00001d028917_T001	0.8127863	0.854323312	Zm00001d028917
Zm00001d038667_T001	0.9689939	0.314262277	Zm00001d038667
Zm00001d053967_T001	0.7325413	-0.336965854	Zm00001d053967
Zm00001d007468_T001	0.7650176	-0.10277035	Zm00001d007468
Zm00001d047538_T003	0.9781672	0.126401571	Zm00001d047538
Zm00001d029932_T001	0.3213694	1.720580978	Zm00001d029932
Zm00001d013021_T001	0.429391	0.805389436	Zm00001d013021
Zm00001d032319_T020	0.9946911	0.21219781	Zm00001d032319
Zm00001d032319_T012	0.9311783	0.012690152	Zm00001d032319
Zm00001d032319_T014	0.2622934	0.997433362	Zm00001d032319
Zm00001d032319_T040	0.7771624	0.479700528	Zm00001d032319
Zm00001d032319_T029	0.6177075	-0.482357343	Zm00001d032319
Zm00001d032319_T017	0.002641253	2.318232169	Zm00001d032319
Zm00001d032319_T002	0.6789723	0.026202723	Zm00001d032319
Zm00001d014756_T004	0.8638849	0.83517728	Zm00001d014756
Zm00001d014756_T002	0.3622612	0.765681546	Zm00001d014756
Zm00001d014756_T001	0.562142	0.51438188	Zm00001d014756
Zm00001d046929_T002	0.9596377	-0.122920042	Zm00001d046929
Zm00001d046929_T001	0.9804433	0.047681469	Zm00001d046929
Zm00001d013445_T003	0.6062927	-0.635172074	Zm00001d013445
Zm00001d013673_T002	0.9979568	0.22168528	Zm00001d013673
Zm00001d030694_T003	0.9998711	0.440214999	Zm00001d030694
Zm00001d026600_T002	0.9145885	-0.011845396	Zm00001d026600
Zm00001d048914_T001	0.9131449	0.659524333	Zm00001d048914
Zm00001d038049_T001	0.5695353	-0.600688824	Zm00001d038049
Zm00001d042064_T001	0.01638171	0.882216665	Zm00001d042064
Zm00001d042064_T007	0.8680274	-0.136558472	Zm00001d042064
Zm00001d042064_T013	0.8817666	0.52756399	Zm00001d042064
Zm00001d042064_T002	0.818597	-0.048178509	Zm00001d042064

Zm00001d042064_T008	0.9124297	-0.019492961	Zm00001d042064
Zm00001d042064_T003	0.477074	-0.366695353	Zm00001d042064
Zm00001d042064_T005	0.9701986	0.140876836	Zm00001d042064
Zm00001d040297_T001	0.9054587	-0.069987795	Zm00001d040297
Zm00001d040681_T001	0.926928	-0.174452663	Zm00001d040681
Zm00001d036409_T001	0.01206564	-2.130642573	Zm00001d036409
Zm00001d026649_T001	0.8439431	0.948307846	Zm00001d026649
Zm00001d026649_T002	0.9448026	0.656699904	Zm00001d026649
Zm00001d046949_T001	0.9211172	-0.045254907	Zm00001d046949
Zm00001d040649_T001	0.9832689	0.511655009	Zm00001d040649
Zm00001d017862_T001	0.9814486	0.187240573	Zm00001d017862
Zm00001d044806_T001	0.3648354	1.088706262	Zm00001d044806
Zm00001d012923_T008	1	0.272374712	Zm00001d012923
Zm00001d012923_T004	0.004527535	-2.302674137	Zm00001d012923
Zm00001d012923_T006	0.2463927	0.28668845	Zm00001d012923
Zm00001d012923_T002	0.8449395	0.765279238	Zm00001d012923
Zm00001d006341_T001	0.894759	-0.161914387	Zm00001d006341
Zm00001d049438_T001	0.2814654	1.528056272	Zm00001d049438
Zm00001d025247_T001	0.01758334	-2.059138668	Zm00001d025247
Zm00001d053761_T001	0.9544149	0.487255707	Zm00001d053761
Zm00001d053761_T009	0.2873151	-0.390940412	Zm00001d053761
Zm00001d053761_T012	0.7137316	-0.298193355	Zm00001d053761
Zm00001d039190_T001	0.7097086	-0.429511188	Zm00001d039190
Zm00001d006860_T002	0.5410776	1.347004624	Zm00001d006860
Zm00001d024861_T002	0.7623518	0.289288115	Zm00001d024861
Zm00001d024861_T004	0.3870314	-0.430671085	Zm00001d024861
Zm00001d024861_T001	0.6156484	0.563113777	Zm00001d024861
Zm00001d024861_T005	0.8892107	0.820447832	Zm00001d024861
Zm00001d028998_T001	0.1763656	-1.283140297	Zm00001d028998
Zm00001d051448_T001	1	0.339969911	Zm00001d051448
Zm00001d022228_T001	1	0.146067764	Zm00001d022228
Zm00001d037701_T001	0.1505056	-0.85714885	Zm00001d037701
Zm00001d033147_T001	0.8643636	-0.370385565	Zm00001d033147
Zm00001d050558_T001	0.2803683	-1.191208099	Zm00001d050558
Zm00001d034023_T001	0.3487622	-0.566152442	Zm00001d034023
Zm00001d034023_T002	0.9311783	0.034677225	Zm00001d034023
Zm00001d044054_T002	0.9324286	-0.143965518	Zm00001d044054
Zm00001d003250_T001	0.9100954	0.732717754	Zm00001d003250
Zm00001d013417_T006	0.9904282	0.240714491	Zm00001d013417
Zm00001d013417_T003	0.5805314	-0.242400228	Zm00001d013417
Zm00001d013417_T002	0.5044186	1.009783741	Zm00001d013417
Zm00001d013417_T005	0.352878	-0.355557774	Zm00001d013417
Zm00001d018362_T003	0.6925804	-0.055675798	Zm00001d018362
Zm00001d028505_T001	0.3919095	1.509333946	Zm00001d028505
Zm00001d028505_T002	0.9100954	0.457099108	Zm00001d028505
Zm00001d022306_T001	0.6139644	-0.415120879	Zm00001d022306
Zm00001d022306_T002	0.9173494	-0.115268019	Zm00001d022306
Zm00001d014232_T008	0.4437212	-0.196873541	Zm00001d014232
Zm00001d014232_T021	0.1159542	-1.126489492	Zm00001d014232
Zm00001d014232_T023	0.06555672	0.699710799	Zm00001d014232
Zm00001d014232_T002	0.8883002	0.831116778	Zm00001d014232
Zm00001d014232_T007	0.6702957	0.879850005	Zm00001d014232
Zm00001d014232_T018	0.06547779	0.351984572	Zm00001d014232
Zm00001d014232_T005	0.6330708	0.207957156	Zm00001d014232

Zm00001d050150_T001	0.2717432	0.605216527	Zm00001d050150
Zm00001d038270_T001	0.01489828	2.900348875	Zm00001d038270
Zm00001d006065_T001	0.2550996	-0.362658073	Zm00001d006065
Zm00001d006065_T004	0.3355093	-1.577611583	Zm00001d006065
Zm00001d006065_T002	0.900661	1.185777318	Zm00001d006065
Zm00001d046519_T001	0.7923921	-0.232732189	Zm00001d046519
Zm00001d023578_T001	0.9239172	-0.01994891	Zm00001d023578
Zm00001d029559_T001	0.8412913	-0.613101931	Zm00001d029559
Zm00001d051065_T007	0.8880053	-0.203637089	Zm00001d051065
Zm00001d051065_T008	0.5112835	0.795132585	Zm00001d051065
Zm00001d051065_T004	0.8310488	-0.041556818	Zm00001d051065
Zm00001d051065_T003	0.9988954	0.178593132	Zm00001d051065
Zm00001d033909_T001	0.000968584	-3.408745462	Zm00001d033909
Zm00001d044212_T002	0.9870394	0.285255379	Zm00001d044212
Zm00001d044212_T004	0.9311499	0.587614285	Zm00001d044212
Zm00001d044212_T001	0.772948	-0.341745214	Zm00001d044212
Zm00001d044646_T041	3.97E-08	1.029281531	Zm00001d044646
Zm00001d044646_T055	0.04818964	0.373351604	Zm00001d044646
Zm00001d044646_T052	0.6330708	-0.305061154	Zm00001d044646
Zm00001d044646_T038	0.002596184	-0.628661701	Zm00001d044646
Zm00001d044646_T053	0.9029876	-0.507196225	Zm00001d044646
Zm00001d044646_T059	1	0.282252009	Zm00001d044646
Zm00001d044646_T037	0.557003	0.520275136	Zm00001d044646
Zm00001d044646_T057	0.6877528	1.135595233	Zm00001d044646
Zm00001d044646_T003	0.9887584	0.09459223	Zm00001d044646
Zm00001d030179_T002	0.4676599	-0.844995171	Zm00001d030179
Zm00001d030179_T004	0.6050512	-1.122576944	Zm00001d030179
Zm00001d049905_T001	0.8694165	-0.092067886	Zm00001d049905
Zm00001d004019_T001	0.9503347	0.060905546	Zm00001d004019
Zm00001d004019_T002	0.01193426	-1.541942717	Zm00001d004019
Zm00001d053662_T002	0.9451281	0.01611439	Zm00001d053662
Zm00001d027757_T001	0.972985	0.133990364	Zm00001d027757
Zm00001d045076_T001	0.6127874	-0.035235328	Zm00001d045076
Zm00001d045076_T005	0.8093695	-0.210393239	Zm00001d045076
Zm00001d045076_T009	0.9924805	0.042054231	Zm00001d045076
Zm00001d045076_T004	0.9832092	0.232716923	Zm00001d045076
Zm00001d045076_T006	0.4430193	-0.508163236	Zm00001d045076
Zm00001d045076_T003	0.2369538	-0.614468624	Zm00001d045076
Zm00001d045076_T002	0.9278769	-0.009636491	Zm00001d045076
Zm00001d011097_T001	0.9003815	-0.158899856	Zm00001d011097
Zm00001d008791_T005	2.96E-11	-3.734030707	Zm00001d008791
Zm00001d008791_T002	0.8031436	1.051178487	Zm00001d008791
Zm00001d002150_T001	0.6012747	-0.54435681	Zm00001d002150
Zm00001d035921_T001	0.3255715	-0.517537305	Zm00001d035921
Zm00001d035921_T004	0.5884523	-0.515954583	Zm00001d035921
Zm00001d008236_T013	0.7743017	1.055090914	Zm00001d008236
Zm00001d008236_T027	0.5437049	-0.140564955	Zm00001d008236
Zm00001d008236_T019	0.4882469	0.702993717	Zm00001d008236
Zm00001d008236_T016	0.4191028	0.340853503	Zm00001d008236
Zm00001d008236_T007	0.909729	0.481818433	Zm00001d008236
Zm00001d008236_T028	0.9887584	0.20104543	Zm00001d008236
Zm00001d008236_T009	0.08916797	1.248838538	Zm00001d008236
Zm00001d008236_T012	0.6318256	0.714613616	Zm00001d008236
Zm00001d008236_T011	1	0.03462036	Zm00001d008236

Zm00001d038067_T001	0.7262357	-0.489784207	Zm00001d038067
Zm00001d036371_T001	0.954984	0.148033151	Zm00001d036371
Zm00001d000102_T001	0.9659036	-0.00703784	Zm00001d000102
Zm00001d024041_T004	0.6891365	0.336926242	Zm00001d024041
Zm00001d024041_T012	0.7146325	0.985771957	Zm00001d024041
Zm00001d024041_T016	0.7721703	0.93788669	Zm00001d024041
Zm00001d024041_T002	0.9747606	0.536401232	Zm00001d024041
Zm00001d038357_T001	0.9984041	0.338761255	Zm00001d038357
Zm00001d038357_T004	0.8676122	0.818691411	Zm00001d038357
Zm00001d038357_T003	0.1191963	2.081274772	Zm00001d038357
Zm00001d038357_T002	0.006733831	2.71204104	Zm00001d038357
Zm00001d018574_T001	0.3412344	-0.856571324	Zm00001d018574
Zm00001d014797_T002	0.6057336	0.79093504	Zm00001d014797
Zm00001d014797_T007	0.3394879	1.563838007	Zm00001d014797
Zm00001d014797_T003	0.8884142	-0.084014459	Zm00001d014797
Zm00001d014797_T001	0.8633864	0.905754076	Zm00001d014797
Zm00001d006500_T001	0.4137215	-0.770788847	Zm00001d006500
Zm00001d044091_T005	0.9749712	0.447961826	Zm00001d044091
Zm00001d044091_T001	1	0.373970273	Zm00001d044091
Zm00001d044091_T002	0.8896831	0.030181112	Zm00001d044091
Zm00001d044091_T004	0.7131979	0.443230519	Zm00001d044091
Zm00001d037095_T001	0.2575624	1.479142972	Zm00001d037095
Zm00001d002455_T002	0.9822138	0.046134571	Zm00001d002455
Zm00001d002455_T004	0.8197974	-0.212036702	Zm00001d002455
Zm00001d015550_T001	0.6048646	-0.571853175	Zm00001d015550
Zm00001d041976_T001	0.03771189	0.216233067	Zm00001d041976
Zm00001d051063_T001	0.8693222	-0.438576522	Zm00001d051063
Zm00001d053852_T001	0.9513815	0.70451464	Zm00001d053852
Zm00001d043082_T001	0.9975882	0.30978027	Zm00001d043082
Zm00001d007184_T001	0.9529184	0.5605032	Zm00001d007184
Zm00001d047667_T001	0.645777	-0.254737685	Zm00001d047667
Zm00001d047667_T002	0.5936419	-0.539021331	Zm00001d047667
Zm00001d029848_T003	0.9570177	0.109556309	Zm00001d029848
Zm00001d029848_T005	0.238984	-0.672970594	Zm00001d029848
Zm00001d029848_T007	0.5408146	-0.307140955	Zm00001d029848
Zm00001d002191_T001	0.9121108	-0.030227784	Zm00001d002191
Zm00001d053009_T006	0.4685762	0.861898683	Zm00001d053009
Zm00001d053009_T002	0.9561521	0.65270574	Zm00001d053009
Zm00001d042505_T016	1.29E-05	1.944672247	Zm00001d042505
Zm00001d042505_T031	0.9975882	0.38794521	Zm00001d042505
Zm00001d042505_T026	0.006079915	-0.874798924	Zm00001d042505
Zm00001d042505_T001	0.003938874	2.171048828	Zm00001d042505
Zm00001d042505_T035	0.000275609	0.536544849	Zm00001d042505
Zm00001d042505_T020	0.4540782	0.374109965	Zm00001d042505
Zm00001d053800_T002	0.9363242	0.485640592	Zm00001d053800
Zm00001d018276_T003	0.04539599	-1.190300581	Zm00001d018276
Zm00001d018276_T004	0.6572222	1.463677241	Zm00001d018276
Zm00001d018276_T006	0.5567925	-0.471205405	Zm00001d018276
Zm00001d004557_T001	0.1116614	-0.462927641	Zm00001d004557
Zm00001d026029_T001	0.5796852	-0.501865354	Zm00001d026029
Zm00001d021731_T001	0.7912425	-0.183288017	Zm00001d021731
Zm00001d007311_T002	0.7037831	-0.330237349	Zm00001d007311
Zm00001d007311_T003	0.9037324	0.797105906	Zm00001d007311
Zm00001d007311_T001	0.9908081	0.454282772	Zm00001d007311

Zm00001d047262_T001	0.908501	-0.142839243	Zm00001d047262
Zm00001d004868_T002	0.01193426	-1.29410286	Zm00001d004868
Zm00001d004868_T001	0.5890988	0.866956089	Zm00001d004868
Zm00001d004868_T004	0.2205165	0.668681788	Zm00001d004868
Zm00001d026344_T002	0.8762906	0.585233854	Zm00001d026344
Zm00001d026344_T001	0.9749728	0.217962103	Zm00001d026344
Zm00001d013920_T008	1.13E-10	1.841729357	Zm00001d013920
Zm00001d013920_T016	0.9995663	0.109755928	Zm00001d013920
Zm00001d013920_T002	0.4274324	0.530945616	Zm00001d013920
Zm00001d013920_T018	0.2390372	0.863502928	Zm00001d013920
Zm00001d013920_T012	0.7020458	0.321246476	Zm00001d013920
Zm00001d013920_T014	0.7251353	0.396920904	Zm00001d013920
Zm00001d013920_T005	0.8050292	0.608182223	Zm00001d013920
Zm00001d013920_T007	0.9834756	0.355918332	Zm00001d013920
Zm00001d013920_T003	0.9326774	0.771102238	Zm00001d013920
Zm00001d052076_T004	0.9419336	0.318993814	Zm00001d052076
Zm00001d052076_T013	0.9895224	0.158488254	Zm00001d052076
Zm00001d052076_T018	0.1412338	0.637394802	Zm00001d052076
Zm00001d052076_T022	0.07320084	0.364807874	Zm00001d052076
Zm00001d052076_T010	0.5455802	-0.222645076	Zm00001d052076
Zm00001d052076_T003	0.2870002	0.614133706	Zm00001d052076
Zm00001d052076_T002	0.7920826	-0.230150762	Zm00001d052076
Zm00001d029654_T004	0.7169281	1.144618081	Zm00001d029654
Zm00001d029654_T005	0.06865764	1.7711457	Zm00001d029654
Zm00001d029654_T006	0.646433	0.751644288	Zm00001d029654
Zm00001d042482_T001	0.9048001	0.815541576	Zm00001d042482
Zm00001d038091_T007	0.9889811	0.215789631	Zm00001d038091
Zm00001d038091_T005	0.1785712	0.753846414	Zm00001d038091
Zm00001d038091_T006	0.9951418	0.240624393	Zm00001d038091
Zm00001d051823_T002	0.9788505	0.192812978	Zm00001d051823
Zm00001d011108_T003	0.9160053	0.027505341	Zm00001d011108
Zm00001d011108_T005	0.9988954	0.207886257	Zm00001d011108
Zm00001d052838_T001	0.6354704	-0.612923863	Zm00001d052838
Zm00001d038747_T004	0.4212568	-0.349231073	Zm00001d038747
Zm00001d038747_T001	0.6597828	-0.40840247	Zm00001d038747
Zm00001d038747_T008	0.4723494	-0.116325895	Zm00001d038747
Zm00001d038747_T007	0.4146004	0.475327888	Zm00001d038747
Zm00001d040567_T001	0.06372667	2.477089191	Zm00001d040567
Zm00001d023230_T009	0.2840392	-1.207797473	Zm00001d023230
Zm00001d023230_T003	0.5992684	-0.578572317	Zm00001d023230
Zm00001d023230_T006	0.8267985	0.176672466	Zm00001d023230
Zm00001d023230_T012	0.9160053	0.147173346	Zm00001d023230
Zm00001d023230_T013	0.07622214	0.648663189	Zm00001d023230
Zm00001d023230_T016	0.8461657	0.65957178	Zm00001d023230
Zm00001d023230_T002	0.2297956	1.361929592	Zm00001d023230
Zm00001d035390_T001	0.5415171	-0.994217015	Zm00001d035390
Zm00001d025977_T001	0.8281834	-0.159058075	Zm00001d025977
Zm00001d006046_T015	0.5861211	0.639422819	Zm00001d006046
Zm00001d006046_T004	0.9821324	0.160503331	Zm00001d006046
Zm00001d006046_T008	0.4546448	0.937822026	Zm00001d006046
Zm00001d006046_T006	0.9160053	-0.075638505	Zm00001d006046
Zm00001d006046_T014	0.9196877	-0.005727425	Zm00001d006046
Zm00001d006046_T010	0.9303773	0.022511202	Zm00001d006046
Zm00001d006046_T005	0.8079397	-0.213146985	Zm00001d006046

Zm00001d006046_T007	0.9324801	-0.004106046	Zm00001d006046
Zm00001d006046_T012	0.1961983	0.964660485	Zm00001d006046
Zm00001d010784_T002	0.9860836	0.153268873	Zm00001d010784
Zm00001d010790_T028	2.49E-06	1.100120583	Zm00001d010790
Zm00001d010790_T026	0.7497503	-0.273782284	Zm00001d010790
Zm00001d010790_T002	0.1816976	-0.977743272	Zm00001d010790
Zm00001d010790_T039	0.3151393	1.006114798	Zm00001d010790
Zm00001d010790_T077	0.000665293	0.271752343	Zm00001d010790
Zm00001d010790_T027	0.9524127	-0.057273375	Zm00001d010790
Zm00001d010790_T011	0.9489956	-0.216811615	Zm00001d010790
Zm00001d013836_T007	0.1189054	1.099020505	Zm00001d013836
Zm00001d013836_T005	0.2387612	0.864516544	Zm00001d013836
Zm00001d013836_T006	0.9878022	0.18140088	Zm00001d013836
Zm00001d013836_T001	0.9836566	0.493247254	Zm00001d013836
Zm00001d013836_T008	0.2304095	0.39151236	Zm00001d013836
Zm00001d033385_T001	0.1928254	1.732019321	Zm00001d033385
Zm00001d006881_T011	0.9279367	-0.043325852	Zm00001d006881
Zm00001d006881_T018	1	0.257897808	Zm00001d006881
Zm00001d006881_T014	1	0.176967042	Zm00001d006881
Zm00001d006881_T016	0.6398988	-0.272147007	Zm00001d006881
Zm00001d022028_T001	0.06364017	-1.5525366	Zm00001d022028
Zm00001d022028_T011	0.9889811	0.391810654	Zm00001d022028
Zm00001d045179_T008	0.9596377	0.022543626	Zm00001d045179
Zm00001d045179_T021	0.2357885	-0.678701789	Zm00001d045179
Zm00001d008904_T006	0.9486412	0.145618982	Zm00001d008904
Zm00001d008904_T009	0.5748211	0.745699693	Zm00001d008904
Zm00001d008904_T010	0.9833513	0.162954467	Zm00001d008904
Zm00001d008904_T005	0.9870482	0.459544121	Zm00001d008904
Zm00001d008904_T008	0.6318496	-0.428962575	Zm00001d008904
Zm00001d042842_T006	0.7454635	0.90594416	Zm00001d042842
Zm00001d042842_T002	0.3296668	0.638125842	Zm00001d042842
Zm00001d042842_T007	0.4574813	-0.669516618	Zm00001d042842
Zm00001d027846_T001	0.04570411	-1.690133658	Zm00001d027846
Zm00001d042451_T006	0.8945288	0.24367469	Zm00001d042451
Zm00001d042451_T004	0.08924544	-0.803995786	Zm00001d042451
Zm00001d042451_T002	0.877725	0.909084997	Zm00001d042451
Zm00001d018034_T001	0.8470623	-0.147955484	Zm00001d018034
Zm00001d002942_T003	0.8368466	-0.306750093	Zm00001d002942
Zm00001d002942_T001	1	0.334698271	Zm00001d002942
Zm00001d002942_T002	0.9845237	0.101621306	Zm00001d002942
Zm00001d048616_T001	0.5924463	1.284784568	Zm00001d048616
Zm00001d008222_T002	0.0324581	-1.105712995	Zm00001d008222
Zm00001d008222_T001	0.02134347	-1.181212266	Zm00001d008222
Zm00001d053913_T001	0.904995	-0.072903546	Zm00001d053913
Zm00001d025706_T003	0.6632799	-0.282737083	Zm00001d025706
Zm00001d025706_T001	0.8412913	-0.077479191	Zm00001d025706
Zm00001d025706_T002	0.9968176	0.070852233	Zm00001d025706
Zm00001d010141_T001	0.6486663	0.48031341	Zm00001d010141
Zm00001d010141_T012	0.8344343	-0.148652872	Zm00001d010141
Zm00001d010141_T006	0.6156484	0.989905973	Zm00001d010141
Zm00001d020068_T002	0.9817074	-0.237124734	Zm00001d020068
Zm00001d011594_T001	0.3649839	-1.193068745	Zm00001d011594
Zm00001d041889_T027	0.8050292	0.376722924	Zm00001d041889
Zm00001d041889_T153	0.08778756	-0.971618432	Zm00001d041889

Zm00001d041889_T170	0.9619176	0.569821517	Zm00001d041889
Zm00001d038193_T005	0.8786231	-0.226195319	Zm00001d038193
Zm00001d038193_T007	0.306157	0.356189385	Zm00001d038193
Zm00001d038193_T006	0.9650061	0.457535206	Zm00001d038193
Zm00001d038193_T009	0.2726876	-0.766631919	Zm00001d038193
Zm00001d038193_T003	0.9451369	0.195810882	Zm00001d038193
Zm00001d038193_T001	0.9419336	0.167700563	Zm00001d038193
Zm00001d046626_T001	0.9544587	0.673510156	Zm00001d046626
Zm00001d007240_T001	0.9244812	0.544296115	Zm00001d007240
Zm00001d015225_T001	0.8643636	-0.085975176	Zm00001d015225
Zm00001d038897_T002	0.9701986	0.345891024	Zm00001d038897
Zm00001d015986_T001	0.9968176	0.196149924	Zm00001d015986
Zm00001d015986_T002	0.9832092	0.112882173	Zm00001d015986
Zm00001d021048_T001	0.9596377	-0.036511033	Zm00001d021048
Zm00001d033621_T001	0.7145827	-0.53872422	Zm00001d033621
Zm00001d012537_T005	0.3193521	-0.475947623	Zm00001d012537
Zm00001d012537_T004	0.9260032	0.023637305	Zm00001d012537
Zm00001d012537_T001	0.9782953	0.072591715	Zm00001d012537
Zm00001d019216_T001	0.9524127	-0.260316217	Zm00001d019216
Zm00001d033632_T002	0.4604152	0.84001808	Zm00001d033632
Zm00001d033632_T007	0.462123	0.69699563	Zm00001d033632
Zm00001d033632_T009	0.8698746	-0.069859134	Zm00001d033632
Zm00001d033632_T008	0.01578834	1.395179878	Zm00001d033632
Zm00001d043225_T001	0.9177458	-0.115729381	Zm00001d043225
Zm00001d012439_T001	0.7946251	-0.17979153	Zm00001d012439
Zm00001d048416_T001	0.03463105	2.434905887	Zm00001d048416
Zm00001d046531_T002	0.9628432	0.404902454	Zm00001d046531
Zm00001d046531_T005	0.8354517	-0.083741439	Zm00001d046531
Zm00001d046531_T001	0.1244517	-0.880084942	Zm00001d046531
Zm00001d046531_T006	0.8111431	1.067576708	Zm00001d046531
Zm00001d049588_T001	0.7565187	-0.213494812	Zm00001d049588
Zm00001d040670_T005	0.9860836	0.132755497	Zm00001d040670
Zm00001d040670_T004	0.9928864	0.451378906	Zm00001d040670
Zm00001d040670_T007	0.6260085	-0.301272927	Zm00001d040670
Zm00001d053349_T001	0.9730613	-0.270027612	Zm00001d053349
Zm00001d008392_T001	1	0.09808553	Zm00001d008392
Zm00001d036635_T001	0.9173494	0.37268287	Zm00001d036635
Zm00001d011038_T001	0.9792387	0.187073092	Zm00001d011038
Zm00001d010635_T001	0.9747606	0.336660477	Zm00001d010635
Zm00001d012019_T001	0.6343135	-0.309390147	Zm00001d012019
Zm00001d030744_T001	0.9963508	0.411698886	Zm00001d030744
Zm00001d010492_T007	0.6925804	0.644810765	Zm00001d010492
Zm00001d010492_T011	0.7545506	0.197513173	Zm00001d010492
Zm00001d010492_T002	0.4580645	0.956168754	Zm00001d010492
Zm00001d010492_T010	0.9979393	0.40144494	Zm00001d010492
Zm00001d010492_T001	0.772442	-0.19033142	Zm00001d010492
Zm00001d010492_T003	0.8430261	-0.020481279	Zm00001d010492
Zm00001d010492_T006	0.6510516	-0.384594021	Zm00001d010492
Zm00001d024573_T001	0.896517	-0.055913472	Zm00001d024573
Zm00001d028288_T001	0.9311783	0.229596863	Zm00001d028288
Zm00001d018755_T001	0.7785078	-0.387187865	Zm00001d018755
Zm00001d029747_T001	0.6812088	-0.490914057	Zm00001d029747
Zm00001d021162_T001	0.8312604	-0.183552194	Zm00001d021162
Zm00001d017467_T001	0.7743716	-0.32709149	Zm00001d017467

Zm00001d014773_T002	0.9145405	0.76261221	Zm00001d014773
Zm00001d014773_T001	1	0.365920427	Zm00001d014773
Zm00001d011189_T001	0.9804228	-0.182926284	Zm00001d011189
Zm00001d024203_T002	0.9791729	0.276038259	Zm00001d024203
Zm00001d024203_T001	0.4748531	-0.338211024	Zm00001d024203
Zm00001d041785_T001	1	0.242145059	Zm00001d041785
Zm00001d041785_T002	0.9701986	0.709943521	Zm00001d041785
Zm00001d041785_T011	0.6865286	1.501187872	Zm00001d041785
Zm00001d041785_T009	0.001916278	-3.328402511	Zm00001d041785
Zm00001d039956_T001	0.9693993	0.232228062	Zm00001d039956
Zm00001d017377_T001	0.598789	-0.550704865	Zm00001d017377
Zm00001d040154_T001	0.5954619	1.168418572	Zm00001d040154
Zm00001d053939_T001	0.9673011	-0.191780363	Zm00001d053939
Zm00001d049100_T001	0.8929316	0.884247012	Zm00001d049100
Zm00001d012766_T001	0.9159727	-0.112504225	Zm00001d012766
Zm00001d006028_T001	0.3113971	-0.709007676	Zm00001d006028
Zm00001d040534_T001	0.6275799	0.820638286	Zm00001d040534
Zm00001d030152_T001	0.9903234	0.218185028	Zm00001d030152
Zm00001d040518_T001	0.4723494	-0.904443045	Zm00001d040518
Zm00001d006019_T001	0.9447472	-0.446127966	Zm00001d006019
Zm00001d047780_T012	0.9722469	0.621590732	Zm00001d047780
Zm00001d047780_T010	0.001148838	0.548163264	Zm00001d047780
Zm00001d047780_T011	6.64E-07	0.712846914	Zm00001d047780
Zm00001d047780_T002	0.02125339	2.024161099	Zm00001d047780
Zm00001d044665_T001	0.9715713	0.566445299	Zm00001d044665
Zm00001d044665_T002	0.02606139	-0.975964638	Zm00001d044665
Zm00001d015202_T001	0.7146325	1.082822238	Zm00001d015202
Zm00001d043491_T001	0.8158142	-0.317924307	Zm00001d043491
Zm00001d025274_T001	0.7837924	-0.28123359	Zm00001d025274
Zm00001d051020_T001	0.9830387	0.145767332	Zm00001d051020
Zm00001d020791_T001	0.268136	-1.148180117	Zm00001d020791
Zm00001d045528_T001	0.7439474	-0.481261356	Zm00001d045528
Zm00001d027799_T002	0.3909958	-0.73318461	Zm00001d027799
Zm00001d027799_T001	0.9721766	0.535964458	Zm00001d027799
Zm00001d031279_T001	0.8920808	-0.108021015	Zm00001d031279
Zm00001d047479_T007	4.27E-05	-1.081670433	Zm00001d047479
Zm00001d047479_T008	0.7336597	-0.55777872	Zm00001d047479
Zm00001d047479_T012	0.1400536	-1.414724603	Zm00001d047479
Zm00001d047479_T016	0.1292305	-1.203551897	Zm00001d047479
Zm00001d047479_T017	0.9509252	-0.165489302	Zm00001d047479
Zm00001d047479_T013	0.9791729	0.382771732	Zm00001d047479
Zm00001d047479_T003	0.9673473	-0.0537807	Zm00001d047479
Zm00001d008858_T002	0.9389552	-0.433472433	Zm00001d008858
Zm00001d008858_T001	0.7812261	-0.308308613	Zm00001d008858
Zm00001d054078_T002	0.9945118	0.205612564	Zm00001d054078
Zm00001d028045_T010	0.965919	0.593481716	Zm00001d028045
Zm00001d028045_T001	0.9789987	0.307503568	Zm00001d028045
Zm00001d028045_T012	1	0.147969995	Zm00001d028045
Zm00001d028045_T013	0.7190231	-0.485372463	Zm00001d028045
Zm00001d054084_T001	0.5563797	-0.743809981	Zm00001d054084
Zm00001d013861_T001	0.9452999	0.007833158	Zm00001d013861
Zm00001d032049_T002	0.9766874	0.182719401	Zm00001d032049
Zm00001d032049_T006	0.01362109	-1.929726429	Zm00001d032049
Zm00001d027700_T001	0.5273755	-0.681430244	Zm00001d027700

Zm00001d034682_T001	0.9997455	0.309928691	Zm00001d034682
Zm00001d034682_T002	0.731653	-0.401205448	Zm00001d034682
Zm00001d050195_T001	0.3615897	-1.149758914	Zm00001d050195
Zm00001d016708_T002	0.9468268	0.711559711	Zm00001d016708
Zm00001d016708_T001	0.1068909	1.258389152	Zm00001d016708
Zm00001d020826_T001	0.929519	0.746322081	Zm00001d020826
Zm00001d042712_T001	0.8987811	0.731149641	Zm00001d042712
Zm00001d029448_T001	0.9956349	0.267720662	Zm00001d029448
Zm00001d042267_T005	0.1512258	2.15468581	Zm00001d042267
Zm00001d042267_T004	0.2510942	1.849993291	Zm00001d042267
Zm00001d042267_T006	0.9914912	0.468563288	Zm00001d042267
Zm00001d042267_T011	0.06964423	2.130034049	Zm00001d042267
Zm00001d051759_T001	0.8527068	0.880480299	Zm00001d051759
Zm00001d051759_T003	0.07595506	0.988585136	Zm00001d051759
Zm00001d051759_T002	0.983226	0.110180729	Zm00001d051759
Zm00001d018389_T001	0.9970835	0.340709813	Zm00001d018389
Zm00001d051676_T006	0.8340906	-0.326746008	Zm00001d051676
Zm00001d051676_T003	0.9791729	0.521976789	Zm00001d051676
Zm00001d051676_T002	0.9076857	-0.026102711	Zm00001d051676
Zm00001d051676_T001	0.1296685	0.591904288	Zm00001d051676
Zm00001d051676_T005	1	0.204712923	Zm00001d051676
Zm00001d006688_T001	0.6217057	1.294087974	Zm00001d006688
Zm00001d033104_T016	0.007563791	1.649561168	Zm00001d033104
Zm00001d033104_T001	0.8099107	-0.137786215	Zm00001d033104
Zm00001d033104_T007	3.06E-05	1.856560847	Zm00001d033104
Zm00001d033104_T010	0.9942835	-0.050672116	Zm00001d033104
Zm00001d046682_T001	0.9210955	-0.394719236	Zm00001d046682
Zm00001d027801_T016	0.449035	-0.374719042	Zm00001d027801
Zm00001d027801_T019	0.754061	0.230434862	Zm00001d027801
Zm00001d002166_T037	0.06824895	-1.661616691	Zm00001d002166
Zm00001d002166_T012	0.9224387	0.88433986	Zm00001d002166
Zm00001d002166_T013	7.75E-06	0.840060427	Zm00001d002166
Zm00001d002166_T034	0.9852645	0.068500425	Zm00001d002166
Zm00001d002166_T026	0.06635247	1.032339673	Zm00001d002166
Zm00001d002166_T010	0.05223114	0.577470726	Zm00001d002166
Zm00001d038248_T005	0.8300901	1.813363271	Zm00001d038248
Zm00001d038248_T003	0.9312703	-0.18814452	Zm00001d038248
Zm00001d038248_T009	0.8659224	0.048970958	Zm00001d038248
Zm00001d038248_T004	0.9706887	0.512038551	Zm00001d038248
Zm00001d038248_T001	0.772948	-0.408851772	Zm00001d038248
Zm00001d038248_T006	0.4039335	-1.153191989	Zm00001d038248
Zm00001d038248_T002	0.9420245	0.139603882	Zm00001d038248
Zm00001d038248_T008	0.8806413	-0.342108638	Zm00001d038248
Zm00001d023524_T001	1	0.333850394	Zm00001d023524
Zm00001d041267_T002	0.9913436	0.380693744	Zm00001d041267
Zm00001d041267_T001	0.9664406	0.201987355	Zm00001d041267
Zm00001d025759_T001	0.4419256	-0.789594503	Zm00001d025759
Zm00001d030010_T001	0.9979568	0.294755233	Zm00001d030010
Zm00001d003602_T001	0.9928864	0.32975321	Zm00001d003602
Zm00001d026531_T002	0.1922398	1.352229224	Zm00001d026531
Zm00001d026531_T001	0.6322656	1.12284978	Zm00001d026531
Zm00001d036241_T001	0.9078934	-0.056823007	Zm00001d036241
Zm00001d028336_T001	0.9981278	0.213554396	Zm00001d028336
Zm00001d008788_T008	0.6412029	-0.405798497	Zm00001d008788

Zm00001d008788_T003	0.3858421	-0.549992146	Zm00001d008788
Zm00001d008788_T004	2.99E-12	2.590213637	Zm00001d008788
Zm00001d032887_T001	0.9984041	0.287154337	Zm00001d032887
Zm00001d014021_T001	0.9832092	0.154638416	Zm00001d014021
Zm00001d049636_T001	0.9269932	0.038966763	Zm00001d049636
Zm00001d021529_T001	0.6588175	-0.732595171	Zm00001d021529
Zm00001d040726_T001	0.9016285	0.495106046	Zm00001d040726
Zm00001d011392_T004	0.3648354	0.763661069	Zm00001d011392
Zm00001d011392_T001	0.5314411	-0.071510905	Zm00001d011392
Zm00001d011392_T002	0.8647028	1.07447261	Zm00001d011392
Zm00001d010754_T001	0.9130008	-0.015482976	Zm00001d010754
Zm00001d014109_T007	0.9827298	0.206259161	Zm00001d014109
Zm00001d014109_T011	0.5910981	-0.362069492	Zm00001d014109
Zm00001d014109_T002	0.960704	0.156484865	Zm00001d014109
Zm00001d014109_T006	0.6626947	-0.258012718	Zm00001d014109
Zm00001d014109_T010	0.9180662	0.269636633	Zm00001d014109
Zm00001d017365_T001	0.8252955	0.731442394	Zm00001d017365
Zm00001d029287_T001	0.7613191	-0.412084953	Zm00001d029287
Zm00001d013097_T001	0.3396442	1.550420476	Zm00001d013097
Zm00001d036875_T001	0.9902022	0.188327564	Zm00001d036875
Zm00001d035243_T001	0.1375053	-1.356721851	Zm00001d035243
Zm00001d002364_T001	0.02171544	2.283684659	Zm00001d002364
Zm00001d005793_T001	1	0.248401723	Zm00001d005793
Zm00001d028469_T001	0.7194121	-1.005680177	Zm00001d028469
Zm00001d010343_T007	0.9419336	0.391843032	Zm00001d010343
Zm00001d010343_T006	1	0.05983143	Zm00001d010343
Zm00001d010343_T003	0.7082783	0.722500622	Zm00001d010343
Zm00001d030862_T001	0.906282	-0.123833085	Zm00001d030862
Zm00001d034425_T001	0.5843839	-0.77634315	Zm00001d034425
Zm00001d005110_T001	1	0.292328158	Zm00001d005110
Zm00001d038106_T009	0.1238108	0.785251945	Zm00001d038106
Zm00001d038106_T010	0.9160053	0.352811143	Zm00001d038106
Zm00001d038106_T011	0.622153	0.242352993	Zm00001d038106
Zm00001d038106_T004	0.8513643	-0.091740023	Zm00001d038106
Zm00001d039660_T009	0.6733323	-0.08490053	Zm00001d039660
Zm00001d039660_T010	0.9881407	0.467039033	Zm00001d039660
Zm00001d038332_T001	0.9399441	0.035060857	Zm00001d038332
Zm00001d024583_T003	0.003503628	1.770459784	Zm00001d024583
Zm00001d024583_T001	0.9396782	0.126258896	Zm00001d024583
Zm00001d024583_T011	0.9046076	0.031911729	Zm00001d024583
Zm00001d050944_T001	0.9201861	-0.382730086	Zm00001d050944
Zm00001d014966_T001	0.9566139	0.113139595	Zm00001d014966
Zm00001d043988_T001	0.3097848	-1.215139038	Zm00001d043988
Zm00001d043822_T003	0.7545745	-0.141642203	Zm00001d043822
Zm00001d006084_T008	0.910614	-0.010514402	Zm00001d006084
Zm00001d006084_T010	0.6436915	0.15884071	Zm00001d006084
Zm00001d006084_T007	0.8894168	-0.068261443	Zm00001d006084
Zm00001d003745_T001	0.6165106	-0.405047467	Zm00001d003745
Zm00001d046231_T009	0.4837004	0.965850927	Zm00001d046231
Zm00001d046231_T008	0.201789	0.569027774	Zm00001d046231
Zm00001d046231_T012	0.4332539	0.774099723	Zm00001d046231
Zm00001d046231_T004	0.7313576	0.665106101	Zm00001d046231
Zm00001d046231_T011	0.2338394	0.591432865	Zm00001d046231
Zm00001d046231_T005	0.6215505	0.595895473	Zm00001d046231

Zm00001d046231_T010	0.885586	-0.088754673	Zm00001d046231
Zm00001d046231_T001	1.09E-06	-1.486029255	Zm00001d046231
Zm00001d027877_T001	0.2935147	-1.063199918	Zm00001d027877
Zm00001d033291_T007	0.90591	-0.068760813	Zm00001d033291
Zm00001d033291_T008	0.9979568	0.338108426	Zm00001d033291
Zm00001d033291_T004	0.3319631	-0.222629235	Zm00001d033291
Zm00001d033291_T005	0.9903234	0.061707759	Zm00001d033291
Zm00001d033291_T006	0.7169281	0.705411321	Zm00001d033291
Zm00001d006878_T001	0.8641671	-0.537581484	Zm00001d006878
Zm00001d010319_T001	0.8911076	-0.069595393	Zm00001d010319
Zm00001d023279_T001	0.9543237	0.09261766	Zm00001d023279
Zm00001d016977_T003	0.3183965	-1.049825253	Zm00001d016977
Zm00001d016977_T002	0.6993528	-0.469781839	Zm00001d016977
Zm00001d016660_T001	0.9123976	-0.298724922	Zm00001d016660
Zm00001d048639_T001	0.957148	0.383405199	Zm00001d048639
Zm00001d021620_T001	0.9249526	-0.118880931	Zm00001d021620
Zm00001d034890_T001	0.9953345	0.025000233	Zm00001d034890
Zm00001d009186_T001	0.03189684	2.501208341	Zm00001d009186
Zm00001d048098_T004	0.9279367	0.25439481	Zm00001d048098
Zm00001d048098_T001	0.9269932	0.465712663	Zm00001d048098
Zm00001d048098_T002	0.9723861	0.380575282	Zm00001d048098
Zm00001d048098_T003	0.9614723	-0.080293967	Zm00001d048098
Zm00001d052108_T003	0.09351605	-0.519004825	Zm00001d052108
Zm00001d052108_T007	0.8922572	-0.496769541	Zm00001d052108
Zm00001d052108_T005	0.8315554	0.944776697	Zm00001d052108
Zm00001d052108_T001	1	0.285884569	Zm00001d052108
Zm00001d039789_T001	0.00090062	1.301667247	Zm00001d039789
Zm00001d043915_T002	0.1044529	1.438897506	Zm00001d043915
Zm00001d043915_T001	0.7610893	0.779097754	Zm00001d043915
Zm00001d026654_T001	0.9460688	0.547687641	Zm00001d026654
Zm00001d032016_T007	0.9981278	0.288940812	Zm00001d032016
Zm00001d032016_T014	0.4287151	0.782187815	Zm00001d032016
Zm00001d032016_T017	0.8328444	0.747282651	Zm00001d032016
Zm00001d032016_T023	0.3941547	0.982367331	Zm00001d032016
Zm00001d032016_T011	0.02184252	0.75034846	Zm00001d032016
Zm00001d032016_T003	0.000421721	0.596645477	Zm00001d032016
Zm00001d032016_T006	0.7335667	0.399159695	Zm00001d032016
Zm00001d027515_T002	0.9652551	0.422997058	Zm00001d027515
Zm00001d027515_T011	0.6019545	-0.236598985	Zm00001d027515
Zm00001d031620_T002	0.8669919	-0.177221186	Zm00001d031620
Zm00001d045611_T006	0.7555823	0.199121274	Zm00001d045611
Zm00001d045611_T004	0.4386149	-0.284284328	Zm00001d045611
Zm00001d045611_T001	0.4557081	1.603409865	Zm00001d045611
Zm00001d003435_T001	0.9281046	-0.329637553	Zm00001d003435
Zm00001d051397_T001	0.578403	1.329693622	Zm00001d051397
Zm00001d032552_T007	0.9969363	-0.127528308	Zm00001d032552
Zm00001d032552_T004	0.1414674	1.458913924	Zm00001d032552
Zm00001d032552_T009	0.9517262	0.066033511	Zm00001d032552
Zm00001d032552_T008	0.8693951	-0.051608426	Zm00001d032552
Zm00001d032552_T002	0.9781672	0.470208681	Zm00001d032552
Zm00001d032552_T003	0.293036	-0.904536105	Zm00001d032552
Zm00001d032552_T001	0.09854607	-1.450522064	Zm00001d032552
Zm00001d046960_T005	0.2416135	-1.645863993	Zm00001d046960
Zm00001d046960_T003	0.7440232	0.451114655	Zm00001d046960

Zm00001d046960_T004	0.9988954	0.287665079	Zm00001d046960
Zm00001d021762_T002	0.9469426	0.047223767	Zm00001d021762
Zm00001d021762_T001	1	0.232073564	Zm00001d021762
Zm00001d027541_T001	0.9724251	0.417926219	Zm00001d027541
Zm00001d021781_T001	0.4049446	-0.619790854	Zm00001d021781
Zm00001d037799_T001	0.6107221	-0.612917621	Zm00001d037799
Zm00001d020019_T003	0.7485406	-0.131081646	Zm00001d020019
Zm00001d020019_T001	1	0.377801873	Zm00001d020019
Zm00001d020019_T005	0.4283558	-0.185834269	Zm00001d020019
Zm00001d033788_T003	1.31E-08	-4.068548356	Zm00001d033788
Zm00001d033788_T001	0.8950139	0.747121897	Zm00001d033788
Zm00001d033788_T002	0.9942835	0.121263342	Zm00001d033788
Zm00001d033788_T004	1	0.185534192	Zm00001d033788
Zm00001d036543_T001	0.9469426	0.732569048	Zm00001d036543
Zm00001d008370_T001	0.444013	1.296449196	Zm00001d008370
Zm00001d027950_T003	0.9988864	0.129290919	Zm00001d027950
Zm00001d027950_T002	0.9975882	0.181066511	Zm00001d027950
Zm00001d032310_T001	0.9249526	0.061907616	Zm00001d032310
Zm00001d032699_T020	0.07108876	1.185476443	Zm00001d032699
Zm00001d032699_T006	0.9832092	0.09993608	Zm00001d032699
Zm00001d032699_T018	3.30E-09	1.28249172	Zm00001d032699
Zm00001d032699_T022	0.9196677	-0.018994903	Zm00001d032699
Zm00001d032699_T005	0.9932384	0.472477999	Zm00001d032699
Zm00001d018605_T001	0.7439782	-0.626413133	Zm00001d018605
Zm00001d004698_T001	0.9049007	0.778287965	Zm00001d004698
Zm00001d033397_T001	0.9717213	0.121078858	Zm00001d033397
Zm00001d033397_T004	0.9825858	0.202694556	Zm00001d033397
Zm00001d045084_T003	0.906534	-0.005468653	Zm00001d045084
Zm00001d045084_T005	1	0.147038482	Zm00001d045084
Zm00001d045084_T004	0.2807387	0.63815653	Zm00001d045084
Zm00001d031318_T001	0.8026836	0.997650924	Zm00001d031318
Zm00001d031318_T003	0.5718169	-0.622738204	Zm00001d031318
Zm00001d031318_T002	0.5239916	0.766067191	Zm00001d031318
Zm00001d051211_T025	0.4748531	-1.955182953	Zm00001d051211
Zm00001d051211_T002	1	0.163692781	Zm00001d051211
Zm00001d051211_T016	0.2945432	2.403976925	Zm00001d051211
Zm00001d051211_T030	0.9419336	-0.266127176	Zm00001d051211
Zm00001d051211_T005	0.9753213	1.243830846	Zm00001d051211
Zm00001d051211_T022	0.8863456	-0.047382525	Zm00001d051211
Zm00001d051211_T019	0.2919864	-0.935704786	Zm00001d051211
Zm00001d051211_T010	0.4812187	-0.224782392	Zm00001d051211
Zm00001d051211_T027	0.9609806	0.58754631	Zm00001d051211
Zm00001d034551_T001	0.8412913	0.931484786	Zm00001d034551
Zm00001d026051_T001	0.9260032	0.381856106	Zm00001d026051
Zm00001d026051_T002	0.1102125	2.133188491	Zm00001d026051
Zm00001d038218_T001	0.852305	0.908174701	Zm00001d038218
Zm00001d029808_T001	0.889323	0.793907875	Zm00001d029808
Zm00001d008548_T001	6.35E-05	-3.192628269	Zm00001d008548
Zm00001d049762_T013	0.8412913	0.199789436	Zm00001d049762
Zm00001d049762_T006	0.9209394	0.422011358	Zm00001d049762
Zm00001d049762_T008	0.7412537	-0.404310948	Zm00001d049762
Zm00001d049762_T010	0.3042782	0.457066583	Zm00001d049762
Zm00001d020607_T001	0.9694613	0.017291602	Zm00001d020607
Zm00001d043462_T009	0.02558382	1.192722656	Zm00001d043462

Zm00001d043462_T010	0.3782078	1.384437897	Zm00001d043462
Zm00001d043462_T008	0.8173759	0.979567468	Zm00001d043462
Zm00001d002286_T001	0.5257585	-0.667137505	Zm00001d002286
Zm00001d037530_T001	0.05628552	-1.562995416	Zm00001d037530
Zm00001d037530_T002	0.9945118	0.414113022	Zm00001d037530
Zm00001d040596_T001	0.9377241	-0.237649131	Zm00001d040596
Zm00001d040596_T002	0.9666757	0.37694652	Zm00001d040596
Zm00001d042493_T001	0.9567316	0.625975782	Zm00001d042493
Zm00001d012217_T001	0.9942835	-0.094325136	Zm00001d012217
Zm00001d037650_T005	0.03230314	1.770769268	Zm00001d037650
Zm00001d037650_T001	0.5060657	1.385057368	Zm00001d037650
Zm00001d037650_T002	0.04082476	1.816931291	Zm00001d037650
Zm00001d030094_T004	0.6606202	1.332808065	Zm00001d030094
Zm00001d030094_T001	0.8966978	0.778904874	Zm00001d030094
Zm00001d030094_T005	0.000933648	-2.643050291	Zm00001d030094
Zm00001d005631_T001	0.9747714	0.271729524	Zm00001d005631
Zm00001d023208_T002	0.9403937	0.098352294	Zm00001d023208
Zm00001d044465_T001	0.9994302	0.344332147	Zm00001d044465
Zm00001d003187_T002	0.002772078	-1.799284757	Zm00001d003187
Zm00001d003187_T001	9.89E-05	-1.807457095	Zm00001d003187
Zm00001d050256_T001	1	0.20141061	Zm00001d050256
Zm00001d034763_T001	0.9549733	-0.455428687	Zm00001d034763
Zm00001d019169_T005	0.8647916	0.820026384	Zm00001d019169
Zm00001d019169_T007	0.5408146	-0.344403077	Zm00001d019169
Zm00001d019169_T002	0.2093834	1.216618064	Zm00001d019169
Zm00001d026331_T009	0.8753765	0.176282056	Zm00001d026331
Zm00001d026331_T018	0.9033985	0.110381223	Zm00001d026331
Zm00001d026331_T006	0.1817182	2.286140459	Zm00001d026331
Zm00001d026331_T031	0.9886764	0.283376155	Zm00001d026331
Zm00001d013030_T002	0.9046076	0.764330612	Zm00001d013030
Zm00001d013030_T001	0.9300016	0.034294747	Zm00001d013030
Zm00001d041985_T001	0.967161	0.568228095	Zm00001d041985
Zm00001d022599_T001	0.7862641	-0.19503886	Zm00001d022599
Zm00001d046665_T001	0.5269468	-0.929685031	Zm00001d046665
Zm00001d026445_T001	0.9515686	0.689460016	Zm00001d026445
Zm00001d026445_T002	1	0.262433458	Zm00001d026445
Zm00001d041530_T001	0.9788109	0.061456855	Zm00001d041530
Zm00001d009005_T001	0.7209242	-0.073264178	Zm00001d009005
Zm00001d009005_T002	1	0.408343929	Zm00001d009005
Zm00001d009005_T003	0.5369152	0.401356716	Zm00001d009005
Zm00001d028715_T002	0.9959972	0.136340812	Zm00001d028715
Zm00001d028715_T001	0.9988864	0.284206357	Zm00001d028715
Zm00001d046811_T001	0.8971598	-0.169077123	Zm00001d046811
Zm00001d009436_T003	0.923446	0.724044256	Zm00001d009436
Zm00001d049526_T001	0.9776209	0.167414712	Zm00001d049526
Zm00001d049526_T003	0.8514567	0.508610865	Zm00001d049526
Zm00001d053427_T009	0.9745948	0.204736528	Zm00001d053427
Zm00001d053427_T001	0.7296512	0.305653272	Zm00001d053427
Zm00001d053427_T011	0.04292993	1.309320058	Zm00001d053427
Zm00001d053427_T005	0.4723494	0.317385359	Zm00001d053427
Zm00001d053427_T012	1	0.244908916	Zm00001d053427
Zm00001d009446_T001	0.7641977	-0.422957674	Zm00001d009446
Zm00001d013323_T001	0.774379	-0.499268844	Zm00001d013323
Zm00001d027707_T001	0.9432413	0.12949028	Zm00001d027707

Zm00001d023337_T003	0.5103238	0.745023931	Zm00001d023337
Zm00001d023337_T027	0.989787	0.261651816	Zm00001d023337
Zm00001d023337_T024	0.4051828	-0.242179994	Zm00001d023337
Zm00001d022388_T012	3.52E-05	1.567492635	Zm00001d022388
Zm00001d022388_T004	0.5437049	0.182117675	Zm00001d022388
Zm00001d022388_T018	0.3183941	-0.781799152	Zm00001d022388
Zm00001d022388_T003	0.008251933	2.094079502	Zm00001d022388
Zm00001d022388_T017	0.902752	0.851809049	Zm00001d022388
Zm00001d022388_T014	0.2386177	1.776848618	Zm00001d022388
Zm00001d022388_T016	1.75E-15	3.447814603	Zm00001d022388
Zm00001d022388_T011	0.4242219	-0.676279204	Zm00001d022388
Zm00001d022388_T019	0.1213982	0.419233889	Zm00001d022388
Zm00001d013300_T001	0.6903979	-0.531146649	Zm00001d013300
Zm00001d013300_T003	0.6292709	-0.226606652	Zm00001d013300
Zm00001d013300_T002	0.842741	-0.378069922	Zm00001d013300
Zm00001d026319_T004	0.960704	0.0667478	Zm00001d026319
Zm00001d026319_T003	0.9621967	0.442013059	Zm00001d026319
Zm00001d034460_T007	0.9400055	0.041234264	Zm00001d034460
Zm00001d034460_T011	1	0.134857185	Zm00001d034460
Zm00001d034460_T014	0.293343	0.797196971	Zm00001d034460
Zm00001d034460_T016	0.94555	0.558459512	Zm00001d034460
Zm00001d034460_T010	0.9596377	0.308470088	Zm00001d034460
Zm00001d034460_T015	0.9722469	0.481689021	Zm00001d034460
Zm00001d007465_T001	0.2178312	-0.943304859	Zm00001d007465
Zm00001d007465_T002	0.9067873	-0.041670148	Zm00001d007465
Zm00001d042558_T003	0.9791729	0.067683474	Zm00001d042558
Zm00001d042558_T002	1.98E-05	0.56673383	Zm00001d042558
Zm00001d026573_T001	0.995534	0.315583004	Zm00001d026573
Zm00001d053976_T001	0.7942213	-0.431676588	Zm00001d053976
Zm00001d027423_T001	0.90276	-0.115499969	Zm00001d027423
Zm00001d027423_T002	0.9857423	0.270379441	Zm00001d027423
Zm00001d044195_T004	0.8939109	0.345882044	Zm00001d044195
Zm00001d044195_T015	0.8716622	-0.163400206	Zm00001d044195
Zm00001d044195_T003	0.9404125	0.116670972	Zm00001d044195
Zm00001d044195_T020	0.001550358	-2.495727588	Zm00001d044195
Zm00001d044195_T007	0.2980621	1.045818453	Zm00001d044195
Zm00001d044195_T011	0.8953011	0.164235801	Zm00001d044195
Zm00001d044195_T017	0.9791729	0.370528691	Zm00001d044195
Zm00001d039683_T004	0.8825173	0.337379203	Zm00001d039683
Zm00001d039683_T006	0.852831	-0.102194943	Zm00001d039683
Zm00001d039683_T001	0.9747714	0.214296929	Zm00001d039683
Zm00001d039683_T009	0.9779656	0.166617545	Zm00001d039683
Zm00001d008569_T002	0.8095986	-0.284935275	Zm00001d008569
Zm00001d008569_T004	0.9894615	0.238788844	Zm00001d008569
Zm00001d053580_T001	0.7811868	-0.081097997	Zm00001d053580
Zm00001d053580_T005	9.73E-05	-2.362590426	Zm00001d053580
Zm00001d053580_T003	0.8585804	0.951505212	Zm00001d053580
Zm00001d003614_T001	0.8720862	0.883789695	Zm00001d003614
Zm00001d020770_T001	0.7233733	-0.781226601	Zm00001d020770
Zm00001d020770_T003	0.9977752	0.336455264	Zm00001d020770
Zm00001d027443_T001	0.7814026	0.877381184	Zm00001d027443
Zm00001d051339_T001	0.7483459	0.931536686	Zm00001d051339
Zm00001d020331_T002	0.3545411	-1.345509096	Zm00001d020331
Zm00001d015449_T001	0.8414741	-0.237158367	Zm00001d015449

Zm00001d035111_T001	0.9812314	0.131865269	Zm00001d035111
Zm00001d034000_T008	0.001167074	0.754967866	Zm00001d034000
Zm00001d034000_T002	0.8716622	0.847649529	Zm00001d034000
Zm00001d034000_T005	0.03734827	1.15058746	Zm00001d034000
Zm00001d034000_T007	0.5437812	-0.61445909	Zm00001d034000
Zm00001d007679_T001	0.07811455	1.936347031	Zm00001d007679
Zm00001d041067_T001	0.01840305	-1.903509585	Zm00001d041067
Zm00001d015692_T001	0.9630058	0.599016442	Zm00001d015692
Zm00001d034782_T001	0.8686839	0.9193608	Zm00001d034782
Zm00001d028007_T002	0.9720425	0.13852069	Zm00001d028007
Zm00001d028007_T004	0.7540032	0.533499502	Zm00001d028007
Zm00001d007836_T001	0.5804156	-0.870796749	Zm00001d007836
Zm00001d008582_T001	0.9696449	0.278330086	Zm00001d008582
Zm00001d043277_T012	0.7927202	0.649886334	Zm00001d043277
Zm00001d043277_T008	1.22E-09	1.189341451	Zm00001d043277
Zm00001d043277_T016	0.006370542	-0.344478972	Zm00001d043277
Zm00001d052640_T001	1	0.122642909	Zm00001d052640
Zm00001d035052_T001	0.9252088	-0.12641604	Zm00001d035052
Zm00001d006496_T002	0.956479	0.02796667	Zm00001d006496
Zm00001d006496_T003	0.9497169	0.111542348	Zm00001d006496
Zm00001d006496_T001	0.1159519	-0.661452185	Zm00001d006496
Zm00001d047090_T002	0.973825	0.521088924	Zm00001d047090
Zm00001d052881_T001	0.9863483	0.00517151	Zm00001d052881
Zm00001d041692_T001	0.9804228	0.052668414	Zm00001d041692
Zm00001d041692_T002	0.9688821	0.583197627	Zm00001d041692
Zm00001d028946_T001	0.954492	0.091545809	Zm00001d028946
Zm00001d012605_T002	0.727861	-0.57298614	Zm00001d012605
Zm00001d012605_T001	1	0.286764848	Zm00001d012605
Zm00001d041610_T001	0.9804228	0.15676623	Zm00001d041610
Zm00001d041082_T001	0.7194777	-0.184227042	Zm00001d041082
Zm00001d013702_T001	0.8427421	0.652703868	Zm00001d013702
Zm00001d043012_T001	0.3713446	-0.166051626	Zm00001d043012
Zm00001d043012_T008	0.8751967	-0.027332722	Zm00001d043012
Zm00001d043012_T018	0.5591883	1.04274264	Zm00001d043012
Zm00001d043012_T015	0.989787	0.185481749	Zm00001d043012
Zm00001d043012_T016	0.9835181	0.126558991	Zm00001d043012
Zm00001d006388_T004	0.6118255	1.489996537	Zm00001d006388
Zm00001d006388_T001	0.7721703	-0.50489476	Zm00001d006388
Zm00001d006388_T006	0.004411808	-3.493378295	Zm00001d006388
Zm00001d052972_T001	0.997764	0.213554179	Zm00001d052972
Zm00001d028608_T010	0.5541374	0.572573413	Zm00001d028608
Zm00001d028608_T002	0.8309341	1.013642999	Zm00001d028608
Zm00001d028608_T005	0.4952796	0.454597426	Zm00001d028608
Zm00001d002668_T003	0.7619918	1.085236015	Zm00001d002668
Zm00001d002668_T008	1	0.283672527	Zm00001d002668
Zm00001d036671_T001	0.8160618	-0.222493259	Zm00001d036671
Zm00001d037808_T001	0.8533785	0.909922929	Zm00001d037808
Zm00001d037808_T007	4.40E-11	-4.310393695	Zm00001d037808
Zm00001d037808_T004	0.7400422	1.112157827	Zm00001d037808
Zm00001d037808_T003	0.6926364	0.288682226	Zm00001d037808
Zm00001d019364_T001	0.006499341	-2.667898676	Zm00001d019364
Zm00001d013721_T002	0.8232235	-0.671937217	Zm00001d013721
Zm00001d013721_T007	0.997449	0.189009769	Zm00001d013721
Zm00001d013721_T001	0.8519292	-0.289897379	Zm00001d013721

Zm00001d013721_T009	0.8613703	-0.262627371	Zm00001d013721
Zm00001d013721_T003	0.9380783	-0.545887703	Zm00001d013721
Zm00001d013721_T004	0.687835	-0.89922364	Zm00001d013721
Zm00001d052327_T008	0.2966369	0.652902364	Zm00001d052327
Zm00001d052327_T018	0.658756	0.597244836	Zm00001d052327
Zm00001d052327_T022	0.9701986	0.129665645	Zm00001d052327
Zm00001d052327_T029	0.1637824	-1.055421507	Zm00001d052327
Zm00001d052327_T026	0.745806	0.237236163	Zm00001d052327
Zm00001d052327_T034	0.5748211	0.381407861	Zm00001d052327
Zm00001d009481_T001	0.5480873	1.016024635	Zm00001d009481
Zm00001d046998_T001	0.6305532	-0.850077284	Zm00001d046998
Zm00001d033128_T002	0.9779656	0.220253772	Zm00001d033128
Zm00001d033128_T001	0.8488494	-0.386747497	Zm00001d033128
Zm00001d036107_T001	0.993158	0.092451984	Zm00001d036107
Zm00001d046893_T001	0.7191935	-0.331414343	Zm00001d046893
Zm00001d018445_T001	0.2526212	-1.009507579	Zm00001d018445
Zm00001d018445_T004	0.9982897	0.413618877	Zm00001d018445
Zm00001d018445_T003	0.000667968	-0.923036292	Zm00001d018445
Zm00001d018445_T002	0.7594555	-0.076434256	Zm00001d018445
Zm00001d023929_T002	0.530398	-0.158406379	Zm00001d023929
Zm00001d023929_T001	0.8122182	-0.20015373	Zm00001d023929
Zm00001d003275_T001	0.9584416	0.022457839	Zm00001d003275
Zm00001d020414_T001	0.0147135	1.227726615	Zm00001d020414
Zm00001d020414_T004	0.6361249	0.989137966	Zm00001d020414
Zm00001d007077_T001	0.9781672	-0.042011634	Zm00001d007077
Zm00001d024364_T004	0.9501111	0.713676328	Zm00001d024364
Zm00001d024364_T001	0.9907928	0.534735263	Zm00001d024364
Zm00001d024364_T002	0.3395336	0.744905334	Zm00001d024364
Zm00001d024364_T003	0.2083072	0.444974826	Zm00001d024364
Zm00001d025067_T001	0.2624975	-0.541378125	Zm00001d025067
Zm00001d041430_T001	0.9945118	0.002413926	Zm00001d041430
Zm00001d010055_T001	1	0.342517912	Zm00001d010055
Zm00001d010055_T003	0.8689882	0.811925923	Zm00001d010055
Zm00001d010055_T002	0.06405891	-2.373623476	Zm00001d010055
Zm00001d010055_T005	0.705921	0.792077893	Zm00001d010055
Zm00001d025044_T005	0.2553148	1.513604989	Zm00001d025044
Zm00001d025044_T040	0.9410845	-0.014719224	Zm00001d025044
Zm00001d035672_T001	0.9182861	0.390395177	Zm00001d035672
Zm00001d032103_T001	0.9791729	0.376503106	Zm00001d032103
Zm00001d019669_T001	0.3015167	1.734156932	Zm00001d019669
Zm00001d053564_T006	0.8613703	0.911534244	Zm00001d053564
Zm00001d053564_T002	0.8032834	-0.189836344	Zm00001d053564
Zm00001d019978_T001	1	0.433809469	Zm00001d019978
Zm00001d019978_T006	3.65E-08	1.402292028	Zm00001d019978
Zm00001d019978_T009	0.9344122	0.632660402	Zm00001d019978
Zm00001d019978_T010	0.01048202	1.206325889	Zm00001d019978
Zm00001d019978_T003	0.5757967	0.581384861	Zm00001d019978
Zm00001d049399_T002	0.3836885	-0.892647953	Zm00001d049399
Zm00001d049399_T001	0.9173494	0.033349247	Zm00001d049399
Zm00001d048665_T002	0.7312547	1.037309792	Zm00001d048665
Zm00001d016619_T002	0.9979568	0.421219183	Zm00001d016619
Zm00001d016619_T001	0.9361945	0.002579623	Zm00001d016619
Zm00001d020666_T020	0.5275964	-0.647046532	Zm00001d020666
Zm00001d020666_T013	0.8581713	0.607315941	Zm00001d020666

Zm00001d020666_T015	0.4377468	0.508829758	Zm00001d020666
Zm00001d025938_T001	0.9022629	-0.13461373	Zm00001d025938
Zm00001d052609_T001	0.8641671	-0.398980986	Zm00001d052609
Zm00001d037619_T001	0.4594105	1.39908462	Zm00001d037619
Zm00001d037619_T002	0.5604174	1.032151341	Zm00001d037619
Zm00001d029428_T001	1	0.169459654	Zm00001d029428
Zm00001d026488_T001	0.8883561	0.430288225	Zm00001d026488
Zm00001d007302_T008	0.6636779	-0.36658734	Zm00001d007302
Zm00001d007302_T009	1	0.342500492	Zm00001d007302
Zm00001d007302_T013	0.6082229	1.190245564	Zm00001d007302
Zm00001d007302_T002	0.4137215	-0.316526401	Zm00001d007302
Zm00001d013625_T001	0.4237296	-0.808260282	Zm00001d013625
Zm00001d008913_T001	0.9824348	0.404845056	Zm00001d008913
Zm00001d048603_T001	0.9554972	0.621629125	Zm00001d048603
Zm00001d004664_T001	0.002306494	2.785293718	Zm00001d004664
Zm00001d051584_T001	0.9766874	0.18468727	Zm00001d051584
Zm00001d013204_T002	0.92623	-0.076415708	Zm00001d013204
Zm00001d020811_T001	0.6086477	1.264801524	Zm00001d020811
Zm00001d049496_T002	0.960043	0.550459644	Zm00001d049496
Zm00001d049496_T001	0.9760472	0.000393372	Zm00001d049496
Zm00001d010862_T006	0.7602652	-0.332327125	Zm00001d010862
Zm00001d010862_T009	0.9352321	-0.03391413	Zm00001d010862
Zm00001d010862_T001	0.9884825	0.469543605	Zm00001d010862
Zm00001d010862_T007	0.5693266	-0.816141888	Zm00001d010862
Zm00001d049965_T001	0.8878424	-0.175612701	Zm00001d049965
Zm00001d016545_T001	0.5886552	-0.712685927	Zm00001d016545
Zm00001d013566_T001	0.9530672	0.653574764	Zm00001d013566
Zm00001d002156_T001	0.79132	-0.487758763	Zm00001d002156
Zm00001d028296_T001	0.9689939	0.109477966	Zm00001d028296
Zm00001d045582_T002	0.9452999	0.396071319	Zm00001d045582
Zm00001d045582_T001	0.9264969	0.711687286	Zm00001d045582
Zm00001d049294_T001	0.9368844	0.012868344	Zm00001d049294
Zm00001d037412_T001	0.9530248	0.052963199	Zm00001d037412
Zm00001d039731_T002	0.5742833	1.042591636	Zm00001d039731
Zm00001d000165_T001	0.7333	-0.69188512	Zm00001d000165
Zm00001d010529_T001	0.9171653	0.669383253	Zm00001d010529
Zm00001d042582_T001	0.9460688	-0.019160595	Zm00001d042582
Zm00001d042582_T002	0.9804228	0.10612167	Zm00001d042582
Zm00001d038847_T001	0.955337	0.53842852	Zm00001d038847
Zm00001d011425_T001	0.02229829	2.039409545	Zm00001d011425
Zm00001d038921_T001	0.01531128	2.194487011	Zm00001d038921
Zm00001d013040_T007	0.9463326	0.292665738	Zm00001d013040
Zm00001d013040_T008	0.9196877	-0.028938984	Zm00001d013040
Zm00001d013040_T002	0.8587779	0.544362412	Zm00001d013040
Zm00001d013040_T005	0.7137316	0.920083087	Zm00001d013040
Zm00001d033169_T001	0.6486478	-0.532252192	Zm00001d033169
Zm00001d037125_T003	0.4283558	0.522218701	Zm00001d037125
Zm00001d037125_T007	0.926743	0.029186571	Zm00001d037125
Zm00001d037125_T010	0.6183146	0.849225314	Zm00001d037125
Zm00001d037125_T001	0.9690925	0.409243898	Zm00001d037125
Zm00001d036337_T008	0.06964423	1.28154184	Zm00001d036337
Zm00001d036337_T005	0.1669096	0.693346504	Zm00001d036337
Zm00001d036337_T007	0.9914036	0.486611075	Zm00001d036337
Zm00001d036337_T010	0.9919298	0.27855434	Zm00001d036337

Zm00001d036337_T001	0.02746855	2.251097814	Zm00001d036337
Zm00001d036337_T003	0.9907696	0.568315592	Zm00001d036337
Zm00001d009160_T001	0.000484721	-2.922530206	Zm00001d009160
Zm00001d030034_T001	0.9616398	0.092006811	Zm00001d030034
Zm00001d030034_T003	0.2724511	-1.286820463	Zm00001d030034
Zm00001d030034_T002	0.9416622	0.002383764	Zm00001d030034
Zm00001d043478_T001	0.1721616	-1.016947735	Zm00001d043478
Zm00001d039514_T003	0.1722301	-0.681650648	Zm00001d039514
Zm00001d039514_T001	0.7289978	-0.034028136	Zm00001d039514
Zm00001d039514_T002	0.929138	0.061618509	Zm00001d039514
Zm00001d016083_T001	0.7882239	-0.33746898	Zm00001d016083
Zm00001d017274_T003	0.02943756	-0.640585473	Zm00001d017274
Zm00001d017274_T005	0.9834026	0.472552371	Zm00001d017274
Zm00001d017274_T002	0.9213556	0.032961654	Zm00001d017274
Zm00001d017274_T004	0.718063	-0.316044008	Zm00001d017274
Zm00001d017274_T001	0.9709496	0.232504259	Zm00001d017274
Zm00001d019949_T001	0.9898682	0.529578862	Zm00001d019949
Zm00001d015251_T001	0.8857953	-0.110693184	Zm00001d015251
Zm00001d015201_T001	0.06650348	-0.491240404	Zm00001d015201
Zm00001d048898_T001	0.9907928	0.211279144	Zm00001d048898
Zm00001d045314_T001	0.9979568	0.244972494	Zm00001d045314
Zm00001d009097_T001	0.3596674	-0.752788964	Zm00001d009097
Zm00001d012485_T005	0.09458322	-0.738024589	Zm00001d012485
Zm00001d016877_T003	0.6966097	0.851049085	Zm00001d016877
Zm00001d016877_T004	0.9469426	-0.049643752	Zm00001d016877
Zm00001d016877_T002	0.9702242	0.081018258	Zm00001d016877
Zm00001d039638_T001	0.8557307	0.776447995	Zm00001d039638
Zm00001d006257_T009	0.4760609	-0.217266787	Zm00001d006257
Zm00001d047708_T001	0.5296936	1.156885174	Zm00001d047708
Zm00001d010403_T001	0.8786965	-0.138354731	Zm00001d010403
Zm00001d010403_T002	0.909729	-0.059019585	Zm00001d010403
Zm00001d017931_T001	0.9468268	-0.00716083	Zm00001d017931
Zm00001d014000_T001	0.9942835	0.095480184	Zm00001d014000
Zm00001d032245_T003	0.9785107	0.173608654	Zm00001d032245
Zm00001d032245_T001	0.6716936	-0.223495343	Zm00001d032245
Zm00001d017960_T001	0.8676122	-0.136091002	Zm00001d017960
Zm00001d017988_T001	0.940651	0.024005247	Zm00001d017988
Zm00001d017988_T005	0.8165501	0.643373029	Zm00001d017988
Zm00001d012463_T001	0.7626242	-0.298767632	Zm00001d012463
Zm00001d033377_T001	0.6357839	0.879381809	Zm00001d033377
Zm00001d033214_T001	0.957148	0.009125429	Zm00001d033214
Zm00001d011878_T001	0.7489032	0.89020602	Zm00001d011878
Zm00001d012831_T001	0.8958197	-0.038778888	Zm00001d012831
Zm00001d000110_T001	0.9223663	0.929533244	Zm00001d000110
Zm00001d000110_T005	0.5776288	0.825163502	Zm00001d000110
Zm00001d000110_T003	0.9184323	0.678530312	Zm00001d000110
Zm00001d000110_T006	0.2566009	-1.25977879	Zm00001d000110
Zm00001d000110_T002	0.9979568	0.232217771	Zm00001d000110
Zm00001d000110_T004	0.004536195	1.305428095	Zm00001d000110
Zm00001d039710_T004	0.7923921	-0.600727373	Zm00001d039710
Zm00001d039710_T003	0.8292199	-0.530934524	Zm00001d039710
Zm00001d039710_T005	0.6436915	-0.068864246	Zm00001d039710
Zm00001d039710_T002	0.7626242	0.65884961	Zm00001d039710
Zm00001d039710_T001	0.9804228	0.242028759	Zm00001d039710

Zm00001d006054_T001	0.949277	0.388319694	Zm00001d006054
Zm00001d036668_T002	0.5395369	1.042938492	Zm00001d036668
Zm00001d036668_T005	0.836612	-0.221430298	Zm00001d036668
Zm00001d036668_T006	0.9822168	0.364374033	Zm00001d036668
Zm00001d036668_T004	0.6084447	0.944749493	Zm00001d036668
Zm00001d019936_T001	0.8046598	0.82162285	Zm00001d019936
Zm00001d001766_T001	0.9524127	0.432668992	Zm00001d001766
Zm00001d052439_T003	0.9469426	0.633366238	Zm00001d052439
Zm00001d053552_T001	0.9726706	0.611696895	Zm00001d053552
Zm00001d037735_T001	0.9867452	0.343922381	Zm00001d037735
Zm00001d048126_T005	0.5179617	1.007702456	Zm00001d048126
Zm00001d048126_T008	0.7594555	0.939362785	Zm00001d048126
Zm00001d048126_T004	0.6217057	0.634640525	Zm00001d048126
Zm00001d048126_T006	0.5489602	1.416808507	Zm00001d048126
Zm00001d048126_T010	0.2172191	-2.200566512	Zm00001d048126
Zm00001d049823_T001	0.9813716	0.055986114	Zm00001d049823
Zm00001d044783_T001	0.7091158	-0.641018575	Zm00001d044783
Zm00001d015249_T002	0.8570642	0.908218877	Zm00001d015249
Zm00001d010726_T001	0.9407613	0.743582437	Zm00001d010726
Zm00001d005445_T001	0.9855106	0.458357674	Zm00001d005445
Zm00001d015426_T001	0.8801107	0.874978772	Zm00001d015426
Zm00001d037858_T001	0.9781672	0.566241501	Zm00001d037858
Zm00001d023646_T003	1	0.123209255	Zm00001d023646
Zm00001d023646_T001	1	0.224383708	Zm00001d023646
Zm00001d022396_T002	0.000678421	1.385840484	Zm00001d022396
Zm00001d022396_T001	0.6507659	-0.5078469	Zm00001d022396
Zm00001d046633_T001	0.9262419	0.582713851	Zm00001d046633
Zm00001d046633_T002	0.8842271	-0.331553983	Zm00001d046633
Zm00001d049921_T001	0.8830977	-0.18907189	Zm00001d049921
Zm00001d041792_T001	0.3118193	-1.435414132	Zm00001d041792
Zm00001d018749_T001	0.8593759	0.829953493	Zm00001d018749
Zm00001d033863_T001	0.5537579	-0.415205328	Zm00001d033863
Zm00001d014319_T001	0.6317099	-0.587446042	Zm00001d014319
Zm00001d013080_T001	1	0.337301353	Zm00001d013080
Zm00001d012239_T002	0.9203655	0.642860772	Zm00001d012239
Zm00001d012239_T003	0.758035	-0.296476704	Zm00001d012239
Zm00001d034968_T004	0.8716622	0.470370006	Zm00001d034968
Zm00001d034968_T001	0.9463296	0.354738837	Zm00001d034968
Zm00001d009715_T001	0.7109402	-0.053330465	Zm00001d009715
Zm00001d013400_T002	0.01049064	-1.042593609	Zm00001d013400
Zm00001d049059_T001	0.8741014	-0.121972737	Zm00001d049059
Zm00001d028667_T001	0.4022327	-1.041928628	Zm00001d028667
Zm00001d012719_T001	0.8123836	-0.206815063	Zm00001d012719
Zm00001d018349_T003	0.9706293	0.582174286	Zm00001d018349
Zm00001d018349_T006	0.9907928	0.471330543	Zm00001d018349
Zm00001d018349_T004	0.1296685	1.239183708	Zm00001d018349
Zm00001d018349_T008	0.04891776	-2.496631179	Zm00001d018349
Zm00001d018349_T001	0.5014025	0.997645135	Zm00001d018349
Zm00001d043600_T001	0.9375274	0.663042212	Zm00001d043600
Zm00001d014736_T001	0.7523559	-0.190709129	Zm00001d014736
Zm00001d019249_T004	0.9782953	0.21678131	Zm00001d019249
Zm00001d019249_T007	0.8369406	-0.541570963	Zm00001d019249
Zm00001d019249_T003	0.7548788	-0.157726272	Zm00001d019249
Zm00001d015914_T005	0.9766291	0.132325724	Zm00001d015914

Zm00001d045729_T002	1	0.107916355	Zm00001d045729
Zm00001d045729_T001	0.9979568	0.057391917	Zm00001d045729
Zm00001d032882_T001	0.9334107	-0.211595226	Zm00001d032882
Zm00001d023439_T002	0.8740066	0.00480114	Zm00001d023439
Zm00001d023439_T001	0.9356676	0.005648703	Zm00001d023439
Zm00001d037693_T002	0.002378541	-1.22035799	Zm00001d037693
Zm00001d037693_T003	0.5978014	-0.397461699	Zm00001d037693
Zm00001d012573_T001	0.9766874	0.034834272	Zm00001d012573
Zm00001d034252_T003	0.9429712	-0.03543745	Zm00001d034252
Zm00001d034252_T001	0.8772264	-0.398472084	Zm00001d034252
Zm00001d036178_T001	0.7109402	-0.348156461	Zm00001d036178
Zm00001d036178_T002	0.2853231	-0.499737177	Zm00001d036178
Zm00001d034157_T006	0.8913084	0.322070713	Zm00001d034157
Zm00001d034157_T016	0.7548788	0.609906397	Zm00001d034157
Zm00001d034157_T017	0.123021	0.487230057	Zm00001d034157
Zm00001d034157_T022	0.9429712	-0.005280632	Zm00001d034157
Zm00001d034157_T019	0.9419336	0.114694939	Zm00001d034157
Zm00001d034157_T010	0.2642794	0.230369703	Zm00001d034157
Zm00001d034157_T013	0.941809	0.466563852	Zm00001d034157
Zm00001d034157_T003	0.000329177	-1.013954772	Zm00001d034157
Zm00001d034157_T001	0.378904	0.555527844	Zm00001d034157
Zm00001d034157_T018	0.2508936	0.418593017	Zm00001d034157
Zm00001d034157_T008	0.004006518	0.959610843	Zm00001d034157
Zm00001d017700_T025	0.000140887	2.261291814	Zm00001d017700
Zm00001d017700_T024	0.9747695	0.476745268	Zm00001d017700
Zm00001d017700_T013	0.01035085	2.184945396	Zm00001d017700
Zm00001d017700_T005	1.36E-08	0.982612571	Zm00001d017700
Zm00001d053401_T012	0.9419336	0.081740784	Zm00001d053401
Zm00001d053401_T003	0.7358211	0.248132359	Zm00001d053401
Zm00001d053401_T005	0.1090245	1.887866732	Zm00001d053401
Zm00001d053401_T007	1	0.101359828	Zm00001d053401
Zm00001d033539_T002	0.05003329	2.325532781	Zm00001d033539
Zm00001d018522_T001	1	0.392949149	Zm00001d018522
Zm00001d018522_T004	0.9524127	0.081603501	Zm00001d018522
Zm00001d018522_T003	0.6494559	-0.055855764	Zm00001d018522
Zm00001d018522_T002	1.16E-12	-2.492442092	Zm00001d018522
Zm00001d016824_T002	1	0.23420822	Zm00001d016824
Zm00001d016824_T001	0.8730347	-0.102257148	Zm00001d016824
Zm00001d008212_T002	0.954492	0.636466837	Zm00001d008212
Zm00001d004818_T001	0.9990127	0.141122014	Zm00001d004818
Zm00001d036255_T001	0.9228108	0.696047343	Zm00001d036255
Zm00001d037512_T003	1	0.311086809	Zm00001d037512
Zm00001d037512_T002	0.3721899	-0.537180316	Zm00001d037512
Zm00001d030061_T030	0.2566009	1.100235828	Zm00001d030061
Zm00001d030061_T028	0.1365976	-0.569649777	Zm00001d030061
Zm00001d030061_T014	0.9580487	-0.360060666	Zm00001d030061
Zm00001d030061_T001	0.04667204	1.551931902	Zm00001d030061
Zm00001d030061_T002	0.8412913	0.172162885	Zm00001d030061
Zm00001d030061_T026	1.52E-06	0.443028011	Zm00001d030061
Zm00001d048313_T001	1	0.19930591	Zm00001d048313
Zm00001d031152_T001	1	0.113508258	Zm00001d031152
Zm00001d002891_T001	0.9936409	0.408635859	Zm00001d002891
Zm00001d038719_T001	0.9529094	0.130086582	Zm00001d038719
Zm00001d033744_T001	1	0.18452979	Zm00001d033744

Zm00001d014088_T002	0.9160053	0.039483813	Zm00001d014088
Zm00001d017336_T006	0.9463326	0.289583164	Zm00001d017336
Zm00001d017336_T004	0.9353691	0.644243171	Zm00001d017336
Zm00001d017336_T005	0.796163	0.894456216	Zm00001d017336
Zm00001d017336_T002	0.7697196	-0.156638748	Zm00001d017336
Zm00001d049433_T001	0.08358949	1.927632533	Zm00001d049433
Zm00001d034662_T001	0.9799748	0.207731999	Zm00001d034662
Zm00001d043789_T006	0.01026997	-0.810053915	Zm00001d043789
Zm00001d043789_T001	0.5579579	-0.125878604	Zm00001d043789
Zm00001d043789_T012	0.05692626	-0.27493146	Zm00001d043789
Zm00001d043789_T005	0.4758333	0.067430269	Zm00001d043789
Zm00001d009024_T001	0.8854695	-0.561457048	Zm00001d009024
Zm00001d011658_T001	0.9897446	0.41251822	Zm00001d011658
Zm00001d025616_T001	0.8865624	-0.394851493	Zm00001d025616
Zm00001d002530_T001	0.7825891	0.912366364	Zm00001d002530
Zm00001d018871_T001	0.4235631	1.288466666	Zm00001d018871
Zm00001d016822_T008	0.5057074	0.642258665	Zm00001d016822
Zm00001d016822_T021	0.4540782	0.56711866	Zm00001d016822
Zm00001d016822_T001	0.404647	1.019087123	Zm00001d016822
Zm00001d016822_T006	0.5123915	-0.638763855	Zm00001d016822
Zm00001d016822_T003	0.581703	1.321910369	Zm00001d016822
Zm00001d025620_T005	0.5069619	0.898802107	Zm00001d025620
Zm00001d025620_T001	0.9732421	0.396571949	Zm00001d025620
Zm00001d025620_T006	0.6844831	-0.743620224	Zm00001d025620
Zm00001d017042_T002	0.002593184	1.894673936	Zm00001d017042
Zm00001d017042_T003	0.2258138	1.92844833	Zm00001d017042
Zm00001d017042_T001	0.001005513	-3.395433605	Zm00001d017042
Zm00001d033545_T001	0.1698704	-0.771161158	Zm00001d033545
Zm00001d023291_T001	0.8289284	0.916236304	Zm00001d023291
Zm00001d044027_T001	0.7889615	2.042408467	Zm00001d044027
Zm00001d041945_T005	0.9813678	0.540120743	Zm00001d041945
Zm00001d041945_T002	0.9139882	-0.084232027	Zm00001d041945
Zm00001d041945_T001	0.01692334	2.311298907	Zm00001d041945
Zm00001d041945_T006	0.0006035	1.685932952	Zm00001d041945
Zm00001d045232_T001	0.005958271	2.139863609	Zm00001d045232
Zm00001d026096_T001	0.9979568	0.168921781	Zm00001d026096
Zm00001d005671_T002	0.9223663	0.548796976	Zm00001d005671
Zm00001d005671_T001	0.9868821	0.123387838	Zm00001d005671
Zm00001d048730_T001	0.9946911	0.160475775	Zm00001d048730
Zm00001d006580_T001	0.8865624	0.502763895	Zm00001d006580
Zm00001d006580_T010	0.9921225	0.496363905	Zm00001d006580
Zm00001d027638_T001	0.7109402	-0.31456598	Zm00001d027638
Zm00001d038312_T001	0.009563045	1.782189556	Zm00001d038312
Zm00001d007032_T001	0.9659036	-0.301875514	Zm00001d007032
Zm00001d007032_T002	0.9704269	-0.344030589	Zm00001d007032
Zm00001d006355_T018	0.7830878	0.960487008	Zm00001d006355
Zm00001d006355_T001	0.6284759	0.048669868	Zm00001d006355
Zm00001d006355_T021	0.1440511	0.642292072	Zm00001d006355
Zm00001d006355_T012	0.7428299	-0.158714788	Zm00001d006355
Zm00001d015704_T001	0.9898682	-0.076579958	Zm00001d015704
Zm00001d015965_T001	0.9363242	0.02958979	Zm00001d015965
Zm00001d029604_T001	0.2719671	-0.978860154	Zm00001d029604
Zm00001d014489_T010	0.9969363	0.519476107	Zm00001d014489
Zm00001d014489_T006	0.4374242	0.47715278	Zm00001d014489

Zm00001d014489_T005	0.9878764	0.159361252	Zm00001d014489
Zm00001d014489_T001	0.7025218	1.071104752	Zm00001d014489
Zm00001d013428_T003	0.9988864	0.497687849	Zm00001d013428
Zm00001d013428_T002	0.9970835	0.385887767	Zm00001d013428
Zm00001d032282_T012	0.9979568	0.483993914	Zm00001d032282
Zm00001d032282_T005	0.08184453	1.042454051	Zm00001d032282
Zm00001d032282_T006	1.52E-06	0.799357837	Zm00001d032282
Zm00001d032282_T003	0.002378541	0.960136464	Zm00001d032282
Zm00001d043206_T001	0.9762137	0.376172058	Zm00001d043206
Zm00001d037573_T001	0.9344122	0.526338879	Zm00001d037573
Zm00001d037573_T002	0.9791729	-0.085571079	Zm00001d037573
Zm00001d010066_T002	0.956053	0.665081386	Zm00001d010066
Zm00001d010066_T001	0.9946911	0.31275557	Zm00001d010066
Zm00001d010066_T003	0.7414776	1.087509089	Zm00001d010066
Zm00001d043835_T006	0.5864542	0.797870964	Zm00001d043835
Zm00001d043835_T010	0.06429032	-1.341525047	Zm00001d043835
Zm00001d043835_T009	0.8412913	0.645387204	Zm00001d043835
Zm00001d043835_T008	0.8720862	0.432651947	Zm00001d043835
Zm00001d039144_T002	0.8676122	0.947103121	Zm00001d039144
Zm00001d022636_T001	0.9741332	0.182902968	Zm00001d022636
Zm00001d023398_T001	0.4332539	-0.516989444	Zm00001d023398
Zm00001d025825_T001	0.9970835	0.437119852	Zm00001d025825
Zm00001d023566_T002	0.8580835	-0.100875791	Zm00001d023566
Zm00001d023566_T003	0.4809727	0.884857329	Zm00001d023566
Zm00001d042769_T001	0.6136711	1.036710277	Zm00001d042769
Zm00001d043128_T001	0.1576715	1.423170709	Zm00001d043128
Zm00001d040285_T001	0.7831859	0.747579727	Zm00001d040285
Zm00001d020219_T001	0.7470071	-0.303092256	Zm00001d020219
Zm00001d020219_T004	0.3263649	-0.374081847	Zm00001d020219
Zm00001d020219_T002	1	0.500419151	Zm00001d020219
Zm00001d018504_T001	0.9216521	-0.008261988	Zm00001d018504
Zm00001d050929_T004	0.85068	0.553695777	Zm00001d050929
Zm00001d045650_T001	0.9889811	-0.132775315	Zm00001d045650
Zm00001d015546_T001	0.7743716	0.96049374	Zm00001d015546
Zm00001d053445_T001	0.001122214	-2.838837486	Zm00001d053445
Zm00001d044596_T001	0.5016312	1.352738714	Zm00001d044596
Zm00001d005159_T006	0.9771927	1.411196675	Zm00001d005159
Zm00001d005159_T001	0.0665061	-1.390474772	Zm00001d005159
Zm00001d051016_T002	0.963405	0.067141107	Zm00001d051016
Zm00001d038267_T001	0.594851	-0.708910371	Zm00001d038267
Zm00001d021629_T003	0.4557081	-0.75037798	Zm00001d021629
Zm00001d021629_T001	0.9139882	0.488388264	Zm00001d021629
Zm00001d021598_T007	0.9732421	0.03625777	Zm00001d021598
Zm00001d021598_T010	0.811177	0.615790468	Zm00001d021598
Zm00001d021598_T002	0.1868442	1.452159848	Zm00001d021598
Zm00001d021598_T008	0.2294707	0.646408289	Zm00001d021598
Zm00001d021598_T005	0.9988063	0.362981748	Zm00001d021598
Zm00001d021598_T001	0.9914064	0.205568435	Zm00001d021598
Zm00001d021598_T003	1	0.248162347	Zm00001d021598
Zm00001d011716_T001	0.9907696	0.275379184	Zm00001d011716
Zm00001d009030_T001	0.9085393	-0.075168944	Zm00001d009030
Zm00001d021646_T005	0.8770116	-0.060399458	Zm00001d021646
Zm00001d021646_T004	0.5328692	-0.222784114	Zm00001d021646
Zm00001d021646_T014	2.06E-06	2.64975754	Zm00001d021646

Zm00001d033730_T001	0.9031961	-0.568483879	Zm00001d033730
Zm00001d024841_T002	0.9954426	0.337732837	Zm00001d024841
Zm00001d024841_T001	0.9822138	0.170471537	Zm00001d024841
Zm00001d025178_T003	0.5705692	-0.737854328	Zm00001d025178
Zm00001d025178_T002	1	0.196270518	Zm00001d025178
Zm00001d025178_T004	0.9855106	0.166858802	Zm00001d025178
Zm00001d025178_T001	0.9596377	0.044784428	Zm00001d025178
Zm00001d040040_T001	0.7367029	-0.301949162	Zm00001d040040
Zm00001d034547_T001	0.757295	-0.33320916	Zm00001d034547
Zm00001d005834_T001	0.6086477	-0.641049215	Zm00001d005834
Zm00001d053591_T002	0.9184323	-0.031483546	Zm00001d053591
Zm00001d017270_T001	0.3685662	-0.912989977	Zm00001d017270
Zm00001d029151_T002	0.5467906	1.357607849	Zm00001d029151
Zm00001d029151_T001	0.7921111	0.478133666	Zm00001d029151
Zm00001d029151_T003	0.2208276	0.720979883	Zm00001d029151
Zm00001d031196_T001	0.5123915	-1.057506496	Zm00001d031196
Zm00001d021487_T001	0.9781672	0.443387136	Zm00001d021487
Zm00001d008248_T001	0.3395336	0.614621015	Zm00001d008248
Zm00001d008248_T005	0.9166509	0.811926888	Zm00001d008248
Zm00001d008248_T003	0.04241009	2.043314069	Zm00001d008248
Zm00001d008248_T002	0.01477296	2.656045773	Zm00001d008248
Zm00001d050244_T005	0.9975532	0.188996078	Zm00001d050244
Zm00001d050244_T017	0.8158535	-0.23951485	Zm00001d050244
Zm00001d050244_T002	0.6126124	1.241349535	Zm00001d050244
Zm00001d050244_T011	0.9070578	0.374104397	Zm00001d050244
Zm00001d050244_T006	0.08504815	0.563681931	Zm00001d050244
Zm00001d027596_T005	0.074741	2.283853108	Zm00001d027596
Zm00001d027596_T007	0.000136164	2.117276134	Zm00001d027596
Zm00001d027596_T008	0.01763173	0.961828207	Zm00001d027596
Zm00001d027596_T003	0.6235378	1.252264184	Zm00001d027596
Zm00001d027596_T002	0.3774483	1.407589274	Zm00001d027596
Zm00001d023838_T008	0.8535962	0.015255798	Zm00001d023838
Zm00001d023838_T013	0.001992364	-1.864279105	Zm00001d023838
Zm00001d023838_T017	0.3840178	0.486543437	Zm00001d023838
Zm00001d023838_T019	0.9279367	1.310278284	Zm00001d023838
Zm00001d023838_T005	0.5750164	0.446773684	Zm00001d023838
Zm00001d023838_T001	0.7993434	1.016216452	Zm00001d023838
Zm00001d017851_T001	0.9486412	0.22557544	Zm00001d017851
Zm00001d006312_T001	0.8742014	-0.547745545	Zm00001d006312
Zm00001d011796_T008	0.9104115	0.440320907	Zm00001d011796
Zm00001d049326_T008	0.9706887	0.282657011	Zm00001d049326
Zm00001d049326_T003	0.7853478	1.152819949	Zm00001d049326
Zm00001d049326_T002	0.8808619	0.40275012	Zm00001d049326
Zm00001d039926_T001	0.965836	0.57628932	Zm00001d039926
Zm00001d012338_T001	0.5626026	-0.570173655	Zm00001d012338
Zm00001d048110_T001	1	0.397172304	Zm00001d048110
Zm00001d020937_T001	0.8590142	0.482330814	Zm00001d020937
Zm00001d039039_T002	0.3655498	-0.847818993	Zm00001d039039
Zm00001d026012_T001	0.8933745	-0.334470022	Zm00001d026012
Zm00001d053513_T001	0.5450635	-0.552638197	Zm00001d053513
Zm00001d032604_T003	0.9980559	0.410645056	Zm00001d032604
Zm00001d032604_T006	0.6354554	0.428444387	Zm00001d032604
Zm00001d025694_T002	0.3783227	-1.006582463	Zm00001d025694
Zm00001d025694_T001	0.9100954	-0.119388867	Zm00001d025694

Zm00001d035115_T001	7.46E-14	-3.460140482	Zm00001d035115
Zm00001d035115_T008	0.8412913	-0.094835312	Zm00001d035115
Zm00001d035115_T006	0.8142464	-0.166754525	Zm00001d035115
Zm00001d035115_T004	0.8158142	0.0016196	Zm00001d035115
Zm00001d035115_T005	0.11565	2.499257365	Zm00001d035115
Zm00001d035115_T007	0.9979568	0.052172701	Zm00001d035115
Zm00001d049369_T001	0.4516888	-0.695265282	Zm00001d049369
Zm00001d049369_T002	0.431797	-0.823727718	Zm00001d049369
Zm00001d019034_T001	0.98721	-0.127725537	Zm00001d019034
Zm00001d025830_T003	0.1079274	1.026716064	Zm00001d025830
Zm00001d025830_T002	9.26E-12	1.704115519	Zm00001d025830
Zm00001d025830_T009	0.9635696	0.646050653	Zm00001d025830
Zm00001d025830_T059	0.01195326	0.387175136	Zm00001d025830
Zm00001d025830_T065	0.2432261	0.731931899	Zm00001d025830
Zm00001d034733_T003	0.8412913	0.41833597	Zm00001d034733
Zm00001d034733_T001	0.9797082	0.552875627	Zm00001d034733
Zm00001d017361_T001	0.9580985	0.076251865	Zm00001d017361
Zm00001d049709_T002	0.7934233	0.254331921	Zm00001d049709
Zm00001d049709_T001	0.9048001	0.82452744	Zm00001d049709
Zm00001d049709_T004	1	0.080304521	Zm00001d049709
Zm00001d012988_T002	0.8486887	-0.424958151	Zm00001d012988
Zm00001d030952_T002	0.6377685	0.498966376	Zm00001d030952
Zm00001d030952_T003	0.9446484	-0.030092589	Zm00001d030952
Zm00001d044838_T001	0.9840918	0.191406376	Zm00001d044838
Zm00001d044838_T002	0.9779656	0.08543158	Zm00001d044838
Zm00001d019981_T001	0.9825014	0.407103538	Zm00001d019981
Zm00001d019981_T003	0.9031129	0.692320782	Zm00001d019981
Zm00001d019981_T002	0.9449404	-0.002999032	Zm00001d019981
Zm00001d016911_T001	0.9312703	0.747546472	Zm00001d016911
Zm00001d008846_T007	0.5473404	0.831316977	Zm00001d008846
Zm00001d008846_T003	0.4022577	0.162529864	Zm00001d008846
Zm00001d008846_T001	0.726898	1.205257988	Zm00001d008846
Zm00001d009448_T001	0.000304177	1.80184416	Zm00001d009448
Zm00001d002756_T001	0.9849234	0.114480844	Zm00001d002756
Zm00001d046919_T001	0.5380217	1.434557063	Zm00001d046919
Zm00001d006328_T001	0.9543285	0.701945213	Zm00001d006328
Zm00001d053101_T001	0.909602	-0.075865785	Zm00001d053101
Zm00001d038299_T001	0.8885217	-0.037880809	Zm00001d038299
Zm00001d038299_T005	0.9869996	0.470170236	Zm00001d038299
Zm00001d038299_T002	0.9971866	0.312770464	Zm00001d038299
Zm00001d034371_T001	0.01524559	0.630081529	Zm00001d034371
Zm00001d034371_T002	0.9804228	0.277410844	Zm00001d034371
Zm00001d040061_T002	0.8802472	0.855011589	Zm00001d040061
Zm00001d039115_T001	0.5843839	-0.74345222	Zm00001d039115
Zm00001d044205_T001	0.5850461	-0.5905295	Zm00001d044205
Zm00001d035918_T001	0.8987811	-0.185066743	Zm00001d035918
Zm00001d020363_T003	0.1380877	0.826677233	Zm00001d020363
Zm00001d020363_T002	0.9173494	0.731838372	Zm00001d020363
Zm00001d020363_T001	0.266228	-0.883170632	Zm00001d020363
Zm00001d033928_T001	0.9992767	0.314129479	Zm00001d033928
Zm00001d033928_T008	0.9532189	0.200125359	Zm00001d033928
Zm00001d033928_T006	0.9419336	0.015478553	Zm00001d033928
Zm00001d033928_T010	0.9125205	0.295424257	Zm00001d033928
Zm00001d007793_T001	0.8815352	-0.184372822	Zm00001d007793

Zm00001d040638_T001	0.2934482	-1.369731084	Zm00001d040638
Zm00001d036423_T001	0.5292925	-0.393743901	Zm00001d036423
Zm00001d036423_T002	0.9524127	-0.013442319	Zm00001d036423
Zm00001d028255_T001	0.9210327	-0.016151246	Zm00001d028255
Zm00001d028255_T006	0.8886159	0.356868858	Zm00001d028255
Zm00001d034883_T003	0.961399	0.124274708	Zm00001d034883
Zm00001d034883_T004	0.9950703	0.079271309	Zm00001d034883
Zm00001d006861_T005	0.2587036	-0.586349516	Zm00001d006861
Zm00001d006861_T014	0.4178252	0.424794555	Zm00001d006861
Zm00001d006861_T011	0.03490439	1.104548455	Zm00001d006861
Zm00001d006861_T019	0.07482195	0.581620101	Zm00001d006861
Zm00001d006861_T009	0.7730711	0.739367543	Zm00001d006861
Zm00001d006861_T035	0.9524127	0.03743468	Zm00001d006861
Zm00001d006861_T007	0.4891923	0.511563782	Zm00001d006861
Zm00001d006861_T028	0.9909153	0.12367836	Zm00001d006861
Zm00001d006861_T008	0.000789023	1.237065537	Zm00001d006861
Zm00001d006861_T015	1.58E-05	0.635066808	Zm00001d006861
Zm00001d006861_T004	0.8888255	0.374738007	Zm00001d006861
Zm00001d006861_T001	0.9468941	0.189221126	Zm00001d006861
Zm00001d006861_T013	0.9979568	0.01430271	Zm00001d006861
Zm00001d006861_T034	0.0269591	0.685202202	Zm00001d006861
Zm00001d038171_T001	0.9965998	0.468523982	Zm00001d038171
Zm00001d014610_T001	0.299369	-0.631586814	Zm00001d014610
Zm00001d016841_T002	0.9968176	0.406659676	Zm00001d016841
Zm00001d016841_T001	0.9606343	0.422463649	Zm00001d016841
Zm00001d026032_T001	0.8886159	0.618781104	Zm00001d026032
Zm00001d043941_T001	0.749176	-0.351881318	Zm00001d043941
Zm00001d011846_T001	0.2388346	-1.221366423	Zm00001d011846
Zm00001d025904_T001	0.9944178	0.252100043	Zm00001d025904
Zm00001d010877_T003	0.957148	0.353163048	Zm00001d010877
Zm00001d046456_T007	0.305747	1.242039369	Zm00001d046456
Zm00001d046456_T006	0.9673473	0.231817424	Zm00001d046456
Zm00001d046456_T002	0.1079908	0.988325501	Zm00001d046456
Zm00001d046456_T008	0.965919	0.537798281	Zm00001d046456
Zm00001d046456_T001	0.9380783	0.695952826	Zm00001d046456
Zm00001d022338_T005	0.7838436	0.982453356	Zm00001d022338
Zm00001d022338_T004	0.745469	-0.291741899	Zm00001d022338
Zm00001d022338_T001	0.6158618	1.112866903	Zm00001d022338
Zm00001d022338_T006	0.07117706	0.241550223	Zm00001d022338
Zm00001d022338_T002	0.00919548	2.207945457	Zm00001d022338
Zm00001d009826_T001	0.2937923	-0.967725903	Zm00001d009826
Zm00001d049956_T002	0.923446	-0.02771803	Zm00001d049956
Zm00001d013029_T002	0.9173494	-0.039223826	Zm00001d013029
Zm00001d025656_T001	0.9845237	0.09353666	Zm00001d025656
Zm00001d011997_T001	0.6470421	-0.299166056	Zm00001d011997
Zm00001d011997_T003	0.6916675	0.485120809	Zm00001d011997
Zm00001d033004_T006	0.02334998	0.836644273	Zm00001d033004
Zm00001d033004_T004	0.8536822	0.952483865	Zm00001d033004
Zm00001d033004_T001	0.2160779	1.628770159	Zm00001d033004
Zm00001d033004_T005	0.4178453	2.02921883	Zm00001d033004
Zm00001d033004_T002	0.07326802	-1.565149632	Zm00001d033004
Zm00001d052898_T001	0.7146325	0.677602743	Zm00001d052898
Zm00001d015183_T003	0.02947584	0.556904462	Zm00001d015183
Zm00001d015183_T001	0.7626242	0.752418397	Zm00001d015183

Zm00001d015183_T009	0.9455574	0.561424003	Zm00001d015183
Zm00001d005698_T001	0.9804281	0.365691669	Zm00001d005698
Zm00001d041375_T001	0.5100873	-0.720669823	Zm00001d041375
Zm00001d046444_T003	0.6974118	-0.373797718	Zm00001d046444
Zm00001d046444_T001	0.8061273	-0.072595924	Zm00001d046444
Zm00001d006553_T001	0.7294039	-0.285930824	Zm00001d006553
Zm00001d052136_T001	0.9242613	-0.052995586	Zm00001d052136
Zm00001d045323_T003	0.255403	0.324136001	Zm00001d045323
Zm00001d045323_T009	0.9964253	0.03601921	Zm00001d045323
Zm00001d045323_T001	0.9184323	-0.033442281	Zm00001d045323
Zm00001d045323_T007	0.3452041	-0.931784097	Zm00001d045323
Zm00001d045323_T002	0.1983104	0.509056422	Zm00001d045323
Zm00001d026398_T006	0.9587507	0.621533829	Zm00001d026398
Zm00001d026398_T007	0.9691383	0.698228632	Zm00001d026398
Zm00001d026398_T009	0.9529094	0.484853369	Zm00001d026398
Zm00001d026398_T004	0.08857859	-0.864476847	Zm00001d026398
Zm00001d026398_T010	0.8032964	0.185278811	Zm00001d026398
Zm00001d026398_T002	0.9419336	0.180586344	Zm00001d026398
Zm00001d018199_T073	0.2279577	-0.93058402	Zm00001d018199
Zm00001d018199_T002	0.5018536	0.476851777	Zm00001d018199
Zm00001d018199_T062	0.000536607	0.527221737	Zm00001d018199
Zm00001d018199_T018	0.06063215	0.689329318	Zm00001d018199
Zm00001d018199_T011	0.1307765	0.448602506	Zm00001d018199
Zm00001d018199_T017	0.9213556	0.079342791	Zm00001d018199
Zm00001d018199_T050	0.09891885	1.454369023	Zm00001d018199
Zm00001d018199_T069	0.9463296	-0.042679559	Zm00001d018199
Zm00001d018199_T013	0.8937675	0.228507212	Zm00001d018199
Zm00001d018199_T070	0.0483012	-0.373616311	Zm00001d018199
Zm00001d032679_T001	0.9402255	0.730973303	Zm00001d032679
Zm00001d023243_T004	0.6876043	-0.369234473	Zm00001d023243
Zm00001d050225_T001	0.9722469	-0.026930568	Zm00001d050225
Zm00001d011447_T005	0.9867452	0.73098328	Zm00001d011447
Zm00001d011447_T002	0.8794273	0.877714947	Zm00001d011447
Zm00001d011447_T001	6.81E-06	0.72951566	Zm00001d011447
Zm00001d013241_T003	0.07111099	1.123834661	Zm00001d013241
Zm00001d013241_T004	0.1922848	1.793687154	Zm00001d013241
Zm00001d013241_T006	0.7771624	0.309366785	Zm00001d013241
Zm00001d013241_T011	0.001010267	1.579876789	Zm00001d013241
Zm00001d013241_T002	0.7460241	1.161776709	Zm00001d013241
Zm00001d038698_T009	0.4964992	0.641362042	Zm00001d038698
Zm00001d038698_T007	0.08690086	1.373397665	Zm00001d038698
Zm00001d038698_T002	0.4982725	1.182093329	Zm00001d038698
Zm00001d038698_T006	9.96E-05	2.463360595	Zm00001d038698
Zm00001d038698_T008	2.70E-08	1.214475234	Zm00001d038698
Zm00001d029391_T003	0.08548693	0.540534167	Zm00001d029391
Zm00001d029391_T002	0.8883561	0.341107258	Zm00001d029391
Zm00001d007910_T001	0.4344214	-0.876727488	Zm00001d007910
Zm00001d033374_T001	0.9979568	0.059359606	Zm00001d033374
Zm00001d021518_T002	0.8767824	-0.154028692	Zm00001d021518
Zm00001d053706_T002	0.5600665	1.07681171	Zm00001d053706
Zm00001d023801_T004	0.9253672	0.756133501	Zm00001d023801
Zm00001d010497_T001	0.9730613	0.169845743	Zm00001d010497
Zm00001d048282_T003	0.4929873	-0.637758657	Zm00001d048282
Zm00001d048282_T002	0.7517012	0.993310156	Zm00001d048282

Zm00001d046472_T001	0.9692803	-0.079880237	Zm00001d046472
Zm00001d046472_T002	0.7489032	0.977379157	Zm00001d046472
Zm00001d032157_T001	0.9745084	0.457821022	Zm00001d032157
Zm00001d018863_T002	0.8807115	-0.044930832	Zm00001d018863
Zm00001d018863_T004	0.429391	0.356918038	Zm00001d018863
Zm00001d027967_T001	0.629722	-0.648177206	Zm00001d027967
Zm00001d046397_T001	0.842741	-0.180141733	Zm00001d046397
Zm00001d009236_T001	0.9184323	0.710306345	Zm00001d009236
Zm00001d009236_T002	0.6445434	-0.215844335	Zm00001d009236
Zm00001d009236_T004	1	0.228518472	Zm00001d009236
Zm00001d009236_T003	0.8566036	-0.259275269	Zm00001d009236
Zm00001d019180_T001	0.9380783	-0.235006166	Zm00001d019180
Zm00001d021347_T002	0.9570177	0.697947771	Zm00001d021347
Zm00001d021347_T005	0.967161	0.117972332	Zm00001d021347
Zm00001d021347_T001	0.4827203	0.245973056	Zm00001d021347
Zm00001d021347_T016	0.5867727	0.43209166	Zm00001d021347
Zm00001d051442_T002	0.8139802	-0.150975334	Zm00001d051442
Zm00001d030936_T001	0.586618	-0.748435143	Zm00001d030936
Zm00001d007027_T001	0.352878	-1.015509508	Zm00001d007027
Zm00001d045183_T001	0.4899462	-0.693627407	Zm00001d045183
Zm00001d051864_T015	0.9690782	0.157494794	Zm00001d051864
Zm00001d051864_T006	0.9224443	-0.030062613	Zm00001d051864
Zm00001d051864_T001	0.338859	0.432492872	Zm00001d051864
Zm00001d038907_T001	0.7325823	-0.37411584	Zm00001d038907
Zm00001d021032_T004	0.9361945	-0.066801145	Zm00001d021032
Zm00001d021032_T001	0.9527801	0.51271886	Zm00001d021032
Zm00001d021032_T002	0.9673473	-0.216744237	Zm00001d021032
Zm00001d045199_T024	0.6893736	-0.520097767	Zm00001d045199
Zm00001d045199_T004	8.43E-08	2.571647932	Zm00001d045199
Zm00001d045199_T022	0.8971712	0.901660307	Zm00001d045199
Zm00001d045199_T017	0.6158612	-0.456950115	Zm00001d045199
Zm00001d045199_T005	0.7147393	0.536671286	Zm00001d045199
Zm00001d026512_T001	0.9580985	-0.053459401	Zm00001d026512
Zm00001d052801_T023	0.01141862	-0.773422212	Zm00001d052801
Zm00001d052801_T016	0.000114044	0.580385394	Zm00001d052801
Zm00001d052801_T024	0.000475302	0.782834768	Zm00001d052801
Zm00001d052801_T009	0.7497503	0.648923263	Zm00001d052801
Zm00001d052801_T001	0.9257893	0.739175665	Zm00001d052801
Zm00001d034878_T001	1	0.237393353	Zm00001d034878
Zm00001d013847_T002	0.9605054	0.425431079	Zm00001d013847
Zm00001d013847_T003	0.4344214	-0.861939005	Zm00001d013847
Zm00001d013847_T001	0.7190231	0.421272721	Zm00001d013847
Zm00001d047979_T005	0.9894615	0.168172343	Zm00001d047979
Zm00001d007675_T006	0.4653557	1.006988398	Zm00001d007675
Zm00001d007675_T002	0.9704112	0.490195535	Zm00001d007675
Zm00001d007675_T007	0.4339887	-0.711323993	Zm00001d007675
Zm00001d050140_T001	0.5304385	-0.664263621	Zm00001d050140
Zm00001d035519_T001	0.9981361	0.263902903	Zm00001d035519
Zm00001d036382_T001	0.7481676	-0.299073218	Zm00001d036382
Zm00001d020748_T001	0.9403937	0.061364055	Zm00001d020748
Zm00001d020748_T002	0.6648793	-0.457474264	Zm00001d020748
Zm00001d044469_T008	0.9979568	-0.051911476	Zm00001d044469
Zm00001d044469_T002	0.8587779	-0.159612268	Zm00001d044469
Zm00001d044469_T006	0.6985759	0.728105393	Zm00001d044469

Zm00001d011891_T001	0.9770343	0.18055985	Zm00001d011891
Zm00001d044228_T001	0.9184323	0.763558229	Zm00001d044228
Zm00001d024339_T002	0.9969202	0.486837751	Zm00001d024339
Zm00001d024339_T003	0.9228108	0.672201235	Zm00001d024339
Zm00001d024339_T001	0.8321156	0.377483216	Zm00001d024339
Zm00001d033488_T001	0.851347	-0.258691438	Zm00001d033488
Zm00001d053313_T002	0.9892483	0.275614628	Zm00001d053313
Zm00001d053313_T006	0.6062627	-0.284067839	Zm00001d053313
Zm00001d053313_T003	0.2629406	0.522112002	Zm00001d053313
Zm00001d053313_T005	5.19E-10	1.42617595	Zm00001d053313
Zm00001d039013_T001	0.74513	-0.324893912	Zm00001d039013
Zm00001d039013_T002	0.8884142	-0.153337883	Zm00001d039013
Zm00001d031476_T001	0.845417	-0.320469453	Zm00001d031476
Zm00001d012933_T001	0.745718	-0.440880303	Zm00001d012933
Zm00001d025765_T001	0.822299	0.791077208	Zm00001d025765
Zm00001d036987_T001	0.6808567	1.171977201	Zm00001d036987
Zm00001d017117_T001	0.9644829	0.129324105	Zm00001d017117
Zm00001d052396_T001	0.7914025	0.415706222	Zm00001d052396
Zm00001d052167_T001	1.71E-06	4.18398872	Zm00001d052167
Zm00001d051394_T001	0.7949312	-0.54281304	Zm00001d051394
Zm00001d007062_T001	0.9922753	0.441699216	Zm00001d007062
Zm00001d050637_T002	0.4753908	-0.641621257	Zm00001d050637
Zm00001d048400_T001	0.4163478	1.160444463	Zm00001d048400
Zm00001d027901_T001	1.37E-05	4.425364509	Zm00001d027901
Zm00001d014100_T001	0.4453003	-1.148898233	Zm00001d014100
Zm00001d038142_T002	0.8121646	0.299102173	Zm00001d038142
Zm00001d038142_T011	0.9841968	0.182673363	Zm00001d038142
Zm00001d038142_T017	0.9138633	0.177735537	Zm00001d038142
Zm00001d038142_T020	0.7746801	0.21080571	Zm00001d038142
Zm00001d038142_T027	0.9895224	0.082207426	Zm00001d038142
Zm00001d038142_T004	0.8125642	0.702957247	Zm00001d038142
Zm00001d038142_T023	0.9779851	0.311486064	Zm00001d038142
Zm00001d038142_T013	4.03E-06	-0.573741831	Zm00001d038142
Zm00001d038142_T016	0.9933044	0.19490017	Zm00001d038142
Zm00001d029444_T001	0.8800222	2.038685838	Zm00001d029444
Zm00001d009813_T004	0.7412991	-0.110769624	Zm00001d009813
Zm00001d009813_T005	0.5373511	-0.769111472	Zm00001d009813
Zm00001d009813_T001	0.9945076	0.468157762	Zm00001d009813
Zm00001d009813_T003	0.9361945	0.371239284	Zm00001d009813
Zm00001d016669_T001	0.3436517	1.59072654	Zm00001d016669
Zm00001d041255_T001	0.9912134	0.469008701	Zm00001d041255
Zm00001d034520_T002	0.2339788	-0.8159755	Zm00001d034520
Zm00001d034520_T003	0.9567316	0.548553888	Zm00001d034520
Zm00001d040190_T001	0.9330832	-0.027000777	Zm00001d040190
Zm00001d013609_T006	0.7552081	-0.212667054	Zm00001d013609
Zm00001d013609_T003	0.967161	0.255379012	Zm00001d013609
Zm00001d013609_T001	0.00793737	0.398051252	Zm00001d013609
Zm00001d013609_T007	0.9991964	0.336754464	Zm00001d013609
Zm00001d013047_T001	1	0.868660405	Zm00001d013047
Zm00001d013047_T005	0.878248	0.084820411	Zm00001d013047
Zm00001d013047_T004	0.9079973	0.742846002	Zm00001d013047
Zm00001d013047_T002	0.972985	0.802274471	Zm00001d013047
Zm00001d047802_T001	1	0.234078339	Zm00001d047802
Zm00001d011373_T007	0.5928377	-0.111071656	Zm00001d011373

Zm00001d011373_T013	2.23E-11	-2.307986478	Zm00001d011373
Zm00001d011373_T005	0.06370899	2.137115843	Zm00001d011373
Zm00001d011373_T011	0.000277524	0.73898453	Zm00001d011373
Zm00001d011373_T001	0.5554844	-0.062389749	Zm00001d011373
Zm00001d011373_T006	0.2877465	1.764889831	Zm00001d011373
Zm00001d011286_T001	0.9979568	-0.043306458	Zm00001d011286
Zm00001d011286_T002	0.9982897	0.071159996	Zm00001d011286
Zm00001d022546_T001	1	0.375537161	Zm00001d022546
Zm00001d022546_T004	0.008135026	2.358401267	Zm00001d022546
Zm00001d051492_T001	0.7688599	-0.487400269	Zm00001d051492
Zm00001d049082_T002	1	0.287257581	Zm00001d049082
Zm00001d049082_T004	0.9912983	0.11267137	Zm00001d049082
Zm00001d049082_T006	0.1947403	0.301249726	Zm00001d049082
Zm00001d027790_T001	0.8774597	0.652296259	Zm00001d027790
Zm00001d033528_T003	0.9380783	0.770689135	Zm00001d033528
Zm00001d033528_T007	0.09126143	1.46973796	Zm00001d033528
Zm00001d033528_T006	1	0.41020835	Zm00001d033528
Zm00001d033528_T002	0.4842719	0.932188642	Zm00001d033528
Zm00001d006776_T001	0.5704509	-0.490485088	Zm00001d006776
Zm00001d003612_T001	0.9614723	0.468194217	Zm00001d003612
Zm00001d004478_T002	0.5549472	-0.352972798	Zm00001d004478
Zm00001d022439_T001	0.000275609	2.343080585	Zm00001d022439
Zm00001d013005_T004	0.9493302	0.235416889	Zm00001d013005
Zm00001d013005_T001	0.6215812	-0.039046253	Zm00001d013005
Zm00001d013005_T003	0.9037807	0.01290605	Zm00001d013005
Zm00001d007758_T006	0.9078934	0.155666858	Zm00001d007758
Zm00001d007758_T003	0.9269932	0.806466913	Zm00001d007758
Zm00001d007758_T008	0.6518228	1.157538394	Zm00001d007758
Zm00001d007758_T001	0.9846714	0.335935578	Zm00001d007758
Zm00001d007758_T013	0.8945288	0.353417409	Zm00001d007758
Zm00001d024409_T001	0.7139898	0.815885329	Zm00001d024409
Zm00001d032168_T003	0.5057074	1.751051632	Zm00001d032168
Zm00001d017571_T001	0.07962716	1.891881316	Zm00001d017571
Zm00001d038810_T001	0.1591955	1.527171468	Zm00001d038810
Zm00001d039706_T001	0.998023	0.320588257	Zm00001d039706
Zm00001d049109_T001	0.9979568	0.163071601	Zm00001d049109
Zm00001d017772_T001	0.8720862	-0.176509811	Zm00001d017772
Zm00001d017772_T003	0.8001345	-0.137541267	Zm00001d017772
Zm00001d005391_T001	0.4846349	-0.7774548	Zm00001d005391
Zm00001d042601_T001	1	0.258962213	Zm00001d042601
Zm00001d049383_T002	0.9693993	0.168436759	Zm00001d049383
Zm00001d049383_T001	0.4056147	0.868867748	Zm00001d049383
Zm00001d020071_T001	0.9737201	0.466130205	Zm00001d020071
Zm00001d040527_T001	0.3475256	1.478944264	Zm00001d040527
Zm00001d029035_T001	0.8494782	0.356091208	Zm00001d029035
Zm00001d011518_T001	0.9979568	0.394854059	Zm00001d011518
Zm00001d051383_T001	0.9107146	-0.10848515	Zm00001d051383
Zm00001d016664_T001	0.1087454	2.200823018	Zm00001d016664
Zm00001d027544_T002	0.9760472	0.438392676	Zm00001d027544
Zm00001d018177_T001	0.9730613	0.503758105	Zm00001d018177
Zm00001d016926_T001	0.8321156	0.672384778	Zm00001d016926
Zm00001d016926_T002	0.5715414	-0.109986959	Zm00001d016926
Zm00001d031562_T001	0.9524127	-0.383574215	Zm00001d031562
Zm00001d024425_T004	0.9563232	0.664958534	Zm00001d024425

Zm00001d024425_T007	0.004726961	1.989400407	Zm00001d024425
Zm00001d024425_T005	0.05663136	2.06409853	Zm00001d024425
Zm00001d048442_T013	0.2895404	-0.610016742	Zm00001d048442
Zm00001d048442_T001	0.8252955	0.337563984	Zm00001d048442
Zm00001d048442_T010	0.6113292	-0.455512028	Zm00001d048442
Zm00001d048442_T002	0.9946911	0.124916694	Zm00001d048442
Zm00001d048442_T004	0.06756891	1.961597556	Zm00001d048442
Zm00001d048442_T005	0.07827475	-0.821194512	Zm00001d048442
Zm00001d041036_T001	0.8884142	-0.102817712	Zm00001d041036
Zm00001d032272_T001	0.8647028	-0.145183153	Zm00001d032272
Zm00001d044088_T003	0.5418433	1.106908674	Zm00001d044088
Zm00001d044088_T004	0.5902617	0.455030649	Zm00001d044088
Zm00001d044088_T002	0.586618	0.847533592	Zm00001d044088
Zm00001d044088_T001	0.9907928	0.269609936	Zm00001d044088
Zm00001d001806_T003	0.5081017	0.719946651	Zm00001d001806
Zm00001d016681_T001	0.8584358	0.876169892	Zm00001d016681
Zm00001d037648_T004	0.02793163	1.778124692	Zm00001d037648
Zm00001d037648_T012	0.9895224	0.058822111	Zm00001d037648
Zm00001d037648_T011	0.1987588	1.412032186	Zm00001d037648
Zm00001d037648_T010	0.8086794	0.416848491	Zm00001d037648
Zm00001d037648_T013	0.2522685	-1.055653957	Zm00001d037648
Zm00001d041571_T001	0.6186951	-0.59610671	Zm00001d041571
Zm00001d002595_T003	0.9840918	0.051262223	Zm00001d002595
Zm00001d026435_T001	0.352878	-0.82608152	Zm00001d026435
Zm00001d010603_T001	0.96672	-0.050489429	Zm00001d010603
Zm00001d010603_T002	0.9768802	0.102801188	Zm00001d010603
Zm00001d034900_T003	0.6394238	0.345545693	Zm00001d034900
Zm00001d034900_T001	0.9252088	0.608713809	Zm00001d034900
Zm00001d034900_T002	1	0.147785049	Zm00001d034900
Zm00001d034900_T004	0.9879601	0.343858321	Zm00001d034900
Zm00001d045785_T001	0.9887584	0.224955811	Zm00001d045785
Zm00001d010667_T001	0.9209394	-0.0223562	Zm00001d010667
Zm00001d032040_T001	0.9664736	0.090159807	Zm00001d032040
Zm00001d047882_T002	0.6420014	0.974426264	Zm00001d047882
Zm00001d047882_T005	0.6158972	0.447769558	Zm00001d047882
Zm00001d047882_T007	0.458869	-0.797407406	Zm00001d047882
Zm00001d047882_T003	0.1153967	0.872730688	Zm00001d047882
Zm00001d047882_T001	0.9628835	0.052058796	Zm00001d047882
Zm00001d033850_T001	0.997764	0.362973105	Zm00001d033850
Zm00001d033850_T019	0.3722505	-0.849833131	Zm00001d033850
Zm00001d033850_T013	0.8713735	1.393298581	Zm00001d033850
Zm00001d033850_T015	0.6030192	-0.798530026	Zm00001d033850
Zm00001d033850_T017	0.01907333	-0.671051325	Zm00001d033850
Zm00001d033850_T024	0.2622146	-1.130755227	Zm00001d033850
Zm00001d033850_T003	0.1297572	-1.145830521	Zm00001d033850
Zm00001d033850_T018	0.8716622	2.758793988	Zm00001d033850
Zm00001d025773_T002	0.9988063	0.373830509	Zm00001d025773
Zm00001d002859_T001	0.8355459	-0.239945386	Zm00001d002859
Zm00001d018151_T003	0.8801704	-0.066661394	Zm00001d018151
Zm00001d002473_T001	0.8356641	0.822339523	Zm00001d002473
Zm00001d007043_T002	0.5269468	1.689168373	Zm00001d007043
Zm00001d007043_T004	0.6594369	-0.548289502	Zm00001d007043
Zm00001d023825_T003	0.917315	-0.08962872	Zm00001d023825
Zm00001d023825_T006	0.9946911	0.355963536	Zm00001d023825

Zm00001d023825_T001	0.9570268	0.265338906	Zm00001d023825
Zm00001d041716_T005	0.6165884	1.138493711	Zm00001d041716
Zm00001d041716_T008	0.2398264	0.862911708	Zm00001d041716
Zm00001d041716_T001	0.3963655	1.003992286	Zm00001d041716
Zm00001d041716_T009	0.00602687	-1.819863618	Zm00001d041716
Zm00001d041716_T006	0.9824348	0.187608889	Zm00001d041716
Zm00001d027651_T001	0.9469426	0.242426281	Zm00001d027651
Zm00001d033028_T002	0.2438204	1.380416258	Zm00001d033028
Zm00001d033028_T001	0.7294039	1.216576878	Zm00001d033028
Zm00001d033028_T003	0.9842144	0.273893205	Zm00001d033028
Zm00001d030159_T002	0.9693993	0.659807225	Zm00001d030159
Zm00001d030159_T001	0.7646718	-0.284124945	Zm00001d030159
Zm00001d014721_T001	0.8728536	-0.108782797	Zm00001d014721
Zm00001d052885_T001	0.556323	1.258286363	Zm00001d052885
Zm00001d052885_T002	0.7077401	-0.756696965	Zm00001d052885
Zm00001d052885_T004	0.02653131	-1.380771348	Zm00001d052885
Zm00001d022626_T001	0.9596377	0.100297824	Zm00001d022626
Zm00001d028064_T001	0.6215505	-0.949879136	Zm00001d028064
Zm00001d047940_T012	0.8987811	-0.093114096	Zm00001d047940
Zm00001d047940_T008	0.9666757	-0.016180096	Zm00001d047940
Zm00001d047940_T011	0.9954444	0.145152876	Zm00001d047940
Zm00001d014224_T001	0.4812187	-0.92159512	Zm00001d014224
Zm00001d051606_T001	0.5507795	-0.655809493	Zm00001d051606
Zm00001d011720_T002	0.0166836	0.976996383	Zm00001d011720
Zm00001d011720_T001	0.9275987	0.065668102	Zm00001d011720
Zm00001d011720_T004	0.9823234	0.21009633	Zm00001d011720
Zm00001d036792_T001	0.6217057	-0.686137394	Zm00001d036792
Zm00001d021710_T014	0.9975882	0.285479553	Zm00001d021710
Zm00001d021710_T013	0.9706293	0.030904696	Zm00001d021710
Zm00001d021710_T005	0.960704	0.319414433	Zm00001d021710
Zm00001d021710_T001	1	0.24913557	Zm00001d021710
Zm00001d021710_T011	1	0.383642505	Zm00001d021710
Zm00001d044506_T012	0.9968176	0.404429541	Zm00001d044506
Zm00001d044506_T013	0.4233271	1.162772757	Zm00001d044506
Zm00001d044506_T009	0.967161	0.471429621	Zm00001d044506
Zm00001d044506_T011	0.9979568	0.124867887	Zm00001d044506
Zm00001d044506_T014	0.1296685	-1.369579293	Zm00001d044506
Zm00001d044506_T004	0.9616398	-0.293580429	Zm00001d044506
Zm00001d044506_T002	0.03465657	1.182374825	Zm00001d044506
Zm00001d044506_T003	0.772948	0.243775598	Zm00001d044506
Zm00001d044506_T006	0.9429978	-0.043771075	Zm00001d044506
Zm00001d037097_T001	0.4843786	-0.827021222	Zm00001d037097
Zm00001d002028_T001	0.8580945	-0.632672278	Zm00001d002028
Zm00001d017425_T001	0.9469426	-0.507785448	Zm00001d017425
Zm00001d023304_T002	0.9693993	0.406110215	Zm00001d023304
Zm00001d023304_T006	0.2964998	-0.460313973	Zm00001d023304
Zm00001d023304_T003	0.9792387	0.059852346	Zm00001d023304
Zm00001d023304_T004	3.91E-09	1.367180295	Zm00001d023304
Zm00001d047570_T001	0.9942502	0.197723825	Zm00001d047570
Zm00001d049746_T003	6.72E-09	2.261256423	Zm00001d049746
Zm00001d049746_T002	1	0.223259008	Zm00001d049746
Zm00001d049746_T006	0.9231466	0.026142754	Zm00001d049746
Zm00001d049746_T004	0.8922572	-0.108829028	Zm00001d049746
Zm00001d017147_T001	0.4246306	-0.944609692	Zm00001d017147

Zm00001d017147_T002	0.208139	-1.133115857	Zm00001d017147
Zm00001d035443_T001	0.9833133	0.232177889	Zm00001d035443
Zm00001d039468_T001	0.2625487	-1.363541798	Zm00001d039468
Zm00001d044213_T001	0.961399	0.156359006	Zm00001d044213
Zm00001d044213_T002	0.9627389	0.581127524	Zm00001d044213
Zm00001d028555_T001	0.7118596	-0.884035688	Zm00001d028555
Zm00001d007259_T001	0.9501111	0.4766489	Zm00001d007259
Zm00001d005485_T001	0.7213609	-0.433168097	Zm00001d005485
Zm00001d012693_T003	0.6336913	-0.418120993	Zm00001d012693
Zm00001d043592_T003	0.8720321	-0.139763252	Zm00001d043592
Zm00001d043592_T005	0.5899787	0.893031575	Zm00001d043592
Zm00001d043592_T001	0.8027057	0.386283883	Zm00001d043592
Zm00001d043592_T004	0.5064329	1.096349884	Zm00001d043592
Zm00001d043592_T002	0.7830446	-0.059540702	Zm00001d043592
Zm00001d033636_T001	0.90276	2.170237359	Zm00001d033636
Zm00001d006702_T002	0.2007953	0.452620668	Zm00001d006702
Zm00001d006702_T001	0.9853728	0.278635313	Zm00001d006702
Zm00001d013307_T003	0.8247949	-0.21370703	Zm00001d013307
Zm00001d013307_T002	0.9619942	0.093505388	Zm00001d013307
Zm00001d014360_T002	0.3583014	1.100044222	Zm00001d014360
Zm00001d014360_T001	0.9666757	0.546258036	Zm00001d014360
Zm00001d020486_T001	0.9469426	-0.238581963	Zm00001d020486
Zm00001d027718_T001	0.000974606	-1.582931745	Zm00001d027718
Zm00001d022280_T001	0.9909466	0.369026401	Zm00001d022280
Zm00001d002523_T001	0.8993058	0.540582673	Zm00001d002523
Zm00001d032368_T001	0.7071453	0.604078084	Zm00001d032368
Zm00001d051908_T002	0.07074972	-1.067419959	Zm00001d051908
Zm00001d051908_T001	0.1505056	-1.313391273	Zm00001d051908
Zm00001d048958_T003	0.8625537	0.17918944	Zm00001d048958
Zm00001d048958_T002	0.992451	0.366669459	Zm00001d048958
Zm00001d017053_T001	0.9855117	0.193952319	Zm00001d017053
Zm00001d017053_T006	0.7831859	0.704840984	Zm00001d017053
Zm00001d013511_T002	0.1059493	-1.719526867	Zm00001d013511
Zm00001d013511_T001	0.8348638	-0.136551392	Zm00001d013511
Zm00001d030212_T001	0.9693993	0.419087947	Zm00001d030212
Zm00001d005269_T003	0.9804228	0.275412943	Zm00001d005269
Zm00001d005269_T005	0.8585804	0.779437092	Zm00001d005269
Zm00001d005269_T004	0.9400905	0.168235515	Zm00001d005269
Zm00001d020102_T001	0.9979568	-0.054890971	Zm00001d020102
Zm00001d021201_T001	0.9416089	0.692395413	Zm00001d021201
Zm00001d021201_T003	0.9855117	0.247569538	Zm00001d021201
Zm00001d038080_T003	1	0.148402669	Zm00001d038080
Zm00001d038080_T001	0.8964117	0.499927702	Zm00001d038080
Zm00001d016402_T082	0.8527068	0.95109228	Zm00001d016402
Zm00001d016402_T020	0.6004941	0.307211893	Zm00001d016402
Zm00001d016402_T081	0.5380217	0.761190235	Zm00001d016402
Zm00001d016402_T025	0.5784042	0.722645727	Zm00001d016402
Zm00001d016402_T024	2.28E-13	-3.089789182	Zm00001d016402
Zm00001d016402_T005	0.1840775	1.894041539	Zm00001d016402
Zm00001d016402_T040	0.8945288	0.235450446	Zm00001d016402
Zm00001d004187_T001	0.9972218	0.246947796	Zm00001d004187
Zm00001d039973_T003	0.7840446	-0.167447688	Zm00001d039973
Zm00001d039973_T007	0.827438	0.749144549	Zm00001d039973
Zm00001d039973_T004	0.8585804	0.874907042	Zm00001d039973

Zm00001d013508_T001	0.9033985	-0.438431008	Zm00001d013508
Zm00001d006595_T002	0.9326482	0.042074528	Zm00001d006595
Zm00001d006595_T001	0.9908081	0.296755604	Zm00001d006595
Zm00001d018096_T001	1	0.067036663	Zm00001d018096
Zm00001d003334_T003	0.6486478	0.68771569	Zm00001d003334
Zm00001d003334_T001	0.01434908	1.072227246	Zm00001d003334
Zm00001d003334_T002	0.9695575	0.517220563	Zm00001d003334
Zm00001d024598_T001	0.7714201	-0.173978139	Zm00001d024598
Zm00001d041854_T004	0.9377241	0.650813734	Zm00001d041854
Zm00001d041854_T005	0.9797926	0.04403173	Zm00001d041854
Zm00001d041854_T001	0.9001906	0.296915392	Zm00001d041854
Zm00001d010805_T001	0.8112903	0.777500095	Zm00001d010805
Zm00001d034512_T002	0.8413741	-0.431023404	Zm00001d034512
Zm00001d030849_T001	0.8619072	-0.124812181	Zm00001d030849
Zm00001d005699_T001	0.8465879	-0.191207714	Zm00001d005699
Zm00001d033610_T001	0.9747606	0.20780215	Zm00001d033610
Zm00001d014446_T001	0.1301479	1.585109889	Zm00001d014446
Zm00001d005612_T001	0.9979568	-0.12904847	Zm00001d005612
Zm00001d053727_T005	0.6602663	0.975672448	Zm00001d053727
Zm00001d053727_T009	0.05547735	-0.866108362	Zm00001d053727
Zm00001d046553_T004	0.4146004	-0.316523336	Zm00001d046553
Zm00001d046553_T001	0.9870394	0.092595131	Zm00001d046553
Zm00001d038973_T001	0.9221549	-0.071322223	Zm00001d038973
Zm00001d023969_T002	0.8012608	-0.201906771	Zm00001d023969
Zm00001d014079_T001	0.772948	-0.21333373	Zm00001d014079
Zm00001d036481_T001	0.5005872	-0.592724682	Zm00001d036481
Zm00001d036481_T003	0.9706887	0.123713702	Zm00001d036481
Zm00001d036481_T006	0.9781672	0.486776434	Zm00001d036481
Zm00001d036481_T004	0.3192778	1.091032921	Zm00001d036481
Zm00001d036481_T002	0.9770343	0.493983176	Zm00001d036481
Zm00001d036481_T007	0.8062829	0.765922778	Zm00001d036481
Zm00001d036481_T005	0.927255	0.79467614	Zm00001d036481
Zm00001d017351_T001	0.8720862	-0.384835148	Zm00001d017351
Zm00001d047053_T011	0.3939786	-0.759820301	Zm00001d047053
Zm00001d047053_T008	1	0.319917875	Zm00001d047053
Zm00001d038880_T001	0.8453095	0.973594783	Zm00001d038880
Zm00001d038880_T004	0.1477236	1.20902284	Zm00001d038880
Zm00001d038880_T009	0.2036868	-0.827665974	Zm00001d038880
Zm00001d038880_T006	0.8457647	-0.135404393	Zm00001d038880
Zm00001d038880_T003	0.9872831	0.205103066	Zm00001d038880
Zm00001d038880_T007	0.9571829	0.144800919	Zm00001d038880
Zm00001d038880_T002	0.3870314	-0.554447379	Zm00001d038880
Zm00001d008187_T001	0.9781672	0.14996092	Zm00001d008187
Zm00001d048054_T004	0.8896831	-0.029629321	Zm00001d048054
Zm00001d048054_T002	0.850642	-0.122056178	Zm00001d048054
Zm00001d048054_T003	0.6466858	-0.717011245	Zm00001d048054
Zm00001d017790_T001	0.9706887	0.18164874	Zm00001d017790
Zm00001d032497_T001	0.9107146	-0.318441248	Zm00001d032497
Zm00001d005361_T001	0.3919325	1.35886144	Zm00001d005361
Zm00001d005361_T010	0.5571558	0.787512572	Zm00001d005361
Zm00001d005361_T021	1	0.463753808	Zm00001d005361
Zm00001d005361_T018	0.9479007	0.121974201	Zm00001d005361
Zm00001d005361_T004	0.6005005	0.548141469	Zm00001d005361
Zm00001d005361_T007	0.08520203	-0.938528719	Zm00001d005361

Zm00001d005361_T011	0.2226091	1.053311225	Zm00001d005361
Zm00001d005361_T012	0.2506983	0.856549061	Zm00001d005361
Zm00001d025319_T001	0.9242247	-0.075832215	Zm00001d025319
Zm00001d021294_T001	0.8819727	-0.166532714	Zm00001d021294
Zm00001d006242_T009	0.8031436	-0.217810402	Zm00001d006242
Zm00001d006242_T003	0.9852645	0.571274417	Zm00001d006242
Zm00001d006242_T001	0.7220539	-0.111605405	Zm00001d006242
Zm00001d006242_T004	0.8701267	-0.03115638	Zm00001d006242
Zm00001d013176_T003	0.9344122	0.000874186	Zm00001d013176
Zm00001d013176_T001	0.9894615	0.195477768	Zm00001d013176
Zm00001d003493_T002	0.9822138	-0.145330382	Zm00001d003493
Zm00001d018738_T001	0.000461316	-3.419589997	Zm00001d018738
Zm00001d003174_T001	0.7799982	-0.392026588	Zm00001d003174
Zm00001d051188_T001	0.9942835	0.119422052	Zm00001d051188
Zm00001d036710_T002	1	0.319659864	Zm00001d036710
Zm00001d036710_T001	0.9740325	0.505024578	Zm00001d036710
Zm00001d032722_T001	0.9001906	-0.123183219	Zm00001d032722
Zm00001d028229_T001	0.8227873	-0.090706575	Zm00001d028229
Zm00001d053765_T006	0.5418278	0.775610322	Zm00001d053765
Zm00001d053765_T003	0.945968	0.73964113	Zm00001d053765
Zm00001d053765_T005	0.8660897	1.72633111	Zm00001d053765
Zm00001d053765_T004	0.5440777	-0.632019082	Zm00001d053765
Zm00001d053765_T007	0.9782953	1.280935111	Zm00001d053765
Zm00001d038180_T001	0.8720321	0.590800393	Zm00001d038180
Zm00001d043607_T001	0.8950139	0.844765847	Zm00001d043607
Zm00001d024531_T001	0.8555166	0.980951686	Zm00001d024531
Zm00001d024531_T006	0.8743763	-0.082706008	Zm00001d024531
Zm00001d024531_T005	0.4730617	-0.550920322	Zm00001d024531
Zm00001d024531_T002	0.9014695	0.810945501	Zm00001d024531
Zm00001d039991_T001	0.9523689	0.486352041	Zm00001d039991
Zm00001d048323_T001	0.8983195	0.718602997	Zm00001d048323
Zm00001d018796_T001	0.7009673	-0.584788417	Zm00001d018796
Zm00001d042260_T001	0.6412388	-0.409667538	Zm00001d042260
Zm00001d022062_T001	0.234686	-1.416825311	Zm00001d022062
Zm00001d035064_T001	0.8799482	-0.140407604	Zm00001d035064
Zm00001d052002_T001	0.6240422	-0.823908827	Zm00001d052002
Zm00001d025946_T001	0.9823234	0.073656288	Zm00001d025946
Zm00001d025946_T003	0.385062	1.56925673	Zm00001d025946
Zm00001d025946_T007	0.4915744	0.347728456	Zm00001d025946
Zm00001d025946_T004	0.9693993	0.12387682	Zm00001d025946
Zm00001d015964_T001	1	0.169434533	Zm00001d015964
Zm00001d038700_T001	0.9690782	0.5156588	Zm00001d038700
Zm00001d040696_T003	0.906282	0.449943297	Zm00001d040696
Zm00001d018479_T001	0.9824078	0.244308263	Zm00001d018479
Zm00001d044184_T001	0.9469426	0.083851392	Zm00001d044184
Zm00001d011366_T006	0.9791729	0.177448722	Zm00001d011366
Zm00001d011366_T002	0.4155898	1.306178971	Zm00001d011366
Zm00001d011366_T003	0.3457862	-0.630092128	Zm00001d011366
Zm00001d037975_T048	2.31E-08	1.449345008	Zm00001d037975
Zm00001d037975_T018	0.6107148	0.452966359	Zm00001d037975
Zm00001d037975_T004	0.2044538	-0.490831976	Zm00001d037975
Zm00001d037975_T008	0.8092097	0.350159086	Zm00001d037975
Zm00001d037975_T001	0.7425196	-0.130858636	Zm00001d037975
Zm00001d037975_T012	0.9666757	0.14761869	Zm00001d037975

Zm00001d037975_T037	0.09964953	0.360004833	Zm00001d037975
Zm00001d011706_T003	0.9872831	0.498968984	Zm00001d011706
Zm00001d044030_T001	0.6007866	-0.550347956	Zm00001d044030
Zm00001d041663_T001	0.001606888	-1.715839111	Zm00001d041663
Zm00001d028347_T001	0.07701001	1.260076221	Zm00001d028347
Zm00001d042469_T006	1	-0.092639074	Zm00001d042469
Zm00001d015777_T001	0.9050121	0.479917597	Zm00001d015777
Zm00001d023377_T001	0.4580753	-0.678172507	Zm00001d023377
Zm00001d007012_T001	0.9968176	0.345788867	Zm00001d007012
Zm00001d014835_T001	0.957768	0.028800456	Zm00001d014835
Zm00001d048050_T004	0.2821922	-0.723348669	Zm00001d048050
Zm00001d048050_T001	0.9532189	-0.025170887	Zm00001d048050
Zm00001d048050_T003	0.7411827	-0.411545196	Zm00001d048050
Zm00001d048050_T005	0.1301872	-2.050510424	Zm00001d048050
Zm00001d017175_T002	0.8366973	-0.179311261	Zm00001d017175
Zm00001d017175_T001	0.9356767	0.017766834	Zm00001d017175
Zm00001d021021_T002	0.9781672	-0.123308458	Zm00001d021021
Zm00001d021021_T003	0.9893214	0.10507081	Zm00001d021021
Zm00001d004676_T003	0.9825858	0.208740886	Zm00001d004676
Zm00001d004676_T002	0.8470633	0.956489616	Zm00001d004676
Zm00001d042479_T002	0.7489032	-0.340534497	Zm00001d042479
Zm00001d017380_T001	0.9400055	-0.036473231	Zm00001d017380
Zm00001d002503_T002	0.9701986	0.237990923	Zm00001d002503
Zm00001d002503_T003	0.09030826	2.561622204	Zm00001d002503
Zm00001d002503_T001	0.9570177	0.828957984	Zm00001d002503
Zm00001d045295_T001	0.8590013	0.94722401	Zm00001d045295
Zm00001d045295_T002	0.1054046	1.995575745	Zm00001d045295
Zm00001d007815_T003	0.7492661	-0.754618677	Zm00001d007815
Zm00001d043036_T001	0.9486412	-0.067868197	Zm00001d043036
Zm00001d041649_T010	0.02141717	-1.664923444	Zm00001d041649
Zm00001d041649_T006	0.3015167	1.246333215	Zm00001d041649
Zm00001d041649_T003	0.1902889	-1.307406794	Zm00001d041649
Zm00001d041649_T001	0.3795013	0.632577489	Zm00001d041649
Zm00001d041649_T005	0.8660897	0.276802231	Zm00001d041649
Zm00001d041649_T004	0.9400055	-0.04682125	Zm00001d041649
Zm00001d041649_T008	0.7081196	0.89408955	Zm00001d041649
Zm00001d052248_T004	0.3987447	1.150244079	Zm00001d052248
Zm00001d052248_T009	0.7102433	0.621352212	Zm00001d052248
Zm00001d052248_T018	0.8842271	0.295505146	Zm00001d052248
Zm00001d052248_T001	0.4492411	-0.752885716	Zm00001d052248
Zm00001d052248_T016	0.9514141	-0.328861756	Zm00001d052248
Zm00001d006113_T011	0.6095559	-0.620189549	Zm00001d006113
Zm00001d006113_T001	0.926133	0.67287959	Zm00001d006113
Zm00001d013814_T001	0.923446	0.008779668	Zm00001d013814
Zm00001d048081_T001	0.9628835	0.188208714	Zm00001d048081
Zm00001d048503_T001	0.8492658	0.972309099	Zm00001d048503
Zm00001d003485_T001	0.00067674	4.417437932	Zm00001d003485
Zm00001d016839_T001	0.7169281	-0.637797141	Zm00001d016839
Zm00001d047854_T005	0.4925065	-0.131293198	Zm00001d047854
Zm00001d047854_T003	0.2982359	1.361176527	Zm00001d047854
Zm00001d047854_T007	0.9797082	0.08196663	Zm00001d047854
Zm00001d047854_T006	0.004173226	1.253073389	Zm00001d047854
Zm00001d047854_T002	0.8959807	0.785059886	Zm00001d047854
Zm00001d047854_T004	0.05279322	1.071418614	Zm00001d047854

Zm00001d024885_T001	0.2375426	-0.535734833	Zm00001d024885
Zm00001d021453_T004	0.4578502	-0.257634612	Zm00001d021453
Zm00001d021453_T001	1	0.277353017	Zm00001d021453
Zm00001d008451_T001	0.72524	-0.672947102	Zm00001d008451
Zm00001d030698_T001	0.2810969	-1.36135904	Zm00001d030698
Zm00001d024511_T002	0.7853827	-0.3788948	Zm00001d024511
Zm00001d007318_T005	0.3636519	-0.362017399	Zm00001d007318
Zm00001d007318_T007	0.09673953	-0.829393026	Zm00001d007318
Zm00001d007318_T001	0.6156311	0.627305862	Zm00001d007318
Zm00001d007318_T009	0.2980719	2.072142244	Zm00001d007318
Zm00001d039884_T002	0.6511883	-0.248866271	Zm00001d039884
Zm00001d039884_T001	0.9889811	0.126868574	Zm00001d039884
Zm00001d053375_T001	0.8097523	-0.355907013	Zm00001d053375
Zm00001d002090_T001	0.9175896	0.626605834	Zm00001d002090
Zm00001d026126_T001	7.37E-05	3.857681367	Zm00001d026126
Zm00001d027506_T003	0.854634	0.834341991	Zm00001d027506
Zm00001d038868_T002	0.9550893	0.61256768	Zm00001d038868
Zm00001d038868_T004	0.9846714	0.081052902	Zm00001d038868
Zm00001d009723_T001	0.9988864	0.36233209	Zm00001d009723
Zm00001d028180_T001	0.9410688	0.087043266	Zm00001d028180
Zm00001d028180_T011	0.8533785	-0.174907447	Zm00001d028180
Zm00001d028574_T001	5.33E-05	3.074863829	Zm00001d028574
Zm00001d003935_T005	0.9177099	0.054455593	Zm00001d003935
Zm00001d003935_T012	0.9673473	0.417788459	Zm00001d003935
Zm00001d003935_T009	0.3543848	-1.018231832	Zm00001d003935
Zm00001d003935_T011	0.9257893	0.334250853	Zm00001d003935
Zm00001d003935_T007	0.3692327	-0.664826797	Zm00001d003935
Zm00001d003935_T010	0.9311783	0.568451674	Zm00001d003935
Zm00001d042314_T009	0.8179463	-0.35678475	Zm00001d042314
Zm00001d042314_T004	0.2859854	1.925689541	Zm00001d042314
Zm00001d042314_T010	0.9231466	0.747013171	Zm00001d042314
Zm00001d042314_T011	5.34E-08	1.738832764	Zm00001d042314
Zm00001d015400_T004	0.8986911	0.442455747	Zm00001d015400
Zm00001d015400_T002	1	0.12954489	Zm00001d015400
Zm00001d015400_T003	0.7993434	0.318311504	Zm00001d015400
Zm00001d015464_T008	0.956479	-0.006398627	Zm00001d015464
Zm00001d015464_T003	0.907891	0.065079824	Zm00001d015464
Zm00001d015464_T006	0.850981	-0.080477847	Zm00001d015464
Zm00001d015464_T005	0.7594555	-0.195961778	Zm00001d015464
Zm00001d046385_T001	1	0.320067283	Zm00001d046385
Zm00001d046385_T002	0.9449404	0.468343746	Zm00001d046385
Zm00001d021535_T001	0.8412913	-0.099523892	Zm00001d021535
Zm00001d021535_T003	0.7261417	-0.123060421	Zm00001d021535
Zm00001d040281_T001	0.9449404	-0.174316306	Zm00001d040281
Zm00001d038720_T010	0.7761227	0.788602527	Zm00001d038720
Zm00001d038720_T006	0.9237037	0.00980607	Zm00001d038720
Zm00001d038720_T005	0.9620888	0.085229127	Zm00001d038720
Zm00001d038720_T007	0.4120397	0.758175844	Zm00001d038720
Zm00001d038720_T001	0.65291	0.819350001	Zm00001d038720
Zm00001d038720_T004	0.02678613	0.818640812	Zm00001d038720
Zm00001d038720_T008	0.9429712	0.038554188	Zm00001d038720
Zm00001d038720_T009	0.006621309	0.798641652	Zm00001d038720
Zm00001d038720_T003	0.7823776	0.61243954	Zm00001d038720
Zm00001d033153_T002	0.9441469	0.662507078	Zm00001d033153

Zm00001d033153_T006	0.7703259	0.560774588	Zm00001d033153
Zm00001d023311_T033	0.05606802	0.892240232	Zm00001d023311
Zm00001d023311_T030	0.263915	-0.541565993	Zm00001d023311
Zm00001d023311_T007	0.02263356	2.362588447	Zm00001d023311
Zm00001d023311_T040	0.2233834	-0.368978675	Zm00001d023311
Zm00001d023311_T010	0.06382188	1.278890214	Zm00001d023311
Zm00001d023311_T037	0.9391166	0.478138088	Zm00001d023311
Zm00001d023311_T001	0.3870314	1.413371695	Zm00001d023311
Zm00001d023311_T034	0.9576728	0.15019631	Zm00001d023311
Zm00001d023311_T013	0.9580876	0.148520367	Zm00001d023311
Zm00001d023311_T035	0.7238616	-0.280234305	Zm00001d023311
Zm00001d023311_T029	1.22E-14	-3.265217154	Zm00001d023311
Zm00001d023311_T022	0.6436915	1.214355075	Zm00001d023311
Zm00001d023311_T041	0.7521077	-0.138757698	Zm00001d023311
Zm00001d005028_T001	0.3720939	1.369539927	Zm00001d005028
Zm00001d048794_T003	0.9791729	0.450545449	Zm00001d048794
Zm00001d048794_T004	1	0.3500788	Zm00001d048794
Zm00001d048794_T001	0.9781672	0.450321402	Zm00001d048794
Zm00001d023934_T003	0.9643773	0.009269807	Zm00001d023934
Zm00001d023934_T001	0.8337863	-0.23928753	Zm00001d023934
Zm00001d023934_T002	0.8119202	0.692191457	Zm00001d023934
Zm00001d015997_T002	0.8364557	-0.476881259	Zm00001d015997
Zm00001d021778_T001	0.9681793	-0.375755249	Zm00001d021778
Zm00001d046537_T002	0.6343135	0.406277498	Zm00001d046537
Zm00001d011750_T004	0.9871621	0.499002353	Zm00001d011750
Zm00001d011750_T003	0.9180662	0.732860283	Zm00001d011750
Zm00001d011750_T001	0.01716409	-2.067177373	Zm00001d011750
Zm00001d011750_T002	0.9975882	0.401335416	Zm00001d011750
Zm00001d005980_T003	0.9706887	0.120712905	Zm00001d005980
Zm00001d005980_T002	1	0.281173248	Zm00001d005980
Zm00001d031327_T004	0.7136625	0.928296483	Zm00001d031327
Zm00001d031327_T025	0.6275807	0.345197716	Zm00001d031327
Zm00001d031327_T011	0.9177099	0.008734449	Zm00001d031327
Zm00001d031327_T008	0.9699039	0.627110842	Zm00001d031327
Zm00001d043558_T001	0.5901362	-0.722333853	Zm00001d043558
Zm00001d014271_T001	0.9706887	0.387093219	Zm00001d014271
Zm00001d034495_T001	0.6053962	-0.579884684	Zm00001d034495
Zm00001d043610_T001	0.9377241	0.911975359	Zm00001d043610
Zm00001d037273_T001	0.9894615	0.280566916	Zm00001d037273
Zm00001d018930_T006	0.9503115	0.023229657	Zm00001d018930
Zm00001d028071_T001	1	0.199520511	Zm00001d028071
Zm00001d037190_T001	0.9469426	-0.010426357	Zm00001d037190
Zm00001d039294_T001	0.923446	-0.008804225	Zm00001d039294
Zm00001d039294_T002	0.5430102	-0.574159564	Zm00001d039294
Zm00001d034415_T001	0.9533693	0.469388123	Zm00001d034415
Zm00001d037160_T002	0.684563	-0.386695995	Zm00001d037160
Zm00001d027899_T001	0.000359435	3.161431461	Zm00001d027899
Zm00001d049548_T009	0.9890855	-0.149688214	Zm00001d049548
Zm00001d049548_T006	0.81024	-0.023774119	Zm00001d049548
Zm00001d049548_T001	0.4341146	0.847367491	Zm00001d049548
Zm00001d049548_T007	0.06165837	1.379310243	Zm00001d049548
Zm00001d049548_T008	0.8031436	0.358892288	Zm00001d049548
Zm00001d049548_T005	0.6532906	0.857290734	Zm00001d049548
Zm00001d033876_T003	0.1740006	1.001640533	Zm00001d033876

Zm00001d033876_T004	0.8950139	0.87966897	Zm00001d033876
Zm00001d033876_T005	0.7334664	-0.156592737	Zm00001d033876
Zm00001d034586_T002	0.8819513	-0.224513172	Zm00001d034586
Zm00001d036262_T001	0.9791729	0.349533483	Zm00001d036262
Zm00001d025031_T003	0.000241971	1.197185466	Zm00001d025031
Zm00001d025031_T002	0.6872622	-0.301410862	Zm00001d025031
Zm00001d025031_T005	0.8537544	0.318220499	Zm00001d025031
Zm00001d025031_T004	0.01089689	1.065422896	Zm00001d025031
Zm00001d031893_T001	0.7074842	-0.328758458	Zm00001d031893
Zm00001d039598_T001	1	0.352917656	Zm00001d039598
Zm00001d017345_T002	0.9609506	0.602417533	Zm00001d017345
Zm00001d017345_T001	0.8861059	-0.011928538	Zm00001d017345
Zm00001d035186_T001	0.9463296	0.091866885	Zm00001d035186
Zm00001d035186_T002	0.8702994	0.413272442	Zm00001d035186
Zm00001d027453_T001	0.8888255	0.340411855	Zm00001d027453
Zm00001d018954_T002	0.8446194	0.72348941	Zm00001d018954
Zm00001d022192_T001	0.2587036	-1.025834947	Zm00001d022192
Zm00001d022192_T002	0.08218661	-1.292269107	Zm00001d022192
Zm00001d020561_T007	0.9730613	0.048073924	Zm00001d020561
Zm00001d036758_T001	1	0.349795057	Zm00001d036758
Zm00001d035195_T001	0.9563257	-0.106522303	Zm00001d035195
Zm00001d035254_T002	0.9984041	0.391779009	Zm00001d035254
Zm00001d035254_T001	0.1758861	0.762442813	Zm00001d035254
Zm00001d053301_T001	0.8559428	0.924016155	Zm00001d053301
Zm00001d053301_T003	0.9988864	0.289540762	Zm00001d053301
Zm00001d022324_T001	0.9673094	0.097206996	Zm00001d022324
Zm00001d007470_T001	0.01598815	-1.565897354	Zm00001d007470
Zm00001d008356_T005	0.9965998	0.04805145	Zm00001d008356
Zm00001d008356_T004	0.8337863	-0.682033786	Zm00001d008356
Zm00001d008356_T003	0.7598721	0.284307417	Zm00001d008356
Zm00001d047349_T001	0.9771057	0.559008878	Zm00001d047349
Zm00001d020886_T001	0.6070742	1.258576544	Zm00001d020886
Zm00001d047717_T011	0.4657833	0.691702597	Zm00001d047717
Zm00001d047717_T018	0.5597598	0.772052129	Zm00001d047717
Zm00001d047717_T007	0.09603243	0.410884163	Zm00001d047717
Zm00001d047717_T019	0.0706417	0.826755409	Zm00001d047717
Zm00001d047717_T026	0.4825809	0.183192797	Zm00001d047717
Zm00001d047717_T014	0.877725	0.910152608	Zm00001d047717
Zm00001d047717_T017	0.9468268	0.211404855	Zm00001d047717
Zm00001d047717_T003	0.9640775	0.314637851	Zm00001d047717
Zm00001d019348_T007	0.9907928	0.41253415	Zm00001d019348
Zm00001d019348_T001	0.9593283	0.588943514	Zm00001d019348
Zm00001d019348_T004	1	0.206922717	Zm00001d019348
Zm00001d019348_T005	0.8716622	0.795605017	Zm00001d019348
Zm00001d026549_T006	0.9966965	0.19950218	Zm00001d026549
Zm00001d026549_T008	0.9311674	0.410903973	Zm00001d026549
Zm00001d049176_T001	0.3780737	-1.394890344	Zm00001d049176
Zm00001d027278_T009	4.74E-12	1.413823194	Zm00001d027278
Zm00001d027278_T074	0.002296034	0.89040892	Zm00001d027278
Zm00001d027278_T114	0.843248	-0.021099404	Zm00001d027278
Zm00001d027278_T054	0.9889811	-0.029488466	Zm00001d027278
Zm00001d027278_T116	0.9282104	0.862742815	Zm00001d027278
Zm00001d027278_T122	2.23E-08	-2.401851384	Zm00001d027278
Zm00001d027278_T008	0.4344214	0.276168116	Zm00001d027278

Zm00001d010522_T003	0.9215988	0.205284392	Zm00001d010522
Zm00001d010522_T001	0.9160053	0.841768365	Zm00001d010522
Zm00001d004401_T001	0.8987811	-0.182437782	Zm00001d004401
Zm00001d021430_T013	0.7249455	0.513935546	Zm00001d021430
Zm00001d021430_T019	0.6619605	-0.548203553	Zm00001d021430
Zm00001d043149_T003	0.9894005	0.418518624	Zm00001d043149
Zm00001d043149_T001	0.9131638	-0.111280099	Zm00001d043149
Zm00001d031077_T001	0.3319365	-0.893158895	Zm00001d031077
Zm00001d029740_T001	0.9804433	-0.000419489	Zm00001d029740
Zm00001d042180_T001	0.9781672	0.558824168	Zm00001d042180
Zm00001d044111_T001	0.6869898	1.148615379	Zm00001d044111
Zm00001d014952_T001	0.8826944	-0.171152634	Zm00001d014952
Zm00001d013252_T003	0.459724	-1.030862376	Zm00001d013252
Zm00001d013252_T001	0.9272378	0.130680823	Zm00001d013252
Zm00001d052930_T002	0.2604053	-0.309662909	Zm00001d052930
Zm00001d052930_T004	0.8817666	0.854085476	Zm00001d052930
Zm00001d052930_T003	0.9968176	0.342919165	Zm00001d052930
Zm00001d052930_T006	0.754061	0.201010356	Zm00001d052930
Zm00001d038513_T002	0.9907928	0.259018074	Zm00001d038513
Zm00001d038513_T001	0.7952435	0.543347339	Zm00001d038513
Zm00001d016237_T001	0.9867452	0.247788031	Zm00001d016237
Zm00001d002822_T001	0.9848773	0.488710638	Zm00001d002822
Zm00001d006788_T001	0.9205739	-0.044871519	Zm00001d006788
Zm00001d016832_T002	0.9717213	0.497680917	Zm00001d016832
Zm00001d016832_T001	0.7771984	-1.35151563	Zm00001d016832
Zm00001d006797_T010	1	0.218473641	Zm00001d006797
Zm00001d006797_T009	0.5718169	-0.579244916	Zm00001d006797
Zm00001d006797_T007	0.6398988	0.677004817	Zm00001d006797
Zm00001d006797_T001	0.8988765	0.717787374	Zm00001d006797
Zm00001d018337_T001	0.8019448	-0.419402598	Zm00001d018337
Zm00001d041640_T001	0.9666757	0.462400066	Zm00001d041640
Zm00001d017296_T006	0.1942389	0.631239156	Zm00001d017296
Zm00001d017296_T005	0.9979568	0.104819023	Zm00001d017296
Zm00001d017296_T002	0.7842749	0.9744654	Zm00001d017296
Zm00001d028113_T001	0.9946911	0.34138944	Zm00001d028113
Zm00001d035491_T002	0.960704	0.30611794	Zm00001d035491
Zm00001d035491_T001	1	0.193582128	Zm00001d035491
Zm00001d017699_T001	0.06097768	2.360428072	Zm00001d017699
Zm00001d006524_T001	0.7450257	0.93021818	Zm00001d006524
Zm00001d033597_T001	0.9377241	0.326619901	Zm00001d033597
Zm00001d033597_T006	0.8575285	0.851988925	Zm00001d033597
Zm00001d033597_T004	0.9235467	0.751927404	Zm00001d033597
Zm00001d033597_T010	0.923446	0.29377097	Zm00001d033597
Zm00001d033597_T007	1.52E-06	0.596997179	Zm00001d033597
Zm00001d032931_T003	0.7614965	0.363664867	Zm00001d032931
Zm00001d032931_T001	0.9673473	0.028893743	Zm00001d032931
Zm00001d032931_T004	0.935477	0.044233215	Zm00001d032931
Zm00001d031090_T001	0.9979568	0.476772546	Zm00001d031090
Zm00001d031090_T002	0.3411233	1.113491299	Zm00001d031090
Zm00001d003198_T021	1.06E-06	1.216157662	Zm00001d003198
Zm00001d003198_T025	0.6361629	0.200704172	Zm00001d003198
Zm00001d003198_T017	0.4083835	-0.516748307	Zm00001d003198
Zm00001d003198_T024	0.7481738	-0.286273303	Zm00001d003198
Zm00001d003198_T004	1	0.160029829	Zm00001d003198

Zm00001d003198_T001	0.9050121	0.148400389	Zm00001d003198
Zm00001d021913_T002	0.9894218	-0.137607787	Zm00001d021913
Zm00001d004669_T005	0.111282	1.574798857	Zm00001d004669
Zm00001d004669_T011	0.217152	0.596716739	Zm00001d004669
Zm00001d004669_T014	0.9561521	0.14269673	Zm00001d004669
Zm00001d004669_T006	2.80E-08	1.42643585	Zm00001d004669
Zm00001d004669_T010	0.9720956	0.771100336	Zm00001d004669
Zm00001d004669_T020	0.691575	-0.404042501	Zm00001d004669
Zm00001d018439_T005	0.08771872	-0.655053204	Zm00001d018439
Zm00001d018439_T011	0.9806244	0.634190439	Zm00001d018439
Zm00001d018439_T006	0.3808423	0.347564157	Zm00001d018439
Zm00001d018439_T008	0.0142963	1.33799075	Zm00001d018439
Zm00001d018439_T009	0.728046	0.575159432	Zm00001d018439
Zm00001d018439_T001	0.9907928	0.503031215	Zm00001d018439
Zm00001d011642_T002	0.8826944	0.875776481	Zm00001d011642
Zm00001d011642_T001	0.355126	-0.879095582	Zm00001d011642
Zm00001d011642_T004	0.6478827	0.494477316	Zm00001d011642
Zm00001d004611_T001	0.9690782	0.465512295	Zm00001d004611
Zm00001d008757_T001	0.8720862	-0.122608727	Zm00001d008757
Zm00001d022450_T001	0.8989472	0.783727356	Zm00001d022450
Zm00001d050196_T001	0.9766874	0.501635839	Zm00001d050196
Zm00001d050196_T002	0.2910059	1.278140481	Zm00001d050196
Zm00001d009394_T002	0.5425833	-0.800265592	Zm00001d009394
Zm00001d037741_T001	0.9979568	0.232959718	Zm00001d037741
Zm00001d003089_T002	0.9899643	0.305109468	Zm00001d003089
Zm00001d047452_T001	0.5668473	1.179055376	Zm00001d047452
Zm00001d008749_T004	0.9543237	-0.097005801	Zm00001d008749
Zm00001d008749_T001	0.8030099	-0.19312065	Zm00001d008749
Zm00001d008749_T003	0.9363242	-0.089979459	Zm00001d008749
Zm00001d034312_T001	0.1562781	1.733464055	Zm00001d034312
Zm00001d050580_T003	0.775604	0.313608744	Zm00001d050580
Zm00001d022445_T001	2.12E-05	-1.26168596	Zm00001d022445
Zm00001d022445_T002	0.8735403	-0.093699245	Zm00001d022445
Zm00001d027239_T003	0.9990357	0.328870498	Zm00001d027239
Zm00001d027239_T001	0.9400453	0.533580505	Zm00001d027239
Zm00001d027239_T004	0.8640292	-0.136165792	Zm00001d027239
Zm00001d027239_T002	1	0.360588675	Zm00001d027239
Zm00001d032479_T001	0.07565341	-1.71026339	Zm00001d032479
Zm00001d014158_T003	0.9782674	0.496871317	Zm00001d014158
Zm00001d014158_T007	0.9410688	0.43638455	Zm00001d014158
Zm00001d014158_T002	0.8825751	-0.051021679	Zm00001d014158
Zm00001d014158_T004	0.01102494	1.51339207	Zm00001d014158
Zm00001d019044_T001	0.4483079	1.35654365	Zm00001d019044
Zm00001d024729_T001	0.9219195	0.017414206	Zm00001d024729
Zm00001d020743_T001	0.7622306	0.50324575	Zm00001d020743
Zm00001d007827_T001	0.8989951	0.675910404	Zm00001d007827
Zm00001d000196_T001	1	0.233539428	Zm00001d000196
Zm00001d008528_T001	0.5652959	-0.450021631	Zm00001d008528
Zm00001d025789_T007	0.005601632	-1.97117956	Zm00001d025789
Zm00001d025789_T003	0.6812088	-0.331347766	Zm00001d025789
Zm00001d025789_T002	0.4475228	1.187800363	Zm00001d025789
Zm00001d034984_T001	0.9532189	0.393895325	Zm00001d034984
Zm00001d022502_T009	0.007149287	2.435843148	Zm00001d022502
Zm00001d022502_T015	0.4357391	1.043722843	Zm00001d022502

Zm00001d022502_T013	0.906282	-0.008250961	Zm00001d022502
Zm00001d022502_T017	0.8864089	0.125076124	Zm00001d022502
Zm00001d022502_T018	0.07445447	0.587850264	Zm00001d022502
Zm00001d022502_T019	0.9224443	0.017490699	Zm00001d022502
Zm00001d029715_T001	0.9627389	0.564154252	Zm00001d029715
Zm00001d039300_T004	0.9907928	0.494781849	Zm00001d039300
Zm00001d039300_T006	0.8835073	-0.068197969	Zm00001d039300
Zm00001d039300_T003	0.9100954	0.368205666	Zm00001d039300
Zm00001d032438_T001	0.1448848	2.072812209	Zm00001d032438
Zm00001d032855_T019	0.805584	0.876457153	Zm00001d032855
Zm00001d032855_T001	0.9486412	0.668825459	Zm00001d032855
Zm00001d032855_T014	2.02E-06	0.766623411	Zm00001d032855
Zm00001d032855_T005	0.01763469	1.367812353	Zm00001d032855
Zm00001d032855_T006	2.18E-05	0.379550518	Zm00001d032855
Zm00001d032855_T003	0.000776638	0.86037005	Zm00001d032855
Zm00001d053062_T001	0.704688	-0.323907565	Zm00001d053062
Zm00001d050375_T002	0.9988864	0.418116679	Zm00001d050375
Zm00001d053825_T002	0.7565187	1.1040813	Zm00001d053825
Zm00001d053825_T004	0.8890998	1.22012739	Zm00001d053825
Zm00001d030172_T003	0.6166973	-0.317864818	Zm00001d030172
Zm00001d030172_T006	0.9123762	-0.000496226	Zm00001d030172
Zm00001d030172_T001	0.7541349	0.373277516	Zm00001d030172
Zm00001d030172_T004	0.8553094	-0.374385154	Zm00001d030172
Zm00001d030172_T005	0.976488	0.488828491	Zm00001d030172
Zm00001d001999_T006	0.9213679	0.282612153	Zm00001d001999
Zm00001d001999_T004	0.473709	-1.19454827	Zm00001d001999
Zm00001d001999_T007	0.7993434	-0.255842085	Zm00001d001999
Zm00001d001999_T008	0.8079251	-0.443836587	Zm00001d001999
Zm00001d001999_T001	0.9781672	0.504246136	Zm00001d001999
Zm00001d001999_T003	0.9722469	-0.019986077	Zm00001d001999
Zm00001d001999_T002	0.9693993	0.283130302	Zm00001d001999
Zm00001d038436_T001	0.03078523	-2.021394949	Zm00001d038436
Zm00001d010962_T001	0.1101754	-1.375670642	Zm00001d010962
Zm00001d009707_T001	0.9867657	0.115362093	Zm00001d009707
Zm00001d020618_T003	0.9979568	0.343578138	Zm00001d020618
Zm00001d050330_T003	0.9105127	-0.178006506	Zm00001d050330
Zm00001d050330_T005	0.9529184	-0.040802363	Zm00001d050330
Zm00001d050330_T004	0.9192642	0.540555625	Zm00001d050330
Zm00001d050330_T006	0.8705753	0.688590786	Zm00001d050330
Zm00001d028453_T003	0.000873857	-0.721294972	Zm00001d028453
Zm00001d028453_T002	0.8863456	0.597344808	Zm00001d028453
Zm00001d028453_T004	0.965989	-0.288213423	Zm00001d028453
Zm00001d029975_T003	0.7213635	1.104481092	Zm00001d029975
Zm00001d036927_T002	0.6808567	0.859032614	Zm00001d036927
Zm00001d036927_T001	0.843747	-0.103085976	Zm00001d036927
Zm00001d042394_T002	0.2488799	1.661110379	Zm00001d042394
Zm00001d042394_T007	0.0373054	-1.661392123	Zm00001d042394
Zm00001d042394_T006	0.9237037	0.027071246	Zm00001d042394
Zm00001d020185_T006	0.1843123	1.282345232	Zm00001d020185
Zm00001d020185_T003	0.9693993	0.673075945	Zm00001d020185
Zm00001d020185_T007	0.004389276	1.069429701	Zm00001d020185
Zm00001d020185_T004	0.4406059	-0.758760375	Zm00001d020185
Zm00001d020185_T005	0.1090245	2.326206978	Zm00001d020185
Zm00001d020185_T008	0.341321	0.731752977	Zm00001d020185

Zm00001d020185_T001	0.2141927	1.797864057	Zm00001d020185
Zm00001d044332_T001	0.9894615	0.057594549	Zm00001d044332
Zm00001d018632_T001	0.7186483	-0.384569366	Zm00001d018632
Zm00001d000238_T001	0.7942213	-0.222240823	Zm00001d000238
Zm00001d050728_T001	0.8926921	0.284546542	Zm00001d050728
Zm00001d011581_T001	1	0.216050093	Zm00001d011581
Zm00001d044953_T001	0.9664406	-0.113889179	Zm00001d044953
Zm00001d012224_T001	0.7897201	1.043534434	Zm00001d012224
Zm00001d012224_T008	0.01214359	0.893422291	Zm00001d012224
Zm00001d012224_T009	0.3547256	-0.754485053	Zm00001d012224
Zm00001d002271_T001	0.03201522	-0.887987798	Zm00001d002271
Zm00001d034700_T001	0.9563232	0.064706873	Zm00001d034700
Zm00001d024048_T004	0.9666757	0.147071468	Zm00001d024048
Zm00001d024048_T006	0.4166993	-0.894527175	Zm00001d024048
Zm00001d009177_T001	0.9856038	0.413992678	Zm00001d009177
Zm00001d038075_T001	4.03E-05	-2.70749134	Zm00001d038075
Zm00001d020655_T001	0.3222183	-1.042682655	Zm00001d020655
Zm00001d047239_T002	0.7483641	0.598815289	Zm00001d047239
Zm00001d047239_T010	0.3668531	0.653875337	Zm00001d047239
Zm00001d047239_T003	0.2719671	1.222849942	Zm00001d047239
Zm00001d047239_T011	0.8616387	-0.099875439	Zm00001d047239
Zm00001d028533_T001	0.9902022	0.281537308	Zm00001d028533
Zm00001d024344_T001	0.7332529	-0.563095026	Zm00001d024344
Zm00001d052273_T002	0.5537557	1.14794151	Zm00001d052273
Zm00001d052273_T001	0.4744959	0.618717193	Zm00001d052273
Zm00001d012924_T001	0.8864089	-0.070499707	Zm00001d012924
Zm00001d029820_T001	0.9568663	0.072033762	Zm00001d029820
Zm00001d027892_T001	0.5471959	1.402693325	Zm00001d027892
Zm00001d037610_T001	0.00904658	-2.261870294	Zm00001d037610
Zm00001d050816_T001	0.9419336	0.554740419	Zm00001d050816
Zm00001d050816_T002	0.7680039	-0.410204152	Zm00001d050816
Zm00001d038489_T001	1	0.364287725	Zm00001d038489
Zm00001d001936_T001	0.9857423	0.138536932	Zm00001d001936
Zm00001d039717_T001	0.5910253	-0.163689604	Zm00001d039717
Zm00001d039717_T003	0.9791729	0.512892034	Zm00001d039717
Zm00001d039717_T002	0.9768802	-0.026165734	Zm00001d039717
Zm00001d039717_T006	0.9833513	0.123901195	Zm00001d039717
Zm00001d052997_T001	0.9968176	0.467073323	Zm00001d052997
Zm00001d029579_T001	0.6156484	1.087799617	Zm00001d029579
Zm00001d013645_T001	0.9404125	-0.271071147	Zm00001d013645
Zm00001d029241_T001	0.4146004	-0.692754598	Zm00001d029241
Zm00001d052918_T001	1	0.240294987	Zm00001d052918
Zm00001d040593_T001	0.7403995	-0.209574803	Zm00001d040593
Zm00001d004929_T001	0.4378952	-0.586922167	Zm00001d004929
Zm00001d004525_T001	0.9469426	0.494604498	Zm00001d004525
Zm00001d004525_T003	0.8857953	0.868009259	Zm00001d004525
Zm00001d040606_T007	0.9618822	0.509663418	Zm00001d040606
Zm00001d040606_T008	0.9130008	0.059378652	Zm00001d040606
Zm00001d040606_T002	0.02622126	0.654153277	Zm00001d040606
Zm00001d040606_T001	0.5651392	1.086332385	Zm00001d040606
Zm00001d049357_T001	0.9580876	0.670587782	Zm00001d049357
Zm00001d003999_T001	0.9524127	-0.464737896	Zm00001d003999
Zm00001d010482_T001	0.589218	0.931899083	Zm00001d010482
Zm00001d039606_T001	0.7059883	0.879606189	Zm00001d039606

Zm00001d039606_T002	0.6976102	-0.158780058	Zm00001d039606
Zm00001d047255_T002	0.9354647	0.276928294	Zm00001d047255
Zm00001d047255_T001	0.9767085	0.569230309	Zm00001d047255
Zm00001d051288_T001	0.467149	-0.772232507	Zm00001d051288
Zm00001d021505_T001	0.8991659	-0.138717024	Zm00001d021505
Zm00001d045512_T001	1	0.165421576	Zm00001d045512
Zm00001d050963_T001	0.9797082	0.222762985	Zm00001d050963
Zm00001d002598_T001	0.9965026	0.292150207	Zm00001d002598
Zm00001d022327_T001	0.0103436	2.808653661	Zm00001d022327
Zm00001d019218_T001	0.9515686	0.024230476	Zm00001d019218
Zm00001d009074_T011	0.9325478	0.16266045	Zm00001d009074
Zm00001d009074_T002	0.9701986	0.080255271	Zm00001d009074
Zm00001d009074_T010	0.9914036	0.161761263	Zm00001d009074
Zm00001d009074_T009	0.09418643	-0.374261716	Zm00001d009074
Zm00001d050411_T001	0.6925804	-0.722853964	Zm00001d050411
Zm00001d022468_T007	0.04263685	0.615882332	Zm00001d022468
Zm00001d022468_T002	3.48E-07	0.453335615	Zm00001d022468
Zm00001d022468_T005	0.9377241	0.671335916	Zm00001d022468
Zm00001d022468_T006	9.73E-05	0.623594573	Zm00001d022468
Zm00001d002510_T020	0.6201695	-0.243295336	Zm00001d002510
Zm00001d002510_T001	0.000970471	0.448789809	Zm00001d002510
Zm00001d040234_T004	0.2341968	-0.175945799	Zm00001d040234
Zm00001d040234_T016	0.6298924	-0.261668694	Zm00001d040234
Zm00001d040234_T008	0.9584734	0.518559386	Zm00001d040234
Zm00001d040234_T011	6.53E-10	1.634654015	Zm00001d040234
Zm00001d040234_T015	0.2575503	0.601087328	Zm00001d040234
Zm00001d012662_T001	0.6857179	-0.168302612	Zm00001d012662
Zm00001d012662_T002	0.91334	0.790305146	Zm00001d012662
Zm00001d041923_T001	0.4296223	-0.943276305	Zm00001d041923
Zm00001d041923_T002	0.9106506	0.786639885	Zm00001d041923
Zm00001d041923_T003	0.9960479	0.188482349	Zm00001d041923
Zm00001d003006_T001	0.8487418	0.867882291	Zm00001d003006
Zm00001d032328_T004	0.7020458	0.378580383	Zm00001d032328
Zm00001d032328_T001	0.2908902	-0.998985519	Zm00001d032328
Zm00001d032328_T002	0.9050121	0.024410243	Zm00001d032328
Zm00001d032328_T005	0.9053735	-0.116127834	Zm00001d032328
Zm00001d011139_T002	0.8293703	-0.170212743	Zm00001d011139
Zm00001d011139_T004	0.817321	-0.295875376	Zm00001d011139
Zm00001d045459_T001	0.04237526	2.462213649	Zm00001d045459
Zm00001d028924_T001	0.9907928	0.133118108	Zm00001d028924
Zm00001d006673_T003	0.9745777	0.345892711	Zm00001d006673
Zm00001d030087_T001	0.9696556	0.571911893	Zm00001d030087
Zm00001d013526_T002	0.2592155	-0.807845418	Zm00001d013526
Zm00001d013526_T001	0.8262962	0.534583867	Zm00001d013526
Zm00001d013526_T004	0.6208075	-0.233731436	Zm00001d013526
Zm00001d013526_T005	0.9947739	0.254327711	Zm00001d013526
Zm00001d033040_T001	0.9709778	0.090400816	Zm00001d033040
Zm00001d017207_T002	0.05187496	-1.572744774	Zm00001d017207
Zm00001d048957_T001	0.1197922	-1.380492111	Zm00001d048957
Zm00001d048957_T003	1	0.059561009	Zm00001d048957
Zm00001d042632_T001	0.9956119	0.313980794	Zm00001d042632
Zm00001d036835_T004	0.9160957	0.643569836	Zm00001d036835
Zm00001d036835_T001	0.7262357	1.011866502	Zm00001d036835
Zm00001d034582_T009	0.633563	0.797606515	Zm00001d034582

Zm00001d034582_T021	0.7137782	-0.424094945	Zm00001d034582
Zm00001d034582_T016	0.07297249	0.938524853	Zm00001d034582
Zm00001d034582_T015	0.1453636	0.474916682	Zm00001d034582
Zm00001d034582_T001	0.05995189	-0.426999026	Zm00001d034582
Zm00001d034582_T012	0.9396569	0.379084394	Zm00001d034582
Zm00001d029359_T001	0.9160957	0.773010749	Zm00001d029359
Zm00001d029359_T002	0.2377811	-0.645156407	Zm00001d029359
Zm00001d034776_T002	0.9619176	0.233629918	Zm00001d034776
Zm00001d034776_T008	5.65E-07	-4.985597205	Zm00001d034776
Zm00001d031701_T002	0.05781038	-1.271406578	Zm00001d031701
Zm00001d031701_T001	0.6797625	-1.047694908	Zm00001d031701
Zm00001d045893_T001	1	0.435347618	Zm00001d045893
Zm00001d049761_T037	0.000870951	0.944821097	Zm00001d049761
Zm00001d049761_T019	0.0313238	1.041917579	Zm00001d049761
Zm00001d049761_T036	2.50E-07	0.783609615	Zm00001d049761
Zm00001d049761_T006	0.9884825	0.536962118	Zm00001d049761
Zm00001d051543_T001	0.7481738	-0.679701831	Zm00001d051543
Zm00001d021465_T001	0.6955134	-0.698274897	Zm00001d021465
Zm00001d049678_T001	0.5578039	-0.365624522	Zm00001d049678
Zm00001d046405_T001	0.8643636	-0.220094246	Zm00001d046405
Zm00001d017448_T006	0.8884142	-0.116965945	Zm00001d017448
Zm00001d017448_T007	0.9970835	0.137513984	Zm00001d017448
Zm00001d017448_T008	0.8751967	0.807492724	Zm00001d017448
Zm00001d017448_T010	0.1048346	-1.698598963	Zm00001d017448
Zm00001d017448_T009	0.8743763	0.575045729	Zm00001d017448
Zm00001d017448_T005	0.8883561	-0.150053066	Zm00001d017448
Zm00001d007195_T019	0.04693522	0.279774687	Zm00001d007195
Zm00001d007195_T012	0.05328006	1.886144122	Zm00001d007195
Zm00001d007195_T009	0.7217563	0.41390246	Zm00001d007195
Zm00001d007195_T001	0.3070924	-0.335407747	Zm00001d007195
Zm00001d028696_T001	0.9709887	0.036129014	Zm00001d028696
Zm00001d028696_T003	0.9031129	-0.102395622	Zm00001d028696
Zm00001d029107_T001	0.09177048	-1.983849664	Zm00001d029107
Zm00001d032166_T008	0.7831859	0.18771577	Zm00001d032166
Zm00001d032166_T013	0.8819981	-0.043944059	Zm00001d032166
Zm00001d032166_T014	0.1700114	0.416030479	Zm00001d032166
Zm00001d032166_T003	0.9925955	0.336141557	Zm00001d032166
Zm00001d032166_T010	0.8704382	0.935629189	Zm00001d032166
Zm00001d032166_T001	0.9419336	0.047093492	Zm00001d032166
Zm00001d032166_T005	0.7814026	0.707228248	Zm00001d032166
Zm00001d019069_T002	0.9419336	-0.044499277	Zm00001d019069
Zm00001d025867_T002	0.9269932	-0.271928368	Zm00001d025867
Zm00001d023833_T003	0.9975882	0.258352177	Zm00001d023833
Zm00001d039331_T001	1	0.164094604	Zm00001d039331
Zm00001d012694_T005	0.9766874	0.255507956	Zm00001d012694
Zm00001d012694_T004	0.9765009	0.536766422	Zm00001d012694
Zm00001d012694_T001	0.9657993	0.159604739	Zm00001d012694
Zm00001d033110_T001	1	0.31731008	Zm00001d033110
Zm00001d048412_T002	0.9616027	0.118590671	Zm00001d048412
Zm00001d048412_T003	0.9748076	0.10609408	Zm00001d048412
Zm00001d006175_T001	0.888802	-0.179494394	Zm00001d006175
Zm00001d034880_T001	0.9242611	-0.047734396	Zm00001d034880
Zm00001d011787_T001	0.5622534	1.263041748	Zm00001d011787
Zm00001d028328_T001	0.926928	0.216038475	Zm00001d028328

Zm00001d010235_T016	0.9804433	0.150870253	Zm00001d010235
Zm00001d010235_T008	0.4146004	-0.130784959	Zm00001d010235
Zm00001d010235_T019	1	0.126692575	Zm00001d010235
Zm00001d010235_T010	0.4357247	0.401742531	Zm00001d010235
Zm00001d010235_T013	0.7136625	0.015914758	Zm00001d010235
Zm00001d010235_T015	0.9180662	-0.023963564	Zm00001d010235
Zm00001d010235_T014	0.4357391	0.851292597	Zm00001d010235
Zm00001d010235_T001	0.5718096	-0.6323763	Zm00001d010235
Zm00001d010235_T009	6.53E-10	1.048048846	Zm00001d010235
Zm00001d010235_T020	0.8557307	0.323290986	Zm00001d010235
Zm00001d012661_T013	0.9919298	0.290862007	Zm00001d012661
Zm00001d012661_T005	0.1519795	1.291836281	Zm00001d012661
Zm00001d012661_T008	0.1448848	-0.814621934	Zm00001d012661
Zm00001d012661_T001	0.000101475	-1.157747875	Zm00001d012661
Zm00001d017215_T002	0.9044906	-0.144726565	Zm00001d017215
Zm00001d017215_T001	0.9760472	0.001760231	Zm00001d017215
Zm00001d022119_T001	0.885791	-0.074090029	Zm00001d022119
Zm00001d020519_T002	0.9942835	0.223671354	Zm00001d020519
Zm00001d035700_T001	0.9988864	0.411572552	Zm00001d035700
Zm00001d032435_T001	1	0.361324396	Zm00001d032435
Zm00001d050182_T001	0.6511883	-0.599465056	Zm00001d050182
Zm00001d040504_T001	0.4895863	-0.838416446	Zm00001d040504
Zm00001d030276_T001	0.7923921	-0.42861209	Zm00001d030276
Zm00001d049940_T001	0.130429	1.540684781	Zm00001d049940
Zm00001d049940_T002	0.9237037	0.699847574	Zm00001d049940
Zm00001d010109_T008	0.9450101	0.578219303	Zm00001d010109
Zm00001d010109_T002	0.9718747	0.259783188	Zm00001d010109
Zm00001d010109_T004	0.7912425	0.887319072	Zm00001d010109
Zm00001d047821_T001	0.9664736	0.055093119	Zm00001d047821
Zm00001d023682_T006	0.910614	0.005291486	Zm00001d023682
Zm00001d052164_T006	7.95E-07	5.111335923	Zm00001d052164
Zm00001d052164_T001	0.009800589	2.381541494	Zm00001d052164
Zm00001d052164_T002	0.001797502	3.061356948	Zm00001d052164
Zm00001d052164_T005	0.002381753	3.328791763	Zm00001d052164
Zm00001d016175_T001	0.9846446	0.232096625	Zm00001d016175
Zm00001d017654_T001	0.002644703	0.518083712	Zm00001d017654
Zm00001d017654_T002	0.6453183	-0.085584729	Zm00001d017654
Zm00001d017654_T008	0.8638849	0.334180388	Zm00001d017654
Zm00001d017654_T005	0.9618822	0.605864016	Zm00001d017654
Zm00001d038465_T001	0.03706504	-1.195544795	Zm00001d038465
Zm00001d015700_T001	0.9946911	-0.026780937	Zm00001d015700
Zm00001d048021_T001	0.5779179	1.327242216	Zm00001d048021
Zm00001d036196_T001	0.9130008	-0.083223282	Zm00001d036196
Zm00001d024698_T002	0.9817074	0.222241291	Zm00001d024698
Zm00001d032556_T001	0.405609	-1.115244037	Zm00001d032556
Zm00001d029247_T001	0.3857715	-0.72269677	Zm00001d029247
Zm00001d039883_T001	0.9210327	-0.345334082	Zm00001d039883
Zm00001d032876_T001	0.9402255	-0.316591874	Zm00001d032876
Zm00001d045319_T001	0.9822138	0.460724546	Zm00001d045319
Zm00001d031817_T007	0.9950703	0.219261329	Zm00001d031817
Zm00001d031817_T001	0.9860836	0.166295328	Zm00001d031817
Zm00001d031817_T006	0.6725985	1.239363979	Zm00001d031817
Zm00001d031817_T008	0.5292925	-1.015161063	Zm00001d031817
Zm00001d031817_T003	1	0.331810459	Zm00001d031817

Zm00001d005752_T001	0.1223186	-1.463378715	Zm00001d005752
Zm00001d035054_T002	0.9930712	-0.031079501	Zm00001d035054
Zm00001d035054_T004	0.9932943	0.136075124	Zm00001d035054
Zm00001d014626_T003	0.6097901	-0.136956475	Zm00001d014626
Zm00001d014626_T002	0.9962972	0.248070714	Zm00001d014626
Zm00001d019591_T001	0.002158908	-2.749952586	Zm00001d019591
Zm00001d019591_T011	0.6395973	0.412949577	Zm00001d019591
Zm00001d019591_T006	0.9311783	0.434789386	Zm00001d019591
Zm00001d019591_T014	0.9866228	0.322817332	Zm00001d019591
Zm00001d019591_T009	0.08939768	0.953801045	Zm00001d019591
Zm00001d019591_T035	0.1902959	0.510445474	Zm00001d019591
Zm00001d042178_T001	0.5593498	-0.822965378	Zm00001d042178
Zm00001d024027_T001	0.466628	-0.852755669	Zm00001d024027
Zm00001d032301_T002	0.5478438	-0.247487257	Zm00001d032301
Zm00001d032301_T005	0.9633184	0.070144545	Zm00001d032301
Zm00001d022104_T001	0.9919298	0.254845688	Zm00001d022104
Zm00001d010360_T001	0.02685638	-1.625264988	Zm00001d010360
Zm00001d030856_T001	0.3002348	1.286183213	Zm00001d030856
Zm00001d030173_T001	0.9894615	0.112038237	Zm00001d030173
Zm00001d030173_T017	0.05077923	0.737224413	Zm00001d030173
Zm00001d030173_T003	0.7930026	0.132039039	Zm00001d030173
Zm00001d030173_T013	0.5490269	0.589786078	Zm00001d030173
Zm00001d030173_T006	0.6014554	0.370631519	Zm00001d030173
Zm00001d030173_T005	0.8929316	0.870724232	Zm00001d030173
Zm00001d030173_T009	0.5348533	0.267834702	Zm00001d030173
Zm00001d015434_T001	0.3060812	-1.431849541	Zm00001d015434
Zm00001d052901_T002	0.9895224	0.342703755	Zm00001d052901
Zm00001d052901_T004	0.4672873	0.454025624	Zm00001d052901
Zm00001d052901_T003	0.3893584	-0.819919002	Zm00001d052901
Zm00001d052901_T001	0.8069976	0.947922607	Zm00001d052901
Zm00001d040124_T001	1	0.207604493	Zm00001d040124
Zm00001d038407_T001	0.8420475	0.408937336	Zm00001d038407
Zm00001d038407_T002	0.9781672	0.027582664	Zm00001d038407
Zm00001d024545_T001	0.8207831	0.561424741	Zm00001d024545
Zm00001d019510_T001	0.7697196	0.717257747	Zm00001d019510
Zm00001d037770_T001	0.885791	-0.122847175	Zm00001d037770
Zm00001d006022_T001	0.1758861	-1.150270194	Zm00001d006022
Zm00001d010812_T001	0.001371047	3.164746122	Zm00001d010812
Zm00001d038850_T001	0.8102147	-0.294887498	Zm00001d038850
Zm00001d049169_T001	0.4178252	-0.774934186	Zm00001d049169
Zm00001d027313_T001	0.8618945	-0.250946925	Zm00001d027313
Zm00001d031269_T002	0.2770216	-0.577898823	Zm00001d031269
Zm00001d031269_T005	0.9766874	0.527575543	Zm00001d031269
Zm00001d031269_T003	0.940051	0.029819306	Zm00001d031269
Zm00001d031269_T010	1.58E-09	-4.022499058	Zm00001d031269
Zm00001d025208_T002	0.9994624	0.189093646	Zm00001d025208
Zm00001d025208_T001	0.7137316	-0.301214338	Zm00001d025208
Zm00001d003175_T001	0.836612	-0.320740637	Zm00001d003175
Zm00001d045036_T001	0.7074815	-0.488661882	Zm00001d045036
Zm00001d052660_T019	0.7102433	-0.700671696	Zm00001d052660
Zm00001d052660_T008	0.1103167	1.101851568	Zm00001d052660
Zm00001d052660_T015	0.2136911	1.540628501	Zm00001d052660
Zm00001d052660_T001	0.5595771	0.665610679	Zm00001d052660
Zm00001d052660_T003	0.8238338	0.965548193	Zm00001d052660

Zm00001d052660_T007	1	0.196653919	Zm00001d052660
Zm00001d052660_T014	0.9986486	-0.115794395	Zm00001d052660
Zm00001d052660_T005	0.3285918	0.296155551	Zm00001d052660
Zm00001d052660_T006	0.6952911	0.854280034	Zm00001d052660
Zm00001d031222_T001	0.9795655	0.535207244	Zm00001d031222
Zm00001d045296_T001	0.8332116	-0.748459953	Zm00001d045296
Zm00001d026664_T001	0.9745777	-0.04605275	Zm00001d026664
Zm00001d008388_T004	0.000270911	-2.545725751	Zm00001d008388
Zm00001d008388_T001	0.8609458	0.84860336	Zm00001d008388
Zm00001d008388_T003	0.9221549	1.311189773	Zm00001d008388
Zm00001d031081_T001	0.4531805	-0.328231647	Zm00001d031081
Zm00001d031081_T004	0.9860836	0.237996082	Zm00001d031081
Zm00001d031081_T002	0.7760798	-0.417257971	Zm00001d031081
Zm00001d017490_T001	0.02334998	-1.195979532	Zm00001d017490
Zm00001d005716_T002	0.8667498	-0.531359579	Zm00001d005716
Zm00001d005716_T001	0.9791729	-0.090711067	Zm00001d005716
Zm00001d038115_T002	0.998484	0.397226662	Zm00001d038115
Zm00001d026440_T001	0.9986486	-0.332617599	Zm00001d026440
Zm00001d010054_T002	1	0.209134483	Zm00001d010054
Zm00001d010054_T006	0.9693993	-0.151610435	Zm00001d010054
Zm00001d010054_T001	0.9048001	0.778272878	Zm00001d010054
Zm00001d010054_T004	1	0.244007539	Zm00001d010054
Zm00001d028194_T001	0.5890036	-0.40277916	Zm00001d028194
Zm00001d028194_T004	0.5695353	2.227856355	Zm00001d028194
Zm00001d028194_T005	0.4715806	-1.278496217	Zm00001d028194
Zm00001d028194_T003	0.2153746	0.454520796	Zm00001d028194
Zm00001d047726_T001	0.9887889	0.476150874	Zm00001d047726
Zm00001d028522_T001	0.8799679	-0.114111698	Zm00001d028522
Zm00001d006605_T001	0.974657	0.138000255	Zm00001d006605
Zm00001d015741_T001	0.7888267	-0.483405985	Zm00001d015741
Zm00001d021267_T001	0.8923684	0.485498407	Zm00001d021267
Zm00001d021267_T002	1	0.109995807	Zm00001d021267
Zm00001d012394_T001	0.6761587	1.176090158	Zm00001d012394
Zm00001d008271_T003	0.8591203	-0.131980031	Zm00001d008271
Zm00001d025286_T001	0.8744797	-0.193411743	Zm00001d025286
Zm00001d003257_T001	0.9984041	0.307358507	Zm00001d003257
Zm00001d031311_T001	0.9173494	-0.365512354	Zm00001d031311
Zm00001d008957_T001	0.7686793	-0.266614714	Zm00001d008957
Zm00001d008564_T001	0.9956119	0.25494129	Zm00001d008564
Zm00001d044192_T001	0.9942835	0.23896806	Zm00001d044192
Zm00001d044192_T002	0.9659036	-0.423915893	Zm00001d044192
Zm00001d028294_T001	0.6967776	-0.411374775	Zm00001d028294
Zm00001d011931_T001	0.7375754	0.859710537	Zm00001d011931
Zm00001d011931_T002	0.8517568	-0.200395177	Zm00001d011931
Zm00001d031981_T008	0.641088	0.531822266	Zm00001d031981
Zm00001d031981_T006	0.4767903	0.91406704	Zm00001d031981
Zm00001d031981_T003	0.9889811	0.515405961	Zm00001d031981
Zm00001d031981_T007	0.8492405	0.464090139	Zm00001d031981
Zm00001d031981_T001	0.587212	0.326342198	Zm00001d031981
Zm00001d002848_T002	0.8955831	0.38531873	Zm00001d002848
Zm00001d002848_T001	0.8412913	-0.248206314	Zm00001d002848
Zm00001d043016_T001	0.9466531	-0.002388961	Zm00001d043016
Zm00001d013898_T001	0.1757415	-0.626030819	Zm00001d013898
Zm00001d053575_T012	0.5706512	0.742631444	Zm00001d053575

Zm00001d053575_T007	0.02913084	1.835827441	Zm00001d053575
Zm00001d053575_T011	0.1111506	0.52281696	Zm00001d053575
Zm00001d053575_T010	0.8906466	0.805553435	Zm00001d053575
Zm00001d053575_T003	0.08048901	0.911281982	Zm00001d053575
Zm00001d053575_T005	0.002805118	1.90704686	Zm00001d053575
Zm00001d017458_T002	0.9708541	0.493796094	Zm00001d017458
Zm00001d045202_T004	0.8665839	0.528216658	Zm00001d045202
Zm00001d045202_T002	0.7137316	1.131941496	Zm00001d045202
Zm00001d045202_T005	0.002583044	-1.755338626	Zm00001d045202
Zm00001d045202_T001	0.6466858	-0.327594149	Zm00001d045202
Zm00001d006625_T002	0.9706293	0.135848228	Zm00001d006625
Zm00001d006625_T006	0.02207954	1.434316866	Zm00001d006625
Zm00001d018502_T001	0.1026969	-1.047773193	Zm00001d018502
Zm00001d037655_T001	0.8412913	-0.496760018	Zm00001d037655
Zm00001d048432_T002	0.8021694	0.442538765	Zm00001d048432
Zm00001d048432_T001	0.9669771	0.166106469	Zm00001d048432
Zm00001d033665_T002	0.9970835	-0.120796937	Zm00001d033665
Zm00001d051695_T001	0.2836527	1.532945305	Zm00001d051695
Zm00001d005766_T001	8.18E-06	4.520131565	Zm00001d005766
Zm00001d020365_T002	1	0.457761814	Zm00001d020365
Zm00001d020365_T005	0.9529184	0.325079004	Zm00001d020365
Zm00001d020365_T003	0.2725766	1.409369705	Zm00001d020365
Zm00001d002403_T001	0.907891	-0.611601227	Zm00001d002403
Zm00001d052321_T002	0.9701986	-0.128389834	Zm00001d052321
Zm00001d052321_T001	0.9580985	-0.204369156	Zm00001d052321
Zm00001d047969_T001	0.9682673	0.653863405	Zm00001d047969
Zm00001d010199_T001	0.9369909	0.417341593	Zm00001d010199
Zm00001d010199_T003	0.9404125	0.732916405	Zm00001d010199
Zm00001d010199_T004	0.1966199	1.091085879	Zm00001d010199
Zm00001d007901_T001	0.2073293	1.870492356	Zm00001d007901
Zm00001d002451_T001	0.7737238	0.875451626	Zm00001d002451
Zm00001d016581_T002	0.1310827	0.709231707	Zm00001d016581
Zm00001d016581_T018	0.5092234	-0.393141957	Zm00001d016581
Zm00001d017352_T007	0.4860327	0.580249681	Zm00001d017352
Zm00001d017352_T003	0.5096243	-0.459524946	Zm00001d017352
Zm00001d017352_T008	0.7976574	-0.289907856	Zm00001d017352
Zm00001d017352_T012	0.6275799	1.851004684	Zm00001d017352
Zm00001d017352_T002	1	0.360720921	Zm00001d017352
Zm00001d017352_T006	1	0.290523029	Zm00001d017352
Zm00001d022142_T004	0.9709887	0.537059174	Zm00001d022142
Zm00001d040116_T001	0.897685	-0.13586715	Zm00001d040116
Zm00001d053589_T002	0.995055	0.465518247	Zm00001d053589
Zm00001d053589_T005	0.4149257	-1.279171053	Zm00001d053589
Zm00001d053589_T003	0.5717645	0.158768655	Zm00001d053589
Zm00001d006646_T001	0.8894168	0.62405534	Zm00001d006646
Zm00001d021516_T001	1.14E-07	-2.131998669	Zm00001d021516
Zm00001d021516_T007	0.8357705	1.382812241	Zm00001d021516
Zm00001d021516_T004	0.6360579	1.394956498	Zm00001d021516
Zm00001d021516_T011	0.9543237	0.041224174	Zm00001d021516
Zm00001d021516_T010	0.7260742	0.365426488	Zm00001d021516
Zm00001d047110_T003	0.92946	0.219203157	Zm00001d047110
Zm00001d047110_T005	0.5433356	1.375652069	Zm00001d047110
Zm00001d047110_T004	0.07487237	1.799902303	Zm00001d047110
Zm00001d043489_T001	0.9845237	0.169252677	Zm00001d043489

Zm00001d039166_T003	0.3260197	-0.721889856	Zm00001d039166
Zm00001d039166_T004	0.000134356	-2.4338919	Zm00001d039166
Zm00001d039166_T001	0.4876864	-0.72719096	Zm00001d039166
Zm00001d051896_T001	0.6395973	-0.520142326	Zm00001d051896
Zm00001d014649_T001	1	0.096881989	Zm00001d014649
Zm00001d045261_T011	0.998484	0.37058577	Zm00001d045261
Zm00001d049999_T002	0.3472469	-1.196984948	Zm00001d049999
Zm00001d007820_T002	0.01640001	-1.949430359	Zm00001d007820
Zm00001d040760_T002	0.9792387	0.56048211	Zm00001d040760
Zm00001d046330_T001	0.2038697	-1.023737259	Zm00001d046330
Zm00001d047392_T002	0.9979568	0.343585978	Zm00001d047392
Zm00001d047392_T006	0.9735632	0.33871439	Zm00001d047392
Zm00001d047392_T001	0.9311783	0.479719124	Zm00001d047392
Zm00001d049500_T002	0.6847424	-0.576621008	Zm00001d049500
Zm00001d049500_T003	0.9857423	0.094709239	Zm00001d049500
Zm00001d017865_T006	0.006902401	1.489304958	Zm00001d017865
Zm00001d017865_T002	0.9614723	0.028150592	Zm00001d017865
Zm00001d017865_T001	0.1517439	1.863753541	Zm00001d017865
Zm00001d017865_T005	0.000118746	0.971846035	Zm00001d017865
Zm00001d029613_T001	0.005389852	1.738987709	Zm00001d029613
Zm00001d036490_T001	0.9692063	0.152237881	Zm00001d036490
Zm00001d019670_T010	5.43E-09	1.140931519	Zm00001d019670
Zm00001d019670_T025	0.06758572	0.255344759	Zm00001d019670
Zm00001d019670_T012	0.5495068	-0.529624462	Zm00001d019670
Zm00001d019670_T001	0.005458031	2.682306634	Zm00001d019670
Zm00001d019670_T013	0.2546519	2.153659307	Zm00001d019670
Zm00001d019670_T005	0.09102453	-0.883836798	Zm00001d019670
Zm00001d019670_T009	0.161363	1.396129494	Zm00001d019670
Zm00001d010988_T001	0.6742084	-0.779863826	Zm00001d010988
Zm00001d028754_T001	0.3870314	1.398430187	Zm00001d028754
Zm00001d049006_T001	0.9753621	-0.169293958	Zm00001d049006
Zm00001d000298_T002	0.6850013	-0.514572242	Zm00001d000298
Zm00001d045746_T001	0.4530974	1.028785556	Zm00001d045746
Zm00001d036139_T001	0.2292281	-1.421320535	Zm00001d036139
Zm00001d031531_T002	0.6097645	-0.399194834	Zm00001d031531
Zm00001d037005_T001	1	0.123596873	Zm00001d037005
Zm00001d026164_T003	0.9588604	0.535343074	Zm00001d026164
Zm00001d026164_T007	0.0884706	-1.294444845	Zm00001d026164
Zm00001d026164_T005	0.9909024	0.163831958	Zm00001d026164
Zm00001d026164_T004	0.7169281	-0.304699868	Zm00001d026164
Zm00001d026164_T006	0.9614996	0.454105288	Zm00001d026164
Zm00001d025463_T001	0.745469	0.418774886	Zm00001d025463
Zm00001d030091_T001	0.9730261	0.105497959	Zm00001d030091
Zm00001d034597_T003	0.983226	0.249924191	Zm00001d034597
Zm00001d032978_T001	0.956184	0.068184526	Zm00001d032978
Zm00001d023459_T001	0.9581123	0.493380333	Zm00001d023459
Zm00001d052177_T001	0.620923	-1.030330511	Zm00001d052177
Zm00001d021846_T018	0.1501156	0.664396921	Zm00001d021846
Zm00001d021846_T010	1	0.410842602	Zm00001d021846
Zm00001d021846_T012	0.147668	1.407715399	Zm00001d021846
Zm00001d021846_T015	0.006622774	1.709770375	Zm00001d021846
Zm00001d021846_T001	0.9521443	-0.128705652	Zm00001d021846
Zm00001d021846_T002	0.2828825	1.736875081	Zm00001d021846
Zm00001d021846_T020	0.7989126	0.217473027	Zm00001d021846

Zm00001d002340_T001	0.9311027	-0.121478157	Zm00001d002340
Zm00001d002340_T003	0.9822138	0.218382489	Zm00001d002340
Zm00001d021504_T001	0.9573272	0.274257183	Zm00001d021504
Zm00001d007839_T001	0.9410845	0.032089824	Zm00001d007839
Zm00001d002296_T003	0.9380783	-0.01466202	Zm00001d002296
Zm00001d012862_T001	0.3222183	0.888115203	Zm00001d012862
Zm00001d023237_T001	0.9486412	0.036409828	Zm00001d023237
Zm00001d017976_T001	0.7238616	0.828803974	Zm00001d017976
Zm00001d043432_T001	0.9983524	0.263410826	Zm00001d043432
Zm00001d052282_T097	0.1264206	-1.078725361	Zm00001d052282
Zm00001d052282_T064	2.50E-07	0.501586193	Zm00001d052282
Zm00001d052282_T082	0.1737031	0.939889342	Zm00001d052282
Zm00001d052282_T095	1	0.455556755	Zm00001d052282
Zm00001d052282_T017	2.63E-13	2.065963862	Zm00001d052282
Zm00001d052282_T006	0.9944247	0.110595591	Zm00001d052282
Zm00001d052282_T045	1	0.252053715	Zm00001d052282
Zm00001d052282_T088	0.8842271	-0.017462154	Zm00001d052282
Zm00001d047458_T001	0.7945048	-0.699468283	Zm00001d047458
Zm00001d044836_T001	0.03420931	-1.476572643	Zm00001d044836
Zm00001d047992_T002	0.9375054	-0.089961376	Zm00001d047992
Zm00001d047992_T004	0.9768802	-0.143449486	Zm00001d047992
Zm00001d047992_T003	0.6906858	0.550165604	Zm00001d047992
Zm00001d047992_T005	0.7717836	0.559505303	Zm00001d047992
Zm00001d047992_T001	0.9361702	0.028184253	Zm00001d047992
Zm00001d028782_T002	0.9718605	0.131580567	Zm00001d028782
Zm00001d040777_T001	0.200223	1.879001439	Zm00001d040777
Zm00001d012044_T002	0.2251209	-0.786856817	Zm00001d012044
Zm00001d012044_T001	0.9916733	-0.086583054	Zm00001d012044
Zm00001d007069_T005	0.9851204	-0.209096433	Zm00001d007069
Zm00001d007069_T004	0.9809965	-0.02409144	Zm00001d007069
Zm00001d007069_T002	0.6101277	-0.519363505	Zm00001d007069
Zm00001d023217_T002	0.9487214	0.676682996	Zm00001d023217
Zm00001d023217_T004	0.759025	0.799136413	Zm00001d023217
Zm00001d042625_T001	0.850981	-0.684160792	Zm00001d042625
Zm00001d046039_T024	5.94E-13	1.842029235	Zm00001d046039
Zm00001d046039_T023	0.6086477	0.463314049	Zm00001d046039
Zm00001d046039_T007	0.3764439	0.190494698	Zm00001d046039
Zm00001d046039_T016	0.6318866	-0.37795276	Zm00001d046039
Zm00001d046039_T036	8.81E-08	0.739782652	Zm00001d046039
Zm00001d046039_T018	0.6702695	0.38182687	Zm00001d046039
Zm00001d046039_T038	0.08080868	0.837452113	Zm00001d046039
Zm00001d046039_T012	4.64E-09	1.156288033	Zm00001d046039
Zm00001d046039_T015	0.5126207	0.256585634	Zm00001d046039
Zm00001d029763_T001	0.6340221	-0.875828779	Zm00001d029763
Zm00001d016203_T004	0.9988063	0.263489337	Zm00001d016203
Zm00001d016203_T001	0.1818739	0.690890944	Zm00001d016203
Zm00001d016203_T003	0.9832092	0.027324313	Zm00001d016203
Zm00001d052921_T002	0.7594555	-0.311518328	Zm00001d052921
Zm00001d052921_T006	0.4193179	-1.429777492	Zm00001d052921
Zm00001d052921_T001	1	0.158107585	Zm00001d052921
Zm00001d024092_T001	1	-0.002770089	Zm00001d024092
Zm00001d011174_T003	0.4521275	0.676141371	Zm00001d011174
Zm00001d011174_T001	0.9307579	0.763139709	Zm00001d011174
Zm00001d011174_T004	0.03896328	0.442529222	Zm00001d011174

Zm00001d011174_T002	0.5880139	-0.492117599	Zm00001d011174
Zm00001d031481_T001	0.900661	-0.084957797	Zm00001d031481
Zm00001d038217_T004	0.9779656	0.499273606	Zm00001d038217
Zm00001d002444_T001	0.8031436	-0.291931172	Zm00001d002444
Zm00001d009792_T001	0.9902022	0.133574964	Zm00001d009792
Zm00001d013979_T001	0.8545986	0.962435945	Zm00001d013979
Zm00001d036090_T003	0.08500844	1.587884523	Zm00001d036090
Zm00001d036090_T008	0.9410845	0.060776098	Zm00001d036090
Zm00001d036090_T005	0.8746701	0.233729038	Zm00001d036090
Zm00001d036090_T006	0.7082783	0.41155861	Zm00001d036090
Zm00001d029387_T004	0.4085315	1.555356692	Zm00001d029387
Zm00001d029387_T002	0.004825465	4.224446781	Zm00001d029387
Zm00001d029387_T003	0.9160053	0.705331934	Zm00001d029387
Zm00001d029387_T001	0.9728551	0.630594513	Zm00001d029387
Zm00001d029387_T005	0.7901736	0.701776925	Zm00001d029387
Zm00001d018488_T001	0.3002348	1.534927446	Zm00001d018488
Zm00001d018488_T003	0.1527884	1.711219848	Zm00001d018488
Zm00001d038722_T001	0.9855106	0.071773478	Zm00001d038722
Zm00001d023429_T001	0.9706293	0.179995172	Zm00001d023429
Zm00001d041870_T001	0.9914064	0.244767003	Zm00001d041870
Zm00001d014186_T017	0.9856038	1.057977298	Zm00001d014186
Zm00001d014186_T020	0.9563232	0.0848629	Zm00001d014186
Zm00001d014186_T019	0.4181514	1.029363787	Zm00001d014186
Zm00001d014186_T014	0.9993872	0.119173749	Zm00001d014186
Zm00001d014186_T007	0.3969484	1.546968788	Zm00001d014186
Zm00001d014186_T013	0.06769495	-1.057032421	Zm00001d014186
Zm00001d014186_T016	0.1476143	1.667541562	Zm00001d014186
Zm00001d014186_T002	0.03694055	1.291660885	Zm00001d014186
Zm00001d014186_T011	0.7294689	0.449919468	Zm00001d014186
Zm00001d014186_T022	0.9015971	0.989583859	Zm00001d014186
Zm00001d014186_T001	0.1941473	1.289805309	Zm00001d014186
Zm00001d014186_T021	0.9223663	0.684944838	Zm00001d014186
Zm00001d007060_T001	0.3437881	0.408664322	Zm00001d007060
Zm00001d007060_T004	0.9984041	0.306090646	Zm00001d007060
Zm00001d013178_T001	0.0924475	2.114574238	Zm00001d013178
Zm00001d016008_T006	0.7400835	0.70608606	Zm00001d016008
Zm00001d016008_T024	0.2966369	0.956249622	Zm00001d016008
Zm00001d016008_T014	0.1940857	0.637528098	Zm00001d016008
Zm00001d016008_T027	0.2447447	-0.77209126	Zm00001d016008
Zm00001d016008_T015	5.60E-09	1.246693539	Zm00001d016008
Zm00001d016008_T004	0.1349847	0.779482603	Zm00001d016008
Zm00001d016008_T005	0.2343475	1.715496888	Zm00001d016008
Zm00001d039006_T007	0.906282	0.292082762	Zm00001d039006
Zm00001d039006_T004	0.9975882	0.572986654	Zm00001d039006
Zm00001d039006_T003	0.4764719	1.367832195	Zm00001d039006
Zm00001d039006_T008	0.000618797	-2.21137947	Zm00001d039006
Zm00001d048324_T002	0.9776645	0.121979825	Zm00001d048324
Zm00001d002243_T005	0.8935288	0.330483819	Zm00001d002243
Zm00001d002243_T004	0.9596377	0.621035236	Zm00001d002243
Zm00001d002243_T001	0.007508833	-0.815103627	Zm00001d002243
Zm00001d014943_T041	0.9898682	0.284378025	Zm00001d014943
Zm00001d014943_T001	0.02255599	1.876753112	Zm00001d014943
Zm00001d014943_T042	0.3118193	-0.725871166	Zm00001d014943
Zm00001d039471_T002	0.6659845	-0.39844497	Zm00001d039471

Zm00001d039471_T001	1	0.169477972	Zm00001d039471
Zm00001d039076_T003	0.8896831	-0.485394759	Zm00001d039076
Zm00001d039076_T001	1	0.008269882	Zm00001d039076
Zm00001d017820_T001	1	0.345450206	Zm00001d017820
Zm00001d046054_T001	0.9647033	0.331124539	Zm00001d046054
Zm00001d027384_T001	0.9618822	0.113944188	Zm00001d027384
Zm00001d042454_T001	0.9326774	-0.433054795	Zm00001d042454
Zm00001d008628_T001	0.9975882	0.48945734	Zm00001d008628
Zm00001d008628_T002	0.9942835	0.352689858	Zm00001d008628
Zm00001d038909_T001	0.4592964	1.435819048	Zm00001d038909
Zm00001d034439_T001	0.7624897	-0.247983571	Zm00001d034439
Zm00001d040711_T001	0.9249382	-0.149272044	Zm00001d040711
Zm00001d030868_T004	0.4052048	1.083129402	Zm00001d030868
Zm00001d030868_T008	0.6201695	0.31698042	Zm00001d030868
Zm00001d030868_T014	0.4297509	-0.365346922	Zm00001d030868
Zm00001d030868_T006	0.7923475	0.739933708	Zm00001d030868
Zm00001d030868_T010	0.5235287	-0.952147056	Zm00001d030868
Zm00001d030868_T007	0.9621967	0.166861058	Zm00001d030868
Zm00001d030868_T005	0.7146325	0.840512656	Zm00001d030868
Zm00001d030868_T001	0.9662615	0.094826214	Zm00001d030868
Zm00001d030868_T003	0.6588175	0.960153804	Zm00001d030868
Zm00001d030868_T009	0.4450021	0.486831365	Zm00001d030868
Zm00001d030868_T012	0.4359356	0.348532842	Zm00001d030868
Zm00001d018320_T001	0.4876603	-1.043170312	Zm00001d018320
Zm00001d003846_T002	0.7831859	0.513595761	Zm00001d003846
Zm00001d003846_T001	0.9375054	-0.001035444	Zm00001d003846
Zm00001d036031_T006	0.6486478	-1.283840123	Zm00001d036031
Zm00001d036031_T016	0.6325449	-0.58865689	Zm00001d036031
Zm00001d036031_T009	0.03467054	3.258630543	Zm00001d036031
Zm00001d036031_T012	0.9979568	0.144502083	Zm00001d036031
Zm00001d036031_T003	0.000727373	-0.908916627	Zm00001d036031
Zm00001d036031_T005	0.9621967	0.164712787	Zm00001d036031
Zm00001d003626_T001	0.4737762	-0.744846608	Zm00001d003626
Zm00001d029193_T001	0.8757833	-0.07124728	Zm00001d029193
Zm00001d031967_T002	0.9771927	0.504834651	Zm00001d031967
Zm00001d031967_T004	0.9356767	0.674083468	Zm00001d031967
Zm00001d039454_T001	0.8514567	-0.212366854	Zm00001d039454
Zm00001d016647_T001	0.9469426	-0.23071462	Zm00001d016647
Zm00001d031599_T001	0.8751967	0.532522645	Zm00001d031599
Zm00001d029716_T001	0.8862467	-0.455336185	Zm00001d029716
Zm00001d003672_T001	0.772948	-0.552724631	Zm00001d003672
Zm00001d014834_T001	0.9429712	0.076786832	Zm00001d014834
Zm00001d047904_T001	0.9932006	0.278510682	Zm00001d047904
Zm00001d048253_T001	0.900661	-0.091276495	Zm00001d048253
Zm00001d048253_T002	0.8203654	-0.097960287	Zm00001d048253
Zm00001d005776_T001	0.9160053	0.051630982	Zm00001d005776
Zm00001d005776_T004	0.9118093	-0.019042367	Zm00001d005776
Zm00001d005776_T003	0.05092897	-0.437608278	Zm00001d005776
Zm00001d022301_T001	0.5089577	-1.068535899	Zm00001d022301
Zm00001d030893_T027	0.9745948	0.145994713	Zm00001d030893
Zm00001d030893_T020	0.02575643	1.911315223	Zm00001d030893
Zm00001d030893_T018	0.4604152	0.728538057	Zm00001d030893
Zm00001d030893_T026	0.4046727	1.217868486	Zm00001d030893
Zm00001d030893_T004	0.2800303	1.753527519	Zm00001d030893

Zm00001d030893_T001	0.004213273	1.18435914	Zm00001d030893
Zm00001d030893_T025	6.45E-11	1.805600475	Zm00001d030893
Zm00001d024588_T002	0.9064794	-0.03267179	Zm00001d024588
Zm00001d024588_T001	1	0.300324736	Zm00001d024588
Zm00001d002830_T001	0.5651087	1.311686296	Zm00001d002830
Zm00001d035926_T001	0.9979568	0.476130457	Zm00001d035926
Zm00001d019397_T006	0.98291	0.194098087	Zm00001d019397
Zm00001d026094_T001	0.5234084	-0.537130883	Zm00001d026094
Zm00001d053234_T001	0.9706887	0.404714126	Zm00001d053234
Zm00001d026127_T001	0.3896536	0.657970934	Zm00001d026127
Zm00001d035898_T001	0.8397564	-0.240850072	Zm00001d035898
Zm00001d002943_T008	0.945968	0.505063964	Zm00001d002943
Zm00001d002943_T012	0.9786754	0.599999374	Zm00001d002943
Zm00001d002943_T013	0.7594555	-0.305716301	Zm00001d002943
Zm00001d002943_T011	0.1191683	-1.338259676	Zm00001d002943
Zm00001d002943_T002	0.9833565	0.586690135	Zm00001d002943
Zm00001d053374_T001	0.9956349	0.192023971	Zm00001d053374
Zm00001d019684_T002	0.8933301	-0.181168975	Zm00001d019684
Zm00001d053859_T001	0.1701649	1.929516118	Zm00001d053859
Zm00001d010284_T001	0.8054763	-0.316049313	Zm00001d010284
Zm00001d012593_T001	0.972861	0.350876758	Zm00001d012593
Zm00001d020681_T001	0.6844831	-0.736306622	Zm00001d020681
Zm00001d032704_T001	0.9959972	0.08331999	Zm00001d032704
Zm00001d032704_T055	0.9237473	0.479768974	Zm00001d032704
Zm00001d032704_T057	0.02028841	0.699168605	Zm00001d032704
Zm00001d032704_T052	0.007202394	1.184364701	Zm00001d032704
Zm00001d032704_T003	0.9050121	0.558473626	Zm00001d032704
Zm00001d032704_T004	1.52E-06	0.591242041	Zm00001d032704
Zm00001d032704_T013	0.6486663	-0.440988359	Zm00001d032704
Zm00001d003313_T001	0.9160053	-0.187900271	Zm00001d003313
Zm00001d020560_T003	0.9918302	0.196661809	Zm00001d020560
Zm00001d020560_T001	0.918657	0.052945854	Zm00001d020560
Zm00001d042044_T001	0.9846714	0.122707885	Zm00001d042044
Zm00001d042044_T002	0.9442514	0.006270947	Zm00001d042044
Zm00001d042709_T001	0.9927201	0.443927415	Zm00001d042709
Zm00001d020419_T005	1	0.383295606	Zm00001d020419
Zm00001d020419_T007	0.9518042	0.574695079	Zm00001d020419
Zm00001d020419_T004	0.5220629	-0.26769826	Zm00001d020419
Zm00001d020419_T006	0.2329183	0.723372008	Zm00001d020419
Zm00001d012602_T001	0.9078934	0.616200251	Zm00001d012602
Zm00001d045858_T001	0.9822138	-0.288827692	Zm00001d045858
Zm00001d049362_T008	0.5122191	-0.457267807	Zm00001d049362
Zm00001d049362_T002	0.9367198	0.646091049	Zm00001d049362
Zm00001d049362_T009	0.589371	-0.566976138	Zm00001d049362
Zm00001d049362_T001	0.1432928	-1.377999715	Zm00001d049362
Zm00001d049362_T003	0.9614723	0.344614932	Zm00001d049362
Zm00001d049362_T004	0.9483904	0.36927335	Zm00001d049362
Zm00001d027454_T001	0.9695609	0.033963598	Zm00001d027454
Zm00001d020794_T001	0.9326774	-0.274712552	Zm00001d020794
Zm00001d003945_T002	0.20922	-1.110951561	Zm00001d003945
Zm00001d002708_T001	0.9828087	0.522506813	Zm00001d002708
Zm00001d006774_T001	0.9469426	0.345691562	Zm00001d006774
Zm00001d006638_T001	0.9356767	0.047717764	Zm00001d006638
Zm00001d047640_T002	0.9610121	-0.108276624	Zm00001d047640

Zm00001d047640_T004	0.9422098	0.516274708	Zm00001d047640
Zm00001d047640_T003	0.9947387	-0.096826423	Zm00001d047640
Zm00001d023836_T001	0.9181664	-0.273874023	Zm00001d023836
Zm00001d010356_T001	0.05703555	-1.292440113	Zm00001d010356
Zm00001d047614_T001	0.9843847	0.096420146	Zm00001d047614
Zm00001d053981_T001	0.6336903	-0.371316398	Zm00001d053981
Zm00001d053981_T005	0.9842144	0.077443226	Zm00001d053981
Zm00001d053981_T004	0.9648856	0.052852421	Zm00001d053981
Zm00001d050317_T001	0.7830878	-0.369461417	Zm00001d050317
Zm00001d027503_T013	0.593486	0.792805236	Zm00001d027503
Zm00001d027503_T029	1	0.172558183	Zm00001d027503
Zm00001d027503_T019	0.7805748	0.365846771	Zm00001d027503
Zm00001d027503_T042	0.3655498	-0.330240831	Zm00001d027503
Zm00001d027503_T043	0.430797	-0.304403639	Zm00001d027503
Zm00001d027503_T033	0.7934233	0.814847585	Zm00001d027503
Zm00001d027503_T021	0.9942835	0.225942172	Zm00001d027503
Zm00001d027503_T001	0.7619918	-0.393554423	Zm00001d027503
Zm00001d000072_T001	1	0.27054329	Zm00001d000072
Zm00001d023273_T001	0.9722469	0.05051984	Zm00001d023273
Zm00001d014250_T001	1	0.021882049	Zm00001d014250
Zm00001d004591_T003	0.9782953	0.257307496	Zm00001d004591
Zm00001d004591_T001	0.850642	-0.220290638	Zm00001d004591
Zm00001d004591_T004	0.8987811	-0.237711115	Zm00001d004591
Zm00001d004591_T002	0.9053391	0.49591188	Zm00001d004591
Zm00001d004591_T005	0.3381821	-0.97258538	Zm00001d004591
Zm00001d043922_T001	0.9930712	0.268306958	Zm00001d043922
Zm00001d015754_T052	0.09367236	-1.322459707	Zm00001d015754
Zm00001d015754_T053	0.7194777	0.395584322	Zm00001d015754
Zm00001d015754_T021	5.68E-10	1.073007372	Zm00001d015754
Zm00001d015754_T050	0.587235	1.035616286	Zm00001d015754
Zm00001d015754_T044	0.9070578	0.299819933	Zm00001d015754
Zm00001d018386_T005	0.4859586	-0.622416068	Zm00001d018386
Zm00001d018386_T003	0.9987435	0.262022314	Zm00001d018386
Zm00001d018386_T001	0.9770343	0.216836301	Zm00001d018386
Zm00001d023729_T003	0.7169281	0.472301278	Zm00001d023729
Zm00001d023729_T002	0.8218874	0.885811749	Zm00001d023729
Zm00001d023729_T001	0.9975882	0.405740967	Zm00001d023729
Zm00001d041951_T001	0.9611506	0.128568382	Zm00001d041951
Zm00001d013151_T001	0.01026064	-1.380220967	Zm00001d013151
Zm00001d050565_T001	0.9706887	0.102367053	Zm00001d050565
Zm00001d033637_T001	0.9766874	-0.109108392	Zm00001d033637
Zm00001d049707_T020	0.1990789	0.567335236	Zm00001d049707
Zm00001d049707_T018	0.6515872	0.904731348	Zm00001d049707
Zm00001d049707_T022	1.69E-05	-3.395054519	Zm00001d049707
Zm00001d049707_T021	1	0.353875838	Zm00001d049707
Zm00001d049707_T006	0.9173494	0.272644223	Zm00001d049707
Zm00001d049707_T002	0.5014025	1.229719446	Zm00001d049707
Zm00001d008600_T003	0.8033671	-0.195650087	Zm00001d008600
Zm00001d008600_T001	0.8863456	0.768023103	Zm00001d008600
Zm00001d044203_T003	0.6098798	0.85056723	Zm00001d044203
Zm00001d044203_T001	0.8884927	-0.050155878	Zm00001d044203
Zm00001d008906_T008	0.04992561	2.060458863	Zm00001d008906
Zm00001d008906_T010	0.09332567	-1.396864411	Zm00001d008906
Zm00001d008906_T003	0.09134785	-0.981218192	Zm00001d008906

Zm00001d008906_T001	0.09313927	2.04308561	Zm00001d008906
Zm00001d008906_T009	0.8863456	0.405675273	Zm00001d008906
Zm00001d033109_T008	6.57E-05	-2.397716874	Zm00001d033109
Zm00001d033109_T004	0.944802	0.637272868	Zm00001d033109
Zm00001d016439_T003	0.9904931	0.489974588	Zm00001d016439
Zm00001d022384_T001	0.851347	0.766856012	Zm00001d022384
Zm00001d022384_T003	0.9031129	0.669622168	Zm00001d022384
Zm00001d022384_T002	0.3394823	-0.675297362	Zm00001d022384
Zm00001d047785_T001	0.006393466	1.242985492	Zm00001d047785
Zm00001d012750_T002	0.7624517	1.040859011	Zm00001d012750
Zm00001d035222_T001	0.9514141	-0.073844667	Zm00001d035222
Zm00001d053994_T008	0.2752586	1.243442239	Zm00001d053994
Zm00001d053994_T001	0.01573827	1.039758081	Zm00001d053994
Zm00001d053994_T006	0.8529914	0.752380675	Zm00001d053994
Zm00001d053994_T007	0.9693993	0.608467986	Zm00001d053994
Zm00001d013442_T088	0.9441469	0.226748298	Zm00001d013442
Zm00001d013442_T008	4.90E-06	1.179333491	Zm00001d013442
Zm00001d013442_T012	0.603064	0.418758001	Zm00001d013442
Zm00001d013442_T078	0.715186	1.219510542	Zm00001d013442
Zm00001d045668_T001	0.9252088	-0.867806654	Zm00001d045668
Zm00001d014889_T001	0.9139882	-0.318002485	Zm00001d014889
Zm00001d005313_T001	0.9664406	0.089710481	Zm00001d005313
Zm00001d023521_T002	0.05895565	-0.662739117	Zm00001d023521
Zm00001d023521_T007	0.7003081	-0.218829948	Zm00001d023521
Zm00001d023521_T001	0.02253332	2.435951665	Zm00001d023521
Zm00001d023521_T004	0.4153853	0.898398589	Zm00001d023521
Zm00001d023521_T005	0.8660897	-0.091149481	Zm00001d023521
Zm00001d039347_T001	0.6733323	1.00219899	Zm00001d039347
Zm00001d038274_T002	0.1966199	-1.087197013	Zm00001d038274
Zm00001d038274_T001	0.567755	-0.533790521	Zm00001d038274
Zm00001d021297_T008	0.8510515	0.947350318	Zm00001d021297
Zm00001d021297_T015	0.5636783	0.714500003	Zm00001d021297
Zm00001d021297_T014	0.437339	1.601951242	Zm00001d021297
Zm00001d021297_T013	0.9213679	0.58120352	Zm00001d021297
Zm00001d021297_T001	0.2465271	1.302279067	Zm00001d021297
Zm00001d021297_T011	0.5314729	0.812264418	Zm00001d021297
Zm00001d021297_T016	0.01170181	-2.013354144	Zm00001d021297
Zm00001d040331_T001	0.9377241	0.040696863	Zm00001d040331
Zm00001d002053_T001	0.9493302	0.104827387	Zm00001d002053
Zm00001d047828_T001	0.7382728	1.183105519	Zm00001d047828
Zm00001d047828_T005	0.006079915	-1.727290008	Zm00001d047828
Zm00001d047828_T011	0.1327235	-1.786577532	Zm00001d047828
Zm00001d047828_T003	0.4319576	0.01532625	Zm00001d047828
Zm00001d046827_T002	0.424524	-0.465737236	Zm00001d046827
Zm00001d038784_T002	0.8529214	0.738327002	Zm00001d038784
Zm00001d028971_T001	0.9804228	0.197026728	Zm00001d028971
Zm00001d038471_T001	0.1017103	2.21243407	Zm00001d038471
Zm00001d028391_T001	0.6843488	1.235205948	Zm00001d028391
Zm00001d051254_T006	1	0.187049615	Zm00001d051254
Zm00001d023968_T001	0.9846714	0.28277291	Zm00001d023968
Zm00001d023968_T003	0.8162418	-0.340219944	Zm00001d023968
Zm00001d017099_T001	0.907891	-0.082615776	Zm00001d017099
Zm00001d044831_T003	0.3517338	1.072970928	Zm00001d044831
Zm00001d044831_T002	0.9201237	-0.078446082	Zm00001d044831

Zm00001d044831_T001	0.9925447	0.303404261	Zm00001d044831
Zm00001d041351_T001	0.7073686	-0.384951591	Zm00001d041351
Zm00001d022472_T001	0.3224744	-1.178475779	Zm00001d022472
Zm00001d020251_T001	0.6844831	0.720415407	Zm00001d020251
Zm00001d032280_T002	0.9290639	0.003031723	Zm00001d032280
Zm00001d035479_T001	0.3865855	1.180139765	Zm00001d035479
Zm00001d040933_T001	0.961473	0.246425374	Zm00001d040933
Zm00001d048271_T001	0.9651683	0.017729124	Zm00001d048271
Zm00001d054107_T006	0.6843488	0.710617198	Zm00001d054107
Zm00001d054107_T004	0.9609806	0.059116454	Zm00001d054107
Zm00001d054107_T002	0.9419336	0.101409604	Zm00001d054107
Zm00001d042848_T003	0.9847267	0.503264359	Zm00001d042848
Zm00001d042848_T001	0.2830537	1.749499892	Zm00001d042848
Zm00001d042848_T002	0.9701986	0.260490804	Zm00001d042848
Zm00001d024645_T001	0.9987435	0.176658607	Zm00001d024645
Zm00001d025185_T001	0.9315414	0.006852358	Zm00001d025185
Zm00001d002029_T001	0.6028805	0.840720023	Zm00001d002029
Zm00001d002029_T003	0.9979568	0.212875545	Zm00001d002029
Zm00001d002029_T002	0.7047956	-0.618262282	Zm00001d002029
Zm00001d046510_T001	0.6727562	-0.6909587	Zm00001d046510
Zm00001d043611_T001	0.9785005	0.438075117	Zm00001d043611
Zm00001d048662_T001	0.007507276	-2.231632461	Zm00001d048662
Zm00001d046687_T001	0.0678675	-2.011329355	Zm00001d046687
Zm00001d052886_T003	1.18E-05	2.096070592	Zm00001d052886
Zm00001d052886_T004	0.000339438	2.134287789	Zm00001d052886
Zm00001d052886_T001	0.09524773	2.055699852	Zm00001d052886
Zm00001d052886_T002	0.04127541	1.891177845	Zm00001d052886
Zm00001d032371_T001	0.9469426	0.644204414	Zm00001d032371
Zm00001d032371_T002	1	0.17433584	Zm00001d032371
Zm00001d027874_T009	0.7712474	-0.362876441	Zm00001d027874
Zm00001d027874_T005	0.7056782	-0.34439464	Zm00001d027874
Zm00001d027874_T004	0.02091574	-1.582063698	Zm00001d027874
Zm00001d027874_T008	0.7273213	0.440911385	Zm00001d027874
Zm00001d027874_T007	0.2662994	-0.844012037	Zm00001d027874
Zm00001d027874_T003	0.1272455	-1.317920714	Zm00001d027874
Zm00001d027874_T006	0.803912	0.360352129	Zm00001d027874
Zm00001d027874_T010	0.4976887	-0.691303359	Zm00001d027874
Zm00001d035337_T001	0.9948667	0.327737554	Zm00001d035337
Zm00001d035337_T002	0.8535962	0.918051945	Zm00001d035337
Zm00001d022387_T036	0.7029928	-0.4052588	Zm00001d022387
Zm00001d022387_T013	0.8983195	0.457358276	Zm00001d022387
Zm00001d022387_T019	0.8774597	0.328178925	Zm00001d022387
Zm00001d043827_T002	0.9756435	0.16207996	Zm00001d043827
Zm00001d043827_T004	0.9851204	0.142718642	Zm00001d043827
Zm00001d043827_T003	0.6214202	0.35875435	Zm00001d043827
Zm00001d006683_T001	0.9470929	0.064642845	Zm00001d006683
Zm00001d039591_T001	0.1090118	-1.792213058	Zm00001d039591
Zm00001d043813_T001	0.880541	0.816636693	Zm00001d043813
Zm00001d043813_T004	0.3394823	0.684803647	Zm00001d043813
Zm00001d043813_T002	0.8616387	-0.125429942	Zm00001d043813
Zm00001d043813_T003	0.7093971	0.69484899	Zm00001d043813
Zm00001d038203_T001	0.7945048	-0.480821144	Zm00001d038203
Zm00001d008842_T001	0.8545986	-0.154485248	Zm00001d008842
Zm00001d005945_T001	0.30546	-0.919459856	Zm00001d005945

Zm00001d048355_T001	0.8987811	-0.021719313	Zm00001d048355
Zm00001d047443_T003	0.9791729	-0.06472314	Zm00001d047443
Zm00001d047443_T001	0.9833565	0.274225286	Zm00001d047443
Zm00001d009701_T001	0.000347145	-2.559508282	Zm00001d009701
Zm00001d025149_T001	0.8817666	0.765558729	Zm00001d025149
Zm00001d035990_T005	0.121866	0.850898883	Zm00001d035990
Zm00001d035990_T017	0.6910721	-0.4026078	Zm00001d035990
Zm00001d035990_T006	0.9872831	0.305636388	Zm00001d035990
Zm00001d035990_T007	0.08432645	0.659600735	Zm00001d035990
Zm00001d035990_T013	0.01050386	0.787732356	Zm00001d035990
Zm00001d035990_T009	0.9361945	0.493223806	Zm00001d035990
Zm00001d035990_T002	0.8206098	0.26777423	Zm00001d035990
Zm00001d029755_T001	0.5555383	0.859332524	Zm00001d029755
Zm00001d009474_T001	0.01056074	1.886129662	Zm00001d009474
Zm00001d025011_T002	0.2903147	1.567665731	Zm00001d025011
Zm00001d025011_T003	0.1260325	1.476051525	Zm00001d025011
Zm00001d025011_T001	0.04539508	2.492272802	Zm00001d025011
Zm00001d004763_T001	0.9825858	0.131726529	Zm00001d004763
Zm00001d024854_T001	0.9176356	-0.024660878	Zm00001d024854
Zm00001d043407_T003	0.9984041	0.291194508	Zm00001d043407
Zm00001d043407_T004	0.01128066	-2.635888751	Zm00001d043407
Zm00001d009783_T002	0.183118	-0.394854012	Zm00001d009783
Zm00001d009783_T004	0.9653972	0.602733222	Zm00001d009783
Zm00001d009783_T001	0.9979568	0.169396576	Zm00001d009783
Zm00001d009783_T006	1	0.29590216	Zm00001d009783
Zm00001d002169_T011	1	0.072061682	Zm00001d002169
Zm00001d002169_T001	0.830404	0.745911148	Zm00001d002169
Zm00001d002169_T005	0.05893106	-0.965455715	Zm00001d002169
Zm00001d002169_T008	0.9173494	0.047439538	Zm00001d002169
Zm00001d002169_T004	0.02873645	1.036968137	Zm00001d002169
Zm00001d019597_T001	1	0.179209857	Zm00001d019597
Zm00001d021890_T015	0.01149774	1.783068741	Zm00001d021890
Zm00001d021890_T031	0.9186944	0.168857536	Zm00001d021890
Zm00001d021890_T037	0.5354383	0.887666743	Zm00001d021890
Zm00001d021890_T024	0.1394173	2.11990096	Zm00001d021890
Zm00001d021890_T053	0.3198828	-1.332665646	Zm00001d021890
Zm00001d021890_T017	6.76E-06	1.38580391	Zm00001d021890
Zm00001d021890_T011	0.7945048	-0.387282544	Zm00001d021890
Zm00001d021890_T052	0.9763286	0.316302066	Zm00001d021890
Zm00001d021890_T018	0.7727043	-0.346799199	Zm00001d021890
Zm00001d021890_T036	0.3431817	1.489173932	Zm00001d021890
Zm00001d021890_T029	0.9942835	0.096808816	Zm00001d021890
Zm00001d021890_T044	0.2476332	1.126223171	Zm00001d021890
Zm00001d021890_T032	0.4616313	0.295572495	Zm00001d021890
Zm00001d023828_T001	0.9968176	-0.13152627	Zm00001d023828
Zm00001d028443_T001	0.0136395	-1.504818538	Zm00001d028443
Zm00001d028443_T002	0.8807115	0.829485894	Zm00001d028443
Zm00001d052420_T001	0.1356597	2.109545477	Zm00001d052420
Zm00001d005248_T001	0.9984041	0.435712671	Zm00001d005248
Zm00001d005248_T005	0.3937219	-0.451072168	Zm00001d005248
Zm00001d005196_T001	0.9708782	-0.023934761	Zm00001d005196
Zm00001d037728_T001	0.5945614	-0.356266608	Zm00001d037728
Zm00001d044445_T001	0.929519	-0.113122035	Zm00001d044445
Zm00001d050606_T009	0.8595871	0.113778572	Zm00001d050606

Zm00001d050606_T021	1	0.38567718	Zm00001d050606
Zm00001d050606_T022	0.005601632	0.717719375	Zm00001d050606
Zm00001d050606_T035	7.89E-05	3.34232861	Zm00001d050606
Zm00001d050606_T016	0.6076098	1.583087701	Zm00001d050606
Zm00001d017317_T001	0.7942213	-0.299755634	Zm00001d017317
Zm00001d004448_T001	0.9196877	-0.012475771	Zm00001d004448
Zm00001d043773_T007	0.1818039	1.01781067	Zm00001d043773
Zm00001d043773_T001	0.6652036	-1.08827371	Zm00001d043773
Zm00001d043773_T006	0.7458694	0.657883591	Zm00001d043773
Zm00001d043773_T004	0.6158138	1.185378299	Zm00001d043773
Zm00001d012801_T002	0.9444056	0.076863565	Zm00001d012801
Zm00001d027242_T005	0.2130588	1.211555966	Zm00001d027242
Zm00001d027242_T008	0.1292507	-0.957979134	Zm00001d027242
Zm00001d027242_T004	0.9113881	0.007768181	Zm00001d027242
Zm00001d027242_T009	0.8585804	0.609412603	Zm00001d027242
Zm00001d027242_T006	0.9763597	0.453655284	Zm00001d027242
Zm00001d027242_T007	0.5292925	0.550784017	Zm00001d027242
Zm00001d027242_T011	0.7287918	-0.274884078	Zm00001d027242
Zm00001d027242_T010	0.5719089	0.904741536	Zm00001d027242
Zm00001d051887_T001	0.6156311	0.909885135	Zm00001d051887
Zm00001d051887_T002	0.9160053	0.765602624	Zm00001d051887
Zm00001d021306_T001	0.9906533	0.219577233	Zm00001d021306
Zm00001d044300_T001	0.0520257	2.453608655	Zm00001d044300
Zm00001d043864_T002	0.9031129	-0.18883621	Zm00001d043864
Zm00001d043864_T001	0.8533785	0.93571445	Zm00001d043864
Zm00001d043864_T003	0.9356767	0.602003975	Zm00001d043864
Zm00001d038797_T001	0.9029876	0.447537491	Zm00001d038797
Zm00001d038797_T003	0.4354839	0.70979188	Zm00001d038797
Zm00001d009010_T001	0.9907928	0.444636288	Zm00001d009010
Zm00001d009010_T002	0.9264959	-0.195457183	Zm00001d009010
Zm00001d002630_T002	0.3870314	1.505432638	Zm00001d002630
Zm00001d002630_T001	0.6470421	0.903938312	Zm00001d002630
Zm00001d028256_T002	0.9778436	0.282594405	Zm00001d028256
Zm00001d028256_T001	0.9048001	-0.052469832	Zm00001d028256
Zm00001d014945_T001	0.01195333	-2.23373117	Zm00001d014945
Zm00001d003004_T001	0.9942835	0.053689381	Zm00001d003004
Zm00001d025221_T001	0.497443	-0.880079737	Zm00001d025221
Zm00001d040829_T006	0.967161	0.072031808	Zm00001d040829
Zm00001d040829_T008	0.5848141	-0.688216042	Zm00001d040829
Zm00001d040829_T003	0.8585804	0.929969012	Zm00001d040829
Zm00001d022166_T001	0.9081066	0.612520366	Zm00001d022166
Zm00001d022166_T002	0.966464	0.469569669	Zm00001d022166
Zm00001d020152_T002	1	0.315879803	Zm00001d020152
Zm00001d020152_T001	1	0.331528925	Zm00001d020152
Zm00001d039111_T011	0.7315461	0.771838651	Zm00001d039111
Zm00001d039111_T008	0.6952911	-0.279421677	Zm00001d039111
Zm00001d039111_T009	0.9689939	0.482325314	Zm00001d039111
Zm00001d039111_T007	0.7328112	-0.46633734	Zm00001d039111
Zm00001d039111_T010	0.4302857	-0.86439652	Zm00001d039111
Zm00001d039111_T003	0.8625761	-0.140681237	Zm00001d039111
Zm00001d031208_T001	0.852305	0.71401657	Zm00001d031208
Zm00001d031635_T001	0.1916695	1.964374426	Zm00001d031635
Zm00001d009147_T002	0.9696556	-0.04931193	Zm00001d009147
Zm00001d009147_T001	0.9719113	0.076303451	Zm00001d009147

Zm00001d047847_T006	0.997764	0.216146166	Zm00001d047847
Zm00001d047847_T007	0.9692063	0.057982879	Zm00001d047847
Zm00001d047847_T005	0.2627228	-1.019451152	Zm00001d047847
Zm00001d047847_T010	0.4698441	-0.716092954	Zm00001d047847
Zm00001d047847_T011	0.9567316	0.2128663	Zm00001d047847
Zm00001d047847_T009	0.9979568	0.344443012	Zm00001d047847
Zm00001d047847_T002	0.5186609	-0.586884627	Zm00001d047847
Zm00001d018277_T004	0.1107948	1.349976904	Zm00001d018277
Zm00001d018277_T001	0.7717836	1.013403008	Zm00001d018277
Zm00001d018277_T002	0.8857065	0.592737199	Zm00001d018277
Zm00001d037233_T001	0.5623908	0.013395372	Zm00001d037233
Zm00001d037233_T002	0.007698854	1.105022231	Zm00001d037233
Zm00001d037233_T003	0.5996455	1.01103058	Zm00001d037233
Zm00001d037233_T004	0.7123806	0.237149091	Zm00001d037233
Zm00001d019062_T007	0.9444293	0.816407692	Zm00001d019062
Zm00001d019062_T008	0.7432107	-0.31590651	Zm00001d019062
Zm00001d019062_T001	0.7511561	1.09533311	Zm00001d019062
Zm00001d019062_T003	0.1750389	2.275211534	Zm00001d019062
Zm00001d022563_T001	0.9037324	0.448548972	Zm00001d022563
Zm00001d018813_T001	0.5069673	-0.758512369	Zm00001d018813
Zm00001d021554_T001	0.9627389	-0.034725294	Zm00001d021554
Zm00001d036831_T007	0.836992	0.770498825	Zm00001d036831
Zm00001d036831_T001	0.06744112	1.875429583	Zm00001d036831
Zm00001d036831_T012	0.8514567	0.832985059	Zm00001d036831
Zm00001d036831_T008	0.003288625	2.908764445	Zm00001d036831
Zm00001d036831_T010	0.9903234	-0.033798324	Zm00001d036831
Zm00001d048611_T001	0.9887889	0.374526723	Zm00001d048611
Zm00001d006416_T001	1	0.128642032	Zm00001d006416
Zm00001d004506_T001	0.3479706	-0.693944245	Zm00001d004506
Zm00001d053042_T001	0.7418111	-0.974963956	Zm00001d053042
Zm00001d002781_T003	0.8790359	0.881612745	Zm00001d002781
Zm00001d002781_T014	0.7312547	1.01796306	Zm00001d002781
Zm00001d002781_T011	0.6984737	0.292875876	Zm00001d002781
Zm00001d002781_T027	0.2097738	-1.189595244	Zm00001d002781
Zm00001d002781_T023	0.9887889	0.311394945	Zm00001d002781
Zm00001d003144_T001	0.3193819	-0.647064825	Zm00001d003144
Zm00001d042968_T003	0.9627507	-0.170271633	Zm00001d042968
Zm00001d042968_T001	0.9690925	0.372392836	Zm00001d042968
Zm00001d042968_T002	0.9493302	-0.16502083	Zm00001d042968
Zm00001d020869_T001	0.9840043	0.317586757	Zm00001d020869
Zm00001d053345_T001	0.9708651	-0.006545328	Zm00001d053345
Zm00001d039852_T001	0.98291	0.484906168	Zm00001d039852
Zm00001d049011_T001	0.5573674	-0.600764813	Zm00001d049011
Zm00001d024940_T001	0.4081094	-0.747259658	Zm00001d024940
Zm00001d048536_T011	0.973788	0.141086128	Zm00001d048536
Zm00001d048536_T004	1	0.245836207	Zm00001d048536
Zm00001d048536_T001	0.9666757	0.425095143	Zm00001d048536
Zm00001d048536_T006	0.6946859	0.360891508	Zm00001d048536
Zm00001d048536_T012	0.8926364	-0.182735538	Zm00001d048536
Zm00001d011242_T003	0.9279014	-0.031925212	Zm00001d011242
Zm00001d005846_T003	0.9970835	0.200812661	Zm00001d005846
Zm00001d030303_T001	0.652583	-0.449931857	Zm00001d030303
Zm00001d033272_T008	0.8676122	0.447488256	Zm00001d033272
Zm00001d033272_T007	0.6131963	0.470714246	Zm00001d033272

Zm00001d033272_T006	0.9846714	0.323484282	Zm00001d033272
Zm00001d033272_T001	0.1633816	1.432938558	Zm00001d033272
Zm00001d033272_T003	0.8603652	0.878748849	Zm00001d033272
Zm00001d033272_T009	0.005578393	0.960318853	Zm00001d033272
Zm00001d013712_T005	0.9616224	0.097181981	Zm00001d013712
Zm00001d013712_T001	0.004393443	-2.626538044	Zm00001d013712
Zm00001d013712_T002	0.9781672	0.468355973	Zm00001d013712
Zm00001d052720_T008	0.9804228	0.146960877	Zm00001d052720
Zm00001d052720_T001	0.3758079	-0.914805214	Zm00001d052720
Zm00001d052720_T003	0.957148	0.384951446	Zm00001d052720
Zm00001d043217_T001	0.9770343	-0.2360925	Zm00001d043217
Zm00001d041714_T001	0.9573434	0.042952162	Zm00001d041714
Zm00001d024494_T001	0.00213664	2.875864588	Zm00001d024494
Zm00001d045022_T001	0.03347087	2.141021208	Zm00001d045022
Zm00001d051595_T004	0.9895224	0.379901347	Zm00001d051595
Zm00001d051595_T001	0.9822138	0.265741247	Zm00001d051595
Zm00001d043525_T001	0.6500235	0.956826789	Zm00001d043525
Zm00001d038794_T002	0.9404125	0.3730943	Zm00001d038794
Zm00001d036230_T001	0.9945118	0.25689714	Zm00001d036230
Zm00001d004132_T001	0.9380783	0.655256979	Zm00001d004132
Zm00001d011710_T001	0.8001345	-0.494439552	Zm00001d011710
Zm00001d042340_T001	0.9979568	0.087779576	Zm00001d042340
Zm00001d004489_T001	1	0.289704795	Zm00001d004489
Zm00001d039218_T002	0.6182353	-0.331642916	Zm00001d039218
Zm00001d011644_T002	0.01744491	0.448817031	Zm00001d011644
Zm00001d049987_T001	0.2908902	-0.910438552	Zm00001d049987
Zm00001d033557_T004	0.997764	0.269330729	Zm00001d033557
Zm00001d033557_T003	1	0.087755098	Zm00001d033557
Zm00001d033557_T006	3.22E-05	-2.866670949	Zm00001d033557
Zm00001d003039_T002	0.6086477	1.093954389	Zm00001d003039
Zm00001d003039_T001	0.8616387	0.822778543	Zm00001d003039
Zm00001d035008_T002	0.5942507	0.524294232	Zm00001d035008
Zm00001d035008_T004	0.6042252	0.37649254	Zm00001d035008
Zm00001d035008_T003	0.5706593	-0.365787752	Zm00001d035008
Zm00001d035008_T001	0.8593759	-0.083344479	Zm00001d035008
Zm00001d000019_T001	0.9130008	2.025580451	Zm00001d000019
Zm00001d009402_T001	0.425485	-0.772614783	Zm00001d009402
Zm00001d016947_T001	0.000135657	3.099215541	Zm00001d016947
Zm00001d042058_T001	0.9768802	0.263543615	Zm00001d042058
Zm00001d047214_T001	0.7598721	0.959419561	Zm00001d047214
Zm00001d037842_T001	0.9988864	0.283341325	Zm00001d037842
Zm00001d011454_T001	0.2061598	1.733678746	Zm00001d011454
Zm00001d031625_T001	0.9419336	1.858675255	Zm00001d031625
Zm00001d042908_T001	0.8643636	0.73753865	Zm00001d042908
Zm00001d042908_T004	0.9710693	0.154131603	Zm00001d042908
Zm00001d027841_T002	0.9889197	0.237640295	Zm00001d027841
Zm00001d022127_T001	0.7707507	1.141183975	Zm00001d022127
Zm00001d047209_T011	2.92E-07	4.728464004	Zm00001d047209
Zm00001d047209_T020	1.01E-08	3.482334412	Zm00001d047209
Zm00001d047209_T013	0.06279624	1.924770451	Zm00001d047209
Zm00001d047209_T021	1.37E-07	1.240472847	Zm00001d047209
Zm00001d047209_T006	0.4268464	1.243778575	Zm00001d047209
Zm00001d047209_T022	0.5850461	1.379502976	Zm00001d047209
Zm00001d047209_T014	0.000274348	1.694772193	Zm00001d047209

Zm00001d002428_T001	0.9065408	-0.374576564	Zm00001d002428
Zm00001d002428_T002	0.9693993	-0.069111262	Zm00001d002428
Zm00001d003477_T001	0.7799713	-0.215968293	Zm00001d003477
Zm00001d003477_T002	0.4792677	0.55815394	Zm00001d003477
Zm00001d043312_T005	0.8086794	0.322957166	Zm00001d043312
Zm00001d043312_T009	1	0.408184705	Zm00001d043312
Zm00001d043312_T011	0.9956349	0.311614249	Zm00001d043312
Zm00001d043312_T001	0.2970714	1.525725103	Zm00001d043312
Zm00001d037382_T001	0.6414865	0.588692607	Zm00001d037382
Zm00001d021061_T001	0.7659546	-0.420317187	Zm00001d021061
Zm00001d041108_T002	1	0.416167342	Zm00001d041108
Zm00001d041108_T021	0.3458718	0.561862704	Zm00001d041108
Zm00001d041108_T012	0.7812469	0.274963607	Zm00001d041108
Zm00001d041108_T006	0.5092251	-1.07014187	Zm00001d041108
Zm00001d041108_T003	0.7518152	-0.070850076	Zm00001d041108
Zm00001d013028_T001	0.9842144	0.149607142	Zm00001d013028
Zm00001d019039_T002	0.9039795	-0.103498053	Zm00001d019039
Zm00001d013523_T001	0.9310695	-0.017718087	Zm00001d013523
Zm00001d013523_T002	0.08348055	1.803615423	Zm00001d013523
Zm00001d026695_T001	0.65541	-0.444117777	Zm00001d026695
Zm00001d026695_T002	0.9370633	-0.042825033	Zm00001d026695
Zm00001d008501_T001	1	0.227081212	Zm00001d008501
Zm00001d015306_T001	1	0.328952267	Zm00001d015306
Zm00001d040298_T001	0.749631	-0.185150073	Zm00001d040298
Zm00001d032239_T001	0.8885217	-0.193475361	Zm00001d032239
Zm00001d032667_T001	0.4656187	0.475873808	Zm00001d032667
Zm00001d032667_T005	0.9029473	0.857640223	Zm00001d032667
Zm00001d032667_T010	1	0.446198595	Zm00001d032667
Zm00001d032667_T007	0.04500811	-0.370071118	Zm00001d032667
Zm00001d010727_T001	0.4639548	-0.689345834	Zm00001d010727
Zm00001d010997_T001	0.9561521	0.626120204	Zm00001d010997
Zm00001d010210_T002	0.9732421	0.090510659	Zm00001d010210
Zm00001d010210_T001	0.8093695	-0.068825624	Zm00001d010210
Zm00001d016025_T001	0.9404125	0.420908578	Zm00001d016025
Zm00001d052210_T001	0.7952435	-0.339030249	Zm00001d052210
Zm00001d037826_T001	0.8412913	0.414366382	Zm00001d037826
Zm00001d007501_T001	0.9768751	0.122238822	Zm00001d007501
Zm00001d034017_T001	0.9249382	0.788723215	Zm00001d034017
Zm00001d034017_T002	0.9782953	0.561908863	Zm00001d034017
Zm00001d027256_T001	0.3020017	-0.704330055	Zm00001d027256
Zm00001d029922_T001	0.8660897	-0.116923501	Zm00001d029922
Zm00001d029922_T002	0.7056513	-0.862925476	Zm00001d029922
Zm00001d049817_T001	0.9220919	-0.01094386	Zm00001d049817
Zm00001d049817_T007	0.03058431	0.662685644	Zm00001d049817
Zm00001d049817_T006	0.9690925	-0.023894189	Zm00001d049817
Zm00001d049817_T004	0.9968176	0.301746773	Zm00001d049817
Zm00001d011514_T001	1	0.290297622	Zm00001d011514
Zm00001d011514_T002	0.9173494	0.47662307	Zm00001d011514
Zm00001d005547_T003	0.9541864	0.009668969	Zm00001d005547
Zm00001d005547_T001	0.9529094	0.576719332	Zm00001d005547
Zm00001d005547_T002	0.9690925	0.208255759	Zm00001d005547
Zm00001d023384_T001	0.07142513	-1.778810578	Zm00001d023384
Zm00001d041362_T001	0.2066496	1.17715302	Zm00001d041362
Zm00001d041362_T003	0.8580945	-0.028407073	Zm00001d041362

Zm00001d028241_T003	0.885586	0.818930913	Zm00001d028241
Zm00001d028241_T001	0.908501	0.314898378	Zm00001d028241
Zm00001d018414_T001	0.4332539	-0.863722511	Zm00001d018414
Zm00001d003463_T001	0.00010633	3.663179679	Zm00001d003463
Zm00001d002542_T001	0.9221549	0.748621197	Zm00001d002542
Zm00001d038970_T002	1	0.382189566	Zm00001d038970
Zm00001d041142_T031	0.9804228	0.315108346	Zm00001d041142
Zm00001d041142_T005	0.580364	0.467549816	Zm00001d041142
Zm00001d041142_T003	0.7737085	1.001772584	Zm00001d041142
Zm00001d041142_T006	0.7954824	-0.189791384	Zm00001d041142
Zm00001d041142_T001	0.9690925	0.065634915	Zm00001d041142
Zm00001d045951_T001	0.9572871	-0.202015373	Zm00001d045951
Zm00001d022190_T002	0.941922	0.657769061	Zm00001d022190
Zm00001d022190_T012	0.9689939	0.35175962	Zm00001d022190
Zm00001d022190_T006	0.5790787	-0.563903018	Zm00001d022190
Zm00001d022190_T001	0.9954444	0.402751156	Zm00001d022190
Zm00001d022190_T007	0.5186912	0.64309551	Zm00001d022190
Zm00001d022190_T014	0.8660897	-0.425209202	Zm00001d022190
Zm00001d044545_T001	0.9514141	-0.419899867	Zm00001d044545
Zm00001d010385_T001	0.01359289	1.775180021	Zm00001d010385
Zm00001d033148_T001	0.9608605	0.457755478	Zm00001d033148
Zm00001d013908_T003	0.5368112	-0.956319826	Zm00001d013908
Zm00001d013908_T001	0.001618499	0.946208348	Zm00001d013908
Zm00001d013908_T002	0.3060739	1.54533501	Zm00001d013908
Zm00001d007851_T001	0.8321137	0.784726561	Zm00001d007851
Zm00001d011758_T001	0.7567574	-0.269563727	Zm00001d011758
Zm00001d047942_T001	1	0.244358876	Zm00001d047942
Zm00001d047942_T002	0.7972183	0.948981602	Zm00001d047942
Zm00001d036756_T001	1	0.366434091	Zm00001d036756
Zm00001d012996_T001	1	0.236183974	Zm00001d012996
Zm00001d020971_T001	0.9842144	0.372420496	Zm00001d020971
Zm00001d009539_T002	0.9979568	0.48067447	Zm00001d009539
Zm00001d009539_T001	0.9817074	0.213456788	Zm00001d009539
Zm00001d033165_T001	0.9812314	-0.108027783	Zm00001d033165
Zm00001d044575_T001	0.8936314	-0.190607504	Zm00001d044575
Zm00001d038143_T002	0.9326774	0.711515911	Zm00001d038143
Zm00001d038143_T001	1	0.182872188	Zm00001d038143
Zm00001d038541_T002	0.8511208	0.933725067	Zm00001d038541
Zm00001d038541_T001	0.9502794	0.164724001	Zm00001d038541
Zm00001d009578_T001	0.9171732	-0.302894794	Zm00001d009578
Zm00001d018144_T001	0.8160996	-0.099830793	Zm00001d018144
Zm00001d018144_T004	0.9914036	0.438149457	Zm00001d018144
Zm00001d012757_T001	0.9673473	0.112672333	Zm00001d012757
Zm00001d003214_T009	0.000315727	2.04830572	Zm00001d003214
Zm00001d003214_T014	0.3675057	0.861368833	Zm00001d003214
Zm00001d003214_T003	0.000218545	-1.710129684	Zm00001d003214
Zm00001d003214_T004	0.9237921	0.29663665	Zm00001d003214
Zm00001d003214_T015	0.02719955	-0.389884362	Zm00001d003214
Zm00001d003214_T001	0.9872831	0.502581881	Zm00001d003214
Zm00001d003214_T002	1	0.168727297	Zm00001d003214
Zm00001d003214_T010	0.7946251	0.389271234	Zm00001d003214
Zm00001d011377_T002	0.2208276	1.824378586	Zm00001d011377
Zm00001d007092_T001	0.9416622	0.725801297	Zm00001d007092
Zm00001d007092_T002	1	0.20731387	Zm00001d007092

Zm00001d025153_T012	0.9583056	-0.024426969	Zm00001d025153
Zm00001d025153_T005	0.8874701	-0.079388336	Zm00001d025153
Zm00001d025153_T002	0.9835181	0.115701577	Zm00001d025153
Zm00001d025153_T007	7.37E-05	0.692692878	Zm00001d025153
Zm00001d025153_T006	0.9766874	0.105686371	Zm00001d025153
Zm00001d021600_T003	0.1950386	0.576774896	Zm00001d021600
Zm00001d021600_T002	0.9501111	0.11543629	Zm00001d021600
Zm00001d021600_T004	0.7694579	0.953402886	Zm00001d021600
Zm00001d021600_T008	0.7157882	-0.426448986	Zm00001d021600
Zm00001d041917_T001	0.9262099	-0.441534122	Zm00001d041917
Zm00001d015389_T001	0.08002632	0.818167318	Zm00001d015389
Zm00001d015389_T002	1	0.335587095	Zm00001d015389
Zm00001d011445_T006	0.9497509	0.379162639	Zm00001d011445
Zm00001d011445_T002	0.1004743	-0.351738925	Zm00001d011445
Zm00001d011445_T003	0.9706887	0.196780141	Zm00001d011445
Zm00001d014126_T001	0.9702062	0.140343452	Zm00001d014126
Zm00001d034062_T001	0.674301	-0.511151438	Zm00001d034062
Zm00001d031940_T001	0.958561	0.69731979	Zm00001d031940
Zm00001d052835_T001	0.8976475	0.005059236	Zm00001d052835
Zm00001d035618_T001	0.4995495	-0.931265249	Zm00001d035618
Zm00001d036698_T001	0.937572	0.025034285	Zm00001d036698
Zm00001d027621_T001	0.6789826	-0.372586403	Zm00001d027621
Zm00001d031216_T005	0.9050121	-0.115593327	Zm00001d031216
Zm00001d031216_T002	0.9843847	0.190487943	Zm00001d031216
Zm00001d031216_T004	0.3544526	0.742475363	Zm00001d031216
Zm00001d004931_T001	0.9846714	0.547149155	Zm00001d004931
Zm00001d011605_T002	0.9747606	-0.085803911	Zm00001d011605
Zm00001d047535_T001	0.9179118	0.657897907	Zm00001d047535
Zm00001d053515_T007	0.9182861	0.057444781	Zm00001d053515
Zm00001d053515_T006	1.28E-09	1.62970188	Zm00001d053515
Zm00001d053515_T005	1	0.284177278	Zm00001d053515
Zm00001d053515_T009	0.05662343	2.123969967	Zm00001d053515
Zm00001d053515_T014	0.7594555	0.178770234	Zm00001d053515
Zm00001d053515_T011	0.785169	-0.312830901	Zm00001d053515
Zm00001d036013_T004	0.9050366	0.867663492	Zm00001d036013
Zm00001d041982_T001	0.9210327	-0.016059137	Zm00001d041982
Zm00001d022448_T001	0.9441469	0.153273601	Zm00001d022448
Zm00001d051746_T001	0.8127169	-0.164501086	Zm00001d051746
Zm00001d002003_T002	0.7546751	0.851754329	Zm00001d002003
Zm00001d002003_T008	0.8442078	-0.144799415	Zm00001d002003
Zm00001d007129_T001	0.9604309	0.053921148	Zm00001d007129
Zm00001d006904_T001	0.8031436	-0.340995257	Zm00001d006904
Zm00001d026326_T001	0.9180662	0.7689568	Zm00001d026326
Zm00001d016724_T001	0.3495559	-0.837940475	Zm00001d016724
Zm00001d006130_T001	0.04346118	2.515809291	Zm00001d006130
Zm00001d037289_T003	0.8932144	-0.022035553	Zm00001d037289
Zm00001d015338_T001	0.681302	-0.413018843	Zm00001d015338
Zm00001d005925_T003	0.01193426	1.565127056	Zm00001d005925
Zm00001d005925_T004	0.002291221	2.637080886	Zm00001d005925
Zm00001d005925_T002	0.8125642	1.045323651	Zm00001d005925
Zm00001d049464_T001	0.4237296	-0.883026702	Zm00001d049464
Zm00001d017528_T001	0.8720862	0.855663426	Zm00001d017528
Zm00001d047679_T005	0.9745777	0.503324838	Zm00001d047679
Zm00001d047679_T004	0.000584599	1.225588852	Zm00001d047679

Zm00001d053938_T001	0.005570846	2.356869192	Zm00001d053938
Zm00001d026477_T006	0.001736934	2.390387672	Zm00001d026477
Zm00001d026477_T003	0.9702242	0.436579493	Zm00001d026477
Zm00001d026477_T007	0.06759318	1.695777849	Zm00001d026477
Zm00001d026477_T002	0.9889811	0.389798821	Zm00001d026477
Zm00001d023507_T001	0.9573434	0.097513189	Zm00001d023507
Zm00001d013261_T001	0.09348184	2.068666289	Zm00001d013261
Zm00001d027341_T001	0.7667736	0.802576439	Zm00001d027341
Zm00001d006751_T005	0.4812187	1.434113698	Zm00001d006751
Zm00001d006751_T004	0.9766874	0.205286692	Zm00001d006751
Zm00001d006751_T003	0.7573198	0.879707676	Zm00001d006751
Zm00001d006751_T008	0.4080185	-0.873687198	Zm00001d006751
Zm00001d006751_T001	0.9175896	0.013410961	Zm00001d006751
Zm00001d006751_T007	0.02424149	0.446553005	Zm00001d006751
Zm00001d042438_T001	0.06639461	1.680031004	Zm00001d042438
Zm00001d016479_T036	0.7594555	-0.454033798	Zm00001d016479
Zm00001d016479_T035	0.8280859	0.324337551	Zm00001d016479
Zm00001d016479_T034	1	0.327754216	Zm00001d016479
Zm00001d016479_T016	0.718063	1.130138857	Zm00001d016479
Zm00001d051130_T001	0.4748531	-0.256420924	Zm00001d051130
Zm00001d024906_T001	0.7099224	-0.521965569	Zm00001d024906
Zm00001d041220_T001	0.9073468	-0.247154235	Zm00001d041220
Zm00001d047664_T001	0.1058486	2.100593374	Zm00001d047664
Zm00001d012021_T001	0.7030641	-0.465914516	Zm00001d012021
Zm00001d008479_T001	0.3547256	-1.065790331	Zm00001d008479
Zm00001d032209_T002	0.9051982	0.393644925	Zm00001d032209
Zm00001d052977_T001	0.9223663	0.032441537	Zm00001d052977
Zm00001d053171_T001	0.9974023	0.416144873	Zm00001d053171
Zm00001d018107_T001	0.06345236	2.182410301	Zm00001d018107
Zm00001d035065_T011	0.7020458	0.510853485	Zm00001d035065
Zm00001d035065_T001	0.9768996	0.605802818	Zm00001d035065
Zm00001d002275_T045	0.7746801	-0.228488605	Zm00001d002275
Zm00001d002275_T027	5.95E-09	0.889809631	Zm00001d002275
Zm00001d002275_T012	1.11E-06	0.932892419	Zm00001d002275
Zm00001d002275_T037	9.01E-14	2.480440308	Zm00001d002275
Zm00001d002275_T031	0.675716	1.179814019	Zm00001d002275
Zm00001d002275_T042	0.00965634	2.830184057	Zm00001d002275
Zm00001d002275_T002	0.01425967	2.249577133	Zm00001d002275
Zm00001d002275_T014	0.003257352	2.67027673	Zm00001d002275
Zm00001d002275_T028	8.47E-07	0.456579116	Zm00001d002275
Zm00001d045147_T019	0.0960361	1.405024887	Zm00001d045147
Zm00001d045147_T021	0.3865855	0.685886256	Zm00001d045147
Zm00001d045147_T015	0.983226	0.24605947	Zm00001d045147
Zm00001d045147_T011	0.7382905	-0.067607427	Zm00001d045147
Zm00001d045147_T023	0.30546	0.513344786	Zm00001d045147
Zm00001d045147_T001	0.9573272	0.183779123	Zm00001d045147
Zm00001d044329_T002	0.8569937	-0.72151833	Zm00001d044329
Zm00001d044329_T001	0.9399572	0.412906908	Zm00001d044329
Zm00001d044329_T004	0.9469426	0.181560423	Zm00001d044329
Zm00001d004839_T005	0.01328455	-1.091021877	Zm00001d004839
Zm00001d004839_T007	1	0.559321327	Zm00001d004839
Zm00001d004839_T003	2.74E-07	-2.716378616	Zm00001d004839
Zm00001d017958_T003	0.9217388	2.8111118	Zm00001d017958
Zm00001d017958_T001	0.9988864	0.335502576	Zm00001d017958

Zm00001d017958_T002	0.01284021	-0.864518701	Zm00001d017958
Zm00001d017958_T004	2.73E-05	-1.468872505	Zm00001d017958
Zm00001d022072_T002	0.7069334	-0.29474807	Zm00001d022072
Zm00001d022072_T001	0.9160053	0.551935922	Zm00001d022072
Zm00001d030498_T001	0.02462255	-0.761183026	Zm00001d030498
Zm00001d047514_T001	0.8114042	0.87691582	Zm00001d047514
Zm00001d031926_T001	1	0.417538	Zm00001d031926
Zm00001d029768_T001	0.9488326	-0.234771576	Zm00001d029768
Zm00001d015412_T001	0.9466261	-0.147150586	Zm00001d015412
Zm00001d038522_T001	0.1613319	-1.13251111	Zm00001d038522
Zm00001d038522_T002	0.01535282	2.93645232	Zm00001d038522
Zm00001d003352_T004	0.9919298	0.11140301	Zm00001d003352
Zm00001d003352_T001	0.9894615	0.256173671	Zm00001d003352
Zm00001d003352_T003	0.04099654	-1.142056133	Zm00001d003352
Zm00001d040088_T001	0.9451369	-0.030008293	Zm00001d040088
Zm00001d032284_T002	0.8827869	0.420350085	Zm00001d032284
Zm00001d013223_T004	0.6354704	-0.880636082	Zm00001d013223
Zm00001d013223_T006	0.9686323	0.172555297	Zm00001d013223
Zm00001d013223_T001	0.8548987	-0.145391323	Zm00001d013223
Zm00001d047787_T001	0.9404125	-0.138481528	Zm00001d047787
Zm00001d024538_T001	1	0.132376525	Zm00001d024538
Zm00001d011799_T001	0.960704	-0.224840561	Zm00001d011799
Zm00001d014351_T001	0.9791729	0.294239729	Zm00001d014351
Zm00001d021627_T001	0.9825858	0.053424308	Zm00001d021627
Zm00001d034005_T004	0.8310488	-0.156604893	Zm00001d034005
Zm00001d034005_T006	0.1824842	-1.212176608	Zm00001d034005
Zm00001d034005_T001	0.7912425	-0.169900203	Zm00001d034005
Zm00001d034005_T003	0.8964117	0.873376858	Zm00001d034005
Zm00001d006353_T001	0.465408	-0.670260394	Zm00001d006353
Zm00001d002650_T006	0.6158972	0.610259434	Zm00001d002650
Zm00001d002650_T004	0.9232281	0.557829495	Zm00001d002650
Zm00001d002650_T009	0.3919095	0.298462837	Zm00001d002650
Zm00001d002650_T016	0.6444213	1.557692292	Zm00001d002650
Zm00001d002650_T064	0.957148	-0.008029535	Zm00001d002650
Zm00001d002650_T011	0.9416089	0.156900394	Zm00001d002650
Zm00001d002650_T015	0.8112903	-0.10296023	Zm00001d002650
Zm00001d002650_T063	2.20E-05	-3.165503565	Zm00001d002650
Zm00001d022082_T001	0.1057904	2.117967463	Zm00001d022082
Zm00001d025166_T002	0.7146325	1.123475193	Zm00001d025166
Zm00001d025166_T001	0.8842271	1.336342386	Zm00001d025166
Zm00001d027290_T001	0.05839366	-1.84671378	Zm00001d027290
Zm00001d048765_T001	0.3753965	1.617888604	Zm00001d048765
Zm00001d034013_T020	0.9246704	0.319208155	Zm00001d034013
Zm00001d034013_T017	0.000108956	0.654849017	Zm00001d034013
Zm00001d034013_T010	0.2157263	-0.988554488	Zm00001d034013
Zm00001d017106_T001	1	0.413096002	Zm00001d017106
Zm00001d017106_T003	0.8192173	0.630287515	Zm00001d017106
Zm00001d017106_T005	0.5140052	1.026954681	Zm00001d017106
Zm00001d017106_T002	0.366346	0.579599438	Zm00001d017106
Zm00001d010290_T001	1	0.140387763	Zm00001d010290
Zm00001d025235_T002	0.3631774	1.581569435	Zm00001d025235
Zm00001d025235_T020	1.91E-12	-2.184151342	Zm00001d025235
Zm00001d025235_T007	4.95E-14	3.286712945	Zm00001d025235
Zm00001d025235_T021	0.5220513	0.343550907	Zm00001d025235

Zm00001d033600_T002	0.9249526	-0.134384582	Zm00001d033600
Zm00001d033600_T001	0.9493302	0.276097613	Zm00001d033600
Zm00001d027514_T004	0.9580876	0.303862292	Zm00001d027514
Zm00001d027514_T003	0.07600847	-1.427586552	Zm00001d027514
Zm00001d027514_T002	0.9419336	0.703200251	Zm00001d027514
Zm00001d017165_T001	1.62E-09	-1.962353639	Zm00001d017165
Zm00001d017165_T004	0.7297076	0.284985552	Zm00001d017165
Zm00001d017165_T002	0.9050121	0.737399969	Zm00001d017165
Zm00001d005470_T001	0.6625248	0.604087371	Zm00001d005470
Zm00001d042433_T001	0.8720862	-0.229858181	Zm00001d042433
Zm00001d012451_T001	0.9845789	0.212057573	Zm00001d012451
Zm00001d035467_T001	0.5773	1.216727129	Zm00001d035467
Zm00001d010908_T001	0.8811401	0.748254585	Zm00001d010908
Zm00001d015181_T001	0.9936409	0.203143545	Zm00001d015181
Zm00001d002534_T003	0.8535962	-0.112075097	Zm00001d002534
Zm00001d002534_T004	1	0.04624083	Zm00001d002534
Zm00001d002258_T001	0.9966571	0.45070164	Zm00001d002258
Zm00001d002258_T003	0.6135035	-0.409088299	Zm00001d002258
Zm00001d044910_T001	0.2961799	1.082284841	Zm00001d044910
Zm00001d043370_T002	0.8851287	-0.322353055	Zm00001d043370
Zm00001d052028_T038	0.5092251	1.041560129	Zm00001d052028
Zm00001d052028_T044	0.9693993	0.121619253	Zm00001d052028
Zm00001d052028_T002	0.9219195	0.671022421	Zm00001d052028
Zm00001d052028_T042	0.9468268	0.587006372	Zm00001d052028
Zm00001d052028_T028	0.7146325	0.362156985	Zm00001d052028
Zm00001d052028_T013	0.5901362	0.349574449	Zm00001d052028
Zm00001d026286_T001	0.7490886	-0.718122867	Zm00001d026286
Zm00001d004466_T006	0.8884927	0.119748886	Zm00001d004466
Zm00001d004466_T013	0.7297076	0.223750064	Zm00001d004466
Zm00001d004466_T023	0.9920932	0.481003679	Zm00001d004466
Zm00001d004466_T022	0.5901362	0.562137487	Zm00001d004466
Zm00001d004466_T015	0.9927338	0.000620126	Zm00001d004466
Zm00001d004466_T001	0.9073468	0.950816197	Zm00001d004466
Zm00001d028773_T001	0.9849018	-0.022337379	Zm00001d028773
Zm00001d017130_T001	0.9760472	0.101719862	Zm00001d017130
Zm00001d017130_T002	0.9835413	0.133140395	Zm00001d017130
Zm00001d004198_T001	0.7737238	-0.455841358	Zm00001d004198
Zm00001d052247_T001	0.9979568	0.250281163	Zm00001d052247
Zm00001d038034_T001	0.9529184	0.382480868	Zm00001d038034
Zm00001d044510_T031	2.42E-16	-4.876026752	Zm00001d044510
Zm00001d044510_T022	0.6993528	0.315312066	Zm00001d044510
Zm00001d044510_T006	0.884967	0.284715281	Zm00001d044510
Zm00001d044510_T030	0.9054108	0.611431195	Zm00001d044510
Zm00001d044510_T002	0.9273674	0.08596366	Zm00001d044510
Zm00001d044510_T015	4.25E-05	1.496329253	Zm00001d044510
Zm00001d044510_T005	0.8486887	0.201777484	Zm00001d044510
Zm00001d016305_T001	1	0.191330934	Zm00001d016305
Zm00001d016180_T001	0.9100328	-0.058588383	Zm00001d016180
Zm00001d052157_T019	0.3736788	-0.810377467	Zm00001d052157
Zm00001d052157_T004	0.3342103	0.182722803	Zm00001d052157
Zm00001d052157_T006	0.5537579	0.710968461	Zm00001d052157
Zm00001d052157_T009	0.6160016	0.577177384	Zm00001d052157
Zm00001d052157_T015	0.9257893	0.257700522	Zm00001d052157
Zm00001d032766_T002	6.56E-06	0.694140214	Zm00001d032766

Zm00001d025081_T001	0.00013693	-3.326711422	Zm00001d025081
Zm00001d025081_T004	3.03E-09	-3.980751774	Zm00001d025081
Zm00001d009316_T002	0.5209964	-1.107116624	Zm00001d009316
Zm00001d009316_T001	0.9488298	0.194233256	Zm00001d009316
Zm00001d045082_T001	0.2599791	1.52817132	Zm00001d045082
Zm00001d045082_T002	0.8774597	0.861004936	Zm00001d045082
Zm00001d045374_T001	0.5083738	-0.897956431	Zm00001d045374
Zm00001d005802_T001	0.9628835	-0.402717086	Zm00001d005802
Zm00001d039518_T009	0.9899276	0.684695477	Zm00001d039518
Zm00001d039518_T013	0.4246306	-0.871972268	Zm00001d039518
Zm00001d039518_T002	0.9196877	0.925877809	Zm00001d039518
Zm00001d039518_T012	6.01E-15	-4.02262628	Zm00001d039518
Zm00001d039518_T005	0.07821005	1.666628547	Zm00001d039518
Zm00001d043736_T003	0.8092097	0.385603278	Zm00001d043736
Zm00001d043736_T004	0.7369755	-0.335061334	Zm00001d043736
Zm00001d043736_T002	0.9945118	0.245270314	Zm00001d043736
Zm00001d053838_T004	0.000633105	-1.647711967	Zm00001d053838
Zm00001d053838_T001	0.9311027	0.699591928	Zm00001d053838
Zm00001d053838_T002	0.9612591	0.214693262	Zm00001d053838
Zm00001d053838_T005	0.8262962	0.050435765	Zm00001d053838
Zm00001d053838_T003	0.9333149	0.059201657	Zm00001d053838
Zm00001d044101_T001	2.04E-07	-1.352278578	Zm00001d044101
Zm00001d044101_T002	0.4357247	1.298798929	Zm00001d044101
Zm00001d002576_T001	0.4099235	0.981080772	Zm00001d002576
Zm00001d029139_T001	0.9618822	0.049353355	Zm00001d029139
Zm00001d003957_T002	0.9749249	0.029964087	Zm00001d003957
Zm00001d005347_T002	0.8382663	0.481685182	Zm00001d005347
Zm00001d005347_T001	0.9930712	0.481523934	Zm00001d005347
Zm00001d042916_T002	0.007336381	0.341972038	Zm00001d042916
Zm00001d042916_T003	0.9210513	0.655539157	Zm00001d042916
Zm00001d005026_T001	0.7585131	-0.126132359	Zm00001d005026
Zm00001d005026_T002	0.9184323	-0.083228498	Zm00001d005026
Zm00001d005026_T004	0.9673094	0.122852583	Zm00001d005026
Zm00001d035658_T001	0.8515792	-0.179941584	Zm00001d035658
Zm00001d046169_T001	0.6896698	-0.78833536	Zm00001d046169
Zm00001d026509_T001	0.66511	-0.300790049	Zm00001d026509
Zm00001d012472_T001	0.6718185	-0.466800429	Zm00001d012472
Zm00001d043202_T004	0.9380783	0.056687222	Zm00001d043202
Zm00001d043202_T002	0.6639456	-0.397134967	Zm00001d043202
Zm00001d048968_T001	1	0.343280098	Zm00001d048968
Zm00001d021356_T001	0.9991964	1.579457972	Zm00001d021356
Zm00001d037734_T001	0.8267985	0.953961317	Zm00001d037734
Zm00001d037734_T006	0.9311027	0.019284892	Zm00001d037734
Zm00001d037734_T011	0.007404649	0.484036119	Zm00001d037734
Zm00001d037734_T007	1	0.089421971	Zm00001d037734
Zm00001d009383_T007	0.9570177	0.351954504	Zm00001d009383
Zm00001d009383_T002	0.7085447	-0.368225569	Zm00001d009383
Zm00001d036570_T001	0.7904254	-0.30155134	Zm00001d036570
Zm00001d022466_T006	0.850642	-0.259676771	Zm00001d022466
Zm00001d025571_T001	0.9429712	0.045142526	Zm00001d025571
Zm00001d012177_T001	0.9914912	0.242880246	Zm00001d012177
Zm00001d050033_T002	0.7979203	0.806136786	Zm00001d050033
Zm00001d009693_T003	0.7059983	1.138237331	Zm00001d009693
Zm00001d009693_T002	0.3097895	1.619443842	Zm00001d009693

Zm00001d017193_T002	1	0.282109911	Zm00001d017193
Zm00001d017193_T003	0.9020229	-0.036423401	Zm00001d017193
Zm00001d050063_T001	0.835786	-0.115918732	Zm00001d050063
Zm00001d018944_T009	0.101456	0.84482529	Zm00001d018944
Zm00001d018944_T010	0.6661718	0.087133642	Zm00001d018944
Zm00001d018944_T001	0.9398534	0.206645776	Zm00001d018944
Zm00001d025474_T002	0.9633184	0.012277981	Zm00001d025474
Zm00001d025474_T003	0.694844	-0.381877633	Zm00001d025474
Zm00001d025474_T001	0.3480056	-0.809883588	Zm00001d025474
Zm00001d004772_T004	0.9937853	0.357741158	Zm00001d004772
Zm00001d004772_T003	0.7979593	-0.090410028	Zm00001d004772
Zm00001d040201_T001	1	0.371474376	Zm00001d040201
Zm00001d009562_T058	0.04491439	0.826879464	Zm00001d009562
Zm00001d009562_T020	0.5509143	1.163144328	Zm00001d009562
Zm00001d009562_T029	0.9664449	-0.064092471	Zm00001d009562
Zm00001d009562_T039	0.2081632	1.02027472	Zm00001d009562
Zm00001d009562_T067	0.9832092	0.410477943	Zm00001d009562
Zm00001d009562_T087	0.8659224	-1.056829153	Zm00001d009562
Zm00001d009562_T052	0.9390517	-0.129528867	Zm00001d009562
Zm00001d009562_T015	4.38E-13	1.99212445	Zm00001d009562
Zm00001d032267_T010	0.7056513	-0.135766781	Zm00001d032267
Zm00001d032267_T011	0.9257893	0.679094745	Zm00001d032267
Zm00001d032267_T009	0.9653972	0.234671109	Zm00001d032267
Zm00001d053977_T003	0.8995312	0.750219965	Zm00001d053977
Zm00001d053977_T002	0.8376132	0.758302637	Zm00001d053977
Zm00001d053977_T004	0.2232757	-1.679196335	Zm00001d053977
Zm00001d036360_T002	0.9833565	0.441605724	Zm00001d036360
Zm00001d043439_T011	0.001131893	-1.482252133	Zm00001d043439
Zm00001d043439_T006	0.9954649	0.440551393	Zm00001d043439
Zm00001d043439_T004	0.9989936	0.054736602	Zm00001d043439
Zm00001d044075_T001	0.9173494	0.425288468	Zm00001d044075
Zm00001d005694_T001	0.8753765	-0.597938939	Zm00001d005694
Zm00001d040684_T001	0.9108637	-0.02781229	Zm00001d040684
Zm00001d040684_T002	0.9998278	0.182686337	Zm00001d040684
Zm00001d013035_T008	0.1876281	-0.733964851	Zm00001d013035
Zm00001d002186_T002	0.8227589	1.009734392	Zm00001d002186
Zm00001d002186_T001	0.5033336	1.455705808	Zm00001d002186
Zm00001d025690_T001	0.7187138	0.537387238	Zm00001d025690
Zm00001d025690_T003	0.9312703	0.712733723	Zm00001d025690
Zm00001d025690_T004	0.9748733	0.282697007	Zm00001d025690
Zm00001d022427_T001	0.9706887	0.265694809	Zm00001d022427
Zm00001d022427_T005	0.8971642	0.834934084	Zm00001d022427
Zm00001d022427_T008	0.9822138	0.228793515	Zm00001d022427
Zm00001d022427_T007	0.9969123	0.087934276	Zm00001d022427
Zm00001d049189_T001	0.8278986	-0.446108158	Zm00001d049189
Zm00001d041758_T001	0.9469426	0.045368086	Zm00001d041758
Zm00001d000023_T006	0.3066462	0.832498854	Zm00001d000023
Zm00001d000023_T001	0.9303009	0.548282325	Zm00001d000023
Zm00001d000023_T004	0.5570384	0.376840148	Zm00001d000023
Zm00001d048226_T001	0.03591437	2.319730468	Zm00001d048226
Zm00001d008412_T001	0.9211172	0.751281973	Zm00001d008412
Zm00001d006148_T001	0.9921225	0.279625782	Zm00001d006148
Zm00001d003071_T001	0.9797082	0.107501451	Zm00001d003071
Zm00001d046500_T001	0.9141518	-0.037111991	Zm00001d046500

Zm00001d003511_T001	0.9493302	-0.001512111	Zm00001d003511
Zm00001d042030_T001	0.000228899	-2.405055908	Zm00001d042030
Zm00001d010152_T006	0.8957211	-0.095925411	Zm00001d010152
Zm00001d010152_T003	0.7697196	0.935778556	Zm00001d010152
Zm00001d010152_T007	0.01316976	2.701081524	Zm00001d010152
Zm00001d010152_T005	0.3422535	1.783213516	Zm00001d010152
Zm00001d010152_T001	0.9627389	0.156796082	Zm00001d010152
Zm00001d034918_T005	0.466515	-0.434877713	Zm00001d034918
Zm00001d034918_T003	0.9673094	0.140703884	Zm00001d034918
Zm00001d034918_T002	0.8972545	-0.050221406	Zm00001d034918
Zm00001d026064_T004	0.4490888	0.725299152	Zm00001d026064
Zm00001d026064_T002	0.9913436	0.399846944	Zm00001d026064
Zm00001d028705_T001	0.9476864	0.012280704	Zm00001d028705
Zm00001d053969_T002	0.8008364	0.723596695	Zm00001d053969
Zm00001d053969_T003	0.962254	0.120529357	Zm00001d053969
Zm00001d053969_T001	0.8057308	0.691589997	Zm00001d053969
Zm00001d053969_T004	6.92E-09	-2.005022894	Zm00001d053969
Zm00001d007153_T001	0.7723306	-0.628634753	Zm00001d007153
Zm00001d013164_T002	0.9778436	0.225644252	Zm00001d013164
Zm00001d014614_T001	0.9614996	0.903141912	Zm00001d014614
Zm00001d007972_T001	0.9954426	0.296606556	Zm00001d007972
Zm00001d007972_T005	0.6274667	-0.81950401	Zm00001d007972
Zm00001d043011_T001	0.7889949	-0.836296512	Zm00001d043011
Zm00001d018659_T003	0.9706293	-0.794510806	Zm00001d018659
Zm00001d018659_T004	0.9422098	0.568021737	Zm00001d018659
Zm00001d018659_T001	1	0.281057435	Zm00001d018659
Zm00001d018659_T002	0.9702252	0.470645784	Zm00001d018659
Zm00001d027685_T001	1	0.155735376	Zm00001d027685
Zm00001d013240_T002	0.9603416	0.073070621	Zm00001d013240
Zm00001d034498_T001	0.3135261	0.375388867	Zm00001d034498
Zm00001d034498_T007	0.9657993	0.607155401	Zm00001d034498
Zm00001d034498_T004	0.3861376	0.471888342	Zm00001d034498
Zm00001d023810_T001	0.906282	0.022335338	Zm00001d023810
Zm00001d001817_T001	0.9016285	-0.054153633	Zm00001d001817
Zm00001d039040_T001	0.9326774	-0.044848469	Zm00001d039040
Zm00001d018031_T001	0.7059883	-0.389226954	Zm00001d018031
Zm00001d042099_T001	0.7091158	-0.671098545	Zm00001d042099
Zm00001d017852_T001	0.7682041	2.337889956	Zm00001d017852
Zm00001d038645_T001	0.954492	0.611958672	Zm00001d038645
Zm00001d030790_T002	0.3229995	1.707273541	Zm00001d030790
Zm00001d044972_T001	0.9825858	0.103904493	Zm00001d044972
Zm00001d007354_T001	0.9604475	0.628242655	Zm00001d007354
Zm00001d018524_T003	0.6086477	1.186581682	Zm00001d018524
Zm00001d018524_T009	0.8960073	-0.046958278	Zm00001d018524
Zm00001d018524_T005	0.3565903	0.350585136	Zm00001d018524
Zm00001d018524_T002	0.9690925	0.289521808	Zm00001d018524
Zm00001d040468_T001	0.9627389	0.502922424	Zm00001d040468
Zm00001d012499_T001	0.9745314	0.136932961	Zm00001d012499
Zm00001d036880_T001	0.003956993	-2.228831189	Zm00001d036880
Zm00001d036880_T002	0.01183344	-2.023389356	Zm00001d036880
Zm00001d048680_T005	0.9081945	0.804536738	Zm00001d048680
Zm00001d048680_T004	0.9846714	0.098905151	Zm00001d048680
Zm00001d048680_T002	0.9685273	0.163873203	Zm00001d048680
Zm00001d048680_T001	0.995534	0.288329393	Zm00001d048680

Zm00001d048680_T009	0.3551811	-1.532770867	Zm00001d048680
Zm00001d048680_T007	0.9388815	0.504466966	Zm00001d048680
Zm00001d026194_T001	0.9112906	0.7413435	Zm00001d026194
Zm00001d044695_T001	0.9559198	0.063502894	Zm00001d044695
Zm00001d040060_T001	0.9879601	0.183122658	Zm00001d040060
Zm00001d026379_T001	0.9166818	0.001737621	Zm00001d026379
Zm00001d019114_T001	0.4146004	0.650874769	Zm00001d019114
Zm00001d019114_T002	0.907891	-0.066095941	Zm00001d019114
Zm00001d019114_T008	0.9954444	0.220231661	Zm00001d019114
Zm00001d014623_T001	0.2611065	-1.051516925	Zm00001d014623
Zm00001d052932_T001	0.3541791	1.520385704	Zm00001d052932
Zm00001d010241_T002	0.9745314	-0.004407105	Zm00001d010241
Zm00001d010241_T001	0.8175659	-0.117086012	Zm00001d010241
Zm00001d010043_T001	0.8989774	-0.372094001	Zm00001d010043
Zm00001d026176_T002	0.9563232	0.111368921	Zm00001d026176
Zm00001d026176_T001	0.9267763	0.272488194	Zm00001d026176
Zm00001d049459_T002	0.002330117	-2.493036367	Zm00001d049459
Zm00001d049459_T001	0.9554387	0.025721714	Zm00001d049459
Zm00001d052777_T001	0.9968176	0.225489092	Zm00001d052777
Zm00001d052777_T003	0.8825173	0.756199938	Zm00001d052777
Zm00001d052777_T002	0.143307	0.626334453	Zm00001d052777
Zm00001d008585_T001	0.9269932	0.573427943	Zm00001d008585
Zm00001d008585_T005	0.2674463	0.519728477	Zm00001d008585
Zm00001d008585_T002	0.000934168	1.143971947	Zm00001d008585
Zm00001d042290_T001	0.8817666	-0.089649922	Zm00001d042290
Zm00001d030267_T001	0.1090046	-1.551723148	Zm00001d030267
Zm00001d048428_T003	0.8818067	0.563254364	Zm00001d048428
Zm00001d048428_T001	0.967161	0.161947949	Zm00001d048428
Zm00001d040582_T002	0.8730771	0.337569497	Zm00001d040582
Zm00001d008215_T001	1	0.278325656	Zm00001d008215
Zm00001d008215_T002	0.9941267	0.256341078	Zm00001d008215
Zm00001d034351_T001	0.9768468	0.187695863	Zm00001d034351
Zm00001d049171_T002	0.9821463	0.22694995	Zm00001d049171
Zm00001d049171_T001	0.10451	1.011784782	Zm00001d049171
Zm00001d020580_T001	0.6289921	-0.434327838	Zm00001d020580
Zm00001d018440_T003	0.1272455	1.464756065	Zm00001d018440
Zm00001d018440_T002	0.7550413	1.105443575	Zm00001d018440
Zm00001d018440_T001	1	0.355215304	Zm00001d018440
Zm00001d039976_T002	0.6401483	-0.610267496	Zm00001d039976
Zm00001d014580_T003	0.8656506	-0.710832794	Zm00001d014580
Zm00001d014580_T001	0.9846714	0.023539449	Zm00001d014580
Zm00001d035436_T002	0.8063892	0.407330719	Zm00001d035436
Zm00001d035436_T003	0.836612	-0.130971947	Zm00001d035436
Zm00001d015570_T002	0.6673479	1.24697679	Zm00001d015570
Zm00001d015570_T007	0.4343337	-0.752469711	Zm00001d015570
Zm00001d015570_T004	0.1095433	1.473179144	Zm00001d015570
Zm00001d015570_T003	0.8321167	-0.078041808	Zm00001d015570
Zm00001d016149_T001	0.7681379	-0.261572439	Zm00001d016149
Zm00001d018744_T001	0.9770343	1.742078368	Zm00001d018744
Zm00001d018580_T001	0.009927578	1.863060141	Zm00001d018580
Zm00001d007479_T001	0.9107241	-0.063244108	Zm00001d007479
Zm00001d038194_T003	0.6863856	0.223063021	Zm00001d038194
Zm00001d038194_T001	0.6332526	-0.462327733	Zm00001d038194
Zm00001d017703_T004	0.9872831	0.155084674	Zm00001d017703

Zm00001d017703_T003	0.4657202	-0.560683032	Zm00001d017703
Zm00001d017703_T001	0.8751469	-0.138859684	Zm00001d017703
Zm00001d052325_T001	0.9257893	-0.22109422	Zm00001d052325
Zm00001d039643_T001	0.8896281	-0.103276109	Zm00001d039643
Zm00001d023213_T001	0.132854	-1.033179843	Zm00001d023213
Zm00001d042596_T005	0.9486412	0.039180802	Zm00001d042596
Zm00001d037174_T003	0.8348638	-0.192295868	Zm00001d037174
Zm00001d027366_T001	0.8557307	-0.140049243	Zm00001d027366
Zm00001d027366_T002	0.8481079	-0.305973079	Zm00001d027366
Zm00001d028936_T004	0.8175659	0.917761725	Zm00001d028936
Zm00001d028936_T013	0.6217057	0.872986853	Zm00001d028936
Zm00001d028936_T010	0.5501607	0.599942247	Zm00001d028936
Zm00001d028936_T011	0.606752	0.810812921	Zm00001d028936
Zm00001d028936_T009	0.9988063	1.377247518	Zm00001d028936
Zm00001d028936_T003	0.962254	0.661207808	Zm00001d028936
Zm00001d041305_T001	0.5549472	-0.738779386	Zm00001d041305
Zm00001d018233_T005	0.3739063	1.1316594	Zm00001d018233
Zm00001d018233_T014	0.6485803	-0.52784896	Zm00001d018233
Zm00001d053873_T001	0.9984041	0.374835205	Zm00001d053873
Zm00001d051617_T001	0.9193633	-0.304608676	Zm00001d051617
Zm00001d036060_T010	0.6824162	-0.411645414	Zm00001d036060
Zm00001d041962_T002	0.9487214	0.559196153	Zm00001d041962
Zm00001d010823_T003	0.6451042	0.976828839	Zm00001d010823
Zm00001d010823_T001	0.9860836	0.198300394	Zm00001d010823
Zm00001d044784_T001	0.5489602	-0.623846583	Zm00001d044784
Zm00001d035854_T001	0.9219195	0.029711373	Zm00001d035854
Zm00001d046149_T001	0.9732421	-0.214481857	Zm00001d046149
Zm00001d011263_T001	0.8158416	-0.276587651	Zm00001d011263
Zm00001d043076_T002	0.9814486	0.162022022	Zm00001d043076
Zm00001d043076_T001	0.9543425	0.086392087	Zm00001d043076
Zm00001d016694_T003	0.7849294	0.683057295	Zm00001d016694
Zm00001d016694_T007	0.3860341	-0.542971116	Zm00001d016694
Zm00001d032971_T001	0.7602652	0.944309853	Zm00001d032971
Zm00001d028438_T019	0.2497698	1.392654013	Zm00001d028438
Zm00001d028438_T021	0.967161	0.218728781	Zm00001d028438
Zm00001d028438_T001	0.001309444	0.278482489	Zm00001d028438
Zm00001d028438_T002	0.1453476	1.920798975	Zm00001d028438
Zm00001d002480_T002	0.9832092	0.337740303	Zm00001d002480
Zm00001d003983_T002	0.6062627	-0.638820691	Zm00001d003983
Zm00001d003983_T001	1	0.228316586	Zm00001d003983
Zm00001d003983_T003	0.9701986	0.098761244	Zm00001d003983
Zm00001d002731_T001	0.8953011	-0.290948583	Zm00001d002731
Zm00001d045575_T001	0.5598591	-0.544944878	Zm00001d045575
Zm00001d004744_T001	0.8991659	0.721429417	Zm00001d004744
Zm00001d017480_T001	0.1705997	-1.561365377	Zm00001d017480
Zm00001d040186_T001	0.5484972	-0.274996313	Zm00001d040186
Zm00001d013703_T001	0.7680039	-0.160754438	Zm00001d013703
Zm00001d013703_T002	0.9907928	0.226586485	Zm00001d013703
Zm00001d039313_T001	0.9048001	0.436731633	Zm00001d039313
Zm00001d014150_T002	0.050072	-0.80298746	Zm00001d014150
Zm00001d014150_T007	0.2748634	0.277060772	Zm00001d014150
Zm00001d014150_T010	0.113946	1.43920961	Zm00001d014150
Zm00001d014150_T005	0.8987811	0.746439489	Zm00001d014150
Zm00001d048453_T001	0.0136329	2.460133308	Zm00001d048453

Zm00001d019374_T001	0.4340285	-0.657668261	Zm00001d019374
Zm00001d006160_T001	0.769035	-0.252488271	Zm00001d006160
Zm00001d003091_T001	0.4604152	-0.729166188	Zm00001d003091
Zm00001d016475_T001	0.9416089	0.545530406	Zm00001d016475
Zm00001d041766_T001	1	0.199698703	Zm00001d041766
Zm00001d048318_T002	0.05889535	0.824620019	Zm00001d048318
Zm00001d048318_T001	0.9938888	0.438743764	Zm00001d048318
Zm00001d040462_T003	0.974657	0.178642977	Zm00001d040462
Zm00001d040462_T001	0.9493302	0.529709471	Zm00001d040462
Zm00001d027429_T002	0.7194777	-0.463612684	Zm00001d027429
Zm00001d027429_T001	0.8039029	-0.243412548	Zm00001d027429
Zm00001d047402_T001	0.3847344	1.469430803	Zm00001d047402
Zm00001d006790_T009	0.1802911	-0.821564298	Zm00001d006790
Zm00001d006790_T011	0.9037807	0.136968454	Zm00001d006790
Zm00001d006790_T005	0.8227873	0.753970194	Zm00001d006790
Zm00001d006790_T002	0.2574259	-0.735680827	Zm00001d006790
Zm00001d006790_T012	0.9213679	0.321552777	Zm00001d006790
Zm00001d006790_T006	0.8341539	1.148103004	Zm00001d006790
Zm00001d005657_T002	0.9860836	0.390312131	Zm00001d005657
Zm00001d053725_T001	0.9968176	0.450770673	Zm00001d053725
Zm00001d053725_T003	0.5005017	-0.832417645	Zm00001d053725
Zm00001d053834_T005	0.08520203	-1.216269555	Zm00001d053834
Zm00001d053834_T004	0.974657	0.354455309	Zm00001d053834
Zm00001d053834_T001	1	0.370270773	Zm00001d053834
Zm00001d053834_T002	0.000288625	-1.412383166	Zm00001d053834
Zm00001d029861_T003	0.3360774	0.560614141	Zm00001d029861
Zm00001d029861_T004	0.5591127	0.526922006	Zm00001d029861
Zm00001d034112_T001	0.7945048	1.049695003	Zm00001d034112
Zm00001d010887_T006	0.03582846	1.130072702	Zm00001d010887
Zm00001d010887_T009	0.85068	0.767671525	Zm00001d010887
Zm00001d010887_T005	0.3363071	1.226093779	Zm00001d010887
Zm00001d010887_T007	0.9745314	0.216628816	Zm00001d010887
Zm00001d011308_T001	0.9901647	0.420673172	Zm00001d011308
Zm00001d015948_T005	0.5384294	0.989148806	Zm00001d015948
Zm00001d015948_T013	0.3183941	0.517108255	Zm00001d015948
Zm00001d015948_T010	0.7029928	1.058220579	Zm00001d015948
Zm00001d015948_T007	0.9883036	0.189316807	Zm00001d015948
Zm00001d031814_T001	0.8494782	0.761802497	Zm00001d031814
Zm00001d028169_T001	0.9073468	-0.049214834	Zm00001d028169
Zm00001d037916_T001	0.9914036	0.229814359	Zm00001d037916
Zm00001d026035_T001	0.9706887	0.358134686	Zm00001d026035
Zm00001d035211_T001	0.9169863	-0.037005619	Zm00001d035211
Zm00001d006871_T001	0.7193076	-0.46128547	Zm00001d006871
Zm00001d002853_T001	0.9939025	0.517102522	Zm00001d002853
Zm00001d021745_T001	0.9749249	0.452822951	Zm00001d021745
Zm00001d020549_T008	0.9337571	0.02937911	Zm00001d020549
Zm00001d020549_T001	1	0.211549888	Zm00001d020549
Zm00001d020549_T010	0.03339051	0.522401875	Zm00001d020549
Zm00001d020549_T016	0.973825	0.565877871	Zm00001d020549
Zm00001d020549_T015	0.8348638	0.637881488	Zm00001d020549
Zm00001d020178_T002	0.2642046	1.791799068	Zm00001d020178
Zm00001d020178_T005	0.9159727	1.115989533	Zm00001d020178
Zm00001d020178_T001	1.76E-14	-2.764669396	Zm00001d020178
Zm00001d020178_T004	0.3492606	0.898144089	Zm00001d020178

Zm00001d013325_T001	0.9221549	-0.290286118	Zm00001d013325
Zm00001d003400_T001	0.2879626	1.437290981	Zm00001d003400
Zm00001d036667_T001	0.9160053	0.421285472	Zm00001d036667
Zm00001d041984_T001	0.9447869	0.015651562	Zm00001d041984
Zm00001d011839_T001	0.8667734	0.620223924	Zm00001d011839
Zm00001d053010_T001	0.96672	-0.015007404	Zm00001d053010
Zm00001d032408_T002	0.8729308	0.627697961	Zm00001d032408
Zm00001d001939_T002	0.1629598	0.849933057	Zm00001d001939
Zm00001d001939_T003	0.836612	-0.212446214	Zm00001d001939
Zm00001d001939_T005	0.3148012	-1.088167868	Zm00001d001939
Zm00001d001939_T001	0.7697196	-0.199328042	Zm00001d001939
Zm00001d014198_T001	0.000776638	-2.557930999	Zm00001d014198
Zm00001d050234_T001	0.8643636	-0.222568565	Zm00001d050234
Zm00001d025988_T001	0.9274507	0.720306521	Zm00001d025988
Zm00001d034674_T001	0.9860836	0.483357246	Zm00001d034674
Zm00001d053624_T001	0.9897446	0.455188152	Zm00001d053624
Zm00001d040357_T001	0.146054	-0.612182788	Zm00001d040357
Zm00001d040357_T004	1	0.162585371	Zm00001d040357
Zm00001d040357_T003	0.9745777	0.098880974	Zm00001d040357
Zm00001d040357_T002	0.8513643	0.489579423	Zm00001d040357
Zm00001d025418_T001	0.9664736	0.015828388	Zm00001d025418
Zm00001d002817_T022	0.3630583	0.544055734	Zm00001d002817
Zm00001d002817_T039	0.8723809	0.29923898	Zm00001d002817
Zm00001d002817_T004	1.70E-08	1.034716748	Zm00001d002817
Zm00001d002817_T001	0.7297964	-0.095500176	Zm00001d002817
Zm00001d002817_T005	0.7379612	0.141366206	Zm00001d002817
Zm00001d052125_T001	0.08515232	-1.071434522	Zm00001d052125
Zm00001d022367_T001	0.5839692	1.313880437	Zm00001d022367
Zm00001d022367_T002	1	0.323644019	Zm00001d022367
Zm00001d023939_T001	0.7821146	-0.267171549	Zm00001d023939
Zm00001d052852_T001	0.8419717	-0.158861625	Zm00001d052852
Zm00001d052852_T003	0.835786	-0.069143423	Zm00001d052852
Zm00001d052852_T002	0.7103637	-0.161582066	Zm00001d052852
Zm00001d014286_T050	0.9303773	0.607407333	Zm00001d014286
Zm00001d014286_T012	0.2691415	2.349607621	Zm00001d014286
Zm00001d014286_T030	0.9988864	0.180555967	Zm00001d014286
Zm00001d014286_T008	0.2406599	0.296424939	Zm00001d014286
Zm00001d014286_T004	0.8727241	0.279704644	Zm00001d014286
Zm00001d014286_T005	0.9469426	0.151506672	Zm00001d014286
Zm00001d014286_T042	0.1244019	-1.024405138	Zm00001d014286
Zm00001d014286_T053	3.11E-07	0.660323475	Zm00001d014286
Zm00001d051325_T001	0.8730771	-0.183761037	Zm00001d051325
Zm00001d034417_T001	0.2167825	1.736237422	Zm00001d034417
Zm00001d043291_T001	0.8951059	0.80451333	Zm00001d043291
Zm00001d053634_T002	0.9585514	0.525585051	Zm00001d053634
Zm00001d053634_T001	0.9557745	0.05402822	Zm00001d053634
Zm00001d027880_T002	0.9451281	0.389021544	Zm00001d027880
Zm00001d027880_T004	0.9979568	0.215848905	Zm00001d027880
Zm00001d017800_T001	0.9429712	0.374981534	Zm00001d017800
Zm00001d048361_T001	0.9422098	0.68958831	Zm00001d048361
Zm00001d013840_T003	0.1785712	0.727731673	Zm00001d013840
Zm00001d013840_T002	0.9616027	0.640359001	Zm00001d013840
Zm00001d018432_T001	0.629494	-0.484321614	Zm00001d018432
Zm00001d020883_T001	0.9693697	0.25203805	Zm00001d020883

Zm00001d045617_T002	0.7976574	0.571290424	Zm00001d045617
Zm00001d045617_T001	0.884138	-0.066563026	Zm00001d045617
Zm00001d025107_T001	0.9817074	0.103244392	Zm00001d025107
Zm00001d031275_T001	0.9881407	0.489146076	Zm00001d031275
Zm00001d050824_T001	0.8702994	-0.4878042	Zm00001d050824
Zm00001d048819_T001	0.9740325	0.567303048	Zm00001d048819
Zm00001d013059_T022	0.6963657	0.489347756	Zm00001d013059
Zm00001d013059_T012	0.3541203	1.19704208	Zm00001d013059
Zm00001d013059_T002	0.8660064	0.969815651	Zm00001d013059
Zm00001d013059_T017	0.5972733	0.323282542	Zm00001d013059
Zm00001d013059_T018	0.1179183	-0.952531607	Zm00001d013059
Zm00001d013059_T003	0.2754868	1.285799047	Zm00001d013059
Zm00001d019303_T001	0.2510107	-1.653102817	Zm00001d019303
Zm00001d012656_T003	0.8413933	0.856729682	Zm00001d012656
Zm00001d012656_T001	0.9988063	-0.015761422	Zm00001d012656
Zm00001d042017_T002	0.9758933	0.369982598	Zm00001d042017
Zm00001d028354_T001	0.9501013	0.649535146	Zm00001d028354
Zm00001d052872_T001	0.9912983	0.238529983	Zm00001d052872
Zm00001d032991_T001	1	0.391035378	Zm00001d032991
Zm00001d045086_T001	0.9946911	0.247307082	Zm00001d045086
Zm00001d037897_T001	0.9591856	-0.188996152	Zm00001d037897
Zm00001d027447_T002	0.8647028	-0.458773152	Zm00001d027447
Zm00001d027447_T010	0.9706887	0.053941279	Zm00001d027447
Zm00001d027447_T005	0.9662615	0.188066022	Zm00001d027447
Zm00001d027447_T011	0.9791729	0.205388716	Zm00001d027447
Zm00001d027447_T007	0.003939115	-2.484055647	Zm00001d027447
Zm00001d027447_T004	0.9792387	0.269963208	Zm00001d027447
Zm00001d010646_T001	0.9671674	0.156659483	Zm00001d010646
Zm00001d019865_T001	0.9842144	0.053503791	Zm00001d019865
Zm00001d002343_T001	0.8892107	0.765482443	Zm00001d002343
Zm00001d017473_T002	0.9849062	0.224518468	Zm00001d017473
Zm00001d017473_T001	0.7020458	-0.447046474	Zm00001d017473
Zm00001d007602_T001	0.6188181	0.368522396	Zm00001d007602
Zm00001d032069_T001	0.1237647	1.92679818	Zm00001d032069
Zm00001d049638_T015	0.02259034	0.676692007	Zm00001d049638
Zm00001d049638_T013	0.7686793	0.194820629	Zm00001d049638
Zm00001d049638_T008	0.85068	0.834618325	Zm00001d049638
Zm00001d049638_T028	0.005533456	-2.278584112	Zm00001d049638
Zm00001d004517_T007	0.4251028	0.29953255	Zm00001d004517
Zm00001d004517_T005	0.9127452	0.833409997	Zm00001d004517
Zm00001d004517_T008	1	0.097354766	Zm00001d004517
Zm00001d004517_T010	0.6955134	-0.718184379	Zm00001d004517
Zm00001d004517_T006	0.9946911	0.423285276	Zm00001d004517
Zm00001d022188_T001	0.9913523	0.19770109	Zm00001d022188
Zm00001d022188_T002	0.530398	-0.681695586	Zm00001d022188
Zm00001d022188_T003	0.9907928	-0.013927071	Zm00001d022188
Zm00001d032439_T002	0.9973181	0.278745024	Zm00001d032439
Zm00001d032439_T001	0.6556118	-0.407607078	Zm00001d032439
Zm00001d032439_T003	0.8720321	0.598642205	Zm00001d032439
Zm00001d018452_T001	0.9821324	0.501696775	Zm00001d018452
Zm00001d032897_T001	0.5778967	-0.591366399	Zm00001d032897
Zm00001d043740_T001	0.003107062	2.991681809	Zm00001d043740
Zm00001d021301_T009	0.3767085	1.778866068	Zm00001d021301
Zm00001d021301_T020	0.1748968	0.850149084	Zm00001d021301

Zm00001d021301_T001	0.8806413	0.915630966	Zm00001d021301
Zm00001d021301_T008	0.8819727	-0.157247231	Zm00001d021301
Zm00001d021301_T006	0.01924177	-0.512828271	Zm00001d021301
Zm00001d021301_T016	0.07713409	0.451765313	Zm00001d021301
Zm00001d033267_T001	0.8380433	0.576661127	Zm00001d033267
Zm00001d009440_T003	0.7063818	-0.192557639	Zm00001d009440
Zm00001d009440_T004	0.9833133	0.256987727	Zm00001d009440
Zm00001d009440_T001	1	0.285228467	Zm00001d009440
Zm00001d037199_T001	0.9402255	0.689292816	Zm00001d037199
Zm00001d028481_T026	0.4150157	1.291538834	Zm00001d028481
Zm00001d028481_T002	0.5418013	1.362341445	Zm00001d028481
Zm00001d028481_T001	0.277679	0.515140171	Zm00001d028481
Zm00001d028481_T040	0.9734722	0.210988618	Zm00001d028481
Zm00001d028481_T041	0.9298968	0.037424221	Zm00001d028481
Zm00001d004716_T003	0.8859449	0.711459882	Zm00001d004716
Zm00001d004716_T015	0.9880075	0.051615761	Zm00001d004716
Zm00001d004716_T012	9.23E-08	0.95551332	Zm00001d004716
Zm00001d004716_T019	0.889796	0.188876891	Zm00001d004716
Zm00001d004716_T017	0.06997179	0.468876012	Zm00001d004716
Zm00001d004716_T007	0.9189175	0.045701429	Zm00001d004716
Zm00001d052627_T001	0.7997379	-0.246699013	Zm00001d052627
Zm00001d033718_T001	0.9016471	0.643338311	Zm00001d033718
Zm00001d018959_T001	0.947501	0.104988453	Zm00001d018959
Zm00001d028662_T004	0.5345031	0.571753174	Zm00001d028662
Zm00001d028662_T007	1	0.120294227	Zm00001d028662
Zm00001d028662_T001	0.9892558	0.296196927	Zm00001d028662
Zm00001d028662_T005	0.9322451	0.447046064	Zm00001d028662
Zm00001d030902_T004	0.3356906	-0.674572969	Zm00001d030902
Zm00001d030902_T003	0.4243023	-0.837869509	Zm00001d030902
Zm00001d030902_T002	0.4941853	-0.404378178	Zm00001d030902
Zm00001d030902_T001	0.08124051	-1.12725088	Zm00001d030902
Zm00001d033647_T002	0.7492661	0.258100936	Zm00001d033647
Zm00001d033647_T001	0.9732421	0.179366436	Zm00001d033647
Zm00001d033647_T006	0.7812261	-0.223992829	Zm00001d033647
Zm00001d033647_T007	0.9180865	0.337162288	Zm00001d033647
Zm00001d033647_T003	0.2748152	0.243308329	Zm00001d033647
Zm00001d000291_T001	0.7233733	-0.36888639	Zm00001d000291
Zm00001d049007_T001	0.9970972	-0.010159642	Zm00001d049007
Zm00001d039673_T023	0.7458694	-0.321707783	Zm00001d039673
Zm00001d039673_T005	0.004451205	0.879881264	Zm00001d039673
Zm00001d039673_T007	0.3551811	0.66034776	Zm00001d039673
Zm00001d039673_T026	0.956184	0.56272628	Zm00001d039673
Zm00001d039673_T013	0.06767717	0.767038068	Zm00001d039673
Zm00001d039673_T027	0.1220498	-1.654119126	Zm00001d039673
Zm00001d039673_T003	0.8489797	0.945319807	Zm00001d039673
Zm00001d031229_T001	1	0.174882191	Zm00001d031229
Zm00001d002087_T005	0.9706887	0.526137797	Zm00001d002087
Zm00001d002087_T001	0.726958	1.078026905	Zm00001d002087
Zm00001d002087_T004	1	0.122960044	Zm00001d002087
Zm00001d016036_T001	0.9973528	0.200417765	Zm00001d016036
Zm00001d016837_T001	0.9986533	0.3292694	Zm00001d016837
Zm00001d016615_T003	0.8757553	-0.089912877	Zm00001d016615
Zm00001d016615_T002	0.7814026	0.987810374	Zm00001d016615
Zm00001d019125_T002	0.7693357	-0.28559075	Zm00001d019125

Zm00001d019125_T004	0.100527	-0.890422261	Zm00001d019125
Zm00001d052227_T001	0.9780511	0.43247516	Zm00001d052227
Zm00001d039387_T001	0.9469426	0.65369136	Zm00001d039387
Zm00001d039387_T002	0.9689939	-0.039914359	Zm00001d039387
Zm00001d015975_T002	0.9732341	0.510346032	Zm00001d015975
Zm00001d017025_T001	0.3195754	1.634527838	Zm00001d017025
Zm00001d053762_T001	0.9852645	0.218789443	Zm00001d053762
Zm00001d015475_T001	0.7571802	-0.51492634	Zm00001d015475
Zm00001d011649_T001	0.7379612	1.096553586	Zm00001d011649
Zm00001d018400_T001	0.2597699	-1.740665035	Zm00001d018400
Zm00001d044320_T001	0.90591	-0.057179246	Zm00001d044320
Zm00001d047772_T002	0.9771057	0.426886826	Zm00001d047772
Zm00001d047772_T001	0.9740325	0.64322276	Zm00001d047772
Zm00001d047772_T003	0.5092234	-0.161015158	Zm00001d047772
Zm00001d037249_T001	0.8252955	-0.201461797	Zm00001d037249
Zm00001d043692_T002	0.9846599	0.13753925	Zm00001d043692
Zm00001d043692_T001	0.646433	-0.390311193	Zm00001d043692
Zm00001d016588_T005	0.6630763	-0.155388037	Zm00001d016588
Zm00001d016588_T006	0.4957685	-0.639200578	Zm00001d016588
Zm00001d016588_T001	0.1688694	-1.235250551	Zm00001d016588
Zm00001d037245_T001	0.7707943	-0.367028645	Zm00001d037245
Zm00001d038227_T001	0.1616209	0.586883204	Zm00001d038227
Zm00001d038227_T002	0.6769227	1.281587254	Zm00001d038227
Zm00001d011353_T005	0.2153746	2.550167139	Zm00001d011353
Zm00001d011353_T001	0.9659036	0.42140733	Zm00001d011353
Zm00001d011353_T004	1	0.319363546	Zm00001d011353
Zm00001d011353_T007	0.9797082	0.474019147	Zm00001d011353
Zm00001d011353_T002	0.807836	0.93680941	Zm00001d011353
Zm00001d016788_T001	0.5344592	1.228348505	Zm00001d016788
Zm00001d020187_T001	0.9819492	0.052903473	Zm00001d020187
Zm00001d033957_T003	0.0322225	1.003238448	Zm00001d033957
Zm00001d033957_T002	0.06650348	2.138661155	Zm00001d033957
Zm00001d033957_T001	0.004411808	2.744494269	Zm00001d033957
Zm00001d033192_T001	0.313785	-1.093321302	Zm00001d033192
Zm00001d029023_T002	0.9779851	0.469067957	Zm00001d029023
Zm00001d029023_T004	0.4146736	1.032782852	Zm00001d029023
Zm00001d017241_T004	0.945968	0.533783472	Zm00001d017241
Zm00001d017241_T007	0.9860836	0.236543463	Zm00001d017241
Zm00001d017241_T002	0.9785005	0.575383966	Zm00001d017241
Zm00001d017241_T005	0.7959622	-0.165904185	Zm00001d017241
Zm00001d017241_T003	0.9931431	0.014215693	Zm00001d017241
Zm00001d012223_T001	0.7601928	0.9896488	Zm00001d012223
Zm00001d022406_T001	0.983078	0.466083367	Zm00001d022406
Zm00001d022406_T003	0.3937976	0.899639384	Zm00001d022406
Zm00001d051660_T001	0.9544149	-0.396893482	Zm00001d051660
Zm00001d045017_T001	0.7492733	0.714141032	Zm00001d045017
Zm00001d016544_T006	0.424032	-0.563534538	Zm00001d016544
Zm00001d016544_T001	0.9879601	0.11998758	Zm00001d016544
Zm00001d016544_T007	0.9851204	0.119042947	Zm00001d016544
Zm00001d016544_T010	0.9954444	0.176855311	Zm00001d016544
Zm00001d016544_T004	0.09409525	0.958034674	Zm00001d016544
Zm00001d016544_T002	0.9889811	0.197007403	Zm00001d016544
Zm00001d016544_T005	0.4359356	-0.574651336	Zm00001d016544
Zm00001d016544_T003	0.9899187	0.256311659	Zm00001d016544

Zm00001d047471_T018	0.9463326	-0.013343979	Zm00001d047471
Zm00001d047471_T009	3.71E-05	1.572179146	Zm00001d047471
Zm00001d047471_T001	0.5662162	0.959477164	Zm00001d047471
Zm00001d047471_T012	0.4233271	-0.87541644	Zm00001d047471
Zm00001d006169_T001	1.90E-07	3.526922001	Zm00001d006169
Zm00001d036982_T006	0.04351232	1.715511534	Zm00001d036982
Zm00001d036982_T004	0.3374375	1.530320892	Zm00001d036982
Zm00001d036982_T001	0.9768468	0.465071337	Zm00001d036982
Zm00001d012277_T003	0.3097988	0.861596376	Zm00001d012277
Zm00001d012277_T001	0.215373	1.69764984	Zm00001d012277
Zm00001d012277_T002	0.9559189	0.693375719	Zm00001d012277
Zm00001d022637_T001	0.8310488	-0.274784458	Zm00001d022637
Zm00001d011610_T033	0.9469426	0.744817155	Zm00001d011610
Zm00001d011610_T031	0.348986	0.159516187	Zm00001d011610
Zm00001d011610_T020	0.7532282	-0.253768653	Zm00001d011610
Zm00001d011610_T002	2.80E-13	-1.887059997	Zm00001d011610
Zm00001d011610_T043	0.9561521	0.129895316	Zm00001d011610
Zm00001d011610_T001	0.1175789	1.403286474	Zm00001d011610
Zm00001d011610_T074	0.1139375	-0.539906979	Zm00001d011610
Zm00001d011610_T016	3.16E-05	0.924056967	Zm00001d011610
Zm00001d021668_T004	0.5468086	-0.885977433	Zm00001d021668
Zm00001d021668_T009	0.9871153	-0.018310653	Zm00001d021668
Zm00001d021668_T007	0.9907928	-0.124222775	Zm00001d021668
Zm00001d021668_T001	0.8884142	-0.173589724	Zm00001d021668
Zm00001d021668_T002	0.9686323	0.503648798	Zm00001d021668
Zm00001d021668_T006	0.7055225	0.787077805	Zm00001d021668
Zm00001d021668_T003	0.8224736	0.39417755	Zm00001d021668
Zm00001d021668_T005	0.0146133	-1.752472408	Zm00001d021668
Zm00001d006676_T001	0.9706293	0.135470746	Zm00001d006676
Zm00001d003281_T001	0.6911155	0.598381627	Zm00001d003281
Zm00001d022492_T003	0.9112906	0.519559072	Zm00001d022492
Zm00001d022492_T002	0.9469426	0.71213402	Zm00001d022492
Zm00001d027694_T002	0.9524127	0.036578547	Zm00001d027694
Zm00001d041119_T005	0.8716622	-0.095219508	Zm00001d041119
Zm00001d041119_T001	0.8465879	0.940120382	Zm00001d041119
Zm00001d014513_T005	1	0.058604833	Zm00001d014513
Zm00001d014513_T002	0.8817666	-0.388180134	Zm00001d014513
Zm00001d014513_T003	0.9945592	-0.038747173	Zm00001d014513
Zm00001d022283_T001	0.9717919	0.050467744	Zm00001d022283
Zm00001d039268_T001	0.9811162	0.035922125	Zm00001d039268
Zm00001d050335_T001	1	0.303999015	Zm00001d050335
Zm00001d031973_T001	0.7169281	0.827979127	Zm00001d031973
Zm00001d031973_T005	0.8146635	0.886562283	Zm00001d031973
Zm00001d011622_T001	0.7655459	-0.534957238	Zm00001d011622
Zm00001d011622_T012	0.9804228	0.548110279	Zm00001d011622
Zm00001d011622_T026	0.6215812	0.702788531	Zm00001d011622
Zm00001d011622_T013	0.2848861	0.404149843	Zm00001d011622
Zm00001d011622_T008	0.3836885	0.613034292	Zm00001d011622
Zm00001d011622_T006	1.82E-11	1.886271167	Zm00001d011622
Zm00001d011622_T009	0.4344214	0.246845555	Zm00001d011622
Zm00001d011622_T030	0.9160053	-0.010118039	Zm00001d011622
Zm00001d022505_T008	0.1891351	-0.406394635	Zm00001d022505
Zm00001d022505_T003	0.9771927	0.2804446	Zm00001d022505
Zm00001d022505_T005	0.8865624	-0.573059918	Zm00001d022505

Zm00001d000257_T005	0.9766874	0.205243175	Zm00001d000257
Zm00001d000257_T001	0.8704456	0.499361552	Zm00001d000257
Zm00001d021513_T001	0.9817074	0.172560389	Zm00001d021513
Zm00001d003328_T001	0.9988864	0.289302197	Zm00001d003328
Zm00001d042263_T001	0.9946911	0.287545445	Zm00001d042263
Zm00001d035486_T001	0.8751967	-0.192340215	Zm00001d035486
Zm00001d048499_T001	0.9100954	-0.067359639	Zm00001d048499
Zm00001d028144_T001	0.8144326	-0.213327057	Zm00001d028144
Zm00001d038768_T001	0.453061	1.447651666	Zm00001d038768
Zm00001d006387_T004	0.7367876	-0.284597845	Zm00001d006387
Zm00001d003663_T001	0.9851204	0.132932013	Zm00001d003663
Zm00001d036328_T001	0.9845237	0.304446921	Zm00001d036328
Zm00001d031233_T001	1	0.214886058	Zm00001d031233
Zm00001d021900_T001	0.04339872	2.240007373	Zm00001d021900
Zm00001d032656_T001	0.8959807	0.635118365	Zm00001d032656
Zm00001d043786_T001	0.4359356	0.488532955	Zm00001d043786
Zm00001d015795_T001	0.9361945	-0.131139228	Zm00001d015795
Zm00001d040619_T001	0.2962803	-1.438146469	Zm00001d040619
Zm00001d002854_T001	0.9695084	0.438076642	Zm00001d002854
Zm00001d045517_T001	1	0.319680408	Zm00001d045517
Zm00001d051981_T002	0.420772	-0.564562303	Zm00001d051981
Zm00001d051981_T003	0.942537	0.746595863	Zm00001d051981
Zm00001d051981_T005	0.6486663	1.225396878	Zm00001d051981
Zm00001d051981_T001	0.9078287	-0.033944316	Zm00001d051981
Zm00001d051981_T006	0.7020458	-0.441486437	Zm00001d051981
Zm00001d018693_T001	0.9745777	0.18977609	Zm00001d018693
Zm00001d052409_T001	0.5704678	-0.544799374	Zm00001d052409
Zm00001d019394_T001	0.92946	-0.030533396	Zm00001d019394
Zm00001d023298_T001	0.7618555	-0.3087014	Zm00001d023298
Zm00001d033473_T001	0.8808619	-0.144246651	Zm00001d033473
Zm00001d025819_T001	0.6556502	0.657945019	Zm00001d025819
Zm00001d033037_T003	0.6826913	-0.095059524	Zm00001d033037
Zm00001d033037_T017	0.1145356	0.45156526	Zm00001d033037
Zm00001d033037_T060	0.9760472	0.219708814	Zm00001d033037
Zm00001d033037_T007	0.5161654	1.285258937	Zm00001d033037
Zm00001d039487_T001	0.0344758	2.449256365	Zm00001d039487
Zm00001d024268_T001	0.8007432	-0.327943491	Zm00001d024268
Zm00001d017047_T001	0.8155253	-0.334690917	Zm00001d017047
Zm00001d038449_T004	0.1286688	1.511808345	Zm00001d038449
Zm00001d038449_T003	0.9745777	0.51336087	Zm00001d038449
Zm00001d038449_T002	0.9797082	0.223710306	Zm00001d038449
Zm00001d051306_T001	1	0.424217144	Zm00001d051306
Zm00001d051306_T003	0.3742095	-0.202784262	Zm00001d051306
Zm00001d050886_T001	0.9821463	-0.103400663	Zm00001d050886
Zm00001d017553_T001	0.9846714	0.416444853	Zm00001d017553
Zm00001d022302_T001	0.9894218	0.175474511	Zm00001d022302
Zm00001d001862_T001	0.37626	1.384111393	Zm00001d001862
Zm00001d024251_T001	0.96672	0.119957744	Zm00001d024251
Zm00001d043948_T008	0.9115088	0.720019265	Zm00001d043948
Zm00001d043948_T007	0.9216521	0.457977203	Zm00001d043948
Zm00001d043948_T006	0.447037	-0.955759602	Zm00001d043948
Zm00001d025951_T039	0.7613191	-0.272282106	Zm00001d025951
Zm00001d025951_T018	0.03044968	0.311198326	Zm00001d025951
Zm00001d025951_T032	0.000747974	2.252574371	Zm00001d025951

Zm00001d025951_T056	0.001090041	0.78982154	Zm00001d025951
Zm00001d025951_T029	0.2484954	-0.452498028	Zm00001d025951
Zm00001d025951_T019	0.669171	0.208747466	Zm00001d025951
Zm00001d025951_T015	0.5505656	-0.198679671	Zm00001d025951
Zm00001d025951_T030	0.9751332	0.27533896	Zm00001d025951
Zm00001d025951_T031	7.59E-05	3.173339124	Zm00001d025951
Zm00001d025951_T038	0.1197922	0.847233508	Zm00001d025951
Zm00001d025951_T035	0.7018776	0.751766406	Zm00001d025951
Zm00001d025951_T037	0.9037324	-0.017442031	Zm00001d025951
Zm00001d025951_T016	0.01500842	0.754473427	Zm00001d025951
Zm00001d037001_T011	0.4191028	0.49728442	Zm00001d037001
Zm00001d037001_T001	0.2922992	-0.563454234	Zm00001d037001
Zm00001d037001_T008	0.2835468	1.356509934	Zm00001d037001
Zm00001d037001_T002	0.000534889	1.977737255	Zm00001d037001
Zm00001d037001_T005	0.9278769	0.042109932	Zm00001d037001
Zm00001d035875_T004	0.3201123	-0.368198556	Zm00001d035875
Zm00001d035875_T003	0.941809	0.111850189	Zm00001d035875
Zm00001d035875_T002	0.9982897	0.20703057	Zm00001d035875
Zm00001d048696_T001	0.7847374	-0.256900153	Zm00001d048696
Zm00001d006108_T010	0.9889197	0.328466913	Zm00001d006108
Zm00001d006108_T011	0.1255252	0.564045764	Zm00001d006108
Zm00001d006108_T009	0.02765694	-0.551746783	Zm00001d006108
Zm00001d021385_T002	0.9689939	0.582262046	Zm00001d021385
Zm00001d044466_T001	1	0.295781127	Zm00001d044466
Zm00001d033333_T001	0.9782953	0.380363358	Zm00001d033333
Zm00001d036632_T001	0.9666757	0.443281893	Zm00001d036632
Zm00001d036632_T004	0.9797082	0.157556873	Zm00001d036632
Zm00001d004697_T001	0.8053357	-0.203505532	Zm00001d004697
Zm00001d008529_T001	0.9880075	0.381226443	Zm00001d008529
Zm00001d002391_T001	7.04E-07	0.660813916	Zm00001d002391
Zm00001d002391_T006	0.7945048	-0.152894827	Zm00001d002391
Zm00001d002391_T002	0.8962371	-0.067588952	Zm00001d002391
Zm00001d002391_T005	0.9706293	0.028809303	Zm00001d002391
Zm00001d002391_T010	0.9363242	0.059783277	Zm00001d002391
Zm00001d002391_T012	0.708953	-0.095320771	Zm00001d002391
Zm00001d007785_T001	0.04449306	-1.38626784	Zm00001d007785
Zm00001d048880_T001	0.9732421	0.042006373	Zm00001d048880
Zm00001d037234_T001	0.7801274	0.562881086	Zm00001d037234
Zm00001d014324_T006	0.1634112	1.438325032	Zm00001d014324
Zm00001d014324_T002	0.5804257	1.240956046	Zm00001d014324
Zm00001d014324_T001	0.8336184	0.848822347	Zm00001d014324
Zm00001d014324_T007	0.07482195	1.047599176	Zm00001d014324
Zm00001d030500_T001	0.9981361	0.302677221	Zm00001d030500
Zm00001d001994_T001	0.9619517	0.10555502	Zm00001d001994
Zm00001d012684_T001	0.07387502	-2.526762641	Zm00001d012684
Zm00001d008976_T002	0.9501111	0.540626068	Zm00001d008976
Zm00001d027527_T002	0.7777722	-0.32751439	Zm00001d027527
Zm00001d042608_T001	0.9956349	0.468759055	Zm00001d042608
Zm00001d042608_T004	0.002685015	1.055568301	Zm00001d042608
Zm00001d042608_T003	0.6843488	0.967595493	Zm00001d042608
Zm00001d043706_T001	0.3694523	1.131220221	Zm00001d043706
Zm00001d036946_T001	0.1328218	1.566817339	Zm00001d036946
Zm00001d010007_T001	0.07373598	-1.633861745	Zm00001d010007
Zm00001d024418_T005	1	0.092321236	Zm00001d024418

Zm00001d024418_T003	0.5433327	0.770039069	Zm00001d024418
Zm00001d024418_T001	0.7778639	-0.101029756	Zm00001d024418
Zm00001d024418_T002	0.9760472	0.555212568	Zm00001d024418
Zm00001d024418_T004	0.5368684	-0.27193638	Zm00001d024418
Zm00001d021243_T001	1	0.224820047	Zm00001d021243
Zm00001d028787_T002	1	0.203974051	Zm00001d028787
Zm00001d028787_T001	1	0.398462426	Zm00001d028787
Zm00001d034643_T001	0.735792	0.742672714	Zm00001d034643
Zm00001d051021_T004	0.968034	0.407020573	Zm00001d051021
Zm00001d051021_T002	0.9768802	0.211028256	Zm00001d051021
Zm00001d051021_T003	0.9878022	0.404941482	Zm00001d051021
Zm00001d028006_T002	0.1009414	1.340520608	Zm00001d028006
Zm00001d028006_T001	0.5306966	1.380828727	Zm00001d028006
Zm00001d028006_T003	0.1779352	1.021431435	Zm00001d028006
Zm00001d007046_T001	0.7161798	-0.240410438	Zm00001d007046
Zm00001d009930_T001	0.8031754	0.820409284	Zm00001d009930
Zm00001d045386_T001	0.9201237	0.363956769	Zm00001d045386
Zm00001d045386_T002	0.4601319	1.253732763	Zm00001d045386
Zm00001d048953_T001	0.5742833	1.331823922	Zm00001d048953
Zm00001d002905_T004	0.954492	0.645633818	Zm00001d002905
Zm00001d002905_T008	0.8107637	-0.356834301	Zm00001d002905
Zm00001d002905_T002	0.07973344	-0.815083464	Zm00001d002905
Zm00001d002905_T009	0.9441469	-0.056565769	Zm00001d002905
Zm00001d017848_T007	0.08777144	1.800740192	Zm00001d017848
Zm00001d017848_T023	0.8153176	-0.235980238	Zm00001d017848
Zm00001d017848_T009	0.8158142	0.531209269	Zm00001d017848
Zm00001d017848_T005	5.01E-06	2.413184743	Zm00001d017848
Zm00001d017848_T019	0.002477464	-1.203500614	Zm00001d017848
Zm00001d026263_T011	0.6171076	-0.486506546	Zm00001d026263
Zm00001d026263_T001	0.205909	1.357051157	Zm00001d026263
Zm00001d026263_T007	0.001157211	1.24913429	Zm00001d026263
Zm00001d026263_T014	1.12E-05	0.311564382	Zm00001d026263
Zm00001d026263_T013	0.299373	0.845856003	Zm00001d026263
Zm00001d026263_T017	0.9906533	0.296735341	Zm00001d026263
Zm00001d026263_T005	0.02264751	1.954635753	Zm00001d026263
Zm00001d031666_T002	0.826773	-0.262650889	Zm00001d031666
Zm00001d028341_T002	0.6086477	0.62593272	Zm00001d028341
Zm00001d028341_T001	1	0.372320725	Zm00001d028341
Zm00001d048925_T001	0.05725737	-1.632668033	Zm00001d048925
Zm00001d049928_T001	0.8659224	-0.117437222	Zm00001d049928
Zm00001d049928_T002	0.01691523	0.52182716	Zm00001d049928
Zm00001d049928_T007	0.08268915	0.660746547	Zm00001d049928
Zm00001d049928_T005	0.7114257	1.050220151	Zm00001d049928
Zm00001d002454_T001	0.1052631	-0.691969141	Zm00001d002454
Zm00001d016070_T003	1	0.44952388	Zm00001d016070
Zm00001d016070_T005	0.06639461	-1.829435012	Zm00001d016070
Zm00001d016070_T004	0.06138299	2.487219204	Zm00001d016070
Zm00001d016070_T002	0.5436993	-0.495964483	Zm00001d016070
Zm00001d053122_T001	0.7868558	-0.354699715	Zm00001d053122
Zm00001d051552_T001	0.9142822	-0.089701269	Zm00001d051552
Zm00001d040183_T001	0.8509137	0.886109139	Zm00001d040183
Zm00001d015297_T005	0.9812284	0.297301146	Zm00001d015297
Zm00001d015297_T003	0.9824348	0.510598707	Zm00001d015297
Zm00001d038477_T001	0.5549472	0.844883248	Zm00001d038477

Zm00001d009220_T002	0.3919095	1.340753367	Zm00001d009220
Zm00001d047946_T002	0.9980559	0.215987794	Zm00001d047946
Zm00001d040667_T003	0.5328547	-0.314130401	Zm00001d040667
Zm00001d040667_T002	0.8711701	-0.144437196	Zm00001d040667
Zm00001d040667_T004	0.5616664	-0.485529247	Zm00001d040667
Zm00001d032934_T001	0.9208326	0.528479584	Zm00001d032934
Zm00001d032934_T002	0.9573272	-0.396000843	Zm00001d032934
Zm00001d016921_T001	0.1526397	-1.166721282	Zm00001d016921
Zm00001d038382_T001	0.9889811	0.097398943	Zm00001d038382
Zm00001d015401_T001	0.4673489	1.473401149	Zm00001d015401
Zm00001d038069_T001	0.9203655	0.561483273	Zm00001d038069
Zm00001d028177_T016	0.9486412	0.208713045	Zm00001d028177
Zm00001d028177_T007	0.6742644	1.726829873	Zm00001d028177
Zm00001d028177_T004	0.07146096	1.858742755	Zm00001d028177
Zm00001d028177_T002	0.6705583	-0.574151075	Zm00001d028177
Zm00001d028177_T013	0.2688725	0.808605985	Zm00001d028177
Zm00001d041056_T001	0.4431901	1.170817156	Zm00001d041056
Zm00001d041056_T007	0.4701937	0.666014546	Zm00001d041056
Zm00001d041056_T013	0.1191683	0.712550198	Zm00001d041056
Zm00001d041056_T018	0.9979393	0.420229711	Zm00001d041056
Zm00001d014880_T005	0.5548793	1.523969226	Zm00001d014880
Zm00001d014880_T004	0.4342973	0.399341008	Zm00001d014880
Zm00001d014880_T007	0.3667933	-1.094905583	Zm00001d014880
Zm00001d014880_T001	0.8600981	-0.119346891	Zm00001d014880
Zm00001d014880_T002	0.9217658	-0.02412198	Zm00001d014880
Zm00001d014880_T006	0.9695609	-0.007710553	Zm00001d014880
Zm00001d024281_T001	0.2404995	-1.084412414	Zm00001d024281
Zm00001d037073_T001	0.1933431	-1.167854102	Zm00001d037073
Zm00001d013984_T022	0.8843752	0.478907743	Zm00001d013984
Zm00001d013984_T012	8.41E-11	2.144712171	Zm00001d013984
Zm00001d013984_T003	0.2365388	-1.338605376	Zm00001d013984
Zm00001d013984_T018	0.7761738	0.954647156	Zm00001d013984
Zm00001d013984_T001	0.9596377	0.117675512	Zm00001d013984
Zm00001d013984_T019	0.1017596	0.799854473	Zm00001d013984
Zm00001d027848_T004	1	0.339997787	Zm00001d027848
Zm00001d027848_T005	0.1677128	0.716660365	Zm00001d027848
Zm00001d027848_T017	0.9855117	0.07542052	Zm00001d027848
Zm00001d046442_T002	0.8119951	0.455848411	Zm00001d046442
Zm00001d036477_T002	0.9311027	0.689130523	Zm00001d036477
Zm00001d008258_T001	0.6866149	1.035261685	Zm00001d008258
Zm00001d016234_T001	0.9969363	0.305609062	Zm00001d016234
Zm00001d017658_T001	0.2385188	-0.816923116	Zm00001d017658
Zm00001d014789_T002	0.7311884	0.84667229	Zm00001d014789
Zm00001d014789_T001	0.8545272	0.813792768	Zm00001d014789
Zm00001d004288_T001	0.01472471	2.107666422	Zm00001d004288
Zm00001d020921_T001	0.9160053	0.313701149	Zm00001d020921
Zm00001d044857_T001	0.3304942	-1.106445102	Zm00001d044857
Zm00001d030223_T001	0.9613462	-0.060045504	Zm00001d030223
Zm00001d015870_T004	0.8557307	0.46746901	Zm00001d015870
Zm00001d017726_T001	0.9760472	0.158988416	Zm00001d017726
Zm00001d034852_T001	0.9344122	-0.046943112	Zm00001d034852
Zm00001d037110_T005	0.9694074	-0.321819643	Zm00001d037110
Zm00001d037110_T002	0.9906533	0.296068848	Zm00001d037110
Zm00001d037110_T004	0.7071453	0.844623225	Zm00001d037110

Zm00001d037110_T003	0.9486412	0.251332572	Zm00001d037110
Zm00001d008591_T001	0.466515	-0.673278901	Zm00001d008591
Zm00001d037719_T001	0.8380163	-0.239554209	Zm00001d037719
Zm00001d043699_T001	0.5910783	-0.572330968	Zm00001d043699
Zm00001d007491_T001	0.5795936	1.075901044	Zm00001d007491
Zm00001d035285_T003	0.9046076	-0.008833643	Zm00001d035285
Zm00001d040317_T011	0.9547035	0.079104642	Zm00001d040317
Zm00001d040317_T006	0.7339388	0.520770125	Zm00001d040317
Zm00001d040317_T012	0.9515686	0.67248883	Zm00001d040317
Zm00001d040317_T008	0.9053391	0.434699942	Zm00001d040317
Zm00001d008953_T001	0.7594555	-0.776003883	Zm00001d008953
Zm00001d033098_T001	0.955464	0.06733632	Zm00001d033098
Zm00001d036656_T001	0.00290736	2.880813994	Zm00001d036656
Zm00001d036656_T004	0.000239955	2.881649649	Zm00001d036656
Zm00001d009071_T002	0.8955231	-0.172354175	Zm00001d009071
Zm00001d009071_T001	0.9946911	0.268914497	Zm00001d009071
Zm00001d047446_T003	1	0.108236022	Zm00001d047446
Zm00001d047446_T001	0.9596377	0.556068135	Zm00001d047446
Zm00001d039658_T002	0.404073	-1.432696838	Zm00001d039658
Zm00001d039658_T001	0.6446589	-0.543884456	Zm00001d039658
Zm00001d012909_T004	0.7020458	0.723733604	Zm00001d012909
Zm00001d012909_T002	0.08254573	2.20466084	Zm00001d012909
Zm00001d012909_T006	0.02718105	-2.931306669	Zm00001d012909
Zm00001d012909_T001	0.7756749	-0.300761493	Zm00001d012909
Zm00001d037606_T001	0.6633441	0.652931296	Zm00001d037606
Zm00001d037606_T007	0.9954649	0.329716434	Zm00001d037606
Zm00001d037606_T006	0.9824348	0.58905736	Zm00001d037606
Zm00001d037606_T004	0.9303773	0.080300923	Zm00001d037606
Zm00001d037606_T005	0.9380783	0.381064677	Zm00001d037606
Zm00001d037606_T003	0.9919298	0.147248968	Zm00001d037606
Zm00001d039303_T001	0.9197676	-0.231833952	Zm00001d039303
Zm00001d052133_T003	0.8641671	0.421393344	Zm00001d052133
Zm00001d052133_T001	0.96672	0.086361879	Zm00001d052133
Zm00001d036432_T003	0.9252088	0.502462144	Zm00001d036432
Zm00001d036432_T001	0.9334843	-0.005292658	Zm00001d036432
Zm00001d024035_T002	0.9690925	0.05747925	Zm00001d024035
Zm00001d047527_T003	0.9732421	0.607310297	Zm00001d047527
Zm00001d047527_T002	0.01191076	2.28103848	Zm00001d047527
Zm00001d047527_T006	0.9695609	0.113154628	Zm00001d047527
Zm00001d047527_T007	0.2996587	1.03839776	Zm00001d047527
Zm00001d034511_T001	0.3600957	1.109463891	Zm00001d034511
Zm00001d025170_T001	0.9701986	0.17067819	Zm00001d025170
Zm00001d025170_T002	0.8421272	0.577191004	Zm00001d025170
Zm00001d005461_T001	0.9895224	0.266569985	Zm00001d005461
Zm00001d043303_T001	0.9173494	0.045495799	Zm00001d043303
Zm00001d043303_T003	0.3306758	1.798809689	Zm00001d043303
Zm00001d043303_T005	0.960704	-0.097459573	Zm00001d043303
Zm00001d044376_T001	0.9455715	0.33724495	Zm00001d044376
Zm00001d020025_T001	0.7224237	-0.722309185	Zm00001d020025
Zm00001d046595_T003	0.000552545	2.236742746	Zm00001d046595
Zm00001d046595_T006	0.85068	-0.250800971	Zm00001d046595
Zm00001d046595_T001	0.4903503	1.034010246	Zm00001d046595
Zm00001d015984_T002	0.7799762	-0.275998254	Zm00001d015984
Zm00001d015984_T001	0.9404125	0.200909793	Zm00001d015984

Zm00001d011603_T001	0.693358	-0.523712674	Zm00001d011603
Zm00001d051928_T001	0.758035	-0.3010819	Zm00001d051928
Zm00001d020274_T001	0.9024309	-0.217215642	Zm00001d020274
Zm00001d006548_T001	0.8571632	-0.510399206	Zm00001d006548
Zm00001d025165_T001	0.768921	-0.101608364	Zm00001d025165
Zm00001d025165_T003	0.9832612	0.265248311	Zm00001d025165
Zm00001d023723_T001	0.9211172	-0.040940405	Zm00001d023723
Zm00001d036956_T002	0.8536822	-0.209886107	Zm00001d036956
Zm00001d036956_T001	0.2703118	-0.68440209	Zm00001d036956
Zm00001d022067_T001	0.931766	0.406916413	Zm00001d022067
Zm00001d032453_T001	0.9154179	0.780153783	Zm00001d032453
Zm00001d032453_T002	0.9596377	0.234496017	Zm00001d032453
Zm00001d042243_T001	0.08572166	-1.734228649	Zm00001d042243
Zm00001d018390_T001	0.8817666	-0.156483662	Zm00001d018390
Zm00001d036978_T001	0.9621967	0.01333667	Zm00001d036978
Zm00001d027732_T001	0.9160053	0.679100193	Zm00001d027732
Zm00001d020955_T001	0.8830977	0.910320821	Zm00001d020955
Zm00001d006024_T001	0.7561907	-0.199129407	Zm00001d006024
Zm00001d042275_T001	0.9404125	0.606185839	Zm00001d042275
Zm00001d021449_T001	0.3996069	-0.884775562	Zm00001d021449
Zm00001d011177_T004	0.9139882	0.21075418	Zm00001d011177
Zm00001d011177_T008	0.8747821	-0.252127573	Zm00001d011177
Zm00001d011177_T002	0.9989936	0.445577053	Zm00001d011177
Zm00001d011177_T006	0.449035	0.37621353	Zm00001d011177
Zm00001d011177_T005	0.2002317	-1.354713558	Zm00001d011177
Zm00001d039133_T002	0.8817666	-0.12618666	Zm00001d039133
Zm00001d038084_T001	0.9428901	0.045191118	Zm00001d038084
Zm00001d045339_T001	0.9469426	-0.121265236	Zm00001d045339
Zm00001d045339_T002	0.9942767	-0.090393398	Zm00001d045339
Zm00001d028902_T001	1	0.339847168	Zm00001d028902
Zm00001d028902_T004	0.1744241	-0.371627746	Zm00001d028902
Zm00001d018801_T001	0.8591203	-0.686785443	Zm00001d018801
Zm00001d018855_T001	0.5711435	-0.701171914	Zm00001d018855
Zm00001d051287_T001	0.8203096	0.779163776	Zm00001d051287
Zm00001d016873_T001	0.0982823	1.766443409	Zm00001d016873
Zm00001d051572_T001	0.945968	0.064465968	Zm00001d051572
Zm00001d038832_T002	0.6082462	-0.610808646	Zm00001d038832
Zm00001d038832_T001	0.2934661	-0.500592518	Zm00001d038832
Zm00001d005972_T011	0.9447869	0.094567978	Zm00001d005972
Zm00001d005972_T004	0.9861215	0.091336557	Zm00001d005972
Zm00001d005972_T008	0.848247	0.425198742	Zm00001d005972
Zm00001d002193_T003	0.07811455	-1.373112772	Zm00001d002193
Zm00001d049080_T001	0.9709627	0.616395838	Zm00001d049080
Zm00001d035662_T001	0.7471629	0.76646986	Zm00001d035662
Zm00001d001831_T003	0.9753621	0.357692429	Zm00001d001831
Zm00001d001831_T005	0.8700632	-0.091647857	Zm00001d001831
Zm00001d001831_T001	0.9673473	0.803785511	Zm00001d001831
Zm00001d001831_T004	0.1052631	-0.68517238	Zm00001d001831
Zm00001d001831_T002	0.9402255	0.091183614	Zm00001d001831
Zm00001d041690_T001	0.9469426	0.052319062	Zm00001d041690
Zm00001d041690_T002	0.9171885	0.572675084	Zm00001d041690
Zm00001d014116_T002	0.4277454	1.478776767	Zm00001d014116
Zm00001d014116_T001	0.4432408	1.455138794	Zm00001d014116
Zm00001d033055_T001	0.9264959	0.74428725	Zm00001d033055

Zm00001d023784_T001	0.3648128	1.208151034	Zm00001d023784
Zm00001d023784_T003	0.7364361	1.091211506	Zm00001d023784
Zm00001d023784_T004	0.09762222	1.107849515	Zm00001d023784
Zm00001d023784_T014	0.4492383	0.559909839	Zm00001d023784
Zm00001d023784_T005	0.7945048	-0.22119307	Zm00001d023784
Zm00001d023784_T018	0.8129337	0.221180054	Zm00001d023784
Zm00001d003497_T001	0.1343579	2.06039086	Zm00001d003497
Zm00001d033493_T006	0.8465879	0.737627173	Zm00001d033493
Zm00001d033493_T007	0.8730771	0.176149664	Zm00001d033493
Zm00001d033493_T012	0.1166311	0.452327474	Zm00001d033493
Zm00001d028731_T001	0.9513407	0.59441492	Zm00001d028731
Zm00001d054046_T001	0.4283558	-1.032656737	Zm00001d054046
Zm00001d021334_T001	0.08451169	-1.333294015	Zm00001d021334
Zm00001d005063_T001	0.6322903	0.901035107	Zm00001d005063
Zm00001d050914_T001	0.6353817	0.531015974	Zm00001d050914
Zm00001d003377_T001	0.9469426	0.461869548	Zm00001d003377
Zm00001d010039_T002	0.9201237	0.786970373	Zm00001d010039
Zm00001d010039_T003	1	0.399710227	Zm00001d010039
Zm00001d010918_T001	1	0.238484884	Zm00001d010918
Zm00001d010918_T002	0.4348925	-0.913782209	Zm00001d010918
Zm00001d012834_T003	1	0.329754101	Zm00001d012834
Zm00001d048178_T001	0.02151911	-1.236591251	Zm00001d048178
Zm00001d025360_T001	0.000203969	-3.050343056	Zm00001d025360
Zm00001d006510_T001	0.8279795	-0.657534453	Zm00001d006510
Zm00001d047893_T001	0.02834503	-0.72252375	Zm00001d047893
Zm00001d047893_T003	0.6503853	-0.439287886	Zm00001d047893
Zm00001d047893_T004	0.2923172	-0.716366444	Zm00001d047893
Zm00001d047893_T002	0.03011949	-1.005930423	Zm00001d047893
Zm00001d009993_T001	0.002831329	-2.780231974	Zm00001d009993
Zm00001d028786_T001	0.8513643	-0.388888278	Zm00001d028786
Zm00001d028786_T003	1.56E-05	-1.469444246	Zm00001d028786
Zm00001d047436_T001	0.5117107	-0.535102884	Zm00001d047436
Zm00001d050656_T001	0.9361945	-0.354729824	Zm00001d050656
Zm00001d041420_T007	0.9490989	0.852293289	Zm00001d041420
Zm00001d041420_T011	0.007404649	0.676826647	Zm00001d041420
Zm00001d041420_T003	0.9690925	0.661810781	Zm00001d041420
Zm00001d041420_T010	0.5296936	0.453538546	Zm00001d041420
Zm00001d041420_T009	0.00090062	0.653071834	Zm00001d041420
Zm00001d043913_T001	0.9766874	-0.153223315	Zm00001d043913
Zm00001d052391_T002	2.45E-11	-4.627923665	Zm00001d052391
Zm00001d052391_T001	0.9878022	0.255844882	Zm00001d052391
Zm00001d027656_T001	0.878248	-0.080250141	Zm00001d027656
Zm00001d053987_T003	1	0.400952738	Zm00001d053987
Zm00001d053987_T002	6.11E-09	-2.09017203	Zm00001d053987
Zm00001d030101_T001	1	0.294502013	Zm00001d030101
Zm00001d022393_T002	0.8883561	0.335523676	Zm00001d022393
Zm00001d022393_T001	0.9553037	-0.058776373	Zm00001d022393
Zm00001d032795_T001	0.3675057	-0.534625274	Zm00001d032795
Zm00001d003155_T003	0.8337863	-0.011554324	Zm00001d003155
Zm00001d003155_T010	0.6988666	0.575037571	Zm00001d003155
Zm00001d003155_T007	0.331216	0.721376216	Zm00001d003155
Zm00001d003155_T001	0.5120497	-0.091154993	Zm00001d003155
Zm00001d004053_T001	0.480132	-0.117491566	Zm00001d004053
Zm00001d004053_T015	0.5851317	0.171228652	Zm00001d004053

Zm00001d004053_T004	0.3394823	-0.168461627	Zm00001d004053
Zm00001d004053_T028	0.3148382	-0.718550924	Zm00001d004053
Zm00001d012899_T001	0.9939021	0.0593619	Zm00001d012899
Zm00001d015637_T001	1	0.201576241	Zm00001d015637
Zm00001d018192_T001	0.9138633	-0.752108255	Zm00001d018192
Zm00001d046952_T001	0.6952911	-0.427141473	Zm00001d046952
Zm00001d011929_T002	0.6453183	-0.482179705	Zm00001d011929
Zm00001d011929_T004	0.9959972	0.309935762	Zm00001d011929
Zm00001d011929_T001	0.5980883	-0.704305651	Zm00001d011929
Zm00001d011929_T003	0.733411	-0.333642987	Zm00001d011929
Zm00001d013039_T001	0.02115378	-2.211763901	Zm00001d013039
Zm00001d025913_T001	0.7945048	-0.218687697	Zm00001d025913
Zm00001d027593_T001	0.8864089	-0.058903229	Zm00001d027593
Zm00001d053889_T005	0.9015226	0.906041072	Zm00001d053889
Zm00001d053889_T011	0.9868821	0.548593317	Zm00001d053889
Zm00001d002320_T002	0.8518279	0.545000007	Zm00001d002320
Zm00001d002320_T001	0.9468268	0.267263442	Zm00001d002320
Zm00001d035395_T001	0.9950703	0.287117504	Zm00001d035395
Zm00001d006323_T168	0.957768	0.063144132	Zm00001d006323
Zm00001d006323_T171	0.5016312	-0.555797828	Zm00001d006323
Zm00001d006323_T176	0.6690332	-0.765297121	Zm00001d006323
Zm00001d049623_T002	0.726898	-0.655073766	Zm00001d049623
Zm00001d049623_T001	0.9673473	-0.321852287	Zm00001d049623
Zm00001d037680_T001	0.7227713	1.007955913	Zm00001d037680
Zm00001d052077_T001	0.8310341	-0.344215496	Zm00001d052077
Zm00001d019266_T001	0.9037324	-0.036461781	Zm00001d019266
Zm00001d024408_T001	0.9673473	0.592251548	Zm00001d024408
Zm00001d006313_T001	1	0.010408596	Zm00001d006313
Zm00001d015123_T001	0.8933301	-0.110214922	Zm00001d015123
Zm00001d015123_T003	0.684563	-0.295981941	Zm00001d015123
Zm00001d035915_T001	1	0.424393796	Zm00001d035915
Zm00001d004139_T010	0.6232596	1.163312641	Zm00001d004139
Zm00001d004139_T004	0.8646111	-0.151807969	Zm00001d004139
Zm00001d004139_T003	2.99E-14	2.539693806	Zm00001d004139
Zm00001d012893_T001	0.8997535	-0.022409301	Zm00001d012893
Zm00001d012893_T002	0.6386265	0.628262204	Zm00001d012893
Zm00001d032473_T002	0.5812143	0.977030635	Zm00001d032473
Zm00001d032473_T003	0.04894618	1.123813566	Zm00001d032473
Zm00001d032473_T001	0.4463519	0.640409703	Zm00001d032473
Zm00001d022557_T001	0.8781102	0.736368508	Zm00001d022557
Zm00001d053807_T001	0.9380452	-0.034688831	Zm00001d053807
Zm00001d018331_T004	0.9219195	0.047115154	Zm00001d018331
Zm00001d018331_T001	0.7359808	-0.279407751	Zm00001d018331
Zm00001d018331_T003	0.891216	0.759626931	Zm00001d018331
Zm00001d046277_T005	0.08230594	1.637145001	Zm00001d046277
Zm00001d046277_T027	0.001451041	2.179185778	Zm00001d046277
Zm00001d046277_T003	5.15E-07	3.613798291	Zm00001d046277
Zm00001d046277_T022	0.214971	2.047251211	Zm00001d046277
Zm00001d046277_T014	0.5719089	0.158435795	Zm00001d046277
Zm00001d046277_T002	1	0.229460128	Zm00001d046277
Zm00001d002549_T002	0.4490888	-0.892069577	Zm00001d002549
Zm00001d002549_T001	0.2308157	-1.362646412	Zm00001d002549
Zm00001d047958_T001	0.6104835	-0.828466647	Zm00001d047958
Zm00001d003470_T001	0.6575648	1.193660348	Zm00001d003470

Zm00001d019339_T002	0.4907087	-0.564532435	Zm00001d019339
Zm00001d024933_T005	0.7991382	0.574553633	Zm00001d024933
Zm00001d024933_T003	0.989787	0.484312696	Zm00001d024933
Zm00001d045230_T001	0.9734722	0.566980965	Zm00001d045230
Zm00001d034343_T001	0.8027057	-0.23843244	Zm00001d034343
Zm00001d034343_T004	0.989787	0.288452897	Zm00001d034343
Zm00001d034343_T003	0.9701986	0.441543854	Zm00001d034343
Zm00001d026629_T003	0.9616027	-0.036883376	Zm00001d026629
Zm00001d026629_T001	0.01625687	-1.327103906	Zm00001d026629
Zm00001d052565_T001	0.9108303	-0.152006527	Zm00001d052565
Zm00001d038109_T005	0.9422098	0.489263078	Zm00001d038109
Zm00001d019531_T003	0.9654804	0.132390052	Zm00001d019531
Zm00001d019531_T001	0.05786074	-0.656411796	Zm00001d019531
Zm00001d019531_T002	0.8489611	0.903660974	Zm00001d019531
Zm00001d017946_T027	2.04E-07	0.554879546	Zm00001d017946
Zm00001d017946_T010	0.9563232	0.688516736	Zm00001d017946
Zm00001d017946_T001	0.001816546	-0.334321536	Zm00001d017946
Zm00001d017946_T003	0.4790679	0.648946842	Zm00001d017946
Zm00001d017946_T013	9.04E-11	1.31547677	Zm00001d017946
Zm00001d017946_T018	0.9303773	0.239826133	Zm00001d017946
Zm00001d037873_T003	0.1415925	-1.531698075	Zm00001d037873
Zm00001d037873_T002	0.9419336	0.082347844	Zm00001d037873
Zm00001d033992_T002	0.449035	0.428996893	Zm00001d033992
Zm00001d033992_T012	0.7439269	-0.170516744	Zm00001d033992
Zm00001d027486_T001	0.00207886	2.191535644	Zm00001d027486
Zm00001d027486_T002	0.000326107	2.841813926	Zm00001d027486
Zm00001d018705_T001	0.671147	-0.472106696	Zm00001d018705
Zm00001d042136_T004	0.6702673	0.813577084	Zm00001d042136
Zm00001d042136_T002	0.7853827	0.967663774	Zm00001d042136
Zm00001d031870_T001	0.8987811	-0.298584442	Zm00001d031870
Zm00001d049075_T002	1	0.438711407	Zm00001d049075
Zm00001d043276_T001	0.705921	0.761321875	Zm00001d043276
Zm00001d028909_T001	0.9902733	0.384680097	Zm00001d028909
Zm00001d021679_T001	0.9252088	0.107018925	Zm00001d021679
Zm00001d033023_T001	0.941922	-0.360247222	Zm00001d033023
Zm00001d028612_T001	0.7957286	-0.315872019	Zm00001d028612
Zm00001d005772_T002	0.4984211	1.118448952	Zm00001d005772
Zm00001d005772_T003	0.4364135	1.168424062	Zm00001d005772
Zm00001d031529_T010	0.9267784	0.085698785	Zm00001d031529
Zm00001d031529_T018	0.7114961	0.718768869	Zm00001d031529
Zm00001d031529_T009	0.9962521	0.150799279	Zm00001d031529
Zm00001d031529_T019	1	0.116873652	Zm00001d031529
Zm00001d023277_T001	0.6324192	1.198985977	Zm00001d023277
Zm00001d023277_T002	0.8001345	-0.189723411	Zm00001d023277
Zm00001d034523_T001	0.7791747	-0.130571005	Zm00001d034523
Zm00001d035236_T001	0.7663452	-0.37971734	Zm00001d035236
Zm00001d006345_T002	0.9840043	0.146239628	Zm00001d006345
Zm00001d012845_T002	0.9906739	0.286458329	Zm00001d012845
Zm00001d012845_T001	0.9983524	-0.007536393	Zm00001d012845
Zm00001d033217_T009	0.3105424	-1.065050816	Zm00001d033217
Zm00001d033217_T004	0.3682405	1.428662336	Zm00001d033217
Zm00001d033217_T008	7.47E-07	0.847496959	Zm00001d033217
Zm00001d033217_T002	0.5243524	1.215194308	Zm00001d033217
Zm00001d033217_T010	0.6523514	1.651384189	Zm00001d033217

Zm00001d033217_T006	0.2916362	0.386759319	Zm00001d033217
Zm00001d005185_T001	0.9860836	0.083286838	Zm00001d005185
Zm00001d005185_T002	0.7146325	-0.434276227	Zm00001d005185
Zm00001d021488_T004	0.7055235	0.393621891	Zm00001d021488
Zm00001d021488_T007	0.8886159	0.648748485	Zm00001d021488
Zm00001d021488_T011	0.3356906	0.178099346	Zm00001d021488
Zm00001d021488_T013	0.940651	0.14414882	Zm00001d021488
Zm00001d010972_T001	0.8434952	-0.066176605	Zm00001d010972
Zm00001d005584_T006	0.3608099	-1.174536361	Zm00001d005584
Zm00001d005584_T008	1	0.143445339	Zm00001d005584
Zm00001d005584_T003	0.2726876	0.402968878	Zm00001d005584
Zm00001d005584_T007	0.4836377	-0.566444723	Zm00001d005584
Zm00001d005584_T002	0.9825858	0.186238406	Zm00001d005584
Zm00001d040148_T001	0.006754496	2.16925368	Zm00001d040148
Zm00001d005199_T001	0.7995023	-0.194314728	Zm00001d005199
Zm00001d043573_T001	0.9777274	0.224171111	Zm00001d043573
Zm00001d047875_T002	1.27E-08	1.433164868	Zm00001d047875
Zm00001d047875_T001	0.1950386	0.578159749	Zm00001d047875
Zm00001d047875_T008	0.3689855	0.808781595	Zm00001d047875
Zm00001d047875_T005	0.8581713	0.282572481	Zm00001d047875
Zm00001d047875_T006	0.8158416	0.964168541	Zm00001d047875
Zm00001d048382_T001	0.6275813	-0.862010222	Zm00001d048382
Zm00001d035470_T001	0.000672201	1.668390668	Zm00001d035470
Zm00001d035470_T012	0.01477598	-1.199425475	Zm00001d035470
Zm00001d035470_T007	2.91E-15	3.358222296	Zm00001d035470
Zm00001d035470_T003	0.941809	0.231679435	Zm00001d035470
Zm00001d034884_T001	0.9216521	-0.102752128	Zm00001d034884
Zm00001d024947_T005	0.9467359	0.062400571	Zm00001d024947
Zm00001d024947_T002	0.98291	0.334526669	Zm00001d024947
Zm00001d001968_T001	0.9311858	0.725069255	Zm00001d001968
Zm00001d020524_T001	0.9994624	0.363938814	Zm00001d020524
Zm00001d001949_T001	0.9921377	0.213804135	Zm00001d001949
Zm00001d051830_T002	0.8031436	-0.283420848	Zm00001d051830
Zm00001d008902_T001	0.9390517	0.687551664	Zm00001d008902
Zm00001d040286_T003	0.706283	1.044630122	Zm00001d040286
Zm00001d040286_T002	0.9692063	0.098490495	Zm00001d040286
Zm00001d040286_T001	0.8891117	0.685351235	Zm00001d040286
Zm00001d007345_T001	0.7294039	1.131463364	Zm00001d007345
Zm00001d005865_T001	0.9737419	0.095056879	Zm00001d005865
Zm00001d018072_T001	0.9706293	0.627170687	Zm00001d018072
Zm00001d021645_T001	0.6621234	-0.610898637	Zm00001d021645
Zm00001d049233_T001	0.8513569	-0.068860642	Zm00001d049233
Zm00001d004335_T001	0.0830571	2.336127641	Zm00001d004335
Zm00001d028111_T004	0.5574313	1.279209598	Zm00001d028111
Zm00001d028111_T003	0.07996841	-1.39518015	Zm00001d028111
Zm00001d028111_T005	0.941809	0.21170541	Zm00001d028111
Zm00001d028111_T002	0.0161377	0.830303369	Zm00001d028111
Zm00001d008249_T013	0.7458694	0.577182221	Zm00001d008249
Zm00001d008249_T007	0.04824454	-0.440702332	Zm00001d008249
Zm00001d008249_T001	0.827133	0.034960782	Zm00001d008249
Zm00001d008249_T008	0.2916362	-0.168241639	Zm00001d008249
Zm00001d008249_T004	0.6318866	1.454598598	Zm00001d008249
Zm00001d045621_T001	0.9909153	0.513905453	Zm00001d045621
Zm00001d012353_T001	0.8377512	-0.262872165	Zm00001d012353

Zm00001d052494_T002	0.000166376	-0.679382313	Zm00001d052494
Zm00001d052494_T001	0.8383745	-0.160005979	Zm00001d052494
Zm00001d031270_T001	0.9614723	0.476518593	Zm00001d031270
Zm00001d002844_T002	0.7485836	0.69143495	Zm00001d002844
Zm00001d042765_T001	0.6344415	-0.785686058	Zm00001d042765
Zm00001d033160_T001	0.8987811	-0.056716636	Zm00001d033160
Zm00001d023452_T002	0.9422098	0.071073062	Zm00001d023452
Zm00001d023452_T003	0.7195936	0.425373482	Zm00001d023452
Zm00001d018555_T001	0.9932006	0.179950829	Zm00001d018555
Zm00001d025665_T001	0.7860612	-0.147164476	Zm00001d025665
Zm00001d025665_T003	0.9918302	0.482696564	Zm00001d025665
Zm00001d025665_T006	0.3002805	1.287467372	Zm00001d025665
Zm00001d003147_T002	0.5731688	-0.485399392	Zm00001d003147
Zm00001d003147_T001	0.9745948	0.633142839	Zm00001d003147
Zm00001d013517_T001	0.7548788	-0.486380829	Zm00001d013517
Zm00001d029552_T001	0.9824516	0.24785051	Zm00001d029552
Zm00001d023634_T001	0.3800213	0.910246902	Zm00001d023634
Zm00001d007034_T009	0.6717063	1.488763277	Zm00001d007034
Zm00001d007034_T007	0.5778967	0.712632165	Zm00001d007034
Zm00001d007034_T017	0.8665954	-0.03327203	Zm00001d007034
Zm00001d007034_T003	0.4669227	0.342110271	Zm00001d007034
Zm00001d007034_T011	0.9740325	0.679497509	Zm00001d007034
Zm00001d007034_T020	0.780547	1.384110266	Zm00001d007034
Zm00001d007034_T021	0.4988467	0.364917534	Zm00001d007034
Zm00001d007034_T008	0.5448104	0.508446054	Zm00001d007034
Zm00001d020261_T001	0.0318764	0.901077477	Zm00001d020261
Zm00001d002421_T001	0.9022629	0.304621321	Zm00001d002421
Zm00001d037696_T024	0.9249382	0.654475251	Zm00001d037696
Zm00001d037696_T005	0.002004246	1.122275462	Zm00001d037696
Zm00001d037696_T001	0.8050292	0.714036944	Zm00001d037696
Zm00001d037696_T032	0.8246838	0.159445611	Zm00001d037696
Zm00001d037696_T044	0.3071757	0.543976945	Zm00001d037696
Zm00001d052807_T001	0.1580062	-1.314303461	Zm00001d052807
Zm00001d034858_T005	0.9038102	-0.083528081	Zm00001d034858
Zm00001d034858_T002	0.6446589	-0.180053038	Zm00001d034858
Zm00001d034858_T004	0.8449483	-0.199096342	Zm00001d034858
Zm00001d034858_T006	0.06609607	-1.258165136	Zm00001d034858
Zm00001d034858_T003	0.7901736	0.392811616	Zm00001d034858
Zm00001d013786_T001	0.9979568	0.303896494	Zm00001d013786
Zm00001d024624_T004	0.98291	0.211726682	Zm00001d024624
Zm00001d038926_T001	0.8705753	0.558553642	Zm00001d038926
Zm00001d031167_T002	0.9160053	-0.010446008	Zm00001d031167
Zm00001d053356_T002	0.8192173	-0.164345315	Zm00001d053356
Zm00001d053356_T001	0.9768468	0.190710905	Zm00001d053356
Zm00001d017455_T001	0.9979568	0.021889256	Zm00001d017455
Zm00001d012815_T038	0.9950703	0.373760911	Zm00001d012815
Zm00001d012815_T025	0.8486461	0.883624729	Zm00001d012815
Zm00001d012815_T002	0.004411808	1.382494644	Zm00001d012815
Zm00001d011692_T001	0.9419336	-0.006132167	Zm00001d011692
Zm00001d033283_T001	0.3546728	-0.746045909	Zm00001d033283
Zm00001d045946_T001	0.3294423	1.489814773	Zm00001d045946
Zm00001d038916_T001	0.9091907	0.807406435	Zm00001d038916
Zm00001d038916_T002	0.4224883	0.618521818	Zm00001d038916
Zm00001d035887_T025	0.9370449	-0.084186655	Zm00001d035887

Zm00001d035887_T016	0.5507795	0.390789373	Zm00001d035887
Zm00001d017366_T001	0.1603091	-1.004649171	Zm00001d017366
Zm00001d042397_T001	0.9513407	0.658241241	Zm00001d042397
Zm00001d012585_T001	0.4294873	1.053637217	Zm00001d012585
Zm00001d030471_T002	0.1601313	1.100566475	Zm00001d030471
Zm00001d030471_T001	0.9432413	0.712809237	Zm00001d030471
Zm00001d030471_T003	0.3212723	0.668966537	Zm00001d030471
Zm00001d012737_T001	0.9158684	0.688067575	Zm00001d012737
Zm00001d028161_T001	0.06234854	-1.647363053	Zm00001d028161
Zm00001d017516_T001	0.2084257	-1.065113869	Zm00001d017516
Zm00001d029473_T001	0.9205739	-0.116409095	Zm00001d029473
Zm00001d036541_T002	0.8631191	-0.118382478	Zm00001d036541
Zm00001d036541_T001	0.871546	-0.06951591	Zm00001d036541
Zm00001d038431_T001	0.9979568	0.394387396	Zm00001d038431
Zm00001d043875_T001	0.7663252	-0.709072827	Zm00001d043875
Zm00001d049120_T001	0.383181	-0.870306026	Zm00001d049120
Zm00001d006060_T001	0.7309184	-0.542817923	Zm00001d006060
Zm00001d011080_T001	0.01943547	-0.91307746	Zm00001d011080
Zm00001d003195_T001	0.5839931	-0.566395527	Zm00001d003195
Zm00001d026300_T001	0.2247419	-0.272170242	Zm00001d026300
Zm00001d018571_T003	0.5903112	-0.727392171	Zm00001d018571
Zm00001d018571_T001	0.2378089	-1.385804292	Zm00001d018571
Zm00001d018571_T002	0.6816502	-0.482016871	Zm00001d018571
Zm00001d052346_T001	0.002095691	2.734271789	Zm00001d052346
Zm00001d052346_T002	0.02685638	2.254696009	Zm00001d052346
Zm00001d008739_T001	0.9771057	0.229628369	Zm00001d008739
Zm00001d020762_T001	0.8056124	-0.279095261	Zm00001d020762
Zm00001d041620_T006	0.9841968	0.174771513	Zm00001d041620
Zm00001d041620_T011	0.7284649	-0.463257585	Zm00001d041620
Zm00001d041620_T007	0.9747606	0.205282606	Zm00001d041620
Zm00001d041620_T012	0.1983022	-1.312261705	Zm00001d041620
Zm00001d041620_T009	0.4332539	-0.376274976	Zm00001d041620
Zm00001d041620_T008	5.31E-10	2.025174992	Zm00001d041620
Zm00001d041620_T002	0.972985	0.557045089	Zm00001d041620
Zm00001d017793_T003	0.04922677	-1.205344263	Zm00001d017793
Zm00001d017793_T004	0.9860836	0.180381056	Zm00001d017793
Zm00001d017793_T007	0.957148	0.130499221	Zm00001d017793
Zm00001d017793_T001	0.373511	1.513092679	Zm00001d017793
Zm00001d017172_T001	0.9075873	-0.364358311	Zm00001d017172
Zm00001d031281_T001	0.8380736	-0.174922738	Zm00001d031281
Zm00001d047883_T001	0.0200952	2.547328925	Zm00001d047883
Zm00001d027557_T001	0.6012987	-0.702084568	Zm00001d027557
Zm00001d007945_T014	0.5785775	0.378997843	Zm00001d007945
Zm00001d007945_T011	0.8816374	0.694269291	Zm00001d007945
Zm00001d007945_T013	0.5437049	0.304184598	Zm00001d007945
Zm00001d007945_T008	0.6746035	1.273575115	Zm00001d007945
Zm00001d007945_T009	0.006314872	1.563878344	Zm00001d007945
Zm00001d007945_T003	3.44E-06	0.772115902	Zm00001d007945
Zm00001d007945_T006	0.09972704	0.360848827	Zm00001d007945
Zm00001d029663_T001	0.9187004	0.022664861	Zm00001d029663
Zm00001d021534_T001	0.9083252	-0.060972903	Zm00001d021534
Zm00001d010447_T001	0.9810561	0.217194484	Zm00001d010447
Zm00001d010447_T003	0.8486887	-0.151157869	Zm00001d010447
Zm00001d037731_T019	0.3855907	0.874905397	Zm00001d037731

Zm00001d037731_T012	0.2800533	1.032747964	Zm00001d037731
Zm00001d037731_T017	0.002572745	1.465770705	Zm00001d037731
Zm00001d037731_T006	0.357066	0.550681176	Zm00001d037731
Zm00001d037731_T020	0.7564036	-0.658023613	Zm00001d037731
Zm00001d037731_T009	0.5864438	0.232597159	Zm00001d037731
Zm00001d037731_T004	0.9745777	0.071601129	Zm00001d037731
Zm00001d042726_T001	0.941922	-0.19616509	Zm00001d042726
Zm00001d009233_T008	0.01402472	-0.875683117	Zm00001d009233
Zm00001d009233_T002	0.3643104	1.664279097	Zm00001d009233
Zm00001d009233_T001	0.08332133	0.756637901	Zm00001d009233
Zm00001d029380_T003	0.708953	-0.587935177	Zm00001d029380
Zm00001d029380_T001	0.9717213	-0.176388779	Zm00001d029380
Zm00001d052034_T001	0.9139882	0.607696506	Zm00001d052034
Zm00001d052034_T002	1	0.007983865	Zm00001d052034
Zm00001d026357_T001	0.5277554	-0.647373075	Zm00001d026357
Zm00001d028954_T001	0.9988063	0.254039325	Zm00001d028954
Zm00001d028949_T001	0.01356004	2.181220276	Zm00001d028949
Zm00001d028949_T002	0.1574436	2.092920356	Zm00001d028949
Zm00001d028949_T004	0.00095437	1.569224209	Zm00001d028949
Zm00001d028949_T003	0.002075853	2.688250772	Zm00001d028949
Zm00001d017832_T003	0.3675057	0.2876636	Zm00001d017832
Zm00001d017832_T001	0.968971	0.609107862	Zm00001d017832
Zm00001d039963_T001	0.07228609	2.30408545	Zm00001d039963
Zm00001d001869_T001	0.7729753	-0.347447411	Zm00001d001869
Zm00001d020323_T001	0.04515747	2.384264844	Zm00001d020323
Zm00001d032886_T001	0.3415934	-1.222797566	Zm00001d032886
Zm00001d014314_T001	0.1976354	1.270334648	Zm00001d014314
Zm00001d044226_T002	0.9395327	0.52615323	Zm00001d044226
Zm00001d048502_T001	0.7746801	0.697603707	Zm00001d048502
Zm00001d048502_T003	0.8325302	0.567821396	Zm00001d048502
Zm00001d048502_T004	0.908501	-0.020604475	Zm00001d048502
Zm00001d010256_T001	0.7827421	-1.004608501	Zm00001d010256
Zm00001d014991_T005	0.50213	-1.021367763	Zm00001d014991
Zm00001d014991_T004	0.8929316	0.698509366	Zm00001d014991
Zm00001d017640_T001	0.9852645	0.302402874	Zm00001d017640
Zm00001d017640_T007	0.9894615	0.210867034	Zm00001d017640
Zm00001d017640_T004	0.9404125	0.292880079	Zm00001d017640
Zm00001d017640_T005	0.8899623	0.464746451	Zm00001d017640
Zm00001d017111_T002	0.852305	-0.143360857	Zm00001d017111
Zm00001d017111_T001	0.9228108	0.736603741	Zm00001d017111
Zm00001d001818_T001	0.7697196	0.955776785	Zm00001d001818
Zm00001d036648_T001	0.7565187	-0.785269861	Zm00001d036648
Zm00001d047498_T005	0.9657993	0.087817656	Zm00001d047498
Zm00001d047498_T008	1	0.364771329	Zm00001d047498
Zm00001d047498_T023	0.7771984	-0.325432339	Zm00001d047498
Zm00001d047498_T014	0.6414865	-0.003486982	Zm00001d047498
Zm00001d047498_T012	0.6528356	-0.278252742	Zm00001d047498
Zm00001d047498_T006	1	0.306386977	Zm00001d047498
Zm00001d047498_T004	0.745718	-0.400445504	Zm00001d047498
Zm00001d047498_T019	0.8593984	0.232036708	Zm00001d047498
Zm00001d047498_T010	0.851982	1.207121173	Zm00001d047498
Zm00001d047498_T015	0.967161	-0.137651337	Zm00001d047498
Zm00001d013023_T011	0.3822529	1.178548039	Zm00001d013023
Zm00001d013023_T015	0.342013	0.838797589	Zm00001d013023

Zm00001d013023_T018	0.6160016	-0.612715479	Zm00001d013023
Zm00001d013023_T016	0.4054378	0.372593061	Zm00001d013023
Zm00001d013023_T017	0.9974023	-0.048948785	Zm00001d013023
Zm00001d013023_T006	0.6866574	-0.124922247	Zm00001d013023
Zm00001d048123_T001	0.8991659	-0.052102637	Zm00001d048123
Zm00001d013006_T004	0.6129837	-0.305075021	Zm00001d013006
Zm00001d013006_T020	0.3319365	-0.72902751	Zm00001d013006
Zm00001d013006_T007	0.1499909	1.675544737	Zm00001d013006
Zm00001d013006_T024	0.1360197	-0.569012108	Zm00001d013006
Zm00001d042213_T001	0.8498341	-0.130123278	Zm00001d042213
Zm00001d042213_T003	7.04E-07	1.489364621	Zm00001d042213
Zm00001d042213_T002	0.891216	0.715822638	Zm00001d042213
Zm00001d042213_T005	0.7233733	0.496527702	Zm00001d042213
Zm00001d023558_T001	0.8884927	-0.133262893	Zm00001d023558
Zm00001d016806_T001	0.6606202	1.171449275	Zm00001d016806
Zm00001d003684_T001	0.9740325	0.365382981	Zm00001d003684
Zm00001d037340_T001	0.5013235	-1.019839911	Zm00001d037340
Zm00001d021665_T001	0.4316312	1.422888252	Zm00001d021665
Zm00001d009567_T001	0.9448555	-0.23226236	Zm00001d009567
Zm00001d043536_T001	0.7454635	-0.422565308	Zm00001d043536
Zm00001d021623_T004	0.9240572	0.787693158	Zm00001d021623
Zm00001d021623_T005	0.000422585	0.680278311	Zm00001d021623
Zm00001d051055_T001	0.9745777	0.263482323	Zm00001d051055
Zm00001d051055_T002	0.3340257	0.61344769	Zm00001d051055
Zm00001d048621_T001	0.9860836	0.176658766	Zm00001d048621
Zm00001d021909_T001	0.8899659	0.524054589	Zm00001d021909
Zm00001d002592_T001	1.34E-05	2.689982045	Zm00001d002592
Zm00001d035501_T001	0.8710199	-0.149825586	Zm00001d035501
Zm00001d020975_T002	0.7972183	0.770550193	Zm00001d020975
Zm00001d020975_T005	0.9673473	0.124323055	Zm00001d020975
Zm00001d035773_T004	0.9936756	0.484076842	Zm00001d035773
Zm00001d035773_T001	0.9804228	0.116063248	Zm00001d035773
Zm00001d050438_T001	1	0.243981943	Zm00001d050438
Zm00001d016298_T002	0.8720862	-0.014833618	Zm00001d016298
Zm00001d030154_T001	0.8158416	0.649835531	Zm00001d030154
Zm00001d047840_T001	0.525543	-0.627472595	Zm00001d047840
Zm00001d031992_T004	0.8781102	0.774558851	Zm00001d031992
Zm00001d031992_T006	0.5988362	1.014361806	Zm00001d031992
Zm00001d031992_T005	0.9303773	0.779970399	Zm00001d031992
Zm00001d031992_T003	0.918657	0.829996471	Zm00001d031992
Zm00001d031992_T001	2.02E-12	-2.727797317	Zm00001d031992
Zm00001d034558_T001	0.7831859	0.99318961	Zm00001d034558
Zm00001d042344_T005	0.02765504	1.305630654	Zm00001d042344
Zm00001d042344_T010	0.727617	1.859341352	Zm00001d042344
Zm00001d042344_T004	0.8863456	0.890506716	Zm00001d042344
Zm00001d042344_T009	0.000221768	-2.461141568	Zm00001d042344
Zm00001d003616_T001	0.9673516	0.10545461	Zm00001d003616
Zm00001d003616_T008	0.2581894	-1.27411212	Zm00001d003616
Zm00001d003616_T014	1	0.272396452	Zm00001d003616
Zm00001d003616_T007	0.000103032	-4.164100832	Zm00001d003616
Zm00001d003616_T024	0.8936314	0.65649827	Zm00001d003616
Zm00001d003616_T022	0.6645568	-0.455302989	Zm00001d003616
Zm00001d003616_T002	3.48E-07	0.688314273	Zm00001d003616
Zm00001d003616_T025	0.001748277	-1.919670059	Zm00001d003616

Zm00001d003616_T003	0.3182957	0.499320993	Zm00001d003616
Zm00001d003616_T006	0.8660897	-0.100052857	Zm00001d003616
Zm00001d044164_T004	0.4616191	1.154314001	Zm00001d044164
Zm00001d044164_T001	0.8077635	0.114942593	Zm00001d044164
Zm00001d044164_T006	0.7791621	-0.264065197	Zm00001d044164
Zm00001d044164_T010	0.3021408	0.637738948	Zm00001d044164
Zm00001d018422_T001	0.5928377	-0.444916261	Zm00001d018422
Zm00001d006459_T002	0.92623	0.063213515	Zm00001d006459
Zm00001d006459_T008	0.08136102	0.699525713	Zm00001d006459
Zm00001d006459_T006	0.147668	0.865214386	Zm00001d006459
Zm00001d006459_T004	0.5048245	0.989648388	Zm00001d006459
Zm00001d017392_T005	0.6010203	1.342176162	Zm00001d017392
Zm00001d017392_T004	0.5636783	0.857461896	Zm00001d017392
Zm00001d017392_T008	0.582048	1.011276081	Zm00001d017392
Zm00001d017392_T002	2.48E-12	-2.524064813	Zm00001d017392
Zm00001d016745_T001	0.405609	-1.007426651	Zm00001d016745
Zm00001d036135_T008	0.4375527	-0.224954765	Zm00001d036135
Zm00001d036135_T005	0.9290482	0.002287903	Zm00001d036135
Zm00001d035092_T002	0.961399	0.022570477	Zm00001d035092
Zm00001d046624_T012	0.8951107	-0.139424192	Zm00001d046624
Zm00001d046624_T007	0.05874777	2.206336906	Zm00001d046624
Zm00001d046624_T006	0.7812261	1.098439543	Zm00001d046624
Zm00001d035343_T001	0.7289978	-0.438191893	Zm00001d035343
Zm00001d035343_T002	0.3987447	-0.67901374	Zm00001d035343
Zm00001d017751_T001	0.7294039	-0.177554335	Zm00001d017751
Zm00001d008898_T001	0.003015693	-1.752123637	Zm00001d008898
Zm00001d019946_T001	0.941809	-0.003092722	Zm00001d019946
Zm00001d029697_T003	0.9561521	0.285339751	Zm00001d029697
Zm00001d029697_T001	0.9127665	0.302376144	Zm00001d029697
Zm00001d034759_T007	0.2529099	0.812705687	Zm00001d034759
Zm00001d034759_T006	0.8293703	0.213654284	Zm00001d034759
Zm00001d034759_T004	0.7697196	0.186587319	Zm00001d034759
Zm00001d034759_T001	0.9375054	0.306910711	Zm00001d034759
Zm00001d034759_T005	0.9979568	0.444936122	Zm00001d034759
Zm00001d044429_T004	0.2890237	1.170556653	Zm00001d044429
Zm00001d044429_T001	0.5910253	-0.625383976	Zm00001d044429
Zm00001d044429_T003	0.9812314	0.001854396	Zm00001d044429
Zm00001d044429_T005	0.8747821	0.420007768	Zm00001d044429
Zm00001d048445_T002	9.12E-09	-1.779708844	Zm00001d048445
Zm00001d048445_T001	0.5853819	-0.583288917	Zm00001d048445
Zm00001d048445_T003	0.9747714	0.050205176	Zm00001d048445
Zm00001d048445_T006	0.8462243	-0.267317276	Zm00001d048445
Zm00001d051678_T007	0.7379612	-0.291675804	Zm00001d051678
Zm00001d051678_T002	0.6720294	0.522637973	Zm00001d051678
Zm00001d051678_T012	0.8001345	0.334264155	Zm00001d051678
Zm00001d051678_T004	0.11023	1.263773767	Zm00001d051678
Zm00001d006267_T001	0.8267985	0.637142123	Zm00001d006267
Zm00001d006267_T006	0.8913084	-0.061803245	Zm00001d006267
Zm00001d006267_T003	0.6436915	0.296533757	Zm00001d006267
Zm00001d054095_T001	0.4089036	0.845762938	Zm00001d054095
Zm00001d027811_T004	0.9825858	0.524964607	Zm00001d027811
Zm00001d027811_T006	0.001669656	-0.608667759	Zm00001d027811
Zm00001d027811_T003	0.1696197	0.694137688	Zm00001d027811
Zm00001d027811_T002	0.9317232	-0.007706349	Zm00001d027811

Zm00001d027811_T007	0.9979568	0.238398979	Zm00001d027811
Zm00001d026515_T006	0.03493085	1.752232528	Zm00001d026515
Zm00001d026515_T001	0.9774157	0.615735106	Zm00001d026515
Zm00001d026515_T008	0.000142536	-2.6313052	Zm00001d026515
Zm00001d026515_T007	0.9210327	-0.063148379	Zm00001d026515
Zm00001d050220_T002	0.9326774	-0.0662265	Zm00001d050220
Zm00001d052532_T001	0.7002849	-0.430366236	Zm00001d052532
Zm00001d001871_T001	0.7192872	-0.332829716	Zm00001d001871
Zm00001d048140_T001	0.9196877	0.514750165	Zm00001d048140
Zm00001d053555_T001	1	0.34705381	Zm00001d053555
Zm00001d040360_T001	1	0.369011552	Zm00001d040360
Zm00001d051349_T002	0.189099	-1.051522054	Zm00001d051349
Zm00001d042585_T003	0.05522541	2.105354752	Zm00001d042585
Zm00001d042585_T005	0.5072553	1.297283647	Zm00001d042585
Zm00001d042585_T001	0.9965026	0.349628342	Zm00001d042585
Zm00001d042585_T006	0.3217764	-1.427604127	Zm00001d042585
Zm00001d032745_T001	0.972985	0.471919747	Zm00001d032745
Zm00001d018135_T001	0.9706293	-0.001063987	Zm00001d018135
Zm00001d015783_T001	0.9563257	0.52014765	Zm00001d015783
Zm00001d011411_T007	0.9894615	0.028686707	Zm00001d011411
Zm00001d011411_T001	0.5711339	1.377534157	Zm00001d011411
Zm00001d011411_T022	0.8704456	-0.17458167	Zm00001d011411
Zm00001d011411_T005	0.09554992	0.562841578	Zm00001d011411
Zm00001d011411_T008	0.9693993	-0.061695254	Zm00001d011411
Zm00001d034730_T001	0.4397133	1.568827115	Zm00001d034730
Zm00001d034730_T005	5.15E-15	-3.23548605	Zm00001d034730
Zm00001d031797_T001	0.3207142	1.65030113	Zm00001d031797
Zm00001d019875_T001	0.01391558	0.953359503	Zm00001d019875
Zm00001d019875_T002	0.5568148	1.27219128	Zm00001d019875
Zm00001d036148_T003	0.926928	0.191328773	Zm00001d036148
Zm00001d036148_T004	0.9203655	-0.064829598	Zm00001d036148
Zm00001d007965_T003	0.9177108	0.527284745	Zm00001d007965
Zm00001d010901_T002	0.9561521	-0.004477728	Zm00001d010901
Zm00001d010901_T004	0.03752477	-2.298129221	Zm00001d010901
Zm00001d046697_T003	0.1372972	-1.149375377	Zm00001d046697
Zm00001d046697_T001	0.6339643	-0.445347759	Zm00001d046697
Zm00001d004924_T001	0.9249935	-0.040916725	Zm00001d004924
Zm00001d023330_T001	0.1143001	1.560746382	Zm00001d023330
Zm00001d049610_T001	0.8665954	-0.131587047	Zm00001d049610
Zm00001d014073_T003	0.96672	-0.280758122	Zm00001d014073
Zm00001d014073_T001	0.8050292	-0.566744444	Zm00001d014073
Zm00001d014073_T002	0.7292263	-0.787268381	Zm00001d014073
Zm00001d014073_T004	0.9672926	1.453699622	Zm00001d014073
Zm00001d023296_T001	0.9692063	0.099594701	Zm00001d023296
Zm00001d035974_T001	0.8964117	-0.083955139	Zm00001d035974
Zm00001d035974_T002	0.8730771	-0.071159287	Zm00001d035974
Zm00001d031977_T001	0.9719113	0.610359402	Zm00001d031977
Zm00001d036801_T001	0.8992258	-0.032339565	Zm00001d036801
Zm00001d011102_T001	0.9763608	0.2016643	Zm00001d011102
Zm00001d003064_T001	0.4178453	-0.832581383	Zm00001d003064
Zm00001d026642_T001	0.9745948	-0.253799269	Zm00001d026642
Zm00001d042738_T001	0.03715458	-1.743438207	Zm00001d042738
Zm00001d052207_T001	0.7011505	-0.620470955	Zm00001d052207
Zm00001d044593_T014	0.1680022	0.60901953	Zm00001d044593

Zm00001d044593_T008	0.000421721	-1.7576604	Zm00001d044593
Zm00001d044593_T001	0.9804228	0.25172248	Zm00001d044593
Zm00001d044593_T018	0.7082783	-0.240559829	Zm00001d044593
Zm00001d044593_T017	0.8817666	-0.073892577	Zm00001d044593
Zm00001d034034_T013	0.135934	-0.310078654	Zm00001d034034
Zm00001d034034_T005	0.03974992	1.169961184	Zm00001d034034
Zm00001d034034_T007	0.4293866	-0.531370518	Zm00001d034034
Zm00001d034034_T010	1.57E-08	0.800316056	Zm00001d034034
Zm00001d034034_T006	0.9970835	-0.024317494	Zm00001d034034
Zm00001d050501_T007	0.9252088	0.282390587	Zm00001d050501
Zm00001d050501_T010	0.2247608	2.140048931	Zm00001d050501
Zm00001d050501_T009	0.5843839	0.689913747	Zm00001d050501
Zm00001d050501_T004	0.2589117	-1.019248066	Zm00001d050501
Zm00001d050501_T005	0.002900162	-0.966437202	Zm00001d050501
Zm00001d050501_T008	4.03E-06	0.932879577	Zm00001d050501
Zm00001d003545_T001	0.4380979	-2.399350869	Zm00001d003545
Zm00001d015363_T001	0.9706887	0.098033184	Zm00001d015363
Zm00001d031956_T004	0.4037749	-1.142536038	Zm00001d031956
Zm00001d031956_T001	0.9824434	0.262032628	Zm00001d031956
Zm00001d031956_T005	0.9545838	0.323984008	Zm00001d031956
Zm00001d052364_T010	0.9918302	0.207141895	Zm00001d052364
Zm00001d052364_T011	0.9740325	0.560521945	Zm00001d052364
Zm00001d034055_T001	0.1878813	1.471064054	Zm00001d034055
Zm00001d034055_T028	0.5514634	-0.635446314	Zm00001d034055
Zm00001d034055_T004	0.9419336	0.036428795	Zm00001d034055
Zm00001d034055_T002	0.5849395	0.302535345	Zm00001d034055
Zm00001d034055_T018	0.7233733	0.249692959	Zm00001d034055
Zm00001d034055_T016	0.004954152	0.529689776	Zm00001d034055
Zm00001d034055_T023	0.9681793	0.028727884	Zm00001d034055
Zm00001d034055_T003	1	0.0293403	Zm00001d034055
Zm00001d034055_T013	0.4928022	0.804214765	Zm00001d034055
Zm00001d014650_T001	0.6745129	1.039131812	Zm00001d014650
Zm00001d019490_T001	1	0.29621721	Zm00001d019490
Zm00001d042478_T002	0.9693239	0.088127919	Zm00001d042478
Zm00001d042478_T001	0.08348055	-0.678860926	Zm00001d042478
Zm00001d011580_T002	0.4263859	1.472140466	Zm00001d011580
Zm00001d011580_T001	0.1990795	1.432882184	Zm00001d011580
Zm00001d011580_T003	0.4365578	1.047448667	Zm00001d011580
Zm00001d011580_T005	0.9031129	-0.021121788	Zm00001d011580
Zm00001d031824_T002	0.9709778	-0.090530731	Zm00001d031824
Zm00001d031824_T001	0.9835413	0.084446145	Zm00001d031824
Zm00001d031824_T003	0.8624945	-0.314732449	Zm00001d031824
Zm00001d037833_T001	0.2597981	-0.176403065	Zm00001d037833
Zm00001d015313_T002	1	0.2475729	Zm00001d015313
Zm00001d015313_T001	0.7640707	-0.331581399	Zm00001d015313
Zm00001d019498_T001	0.7635481	-0.367679974	Zm00001d019498
Zm00001d023887_T001	0.9936409	0.222554307	Zm00001d023887
Zm00001d018795_T104	0.9194411	0.790529627	Zm00001d018795
Zm00001d018795_T047	0.00017836	1.523646958	Zm00001d018795
Zm00001d018795_T071	0.3146885	0.979415223	Zm00001d018795
Zm00001d018795_T040	0.03890213	0.906578387	Zm00001d018795
Zm00001d018795_T022	0.3756254	0.26059437	Zm00001d018795
Zm00001d018795_T018	2.29E-09	0.916360699	Zm00001d018795
Zm00001d018795_T082	0.06858518	0.811523129	Zm00001d018795

Zm00001d018795_T083	0.006476742	0.525695415	Zm00001d018795
Zm00001d018795_T059	4.03E-06	0.355593074	Zm00001d018795
Zm00001d018795_T062	3.65E-08	0.673799819	Zm00001d018795
Zm00001d018795_T009	0.000307041	0.393804365	Zm00001d018795
Zm00001d018795_T095	0.3469239	1.499436223	Zm00001d018795
Zm00001d015204_T001	0.8508017	-0.327279026	Zm00001d015204
Zm00001d049724_T001	0.8591203	-0.091656816	Zm00001d049724
Zm00001d011832_T001	0.9368844	-0.036991691	Zm00001d011832
Zm00001d009027_T001	0.9404125	0.013592955	Zm00001d009027
Zm00001d009027_T004	0.9988954	0.3504839	Zm00001d009027
Zm00001d009027_T005	0.9173494	-0.266190476	Zm00001d009027
Zm00001d007581_T001	0.6544687	1.033531047	Zm00001d007581
Zm00001d002594_T001	0.1991689	-0.705978331	Zm00001d002594
Zm00001d030345_T001	0.4606419	-0.875097904	Zm00001d030345
Zm00001d028529_T001	0.9088617	-0.109644984	Zm00001d028529
Zm00001d041480_T001	0.8158142	-0.183187438	Zm00001d041480
Zm00001d012866_T006	0.08686142	-1.041421663	Zm00001d012866
Zm00001d012866_T003	0.9979568	0.282566408	Zm00001d012866
Zm00001d012866_T001	1	0.229316118	Zm00001d012866
Zm00001d012866_T002	0.9269932	0.206692528	Zm00001d012866
Zm00001d017101_T001	0.9244812	-0.041177928	Zm00001d017101
Zm00001d016997_T001	0.5741984	-0.824044735	Zm00001d016997
Zm00001d003516_T003	0.8786965	0.526232322	Zm00001d003516
Zm00001d003516_T001	0.3881179	1.06529563	Zm00001d003516
Zm00001d043830_T001	0.9613831	0.45901833	Zm00001d043830
Zm00001d033502_T003	0.2750008	0.77538833	Zm00001d033502
Zm00001d033502_T008	0.9894615	0.027245671	Zm00001d033502
Zm00001d033502_T002	0.9970835	0.450786443	Zm00001d033502
Zm00001d033502_T005	0.7505997	1.183720527	Zm00001d033502
Zm00001d020544_T001	1	0.38214615	Zm00001d020544
Zm00001d034872_T001	0.9740325	0.114023278	Zm00001d034872
Zm00001d034872_T002	0.909044	-0.076618153	Zm00001d034872
Zm00001d029625_T001	1	0.014596284	Zm00001d029625
Zm00001d044570_T065	0.7315461	-0.383479768	Zm00001d044570
Zm00001d044570_T002	1	0.046902224	Zm00001d044570
Zm00001d044570_T025	0.7378574	-0.27318599	Zm00001d044570
Zm00001d044570_T032	0.001309444	1.345673946	Zm00001d044570
Zm00001d044570_T006	0.9657993	0.21935925	Zm00001d044570
Zm00001d044570_T028	3.81E-09	1.313309089	Zm00001d044570
Zm00001d044570_T067	0.3916044	-0.584017896	Zm00001d044570
Zm00001d044570_T064	0.5348533	-0.511480448	Zm00001d044570
Zm00001d044570_T003	0.9855117	0.272670101	Zm00001d044570
Zm00001d033884_T001	0.6745773	-0.332940007	Zm00001d033884
Zm00001d006119_T001	0.8264047	-0.673636809	Zm00001d006119
Zm00001d019764_T001	0.9962521	0.157744031	Zm00001d019764
Zm00001d012851_T001	0.9646144	0.620785912	Zm00001d012851
Zm00001d015600_T002	1	0.25297313	Zm00001d015600
Zm00001d015600_T001	0.5189688	0.490986649	Zm00001d015600
Zm00001d007113_T003	0.9969123	-0.034058201	Zm00001d007113
Zm00001d007113_T002	0.9969363	0.470532941	Zm00001d007113
Zm00001d007113_T001	0.8716588	-0.07963871	Zm00001d007113
Zm00001d048059_T012	0.9903568	0.481246938	Zm00001d048059
Zm00001d048059_T009	0.9693993	0.055417925	Zm00001d048059
Zm00001d048059_T017	0.680648	1.27280883	Zm00001d048059

Zm00001d048059_T014	0.927255	0.192607428	Zm00001d048059
Zm00001d048059_T011	0.2664773	0.425625617	Zm00001d048059
Zm00001d048059_T020	0.4599725	-0.748671083	Zm00001d048059
Zm00001d052066_T001	0.8111574	0.844250722	Zm00001d052066
Zm00001d010855_T006	0.9673473	0.191142348	Zm00001d010855
Zm00001d010855_T001	0.8720862	0.824600876	Zm00001d010855
Zm00001d038371_T002	0.8019448	1.397424968	Zm00001d038371
Zm00001d038371_T001	0.9257893	0.968943986	Zm00001d038371
Zm00001d051451_T001	9.73E-05	2.789157301	Zm00001d051451
Zm00001d033256_T001	0.6445834	-0.39429554	Zm00001d033256
Zm00001d040067_T001	0.3761973	1.667556932	Zm00001d040067
Zm00001d033747_T001	1	0.24808879	Zm00001d033747
Zm00001d028375_T003	0.06650348	0.722998982	Zm00001d028375
Zm00001d028375_T002	0.9778436	-0.019532072	Zm00001d028375
Zm00001d028375_T005	0.5874329	0.574195351	Zm00001d028375
Zm00001d021938_T001	0.9975882	0.35823169	Zm00001d021938
Zm00001d025371_T001	0.8612451	-0.244539005	Zm00001d025371
Zm00001d030132_T001	0.718063	0.650914176	Zm00001d030132
Zm00001d014191_T001	0.7169281	1.016445984	Zm00001d014191
Zm00001d031475_T001	0.9562711	-0.060127622	Zm00001d031475
Zm00001d038304_T001	0.7054529	-0.360924722	Zm00001d038304
Zm00001d011167_T001	0.9121108	-0.150624632	Zm00001d011167
Zm00001d013186_T002	0.705921	0.264107257	Zm00001d013186
Zm00001d053799_T001	0.3388363	0.591112546	Zm00001d053799
Zm00001d006866_T002	0.9780511	0.593482492	Zm00001d006866
Zm00001d006866_T001	1.24E-07	-1.566296141	Zm00001d006866
Zm00001d017762_T002	0.9103625	0.740361305	Zm00001d017762
Zm00001d017762_T004	0.4733392	1.560050375	Zm00001d017762
Zm00001d012336_T001	0.9605953	0.002548306	Zm00001d012336
Zm00001d048511_T010	1	0.049979483	Zm00001d048511
Zm00001d048511_T028	0.9196877	-0.373864121	Zm00001d048511
Zm00001d048511_T001	0.2427568	1.694988434	Zm00001d048511
Zm00001d048511_T012	0.9804228	0.268351104	Zm00001d048511
Zm00001d027929_T001	0.5177762	1.213571857	Zm00001d027929
Zm00001d049894_T011	0.452751	1.687083258	Zm00001d049894
Zm00001d049894_T029	0.8125662	-0.17091356	Zm00001d049894
Zm00001d049894_T027	0.3725038	1.681402683	Zm00001d049894
Zm00001d049894_T014	0.3443331	1.273468513	Zm00001d049894
Zm00001d025940_T001	0.9221995	-0.258072805	Zm00001d025940
Zm00001d020731_T001	0.6725291	0.986421793	Zm00001d020731
Zm00001d038650_T001	0.9561521	0.124788881	Zm00001d038650
Zm00001d048961_T001	0.7568663	-0.409806638	Zm00001d048961
Zm00001d031496_T001	0.2566009	-0.654546814	Zm00001d031496
Zm00001d033625_T003	0.9954444	0.122593566	Zm00001d033625
Zm00001d033625_T021	0.7590802	0.334672041	Zm00001d033625
Zm00001d033625_T058	0.9673473	-0.001492722	Zm00001d033625
Zm00001d029060_T007	0.2950821	1.565424107	Zm00001d029060
Zm00001d026258_T002	0.9545374	0.665066424	Zm00001d026258
Zm00001d026258_T008	0.5332111	-0.583144897	Zm00001d026258
Zm00001d034667_T003	0.7923921	0.644873683	Zm00001d034667
Zm00001d034667_T002	0.956479	0.07752459	Zm00001d034667
Zm00001d034667_T001	0.5966085	-0.44865994	Zm00001d034667
Zm00001d044673_T005	0.9561521	0.218591866	Zm00001d044673
Zm00001d044673_T001	0.9779656	0.224357558	Zm00001d044673

Zm00001d044673_T003	0.8558921	-0.1369721	Zm00001d044673
Zm00001d014760_T010	0.09040161	1.560458502	Zm00001d014760
Zm00001d014760_T003	0.9955906	0.477600535	Zm00001d014760
Zm00001d014760_T011	0.5761765	-0.227849624	Zm00001d014760
Zm00001d014760_T009	0.9644829	0.128313238	Zm00001d014760
Zm00001d009735_T002	0.9760472	0.309009431	Zm00001d009735
Zm00001d009735_T004	0.927255	-0.039534779	Zm00001d009735
Zm00001d002351_T002	3.84E-05	-2.742714599	Zm00001d002351
Zm00001d002351_T001	0.01261341	-1.371211709	Zm00001d002351
Zm00001d008610_T001	0.6969651	-0.486268625	Zm00001d008610
Zm00001d033823_T001	0.1402804	-0.83906596	Zm00001d033823
Zm00001d041387_T001	0.2419534	1.632099951	Zm00001d041387
Zm00001d015853_T001	0.9984041	0.266530598	Zm00001d015853
Zm00001d034719_T002	0.9888172	0.617480457	Zm00001d034719
Zm00001d034719_T003	0.9833513	0.216495076	Zm00001d034719
Zm00001d041472_T001	0.987516	0.222757168	Zm00001d041472
Zm00001d037608_T001	0.08908701	-1.467296059	Zm00001d037608
Zm00001d028757_T003	0.9890668	0.254767478	Zm00001d028757
Zm00001d004595_T003	0.9969123	0.200828655	Zm00001d004595
Zm00001d046388_T004	1	0.337266553	Zm00001d046388
Zm00001d046388_T003	0.8342838	-0.055989798	Zm00001d046388
Zm00001d017653_T001	0.9554972	-0.038704933	Zm00001d017653
Zm00001d006680_T001	0.7624517	-0.301604534	Zm00001d006680
Zm00001d040697_T001	0.9075317	0.722818393	Zm00001d040697
Zm00001d030264_T002	0.9033985	0.71681986	Zm00001d030264
Zm00001d030264_T003	0.9031129	0.476094871	Zm00001d030264
Zm00001d044333_T001	0.9988954	0.317600115	Zm00001d044333
Zm00001d023280_T001	0.6922415	-1.06291555	Zm00001d023280
Zm00001d053003_T003	0.01149774	-1.493981603	Zm00001d053003
Zm00001d053003_T002	0.9988864	0.442260592	Zm00001d053003
Zm00001d053003_T005	0.5015618	0.728980237	Zm00001d053003
Zm00001d033931_T003	0.3187416	-0.765423024	Zm00001d033931
Zm00001d033931_T001	0.2183658	-1.165760495	Zm00001d033931
Zm00001d033931_T006	0.000580616	-1.966542189	Zm00001d033931
Zm00001d033931_T002	0.01537203	-0.856653503	Zm00001d033931
Zm00001d035628_T005	0.09781749	-1.693949289	Zm00001d035628
Zm00001d035628_T004	0.9524127	0.469307191	Zm00001d035628
Zm00001d035628_T002	0.9464035	0.681530276	Zm00001d035628
Zm00001d035628_T003	1	0.161648873	Zm00001d035628
Zm00001d035628_T001	0.9450101	0.082182431	Zm00001d035628
Zm00001d042847_T001	0.6573819	-0.310737501	Zm00001d042847
Zm00001d008425_T003	0.9822138	0.292492154	Zm00001d008425
Zm00001d008425_T001	0.989787	0.092740345	Zm00001d008425
Zm00001d042393_T006	0.8585804	0.184253959	Zm00001d042393
Zm00001d042393_T009	0.9404125	0.002788093	Zm00001d042393
Zm00001d042393_T008	1	0.113796852	Zm00001d042393
Zm00001d049384_T002	0.9664406	-0.135664711	Zm00001d049384
Zm00001d013759_T001	0.8280859	0.416355628	Zm00001d013759
Zm00001d012237_T001	0.7672124	-0.599144298	Zm00001d012237
Zm00001d010062_T002	0.9706887	0.076496374	Zm00001d010062
Zm00001d012974_T021	1	0.206538151	Zm00001d012974
Zm00001d012974_T017	0.9745084	0.114672122	Zm00001d012974
Zm00001d012974_T010	0.8412913	0.205370361	Zm00001d012974
Zm00001d012974_T006	0.000143446	0.835176181	Zm00001d012974

Zm00001d012974_T060	0.006958403	-1.847231277	Zm00001d012974
Zm00001d012974_T052	0.1140589	1.217657749	Zm00001d012974
Zm00001d012974_T054	0.9015226	0.149885027	Zm00001d012974
Zm00001d012974_T005	0.530398	0.344716048	Zm00001d012974
Zm00001d012974_T061	0.9107561	-0.014975459	Zm00001d012974
Zm00001d012974_T070	0.366099	0.552096818	Zm00001d012974
Zm00001d012974_T007	0.835786	0.502967107	Zm00001d012974
Zm00001d020623_T001	0.6547916	-0.805951458	Zm00001d020623
Zm00001d034788_T001	0.9851204	0.303381376	Zm00001d034788
Zm00001d037849_T003	0.9361945	0.742485974	Zm00001d037849
Zm00001d024530_T002	0.9380783	0.047644307	Zm00001d024530
Zm00001d024530_T008	0.572715	-0.498999969	Zm00001d024530
Zm00001d024530_T006	0.1006909	-0.564348158	Zm00001d024530
Zm00001d051692_T001	0.1102686	-1.34096543	Zm00001d051692
Zm00001d014817_T001	0.9410688	-0.017143644	Zm00001d014817
Zm00001d010337_T001	0.9925447	0.214672712	Zm00001d010337
Zm00001d038579_T001	1	0.312221339	Zm00001d038579
Zm00001d037150_T001	0.9734722	0.536351577	Zm00001d037150
Zm00001d022050_T001	0.9804228	0.126870349	Zm00001d022050
Zm00001d022050_T002	0.9252088	-0.01599676	Zm00001d022050
Zm00001d039874_T002	0.9916451	0.185231597	Zm00001d039874
Zm00001d039874_T001	0.7983069	-0.446912702	Zm00001d039874
Zm00001d025305_T002	0.4530417	-1.021775879	Zm00001d025305
Zm00001d025305_T001	0.8922178	0.883251068	Zm00001d025305
Zm00001d045366_T002	0.9878022	0.269055989	Zm00001d045366
Zm00001d011629_T001	0.993158	0.433231032	Zm00001d011629
Zm00001d024873_T001	1	0.315736582	Zm00001d024873
Zm00001d006377_T001	0.9979443	0.289308134	Zm00001d006377
Zm00001d049929_T006	8.91E-05	-1.998479458	Zm00001d049929
Zm00001d049929_T003	0.8728536	1.625314435	Zm00001d049929
Zm00001d049929_T008	1	0.338845726	Zm00001d049929
Zm00001d049929_T001	0.3323541	-0.076856567	Zm00001d049929
Zm00001d039052_T001	0.9221549	0.017978729	Zm00001d039052
Zm00001d032900_T001	0.8140151	-0.159064528	Zm00001d032900
Zm00001d033965_T002	0.0372102	1.515122464	Zm00001d033965
Zm00001d033965_T003	0.903838	0.725891221	Zm00001d033965
Zm00001d033965_T001	0.4635996	1.455226152	Zm00001d033965
Zm00001d052316_T001	0.9852645	0.184669054	Zm00001d052316
Zm00001d052316_T002	0.9619687	0.504305311	Zm00001d052316
Zm00001d023564_T003	0.6002245	0.820369439	Zm00001d023564
Zm00001d023564_T001	0.9071133	-0.033853424	Zm00001d023564
Zm00001d023564_T002	0.8675673	-0.025924911	Zm00001d023564
Zm00001d029583_T001	0.6407184	-0.757545552	Zm00001d029583
Zm00001d011152_T105	0.255403	-0.829997357	Zm00001d011152
Zm00001d011152_T035	0.4701937	0.673759486	Zm00001d011152
Zm00001d011152_T081	0.897685	-0.09592212	Zm00001d011152
Zm00001d011152_T024	0.9563257	0.066381048	Zm00001d011152
Zm00001d011152_T045	0.9717919	0.095045792	Zm00001d011152
Zm00001d011152_T008	0.7740937	-0.185691656	Zm00001d011152
Zm00001d006549_T003	0.5537579	0.192526044	Zm00001d006549
Zm00001d048563_T001	0.8358158	-0.18109788	Zm00001d048563
Zm00001d041715_T009	0.9944178	0.51419114	Zm00001d041715
Zm00001d041715_T002	0.9616398	0.210429877	Zm00001d041715
Zm00001d041715_T010	0.1951804	1.356253346	Zm00001d041715

Zm00001d050353_T008	0.8884142	0.657605802	Zm00001d050353
Zm00001d050353_T002	0.973825	0.213240389	Zm00001d050353
Zm00001d050353_T003	0.8412913	0.216618986	Zm00001d050353
Zm00001d045544_T001	1	0.429111433	Zm00001d045544
Zm00001d047983_T001	0.9881407	0.022520268	Zm00001d047983
Zm00001d047983_T004	0.3865855	-1.210501024	Zm00001d047983
Zm00001d047983_T002	0.9968176	0.37922279	Zm00001d047983
Zm00001d017438_T001	0.02309348	-1.702569543	Zm00001d017438
Zm00001d027871_T001	0.900806	-0.048492908	Zm00001d027871
Zm00001d014849_T003	0.9086266	-0.035696041	Zm00001d014849
Zm00001d014849_T001	0.7673401	0.38782938	Zm00001d014849
Zm00001d016559_T002	0.9887584	0.494031562	Zm00001d016559
Zm00001d016559_T004	0.7132498	0.241957209	Zm00001d016559
Zm00001d038378_T001	0.9954326	0.151261361	Zm00001d038378
Zm00001d038378_T002	0.9988393	0.241896055	Zm00001d038378
Zm00001d033849_T001	0.2725606	-0.450952007	Zm00001d033849
Zm00001d033849_T002	0.4882783	-0.660897063	Zm00001d033849
Zm00001d018535_T001	1	0.444644453	Zm00001d018535
Zm00001d018535_T003	0.9573272	0.704233044	Zm00001d018535
Zm00001d018535_T002	0.9979568	0.298030355	Zm00001d018535
Zm00001d015141_T001	0.976488	0.034586983	Zm00001d015141
Zm00001d015141_T003	0.1106445	-0.554166645	Zm00001d015141
Zm00001d028713_T001	0.7167911	1.154851729	Zm00001d028713
Zm00001d029918_T001	0.7921111	-0.170960324	Zm00001d029918
Zm00001d027606_T001	0.9768802	0.153379559	Zm00001d027606
Zm00001d013863_T010	0.002814932	1.813579971	Zm00001d013863
Zm00001d013863_T008	1	0.442167141	Zm00001d013863
Zm00001d006382_T008	0.414356	0.997957643	Zm00001d006382
Zm00001d006382_T005	0.9334932	0.307841831	Zm00001d006382
Zm00001d006382_T006	1.99E-08	-0.94131816	Zm00001d006382
Zm00001d006382_T010	0.4875212	0.594277171	Zm00001d006382
Zm00001d008488_T001	0.9668789	0.54482556	Zm00001d008488
Zm00001d008488_T004	0.7520438	0.178877847	Zm00001d008488
Zm00001d008488_T009	0.009049317	-2.315526243	Zm00001d008488
Zm00001d008488_T002	0.995534	0.311900616	Zm00001d008488
Zm00001d038594_T002	0.9931019	-0.024587061	Zm00001d038594
Zm00001d038594_T001	0.8844101	0.603285306	Zm00001d038594
Zm00001d038594_T005	0.9103625	0.208717957	Zm00001d038594
Zm00001d048480_T003	0.955977	0.582386934	Zm00001d048480
Zm00001d048480_T001	0.3725038	0.504502455	Zm00001d048480
Zm00001d046190_T003	0.9846714	0.519933448	Zm00001d046190
Zm00001d046190_T001	0.8252955	0.132754539	Zm00001d046190
Zm00001d046190_T002	0.09517642	-1.414798132	Zm00001d046190
Zm00001d030774_T015	0.6889474	-0.426867062	Zm00001d030774
Zm00001d030774_T016	0.5842609	0.194756579	Zm00001d030774
Zm00001d030774_T011	0.9154179	0.107109183	Zm00001d030774
Zm00001d030774_T018	0.8077635	0.93921992	Zm00001d030774
Zm00001d030774_T002	0.06291771	1.431279694	Zm00001d030774
Zm00001d030774_T020	0.6168858	1.070009472	Zm00001d030774
Zm00001d036533_T003	0.8677544	-0.064356208	Zm00001d036533
Zm00001d036533_T007	0.4683268	-0.693587057	Zm00001d036533
Zm00001d036533_T002	0.7389826	0.680558825	Zm00001d036533
Zm00001d036533_T005	0.8646111	0.548744644	Zm00001d036533
Zm00001d011474_T001	0.7336274	-0.534121269	Zm00001d011474

Zm00001d000175_T001	0.8819727	0.615714362	Zm00001d000175
Zm00001d000175_T002	0.9951827	0.23352873	Zm00001d000175
Zm00001d026310_T001	0.8801933	-0.24893637	Zm00001d026310
Zm00001d032375_T002	0.8283699	-0.225351737	Zm00001d032375
Zm00001d032375_T001	0.9291272	0.002740065	Zm00001d032375
Zm00001d032375_T003	0.05613573	1.357029279	Zm00001d032375
Zm00001d051399_T006	0.8557307	0.858126077	Zm00001d051399
Zm00001d051399_T007	0.8989636	0.02626491	Zm00001d051399
Zm00001d051399_T002	0.07482195	-0.257956675	Zm00001d051399
Zm00001d016842_T002	0.8565397	-0.212934955	Zm00001d016842
Zm00001d016842_T001	0.5613135	-0.582038874	Zm00001d016842
Zm00001d021169_T007	0.8139362	-0.15349652	Zm00001d021169
Zm00001d021169_T005	0.8109283	0.952489634	Zm00001d021169
Zm00001d021169_T006	0.6880875	-0.20563394	Zm00001d021169
Zm00001d047901_T001	0.7610869	-0.496062764	Zm00001d047901
Zm00001d047181_T002	0.5702654	0.975712555	Zm00001d047181
Zm00001d047181_T006	0.9777548	0.482932476	Zm00001d047181
Zm00001d047181_T004	0.9562711	0.298222551	Zm00001d047181
Zm00001d047181_T005	1	0.377045451	Zm00001d047181
Zm00001d047181_T008	0.8513643	-0.09723104	Zm00001d047181
Zm00001d006889_T001	1	0.399187722	Zm00001d006889
Zm00001d006889_T002	0.9635696	0.391345285	Zm00001d006889
Zm00001d006889_T003	0.7754644	-0.032259506	Zm00001d006889
Zm00001d006889_T004	0.9671056	0.15336075	Zm00001d006889
Zm00001d040997_T001	0.1665069	0.976702755	Zm00001d040997
Zm00001d026413_T001	0.9739619	-0.309932954	Zm00001d026413
Zm00001d047941_T002	0.9845789	0.233639	Zm00001d047941
Zm00001d047941_T003	0.991538	0.225881838	Zm00001d047941
Zm00001d004564_T011	0.2496086	0.76675293	Zm00001d004564
Zm00001d004564_T019	0.7831859	0.312195084	Zm00001d004564
Zm00001d004564_T008	0.2487632	1.746023591	Zm00001d004564
Zm00001d004564_T012	0.9141981	-0.267087992	Zm00001d004564
Zm00001d004564_T001	0.8219372	0.92536536	Zm00001d004564
Zm00001d004564_T005	0.008047309	1.363737419	Zm00001d004564
Zm00001d004564_T016	0.7448452	-0.263477667	Zm00001d004564
Zm00001d041912_T006	0.9563232	0.275263702	Zm00001d041912
Zm00001d041912_T011	0.9407613	0.110525718	Zm00001d041912
Zm00001d041912_T002	0.9791729	0.481956424	Zm00001d041912
Zm00001d051594_T001	0.5576332	0.873745016	Zm00001d051594
Zm00001d012477_T002	0.6740644	1.236385717	Zm00001d012477
Zm00001d012477_T001	0.5167123	-0.62217882	Zm00001d012477
Zm00001d053750_T001	0.7932535	-0.25831378	Zm00001d053750
Zm00001d017872_T008	0.000974606	0.551688219	Zm00001d017872
Zm00001d017872_T009	0.8894416	1.538524133	Zm00001d017872
Zm00001d017872_T014	0.1683168	1.481326388	Zm00001d017872
Zm00001d017872_T006	1.96E-10	2.082402168	Zm00001d017872
Zm00001d017872_T005	0.9768996	0.222778826	Zm00001d017872
Zm00001d017872_T015	0.4483563	0.596798794	Zm00001d017872
Zm00001d017872_T007	0.4190286	0.411100693	Zm00001d017872
Zm00001d017872_T001	0.9563232	0.493610216	Zm00001d017872
Zm00001d017872_T003	0.9180865	0.930645458	Zm00001d017872
Zm00001d017872_T016	0.3766385	-1.082246288	Zm00001d017872
Zm00001d017872_T021	0.005075492	-0.889432692	Zm00001d017872
Zm00001d006453_T005	0.9162944	0.452334916	Zm00001d006453

Zm00001d006453_T002	0.4763999	-0.74696509	Zm00001d006453
Zm00001d006453_T004	0.8321156	-0.109493707	Zm00001d006453
Zm00001d008975_T004	1	0.101971442	Zm00001d008975
Zm00001d008975_T002	0.909729	0.000678505	Zm00001d008975
Zm00001d008975_T006	0.941809	0.04471646	Zm00001d008975
Zm00001d025687_T002	0.9979568	0.30142165	Zm00001d025687
Zm00001d006182_T001	0.7564036	-0.450813786	Zm00001d006182
Zm00001d001812_T001	0.8849873	-0.12463193	Zm00001d001812
Zm00001d000060_T001	0.01229362	1.940451167	Zm00001d000060
Zm00001d037108_T002	0.9426502	0.695311491	Zm00001d037108
Zm00001d037108_T005	0.9402255	-0.323610433	Zm00001d037108
Zm00001d034416_T001	0.8518279	0.50574568	Zm00001d034416
Zm00001d053547_T001	0.2627267	-1.357398788	Zm00001d053547
Zm00001d053547_T003	0.7718656	-0.518637812	Zm00001d053547
Zm00001d021368_T001	0.9624437	0.446724052	Zm00001d021368
Zm00001d025375_T004	0.9037324	0.012243877	Zm00001d025375
Zm00001d025375_T005	0.1594086	-0.682351776	Zm00001d025375
Zm00001d034063_T002	0.9257893	-0.030415518	Zm00001d034063
Zm00001d001837_T001	0.824385	-0.253099708	Zm00001d001837
Zm00001d023425_T001	0.9543425	0.493675642	Zm00001d023425
Zm00001d026680_T003	1	0.325180296	Zm00001d026680
Zm00001d026680_T001	0.01022151	-1.124239342	Zm00001d026680
Zm00001d026680_T004	0.9543374	0.587907495	Zm00001d026680
Zm00001d026680_T002	0.5936419	0.958792721	Zm00001d026680
Zm00001d017460_T003	0.9768802	-0.090633441	Zm00001d017460
Zm00001d017460_T001	1	0.160804387	Zm00001d017460
Zm00001d005107_T001	0.9311783	0.781347386	Zm00001d005107
Zm00001d005107_T003	0.3685079	-0.831455599	Zm00001d005107
Zm00001d005107_T002	0.974657	0.338955368	Zm00001d005107
Zm00001d009013_T001	0.9908081	0.434554912	Zm00001d009013
Zm00001d040050_T001	0.9747606	0.229262934	Zm00001d040050
Zm00001d033309_T001	0.8213498	0.582637038	Zm00001d033309
Zm00001d033309_T003	0.7289978	-0.186933997	Zm00001d033309
Zm00001d033309_T004	0.9404125	0.085088777	Zm00001d033309
Zm00001d054071_T001	0.956479	0.108808273	Zm00001d054071
Zm00001d013531_T001	1	0.253211585	Zm00001d013531
Zm00001d013531_T002	0.2373727	-1.31780264	Zm00001d013531
Zm00001d030079_T001	0.9695609	0.053240446	Zm00001d030079
Zm00001d044024_T001	0.929138	0.73844938	Zm00001d044024
Zm00001d044024_T002	0.7737085	0.779286393	Zm00001d044024
Zm00001d006219_T004	0.9781672	0.133250584	Zm00001d006219
Zm00001d051343_T001	0.9914064	0.26826254	Zm00001d051343
Zm00001d042061_T001	0.9894615	0.305635307	Zm00001d042061
Zm00001d026415_T001	0.328751	0.413236876	Zm00001d026415
Zm00001d026415_T002	0.9410688	0.118662305	Zm00001d026415
Zm00001d041268_T001	0.9975882	0.173464813	Zm00001d041268
Zm00001d041268_T004	0.9209394	-0.108830325	Zm00001d041268
Zm00001d041268_T002	1	0.293805809	Zm00001d041268
Zm00001d016490_T001	0.9979568	0.249339461	Zm00001d016490
Zm00001d043156_T001	0.9902733	0.460121944	Zm00001d043156
Zm00001d018887_T001	0.8332116	-0.115150256	Zm00001d018887
Zm00001d018887_T002	0.9652551	0.621183224	Zm00001d018887
Zm00001d049333_T002	0.9887584	0.216520173	Zm00001d049333
Zm00001d049333_T001	0.1876281	-0.669054701	Zm00001d049333

Zm00001d019104_T001	0.9206245	0.730900748	Zm00001d019104
Zm00001d043954_T004	0.918657	0.015951479	Zm00001d043954
Zm00001d043954_T003	0.9469426	0.700770597	Zm00001d043954
Zm00001d052625_T001	0.7681941	-0.328400678	Zm00001d052625
Zm00001d029708_T001	0.9747695	0.173869579	Zm00001d029708
Zm00001d053001_T001	0.8412913	0.669176779	Zm00001d053001
Zm00001d003123_T005	0.9657993	0.630503903	Zm00001d003123
Zm00001d003182_T001	0.772948	-0.741248443	Zm00001d003182
Zm00001d048644_T001	0.9529094	0.657756726	Zm00001d048644
Zm00001d019881_T030	0.9046438	0.022942512	Zm00001d019881
Zm00001d019881_T002	8.37E-05	1.810325571	Zm00001d019881
Zm00001d019881_T021	0.9596377	-0.129335807	Zm00001d019881
Zm00001d019881_T001	0.2202395	-0.128151312	Zm00001d019881
Zm00001d019881_T023	0.1475494	1.684345259	Zm00001d019881
Zm00001d019881_T003	4.83E-06	2.350934579	Zm00001d019881
Zm00001d016075_T001	0.9567162	0.126587973	Zm00001d016075
Zm00001d024178_T002	0.956184	0.15497881	Zm00001d024178
Zm00001d037967_T005	0.1240443	0.271530611	Zm00001d037967
Zm00001d037967_T008	0.7697196	-0.046939211	Zm00001d037967
Zm00001d037967_T002	0.9979568	0.242512278	Zm00001d037967
Zm00001d037967_T001	0.8706608	-0.030274156	Zm00001d037967
Zm00001d028820_T001	0.9956349	0.147884645	Zm00001d028820
Zm00001d043655_T006	0.3332132	-0.949827589	Zm00001d043655
Zm00001d043655_T001	0.3295805	1.484005741	Zm00001d043655
Zm00001d043655_T004	0.03317233	0.759283636	Zm00001d043655
Zm00001d043655_T003	0.9330832	-0.040412899	Zm00001d043655
Zm00001d043655_T009	0.941809	-0.01621681	Zm00001d043655
Zm00001d029256_T004	0.8054377	-0.082754156	Zm00001d029256
Zm00001d029256_T005	0.9778436	0.11917888	Zm00001d029256
Zm00001d029256_T002	0.8781102	-0.293223379	Zm00001d029256
Zm00001d029256_T003	0.9937567	0.143061063	Zm00001d029256
Zm00001d008619_T001	0.9751332	0.499034729	Zm00001d008619
Zm00001d008619_T002	0.9814848	0.333591985	Zm00001d008619
Zm00001d036480_T002	0.9290834	0.73232144	Zm00001d036480
Zm00001d036480_T001	0.5954619	-0.544453308	Zm00001d036480
Zm00001d046971_T001	0.9885729	0.238389011	Zm00001d046971
Zm00001d011981_T068	0.533111	1.035226196	Zm00001d011981
Zm00001d011981_T017	0.5473748	1.085080002	Zm00001d011981
Zm00001d011981_T076	0.6076758	-0.882201305	Zm00001d011981
Zm00001d011981_T034	0.9814175	0.053181917	Zm00001d011981
Zm00001d011981_T031	1	0.061217126	Zm00001d011981
Zm00001d011981_T026	0.000736708	0.622402919	Zm00001d011981
Zm00001d011981_T072	1.04E-10	-1.584680238	Zm00001d011981
Zm00001d011981_T062	0.7585131	0.271089057	Zm00001d011981
Zm00001d025016_T002	0.9809771	0.255553053	Zm00001d025016
Zm00001d025016_T004	0.3071359	1.09667964	Zm00001d025016
Zm00001d014973_T013	0.6165884	-0.280741698	Zm00001d014973
Zm00001d014973_T014	0.3129507	-1.272295145	Zm00001d014973
Zm00001d014973_T012	0.8941695	0.396638312	Zm00001d014973
Zm00001d014973_T001	0.8099107	0.438681365	Zm00001d014973
Zm00001d022296_T001	0.524978	-1.117312711	Zm00001d022296
Zm00001d041538_T001	0.584304	-0.600017529	Zm00001d041538
Zm00001d018076_T001	0.8641671	0.667974371	Zm00001d018076
Zm00001d013120_T001	0.4054378	-0.464504733	Zm00001d013120

Zm00001d006929_T001	0.9843847	0.520900396	Zm00001d006929
Zm00001d015723_T001	0.9765009	0.11276574	Zm00001d015723
Zm00001d006810_T001	0.9050121	-0.123647422	Zm00001d006810
Zm00001d048628_T001	0.4464261	1.208586772	Zm00001d048628
Zm00001d048628_T018	0.3357027	-0.871619809	Zm00001d048628
Zm00001d048628_T016	0.997586	0.382168886	Zm00001d048628
Zm00001d008916_T001	0.9269932	-0.488329174	Zm00001d008916
Zm00001d045563_T001	0.9690925	0.113938283	Zm00001d045563
Zm00001d045563_T002	0.9211172	-0.278415754	Zm00001d045563
Zm00001d051229_T005	0.2151537	0.38480468	Zm00001d051229
Zm00001d051229_T004	0.07320084	0.603554801	Zm00001d051229
Zm00001d051229_T002	0.7921111	0.889921668	Zm00001d051229
Zm00001d053856_T001	0.9954444	0.237291302	Zm00001d053856
Zm00001d012327_T001	0.552492	1.283617612	Zm00001d012327
Zm00001d038081_T001	0.4715806	-0.593927634	Zm00001d038081
Zm00001d027293_T008	2.14E-07	1.240680966	Zm00001d027293
Zm00001d027293_T006	0.226067	1.709383857	Zm00001d027293
Zm00001d027293_T007	0.9791729	0.776583746	Zm00001d027293
Zm00001d027293_T002	0.8984709	0.965395895	Zm00001d027293
Zm00001d027293_T011	0.1191683	0.45282924	Zm00001d027293
Zm00001d019917_T001	0.9089465	-0.262689045	Zm00001d019917
Zm00001d003151_T014	0.6948891	0.565162667	Zm00001d003151
Zm00001d003151_T016	0.9981361	0.100455611	Zm00001d003151
Zm00001d010075_T001	0.6088201	0.972341858	Zm00001d010075
Zm00001d007869_T001	0.7984409	-0.278825488	Zm00001d007869
Zm00001d029923_T001	0.163204	-1.75843075	Zm00001d029923
Zm00001d053410_T001	0.342013	1.212524437	Zm00001d053410
Zm00001d012731_T009	0.9217658	0.695209668	Zm00001d012731
Zm00001d012731_T004	0.4274324	1.502728028	Zm00001d012731
Zm00001d012731_T008	0.5795936	1.130321394	Zm00001d012731
Zm00001d023259_T001	0.923446	-0.138413075	Zm00001d023259
Zm00001d025644_T001	0.9730613	0.178578642	Zm00001d025644
Zm00001d006508_T011	0.3715553	0.582769845	Zm00001d006508
Zm00001d006508_T003	1	0.253244965	Zm00001d006508
Zm00001d043854_T003	0.8412913	1.797785248	Zm00001d043854
Zm00001d043854_T004	0.7407514	-0.280565316	Zm00001d043854
Zm00001d043854_T002	0.9836566	0.277677021	Zm00001d043854
Zm00001d012635_T001	0.6183338	-0.091115434	Zm00001d012635
Zm00001d012635_T002	0.649406	0.964477418	Zm00001d012635
Zm00001d010440_T001	0.9895224	0.112465464	Zm00001d010440
Zm00001d008725_T013	0.3183941	0.767230336	Zm00001d008725
Zm00001d008725_T010	0.2247608	0.67060451	Zm00001d008725
Zm00001d008725_T006	0.8591203	-0.152032108	Zm00001d008725
Zm00001d008725_T005	0.908501	-0.016061438	Zm00001d008725
Zm00001d008725_T007	0.3497147	0.563819998	Zm00001d008725
Zm00001d008725_T008	0.6727562	0.895364423	Zm00001d008725
Zm00001d018867_T004	0.931267	0.683833945	Zm00001d018867
Zm00001d018867_T001	0.4270038	1.076697093	Zm00001d018867
Zm00001d018867_T003	0.1349847	-0.920179669	Zm00001d018867
Zm00001d030725_T001	0.225015	-1.107549918	Zm00001d030725
Zm00001d030725_T004	0.0252917	3.342441001	Zm00001d030725
Zm00001d030725_T006	0.8894168	1.954540969	Zm00001d030725
Zm00001d030725_T003	0.011246	-1.718912846	Zm00001d030725
Zm00001d005006_T002	0.9129863	0.656796008	Zm00001d005006

Zm00001d005006_T003	0.9942835	0.408242704	Zm00001d005006
Zm00001d005006_T001	0.9563232	0.509859425	Zm00001d005006
Zm00001d039175_T001	0.4236278	1.272570199	Zm00001d039175
Zm00001d021433_T005	0.09362418	-2.76453446	Zm00001d021433
Zm00001d021433_T007	1	0.207682897	Zm00001d021433
Zm00001d021433_T006	0.8332116	-0.32253077	Zm00001d021433
Zm00001d021433_T003	0.8979716	0.58380446	Zm00001d021433
Zm00001d051309_T001	0.000148816	3.743389406	Zm00001d051309
Zm00001d030995_T005	0.1203427	-0.450619325	Zm00001d030995
Zm00001d030995_T010	0.7109402	-0.312685834	Zm00001d030995
Zm00001d030995_T002	0.9760472	0.110000875	Zm00001d030995
Zm00001d023654_T001	0.7558742	-0.319793178	Zm00001d023654
Zm00001d015520_T001	0.9324462	0.692905232	Zm00001d015520
Zm00001d047204_T005	0.6424657	-0.458143779	Zm00001d047204
Zm00001d047204_T008	0.2903696	-0.464776769	Zm00001d047204
Zm00001d047204_T004	0.7678152	-0.276804242	Zm00001d047204
Zm00001d047204_T007	0.9042547	-0.05421117	Zm00001d047204
Zm00001d047204_T002	0.664701	-0.36322512	Zm00001d047204
Zm00001d011572_T001	0.9827917	0.191372112	Zm00001d011572
Zm00001d035597_T001	0.7711192	-0.268007839	Zm00001d035597
Zm00001d052106_T001	1	0.119405969	Zm00001d052106
Zm00001d030366_T008	0.8583111	0.794386931	Zm00001d030366
Zm00001d030366_T001	0.5762747	-1.172768534	Zm00001d030366
Zm00001d030366_T024	0.9272378	0.585635781	Zm00001d030366
Zm00001d030366_T022	0.8112903	0.303315925	Zm00001d030366
Zm00001d051375_T003	0.8494782	0.547489804	Zm00001d051375
Zm00001d051375_T001	0.9852645	0.419492612	Zm00001d051375
Zm00001d051548_T001	0.9180662	0.299389787	Zm00001d051548
Zm00001d035101_T001	0.6342868	-1.052718801	Zm00001d035101
Zm00001d020714_T001	0.05839599	2.241685759	Zm00001d020714
Zm00001d024832_T005	0.7602652	0.38047499	Zm00001d024832
Zm00001d024832_T003	0.4988467	0.5261707	Zm00001d024832
Zm00001d024832_T012	0.6716447	0.159197887	Zm00001d024832
Zm00001d024832_T001	0.9779851	0.586720382	Zm00001d024832
Zm00001d018515_T002	0.9846714	0.256444876	Zm00001d018515
Zm00001d025776_T001	0.9210513	0.002640158	Zm00001d025776
Zm00001d025776_T002	0.9633575	0.169470228	Zm00001d025776
Zm00001d034727_T001	1	0.120807361	Zm00001d034727
Zm00001d034727_T004	0.7437035	0.910690203	Zm00001d034727
Zm00001d034727_T006	0.9201341	0.739592341	Zm00001d034727
Zm00001d034727_T005	0.0853584	0.727979959	Zm00001d034727
Zm00001d018407_T001	0.935477	-0.039835113	Zm00001d018407
Zm00001d043232_T001	0.9469426	0.417634551	Zm00001d043232
Zm00001d037611_T001	0.9048001	-0.107534107	Zm00001d037611
Zm00001d019960_T001	0.8545272	-0.372499412	Zm00001d019960
Zm00001d029242_T001	0.1830581	-1.200281003	Zm00001d029242
Zm00001d021046_T003	1	0.348647537	Zm00001d021046
Zm00001d021046_T006	0.3306762	-1.44298697	Zm00001d021046
Zm00001d021046_T005	0.9991964	0.141996006	Zm00001d021046
Zm00001d019677_T001	0.9830028	0.209147042	Zm00001d019677
Zm00001d002511_T001	0.6963782	0.751290408	Zm00001d002511
Zm00001d002511_T004	0.9749728	0.51161215	Zm00001d002511
Zm00001d002511_T006	0.009606413	-2.545195946	Zm00001d002511
Zm00001d002511_T010	0.7912425	0.752066992	Zm00001d002511

Zm00001d002511_T002	0.9562711	0.561822234	Zm00001d002511
Zm00001d016786_T001	0.9907928	0.442050077	Zm00001d016786
Zm00001d047562_T002	0.9954444	0.265812811	Zm00001d047562
Zm00001d046004_T001	0.9301174	0.783437762	Zm00001d046004
Zm00001d046004_T003	0.002602753	-0.797140321	Zm00001d046004
Zm00001d046004_T002	0.993158	0.539387648	Zm00001d046004
Zm00001d004524_T001	0.8139802	-0.433702267	Zm00001d004524
Zm00001d012567_T001	0.5549472	1.341267206	Zm00001d012567
Zm00001d012567_T003	0.5748211	1.584604827	Zm00001d012567
Zm00001d012567_T002	0.2829145	1.770148527	Zm00001d012567
Zm00001d009987_T005	0.9942835	-0.174618041	Zm00001d009987
Zm00001d009987_T003	0.9136408	0.66163502	Zm00001d009987
Zm00001d009987_T002	0.9264186	0.328587634	Zm00001d009987
Zm00001d051125_T001	0.9090332	0.482439182	Zm00001d051125
Zm00001d047788_T001	0.8050292	-0.636568098	Zm00001d047788
Zm00001d047788_T002	1	0.281801789	Zm00001d047788
Zm00001d047788_T003	0.530398	-0.840953257	Zm00001d047788
Zm00001d019329_T001	1	0.205487633	Zm00001d019329
Zm00001d025751_T001	0.9921225	0.35449014	Zm00001d025751
Zm00001d025751_T005	0.5035645	1.29829531	Zm00001d025751
Zm00001d025751_T003	0.01637723	1.220513827	Zm00001d025751
Zm00001d025751_T004	0.2594098	1.673018173	Zm00001d025751
Zm00001d021787_T002	0.9995663	0.290108771	Zm00001d021787
Zm00001d021787_T001	0.9804228	0.558676614	Zm00001d021787
Zm00001d021787_T004	0.2606035	1.548405983	Zm00001d021787
Zm00001d033271_T001	0.3768189	-0.921308185	Zm00001d033271
Zm00001d028280_T001	0.6742084	1.208080321	Zm00001d028280
Zm00001d044290_T018	0.01537015	1.842290026	Zm00001d044290
Zm00001d044290_T009	2.05E-11	2.060453491	Zm00001d044290
Zm00001d044290_T020	0.2317185	1.610029071	Zm00001d044290
Zm00001d044290_T032	0.5088344	-0.692116874	Zm00001d044290
Zm00001d044290_T025	0.9501111	-0.008893522	Zm00001d044290
Zm00001d044290_T005	0.08207564	-0.834770458	Zm00001d044290
Zm00001d006647_T007	0.2047087	-1.217127579	Zm00001d006647
Zm00001d006647_T002	0.7128527	-0.223781447	Zm00001d006647
Zm00001d006647_T001	0.9273903	0.453892573	Zm00001d006647
Zm00001d006647_T003	0.281775	0.813372987	Zm00001d006647
Zm00001d006647_T005	1	0.294956192	Zm00001d006647
Zm00001d044303_T004	0.9852645	0.136646111	Zm00001d044303
Zm00001d044303_T011	0.01937267	0.57241844	Zm00001d044303
Zm00001d044303_T009	2.82E-10	1.29982814	Zm00001d044303
Zm00001d044303_T003	0.04407915	1.940605913	Zm00001d044303
Zm00001d044303_T006	0.7222076	1.138988291	Zm00001d044303
Zm00001d005329_T001	0.9016285	-0.166171236	Zm00001d005329
Zm00001d046792_T001	0.9213556	0.695126269	Zm00001d046792
Zm00001d040233_T002	0.9975882	0.239661529	Zm00001d040233
Zm00001d040233_T003	0.9224443	0.009567074	Zm00001d040233
Zm00001d024965_T007	0.9213534	0.463794023	Zm00001d024965
Zm00001d024965_T001	0.9281	-0.030097812	Zm00001d024965
Zm00001d053953_T001	0.9982897	0.121512011	Zm00001d053953
Zm00001d015513_T001	0.8885217	0.81545495	Zm00001d015513
Zm00001d045400_T001	0.2196489	-1.243186946	Zm00001d045400
Zm00001d019089_T001	0.9201237	0.029268556	Zm00001d019089
Zm00001d025788_T001	0.9979568	0.362890731	Zm00001d025788

Zm00001d012228_T003	0.9262947	0.699634669	Zm00001d012228
Zm00001d012228_T004	0.8512136	0.797833346	Zm00001d012228
Zm00001d012228_T001	0.6118682	0.798017983	Zm00001d012228
Zm00001d028654_T002	0.9486412	0.27082287	Zm00001d028654
Zm00001d028654_T003	0.06921822	1.824249392	Zm00001d028654
Zm00001d028654_T001	0.211796	1.787919011	Zm00001d028654
Zm00001d027550_T001	0.9931431	0.264813035	Zm00001d027550
Zm00001d049461_T001	0.4661639	-0.550084607	Zm00001d049461
Zm00001d014865_T001	0.8345405	-0.148043116	Zm00001d014865
Zm00001d028536_T003	0.8929316	-0.068391433	Zm00001d028536
Zm00001d028536_T004	0.957148	0.063025391	Zm00001d028536
Zm00001d028536_T002	0.1929453	-1.069695004	Zm00001d028536
Zm00001d028536_T008	0.01477296	1.893488422	Zm00001d028536
Zm00001d028536_T001	0.9100716	0.012824339	Zm00001d028536
Zm00001d038392_T001	0.8819536	-0.155230087	Zm00001d038392
Zm00001d017675_T001	0.366346	0.807961083	Zm00001d017675
Zm00001d017675_T004	0.9493302	0.710912374	Zm00001d017675
Zm00001d039179_T001	0.9149744	-0.025852831	Zm00001d039179
Zm00001d030357_T011	0.2174934	0.469718966	Zm00001d030357
Zm00001d030357_T013	0.8031436	1.039135416	Zm00001d030357
Zm00001d030357_T009	0.2110854	1.644559587	Zm00001d030357
Zm00001d030357_T002	0.2238491	0.958292798	Zm00001d030357
Zm00001d030357_T001	2.60E-05	-2.753311	Zm00001d030357
Zm00001d024939_T004	0.008335767	-2.138784386	Zm00001d024939
Zm00001d024939_T003	0.9334932	0.829543031	Zm00001d024939
Zm00001d040127_T001	0.7831859	0.730771793	Zm00001d040127
Zm00001d003533_T002	0.04043864	2.390912826	Zm00001d003533
Zm00001d003533_T001	0.09143854	2.171959461	Zm00001d003533
Zm00001d013202_T001	0.000224843	3.894071654	Zm00001d013202
Zm00001d022231_T001	0.9395327	0.007772872	Zm00001d022231
Zm00001d041991_T001	0.9942835	0.320235781	Zm00001d041991
Zm00001d045470_T001	0.1719083	2.053823818	Zm00001d045470
Zm00001d034824_T001	0.9292284	-0.022935147	Zm00001d034824
Zm00001d048457_T001	0.279901	-1.403061602	Zm00001d048457
Zm00001d013087_T001	0.8806256	-0.320292582	Zm00001d013087
Zm00001d000301_T001	0.9055179	-0.028866381	Zm00001d000301
Zm00001d051321_T009	0.9732421	0.551141303	Zm00001d051321
Zm00001d051321_T011	0.9200919	-0.137728613	Zm00001d051321
Zm00001d051321_T004	0.9763597	0.642474623	Zm00001d051321
Zm00001d041883_T001	0.9173494	-0.026983846	Zm00001d041883
Zm00001d043445_T003	1	0.349316244	Zm00001d043445
Zm00001d043445_T001	0.9979568	0.341560805	Zm00001d043445
Zm00001d053697_T003	0.5327484	0.745447398	Zm00001d053697
Zm00001d053697_T023	0.921509	0.548439413	Zm00001d053697
Zm00001d053697_T018	0.009538513	0.563362588	Zm00001d053697
Zm00001d036583_T002	1	0.027178452	Zm00001d036583
Zm00001d036583_T001	0.0585403	0.39076327	Zm00001d036583
Zm00001d029852_T001	0.883637	0.452684673	Zm00001d029852
Zm00001d003875_T001	0.9817074	0.025268007	Zm00001d003875
Zm00001d022267_T002	0.6945741	0.745050296	Zm00001d022267
Zm00001d022267_T012	0.9257893	-0.321567411	Zm00001d022267
Zm00001d022267_T009	0.6876043	-0.305724961	Zm00001d022267
Zm00001d022267_T001	0.3360774	-0.173844096	Zm00001d022267
Zm00001d022267_T008	9.65E-08	0.684316302	Zm00001d022267

Zm00001d048165_T006	0.6244596	1.106665101	Zm00001d048165
Zm00001d048165_T003	0.3188469	1.591277707	Zm00001d048165
Zm00001d048165_T001	0.1883701	2.99069012	Zm00001d048165
Zm00001d011036_T002	0.2982359	-1.339032539	Zm00001d011036
Zm00001d011036_T001	0.6436915	-0.52742049	Zm00001d011036
Zm00001d021812_T001	0.7575375	-0.34123551	Zm00001d021812
Zm00001d040192_T001	0.8280859	-0.17768494	Zm00001d040192
Zm00001d027807_T003	0.6745773	0.254818793	Zm00001d027807
Zm00001d027807_T001	0.6357127	1.218229831	Zm00001d027807
Zm00001d027807_T009	0.550715	-0.4928772	Zm00001d027807
Zm00001d025984_T002	0.2484954	0.611043465	Zm00001d025984
Zm00001d025984_T003	0.05893106	2.414130634	Zm00001d025984
Zm00001d044324_T002	0.9404125	0.729581083	Zm00001d044324
Zm00001d044324_T001	0.806534	-0.139965724	Zm00001d044324
Zm00001d051656_T002	0.9493302	0.077108296	Zm00001d051656
Zm00001d051656_T001	0.02128297	1.662321857	Zm00001d051656
Zm00001d020686_T001	0.8447818	-0.20350911	Zm00001d020686
Zm00001d042774_T001	0.9946911	0.356041539	Zm00001d042774
Zm00001d051929_T001	0.859708	-0.136951485	Zm00001d051929
Zm00001d051929_T002	0.8412913	-0.071952588	Zm00001d051929
Zm00001d007782_T001	0.8580945	0.721052487	Zm00001d007782
Zm00001d003947_T012	0.1768896	0.577289301	Zm00001d003947
Zm00001d003947_T022	0.4131756	1.306463875	Zm00001d003947
Zm00001d003947_T028	0.9293689	-0.056705812	Zm00001d003947
Zm00001d003947_T004	0.718063	0.530497086	Zm00001d003947
Zm00001d003947_T003	0.1406927	0.454278477	Zm00001d003947
Zm00001d003947_T002	0.4178252	1.183040555	Zm00001d003947
Zm00001d003015_T001	0.8487747	0.841129956	Zm00001d003015
Zm00001d032815_T009	1.35E-05	-0.359386268	Zm00001d032815
Zm00001d032815_T015	0.03566678	1.190559616	Zm00001d032815
Zm00001d032815_T013	0.9213679	0.12510211	Zm00001d032815
Zm00001d032815_T002	0.5784042	-0.57235316	Zm00001d032815
Zm00001d032815_T011	0.4344214	1.470381906	Zm00001d032815
Zm00001d032815_T008	0.07875319	0.696771107	Zm00001d032815
Zm00001d032815_T016	0.9518412	0.050048471	Zm00001d032815
Zm00001d032815_T006	0.9396809	0.368416685	Zm00001d032815
Zm00001d021283_T003	0.9741922	0.130444211	Zm00001d021283
Zm00001d021283_T001	0.945968	0.493654436	Zm00001d021283
Zm00001d021283_T004	0.8494782	0.021105456	Zm00001d021283
Zm00001d008171_T001	0.8109592	0.967857004	Zm00001d008171
Zm00001d019505_T001	0.1197922	2.148757852	Zm00001d019505
Zm00001d048340_T002	0.9845488	0.436958482	Zm00001d048340
Zm00001d048340_T003	0.8412913	0.326427645	Zm00001d048340
Zm00001d049713_T001	0.9889811	0.480039391	Zm00001d049713
Zm00001d024800_T001	0.8899623	-0.25267699	Zm00001d024800
Zm00001d048544_T002	1	0.047956557	Zm00001d048544
Zm00001d048544_T001	0.9781672	0.0866483	Zm00001d048544
Zm00001d048544_T003	0.9746429	0.1568297	Zm00001d048544
Zm00001d021141_T001	0.8227428	-0.567926213	Zm00001d021141
Zm00001d022085_T001	1	0.389211706	Zm00001d022085
Zm00001d016381_T001	0.9090332	0.346557293	Zm00001d016381
Zm00001d012679_T001	0.9468268	-0.207088226	Zm00001d012679
Zm00001d012679_T003	0.9048001	-0.386460135	Zm00001d012679
Zm00001d012679_T002	0.9184323	-0.473612987	Zm00001d012679

Zm00001d037308_T002	0.9930712	0.344485933	Zm00001d037308
Zm00001d037308_T004	0.9633184	0.222089437	Zm00001d037308
Zm00001d002742_T004	0.3071359	0.860346801	Zm00001d002742
Zm00001d002742_T001	1	0.363470257	Zm00001d002742
Zm00001d014347_T010	1	0.107583491	Zm00001d014347
Zm00001d014347_T007	0.965836	0.285409232	Zm00001d014347
Zm00001d014347_T009	1	0.248843743	Zm00001d014347
Zm00001d014347_T001	0.6185011	-0.706587542	Zm00001d014347
Zm00001d014347_T008	0.284192	-0.779887912	Zm00001d014347
Zm00001d014347_T006	0.923446	-0.178272089	Zm00001d014347
Zm00001d014347_T005	0.8631191	-0.091659919	Zm00001d014347
Zm00001d014347_T004	0.9293689	-0.055464796	Zm00001d014347
Zm00001d043569_T001	0.9965998	0.434419265	Zm00001d043569
Zm00001d016506_T006	0.3772755	0.762297508	Zm00001d016506
Zm00001d016506_T027	0.6053962	0.524732785	Zm00001d016506
Zm00001d016506_T002	0.6298924	-0.028200414	Zm00001d016506
Zm00001d016506_T008	0.1010357	0.995470793	Zm00001d016506
Zm00001d016506_T015	0.00734409	0.955420418	Zm00001d016506
Zm00001d016506_T019	0.05697743	0.994669208	Zm00001d016506
Zm00001d016506_T014	0.5824385	-0.538964485	Zm00001d016506
Zm00001d047746_T003	0.5705692	0.904892369	Zm00001d047746
Zm00001d047746_T001	0.8878472	-0.079353218	Zm00001d047746
Zm00001d047746_T002	0.000216826	0.558193954	Zm00001d047746
Zm00001d023249_T001	0.2002966	-0.907186914	Zm00001d023249
Zm00001d032923_T003	0.8435976	0.838562901	Zm00001d032923
Zm00001d032923_T005	0.9902022	0.42275854	Zm00001d032923
Zm00001d032923_T001	0.9717919	0.17142478	Zm00001d032923
Zm00001d032923_T004	0.5987102	-0.375068473	Zm00001d032923
Zm00001d018665_T001	0.9355135	0.689907774	Zm00001d018665
Zm00001d011111_T001	0.3222139	-1.060641788	Zm00001d011111
Zm00001d018305_T001	0.8732588	0.664264902	Zm00001d018305
Zm00001d011913_T001	0.9486412	0.732229701	Zm00001d011913
Zm00001d011913_T007	0.3541203	-0.340069429	Zm00001d011913
Zm00001d011913_T008	1	0.200910964	Zm00001d011913
Zm00001d026067_T001	0.7805748	-0.187077935	Zm00001d026067
Zm00001d034898_T001	0.5825908	-0.600473904	Zm00001d034898
Zm00001d012155_T016	9.50E-11	1.627224736	Zm00001d012155
Zm00001d012155_T002	0.6952911	0.479567604	Zm00001d012155
Zm00001d012155_T004	0.8828258	-0.044714149	Zm00001d012155
Zm00001d012155_T011	0.01151883	0.551905694	Zm00001d012155
Zm00001d012155_T014	0.004365318	1.835359148	Zm00001d012155
Zm00001d012155_T022	0.000197484	1.182041242	Zm00001d012155
Zm00001d012155_T023	6.96E-10	1.398594244	Zm00001d012155
Zm00001d012155_T008	0.000174262	3.452492636	Zm00001d012155
Zm00001d012155_T013	0.001892338	1.450151233	Zm00001d012155
Zm00001d014451_T001	0.5411248	-0.139798764	Zm00001d014451
Zm00001d010000_T001	0.9986724	0.182525353	Zm00001d010000
Zm00001d034617_T004	1	0.351916953	Zm00001d034617
Zm00001d052224_T002	0.3450234	-1.244518255	Zm00001d052224
Zm00001d052224_T001	0.7167212	-0.526173571	Zm00001d052224
Zm00001d048741_T129	0.6053962	-0.157148515	Zm00001d048741
Zm00001d048741_T088	0.8757823	0.307237806	Zm00001d048741
Zm00001d048741_T135	5.17E-07	-1.884444114	Zm00001d048741
Zm00001d048741_T086	0.08118697	-0.879308583	Zm00001d048741

Zm00001d048741_T024	0.2110339	1.338874575	Zm00001d048741
Zm00001d048741_T071	0.9050121	0.346674879	Zm00001d048741
Zm00001d048741_T082	1.80E-12	-2.540241138	Zm00001d048741
Zm00001d048741_T132	1	0.084883592	Zm00001d048741
Zm00001d048741_T133	0.8558519	-0.017739521	Zm00001d048741
Zm00001d048741_T008	2.71E-09	1.236094892	Zm00001d048741
Zm00001d048741_T084	0.6165884	0.265669567	Zm00001d048741
Zm00001d018244_T001	0.6876043	-0.395197407	Zm00001d018244
Zm00001d050840_T001	0.1344806	1.793200112	Zm00001d050840
Zm00001d032805_T002	0.8008491	-0.301143302	Zm00001d032805
Zm00001d017625_T001	0.9618192	0.125653526	Zm00001d017625
Zm00001d020359_T002	0.974657	0.173514463	Zm00001d020359
Zm00001d033780_T001	1	0.401935702	Zm00001d033780
Zm00001d033780_T006	0.9807381	0.358149847	Zm00001d033780
Zm00001d033780_T002	1	0.287263535	Zm00001d033780
Zm00001d033780_T004	0.7382728	-0.358111663	Zm00001d033780
Zm00001d033780_T005	0.9703357	0.713968094	Zm00001d033780
Zm00001d000316_T001	1	0.262959769	Zm00001d000316
Zm00001d053908_T001	0.7437035	-0.787484043	Zm00001d053908
Zm00001d023267_T002	0.9571855	0.520698374	Zm00001d023267
Zm00001d023267_T001	0.9396809	0.792739248	Zm00001d023267
Zm00001d015329_T006	0.8950139	0.72404425	Zm00001d015329
Zm00001d015329_T001	5.11E-06	-0.751763468	Zm00001d015329
Zm00001d015329_T002	0.9605054	-0.026525757	Zm00001d015329
Zm00001d015329_T005	0.8611836	-0.126946737	Zm00001d015329
Zm00001d014572_T005	0.002765635	-2.544675974	Zm00001d014572
Zm00001d014572_T002	0.9201237	0.719348294	Zm00001d014572
Zm00001d003829_T001	0.6727656	0.755899021	Zm00001d003829
Zm00001d027749_T001	0.9539896	0.143116744	Zm00001d027749
Zm00001d012261_T001	0.5328547	0.849903848	Zm00001d012261
Zm00001d053783_T001	0.9494803	0.710875879	Zm00001d053783
Zm00001d053783_T002	1	0.281389958	Zm00001d053783
Zm00001d017557_T001	0.5254821	-0.733778605	Zm00001d017557
Zm00001d051239_T001	0.3692327	0.841001416	Zm00001d051239
Zm00001d013139_T001	0.9749249	0.004451067	Zm00001d013139
Zm00001d008847_T001	0.7920384	-0.137976832	Zm00001d008847
Zm00001d008847_T002	0.9979568	1.225608642	Zm00001d008847
Zm00001d005118_T002	0.9747714	0.543495587	Zm00001d005118
Zm00001d005118_T001	0.9127803	0.687487666	Zm00001d005118
Zm00001d053371_T001	0.9527167	0.037119836	Zm00001d053371
Zm00001d003267_T007	0.9139882	-0.323852156	Zm00001d003267
Zm00001d003267_T036	0.9208326	0.981509777	Zm00001d003267
Zm00001d003267_T037	0.9267763	0.08937826	Zm00001d003267
Zm00001d003267_T015	0.9852645	0.242051072	Zm00001d003267
Zm00001d003267_T039	0.3353627	-0.719185942	Zm00001d003267
Zm00001d003267_T032	0.2142641	1.002578677	Zm00001d003267
Zm00001d003267_T033	0.3288849	0.821625837	Zm00001d003267
Zm00001d003267_T044	0.9659036	-0.053024593	Zm00001d003267
Zm00001d003267_T001	0.9694074	0.241418956	Zm00001d003267
Zm00001d003267_T053	0.7602652	0.773912855	Zm00001d003267
Zm00001d003267_T040	0.9022629	-0.036358169	Zm00001d003267
Zm00001d003267_T052	0.7345621	0.459774681	Zm00001d003267
Zm00001d003267_T026	0.5269468	0.533761266	Zm00001d003267
Zm00001d003267_T055	0.9693993	0.017072757	Zm00001d003267

Zm00001d003267_T018	0.988575	0.274433306	Zm00001d003267
Zm00001d042599_T001	0.9954444	0.364173706	Zm00001d042599
Zm00001d039524_T001	2.35E-08	5.626440521	Zm00001d039524
Zm00001d039295_T001	0.1054046	-1.341424442	Zm00001d039295
Zm00001d022531_T001	0.8039937	-0.837122986	Zm00001d022531
Zm00001d006117_T002	0.8537544	0.125557776	Zm00001d006117
Zm00001d006117_T003	0.1191683	-0.46950398	Zm00001d006117
Zm00001d016591_T002	0.9090332	0.837939003	Zm00001d016591
Zm00001d016591_T001	0.2621487	0.644994007	Zm00001d016591
Zm00001d015746_T001	0.9706887	0.164028143	Zm00001d015746
Zm00001d045529_T001	0.8645432	0.141291929	Zm00001d045529
Zm00001d045529_T004	0.9926735	0.134966948	Zm00001d045529
Zm00001d031892_T001	0.09213667	1.926897092	Zm00001d031892
Zm00001d017176_T001	0.6284759	-0.477695356	Zm00001d017176
Zm00001d033459_T001	0.2454012	-1.140918005	Zm00001d033459
Zm00001d038890_T001	0.3795013	-1.216827884	Zm00001d038890
Zm00001d038890_T003	0.9979568	0.235156156	Zm00001d038890
Zm00001d038890_T002	0.9612081	-0.233190462	Zm00001d038890
Zm00001d037720_T001	0.8345927	-0.383854486	Zm00001d037720
Zm00001d009181_T001	0.7743716	0.932275062	Zm00001d009181
Zm00001d009181_T002	0.2391684	0.570216475	Zm00001d009181
Zm00001d013651_T001	1	0.187551255	Zm00001d013651
Zm00001d019582_T001	0.587212	0.643773747	Zm00001d019582
Zm00001d044470_T012	0.6580146	-0.80259702	Zm00001d044470
Zm00001d044470_T006	0.9918302	0.178475529	Zm00001d044470
Zm00001d044470_T001	0.7610341	0.004028816	Zm00001d044470
Zm00001d044470_T002	0.08698389	0.689891105	Zm00001d044470
Zm00001d044470_T003	0.9141981	0.474571568	Zm00001d044470
Zm00001d044470_T011	0.3082157	-1.405855139	Zm00001d044470
Zm00001d013452_T001	0.06821304	1.874594892	Zm00001d013452
Zm00001d013452_T002	0.05275691	-1.446935003	Zm00001d013452
Zm00001d011536_T001	1	0.169521692	Zm00001d011536
Zm00001d003398_T001	0.4115493	-0.781990248	Zm00001d003398
Zm00001d002543_T001	0.7056513	-0.340678841	Zm00001d002543
Zm00001d038338_T001	0.9799496	-0.037531193	Zm00001d038338
Zm00001d027464_T001	0.7973839	0.785943008	Zm00001d027464
Zm00001d005544_T001	0.852305	0.438918322	Zm00001d005544
Zm00001d010615_T001	0.92856	-0.000138963	Zm00001d010615
Zm00001d044762_T001	0.9539896	0.028638891	Zm00001d044762
Zm00001d007103_T002	0.894759	-0.215357972	Zm00001d007103
Zm00001d007103_T001	0.997212	0.354199306	Zm00001d007103
Zm00001d043808_T001	0.8862539	-0.328327211	Zm00001d043808
Zm00001d005442_T001	0.748607	1.082134902	Zm00001d005442
Zm00001d048366_T001	0.8854695	-0.108307631	Zm00001d048366
Zm00001d028427_T001	0.9628835	0.084943842	Zm00001d028427
Zm00001d027665_T001	0.1257207	0.414131322	Zm00001d027665
Zm00001d027665_T007	0.006154785	1.278534713	Zm00001d027665
Zm00001d027665_T009	0.7353983	0.334866193	Zm00001d027665
Zm00001d027665_T023	0.961473	0.126046311	Zm00001d027665
Zm00001d027665_T014	0.0192532	1.000997647	Zm00001d027665
Zm00001d027665_T038	0.8747821	0.724995895	Zm00001d027665
Zm00001d027665_T026	0.4849772	0.421288112	Zm00001d027665
Zm00001d027665_T039	0.2653595	-0.926023894	Zm00001d027665
Zm00001d019541_T001	0.9981361	0.121889003	Zm00001d019541

Zm00001d027612_T001	0.9969363	0.247205719	Zm00001d027612
Zm00001d027612_T003	0.9825858	0.278253509	Zm00001d027612
Zm00001d027285_T001	0.001503204	-1.612178583	Zm00001d027285
Zm00001d002470_T001	0.8720862	-0.267160699	Zm00001d002470
Zm00001d020764_T003	0.9693239	0.262818015	Zm00001d020764
Zm00001d020764_T006	1	0.19439645	Zm00001d020764
Zm00001d020764_T004	0.03578504	2.849771389	Zm00001d020764
Zm00001d020764_T002	0.5819598	1.146389065	Zm00001d020764
Zm00001d023813_T001	0.923446	0.008057362	Zm00001d023813
Zm00001d032652_T001	1	0.237934857	Zm00001d032652
Zm00001d030556_T001	0.7367876	1.125908172	Zm00001d030556
Zm00001d052616_T001	0.9895224	0.230555554	Zm00001d052616
Zm00001d010121_T001	0.9862794	0.171828304	Zm00001d010121
Zm00001d000401_T004	0.212393	-0.860396857	Zm00001d000401
Zm00001d000401_T002	0.9206245	-0.01698555	Zm00001d000401
Zm00001d000401_T001	0.7183895	-0.157712775	Zm00001d000401
Zm00001d002609_T001	0.757295	-0.230594642	Zm00001d002609
Zm00001d014903_T003	0.9918302	0.233851684	Zm00001d014903
Zm00001d014903_T005	0.6511883	0.386655134	Zm00001d014903
Zm00001d014903_T001	0.7759478	1.059843376	Zm00001d014903
Zm00001d035094_T001	0.7018195	-0.784348782	Zm00001d035094
Zm00001d035094_T002	0.4297509	-0.517033061	Zm00001d035094
Zm00001d011792_T005	0.941547	0.680330433	Zm00001d011792
Zm00001d011792_T009	0.975323	0.078901469	Zm00001d011792
Zm00001d011792_T006	0.9994624	0.268282334	Zm00001d011792
Zm00001d008321_T033	0.2988663	-0.452332266	Zm00001d008321
Zm00001d008321_T003	0.01371018	0.596510071	Zm00001d008321
Zm00001d008321_T028	0.9429712	-0.20088121	Zm00001d008321
Zm00001d008321_T008	0.001687576	0.58238696	Zm00001d008321
Zm00001d007884_T001	0.8933301	-0.526642968	Zm00001d007884
Zm00001d007884_T002	0.8086794	0.433310436	Zm00001d007884
Zm00001d000434_T001	0.8987811	0.721424838	Zm00001d000434
Zm00001d014668_T001	1	0.414336624	Zm00001d014668
Zm00001d006777_T002	0.8618945	2.330422692	Zm00001d006777
Zm00001d039368_T003	0.8953011	0.018459251	Zm00001d039368
Zm00001d039368_T001	0.2019121	-0.59926415	Zm00001d039368
Zm00001d052021_T004	0.6053962	-0.182759511	Zm00001d052021
Zm00001d052021_T001	0.1145356	1.519005277	Zm00001d052021
Zm00001d051496_T002	0.184583	1.670559883	Zm00001d051496
Zm00001d036593_T001	0.9832092	0.277101576	Zm00001d036593
Zm00001d037689_T001	0.7141136	1.163064675	Zm00001d037689
Zm00001d048204_T001	1	0.168158812	Zm00001d048204
Zm00001d020528_T001	0.9840918	0.403376511	Zm00001d020528
Zm00001d044157_T001	0.9988864	0.328624033	Zm00001d044157
Zm00001d042335_T004	0.01808074	1.126368552	Zm00001d042335
Zm00001d042335_T010	0.3980033	0.881110978	Zm00001d042335
Zm00001d042335_T018	0.7059983	0.134910814	Zm00001d042335
Zm00001d042335_T007	8.67E-08	-1.763631407	Zm00001d042335
Zm00001d042335_T020	0.718063	0.294081888	Zm00001d042335
Zm00001d042335_T006	0.4418602	1.276369986	Zm00001d042335
Zm00001d042335_T015	0.5597598	1.091867332	Zm00001d042335
Zm00001d042335_T003	0.8783081	0.167319285	Zm00001d042335
Zm00001d008333_T002	0.8922781	0.715045606	Zm00001d008333
Zm00001d008333_T004	0.934325	0.629213791	Zm00001d008333

Zm00001d008333_T007	0.998023	0.291649834	Zm00001d008333
Zm00001d008333_T001	0.8863456	0.798885461	Zm00001d008333
Zm00001d008333_T006	0.04127541	1.111086457	Zm00001d008333
Zm00001d002316_T003	0.9779656	0.672921408	Zm00001d002316
Zm00001d002316_T001	0.9363115	0.708862935	Zm00001d002316
Zm00001d017320_T001	0.9694613	0.045608306	Zm00001d017320
Zm00001d002181_T001	0.2817023	0.842753989	Zm00001d002181
Zm00001d040807_T001	0.323944	-0.933741157	Zm00001d040807
Zm00001d005895_T001	0.9722469	0.139521515	Zm00001d005895
Zm00001d003583_T001	0.4669321	-0.852264889	Zm00001d003583
Zm00001d034813_T001	0.1631405	1.491602477	Zm00001d034813
Zm00001d034813_T005	0.745718	-0.612773627	Zm00001d034813
Zm00001d034813_T003	0.9469426	0.076797947	Zm00001d034813
Zm00001d023506_T002	0.9180865	0.656563306	Zm00001d023506
Zm00001d051916_T005	0.9988864	0.328399602	Zm00001d051916
Zm00001d051916_T008	0.9260032	-0.101324963	Zm00001d051916
Zm00001d051916_T004	0.8557307	0.155829562	Zm00001d051916
Zm00001d051916_T006	0.9469426	0.011475765	Zm00001d051916
Zm00001d051916_T007	0.1401867	-1.268668136	Zm00001d051916
Zm00001d051916_T003	0.6436915	-0.092851025	Zm00001d051916
Zm00001d031328_T001	0.8533785	-0.150974893	Zm00001d031328
Zm00001d021960_T003	0.950155	0.366744904	Zm00001d021960
Zm00001d021960_T001	0.9782953	0.47222602	Zm00001d021960
Zm00001d044410_T001	0.5368684	-0.460060019	Zm00001d044410
Zm00001d024749_T001	0.3756867	1.425951637	Zm00001d024749
Zm00001d016653_T001	0.708674	-0.461799818	Zm00001d016653
Zm00001d031135_T001	0.5775553	-0.543043548	Zm00001d031135
Zm00001d003157_T002	0.5747705	-0.312727328	Zm00001d003157
Zm00001d003157_T001	0.971204	0.245182244	Zm00001d003157
Zm00001d048091_T015	0.9442514	3.22661511	Zm00001d048091
Zm00001d048091_T008	0.9703357	-0.17459609	Zm00001d048091
Zm00001d048091_T002	0.9693239	0.784048238	Zm00001d048091
Zm00001d048091_T013	0.9201237	0.459544616	Zm00001d048091
Zm00001d048091_T014	0.8175659	-1.154703388	Zm00001d048091
Zm00001d043621_T001	0.4860327	-0.953938691	Zm00001d043621
Zm00001d009517_T002	0.6389997	-0.442811861	Zm00001d009517
Zm00001d009517_T001	0.9474116	0.126954397	Zm00001d009517
Zm00001d004512_T001	0.9887584	0.396843442	Zm00001d004512
Zm00001d017906_T004	0.4893321	0.688668288	Zm00001d017906
Zm00001d010481_T001	0.9529184	-0.309982402	Zm00001d010481
Zm00001d007597_T001	1	0.322404027	Zm00001d007597
Zm00001d012627_T001	0.9514141	0.610403722	Zm00001d012627
Zm00001d049430_T001	0.2029212	1.712835176	Zm00001d049430
Zm00001d011426_T002	1.70E-11	-2.056464731	Zm00001d011426
Zm00001d011426_T003	0.1004019	2.732592002	Zm00001d011426
Zm00001d011426_T004	0.9867452	0.553067566	Zm00001d011426
Zm00001d011426_T005	0.8716108	-0.250847674	Zm00001d011426
Zm00001d041650_T001	0.9252088	-0.388917551	Zm00001d041650
Zm00001d014011_T001	0.967161	0.144173575	Zm00001d014011
Zm00001d005302_T001	1	0.250204002	Zm00001d005302
Zm00001d044271_T016	0.9870625	0.438898138	Zm00001d044271
Zm00001d044271_T007	0.6683377	-0.084585694	Zm00001d044271
Zm00001d044271_T001	0.7609047	1.100297662	Zm00001d044271
Zm00001d044271_T017	0.001301872	-2.228517034	Zm00001d044271

Zm00001d018787_T001	0.7379612	-0.162291286	Zm00001d018787
Zm00001d018787_T002	0.9273674	0.591487929	Zm00001d018787
Zm00001d011655_T001	0.004762117	1.608212047	Zm00001d011655
Zm00001d010796_T001	0.9065259	0.831375287	Zm00001d010796
Zm00001d010796_T002	0.6302619	-0.49872226	Zm00001d010796
Zm00001d018523_T012	0.6838482	0.757185598	Zm00001d018523
Zm00001d018523_T006	0.8946287	-0.004204318	Zm00001d018523
Zm00001d018523_T019	0.8892107	0.779563699	Zm00001d018523
Zm00001d018523_T016	0.9975882	0.246635168	Zm00001d018523
Zm00001d018523_T015	0.7214524	0.87744674	Zm00001d018523
Zm00001d018523_T008	0.9666757	0.196315628	Zm00001d018523
Zm00001d018523_T003	1	0.389978482	Zm00001d018523
Zm00001d047769_T001	0.9160957	0.846095894	Zm00001d047769
Zm00001d014332_T001	0.9664406	0.253692815	Zm00001d014332
Zm00001d049371_T011	0.8816665	-0.044936191	Zm00001d049371
Zm00001d049371_T003	1	0.29480895	Zm00001d049371
Zm00001d049371_T004	0.9089465	0.823349253	Zm00001d049371
Zm00001d025118_T001	0.9673473	-0.146113263	Zm00001d025118
Zm00001d025118_T003	1	0.132229011	Zm00001d025118
Zm00001d042924_T001	0.9817074	0.326219527	Zm00001d042924
Zm00001d043647_T001	0.5717645	0.680325985	Zm00001d043647
Zm00001d034050_T003	0.1048346	0.988591898	Zm00001d034050
Zm00001d034050_T002	0.3051425	1.666372534	Zm00001d034050
Zm00001d044227_T001	0.6876043	-0.939945331	Zm00001d044227
Zm00001d016550_T001	1	0.30142868	Zm00001d016550
Zm00001d036763_T001	0.000699734	3.125398696	Zm00001d036763
Zm00001d051119_T001	0.5401027	-0.19105413	Zm00001d051119
Zm00001d006533_T036	0.9281046	-0.004645966	Zm00001d006533
Zm00001d006533_T024	6.01E-15	3.607288579	Zm00001d006533
Zm00001d006533_T031	0.01636598	0.901509889	Zm00001d006533
Zm00001d006533_T009	1.09E-06	0.613960575	Zm00001d006533
Zm00001d006533_T001	1.04E-05	-0.915049273	Zm00001d006533
Zm00001d049551_T001	0.7315461	-0.212450754	Zm00001d049551
Zm00001d026031_T002	0.9921225	-0.129105928	Zm00001d026031
Zm00001d026031_T005	0.9963508	-0.177124939	Zm00001d026031
Zm00001d033060_T001	0.9968176	0.338253774	Zm00001d033060
Zm00001d027626_T007	0.1980319	1.303761202	Zm00001d027626
Zm00001d027626_T002	0.0043297	1.227119095	Zm00001d027626
Zm00001d027626_T005	0.9954426	0.276011942	Zm00001d027626
Zm00001d027626_T008	1	0.136979709	Zm00001d027626
Zm00001d027626_T010	0.9160053	0.287974436	Zm00001d027626
Zm00001d027626_T001	0.01901268	-2.408693605	Zm00001d027626
Zm00001d043243_T001	0.6214202	-0.39487451	Zm00001d043243
Zm00001d013245_T001	0.8236738	0.959889241	Zm00001d013245
Zm00001d039791_T001	0.8079397	0.948537969	Zm00001d039791
Zm00001d014758_T001	0.6780291	-0.752672285	Zm00001d014758
Zm00001d013015_T001	0.8976475	-0.170134324	Zm00001d013015
Zm00001d013015_T002	0.9686323	0.485946433	Zm00001d013015
Zm00001d004015_T001	0.9918302	0.242477716	Zm00001d004015
Zm00001d015376_T002	0.001821067	-1.409832878	Zm00001d015376
Zm00001d015376_T001	0.9514141	0.117258963	Zm00001d015376
Zm00001d012780_T001	0.6019353	-0.587227518	Zm00001d012780
Zm00001d040445_T001	0.7595879	-0.481822378	Zm00001d040445
Zm00001d037257_T001	0.9822138	0.546627429	Zm00001d037257

Zm00001d053404_T007	0.5448225	0.747645217	Zm00001d053404
Zm00001d053404_T002	0.3475256	0.401716907	Zm00001d053404
Zm00001d053404_T009	0.0624735	2.399693621	Zm00001d053404
Zm00001d053404_T001	1	0.443867345	Zm00001d053404
Zm00001d042436_T001	0.9282737	-0.084592453	Zm00001d042436
Zm00001d042047_T001	0.885791	-0.087654224	Zm00001d042047
Zm00001d005674_T001	0.885791	0.821508996	Zm00001d005674
Zm00001d052188_T001	0.8740066	0.881042909	Zm00001d052188
Zm00001d048337_T001	0.003961109	0.650324383	Zm00001d048337
Zm00001d031041_T003	0.9326482	0.083918185	Zm00001d031041
Zm00001d034796_T001	0.7059983	-0.045656944	Zm00001d034796
Zm00001d034796_T006	0.7434928	0.602329518	Zm00001d034796
Zm00001d034796_T007	0.9825388	0.243621059	Zm00001d034796
Zm00001d034796_T003	0.9942091	0.343377841	Zm00001d034796
Zm00001d034796_T005	0.8353818	0.846589984	Zm00001d034796
Zm00001d029516_T003	0.757295	0.382073924	Zm00001d029516
Zm00001d029516_T002	0.3643104	0.800219419	Zm00001d029516
Zm00001d029516_T001	0.7805958	1.060286721	Zm00001d029516
Zm00001d002000_T009	0.0161855	-1.564710773	Zm00001d002000
Zm00001d002000_T007	0.6465371	-0.394854488	Zm00001d002000
Zm00001d002000_T002	0.1557051	-0.978321317	Zm00001d002000
Zm00001d002000_T010	0.3344918	-0.810665447	Zm00001d002000
Zm00001d002000_T008	0.3045236	-0.358585693	Zm00001d002000
Zm00001d002000_T005	0.2694466	-0.71253308	Zm00001d002000
Zm00001d018203_T001	0.7492733	-0.30337276	Zm00001d018203
Zm00001d044176_T001	0.2642046	-0.743739959	Zm00001d044176
Zm00001d020160_T001	0.2715931	-1.392288026	Zm00001d020160
Zm00001d027957_T004	0.8842271	0.61691018	Zm00001d027957
Zm00001d027957_T005	0.3392089	-0.891488246	Zm00001d027957
Zm00001d027957_T003	0.8958882	-0.107679573	Zm00001d027957
Zm00001d039770_T001	0.9828436	0.170002781	Zm00001d039770
Zm00001d040274_T002	0.8068255	-0.208213896	Zm00001d040274
Zm00001d040274_T004	1	0.19791498	Zm00001d040274
Zm00001d036522_T001	0.8968577	-0.059107968	Zm00001d036522
Zm00001d027649_T003	0.3205135	1.184208904	Zm00001d027649
Zm00001d027649_T009	0.8031436	0.594879283	Zm00001d027649
Zm00001d027649_T008	0.4041059	1.524591831	Zm00001d027649
Zm00001d027649_T002	0.1417758	1.128006466	Zm00001d027649
Zm00001d027649_T010	0.1858262	1.666741324	Zm00001d027649
Zm00001d027649_T001	0.776742	0.976154621	Zm00001d027649
Zm00001d036834_T002	0.9196877	0.528547506	Zm00001d036834
Zm00001d036834_T001	0.539175	1.125754101	Zm00001d036834
Zm00001d048239_T001	1	0.267518967	Zm00001d048239
Zm00001d042371_T001	0.3301595	-0.814679653	Zm00001d042371
Zm00001d022428_T002	0.967161	0.313317205	Zm00001d022428
Zm00001d022428_T001	0.9782953	0.240544896	Zm00001d022428
Zm00001d031740_T001	0.9416622	0.040875852	Zm00001d031740
Zm00001d008523_T001	1.60E-07	-5.241753202	Zm00001d008523
Zm00001d037624_T010	0.07400091	0.61786373	Zm00001d037624
Zm00001d037624_T007	0.9497909	0.430015055	Zm00001d037624
Zm00001d037624_T001	0.5006087	-0.140937976	Zm00001d037624
Zm00001d037624_T006	0.5628302	1.321891096	Zm00001d037624
Zm00001d037624_T002	0.6844831	-0.328012877	Zm00001d037624
Zm00001d023868_T004	0.9524127	0.0431855	Zm00001d023868

Zm00001d023868_T001	0.5141531	-0.583557035	Zm00001d023868
Zm00001d023868_T005	0.8613703	-0.085124781	Zm00001d023868
Zm00001d039827_T001	0.9771927	0.042985178	Zm00001d039827
Zm00001d024499_T001	0.4656438	-0.737583095	Zm00001d024499
Zm00001d034779_T002	1	0.122579786	Zm00001d034779
Zm00001d043330_T011	0.4949487	0.390353373	Zm00001d043330
Zm00001d043330_T004	0.6320295	-0.150791792	Zm00001d043330
Zm00001d043330_T001	0.8142464	-0.279383319	Zm00001d043330
Zm00001d045398_T001	0.2387996	-1.080560361	Zm00001d045398
Zm00001d048771_T001	0.6558999	-0.465156266	Zm00001d048771
Zm00001d045139_T001	0.9956349	0.525612513	Zm00001d045139
Zm00001d045139_T003	0.7071453	1.204675534	Zm00001d045139
Zm00001d045139_T002	0.8647028	0.974367931	Zm00001d045139
Zm00001d021628_T001	0.6927383	-0.898583732	Zm00001d021628
Zm00001d011919_T001	0.5333953	1.289383852	Zm00001d011919
Zm00001d007891_T001	0.6667748	-0.595215581	Zm00001d007891
Zm00001d002126_T001	0.004246457	3.238330133	Zm00001d002126
Zm00001d053995_T001	0.2205171	1.183359823	Zm00001d053995
Zm00001d039701_T001	1	0.30724647	Zm00001d039701
Zm00001d038266_T012	0.2476332	0.499279066	Zm00001d038266
Zm00001d038266_T010	0.9299574	0.539488118	Zm00001d038266
Zm00001d038266_T007	0.976488	0.060953551	Zm00001d038266
Zm00001d038266_T003	0.871546	-0.083183124	Zm00001d038266
Zm00001d038266_T011	0.9907928	0.160388657	Zm00001d038266
Zm00001d025713_T004	0.1107948	0.729307818	Zm00001d025713
Zm00001d025713_T002	0.7831859	-0.474067048	Zm00001d025713
Zm00001d025713_T001	0.9908081	0.442176447	Zm00001d025713
Zm00001d038412_T001	1	0.37251806	Zm00001d038412
Zm00001d037298_T001	0.6954199	-0.421805096	Zm00001d037298
Zm00001d052747_T001	0.9659036	0.146025231	Zm00001d052747
Zm00001d022041_T002	0.000442113	2.593085868	Zm00001d022041
Zm00001d022041_T003	0.9633184	0.561700833	Zm00001d022041
Zm00001d022041_T012	0.000779771	0.607321387	Zm00001d022041
Zm00001d022041_T005	0.07457948	2.265158212	Zm00001d022041
Zm00001d038754_T001	0.8368665	-0.233349535	Zm00001d038754
Zm00001d038754_T002	0.7585131	-0.271420096	Zm00001d038754
Zm00001d038754_T004	0.2146312	2.200477979	Zm00001d038754
Zm00001d044096_T002	1	0.183892527	Zm00001d044096
Zm00001d044096_T005	1.69E-05	1.022530287	Zm00001d044096
Zm00001d044096_T001	0.6633441	1.121998947	Zm00001d044096
Zm00001d044096_T003	0.8585804	0.692978189	Zm00001d044096
Zm00001d044096_T004	0.000113535	2.171978809	Zm00001d044096
Zm00001d034143_T001	0.7831859	-0.193872976	Zm00001d034143
Zm00001d042410_T002	0.9210327	-0.143631782	Zm00001d042410
Zm00001d042410_T007	0.3439954	1.180399781	Zm00001d042410
Zm00001d018125_T001	0.5701056	0.981165747	Zm00001d018125
Zm00001d017168_T005	0.002048357	-2.72165284	Zm00001d017168
Zm00001d017168_T002	1	0.337793818	Zm00001d017168
Zm00001d017168_T004	0.8200126	-0.264834857	Zm00001d017168
Zm00001d035569_T001	0.8656238	-0.468670543	Zm00001d035569
Zm00001d030121_T001	0.04916801	-0.670658882	Zm00001d030121
Zm00001d010528_T004	0.02483077	-1.134191326	Zm00001d010528
Zm00001d010528_T001	0.6856739	-0.8446177	Zm00001d010528
Zm00001d010528_T005	0.8383745	-0.433085662	Zm00001d010528

Zm00001d010528_T003	0.8050292	-0.692265526	Zm00001d010528
Zm00001d022616_T001	0.9334932	-0.106624438	Zm00001d022616
Zm00001d022616_T002	1	0.219192278	Zm00001d022616
Zm00001d023368_T007	0.997764	0.429712416	Zm00001d023368
Zm00001d023368_T014	0.5615308	0.518462543	Zm00001d023368
Zm00001d041474_T002	0.9429978	-0.118634631	Zm00001d041474
Zm00001d041474_T003	0.9988864	0.131883984	Zm00001d041474
Zm00001d041474_T001	0.9483517	0.075372499	Zm00001d041474
Zm00001d012701_T012	0.3976906	1.21864967	Zm00001d012701
Zm00001d012701_T010	0.7383611	-0.046438864	Zm00001d012701
Zm00001d012701_T021	5.90E-11	-2.438846528	Zm00001d012701
Zm00001d012701_T023	0.397669	1.815664245	Zm00001d012701
Zm00001d012701_T001	0.8984125	0.811524043	Zm00001d012701
Zm00001d012701_T006	0.8107637	0.524833353	Zm00001d012701
Zm00001d030941_T001	1	0.066664932	Zm00001d030941
Zm00001d031618_T001	0.7100246	-0.439282481	Zm00001d031618
Zm00001d038302_T018	0.9888172	0.365924216	Zm00001d038302
Zm00001d038302_T074	0.7846759	-0.231483592	Zm00001d038302
Zm00001d038302_T026	0.01544152	0.611843802	Zm00001d038302
Zm00001d043682_T008	0.8050292	-0.056720999	Zm00001d043682
Zm00001d043682_T013	0.9945118	0.453265581	Zm00001d043682
Zm00001d043682_T010	0.7912425	0.302013072	Zm00001d043682
Zm00001d043682_T004	0.06820211	-2.016933995	Zm00001d043682
Zm00001d009958_T001	0.9264186	0.357276694	Zm00001d009958
Zm00001d039196_T001	0.9513906	0.652138421	Zm00001d039196
Zm00001d034368_T001	0.9689939	0.606212824	Zm00001d034368
Zm00001d042233_T002	0.9221421	-0.053278165	Zm00001d042233
Zm00001d028297_T004	0.6876043	0.392666679	Zm00001d028297
Zm00001d028297_T010	0.9690925	-0.00343601	Zm00001d028297
Zm00001d006701_T001	0.9375054	0.69613841	Zm00001d006701
Zm00001d024318_T001	0.9817074	0.230837704	Zm00001d024318
Zm00001d024318_T010	0.02312763	2.083460802	Zm00001d024318
Zm00001d024318_T005	0.02507626	0.981999151	Zm00001d024318
Zm00001d040958_T001	0.9086616	-0.026718047	Zm00001d040958
Zm00001d052410_T001	0.5670267	-0.624368625	Zm00001d052410
Zm00001d004855_T004	0.0147663	-2.090339161	Zm00001d004855
Zm00001d004855_T005	0.04818964	-1.632877932	Zm00001d004855
Zm00001d004855_T007	0.7392267	0.33789758	Zm00001d004855
Zm00001d004855_T002	0.2035902	-0.973985781	Zm00001d004855
Zm00001d051362_T001	0.9647033	0.588658252	Zm00001d051362
Zm00001d042820_T003	0.7289978	-0.332991885	Zm00001d042820
Zm00001d042820_T001	0.9706293	0.135991836	Zm00001d042820
Zm00001d012418_T001	0.8902687	0.710428384	Zm00001d012418
Zm00001d017601_T003	0.9982897	0.227861809	Zm00001d017601
Zm00001d017601_T002	0.7548788	0.457641428	Zm00001d017601
Zm00001d017601_T001	0.9850313	0.490466186	Zm00001d017601
Zm00001d007158_T009	0.4386149	1.444462065	Zm00001d007158
Zm00001d007158_T019	1	0.502943688	Zm00001d007158
Zm00001d007158_T007	0.8609127	0.211704282	Zm00001d007158
Zm00001d005170_T009	0.002306279	1.35198342	Zm00001d005170
Zm00001d005170_T001	1	0.379902515	Zm00001d005170
Zm00001d005170_T008	0.02706274	1.246716846	Zm00001d005170
Zm00001d005170_T005	0.9745948	0.303292725	Zm00001d005170
Zm00001d005647_T002	0.987643	0.522510107	Zm00001d005647

Zm00001d005647_T003	0.2923	-0.821298079	Zm00001d005647
Zm00001d000184_T001	0.8609458	0.877958145	Zm00001d000184
Zm00001d014003_T002	0.6358713	-0.33114841	Zm00001d014003
Zm00001d014003_T005	0.6500231	-0.410586748	Zm00001d014003
Zm00001d014003_T006	0.5373511	-0.447799728	Zm00001d014003
Zm00001d014003_T001	0.9469426	0.039821289	Zm00001d014003
Zm00001d014003_T003	0.7194777	-0.399587255	Zm00001d014003
Zm00001d007495_T001	0.8421761	0.675615868	Zm00001d007495
Zm00001d042051_T001	0.3391928	-0.761554202	Zm00001d042051
Zm00001d003751_T001	0.9708651	0.504726494	Zm00001d003751
Zm00001d043395_T003	0.9832612	0.231027334	Zm00001d043395
Zm00001d043395_T002	0.9363242	0.723769951	Zm00001d043395
Zm00001d012178_T001	0.8557307	0.527184018	Zm00001d012178
Zm00001d012747_T016	0.04361477	0.749816893	Zm00001d012747
Zm00001d012747_T009	0.5092251	1.150893239	Zm00001d012747
Zm00001d012747_T018	0.004149465	0.838529724	Zm00001d012747
Zm00001d012747_T001	0.7999494	0.905604549	Zm00001d012747
Zm00001d012167_T001	0.7179988	1.201607999	Zm00001d012167
Zm00001d032761_T001	0.02706902	-0.920477863	Zm00001d032761
Zm00001d032761_T004	0.000259008	-1.385201501	Zm00001d032761
Zm00001d032761_T003	0.9519347	0.327691578	Zm00001d032761
Zm00001d032761_T002	0.7993434	1.00033812	Zm00001d032761
Zm00001d044520_T001	0.9822138	0.110404401	Zm00001d044520
Zm00001d011331_T001	0.8160463	-0.468360145	Zm00001d011331
Zm00001d044745_T015	0.7195936	0.403886518	Zm00001d044745
Zm00001d008892_T001	1	-0.107529969	Zm00001d008892
Zm00001d032464_T061	0.7379612	0.201389551	Zm00001d032464
Zm00001d032464_T031	0.9070578	0.3160629	Zm00001d032464
Zm00001d032464_T003	0.02917075	1.305773253	Zm00001d032464
Zm00001d032464_T060	0.06597925	-0.749812033	Zm00001d032464
Zm00001d032464_T022	0.7923921	1.037266685	Zm00001d032464
Zm00001d032464_T030	0.7341184	0.78404586	Zm00001d032464
Zm00001d024382_T001	0.5526129	-0.680326734	Zm00001d024382
Zm00001d052734_T001	0.8023059	0.605108123	Zm00001d052734
Zm00001d052734_T006	0.1378997	-1.596791998	Zm00001d052734
Zm00001d052734_T002	0.9380783	0.654128732	Zm00001d052734
Zm00001d052734_T003	0.4344214	-0.158120562	Zm00001d052734
Zm00001d040313_T001	0.00023792	2.051542566	Zm00001d040313
Zm00001d044056_T001	0.1258785	0.679987684	Zm00001d044056
Zm00001d044056_T010	0.9791729	0.201590006	Zm00001d044056
Zm00001d013642_T001	0.8830711	-0.224576452	Zm00001d013642
Zm00001d002019_T001	0.9766874	0.175176662	Zm00001d002019
Zm00001d042613_T001	0.8885217	-0.286079076	Zm00001d042613
Zm00001d022475_T001	0.001266433	1.462883959	Zm00001d022475
Zm00001d022475_T008	0.5042696	1.111639203	Zm00001d022475
Zm00001d022475_T014	0.123021	-1.099526255	Zm00001d022475
Zm00001d022475_T012	0.9701986	0.5345324	Zm00001d022475
Zm00001d017717_T002	0.7712604	0.862357722	Zm00001d017717
Zm00001d017717_T003	0.5552199	0.58196271	Zm00001d017717
Zm00001d012745_T003	0.8964117	0.276629938	Zm00001d012745
Zm00001d012745_T001	0.9529184	0.012615696	Zm00001d012745
Zm00001d012745_T004	0.9781672	0.373167288	Zm00001d012745
Zm00001d012745_T002	0.9211172	-0.006207912	Zm00001d012745
Zm00001d037974_T002	0.941809	-0.270404805	Zm00001d037974

Zm00001d043307_T001	0.7541349	-0.326348088	Zm00001d043307
Zm00001d041035_T002	9.93E-11	1.768837041	Zm00001d041035
Zm00001d041035_T005	0.8650248	0.958128923	Zm00001d041035
Zm00001d025240_T009	0.002559064	-2.075003567	Zm00001d025240
Zm00001d025240_T041	0.7921111	-0.299487466	Zm00001d025240
Zm00001d025240_T005	0.9959972	0.265586926	Zm00001d025240
Zm00001d025240_T039	0.0830571	2.560346001	Zm00001d025240
Zm00001d025240_T015	0.780547	0.354884916	Zm00001d025240
Zm00001d025240_T003	0.3097848	1.733112408	Zm00001d025240
Zm00001d035304_T001	0.8687089	-0.257654987	Zm00001d035304
Zm00001d011840_T001	0.9517262	0.313758531	Zm00001d011840
Zm00001d049902_T001	0.9529184	-0.284347188	Zm00001d049902
Zm00001d019956_T002	0.9927338	0.246395269	Zm00001d019956
Zm00001d029339_T001	0.9918302	0.252246533	Zm00001d029339
Zm00001d007026_T001	0.7957286	-0.309707727	Zm00001d007026
Zm00001d028992_T001	0.7106278	-0.395047203	Zm00001d028992
Zm00001d032081_T002	0.190707	-1.438693223	Zm00001d032081
Zm00001d032081_T001	0.8885778	-0.052407514	Zm00001d032081
Zm00001d033152_T001	0.6336913	-0.471104019	Zm00001d033152
Zm00001d044961_T007	0.4646235	-0.763131209	Zm00001d044961
Zm00001d044961_T001	0.9846714	0.263916604	Zm00001d044961
Zm00001d044961_T008	0.8903672	0.785282478	Zm00001d044961
Zm00001d017966_T002	0.7345787	-0.057512506	Zm00001d017966
Zm00001d017966_T001	0.9706887	0.168799077	Zm00001d017966
Zm00001d043776_T006	1	0.263930109	Zm00001d043776
Zm00001d043776_T004	0.9673094	0.401218869	Zm00001d043776
Zm00001d043776_T005	0.9706887	0.54250881	Zm00001d043776
Zm00001d043776_T007	0.9719418	0.570734764	Zm00001d043776
Zm00001d043776_T003	0.08742619	-1.096338022	Zm00001d043776
Zm00001d018485_T002	0.8784569	-0.117475286	Zm00001d018485
Zm00001d048373_T002	0.9429978	0.719197532	Zm00001d048373
Zm00001d048373_T001	0.481901	-0.404553875	Zm00001d048373
Zm00001d037069_T001	0.2496307	0.493106613	Zm00001d037069
Zm00001d043562_T011	0.6169231	0.268262364	Zm00001d043562
Zm00001d043562_T013	0.9176741	-0.354466166	Zm00001d043562
Zm00001d043562_T008	0.8099592	0.898711162	Zm00001d043562
Zm00001d043562_T006	0.3847344	-0.235896967	Zm00001d043562
Zm00001d032605_T004	0.9834026	0.503416327	Zm00001d032605
Zm00001d032605_T001	0.000934168	-0.513639082	Zm00001d032605
Zm00001d032605_T003	0.6577149	1.72274313	Zm00001d032605
Zm00001d032605_T002	0.3395336	1.026628236	Zm00001d032605
Zm00001d033225_T002	1	0.380015255	Zm00001d033225
Zm00001d033225_T001	0.7993434	-0.306216361	Zm00001d033225
Zm00001d025695_T001	0.9870394	0.092761094	Zm00001d025695
Zm00001d042624_T001	0.9198718	-0.137656733	Zm00001d042624
Zm00001d004137_T001	0.9981278	-0.036837515	Zm00001d004137
Zm00001d012512_T001	0.3515304	1.227537667	Zm00001d012512
Zm00001d037864_T004	0.1892576	1.335739731	Zm00001d037864
Zm00001d037864_T001	0.9893214	0.259431137	Zm00001d037864
Zm00001d037864_T016	0.000463427	0.644829704	Zm00001d037864
Zm00001d042658_T002	0.5591883	-0.689278814	Zm00001d042658
Zm00001d042658_T003	0.8422323	-0.155667223	Zm00001d042658
Zm00001d039022_T001	1	0.211758386	Zm00001d039022
Zm00001d018040_T002	0.9648856	0.634279771	Zm00001d018040

Zm00001d018040_T005	0.8537544	-0.080169205	Zm00001d018040
Zm00001d018040_T004	0.9766874	0.342407146	Zm00001d018040
Zm00001d018323_T001	0.2038212	-1.354754813	Zm00001d018323
Zm00001d034546_T001	0.6330708	0.866765475	Zm00001d034546
Zm00001d026523_T001	0.9732421	0.351874293	Zm00001d026523
Zm00001d033891_T001	0.7296512	-0.170062557	Zm00001d033891
Zm00001d048424_T002	0.000248481	-1.087790902	Zm00001d048424
Zm00001d048424_T001	0.7897281	-0.195253202	Zm00001d048424
Zm00001d048424_T008	0.03246552	-1.173436974	Zm00001d048424
Zm00001d048424_T019	0.8376343	0.288935304	Zm00001d048424
Zm00001d048424_T009	0.9420032	1.451186667	Zm00001d048424
Zm00001d048424_T006	5.17E-06	-2.946186528	Zm00001d048424
Zm00001d048424_T005	0.1276214	-1.361708664	Zm00001d048424
Zm00001d048424_T010	0.72524	-0.152258811	Zm00001d048424
Zm00001d048424_T007	0.8673643	0.024291392	Zm00001d048424
Zm00001d030627_T002	0.9257238	-0.226071949	Zm00001d030627
Zm00001d030627_T008	0.5719089	0.205923281	Zm00001d030627
Zm00001d030627_T009	0.1399853	-1.240316612	Zm00001d030627
Zm00001d030627_T006	0.1799392	-1.29154934	Zm00001d030627
Zm00001d030627_T004	0.9805897	0.164395396	Zm00001d030627
Zm00001d030627_T003	0.2087765	-1.096232666	Zm00001d030627
Zm00001d051829_T001	0.9290834	0.078304644	Zm00001d051829
Zm00001d051829_T002	0.145781	-1.039789356	Zm00001d051829
Zm00001d037396_T002	0.09496973	1.889902068	Zm00001d037396
Zm00001d029356_T001	0.4844771	1.469855691	Zm00001d029356
Zm00001d029356_T004	0.9211172	-0.065758093	Zm00001d029356
Zm00001d029356_T003	0.6264601	0.931655754	Zm00001d029356
Zm00001d016273_T001	0.923446	-0.025637673	Zm00001d016273
Zm00001d016273_T003	1	0.147306321	Zm00001d016273
Zm00001d038689_T004	0.9881407	0.460416459	Zm00001d038689
Zm00001d038689_T001	1	0.366583647	Zm00001d038689
Zm00001d025881_T001	0.3497147	0.645769769	Zm00001d025881
Zm00001d025446_T001	0.909729	0.044669867	Zm00001d025446
Zm00001d025446_T004	0.7753159	-0.16173413	Zm00001d025446
Zm00001d025446_T005	0.997764	0.052044843	Zm00001d025446
Zm00001d040639_T001	0.1106445	-1.261098524	Zm00001d040639
Zm00001d031203_T001	0.5861371	1.070193102	Zm00001d031203
Zm00001d014775_T001	0.6053962	-0.432319181	Zm00001d014775
Zm00001d014775_T002	0.9369505	0.054684171	Zm00001d014775
Zm00001d011457_T002	0.893207	-0.081507029	Zm00001d011457
Zm00001d028620_T001	1	0.422813764	Zm00001d028620
Zm00001d039584_T001	0.4003047	-0.485496264	Zm00001d039584
Zm00001d012886_T001	0.675716	-1.018760304	Zm00001d012886
Zm00001d018989_T001	0.3497147	-0.787236742	Zm00001d018989
Zm00001d016049_T002	0.9860836	-0.089592709	Zm00001d016049
Zm00001d038883_T001	0.3131019	1.180632718	Zm00001d038883
Zm00001d021310_T001	1	0.304886774	Zm00001d021310
Zm00001d011669_T001	0.9490989	0.431807831	Zm00001d011669
Zm00001d018872_T016	0.3767092	-1.159311137	Zm00001d018872
Zm00001d018872_T025	0.3870314	0.596557393	Zm00001d018872
Zm00001d018872_T009	0.8883561	0.801530664	Zm00001d018872
Zm00001d018872_T002	0.5507275	0.648558933	Zm00001d018872
Zm00001d018872_T007	0.006260083	1.234366431	Zm00001d018872
Zm00001d044116_T001	1	0.272507707	Zm00001d044116

Zm00001d035035_T001	0.8364213	0.948142759	Zm00001d035035
Zm00001d048398_T001	0.2021453	1.648883065	Zm00001d048398
Zm00001d032158_T002	1	0.300619202	Zm00001d032158
Zm00001d032158_T001	0.8996827	-0.069803737	Zm00001d032158
Zm00001d034882_T001	0.9221549	-0.005129301	Zm00001d034882
Zm00001d043731_T001	0.4608389	-0.511806839	Zm00001d043731
Zm00001d040389_T001	0.9766874	0.507542005	Zm00001d040389
Zm00001d040389_T002	0.9756435	-0.020662615	Zm00001d040389
Zm00001d026350_T001	0.9791729	0.46309725	Zm00001d026350
Zm00001d003031_T005	0.9851204	0.555307974	Zm00001d003031
Zm00001d003031_T001	0.001103033	-0.590041555	Zm00001d003031
Zm00001d029392_T006	0.9446379	0.697246628	Zm00001d029392
Zm00001d029392_T003	0.1084843	1.996203863	Zm00001d029392
Zm00001d029392_T005	0.02168606	1.032019922	Zm00001d029392
Zm00001d029392_T007	2.67E-05	1.091137502	Zm00001d029392
Zm00001d017096_T005	0.8975864	-0.069983085	Zm00001d017096
Zm00001d017096_T022	0.6097901	1.218049627	Zm00001d017096
Zm00001d017096_T016	0.005375707	0.817652746	Zm00001d017096
Zm00001d017096_T025	0.000839604	3.1079675	Zm00001d017096
Zm00001d017096_T033	0.8142704	0.61749441	Zm00001d017096
Zm00001d017096_T017	0.000973999	2.983312888	Zm00001d017096
Zm00001d017096_T009	0.1895778	0.532844609	Zm00001d017096
Zm00001d017096_T012	0.8782162	0.736580742	Zm00001d017096
Zm00001d017096_T011	0.8605736	0.81368269	Zm00001d017096
Zm00001d017096_T021	0.001968686	2.456262594	Zm00001d017096
Zm00001d017096_T019	0.1397589	1.249789806	Zm00001d017096
Zm00001d017096_T014	0.9781672	0.367608463	Zm00001d017096
Zm00001d017096_T010	0.1349847	1.906546921	Zm00001d017096
Zm00001d017096_T001	0.9020229	0.074445089	Zm00001d017096
Zm00001d017096_T004	0.6858852	1.153544298	Zm00001d017096
Zm00001d001928_T051	0.215298	0.577174338	Zm00001d001928
Zm00001d001928_T052	0.5747568	-0.633296806	Zm00001d001928
Zm00001d001928_T035	0.9701986	-0.001627186	Zm00001d001928
Zm00001d051461_T001	0.9237617	0.741566692	Zm00001d051461
Zm00001d021520_T002	0.9195956	-0.088961298	Zm00001d021520
Zm00001d046921_T001	0.8127863	-0.3051477	Zm00001d046921
Zm00001d026141_T001	0.3919095	-1.212617224	Zm00001d026141
Zm00001d044231_T001	0.835786	0.993888397	Zm00001d044231
Zm00001d003258_T001	0.8513569	-0.145892531	Zm00001d003258
Zm00001d049023_T006	0.9689939	0.057076991	Zm00001d049023
Zm00001d049023_T025	0.6436915	0.966424138	Zm00001d049023
Zm00001d049023_T033	0.1456892	-0.16489611	Zm00001d049023
Zm00001d049023_T041	0.9469426	0.459691583	Zm00001d049023
Zm00001d049023_T044	0.8513643	-0.481625022	Zm00001d049023
Zm00001d049023_T043	0.9259902	0.336494703	Zm00001d049023
Zm00001d021187_T001	0.8863456	0.020820148	Zm00001d021187
Zm00001d021187_T002	0.8044803	1.035512737	Zm00001d021187
Zm00001d026397_T001	0.9022629	-0.078238524	Zm00001d026397
Zm00001d038503_T001	0.7970469	0.445215179	Zm00001d038503
Zm00001d009868_T007	0.4179542	-0.701910084	Zm00001d009868
Zm00001d009868_T003	0.8896831	-0.052382274	Zm00001d009868
Zm00001d045130_T001	1	0.32496196	Zm00001d045130
Zm00001d006754_T001	0.4315667	1.356349546	Zm00001d006754
Zm00001d032058_T001	0.9911485	0.139589045	Zm00001d032058

Zm00001d045394_T001	0.9745777	0.567423794	Zm00001d045394
Zm00001d007205_T001	0.8825173	-0.047651591	Zm00001d007205
Zm00001d016896_T001	0.9974734	0.440882551	Zm00001d016896
Zm00001d012915_T002	1	0.308708176	Zm00001d012915
Zm00001d015886_T001	0.9628432	0.153066884	Zm00001d015886
Zm00001d035447_T001	0.7109402	-0.355682961	Zm00001d035447
Zm00001d035447_T002	0.967161	0.076539926	Zm00001d035447
Zm00001d014099_T001	0.2729622	1.24328075	Zm00001d014099
Zm00001d005961_T001	1	0.111008101	Zm00001d005961
Zm00001d022547_T001	0.8050292	0.913223135	Zm00001d022547
Zm00001d046424_T001	1	0.116402923	Zm00001d046424
Zm00001d011387_T001	0.9189175	-0.17199814	Zm00001d011387
Zm00001d010911_T001	0.7746801	-0.530863453	Zm00001d010911
Zm00001d001841_T001	0.9344122	-0.245682351	Zm00001d001841
Zm00001d022259_T001	0.6978306	0.785353487	Zm00001d022259
Zm00001d022259_T002	0.9804228	0.168073435	Zm00001d022259
Zm00001d015195_T001	0.9720425	0.5397384	Zm00001d015195
Zm00001d048272_T015	0.453061	-0.358589676	Zm00001d048272
Zm00001d048272_T001	0.9907928	0.217921215	Zm00001d048272
Zm00001d048272_T002	0.7367876	-0.329122578	Zm00001d048272
Zm00001d048272_T011	0.000435301	2.461490856	Zm00001d048272
Zm00001d048272_T005	0.9228108	0.184715599	Zm00001d048272
Zm00001d048272_T022	0.8922781	-0.051013255	Zm00001d048272
Zm00001d048272_T003	0.05187496	1.233044653	Zm00001d048272
Zm00001d048272_T017	0.3548936	0.577180827	Zm00001d048272
Zm00001d053151_T003	0.718063	-0.320593099	Zm00001d053151
Zm00001d053151_T001	0.003224906	-0.527412498	Zm00001d053151
Zm00001d043795_T002	0.2719671	1.367871527	Zm00001d043795
Zm00001d043795_T001	0.9563232	0.488870799	Zm00001d043795
Zm00001d031676_T005	0.05891266	2.129860025	Zm00001d031676
Zm00001d031676_T009	0.9519877	-0.287174893	Zm00001d031676
Zm00001d031676_T006	0.00734409	1.232645147	Zm00001d031676
Zm00001d031676_T004	0.9689939	0.610979427	Zm00001d031676
Zm00001d011245_T003	0.9928864	0.276020579	Zm00001d011245
Zm00001d025900_T001	0.2677246	-0.949112528	Zm00001d025900
Zm00001d025900_T002	0.4302857	-0.607923383	Zm00001d025900
Zm00001d039513_T001	0.4106548	-0.781944194	Zm00001d039513
Zm00001d044615_T003	0.9272378	-0.152932187	Zm00001d044615
Zm00001d044615_T002	0.8676122	-0.156571521	Zm00001d044615
Zm00001d003621_T009	0.02279034	0.572826302	Zm00001d003621
Zm00001d003621_T015	0.9867505	0.515484708	Zm00001d003621
Zm00001d003621_T010	0.9381687	0.651660022	Zm00001d003621
Zm00001d003621_T001	0.5748211	-0.339940433	Zm00001d003621
Zm00001d003621_T004	0.614631	-0.091265531	Zm00001d003621
Zm00001d003621_T011	0.6288391	-0.372850686	Zm00001d003621
Zm00001d003621_T003	0.8690525	0.004219427	Zm00001d003621
Zm00001d042723_T001	0.9031129	0.821338197	Zm00001d042723
Zm00001d022487_T002	0.8246527	-0.510447772	Zm00001d022487
Zm00001d050483_T004	0.8033798	0.647765709	Zm00001d050483
Zm00001d050483_T001	0.5784042	-0.714923059	Zm00001d050483
Zm00001d050483_T003	0.4178262	1.286290248	Zm00001d050483
Zm00001d041069_T001	0.8553094	0.627685707	Zm00001d041069
Zm00001d014344_T036	0.000169193	1.2929539	Zm00001d014344
Zm00001d014344_T038	0.02888804	0.532429266	Zm00001d014344

Zm00001d014344_T063	0.3380134	0.226872934	Zm00001d014344
Zm00001d014344_T009	0.3033922	0.726723523	Zm00001d014344
Zm00001d014344_T024	0.9356767	0.049090801	Zm00001d014344
Zm00001d014344_T039	0.6014554	0.266029904	Zm00001d014344
Zm00001d014344_T029	0.136979	0.786326458	Zm00001d014344
Zm00001d014344_T034	0.1429392	2.125774737	Zm00001d014344
Zm00001d014344_T041	0.3913208	0.73062369	Zm00001d014344
Zm00001d014344_T001	0.7483641	0.970526197	Zm00001d014344
Zm00001d014344_T030	0.1826126	0.425138749	Zm00001d014344
Zm00001d050610_T002	0.9690925	0.509213414	Zm00001d050610
Zm00001d050610_T001	0.9519347	0.084228146	Zm00001d050610
Zm00001d018147_T003	0.113295	-1.050345309	Zm00001d018147
Zm00001d018147_T002	1	0.378103477	Zm00001d018147
Zm00001d044106_T001	0.4334715	1.441772075	Zm00001d044106
Zm00001d010907_T001	0.9732421	0.147535665	Zm00001d010907
Zm00001d010907_T004	0.8863456	0.031291677	Zm00001d010907
Zm00001d010907_T002	0.4805166	-0.520096979	Zm00001d010907
Zm00001d050903_T001	0.7222076	-0.336084573	Zm00001d050903
Zm00001d012861_T001	0.9022629	0.729933477	Zm00001d012861
Zm00001d001947_T001	0.8412913	0.919824285	Zm00001d001947
Zm00001d017691_T001	0.9979568	0.259557256	Zm00001d017691
Zm00001d005051_T001	0.04714113	-1.319372358	Zm00001d005051
Zm00001d008439_T001	0.9623293	0.186563361	Zm00001d008439
Zm00001d049325_T002	0.9840918	0.053699658	Zm00001d049325
Zm00001d001807_T001	0.9315306	-0.04784165	Zm00001d001807
Zm00001d008200_T001	0.7602652	-0.489396271	Zm00001d008200
Zm00001d028862_T001	0.7444349	0.805126566	Zm00001d028862
Zm00001d027471_T001	0.8886159	-0.190343283	Zm00001d027471
Zm00001d010714_T005	0.9782674	0.49277661	Zm00001d010714
Zm00001d010714_T006	0.8802472	-0.063223167	Zm00001d010714
Zm00001d010714_T001	0.3080118	-0.809393925	Zm00001d010714
Zm00001d010714_T010	0.9451369	-0.025799667	Zm00001d010714
Zm00001d010714_T007	0.2619882	0.58982987	Zm00001d010714
Zm00001d039318_T001	0.9070578	0.59239489	Zm00001d039318
Zm00001d026464_T001	0.8452909	0.966382134	Zm00001d026464
Zm00001d035149_T001	0.153989	1.33115499	Zm00001d035149
Zm00001d032264_T001	0.1155712	-1.218311088	Zm00001d032264
Zm00001d032748_T003	0.9706887	0.210739189	Zm00001d032748
Zm00001d032748_T006	0.9187874	0.536420544	Zm00001d032748
Zm00001d032748_T001	0.424032	-0.249306101	Zm00001d032748
Zm00001d032748_T004	0.3412344	-1.041353404	Zm00001d032748
Zm00001d032748_T002	0.9966965	0.113632635	Zm00001d032748
Zm00001d039740_T002	0.9355135	0.637191776	Zm00001d039740
Zm00001d046489_T003	0.9210235	0.701486603	Zm00001d046489
Zm00001d046489_T001	0.9845237	0.274749962	Zm00001d046489
Zm00001d015446_T010	0.7697196	0.305021056	Zm00001d015446
Zm00001d015446_T013	0.3996069	0.634759606	Zm00001d015446
Zm00001d015446_T004	0.3795013	0.662895659	Zm00001d015446
Zm00001d015446_T017	0.9694613	0.152635189	Zm00001d015446
Zm00001d013505_T001	0.9312703	-0.32920107	Zm00001d013505
Zm00001d013505_T002	0.9851204	1.035940183	Zm00001d013505
Zm00001d047962_T001	0.8827359	-0.41092727	Zm00001d047962
Zm00001d030339_T001	0.8412913	0.802841494	Zm00001d030339
Zm00001d038971_T001	0.8643636	0.928507467	Zm00001d038971

Zm00001d019075_T006	0.684563	1.04303073	Zm00001d019075
Zm00001d019075_T005	0.4444451	0.422517978	Zm00001d019075
Zm00001d019075_T009	0.4723494	0.28128803	Zm00001d019075
Zm00001d019075_T010	0.00914723	-1.690632567	Zm00001d019075
Zm00001d042288_T003	0.8420475	0.833974489	Zm00001d042288
Zm00001d042288_T002	0.889992	-0.157871058	Zm00001d042288
Zm00001d042288_T001	0.9240693	0.39177694	Zm00001d042288
Zm00001d046717_T008	0.04308926	-0.41254888	Zm00001d046717
Zm00001d046717_T001	0.7807878	-0.112909241	Zm00001d046717
Zm00001d046717_T007	0.3655498	1.168143365	Zm00001d046717
Zm00001d046717_T005	0.7328852	0.882804432	Zm00001d046717
Zm00001d025545_T001	0.9868533	0.425720642	Zm00001d025545
Zm00001d018567_T001	0.866232	-0.221799852	Zm00001d018567
Zm00001d032507_T001	0.8413408	0.744710174	Zm00001d032507
Zm00001d011512_T001	0.8132343	0.343481871	Zm00001d011512
Zm00001d011512_T002	1	0.334605867	Zm00001d011512
Zm00001d011512_T007	0.8050292	0.564081321	Zm00001d011512
Zm00001d004976_T001	0.9832092	0.196195093	Zm00001d004976
Zm00001d004976_T003	0.9885392	0.259264372	Zm00001d004976
Zm00001d053287_T001	0.8665954	-0.040239572	Zm00001d053287
Zm00001d020968_T001	0.3864378	1.092958826	Zm00001d020968
Zm00001d010684_T011	0.5592613	0.349714013	Zm00001d010684
Zm00001d010684_T015	0.9859755	0.199103008	Zm00001d010684
Zm00001d010684_T012	0.8849873	0.467141888	Zm00001d010684
Zm00001d013778_T002	0.9493645	0.676433659	Zm00001d013778
Zm00001d013778_T003	0.08577949	-0.852357212	Zm00001d013778
Zm00001d050550_T001	0.8253052	-0.11109218	Zm00001d050550
Zm00001d050550_T002	0.9664736	0.550594892	Zm00001d050550
Zm00001d019750_T001	0.8201926	0.786344842	Zm00001d019750
Zm00001d005843_T001	0.4354411	-0.718015102	Zm00001d005843
Zm00001d052713_T001	0.03547668	1.069091692	Zm00001d052713
Zm00001d013331_T001	0.8690525	0.685799558	Zm00001d013331
Zm00001d011747_T001	0.683785	-0.825119331	Zm00001d011747
Zm00001d000311_T003	0.360671	1.304187267	Zm00001d000311
Zm00001d000311_T001	0.7609047	-0.49163998	Zm00001d000311
Zm00001d014587_T001	0.9887709	0.253164875	Zm00001d014587
Zm00001d023419_T001	0.8858223	0.872059013	Zm00001d023419
Zm00001d005293_T001	0.4964747	-0.516638677	Zm00001d005293
Zm00001d001940_T001	0.8301928	-0.584085372	Zm00001d001940
Zm00001d035297_T001	0.9914036	0.215066474	Zm00001d035297
Zm00001d033707_T001	0.993158	0.284811647	Zm00001d033707
Zm00001d021834_T001	0.9266346	-0.060704422	Zm00001d021834
Zm00001d015493_T001	0.9524127	0.038452332	Zm00001d015493
Zm00001d015493_T005	0.3984377	0.809283911	Zm00001d015493
Zm00001d015493_T002	0.3181781	1.025062247	Zm00001d015493
Zm00001d012939_T001	0.3010905	1.2683392	Zm00001d012939
Zm00001d049789_T001	0.9886764	0.114611788	Zm00001d049789
Zm00001d015203_T007	0.9804228	0.57149603	Zm00001d015203
Zm00001d015203_T013	0.7082783	0.39871483	Zm00001d015203
Zm00001d015203_T023	0.9567316	0.576088543	Zm00001d015203
Zm00001d021248_T003	0.9213679	0.529493467	Zm00001d021248
Zm00001d021248_T001	0.2793723	1.666496191	Zm00001d021248
Zm00001d021248_T002	1	0.285658068	Zm00001d021248
Zm00001d025273_T012	0.139783	2.625485759	Zm00001d025273

Zm00001d025273_T004	0.000171503	0.465283983	Zm00001d025273
Zm00001d025273_T013	1.61E-12	-3.886770975	Zm00001d025273
Zm00001d025273_T001	0.08298636	-1.399840036	Zm00001d025273
Zm00001d019789_T003	0.000952931	2.776274015	Zm00001d019789
Zm00001d019789_T004	0.9791729	0.194857026	Zm00001d019789
Zm00001d019789_T006	0.2706009	-0.706815819	Zm00001d019789
Zm00001d019789_T002	0.3405806	-1.102045224	Zm00001d019789
Zm00001d021908_T001	0.9909153	0.259505112	Zm00001d021908
Zm00001d009954_T001	0.9570177	0.099766453	Zm00001d009954
Zm00001d011767_T003	0.9804228	0.546087745	Zm00001d011767
Zm00001d011767_T001	0.6286012	-0.171168586	Zm00001d011767
Zm00001d014729_T001	0.9535734	-0.093038852	Zm00001d014729
Zm00001d040215_T001	0.2802962	-1.115132905	Zm00001d040215
Zm00001d048352_T001	0.5936419	0.939727514	Zm00001d048352
Zm00001d047395_T003	0.9216521	0.017492616	Zm00001d047395
Zm00001d047395_T005	0.009667038	1.008296618	Zm00001d047395
Zm00001d045760_T018	0.9213679	0.19160484	Zm00001d045760
Zm00001d045760_T008	0.9779656	0.337305886	Zm00001d045760
Zm00001d045760_T010	0.8906466	0.194057515	Zm00001d045760
Zm00001d045760_T016	1	0.190470064	Zm00001d045760
Zm00001d051892_T001	0.9998278	0.361271572	Zm00001d051892
Zm00001d022245_T001	1	0.309333353	Zm00001d022245
Zm00001d020434_T001	0.5822105	-0.792236459	Zm00001d020434
Zm00001d050092_T001	0.2401157	-0.863338592	Zm00001d050092
Zm00001d037744_T001	0.8964117	0.778782697	Zm00001d037744
Zm00001d014962_T004	0.9889197	0.333489347	Zm00001d014962
Zm00001d014962_T001	0.5639395	0.774572502	Zm00001d014962
Zm00001d014962_T003	0.7514991	0.639637073	Zm00001d014962
Zm00001d025005_T001	0.4086899	1.355069434	Zm00001d025005
Zm00001d006920_T001	0.8007432	-0.24427528	Zm00001d006920
Zm00001d031244_T001	0.7054048	-0.768550005	Zm00001d031244
Zm00001d045558_T004	0.7531787	0.992044675	Zm00001d045558
Zm00001d045558_T002	0.9469426	0.736204661	Zm00001d045558
Zm00001d045558_T003	0.8643636	0.824276862	Zm00001d045558
Zm00001d053872_T041	0.8896281	0.202738645	Zm00001d053872
Zm00001d053872_T046	0.7009248	-0.26725522	Zm00001d053872
Zm00001d053872_T006	0.4110909	0.754468266	Zm00001d053872
Zm00001d053872_T047	0.7379612	-0.282350581	Zm00001d053872
Zm00001d020224_T002	0.9979393	0.212988692	Zm00001d020224
Zm00001d020224_T001	0.8883561	-0.2335906	Zm00001d020224
Zm00001d006103_T001	0.9821248	0.196121504	Zm00001d006103
Zm00001d024515_T001	0.8465879	-0.173335133	Zm00001d024515
Zm00001d002356_T001	0.7808406	0.91589909	Zm00001d002356
Zm00001d007930_T004	1	0.20232274	Zm00001d007930
Zm00001d007930_T001	0.9756546	0.081534939	Zm00001d007930
Zm00001d037700_T002	0.8307555	1.049349106	Zm00001d037700
Zm00001d037700_T012	0.00238724	-2.778616785	Zm00001d037700
Zm00001d049234_T001	0.9706293	0.632239756	Zm00001d049234
Zm00001d047680_T001	0.6705583	0.842371719	Zm00001d047680
Zm00001d018756_T001	0.9528476	0.072771687	Zm00001d018756
Zm00001d048950_T001	0.04667616	-1.75837955	Zm00001d048950
Zm00001d006029_T001	0.4949487	-0.810257228	Zm00001d006029
Zm00001d013548_T002	0.9825858	0.311685125	Zm00001d013548
Zm00001d013548_T001	0.8637863	0.502093163	Zm00001d013548

Zm00001d026203_T001	0.112716	1.844959593	Zm00001d026203
Zm00001d019967_T001	0.9766874	-0.098430414	Zm00001d019967
Zm00001d001779_T001	0.8991659	0.589548989	Zm00001d001779
Zm00001d021180_T005	0.286303	1.459617491	Zm00001d021180
Zm00001d021180_T003	0.4959633	-0.438444101	Zm00001d021180
Zm00001d021180_T001	0.9380783	0.758190361	Zm00001d021180
Zm00001d021180_T004	0.005727571	1.358152418	Zm00001d021180
Zm00001d031853_T001	0.954492	0.109649266	Zm00001d031853
Zm00001d028825_T002	0.6184626	0.678614307	Zm00001d028825
Zm00001d028825_T004	0.4812187	0.476004357	Zm00001d028825
Zm00001d028825_T007	0.9050121	0.748334511	Zm00001d028825
Zm00001d028825_T003	0.03331115	2.039886273	Zm00001d028825
Zm00001d045185_T001	0.00053586	2.873710731	Zm00001d045185
Zm00001d020810_T003	0.9568315	0.166649347	Zm00001d020810
Zm00001d020810_T008	0.5713838	0.345808244	Zm00001d020810
Zm00001d034066_T002	0.9793888	0.195089668	Zm00001d034066
Zm00001d034066_T001	0.5058724	-0.695518868	Zm00001d034066
Zm00001d002602_T002	0.9903568	0.165996667	Zm00001d002602
Zm00001d002602_T003	0.5822629	-0.359663461	Zm00001d002602
Zm00001d045090_T001	0.8412913	0.426525393	Zm00001d045090
Zm00001d045518_T001	0.9889811	0.149258826	Zm00001d045518
Zm00001d050433_T001	0.9902022	0.163227016	Zm00001d050433
Zm00001d053890_T008	0.8757395	-0.068072531	Zm00001d053890
Zm00001d053890_T005	0.586618	-0.254628486	Zm00001d053890
Zm00001d053890_T003	1	0.159521207	Zm00001d053890
Zm00001d053890_T001	0.007128408	-1.208848437	Zm00001d053890
Zm00001d018916_T001	0.9839367	0.356390215	Zm00001d018916
Zm00001d044393_T003	0.2509097	0.571113507	Zm00001d044393
Zm00001d044393_T006	0.8585804	-0.255585491	Zm00001d044393
Zm00001d044393_T002	0.9272378	0.719356209	Zm00001d044393
Zm00001d044393_T004	0.3482663	0.884128443	Zm00001d044393
Zm00001d038358_T010	0.6361629	-0.367116037	Zm00001d038358
Zm00001d052904_T002	0.9747606	0.12364485	Zm00001d052904
Zm00001d052904_T001	0.9487214	0.602003483	Zm00001d052904
Zm00001d033633_T002	0.9979568	0.277989336	Zm00001d033633
Zm00001d033633_T001	0.852305	-0.18815012	Zm00001d033633
Zm00001d044364_T002	0.2767835	1.07806747	Zm00001d044364
Zm00001d044364_T001	0.9272378	0.057238385	Zm00001d044364
Zm00001d044364_T005	0.2908902	1.402954582	Zm00001d044364
Zm00001d004999_T001	0.9988864	0.187436964	Zm00001d004999
Zm00001d038166_T003	0.9832612	0.082878701	Zm00001d038166
Zm00001d038166_T001	0.9740325	0.516057596	Zm00001d038166
Zm00001d002349_T010	0.3217566	0.563765524	Zm00001d002349
Zm00001d011802_T001	0.4282459	0.556080221	Zm00001d011802
Zm00001d044649_T021	0.333158	0.274382335	Zm00001d044649
Zm00001d044649_T006	0.9259902	0.148798211	Zm00001d044649
Zm00001d044649_T031	0.02919597	-0.934044319	Zm00001d044649
Zm00001d044649_T012	0.3130839	0.567481899	Zm00001d044649
Zm00001d044649_T028	0.001632575	1.667371601	Zm00001d044649
Zm00001d044649_T030	0.7594555	-0.167659416	Zm00001d044649
Zm00001d017845_T001	0.9747606	0.38651867	Zm00001d017845
Zm00001d022550_T001	0.5715685	-0.560171149	Zm00001d022550
Zm00001d037620_T002	0.7662609	-0.586903516	Zm00001d037620
Zm00001d015004_T003	0.986738	0.090650104	Zm00001d015004

Zm00001d015004_T002	0.9509189	0.034086623	Zm00001d015004
Zm00001d026471_T001	0.8660897	0.651580167	Zm00001d026471
Zm00001d039273_T001	0.7548788	-0.647811495	Zm00001d039273
Zm00001d032777_T001	0.7979593	-0.835758154	Zm00001d032777
Zm00001d049860_T001	0.930095	-0.016131169	Zm00001d049860
Zm00001d045740_T001	0.969237	0.196428019	Zm00001d045740
Zm00001d041192_T007	0.9419336	0.92458228	Zm00001d041192
Zm00001d041192_T003	0.9046076	0.842615207	Zm00001d041192
Zm00001d041192_T005	0.9203655	-0.063261201	Zm00001d041192
Zm00001d041192_T001	0.811032	-0.09188554	Zm00001d041192
Zm00001d041192_T004	0.2202395	-1.495413135	Zm00001d041192
Zm00001d041192_T006	0.01225238	-0.925916013	Zm00001d041192
Zm00001d017962_T003	0.08002632	0.50721672	Zm00001d017962
Zm00001d017962_T002	0.8050292	0.317521401	Zm00001d017962
Zm00001d020312_T035	3.99E-05	0.384386847	Zm00001d020312
Zm00001d010470_T038	0.4900864	0.361782555	Zm00001d010470
Zm00001d010470_T007	0.9812314	0.254963043	Zm00001d010470
Zm00001d010470_T036	1	0.091630298	Zm00001d010470
Zm00001d010470_T010	0.1761471	0.355634225	Zm00001d010470
Zm00001d010470_T002	0.05147956	1.321475969	Zm00001d010470
Zm00001d009797_T001	0.1723862	-1.763217525	Zm00001d009797
Zm00001d011595_T017	0.8505918	0.613763802	Zm00001d011595
Zm00001d011595_T006	0.4586436	0.838513954	Zm00001d011595
Zm00001d011595_T022	0.640996	0.708249662	Zm00001d011595
Zm00001d011595_T012	0.9416089	0.279062213	Zm00001d011595
Zm00001d011504_T003	0.9311783	-0.047732061	Zm00001d011504
Zm00001d011504_T001	0.7984409	0.303900033	Zm00001d011504
Zm00001d009850_T005	0.9129863	0.026716506	Zm00001d009850
Zm00001d009850_T003	0.8239342	0.422572438	Zm00001d009850
Zm00001d009850_T010	0.8189989	-0.155162518	Zm00001d009850
Zm00001d009850_T008	0.9404125	0.115419855	Zm00001d009850
Zm00001d003710_T002	0.3655498	-0.235101428	Zm00001d003710
Zm00001d003710_T005	0.9404125	0.29305842	Zm00001d003710
Zm00001d003710_T007	0.9082698	0.527219215	Zm00001d003710
Zm00001d003710_T010	0.2632848	0.418494322	Zm00001d003710
Zm00001d003710_T006	0.07297249	-1.205560046	Zm00001d003710
Zm00001d002938_T002	0.9879601	0.427019274	Zm00001d002938
Zm00001d002938_T001	0.904288	0.055095692	Zm00001d002938
Zm00001d037115_T001	0.9370633	0.097275776	Zm00001d037115
Zm00001d037115_T003	0.9524127	0.10401767	Zm00001d037115
Zm00001d037115_T006	0.03708148	0.443691204	Zm00001d037115
Zm00001d037115_T009	0.9918302	0.455150167	Zm00001d037115
Zm00001d008250_T001	0.7238616	1.09105612	Zm00001d008250
Zm00001d049725_T001	0.9760472	0.202546218	Zm00001d049725
Zm00001d049725_T006	0.8439431	-0.123314256	Zm00001d049725
Zm00001d046837_T002	0.9193633	-0.076203051	Zm00001d046837
Zm00001d036439_T001	0.9210327	0.649141645	Zm00001d036439
Zm00001d023253_T001	0.3319365	1.234231078	Zm00001d023253
Zm00001d042018_T001	0.9792387	0.23668914	Zm00001d042018
Zm00001d042018_T002	0.9372575	0.711540403	Zm00001d042018
Zm00001d042018_T004	0.02546853	-1.940201969	Zm00001d042018
Zm00001d042018_T005	0.2575503	-1.018493151	Zm00001d042018
Zm00001d025104_T001	0.9210327	-0.571156221	Zm00001d025104
Zm00001d025104_T002	0.9624437	0.286696197	Zm00001d025104

Zm00001d043480_T001	0.745469	1.162249787	Zm00001d043480
Zm00001d039400_T012	0.2717432	0.271027297	Zm00001d039400
Zm00001d021384_T004	0.9068989	-0.017724133	Zm00001d021384
Zm00001d024975_T001	0.6342868	0.992159584	Zm00001d024975
Zm00001d014597_T001	0.9422098	0.025471568	Zm00001d014597
Zm00001d039971_T004	0.9851017	0.510625547	Zm00001d039971
Zm00001d039971_T001	0.9745948	0.147299481	Zm00001d039971
Zm00001d039971_T007	0.7055225	0.410361653	Zm00001d039971
Zm00001d039971_T005	0.6282915	0.441956459	Zm00001d039971
Zm00001d002301_T001	0.8412913	0.491111652	Zm00001d002301
Zm00001d003649_T002	0.02892183	1.658870422	Zm00001d003649
Zm00001d003649_T005	0.5547266	1.152861947	Zm00001d003649
Zm00001d042620_T001	0.9363115	-0.190922706	Zm00001d042620
Zm00001d040023_T001	0.9503115	0.035074487	Zm00001d040023
Zm00001d050823_T003	0.3777631	-1.073347288	Zm00001d050823
Zm00001d050823_T004	1	-0.027663497	Zm00001d050823
Zm00001d050823_T002	0.9596377	-0.127504688	Zm00001d050823
Zm00001d029083_T001	0.8031436	-0.166035796	Zm00001d029083
Zm00001d029083_T002	0.355126	-1.072900591	Zm00001d029083
Zm00001d020585_T001	0.8411786	-0.07743346	Zm00001d020585
Zm00001d054077_T001	0.9046076	-0.195532405	Zm00001d054077
Zm00001d043587_T001	0.9954444	0.159713725	Zm00001d043587
Zm00001d005205_T001	0.9839367	0.225778081	Zm00001d005205
Zm00001d002313_T002	0.1377291	2.006622475	Zm00001d002313
Zm00001d002313_T006	0.1113752	0.702433305	Zm00001d002313
Zm00001d002313_T005	0.05492361	1.088835504	Zm00001d002313
Zm00001d002313_T001	0.2304806	1.196470723	Zm00001d002313
Zm00001d002313_T009	0.9835145	0.408369229	Zm00001d002313
Zm00001d002313_T007	0.003920049	-2.365946404	Zm00001d002313
Zm00001d029490_T027	0.6324192	-1.075372932	Zm00001d029490
Zm00001d029490_T008	0.9325478	0.063227218	Zm00001d029490
Zm00001d029490_T001	0.9931431	0.531777798	Zm00001d029490
Zm00001d029490_T016	0.000284972	1.41472309	Zm00001d029490
Zm00001d029490_T022	0.8001345	0.463277055	Zm00001d029490
Zm00001d029490_T025	0.9791729	0.148855301	Zm00001d029490
Zm00001d029490_T011	0.3270101	0.715888479	Zm00001d029490
Zm00001d037492_T004	0.9377241	0.199815079	Zm00001d037492
Zm00001d037492_T001	0.9833513	0.462248187	Zm00001d037492
Zm00001d042268_T001	0.815049	-0.151091258	Zm00001d042268
Zm00001d031847_T038	0.851347	0.484631744	Zm00001d031847
Zm00001d031847_T047	0.06026596	0.813912699	Zm00001d031847
Zm00001d031847_T049	0.1046742	-1.050128488	Zm00001d031847
Zm00001d031847_T051	0.7612164	-0.306144133	Zm00001d031847
Zm00001d031847_T005	0.9644829	0.34125212	Zm00001d031847
Zm00001d031847_T021	0.001228936	-2.615663074	Zm00001d031847
Zm00001d031847_T039	0.1595905	1.005478875	Zm00001d031847
Zm00001d031847_T011	0.008554004	3.013108228	Zm00001d031847
Zm00001d031847_T043	0.1087456	2.289071634	Zm00001d031847
Zm00001d019230_T004	0.9344122	0.025323502	Zm00001d019230
Zm00001d019230_T002	0.8660897	-0.064974126	Zm00001d019230
Zm00001d033094_T003	0.708953	0.324979346	Zm00001d033094
Zm00001d033094_T001	0.9402253	0.713337386	Zm00001d033094
Zm00001d001799_T003	0.9486412	-0.016865493	Zm00001d001799
Zm00001d000202_T002	0.8908955	-0.231113821	Zm00001d000202

Zm00001d042534_T005	0.9868821	0.040537588	Zm00001d042534
Zm00001d042534_T001	0.8995312	0.254528847	Zm00001d042534
Zm00001d042534_T006	0.8412913	-0.190762167	Zm00001d042534
Zm00001d008221_T001	0.7234221	-0.25797665	Zm00001d008221
Zm00001d020840_T001	0.6801397	-0.797175362	Zm00001d020840
Zm00001d020615_T001	0.9733967	0.616506879	Zm00001d020615
Zm00001d036736_T001	0.6201746	0.936868384	Zm00001d036736
Zm00001d018827_T001	0.9706293	-0.189173714	Zm00001d018827
Zm00001d012003_T001	0.9419336	0.038092717	Zm00001d012003
Zm00001d050965_T001	1	0.148293678	Zm00001d050965
Zm00001d013108_T001	0.8307555	1.016005808	Zm00001d013108
Zm00001d038765_T001	0.8111431	-0.270992566	Zm00001d038765
Zm00001d046625_T001	0.9768802	0.548736045	Zm00001d046625
Zm00001d049060_T001	0.000241971	5.022721524	Zm00001d049060
Zm00001d039064_T001	0.9272378	-0.280802845	Zm00001d039064
Zm00001d013941_T001	0.5714328	0.689225913	Zm00001d013941
Zm00001d013012_T004	0.4302857	1.39506632	Zm00001d013012
Zm00001d013012_T007	0.002602363	-1.229848941	Zm00001d013012
Zm00001d013012_T001	0.9567316	0.243487791	Zm00001d013012
Zm00001d013012_T008	0.5618486	-0.528604684	Zm00001d013012
Zm00001d048243_T001	0.9925955	0.263460807	Zm00001d048243
Zm00001d039321_T009	0.8621066	0.607469676	Zm00001d039321
Zm00001d039321_T015	0.9681383	0.362318864	Zm00001d039321
Zm00001d039321_T003	0.928383	0.066833	Zm00001d039321
Zm00001d039321_T001	0.9730261	0.128131486	Zm00001d039321
Zm00001d039321_T004	0.9257893	0.274944663	Zm00001d039321
Zm00001d039321_T002	0.5480167	0.47148592	Zm00001d039321
Zm00001d039321_T008	0.2002595	0.747677304	Zm00001d039321
Zm00001d029862_T001	0.941922	0.005383849	Zm00001d029862
Zm00001d037155_T001	0.9219195	-0.106232906	Zm00001d037155
Zm00001d026102_T001	0.4011355	1.33236424	Zm00001d026102
Zm00001d028398_T004	0.9945118	0.455099838	Zm00001d028398
Zm00001d028398_T007	0.07701001	0.644438168	Zm00001d028398
Zm00001d048866_T001	0.163796	-0.774885329	Zm00001d048866
Zm00001d053926_T001	0.8751525	-0.446510181	Zm00001d053926
Zm00001d038896_T001	0.8413408	-0.216291903	Zm00001d038896
Zm00001d046174_T001	0.9979568	0.277929131	Zm00001d046174
Zm00001d035028_T001	0.481034	-0.875030959	Zm00001d035028
Zm00001d002857_T002	0.9713036	0.10986832	Zm00001d002857
Zm00001d047931_T001	0.08197566	2.262906436	Zm00001d047931
Zm00001d032292_T004	0.9805029	0.242878928	Zm00001d032292
Zm00001d032292_T001	0.9160053	0.751983796	Zm00001d032292
Zm00001d031486_T002	0.9907928	0.214469669	Zm00001d031486
Zm00001d031486_T001	1	0.372948442	Zm00001d031486
Zm00001d007301_T006	0.9833568	0.01845581	Zm00001d007301
Zm00001d007301_T005	0.8863456	-0.057617472	Zm00001d007301
Zm00001d007301_T003	0.9666757	0.171531312	Zm00001d007301
Zm00001d051456_T001	0.9907928	0.00332901	Zm00001d051456
Zm00001d049662_T001	0.9942835	0.44528161	Zm00001d049662
Zm00001d007776_T002	0.8767824	0.490734401	Zm00001d007776
Zm00001d007776_T006	0.8958882	0.272894843	Zm00001d007776
Zm00001d007776_T003	0.1058437	1.826005733	Zm00001d007776
Zm00001d036641_T001	0.7889631	-0.238967815	Zm00001d036641
Zm00001d049230_T002	0.9804228	0.045087567	Zm00001d049230

Zm00001d049230_T001	1	0.311201908	Zm00001d049230
Zm00001d042331_T001	0.9802412	0.534380904	Zm00001d042331
Zm00001d022240_T013	0.00952161	0.838367176	Zm00001d022240
Zm00001d022240_T001	0.8368233	0.007206875	Zm00001d022240
Zm00001d022240_T010	0.1429392	0.353286102	Zm00001d022240
Zm00001d022240_T008	0.9776426	0.484065882	Zm00001d022240
Zm00001d022240_T009	0.4825966	-0.1987964	Zm00001d022240
Zm00001d044498_T003	0.6042072	1.05614603	Zm00001d044498
Zm00001d044498_T012	0.8746701	0.23590575	Zm00001d044498
Zm00001d044498_T011	0.9722469	0.204404029	Zm00001d044498
Zm00001d028680_T002	0.05103414	1.218421046	Zm00001d028680
Zm00001d028680_T003	0.9906533	0.306352344	Zm00001d028680
Zm00001d028680_T001	0.2398048	-0.433331076	Zm00001d028680
Zm00001d044092_T001	0.6330708	-0.541084448	Zm00001d044092
Zm00001d042307_T001	0.02779977	-2.068953451	Zm00001d042307
Zm00001d040305_T002	0.005677468	0.52176784	Zm00001d040305
Zm00001d040305_T015	2.38E-07	-0.774894506	Zm00001d040305
Zm00001d040305_T022	0.5717645	-0.237031981	Zm00001d040305
Zm00001d025679_T001	0.9909153	0.304091255	Zm00001d025679
Zm00001d038905_T001	0.8537544	1.003023635	Zm00001d038905
Zm00001d041510_T002	0.8535962	0.945814194	Zm00001d041510
Zm00001d050368_T001	0.8192027	-0.370574894	Zm00001d050368
Zm00001d019079_T001	0.894937	0.811875226	Zm00001d019079
Zm00001d048099_T002	0.01582338	-1.049140035	Zm00001d048099
Zm00001d048099_T001	0.02631091	-1.903010202	Zm00001d048099
Zm00001d048099_T005	0.3057459	-0.905475462	Zm00001d048099
Zm00001d053917_T006	0.5426411	0.311187476	Zm00001d053917
Zm00001d053917_T001	0.6838428	-0.30597418	Zm00001d053917
Zm00001d053917_T035	0.9973914	0.319029281	Zm00001d053917
Zm00001d053917_T007	0.7746801	0.731634706	Zm00001d053917
Zm00001d053917_T027	0.02044646	1.076577437	Zm00001d053917
Zm00001d053917_T024	0.6814077	0.379199796	Zm00001d053917
Zm00001d053917_T029	0.4096848	-0.229719064	Zm00001d053917
Zm00001d053917_T002	0.909729	0.1227462	Zm00001d053917
Zm00001d039441_T001	0.9232281	-0.157078076	Zm00001d039441
Zm00001d028812_T013	0.9689939	0.123232548	Zm00001d028812
Zm00001d028812_T018	0.2303643	0.808786207	Zm00001d028812
Zm00001d028812_T019	0.741153	0.276874835	Zm00001d028812
Zm00001d028812_T001	0.9913523	0.211304342	Zm00001d028812
Zm00001d028812_T006	0.8010758	-0.251365337	Zm00001d028812
Zm00001d019525_T001	0.6891365	0.772883998	Zm00001d019525
Zm00001d052928_T001	0.979536	0.221128846	Zm00001d052928
Zm00001d010180_T002	0.9979568	0.178258922	Zm00001d010180
Zm00001d028372_T007	0.2496307	-0.185672357	Zm00001d028372
Zm00001d028372_T014	0.9563232	0.024048836	Zm00001d028372
Zm00001d028372_T011	0.02570104	1.60841305	Zm00001d028372
Zm00001d028372_T008	0.7492661	0.303759767	Zm00001d028372
Zm00001d028372_T013	1	0.359753121	Zm00001d028372
Zm00001d003469_T001	0.9781672	0.154344101	Zm00001d003469
Zm00001d040745_T001	0.9817074	0.129839049	Zm00001d040745
Zm00001d021473_T002	0.8273397	-0.079367609	Zm00001d021473
Zm00001d021473_T004	0.741153	0.244884507	Zm00001d021473
Zm00001d021473_T001	0.9690925	0.573121102	Zm00001d021473
Zm00001d052654_T001	0.8412913	0.541598789	Zm00001d052654

Zm00001d009352_T002	0.9979568	0.470132837	Zm00001d009352
Zm00001d009352_T003	0.8336303	0.564240265	Zm00001d009352
Zm00001d007686_T001	0.8039029	-0.25405442	Zm00001d007686
Zm00001d034829_T001	0.4016945	1.473872608	Zm00001d034829
Zm00001d043618_T001	0.7333	-0.728696701	Zm00001d043618
Zm00001d021395_T004	0.4701937	0.570742371	Zm00001d021395
Zm00001d021395_T001	1	0.285167314	Zm00001d021395
Zm00001d021395_T005	0.9657993	0.18083371	Zm00001d021395
Zm00001d007719_T008	0.08548693	0.776143621	Zm00001d007719
Zm00001d007719_T007	0.2146351	0.352778068	Zm00001d007719
Zm00001d007719_T002	0.5269468	0.268139269	Zm00001d007719
Zm00001d007719_T037	1.03E-06	0.551063737	Zm00001d007719
Zm00001d007719_T045	0.2646806	-0.740259066	Zm00001d007719
Zm00001d007719_T003	0.9627438	0.554965116	Zm00001d007719
Zm00001d007719_T012	0.9664736	0.039995695	Zm00001d007719
Zm00001d009751_T001	0.1414674	1.350091092	Zm00001d009751
Zm00001d006371_T001	0.9791729	0.241139023	Zm00001d006371
Zm00001d034509_T002	0.9311783	-0.004945266	Zm00001d034509
Zm00001d034509_T001	0.9701986	0.1106457	Zm00001d034509
Zm00001d034509_T003	0.3202489	-1.336717222	Zm00001d034509
Zm00001d042795_T005	0.9916733	0.163949064	Zm00001d042795
Zm00001d042795_T007	0.8376343	0.85460448	Zm00001d042795
Zm00001d042795_T001	0.9216521	0.020468939	Zm00001d042795
Zm00001d042795_T004	0.9389075	0.606441702	Zm00001d042795
Zm00001d049140_T005	0.7897201	0.393719998	Zm00001d049140
Zm00001d049140_T028	0.1429392	-0.527994767	Zm00001d049140
Zm00001d049140_T018	0.7933933	0.656333686	Zm00001d049140
Zm00001d049140_T010	0.167432	0.358012007	Zm00001d049140
Zm00001d019534_T001	0.9508258	0.313739582	Zm00001d019534
Zm00001d019534_T002	0.6559024	-0.346411802	Zm00001d019534
Zm00001d019534_T003	0.9893214	0.281404831	Zm00001d019534
Zm00001d038451_T002	0.000274955	2.51011571	Zm00001d038451
Zm00001d038451_T003	0.005246483	2.478572212	Zm00001d038451
Zm00001d005628_T001	0.6324192	-0.713278986	Zm00001d005628
Zm00001d003423_T002	0.9657993	-0.003259221	Zm00001d003423
Zm00001d003423_T001	0.481833	-0.730765308	Zm00001d003423
Zm00001d018938_T001	0.5160512	-0.713022321	Zm00001d018938
Zm00001d035300_T001	0.8896831	0.614956218	Zm00001d035300
Zm00001d001917_T018	0.003831125	-1.588001319	Zm00001d001917
Zm00001d001917_T016	0.2642794	-1.311233846	Zm00001d001917
Zm00001d001917_T021	0.02263356	1.488363037	Zm00001d001917
Zm00001d001917_T002	0.3003804	-0.243262614	Zm00001d001917
Zm00001d001917_T007	0.9956688	0.62655322	Zm00001d001917
Zm00001d001917_T012	0.9845237	0.174394821	Zm00001d001917
Zm00001d010193_T001	0.9326774	0.36311777	Zm00001d010193
Zm00001d008586_T001	0.9037807	0.310729661	Zm00001d008586
Zm00001d020592_T001	0.9322451	-0.433914864	Zm00001d020592
Zm00001d018936_T001	0.9706293	0.139642566	Zm00001d018936
Zm00001d010976_T001	0.9203655	0.796374275	Zm00001d010976
Zm00001d024463_T008	0.001980112	1.215355982	Zm00001d024463
Zm00001d024463_T004	1.84E-10	-1.13335067	Zm00001d024463
Zm00001d024463_T011	0.6470421	-0.647846417	Zm00001d024463
Zm00001d024463_T001	0.9469426	0.048832606	Zm00001d024463
Zm00001d047256_T008	0.6390045	-0.196896701	Zm00001d047256

Zm00001d047256_T005	0.6214202	-0.334375587	Zm00001d047256
Zm00001d047256_T006	0.6556502	0.404039782	Zm00001d047256
Zm00001d047256_T002	0.4163611	1.402397135	Zm00001d047256
Zm00001d047256_T007	0.0797978	-0.868032955	Zm00001d047256
Zm00001d047256_T004	0.9237473	0.252938966	Zm00001d047256
Zm00001d010594_T001	0.9458733	0.312576056	Zm00001d010594
Zm00001d046654_T001	0.5220513	0.870976881	Zm00001d046654
Zm00001d046654_T004	0.7575362	0.973436162	Zm00001d046654
Zm00001d046654_T002	0.7109402	-0.381803133	Zm00001d046654
Zm00001d027323_T001	0.9396128	-0.265019684	Zm00001d027323
Zm00001d044304_T004	0.8865624	0.78952272	Zm00001d044304
Zm00001d005681_T001	0.9514141	-0.29089579	Zm00001d005681
Zm00001d028905_T001	0.8077635	0.991707028	Zm00001d028905
Zm00001d034318_T001	0.9279367	-0.025356529	Zm00001d034318
Zm00001d042809_T002	0.0318764	-1.913952606	Zm00001d042809
Zm00001d042809_T003	0.8865278	-0.051756101	Zm00001d042809
Zm00001d042809_T001	0.5615308	-0.27176917	Zm00001d042809
Zm00001d042809_T008	0.6284759	-0.365209465	Zm00001d042809
Zm00001d042809_T005	0.989787	0.123787431	Zm00001d042809
Zm00001d048796_T002	0.9937853	0.498656436	Zm00001d048796
Zm00001d048796_T005	0.9410648	0.019654281	Zm00001d048796
Zm00001d048796_T004	0.5628469	1.048842011	Zm00001d048796
Zm00001d048615_T001	0.7076199	-0.680163976	Zm00001d048615
Zm00001d022057_T001	0.9979568	0.569923065	Zm00001d022057
Zm00001d035086_T001	0.9524127	0.111109559	Zm00001d035086
Zm00001d035086_T002	0.06609607	-1.303225398	Zm00001d035086
Zm00001d010763_T003	0.7824658	-0.380096306	Zm00001d010763
Zm00001d010763_T002	0.9628812	0.135503228	Zm00001d010763
Zm00001d010763_T004	0.749631	-0.36188192	Zm00001d010763
Zm00001d010763_T001	0.9843639	0.129148802	Zm00001d010763
Zm00001d043766_T008	1.63E-11	1.91373189	Zm00001d043766
Zm00001d043766_T013	0.6012987	0.732613297	Zm00001d043766
Zm00001d043766_T001	0.9940329	0.109404294	Zm00001d043766
Zm00001d043766_T002	0.6286012	0.270898013	Zm00001d043766
Zm00001d043766_T010	0.323944	0.4160031	Zm00001d043766
Zm00001d043766_T006	0.3951093	0.699766832	Zm00001d043766
Zm00001d042508_T001	0.9257238	0.01918817	Zm00001d042508
Zm00001d048604_T001	0.9687311	0.567967164	Zm00001d048604
Zm00001d036688_T001	0.9482354	0.036920819	Zm00001d036688
Zm00001d014329_T001	0.6004941	0.898472641	Zm00001d014329
Zm00001d048133_T001	0.9745948	0.596604785	Zm00001d048133
Zm00001d004413_T002	0.9625435	0.613662795	Zm00001d004413
Zm00001d052264_T001	0.9894615	0.189353555	Zm00001d052264
Zm00001d019122_T001	0.7439239	0.775224121	Zm00001d019122
Zm00001d020697_T001	0.9732421	0.184040719	Zm00001d020697
Zm00001d025125_T003	0.9666757	0.515919087	Zm00001d025125
Zm00001d025125_T004	0.8332116	0.556225354	Zm00001d025125
Zm00001d025125_T001	0.8152254	0.55823277	Zm00001d025125
Zm00001d032072_T001	0.2044538	-0.570188661	Zm00001d032072
Zm00001d047407_T001	0.9840918	0.306408	Zm00001d047407
Zm00001d018859_T041	0.1968397	0.617629936	Zm00001d018859
Zm00001d018859_T004	0.00396837	-1.629830578	Zm00001d018859
Zm00001d018859_T001	0.1179183	1.869152469	Zm00001d018859
Zm00001d018859_T023	0.8175516	0.252153955	Zm00001d018859

Zm00001d018859_T018	0.9747606	0.731255769	Zm00001d018859
Zm00001d031377_T001	1	0.254814824	Zm00001d031377
Zm00001d049563_T003	1	0.400367451	Zm00001d049563
Zm00001d049563_T005	0.9160053	0.377538551	Zm00001d049563
Zm00001d035963_T001	0.8455868	-0.626688467	Zm00001d035963
Zm00001d044936_T001	0.9881957	0.274558128	Zm00001d044936
Zm00001d043669_T009	0.9681793	0.027430181	Zm00001d043669
Zm00001d043669_T008	0.9777274	0.318321447	Zm00001d043669
Zm00001d043669_T006	0.4292753	-1.297011655	Zm00001d043669
Zm00001d043669_T004	0.9877559	0.490269491	Zm00001d043669
Zm00001d023495_T001	0.9954444	0.122846492	Zm00001d023495
Zm00001d015091_T004	0.9940601	0.232546721	Zm00001d015091
Zm00001d015091_T001	0.8891117	-0.086874387	Zm00001d015091
Zm00001d015091_T003	0.3143958	1.704921669	Zm00001d015091
Zm00001d042359_T001	0.9326774	0.47492183	Zm00001d042359
Zm00001d008752_T001	0.5468088	1.208659694	Zm00001d008752
Zm00001d008752_T005	1	0.408831276	Zm00001d008752
Zm00001d008752_T004	0.3912133	-1.022810872	Zm00001d008752
Zm00001d019253_T006	0.6523514	0.818210216	Zm00001d019253
Zm00001d019253_T001	0.9732421	0.141549033	Zm00001d019253
Zm00001d019253_T005	0.9219195	-0.286708248	Zm00001d019253
Zm00001d019253_T003	0.872254	-0.637328195	Zm00001d019253
Zm00001d006628_T004	0.002521412	-1.947371756	Zm00001d006628
Zm00001d006628_T002	0.493096	-0.119399227	Zm00001d006628
Zm00001d006628_T006	0.2064662	1.03369657	Zm00001d006628
Zm00001d006628_T001	0.9563257	0.731356087	Zm00001d006628
Zm00001d032798_T001	0.9252088	-0.04049876	Zm00001d032798
Zm00001d032869_T001	0.9184323	-0.007639913	Zm00001d032869
Zm00001d053986_T001	0.9177108	-0.007346157	Zm00001d053986
Zm00001d028572_T006	0.002782443	-1.410693955	Zm00001d028572
Zm00001d028572_T009	0.009336484	-0.993633891	Zm00001d028572
Zm00001d028572_T003	0.9753288	0.211201428	Zm00001d028572
Zm00001d028572_T002	0.6470701	-0.333650963	Zm00001d028572
Zm00001d028572_T004	0.852305	-0.094097204	Zm00001d028572
Zm00001d028572_T001	0.4671646	-0.755287549	Zm00001d028572
Zm00001d028572_T008	0.9812314	0.35841015	Zm00001d028572
Zm00001d033301_T001	0.2796168	-1.023348597	Zm00001d033301
Zm00001d007567_T001	0.9429712	-0.173492211	Zm00001d007567
Zm00001d017613_T001	0.8503112	-0.125544068	Zm00001d017613
Zm00001d016371_T001	0.9468268	-0.260952235	Zm00001d016371
Zm00001d033533_T004	0.01095096	1.573775901	Zm00001d033533
Zm00001d033533_T005	0.5711435	0.450353301	Zm00001d033533
Zm00001d014719_T021	0.941922	-0.167425148	Zm00001d014719
Zm00001d014719_T017	0.8369406	-0.164037065	Zm00001d014719
Zm00001d014719_T002	0.9673473	0.381667172	Zm00001d014719
Zm00001d014719_T018	0.6182353	0.296619428	Zm00001d014719
Zm00001d014719_T022	0.3486589	1.189593605	Zm00001d014719
Zm00001d014719_T013	1	0.209839903	Zm00001d014719
Zm00001d024772_T001	0.1155336	1.705783862	Zm00001d024772
Zm00001d011185_T001	0.9817074	0.124617431	Zm00001d011185
Zm00001d039299_T001	0.7134188	-0.753290508	Zm00001d039299
Zm00001d028548_T002	0.9219195	-0.321989425	Zm00001d028548
Zm00001d041774_T001	0.02081089	-2.289284949	Zm00001d041774
Zm00001d040019_T001	0.2648796	-0.596499192	Zm00001d040019

Zm00001d050557_T001	0.2061804	-1.38042661	Zm00001d050557
Zm00001d021720_T001	0.9708651	-0.749554998	Zm00001d021720
Zm00001d046391_T001	0.8819727	-0.08571655	Zm00001d046391
Zm00001d001924_T001	0.7664596	0.455517137	Zm00001d001924
Zm00001d043384_T001	0.85155	0.605668485	Zm00001d043384
Zm00001d043257_T004	0.7710268	0.419009624	Zm00001d043257
Zm00001d016803_T001	0.7983502	-0.351171741	Zm00001d016803
Zm00001d008456_T001	0.9668789	0.126820702	Zm00001d008456
Zm00001d019907_T012	0.98291	-0.013086124	Zm00001d019907
Zm00001d019907_T001	0.9109605	0.742230885	Zm00001d019907
Zm00001d038228_T001	0.9860836	0.449384828	Zm00001d038228
Zm00001d010456_T002	0.09891885	1.065463751	Zm00001d010456
Zm00001d010456_T001	0.6227737	-0.573850313	Zm00001d010456
Zm00001d010456_T003	0.9825858	0.278813973	Zm00001d010456
Zm00001d010456_T004	1	0.290390533	Zm00001d010456
Zm00001d043907_T001	0.9085393	-0.159091394	Zm00001d043907
Zm00001d043907_T003	0.827438	0.87723749	Zm00001d043907
Zm00001d034531_T008	0.9768802	0.517947793	Zm00001d034531
Zm00001d034531_T002	0.7488344	1.070338766	Zm00001d034531
Zm00001d024310_T009	0.9894615	0.184215756	Zm00001d024310
Zm00001d024310_T006	0.3595196	-0.16999666	Zm00001d024310
Zm00001d024310_T003	0.893914	0.439216843	Zm00001d024310
Zm00001d017432_T001	0.9224443	-0.098476267	Zm00001d017432
Zm00001d014060_T001	0.3843819	-0.761709003	Zm00001d014060
Zm00001d009676_T009	0.9567316	0.13235778	Zm00001d009676
Zm00001d009676_T006	0.3427966	0.410421838	Zm00001d009676
Zm00001d009676_T003	0.9380783	0.603342108	Zm00001d009676
Zm00001d009676_T011	0.9732421	0.109320721	Zm00001d009676
Zm00001d009676_T007	0.003247529	1.449080766	Zm00001d009676
Zm00001d009676_T005	0.4812187	0.358205041	Zm00001d009676
Zm00001d024522_T001	1.99E-08	5.42804365	Zm00001d024522
Zm00001d039750_T003	0.9851204	0.329530935	Zm00001d039750
Zm00001d039750_T001	0.9628835	0.058991782	Zm00001d039750
Zm00001d029888_T002	0.9416622	0.014242219	Zm00001d029888
Zm00001d032027_T002	0.7286023	-0.401454505	Zm00001d032027
Zm00001d017026_T006	0.9894615	0.342436702	Zm00001d017026
Zm00001d017026_T005	0.923766	-0.097057952	Zm00001d017026
Zm00001d014503_T001	0.9902733	-0.231351801	Zm00001d014503
Zm00001d011855_T007	0.93919	0.564696079	Zm00001d011855
Zm00001d011855_T008	0.1933431	1.715333194	Zm00001d011855
Zm00001d011855_T009	0.6772176	-0.677734591	Zm00001d011855
Zm00001d011855_T006	0.8647028	0.269049987	Zm00001d011855
Zm00001d011855_T001	0.912978	-0.014961549	Zm00001d011855
Zm00001d051742_T001	0.8104836	-0.204216225	Zm00001d051742
Zm00001d031317_T001	1	0.409364251	Zm00001d031317
Zm00001d021131_T002	0.9919298	0.186359601	Zm00001d021131
Zm00001d026655_T001	1	0.17377452	Zm00001d026655
Zm00001d037198_T001	1	0.429187988	Zm00001d037198
Zm00001d037035_T002	0.9479007	0.713255042	Zm00001d037035
Zm00001d052873_T001	1	0.405656254	Zm00001d052873
Zm00001d017911_T001	0.004998382	-2.170410495	Zm00001d017911
Zm00001d013964_T005	0.7100246	1.0548002	Zm00001d013964
Zm00001d013964_T004	0.1049051	2.051757189	Zm00001d013964
Zm00001d013964_T003	0.9232446	0.01311125	Zm00001d013964

Zm00001d048983_T003	0.5525713	-0.608504465	Zm00001d048983
Zm00001d048983_T001	0.7680039	0.95398075	Zm00001d048983
Zm00001d048983_T002	0.6131963	-0.405559103	Zm00001d048983
Zm00001d019696_T001	0.8760042	-0.085190017	Zm00001d019696
Zm00001d049210_T001	0.98291	0.551147445	Zm00001d049210
Zm00001d049210_T012	0.8751525	0.731949391	Zm00001d049210
Zm00001d049210_T007	0.4918762	0.32954898	Zm00001d049210
Zm00001d049210_T014	0.7249357	0.131256831	Zm00001d049210
Zm00001d049210_T010	0.7658902	0.135514958	Zm00001d049210
Zm00001d027311_T001	0.946422	0.069757288	Zm00001d027311
Zm00001d013150_T004	0.1218863	-1.209314817	Zm00001d013150
Zm00001d013150_T001	0.9889811	0.181523736	Zm00001d013150
Zm00001d011131_T001	0.9570177	-0.010835807	Zm00001d011131
Zm00001d031883_T001	0.9770343	0.222504166	Zm00001d031883
Zm00001d007388_T024	0.3919095	1.37693888	Zm00001d007388
Zm00001d007388_T002	0.6113292	0.916063275	Zm00001d007388
Zm00001d007388_T022	0.8751967	-0.031930402	Zm00001d007388
Zm00001d007388_T018	0.518342	0.197970838	Zm00001d007388
Zm00001d007388_T005	0.9711278	0.236160604	Zm00001d007388
Zm00001d007388_T006	0.9706887	0.254864338	Zm00001d007388
Zm00001d007388_T017	0.2982359	0.193064163	Zm00001d007388
Zm00001d007388_T009	1	0.035910953	Zm00001d007388
Zm00001d013146_T001	0.9503115	-0.236876546	Zm00001d013146
Zm00001d008903_T001	0.9870394	0.189931851	Zm00001d008903
Zm00001d040171_T001	0.9469426	0.540880052	Zm00001d040171
Zm00001d043059_T005	1	0.364991274	Zm00001d043059
Zm00001d043059_T003	0.9249382	0.002766011	Zm00001d043059
Zm00001d043059_T007	0.6727656	0.394921253	Zm00001d043059
Zm00001d008595_T001	1	0.32330648	Zm00001d008595
Zm00001d021090_T001	0.9232281	0.775660697	Zm00001d021090
Zm00001d034805_T006	0.8467631	0.792857142	Zm00001d034805
Zm00001d034805_T003	0.8413408	0.29509419	Zm00001d034805
Zm00001d034805_T005	0.9868821	0.466591657	Zm00001d034805
Zm00001d040036_T006	0.002628689	1.121770606	Zm00001d040036
Zm00001d040036_T001	0.0141963	-1.11240837	Zm00001d040036
Zm00001d040036_T024	1	0.40055784	Zm00001d040036
Zm00001d040036_T022	0.1102125	0.751280455	Zm00001d040036
Zm00001d031292_T001	0.4423489	-1.120028149	Zm00001d031292
Zm00001d031875_T001	0.001651001	1.073569011	Zm00001d031875
Zm00001d021899_T001	0.6972481	0.408063574	Zm00001d021899
Zm00001d002082_T004	0.4322189	0.7399466	Zm00001d002082
Zm00001d002082_T001	0.5081671	-0.674156106	Zm00001d002082
Zm00001d046587_T028	5.75E-09	1.325291004	Zm00001d046587
Zm00001d046587_T026	0.02214	-1.346190929	Zm00001d046587
Zm00001d046587_T008	0.148906	0.793430907	Zm00001d046587
Zm00001d046587_T025	0.002146068	0.807050775	Zm00001d046587
Zm00001d046587_T014	0.4339892	-0.600965763	Zm00001d046587
Zm00001d046587_T004	1	0.284548295	Zm00001d046587
Zm00001d006166_T001	0.8008612	1.026572115	Zm00001d006166
Zm00001d006166_T004	0.8885217	-0.084924234	Zm00001d006166
Zm00001d006166_T002	0.803996	0.447304207	Zm00001d006166
Zm00001d010634_T001	0.9384219	0.032566276	Zm00001d010634
Zm00001d023262_T033	0.000197484	0.926369581	Zm00001d023262
Zm00001d023262_T003	3.37E-15	3.452003261	Zm00001d023262

Zm00001d023262_T036	0.5048245	-0.475265376	Zm00001d023262
Zm00001d023262_T023	0.8391674	1.051581344	Zm00001d023262
Zm00001d023262_T018	0.7235209	0.527562778	Zm00001d023262
Zm00001d023262_T070	0.5619008	-0.866825655	Zm00001d023262
Zm00001d023262_T008	0.1935734	0.439293988	Zm00001d023262
Zm00001d023262_T031	0.7610341	-0.368273028	Zm00001d023262
Zm00001d023262_T014	0.7503001	0.681258523	Zm00001d023262
Zm00001d023262_T071	0.7746801	0.698550061	Zm00001d023262
Zm00001d023262_T001	0.9872831	0.207071236	Zm00001d023262
Zm00001d008779_T001	0.8604689	-0.132080042	Zm00001d008779
Zm00001d008769_T002	0.8203654	0.694383558	Zm00001d008769
Zm00001d042691_T001	0.9690782	0.16290664	Zm00001d042691
Zm00001d042691_T003	1	0.125618452	Zm00001d042691
Zm00001d012672_T014	0.8891117	-0.089465169	Zm00001d012672
Zm00001d012672_T002	0.9314224	0.38296107	Zm00001d012672
Zm00001d012672_T005	0.8412913	-0.141382201	Zm00001d012672
Zm00001d012672_T015	0.9547831	0.085066416	Zm00001d012672
Zm00001d012672_T012	0.9817074	0.015986347	Zm00001d012672
Zm00001d012672_T008	0.957148	0.12552111	Zm00001d012672
Zm00001d012672_T013	0.7613191	0.547909494	Zm00001d012672
Zm00001d047617_T001	0.8325937	-0.147562387	Zm00001d047617
Zm00001d008789_T008	0.2760977	0.541062464	Zm00001d008789
Zm00001d008789_T031	0.592105	0.397724405	Zm00001d008789
Zm00001d008789_T026	0.8476927	0.173112683	Zm00001d008789
Zm00001d008789_T035	1.52E-13	2.603842872	Zm00001d008789
Zm00001d008789_T033	0.00150025	0.630552834	Zm00001d008789
Zm00001d008789_T005	0.8987811	-0.096021583	Zm00001d008789
Zm00001d008789_T001	0.9469426	0.085056326	Zm00001d008789
Zm00001d008789_T020	0.453061	1.382276382	Zm00001d008789
Zm00001d031542_T001	0.9851204	0.2624666	Zm00001d031542
Zm00001d002978_T003	0.9390517	0.02867663	Zm00001d002978
Zm00001d002978_T005	0.7788277	-0.303223991	Zm00001d002978
Zm00001d002978_T001	0.990751	0.447018182	Zm00001d002978
Zm00001d002978_T002	0.9726297	0.114399654	Zm00001d002978
Zm00001d035722_T007	0.3550006	0.571316158	Zm00001d035722
Zm00001d035722_T002	0.0324581	-0.294369982	Zm00001d035722
Zm00001d035722_T003	0.9619517	0.124213155	Zm00001d035722
Zm00001d035722_T004	0.8616387	-0.191778903	Zm00001d035722
Zm00001d035722_T001	0.5069673	-0.23188125	Zm00001d035722
Zm00001d005995_T006	0.001036334	1.185461214	Zm00001d005995
Zm00001d005995_T005	0.98291	0.015118311	Zm00001d005995
Zm00001d005995_T003	0.8640519	-0.063812023	Zm00001d005995
Zm00001d005995_T008	0.9979568	0.42836603	Zm00001d005995
Zm00001d005497_T002	0.5822332	-0.281986628	Zm00001d005497
Zm00001d005497_T004	0.9960479	0.401383213	Zm00001d005497
Zm00001d017091_T001	0.9546532	0.632838354	Zm00001d017091
Zm00001d036838_T001	0.8449395	-0.613754645	Zm00001d036838
Zm00001d033222_T001	0.5488962	0.750278387	Zm00001d033222
Zm00001d030614_T002	0.9171732	0.646065925	Zm00001d030614
Zm00001d030614_T005	0.2846967	0.657574139	Zm00001d030614
Zm00001d030614_T001	0.005176924	-1.570315022	Zm00001d030614
Zm00001d030614_T004	0.6395973	-0.233109804	Zm00001d030614
Zm00001d029832_T001	0.4701937	-0.519825357	Zm00001d029832
Zm00001d029832_T002	0.957148	0.536912982	Zm00001d029832

Zm00001d049938_T024	0.7923921	-0.147491517	Zm00001d049938
Zm00001d049938_T013	0.716106	0.790579607	Zm00001d049938
Zm00001d049938_T025	0.2322824	-1.09876287	Zm00001d049938
Zm00001d012610_T001	0.9855117	-0.111109047	Zm00001d012610
Zm00001d029050_T001	0.2687255	-1.15466387	Zm00001d029050
Zm00001d029050_T002	0.6060903	1.19220157	Zm00001d029050
Zm00001d050978_T001	1	0.280506708	Zm00001d050978
Zm00001d036961_T007	0.7885216	0.770358925	Zm00001d036961
Zm00001d036961_T003	0.2619882	0.420296267	Zm00001d036961
Zm00001d036961_T004	0.9537113	0.703539436	Zm00001d036961
Zm00001d036961_T008	0.006876689	-1.08806706	Zm00001d036961
Zm00001d003186_T001	0.5234237	-0.645469899	Zm00001d003186
Zm00001d023542_T001	0.9541864	-0.032399302	Zm00001d023542
Zm00001d012966_T001	0.9979568	0.156800378	Zm00001d012966
Zm00001d012966_T002	0.9257893	0.476189183	Zm00001d012966
Zm00001d031024_T003	0.907891	-0.065841248	Zm00001d031024
Zm00001d031024_T001	1	0.249655645	Zm00001d031024
Zm00001d028486_T001	0.2721567	1.616413351	Zm00001d028486
Zm00001d034324_T001	0.9814486	0.345913554	Zm00001d034324
Zm00001d053630_T001	0.5467906	1.308900872	Zm00001d053630
Zm00001d053630_T002	0.6745773	-0.587295617	Zm00001d053630
Zm00001d015571_T001	0.9852645	0.484938033	Zm00001d015571
Zm00001d033787_T001	0.9160053	0.736235405	Zm00001d033787
Zm00001d034772_T090	1	0.0479315	Zm00001d034772
Zm00001d034772_T040	0.9469426	0.362409676	Zm00001d034772
Zm00001d034772_T088	0.9604838	0.557260392	Zm00001d034772
Zm00001d034772_T013	0.844285	0.411740405	Zm00001d034772
Zm00001d034772_T005	0.6019545	0.420860732	Zm00001d034772
Zm00001d034772_T086	0.6934931	-0.312536327	Zm00001d034772
Zm00001d034772_T031	0.910614	0.373249889	Zm00001d034772
Zm00001d034772_T084	0.7244917	0.839496073	Zm00001d034772
Zm00001d034772_T007	0.1518689	0.408839359	Zm00001d034772
Zm00001d034772_T006	0.04054157	0.564169442	Zm00001d034772
Zm00001d034772_T027	0.9804433	-0.033242947	Zm00001d034772
Zm00001d034772_T039	0.9950703	0.168473622	Zm00001d034772
Zm00001d034772_T107	0.01225238	-1.430074211	Zm00001d034772
Zm00001d034772_T110	3.91E-09	-3.296439215	Zm00001d034772
Zm00001d034772_T062	0.9429712	-0.007885287	Zm00001d034772
Zm00001d038105_T002	0.9843321	0.565319855	Zm00001d038105
Zm00001d038105_T001	0.9573434	0.398144512	Zm00001d038105
Zm00001d007603_T001	0.5695353	0.947971269	Zm00001d007603
Zm00001d021401_T001	0.9673473	-0.054698742	Zm00001d021401
Zm00001d034109_T001	0.3748085	1.59184282	Zm00001d034109
Zm00001d038481_T001	0.9732421	0.142508933	Zm00001d038481
Zm00001d009439_T002	0.9785005	0.325135587	Zm00001d009439
Zm00001d009439_T004	0.9912886	0.514888958	Zm00001d009439
Zm00001d037672_T001	0.9176356	0.047166095	Zm00001d037672
Zm00001d030074_T001	0.9558396	0.063481192	Zm00001d030074
Zm00001d017386_T001	0.9401799	-0.189520238	Zm00001d017386
Zm00001d033139_T001	0.9979568	0.139874562	Zm00001d033139
Zm00001d029174_T001	0.6023446	-0.524662713	Zm00001d029174
Zm00001d032186_T001	0.3893277	1.503523727	Zm00001d032186
Zm00001d029776_T002	1.43E-06	4.342432513	Zm00001d029776
Zm00001d018854_T001	0.7272863	-0.5194104	Zm00001d018854

Zm00001d007434_T001	1.88E-05	-2.711650768	Zm00001d007434
Zm00001d007434_T013	0.009549599	-1.500478644	Zm00001d007434
Zm00001d007434_T008	0.136916	0.731232679	Zm00001d007434
Zm00001d007434_T007	0.917315	0.117144147	Zm00001d007434
Zm00001d007434_T009	0.9043641	1.390706183	Zm00001d007434
Zm00001d007434_T014	0.1565832	-1.126907507	Zm00001d007434
Zm00001d007434_T005	0.9570268	0.570300774	Zm00001d007434
Zm00001d007434_T010	0.9852645	0.9909128	Zm00001d007434
Zm00001d032342_T001	0.2139984	-1.095888278	Zm00001d032342
Zm00001d016440_T001	1	0.356879512	Zm00001d016440
Zm00001d014244_T001	0.06130112	2.044792444	Zm00001d014244
Zm00001d021856_T001	0.9136458	0.582743128	Zm00001d021856
Zm00001d020097_T002	0.9833133	0.470728464	Zm00001d020097
Zm00001d020097_T008	3.28E-10	1.688133335	Zm00001d020097
Zm00001d020097_T009	1	0.255173375	Zm00001d020097
Zm00001d020097_T006	9.30E-10	-1.186924091	Zm00001d020097
Zm00001d020097_T004	0.2767107	1.259942984	Zm00001d020097
Zm00001d046999_T001	0.08435137	1.914978133	Zm00001d046999
Zm00001d046999_T002	0.07832989	1.31793592	Zm00001d046999
Zm00001d024373_T001	0.8125642	-0.35601933	Zm00001d024373
Zm00001d049573_T001	0.1121014	-0.847760802	Zm00001d049573
Zm00001d045706_T002	0.9139422	0.354344003	Zm00001d045706
Zm00001d045706_T003	1	0.387124259	Zm00001d045706
Zm00001d031832_T006	0.9618822	0.653363974	Zm00001d031832
Zm00001d031832_T010	0.8863456	0.737948914	Zm00001d031832
Zm00001d031832_T009	0.166116	0.617383582	Zm00001d031832
Zm00001d031832_T004	0.9024215	0.297746694	Zm00001d031832
Zm00001d031832_T001	0.6222715	-0.619399343	Zm00001d031832
Zm00001d031832_T008	0.2138161	0.524136132	Zm00001d031832
Zm00001d031832_T005	0.004998066	2.101988173	Zm00001d031832
Zm00001d031832_T011	0.8638849	0.719116804	Zm00001d031832
Zm00001d039505_T002	0.7163206	-0.350323763	Zm00001d039505
Zm00001d039505_T001	0.9656741	0.392305692	Zm00001d039505
Zm00001d039505_T003	0.7745894	2.118005469	Zm00001d039505
Zm00001d003302_T001	0.9312703	0.663535682	Zm00001d003302
Zm00001d013720_T001	0.112599	-0.492252869	Zm00001d013720
Zm00001d014366_T002	0.9024309	-0.02001005	Zm00001d014366
Zm00001d002292_T008	0.009256586	1.802854404	Zm00001d002292
Zm00001d002292_T001	0.9967884	0.363534739	Zm00001d002292
Zm00001d002292_T007	0.8179463	0.417279833	Zm00001d002292
Zm00001d002292_T009	0.2513095	1.572418334	Zm00001d002292
Zm00001d002292_T002	0.07862861	1.269989804	Zm00001d002292
Zm00001d002292_T006	0.2142641	1.910828388	Zm00001d002292
Zm00001d049179_T002	1	0.332353191	Zm00001d049179
Zm00001d025699_T001	0.9968023	0.32942258	Zm00001d025699
Zm00001d003176_T001	0.954492	0.171231341	Zm00001d003176
Zm00001d025816_T004	0.2378089	-0.467990703	Zm00001d025816
Zm00001d043890_T002	0.9160053	0.113691132	Zm00001d043890
Zm00001d043890_T001	0.8865624	1.100773827	Zm00001d043890
Zm00001d043890_T004	1.00E-05	-2.907986251	Zm00001d043890
Zm00001d014899_T001	0.9157081	-0.003026764	Zm00001d014899
Zm00001d037513_T001	0.835786	-0.744417159	Zm00001d037513
Zm00001d048651_T001	0.7712604	0.790225664	Zm00001d048651
Zm00001d010425_T005	0.9606343	0.557336416	Zm00001d010425

Zm00001d010425_T002	0.000433089	-3.956899535	Zm00001d010425
Zm00001d010425_T007	0.9771927	0.25463568	Zm00001d010425
Zm00001d010425_T001	0.7853827	0.347506919	Zm00001d010425
Zm00001d010425_T003	0.7358211	-0.177283893	Zm00001d010425
Zm00001d020051_T002	0.8863085	-0.148458407	Zm00001d020051
Zm00001d020051_T001	0.9673473	0.563259426	Zm00001d020051
Zm00001d007162_T002	0.929138	0.013263429	Zm00001d007162
Zm00001d007162_T001	0.6167301	-0.887273466	Zm00001d007162
Zm00001d043081_T001	0.9846446	0.130501846	Zm00001d043081
Zm00001d037951_T001	0.01150765	1.60097033	Zm00001d037951
Zm00001d053684_T006	0.9090332	-0.030919636	Zm00001d053684
Zm00001d053684_T001	0.8763786	0.056220607	Zm00001d053684
Zm00001d038445_T001	0.9778436	0.070927181	Zm00001d038445
Zm00001d008264_T003	0.9782953	0.102763585	Zm00001d008264
Zm00001d030549_T001	0.8409438	-0.191494155	Zm00001d030549
Zm00001d008285_T001	0.8122182	-0.147022644	Zm00001d008285
Zm00001d022478_T001	0.8669288	0.65384593	Zm00001d022478
Zm00001d014056_T001	0.7610341	0.56741579	Zm00001d014056
Zm00001d026257_T019	0.2611065	-0.968844838	Zm00001d026257
Zm00001d026257_T012	0.7163781	1.126492354	Zm00001d026257
Zm00001d026257_T016	0.5864438	0.88076588	Zm00001d026257
Zm00001d026257_T018	0.9410845	-0.103400172	Zm00001d026257
Zm00001d026257_T001	0.1986521	-0.403201052	Zm00001d026257
Zm00001d046378_T001	0.9410845	0.697216452	Zm00001d046378
Zm00001d047044_T016	0.002628042	1.461932104	Zm00001d047044
Zm00001d047044_T014	0.9748076	0.241288121	Zm00001d047044
Zm00001d047044_T013	0.4562375	0.353266278	Zm00001d047044
Zm00001d047044_T010	0.1557051	0.48786873	Zm00001d047044
Zm00001d047044_T002	0.1224674	1.275706598	Zm00001d047044
Zm00001d051684_T007	0.5428594	0.285652696	Zm00001d051684
Zm00001d051684_T002	0.8896281	0.771305745	Zm00001d051684
Zm00001d051684_T006	0.6242825	0.214417175	Zm00001d051684
Zm00001d006330_T001	0.03649979	2.493523867	Zm00001d006330
Zm00001d013915_T001	0.9735632	0.487409122	Zm00001d013915
Zm00001d018257_T001	0.9224443	0.651421714	Zm00001d018257
Zm00001d018257_T008	0.8677544	0.825582256	Zm00001d018257
Zm00001d018257_T005	0.3254356	1.063522987	Zm00001d018257
Zm00001d018257_T003	0.5549472	-0.73808611	Zm00001d018257
Zm00001d018257_T006	0.01806011	1.651195841	Zm00001d018257
Zm00001d018257_T009	0.3456233	-0.679164449	Zm00001d018257
Zm00001d044895_T001	0.7314473	-0.313719512	Zm00001d044895
Zm00001d027883_T001	0.333158	-1.300714468	Zm00001d027883
Zm00001d039250_T001	0.852305	-0.217720411	Zm00001d039250
Zm00001d021582_T001	1	0.344969812	Zm00001d021582
Zm00001d048440_T005	1	0.400397426	Zm00001d048440
Zm00001d048440_T008	0.6318256	1.025032897	Zm00001d048440
Zm00001d048440_T001	0.8591203	0.200880858	Zm00001d048440
Zm00001d017197_T006	0.9640775	0.183471516	Zm00001d017197
Zm00001d017197_T008	0.9486412	0.733326903	Zm00001d017197
Zm00001d017197_T011	0.1375045	-0.975104857	Zm00001d017197
Zm00001d017197_T003	0.3294423	-0.394257554	Zm00001d017197
Zm00001d017197_T013	0.6468965	0.298743019	Zm00001d017197
Zm00001d009496_T001	1	0.137957563	Zm00001d009496
Zm00001d009496_T003	0.9109224	-0.097663702	Zm00001d009496

Zm00001d006915_T001	0.1328918	-1.331120301	Zm00001d006915
Zm00001d016674_T001	0.9269932	0.590995713	Zm00001d016674
Zm00001d037182_T001	0.5824385	1.302511287	Zm00001d037182
Zm00001d015862_T001	0.7287219	-0.679557637	Zm00001d015862
Zm00001d037481_T004	0.2002595	1.51625698	Zm00001d037481
Zm00001d037481_T003	0.8964117	0.828068804	Zm00001d037481
Zm00001d037481_T006	0.003579954	-3.064395783	Zm00001d037481
Zm00001d037481_T005	0.8104836	0.298300271	Zm00001d037481
Zm00001d050163_T001	0.8007432	0.928251549	Zm00001d050163
Zm00001d031792_T001	0.9192339	0.524932987	Zm00001d031792
Zm00001d002105_T001	0.9701986	-0.194428945	Zm00001d002105
Zm00001d044660_T001	0.906046	-0.487318131	Zm00001d044660
Zm00001d021805_T002	0.9493302	0.643900717	Zm00001d021805
Zm00001d021805_T001	0.7249455	-0.797168487	Zm00001d021805
Zm00001d045965_T003	0.5405996	-0.21698213	Zm00001d045965
Zm00001d045965_T001	0.9596377	0.124259101	Zm00001d045965
Zm00001d027939_T001	0.3917302	-0.821501927	Zm00001d027939
Zm00001d048108_T001	0.3719559	1.421698068	Zm00001d048108
Zm00001d018112_T003	0.9090686	-0.105611379	Zm00001d018112
Zm00001d018112_T001	0.9628812	0.170319542	Zm00001d018112
Zm00001d018112_T002	0.6725291	-0.408821767	Zm00001d018112
Zm00001d025130_T002	0.9701986	0.28711419	Zm00001d025130
Zm00001d025130_T001	1	0.110170622	Zm00001d025130
Zm00001d016397_T005	0.961473	0.084327593	Zm00001d016397
Zm00001d016397_T016	0.8187452	0.200452153	Zm00001d016397
Zm00001d009808_T001	0.9706887	0.103117662	Zm00001d009808
Zm00001d042033_T001	0.4844771	1.20377079	Zm00001d042033
Zm00001d033526_T001	0.6466858	-0.917056151	Zm00001d033526
Zm00001d053202_T001	0.07195548	-1.49768764	Zm00001d053202
Zm00001d041827_T001	0.6420323	-0.46958472	Zm00001d041827
Zm00001d025263_T004	0.5208223	0.417235277	Zm00001d025263
Zm00001d025263_T002	0.8902687	0.845411891	Zm00001d025263
Zm00001d025263_T005	0.8749298	0.2734794	Zm00001d025263
Zm00001d016322_T001	0.2714979	-0.503080402	Zm00001d016322
Zm00001d011236_T001	0.3438181	-0.887181157	Zm00001d011236
Zm00001d005094_T001	0.9469426	0.016599583	Zm00001d005094
Zm00001d037902_T001	0.9579209	-0.026188101	Zm00001d037902
Zm00001d037902_T002	0.99543	0.111998818	Zm00001d037902
Zm00001d037902_T003	0.9015226	-0.148850707	Zm00001d037902
Zm00001d023299_T006	0.4995495	0.835395991	Zm00001d023299
Zm00001d023299_T009	0.7155054	0.65596162	Zm00001d023299
Zm00001d023299_T002	0.6338908	0.535361085	Zm00001d023299
Zm00001d053409_T001	0.5525396	-0.523418269	Zm00001d053409
Zm00001d032142_T003	0.8548987	-0.097682828	Zm00001d032142
Zm00001d032142_T004	0.9196877	-0.011147803	Zm00001d032142
Zm00001d007661_T003	0.9563257	0.718614773	Zm00001d007661
Zm00001d007661_T002	0.456189	-0.403407792	Zm00001d007661
Zm00001d052149_T054	0.8884142	0.530805929	Zm00001d052149
Zm00001d052149_T001	0.0361763	-1.398766248	Zm00001d052149
Zm00001d052149_T047	1.57E-11	1.665213828	Zm00001d052149
Zm00001d052149_T041	2.31E-05	1.489650598	Zm00001d052149
Zm00001d052149_T035	0.9160053	0.190854394	Zm00001d052149
Zm00001d052149_T037	0.7288105	0.620477985	Zm00001d052149
Zm00001d032526_T006	0.7289978	0.160584866	Zm00001d032526

Zm00001d032526_T010	0.2604053	-0.85142482	Zm00001d032526
Zm00001d032526_T002	0.8631191	0.732085714	Zm00001d032526
Zm00001d032526_T001	0.7830446	-0.095613412	Zm00001d032526
Zm00001d032526_T004	1	0.348540927	Zm00001d032526
Zm00001d032526_T005	0.2655131	0.953026874	Zm00001d032526
Zm00001d048695_T001	0.3496448	-0.76165052	Zm00001d048695
Zm00001d045045_T001	0.9486412	-0.20404659	Zm00001d045045
Zm00001d002642_T002	0.9781672	0.359022525	Zm00001d002642
Zm00001d002642_T001	1	0.347771773	Zm00001d002642
Zm00001d032989_T008	0.9984041	0.089850007	Zm00001d032989
Zm00001d032989_T002	0.8702486	0.848549225	Zm00001d032989
Zm00001d032989_T005	0.01591506	-1.356753885	Zm00001d032989
Zm00001d032989_T003	0.8492405	0.302814781	Zm00001d032989
Zm00001d032989_T024	0.941809	0.00971555	Zm00001d032989
Zm00001d032989_T004	0.03448305	-0.715242571	Zm00001d032989
Zm00001d032989_T011	0.5047678	0.216827084	Zm00001d032989
Zm00001d052768_T001	0.7743939	-0.467914345	Zm00001d052768
Zm00001d051976_T005	0.5479918	0.775392603	Zm00001d051976
Zm00001d032685_T001	0.8919143	0.884486501	Zm00001d032685
Zm00001d008689_T003	0.9673094	0.09134628	Zm00001d008689
Zm00001d008689_T001	0.7527702	-0.217769489	Zm00001d008689
Zm00001d038981_T001	0.9597485	0.599327322	Zm00001d038981
Zm00001d008268_T002	0.9944247	0.335807171	Zm00001d008268
Zm00001d002135_T001	0.9666757	0.144028105	Zm00001d002135
Zm00001d040448_T002	0.9693993	0.109846316	Zm00001d040448
Zm00001d002965_T001	0.06597925	2.169016995	Zm00001d002965
Zm00001d051870_T001	0.007975205	-1.919810691	Zm00001d051870
Zm00001d012606_T001	0.8098845	-0.271951352	Zm00001d012606
Zm00001d011764_T001	0.5537579	-0.461158241	Zm00001d011764
Zm00001d052256_T001	0.8517668	-0.263447191	Zm00001d052256
Zm00001d050430_T004	0.9821324	0.44007947	Zm00001d050430
Zm00001d050430_T001	1	0.268745003	Zm00001d050430
Zm00001d019497_T024	0.01462387	2.05252556	Zm00001d019497
Zm00001d019497_T004	1	0.491901482	Zm00001d019497
Zm00001d019497_T043	0.2366152	-1.433562604	Zm00001d019497
Zm00001d019497_T034	0.5390482	-0.887453164	Zm00001d019497
Zm00001d019497_T042	5.25E-12	-4.235205148	Zm00001d019497
Zm00001d019497_T041	0.000992527	-2.50359121	Zm00001d019497
Zm00001d039216_T001	0.4146004	-0.910854572	Zm00001d039216
Zm00001d017754_T019	2.86E-15	3.804983114	Zm00001d017754
Zm00001d017754_T045	0.9503115	0.238525429	Zm00001d017754
Zm00001d017754_T021	0.01774747	1.09360577	Zm00001d017754
Zm00001d017754_T013	0.4530974	0.269580501	Zm00001d017754
Zm00001d017754_T038	0.8860576	-0.010626549	Zm00001d017754
Zm00001d017754_T039	0.02202181	1.34682298	Zm00001d017754
Zm00001d017754_T004	0.995534	0.311909212	Zm00001d017754
Zm00001d017754_T012	0.2172819	0.79935569	Zm00001d017754
Zm00001d017754_T034	0.9821324	0.576812874	Zm00001d017754
Zm00001d017754_T024	0.4556297	-1.051713139	Zm00001d017754
Zm00001d045369_T001	1	-0.159101363	Zm00001d045369
Zm00001d009570_T001	0.8637863	-0.341645476	Zm00001d009570
Zm00001d049343_T003	0.9999526	0.207733556	Zm00001d049343
Zm00001d049343_T001	0.9804228	0.365048064	Zm00001d049343
Zm00001d038208_T001	0.8372625	-0.309953703	Zm00001d038208

Zm00001d027460_T001	0.9100954	0.717410001	Zm00001d027460
Zm00001d036959_T003	0.7194777	-0.325750307	Zm00001d036959
Zm00001d036959_T002	0.1619751	-1.330862165	Zm00001d036959
Zm00001d036959_T005	1	1.970660006	Zm00001d036959
Zm00001d036959_T004	0.8350279	-0.080360863	Zm00001d036959
Zm00001d011823_T001	0.9311027	0.628720979	Zm00001d011823
Zm00001d011823_T002	0.060383	1.961774718	Zm00001d011823
Zm00001d045535_T001	0.7626242	-0.700077523	Zm00001d045535
Zm00001d034382_T001	0.1459918	-1.098384909	Zm00001d034382
Zm00001d020036_T006	0.9073468	0.617544193	Zm00001d020036
Zm00001d020036_T011	0.7831859	0.519901837	Zm00001d020036
Zm00001d020036_T005	0.9388815	0.303434441	Zm00001d020036
Zm00001d020036_T014	0.7626242	-0.392726956	Zm00001d020036
Zm00001d020036_T004	0.1062521	1.475956664	Zm00001d020036
Zm00001d020036_T015	0.1308072	-0.906767232	Zm00001d020036
Zm00001d020036_T001	0.01190599	1.341760256	Zm00001d020036
Zm00001d020036_T013	0.5570384	-0.344098014	Zm00001d020036
Zm00001d020036_T009	0.9508258	0.009507415	Zm00001d020036
Zm00001d022163_T001	0.1947403	-1.100136334	Zm00001d022163
Zm00001d042460_T002	0.3120537	0.508304097	Zm00001d042460
Zm00001d014996_T001	0.5428594	-0.465755696	Zm00001d014996
Zm00001d042022_T001	0.5006675	-0.700872242	Zm00001d042022
Zm00001d034739_T001	0.9974232	0.258582178	Zm00001d034739
Zm00001d012402_T001	0.1233916	1.979721875	Zm00001d012402
Zm00001d047068_T001	0.9869996	0.204927211	Zm00001d047068
Zm00001d030106_T067	0.5347419	-0.234493176	Zm00001d030106
Zm00001d030106_T074	0.5380217	-0.229551094	Zm00001d030106
Zm00001d030106_T007	0.8746701	-0.0366404	Zm00001d030106
Zm00001d030106_T008	0.5742778	-0.45844783	Zm00001d030106
Zm00001d030106_T035	0.9543425	0.1833686	Zm00001d030106
Zm00001d030106_T048	0.2928549	-0.648016911	Zm00001d030106
Zm00001d030106_T107	0.03449392	-1.723737918	Zm00001d030106
Zm00001d030106_T082	0.5795936	-0.475052057	Zm00001d030106
Zm00001d030106_T029	0.9423654	0.656496915	Zm00001d030106
Zm00001d030106_T015	0.497443	0.567013844	Zm00001d030106
Zm00001d030106_T028	0.9959972	0.114710111	Zm00001d030106
Zm00001d030106_T032	0.8581713	0.316764927	Zm00001d030106
Zm00001d019145_T001	0.5892648	-0.46333025	Zm00001d019145
Zm00001d048540_T005	0.8015556	-0.075210683	Zm00001d048540
Zm00001d048540_T003	0.8093208	0.321949217	Zm00001d048540
Zm00001d048540_T009	0.8993058	0.504094448	Zm00001d048540
Zm00001d015788_T001	1	0.40659821	Zm00001d015788
Zm00001d015788_T002	0.4825809	0.407817578	Zm00001d015788
Zm00001d015788_T004	0.9249382	0.213428863	Zm00001d015788
Zm00001d049629_T002	0.9671056	0.532280072	Zm00001d049629
Zm00001d049629_T001	0.9732421	0.360341338	Zm00001d049629
Zm00001d024703_T004	0.8775735	0.273532466	Zm00001d024703
Zm00001d024703_T010	0.9037324	0.858489748	Zm00001d024703
Zm00001d024703_T003	0.6789826	1.093989425	Zm00001d024703
Zm00001d024703_T002	0.4167857	0.351212872	Zm00001d024703
Zm00001d024703_T011	0.01161943	-1.558999494	Zm00001d024703
Zm00001d024703_T012	0.9806983	-0.103925259	Zm00001d024703
Zm00001d024703_T005	2.06E-08	0.942071201	Zm00001d024703
Zm00001d027611_T001	0.8863456	-0.097245706	Zm00001d027611

Zm00001d022040_T003	1.53E-12	-1.813647561	Zm00001d022040
Zm00001d022040_T002	0.02081208	2.282091743	Zm00001d022040
Zm00001d008635_T001	0.9656049	0.349054635	Zm00001d008635
Zm00001d005158_T001	0.9705652	0.352888013	Zm00001d005158
Zm00001d021935_T002	0.885791	-0.241576098	Zm00001d021935
Zm00001d021935_T001	0.9703357	0.220761444	Zm00001d021935
Zm00001d018798_T006	0.7020458	0.284125198	Zm00001d018798
Zm00001d018798_T002	0.9881407	0.333219137	Zm00001d018798
Zm00001d013697_T002	1	0.35685805	Zm00001d013697
Zm00001d025327_T001	0.5440777	0.923214583	Zm00001d025327
Zm00001d042347_T012	0.9979393	0.313888131	Zm00001d042347
Zm00001d042347_T011	0.9914064	0.003693201	Zm00001d042347
Zm00001d042347_T013	0.9493302	-0.219388015	Zm00001d042347
Zm00001d042347_T010	0.9669771	2.543617517	Zm00001d042347
Zm00001d042347_T008	0.9936756	0.330203381	Zm00001d042347
Zm00001d042347_T005	0.9291337	0.541799225	Zm00001d042347
Zm00001d042347_T016	0.8726017	0.845610939	Zm00001d042347
Zm00001d042347_T001	0.9823248	0.486594809	Zm00001d042347
Zm00001d042347_T018	0.5448225	-0.158027219	Zm00001d042347
Zm00001d042347_T014	0.5890036	-0.588896224	Zm00001d042347
Zm00001d042347_T006	0.8499233	0.377636777	Zm00001d042347
Zm00001d042347_T007	0.0853967	2.00872827	Zm00001d042347
Zm00001d042347_T015	0.9219195	-0.135541426	Zm00001d042347
Zm00001d007081_T003	0.4557436	0.435680586	Zm00001d007081
Zm00001d007081_T008	0.908501	0.005377278	Zm00001d007081
Zm00001d007081_T001	0.8660897	-0.039828896	Zm00001d007081
Zm00001d007081_T027	0.2537249	0.508808057	Zm00001d007081
Zm00001d007081_T012	3.81E-09	1.344120127	Zm00001d007081
Zm00001d048203_T002	0.9988954	0.33328116	Zm00001d048203
Zm00001d048203_T001	0.6344415	-0.227634641	Zm00001d048203
Zm00001d048203_T003	0.9524127	0.32802498	Zm00001d048203
Zm00001d007810_T006	0.9396809	-0.018011249	Zm00001d007810
Zm00001d007810_T005	0.5097281	-2.176637125	Zm00001d007810
Zm00001d007810_T002	0.005912899	-1.351582573	Zm00001d007810
Zm00001d007810_T007	0.9812314	1.887830398	Zm00001d007810
Zm00001d007810_T008	0.7653096	-0.587554906	Zm00001d007810
Zm00001d031866_T001	0.6603537	-0.251266082	Zm00001d031866
Zm00001d037228_T001	0.4189302	-0.702505684	Zm00001d037228
Zm00001d027812_T001	0.6956747	-0.368727661	Zm00001d027812
Zm00001d019422_T001	0.9924805	0.468139996	Zm00001d019422
Zm00001d011213_T003	1	0.50313896	Zm00001d011213
Zm00001d011213_T001	0.7568663	1.100247596	Zm00001d011213
Zm00001d049521_T001	0.4557958	-1.009307617	Zm00001d049521
Zm00001d014285_T001	0.6812088	-0.740185815	Zm00001d014285
Zm00001d017089_T001	0.9257893	-0.034281078	Zm00001d017089
Zm00001d018047_T003	0.7296232	0.886056438	Zm00001d018047
Zm00001d018047_T006	0.3500209	1.269048567	Zm00001d018047
Zm00001d022335_T003	0.897691	0.829265963	Zm00001d022335
Zm00001d022335_T005	2.00E-09	-1.54588862	Zm00001d022335
Zm00001d024301_T006	0.7814163	-0.294293346	Zm00001d024301
Zm00001d024301_T002	0.9404125	0.113319517	Zm00001d024301
Zm00001d024301_T005	0.8647831	-0.08585263	Zm00001d024301
Zm00001d024301_T001	0.7997379	0.672253924	Zm00001d024301
Zm00001d024301_T003	0.8643636	0.18005257	Zm00001d024301

Zm00001d027265_T002	0.6183146	-0.345921462	Zm00001d027265
Zm00001d027265_T007	0.6085674	0.992591201	Zm00001d027265
Zm00001d027265_T005	0.6745773	0.262620901	Zm00001d027265
Zm00001d027265_T004	0.8449359	0.645046996	Zm00001d027265
Zm00001d006515_T001	0.5410776	-0.755979948	Zm00001d006515
Zm00001d037632_T001	0.5830307	-0.940528484	Zm00001d037632
Zm00001d018211_T001	0.9912886	0.211779203	Zm00001d018211
Zm00001d028896_T017	0.4650584	1.653490515	Zm00001d028896
Zm00001d028896_T009	0.7923921	0.48163248	Zm00001d028896
Zm00001d028896_T007	0.8863456	0.84433383	Zm00001d028896
Zm00001d028896_T015	0.004986224	-2.202428248	Zm00001d028896
Zm00001d028896_T001	0.9078934	0.314735519	Zm00001d028896
Zm00001d021871_T003	0.1624805	1.072444157	Zm00001d021871
Zm00001d021871_T001	0.9160053	0.343366109	Zm00001d021871
Zm00001d048524_T004	0.09409525	0.332845188	Zm00001d048524
Zm00001d048524_T013	1	0.062835242	Zm00001d048524
Zm00001d048524_T001	0.9355135	-0.038416794	Zm00001d048524
Zm00001d039914_T001	1	0.066155194	Zm00001d039914
Zm00001d040536_T005	0.4824723	0.811651698	Zm00001d040536
Zm00001d040536_T001	0.9260032	0.420715382	Zm00001d040536
Zm00001d040536_T003	0.9361945	0.455165053	Zm00001d040536
Zm00001d040536_T004	0.7183895	0.780833092	Zm00001d040536
Zm00001d016255_T001	0.6260085	1.213157481	Zm00001d016255
Zm00001d020283_T001	0.9544149	0.614201358	Zm00001d020283
Zm00001d012980_T001	0.8664689	-0.160236897	Zm00001d012980
Zm00001d012980_T003	0.4386149	1.340543883	Zm00001d012980
Zm00001d029275_T001	0.9825858	0.458128694	Zm00001d029275
Zm00001d042258_T004	0.7907819	0.615256995	Zm00001d042258
Zm00001d042258_T001	0.9766874	0.133929652	Zm00001d042258
Zm00001d044628_T002	0.9778436	0.72941896	Zm00001d044628
Zm00001d044628_T001	0.9252088	-0.024710104	Zm00001d044628
Zm00001d044628_T005	0.8665839	-0.074494688	Zm00001d044628
Zm00001d014915_T001	0.8675673	0.661145039	Zm00001d014915
Zm00001d024574_T001	0.9690925	0.390785219	Zm00001d024574
Zm00001d007888_T003	0.993158	0.234167764	Zm00001d007888
Zm00001d007888_T002	0.5936419	-0.165865049	Zm00001d007888
Zm00001d045085_T001	0.9781672	0.441539943	Zm00001d045085
Zm00001d045085_T002	1	0.419564768	Zm00001d045085
Zm00001d044266_T002	0.03196455	-0.772239398	Zm00001d044266
Zm00001d044266_T001	0.02355239	2.629693217	Zm00001d044266
Zm00001d044266_T010	0.5468086	-0.572204444	Zm00001d044266
Zm00001d044266_T004	0.5186912	0.435945468	Zm00001d044266
Zm00001d044266_T008	0.05368751	0.497149867	Zm00001d044266
Zm00001d010218_T001	0.5415171	1.262908329	Zm00001d010218
Zm00001d041864_T008	0.974657	0.28061391	Zm00001d041864
Zm00001d041864_T006	0.9942835	0.585833531	Zm00001d041864
Zm00001d041864_T005	0.5595771	-1.065573586	Zm00001d041864
Zm00001d041864_T007	0.8102147	1.036189334	Zm00001d041864
Zm00001d041864_T001	0.8361438	-0.254737649	Zm00001d041864
Zm00001d034255_T001	0.9419336	0.017888584	Zm00001d034255
Zm00001d013463_T001	0.8376861	-0.143893771	Zm00001d013463
Zm00001d046945_T001	0.8823012	0.937222285	Zm00001d046945
Zm00001d003917_T007	0.9939021	0.205369317	Zm00001d003917
Zm00001d003917_T001	0.8075512	0.757083009	Zm00001d003917

Zm00001d003917_T011	0.2824677	-0.998909321	Zm00001d003917
Zm00001d003917_T008	0.9311783	0.233072993	Zm00001d003917
Zm00001d003917_T009	0.6517512	0.146676223	Zm00001d003917
Zm00001d028840_T002	0.9709887	0.479886766	Zm00001d028840
Zm00001d028840_T003	0.9881407	0.055715205	Zm00001d028840
Zm00001d028104_T003	0.9932006	0.244930978	Zm00001d028104
Zm00001d034957_T002	0.9257893	0.277956766	Zm00001d034957
Zm00001d049643_T002	0.2617469	1.657912728	Zm00001d049643
Zm00001d049643_T005	0.3367371	1.561431116	Zm00001d049643
Zm00001d049643_T006	0.0655225	-0.903384298	Zm00001d049643
Zm00001d049643_T001	0.3870314	0.35174578	Zm00001d049643
Zm00001d049258_T001	0.8842271	-0.101199381	Zm00001d049258
Zm00001d003247_T002	0.7294039	-0.333065729	Zm00001d003247
Zm00001d003247_T001	0.2221685	-0.636132095	Zm00001d003247
Zm00001d048344_T003	0.5937879	-0.856853646	Zm00001d048344
Zm00001d048344_T002	0.9817074	0.189627781	Zm00001d048344
Zm00001d051112_T001	0.9509252	0.063300084	Zm00001d051112
Zm00001d020642_T001	0.9561521	0.677874309	Zm00001d020642
Zm00001d036395_T014	0.9928864	0.278614517	Zm00001d036395
Zm00001d036395_T013	0.002228826	2.328833557	Zm00001d036395
Zm00001d036395_T001	0.5493605	1.310549012	Zm00001d036395
Zm00001d036395_T010	0.9337571	0.108427891	Zm00001d036395
Zm00001d036395_T011	0.79251	0.234440086	Zm00001d036395
Zm00001d036395_T002	0.9073468	-0.035397585	Zm00001d036395
Zm00001d033068_T001	0.6075235	0.637758904	Zm00001d033068
Zm00001d045065_T002	0.908501	-0.045778382	Zm00001d045065
Zm00001d038250_T001	0.9781672	0.260258989	Zm00001d038250
Zm00001d026492_T001	0.9982897	0.275374769	Zm00001d026492
Zm00001d016766_T001	0.1824072	0.68882504	Zm00001d016766
Zm00001d016766_T006	0.9363115	-0.135672331	Zm00001d016766
Zm00001d045843_T001	0.5677369	-0.607131651	Zm00001d045843
Zm00001d029142_T007	1	0.411432598	Zm00001d029142
Zm00001d029142_T013	0.9016285	-0.019971419	Zm00001d029142
Zm00001d012173_T001	0.9768802	0.502358475	Zm00001d012173
Zm00001d035019_T001	0.7251353	-0.280186056	Zm00001d035019
Zm00001d029878_T001	0.9523689	0.504907445	Zm00001d029878
Zm00001d028776_T001	0.8780771	-0.69024652	Zm00001d028776
Zm00001d026543_T003	0.8379348	-0.177081734	Zm00001d026543
Zm00001d026543_T008	0.9717761	0.445328177	Zm00001d026543
Zm00001d026543_T006	0.9887584	0.145188974	Zm00001d026543
Zm00001d026543_T001	0.7891719	-0.282661339	Zm00001d026543
Zm00001d026543_T010	0.9690782	0.169166158	Zm00001d026543
Zm00001d026543_T009	0.9189175	0.267136622	Zm00001d026543
Zm00001d005177_T001	0.9510902	-0.211310854	Zm00001d005177
Zm00001d042421_T006	0.2719242	1.005951006	Zm00001d042421
Zm00001d042421_T005	0.6809685	0.719663964	Zm00001d042421
Zm00001d042421_T003	0.4786492	0.770857224	Zm00001d042421
Zm00001d042421_T002	0.9822138	0.528858835	Zm00001d042421
Zm00001d036496_T004	0.9832092	0.198791257	Zm00001d036496
Zm00001d036496_T003	0.9672613	0.5606012	Zm00001d036496
Zm00001d036496_T005	0.8424969	0.535009562	Zm00001d036496
Zm00001d005587_T007	0.1116614	-1.253103996	Zm00001d005587
Zm00001d005587_T003	0.9781672	0.40355784	Zm00001d005587
Zm00001d035167_T007	0.1419397	-0.681769686	Zm00001d035167

Zm00001d035167_T005	0.1940277	0.475691863	Zm00001d035167
Zm00001d035167_T006	0.8752644	0.317928962	Zm00001d035167
Zm00001d035167_T001	0.9469426	0.64251374	Zm00001d035167
Zm00001d049208_T003	0.6167332	1.180220594	Zm00001d049208
Zm00001d049208_T034	0.7933933	-0.106959804	Zm00001d049208
Zm00001d049208_T017	0.4422995	0.876979855	Zm00001d049208
Zm00001d049208_T024	0.9352321	0.761554767	Zm00001d049208
Zm00001d014166_T001	0.005636025	0.751942174	Zm00001d014166
Zm00001d038929_T001	0.9561521	0.167915995	Zm00001d038929
Zm00001d038692_T001	0.3913208	-0.703231883	Zm00001d038692
Zm00001d044447_T001	0.9975882	0.211344369	Zm00001d044447
Zm00001d044447_T006	0.9833565	0.508573848	Zm00001d044447
Zm00001d044447_T004	0.3258255	-0.256164222	Zm00001d044447
Zm00001d044447_T007	0.04707027	1.007076821	Zm00001d044447
Zm00001d013330_T001	0.9969123	0.468646422	Zm00001d013330
Zm00001d025628_T001	0.9468268	0.635670236	Zm00001d025628
Zm00001d005705_T002	0.9863483	-0.032308532	Zm00001d005705
Zm00001d005705_T001	0.9361945	0.001831662	Zm00001d005705
Zm00001d017859_T002	0.9563232	0.101830902	Zm00001d017859
Zm00001d017859_T003	0.9846714	0.00424704	Zm00001d017859
Zm00001d030370_T001	0.6709825	0.953285605	Zm00001d030370
Zm00001d013135_T002	0.2679983	-0.818364624	Zm00001d013135
Zm00001d036044_T001	0.7990254	-0.276283542	Zm00001d036044
Zm00001d023803_T001	0.9732421	0.373742443	Zm00001d023803
Zm00001d048143_T003	0.9787803	0.435382754	Zm00001d048143
Zm00001d026038_T001	1	0.187821578	Zm00001d026038
Zm00001d028870_T001	0.9429712	0.101156527	Zm00001d028870
Zm00001d050003_T001	0.9334932	0.264273724	Zm00001d050003
Zm00001d012816_T003	0.00095619	-1.279221178	Zm00001d012816
Zm00001d012816_T001	0.9852645	0.276749022	Zm00001d012816
Zm00001d012816_T002	0.9705226	0.626405365	Zm00001d012816
Zm00001d012816_T005	1	0.414793964	Zm00001d012816
Zm00001d012816_T004	0.6713115	-0.26638048	Zm00001d012816
Zm00001d018191_T001	0.000251052	2.509806067	Zm00001d018191
Zm00001d017534_T001	0.602511	-0.656415994	Zm00001d017534
Zm00001d051917_T001	0.9514141	0.274928867	Zm00001d051917
Zm00001d051917_T005	0.998484	0.307699775	Zm00001d051917
Zm00001d051917_T003	0.5474738	-0.154089625	Zm00001d051917
Zm00001d051917_T007	0.0505667	1.681074131	Zm00001d051917
Zm00001d037892_T001	0.8776881	0.250043383	Zm00001d037892
Zm00001d046304_T001	0.9469426	0.507712453	Zm00001d046304
Zm00001d038364_T002	0.7226402	-0.338608262	Zm00001d038364
Zm00001d038364_T001	0.646433	-0.482032439	Zm00001d038364
Zm00001d027941_T001	0.3450957	-0.226300637	Zm00001d027941
Zm00001d027941_T004	0.9543237	0.658806133	Zm00001d027941
Zm00001d012084_T001	0.9802432	0.450456571	Zm00001d012084
Zm00001d012084_T002	0.9825014	0.204465352	Zm00001d012084
Zm00001d040629_T002	0.9243991	-0.00501142	Zm00001d040629
Zm00001d026014_T001	0.9207666	-0.089886824	Zm00001d026014
Zm00001d012890_T001	0.8710199	-0.191453229	Zm00001d012890
Zm00001d021321_T001	0.000370758	3.25363759	Zm00001d021321
Zm00001d043165_T002	0.2982359	-0.43030711	Zm00001d043165
Zm00001d043165_T003	0.768921	0.472108302	Zm00001d043165
Zm00001d043165_T001	0.08348055	-0.567232182	Zm00001d043165

Zm00001d016733_T001	0.9664406	-0.02893122	Zm00001d016733
Zm00001d043801_T002	0.9536352	0.544204354	Zm00001d043801
Zm00001d013747_T001	0.8187547	-0.256826989	Zm00001d013747
Zm00001d043700_T003	2.66E-10	-3.721089248	Zm00001d043700
Zm00001d043700_T006	0.4236278	0.933510607	Zm00001d043700
Zm00001d043700_T002	0.9303773	0.656909798	Zm00001d043700
Zm00001d043700_T001	0.8523741	0.755499624	Zm00001d043700
Zm00001d027487_T026	0.9666757	0.114934945	Zm00001d027487
Zm00001d027487_T001	0.8221926	-0.592669015	Zm00001d027487
Zm00001d027487_T002	0.258026	0.468370103	Zm00001d027487
Zm00001d027487_T017	0.8877503	0.854492636	Zm00001d027487
Zm00001d027487_T028	0.9073468	-0.013573099	Zm00001d027487
Zm00001d027487_T019	0.005340142	1.353382147	Zm00001d027487
Zm00001d046142_T001	0.8704456	-0.318419052	Zm00001d046142
Zm00001d031062_T001	0.9570177	-0.091692745	Zm00001d031062
Zm00001d033969_T006	0.8995848	0.819427664	Zm00001d033969
Zm00001d033969_T007	0.5717645	0.303837832	Zm00001d033969
Zm00001d033969_T001	0.2887469	-1.032982906	Zm00001d033969
Zm00001d016798_T001	0.9881407	0.272962135	Zm00001d016798
Zm00001d025722_T002	0.9705226	0.12330499	Zm00001d025722
Zm00001d019756_T005	0.9778436	0.430354922	Zm00001d019756
Zm00001d011349_T001	0.9706293	0.153834377	Zm00001d011349
Zm00001d003222_T007	0.8987811	0.818923385	Zm00001d003222
Zm00001d003222_T009	0.1358919	0.863077652	Zm00001d003222
Zm00001d003222_T015	0.5616345	0.480061586	Zm00001d003222
Zm00001d003222_T013	0.4422995	0.862095904	Zm00001d003222
Zm00001d003222_T017	0.9792387	0.224273784	Zm00001d003222
Zm00001d010585_T001	0.2038212	0.949003761	Zm00001d010585
Zm00001d010585_T005	0.1921001	-0.383167466	Zm00001d010585
Zm00001d053884_T001	0.2090746	-1.30441273	Zm00001d053884
Zm00001d036069_T001	0.005601632	-0.894100581	Zm00001d036069
Zm00001d021696_T004	0.9503115	0.11161664	Zm00001d021696
Zm00001d021696_T003	0.9257893	-0.008848174	Zm00001d021696
Zm00001d038820_T001	0.8647916	-0.124843678	Zm00001d038820
Zm00001d021309_T015	0.04391107	-0.925277757	Zm00001d021309
Zm00001d021309_T002	0.1429392	0.838816273	Zm00001d021309
Zm00001d021309_T010	0.844285	0.808018554	Zm00001d021309
Zm00001d021309_T014	0.5229047	0.975193894	Zm00001d021309
Zm00001d021309_T009	0.9262099	-0.044777487	Zm00001d021309
Zm00001d021309_T012	0.8203654	0.609260205	Zm00001d021309
Zm00001d042887_T001	0.03254374	2.538554011	Zm00001d042887
Zm00001d036784_T001	0.03449192	2.061636879	Zm00001d036784
Zm00001d036784_T003	0.1711609	1.892799047	Zm00001d036784
Zm00001d036784_T002	0.4614239	0.387106637	Zm00001d036784
Zm00001d012956_T001	0.03997268	2.448730691	Zm00001d012956
Zm00001d005662_T001	1	0.362122313	Zm00001d005662
Zm00001d005662_T002	0.7619013	-0.343449157	Zm00001d005662
Zm00001d032626_T001	0.4952796	-0.965092778	Zm00001d032626
Zm00001d014456_T001	0.6736783	0.765967771	Zm00001d014456
Zm00001d051389_T001	0.7756749	1.064685884	Zm00001d051389
Zm00001d039903_T001	0.1344806	-0.94061076	Zm00001d039903
Zm00001d039903_T002	0.8807115	0.453661162	Zm00001d039903
Zm00001d029811_T001	0.6745773	-0.482185945	Zm00001d029811
Zm00001d049247_T001	0.9176356	-0.161235591	Zm00001d049247

Zm00001d034756_T001	0.9053391	1.16400385	Zm00001d034756
Zm00001d042108_T001	0.9201237	0.643426734	Zm00001d042108
Zm00001d005355_T001	0.06303158	1.667230909	Zm00001d005355
Zm00001d052985_T002	0.9702242	-0.050675696	Zm00001d052985
Zm00001d009525_T002	0.8955831	-0.210350076	Zm00001d009525
Zm00001d040196_T001	0.9528476	-0.0534369	Zm00001d040196
Zm00001d040196_T002	1	0.098323095	Zm00001d040196
Zm00001d054012_T001	0.7003081	0.587903012	Zm00001d054012
Zm00001d010396_T001	0.4604152	-1.017640582	Zm00001d010396
Zm00001d024740_T001	0.9822168	0.058505366	Zm00001d024740
Zm00001d035663_T001	0.2982359	1.479581565	Zm00001d035663
Zm00001d046255_T014	0.04099654	-0.905295409	Zm00001d046255
Zm00001d046255_T002	0.9835413	0.155153697	Zm00001d046255
Zm00001d046255_T038	0.9219195	0.757500984	Zm00001d046255
Zm00001d046255_T019	0.9979568	0.332663634	Zm00001d046255
Zm00001d046255_T039	0.917315	0.465953603	Zm00001d046255
Zm00001d046255_T008	0.9965998	0.258389769	Zm00001d046255
Zm00001d046255_T027	1.25E-06	1.005484551	Zm00001d046255
Zm00001d023738_T001	1	0.371970803	Zm00001d023738
Zm00001d013672_T001	0.6215642	1.127785786	Zm00001d013672
Zm00001d046529_T001	0.8984125	-0.103774112	Zm00001d046529
Zm00001d050860_T001	0.8097414	-0.255954635	Zm00001d050860
Zm00001d021565_T003	0.9838742	0.49597688	Zm00001d021565
Zm00001d021565_T004	0.9851204	0.342013312	Zm00001d021565
Zm00001d021565_T001	0.8695028	0.980978143	Zm00001d021565
Zm00001d043724_T001	0.9851204	0.376530092	Zm00001d043724
Zm00001d045984_T001	0.9726706	0.426258204	Zm00001d045984
Zm00001d013268_T008	0.194003	0.806050505	Zm00001d013268
Zm00001d013268_T003	0.8411786	-0.321954426	Zm00001d013268
Zm00001d013268_T007	0.2924497	2.366611568	Zm00001d013268
Zm00001d013268_T002	0.9124297	0.163464428	Zm00001d013268
Zm00001d013268_T005	0.366346	-0.539627125	Zm00001d013268
Zm00001d013268_T009	0.01617441	1.663019287	Zm00001d013268
Zm00001d048567_T002	0.4177213	-0.376407669	Zm00001d048567
Zm00001d048567_T003	0.9612081	0.008557397	Zm00001d048567
Zm00001d048567_T001	0.2719671	1.491905071	Zm00001d048567
Zm00001d048567_T008	0.9918302	0.215501402	Zm00001d048567
Zm00001d048567_T006	0.5576332	0.220624572	Zm00001d048567
Zm00001d048567_T007	0.9793659	0.464153093	Zm00001d048567
Zm00001d044146_T001	0.6916675	1.181585178	Zm00001d044146
Zm00001d040873_T001	0.9756546	0.062828886	Zm00001d040873
Zm00001d017713_T001	0.08515007	1.886197002	Zm00001d017713
Zm00001d010126_T003	0.9914064	0.507086177	Zm00001d010126
Zm00001d010126_T007	0.2246498	0.772702961	Zm00001d010126
Zm00001d038854_T003	0.9037324	1.245533495	Zm00001d038854
Zm00001d038854_T006	0.8260686	1.172779327	Zm00001d038854
Zm00001d038854_T004	0.8064667	0.3695352	Zm00001d038854
Zm00001d038854_T005	0.5715832	0.394124743	Zm00001d038854
Zm00001d035762_T001	0.9127452	0.690116423	Zm00001d035762
Zm00001d029948_T001	0.7212391	-0.249545223	Zm00001d029948
Zm00001d047385_T007	0.7885216	-0.047404618	Zm00001d047385
Zm00001d047385_T001	0.06395677	0.955997085	Zm00001d047385
Zm00001d047385_T009	0.03706504	1.122837735	Zm00001d047385
Zm00001d047385_T002	0.7428299	1.064874528	Zm00001d047385

Zm00001d047385_T010	0.4015159	-1.139211509	Zm00001d047385
Zm00001d015012_T001	0.9588604	0.124377666	Zm00001d015012
Zm00001d038801_T001	0.8433701	-0.16411012	Zm00001d038801
Zm00001d011908_T005	0.1924046	1.546877245	Zm00001d011908
Zm00001d011908_T001	0.835901	-0.26125514	Zm00001d011908
Zm00001d011908_T002	0.949733	0.635345556	Zm00001d011908
Zm00001d011908_T003	1.25E-07	-2.538861793	Zm00001d011908
Zm00001d030652_T001	0.9422098	0.725519742	Zm00001d030652
Zm00001d009927_T003	0.6777281	1.147450347	Zm00001d009927
Zm00001d009927_T002	0.1242192	2.126108979	Zm00001d009927
Zm00001d009927_T006	0.006103531	0.963198082	Zm00001d009927
Zm00001d009927_T009	0.002951811	2.781397095	Zm00001d009927
Zm00001d009927_T001	0.08474288	1.703074347	Zm00001d009927
Zm00001d009927_T008	6.39E-07	3.171799516	Zm00001d009927
Zm00001d009927_T005	0.000191577	2.63883663	Zm00001d009927
Zm00001d025754_T001	0.01455282	-1.82719349	Zm00001d025754
Zm00001d019117_T001	0.9303773	-0.044289063	Zm00001d019117
Zm00001d019117_T002	0.9363424	-0.010568032	Zm00001d019117
Zm00001d027307_T002	0.01812238	0.357851613	Zm00001d027307
Zm00001d027307_T072	0.4386149	1.423644751	Zm00001d027307
Zm00001d027307_T039	0.5525396	-0.576517101	Zm00001d027307
Zm00001d027307_T040	0.003495296	1.864295863	Zm00001d027307
Zm00001d047325_T001	0.5196715	0.923879806	Zm00001d047325
Zm00001d016572_T001	0.7146325	-0.351474056	Zm00001d016572
Zm00001d045028_T001	0.8643636	-0.11829355	Zm00001d045028
Zm00001d033711_T001	1	0.081049104	Zm00001d033711
Zm00001d029091_T001	0.07348954	-2.117653321	Zm00001d029091
Zm00001d029091_T008	0.001350714	0.766341754	Zm00001d029091
Zm00001d029091_T015	0.392691	0.464644019	Zm00001d029091
Zm00001d029091_T011	0.8547041	0.703844774	Zm00001d029091
Zm00001d029091_T012	0.2619882	-0.506077424	Zm00001d029091
Zm00001d047502_T001	0.9181664	0.026281975	Zm00001d047502
Zm00001d036571_T004	0.3181489	0.921395269	Zm00001d036571
Zm00001d036571_T002	0.9902022	0.283239768	Zm00001d036571
Zm00001d048185_T001	0.09696536	-1.464473692	Zm00001d048185
Zm00001d017801_T001	0.9067774	0.093635003	Zm00001d017801
Zm00001d014540_T001	0.6946859	0.415426443	Zm00001d014540
Zm00001d011459_T001	0.8832328	-0.079269731	Zm00001d011459
Zm00001d012868_T002	0.991538	0.098362091	Zm00001d012868
Zm00001d041403_T001	0.9180662	0.575154946	Zm00001d041403
Zm00001d048101_T007	0.9822138	0.096915563	Zm00001d048101
Zm00001d048101_T006	0.6958637	0.880025428	Zm00001d048101
Zm00001d048101_T005	0.8520487	-0.093979903	Zm00001d048101
Zm00001d048101_T002	0.3943165	-0.424451419	Zm00001d048101
Zm00001d048101_T004	0.5843839	-0.522130651	Zm00001d048101
Zm00001d048101_T003	0.03676439	-1.755119565	Zm00001d048101
Zm00001d048101_T001	0.9722876	0.064566365	Zm00001d048101
Zm00001d052943_T009	4.38E-10	1.444275326	Zm00001d052943
Zm00001d052943_T024	1	0.47813772	Zm00001d052943
Zm00001d052943_T018	0.9904282	0.330543336	Zm00001d052943
Zm00001d045354_T002	0.9843847	0.219066187	Zm00001d045354
Zm00001d019241_T001	0.9832612	0.197261556	Zm00001d019241
Zm00001d019241_T002	0.9127665	0.64947397	Zm00001d019241
Zm00001d027441_T002	0.6026834	-0.574662344	Zm00001d027441

Zm00001d027441_T001	1	0.372117901	Zm00001d027441
Zm00001d008691_T002	0.9180865	0.041535369	Zm00001d008691
Zm00001d008691_T003	0.9396569	0.070485233	Zm00001d008691
Zm00001d016716_T003	0.8935921	-0.036401633	Zm00001d016716
Zm00001d016716_T002	0.9832612	0.172695847	Zm00001d016716
Zm00001d009502_T001	0.872968	-0.351726138	Zm00001d009502
Zm00001d038273_T003	0.9561521	0.17738303	Zm00001d038273
Zm00001d038273_T001	0.8106492	-0.164470703	Zm00001d038273
Zm00001d000004_T001	0.9907928	0.041979298	Zm00001d000004
Zm00001d021196_T004	0.9126787	1.368208039	Zm00001d021196
Zm00001d021196_T011	0.6958554	1.285457684	Zm00001d021196
Zm00001d021196_T009	1	0.562186997	Zm00001d021196
Zm00001d021196_T012	0.3068305	-1.000227901	Zm00001d021196
Zm00001d021196_T005	0.02255438	-1.312760531	Zm00001d021196
Zm00001d021196_T006	0.9222337	0.672642188	Zm00001d021196
Zm00001d034710_T004	1	0.335223216	Zm00001d034710
Zm00001d034710_T001	0.9860836	0.138393664	Zm00001d034710
Zm00001d034710_T005	0.9872831	0.031401562	Zm00001d034710
Zm00001d017926_T003	0.8054763	-0.271937161	Zm00001d017926
Zm00001d017926_T014	0.05267929	-1.474329177	Zm00001d017926
Zm00001d017926_T011	0.9805897	0.07987352	Zm00001d017926
Zm00001d039492_T001	0.1275396	-1.428496045	Zm00001d039492
Zm00001d017263_T001	0.5044649	-0.725881113	Zm00001d017263
Zm00001d004118_T002	0.8933301	0.5376833	Zm00001d004118
Zm00001d004118_T003	0.002129765	2.644295415	Zm00001d004118
Zm00001d004118_T004	0.3105424	-1.224128273	Zm00001d004118
Zm00001d017812_T003	0.9395327	-0.001302617	Zm00001d017812
Zm00001d017812_T005	0.8999046	-0.260294893	Zm00001d017812
Zm00001d017812_T001	0.9983186	0.129352419	Zm00001d017812
Zm00001d039162_T001	0.9945094	0.327745962	Zm00001d039162
Zm00001d039162_T003	0.9596377	-0.022098837	Zm00001d039162
Zm00001d021571_T001	0.9580985	0.626038068	Zm00001d021571
Zm00001d023410_T001	0.7109402	0.714233686	Zm00001d023410
Zm00001d048583_T001	0.98291	0.271807869	Zm00001d048583
Zm00001d012146_T003	0.7311884	-0.228464643	Zm00001d012146
Zm00001d012146_T012	0.6050512	-0.273620723	Zm00001d012146
Zm00001d028537_T001	0.0462395	-1.350276367	Zm00001d028537
Zm00001d002671_T001	0.9285812	0.423293587	Zm00001d002671
Zm00001d020344_T001	1	0.277565705	Zm00001d020344
Zm00001d053775_T001	0.6086477	-0.481182339	Zm00001d053775
Zm00001d039011_T001	0.4115587	-0.816967423	Zm00001d039011
Zm00001d012597_T005	0.9889811	0.247802311	Zm00001d012597
Zm00001d012597_T001	0.9429978	0.049761422	Zm00001d012597
Zm00001d012597_T004	0.9711278	0.619045121	Zm00001d012597
Zm00001d025894_T002	0.6695932	1.934514028	Zm00001d025894
Zm00001d025894_T003	0.9781672	0.49641768	Zm00001d025894
Zm00001d025894_T001	0.4921668	-0.451627974	Zm00001d025894
Zm00001d025894_T004	0.2262715	1.044861156	Zm00001d025894
Zm00001d037165_T001	0.02334998	1.950570245	Zm00001d037165
Zm00001d019215_T004	0.8988681	-0.00141973	Zm00001d019215
Zm00001d019215_T002	0.196511	-0.999150305	Zm00001d019215
Zm00001d019215_T001	0.9529184	0.616537251	Zm00001d019215
Zm00001d015618_T003	0.8056124	-0.207714098	Zm00001d015618
Zm00001d015618_T001	0.9380783	0.734254884	Zm00001d015618

Zm00001d009837_T002	0.9834601	0.293263331	Zm00001d009837
Zm00001d009837_T001	0.9410688	0.052358124	Zm00001d009837
Zm00001d018343_T001	0.793655	-0.322087203	Zm00001d018343
Zm00001d047594_T001	0.9111683	-0.058647444	Zm00001d047594
Zm00001d047594_T002	0.6159869	-0.482893732	Zm00001d047594
Zm00001d043365_T001	0.9159727	0.790985624	Zm00001d043365
Zm00001d013465_T001	0.9962521	0.317805361	Zm00001d013465
Zm00001d006322_T004	0.9706887	0.131618554	Zm00001d006322
Zm00001d006322_T005	0.7141136	0.596616819	Zm00001d006322
Zm00001d006322_T001	0.9621927	0.151981722	Zm00001d006322
Zm00001d016856_T002	0.9429712	0.033531488	Zm00001d016856
Zm00001d016856_T011	0.000157029	-1.038611297	Zm00001d016856
Zm00001d016856_T003	0.8520487	-0.118166789	Zm00001d016856
Zm00001d016856_T007	0.9706887	0.810427664	Zm00001d016856
Zm00001d016856_T008	0.4003047	0.547317152	Zm00001d016856
Zm00001d016856_T005	0.9404125	0.017132819	Zm00001d016856
Zm00001d053065_T001	0.9781672	0.207896846	Zm00001d053065
Zm00001d053065_T002	0.1785712	-0.657439258	Zm00001d053065
Zm00001d036023_T001	0.8631191	0.76544042	Zm00001d036023
Zm00001d036006_T002	0.9969123	0.240517662	Zm00001d036006
Zm00001d008430_T005	0.9281046	0.379248026	Zm00001d008430
Zm00001d008430_T004	0.5925664	1.052345557	Zm00001d008430
Zm00001d008430_T006	0.9237921	-0.289432647	Zm00001d008430
Zm00001d008430_T003	0.9791729	0.339861448	Zm00001d008430
Zm00001d008430_T002	0.9210327	0.004263527	Zm00001d008430
Zm00001d009017_T004	0.9937567	0.200962314	Zm00001d009017
Zm00001d009017_T007	0.7146325	0.233268152	Zm00001d009017
Zm00001d009017_T006	0.229343	-0.839120332	Zm00001d009017
Zm00001d009017_T002	0.9914912	0.471058994	Zm00001d009017
Zm00001d009017_T003	0.01476527	-1.062000688	Zm00001d009017
Zm00001d026553_T001	1	0.431751298	Zm00001d026553
Zm00001d003505_T001	0.8940288	0.457722895	Zm00001d003505
Zm00001d017943_T001	0.7985128	-0.345877803	Zm00001d017943
Zm00001d029478_T001	0.9907928	0.391020393	Zm00001d029478
Zm00001d018575_T003	0.9798623	0.098204821	Zm00001d018575
Zm00001d039541_T003	0.9909153	0.234305124	Zm00001d039541
Zm00001d039541_T007	1	0.206588273	Zm00001d039541
Zm00001d039541_T006	0.3437881	0.560720102	Zm00001d039541
Zm00001d041025_T001	0.3183965	-0.456882694	Zm00001d041025
Zm00001d013113_T001	0.7924502	0.894125722	Zm00001d013113
Zm00001d049796_T001	0.7685707	-0.312982501	Zm00001d049796
Zm00001d020002_T002	0.9766874	0.037106769	Zm00001d020002
Zm00001d022480_T001	0.9701986	0.107292848	Zm00001d022480
Zm00001d026361_T002	0.9825858	0.266377178	Zm00001d026361
Zm00001d026361_T003	0.8751967	-0.467099562	Zm00001d026361
Zm00001d026361_T001	0.9706293	0.104446487	Zm00001d026361
Zm00001d012563_T001	0.8647484	-0.12671089	Zm00001d012563
Zm00001d031210_T002	0.9804228	0.212038091	Zm00001d031210
Zm00001d031210_T001	0.5988928	0.221690102	Zm00001d031210
Zm00001d049485_T001	0.9689939	0.211994347	Zm00001d049485
Zm00001d002063_T001	0.9979568	0.284762262	Zm00001d002063
Zm00001d029201_T001	0.9708541	0.318510993	Zm00001d029201
Zm00001d020264_T001	0.963405	0.592346234	Zm00001d020264
Zm00001d021705_T001	0.07550872	-1.729413529	Zm00001d021705

Zm00001d013669_T005	0.9673473	0.162905482	Zm00001d013669
Zm00001d013669_T001	0.9293689	0.612857768	Zm00001d013669
Zm00001d013669_T004	0.9825858	0.818594707	Zm00001d013669
Zm00001d019163_T001	0.9252465	0.064157459	Zm00001d019163
Zm00001d028118_T001	0.8686467	-0.243249294	Zm00001d028118
Zm00001d005149_T001	1	0.202103473	Zm00001d005149
Zm00001d028599_T001	0.07463567	1.987774058	Zm00001d028599
Zm00001d018237_T001	0.972861	0.188651535	Zm00001d018237
Zm00001d022437_T001	0.9520547	0.61085851	Zm00001d022437
Zm00001d015269_T003	0.147668	-1.323887599	Zm00001d015269
Zm00001d015269_T002	0.976488	0.199342021	Zm00001d015269
Zm00001d015269_T001	0.08908232	1.030808269	Zm00001d015269
Zm00001d024018_T003	0.04031304	-0.615689793	Zm00001d024018
Zm00001d024018_T001	0.9319754	-0.024850257	Zm00001d024018
Zm00001d024018_T002	0.4754745	0.741624316	Zm00001d024018
Zm00001d040034_T001	0.9519258	0.580335738	Zm00001d040034
Zm00001d032327_T001	0.977115	0.437195788	Zm00001d032327
Zm00001d039180_T001	0.9702242	0.204228002	Zm00001d039180
Zm00001d012615_T001	0.9945118	0.204656669	Zm00001d012615
Zm00001d035318_T001	0.9541039	0.025606933	Zm00001d035318
Zm00001d049522_T001	0.9469426	0.093811927	Zm00001d049522
Zm00001d049522_T002	0.8614638	0.617061623	Zm00001d049522
Zm00001d049522_T005	0.9070578	0.180940973	Zm00001d049522
Zm00001d049522_T006	0.851982	0.860160022	Zm00001d049522
Zm00001d041332_T001	0.255306	-1.726446419	Zm00001d041332
Zm00001d012814_T001	0.9107561	-0.616671801	Zm00001d012814
Zm00001d042643_T001	0.6329403	1.040770191	Zm00001d042643
Zm00001d042643_T002	0.7231383	1.01346246	Zm00001d042643
Zm00001d042118_T001	0.9428901	0.297810869	Zm00001d042118
Zm00001d029454_T010	0.9944696	0.010472635	Zm00001d029454
Zm00001d029454_T007	1	0.199319308	Zm00001d029454
Zm00001d029454_T001	0.2898764	0.830792179	Zm00001d029454
Zm00001d029454_T009	0.9216521	1.108070258	Zm00001d029454
Zm00001d039098_T001	0.9825858	-0.028471684	Zm00001d039098
Zm00001d014007_T001	0.9210513	0.596773807	Zm00001d014007
Zm00001d038510_T001	0.9954426	0.085358212	Zm00001d038510
Zm00001d043598_T004	0.8595436	0.890665198	Zm00001d043598
Zm00001d043598_T009	0.009054511	1.090227607	Zm00001d043598
Zm00001d043598_T005	2.44E-19	5.787983666	Zm00001d043598
Zm00001d043598_T001	0.05546987	1.916962324	Zm00001d043598
Zm00001d043598_T008	0.1285728	-0.900976494	Zm00001d043598
Zm00001d043598_T002	0.009407985	2.825738877	Zm00001d043598
Zm00001d038728_T002	0.2509097	0.590099357	Zm00001d038728
Zm00001d038728_T007	0.003943145	1.24572272	Zm00001d038728
Zm00001d038728_T013	0.965836	0.422064696	Zm00001d038728
Zm00001d038728_T001	0.2202602	0.454462093	Zm00001d038728
Zm00001d038728_T022	8.47E-07	0.736195504	Zm00001d038728
Zm00001d038728_T003	0.002860823	3.271828335	Zm00001d038728
Zm00001d038728_T014	0.06662569	0.651061029	Zm00001d038728
Zm00001d051869_T001	0.9914036	-0.063582608	Zm00001d051869
Zm00001d014530_T001	0.9766874	0.492396717	Zm00001d014530
Zm00001d036903_T003	0.9563257	0.133042507	Zm00001d036903
Zm00001d036903_T008	0.8092097	-0.256140221	Zm00001d036903
Zm00001d036903_T005	0.997586	0.057559594	Zm00001d036903

Zm00001d036903_T001	0.9432712	0.467988068	Zm00001d036903
Zm00001d015184_T001	0.6602663	-0.552451855	Zm00001d015184
Zm00001d053350_T001	0.9740325	0.085625168	Zm00001d053350
Zm00001d053350_T002	0.6858183	0.581813528	Zm00001d053350
Zm00001d028257_T003	0.4545925	-1.921424193	Zm00001d028257
Zm00001d028257_T001	0.7821468	1.926882588	Zm00001d028257
Zm00001d027493_T001	0.9237037	0.169138499	Zm00001d027493
Zm00001d012176_T001	0.5883605	1.12951546	Zm00001d012176
Zm00001d022266_T001	0.7778639	-0.368261216	Zm00001d022266
Zm00001d038685_T003	0.09384546	1.290626982	Zm00001d038685
Zm00001d038685_T006	0.4939103	0.568683696	Zm00001d038685
Zm00001d038685_T004	0.9575224	0.652088983	Zm00001d038685
Zm00001d013845_T001	0.966464	0.152019003	Zm00001d013845
Zm00001d013845_T012	0.9570177	0.021268547	Zm00001d013845
Zm00001d013845_T014	0.3603685	-0.888344013	Zm00001d013845
Zm00001d013845_T006	0.7788618	0.231532997	Zm00001d013845
Zm00001d050521_T003	0.7860927	0.459196744	Zm00001d050521
Zm00001d050521_T001	0.9050121	0.622910597	Zm00001d050521
Zm00001d013768_T001	0.8192173	-0.161193941	Zm00001d013768
Zm00001d013768_T003	0.9419096	0.093571726	Zm00001d013768
Zm00001d021972_T009	0.000635029	0.95909867	Zm00001d021972
Zm00001d021972_T007	0.7138265	0.230176368	Zm00001d021972
Zm00001d021972_T001	0.9693993	0.574242895	Zm00001d021972
Zm00001d021972_T006	0.000391153	0.962105602	Zm00001d021972
Zm00001d009108_T001	0.6896661	-0.439094524	Zm00001d009108
Zm00001d005803_T001	0.8746701	0.264704445	Zm00001d005803
Zm00001d021858_T006	1.24E-07	0.694785725	Zm00001d021858
Zm00001d021858_T001	0.9701986	0.663435208	Zm00001d021858
Zm00001d021858_T007	0.3596787	1.612161242	Zm00001d021858
Zm00001d021858_T003	0.2642794	1.430302061	Zm00001d021858
Zm00001d027599_T001	0.9942835	0.086898274	Zm00001d027599
Zm00001d018025_T001	0.8095248	1.000350087	Zm00001d018025
Zm00001d042059_T001	1	0.427990891	Zm00001d042059
Zm00001d006907_T002	0.8660897	-0.352357142	Zm00001d006907
Zm00001d006907_T003	0.8398749	-0.545332039	Zm00001d006907
Zm00001d048927_T001	0.03648535	1.112282515	Zm00001d048927
Zm00001d047881_T005	5.11E-05	-1.224660686	Zm00001d047881
Zm00001d047881_T001	0.9661155	0.690072323	Zm00001d047881
Zm00001d047881_T006	0.4236325	1.009275587	Zm00001d047881
Zm00001d047881_T003	0.4677796	0.862709224	Zm00001d047881
Zm00001d039363_T001	1	0.409158916	Zm00001d039363
Zm00001d044087_T001	0.9116127	-0.316306195	Zm00001d044087
Zm00001d044307_T001	0.9410688	0.054120608	Zm00001d044307
Zm00001d044307_T002	0.5570384	-0.280066957	Zm00001d044307
Zm00001d025823_T001	0.9543237	0.395613099	Zm00001d025823
Zm00001d040164_T002	0.9166771	-0.360683832	Zm00001d040164
Zm00001d042600_T001	0.8807115	-0.416883867	Zm00001d042600
Zm00001d021577_T001	0.3439954	-0.922960464	Zm00001d021577
Zm00001d028948_T001	0.9310389	0.616386769	Zm00001d028948
Zm00001d045173_T001	0.8412913	-0.339189839	Zm00001d045173
Zm00001d018873_T001	0.9050121	-0.075654395	Zm00001d018873
Zm00001d018873_T003	0.6568375	-0.214232197	Zm00001d018873
Zm00001d016034_T002	0.9907928	0.327456102	Zm00001d016034
Zm00001d002362_T001	0.8187452	0.456268748	Zm00001d002362

Zm00001d039417_T056	0.9046076	-0.13674324	Zm00001d039417
Zm00001d039417_T170	0.9264969	0.019263514	Zm00001d039417
Zm00001d039417_T110	0.8450197	0.450511903	Zm00001d039417
Zm00001d039417_T156	0.4786492	0.438307468	Zm00001d039417
Zm00001d039417_T053	4.77E-10	1.296355818	Zm00001d039417
Zm00001d039417_T160	0.9825858	0.129901824	Zm00001d039417
Zm00001d039417_T028	0.01993352	0.941430768	Zm00001d039417
Zm00001d039417_T161	0.9664406	-0.038477554	Zm00001d039417
Zm00001d039417_T139	0.2238491	0.394290739	Zm00001d039417
Zm00001d039417_T176	0.8377512	-0.138083073	Zm00001d039417
Zm00001d039417_T133	0.5327463	0.418702224	Zm00001d039417
Zm00001d039417_T136	0.9932006	0.167100549	Zm00001d039417
Zm00001d039417_T031	0.2398048	-0.414463676	Zm00001d039417
Zm00001d039417_T047	0.9562711	-0.023421278	Zm00001d039417
Zm00001d029756_T001	0.9240693	-0.291845581	Zm00001d029756
Zm00001d032750_T001	0.1336234	-0.596975648	Zm00001d032750
Zm00001d016465_T002	0.9825858	0.113869363	Zm00001d016465
Zm00001d039474_T003	0.01942718	1.329653144	Zm00001d039474
Zm00001d039474_T008	0.3563562	-0.487616464	Zm00001d039474
Zm00001d039474_T014	0.8452909	0.47014576	Zm00001d039474
Zm00001d052742_T003	0.9614723	0.420759846	Zm00001d052742
Zm00001d052742_T006	0.7135988	-0.573917236	Zm00001d052742
Zm00001d052742_T009	0.8518279	-0.08704131	Zm00001d052742
Zm00001d052742_T010	0.5437049	0.623172631	Zm00001d052742
Zm00001d052742_T008	0.7142696	0.931300889	Zm00001d052742
Zm00001d042137_T001	0.8530796	0.769837413	Zm00001d042137
Zm00001d042137_T003	0.6843488	0.683959314	Zm00001d042137
Zm00001d042137_T002	0.2329183	-0.662340827	Zm00001d042137
Zm00001d031911_T002	0.9506587	0.15020003	Zm00001d031911
Zm00001d031911_T001	0.9754972	0.423741691	Zm00001d031911
Zm00001d010892_T016	0.9693993	0.127028531	Zm00001d010892
Zm00001d010892_T019	0.07256374	-0.840829407	Zm00001d010892
Zm00001d010892_T002	0.7639258	0.186573718	Zm00001d010892
Zm00001d010892_T017	0.9237921	0.653846872	Zm00001d010892
Zm00001d010892_T026	0.8987811	-0.360257138	Zm00001d010892
Zm00001d010892_T015	0.9893214	0.147285148	Zm00001d010892
Zm00001d051018_T002	0.1011573	-1.554127247	Zm00001d051018
Zm00001d033189_T001	0.9746429	0.03718406	Zm00001d033189
Zm00001d041847_T002	0.9975882	0.287859183	Zm00001d041847
Zm00001d041847_T003	0.9942835	0.091486096	Zm00001d041847
Zm00001d029872_T002	0.3391928	1.684108283	Zm00001d029872
Zm00001d029872_T003	0.8276231	1.016457961	Zm00001d029872
Zm00001d029872_T006	0.000137405	2.789614695	Zm00001d029872
Zm00001d034876_T001	0.8446194	-0.130139201	Zm00001d034876
Zm00001d049232_T002	0.8752115	-0.105798676	Zm00001d049232
Zm00001d033563_T003	0.5512562	-0.305034562	Zm00001d033563
Zm00001d033563_T001	0.8256454	0.582790022	Zm00001d033563
Zm00001d046592_T003	0.9912886	0.336727296	Zm00001d046592
Zm00001d046592_T001	0.997449	0.208264888	Zm00001d046592
Zm00001d046592_T002	0.9706887	0.249923386	Zm00001d046592
Zm00001d005590_T001	0.006919847	-1.29415339	Zm00001d005590
Zm00001d005590_T002	0.8677129	-0.083233466	Zm00001d005590
Zm00001d007706_T001	1	0.294011101	Zm00001d007706
Zm00001d034770_T003	4.68E-05	2.956766057	Zm00001d034770

Zm00001d034770_T004	0.1757415	0.432005373	Zm00001d034770
Zm00001d034770_T030	1.16E-06	0.673284928	Zm00001d034770
Zm00001d034770_T045	0.4702812	1.564851424	Zm00001d034770
Zm00001d034770_T009	0.009100825	2.185530694	Zm00001d034770
Zm00001d034770_T020	0.9356767	0.053571404	Zm00001d034770
Zm00001d034770_T002	0.8310488	0.010088692	Zm00001d034770
Zm00001d034770_T017	0.4661639	0.441628402	Zm00001d034770
Zm00001d015103_T002	0.6786506	-0.681560818	Zm00001d015103
Zm00001d015103_T006	0.1140834	-0.498244309	Zm00001d015103
Zm00001d015103_T003	0.1197922	-0.64756377	Zm00001d015103
Zm00001d015103_T005	0.6260085	0.399632538	Zm00001d015103
Zm00001d015103_T007	1	0.237617256	Zm00001d015103
Zm00001d015103_T001	0.9984041	0.262835836	Zm00001d015103
Zm00001d010038_T001	0.5912135	0.973429555	Zm00001d010038
Zm00001d017371_T008	0.9988864	0.342375526	Zm00001d017371
Zm00001d017371_T005	0.9531608	0.649629323	Zm00001d017371
Zm00001d006344_T004	0.7594555	0.665049249	Zm00001d006344
Zm00001d006344_T002	0.997586	0.47442517	Zm00001d006344
Zm00001d006344_T005	0.9748733	0.370834762	Zm00001d006344
Zm00001d006344_T008	1	0.340499979	Zm00001d006344
Zm00001d050920_T002	0.7954824	-0.405197313	Zm00001d050920
Zm00001d050920_T001	0.8899623	-0.072771003	Zm00001d050920
Zm00001d026206_T001	0.8613022	-0.174393205	Zm00001d026206
Zm00001d050165_T001	0.8537544	-0.140475771	Zm00001d050165
Zm00001d027468_T002	0.5571558	0.008145599	Zm00001d027468
Zm00001d027468_T001	0.9513407	0.106757538	Zm00001d027468
Zm00001d001907_T001	0.2090746	1.70428539	Zm00001d001907
Zm00001d043900_T007	0.9887435	0.134536953	Zm00001d043900
Zm00001d043900_T003	0.1343369	0.591269374	Zm00001d043900
Zm00001d043900_T008	0.000858087	-1.113384497	Zm00001d043900
Zm00001d043900_T009	0.3725038	0.233575095	Zm00001d043900
Zm00001d043900_T018	0.9539869	0.281383882	Zm00001d043900
Zm00001d043900_T020	1	0.223177883	Zm00001d043900
Zm00001d043900_T006	7.79E-11	1.580079165	Zm00001d043900
Zm00001d043900_T019	0.1920062	0.462771539	Zm00001d043900
Zm00001d043900_T012	0.7979593	1.064869732	Zm00001d043900
Zm00001d039112_T001	0.6812088	-0.552864551	Zm00001d039112
Zm00001d021737_T001	0.9745777	0.203287577	Zm00001d021737
Zm00001d048104_T002	0.9269932	-0.003213803	Zm00001d048104
Zm00001d019968_T001	0.63821	0.924802392	Zm00001d019968
Zm00001d003975_T002	0.9486412	0.410775095	Zm00001d003975
Zm00001d003975_T001	0.9867452	0.160539178	Zm00001d003975
Zm00001d021551_T004	1	0.296277269	Zm00001d021551
Zm00001d039581_T001	0.6976102	-0.470777031	Zm00001d039581
Zm00001d041462_T001	0.8819727	0.84580666	Zm00001d041462
Zm00001d047848_T002	0.05001771	-1.462013909	Zm00001d047848
Zm00001d047848_T001	0.9907928	0.502870111	Zm00001d047848
Zm00001d010395_T002	1	0.272677157	Zm00001d010395
Zm00001d010395_T003	0.9493302	0.18339202	Zm00001d010395
Zm00001d044061_T001	0.6688463	-0.449247354	Zm00001d044061
Zm00001d033815_T001	0.941922	0.537396885	Zm00001d033815
Zm00001d024171_T001	0.7681379	-0.340749855	Zm00001d024171
Zm00001d024171_T002	0.9804228	0.086852973	Zm00001d024171
Zm00001d013019_T011	0.8740066	0.369275464	Zm00001d013019

Zm00001d013019_T014	0.9562711	-0.014675208	Zm00001d013019
Zm00001d013019_T002	0.6189382	1.352990506	Zm00001d013019
Zm00001d013019_T020	0.8964117	-0.041326257	Zm00001d013019
Zm00001d043195_T001	0.9977426	0.457583164	Zm00001d043195
Zm00001d046539_T002	0.9902733	0.48059044	Zm00001d046539
Zm00001d046539_T004	0.9493302	0.23349847	Zm00001d046539
Zm00001d021965_T001	0.7518152	-0.392105423	Zm00001d021965
Zm00001d028941_T001	0.08776846	2.00034786	Zm00001d028941
Zm00001d010638_T001	0.9734722	0.49858056	Zm00001d010638
Zm00001d004133_T001	0.909186	-0.08870011	Zm00001d004133
Zm00001d032804_T001	0.7560147	-0.692541831	Zm00001d032804
Zm00001d013222_T001	0.8008612	-0.54173626	Zm00001d013222
Zm00001d042793_T006	0.960712	0.130535608	Zm00001d042793
Zm00001d042793_T009	0.7682607	0.68882125	Zm00001d042793
Zm00001d042793_T011	3.73E-09	-1.018974368	Zm00001d042793
Zm00001d042793_T005	1.53E-10	1.337825335	Zm00001d042793
Zm00001d042793_T012	0.954492	0.458818486	Zm00001d042793
Zm00001d037038_T001	0.6250123	0.751050127	Zm00001d037038
Zm00001d002716_T002	0.9565533	0.177669032	Zm00001d002716
Zm00001d002716_T001	0.09247444	-1.293279412	Zm00001d002716
Zm00001d015152_T001	0.8173136	0.900547651	Zm00001d015152
Zm00001d015152_T002	0.9609806	0.116302835	Zm00001d015152
Zm00001d015152_T011	0.96672	0.074379566	Zm00001d015152
Zm00001d015152_T004	0.5549472	0.341126463	Zm00001d015152
Zm00001d017602_T001	0.1213982	-1.053759487	Zm00001d017602
Zm00001d025757_T002	0.6086477	1.201555565	Zm00001d025757
Zm00001d025757_T003	0.8637318	-0.085176989	Zm00001d025757
Zm00001d002884_T001	0.3550006	-1.386915876	Zm00001d002884
Zm00001d029336_T008	0.03277874	2.121115822	Zm00001d029336
Zm00001d029336_T010	0.2447447	0.394206643	Zm00001d029336
Zm00001d008406_T001	0.9786754	0.523177123	Zm00001d008406
Zm00001d026421_T001	1	0.11385134	Zm00001d026421
Zm00001d026421_T015	0.5092251	-0.32680052	Zm00001d026421
Zm00001d008977_T006	0.7296232	0.381279239	Zm00001d008977
Zm00001d008977_T004	0.9796529	0.184210404	Zm00001d008977
Zm00001d008977_T001	0.9946911	0.350244026	Zm00001d008977
Zm00001d008977_T008	0.7628188	-0.155635741	Zm00001d008977
Zm00001d032101_T001	0.352878	1.287590961	Zm00001d032101
Zm00001d032101_T002	0.4023858	0.434965108	Zm00001d032101
Zm00001d032101_T003	0.9690925	0.54015464	Zm00001d032101
Zm00001d053962_T002	0.9909466	0.406050699	Zm00001d053962
Zm00001d053962_T001	0.9950761	0.21125578	Zm00001d053962
Zm00001d039139_T001	0.281775	-0.840776756	Zm00001d039139
Zm00001d029030_T001	0.9953345	-0.044148305	Zm00001d029030
Zm00001d029669_T001	0.9673094	0.033487626	Zm00001d029669
Zm00001d021992_T001	0.01122327	2.442714485	Zm00001d021992
Zm00001d011697_T001	0.8836975	0.750873693	Zm00001d011697
Zm00001d039315_T014	0.630204	0.710465754	Zm00001d039315
Zm00001d039315_T003	0.9022629	0.656986453	Zm00001d039315
Zm00001d039315_T013	0.7616771	-0.22506559	Zm00001d039315
Zm00001d040193_T022	0.02530258	0.684437284	Zm00001d040193
Zm00001d040193_T047	0.003795493	-0.752860788	Zm00001d040193
Zm00001d040193_T011	0.6934931	0.216220085	Zm00001d040193
Zm00001d040193_T008	0.9354647	0.368493682	Zm00001d040193

Zm00001d040193_T063	0.9965026	0.23830475	Zm00001d040193
Zm00001d037945_T001	0.7847374	-0.416835701	Zm00001d037945
Zm00001d032036_T001	0.2694259	-1.188117252	Zm00001d032036
Zm00001d011928_T002	0.428401	-1.022143156	Zm00001d011928
Zm00001d011928_T001	0.745718	-0.356437699	Zm00001d011928
Zm00001d008460_T003	0.9184323	0.567394539	Zm00001d008460
Zm00001d012199_T009	0.8513643	0.576111088	Zm00001d012199
Zm00001d012199_T010	0.02539639	-2.393341812	Zm00001d012199
Zm00001d012199_T008	0.9770343	0.269079403	Zm00001d012199
Zm00001d012199_T003	0.6149869	1.22522448	Zm00001d012199
Zm00001d012199_T007	0.5186293	-0.127236772	Zm00001d012199
Zm00001d011357_T003	0.7697196	-0.236177054	Zm00001d011357
Zm00001d011357_T004	0.001372749	-2.295764868	Zm00001d011357
Zm00001d053886_T006	0.9994997	0.357194905	Zm00001d053886
Zm00001d053886_T004	0.7071453	0.462298057	Zm00001d053886
Zm00001d053886_T001	0.3962489	1.270170526	Zm00001d053886
Zm00001d042888_T001	0.9563232	-0.040500844	Zm00001d042888
Zm00001d028615_T001	0.9224387	0.026547107	Zm00001d028615
Zm00001d014783_T001	0.9889811	0.500846022	Zm00001d014783
Zm00001d003508_T001	0.9404125	0.739216277	Zm00001d003508
Zm00001d045733_T001	0.9160053	-0.204945242	Zm00001d045733
Zm00001d013934_T001	1	0.357217354	Zm00001d013934
Zm00001d013934_T002	0.4137215	-0.836740255	Zm00001d013934
Zm00001d008367_T001	0.8757823	-0.114016645	Zm00001d008367
Zm00001d008367_T002	0.8752644	-0.147540799	Zm00001d008367
Zm00001d029396_T002	1	0.146859093	Zm00001d029396
Zm00001d012896_T008	0.9825858	0.279319509	Zm00001d012896
Zm00001d012896_T003	0.969231	0.271723864	Zm00001d012896
Zm00001d012896_T005	0.9173494	-0.044676202	Zm00001d012896
Zm00001d012896_T001	0.7488344	-0.279576351	Zm00001d012896
Zm00001d012896_T004	0.9907928	0.056491512	Zm00001d012896
Zm00001d043512_T001	0.9972036	0.242857146	Zm00001d043512
Zm00001d032419_T001	0.8039111	-0.343585204	Zm00001d032419
Zm00001d036787_T001	1	-0.093481278	Zm00001d036787
Zm00001d034607_T001	0.8847217	-0.128322925	Zm00001d034607
Zm00001d034607_T003	0.7945048	-0.261099777	Zm00001d034607
Zm00001d007500_T049	0.967161	0.118557586	Zm00001d007500
Zm00001d007500_T051	7.51E-06	-2.751924427	Zm00001d007500
Zm00001d007500_T005	0.6436915	0.453121584	Zm00001d007500
Zm00001d007500_T008	0.05901609	2.029494024	Zm00001d007500
Zm00001d007500_T048	0.9898682	0.468268298	Zm00001d007500
Zm00001d007500_T046	0.8637863	0.415704957	Zm00001d007500
Zm00001d006555_T001	0.6511883	-0.452972836	Zm00001d006555
Zm00001d034018_T018	0.9840043	0.029336572	Zm00001d034018
Zm00001d034018_T022	0.3847344	0.76225716	Zm00001d034018
Zm00001d034018_T015	0.9833513	0.481251222	Zm00001d034018
Zm00001d034018_T002	0.01474121	1.167081308	Zm00001d034018
Zm00001d034018_T014	0.007837059	0.755012109	Zm00001d034018
Zm00001d034018_T020	0.1755327	0.365449618	Zm00001d034018
Zm00001d034018_T010	8.86E-11	1.811555395	Zm00001d034018
Zm00001d037381_T001	0.9221549	-0.348150412	Zm00001d037381
Zm00001d019816_T001	0.5715685	-0.76784891	Zm00001d019816
Zm00001d042507_T001	0.998484	0.436229419	Zm00001d042507
Zm00001d028282_T001	0.7326956	-0.398230971	Zm00001d028282

Zm00001d002663_T009	3.20E-11	-2.538267382	Zm00001d002663
Zm00001d002663_T007	0.7740061	0.903405347	Zm00001d002663
Zm00001d002663_T010	0.957148	0.247556391	Zm00001d002663
Zm00001d002663_T002	0.1387725	2.059562392	Zm00001d002663
Zm00001d002663_T006	0.965919	0.129266442	Zm00001d002663
Zm00001d014291_T001	0.8884173	-0.046561162	Zm00001d014291
Zm00001d020958_T002	3.69E-05	-3.214155645	Zm00001d020958
Zm00001d005822_T003	0.8641671	-0.085601706	Zm00001d005822
Zm00001d005822_T001	0.9514141	0.100676193	Zm00001d005822
Zm00001d032666_T002	0.07216589	-1.138905406	Zm00001d032666
Zm00001d032666_T001	0.8650248	0.781567667	Zm00001d032666
Zm00001d035543_T002	0.06542281	1.975256163	Zm00001d035543
Zm00001d035543_T003	0.4050212	0.318549944	Zm00001d035543
Zm00001d035543_T001	0.2460878	1.767441303	Zm00001d035543
Zm00001d052001_T014	0.005368589	-1.571672085	Zm00001d052001
Zm00001d052001_T004	0.2221685	0.413675899	Zm00001d052001
Zm00001d052001_T003	0.7697196	0.790386097	Zm00001d052001
Zm00001d016857_T001	0.04103975	-1.71164179	Zm00001d016857
Zm00001d022418_T003	0.4812187	-0.471960137	Zm00001d022418
Zm00001d022418_T002	0.9760472	-0.281609212	Zm00001d022418
Zm00001d002374_T001	0.03198968	-2.185936978	Zm00001d002374
Zm00001d003376_T002	0.9580985	0.35510524	Zm00001d003376
Zm00001d003376_T004	0.9988864	0.263396902	Zm00001d003376
Zm00001d003376_T006	0.9419336	0.531752919	Zm00001d003376
Zm00001d021695_T001	0.9909153	0.316857472	Zm00001d021695
Zm00001d000053_T001	1	0.33988412	Zm00001d000053
Zm00001d000053_T002	0.4226019	0.940717634	Zm00001d000053
Zm00001d002624_T004	0.9363242	0.296592777	Zm00001d002624
Zm00001d002624_T006	0.9419336	-0.029326179	Zm00001d002624
Zm00001d002624_T001	0.9335862	0.062595167	Zm00001d002624
Zm00001d003041_T001	0.9544149	0.607844385	Zm00001d003041
Zm00001d014493_T001	0.8863456	0.051709538	Zm00001d014493
Zm00001d042357_T002	0.7448452	-0.339282645	Zm00001d042357
Zm00001d037272_T001	0.9374953	0.694106483	Zm00001d037272
Zm00001d023241_T001	0.9981392	0.073748707	Zm00001d023241
Zm00001d044463_T001	0.7248132	0.712360898	Zm00001d044463
Zm00001d024633_T001	0.9817074	0.197250956	Zm00001d024633
Zm00001d024633_T011	0.8557049	0.565557107	Zm00001d024633
Zm00001d024633_T003	0.002613826	3.041965083	Zm00001d024633
Zm00001d024633_T004	1	0.370499906	Zm00001d024633
Zm00001d011269_T001	0.5793913	1.087771564	Zm00001d011269
Zm00001d051529_T002	0.9804228	0.314989441	Zm00001d051529
Zm00001d051529_T003	0.9732421	0.1928701	Zm00001d051529
Zm00001d020500_T001	0.9628812	0.606965723	Zm00001d020500
Zm00001d030960_T001	0.3294423	1.323439875	Zm00001d030960
Zm00001d030960_T031	0.9693993	0.858192005	Zm00001d030960
Zm00001d030960_T033	0.03848759	-1.465528382	Zm00001d030960
Zm00001d035031_T001	0.9730261	0.561306372	Zm00001d035031
Zm00001d035031_T006	0.5646517	1.161328451	Zm00001d035031
Zm00001d035031_T002	0.9664736	0.069770882	Zm00001d035031
Zm00001d035031_T004	0.5012564	-0.671101807	Zm00001d035031
Zm00001d035031_T005	0.9770343	0.359173522	Zm00001d035031
Zm00001d008665_T001	0.6205456	1.107492978	Zm00001d008665
Zm00001d034562_T002	0.8896831	0.890273519	Zm00001d034562

Zm00001d034562_T001	0.757295	1.014532541	Zm00001d034562
Zm00001d003068_T001	0.01128472	-1.331491095	Zm00001d003068
Zm00001d011078_T003	0.9909153	0.490757084	Zm00001d011078
Zm00001d025548_T006	0.7912425	-0.402919826	Zm00001d025548
Zm00001d025548_T001	0.7626242	1.08733238	Zm00001d025548
Zm00001d025548_T007	0.7223433	0.542226707	Zm00001d025548
Zm00001d025548_T002	0.08243644	1.259686557	Zm00001d025548
Zm00001d025548_T008	0.094843	1.237687985	Zm00001d025548
Zm00001d044395_T002	0.6572222	1.009358827	Zm00001d044395
Zm00001d044395_T001	0.9038102	0.737075292	Zm00001d044395
Zm00001d044395_T004	0.1916757	1.769350877	Zm00001d044395
Zm00001d044395_T005	0.9988864	0.106885103	Zm00001d044395
Zm00001d044395_T007	0.000294231	2.44816088	Zm00001d044395
Zm00001d025727_T007	0.9996112	0.489019811	Zm00001d025727
Zm00001d025727_T010	0.9303773	0.440603967	Zm00001d025727
Zm00001d025727_T006	0.9260032	0.354843365	Zm00001d025727
Zm00001d025727_T001	0.000196681	-3.230916821	Zm00001d025727
Zm00001d002519_T001	0.5711339	-0.653518275	Zm00001d002519
Zm00001d010604_T001	0.9959972	0.290214559	Zm00001d010604
Zm00001d010604_T006	0.8959807	0.753289064	Zm00001d010604
Zm00001d010604_T008	0.9880075	0.269618147	Zm00001d010604
Zm00001d010604_T002	0.7406865	0.221533103	Zm00001d010604
Zm00001d022565_T001	0.6721308	1.19805381	Zm00001d022565
Zm00001d022565_T002	0.01535282	0.555333885	Zm00001d022565
Zm00001d022498_T009	0.1827657	-1.460138822	Zm00001d022498
Zm00001d022498_T006	0.9984686	0.337286772	Zm00001d022498
Zm00001d022498_T005	0.9791729	0.175711495	Zm00001d022498
Zm00001d022498_T007	0.5006087	-0.488440173	Zm00001d022498
Zm00001d022498_T002	0.8580945	0.09625735	Zm00001d022498
Zm00001d047043_T001	0.9666757	0.050269192	Zm00001d047043
Zm00001d006939_T001	0.9441446	0.55679148	Zm00001d006939
Zm00001d010733_T004	0.9991964	0.00897557	Zm00001d010733
Zm00001d010733_T003	1	0.32335955	Zm00001d010733
Zm00001d010733_T005	0.9689939	0.555934505	Zm00001d010733
Zm00001d016933_T001	0.268975	1.791042475	Zm00001d016933
Zm00001d022081_T001	0.8658477	-0.095768288	Zm00001d022081
Zm00001d022081_T003	0.961399	0.495359537	Zm00001d022081
Zm00001d013699_T001	0.8894168	-0.025035254	Zm00001d013699
Zm00001d044518_T001	0.961473	0.474802523	Zm00001d044518
Zm00001d053576_T001	0.6362891	-0.44326073	Zm00001d053576
Zm00001d053576_T002	0.9931613	0.471354302	Zm00001d053576
Zm00001d037855_T001	0.7759478	0.322340777	Zm00001d037855
Zm00001d046938_T001	0.9263167	0.701124955	Zm00001d046938
Zm00001d022113_T004	0.9130008	0.026226658	Zm00001d022113
Zm00001d052221_T002	0.9237037	0.652596247	Zm00001d052221
Zm00001d052221_T003	0.8239342	-0.233151776	Zm00001d052221
Zm00001d052221_T004	0.8031436	-0.322088928	Zm00001d052221
Zm00001d025639_T006	0.9746429	0.531039674	Zm00001d025639
Zm00001d025639_T015	0.8884419	0.179074074	Zm00001d025639
Zm00001d025639_T009	0.4358046	0.947481855	Zm00001d025639
Zm00001d025639_T004	0.7911642	0.682728264	Zm00001d025639
Zm00001d025639_T012	0.8680274	-0.250010923	Zm00001d025639
Zm00001d045873_T002	0.954492	0.504318298	Zm00001d045873
Zm00001d023451_T001	0.2146312	0.985083948	Zm00001d023451

Zm00001d027921_T002	0.9139882	0.763281507	Zm00001d027921
Zm00001d027921_T003	0.9778436	0.418401995	Zm00001d027921
Zm00001d009055_T002	0.8561115	-0.126144695	Zm00001d009055
Zm00001d009055_T001	0.9390501	0.689028667	Zm00001d009055
Zm00001d018256_T001	0.1140834	-1.205109476	Zm00001d018256
Zm00001d013646_T001	0.8028573	-0.210922216	Zm00001d013646
Zm00001d020093_T002	0.8162418	0.635730861	Zm00001d020093
Zm00001d020093_T001	0.9336646	0.384295516	Zm00001d020093
Zm00001d009872_T008	0.9970835	0.343530468	Zm00001d009872
Zm00001d009872_T003	0.7353854	0.831944293	Zm00001d009872
Zm00001d020402_T002	0.01306269	-2.219289668	Zm00001d020402
Zm00001d020402_T001	0.7159087	-0.396218639	Zm00001d020402
Zm00001d006354_T002	0.1642615	0.455749003	Zm00001d006354
Zm00001d021626_T002	0.8413408	-0.402676837	Zm00001d021626
Zm00001d021626_T001	0.3394823	-0.329839513	Zm00001d021626
Zm00001d028361_T001	0.1244517	-1.123903517	Zm00001d028361
Zm00001d013251_T001	0.6735573	1.143476461	Zm00001d013251
Zm00001d034159_T003	0.7252858	-0.403157991	Zm00001d034159
Zm00001d034159_T002	0.3579474	-0.802973175	Zm00001d034159
Zm00001d011424_T007	0.09083989	1.39391931	Zm00001d011424
Zm00001d011424_T005	1	0.199327386	Zm00001d011424
Zm00001d011424_T001	0.9306419	0.71431314	Zm00001d011424
Zm00001d011424_T012	0.1417015	0.922208781	Zm00001d011424
Zm00001d011531_T002	0.8783081	-0.235554237	Zm00001d011531
Zm00001d011531_T001	0.8125642	-0.237045734	Zm00001d011531
Zm00001d014438_T003	0.9023635	0.596859289	Zm00001d014438
Zm00001d014438_T001	0.8598212	-0.131524319	Zm00001d014438
Zm00001d047324_T001	0.8580945	-0.132478161	Zm00001d047324
Zm00001d029725_T001	1	0.25342101	Zm00001d029725
Zm00001d018009_T011	0.9979568	0.319558557	Zm00001d018009
Zm00001d018009_T002	0.9979393	0.369335399	Zm00001d018009
Zm00001d028774_T001	0.9563232	0.149247798	Zm00001d028774
Zm00001d011063_T001	0.8537544	0.585662334	Zm00001d011063
Zm00001d023437_T001	0.8990393	0.919897534	Zm00001d023437
Zm00001d033345_T001	0.9745777	0.093907015	Zm00001d033345
Zm00001d033345_T005	0.1786485	0.375067238	Zm00001d033345
Zm00001d033345_T009	0.3748085	-1.073047669	Zm00001d033345
Zm00001d033345_T007	0.9984041	0.416082569	Zm00001d033345
Zm00001d025167_T013	0.05380117	1.198075038	Zm00001d025167
Zm00001d025167_T005	0.03925128	1.612651597	Zm00001d025167
Zm00001d025167_T014	0.8993746	0.736122123	Zm00001d025167
Zm00001d036617_T001	0.9344122	0.36233566	Zm00001d036617
Zm00001d002597_T001	0.8494782	-0.151641784	Zm00001d002597
Zm00001d002597_T003	0.9416089	0.545038923	Zm00001d002597
Zm00001d002597_T002	0.9966965	0.46223936	Zm00001d002597
Zm00001d012060_T001	0.9770343	0.219838149	Zm00001d012060
Zm00001d012060_T004	0.6721904	0.636736175	Zm00001d012060
Zm00001d011826_T001	0.9957676	-0.00961059	Zm00001d011826
Zm00001d003650_T001	0.9782953	0.026484016	Zm00001d003650
Zm00001d034664_T001	0.9768468	0.547060928	Zm00001d034664
Zm00001d012029_T001	0.9701986	0.104688147	Zm00001d012029
Zm00001d023238_T002	0.9404125	0.082047523	Zm00001d023238
Zm00001d023238_T003	0.4085315	-0.312833306	Zm00001d023238
Zm00001d023238_T010	0.4978708	1.185171098	Zm00001d023238

Zm00001d023238_T008	0.9037324	0.339096987	Zm00001d023238
Zm00001d043898_T001	0.8518279	-0.534566415	Zm00001d043898
Zm00001d015508_T001	1	0.107738175	Zm00001d015508
Zm00001d048766_T004	0.9894615	0.272107264	Zm00001d048766
Zm00001d019645_T001	0.6086477	-0.641491076	Zm00001d019645
Zm00001d054015_T001	0.1429392	-1.36346656	Zm00001d054015
Zm00001d039073_T001	0.9822138	0.145136298	Zm00001d039073
Zm00001d052906_T001	0.4413665	-0.696875079	Zm00001d052906
Zm00001d024732_T001	0.9868533	0.275430316	Zm00001d024732
Zm00001d045193_T001	0.9514141	0.063937218	Zm00001d045193
Zm00001d002259_T005	0.936423	-0.328270073	Zm00001d002259
Zm00001d002259_T004	0.9091175	-0.016132399	Zm00001d002259
Zm00001d002259_T001	0.2566009	-1.283567487	Zm00001d002259
Zm00001d042270_T002	0.8412913	-0.066842477	Zm00001d042270
Zm00001d006789_T002	0.6258249	0.684222829	Zm00001d006789
Zm00001d006789_T003	0.3497147	-0.36646943	Zm00001d006789
Zm00001d006789_T013	0.001282657	1.255438929	Zm00001d006789
Zm00001d006789_T019	0.00107198	2.603534719	Zm00001d006789
Zm00001d006789_T010	0.9374853	0.090777744	Zm00001d006789
Zm00001d006789_T024	0.4228909	-0.698759348	Zm00001d006789
Zm00001d006789_T035	0.6812088	0.692965916	Zm00001d006789
Zm00001d006789_T026	0.7497503	0.221085107	Zm00001d006789
Zm00001d026476_T001	1	0.234966309	Zm00001d026476
Zm00001d033375_T001	0.007072787	1.448244169	Zm00001d033375
Zm00001d033375_T015	0.000100728	1.150838287	Zm00001d033375
Zm00001d033375_T005	0.02913084	0.364798697	Zm00001d033375
Zm00001d033375_T006	0.2158642	0.398267728	Zm00001d033375
Zm00001d033375_T004	2.18E-06	-0.229684957	Zm00001d033375
Zm00001d033375_T043	0.08001694	0.421379644	Zm00001d033375
Zm00001d033375_T012	0.002930215	0.564475068	Zm00001d033375
Zm00001d033375_T013	0.995534	0.503625191	Zm00001d033375
Zm00001d016165_T041	2.50E-07	-0.934623293	Zm00001d016165
Zm00001d016165_T039	0.1317675	-0.35914991	Zm00001d016165
Zm00001d016165_T086	0.9487604	0.073338671	Zm00001d016165
Zm00001d003673_T001	0.9284215	0.730925541	Zm00001d003673
Zm00001d008722_T001	0.737932	-0.725392798	Zm00001d008722
Zm00001d007778_T002	0.919888	0.040298886	Zm00001d007778
Zm00001d007778_T001	0.9419336	0.082442402	Zm00001d007778
Zm00001d048501_T001	0.000319487	1.387695798	Zm00001d048501
Zm00001d048501_T008	1.70E-05	0.588686551	Zm00001d048501
Zm00001d048501_T010	0.4941853	0.415561245	Zm00001d048501
Zm00001d048501_T003	0.8964117	-0.110181186	Zm00001d048501
Zm00001d048501_T011	0.5211546	0.991012684	Zm00001d048501
Zm00001d010499_T001	0.4471095	1.170509079	Zm00001d010499
Zm00001d006567_T001	0.8630547	0.795886119	Zm00001d006567
Zm00001d006567_T005	0.2853109	-1.770789625	Zm00001d006567
Zm00001d006567_T003	0.9543237	0.273783276	Zm00001d006567
Zm00001d006567_T004	0.4063448	0.890128407	Zm00001d006567
Zm00001d032896_T001	0.5613757	-0.766492251	Zm00001d032896
Zm00001d038459_T002	0.5588038	1.124296277	Zm00001d038459
Zm00001d050076_T001	0.9969123	0.172336121	Zm00001d050076
Zm00001d048843_T001	0.6445834	-0.622066402	Zm00001d048843
Zm00001d006720_T001	0.5716153	-0.91492015	Zm00001d006720
Zm00001d043126_T001	0.0600926	0.521982917	Zm00001d043126

Zm00001d043126_T007	0.4929873	0.245002064	Zm00001d043126
Zm00001d043126_T005	0.9781672	0.535206064	Zm00001d043126
Zm00001d022581_T002	0.8688441	-0.069048878	Zm00001d022581
Zm00001d022581_T003	0.6123019	-0.207710382	Zm00001d022581
Zm00001d022342_T001	0.9651683	0.569832949	Zm00001d022342
Zm00001d011507_T001	0.4825966	-0.827661001	Zm00001d011507
Zm00001d014737_T001	0.3544526	-0.530598869	Zm00001d014737
Zm00001d002685_T001	0.8984709	-0.515293926	Zm00001d002685
Zm00001d041607_T001	1	0.274891925	Zm00001d041607
Zm00001d029545_T001	0.9901182	0.244969535	Zm00001d029545
Zm00001d029545_T003	0.830457	-0.224689133	Zm00001d029545
Zm00001d053275_T001	0.9604838	0.069686921	Zm00001d053275
Zm00001d011099_T006	0.9813678	1.875631294	Zm00001d011099
Zm00001d011099_T016	0.3376079	1.001093373	Zm00001d011099
Zm00001d011099_T002	0.09905547	2.590401889	Zm00001d011099
Zm00001d011099_T013	0.9221995	0.805761511	Zm00001d011099
Zm00001d029976_T008	0.004890922	2.451047354	Zm00001d029976
Zm00001d029976_T004	0.8412913	0.853000193	Zm00001d029976
Zm00001d029976_T009	7.36E-05	2.332917836	Zm00001d029976
Zm00001d029976_T003	0.00207132	1.000926113	Zm00001d029976
Zm00001d029976_T010	0.005900643	1.338129181	Zm00001d029976
Zm00001d029976_T005	0.8483996	0.82826282	Zm00001d029976
Zm00001d029976_T007	0.002796955	1.480316719	Zm00001d029976
Zm00001d053689_T001	0.1134387	-2.391656027	Zm00001d053689
Zm00001d038333_T001	0.1889665	-0.495544475	Zm00001d038333
Zm00001d023378_T001	0.9390517	0.598331223	Zm00001d023378
Zm00001d048877_T003	0.4331173	0.617088949	Zm00001d048877
Zm00001d048877_T001	0.06461983	1.938681743	Zm00001d048877
Zm00001d048877_T004	7.02E-15	-3.953429612	Zm00001d048877
Zm00001d048877_T002	0.7483641	1.069418515	Zm00001d048877
Zm00001d002799_T001	0.7169281	-0.345884913	Zm00001d002799
Zm00001d035383_T001	0.7946251	-0.541988118	Zm00001d035383
Zm00001d013302_T005	0.2916362	-1.051723649	Zm00001d013302
Zm00001d013302_T002	0.9201341	0.705751421	Zm00001d013302
Zm00001d013302_T004	0.9173494	-0.118017531	Zm00001d013302
Zm00001d013568_T001	0.3903214	-1.212772382	Zm00001d013568
Zm00001d036709_T001	0.9303773	-0.16109223	Zm00001d036709
Zm00001d007420_T001	0.282899	-1.298433409	Zm00001d007420
Zm00001d008614_T006	0.852305	0.420993909	Zm00001d008614
Zm00001d008614_T001	0.9894615	0.49049278	Zm00001d008614
Zm00001d008614_T007	0.9766874	-0.0376397	Zm00001d008614
Zm00001d008614_T004	0.6169231	0.247718085	Zm00001d008614
Zm00001d043240_T003	0.9701986	0.173518199	Zm00001d043240
Zm00001d043240_T005	0.4478151	-1.276197243	Zm00001d043240
Zm00001d043240_T004	0.7379612	-0.347753064	Zm00001d043240
Zm00001d043240_T002	0.6955134	-0.219005042	Zm00001d043240
Zm00001d031925_T001	0.9380783	0.004414225	Zm00001d031925
Zm00001d032304_T003	0.8641671	-0.11174645	Zm00001d032304
Zm00001d012865_T027	0.5231196	0.421805592	Zm00001d012865
Zm00001d012865_T014	0.5437196	1.131292638	Zm00001d012865
Zm00001d012865_T031	0.8992258	-0.111832032	Zm00001d012865
Zm00001d012865_T026	0.8883561	-0.1548443	Zm00001d012865
Zm00001d012865_T010	0.987516	0.528294987	Zm00001d012865
Zm00001d012865_T007	0.9570268	0.208079992	Zm00001d012865

Zm00001d012865_T012	0.7626242	-0.267939043	Zm00001d012865
Zm00001d012865_T006	0.9982897	0.372005625	Zm00001d012865
Zm00001d047069_T001	0.2917536	1.599497971	Zm00001d047069
Zm00001d019182_T001	0.9782953	0.204917001	Zm00001d019182
Zm00001d008279_T001	0.6258249	-0.463912605	Zm00001d008279
Zm00001d012090_T001	0.7289978	0.584715623	Zm00001d012090
Zm00001d045146_T005	0.08760185	-2.066137489	Zm00001d045146
Zm00001d045146_T006	0.7930026	0.237306808	Zm00001d045146
Zm00001d045146_T003	0.7626242	-0.195681863	Zm00001d045146
Zm00001d045146_T002	0.9768802	0.499279997	Zm00001d045146
Zm00001d025567_T001	0.04535247	-1.716759215	Zm00001d025567
Zm00001d051873_T007	0.04883176	0.563791813	Zm00001d051873
Zm00001d051873_T004	0.8717403	0.347175414	Zm00001d051873
Zm00001d051873_T008	0.4378952	0.830977138	Zm00001d051873
Zm00001d051873_T003	0.7746801	-0.245635105	Zm00001d051873
Zm00001d051873_T005	0.3756254	0.650305477	Zm00001d051873
Zm00001d051873_T001	1	0.257582357	Zm00001d051873
Zm00001d051873_T006	0.3812385	0.779508442	Zm00001d051873
Zm00001d033830_T002	0.9979568	0.427273596	Zm00001d033830
Zm00001d009912_T004	0.9887889	0.148466564	Zm00001d009912
Zm00001d009912_T003	0.9822168	0.228363529	Zm00001d009912
Zm00001d009912_T002	0.1616209	1.192956224	Zm00001d009912
Zm00001d031239_T013	0.9265089	0.233126214	Zm00001d031239
Zm00001d031239_T001	0.9524127	0.07105673	Zm00001d031239
Zm00001d031239_T007	0.01628133	0.765371882	Zm00001d031239
Zm00001d012850_T001	0.6173709	1.241727082	Zm00001d012850
Zm00001d017817_T001	0.01048202	-2.183783776	Zm00001d017817
Zm00001d029352_T001	0.9884825	0.303407594	Zm00001d029352
Zm00001d029352_T004	0.4510869	0.405398514	Zm00001d029352
Zm00001d048982_T001	0.967161	0.330008304	Zm00001d048982
Zm00001d023734_T004	0.6983911	-0.489264674	Zm00001d023734
Zm00001d023734_T003	0.9179118	-0.115915439	Zm00001d023734
Zm00001d023734_T005	0.005618118	-2.737465065	Zm00001d023734
Zm00001d053245_T002	0.8310488	0.897345379	Zm00001d053245
Zm00001d054081_T001	0.5908824	1.125983238	Zm00001d054081
Zm00001d041488_T004	0.987516	0.207230837	Zm00001d041488
Zm00001d041488_T005	0.7428653	1.081681564	Zm00001d041488
Zm00001d021836_T001	0.2662835	-1.238227875	Zm00001d021836
Zm00001d044288_T001	0.9979568	0.262354484	Zm00001d044288
Zm00001d044578_T001	0.9887769	0.170371763	Zm00001d044578
Zm00001d023933_T001	0.1876744	-1.170847491	Zm00001d023933
Zm00001d013053_T003	0.1029879	-0.840105337	Zm00001d013053
Zm00001d013053_T001	0.05881897	-1.589133661	Zm00001d013053
Zm00001d006390_T001	0.7236841	0.86448813	Zm00001d006390
Zm00001d034799_T003	0.9804228	0.547027545	Zm00001d034799
Zm00001d034799_T006	0.8022617	-0.301909333	Zm00001d034799
Zm00001d034799_T004	0.869008	-0.155686592	Zm00001d034799
Zm00001d008189_T008	0.9682673	0.711227639	Zm00001d008189
Zm00001d008189_T003	0.9771927	0.393474508	Zm00001d008189
Zm00001d008189_T006	0.5910981	-0.497891054	Zm00001d008189
Zm00001d008189_T002	0.2660558	-0.263608321	Zm00001d008189
Zm00001d008189_T001	0.836612	0.722689784	Zm00001d008189
Zm00001d008189_T005	2.62E-17	5.772725694	Zm00001d008189
Zm00001d004270_T003	0.3358121	-1.075295122	Zm00001d004270

Zm00001d004270_T001	0.8941695	-0.138831524	Zm00001d004270
Zm00001d007164_T001	0.6841106	0.913274592	Zm00001d007164
Zm00001d001870_T001	0.9514141	0.557473836	Zm00001d001870
Zm00001d028388_T001	0.8648059	0.431592163	Zm00001d028388
Zm00001d008960_T001	0.4366553	-0.730578786	Zm00001d008960
Zm00001d031416_T001	0.7020458	-0.232842051	Zm00001d031416
Zm00001d011217_T001	0.9823248	-0.002170471	Zm00001d011217
Zm00001d019036_T074	0.1763213	-1.197415838	Zm00001d019036
Zm00001d019036_T007	0.03938203	0.751019117	Zm00001d019036
Zm00001d019036_T033	0.4132629	-0.35229448	Zm00001d019036
Zm00001d019036_T015	0.1701649	0.930987219	Zm00001d019036
Zm00001d019036_T047	0.02483077	0.671566935	Zm00001d019036
Zm00001d019036_T044	0.6441703	0.299482141	Zm00001d019036
Zm00001d019036_T073	0.8580945	-0.145451805	Zm00001d019036
Zm00001d019036_T056	0.9827298	0.231619791	Zm00001d019036
Zm00001d019036_T024	0.4044152	0.519396941	Zm00001d019036
Zm00001d019036_T045	0.1203427	0.64096311	Zm00001d019036
Zm00001d019036_T011	0.9037002	0.025460554	Zm00001d019036
Zm00001d019036_T041	0.2153407	0.468094962	Zm00001d019036
Zm00001d019036_T022	0.9173494	-0.086862559	Zm00001d019036
Zm00001d019036_T040	0.1802911	0.548588281	Zm00001d019036
Zm00001d019036_T027	0.8883561	0.306281994	Zm00001d019036
Zm00001d019036_T042	0.2451289	0.746023873	Zm00001d019036
Zm00001d011138_T002	0.9779656	0.592592164	Zm00001d011138
Zm00001d011138_T004	0.7085991	-0.331668576	Zm00001d011138
Zm00001d041626_T001	0.9552021	0.592325848	Zm00001d041626
Zm00001d052386_T001	0.2461544	0.797928137	Zm00001d052386
Zm00001d037410_T001	0.908501	-0.104912862	Zm00001d037410
Zm00001d004088_T002	0.851347	0.540785579	Zm00001d004088
Zm00001d004088_T004	0.8807115	-0.702118706	Zm00001d004088
Zm00001d039946_T001	0.6502105	1.181602801	Zm00001d039946
Zm00001d029526_T001	0.9533693	0.607176923	Zm00001d029526
Zm00001d016935_T001	0.9090332	-0.175291584	Zm00001d016935
Zm00001d049455_T003	0.9673473	0.648408292	Zm00001d049455
Zm00001d044488_T002	0.9127665	0.683633049	Zm00001d044488
Zm00001d044488_T005	0.266766	-0.915817353	Zm00001d044488
Zm00001d038982_T012	1	0.085231509	Zm00001d038982
Zm00001d038982_T011	0.2047087	1.140006185	Zm00001d038982
Zm00001d038982_T003	0.8865278	-0.044491685	Zm00001d038982
Zm00001d038982_T024	0.4193179	1.604294172	Zm00001d038982
Zm00001d038257_T002	0.7889615	-0.079962058	Zm00001d038257
Zm00001d038257_T020	0.8264567	-0.193776455	Zm00001d038257
Zm00001d038257_T021	0.5589324	0.502378537	Zm00001d038257
Zm00001d038257_T006	0.9377241	0.420864675	Zm00001d038257
Zm00001d046574_T001	0.8513643	1.125200555	Zm00001d046574
Zm00001d012878_T001	0.6720294	-0.560450949	Zm00001d012878
Zm00001d025640_T001	0.9031129	-0.014543404	Zm00001d025640
Zm00001d025640_T002	1	0.179164752	Zm00001d025640
Zm00001d023553_T001	0.6354704	0.377617305	Zm00001d023553
Zm00001d021958_T006	1	0.273082469	Zm00001d021958
Zm00001d021958_T001	1	0.047610438	Zm00001d021958
Zm00001d049043_T001	0.9455514	0.039554021	Zm00001d049043
Zm00001d053988_T001	0.2304806	-1.713546882	Zm00001d053988
Zm00001d016085_T001	0.3221433	-1.288871758	Zm00001d016085

Zm00001d027918_T004	0.6906858	0.245827049	Zm00001d027918
Zm00001d027918_T007	0.9797082	0.044861912	Zm00001d027918
Zm00001d035741_T002	0.9528476	0.383757072	Zm00001d035741
Zm00001d012085_T003	0.9112545	-0.028048321	Zm00001d012085
Zm00001d012085_T004	1.65E-12	2.887005377	Zm00001d012085
Zm00001d012085_T001	0.8032834	-0.377940241	Zm00001d012085
Zm00001d012085_T002	0.9582109	0.604942259	Zm00001d012085
Zm00001d044366_T003	0.5717645	0.996314425	Zm00001d044366
Zm00001d044366_T002	0.9368844	0.608673263	Zm00001d044366
Zm00001d030385_T001	0.3615735	-0.391569811	Zm00001d030385
Zm00001d030385_T007	0.9720425	0.146433048	Zm00001d030385
Zm00001d030385_T005	0.5023988	0.617880116	Zm00001d030385
Zm00001d030385_T003	0.9918302	0.174467079	Zm00001d030385
Zm00001d032400_T001	0.9722469	0.003243527	Zm00001d032400
Zm00001d045434_T005	0.9970835	0.275496235	Zm00001d045434
Zm00001d045434_T003	0.9210235	0.694955242	Zm00001d045434
Zm00001d045434_T001	0.1547415	0.702013033	Zm00001d045434
Zm00001d053079_T001	0.000837816	2.473215784	Zm00001d053079
Zm00001d025610_T001	0.9989936	0.077505806	Zm00001d025610
Zm00001d030527_T001	0.9843321	0.527281333	Zm00001d030527
Zm00001d030527_T002	0.9024215	0.466686081	Zm00001d030527
Zm00001d013825_T001	0.8881183	0.49968569	Zm00001d013825
Zm00001d039548_T001	0.9399441	-0.038714633	Zm00001d039548
Zm00001d006461_T002	0.8987811	0.379106835	Zm00001d006461
Zm00001d006461_T001	0.9293689	0.078319304	Zm00001d006461
Zm00001d006461_T005	0.9722469	0.399490015	Zm00001d006461
Zm00001d008491_T002	1	0.340747527	Zm00001d008491
Zm00001d010622_T005	0.9887584	0.375848262	Zm00001d010622
Zm00001d010622_T004	0.8027057	-0.341077709	Zm00001d010622
Zm00001d010622_T001	0.9851204	0.10434457	Zm00001d010622
Zm00001d037905_T002	0.9773181	0.173377285	Zm00001d037905
Zm00001d037905_T003	0.8620331	1.338757529	Zm00001d037905
Zm00001d037905_T001	0.1402257	1.256284275	Zm00001d037905
Zm00001d037905_T015	0.9272378	-0.077550369	Zm00001d037905
Zm00001d037905_T014	0.8806256	-0.085839448	Zm00001d037905
Zm00001d037905_T012	0.9968176	0.922220155	Zm00001d037905
Zm00001d037905_T006	0.4298304	1.338899381	Zm00001d037905
Zm00001d037905_T004	1.82E-07	-2.757247296	Zm00001d037905
Zm00001d042851_T009	0.6761847	1.163960362	Zm00001d042851
Zm00001d042851_T008	0.04489514	0.969227969	Zm00001d042851
Zm00001d042851_T003	1	0.378279258	Zm00001d042851
Zm00001d014696_T001	0.8922781	-0.084514037	Zm00001d014696
Zm00001d014696_T003	0.9160053	0.332576115	Zm00001d014696
Zm00001d014696_T002	0.9539869	0.056967605	Zm00001d014696
Zm00001d034828_T001	0.9862019	0.126943484	Zm00001d034828
Zm00001d046450_T001	1	0.34037515	Zm00001d046450
Zm00001d032470_T001	0.9209394	0.070969457	Zm00001d032470
Zm00001d032470_T002	0.5327484	0.966205376	Zm00001d032470
Zm00001d040094_T004	0.1330942	-1.034358286	Zm00001d040094
Zm00001d040094_T003	0.8643636	-0.062901747	Zm00001d040094
Zm00001d025580_T001	0.9822138	0.50725747	Zm00001d025580
Zm00001d048349_T002	0.8341208	-0.187978663	Zm00001d048349
Zm00001d029123_T002	0.9377241	-0.008230772	Zm00001d029123
Zm00001d029123_T005	0.9321001	-0.03635628	Zm00001d029123

Zm00001d029123_T001	0.7853827	0.363644867	Zm00001d029123
Zm00001d048632_T001	0.9988864	0.463123423	Zm00001d048632
Zm00001d048632_T005	0.8660897	0.186396056	Zm00001d048632
Zm00001d032353_T001	1	0.273721104	Zm00001d032353
Zm00001d049692_T001	0.9573272	-0.062359846	Zm00001d049692
Zm00001d049692_T003	1	0.202868505	Zm00001d049692
Zm00001d048467_T001	0.7682041	-0.274891191	Zm00001d048467
Zm00001d028685_T001	0.146312	-1.189362783	Zm00001d028685
Zm00001d038964_T001	1	0.292335363	Zm00001d038964
Zm00001d039160_T001	0.888502	0.521837142	Zm00001d039160
Zm00001d039160_T010	0.9567316	0.015583917	Zm00001d039160
Zm00001d039160_T009	0.6088201	-0.430370684	Zm00001d039160
Zm00001d039160_T025	6.53E-06	2.967305478	Zm00001d039160
Zm00001d039160_T032	0.001774985	0.784908192	Zm00001d039160
Zm00001d039160_T021	2.05E-05	2.009691285	Zm00001d039160
Zm00001d014613_T001	0.8348638	-0.467694922	Zm00001d014613
Zm00001d014419_T001	1	0.380529294	Zm00001d014419
Zm00001d052258_T001	0.9694613	0.113627438	Zm00001d052258
Zm00001d021195_T002	0.6086477	1.241047922	Zm00001d021195
Zm00001d006757_T009	0.8054377	-0.193954214	Zm00001d006757
Zm00001d034067_T001	0.1731797	-0.877382375	Zm00001d034067
Zm00001d045405_T001	0.9219195	-0.082761928	Zm00001d045405
Zm00001d026254_T001	0.7877461	-0.497929884	Zm00001d026254
Zm00001d020541_T005	0.4757598	0.274104213	Zm00001d020541
Zm00001d020541_T010	0.261866	-0.295295794	Zm00001d020541
Zm00001d020541_T012	0.3071359	-0.831686316	Zm00001d020541
Zm00001d020541_T014	0.8160463	-0.061467857	Zm00001d020541
Zm00001d020541_T008	0.1592566	1.179327739	Zm00001d020541
Zm00001d020541_T011	0.9792387	0.046045105	Zm00001d020541
Zm00001d020541_T013	0.9580876	0.120639981	Zm00001d020541
Zm00001d013371_T004	0.9753621	0.310963111	Zm00001d013371
Zm00001d013371_T001	0.8142464	-0.149833016	Zm00001d013371
Zm00001d013371_T008	0.8593984	0.424723051	Zm00001d013371
Zm00001d013371_T002	0.9657307	0.60907317	Zm00001d013371
Zm00001d013371_T009	7.79E-11	2.740931427	Zm00001d013371
Zm00001d020175_T002	0.09320356	1.533081012	Zm00001d020175
Zm00001d020175_T001	0.9771927	0.616732096	Zm00001d020175
Zm00001d036902_T001	0.7853827	-0.223933186	Zm00001d036902
Zm00001d025756_T001	1	0.016026776	Zm00001d025756
Zm00001d036919_T001	0.8224042	0.066083191	Zm00001d036919
Zm00001d036919_T016	0.02676713	-1.879944309	Zm00001d036919
Zm00001d036919_T004	0.9954444	0.481282445	Zm00001d036919
Zm00001d014625_T001	0.9974734	0.284981832	Zm00001d014625
Zm00001d021667_T001	0.8788331	-0.142746948	Zm00001d021667
Zm00001d047612_T002	0.9451281	0.123851362	Zm00001d047612
Zm00001d047612_T001	0.9440782	0.060916925	Zm00001d047612
Zm00001d047403_T001	0.5957674	1.183023573	Zm00001d047403
Zm00001d043286_T001	0.8670002	0.029690904	Zm00001d043286
Zm00001d043286_T004	0.8958317	0.435807655	Zm00001d043286
Zm00001d043286_T006	0.8868226	0.765178734	Zm00001d043286
Zm00001d043286_T011	0.000815476	-1.984667924	Zm00001d043286
Zm00001d043286_T012	0.6138326	-0.448617925	Zm00001d043286
Zm00001d043286_T002	0.8616387	0.312214558	Zm00001d043286
Zm00001d006153_T009	0.9869996	-0.244955806	Zm00001d006153

Zm00001d006153_T003	0.2684967	1.811995398	Zm00001d006153
Zm00001d006153_T007	0.05301273	1.947459883	Zm00001d006153
Zm00001d006153_T008	0.2640227	1.579812988	Zm00001d006153
Zm00001d006153_T002	0.03585832	0.701851945	Zm00001d006153
Zm00001d017746_T001	0.9274507	0.659045249	Zm00001d017746
Zm00001d044878_T002	0.9219195	0.589564381	Zm00001d044878
Zm00001d034433_T001	0.01686346	-2.490030655	Zm00001d034433
Zm00001d034433_T005	0.8728536	-0.198889958	Zm00001d034433
Zm00001d034433_T007	0.4108283	0.572968418	Zm00001d034433
Zm00001d034433_T004	0.6523514	0.94877398	Zm00001d034433
Zm00001d034433_T010	0.9689939	0.524387234	Zm00001d034433
Zm00001d034433_T002	0.8704456	0.92741896	Zm00001d034433
Zm00001d031688_T001	0.9781938	0.16356362	Zm00001d031688
Zm00001d036965_T001	0.9963675	0.166879055	Zm00001d036965
Zm00001d036965_T003	0.7149448	1.010010499	Zm00001d036965
Zm00001d036215_T001	0.050072	1.114943693	Zm00001d036215
Zm00001d036215_T003	0.9846714	0.521441287	Zm00001d036215
Zm00001d036215_T002	0.6805241	-0.385202776	Zm00001d036215
Zm00001d017306_T002	0.8985569	0.562195409	Zm00001d017306
Zm00001d042810_T001	0.6053962	0.338884405	Zm00001d042810
Zm00001d042810_T002	0.9213556	0.191567617	Zm00001d042810
Zm00001d007279_T001	0.9493302	0.09491523	Zm00001d007279
Zm00001d006872_T001	0.9975882	0.349680409	Zm00001d006872
Zm00001d000435_T017	0.8193651	-0.336191118	Zm00001d000435
Zm00001d000435_T001	0.9906533	0.109816408	Zm00001d000435
Zm00001d000435_T009	0.651814	-0.280106585	Zm00001d000435
Zm00001d000435_T014	0.9390517	0.73081627	Zm00001d000435
Zm00001d000435_T002	0.9904282	0.211308469	Zm00001d000435
Zm00001d000435_T006	0.9288479	0.025226635	Zm00001d000435
Zm00001d000435_T007	0.5526067	0.148798022	Zm00001d000435
Zm00001d000435_T004	0.9887584	0.272316025	Zm00001d000435
Zm00001d000435_T012	5.34E-08	0.877057105	Zm00001d000435
Zm00001d046399_T001	0.9607621	0.650171876	Zm00001d046399
Zm00001d045698_T001	0.4882783	-1.366282771	Zm00001d045698
Zm00001d036567_T001	0.7813925	-0.675242799	Zm00001d036567
Zm00001d030028_T001	0.5626372	1.401538201	Zm00001d030028
Zm00001d049487_T001	0.9091907	0.047487325	Zm00001d049487
Zm00001d040123_T044	0.1112801	1.968532094	Zm00001d040123
Zm00001d040123_T006	0.9326774	-0.048535756	Zm00001d040123
Zm00001d040123_T010	0.6108895	1.059055284	Zm00001d040123
Zm00001d040123_T005	0.1626072	0.84427529	Zm00001d040123
Zm00001d040123_T019	0.07985658	-0.418705902	Zm00001d040123
Zm00001d040123_T001	0.5695353	1.285940423	Zm00001d040123
Zm00001d040123_T003	0.1668576	-0.693750611	Zm00001d040123
Zm00001d040123_T014	5.43E-09	0.794105617	Zm00001d040123
Zm00001d034455_T001	0.9954426	0.277054601	Zm00001d034455
Zm00001d052451_T001	0.5496315	-0.617647035	Zm00001d052451
Zm00001d025020_T001	0.6260085	0.787675046	Zm00001d025020
Zm00001d025020_T002	1	0.130321209	Zm00001d025020
Zm00001d025020_T004	0.8605736	0.510964471	Zm00001d025020
Zm00001d025020_T003	0.9771927	0.05611515	Zm00001d025020
Zm00001d006823_T001	0.9293689	0.064643158	Zm00001d006823
Zm00001d053018_T002	0.190471	-1.176089218	Zm00001d053018
Zm00001d053018_T004	0.960704	0.119084487	Zm00001d053018

Zm00001d053018_T001	0.8730771	0.31172509	Zm00001d053018
Zm00001d032399_T001	0.9521263	0.432228828	Zm00001d032399
Zm00001d034986_T001	0.6386998	-0.283270516	Zm00001d034986
Zm00001d047464_T016	0.5369152	0.629520899	Zm00001d047464
Zm00001d047464_T017	0.7333809	-0.188192662	Zm00001d047464
Zm00001d047464_T028	0.11023	-0.089369679	Zm00001d047464
Zm00001d047464_T026	0.9927847	0.573958993	Zm00001d047464
Zm00001d047464_T001	0.08939828	2.336955868	Zm00001d047464
Zm00001d047464_T027	0.9878022	0.867444924	Zm00001d047464
Zm00001d034004_T056	0.4876711	1.288515484	Zm00001d034004
Zm00001d034004_T015	0.7725069	-0.0139754	Zm00001d034004
Zm00001d003617_T001	0.67913	0.791201354	Zm00001d003617
Zm00001d027314_T003	0.9196877	-0.339202212	Zm00001d027314
Zm00001d027314_T002	0.712565	1.068556337	Zm00001d027314
Zm00001d027314_T001	0.9907928	0.119995275	Zm00001d027314
Zm00001d013421_T003	0.9469426	0.305786072	Zm00001d013421
Zm00001d013421_T008	0.8667916	0.314017533	Zm00001d013421
Zm00001d013421_T001	1	0.197940435	Zm00001d013421
Zm00001d013421_T007	0.9840918	0.503623255	Zm00001d013421
Zm00001d013421_T005	0.7757173	0.687163341	Zm00001d013421
Zm00001d013421_T002	0.9269932	0.159173454	Zm00001d013421
Zm00001d005563_T003	0.9706887	0.059622272	Zm00001d005563
Zm00001d005563_T006	0.9262099	0.475631368	Zm00001d005563
Zm00001d005563_T008	0.9941329	0.165113178	Zm00001d005563
Zm00001d003181_T005	0.9257893	0.036115555	Zm00001d003181
Zm00001d003181_T003	0.6519429	-0.792047455	Zm00001d003181
Zm00001d003181_T002	0.7921111	-0.256357302	Zm00001d003181
Zm00001d013857_T007	0.007004906	0.983430149	Zm00001d013857
Zm00001d013857_T005	0.000294231	1.347393426	Zm00001d013857
Zm00001d013857_T008	0.923446	0.039558139	Zm00001d013857
Zm00001d013857_T009	0.9910068	0.190173264	Zm00001d013857
Zm00001d013857_T004	0.9717919	0.078005478	Zm00001d013857
Zm00001d013857_T006	0.957265	0.091979323	Zm00001d013857
Zm00001d027619_T002	0.9965998	0.428790147	Zm00001d027619
Zm00001d034596_T001	0.8379797	-0.602415159	Zm00001d034596
Zm00001d006168_T006	0.7481738	0.52424143	Zm00001d006168
Zm00001d006168_T009	0.3777631	0.595484577	Zm00001d006168
Zm00001d038178_T001	0.8835073	0.49418584	Zm00001d038178
Zm00001d029839_T004	0.8107637	-0.057672984	Zm00001d029839
Zm00001d029839_T005	0.5363283	-0.502207664	Zm00001d029839
Zm00001d029839_T007	0.000463721	-1.279409689	Zm00001d029839
Zm00001d029839_T003	6.81E-10	-2.12024455	Zm00001d029839
Zm00001d029839_T002	0.5408146	1.258999762	Zm00001d029839
Zm00001d031223_T002	0.2665463	-1.157984991	Zm00001d031223
Zm00001d031223_T001	0.9402255	0.080589508	Zm00001d031223
Zm00001d031223_T003	0.925138	-0.015651814	Zm00001d031223
Zm00001d040146_T002	0.9570268	0.027208417	Zm00001d040146
Zm00001d040146_T001	0.9864707	0.352098966	Zm00001d040146
Zm00001d050928_T002	0.9099617	0.356455664	Zm00001d050928
Zm00001d050928_T007	0.470047	-0.380071626	Zm00001d050928
Zm00001d050928_T005	0.9032606	0.368765559	Zm00001d050928
Zm00001d050928_T001	0.5606978	0.261049459	Zm00001d050928
Zm00001d050928_T003	0.9469426	0.078577794	Zm00001d050928
Zm00001d020705_T001	0.1106445	1.358521795	Zm00001d020705

Zm00001d013995_T001	0.1572841	1.803526779	Zm00001d013995
Zm00001d035652_T001	0.1389574	1.78388517	Zm00001d035652
Zm00001d012664_T001	0.6871989	-0.461563294	Zm00001d012664
Zm00001d007684_T001	0.8549534	-0.345163089	Zm00001d007684
Zm00001d038879_T005	0.9032233	0.906565231	Zm00001d038879
Zm00001d038879_T004	0.9687599	0.159527977	Zm00001d038879
Zm00001d038879_T006	0.3547256	1.104433947	Zm00001d038879
Zm00001d022534_T001	0.9945118	0.336842953	Zm00001d022534
Zm00001d018134_T002	0.9216521	-0.056238837	Zm00001d018134
Zm00001d018134_T001	0.9824516	-0.096526358	Zm00001d018134
Zm00001d033154_T001	0.01873517	-1.762723686	Zm00001d033154
Zm00001d051241_T002	0.9910206	0.423735813	Zm00001d051241
Zm00001d051241_T003	0.7020458	0.34653197	Zm00001d051241
Zm00001d049584_T001	0.961399	0.444869915	Zm00001d049584
Zm00001d033646_T008	0.9160053	0.227989495	Zm00001d033646
Zm00001d033646_T009	0.4878408	-0.532576986	Zm00001d033646
Zm00001d033646_T003	0.9173494	0.666384338	Zm00001d033646
Zm00001d033646_T013	1	0.992611363	Zm00001d033646
Zm00001d019450_T002	0.3692804	-0.808827706	Zm00001d019450
Zm00001d021736_T001	0.9068989	0.794975886	Zm00001d021736
Zm00001d018050_T009	0.4604152	-0.506643161	Zm00001d018050
Zm00001d018050_T005	0.9537771	0.480907352	Zm00001d018050
Zm00001d018050_T004	3.44E-06	-0.554858031	Zm00001d018050
Zm00001d018050_T007	0.7522322	0.568594709	Zm00001d018050
Zm00001d018050_T006	0.6364634	-0.895397859	Zm00001d018050
Zm00001d018050_T008	0.6333088	1.239117406	Zm00001d018050
Zm00001d009500_T001	0.7626242	0.672135982	Zm00001d009500
Zm00001d009500_T002	0.9419829	0.070567652	Zm00001d009500
Zm00001d031846_T001	0.9942835	-0.141885477	Zm00001d031846
Zm00001d053612_T003	0.5571558	1.124748379	Zm00001d053612
Zm00001d002175_T001	0.95677	0.169449113	Zm00001d002175
Zm00001d050660_T001	0.9237037	0.484858488	Zm00001d050660
Zm00001d017919_T001	0.003224906	-1.151555043	Zm00001d017919
Zm00001d009683_T018	0.3847344	-0.650235953	Zm00001d009683
Zm00001d009683_T003	1	0.491304599	Zm00001d009683
Zm00001d009683_T002	0.08327624	0.788447722	Zm00001d009683
Zm00001d009683_T005	4.14E-09	-1.405377469	Zm00001d009683
Zm00001d009683_T008	0.1968397	0.784470595	Zm00001d009683
Zm00001d009683_T006	0.954492	0.206926894	Zm00001d009683
Zm00001d009683_T015	0.5205106	1.278791961	Zm00001d009683
Zm00001d009683_T011	0.3701709	-0.804701725	Zm00001d009683
Zm00001d009683_T014	0.2905078	-0.387536763	Zm00001d009683
Zm00001d021407_T001	0.9965998	0.262607559	Zm00001d021407
Zm00001d011136_T003	0.7688599	-0.728295205	Zm00001d011136
Zm00001d011136_T001	0.7097086	0.640284862	Zm00001d011136
Zm00001d011136_T005	0.7784822	-0.858308221	Zm00001d011136
Zm00001d002964_T001	0.4442914	1.269230131	Zm00001d002964
Zm00001d013614_T001	0.9804228	0.155444263	Zm00001d013614
Zm00001d037151_T001	0.8090828	-0.25556513	Zm00001d037151
Zm00001d041098_T001	0.9679145	0.66229758	Zm00001d041098
Zm00001d015182_T001	0.990751	0.162752569	Zm00001d015182
Zm00001d011499_T001	0.3336022	-1.019004513	Zm00001d011499
Zm00001d022353_T001	0.9689939	0.105567224	Zm00001d022353
Zm00001d051611_T001	0.9512833	0.584632843	Zm00001d051611

Zm00001d044461_T001	0.9895089	0.037042383	Zm00001d044461
Zm00001d010236_T001	0.9959972	-0.06773065	Zm00001d010236
Zm00001d013819_T005	0.708953	0.296396428	Zm00001d013819
Zm00001d013819_T002	0.4395938	-0.094564284	Zm00001d013819
Zm00001d013819_T001	0.8720862	0.481072946	Zm00001d013819
Zm00001d013819_T004	0.7798168	-0.226042918	Zm00001d013819
Zm00001d034078_T001	7.11E-05	0.221262233	Zm00001d034078
Zm00001d006055_T002	0.6970851	-0.304113789	Zm00001d006055
Zm00001d006055_T001	0.3912133	-0.416956823	Zm00001d006055
Zm00001d014463_T001	0.9224443	0.546401457	Zm00001d014463
Zm00001d023399_T001	0.7235553	-0.376329807	Zm00001d023399
Zm00001d034863_T001	0.9687311	0.158223179	Zm00001d034863
Zm00001d017060_T002	0.9974734	-0.083865111	Zm00001d017060
Zm00001d017060_T001	0.9895224	0.024339346	Zm00001d017060
Zm00001d008970_T001	0.6364634	-0.610645767	Zm00001d008970
Zm00001d009138_T001	0.6611576	-0.52943533	Zm00001d009138
Zm00001d030026_T032	1.06E-11	1.377428605	Zm00001d030026
Zm00001d030026_T041	0.4898733	0.700962021	Zm00001d030026
Zm00001d030026_T036	1	0.157778314	Zm00001d030026
Zm00001d030026_T039	4.38E-10	1.113717958	Zm00001d030026
Zm00001d030026_T004	7.87E-07	1.86147485	Zm00001d030026
Zm00001d030026_T043	2.05E-09	-1.011439322	Zm00001d030026
Zm00001d030026_T052	0.9503014	0.122711627	Zm00001d030026
Zm00001d030026_T051	0.3431817	-0.790285264	Zm00001d030026
Zm00001d030026_T015	1	0.025748224	Zm00001d030026
Zm00001d030026_T042	0.001532887	1.291073869	Zm00001d030026
Zm00001d030901_T001	0.9939021	0.107205124	Zm00001d030901
Zm00001d017216_T001	0.9613831	0.171968709	Zm00001d017216
Zm00001d002589_T001	0.9766874	0.370789952	Zm00001d002589
Zm00001d033330_T001	0.3342103	0.674928974	Zm00001d033330
Zm00001d025709_T001	0.2460097	-0.978704866	Zm00001d025709
Zm00001d037713_T002	0.8031436	-0.264448158	Zm00001d037713
Zm00001d029443_T014	0.004072516	0.652318562	Zm00001d029443
Zm00001d029443_T006	2.79E-09	3.16029675	Zm00001d029443
Zm00001d038608_T001	0.8720862	0.716955241	Zm00001d038608
Zm00001d045125_T001	0.5422838	-0.617344292	Zm00001d045125
Zm00001d006221_T002	0.2518892	1.108800061	Zm00001d006221
Zm00001d006221_T001	0.9732341	0.122790327	Zm00001d006221
Zm00001d009938_T007	0.7236841	-0.249207054	Zm00001d009938
Zm00001d009938_T006	0.7854095	0.844890837	Zm00001d009938
Zm00001d043556_T001	0.9902022	0.290699377	Zm00001d043556
Zm00001d004552_T006	1	0.328192451	Zm00001d004552
Zm00001d004552_T013	0.617335	1.081768936	Zm00001d004552
Zm00001d004552_T008	0.8267985	0.412815413	Zm00001d004552
Zm00001d004552_T005	1	0.170231394	Zm00001d004552
Zm00001d004552_T011	0.7042561	0.867182119	Zm00001d004552
Zm00001d004552_T009	0.9024215	0.827277551	Zm00001d004552
Zm00001d004552_T010	0.7778639	-0.071448887	Zm00001d004552
Zm00001d013078_T001	0.9627389	0.0344892	Zm00001d013078
Zm00001d008762_T010	0.07256374	-1.595603608	Zm00001d008762
Zm00001d008762_T001	0.04916801	-0.97557108	Zm00001d008762
Zm00001d008762_T007	0.8288568	-0.252595065	Zm00001d008762
Zm00001d024823_T001	0.8676122	-0.078730568	Zm00001d024823
Zm00001d012255_T001	0.9899727	0.286965315	Zm00001d012255

Zm00001d027851_T001	0.8580945	0.803621052	Zm00001d027851
Zm00001d030892_T002	0.9296811	0.383324131	Zm00001d030892
Zm00001d030892_T001	0.9949083	0.321099061	Zm00001d030892
Zm00001d030892_T003	0.9302759	0.566599123	Zm00001d030892
Zm00001d034651_T001	0.1925804	1.722112372	Zm00001d034651
Zm00001d001769_T001	0.7984948	0.792681318	Zm00001d001769
Zm00001d014844_T026	0.135934	-0.60193154	Zm00001d014844
Zm00001d014844_T011	0.7358894	0.509956525	Zm00001d014844
Zm00001d014844_T005	0.8863456	0.115094915	Zm00001d014844
Zm00001d014844_T030	0.2319143	0.844624926	Zm00001d014844
Zm00001d014844_T012	0.9448309	0.13580652	Zm00001d014844
Zm00001d014844_T021	0.8250026	0.69222643	Zm00001d014844
Zm00001d014844_T009	0.3547256	-1.236610649	Zm00001d014844
Zm00001d023534_T001	0.926133	-0.058878949	Zm00001d023534
Zm00001d023534_T002	1	0.136261732	Zm00001d023534
Zm00001d036724_T001	0.9914036	-0.011491194	Zm00001d036724
Zm00001d027326_T001	0.9693993	0.594167562	Zm00001d027326
Zm00001d027326_T002	0.5448104	-0.907411156	Zm00001d027326
Zm00001d038114_T004	0.9701986	0.235800684	Zm00001d038114
Zm00001d038114_T003	0.588923	-0.863924233	Zm00001d038114
Zm00001d038114_T001	0.9919298	0.262239623	Zm00001d038114
Zm00001d046599_T001	0.9919493	0.18227702	Zm00001d046599
Zm00001d038373_T006	0.4898849	-0.471103015	Zm00001d038373
Zm00001d042960_T001	0.9804433	0.394499471	Zm00001d042960
Zm00001d045679_T001	0.1785712	0.821900619	Zm00001d045679
Zm00001d020472_T001	0.6386998	-0.972771148	Zm00001d020472
Zm00001d049630_T002	0.6070742	-0.420516036	Zm00001d049630
Zm00001d049630_T001	0.5563797	-0.266052505	Zm00001d049630
Zm00001d040526_T001	1	0.277136359	Zm00001d040526
Zm00001d019555_T002	0.9868821	0.138977293	Zm00001d019555
Zm00001d039252_T002	0.9895992	0.300002801	Zm00001d039252
Zm00001d039252_T001	0.6077603	0.715822762	Zm00001d039252
Zm00001d039252_T003	0.9529184	-0.043538077	Zm00001d039252
Zm00001d040652_T001	0.8964117	0.583958551	Zm00001d040652
Zm00001d021727_T001	0.2502162	1.04065183	Zm00001d021727
Zm00001d042308_T003	0.7626242	-0.5964034	Zm00001d042308
Zm00001d042308_T001	0.6597194	-0.341636884	Zm00001d042308
Zm00001d027523_T006	1	0.150970021	Zm00001d027523
Zm00001d027523_T001	0.9804228	0.200107653	Zm00001d027523
Zm00001d027523_T005	0.9486412	0.652219632	Zm00001d027523
Zm00001d027523_T003	0.7408813	0.559260576	Zm00001d027523
Zm00001d027523_T007	0.6664213	-0.749879934	Zm00001d027523
Zm00001d024674_T001	0.5753523	-0.705895973	Zm00001d024674
Zm00001d003659_T006	0.3301661	-1.178827348	Zm00001d003659
Zm00001d003659_T002	0.8557307	-0.184016402	Zm00001d003659
Zm00001d003659_T001	0.9608605	0.006773672	Zm00001d003659
Zm00001d045576_T001	0.9722469	0.550901557	Zm00001d045576
Zm00001d010602_T001	0.5006675	1.471078725	Zm00001d010602
Zm00001d027416_T001	0.7146325	-0.322258599	Zm00001d027416
Zm00001d020694_T002	0.6053962	1.323209502	Zm00001d020694
Zm00001d020694_T003	0.3996069	0.967529656	Zm00001d020694
Zm00001d020694_T001	1	0.507080528	Zm00001d020694
Zm00001d047290_T001	0.1191683	2.123469516	Zm00001d047290
Zm00001d047290_T002	0.000247069	3.422142821	Zm00001d047290

Zm00001d008993_T023	0.7982735	-0.343196719	Zm00001d008993
Zm00001d008993_T003	0.9846714	0.298069447	Zm00001d008993
Zm00001d008993_T018	0.897685	-0.189268024	Zm00001d008993
Zm00001d008993_T010	0.6530894	0.524494598	Zm00001d008993
Zm00001d008993_T014	0.5081812	0.356306049	Zm00001d008993
Zm00001d008993_T022	0.5101993	-0.816332167	Zm00001d008993
Zm00001d008993_T008	0.873307	-0.225402591	Zm00001d008993
Zm00001d008993_T013	0.9337127	0.268486631	Zm00001d008993
Zm00001d008993_T011	0.7680039	0.59949154	Zm00001d008993
Zm00001d031310_T001	0.9179791	-0.164214602	Zm00001d031310
Zm00001d051234_T001	0.8585804	-0.140283475	Zm00001d051234
Zm00001d003418_T001	0.9275641	0.586210561	Zm00001d003418
Zm00001d044547_T001	0.4395938	-0.13015411	Zm00001d044547
Zm00001d044547_T011	0.8625537	0.140245524	Zm00001d044547
Zm00001d006419_T002	0.7817347	-0.189584868	Zm00001d006419
Zm00001d027459_T001	0.9740325	0.009003171	Zm00001d027459
Zm00001d031725_T001	0.3547256	1.503407291	Zm00001d031725
Zm00001d031028_T001	0.8665954	-0.179181679	Zm00001d031028
Zm00001d031028_T006	0.463923	1.390039104	Zm00001d031028
Zm00001d031028_T002	0.9861215	-0.002623192	Zm00001d031028
Zm00001d031028_T004	0.9695609	0.090878399	Zm00001d031028
Zm00001d032977_T004	0.9619517	-0.056298001	Zm00001d032977
Zm00001d002026_T001	1	0.27224395	Zm00001d002026
Zm00001d002026_T002	1	0.338714248	Zm00001d002026
Zm00001d034493_T001	0.9851204	0.220792164	Zm00001d034493
Zm00001d006645_T020	0.5408146	0.310705555	Zm00001d006645
Zm00001d006645_T014	0.7408813	-0.242811127	Zm00001d006645
Zm00001d006645_T003	0.9998515	0.228905623	Zm00001d006645
Zm00001d006645_T019	0.333158	0.639627281	Zm00001d006645
Zm00001d006645_T024	0.4124693	0.671953939	Zm00001d006645
Zm00001d006645_T018	0.8730771	0.299871772	Zm00001d006645
Zm00001d006645_T013	0.9690925	0.021920005	Zm00001d006645
Zm00001d006645_T009	0.9468268	0.280944318	Zm00001d006645
Zm00001d006645_T036	0.9124297	-0.159679258	Zm00001d006645
Zm00001d018369_T002	0.3431817	0.624265586	Zm00001d018369
Zm00001d039310_T001	0.005921748	-1.778234569	Zm00001d039310
Zm00001d004407_T004	0.9320948	0.538185535	Zm00001d004407
Zm00001d004407_T005	0.6436915	-0.409064521	Zm00001d004407
Zm00001d004407_T006	0.0755688	-1.71434934	Zm00001d004407
Zm00001d004407_T003	1	0.312200076	Zm00001d004407
Zm00001d050964_T001	0.8751525	-0.245301436	Zm00001d050964
Zm00001d021436_T001	0.9596377	-0.16843465	Zm00001d021436
Zm00001d021436_T002	0.9223923	-0.092573899	Zm00001d021436
Zm00001d019191_T005	0.98291	-0.0601909	Zm00001d019191
Zm00001d019191_T001	0.9962521	0.019771588	Zm00001d019191
Zm00001d019191_T007	1.20E-07	-1.400551194	Zm00001d019191
Zm00001d019191_T004	0.9759623	0.591900973	Zm00001d019191
Zm00001d048837_T001	0.7311884	-0.394055126	Zm00001d048837
Zm00001d050496_T001	0.9429712	0.010277747	Zm00001d050496
Zm00001d047796_T001	0.2196489	1.931747915	Zm00001d047796
Zm00001d047796_T003	0.1276369	1.89668172	Zm00001d047796
Zm00001d019699_T005	0.9765009	0.146938539	Zm00001d019699
Zm00001d019699_T007	0.7416009	-0.626113331	Zm00001d019699
Zm00001d019699_T003	0.9819415	0.459827321	Zm00001d019699

Zm00001d013074_T008	0.9988864	0.196917268	Zm00001d013074
Zm00001d013074_T005	0.8836975	0.349960654	Zm00001d013074
Zm00001d013074_T002	0.8555654	-0.17645795	Zm00001d013074
Zm00001d051700_T001	0.8677544	-0.49212625	Zm00001d051700
Zm00001d039901_T003	0.9833133	0.541417663	Zm00001d039901
Zm00001d044920_T001	1	0.418256822	Zm00001d044920
Zm00001d044920_T002	0.9664406	0.057439683	Zm00001d044920
Zm00001d013056_T005	0.7494913	-0.409676722	Zm00001d013056
Zm00001d013056_T002	0.7536836	-0.284301445	Zm00001d013056
Zm00001d003288_T002	0.9664406	0.596145637	Zm00001d003288
Zm00001d003288_T001	0.7250499	1.46175518	Zm00001d003288
Zm00001d003288_T003	0.6678281	-0.815489144	Zm00001d003288
Zm00001d039283_T002	0.9232446	-0.097098462	Zm00001d039283
Zm00001d039283_T004	0.07146096	-0.879730672	Zm00001d039283
Zm00001d039283_T003	0.08432998	-1.495342492	Zm00001d039283
Zm00001d024544_T002	0.9849414	0.260073679	Zm00001d024544
Zm00001d024544_T003	0.9419336	0.590117154	Zm00001d024544
Zm00001d010001_T001	0.07548051	-2.090399235	Zm00001d010001
Zm00001d018976_T078	0.3486589	0.962138593	Zm00001d018976
Zm00001d018976_T015	0.7169281	-0.440981129	Zm00001d018976
Zm00001d018976_T062	0.00919548	-1.71838908	Zm00001d018976
Zm00001d018976_T002	0.7727409	-0.143197419	Zm00001d018976
Zm00001d018976_T055	1.65E-14	2.956206531	Zm00001d018976
Zm00001d019260_T001	0.9902733	-0.226951568	Zm00001d019260
Zm00001d017720_T001	4.62E-07	-0.516214968	Zm00001d017720
Zm00001d017720_T010	0.3302389	-0.789852698	Zm00001d017720
Zm00001d017720_T009	0.004806727	1.326797249	Zm00001d017720
Zm00001d017720_T007	0.5550339	0.773866234	Zm00001d017720
Zm00001d049529_T002	0.7846759	-0.674553937	Zm00001d049529
Zm00001d015469_T002	0.7568663	0.434278607	Zm00001d015469
Zm00001d015469_T001	0.772948	0.234795704	Zm00001d015469
Zm00001d006311_T001	0.8716627	-0.084858387	Zm00001d006311
Zm00001d006311_T002	0.9493302	0.050076236	Zm00001d006311
Zm00001d017702_T001	0.8244021	-0.163610634	Zm00001d017702
Zm00001d049718_T004	0.4898849	-0.593864326	Zm00001d049718
Zm00001d049718_T002	0.9736405	0.447371307	Zm00001d049718
Zm00001d005641_T001	0.9745948	0.159394277	Zm00001d005641
Zm00001d005641_T002	1	0.233463566	Zm00001d005641
Zm00001d041548_T002	0.8092097	-0.200547125	Zm00001d041548
Zm00001d034715_T001	0.5706378	-1.098023303	Zm00001d034715
Zm00001d021546_T002	0.003214349	-0.517716593	Zm00001d021546
Zm00001d021546_T004	0.8864089	0.005058327	Zm00001d021546
Zm00001d021546_T003	0.8991659	0.861633257	Zm00001d021546
Zm00001d021546_T001	0.3949594	0.447701815	Zm00001d021546
Zm00001d002730_T001	0.7799762	-0.488038339	Zm00001d002730
Zm00001d052320_T003	0.9685273	0.279869232	Zm00001d052320
Zm00001d052320_T004	0.997764	0.414331216	Zm00001d052320
Zm00001d052320_T002	0.9992962	0.4094279	Zm00001d052320
Zm00001d012961_T001	0.9390501	0.016406118	Zm00001d012961
Zm00001d009396_T001	0.9832092	0.056831216	Zm00001d009396
Zm00001d009396_T002	0.9188466	0.028482782	Zm00001d009396
Zm00001d043029_T001	0.9226671	0.043207236	Zm00001d043029
Zm00001d018461_T001	0.5134498	1.391354525	Zm00001d018461
Zm00001d021464_T002	0.07220675	0.719783051	Zm00001d021464

Zm00001d021464_T006	0.9770343	0.195246723	Zm00001d021464
Zm00001d007175_T001	0.006155769	2.580935866	Zm00001d007175
Zm00001d033666_T001	0.9984041	0.171656348	Zm00001d033666
Zm00001d034894_T001	0.9942835	0.486107698	Zm00001d034894
Zm00001d036301_T012	0.9182861	0.18266823	Zm00001d036301
Zm00001d036301_T009	0.404976	-0.897427459	Zm00001d036301
Zm00001d036301_T002	0.9429712	-0.100727086	Zm00001d036301
Zm00001d036301_T014	0.4760609	0.403481486	Zm00001d036301
Zm00001d036301_T008	0.6985673	-0.694348692	Zm00001d036301
Zm00001d036301_T006	0.9139882	-0.018923895	Zm00001d036301
Zm00001d036301_T007	5.43E-11	1.111701261	Zm00001d036301
Zm00001d007900_T005	0.7791621	-0.657742737	Zm00001d007900
Zm00001d007900_T003	0.9832092	0.202807856	Zm00001d007900
Zm00001d022155_T001	0.8155056	1.022699162	Zm00001d022155
Zm00001d047968_T001	0.750563	-0.96683942	Zm00001d047968
Zm00001d043058_T001	0.7622306	-0.203935239	Zm00001d043058
Zm00001d024169_T001	0.5467906	-0.35212406	Zm00001d024169
Zm00001d024169_T002	0.8751525	0.567536061	Zm00001d024169
Zm00001d043401_T001	0.7988381	0.970038457	Zm00001d043401
Zm00001d023865_T001	0.9573434	0.038021002	Zm00001d023865
Zm00001d024764_T001	0.8891117	-0.094343382	Zm00001d024764
Zm00001d029488_T001	0.07761644	2.11554151	Zm00001d029488
Zm00001d035023_T001	0.8520487	-0.134821216	Zm00001d035023
Zm00001d053514_T001	0.888502	0.8014325	Zm00001d053514
Zm00001d029483_T001	0.909729	-0.46362866	Zm00001d029483
Zm00001d029511_T002	0.6324192	-0.51621036	Zm00001d029511
Zm00001d042861_T001	0.9449404	0.600966411	Zm00001d042861
Zm00001d052276_T001	1	0.129503654	Zm00001d052276
Zm00001d052276_T004	0.6962415	0.633191411	Zm00001d052276
Zm00001d033986_T001	0.7712474	0.668500046	Zm00001d033986
Zm00001d033986_T003	1	0.202776231	Zm00001d033986
Zm00001d004386_T001	1	0.226254914	Zm00001d004386
Zm00001d013212_T001	0.6801397	-0.507939344	Zm00001d013212
Zm00001d032377_T007	0.000818754	1.578438281	Zm00001d032377
Zm00001d032377_T005	0.3462656	0.892201948	Zm00001d032377
Zm00001d032377_T001	0.7577723	0.845639481	Zm00001d032377
Zm00001d006717_T008	0.01537015	-0.722002759	Zm00001d006717
Zm00001d006717_T010	0.9469426	0.894660525	Zm00001d006717
Zm00001d006717_T002	0.7520438	-0.192937305	Zm00001d006717
Zm00001d015293_T001	0.007202394	0.873734603	Zm00001d015293
Zm00001d015293_T003	0.8817666	-0.083730429	Zm00001d015293
Zm00001d015293_T002	0.3999554	1.551166875	Zm00001d015293
Zm00001d015293_T005	0.8537544	-0.11172799	Zm00001d015293
Zm00001d009023_T002	0.8926893	-0.520521178	Zm00001d009023
Zm00001d009023_T001	0.7737238	-0.413269884	Zm00001d009023
Zm00001d046688_T003	0.7130452	-0.374902508	Zm00001d046688
Zm00001d046688_T002	0.03495473	-2.079381851	Zm00001d046688
Zm00001d046688_T001	0.3432136	-0.660503287	Zm00001d046688
Zm00001d051939_T001	0.8929316	-0.088296792	Zm00001d051939
Zm00001d007653_T002	0.7122217	-0.211738594	Zm00001d007653
Zm00001d007653_T001	0.6566544	-0.326399927	Zm00001d007653
Zm00001d007653_T003	0.9361945	0.448807246	Zm00001d007653
Zm00001d051609_T008	0.7289978	-0.329878033	Zm00001d051609
Zm00001d051609_T004	0.8414741	0.98616662	Zm00001d051609

Zm00001d051609_T001	0.9970835	0.70330209	Zm00001d051609
Zm00001d051609_T007	0.530398	0.837205321	Zm00001d051609
Zm00001d051609_T006	0.9979568	0.4799711	Zm00001d051609
Zm00001d025590_T001	0.9083745	0.226589086	Zm00001d025590
Zm00001d025590_T006	0.9193633	0.6473702	Zm00001d025590
Zm00001d025590_T022	0.06586321	0.786579629	Zm00001d025590
Zm00001d025590_T009	0.6364634	1.126490214	Zm00001d025590
Zm00001d025590_T011	9.56E-13	-2.348847362	Zm00001d025590
Zm00001d025590_T005	0.004082558	0.781982205	Zm00001d025590
Zm00001d008255_T001	0.954759	0.061360138	Zm00001d008255
Zm00001d039302_T001	1	0.422331344	Zm00001d039302
Zm00001d032422_T004	0.1752559	-1.406654468	Zm00001d032422
Zm00001d052019_T002	0.768921	0.977361344	Zm00001d052019
Zm00001d052019_T001	0.5704041	-0.292472696	Zm00001d052019
Zm00001d036655_T003	0.9322451	0.410382825	Zm00001d036655
Zm00001d036655_T006	0.4530974	0.402584233	Zm00001d036655
Zm00001d051167_T001	0.9043641	-0.274936068	Zm00001d051167
Zm00001d032366_T001	1	0.136141234	Zm00001d032366
Zm00001d003312_T005	0.5582757	-0.801969038	Zm00001d003312
Zm00001d003312_T004	0.8329158	-0.267838441	Zm00001d003312
Zm00001d042977_T002	0.9528476	0.494438659	Zm00001d042977
Zm00001d042977_T001	0.9979568	0.399998311	Zm00001d042977
Zm00001d053165_T001	0.1974301	-1.589838234	Zm00001d053165
Zm00001d027996_T002	0.9554387	0.106768824	Zm00001d027996
Zm00001d027996_T001	0.3015167	-0.394481149	Zm00001d027996
Zm00001d027996_T003	0.9931431	0.227630964	Zm00001d027996
Zm00001d020954_T001	0.6086477	-0.840639358	Zm00001d020954
Zm00001d045501_T001	0.6231523	-0.55042894	Zm00001d045501
Zm00001d045501_T003	0.5335622	0.75782233	Zm00001d045501
Zm00001d045501_T006	0.3748743	0.62472898	Zm00001d045501
Zm00001d009964_T001	0.9701986	0.14409299	Zm00001d009964
Zm00001d028456_T001	0.9813293	0.252244375	Zm00001d028456
Zm00001d022513_T001	0.581521	-0.748516784	Zm00001d022513
Zm00001d022513_T002	0.8412913	-0.240014478	Zm00001d022513
Zm00001d027715_T004	0.9363242	0.37374117	Zm00001d027715
Zm00001d044330_T006	0.8720321	0.299464074	Zm00001d044330
Zm00001d044330_T002	0.919888	0.716578844	Zm00001d044330
Zm00001d050428_T002	0.9812314	0.452477907	Zm00001d050428
Zm00001d007059_T002	0.9979568	0.197941614	Zm00001d007059
Zm00001d035666_T002	0.8643636	-0.06401966	Zm00001d035666
Zm00001d035666_T001	0.6502105	-1.040054855	Zm00001d035666
Zm00001d053740_T001	0.5954619	0.753827674	Zm00001d053740
Zm00001d040228_T001	0.9979568	0.24394899	Zm00001d040228
Zm00001d020400_T001	0.3353806	1.639316503	Zm00001d020400
Zm00001d040218_T018	0.3497147	0.340581264	Zm00001d040218
Zm00001d040218_T035	0.3229035	1.279578389	Zm00001d040218
Zm00001d040218_T001	0.9419336	0.474419245	Zm00001d040218
Zm00001d040218_T019	0.8037772	-0.191845714	Zm00001d040218
Zm00001d040218_T026	0.8730771	0.896801202	Zm00001d040218
Zm00001d050406_T008	0.7490886	0.684045563	Zm00001d050406
Zm00001d006348_T001	0.7969526	-0.29497638	Zm00001d006348
Zm00001d043938_T001	0.9805897	0.135946622	Zm00001d043938
Zm00001d031822_T001	0.9614723	0.249402403	Zm00001d031822
Zm00001d011776_T014	0.8892594	0.340415639	Zm00001d011776

Zm00001d011776_T004	0.8755938	0.256707037	Zm00001d011776
Zm00001d011776_T007	0.950155	-0.008882521	Zm00001d011776
Zm00001d011776_T011	0.9955906	0.059690012	Zm00001d011776
Zm00001d011776_T005	0.5159743	0.804582796	Zm00001d011776
Zm00001d050336_T001	0.9817074	0.155394292	Zm00001d050336
Zm00001d050336_T007	0.9673473	0.12347357	Zm00001d050336
Zm00001d013879_T001	0.2879626	-1.590492559	Zm00001d013879
Zm00001d012525_T071	0.9223663	0.195085815	Zm00001d012525
Zm00001d012525_T021	0.003082396	0.396692627	Zm00001d012525
Zm00001d012525_T072	0.8740066	0.256700065	Zm00001d012525
Zm00001d012525_T095	0.6143693	1.049061617	Zm00001d012525
Zm00001d012525_T097	0.5954619	1.362933471	Zm00001d012525
Zm00001d012525_T098	0.09658809	-1.698177249	Zm00001d012525
Zm00001d012525_T003	0.03867058	1.884157255	Zm00001d012525
Zm00001d021203_T002	0.9857423	0.268387515	Zm00001d021203
Zm00001d021203_T001	0.9145405	-0.06889439	Zm00001d021203
Zm00001d010092_T002	1	0.231228785	Zm00001d010092
Zm00001d010092_T005	0.9781672	0.043707056	Zm00001d010092
Zm00001d031061_T002	0.7512335	-0.244314383	Zm00001d031061
Zm00001d015211_T001	1	0.279554225	Zm00001d015211
Zm00001d045182_T009	0.389058	0.489059142	Zm00001d045182
Zm00001d045182_T008	0.9945118	0.330268725	Zm00001d045182
Zm00001d013175_T001	0.2563583	-1.593477601	Zm00001d013175
Zm00001d008865_T002	0.9869501	0.390404834	Zm00001d008865
Zm00001d008865_T001	0.498526	0.796609621	Zm00001d008865
Zm00001d008865_T003	0.6044465	-0.815298431	Zm00001d008865
Zm00001d049641_T002	0.0844934	-0.739794295	Zm00001d049641
Zm00001d049641_T003	0.6533556	-1.085502417	Zm00001d049641
Zm00001d049641_T005	0.9902733	0.556546056	Zm00001d049641
Zm00001d049641_T007	4.66E-13	-4.700278702	Zm00001d049641
Zm00001d049641_T004	0.6625969	0.0805543	Zm00001d049641
Zm00001d049641_T001	0.8537544	0.309759798	Zm00001d049641
Zm00001d049641_T006	0.9419336	0.528229913	Zm00001d049641
Zm00001d047427_T002	0.897685	-0.341220415	Zm00001d047427
Zm00001d045798_T001	1	0.354630043	Zm00001d045798
Zm00001d013947_T001	5.20E-08	5.144506081	Zm00001d013947
Zm00001d041866_T001	0.3698673	-0.650435099	Zm00001d041866
Zm00001d007202_T013	0.1094786	1.569244796	Zm00001d007202
Zm00001d007202_T007	0.9825858	0.258636237	Zm00001d007202
Zm00001d007202_T023	0.9451369	0.087672418	Zm00001d007202
Zm00001d042802_T006	0.9825858	0.238531838	Zm00001d042802
Zm00001d042802_T007	0.9529184	0.30508292	Zm00001d042802
Zm00001d025953_T003	0.305747	-0.885430756	Zm00001d025953
Zm00001d025953_T001	0.4196849	-0.741129831	Zm00001d025953
Zm00001d045297_T001	0.9914912	0.256543698	Zm00001d045297
Zm00001d006320_T003	0.9760472	0.330134457	Zm00001d006320
Zm00001d006320_T001	0.8605736	-0.303939731	Zm00001d006320
Zm00001d006320_T004	0.9404125	0.371025308	Zm00001d006320
Zm00001d034044_T001	0.9804228	0.538389657	Zm00001d034044
Zm00001d010406_T002	0.8423784	0.844206866	Zm00001d010406
Zm00001d010406_T007	0.9765009	-0.009606821	Zm00001d010406
Zm00001d052675_T011	0.4569211	-0.364538036	Zm00001d052675
Zm00001d052675_T013	0.4466558	1.058262011	Zm00001d052675
Zm00001d052675_T014	0.2421686	-0.787521867	Zm00001d052675

Zm00001d052675_T010	0.9252088	0.306907617	Zm00001d052675
Zm00001d052675_T003	0.3287748	-0.447652669	Zm00001d052675
Zm00001d052675_T012	0.8062829	0.609215986	Zm00001d052675
Zm00001d052675_T016	0.1489084	-0.26325765	Zm00001d052675
Zm00001d004020_T004	0.9469426	0.036679212	Zm00001d004020
Zm00001d026139_T003	1	0.313482861	Zm00001d026139
Zm00001d026139_T002	0.9722469	0.35215006	Zm00001d026139
Zm00001d026139_T001	0.5974852	0.894888476	Zm00001d026139
Zm00001d027448_T004	0.9529184	0.590644761	Zm00001d027448
Zm00001d027448_T008	0.02242615	1.337861771	Zm00001d027448
Zm00001d027448_T007	0.8660064	-0.420338799	Zm00001d027448
Zm00001d003462_T001	0.9770343	0.573104535	Zm00001d003462
Zm00001d026305_T011	0.41048	0.324454791	Zm00001d026305
Zm00001d026305_T005	0.08002632	-0.534725658	Zm00001d026305
Zm00001d026305_T004	0.6503853	-0.197152335	Zm00001d026305
Zm00001d026148_T001	1	0.177260639	Zm00001d026148
Zm00001d053667_T001	0.9835181	0.180640968	Zm00001d053667
Zm00001d027257_T006	0.9182861	0.330106906	Zm00001d027257
Zm00001d027257_T005	0.9969363	0.312104469	Zm00001d027257
Zm00001d027257_T003	0.1475494	-0.666665504	Zm00001d027257
Zm00001d027257_T007	0.85155	0.490299243	Zm00001d027257
Zm00001d027257_T001	0.310891	1.125351689	Zm00001d027257
Zm00001d021126_T002	0.9333149	-0.019308714	Zm00001d021126
Zm00001d021126_T005	0.002685015	2.237260381	Zm00001d021126
Zm00001d021126_T001	0.8569937	0.86997142	Zm00001d021126
Zm00001d021126_T004	0.8807115	-0.146349417	Zm00001d021126
Zm00001d044841_T001	0.9400285	-0.025935044	Zm00001d044841
Zm00001d020497_T001	0.9460688	0.490716845	Zm00001d020497
Zm00001d047896_T001	0.9364913	0.086784466	Zm00001d047896
Zm00001d047896_T007	0.6331067	1.240127188	Zm00001d047896
Zm00001d047896_T002	0.9745948	0.611347741	Zm00001d047896
Zm00001d047896_T003	0.9588604	0.608540564	Zm00001d047896
Zm00001d047896_T004	0.04663752	2.013279483	Zm00001d047896
Zm00001d047896_T009	0.4988467	0.714893127	Zm00001d047896
Zm00001d011476_T001	0.9872831	0.081817315	Zm00001d011476
Zm00001d025124_T001	0.313785	0.852359459	Zm00001d025124
Zm00001d017092_T001	0.8277395	-0.208725653	Zm00001d017092
Zm00001d041972_T001	0.9804228	0.255443216	Zm00001d041972
Zm00001d041972_T003	0.001497347	-0.922744476	Zm00001d041972
Zm00001d041972_T002	0.5850461	-0.499722745	Zm00001d041972
Zm00001d011930_T001	0.001750147	2.758551665	Zm00001d011930
Zm00001d019766_T001	0.7114961	0.802081672	Zm00001d019766
Zm00001d053395_T001	0.8716588	-0.081922322	Zm00001d053395
Zm00001d038908_T006	0.002146068	2.797063671	Zm00001d038908
Zm00001d038908_T001	0.9846714	0.106083569	Zm00001d038908
Zm00001d038908_T003	0.3097895	1.413419106	Zm00001d038908
Zm00001d038908_T004	0.3861385	1.570911666	Zm00001d038908
Zm00001d047993_T002	1.96E-06	3.561940566	Zm00001d047993
Zm00001d047993_T001	1.90E-05	4.402660091	Zm00001d047993
Zm00001d040004_T001	0.004509663	-2.377871287	Zm00001d040004
Zm00001d044849_T001	0.4193179	1.202250552	Zm00001d044849
Zm00001d005775_T003	0.03619664	1.66804154	Zm00001d005775
Zm00001d005775_T009	0.9112426	0.83777384	Zm00001d005775
Zm00001d005775_T005	0.4146004	1.63037173	Zm00001d005775

Zm00001d011903_T001	0.02300732	-1.634105436	Zm00001d011903
Zm00001d037313_T005	0.9979393	0.237475293	Zm00001d037313
Zm00001d037313_T013	0.9417709	0.494089326	Zm00001d037313
Zm00001d037313_T019	0.8416722	0.580829202	Zm00001d037313
Zm00001d037313_T006	0.1527884	-0.523421266	Zm00001d037313
Zm00001d037313_T023	0.6445434	0.735639087	Zm00001d037313
Zm00001d037313_T015	8.55E-11	1.380836939	Zm00001d037313
Zm00001d037313_T022	0.909729	-0.239880616	Zm00001d037313
Zm00001d007534_T001	0.8158416	0.57291534	Zm00001d007534
Zm00001d048369_T001	0.9706887	0.123350937	Zm00001d048369
Zm00001d030157_T001	0.9837576	0.161384713	Zm00001d030157
Zm00001d011543_T001	0.6891365	-0.895288022	Zm00001d011543
Zm00001d011222_T001	0.9586437	0.613478858	Zm00001d011222
Zm00001d011222_T007	0.357066	-0.724745795	Zm00001d011222
Zm00001d020487_T001	0.900661	-0.075306053	Zm00001d020487
Zm00001d031093_T002	0.157381	-0.72736299	Zm00001d031093
Zm00001d031093_T001	1	0.267029231	Zm00001d031093
Zm00001d031093_T003	1	0.282243174	Zm00001d031093
Zm00001d013603_T004	0.7364361	0.5715072	Zm00001d013603
Zm00001d013603_T001	0.6755118	0.786961921	Zm00001d013603
Zm00001d004153_T002	0.8593984	-0.313756659	Zm00001d004153
Zm00001d025225_T001	0.1439251	1.84471018	Zm00001d025225
Zm00001d006433_T005	0.9050121	1.013586502	Zm00001d006433
Zm00001d006433_T008	0.000190083	0.813884671	Zm00001d006433
Zm00001d006433_T014	6.56E-12	-4.288403225	Zm00001d006433
Zm00001d006433_T004	0.001716737	0.933269881	Zm00001d006433
Zm00001d006433_T003	0.7577723	0.107478592	Zm00001d006433
Zm00001d006433_T011	0.7074815	-0.119858816	Zm00001d006433
Zm00001d006433_T001	0.9817074	0.533111414	Zm00001d006433
Zm00001d006433_T006	0.06729916	1.584354808	Zm00001d006433
Zm00001d032313_T001	0.9745948	-0.102532604	Zm00001d032313
Zm00001d050359_T001	0.9416622	0.028353252	Zm00001d050359
Zm00001d037359_T001	0.7614965	1.053078505	Zm00001d037359
Zm00001d048663_T001	0.7649713	0.677765121	Zm00001d048663
Zm00001d017222_T001	0.991869	-0.024670872	Zm00001d017222
Zm00001d013676_T004	0.9825858	0.514916358	Zm00001d013676
Zm00001d013676_T001	0.7020458	1.099345356	Zm00001d013676
Zm00001d038764_T001	0.8276231	0.893233187	Zm00001d038764
Zm00001d034671_T001	0.9135067	-0.017581415	Zm00001d034671
Zm00001d034640_T017	0.9894615	0.240067287	Zm00001d034640
Zm00001d034640_T016	0.9732421	0.066149982	Zm00001d034640
Zm00001d034640_T031	0.8363027	0.16345628	Zm00001d034640
Zm00001d034640_T003	0.9880075	0.270566777	Zm00001d034640
Zm00001d034640_T028	0.6012987	0.377537732	Zm00001d034640
Zm00001d016230_T001	1	0.291302408	Zm00001d016230
Zm00001d015085_T001	0.8926921	-0.380481323	Zm00001d015085
Zm00001d000213_T003	0.9760472	0.155307737	Zm00001d000213
Zm00001d011737_T001	0.001685107	-1.075778688	Zm00001d011737
Zm00001d043632_T001	0.9825014	0.245917855	Zm00001d043632
Zm00001d043632_T003	0.1054869	-1.470347016	Zm00001d043632
Zm00001d012751_T091	0.9975882	0.373516347	Zm00001d012751
Zm00001d012751_T010	0.02342941	0.490838152	Zm00001d012751
Zm00001d012751_T008	0.4693129	0.168923436	Zm00001d012751
Zm00001d012751_T021	2.74E-14	-2.856209639	Zm00001d012751

Zm00001d012751_T015	7.33E-08	0.679749171	Zm00001d012751
Zm00001d012751_T025	0.03297091	0.428705452	Zm00001d012751
Zm00001d012751_T003	0.9530658	-0.083436411	Zm00001d012751
Zm00001d012751_T019	1.75E-15	3.384418472	Zm00001d012751
Zm00001d012280_T001	0.6436915	-0.485655155	Zm00001d012280
Zm00001d051636_T002	0.7649515	0.822177644	Zm00001d051636
Zm00001d051636_T003	0.5455802	1.059240694	Zm00001d051636
Zm00001d051636_T001	0.6492064	0.90708001	Zm00001d051636
Zm00001d000205_T002	0.959308	0.110551277	Zm00001d000205
Zm00001d000205_T004	0.3757326	0.703047688	Zm00001d000205
Zm00001d051668_T003	0.6343135	-0.461301917	Zm00001d051668
Zm00001d046834_T001	0.9991964	-0.051846488	Zm00001d046834
Zm00001d008875_T001	0.9130008	-0.009912418	Zm00001d008875
Zm00001d035714_T002	0.9618822	0.62276829	Zm00001d035714
Zm00001d035714_T004	0.9537771	-0.036248973	Zm00001d035714
Zm00001d035714_T001	0.6926364	-0.322204848	Zm00001d035714
Zm00001d022182_T001	0.7979593	0.510138626	Zm00001d022182
Zm00001d022182_T002	0.9469426	0.572138071	Zm00001d022182
Zm00001d016519_T002	0.9048001	-0.005923664	Zm00001d016519
Zm00001d002160_T001	0.2262715	0.066824227	Zm00001d002160
Zm00001d006620_T001	0.8987811	-0.14505038	Zm00001d006620
Zm00001d006620_T002	0.6727562	0.888501617	Zm00001d006620
Zm00001d010274_T001	0.9778436	0.081914802	Zm00001d010274
Zm00001d012020_T001	1	0.221706648	Zm00001d012020
Zm00001d044478_T026	0.8958882	-0.031505064	Zm00001d044478
Zm00001d044478_T033	0.3285918	-0.21467123	Zm00001d044478
Zm00001d044478_T009	0.001935712	0.583233269	Zm00001d044478
Zm00001d044478_T015	0.001345215	3.278683119	Zm00001d044478
Zm00001d044478_T059	0.04441707	-1.576697662	Zm00001d044478
Zm00001d044478_T050	0.8174507	0.284685764	Zm00001d044478
Zm00001d044478_T029	0.8376861	0.071246582	Zm00001d044478
Zm00001d044478_T061	2.15E-14	-3.194177768	Zm00001d044478
Zm00001d044478_T031	0.09879412	0.505909653	Zm00001d044478
Zm00001d007045_T001	0.9113566	0.77204885	Zm00001d007045
Zm00001d018495_T004	0.9614276	0.019834289	Zm00001d018495
Zm00001d018495_T002	1	0.266686978	Zm00001d018495
Zm00001d047039_T001	0.8825173	-0.120568231	Zm00001d047039
Zm00001d013294_T001	0.6260085	-0.441045474	Zm00001d013294
Zm00001d018411_T001	0.9404125	-0.014599584	Zm00001d018411
Zm00001d018411_T003	0.8572948	0.389692144	Zm00001d018411
Zm00001d020118_T001	1	0.278852517	Zm00001d020118
Zm00001d043038_T002	0.9043641	0.618919578	Zm00001d043038
Zm00001d043038_T001	0.2523459	1.716047271	Zm00001d043038
Zm00001d014325_T001	0.5004914	-0.4432327	Zm00001d014325
Zm00001d000071_T001	0.9419336	0.389591879	Zm00001d000071
Zm00001d011730_T001	0.961473	-0.033184088	Zm00001d011730
Zm00001d029580_T001	0.6044228	1.112619587	Zm00001d029580
Zm00001d029418_T002	0.8700564	-0.491701645	Zm00001d029418
Zm00001d029418_T003	0.8488494	-0.093830771	Zm00001d029418
Zm00001d019701_T001	1	0.212262285	Zm00001d019701
Zm00001d042498_T001	0.9760472	0.093562432	Zm00001d042498
Zm00001d021901_T001	0.8705378	0.433874461	Zm00001d021901
Zm00001d008298_T001	0.9722727	0.500693604	Zm00001d008298
Zm00001d048198_T001	0.873727	0.392320845	Zm00001d048198

Zm00001d048198_T002	0.9211172	-0.481133765	Zm00001d048198
Zm00001d016758_T001	0.7501444	-0.521695411	Zm00001d016758
Zm00001d030969_T001	0.1145356	-1.178208184	Zm00001d030969
Zm00001d018634_T001	0.8799382	-0.166189484	Zm00001d018634
Zm00001d020227_T024	0.000139328	1.979149911	Zm00001d020227
Zm00001d020227_T005	1.46E-09	1.337366053	Zm00001d020227
Zm00001d020227_T026	0.9487214	0.346760827	Zm00001d020227
Zm00001d020227_T003	0.852305	-0.270631516	Zm00001d020227
Zm00001d020227_T032	0.3554614	-0.67961211	Zm00001d020227
Zm00001d020227_T039	0.01023328	0.72551359	Zm00001d020227
Zm00001d020227_T034	6.85E-14	3.048279298	Zm00001d020227
Zm00001d008905_T001	0.1310911	-1.331680618	Zm00001d008905
Zm00001d045370_T001	0.001410393	-3.007504084	Zm00001d045370
Zm00001d041954_T002	4.62E-07	1.271878875	Zm00001d041954
Zm00001d041954_T005	0.8292199	-0.118915824	Zm00001d041954
Zm00001d041954_T001	2.53E-06	-2.106939002	Zm00001d041954
Zm00001d041954_T004	0.9913436	0.258594913	Zm00001d041954
Zm00001d018241_T001	0.9208929	-0.095041621	Zm00001d018241
Zm00001d013743_T001	0.02454213	-2.271332062	Zm00001d013743
Zm00001d007892_T002	0.6062892	-0.464852647	Zm00001d007892
Zm00001d007892_T001	0.9375054	0.013987052	Zm00001d007892
Zm00001d004534_T001	0.9931431	0.227141354	Zm00001d004534
Zm00001d025853_T003	0.9468268	0.542198958	Zm00001d025853
Zm00001d025853_T001	1	0.293123592	Zm00001d025853
Zm00001d040257_T010	0.6507629	-0.477231718	Zm00001d040257
Zm00001d040257_T007	0.7877461	1.844594936	Zm00001d040257
Zm00001d040257_T006	0.1882298	0.906913074	Zm00001d040257
Zm00001d040257_T003	0.7888267	-0.013842328	Zm00001d040257
Zm00001d007650_T003	0.937572	0.111187922	Zm00001d007650
Zm00001d007650_T002	0.9907928	0.347741824	Zm00001d007650
Zm00001d047063_T004	0.947501	0.083231116	Zm00001d047063
Zm00001d047063_T003	0.9171885	0.480161773	Zm00001d047063
Zm00001d013926_T001	0.9846714	0.223566349	Zm00001d013926
Zm00001d031588_T001	0.8640519	0.442890547	Zm00001d031588
Zm00001d029567_T002	0.9524127	0.474141742	Zm00001d029567
Zm00001d029567_T001	0.9918302	0.0755793	Zm00001d029567
Zm00001d016834_T001	0.9897446	-0.188985291	Zm00001d016834
Zm00001d003007_T001	0.9673473	0.538971184	Zm00001d003007
Zm00001d031280_T001	0.7146325	-0.338573501	Zm00001d031280
Zm00001d053923_T002	0.9410845	0.633029894	Zm00001d053923
Zm00001d053923_T005	0.9037002	-0.010449758	Zm00001d053923
Zm00001d028558_T004	0.9429978	0.019743013	Zm00001d028558
Zm00001d028558_T001	0.9988864	0.2779368	Zm00001d028558
Zm00001d028558_T005	0.000823508	1.100525674	Zm00001d028558
Zm00001d018210_T001	0.9693993	0.019279029	Zm00001d018210
Zm00001d043568_T001	0.6910922	0.652876422	Zm00001d043568
Zm00001d020371_T001	0.3840837	1.540450453	Zm00001d020371
Zm00001d020371_T003	0.09603243	-1.241266301	Zm00001d020371
Zm00001d046508_T001	0.9979568	0.492510425	Zm00001d046508
Zm00001d048325_T001	0.923684	-0.410176898	Zm00001d048325
Zm00001d028416_T002	0.9791066	0.579336093	Zm00001d028416
Zm00001d027439_T001	0.7008849	1.12858205	Zm00001d027439
Zm00001d006815_T001	0.7421355	-0.879548871	Zm00001d006815
Zm00001d023592_T001	0.06641903	2.207749534	Zm00001d023592

Zm00001d012932_T001	0.9740325	0.165172137	Zm00001d012932
Zm00001d021448_T002	0.6745773	-0.230211564	Zm00001d021448
Zm00001d003780_T001	0.8162418	-0.470970876	Zm00001d003780
Zm00001d019277_T001	0.6020989	-0.847166529	Zm00001d019277
Zm00001d040266_T001	0.9956349	0.152294196	Zm00001d040266
Zm00001d025059_T001	0.8990393	-0.220971655	Zm00001d025059
Zm00001d028369_T001	0.9825388	0.270147981	Zm00001d028369
Zm00001d053070_T001	0.9909153	0.442196211	Zm00001d053070
Zm00001d024160_T001	0.5005017	-0.539666501	Zm00001d024160
Zm00001d024160_T003	0.7759327	-0.267706912	Zm00001d024160
Zm00001d024160_T002	0.7748675	-0.273792075	Zm00001d024160
Zm00001d021954_T012	0.9003815	-0.269162998	Zm00001d021954
Zm00001d021954_T006	0.05692626	0.394365253	Zm00001d021954
Zm00001d021954_T003	0.8641671	0.872920351	Zm00001d021954
Zm00001d013509_T001	0.9002941	-0.610480603	Zm00001d013509
Zm00001d021089_T001	0.7440232	-0.421076019	Zm00001d021089
Zm00001d017036_T003	0.2664773	-1.275412354	Zm00001d017036
Zm00001d017036_T005	0.0419536	-1.666462967	Zm00001d017036
Zm00001d017036_T004	3.61E-05	3.072976408	Zm00001d017036
Zm00001d025912_T001	0.8926893	-0.10911866	Zm00001d025912
Zm00001d038176_T001	0.9037002	0.411983299	Zm00001d038176
Zm00001d043779_T002	0.907891	0.441348291	Zm00001d043779
Zm00001d043779_T001	0.9431783	-0.020833815	Zm00001d043779
Zm00001d043779_T003	0.9819191	0.116714099	Zm00001d043779
Zm00001d032794_T001	0.7847557	-0.263596385	Zm00001d032794
Zm00001d041065_T001	0.757295	0.672951364	Zm00001d041065
Zm00001d001941_T001	0.6427032	-0.393280459	Zm00001d001941
Zm00001d050553_T001	0.8030099	-0.377280166	Zm00001d050553
Zm00001d028867_T003	0.9894615	-0.019035533	Zm00001d028867
Zm00001d028867_T002	0.9374853	-0.362880779	Zm00001d028867
Zm00001d028995_T001	0.9529184	0.661942048	Zm00001d028995
Zm00001d053104_T005	0.9988954	0.321352826	Zm00001d053104
Zm00001d053104_T004	0.8672229	-0.176499031	Zm00001d053104
Zm00001d053104_T001	0.05065135	0.949707547	Zm00001d053104
Zm00001d053104_T006	0.9791729	0.001278126	Zm00001d053104
Zm00001d053545_T003	0.9312703	0.017039241	Zm00001d053545
Zm00001d011654_T001	0.001570084	3.117998113	Zm00001d011654
Zm00001d009883_T098	0.5902617	-0.306045607	Zm00001d009883
Zm00001d009883_T080	0.85068	-0.084808036	Zm00001d009883
Zm00001d009883_T021	0.907891	0.436567759	Zm00001d009883
Zm00001d009883_T090	0.896517	0.259867664	Zm00001d009883
Zm00001d009883_T085	0.8998284	-0.319197269	Zm00001d009883
Zm00001d009883_T055	0.9822138	-0.003347816	Zm00001d009883
Zm00001d009883_T102	1	0.245243083	Zm00001d009883
Zm00001d009883_T086	0.08984708	-0.821271811	Zm00001d009883
Zm00001d009883_T031	0.9449404	0.286099986	Zm00001d009883
Zm00001d009883_T008	1.09E-06	-0.898044473	Zm00001d009883
Zm00001d009883_T002	0.7614252	-0.197657619	Zm00001d009883
Zm00001d009883_T050	8.50E-09	0.804893556	Zm00001d009883
Zm00001d032362_T001	0.9203655	0.660383109	Zm00001d032362
Zm00001d032362_T002	0.7289263	1.010941463	Zm00001d032362
Zm00001d032362_T003	0.03353728	2.025937395	Zm00001d032362
Zm00001d004979_T010	0.5247476	0.885071632	Zm00001d004979
Zm00001d004979_T003	0.03045399	1.156222922	Zm00001d004979

Zm00001d004979_T017	0.9914036	0.3326597	Zm00001d004979
Zm00001d036719_T001	1	0.241702238	Zm00001d036719
Zm00001d030613_T003	0.9390517	0.574057644	Zm00001d030613
Zm00001d022258_T003	0.925875	0.191010724	Zm00001d022258
Zm00001d022258_T002	0.7775347	1.088568262	Zm00001d022258
Zm00001d022258_T005	2.24E-13	-3.759860034	Zm00001d022258
Zm00001d043275_T005	0.4344214	-0.26513906	Zm00001d043275
Zm00001d043275_T009	0.8770159	-0.023598098	Zm00001d043275
Zm00001d043275_T008	0.01526086	0.913199656	Zm00001d043275
Zm00001d043275_T003	1	0.27981818	Zm00001d043275
Zm00001d043275_T001	0.7749749	-0.170465409	Zm00001d043275
Zm00001d043275_T007	0.7951038	1.036892915	Zm00001d043275
Zm00001d043275_T011	0.8290178	0.306170216	Zm00001d043275
Zm00001d003252_T006	0.8439431	0.931713032	Zm00001d003252
Zm00001d003252_T002	0.9067873	0.677384113	Zm00001d003252
Zm00001d003252_T001	0.6821817	0.225235304	Zm00001d003252
Zm00001d003252_T004	0.9732421	0.272523757	Zm00001d003252
Zm00001d038548_T001	0.8929316	0.240175832	Zm00001d038548
Zm00001d046488_T003	0.6761847	-0.300161679	Zm00001d046488
Zm00001d046488_T002	0.8095986	-0.204474329	Zm00001d046488
Zm00001d046488_T001	0.8684855	-0.015125809	Zm00001d046488
Zm00001d042386_T001	0.9189175	-0.174337686	Zm00001d042386
Zm00001d042386_T003	0.9880075	-0.118839015	Zm00001d042386
Zm00001d003220_T002	0.9804228	0.191486346	Zm00001d003220
Zm00001d024390_T001	0.954492	-0.173311743	Zm00001d024390
Zm00001d035038_T005	0.9493302	0.360964016	Zm00001d035038
Zm00001d037532_T001	0.9988864	0.325516599	Zm00001d037532
Zm00001d008997_T004	0.9198491	-0.209452571	Zm00001d008997
Zm00001d008997_T002	0.8740066	0.186048236	Zm00001d008997
Zm00001d008997_T001	0.5831618	-0.103079066	Zm00001d008997
Zm00001d008997_T003	0.3097895	0.560602583	Zm00001d008997
Zm00001d008997_T006	0.9359529	-0.194565078	Zm00001d008997
Zm00001d029938_T005	0.9779851	0.044033779	Zm00001d029938
Zm00001d029938_T010	0.9745314	0.139077833	Zm00001d029938
Zm00001d029938_T002	0.467848	1.373812724	Zm00001d029938
Zm00001d029938_T003	0.02815838	0.380270262	Zm00001d029938
Zm00001d045427_T001	0.6876043	-0.557274767	Zm00001d045427
Zm00001d049355_T004	0.9580876	0.340234203	Zm00001d049355
Zm00001d049355_T001	0.2873089	-0.682666746	Zm00001d049355
Zm00001d013976_T001	0.8142464	-0.150833211	Zm00001d013976
Zm00001d013976_T003	0.1178643	0.548022678	Zm00001d013976
Zm00001d013976_T005	0.33771	0.665810411	Zm00001d013976
Zm00001d049081_T001	0.5748211	0.913977333	Zm00001d049081
Zm00001d028955_T001	0.9554972	-0.118027824	Zm00001d028955
Zm00001d046973_T005	0.9215988	0.022392168	Zm00001d046973
Zm00001d046973_T002	0.9203655	0.778443331	Zm00001d046973
Zm00001d046973_T006	0.587212	0.247524235	Zm00001d046973
Zm00001d052230_T001	0.9303773	0.69786502	Zm00001d052230
Zm00001d052230_T002	0.6086477	0.770432295	Zm00001d052230
Zm00001d049815_T002	0.90641	-0.39972191	Zm00001d049815
Zm00001d010919_T001	0.9797082	0.541809992	Zm00001d010919
Zm00001d029686_T001	0.7440232	-0.27037827	Zm00001d029686
Zm00001d026301_T002	1.11E-11	2.164264922	Zm00001d026301
Zm00001d026301_T001	0.9016285	-0.01852084	Zm00001d026301

Zm00001d026301_T004	5.10E-11	-1.605910997	Zm00001d026301
Zm00001d026301_T006	0.9613188	0.015949079	Zm00001d026301
Zm00001d026301_T003	0.5514634	0.741713334	Zm00001d026301
Zm00001d025222_T001	0.9766874	0.212516236	Zm00001d025222
Zm00001d033616_T039	5.34E-08	0.961209492	Zm00001d033616
Zm00001d033616_T044	0.8641671	-0.076340579	Zm00001d033616
Zm00001d033616_T043	0.3097848	1.301052453	Zm00001d033616
Zm00001d033616_T042	0.4146004	-0.095930009	Zm00001d033616
Zm00001d033616_T005	0.3686912	0.866077589	Zm00001d033616
Zm00001d033616_T004	2.82E-12	2.158789941	Zm00001d033616
Zm00001d033616_T040	0.9745777	0.100157465	Zm00001d033616
Zm00001d033616_T027	0.1587915	0.726619716	Zm00001d033616
Zm00001d033616_T041	0.8955831	-0.062012083	Zm00001d033616
Zm00001d033616_T013	0.2265356	0.430549705	Zm00001d033616
Zm00001d031287_T001	0.8224736	0.913036521	Zm00001d031287
Zm00001d016874_T001	0.8660897	0.607599536	Zm00001d016874
Zm00001d051286_T008	0.5817672	0.82459026	Zm00001d051286
Zm00001d051286_T015	0.5466134	-0.622634827	Zm00001d051286
Zm00001d051286_T006	0.9139882	0.7049012	Zm00001d051286
Zm00001d051286_T010	0.5519436	0.389599208	Zm00001d051286
Zm00001d044104_T001	0.9091175	-0.085266211	Zm00001d044104
Zm00001d036797_T002	0.4786492	-0.331609111	Zm00001d036797
Zm00001d035309_T001	0.9668789	0.319447246	Zm00001d035309
Zm00001d002240_T001	0.9683523	0.514038841	Zm00001d002240
Zm00001d012916_T001	0.9561521	0.655606971	Zm00001d012916
Zm00001d012916_T007	0.01296494	0.888391125	Zm00001d012916
Zm00001d010945_T002	0.9979568	0.366296436	Zm00001d010945
Zm00001d031535_T001	0.7211861	0.73075303	Zm00001d031535
Zm00001d008411_T002	0.3793662	0.383927272	Zm00001d008411
Zm00001d008411_T011	0.6465648	-0.418963819	Zm00001d008411
Zm00001d008411_T001	0.6062927	0.868267522	Zm00001d008411
Zm00001d049370_T003	0.8740066	0.777513405	Zm00001d049370
Zm00001d005190_T001	0.7297076	-0.303912003	Zm00001d005190
Zm00001d020674_T001	0.772948	0.689174562	Zm00001d020674
Zm00001d005685_T001	0.9717989	0.357557609	Zm00001d005685
Zm00001d020537_T001	1	0.38017167	Zm00001d020537
Zm00001d045096_T001	0.9441918	-0.296477769	Zm00001d045096
Zm00001d017780_T004	0.001591827	2.206673346	Zm00001d017780
Zm00001d017780_T002	0.002825	3.226006847	Zm00001d017780
Zm00001d017780_T001	0.1272455	2.196926174	Zm00001d017780
Zm00001d044411_T001	0.9937567	0.08840033	Zm00001d044411
Zm00001d013360_T002	0.9864331	0.452081962	Zm00001d013360
Zm00001d013360_T003	0.9756546	0.370773903	Zm00001d013360
Zm00001d013360_T004	0.965836	0.05904833	Zm00001d013360
Zm00001d046364_T001	0.1098307	2.136552998	Zm00001d046364
Zm00001d013983_T001	0.8537544	0.851846093	Zm00001d013983
Zm00001d009603_T002	0.9782953	0.133964407	Zm00001d009603
Zm00001d009603_T001	0.93919	0.728685205	Zm00001d009603
Zm00001d009603_T004	0.4579981	0.580705737	Zm00001d009603
Zm00001d009603_T003	1	0.330745884	Zm00001d009603
Zm00001d043665_T001	0.9890668	-0.017124901	Zm00001d043665
Zm00001d044043_T001	0.9647033	0.112598304	Zm00001d044043
Zm00001d031168_T001	0.4035077	-1.023034209	Zm00001d031168
Zm00001d011278_T002	0.9508107	-0.197742849	Zm00001d011278

Zm00001d011278_T001	0.9894615	-0.0587072	Zm00001d011278
Zm00001d031205_T003	0.9519258	0.445707859	Zm00001d031205
Zm00001d031205_T001	0.9969123	0.042392512	Zm00001d031205
Zm00001d036072_T001	0.006436882	-0.606012028	Zm00001d036072
Zm00001d050393_T002	0.6698357	-0.650151266	Zm00001d050393
Zm00001d021521_T001	0.9851204	-0.171231979	Zm00001d021521
Zm00001d018325_T001	0.8753591	0.748117011	Zm00001d018325
Zm00001d032409_T001	0.8924711	-0.057222218	Zm00001d032409
Zm00001d021732_T001	1	0.138816155	Zm00001d021732
Zm00001d003038_T001	0.949733	0.066357518	Zm00001d003038
Zm00001d012883_T001	0.3385848	-0.898915348	Zm00001d012883
Zm00001d025017_T001	0.9360579	0.629127287	Zm00001d025017
Zm00001d011691_T006	0.728046	0.121672151	Zm00001d011691
Zm00001d011691_T002	0.8886159	-0.145207815	Zm00001d011691
Zm00001d012517_T001	0.8929316	-0.082978214	Zm00001d012517
Zm00001d038793_T001	0.1966199	1.09085669	Zm00001d038793
Zm00001d030677_T005	0.8676122	-0.15643721	Zm00001d030677
Zm00001d030677_T008	0.7458694	-0.188749007	Zm00001d030677
Zm00001d042380_T001	1	0.162558232	Zm00001d042380
Zm00001d004070_T003	0.8955831	-0.031266289	Zm00001d004070
Zm00001d004070_T001	0.9173494	0.00109847	Zm00001d004070
Zm00001d004070_T005	0.843874	-0.498656796	Zm00001d004070
Zm00001d015623_T001	0.9902733	0.389659282	Zm00001d015623
Zm00001d030031_T002	0.9855117	0.523998632	Zm00001d030031
Zm00001d048521_T003	0.01102023	1.094578956	Zm00001d048521
Zm00001d048521_T002	0.5535159	1.143849978	Zm00001d048521
Zm00001d042437_T001	0.02879806	2.626731082	Zm00001d042437
Zm00001d042437_T008	0.2433232	1.833194471	Zm00001d042437
Zm00001d046221_T052	0.644634	-0.420546887	Zm00001d046221
Zm00001d046221_T021	0.98291	-0.555397413	Zm00001d046221
Zm00001d046221_T002	0.9570268	0.34117067	Zm00001d046221
Zm00001d046221_T014	0.01472471	1.730671277	Zm00001d046221
Zm00001d046221_T053	0.9832092	0.160274908	Zm00001d046221
Zm00001d043991_T001	0.9361945	-0.046461298	Zm00001d043991
Zm00001d027963_T001	0.9664406	0.061773942	Zm00001d027963
Zm00001d052436_T001	0.9825858	0.206754119	Zm00001d052436
Zm00001d009861_T001	0.8993062	-0.559809552	Zm00001d009861
Zm00001d017212_T001	0.9160053	0.630240206	Zm00001d017212
Zm00001d021533_T001	0.07204629	-1.455097639	Zm00001d021533
Zm00001d004120_T001	0.9486412	-0.158556917	Zm00001d004120
Zm00001d037868_T001	0.000475705	2.432534481	Zm00001d037868
Zm00001d017547_T001	0.5925664	1.278542972	Zm00001d017547
Zm00001d053179_T003	0.4274324	-0.799246431	Zm00001d053179
Zm00001d053179_T002	0.6503892	1.213254621	Zm00001d053179
Zm00001d053179_T004	0.5186912	0.813872371	Zm00001d053179
Zm00001d053179_T001	0.65541	0.892336159	Zm00001d053179
Zm00001d048683_T001	0.7561027	-0.250032433	Zm00001d048683
Zm00001d032083_T001	0.4949998	1.327743193	Zm00001d032083
Zm00001d052144_T001	0.9488298	0.03698509	Zm00001d052144
Zm00001d042909_T001	0.2661465	1.469606992	Zm00001d042909
Zm00001d014549_T001	0.9979568	0.177138669	Zm00001d014549
Zm00001d000228_T066	0.6343135	-0.211076453	Zm00001d000228
Zm00001d000228_T042	0.003658349	-3.288127237	Zm00001d000228
Zm00001d000228_T026	0.3706063	0.622991404	Zm00001d000228

Zm00001d000228_T043	0.2731207	-0.937695083	Zm00001d000228
Zm00001d000228_T035	0.5042696	-0.77402492	Zm00001d000228
Zm00001d000228_T025	0.9447869	-0.278054379	Zm00001d000228
Zm00001d000228_T001	0.3746954	-0.599743964	Zm00001d000228
Zm00001d000228_T040	4.75E-12	2.167081783	Zm00001d000228
Zm00001d000228_T033	0.7990254	-0.403738077	Zm00001d000228
Zm00001d000228_T051	0.7273213	-0.312918754	Zm00001d000228
Zm00001d000228_T032	0.4471095	0.778873659	Zm00001d000228
Zm00001d000228_T012	0.9666757	0.601417717	Zm00001d000228
Zm00001d000228_T060	0.7369755	-0.210293883	Zm00001d000228
Zm00001d000228_T028	0.9344642	0.047247665	Zm00001d000228
Zm00001d012748_T018	0.9172003	1.014847407	Zm00001d012748
Zm00001d012748_T023	0.05289783	2.000739144	Zm00001d012748
Zm00001d012748_T002	0.9020229	0.95082403	Zm00001d012748
Zm00001d012748_T021	0.3221433	1.727971454	Zm00001d012748
Zm00001d012748_T001	0.007698854	1.41600008	Zm00001d012748
Zm00001d012748_T003	1.86E-15	4.026054559	Zm00001d012748
Zm00001d013796_T008	0.9209394	-0.039369043	Zm00001d013796
Zm00001d013796_T024	4.34E-08	0.715088646	Zm00001d013796
Zm00001d013796_T006	0.8751967	-0.019814786	Zm00001d013796
Zm00001d013796_T052	0.6026834	-0.253485046	Zm00001d013796
Zm00001d013796_T048	0.2461415	0.163097672	Zm00001d013796
Zm00001d013796_T001	1.48E-05	0.419120556	Zm00001d013796
Zm00001d021442_T011	0.03180938	1.067051024	Zm00001d021442
Zm00001d021442_T005	0.6504599	0.95849939	Zm00001d021442
Zm00001d021442_T010	0.1285728	-0.356303915	Zm00001d021442
Zm00001d021442_T007	0.9618671	0.573587115	Zm00001d021442
Zm00001d021442_T008	0.9390501	0.290173666	Zm00001d021442
Zm00001d021442_T015	0.04208336	2.457210474	Zm00001d021442
Zm00001d021442_T004	0.9529184	0.723828428	Zm00001d021442
Zm00001d021442_T013	0.000441925	0.923098856	Zm00001d021442
Zm00001d021442_T003	3.77E-06	-2.680146018	Zm00001d021442
Zm00001d049336_T001	0.9673473	0.220600977	Zm00001d049336
Zm00001d018810_T002	0.8377512	-0.150366456	Zm00001d018810
Zm00001d038448_T003	0.01084676	2.843652882	Zm00001d038448
Zm00001d038448_T002	6.98E-05	4.010845945	Zm00001d038448
Zm00001d052022_T006	0.6914102	0.499663566	Zm00001d052022
Zm00001d020014_T009	0.9734722	0.197220561	Zm00001d020014
Zm00001d020014_T002	0.3744576	-0.520037551	Zm00001d020014
Zm00001d020014_T010	0.631264	0.900308253	Zm00001d020014
Zm00001d020014_T012	0.3780662	-0.741077854	Zm00001d020014
Zm00001d053866_T001	0.5807146	-0.174605858	Zm00001d053866
Zm00001d038997_T001	0.000227529	-4.614551951	Zm00001d038997
Zm00001d005052_T004	0.9700977	0.541743429	Zm00001d005052
Zm00001d005052_T003	0.3163102	0.84619477	Zm00001d005052
Zm00001d005052_T002	0.000267083	0.551276382	Zm00001d005052
Zm00001d008634_T001	0.8494782	-0.361080828	Zm00001d008634
Zm00001d021060_T001	0.8781102	-0.404334515	Zm00001d021060
Zm00001d009899_T001	0.5695353	-0.583997274	Zm00001d009899
Zm00001d051520_T001	0.1947403	-0.947159115	Zm00001d051520
Zm00001d012889_T001	0.3847344	-1.181522448	Zm00001d012889
Zm00001d049019_T004	0.06964423	1.435262449	Zm00001d049019
Zm00001d049019_T007	0.8810243	1.285117639	Zm00001d049019
Zm00001d049019_T008	0.3668531	-0.728222782	Zm00001d049019

Zm00001d049019_T006	0.3632201	1.043727354	Zm00001d049019
Zm00001d049019_T002	0.9690925	0.672914474	Zm00001d049019
Zm00001d003994_T001	0.07220675	-0.653343641	Zm00001d003994
Zm00001d027581_T001	0.9410688	-0.118107814	Zm00001d027581
Zm00001d023946_T001	0.9768514	0.459029496	Zm00001d023946
Zm00001d023946_T002	0.9037807	0.42518442	Zm00001d023946
Zm00001d019507_T036	0.08647754	0.924016708	Zm00001d019507
Zm00001d019507_T010	0.000479665	1.01117963	Zm00001d019507
Zm00001d019507_T032	1.09E-16	4.689461216	Zm00001d019507
Zm00001d019507_T011	0.9899643	0.567961605	Zm00001d019507
Zm00001d019507_T023	0.2796168	-0.862257785	Zm00001d019507
Zm00001d019507_T001	0.9765009	0.870174722	Zm00001d019507
Zm00001d019507_T006	0.07756837	2.611040932	Zm00001d019507
Zm00001d048608_T001	0.9605339	0.545589851	Zm00001d048608
Zm00001d031232_T009	0.8021694	-0.08173445	Zm00001d031232
Zm00001d031232_T024	0.8267985	-0.402577038	Zm00001d031232
Zm00001d031232_T067	0.2719671	0.781287314	Zm00001d031232
Zm00001d031232_T020	0.001248791	0.622599707	Zm00001d031232
Zm00001d031232_T004	0.8670002	-0.07183455	Zm00001d031232
Zm00001d031232_T056	8.17E-11	1.544408207	Zm00001d031232
Zm00001d031232_T043	0.9093204	-0.303060271	Zm00001d031232
Zm00001d031232_T022	0.001684569	1.046224887	Zm00001d031232
Zm00001d031232_T003	0.6004941	0.793223018	Zm00001d031232
Zm00001d020411_T001	0.4051828	1.416060073	Zm00001d020411
Zm00001d042764_T001	0.8883116	-0.090374785	Zm00001d042764
Zm00001d008620_T001	0.9833133	0.323774043	Zm00001d008620
Zm00001d043083_T006	0.2183658	0.934346035	Zm00001d043083
Zm00001d043083_T008	0.3205135	0.969683336	Zm00001d043083
Zm00001d043083_T001	0.9872831	0.411059892	Zm00001d043083
Zm00001d043083_T002	0.9192339	0.809436005	Zm00001d043083
Zm00001d043083_T003	0.07223423	1.864493625	Zm00001d043083
Zm00001d029072_T007	0.5667994	0.573357822	Zm00001d029072
Zm00001d029072_T008	0.9493688	0.51554603	Zm00001d029072
Zm00001d029072_T002	1	0.253095263	Zm00001d029072
Zm00001d029072_T013	1	0.827436779	Zm00001d029072
Zm00001d029072_T012	0.6115361	-0.169955126	Zm00001d029072
Zm00001d029072_T011	0.1238487	0.955048081	Zm00001d029072
Zm00001d029072_T003	0.09049554	0.4931162	Zm00001d029072
Zm00001d002737_T003	0.9907928	0.312738171	Zm00001d002737
Zm00001d022588_T003	0.9867452	0.472916391	Zm00001d022588
Zm00001d022588_T006	0.918657	0.338149831	Zm00001d022588
Zm00001d034840_T002	0.9887584	0.161382282	Zm00001d034840
Zm00001d034189_T001	0.1099527	1.39580388	Zm00001d034189
Zm00001d041950_T001	0.9979568	0.418223405	Zm00001d041950
Zm00001d014948_T001	0.995534	0.288855613	Zm00001d014948
Zm00001d021313_T001	0.976488	0.143839828	Zm00001d021313
Zm00001d025752_T006	0.07701001	1.369384496	Zm00001d025752
Zm00001d025752_T002	0.5440777	1.233345245	Zm00001d025752
Zm00001d025752_T004	0.5664363	1.308264295	Zm00001d025752
Zm00001d026359_T008	0.9410688	0.266229063	Zm00001d026359
Zm00001d026359_T007	0.1252127	0.491343957	Zm00001d026359
Zm00001d026359_T004	0.9780508	0.968953774	Zm00001d026359
Zm00001d026359_T002	0.1799314	1.452658103	Zm00001d026359
Zm00001d037807_T003	0.7943953	0.327876477	Zm00001d037807

Zm00001d037807_T001	0.9135335	-0.05765523	Zm00001d037807
Zm00001d037807_T006	0.4880931	-0.899373159	Zm00001d037807
Zm00001d037807_T004	0.04798446	2.3681619	Zm00001d037807
Zm00001d013785_T001	0.9909466	-0.10587026	Zm00001d013785
Zm00001d009552_T001	0.9373421	0.398945276	Zm00001d009552
Zm00001d009552_T003	1	0.147078445	Zm00001d009552
Zm00001d009552_T005	0.5549472	-1.23898263	Zm00001d009552
Zm00001d009552_T004	0.6217057	0.407989434	Zm00001d009552
Zm00001d043233_T001	0.9988864	0.346675722	Zm00001d043233
Zm00001d037784_T001	0.9975882	0.37222552	Zm00001d037784
Zm00001d026563_T001	0.1252127	1.96386894	Zm00001d026563
Zm00001d028584_T001	0.4386149	-1.218002982	Zm00001d028584
Zm00001d010590_T001	0.728046	-0.34072435	Zm00001d010590
Zm00001d016941_T010	0.9396809	0.239086282	Zm00001d016941
Zm00001d016941_T011	0.4360636	-1.106530394	Zm00001d016941
Zm00001d016941_T006	0.8535185	0.862421358	Zm00001d016941
Zm00001d016941_T008	0.8892107	0.591906691	Zm00001d016941
Zm00001d018032_T002	0.9493302	0.593914521	Zm00001d018032
Zm00001d018032_T003	0.9562711	0.029031675	Zm00001d018032
Zm00001d001879_T024	0.6034366	0.904762408	Zm00001d001879
Zm00001d001879_T014	0.7857363	-0.042370547	Zm00001d001879
Zm00001d001879_T039	0.07694878	0.53580628	Zm00001d001879
Zm00001d001879_T032	0.8990393	-0.016502903	Zm00001d001879
Zm00001d001879_T022	0.8350279	-0.044525762	Zm00001d001879
Zm00001d001879_T038	0.6845507	-0.541712669	Zm00001d001879
Zm00001d026193_T001	0.847053	0.920869355	Zm00001d026193
Zm00001d044705_T001	0.9822138	0.180606614	Zm00001d044705
Zm00001d019993_T001	0.9023635	-0.091589906	Zm00001d019993
Zm00001d044971_T014	0.01328455	-1.144585188	Zm00001d044971
Zm00001d044971_T016	0.5651392	-0.966238715	Zm00001d044971
Zm00001d044971_T001	1.98E-05	0.466499808	Zm00001d044971
Zm00001d044971_T006	0.2324372	1.789076717	Zm00001d044971
Zm00001d002132_T001	0.003908299	-2.538556714	Zm00001d002132
Zm00001d043201_T001	0.9791729	0.082486597	Zm00001d043201
Zm00001d010216_T003	0.08857859	-0.962850925	Zm00001d010216
Zm00001d010216_T004	0.9984041	0.316428106	Zm00001d010216
Zm00001d006205_T001	0.9791729	0.209686536	Zm00001d006205
Zm00001d031796_T001	4.98E-09	5.238569949	Zm00001d031796
Zm00001d047423_T002	0.909729	-0.142205236	Zm00001d047423
Zm00001d047423_T003	0.4431901	-0.609772121	Zm00001d047423
Zm00001d040737_T001	0.001627225	2.100746013	Zm00001d040737
Zm00001d013262_T002	0.9474116	0.395846803	Zm00001d013262
Zm00001d013262_T001	0.907891	-0.012001763	Zm00001d013262
Zm00001d013262_T004	0.7876948	0.378722301	Zm00001d013262
Zm00001d013262_T005	0.9257893	-0.129449368	Zm00001d013262
Zm00001d013262_T003	0.2398048	0.485360813	Zm00001d013262
Zm00001d030667_T001	0.8955831	0.455311325	Zm00001d030667
Zm00001d025712_T001	0.8661044	-0.052952066	Zm00001d025712
Zm00001d025712_T006	0.9570268	0.486365293	Zm00001d025712
Zm00001d003501_T001	0.8262962	0.725215929	Zm00001d003501
Zm00001d005735_T001	0.6838553	0.828456371	Zm00001d005735
Zm00001d022361_T001	0.8640519	0.900115281	Zm00001d022361
Zm00001d002272_T003	0.0372102	2.538063383	Zm00001d002272
Zm00001d002272_T013	1	0.424223634	Zm00001d002272

Zm00001d002272_T011	0.9822138	0.110840905	Zm00001d002272
Zm00001d002272_T009	0.90276	0.353964921	Zm00001d002272
Zm00001d002272_T008	0.3967188	1.262312435	Zm00001d002272
Zm00001d034400_T005	0.9911688	0.247019127	Zm00001d034400
Zm00001d034400_T003	0.9984041	0.408104321	Zm00001d034400
Zm00001d050697_T001	0.6086477	-0.556941883	Zm00001d050697
Zm00001d046812_T001	0.2461465	1.435826564	Zm00001d046812
Zm00001d043680_T001	0.7697196	0.972780475	Zm00001d043680
Zm00001d016228_T002	0.9429712	0.079407202	Zm00001d016228
Zm00001d016228_T005	1	0.316191173	Zm00001d016228
Zm00001d011255_T001	1	0.201749261	Zm00001d011255
Zm00001d011606_T001	0.7626242	-0.071951277	Zm00001d011606
Zm00001d011606_T002	0.7640001	-0.308827353	Zm00001d011606
Zm00001d037297_T002	1	0.151729442	Zm00001d037297
Zm00001d037297_T003	0.8197974	0.69230334	Zm00001d037297
Zm00001d037297_T004	0.9914912	0.72588814	Zm00001d037297
Zm00001d037297_T001	0.6340221	-0.340324274	Zm00001d037297
Zm00001d037297_T008	0.760208	-0.179267005	Zm00001d037297
Zm00001d011471_T001	0.7486045	-0.282238573	Zm00001d011471
Zm00001d002121_T001	0.006513324	-1.817438133	Zm00001d002121
Zm00001d048773_T001	0.3900878	-0.928906275	Zm00001d048773
Zm00001d015505_T001	0.9242611	-0.052207543	Zm00001d015505
Zm00001d010816_T001	0.3404511	1.455152472	Zm00001d010816
Zm00001d052192_T001	0.9469426	0.636484932	Zm00001d052192
Zm00001d014175_T013	1	0.024309357	Zm00001d014175
Zm00001d014175_T005	0.5495068	0.788339411	Zm00001d014175
Zm00001d014175_T011	0.9213679	0.715653471	Zm00001d014175
Zm00001d037401_T002	0.03347087	0.784721783	Zm00001d037401
Zm00001d037401_T001	0.666332	1.234067863	Zm00001d037401
Zm00001d021136_T001	1	0.353221507	Zm00001d021136
Zm00001d010244_T001	0.878248	0.877329441	Zm00001d010244
Zm00001d034777_T011	0.811516	0.441645948	Zm00001d034777
Zm00001d034777_T005	0.996879	0.42729091	Zm00001d034777
Zm00001d034777_T008	0.002951811	1.39308626	Zm00001d034777
Zm00001d034777_T013	0.5061365	0.414329837	Zm00001d034777
Zm00001d034777_T007	0.2380337	1.160963559	Zm00001d034777
Zm00001d034777_T014	0.9887584	0.143979799	Zm00001d034777
Zm00001d034777_T001	0.4941853	0.469646841	Zm00001d034777
Zm00001d034777_T015	0.7434928	-0.775435988	Zm00001d034777
Zm00001d046927_T001	0.006990354	-2.509833082	Zm00001d046927
Zm00001d010026_T001	0.9928864	0.298266429	Zm00001d010026
Zm00001d010026_T002	0.9979568	0.296712838	Zm00001d010026
Zm00001d018206_T001	0.000425577	3.854302506	Zm00001d018206
Zm00001d006335_T003	0.9939021	0.250485659	Zm00001d006335
Zm00001d006335_T001	0.6184626	0.541353956	Zm00001d006335
Zm00001d006335_T004	0.8323635	-0.122556151	Zm00001d006335
Zm00001d012204_T001	0.9921225	0.193711439	Zm00001d012204
Zm00001d053150_T019	0.7285296	-0.549269626	Zm00001d053150
Zm00001d053150_T018	0.9880003	-0.125429497	Zm00001d053150
Zm00001d053150_T007	0.07142513	1.403925546	Zm00001d053150
Zm00001d053150_T017	0.9203655	-0.105656446	Zm00001d053150
Zm00001d053150_T008	0.9269932	0.005800736	Zm00001d053150
Zm00001d045605_T001	0.4359356	0.657823753	Zm00001d045605
Zm00001d008493_T001	0.7509844	-0.297329594	Zm00001d008493

Zm00001d008493_T010	0.4187707	0.558821848	Zm00001d008493
Zm00001d008493_T002	0.3989002	0.582506671	Zm00001d008493
Zm00001d003369_T001	0.0606758	2.338729104	Zm00001d003369
Zm00001d003369_T002	0.195109	-0.28524974	Zm00001d003369
Zm00001d003369_T003	0.5615573	0.867669722	Zm00001d003369
Zm00001d003369_T004	0.8125642	-0.194572835	Zm00001d003369
Zm00001d042343_T001	0.8728996	0.85633427	Zm00001d042343
Zm00001d024519_T008	0.9693382	0.10496691	Zm00001d024519
Zm00001d024519_T017	0.8300193	0.868266736	Zm00001d024519
Zm00001d024519_T001	0.7631455	-0.319967168	Zm00001d024519
Zm00001d024519_T015	0.9083252	0.652332703	Zm00001d024519
Zm00001d024519_T004	0.7458694	-0.253029621	Zm00001d024519
Zm00001d024519_T012	1	0.09925366	Zm00001d024519
Zm00001d016242_T002	0.997449	0.173663506	Zm00001d016242
Zm00001d016242_T001	0.1692579	-1.002577434	Zm00001d016242
Zm00001d009669_T004	0.57162	-0.203104919	Zm00001d009669
Zm00001d009669_T001	0.4963648	-0.716194597	Zm00001d009669
Zm00001d009669_T003	0.8654191	0.892177912	Zm00001d009669
Zm00001d009669_T010	0.1548815	1.984572514	Zm00001d009669
Zm00001d009669_T006	0.6023541	0.416391314	Zm00001d009669
Zm00001d044225_T001	0.8346952	-0.197644539	Zm00001d044225
Zm00001d032703_T002	0.09289352	-1.894445109	Zm00001d032703
Zm00001d032703_T001	0.9090332	0.386031176	Zm00001d032703
Zm00001d032703_T007	0.3124347	0.937058695	Zm00001d032703
Zm00001d032703_T013	0.001297994	2.038892738	Zm00001d032703
Zm00001d032703_T006	0.1813603	0.693738992	Zm00001d032703
Zm00001d032703_T018	0.52848	-0.086901498	Zm00001d032703
Zm00001d032703_T011	0.7037831	0.906570902	Zm00001d032703
Zm00001d032703_T009	0.3202352	1.736176173	Zm00001d032703
Zm00001d049986_T001	0.4647287	0.782817931	Zm00001d049986
Zm00001d005675_T005	0.2935147	-0.279201795	Zm00001d005675
Zm00001d005675_T009	0.1848367	0.56837378	Zm00001d005675
Zm00001d005675_T003	0.963455	-0.321097185	Zm00001d005675
Zm00001d005675_T001	0.9201237	0.560535937	Zm00001d005675
Zm00001d005675_T004	0.9748107	-0.003636819	Zm00001d005675
Zm00001d012276_T001	0.8489797	-0.302143725	Zm00001d012276
Zm00001d029084_T001	1	0.369977451	Zm00001d029084
Zm00001d029084_T002	0.9213679	-0.077672024	Zm00001d029084
Zm00001d029084_T005	0.8686467	0.417130917	Zm00001d029084
Zm00001d008178_T028	0.02165489	-1.787687113	Zm00001d008178
Zm00001d008178_T019	0.9979568	0.286978282	Zm00001d008178
Zm00001d008178_T026	0.9370449	0.329897013	Zm00001d008178
Zm00001d008178_T022	0.9745777	0.274463793	Zm00001d008178
Zm00001d008178_T001	0.6778694	1.233558807	Zm00001d008178
Zm00001d008178_T007	0.9894615	0.297933789	Zm00001d008178
Zm00001d008178_T003	0.2986892	0.63848493	Zm00001d008178
Zm00001d008178_T012	0.01215579	3.050632203	Zm00001d008178
Zm00001d021979_T003	0.6113292	1.297712503	Zm00001d021979
Zm00001d021979_T005	0.1763656	0.321174663	Zm00001d021979
Zm00001d021979_T006	0.8831529	0.802707963	Zm00001d021979
Zm00001d042052_T009	0.6292709	1.162174572	Zm00001d042052
Zm00001d042052_T001	0.8862467	-0.387603171	Zm00001d042052
Zm00001d042052_T007	0.4988467	0.231567181	Zm00001d042052
Zm00001d042052_T002	0.8677544	0.214740216	Zm00001d042052

Zm00001d003683_T003	0.9116127	-0.034319968	Zm00001d003683
Zm00001d003683_T001	0.1237647	-0.287817071	Zm00001d003683
Zm00001d043418_T050	0.8310488	0.785475706	Zm00001d043418
Zm00001d043418_T114	0.1456892	0.882698746	Zm00001d043418
Zm00001d043418_T080	0.05599027	0.614777176	Zm00001d043418
Zm00001d043418_T036	0.3838671	0.217294195	Zm00001d043418
Zm00001d043418_T032	0.07233484	0.909204016	Zm00001d043418
Zm00001d043418_T003	0.01325275	1.091674473	Zm00001d043418
Zm00001d043418_T010	0.35976	0.773992884	Zm00001d043418
Zm00001d043418_T008	0.1763656	-0.293156748	Zm00001d043418
Zm00001d043418_T136	0.2389955	-1.037855936	Zm00001d043418
Zm00001d043418_T031	0.06540092	0.919320311	Zm00001d043418
Zm00001d043418_T075	0.08392805	0.538380861	Zm00001d043418
Zm00001d043418_T053	0.9666757	-0.070193829	Zm00001d043418
Zm00001d043418_T051	0.9019184	0.541459088	Zm00001d043418
Zm00001d043418_T125	0.9889811	-0.047569909	Zm00001d043418
Zm00001d043418_T083	0.9868821	-0.011821186	Zm00001d043418
Zm00001d043418_T066	0.5936419	0.283687273	Zm00001d043418
Zm00001d043418_T079	4.26E-09	0.90092109	Zm00001d043418
Zm00001d043418_T139	0.4259365	-1.137599483	Zm00001d043418
Zm00001d022332_T001	0.9781672	0.007609501	Zm00001d022332
Zm00001d012479_T001	0.3808423	0.873749941	Zm00001d012479
Zm00001d012479_T003	0.8172581	-0.043844985	Zm00001d012479
Zm00001d012479_T004	0.331885	-0.59199477	Zm00001d012479
Zm00001d036947_T001	0.8720862	0.293572779	Zm00001d036947
Zm00001d037898_T004	0.919888	-0.117735301	Zm00001d037898
Zm00001d037898_T002	0.2790987	0.998205778	Zm00001d037898
Zm00001d037898_T006	0.9529184	-0.068946269	Zm00001d037898
Zm00001d034365_T001	0.3692327	1.412025396	Zm00001d034365
Zm00001d009504_T003	0.7505198	1.087773567	Zm00001d009504
Zm00001d009504_T004	0.9974023	0.237928541	Zm00001d009504
Zm00001d016632_T001	0.08348055	0.98942136	Zm00001d016632
Zm00001d038057_T003	0.8012608	-0.356239547	Zm00001d038057
Zm00001d038057_T001	0.9855117	-0.241242775	Zm00001d038057
Zm00001d045049_T002	0.9942835	0.213635541	Zm00001d045049
Zm00001d030116_T001	0.9129428	0.657198961	Zm00001d030116
Zm00001d019809_T001	0.5718096	-0.613818546	Zm00001d019809
Zm00001d031157_T001	0.2786734	-1.282551052	Zm00001d031157
Zm00001d029803_T005	0.606598	1.238646882	Zm00001d029803
Zm00001d029803_T009	0.4403985	0.769909693	Zm00001d029803
Zm00001d048294_T001	0.001131005	3.615368426	Zm00001d048294
Zm00001d049020_T001	0.9486412	0.031060006	Zm00001d049020
Zm00001d032763_T004	1	0.158007302	Zm00001d032763
Zm00001d032763_T003	0.9694613	0.230877014	Zm00001d032763
Zm00001d032763_T008	0.6727631	-0.466740058	Zm00001d032763
Zm00001d032763_T002	0.9091175	0.638471652	Zm00001d032763
Zm00001d051846_T004	0.9613462	0.157588587	Zm00001d051846
Zm00001d051846_T009	1	0.022323171	Zm00001d051846
Zm00001d051846_T014	0.8412913	0.203593107	Zm00001d051846
Zm00001d051846_T007	0.04016634	0.669930111	Zm00001d051846
Zm00001d013301_T001	0.000973999	-0.461218732	Zm00001d013301
Zm00001d013301_T004	0.007772415	2.067071869	Zm00001d013301
Zm00001d013301_T003	0.8891117	0.015070396	Zm00001d013301
Zm00001d013301_T002	0.894759	-0.038182023	Zm00001d013301

Zm00001d017252_T010	0.768921	0.639466802	Zm00001d017252
Zm00001d017252_T002	0.9326774	0.696849957	Zm00001d017252
Zm00001d017252_T008	0.965919	-0.033732611	Zm00001d017252
Zm00001d017252_T009	0.8332116	-0.310810368	Zm00001d017252
Zm00001d006497_T005	0.7960956	0.607135016	Zm00001d006497
Zm00001d006497_T001	0.8127863	-0.156186798	Zm00001d006497
Zm00001d006497_T008	0.07730424	0.857430372	Zm00001d006497
Zm00001d004300_T003	0.7846964	1.056495488	Zm00001d004300
Zm00001d004300_T012	0.577886	-0.920135748	Zm00001d004300
Zm00001d004300_T005	0.3254084	0.617564261	Zm00001d004300
Zm00001d004300_T009	0.8119951	0.131350915	Zm00001d004300
Zm00001d004300_T006	0.9979568	0.733644295	Zm00001d004300
Zm00001d018081_T001	0.522218	-0.843411222	Zm00001d018081
Zm00001d042856_T002	0.9226373	-0.399502861	Zm00001d042856
Zm00001d052371_T001	0.6451042	-0.638545915	Zm00001d052371
Zm00001d052154_T001	0.9706887	0.336492908	Zm00001d052154
Zm00001d042670_T033	1	0.057279605	Zm00001d042670
Zm00001d042670_T025	0.3761866	-0.568527092	Zm00001d042670
Zm00001d042670_T004	9.63E-11	1.468390403	Zm00001d042670
Zm00001d042670_T041	0.007698854	-1.106150607	Zm00001d042670
Zm00001d042670_T012	0.2627766	0.682474636	Zm00001d042670
Zm00001d028025_T001	0.6445434	-0.601658597	Zm00001d028025
Zm00001d054088_T001	0.3097848	1.910478844	Zm00001d054088
Zm00001d054088_T012	0.9272378	0.111981047	Zm00001d054088
Zm00001d054088_T002	0.5980883	1.113362051	Zm00001d054088
Zm00001d054088_T003	0.9605339	0.955778227	Zm00001d054088
Zm00001d021350_T003	0.9592583	0.025972109	Zm00001d021350
Zm00001d048962_T001	1	0.422498378	Zm00001d048962
Zm00001d016388_T001	0.8580945	-0.400407421	Zm00001d016388
Zm00001d018366_T001	0.9970835	0.255402794	Zm00001d018366
Zm00001d040082_T002	0.8158142	0.885412243	Zm00001d040082
Zm00001d040082_T012	0.8248596	0.862302348	Zm00001d040082
Zm00001d021869_T001	0.9781672	-0.059108951	Zm00001d021869
Zm00001d008395_T001	0.8520487	-0.422052099	Zm00001d008395
Zm00001d002656_T004	0.497443	0.481458011	Zm00001d002656
Zm00001d002656_T007	0.9273903	-0.322264853	Zm00001d002656
Zm00001d002656_T003	0.896517	0.743352572	Zm00001d002656
Zm00001d008320_T003	0.6502105	1.072398533	Zm00001d008320
Zm00001d008320_T001	0.9519347	0.430423156	Zm00001d008320
Zm00001d008320_T005	0.6988664	0.661967894	Zm00001d008320
Zm00001d008320_T006	0.954492	0.07829616	Zm00001d008320
Zm00001d008320_T004	0.8050292	0.902880534	Zm00001d008320
Zm00001d008320_T002	0.325118	0.68632822	Zm00001d008320
Zm00001d021375_T001	0.9979568	-0.09300751	Zm00001d021375
Zm00001d021375_T003	7.59E-05	-0.896335339	Zm00001d021375
Zm00001d021375_T002	0.06044655	1.109242041	Zm00001d021375
Zm00001d022053_T001	0.9826672	0.242604661	Zm00001d022053
Zm00001d005649_T001	0.9819471	0.208830161	Zm00001d005649
Zm00001d005649_T002	0.9748076	0.153835091	Zm00001d005649
Zm00001d027726_T002	0.9160053	0.828816615	Zm00001d027726
Zm00001d027726_T007	0.1197456	0.780695167	Zm00001d027726
Zm00001d027726_T011	0.9213679	0.035056441	Zm00001d027726
Zm00001d027726_T013	0.139268	0.602287286	Zm00001d027726
Zm00001d012450_T001	0.9747714	0.056371196	Zm00001d012450

Zm00001d007037_T003	0.1889053	1.675534878	Zm00001d007037
Zm00001d007037_T002	0.3097848	1.666533887	Zm00001d007037
Zm00001d007037_T001	0.5079763	1.18474026	Zm00001d007037
Zm00001d036364_T001	0.6847424	-0.346873681	Zm00001d036364
Zm00001d031991_T001	0.9988864	-0.026112542	Zm00001d031991
Zm00001d005275_T001	1	0.325545775	Zm00001d005275
Zm00001d014796_T001	0.8238535	-0.238345391	Zm00001d014796
Zm00001d048220_T001	6.88E-08	4.526765037	Zm00001d048220
Zm00001d012789_T001	0.7345787	0.846862981	Zm00001d012789
Zm00001d002996_T001	0.5988928	0.789131556	Zm00001d002996
Zm00001d029133_T009	0.9851204	0.548813847	Zm00001d029133
Zm00001d029133_T023	0.8380736	-0.25184459	Zm00001d029133
Zm00001d029133_T005	0.001494715	-1.067539578	Zm00001d029133
Zm00001d029133_T008	0.3195525	1.280509064	Zm00001d029133
Zm00001d029133_T010	0.139268	0.334211323	Zm00001d029133
Zm00001d016311_T006	0.6806964	0.500383828	Zm00001d016311
Zm00001d016311_T001	0.5001427	-0.226756804	Zm00001d016311
Zm00001d016311_T002	0.9016285	0.823910362	Zm00001d016311
Zm00001d016311_T007	0.7425238	0.228211572	Zm00001d016311
Zm00001d036888_T006	0.9563232	0.380872397	Zm00001d036888
Zm00001d036888_T003	0.998484	0.378266461	Zm00001d036888
Zm00001d051139_T001	0.6019545	-0.501038954	Zm00001d051139
Zm00001d051139_T003	0.7758624	-0.10067794	Zm00001d051139
Zm00001d031032_T001	0.9272378	0.36809484	Zm00001d031032
Zm00001d046783_T001	0.8050292	-0.201110454	Zm00001d046783
Zm00001d008274_T002	5.53E-06	-0.656389313	Zm00001d008274
Zm00001d032475_T001	0.000190425	3.95509365	Zm00001d032475
Zm00001d019462_T001	0.8588584	-0.136135744	Zm00001d019462
Zm00001d040066_T001	0.9371235	0.065414083	Zm00001d040066
Zm00001d025323_T009	0.6231523	1.36106181	Zm00001d025323
Zm00001d025323_T011	2.14E-08	1.534687517	Zm00001d025323
Zm00001d025323_T002	1	-0.011089342	Zm00001d025323
Zm00001d025323_T010	0.0164348	2.094388149	Zm00001d025323
Zm00001d025323_T004	0.04031405	2.546870811	Zm00001d025323
Zm00001d025323_T001	0.00012109	1.459667231	Zm00001d025323
Zm00001d025323_T012	0.000326064	2.000572513	Zm00001d025323
Zm00001d028355_T001	0.9792387	0.099226223	Zm00001d028355
Zm00001d028355_T006	0.6827367	0.446442488	Zm00001d028355
Zm00001d028355_T004	0.956053	0.071798026	Zm00001d028355
Zm00001d038377_T001	0.8050292	0.90962033	Zm00001d038377
Zm00001d045153_T001	0.9363242	0.372992102	Zm00001d045153
Zm00001d045153_T002	0.989392	0.502852432	Zm00001d045153
Zm00001d022421_T012	0.4793027	-0.752142084	Zm00001d022421
Zm00001d022421_T001	0.2502162	1.414539868	Zm00001d022421
Zm00001d022421_T002	0.6313899	1.284501758	Zm00001d022421
Zm00001d043686_T009	0.2703118	1.035974906	Zm00001d043686
Zm00001d043686_T004	0.9749728	0.296620945	Zm00001d043686
Zm00001d043686_T010	0.9867452	0.167079483	Zm00001d043686
Zm00001d026603_T002	0.9887584	0.104003417	Zm00001d026603
Zm00001d012809_T001	0.9918302	0.242427193	Zm00001d012809
Zm00001d014759_T001	0.9993872	0.335995344	Zm00001d014759
Zm00001d048191_T002	0.9819191	0.507037152	Zm00001d048191
Zm00001d048191_T001	0.7294039	-0.294085013	Zm00001d048191
Zm00001d027723_T001	0.9541864	0.109568266	Zm00001d027723

Zm00001d027723_T002	0.5718096	0.346558915	Zm00001d027723
Zm00001d047720_T001	0.986738	0.009626385	Zm00001d047720
Zm00001d035129_T001	0.9894615	0.376844626	Zm00001d035129
Zm00001d037288_T001	0.9804433	-0.017908297	Zm00001d037288
Zm00001d024134_T001	0.6357777	-0.430721924	Zm00001d024134
Zm00001d005346_T001	0.9907928	0.346525029	Zm00001d005346
Zm00001d034489_T001	0.9945118	0.263239166	Zm00001d034489
Zm00001d024710_T034	0.2907439	0.657098777	Zm00001d024710
Zm00001d024710_T011	0.0146133	1.091888311	Zm00001d024710
Zm00001d024710_T040	0.9914912	0.34766062	Zm00001d024710
Zm00001d024710_T039	0.923446	-0.054617426	Zm00001d024710
Zm00001d024710_T004	0.1928254	0.479878901	Zm00001d024710
Zm00001d024710_T025	0.3986945	0.500353264	Zm00001d024710
Zm00001d024710_T024	0.6761259	-0.297191604	Zm00001d024710
Zm00001d035066_T001	0.9085181	-0.146252092	Zm00001d035066
Zm00001d008338_T001	0.3461501	0.361231375	Zm00001d008338
Zm00001d008338_T002	0.8294797	0.73615132	Zm00001d008338
Zm00001d044093_T001	0.8645268	0.791464722	Zm00001d044093
Zm00001d021370_T001	1	0.030690785	Zm00001d021370
Zm00001d030557_T001	0.2638317	-1.055842094	Zm00001d030557
Zm00001d046961_T001	0.02135948	-2.286799819	Zm00001d046961
Zm00001d005513_T001	0.9469426	0.024467887	Zm00001d005513
Zm00001d035108_T001	0.9748733	0.172076301	Zm00001d035108
Zm00001d038589_T006	0.9487214	0.231980292	Zm00001d038589
Zm00001d038589_T008	0.9180865	0.210471106	Zm00001d038589
Zm00001d038589_T001	0.7626242	0.977911141	Zm00001d038589
Zm00001d038589_T007	0.6946859	0.336510226	Zm00001d038589
Zm00001d014162_T001	0.9296811	0.468429584	Zm00001d014162
Zm00001d042540_T009	0.1443426	-1.031151045	Zm00001d042540
Zm00001d042540_T015	0.00601412	-2.28221241	Zm00001d042540
Zm00001d042540_T007	0.03444594	-1.604048527	Zm00001d042540
Zm00001d006090_T003	0.9903234	0.242039242	Zm00001d006090
Zm00001d019320_T001	0.9395824	-0.098711294	Zm00001d019320
Zm00001d037832_T015	0.3497147	0.379682486	Zm00001d037832
Zm00001d037832_T010	0.002644703	1.222030469	Zm00001d037832
Zm00001d037832_T004	0.9303773	0.057405728	Zm00001d037832
Zm00001d037832_T003	0.7545745	0.430656559	Zm00001d037832
Zm00001d011103_T001	0.9370449	-0.160990223	Zm00001d011103
Zm00001d041955_T001	0.4326521	1.324463326	Zm00001d041955
Zm00001d031321_T001	0.8125642	-0.2338842	Zm00001d031321
Zm00001d013999_T003	0.06597925	-3.074808569	Zm00001d013999
Zm00001d013999_T002	0.5830262	-0.321518521	Zm00001d013999
Zm00001d013999_T001	0.9235467	-0.224284139	Zm00001d013999
Zm00001d019449_T004	0.9944178	0.31481401	Zm00001d019449
Zm00001d034241_T001	0.9192339	0.716793366	Zm00001d034241
Zm00001d045596_T001	0.4758968	-0.711546612	Zm00001d045596
Zm00001d039169_T001	0.8471986	0.749117006	Zm00001d039169
Zm00001d031441_T002	0.9673473	0.178586693	Zm00001d031441
Zm00001d031441_T001	0.2268427	-0.362340864	Zm00001d031441
Zm00001d053687_T002	0.9777274	0.548917644	Zm00001d053687
Zm00001d053687_T003	0.5443214	0.409181792	Zm00001d053687
Zm00001d040362_T001	0.3356906	-0.836817727	Zm00001d040362
Zm00001d046423_T001	0.9956349	0.388860448	Zm00001d046423
Zm00001d044129_T011	0.8743763	0.959417339	Zm00001d044129

Zm00001d036392_T001	0.92623	-0.411865501	Zm00001d036392
Zm00001d035093_T035	0.7599	-0.260981511	Zm00001d035093
Zm00001d035093_T012	0.004835316	0.475267107	Zm00001d035093
Zm00001d035093_T006	0.4127422	1.22734923	Zm00001d035093
Zm00001d035093_T026	0.4191028	0.203417661	Zm00001d035093
Zm00001d035093_T022	0.01714986	0.59443914	Zm00001d035093
Zm00001d035093_T003	0.2603326	0.344547697	Zm00001d035093
Zm00001d020650_T001	0.9249382	-0.016931563	Zm00001d020650
Zm00001d037225_T001	0.01033728	1.744233207	Zm00001d037225
Zm00001d009878_T001	0.9781672	0.535544775	Zm00001d009878
Zm00001d023841_T008	0.6821817	0.647656	Zm00001d023841
Zm00001d044076_T001	0.9380783	0.022558257	Zm00001d044076
Zm00001d008552_T001	0.9778436	0.033491572	Zm00001d008552
Zm00001d036305_T015	0.5983973	0.503169884	Zm00001d036305
Zm00001d036305_T019	0.7071453	-0.374620581	Zm00001d036305
Zm00001d036305_T006	0.1303329	0.637508392	Zm00001d036305
Zm00001d036305_T010	0.2611065	1.671326429	Zm00001d036305
Zm00001d036305_T016	0.9498304	-0.009485994	Zm00001d036305
Zm00001d031825_T003	0.6719261	0.805851709	Zm00001d031825
Zm00001d031825_T001	0.2748634	-0.626195656	Zm00001d031825
Zm00001d031825_T002	0.9817074	0.345136788	Zm00001d031825
Zm00001d039642_T001	0.9380783	0.029155599	Zm00001d039642
Zm00001d008651_T001	1	0.3146998	Zm00001d008651
Zm00001d050340_T001	0.960704	0.137694522	Zm00001d050340
Zm00001d007763_T001	0.04091628	-1.181466637	Zm00001d007763
Zm00001d007763_T002	0.6645568	-0.731178381	Zm00001d007763
Zm00001d039086_T001	0.9400285	0.072252818	Zm00001d039086
Zm00001d018431_T001	0.6367667	-0.411552401	Zm00001d018431
Zm00001d049948_T001	0.8214774	0.968124294	Zm00001d049948
Zm00001d047745_T001	0.945968	-0.294716564	Zm00001d047745
Zm00001d047632_T002	0.003774627	1.450429496	Zm00001d047632
Zm00001d047632_T001	0.8092097	0.808944594	Zm00001d047632
Zm00001d019091_T001	0.7866157	-0.394381344	Zm00001d019091
Zm00001d019091_T002	0.9165176	-0.126104744	Zm00001d019091
Zm00001d024907_T001	0.9962521	0.268910809	Zm00001d024907
Zm00001d024571_T001	0.7712604	-0.670627514	Zm00001d024571
Zm00001d013334_T005	0.8849109	0.604034179	Zm00001d013334
Zm00001d013334_T006	1	0.240803353	Zm00001d013334
Zm00001d013334_T001	0.5876806	0.713817969	Zm00001d013334
Zm00001d013334_T007	0.198071	0.546522259	Zm00001d013334
Zm00001d016787_T001	0.9798623	-0.256123127	Zm00001d016787
Zm00001d013453_T001	0.9224378	0.725937272	Zm00001d013453
Zm00001d003742_T001	0.09840393	2.152048866	Zm00001d003742
Zm00001d003742_T008	0.4364526	1.436208872	Zm00001d003742
Zm00001d003742_T003	0.03273708	0.855498717	Zm00001d003742
Zm00001d039994_T001	0.5717645	-0.447964438	Zm00001d039994
Zm00001d015974_T003	0.8581713	-0.179706577	Zm00001d015974
Zm00001d015974_T001	0.9954444	0.214503484	Zm00001d015974
Zm00001d014046_T002	0.8377512	0.090306037	Zm00001d014046
Zm00001d014046_T001	0.6488822	-0.58283894	Zm00001d014046
Zm00001d042590_T001	0.9804228	0.230380495	Zm00001d042590
Zm00001d027276_T001	0.8437109	-0.086322198	Zm00001d027276
Zm00001d022407_T004	0.941922	0.66095046	Zm00001d022407
Zm00001d022407_T005	0.9950703	0.074431153	Zm00001d022407

Zm00001d022407_T001	0.7639258	0.333309198	Zm00001d022407
Zm00001d022407_T006	0.9344642	0.063970591	Zm00001d022407
Zm00001d034608_T004	0.3675057	1.567899456	Zm00001d034608
Zm00001d034608_T002	0.9416622	0.574379665	Zm00001d034608
Zm00001d034608_T003	0.9404125	0.389894138	Zm00001d034608
Zm00001d034608_T001	1	0.410669887	Zm00001d034608
Zm00001d022594_T007	0.711139	-0.374183683	Zm00001d022594
Zm00001d022594_T002	0.9909466	0.113991618	Zm00001d022594
Zm00001d022594_T003	0.8179463	-0.083589891	Zm00001d022594
Zm00001d022594_T004	0.5690651	1.308993969	Zm00001d022594
Zm00001d022594_T005	0.839321	-0.255127929	Zm00001d022594
Zm00001d015067_T001	0.9528476	0.03750433	Zm00001d015067
Zm00001d009694_T001	0.2955231	-0.902167668	Zm00001d009694
Zm00001d011309_T016	0.919888	0.216402587	Zm00001d011309
Zm00001d011309_T031	0.890296	1.182230069	Zm00001d011309
Zm00001d011309_T001	0.4860327	1.274471387	Zm00001d011309
Zm00001d011309_T022	0.9986486	0.258915843	Zm00001d011309
Zm00001d011309_T028	0.6929566	-0.556553715	Zm00001d011309
Zm00001d011309_T018	0.4373102	1.143647984	Zm00001d011309
Zm00001d011309_T027	0.01328455	1.00473281	Zm00001d011309
Zm00001d025205_T001	0.2448919	-1.108602206	Zm00001d025205
Zm00001d012675_T002	0.9845237	0.536659051	Zm00001d012675
Zm00001d012675_T001	0.9988762	0.266826734	Zm00001d012675
Zm00001d012675_T003	0.878248	-0.535962913	Zm00001d012675
Zm00001d004521_T002	0.8001345	-0.00164085	Zm00001d004521
Zm00001d004521_T004	0.9919298	0.277060182	Zm00001d004521
Zm00001d021784_T001	0.6869898	-0.469991892	Zm00001d021784
Zm00001d023651_T001	0.7831859	1.000370233	Zm00001d023651
Zm00001d031450_T001	0.9180662	0.406607664	Zm00001d031450
Zm00001d031450_T002	0.9561521	0.092354823	Zm00001d031450
Zm00001d034448_T001	0.9781672	0.420851565	Zm00001d034448
Zm00001d024781_T001	1	0.199661655	Zm00001d024781
Zm00001d048852_T001	0.9945118	-0.070940872	Zm00001d048852
Zm00001d007418_T001	0.7667736	-0.35944139	Zm00001d007418
Zm00001d023326_T013	3.69E-15	3.414686124	Zm00001d023326
Zm00001d023326_T005	0.925138	0.073435047	Zm00001d023326
Zm00001d023326_T006	0.7175167	-0.262429123	Zm00001d023326
Zm00001d023326_T012	0.3642154	-1.170492533	Zm00001d023326
Zm00001d023326_T002	0.9892551	0.573489802	Zm00001d023326
Zm00001d023326_T001	0.2715931	0.658201069	Zm00001d023326
Zm00001d011648_T001	0.8643636	0.831374847	Zm00001d011648
Zm00001d027856_T001	0.8084847	0.93937951	Zm00001d027856
Zm00001d007788_T001	0.9302759	0.535506246	Zm00001d007788
Zm00001d026588_T007	0.941922	0.013580577	Zm00001d026588
Zm00001d026588_T001	0.7454635	-0.508718257	Zm00001d026588
Zm00001d026588_T005	0.4657833	-1.322937797	Zm00001d026588
Zm00001d026588_T008	0.8031436	-0.098111374	Zm00001d026588
Zm00001d026588_T002	0.3643344	-1.085045421	Zm00001d026588
Zm00001d008284_T007	0.01555155	0.634148335	Zm00001d008284
Zm00001d008284_T004	0.9690925	0.117538265	Zm00001d008284
Zm00001d008284_T006	0.9791066	1.546344781	Zm00001d008284
Zm00001d007896_T001	0.9887889	0.376817584	Zm00001d007896
Zm00001d027572_T003	0.995534	0.503813267	Zm00001d027572
Zm00001d027572_T002	1	0.228567879	Zm00001d027572

Zm00001d027572_T004	0.09421101	-0.841093381	Zm00001d027572
Zm00001d027572_T001	0.852305	0.935476805	Zm00001d027572
Zm00001d049184_T001	0.9921225	0.222503125	Zm00001d049184
Zm00001d021815_T002	0.9216521	0.017212463	Zm00001d021815
Zm00001d021815_T001	0.9781672	0.234440003	Zm00001d021815
Zm00001d032531_T002	0.8971642	1.083990856	Zm00001d032531
Zm00001d032531_T001	0.9887584	0.278402701	Zm00001d032531
Zm00001d032531_T003	1	0.130856723	Zm00001d032531
Zm00001d032531_T005	0.885791	0.66527574	Zm00001d032531
Zm00001d010987_T002	0.9909153	0.207370777	Zm00001d010987
Zm00001d010987_T001	0.9855117	0.281222017	Zm00001d010987
Zm00001d031891_T001	0.7234221	1.810153513	Zm00001d031891
Zm00001d031891_T013	0.5539833	-0.071177527	Zm00001d031891
Zm00001d031891_T012	0.218911	2.114954712	Zm00001d031891
Zm00001d031891_T021	0.02936899	-2.033579293	Zm00001d031891
Zm00001d031891_T019	0.2800967	-0.957960726	Zm00001d031891
Zm00001d031891_T002	0.9269932	0.71326532	Zm00001d031891
Zm00001d031891_T022	0.941809	0.088936025	Zm00001d031891
Zm00001d031891_T010	0.9760472	0.134518993	Zm00001d031891
Zm00001d019734_T001	0.6789826	-0.719468375	Zm00001d019734
Zm00001d028043_T001	0.8532509	-0.156260376	Zm00001d028043
Zm00001d028195_T003	0.9711278	0.108210752	Zm00001d028195
Zm00001d022527_T016	0.7307299	-0.16144165	Zm00001d022527
Zm00001d022527_T008	1	0.18107435	Zm00001d022527
Zm00001d022527_T020	0.8264567	-0.128673382	Zm00001d022527
Zm00001d022527_T001	0.000182193	-0.645066147	Zm00001d022527
Zm00001d022527_T018	8.84E-12	1.735101562	Zm00001d022527
Zm00001d002693_T001	0.7888267	-0.11505664	Zm00001d002693
Zm00001d041612_T001	1	0.433590321	Zm00001d041612
Zm00001d040603_T003	0.6878307	-0.868045207	Zm00001d040603
Zm00001d040603_T005	0.7565187	1.036564753	Zm00001d040603
Zm00001d040603_T001	0.8695755	0.861235751	Zm00001d040603
Zm00001d040603_T004	0.8486887	0.923298468	Zm00001d040603
Zm00001d040603_T002	0.7097086	0.581159981	Zm00001d040603
Zm00001d048092_T008	0.8987811	0.772464024	Zm00001d048092
Zm00001d048092_T009	0.9249526	0.025205753	Zm00001d048092
Zm00001d048092_T011	0.9690925	0.229886379	Zm00001d048092
Zm00001d048092_T004	1	0.094195583	Zm00001d048092
Zm00001d048092_T010	0.004479976	-1.838046232	Zm00001d048092
Zm00001d009248_T015	1	0.316549747	Zm00001d009248
Zm00001d009248_T004	0.5890036	1.00428433	Zm00001d009248
Zm00001d020595_T001	0.852305	-0.159598301	Zm00001d020595
Zm00001d051232_T004	0.6974118	0.984420957	Zm00001d051232
Zm00001d051232_T014	0.9048001	0.204070676	Zm00001d051232
Zm00001d051232_T015	0.9040261	-0.099433471	Zm00001d051232
Zm00001d051232_T007	0.5719089	0.177769582	Zm00001d051232
Zm00001d012921_T002	1	0.380753305	Zm00001d012921
Zm00001d043118_T002	0.9979393	0.25584936	Zm00001d043118
Zm00001d043118_T001	0.09669157	2.844219823	Zm00001d043118
Zm00001d004293_T001	0.9979568	0.235387804	Zm00001d004293
Zm00001d022277_T001	0.9747606	0.584172841	Zm00001d022277
Zm00001d021286_T001	0.8214774	0.922611944	Zm00001d021286
Zm00001d038928_T002	0.9804228	0.138344621	Zm00001d038928
Zm00001d052324_T001	0.4319576	-0.629609037	Zm00001d052324

Zm00001d048146_T001	0.7589715	-0.267086203	Zm00001d048146
Zm00001d038340_T001	0.8557307	0.914365069	Zm00001d038340
Zm00001d046746_T001	0.8446194	-0.393906155	Zm00001d046746
Zm00001d013217_T001	0.9931431	0.467932513	Zm00001d013217
Zm00001d022373_T001	0.957768	0.104145848	Zm00001d022373
Zm00001d031898_T001	0.4815614	1.458590425	Zm00001d031898
Zm00001d031898_T004	0.7577723	0.511092927	Zm00001d031898
Zm00001d031898_T010	0.5415171	0.628950561	Zm00001d031898
Zm00001d031898_T005	0.909044	0.056713824	Zm00001d031898
Zm00001d031898_T008	0.8280859	-0.046761495	Zm00001d031898
Zm00001d031898_T012	0.9352862	0.018420712	Zm00001d031898
Zm00001d018653_T001	1	0.382205941	Zm00001d018653
Zm00001d010053_T001	0.9954444	0.354001477	Zm00001d010053
Zm00001d010053_T002	0.8512136	0.637520732	Zm00001d010053
Zm00001d028625_T002	0.9518042	0.033787313	Zm00001d028625
Zm00001d028625_T001	0.3242799	-0.85038514	Zm00001d028625
Zm00001d018660_T001	0.9768802	0.147995116	Zm00001d018660
Zm00001d013451_T001	0.7948034	-0.660109531	Zm00001d013451
Zm00001d012603_T001	0.3015167	-1.186474317	Zm00001d012603
Zm00001d005902_T001	0.6466858	1.135758944	Zm00001d005902
Zm00001d005902_T003	0.9524127	0.602349758	Zm00001d005902
Zm00001d028751_T001	0.4235631	1.587518157	Zm00001d028751
Zm00001d034418_T001	0.6026834	1.146265626	Zm00001d034418
Zm00001d035835_T001	0.100527	-1.451039875	Zm00001d035835
Zm00001d043009_T002	0.669171	-0.279200641	Zm00001d043009
Zm00001d029238_T001	1	0.017054388	Zm00001d029238
Zm00001d049158_T001	0.9887889	0.451803309	Zm00001d049158
Zm00001d043851_T002	0.0404945	0.642060032	Zm00001d043851
Zm00001d043851_T001	0.1124503	1.59437612	Zm00001d043851
Zm00001d051687_T001	0.5635628	-0.616614128	Zm00001d051687
Zm00001d041481_T001	0.8142464	-0.217827699	Zm00001d041481
Zm00001d030588_T001	0.7481233	0.974151088	Zm00001d030588
Zm00001d017441_T001	0.9309468	-0.189965691	Zm00001d017441
Zm00001d002835_T006	0.1777154	1.753648234	Zm00001d002835
Zm00001d002835_T007	0.8207831	-0.033784964	Zm00001d002835
Zm00001d002835_T001	0.997764	0.562541529	Zm00001d002835
Zm00001d002835_T005	0.8964117	-0.056775792	Zm00001d002835
Zm00001d002835_T008	0.9709887	0.186275311	Zm00001d002835
Zm00001d002835_T004	0.9792058	0.578029373	Zm00001d002835
Zm00001d002835_T009	0.971204	0.333313222	Zm00001d002835
Zm00001d014664_T001	0.9503115	0.119430821	Zm00001d014664
Zm00001d014664_T003	0.4610015	-0.224789762	Zm00001d014664
Zm00001d007753_T001	0.5057074	-0.768707432	Zm00001d007753
Zm00001d010650_T001	0.9988864	-0.803553583	Zm00001d010650
Zm00001d042638_T002	0.2341185	-1.352048426	Zm00001d042638
Zm00001d033193_T006	0.8643636	0.65419315	Zm00001d033193
Zm00001d039384_T001	0.9865337	0.234414451	Zm00001d039384
Zm00001d040660_T001	0.003102858	2.568574293	Zm00001d040660
Zm00001d040660_T002	0.0086703	2.321376316	Zm00001d040660
Zm00001d020188_T003	0.8245192	-0.198389572	Zm00001d020188
Zm00001d020188_T002	0.000619347	-2.564594974	Zm00001d020188
Zm00001d020188_T001	0.172874	0.771471494	Zm00001d020188
Zm00001d004541_T009	0.8609458	0.709953981	Zm00001d004541
Zm00001d004541_T011	0.7958376	-0.23611343	Zm00001d004541

Zm00001d002086_T001	0.949277	0.091046802	Zm00001d002086
Zm00001d003984_T001	0.9833565	0.249043782	Zm00001d003984
Zm00001d047504_T001	0.9533693	0.049010866	Zm00001d047504
Zm00001d003678_T008	0.1235256	-0.524825063	Zm00001d003678
Zm00001d003678_T005	0.9322451	0.409420786	Zm00001d003678
Zm00001d003678_T006	0.08207564	-1.173879187	Zm00001d003678
Zm00001d003678_T001	1	0.276859019	Zm00001d003678
Zm00001d000345_T001	0.8935921	-0.559515797	Zm00001d000345
Zm00001d043615_T001	0.8369406	0.677867556	Zm00001d043615
Zm00001d047829_T001	0.9958181	0.218606046	Zm00001d047829
Zm00001d043075_T026	0.1021399	-0.467320743	Zm00001d043075
Zm00001d043075_T008	0.3128816	0.273577666	Zm00001d043075
Zm00001d043075_T011	0.05922365	1.843023981	Zm00001d043075
Zm00001d043075_T010	8.51E-12	2.195957309	Zm00001d043075
Zm00001d043075_T006	0.1341567	1.081664894	Zm00001d043075
Zm00001d043075_T028	0.001363155	-2.22852697	Zm00001d043075
Zm00001d043075_T019	0.05122384	2.071082987	Zm00001d043075
Zm00001d043075_T015	0.8197974	0.684017287	Zm00001d043075
Zm00001d043075_T005	0.00238057	1.408858915	Zm00001d043075
Zm00001d043075_T007	0.1417015	0.738511262	Zm00001d043075
Zm00001d043075_T013	0.2808297	1.63861556	Zm00001d043075
Zm00001d039406_T001	0.9740325	0.363477398	Zm00001d039406
Zm00001d048667_T003	0.7697202	0.671844546	Zm00001d048667
Zm00001d048667_T012	0.9846714	0.04880584	Zm00001d048667
Zm00001d023695_T004	0.7195936	0.475352642	Zm00001d023695
Zm00001d023695_T001	0.9984041	0.387190138	Zm00001d023695
Zm00001d032339_T001	0.00113879	2.713988963	Zm00001d032339
Zm00001d036151_T001	0.9248904	0.027590072	Zm00001d036151
Zm00001d008224_T002	0.8609329	-0.102891706	Zm00001d008224
Zm00001d008224_T004	0.9732421	0.166946804	Zm00001d008224
Zm00001d044431_T001	0.956479	0.65366539	Zm00001d044431
Zm00001d027701_T002	0.9374853	0.705176784	Zm00001d027701
Zm00001d027701_T004	0.05256956	0.715959826	Zm00001d027701
Zm00001d027701_T003	0.9797082	0.096313315	Zm00001d027701
Zm00001d027701_T001	0.9513815	0.688588378	Zm00001d027701
Zm00001d037213_T001	0.9833133	0.259832956	Zm00001d037213
Zm00001d003632_T003	0.8114815	-0.186796157	Zm00001d003632
Zm00001d003632_T001	0.4263859	-0.894354702	Zm00001d003632
Zm00001d017622_T001	0.07537729	-1.791658034	Zm00001d017622
Zm00001d010756_T001	0.9529184	-0.115814996	Zm00001d010756
Zm00001d034834_T001	9.78E-06	2.113429099	Zm00001d034834
Zm00001d034834_T002	0.9979393	0.444310268	Zm00001d034834
Zm00001d034834_T004	0.8530103	0.436843557	Zm00001d034834
Zm00001d034834_T010	0.03107792	-1.985380574	Zm00001d034834
Zm00001d034834_T005	0.4457952	0.863282965	Zm00001d034834
Zm00001d024601_T003	0.9975882	0.316332286	Zm00001d024601
Zm00001d024601_T004	0.5677185	1.17744358	Zm00001d024601
Zm00001d024601_T002	0.1887255	-0.800347678	Zm00001d024601
Zm00001d008618_T005	0.9984041	0.448072088	Zm00001d008618
Zm00001d008618_T004	0.6890838	0.823853506	Zm00001d008618
Zm00001d027231_T001	0.1712254	-0.883357558	Zm00001d027231
Zm00001d027231_T005	1	0.355375485	Zm00001d027231
Zm00001d027231_T003	1	0.245213416	Zm00001d027231
Zm00001d052632_T007	0.9524127	0.024529076	Zm00001d052632

Zm00001d052632_T012	0.9619942	0.058526952	Zm00001d052632
Zm00001d052632_T010	0.6474877	1.043506552	Zm00001d052632
Zm00001d016121_T001	0.08754737	1.832388971	Zm00001d016121
Zm00001d000320_T001	0.681302	-0.429753403	Zm00001d000320
Zm00001d019706_T013	0.9824348	-0.107554758	Zm00001d019706
Zm00001d019706_T005	0.9842144	0.27577582	Zm00001d019706
Zm00001d019706_T002	0.90276	0.769011963	Zm00001d019706
Zm00001d019706_T007	0.9380783	0.011763163	Zm00001d019706
Zm00001d032970_T017	0.802225	0.196033566	Zm00001d032970
Zm00001d032970_T008	0.9160053	-0.244920973	Zm00001d032970
Zm00001d047517_T001	0.91126	0.646671549	Zm00001d047517
Zm00001d013086_T003	0.924619	-0.000577493	Zm00001d013086
Zm00001d013086_T001	0.7439474	-0.62162876	Zm00001d013086
Zm00001d020571_T004	0.9822138	0.323186488	Zm00001d020571
Zm00001d020571_T002	1	0.774322959	Zm00001d020571
Zm00001d020571_T003	0.3146527	-0.961763958	Zm00001d020571
Zm00001d027767_T001	0.8213533	0.67332073	Zm00001d027767
Zm00001d048473_T002	0.9161657	-0.481143508	Zm00001d048473
Zm00001d048473_T003	0.4385251	1.284823761	Zm00001d048473
Zm00001d048473_T001	0.6896661	0.856029226	Zm00001d048473
Zm00001d048473_T004	0.007983482	0.835628787	Zm00001d048473
Zm00001d037205_T001	0.9598922	-0.049137271	Zm00001d037205
Zm00001d030620_T001	0.8958317	0.775206858	Zm00001d030620
Zm00001d030620_T003	0.682021	1.184045192	Zm00001d030620
Zm00001d030620_T002	0.7697196	-0.286049728	Zm00001d030620
Zm00001d037687_T001	0.9771927	0.435126072	Zm00001d037687
Zm00001d002039_T001	0.991917	0.327179228	Zm00001d002039
Zm00001d002457_T001	0.9160053	-0.101106784	Zm00001d002457
Zm00001d044747_T001	0.3590283	0.340886171	Zm00001d044747
Zm00001d009548_T003	0.9419336	0.246942194	Zm00001d009548
Zm00001d009548_T006	0.9914912	0.251473836	Zm00001d009548
Zm00001d042131_T007	0.6557732	-0.27589429	Zm00001d042131
Zm00001d042131_T001	0.9319754	0.000612504	Zm00001d042131
Zm00001d030860_T005	0.3695381	-0.622945578	Zm00001d030860
Zm00001d030860_T003	0.6470421	0.766697705	Zm00001d030860
Zm00001d043097_T001	0.649125	0.68876345	Zm00001d043097
Zm00001d034933_T001	0.9429712	-0.018382338	Zm00001d034933
Zm00001d032164_T001	0.9486412	-0.015533024	Zm00001d032164
Zm00001d011781_T001	0.92946	-0.008691985	Zm00001d011781
Zm00001d050850_T001	1	0.423370323	Zm00001d050850
Zm00001d050850_T003	0.9975882	0.333867588	Zm00001d050850
Zm00001d050850_T004	0.9260032	-0.15413246	Zm00001d050850
Zm00001d050850_T005	0.6335251	-0.779404766	Zm00001d050850
Zm00001d040798_T001	0.9954444	0.248618682	Zm00001d040798
Zm00001d034761_T002	0.6625248	-0.261760467	Zm00001d034761
Zm00001d034761_T001	0.7923921	-0.2640711	Zm00001d034761
Zm00001d040163_T001	0.9760472	0.171814532	Zm00001d040163
Zm00001d040163_T003	0.8412913	0.253907405	Zm00001d040163
Zm00001d040163_T002	0.3879539	1.570177083	Zm00001d040163
Zm00001d016414_T001	0.1862644	-1.213385637	Zm00001d016414
Zm00001d038397_T003	0.7458694	1.423649811	Zm00001d038397
Zm00001d038397_T006	0.9706887	0.455643305	Zm00001d038397
Zm00001d038397_T007	0.313739	1.664385313	Zm00001d038397
Zm00001d038397_T005	0.000175579	-2.245597383	Zm00001d038397

Zm00001d038397_T004	0.9563232	0.328584762	Zm00001d038397
Zm00001d038397_T001	0.8530103	-0.304593722	Zm00001d038397
Zm00001d011611_T005	0.923446	0.723599695	Zm00001d011611
Zm00001d011611_T004	1.02E-05	0.696933642	Zm00001d011611
Zm00001d011611_T008	0.71455	-0.849420308	Zm00001d011611
Zm00001d011611_T002	0.02980711	-0.77794986	Zm00001d011611
Zm00001d011611_T001	0.08737888	1.058283813	Zm00001d011611
Zm00001d011611_T006	0.1402804	1.483542841	Zm00001d011611
Zm00001d000302_T001	0.9220919	0.005736533	Zm00001d000302
Zm00001d011569_T001	0.9804228	0.400624799	Zm00001d011569
Zm00001d038717_T001	0.2205165	-1.367880476	Zm00001d038717
Zm00001d028693_T001	0.8660897	0.454711785	Zm00001d028693
Zm00001d028693_T004	0.8489156	-0.292744844	Zm00001d028693
Zm00001d028693_T003	0.5709544	-0.117459271	Zm00001d028693
Zm00001d052628_T001	0.6885591	-0.399065818	Zm00001d052628
Zm00001d020965_T006	0.4289534	0.590649226	Zm00001d020965
Zm00001d020965_T015	0.2154325	1.13844839	Zm00001d020965
Zm00001d020965_T002	0.1074904	1.612762233	Zm00001d020965
Zm00001d020965_T017	0.541024	-0.975745896	Zm00001d020965
Zm00001d020965_T016	0.0136018	0.909032915	Zm00001d020965
Zm00001d020965_T008	0.000197484	1.11206652	Zm00001d020965
Zm00001d020965_T003	0.9914912	0.50146648	Zm00001d020965
Zm00001d018318_T024	0.5064329	0.754930351	Zm00001d018318
Zm00001d018318_T010	0.3347855	-0.406275227	Zm00001d018318
Zm00001d018318_T055	0.9804228	-0.040442138	Zm00001d018318
Zm00001d018318_T032	0.9912886	0.66940629	Zm00001d018318
Zm00001d018318_T017	0.03058431	0.616449197	Zm00001d018318
Zm00001d018318_T002	0.2622146	0.506081585	Zm00001d018318
Zm00001d033913_T001	0.5658691	-0.628083434	Zm00001d033913
Zm00001d022189_T007	0.9771927	0.341782823	Zm00001d022189
Zm00001d022189_T004	0.7438304	1.278440137	Zm00001d022189
Zm00001d022189_T008	0.9061819	-0.480432968	Zm00001d022189
Zm00001d022189_T003	0.98291	0.077241764	Zm00001d022189
Zm00001d022189_T001	1	0.268059272	Zm00001d022189
Zm00001d022189_T006	0.9962521	0.33245209	Zm00001d022189
Zm00001d034072_T001	1	0.328067396	Zm00001d034072
Zm00001d049597_T001	0.3713446	-0.367050865	Zm00001d049597
Zm00001d049597_T002	0.1034632	2.129447373	Zm00001d049597
Zm00001d020929_T002	0.8089972	0.543025485	Zm00001d020929
Zm00001d050304_T001	0.7480195	-0.14891728	Zm00001d050304
Zm00001d050304_T002	0.564657	0.935893805	Zm00001d050304
Zm00001d036417_T003	1	0.279044211	Zm00001d036417
Zm00001d036417_T001	0.941809	0.059967254	Zm00001d036417
Zm00001d036417_T006	0.9825858	0.255224824	Zm00001d036417
Zm00001d036417_T005	0.9356767	-0.422122326	Zm00001d036417
Zm00001d051544_T004	0.7933933	0.728945555	Zm00001d051544
Zm00001d051544_T002	0.8580945	-0.068308981	Zm00001d051544
Zm00001d030682_T001	0.01080912	2.329098094	Zm00001d030682
Zm00001d013730_T001	0.909739	-0.134983412	Zm00001d013730
Zm00001d053853_T004	0.987516	0.295354121	Zm00001d053853
Zm00001d053853_T001	0.8740066	-0.180884618	Zm00001d053853
Zm00001d053853_T003	0.9363242	0.388052363	Zm00001d053853
Zm00001d046234_T002	0.2151243	-1.19259864	Zm00001d046234
Zm00001d027421_T001	0.9804228	0.042396527	Zm00001d027421

Zm00001d006082_T001	0.8581713	0.755536923	Zm00001d006082
Zm00001d036702_T001	0.8929316	0.04374069	Zm00001d036702
Zm00001d028440_T001	0.8050292	-0.409343633	Zm00001d028440
Zm00001d037493_T001	0.5006087	0.600004281	Zm00001d037493
Zm00001d052165_T001	0.000110548	4.161401272	Zm00001d052165
Zm00001d025864_T001	0.9744451	0.533585687	Zm00001d025864
Zm00001d025864_T003	0.8828258	-0.187927702	Zm00001d025864
Zm00001d043337_T002	0.8112903	-0.597803289	Zm00001d043337
Zm00001d044023_T038	0.2572389	-0.53816785	Zm00001d044023
Zm00001d044023_T020	0.05702114	0.33649196	Zm00001d044023
Zm00001d044023_T003	0.9609153	0.071268323	Zm00001d044023
Zm00001d044023_T042	0.1910962	-1.448322012	Zm00001d044023
Zm00001d044023_T017	0.3591255	1.116544592	Zm00001d044023
Zm00001d052103_T001	0.9121108	0.788149571	Zm00001d052103
Zm00001d007280_T001	0.03674033	-2.003709513	Zm00001d007280
Zm00001d049637_T002	0.4177213	-0.654915821	Zm00001d049637
Zm00001d049637_T001	1	0.233789247	Zm00001d049637
Zm00001d006420_T002	0.9812314	0.259047104	Zm00001d006420
Zm00001d044823_T005	0.7017973	0.705652102	Zm00001d044823
Zm00001d044823_T003	0.6436915	-0.611474587	Zm00001d044823
Zm00001d044823_T011	0.01856152	1.985651936	Zm00001d044823
Zm00001d044823_T004	0.139268	1.218834732	Zm00001d044823
Zm00001d013397_T002	0.98291	0.364688254	Zm00001d013397
Zm00001d013397_T001	0.9843321	0.139907585	Zm00001d013397
Zm00001d031849_T001	0.8660897	-0.212944004	Zm00001d031849
Zm00001d031849_T004	0.7933933	-0.127669921	Zm00001d031849
Zm00001d031849_T002	0.9442514	0.691140693	Zm00001d031849
Zm00001d008819_T001	0.6042072	-0.383338743	Zm00001d008819
Zm00001d024808_T002	1	0.307781365	Zm00001d024808
Zm00001d022230_T002	0.01014068	-0.835964727	Zm00001d022230
Zm00001d022230_T001	0.9706293	0.610035363	Zm00001d022230
Zm00001d048360_T001	0.9177108	-0.11529709	Zm00001d048360
Zm00001d050577_T001	0.1732158	-1.034221748	Zm00001d050577
Zm00001d028921_T004	0.9376374	0.050027245	Zm00001d028921
Zm00001d028921_T003	0.2718748	-0.568019875	Zm00001d028921
Zm00001d010052_T001	0.09028577	-1.837978468	Zm00001d010052
Zm00001d008808_T001	0.5945614	-0.700183328	Zm00001d008808
Zm00001d008808_T002	0.9979568	0.286406895	Zm00001d008808
Zm00001d001982_T001	1	-0.106530837	Zm00001d001982
Zm00001d034787_T001	2.11E-05	4.529328486	Zm00001d034787
Zm00001d026060_T004	0.2372526	1.750834874	Zm00001d026060
Zm00001d026060_T002	0.2512831	1.398605628	Zm00001d026060
Zm00001d052331_T001	0.3319365	-0.959197321	Zm00001d052331
Zm00001d030222_T001	0.796163	0.709845644	Zm00001d030222
Zm00001d030222_T002	0.6727562	0.88781005	Zm00001d030222
Zm00001d051033_T004	0.967517	0.446860045	Zm00001d051033
Zm00001d051033_T002	0.9785005	1.018194749	Zm00001d051033
Zm00001d051033_T001	0.4893321	-0.227732901	Zm00001d051033
Zm00001d051033_T003	0.9637489	0.009052649	Zm00001d051033
Zm00001d002167_T002	0.8412913	-0.194486728	Zm00001d002167
Zm00001d002167_T001	0.9765009	0.57023716	Zm00001d002167
Zm00001d036010_T001	0.9362766	0.015159626	Zm00001d036010
Zm00001d036010_T003	1	0.436845599	Zm00001d036010
Zm00001d038533_T002	0.9747606	0.402333647	Zm00001d038533

Zm00001d038533_T010	0.8751967	-0.226439954	Zm00001d038533
Zm00001d038533_T006	0.9586437	0.405028414	Zm00001d038533
Zm00001d038533_T001	0.9867591	0.084376864	Zm00001d038533
Zm00001d002295_T001	0.5256861	-0.7624397	Zm00001d002295
Zm00001d004400_T001	0.9356656	0.669347488	Zm00001d004400
Zm00001d004400_T002	0.405448	-0.582797961	Zm00001d004400
Zm00001d003160_T001	0.9048001	-0.07076563	Zm00001d003160
Zm00001d035874_T001	4.59E-05	-3.984727719	Zm00001d035874
Zm00001d035874_T002	0.9186216	0.416917645	Zm00001d035874
Zm00001d035874_T003	0.9909466	0.430567317	Zm00001d035874
Zm00001d000191_T006	0.3097895	0.724130056	Zm00001d000191
Zm00001d000191_T018	0.5705692	0.408877809	Zm00001d000191
Zm00001d000191_T012	0.006370542	1.052551301	Zm00001d000191
Zm00001d000191_T011	0.6044465	1.21608224	Zm00001d000191
Zm00001d000191_T002	0.4582173	-0.309846112	Zm00001d000191
Zm00001d001974_T002	0.8885217	-0.112316386	Zm00001d001974
Zm00001d001974_T001	0.9448555	0.059783696	Zm00001d001974
Zm00001d009979_T002	0.9522359	0.533967489	Zm00001d009979
Zm00001d015549_T001	0.9950703	0.386444339	Zm00001d015549
Zm00001d015549_T002	0.9619176	0.092405077	Zm00001d015549
Zm00001d036410_T003	0.7167255	1.052715851	Zm00001d036410
Zm00001d036410_T004	7.66E-09	-2.063583313	Zm00001d036410
Zm00001d036410_T001	0.9325997	-0.013787252	Zm00001d036410
Zm00001d053082_T005	0.9673473	0.229775092	Zm00001d053082
Zm00001d053082_T012	0.640996	0.316247205	Zm00001d053082
Zm00001d053082_T001	1.70E-11	2.681752321	Zm00001d053082
Zm00001d053082_T002	0.106833	-1.289891272	Zm00001d053082
Zm00001d053082_T008	0.3020017	0.672509761	Zm00001d053082
Zm00001d053082_T007	0.1353026	0.799701851	Zm00001d053082
Zm00001d053082_T006	0.9673473	0.491389778	Zm00001d053082
Zm00001d031589_T001	0.8125642	0.889624494	Zm00001d031589
Zm00001d045825_T001	0.9717989	0.150201209	Zm00001d045825
Zm00001d043155_T002	0.7097086	-0.311732485	Zm00001d043155
Zm00001d020772_T001	0.1923782	1.685764882	Zm00001d020772
Zm00001d021840_T001	1	0.270113991	Zm00001d021840
Zm00001d042530_T002	0.8366185	0.587298141	Zm00001d042530
Zm00001d042530_T003	1	0.316427599	Zm00001d042530
Zm00001d010869_T003	0.4676599	-0.74870621	Zm00001d010869
Zm00001d010869_T001	0.8545272	-0.095635536	Zm00001d010869
Zm00001d006606_T006	0.9690925	0.124663542	Zm00001d006606
Zm00001d006606_T004	0.164302	0.711730181	Zm00001d006606
Zm00001d006606_T005	0.9979568	0.166094487	Zm00001d006606
Zm00001d006606_T001	0.9768468	0.129196144	Zm00001d006606
Zm00001d024894_T030	0.5292925	-0.519920263	Zm00001d024894
Zm00001d040221_T005	0.745806	-0.388460457	Zm00001d040221
Zm00001d040221_T003	0.9322451	-0.000913114	Zm00001d040221
Zm00001d040221_T006	1	0.268181339	Zm00001d040221
Zm00001d040221_T002	1	0.302183307	Zm00001d040221
Zm00001d020820_T001	0.7561907	-0.369151287	Zm00001d020820
Zm00001d051693_T001	0.9269932	0.008280353	Zm00001d051693
Zm00001d009653_T002	0.8707768	-0.326674005	Zm00001d009653
Zm00001d009653_T007	0.8536822	-0.754127916	Zm00001d009653
Zm00001d009653_T009	0.1145356	-0.837310278	Zm00001d009653
Zm00001d009653_T005	0.9653099	-0.335660987	Zm00001d009653

Zm00001d009653_T008	0.5541374	-0.211940891	Zm00001d009653
Zm00001d009653_T003	0.7425196	-0.552429819	Zm00001d009653
Zm00001d006540_T001	0.2049667	-1.211719179	Zm00001d006540
Zm00001d022462_T001	0.65291	-0.746273208	Zm00001d022462
Zm00001d036057_T002	0.9709496	0.051073583	Zm00001d036057
Zm00001d036057_T003	0.2948566	-0.371046226	Zm00001d036057
Zm00001d039154_T009	0.9833133	0.150366258	Zm00001d039154
Zm00001d039154_T004	0.9361945	0.332008021	Zm00001d039154
Zm00001d039154_T002	0.963405	0.597246829	Zm00001d039154
Zm00001d039154_T008	0.389754	-1.593514389	Zm00001d039154
Zm00001d027404_T001	1	0.021388631	Zm00001d027404
Zm00001d038712_T001	0.7285637	0.805373656	Zm00001d038712
Zm00001d027349_T001	0.9732421	0.122257019	Zm00001d027349
Zm00001d043707_T001	0.7890231	0.620383928	Zm00001d043707
Zm00001d043652_T004	0.2899035	1.72654138	Zm00001d043652
Zm00001d043652_T001	0.1565121	0.675493245	Zm00001d043652
Zm00001d037625_T002	0.7434925	0.911188761	Zm00001d037625
Zm00001d037625_T003	0.6413696	-0.46978401	Zm00001d037625
Zm00001d037625_T004	0.9973528	0.387985659	Zm00001d037625
Zm00001d037625_T005	1	0.395003095	Zm00001d037625
Zm00001d037625_T006	0.4179542	0.928276563	Zm00001d037625
Zm00001d048155_T001	1	0.25747918	Zm00001d048155
Zm00001d048155_T002	4.96E-10	-3.669128074	Zm00001d048155
Zm00001d053919_T003	0.8860576	-0.345085899	Zm00001d053919
Zm00001d053919_T005	0.7984409	-0.679608899	Zm00001d053919
Zm00001d053919_T006	0.6847424	0.887309967	Zm00001d053919
Zm00001d053919_T004	1	0.265163546	Zm00001d053919
Zm00001d051586_T001	0.9482354	0.096079524	Zm00001d051586
Zm00001d007197_T001	0.9429978	0.570550696	Zm00001d007197
Zm00001d007197_T002	0.8169226	0.873491988	Zm00001d007197
Zm00001d003129_T001	0.8533785	0.867015896	Zm00001d003129
Zm00001d000147_T003	0.8854695	-0.030899852	Zm00001d000147
Zm00001d003280_T003	0.9272403	-0.301942804	Zm00001d003280
Zm00001d017375_T001	0.9596377	0.528828149	Zm00001d017375
Zm00001d022252_T001	0.9735632	0.506949686	Zm00001d022252
Zm00001d013632_T001	0.7275347	-0.361313424	Zm00001d013632
Zm00001d003329_T017	0.000214449	0.607582535	Zm00001d003329
Zm00001d003329_T001	0.005747559	0.431948123	Zm00001d003329
Zm00001d003329_T004	0.9930712	0.540767591	Zm00001d003329
Zm00001d003329_T009	5.18E-07	-0.478835532	Zm00001d003329
Zm00001d003329_T021	0.07730424	0.907707034	Zm00001d003329
Zm00001d003329_T012	0.5277554	0.675763155	Zm00001d003329
Zm00001d035200_T001	0.9654804	0.239405306	Zm00001d035200
Zm00001d023927_T001	1	0.297464759	Zm00001d023927
Zm00001d030314_T001	0.9804228	-0.063504879	Zm00001d030314
Zm00001d011771_T001	0.3105424	-0.942347285	Zm00001d011771
Zm00001d028143_T002	0.0625605	-0.681927794	Zm00001d028143
Zm00001d028143_T001	0.6410529	-0.168183312	Zm00001d028143
Zm00001d028143_T003	0.9287119	0.688693478	Zm00001d028143
Zm00001d028143_T004	1	0.343931161	Zm00001d028143
Zm00001d022205_T012	0.2981646	-0.994657366	Zm00001d022205
Zm00001d022205_T003	0.4744745	1.253573179	Zm00001d022205
Zm00001d022205_T013	0.541024	1.297103687	Zm00001d022205
Zm00001d022205_T010	0.9112906	0.039485502	Zm00001d022205

Zm00001d022205_T005	9.11E-05	2.503750745	Zm00001d022205
Zm00001d047017_T001	0.2906834	1.740010094	Zm00001d047017
Zm00001d022554_T001	0.9732421	0.569213089	Zm00001d022554
Zm00001d022554_T002	0.9877195	0.292258334	Zm00001d022554
Zm00001d026223_T001	0.940651	-0.429069275	Zm00001d026223
Zm00001d047182_T001	0.7199158	-0.38947623	Zm00001d047182
Zm00001d047923_T002	0.8955128	0.90320096	Zm00001d047923
Zm00001d047923_T007	0.9975882	0.476354655	Zm00001d047923
Zm00001d047923_T008	0.6565447	0.562104328	Zm00001d047923
Zm00001d047923_T021	0.705921	0.682473667	Zm00001d047923
Zm00001d045188_T001	0.9396809	0.676851055	Zm00001d045188
Zm00001d029435_T001	0.9833513	0.194656632	Zm00001d029435
Zm00001d027365_T001	0.4885085	-0.671243197	Zm00001d027365
Zm00001d008428_T003	0.7722321	0.375274611	Zm00001d008428
Zm00001d008428_T002	0.9993872	0.125714787	Zm00001d008428
Zm00001d017461_T001	0.67208	1.065083567	Zm00001d017461
Zm00001d007310_T001	0.1205125	-1.28591709	Zm00001d007310
Zm00001d020808_T003	0.9062137	0.574455052	Zm00001d020808
Zm00001d020808_T001	0.3353806	0.596113336	Zm00001d020808
Zm00001d020808_T002	0.8922781	0.773459495	Zm00001d020808
Zm00001d022627_T003	0.8543794	-0.139511748	Zm00001d022627
Zm00001d030742_T002	0.8640519	0.535563632	Zm00001d030742
Zm00001d006501_T001	0.8591203	-0.521878377	Zm00001d006501
Zm00001d025960_T004	0.6162619	0.942490386	Zm00001d025960
Zm00001d025960_T002	0.9416622	0.265878704	Zm00001d025960
Zm00001d038578_T001	0.955289	-0.260083431	Zm00001d038578
Zm00001d011956_T007	0.957148	0.494365954	Zm00001d011956
Zm00001d011956_T001	0.9902733	0.299264897	Zm00001d011956
Zm00001d021362_T001	0.7997379	-0.280703825	Zm00001d021362
Zm00001d000179_T001	0.000403041	3.431764379	Zm00001d000179
Zm00001d036977_T001	0.7379612	1.150875188	Zm00001d036977
Zm00001d017249_T001	0.6898216	-0.460978349	Zm00001d017249
Zm00001d032935_T006	1	0.090637349	Zm00001d032935
Zm00001d039560_T001	0.9942835	0.187612398	Zm00001d039560
Zm00001d021961_T001	0.827438	-0.216249201	Zm00001d021961
Zm00001d052078_T002	0.9925955	0.292708234	Zm00001d052078
Zm00001d052078_T004	0.9000138	0.592681195	Zm00001d052078
Zm00001d048902_T001	0.757578	-0.358426338	Zm00001d048902
Zm00001d048902_T004	0.6674327	1.211312454	Zm00001d048902
Zm00001d048902_T010	0.6957026	0.260310622	Zm00001d048902
Zm00001d054072_T003	0.923446	-0.020851484	Zm00001d054072
Zm00001d054072_T002	0.5936419	0.297514514	Zm00001d054072
Zm00001d054072_T001	0.03159677	-0.789168036	Zm00001d054072
Zm00001d016756_T001	0.9942835	0.096958518	Zm00001d016756
Zm00001d047608_T006	0.9891311	0.412113503	Zm00001d047608
Zm00001d047608_T002	0.9778436	0.497085183	Zm00001d047608
Zm00001d047608_T005	0.8730771	-0.410960148	Zm00001d047608
Zm00001d047608_T003	0.9833513	0.542003008	Zm00001d047608
Zm00001d042714_T001	0.8412913	-0.180293085	Zm00001d042714
Zm00001d031049_T001	0.06119341	2.287485097	Zm00001d031049
Zm00001d031049_T002	0.07754764	2.248585911	Zm00001d031049
Zm00001d014788_T002	0.8377512	0.876183534	Zm00001d014788
Zm00001d014788_T006	0.5503027	1.335699508	Zm00001d014788
Zm00001d026162_T001	0.9894615	0.234905106	Zm00001d026162

Zm00001d020861_T001	0.7997379	0.479863331	Zm00001d020861
Zm00001d029195_T001	0.9037324	-0.038397407	Zm00001d029195
Zm00001d011062_T001	0.8257487	-0.219914021	Zm00001d011062
Zm00001d013274_T007	0.7746801	0.887922475	Zm00001d013274
Zm00001d013274_T076	0.2269983	0.251061309	Zm00001d013274
Zm00001d013274_T006	1	0.198731078	Zm00001d013274
Zm00001d013274_T036	0.2802804	0.703987802	Zm00001d013274
Zm00001d013274_T034	0.1168484	0.486725982	Zm00001d013274
Zm00001d013274_T045	0.3520495	-1.364545833	Zm00001d013274
Zm00001d037605_T001	0.4806969	-0.889748021	Zm00001d037605
Zm00001d012645_T002	0.9370449	0.522291365	Zm00001d012645
Zm00001d040960_T007	0.5570384	0.424903811	Zm00001d040960
Zm00001d040960_T005	0.5220513	1.40258169	Zm00001d040960
Zm00001d040960_T003	0.3870314	0.394695625	Zm00001d040960
Zm00001d040960_T006	0.008916495	-1.620266724	Zm00001d040960
Zm00001d040960_T001	0.9906533	0.398066244	Zm00001d040960
Zm00001d013940_T008	0.4074201	-1.169040939	Zm00001d013940
Zm00001d013940_T005	0.9971866	0.125278252	Zm00001d013940
Zm00001d013940_T007	1	0.159862267	Zm00001d013940
Zm00001d013940_T001	0.9907928	0.295599721	Zm00001d013940
Zm00001d025433_T003	0.8591654	-0.121728004	Zm00001d025433
Zm00001d020063_T001	0.9979568	0.305597866	Zm00001d020063
Zm00001d020063_T004	0.85068	0.468434681	Zm00001d020063
Zm00001d020063_T002	0.9954444	0.21984053	Zm00001d020063
Zm00001d017886_T002	0.9239352	0.532755176	Zm00001d017886
Zm00001d018470_T001	0.9860836	0.19482216	Zm00001d018470
Zm00001d027625_T001	0.01189263	1.999419336	Zm00001d027625
Zm00001d027625_T002	0.9053391	0.856600479	Zm00001d027625
Zm00001d032423_T003	2.37E-09	-4.790667212	Zm00001d032423
Zm00001d032423_T001	0.00224098	-2.663489454	Zm00001d032423
Zm00001d032423_T006	0.2509398	-1.130474143	Zm00001d032423
Zm00001d032423_T005	0.02175173	-2.268530182	Zm00001d032423
Zm00001d047762_T010	0.8746701	-0.07019537	Zm00001d047762
Zm00001d047762_T020	0.7353854	0.382328044	Zm00001d047762
Zm00001d047762_T003	0.02339581	-1.662457784	Zm00001d047762
Zm00001d047762_T024	0.1863747	0.3379942	Zm00001d047762
Zm00001d047762_T009	0.003185403	2.499180147	Zm00001d047762
Zm00001d047762_T002	1	0.13818494	Zm00001d047762
Zm00001d047762_T034	0.7187348	0.658473766	Zm00001d047762
Zm00001d047762_T028	0.6053514	0.596161527	Zm00001d047762
Zm00001d031023_T001	0.6896661	-0.314442259	Zm00001d031023
Zm00001d024979_T001	0.9727583	0.053034961	Zm00001d024979
Zm00001d040318_T002	0.2035902	1.952122727	Zm00001d040318
Zm00001d038618_T001	0.7577723	-0.235821066	Zm00001d038618
Zm00001d037847_T001	0.9666757	0.606886635	Zm00001d037847
Zm00001d049995_T001	0.6619605	1.271671023	Zm00001d049995
Zm00001d035411_T001	0.9432413	-0.165257299	Zm00001d035411
Zm00001d009745_T003	0.6254218	-0.263108637	Zm00001d009745
Zm00001d009745_T026	0.1587104	0.908502736	Zm00001d009745
Zm00001d009745_T018	0.01493221	0.825647668	Zm00001d009745
Zm00001d009745_T001	0.000224843	0.778249726	Zm00001d009745
Zm00001d009745_T030	0.5037407	-0.701768244	Zm00001d009745
Zm00001d009745_T028	0.923446	0.53238674	Zm00001d009745
Zm00001d001804_T005	0.9686323	0.466385388	Zm00001d001804

Zm00001d043360_T001	0.8545986	-0.661553663	Zm00001d043360
Zm00001d045338_T001	0.972985	0.512248695	Zm00001d045338
Zm00001d042468_T002	0.4086684	1.269170382	Zm00001d042468
Zm00001d042468_T025	0.8000103	-0.27809681	Zm00001d042468
Zm00001d018391_T004	0.9210327	0.46038957	Zm00001d018391
Zm00001d018391_T001	0.9833565	0.433717509	Zm00001d018391
Zm00001d018391_T003	1	0.393875359	Zm00001d018391
Zm00001d018391_T002	0.7187653	0.919446633	Zm00001d018391
Zm00001d042944_T001	0.000455444	-2.133019312	Zm00001d042944
Zm00001d040622_T001	0.961399	0.317088797	Zm00001d040622
Zm00001d018882_T001	0.4723494	-1.066969254	Zm00001d018882
Zm00001d042264_T010	0.9975882	0.322559336	Zm00001d042264
Zm00001d042264_T005	0.9170869	0.68751973	Zm00001d042264
Zm00001d042264_T012	5.53E-06	0.602951491	Zm00001d042264
Zm00001d033496_T001	0.5978467	0.78844313	Zm00001d033496
Zm00001d009779_T002	0.5437196	0.604534142	Zm00001d009779
Zm00001d009779_T001	0.9689939	0.63404731	Zm00001d009779
Zm00001d021642_T002	0.3545411	-0.603607616	Zm00001d021642
Zm00001d021642_T003	0.942907	-0.221062276	Zm00001d021642
Zm00001d050352_T001	0.6329087	0.814226818	Zm00001d050352
Zm00001d006839_T002	0.9832092	0.460244856	Zm00001d006839
Zm00001d024043_T001	0.429391	1.253833578	Zm00001d024043
Zm00001d036447_T001	0.9988063	0.183875925	Zm00001d036447
Zm00001d036447_T002	0.9207666	0.532013349	Zm00001d036447
Zm00001d036447_T005	0.006217153	-1.261055512	Zm00001d036447
Zm00001d036447_T004	0.8412913	-0.10641199	Zm00001d036447
Zm00001d026317_T004	0.8802656	-0.133984204	Zm00001d026317
Zm00001d012599_T001	0.9753213	0.383659414	Zm00001d012599
Zm00001d029619_T001	0.9545741	0.473819655	Zm00001d029619
Zm00001d002410_T001	0.3602097	1.458878063	Zm00001d002410
Zm00001d012219_T001	0.9894615	0.089925207	Zm00001d012219
Zm00001d027330_T001	0.9822138	0.303595016	Zm00001d027330
Zm00001d040693_T003	0.5945614	0.975584819	Zm00001d040693
Zm00001d040693_T005	0.8835073	0.608569366	Zm00001d040693
Zm00001d040693_T004	0.935477	0.539221538	Zm00001d040693
Zm00001d040693_T014	0.2461465	-1.422498528	Zm00001d040693
Zm00001d040693_T009	0.9361945	0.484520422	Zm00001d040693
Zm00001d040693_T006	0.9586882	0.606271094	Zm00001d040693
Zm00001d040693_T001	0.7949312	0.820438657	Zm00001d040693
Zm00001d042202_T001	0.5423554	-0.538985569	Zm00001d042202
Zm00001d036482_T001	0.9706293	0.172418646	Zm00001d036482
Zm00001d038851_T001	0.8660897	-0.152300886	Zm00001d038851
Zm00001d041079_T001	0.5004602	1.037154824	Zm00001d041079
Zm00001d002532_T004	0.5539906	-0.450015077	Zm00001d002532
Zm00001d002532_T001	0.06614768	-0.898803031	Zm00001d002532
Zm00001d002532_T003	0.9846446	0.837744421	Zm00001d002532
Zm00001d037067_T001	1	0.177543098	Zm00001d037067
Zm00001d037067_T002	0.9942835	0.384892251	Zm00001d037067
Zm00001d017639_T001	0.6887933	0.729738377	Zm00001d017639
Zm00001d035651_T001	0.8747821	-0.10006416	Zm00001d035651
Zm00001d045492_T004	0.6194652	-0.440779655	Zm00001d045492
Zm00001d045492_T002	0.8913084	-0.05004053	Zm00001d045492
Zm00001d045492_T003	0.9987435	0.081742931	Zm00001d045492
Zm00001d054016_T011	0.7309251	0.23984518	Zm00001d054016

Zm00001d054016_T002	1.81E-10	1.792411154	Zm00001d054016
Zm00001d054016_T005	0.8296601	-0.157032135	Zm00001d054016
Zm00001d023881_T003	0.7167212	0.307734976	Zm00001d023881
Zm00001d023881_T002	0.9937853	0.219054284	Zm00001d023881
Zm00001d023881_T006	0.9380783	-0.035194314	Zm00001d023881
Zm00001d023881_T005	0.5539906	0.736325525	Zm00001d023881
Zm00001d023881_T001	0.9959972	0.105613125	Zm00001d023881
Zm00001d024717_T001	0.745469	0.840147443	Zm00001d024717
Zm00001d031040_T019	0.4879435	0.886588182	Zm00001d031040
Zm00001d031040_T005	0.973788	0.512417573	Zm00001d031040
Zm00001d031040_T004	0.2750324	0.237262065	Zm00001d031040
Zm00001d033800_T001	0.9491569	0.265205546	Zm00001d033800
Zm00001d033800_T002	0.9956119	0.496723135	Zm00001d033800
Zm00001d050248_T002	0.9781672	0.345738558	Zm00001d050248
Zm00001d017334_T003	0.06811438	1.522233252	Zm00001d017334
Zm00001d017334_T006	0.9970835	0.037116952	Zm00001d017334
Zm00001d017334_T002	0.9782953	0.400867593	Zm00001d017334
Zm00001d017334_T001	0.007616066	2.158834228	Zm00001d017334
Zm00001d017334_T004	0.9819471	0.295000183	Zm00001d017334
Zm00001d004042_T003	0.8951107	0.372103165	Zm00001d004042
Zm00001d014717_T001	0.9719113	0.124982593	Zm00001d014717
Zm00001d019228_T001	0.8862467	-0.134445286	Zm00001d019228
Zm00001d038833_T001	0.893908	0.696309919	Zm00001d038833
Zm00001d051945_T002	0.9983524	0.325091816	Zm00001d051945
Zm00001d051945_T001	0.909186	0.776295283	Zm00001d051945
Zm00001d043022_T001	0.9596377	0.623562163	Zm00001d043022
Zm00001d027449_T001	0.9732421	0.382104806	Zm00001d027449
Zm00001d040463_T001	0.6044465	-0.36097446	Zm00001d040463
Zm00001d052523_T001	0.9090686	0.68538618	Zm00001d052523
Zm00001d031607_T001	0.7666773	0.552153351	Zm00001d031607
Zm00001d010401_T001	0.8489797	-0.355536117	Zm00001d010401
Zm00001d005473_T004	0.9945118	0.413205593	Zm00001d005473
Zm00001d005473_T008	0.03110152	1.694821549	Zm00001d005473
Zm00001d018342_T002	1	0.408368309	Zm00001d018342
Zm00001d018342_T003	0.9224378	0.642414982	Zm00001d018342
Zm00001d034254_T002	0.923446	-0.007238888	Zm00001d034254
Zm00001d034254_T001	0.8039111	-0.54563858	Zm00001d034254
Zm00001d002139_T001	0.8535962	0.6541501	Zm00001d002139
Zm00001d026030_T016	0.6843488	-0.498651225	Zm00001d026030
Zm00001d026030_T004	0.9216521	0.7227919	Zm00001d026030
Zm00001d022544_T002	0.9930712	0.18968417	Zm00001d022544
Zm00001d007112_T002	0.1329691	1.606416785	Zm00001d007112
Zm00001d043675_T002	1	0.273093263	Zm00001d043675
Zm00001d043675_T001	0.4282459	-0.45474591	Zm00001d043675
Zm00001d040075_T001	0.989787	0.316503673	Zm00001d040075
Zm00001d040075_T002	0.2897085	-0.730406794	Zm00001d040075
Zm00001d006321_T001	0.8747436	-0.24441169	Zm00001d006321
Zm00001d026253_T006	0.9982897	0.123241129	Zm00001d026253
Zm00001d026253_T001	0.1453031	0.789974813	Zm00001d026253
Zm00001d026253_T007	1	0.070969005	Zm00001d026253
Zm00001d026253_T004	1.16E-08	1.109853097	Zm00001d026253
Zm00001d046835_T011	0.9640775	0.173920422	Zm00001d046835
Zm00001d046835_T001	0.8700497	0.691537692	Zm00001d046835
Zm00001d042939_T003	0.0165698	0.583515458	Zm00001d042939

Zm00001d042939_T001	1	0.34709412	Zm00001d042939
Zm00001d037509_T001	1	0.197674615	Zm00001d037509
Zm00001d005015_T001	0.9814079	0.178097161	Zm00001d005015
Zm00001d020205_T002	0.9766874	0.100999321	Zm00001d020205
Zm00001d020205_T001	0.964271	0.0220698	Zm00001d020205
Zm00001d011451_T001	0.00087074	2.990161747	Zm00001d011451
Zm00001d051565_T003	0.9588604	0.257454074	Zm00001d051565
Zm00001d051565_T026	0.09964953	0.438528865	Zm00001d051565
Zm00001d051565_T008	0.7289978	0.20160985	Zm00001d051565
Zm00001d051565_T011	0.006892549	1.005843576	Zm00001d051565
Zm00001d047166_T002	0.9633184	0.619910221	Zm00001d047166
Zm00001d047166_T003	0.9244812	0.022784116	Zm00001d047166
Zm00001d047166_T001	0.990751	0.511735932	Zm00001d047166
Zm00001d015780_T004	0.8899623	0.235552525	Zm00001d015780
Zm00001d015780_T001	0.906282	-0.072113887	Zm00001d015780
Zm00001d015780_T006	0.8680274	0.805821661	Zm00001d015780
Zm00001d035761_T001	0.954492	-0.021572041	Zm00001d035761
Zm00001d046967_T001	1	-0.08131645	Zm00001d046967
Zm00001d018477_T008	0.8307578	0.264460492	Zm00001d018477
Zm00001d018477_T010	7.18E-12	-2.02210976	Zm00001d018477
Zm00001d018477_T011	0.008110248	2.185937921	Zm00001d018477
Zm00001d018477_T003	0.9760472	0.109200444	Zm00001d018477
Zm00001d018477_T002	0.9312703	0.314525257	Zm00001d018477
Zm00001d043089_T001	0.05522144	1.833756503	Zm00001d043089
Zm00001d023950_T001	0.9666757	-0.093764106	Zm00001d023950
Zm00001d015301_T001	0.6432857	-0.905508169	Zm00001d015301
Zm00001d002593_T001	0.6020989	-0.539492815	Zm00001d002593
Zm00001d052054_T001	0.02638727	-1.494829741	Zm00001d052054
Zm00001d006470_T001	0.4793027	0.853479864	Zm00001d006470
Zm00001d006470_T002	0.000929456	-1.049035363	Zm00001d006470
Zm00001d006470_T005	0.9545564	0.068311908	Zm00001d006470
Zm00001d006470_T006	0.9588604	0.072040577	Zm00001d006470
Zm00001d036929_T002	0.9573272	0.583626362	Zm00001d036929
Zm00001d016200_T007	0.7975103	-0.228816974	Zm00001d016200
Zm00001d016200_T005	0.5549472	-0.343299858	Zm00001d016200
Zm00001d016200_T001	0.7137316	-0.590058263	Zm00001d016200
Zm00001d009520_T002	0.001006918	-0.872979879	Zm00001d009520
Zm00001d009520_T003	0.9422098	-0.293196987	Zm00001d009520
Zm00001d009520_T001	0.9939021	0.49131058	Zm00001d009520
Zm00001d003905_T001	0.930878	-0.200249656	Zm00001d003905
Zm00001d048585_T002	0.919888	-0.054168262	Zm00001d048585
Zm00001d048585_T003	1	0.345785012	Zm00001d048585
Zm00001d029087_T008	0.9596377	0.233478338	Zm00001d029087
Zm00001d012726_T001	0.3713446	2.734521712	Zm00001d012726
Zm00001d011490_T008	0.9771927	0.513661565	Zm00001d011490
Zm00001d011490_T002	0.1709638	1.222071315	Zm00001d011490
Zm00001d011490_T005	0.1544336	0.979644381	Zm00001d011490
Zm00001d033328_T001	1	0.396831737	Zm00001d033328
Zm00001d048462_T001	1	0.019432322	Zm00001d048462
Zm00001d048729_T001	0.8310488	0.688475373	Zm00001d048729
Zm00001d037710_T001	0.2038697	0.722424057	Zm00001d037710
Zm00001d006034_T004	0.9869996	0.240727996	Zm00001d006034
Zm00001d006034_T001	0.03992146	-2.108437483	Zm00001d006034
Zm00001d006034_T006	1	0.28621771	Zm00001d006034

Zm00001d006034_T005	0.5322043	-0.341032737	Zm00001d006034
Zm00001d014734_T001	0.9804228	0.084807546	Zm00001d014734
Zm00001d035095_T001	0.792688	-0.237553956	Zm00001d035095
Zm00001d003512_T001	0.4859586	1.357567712	Zm00001d003512
Zm00001d003277_T003	0.001774985	0.752739036	Zm00001d003277
Zm00001d003277_T001	1.62E-09	1.668394327	Zm00001d003277
Zm00001d003277_T002	0.9840043	0.274648303	Zm00001d003277
Zm00001d003277_T006	0.8704049	0.058432922	Zm00001d003277
Zm00001d003277_T004	0.7624897	1.138207123	Zm00001d003277
Zm00001d021512_T005	0.8807115	0.772706914	Zm00001d021512
Zm00001d021512_T003	0.6099833	-0.479765869	Zm00001d021512
Zm00001d021512_T002	0.8127169	0.936331095	Zm00001d021512
Zm00001d021512_T001	0.9201237	-0.062588905	Zm00001d021512
Zm00001d021512_T004	0.9628835	0.416185401	Zm00001d021512
Zm00001d003378_T001	0.3263146	-0.213536942	Zm00001d003378
Zm00001d003378_T002	0.9497937	0.622449043	Zm00001d003378
Zm00001d021404_T003	0.4612153	0.455247656	Zm00001d021404
Zm00001d021404_T010	0.8310328	0.430167102	Zm00001d021404
Zm00001d021404_T005	0.4146004	0.417004656	Zm00001d021404
Zm00001d021404_T008	4.06E-11	-1.992582222	Zm00001d021404
Zm00001d021404_T002	0.1234187	-0.656318255	Zm00001d021404
Zm00001d043807_T005	0.002259866	-1.732132388	Zm00001d043807
Zm00001d043807_T003	0.9954752	0.457788593	Zm00001d043807
Zm00001d043807_T001	0.4538873	-0.485511125	Zm00001d043807
Zm00001d014655_T001	0.9939021	0.325556766	Zm00001d014655
Zm00001d031136_T003	0.8667498	-0.175333842	Zm00001d031136
Zm00001d031136_T002	0.7547798	0.36845171	Zm00001d031136
Zm00001d024458_T001	0.03126852	2.517581326	Zm00001d024458
Zm00001d044754_T006	0.6996313	-0.540196455	Zm00001d044754
Zm00001d044754_T005	0.7523526	0.664146033	Zm00001d044754
Zm00001d044754_T002	0.9832092	0.572980761	Zm00001d044754
Zm00001d044754_T003	0.8580945	-0.172830943	Zm00001d044754
Zm00001d007424_T006	0.9860836	-0.191406829	Zm00001d007424
Zm00001d007424_T007	0.7628188	0.71070344	Zm00001d007424
Zm00001d007424_T003	2.50E-07	-1.235610784	Zm00001d007424
Zm00001d007424_T005	0.1921001	0.555570372	Zm00001d007424
Zm00001d007424_T002	0.9932384	0.282935349	Zm00001d007424
Zm00001d007424_T004	0.5954407	0.967068528	Zm00001d007424
Zm00001d011585_T001	0.9877195	0.368609169	Zm00001d011585
Zm00001d029140_T002	0.9272378	-0.02155293	Zm00001d029140
Zm00001d013167_T001	0.9833513	0.058045476	Zm00001d013167
Zm00001d033063_T001	0.9936409	0.299017859	Zm00001d033063
Zm00001d029681_T001	0.1189054	-1.933483751	Zm00001d029681
Zm00001d038003_T001	0.01286019	-2.492126783	Zm00001d038003
Zm00001d018677_T001	6.91E-05	-2.864634073	Zm00001d018677
Zm00001d040232_T001	0.9116059	-0.150094945	Zm00001d040232
Zm00001d028151_T001	0.8806413	0.879234638	Zm00001d028151
Zm00001d038849_T004	0.8858223	0.321866051	Zm00001d038849
Zm00001d038849_T002	0.9659036	0.452073168	Zm00001d038849
Zm00001d038849_T003	0.983226	-0.099064952	Zm00001d038849
Zm00001d038849_T005	0.8449719	-0.255859923	Zm00001d038849
Zm00001d050666_T001	0.8842271	-0.186117397	Zm00001d050666
Zm00001d040752_T001	0.9822138	0.115800111	Zm00001d040752
Zm00001d032719_T002	0.5480167	0.867535546	Zm00001d032719

Zm00001d032719_T001	0.7074815	1.025733533	Zm00001d032719
Zm00001d032719_T004	0.9201237	-0.008634164	Zm00001d032719
Zm00001d032719_T003	0.935477	-0.077475616	Zm00001d032719
Zm00001d047651_T001	0.8192173	0.908059292	Zm00001d047651
Zm00001d007489_T001	0.9666757	0.115481303	Zm00001d007489
Zm00001d030942_T001	0.8781102	-0.070711641	Zm00001d030942
Zm00001d011779_T009	0.9400285	0.034702992	Zm00001d011779
Zm00001d011779_T015	0.7273998	-0.259961757	Zm00001d011779
Zm00001d025574_T001	1	0.370291963	Zm00001d025574
Zm00001d008596_T003	0.7602652	0.754109846	Zm00001d008596
Zm00001d008596_T002	0.9330832	0.757555201	Zm00001d008596
Zm00001d008596_T004	0.07193039	1.143424684	Zm00001d008596
Zm00001d049273_T001	1	0.460452057	Zm00001d049273
Zm00001d031050_T002	0.7187348	-0.342081637	Zm00001d031050
Zm00001d031050_T001	0.5790787	-0.504378292	Zm00001d031050
Zm00001d016748_T001	0.2178312	-1.392960625	Zm00001d016748
Zm00001d004856_T001	0.956053	0.082250727	Zm00001d004856
Zm00001d018113_T002	0.2748634	-0.185873854	Zm00001d018113
Zm00001d018113_T001	0.2663896	0.482270821	Zm00001d018113
Zm00001d018113_T022	0.06500622	-0.7220332	Zm00001d018113
Zm00001d018113_T011	0.02764265	1.781327689	Zm00001d018113
Zm00001d018113_T017	0.9942835	0.450448185	Zm00001d018113
Zm00001d018113_T023	0.4574813	0.991330367	Zm00001d018113
Zm00001d018696_T001	0.9112906	0.003643543	Zm00001d018696
Zm00001d034808_T001	0.9720956	-0.090992067	Zm00001d034808
Zm00001d016956_T012	0.02930817	0.616541881	Zm00001d016956
Zm00001d016956_T002	0.9384219	0.471101431	Zm00001d016956
Zm00001d016956_T008	0.3247773	0.850378793	Zm00001d016956
Zm00001d016956_T009	0.9969123	0.131967869	Zm00001d016956
Zm00001d016956_T006	0.9344122	-0.08302946	Zm00001d016956
Zm00001d002146_T001	0.9519347	0.467818397	Zm00001d002146
Zm00001d044389_T002	0.9160053	0.620902059	Zm00001d044389
Zm00001d044389_T003	0.9887584	0.116512763	Zm00001d044389
Zm00001d044389_T001	0.8901666	-0.130262785	Zm00001d044389
Zm00001d029442_T001	0.1026268	-1.349592638	Zm00001d029442
Zm00001d039650_T002	0.9540138	0.11943478	Zm00001d039650
Zm00001d039650_T001	0.8648059	0.347826925	Zm00001d039650
Zm00001d031182_T002	0.9816835	0.248400779	Zm00001d031182
Zm00001d031182_T001	0.9782953	0.26713966	Zm00001d031182
Zm00001d017276_T001	0.1442381	1.913942073	Zm00001d017276
Zm00001d033792_T001	0.9596377	-0.061939008	Zm00001d033792
Zm00001d007635_T004	0.9706293	0.590369385	Zm00001d007635
Zm00001d042584_T001	1	0.209506526	Zm00001d042584
Zm00001d042584_T004	0.6007866	0.740913094	Zm00001d042584
Zm00001d010724_T001	0.9272378	0.005300998	Zm00001d010724
Zm00001d014864_T009	0.06026596	0.952277221	Zm00001d014864
Zm00001d014864_T004	0.9817085	0.4179242	Zm00001d014864
Zm00001d014864_T003	0.2044538	0.292587649	Zm00001d014864
Zm00001d014864_T001	0.09126143	0.540290171	Zm00001d014864
Zm00001d014864_T010	0.969231	0.169259588	Zm00001d014864
Zm00001d013868_T001	0.1614401	0.780978969	Zm00001d013868
Zm00001d013868_T004	0.3071359	1.383774381	Zm00001d013868
Zm00001d013868_T002	0.1374691	1.470262236	Zm00001d013868
Zm00001d026010_T010	0.7432117	1.18171121	Zm00001d026010

Zm00001d026010_T007	0.5549906	-0.336789011	Zm00001d026010
Zm00001d026010_T011	0.8000033	-0.16802914	Zm00001d026010
Zm00001d026010_T003	0.7865309	0.767137788	Zm00001d026010
Zm00001d026010_T009	6.27E-13	2.637976029	Zm00001d026010
Zm00001d026010_T012	1.06E-16	-4.816861321	Zm00001d026010
Zm00001d026010_T006	0.9326774	0.440388681	Zm00001d026010
Zm00001d014896_T001	1	0.38061296	Zm00001d014896
Zm00001d035551_T005	0.9979568	0.107058536	Zm00001d035551
Zm00001d035551_T008	0.04606011	-1.184906023	Zm00001d035551
Zm00001d035551_T003	0.7784714	-0.411689948	Zm00001d035551
Zm00001d035551_T002	0.7294039	-0.407758194	Zm00001d035551
Zm00001d035551_T004	0.4332539	-0.854378432	Zm00001d035551
Zm00001d035551_T001	0.8489797	-0.157016773	Zm00001d035551
Zm00001d044019_T014	0.2039461	0.718181193	Zm00001d044019
Zm00001d044019_T016	0.9608605	0.100366172	Zm00001d044019
Zm00001d044019_T001	0.8211357	-0.061568938	Zm00001d044019
Zm00001d044019_T004	0.009172744	-0.310217969	Zm00001d044019
Zm00001d044019_T003	0.9016285	0.660563892	Zm00001d044019
Zm00001d008891_T001	0.4329511	1.065188268	Zm00001d008891
Zm00001d044995_T001	0.4903572	1.497029754	Zm00001d044995
Zm00001d009760_T001	0.7106278	-0.283532809	Zm00001d009760
Zm00001d030299_T002	0.9966965	0.175636999	Zm00001d030299
Zm00001d030299_T001	0.8377512	-0.174222766	Zm00001d030299
Zm00001d012709_T001	0.9914064	0.431562896	Zm00001d012709
Zm00001d022305_T011	0.6160016	0.45850329	Zm00001d022305
Zm00001d022305_T010	0.9206245	0.022146551	Zm00001d022305
Zm00001d022305_T003	0.6450309	1.257120751	Zm00001d022305
Zm00001d019561_T010	0.8865624	0.113124699	Zm00001d019561
Zm00001d019561_T009	0.6044867	1.111584202	Zm00001d019561
Zm00001d019561_T002	0.1842201	-0.110885202	Zm00001d019561
Zm00001d019561_T054	8.81E-08	0.829434124	Zm00001d019561
Zm00001d019561_T022	0.6899653	0.858018734	Zm00001d019561
Zm00001d019561_T007	0.9446379	0.109628845	Zm00001d019561
Zm00001d019561_T012	0.9970835	0.156088013	Zm00001d019561
Zm00001d019561_T024	0.6466858	0.36002215	Zm00001d019561
Zm00001d019561_T018	0.004443898	0.795296462	Zm00001d019561
Zm00001d019536_T003	0.9514141	0.102479615	Zm00001d019536
Zm00001d019536_T001	0.9686323	0.323273218	Zm00001d019536
Zm00001d019536_T002	0.5082706	0.961793677	Zm00001d019536
Zm00001d018790_T001	0.774379	-0.368103067	Zm00001d018790
Zm00001d027393_T001	0.9791729	0.261317639	Zm00001d027393
Zm00001d031951_T007	0.2199909	1.773184644	Zm00001d031951
Zm00001d031951_T003	0.6014554	-0.345472635	Zm00001d031951
Zm00001d031951_T005	0.8581713	-0.469003961	Zm00001d031951
Zm00001d040513_T001	0.9312703	0.569490085	Zm00001d040513
Zm00001d041718_T006	0.5646517	0.340961661	Zm00001d041718
Zm00001d041718_T005	0.02518239	1.008192703	Zm00001d041718
Zm00001d041718_T002	1	0.29913222	Zm00001d041718
Zm00001d006049_T001	0.9828518	0.428570143	Zm00001d006049
Zm00001d003750_T001	0.401517	1.195070823	Zm00001d003750
Zm00001d024319_T001	0.9804228	0.096521494	Zm00001d024319
Zm00001d033525_T001	0.9846714	0.191489171	Zm00001d033525
Zm00001d041181_T001	0.6102438	-0.487374915	Zm00001d041181
Zm00001d006212_T002	0.03256577	-0.67940588	Zm00001d006212

Zm00001d006212_T004	0.6911353	-0.336296792	Zm00001d006212
Zm00001d006212_T003	0.2020154	-1.029599265	Zm00001d006212
Zm00001d031303_T007	0.9664406	0.204290515	Zm00001d031303
Zm00001d049091_T001	0.9939021	0.419825704	Zm00001d049091
Zm00001d007912_T013	0.8267006	0.222727259	Zm00001d007912
Zm00001d007912_T009	0.8885217	0.400510793	Zm00001d007912
Zm00001d007912_T003	1	0.443655737	Zm00001d007912
Zm00001d020691_T001	0.8384064	-0.17938844	Zm00001d020691
Zm00001d040390_T007	0.9928593	0.199725558	Zm00001d040390
Zm00001d040390_T006	0.6235378	-0.379688279	Zm00001d040390
Zm00001d040390_T004	0.8676122	-0.416858899	Zm00001d040390
Zm00001d040390_T005	0.9706293	0.427568533	Zm00001d040390
Zm00001d031741_T001	0.8028573	-0.531131096	Zm00001d031741
Zm00001d011386_T001	0.9907928	0.142121459	Zm00001d011386
Zm00001d011386_T005	0.7594555	0.477036018	Zm00001d011386
Zm00001d035526_T003	0.1375907	-0.291446833	Zm00001d035526
Zm00001d035526_T016	0.9641291	0.135805715	Zm00001d035526
Zm00001d035526_T015	0.997764	0.120841931	Zm00001d035526
Zm00001d035526_T002	0.01206564	1.60514593	Zm00001d035526
Zm00001d043891_T001	0.9701986	0.242922455	Zm00001d043891
Zm00001d033776_T008	0.3394879	0.831452617	Zm00001d033776
Zm00001d033776_T009	0.01758334	0.626642421	Zm00001d033776
Zm00001d033776_T002	0.9514141	0.656769452	Zm00001d033776
Zm00001d033776_T004	0.7888267	0.823824923	Zm00001d033776
Zm00001d033776_T003	1	0.22930218	Zm00001d033776
Zm00001d031764_T003	1	0.390440155	Zm00001d031764
Zm00001d031764_T005	0.9760516	0.343087274	Zm00001d031764
Zm00001d031764_T006	0.8849109	0.557763241	Zm00001d031764
Zm00001d016063_T001	0.8557307	-0.114188268	Zm00001d016063
Zm00001d016063_T007	0.5243524	-0.207054292	Zm00001d016063
Zm00001d016063_T009	0.01983628	0.594524397	Zm00001d016063
Zm00001d016063_T008	0.01493221	0.501056776	Zm00001d016063
Zm00001d016063_T002	0.6250286	-0.175173951	Zm00001d016063
Zm00001d002489_T001	0.7383872	-0.382990129	Zm00001d002489
Zm00001d047491_T003	0.7651217	0.460701545	Zm00001d047491
Zm00001d047491_T001	0.8987811	0.325572716	Zm00001d047491
Zm00001d047491_T010	0.8412913	0.268094043	Zm00001d047491
Zm00001d047491_T006	0.9932384	0.28004267	Zm00001d047491
Zm00001d047491_T012	0.08348055	1.510202909	Zm00001d047491
Zm00001d011571_T001	0.05495233	2.236934082	Zm00001d011571
Zm00001d013838_T003	0.9979568	0.03088377	Zm00001d013838
Zm00001d013838_T001	0.9846714	0.531795751	Zm00001d013838
Zm00001d027458_T001	0.9570268	-0.265032781	Zm00001d027458
Zm00001d031887_T001	0.8192173	0.632333147	Zm00001d031887
Zm00001d025136_T002	0.9223663	0.046576185	Zm00001d025136
Zm00001d000402_T001	0.8643636	-0.137047177	Zm00001d000402
Zm00001d041880_T012	0.9416622	-0.018886093	Zm00001d041880
Zm00001d041880_T001	0.9180662	0.860049905	Zm00001d041880
Zm00001d041880_T011	0.9498304	0.471453343	Zm00001d041880
Zm00001d041880_T005	0.1926277	1.908025632	Zm00001d041880
Zm00001d041880_T003	0.7412991	0.38032854	Zm00001d041880
Zm00001d041880_T010	0.9007927	0.9138415	Zm00001d041880
Zm00001d036989_T001	0.9862019	0.268557549	Zm00001d036989
Zm00001d024916_T001	0.4665568	1.487745533	Zm00001d024916

Zm00001d041790_T001	0.7439594	0.736115229	Zm00001d041790
Zm00001d048205_T002	1	0.341395281	Zm00001d048205
Zm00001d048205_T005	0.9619533	0.032909236	Zm00001d048205
Zm00001d048205_T004	0.4532331	1.269411852	Zm00001d048205
Zm00001d042349_T001	0.7876948	-0.236000237	Zm00001d042349
Zm00001d048823_T001	0.5335596	-0.400369006	Zm00001d048823
Zm00001d048823_T006	0.5455802	0.509909901	Zm00001d048823
Zm00001d048823_T007	0.956479	0.678152112	Zm00001d048823
Zm00001d038653_T001	0.9932006	0.091340952	Zm00001d038653
Zm00001d038653_T002	0.7619918	0.381306734	Zm00001d038653
Zm00001d034131_T001	0.9132406	0.51970932	Zm00001d034131
Zm00001d008925_T001	0.998322	0.315923032	Zm00001d008925
Zm00001d019459_T001	0.8638849	-0.382587762	Zm00001d019459
Zm00001d003196_T001	0.9970835	0.228058595	Zm00001d003196
Zm00001d026283_T002	1	0.308503562	Zm00001d026283
Zm00001d026283_T001	0.1889053	-0.89377929	Zm00001d026283
Zm00001d012620_T025	0.06202197	1.962717123	Zm00001d012620
Zm00001d012620_T012	0.1006033	0.605462845	Zm00001d012620
Zm00001d012620_T001	0.9628812	0.417523156	Zm00001d012620
Zm00001d012620_T018	0.009821311	1.133825634	Zm00001d012620
Zm00001d012620_T017	0.3837834	-1.197235595	Zm00001d012620
Zm00001d012620_T016	0.8645432	-0.270383519	Zm00001d012620
Zm00001d012620_T007	0.3083073	0.509111452	Zm00001d012620
Zm00001d052260_T001	0.7702714	-0.288603908	Zm00001d052260
Zm00001d031510_T001	0.09671284	2.106403326	Zm00001d031510
Zm00001d017157_T001	0.8827359	0.773980856	Zm00001d017157
Zm00001d017157_T002	0.9706887	0.372990474	Zm00001d017157
Zm00001d013914_T001	0.9628835	0.031299648	Zm00001d013914
Zm00001d046281_T001	0.98291	-0.107227745	Zm00001d046281
Zm00001d014950_T001	0.8453901	0.465549327	Zm00001d014950
Zm00001d014950_T002	0.9722469	0.17319428	Zm00001d014950
Zm00001d048189_T001	0.7430929	-0.153578695	Zm00001d048189
Zm00001d010259_T002	0.9690782	0.31995138	Zm00001d010259
Zm00001d039251_T001	0.592105	-0.940755452	Zm00001d039251
Zm00001d026514_T004	0.4784037	1.189388236	Zm00001d026514
Zm00001d026514_T001	0.7177635	-0.45356382	Zm00001d026514
Zm00001d026514_T002	0.785055	0.984331397	Zm00001d026514
Zm00001d012465_T001	0.9954444	0.208785146	Zm00001d012465
Zm00001d018156_T001	0.6292709	-0.232250528	Zm00001d018156
Zm00001d054066_T001	0.935477	0.756648013	Zm00001d054066
Zm00001d038301_T002	0.9367198	0.041620492	Zm00001d038301
Zm00001d017395_T001	0.9003815	-0.087967857	Zm00001d017395
Zm00001d017395_T002	0.9846714	0.102717006	Zm00001d017395
Zm00001d015247_T001	0.9363242	-0.030457029	Zm00001d015247
Zm00001d025721_T002	0.897685	-0.136601345	Zm00001d025721
Zm00001d035462_T001	0.8380736	-0.173923785	Zm00001d035462
Zm00001d041709_T001	0.7697196	0.948463848	Zm00001d041709
Zm00001d047835_T001	0.7686657	-0.012779409	Zm00001d047835
Zm00001d047835_T004	0.9138633	-0.012126079	Zm00001d047835
Zm00001d047835_T002	0.9123976	0.357942222	Zm00001d047835
Zm00001d047835_T003	0.249617	0.950805104	Zm00001d047835
Zm00001d046571_T002	0.9192339	-0.032212012	Zm00001d046571
Zm00001d012443_T001	0.9706887	3.006941889	Zm00001d012443
Zm00001d037453_T002	0.3823269	-0.951064706	Zm00001d037453

Zm00001d037453_T004	0.2948251	-0.54454995	Zm00001d037453
Zm00001d037453_T001	0.1070785	-0.650348055	Zm00001d037453
Zm00001d037453_T003	0.741153	-0.180498148	Zm00001d037453
Zm00001d004138_T001	0.112368	2.119339926	Zm00001d004138
Zm00001d016586_T002	0.2344735	1.825134069	Zm00001d016586
Zm00001d047558_T002	0.8488494	0.58815619	Zm00001d047558
Zm00001d047558_T004	1	0.30249634	Zm00001d047558
Zm00001d047558_T001	0.9565533	0.13148805	Zm00001d047558
Zm00001d046182_T001	0.7640001	1.095760148	Zm00001d046182
Zm00001d021215_T001	0.9745777	0.391354488	Zm00001d021215
Zm00001d021215_T002	0.8819727	0.198082516	Zm00001d021215
Zm00001d021215_T004	0.05995189	1.262793026	Zm00001d021215
Zm00001d017682_T001	0.9561521	0.055305805	Zm00001d017682
Zm00001d027546_T001	0.8062829	1.008263166	Zm00001d027546
Zm00001d035113_T011	5.37E-12	2.594978441	Zm00001d035113
Zm00001d035113_T013	0.1887981	-1.003574416	Zm00001d035113
Zm00001d035113_T012	0.724133	-0.474772957	Zm00001d035113
Zm00001d035113_T001	0.002758715	0.965899342	Zm00001d035113
Zm00001d035113_T008	0.3226688	0.536711541	Zm00001d035113
Zm00001d031957_T001	0.003322704	-1.436741045	Zm00001d031957
Zm00001d033226_T002	0.4701937	-0.674816402	Zm00001d033226
Zm00001d046979_T001	0.5006087	1.284304162	Zm00001d046979
Zm00001d010225_T001	0.9931431	0.254538719	Zm00001d010225
Zm00001d025374_T001	0.1507622	-1.605671676	Zm00001d025374
Zm00001d003502_T001	0.7565187	-0.829108537	Zm00001d003502
Zm00001d003502_T004	0.8434952	-0.085936795	Zm00001d003502
Zm00001d003502_T006	0.1684497	-1.756406717	Zm00001d003502
Zm00001d003502_T005	0.8112443	-0.723623963	Zm00001d003502
Zm00001d024423_T010	0.8342677	-0.174791234	Zm00001d024423
Zm00001d024423_T008	0.9933044	-0.019207462	Zm00001d024423
Zm00001d041445_T010	0.1273888	1.631119783	Zm00001d041445
Zm00001d041445_T011	0.6331067	-0.697636845	Zm00001d041445
Zm00001d041445_T002	1	0.427241432	Zm00001d041445
Zm00001d052038_T001	0.9706887	0.489481232	Zm00001d052038
Zm00001d032220_T004	0.9550193	0.732735257	Zm00001d032220
Zm00001d032220_T019	0.2073192	1.490059919	Zm00001d032220
Zm00001d032220_T014	0.9649717	0.518763936	Zm00001d032220
Zm00001d032220_T008	0.9918302	0.068791298	Zm00001d032220
Zm00001d032220_T025	0.2649044	0.950386711	Zm00001d032220
Zm00001d032220_T028	0.2572096	0.99722293	Zm00001d032220
Zm00001d032220_T001	0.2329183	0.570352888	Zm00001d032220
Zm00001d032220_T020	0.7869719	0.590987679	Zm00001d032220
Zm00001d032220_T018	0.1304309	0.812171496	Zm00001d032220
Zm00001d032220_T006	0.8187452	0.485929634	Zm00001d032220
Zm00001d032220_T010	0.9543425	0.134450872	Zm00001d032220
Zm00001d042480_T007	0.1250366	0.582147566	Zm00001d042480
Zm00001d042480_T004	0.7169281	-0.35352263	Zm00001d042480
Zm00001d020309_T001	0.9965998	0.467248278	Zm00001d020309
Zm00001d020309_T002	0.01663882	0.957447151	Zm00001d020309
Zm00001d020309_T003	1	-0.038353602	Zm00001d020309
Zm00001d005976_T012	0.4347196	-0.789954735	Zm00001d005976
Zm00001d005976_T001	0.5010043	1.226964521	Zm00001d005976
Zm00001d005976_T009	0.3198828	-0.465940492	Zm00001d005976
Zm00001d048407_T001	0.6050512	-0.996088135	Zm00001d048407

Zm00001d027943_T006	0.3159722	1.479191038	Zm00001d027943
Zm00001d027943_T028	0.5539906	-0.332107055	Zm00001d027943
Zm00001d027943_T012	0.6824162	-0.131948822	Zm00001d027943
Zm00001d027943_T002	0.2516736	0.904606317	Zm00001d027943
Zm00001d014098_T002	0.6272189	0.329119574	Zm00001d014098
Zm00001d015955_T007	0.9326774	0.553107066	Zm00001d015955
Zm00001d015955_T002	0.91126	0.038055058	Zm00001d015955
Zm00001d015955_T004	0.9832092	0.549301875	Zm00001d015955
Zm00001d035646_T001	0.7063456	-0.70564551	Zm00001d035646
Zm00001d008662_T001	1	0.21156863	Zm00001d008662
Zm00001d043181_T001	0.9132688	0.627367278	Zm00001d043181
Zm00001d037870_T003	0.8603905	-0.092886912	Zm00001d037870
Zm00001d037870_T007	2.80E-08	1.406054535	Zm00001d037870
Zm00001d037870_T001	0.9724085	0.138080277	Zm00001d037870
Zm00001d039010_T001	0.9926496	-0.05080554	Zm00001d039010
Zm00001d034635_T001	0.7123806	1.039198901	Zm00001d034635
Zm00001d020728_T011	0.08776846	0.443434201	Zm00001d020728
Zm00001d027351_T001	0.8988681	0.001345879	Zm00001d027351
Zm00001d000154_T001	0.718063	0.678719238	Zm00001d000154
Zm00001d044908_T001	0.08695175	2.22244587	Zm00001d044908
Zm00001d039764_T002	0.9124297	-0.149852104	Zm00001d039764
Zm00001d039764_T001	1	0.350302926	Zm00001d039764
Zm00001d014284_T001	0.5980883	-0.331838912	Zm00001d014284
Zm00001d018491_T001	0.08045219	2.106673569	Zm00001d018491
Zm00001d029202_T001	0.7511561	-0.460452807	Zm00001d029202
Zm00001d012847_T001	0.9745748	0.281164565	Zm00001d012847
Zm00001d035265_T003	0.6729605	0.550785948	Zm00001d035265
Zm00001d023396_T001	0.7685707	-0.35512579	Zm00001d023396
Zm00001d050457_T002	0.9942835	0.472094213	Zm00001d050457
Zm00001d050457_T001	0.9160053	-0.011865511	Zm00001d050457
Zm00001d040323_T001	0.965836	-1.001292757	Zm00001d040323
Zm00001d039263_T001	0.8998563	-0.432480235	Zm00001d039263
Zm00001d042256_T004	0.997764	0.345176409	Zm00001d042256
Zm00001d042256_T005	0.99543	0.201805447	Zm00001d042256
Zm00001d014152_T004	0.9285764	0.704306148	Zm00001d014152
Zm00001d038401_T001	0.5423036	0.45063582	Zm00001d038401
Zm00001d050925_T004	0.3418186	0.569363573	Zm00001d050925
Zm00001d050925_T007	2.93E-06	0.841674563	Zm00001d050925
Zm00001d050925_T005	1	0.445086298	Zm00001d050925
Zm00001d050925_T003	0.000773216	1.262315687	Zm00001d050925
Zm00001d011427_T014	0.000484721	1.456136142	Zm00001d011427
Zm00001d011427_T052	0.9363242	0.410957699	Zm00001d011427
Zm00001d011427_T050	0.5471937	1.055931875	Zm00001d011427
Zm00001d011427_T001	0.6741927	0.851254158	Zm00001d011427
Zm00001d011427_T054	2.44E-10	-1.099528069	Zm00001d011427
Zm00001d011427_T049	0.7520186	0.50016753	Zm00001d011427
Zm00001d011427_T056	2.10E-05	-0.829898962	Zm00001d011427
Zm00001d011427_T047	0.01322602	0.689215879	Zm00001d011427
Zm00001d022430_T001	0.9515686	0.542125945	Zm00001d022430
Zm00001d037747_T001	0.9080042	-0.19452133	Zm00001d037747
Zm00001d033568_T001	0.1701649	1.470291289	Zm00001d033568
Zm00001d021574_T003	0.2619882	0.585626045	Zm00001d021574
Zm00001d021574_T004	0.9781672	0.167846423	Zm00001d021574
Zm00001d021574_T005	0.02012514	1.171171938	Zm00001d021574

Zm00001d008539_T001	0.08012468	2.373294059	Zm00001d008539
Zm00001d026395_T001	0.9872831	0.194701085	Zm00001d026395
Zm00001d004473_T001	0.9781672	0.572823174	Zm00001d004473
Zm00001d044460_T001	0.8411786	-0.334772535	Zm00001d044460
Zm00001d039918_T003	0.9777274	0.549195457	Zm00001d039918
Zm00001d039918_T001	0.9790102	0.192370412	Zm00001d039918
Zm00001d049571_T001	0.9915941	0.23852848	Zm00001d049571
Zm00001d020428_T001	0.8805297	0.576633521	Zm00001d020428
Zm00001d044860_T006	0.9682673	0.633323689	Zm00001d044860
Zm00001d044860_T002	0.3704034	0.562740558	Zm00001d044860
Zm00001d044860_T003	0.9984041	0.379339298	Zm00001d044860
Zm00001d053862_T002	0.9689939	0.210835718	Zm00001d053862
Zm00001d011246_T002	0.5282092	0.818442748	Zm00001d011246
Zm00001d039401_T001	0.6436915	1.174972807	Zm00001d039401
Zm00001d039401_T002	0.63483	1.177930137	Zm00001d039401
Zm00001d013532_T001	0.995534	0.204006969	Zm00001d013532
Zm00001d013532_T003	0.9573434	-0.281763062	Zm00001d013532
Zm00001d013532_T002	0.7952206	-0.616059071	Zm00001d013532
Zm00001d051591_T001	0.8720321	-0.730609665	Zm00001d051591
Zm00001d034522_T003	0.8412913	0.905124735	Zm00001d034522
Zm00001d034522_T001	2.95E-09	2.087664566	Zm00001d034522
Zm00001d047469_T001	0.8503172	-0.315036383	Zm00001d047469
Zm00001d029343_T002	0.9862329	0.203886155	Zm00001d029343
Zm00001d047242_T002	0.8951107	0.822536975	Zm00001d047242
Zm00001d047242_T004	0.4068508	-0.896580092	Zm00001d047242
Zm00001d044877_T005	0.7142338	-0.301218137	Zm00001d044877
Zm00001d044877_T001	0.002844203	0.89373355	Zm00001d044877
Zm00001d044877_T004	0.9232446	0.4567636	Zm00001d044877
Zm00001d053885_T001	0.9883036	0.417141489	Zm00001d053885
Zm00001d009961_T001	0.2616476	1.633925064	Zm00001d009961
Zm00001d053715_T001	0.7979203	-0.279783435	Zm00001d053715
Zm00001d002734_T001	0.852305	-0.332109971	Zm00001d002734
Zm00001d018821_T001	0.6097645	-0.19238676	Zm00001d018821
Zm00001d002591_T001	0.9912886	0.096524171	Zm00001d002591
Zm00001d005393_T002	0.7602652	-0.031826161	Zm00001d005393
Zm00001d005393_T007	0.9967276	0.379849022	Zm00001d005393
Zm00001d005393_T004	0.8021989	-0.268071256	Zm00001d005393
Zm00001d005393_T001	0.4351411	0.622191187	Zm00001d005393
Zm00001d049088_T001	0.9944247	0.389058558	Zm00001d049088
Zm00001d013065_T002	0.9931431	0.182190819	Zm00001d013065
Zm00001d013065_T006	0.1090404	1.681292528	Zm00001d013065
Zm00001d013065_T001	0.9766874	0.518469725	Zm00001d013065
Zm00001d013065_T005	1	0.146164761	Zm00001d013065
Zm00001d013470_T008	0.02037655	-0.538625303	Zm00001d013470
Zm00001d013470_T014	0.9715541	0.16754493	Zm00001d013470
Zm00001d013470_T020	0.3827309	0.810827908	Zm00001d013470
Zm00001d013470_T029	0.3870314	-0.636073136	Zm00001d013470
Zm00001d013470_T009	0.04919818	2.004214938	Zm00001d013470
Zm00001d013470_T015	0.3358121	0.946188141	Zm00001d013470
Zm00001d013470_T001	5.42E-10	-2.815127062	Zm00001d013470
Zm00001d013470_T005	0.01810291	0.367369949	Zm00001d013470
Zm00001d012837_T001	0.6829189	-0.403659399	Zm00001d012837
Zm00001d052200_T001	0.9529184	0.123279433	Zm00001d052200
Zm00001d052200_T003	0.1928753	-1.076512231	Zm00001d052200

Zm00001d052200_T005	0.615315	-0.530516772	Zm00001d052200
Zm00001d052200_T002	0.01141862	-1.092649688	Zm00001d052200
Zm00001d042958_T001	0.9447869	0.592064811	Zm00001d042958
Zm00001d002758_T002	0.9979568	0.199884635	Zm00001d002758
Zm00001d019263_T002	0.9048001	-0.363522235	Zm00001d019263
Zm00001d019263_T001	0.8957792	0.723966041	Zm00001d019263
Zm00001d041823_T010	4.62E-07	-1.334636124	Zm00001d041823
Zm00001d041823_T003	0.1424006	1.210136412	Zm00001d041823
Zm00001d028657_T002	0.8684295	0.703759095	Zm00001d028657
Zm00001d028657_T001	0.5257324	1.334017181	Zm00001d028657
Zm00001d025619_T001	0.7506677	-0.418476026	Zm00001d025619
Zm00001d037333_T007	1	0.42144461	Zm00001d037333
Zm00001d037333_T015	0.9561521	0.231784592	Zm00001d037333
Zm00001d037333_T019	0.9486412	0.006734463	Zm00001d037333
Zm00001d037333_T027	0.7703259	0.601358742	Zm00001d037333
Zm00001d037333_T011	0.3353806	0.725555356	Zm00001d037333
Zm00001d037333_T010	0.000286648	-0.795742406	Zm00001d037333
Zm00001d037333_T001	0.7723306	-0.032131302	Zm00001d037333
Zm00001d037333_T028	0.6201573	0.993498977	Zm00001d037333
Zm00001d028385_T002	0.9443082	0.068243326	Zm00001d028385
Zm00001d028385_T001	0.1161894	1.38978254	Zm00001d028385
Zm00001d031677_T001	0.8825751	-0.107854994	Zm00001d031677
Zm00001d034305_T001	0.9596377	0.305828398	Zm00001d034305
Zm00001d021050_T001	0.9419336	0.603124441	Zm00001d021050
Zm00001d021050_T002	0.9851204	0.193319116	Zm00001d021050
Zm00001d021050_T003	0.8032834	-0.133731589	Zm00001d021050
Zm00001d042499_T001	0.2611065	1.668403766	Zm00001d042499
Zm00001d005085_T020	0.9160053	-0.078289758	Zm00001d005085
Zm00001d005085_T028	0.2566009	-1.372439692	Zm00001d005085
Zm00001d005085_T022	0.8453277	0.937537246	Zm00001d005085
Zm00001d005085_T023	0.126457	1.70951175	Zm00001d005085
Zm00001d043534_T001	0.9395327	0.026011285	Zm00001d043534
Zm00001d025514_T001	0.9988063	0.208142425	Zm00001d025514
Zm00001d014499_T005	0.6275807	-0.198811064	Zm00001d014499
Zm00001d014499_T007	0.5463645	-0.575676256	Zm00001d014499
Zm00001d014499_T002	1	0.240990108	Zm00001d014499
Zm00001d014499_T006	0.9573434	0.074719654	Zm00001d014499
Zm00001d011829_T004	0.9956491	0.253221061	Zm00001d011829
Zm00001d030954_T001	0.9846714	0.235425522	Zm00001d030954
Zm00001d020157_T026	0.7008177	0.595813914	Zm00001d020157
Zm00001d020157_T028	0.9400285	0.416751812	Zm00001d020157
Zm00001d020157_T004	0.5048676	0.653195005	Zm00001d020157
Zm00001d020157_T014	0.9835413	0.156533765	Zm00001d020157
Zm00001d020157_T023	0.399447	0.699097263	Zm00001d020157
Zm00001d020157_T040	0.6517512	0.049420039	Zm00001d020157
Zm00001d020157_T007	0.03178132	0.645722169	Zm00001d020157
Zm00001d020157_T017	0.9709627	0.303786357	Zm00001d020157
Zm00001d017997_T003	0.708953	0.283664553	Zm00001d017997
Zm00001d017997_T006	0.6619288	0.58242748	Zm00001d017997
Zm00001d017997_T001	0.900661	0.300601015	Zm00001d017997
Zm00001d017997_T011	0.8917959	0.255319131	Zm00001d017997
Zm00001d017997_T007	0.7530619	0.904714844	Zm00001d017997
Zm00001d017997_T017	0.5455802	-0.280476924	Zm00001d017997
Zm00001d017997_T010	0.08857859	-1.135425888	Zm00001d017997

Zm00001d041697_T001	0.9493688	0.638171371	Zm00001d041697
Zm00001d038108_T004	0.3648128	-0.970315727	Zm00001d038108
Zm00001d038108_T002	0.900661	-0.09112613	Zm00001d038108
Zm00001d038108_T001	0.9768468	0.428997732	Zm00001d038108
Zm00001d038108_T003	0.989684	0.028302479	Zm00001d038108
Zm00001d014025_T005	0.1491816	2.094531575	Zm00001d014025
Zm00001d014025_T008	0.04527883	1.504024706	Zm00001d014025
Zm00001d014025_T006	0.8914474	0.586113198	Zm00001d014025
Zm00001d014025_T007	0.9082423	0.726496755	Zm00001d014025
Zm00001d014025_T003	0.878248	0.602405972	Zm00001d014025
Zm00001d014025_T010	0.843874	0.928954365	Zm00001d014025
Zm00001d005161_T002	0.8864089	0.383341869	Zm00001d005161
Zm00001d044663_T003	0.9192339	0.437158688	Zm00001d044663
Zm00001d044663_T002	0.9555571	0.610601305	Zm00001d044663
Zm00001d014993_T001	0.7614252	1.184640099	Zm00001d014993
Zm00001d014993_T004	1.41E-06	-4.499061238	Zm00001d014993
Zm00001d014993_T003	0.9257893	1.94370097	Zm00001d014993
Zm00001d021071_T006	0.3938192	1.191104553	Zm00001d021071
Zm00001d021071_T002	1	0.4305867	Zm00001d021071
Zm00001d013777_T001	0.9215966	0.022416998	Zm00001d013777
Zm00001d046986_T001	0.1912529	1.998010372	Zm00001d046986
Zm00001d039856_T001	0.9379764	0.69521021	Zm00001d039856
Zm00001d039856_T003	0.9047923	0.521829846	Zm00001d039856
Zm00001d039856_T007	0.9979393	0.417466337	Zm00001d039856
Zm00001d039856_T006	0.9945118	0.175375116	Zm00001d039856
Zm00001d012813_T001	0.579551	-0.597472493	Zm00001d012813
Zm00001d006310_T001	0.9561521	0.559983412	Zm00001d006310
Zm00001d006310_T004	0.8337863	0.739562227	Zm00001d006310
Zm00001d006310_T006	0.4530417	0.758018287	Zm00001d006310
Zm00001d006310_T003	0.938699	5.54E-05	Zm00001d006310
Zm00001d042777_T013	0.1932887	1.016617673	Zm00001d042777
Zm00001d042777_T004	0.9293689	0.028806339	Zm00001d042777
Zm00001d042777_T001	0.1327235	-0.130986207	Zm00001d042777
Zm00001d042777_T002	0.9363242	-0.000815557	Zm00001d042777
Zm00001d042777_T008	0.8019809	-0.248643128	Zm00001d042777
Zm00001d042777_T014	0.4614239	0.265928168	Zm00001d042777
Zm00001d042777_T003	0.8926893	-0.032992402	Zm00001d042777
Zm00001d025156_T001	0.277679	0.96340925	Zm00001d025156
Zm00001d011873_T002	0.9554387	-0.071271583	Zm00001d011873
Zm00001d011873_T001	0.9979568	0.194308918	Zm00001d011873
Zm00001d045074_T001	0.8208116	0.904561449	Zm00001d045074
Zm00001d045074_T002	0.6470421	1.001848134	Zm00001d045074
Zm00001d038258_T001	0.9846714	-0.063233895	Zm00001d038258
Zm00001d042287_T001	0.9782953	0.275539618	Zm00001d042287
Zm00001d000310_T001	0.9218788	0.294012236	Zm00001d000310
Zm00001d028098_T003	1	0.314185166	Zm00001d028098
Zm00001d028098_T001	0.9822138	0.275361692	Zm00001d028098
Zm00001d033754_T001	1.02E-05	1.626537169	Zm00001d033754
Zm00001d015818_T002	0.9673094	0.173032797	Zm00001d015818
Zm00001d019542_T021	4.79E-09	-0.820340193	Zm00001d019542
Zm00001d019542_T019	0.9805897	0.271326095	Zm00001d019542
Zm00001d019542_T007	0.000196681	1.055884933	Zm00001d019542
Zm00001d019542_T020	0.6897862	0.584665077	Zm00001d019542
Zm00001d019542_T003	0.6209052	1.275025593	Zm00001d019542

Zm00001d049363_T001	0.9627389	0.094832691	Zm00001d049363
Zm00001d015651_T001	0.991917	0.406618524	Zm00001d015651
Zm00001d035088_T002	0.3987447	0.859178362	Zm00001d035088
Zm00001d035088_T003	1	0.425296735	Zm00001d035088
Zm00001d035088_T001	0.7356029	0.706458667	Zm00001d035088
Zm00001d024723_T002	0.2894145	1.794917132	Zm00001d024723
Zm00001d018190_T001	0.7686246	0.757048983	Zm00001d018190
Zm00001d029940_T001	0.9946911	-0.194719304	Zm00001d029940
Zm00001d028630_T001	0.4267333	1.129951632	Zm00001d028630
Zm00001d028630_T005	0.3396442	1.68028589	Zm00001d028630
Zm00001d002546_T001	0.787884	-0.643401548	Zm00001d002546
Zm00001d004443_T001	0.00709523	2.697461648	Zm00001d004443
Zm00001d023808_T002	0.9988864	0.264887098	Zm00001d023808
Zm00001d023808_T001	0.6474065	-0.146127408	Zm00001d023808
Zm00001d024646_T001	0.9599906	0.634478623	Zm00001d024646
Zm00001d023317_T002	0.9817074	0.131311374	Zm00001d023317
Zm00001d037122_T013	0.98291	0.259430919	Zm00001d037122
Zm00001d037122_T005	0.2800967	1.343561889	Zm00001d037122
Zm00001d037122_T011	0.1594022	-0.300579862	Zm00001d037122
Zm00001d037122_T007	0.7079552	1.133710416	Zm00001d037122
Zm00001d011732_T002	0.9942091	0.325293643	Zm00001d011732
Zm00001d011732_T001	0.9468268	-0.085996592	Zm00001d011732
Zm00001d013082_T001	0.8566036	-0.63728975	Zm00001d013082
Zm00001d005179_T001	0.7624517	-0.46522776	Zm00001d005179
Zm00001d044672_T003	0.9043641	-0.047570845	Zm00001d044672
Zm00001d044672_T010	0.8883561	-0.490614	Zm00001d044672
Zm00001d036637_T001	0.6309534	-0.272583339	Zm00001d036637
Zm00001d020329_T001	0.447037	-1.001984617	Zm00001d020329
Zm00001d020329_T003	0.9956349	0.398348968	Zm00001d020329
Zm00001d020329_T002	0.9147333	-0.155864982	Zm00001d020329
Zm00001d020345_T009	0.9870394	0.199555811	Zm00001d020345
Zm00001d020345_T008	0.8598212	-0.087055911	Zm00001d020345
Zm00001d020345_T005	0.836612	0.813258197	Zm00001d020345
Zm00001d020345_T010	0.6416932	-0.352394734	Zm00001d020345
Zm00001d020345_T001	0.519412	1.435679509	Zm00001d020345
Zm00001d023557_T002	0.8514567	-0.181139782	Zm00001d023557
Zm00001d023557_T001	0.9912886	0.253703521	Zm00001d023557
Zm00001d023557_T003	0.9913523	0.386231899	Zm00001d023557
Zm00001d005813_T001	0.8001345	0.699003477	Zm00001d005813
Zm00001d005813_T002	0.348832	1.163487806	Zm00001d005813
Zm00001d052215_T001	0.9429712	0.653842251	Zm00001d052215
Zm00001d043905_T006	1	0.182997141	Zm00001d043905
Zm00001d043905_T001	0.9856605	0.346759621	Zm00001d043905
Zm00001d043905_T002	0.007316201	-1.636606132	Zm00001d043905
Zm00001d043905_T007	0.9477983	-0.053095272	Zm00001d043905
Zm00001d020503_T001	1.97E-05	4.749834404	Zm00001d020503
Zm00001d009753_T003	0.9951827	0.493254446	Zm00001d009753
Zm00001d009753_T011	0.7737238	0.484851199	Zm00001d009753
Zm00001d034718_T001	0.9619942	0.106442589	Zm00001d034718
Zm00001d052242_T001	0.811177	-0.61536245	Zm00001d052242
Zm00001d025696_T001	0.9543237	0.539754091	Zm00001d025696
Zm00001d023785_T001	0.8483996	-0.247329912	Zm00001d023785
Zm00001d039089_T001	0.9706887	0.152484213	Zm00001d039089
Zm00001d003040_T002	0.9616115	0.150129612	Zm00001d003040

Zm00001d032070_T001	0.7194777	-0.33690743	Zm00001d032070
Zm00001d046029_T001	0.8984709	-0.04882218	Zm00001d046029
Zm00001d002815_T001	0.6022016	-0.648091133	Zm00001d002815
Zm00001d008390_T006	0.01482443	1.521762681	Zm00001d008390
Zm00001d008390_T025	1	0.418596483	Zm00001d008390
Zm00001d008390_T009	0.01153328	-1.494875302	Zm00001d008390
Zm00001d008390_T004	0.5141251	1.217071334	Zm00001d008390
Zm00001d008390_T012	0.9647033	0.282022676	Zm00001d008390
Zm00001d002954_T002	0.9705652	0.112635872	Zm00001d002954
Zm00001d037757_T001	0.5620225	-0.873385447	Zm00001d037757
Zm00001d019299_T008	0.9745084	0.184550603	Zm00001d019299
Zm00001d019299_T004	0.7292566	0.861435917	Zm00001d019299
Zm00001d019299_T001	0.9666757	0.117800256	Zm00001d019299
Zm00001d032287_T001	1.88E-05	3.540293731	Zm00001d032287
Zm00001d017535_T001	0.8520487	-0.335290235	Zm00001d017535
Zm00001d037695_T012	0.03698597	1.17061986	Zm00001d037695
Zm00001d037695_T004	0.6272189	0.489190419	Zm00001d037695
Zm00001d037695_T009	0.6846303	0.630532129	Zm00001d037695
Zm00001d037695_T006	0.8892107	0.36205044	Zm00001d037695
Zm00001d037695_T003	0.822299	0.790825141	Zm00001d037695
Zm00001d043164_T001	1	0.341817123	Zm00001d043164
Zm00001d004152_T002	0.9404125	0.319504418	Zm00001d004152
Zm00001d004152_T003	0.9563232	0.134169645	Zm00001d004152
Zm00001d045987_T002	0.4566521	-0.510391712	Zm00001d045987
Zm00001d045987_T001	0.4760609	-0.398142941	Zm00001d045987
Zm00001d052974_T001	0.9404125	0.869733182	Zm00001d052974
Zm00001d052974_T003	0.9486412	0.833611676	Zm00001d052974
Zm00001d052974_T019	1	0.106417599	Zm00001d052974
Zm00001d033994_T002	0.9666757	0.146634432	Zm00001d033994
Zm00001d026268_T001	0.9232281	-0.018410326	Zm00001d026268
Zm00001d027286_T001	0.983078	0.374127703	Zm00001d027286
Zm00001d029785_T002	0.9073468	0.778101235	Zm00001d029785
Zm00001d001771_T001	0.4604152	-0.74165872	Zm00001d001771
Zm00001d017860_T002	0.4175448	1.484062475	Zm00001d017860
Zm00001d017860_T001	0.1386783	1.802265085	Zm00001d017860
Zm00001d017860_T008	0.8656238	0.284210584	Zm00001d017860
Zm00001d017860_T006	0.8086794	0.319337758	Zm00001d017860
Zm00001d026143_T001	0.8884927	-0.356541189	Zm00001d026143
Zm00001d048333_T001	0.8716588	0.783370179	Zm00001d048333
Zm00001d015059_T001	0.941547	0.07410417	Zm00001d015059
Zm00001d003347_T003	0.9292941	0.369187991	Zm00001d003347
Zm00001d003347_T001	0.989787	0.22265979	Zm00001d003347
Zm00001d013927_T001	0.9231466	0.027971825	Zm00001d013927
Zm00001d006489_T001	0.4153808	-0.977755129	Zm00001d006489
Zm00001d005462_T001	0.04449306	-0.736631312	Zm00001d005462
Zm00001d005462_T006	1	0.297159095	Zm00001d005462
Zm00001d005462_T007	0.1523883	1.334561489	Zm00001d005462
Zm00001d005462_T004	0.4927306	0.72135724	Zm00001d005462
Zm00001d005462_T002	3.80E-08	0.973395722	Zm00001d005462
Zm00001d051128_T001	0.491239	0.934165595	Zm00001d051128
Zm00001d023312_T001	0.9945118	0.255987184	Zm00001d023312
Zm00001d053709_T001	0.9959972	0.240398005	Zm00001d053709
Zm00001d028727_T001	1	0.419765212	Zm00001d028727
Zm00001d036455_T001	0.1850398	-0.989372276	Zm00001d036455

Zm00001d025228_T003	0.9773181	0.019744489	Zm00001d025228
Zm00001d047472_T002	0.8580945	-0.121878637	Zm00001d047472
Zm00001d047472_T003	0.9206245	0.028657176	Zm00001d047472
Zm00001d047472_T007	3.29E-05	-3.453887689	Zm00001d047472
Zm00001d001997_T001	0.8865624	0.618087864	Zm00001d001997
Zm00001d038703_T001	0.1054712	2.124636706	Zm00001d038703
Zm00001d045804_T006	0.7253704	-0.280690764	Zm00001d045804
Zm00001d045804_T012	1	-0.003922418	Zm00001d045804
Zm00001d045804_T009	0.3919095	-1.070002239	Zm00001d045804
Zm00001d045804_T013	1.68E-12	2.970327147	Zm00001d045804
Zm00001d045804_T004	0.9704407	0.445050253	Zm00001d045804
Zm00001d045804_T014	0.6548742	-0.331914794	Zm00001d045804
Zm00001d039977_T001	0.4604152	-1.12222741	Zm00001d039977
Zm00001d048891_T001	0.6134001	-0.231923237	Zm00001d048891
Zm00001d048891_T004	0.1420257	-0.382972553	Zm00001d048891
Zm00001d048891_T008	0.8517568	-0.097087748	Zm00001d048891
Zm00001d003852_T001	0.09644783	-1.321683728	Zm00001d003852
Zm00001d037973_T001	0.005816556	-1.385765421	Zm00001d037973
Zm00001d022009_T001	0.9968176	0.32930115	Zm00001d022009
Zm00001d045381_T001	0.9770343	0.156005006	Zm00001d045381
Zm00001d017097_T001	0.8812378	-0.143049828	Zm00001d017097
Zm00001d033056_T001	0.9468941	0.084864169	Zm00001d033056
Zm00001d033056_T004	0.9165293	0.573089046	Zm00001d033056
Zm00001d012063_T001	0.888622	-0.734335798	Zm00001d012063
Zm00001d029577_T003	0.3205135	-0.680126457	Zm00001d029577
Zm00001d043705_T006	0.9160053	-0.474943329	Zm00001d043705
Zm00001d041900_T002	0.7979593	0.415296613	Zm00001d041900
Zm00001d041900_T014	0.9311783	0.078443332	Zm00001d041900
Zm00001d041900_T006	0.02511518	-1.251160851	Zm00001d041900
Zm00001d041900_T001	0.795829	-0.089547177	Zm00001d041900
Zm00001d012504_T002	0.8836195	-0.131745071	Zm00001d012504
Zm00001d012504_T001	0.9487214	0.395197508	Zm00001d012504
Zm00001d023418_T001	0.480132	-0.233917289	Zm00001d023418
Zm00001d039444_T001	0.6107148	1.260396133	Zm00001d039444
Zm00001d052482_T001	0.9887584	0.390657332	Zm00001d052482
Zm00001d051736_T001	0.9493302	-0.097339611	Zm00001d051736
Zm00001d006541_T005	0.9998278	-0.050017923	Zm00001d006541
Zm00001d006541_T001	0.885791	-0.209478831	Zm00001d006541
Zm00001d007010_T001	0.9849018	0.192153955	Zm00001d007010
Zm00001d027928_T001	0.004041229	2.959059574	Zm00001d027928
Zm00001d038269_T001	0.8926643	-0.061419837	Zm00001d038269
Zm00001d037607_T001	0.3207232	-1.036199159	Zm00001d037607
Zm00001d048256_T002	0.9186177	-0.047550392	Zm00001d048256
Zm00001d048256_T001	0.8958197	-0.031215804	Zm00001d048256
Zm00001d010967_T001	0.8859977	0.935278121	Zm00001d010967
Zm00001d047839_T001	0.522218	-0.40676869	Zm00001d047839
Zm00001d047839_T003	4.64E-09	1.237918614	Zm00001d047839
Zm00001d047178_T009	0.9469426	0.511141504	Zm00001d047178
Zm00001d047178_T011	0.8449719	-0.482039126	Zm00001d047178
Zm00001d047178_T010	0.9975532	0.190887044	Zm00001d047178
Zm00001d047178_T006	0.8688441	0.235050542	Zm00001d047178
Zm00001d003491_T011	0.4625124	-0.661972782	Zm00001d003491
Zm00001d003491_T009	0.5044649	0.599116843	Zm00001d003491
Zm00001d003491_T001	0.8730771	-0.066170926	Zm00001d003491

Zm00001d003491_T007	0.8636447	0.712409355	Zm00001d003491
Zm00001d003491_T010	0.3584516	0.728005386	Zm00001d003491
Zm00001d034045_T006	0.09554992	1.434972825	Zm00001d034045
Zm00001d034045_T004	0.5718169	0.640146789	Zm00001d034045
Zm00001d002856_T001	0.1789557	-1.428091983	Zm00001d002856
Zm00001d018720_T002	0.4879596	1.181708717	Zm00001d018720
Zm00001d037237_T002	0.8926364	0.672161243	Zm00001d037237
Zm00001d037237_T001	0.6019353	0.735414072	Zm00001d037237
Zm00001d016995_T001	0.1813603	0.993793681	Zm00001d016995
Zm00001d012101_T007	1.90E-08	-2.808203474	Zm00001d012101
Zm00001d012101_T003	0.04606011	1.320092622	Zm00001d012101
Zm00001d012101_T001	0.772948	1.102309033	Zm00001d012101
Zm00001d012101_T008	0.2002317	2.278847004	Zm00001d012101
Zm00001d021638_T001	0.07739097	2.10010716	Zm00001d021638
Zm00001d013652_T003	0.866232	0.791826393	Zm00001d013652
Zm00001d013652_T007	0.9822138	0.270962359	Zm00001d013652
Zm00001d013652_T010	0.7850531	0.386312006	Zm00001d013652
Zm00001d005423_T001	0.9760472	0.047425347	Zm00001d005423
Zm00001d035879_T001	0.9771271	-0.149987716	Zm00001d035879
Zm00001d052298_T002	0.8453901	-0.177742717	Zm00001d052298
Zm00001d004169_T002	0.677308	-0.403350711	Zm00001d004169
Zm00001d004169_T001	0.900806	0.803118373	Zm00001d004169
Zm00001d011635_T001	0.5609886	1.474420465	Zm00001d011635
Zm00001d026142_T001	0.9130008	-0.334084144	Zm00001d026142
Zm00001d035156_T009	0.9037807	-0.235566868	Zm00001d035156
Zm00001d035156_T004	0.9822138	0.524232143	Zm00001d035156
Zm00001d035156_T005	0.5526129	-0.538403977	Zm00001d035156
Zm00001d008758_T001	0.8695779	0.819907926	Zm00001d008758
Zm00001d021249_T003	0.864155	-0.17562813	Zm00001d021249
Zm00001d021249_T004	0.1802911	-1.233343069	Zm00001d021249
Zm00001d015686_T001	1	-0.306722578	Zm00001d015686
Zm00001d017449_T001	0.9760472	0.10385415	Zm00001d017449
Zm00001d051986_T002	0.9760472	-0.069995644	Zm00001d051986
Zm00001d042846_T001	0.946422	-0.221798823	Zm00001d042846
Zm00001d028576_T001	0.7024944	0.949595661	Zm00001d028576
Zm00001d002827_T002	0.9125205	0.57081919	Zm00001d002827
Zm00001d002827_T009	0.3002348	1.294770711	Zm00001d002827
Zm00001d002827_T003	0.768921	-0.566695031	Zm00001d002827
Zm00001d002827_T007	0.5783955	0.355039943	Zm00001d002827
Zm00001d002827_T006	0.8767824	0.242199731	Zm00001d002827
Zm00001d003590_T001	0.9770343	0.016665379	Zm00001d003590
Zm00001d003590_T002	1	0.322497346	Zm00001d003590
Zm00001d003590_T003	1	0.32042408	Zm00001d003590
Zm00001d043762_T001	0.7158558	1.11697704	Zm00001d043762
Zm00001d014885_T001	0.7904511	-0.309676986	Zm00001d014885
Zm00001d022246_T002	0.9517262	0.099440917	Zm00001d022246
Zm00001d022246_T003	0.8055708	-0.319912233	Zm00001d022246
Zm00001d047852_T001	0.991324	0.240207616	Zm00001d047852
Zm00001d015557_T001	0.9856605	0.13190301	Zm00001d015557
Zm00001d026089_T001	0.9216521	-0.021739107	Zm00001d026089
Zm00001d046716_T002	0.9914912	0.197444281	Zm00001d046716
Zm00001d046716_T003	1	0.293830368	Zm00001d046716
Zm00001d046716_T004	0.9673473	-0.4834278	Zm00001d046716
Zm00001d027508_T002	0.9822138	0.202448615	Zm00001d027508

Zm00001d038761_T002	0.6162619	1.115612785	Zm00001d038761
Zm00001d038206_T002	0.9706293	0.422593014	Zm00001d038206
Zm00001d032545_T001	0.9550893	0.117332817	Zm00001d032545
Zm00001d051794_T001	0.8884927	-0.504307024	Zm00001d051794
Zm00001d051794_T002	0.8717403	0.301340384	Zm00001d051794
Zm00001d051794_T004	0.9344642	0.426364956	Zm00001d051794
Zm00001d013816_T001	0.8865624	-0.117118039	Zm00001d013816
Zm00001d033655_T004	0.7085932	-0.220312473	Zm00001d033655
Zm00001d033655_T002	0.8247952	0.666907446	Zm00001d033655
Zm00001d033655_T003	0.9311027	0.692870925	Zm00001d033655
Zm00001d033655_T005	0.5185832	1.058838204	Zm00001d033655
Zm00001d018041_T001	0.9860836	0.206830756	Zm00001d018041
Zm00001d047633_T002	1	0.315630253	Zm00001d047633
Zm00001d047633_T001	0.7174573	-0.34289693	Zm00001d047633
Zm00001d042211_T001	0.5002632	1.289478814	Zm00001d042211
Zm00001d052362_T004	0.9168576	0.643296006	Zm00001d052362
Zm00001d052362_T001	0.9822138	0.374097476	Zm00001d052362
Zm00001d052362_T003	0.9988393	0.285691214	Zm00001d052362
Zm00001d047880_T001	0.9706887	0.538462673	Zm00001d047880
Zm00001d047880_T002	0.1217329	0.829576239	Zm00001d047880
Zm00001d038297_T001	0.6600822	0.985798014	Zm00001d038297
Zm00001d033745_T001	0.8677531	0.926000078	Zm00001d033745
Zm00001d009673_T001	0.9532563	-0.095100436	Zm00001d009673
Zm00001d033941_T001	0.8517246	-0.110805269	Zm00001d033941
Zm00001d028227_T004	0.6391307	0.588909389	Zm00001d028227
Zm00001d028227_T007	0.000196768	-1.289989912	Zm00001d028227
Zm00001d028227_T002	0.9690925	0.619218798	Zm00001d028227
Zm00001d028227_T009	0.9693382	0.008138441	Zm00001d028227
Zm00001d005622_T001	0.7224046	0.950628318	Zm00001d005622
Zm00001d023413_T001	0.001405609	1.45015102	Zm00001d023413
Zm00001d037111_T001	0.5630233	-0.606473029	Zm00001d037111
Zm00001d037547_T001	1	0.315169873	Zm00001d037547
Zm00001d021895_T001	0.8790359	0.342801542	Zm00001d021895
Zm00001d030507_T002	0.146312	-0.997249414	Zm00001d030507
Zm00001d030507_T003	0.7801274	-0.320765759	Zm00001d030507
Zm00001d030507_T001	0.9702242	0.123680139	Zm00001d030507
Zm00001d048564_T003	0.3845866	-0.002051486	Zm00001d048564
Zm00001d048564_T005	0.3455702	0.554052377	Zm00001d048564
Zm00001d048564_T010	0.7292566	-0.466824715	Zm00001d048564
Zm00001d048564_T002	1	0.147773612	Zm00001d048564
Zm00001d048564_T006	0.901721	-0.018528046	Zm00001d048564
Zm00001d048564_T007	0.4151638	1.343274413	Zm00001d048564
Zm00001d039565_T001	0.5636783	-0.701847906	Zm00001d039565
Zm00001d048886_T001	0.9986724	-0.178273545	Zm00001d048886
Zm00001d052269_T001	0.2546213	-0.98099207	Zm00001d052269
Zm00001d052018_T001	0.991004	0.256102345	Zm00001d052018
Zm00001d003641_T002	0.9550193	-0.072978419	Zm00001d003641
Zm00001d010928_T001	0.9945118	0.453479363	Zm00001d010928
Zm00001d001963_T001	0.000669937	3.562007022	Zm00001d001963
Zm00001d012033_T002	0.9596377	0.623135448	Zm00001d012033
Zm00001d022295_T001	0.3243413	-0.763371724	Zm00001d022295
Zm00001d052988_T011	0.6300614	0.749765117	Zm00001d052988
Zm00001d052988_T010	0.8412913	0.971718387	Zm00001d052988
Zm00001d003522_T001	0.6107449	1.054319598	Zm00001d003522

Zm00001d006365_T001	0.9090981	0.713090488	Zm00001d006365
Zm00001d034802_T001	0.9562711	-0.030164015	Zm00001d034802
Zm00001d037968_T002	0.8187452	-0.628437035	Zm00001d037968
Zm00001d020172_T001	0.9902022	0.170213479	Zm00001d020172
Zm00001d044489_T001	0.4977061	-0.991555016	Zm00001d044489
Zm00001d049539_T001	0.7288105	-0.315576364	Zm00001d049539
Zm00001d007048_T001	0.9872831	0.274703284	Zm00001d007048
Zm00001d037271_T001	1	0.41110153	Zm00001d037271
Zm00001d043809_T002	2.93E-06	1.529470493	Zm00001d043809
Zm00001d043809_T013	0.8807115	-0.021918465	Zm00001d043809
Zm00001d043809_T008	0.9984041	0.236758739	Zm00001d043809
Zm00001d039987_T002	0.8549534	0.328393203	Zm00001d039987
Zm00001d050339_T002	1	0.313483565	Zm00001d050339
Zm00001d050339_T001	0.5690605	1.260393775	Zm00001d050339
Zm00001d050339_T004	0.9303773	-0.3802095	Zm00001d050339
Zm00001d044641_T004	0.9988864	0.265419483	Zm00001d044641
Zm00001d044641_T009	0.005716143	-1.801491048	Zm00001d044641
Zm00001d054033_T001	0.9608605	-0.175201708	Zm00001d054033
Zm00001d014972_T003	0.9988864	0.210432029	Zm00001d014972
Zm00001d014972_T001	0.9050121	-0.41366546	Zm00001d014972
Zm00001d034713_T001	0.8412913	-0.119724188	Zm00001d034713
Zm00001d017882_T002	0.5754586	-0.620660837	Zm00001d017882
Zm00001d017882_T001	0.6115361	-0.344239906	Zm00001d017882
Zm00001d017882_T005	0.174228	-1.561730716	Zm00001d017882
Zm00001d017882_T004	0.4007245	-0.662464515	Zm00001d017882
Zm00001d017882_T003	0.05459656	-1.784932832	Zm00001d017882
Zm00001d044931_T001	1	0.35901195	Zm00001d044931
Zm00001d018123_T001	0.9954444	0.38043171	Zm00001d018123
Zm00001d030310_T001	0.9196677	0.019545652	Zm00001d030310
Zm00001d027455_T001	0.6207301	1.205699979	Zm00001d027455
Zm00001d030038_T001	0.7526414	0.700941696	Zm00001d030038
Zm00001d002925_T005	0.9775586	0.571740607	Zm00001d002925
Zm00001d002925_T007	0.4860327	0.466368374	Zm00001d002925
Zm00001d002925_T006	0.6786506	0.540009997	Zm00001d002925
Zm00001d002925_T004	0.909729	-0.025211488	Zm00001d002925
Zm00001d051608_T002	0.9293689	0.585596039	Zm00001d051608
Zm00001d051608_T019	0.5579393	-0.939010554	Zm00001d051608
Zm00001d051608_T003	0.8860574	0.479279268	Zm00001d051608
Zm00001d051608_T009	0.9944247	0.362067401	Zm00001d051608
Zm00001d051608_T017	0.718063	1.142673303	Zm00001d051608
Zm00001d051608_T014	1	0.176433646	Zm00001d051608
Zm00001d051608_T010	0.744567	0.192998545	Zm00001d051608
Zm00001d051608_T011	0.9073468	0.192015718	Zm00001d051608
Zm00001d051608_T001	0.4146004	0.638942631	Zm00001d051608
Zm00001d051608_T020	0.4503499	-0.532608146	Zm00001d051608
Zm00001d051608_T004	0.9429712	0.285759992	Zm00001d051608
Zm00001d051608_T018	0.9909466	0.2727273	Zm00001d051608
Zm00001d012328_T002	0.6745773	0.350726892	Zm00001d012328
Zm00001d028493_T001	0.8031436	0.672236944	Zm00001d028493
Zm00001d028493_T002	0.9778436	0.501171958	Zm00001d028493
Zm00001d038593_T001	0.9363242	-0.140859903	Zm00001d038593
Zm00001d010507_T001	0.9273903	-0.204206204	Zm00001d010507
Zm00001d015839_T001	0.9682046	0.139367249	Zm00001d015839
Zm00001d015839_T003	0.9822138	0.2669222	Zm00001d015839

Zm00001d015839_T004	0.3161027	-0.86392194	Zm00001d015839
Zm00001d010803_T001	0.7997379	-0.548970574	Zm00001d010803
Zm00001d049744_T001	3.38E-08	1.251747134	Zm00001d049744
Zm00001d018209_T001	0.9374853	-0.244855261	Zm00001d018209
Zm00001d052016_T001	1	0.527959909	Zm00001d052016
Zm00001d039911_T002	0.453717	-0.46717685	Zm00001d039911
Zm00001d039911_T001	0.9928864	0.237845616	Zm00001d039911
Zm00001d005146_T001	0.1721794	-2.006229026	Zm00001d005146
Zm00001d011854_T055	0.3513513	1.043712715	Zm00001d011854
Zm00001d011854_T031	0.000585379	2.779291373	Zm00001d011854
Zm00001d011854_T006	0.8419717	-0.244861895	Zm00001d011854
Zm00001d011854_T039	0.962254	0.2261614	Zm00001d011854
Zm00001d011854_T002	0.6007475	-0.367852469	Zm00001d011854
Zm00001d011854_T022	1	0.342114991	Zm00001d011854
Zm00001d011854_T050	0.1928401	0.873136634	Zm00001d011854
Zm00001d010520_T001	0.7203247	1.149410817	Zm00001d010520
Zm00001d005874_T001	0.3067075	-0.836124587	Zm00001d005874
Zm00001d022210_T001	0.8819727	0.608127963	Zm00001d022210
Zm00001d046958_T001	0.424032	1.144789645	Zm00001d046958
Zm00001d002937_T007	0.6496559	-0.383039401	Zm00001d002937
Zm00001d002937_T001	0.9982897	0.298054081	Zm00001d002937
Zm00001d002937_T020	0.9469426	0.494367255	Zm00001d002937
Zm00001d002937_T036	0.8747409	-0.141594696	Zm00001d002937
Zm00001d002937_T025	0.6311132	-0.242349013	Zm00001d002937
Zm00001d002937_T012	0.9822138	0.122206755	Zm00001d002937
Zm00001d002937_T021	0.5567755	0.837101915	Zm00001d002937
Zm00001d002937_T034	0.9782953	0.114187703	Zm00001d002937
Zm00001d002937_T006	0.08572166	-0.367683068	Zm00001d002937
Zm00001d002937_T016	0.3784952	0.792351422	Zm00001d002937
Zm00001d002937_T002	0.718063	-0.17775549	Zm00001d002937
Zm00001d041593_T001	0.9832092	0.542094274	Zm00001d041593
Zm00001d040399_T001	0.8383509	-0.220560178	Zm00001d040399
Zm00001d032499_T001	0.9112906	-0.351085401	Zm00001d032499
Zm00001d035445_T003	0.7791621	1.015151558	Zm00001d035445
Zm00001d035445_T001	0.4748531	-0.387206092	Zm00001d035445
Zm00001d035445_T002	1	0.404867192	Zm00001d035445
Zm00001d036340_T001	0.9213556	-0.022295808	Zm00001d036340
Zm00001d010527_T001	0.8791987	-0.170037344	Zm00001d010527
Zm00001d014559_T001	0.9843847	0.063776769	Zm00001d014559
Zm00001d051308_T002	0.6972481	0.899633956	Zm00001d051308
Zm00001d051308_T020	0.01004572	1.040571277	Zm00001d051308
Zm00001d051308_T014	0.9561521	0.247216411	Zm00001d051308
Zm00001d051308_T019	0.9817074	0.233577628	Zm00001d051308
Zm00001d051308_T021	0.08416207	0.964738404	Zm00001d051308
Zm00001d051308_T008	0.188277	0.512933624	Zm00001d051308
Zm00001d051308_T018	0.8001345	0.287998059	Zm00001d051308
Zm00001d051308_T010	1.04E-06	1.959218764	Zm00001d051308
Zm00001d037099_T002	0.6235378	-0.336360363	Zm00001d037099
Zm00001d037099_T052	0.000555512	0.865948853	Zm00001d037099
Zm00001d037099_T024	0.001887014	0.380861863	Zm00001d037099
Zm00001d037099_T015	0.6061607	0.443690964	Zm00001d037099
Zm00001d037099_T012	4.59E-06	2.848417172	Zm00001d037099
Zm00001d037099_T034	0.218911	0.530231739	Zm00001d037099
Zm00001d037099_T010	0.9124169	-0.054215948	Zm00001d037099

Zm00001d037099_T035	0.9766874	0.189200936	Zm00001d037099
Zm00001d035032_T001	0.9931431	0.549460694	Zm00001d035032
Zm00001d011782_T002	0.6670879	-0.432567499	Zm00001d011782
Zm00001d009171_T001	0.3428555	1.299289393	Zm00001d009171
Zm00001d047951_T002	0.7136625	1.169841325	Zm00001d047951
Zm00001d047951_T003	0.02219849	1.952772412	Zm00001d047951
Zm00001d047951_T004	0.9355135	-0.004001514	Zm00001d047951
Zm00001d047951_T008	0.9421089	0.557433492	Zm00001d047951
Zm00001d034505_T004	0.9509252	0.260571931	Zm00001d034505
Zm00001d034505_T005	0.9193633	2.120943634	Zm00001d034505
Zm00001d034505_T007	0.9180865	0.360624306	Zm00001d034505
Zm00001d034505_T003	0.8061924	-0.30803195	Zm00001d034505
Zm00001d034505_T002	0.2589628	-0.63978869	Zm00001d034505
Zm00001d042293_T001	0.453061	-0.620416743	Zm00001d042293
Zm00001d042293_T002	0.7654499	-0.112814486	Zm00001d042293
Zm00001d048049_T071	1	0.508304437	Zm00001d048049
Zm00001d048049_T090	0.9356767	0.035215203	Zm00001d048049
Zm00001d048049_T108	0.9913523	0.377731152	Zm00001d048049
Zm00001d048049_T080	0.4332539	0.317518271	Zm00001d048049
Zm00001d048049_T007	0.3116099	1.553600633	Zm00001d048049
Zm00001d048049_T045	0.0788527	0.670479314	Zm00001d048049
Zm00001d048049_T022	0.3431817	0.340555968	Zm00001d048049
Zm00001d048049_T018	0.415447	0.607084808	Zm00001d048049
Zm00001d048049_T062	0.631455	0.686959314	Zm00001d048049
Zm00001d048049_T082	0.9396809	0.176118425	Zm00001d048049
Zm00001d048049_T105	0.8784177	-0.168731343	Zm00001d048049
Zm00001d053022_T001	0.9654566	0.191735667	Zm00001d053022
Zm00001d053022_T005	0.6338908	0.87824383	Zm00001d053022
Zm00001d032540_T001	0.01591851	-0.617693722	Zm00001d032540
Zm00001d032540_T008	0.3163699	0.673741293	Zm00001d032540
Zm00001d032540_T009	0.9881407	0.479772687	Zm00001d032540
Zm00001d032540_T013	0.9779656	0.54284142	Zm00001d032540
Zm00001d032540_T003	0.0140415	1.09125113	Zm00001d032540
Zm00001d017955_T001	0.06023062	-1.96413055	Zm00001d017955
Zm00001d051876_T005	0.7293985	1.066913449	Zm00001d051876
Zm00001d051876_T001	0.1150628	1.228485247	Zm00001d051876
Zm00001d051876_T004	0.9352321	0.183666456	Zm00001d051876
Zm00001d029055_T002	0.9852645	0.301043336	Zm00001d029055
Zm00001d029055_T005	0.683785	-0.295845486	Zm00001d029055
Zm00001d029055_T011	0.8953011	-0.047343544	Zm00001d029055
Zm00001d029055_T012	0.3973152	1.44485449	Zm00001d029055
Zm00001d029055_T010	7.20E-05	1.561789012	Zm00001d029055
Zm00001d029055_T014	0.1178643	0.262263537	Zm00001d029055
Zm00001d016771_T002	0.8557049	-0.115375086	Zm00001d016771
Zm00001d046311_T001	0.2007953	1.187791843	Zm00001d046311
Zm00001d040702_T001	0.7613191	-0.329829426	Zm00001d040702
Zm00001d048341_T001	0.1152548	-0.730357377	Zm00001d048341
Zm00001d050023_T001	0.9804228	0.246734176	Zm00001d050023
Zm00001d004581_T001	0.8701267	0.504109521	Zm00001d004581
Zm00001d017584_T002	0.9588604	0.641899985	Zm00001d017584
Zm00001d035604_T001	0.4377468	-0.920938847	Zm00001d035604
Zm00001d012590_T001	0.9922415	0.219834792	Zm00001d012590
Zm00001d039698_T002	0.5572513	1.381696811	Zm00001d039698
Zm00001d050955_T018	0.6001859	-0.323654776	Zm00001d050955

Zm00001d050955_T022	0.9717989	0.289478328	Zm00001d050955
Zm00001d050955_T021	0.9802412	0.278725811	Zm00001d050955
Zm00001d052271_T005	0.8553094	0.518650668	Zm00001d052271
Zm00001d052271_T004	0.9883036	0.118192354	Zm00001d052271
Zm00001d052271_T001	0.9907928	0.299500025	Zm00001d052271
Zm00001d007825_T004	0.8644019	0.372281651	Zm00001d007825
Zm00001d007825_T006	0.7591483	-0.364387744	Zm00001d007825
Zm00001d007825_T007	0.9895224	0.329545223	Zm00001d007825
Zm00001d013481_T001	0.9269932	-0.048242219	Zm00001d013481
Zm00001d045555_T001	0.5513525	-0.787436269	Zm00001d045555
Zm00001d045555_T003	0.8449222	-0.560614351	Zm00001d045555
Zm00001d020006_T001	0.9500446	0.479560895	Zm00001d020006
Zm00001d002677_T001	1	0.017941544	Zm00001d002677
Zm00001d037498_T001	0.9303773	-0.005178551	Zm00001d037498
Zm00001d037498_T002	0.9988864	0.194997457	Zm00001d037498
Zm00001d002727_T001	0.2308305	1.149040025	Zm00001d002727
Zm00001d002727_T002	0.8667498	-0.231051618	Zm00001d002727
Zm00001d012260_T001	0.9321001	0.010158092	Zm00001d012260
Zm00001d029875_T001	0.9529184	0.584893905	Zm00001d029875
Zm00001d018630_T001	0.9727583	0.138255701	Zm00001d018630
Zm00001d052683_T001	0.05957342	-1.619879264	Zm00001d052683
Zm00001d032443_T001	0.8605736	0.746088915	Zm00001d032443
Zm00001d036812_T001	0.9291214	0.443324827	Zm00001d036812
Zm00001d036228_T001	0.9334932	0.281357366	Zm00001d036228
Zm00001d033805_T001	0.9968176	0.33383343	Zm00001d033805
Zm00001d041545_T001	0.9257893	0.436344483	Zm00001d041545
Zm00001d030181_T001	0.6955963	-0.51036569	Zm00001d030181
Zm00001d043953_T001	0.9969123	0.285718311	Zm00001d043953
Zm00001d028535_T001	0.9219195	0.749827697	Zm00001d028535
Zm00001d036222_T003	0.38637	1.507503844	Zm00001d036222
Zm00001d053377_T001	0.9897942	0.035603073	Zm00001d053377
Zm00001d039521_T004	0.01670316	-1.076195978	Zm00001d039521
Zm00001d039521_T005	0.9989936	0.300868166	Zm00001d039521
Zm00001d039521_T003	1	0.34956925	Zm00001d039521
Zm00001d053635_T001	0.7712604	-0.522056765	Zm00001d053635
Zm00001d052878_T001	0.7003081	-0.420565382	Zm00001d052878
Zm00001d027329_T010	0.5980883	-0.14255625	Zm00001d027329
Zm00001d027329_T003	0.9253672	0.069480421	Zm00001d027329
Zm00001d027329_T005	0.3603685	0.437575677	Zm00001d027329
Zm00001d005748_T001	0.1122861	-1.343782329	Zm00001d005748
Zm00001d020915_T002	0.04558476	2.405878357	Zm00001d020915
Zm00001d016561_T001	0.934325	0.170710838	Zm00001d016561
Zm00001d016382_T008	0.557673	0.618571092	Zm00001d016382
Zm00001d016382_T013	0.3881179	-0.472754201	Zm00001d016382
Zm00001d016382_T012	0.9832092	0.497012133	Zm00001d016382
Zm00001d016382_T005	0.90591	0.789943655	Zm00001d016382
Zm00001d042787_T002	0.7807489	0.508620425	Zm00001d042787
Zm00001d042787_T001	0.967161	0.125499158	Zm00001d042787
Zm00001d013071_T001	0.9918302	0.350111137	Zm00001d013071
Zm00001d013615_T001	0.6360579	-0.499908025	Zm00001d013615
Zm00001d030023_T001	0.6075235	0.721402531	Zm00001d030023
Zm00001d010308_T001	0.4957869	1.468208148	Zm00001d010308
Zm00001d016443_T003	0.9419336	-0.02512296	Zm00001d016443
Zm00001d016443_T001	0.7169281	-0.294824534	Zm00001d016443

Zm00001d016443_T002	0.9416089	-0.027059547	Zm00001d016443
Zm00001d004959_T001	0.4562375	-0.577498567	Zm00001d004959
Zm00001d004959_T002	0.9668789	0.017569403	Zm00001d004959
Zm00001d025522_T001	0.4789365	-0.680663748	Zm00001d025522
Zm00001d025522_T002	0.7009248	-0.23850776	Zm00001d025522
Zm00001d039674_T001	0.9534335	0.088142413	Zm00001d039674
Zm00001d034537_T003	0.9974734	0.302352616	Zm00001d034537
Zm00001d034537_T005	0.1191683	-0.298709897	Zm00001d034537
Zm00001d034537_T001	0.9990127	0.2310274	Zm00001d034537
Zm00001d034537_T002	0.9850116	0.153509586	Zm00001d034537
Zm00001d012226_T001	0.5825098	-0.438060584	Zm00001d012226
Zm00001d034117_T028	1	0.117183826	Zm00001d034117
Zm00001d034117_T006	0.7059983	-0.04649672	Zm00001d034117
Zm00001d034117_T014	0.8883561	0.205439244	Zm00001d034117
Zm00001d034117_T022	0.630204	-0.250056328	Zm00001d034117
Zm00001d034117_T026	0.04207703	-0.825856257	Zm00001d034117
Zm00001d034117_T009	0.000106198	0.742329666	Zm00001d034117
Zm00001d048315_T004	0.7400422	1.119670805	Zm00001d048315
Zm00001d048315_T001	0.8591203	-0.156222881	Zm00001d048315
Zm00001d048315_T005	0.923446	0.649060952	Zm00001d048315
Zm00001d048315_T003	0.9472494	0.082534301	Zm00001d048315
Zm00001d052798_T001	0.9451369	0.401686732	Zm00001d052798
Zm00001d015474_T001	0.9782953	0.222238803	Zm00001d015474
Zm00001d049786_T001	0.9652801	0.118227046	Zm00001d049786
Zm00001d047982_T001	0.366346	0.931563857	Zm00001d047982
Zm00001d018963_T001	0.6816502	0.608071606	Zm00001d018963
Zm00001d013045_T001	0.9487214	-0.270823073	Zm00001d013045
Zm00001d005338_T001	0.1236256	-1.707163959	Zm00001d005338
Zm00001d005338_T005	0.9177108	0.814642882	Zm00001d005338
Zm00001d005338_T003	0.9782953	0.453209791	Zm00001d005338
Zm00001d031720_T001	0.9749712	0.101895035	Zm00001d031720
Zm00001d025983_T002	0.4426726	0.458376096	Zm00001d025983
Zm00001d028485_T001	0.8900518	0.614226551	Zm00001d028485
Zm00001d028485_T004	0.8173136	0.379162867	Zm00001d028485
Zm00001d028485_T002	0.9914912	0.212304527	Zm00001d028485
Zm00001d052725_T001	0.7736571	1.011651291	Zm00001d052725
Zm00001d049381_T001	0.3064615	1.273688778	Zm00001d049381
Zm00001d007606_T001	0.02008212	-1.857333273	Zm00001d007606
Zm00001d020054_T001	0.6538118	-0.822613417	Zm00001d020054
Zm00001d017383_T001	1	0.275650762	Zm00001d017383
Zm00001d037836_T019	0.6110258	0.573862055	Zm00001d037836
Zm00001d037836_T009	0.4811647	1.434177625	Zm00001d037836
Zm00001d037836_T011	0.06202693	-0.230091315	Zm00001d037836
Zm00001d026124_T003	0.7138265	0.822769222	Zm00001d026124
Zm00001d026124_T002	0.9894615	0.34862032	Zm00001d026124
Zm00001d046805_T001	0.01640001	2.476653518	Zm00001d046805
Zm00001d012118_T002	0.9419336	0.708427508	Zm00001d012118
Zm00001d012118_T001	0.9819191	0.250941649	Zm00001d012118
Zm00001d009787_T001	0.6171774	-0.615854551	Zm00001d009787
Zm00001d047203_T001	0.6450309	-0.553504798	Zm00001d047203
Zm00001d050874_T001	0.8605736	-0.211192888	Zm00001d050874
Zm00001d002422_T002	0.4191028	-0.257771403	Zm00001d002422
Zm00001d002422_T004	1	0.340692935	Zm00001d002422
Zm00001d041204_T001	0.9173494	0.759945509	Zm00001d041204

Zm00001d043543_T005	0.4216891	1.551525532	Zm00001d043543
Zm00001d043543_T002	0.2669525	1.847759235	Zm00001d043543
Zm00001d043543_T003	0.003015693	0.983331667	Zm00001d043543
Zm00001d043543_T007	0.04031304	0.518187742	Zm00001d043543
Zm00001d002845_T001	0.3757326	1.655823375	Zm00001d002845
Zm00001d041918_T001	0.9264969	0.742401102	Zm00001d041918
Zm00001d016144_T006	0.4621499	0.670329835	Zm00001d016144
Zm00001d016144_T003	0.9839367	0.293923394	Zm00001d016144
Zm00001d014564_T001	0.4319576	-0.533231911	Zm00001d014564
Zm00001d048814_T001	0.6330708	0.824727193	Zm00001d048814
Zm00001d050708_T005	0.5448225	-1.09993956	Zm00001d050708
Zm00001d050708_T004	0.957148	0.044572929	Zm00001d050708
Zm00001d050708_T002	0.9972036	0.348686095	Zm00001d050708
Zm00001d050708_T001	1	0.217002571	Zm00001d050708
Zm00001d030623_T002	0.7418111	0.886817217	Zm00001d030623
Zm00001d050166_T001	0.9410688	-0.025032731	Zm00001d050166
Zm00001d049798_T001	0.9929973	0.286133253	Zm00001d049798
Zm00001d049798_T005	0.6996374	-0.811050013	Zm00001d049798
Zm00001d041325_T001	0.965836	0.059070802	Zm00001d041325
Zm00001d052698_T001	0.9851204	0.200186144	Zm00001d052698
Zm00001d018514_T010	0.9561521	0.054775291	Zm00001d018514
Zm00001d018514_T011	0.8706608	-0.139332717	Zm00001d018514
Zm00001d018514_T008	0.7877461	0.726085577	Zm00001d018514
Zm00001d018514_T005	0.9709778	0.269458597	Zm00001d018514
Zm00001d018514_T015	0.8716622	-0.268462813	Zm00001d018514
Zm00001d018514_T014	1	0.340895014	Zm00001d018514
Zm00001d035310_T001	0.8637863	-0.324189523	Zm00001d035310
Zm00001d008201_T001	0.9728551	0.025861557	Zm00001d008201
Zm00001d009904_T001	0.9363242	-0.054512498	Zm00001d009904
Zm00001d021272_T001	0.9988864	0.126639143	Zm00001d021272
Zm00001d048909_T001	0.2029262	-1.590672232	Zm00001d048909
Zm00001d029815_T001	1	0.050133474	Zm00001d029815
Zm00001d053732_T001	0.9621967	0.148838848	Zm00001d053732
Zm00001d032828_T001	0.9524127	0.243766123	Zm00001d032828
Zm00001d044528_T001	1	0.287013681	Zm00001d044528
Zm00001d008526_T001	0.1272455	-0.71849166	Zm00001d008526
Zm00001d037920_T002	0.8379797	-0.143692684	Zm00001d037920
Zm00001d026431_T001	0.9939021	0.195351919	Zm00001d026431
Zm00001d011239_T001	1	0.352707724	Zm00001d011239
Zm00001d052348_T001	0.004352965	1.937682656	Zm00001d052348
Zm00001d023936_T001	0.8236738	-0.408871999	Zm00001d023936
Zm00001d040475_T001	0.8886159	-0.050635981	Zm00001d040475
Zm00001d025745_T001	0.7629097	-0.387721862	Zm00001d025745
Zm00001d043612_T001	0.8746701	0.618680877	Zm00001d043612
Zm00001d012618_T001	0.9979568	-0.166129697	Zm00001d012618
Zm00001d041300_T001	0.6210638	-0.391458744	Zm00001d041300
Zm00001d035233_T001	0.04322802	-1.793694771	Zm00001d035233
Zm00001d021024_T001	0.5024891	-0.614883229	Zm00001d021024
Zm00001d016683_T001	0.8915153	0.515346913	Zm00001d016683
Zm00001d010838_T001	0.7257922	-0.37148805	Zm00001d010838
Zm00001d028784_T001	0.8367125	0.91882294	Zm00001d028784
Zm00001d011266_T001	0.9031129	0.415306992	Zm00001d011266
Zm00001d011266_T003	1	0.281306289	Zm00001d011266
Zm00001d039091_T001	0.9237921	-0.077054297	Zm00001d039091

Zm00001d039091_T003	0.08416207	0.847748836	Zm00001d039091
Zm00001d013377_T001	0.7825891	0.669440208	Zm00001d013377
Zm00001d009391_T001	0.581703	-0.31154987	Zm00001d009391
Zm00001d050153_T003	0.8050292	-0.35016242	Zm00001d050153
Zm00001d050153_T002	0.9249382	0.750430323	Zm00001d050153
Zm00001d050153_T001	0.7658741	0.560854845	Zm00001d050153
Zm00001d037688_T001	0.6843488	1.087160327	Zm00001d037688
Zm00001d033669_T009	0.7056513	1.193168718	Zm00001d033669
Zm00001d033669_T011	0.9760472	0.063632944	Zm00001d033669
Zm00001d033669_T012	0.8730771	0.840543285	Zm00001d033669
Zm00001d006623_T001	0.02252111	-2.170239248	Zm00001d006623
Zm00001d033999_T001	0.8665954	-0.147071022	Zm00001d033999
Zm00001d020708_T004	0.5956992	0.863476701	Zm00001d020708
Zm00001d020708_T008	0.9820148	0.437999381	Zm00001d020708
Zm00001d020708_T009	0.8342838	0.368247305	Zm00001d020708
Zm00001d020708_T002	0.3455702	1.207769128	Zm00001d020708
Zm00001d020708_T010	0.2536508	1.888576256	Zm00001d020708
Zm00001d036214_T001	0.6912243	0.932357376	Zm00001d036214
Zm00001d013348_T003	0.9981278	0.255969395	Zm00001d013348
Zm00001d013348_T001	0.9988864	0.260471006	Zm00001d013348
Zm00001d013348_T002	0.9628812	0.011775229	Zm00001d013348
Zm00001d028526_T001	0.9529184	0.092954057	Zm00001d028526
Zm00001d042303_T001	0.9768468	0.21122385	Zm00001d042303
Zm00001d042303_T002	0.9825014	0.358304461	Zm00001d042303
Zm00001d036739_T001	0.9561521	0.147204267	Zm00001d036739
Zm00001d035698_T003	0.9804228	0.284887236	Zm00001d035698
Zm00001d035698_T002	0.9441446	0.075660307	Zm00001d035698
Zm00001d042569_T001	1	0.392183062	Zm00001d042569
Zm00001d041760_T001	0.8412913	-0.385278167	Zm00001d041760
Zm00001d017778_T001	0.9907928	0.197659955	Zm00001d017778
Zm00001d042182_T001	0.9954426	0.52536683	Zm00001d042182
Zm00001d037935_T001	0.2329183	1.424225573	Zm00001d037935
Zm00001d037009_T001	0.4194004	-0.454115347	Zm00001d037009
Zm00001d035323_T001	0.9990127	0.422388097	Zm00001d035323
Zm00001d008214_T001	0.9931431	0.225109949	Zm00001d008214
Zm00001d043108_T002	0.9487214	0.052972443	Zm00001d043108
Zm00001d043108_T001	0.945968	0.081853085	Zm00001d043108
Zm00001d049674_T001	0.8008364	-0.724406492	Zm00001d049674
Zm00001d049674_T002	0.7548283	-0.728746033	Zm00001d049674
Zm00001d012761_T005	0.005053822	1.247769959	Zm00001d012761
Zm00001d012761_T002	0.7414776	0.891833776	Zm00001d012761
Zm00001d012761_T008	0.923446	0.011544266	Zm00001d012761
Zm00001d012761_T007	1.52E-08	-1.322285344	Zm00001d012761
Zm00001d028836_T001	0.7102433	-0.711712162	Zm00001d028836
Zm00001d044988_T001	0.6769189	0.896516012	Zm00001d044988
Zm00001d045764_T001	0.957148	0.617526089	Zm00001d045764
Zm00001d008785_T001	0.9559227	0.551920195	Zm00001d008785
Zm00001d028769_T004	0.5088062	0.537639922	Zm00001d028769
Zm00001d048693_T001	0.852305	-0.096800322	Zm00001d048693
Zm00001d031075_T001	0.03577387	-1.77712344	Zm00001d031075
Zm00001d028455_T001	5.11E-06	-0.795824096	Zm00001d028455
Zm00001d033586_T001	0.705921	-0.462825621	Zm00001d033586
Zm00001d008946_T001	0.1231877	-1.694699293	Zm00001d008946
Zm00001d010450_T001	0.3236887	-0.750883582	Zm00001d010450

Zm00001d052339_T001	0.9722469	0.199702775	Zm00001d052339
Zm00001d017029_T002	0.4466862	1.48917214	Zm00001d017029
Zm00001d017029_T001	0.9469426	0.161910907	Zm00001d017029
Zm00001d017029_T003	7.88E-06	0.994269706	Zm00001d017029
Zm00001d017029_T006	0.000276863	0.893366414	Zm00001d017029
Zm00001d050227_T001	1	0.421860157	Zm00001d050227
Zm00001d019111_T010	0.5943719	-0.844930367	Zm00001d019111
Zm00001d019111_T003	0.9840918	0.134875326	Zm00001d019111
Zm00001d034616_T002	3.91E-07	1.752753463	Zm00001d034616
Zm00001d034616_T001	0.9172003	0.789200687	Zm00001d034616
Zm00001d032401_T001	0.5361942	-0.923655464	Zm00001d032401
Zm00001d013317_T002	0.0496363	2.185527811	Zm00001d013317
Zm00001d013317_T001	0.8229103	0.689621988	Zm00001d013317
Zm00001d025268_T001	0.9770343	0.572137848	Zm00001d025268
Zm00001d025268_T007	0.6075318	0.534279505	Zm00001d025268
Zm00001d025268_T008	0.9008366	0.264842411	Zm00001d025268
Zm00001d000295_T001	0.9468268	0.126391579	Zm00001d000295
Zm00001d023552_T005	0.9979568	0.057893421	Zm00001d023552
Zm00001d023552_T001	0.9694613	-0.171785255	Zm00001d023552
Zm00001d035964_T006	0.9321001	0.192253789	Zm00001d035964
Zm00001d035964_T003	0.2163442	1.981221977	Zm00001d035964
Zm00001d035964_T005	2.27E-05	-4.285863946	Zm00001d035964
Zm00001d051620_T001	0.9568663	-0.099653843	Zm00001d051620
Zm00001d005504_T001	0.9979568	0.241466259	Zm00001d005504
Zm00001d048137_T003	0.3541203	0.565334106	Zm00001d048137
Zm00001d048137_T005	1	0.363550973	Zm00001d048137
Zm00001d048137_T002	0.9791729	0.107146593	Zm00001d048137
Zm00001d044752_T001	0.09537414	2.095688799	Zm00001d044752
Zm00001d028665_T001	0.7561299	-0.692057517	Zm00001d028665
Zm00001d019522_T001	0.9782953	0.535680678	Zm00001d019522
Zm00001d019522_T003	0.7568663	0.679483945	Zm00001d019522
Zm00001d025557_T001	7.50E-09	-4.126900203	Zm00001d025557
Zm00001d039975_T001	0.9619942	0.154695371	Zm00001d039975
Zm00001d001898_T009	0.9779656	0.108743219	Zm00001d001898
Zm00001d001898_T011	0.004573701	1.199960446	Zm00001d001898
Zm00001d001898_T006	0.9552021	0.586201283	Zm00001d001898
Zm00001d001898_T001	0.5802127	1.160612282	Zm00001d001898
Zm00001d001898_T007	0.9909153	0.180518991	Zm00001d001898
Zm00001d019553_T001	0.9756551	0.222445918	Zm00001d019553
Zm00001d044986_T001	0.8595871	1.041601571	Zm00001d044986
Zm00001d007781_T001	0.1781613	-0.782725076	Zm00001d007781
Zm00001d048509_T003	0.7972819	0.788808246	Zm00001d048509
Zm00001d048509_T004	0.9749249	0.517527769	Zm00001d048509
Zm00001d048509_T001	0.7737238	-0.212134123	Zm00001d048509
Zm00001d048509_T002	0.8857953	0.768039988	Zm00001d048509
Zm00001d006917_T001	0.8231712	0.961311695	Zm00001d006917
Zm00001d006917_T002	7.65E-10	-2.634337843	Zm00001d006917
Zm00001d035256_T002	0.7541349	-0.283003037	Zm00001d035256
Zm00001d045212_T001	0.7827148	0.780866568	Zm00001d045212
Zm00001d045212_T003	0.935477	0.360290259	Zm00001d045212
Zm00001d045212_T002	0.9567316	0.512776086	Zm00001d045212
Zm00001d047340_T001	0.6892429	1.09873796	Zm00001d047340
Zm00001d047340_T003	0.6556513	0.979311199	Zm00001d047340
Zm00001d006165_T001	0.8449395	-0.339474977	Zm00001d006165

Zm00001d006726_T001	0.9690925	0.10927342	Zm00001d006726
Zm00001d042746_T003	0.8884016	0.821479951	Zm00001d042746
Zm00001d042746_T001	0.8188896	0.768016745	Zm00001d042746
Zm00001d042746_T002	0.9019013	-0.038485332	Zm00001d042746
Zm00001d047282_T001	0.6993528	-0.415057861	Zm00001d047282
Zm00001d003540_T001	0.908501	-0.155393252	Zm00001d003540
Zm00001d015715_T002	0.001236951	-2.640213602	Zm00001d015715
Zm00001d015715_T001	0.00324374	-2.087991308	Zm00001d015715
Zm00001d013782_T001	0.3400339	-0.320883797	Zm00001d013782
Zm00001d039043_T006	0.000820562	-1.083885644	Zm00001d039043
Zm00001d039043_T004	0.7682041	1.506910159	Zm00001d039043
Zm00001d039043_T003	0.9942835	0.033202581	Zm00001d039043
Zm00001d039043_T013	0.8520487	-0.290837773	Zm00001d039043
Zm00001d039043_T001	0.3778027	1.45423765	Zm00001d039043
Zm00001d039043_T002	0.9988095	0.353795472	Zm00001d039043
Zm00001d029784_T001	0.2378089	1.117012707	Zm00001d029784
Zm00001d002873_T005	0.9662615	-0.122625347	Zm00001d002873
Zm00001d002873_T003	1	-0.16955501	Zm00001d002873
Zm00001d006456_T001	0.8457647	-0.120923318	Zm00001d006456
Zm00001d006456_T004	0.1244317	0.499974231	Zm00001d006456
Zm00001d006456_T003	0.9870482	0.287051413	Zm00001d006456
Zm00001d016844_T001	0.9469426	0.507989982	Zm00001d016844
Zm00001d046155_T001	0.9690925	0.123826258	Zm00001d046155
Zm00001d008984_T003	0.9107146	0.675050108	Zm00001d008984
Zm00001d008984_T004	0.9335676	0.52237594	Zm00001d008984
Zm00001d008984_T002	0.9979568	0.326816001	Zm00001d008984
Zm00001d008413_T001	0.9050121	0.78183872	Zm00001d008413
Zm00001d006040_T001	0.8421761	0.971917495	Zm00001d006040
Zm00001d036296_T008	0.3966246	0.426749957	Zm00001d036296
Zm00001d036296_T012	0.9494694	0.678259229	Zm00001d036296
Zm00001d036296_T001	0.9849414	0.857550194	Zm00001d036296
Zm00001d021426_T002	0.9690782	0.093135985	Zm00001d021426
Zm00001d021426_T001	0.9944247	0.416906305	Zm00001d021426
Zm00001d017560_T002	0.9984041	0.448149651	Zm00001d017560
Zm00001d017560_T001	0.8572948	0.382122259	Zm00001d017560
Zm00001d017560_T003	0.9722469	0.305974618	Zm00001d017560
Zm00001d048238_T001	0.6726176	-0.639752011	Zm00001d048238
Zm00001d019549_T001	0.9354647	-0.157075211	Zm00001d019549
Zm00001d022169_T025	0.8660897	-0.117445025	Zm00001d022169
Zm00001d022169_T001	0.04054157	1.997399483	Zm00001d022169
Zm00001d022169_T008	0.7594555	-0.182187485	Zm00001d022169
Zm00001d022169_T027	0.9747606	0.138537043	Zm00001d022169
Zm00001d028730_T003	0.9690925	0.160254535	Zm00001d028730
Zm00001d028730_T005	0.01067552	0.838739312	Zm00001d028730
Zm00001d028730_T001	0.989787	0.265687668	Zm00001d028730
Zm00001d028730_T004	0.366099	-0.568251398	Zm00001d028730
Zm00001d030398_T006	0.8505918	0.262452048	Zm00001d030398
Zm00001d030398_T005	0.2360889	-0.46766742	Zm00001d030398
Zm00001d030398_T001	0.5208234	-0.561506235	Zm00001d030398
Zm00001d030398_T003	0.1479646	0.730563023	Zm00001d030398
Zm00001d043729_T001	0.708571	0.732116309	Zm00001d043729
Zm00001d027682_T002	0.8964439	-0.057539139	Zm00001d027682
Zm00001d027682_T001	0.4236198	-0.773537131	Zm00001d027682
Zm00001d015307_T001	0.5554844	1.322567134	Zm00001d015307

Zm00001d029303_T001	0.9893214	0.232460023	Zm00001d029303
Zm00001d022374_T001	0.6608119	1.193535851	Zm00001d022374
Zm00001d029515_T004	1	0.223090955	Zm00001d029515
Zm00001d029515_T001	0.9683047	0.262710654	Zm00001d029515
Zm00001d029515_T010	1	0.261594544	Zm00001d029515
Zm00001d029515_T003	0.9708651	0.209722156	Zm00001d029515
Zm00001d029515_T002	0.9378338	-0.03386863	Zm00001d029515
Zm00001d029515_T005	0.9931009	0.036238254	Zm00001d029515
Zm00001d039681_T002	0.8929316	-0.253440855	Zm00001d039681
Zm00001d052689_T002	0.6922261	-0.362627949	Zm00001d052689
Zm00001d006844_T003	0.9518042	0.606885703	Zm00001d006844
Zm00001d006844_T002	0.02151911	-1.385680349	Zm00001d006844
Zm00001d016066_T001	0.6422833	-0.548906667	Zm00001d016066
Zm00001d025264_T001	0.7941326	0.764802161	Zm00001d025264
Zm00001d024537_T001	0.923446	-0.293700765	Zm00001d024537
Zm00001d003925_T001	0.9781672	0.544656933	Zm00001d003925
Zm00001d002006_T007	0.9148331	0.812560267	Zm00001d002006
Zm00001d002006_T002	0.8660897	0.642445268	Zm00001d002006
Zm00001d002006_T011	0.01234228	1.417453876	Zm00001d002006
Zm00001d002006_T006	0.1179851	2.321944199	Zm00001d002006
Zm00001d002006_T001	0.1678876	2.182666411	Zm00001d002006
Zm00001d002006_T013	5.11E-06	1.120078339	Zm00001d002006
Zm00001d002006_T016	1.65E-14	4.33867938	Zm00001d002006
Zm00001d009583_T001	0.1010062	-1.634609084	Zm00001d009583
Zm00001d027345_T001	0.8376861	-0.582900728	Zm00001d027345
Zm00001d018219_T001	0.9257893	0.036038162	Zm00001d018219
Zm00001d047081_T001	0.2244109	1.842596847	Zm00001d047081
Zm00001d035759_T001	0.9596377	1.177947956	Zm00001d035759
Zm00001d039719_T005	0.8498341	0.362497794	Zm00001d039719
Zm00001d039719_T003	0.9429712	0.616039297	Zm00001d039719
Zm00001d039719_T004	0.4331173	-0.222911449	Zm00001d039719
Zm00001d020448_T001	0.9937567	0.126462461	Zm00001d020448
Zm00001d022289_T001	0.9768468	0.148760191	Zm00001d022289
Zm00001d010329_T001	0.9321001	-0.041319533	Zm00001d010329
Zm00001d049507_T002	0.9566139	0.504511523	Zm00001d049507
Zm00001d049507_T001	0.9535734	-0.239359797	Zm00001d049507
Zm00001d033556_T007	0.9921225	0.49560258	Zm00001d033556
Zm00001d005002_T004	0.06783865	-1.543496029	Zm00001d005002
Zm00001d005002_T002	0.06357073	-1.339134306	Zm00001d005002
Zm00001d032760_T001	0.7146325	-0.786172423	Zm00001d032760
Zm00001d034432_T002	0.9988864	0.329995301	Zm00001d034432
Zm00001d034432_T001	0.9469426	0.04151166	Zm00001d034432
Zm00001d018912_T003	0.6995506	0.925368047	Zm00001d018912
Zm00001d019922_T005	0.6910922	0.355016208	Zm00001d019922
Zm00001d019922_T004	0.2264723	0.41071353	Zm00001d019922
Zm00001d019922_T003	0.7289978	1.007576207	Zm00001d019922
Zm00001d019922_T007	0.745469	-0.627567566	Zm00001d019922
Zm00001d019922_T010	1	0.055020643	Zm00001d019922
Zm00001d034956_T001	0.9828518	0.378457739	Zm00001d034956
Zm00001d037950_T001	0.8152824	0.495567098	Zm00001d037950
Zm00001d037950_T005	0.7831859	0.868001212	Zm00001d037950
Zm00001d037950_T007	0.6318496	0.184676582	Zm00001d037950
Zm00001d037950_T017	8.77E-05	1.809616828	Zm00001d037950
Zm00001d037950_T010	0.8518279	-0.112245173	Zm00001d037950

Zm00001d023426_T001	0.9840043	0.405804176	Zm00001d023426
Zm00001d023426_T005	0.9290834	-0.044861977	Zm00001d023426
Zm00001d006001_T004	1	0.243503399	Zm00001d006001
Zm00001d006001_T006	0.9179118	0.210762424	Zm00001d006001
Zm00001d006001_T007	0.9894615	0.326746808	Zm00001d006001
Zm00001d006001_T001	0.9771927	0.238015206	Zm00001d006001
Zm00001d027275_T001	0.1710536	1.307691969	Zm00001d027275
Zm00001d012539_T001	0.8706608	-0.292635818	Zm00001d012539
Zm00001d034871_T001	0.9588604	0.109143534	Zm00001d034871
Zm00001d043080_T001	0.8328744	-0.202674221	Zm00001d043080
Zm00001d019054_T004	0.4583992	-0.367646193	Zm00001d019054
Zm00001d019054_T001	0.9384219	0.079801736	Zm00001d019054
Zm00001d053616_T002	1	0.378781823	Zm00001d053616
Zm00001d053616_T003	0.4437212	1.065944759	Zm00001d053616
Zm00001d047207_T001	0.9702252	-0.060022887	Zm00001d047207
Zm00001d029561_T008	0.6486663	-0.460735941	Zm00001d029561
Zm00001d029561_T001	0.6774508	1.188874899	Zm00001d029561
Zm00001d043216_T001	0.9486412	0.066698524	Zm00001d043216
Zm00001d018235_T001	0.6926364	-0.62604999	Zm00001d018235
Zm00001d026192_T011	0.9993872	0.243124067	Zm00001d026192
Zm00001d039047_T010	0.7222527	0.834207569	Zm00001d039047
Zm00001d039047_T011	0.1312124	0.546142403	Zm00001d039047
Zm00001d039047_T005	0.4799121	-0.204844041	Zm00001d039047
Zm00001d039047_T014	0.7020458	-0.327069385	Zm00001d039047
Zm00001d039047_T003	0.8412913	-0.087775437	Zm00001d039047
Zm00001d039047_T008	0.8585804	-0.169720263	Zm00001d039047
Zm00001d039047_T012	0.9817074	0.161232682	Zm00001d039047
Zm00001d033321_T004	0.8256776	0.423062845	Zm00001d033321
Zm00001d033321_T002	0.9711278	0.198509813	Zm00001d033321
Zm00001d033321_T003	0.8536822	0.710275359	Zm00001d033321
Zm00001d026647_T001	0.995534	0.144186207	Zm00001d026647
Zm00001d026647_T007	0.6380638	-0.465344669	Zm00001d026647
Zm00001d026647_T010	0.9322451	0.302308527	Zm00001d026647
Zm00001d026647_T009	0.9292973	-0.064073075	Zm00001d026647
Zm00001d011202_T001	0.5408189	1.379761094	Zm00001d011202
Zm00001d043442_T001	0.001645855	3.457470443	Zm00001d043442
Zm00001d027727_T001	0.74819	-0.41182244	Zm00001d027727
Zm00001d041701_T001	1	0.187622776	Zm00001d041701
Zm00001d052935_T001	0.04663752	-1.666214006	Zm00001d052935
Zm00001d021583_T009	0.8893434	-0.17940706	Zm00001d021583
Zm00001d021583_T011	0.923446	-0.085327606	Zm00001d021583
Zm00001d021583_T001	0.328324	0.943633786	Zm00001d021583
Zm00001d021583_T008	0.5377194	0.442131276	Zm00001d021583
Zm00001d048516_T004	0.9177099	-0.24013673	Zm00001d048516
Zm00001d048516_T001	0.9400055	0.040131231	Zm00001d048516
Zm00001d021870_T001	0.4878494	1.056944912	Zm00001d021870
Zm00001d010690_T001	0.8744797	0.717613502	Zm00001d010690
Zm00001d008305_T002	0.6955963	1.042719426	Zm00001d008305
Zm00001d008305_T001	0.7146325	1.010996277	Zm00001d008305
Zm00001d011258_T013	0.8643636	-0.142464232	Zm00001d011258
Zm00001d011258_T009	0.9099617	-0.044573681	Zm00001d011258
Zm00001d011258_T002	2.14E-07	1.198259164	Zm00001d011258
Zm00001d011258_T012	0.9463296	0.425757925	Zm00001d011258
Zm00001d011258_T008	0.5048676	-0.576700898	Zm00001d011258

Zm00001d011258_T007	0.03157688	1.282283484	Zm00001d011258
Zm00001d011258_T016	0.9515686	0.51151526	Zm00001d011258
Zm00001d011258_T011	0.9881407	0.171647833	Zm00001d011258
Zm00001d041653_T001	0.8080206	-0.192928022	Zm00001d041653
Zm00001d022276_T004	0.3678033	0.604837933	Zm00001d022276
Zm00001d022276_T002	0.5180698	0.22374261	Zm00001d022276
Zm00001d022276_T006	0.2635556	-0.616553509	Zm00001d022276
Zm00001d017868_T004	0.001679745	1.799352116	Zm00001d017868
Zm00001d017868_T001	0.6389363	1.188418397	Zm00001d017868
Zm00001d017868_T009	9.55E-05	2.004843341	Zm00001d017868
Zm00001d017868_T006	8.52E-06	0.624422184	Zm00001d017868
Zm00001d010248_T001	0.8799676	-0.142058718	Zm00001d010248
Zm00001d052471_T001	0.9050121	0.415293679	Zm00001d052471
Zm00001d013419_T001	0.9913523	0.430006443	Zm00001d013419
Zm00001d002579_T002	0.9375054	0.050984852	Zm00001d002579
Zm00001d015133_T002	0.9180865	0.618419955	Zm00001d015133
Zm00001d026113_T008	0.2984976	-1.131270674	Zm00001d026113
Zm00001d026113_T004	0.6761587	0.848938297	Zm00001d026113
Zm00001d026113_T005	0.1513922	1.40584867	Zm00001d026113
Zm00001d007692_T002	0.8941695	0.885026259	Zm00001d007692
Zm00001d007692_T003	0.7123806	1.103275106	Zm00001d007692
Zm00001d007692_T004	0.5983973	0.42954861	Zm00001d007692
Zm00001d007692_T001	0.6217057	0.18320804	Zm00001d007692
Zm00001d007692_T008	0.9884825	0.283217104	Zm00001d007692
Zm00001d035670_T001	0.9988864	0.14996031	Zm00001d035670
Zm00001d000428_T001	0.9797082	0.260919978	Zm00001d000428
Zm00001d050107_T001	0.9320554	0.022540568	Zm00001d050107
Zm00001d033843_T007	0.9662615	0.208628844	Zm00001d033843
Zm00001d018927_T001	0.9766874	0.118292787	Zm00001d018927
Zm00001d029611_T001	0.8569937	-0.147419812	Zm00001d029611
Zm00001d053090_T001	0.9782953	0.21306494	Zm00001d053090
Zm00001d018104_T001	0.8894168	-0.081176347	Zm00001d018104
Zm00001d051135_T002	0.8031436	0.997287105	Zm00001d051135
Zm00001d010564_T011	0.3795013	0.45269684	Zm00001d010564
Zm00001d010564_T002	0.6792923	0.894997983	Zm00001d010564
Zm00001d010564_T006	0.8575897	0.558780851	Zm00001d010564
Zm00001d010564_T013	0.9766874	0.567391464	Zm00001d010564
Zm00001d008837_T001	0.9561521	0.091726468	Zm00001d008837
Zm00001d012653_T001	0.02305487	1.233127024	Zm00001d012653
Zm00001d012653_T004	0.9596377	0.483253708	Zm00001d012653
Zm00001d012653_T005	0.4799121	1.016214774	Zm00001d012653
Zm00001d006758_T006	0.005709369	1.955434626	Zm00001d006758
Zm00001d006758_T005	0.7613827	-0.167888457	Zm00001d006758
Zm00001d006758_T011	1	0.35028893	Zm00001d006758
Zm00001d032187_T001	0.000186139	3.91458543	Zm00001d032187
Zm00001d033917_T001	0.8031436	1.067910371	Zm00001d033917
Zm00001d020339_T001	0.5767875	1.388813817	Zm00001d020339
Zm00001d048570_T001	0.9396809	0.410095887	Zm00001d048570
Zm00001d050698_T001	0.745718	-0.416504329	Zm00001d050698
Zm00001d007124_T003	0.9745948	0.106795517	Zm00001d007124
Zm00001d007124_T002	0.1272455	-1.200829198	Zm00001d007124
Zm00001d038355_T001	0.8873864	-0.05339309	Zm00001d038355
Zm00001d040270_T018	0.8412913	0.2383345	Zm00001d040270
Zm00001d040270_T014	0.9940329	0.084009706	Zm00001d040270

Zm00001d040270_T001	0.3374375	-0.821795511	Zm00001d040270
Zm00001d040270_T006	0.7029164	1.144512643	Zm00001d040270
Zm00001d040270_T013	0.1616209	1.749134953	Zm00001d040270
Zm00001d049656_T001	0.9326774	-0.063380494	Zm00001d049656
Zm00001d017959_T004	0.92946	0.485772465	Zm00001d017959
Zm00001d017959_T002	1	0.489780682	Zm00001d017959
Zm00001d043737_T001	0.9971866	0.194113239	Zm00001d043737
Zm00001d049982_T004	0.9711721	0.558854911	Zm00001d049982
Zm00001d049982_T008	0.9845237	0.122168378	Zm00001d049982
Zm00001d049982_T007	0.4014435	-1.207506184	Zm00001d049982
Zm00001d019994_T001	0.9563232	0.624428959	Zm00001d019994
Zm00001d037717_T002	0.9995663	0.355120596	Zm00001d037717
Zm00001d018005_T002	0.1768179	-0.269072777	Zm00001d018005
Zm00001d018005_T004	0.3739063	-0.57473588	Zm00001d018005
Zm00001d018005_T007	0.9016285	1.516098174	Zm00001d018005
Zm00001d008455_T001	0.5954407	-0.546473813	Zm00001d008455
Zm00001d038830_T005	0.006621309	1.585743413	Zm00001d038830
Zm00001d038830_T003	0.5092251	1.205391437	Zm00001d038830
Zm00001d038830_T004	0.9404125	0.068659599	Zm00001d038830
Zm00001d011463_T001	0.2443227	-1.314638638	Zm00001d011463
Zm00001d006602_T005	0.0797978	0.921815139	Zm00001d006602
Zm00001d006602_T003	0.6727656	0.853401358	Zm00001d006602
Zm00001d006602_T002	0.9760472	0.217680905	Zm00001d006602
Zm00001d019467_T004	0.9979568	0.092819129	Zm00001d019467
Zm00001d019467_T001	0.989787	0.205435555	Zm00001d019467
Zm00001d019467_T003	0.9581123	0.598011858	Zm00001d019467
Zm00001d010614_T005	0.7451122	-0.568754578	Zm00001d010614
Zm00001d010614_T009	0.000165295	-1.836349669	Zm00001d010614
Zm00001d010614_T008	0.8653965	-0.074237467	Zm00001d010614
Zm00001d010614_T001	0.6377685	-0.879849676	Zm00001d010614
Zm00001d010614_T003	0.9210327	-0.056689235	Zm00001d010614
Zm00001d009404_T001	0.9822138	0.133065133	Zm00001d009404
Zm00001d032857_T002	0.9732421	0.230937387	Zm00001d032857
Zm00001d032857_T003	0.8840817	0.729046945	Zm00001d032857
Zm00001d009475_T001	0.3812783	-0.931074808	Zm00001d009475
Zm00001d015175_T001	0.9659036	0.560831177	Zm00001d015175
Zm00001d015175_T002	0.2496307	-0.26849306	Zm00001d015175
Zm00001d039050_T001	0.525543	-0.817567162	Zm00001d039050
Zm00001d037684_T001	0.3845928	0.342185344	Zm00001d037684
Zm00001d042435_T003	0.9928864	0.172247227	Zm00001d042435
Zm00001d042435_T001	0.8786965	-0.033465082	Zm00001d042435
Zm00001d042435_T005	0.9986486	0.204729356	Zm00001d042435
Zm00001d042435_T004	0.9791729	-0.130847537	Zm00001d042435
Zm00001d026469_T002	0.9832092	0.39621591	Zm00001d026469
Zm00001d040383_T004	0.9278539	0.314995022	Zm00001d040383
Zm00001d040383_T005	0.9849018	0.179026333	Zm00001d040383
Zm00001d040383_T002	0.4550144	-0.547941064	Zm00001d040383
Zm00001d040383_T003	0.8660897	0.69265848	Zm00001d040383
Zm00001d011003_T001	0.3780662	-0.752454832	Zm00001d011003
Zm00001d005246_T001	0.4146004	0.799272139	Zm00001d005246
Zm00001d026278_T001	0.5490269	-1.005437276	Zm00001d026278
Zm00001d010651_T001	0.5719089	0.999856882	Zm00001d010651
Zm00001d011444_T002	0.8157787	-0.198081612	Zm00001d011444
Zm00001d011444_T005	0.06691707	-1.63898389	Zm00001d011444

Zm00001d045935_T001	0.7626242	-0.383826522	Zm00001d045935
Zm00001d018596_T003	0.9321001	0.072588279	Zm00001d018596
Zm00001d018596_T004	0.0626471	0.93592429	Zm00001d018596
Zm00001d018596_T011	0.8350279	0.241707997	Zm00001d018596
Zm00001d018596_T001	0.8505918	-0.0257523	Zm00001d018596
Zm00001d018596_T009	1.06E-07	-0.76695057	Zm00001d018596
Zm00001d018596_T005	0.357909	0.630583955	Zm00001d018596
Zm00001d018596_T017	0.7898216	0.003680137	Zm00001d018596
Zm00001d018596_T006	0.6639456	0.592741092	Zm00001d018596
Zm00001d009666_T001	0.5865893	-0.586118986	Zm00001d009666
Zm00001d003495_T007	0.8513569	0.660928444	Zm00001d003495
Zm00001d003495_T010	0.2982377	1.168567398	Zm00001d003495
Zm00001d003495_T003	0.5464623	1.127262748	Zm00001d003495
Zm00001d003495_T002	0.05032459	1.655633264	Zm00001d003495
Zm00001d003495_T005	0.3675057	0.385356741	Zm00001d003495
Zm00001d003495_T001	0.9975882	0.110230333	Zm00001d003495
Zm00001d003495_T009	0.000116863	1.341963806	Zm00001d003495
Zm00001d003495_T006	0.6910922	0.873984943	Zm00001d003495
Zm00001d024602_T001	0.8665954	-0.138165063	Zm00001d024602
Zm00001d053232_T002	0.9706887	0.60070213	Zm00001d053232
Zm00001d053232_T006	0.9237037	-0.094642442	Zm00001d053232
Zm00001d053232_T010	0.941922	0.317338481	Zm00001d053232
Zm00001d053232_T009	0.200223	0.411163099	Zm00001d053232
Zm00001d019988_T001	0.9845237	0.563930443	Zm00001d019988
Zm00001d002824_T001	0.732047	1.123812118	Zm00001d002824
Zm00001d002824_T002	0.3381821	-0.088807344	Zm00001d002824
Zm00001d038134_T002	0.8485027	1.296538963	Zm00001d038134
Zm00001d038134_T008	1	0.320681275	Zm00001d038134
Zm00001d038134_T004	0.9170869	0.607305978	Zm00001d038134
Zm00001d023283_T001	0.9929419	0.22010765	Zm00001d023283
Zm00001d044170_T001	0.9375054	0.685783859	Zm00001d044170
Zm00001d047676_T004	0.3273212	0.452107403	Zm00001d047676
Zm00001d047676_T001	0.2645495	1.827611004	Zm00001d047676
Zm00001d047676_T003	0.2991114	1.614698717	Zm00001d047676
Zm00001d047676_T005	0.002095691	2.479904118	Zm00001d047676
Zm00001d047676_T002	0.9089821	0.790687962	Zm00001d047676
Zm00001d006534_T002	0.9422823	0.493569184	Zm00001d006534
Zm00001d006534_T001	0.9988864	0.273109673	Zm00001d006534
Zm00001d017501_T002	0.2601501	1.408981569	Zm00001d017501
Zm00001d017501_T001	1	0.270249975	Zm00001d017501
Zm00001d034405_T006	0.5837214	0.351567536	Zm00001d034405
Zm00001d034405_T010	0.7051788	-0.097661382	Zm00001d034405
Zm00001d035812_T002	0.9870394	0.474216082	Zm00001d035812
Zm00001d035812_T005	0.6038555	0.789406029	Zm00001d035812
Zm00001d035812_T001	0.2766589	-0.93742751	Zm00001d035812
Zm00001d035812_T006	0.6398988	-0.20031287	Zm00001d035812
Zm00001d011923_T004	0.9628432	0.13447341	Zm00001d011923
Zm00001d018322_T001	0.5899787	-0.843044547	Zm00001d018322
Zm00001d033882_T001	0.9184323	0.551742965	Zm00001d033882
Zm00001d022613_T001	0.7020458	-0.438781744	Zm00001d022613
Zm00001d036246_T001	0.6873614	-0.664390998	Zm00001d036246
Zm00001d005550_T001	1	0.259946697	Zm00001d005550
Zm00001d000005_T001	0.8751967	0.589317223	Zm00001d000005
Zm00001d000005_T003	0.3116357	-0.905626326	Zm00001d000005

Zm00001d023753_T001	0.4208649	-1.178005853	Zm00001d023753
Zm00001d022073_T001	1	0.249001092	Zm00001d022073
Zm00001d030096_T002	0.4121688	-0.883554781	Zm00001d030096
Zm00001d030096_T001	0.9887584	0.231405912	Zm00001d030096
Zm00001d047159_T001	0.0797978	-1.80426671	Zm00001d047159
Zm00001d047548_T001	0.02671151	-2.141207592	Zm00001d047548
Zm00001d005157_T001	0.6951496	-0.489805774	Zm00001d005157
Zm00001d005157_T012	0.9380783	-0.041587064	Zm00001d005157
Zm00001d005157_T010	0.07726517	0.621913195	Zm00001d005157
Zm00001d005157_T032	0.04646514	0.445117872	Zm00001d005157
Zm00001d005157_T002	0.850642	0.881636423	Zm00001d005157
Zm00001d028808_T001	0.000628766	3.390090356	Zm00001d028808
Zm00001d038319_T002	0.9274507	0.514327565	Zm00001d038319
Zm00001d038319_T001	0.9970835	0.414390468	Zm00001d038319
Zm00001d042808_T001	0.8585804	-0.662926651	Zm00001d042808
Zm00001d008656_T002	0.8868226	-0.044864159	Zm00001d008656
Zm00001d008656_T003	0.9242613	0.619215176	Zm00001d008656
Zm00001d052978_T001	0.9843847	0.075345276	Zm00001d052978
Zm00001d021929_T001	0.3177865	1.574344003	Zm00001d021929
Zm00001d000098_T004	0.989787	0.166061987	Zm00001d000098
Zm00001d000098_T002	0.7914025	0.576351167	Zm00001d000098
Zm00001d000098_T006	0.4073276	-0.213408902	Zm00001d000098
Zm00001d008444_T001	0.003659006	-0.974170491	Zm00001d008444
Zm00001d015802_T001	0.9187004	0.625504568	Zm00001d015802
Zm00001d014187_T011	0.07256374	1.54923162	Zm00001d014187
Zm00001d014187_T015	0.3343236	0.333625087	Zm00001d014187
Zm00001d014187_T016	0.8968577	-0.060856016	Zm00001d014187
Zm00001d014187_T007	1	0.43569709	Zm00001d014187
Zm00001d008716_T002	1	0.292220701	Zm00001d008716
Zm00001d008716_T001	0.9429712	0.103621717	Zm00001d008716
Zm00001d025421_T002	0.8585804	-0.156427372	Zm00001d025421
Zm00001d005926_T001	0.5843839	-0.768651152	Zm00001d005926
Zm00001d036673_T002	0.9822138	0.456720925	Zm00001d036673
Zm00001d036673_T001	0.4565762	0.500621005	Zm00001d036673
Zm00001d008692_T001	0.9075969	0.70084686	Zm00001d008692
Zm00001d026295_T002	1.50E-09	0.918030352	Zm00001d026295
Zm00001d026295_T003	0.6821817	-0.224988024	Zm00001d026295
Zm00001d026295_T004	0.9706293	0.094625248	Zm00001d026295
Zm00001d026295_T005	0.5549472	-0.250044436	Zm00001d026295
Zm00001d029215_T030	0.3439954	-0.570576909	Zm00001d029215
Zm00001d029215_T011	0.6201695	-0.116724011	Zm00001d029215
Zm00001d022005_T001	0.9031129	-0.193617492	Zm00001d022005
Zm00001d005270_T001	0.9832612	0.370388891	Zm00001d005270
Zm00001d005270_T002	0.941922	0.371966317	Zm00001d005270
Zm00001d014900_T007	0.03485146	0.319325666	Zm00001d014900
Zm00001d014900_T062	1	0.278884071	Zm00001d014900
Zm00001d014900_T044	0.7676077	0.150828899	Zm00001d014900
Zm00001d014900_T024	0.2894393	0.349480666	Zm00001d014900
Zm00001d014900_T001	0.02081089	0.319030363	Zm00001d014900
Zm00001d014900_T058	0.9722469	0.27943136	Zm00001d014900
Zm00001d014900_T040	0.002528307	1.932788005	Zm00001d014900
Zm00001d014900_T059	0.9690925	0.148277829	Zm00001d014900
Zm00001d040937_T003	0.965836	0.095637796	Zm00001d040937
Zm00001d032215_T002	0.8881012	-0.334272776	Zm00001d032215

Zm00001d005894_T001	0.9363242	0.684942558	Zm00001d005894
Zm00001d053053_T002	0.6503853	0.619418974	Zm00001d053053
Zm00001d017279_T001	0.2171279	1.947833816	Zm00001d017279
Zm00001d009140_T001	0.9501111	0.024097849	Zm00001d009140
Zm00001d039026_T001	0.997764	0.218940832	Zm00001d039026
Zm00001d038904_T001	0.7194918	-0.398763475	Zm00001d038904
Zm00001d011193_T001	0.9970835	0.378798475	Zm00001d011193
Zm00001d032472_T001	0.9979568	0.346081279	Zm00001d032472
Zm00001d032472_T003	0.7819535	-0.655221478	Zm00001d032472
Zm00001d032472_T004	3.85E-12	3.143874364	Zm00001d032472
Zm00001d032472_T002	0.9561521	0.123290958	Zm00001d032472
Zm00001d021566_T002	0.8252955	0.102803186	Zm00001d021566
Zm00001d021566_T009	0.6709825	0.208948704	Zm00001d021566
Zm00001d021566_T005	0.9613831	0.148496577	Zm00001d021566
Zm00001d044701_T002	0.6096177	1.188733154	Zm00001d044701
Zm00001d044701_T001	0.3397699	1.367373999	Zm00001d044701
Zm00001d053066_T001	0.9550374	0.473413471	Zm00001d053066
Zm00001d029104_T007	0.9851204	0.078932175	Zm00001d029104
Zm00001d029104_T002	0.9881407	0.514091938	Zm00001d029104
Zm00001d034817_T004	0.7533768	0.99075145	Zm00001d034817
Zm00001d034817_T002	0.8922781	1.390747209	Zm00001d034817
Zm00001d034817_T003	0.4393826	1.46464213	Zm00001d034817
Zm00001d040959_T001	0.8643636	0.768596603	Zm00001d040959
Zm00001d040959_T002	0.9721407	0.119012563	Zm00001d040959
Zm00001d014617_T001	0.000760374	-2.270329831	Zm00001d014617
Zm00001d043879_T001	0.6716118	1.186620362	Zm00001d043879
Zm00001d015445_T018	0.862753	-0.108986043	Zm00001d015445
Zm00001d015445_T006	0.92856	-0.048416047	Zm00001d015445
Zm00001d015445_T005	0.9979568	0.124115642	Zm00001d015445
Zm00001d015445_T001	0.7146325	0.431999098	Zm00001d015445
Zm00001d011812_T001	0.8842271	-0.086529486	Zm00001d011812
Zm00001d028174_T001	0.7137316	-0.45742356	Zm00001d028174
Zm00001d024755_T005	0.02184252	0.81972122	Zm00001d024755
Zm00001d024755_T001	0.9968176	0.419435269	Zm00001d024755
Zm00001d024755_T009	0.03355314	1.685573263	Zm00001d024755
Zm00001d024755_T003	5.11E-07	3.324214892	Zm00001d024755
Zm00001d024755_T007	0.02673031	1.574014956	Zm00001d024755
Zm00001d024755_T002	0.2484954	1.866234345	Zm00001d024755
Zm00001d024755_T004	0.003590947	1.545357274	Zm00001d024755
Zm00001d013147_T001	0.9469426	0.057699189	Zm00001d013147
Zm00001d010021_T007	1.31E-08	-1.255076171	Zm00001d010021
Zm00001d010021_T002	5.28E-09	1.603082865	Zm00001d010021
Zm00001d010021_T004	0.9823248	0.504493171	Zm00001d010021
Zm00001d038991_T001	0.7610341	-0.29225991	Zm00001d038991
Zm00001d018334_T002	0.9778436	-0.118881054	Zm00001d018334
Zm00001d018334_T001	0.8360354	-0.409573119	Zm00001d018334
Zm00001d022264_T001	0.9771927	-0.16064697	Zm00001d022264
Zm00001d042492_T001	0.3649839	-0.71921243	Zm00001d042492
Zm00001d045208_T001	1	0.248531678	Zm00001d045208
Zm00001d014123_T001	0.757295	-0.411833195	Zm00001d014123
Zm00001d012553_T002	0.9877579	0.549562155	Zm00001d012553
Zm00001d012553_T001	0.9727064	0.509753017	Zm00001d012553
Zm00001d012553_T008	0.4146004	-0.861156187	Zm00001d012553
Zm00001d012553_T004	0.8498341	0.931767756	Zm00001d012553

Zm00001d012553_T006	0.8383745	-0.154919399	Zm00001d012553
Zm00001d012553_T005	0.9847267	0.267615771	Zm00001d012553
Zm00001d012553_T007	0.8192001	0.769898658	Zm00001d012553
Zm00001d052810_T002	0.8153984	-0.237026598	Zm00001d052810
Zm00001d015095_T001	0.9907928	0.210987418	Zm00001d015095
Zm00001d008208_T001	0.9040654	-0.337223053	Zm00001d008208
Zm00001d005035_T001	0.9230476	0.002836495	Zm00001d005035
Zm00001d000329_T001	0.7613827	-0.296468818	Zm00001d000329
Zm00001d044653_T001	0.9322451	-0.126656657	Zm00001d044653
Zm00001d018360_T002	0.923446	-0.033835827	Zm00001d018360
Zm00001d018360_T001	0.9690782	0.162059487	Zm00001d018360
Zm00001d012560_T002	0.9860492	0.248259857	Zm00001d012560
Zm00001d044808_T001	0.9792387	0.443236423	Zm00001d044808
Zm00001d044109_T003	0.5467603	-0.451126168	Zm00001d044109
Zm00001d049162_T001	0.1551001	-1.249867736	Zm00001d049162
Zm00001d047531_T003	0.3497147	0.610039951	Zm00001d047531
Zm00001d047531_T002	0.7287219	-0.229398998	Zm00001d047531
Zm00001d013911_T003	0.843747	0.967062145	Zm00001d013911
Zm00001d037338_T001	0.9988063	0.325480321	Zm00001d037338
Zm00001d046483_T003	0.9969363	0.257898019	Zm00001d046483
Zm00001d025920_T001	0.7914025	-0.241215906	Zm00001d025920
Zm00001d022357_T006	0.3207142	1.282179218	Zm00001d022357
Zm00001d022357_T007	0.4246306	1.145358643	Zm00001d022357
Zm00001d022357_T002	1	0.418167679	Zm00001d022357
Zm00001d022357_T009	0.6945741	1.032667932	Zm00001d022357
Zm00001d012395_T005	0.8613703	0.588964623	Zm00001d012395
Zm00001d012395_T002	0.9334729	0.572546193	Zm00001d012395
Zm00001d012395_T006	0.9745433	0.525233078	Zm00001d012395
Zm00001d012395_T003	0.9493131	0.094415386	Zm00001d012395
Zm00001d012395_T001	0.6675417	-0.375644474	Zm00001d012395
Zm00001d023469_T003	0.2206757	0.914151225	Zm00001d023469
Zm00001d007141_T002	0.9946911	0.310707481	Zm00001d007141
Zm00001d007141_T003	0.146312	-0.712803769	Zm00001d007141
Zm00001d007141_T001	0.7336193	-0.260741335	Zm00001d007141
Zm00001d017540_T001	0.6338908	-0.605592077	Zm00001d017540
Zm00001d017765_T001	0.07220675	-1.3951914	Zm00001d017765
Zm00001d017765_T003	0.6863856	-0.177803541	Zm00001d017765
Zm00001d017765_T002	0.9862402	0.240850203	Zm00001d017765
Zm00001d048195_T001	0.4898707	-0.761427066	Zm00001d048195
Zm00001d038047_T001	0.9404125	0.284454699	Zm00001d038047
Zm00001d052423_T002	0.9914326	0.200836761	Zm00001d052423
Zm00001d040561_T001	0.7928239	-0.503972639	Zm00001d040561
Zm00001d052864_T001	0.9766874	-0.160072934	Zm00001d052864
Zm00001d018788_T001	0.2619882	1.464152931	Zm00001d018788
Zm00001d012806_T002	0.9451369	0.081448916	Zm00001d012806
Zm00001d003259_T011	0.9252088	0.205604082	Zm00001d003259
Zm00001d003259_T003	0.9493302	0.668662004	Zm00001d003259
Zm00001d003259_T013	0.9619687	-0.082936841	Zm00001d003259
Zm00001d052792_T001	0.91922	0.772628019	Zm00001d052792
Zm00001d027428_T001	0.6231219	-0.442581756	Zm00001d027428
Zm00001d017505_T001	7.60E-05	1.057133242	Zm00001d017505
Zm00001d017505_T014	0.1197922	1.996598963	Zm00001d017505
Zm00001d017505_T009	0.001647574	2.636351671	Zm00001d017505
Zm00001d017505_T039	0.7016389	-0.237505137	Zm00001d017505

Zm00001d017505_T006	3.13E-07	1.259991559	Zm00001d017505
Zm00001d017505_T021	0.6925804	-0.234422773	Zm00001d017505
Zm00001d017505_T023	0.9867452	0.094785145	Zm00001d017505
Zm00001d017505_T012	0.02203454	0.544672476	Zm00001d017505
Zm00001d017505_T017	0.9690782	0.120716827	Zm00001d017505
Zm00001d017505_T015	0.5377194	0.670553543	Zm00001d017505
Zm00001d044418_T001	0.8353818	-0.112192832	Zm00001d044418
Zm00001d023943_T007	0.9048001	1.617328605	Zm00001d023943
Zm00001d023943_T010	0.9825014	-0.083328331	Zm00001d023943
Zm00001d023943_T002	0.9228108	0.335717654	Zm00001d023943
Zm00001d023943_T005	0.7439474	-0.095503938	Zm00001d023943
Zm00001d023943_T009	0.9129863	-0.044644341	Zm00001d023943
Zm00001d013013_T003	0.9907928	0.116532689	Zm00001d013013
Zm00001d008502_T001	0.9361945	-0.006718367	Zm00001d008502
Zm00001d004396_T023	0.907891	-0.112041267	Zm00001d004396
Zm00001d004396_T002	0.8704891	0.808375376	Zm00001d004396
Zm00001d004396_T014	0.7771624	0.480231971	Zm00001d004396
Zm00001d004396_T016	0.000306523	2.697358866	Zm00001d004396
Zm00001d004396_T029	0.8987811	0.133498247	Zm00001d004396
Zm00001d004396_T021	2.95E-09	0.863829688	Zm00001d004396
Zm00001d047763_T001	0.9410845	0.65540548	Zm00001d047763
Zm00001d043220_T001	0.6662471	-0.791389944	Zm00001d043220
Zm00001d035512_T002	0.9607112	0.674772784	Zm00001d035512
Zm00001d035512_T003	0.3098034	-0.876687528	Zm00001d035512
Zm00001d035512_T004	0.5882388	-0.893969332	Zm00001d035512
Zm00001d035512_T001	0.9865262	0.608436032	Zm00001d035512
Zm00001d005403_T004	0.53504	-0.414975124	Zm00001d005403
Zm00001d005403_T001	0.90276	0.751443716	Zm00001d005403
Zm00001d005403_T006	0.6769189	-0.695536222	Zm00001d005403
Zm00001d053298_T001	0.9717919	0.5535256	Zm00001d053298
Zm00001d025908_T001	0.9760472	0.084129792	Zm00001d025908
Zm00001d039923_T001	0.9979568	0.181240645	Zm00001d039923
Zm00001d025131_T003	0.9050121	0.541974011	Zm00001d025131
Zm00001d025131_T001	1	0.348837843	Zm00001d025131
Zm00001d031745_T001	0.9368844	-0.024416874	Zm00001d031745
Zm00001d021951_T001	0.9543425	0.113634642	Zm00001d021951
Zm00001d048474_T004	0.9956182	0.175576722	Zm00001d048474
Zm00001d048474_T001	0.7192872	-0.396440798	Zm00001d048474
Zm00001d018392_T006	0.9766874	0.150840943	Zm00001d018392
Zm00001d018392_T002	0.5695353	1.009772182	Zm00001d018392
Zm00001d049095_T002	0.3301595	0.748600167	Zm00001d049095
Zm00001d049095_T004	0.9040512	0.94098306	Zm00001d049095
Zm00001d049095_T007	0.9455514	1.200156907	Zm00001d049095
Zm00001d017544_T002	0.9804228	0.024178299	Zm00001d017544
Zm00001d017544_T003	0.956479	0.134718933	Zm00001d017544
Zm00001d032508_T001	0.986738	0.161658529	Zm00001d032508
Zm00001d030532_T001	0.8412913	-0.232131874	Zm00001d030532
Zm00001d030532_T002	0.8633306	-0.051841601	Zm00001d030532
Zm00001d038121_T002	0.9506104	0.056611577	Zm00001d038121
Zm00001d038121_T001	1	0.200099601	Zm00001d038121
Zm00001d003774_T003	0.9668789	0.495880556	Zm00001d003774
Zm00001d003774_T004	0.9863483	0.114669414	Zm00001d003774
Zm00001d041569_T009	0.006076389	-2.119654218	Zm00001d041569
Zm00001d041569_T005	0.930571	2.099106726	Zm00001d041569

Zm00001d041569_T003	0.9732421	0.408516525	Zm00001d041569
Zm00001d041569_T006	0.6271198	-0.822812185	Zm00001d041569
Zm00001d041569_T001	0.9468268	-0.100385189	Zm00001d041569
Zm00001d037867_T004	2.71E-05	0.660554217	Zm00001d037867
Zm00001d037867_T008	0.3865855	0.755179214	Zm00001d037867
Zm00001d037867_T011	0.4451552	0.356755534	Zm00001d037867
Zm00001d037867_T002	0.004389276	1.20210402	Zm00001d037867
Zm00001d037867_T007	0.001280491	2.15281067	Zm00001d037867
Zm00001d037867_T001	0.2135786	-1.901026119	Zm00001d037867
Zm00001d037867_T026	0.003000573	1.759950593	Zm00001d037867
Zm00001d037867_T005	0.04832468	-0.539810798	Zm00001d037867
Zm00001d009016_T002	0.754061	-0.30228224	Zm00001d009016
Zm00001d046767_T003	0.7439594	-0.307883973	Zm00001d046767
Zm00001d046767_T001	0.3372969	-0.980759539	Zm00001d046767
Zm00001d046767_T004	0.6250123	-0.596301724	Zm00001d046767
Zm00001d046767_T005	0.003343235	-1.878237561	Zm00001d046767
Zm00001d004465_T001	0.9621927	-0.112330817	Zm00001d004465
Zm00001d004465_T002	0.8262962	0.438633354	Zm00001d004465
Zm00001d008399_T001	0.8342677	-0.141234646	Zm00001d008399
Zm00001d043134_T006	1	0.100177353	Zm00001d043134
Zm00001d043134_T005	1	0.30606201	Zm00001d043134
Zm00001d043134_T003	0.9543237	0.53782141	Zm00001d043134
Zm00001d029973_T001	0.8574951	-0.153429428	Zm00001d029973
Zm00001d026391_T004	0.7103053	-0.355112649	Zm00001d026391
Zm00001d026391_T001	0.9942835	0.074356826	Zm00001d026391
Zm00001d026391_T002	0.8759538	-0.287914358	Zm00001d026391
Zm00001d006250_T001	0.8509137	-0.222219441	Zm00001d006250
Zm00001d017347_T001	0.9429712	-0.114618163	Zm00001d017347
Zm00001d051842_T001	0.9537113	0.010894842	Zm00001d051842
Zm00001d031204_T003	0.8195759	-0.319271059	Zm00001d031204
Zm00001d031204_T002	0.7825891	-0.361600764	Zm00001d031204
Zm00001d038558_T003	0.9763286	-0.130602516	Zm00001d038558
Zm00001d038558_T002	0.6171076	-0.622474516	Zm00001d038558
Zm00001d038558_T001	0.9654566	0.281379353	Zm00001d038558
Zm00001d008621_T001	0.754061	-0.22157128	Zm00001d008621
Zm00001d031833_T001	0.02561293	2.55889543	Zm00001d031833
Zm00001d022549_T001	0.9612081	0.418892869	Zm00001d022549
Zm00001d021684_T003	0.7997379	-0.117952239	Zm00001d021684
Zm00001d021684_T001	0.9442599	0.080119482	Zm00001d021684
Zm00001d017476_T001	0.9792577	0.218549192	Zm00001d017476
Zm00001d007232_T001	0.9902022	0.24921673	Zm00001d007232
Zm00001d002369_T003	1	0.348899676	Zm00001d002369
Zm00001d002369_T002	0.9252465	0.655896046	Zm00001d002369
Zm00001d032190_T010	0.002796955	-1.754717514	Zm00001d032190
Zm00001d032190_T007	0.9428901	0.660423053	Zm00001d032190
Zm00001d032190_T008	0.3254084	0.799764615	Zm00001d032190
Zm00001d032190_T009	0.6217057	1.081417788	Zm00001d032190
Zm00001d032190_T003	0.741153	0.953782777	Zm00001d032190
Zm00001d026354_T001	0.9468268	-0.240989011	Zm00001d026354
Zm00001d028026_T003	0.8412913	-0.148613024	Zm00001d028026
Zm00001d053659_T001	0.277679	0.880885554	Zm00001d053659
Zm00001d030426_T004	1	0.13998047	Zm00001d030426
Zm00001d036448_T013	0.5641023	1.281291812	Zm00001d036448
Zm00001d036448_T014	0.7640001	1.105717659	Zm00001d036448

Zm00001d036448_T015	0.5954619	0.450770359	Zm00001d036448
Zm00001d036448_T010	0.8643636	-0.21118006	Zm00001d036448
Zm00001d036448_T001	3.88E-10	-1.516421568	Zm00001d036448
Zm00001d036448_T004	0.803912	0.24199464	Zm00001d036448
Zm00001d002426_T001	0.9227526	-0.029649759	Zm00001d002426
Zm00001d041515_T005	0.9252088	-0.032977835	Zm00001d041515
Zm00001d041515_T003	0.006223984	0.470107024	Zm00001d041515
Zm00001d041515_T001	0.9781672	0.082156916	Zm00001d041515
Zm00001d044501_T001	0.9469426	-0.085219294	Zm00001d044501
Zm00001d028221_T001	0.8847217	-0.085234864	Zm00001d028221
Zm00001d026165_T004	0.7332529	-0.180875934	Zm00001d026165
Zm00001d026165_T006	0.7253704	-0.663499279	Zm00001d026165
Zm00001d026165_T001	0.7594555	1.041831866	Zm00001d026165
Zm00001d028676_T001	0.7697196	0.889900203	Zm00001d028676
Zm00001d014771_T001	0.971204	0.594534657	Zm00001d014771
Zm00001d012888_T002	0.9791729	0.053431416	Zm00001d012888
Zm00001d012888_T001	0.8641671	-0.398662865	Zm00001d012888
Zm00001d009926_T006	0.9659036	0.097908205	Zm00001d009926
Zm00001d009926_T003	0.9792574	0.489212023	Zm00001d009926
Zm00001d014091_T001	0.9599154	0.019235592	Zm00001d014091
Zm00001d033905_T001	0.05163354	-1.476668953	Zm00001d033905
Zm00001d042146_T002	0.3125489	1.648965472	Zm00001d042146
Zm00001d042146_T004	3.15E-05	1.950849503	Zm00001d042146
Zm00001d042146_T001	6.19E-06	1.223173468	Zm00001d042146
Zm00001d042146_T006	0.9702493	0.122237609	Zm00001d042146
Zm00001d042146_T005	0.1555784	1.951533953	Zm00001d042146
Zm00001d041852_T002	0.9954444	0.528012003	Zm00001d041852
Zm00001d041852_T009	0.8993399	-0.400443805	Zm00001d041852
Zm00001d041852_T005	0.9913523	0.551460752	Zm00001d041852
Zm00001d041852_T006	0.9573434	0.280513732	Zm00001d041852
Zm00001d041852_T007	0.9825858	-0.172105314	Zm00001d041852
Zm00001d049737_T001	0.8021989	-0.226389082	Zm00001d049737
Zm00001d045029_T002	0.9580985	-0.092828018	Zm00001d045029
Zm00001d028569_T009	0.9252088	0.466735383	Zm00001d028569
Zm00001d028569_T007	0.9040872	0.518363869	Zm00001d028569
Zm00001d028569_T004	1.12E-08	1.592107984	Zm00001d028569
Zm00001d028569_T003	3.15E-08	-1.240806935	Zm00001d028569
Zm00001d028569_T005	0.5698311	1.297974182	Zm00001d028569
Zm00001d028569_T008	0.888502	-0.200671349	Zm00001d028569
Zm00001d028569_T017	0.1892576	0.967421324	Zm00001d028569
Zm00001d020978_T013	0.9766874	0.427509135	Zm00001d020978
Zm00001d020978_T031	0.910614	0.213829488	Zm00001d020978
Zm00001d014226_T001	0.1562235	-0.783641716	Zm00001d014226
Zm00001d044452_T003	0.9404125	-0.032573644	Zm00001d044452
Zm00001d044452_T002	0.4578502	0.565143594	Zm00001d044452
Zm00001d045647_T002	0.2205801	1.699101485	Zm00001d045647
Zm00001d045647_T001	0.9228865	0.256367834	Zm00001d045647
Zm00001d018711_T001	0.9981361	0.404681088	Zm00001d018711
Zm00001d012580_T001	0.4849772	-0.585227649	Zm00001d012580
Zm00001d006807_T001	0.9822138	0.107285063	Zm00001d006807
Zm00001d014472_T002	0.7369802	0.848054399	Zm00001d014472
Zm00001d014472_T003	0.9702242	0.581550587	Zm00001d014472
Zm00001d014472_T008	0.8494782	0.750989163	Zm00001d014472
Zm00001d014472_T004	0.7751061	-0.17918019	Zm00001d014472

Zm00001d014472_T001	0.5269468	0.226417264	Zm00001d014472
Zm00001d016271_T001	0.1296685	-1.547502969	Zm00001d016271
Zm00001d005368_T003	1	0.387995567	Zm00001d005368
Zm00001d005368_T001	0.891216	0.292595221	Zm00001d005368
Zm00001d036003_T001	3.36E-08	4.220881606	Zm00001d036003
Zm00001d032554_T001	0.9822168	0.46591542	Zm00001d032554
Zm00001d036602_T002	0.7626242	0.977327308	Zm00001d036602
Zm00001d036602_T001	0.9979568	0.393884953	Zm00001d036602
Zm00001d040392_T001	1	0.195918616	Zm00001d040392
Zm00001d018620_T001	0.174924	-1.36820425	Zm00001d018620
Zm00001d032501_T002	0.4165752	1.478548378	Zm00001d032501
Zm00001d032501_T001	0.8093695	0.779940811	Zm00001d032501
Zm00001d041759_T005	0.5443214	-0.704459393	Zm00001d041759
Zm00001d041759_T001	0.9579685	0.595855452	Zm00001d041759
Zm00001d021613_T001	0.8647916	-0.086477631	Zm00001d021613
Zm00001d021677_T001	0.01606913	2.179295942	Zm00001d021677
Zm00001d018484_T001	0.9062902	0.616527404	Zm00001d018484
Zm00001d018484_T002	0.9724404	0.280565453	Zm00001d018484
Zm00001d018484_T005	0.9091907	-0.188463771	Zm00001d018484
Zm00001d018484_T004	0.9556568	0.012408731	Zm00001d018484
Zm00001d018484_T009	0.8250026	0.8097728	Zm00001d018484
Zm00001d012732_T001	0.8142704	-0.320690189	Zm00001d012732
Zm00001d047937_T001	0.7051648	1.081798121	Zm00001d047937
Zm00001d033335_T001	0.953881	0.32669074	Zm00001d033335
Zm00001d050383_T001	1	0.380054444	Zm00001d050383
Zm00001d043392_T016	1.65E-09	-2.036705007	Zm00001d043392
Zm00001d043392_T009	8.64E-05	0.363507753	Zm00001d043392
Zm00001d043392_T002	0.02334998	1.014283418	Zm00001d043392
Zm00001d043392_T006	0.6894164	0.80476302	Zm00001d043392
Zm00001d043392_T004	0.0169289	1.709750403	Zm00001d043392
Zm00001d043392_T013	0.9167538	0.751611318	Zm00001d043392
Zm00001d038337_T001	0.9303773	0.665035313	Zm00001d038337
Zm00001d038337_T003	0.7778639	0.85492401	Zm00001d038337
Zm00001d016582_T001	0.9466261	-0.330142803	Zm00001d016582
Zm00001d018086_T001	0.9745777	0.017222613	Zm00001d018086
Zm00001d026020_T002	0.9814175	0.334455656	Zm00001d026020
Zm00001d047187_T001	0.9213679	-0.194862858	Zm00001d047187
Zm00001d029424_T001	0.9422098	-0.17957248	Zm00001d029424
Zm00001d028286_T001	0.5929798	-0.523013749	Zm00001d028286
Zm00001d030990_T001	0.318421	-0.737756463	Zm00001d030990
Zm00001d038165_T003	0.6604543	1.269994348	Zm00001d038165
Zm00001d038165_T002	0.9771927	0.114530402	Zm00001d038165
Zm00001d051507_T005	0.289786	1.109988543	Zm00001d051507
Zm00001d051507_T001	0.6201695	-0.353637499	Zm00001d051507
Zm00001d051507_T028	0.9573434	0.051590124	Zm00001d051507
Zm00001d051507_T011	0.8585804	-0.050788814	Zm00001d051507
Zm00001d051507_T003	0.90276	-0.015425359	Zm00001d051507
Zm00001d051507_T037	0.07023397	1.014755757	Zm00001d051507
Zm00001d051507_T052	0.00014851	0.476759585	Zm00001d051507
Zm00001d000357_T010	0.000477176	1.144770998	Zm00001d000357
Zm00001d000357_T011	0.2525169	1.490550414	Zm00001d000357
Zm00001d000357_T009	0.000512822	1.07175116	Zm00001d000357
Zm00001d000357_T003	0.02147519	2.385530855	Zm00001d000357
Zm00001d046186_T001	0.8994124	-0.114115853	Zm00001d046186

Zm00001d003556_T002	0.08686142	1.070029516	Zm00001d003556
Zm00001d003556_T001	0.8987811	-0.018028171	Zm00001d003556
Zm00001d003556_T006	0.9252088	0.168725961	Zm00001d003556
Zm00001d045953_T013	0.000190083	0.918955122	Zm00001d045953
Zm00001d045953_T016	0.03949883	1.003117868	Zm00001d045953
Zm00001d045953_T003	0.7796003	-0.212770658	Zm00001d045953
Zm00001d002850_T001	0.9999526	0.126776649	Zm00001d002850
Zm00001d023417_T001	0.4137215	-0.74584087	Zm00001d023417
Zm00001d015988_T002	0.8412913	0.405769656	Zm00001d015988
Zm00001d015988_T003	0.9696556	0.052856022	Zm00001d015988
Zm00001d032776_T001	0.5279284	1.497615377	Zm00001d032776
Zm00001d032776_T003	0.6187525	-1.231479845	Zm00001d032776
Zm00001d032776_T002	0.4137646	1.103088846	Zm00001d032776
Zm00001d032776_T004	0.9463326	0.797032895	Zm00001d032776
Zm00001d026120_T001	0.8873974	-0.12187409	Zm00001d026120
Zm00001d028208_T018	0.9690925	0.602670741	Zm00001d028208
Zm00001d028208_T031	0.9781672	0.258924829	Zm00001d028208
Zm00001d028208_T008	0.5005017	-0.340726901	Zm00001d028208
Zm00001d028208_T001	0.7418111	1.069835596	Zm00001d028208
Zm00001d028208_T013	0.1375053	0.954692142	Zm00001d028208
Zm00001d028208_T041	0.9854536	0.277319967	Zm00001d028208
Zm00001d028208_T007	0.8498341	0.464315356	Zm00001d028208
Zm00001d028208_T009	0.8412913	-0.122980771	Zm00001d028208
Zm00001d034181_T001	0.8224736	-0.158137393	Zm00001d034181
Zm00001d048892_T001	0.9237037	-0.110837496	Zm00001d048892
Zm00001d044244_T001	0.4698008	-1.067443152	Zm00001d044244
Zm00001d010168_T001	0.3335948	1.281346562	Zm00001d010168
Zm00001d012325_T001	1	0.23714237	Zm00001d012325
Zm00001d007079_T001	0.8967568	-0.128040648	Zm00001d007079
Zm00001d043666_T001	0.9682673	0.172758091	Zm00001d043666
Zm00001d006885_T001	0.9311027	0.017426786	Zm00001d006885
Zm00001d006885_T002	0.6746512	1.107273367	Zm00001d006885
Zm00001d031852_T001	0.7378574	-0.757924026	Zm00001d031852
Zm00001d049127_T001	0.9341847	0.630092602	Zm00001d049127
Zm00001d029707_T001	0.01144665	-1.781760737	Zm00001d029707
Zm00001d037961_T008	0.002863375	1.51489413	Zm00001d037961
Zm00001d037961_T012	0.7688599	-1.904946317	Zm00001d037961
Zm00001d037961_T004	0.9745777	0.136383538	Zm00001d037961
Zm00001d037961_T011	0.08092203	3.186891297	Zm00001d037961
Zm00001d033634_T001	0.6961049	-0.640103413	Zm00001d033634
Zm00001d029164_T001	0.0161978	-1.298112943	Zm00001d029164
Zm00001d030999_T001	0.5431596	-0.621226011	Zm00001d030999
Zm00001d030999_T002	0.816571	-0.172903888	Zm00001d030999
Zm00001d001993_T001	0.01910192	1.70081013	Zm00001d001993
Zm00001d001993_T002	0.1014575	1.114197809	Zm00001d001993
Zm00001d004898_T005	0.9563257	-0.028391703	Zm00001d004898
Zm00001d004898_T012	0.4479563	1.277621907	Zm00001d004898
Zm00001d004898_T006	0.98291	-0.026802447	Zm00001d004898
Zm00001d004898_T004	0.8050292	1.042823666	Zm00001d004898
Zm00001d004898_T008	0.1708958	1.430308656	Zm00001d004898
Zm00001d004898_T001	0.01220849	-1.574277897	Zm00001d004898
Zm00001d004898_T003	0.5006087	0.457641386	Zm00001d004898
Zm00001d022643_T001	0.4627153	-0.672717783	Zm00001d022643
Zm00001d003730_T002	0.715186	-0.467735224	Zm00001d003730

Zm00001d003730_T003	1	1.523756117	Zm00001d003730
Zm00001d003730_T004	0.880737	1.813738898	Zm00001d003730
Zm00001d003730_T001	0.8453901	-0.304083985	Zm00001d003730
Zm00001d046126_T001	0.284192	-0.757023164	Zm00001d046126
Zm00001d038538_T001	0.8857953	0.738515733	Zm00001d038538
Zm00001d028084_T007	0.6127874	0.214807143	Zm00001d028084
Zm00001d028084_T005	1.40E-10	1.439941479	Zm00001d028084
Zm00001d028084_T003	0.9842563	0.174479822	Zm00001d028084
Zm00001d028084_T006	0.003015693	0.820106228	Zm00001d028084
Zm00001d028084_T013	0.4332539	-0.661074506	Zm00001d028084
Zm00001d053308_T002	0.1122861	1.305480475	Zm00001d053308
Zm00001d053308_T011	0.9859755	0.34222197	Zm00001d053308
Zm00001d053308_T001	0.9368195	0.745185536	Zm00001d053308
Zm00001d053308_T004	0.9817074	0.48622876	Zm00001d053308
Zm00001d053308_T010	0.1487473	1.826780066	Zm00001d053308
Zm00001d003190_T001	0.5196622	-0.78525604	Zm00001d003190
Zm00001d019269_T001	0.5257585	-0.551450452	Zm00001d019269
Zm00001d011775_T001	0.9819415	0.231394464	Zm00001d011775
Zm00001d011775_T004	0.9862402	0.197762675	Zm00001d011775
Zm00001d051893_T001	0.9446484	0.082162944	Zm00001d051893
Zm00001d051893_T005	0.7454635	-0.159786408	Zm00001d051893
Zm00001d035989_T001	0.3978642	1.368838735	Zm00001d035989
Zm00001d023529_T001	0.957768	0.35681669	Zm00001d023529
Zm00001d014679_T001	0.7213008	-0.827961256	Zm00001d014679
Zm00001d040214_T001	0.4836377	-0.334483697	Zm00001d040214
Zm00001d018278_T001	0.4038198	1.485499979	Zm00001d018278
Zm00001d043944_T001	0.6044465	-0.602748043	Zm00001d043944
Zm00001d020416_T004	1	0.112570762	Zm00001d020416
Zm00001d014269_T001	0.9562711	-0.445319651	Zm00001d014269
Zm00001d006176_T001	1	0.341709416	Zm00001d006176
Zm00001d011680_T001	0.9907928	0.33567451	Zm00001d011680
Zm00001d011680_T003	0.9779656	0.087861715	Zm00001d011680
Zm00001d006183_T001	0.9695969	0.120930132	Zm00001d006183
Zm00001d035331_T001	0.750706	-0.504411543	Zm00001d035331
Zm00001d018727_T001	0.6236903	-0.765839917	Zm00001d018727
Zm00001d025767_T001	0.8641671	-0.050181875	Zm00001d025767
Zm00001d025767_T002	0.8984709	-0.076234699	Zm00001d025767
Zm00001d012768_T002	0.7414776	-0.696274155	Zm00001d012768
Zm00001d034680_T001	0.5210845	-0.893378392	Zm00001d034680
Zm00001d005458_T003	0.5112002	0.736908638	Zm00001d005458
Zm00001d024784_T001	0.7513713	-0.340638667	Zm00001d024784
Zm00001d053133_T001	0.9524127	0.004438306	Zm00001d053133
Zm00001d021878_T007	0.8842271	-0.413101633	Zm00001d021878
Zm00001d021878_T003	0.923446	0.770637015	Zm00001d021878
Zm00001d021878_T005	0.5537579	0.916192205	Zm00001d021878
Zm00001d027385_T002	0.9804228	0.009186976	Zm00001d027385
Zm00001d027385_T001	0.8450197	-0.306423377	Zm00001d027385
Zm00001d023698_T003	0.9745777	0.49301944	Zm00001d023698
Zm00001d023698_T002	0.9967884	0.282262528	Zm00001d023698
Zm00001d011211_T001	0.9997235	0.349270056	Zm00001d011211
Zm00001d051268_T009	0.174228	0.797639863	Zm00001d051268
Zm00001d051268_T007	0.9016285	1.251617089	Zm00001d051268
Zm00001d051268_T004	0.9975882	0.583733572	Zm00001d051268
Zm00001d051268_T002	0.6201695	-0.103354057	Zm00001d051268

Zm00001d029321_T001	0.9902022	0.250412696	Zm00001d029321
Zm00001d017889_T001	0.9055179	-0.080710635	Zm00001d017889
Zm00001d042535_T001	0.8548577	-0.201357673	Zm00001d042535
Zm00001d022151_T001	0.4714737	-0.651898091	Zm00001d022151
Zm00001d023576_T001	0.7293985	0.03096645	Zm00001d023576
Zm00001d023576_T007	0.6993528	-0.436185781	Zm00001d023576
Zm00001d023576_T005	0.5843637	1.608110708	Zm00001d023576
Zm00001d023576_T003	0.9139882	0.806938811	Zm00001d023576
Zm00001d023576_T009	0.000234904	2.29022225	Zm00001d023576
Zm00001d024514_T001	0.7771624	0.618885049	Zm00001d024514
Zm00001d037028_T001	0.9563232	-0.204369506	Zm00001d037028
Zm00001d048214_T001	0.04476943	-1.736049529	Zm00001d048214
Zm00001d005356_T001	0.3742618	1.470877011	Zm00001d005356
Zm00001d037100_T119	0.8645432	0.844420616	Zm00001d037100
Zm00001d037100_T114	1	0.043185795	Zm00001d037100
Zm00001d037100_T109	2.14E-07	0.808814739	Zm00001d037100
Zm00001d037100_T008	0.9671056	-0.047491943	Zm00001d037100
Zm00001d037100_T048	0.225505	0.525706045	Zm00001d037100
Zm00001d037100_T110	1.27E-08	0.732445138	Zm00001d037100
Zm00001d037100_T014	0.772948	-0.125994538	Zm00001d037100
Zm00001d037100_T124	0.7912955	-0.31537726	Zm00001d037100
Zm00001d037100_T125	0.5369152	0.530233153	Zm00001d037100
Zm00001d007300_T005	0.816571	0.810080781	Zm00001d007300
Zm00001d007300_T011	0.006621309	-1.12242355	Zm00001d007300
Zm00001d007300_T008	0.09496973	0.382505292	Zm00001d007300
Zm00001d017606_T001	0.9264969	0.431159756	Zm00001d017606
Zm00001d002359_T005	0.481901	0.753372782	Zm00001d002359
Zm00001d002359_T001	0.09863512	2.064336376	Zm00001d002359
Zm00001d002359_T007	0.01118874	-1.418339122	Zm00001d002359
Zm00001d002359_T008	1.10E-13	-2.874040076	Zm00001d002359
Zm00001d034688_T001	0.7853478	-0.591506661	Zm00001d034688
Zm00001d011092_T002	0.9564329	-0.16048987	Zm00001d011092
Zm00001d011092_T001	0.9310389	-0.205770423	Zm00001d011092
Zm00001d013430_T001	0.9109605	0.004105572	Zm00001d013430
Zm00001d038173_T003	0.4860327	0.91693234	Zm00001d038173
Zm00001d038173_T002	0.7194569	0.790302985	Zm00001d038173
Zm00001d038173_T001	0.8514567	0.647681194	Zm00001d038173
Zm00001d007181_T001	0.9931431	0.14500291	Zm00001d007181
Zm00001d053747_T004	0.9763597	0.543865786	Zm00001d053747
Zm00001d053747_T003	0.7425238	0.74394497	Zm00001d053747
Zm00001d004073_T003	0.8075512	0.966697158	Zm00001d004073
Zm00001d038126_T001	0.9740325	0.069342795	Zm00001d038126
Zm00001d014795_T001	0.8817916	-0.172950301	Zm00001d014795
Zm00001d014795_T002	0.7989126	-0.684368906	Zm00001d014795
Zm00001d002246_T004	1	0.153260539	Zm00001d002246
Zm00001d002246_T002	0.9687311	0.598160115	Zm00001d002246
Zm00001d043841_T005	0.457214	-1.055380143	Zm00001d043841
Zm00001d043841_T010	0.9988864	0.318482529	Zm00001d043841
Zm00001d043841_T007	0.1272455	0.490635165	Zm00001d043841
Zm00001d043841_T001	0.328751	-0.209473985	Zm00001d043841
Zm00001d043841_T002	0.6028805	1.000916313	Zm00001d043841
Zm00001d043841_T006	0.5283591	0.352088347	Zm00001d043841
Zm00001d047759_T003	0.9375274	0.495792164	Zm00001d047759
Zm00001d002282_T001	0.9476864	0.093119248	Zm00001d002282

Zm00001d030989_T001	1	0.411504843	Zm00001d030989
Zm00001d040641_T005	0.9690925	0.584784047	Zm00001d040641
Zm00001d048541_T003	0.8693754	0.019779727	Zm00001d048541
Zm00001d048541_T010	0.08806919	0.328698445	Zm00001d048541
Zm00001d048541_T048	0.9061737	0.035511609	Zm00001d048541
Zm00001d048541_T006	0.5579874	1.200114972	Zm00001d048541
Zm00001d048541_T007	3.07E-05	1.528818458	Zm00001d048541
Zm00001d048541_T018	0.9232281	0.732608123	Zm00001d048541
Zm00001d030317_T001	0.7448423	0.83614463	Zm00001d030317
Zm00001d026405_T001	0.9970835	0.417520561	Zm00001d026405
Zm00001d028671_T002	0.2950499	0.683002729	Zm00001d028671
Zm00001d028671_T015	0.001874253	1.703272703	Zm00001d028671
Zm00001d028671_T005	0.9747606	0.579215425	Zm00001d028671
Zm00001d028671_T018	0.01028758	1.307297841	Zm00001d028671
Zm00001d028671_T001	0.7853827	0.257021041	Zm00001d028671
Zm00001d036351_T023	0.9296811	0.006087697	Zm00001d036351
Zm00001d036351_T216	0.1454721	1.548972143	Zm00001d036351
Zm00001d036351_T218	0.003093763	-2.188095524	Zm00001d036351
Zm00001d036351_T009	0.009186909	1.385735505	Zm00001d036351
Zm00001d036351_T188	0.9664736	0.249791365	Zm00001d036351
Zm00001d039272_T003	0.9419336	-0.007411153	Zm00001d039272
Zm00001d039272_T002	0.9180662	0.003060307	Zm00001d039272
Zm00001d028371_T001	0.4096848	-1.356856096	Zm00001d028371
Zm00001d032715_T001	0.5815344	1.074142291	Zm00001d032715
Zm00001d051203_T001	0.9404125	-0.011891595	Zm00001d051203
Zm00001d030867_T001	0.925875	0.523027845	Zm00001d030867
Zm00001d006834_T003	0.5652791	0.861611764	Zm00001d006834
Zm00001d006834_T001	0.8883561	-0.192450243	Zm00001d006834
Zm00001d044648_T007	0.8802125	1.147521886	Zm00001d044648
Zm00001d044648_T004	0.9631478	0.393358139	Zm00001d044648
Zm00001d044648_T006	0.8941695	1.069207323	Zm00001d044648
Zm00001d008793_T001	0.8997535	-0.244139253	Zm00001d008793
Zm00001d052329_T001	1	0.110056386	Zm00001d052329
Zm00001d052329_T003	0.9818386	0.311466635	Zm00001d052329
Zm00001d052329_T002	0.972985	0.117304497	Zm00001d052329
Zm00001d048970_T012	0.9666757	-0.078828741	Zm00001d048970
Zm00001d048970_T004	0.9950703	0.359349112	Zm00001d048970
Zm00001d048970_T011	0.02388832	0.486730949	Zm00001d048970
Zm00001d048970_T017	0.7194121	0.854968248	Zm00001d048970
Zm00001d048970_T016	0.9469426	-0.041855091	Zm00001d048970
Zm00001d048970_T014	0.9730261	0.106395579	Zm00001d048970
Zm00001d049493_T001	0.9786754	0.106055956	Zm00001d049493
Zm00001d019317_T002	0.5868947	0.453234507	Zm00001d019317
Zm00001d019317_T017	0.9377241	0.771940969	Zm00001d019317
Zm00001d019317_T004	0.1626016	1.026258854	Zm00001d019317
Zm00001d019317_T011	0.6035753	1.367386285	Zm00001d019317
Zm00001d019317_T010	0.8332116	1.086463019	Zm00001d019317
Zm00001d012289_T001	0.9609153	0.076442346	Zm00001d012289
Zm00001d003468_T001	1	0.424797	Zm00001d003468
Zm00001d014330_T051	0.7289978	-0.089243192	Zm00001d014330
Zm00001d014330_T019	0.4423489	0.147809737	Zm00001d014330
Zm00001d014330_T030	0.6182122	0.618650849	Zm00001d014330
Zm00001d014330_T001	0.9894615	0.47294676	Zm00001d014330
Zm00001d014330_T021	0.3676545	0.969824188	Zm00001d014330

Zm00001d014330_T037	0.437589	1.254551093	Zm00001d014330
Zm00001d014330_T023	0.9311499	0.157602721	Zm00001d014330
Zm00001d014330_T035	0.9283222	-0.106580714	Zm00001d014330
Zm00001d014330_T003	0.9575224	0.185782813	Zm00001d014330
Zm00001d014330_T053	0.007583227	0.636739243	Zm00001d014330
Zm00001d014330_T047	0.9228108	0.027092244	Zm00001d014330
Zm00001d014330_T009	0.9965998	0.271267876	Zm00001d014330
Zm00001d014330_T024	0.02025634	-0.567136497	Zm00001d014330
Zm00001d014330_T059	0.9327976	0.568582965	Zm00001d014330
Zm00001d014330_T028	0.90276	0.305436588	Zm00001d014330
Zm00001d017783_T003	0.9561521	0.587536592	Zm00001d017783
Zm00001d004383_T002	0.8280859	0.164798105	Zm00001d004383
Zm00001d004383_T001	0.9469426	0.079836632	Zm00001d004383
Zm00001d004383_T007	0.4647287	-0.639583438	Zm00001d004383
Zm00001d028712_T019	0.2698271	0.277287828	Zm00001d028712
Zm00001d028712_T013	0.9528476	0.610104591	Zm00001d028712
Zm00001d028712_T018	0.8906466	0.425727131	Zm00001d028712
Zm00001d028712_T011	1	0.126266396	Zm00001d028712
Zm00001d028712_T012	0.8595871	-0.100733452	Zm00001d028712
Zm00001d028712_T015	0.7329635	-0.30839158	Zm00001d028712
Zm00001d038089_T002	1	0.233916034	Zm00001d038089
Zm00001d038089_T001	0.4654262	1.103218255	Zm00001d038089
Zm00001d031872_T001	1	0.236909284	Zm00001d031872
Zm00001d038745_T007	0.169403	2.060168581	Zm00001d038745
Zm00001d038745_T002	0.1405321	-0.162902814	Zm00001d038745
Zm00001d038745_T001	0.2082029	1.458643477	Zm00001d038745
Zm00001d038745_T004	0.9704407	0.616595927	Zm00001d038745
Zm00001d013196_T001	0.9771927	0.545520662	Zm00001d013196
Zm00001d044540_T004	0.86843	0.82168393	Zm00001d044540
Zm00001d044540_T005	0.8612451	-0.153116228	Zm00001d044540
Zm00001d038092_T002	0.9867452	0.258733279	Zm00001d038092
Zm00001d015852_T006	0.9152076	0.509457649	Zm00001d015852
Zm00001d015852_T004	0.7957286	-0.134732494	Zm00001d015852
Zm00001d015852_T002	0.5147606	-0.377332623	Zm00001d015852
Zm00001d015852_T005	0.7429809	-0.326710717	Zm00001d015852
Zm00001d048703_T001	0.9561521	0.109691034	Zm00001d048703
Zm00001d029402_T001	0.7897201	-0.243301432	Zm00001d029402
Zm00001d016101_T003	0.7489032	0.612300839	Zm00001d016101
Zm00001d016101_T007	0.1057724	0.48747874	Zm00001d016101
Zm00001d017282_T001	0.9596377	0.608321826	Zm00001d017282
Zm00001d017282_T003	0.9946831	0.291281468	Zm00001d017282
Zm00001d020862_T001	0.9921225	0.386234831	Zm00001d020862
Zm00001d050251_T006	0.8226563	0.914594949	Zm00001d050251
Zm00001d050251_T004	0.8888255	0.322627375	Zm00001d050251
Zm00001d046855_T001	0.5455802	-0.652429097	Zm00001d046855
Zm00001d041343_T001	0.9984041	0.472802881	Zm00001d041343
Zm00001d041343_T002	0.7126069	-0.394632701	Zm00001d041343
Zm00001d052735_T015	0.7314608	-0.787956264	Zm00001d052735
Zm00001d052735_T011	0.1272455	0.561290527	Zm00001d052735
Zm00001d052735_T002	0.2673896	0.867941771	Zm00001d052735
Zm00001d052735_T012	0.9257238	0.256859429	Zm00001d052735
Zm00001d052735_T013	0.8912963	0.835151482	Zm00001d052735
Zm00001d052735_T001	0.577931	-0.524999298	Zm00001d052735
Zm00001d019233_T001	0.9975882	0.345617891	Zm00001d019233

Zm00001d004435_T002	0.9160053	0.381656454	Zm00001d004435
Zm00001d019361_T001	1	0.29514235	Zm00001d019361
Zm00001d030870_T001	0.6946859	0.809353721	Zm00001d030870
Zm00001d037540_T001	0.9979568	0.332836443	Zm00001d037540
Zm00001d007594_T001	0.96672	-0.024323201	Zm00001d007594
Zm00001d007594_T003	0.5627844	-0.205784262	Zm00001d007594
Zm00001d007594_T002	0.9867452	0.098634407	Zm00001d007594
Zm00001d033455_T001	0.0483012	-1.810069018	Zm00001d033455
Zm00001d013095_T001	0.8974665	-0.101261372	Zm00001d013095
Zm00001d024587_T001	0.9216552	0.048193036	Zm00001d024587
Zm00001d012712_T001	0.9887889	0.387435478	Zm00001d012712
Zm00001d009946_T001	0.9640775	0.431064633	Zm00001d009946
Zm00001d007555_T001	0.4972742	0.502438162	Zm00001d007555
Zm00001d038191_T001	0.6530894	-0.690253621	Zm00001d038191
Zm00001d050315_T001	0.9404125	0.104117251	Zm00001d050315
Zm00001d012529_T001	1	0.37843391	Zm00001d012529
Zm00001d053664_T001	1	0.360363194	Zm00001d053664
Zm00001d011596_T001	0.9404125	0.034599509	Zm00001d011596
Zm00001d039808_T001	0.5917805	0.824192316	Zm00001d039808
Zm00001d006883_T001	0.1983605	-1.288229721	Zm00001d006883
Zm00001d013456_T001	0.9817074	0.187064553	Zm00001d013456
Zm00001d002386_T001	0.2280003	-2.432269723	Zm00001d002386
Zm00001d002386_T003	0.9193648	0.709232766	Zm00001d002386
Zm00001d002386_T002	0.6967776	0.615426795	Zm00001d002386
Zm00001d037760_T007	0.4219823	0.537079821	Zm00001d037760
Zm00001d037760_T001	0.1411082	-0.295995479	Zm00001d037760
Zm00001d037760_T005	0.9514141	0.639517506	Zm00001d037760
Zm00001d002944_T001	0.983226	0.254132343	Zm00001d002944
Zm00001d054009_T001	0.9019144	-0.206048621	Zm00001d054009
Zm00001d026585_T006	0.9810886	0.438205835	Zm00001d026585
Zm00001d026585_T007	0.5494973	1.405998201	Zm00001d026585
Zm00001d026585_T001	0.02985073	-0.301242376	Zm00001d026585
Zm00001d026585_T010	0.05477047	1.009102374	Zm00001d026585
Zm00001d026585_T004	0.006779326	1.19628421	Zm00001d026585
Zm00001d026585_T002	0.8409792	0.97444659	Zm00001d026585
Zm00001d032643_T001	0.03529405	-1.967304317	Zm00001d032643
Zm00001d024454_T001	0.8002807	0.855889062	Zm00001d024454
Zm00001d051650_T001	0.9979568	0.431508375	Zm00001d051650
Zm00001d019577_T002	0.6577149	-0.663075426	Zm00001d019577
Zm00001d019577_T001	0.8158416	-0.504544959	Zm00001d019577
Zm00001d028303_T002	0.772948	-0.201865525	Zm00001d028303
Zm00001d024291_T004	0.7412102	-0.18298933	Zm00001d024291
Zm00001d024291_T006	0.3148171	-0.351843701	Zm00001d024291
Zm00001d034577_T003	0.2662346	-0.448238207	Zm00001d034577
Zm00001d034577_T004	0.9822138	0.414350636	Zm00001d034577
Zm00001d021519_T002	0.3648156	-1.299651069	Zm00001d021519
Zm00001d021519_T001	0.9230223	0.758034461	Zm00001d021519
Zm00001d028762_T011	0.9778436	0.179184172	Zm00001d028762
Zm00001d028762_T002	0.5128433	1.274470272	Zm00001d028762
Zm00001d028762_T010	0.05615106	0.472968599	Zm00001d028762
Zm00001d042158_T001	1	0.394187127	Zm00001d042158
Zm00001d042158_T007	1	0.562557336	Zm00001d042158
Zm00001d042158_T006	0.7167212	-0.066064199	Zm00001d042158
Zm00001d035102_T001	0.8031754	0.582728552	Zm00001d035102

Zm00001d034077_T001	0.7799762	-0.467401034	Zm00001d034077
Zm00001d051749_T002	0.462904	-0.9092105	Zm00001d051749
Zm00001d051749_T001	0.8478451	-0.183941461	Zm00001d051749
Zm00001d022200_T004	0.9744451	0.123368868	Zm00001d022200
Zm00001d022200_T002	0.9968176	0.380497915	Zm00001d022200
Zm00001d052961_T001	0.811177	0.314270639	Zm00001d052961
Zm00001d039377_T001	0.4503499	-0.978425038	Zm00001d039377
Zm00001d044339_T001	0.9451369	0.587067505	Zm00001d044339
Zm00001d044339_T006	0.6039618	-0.444607949	Zm00001d044339
Zm00001d044339_T002	0.9870466	0.293859496	Zm00001d044339
Zm00001d044339_T003	0.9701986	0.301630975	Zm00001d044339
Zm00001d012275_T001	0.9822138	0.449725506	Zm00001d012275
Zm00001d008288_T001	0.000281578	-4.38046484	Zm00001d008288
Zm00001d041987_T001	0.8894168	-0.116237455	Zm00001d041987
Zm00001d029342_T011	0.7526414	0.442923721	Zm00001d029342
Zm00001d029342_T007	0.9543425	0.127447583	Zm00001d029342
Zm00001d029342_T006	0.9813293	0.199628015	Zm00001d029342
Zm00001d029342_T013	0.06650348	0.372166725	Zm00001d029342
Zm00001d029342_T014	0.8158416	0.467170706	Zm00001d029342
Zm00001d029342_T004	1	0.28825587	Zm00001d029342
Zm00001d029342_T009	0.203661	0.791220722	Zm00001d029342
Zm00001d029342_T003	0.907938	-0.055405967	Zm00001d029342
Zm00001d006286_T001	0.5279144	1.41269563	Zm00001d006286
Zm00001d007801_T001	0.9717919	0.213636172	Zm00001d007801
Zm00001d018465_T003	0.2247419	0.298195249	Zm00001d018465
Zm00001d039007_T004	0.9508258	0.012827654	Zm00001d039007
Zm00001d039007_T005	0.3464158	-1.065736844	Zm00001d039007
Zm00001d039007_T001	0.9840918	0.078987343	Zm00001d039007
Zm00001d036760_T001	0.7434928	-0.410302023	Zm00001d036760
Zm00001d022597_T001	0.3343236	1.508422386	Zm00001d022597
Zm00001d048431_T001	0.941922	-0.125428859	Zm00001d048431
Zm00001d006607_T194	0.5097225	0.193813609	Zm00001d006607
Zm00001d006607_T246	0.961473	0.65223327	Zm00001d006607
Zm00001d006607_T250	0.6032587	-0.534734315	Zm00001d006607
Zm00001d006607_T110	0.9596377	0.194308724	Zm00001d006607
Zm00001d006607_T055	0.000146005	1.232079836	Zm00001d006607
Zm00001d006607_T085	0.08601829	0.399796402	Zm00001d006607
Zm00001d006607_T155	0.8470633	0.112002783	Zm00001d006607
Zm00001d006607_T203	0.002397973	0.869983626	Zm00001d006607
Zm00001d006607_T044	0.9613188	0.047588203	Zm00001d006607
Zm00001d006607_T210	0.961399	-0.023783468	Zm00001d006607
Zm00001d006607_T233	0.6235378	-0.310403098	Zm00001d006607
Zm00001d048552_T013	0.9176356	-0.007852539	Zm00001d048552
Zm00001d048552_T002	0.1155712	1.190449246	Zm00001d048552
Zm00001d048552_T001	0.8269623	0.513723985	Zm00001d048552
Zm00001d048552_T008	0.05001771	0.705438941	Zm00001d048552
Zm00001d042883_T003	0.2317185	0.604266403	Zm00001d042883
Zm00001d042883_T011	0.3355093	0.680764233	Zm00001d042883
Zm00001d042883_T010	0.9979568	0.239300448	Zm00001d042883
Zm00001d022174_T001	0.9173494	-0.121452272	Zm00001d022174
Zm00001d018029_T001	0.9817074	-0.056413057	Zm00001d018029
Zm00001d015698_T001	0.9956119	0.112163588	Zm00001d015698
Zm00001d053087_T001	0.587212	1.187162588	Zm00001d053087
Zm00001d043921_T001	0.8030992	0.903976009	Zm00001d043921

Zm00001d048402_T001	0.7828357	-0.44831129	Zm00001d048402
Zm00001d018668_T004	0.8847217	-0.533967668	Zm00001d018668
Zm00001d018668_T002	0.76221	-0.686700863	Zm00001d018668
Zm00001d016602_T002	0.9244812	0.785971416	Zm00001d016602
Zm00001d052310_T002	1	0.267006681	Zm00001d052310
Zm00001d052310_T001	0.9840918	0.104518753	Zm00001d052310
Zm00001d014335_T001	0.960712	0.43359449	Zm00001d014335
Zm00001d026706_T009	0.9845789	0.279857167	Zm00001d026706
Zm00001d026706_T005	0.6432444	-0.334220128	Zm00001d026706
Zm00001d026706_T001	0.6394238	-0.411921209	Zm00001d026706
Zm00001d026706_T011	0.04971279	2.858852846	Zm00001d026706
Zm00001d026706_T008	0.4588441	1.087942817	Zm00001d026706
Zm00001d034462_T001	0.6962193	-0.543078338	Zm00001d034462
Zm00001d013329_T001	0.7853478	-0.297966635	Zm00001d013329
Zm00001d042821_T001	0.698243	-0.418929908	Zm00001d042821
Zm00001d025012_T001	0.9791729	0.172135617	Zm00001d025012
Zm00001d025012_T020	0.01901268	2.216991076	Zm00001d025012
Zm00001d025012_T004	0.8377512	0.284558975	Zm00001d025012
Zm00001d025012_T031	0.9106973	-0.005693393	Zm00001d025012
Zm00001d025012_T003	0.06348214	1.795176923	Zm00001d025012
Zm00001d023820_T001	0.9666757	0.457140751	Zm00001d023820
Zm00001d022575_T001	0.5259724	1.362877567	Zm00001d022575
Zm00001d029185_T001	0.2319985	-1.280636979	Zm00001d029185
Zm00001d045445_T001	0.9969123	0.324348331	Zm00001d045445
Zm00001d045445_T002	0.1670357	-0.854451755	Zm00001d045445
Zm00001d034443_T002	0.9817074	0.201499039	Zm00001d034443
Zm00001d034443_T001	0.8888255	0.807860573	Zm00001d034443
Zm00001d021063_T001	1	-0.049543546	Zm00001d021063
Zm00001d016518_T001	0.8595871	-0.037240269	Zm00001d016518
Zm00001d002458_T001	0.8832294	-0.449264733	Zm00001d002458
Zm00001d030678_T002	0.8701192	-0.126489013	Zm00001d030678
Zm00001d030678_T001	0.8899623	0.754370838	Zm00001d030678
Zm00001d030678_T004	0.8087738	2.009483183	Zm00001d030678
Zm00001d030678_T003	5.63E-05	-1.498999157	Zm00001d030678
Zm00001d034784_T006	0.7568663	0.935643577	Zm00001d034784
Zm00001d034784_T010	0.685028	-0.579736526	Zm00001d034784
Zm00001d034784_T008	0.240891	1.562556624	Zm00001d034784
Zm00001d034784_T001	0.4521275	1.347416072	Zm00001d034784
Zm00001d034784_T009	0.4373578	0.531684852	Zm00001d034784
Zm00001d052527_T001	0.01956599	-2.041490924	Zm00001d052527
Zm00001d027312_T002	0.900661	0.519878631	Zm00001d027312
Zm00001d015932_T001	0.9887584	0.199355456	Zm00001d015932
Zm00001d047260_T001	0.103251	-1.608296548	Zm00001d047260
Zm00001d048026_T001	0.7011505	0.847730835	Zm00001d048026
Zm00001d048026_T002	0.9597485	0.495696355	Zm00001d048026
Zm00001d046996_T001	0.9979393	-0.041468337	Zm00001d046996
Zm00001d025152_T001	0.9640775	0.224062256	Zm00001d025152
Zm00001d007677_T001	0.9791066	0.584702001	Zm00001d007677
Zm00001d014183_T004	0.9942091	0.347806973	Zm00001d014183
Zm00001d014183_T008	0.9950761	0.385375179	Zm00001d014183
Zm00001d010217_T001	0.8935546	-0.255334584	Zm00001d010217
Zm00001d010710_T009	1	0.210604341	Zm00001d010710
Zm00001d010710_T004	0.9692664	0.613436891	Zm00001d010710
Zm00001d010710_T007	0.9887584	0.225631998	Zm00001d010710

Zm00001d010710_T008	0.007525957	1.528709044	Zm00001d010710
Zm00001d010710_T006	0.3536779	0.4327499	Zm00001d010710
Zm00001d015977_T001	0.283017	-1.002701926	Zm00001d015977
Zm00001d036829_T015	0.4237296	-0.607056428	Zm00001d036829
Zm00001d036829_T002	0.9377241	0.002581511	Zm00001d036829
Zm00001d036829_T001	0.9766874	0.52542334	Zm00001d036829
Zm00001d042259_T003	0.9988864	0.29284443	Zm00001d042259
Zm00001d042259_T004	0.9941329	0.118913084	Zm00001d042259
Zm00001d012244_T001	0.9927201	0.241181171	Zm00001d012244
Zm00001d040988_T001	0.9451281	0.548028885	Zm00001d040988
Zm00001d022107_T002	0.7680039	-0.490991435	Zm00001d022107
Zm00001d017788_T001	0.2494451	-0.594837618	Zm00001d017788
Zm00001d004689_T001	0.7626242	1.058837	Zm00001d004689
Zm00001d026452_T001	0.9483904	0.148605253	Zm00001d026452
Zm00001d022237_T001	0.7761738	-0.337975276	Zm00001d022237
Zm00001d027445_T004	0.2766427	0.475180117	Zm00001d027445
Zm00001d027445_T002	0.9442138	0.013407538	Zm00001d027445
Zm00001d020361_T001	0.9988954	-0.019761009	Zm00001d020361
Zm00001d034475_T001	0.768921	-0.472774508	Zm00001d034475
Zm00001d046663_T001	0.9441446	-0.201360827	Zm00001d046663
Zm00001d031344_T001	0.000339742	-3.245116962	Zm00001d031344
Zm00001d031344_T002	9.83E-11	-4.459464502	Zm00001d031344
Zm00001d003083_T004	0.8187452	0.254155367	Zm00001d003083
Zm00001d003083_T001	1	0.404954658	Zm00001d003083
Zm00001d025413_T003	0.9419829	0.04224146	Zm00001d025413
Zm00001d025413_T002	0.2135821	-0.674385455	Zm00001d025413
Zm00001d025413_T001	0.8580945	-0.395997325	Zm00001d025413
Zm00001d048721_T001	0.9561521	0.058505784	Zm00001d048721
Zm00001d025304_T004	0.9745777	0.081978108	Zm00001d025304
Zm00001d025304_T003	0.1315154	-1.154515526	Zm00001d025304
Zm00001d025304_T002	0.8917959	0.447149147	Zm00001d025304
Zm00001d035041_T002	0.925875	0.769207	Zm00001d035041
Zm00001d032073_T001	1	0.019067786	Zm00001d032073
Zm00001d038913_T001	0.2291495	-1.190704212	Zm00001d038913
Zm00001d032010_T008	0.803794	-0.16313915	Zm00001d032010
Zm00001d032010_T002	0.8581713	0.203262923	Zm00001d032010
Zm00001d032010_T003	0.606752	-0.197539655	Zm00001d032010
Zm00001d032010_T001	0.9744451	0.204734189	Zm00001d032010
Zm00001d003981_T001	0.002666247	3.243776826	Zm00001d003981
Zm00001d019148_T002	0.9690925	0.342246962	Zm00001d019148
Zm00001d019148_T004	0.3941547	1.122158504	Zm00001d019148
Zm00001d019148_T001	0.9902733	0.262651954	Zm00001d019148
Zm00001d003143_T002	0.9244812	0.433667614	Zm00001d003143
Zm00001d003143_T009	0.8987811	-0.396230818	Zm00001d003143
Zm00001d003143_T001	0.8032834	0.361614118	Zm00001d003143
Zm00001d003143_T004	0.2250035	-0.444928212	Zm00001d003143
Zm00001d003143_T006	0.8964117	0.488020702	Zm00001d003143
Zm00001d024904_T001	0.9563232	0.062263001	Zm00001d024904
Zm00001d015435_T020	0.01594071	1.118808899	Zm00001d015435
Zm00001d015435_T001	0.6182279	-0.301592422	Zm00001d015435
Zm00001d015435_T002	0.9969123	0.463397051	Zm00001d015435
Zm00001d005791_T001	0.3675057	-0.780977	Zm00001d005791
Zm00001d006408_T003	0.7648638	0.812965731	Zm00001d006408
Zm00001d006408_T002	1	0.314230524	Zm00001d006408

Zm00001d041871_T001	0.9596377	0.035043517	Zm00001d041871
Zm00001d041871_T002	0.9770538	0.544320186	Zm00001d041871
Zm00001d020605_T001	0.5844177	-1.011748645	Zm00001d020605
Zm00001d037787_T001	0.9747606	0.20526257	Zm00001d037787
Zm00001d031627_T003	0.7548788	0.334417901	Zm00001d031627
Zm00001d031627_T001	0.8616387	-0.318353721	Zm00001d031627
Zm00001d020687_T003	0.7945048	-0.143976053	Zm00001d020687
Zm00001d020687_T001	0.852305	0.914844587	Zm00001d020687
Zm00001d020687_T004	0.720853	-0.582299464	Zm00001d020687
Zm00001d001784_T001	0.9781672	0.247605559	Zm00001d001784
Zm00001d032300_T001	0.9878022	0.26260378	Zm00001d032300
Zm00001d016219_T001	0.8643636	0.683599438	Zm00001d016219
Zm00001d045655_T013	0.1794817	1.024209753	Zm00001d045655
Zm00001d045655_T004	0.4357601	-0.551947111	Zm00001d045655
Zm00001d017676_T001	0.9596377	0.130330146	Zm00001d017676
Zm00001d024675_T001	0.8125885	-0.45393619	Zm00001d024675
Zm00001d014385_T001	0.933055	0.057098499	Zm00001d014385
Zm00001d011963_T002	1	0.354162522	Zm00001d011963
Zm00001d011963_T001	0.3870314	0.351689931	Zm00001d011963
Zm00001d011963_T003	0.8084847	0.823511673	Zm00001d011963
Zm00001d012687_T001	0.9613849	0.240616191	Zm00001d012687
Zm00001d045312_T001	0.3111248	1.694772061	Zm00001d045312
Zm00001d045312_T003	0.5479918	1.136544112	Zm00001d045312
Zm00001d008799_T001	0.2064251	1.812546639	Zm00001d008799
Zm00001d034755_T001	0.8891117	0.48222368	Zm00001d034755
Zm00001d021400_T001	0.4030962	1.643594308	Zm00001d021400
Zm00001d021400_T002	0.935477	0.062778527	Zm00001d021400
Zm00001d050755_T002	0.9419096	0.665662435	Zm00001d050755
Zm00001d050755_T004	0.1092904	-1.995467044	Zm00001d050755
Zm00001d050755_T001	0.5166741	1.346280894	Zm00001d050755
Zm00001d050755_T006	0.4193179	1.028561071	Zm00001d050755
Zm00001d036237_T002	0.9965026	0.347725891	Zm00001d036237
Zm00001d028655_T003	0.9363242	-0.366391374	Zm00001d028655
Zm00001d028655_T001	0.9846871	0.543660433	Zm00001d028655
Zm00001d022124_T001	0.4859645	-0.694700512	Zm00001d022124
Zm00001d017185_T001	0.9311783	0.070867824	Zm00001d017185
Zm00001d053719_T003	0.9979568	0.242706928	Zm00001d053719
Zm00001d042168_T001	0.9492285	0.697011156	Zm00001d042168
Zm00001d006235_T001	0.9715915	0.097414369	Zm00001d006235
Zm00001d008954_T003	0.3337903	0.909179729	Zm00001d008954
Zm00001d008954_T006	0.4604152	0.821671886	Zm00001d008954
Zm00001d008954_T005	0.03057712	-0.76154262	Zm00001d008954
Zm00001d011158_T012	8.04E-11	1.66519565	Zm00001d011158
Zm00001d011158_T007	0.9361945	0.058500151	Zm00001d011158
Zm00001d023471_T001	0.9914036	0.158364571	Zm00001d023471
Zm00001d037197_T001	0.5472547	-1.324435013	Zm00001d037197
Zm00001d029285_T004	0.8411786	0.940162475	Zm00001d029285
Zm00001d029285_T003	0.9216521	0.446587274	Zm00001d029285
Zm00001d008726_T004	0.9363242	0.21177623	Zm00001d008726
Zm00001d008726_T002	0.8643636	-0.298198049	Zm00001d008726
Zm00001d008726_T003	0.9562711	0.532411149	Zm00001d008726
Zm00001d034488_T001	0.3232802	1.688894138	Zm00001d034488
Zm00001d020234_T002	0.5103173	-0.595920561	Zm00001d020234
Zm00001d020234_T001	0.9296811	-0.007881508	Zm00001d020234

Zm00001d053900_T001	0.9176222	-0.010176817	Zm00001d053900
Zm00001d005323_T005	0.8865624	-0.156039794	Zm00001d005323
Zm00001d005323_T015	0.9928864	0.136278272	Zm00001d005323
Zm00001d005323_T002	0.802225	0.850610628	Zm00001d005323
Zm00001d005323_T009	0.01624246	1.518283056	Zm00001d005323
Zm00001d005323_T001	0.9720956	0.180823245	Zm00001d005323
Zm00001d043270_T002	0.2058947	1.793297881	Zm00001d043270
Zm00001d043270_T001	0.9013602	0.699755077	Zm00001d043270
Zm00001d013834_T001	0.003542199	3.255129613	Zm00001d013834
Zm00001d048529_T003	0.2801949	-1.181338907	Zm00001d048529
Zm00001d048529_T001	0.9994451	0.326077515	Zm00001d048529
Zm00001d048529_T002	0.9753621	0.235630651	Zm00001d048529
Zm00001d022103_T002	1	0.452372161	Zm00001d022103
Zm00001d022103_T001	0.8162418	0.287484358	Zm00001d022103
Zm00001d003014_T001	0.9262099	-0.30108885	Zm00001d003014
Zm00001d036250_T001	0.8409438	-0.229264144	Zm00001d036250
Zm00001d039709_T001	0.9887889	0.221356511	Zm00001d039709
Zm00001d012290_T001	0.5344384	-1.051231598	Zm00001d012290
Zm00001d020512_T002	0.6500707	-0.366944	Zm00001d020512
Zm00001d023455_T001	0.9309796	0.49847133	Zm00001d023455
Zm00001d047117_T001	0.2804268	-1.121111864	Zm00001d047117
Zm00001d021412_T001	0.7078283	-0.35926309	Zm00001d021412
Zm00001d049320_T001	0.9822138	0.261171561	Zm00001d049320
Zm00001d043018_T001	0.9279367	-0.488654058	Zm00001d043018
Zm00001d001827_T004	0.4987058	0.953303463	Zm00001d001827
Zm00001d001827_T006	0.9673094	0.628182856	Zm00001d001827
Zm00001d001827_T010	0.8020448	-0.248930839	Zm00001d001827
Zm00001d031805_T001	0.9290482	-0.315816374	Zm00001d031805
Zm00001d031702_T002	0.9068989	0.535054152	Zm00001d031702
Zm00001d031316_T011	0.8077635	0.251193877	Zm00001d031316
Zm00001d031316_T022	0.7378574	-0.397440697	Zm00001d031316
Zm00001d031316_T021	0.9986724	0.362417416	Zm00001d031316
Zm00001d031316_T009	0.9396569	0.504482647	Zm00001d031316
Zm00001d031316_T006	0.002796955	-1.663550703	Zm00001d031316
Zm00001d049211_T001	1	0.403692361	Zm00001d049211
Zm00001d008777_T009	0.07730424	-0.369328318	Zm00001d008777
Zm00001d008777_T001	0.9611506	0.180759985	Zm00001d008777
Zm00001d052007_T001	0.9778436	-0.04607932	Zm00001d052007
Zm00001d048535_T033	0.6224298	1.341712512	Zm00001d048535
Zm00001d048535_T010	0.9911688	0.030037111	Zm00001d048535
Zm00001d048535_T021	0.1500946	1.363929117	Zm00001d048535
Zm00001d048535_T015	0.008068237	0.661532921	Zm00001d048535
Zm00001d048535_T035	0.2662963	0.242514833	Zm00001d048535
Zm00001d048535_T026	6.67E-10	1.098062406	Zm00001d048535
Zm00001d013812_T001	0.7810009	-0.300717542	Zm00001d013812
Zm00001d035474_T001	0.8097969	-0.411370754	Zm00001d035474
Zm00001d052696_T001	0.9887889	0.471409315	Zm00001d052696
Zm00001d032889_T001	0.8646111	-0.190096356	Zm00001d032889
Zm00001d044917_T001	0.9985916	0.250499429	Zm00001d044917
Zm00001d044917_T002	1	0.253304506	Zm00001d044917
Zm00001d013553_T001	0.4241685	-1.041436288	Zm00001d013553
Zm00001d005879_T001	0.9782953	-0.52656238	Zm00001d005879
Zm00001d009895_T001	0.7727043	0.84034938	Zm00001d009895
Zm00001d043752_T001	0.6338908	1.201705511	Zm00001d043752

Zm00001d031660_T015	0.2015088	0.782610058	Zm00001d031660
Zm00001d031660_T007	0.03276415	0.858979533	Zm00001d031660
Zm00001d031660_T018	0.002863669	0.69848642	Zm00001d031660
Zm00001d031660_T009	0.8609127	0.555910245	Zm00001d031660
Zm00001d031660_T016	0.9086526	-0.451962177	Zm00001d031660
Zm00001d031660_T019	0.9031129	-0.050349491	Zm00001d031660
Zm00001d031660_T013	0.9529184	0.10634168	Zm00001d031660
Zm00001d031660_T002	1.00E-13	3.213266961	Zm00001d031660
Zm00001d048672_T002	0.9184323	-0.013125932	Zm00001d048672
Zm00001d012819_T001	0.9849018	0.114272548	Zm00001d012819
Zm00001d024523_T032	0.05256028	-0.364238908	Zm00001d024523
Zm00001d024523_T020	0.132128	1.047018988	Zm00001d024523
Zm00001d024523_T004	0.3854293	-0.722701319	Zm00001d024523
Zm00001d024523_T006	0.2836575	1.604887113	Zm00001d024523
Zm00001d024523_T002	0.1512123	1.97276721	Zm00001d024523
Zm00001d024523_T025	0.9201237	-0.017604678	Zm00001d024523
Zm00001d012017_T002	0.8239318	-0.354505654	Zm00001d012017
Zm00001d026605_T001	0.1616209	-0.573338566	Zm00001d026605
Zm00001d035862_T002	0.954492	0.139805275	Zm00001d035862
Zm00001d035862_T005	0.9363242	-0.043692354	Zm00001d035862
Zm00001d035862_T003	0.7017973	0.973880404	Zm00001d035862
Zm00001d020384_T001	0.8412913	-0.116901541	Zm00001d020384
Zm00001d035087_T001	0.5117928	-1.110203793	Zm00001d035087
Zm00001d020583_T001	0.969231	0.393386832	Zm00001d020583
Zm00001d048119_T002	0.8695779	0.768711806	Zm00001d048119
Zm00001d048119_T027	0.4503499	-0.628057842	Zm00001d048119
Zm00001d048119_T018	0.3054467	0.914720687	Zm00001d048119
Zm00001d048119_T001	0.3982591	0.860441137	Zm00001d048119
Zm00001d048119_T013	0.4044152	0.293265584	Zm00001d048119
Zm00001d048119_T034	0.3614644	-0.239739097	Zm00001d048119
Zm00001d048119_T040	0.006510461	-1.957796796	Zm00001d048119
Zm00001d048119_T039	0.8307555	-0.331900474	Zm00001d048119
Zm00001d030683_T001	0.7422564	1.026044174	Zm00001d030683
Zm00001d030683_T002	0.9702242	0.113203928	Zm00001d030683
Zm00001d044197_T001	0.9514141	0.557451143	Zm00001d044197
Zm00001d026177_T001	0.9870482	0.119128967	Zm00001d026177
Zm00001d031760_T001	0.8929316	0.709531311	Zm00001d031760
Zm00001d024300_T002	0.9046076	-0.030083233	Zm00001d024300
Zm00001d024300_T008	0.001782387	-1.450685587	Zm00001d024300
Zm00001d024300_T001	0.9700977	0.558909204	Zm00001d024300
Zm00001d036922_T001	0.2002595	1.736036116	Zm00001d036922
Zm00001d002867_T001	0.8293703	0.473688982	Zm00001d002867
Zm00001d049945_T001	0.2294707	-1.081003203	Zm00001d049945
Zm00001d049945_T004	0.9544149	0.015418334	Zm00001d049945
Zm00001d049945_T010	0.6937558	0.624845055	Zm00001d049945
Zm00001d049945_T005	0.6934931	-0.039817893	Zm00001d049945
Zm00001d049945_T006	0.6021328	-0.7995577	Zm00001d049945
Zm00001d049945_T003	0.9081066	-0.141654513	Zm00001d049945
Zm00001d022242_T001	0.5761765	-0.453454498	Zm00001d022242
Zm00001d022242_T002	0.9621967	0.491100219	Zm00001d022242
Zm00001d037779_T001	0.8503172	-0.223483427	Zm00001d037779
Zm00001d024416_T001	0.9361945	0.061157815	Zm00001d024416
Zm00001d017802_T001	0.9975882	0.447372602	Zm00001d017802
Zm00001d041969_T001	0.9657993	-0.001336816	Zm00001d041969

Zm00001d008567_T004	0.8844101	0.063401477	Zm00001d008567
Zm00001d008567_T007	0.6611576	0.40584037	Zm00001d008567
Zm00001d008567_T001	0.5472547	0.56864015	Zm00001d008567
Zm00001d008567_T006	0.9319754	0.723970831	Zm00001d008567
Zm00001d008567_T009	0.956479	0.704069203	Zm00001d008567
Zm00001d008567_T011	0.6523514	-0.878144033	Zm00001d008567
Zm00001d026536_T002	0.6338908	-0.39804833	Zm00001d026536
Zm00001d026536_T004	0.9429978	0.584907063	Zm00001d026536
Zm00001d053578_T001	0.01500842	1.218959898	Zm00001d053578
Zm00001d037494_T001	0.7227048	-0.821919746	Zm00001d037494
Zm00001d040303_T001	0.9947747	0.324487794	Zm00001d040303
Zm00001d018122_T001	0.9828128	0.421687237	Zm00001d018122
Zm00001d024225_T066	0.06242909	-1.963493288	Zm00001d024225
Zm00001d024225_T061	0.002132976	-0.878794895	Zm00001d024225
Zm00001d024225_T036	2.10E-09	-0.80757524	Zm00001d024225
Zm00001d024225_T063	0.4531805	0.227618518	Zm00001d024225
Zm00001d024225_T001	0.1003679	1.750246115	Zm00001d024225
Zm00001d024225_T057	0.1979906	2.011386292	Zm00001d024225
Zm00001d024225_T032	1.51E-11	1.463464012	Zm00001d024225
Zm00001d024225_T020	0.3076698	0.347450139	Zm00001d024225
Zm00001d024225_T046	0.4669227	0.341174021	Zm00001d024225
Zm00001d008516_T001	0.9903568	0.083915912	Zm00001d008516
Zm00001d021536_T001	0.965836	-0.158987842	Zm00001d021536
Zm00001d021536_T003	0.9210327	-0.013938738	Zm00001d021536
Zm00001d013705_T001	0.5463608	-0.478072834	Zm00001d013705
Zm00001d034419_T001	1	0.163488792	Zm00001d034419
Zm00001d043328_T003	0.98291	0.439366516	Zm00001d043328
Zm00001d043328_T001	0.7146325	-0.128786041	Zm00001d043328
Zm00001d025837_T001	1	0.374453075	Zm00001d025837
Zm00001d041550_T001	0.1718704	-1.139275727	Zm00001d041550
Zm00001d041550_T004	0.01048202	-2.145699782	Zm00001d041550
Zm00001d041550_T005	0.9910433	1.127163038	Zm00001d041550
Zm00001d041550_T002	0.9194209	0.044393412	Zm00001d041550
Zm00001d001983_T003	0.8581713	0.789602399	Zm00001d001983
Zm00001d001983_T002	0.9909466	0.49239044	Zm00001d001983
Zm00001d001983_T001	0.9999526	0.307727606	Zm00001d001983
Zm00001d020651_T001	0.929138	0.450999605	Zm00001d020651
Zm00001d026554_T001	1	0.224085734	Zm00001d026554
Zm00001d017475_T002	0.8720321	-0.058849865	Zm00001d017475
Zm00001d017475_T003	0.05103414	1.044766794	Zm00001d017475
Zm00001d038600_T001	0.9609806	0.552490367	Zm00001d038600
Zm00001d002267_T001	0.8672898	-0.116892796	Zm00001d002267
Zm00001d024889_T003	0.5705692	1.129486517	Zm00001d024889
Zm00001d024889_T001	0.9220919	-0.015107257	Zm00001d024889
Zm00001d024889_T004	0.000124973	-2.039673065	Zm00001d024889
Zm00001d024889_T002	0.7406865	-0.288825316	Zm00001d024889
Zm00001d032821_T001	0.850642	-0.310691279	Zm00001d032821
Zm00001d049872_T001	0.7105846	0.705311036	Zm00001d049872
Zm00001d051799_T001	0.2140375	-1.233087839	Zm00001d051799
Zm00001d028660_T007	0.4685762	-0.216110957	Zm00001d028660
Zm00001d028660_T012	0.3024505	0.556424777	Zm00001d028660
Zm00001d028660_T002	0.03189604	0.952513479	Zm00001d028660
Zm00001d028660_T015	0.4844771	0.665771724	Zm00001d028660
Zm00001d028660_T008	0.1387257	0.584176887	Zm00001d028660

Zm00001d028660_T005	0.3344805	-0.794191088	Zm00001d028660
Zm00001d047130_T001	0.9782953	0.06291716	Zm00001d047130
Zm00001d027539_T001	0.8688441	-0.192280582	Zm00001d027539
Zm00001d027539_T002	2.48E-12	-4.965276973	Zm00001d027539
Zm00001d023544_T006	0.2808487	1.113088447	Zm00001d023544
Zm00001d023544_T003	0.3943094	0.959575336	Zm00001d023544
Zm00001d037941_T001	3.50E-05	-3.549849055	Zm00001d037941
Zm00001d037941_T002	7.80E-08	-3.273246105	Zm00001d037941
Zm00001d011005_T002	0.8125642	-0.332511943	Zm00001d011005
Zm00001d011005_T001	0.9189175	0.008331199	Zm00001d011005
Zm00001d011005_T003	0.6578675	-0.524011024	Zm00001d011005
Zm00001d011005_T004	0.7820223	0.550303357	Zm00001d011005
Zm00001d020658_T001	0.9760472	-0.211927104	Zm00001d020658
Zm00001d039385_T001	0.6926364	-0.173102861	Zm00001d039385
Zm00001d039385_T003	5.16E-08	0.545234693	Zm00001d039385
Zm00001d039385_T007	0.1628238	-0.268511947	Zm00001d039385
Zm00001d039385_T029	0.06026596	0.353959244	Zm00001d039385
Zm00001d041989_T001	0.9702162	0.136094221	Zm00001d041989
Zm00001d042589_T001	0.9400285	-0.279426971	Zm00001d042589
Zm00001d034316_T001	0.9720837	0.2314899	Zm00001d034316
Zm00001d024875_T001	0.9988864	0.18419369	Zm00001d024875
Zm00001d018454_T012	1	0.337352144	Zm00001d018454
Zm00001d018454_T005	0.5501607	1.279491897	Zm00001d018454
Zm00001d018454_T013	0.6587465	0.811844858	Zm00001d018454
Zm00001d018454_T008	0.7497503	1.086655961	Zm00001d018454
Zm00001d018454_T007	0.9950761	0.121490636	Zm00001d018454
Zm00001d018454_T003	0.947501	0.711124163	Zm00001d018454
Zm00001d018454_T010	0.7831859	-0.122032356	Zm00001d018454
Zm00001d042490_T001	0.9262099	0.046665807	Zm00001d042490
Zm00001d042490_T005	0.9867452	0.249782957	Zm00001d042490
Zm00001d014585_T003	0.956184	0.11329602	Zm00001d014585
Zm00001d014585_T001	0.8720862	0.33667901	Zm00001d014585
Zm00001d014327_T002	0.9100716	-0.222616751	Zm00001d014327
Zm00001d014327_T001	0.8012608	-0.227294374	Zm00001d014327
Zm00001d014327_T008	1	0.071580451	Zm00001d014327
Zm00001d014327_T005	0.9970835	0.072523064	Zm00001d014327
Zm00001d014327_T003	0.9986486	0.290426677	Zm00001d014327
Zm00001d001763_T001	0.9541864	-0.209246923	Zm00001d001763
Zm00001d032030_T001	0.005641901	-2.620562814	Zm00001d032030
Zm00001d025343_T001	0.9706887	0.458620408	Zm00001d025343
Zm00001d005898_T001	0.9003815	0.298690517	Zm00001d005898
Zm00001d037601_T001	0.9296811	0.591247099	Zm00001d037601
Zm00001d012770_T005	0.9732421	1.117451074	Zm00001d012770
Zm00001d012770_T001	0.02632035	-0.737305389	Zm00001d012770
Zm00001d012770_T004	0.9954444	0.234254665	Zm00001d012770
Zm00001d012770_T006	0.366346	0.444911985	Zm00001d012770
Zm00001d012770_T003	0.004479976	-2.434537662	Zm00001d012770
Zm00001d031453_T001	0.9692109	0.076096995	Zm00001d031453
Zm00001d051599_T009	0.004954152	0.993190587	Zm00001d051599
Zm00001d051599_T001	1	0.45298793	Zm00001d051599
Zm00001d051599_T007	0.9748076	0.686444535	Zm00001d051599
Zm00001d051599_T008	0.7788618	0.200077989	Zm00001d051599
Zm00001d051599_T010	0.1145356	1.171589847	Zm00001d051599
Zm00001d045673_T003	1	0.314059814	Zm00001d045673

Zm00001d045673_T006	0.9895224	0.133062251	Zm00001d045673
Zm00001d049809_T001	1	0.277158732	Zm00001d049809
Zm00001d037243_T001	0.01423233	-2.202752002	Zm00001d037243
Zm00001d032024_T001	0.8453498	-0.270068698	Zm00001d032024
Zm00001d032024_T002	0.3989592	-0.87337107	Zm00001d032024
Zm00001d043096_T001	0.9602735	-0.139326923	Zm00001d043096
Zm00001d052434_T003	0.9811162	0.179890933	Zm00001d052434
Zm00001d052434_T001	0.1006821	-0.665265874	Zm00001d052434
Zm00001d052434_T002	0.8668127	-0.12042653	Zm00001d052434
Zm00001d038619_T001	1	-0.200288822	Zm00001d038619
Zm00001d053627_T002	0.9541864	0.608276218	Zm00001d053627
Zm00001d041793_T002	0.9849018	0.051081775	Zm00001d041793
Zm00001d041793_T005	0.1685404	-1.372813181	Zm00001d041793
Zm00001d041793_T003	0.8717403	-0.458985305	Zm00001d041793
Zm00001d037247_T003	0.9987435	0.240095875	Zm00001d037247
Zm00001d037247_T017	0.845508	0.507910861	Zm00001d037247
Zm00001d037247_T009	0.9773181	0.222148111	Zm00001d037247
Zm00001d037247_T015	0.9180865	0.668187414	Zm00001d037247
Zm00001d049187_T001	0.04827422	2.548560527	Zm00001d049187
Zm00001d039576_T001	0.9312703	-0.010881681	Zm00001d039576
Zm00001d004857_T004	0.9531523	0.383322937	Zm00001d004857
Zm00001d004857_T002	0.9363242	0.698346227	Zm00001d004857
Zm00001d014663_T001	0.9986486	0.2872959	Zm00001d014663
Zm00001d034869_T001	0.1124682	-1.658121937	Zm00001d034869
Zm00001d033240_T003	0.000866567	-2.588616143	Zm00001d033240
Zm00001d033240_T002	0.5954124	0.112070764	Zm00001d033240
Zm00001d033240_T001	0.8107637	1.401313179	Zm00001d033240
Zm00001d031273_T001	0.9363424	-0.261307481	Zm00001d031273
Zm00001d009271_T002	0.995534	0.129018689	Zm00001d009271
Zm00001d009271_T004	0.9666757	0.031046469	Zm00001d009271
Zm00001d009271_T001	0.9865262	0.288926003	Zm00001d009271
Zm00001d047710_T004	0.9768802	0.224037021	Zm00001d047710
Zm00001d047710_T001	0.8323635	-0.181427293	Zm00001d047710
Zm00001d047710_T018	0.894759	0.311670137	Zm00001d047710
Zm00001d047710_T025	0.3808423	0.446521898	Zm00001d047710
Zm00001d047710_T005	0.7138265	0.416954592	Zm00001d047710
Zm00001d047710_T014	0.05356323	0.625450753	Zm00001d047710
Zm00001d047710_T022	0.592105	-0.232652056	Zm00001d047710
Zm00001d047710_T013	0.002206201	0.797004713	Zm00001d047710
Zm00001d047710_T008	0.05435502	0.574403412	Zm00001d047710
Zm00001d047710_T016	0.9846446	-0.036839824	Zm00001d047710
Zm00001d026560_T001	1	0.11985905	Zm00001d026560
Zm00001d006914_T001	0.9719113	0.230389812	Zm00001d006914
Zm00001d045309_T001	0.9475886	0.095564498	Zm00001d045309
Zm00001d008642_T003	0.9988864	0.404672706	Zm00001d008642
Zm00001d008642_T001	0.8817666	0.781095327	Zm00001d008642
Zm00001d028968_T001	0.1421277	1.772638861	Zm00001d028968
Zm00001d010190_T001	0.9931431	0.265355744	Zm00001d010190
Zm00001d010190_T002	0.8537544	0.923370686	Zm00001d010190
Zm00001d045268_T001	0.7752307	-0.772260186	Zm00001d045268
Zm00001d006319_T002	0.5380217	-0.326146484	Zm00001d006319
Zm00001d033797_T002	8.45E-05	3.578170087	Zm00001d033797
Zm00001d033797_T001	0.007367894	1.724201335	Zm00001d033797
Zm00001d035194_T001	0.8032546	1.005621159	Zm00001d035194

Zm00001d021128_T001	0.9745433	0.147001159	Zm00001d021128
Zm00001d021592_T001	0.9846714	0.260459047	Zm00001d021592
Zm00001d016522_T001	0.9103625	0.857588079	Zm00001d016522
Zm00001d048623_T001	0.006756568	-2.306284688	Zm00001d048623
Zm00001d048623_T002	0.04327008	-1.791577868	Zm00001d048623
Zm00001d016052_T001	0.7020458	1.13870105	Zm00001d016052
Zm00001d017666_T001	0.101625	2.185949437	Zm00001d017666
Zm00001d001773_T001	1	0.177404834	Zm00001d001773
Zm00001d024518_T001	0.9954444	-0.056527258	Zm00001d024518
Zm00001d019060_T001	0.08778756	2.223975417	Zm00001d019060
Zm00001d034191_T002	0.4545925	0.934094814	Zm00001d034191
Zm00001d034191_T003	0.4445976	1.224945017	Zm00001d034191
Zm00001d034191_T001	0.7646718	-0.270116037	Zm00001d034191
Zm00001d002482_T001	0.009394971	1.739123354	Zm00001d002482
Zm00001d047853_T003	0.7545865	0.352021285	Zm00001d047853
Zm00001d047853_T002	0.9832092	0.103203206	Zm00001d047853
Zm00001d047853_T001	0.6027188	0.877457153	Zm00001d047853
Zm00001d050584_T017	0.7946251	1.04420625	Zm00001d050584
Zm00001d016662_T003	0.9779656	0.536551557	Zm00001d016662
Zm00001d016662_T004	0.8728996	0.702252933	Zm00001d016662
Zm00001d019090_T008	0.3273212	1.23585429	Zm00001d019090
Zm00001d019090_T010	0.8513643	0.366844328	Zm00001d019090
Zm00001d019090_T004	0.9846446	0.243515504	Zm00001d019090
Zm00001d046545_T007	0.9463296	-0.176052132	Zm00001d046545
Zm00001d046545_T001	0.8388595	0.58601929	Zm00001d046545
Zm00001d051214_T001	0.9100328	-0.054433821	Zm00001d051214
Zm00001d051214_T002	1	0.072109793	Zm00001d051214
Zm00001d039059_T001	1	0.297258257	Zm00001d039059
Zm00001d027427_T002	0.03117416	-1.79375409	Zm00001d027427
Zm00001d027427_T001	0.9563232	-0.009900629	Zm00001d027427
Zm00001d041201_T001	0.3423638	-0.799189313	Zm00001d041201
Zm00001d047666_T005	0.9894615	0.241084446	Zm00001d047666
Zm00001d020932_T001	0.9747606	0.549262594	Zm00001d020932
Zm00001d022130_T001	0.1639346	1.513283615	Zm00001d022130
Zm00001d007264_T001	0.03285779	-1.672730483	Zm00001d007264
Zm00001d010969_T001	0.453061	-0.751567688	Zm00001d010969
Zm00001d038675_T098	0.9580985	0.07167772	Zm00001d038675
Zm00001d038675_T120	0.000249454	1.420056167	Zm00001d038675
Zm00001d038675_T022	0.9444056	0.095525206	Zm00001d038675
Zm00001d038675_T187	0.008747332	-1.114717977	Zm00001d038675
Zm00001d038675_T231	0.3780737	0.453973932	Zm00001d038675
Zm00001d038675_T544	2.50E-11	-2.059264445	Zm00001d038675
Zm00001d038675_T201	0.02108144	1.774945124	Zm00001d038675
Zm00001d038675_T236	0.139394	1.211531578	Zm00001d038675
Zm00001d038675_T063	7.41E-09	0.882596993	Zm00001d038675
Zm00001d038675_T533	0.7020458	-0.140875382	Zm00001d038675
Zm00001d038675_T115	0.3045236	0.375442672	Zm00001d038675
Zm00001d038675_T182	7.88E-13	1.873867921	Zm00001d038675
Zm00001d038675_T589	0.0422188	-1.259861932	Zm00001d038675
Zm00001d038675_T192	0.003719716	0.960661402	Zm00001d038675
Zm00001d038675_T226	0.7565187	0.011284388	Zm00001d038675
Zm00001d038675_T583	0.404311	-0.76121934	Zm00001d038675
Zm00001d038675_T034	0.0294937	1.792719916	Zm00001d038675
Zm00001d038675_T152	0.7573198	-0.13630414	Zm00001d038675

Zm00001d038675_T167	0.3370563	0.838280131	Zm00001d038675
Zm00001d038675_T096	0.6681963	0.440637617	Zm00001d038675
Zm00001d038675_T199	0.9474286	0.450545496	Zm00001d038675
Zm00001d038675_T571	0.5189688	0.447444037	Zm00001d038675
Zm00001d038675_T197	1.53E-10	1.165428411	Zm00001d038675
Zm00001d038675_T056	1.79E-09	1.075638357	Zm00001d038675
Zm00001d038675_T081	0.6466858	-0.40642792	Zm00001d038675
Zm00001d038675_T579	0.9596377	0.032626148	Zm00001d038675
Zm00001d038675_T181	0.1805724	-0.343515689	Zm00001d038675
Zm00001d038675_T227	0.08864449	-0.099636478	Zm00001d038675
Zm00001d038675_T252	0.2055034	0.309985435	Zm00001d038675
Zm00001d038675_T031	0.8044932	0.178202422	Zm00001d038675
Zm00001d038675_T101	0.6117378	-0.175969065	Zm00001d038675
Zm00001d038675_T586	0.3497147	0.467439757	Zm00001d038675
Zm00001d038675_T060	0.7941326	0.397055161	Zm00001d038675
Zm00001d038675_T578	0.2367311	-0.992104989	Zm00001d038675
Zm00001d038675_T065	0.006217153	1.666286691	Zm00001d038675
Zm00001d038675_T556	0.00081066	0.6352231	Zm00001d038675
Zm00001d038675_T015	0.6384824	0.791348981	Zm00001d038675
Zm00001d038675_T510	1	0.43840244	Zm00001d038675
Zm00001d038675_T055	0.2288219	0.858702302	Zm00001d038675
Zm00001d038675_T584	0.4422995	0.853837906	Zm00001d038675
Zm00001d038675_T228	0.02237672	-0.672632241	Zm00001d038675
Zm00001d038675_T117	0.4701937	0.505695536	Zm00001d038675
Zm00001d038675_T276	0.09637979	0.920564016	Zm00001d038675
Zm00001d038675_T582	0.9380783	0.203058906	Zm00001d038675
Zm00001d038675_T088	0.004597139	0.667540922	Zm00001d038675
Zm00001d038675_T585	0.5411872	0.573421884	Zm00001d038675
Zm00001d038675_T105	0.7184566	0.351379001	Zm00001d038675
Zm00001d038675_T177	0.6993528	-0.152232224	Zm00001d038675
Zm00001d019754_T005	0.8597376	0.366958449	Zm00001d019754
Zm00001d019754_T003	0.9747606	0.039650407	Zm00001d019754
Zm00001d019754_T002	0.9326774	0.695937917	Zm00001d019754
Zm00001d032830_T013	0.7440932	0.879751233	Zm00001d032830
Zm00001d032830_T003	0.9613188	0.41056635	Zm00001d032830
Zm00001d032830_T014	0.9867657	0.325065969	Zm00001d032830
Zm00001d032830_T006	1	-0.023876496	Zm00001d032830
Zm00001d032830_T007	0.9474116	0.028363372	Zm00001d032830
Zm00001d032830_T001	0.00309982	-1.601134122	Zm00001d032830
Zm00001d032830_T004	0.9951419	0.164153502	Zm00001d032830
Zm00001d032830_T009	0.05001771	2.031414547	Zm00001d032830
Zm00001d032830_T010	0.5298301	-1.264625039	Zm00001d032830
Zm00001d032830_T005	0.9404125	0.574145117	Zm00001d032830
Zm00001d013811_T001	0.7249016	-0.363573789	Zm00001d013811
Zm00001d041996_T001	0.9676171	0.57918975	Zm00001d041996
Zm00001d009193_T002	0.2509097	-1.407356905	Zm00001d009193
Zm00001d009193_T001	0.9988864	0.123355849	Zm00001d009193
Zm00001d031662_T001	0.9469426	0.053853951	Zm00001d031662
Zm00001d022006_T001	0.7222076	-0.315988484	Zm00001d022006
Zm00001d017243_T001	2.92E-06	2.461608986	Zm00001d017243
Zm00001d048046_T003	0.9833565	0.04218179	Zm00001d048046
Zm00001d048046_T001	0.9749249	0.604676519	Zm00001d048046
Zm00001d048046_T002	0.7146325	0.692297676	Zm00001d048046
Zm00001d032331_T001	0.6386998	-0.365425753	Zm00001d032331

Zm00001d010262_T001	0.8770116	-0.169289273	Zm00001d010262
Zm00001d037927_T001	0.9894615	0.189786005	Zm00001d037927
Zm00001d038220_T001	0.8891117	-0.259422206	Zm00001d038220
Zm00001d028038_T006	0.9979568	0.278990874	Zm00001d028038
Zm00001d028038_T005	0.7437035	-0.164315108	Zm00001d028038
Zm00001d028038_T004	0.1204618	0.335210167	Zm00001d028038
Zm00001d047351_T003	0.6002185	1.092573649	Zm00001d047351
Zm00001d047351_T002	0.7497503	0.874665988	Zm00001d047351
Zm00001d047351_T001	0.8896281	-0.063149261	Zm00001d047351
Zm00001d014243_T001	0.9196877	0.607413253	Zm00001d014243
Zm00001d014243_T002	0.9870394	0.186834995	Zm00001d014243
Zm00001d013346_T001	0.7881764	-0.216813757	Zm00001d013346
Zm00001d011526_T001	0.9543425	0.073219006	Zm00001d011526
Zm00001d011526_T002	0.8197974	-0.200771055	Zm00001d011526
Zm00001d033675_T001	0.5872455	1.144431279	Zm00001d033675
Zm00001d013380_T005	0.9666757	0.092564358	Zm00001d013380
Zm00001d013380_T006	4.38E-07	0.51680141	Zm00001d013380
Zm00001d013380_T011	0.4849772	0.630147885	Zm00001d013380
Zm00001d013380_T033	0.04298108	0.542946982	Zm00001d013380
Zm00001d013380_T001	0.9737201	0.519310599	Zm00001d013380
Zm00001d005736_T019	0.7520438	0.263144742	Zm00001d005736
Zm00001d005736_T030	0.2629406	0.424949043	Zm00001d005736
Zm00001d005736_T007	0.2055489	1.284619583	Zm00001d005736
Zm00001d005736_T020	0.07692255	1.343999667	Zm00001d005736
Zm00001d005736_T036	1.42E-08	-2.361880022	Zm00001d005736
Zm00001d005736_T015	0.3795013	1.457013996	Zm00001d005736
Zm00001d012954_T002	0.9782953	0.316639601	Zm00001d012954
Zm00001d018931_T001	0.9944247	0.2950343	Zm00001d018931
Zm00001d015326_T003	0.4246306	-0.356900919	Zm00001d015326
Zm00001d015326_T004	0.5065034	0.318007743	Zm00001d015326
Zm00001d015326_T007	0.4101453	1.199027783	Zm00001d015326
Zm00001d012246_T001	0.2153703	-0.692640931	Zm00001d012246
Zm00001d046898_T001	0.7074924	-0.401085615	Zm00001d046898
Zm00001d004172_T015	0.8214332	-0.249807337	Zm00001d004172
Zm00001d004172_T007	0.2573404	0.611971222	Zm00001d004172
Zm00001d004172_T006	1	0.221978031	Zm00001d004172
Zm00001d004172_T005	0.7664596	0.999049648	Zm00001d004172
Zm00001d044420_T001	0.9740325	0.520397737	Zm00001d044420
Zm00001d032867_T004	0.9377241	0.309249129	Zm00001d032867
Zm00001d032867_T003	0.7990254	0.458514687	Zm00001d032867
Zm00001d032867_T002	1	0.06566369	Zm00001d032867
Zm00001d029818_T022	0.9317791	-0.035679589	Zm00001d029818
Zm00001d029818_T005	0.2982359	-0.511741076	Zm00001d029818
Zm00001d029818_T001	0.1498804	1.725245545	Zm00001d029818
Zm00001d037041_T001	0.9269932	-0.014377145	Zm00001d037041
Zm00001d017913_T002	0.2917536	1.206139555	Zm00001d017913
Zm00001d017913_T003	0.9609806	0.185266229	Zm00001d017913
Zm00001d017913_T007	0.07932981	1.933882358	Zm00001d017913
Zm00001d017913_T004	0.001017881	0.852606983	Zm00001d017913
Zm00001d017913_T001	0.001760922	-2.394048899	Zm00001d017913
Zm00001d000224_T006	0.4952796	-0.433461019	Zm00001d000224
Zm00001d000224_T003	0.09191856	-0.757042341	Zm00001d000224
Zm00001d048472_T001	0.3510348	1.648522455	Zm00001d048472
Zm00001d010169_T002	0.4396896	-0.641888974	Zm00001d010169

Zm00001d002456_T002	0.3318423	0.529139957	Zm00001d002456
Zm00001d002456_T001	1	0.349723231	Zm00001d002456
Zm00001d002851_T001	0.9580876	0.453891259	Zm00001d002851
Zm00001d011967_T001	0.7188446	-0.221283801	Zm00001d011967
Zm00001d026705_T001	1	0.262408823	Zm00001d026705
Zm00001d014727_T004	1	0.190603915	Zm00001d014727
Zm00001d014727_T001	1	0.267811477	Zm00001d014727
Zm00001d023215_T001	0.000326064	-0.938828754	Zm00001d023215
Zm00001d045322_T001	0.9681793	0.126465121	Zm00001d045322
Zm00001d030020_T006	0.7858094	-0.494388257	Zm00001d030020
Zm00001d030020_T003	0.1106602	-1.771463903	Zm00001d030020
Zm00001d030020_T001	0.9868533	0.297142328	Zm00001d030020
Zm00001d030020_T004	0.7091158	0.944265984	Zm00001d030020
Zm00001d030020_T002	0.9326774	0.302416201	Zm00001d030020
Zm00001d001897_T001	0.9666757	0.095437244	Zm00001d001897
Zm00001d036161_T016	0.9539896	0.170129295	Zm00001d036161
Zm00001d036161_T004	0.3547256	0.322271692	Zm00001d036161
Zm00001d036161_T018	3.57E-10	1.642868384	Zm00001d036161
Zm00001d036161_T001	0.9664019	0.660179337	Zm00001d036161
Zm00001d036161_T014	5.34E-08	0.981950192	Zm00001d036161
Zm00001d036161_T010	0.2598343	-0.746805181	Zm00001d036161
Zm00001d020531_T003	0.3207142	1.12054436	Zm00001d020531
Zm00001d020531_T004	1	0.327624717	Zm00001d020531
Zm00001d020531_T002	0.09143854	2.33552104	Zm00001d020531
Zm00001d020531_T001	0.8783081	0.503314861	Zm00001d020531
Zm00001d054034_T004	0.6398988	-0.367231937	Zm00001d054034
Zm00001d054034_T001	0.9863483	0.191098393	Zm00001d054034
Zm00001d054034_T003	0.8757823	0.011193101	Zm00001d054034
Zm00001d028879_T001	0.9076857	0.576098372	Zm00001d028879
Zm00001d012326_T001	0.7414776	1.023918971	Zm00001d012326
Zm00001d048450_T001	0.9778436	0.009995098	Zm00001d048450
Zm00001d048450_T006	0.9450101	0.484665279	Zm00001d048450
Zm00001d048450_T002	0.5428594	-0.623060772	Zm00001d048450
Zm00001d048450_T004	0.755035	0.682945627	Zm00001d048450
Zm00001d016307_T001	0.5208223	-0.644618177	Zm00001d016307
Zm00001d012501_T002	0.3754417	-0.762562498	Zm00001d012501
Zm00001d012501_T007	0.9894615	0.211109761	Zm00001d012501
Zm00001d012501_T003	0.6445434	1.180606275	Zm00001d012501
Zm00001d012501_T004	0.9033985	0.306188395	Zm00001d012501
Zm00001d010009_T005	0.7055235	0.342253352	Zm00001d010009
Zm00001d010009_T001	0.9232281	-0.138218339	Zm00001d010009
Zm00001d043293_T001	0.8086794	0.691466941	Zm00001d043293
Zm00001d025981_T004	0.8398375	0.913602433	Zm00001d025981
Zm00001d025981_T009	0.4137215	1.11118291	Zm00001d025981
Zm00001d025981_T020	0.9942835	0.205775344	Zm00001d025981
Zm00001d025981_T002	0.359111	-0.435054112	Zm00001d025981
Zm00001d042778_T002	0.6343135	1.653613966	Zm00001d042778
Zm00001d049126_T001	0.9628812	0.568868499	Zm00001d049126
Zm00001d052372_T001	0.8370558	-0.344894527	Zm00001d052372
Zm00001d052372_T002	0.8819727	-0.403884961	Zm00001d052372
Zm00001d032932_T001	0.5137998	-0.653010374	Zm00001d032932
Zm00001d009398_T003	0.9804228	0.010297671	Zm00001d009398
Zm00001d009398_T001	1	0.240000097	Zm00001d009398
Zm00001d017728_T001	0.9791066	0.443009349	Zm00001d017728

Zm00001d011979_T001	0.9509189	0.735481473	Zm00001d011979
Zm00001d038870_T001	0.02815853	-1.889852092	Zm00001d038870
Zm00001d047944_T001	0.02424821	2.278011857	Zm00001d047944
Zm00001d026265_T001	0.2021231	2.456379667	Zm00001d026265
Zm00001d026265_T002	0.000150852	-2.427326235	Zm00001d026265
Zm00001d026265_T005	0.419729	-0.691870899	Zm00001d026265
Zm00001d047898_T007	0.9732421	0.386158293	Zm00001d047898
Zm00001d047898_T008	0.941922	0.199969161	Zm00001d047898
Zm00001d047898_T002	0.9981278	0.223803264	Zm00001d047898
Zm00001d047898_T005	0.9216521	0.02944931	Zm00001d047898
Zm00001d037637_T001	0.3827249	-1.2035791	Zm00001d037637
Zm00001d026061_T001	0.3532541	-0.923440607	Zm00001d026061
Zm00001d037626_T002	0.215622	-1.116290428	Zm00001d037626
Zm00001d037626_T001	0.9416622	0.67964389	Zm00001d037626
Zm00001d032632_T006	0.9493302	0.123862095	Zm00001d032632
Zm00001d032632_T001	0.635589	0.938710054	Zm00001d032632
Zm00001d032632_T007	0.5221985	-0.823719645	Zm00001d032632
Zm00001d032632_T003	0.7141136	-0.021428906	Zm00001d032632
Zm00001d032632_T008	0.6821817	-0.831819707	Zm00001d032632
Zm00001d040210_T001	0.7812469	2.247835329	Zm00001d040210
Zm00001d011708_T001	0.9766874	0.157632585	Zm00001d011708
Zm00001d006950_T001	0.8419067	-0.724091235	Zm00001d006950
Zm00001d046930_T001	0.9032802	-0.254060737	Zm00001d046930
Zm00001d018733_T001	0.8816665	-0.00026961	Zm00001d018733
Zm00001d018733_T003	0.9118093	0.419443895	Zm00001d018733
Zm00001d018733_T006	0.9979568	0.138779211	Zm00001d018733
Zm00001d018733_T008	0.9422098	0.512704252	Zm00001d018733
Zm00001d018733_T005	0.9160053	0.019298758	Zm00001d018733
Zm00001d025763_T002	0.8135961	-0.230688023	Zm00001d025763
Zm00001d025763_T004	0.9812314	0.453275462	Zm00001d025763
Zm00001d025763_T006	0.6939449	-0.561023843	Zm00001d025763
Zm00001d025763_T008	0.5636783	-0.48135688	Zm00001d025763
Zm00001d025763_T001	0.08806919	-1.994258475	Zm00001d025763
Zm00001d025763_T005	0.1234187	-0.787360839	Zm00001d025763
Zm00001d025763_T003	0.9970835	0.382920716	Zm00001d025763
Zm00001d020887_T001	0.3495559	1.586899957	Zm00001d020887
Zm00001d002864_T001	0.6896661	1.215501051	Zm00001d002864
Zm00001d002901_T001	0.7183015	-0.556312119	Zm00001d002901
Zm00001d039422_T009	0.9907928	0.196873688	Zm00001d039422
Zm00001d039422_T007	0.9686323	0.181693878	Zm00001d039422
Zm00001d017429_T001	0.7912975	1.100501414	Zm00001d017429
Zm00001d012314_T002	0.8533785	-0.077337914	Zm00001d012314
Zm00001d012314_T005	1	0.370575584	Zm00001d012314
Zm00001d024796_T003	0.9031129	0.660601002	Zm00001d024796
Zm00001d024796_T004	0.9695609	0.137364572	Zm00001d024796
Zm00001d024796_T005	0.1538461	0.362055328	Zm00001d024796
Zm00001d016098_T002	0.1906129	-1.017689209	Zm00001d016098
Zm00001d014595_T001	0.9628432	0.068295629	Zm00001d014595
Zm00001d052937_T001	0.01232703	-1.901837486	Zm00001d052937
Zm00001d053155_T007	0.04008706	0.626444943	Zm00001d053155
Zm00001d053155_T004	0.9711278	0.171178984	Zm00001d053155
Zm00001d053155_T016	6.27E-13	2.701825138	Zm00001d053155
Zm00001d053155_T012	0.9860108	0.587707411	Zm00001d053155
Zm00001d020023_T002	0.757295	0.865421762	Zm00001d020023

Zm00001d017086_T001	0.2910059	-1.493261241	Zm00001d017086
Zm00001d018929_T007	0.9218473	0.03222935	Zm00001d018929
Zm00001d018929_T008	0.04312067	-0.508968203	Zm00001d018929
Zm00001d018929_T010	3.27E-08	1.266830101	Zm00001d018929
Zm00001d018929_T001	0.9429712	0.113098182	Zm00001d018929
Zm00001d018929_T006	0.9656136	0.099405464	Zm00001d018929
Zm00001d052011_T006	0.9671056	0.213326694	Zm00001d052011
Zm00001d052011_T002	0.8687321	-0.076687458	Zm00001d052011
Zm00001d017050_T006	0.9727583	0.136969526	Zm00001d017050
Zm00001d017050_T001	0.000275609	0.529545401	Zm00001d017050
Zm00001d017050_T012	0.9886764	0.072685774	Zm00001d017050
Zm00001d017050_T007	0.6589566	0.299010818	Zm00001d017050
Zm00001d003246_T005	0.7959622	0.671973166	Zm00001d003246
Zm00001d003246_T002	0.989787	-0.120678945	Zm00001d003246
Zm00001d027431_T002	0.3750526	1.622476084	Zm00001d027431
Zm00001d036437_T001	0.9100377	-0.501981314	Zm00001d036437
Zm00001d018464_T001	0.8537544	-0.373413466	Zm00001d018464
Zm00001d007216_T001	0.5599292	1.163830933	Zm00001d007216
Zm00001d006421_T001	0.9616027	0.233503455	Zm00001d006421
Zm00001d033591_T002	0.5954619	-0.446897805	Zm00001d033591
Zm00001d033591_T001	0.8010758	0.817526036	Zm00001d033591
Zm00001d046383_T001	0.8030099	-0.225747769	Zm00001d046383
Zm00001d007015_T001	0.9524127	0.50526629	Zm00001d007015
Zm00001d029620_T001	0.9237921	0.748293588	Zm00001d029620
Zm00001d036485_T003	0.7480195	0.787806296	Zm00001d036485
Zm00001d036485_T002	0.8598212	0.599833179	Zm00001d036485
Zm00001d041346_T001	0.9037324	-0.033903111	Zm00001d041346
Zm00001d037544_T001	0.6280901	1.31747503	Zm00001d037544
Zm00001d011953_T002	0.4052555	1.607803705	Zm00001d011953
Zm00001d011953_T004	0.6587378	0.298411724	Zm00001d011953
Zm00001d011953_T003	0.9887196	0.307155417	Zm00001d011953
Zm00001d012900_T001	0.04721739	2.169580629	Zm00001d012900
Zm00001d041280_T001	0.8008683	0.998593644	Zm00001d041280
Zm00001d041280_T002	0.3941547	0.465554617	Zm00001d041280
Zm00001d018218_T001	0.9706887	0.420826334	Zm00001d018218
Zm00001d044930_T001	0.996299	0.350665232	Zm00001d044930
Zm00001d051419_T003	0.02021864	-2.815788641	Zm00001d051419
Zm00001d051419_T001	0.7682041	0.393319472	Zm00001d051419
Zm00001d051419_T002	1	0.374609049	Zm00001d051419
Zm00001d006079_T001	0.6988666	1.055454909	Zm00001d006079
Zm00001d027347_T002	0.9928864	0.340931844	Zm00001d027347
Zm00001d027347_T003	0.9936409	0.349080592	Zm00001d027347
Zm00001d003062_T001	0.7470071	-0.571378388	Zm00001d003062
Zm00001d003419_T001	0.005206629	3.146493492	Zm00001d003419
Zm00001d020272_T001	0.3130813	1.477582075	Zm00001d020272
Zm00001d028577_T001	0.2432261	1.539128347	Zm00001d028577
Zm00001d049276_T003	0.9939021	0.188349747	Zm00001d049276
Zm00001d049276_T005	0.6632799	-0.320783301	Zm00001d049276
Zm00001d053524_T002	0.8533087	-0.137321599	Zm00001d053524
Zm00001d053524_T001	0.9429712	0.121998737	Zm00001d053524
Zm00001d010207_T002	0.9046104	0.236024445	Zm00001d010207
Zm00001d005856_T005	0.8720862	0.683653741	Zm00001d005856
Zm00001d005856_T003	0.2704636	-0.899106559	Zm00001d005856
Zm00001d005856_T004	0.6673107	0.938436941	Zm00001d005856

Zm00001d054076_T011	0.0299214	1.559382839	Zm00001d054076
Zm00001d054076_T044	0.7912425	0.694025963	Zm00001d054076
Zm00001d054076_T002	0.7055235	-0.224514081	Zm00001d054076
Zm00001d054076_T040	0.04000108	-1.235409275	Zm00001d054076
Zm00001d054076_T014	0.9822138	-0.005224783	Zm00001d054076
Zm00001d036387_T001	0.8987811	-0.0328443	Zm00001d036387
Zm00001d014253_T001	7.15E-07	4.573160869	Zm00001d014253
Zm00001d003923_T005	0.9293689	1.393998694	Zm00001d003923
Zm00001d003923_T003	1	0.274505381	Zm00001d003923
Zm00001d003923_T001	1	0.407620927	Zm00001d003923
Zm00001d032621_T003	0.9884825	0.233308387	Zm00001d032621
Zm00001d032621_T001	0.1070785	1.068384136	Zm00001d032621
Zm00001d006026_T001	0.9825858	0.179262954	Zm00001d006026
Zm00001d046323_T001	0.8647916	-0.129924707	Zm00001d046323
Zm00001d024938_T002	0.897685	0.701958393	Zm00001d024938
Zm00001d024938_T005	0.9894796	0.137165539	Zm00001d024938
Zm00001d024938_T003	0.9682673	-0.045365776	Zm00001d024938
Zm00001d014496_T001	0.6354704	-0.464943585	Zm00001d014496
Zm00001d022069_T001	0.9745948	0.499219664	Zm00001d022069
Zm00001d017513_T001	0.7511561	-0.264031152	Zm00001d017513
Zm00001d024593_T001	0.4446973	-0.828367597	Zm00001d024593
Zm00001d004099_T006	1.39E-09	1.93582664	Zm00001d004099
Zm00001d004099_T001	0.9723695	0.085600285	Zm00001d004099
Zm00001d004099_T003	0.9778436	0.398878803	Zm00001d004099
Zm00001d004099_T004	0.6367762	-0.316964415	Zm00001d004099
Zm00001d002564_T001	0.001416617	1.754354398	Zm00001d002564
Zm00001d039656_T005	0.9562711	0.357533223	Zm00001d039656
Zm00001d039656_T003	1	0.36527603	Zm00001d039656
Zm00001d039656_T001	0.850642	0.705688358	Zm00001d039656
Zm00001d042214_T001	0.01506602	2.660829122	Zm00001d042214
Zm00001d031488_T005	0.6156484	-0.257963583	Zm00001d031488
Zm00001d049664_T002	0.9262099	-0.321511448	Zm00001d049664
Zm00001d003563_T001	0.5501607	-0.571579335	Zm00001d003563
Zm00001d050323_T001	0.9049007	0.669097793	Zm00001d050323
Zm00001d002168_T001	0.7691507	1.056567182	Zm00001d002168
Zm00001d032363_T001	0.8786965	0.708709159	Zm00001d032363
Zm00001d051550_T001	0.5346213	-0.466739991	Zm00001d051550
Zm00001d028109_T005	0.9543237	0.133231734	Zm00001d028109
Zm00001d028109_T001	0.2419153	0.491575506	Zm00001d028109
Zm00001d047096_T001	0.6284759	-0.578849391	Zm00001d047096
Zm00001d025943_T001	0.9326774	0.005825672	Zm00001d025943
Zm00001d027376_T004	0.8031436	0.234915972	Zm00001d027376
Zm00001d027376_T003	0.2324687	-0.918463773	Zm00001d027376
Zm00001d027376_T008	0.9160957	-0.003491372	Zm00001d027376
Zm00001d027376_T001	0.7079663	0.997664913	Zm00001d027376
Zm00001d042575_T001	0.9979568	0.239131813	Zm00001d042575
Zm00001d002520_T001	0.2903147	-1.065691689	Zm00001d002520
Zm00001d012030_T001	0.9878022	0.260012811	Zm00001d012030
Zm00001d012030_T003	0.9894615	0.092193346	Zm00001d012030
Zm00001d042282_T002	0.9780511	0.565290932	Zm00001d042282
Zm00001d042282_T004	0.8031436	-0.702852346	Zm00001d042282
Zm00001d042282_T001	0.586985	-0.003727093	Zm00001d042282
Zm00001d042282_T003	0.9852645	0.521457079	Zm00001d042282
Zm00001d022160_T001	0.4569211	1.248967265	Zm00001d022160

Zm00001d022160_T003	0.8645432	0.67330581	Zm00001d022160
Zm00001d022160_T002	2.48E-11	-4.344683695	Zm00001d022160
Zm00001d040827_T004	0.6625969	0.348425691	Zm00001d040827
Zm00001d040827_T001	0.001423487	-1.576133117	Zm00001d040827
Zm00001d040827_T005	0.9832092	0.154739571	Zm00001d040827
Zm00001d002095_T001	1	0.345478585	Zm00001d002095
Zm00001d022490_T005	0.5877187	-0.551572458	Zm00001d022490
Zm00001d022490_T004	0.7020458	0.640696175	Zm00001d022490
Zm00001d022490_T003	0.01911835	1.454552181	Zm00001d022490
Zm00001d022490_T001	0.9262419	0.7891004	Zm00001d022490
Zm00001d048053_T002	0.6354704	-0.314783744	Zm00001d048053
Zm00001d048053_T001	0.9222107	0.044794841	Zm00001d048053
Zm00001d048053_T004	0.6926729	-0.844454759	Zm00001d048053
Zm00001d048053_T003	0.7458694	-0.465324771	Zm00001d048053
Zm00001d022621_T028	0.9085393	-0.038785867	Zm00001d022621
Zm00001d022621_T017	0.3381821	1.282010348	Zm00001d022621
Zm00001d022621_T013	0.967161	0.144774648	Zm00001d022621
Zm00001d022621_T001	0.8727241	0.433438687	Zm00001d022621
Zm00001d022621_T003	0.7614965	0.318790006	Zm00001d022621
Zm00001d022621_T019	0.005500518	-2.608759239	Zm00001d022621
Zm00001d030955_T003	0.718063	-0.277490326	Zm00001d030955
Zm00001d030955_T001	0.9188198	0.722893554	Zm00001d030955
Zm00001d048116_T001	0.5045621	-0.923902908	Zm00001d048116
Zm00001d028907_T035	0.6414865	0.188289528	Zm00001d028907
Zm00001d028907_T001	0.7006089	-0.293800555	Zm00001d028907
Zm00001d028907_T007	0.6450309	-0.08506332	Zm00001d028907
Zm00001d028907_T018	0.8050292	-0.157938392	Zm00001d028907
Zm00001d028907_T031	0.9781672	0.132868359	Zm00001d028907
Zm00001d028907_T047	0.9851204	0.137653844	Zm00001d028907
Zm00001d028907_T014	7.83E-05	-1.523353964	Zm00001d028907
Zm00001d049505_T001	0.9944899	0.171640802	Zm00001d049505
Zm00001d053569_T001	0.9902022	0.260158046	Zm00001d053569
Zm00001d013542_T006	0.5535826	-0.686551227	Zm00001d013542
Zm00001d013542_T001	0.9666757	0.477397809	Zm00001d013542
Zm00001d013542_T005	0.01442294	-1.289931744	Zm00001d013542
Zm00001d022247_T001	0.9410845	0.704980312	Zm00001d022247
Zm00001d052138_T004	0.9171732	0.628779342	Zm00001d052138
Zm00001d039271_T001	1	0.381917963	Zm00001d039271
Zm00001d021246_T002	0.972985	0.536588536	Zm00001d021246
Zm00001d021246_T001	0.9849018	0.009597276	Zm00001d021246
Zm00001d021879_T001	0.3321759	-0.86783697	Zm00001d021879
Zm00001d003084_T001	1	0.314991303	Zm00001d003084
Zm00001d025126_T001	0.9910452	0.0736029	Zm00001d025126
Zm00001d008826_T009	0.01067914	-2.199485473	Zm00001d008826
Zm00001d008826_T015	1.24E-19	5.910344573	Zm00001d008826
Zm00001d008826_T014	0.000149326	2.632281777	Zm00001d008826
Zm00001d008826_T068	0.6354554	1.269467892	Zm00001d008826
Zm00001d008826_T041	0.000142536	2.002638609	Zm00001d008826
Zm00001d008826_T022	5.25E-06	2.582279583	Zm00001d008826
Zm00001d008826_T006	0.7697196	0.376644091	Zm00001d008826
Zm00001d008826_T017	0.7875308	1.078098787	Zm00001d008826
Zm00001d008826_T048	1	0.418044967	Zm00001d008826
Zm00001d008826_T101	0.01936948	0.930512712	Zm00001d008826
Zm00001d008826_T087	0.04404322	0.254457728	Zm00001d008826

Zm00001d039625_T001	0.9248904	0.039234647	Zm00001d039625
Zm00001d052266_T001	0.9177108	-0.094785749	Zm00001d052266
Zm00001d052585_T001	0.09313388	1.807020492	Zm00001d052585
Zm00001d033585_T001	0.65066	1.274444782	Zm00001d033585
Zm00001d042996_T001	0.9681793	0.134109069	Zm00001d042996
Zm00001d003271_T001	0.6316155	-0.726700085	Zm00001d003271
Zm00001d005029_T001	0.9918302	0.269585566	Zm00001d005029
Zm00001d006517_T001	0.8896831	0.760988964	Zm00001d006517
Zm00001d029571_T001	0.745469	0.852986307	Zm00001d029571
Zm00001d046214_T003	0.8990587	0.867488925	Zm00001d046214
Zm00001d010808_T001	0.9971866	0.375257678	Zm00001d010808
Zm00001d003193_T004	1	0.245278624	Zm00001d003193
Zm00001d003193_T001	0.4763999	-0.708602941	Zm00001d003193
Zm00001d003193_T003	0.5901362	-0.645578382	Zm00001d003193
Zm00001d003193_T002	0.7648433	-0.287866487	Zm00001d003193
Zm00001d004051_T001	0.9664406	-0.199285683	Zm00001d004051
Zm00001d009811_T005	3.64E-10	-1.820950172	Zm00001d009811
Zm00001d009811_T001	0.9130326	0.682756486	Zm00001d009811
Zm00001d045184_T001	0.004070328	2.808367297	Zm00001d045184
Zm00001d009990_T003	0.8851966	-0.154078218	Zm00001d009990
Zm00001d052393_T001	0.4438025	0.886916576	Zm00001d052393
Zm00001d014518_T001	0.633194	-1.245063979	Zm00001d014518
Zm00001d039970_T001	0.8160618	-0.323631669	Zm00001d039970
Zm00001d035601_T001	0.9745948	0.124434769	Zm00001d035601
Zm00001d023673_T001	0.9224443	0.705831452	Zm00001d023673
Zm00001d012144_T001	0.8494747	0.894469957	Zm00001d012144
Zm00001d043768_T001	0.7527904	1.022276875	Zm00001d043768
Zm00001d043768_T002	0.9315306	-0.04721059	Zm00001d043768
Zm00001d016722_T002	0.4647353	-0.478774197	Zm00001d016722
Zm00001d016722_T004	0.9962521	0.114748413	Zm00001d016722
Zm00001d052079_T001	0.9971866	0.20361056	Zm00001d052079
Zm00001d052079_T007	0.9833565	0.10624969	Zm00001d052079
Zm00001d052079_T003	0.04732668	0.474495026	Zm00001d052079
Zm00001d052079_T008	0.2038697	0.802720066	Zm00001d052079
Zm00001d052079_T041	0.9561521	-0.000493961	Zm00001d052079
Zm00001d052079_T004	0.3343236	0.586480421	Zm00001d052079
Zm00001d020807_T003	0.965989	0.619243082	Zm00001d020807
Zm00001d005037_T001	0.7945048	-0.57253036	Zm00001d005037
Zm00001d048074_T002	0.8597935	0.122250113	Zm00001d048074
Zm00001d048074_T003	0.2638258	1.649118826	Zm00001d048074
Zm00001d048074_T001	0.5092251	1.197554603	Zm00001d048074
Zm00001d043727_T001	0.9390517	0.007761051	Zm00001d043727
Zm00001d011335_T001	0.9355135	0.010382012	Zm00001d011335
Zm00001d019426_T004	0.9888172	0.046798345	Zm00001d019426
Zm00001d019426_T001	0.923446	-0.086897782	Zm00001d019426
Zm00001d019426_T002	0.5101057	-0.625781553	Zm00001d019426
Zm00001d020821_T005	0.9745948	0.444139202	Zm00001d020821
Zm00001d020821_T003	0.2836969	0.318527449	Zm00001d020821
Zm00001d045389_T004	0.07120296	-0.834209576	Zm00001d045389
Zm00001d045389_T010	1	0.203801394	Zm00001d045389
Zm00001d045389_T003	0.3449255	0.674046512	Zm00001d045389
Zm00001d045389_T011	0.8720862	0.226970083	Zm00001d045389
Zm00001d045389_T008	0.9475709	-0.025669804	Zm00001d045389
Zm00001d033233_T001	0.910614	-0.001723391	Zm00001d033233

Zm00001d033233_T002	0.7861057	-0.390777297	Zm00001d033233
Zm00001d048722_T001	0.9753621	0.195398127	Zm00001d048722
Zm00001d013282_T001	0.7285296	-0.8821065	Zm00001d013282
Zm00001d013282_T002	0.6384824	-0.64954664	Zm00001d013282
Zm00001d022465_T002	0.8609127	0.601672526	Zm00001d022465
Zm00001d022465_T003	0.9141981	-0.090530402	Zm00001d022465
Zm00001d024596_T005	0.9968176	0.080861174	Zm00001d024596
Zm00001d024596_T003	0.5890036	0.361733663	Zm00001d024596
Zm00001d024596_T006	0.8842271	-0.100806568	Zm00001d024596
Zm00001d044284_T001	0.9343321	0.109203068	Zm00001d044284
Zm00001d005456_T001	0.1296276	1.841383369	Zm00001d005456
Zm00001d015007_T004	1	0.216164005	Zm00001d015007
Zm00001d030841_T001	0.5270926	-0.614005335	Zm00001d030841
Zm00001d052766_T002	0.8987811	-0.044441538	Zm00001d052766
Zm00001d024952_T002	0.1156933	-0.583137216	Zm00001d024952
Zm00001d024952_T005	1	0.409569331	Zm00001d024952
Zm00001d024952_T006	0.9118093	0.866548546	Zm00001d024952
Zm00001d024952_T013	0.9216521	0.457818529	Zm00001d024952
Zm00001d024952_T007	0.3919325	-0.166239541	Zm00001d024952
Zm00001d024952_T004	0.7945048	1.009599953	Zm00001d024952
Zm00001d024952_T015	1	0.367532873	Zm00001d024952
Zm00001d044775_T002	0.9108174	-0.173491099	Zm00001d044775
Zm00001d005267_T001	0.4848966	-1.896009332	Zm00001d005267
Zm00001d019948_T002	0.8660897	0.714304415	Zm00001d019948
Zm00001d019948_T001	0.1459538	1.406774039	Zm00001d019948
Zm00001d021715_T001	0.9111683	-0.035470372	Zm00001d021715
Zm00001d053863_T001	0.8109592	-0.533541997	Zm00001d053863
Zm00001d034447_T002	0.684918	0.366374499	Zm00001d034447
Zm00001d034447_T001	0.9501111	-0.088044521	Zm00001d034447
Zm00001d033683_T001	0.9954444	0.224132969	Zm00001d033683
Zm00001d033683_T002	0.001075711	-2.898998394	Zm00001d033683
Zm00001d033683_T003	0.9825858	0.306219484	Zm00001d033683
Zm00001d047673_T001	0.8984125	-0.077163407	Zm00001d047673
Zm00001d047673_T002	0.6609807	0.437681887	Zm00001d047673
Zm00001d020613_T001	0.745718	-0.353663883	Zm00001d020613
Zm00001d004997_T001	0.9893167	0.296673283	Zm00001d004997
Zm00001d017887_T001	0.000948121	2.222249511	Zm00001d017887
Zm00001d017887_T010	0.4597444	-0.433454357	Zm00001d017887
Zm00001d017887_T011	0.8229103	-0.144404577	Zm00001d017887
Zm00001d017887_T006	0.960704	0.062328085	Zm00001d017887
Zm00001d004539_T001	0.9791729	0.638015172	Zm00001d004539
Zm00001d053282_T001	0.7907437	-0.228361559	Zm00001d053282
Zm00001d032788_T001	0.8727241	0.564354039	Zm00001d032788
Zm00001d027624_T001	0.941547	0.761760039	Zm00001d027624
Zm00001d027624_T002	0.9851204	1.118979235	Zm00001d027624
Zm00001d012699_T004	1	0.416583754	Zm00001d012699
Zm00001d012699_T005	0.6644959	1.087491877	Zm00001d012699
Zm00001d036630_T001	0.9979568	0.405128277	Zm00001d036630
Zm00001d025808_T001	0.9979393	0.427346844	Zm00001d025808
Zm00001d026039_T004	0.963405	0.076990005	Zm00001d026039
Zm00001d003161_T001	0.6639639	-0.334007938	Zm00001d003161
Zm00001d037875_T003	0.8923684	-0.072544171	Zm00001d037875
Zm00001d037875_T006	0.3675057	-1.090018	Zm00001d037875
Zm00001d009932_T001	0.973825	0.289470071	Zm00001d009932

Zm00001d019627_T002	0.8492405	-0.096988012	Zm00001d019627
Zm00001d010523_T004	0.8554968	0.019130071	Zm00001d010523
Zm00001d010523_T006	0.9269932	0.727180627	Zm00001d010523
Zm00001d010523_T007	1	0.057734011	Zm00001d010523
Zm00001d010523_T005	0.7183895	0.895628184	Zm00001d010523
Zm00001d008527_T001	0.5714373	-0.730550899	Zm00001d008527
Zm00001d044527_T001	0.9580487	0.508475286	Zm00001d044527
Zm00001d049178_T001	0.8920456	0.779147903	Zm00001d049178
Zm00001d003333_T010	0.7566503	0.124020687	Zm00001d003333
Zm00001d003333_T002	0.812161	1.026065726	Zm00001d003333
Zm00001d003333_T008	0.000799898	0.550175199	Zm00001d003333
Zm00001d041979_T001	0.9294749	-0.340940801	Zm00001d041979
Zm00001d048637_T001	0.148906	0.669567252	Zm00001d048637
Zm00001d047587_T001	0.9497909	0.495107508	Zm00001d047587
Zm00001d047587_T005	0.8751525	0.093160661	Zm00001d047587
Zm00001d047587_T002	1	0.153133168	Zm00001d047587
Zm00001d047587_T010	0.8787096	0.771235051	Zm00001d047587
Zm00001d047587_T008	0.9468268	0.064217591	Zm00001d047587
Zm00001d047587_T009	0.9942835	0.215237515	Zm00001d047587
Zm00001d026291_T013	5.80E-05	1.960377463	Zm00001d026291
Zm00001d026291_T012	0.5127506	0.714962562	Zm00001d026291
Zm00001d026291_T007	0.6394238	-0.414628561	Zm00001d026291
Zm00001d030612_T001	0.9802432	0.175571822	Zm00001d030612
Zm00001d008633_T001	0.997764	0.122385389	Zm00001d008633
Zm00001d051384_T001	0.8585804	-0.24955667	Zm00001d051384
Zm00001d044723_T002	0.9894615	0.273291856	Zm00001d044723
Zm00001d044723_T001	0.16474	-0.483996985	Zm00001d044723
Zm00001d047775_T001	0.9861428	0.001505343	Zm00001d047775
Zm00001d025252_T001	0.9609506	0.57002587	Zm00001d025252
Zm00001d038122_T001	0.839321	-0.160268474	Zm00001d038122
Zm00001d037214_T009	0.9053391	0.759131762	Zm00001d037214
Zm00001d037214_T002	0.00238057	2.52344656	Zm00001d037214
Zm00001d037214_T010	3.12E-09	0.888099026	Zm00001d037214
Zm00001d037214_T007	1	-0.030891275	Zm00001d037214
Zm00001d037214_T006	0.005033695	2.090514266	Zm00001d037214
Zm00001d037214_T001	0.8988878	-0.071847586	Zm00001d037214
Zm00001d052147_T001	0.9219195	0.781010524	Zm00001d052147
Zm00001d053718_T001	0.9139882	0.040437358	Zm00001d053718
Zm00001d042487_T001	0.7313444	0.659462246	Zm00001d042487
Zm00001d050905_T001	0.9196877	-0.142277216	Zm00001d050905
Zm00001d031256_T031	0.5890036	0.479645093	Zm00001d031256
Zm00001d031256_T002	0.2551003	1.204719769	Zm00001d031256
Zm00001d031256_T021	0.9377241	0.088138272	Zm00001d031256
Zm00001d031256_T017	0.9988864	0.024447129	Zm00001d031256
Zm00001d031256_T001	0.9840918	0.071745676	Zm00001d031256
Zm00001d008310_T002	0.9573434	0.533218537	Zm00001d008310
Zm00001d008310_T003	0.8050292	0.323754487	Zm00001d008310
Zm00001d008310_T004	0.1272455	-1.787727805	Zm00001d008310
Zm00001d043288_T001	0.908501	0.830923095	Zm00001d043288
Zm00001d021410_T001	0.01989623	2.75364622	Zm00001d021410
Zm00001d034525_T001	0.9702242	0.108951474	Zm00001d034525
Zm00001d012098_T003	0.9184323	0.588467482	Zm00001d012098
Zm00001d012098_T002	0.6767305	1.079360712	Zm00001d012098
Zm00001d012098_T001	0.3288311	-0.90124298	Zm00001d012098

Zm00001d013493_T001	0.8749606	0.618642307	Zm00001d013493
Zm00001d013493_T005	0.9968176	0.102077826	Zm00001d013493
Zm00001d013493_T006	0.1911183	-0.948319771	Zm00001d013493
Zm00001d013493_T003	0.8236738	-0.340855083	Zm00001d013493
Zm00001d026653_T001	0.8520487	0.679165151	Zm00001d026653
Zm00001d026653_T006	0.0153026	-0.866534553	Zm00001d026653
Zm00001d026653_T007	0.9588436	0.838740763	Zm00001d026653
Zm00001d026653_T005	1	0.198257287	Zm00001d026653
Zm00001d047804_T001	0.9892044	0.28823283	Zm00001d047804
Zm00001d034518_T003	0.8027057	-0.324574873	Zm00001d034518
Zm00001d034518_T001	0.9055515	-0.033083832	Zm00001d034518
Zm00001d034518_T004	0.3451683	-1.052787769	Zm00001d034518
Zm00001d011384_T001	0.9213556	-0.22065364	Zm00001d011384
Zm00001d039745_T001	0.2090746	-0.906783578	Zm00001d039745
Zm00001d028404_T001	0.02545254	-2.516168559	Zm00001d028404
Zm00001d050726_T001	0.02555124	1.190876882	Zm00001d050726
Zm00001d050726_T002	0.08584965	-1.311620566	Zm00001d050726
Zm00001d050726_T003	0.7561907	1.008493979	Zm00001d050726
Zm00001d040788_T001	0.8645515	0.915575686	Zm00001d040788
Zm00001d030108_T014	0.1116166	1.986558833	Zm00001d030108
Zm00001d030108_T025	0.4502273	0.739120177	Zm00001d030108
Zm00001d015775_T001	0.9218473	0.486858903	Zm00001d015775
Zm00001d001966_T008	0.9690782	0.341543997	Zm00001d001966
Zm00001d001966_T002	0.9073468	0.82680525	Zm00001d001966
Zm00001d001966_T001	0.09893999	-0.556993569	Zm00001d001966
Zm00001d001966_T005	0.9846714	0.140737853	Zm00001d001966
Zm00001d001966_T003	0.5231196	-1.219992604	Zm00001d001966
Zm00001d027872_T009	0.8892107	-0.020043611	Zm00001d027872
Zm00001d027872_T007	0.972985	0.151914753	Zm00001d027872
Zm00001d027872_T005	0.9722469	0.40471792	Zm00001d027872
Zm00001d027872_T003	0.528476	0.768101385	Zm00001d027872
Zm00001d018074_T001	0.90276	-0.047507049	Zm00001d018074
Zm00001d041513_T001	0.9942835	0.101662574	Zm00001d041513
Zm00001d026335_T003	0.9782953	0.32050843	Zm00001d026335
Zm00001d026335_T010	0.9618822	0.321780619	Zm00001d026335
Zm00001d026335_T006	0.7481233	-0.780338246	Zm00001d026335
Zm00001d026335_T002	0.9040442	0.530698318	Zm00001d026335
Zm00001d026129_T007	0.7544299	-0.608366921	Zm00001d026129
Zm00001d026129_T001	0.8557049	-0.212276255	Zm00001d026129
Zm00001d022589_T003	0.9498304	0.623757671	Zm00001d022589
Zm00001d022589_T001	0.4799121	1.272993604	Zm00001d022589
Zm00001d022589_T004	0.8764225	0.8214042	Zm00001d022589
Zm00001d005498_T004	0.9487214	0.647949674	Zm00001d005498
Zm00001d013060_T001	0.9420032	0.625146659	Zm00001d013060
Zm00001d013060_T003	0.3306249	-0.278227653	Zm00001d013060
Zm00001d002842_T003	0.6405312	1.053255025	Zm00001d002842
Zm00001d002842_T001	0.9326774	-0.076813532	Zm00001d002842
Zm00001d002842_T002	0.9325478	0.49727028	Zm00001d002842
Zm00001d019283_T006	0.9493302	-0.326796399	Zm00001d019283
Zm00001d019283_T004	0.9709887	1.085452705	Zm00001d019283
Zm00001d019283_T001	0.05503591	-1.135825064	Zm00001d019283
Zm00001d019283_T007	0.9672613	0.176717001	Zm00001d019283
Zm00001d019283_T010	0.1218964	3.149483785	Zm00001d019283
Zm00001d019283_T008	0.8038564	2.20164274	Zm00001d019283

Zm00001d006198_T002	0.7232875	0.788103945	Zm00001d006198
Zm00001d006198_T003	0.8097414	-0.165559992	Zm00001d006198
Zm00001d006198_T004	0.9966571	0.186006983	Zm00001d006198
Zm00001d039916_T003	0.9196877	-0.044610313	Zm00001d039916
Zm00001d039916_T009	0.09695879	-1.533773878	Zm00001d039916
Zm00001d039916_T010	0.1217329	0.001343855	Zm00001d039916
Zm00001d039916_T005	0.9529184	0.218444196	Zm00001d039916
Zm00001d039916_T002	0.9627389	0.638622879	Zm00001d039916
Zm00001d039916_T006	0.8409106	1.930750317	Zm00001d039916
Zm00001d024248_T001	0.9846714	0.201141794	Zm00001d024248
Zm00001d009541_T001	0.9781725	0.54901463	Zm00001d009541
Zm00001d017994_T001	0.9884825	0.070661677	Zm00001d017994
Zm00001d002046_T008	0.1410912	1.437979716	Zm00001d002046
Zm00001d002046_T009	0.718063	1.130957225	Zm00001d002046
Zm00001d002046_T002	0.2387791	0.436030565	Zm00001d002046
Zm00001d002046_T006	0.1231755	0.664537266	Zm00001d002046
Zm00001d047356_T001	0.9955906	-0.142847762	Zm00001d047356
Zm00001d047356_T002	0.9894615	-0.196040865	Zm00001d047356
Zm00001d009211_T002	0.9824348	0.185878412	Zm00001d009211
Zm00001d009211_T001	0.9894615	0.351785997	Zm00001d009211
Zm00001d009211_T003	0.9260032	1.758547849	Zm00001d009211
Zm00001d032485_T001	0.9944696	0.117115929	Zm00001d032485
Zm00001d032485_T002	0.6321607	1.258928193	Zm00001d032485
Zm00001d034754_T001	0.9825858	0.421470411	Zm00001d034754
Zm00001d035476_T002	0.9970835	0.29644003	Zm00001d035476
Zm00001d012938_T003	0.9584734	0.147340515	Zm00001d012938
Zm00001d012938_T004	0.8325146	0.699963191	Zm00001d012938
Zm00001d012938_T001	0.3440588	-0.797341499	Zm00001d012938
Zm00001d012938_T002	0.8593483	0.710440636	Zm00001d012938
Zm00001d032279_T006	0.3515082	1.700084291	Zm00001d032279
Zm00001d032279_T001	0.9954444	0.335253239	Zm00001d032279
Zm00001d038060_T001	0.7979593	-0.238960724	Zm00001d038060
Zm00001d050394_T001	0.7297076	1.061319957	Zm00001d050394
Zm00001d027299_T015	0.04573669	-1.273637816	Zm00001d027299
Zm00001d027299_T001	0.5954124	-0.695735488	Zm00001d027299
Zm00001d027299_T003	0.8022394	0.458243207	Zm00001d027299
Zm00001d027299_T008	0.08311592	0.409898018	Zm00001d027299
Zm00001d027299_T010	6.54E-15	2.714448638	Zm00001d027299
Zm00001d040056_T004	0.7945048	0.390150297	Zm00001d040056
Zm00001d040056_T003	0.9368844	0.746951755	Zm00001d040056
Zm00001d005867_T002	0.9611351	0.240827466	Zm00001d005867
Zm00001d007450_T001	0.710892	-0.463504107	Zm00001d007450
Zm00001d052009_T008	0.3380134	0.379796608	Zm00001d052009
Zm00001d052009_T002	0.9089718	0.030922106	Zm00001d052009
Zm00001d052009_T005	0.9579944	0.442718357	Zm00001d052009
Zm00001d004730_T001	0.6354554	-0.883278079	Zm00001d004730
Zm00001d035356_T002	0.5523491	-0.452247025	Zm00001d035356
Zm00001d035356_T001	0.9942835	0.366164562	Zm00001d035356
Zm00001d041229_T005	0.7509844	0.835328536	Zm00001d041229
Zm00001d041229_T006	0.03648535	-0.429771108	Zm00001d041229
Zm00001d041229_T001	0.3486589	0.866329916	Zm00001d041229
Zm00001d041229_T004	0.6014554	0.265137672	Zm00001d041229
Zm00001d029475_T001	0.08717153	-1.771587629	Zm00001d029475
Zm00001d029495_T001	0.3544526	-0.916321248	Zm00001d029495

Zm00001d024274_T005	0.6910922	0.223079935	Zm00001d024274
Zm00001d024274_T007	1	0.208790942	Zm00001d024274
Zm00001d024274_T001	0.09739046	-1.875590587	Zm00001d024274
Zm00001d024274_T002	0.9500229	0.092521472	Zm00001d024274
Zm00001d044008_T005	0.2237488	1.680631198	Zm00001d044008
Zm00001d044008_T001	7.74E-12	1.620733799	Zm00001d044008
Zm00001d044008_T011	0.8588584	-0.102198189	Zm00001d044008
Zm00001d009231_T001	0.9889811	0.109117103	Zm00001d009231
Zm00001d030593_T005	0.7358894	0.869084967	Zm00001d030593
Zm00001d030593_T002	0.6162619	0.68236504	Zm00001d030593
Zm00001d030593_T003	0.815049	0.49800328	Zm00001d030593
Zm00001d030593_T001	1	0.430421457	Zm00001d030593
Zm00001d035169_T001	0.8031436	-0.281719575	Zm00001d035169
Zm00001d009746_T001	1	0.310146112	Zm00001d009746
Zm00001d038914_T001	0.8497683	-0.389210228	Zm00001d038914
Zm00001d017810_T007	0.9380783	0.382743987	Zm00001d017810
Zm00001d017810_T011	0.5593597	-0.48748021	Zm00001d017810
Zm00001d017810_T013	0.2408958	-0.705859044	Zm00001d017810
Zm00001d017810_T004	0.3354449	1.342862095	Zm00001d017810
Zm00001d017810_T025	0.1338921	1.466060051	Zm00001d017810
Zm00001d017810_T014	0.9529094	0.133016185	Zm00001d017810
Zm00001d017810_T023	0.9747606	0.179447636	Zm00001d017810
Zm00001d017810_T022	0.7923921	-0.267615166	Zm00001d017810
Zm00001d017810_T021	0.9817074	0.186229352	Zm00001d017810
Zm00001d018341_T001	0.3442322	0.277313232	Zm00001d018341
Zm00001d018341_T010	0.6927383	-0.501281094	Zm00001d018341
Zm00001d018341_T008	0.9781672	0.03341112	Zm00001d018341
Zm00001d018341_T003	0.9968176	0.264314314	Zm00001d018341
Zm00001d049038_T006	0.8203654	0.679571122	Zm00001d049038
Zm00001d049038_T017	0.9913523	0.3205591	Zm00001d049038
Zm00001d049038_T015	1.16E-11	2.942039882	Zm00001d049038
Zm00001d049038_T002	0.7626242	1.010209544	Zm00001d049038
Zm00001d049038_T005	0.9544587	0.149444474	Zm00001d049038
Zm00001d038272_T004	0.3225834	1.442327049	Zm00001d038272
Zm00001d038272_T001	0.9889811	0.494358669	Zm00001d038272
Zm00001d038272_T006	0.1786485	1.479119408	Zm00001d038272
Zm00001d038272_T003	0.5718169	1.018756467	Zm00001d038272
Zm00001d020042_T001	0.9173494	0.314683828	Zm00001d020042
Zm00001d047592_T006	0.889025	0.616650028	Zm00001d047592
Zm00001d047592_T001	0.7897201	0.549987736	Zm00001d047592
Zm00001d047592_T008	0.8545272	-0.069891385	Zm00001d047592
Zm00001d047592_T002	0.366346	-1.027177668	Zm00001d047592
Zm00001d047592_T005	0.9685273	0.08487939	Zm00001d047592
Zm00001d047592_T009	0.967161	0.019629624	Zm00001d047592
Zm00001d047592_T003	0.1580062	1.090031195	Zm00001d047592
Zm00001d038924_T001	0.894759	0.685814379	Zm00001d038924
Zm00001d020755_T001	0.2153407	-0.405294353	Zm00001d020755
Zm00001d010944_T001	0.9216521	0.007329246	Zm00001d010944
Zm00001d038447_T001	0.000368451	3.724451572	Zm00001d038447
Zm00001d021232_T001	0.9995663	0.23839672	Zm00001d021232
Zm00001d000012_T001	0.003148726	1.487517707	Zm00001d000012
Zm00001d018197_T001	0.9860108	0.436361747	Zm00001d018197
Zm00001d031338_T002	0.9819415	0.169337763	Zm00001d031338
Zm00001d031338_T001	0.9400285	0.749479163	Zm00001d031338

Zm00001d017150_T001	0.932111	0.713224058	Zm00001d017150
Zm00001d017150_T002	0.5718096	0.768519413	Zm00001d017150
Zm00001d017150_T004	0.6182379	-0.795109744	Zm00001d017150
Zm00001d017150_T003	0.8647028	-0.103023081	Zm00001d017150
Zm00001d053748_T001	0.147668	1.667021343	Zm00001d053748
Zm00001d053748_T002	0.4198364	1.154829815	Zm00001d053748
Zm00001d028093_T003	0.000476965	2.145586514	Zm00001d028093
Zm00001d028093_T001	0.07865132	2.148317089	Zm00001d028093
Zm00001d002333_T002	0.7866157	-0.036126774	Zm00001d002333
Zm00001d002333_T008	6.25E-07	-0.829353571	Zm00001d002333
Zm00001d021332_T001	0.9760472	0.349124278	Zm00001d021332
Zm00001d021332_T002	0.9563257	0.220235244	Zm00001d021332
Zm00001d021332_T004	1	0.342365532	Zm00001d021332
Zm00001d021332_T005	0.7697196	0.441976047	Zm00001d021332
Zm00001d021332_T003	0.9804228	0.459589741	Zm00001d021332
Zm00001d020311_T001	1	0.028127104	Zm00001d020311
Zm00001d010915_T001	0.7831859	-0.286276674	Zm00001d010915
Zm00001d050947_T001	0.916914	0.549946994	Zm00001d050947
Zm00001d050947_T002	0.0191703	0.746115713	Zm00001d050947
Zm00001d050947_T003	0.6107148	-0.084483581	Zm00001d050947
Zm00001d050947_T006	0.9830387	0.239867404	Zm00001d050947
Zm00001d041575_T002	0.8660897	-0.12754308	Zm00001d041575
Zm00001d041575_T003	0.7909906	-0.280391918	Zm00001d041575
Zm00001d020003_T002	1	0.151266695	Zm00001d020003
Zm00001d020003_T001	0.9887889	0.237494724	Zm00001d020003
Zm00001d020003_T003	0.5754586	-0.837551142	Zm00001d020003
Zm00001d047271_T002	0.7952435	-0.395725314	Zm00001d047271
Zm00001d047271_T003	0.9486412	0.039662223	Zm00001d047271
Zm00001d038070_T001	0.9945076	-0.327075617	Zm00001d038070
Zm00001d017932_T001	0.7499604	-0.700101154	Zm00001d017932
Zm00001d017932_T002	0.7114361	0.648793528	Zm00001d017932
Zm00001d033854_T001	0.985857	0.210126868	Zm00001d033854
Zm00001d019165_T002	0.9412467	0.631598119	Zm00001d019165
Zm00001d017531_T001	0.9196877	-0.018335371	Zm00001d017531
Zm00001d002999_T001	0.000105547	3.51827414	Zm00001d002999
Zm00001d003395_T005	0.3373089	-1.759953799	Zm00001d003395
Zm00001d003395_T001	0.9303773	0.09181904	Zm00001d003395
Zm00001d003395_T003	0.9408833	-0.004845498	Zm00001d003395
Zm00001d053891_T001	0.3468213	0.64727394	Zm00001d053891
Zm00001d012137_T010	0.7735802	0.87571885	Zm00001d012137
Zm00001d012137_T009	0.3097848	-0.206890051	Zm00001d012137
Zm00001d012137_T001	0.9965998	0.280652028	Zm00001d012137
Zm00001d012137_T015	0.63821	-0.731982797	Zm00001d012137
Zm00001d039822_T001	0.6044228	-0.273931645	Zm00001d039822
Zm00001d018420_T009	0.9779656	0.222130366	Zm00001d018420
Zm00001d018420_T011	0.6436915	0.913359556	Zm00001d018420
Zm00001d018420_T001	0.9465969	0.483409331	Zm00001d018420
Zm00001d018420_T005	0.8444973	0.559829927	Zm00001d018420
Zm00001d048404_T004	0.9804228	0.421304543	Zm00001d048404
Zm00001d048404_T003	0.5902617	-0.083162662	Zm00001d048404
Zm00001d048404_T020	3.69E-12	-1.914016962	Zm00001d048404
Zm00001d048404_T013	6.73E-11	1.567546463	Zm00001d048404
Zm00001d048404_T002	0.2048303	0.359030479	Zm00001d048404
Zm00001d048404_T011	3.55E-12	1.961619704	Zm00001d048404

Zm00001d051946_T001	0.9902022	0.475065784	Zm00001d051946
Zm00001d049018_T003	0.8921702	-0.093589574	Zm00001d049018
Zm00001d049018_T001	0.9797082	0.474889373	Zm00001d049018
Zm00001d047555_T001	0.8598316	-0.192998198	Zm00001d047555
Zm00001d031534_T001	0.4520593	1.566842202	Zm00001d031534
Zm00001d033393_T001	0.9921377	0.348692201	Zm00001d033393
Zm00001d015407_T001	0.9968176	0.265071045	Zm00001d015407
Zm00001d018803_T001	0.8746701	-0.089226282	Zm00001d018803
Zm00001d016731_T002	0.7473143	-0.588715839	Zm00001d016731
Zm00001d016731_T001	0.5750109	1.337801196	Zm00001d016731
Zm00001d016604_T001	0.9907928	0.224875927	Zm00001d016604
Zm00001d027267_T002	0.9078934	0.018837612	Zm00001d027267
Zm00001d003830_T001	0.1552605	1.970511993	Zm00001d003830
Zm00001d042239_T001	0.009062656	2.419777145	Zm00001d042239
Zm00001d029718_T001	0.9701986	-0.154572212	Zm00001d029718
Zm00001d034978_T001	0.7847088	-0.553497821	Zm00001d034978
Zm00001d008996_T002	0.9540138	0.45985237	Zm00001d008996
Zm00001d008996_T001	0.8886272	-0.072065943	Zm00001d008996
Zm00001d050224_T001	0.9914064	0.427114239	Zm00001d050224
Zm00001d023779_T003	0.06677029	-0.6433754	Zm00001d023779
Zm00001d023779_T001	0.7458694	-0.33113526	Zm00001d023779
Zm00001d025724_T001	0.8819727	-0.130421208	Zm00001d025724
Zm00001d003368_T008	0.7000765	0.792425234	Zm00001d003368
Zm00001d003368_T004	0.02094954	-0.999304185	Zm00001d003368
Zm00001d003368_T002	0.02530258	0.485542037	Zm00001d003368
Zm00001d005819_T001	0.8014743	-0.207059158	Zm00001d005819
Zm00001d002736_T001	0.03699476	2.385362365	Zm00001d002736
Zm00001d037465_T013	0.4206129	0.982534766	Zm00001d037465
Zm00001d037465_T009	0.9846714	0.266186944	Zm00001d037465
Zm00001d037465_T002	0.05577212	1.803350621	Zm00001d037465
Zm00001d023582_T002	1	0.304346997	Zm00001d023582
Zm00001d021698_T001	0.9686323	0.132695836	Zm00001d021698
Zm00001d007667_T002	0.8636447	-0.279723023	Zm00001d007667
Zm00001d007667_T001	0.6611576	0.404634681	Zm00001d007667
Zm00001d034341_T006	0.9817074	0.366137776	Zm00001d034341
Zm00001d034341_T001	0.7771984	0.700099507	Zm00001d034341
Zm00001d034341_T002	0.8409438	-0.203291137	Zm00001d034341
[truncated: 1,096,629 more chars]
