# Supplementary material for: High-resolution profile of transcriptomes reveals a role of alternative splicing for modulating response to nitrogen in maize
Source: BMC Genomics. 2020 May 11;21:353. doi: 10.1186/s12864-020-6769-8 (PMC7216474; doi:10.1186/s12864-020-6769-8)
Supplement: Supplementary file 10 — Additional file 10: Table S4. The GO enrichment analysis of DEGs. [file 12864_2020_6769_MOESM10_ESM.pdf]

Supplemental Table S4: The GO enrichment analysis of DEGs.

GO term	Ontology	Description	Number in input list	Number in BG/Ref	*p*- Value	FDR	Enrichment Factor
GO:0001101	P	response to acid chemical	54	421	0.00039	0.044	0.128266033
GO:0006082	P	organic acid metabolic process	124	1144	0.00015	0.021	0.108391608
GO:0006325	P	chromatin organization	60	442	0.000048	0.01	0.135746606
GO:0006351	P	transcription, DNA-templated	271	2807	0.000069	0.013	0.096544353
GO:0006355	P	regulation of transcription, DNA-templated	253	2596	0.000071	0.013	0.097457627
GO:0006464	P	cellular protein modification process	379	3847	0.0000003	0.00018	0.098518326
GO:0006468	P	protein phosphorylation	188	1855	0.0001	0.017	0.101347709
GO:0006541	P	glutamine metabolic process	15	48	0.000025	0.006	0.3125
GO:0007017	P	microtubule-based process	44	311	0.00021	0.028	0.1414791
GO:0007154	P	cell communication	160	1452	0.0000074	0.0028	0.110192837
GO:0007165	P	signal transduction	153	1321	0.00000098	0.00054	0.115821347
GO:0007623	P	circadian rhythm	12	41	0.00026	0.032	0.292682927
GO:0009064	P	glutamine family amino acid metabolic process	20	99	0.00025	0.032	0.202020202
GO:0009719	P	response to endogenous stimulus	93	711	0.0000019	0.00079	0.130801688
GO:0009725	P	response to hormone	92	699	0.0000017	0.00075	0.131616595
GO:0009753	P	response to jasmonic acid	19	87	0.00015	0.021	0.218390805
GO:0009755	P	hormone-mediated signaling pathway	55	405	0.000096	0.016	0.135802469
GO:0009889	P	regulation of biosynthetic process	282	2874	0.000017	0.0048	0.098121086
GO:0009987	P	intracellular signal transduction	1408	16613	1.9E-09	0.0000048	0.084752904
GO:0010033	P	response to organic substance	117	897	0.00000011	0.000081	0.130434783
GO:0010467	P	gene expression	458	4995	0.00002	0.0052	0.091691692
GO:0010468	P	regulation of gene expression	318	3001	6.1E-09	0.0000095	0.105964678
GO:0010556	P	regulation of macromolecule biosynthetic process	280	2851	0.000017	0.0048	0.098211154
GO:0010629	P	negative regulation of gene expression	51	381	0.00023	0.03	0.133858268
GO:0016192	P	vesicle-mediated transport	72	597	0.00025	0.032	0.120603015
GO:0016569	P	covalent chromatin modification	43	278	0.000041	0.0089	0.154676259
GO:0018130	P	heterocycle biosynthetic process	320	3382	0.000059	0.012	0.094618569
GO:0019219	P	regulation of nucleobase-containing compound metabolic process	272	2764	0.00002	0.0052	0.098408104
GO:0019222	P	regulation of metabolic process	362	3582	5.5E-08	0.000047	0.10106086
GO:0019438	P	aromatic compound biosynthetic process	323	3450	0.00011	0.017	0.093623188
GO:0023014	P	signal transduction by protein phosphorylation	22	89	0.000009	0.0032	0.247191011

GO:0023052	P	signaling	154	1335	0.0000011	0.00054	0.115355805
GO:0031323	P	regulation of cellular metabolic process	329	3404	0.0000095	0.0032	0.096650999
GO:0031326	P	regulation of cellular biosynthetic process	282	2871	0.000015	0.0048	0.098223615
GO:0032774	P	RNA biosynthetic process	275	2841	0.000051	0.01	0.096796902
GO:0032870	P	cellular response to hormone stimulus	57	428	0.00012	0.017	0.13317757
GO:0034654	P	nucleobase-containing compound biosynthetic process	303	3211	0.00011	0.017	0.094363127
GO:0035556	P	intracellular signal transduction	79	485	4.6E-09	0.0000088	0.162886598
GO:0036211	P	protein modification process	379	3847	0.0000003	0.00018	0.098518326
GO:0042221	P	response to chemical	160	1493	0.000028	0.0064	0.107166778
GO:0043412	P	macromolecule modification	403	4323	0.000019	0.0052	0.093222299
GO:0043436	P	oxoacid metabolic process	111	1011	0.00021	0.028	0.109792285
GO:0044237	P	cellular metabolic process	1130	13654	0.00011	0.017	0.082759631
GO:0044238	P	primary metabolic process	1116	13559	0.0003	0.036	0.082306955
GO:0044267	P	cellular protein metabolic process	485	5508	0.00038	0.044	0.08805374
GO:0044699	P	single-organism process	870	9791	5.4E-08	0.000047	0.088857114
GO:0044700	P	single organism signaling	154	1334	0.0000011	0.00054	0.115442279
GO:0044763	P	single-organism cellular process	650	6774	1.4E-10	0.00000071	0.095955123
GO:0048511	P	rhythmic process	12	41	0.00026	0.032	0.292682927
GO:0050789	P	regulation of biological process	527	5307	1.8E-10	0.00000071	0.099302808
GO:0050794	P	regulation of cellular process	484	4991	2.6E-08	0.000028	0.096974554
GO:0050896	P	response to stimulus	355	3848	0.00014	0.021	0.092255717
GO:0051171	P	regulation of nitrogen compound metabolic process	298	2956	0.0000012	0.00056	0.100811908
GO:0051252	P	regulation of RNA metabolic process	265	2687	0.000022	0.0055	0.098623
GO:0051641	P	cellular localization	114	1061	0.00036	0.042	0.107445806
GO:0060255	P	regulation of macromolecule metabolic process	351	3482	0.00000012	0.000081	0.100804136
GO:0065007	P	biological regulation	591	6244	1.1E-08	0.000014	0.094650865
GO:0070887	P	cellular response to chemical stimulus	80	686	0.00031	0.036	0.116618076
GO:0071229	P	cellular response to acid chemical	35	226	0.0002	0.028	0.154867257
GO:0071310	P	cellular response to organic substance	72	555	0.000034	0.0075	0.12972973
GO:0071495	P	cellular response to endogenous stimulus	57	433	0.00015	0.021	0.131639723
GO:0071704	P	organic substance metabolic process	1187	14289	0.000026	0.0062	0.083070894
GO:0080090	P	regulation of primary metabolic process	326	3368	0.0000094	0.0032	0.096793349
GO:0097659	P	nucleic acid-templated transcription	272	2829	0.000086	0.016	0.096147048
GO:1901362	P	organic cyclic compound biosynthetic process	332	3555	0.0001	0.017	0.093389592
GO:1901700	P	response to oxygen-containing compound	77	662	0.00042	0.047	0.116314199

GO:1903506	P	regulation of nucleic acid-templated transcription	254	2619	0.000091	0.016	0.096983582
GO:2000112	P	regulation of cellular macromolecule biosynthetic process	279	2823	0.000011	0.0037	0.098831031
GO:2001141	P	regulation of RNA biosynthetic process	254	2619	0.000091	0.016	0.096983582
GO:0000166	F	nucleotide binding	492	4252	3.7E-20	3.1E-17	0.115710254
GO:0001882	F	nucleoside binding	449	3843	5.8E-19	1.5E-16	0.116835805
GO:0001883	F	purine nucleoside binding	447	3829	8.2E-19	1.6E-16	0.116740663
GO:0003676	F	nucleic acid binding	473	5124	0.0000074	0.00063	0.092310695
GO:0003677	F	DNA binding	271	2927	0.00077	0.038	0.092586266
GO:0003682	F	chromatin binding	34	162	0.000001	0.000094	0.209876543
GO:0003723	F	RNA binding	166	1586	0.000061	0.0041	0.104665826
GO:0003729	F	mRNA binding	35	214	0.000078	0.0049	0.163551402
GO:0003779	F	actin binding	22	119	0.00037	0.021	0.18487395
GO:0004672	F	protein kinase activity	183	1745	0.000023	0.0017	0.10487106
GO:0004674	F	protein serine/threonine kinase activity	138	1104	7.6E-08	0.0000077	0.125
GO:0004702	F	receptor signaling protein serine/threonine kinase activity	8	21	0.00069	0.034	0.380952381
GO:0004707	F	MAP kinase activity	8	20	0.00053	0.028	0.4
GO:0005057	F	receptor signaling protein activity	8	21	0.00069	0.034	0.380952381
GO:0005488	F	binding	1485	15954	7.5E-29	1.9E-25	0.093080105
GO:0005515	F	protein binding	544	5711	2.5E-08	0.0000031	0.095254771
GO:0005516	F	calmodulin binding	26	156	0.00048	0.026	0.166666667
GO:0005524	F	ATP binding	392	3341	1E-16	1.4E-14	0.117330141
GO:0008092	F	cytoskeletal protein binding	49	361	0.00023	0.014	0.135734072
GO:0015399	F	primary active transmembrane transporter activity	45	324	0.00025	0.015	0.138888889
GO:0015405	F	P-P-bond-hydrolysis-driven transmembrane transporter activity	45	323	0.00024	0.014	0.139318885
GO:0016462	F	pyrophosphatase activity	174	1631	0.000016	0.0012	0.106683017
GO:0016773	F	phosphotransferase activity, alcohol group as acceptor	207	1956	0.0000039	0.00035	0.105828221
GO:0016817	F	hydrolase activity, acting on acid anhydrides	177	1651	0.00001	0.00084	0.107207753
GO:0016818	F	hydrolase activity, acting on acid anhydrides, in phosphorus-containing anhydrides	177	1641	0.0000075	0.00063	0.10786106
GO:0016820	F	hydrolase activity, acting on acid anhydrides, catalyzing transmembrane movement of substances	43	283	0.00006	0.0041	0.151943463

GO:0016887	F	ATPase activity	109	729	4.7E-10	0.000000063	0.14951989
GO:0017076	F	purine nucleotide binding	449	3841	5.3E-19	1.5E-16	0.116896641
GO:0017111	F	nucleoside-triphosphatase activity	169	1563	0.000011	0.00085	0.1081254
GO:0030554	F	adenyl nucleotide binding	411	3494	1E-17	1.5E-15	0.117630223
GO:0032403	F	protein complex binding	27	166	0.00052	0.028	0.162650602
GO:0032549	F	ribonucleoside binding	448	3842	8.9E-19	1.6E-16	0.116605934
GO:0032550	F	purine ribonucleoside binding	447	3829	8.2E-19	1.6E-16	0.116740663
GO:0032553	F	ribonucleotide binding	454	3872	1.9E-19	7.2E-17	0.117252066
GO:0032555	F	purine ribonucleotide binding	447	3829	8.2E-19	1.6E-16	0.116740663
GO:0032559	F	adenyl ribonucleotide binding	410	3483	1E-17	1.5E-15	0.117714614
GO:0035639	F	purine ribonucleoside triphosphate binding	429	3686	8.4E-18	1.4E-15	0.116386327
GO:0036094	F	small molecule binding	498	4327	5.3E-20	3.1E-17	0.115091287
GO:0042393	F	histone binding	20	112	0.00098	0.047	0.178571429
GO:0042623	F	ATPase activity, coupled	83	551	4.2E-08	0.0000046	0.150635209
GO:0042626	F	ATPase activity, coupled to transmembrane movement of substances	43	283	0.00006	0.0041	0.151943463
GO:0043167	F	ion binding	355	3477	2.8E-08	0.0000034	0.102099511
GO:0043169	F	cation binding	336	3260	0.00000003	0.0000035	0.103067485
GO:0043492	F	ATPase activity, coupled to movement of substances	47	306	0.000022	0.0016	0.153594771
GO:0044822	F	poly(A) RNA binding	35	214	0.000078	0.0049	0.163551402
GO:0044877	F	macromolecular complex binding	66	406	8.8E-08	0.0000085	0.162561576
GO:0046872	F	metal ion binding	334	3250	4.4E-08	0.0000046	0.102769231
GO:0051015	F	actin filament binding	15	62	0.00028	0.016	0.241935484
GO:0097159	F	organic cyclic compound binding	947	9627	6.3E-20	3.1E-17	0.09836917
GO:0097367	F	carbohydrate derivative binding	457	3909	2.3E-19	7.2E-17	0.116909696
GO:1901265	F	nucleoside phosphate binding	492	4252	3.7E-20	3.1E-17	0.115710254
GO:1901363	F	heterocyclic compound binding	942	9607	2.2E-19	7.2E-17	0.098053503
GO:0005622	C	intracellular	1181	13573	9.6E-10	0.00000063	0.087010978
GO:0005623	C	cell	1319	15669	0.00000013	0.000038	0.084178952
GO:0005634	C	nucleus	540	5408	4.4E-11	0.000000058	0.099852071
GO:0005654	C	nucleoplasm	60	473	0.00025	0.027	0.126849894
GO:0008023	C	transcription elongation factor complex	13	47	0.00024	0.027	0.276595745
GO:0016020	C	membrane	876	10325	0.000068	0.015	0.084842615
GO:0030660	C	Golgi-associated vesicle membrane	15	67	0.00057	0.047	0.223880597
GO:0043226	C	organelle	949	11307	0.00012	0.017	0.083930309

GO:0043227	C	membrane-bounded organelle	871	10302	0.00012	0.017	0.08454669
GO:0043229	C	intracellular organelle	947	11291	0.00013	0.017	0.083872111
GO:0043231	C	intracellular membrane-bounded organelle	870	10295	0.00013	0.017	0.084507042
GO:0043234	C	protein complex	278	2994	0.00056	0.047	0.092852371
GO:0044424	C	intracellular part	1151	13331	1.5E-08	0.0000065	0.08634011
GO:0044428	C	nuclear part	149	1449	0.0003	0.03	0.102829538
GO:0044431	C	Golgi apparatus part	64	535	0.00065	0.047	0.119626168
GO:0044433	C	cytoplasmic vesicle part	25	150	0.00061	0.047	0.166666667
GO:0044451	C	nucleoplasm part	52	410	0.00062	0.047	0.126829268
GO:0044464	C	cell part	1307	15517	0.00000014	0.000038	0.084230199
