# Supplementary material for: High-resolution profile of transcriptomes reveals a role of alternative splicing for modulating response to nitrogen in maize
Source: BMC Genomics. 2020 May 11;21:353. doi: 10.1186/s12864-020-6769-8 (PMC7216474; doi:10.1186/s12864-020-6769-8)
Supplement: Supplementary file 11 — Additional file 11: Table S5. The distribution of the read lengths generated from Sequel system. [file 12864_2020_6769_MOESM11_ESM.pdf]

Supplemental table S5: The distribution of the read lengths generated from *Sequel* system.

| | Number of reads | |
|---|---|---|
| Range of Length | Untreated | Treated |
| 0-1 kb | 2992340 | 3973922 |
| 1-2 kb | 3188304 | 4056753 |
| 2-3 kb | 1399920 | 854655 |
| 3-5 kb | 241049 | 179574 |
| >5 kb | 29801 | 27148 |
| total | 7851414 | 9092052 |
