# Supplementary material for: High-resolution profile of transcriptomes reveals a role of alternative splicing for modulating response to nitrogen in maize
Source: BMC Genomics. 2020 May 11;21:353. doi: 10.1186/s12864-020-6769-8 (PMC7216474; doi:10.1186/s12864-020-6769-8)
Supplement: Supplementary file 12 — Additional file 12: Table S6. GO enrichment analysis of non-DEGs that experienced AS specifally after nitrate supply. [file 12864_2020_6769_MOESM12_ESM.pdf]

Supplemental Table S6: GO enrichment analysis of non-DEGs that experienced AS specifally after nitrate supply.

GO term	Ontology	Description	Number in input list	Number in BG/Ref	p_Value	FDR	Enrichment Factor
GO:0034050	P	host programmed cell death induced by symbiont	7	20	4.9E-06	0.0088	0.35
GO:0009626	P	plant-type hypersensitive response	7	20	4.9E-06	0.0088	0.35
GO:0002376	P	immune system process	13	102	0.000012	0.014	0.12745098
GO:0008219	P	cell death	9	57	0.000058	0.021	0.157894737
GO:0045087	P	innate immune response	12	95	0.000027	0.021	0.126315789
GO:0033036	P	macromolecule localization	58	1205	0.000037	0.021	0.04813278
GO:0007264	P	small GTPase mediated signal transduction	13	120	0.000054	0.021	0.108333333
GO:0045184	P	establishment of protein localization	45	869	0.000057	0.021	0.051783659
GO:0006955	P	immune response	12	96	0.00003	0.021	0.125
GO:0015031	P	protein transport	45	861	0.000047	0.021	0.052264808
GO:0008104	P	protein localization	49	986	0.000072	0.023	0.04969574
GO:0035601	P	protein deacylation	7	34	0.000091	0.027	0.205882353
GO:0012501	P	programmed cell death	8	49	0.00012	0.034	0.163265306
GO:0098732	P	macromolecule deacylation	7	37	0.00014	0.037	0.189189189
GO:0005737	C	cytoplasm	286	8466	0.000015	0.0062	0.033782188
GO:0016023	C	cytoplasmic, membrane-bounded vesicle	18	209	0.000038	0.0062	0.086124402
GO:0031988	C	membrane-bounded vesicle	18	211	0.000043	0.0062	0.085308057
GO:0097708	C	intracellular vesicle	18	211	0.000043	0.0062	0.085308057
GO:0031410	C	cytoplasmic vesicle	18	210	0.000041	0.0062	0.085714286
GO:0031982	C	vesicle	18	229	0.00011	0.014	0.07860262
