# Supplementary material for: High-resolution profile of transcriptomes reveals a role of alternative splicing for modulating response to nitrogen in maize
Source: BMC Genomics. 2020 May 11;21:353. doi: 10.1186/s12864-020-6769-8 (PMC7216474; doi:10.1186/s12864-020-6769-8)
Supplement: Supplementary file 13 — Additional file 13: Table S7. The list of ZmNLP6’s putative targets that associated with N metabolism. [file 12864_2020_6769_MOESM13_ESM.pdf]

Supplemental Table S7: The list of ZmNLP6's putative targets that associated with N metabolism. Some genes were classfied into mutiple pathways.

**Gene ID and GO terms**	**Annotation**
**Cellular nitrogen compound metabolic process (GO:0034641)**	
Zm00001d042593	ap2 erf domain-containing transcription factor
Zm00001d041083	fidgetin-like protein 1-like
Zm00001d002125	#N/A
Zm00001d040148	hlh dna-binding domain superfamily protein
Zm00001d033817	translational activator gcn1-like
Zm00001d051542	dna topoisomerase 2-like
Zm00001d005962	bzip protein
Zm00001d045263	gdsl esterase lipase at4g01130-like
Zm00001d002006	autoinhibited h+ atpase
Zm00001d017720	dna topoisomerase 2-like
Zm00001d026010	regulator of nonsense transcripts-like protein
Zm00001d018657	low quality protein: nipped-b-like
Zm00001d046581	#N/A
Zm00001d020881	probable wrky transcription factor 2-like
Zm00001d047290	ninja-family protein 6
Zm00001d029375	intron-binding protein aquarius-like
Zm00001d053559	#N/A
Zm00001d034810	peptide chain release factor 1-like
Zm00001d024953	breast cancer type 2 susceptibility protein
Zm00001d039729	#N/A
Zm00001d007945	uncharacterized protein LOC100277946
Zm00001d005473	mrna-decapping enzyme-like
Zm00001d021946	scarecrow-like transcription factor pat1-like
Zm00001d017308	b-block binding subunit of tfiiic
Zm00001d042443	btb poz and taz domain-containing protein 2-like
Zm00001d006438	regulator of nonsense transcripts 1 homolog
Zm00001d047471	dna helicase ino80-like
Zm00001d053917	dna repair endonuclease uvh1-like
Zm00001d046221	uncharacterized loc101205105

Zm00001d044138	siroheme synthase-like
Zm00001d021318	dna-(apurinic or apyrimidinic site) lyase 2-like
Zm00001d021947	probable wrky transcription factor 20-like
Zm00001d014422	lysine-specific demethylase 5d-like
Zm00001d048655	splicing factor u2af large subunit b-like
Zm00001d041582	nuclear export mediator factor nemf-like
Zm00001d015212	atp-dependent dna helicase q-like 4a-like
Zm00001d027633	protein pir-like
Zm00001d029529	ninja-family protein 6
Zm00001d032567	mediator of rna polymerase ii transcription subunit 16-like
Zm00001d043927	uncharacterized loc101208571
Zm00001d029180	dna helicase ino80-like
Zm00001d032815	protein flug-like
Zm00001d012156	uncharacterized partial
Zm00001d029055	dna-directed rna polymerase i subunit rpa1-like
Zm00001d035113	hypothetical protein SORBIDRAFT_07g001795, partial
Zm00001d041453	#N/A
Zm00001d044358	regulator of telomere elongation helicase 1-like
Zm00001d019115	#N/A
Zm00001d028372	transcription factor hbp-1a
Zm00001d045635	chromatin remodeling complex subunit
Zm00001d042830	myb dna-binding domain superfamily protein
Zm00001d035922	pentatricopeptide repeat-containing protein at1g11290-like
Zm00001d049295	auxin response factor 2-like
Zm00001d019670	chromosome-associated kinesin kif4a-like
Zm00001d010021	probable exonuclease mut-7 homolog
Zm00001d014568	dna repair protein rev1-like
Zm00001d049543	protein lhy-like
Zm00001d011652	uncharacterized protein LOC100383427
Zm00001d009631	#N/A
Zm00001d022491	dna polymerase zeta subunit
Zm00001d022490	xylose isomerase-like

Zm00001d043277	replication factor c dna polymerase iii gamma-tau
Zm00001d034160	myb-like dna-binding shaqkyf class family protein
Zm00001d049269	rna polymerase ii c-terminal domain phosphatase-like 3-like
Zm00001d038475	low quality protein: midasin-like
Zm00001d046664	#N/A
Zm00001d010719	suppressor of
Zm00001d042713	dna polymerase alpha catalytic subunit-like
Zm00001d052233	#N/A
Zm00001d003214	protoporphyrinogen mitochondrial-like
Zm00001d039165	#N/A
Zm00001d024463	thyroid adenoma-associated protein homolog
Zm00001d045046	bhlh transcription factor ptf1
Zm00001d053300	dna binding
Zm00001d025015	glucose-6-phosphate 1- chloroplastic-like
Zm00001d011309	transcriptional corepressor leunig-like
Zm00001d023796	#N/A
Zm00001d049379	general transcription factor 3c polypeptide 3-like
Zm00001d025830	dicer-like protein 4-like
Zm00001d023262	protein nap1-like
Zm00001d037313	probable lysine-specific demethylase jmj14-like
Zm00001d037377	sister chromatid cohesion protein pds5 homolog b-like
Zm00001d010866	atp-dependent dna helicase q-like sim-like
Zm00001d027278	#N/A
Zm00001d006227	protein xap5 circadian timekeeper-like
Zm00001d044171	wrky transcription factor 22-like
Zm00001d016286	uncharacterized loc101219624
Zm00001d044573	#N/A
Zm00001d044570	#N/A
Zm00001d045603	mediator of rna polymerase ii transcription subunit 13-like
Zm00001d003198	probable serine threonine-protein kinase gcn2-like
Zm00001d034962	pentatricopeptide repeat-containing protein chloroplastic-like
Zm00001d005507	mediator of rna polymerase ii transcription subunit 14-like

Zm00001d048095	activating signal cointegrator 1 complex subunit 3
Zm00001d019108	protein rrp5 homolog
Zm00001d018976	#N/A
Zm00001d014297	histidine kinase 2-like
Zm00001d002483	transducin beta-like protein 3-like
Zm00001d026490	plasma-membrane h+atpase2
Zm00001d044940	bzip transcription factor family protein
Zm00001d033616	pescadillo homolog
Zm00001d023741	alanyl-trna synthetase
Zm00001d039417	progestin and adipoq receptor-like protein 1-like
Zm00001d008570	hypothetical protein SORBIDRAFT_03g007000
Zm00001d049187	6-phosphogluconate decarboxylating-like
Zm00001d024567	uncharacterized loc101203806
Zm00001d009562	PREDICTED: uncharacterized protein LOC100835009
Zm00001d018206	nitrate reductase
Zm00001d002462	#N/A
Zm00001d021844	deoxyribonuclease tatdn1-like
Zm00001d004669	#N/A
Zm00001d032532	#N/A
Zm00001d048541	mediator of rna polymerase ii transcription subunit 12-like
Zm00001d006296	valyl-trna synthetase-like
**Negative regulation of nitrogen compound metabolic process (GO:0051172)**	
Zm00001d047290	ninja-family protein 6
Zm00001d014422	lysine-specific demethylase 5d-like
Zm00001d041453	#N/A
Zm00001d041083	fidgetin-like protein 1-like
Zm00001d043927	uncharacterized loc101208571
Zm00001d045635	chromatin remodeling complex subunit
Zm00001d027278	#N/A
Zm00001d006227	protein xap5 circadian timekeeper-like
Zm00001d044358	regulator of telomere elongation helicase 1-like
Zm00001d037313	probable lysine-specific demethylase jmj14-like

Zm00001d029529	ninja-family protein 6

Regulation of nitrogen compound metabolic process (GO:0051171)

Zm00001d046221	uncharacterized loc101205105
Zm00001d042593	ap2 erf domain-containing transcription factor
Zm00001d041083	fidgetin-like protein 1-like
Zm00001d020881	probable wrky transcription factor 2-like
Zm00001d040148	hlh dna-binding domain superfamily protein
Zm00001d033817	translational activator gcn1-like
Zm00001d027278	#N/A
Zm00001d042830	myb dna-binding domain superfamily protein
Zm00001d006227	protein xap5 circadian timekeeper-like
Zm00001d005962	bzip protein
Zm00001d048541	mediator of rna polymerase ii transcription subunit 12-like
Zm00001d044171	wrky transcription factor 22-like
Zm00001d041582	nuclear export mediator factor nemf-like
Zm00001d049543	protein lhy-like
Zm00001d010719	suppressor of
Zm00001d027633	protein pir-like
Zm00001d029529	ninja-family protein 6
Zm00001d042443	btb poz and taz domain-containing protein 2-like
Zm00001d032567	mediator of rna polymerase ii transcription subunit 16-like
Zm00001d045603	mediator of rna polymerase ii transcription subunit 13-like
Zm00001d006296	valyl-trna synthetase-like
Zm00001d003198	probable serine threonine-protein kinase gcn2-like
Zm00001d044940	bzip transcription factor family protein
Zm00001d029180	dna helicase ino80-like
Zm00001d005507	mediator of rna polymerase ii transcription subunit 14-like
Zm00001d018657	low quality protein: nipped-b-like
Zm00001d024953	breast cancer type 2 susceptibility protein
Zm00001d046581	#N/A
Zm00001d014297	histidine kinase 2-like
Zm00001d047290	ninja-family protein 6

Zm00001d010866	atp-dependent dna helicase q-like sim-like
Zm00001d041453	#N/A
Zm00001d034160	myb-like dna-binding shaqkyf class family protein
Zm00001d043927	uncharacterized loc101208571
Zm00001d049269	rna polymerase ii c-terminal domain phosphatase-like 3-like
Zm00001d035113	hypothetical protein SORBIDRAFT_07g001795, partial
Zm00001d019670	chromosome-associated kinesin kif4a-like
Zm00001d046664	#N/A
Zm00001d008570	hypothetical protein SORBIDRAFT_03g007000
Zm00001d044358	regulator of telomere elongation helicase 1-like
Zm00001d045046	bhlh transcription factor ptf1
Zm00001d009562	PREDICTED: uncharacterized protein LOC100835009
Zm00001d039729	#N/A
Zm00001d053300	dna binding
Zm00001d047471	dna helicase ino80-like
Zm00001d028372	transcription factor hbp-1a
Zm00001d045635	chromatin remodeling complex subunit
Zm00001d021946	scarecrow-like transcription factor pat1-like
Zm00001d021947	probable wrky transcription factor 20-like
Zm00001d011309	transcriptional corepressor leunig-like
Zm00001d035922	pentatricopeptide repeat-containing protein at1g11290-like
Zm00001d014422	lysine-specific demethylase 5d-like
Zm00001d049295	auxin response factor 2-like
Zm00001d033616	pescadillo homolog
Zm00001d023262	protein nap1-like
Zm00001d037313	probable lysine-specific demethylase jmj14-like
**Nitrogen compound metabolic process (GO:0006807)**	
Zm00001d042593	ap2 erf domain-containing transcription factor
Zm00001d041083	fidgetin-like protein 1-like
Zm00001d002128	#N/A
Zm00001d002125	#N/A
Zm00001d040148	hlh dna-binding domain superfamily protein

Zm00001d033817	translational activator gcn1-like
Zm00001d052164	ferredoxin--nitrite chloroplastic-like
Zm00001d051542	dna topoisomerase 2-like
Zm00001d005962	bzip protein
Zm00001d045263	gdsl esterase lipase at4g01130-like
Zm00001d002006	autoinhibited h+ atpase
Zm00001d017720	dna topoisomerase 2-like
Zm00001d026010	regulator of nonsense transcripts-like protein
Zm00001d018657	low quality protein: nipped-b-like
Zm00001d046581	#N/A
Zm00001d020881	probable wrky transcription factor 2-like
Zm00001d047290	ninja-family protein 6
Zm00001d029375	intron-binding protein aquarius-like
Zm00001d053559	#N/A
Zm00001d034810	peptide chain release factor 1-like
Zm00001d024953	breast cancer type 2 susceptibility protein
Zm00001d039729	#N/A
Zm00001d005473	mrna-decapping enzyme-like
Zm00001d021946	scarecrow-like transcription factor pat1-like
Zm00001d017308	b-block binding subunit of tfiiic
Zm00001d042443	btb poz and taz domain-containing protein 2-like
Zm00001d006438	regulator of nonsense transcripts 1 homolog
Zm00001d047471	dna helicase ino80-like
Zm00001d053917	dna repair endonuclease uvh1-like
Zm00001d046221	uncharacterized loc101205105
Zm00001d044138	siroheme synthase-like
Zm00001d021318	dna-(apurinic or apyrimidinic site) lyase 2-like
Zm00001d021947	probable wrky transcription factor 20-like
Zm00001d008570	hypothetical protein SORBIDRAFT_03g007000
Zm00001d014422	lysine-specific demethylase 5d-like
Zm00001d047981	glutamate decarboxylase 1
Zm00001d041582	nuclear export mediator factor nemf-like

Zm00001d015212	atp-dependent dna helicase q-like 4a-like
Zm00001d027633	protein pir-like
Zm00001d029529	ninja-family protein 6
Zm00001d032567	mediator of rna polymerase ii transcription subunit 16-like
Zm00001d043927	uncharacterized loc101208571
Zm00001d029180	dna helicase ino80-like
Zm00001d032815	protein flug-like
Zm00001d012156	uncharacterized partial
Zm00001d029055	dna-directed rna polymerase i subunit rpa1-like
Zm00001d035113	hypothetical protein SORBIDRAFT_07g001795, partial
Zm00001d041453	#N/A
Zm00001d044358	regulator of telomere elongation helicase 1-like
Zm00001d019115	#N/A
Zm00001d028372	transcription factor hbp-1a
Zm00001d045635	chromatin remodeling complex subunit
Zm00001d042830	myb dna-binding domain superfamily protein
Zm00001d035922	pentatricopeptide repeat-containing protein at1g11290-like
Zm00001d049295	auxin response factor 2-like
Zm00001d019670	chromosome-associated kinesin kif4a-like
Zm00001d010021	probable exonuclease mut-7 homolog
Zm00001d014568	dna repair protein rev1-like
Zm00001d049543	protein lhy-like
Zm00001d026501	uncharacterized loc101213606
Zm00001d011652	uncharacterized protein LOC100383427
Zm00001d009631	#N/A
Zm00001d022491	dna polymerase zeta subunit
Zm00001d022490	xylose isomerase-like
Zm00001d043277	replication factor c dna polymerase iii gamma-tau
Zm00001d034160	myb-like dna-binding shaqkyf class family protein
Zm00001d049269	rna polymerase ii c-terminal domain phosphatase-like 3-like
Zm00001d038475	low quality protein: midasin-like
Zm00001d046664	#N/A

Zm00001d010719	suppressor of
Zm00001d042713	dna polymerase alpha catalytic subunit-like
Zm00001d052233	#N/A
Zm00001d003214	protoporphyrinogen mitochondrial-like
Zm00001d039165	#N/A
Zm00001d024463	thyroid adenoma-associated protein homolog
Zm00001d045046	bhlh transcription factor ptf1
Zm00001d033539	glutamine amidotransferase ylr126c-like
Zm00001d053300	dna binding
Zm00001d048655	splicing factor u2af large subunit b-like
Zm00001d025015	glucose-6-phosphate 1- chloroplastic-like
Zm00001d011309	transcriptional corepressor leunig-like
Zm00001d023796	#N/A
Zm00001d049379	general transcription factor 3c polypeptide 3-like
Zm00001d025830	dicer-like protein 4-like
Zm00001d023262	protein nap1-like
Zm00001d022388	ferredoxin-dependent glutamate synthase chloroplastic-like
Zm00001d037313	probable lysine-specific demethylase jmj14-like
Zm00001d037377	sister chromatid cohesion protein pds5 homolog b-like
Zm00001d010866	atp-dependent dna helicase q-like sim-like
Zm00001d027278	#N/A
Zm00001d006227	protein xap5 circadian timekeeper-like
Zm00001d044171	wrky transcription factor 22-like
Zm00001d016286	uncharacterized loc101219624
Zm00001d044573	#N/A
Zm00001d044570	#N/A
Zm00001d045603	mediator of rna polymerase ii transcription subunit 13-like
Zm00001d006296	valyl-trna synthetase-like
Zm00001d039487	1-aminocyclopropane-1-carboxylate synthase 7-like
Zm00001d028154	serine acetyltransferase mitochondrial-like
Zm00001d034962	pentatricopeptide repeat-containing protein chloroplastic-like
Zm00001d005507	mediator of rna polymerase ii transcription subunit 14-like

Zm00001d048095	activating signal cointegrator 1 complex subunit 3
Zm00001d019108	protein rrp5 homolog
Zm00001d005151	aspartokinase chloroplastic-like
Zm00001d018976	#N/A
Zm00001d014297	histidine kinase 2-like
Zm00001d002483	transducin beta-like protein 3-like
Zm00001d026490	plasma-membrane h+atpase2
Zm00001d044940	bzip transcription factor family protein
Zm00001d007945	uncharacterized protein LOC100277946
Zm00001d023741	alanyl-trna synthetase
Zm00001d039417	progestin and adipoq receptor-like protein 1-like
Zm00001d049187	6-phosphogluconate decarboxylating-like
Zm00001d024567	uncharacterized loc101203806
Zm00001d047896	chorismate synthase chloroplastic precursor
Zm00001d009562	PREDICTED: uncharacterized protein LOC100835009
Zm00001d018206	nitrate reductase
Zm00001d002462	#N/A
Zm00001d021844	deoxyribonuclease tatdn1-like
Zm00001d004669	#N/A
Zm00001d032532	#N/A
Zm00001d048541	mediator of rna polymerase ii transcription subunit 12-like
Zm00001d003198	probable serine threonine-protein kinase gcn2-like
Zm00001d033616	pescadillo homolog
**Organonitrogen compound metabolic process (GO:1901564)**	
Zm00001d044138	siroheme synthase-like
Zm00001d003214	protoporphyrinogen mitochondrial-like
Zm00001d002128	#N/A
Zm00001d033817	translational activator gcn1-like
Zm00001d027278	#N/A
Zm00001d006227	protein xap5 circadian timekeeper-like
Zm00001d045263	gdsl esterase lipase at4g01130-like
Zm00001d041582	nuclear export mediator factor nemf-like

Zm00001d027633	protein pir-like
Zm00001d009631	#N/A
Zm00001d049187	6-phosphogluconate decarboxylating-like
Zm00001d006296	valyl-trna synthetase-like
Zm00001d039487	1-aminocyclopropane-1-carboxylate synthase 7-like
Zm00001d028154	serine acetyltransferase mitochondrial-like
Zm00001d032815	protein flug-like
Zm00001d005151	aspartokinase chloroplastic-like
Zm00001d046581	#N/A
Zm00001d014297	histidine kinase 2-like
Zm00001d026501	uncharacterized loc101213606
Zm00001d026490	plasma-membrane h+atpase2
Zm00001d007945	uncharacterized protein LOC100277946
Zm00001d023741	alanyl-trna synthetase
Zm00001d002462	#N/A
Zm00001d047981	glutamate decarboxylase 1
Zm00001d052233	#N/A
Zm00001d034810	peptide chain release factor 1-like
Zm00001d047896	chorismate synthase chloroplastic precursor
Zm00001d033539	glutamine amidotransferase ylr126c-like
Zm00001d039729	#N/A
Zm00001d003198	probable serine threonine-protein kinase gcn2-like
Zm00001d025015	glucose-6-phosphate 1- chloroplastic-like
Zm00001d032532	#N/A
Zm00001d002006	autoinhibited h+ atpase
Zm00001d022490	xylose isomerase-like
Zm00001d022388	ferredoxin-dependent glutamate synthase chloroplastic-like
**Organonitrogen compound biosynthetic process (GO:1901566)**	
Zm00001d044138	siroheme synthase-like
Zm00001d045263	gdsl esterase lipase at4g01130-like
Zm00001d002128	#N/A
Zm00001d033817	translational activator gcn1-like

Zm00001d027278	#N/A
Zm00001d006227	protein xap5 circadian timekeeper-like
Zm00001d026501	uncharacterized loc101213606
Zm00001d041582	nuclear export mediator factor nemf-like
Zm00001d002006	autoinhibited h+ atpase
Zm00001d009631	#N/A
Zm00001d039487	1-aminocyclopropane-1-carboxylate synthase 7-like
Zm00001d028154	serine acetyltransferase mitochondrial-like
Zm00001d027633	protein pir-like
Zm00001d032815	protein flug-like
Zm00001d005151	aspartokinase chloroplastic-like
Zm00001d046581	#N/A
Zm00001d026490	plasma-membrane h+atpase2
Zm00001d023741	alanyl-trna synthetase
Zm00001d002462	#N/A
Zm00001d034810	peptide chain release factor 1-like
Zm00001d047896	chorismate synthase chloroplastic precursor
Zm00001d039729	#N/A
Zm00001d003214	protoporphyrinogen mitochondrial-like
Zm00001d003198	probable serine threonine-protein kinase gcn2-like
Zm00001d032532	#N/A
Zm00001d006296	valyl-trna synthetase-like
Zm00001d022388	ferredoxin-dependent glutamate synthase chloroplastic-like
**Cellular nitrogen compound biosynthetic process (GO:0044271)**	
Zm00001d046581	#N/A
Zm00001d044138	siroheme synthase-like
Zm00001d042593	ap2 erf domain-containing transcription factor
Zm00001d046221	uncharacterized loc101205105
Zm00001d002125	0
Zm00001d040148	hlh dna-binding domain superfamily protein
Zm00001d033817	translational activator gcn1-like
Zm00001d027278	#N/A

Zm00001d019670	chromosome-associated kinesin kif4a-like
Zm00001d014568	dna repair protein rev1-like
Zm00001d006227	protein xap5 circadian timekeeper-like
Zm00001d005962	bzip protein
Zm00001d048541	mediator of rna polymerase ii transcription subunit 12-like
Zm00001d045263	gdsl esterase lipase at4g01130-like
Zm00001d044171	wrky transcription factor 22-like
Zm00001d002006	autoinhibited h+ atpase
Zm00001d049543	protein lhy-like
Zm00001d010719	suppressor of
Zm00001d027633	protein pir-like
Zm00001d026490	plasma-membrane h+atpase2
Zm00001d042443	btb poz and taz domain-containing protein 2-like
Zm00001d032567	mediator of rna polymerase ii transcription subunit 16-like
Zm00001d045603	mediator of rna polymerase ii transcription subunit 13-like
Zm00001d023796	#N/A
Zm00001d022491	dna polymerase zeta subunit
Zm00001d003198	probable serine threonine-protein kinase gcn2-like
Zm00001d044940	bzip transcription factor family protein
Zm00001d034962	pentatricopeptide repeat-containing protein chloroplastic-like
Zm00001d029180	dna helicase ino80-like
Zm00001d017308	b-block binding subunit of tfiiic
Zm00001d032532	#N/A
Zm00001d042830	myb dna-binding domain superfamily protein
Zm00001d018657	low quality protein: nipped-b-like
Zm00001d035113	hypothetical protein SORBIDRAFT_07g001795, partial
Zm00001d024953	breast cancer type 2 susceptibility protein
Zm00001d009631	#N/A
Zm00001d005507	mediator of rna polymerase ii transcription subunit 14-like
Zm00001d020881	probable wrky transcription factor 2-like
Zm00001d047290	ninja-family protein 6
Zm00001d010866	atp-dependent dna helicase q-like sim-like

Zm00001d041453	#N/A
Zm00001d034160	myb-like dna-binding shaqkyf class family protein
Zm00001d043927	uncharacterized loc101208571
Zm00001d049269	rna polymerase ii c-terminal domain phosphatase-like 3-like
Zm00001d043277	replication factor c dna polymerase iii gamma-tau
Zm00001d034810	peptide chain release factor 1-like
Zm00001d046664	#N/A
Zm00001d023741	alanyl-trna synthetase
Zm00001d002462	#N/A
Zm00001d008570	hypothetical protein SORBIDRAFT_03g007000
Zm00001d042713	dna polymerase alpha catalytic subunit-like
Zm00001d003214	protoporphyrinogen mitochondrial-like
Zm00001d024567	uncharacterized loc101203806
Zm00001d029055	dna-directed rna polymerase i subunit rpa1-like
Zm00001d045046	bhlh transcription factor ptf1
Zm00001d009562	PREDICTED: uncharacterized protein LOC100835009
Zm00001d039729	#N/A
Zm00001d018206	nitrate reductase
Zm00001d053300	dna binding
Zm00001d047471	dna helicase ino80-like
Zm00001d028372	transcription factor hbp-1a
Zm00001d045635	chromatin remodeling complex subunit
Zm00001d021946	scarecrow-like transcription factor pat1-like
Zm00001d021947	probable wrky transcription factor 20-like
Zm00001d011309	transcriptional corepressor leunig-like
Zm00001d035922	pentatricopeptide repeat-containing protein at1g11290-like
Zm00001d049379	general transcription factor 3c polypeptide 3-like
Zm00001d014422	lysine-specific demethylase 5d-like
Zm00001d049295	auxin response factor 2-like
Zm00001d041582	nuclear export mediator factor nemf-like
Zm00001d033616	pescadillo homolog
Zm00001d029529	ninja-family protein 6

Zm00001d006296	valyl-trna synthetase-like
Zm00001d025830	dicer-like protein 4-like
Zm00001d023262	protein nap1-like
Zm00001d037313	probable lysine-specific demethylase jmj14-like
**Cellular nitrogen compound catabolic process (GO:0044270)**	
Zm00001d046221	uncharacterized loc101205105
Zm00001d023262	protein nap1-like
Zm00001d043927	uncharacterized loc101208571
Zm00001d010866	atp-dependent dna helicase q-like sim-like
Zm00001d011309	transcriptional corepressor leunig-like
Zm00001d018657	low quality protein: nipped-b-like
Zm00001d019670	chromosome-associated kinesin kif4a-like
Zm00001d024953	breast cancer type 2 susceptibility protein
Zm00001d045046	bhlh transcription factor ptf1
Zm00001d039729	#N/A
**Positive regulation of nitrogen compound metabolic process (GO:0051173)**	
Zm00001d046221	uncharacterized loc101205105
Zm00001d023262	protein nap1-like
Zm00001d043927	uncharacterized loc101208571
Zm00001d010866	atp-dependent dna helicase q-like sim-like
Zm00001d011309	transcriptional corepressor leunig-like
Zm00001d018657	low quality protein: nipped-b-like
Zm00001d019670	chromosome-associated kinesin kif4a-like
Zm00001d024953	breast cancer type 2 susceptibility protein
Zm00001d045046	bhlh transcription factor ptf1
Zm00001d039729	#N/A
