# Supplementary material for: High-resolution profile of transcriptomes reveals a role of alternative splicing for modulating response to nitrogen in maize
Source: BMC Genomics. 2020 May 11;21:353. doi: 10.1186/s12864-020-6769-8 (PMC7216474; doi:10.1186/s12864-020-6769-8)
Supplement: Supplementary file 14 — Additional file 14: Table S8. The primers used in this study. [file 12864_2020_6769_MOESM14_ESM.pdf]

Supplemental Table S8: The primers used in this study.

Label	Sequence	Gene	Gene_id	Application
P01	GATCAAAGGATACGCATACT	*NR2*	Zm00001d018206	qPCR
P02	TCGACGTCGACGGACCAGAA			
P03	CTGGACCGGATGCCCCAACA	*NIR2*	Zm00001d052164	qPCR
P04	CGACGCGGCCGCCCACGAAG			
P05	GGGTCGCCCGCTACATTCTC	*GS3*	Zm00001d017958	qPCR
P06	GCTTCTCGATCGCCTCCTTG			
P07	GCACGCTACCTGTGGTGTTCG	*NRT1*	Zm00001d054060	qPCR
P08	TTGCTCTTCTCGTCGTCGTTCC			
P09	TGCTCCATTTCCACGGCATCGCTTT		Zm00001d052405	PCR validation
P10	CACGCACGCATGCATAATTGACCGA			
P11	GCTAAAAGCCTTGGTGTTTGTCCTA	*ZmNLP6*	Zm00001d039266	PCR validation
P12	GGAAAGTGGTAGTTGTGGTTCTTGG			
P13	GGATCAGCGTGGACCAGTTCTTCGA		Zm00001d046718	PCR validation
P14	GTACGGCAGACTCCTGCGTGGCTGT			
P15	TCGAATCAAGGATCCACGCGTAATGGACCTCGACCCGGCCTATCC	*ZmNLP6*	Zm00001d039266	Vector contruction
P16	TTCGAGCTCGGTACCACGCGTTCAACCGGAGCTTCCACAAGAACTG			
P17	TCGAATCAAGGATCCACGCGTAATGAAGAGGTGTCTTCGGAGCCTAA			
P18	TTCGAGCTCGGTACCACGCGTTCAGACCTTATTGATTTTTCGAGAT			
