# Supplementary material for: High-resolution profile of transcriptomes reveals a role of alternative splicing for modulating response to nitrogen in maize
Source: BMC Genomics. 2020 May 11;21:353. doi: 10.1186/s12864-020-6769-8 (PMC7216474; doi:10.1186/s12864-020-6769-8)
Supplement: Supplementary file 8 — Additional file 8: Table S2. Transcriptional abundance (log2 TPM + 1) of genes in RNA-Seq analysis. [file 12864_2020_6769_MOESM8_ESM.pdf]

Supplemental Table S2: Transcriptional abundance (log2 TPM+1) of genes based on RNA-Seq analysis. RNA was extracted from N-starved root tissues. Untreated: the N-starved roots, Treated: the N-starved roots treated with 30 min of nitrate supply. Each treatment has three replicates.

Tracking_id	Untreated_1	Untreated_2	Untreated_3	Treated_1	Treated_2	Treated_3
GRMZM5G859187_T01	2.449783209	3.226750962	1.999059778	3.172014754	3.162269664	3.068689725
GRMZM5G862955_T01	4.363893225	3.455715175	4.494104124	3.912734834	3.917845886	3.887519617
GRMZM5G862955_T02	2.071221729	1.209781483	2.197113032	2.660734705	2.687190615	2.40552191
GRMZM5G855343_T01	5.864934146	6.930440029	5.887566772	6.05301582	5.86486097	5.932897468
GRMZM5G889299_T01	1.501335191	1.031613984	1.631176008	1.362309409	1.523149412	1.080656981
GRMZM5G877040_T01	2.678543184	4.14785127	2.975413014	3.192615232	2.712402308	2.808914868
GRMZM5G884538_T01	2.602771363	2.655326409	2.810726885	3.372883443	3.677061665	3.530608156
GRMZM5G818111_T02	3.155979411	3.325708625	3.389783337	4.300191244	4.087470055	4.233362289
GRMZM5G816772_T01	1.171062679	1.025466145	1.773253694	2.117409135	1.852728393	1.717781034
GRMZM5G899836_T01	3.719989459	4.022901201	3.58567045	4.501309201	4.673906823	4.445093333
GRMZM5G849223_T01	2.519874989	2.012688804	2.484882365	1.876334628	2.045722186	2.002833554
GRMZM5G807592_T01	4.040444126	3.048501958	4.134604649	4.439151159	4.409079077	4.322213504
GRMZM5G844628_T01	4.423207634	2.254479457	4.648721976	4.68842573	4.536104886	4.420997231
GRMZM5G825253_T01	6.135581287	5.521095758	6.099258505	6.070939251	5.885637932	6.005584647
GRMZM5G889036_T01	2.325450603	1.667769077	2.141914372	2.304778821	1.865519792	1.983469392
GRMZM5G881135_T01	4.786805956	5.193450491	4.692309963	4.370153607	4.182841564	4.595206689
GRMZM5G861791_T01	3.601261503	2.803628579	3.75247972	2.892732763	2.921406052	2.72513669
GRMZM5G894515_T01	0.954477748	0.632958682	1.348358777	0.871793981	0.748426024	0.886789699
GRMZM5G853409_T01	0.732501054	0.410890288	0.491702279	0.682243372	1.026828382	0.816081923
GRMZM5G899892_T01	4.0028709	2.842546662	3.772782601	4.624534925	5.452157506	5.024195637
GRMZM5G839924_T01	3.213337949	3.482291228	3.365612199	3.798421772	3.90708073	4.038319669
GRMZM5G830210_T01	8.853674993	8.158367325	8.652680511	8.027757165	8.029846994	7.609152079
GRMZM5G834128_T01	1.247260986	0.982485109	1.141668207	1.78339421	1.667794504	1.518026959
GRMZM5G834666_T01	3.489583267	2.468438128	3.50062298	2.615625341	2.713035675	3.267409351
GRMZM5G892247_T01	1.726363464	1.329728684	2.029689281	0.825259731	0.895650843	0.701185288
GRMZM5G809869_T01	4.257877258	4.366012447	4.353872032	3.029476739	3.012350018	3.424318824
GRMZM5G808402_T01	0.114207087	0.105649921	0.69950366	0.946321384	0.917111673	0.667185474
GRMZM5G892769_T01	3.719370201	3.324104201	4.089220161	3.729620852	3.782510997	3.714926639

GRMZM5G853305_T01	6.926714657	7.21434402	6.480597721	7.330390745	6.958202691	6.852362337
GRMZM5G858556_T01	5.686503273	6.264993712	5.491137952	5.200658929	5.23696511	5.148199111
GRMZM5G884912_T01	3.279725936	3.675263357	2.983566798	3.977019757	3.769707642	3.933712652
GRMZM5G852144_T01	1.785568546	0.606722685	1.572737874	1.311363606	1.064408312	1.086924361
GRMZM5G856027_T02	0.666193036	0.633060084	0.997947028	2.126475027	1.319671687	1.417109884
GRMZM5G867512_T01	2.845341508	2.218572884	2.978424648	2.533704122	2.521784465	2.424750137
GRMZM5G851769_T01	0.969888345	0.53187997	0.751219466	1.046033488	1.149297091	1.202667213
GRMZM5G838396_T01	6.464901298	5.799068676	6.479780862	5.804365345	5.905108777	5.873008174
GRMZM5G898772_T01	5.311886916	5.953072339	5.108602026	4.719015007	4.777193443	4.969297364
GRMZM5G854065_T01	3.06260512	2.468208454	2.397004194	2.875533565	3.086592039	2.719392435
GRMZM5G884707_T01	1.769000262	1.184287278	1.019372423	2.730684513	2.445164133	2.673416444
GRMZM5G815453_T01	2.210218649	1.587546937	2.598167941	1.881828259	1.863787832	1.351147162
GRMZM5G827309_T01	1.572500209	1.180858847	1.698688333	1.083627988	0.983884873	1.078669946
GRMZM5G811749_T01	0.833558865	0.915683039	1.103147792	1.077032391	1.137045723	1.038083542
GRMZM5G879235_T01	2.094683337	1.763719203	2.610725807	3.062431222	2.857723476	2.965180119
GRMZM5G804776_T01	7.495851854	7.427463891	7.037374292	7.294803996	7.370926491	7.337116982
GRMZM5G813608_T01	1.155804919	0.607579827	1.193368679	1.012683798	1.149369287	1.341582761
GRMZM5G800980_T01	4.028986961	3.386210158	4.142524781	4.129184086	4.016089948	4.273523424
GRMZM5G856777_T01	1.180977841	0.384277525	0.944981351	0.990629628	0.953996739	0.961739628
GRMZM5G800101_T01	4.944149852	4.04576071	4.900675418	4.5015308	4.408653717	4.351235661
GRMZM5G804358_T02	1.5464184	0.892538688	1.535233316	1.944687186	1.675274424	2.221442506
GRMZM5G851130_T01	2.150613289	1.83442726	2.357371269	2.167716951	2.211458643	2.036612345
GRMZM5G804708_T01	3.018445102	2.440732456	3.212628633	3.330562415	3.26773878	3.290462447
GRMZM5G845024_T01	9.210970458	8.866618146	9.028224211	8.200072784	8.187016329	8.351737763
GRMZM5G845244_T01	8.274652245	7.79908693	8.081435023	7.360735149	7.481349677	7.292791401
GRMZM5G831540_T01	1.21778845	0.667459819	1.006675032	1.706291833	1.812457562	1.75469
GRMZM5G801958_T01	1.230137211	0.599963291	1.088440827	1.279216313	1.313434522	1.373204408
GRMZM5G844030_T01	0.667762265	0.318587565	0.355908226	0.729639775	0.601363731	0.616192055
GRMZM5G827951_T01	3.481034225	2.792828705	3.393384073	3.629229308	3.762613728	3.597911791
Zm00001d035916_T001	1.887717097	1.905093995	1.878802231	2.562136165	2.510486511	2.441587853
Zm00001d048284_T003	1.731905277	2.139684934	1.777234955	1.314191857	0	1.649384149
Zm00001d028803_T002	1.451941672	0.604305743	1.411591658	2.814589797	3.039628686	3.063003607

Zm00001d028803_T001	0.700348271	0.564105945	0.674310589	1.990002549	1.34117112	1.182408179
Zm00001d027450_T007	0.353378626	0.045895839	0.300837133	0.590687283	0.561789484	0.690644154
Zm00001d027450_T001	3.68125067	2.76369265	3.937354468	3.436524067	4.031475834	3.9632294
Zm00001d027450_T006	1.926274264	2.866248875	1.982313146	1.926854911	2.141580584	0.906500438
Zm00001d021010_T001	5.876295921	5.746772942	5.868549654	4.622115326	4.152230561	4.152963383
Zm00001d011700_T013	2.100427705	0	2.844930126	3.179412956	3.1055336	2.809023155
Zm00001d011700_T007	2.214430413	2.303245354	2.445809852	1.313107672	2.07152998	2.112142294
Zm00001d011700_T001	0.223065385	0.054848477	0.332172856	0.358962201	1.057227742	1.065812856
Zm00001d011700_T012	0.316965933	0	0.051061602	0.757842533	1.099569913	1.204186426
Zm00001d011700_T010	0	0.30642791	0.29342699	1.520945673	0.996349696	1.15309581
Zm00001d011700_T002	2.113269426	1.342476806	1.531523196	2.989893227	2.712238749	2.708921311
Zm00001d011700_T006	0	0	0	1.066235261	0.998339224	0.809373283
Zm00001d025899_T002	3.552034064	2.783856195	3.728054156	3.571117946	3.606327684	3.638591608
Zm00001d025899_T001	1.425784761	0.804397253	0.860184148	0.810197115	0.87703588	0.907536778
Zm00001d028830_T001	3.260721536	2.611707329	3.256972438	3.449107828	3.343168301	3.526638514
Zm00001d028830_T002	1.491452933	0.576830718	1.205245483	2.431810939	1.920922841	1.782159427
Zm00001d028830_T006	0	0.199172909	0.657591533	0.49700016	0.498110932	0.064624909
Zm00001d028830_T011	0	0	0	0.918378601	1.233959825	0.616897783
Zm00001d028830_T010	0	0	0.522112009	1.188383199	1.667987015	0
Zm00001d019765_T001	3.43645504	2.988419116	3.410057477	4.091370557	4.19504312	4.187679476
Zm00001d031523_T001	1.410587503	0.930782746	0.897577338	1.358047362	1.45735842	1.385795461
Zm00001d000002_T001	0	0.361962577	0.500836718	0.519624428	0	0
Zm00001d000002_T011	1.567161846	1.086203583	1.514007598	1.128274262	1.124643111	0.644186702
Zm00001d000002_T028	0.525676025	0	0	0.715029691	0.712677652	0.568743302
Zm00001d015010_T001	1.03011851	1.044658502	0.940404329	0.917756349	0.822890008	1.183657373
Zm00001d038020_T001	3.480473367	3.036449353	3.736034828	3.447804509	3.690616668	3.647526696
Zm00001d038020_T002	2.914991911	1.804133293	3.043990458	2.580805885	2.553477487	2.776833155
Zm00001d038678_T001	1.20622123	1.131705369	1.533479625	1.466920458	1.369014131	1.417626256
Zm00001d005036_T001	3.549361133	2.770547878	3.585705658	3.246628655	2.812100788	3.180341396
Zm00001d005036_T008	1.588013458	1.269669348	1.347359512	2.416565766	1.926887162	1.820561724
Zm00001d005036_T009	0.609724709	0.393120712	0.836531737	1.845427023	1.525613389	1.565812589
Zm00001d039768_T002	0.646216123	0.565957805	0.687412806	0.954120357	1.02674058	0.749509382

Zm00001d039768_T003	3.002475163	2.010109886	2.102392745	0.559695416	1.564529869	0.646663135
Zm00001d039768_T001	4.265317467	3.661318984	4.691536342	4.570686847	4.523521435	4.638978859
Zm00001d045551_T035	0	0	1.000437792	1.488155062	1.056823495	1.565033147
Zm00001d045551_T068	2.115774715	1.62930952	1.757574609	1.703180453	1.950564342	0.999853559
Zm00001d045551_T008	0.767458189	0.250420765	0.405801802	0	0.205346216	0
Zm00001d032787_T001	4.854506486	4.613176888	4.999630397	5.367511134	5.255578795	5.309751615
Zm00001d004127_T001	1.33214879	0.726965456	1.230407165	2.027347648	2.083452691	2.080534534
Zm00001d053526_T001	3.926163041	3.81732507	3.153103594	3.285439506	2.945698337	2.677768231
Zm00001d050493_T001	5.596027986	5.605047867	5.649310938	5.631492209	5.640685762	5.743031314
Zm00001d040071_T001	2.871339603	2.327769843	2.81941988	2.184544685	2.53494782	2.414255707
Zm00001d038061_T001	4.551063943	4.502182531	4.486370003	4.794853132	5.027608981	4.795661038
Zm00001d005089_T003	4.236641013	3.762259189	4.482321242	3.701919054	3.624238371	3.52819747
Zm00001d005089_T001	2.405254763	1.837902079	2.210044048	1.837187596	1.733840653	1.538831143
Zm00001d044774_T007	0.395248199	0.070607777	0.088976029	0.681032701	0.959851003	1.400519896
Zm00001d044774_T003	0.839165484	0	0.938062171	1.711348647	1.359319314	0
Zm00001d044774_T010	2.276802443	1.934244355	1.998703517	0	0	1.555499611
Zm00001d044774_T008	0	0.963656854	0.236901566	0.738924309	0.762626909	0
Zm00001d044774_T006	2.619268616	0.982860362	2.238287332	2.478005458	3.029227232	2.874688391
Zm00001d044774_T009	1.361676855	0.969460339	1.042335456	1.891655987	1.667198193	1.469429137
Zm00001d043849_T001	4.765017087	4.376021312	4.34879195	1.133827185	1.659680741	2.300003324
Zm00001d018716_T001	4.829521162	4.234780574	4.847716606	3.612043047	3.632922981	3.752259405
Zm00001d025526_T002	2.189002502	2.672183854	0	0	0	2.209920255
Zm00001d025526_T001	3.143186535	2.341646233	2.828509118	2.945480357	3.134819091	3.140472909
Zm00001d025526_T004	3.515756851	2.354395174	3.604736993	3.482289808	3.297071065	3.222050565
Zm00001d025526_T003	0	0	2.176337455	1.081869973	1.39409812	0
Zm00001d050985_T003	0.326247399	3.27323911	0	1.723301234	1.228089079	1.184593241
Zm00001d050985_T001	2.049716491	1.622655838	2.374111915	3.409072082	3.276071388	3.179741927
Zm00001d050985_T005	4.758625541	2.2931256	4.766704022	5.048392141	5.00032475	4.890708942
Zm00001d050985_T004	2.816347597	2.384936709	2.782170543	2.761717166	2.380396438	2.265796925
Zm00001d013466_T003	2.193197705	1.882546019	2.320173288	2.137142149	1.908938154	2.414436762
Zm00001d013466_T005	3.95024154	2.884750485	4.093470258	4.439747855	4.317055686	4.329349027
Zm00001d013466_T001	0.634857158	0.875851606	0	0.473438857	2.012774605	1.280088061

Zm00001d035168_T001	5.651625038	5.706127802	5.750893461	6.10204914	6.190608184	6.171882072
Zm00001d035168_T003	0	0.695822817	1.236999501	1.673331635	0.688196475	2.27122396
Zm00001d035168_T004	1.510772078	1.026893522	1.352114422	1.575247941	1.856336779	1.479156044
Zm00001d047499_T001	4.598389445	4.190121921	4.261424377	2.979677476	3.263003147	2.913075713
Zm00001d003430_T001	2.771738978	2.308990744	2.840098007	2.400946635	2.819747264	2.664250489
Zm00001d018544_T002	3.124050323	2.556591763	3.279563708	3.258595655	2.994187048	3.084823181
Zm00001d037691_T002	2.810814681	1.88924612	2.506182741	1.660310747	1.896553191	2.226265902
Zm00001d026652_T001	3.04475029	2.175389826	3.145171364	2.85674532	2.846655378	2.662057348
Zm00001d040057_T001	1.318123601	1.358023161	0.968891433	2.699654678	2.959537043	2.920187715
Zm00001d008193_T002	1.250646935	1.072371328	1.241014592	0.960298923	1.197227546	1.302613263
Zm00001d039014_T001	7.397681957	7.32682377	7.328316924	6.780773834	6.847185977	6.825517539
Zm00001d004477_T001	1.357802519	1.536035488	2.068460667	1.815151985	1.305969354	1.929946997
Zm00001d004477_T003	1.62165572	0.497247526	1.695541787	0.640904072	1.130318088	0.766008212
Zm00001d004477_T004	2.513770414	0.939716296	2.227437351	1.397187974	1.670926877	1.744233444
Zm00001d004477_T002	3.753806572	3.701411989	3.905939546	3.158193203	3.073679555	3.310260421
Zm00001d052767_T002	4.516197301	3.552021518	4.375888182	3.544192317	3.614813714	3.494883546
Zm00001d035825_T001	1.066020973	0.659372942	0.675418514	0.750037032	1.204031133	0.459101072
Zm00001d024935_T002	2.343971429	1.943890579	2.222678768	1.949769854	1.770355468	1.769934615
Zm00001d018825_T004	2.105394472	1.707307884	1.582238527	0.774994898	1.06106556	0.275203765
Zm00001d018825_T003	3.458728593	2.580596316	3.56131416	3.240146539	2.665009305	2.867299104
Zm00001d018825_T002	2.630095557	2.42887749	2.607997794	0.307105559	1.958971445	1.496279796
Zm00001d018825_T001	1.44570234	2.028160331	2.251090986	1.664858256	0.740954321	0.47050667
Zm00001d000018_T001	0.584104323	1.014513838	0.638855114	3.239330519	3.091390616	2.812093598
Zm00001d041076_T001	3.270139736	3.107706614	3.065265887	3.76390634	3.475260279	3.479151775
Zm00001d020043_T003	2.670155981	1.474611518	2.493161572	2.951737912	2.496120235	2.813893832
Zm00001d020043_T002	0	1.250933689	1.133519468	0.509583361	1.397493037	0.233662315
Zm00001d007790_T001	3.561352778	3.169175086	3.641402908	3.407648738	3.262855711	3.144936518
Zm00001d000447_T001	2.509188339	2.645506151	2.495972164	0.437222411	0.654762722	0.509361412
Zm00001d022022_T001	3.47141588	3.34580667	3.327785361	2.825183599	2.385517182	2.328612749
Zm00001d016946_T001	3.197780802	4.031786432	2.330125813	4.709492522	3.90036604	4.211930645
Zm00001d009542_T001	1.562746012	1.027527773	0	2.802554104	2.955623407	2.707897785
Zm00001d037874_T001	1.912210284	2.011519612	1.923114183	2.682381824	3.05219363	2.909649659

Zm00001d045359_T001	2.251135535	2.770062999	1.735335005	1.612201497	1.349596271	1.818124533
Zm00001d045359_T003	5.33198046	4.481797947	5.248957647	4.806847107	4.816624536	4.915378728
Zm00001d045359_T002	1.153076348	1.285731856	2.010089113	1.60506331	1.98012038	1.348714559
Zm00001d033295_T001	5.728282281	5.077607985	5.688868944	5.83207028	5.828535394	5.765433781
Zm00001d009230_T001	1.986226695	1.522348076	2.032028177	2.283343393	2.617609278	2.295925164
Zm00001d031930_T001	2.396650262	2.968210956	2.58051359	1.851545576	1.994775932	1.806725357
Zm00001d003800_T002	0	2.241132998	2.435662994	1.866484699	0	0
Zm00001d003800_T008	1.043313705	0.222185071	0.245964113	1.309826942	0.992189787	0.271363521
Zm00001d003800_T003	4.302123437	3.739768407	3.937966256	3.169952573	3.725008064	3.503258405
Zm00001d003800_T004	4.250219198	2.603029027	4.223563955	3.919089854	3.737593643	4.055732226
Zm00001d003800_T010	3.482843638	2.947417988	3.630543979	3.16781793	3.224607884	3.537069145
Zm00001d003800_T006	3.146022194	2.511157556	3.544152618	3.567701926	3.340208459	3.520154304
Zm00001d003800_T001	2.629372475	2.073682125	0	0	2.250011689	1.921375773
Zm00001d026306_T008	2.444196869	0	1.325482553	2.716858797	2.38625169	2.100624161
Zm00001d026306_T010	0.811971571	0.406013047	0.613390205	1.649924427	1.823933965	2.129874483
Zm00001d026306_T007	5.683992682	3.548764955	5.663253978	4.460279412	4.865537261	4.844521088
Zm00001d026306_T012	1.844056178	0.75162225	1.46253558	2.361119281	2.598556012	2.174640031
Zm00001d026306_T009	2.577591005	2.122594536	3.261711329	5.288728934	5.068437407	4.997451981
Zm00001d026306_T001	2.404678909	4.231510977	2.767331268	0	0.636860768	0.988456382
Zm00001d043287_T001	1.287892454	0.474066334	1.021808028	1.074018976	0	0.670466927
Zm00001d043287_T004	0	0	0.873399816	0	0.876574702	0.674556466
Zm00001d010149_T002	3.034570358	2.563271393	3.379121449	1.755768666	0	0
Zm00001d010149_T003	3.747187364	3.478488454	3.859722421	4.410942102	4.535831007	4.570890636
Zm00001d010149_T014	0	1.739690434	0	3.851978256	3.887141736	4.040541443
Zm00001d010149_T005	4.548173895	2.988402573	4.376675944	3.536735283	2.753435741	3.954962504
Zm00001d010149_T011	3.173598392	0.518365933	2.526528848	2.713080339	3.351623524	0
Zm00001d011560_T027	0.347850445	0.043019669	0.668202651	0.652865504	0.472850609	0.384494155
Zm00001d011560_T061	2.518693751	2.196791775	2.788149037	1.000945377	1.753419695	2.038461116
Zm00001d011560_T049	0	0	0.108296953	0.743051301	1.000693048	0.629461073
Zm00001d011560_T028	1.597031001	0	1.627491978	1.267148031	1.409205716	1.732689029
Zm00001d011560_T014	0.643559772	0.785850986	0.502765433	0.70782602	1.778702709	1.717140971
Zm00001d011560_T055	0	0.195683978	0.991481031	0.854811148	0.512821544	0

Zm00001d011560_T029	0.682689248	0.7916874	0.101313901	0.428506906	0	0.124489602
Zm00001d044696_T006	2.53737957	0	2.16920604	1.885219797	2.07440576	2.311254895
Zm00001d044696_T002	2.644286857	2.643558849	2.701366297	2.868255907	2.664063852	2.845756988
Zm00001d044696_T001	1.296407481	0	1.301406201	1.558055011	2.055654841	1.180970205
Zm00001d034282_T004	1.301368153	1.272781674	0	3.089540014	2.384022445	2.36385623
Zm00001d034282_T001	0.308169793	0	0.715128122	0	0.919869387	1.252646966
Zm00001d034282_T009	2.510793089	0.773144799	2.60179705	1.025675204	2.419979183	2.398524129
Zm00001d034282_T002	1.485583969	1.452921678	2.214475487	0	0	0.335947386
Zm00001d022562_T001	0.879770059	0.573696382	0.865575218	0.241388699	0.259075996	0.543781919
Zm00001d046866_T001	5.24851514	4.75104557	5.389443427	5.057709457	5.086352345	4.935093606
Zm00001d014495_T001	2.600191479	2.117948988	2.477129155	2.248906999	2.555491021	2.208670823
Zm00001d025091_T001	1.430978885	0.911140089	1.638025631	1.051471643	1.110186153	1.401511966
Zm00001d043371_T001	5.744406225	6.437071134	5.820506455	4.852514978	4.071752374	4.375590401
Zm00001d018562_T001	1.232721533	1.353275859	2.310618743	1.471630738	2.658991299	3.18700424
Zm00001d018562_T002	1.163104514	0.58150744	1.282061363	2.633662302	2.638136642	2.670758548
Zm00001d018562_T006	1.732108964	1.071499776	0	3.514088418	3.22005238	2.662068973
Zm00001d043223_T001	5.552866566	4.891964809	5.613537399	5.162820216	5.072833612	5.132994539
Zm00001d036780_T001	1.336988279	2.021558256	2.588119988	7.315646824	7.355535556	7.127722197
Zm00001d023710_T002	1.278408867	0.858387678	1.051814066	1.01040605	1.301104131	1.32762931
Zm00001d014138_T013	2.681773751	0	2.35035778	0	2.627719355	0
Zm00001d014138_T010	0.742020012	0.387612895	1.047134908	0.948332466	1.322598504	1.003584248
Zm00001d014138_T005	0.820185499	2.192380725	0	2.691647387	0.722977435	2.934224159
Zm00001d014138_T002	4.408344076	3.236272179	4.362609943	4.731557827	4.725729597	4.401490258
Zm00001d014138_T007	0.559752185	0.062244695	0.243544789	0.276359685	0.823680892	0.717104111
Zm00001d014138_T001	4.892468453	5.415838897	5.059059561	3.57929385	3.591034512	3.693120703
Zm00001d014138_T020	0.508968104	0.289993772	0.545145008	0	0.379075274	0.489541908
Zm00001d014138_T009	1.606148921	0.70009787	1.624091534	2.933983672	2.359362893	2.573660759
Zm00001d009118_T001	7.470256742	7.451722782	7.546643008	5.23035025	5.205437557	5.113146257
Zm00001d004339_T003	1.05240546	0.754706246	1.066996382	1.353856792	0.955445981	0.824079431
Zm00001d017686_T004	1.632917747	1.241385038	1.513356324	1.672064567	0.971796986	1.472733659
Zm00001d017686_T003	0.947827715	0.616354874	0.946764541	1.4432332	1.825271131	1.566026509
Zm00001d026334_T018	0	0.241159242	1.620715895	1.02837678	0	1.636546205

Zm00001d026334_T001	2.05949684	1.677983988	1.965600347	3.115343263	3.308641045	3.00799038
Zm00001d026334_T009	0	0.24177064	0	1.790830412	1.482550422	1.193707412
Zm00001d026334_T022	3.018598035	2.709125621	2.98238124	2.979165278	2.562504986	3.212881811
Zm00001d026334_T005	0	0	0	0.605154836	0.472105082	0.894470117
Zm00001d026334_T004	0.446320819	0	0.358869955	0.644860418	0	0.408028117
Zm00001d026334_T016	0.561762118	0.097306046	0.28013202	0.552068135	0.667028293	0.654849775
Zm00001d001967_T004	0.506483126	0.15155752	0.449521229	0.395742845	0.4099774	0.737249777
Zm00001d001967_T001	2.252379325	1.24784672	2.316824345	2.220719368	2.27380082	2.15045472
Zm00001d001967_T003	4.13341384	3.877546185	4.323407469	3.957640052	4.052051088	4.027846032
Zm00001d052584_T029	4.465650246	3.843342526	4.442011114	3.999037672	4.120941191	4.041548331
Zm00001d052584_T001	0	0	1.367831201	1.888888948	3.122000574	2.015993107
Zm00001d052584_T028	2.945139276	1.707626386	2.422021695	3.747960926	3.125319482	3.616570839
Zm00001d052584_T017	0.535264675	0.318017135	1.451767637	1.612227924	1.137938234	1.137427452
Zm00001d052584_T025	0	0.144843875	0	0.913889322	0.920061536	0.848378369
Zm00001d023349_T001	1.550244163	1.138895708	1.491847454	0.89461679	0.868754711	0.997007635
Zm00001d000116_T002	0.830725868	0.954779228	1.622648342	1.197026825	1.486308663	1.851397256
Zm00001d000116_T001	3.187523266	2.878524664	3.745732777	5.133084288	4.94109936	5.069256711
Zm00001d000116_T004	0.469544272	0.504961006	0.40306153	1.299830176	1.977202245	0.652964599
Zm00001d000116_T005	5.96771468	5.10968914	5.869851053	6.277952788	6.417595861	6.349793656
Zm00001d008535_T001	2.364281623	1.896636497	2.386171661	1.969074952	2.152034111	2.054457455
Zm00001d002155_T001	6.420206102	6.517193161	6.510986391	6.268210621	6.10604432	6.200956969
Zm00001d013061_T002	1.606845368	1.655890334	1.60261128	2.010326196	2.044217488	2.239448758
Zm00001d013061_T001	5.992408528	6.82763446	5.932203364	6.125332316	6.326410665	6.054037992
Zm00001d013061_T003	3.98805158	3.39572667	3.781110499	3.43866785	3.474290274	3.351237923
Zm00001d017404_T001	2.554477115	2.985270164	3.179277748	2.008699498	2.187216023	1.343410096
Zm00001d029551_T002	4.622013269	0	4.839533416	4.419846139	4.681109298	4.327345474
Zm00001d052947_T001	1.317921655	0.832231387	1.436401202	1.910560756	1.654592267	2.003136254
Zm00001d041576_T002	4.937060628	4.615659767	5.307317295	5.704662515	5.674084138	5.76450845
Zm00001d041576_T001	4.936390269	5.026564651	4.984958726	5.231755834	5.3854553	5.275696671
Zm00001d041576_T003	4.969233577	3.777078268	4.972366908	5.414822916	5.637717957	5.333439048
Zm00001d052450_T002	6.016446577	5.674624087	6.074870859	6.186402403	6.399752943	6.346040079
Zm00001d052450_T001	1.142014722	0.620414684	0.951885404	1.010446155	0.757461964	1.158643373

Zm00001d052450_T003	3.925131452	3.651586523	4.151383548	4.230482639	4.408551281	4.390382709
Zm00001d026290_T001	2.429861503	2.056529079	2.526851312	3.068254622	2.924766337	3.004166612
Zm00001d012145_T001	1.729881187	1.256248022	1.9279738	1.466792065	1.474357644	1.517731236
Zm00001d052394_T001	1.138079175	1.055040129	1.232029516	2.166260073	1.713267781	1.451605713
Zm00001d032518_T001	1.642079543	1.08941137	1.565192092	1.481861028	1.4237508	1.512361919
Zm00001d026053_T001	4.201906458	4.356577047	3.937251819	6.405234933	6.343375219	6.449917531
Zm00001d008592_T002	0.891451851	0.457279086	0.721657014	0.949753795	0.607898875	0.718604932
Zm00001d014937_T001	7.030070349	7.418842615	6.849946596	6.630119648	6.476624887	6.639396281
Zm00001d002970_T001	1.002508108	0.91556985	1.20478365	0.425503348	0.930579153	0.668362428
Zm00001d039907_T001	6.196602953	5.905111835	6.108102585	3.633312046	3.83808821	3.844584507
Zm00001d014673_T002	1.437853588	0.598856819	2.215421094	2.476478901	3.478170531	3.260067822
Zm00001d014673_T001	7.901732389	7.091747482	7.907174773	7.423097038	7.415227875	7.415496527
Zm00001d026369_T004	4.48994395	3.903455878	4.543402526	4.236098406	4.179492738	3.953462863
Zm00001d026369_T001	4.5345059	5.129172667	5.122983958	5.517816634	5.874775527	5.726400769
Zm00001d026369_T005	5.198482367	0	4.80581232	4.431692018	3.798871404	4.014841763
Zm00001d019115_T034	1.607040503	1.002865231	0	0.61063676	0	0
Zm00001d019115_T001	3.538275674	3.118417373	3.751867294	3.178832697	2.926263066	2.785300282
Zm00001d019115_T024	0	0	0	2.194892983	2.186356661	1.330703523
Zm00001d019115_T042	0.048976877	0.117281455	0.403108444	0.193278462	0.321542555	0.340006162
Zm00001d019115_T009	0.854369143	0	1.182649715	0	0	1.978484872
Zm00001d019115_T047	0.23727613	0.063337264	0.279695929	0.494075571	0.400505141	0.3455508
Zm00001d019115_T003	0	0.338503144	0.931990093	1.083255585	1.186577254	0
Zm00001d019115_T033	0	0	0.198784549	0.857455559	0.650341797	0.932275896
Zm00001d033684_T002	4.933760658	4.365182437	4.885211449	5.645195457	5.576045414	5.639876427
Zm00001d033684_T001	0.650442903	0.49590797	0.558720212	0.889024115	0.807567601	0.973374695
Zm00001d004165_T001	1.892305142	1.476678233	1.809440375	2.193973864	2.28253351	2.037723789
Zm00001d034340_T001	3.415297882	2.891301744	3.638029339	3.209230472	2.971905665	3.09091896
Zm00001d013310_T001	3.756039275	2.97140836	3.513469269	3.169150387	3.13750959	3.254025106
Zm00001d038610_T001	2.959799083	3.140615894	2.559886757	2.420029864	1.949260375	2.409232876
Zm00001d038961_T007	0	0	0	0.907004468	0.700069453	0.907103714
Zm00001d038961_T012	1.624376537	0.985834834	1.48232221	1.410101721	1.714773475	1.439063503
Zm00001d050100_T001	8.758519403	8.301692393	8.650278142	7.984180396	8.195413609	8.212520981

Zm00001d031543_T012	1.701234535	1.167243587	1.778665708	2.820269454	2.474550001	2.926936866
Zm00001d031543_T023	4.727640926	4.111250038	4.79937763	4.55353917	4.58558519	4.682955437
Zm00001d031543_T015	0	0	0.952225453	1.511772638	1.763766362	1.273099283
Zm00001d031543_T020	2.183137431	0	2.107960985	3.425015847	3.482004615	3.155554151
Zm00001d031543_T022	0.064037124	0.073175846	0	0.508307958	0.606292502	0.51994542
Zm00001d031543_T008	0.647793409	0.499436199	0.067671755	0.88188228	0.850733026	0.745237332
Zm00001d040578_T001	1.92603093	1.157508791	1.885758643	0.531651445	0.42661901	0.464130815
Zm00001d015495_T016	2.759223598	1.942246116	2.46494528	2.793508061	2.335223689	2.366861279
Zm00001d015495_T014	0	0	0	0.322430065	0.307248979	0.385058788
Zm00001d015495_T003	1.372038845	0	1.590408961	2.074384522	1.89986703	2.427295343
Zm00001d033170_T002	4.280805803	3.989511898	4.289862419	4.222220009	4.106847866	4.112684455
Zm00001d033170_T001	2.296868499	1.424234159	2.125299642	1.525958602	1.056953865	0.989237122
Zm00001d006596_T001	4.701314669	4.053466926	4.828471849	4.442547646	4.360607913	4.300625139
Zm00001d022578_T001	3.12280456	2.422721712	3.203215098	3.04198044	2.904507605	2.9501931
Zm00001d033523_T001	4.463206798	4.091538548	4.471542236	4.863648198	4.910650117	4.950968372
Zm00001d039185_T003	1.729377439	0.970805073	1.300862898	0.70327881	0.639312748	0.505536308
Zm00001d051355_T001	2.47012762	2.482737818	1.72079284	2.539972696	2.343319979	1.667668723
Zm00001d005251_T001	6.056966043	6.025669889	6.289543924	8.569319699	8.687399368	8.635121009
Zm00001d047554_T001	4.585869911	4.161793783	4.836777113	5.334633559	5.400644454	5.363636863
Zm00001d014526_T001	5.138784699	4.023725172	5.141930512	5.539619748	5.530075488	5.479270179
Zm00001d014526_T004	0	0	0.950807305	1.326265811	1.523605111	2.005394239
Zm00001d014526_T005	1.103069887	0.518057687	1.033585103	1.752306725	1.567621864	1.717612162
Zm00001d038275_T005	1.998267365	1.036887298	1.948076	1.831668927	1.707461622	1.531361497
Zm00001d038275_T002	2.361468001	1.330686315	2.145849264	1.693194765	1.77215128	2.240875116
Zm00001d013007_T001	8.143771881	7.657503424	8.210262135	8.448584348	8.464845653	8.381093825
Zm00001d013007_T002	3.253166817	3.562684722	3.746742501	3.980387483	3.967791396	3.852453454
Zm00001d035492_T008	1.187457389	0.513176401	1.162305439	1.737204773	2.038614225	1.555013126
Zm00001d035492_T001	0	0.5284613	0.344261531	2.233510776	0.980486011	1.67065319
Zm00001d035492_T007	2.800592644	0	2.399281035	4.34358861	4.437983451	0
Zm00001d035492_T002	4.586681169	3.500293707	4.528396035	4.181471926	4.564383047	4.598559525
Zm00001d035492_T003	5.055418498	4.636654663	5.041389137	4.624216844	4.375012246	4.877455289
Zm00001d035492_T009	3.887776258	3.323638406	4.256547252	4.712923953	4.790171276	4.894765243

Zm00001d003249_T001	1.629799561	1.06695231	1.32403808	1.912863667	1.796304534	1.851643914
Zm00001d042143_T001	4.102063548	3.688806435	4.306797609	2.693678078	2.255162302	2.24605231
Zm00001d043168_T001	2.715063527	2.850258361	2.76593822	1.294283137	1.510112757	1.875842565
Zm00001d029290_T001	0.751407161	0.364242956	0.757131658	1.603278098	1.437323099	1.128990161
Zm00001d004731_T001	1.740915906	3.554517636	2.336613313	1.475140929	2.631159241	1.916439956
Zm00001d004731_T002	5.432363271	3.877855343	5.413097123	5.196173722	5.145598472	5.16906538
Zm00001d034731_T001	3.499803801	2.868180232	3.698144786	2.79831953	2.724599198	2.944498851
Zm00001d042215_T002	2.727291193	2.157516552	2.696773059	3.269359811	3.223011308	3.202898488
Zm00001d038923_T001	7.789630578	7.208122275	7.898429753	7.182945164	7.242761218	7.205064882
Zm00001d051075_T002	1.390824492	0.978088705	1.124297692	2.464561368	2.307309316	2.206332211
Zm00001d051075_T010	3.379657694	2.444209328	3.478813423	3.34645629	3.427034076	3.392122989
Zm00001d032633_T004	2.16442749	1.913418331	1.997285903	2.707656421	2.780669783	2.811144229
Zm00001d032633_T005	0.887068995	0.571348678	1.07862213	1.648079292	1.575707807	1.347175794
Zm00001d018194_T001	4.126302052	3.558359973	4.085422447	4.011658523	3.889357889	3.927761846
Zm00001d019250_T001	2.990896788	2.558672218	2.802044906	1.776792105	1.75077113	1.57607995
Zm00001d019166_T004	0.65380388	0.337832096	0.645262651	0.997703567	1.024812551	1.182555013
Zm00001d019166_T001	2.610332964	1.59787533	2.705768502	2.988769019	3.003868265	2.886073462
Zm00001d019166_T003	0.853339401	0.290154242	0.396320632	1.202023336	0.893959359	0.481323744
Zm00001d019166_T009	1.92874097	2.562648825	1.89001742	1.821160376	1.558034433	2.22926428
Zm00001d022099_T001	3.678297309	3.71026589	3.884170226	2.526363081	2.776709372	3.372064484
Zm00001d017539_T002	0.746060757	0.486125335	0.752374384	0.18801092	0.528428292	0.813740564
Zm00001d025654_T001	5.707618628	6.363728769	5.906216916	6.784449711	6.786950275	6.772671045
Zm00001d043131_T001	2.504387	1.595619663	2.520915509	2.357506401	2.155980058	2.115741761
Zm00001d019314_T001	0.589311915	0	0.734482022	1.111695734	0.971410015	1.08605884
Zm00001d019314_T011	0.234321049	0.460868352	0.309282144	0.465312109	0.69224685	0.584457469
Zm00001d019314_T008	2.578082742	1.608247665	2.769838372	2.912778418	3.197480296	3.094729103
Zm00001d019314_T005	3.158586342	1.329602405	1.859846338	2.288261099	1.746691129	2.030753464
Zm00001d045644_T001	1.403874672	0.933899662	1.501635822	1.348448864	1.337902868	1.108424092
Zm00001d015905_T001	7.229281808	6.58352987	7.189877621	6.736710016	6.741744645	6.682327504
Zm00001d019149_T001	1.371935169	1.705358681	1.797386577	1.367307317	2.01284074	1.304438613
Zm00001d019149_T008	0.383113133	0.390903163	1.008853289	2.182472381	2.579642383	1.218137002
Zm00001d019149_T005	5.244191758	5.164567235	5.213721478	5.784139677	5.509623389	5.733927264

Zm00001d019149_T007	5.175817541	0	5.483824707	5.300658851	5.465098202	4.837655481
Zm00001d019149_T003	4.766492283	3.661514641	4.469795219	5.255234716	5.374814944	5.735723783
Zm00001d019149_T002	0.448601812	0.37951783	0.14860877	0	0.659055941	0.690199837
Zm00001d002819_T002	4.331024404	3.528121188	4.346422816	4.106095759	4.208786296	4.139665454
Zm00001d023760_T001	4.494944011	4.238155925	4.48191184	4.129835505	4.078135689	4.186226144
Zm00001d005334_T001	8.110449771	7.703468298	8.182448519	7.597659334	7.567676595	7.532051854
Zm00001d043413_T001	8.517132381	3.921994658	8.469356506	8.446619435	8.444096586	8.413451829
Zm00001d052853_T001	2.496735114	1.789613247	2.512287071	2.806262801	2.848048001	2.947891473
Zm00001d030958_T001	1.765838529	1.186525912	1.602086728	2.043413453	2.232683164	1.97333722
Zm00001d012833_T002	4.966136859	4.502209332	4.910274313	4.183305551	4.245929288	4.245497564
Zm00001d048801_T001	6.072112941	5.944151047	5.762785385	4.993222464	4.98945731	5.267798433
Zm00001d039323_T001	1.438745286	1.071922579	1.428551927	1.187365535	1.312374166	1.070236119
Zm00001d017472_T004	2.635555905	1.367235737	2.317596688	3.095926951	3.076635537	2.846621684
Zm00001d017472_T002	3.662795676	2.82050352	3.768468389	4.02485146	4.006494529	3.822434238
Zm00001d032773_T002	0.342104117	0.107113355	0.275429063	0.391130902	0.361607802	0.673314899
Zm00001d032773_T003	3.476840088	2.536218801	3.442268223	3.600864885	3.629585335	3.637057335
Zm00001d028240_T001	2.016198194	1.915459329	2.372718128	1.674406414	1.861513955	1.692078534
Zm00001d015260_T005	3.435901774	3.406600069	3.894161006	3.006452071	1.385906979	3.44413245
Zm00001d015260_T003	4.627203387	3.830003358	4.415620028	4.859503677	5.189221149	4.899828032
Zm00001d015260_T001	1.033286963	0.643496972	1.152475499	1.138593658	1.277489089	0.922270172
Zm00001d015260_T004	1.081854978	0.646276962	1.118913173	1.418671396	0.274093449	1.197341423
Zm00001d039324_T001	3.852365159	3.666454276	3.961724906	4.613826703	4.448968328	4.352166483
Zm00001d002390_T015	0.14130068	0	0.507288245	0.866393698	1.071422203	0.691269721
Zm00001d002390_T001	2.689787805	1.378926061	0	2.435972027	2.830247616	2.885367414
Zm00001d002390_T004	2.004665697	1.957740796	1.946932561	0.375240769	0	0
Zm00001d002390_T019	0.725498615	0.224628102	0.578500164	1.143128889	1.215491609	1.203407912
Zm00001d002390_T018	1.325137117	0.165038503	1.373421037	1.623841584	1.58229297	1.534087507
Zm00001d003394_T001	1.503482057	2.971083474	2.400243896	2.022343309	2.53758582	1.682901799
Zm00001d003394_T003	4.75385951	3.398827404	4.552096117	4.03891583	4.020554294	4.038140624
Zm00001d003394_T002	1.980854474	0.822443008	2.353324138	2.260433709	2.027339509	2.077579019
Zm00001d049190_T008	2.296970666	0	1.965619923	0	2.261737501	2.327105858
Zm00001d049190_T001	1.930116987	1.677519969	2.480258917	2.810807896	3.017820203	2.770856314

Zm00001d049190_T007	3.58321807	2.777029333	3.452201499	3.047642731	2.490340873	2.322739527
Zm00001d049190_T006	0.340907449	0.072561353	0.143243198	0.392839447	0.62747797	0.310841486
Zm00001d012765_T008	1.053650769	0.600450555	1.205010204	1.275063085	1.4314502	1.787771784
Zm00001d012765_T005	2.979517616	2.232172921	3.333693927	3.550622316	3.435157441	3.304541706
Zm00001d044285_T009	1.753625928	1.063551952	1.329075936	0.558583082	0.60979845	0.650022805
Zm00001d044285_T012	3.637943699	2.188614876	3.59544303	2.637248774	2.441545361	2.399875996
Zm00001d044285_T013	1.075272478	1.082157559	1.203977903	0.837606641	1.039141202	0.752492565
Zm00001d029717_T001	1.867219622	2.609249552	2.113802831	2.210910921	2.102274481	2.057389964
Zm00001d039842_T001	7.302015773	7.37777717	7.171474285	7.068929482	6.860276958	7.00016128
Zm00001d010031_T006	0.951303554	0.962227689	1.526365836	0	1.333403124	1.163337064
Zm00001d010031_T001	2.934571982	3.426632159	3.725319955	2.551505436	2.658281645	2.3946195
Zm00001d010031_T002	0.761184834	0.279389278	0.171387473	1.363579292	0	0.360206934
Zm00001d050916_T001	4.216149453	5.774245111	3.660505857	4.906482448	5.0864343	4.827357746
Zm00001d050916_T003	2.133492507	5.246887069	3.271444502	1.811337441	2.163631081	1.524314972
Zm00001d050916_T002	6.363947088	6.588159935	6.431757262	6.027756811	5.918530544	5.962984773
Zm00001d050916_T004	1.302580511	0.896188908	0.874990492	1.040083734	0.945410686	1.126536811
Zm00001d035270_T012	0.556001633	0.417927567	0.727731422	0.787027015	0.865645686	0.720340638
Zm00001d035270_T008	0.588019696	0.304423426	0.624846738	0	0	0
Zm00001d035270_T011	3.660116182	2.65403494	2.938687394	1.660764364	1.62503384	1.395611249
Zm00001d035270_T003	3.493715855	2.366864356	3.602714125	3.074976838	3.233503413	3.125271038
Zm00001d035270_T001	0	0	0	0.59076871	0.897145146	0.543575069
Zm00001d035270_T005	3.129829489	3.386134128	2.601470706	3.177994309	2.493569971	3.139552007
Zm00001d035270_T004	1.646591714	1.991096038	2.906341305	2.660046224	2.560773395	2.804281596
Zm00001d017853_T005	2.195449969	1.462809862	2.746740781	2.725811163	2.467695532	2.32815918
Zm00001d017853_T003	3.062070408	2.128689989	2.753838121	2.757992898	3.394477237	2.933900606
Zm00001d001915_T001	6.821252503	6.6384529	7.04886143	6.713589918	6.746000314	6.80543922
Zm00001d001915_T002	3.17300907	3.6776706	2.88955395	3.276981056	2.725029775	2.641658388
Zm00001d038655_T041	0.077386578	0	0.123549664	0.418263363	0.486187133	0.384788101
Zm00001d038655_T002	1.587018318	0.892203706	2.206057714	2.766757125	2.474471607	2.731578408
Zm00001d038655_T005	0.49154221	0	0	0.836307126	0.881056876	0
Zm00001d038655_T044	3.888173168	3.281600188	3.759365084	3.126178462	2.463127524	3.41724048
Zm00001d038655_T035	0	0.086454979	0.470255717	1.085253997	0	1.179899538

Zm00001d038655_T037	2.73207357	1.921211225	2.468834301	3.172630722	2.532104966	3.106720839
Zm00001d038655_T032	0.298055307	0	0.106572111	0.850717027	1.291812757	0.291795669
Zm00001d027816_T001	6.009035894	5.545976923	6.137497786	6.218508487	6.135550876	6.179013963
Zm00001d010912_T001	2.757575676	3.05503388	2.757072331	1.671062342	1.931241672	1.697793387
Zm00001d012437_T001	5.765756809	5.359994811	5.870429627	5.896982194	6.052395678	5.968279604
Zm00001d053041_T002	1.141938234	0.765464307	0.971070042	0.727699188	0.377785654	0.701857745
Zm00001d053041_T004	0	0	1.229303662	1.164712919	1.025734018	0.987065415
Zm00001d053041_T003	0.92099218	0.951079704	0.380265096	0.806266307	0.901606437	0
Zm00001d020260_T001	4.546487609	3.746172145	4.643412753	5.083875154	5.095465638	5.151177198
Zm00001d018804_T002	1.93336075	1.410976554	1.805754292	2.121517106	1.992089697	2.170990279
Zm00001d050242_T002	4.486077761	3.994054864	4.581750554	4.142003118	4.103898836	4.016330147
Zm00001d050242_T001	0.775863027	0.265798125	0.56509897	0.954623657	0.7509846	0.803817222
Zm00001d044382_T001	5.643681815	5.001571411	5.70890639	5.182828934	4.901393036	5.132216412
Zm00001d006780_T020	4.908349288	4.544806334	5.045088719	4.54126931	4.374833059	4.73591594
Zm00001d006780_T007	0.371381547	0	0.29246491	0.549240364	0.900335509	0.801013758
Zm00001d006780_T019	1.209646752	0.307849323	1.036272625	0.710609195	0.85865025	0
Zm00001d006780_T018	0	0.087241498	0	0.592256571	0.68415086	0.790168982
Zm00001d006780_T002	4.160996402	3.101835107	3.932792352	4.844631806	5.037936086	4.935589066
Zm00001d048460_T004	0	0	1.458223446	1.54400012	1.659372942	2.257920316
Zm00001d048460_T003	2.237435228	1.965831927	2.016071579	2.771348957	3.03318616	2.727123867
Zm00001d048460_T005	1.73740901	0	1.540236117	2.752898846	1.792362712	2.142600012
Zm00001d038311_T001	3.677940366	3.10818369	3.734564395	4.191827684	4.246495878	4.075174908
Zm00001d052661_T001	4.343578164	5.141196274	4.520372398	3.557473811	3.783352613	3.573589872
Zm00001d005137_T027	0.782862271	0	0.637298629	1.619146281	2.124854821	2.16302817
Zm00001d005137_T013	0	0	0.062885696	0.459400141	0.505377767	0.792408943
Zm00001d005137_T009	0.812545869	0.170334028	1.432459186	2.627474468	2.699933576	2.390306692
Zm00001d005137_T001	0.908498593	1.057127211	0.527098368	1.205426922	1.131430786	1.331445542
Zm00001d005137_T007	0.579436025	0	1.079775406	1.166092606	0.958695708	1.05932551
Zm00001d005137_T011	0.203194891	0.32709292	0.349932505	0.968177035	1.331561341	1.019226512
Zm00001d005137_T010	2.608646516	1.981615228	2.623192869	2.550039222	2.349735812	1.974032911
Zm00001d008210_T004	5.868519579	3.98883372	5.868367091	5.584127209	5.734121748	5.550893463
Zm00001d004094_T003	0.506675055	0.139639753	0.667210912	0.21085888	0.383958046	0.442089964

Zm00001d004982_T003	0.551252186	0	0.791083893	1.110902399	0.886499423	1.050007074
Zm00001d004982_T001	4.457592585	3.187206044	4.180005479	4.19314857	4.133406524	4.138216744
Zm00001d004982_T005	2.43972286	2.290587479	2.66726156	2.805204529	2.6990514	2.667015799
Zm00001d028759_T001	6.92883885	5.938939868	6.952235799	6.499621654	6.560535936	6.498795446
Zm00001d044185_T005	3.410134835	3.351350307	4.119123982	4.893006153	4.811692864	4.692577452
Zm00001d044185_T003	2.83029914	1.943340007	2.933789404	3.833409392	3.791820113	3.860240376
Zm00001d005650_T001	1.430199083	0.93564606	1.204733578	2.023554862	2.224737367	2.450168433
Zm00001d005650_T007	1.821463283	1.085094199	1.773278596	1.133343225	0	1.083265798
Zm00001d017164_T003	0.329905454	0	0	0	0.449464179	0.583183042
Zm00001d017164_T007	2.021638557	1.306242405	2.116645882	2.382770437	2.572203558	2.190246634
Zm00001d023914_T006	3.158636103	1.374021747	3.183634667	3.472372741	3.522786456	3.329247189
Zm00001d023914_T003	0.204367372	0.354518736	0.698048635	1.332570459	1.103028247	0.570997164
Zm00001d030191_T001	4.516649844	3.988525641	4.685915487	2.742161255	2.80606045	2.764744655
Zm00001d029149_T001	4.410169306	3.636110669	4.403579273	4.272316328	4.149444325	4.295106515
Zm00001d017657_T001	4.526951566	4.945545576	4.627821301	3.55262459	3.617766178	3.246898885
Zm00001d018618_T001	4.481488955	3.694617699	4.439417622	4.665075157	4.685152953	4.768872842
Zm00001d037505_T011	0	0	0.497720675	0.400450491	0.683165535	0.527225509
Zm00001d037505_T015	2.48082632	2.100411556	1.729909023	2.338152823	2.546019752	0.679859739
Zm00001d037505_T001	5.778388955	4.981787091	5.703591087	6.037507003	5.989690746	5.971336642
Zm00001d037505_T013	0.883566861	0.306018367	0.56786082	0.066137423	0.257080639	0.735274342
Zm00001d037505_T009	0.59018521	0.284711928	0.571216627	0.619169763	0.895912148	0
Zm00001d030697_T002	2.205792233	1.615129048	1.966404995	1.531409909	1.11349248	0.625642689
Zm00001d046493_T001	1.435582726	1.721188455	1.13418479	0.62409247	0.738908749	0.80007944
Zm00001d016308_T001	3.156449851	3.127574514	3.489942473	2.432221574	3.272893621	2.657069216
Zm00001d016308_T003	3.754663493	3.179838247	3.79470656	3.999494517	4.000381002	4.123087827
Zm00001d037485_T001	7.590075134	8.151718596	7.634291485	7.375706185	7.323916966	7.390723309
Zm00001d033596_T001	6.640386133	6.186922041	6.579713425	6.426002479	6.354159339	6.231091798
Zm00001d038389_T002	0.650803153	2.537915013	0	2.166316641	2.481994027	2.295057645
Zm00001d038389_T004	3.489435038	0	3.560285628	4.079720033	4.02812664	3.740754578
Zm00001d022016_T001	4.133132922	3.344479993	4.194516628	4.155960069	4.353610249	4.204610737
Zm00001d022016_T003	2.848691037	2.276353431	2.998345001	2.2927685	2.687381675	2.725420958
Zm00001d022016_T002	1.142299717	0.483594472	1.118557078	0.992997504	0.817821333	0.942813992

Zm00001d033837_T001	1.227054678	1.941998702	0	1.842488131	1.096372514	0.614251878
Zm00001d033837_T003	2.580530473	2.246078464	3.319773842	3.823240761	3.976109996	3.724390192
Zm00001d025622_T001	4.620099976	5.470138133	4.508394233	4.578223466	4.546672191	4.582503798
Zm00001d035619_T001	4.654366395	5.966027122	4.955553341	4.16650031	3.794982955	3.663644059
Zm00001d018473_T001	1.443146189	0.911948464	1.515166431	1.763779107	1.805614395	1.959682627
Zm00001d039238_T001	5.850302174	5.343475727	5.471377704	5.520366551	5.465133311	5.541900985
Zm00001d052981_T001	3.563871978	2.750736835	3.48371622	2.883282388	2.970541343	2.960796181
Zm00001d000163_T001	6.133671239	5.700019639	6.309441447	6.715465453	6.7299061	6.694063335
Zm00001d036892_T001	3.573635797	4.263864468	3.714980912	3.101984156	3.027112285	3.443598828
Zm00001d028117_T003	8.454769838	7.059151432	8.430071136	8.091432287	8.102288945	8.0513203
Zm00001d020732_T001	2.520412693	2.615665593	2.845183916	0.448655725	0.479827593	0.818682073
Zm00001d011904_T001	3.97489462	3.296741954	4.056757526	4.111376082	4.192218631	4.24263078
Zm00001d011904_T003	1.175147072	1.022903242	0.634283788	0.780657816	0.529779976	1.17105435
Zm00001d045496_T001	3.498954042	3.275490746	3.607269941	3.020311062	2.966574988	3.170088017
Zm00001d026287_T003	1.372938729	0.398759696	0.825314284	1.204215855	1.591103468	1.339144798
Zm00001d026287_T006	5.394638397	3.566127488	5.372949348	5.062568598	5.482429092	5.265097674
Zm00001d026287_T001	3.494882139	3.887749651	3.477258186	3.571280834	3.345397511	3.374077268
Zm00001d026287_T005	3.36673821	3.150901626	2.928794778	3.471334845	2.186963211	3.390686668
Zm00001d010173_T006	0.735243143	0.602255423	0.940326143	1.241643134	1.646516143	1.153333871
Zm00001d010173_T008	3.863008623	3.76428808	4.016511118	3.386681015	2.645071466	3.135870879
Zm00001d010173_T005	0.710222131	0.168251788	0.802983949	0.632694446	1.072116779	0.847526351
Zm00001d010173_T004	2.711607675	1.684910726	2.621355643	2.977248779	2.994504782	3.040760952
Zm00001d019206_T001	1.957611917	1.568131363	1.951729148	1.505420961	1.50043496	1.751465007
Zm00001d008409_T001	2.265784925	1.531076482	2.17254544	2.136076189	2.25368527	2.20522421
Zm00001d021127_T008	3.434137528	2.72173881	3.442277514	2.569654407	2.776947876	2.688799163
Zm00001d021127_T006	0.261329817	0.422949864	0.155374912	0.574928852	0.479808972	0.219648693
Zm00001d021127_T001	2.388070203	1.456020205	2.352476907	2.578354756	2.53039691	2.552279921
Zm00001d010673_T001	5.120729321	4.493760745	5.183075999	4.826875478	4.777213331	4.780992593
Zm00001d039255_T007	1.51480651	0	1.149698352	2.161294685	2.455961308	1.26033404
Zm00001d039255_T003	2.839833245	2.545097549	3.043432352	2.560028655	2.846870363	2.878711022
Zm00001d039255_T005	1.567627704	0.781382377	1.747527473	1.936019744	1.364960647	1.21772208
Zm00001d039255_T004	0.65956384	0.493174897	0.638975558	0.664105961	0.793412337	1.17542943

Zm00001d002250_T015	1.748380509	1.432648393	2.030934223	0.195375318	0.676084126	0.351102486
Zm00001d002250_T003	1.631557397	1.326571862	2.039252471	1.891152011	2.103430833	1.96446998
Zm00001d002250_T014	1.419348497	0	0.831936407	2.246236291	2.009678245	1.720539834
Zm00001d002250_T001	0.369121368	0.648607175	0.802250303	0.424182551	0.237889175	0.09854218
Zm00001d011180_T002	0.577284279	0.25125372	0.649665119	0.874905533	0.488639427	0.799629459
Zm00001d011180_T003	6.292701571	5.697072787	6.315025263	6.193203344	6.123744218	6.223122723
Zm00001d041672_T001	7.729147047	7.430269481	7.580040075	7.285007172	7.178881535	7.323887954
Zm00001d033853_T002	1.079409516	0.230827677	0.684629357	0.939312347	0.832960477	1.293889549
Zm00001d033853_T003	2.788598436	1.378665318	2.846576957	2.807716581	3.852396822	3.288038239
Zm00001d033853_T001	2.855654491	2.89907313	2.996574224	3.202529309	2.051343213	2.398519478
Zm00001d015553_T001	3.55198105	2.813394985	3.799141717	3.91462709	3.857926072	3.899104751
Zm00001d025236_T001	3.135388918	2.998174197	3.252080138	3.147309141	2.966823943	3.003880855
Zm00001d021824_T001	1.121340385	0.462554949	1.248435253	2.325701574	2.143833214	1.936434039
Zm00001d006841_T001	1.984902098	1.627640921	2.006519573	2.808383815	2.845498682	2.768936765
Zm00001d006750_T001	1.826589162	2.436614943	2.319632673	1.31686633	1.386208364	1.248942206
Zm00001d019567_T001	3.443638343	3.761678344	3.61334211	3.428840249	3.598062389	3.863935577
Zm00001d023293_T001	6.768934292	6.460482256	6.739733741	5.906961887	5.799120238	5.531303475
Zm00001d010870_T001	5.78353159	5.354139834	5.800212651	5.994338206	5.954674252	5.959001317
Zm00001d026610_T001	3.629310919	3.106750148	3.673699895	3.460292782	3.35163582	3.473671855
Zm00001d047856_T001	5.561959048	5.10792095	5.566373025	5.553870211	5.481008707	5.513193301
Zm00001d022131_T002	0	0.123966754	0.050685571	1.008795933	0.803989107	1.240190359
Zm00001d022131_T001	0.708626932	0.73060257	0.356789497	1.531093454	1.43376442	1.356520214
Zm00001d018794_T001	4.578897363	5.642307665	4.744221063	4.491295403	3.910794627	4.150162881
Zm00001d018794_T004	3.882909068	2.779720301	4.096453902	3.949719157	4.171269287	4.365948289
Zm00001d018794_T005	0.880367372	0.609788051	1.09111079	1.522775912	1.372211064	1.460988889
Zm00001d018794_T002	0.617800611	0.339567278	0.816900296	1.472654127	1.220697083	1.189824559
Zm00001d039145_T001	6.22011756	6.357805158	6.261888549	6.21192288	6.24306302	6.269014514
Zm00001d011833_T001	2.46313485	2.2329953	2.908473614	2.396832444	2.217551486	2.236463228
Zm00001d050062_T002	1.309351419	0.732122426	1.183842182	1.303653932	1.37070777	1.277662261
Zm00001d003308_T001	2.711280799	2.053473182	2.597724712	2.662168577	2.367316544	2.59114848
Zm00001d027969_T005	1.289968992	0.560935988	1.129771637	1.288103373	1.106448159	1.083144594
Zm00001d027969_T010	2.251893266	1.798854715	2.453252062	3.037288734	2.910973986	2.956151094

Zm00001d009131_T003	2.051863474	1.187470058	0.940110357	0.844636325	0.309590655	0.735828014
Zm00001d009131_T004	0.516692954	0.471575596	0	0	0	0.771127489
Zm00001d009131_T006	3.118782471	2.673781856	3.285969715	3.049773288	3.016653204	2.992748856
Zm00001d009131_T005	0.222436114	0	0.643048054	0.892077777	1.035388133	0.669103889
Zm00001d009131_T002	2.271405356	2.431762543	2.349838549	3.161686497	2.597148789	2.778476901
Zm00001d025570_T005	0.710495467	0.279920526	0.810943179	0.931357043	1.104937769	0.930114337
Zm00001d025570_T006	2.913295772	0	2.324080144	3.613637258	3.546572124	3.695213701
Zm00001d025570_T004	3.131699937	2.695515062	3.187226001	3.043767241	2.805959986	2.680454666
Zm00001d047636_T002	0.624546388	0.22510461	0.342884662	1.546265771	1.697719117	1.46606481
Zm00001d047636_T006	2.404475917	1.805176457	2.232498675	2.757918251	2.921386628	2.796810986
Zm00001d047636_T003	0.917612773	0.367363237	1.11046481	1.344545042	0.474761019	1.049940879
Zm00001d008218_T003	0.580963584	0.860837289	1.164727076	1.282710822	1.337689402	1.147682467
Zm00001d008218_T012	4.266321656	2.918921039	4.522689803	4.140919892	4.155530918	4.417490159
Zm00001d008218_T005	3.853683928	3.137726468	3.888711887	4.267990857	4.19287029	4.194416949
Zm00001d013163_T001	4.048544068	3.504977018	4.23364506	5.337294634	5.511981696	5.545084324
Zm00001d036742_T002	1.634909186	0.668234427	0.901634238	1.911207982	1.739716354	1.619698939
Zm00001d036742_T005	2.996409718	1.95558956	3.088796293	3.539704388	3.737546703	3.705915439
Zm00001d036742_T001	2.3430567	2.237842074	2.514783286	2.491569403	2.231913739	1.699069617
Zm00001d002314_T001	3.619309588	3.392733958	3.806999151	3.440841003	3.50424982	3.309307905
Zm00001d053183_T001	1.589198281	0.897859974	1.335747349	1.045936366	1.576956937	1.1229397
Zm00001d038221_T001	3.125673582	2.695425529	3.31470334	3.485530775	3.529642316	3.220687024
Zm00001d026479_T005	1.059965732	0	0.618606097	0	0.523288947	1.025376134
Zm00001d026479_T007	4.450550915	3.705563606	4.479906666	4.922840352	4.800565777	4.958209049
Zm00001d034223_T001	6.806049723	7.137055829	6.851346442	6.400210434	6.408051545	6.429489966
Zm00001d051053_T001	2.266233939	2.517081137	2.167929497	1.796580727	1.149230745	1.613045015
Zm00001d032123_T001	6.08514299	6.572073588	6.065861787	6.071151694	6.035209955	6.094875617
Zm00001d020641_T001	3.786650723	3.272761523	3.601237966	3.733896931	4.077708381	3.78307136
Zm00001d051803_T001	7.447575519	7.074201717	7.203501598	7.140972852	7.147617736	7.297475882
Zm00001d039165_T035	2.591652611	1.770681915	2.987489504	2.366471058	3.74643725	2.990345533
Zm00001d039165_T034	5.042745226	4.514948558	5.198433451	3.955038133	3.548004491	4.267985241
Zm00001d039165_T012	0.68473527	0	0.927602956	1.375337534	0.936767952	1.024996892
Zm00001d039165_T017	4.164363124	3.315024321	3.900929585	3.801618817	3.115971922	3.536599777

Zm00001d039165_T023	2.552742003	1.84498896	2.747833782	3.22343398	3.368674354	3.165854391
Zm00001d039165_T020	0.66591754	1.47754937	0	1.456950674	1.50855745	1.771668419
Zm00001d039165_T011	0.055316452	0.155983619	0	0.428945273	0.709813794	0.349302993
Zm00001d039165_T003	0.63328705	0	0.425051038	0.932704494	0	0.838151459
Zm00001d039165_T018	0.282796802	0.207336901	0.463568054	0.240427908	0.448644097	0.568560429
Zm00001d039165_T016	0.844405741	0.287727596	0.775015132	0.940938738	1.359508239	1.448700651
Zm00001d039165_T015	0.361487652	0.081353263	0.358093512	0.808210393	0.895162223	0.729034982
Zm00001d039165_T029	0	0.153994657	0.07659193	0.890980479	1.616703507	1.38721694
Zm00001d039165_T010	1.381597353	0	1.685065074	1.363650494	1.878069654	1.628628035
Zm00001d039165_T046	1.16405123	0	0	0.451542943	0	1.100671589
Zm00001d048568_T005	1.440906499	0.60266496	1.144943695	1.574924978	0.957049093	1.585532253
Zm00001d034919_T001	6.022336784	5.227367379	6.116605214	5.924618184	5.983089189	5.979836426
Zm00001d040724_T002	0.291496295	0.173494618	0.164893093	0.898031077	0.695329308	0.820092347
Zm00001d040724_T001	4.028604777	3.031724424	3.850811022	4.005916427	4.160338493	4.112497224
Zm00001d040467_T002	3.956177027	4.532945399	3.203083825	3.597226275	3.48712949	3.797170939
Zm00001d040467_T001	1.62845913	1.301916532	1.780149231	1.542096558	1.705323289	1.479153457
Zm00001d019142_T001	6.054369105	6.524911186	5.969135866	6.00708337	5.705656051	6.118547111
Zm00001d044600_T013	1.197797469	0.278284559	0.637958881	2.112017485	2.608095523	1.558529696
Zm00001d044600_T007	0.894095217	0.11609589	0.461594564	1.022009803	0.980046511	0.646535036
Zm00001d044600_T112	2.969735316	0	3.213451183	3.907344396	3.827962779	3.72144255
Zm00001d044600_T006	1.882642658	3.263301881	1.322000805	1.82386632	2.013947067	1.510948758
Zm00001d044600_T010	3.671905878	3.95705959	3.96943842	0	0	2.805728088
Zm00001d044600_T004	4.655859488	3.433770428	4.909555436	4.761716262	4.842312072	4.939037764
Zm00001d044600_T026	1.585776913	1.560129444	1.892440378	2.287708095	1.803776727	2.164165503
Zm00001d044600_T114	3.322446939	2.899243122	3.073723251	2.00040534	1.378269674	1.959431509
Zm00001d004568_T001	6.683759101	6.54419142	6.706732107	6.359997375	6.402860577	6.299343781
Zm00001d030295_T001	0.572166928	0.673832273	1.248242746	1.287287292	1.136104743	1.254577113
Zm00001d007485_T001	1.046181604	0.598105039	1.284081141	1.324194807	1.514153576	1.517068031
Zm00001d012632_T001	3.200711496	3.66851799	3.155117106	2.728727267	3.04587278	3.183314207
Zm00001d035777_T001	2.807535248	2.518296181	2.503606463	2.486529037	2.689547864	1.946871193
Zm00001d025784_T002	1.558257347	2.645590766	1.161224051	1.08777986	1.028180845	0.790415073
Zm00001d025784_T004	2.746477237	3.371395627	2.303149477	1.445692277	1.96023293	1.710317363

Zm00001d025784_T003	6.470121616	6.634750626	6.303766449	5.981972447	5.773311594	5.6967663
Zm00001d025784_T001	4.060054649	3.407161694	3.895619437	3.670132068	3.449033721	4.050683829
Zm00001d025784_T008	6.256754445	5.160752625	6.414844526	5.790038464	5.812175611	5.945792078
Zm00001d010821_T001	0.347646382	0	0	1.05330322	0.666043929	0.128635225
Zm00001d010821_T007	1.604683864	1.104277609	1.420308842	0	1.608690511	0.956895254
Zm00001d010821_T002	0	0.386307708	0.634329331	0.608343721	0	1.246621511
Zm00001d047528_T004	0.75771455	0.162396969	0.38809363	1.032696141	0.998575357	0.746438325
Zm00001d047528_T001	2.543649053	1.746406076	2.286530187	3.808651013	3.657582616	3.891902953
Zm00001d047528_T002	1.760034396	1.639102014	2.09259851	0.982332498	0.927065859	1.320060616
Zm00001d029540_T001	3.531991973	3.089973152	3.446930301	3.215288597	3.180221713	3.168802308
Zm00001d028374_T001	0.734710054	0.543668106	0.700990944	3.423839123	3.409749495	3.373564835
Zm00001d018637_T005	2.936801113	2.500495894	3.041107329	2.756954738	2.653038916	2.662839189
Zm00001d018637_T003	1.00462651	0.501601177	0.979175158	0.638752266	0.745035922	0.522545941
Zm00001d018637_T001	1.299448649	0.078765572	1.046014623	1.1553924	1.167765756	1.09300706
Zm00001d012857_T006	4.828934447	7.856019656	5.565654931	5.264989435	4.979364188	4.496938823
Zm00001d012857_T007	1.00272124	1.718520328	1.72473539	2.309131648	1.349953447	1.652567261
Zm00001d012857_T002	5.792602052	5.374699298	5.843248973	5.723732881	5.648383901	5.508808102
Zm00001d012857_T001	2.505525638	3.234995878	2.403341082	2.635730017	2.903051	2.163960128
Zm00001d012857_T003	6.800617686	5.887150755	6.472738824	5.911244277	6.04962859	6.183593502
Zm00001d033071_T001	3.583602962	2.861380553	3.500126046	2.56320329	2.610842955	2.628216236
Zm00001d040555_T001	4.724506868	4.132324801	4.722762457	5.165172117	5.131630473	5.133582431
Zm00001d031132_T002	7.985198727	7.506902819	8.147852288	8.08010349	8.114253441	8.019674691
Zm00001d031132_T003	1.534492454	2.323171162	1.439364626	1.076086328	1.310558254	1.255226368
Zm00001d039997_T001	5.303312728	5.23566978	5.278461836	6.122221827	6.158015726	6.186073581
Zm00001d003546_T001	3.472255492	3.260606081	3.799727049	3.873869783	3.671644265	3.817280449
Zm00001d052124_T002	3.243884738	2.380021778	3.420235801	3.557354455	3.691178807	3.511570886
Zm00001d052124_T004	2.515437701	4.239159177	2.407547726	1.460610474	0	2.028557483
Zm00001d028960_T004	2.037072914	1.057180597	2.078748838	3.968512621	4.136872945	3.945191164
Zm00001d028960_T009	0	0	0.095936569	0.653317798	0.8731982	0.560089814
Zm00001d028960_T006	0.627460227	0.401275483	1.061466546	2.754377874	2.879380535	2.792193185
Zm00001d028960_T002	0.402411124	0.521042694	0.482823507	2.836065104	2.552846006	2.846889815
Zm00001d028960_T007	0	0	0.060869174	0.543510731	0.495154818	0.832701287

Zm00001d028960_T005	3.048527939	2.405983105	2.68225079	3.000340256	3.002184929	2.818949109
Zm00001d037778_T001	1.56169712	1.990675005	1.431119065	2.148662781	2.517710074	1.857580169
Zm00001d010982_T001	6.113849661	5.382933186	6.145543694	5.718838883	5.792776345	5.817390029
Zm00001d041647_T007	1.449067406	0.165286823	1.379120201	1.123463583	1.534566657	0.680653821
Zm00001d041647_T004	2.578947405	2.454764961	2.48074439	3.260891314	2.871493772	3.01762372
Zm00001d041647_T009	4.446801704	3.277640542	4.608394467	4.100602465	4.054985796	4.063982179
Zm00001d041647_T002	0.947087093	0.220094646	0.399086854	0.637528639	0.560668005	1.08142485
Zm00001d025996_T001	2.415898982	2.398808665	2.75609843	2.268691888	2.450699763	2.17352404
Zm00001d019335_T013	0.65913908	0.224190959	0.541666485	0.10823271	0.508954925	0.498866626
Zm00001d019335_T017	0	0	0	1.386724879	1.183151726	0.818556921
Zm00001d019335_T018	0.277257565	0	0.452311823	1.156094324	1.376861569	0.995656004
Zm00001d019335_T012	1.052177976	0.684876175	1.57271411	1.880452011	1.577093298	1.962931717
Zm00001d018525_T005	4.418203309	1.333642984	4.678650107	3.869857196	4.032389089	4.035364025
Zm00001d018525_T003	1.319511	0.66898049	1.910023802	1.604583295	1.201106868	1.35020585
Zm00001d018525_T001	3.07547701	3.448610533	1.757779823	2.860840466	2.610365331	2.792328766
Zm00001d018525_T008	0.365614158	0.849681471	0.930588236	1.076602875	0.376352625	0.451263345
Zm00001d036985_T001	5.800754148	6.254046145	5.972179993	5.271769276	5.353249284	5.072603282
Zm00001d037055_T001	0.51457072	0.382942763	0	0.090131225	0.618336398	0.102658131
Zm00001d037055_T004	3.202464896	2.616615298	3.295978045	3.19582427	2.640722256	2.504011408
Zm00001d037055_T002	2.357371832	1.726039479	2.171879971	2.943108914	2.969299712	3.234631758
Zm00001d037055_T003	0.874244565	0.204497589	0.386536174	1.228175913	1.27789967	1.012012226
Zm00001d019947_T010	2.203372515	1.553058661	2.653558796	2.059897571	3.175816943	2.984403602
Zm00001d019947_T004	0.086584057	0.03088268	0.364278821	0.546500882	0.221137143	0.340326405
Zm00001d019947_T024	1.589912542	0.134880682	0.775590002	0.660004909	0.993241035	0.589436567
Zm00001d019947_T012	1.23401748	1.222935597	0.767947121	1.650912497	1.652826048	1.625053017
Zm00001d046691_T002	0.790691979	0.518342767	0.090150199	0.359404221	0.288213252	0.225411912
Zm00001d046691_T004	0.324840551	0.374274983	0.728989721	1.040967456	1.511867243	1.248978627
Zm00001d007639_T002	2.860147986	1.531434863	2.61655202	2.342601393	2.37933428	2.537506553
Zm00001d049619_T003	6.592081499	6.094213264	6.615527429	5.868754464	6.222866662	6.038184575
Zm00001d049619_T001	4.921213701	0.757618982	4.719472772	3.977601311	2.263941816	3.178826484
Zm00001d049619_T002	4.714441876	5.885584467	5.551084498	4.069361987	0	0
Zm00001d012958_T001	2.073921321	1.591856033	2.290148343	2.307366153	2.517381781	2.674627646

Zm00001d013485_T007	2.458137334	1.603166508	2.77053773	2.390784604	2.369916741	2.101644698
Zm00001d013485_T002	2.830378248	2.971132781	3.036928431	4.010307664	4.377097922	4.026730932
Zm00001d013485_T008	3.797122681	3.385213443	3.84891483	2.66836674	0	2.53267977
Zm00001d013485_T003	1.858137395	0.14964568	2.077306927	1.942851892	1.528232233	1.871866905
Zm00001d047042_T003	1.253883404	0.877597421	1.114854727	1.496763487	1.084948665	1.245103751
Zm00001d047042_T004	5.450600269	4.708455826	5.487588187	5.366014232	5.328198611	5.375664904
Zm00001d047042_T001	2.917956988	5.046678195	2.892632135	2.790186502	3.014820523	2.888827398
Zm00001d047042_T002	1.466438141	0	1.606977513	1.404710786	1.412533333	1.162777849
Zm00001d047566_T004	0.59054741	0.171006938	0	0.892339729	0.291512797	0
Zm00001d047566_T011	1.239369322	0.994789687	1.580252597	1.144370776	0.377308136	1.596952788
Zm00001d047566_T005	0.517027705	0.191749608	0.827221503	0.783434862	0.533476634	0.57348699
Zm00001d047566_T015	3.681110085	4.799078963	3.055966783	0	0	2.651757334
Zm00001d047566_T008	3.191077934	1.933565463	3.177078075	3.591194688	3.420132162	3.269236666
Zm00001d016861_T005	1.377190398	0.761178025	1.159168664	1.070319939	0	0
Zm00001d016861_T002	6.02399665	5.698578912	6.24074671	5.825248001	5.762450502	5.742004863
Zm00001d016861_T006	1.498096121	0.902746616	1.670036752	1.798593472	2.213105542	1.92843741
Zm00001d016861_T004	0	0	0	1.65236855	1.867879863	1.514587888
Zm00001d010255_T001	3.897528936	3.143185392	3.909688153	4.161419174	4.193320653	4.231151738
Zm00001d037982_T008	1.729928159	1.1184089	1.362946734	0	0	0
Zm00001d037982_T001	2.247132141	1.64077176	2.586118598	2.896797092	2.919677212	2.785880064
Zm00001d034927_T001	0.186980953	0.197665388	0	0.595877892	0.17583626	0.477044164
Zm00001d034927_T007	0.558182393	0.333819848	0.381267866	0.100748951	0.565475321	0.474255354
Zm00001d015243_T001	0.991642835	0.551869358	0.458715864	1.141946733	1.229616838	1.175570588
Zm00001d015243_T013	1.212627855	0	0	1.057209716	0.509879239	0
Zm00001d015243_T003	0	0	1.408386878	1.189557655	1.11720564	1.855479483
Zm00001d015243_T009	2.320014101	1.688054999	2.47936225	1.42512894	0	0.220567701
Zm00001d008513_T001	3.873369398	3.120586343	3.873817135	3.883883332	3.980299642	3.975537745
Zm00001d008513_T002	1.884428343	1.408533075	2.714601613	2.218779618	2.458358904	1.845009039
Zm00001d016807_T001	0.317301753	0.329372781	0.434056507	0.564077647	0.73885861	0.586010478
Zm00001d049026_T011	0	1.538549586	1.979675647	1.194044805	2.101873253	0
Zm00001d049026_T007	2.500995249	0	1.328977166	1.475402448	0	1.511281808
Zm00001d049026_T012	1.560180325	0.878279616	2.27575473	1.945828285	1.383695446	2.120397729

Zm00001d049026_T006	0.550722409	0.049485794	0.17340379	0.344005813	0.435945366	0.107015571
Zm00001d049026_T013	0.267260117	0.062040178	0.472680118	0.333703086	0	0.351496038
Zm00001d000030_T001	5.657739744	4.786317728	5.5763752	5.342249328	5.350270429	5.242119088
Zm00001d029564_T001	1.242908541	0.924216209	1.047967574	0.499651517	0.740905979	0.411775488
Zm00001d035346_T118	0	0	0.34425244	0.767859007	0.142068326	0.491483719
Zm00001d035346_T066	0.589637905	0	0.426873377	0.326249701	0.97722936	0.514469728
Zm00001d035346_T017	0.495457763	0.5341433	1.043114187	0.859982266	1.141513883	1.24168035
Zm00001d035346_T101	0.200720283	0.057961753	0.423000439	0	0.445200161	0.922180338
Zm00001d035346_T216	0.509746506	0.399444565	0.357720896	1.102256338	0.476965388	0.521568406
Zm00001d035346_T015	0.75338803	0.2869722	0.48311357	0.996115368	0	1.291845755
Zm00001d035346_T126	0	0	0.738553416	0.845604057	0	0.639818385
Zm00001d035346_T156	1.011601547	0.488372069	1.232580332	1.438098527	1.692281671	1.580819389
Zm00001d035346_T026	1.554604077	0.976954525	1.533903674	1.254194919	0.679018363	1.282639663
Zm00001d035346_T107	0.384260944	0.27340608	0.426188525	0.594100385	0	0.673969726
Zm00001d035346_T149	0.044823541	0.070975905	0.178886703	0.666513017	0.585961462	0.850662627
Zm00001d035346_T001	3.382295396	5.056461883	3.943638759	2.865606494	2.913961489	3.141944118
Zm00001d035346_T157	0.674673063	0	0	0	0.659592152	0.984256845
Zm00001d035346_T041	1.284281957	1.317617815	1.18109937	1.098585298	1.562582402	0.833697293
Zm00001d035346_T142	1.001748208	0.277834818	0	0.280095189	0.879127004	0.457813844
Zm00001d035346_T145	0.231254211	0.078974561	0.383053395	0.484261896	0.552507906	0.492671545
Zm00001d035346_T160	1.155647567	0.253065888	0.84514316	0.709907289	1.746817514	0.8209893
Zm00001d035346_T094	1.037197348	0	0.966604702	1.269595149	1.181790172	0.956342189
Zm00001d035346_T172	2.816342272	3.316441059	3.107372517	2.83937292	2.24380585	2.063756945
Zm00001d035346_T130	0.672206262	0	0.253886429	1.01122654	1.190758332	0.769164918
Zm00001d035346_T097	0	0.059268049	0.224452767	0.554272529	0.40471896	0.558328378
Zm00001d035346_T062	1.887860171	1.213691932	2.509803248	0.854405849	0.960697781	0.585575035
Zm00001d035346_T052	0	0.163220471	0.295547892	0.445687511	0	0.458916353
Zm00001d035346_T117	2.673827978	0.380038961	1.323057571	0	1.504851205	0
Zm00001d035346_T027	0.835079224	0.192778701	0	0.621767295	0.594491223	0
Zm00001d035346_T061	0.562099277	0.669233628	0.44283524	0.951870488	0	0.969738079
Zm00001d035346_T171	0.550161894	0.812752035	1.018768041	0	0.54970071	0
Zm00001d035346_T068	0.64753556	0.15348499	0.516515464	0.638629023	0.55065839	0.808519325

Zm00001d035346_T064	0.521825663	0.283898073	0.301409713	0.902122215	0.741235705	0.631642602
Zm00001d035346_T036	0.137250374	0.121353648	0.083269202	0.432915591	0.861519513	0.432572499
Zm00001d053433_T001	4.490657922	4.275417442	4.317995869	2.040193878	3.178210128	2.426795569
Zm00001d011786_T001	6.811618414	6.980338684	6.878083979	6.922174616	6.947996744	6.879018644
Zm00001d042520_T004	1.522207445	0.400599134	1.364606048	1.634551915	1.761763964	1.928862224
Zm00001d042520_T002	2.274886917	2.266948113	3.11861372	3.040797767	3.218523598	2.835911951
Zm00001d042520_T005	2.075496007	0.203875196	0	1.241472295	0.831510037	1.783943547
Zm00001d035770_T002	0.708278188	0.581959534	0.884565101	0.959003729	0.966292393	0.84373547
Zm00001d035770_T001	7.057499596	8.030028071	7.254365845	6.900557434	7.005075513	6.965349795
Zm00001d041772_T002	1.788670678	2.631776219	0	2.1744371	1.674379746	2.074899623
Zm00001d041772_T003	6.301358312	6.130411587	6.189618199	6.341215286	6.378132485	6.18316479
Zm00001d044035_T001	8.81784594	7.965139498	8.841050855	8.166867413	8.191864169	8.142821967
Zm00001d019297_T001	1.49187464	0.800049611	1.55077904	1.687764838	1.397775586	1.717278308
Zm00001d017153_T005	3.957187312	3.260709946	4.05273184	3.670813716	3.853543712	3.798185236
Zm00001d017153_T010	0.381848156	0	0.872455285	0.909532731	0.875148598	0.98699918
Zm00001d017153_T006	0.274890196	0.076554991	0.401380349	0	0.264422332	0.50871767
Zm00001d009403_T001	4.455019281	4.936108673	4.648457447	4.733669475	4.378492965	4.447422687
Zm00001d024606_T002	3.647047131	2.985028016	3.761778004	3.535442362	3.78380728	3.602381337
Zm00001d050899_T001	6.019959322	6.245292132	6.012431079	5.130254242	5.307551513	5.242572326
Zm00001d050899_T002	4.566399635	6.138320055	4.89204362	3.945626242	3.999601942	4.068617332
Zm00001d037829_T024	0	0.996908602	0.75107718	1.378818441	1.131000038	1.377500828
Zm00001d037829_T014	2.247945737	1.936574247	2.341342224	3.920714708	4.028946131	3.975543798
Zm00001d037829_T009	0.689529973	0.464408977	0.777978905	1.660388338	1.127361222	2.100359746
Zm00001d037829_T004	0.935861006	0	0.935253041	2.122017974	1.466819728	2.133286665
Zm00001d037829_T008	4.959995257	3.423871655	4.903140538	5.608109514	5.70243198	5.498304074
Zm00001d037829_T010	0.169638999	0.018768041	0	0.714032698	0.60536632	0.523506756
Zm00001d037829_T005	1.201525341	0	0.774698941	0	1.136445378	0.589476837
Zm00001d037829_T006	0.636831078	0.064482814	0.864132646	0.696150542	0	0.880486492
Zm00001d043146_T002	2.275991857	1.903019754	2.282765672	2.54929237	2.126312791	2.123171199
Zm00001d043146_T001	1.476893864	0.909157884	1.785475643	1.868011875	1.626807298	1.448752446
Zm00001d027342_T001	2.33609654	2.852717408	0.355117779	1.578988452	2.978345384	1.296271203
Zm00001d027342_T002	7.223810309	7.638467555	7.231517505	6.742858785	6.767600087	6.77942384

Zm00001d013569_T001	1.435744325	1.370737338	1.145818294	1.346445794	1.980079789	2.230176877
Zm00001d040509_T001	7.68575931	7.798752549	7.59862036	7.709013286	7.76945273	7.731231082
Zm00001d022603_T001	4.870842678	4.833880122	4.956715382	4.166430975	3.994530849	4.063415273
Zm00001d025564_T001	5.557134986	6.51178225	5.456791006	6.557413537	6.690993248	6.769124695
Zm00001d043429_T005	3.245100556	3.328598676	2.903360912	2.862071251	2.850823821	2.715182168
Zm00001d025822_T001	5.083540512	6.405380395	4.69700863	5.033855217	5.179254734	4.757536155
Zm00001d026518_T001	4.737312412	4.671146208	4.617570608	3.833323061	4.0005081	4.056751891
Zm00001d028447_T001	2.291174752	1.781507438	2.062414643	1.726847344	1.544474505	1.710202731
Zm00001d008851_T002	1.880186323	2.115880228	1.359151172	1.779348402	1.939061796	1.291680759
Zm00001d008851_T003	5.390446305	5.255002955	5.497921979	5.318128302	5.180770072	5.361735555
Zm00001d047435_T002	3.092280688	2.409408045	2.987004457	3.01415173	2.986869794	3.126526734
Zm00001d047435_T007	0.432102085	0.19835082	0.348708328	0.200017137	0.232076193	0.358994822
Zm00001d047435_T010	1.123823765	0	1.413389903	1.192471313	1.164804939	0.95913805
Zm00001d047435_T008	1.114261068	0.379168455	0.993201174	1.283451857	1.22992687	0.994148843
Zm00001d054062_T001	0.985215506	0.402977518	0.8369962	0.965429684	1.31215224	1.357889767
Zm00001d044060_T001	4.55612386	3.766646254	4.565503654	4.615817585	4.614232969	4.547874357
Zm00001d016299_T001	4.010872734	3.352819813	4.153714557	4.125970334	4.155696134	4.111203958
Zm00001d014414_T001	2.197006374	1.535269155	2.113137712	3.148284339	3.155612759	3.340332958
Zm00001d048547_T001	4.51505772	3.957902994	4.300648565	4.254110242	4.138561304	4.273548488
Zm00001d032613_T001	3.346101369	2.866905803	3.504353437	3.971854085	3.994565062	3.903241641
Zm00001d047412_T004	2.774147758	0	2.620354153	1.9548611	2.989034345	2.650593401
Zm00001d047412_T001	2.037770169	2.228310465	1.924824283	2.739782878	1.908961204	2.852601566
Zm00001d047412_T002	0.682397107	0	0.909942033	2.119911566	2.174382768	1.663847368
Zm00001d014702_T002	0.331220224	0.165665012	0.430805513	0.648635703	0.919414837	0.740558045
Zm00001d014702_T001	4.097924498	4.452992095	4.049803689	4.13493332	3.64754995	3.701905528
Zm00001d047814_T003	0.7904084	0.531867996	0.515564108	1.274696417	1.233732251	1.289275571
Zm00001d047814_T001	2.535703875	1.660934092	2.554307652	3.695422745	3.946795228	3.713866685
Zm00001d021253_T001	1.064026773	0.85184297	1.868063649	1.756927633	2.082223996	1.700326518
Zm00001d028661_T001	4.695476643	4.793119666	4.80038608	4.126604043	4.61645992	4.25129031
Zm00001d047373_T005	0.447640553	0.321188091	0.209719109	0.855646917	1.201404266	1.006114152
Zm00001d047373_T009	3.653531396	4.815658218	3.530320994	1.972383131	2.89422097	1.395984623
Zm00001d047373_T001	3.591331266	3.123730749	3.986127739	4.930777212	4.623011734	4.606573166

Zm00001d047373_T002	2.913366436	0	2.050739892	0	3.127397543	3.54876816
Zm00001d049979_T001	1.906453104	1.852249788	1.433218001	2.821153231	2.992945494	3.209511384
Zm00001d053763_T001	0.34252175	0.745921401	1.703820981	1.830535235	1.946685202	2.506021224
Zm00001d053763_T002	2.844827915	1.496426557	2.12408921	2.646764269	3.151129006	2.621783468
Zm00001d020882_T001	2.919254453	2.076768062	2.598182713	3.356006156	3.646465683	3.61276885
Zm00001d008266_T001	5.448101666	4.802986843	5.270909487	4.267591228	4.129014568	4.12837078
Zm00001d049145_T001	3.211821185	2.455587096	3.285910106	3.965132178	3.978588198	4.117118015
Zm00001d035600_T001	1.419125709	0.711020832	1.076362071	1.517564455	1.355610591	1.40615513
Zm00001d043547_T001	3.562060801	3.133899502	3.624477374	3.665342512	3.94247913	3.869556387
Zm00001d021219_T001	2.578815566	2.435839249	2.775998046	3.860019326	3.881685858	3.773036635
Zm00001d018986_T001	4.949271162	4.130740886	4.903749268	4.774465336	4.82443407	4.654925598
Zm00001d032822_T001	8.236479043	8.893906456	8.335235027	7.344989167	7.300637529	7.346273645
Zm00001d018510_T001	3.919623254	3.930865519	3.918577818	3.538210851	4.268611725	3.510526767
Zm00001d007909_T001	1.089758463	0.236512211	0.648491213	1.64394021	1.522378712	1.784390489
Zm00001d053554_T001	4.496108791	4.24694174	4.610021425	0.706288296	0.916590527	1.038616683
Zm00001d041329_T001	2.635218069	2.424186583	2.382287371	2.927699847	2.851983051	2.818650786
Zm00001d014984_T001	4.328974079	3.918731505	4.280872154	4.591092395	4.724804627	4.671230522
Zm00001d040342_T001	3.250224429	2.358430022	3.321665933	3.2410593	3.082021099	3.179310397
Zm00001d023543_T001	3.137782035	3.053263767	3.500473586	3.869084716	3.941398202	3.982764641
Zm00001d023543_T003	4.91351685	4.273918058	5.146547904	5.650501496	5.669643635	5.649599049
Zm00001d009858_T005	3.198165345	3.843278298	3.151438023	3.577333834	3.868982721	3.655658312
Zm00001d009858_T002	8.171304014	7.352694498	8.256604171	8.402423527	8.460329798	8.424027926
Zm00001d009858_T004	0.618214219	0.739108424	1.557605165	1.962559757	2.376303999	1.349031743
Zm00001d022500_T001	2.229557469	1.75227889	2.154194649	2.113741499	2.28173593	2.027712121
Zm00001d032447_T001	5.539226477	5.25485708	5.460544098	2.096801919	2.635228054	1.643860806
Zm00001d014488_T002	3.122188482	3.285101071	3.08698939	3.887868747	3.618992936	3.187034022
Zm00001d007821_T001	1.315506527	0.731089376	1.077599184	1.841744121	1.948626263	1.649429219
Zm00001d007821_T002	2.544225213	1.921801148	2.386362069	2.646838445	2.774269652	2.668535349
Zm00001d022179_T003	5.965617269	4.748969575	6.023990265	6.450621547	6.384406066	6.397789223
Zm00001d022179_T001	2.123935642	0	1.914620393	2.433755609	1.777072059	0
Zm00001d022179_T004	1.00208463	0.499124901	1.267554974	2.226923707	2.650009244	2.495715115
Zm00001d016277_T001	0.879068174	0.635785032	1.060746074	1.488175632	1.422285755	1.54467826

Zm00001d016277_T003	3.280700554	3.229489945	3.313341931	3.383540213	3.185149141	3.201611907
Zm00001d016277_T002	2.011580438	1.281035274	1.83138275	2.23511003	2.565281552	2.125108832
Zm00001d047732_T001	2.002189791	1.204833094	1.905679378	3.496181095	3.568885791	3.488334789
Zm00001d037246_T003	2.261031878	2.219003056	2.512383918	1.720655399	2.13448415	1.337394264
Zm00001d037246_T004	0.800820815	0.844502157	1.298421366	2.021930943	2.507418666	2.032075093
Zm00001d037246_T002	6.662319025	6.200805014	6.685114903	6.343981038	6.32716316	6.404723251
Zm00001d037246_T008	1.801104011	1.51357968	2.080761684	0.607262602	0	1.276898599
Zm00001d005687_T002	4.647880884	3.879417296	5.002599962	3.238661994	3.208449056	2.324778063
Zm00001d005687_T001	0.260340063	0.125702673	0.371963597	0.196149992	0.257108404	0.326799632
Zm00001d005687_T003	3.652936958	2.22363971	2.406205754	2.963421409	2.961899433	3.650660373
Zm00001d022161_T001	2.970577786	2.283551127	3.005990958	3.096503744	2.887183272	2.889497295
Zm00001d032433_T001	2.555010425	2.508609422	2.748684275	4.11306468	4.472484002	4.268166221
Zm00001d040126_T001	5.474576347	5.392044247	5.6681816	6.574133487	6.67703045	6.61051809
Zm00001d021718_T001	6.879071802	6.42512758	7.013880458	6.373897425	6.477231079	6.440125766
Zm00001d008794_T001	5.470891769	4.700129838	5.597242934	6.307328189	6.387763745	6.373091208
Zm00001d036540_T001	5.204604321	4.540084321	5.213468836	5.385648978	5.405545429	5.282209296
Zm00001d017387_T002	3.250147252	2.794920072	3.471213738	3.066779624	3.3562058	3.344845111
Zm00001d017387_T012	0.236578336	0	0	0.285650785	0	0.559286224
Zm00001d023904_T001	2.355470771	3.480721774	2.204215542	2.08511834	2.246679574	2.187517249
Zm00001d023904_T002	6.246545566	5.47318523	5.981894495	5.729539121	5.890773621	5.745299299
Zm00001d023904_T003	0	5.444686611	4.474850296	4.541147738	3.811142482	4.329283002
Zm00001d037196_T003	1.245515134	1.083749151	1.451143048	1.623127069	1.457627384	1.32190328
Zm00001d003172_T001	5.935484665	6.117991363	5.821528617	5.677238508	5.656081407	5.625358814
Zm00001d041495_T004	3.889750081	3.445311816	3.646502204	3.255268221	3.247967908	2.622634053
Zm00001d041495_T003	4.514023194	3.791336913	4.921374202	3.980853195	4.070971784	4.344378508
Zm00001d007490_T001	3.064840439	2.373210813	2.85259857	2.533302194	2.927127507	2.904722809
Zm00001d033241_T001	4.516557947	3.73491822	4.541889775	4.851215433	4.847188771	4.95269345
Zm00001d043757_T001	6.947370762	6.358761461	6.988172985	6.468092365	6.459256681	6.543855213
Zm00001d037242_T001	4.804282525	4.028349902	4.889661606	5.19323627	5.211478618	5.24444117
Zm00001d017914_T001	6.441390353	5.979968044	6.371311632	5.819423712	5.837996913	5.828194737
Zm00001d032866_T001	2.401560023	1.828874041	2.482088539	2.40083163	2.523080392	2.42980367
Zm00001d009075_T001	1.262209429	0.864348213	1.519689337	2.625050912	2.773427572	2.760451546

Zm00001d028531_T001	5.615328098	6.023260831	5.921355943	6.024347009	6.016712209	5.97345212
Zm00001d006410_T012	2.64869644	5.102182249	2.403293631	1.030275332	1.278137635	0.75470026
Zm00001d006410_T009	0.119981597	0	0.261369537	0.274822228	0.311067102	0.292639239
Zm00001d006410_T011	4.781005502	3.909245267	4.720575019	4.144462635	4.196656455	4.488800204
Zm00001d006410_T001	0.237785184	0.311384533	0.354402509	0.343443072	0.593504846	0.654919413
Zm00001d006410_T006	2.938109609	2.501077311	3.023024822	2.968158967	2.242366534	2.648550115
Zm00001d037939_T003	3.106346477	2.223830586	3.073124076	3.463495732	3.599464794	3.301258101
Zm00001d037939_T004	2.688760901	2.08498267	2.505075882	2.394923186	1.88877987	2.625429247
Zm00001d049888_T003	4.17970682	3.196988597	4.225598312	4.11278886	4.369597067	4.331099883
Zm00001d049888_T001	1.120886713	1.92328498	1.248578554	1.285068363	1.337103063	1.353062961
Zm00001d015566_T003	3.282291821	2.499758143	3.354459354	3.43697013	3.652563016	3.609094797
Zm00001d002266_T001	0	0.076430485	0.141065203	1.106635758	0.862467261	1.419367915
Zm00001d002266_T003	3.263909826	2.637600741	3.277359791	4.736249678	4.595931822	4.421692158
Zm00001d002266_T010	0.949113549	0.475516589	0.345109057	1.699360623	1.74718543	2.28364239
Zm00001d002266_T007	0	0.101332729	0.195384138	0.897215638	0.9556394	0.687738864
Zm00001d002266_T026	0.440428163	0.329589777	0.548784802	0.88606468	1.216521294	1.269415619
Zm00001d002266_T006	0.29460487	0	0.78881014	2.124167974	2.179835859	2.009327463
Zm00001d002266_T027	4.078662347	4.066338088	4.399536371	4.61367238	4.815250786	4.871619048
Zm00001d014391_T003	2.659049774	1.592937278	2.555653475	2.576304689	2.596299978	2.538724386
Zm00001d014391_T004	4.040455349	3.295138629	3.8719819	4.975655176	4.90149933	4.947681023
Zm00001d014391_T011	0.364548901	0	0.431611193	0.775125572	0.6595474	0.711951211
Zm00001d014391_T006	0.746780545	0.195657524	0.685078983	1.556220446	1.26981953	2.137330718
Zm00001d014391_T007	1.451121415	0.987896374	1.373668811	1.978317192	1.362550122	1.809157988
Zm00001d014391_T008	0.061589585	0.073761291	0.488965331	0.638857894	0.368010631	0.533346055
Zm00001d014391_T001	0.447451186	0.258552693	0	0.686824103	0.194292596	0.693937844
Zm00001d017000_T001	4.611577624	4.069706119	4.691008971	4.512748995	4.428355884	4.581987303
Zm00001d029280_T001	5.341391047	4.812128314	5.426235599	5.415149191	5.47303011	5.527751788
Zm00001d051336_T006	2.458973031	2.293341642	2.909970057	4.071092728	4.152615189	3.973865263
Zm00001d051336_T004	0	4.377317021	0	2.840742581	3.198253684	3.237709174
Zm00001d051336_T003	6.523503228	5.624861795	6.371584388	7.196423548	7.100550377	7.089478903
Zm00001d051336_T002	0.384903011	0.242329336	0.281497668	2.206924172	2.205571744	2.047167023
Zm00001d033275_T008	5.671057953	4.861906	5.620826077	5.989931023	6.077128639	5.801651562

Zm00001d033275_T007	2.522946358	1.348264718	2.816844606	3.159610135	3.323811313	3.261675378
Zm00001d033275_T005	0.365541374	0	0	1.593835625	1.066817331	1.776565577
Zm00001d033275_T001	1.545462349	1.268260709	1.637963052	1.741248219	1.67977103	1.364231188
Zm00001d018045_T001	2.278018658	1.998104858	2.121978203	1.10150553	0.813095306	1.195506349
Zm00001d047939_T001	4.359671912	3.687431619	4.507049573	5.091882973	5.213749236	5.104116003
Zm00001d036683_T001	3.854046629	3.625151	4.008124162	2.559796472	2.914292797	2.235754933
Zm00001d038487_T006	0.260005175	0.222512778	0.282526394	0.691811064	0.35888683	0.74764777
Zm00001d038487_T004	1.025761653	0.537578365	0	0	0.405429327	1.195886776
Zm00001d038487_T003	3.086124928	2.258129933	3.406062451	3.488145419	3.547826495	3.271614517
Zm00001d016438_T001	3.617630904	2.959123579	3.495313975	4.63861993	4.777383418	4.716781496
Zm00001d035238_T001	3.122232721	1.920464813	2.821409197	1.140899693	2.092932325	1.560356437
Zm00001d053754_T001	5.558285515	6.074889736	5.423265732	5.185403382	4.921153325	5.313402377
Zm00001d036925_T001	5.659891686	5.404264072	5.679030162	0.622973444	0.790651113	0.488145805
Zm00001d049579_T004	4.32062793	3.691631532	4.223301754	4.773058374	4.696180706	4.920423985
Zm00001d049579_T001	0.715251151	0.617278733	0.825025214	0.880306242	1.224037804	0.730437367
Zm00001d027947_T001	0.068735464	0	0.127359901	1.578761474	1.593614795	1.458644479
Zm00001d024220_T001	1.135050826	1.029274062	1.293893668	1.004906908	0.733139114	0.832634052
Zm00001d038607_T001	6.642943541	6.853889399	6.547807127	5.456084227	5.437791082	5.545494969
Zm00001d030782_T001	0.213663318	0.131503881	0.166450659	0.434897728	0.373485629	0.417509602
Zm00001d053084_T001	3.855304354	4.439416359	3.782997161	3.714247166	3.806569986	3.834099548
Zm00001d044191_T004	0.512841766	0.507749995	0	1.699088279	1.173149825	2.002297465
Zm00001d044191_T007	2.042115836	1.886020184	2.857308443	3.641661741	3.236704606	3.665204804
Zm00001d044191_T009	0.447948352	0.130050491	0.267336833	1.133831129	1.025072748	0
Zm00001d026651_T001	5.590242768	6.068496783	5.618784204	5.559507629	5.495360669	5.318164247
Zm00001d033469_T001	4.081146409	3.303610098	4.063860811	3.888341332	3.613029215	4.01057398
Zm00001d039094_T001	0.734853097	0.717705153	0.744985995	1.54145487	1.61591814	1.661099694
Zm00001d011162_T003	1.637989475	2.251791491	1.977005831	2.560030123	2.577676558	2.593734774
Zm00001d011162_T001	6.00814122	6.848685654	5.966711374	5.998215658	5.964799546	6.063478739
Zm00001d011162_T002	8.090735312	7.71880243	8.139150836	8.194803074	8.275632856	8.250817141
Zm00001d021393_T001	2.858008257	2.176209459	2.993456447	3.649071731	3.469580869	3.610338516
Zm00001d017423_T001	2.688974126	2.170499721	2.761130995	2.446853832	2.606093239	2.461575444
Zm00001d015586_T003	3.459785691	3.248271593	3.496919271	3.178457148	3.345261383	3.076945883

Zm00001d034429_T002	1.359897257	0.705686901	1.621649625	1.763494862	1.79377522	1.714794572
Zm00001d034429_T003	6.96335164	6.588681869	6.917141349	6.57800351	6.567500941	6.667451744
Zm00001d042588_T001	3.692719546	3.832300262	3.760891245	2.820845359	3.206129782	2.98489408
Zm00001d039189_T012	3.816439353	0	3.841236593	4.308100679	2.786985419	3.818290733
Zm00001d039189_T001	1.648908998	0.445969251	1.335870804	3.162915096	3.400098633	3.181362117
Zm00001d039189_T015	3.87588718	3.989742851	4.18621172	2.890569458	2.501389972	0
Zm00001d039189_T007	2.151984771	3.282049498	1.89860811	0.551142899	1.397582298	2.246728211
Zm00001d039189_T005	0	0	0	3.69730311	3.192304021	2.88374475
Zm00001d039189_T010	3.00547742	0	3.136623867	0	3.935616361	3.470842291
Zm00001d039189_T014	1.655916888	0.927927544	1.478547344	1.771456297	1.963712334	1.467483478
Zm00001d015030_T001	2.701513571	2.229001805	2.664011271	2.250470792	2.33950685	2.37208427
Zm00001d042080_T001	1.40047727	0.966912516	1.364504078	2.199504315	1.880488451	1.922654947
Zm00001d047228_T001	0.390590652	0.246896909	0.432001567	0.440911813	0.17138363	0.564827858
Zm00001d000417_T001	4.623812853	3.294154895	4.091281011	4.520855809	4.258712628	4.159949704
Zm00001d001896_T001	0	1.421678425	1.647598645	1.649243414	1.217935448	0.873740767
Zm00001d001896_T002	4.929466213	4.431556707	4.924137141	4.313641595	4.432550319	4.685076403
Zm00001d001896_T004	0.848522593	1.287199215	1.542727066	1.356175932	1.478370276	1.235493637
Zm00001d015325_T001	3.440110513	2.987016467	3.59024139	4.381911473	4.307794326	4.372062533
Zm00001d044944_T001	1.1768506	0.71293116	1.323367763	1.566004095	1.507158319	1.445439619
Zm00001d042291_T002	2.856304107	2.365956475	3.079540251	2.930807153	2.845255588	2.823114883
Zm00001d042291_T001	2.254750034	1.359861845	2.304426055	2.80256982	2.692399076	2.779390429
Zm00001d011877_T001	2.196787999	1.664000344	2.006540757	2.538772055	2.420590193	2.516973263
Zm00001d011877_T007	3.471732303	2.981032641	3.37070247	2.7927879	2.567613346	2.261147751
Zm00001d014080_T001	1.81521594	1.529817449	1.852441968	1.254125968	1.404196312	1.090390746
Zm00001d053367_T001	3.139812283	2.937939052	3.093191343	2.551369341	2.565326645	2.325524575
Zm00001d000270_T009	3.63906079	2.499451507	3.650603281	2.778437581	2.813729894	2.628687754
Zm00001d000270_T010	3.948186854	4.825976024	3.852089952	2.987408553	3.06507714	3.27587732
Zm00001d000270_T003	0.357992206	0.140955302	0	0	0.570500819	0.793134288
Zm00001d000270_T006	1.979273586	1.302709761	1.959790553	0.969468444	0.598741553	1.073733875
Zm00001d000270_T007	3.047445033	1.829358432	2.84307273	2.157970813	2.272669718	2.187002181
Zm00001d000270_T001	0.334110568	0	0.075657214	0.22923105	0.422516129	0.535726516
Zm00001d000270_T002	0	0.126908118	0.658805579	0.834249253	0.468381039	0.418354047

Zm00001d000270_T008	2.509607428	1.654158707	2.332710798	1.995955873	1.699447691	1.749895057
Zm00001d000270_T011	4.285463477	4.406312983	4.96396599	3.639954719	3.338130711	3.608199753
Zm00001d000270_T004	0.381951122	0	0.387359232	0.601782077	0.384061967	0
Zm00001d031064_T002	2.487213632	1.218804102	2.633854075	2.27963294	2.180876346	0.987884735
Zm00001d031064_T003	0.970859543	1.191740134	0	0	0.470866884	1.451363057
Zm00001d031064_T006	0.970635761	0.549003727	1.079160304	1.579282504	1.650935926	1.708860852
Zm00001d045669_T001	5.502587876	5.032923192	4.893296676	5.651889991	5.435277578	4.95533438
Zm00001d043691_T001	1.249147973	0.81437651	1.283205283	0.776005418	1.292182759	1.101662177
Zm00001d034317_T001	4.360634809	3.875098455	4.39975079	4.184976715	4.192449929	4.318964643
Zm00001d023498_T001	3.969156293	3.348572937	4.040562221	3.815630861	3.735259176	3.702629842
Zm00001d032875_T016	2.711400413	0	0	3.456102237	3.064103538	3.147794951
Zm00001d032875_T009	4.31023046	0	4.425362553	4.072942698	4.180659972	3.845826423
Zm00001d032875_T017	0.37456768	0.246809371	0.282703114	0.346901307	0.631245867	0.507323769
Zm00001d032875_T001	2.548245486	2.340831129	2.593495762	2.374824715	2.951471233	2.817417582
Zm00001d032875_T004	2.337655153	3.280535169	2.854123948	1.252375086	0	2.17511353
Zm00001d034965_T001	2.421874436	1.840159455	2.506963408	2.439781094	2.516363672	2.683386326
Zm00001d003406_T002	1.898666536	1.359694891	1.989066326	1.077957332	0.985370728	1.258983166
Zm00001d023608_T001	2.715167668	1.750657952	2.671308547	3.253447322	3.263432445	3.329861264
Zm00001d032405_T001	4.579927254	3.770610564	4.453264443	4.007968052	3.967025899	4.000104321
Zm00001d011527_T001	2.119297406	1.527274561	2.543495636	4.048762363	4.067797363	3.99540293
Zm00001d006593_T002	0.971704299	0.532410725	1.095123713	1.913797433	2.569941378	2.117962615
Zm00001d006593_T012	1.514034875	0	0	3.30195281	3.18302068	3.006024541
Zm00001d010572_T015	0.805319699	0	0	2.978698288	3.137530574	2.046493854
Zm00001d010572_T021	1.364445806	0.365566009	1.456666866	1.613260061	1.387840603	1.926583212
Zm00001d010572_T002	0	0.441568466	1.27956876	0	0	1.660752501
Zm00001d010572_T032	4.127152274	2.856979915	4.295091671	4.123584674	4.196891291	4.108254125
Zm00001d010572_T001	0	0	0	1.057240221	0.889787358	1.894813496
Zm00001d010572_T031	3.015260086	2.492676928	3.231073841	2.984035679	2.964852164	2.764765245
Zm00001d010572_T013	0.764674814	0.152968655	0.401845599	0.545559218	0.283587573	0.597873424
Zm00001d023218_T180	5.011775867	3.924817348	4.967118472	5.106436182	4.988315628	4.816755589
Zm00001d023218_T132	0.174625011	0	0.113028329	0.518637861	0.375466545	0.435741659
Zm00001d023218_T175	1.321862884	0	0	1.71958125	2.672862271	2.729489689

Zm00001d023218_T039	1.620681649	1.267040133	1.132355733	1.946474109	1.255344823	1.214527709
Zm00001d023218_T197	5.520934833	6.094591062	5.658201741	4.754715277	5.191239919	5.01909687
Zm00001d023218_T038	0	0	1.11364583	3.690430729	3.818874074	3.926604559
Zm00001d008262_T001	2.217486345	1.936948444	2.438789192	2.507764454	2.562934752	2.310159191
Zm00001d039517_T004	0	0.648655029	1.474078278	2.245969283	2.216741979	1.759794144
Zm00001d039517_T001	0.619718186	0.294016639	0.402142582	0.70005258	0.262069882	0.469174349
Zm00001d036560_T001	3.949608607	3.406185341	4.046767891	4.270278056	4.225256109	4.357058378
Zm00001d006794_T002	1.646203679	0.769756508	1.451759199	1.006662108	1.039435327	0.770832428
Zm00001d006794_T001	3.318082665	3.239240993	2.878965019	2.915265801	2.80874954	2.818658557
Zm00001d048029_T002	1.325982713	0.958698677	1.118541131	0.737025609	1.604197079	0.608434566
Zm00001d048029_T001	0.984222569	0.908485527	1.142987787	2.195719564	1.272481314	2.112837224
Zm00001d031194_T001	3.403884349	3.633525139	3.346690303	3.208189011	2.419020777	2.682186954
Zm00001d023539_T001	3.320864148	2.553241544	3.184884544	2.982038913	3.148934592	3.049140726
Zm00001d048647_T001	3.461077319	2.823278574	3.413887303	2.776700951	2.78089063	2.924373002
Zm00001d000299_T001	2.210517292	2.441822866	2.3705164	2.395667453	2.814958873	1.88228189
Zm00001d000299_T003	4.633148282	3.733776349	4.624531767	4.952484771	4.89012548	4.949364881
Zm00001d000299_T002	2.180193701	1.93902342	2.126972196	2.872754703	2.678827094	2.963000546
Zm00001d007068_T001	5.442299579	4.659869144	5.494933006	5.717011218	5.669009894	5.613850125
Zm00001d043104_T001	4.957988217	3.959770526	4.917354176	5.247011681	5.346685268	5.279002455
Zm00001d042512_T005	6.234757031	0	6.174267923	6.558622539	6.56446931	6.533595615
Zm00001d042512_T002	3.973097287	4.192854986	4.301270833	4.402372169	4.311488802	3.967969247
Zm00001d042512_T007	3.19850814	6.29394199	2.414168555	3.499209494	0	0
Zm00001d042512_T004	0.702864071	0.295563173	0.300541972	0.66513168	0.765075557	0.83775356
Zm00001d051272_T001	5.717325418	5.188619069	5.760968346	5.753435313	5.813716685	5.770037302
Zm00001d021145_T008	0	0.146080375	0	0.533320138	0.519738142	0.333882804
Zm00001d021145_T009	1.767063193	1.586282924	1.121028667	1.735193309	1.274820439	0.272762567
Zm00001d021145_T003	2.570223433	1.270500836	2.827937077	3.8034706	3.881832174	3.899389408
Zm00001d021145_T010	4.251157127	4.040794086	4.132973817	2.997630954	2.99403911	2.975966703
Zm00001d021145_T002	0.335879948	0.211265188	0.655322516	0	0.196158807	0.182854989
Zm00001d043292_T003	2.876639527	2.285281175	3.010339624	3.135103868	3.198256514	3.345360322
Zm00001d008781_T001	3.027808354	2.149431399	2.833275491	2.453271029	2.292808542	2.820959696
Zm00001d005828_T003	2.52200878	1.902576457	2.256354314	2.19793677	2.204567075	2.098068813

Zm00001d008951_T002	6.087966042	5.418915735	6.066374364	6.256532948	6.235132994	6.124651402
Zm00001d044390_T001	5.132043218	4.451813192	5.194663849	5.990842457	5.924880232	6.01505442
Zm00001d049727_T001	2.273120176	1.712502899	2.178840503	3.03880313	3.211415655	3.113052674
Zm00001d028073_T002	1.072599766	0	0.669937463	1.284214429	2.688712121	2.148408653
Zm00001d028073_T005	2.504426664	1.123716514	2.290172235	2.939188018	2.170903138	2.609570832
Zm00001d028073_T009	2.87507761	4.544709785	2.99376275	3.046128857	2.709881931	3.579660725
Zm00001d028073_T001	0	0.462728734	0.123176166	0.656876158	0.213884751	0.8497231
Zm00001d034322_T001	3.342935564	2.840110901	3.588994124	2.219157986	2.303269907	2.18818061
Zm00001d052050_T004	0.922456673	0.666001194	0.753699515	1.20107612	1.511352153	1.300938434
Zm00001d052050_T005	0.7919099	0.355200114	0.881085859	1.676755747	1.763913774	1.601293361
Zm00001d052050_T001	0.958884981	0.187888072	0.486301453	1.189638617	1.172847184	0.961007598
Zm00001d052050_T002	5.13530781	4.204122479	5.209883342	5.379213275	5.404775662	5.411228879
Zm00001d049295_T008	1.066253864	0.084307869	2.048988033	3.135581492	3.753145171	3.202256595
Zm00001d049295_T009	1.260405706	0.887005027	1.63277307	1.009294136	1.131555244	1.003428095
Zm00001d049295_T001	2.846130816	1.59358946	2.413998836	2.737213212	2.9841387	2.960179541
Zm00001d049295_T002	5.046544798	4.468302179	5.33937861	5.001304643	5.325896787	5.206214938
Zm00001d049295_T007	3.575401891	2.104865663	2.037560395	3.409283664	0	1.797857174
Zm00001d049295_T006	2.440412481	2.071650791	3.026207813	4.166012482	4.028925892	4.109108693
Zm00001d049295_T003	0.686097076	0.251303415	0.66917375	1.15833121	0.696779067	0.745004072
Zm00001d010488_T008	0.490238426	0	1.016009159	0.615484099	1.465325171	0.605174753
Zm00001d010488_T002	2.592014443	1.768400739	1.983120572	3.015338597	2.816642301	2.973375246
Zm00001d010488_T009	0.836711078	0.686335574	0.883616125	0.79522833	0.591003384	0
Zm00001d010454_T001	4.075571137	3.186870692	4.174168373	4.387582844	4.457729875	4.338430904
Zm00001d021716_T001	1.752949356	1.217617865	1.884976859	1.952936986	2.064121305	2.168381454
Zm00001d040423_T001	0.30880586	0.214966546	0.590781163	1.169994249	1.337680269	1.618871517
Zm00001d018333_T001	4.778054467	4.09081296	4.887930241	5.485228218	5.369977165	5.296925235
Zm00001d024314_T001	0.883526197	0.575708291	0.808808426	0.937806884	1.055219259	1.19821376
Zm00001d038937_T001	3.965877445	3.219175027	4.089682459	3.53576371	3.530420758	3.668684879
Zm00001d013635_T001	4.558063585	3.833020104	4.54167268	4.23254219	4.199463404	4.309122988
Zm00001d008565_T004	1.860503207	0.598732026	1.747890483	1.834860452	2.216690147	1.729812465
Zm00001d008565_T002	1.240246544	1.344840425	1.527710446	2.072011448	1.261802179	1.989694538
Zm00001d049767_T001	3.771637469	3.063476884	3.873868701	4.010435412	4.076230535	3.986048259

Zm00001d026214_T003	5.766479323	5.157293168	5.6676849	5.854771685	5.734221785	5.796415433
Zm00001d026214_T001	0.640921651	0.07983069	0.228715274	0	0.295946316	0.434310633
Zm00001d018028_T001	5.693085707	5.125405866	5.656196646	6.106603746	6.163623051	6.030616092
Zm00001d038521_T001	0.353300705	0.324509939	0.243314456	2.860621594	2.998549001	3.060287813
Zm00001d002820_T001	5.164324785	6.117673248	5.209212025	4.803297981	4.927832422	5.174893414
Zm00001d002713_T001	3.179859102	2.763667795	3.219544928	2.753512547	2.892139188	2.820250253
Zm00001d047347_T034	0.74457791	0.342847123	0	0.360544075	0.793457287	0.824999155
Zm00001d047347_T007	2.876742063	1.799844937	2.677667894	2.670758774	2.669905498	2.151384117
Zm00001d049349_T001	2.176735095	1.487217234	2.178498261	1.651931567	1.461929776	1.524556195
Zm00001d048944_T006	0.234931674	0	0.313309712	0.461168103	0.058727943	0.315765609
Zm00001d048944_T002	1.116141148	1.723593072	0	1.0505811	0.226277927	0.724939218
Zm00001d048944_T007	2.528315011	0	2.839814908	2.068053363	2.38435683	2.321035079
Zm00001d000223_T002	2.022805971	1.206650725	2.03296338	2.06204536	2.309212575	2.284556478
Zm00001d014029_T001	1.789014716	1.493003716	2.102026163	2.543240729	2.618097904	2.555444638
Zm00001d032809_T001	2.764034625	3.764488201	3.396322276	4.168570396	4.079706126	3.868731503
Zm00001d014338_T001	5.122415462	4.811641082	5.132744174	4.681494525	4.721947271	4.793932236
Zm00001d029165_T003	5.016908326	4.036176866	5.011864137	5.344517098	5.509481417	5.390110645
Zm00001d029165_T004	2.172889417	2.735372701	2.054265031	3.325133806	2.810327313	3.1685363
Zm00001d029165_T002	3.48832849	2.279819224	3.845545449	4.065706029	4.07448754	4.234790919
Zm00001d005917_T002	0.647702246	0.130592225	0.138617903	0.653532428	0.591284943	0.607769181
Zm00001d005917_T003	2.457920457	1.580504907	2.455556849	2.034267491	1.969519281	1.939924245
Zm00001d005917_T004	0.455022307	0.757887751	0.666088481	0.755389259	0.795332245	0.694064477
Zm00001d041214_T004	1.079466181	1.504099657	1.946133824	0	0	0
Zm00001d041214_T001	4.611936086	3.778836937	4.747246024	4.165856481	4.647111044	4.576793056
Zm00001d041214_T003	1.730154303	0.132672909	0.709257987	3.344043463	3.137710732	3.465318379
Zm00001d013109_T002	4.105491193	3.130195666	4.26330436	6.820971167	6.785948964	6.769127526
Zm00001d013109_T004	3.465694515	5.448963969	3.629232106	5.166004767	4.872503994	5.038827312
Zm00001d013109_T001	0.267743124	0.237728903	0	1.566358289	1.392444924	1.661874531
Zm00001d004585_T001	3.263609566	3.126894246	3.126830661	3.220156728	3.160879701	2.898624555
Zm00001d016991_T001	4.925854999	5.104008703	5.095282531	4.486176126	4.704048818	4.471409702
Zm00001d033517_T001	2.516756482	1.892173002	2.341288988	1.098275362	0.969248131	1.311911686
Zm00001d028880_T001	6.164467818	6.36900008	6.32335254	5.335027554	5.352287765	5.736466714

Zm00001d043909_T002	0.275516074	0.097370777	0.173082648	1.003486385	0.751258036	0.75455062
Zm00001d043909_T022	1.997139187	0.997066907	2.588766916	2.839936214	3.066823529	2.728789947
Zm00001d043909_T029	1.508848469	1.894559364	1.042460137	1.376887122	1.608706595	2.104983044
Zm00001d043909_T003	1.101877286	0.11428306	0.841631822	0	1.912227149	1.496088525
Zm00001d023390_T001	1.379486223	1.315514642	0.965746613	2.288567642	2.162348626	2.113881491
Zm00001d053438_T002	3.750143503	2.971187607	3.871817238	2.838157915	3.056443761	2.925483373
Zm00001d048319_T001	2.152737671	1.661951125	1.914332602	1.887061194	2.610437623	2.210128857
Zm00001d011760_T001	1.190197003	0.85355978	0.572000015	1.514992287	1.474391912	1.711739373
Zm00001d006309_T007	5.279464238	3.198981256	5.313510418	5.380870754	5.302968447	5.412274868
Zm00001d006309_T012	2.894041457	3.300990253	2.822210684	2.407043511	2.274736666	1.965336587
Zm00001d006309_T014	5.310903091	4.368873719	5.463167195	4.588956658	4.673846008	4.630494297
Zm00001d006309_T004	1.445635605	1.636795356	1.94412642	2.25709603	1.667646017	2.386559625
Zm00001d006309_T003	1.477388242	4.925282829	2.369244513	1.82278031	2.676480456	1.818799035
Zm00001d006309_T002	0.613828648	0	0.58384446	0.96503435	0.570696075	0.614293346
Zm00001d006309_T017	6.200585174	5.852683206	5.958569859	5.483045059	5.126804276	5.439261787
Zm00001d052335_T003	5.571460268	4.844148298	5.352266963	5.330006887	5.346269265	5.427447883
Zm00001d052335_T001	2.759125147	3.524171397	3.277810424	2.716540572	2.557077716	3.048801328
Zm00001d051661_T001	6.372764368	6.296200434	6.337359508	6.774058123	6.702617265	6.910898864
Zm00001d006101_T001	5.068918059	5.137930121	5.199022495	8.190563459	8.281860804	8.329808028
Zm00001d020366_T001	1.137337604	0.845890638	1.067481774	1.323435212	1.306781936	1.400038911
Zm00001d013923_T001	3.108955445	2.433856004	3.054484198	2.418008014	2.443706629	2.698094877
Zm00001d039231_T002	1.1660251	0.839831432	0.888853498	1.038715001	0.478986297	0.919914376
Zm00001d039231_T001	1.216844705	0.793671192	1.518882049	1.962222135	2.026837587	2.115811662
Zm00001d039231_T005	2.540328626	3.288400646	2.067813193	1.710510455	2.156519263	0
Zm00001d006078_T001	2.981236546	2.702102298	2.388757134	3.267464785	3.34027769	3.644169625
Zm00001d002485_T001	2.936180538	2.499150931	3.041702777	3.529435802	3.891111366	3.723731325
Zm00001d002485_T002	2.388458209	0	2.203197084	2.073913783	0	0
Zm00001d003087_T001	0	0	1.068337181	2.170133376	2.11659199	2.503111829
Zm00001d003087_T027	2.765834499	0	3.099743431	3.257557712	2.361294775	3.659752429
Zm00001d003087_T041	2.243860981	2.223280745	2.466025643	0	1.248505083	0
Zm00001d003087_T043	0.299840138	0.379877098	0.793353236	0	1.258213168	1.553555635
Zm00001d003087_T022	1.746842016	1.682793056	0	2.109052261	2.732630859	1.01E-05

Zm00001d003087_T047	3.618438363	3.88961469	3.884807619	0	1.753707216	0
Zm00001d050449_T001	2.496947768	3.028791551	2.46536514	2.29426549	1.805134762	2.294345195
Zm00001d016446_T001	9.326102118	8.74763656	9.401543819	9.974140348	9.970153109	9.958693845
Zm00001d018883_T001	4.268437762	3.484097261	4.46451641	4.386710396	4.427842804	4.497457231
Zm00001d020552_T001	7.375768833	6.84032767	7.364156932	9.021186461	9.082243152	9.074031616
Zm00001d048252_T001	6.268279819	5.56914739	6.247405809	5.993346435	5.970757111	5.797034878
Zm00001d048252_T003	1.371875524	1.184176175	1.348357077	0.969643786	0.404204488	1.16802388
Zm00001d048252_T005	5.818683888	7.022572783	5.570814454	5.011652307	4.817699841	4.879519733
Zm00001d048769_T001	4.901595915	4.369570058	4.954337501	4.283479564	4.343277334	4.303051766
Zm00001d048769_T002	1.378486653	0	0.772623002	0.398287976	1.073640656	1.2260146
Zm00001d019254_T001	2.884420528	2.536860086	3.240085007	3.155308198	3.197190896	2.986867792
Zm00001d047986_T029	2.023939095	0	2.171847954	1.795736199	1.870833408	0
Zm00001d047986_T013	0	0	0.353826876	0.383551134	1.360325504	1.041782665
Zm00001d047986_T025	2.702907943	0	1.829273178	1.711823495	0	0
Zm00001d047986_T010	2.208378352	0	0	3.351326134	2.977250245	3.453891538
Zm00001d047986_T024	0.364857021	0	0.680914818	0.914124377	1.834402583	0
Zm00001d047986_T042	3.49104278	2.519426162	3.41774047	2.258652485	2.57373152	2.973693555
Zm00001d047986_T015	0	0	0	2.309021314	2.627247754	0.921478225
Zm00001d047986_T017	0.727043029	2.879239167	0.890756406	1.535399062	0	0.961778146
Zm00001d047986_T046	0	0	0	0.592154174	0.379626507	0.767697168
Zm00001d047986_T023	0.463422621	0.078910365	0.405492502	0.908903578	1.035893422	0.976692833
Zm00001d050324_T004	3.815003668	3.656387335	3.617344683	2.496938056	2.626390773	3.058690197
Zm00001d050324_T002	5.89649681	5.377695993	5.983189243	6.195999755	6.171684757	6.054897781
Zm00001d023728_T001	1.484813036	0.835908718	1.39869732	1.460754619	1.565646889	1.379201732
Zm00001d008251_T002	2.093082466	1.530661087	2.050023796	0.987286663	1.153699643	1.116081913
Zm00001d000029_T002	1.848280208	1.187578373	1.809667563	2.415523425	2.497029805	2.548320218
Zm00001d000029_T004	1.891464295	0.903921361	1.943913452	2.640574426	2.001110087	2.012197136
Zm00001d000029_T003	6.038756126	5.372804444	5.959848709	6.156013117	6.224858101	6.15195513
Zm00001d011639_T001	2.10568981	1.954476259	2.103729929	4.014142623	3.901293058	4.170890162
Zm00001d051219_T001	4.280456108	3.53340873	4.366339142	4.273398918	4.116670332	4.107727283
Zm00001d029651_T001	1.200526325	0.929028175	1.315664764	2.029060221	1.965727037	1.637435434
Zm00001d043726_T006	0	0	0	3.162240979	2.67289055	2.251276447

Zm00001d043726_T002	1.91556985	2.052151886	1.43647263	0	0	2.82459028
Zm00001d043726_T003	0.55415168	0.422051037	0.603252932	0.478711967	0.7432211	0.320086041
Zm00001d043726_T001	4.807332921	3.671575429	4.842596241	5.294131289	5.313135613	5.253020301
Zm00001d019600_T001	2.901040066	3.460584658	4.182384898	4.845421051	4.573574846	4.48907532
Zm00001d019600_T006	4.459062241	0	3.743755254	0	2.059048568	3.508593704
Zm00001d019600_T002	3.378569746	3.261219527	3.262429098	2.757983301	3.117529489	2.070089766
Zm00001d006474_T001	8.046005431	8.527575622	8.099440277	8.326380916	8.359976614	8.313025775
Zm00001d015968_T002	1.532469076	1.275300922	1.479009586	2.070261197	2.641069214	2.211237461
Zm00001d015968_T005	3.826544013	2.760285893	4.109325286	2.623827775	3.958268823	2.750423852
Zm00001d015968_T001	0.426264755	0.271897732	0.714809075	1.646477895	1.568885791	0.740008777
Zm00001d015968_T004	1.328904807	0.849875194	0.595448281	2.085287655	1.975364219	1.904712214
Zm00001d015968_T003	4.582905555	3.880058458	4.587213032	5.141333259	4.762645723	5.023541911
Zm00001d026128_T001	2.132829169	1.488035231	2.009616622	2.103470783	2.09645348	2.014398859
Zm00001d040411_T002	4.626221431	4.002622058	4.672001354	5.123385112	5.106610132	4.990775193
Zm00001d017445_T002	2.800864291	1.231592153	2.63320729	3.09485356	3.043986435	3.170757997
Zm00001d017445_T001	4.60851068	4.195158035	4.751218341	5.031585402	5.095617878	5.16875775
Zm00001d008289_T003	3.194738892	2.907103137	3.336182396	1.992746681	2.895476161	3.230008058
Zm00001d008289_T007	3.485607282	2.432024559	3.515522857	4.263473685	4.225833265	4.151312996
Zm00001d008289_T001	4.34048237	2.307478946	4.538843059	2.933627399	0	0
Zm00001d008289_T004	0.405725573	0.100623825	0.462531916	0.544221255	0.647030778	0.392105262
Zm00001d010676_T001	2.181570754	1.96053877	2.348782533	5.754896559	5.883789658	5.451908911
Zm00001d018758_T004	3.835565983	0	3.416851494	3.375721613	3.908610897	3.853379527
Zm00001d018758_T005	5.305039872	4.901655571	5.242526482	5.087383745	5.228493143	5.186853859
Zm00001d018758_T002	4.322700668	1.480227374	4.663411799	5.586033034	5.48800855	5.437369611
Zm00001d004621_T001	2.278327979	1.63269305	2.319740177	3.052692373	3.231052023	3.070667879
Zm00001d032210_T002	1.633364712	1.002420974	1.729136379	1.885431454	2.190260859	1.904220102
Zm00001d032210_T005	2.37819142	2.63161746	2.836202281	2.342305836	1.84195018	2.633100317
Zm00001d022341_T002	1.810207811	1.401575313	1.731064605	1.769118359	1.753869348	1.716959736
Zm00001d022341_T001	6.691361006	5.823456614	6.714499561	6.800682354	6.760982167	6.749232128
Zm00001d018724_T007	0.349249767	0.205747825	0.511304076	0.802021117	0.63640514	0.582025071
Zm00001d018724_T002	3.003381678	2.661230839	3.053315038	3.789560771	3.8635478	3.721015387
Zm00001d018724_T009	3.897740441	2.52567377	4.022547318	3.603898332	3.818646492	3.703746578

Zm00001d014094_T001	3.670465567	3.175759948	3.498007121	1.545810751	2.741274327	1.910801342
Zm00001d014094_T002	3.351043812	3.097309586	3.638635162	3.471422774	3.452190032	3.476461921
Zm00001d014094_T003	0.677256552	0.263070473	0.693958356	0.905078973	0.472371311	0.584665275
Zm00001d036388_T001	1.454126398	1.554519109	1.828255831	0.583997496	1.05027461	0.572659789
Zm00001d036388_T002	2.271900719	1.06821334	1.921077898	1.602083877	1.257495862	2.042521773
Zm00001d038796_T001	3.252920157	2.827580046	3.403238268	3.541344962	3.493164391	3.418007204
Zm00001d013583_T001	6.785653153	6.600413534	6.953035777	7.256979106	7.179439531	7.198536349
Zm00001d032620_T001	6.050951675	6.395393608	5.986515993	7.25684009	7.155468218	7.266774062
Zm00001d032268_T001	3.282712453	2.714373898	3.36858175	3.692881986	3.841564297	3.585810075
Zm00001d012007_T005	4.485743863	4.080579558	4.833619277	3.601126456	3.420031885	3.223124249
Zm00001d012007_T002	1.759495477	1.194926068	1.724091419	1.134170987	1.395173603	1.385304571
Zm00001d012007_T006	3.001489535	1.621774327	2.572044899	3.257056041	3.477604932	3.327860937
Zm00001d012007_T001	4.250505355	3.354468805	4.269875545	3.76802464	3.856702201	3.933329871
Zm00001d053982_T001	3.994378421	3.225934883	3.841357176	3.640422417	3.758617333	3.801627714
Zm00001d011917_T001	3.989984844	3.93879775	4.189268164	4.487639152	4.33637566	4.355557667
Zm00001d017271_T005	6.876885726	6.143826807	6.922322556	7.280302788	7.213537785	7.19098086
Zm00001d017271_T011	2.863691308	3.48377838	2.319045309	1.57196993	2.156691727	1.056235991
Zm00001d017271_T001	6.956118073	5.267140576	6.902560624	6.537711698	6.704072757	6.755401397
Zm00001d017271_T002	0.844187173	0.466754484	0.452106195	0.887221883	0.39353372	0.53643591
Zm00001d017271_T003	6.671969308	8.645892227	6.687612151	5.166987692	5.49539118	5.213719651
Zm00001d017271_T004	2.210065874	1.499567332	2.176735733	1.470959525	1.821680014	1.589609144
Zm00001d039657_T001	4.69010377	5.53343312	4.924648443	3.133545278	3.146773324	3.573806519
Zm00001d018487_T003	2.002199875	1.41500089	1.687699007	1.62777771	2.138833469	1.983087729
Zm00001d018487_T001	6.062440742	5.122670855	6.086048986	6.57802897	6.60627346	6.564552291
Zm00001d018487_T002	1.009835871	0.068553877	0.780588111	0.977285053	0.433001085	0.621939798
Zm00001d018487_T004	6.745534649	6.648221996	6.844787187	6.92495749	7.083017783	6.83874674
Zm00001d049914_T002	2.732565307	2.20305453	3.03343886	2.83398726	2.632148606	2.72595073
Zm00001d040441_T001	4.437438028	5.152761761	4.545952497	5.005151941	5.024429925	5.146824146
Zm00001d045141_T001	1.747909812	1.793598784	1.464406886	1.737399059	1.241289841	1.599747681
Zm00001d013118_T001	3.890847537	4.305364943	4.137602214	3.36038296	3.353864835	3.687080626
Zm00001d027504_T003	0.631974062	0.139292667	0.659640557	0.133710813	0.412007546	0.679439112
Zm00001d027504_T002	3.888546006	2.97703808	3.962893045	3.772435092	3.673700912	3.554043717

Zm00001d011626_T001	3.657736715	3.52178233	3.555805605	5.423119306	5.403652515	5.346770107
Zm00001d040185_T001	5.215795762	4.803074956	5.067114604	5.529741502	5.539141499	5.490906153
Zm00001d014219_T001	4.729022253	5.565535884	4.641739185	2.064728712	2.356843291	0.83254089
Zm00001d027922_T002	1.603060609	0.886878643	1.692728918	1.156678785	1.40113565	0.964591595
Zm00001d027922_T003	1.136596964	0.508311001	0.685186656	0.997424656	1.269604125	1.104244056
Zm00001d027922_T001	4.65440283	4.398480623	4.609248784	4.675322305	4.598469664	4.564657595
Zm00001d036550_T001	5.328326125	5.250391771	5.432027199	6.255593882	6.348336148	6.344008779
Zm00001d008820_T002	5.869224787	5.878873355	5.846234515	5.356662326	5.21807464	5.326400363
Zm00001d008820_T004	5.464448481	3.959624858	5.703052928	5.210024638	5.306929689	4.859189766
Zm00001d008820_T003	3.330573028	2.709289312	2.042927246	2.726714838	2.847245909	2.727908913
Zm00001d008820_T001	1.690938651	2.660533238	2.30710206	1.909151354	2.061992499	2.527329367
Zm00001d008820_T005	6.098202479	4.767523393	6.295346876	6.057533994	6.002549603	6.058058091
Zm00001d034853_T001	3.530101464	3.542042187	3.771429463	3.4319882	3.368649772	3.476505731
Zm00001d033945_T001	3.86492115	3.588180087	4.063821905	3.17478542	3.3782712	3.303254415
Zm00001d033945_T008	0.274799571	0.028880288	0.322469293	0	0.063796974	0.295139952
Zm00001d033945_T003	1.451283393	0.600232639	1.375967464	1.228777448	1.459026033	1.511216014
Zm00001d019053_T001	2.453508097	1.842592319	2.480873615	2.138503555	2.4033539	2.163438833
Zm00001d016483_T001	3.804067193	3.224211955	4.14474576	3.937778265	3.82835252	4.422515187
Zm00001d016483_T002	3.184646556	2.302154371	2.613330557	3.900000979	3.940591414	3.566614624
Zm00001d016483_T003	0.800632815	0.17243759	0.612524244	0.650949249	0.875258712	0.999836245
Zm00001d014449_T002	4.223350491	3.574025919	4.24015967	4.427941735	4.590694288	4.478541261
Zm00001d025218_T001	2.305459288	1.900351354	2.130271955	2.617819179	2.617253809	2.647184396
Zm00001d022243_T004	3.427354886	2.921076374	3.305254247	3.26317957	3.638526622	2.877375776
Zm00001d022243_T006	4.46523602	3.95927208	4.454422025	3.736717213	3.484671404	3.997586337
Zm00001d022243_T003	1.738564224	0.670165954	1.11061915	1.46567101	1.343980807	1.624705927
Zm00001d022243_T005	1.037231794	1.533847374	0.815458205	1.124370489	0.930911396	1.028499137
Zm00001d009677_T001	1.149399856	0.772762335	1.154863789	1.720351583	1.872633761	1.587170538
Zm00001d023302_T001	1.276136915	0.781018043	1.253292854	1.568844943	1.79384221	1.688157974
Zm00001d047994_T001	2.934101655	2.421381936	2.801137957	3.907750666	3.739616778	3.602680161
Zm00001d047994_T007	2.066803557	1.06456352	1.856984495	2.018360706	1.959448573	1.823052297
Zm00001d005859_T001	2.074817096	1.77903526	2.215537892	1.97329901	2.211985295	1.86642456
Zm00001d009810_T001	3.735173376	3.144368492	3.873184515	4.703015292	4.705632609	4.758325353

Zm00001d006111_T001	7.720502521	8.846438079	7.706902499	8.312770693	8.370706149	8.33486363
Zm00001d007640_T001	3.953170795	3.262861122	3.885785092	3.834363948	3.740088028	3.752651303
Zm00001d007640_T002	2.371377643	1.512243071	2.570630021	2.172176413	2.767681067	2.838180309
Zm00001d006951_T001	5.924291971	6.139849663	6.054110035	6.158664315	6.187074693	6.006462102
Zm00001d020910_T001	10.45978294	10.06611705	10.53755064	9.716335233	9.750030937	9.709006098
Zm00001d024338_T004	5.003436551	3.560880488	4.990311402	4.864333057	4.920096798	4.959605339
Zm00001d024338_T008	1.556490727	2.004904032	1.085523235	0.90815269	0.971602778	1.111713092
Zm00001d051227_T001	5.262090635	4.509632509	5.059089637	5.827516281	5.801843411	5.673313938
Zm00001d041669_T001	2.729955559	2.514120492	2.924822193	3.175822371	3.137948887	3.241883494
Zm00001d032298_T001	4.65887971	3.982306939	4.885788459	3.92121951	3.918107115	4.045466736
Zm00001d019194_T008	1.827847065	1.049062638	1.632780514	2.350577583	2.400373431	2.022359645
Zm00001d019194_T001	0.126431083	0.111220988	0.274085097	0.304498192	0.298025972	0.249186816
Zm00001d019194_T004	1.656147153	1.492505944	1.790971333	1.339616303	1.48655478	1.845359174
Zm00001d019194_T002	0	0.525210955	0	0.804564113	0.570846627	1.152907019
Zm00001d019194_T005	0	0.292833568	0.188271779	0	0.832653494	0.419231443
Zm00001d008900_T001	5.858212927	5.242337873	5.927668515	5.63940971	5.635004703	5.557984027
Zm00001d017864_T001	3.132118134	2.465063125	3.173551383	2.815109552	3.074177779	2.88029135
Zm00001d041352_T001	8.062833524	7.447863997	8.098060916	8.055930722	8.126824374	8.056006769
Zm00001d041352_T002	5.509730547	6.787995568	5.628510807	5.137026167	4.131396212	3.82698761
Zm00001d052955_T001	1.930082915	1.192636692	1.888776364	1.860169048	2.011187888	1.916582502
Zm00001d048356_T001	5.708259341	5.448823199	5.751819665	5.391530974	5.492003513	5.438532327
Zm00001d005790_T001	5.654290685	5.464115585	5.615282157	6.140443767	6.163794285	6.229777053
Zm00001d002475_T001	4.306910738	3.59755072	4.17349142	4.247979981	4.115512563	4.113628663
Zm00001d015427_T001	7.403274668	8.119830324	7.317694676	7.572892677	7.611053282	7.535869295
Zm00001d053283_T007	2.230759752	0.903090731	2.041185147	2.032466938	2.075936461	2.022068056
Zm00001d053283_T001	2.635907125	2.432341321	2.271571193	2.194852965	1.678365824	2.070218255
Zm00001d053283_T006	0	0	0.413376362	1.189971904	0.818769591	0.99047134
Zm00001d053283_T008	1.880380696	0.714291244	1.452660268	0	0	0
Zm00001d053283_T003	2.440467499	1.620389346	0	2.993024142	0	3.33821716
Zm00001d053283_T002	2.006408982	1.568822573	2.08812056	2.579520744	2.822814566	2.110096597
Zm00001d053283_T004	0	0	2.708936757	1.425786909	3.212000708	0
Zm00001d037002_T003	0	0	1.911938496	0	1.760829412	1.966531984

Zm00001d011964_T002	4.59310316	4.033264935	4.752644613	4.863073863	5.099747007	4.999494247
Zm00001d011964_T001	1.207127583	0.512491883	1.861567948	2.447155186	2.49633119	2.658623492
Zm00001d024265_T001	4.615435838	4.148163187	4.692804283	2.903932348	3.158203546	3.08951815
Zm00001d052433_T001	5.297005417	4.854529226	5.361449647	6.028440524	6.007310899	5.977010275
Zm00001d027938_T001	4.745780957	4.068236303	4.886779117	5.405272738	5.487264159	5.519002775
Zm00001d050747_T001	1.856664259	1.120448825	1.82138001	0	1.016802227	2.285113355
Zm00001d050747_T007	0	0	1.061975915	0.345855058	0.345244206	0.443633171
Zm00001d046315_T001	5.519114807	6.125905179	5.531636007	5.503652	5.414616915	5.696981877
Zm00001d027268_T001	5.331103682	4.204075515	5.404776683	4.262559591	4.177997975	4.363106442
Zm00001d014594_T001	1.317426796	0	2.365462147	1.009075522	2.71591467	1.234478635
Zm00001d014594_T002	3.991866283	3.378872807	3.898850407	4.654442358	4.386167245	4.541672866
Zm00001d041839_T002	2.601052983	1.668795371	2.052171019	2.630686201	2.571772663	2.488785939
Zm00001d041839_T001	3.247818776	2.741490501	3.677278656	3.075225748	3.265715626	3.350806969
Zm00001d020257_T001	2.527567334	2.224985189	2.945536728	3.006707881	2.92302345	2.929631384
Zm00001d029288_T001	1.805576426	1.615374391	1.936872708	1.522582598	1.193941388	1.475057378
Zm00001d043050_T001	4.38303604	3.556150537	3.856973443	5.208182806	5.262375425	5.23699996
Zm00001d049277_T001	3.437404336	3.017153513	3.825537459	2.801917682	2.647804229	2.986460632
Zm00001d053808_T001	3.00694229	2.319367348	3.296870994	2.693176696	2.651559201	2.757898842
Zm00001d034519_T001	4.113135294	2.918830806	4.187539022	4.824312116	4.849361311	4.723395662
Zm00001d034519_T002	3.725711277	4.605676793	2.93112024	2.082883085	1.363122846	2.084046571
Zm00001d020612_T011	1.867710285	3.312628147	1.714088984	2.197698409	2.24600882	1.988084037
Zm00001d020612_T001	3.066333954	2.209275864	2.836712694	3.19608498	3.048227116	3.358332116
Zm00001d008878_T001	5.603152054	7.091160637	5.579071568	4.600920949	4.867722589	4.700522393
Zm00001d006199_T001	7.781763146	7.046706676	7.783258244	6.029815016	6.070529597	6.089299983
Zm00001d042609_T001	6.189277318	5.559832348	6.371389249	5.943401713	5.875642494	5.88246703
Zm00001d032775_T002	5.194439683	4.662362353	5.265464014	5.347993237	5.402768113	5.322813061
Zm00001d032775_T001	0.935351117	0.431932056	0.910296718	1.164678168	1.452977011	1.25521005
Zm00001d041732_T001	3.113870159	2.765090625	3.228686649	2.850501414	2.571161522	2.726162672
Zm00001d008882_T002	1.27361495	1.120048637	1.324159084	1.076262864	1.142193177	1.328434958
Zm00001d020409_T002	1.647423657	2.36326501	1.801411985	1.81230599	2.299402636	2.147533669
Zm00001d020409_T003	3.414094119	0	3.668844943	4.298910912	3.133091811	3.119963343
Zm00001d020409_T001	4.728649022	4.059815046	4.587155953	4.823328156	5.124062817	5.003119024

Zm00001d006704_T002	0.356923556	0.12939645	0.433290655	1.695683868	1.723425316	1.651752054
Zm00001d006704_T003	2.554768594	2.076589536	2.260443043	4.278855976	4.295533346	4.329146134
Zm00001d006704_T008	4.009739685	3.243092924	4.042762515	5.211245678	5.14997907	4.993928464
Zm00001d049736_T001	2.406736925	1.490605556	2.474664209	2.4496958	2.313610688	2.221342582
Zm00001d002933_T023	1.391315707	0.3634694	0.783219434	0	0	0
Zm00001d002933_T012	0.635192525	0.317346909	0	1.187405445	0.817601975	0
Zm00001d002933_T025	1.974020792	1.545014492	1.488301622	0.94503006	1.140276079	1.449236484
Zm00001d047910_T001	5.026018447	4.448139174	5.384004687	5.649397142	5.705913974	5.526470538
Zm00001d047910_T003	3.78765792	1.138876054	2.332528069	0.989924463	0	0.842616856
Zm00001d040934_T001	5.272531478	4.371110163	5.020404057	5.565398335	5.892641824	5.349245449
Zm00001d041380_T001	0.859282614	0.850185737	1.292590948	0.939492145	0.812043063	1.064619391
Zm00001d010728_T001	2.504718009	1.851172548	1.948308167	2.652204697	1.513855037	2.563572815
Zm00001d010728_T005	4.827918943	3.95414158	4.764390108	5.064718496	5.179694441	5.184207404
Zm00001d010728_T009	1.494508288	2.939691664	1.73390181	1.760493489	1.505463646	1.641096504
Zm00001d010728_T010	1.067455615	1.886180989	0.215780467	1.628509524	1.747849246	1.688413588
Zm00001d014321_T003	4.011893202	3.255943746	3.805637299	4.118527842	3.913037884	4.090421319
Zm00001d014321_T009	0	0	1.065997544	0.74299872	0.944692807	0
Zm00001d014321_T011	0	0	0.901780185	1.32569553	1.277114703	0
Zm00001d014321_T008	0.823274089	0.281383716	0	0.477963248	1.190559869	1.549400073
Zm00001d014321_T006	1.237851861	0.775970032	1.932048317	1.770861599	1.840513992	0.658940816
Zm00001d014321_T002	0.904434	0.442453095	0	0	0	1.563677505
Zm00001d016332_T001	7.861051623	7.159757702	7.898897447	7.231972358	7.273283279	7.21099489
Zm00001d015459_T002	6.897152397	6.101895077	7.011575328	7.602443139	7.693609637	7.656005817
Zm00001d015459_T004	2.443668177	1.141873511	2.23755913	2.353174497	2.408253169	2.467117462
Zm00001d015459_T003	0.883808472	0.450553027	1.319219057	1.766674025	1.037568478	1.555339594
Zm00001d048345_T003	4.159584141	3.426175909	4.141408838	4.723365843	4.625041207	4.613997036
Zm00001d053874_T001	0.958873848	0	0.51351298	1.060814541	0	2.027573774
Zm00001d053874_T002	1.265097787	1.199602623	1.781976531	1.569744805	2.20975934	0
Zm00001d021498_T022	0.373954395	0.323662902	1.136115246	1.214911854	1.332700487	1.129363476
Zm00001d021498_T013	0.280161722	0.118597608	0.348368409	0.556552036	0.622332539	0.454383332
Zm00001d021498_T020	3.572934767	0	3.232367277	3.418154724	3.028178193	3.406276788
Zm00001d021498_T014	2.333790086	0	2.300927309	2.706234358	2.713311558	2.20988034

Zm00001d021498_T003	2.125995205	0	2.203903692	2.926421535	0	3.163117398
Zm00001d018356_T003	2.030535252	1.77847564	0	2.15491303	2.064924573	0
Zm00001d018356_T002	2.036156905	1.167942339	2.79399095	2.679887881	2.395029063	1.894988064
Zm00001d048157_T001	7.597466509	7.027048117	7.542763315	6.949864778	6.965002906	6.926239852
Zm00001d038007_T003	2.831404641	2.968998673	1.969120275	2.653035704	3.230026509	3.091504867
Zm00001d038007_T001	4.717282147	3.955057575	4.697697491	4.589462754	4.604372941	4.459965973
Zm00001d005614_T002	3.715514751	3.155265285	3.931556272	3.593255755	3.475670664	3.628731025
Zm00001d025845_T001	2.151139238	1.534755384	2.125687797	2.440813767	2.241412802	2.417062073
Zm00001d054037_T001	0.851570361	0.397282731	0.979285661	1.127847192	1.407666004	1.525462051
Zm00001d054037_T002	3.493600456	2.907204684	3.654387878	4.049724331	4.143500441	4.15899593
Zm00001d043382_T004	7.129944166	4.920272527	7.17430566	6.339279937	6.400338452	6.32386021
Zm00001d043382_T001	6.045968224	5.712701283	6.000886647	5.538148416	5.489818552	5.436875513
Zm00001d020970_T001	1.650800396	1.860716536	2.395536123	2.061389469	2.6682603	2.626038865
Zm00001d040545_T001	1.985743409	2.593507953	2.433075085	0.464812529	0.957924996	1.046904497
Zm00001d037072_T001	4.528180401	5.480413908	4.1526203	3.558810074	4.232845226	2.835141493
Zm00001d043263_T004	0.189938389	0.066652722	0.147662281	0.272886755	0.307397048	0.291228692
Zm00001d043263_T001	1.927347329	0.884843266	1.670320528	1.751710077	1.542705773	1.920784914
Zm00001d027375_T001	5.116687547	5.272023152	5.25271716	4.023403873	4.014352883	3.225726704
Zm00001d018119_T001	2.777382681	2.282563163	2.929878276	2.041589944	1.855297277	1.812036078
Zm00001d046981_T001	0.919356102	0.711124824	1.136356125	1.358766454	1.131056688	1.16531577
Zm00001d034501_T001	5.840027137	5.295908638	5.990752506	6.56395818	6.678654783	6.65027909
Zm00001d002094_T001	3.207207096	3.180872528	3.309210246	1.615552838	1.501034497	1.710455792
Zm00001d039624_T001	8.057320104	7.632848456	8.067320092	7.12536844	7.104785178	7.092006956
Zm00001d021296_T003	2.322402952	1.453044461	2.750704468	2.986155688	2.969311502	2.585159896
Zm00001d021296_T004	3.449331449	3.595434556	3.52895157	2.802481727	2.724059977	3.046236968
Zm00001d051102_T001	1.688161555	0.806512142	1.866484699	0.213939482	0.602478735	0.32629918
Zm00001d020741_T001	4.294103418	4.073262838	4.387862861	2.664821857	2.645227829	2.657773065
Zm00001d015776_T001	1.218633637	1.805169439	1.56215644	2.226784095	2.203549491	1.678836782
Zm00001d015776_T002	3.406225486	2.372382706	3.193953527	2.927322105	2.860457915	2.806600814
Zm00001d042872_T001	2.822965854	3.172640482	2.800452453	2.800923293	2.357335232	2.889677764
Zm00001d050970_T006	4.117515027	1.362508042	3.554812544	3.39991823	3.101248338	2.872268314
Zm00001d050970_T017	2.747333687	1.418268761	2.864229933	2.675583365	2.634120424	2.55877555

Zm00001d050970_T011	1.58653226	0	0.707884316	1.892317966	0	2.159536189
Zm00001d050970_T001	0.330347286	0.97128419	1.086037773	1.332652373	1.71610416	1.575632302
Zm00001d050970_T018	1.300892176	1.074558184	1.636484955	1.672319427	1.407506667	0.859116397
Zm00001d050970_T012	1.526701856	0	1.673409886	0	1.614888845	0
Zm00001d050970_T007	2.384030737	1.43733322	1.653880446	1.248987125	0	1.858496293
Zm00001d050970_T021	0	0	1.506086493	0.721158263	2.297189068	0
Zm00001d050970_T008	0	5.915711598	3.930684264	0.79215986	1.558467001	2.166349745
Zm00001d050970_T022	0.888266703	0.64117791	1.062338653	1.223756772	2.167867855	1.18391521
Zm00001d050970_T005	2.816641277	1.110230261	2.891494042	2.958608114	2.83600833	3.105901303
Zm00001d050970_T003	1.486979979	2.605439811	1.394810443	2.596876241	1.918697258	2.384951348
Zm00001d033108_T001	4.822087368	3.722428208	4.672783821	4.346567557	4.5819005	4.518329169
Zm00001d033108_T006	3.722128372	2.793305786	3.852598969	3.535755003	3.275638982	3.356301878
Zm00001d033108_T003	1.265799925	0	1.238596118	1.134501564	1.748335421	1.271076016
Zm00001d031291_T004	2.611733529	2.191533279	2.493478771	2.741086388	2.852243395	2.713434743
Zm00001d038871_T009	3.993244931	3.784268296	4.253217808	3.369491471	3.501816171	2.875412856
Zm00001d038871_T007	4.006191815	2.969036629	3.782307489	4.37446196	4.176141467	4.212856371
Zm00001d038871_T005	1.610021543	0.925928801	1.294469598	1.079441604	1.753882609	1.82022186
Zm00001d035954_T001	3.748592937	3.176669839	3.784765173	3.459154988	3.300064844	3.414385479
Zm00001d011329_T008	0.9469494	0.793464778	1.482161097	0.780544439	1.325230393	1.577027053
Zm00001d011329_T002	2.333929733	1.324638967	1.942860523	2.404990291	2.263451224	2.350050791
Zm00001d011329_T007	2.247783237	1.133967213	1.883731067	2.194459343	2.269309686	1.636372192
Zm00001d011329_T005	0.413185694	0.739391897	0.371160707	0.92187273	0.748076463	0.964352051
Zm00001d039639_T001	1.867043638	1.493213333	1.790437594	2.048940618	1.70403487	2.003551148
Zm00001d021891_T001	5.872448734	5.329427822	5.890528821	5.707058882	5.815810795	5.813362043
Zm00001d021891_T003	1.519556498	1.040110394	2.124381077	0.568364884	1.079548784	1.299734071
Zm00001d021891_T006	4.693597908	6.452546248	4.237597139	3.924613671	3.34883252	4.473348907
Zm00001d021891_T005	7.246831988	0	7.344832402	6.967272228	6.924464125	6.843506726
Zm00001d012953_T001	1.516662701	1.038380692	1.583854567	3.458660226	3.315406244	3.449048252
Zm00001d041537_T001	1.307859812	1.138042466	1.248547587	1.117626563	1.17970276	0.677466748
Zm00001d006369_T002	1.700606617	1.393955943	1.881107792	1.74034389	2.023773778	2.304398309
Zm00001d006369_T001	1.804193608	1.787874149	1.124770141	2.042503563	0.969747655	1.342702648
Zm00001d027903_T001	1.221691514	0.87548835	1.428097892	1.02167089	0.782955343	0.784352377

Zm00001d004908_T001	4.932924555	6.065235358	5.129405971	4.684921439	4.73923985	4.834365314
Zm00001d028237_T005	1.915822602	1.383001952	2.006597125	1.266978987	1.24995376	1.063812157
Zm00001d012557_T001	6.736083076	6.433996606	6.82794189	6.854983956	6.918791077	6.897041787
Zm00001d052842_T001	5.534358293	5.243876629	5.597787154	6.776115677	6.748363547	6.857162282
Zm00001d030346_T001	6.012072785	6.099164039	6.247399163	6.162858575	6.023092707	6.048029804
Zm00001d027477_T001	3.705719188	4.001924353	3.664548261	3.039862192	3.068225209	3.389918495
Zm00001d012738_T001	1.665310446	1.426631354	1.652169354	1.694633298	1.350414643	1.703653125
Zm00001d011819_T001	2.442056779	1.282187719	2.340419274	0	1.931271556	1.407472948
Zm00001d011819_T002	0.887854324	0.768924489	1.44969131	0.500798994	0.135442934	0.640413624
Zm00001d020088_T054	2.992904719	3.664446773	3.259546535	3.703434196	2.151637071	2.855044662
Zm00001d020088_T015	2.275166212	1.498852333	2.348182836	2.085821989	2.234934125	1.877691645
Zm00001d020088_T006	0	0.740936193	1.256961119	2.293534993	1.510999378	1.343069496
Zm00001d020088_T004	1.068730649	0	0	0.843257893	0.233211945	0.898947411
Zm00001d020088_T037	0.274836537	0.198245197	0.508328244	1.015101445	0	0.859927419
Zm00001d020088_T014	0	0	0	1.384259839	1.766336068	1.784308819
Zm00001d038673_T004	4.921373535	4.17966813	4.890301297	4.813588357	4.80197209	4.758450456
Zm00001d038673_T003	0.771173138	0.493096997	0.865689232	0.397345169	0.426936693	0
Zm00001d033284_T001	2.24610553	2.208348696	2.1955706	3.168486398	3.504044343	3.293977219
Zm00001d053801_T001	3.298589258	2.629359185	3.2942249	3.656682959	3.833164252	3.783140212
Zm00001d000396_T002	4.636501892	3.609815822	4.798133226	4.980464442	4.731830408	4.924702451
Zm00001d000396_T001	4.342455756	3.884902252	4.000490433	4.793189652	4.862365938	4.692541466
Zm00001d027483_T001	4.503416098	4.420665978	4.208729575	7.011150992	6.999915915	7.05220999
Zm00001d007743_T001	1.011843389	0.421800127	1.003298555	0.753698659	0.937955995	1.228625395
Zm00001d015856_T015	0.32084971	0.205580189	0.205007075	0.879892364	0.911710754	0.847207216
Zm00001d015856_T003	5.268863612	4.48970907	5.224664563	5.762336775	5.847397863	5.875009519
Zm00001d015856_T011	1.14396085	0	1.701044635	1.15770668	1.253304353	1.373948272
Zm00001d015856_T012	0.771266122	0.788577139	0.863898815	1.879519929	2.016315526	1.722949028
Zm00001d015856_T021	4.715837649	6.966463519	4.586280221	4.273224188	3.861324469	0
Zm00001d015856_T020	5.780408927	3.802306973	5.992107127	5.760667101	5.664917887	5.947193534
Zm00001d042572_T001	8.709572478	9.994479533	8.56363644	7.391787874	7.198529238	7.43973569
Zm00001d021391_T001	2.302924081	2.606892023	2.516251205	1.670514061	1.864555031	1.256734729
Zm00001d010185_T001	2.953786527	2.718326771	3.235526723	2.685478906	2.781867663	2.534174184

Zm00001d044533_T003	1.104255464	0.87558507	0.874774937	1.211355533	0.921855215	1.36325744
Zm00001d044533_T001	6.142838688	5.141755743	6.264270893	4.997823451	5.098288797	4.972466813
Zm00001d044533_T002	5.515416475	5.246074967	5.685685412	4.416588667	4.267263263	4.316434542
Zm00001d052218_T001	1.9030483	1.20509531	1.74289312	2.877410919	2.838936039	2.814496898
Zm00001d001952_T001	3.302760201	2.508539956	3.402698714	3.18944854	3.105288524	3.096795174
Zm00001d018330_T003	3.052291027	2.214568428	2.929336913	2.59530123	2.563043882	2.587529415
Zm00001d020974_T001	6.889590538	5.987921083	6.883341255	7.36006878	7.441181254	7.36248069
Zm00001d018416_T002	0.704617922	0.645898062	1.000116854	0.791013023	0.777495886	0.779446323
Zm00001d018416_T004	0.690063922	0.453009682	0.142145461	0.479757245	0.642492718	0.238214559
Zm00001d028915_T006	0.688123948	0.368682316	0.950770733	0.76664265	0.89624782	0.828515414
Zm00001d028915_T010	1.007320386	0.580575816	0.694400626	0.864161972	0.25583546	0.362323999
Zm00001d028915_T005	0.077936147	0	0.244502289	0.215302111	0.478011932	0.675843024
Zm00001d028915_T025	2.435407506	0.933784873	1.571848127	0	0	0
Zm00001d028915_T019	1.490947441	0	2.143663123	1.772129316	2.158773583	1.834173593
Zm00001d028915_T007	2.710404434	2.16480687	2.701035095	2.724436143	2.790296621	2.903018597
Zm00001d028915_T014	0.736898363	0.547188215	0.865158676	0.928795536	0.765032262	0.57269762
Zm00001d028915_T002	0.397024189	0.27195628	0.344869392	0.677610169	0.267334436	0.734359755
Zm00001d028915_T009	2.371775181	1.091876501	2.018747392	2.227247876	2.4653061	2.349944957
Zm00001d028915_T017	0	1.454479143	0	0.917113965	1.578231559	1.31711243
Zm00001d029180_T021	0.811306611	0	0.637660321	1.785767724	2.340946177	0.393169048
Zm00001d029180_T007	0	0	0	1.123272861	0.876616348	1.78461328
Zm00001d029180_T002	2.584647478	1.837989246	2.616941056	3.1855377	2.922169489	3.036358793
Zm00001d029180_T025	3.649689948	3.502525375	3.699675442	3.343564876	3.309568829	3.123864975
Zm00001d028330_T167	0.235366801	0	0.938376134	0.548750284	0.762118306	0.807995336
Zm00001d028330_T201	0	0.228370501	0.265127199	0.345816462	0.604223178	0.305056476
Zm00001d028330_T105	1.205471965	1.025763779	1.39409812	1.813110496	1.113144381	1.592778012
Zm00001d028330_T128	4.205696848	3.50236523	4.441114592	3.528667704	3.412994324	3.678091629
Zm00001d028330_T150	0.191740766	0.098446509	0.509061373	0.463164153	0.461547418	0.878215257
Zm00001d028330_T120	0.490607096	0	0	0	2.057223582	1.849809155
Zm00001d028330_T001	0	0.819113068	0.700845391	1.01000351	0.50571109	0
Zm00001d028330_T079	0.230078786	0.381072906	0.390647877	0	0.438650553	1.043958972
Zm00001d028330_T022	1.311505441	0	0	1.937098774	0	1.546601633

Zm00001d028330_T206	0.419279992	0.040269641	0.451088172	0.075881711	0.399302366	0.377713481
Zm00001d028330_T187	0	0	0.229666669	0.577430278	0	0.559166773
Zm00001d028330_T214	0.642366098	0.525407425	1.342859067	1.464830822	1.880265878	1.353721318
Zm00001d028330_T092	1.875532386	0	0.698839881	1.476291468	2.450569654	0
Zm00001d028330_T162	2.068764351	0	1.210363302	1.056324097	0	0
Zm00001d028330_T037	0.338511131	0	0.649243874	0	0	0.63874022
Zm00001d028330_T118	1.195485561	0	0.645341977	0.94759734	1.4360776	1.338244691
Zm00001d028330_T226	0.069883609	0.031496831	0	1.219086101	0.293208017	0.639768385
Zm00001d028330_T216	0	0.174603282	0.733492312	0.989564122	0.842234672	0
Zm00001d028330_T153	0.473947923	0.230820301	0.699613816	0.831516522	1.257547153	0.834312367
Zm00001d028330_T039	0.702496201	0.612784653	0.876617134	2.176791891	0.800480409	1.874546366
Zm00001d028330_T129	0.48958301	0.238312404	0.467349396	0.641595043	0	1.61742074
Zm00001d028330_T024	2.362418268	1.551157668	2.324792174	2.383411508	1.384021063	1.585106282
Zm00001d028330_T207	2.917179477	2.336669864	2.560600512	2.529437176	2.432711456	2.61246456
Zm00001d028330_T025	0.629031088	0.723199427	0.690514538	1.108742559	0	0
Zm00001d028330_T224	0.141117535	0.277294468	0	0.387681266	0.627024974	0.575331696
Zm00001d028330_T065	0	0.734722191	0.789239299	1.668690551	0	1.540008417
Zm00001d014779_T001	2.744794881	3.467834407	2.573222783	2.487613171	2.420353851	1.865003036
Zm00001d014779_T003	4.009636506	4.000278594	4.493007111	3.471815007	3.188997915	3.137012268
Zm00001d043528_T001	2.789472402	2.41943318	2.707102876	4.335991605	4.29195557	4.311692522
Zm00001d046073_T001	1.95252554	1.219317236	2.052854062	2.132923574	2.403390169	2.259981983
Zm00001d034510_T005	0	0.27616789	0.438498328	1.232401048	0	2.216962009
Zm00001d034510_T015	0.677883441	0	0	1.342584327	2.043518094	0
Zm00001d034510_T013	3.507095382	2.734625522	3.41866492	3.185798372	2.962951885	2.950228007
Zm00001d034510_T014	1.525348787	1.076178704	1.42705366	1.229778629	1.774310562	1.234690765
Zm00001d034510_T001	0.614347064	0.387416586	0.797889538	0.877683008	0.79078955	1.172298685
Zm00001d034510_T008	0.601646128	0	0.312510674	1.477773677	1.35794155	1.094221879
Zm00001d001847_T003	0.184681463	0.17528698	0.067569884	0.413093601	0.30084416	0.164561041
Zm00001d001847_T005	0.476036333	0	0.573095268	0.647688433	0.52550565	0.845438665
Zm00001d001847_T001	2.371029917	1.837061617	2.574885753	2.260470745	1.597221745	2.061400875
Zm00001d001847_T004	1.001939121	0.762633712	1.814036827	1.098361612	2.36906021	2.401965982
Zm00001d027847_T002	2.103477833	1.542309565	2.226280702	2.121161321	1.977220199	2.134951961

Zm00001d015636_T001	3.13788382	2.649361384	3.280721931	3.835583869	3.839225359	3.868145851
Zm00001d002334_T001	5.286615755	4.359247969	5.277124116	4.890701355	4.92591238	4.928943307
Zm00001d007512_T001	0.950498276	0.503160435	0.955608901	0.683508723	1.123831048	0.444835601
Zm00001d008625_T001	2.888281513	2.134094411	3.06921374	3.170755755	3.084883204	3.180204842
Zm00001d046194_T023	0.140735472	0.1460543	0.752299873	1.0024577	1.168827495	0.909443637
Zm00001d046194_T001	2.369177497	1.362825574	2.32020391	2.730524101	2.659970669	2.612860597
Zm00001d046194_T002	0	0.271547589	0.090510665	0.524460914	0.39036723	0.449686029
Zm00001d046194_T007	0.3742338	0.122295018	0.367499672	1.04555061	0.943922076	0.971955864
Zm00001d023318_T023	0.232709869	0.035836428	0.103328434	0.463570146	0.296509094	0.457402025
Zm00001d023318_T007	0.298993732	0.20239766	0.285054154	0.321392467	0.49996066	0.521846764
Zm00001d023318_T006	1.007266558	0.501407552	0.905484918	1.179391935	1.230126756	1.385920228
Zm00001d011763_T002	1.473986875	1.957303227	1.575521942	1.420643007	1.359026881	1.787159112
Zm00001d011763_T005	1.744192533	1.280443858	1.955169941	2.198279148	1.845345925	2.008675837
Zm00001d044409_T001	5.250305771	4.784072471	5.3189731	2.225608106	1.964295856	1.625903079
Zm00001d050307_T001	4.214381529	3.655567186	4.210657708	4.383045236	4.270685014	4.357116526
Zm00001d050307_T002	3.887473411	3.513226817	4.222041057	4.355695575	4.325119195	3.997828915
Zm00001d033149_T001	3.054233942	3.071263439	3.08846118	2.664637342	2.463933142	2.468492869
Zm00001d028009_T001	4.183625854	3.41875396	4.19362794	4.483304054	4.319752892	4.445672548
Zm00001d021881_T001	3.944296622	3.110017396	4.011457353	4.593671201	4.645403833	4.51593393
Zm00001d025707_T001	3.65411115	3.21872569	3.941620649	5.697069867	5.565377221	5.708057117
Zm00001d005554_T001	1.966034299	1.559323917	2.378224443	2.335712196	2.005396754	2.428123895
Zm00001d034033_T002	4.89163504	3.784841061	4.629491324	5.089690466	5.024015717	5.012093328
Zm00001d034033_T007	1.536949083	2.148520592	2.66567496	0	0	0
Zm00001d034033_T005	0	0	0.947757409	0.703406398	0.684400452	1.048558454
Zm00001d034033_T013	2.751645814	1.682737782	2.508670772	2.187660396	1.741831524	2.037469379
Zm00001d005971_T001	1.85554606	1.812343782	1.445599588	1.928125447	1.06559299	1.38718109
Zm00001d042725_T002	6.789994595	7.903307117	6.890924136	6.747355629	6.673732849	6.729098057
Zm00001d019420_T001	0.681479851	0.487510017	0.455307491	0.634292153	0.737022147	0.760737882
Zm00001d025887_T001	5.163875136	4.254670151	5.120870502	4.213347204	3.941549008	3.971192666
Zm00001d049511_T001	1.237498251	0.940818495	1.109048917	0.836090561	0.954404788	0.914138158
Zm00001d033054_T001	5.428727379	5.396371362	5.463306996	5.76091519	5.694418429	5.807112709
Zm00001d019221_T001	8.275704585	7.694596082	8.402346176	8.13035917	8.203543105	8.141603742

Zm00001d020947_T002	1.095740823	0.677740949	1.189243253	1.656354954	1.559687096	1.173116557
Zm00001d020947_T001	4.451516502	3.806770203	4.690104999	4.62285839	4.738363501	4.706732715
Zm00001d029420_T002	1.102939589	0.390896562	0.477563358	1.705153838	1.7910026	1.726066081
Zm00001d029420_T001	4.517998624	3.679585152	4.683752589	4.91600903	4.979527128	4.926346193
Zm00001d031101_T002	6.343207928	5.618922511	6.316618633	6.454655006	6.388790655	6.443363524
Zm00001d031101_T003	1.810241544	1.655242821	2.048739437	1.676439384	2.243546922	2.174802993
Zm00001d036936_T002	0.838854173	0.980398999	0.984555812	1.502685503	1.419254639	1.032560739
Zm00001d048258_T024	0	0.02857481	0.389197831	0.506411019	1.036857063	0.554215544
Zm00001d048258_T003	0.145429649	0	0.36934252	0.839155807	1.047476966	0.569035074
Zm00001d048258_T023	0.223842631	0.239104707	0.329463487	0.523017895	0.278654478	0.259825653
Zm00001d048258_T011	3.240413254	2.152146094	2.527241777	4.396480387	4.456666472	4.490473284
Zm00001d048258_T018	1.990206643	0	2.081419396	1.889658492	1.600990445	2.168259812
Zm00001d048258_T001	2.77665295	2.900209707	3.442311888	0	0	0
Zm00001d048258_T021	2.470107832	0	2.606299136	3.675990557	3.131757054	2.963878947
Zm00001d048258_T013	0.339232041	0	0.250252175	1.10469427	1.020115274	1.199180449
Zm00001d012741_T001	2.425604856	1.222034795	2.030504883	2.754597224	2.226395699	2.770010335
Zm00001d012741_T003	8.825615645	9.835497433	8.943711879	8.948610787	9.029180936	8.995472537
Zm00001d012741_T002	3.251399014	3.971504376	2.864751125	3.233496664	3.252234584	2.41772913
Zm00001d024759_T001	3.507586243	2.922923581	3.704773938	3.698501432	3.745145883	3.705251286
Zm00001d039434_T001	1.729435305	1.442938189	1.295767098	1.200542646	1.11544681	1.29362592
Zm00001d034025_T001	5.735096834	1.448869243	6.080317371	4.99452922	5.758306589	6.157993179
Zm00001d036830_T004	1.031662677	0.873302162	1.419425629	1.607471883	1.607415068	2.071932873
Zm00001d036830_T007	3.094376109	2.476863283	3.001188291	2.795143945	2.548433665	2.698744999
Zm00001d036830_T001	0.522379214	0	0.682313499	0.686428811	0.808886663	1.006134982
Zm00001d003034_T001	2.965185292	2.563499357	3.120622835	3.10385831	2.97327127	3.113368121
Zm00001d047247_T001	5.603312642	5.432941307	5.368406583	5.761762741	5.654711806	5.880556186
Zm00001d002541_T001	1.550892786	0.973321054	1.722166962	2.373257872	2.194594239	2.078127831
Zm00001d034207_T001	3.322277473	3.048267055	3.558855737	3.141094967	2.898415598	2.868858994
Zm00001d020351_T001	1.177211735	1.113787832	1.557976616	2.437839742	2.578877865	2.488719625
Zm00001d007050_T001	2.744936928	2.237293876	2.812038954	5.432832932	5.450061906	5.479391322
Zm00001d002405_T001	6.637336527	6.726787985	6.809897607	5.464678525	5.518743282	5.423790459
Zm00001d045324_T002	2.382642355	2.252937561	2.848066835	2.743934984	2.761823736	2.931692322

Zm00001d004392_T001	2.382831846	2.078042755	2.020114562	2.244780223	2.359613936	2.137580247
Zm00001d031949_T001	2.233969025	3.072833312	2.345303543	2.362469047	2.312019766	2.31917396
Zm00001d017487_T004	1.77161349	1.012221813	1.699118048	1.986138937	2.071720115	1.399083025
Zm00001d017487_T003	0	1.844391278	0	0	1.255398608	1.074781481
Zm00001d017487_T006	1.969660361	0.514095995	2.125034419	1.017748062	0	0
Zm00001d020530_T001	5.284131311	4.612156666	5.268170938	4.582812185	4.57891336	4.632402415
Zm00001d039192_T001	1.549804687	1.662837594	1.988045124	1.767330632	1.329046651	1.305062315
Zm00001d039192_T003	2.31922773	0.950605772	2.811179379	2.103462054	1.77387483	2.004992454
Zm00001d039192_T002	2.702807567	2.207685488	2.681320845	3.283118883	3.412304206	3.408042659
Zm00001d028272_T002	3.461287435	2.838588577	3.313950916	3.415376177	3.395037292	3.403845503
Zm00001d028272_T003	0.298151524	0.618325121	0.581519974	0.716081326	1.029212564	0.828342364
Zm00001d028511_T035	0.787103098	0.251263417	0.793375711	1.001281986	0.853219616	0.98382141
Zm00001d028511_T025	0.586343941	0.515708416	1.053921104	0.745909357	0.650634986	1.101766374
Zm00001d028511_T008	3.096760941	2.867011801	3.671566938	3.492315326	4.241340186	4.051012166
Zm00001d016891_T001	4.615136995	3.71191587	4.7363897	4.500489584	4.492345837	4.457892623
Zm00001d038554_T001	1.597712796	2.317425928	1.958647458	1.456264183	1.650146457	1.338341687
Zm00001d032504_T001	1.531879471	1.041052994	1.468700605	0.854928408	0.800291537	0.772783445
Zm00001d004936_T002	7.608948103	7.076882419	7.6066147	7.630251324	7.632637735	7.620429993
Zm00001d004936_T001	5.341775908	5.148297763	5.396442951	5.660294886	5.782801661	5.668346996
Zm00001d043336_T001	1.572134905	1.177260858	1.710746714	2.513786834	2.115586303	1.395808091
Zm00001d043336_T003	0.785505356	0	0	1.876854019	1.54889772	0
Zm00001d010654_T001	5.672563862	5.442265867	5.661729166	5.490759343	5.624279612	5.697813539
Zm00001d031638_T001	2.830469927	2.173117517	2.811370943	2.929762409	3.101606042	2.907645793
Zm00001d039020_T001	6.51844031	5.765183869	6.628090457	6.041252796	5.996723303	5.945030482
Zm00001d039101_T001	1.827832436	1.488317563	2.001330273	1.6358296	1.558485614	1.744192102
Zm00001d026676_T005	1.687750508	0.876270575	1.001895177	0.720254821	0.943606318	0.692045048
Zm00001d026676_T001	1.823060044	2.035899403	1.528885827	1.637010138	1.373442197	1.70174776
Zm00001d026676_T004	1.874739926	1.663105934	2.130575093	1.137031292	0.826007814	0
Zm00001d019106_T004	0.327955186	0.093998191	0.078072823	0.979046349	0.584993278	0.999326104
Zm00001d019106_T003	5.176740679	4.239379328	5.15704367	5.564782871	5.564984235	5.523934092
Zm00001d053036_T003	3.711094971	4.018946748	4.315035555	4.087329853	4.11435628	4.023335115
Zm00001d053036_T002	1.574954033	1.416969957	1.441693813	2.274988567	2.148104031	2.351670445

Zm00001d053036_T001	2.964258145	2.494006426	2.5828645	3.125949924	3.087890664	2.835775958
Zm00001d012456_T001	1.603034014	0.840025676	1.591788548	1.429459569	1.710518389	1.476913559
Zm00001d012456_T003	0	0	0.054126081	1.215592871	1.28061252	1.242512878
Zm00001d012456_T004	0	0.249123695	0	1.448161975	0.869053318	1.436792947
Zm00001d012456_T002	0	0	0.050315014	0.773822516	0.498573587	0.465303749
Zm00001d048813_T001	1.863728372	2.126973187	1.461941298	1.976375001	1.413395861	1.723531477
Zm00001d045945_T003	3.508140845	2.055750617	3.247121809	3.329095316	3.61315938	3.09097128
Zm00001d045945_T001	2.088655881	1.137568444	1.941247568	1.686593305	1.163280379	0.410070778
Zm00001d045945_T005	2.554150206	0.789024735	2.973281007	2.346705335	0	0
Zm00001d045945_T002	2.429662023	2.909494905	2.415849778	1.228331708	2.235871016	2.940714964
Zm00001d045945_T004	2.123265576	1.647615682	1.962717806	2.06289122	1.503967409	1.794118043
Zm00001d005097_T001	2.651550017	2.994548046	1.74349558	3.691561299	3.898769066	3.988665057
Zm00001d028672_T002	2.052517102	0	1.913204228	2.088701669	1.614163754	0.650506321
Zm00001d028672_T001	4.502530722	3.967847271	4.575719665	4.448581924	4.453703913	4.650875571
Zm00001d034244_T001	6.384988997	6.027269832	6.394958348	6.577967933	6.597718233	6.529219292
Zm00001d041414_T001	1.060757831	0.464877338	0.984504773	0.776141898	0.980129887	0.969723346
Zm00001d020827_T001	4.14829996	3.374961558	4.275191024	4.200861891	4.104074015	4.136792821
Zm00001d024750_T001	3.02933379	4.210635423	3.072770899	3.502742141	3.29584202	3.398074776
Zm00001d010220_T004	3.868008812	6.179252743	4.018239444	3.881910757	2.859859057	3.295586976
Zm00001d010220_T001	5.535096736	4.753081347	5.606706728	4.678469831	5.213490066	5.028321875
Zm00001d010220_T003	1.360637337	1.886838462	1.100880792	0.854795193	1.510992291	0
Zm00001d036790_T011	2.296817119	1.589182458	2.540593471	0	2.18106692	0
Zm00001d036790_T010	2.287213698	1.72849524	2.544467581	1.700695829	2.103149471	1.212061894
Zm00001d036790_T017	0.766983503	0.357236689	0.582408595	0.815238487	0.984342166	1.0763046
Zm00001d032468_T001	8.524684054	9.374177513	8.456908702	8.137575744	8.277933518	8.221830083
Zm00001d033080_T001	0.31609939	0.231051408	0.51819973	0.822234959	0.898743144	0.7842284
Zm00001d001830_T001	4.049726247	3.508201328	3.925330119	3.977358605	3.824073421	3.856404213
Zm00001d033652_T001	5.729842585	4.820965872	5.782334567	5.130626077	5.141849974	5.037097565
Zm00001d004480_T001	3.863719255	3.948601034	3.648249483	3.655955917	3.705769497	3.635270317
Zm00001d003207_T013	4.327532597	3.348323506	4.446419616	4.464625142	4.559083053	4.496915438
Zm00001d003207_T011	0.165320272	0.310244713	0	0.671709135	0.527256542	0.789218428
Zm00001d003207_T012	1.287354676	0.390227437	1.128561324	1.753385463	1.857199539	1.81625645

Zm00001d003207_T001	2.157803998	2.542257059	2.183185717	3.428551525	3.116995133	3.222401181
Zm00001d027934_T004	3.236915492	0	3.240800947	1.833598937	2.829287185	0
Zm00001d027934_T005	0.876463118	0.546327022	0.928020054	1.288899508	0.887278822	1.323375834
Zm00001d027934_T002	4.539058967	3.46551103	4.452486319	4.878990759	4.799306699	5.039505076
Zm00001d011274_T001	5.272560665	5.750587614	5.239550567	6.013195649	6.112390087	6.198840465
Zm00001d045568_T001	3.070441711	4.401857952	2.868327627	2.821520836	1.55443512	2.544097335
Zm00001d004121_T001	3.97865299	3.834392065	4.15301715	5.185382963	4.233033277	4.666795158
Zm00001d011166_T002	2.207421557	1.810374001	2.222364701	2.545145996	2.516685138	2.030047511
Zm00001d011166_T003	0.85501854	0.819599521	1.368799633	0.857563847	1.777088055	1.401255273
Zm00001d011166_T001	3.055604869	2.520137076	2.835937006	3.48719176	3.128865812	3.118488805
Zm00001d033777_T002	0.208411129	0.021455564	0	0.863306538	0.76544309	0.578016059
Zm00001d033777_T001	2.757836561	1.99803046	2.916879778	3.611249825	3.545099155	3.596289481
Zm00001d019552_T002	2.894235719	2.226492192	3.224612205	1.531768207	1.621905111	1.518941453
Zm00001d019552_T005	3.259652435	1.49259208	3.74307188	1.729569737	1.961630736	2.049354399
Zm00001d008606_T002	0.864019302	0.407724731	0.686296126	1.018331504	1.581758085	1.581692536
Zm00001d008606_T004	0.717380534	0.535497605	0.803027774	1.416331737	1.021188316	1.126292304
Zm00001d008606_T003	4.852008229	4.333450927	4.829899729	4.619960931	4.676681232	4.773310407
Zm00001d026084_T001	4.522113391	3.768590446	4.529536501	4.851151605	4.867519684	4.953124083
Zm00001d006744_T001	4.26998211	3.716616733	4.294890149	3.563227578	3.724707564	3.612399919
Zm00001d039595_T002	2.075801635	1.271233822	2.032823421	1.810932899	2.036311665	1.420139036
Zm00001d052033_T001	1.116227002	0.781420987	1.280817975	0.476146274	1.070484817	0.544435927
Zm00001d007145_T001	5.478766384	5.004401793	5.627650988	5.208259256	5.141451062	5.01336636
Zm00001d005645_T001	5.77982786	5.433074918	5.835351353	5.606864729	5.59512396	5.652973774
Zm00001d005645_T002	3.466255665	2.683080623	3.336399513	3.163769373	3.197589121	3.396137838
Zm00001d004075_T004	0.639340535	0.753510407	0.841459533	0.690266001	0.555121139	0.747949329
Zm00001d004075_T001	1.121682535	2.138103429	1.882874644	1.708818484	0.242043916	0.410359559
Zm00001d004075_T003	3.982976057	2.771966688	4.015182463	3.446981234	3.604767588	3.561271752
Zm00001d008926_T001	2.826994827	2.260729664	3.046263166	2.672072263	2.408091994	2.234045693
Zm00001d051420_T001	7.733657986	7.211212537	7.835793179	7.262588867	7.303315459	7.201573035
Zm00001d051420_T002	0.220547891	0.976235309	1.219426904	2.171911987	2.138301048	2.027843025
Zm00001d017983_T001	2.816580047	2.179902404	3.207509643	5.254137384	5.361397674	5.312319886
Zm00001d037675_T001	2.141839187	1.459481981	1.830323481	1.777997835	2.273831547	1.735561602

Zm00001d038804_T007	3.781079436	3.479886947	3.764368769	4.192816174	4.302813855	4.261770969
Zm00001d038804_T011	4.70559081	2.889190232	4.534802997	4.184784991	4.405357502	4.364964918
Zm00001d038125_T001	3.022584245	2.374755731	3.203566875	4.119606842	4.050936617	4.181374999
Zm00001d039335_T001	1.928738696	1.850665827	2.111250372	1.366101159	1.466822859	1.424701234
Zm00001d027411_T001	6.45650168	5.915368855	6.587354489	5.707848406	5.787581318	5.735683443
Zm00001d048561_T001	5.029236863	4.629160692	5.196813213	4.880371095	4.920630112	4.933219852
Zm00001d018627_T001	6.880586796	6.169263514	6.976215921	6.620000065	6.612116626	6.705770382
Zm00001d034841_T002	4.244857611	3.322383193	4.247497526	3.472961573	3.688068878	3.858336849
Zm00001d027613_T001	2.507318948	4.067489518	3.171714916	3.829280689	3.510824857	3.314278301
Zm00001d027613_T002	3.28074212	3.334054346	3.433910204	3.749704485	3.438420479	3.867004385
Zm00001d037388_T002	0.184078398	0.225627847	0	0.433495776	0.534511876	0.379809465
Zm00001d037388_T003	2.573660274	2.048893202	2.643598328	2.295007964	2.083041767	2.03422276
Zm00001d037388_T001	0.500124898	0.050046093	0.477056602	0.367059007	0.065255201	0.410849052
Zm00001d033579_T001	4.700299936	4.560624294	4.871063604	4.969312561	4.883014386	4.789322778
Zm00001d038954_T006	3.418618104	4.114405674	4.870574672	3.889887795	4.527873806	4.382384219
Zm00001d038954_T007	4.215003136	0	0	3.499558021	0	2.226421596
Zm00001d038954_T003	1.435208258	0.804400557	0.682926512	0.286057867	1.767500987	1.379603219
Zm00001d036764_T006	1.161300168	0.754204251	1.344374033	0.63268421	1.148759084	0
Zm00001d036764_T004	1.48805786	0.852117924	1.0266117	0.96196035	0.381569114	0
Zm00001d029040_T002	4.539494346	4.655012011	4.442180556	5.053889704	4.964380425	4.937439972
Zm00001d029040_T003	5.036966223	2.13372462	5.184754171	4.438908727	4.54977666	4.742153761
Zm00001d033339_T003	3.432648393	4.740811611	3.358486428	3.25239946	3.127638067	3.189648737
Zm00001d033339_T002	3.912170803	3.008702008	3.923291446	4.238624853	3.984622797	4.320926517
Zm00001d033339_T001	0.786843063	0.187390241	0.304424594	0.689127369	1.239365655	1.032853388
Zm00001d053705_T001	0.87436183	1.115660539	1.275058912	1.117563399	0.92051893	0.86473013
Zm00001d003778_T001	1.3735118	0.919826683	1.36227574	1.263855907	1.535901161	1.448098526
Zm00001d013522_T001	2.902850206	1.987035573	2.795805175	3.001838445	2.796171818	2.930366636
Zm00001d002618_T001	0.771728415	1.27643711	0.401654493	5.075050069	5.365295	5.29041751
Zm00001d028162_T006	1.444062195	0	1.531074485	2.848522192	1.900586306	0
Zm00001d028162_T001	2.728363539	2.396026286	2.138592348	1.649923508	2.584400462	2.97165593
Zm00001d028162_T008	3.448186429	2.069924499	3.551652713	3.018494598	3.309803955	3.357554397
Zm00001d031200_T001	3.892624073	3.514571478	3.453537465	4.309942888	4.235796589	4.102211902

Zm00001d044630_T004	1.25358937	0.961875917	1.722371153	1.587418761	1.564640586	1.867066972
Zm00001d044630_T002	0.415553171	0.298150351	0.330387446	0.865412892	1.022531161	0.756853357
Zm00001d049166_T001	9.023513809	4.177935567	9.239678265	8.827684175	8.887983899	8.63205405
Zm00001d006443_T002	0.497088072	0.310113227	0.525867421	0.773513659	0.612616713	0.394531702
Zm00001d039628_T001	2.621590786	2.231964719	2.459445259	3.451617977	3.406112806	3.292564593
Zm00001d032550_T002	0.691381401	0.345306665	0.872830335	0.629510499	0.681217156	0.717726207
Zm00001d032550_T001	4.886009352	4.081811443	4.747893114	3.77153067	3.814383586	4.154314566
Zm00001d039488_T001	0.40539447	0.16520963	0.580310492	1.070071213	1.091022751	0.726163326
Zm00001d039488_T002	2.164734155	1.534918695	1.959956692	2.588670028	2.51341065	2.545147232
Zm00001d051596_T001	5.078384439	5.168611992	4.303889804	4.427431121	4.691269498	4.327623275
Zm00001d046120_T001	5.419753628	6.139886264	5.359413885	5.730386307	5.768374245	5.612489065
Zm00001d037993_T002	6.25803329	5.895111679	6.329297893	6.251836927	6.272739524	6.223786866
Zm00001d037993_T004	0.928565891	0.611476471	1.187171039	1.097554841	1.258410982	0.928361984
Zm00001d008546_T001	6.203588623	5.8179299	6.323483599	5.885919721	5.840819634	5.959468558
Zm00001d003833_T001	4.74842334	4.501365002	5.027477878	5.209706946	5.234607426	5.229746487
Zm00001d052951_T001	4.887559339	4.138578505	4.972094271	4.625577409	4.629355629	4.787069133
Zm00001d021562_T001	2.189048694	1.799637332	2.153764486	5.103991966	5.145734795	5.159050846
Zm00001d043863_T001	1.318150795	0.974444143	1.570864595	2.057461363	2.370429634	2.252234584
Zm00001d043863_T004	4.535177566	4.340527519	4.514495482	4.646418621	4.317743265	4.501445196
Zm00001d009146_T009	8.522854756	7.464052515	8.581536608	9.037866587	9.099729186	9.063010739
Zm00001d009146_T001	1.875084886	3.613063055	1.519009916	1.492444929	1.784979638	1.715182168
Zm00001d009146_T005	3.970656466	5.394775775	4.326698981	5.122692471	5.144890116	4.738231346
Zm00001d009146_T010	3.990163157	3.462430486	4.05223772	4.097137798	4.485909341	4.622216848
Zm00001d009146_T004	5.907108642	3.496292709	5.499585573	6.025164417	6.125360752	6.304815009
Zm00001d009146_T007	0	0	2.279757429	1.220161527	0	1.736176657
Zm00001d026152_T001	2.878790266	2.247025471	2.65099381	2.036144945	2.443173778	1.806040234
Zm00001d006099_T001	0.178616493	0.162821022	0.242925609	0.34306324	0.252587629	0.315312331
Zm00001d018819_T001	0	0.025863685	0.052397808	1.07357211	1.081672991	1.476269171
Zm00001d047838_T001	3.135306004	2.371708839	3.134117417	2.967445866	2.821507162	2.69460611
Zm00001d005454_T001	1.110031098	0.985708076	1.252339962	1.121519427	1.360065873	1.268340966
Zm00001d024533_T001	3.291088091	2.584003511	3.328044832	3.762042277	3.811676528	3.676387139
Zm00001d015829_T001	4.766018021	3.559123322	4.565226097	3.839478945	3.3486167	3.551486118

Zm00001d015829_T003	3.505416388	2.961388478	3.66759334	4.953128554	5.135356328	5.007117129
Zm00001d005080_T002	2.00716392	1.514405594	2.096510153	2.36789269	2.213190793	2.323732643
Zm00001d036642_T001	5.102818515	4.924635332	5.114686182	4.32598782	4.316886671	4.110696815
Zm00001d023367_T001	0.75380561	0.249241438	0.53480169	0.677854583	0.741053588	0.616816877
Zm00001d023367_T002	3.568911078	2.723768671	3.575307126	3.455986287	3.448284371	3.421366316
Zm00001d020296_T001	3.003289919	2.267767989	2.904903888	2.858589386	2.989473296	2.897447617
Zm00001d003544_T003	1.249680765	1.352486229	0.968353271	1.609309133	1.746312766	1.800819573
Zm00001d021069_T001	3.223978359	2.827010076	3.325575519	3.648756369	4.028421428	4.041614299
Zm00001d021069_T002	2.802794783	1.724628882	2.713227963	3.678321988	3.480908762	3.53649472
Zm00001d038582_T002	3.555840568	3.272482956	3.643191012	3.09073608	3.302613995	3.287720505
Zm00001d040883_T001	3.265519111	2.576696025	3.300927309	3.539561593	3.563309715	3.685819078
Zm00001d032693_T001	2.297131535	1.950626299	2.596853825	2.564681066	2.636229485	2.725850418
Zm00001d028154_T001	2.949120274	3.310116573	2.840588514	6.411462567	6.447599627	6.465845024
Zm00001d018521_T001	9.52478213	5.713825948	9.526603192	9.244402015	9.382404841	9.40325888
Zm00001d026557_T001	2.619691662	2.235288058	2.697088332	3.97366316	4.117022736	4.060360798
Zm00001d006438_T020	1.801338723	0.33235964	1.484167011	3.56231387	3.325905168	3.329957821
Zm00001d006438_T073	1.573200966	0	2.285943091	2.591557113	0	3.451559163
Zm00001d006438_T080	4.102422897	3.839181209	4.284265593	3.866647597	3.421987776	3.515854348
Zm00001d006438_T078	0.666157579	0.621267483	1.001400898	1.620850059	1.667057368	1.126508398
Zm00001d004925_T005	2.883416713	1.92013854	2.758340065	2.832814283	2.904607408	2.699060731
Zm00001d004925_T001	1.169120074	0.631967545	1.541108389	2.374658927	2.436320708	2.406908044
Zm00001d008649_T004	1.052473629	0.829556527	1.65945378	1.945338765	1.974848285	1.763706033
Zm00001d008649_T001	2.033758461	1.504442428	1.990417243	2.337917136	2.064343464	2.27860304
Zm00001d049099_T008	5.833125888	4.861881838	5.982724102	6.277022766	6.205566603	6.089139763
Zm00001d049099_T002	0.893823489	0.830337275	1.551557852	0	0	1.144520879
Zm00001d049099_T003	4.034257101	4.245555901	4.091219476	2.877613126	3.170545547	3.275323124
Zm00001d049099_T004	6.999305401	6.24441805	7.003247489	6.236226231	6.391275988	6.379842069
Zm00001d049099_T006	1.495850679	0.977940755	1.097457081	1.15793299	1.201501504	0.883717775
Zm00001d009568_T001	5.061427835	4.522940146	4.537188575	4.780972705	4.681040856	4.626125565
Zm00001d044153_T001	4.437656075	3.74192782	4.51318632	4.030408388	4.127197847	4.248037002
Zm00001d050193_T001	7.859967716	7.482164026	7.994404038	7.191142758	7.276240431	7.294500954
Zm00001d009205_T005	0	0	0	4.126153924	4.026432524	3.343041345

Zm00001d009205_T003	0.588625178	0.394056402	0.896133095	1.147106075	0.925580217	0.713036775
Zm00001d009205_T006	1.417525264	0.669922954	1.56193706	2.46685026	2.51615487	2.392119004
Zm00001d009205_T001	2.814995365	3.546024322	3.097041022	2.908166528	3.282585402	3.526755181
Zm00001d052041_T001	2.281073862	2.529320476	2.115233714	2.333443771	2.097303686	1.657286948
Zm00001d039510_T001	2.63432724	2.23128002	3.010449735	2.024963925	2.464489749	2.555792601
Zm00001d035888_T001	6.760340243	6.823558275	6.765944345	8.003099646	8.093604172	8.217925772
Zm00001d035659_T001	7.061871989	7.166723026	7.081482607	8.229658547	8.272323649	8.304024707
Zm00001d024342_T001	5.034411669	4.100786537	5.062470209	5.019795749	5.036675506	4.951619078
Zm00001d002147_T002	1.884868267	1.293938386	2.028543339	1.69676349	1.348896949	1.948250596
Zm00001d029205_T002	1.718561973	1.019412991	1.581980255	1.446320819	1.518826166	1.463527774
Zm00001d040252_T001	2.511896331	3.093689803	2.841928851	2.478755449	2.978513793	3.374142665
Zm00001d048512_T002	0	0.060073677	0	0.59110682	0.478592902	0.401003452
Zm00001d048512_T003	0.843913114	0.471783668	0.915047382	1.274669584	1.196279694	0
Zm00001d048512_T010	1.824130768	0.536163337	1.478396683	0	0	0.954568571
Zm00001d024527_T001	2.746674362	2.208835604	2.766311683	4.092610095	4.176229968	4.133083918
Zm00001d012329_T001	6.235346254	5.350064975	6.240051834	6.017686805	6.006965598	5.936097834
Zm00001d015114_T001	2.833486812	2.074021032	2.929440126	3.07685954	3.075127661	3.144918251
Zm00001d039670_T008	0.446239288	0.118784963	0.485260908	1.338984554	1.524007993	1.846271855
Zm00001d039670_T007	0.53593947	0.259453283	1.515668051	1.630661276	1.098769207	1.830540508
Zm00001d039670_T013	0	0	2.496573813	3.227964516	2.113236415	3.111927375
Zm00001d039670_T003	2.79316426	2.704422269	3.347307064	3.757669647	3.870273568	4.015154892
Zm00001d039670_T004	1.783893692	1.702169028	2.160273218	2.411736446	2.222425294	2.908717237
Zm00001d039670_T009	5.361282772	4.391506125	5.226832483	5.432245999	5.586761681	5.002043977
Zm00001d039670_T005	0.592927234	0	0	0.720322249	0.379724087	0.936461931
Zm00001d029603_T002	1.124239453	0.65682766	1.035960968	1.216054984	0.97373249	1.263516426
Zm00001d029603_T005	4.148487076	2.994875835	4.104062857	4.211316825	4.282780125	4.253261535
Zm00001d029603_T003	1.412519785	2.887187953	2.478297268	1.718714954	1.127542159	1.176179134
Zm00001d010538_T010	6.341956509	5.688392326	5.912809453	5.278226044	5.479351093	5.301233404
Zm00001d010538_T005	6.41484248	6.091051089	6.581800603	6.576091442	6.594103745	6.57037701
Zm00001d010538_T006	3.184464865	2.232967374	3.05985951	3.546016417	3.060769588	3.22307002
Zm00001d030233_T001	1.398691848	0.653279272	1.140844083	0.413659067	0.393924651	0.259914814
Zm00001d051990_T005	0	0.334599166	0.528050154	1.104540636	0	0.53799376

Zm00001d051990_T002	0.44530824	0.174370622	0	0.08422758	0.645077232	0.454920216
Zm00001d003913_T002	2.947718328	3.228574604	2.725579758	2.839659129	3.413471145	3.076250377
Zm00001d007307_T002	0.718788151	2.340154896	1.383525698	0.902140743	1.537914765	1.439866713
Zm00001d007307_T004	0.833942544	0.586451555	1.086196788	1.405971127	1.599642959	1.181665521
Zm00001d007307_T009	0	0.178849743	0	1.702639371	1.297371632	1.221269568
Zm00001d007307_T011	4.427176317	3.215086489	4.286654231	4.927254492	4.640071079	4.929756729
Zm00001d007307_T008	0.222288956	0.149284082	0.313190117	0.455092819	0.458788296	0.332619722
Zm00001d007307_T005	0.834842254	0	0	0.848656388	1.356644158	1.483926159
Zm00001d007307_T010	1.675422128	1.124433355	1.941552951	2.665564911	2.435027657	2.682247193
Zm00001d035500_T001	5.434187079	5.592861444	5.404321507	5.447614007	5.359881799	5.515028127
Zm00001d035500_T002	1.909411761	1.8594508	1.830358776	2.075343682	1.291156182	1.388825451
Zm00001d035500_T003	2.313051641	3.816495776	3.071670696	2.407598573	2.076757803	1.968483407
Zm00001d052253_T001	4.005292455	3.542316995	4.045694232	4.932950956	5.052895264	4.7985365
Zm00001d045190_T002	1.3126553	0.714998051	1.342566123	0.8265432	0.615075359	0.896752344
Zm00001d029231_T001	3.488424131	2.931553341	3.4999298	3.757091538	4.096245827	3.642094565
Zm00001d031053_T001	2.417867364	2.019853114	2.623485073	2.08605884	2.098635825	2.01275244
Zm00001d005295_T001	5.620912153	5.126646124	5.547397541	5.042829346	5.005697345	5.065742084
Zm00001d039397_T001	1.996450575	1.986974432	1.992844188	1.610203017	2.0385763	1.828497135
Zm00001d013415_T001	2.438368711	1.919416363	2.809486063	2.414897281	2.520204728	2.468631276
Zm00001d011429_T003	0.548545131	0.352063559	0.375693398	3.418390211	3.329800715	3.525382366
Zm00001d036611_T001	1.823636059	1.094578627	0	0.823606714	0.9051537	0
Zm00001d036611_T004	0.780065639	0.45488443	0.927716722	0	0	0.673665866
Zm00001d036611_T007	0	0	0.677369322	1.40306644	1.224363239	1.367131715
Zm00001d036611_T006	0	0.148574931	0.8691947	1.461480364	0.71740949	1.174655049
Zm00001d036611_T005	0	0.043152693	0.769833507	0.310395966	0.971707242	0.816824131
Zm00001d003099_T003	2.404243461	1.745853009	2.271991826	3.521577325	3.620064483	3.511944515
Zm00001d003099_T002	0	0.69692236	0.720163746	1.39997003	1.119148308	1.393672096
Zm00001d019599_T005	2.031779464	2.404592539	1.717185289	1.743349513	1.501918053	1.20669323
Zm00001d019599_T001	3.609622126	5.715909263	2.971345449	3.031612396	3.691833058	2.628537753
Zm00001d019599_T003	4.234985152	4.238242155	4.553639737	4.100273111	4.204104712	4.288567642
Zm00001d017529_T001	5.51024764	5.808871891	5.477952275	5.514289755	5.551716504	5.624204003
Zm00001d048076_T001	1.187603075	0.638173953	1.24761525	0.689137212	1.178095049	1.299220622

Zm00001d016909_T001	5.822675217	5.592344159	5.834481163	4.860659433	4.95230803	5.084526687
Zm00001d002899_T001	7.163748988	7.039480533	7.219265449	6.165160758	6.295773121	6.229898788
Zm00001d042660_T001	4.124226134	3.480732759	4.183313015	4.148478128	4.079556207	4.073915582
Zm00001d036079_T001	4.242588635	4.102400556	4.30321342	4.123962617	4.020056941	4.024731895
Zm00001d028088_T003	3.512019761	2.691851478	3.584611282	3.66235808	3.725279049	3.618802113
Zm00001d028088_T002	0.269124135	0.107722681	0.519139283	1.478136223	1.49531244	1.530502787
Zm00001d028986_T001	1.122112096	0.846052768	1.161003418	1.018543736	1.072701282	0.793731945
Zm00001d028986_T002	0.708597799	0.534149277	0.807248571	0.852972028	0.945675866	0.87895992
Zm00001d040308_T001	2.477045201	2.754763318	2.809308862	2.185340738	2.207636142	1.954173972
Zm00001d046091_T001	1.640162321	0	1.751268321	1.239519025	1.219460361	0
Zm00001d048234_T001	2.757412039	2.687663627	2.755663997	0.964846621	1.12177933	0.85687019
Zm00001d011312_T001	2.708059408	2.038622301	2.810361661	3.274812092	3.22161063	2.998388331
Zm00001d038147_T001	9.047348292	8.554668194	9.08675626	9.059950679	9.084402774	9.052684326
Zm00001d026333_T001	4.570711374	3.94960954	4.622699466	4.209344029	4.324247814	4.109115046
Zm00001d041232_T001	2.874111744	1.954708516	2.810675274	2.705867567	2.886140009	2.842307496
Zm00001d048731_T011	1.423781989	0.917368356	1.693848213	1.677882539	1.380621409	1.240720364
Zm00001d034970_T005	1.989672742	1.58790404	2.355740532	2.936769837	2.590455431	2.758434519
Zm00001d034970_T004	1.406699923	1.145972811	1.714961137	1.374085756	1.2417743	1.465950963
Zm00001d034970_T002	2.815618873	2.058391659	2.416287414	2.708859969	2.994450473	2.865103234
Zm00001d034970_T003	0	0.059017409	0.798039728	0.829130265	0.533610191	0.128980926
Zm00001d014575_T012	0.692352205	1.059264587	0.67307062	1.216036974	0.79413801	1.460301695
Zm00001d014575_T007	0.634998374	0.220796743	0.648816985	0.644418385	0	0.706995499
Zm00001d014575_T004	1.371339724	2.485506946	1.925553253	0	1.864799847	0
Zm00001d014575_T005	0	0.34279252	0	0.950584124	0	1.223819159
Zm00001d014575_T011	0.393347002	0.207538067	0.643626264	0	0.773853736	0.653206802
Zm00001d014575_T008	4.694800257	3.350838927	4.022569599	4.89358651	4.881531551	4.825277899
Zm00001d026643_T009	0	0	1.591148001	1.557072323	1.635041573	0
Zm00001d026643_T005	0	0	1.25635673	1.204395545	1.817982147	1.216698217
Zm00001d026643_T006	0.775302596	0.769134443	0	0	0	1.042353668
Zm00001d007154_T001	7.968024654	7.752777775	8.014529253	7.411014488	7.420766084	7.516982967
Zm00001d010325_T001	2.685536317	1.679035033	2.512020772	2.158492794	2.461632279	2.151386715
Zm00001d030900_T001	4.135155513	3.154389428	4.148273925	3.545436277	3.577690574	3.539658983

Zm00001d031868_T001	4.712865752	5.636727418	4.287650331	4.538586832	4.594456169	4.887711152
Zm00001d049652_T007	4.180184867	3.123635078	4.234397315	4.13962502	4.066488094	3.990841799
Zm00001d049652_T003	2.374896754	2.552329604	2.338442101	2.935087051	3.109929334	2.771183071
Zm00001d002034_T005	4.905886756	4.621100587	4.96264466	4.775524161	4.851224979	4.616876087
Zm00001d002034_T001	4.203737016	3.294898308	3.901676422	3.954619749	4.055737344	3.986628188
Zm00001d002034_T004	1.161476254	2.491974919	1.305968187	0.724993335	1.001125584	0.327409144
Zm00001d052378_T001	2.907393143	2.312811527	2.926619834	2.889456604	2.806001658	2.727835088
Zm00001d020444_T004	0.535840958	0.620963573	0.82642605	0.557482634	0.275255027	0.069696668
Zm00001d020444_T003	3.540553301	3.011039894	3.739558995	3.32977747	3.452878728	3.413453678
Zm00001d047637_T001	0.594849478	0.437368379	0.847818963	0.888936861	1.006631951	1.076447588
Zm00001d006351_T001	0.390533424	0.137002428	0.493812292	0.905359372	0.742852171	0.883478495
Zm00001d003269_T001	2.790451148	4.227288469	2.751131825	2.448534946	3.012832875	2.87291266
Zm00001d017857_T003	1.723389491	0.826695317	1.266158063	1.980592027	2.55919223	2.340098766
Zm00001d017857_T001	8.751405607	7.97232827	8.727752068	8.767854975	8.778853426	8.750972736
Zm00001d017857_T004	1.798727831	0.549451333	0.809780731	0.705827544	1.540814881	0
Zm00001d028785_T001	5.424942708	5.039853553	5.345146501	5.740498896	5.845346226	5.679260512
Zm00001d028785_T002	3.181086167	4.918097523	3.786561653	3.246810435	4.191613024	4.096370237
Zm00001d033292_T003	3.428404529	2.556431623	3.568416083	3.33246176	3.47127735	3.49702725
Zm00001d033292_T004	5.555816155	4.821026507	5.649519123	5.783324794	5.783025143	5.739606814
Zm00001d033292_T002	0.684728987	0.279851606	0.533345059	0.688930499	0.675861085	0.843095449
Zm00001d033292_T001	0.901903727	0	1.484155665	0.272928547	1.101387866	0.249906444
Zm00001d036142_T001	4.005820428	4.562125905	3.465362005	3.299167421	4.141746098	3.941078791
Zm00001d042929_T001	5.632080013	5.23146432	5.547961166	5.745711052	5.7386251	5.78466455
Zm00001d052998_T003	2.019396977	1.341849723	1.782083083	1.859470281	2.093241044	1.984278358
Zm00001d040730_T002	2.809613856	2.260932853	2.978162491	4.523032892	4.493856154	4.278693799
Zm00001d045931_T001	4.470800066	4.839294555	4.01262722	5.938645856	6.105465777	5.953781476
Zm00001d045318_T001	4.626368285	4.868185369	4.091827936	3.961034277	3.750786348	4.180854313
Zm00001d012307_T003	0.597357635	0.659076954	1.274856212	1.012219667	1.000305819	0.76039044
Zm00001d037367_T001	3.308626485	2.382670019	3.378297978	4.157560368	4.21254506	4.209447751
Zm00001d037367_T002	0.323870386	0.120069213	0.934271157	0.774866742	0.599865247	0.192464369
Zm00001d025941_T003	1.318603182	0.636657563	1.220747842	2.299392672	2.294113713	1.972040445
Zm00001d025941_T001	2.599752917	2.531926618	2.803219182	2.682765644	3.0957874	3.088148213

Zm00001d025941_T002	2.727499888	4.337558681	3.195939517	3.740890117	3.559826201	3.561749412
Zm00001d007934_T001	2.941734723	2.27264643	3.115293651	2.814535248	2.925930319	2.894157319
Zm00001d007934_T002	1.833049157	0.369397244	0.993919228	1.878299237	1.351472293	1.590680098
Zm00001d038351_T001	1.231281249	0.701625348	1.094828568	2.632310332	2.633019385	2.575644645
Zm00001d038351_T003	6.752279733	6.849883974	6.805130904	6.477155632	6.561376883	6.539570293
Zm00001d009452_T020	3.891961748	0	2.585371928	2.110420375	3.054179057	3.713222353
Zm00001d009452_T013	0	0.186965745	0	1.383969655	0.565314458	0.599932832
Zm00001d009452_T014	1.694271793	0	3.52965993	3.817814581	3.814983065	2.616302171
Zm00001d009452_T021	0.114357696	0	0.224697243	0.959718232	0.811672418	0.784061684
Zm00001d009452_T008	3.10382609	0.874866198	2.478426972	3.376787127	2.967855477	3.130358784
Zm00001d028425_T005	0.952204575	0.670048086	0.747444977	0.247910505	0.85687258	0
Zm00001d028425_T007	4.107562423	3.527187969	4.029234831	4.154870106	3.957811731	4.291129875
Zm00001d001857_T001	2.807858335	2.375219079	2.895525407	0.405789824	0.275836657	1.005116092
Zm00001d050915_T003	0.991239379	0.691699418	1.182952213	0.912222166	1.29849703	1.439766735
Zm00001d050915_T001	5.787987369	5.358524792	5.882179699	6.058462078	6.159454973	6.064057415
Zm00001d037551_T001	2.440318655	1.647935667	2.654721485	2.453675865	2.442049876	2.226295501
Zm00001d004875_T006	1.978666077	0.71133543	1.811860218	1.849188641	1.786426992	2.079098164
Zm00001d004875_T002	2.474195899	1.468011339	2.34448255	2.915180694	2.981703811	2.95386529
Zm00001d004875_T004	3.313572381	2.765392804	3.394295855	2.520233649	2.759434543	2.714123062
Zm00001d004875_T005	0	0.324479984	0.397244391	0.520433312	0.734778543	0.621366897
Zm00001d004875_T009	0.783101228	0.099666857	0.52110402	1.542233282	1.182063603	1.329408375
Zm00001d032609_T005	0.412700244	0.36653876	0.515618604	0.562701083	0.844285211	1.10738524
Zm00001d032609_T004	3.645337366	4.001811698	3.632124288	4.141756642	4.17497664	4.396697659
Zm00001d032609_T002	2.763776345	2.106786489	2.614666033	3.27047213	3.062479405	3.202151727
Zm00001d006254_T001	3.78791008	2.934547827	3.738329018	3.202724565	3.116890034	3.005273859
Zm00001d005010_T001	3.107823429	3.465038955	3.239115249	4.849251906	4.68969909	4.812672314
Zm00001d017509_T001	4.525366015	5.125406568	4.302919111	4.303997903	4.425003557	4.391187694
Zm00001d018259_T004	2.687703709	2.920125389	2.941333405	3.089237961	3.080920258	3.137754006
Zm00001d018259_T002	4.077424094	4.331867919	3.80535138	4.124016483	4.115984984	3.977714224
Zm00001d049554_T001	3.160722578	2.333286328	3.177749282	4.188846436	4.163183189	4.212436113
Zm00001d009608_T001	5.413325888	4.825087461	5.461542769	5.274438874	5.413133723	5.366913476
Zm00001d000424_T001	5.1276913	4.597577237	5.151364145	5.660265674	5.687547768	5.740754551

Zm00001d030920_T001	2.271346188	2.65439819	2.412702141	2.684768029	2.186736339	2.388233906
Zm00001d030920_T003	2.1044722	2.278794809	2.594655315	2.046360091	1.605999639	1.978062339
Zm00001d030920_T002	2.227585833	3.459475161	2.597757125	2.423485126	3.208643357	3.010661161
Zm00001d011231_T004	0	0	0.111210302	0.696045464	0.82692631	1.009250416
Zm00001d011231_T007	2.734275202	0.439443355	2.048406397	3.861346903	3.838762319	3.936757213
Zm00001d011231_T008	3.26013935	3.103228043	3.217954054	3.189388443	2.753092742	2.829336104
Zm00001d011231_T001	2.903378845	2.470891086	3.268760146	3.738245022	3.917333122	3.964634657
Zm00001d033606_T001	3.292319424	3.45159912	3.691857172	3.578955615	3.510022343	4.060264291
Zm00001d042212_T012	0.355986007	0	0.334684968	0.750044752	1.417349728	0.566962216
Zm00001d042212_T006	0.792119869	0	0	1.427904335	0.938605728	1.365342038
Zm00001d042212_T005	2.931989148	2.228746054	3.112095909	3.228037654	2.991985429	3.117251362
Zm00001d042212_T011	0.37968306	0.276262004	0.32392571	0.485721519	0.668489511	0.404244824
Zm00001d042212_T010	0.385228908	0.359460448	0	0.893727207	1.337016262	1.21722265
Zm00001d020799_T016	0.262727801	0	0.504182052	0.66904582	1.239427372	0.536003151
Zm00001d020799_T010	1.843281615	1.895505825	2.03061788	1.699806125	0	1.642756553
Zm00001d020799_T017	0	0.090217963	0.443352036	0.668571201	0.350187176	0.669664495
Zm00001d020799_T006	1.104958562	0.551228557	1.011106288	1.71428245	1.774246033	1.391089648
Zm00001d035562_T007	3.182714065	2.530336727	3.714958061	3.217829233	2.630080642	3.146553239
Zm00001d035562_T004	1.166439732	0.795109441	1.26529346	0	0.518076828	1.084738499
Zm00001d022617_T001	3.135564091	3.301981627	3.303711059	2.93970163	2.772763391	2.698723889
Zm00001d015355_T001	3.383840435	3.956281591	3.27668797	3.444871902	3.509063907	3.414768908
Zm00001d030018_T001	3.655886214	4.224553556	3.997297918	3.603306231	3.469617855	3.369918276
Zm00001d017110_T002	2.05328584	1.567220756	1.976832848	2.101044232	2.040155293	2.411497836
Zm00001d017110_T003	0.579951504	0.036864797	0.404519515	0.763154884	0	0.637996891
Zm00001d021422_T001	10.52620553	9.831740048	10.34261769	9.484676094	9.523243787	9.552844492
Zm00001d005583_T001	3.91231358	4.856432899	3.78886421	4.381770511	3.300289369	4.079131795
Zm00001d051178_T001	5.362149578	4.659792096	5.372724187	5.42059282	5.406329924	5.459627845
Zm00001d052351_T001	2.213549789	1.819873743	2.063731408	4.785957096	4.076202653	4.627437638
Zm00001d013359_T003	2.389589113	2.791393597	2.248187175	1.696633088	1.891627993	2.221443125
Zm00001d013359_T002	2.885253578	3.047331078	2.946321945	2.294536353	2.380334925	2.433331788
Zm00001d002703_T002	3.039302311	1.322349877	2.905557314	2.069535137	2.439374467	2.097926604
Zm00001d002703_T001	2.460465529	2.306184357	2.721983734	3.679402853	3.596891742	3.457936343

Zm00001d007107_T001	0.329666694	0.11083894	0.333368773	0.240035839	0.332806449	0.377719033
Zm00001d004707_T010	1.48134803	1.425111748	1.52314389	0.429539911	0.958912442	0.647942573
Zm00001d004707_T004	0	0	0.892974592	1.674286632	1.112106921	0.631256112
Zm00001d004707_T001	1.43246667	0.367393433	1.025283282	1.552751346	1.496950579	1.837982789
Zm00001d052206_T001	4.26139534	4.097770563	4.143623291	4.756678111	4.739417228	4.75070779
Zm00001d038195_T001	3.872696892	3.355661751	3.916020547	5.121371139	5.146115332	5.121722646
Zm00001d013992_T002	1.298545124	0.167575022	1.671996204	0.230682602	0.977314365	0.981092756
Zm00001d013992_T001	1.842517095	1.5602767	1.382821058	1.793344911	1.81082969	1.613234125
Zm00001d027675_T002	4.556112883	4.777682612	4.582963946	4.884335243	4.925477249	4.680010857
Zm00001d022366_T001	4.439802699	3.6484009	4.508971906	4.650902906	4.592971711	4.504801449
Zm00001d022366_T004	0.423129562	0.08209623	0.4396125	0.68444085	0.652266202	0.503056604
Zm00001d004417_T001	1.525471573	1.837817732	1.707975728	0.847210423	0.518049627	0.676163581
Zm00001d029673_T001	4.548869615	4.349868972	4.837084936	4.633542809	4.525449647	4.540036757
Zm00001d029673_T003	3.700440273	2.304507829	3.646521443	3.496440235	4.142267527	3.740421503
Zm00001d020852_T001	3.324457085	2.478271378	3.356047158	2.455590515	2.325670205	2.249730211
Zm00001d021329_T001	3.595352556	2.968744011	3.594280647	4.528664141	4.477125916	4.510960907
Zm00001d005830_T001	1.425612912	2.092434448	1.408808581	0.385105186	0	0.630488415
Zm00001d010627_T001	2.489585579	2.991266957	2.616075812	1.503354842	1.867862867	1.074758879
Zm00001d032572_T002	0	0.156223146	1.772642847	1.383357589	0	1.188317361
Zm00001d032572_T001	2.672290458	2.377085703	2.514218731	2.622053929	2.706048211	2.928886284
Zm00001d004013_T001	2.128984554	1.680484147	2.350197928	1.755399515	2.272037526	1.665191726
Zm00001d044940_T002	3.11728478	2.29020114	2.870915847	3.464924506	3.474066074	3.663993515
Zm00001d044940_T005	0.325884882	0.117514196	0.308853612	1.987571542	2.152254497	1.986240532
Zm00001d017100_T001	1.294354901	1.569582473	1.066483961	0.737698009	1.209612443	1.217501235
Zm00001d017100_T004	4.705831414	4.926486578	4.328291259	3.924971702	3.748391673	3.221038745
Zm00001d017100_T006	3.461908697	3.372944021	4.011188425	4.490550043	4.533509215	4.588522823
Zm00001d017100_T003	1.008459638	0	0.949683584	1.197880485	1.186615918	0.532645113
Zm00001d017100_T002	2.066431951	2.721623332	2.233128483	1.975408978	1.569004927	2.633077992
Zm00001d017100_T005	0.452806263	0.599212087	0.47504129	0.469816184	0.703004103	0.847840606
Zm00001d045373_T001	0.959525358	0.458984576	0.920124817	1.166324033	1.408810754	0.650324332
Zm00001d032112_T001	3.379987965	2.563595999	3.174856831	3.323916201	3.381759992	3.33199407
Zm00001d033111_T001	1.977939657	0.913870945	1.804006047	1.787695319	2.029461367	2.070097668

Zm00001d033111_T003	2.182938869	1.84792998	2.456140622	2.604818105	2.618329115	2.387742742
Zm00001d021948_T001	5.34654061	4.808217396	5.357021136	6.449023516	6.543715704	6.475108414
Zm00001d033847_T007	0.8775134	0.528895326	1.162422106	1.507697231	1.886740543	0.827012516
Zm00001d033847_T003	5.300255209	4.482193308	5.39862321	5.327475579	5.474807475	5.248027663
Zm00001d012729_T010	3.022351122	2.497628459	2.679010479	2.166240145	2.365763635	2.42691201
Zm00001d012729_T001	2.47267492	3.531108555	2.784448284	1.97072042	1.808891192	2.033852883
Zm00001d012729_T017	3.428720211	3.914043509	3.886149083	3.110144551	2.944811979	3.759448818
Zm00001d012729_T011	2.555176615	2.834409055	2.927596698	2.197667589	2.101349539	2.312981672
Zm00001d012729_T015	3.498832042	4.66454297	3.079344316	3.072798334	3.218556146	2.637693472
Zm00001d047238_T001	1.493193346	1.668213546	1.694988474	1.301996109	1.51607467	1.333164941
Zm00001d047965_T001	4.864712106	3.903508805	4.539388817	4.807190652	4.74132093	4.756257145
Zm00001d024961_T001	1.067331699	0.689978968	0.83328035	0.612808238	0.537392492	0.732250094
Zm00001d026623_T001	7.130383466	6.346859495	7.006102951	6.899113261	6.841614903	6.945509725
Zm00001d040581_T001	7.557955004	6.953848788	7.523048791	6.928300402	6.93689952	6.870829858
Zm00001d014752_T001	4.311593426	4.83311946	4.719808144	4.461883558	4.090983132	4.318623716
Zm00001d029558_T001	9.831565227	9.831994694	9.795481881	7.698250794	7.544461105	7.580860066
Zm00001d046226_T006	0	0	0.57708121	0.639259952	1.284413449	1.2654591
Zm00001d046226_T011	0.769968034	1.320790226	1.201967524	0.575703451	0.809124641	0.708301146
Zm00001d046226_T002	1.285059483	0.916981022	1.377169293	2.327868121	0.717691117	2.043668217
Zm00001d046226_T001	0.309867677	0.18040378	0	1.068509853	2.11598174	1.239834878
Zm00001d046226_T010	3.788925479	2.690004902	3.735376601	3.767420897	3.65598018	3.533424928
Zm00001d014922_T001	1.068181002	0.922544206	1.230695512	0.836266724	1.235423174	1.239439593
Zm00001d020838_T001	2.786167253	2.577403207	2.372012926	2.763144896	2.616054869	2.361045989
Zm00001d020838_T002	0.386597975	0.218400531	0.595739476	0.530451348	0.72140941	0.685413642
Zm00001d038775_T002	5.556036898	4.708556362	5.604650036	5.060505505	5.008086817	4.997825212
Zm00001d028816_T001	4.897768994	5.097751057	5.009846663	3.641195023	3.665143962	3.607556629
Zm00001d038843_T001	4.643952616	4.178265512	4.783048409	4.538942918	4.337794141	4.470881847
Zm00001d006651_T001	6.153436591	4.803135315	6.027742305	6.202936266	6.272822125	6.544751895
Zm00001d006651_T005	2.840106872	4.674032457	3.121843303	2.803830445	2.763551588	2.457879231
Zm00001d006651_T007	4.897794982	0	5.101461576	4.101254726	3.980718596	0
Zm00001d026690_T001	5.982461082	5.336310135	5.824442956	5.304517649	5.287779301	5.236513474
Zm00001d026690_T002	5.102663968	5.139837898	5.181436662	3.282392209	3.589420507	3.289655666

Zm00001d030131_T001	4.715571144	4.068821435	4.624560015	6.708434002	6.708393526	6.581288407
Zm00001d030131_T005	1.024723209	0.414002355	0.665511929	4.003245656	4.290396461	4.288660064
Zm00001d030131_T006	0.445549798	0.2204154	0.47612242	4.26652196	4.19315204	4.158638931
Zm00001d050250_T003	1.935043664	2.580631769	1.924654428	1.980203752	2.168283884	2.198509869
Zm00001d006619_T001	2.78582128	2.004731486	2.76176992	3.757547835	3.824699128	3.779575019
Zm00001d044494_T009	0.229063659	0	0	0.624167353	0.418083058	0.274274783
Zm00001d044494_T026	0.152719502	0.188208468	0.247723396	0.470978261	0.545416878	0.573754541
Zm00001d044494_T013	1.896789537	0	0	2.838981004	2.3735466	2.877253258
Zm00001d044494_T025	0.768071658	0.323371222	0.865769194	0.574310811	0.314437832	0.866986291
Zm00001d044494_T010	2.559279125	1.587878605	3.135408292	2.43198419	2.703401968	2.940296634
Zm00001d044494_T024	0.722802614	0.666061204	1.000023083	0.35988208	0.379043103	0.707365759
Zm00001d044494_T022	1.921028754	1.831912903	1.307204672	1.052648209	1.064706297	1.808221515
Zm00001d044494_T014	0.282023395	0.069707665	0.255974403	0.336036537	0.331461021	0.499499471
Zm00001d044494_T017	2.304514838	2.507512542	2.67543229	1.627206657	1.65946337	1.953265984
Zm00001d044494_T002	1.457087303	1.220758365	0.90032005	1.207086341	0.663067668	1.177898025
Zm00001d044494_T001	0	0.254803237	0.752845339	0.618962171	0.444358566	0.785873579
Zm00001d044494_T016	1.318217332	0.482371263	1.012933324	0.63604499	0.835928924	0.759155834
Zm00001d044494_T003	1.706631333	1.266450747	1.877198867	1.383783353	1.668839384	0
Zm00001d044494_T021	1.086029618	0.394426337	0.613410952	2.689780873	2.530686304	2.764631506
Zm00001d044494_T005	2.219964591	1.543171674	1.776715477	1.399352137	1.682610151	0.85599448
Zm00001d027645_T001	3.950229314	3.117684904	4.001651932	2.121222338	2.112818212	2.250169996
Zm00001d053107_T002	5.592809251	4.85155497	5.722311254	5.368961627	5.42206443	5.403520001
Zm00001d053107_T003	0.483259096	0.385927957	1.147327541	1.10844818	1.032605874	0.851289704
Zm00001d053107_T001	0.124226216	1.119079897	0.125628621	0.502392728	1.103359994	1.137430076
Zm00001d003847_T001	2.968555105	2.172358218	3.043716331	3.785212698	3.765657052	3.948884406
Zm00001d007835_T001	3.260254843	2.099083077	3.035565145	2.529199517	2.586287008	2.935849506
Zm00001d007835_T004	2.69203076	3.380461273	3.017736661	0	0	0
Zm00001d007835_T002	5.963802323	5.173911906	6.000605579	5.401113151	5.329935835	5.290615419
Zm00001d045838_T001	1.555894667	1.216528123	1.811487882	1.005347525	1.454733382	1.77928746
Zm00001d016474_T001	5.881891038	4.982099534	5.980295597	5.57041232	5.638452654	5.540733653
Zm00001d053593_T001	10.12991954	11.7461351	10.14055757	10.13117433	10.14939397	10.18386054
Zm00001d047113_T001	6.814562215	7.586664935	6.54316298	5.750793636	5.375133708	5.757442362

Zm00001d054003_T013	0.264296212	0	0.667511594	0	0.593833713	0.759836744
Zm00001d054003_T021	2.443750383	1.610270118	2.467353309	2.487284131	2.766135673	2.872299642
Zm00001d054003_T008	0.112838892	0.151056086	0.257177213	0.513179433	0.375143997	0.582556003
Zm00001d054003_T010	0.763475315	0	0	2.360183893	1.503679474	0
Zm00001d054003_T001	4.634357737	4.172345655	4.583412093	4.340653414	4.262621075	4.291450175
Zm00001d034409_T001	4.239891688	3.692171512	4.380445341	4.40388026	4.487478825	4.291025598
Zm00001d003427_T001	3.525956723	2.717742655	3.597882122	3.1816307	3.257306521	3.185547373
Zm00001d008575_T002	1.000646904	1.003767004	0.534644341	0.997545331	0.95049305	0.908833655
Zm00001d043068_T001	0.579338508	0.505749701	0.926948248	0.669576518	1.088180276	1.214561279
Zm00001d040783_T001	5.213756078	5.825817727	5.127163874	5.012429001	4.725622181	4.845572094
Zm00001d017128_T002	2.126022968	1.303999875	2.111923704	2.674624934	2.979063	2.79021153
Zm00001d017128_T005	0.174094451	0	0.590038579	0.625698792	1.080180097	0
Zm00001d017128_T008	2.735662974	0	2.780167502	2.702172796	2.256377565	2.465780438
Zm00001d017128_T001	0.822385083	1.815457795	1.122752885	1.992561074	2.182701513	2.285001168
Zm00001d037268_T008	0.485414461	0.149073325	0.445043327	0.511059108	0.46158723	0.548864684
Zm00001d037268_T002	1.101085257	0.641103905	1.089615433	1.927129214	1.699273994	1.607409386
Zm00001d037268_T007	1.688818182	1.461008278	0.832877055	1.116523125	1.341245714	1.283160824
Zm00001d037268_T004	0.517473247	0.175960138	0.474011285	0.427793872	0.659654256	0.499490287
Zm00001d047126_T014	2.371043024	1.143151099	1.597109686	0.979322981	0.75080371	1.050693928
Zm00001d047126_T004	2.918951752	2.298725757	3.089102169	2.752309937	3.31892373	3.244391923
Zm00001d047126_T005	0	0.192221946	0.53273088	1.36740909	1.000336109	1.495434738
Zm00001d004677_T001	6.7482957	6.08297043	6.700553298	5.848990922	5.996564146	6.059425931
Zm00001d039528_T001	2.630600008	1.546188709	2.503288176	2.849935627	2.898174293	2.934113359
Zm00001d020960_T001	3.087412601	2.13795954	3.073939311	4.127939366	4.183502156	4.087729121
Zm00001d014508_T003	0.199401603	0.222029229	0.217400727	1.359580204	1.43728421	1.107863928
Zm00001d034591_T004	0.57710732	0.228513345	0.435271209	0.632573476	0.43346266	0.78825054
Zm00001d034591_T001	3.186689756	2.292810603	3.180166643	3.700078777	3.667999614	3.485855321
Zm00001d029733_T001	4.288715426	3.756156088	4.107122898	4.26479995	4.406310398	4.215805545
Zm00001d041983_T015	0.35790215	0	0.388225914	0.547143785	0.490698478	1.062675053
Zm00001d041983_T001	1.38355224	1.093318805	2.134968713	1.559437482	1.710293556	0
Zm00001d041983_T023	0.222125704	0	0.102617823	0.594711918	0.802421548	1.441131262
Zm00001d041983_T019	0.557297346	0.243220606	0.349356218	0.933740313	0.772504769	0.77179348

Zm00001d041983_T025	0.629184069	0	0.404776717	1.02987194	0.504803419	0
Zm00001d041983_T020	0.882604707	0.703657111	0.575355901	1.116032659	1.28475397	0
Zm00001d041983_T021	0.912851407	0.265186023	0.205712798	0.228904865	0.517563951	0.292016034
Zm00001d041983_T018	0.251464611	0.20479429	0.335228263	0.36527372	0.464064927	0.214875806
Zm00001d041983_T012	0.596133688	0.398285787	0.39163466	0.888362571	0	0.869606131
Zm00001d043981_T007	0.657522939	0	0.357421382	0.422534428	0.303563341	0.271577468
Zm00001d043981_T005	4.23328244	3.046619923	4.202592464	4.344108958	4.18936219	4.254833616
Zm00001d025065_T001	1.208299372	0.536850639	0.655273967	0.317246174	0.339940053	0.409838408
Zm00001d035201_T001	8.588490022	7.88684677	8.619123485	7.997428004	8.031779728	8.044946855
Zm00001d035201_T002	0.796817007	0.179005222	0.784297091	0.786280189	0.601606197	0.537911287
Zm00001d043911_T001	5.145859492	4.522216423	5.210284002	6.315836258	6.328270539	6.284348482
Zm00001d035922_T001	0.742808204	0.944865191	1.398310417	0.263879337	0	0
Zm00001d035922_T012	0	0.37860595	0.699595161	0.921667113	0.728478697	0
Zm00001d035922_T008	0	0	0	1.661656123	1.643128888	2.229395653
Zm00001d035922_T004	1.577674383	0	1.00940594	1.171155576	1.594769236	0
Zm00001d031809_T002	4.329236208	3.961411538	4.325520762	4.327852891	4.315161086	4.335952173
Zm00001d030164_T120	0.849542167	0	0	2.473538606	2.073029104	2.624515507
Zm00001d030164_T050	0.513218857	0	0.147681816	1.002824915	0	0.954682463
Zm00001d030164_T096	0.365685819	0.03124979	0.149827746	0.544402294	0.724888591	0.704628544
Zm00001d030164_T024	0.402268126	0.194827126	0.422945559	1.143134115	0.761861385	1.286277928
Zm00001d030164_T068	0.149929172	0.047028784	0.196516401	0.487196135	0.183617359	0.648203111
Zm00001d030164_T059	1.163388594	1.25969672	0.772595978	2.119795393	1.716339066	1.709759987
Zm00001d030164_T121	4.592698014	3.716453761	4.61541047	3.856406504	3.987994391	3.866816905
Zm00001d030164_T056	0.188427512	0.189079382	0.106212956	0.364179069	1.172771034	0.572367779
Zm00001d030164_T040	0.439892238	0.041685958	0.160148308	0.441832956	0.330783248	0.540355658
Zm00001d030164_T115	0.228758365	0	0	0.540152284	0.430678146	0
Zm00001d006375_T001	1.27999479	0.810857652	0.978138507	2.025662094	2.07298453	1.664281668
Zm00001d043469_T004	0	0	0	2.121921195	3.386114395	3.278931558
Zm00001d043469_T005	3.663761768	2.408961255	3.259694159	5.078939219	4.99425662	4.914538549
Zm00001d043469_T015	0.585177927	0.28892903	0.651600529	1.933440826	1.918920849	2.03562778
Zm00001d043469_T002	0.834131109	1.194885106	1.719858512	3.503279907	3.58214409	3.651939601
Zm00001d053640_T001	2.953815389	2.204064009	2.895030927	2.783820582	2.69100858	2.671334365

Zm00001d035079_T001	7.377726676	8.132302615	7.196957132	2.963461177	3.055191489	3.062048643
Zm00001d048556_T002	4.010335059	3.409235592	4.144826259	3.884645098	4.110135528	3.90844383
Zm00001d048556_T001	0.853001581	0.37021116	0.893707018	0.926456453	1.039471123	0.980591296
Zm00001d028183_T002	4.185480771	3.919130385	4.265527287	4.683900045	4.504301058	4.564705878
Zm00001d028183_T008	5.069458876	3.631878868	4.931351369	4.072054851	4.351800529	4.038005732
Zm00001d006170_T001	3.613590467	4.163832156	3.515491444	4.597008229	4.897693883	4.861017159
Zm00001d038779_T002	2.5543693	2.278526326	1.807939301	2.241069829	2.158327008	2.392534223
Zm00001d038779_T005	0.446166224	0.3522783	0.676416365	1.031328142	1.247278003	0.979461277
Zm00001d038779_T004	1.132570263	0.384847767	1.430313645	2.174038826	2.455541595	2.350899731
Zm00001d038779_T001	3.185099335	2.464615733	3.304717648	3.546029015	3.274389299	3.331789042
Zm00001d051564_T002	4.925731926	5.355091238	4.593721092	4.327264265	4.189836338	4.631565429
Zm00001d051564_T001	0.9267942	0.514491947	0.94643518	0.794090586	0.927257811	1.13883609
Zm00001d014977_T007	2.084074473	0	2.01050058	3.579901195	3.474996009	3.612947856
Zm00001d014977_T035	2.360858108	1.259683465	2.527425709	1.468455072	1.325385263	0
Zm00001d014977_T005	5.024646802	3.629647695	5.026380555	4.808274965	4.585175884	4.715943655
Zm00001d014977_T028	0.620282357	0	0	2.456465801	3.009216908	2.50113567
Zm00001d014977_T002	0.242553729	1.112399888	0.145295294	0.609396607	0.811220284	0.829942896
Zm00001d042804_T003	0.341427911	0.421290635	0.307531113	2.775265507	2.682267872	2.069088599
Zm00001d042804_T002	2.956705162	2.202984657	2.863889303	4.022644606	3.978504732	3.785078643
Zm00001d042804_T004	0.281533276	0.109368584	0.222159098	1.595471197	1.673563208	1.409412118
Zm00001d041914_T004	1.895882685	0.488039859	1.704170804	1.060647171	1.634649488	1.117322021
Zm00001d041914_T001	0.633132643	1.103514425	1.001715786	1.00252755	0.702012951	1.066010637
Zm00001d041914_T003	0.057479386	0	0.053622968	0.292441351	0.312023833	0.307792213
Zm00001d051183_T011	0.147554183	0	0	0.372196574	0.358532423	0.975831189
Zm00001d051183_T019	1.561735239	0	1.813094895	2.030479105	2.10301918	0
Zm00001d051183_T003	0	0	0.472187244	1.233752494	0.562477389	1.197533923
Zm00001d051183_T001	3.101356095	2.50167327	2.965975585	2.5252295	3.100205145	3.180243359
Zm00001d049966_T001	7.480487238	3.797575208	7.072631008	7.662333988	7.780577095	7.739727909
Zm00001d041997_T001	0.929672851	1.123050321	1.277872897	1.489316341	1.380153142	1.449924743
Zm00001d028888_T001	7.310427151	6.556225811	7.296575002	7.584705836	7.64357014	7.658539544
Zm00001d029850_T001	3.267229998	2.454371749	3.165595878	2.786529662	2.846983659	2.949987933
Zm00001d022194_T001	2.05599211	1.928165251	2.218050182	1.57237069	1.694807109	1.816778266

Zm00001d002013_T001	3.689845939	3.203748368	4.003329502	2.237987654	2.553958348	2.454893377
Zm00001d004651_T001	3.527631761	2.826873038	3.810421715	3.912763188	3.818386763	3.862893208
Zm00001d000360_T001	0.902797544	0.475284148	0.867452915	0.577131496	0.575880583	0.394807151
Zm00001d024306_T001	4.096144773	3.45302391	4.118629583	4.51702216	4.649587812	4.526501493
Zm00001d019001_T001	1.157553415	0.932848087	1.309044604	1.317680324	0.957011192	1.4747714
Zm00001d034534_T002	2.316877913	1.447973735	2.10281734	3.185441443	3.133466367	3.105109637
Zm00001d034534_T003	2.881513491	2.49273409	2.651510065	3.654702584	3.641384642	3.603507661
Zm00001d023962_T001	2.880607757	3.349568248	3.193799809	2.819749716	2.951010863	2.93950136
Zm00001d023962_T003	5.110907743	4.180887403	4.987641571	4.913139829	4.815438786	4.851355525
Zm00001d023962_T002	1.195313578	0.696594811	1.310710636	0.791658231	0.747255048	0.839213062
Zm00001d023962_T004	1.858371365	0	2.229912108	1.302249437	1.522858753	2.109894074
Zm00001d007085_T001	3.153292683	2.722021562	3.069769695	2.217188521	2.544419853	2.31553435
Zm00001d042154_T027	1.035963079	0.863149509	1.019625064	1.018617084	1.21049672	1.420829998
Zm00001d042154_T026	0.915533138	0.295323363	0.6097266	1.451670063	0	0.840737144
Zm00001d042154_T001	3.411209147	2.629105015	2.861186979	3.92248398	4.02242552	3.658930309
Zm00001d042154_T025	1.182736785	0.594624026	1.462772178	0	1.357382531	0
Zm00001d042154_T020	0.823698009	0.075104037	1.322379299	0.960413847	0.654513448	1.354000719
Zm00001d042154_T008	1.210717392	0	1.432419097	0	3.068882471	2.487012405
Zm00001d042154_T003	2.14184932	2.31815282	3.51743331	1.159755755	0.898129394	1.328020697
Zm00001d042154_T015	1.469172786	0.955254023	0.867912274	1.315595211	1.455951842	1.706336486
Zm00001d002479_T001	2.886246359	2.875899955	2.996351684	1.407103079	1.715226986	1.626054523
Zm00001d011927_T002	2.490550877	1.832850732	2.327001202	2.891908881	2.709470851	2.805118248
Zm00001d020337_T001	2.978389136	2.288746871	3.027226255	3.351298003	3.633043921	3.52055262
Zm00001d005767_T001	4.512439545	3.923095162	4.416412003	4.40188682	4.514373715	4.458061917
Zm00001d021746_T001	3.075355663	2.916975482	3.080613325	2.277339406	1.915883776	1.959495683
Zm00001d002796_T002	2.484939835	2.305348671	2.830235039	2.499065951	2.522038419	2.432970361
Zm00001d002796_T006	2.372417255	0.684715524	1.747683428	0.865778694	0	1.426987129
Zm00001d039267_T008	4.842825355	3.705029391	4.971752758	5.095208421	4.826549657	4.755572406
Zm00001d039267_T013	2.548256092	2.027145203	2.254334625	2.400309758	2.554918608	2.490098225
Zm00001d039267_T012	0.543384027	0	0.670214005	0.892546459	0.865511874	0.963276572
Zm00001d039267_T004	4.142616512	3.680848223	4.023379653	5.340212875	5.55814914	5.431412958
Zm00001d039267_T010	0.693068055	0.441300741	1.677098659	1.803755239	1.20588855	2.327443637

Zm00001d039267_T014	1.510221652	0.267166613	1.726700453	2.048530903	2.011040789	2.2390989
Zm00001d017761_T001	4.878505046	4.428305429	4.88526588	5.728395649	5.79693752	5.652069516
Zm00001d017761_T002	1.9655442	2.826116656	3.789374324	1.354541868	1.030319126	0.751378879
Zm00001d013890_T012	0	0	1.357991643	0.946853606	0	2.196875479
Zm00001d013890_T004	1.70885688	1.243236451	0	2.549537092	1.787640161	1.479086699
Zm00001d013890_T015	0	0.057362928	0	0.614696654	0.809342822	0.648162606
Zm00001d013890_T011	0.120625323	0.298883499	0.93141151	0.833750717	1.060222434	0.200788069
Zm00001d013890_T025	1.732287871	2.125758539	2.658864519	1.823498701	2.208675504	2.027672139
Zm00001d030516_T001	1.65359571	1.164025477	1.680135286	1.585500526	1.260942786	1.370855604
Zm00001d028307_T001	3.422569411	2.740855478	3.358005573	4.884041394	4.951564576	4.741892667
Zm00001d030016_T001	1.296614223	0.973700902	1.564561573	2.538893455	2.753271848	2.561782887
Zm00001d033090_T001	5.057983191	4.222354808	5.2003179	5.407782538	5.298360986	5.328713339
Zm00001d033090_T002	1.871314958	1.712233466	1.387615101	2.229675897	2.401709916	1.905840701
Zm00001d015348_T002	5.763600026	4.996145294	5.739353819	5.384690068	5.459995045	5.42070754
Zm00001d001788_T022	1.432000498	0	0	1.516843198	1.914933378	2.235009516
Zm00001d001788_T033	0	0.296739311	1.040840542	0.938723898	1.192500982	1.194893929
Zm00001d001788_T027	0.382237838	0.035160765	0.543386007	0.501527806	0.200966303	0.47870472
Zm00001d001788_T012	0	0.227150818	0	0.288443615	0.80962435	0.935678493
Zm00001d001788_T024	3.993101789	3.010616408	3.82359846	4.226835565	4.089170406	3.831641568
Zm00001d001788_T001	0.190031976	0.892951285	1.520769711	1.99184851	2.313616202	1.631325502
Zm00001d016553_T001	3.168949249	2.715627425	3.005969767	1.752772137	2.222910874	1.416851089
Zm00001d016553_T002	0.98476578	1.438316808	0.429834461	0.265934912	0.428247466	1.439227905
Zm00001d016553_T003	6.267191844	5.268450451	6.312119559	6.376716484	6.355177205	6.319085712
Zm00001d027743_T004	0	0	0	1.574432406	1.303912813	2.116078252
Zm00001d027743_T012	3.586818416	2.47563513	3.594058093	3.583835075	3.082057387	3.220105329
Zm00001d027743_T001	0.413207362	0.058326194	0	1.043619578	1.004128131	0.938809697
Zm00001d027743_T002	0.79492319	0.825218205	1.150070897	2.228856854	2.819917058	1.894865869
Zm00001d044553_T014	0.180673655	0.147532041	0.328227555	0	0.395059508	0.281306555
Zm00001d044553_T009	1.284968309	2.217059134	1.86938977	2.77293775	3.372103081	3.245411387
Zm00001d044553_T008	3.41935888	0	3.557350166	3.002105519	0	1.882111648
Zm00001d029997_T001	4.963343397	4.210189264	4.532748269	5.14207041	5.044206251	3.241995883
Zm00001d029997_T003	3.829458396	5.501666869	4.444517927	0	0	4.878453646

Zm00001d027944_T001	6.00895462	5.848596202	5.713210088	5.801529425	5.567540215	6.055469865
Zm00001d002481_T001	0	0.881121108	0.811945275	1.351808089	1.045089243	1.074343758
Zm00001d002481_T002	1.496557452	0.70168212	1.522608208	1.87078489	1.253347926	1.329144268
Zm00001d002481_T003	0.427867872	0.181998112	0.524597314	1.42727469	1.493893739	1.407795962
Zm00001d000313_T001	3.346162941	2.780555147	3.392277302	3.659689302	3.667363078	3.788339401
Zm00001d040372_T001	0.839980543	0.460390299	0.896515216	1.124406885	1.448067858	1.229251973
Zm00001d040372_T002	2.698965419	1.447305177	2.456461333	2.697643064	2.807653529	2.817275946
Zm00001d040372_T003	0	2.654742104	0	2.129908104	0	1.678695285
Zm00001d018664_T005	3.899738481	2.96421156	3.809542347	4.180389696	4.166448811	4.004937011
Zm00001d018664_T007	2.131284583	1.939139675	2.29418578	2.255919734	1.886736642	2.157828245
Zm00001d002880_T001	3.699408822	3.014668617	3.59463358	3.863142271	3.914175958	3.814705246
Zm00001d024546_T001	3.028606633	2.450858886	3.03599562	3.060038388	2.890039217	2.951507601
Zm00001d039788_T001	4.69570892	4.517871989	4.798575483	5.02350412	5.061594294	4.852520221
Zm00001d051430_T001	5.333843171	5.955105969	5.195945028	4.701927313	4.573066176	4.748198701
Zm00001d010851_T001	2.882547388	2.461818484	2.952745067	2.984225122	3.280449203	3.305136755
Zm00001d050993_T001	4.808126763	4.297271548	5.012742966	4.754072363	4.904264136	4.684928506
Zm00001d013343_T001	0.736551193	0.668428694	1.100680334	1.098404735	0.729110704	0.822541719
Zm00001d037173_T001	6.74332848	6.327986649	6.601583007	7.180793599	6.989843035	7.030866672
Zm00001d038494_T003	0.286995849	0	0.259077201	1.069246398	1.630419917	0.808397408
Zm00001d038494_T012	1.106910412	0.649954769	0.602917656	2.881288922	2.566614015	2.520735773
Zm00001d038494_T016	3.460863766	3.770045022	3.783691322	2.037944078	1.579990109	2.304622306
Zm00001d038494_T011	2.819164583	0	2.108736873	2.91249754	3.683362519	3.141987429
Zm00001d038494_T015	3.11165284	2.441112931	3.3500764	4.118604252	3.721868275	4.105686374
Zm00001d032536_T006	5.279367956	4.034808909	5.241551539	5.931344821	5.878480989	5.893217837
Zm00001d032536_T007	0.314937776	0	0.539623377	1.311507766	1.420549772	1.363037036
Zm00001d032536_T004	0.341258241	0.454443346	0.399189691	1.613260061	2.43517358	1.941522904
Zm00001d032536_T005	1.13623208	1.6202481	0.647313588	2.358086196	2.633583261	2.287792891
Zm00001d032536_T001	3.993217844	4.44519228	4.579413758	4.900032629	4.892838289	5.236499966
Zm00001d009324_T002	3.161209053	2.219685861	3.214742793	4.006948661	4.034582154	3.643279227
Zm00001d009324_T001	3.20564931	3.208206963	2.37769516	0	0	2.03941427
Zm00001d038918_T001	2.674418618	2.335084739	2.754952903	2.266019498	2.318414898	1.756403352
Zm00001d015431_T001	10.49258751	13.41340697	10.39912411	10.46038034	10.43601671	10.38112098

Zm00001d005713_T001	5.979711082	5.268634255	5.978233894	6.300335445	6.17268768	6.279251866
Zm00001d052113_T002	3.224435325	2.296999436	3.156933725	3.211013596	3.120330207	3.188940647
Zm00001d043274_T011	1.296694093	0.794503205	1.290184328	2.344816569	2.396871343	2.303506939
Zm00001d043274_T008	1.269213921	0	1.708032693	2.997598801	2.973899051	2.870997295
Zm00001d043274_T002	1.750954167	2.359845263	2.01271383	0	0	0
Zm00001d043274_T019	0.562026966	0	0.241897524	2.330162537	0.968133525	2.695225284
Zm00001d043274_T001	2.328903371	0.496371331	1.419593902	2.632470644	2.939606481	0
Zm00001d039580_T001	4.875182272	4.06737051	4.674686676	4.800372256	5.074613198	5.116282738
Zm00001d039580_T002	2.4832013	3.338800622	2.289717629	2.193383819	2.01752538	2.70807133
Zm00001d045888_T001	9.624512702	5.648244247	9.767016515	9.308747385	9.244017992	8.912539557
Zm00001d025835_T001	0.775559663	0.785840108	0.93231672	0.481717428	0.442273665	0.694600316
Zm00001d047702_T001	4.558644914	5.796999874	4.643017163	4.516135198	4.848975353	4.514320562
Zm00001d015560_T001	3.505107395	2.789284795	3.482746852	2.660685881	2.400773988	2.517523639
Zm00001d013737_T002	5.509751383	5.144380729	5.567827638	5.302131883	5.366144708	5.270265309
Zm00001d013737_T001	4.314165458	3.634742293	4.230935859	4.400924509	4.482852283	4.274503059
Zm00001d010315_T010	0	0	0	2.928065738	2.242706455	2.514842607
Zm00001d010315_T002	3.815868959	2.68206377	3.424475912	3.565672842	3.677140841	3.81028299
Zm00001d010315_T003	1.664435053	1.680351365	2.644207934	2.760444733	3.079447065	2.640429358
Zm00001d010315_T007	0.350467827	0.168086157	0.340128113	0.705125963	0.695392565	0.608867895
Zm00001d010315_T008	0.879403866	0.231964412	0.929140313	1.976485352	2.004204733	1.448047765
Zm00001d049324_T001	1.570286591	0.932163245	1.836394389	1.58225539	1.830472767	1.68214402
Zm00001d019327_T001	6.72185786	6.046969129	6.720896937	6.485316431	6.390060761	6.350283072
Zm00001d025380_T003	0.776738213	0.411969595	0.507490215	1.267364402	1.155698077	1.190936547
Zm00001d025380_T001	0.631589524	0.706459825	0.381298879	1.219408937	1.544144084	1.796342747
Zm00001d025380_T004	4.797182095	5.067852378	4.478493566	3.214179527	3.537307996	3.409315031
Zm00001d025380_T002	1.843097862	1.168317264	1.961797775	2.333028942	2.032473991	2.110089246
Zm00001d012240_T004	2.879950572	2.422138253	3.070702737	3.151357974	3.118461894	3.132244014
Zm00001d012240_T001	1.066509449	0.895391042	1.378722463	1.896554353	2.043730502	2.24255251
Zm00001d039614_T001	4.765811378	4.127689237	4.980718551	5.073637829	5.098208102	5.099009204
Zm00001d045477_T001	3.39073096	2.839436415	3.475008595	2.735740298	2.698365408	2.794562676
Zm00001d017240_T001	7.031235617	6.614264189	7.07094262	7.039846799	7.05346862	6.998470305
Zm00001d033451_T004	1.590384048	0.648115656	1.659706311	2.553436446	2.013172798	2.121329775

Zm00001d033451_T001	1.598836815	1.337779586	1.424644269	0	0.780126128	0
Zm00001d039016_T001	5.794458887	5.322065721	5.949262243	5.525602302	5.560167482	5.702549559
Zm00001d034420_T001	5.663552042	7.018180323	5.684775798	4.934473758	5.1708554	4.490983178
Zm00001d034420_T009	6.816870135	5.576544782	6.802633908	6.376026888	6.613594961	6.182361894
Zm00001d034420_T011	0.565377831	0.148150575	0.436443313	1.348133252	1.337051097	1.413004753
Zm00001d034420_T004	1.722083878	0.916076074	2.142929317	2.312411634	1.790847089	1.795503482
Zm00001d034420_T008	0	1.544141611	1.132712387	1.070045101	1.788202504	1.897662134
Zm00001d034420_T005	1.045308061	0.452980172	0.380272855	1.506819239	0.817670733	0.722220308
Zm00001d034420_T007	4.670488795	4.304872473	4.670358319	6.371760739	6.18694127	6.594513885
Zm00001d034420_T002	0.391951344	0	1.489387252	2.304002797	2.366871068	1.612857295
Zm00001d034420_T003	1.721360409	1.172062453	1.770067017	2.192060307	2.302995709	2.144381545
Zm00001d048135_T002	3.717077234	5.291929645	3.789922015	2.969385554	2.800782099	3.154869296
Zm00001d048135_T001	3.573592902	1.515322386	3.340882247	3.21490191	3.29351557	3.30975187
Zm00001d048135_T003	3.293078649	4.239616171	3.827791593	3.338487456	3.490298252	3.795713552
Zm00001d016274_T011	0.509594508	0	0.511869772	1.126320061	1.28197178	1.856823191
Zm00001d016274_T009	3.444276925	0	2.703934351	3.636168338	3.559333096	3.400883944
Zm00001d016274_T010	0.689414572	0.074439671	2.992708076	1.527669415	1.680213168	0
Zm00001d016274_T006	2.919833356	0	2.655360531	4.593100769	4.360349105	4.529598217
Zm00001d016274_T001	5.513641987	5.003854102	5.676646737	5.212661041	5.289072903	5.491333793
Zm00001d019733_T003	0.743105604	0.341221799	0.840669477	1.200749155	1.051444496	1.141417749
Zm00001d019733_T001	0.447068146	0.285437731	0.523559949	0.689037886	0.703906895	0.605161475
Zm00001d052948_T001	4.944368268	4.198829408	4.954217532	4.945181564	4.865476439	4.863202994
Zm00001d034736_T003	1.331037307	1.285534201	1.016673169	1.599492049	1.510310788	1.048736298
Zm00001d034736_T001	0.204546418	0.144310078	0.237086412	0.259066351	0.173594394	0.157759058
Zm00001d023978_T001	4.922725772	6.170627263	5.224729998	4.951840002	5.298304636	4.936593091
Zm00001d042879_T003	1.5342494	0.876140102	1.756599339	1.383022419	1.803620933	1.672005711
Zm00001d042879_T002	3.814432501	3.07893017	3.873537229	4.081722835	4.154806527	4.116176589
Zm00001d009336_T001	1.682742725	2.247478841	1.896052085	3.269408288	3.602221448	3.295544807
Zm00001d042755_T001	2.916690071	2.489142645	3.165017914	2.52954862	2.907820353	2.669535249
Zm00001d010008_T001	2.705400708	2.337084504	2.660304814	2.125190516	2.060306146	2.236005158
Zm00001d000288_T001	2.609746926	2.338635205	2.213271995	1.810780756	1.960035305	2.183191117
Zm00001d000288_T002	0.203286368	0.443872889	1.167948759	1.652532385	0.99625351	1.294136069

Zm00001d002974_T001	4.144091331	3.393326681	4.406481432	2.689817543	2.744600943	2.929995649
Zm00001d007272_T001	2.588304717	2.371440097	2.92124246	1.619042957	1.459327741	1.795628572
Zm00001d006107_T001	4.714595734	3.996855558	4.760427381	4.846883247	4.959354069	4.806021256
Zm00001d041450_T004	0.51482519	0	0	1.6978134	2.457183198	2.071062772
Zm00001d041450_T001	1.98821786	1.351108142	2.091462891	2.494534913	2.779297757	2.722407223
Zm00001d041450_T002	0.53989232	0.56621214	0.489219218	1.648476027	1.005997783	1.414767149
Zm00001d041450_T003	0.615069707	0.334933194	0.580300843	1.640649152	1.380599245	1.545357565
Zm00001d020238_T005	0.604901584	0	0.596771071	0.644405463	0.614009643	0.620456914
Zm00001d014961_T001	5.286925455	4.319870644	5.295288094	5.726204533	5.645302228	5.633656258
Zm00001d005381_T001	4.348993232	5.111215603	4.471234487	2.805946164	2.908119248	2.713825783
Zm00001d006231_T001	1.13012893	0.896836802	1.235590441	1.922670549	1.809107762	1.944938635
Zm00001d040461_T001	2.703073893	2.496381047	2.058069155	3.224155451	3.153070509	3.634453406
Zm00001d004555_T001	0.585755765	0.439027321	0.560928164	1.203474945	0.88087983	0.809868791
Zm00001d029093_T001	3.824008533	2.979403838	4.021833429	4.211514556	4.212048349	4.092473859
Zm00001d027271_T007	2.81195452	1.636323001	3.028326738	2.814143089	2.84842925	3.033172766
Zm00001d027271_T001	1.595340381	1.253844687	1.668583908	2.200909195	2.11087735	2.111528823
Zm00001d027271_T004	1.114191092	0.833064949	1.33088763	0.917876239	1.40033572	0.95484026
Zm00001d027271_T008	0.592268054	0.21872786	0.643803559	0.931005985	1.08399417	0.914709934
Zm00001d027271_T003	1.501266903	1.911680079	1.718594411	0.995834703	0	0.948558986
Zm00001d027271_T014	3.198240167	2.230593767	2.892946429	2.448988937	1.904389296	2.380207457
Zm00001d027271_T005	0.935373749	0.285837773	0.863978084	2.264522019	2.383226517	2.390809637
Zm00001d045610_T003	3.212490588	2.534660525	3.27045718	3.302134626	2.877613714	3.050096084
Zm00001d045610_T001	3.257977775	2.61205284	3.136246192	2.884017014	3.18277158	3.255463721
Zm00001d004109_T006	0.786889889	0.723798809	1.268291854	1.216767739	0	1.227888295
Zm00001d025034_T002	2.226147194	1.657080652	2.336248249	2.14635577	2.046464518	2.222018406
Zm00001d034722_T001	6.890636952	8.64991974	6.723684177	7.048987913	7.024233188	7.092329014
Zm00001d042123_T002	2.354881746	1.876351917	1.486114005	1.303879505	1.230522142	0
Zm00001d011013_T001	4.561584711	3.627526064	4.593975359	5.014968219	5.008342383	5.025708553
Zm00001d034865_T001	2.508949349	1.715666298	2.436033612	2.410983607	2.486717462	2.47603374
Zm00001d006089_T001	0.67520802	0	0.75089973	1.099876206	1.248655663	1.127121478
Zm00001d051545_T003	1.543952623	0.99753377	2.03855207	3.282443215	3.258843909	3.047658608
Zm00001d051545_T001	3.703005433	3.12803698	3.623306082	3.58855982	3.410418177	3.834055039

Zm00001d051545_T005	1.193754715	0.338301178	0.811664199	1.274921199	1.407042151	1.467760994
Zm00001d012660_T001	0	2.793768978	0.823849612	1.33888646	0	0.801328375
Zm00001d012660_T002	5.033340006	3.882448492	5.052505799	6.089919186	6.179713885	6.085529289
Zm00001d009891_T001	3.153527955	3.137659753	3.31754633	3.228669566	3.46753969	3.039761847
Zm00001d013314_T003	3.240321047	2.456459493	3.123487752	3.083570976	3.011955353	2.798516334
Zm00001d013314_T011	1.200081188	0.150654553	0	1.305565518	1.013884555	0
Zm00001d013314_T012	2.345888545	1.257543532	2.375737318	2.333551109	1.986314085	2.781550871
Zm00001d013314_T013	0.531761221	0	0	2.868528147	3.195202534	2.825098709
Zm00001d007194_T001	8.837420558	8.392290054	8.548130852	8.55345272	8.684861241	8.697043188
Zm00001d008387_T002	9.072568697	8.973437976	9.116944429	8.353430867	8.344692927	8.39706402
Zm00001d041467_T001	2.335890521	2.310801651	2.137623853	2.224618842	2.21674322	2.769595093
Zm00001d021652_T001	8.161113537	8.511200834	8.05394498	7.481515255	7.330370531	7.395920842
Zm00001d028463_T001	0.939386074	1.570032001	1.31399168	0.955571706	0.994634752	1.328012078
Zm00001d028463_T003	3.275515776	2.231805323	3.302580219	2.953526745	2.852031807	2.71164974
Zm00001d050834_T001	4.879780006	4.397661902	5.051793754	3.600826359	3.748288937	3.747930859
Zm00001d013557_T001	1.954134134	2.952191899	1.842969985	1.574804878	1.61507112	2.401636473
Zm00001d017660_T006	0.12397205	0.09998457	0.476202279	1.079083141	1.282107042	0.663473056
Zm00001d017660_T001	0.864871937	0.459725374	1.014765903	1.438347684	1.290163681	1.266962801
Zm00001d017660_T011	0.281843032	0.080483029	0.34122066	0.810691518	0.067502425	0.499722942
Zm00001d017660_T004	0	0	0.061136086	1.034962934	0.555520716	0.907088327
Zm00001d017660_T002	1.712518747	0.835165753	1.32280095	0	0	1.381387486
Zm00001d017660_T012	1.638113236	1.10611176	1.609004585	0.930615484	0.663874687	0.973468744
Zm00001d017660_T003	0	0	0	1.240474315	1.520331221	1.393251437
Zm00001d048620_T001	2.025738979	1.384245469	1.889645254	2.149947701	2.128742771	2.002313669
Zm00001d041455_T001	4.099191586	2.844233983	3.977406875	3.039543415	3.082239156	3.08128048
Zm00001d041455_T002	1.722128482	2.682613521	1.912097972	0.893287647	1.40008319	0.523882073
Zm00001d025106_T005	1.902414768	1.09415971	2.191540859	1.950660263	1.829113618	0.681883695
Zm00001d025106_T001	0	0.381710856	0	0	0.761699723	1.245463413
Zm00001d025106_T004	1.691899036	1.826257631	0.786224977	1.383986238	0.634583046	1.815776654
Zm00001d025106_T008	2.713277901	2.368644046	2.517057445	2.414082209	2.491565298	0
Zm00001d024883_T001	4.728815791	3.908352747	4.623178586	3.873291529	4.034282724	4.002606488
Zm00001d028263_T001	0.564137171	0.299735829	0.739835144	1.161627813	0.689280372	0.742282216

Zm00001d036877_T001	3.202839732	2.841392503	3.167599748	3.614375807	3.436489288	3.720713945
Zm00001d040650_T001	1.86025925	1.46197272	2.032544166	1.238565548	1.390538927	1.270327998
Zm00001d049313_T004	1.874243384	0.606277339	1.857198743	2.010524928	2.028973616	1.8758406
Zm00001d049313_T003	0.291749706	1.086436635	0.98021399	0.805621823	0.262787924	0.277064699
Zm00001d049313_T006	0.808329854	0.293866013	0.511069232	1.242589702	1.294563113	0.922449061
Zm00001d034668_T002	1.825161613	1.697072314	2.135834935	1.424997311	2.123979993	1.948451338
Zm00001d017002_T001	2.824668887	2.397042267	2.086368354	0.516472096	1.926149375	1.032909795
Zm00001d053932_T001	1.74089562	3.669499307	1.58300825	1.39025991	1.189224273	1.489166286
Zm00001d053932_T003	6.348858557	5.292575598	6.517022538	6.16893788	6.264800457	6.164452713
Zm00001d051598_T001	5.145187182	4.799677581	5.173301282	4.615343725	4.775003276	4.607695573
Zm00001d010234_T001	3.538693226	2.929258693	3.588864039	4.307123414	4.321802214	4.430309697
Zm00001d024679_T002	6.634713982	6.05557049	6.781592454	6.579913199	6.662585325	6.605183155
Zm00001d024679_T001	1.188864867	1.593531139	0.775849546	1.700958995	1.085799219	1.591645431
Zm00001d000124_T004	3.117260839	2.343172708	2.755871832	2.297902462	1.48852632	2.783428785
Zm00001d000124_T002	0	0	0	1.467938326	1.689457513	2.008586565
Zm00001d000124_T003	0.768933802	0.351065161	0.796151671	2.317939594	2.179393528	1.719552338
Zm00001d000124_T005	1.352753441	1.064960777	2.103820217	3.890014306	4.105712519	3.501619136
Zm00001d048539_T001	1.403549688	0.599622489	1.344957425	1.445083597	1.413138565	1.652701707
Zm00001d048539_T002	5.656547739	4.796608032	5.631165645	5.516267155	5.526504372	5.580063607
Zm00001d021757_T001	5.47317094	4.87121169	5.589339034	5.047110123	4.931453966	4.917650816
Zm00001d034611_T001	2.347559647	2.63901169	2.300740217	4.138394441	3.788623283	3.893405996
Zm00001d043329_T002	5.15267562	4.106787578	5.19928192	5.596901728	5.582063349	5.5371329
Zm00001d043329_T003	0.407047047	0.033818356	0.164827461	0.527446729	0.270554059	0.409646186
Zm00001d021764_T004	0.737972244	0.260034089	0.667788602	0.83586588	0	0
Zm00001d021764_T005	0.471904337	0.439307173	0.701310399	0	0.624258143	0.223397835
Zm00001d021764_T001	4.182712476	3.852546837	4.383013223	4.220037981	4.101857205	4.136127225
Zm00001d030618_T001	9.186293747	8.244417303	9.147398149	9.398421762	9.393150547	9.252786765
Zm00001d037727_T001	6.00039236	5.221315357	6.067238517	6.078289727	6.062149868	6.013871293
Zm00001d025992_T001	2.423882543	1.854535509	2.497644042	2.671142074	2.62912134	2.311922145
Zm00001d002284_T001	4.520879689	3.688056454	4.533967321	4.368762553	4.485736329	4.441598343
Zm00001d002284_T004	1.214145948	0.822192529	0.746301586	2.583094926	2.471782628	2.545217923
Zm00001d002284_T002	0.615895546	1.318633259	1.3441297	0.910119386	0.934404025	1.009896768

Zm00001d045215_T001	2.42289256	1.812848126	2.411483464	2.821668791	2.917758067	2.668005629
Zm00001d019957_T003	0.948460309	0.662842606	0.912398473	1.413784696	1.272390537	1.532678523
Zm00001d038329_T001	3.935373938	4.075109344	4.095476102	4.110355642	3.99424372	4.166776252
Zm00001d021291_T003	3.244903343	3.120912585	3.431408345	2.192184063	2.448465699	2.369610257
Zm00001d021291_T006	4.114162851	2.490049691	4.091435471	3.669288854	3.259317236	3.519751475
Zm00001d006401_T001	3.198732854	2.913227402	3.099201098	2.769246181	2.908523571	2.878778301
Zm00001d047952_T001	2.120682714	1.969926278	2.478193704	1.876886622	1.64669631	2.084119045
Zm00001d032170_T001	2.662911404	3.246075575	2.945027812	3.87210467	3.804594365	3.784000208
Zm00001d051360_T001	1.665523299	1.605923807	1.788470647	2.564558647	2.644342238	2.389872939
Zm00001d050131_T003	1.497371705	0.811407731	1.122479905	1.708189885	1.843730647	1.77562624
Zm00001d050131_T002	3.263011112	2.418015302	3.29073948	3.725765474	3.765680918	3.761024692
Zm00001d050131_T001	0.61906738	0.549218674	0.449389165	0.872377268	0.92083902	0.808905604
Zm00001d020267_T001	7.362510953	7.519304961	7.380096772	6.513290056	6.433796695	6.634327312
Zm00001d051157_T001	5.730346143	5.193925189	5.814626299	5.66308347	5.632570743	5.686563892
Zm00001d022043_T001	3.722247418	3.032369604	3.75463581	3.644526368	3.60535423	3.558030145
Zm00001d043425_T002	2.146028224	1.419167788	2.147859572	2.641950574	2.505373448	2.533225185
Zm00001d039871_T001	4.626760232	3.792861077	4.653137826	4.945382733	4.846521498	4.773830954
Zm00001d009007_T001	1.390640724	0.91326513	1.256014875	1.507797684	1.496172401	1.731069386
Zm00001d017564_T009	0.973282109	0.13275318	0	0.3943978	1.167179989	0.838298323
Zm00001d017564_T007	1.361913183	0.710063396	1.559811398	1.237983984	0.916680709	1.706036714
Zm00001d017564_T004	0.422744313	0.21228295	0.257880802	0.676863142	0.540087794	0.598863487
Zm00001d017564_T003	4.471609345	3.836439937	4.417763554	4.220459126	4.015988827	4.048325915
Zm00001d014888_T001	3.63612146	4.101285743	3.290531747	2.919228133	2.187039566	2.101512254
Zm00001d021343_T001	1.832068912	1.384358765	1.959652213	2.323758579	2.063371782	2.037472541
Zm00001d041072_T001	5.980423038	5.182676448	5.957330974	5.693877338	5.547255752	5.601694021
Zm00001d041072_T002	2.06821506	0.116843801	1.726539162	2.677099787	3.084775059	2.748593474
Zm00001d006209_T001	0.743652833	0.720918451	1.042917443	0.942597827	0.583267782	1.150254859
Zm00001d024998_T095	0	0.126955681	0.350368247	0.524726682	0.646161735	0.953894709
Zm00001d024998_T134	1.260236473	0.547290892	0.922923201	1.889696257	0	2.045721487
Zm00001d024998_T055	3.485885454	2.550629334	3.485879788	3.017913715	2.862495827	2.770014988
Zm00001d024998_T005	0.029797749	0.148197443	0.367703183	0.428397561	0.383752393	0.477713589
Zm00001d024998_T146	0	0	1.306935301	2.508405833	1.711479929	2.091882254

Zm00001d024998_T001	0.050631247	0	0	1.191959274	0.565544536	0.491051633
Zm00001d011564_T001	0.160028229	0.119340239	0.674900805	1.549813556	1.481648722	1.455719909
Zm00001d025182_T007	1.814282982	1.672266467	1.890566928	0	1.811265093	2.290375153
Zm00001d044525_T002	2.301576819	2.869931007	2.135356902	1.117830663	1.869585997	1.601122176
Zm00001d044525_T001	3.230384722	2.635109387	3.446265363	3.575856144	3.540967096	3.197425566
Zm00001d022491_T004	0.978666443	0.18995483	0.758506155	1.215673627	1.538422445	0
Zm00001d022491_T009	2.454019768	1.836286925	2.346068171	0	0	0
Zm00001d022491_T001	0	0	0	1.307190096	1.939317989	2.292738173
Zm00001d022491_T008	0	0.239664436	0.913167073	2.456727311	2.354645391	1.615363561
Zm00001d014705_T002	3.133971158	2.551325286	3.230092778	3.255272754	3.466419347	3.32016231
Zm00001d026042_T001	0.960650339	0.580164785	1.072750666	0.116110532	0.084550072	0
Zm00001d009950_T001	2.185355329	2.832455217	2.198810003	2.702725577	2.737384564	2.560798824
Zm00001d009950_T006	3.642792474	0	3.698656555	3.629292382	3.671083747	3.655983614
Zm00001d003572_T003	0.358428897	0.230756371	0.262350166	1.315023596	0.945513318	0.778144642
Zm00001d003572_T001	2.285546925	1.084491566	2.742996349	2.188392695	2.0318091	2.255561159
Zm00001d003572_T004	3.64661533	2.298861217	3.532558095	3.983843021	4.09871119	4.011776136
Zm00001d017829_T005	1.544709909	1.053444335	1.487468856	3.000189161	2.769821027	2.859913509
Zm00001d017829_T003	3.907334685	3.024997778	4.157922564	4.154447922	4.20727528	4.264504829
Zm00001d051927_T001	1.887534629	1.908656912	1.870427852	1.716233692	2.023713109	1.777380577
Zm00001d051927_T003	3.044499563	3.230753143	2.958831542	2.753633629	3.01642536	2.713539221
Zm00001d051927_T002	1.267268508	1.173277133	1.346744191	1.355052923	1.230829521	0.978349411
Zm00001d033742_T001	2.82737172	2.044763752	2.578389781	3.448486183	3.212374943	3.390171708
Zm00001d003855_T005	3.831092056	3.187051447	3.948365176	4.859039247	4.894507078	5.026675876
Zm00001d003855_T008	0.758591431	0.505581022	0.982469776	0.439777371	1.42515043	1.044233928
Zm00001d003855_T007	0	0.107844515	0.469491134	1.242337263	1.241464973	1.195365868
Zm00001d003855_T002	0	0.091117562	0	1.514041947	1.478985261	0.973598052
Zm00001d053954_T002	0.313159926	0.104022588	0.312589668	0.360726099	0.403182631	0.344691037
Zm00001d053954_T003	3.882015854	3.114780449	4.031292496	4.182613964	3.953113976	3.593056595
Zm00001d053954_T001	2.509416396	2.506284569	2.577512215	2.104479916	2.304435108	3.03009714
Zm00001d046751_T001	5.988539819	5.094779934	6.05034314	6.192952998	6.160258149	6.134532634
Zm00001d027888_T002	3.543504792	2.782375643	3.754597438	3.800055618	3.828063551	3.710177488
Zm00001d039453_T001	0.527728959	0.248657484	1.388212135	1.163621099	1.165652793	1.01676729

Zm00001d039453_T002	5.328453305	4.631633348	5.234336456	5.335833583	5.362102821	5.371766435
Zm00001d032843_T001	3.732893472	3.458062901	3.822821091	3.807137986	3.873251363	3.85574976
Zm00001d024252_T001	1.286424616	1.093687268	1.277621797	1.105661988	0.583320742	0.715498936
Zm00001d011684_T001	0.531243205	0	0	1.377478062	1.238663369	1.764827228
Zm00001d011684_T004	0.084554154	0.097175226	0.063382828	1.371966384	1.758215751	1.323942422
Zm00001d011684_T003	0	0	0	1.410568509	1.568351749	1.2722896
Zm00001d011684_T002	1.221900585	0.774864212	1.38436816	4.577279082	4.652305903	4.639074338
Zm00001d011684_T007	4.050633249	3.191719608	3.915941506	6.013631867	6.025347273	5.992916567
Zm00001d019624_T003	3.084619459	2.301838999	2.874687407	3.312987479	3.333182406	3.214934384
Zm00001d019624_T001	0.123725783	0	0.635508311	0	0.708865265	0.935154204
Zm00001d004910_T001	4.85930836	5.123545815	4.295246651	4.964052242	5.018714327	4.753118101
Zm00001d039910_T001	4.181238861	3.555596667	4.194970338	3.655050549	3.561694554	3.713989822
Zm00001d037352_T001	3.969010189	3.185672323	3.856406006	4.076757974	3.735392091	3.989540417
Zm00001d037352_T002	2.402566113	1.412673692	2.282134034	2.358135158	2.593941009	2.409806915
Zm00001d037352_T003	0.770090705	0.447159154	0.642097107	0.729169886	0.604830437	0.785340462
Zm00001d050024_T009	0.162804268	0.045438777	0.091529239	0.278081122	0.418164036	0.244581443
Zm00001d050024_T003	0	0	0.328159755	0.603186453	0.342591154	0.342872149
Zm00001d029263_T001	4.448407021	4.499560699	4.571403837	3.996768443	4.132353759	3.756973733
Zm00001d035440_T001	9.503566393	8.744074255	9.320087776	8.858668854	8.937388943	9.064803871
Zm00001d038476_T001	4.013270722	3.463149371	4.168431841	2.881359996	3.037937228	2.770215902
Zm00001d052044_T003	1.545663493	0.546152153	1.591758873	0.810026791	0.982743562	0.27641567
Zm00001d052044_T001	2.31177977	1.732030359	2.472255882	1.720541148	1.904055898	2.313245271
Zm00001d037015_T001	2.129946669	1.534597532	2.110072203	2.454702591	2.229351966	2.222085198
Zm00001d032232_T010	2.301448931	3.750616687	3.226696253	2.199961561	3.613460459	2.174284964
Zm00001d032232_T009	3.015401046	2.250230039	2.539979146	2.636385418	2.838569216	2.729924898
Zm00001d032232_T011	0.135102719	0.02428847	0.383104283	0.418405864	0.346981844	0.254560182
Zm00001d032232_T006	3.914191843	4.048253801	4.233287119	3.628543469	3.618495569	3.875822518
Zm00001d032232_T004	3.67942042	3.390014694	3.243131321	3.635693339	3.278141204	3.567992205
Zm00001d014200_T001	3.068680268	3.445665001	3.192179801	2.523863761	2.706675533	2.582990673
Zm00001d005769_T003	0.928167905	0.157549535	0.96207292	0.607547634	1.033517445	1.255233621
Zm00001d005769_T001	3.135775682	2.385786352	3.118245422	2.800586018	2.970494224	2.642752625
Zm00001d033680_T001	5.74847712	5.05945401	5.768041292	6.320541468	6.397439779	6.496021635

Zm00001d005680_T001	2.003016405	1.869585997	2.257211314	2.406721145	2.436745517	2.58287052
Zm00001d041711_T002	4.55938376	4.299872439	4.891231449	3.89888097	4.103390126	4.059928711
Zm00001d041711_T005	3.248568687	5.579934734	2.567987096	0	0	0
Zm00001d020713_T001	0.703000558	1.255374435	1.363238934	0.917000124	0.693561446	1.329310776
Zm00001d007417_T001	0.753724328	0.602600354	0.702407543	1.043413803	0.945971702	1.049453033
Zm00001d024093_T001	1.763104305	1.59255128	1.66869282	2.36848481	2.554025661	2.543880138
Zm00001d029325_T002	4.615298344	4.197953199	4.528974442	4.097642482	3.939461678	4.153615747
Zm00001d030985_T001	2.413642009	1.746106345	2.433117828	1.522221509	1.970122921	1.752284457
Zm00001d020222_T001	3.140004431	2.902278148	3.180043284	2.65198	2.510253813	2.707305013
Zm00001d005106_T008	4.04611174	5.261840119	4.500714303	4.01183552	4.209987064	3.394048442
Zm00001d005106_T004	0.810413489	0.39287241	1.859762071	0.854933194	0.870614075	0.857945972
Zm00001d005106_T002	1.59173877	1.604544393	2.768374907	2.71099968	3.088577868	2.110333841
Zm00001d005106_T007	1.989715607	0.727708771	1.501837567	1.187799413	1.06644814	1.560802492
Zm00001d005106_T003	2.882185227	1.893513268	2.606664419	3.364867244	3.158890536	3.747673762
Zm00001d014920_T034	2.407379402	0.242654939	2.604731538	3.403904386	3.284237531	3.410840642
Zm00001d014920_T026	2.401968166	0	2.41701588	2.73045954	2.475394147	2.428053124
Zm00001d014920_T003	1.056734032	1.447674403	0.925912854	1.532004695	2.120317764	1.957059869
Zm00001d014920_T038	0.714963773	0.26698678	0.963022749	1.109173983	1.430967113	1.176860172
Zm00001d014920_T007	0.414452729	0	0.355564362	1.432620067	1.397681957	0.728484792
Zm00001d014920_T008	4.118908939	3.866252436	3.671007636	3.894286269	3.956891259	3.895668193
Zm00001d014920_T032	0.153749572	0.027936774	0.352834217	0.588578169	0.236546498	0.287117636
Zm00001d014920_T001	6.059022321	6.704415463	6.014234364	6.149268086	6.060864269	6.016544167
Zm00001d024788_T002	3.210804187	2.782859756	2.821106279	2.90104586	3.352519139	3.050835124
Zm00001d024788_T003	0	0.14618728	0.959368073	1.117651163	0.658301984	1.038826654
Zm00001d024788_T001	1.483380872	0	2.060294039	1.213755378	0.859736632	1.960309303
Zm00001d005384_T001	2.821384705	4.24716952	2.500524958	2.730604961	2.309874951	2.556769295
Zm00001d049958_T001	1.577800527	1.599500619	1.560203808	1.457482927	1.060834597	1.685116669
Zm00001d049958_T003	0.69589763	0	0.798457026	1.171989463	1.564976098	1.299095163
Zm00001d049958_T005	3.483426923	1.722697274	3.379935582	3.228588764	3.412529404	3.017976409
Zm00001d020463_T002	4.497631525	3.824868848	4.68346218	4.840868491	4.756357874	4.797064665
Zm00001d051082_T001	3.785464552	3.420355064	3.319966717	3.816739771	3.489971108	3.43686289
Zm00001d031009_T001	3.128396518	2.019203022	3.288040307	3.039569207	3.093779876	2.990050125

Zm00001d052939_T001	3.936797062	3.093122028	3.989207779	2.361940967	2.246661639	2.422389912
Zm00001d008599_T001	2.1391525	2.955457884	1.739774238	1.926054088	1.680108274	1.651624406
Zm00001d044270_T001	3.843778577	3.973631387	3.786367499	4.139071438	4.126800436	4.034377992
Zm00001d052030_T001	1.516262301	1.258940968	1.518969645	1.848291427	1.400113802	1.647252794
Zm00001d017947_T001	3.140986212	3.428378264	3.389899364	2.8767839	2.891946394	2.906723618
Zm00001d018531_T003	1.361188358	0	1.955657253	0	0.44758237	1.290604287
Zm00001d018531_T008	1.280787099	0.28147749	1.458113705	0.847789304	0.679367044	1.582819951
Zm00001d018531_T007	3.157081071	3.133815679	3.028789783	3.5972165	3.569989849	3.044295838
Zm00001d018531_T005	1.109892069	1.047784052	1.412395129	1.287175569	1.38843589	1.453058161
Zm00001d018531_T002	1.641732828	0	1.387685126	0.96864007	0.996840648	1.273769495
Zm00001d024148_T001	1.129169575	0.797459625	1.320621576	0.311456614	0.283295975	0.308836142
Zm00001d025538_T002	1.405633002	1.047502094	2.41309306	2.909858531	3.097204897	3.046815813
Zm00001d053724_T001	3.860671549	3.791559989	3.760505837	3.755130175	3.734914103	3.642941
Zm00001d023856_T001	8.735852067	9.483182678	8.365369966	8.609880412	8.780227239	8.804082062
Zm00001d046953_T003	1.849310764	1.320246086	1.837722886	2.832227944	2.81132922	2.685255743
Zm00001d046953_T009	1.033568189	0.493909618	0.665851159	1.766335644	1.270316634	1.323932049
Zm00001d046953_T011	1.482494147	1.195171818	0.236841578	1.171678253	0.849390037	0.776451036
Zm00001d046953_T005	2.644163163	0.655496549	2.986219777	3.207374075	3.344265793	3.457169011
Zm00001d046953_T001	0.999885301	1.627766973	0.788638107	0.980181081	1.246100361	1.18290392
Zm00001d033481_T001	2.812225268	2.859453782	2.715924332	2.799118606	2.708033134	2.859859852
Zm00001d043975_T001	1.981257921	1.593255277	1.899255692	2.240768287	2.131555902	2.006508084
Zm00001d010016_T002	7.31665019	6.194293404	7.323471043	6.783283576	6.803674166	6.855174763
Zm00001d027751_T001	2.351140093	1.483684236	2.223938368	2.060425825	2.186664405	2.211453348
Zm00001d036465_T001	6.075645	7.213731683	6.204074889	7.014982452	6.988600167	6.845215549
Zm00001d024200_T001	6.009967915	6.632991665	6.135539528	4.502110081	4.551876923	4.810668951
Zm00001d016599_T002	3.857733626	3.069094959	3.891948532	3.932619559	3.912093563	3.762411115
Zm00001d052245_T008	1.701059722	2.201045372	1.495416829	2.357301165	2.001852313	1.765336574
Zm00001d052245_T004	0.674893576	0.299474439	0.75135574	1.191870864	1.505872642	1.110860984
Zm00001d033248_T001	1.140265606	0.359834863	1.145021328	1.647904823	1.755750296	1.684899508
Zm00001d033248_T002	4.170111017	3.568477009	4.444080886	4.479951599	4.795958699	4.710073152
Zm00001d033248_T006	4.125934059	4.776605843	2.750639946	3.914539649	2.958391148	0
Zm00001d033248_T003	3.453543391	1.75079728	3.335402361	2.939093588	2.37104414	3.553594584

Zm00001d006025_T002	1.134603417	0.326577614	1.290539414	2.057569842	2.182163424	1.860147986
Zm00001d039965_T001	5.947327509	5.247053127	5.948593045	5.709418162	5.749802	5.693041145
Zm00001d049134_T002	5.703143595	5.801366145	5.732936034	5.535106474	5.755223558	5.601658131
Zm00001d002384_T002	2.964585865	1.277309348	2.60978167	2.61802388	2.652468368	2.66817133
Zm00001d002384_T001	2.178387668	2.094143829	2.229057812	1.373576945	1.354116414	1.568677159
Zm00001d023516_T001	3.264094998	3.98316427	2.714292123	1.357884701	2.135105675	1.722996664
Zm00001d042329_T002	1.667329012	1.177539613	1.733037564	2.386459471	2.517531702	2.291740278
Zm00001d042329_T003	4.634375628	6.587770692	5.238688359	5.17631595	4.505947951	5.009102402
Zm00001d042329_T001	6.30100361	5.799587758	6.250839007	5.647835075	5.866011577	5.762850934
Zm00001d051914_T001	2.82562039	2.018550501	2.455058879	1.662833494	1.690316517	1.73458347
Zm00001d010333_T001	2.136423065	2.316497103	2.218318377	1.643559772	2.005430171	2.223058896
Zm00001d005312_T001	4.79691375	4.478425418	5.135335763	4.818851583	4.833993987	4.780393361
Zm00001d035838_T002	5.85083097	6.120583668	5.916824283	4.893042472	4.897229145	4.848346567
Zm00001d033553_T044	0.062696464	0	0.309782714	0.233382538	0.46708015	0.456986935
Zm00001d033553_T037	0.343596568	0.249848208	0.250762735	0.294290784	0.559041436	0.059088036
Zm00001d033553_T027	1.51526687	0.86673077	1.733882726	1.051635209	1.147321679	0
Zm00001d027389_T001	8.204863165	4.332105886	7.957810176	8.280104968	8.279842299	8.293959737
Zm00001d013192_T001	3.35940647	2.605343087	3.339272239	3.558603775	3.693472015	3.482747239
Zm00001d037970_T039	1.484338728	1.62540236	1.704557281	1.592592898	0	2.427569435
Zm00001d037970_T018	1.250865189	0.476487455	0.961617402	3.13330212	2.935097237	2.702625853
Zm00001d037970_T005	0.493208721	0.170221204	0.090708476	0.694314144	0.400535744	0.403121536
Zm00001d037970_T041	3.175407073	2.217127707	3.367377636	3.081870825	3.068822466	2.858989335
Zm00001d006762_T001	3.250972636	2.334554261	3.089652039	3.442148236	3.331896073	3.068711047
Zm00001d006762_T007	0.774640754	0.090658349	0.442935005	0.589292737	0.335021263	0.525014458
Zm00001d006762_T003	0	0	0	2.334513072	2.339737148	2.483931833
Zm00001d042043_T001	4.540680501	4.006564723	4.650217644	4.742387159	4.701186563	4.646170493
Zm00001d020275_T001	2.373764012	1.849854382	2.188566455	3.800178346	3.658127261	3.578215857
Zm00001d011604_T062	0.093933309	0.169536383	0	1.275193036	1.157612266	0.602968949
Zm00001d011604_T027	0.685523085	0.943956573	0	0	2.106363396	0
Zm00001d011604_T015	2.914986364	2.479987415	2.90381245	1.698225147	2.373591143	0
Zm00001d011604_T040	0	0.150423199	0	2.389613065	2.369689815	2.192996422
Zm00001d011604_T074	1.17360143	0.441775601	1.290682131	1.41175434	1.22236501	0.859244442

Zm00001d011604_T029	0.932488321	0	0	1.545201363	1.463655411	1.460393969
Zm00001d011604_T065	0	0	0.187750014	0.497246504	0.532458603	0.710697349
Zm00001d011604_T054	1.390643475	1.032425325	1.858509422	0.835419649	0	1.463411634
Zm00001d011604_T044	0	0	0.632450635	1.661444066	0	1.482341831
Zm00001d011604_T010	0	0	0	2.055033533	2.252143735	2.704384641
Zm00001d011604_T058	1.778750642	1.062089928	1.913648102	2.493059582	2.255380478	2.347333146
Zm00001d011604_T020	2.129709332	1.81295037	1.407470229	1.425854568	1.852677663	0
Zm00001d011604_T024	1.3213255	0	1.799062432	1.615691249	1.654994546	2.705855406
Zm00001d011604_T072	2.397136759	2.773826735	3.062623251	1.661123415	1.70951739	1.888557014
Zm00001d011604_T014	0	0.433944378	0	1.221100631	1.01549971	0.952998469
Zm00001d011604_T018	0.554696896	0	0.571551585	1.233450033	0.820077638	0
Zm00001d011604_T053	0	0	0.878770848	1.163255901	1.182493993	0
Zm00001d011604_T060	2.411990468	1.440770986	1.679869195	0.988898417	1.329194222	1.678615518
Zm00001d011604_T037	1.234070839	0	1.108751925	0	0	0.415948995
Zm00001d041471_T002	0.883623944	0.697659965	0.95177129	0.76417033	1.014083869	0.64497298
Zm00001d041471_T001	2.377702933	1.557991806	2.555780578	2.708432484	2.307829801	2.672613842
Zm00001d032734_T004	0	0	0	0.535379154	0.695159124	0.297778355
Zm00001d032734_T007	1.094376616	1.004378421	1.385568535	1.987801805	2.398689386	1.774785373
Zm00001d032734_T008	2.252457745	1.272796004	2.549795324	2.844159247	2.700055466	2.743147408
Zm00001d032734_T001	3.06759759	2.088123953	2.739348472	3.696324059	3.565438763	3.715306182
Zm00001d032734_T003	0.218508409	0	0.407540928	0.236971345	0.109527722	0.200203002
Zm00001d020195_T001	3.871063752	3.57214376	3.962215008	1.754024619	1.83344876	1.298068801
Zm00001d048831_T001	2.618884904	2.004469032	2.766306594	2.288316034	2.266932527	2.261278359
Zm00001d038085_T001	6.279217316	5.715588989	6.388958614	6.348376979	6.355959904	6.263145008
Zm00001d047364_T001	3.989173348	3.728099991	3.765776059	4.172820068	4.297918158	4.0938887
Zm00001d041818_T001	3.172163609	2.635375736	3.10309927	3.402179837	3.809078634	3.783358166
Zm00001d008377_T001	7.034211059	6.994244886	7.15195096	6.648974769	6.54151501	6.581204002
Zm00001d017491_T002	2.634335372	3.246517843	2.144100551	2.292735229	2.040833179	2.039212457
Zm00001d017491_T004	2.505615318	2.40610641	2.190617388	1.695416174	1.646692623	1.940678699
Zm00001d018800_T001	0.501003914	0.281663833	0.656970403	0.391251908	0.29196183	0.269216315
Zm00001d028744_T001	1.957238583	1.448044064	2.443617259	1.180634832	0.881653657	0.94689402
Zm00001d024713_T058	0.828154666	0.956545153	1.321807481	1.967145731	1.909738932	2.287758619

Zm00001d024713_T004	0	0.534623427	0.217772944	1.116209699	0	0.356119014
Zm00001d024713_T027	0.878037078	0.581098603	0.19784904	1.362897935	0.855846218	0
Zm00001d024713_T053	0.098998891	0.052029083	0.423067154	1.34223272	1.179222482	1.504347843
Zm00001d024713_T026	0.949316038	0	0.84534472	1.252442909	0	0.898708323
Zm00001d024713_T032	0.371400506	0	0.449515947	0.395842631	1.210093936	0.576367339
Zm00001d024713_T014	2.117016085	1.887720606	2.321139591	0	0	0.39170724
Zm00001d024713_T019	1.685081674	0	1.27503924	2.288378349	1.936000893	1.736273223
Zm00001d024713_T045	1.190790564	0.233845122	0.961702591	0.63108472	1.251507633	0.835468968
Zm00001d024713_T013	0	1.297026444	0	2.166471548	2.658912491	2.783895997
Zm00001d024713_T042	0	0	0	2.099817981	1.765536019	1.802776796
Zm00001d002318_T002	6.242824114	6.715126543	6.216838382	7.618295744	7.620235077	7.676434306
Zm00001d002318_T001	5.355272964	6.627556924	5.445000539	6.805597822	6.861499576	6.949947046
Zm00001d045744_T003	2.978256229	2.282634622	2.983684078	3.525671139	3.426176848	3.287474659
Zm00001d036534_T001	4.412988094	3.496325948	4.298490651	3.236205582	3.477799576	3.373473101
Zm00001d017849_T001	5.106240097	4.295416162	5.085360959	5.238680258	5.307700244	5.350665976
Zm00001d038130_T013	0.538707006	0.284403973	0.406254743	0.529959838	0.890579769	0.754820816
Zm00001d038130_T007	2.475094224	1.90307183	2.829254503	2.982160335	3.114185594	3.058618858
Zm00001d038130_T006	0.384942785	0.083998934	0.360529468	0.294770708	0.642518596	0.792403945
Zm00001d038130_T002	2.044118145	0	1.782768774	1.58608352	0.983280762	2.089357468
Zm00001d051061_T001	6.040810258	6.318100258	6.023521108	6.069435782	5.994412366	5.873172651
Zm00001d032088_T001	6.461953326	5.9645617	6.395824727	7.365053379	7.320289435	7.443012055
Zm00001d038658_T001	6.298167144	5.700617242	6.356281082	6.591456192	6.561683482	6.503060024
Zm00001d027339_T002	4.610814968	4.684075996	4.598085859	4.444786447	4.466923393	4.034232622
Zm00001d038772_T001	5.98177013	5.752324336	6.158501397	3.008440455	3.390607297	3.208934526
Zm00001d034887_T001	4.941588631	4.165300895	4.880061202	5.280355845	5.119187701	5.241532587
Zm00001d008631_T001	0.362525996	0.727903033	0.833340267	0.813940815	0	0.835945088
Zm00001d008631_T003	0.679665203	0.389079958	0.684878867	1.025013907	1.184821085	0.905254614
Zm00001d015361_T001	4.106828356	3.201149693	4.1110966	4.402834849	4.346382391	4.315202105
Zm00001d028549_T001	3.3758164	2.668765197	3.291009385	3.164988158	3.131323111	3.04824665
Zm00001d018730_T001	4.414900459	3.94803431	4.564233038	5.008705772	5.049022852	4.851297501
Zm00001d043484_T006	1.589633591	1.262350166	1.55375762	2.200178828	2.461619445	2.628377937
Zm00001d043484_T023	1.713943867	1.105703553	1.973011298	1.95028214	1.588029774	1.577087012

Zm00001d043484_T009	0.976654712	0.499146337	0.987455487	0	0	0
Zm00001d043484_T005	0	0	1.797870867	2.068224349	1.941390314	2.572929675
Zm00001d043484_T012	2.187423498	0	1.125153147	2.090740652	0	0
Zm00001d043484_T011	1.533691416	0	0.958411379	0.817507019	0	0
Zm00001d043484_T002	1.801698376	2.085060877	2.004607815	2.019811491	2.221235844	0
Zm00001d043484_T022	1.959464153	0.249947694	2.32560286	1.204537654	1.93871035	0.863462758
Zm00001d043484_T010	0.596024884	0.245199913	0.128938708	0.315409722	0.803153453	0.404700433
Zm00001d038707_T001	1.094858288	0.507042594	0.985486586	2.687056656	2.824436514	2.891286772
Zm00001d052916_T001	2.432936164	1.795195907	2.213239017	2.889987642	2.741438725	2.436056006
Zm00001d021828_T003	0.239719419	0.017287695	0.176099329	0.370935372	0.48052468	0.38526315
Zm00001d021828_T001	3.095495766	1.913316071	2.843292069	3.67149323	3.617028775	3.649747422
Zm00001d021648_T059	0.639870235	0.742256343	0.769649888	0.83987254	0.360546323	0.445208638
Zm00001d021648_T055	0	0.392193208	0.922583023	0	0	1.166293178
Zm00001d040612_T020	0.184045381	0	0.188447769	0.629634523	0.923088314	0.883460508
Zm00001d040612_T001	0	1.304452048	0.439264613	2.605284765	1.342146227	1.632070879
Zm00001d040612_T015	2.296364602	0	2.542434382	2.408711318	2.810272397	2.894364177
Zm00001d040612_T002	2.110146054	1.53742281	1.35784924	1.063134974	1.684800788	0.778965477
Zm00001d040612_T007	0.171235015	0.172825428	0.591532938	0.498776781	0.582970207	0.544993727
Zm00001d018284_T001	5.288827842	4.708437009	5.346528838	3.807394183	4.426982569	4.64591074
Zm00001d005062_T003	3.746635778	2.771185607	3.710544728	4.105022448	4.25152619	4.224510031
Zm00001d005062_T001	0.143136075	0.368719188	0	1.290440919	0.501252634	1.428830603
Zm00001d012510_T001	2.724995517	1.692577173	2.310010532	3.153733686	3.084106796	3.074938488
Zm00001d024430_T006	3.669391703	1.595649259	3.754277167	3.314233484	2.609274378	3.952048914
Zm00001d024430_T016	0.814815355	0.351379567	0.531688371	0.21768362	0.237437676	0.205432553
Zm00001d024430_T003	4.631872351	4.251904548	4.862209252	5.08306441	5.247972009	4.930630811
Zm00001d024231_T001	3.33370838	2.913368543	3.403600131	4.091402126	4.007154678	4.078325469
Zm00001d050848_T001	6.139571673	5.652468511	6.1879361	5.823909642	5.855643678	5.945941419
Zm00001d026245_T001	3.301586915	3.229683894	3.196295748	2.946232099	3.191449265	2.963742847
Zm00001d010462_T001	3.230608983	2.241583038	3.318704401	3.888396181	3.818903618	3.666159397
Zm00001d025762_T001	4.715348634	5.12493714	4.667361431	4.609444834	4.670168107	4.538152297
Zm00001d015690_T003	1.206855737	1.363709359	0.849671865	0.881394456	1.269941579	0.993993114
Zm00001d015690_T005	1.15949098	0.671390304	1.639567441	1.110060506	0.499287193	0.485126922

Zm00001d019461_T001	1.626571368	1.096962112	1.524659492	1.518789917	1.343914311	1.198297381
Zm00001d045872_T002	3.78145624	3.351444447	3.805333631	4.220981729	4.301723862	4.184852656
Zm00001d045872_T003	0.259994332	0.073614609	0.166995603	0.266930428	0.284774695	0.211653941
Zm00001d033099_T001	6.103676035	5.769183938	6.145065138	5.749758136	5.814180474	5.659255473
Zm00001d015229_T001	2.774610605	2.722079068	3.209176186	3.139976445	3.367522177	3.048924929
Zm00001d039880_T001	3.38516332	3.133837046	3.187576314	3.393472628	3.544439884	3.64605422
Zm00001d023421_T002	2.755244605	1.960546183	2.799211671	3.093200303	3.166882839	2.497340533
Zm00001d030379_T001	1.428363255	1.125721846	1.679502619	1.489926678	1.498444916	1.674363474
Zm00001d046935_T001	1.599273988	1.14125882	1.768704342	2.12201002	2.140909507	1.963124135
Zm00001d048707_T001	5.952873008	5.241447087	5.974998617	6.301326189	6.35184778	6.367787126
Zm00001d013232_T001	1.79785593	1.782031486	2.19640781	2.440186277	2.318155424	2.569925099
Zm00001d029722_T013	1.404712421	0.580348123	1.088603647	0.355768435	1.02262206	0.275429063
Zm00001d029722_T001	3.189347323	2.760155997	3.381483841	3.585979122	3.448491601	3.664176064
Zm00001d029722_T014	0.58771926	0	0.435928302	0.928968313	0.745278644	0.219670994
Zm00001d029722_T005	0.143896217	0.253006568	0	1.005439513	0.485697822	1.079272967
Zm00001d029722_T010	2.971923686	1.974320051	2.968906359	2.937596183	2.990731452	2.942743254
Zm00001d029722_T017	0.265843722	0.270629404	0.479203653	0.732781488	1.255311584	1.008540672
Zm00001d029722_T012	0.456690519	0.039612896	0.073225213	0	0.584999049	0.41607983
Zm00001d015589_T001	3.587654941	3.493506567	3.757843919	4.631626714	4.411743563	4.607189085
Zm00001d016062_T001	4.432026965	4.924570633	4.410917348	4.513426252	4.606513532	4.30395408
Zm00001d035978_T001	2.218617209	1.767024196	1.854568621	1.117625898	1.56402544	1.33915107
Zm00001d003242_T001	5.25499147	4.633369653	5.232878181	5.086712148	5.08007484	5.112331198
Zm00001d004513_T001	3.290946741	2.309129611	3.247426432	2.866427725	3.012149212	2.991834545
Zm00001d020280_T001	3.650849097	2.789672919	3.670034145	4.168343422	3.999962039	4.1771343
Zm00001d037484_T002	0.4537525	0.6359308	1.172760155	1.70420401	0.870567521	0.706696749
Zm00001d037484_T003	3.559566332	3.252143432	4.159227691	5.027993904	4.78536233	4.985917149
Zm00001d037484_T001	5.397739654	4.486051881	5.349125434	4.699224568	4.973534914	4.881604865
Zm00001d028358_T007	0.500745975	0.132138539	0	0.926379784	0.792051549	0
Zm00001d028358_T010	2.092008817	1.410751384	2.180584869	2.270911318	2.314793089	2.52621057
Zm00001d049612_T001	1.758418742	0	2.64399699	2.444252272	2.216968215	2.120732141
Zm00001d020322_T001	0.872864211	0.616213703	0.913336371	2.375889641	2.37825025	2.23683056
Zm00001d012195_T002	3.414062314	3.040916096	3.405457783	3.458077473	3.617616447	3.450798459

Zm00001d012195_T001	2.670824477	1.65234239	2.805161596	2.534885337	2.680590814	2.304552511
Zm00001d012195_T007	1.76197709	1.616280525	2.330435637	2.322468427	1.802721798	0.551685317
Zm00001d012195_T003	0.463058451	0	0	0	1.277189112	1.94140947
Zm00001d040246_T001	3.858004675	3.963587963	3.972816243	4.241891653	4.180572776	4.287591308
Zm00001d006143_T001	0.882912979	0.5689174	1.21564878	1.97603693	1.945313666	2.142902858
Zm00001d033602_T004	3.581101496	2.72630155	3.708776336	3.375665599	3.06020704	3.013294665
Zm00001d033602_T002	4.574483996	4.051202762	4.631292147	4.395596306	4.448948974	4.619928427
Zm00001d033602_T003	0.422233001	0.316335772	0.421169967	0.912306489	1.177255754	1.414228642
Zm00001d050273_T001	0.963951244	0.481967428	0.74454605	0.720941208	0.723289437	0.581937366
Zm00001d013829_T001	0	0	0.497626671	1.851105368	1.851414047	1.890854442
Zm00001d013829_T020	2.990772289	2.339292195	2.919058	2.931331322	2.947243852	2.917286046
Zm00001d013829_T017	0	0	0.044949406	0.789261838	0.908848254	0.414960313
Zm00001d013829_T003	0.812346247	0.390003959	0	0	0.199778492	0
Zm00001d019409_T001	0.848064231	0.806093868	1.331255773	0.16577562	0.6906665	0.520341782
Zm00001d019186_T001	7.05523578	6.791370023	7.104710894	7.002867728	6.988523448	6.910705239
Zm00001d051838_T001	4.731115614	3.433794593	4.59477049	4.577331175	4.543356185	4.436743918
Zm00001d051838_T002	2.707362666	4.372098065	3.302352394	0	0	0
Zm00001d014916_T001	1.912623808	1.339067815	1.570946663	1.546487547	1.62064224	1.639166858
Zm00001d021216_T001	1.339648225	1.100222137	1.382716494	1.100254438	1.027919793	0.873190324
Zm00001d018339_T001	4.04492843	3.091273478	4.031195524	4.563644439	4.631098751	4.51426324
Zm00001d048601_T001	3.963850655	3.297882072	4.06255582	5.495725186	5.596316946	5.603340151
Zm00001d020872_T001	9.041917218	5.330412689	8.844405493	8.611348179	8.671043317	8.6934227
Zm00001d030040_T001	4.163309769	3.506408353	4.232654771	3.921022468	3.831608129	3.760801421
Zm00001d028770_T001	4.098197783	3.402295144	4.248538479	4.936045146	4.813815149	4.937005338
Zm00001d028770_T002	1.526687835	1.484267053	1.494545153	3.066153787	3.045332528	2.366375095
Zm00001d028770_T003	0.756726994	0.161191152	1.155911106	2.207755133	1.770771548	2.399596045
Zm00001d033390_T001	1.812376233	2.991808611	2.068278358	2.371520948	2.534891561	2.979571575
Zm00001d011718_T001	1.566510269	0.801797688	1.109920811	1.531886955	1.407942218	1.304931523
Zm00001d011718_T003	5.617935546	5.684360334	5.662780868	5.808079952	5.839906594	5.8706912
Zm00001d014733_T001	9.925938129	11.14963503	9.86674916	9.06265968	9.152083607	9.279374333
Zm00001d014017_T001	4.165232306	5.32203907	3.846439659	4.844843579	4.653189315	4.816394295
Zm00001d014017_T003	3.397103482	4.277681378	3.109666619	2.5127495	2.268887373	2.602546672

Zm00001d014017_T002	1.282139669	0.625003905	1.351026704	0.992968509	1.081440531	0.962138088
Zm00001d014017_T004	6.238221018	6.752594655	6.204413936	5.507560175	5.678346864	5.440222828
Zm00001d043855_T001	5.152332184	4.641792584	5.233372567	3.899734808	3.982877881	3.991144394
Zm00001d043855_T004	3.96701898	4.292621793	3.28479764	1.26413284	0.511444762	1.362260027
Zm00001d043855_T003	6.2801465	6.302137405	6.404129979	4.995261585	5.15901822	5.107731635
Zm00001d025401_T001	7.950612362	8.140105901	7.882960572	8.201751487	8.218268971	8.271871145
Zm00001d027435_T001	3.520156693	3.382093519	3.856772904	3.961973496	4.250884966	4.002053116
Zm00001d027435_T002	5.049600232	3.842428492	5.050760262	4.568332898	4.512876459	4.712576522
Zm00001d049431_T001	2.189454865	0.894568677	2.11167871	3.290651908	3.509961051	3.409861076
Zm00001d045926_T002	5.579469032	5.09885871	5.537676417	5.019787211	5.148330877	4.853529539
Zm00001d044945_T001	3.685406129	3.015770511	3.61811964	3.185404333	3.017936335	3.101565535
Zm00001d036749_T002	1.782313361	1.19760752	2.16941548	1.762487869	2.036561357	2.20973595
Zm00001d036749_T001	1.62476347	1.181086644	1.770740685	2.588698807	2.408086014	2.577401756
Zm00001d002893_T001	1.360469911	0.6858128	0.925976639	1.199365165	0.864917882	1.252470764
Zm00001d027925_T001	8.867444822	7.968332354	8.971649453	8.735445089	8.764875319	8.745298795
Zm00001d046496_T001	5.1227598	6.304724108	5.117750639	4.112664107	3.997632489	4.247915592
Zm00001d004410_T001	4.266534703	4.655356695	4.042578329	3.152217336	3.472724172	3.169666736
Zm00001d038310_T003	0.477830654	0.328884706	0.564514749	1.49619746	1.241898744	0.900163132
Zm00001d038310_T001	2.558465287	1.799084404	2.727515354	1.805398126	2.175780703	2.198000598
Zm00001d038310_T004	2.620249977	1.897537842	2.667881001	2.79230794	2.826166498	2.813222574
Zm00001d038310_T002	2.338101466	1.870402205	2.545158108	2.332625737	2.476011958	2.746362432
Zm00001d009000_T001	8.128354024	8.833771345	8.063164658	7.506435124	7.555807721	7.367087776
Zm00001d046758_T001	2.840873878	2.648416432	3.0063474	2.795232071	2.791214577	2.868114235
Zm00001d040694_T001	0.036285287	0.046067724	0.03459888	1.706786469	1.923219174	1.833877391
Zm00001d034250_T002	2.207851007	2.284589938	2.038100403	2.805450751	2.077302483	1.979317859
Zm00001d034250_T004	0.57887207	0.533203502	0.795313957	0.665968461	0.289600783	0.678106169
Zm00001d034250_T001	3.284969641	1.864769742	3.527698687	4.202399149	4.447141363	4.418093585
Zm00001d034250_T005	0.31954626	0.087963794	0.286754611	0.404590359	0.589042439	0.479361992
Zm00001d043147_T001	1.871659949	1.435831784	1.772907143	1.712198242	2.054614432	1.677515008
Zm00001d043147_T002	2.740162956	1.959300183	2.946647402	3.071520884	3.290515086	3.035195436
Zm00001d046644_T001	1.350462169	0.875261858	1.363409964	1.713972012	1.768232608	2.014129586
Zm00001d043244_T001	1.426711317	0.828717686	0.863334294	1.315617237	1.916741082	1.413468978

Zm00001d003644_T019	2.573209693	1.732240541	1.38646223	1.625041324	0.978320853	0.687284692
Zm00001d003644_T018	0	0.675862891	2.173768986	1.745720086	2.144856271	2.372206048
Zm00001d003644_T002	0.854713025	0.178194508	0.366574568	1.648861152	1.408377639	1.221920377
Zm00001d048865_T001	1.310584427	1.029223874	1.051326854	0.778308678	0.679490459	0.648247297
Zm00001d006061_T007	1.666314401	0.920253659	1.518059198	2.021326211	1.478934023	1.988815546
Zm00001d006061_T005	4.017981485	3.695503703	4.038918287	4.123077478	3.759345375	3.89635866
Zm00001d006061_T004	1.74785526	1.033332076	1.521757334	1.958268452	2.742086646	1.083376779
Zm00001d034130_T001	1.753100018	2.011246223	1.564318167	5.182115859	5.332896653	5.112381078
Zm00001d014343_T001	3.027247845	2.386487614	3.131479517	3.39477917	2.746492716	2.916662559
Zm00001d014343_T006	6.28464603	5.056984809	6.287922384	5.803025138	5.907054212	5.794755549
Zm00001d014343_T008	0	0	0	2.769977129	3.294004724	3.030102085
Zm00001d014343_T005	2.953567903	0.573952264	2.715772812	4.016746791	4.09439469	4.120627231
Zm00001d033216_T001	3.925034104	4.89883029	4.016111704	3.175401164	3.402329393	3.445114461
Zm00001d016278_T001	5.34976783	6.175876649	5.11412961	4.61171205	4.763719203	4.469854726
Zm00001d031929_T010	3.439296001	2.717158084	4.364549111	2.252129199	2.921732419	2.632489489
Zm00001d031929_T008	4.034243012	0	2.459003993	3.752479827	4.243969789	3.94964082
Zm00001d031929_T009	5.069496728	4.706749068	4.727154697	3.785802452	2.5001759	2.668942833
Zm00001d031929_T004	1.577598497	3.267967493	0.678829121	2.829861532	0	2.686899832
Zm00001d031929_T001	2.222679386	1.002770919	2.469964359	1.779103779	1.755376439	1.553204182
Zm00001d014302_T003	3.93590748	2.8815501	4.018169995	4.385855017	4.375151297	4.294027082
Zm00001d014302_T001	0	1.877238138	0.286829115	1.776979449	1.254224555	2.530274044
Zm00001d040048_T001	3.381779508	4.290043479	3.329980059	5.160910187	5.046162173	5.32498625
Zm00001d041775_T001	5.188017094	4.60935157	5.069017343	5.044687875	5.147508434	5.095030305
Zm00001d031436_T003	1.768783091	1.192011686	1.664304427	1.403756356	1.909450933	1.413230655
Zm00001d043410_T001	5.340703683	4.363546494	5.438201516	5.299933379	5.297364075	5.287964979
Zm00001d033308_T001	3.202260827	2.748613223	3.428261002	3.871321365	3.80391484	4.0310666
Zm00001d021485_T006	0.654726067	0.437912697	0.569505674	1.174211447	0.853293884	1.097341783
Zm00001d021485_T004	0	2.032374894	0	2.852835238	3.320905728	2.69635511
Zm00001d021485_T007	0.207006977	0.110154742	0.211572959	0.489728912	0.677108585	0.4948385
Zm00001d021485_T003	5.364687108	3.43317633	5.556901632	5.297829408	5.250467836	5.356265145
Zm00001d038156_T007	0	0	0	2.948419939	2.170327617	3.051611023
Zm00001d038156_T008	0.620743104	0	0	2.345348682	2.185619522	2.114854727

Zm00001d038156_T001	0.349372072	0.152404107	0.389322304	0.334759326	0	0.758439636
Zm00001d038156_T017	0	1.087707909	0	1.711323535	0	1.816301512
Zm00001d038156_T016	1.357402238	0.222389124	2.020254338	0	1.557490476	0
Zm00001d038156_T014	0.544954174	0.175698321	0.529803959	1.19173445	1.421006727	1.099515379
Zm00001d038156_T003	2.767825115	1.406064765	2.690216155	2.811996018	3.430996943	3.182158656
Zm00001d038156_T005	4.755848551	4.437577915	4.945801604	4.537104938	4.619843935	4.470569532
Zm00001d028365_T006	2.116133494	2.790754942	2.650775816	2.683538142	2.509799702	2.395197189
Zm00001d028365_T003	1.877044128	1.319450304	1.364158333	1.091897483	1.700313643	1.306271574
Zm00001d028365_T007	2.133622048	1.666201218	2.633896611	2.120653189	2.023279486	1.665094377
Zm00001d028365_T002	0.41969313	0.085628604	1.323235738	1.297627535	0.193668307	1.011045442
Zm00001d028365_T001	0.260232859	0.076564568	0.499413747	0.563398315	1.036845812	0.43975929
Zm00001d053733_T001	1.035925788	1.180708659	1.100587496	1.947586867	2.203205542	2.344084806
Zm00001d005732_T009	0	0.286979295	1.034825632	0	0	0.736533875
Zm00001d005732_T006	4.763052928	4.006941751	4.840571898	4.584060595	4.6778977	4.70280729
Zm00001d047913_T001	8.625481982	8.609411767	8.442711104	7.653951649	7.682490121	7.767568911
Zm00001d042735_T001	6.845816502	7.837743319	7.075666947	5.118010681	5.138734331	5.19456745
Zm00001d049113_T001	4.512584608	3.862591042	4.63649818	3.417396855	3.656673922	3.45680155
Zm00001d008744_T008	0	0.78151667	0	1.49052957	0.454043201	1.230780345
Zm00001d008744_T007	3.359546049	0	4.123711797	4.151259896	4.12305297	4.516515337
Zm00001d008744_T009	1.339551316	0	1.105169827	1.893207642	2.606917126	1.893802913
Zm00001d008744_T003	4.328729134	3.77066458	3.84786816	3.277706444	3.366557363	2.439630318
Zm00001d008744_T001	3.623678321	3.004105833	3.828739922	4.580344806	4.751376094	4.645849367
Zm00001d033316_T001	5.879334729	5.431236596	5.996131122	6.704559148	6.800332829	6.806274686
Zm00001d032308_T001	2.233527955	1.948543288	2.951068324	2.475286483	2.815113037	2.706366327
Zm00001d032308_T002	4.096929401	3.524617623	4.197487216	4.123182458	3.951250206	4.16369242
Zm00001d011656_T001	1.649668798	1.559355246	1.760437281	7.145830469	7.143673438	7.152719168
Zm00001d042598_T003	1.743532202	2.262759967	1.614364498	3.195703346	3.059826292	3.533280388
Zm00001d042598_T002	5.228299766	4.237278042	5.157042052	5.436846271	5.533585493	5.341207457
Zm00001d005516_T001	0.501841642	0.699583613	0.815754935	0.378133144	0.764105771	0.427262889
Zm00001d005516_T004	2.965370206	2.323742441	3.176155672	3.242023178	2.97987188	3.333380653
Zm00001d005516_T005	0.612103338	0.044105901	0	0.772839174	0.549881063	1.126746261
Zm00001d007774_T001	2.137896932	1.780328579	2.26062008	1.624817735	1.90978923	2.012404193

Zm00001d018995_T001	1.40263869	1.026212949	1.020069746	0.697716893	0.556459826	0.603113322
Zm00001d044426_T001	2.81428852	2.156564242	2.819571319	2.563744366	2.778735505	2.677814
Zm00001d049560_T001	4.966177429	4.229699274	5.180679701	5.198228456	5.170761121	5.10226289
Zm00001d002601_T001	8.962474859	8.78276504	9.065041273	7.895265051	7.942435577	7.897916627
Zm00001d010230_T006	0.085356667	0.115633908	0.311735602	0.974817823	1.266158063	0.864977292
Zm00001d010230_T002	1.391476842	2.039635003	1.34278342	0.810670133	0	0
Zm00001d010230_T003	2.863986407	1.456777768	2.726417091	4.08821183	4.195888744	4.343473986
Zm00001d010230_T004	0.706977824	0.302889874	0.550059422	2.34934178	2.041370211	2.030417305
Zm00001d021137_T001	3.245449418	2.59813101	3.26922245	3.237861649	3.237345431	3.136588598
Zm00001d011095_T001	6.036976418	5.180968256	5.945319051	5.882031021	6.052427024	6.051518039
Zm00001d032618_T001	3.333990682	3.27249057	3.775581575	3.399003294	3.42533442	3.36305891
Zm00001d015415_T004	4.633134561	4.107001924	4.502616388	4.83277424	4.83960693	4.675964483
Zm00001d015415_T008	0.734577401	0.510553857	0.655892166	1.338195619	1.198311841	1.122911216
Zm00001d015415_T007	1.919884256	2.378520224	2.681270913	2.260030475	2.090734556	2.179970856
Zm00001d015415_T003	2.103782961	1.748578877	2.05644168	1.945945117	1.727312326	2.087550758
Zm00001d030012_T001	1.026976358	1.366972872	1.326906603	2.423499379	2.492331863	2.275336684
Zm00001d030012_T002	2.847694911	1.876688635	2.808804308	3.840427984	3.667626604	3.699888503
Zm00001d045493_T002	3.824779562	3.457880544	3.638163872	3.874570169	3.638346127	4.001054495
Zm00001d027636_T001	4.183150058	4.495046955	3.951934627	4.01554565	4.044917766	4.154195865
Zm00001d020431_T002	1.584047542	1.117486269	2.212091163	2.460010417	2.579699579	2.305880076
Zm00001d020431_T003	0.942938572	0.544766281	0.592677566	1.174357838	1.187878573	1.46975993
Zm00001d039142_T002	2.957713313	1.303165262	2.984894262	1.7671628	2.79879625	1.872441888
Zm00001d039142_T001	3.515837195	3.284341633	3.720097953	3.574454083	3.549448992	3.660236462
Zm00001d016625_T001	11.90620601	12.07127527	11.95185395	10.21959451	10.21457123	10.20061104
Zm00001d006358_T001	2.926249591	2.5822313	3.208091287	1.971639562	2.150769567	1.977594259
Zm00001d041173_T001	7.866791737	7.001972596	7.921674777	7.071839924	7.074524029	7.058454459
Zm00001d017495_T001	3.091647372	2.912657912	3.277506943	2.44688479	2.747126123	2.66695333
Zm00001d002159_T001	9.500326723	11.76358366	9.698873181	6.415666332	5.795719006	5.916639965
Zm00001d016606_T037	5.081233262	0	4.341237457	4.72988347	4.785877972	4.581013253
Zm00001d016606_T005	0	0.116247633	0.395553123	0.532477555	0.905810671	0.150433598
Zm00001d016606_T022	0.352488489	0.222156624	0.315674037	0.303296798	0.153509634	0.315824721
Zm00001d016606_T020	2.662108861	0	0	2.82040855	4.444107795	1.144666399

Zm00001d016606_T024	0.400620991	2.976994654	1.113213074	1.476067449	1.172194338	2.070680929
Zm00001d016606_T001	0	0	1.093541247	1.787498914	0.246470111	0.820686293
Zm00001d016606_T017	0.494165709	0.172258356	0.316623085	0.323345856	0.540264392	0.745732992
Zm00001d016606_T026	1.27555183	0.77429327	1.640265069	0.991726269	1.242269573	1.361069297
Zm00001d016606_T011	0.880346996	0.272715994	0.661017123	0.887650819	1.037594481	0.406990468
Zm00001d016606_T010	0.413266948	0	0.473829504	0.673323041	0	1.023735816
Zm00001d017167_T001	5.239247829	4.858499476	5.008024544	4.994019417	5.033984684	5.174358996
Zm00001d032094_T001	4.391153385	4.169854147	4.596591895	5.93803584	5.854093919	5.994572823
Zm00001d013469_T006	1.482886995	2.007218111	2.214435698	1.188258484	0.491765891	0
Zm00001d013469_T001	2.476984564	1.002389287	2.221079899	2.904097144	2.966034115	2.944312179
Zm00001d027827_T001	2.037266591	1.417063964	1.963673868	1.810146102	1.812469063	1.667805856
Zm00001d010375_T001	0.318582938	0.271771069	0.34260822	2.521567652	2.430655669	2.552281151
Zm00001d042549_T002	2.72149472	1.781141037	2.59338437	3.336929171	3.100329298	3.125577048
Zm00001d042549_T003	0.562512557	0.414187502	0.297552999	0.236752204	0.502577056	0.330148766
Zm00001d032740_T001	2.309893572	1.853440013	2.45247604	2.732032096	2.946907491	2.870102495
Zm00001d044622_T002	0.833394515	0.650336282	0.714087225	0	0	0
Zm00001d044622_T001	3.686405951	2.721687634	3.455431387	2.948082917	2.904342278	2.919044076
Zm00001d044622_T003	5.192938839	4.945668423	5.287089814	3.74247819	3.70707548	3.274336665
Zm00001d042197_T001	2.674299967	2.404784346	2.643478731	2.081866906	2.683485143	2.084467414
Zm00001d048112_T001	5.257195471	5.401632548	4.982755881	4.929805197	5.249910274	5.024384445
Zm00001d040142_T001	4.210893782	3.150325707	4.109381289	4.092010001	3.989827121	4.057023206
Zm00001d040142_T002	0.42234712	0.543646332	0.870814474	1.801944162	1.842633348	1.382975401
Zm00001d040142_T004	1.434193185	0.068395658	1.506669978	1.079905765	1.635143293	1.748671181
Zm00001d040142_T005	0.986585686	0.24042913	0.492510559	0.706926562	0.907497554	1.18670465
Zm00001d022517_T001	6.206773158	5.546222918	6.267221064	6.624336659	6.575552422	6.539373881
Zm00001d001946_T001	5.827589191	5.370220997	5.805824751	5.385944552	5.400558286	5.550449721
Zm00001d015346_T001	1.112845562	0.46223559	0.872482865	0.774181923	0.93429758	0.981127837
Zm00001d002512_T001	2.410143521	2.545661023	2.465183834	3.119248095	2.977751951	3.262947992
Zm00001d046922_T001	3.949986813	3.760069003	4.06407956	3.586405196	3.586804608	3.609548375
Zm00001d021439_T001	4.334498342	4.15338811	4.120965652	4.367505753	4.517833889	4.037189352
Zm00001d031975_T001	7.140399224	7.647724016	7.257008185	7.020896868	6.892193601	6.922410966
Zm00001d008934_T001	2.482349835	1.759771565	2.23169352	1.841646312	1.704159292	1.881624685

Zm00001d049252_T001	3.686268557	3.243050563	3.822415474	4.80549868	4.746600094	4.695090622
Zm00001d014316_T001	3.112679785	2.59553493	3.149649582	3.399537123	3.193137764	3.488795963
Zm00001d039258_T001	1.613968638	1.152811642	1.643534837	1.395440155	1.743734253	1.694760316
Zm00001d032733_T002	0.088680301	0.135118483	0.158401663	0.547027274	0.333278312	0.419078234
Zm00001d042274_T001	4.503935999	4.344998742	4.721492095	4.892913702	4.690179495	4.705002841
Zm00001d033167_T001	6.418732442	5.595105008	6.454181372	5.833629522	5.855551492	5.747592644
Zm00001d011112_T001	1.820106647	1.147079367	1.479934143	1.988802824	1.590482738	1.748260702
Zm00001d037034_T002	4.564680274	3.43135044	4.598671233	4.816726973	4.759844945	4.857382003
Zm00001d037034_T001	2.554355055	3.194664204	2.742939887	2.369070543	2.608948775	2.027396132
Zm00001d037617_T001	2.752897562	2.039183324	2.397535477	3.744940048	4.004533392	3.920684414
Zm00001d011898_T001	3.657172597	3.140539823	3.761648668	4.475694851	4.472439031	4.448814744
Zm00001d047501_T001	3.034530214	2.54112227	3.105665173	2.742155217	2.79734092	3.036636608
Zm00001d034795_T001	6.298876005	4.918844494	6.318569128	5.937342556	5.899381408	5.928867647
Zm00001d048969_T004	0.721026984	0	0.892334289	0.656479896	0.328368891	0
Zm00001d048969_T002	0.536414026	0.086430521	0.182059152	0.695246446	0.698295835	0.36989525
Zm00001d046454_T001	4.756047337	3.558126542	4.554768287	5.493869472	4.931253777	5.046468447
Zm00001d046454_T008	3.040131791	1.822198649	2.564517431	4.412933576	4.990697194	4.202682727
Zm00001d046454_T004	0	0	0.255262631	1.255356305	2.166201896	0.68667801
Zm00001d046454_T006	2.053307043	2.751619466	3.318937609	3.889916894	3.73142005	4.470392914
Zm00001d011805_T001	3.287034426	2.345743239	3.072510933	3.159956726	3.065110582	3.278520379
Zm00001d005143_T001	3.838067934	3.400872334	3.581726637	3.763907402	3.861762959	3.849508339
Zm00001d010451_T001	6.850227514	6.419330141	6.88991221	6.003629017	6.078388858	6.127035052
Zm00001d017714_T001	1.450737764	1.001869963	0.834663489	2.839773194	2.67000207	3.177139962
Zm00001d017714_T002	4.108769151	3.083872517	4.113456807	5.994648486	6.089518362	6.054557456
Zm00001d042930_T001	7.833430544	8.446379296	7.87371238	7.5728633	7.612602744	7.457445891
Zm00001d048309_T001	1.838078425	1.654056944	1.830318613	1.853936182	1.8195002	1.5302196
Zm00001d000112_T001	5.085434557	4.281442993	4.987144657	7.920454844	7.959324083	7.910745672
Zm00001d000112_T003	1.190965615	0.194390945	1.312386365	2.697433341	3.029274945	2.610274135
Zm00001d000112_T002	0.496939845	0.310254022	1.01275101	2.289754805	2.293863365	2.750379257
Zm00001d000112_T004	2.342781713	1.591464481	2.687382571	5.487624425	5.427430987	5.541954216
Zm00001d007845_T001	0.843062476	0.651363567	0.844336638	0.423325375	0.243973672	0.491102956
Zm00001d040334_T001	6.287394499	5.478190403	6.169878794	6.548925825	6.318714395	6.487158648

Zm00001d035693_T001	5.327107871	5.520566794	5.334351633	4.976539012	4.961054935	4.94860024
Zm00001d006494_T007	1.311401972	0	1.610539439	1.609927016	1.759640345	1.744154205
Zm00001d006494_T003	2.906359201	2.378733559	2.80029133	2.903874521	2.739776398	3.12732556
Zm00001d006494_T006	1.58831959	1.417598713	1.200371264	1.394100865	1.376166449	1.48131186
Zm00001d006494_T001	0.561595952	0	1.377405318	2.007067376	2.121513459	1.813598156
Zm00001d033039_T001	4.218200564	3.257955304	4.320628579	3.778027913	3.749867068	3.850761325
Zm00001d043376_T001	2.530797901	2.700940136	1.847026769	0	0.904172693	0
Zm00001d043376_T004	0.45673467	0.306462908	0.709000303	0.629531948	0.502231805	0.266343996
Zm00001d043376_T006	3.021129316	1.050482194	3.341041569	4.017026981	3.972648913	3.986246359
Zm00001d043376_T003	1.050633336	0.573985216	1.120923198	1.758086522	1.899853499	2.041965901
Zm00001d053014_T001	2.66382005	1.917386306	2.000117935	2.521457851	2.721429316	2.455433754
Zm00001d053014_T003	0.816820855	0	1.66230669	2.117975576	1.988914773	2.125499022
Zm00001d053014_T002	0.534947074	0	0.600446749	0.660515212	0	1.170829448
Zm00001d046168_T002	1.940991717	1.580110261	1.44991735	2.252715987	2.271030285	2.013880983
Zm00001d046168_T001	1.11636276	1.521959298	1.681866608	1.871547195	2.39369049	2.467228607
Zm00001d003322_T009	0.357794076	0	0.36525356	0.407720381	0.420179468	0.344616053
Zm00001d003322_T006	2.25897985	1.2315258	2.071838851	2.001198382	1.992947125	2.111855283
Zm00001d003322_T003	1.015982763	0	1.267195384	0.337965646	0.877497695	0.630602104
Zm00001d028428_T001	4.462372766	3.661942463	4.593159825	5.183273112	5.402332292	5.279494845
Zm00001d028428_T002	1.143718625	0.996126217	0	1.95519152	1.28101687	2.074174524
Zm00001d009705_T001	0.300674334	0.198534385	0.494736117	0.813228321	0.93817135	0.978831886
Zm00001d002067_T001	4.652788081	4.313186561	4.577926782	4.67266464	4.394989634	4.707650348
Zm00001d043535_T001	3.485097031	3.154720104	3.554939599	5.021592189	5.032171742	5.148590956
Zm00001d012121_T001	0.815065477	2.538603468	1.621029707	1.892846399	2.243958444	1.563244055
Zm00001d012121_T002	4.853230447	4.181209119	4.712972967	5.100724145	4.977773744	5.06598794
Zm00001d009373_T001	6.727647991	6.025937061	6.744359264	6.075616401	6.216001672	6.113308438
Zm00001d016081_T002	1.729774188	1.053155833	1.730952913	2.208905817	2.074048442	2.150770542
Zm00001d014415_T002	4.284413523	4.152020721	3.868396182	4.704982154	4.604410127	4.598721607
Zm00001d017398_T001	5.841205791	5.048997054	5.900299663	5.895193494	5.674831955	5.832691339
Zm00001d017398_T005	1.76457546	1.009825125	1.496720544	2.397194	2.667777023	2.29531484
Zm00001d017398_T002	3.635503087	2.609670109	3.794408692	3.366106196	3.962204364	3.676367617
Zm00001d022049_T001	3.45466601	2.748958147	3.489465353	3.416459836	3.375734261	3.444376325

Zm00001d022049_T007	1.880616572	1.538998958	2.180073369	1.704289016	2.133524071	1.302373453
Zm00001d033061_T002	1.706461151	1.288290634	1.737839458	1.436028013	1.382779012	1.055246335
Zm00001d029868_T001	1.315253777	0.976049032	1.448481293	1.913610575	1.711072829	1.384681469
Zm00001d029868_T004	0	0	0	1.2290255	1.94856833	0.9614233
Zm00001d029868_T002	2.847763652	1.94038967	2.807666511	1.932401389	2.020321198	2.586235838
Zm00001d031412_T008	1.936438186	1.580812155	1.958874219	2.171456331	2.443916108	2.446668332
Zm00001d031412_T006	3.893712066	3.250131634	4.28616805	3.847315473	3.890618386	3.64091876
Zm00001d031412_T001	2.518881294	2.557171357	2.138871468	2.395539688	2.311909942	2.695771603
Zm00001d031412_T007	4.579530459	3.093840035	4.474342197	3.889358862	3.85816535	3.9914784
Zm00001d045138_T002	2.575406006	1.497990904	2.599655811	1.342062006	2.219879429	1.452029209
Zm00001d045138_T001	2.294681322	1.769862698	2.104545332	2.175275801	2.414520922	2.240499044
Zm00001d012854_T004	4.389731377	4.502751114	4.400682875	4.178125653	4.350934515	4.342853664
Zm00001d012854_T006	2.952779726	1.311030471	2.623849776	2.586456599	2.629972971	2.587247362
Zm00001d051052_T001	6.230966399	5.529728105	6.295163045	5.431573889	5.431888042	5.35791105
Zm00001d045430_T001	4.800611694	4.41387485	4.925054334	4.302476693	4.1183471	4.178843928
Zm00001d041965_T001	5.534178636	5.478922345	5.661691804	5.328595516	5.188505293	5.290239521
Zm00001d018429_T001	4.516191501	3.782616566	4.354193163	2.278820376	2.459906843	2.432642781
Zm00001d010495_T001	2.810536675	2.211378274	2.923038478	2.66471652	2.708787369	3.083090968
Zm00001d010495_T003	0.39374018	0.133804174	0.296982409	0.568833758	0.51412226	0.615400288
Zm00001d006573_T019	1.883102291	0.954159079	1.644653261	1.805658552	2.27538943	0
Zm00001d006573_T059	4.71865304	4.190527869	4.538955951	4.345877052	4.593252588	4.421440781
Zm00001d006573_T049	3.608254645	1.337687689	3.602718875	3.899835136	4.254612636	3.948862079
Zm00001d006573_T054	0	0	0	3.145964824	0.673853977	3.050365693
Zm00001d006573_T002	0.11168572	0.200461666	0.161081482	0.550535267	0.638093305	0.551079883
Zm00001d006573_T001	0.554844217	0	0.741259008	3.443037152	2.940807597	3.424244104
Zm00001d018528_T001	1.299255796	0.751378879	1.315243921	1.348299849	1.389664824	1.140414828
Zm00001d006753_T001	2.32384157	1.766475586	2.248973164	3.814009542	3.784916422	3.82815233
Zm00001d006753_T005	1.392961408	0	1.74217182	2.705608392	2.205554854	2.32811666
Zm00001d051460_T001	1.815070807	1.919194375	1.852171471	4.71949725	4.86743967	4.795190399
Zm00001d009888_T001	3.172083741	2.452596225	2.758326419	3.307445721	3.104952023	3.287118966
Zm00001d014428_T001	6.872359734	6.445413099	6.78358147	6.853713104	6.933870494	6.88591844
Zm00001d043870_T001	2.734207123	2.064805612	2.648216919	2.66987716	2.608190407	2.432397716

Zm00001d015234_T003	3.418525818	2.258287653	3.655487962	2.947819675	2.831184298	0
Zm00001d015234_T006	0.675348052	0.179223119	0.851324889	0.672849828	0.751870721	0.649551082
Zm00001d015234_T010	1.929549014	0.76041088	1.512275945	2.138859675	2.192843705	2.687582787
Zm00001d015234_T005	1.957190284	0.619226117	1.13244326	1.94092709	1.730634739	2.527070336
Zm00001d050943_T001	0	0.066528736	0.133526702	1.097158367	1.192857589	0.785573986
Zm00001d050943_T002	3.472909081	2.648491444	3.559154533	3.837356662	3.833874659	3.854738454
Zm00001d032090_T001	2.024698036	1.685625344	1.951239387	2.018641295	2.473312341	1.736239447
Zm00001d032090_T002	1.725101125	1.295661319	1.79314969	2.407230096	2.464505693	2.446742958
Zm00001d023846_T008	5.080634727	4.412245935	5.085715571	5.136214974	5.165527866	4.865766868
Zm00001d023846_T006	1.510546768	2.020218773	1.330898526	1.550949903	1.794039001	0.816823312
Zm00001d023846_T002	1.951934999	0	2.312207144	1.51309098	1.941948389	0
Zm00001d023846_T001	0	2.906043196	0	2.063331396	1.61516484	2.745709331
Zm00001d013529_T002	5.83876731	5.200476065	6.048112853	6.486298235	6.542319022	6.47449239
Zm00001d013402_T004	0.822595558	1.224515124	0.810058882	0.427859293	0	0.326971012
Zm00001d013402_T001	2.434453693	1.317960426	2.661711982	2.133333689	2.01452812	2.253956904
Zm00001d053867_T001	3.064862163	2.140902964	3.051515146	3.73126602	3.624056314	3.667866018
Zm00001d012295_T001	1.568757406	1.094922454	1.891321578	1.198431918	0.454643377	1.426167051
Zm00001d012295_T005	2.793924591	3.422282121	3.209528852	3.652971824	3.682212017	3.391118939
Zm00001d012295_T007	3.133658869	2.109538754	2.97544915	3.545189499	3.40478598	3.475025202
Zm00001d012295_T006	2.991818948	2.295799418	2.586460202	2.665731801	2.767978892	2.511555833
Zm00001d012295_T004	0.703778465	0.256368204	0.30294133	0.949939765	1.113728499	1.093304606
Zm00001d021447_T001	4.638795178	4.017744232	4.484851953	3.818766013	3.947393954	3.823403011
Zm00001d018070_T001	3.048891633	2.757804566	3.418062003	3.359386649	3.223443866	3.178661266
Zm00001d014609_T003	0.899344257	0.609087349	1.029372312	0.79652217	1.129492728	0.885917135
Zm00001d014609_T001	4.427429579	3.388168592	4.326717602	4.731437049	4.608039383	4.641652956
Zm00001d050868_T007	4.620952375	1.794550616	4.857734721	4.066018217	4.328437471	3.977488389
Zm00001d050868_T004	4.868644594	3.966619744	4.706316536	5.281585167	5.240242422	5.168606257
Zm00001d050868_T006	1.62288023	0.950095094	1.831307345	2.637186626	2.207604908	2.185791396
Zm00001d050868_T001	1.592081439	0.765561054	1.127977241	3.094404656	2.57394863	2.595799139
Zm00001d033966_T001	5.841742888	6.111244779	5.788222659	6.260654025	6.391393562	6.31617435
Zm00001d040197_T001	1.82755891	2.036132282	1.858638316	1.660794935	1.842314335	2.05047906
Zm00001d019343_T001	2.845788495	2.686002473	2.745948284	2.623628343	2.96679737	2.875585266

Zm00001d030598_T005	3.533819099	2.913328711	3.492528761	4.040384593	3.937448541	4.025793008
Zm00001d030598_T001	1.566085465	0.424347045	1.249415607	1.491223046	2.095146674	1.531365989
Zm00001d034856_T005	5.564979573	4.677605039	5.421431288	4.657214107	4.516928523	4.779313098
Zm00001d034856_T003	5.382497453	4.791969634	5.382790215	5.062180527	5.071506597	4.911300467
Zm00001d017965_T001	1.913870179	1.335997677	2.295846721	1.85662044	1.926810517	2.078093665
Zm00001d031863_T027	2.731825141	1.457316389	2.710986019	2.673508032	2.477097284	2.369194531
Zm00001d031863_T012	0.99443418	0.220898252	0.850687427	1.290794762	0.623228227	1.213168081
Zm00001d031863_T018	0.973084426	0.803868459	1.100257802	2.340601291	2.797256036	2.784384207
Zm00001d010970_T001	0.241823103	0.177314444	0.185299502	0.35511214	0.28645892	0.304466651
Zm00001d010970_T004	3.185846886	2.509440213	3.435816586	3.491463837	3.42274512	3.22695961
Zm00001d010970_T002	1.215437558	0.90659741	1.182431699	1.083954017	1.145559427	1.170537215
Zm00001d036400_T001	5.297953107	5.235019054	5.267746906	4.928167242	5.079575705	5.06132859
Zm00001d017119_T001	2.340938773	1.347351574	2.663159687	3.373057511	3.185354377	3.535849416
Zm00001d017119_T004	6.924364094	6.863813869	6.974790215	6.820760979	6.747786878	6.626713865
Zm00001d017119_T003	5.594325321	4.709979722	5.457887634	6.273570568	6.256729956	6.24135918
Zm00001d024229_T006	1.097513041	1.131767259	1.565580604	1.688590836	1.709188717	1.844393688
Zm00001d024229_T001	0.637663103	0.150961236	0.804564113	1.146303954	0.981138799	0
Zm00001d024229_T004	1.604913441	0.459109468	0.517843084	1.633374013	1.100265877	1.151979577
Zm00001d024229_T002	0.9634904	0.604855102	1.231300913	1.45796352	1.647740922	1.252031075
Zm00001d042114_T001	5.137798058	5.565200502	5.183098713	5.185373884	5.249729149	5.014711771
Zm00001d046555_T001	2.667833781	2.733838701	2.360932533	3.252330423	3.13702834	3.182432335
Zm00001d021532_T001	1.053148186	0.762286726	1.243355894	1.451590417	1.702242182	1.170996045
Zm00001d038113_T001	1.546037538	0.903491066	1.626349418	1.355435814	1.575832672	1.501938938
Zm00001d014826_T001	2.343445915	2.145299534	2.574757174	1.880757609	1.346217132	1.604062307
Zm00001d010131_T001	1.712493214	1.245363009	1.866395676	3.342601251	3.326610833	3.375260095
Zm00001d009125_T015	4.201143732	0	4.204233622	4.124263113	4.125148435	4.491547661
Zm00001d009125_T003	3.021015396	2.37447448	2.963731567	0	0	0
Zm00001d009125_T002	0	0	0	3.877839347	3.870331772	2.687098099
Zm00001d009125_T005	0	2.13671376	0	3.597783817	3.716651849	3.867248489
Zm00001d009125_T010	0	0.113949816	0	1.320752685	0.628550584	0.954451691
Zm00001d013849_T003	1.661356955	0.670722523	2.050179863	1.851258917	1.701635993	1.842684432
Zm00001d013849_T004	1.361245077	1.388869524	1.860102286	1.404709152	1.373507345	0.791222289

Zm00001d013849_T001	1.079170546	0.85761321	0.743558914	0.552136028	0.782687012	1.079278429
Zm00001d052143_T013	0.203930308	0	0.121727618	0.606675315	0.52195327	0.311721654
Zm00001d052143_T016	0.824270104	0.621651969	0.956184556	0.912870563	1.153991416	1.392383373
Zm00001d047800_T001	4.210448794	3.526674691	4.182625087	5.226844773	5.214485978	5.084633957
Zm00001d048508_T001	4.432677186	4.604236168	4.420353582	2.918496722	3.073228813	3.588731543
Zm00001d011886_T001	1.737893526	2.106736247	1.755575984	2.933258384	2.44532069	2.471716568
Zm00001d013506_T001	2.580936096	1.953078207	2.643214798	2.342425034	2.538403324	2.574682345
Zm00001d032229_T004	3.778574041	3.084252926	3.795414022	3.435273343	3.310231624	3.243322226
Zm00001d024102_T002	3.387058363	2.861750356	3.565027296	3.985595605	3.611309441	3.940894024
Zm00001d024102_T001	0	0.902146146	0	1.440821473	2.344649853	1.276223883
Zm00001d051814_T002	0	2.874282736	1.918835385	3.955923067	1.784485138	1.879153281
Zm00001d051814_T001	3.51304056	0	3.079639744	3.119481847	4.482904674	4.211807326
Zm00001d042370_T020	0.417801218	0.410259686	0.57088839	0.539871482	0.522783946	0.747220669
Zm00001d042370_T019	3.702174015	2.506849698	3.928468395	3.547173282	3.802583157	3.680287445
Zm00001d042370_T004	3.641939595	2.891957083	3.46386646	3.634564577	3.643087663	3.561393839
Zm00001d002435_T001	1.908167297	1.585819688	2.214517451	4.590778049	4.501292002	4.518321992
Zm00001d006678_T001	4.162144765	3.453444751	4.22508301	4.96918333	4.822822926	4.923612965
Zm00001d050643_T001	3.882694399	3.244705493	3.979050009	4.016931274	3.789556076	3.742203409
Zm00001d031669_T001	3.201668987	2.727406219	3.543399618	3.180755435	3.162724364	3.133673499
Zm00001d031669_T002	0.599888093	0.15922874	0.469135788	0.577281379	0.501599138	0.501143569
Zm00001d016407_T001	1.688269895	1.90304097	2.437832287	2.175993979	2.396612179	1.774663101
Zm00001d016407_T004	2.5686436	0.633840362	1.620987025	2.115832964	1.283563868	2.366766181
Zm00001d016407_T006	0.682416885	0.371387123	0.59072177	0.469060748	0.76805895	0.21678636
Zm00001d015999_T001	0.800641097	0.538948317	0.80235206	0.848649979	0.899188781	0.984682671
Zm00001d042667_T001	4.367105988	3.4781515	4.295741462	4.701047242	4.580856464	4.677602389
Zm00001d028504_T001	1.746751743	1.522877329	1.646993005	0.755220886	1.237670173	1.359292885
Zm00001d017330_T001	5.216477487	5.420189272	5.270291962	5.091120567	5.128308333	5.204416283
Zm00001d014178_T013	1.316800311	0.648549195	1.20111126	2.18329086	2.088093076	1.728846966
Zm00001d014178_T006	1.083724647	0.563191329	0	0.900161586	1.197903125	0.910083303
Zm00001d014178_T003	0.220989852	0.577393538	0.792200682	1.146819422	1.343977965	1.011534282
Zm00001d014178_T008	2.924918321	2.041346729	3.007138977	3.807477546	3.67254279	3.589169751
Zm00001d014178_T002	0.734573933	0.294655447	0.673872968	1.172171931	1.29294132	1.457539661

Zm00001d014178_T007	1.243018259	1.543792806	1.669179647	2.194628907	2.765401503	2.43204247
Zm00001d014178_T009	1.297042296	0.390006161	1.264896684	1.833339458	1.781428122	1.976922274
Zm00001d007207_T006	1.471127618	1.116686798	0.823349915	1.334888013	0.368961627	1.09704844
Zm00001d007207_T005	1.64981225	0.401777896	1.739829097	1.716771456	2.145486387	2.169075164
Zm00001d007207_T003	4.140481335	3.531829204	4.108231706	4.262374222	4.273209863	4.194677282
Zm00001d033567_T003	0.260754349	0.832433949	0	0.737278337	1.311855318	0.301766729
Zm00001d033567_T001	3.044042409	2.144784175	3.216011977	3.285862328	3.101784692	3.507690382
Zm00001d044467_T001	5.173652314	5.438976772	4.920948653	4.905233141	4.935778378	5.007935769
Zm00001d005993_T001	1.900027459	2.225292825	1.62604237	2.668517649	2.31002566	2.458544744
Zm00001d031749_T003	3.927659745	3.328166363	3.957008406	4.275210248	4.175091169	4.248846066
Zm00001d031749_T001	3.477742468	2.574831029	3.54922459	2.047878607	0.711413849	1.79867102
Zm00001d031749_T007	0.712001413	0.287906043	0.12341458	0.67474627	1.031587166	0.878344757
Zm00001d031749_T004	0.937089732	0.924147785	0	1.097020789	1.656758114	1.387450776
Zm00001d039367_T001	3.940124832	4.275348231	3.682303048	4.493816006	4.637628213	4.408390072
Zm00001d034644_T001	9.195953344	8.283579013	9.151850843	9.267732517	9.259811636	9.188765388
Zm00001d034644_T004	3.00949319	7.155197301	2.625823378	3.082442874	2.255647563	2.658154574
Zm00001d016568_T001	7.58710771	8.395337084	7.609155256	7.304421747	7.347282195	7.445097059
Zm00001d039319_T008	1.505809649	0.832224905	1.583470945	1.366597507	1.753298594	1.503310059
Zm00001d039319_T006	2.869537436	0	1.783497305	3.149302945	2.675603688	2.533071403
Zm00001d039319_T001	2.242729012	2.199777236	2.949588718	1.550143174	2.170369604	2.398140464
Zm00001d039319_T007	0.619181973	0.190230512	0.345855058	1.144815167	0.875817801	1.259643095
Zm00001d005743_T001	3.587808773	2.778989649	3.612094371	4.066743896	3.998532303	4.073017361
Zm00001d005743_T002	0.768603572	0.057522362	0.968199158	0.871944555	0.973387921	1.2393626
Zm00001d021199_T001	4.417260061	3.508786239	4.671168858	4.68530963	4.649640059	4.642770297
Zm00001d016197_T001	2.337017119	1.698797219	2.45687237	2.563284574	2.461247746	2.504257067
Zm00001d017769_T002	1.025143638	0.417274106	0.636168454	0.447013115	0.374209313	0.362075952
Zm00001d017769_T001	4.110479008	2.909755058	4.263080842	3.993513872	4.014237534	3.694380009
Zm00001d002573_T001	5.031843543	4.350901145	5.033022383	6.132622594	6.159205929	6.189754379
Zm00001d021528_T003	1.082740053	0.754562592	1.336467927	1.223136454	0.946531002	0.900190188
Zm00001d021528_T001	1.579966463	2.055334502	2.124209339	2.573170421	2.032266267	1.938089655
Zm00001d021528_T002	2.941200985	1.600298778	2.645774737	1.506068716	2.376421809	2.772895112
Zm00001d006697_T001	4.362892886	3.482089959	4.431540795	4.753570362	4.847970206	4.841766358

Zm00001d047918_T001	2.442694601	2.014555614	2.609771034	2.799907295	2.649446924	2.755338835
Zm00001d032797_T001	2.537627812	1.944411703	2.635061546	1.823108156	2.050409752	2.259759682
Zm00001d028513_T004	4.377648661	4.115598038	4.54312415	3.530952675	3.976815347	3.442423236
Zm00001d028513_T002	3.973409505	2.927032853	3.933518158	3.731115885	3.406801947	3.807875538
Zm00001d028513_T001	2.827157265	2.907782675	3.1131944	3.130429298	3.438604648	3.12605073
Zm00001d046970_T001	0	0.195033825	0.718688216	0.712849303	0.582551186	0.372650159
Zm00001d046970_T002	0.487569698	0.262547414	0.266303213	0.402298691	0.577733831	0.751875861
Zm00001d030626_T005	3.292126499	2.108854942	3.403370126	3.637238339	3.532007314	3.7872285
Zm00001d030626_T003	2.523417679	2.233456475	2.612729933	2.904309326	3.033850593	2.824910385
Zm00001d020765_T001	4.543316711	3.953136052	4.741075329	4.285096705	4.381306633	4.354110065
Zm00001d046778_T001	2.283681501	2.292291739	2.975732708	2.973813294	2.953540899	3.024103507
Zm00001d046778_T002	2.314931977	0.710913306	1.186373144	2.255849053	2.502579093	2.428372904
Zm00001d003200_T003	4.356247148	3.738135614	4.420530774	5.615018047	5.536283806	5.564799787
Zm00001d043654_T001	3.199445264	2.965680315	3.68555123	3.81772414	4.050186046	3.821391134
Zm00001d021673_T001	5.756803704	5.628263868	5.927592574	5.14832986	5.402099594	5.317601536
Zm00001d034529_T004	2.296237719	2.653158181	2.353142308	2.33741082	2.614213707	2.505529449
Zm00001d034529_T001	3.246343932	3.207690017	3.377705986	3.24814375	3.053636347	3.125079729
Zm00001d034529_T002	2.986378349	2.594327829	3.168362358	2.507411308	2.765277372	3.038083191
Zm00001d048100_T006	0.154541733	0.350996164	0.486467253	0.469375474	0.428954918	0.508233914
Zm00001d048100_T001	6.358611504	5.532129596	6.387266372	6.345536749	6.475237237	6.32288059
Zm00001d041404_T001	3.369089393	3.712927199	3.213842146	3.022908567	3.811906342	3.190207277
Zm00001d043998_T006	0.846623288	5.99819925	2.304198229	0	0	2.593766081
Zm00001d043998_T002	6.665609449	4.349345036	6.570699596	6.419123282	6.429590324	6.435181399
Zm00001d043998_T004	3.905451993	3.051392985	3.79710265	3.70698854	3.609032488	3.705672748
Zm00001d043998_T003	8.664197598	9.0253212	8.722737228	8.393637687	8.412742122	8.357538827
Zm00001d043998_T001	3.623731105	6.412241548	4.286153334	4.185291572	4.021782537	3.745505943
Zm00001d011458_T004	2.28217111	2.120964325	1.963134126	2.04546394	1.905011561	1.934263984
Zm00001d011458_T003	0.836610908	0.232641111	0.592436466	0.533527467	0.565952932	0.576887784
Zm00001d020898_T010	6.234805152	5.00814048	6.38506281	5.331179299	4.971377088	5.504991028
Zm00001d020898_T003	4.947122262	4.242417832	4.804725741	5.077820346	5.441181702	4.846825845
Zm00001d005798_T001	6.965262662	6.23548458	6.963892468	6.149473916	6.261005185	6.085085869
Zm00001d007575_T001	1.860199248	1.74526057	2.167617731	1.643303936	1.746063337	1.709247398

Zm00001d031227_T001	6.425945011	6.581067697	6.416239457	6.26308751	6.290858058	6.238334418
Zm00001d034360_T001	2.327937946	2.039808337	2.566384594	2.262898245	2.242033242	1.973353753
Zm00001d008974_T001	0.870304742	0.429051362	1.150054645	1.407091111	0.998846826	1.534875878
Zm00001d050417_T001	2.322772401	2.028342829	1.631523406	3.699552077	3.779377191	3.64830011
Zm00001d002762_T001	5.358934499	5.939606951	5.464691756	5.680642035	5.800071801	5.630730315
Zm00001d036234_T001	1.567891478	1.80998647	2.059097383	1.938565081	1.959406654	1.689212373
Zm00001d007942_T001	4.870243429	4.102667369	4.925047828	5.133281444	5.129115984	5.065957619
Zm00001d018691_T001	2.447224496	1.864299872	2.495598724	2.38533329	2.513957335	2.381764006
Zm00001d038325_T001	6.073606019	5.487907706	6.066654552	6.749663541	6.782106731	6.777919614
Zm00001d028598_T001	5.099457141	4.786170129	5.157920948	2.903829992	3.999895762	2.808363014
Zm00001d020017_T001	1.743750623	1.362142738	1.928839869	1.377224831	1.429027251	0.896935975
Zm00001d005708_T001	2.436900325	1.876517339	2.525468065	2.49041199	2.54922804	2.639571145
Zm00001d040204_T001	3.942331424	3.131959	4.026586737	3.989427608	4.036819795	3.881033376
Zm00001d038006_T004	0.607912128	0.080787261	0.474864829	1.173281611	1.034255163	1.241790771
Zm00001d038006_T001	3.722454767	2.896016424	3.872164277	3.479373892	3.561956116	3.461160898
Zm00001d050178_T001	2.94660249	2.319392206	3.035523093	3.414963424	3.654031961	3.613048552
Zm00001d031115_T002	2.510476637	1.932046427	2.700223076	2.786422601	2.917480449	2.759946862
Zm00001d031115_T001	1.630002337	0.874889012	1.072730776	1.501749435	1.291126706	1.828493073
Zm00001d031115_T004	0.332786975	0.308827989	0.566166343	0.581586495	0.922569323	0.778765358
Zm00001d030601_T001	4.453491833	3.662276379	4.245352284	4.392007004	4.460685759	4.284133421
Zm00001d009941_T002	5.390833638	4.980585996	5.472819488	5.601383403	5.725176181	5.547556405
Zm00001d032032_T001	0.947750678	0.540860498	0.755778919	1.895569805	1.709936395	1.735120506
Zm00001d032032_T002	1.929628922	1.418500856	1.942261132	1.13731924	2.034924913	1.628223937
Zm00001d045027_T001	4.464478051	3.761671323	4.458070187	4.767257261	4.740173158	4.782992707
Zm00001d016885_T002	2.906724965	2.505347023	3.230626812	2.896026696	2.726816398	3.355481343
Zm00001d021596_T001	4.609538565	3.730963996	4.618290524	4.37057101	4.428265894	4.411516344
Zm00001d006010_T001	8.708654746	8.27227785	8.786509752	7.42357402	7.502501086	7.448707852
Zm00001d007097_T001	8.950508394	9.945125026	8.928143024	7.31447812	7.18832292	7.048295385
Zm00001d029963_T001	7.858020588	7.274765184	8.00240683	7.170555572	7.208815407	7.197070932
Zm00001d041584_T001	0.531329055	0.460943819	0.510370546	0.446367406	0.550998154	0.936575755
Zm00001d052197_T013	3.370835801	0	3.389029142	3.911341554	3.741913263	3.917526277
Zm00001d052197_T014	0.378475001	0.227350473	0.373177119	0.446069859	0.672497758	0.289474484

Zm00001d052197_T003	2.163910228	2.23248793	1.587416361	1.080099577	1.999131236	1.210486745
Zm00001d052197_T005	0.607166	0.215043609	0.522405329	0	0	0
Zm00001d052197_T010	0.361085584	0	0	1.437715658	1.406674348	0.747006641
Zm00001d011001_T001	6.522133687	5.947412658	6.521600597	5.902344626	5.905319752	5.904467311
Zm00001d013027_T002	4.182814953	3.396766149	4.260598705	4.244476639	4.229014191	4.178896898
Zm00001d021015_T002	1.385481841	1.024648752	1.231841565	1.509534211	1.646395405	1.473518345
Zm00001d021015_T008	0.34768493	0.119312348	0.285015081	0.254226369	0.309868841	0.284343558
Zm00001d021015_T006	0.22354488	0.120142223	0.301120524	0.92902969	0.785647635	0.886848215
Zm00001d021015_T011	0.359055565	0.06085396	0.137420894	0.325273574	0.348698132	0
Zm00001d004164_T001	1.328402786	0.75777257	1.218721041	0.358704574	0.573266902	0.416902419
Zm00001d044065_T001	2.447897586	2.122223442	2.438848528	1.215514595	1.375149002	1.183513828
Zm00001d018828_T003	2.199388096	1.717963481	0	1.131898929	0	0
Zm00001d018828_T005	0	0	0	1.70055069	0.680883321	1.194349336
Zm00001d018828_T002	1.467173557	0.903783342	2.621692053	3.073519155	3.690640467	3.475587403
Zm00001d014689_T024	3.191736503	2.534897287	3.512181999	3.118980264	2.816883303	3.25895619
Zm00001d014689_T006	0.756283783	0.755990795	0.508443865	1.593191683	1.566407489	1.712563207
Zm00001d014689_T007	0.52476579	0	0	0.821431852	1.030278864	0
Zm00001d014689_T015	0.13437736	0	0.485837937	0.470833573	0.867872748	0.36699972
Zm00001d034750_T001	0.49796894	0.309334538	0.560180814	0.579800933	0.557892338	0.680063253
Zm00001d034750_T007	0.140892497	0.160326471	1.02525422	0.711989083	1.271802138	0.135220949
Zm00001d034750_T003	3.356837659	2.699047623	3.277287772	3.733813544	3.500231999	3.62072821
Zm00001d034750_T006	3.963747933	3.053005303	3.725850527	2.431268861	2.599988277	2.921161324
Zm00001d015156_T004	1.760020339	0.976606325	1.569849777	2.524896647	2.345219505	2.571753978
Zm00001d015156_T002	2.946741713	2.514422512	2.612257653	1.677603404	1.713530863	0
Zm00001d041635_T003	5.340925852	4.506312945	5.584258113	4.275034992	4.294874714	4.258833509
Zm00001d041635_T002	5.291374325	3.850244457	5.32594207	5.554226444	5.519304937	5.542808893
Zm00001d041635_T004	2.801553739	0	0	3.123908998	3.646052953	3.409463578
Zm00001d037385_T002	2.971143636	2.961722036	3.127841911	3.495284294	3.018430146	2.671444655
Zm00001d039037_T001	4.281600856	3.189368674	4.162151293	5.254356963	5.262031196	5.169293404
Zm00001d011679_T001	8.78634908	8.095210109	8.798289471	9.143197989	9.163096919	9.173541412
Zm00001d024397_T001	2.549578738	2.458127095	3.413546696	3.457038959	2.830385347	3.382558943
Zm00001d041422_T001	4.084145244	3.940647224	3.904996633	1.91857133	2.182751402	2.059062071

Zm00001d027472_T001	4.587388215	3.694805104	4.61779879	4.692999565	4.643754851	4.680146766
Zm00001d027472_T003	1.199091224	0.544684194	0.786697556	1.241704143	1.471840363	1.104995452
Zm00001d027472_T002	1.420991103	0.869114928	1.602893433	3.660656677	3.788783001	3.663926249
Zm00001d025237_T001	3.297279766	2.506556753	3.326936364	4.685066588	4.49287128	4.450126655
Zm00001d050000_T001	0.790716998	0.494487295	1.177070737	0.835422883	0.981608653	1.051164645
Zm00001d031211_T004	0.79031247	0.607504078	0.918843397	0.50493864	0.63051544	0.415452575
Zm00001d033836_T001	2.576291385	1.97810079	2.447670701	3.001173876	2.843424139	2.449556621
Zm00001d028611_T001	2.143815259	1.46712555	2.093011456	2.208705778	2.451425571	2.262270477
Zm00001d023395_T001	7.213384739	7.391062041	7.154014451	5.256801405	5.360182523	5.200080442
Zm00001d032808_T001	2.004160858	1.406030468	1.9160906	2.823965137	2.048823469	2.830275609
Zm00001d033955_T002	4.063519595	3.497294158	4.002135681	3.701020342	3.652806663	3.636355138
Zm00001d028097_T003	0.918333564	0.408614048	1.195556742	1.26225093	1.181217071	1.182726617
Zm00001d028097_T002	4.227565348	3.849930825	4.259308573	4.3511994	4.386436225	4.394500423
Zm00001d022561_T001	2.457092558	3.127776552	2.482274704	3.431607182	3.56463327	3.4405855
Zm00001d022561_T004	2.454191426	1.806707213	2.175792517	2.551701803	2.736365452	2.809456016
Zm00001d042884_T003	3.994731136	2.619038495	4.025831004	5.001197706	5.123454519	4.965691995
Zm00001d042884_T002	3.798508039	3.314442473	3.867767209	2.931500014	2.748249536	2.628566915
Zm00001d042884_T001	2.580792622	2.040910661	2.348876842	2.655511432	2.463472844	2.645241435
Zm00001d025470_T002	0.884755759	0.552689875	0.747065953	1.518596068	1.427000006	1.436405467
Zm00001d025470_T006	0.973383512	0	0	1.114872046	1.358107582	1.024662935
Zm00001d025470_T010	2.469785713	2.180865528	2.527659908	2.525460046	2.740561283	2.441649996
Zm00001d028641_T002	5.390908871	6.422938413	5.4029425	5.223282869	5.352774553	5.187542863
Zm00001d028641_T001	1.991270948	2.00965854	2.265254447	2.067559215	2.382693257	2.197555625
Zm00001d036018_T001	6.775549102	6.543105462	6.653090837	6.912684172	6.728840614	6.790039012
Zm00001d006551_T001	4.225265751	3.636811245	4.31687603	5.549213436	5.685151355	5.608323552
Zm00001d005429_T024	2.192079566	1.212095522	2.322958388	2.565747043	2.29843163	2.304553095
Zm00001d005429_T003	0.934629724	0.092929958	0.853358566	1.433670427	1.324503602	0.976895154
Zm00001d005429_T001	0	0	0.318090042	0.627907485	0.574440641	1.166837539
Zm00001d012335_T001	1.394426337	1.370239622	1.72143216	0.974618147	1.14007382	0.955817919
Zm00001d024468_T002	3.444824605	4.297154505	0	0	1.684845213	0
Zm00001d024468_T003	3.735706618	2.810948524	4.423305136	4.225772737	4.021988667	4.207998318
Zm00001d035960_T028	2.939061796	2.153689592	2.986937491	2.14692334	2.023784421	2.659000435

Zm00001d035960_T029	0.409721123	0.204742966	0.424408322	0.815604119	0.789647465	0.572101912
Zm00001d035960_T007	1.831938028	0.645099373	1.633617203	2.462888892	2.754434318	2.312637005
Zm00001d003049_T005	4.134375224	3.097484724	4.108012607	4.703173143	4.730591648	4.530926027
Zm00001d003049_T006	2.702016277	2.075698283	2.653956549	3.078852825	3.544568597	3.572421505
Zm00001d003049_T007	0.669327976	0.109819207	0.358040608	1.555252707	1.291340101	1.173373726
Zm00001d003049_T004	0.475467821	0.778328025	1.006159404	1.30190951	0.832804159	0.444608764
Zm00001d004620_T001	4.509834912	3.934783314	4.621316898	4.706050643	4.834293807	4.815438786
Zm00001d041136_T002	0.508564549	0.404225201	1.06537516	0.244330571	2.05361845	1.264243959
Zm00001d041136_T001	1.062188041	1.279263271	1.409493041	2.231640994	0.983622247	1.668836662
Zm00001d041136_T004	1.85119534	0	0.911530528	1.385037797	1.564176202	1.495757064
Zm00001d041826_T001	1.311717004	1.230275577	0.898216093	1.942414287	1.68524049	1.851480413
Zm00001d005684_T001	3.78626053	3.19081379	3.8777099	3.696324727	3.642311217	3.503125062
Zm00001d010702_T001	2.90057085	2.469095663	2.636884195	2.348417136	2.547195867	2.45765286
Zm00001d045580_T001	1.626767123	1.173575847	1.781379859	1.264618697	1.361806531	1.009808646
Zm00001d007828_T001	1.016017719	0.788339923	1.378285213	0.951262518	1.183909495	1.109775755
Zm00001d037949_T001	6.647219473	5.966934104	6.680731956	4.861700886	4.875043491	5.033044635
Zm00001d049954_T001	7.361535806	6.707588302	7.507037074	7.682538466	7.706567089	7.714971423
Zm00001d017204_T003	2.510770813	2.309063236	2.289870753	2.418328138	2.321532165	1.721863464
Zm00001d038857_T001	4.326929463	3.466750961	4.298842379	4.130904354	4.035719353	4.065509326
Zm00001d003372_T004	1.887136471	0.934191882	1.549460698	1.14419259	0	1.349249767
Zm00001d003372_T002	1.516325848	1.124385709	1.677786494	2.02826573	2.106615324	1.701357424
Zm00001d020939_T001	3.919049512	3.349259535	3.985666094	5.437619259	5.403056893	5.401244486
Zm00001d005696_T001	2.029471615	1.451693798	2.291870503	2.156164239	2.225344348	2.24762406
Zm00001d025658_T004	0.257639472	0.044841723	0.349578156	0.521640764	0.34664946	0.046727125
Zm00001d025658_T001	4.877076137	4.186965508	4.573652883	4.845496073	4.839018406	4.581797907
Zm00001d025658_T006	0	0	3.326230139	1.746619763	2.480645654	3.761412089
Zm00001d025658_T002	0.13874764	0.063096998	0.267379985	0.775593373	0.833833279	0.816461267
Zm00001d024005_T001	3.299140746	2.546316649	3.18037975	2.739019611	2.579543914	2.492154941
Zm00001d014992_T001	6.371298562	2.026865908	6.519338233	5.660205223	6.615659664	5.637955346
Zm00001d020476_T001	3.309378925	2.467464697	3.120738775	2.909415602	3.159602708	3.0879397
Zm00001d020476_T004	4.005386513	3.785338578	3.844413474	3.922408623	3.633228452	3.924369487
Zm00001d020476_T005	2.848619335	3.210536929	2.646617058	2.221425492	1.975244976	2.448337239

Zm00001d044815_T001	2.180314659	1.278263146	1.870317369	2.507520153	1.791109739	1.918207604
Zm00001d026250_T001	1.321790167	1.001108285	1.17415263	0.58704425	0.763386077	0.891755906
Zm00001d051615_T002	3.220343422	1.825052495	3.121944562	2.413490913	2.584620541	2.926142922
Zm00001d051615_T001	4.536053957	3.745051201	4.509229836	3.838257271	3.794475445	3.702017275
Zm00001d007885_T001	2.105394137	3.27802312	2.480446869	3.828924396	3.618088152	3.524578134
Zm00001d032270_T001	3.554172067	2.830084511	3.377293002	3.896728711	4.10282053	4.045830329
Zm00001d028349_T001	7.864718791	7.272338772	7.948821743	7.3827664	7.454146925	7.389103945
Zm00001d010948_T001	3.42791211	2.911497356	3.558902378	2.453971058	2.192818146	2.098860815
Zm00001d002778_T001	4.406300805	3.658066349	4.466797154	4.709488331	4.634241031	4.785637487
Zm00001d001979_T001	6.096002965	5.285940133	6.16649798	6.886917932	6.797187103	6.853370731
Zm00001d001979_T002	3.526938049	3.310482492	3.584627396	4.457579649	4.607870417	4.427940193
Zm00001d052237_T002	3.015896261	1.598682011	3.03419511	3.316293481	3.367769429	3.399416536
Zm00001d052237_T001	2.277016776	2.421228686	2.024691299	2.189097099	2.103758124	2.276492795
Zm00001d043253_T002	3.215107151	2.251688497	3.330982255	3.597052342	3.715457971	3.668131155
Zm00001d010120_T005	4.619246663	3.86417178	4.337736421	4.481320773	4.298695042	4.359588075
Zm00001d010120_T006	4.813812994	5.275788954	5.576923691	4.36271885	5.278978006	4.994666021
Zm00001d010120_T007	4.332287665	3.831777445	0	3.904988544	0	0
Zm00001d047274_T001	2.561501131	2.09225176	2.551111883	2.208917051	2.644113313	2.55243462
Zm00001d045956_T025	2.161282107	1.713268221	1.756005318	2.693051993	2.056880707	2.151656553
Zm00001d045956_T029	1.166780349	1.678027722	1.08700518	0.906189468	0	0
Zm00001d045956_T028	4.601403016	3.675631575	4.677197965	4.690915862	4.742567545	4.592489628
Zm00001d045956_T017	0	0	0	2.170289156	2.531164088	2.760171969
Zm00001d045956_T001	0	0.223130892	1.245298503	3.280475484	3.162725814	3.159701351
Zm00001d045956_T008	0.836046112	0.535146205	1.051782054	0.875406565	1.053859953	1.091067448
Zm00001d023749_T005	0	0	0.217267947	0.485451559	0.459575356	0.685283556
Zm00001d023749_T006	0.557357151	0.704894092	0.611303657	0.896141623	0.814109857	0.439278445
Zm00001d023749_T001	4.347276232	4.485325706	4.264446504	4.228667488	3.663326742	3.889230444
Zm00001d023749_T008	1.113717166	0.825442921	1.155484369	1.423487815	1.146958847	1.540013874
Zm00001d023749_T007	4.741840367	0	4.788324574	5.412889757	5.635905675	5.598487737
Zm00001d023749_T010	0.222993694	0.167684199	0.290721642	0.636458041	0.588066724	0.627729159
Zm00001d019983_T001	3.688726554	2.761846283	3.664509236	3.496904064	3.469273612	3.480469748
Zm00001d045304_T001	1.947827715	1.679547208	1.741239589	2.162816511	1.951457991	1.79978444

Zm00001d016802_T002	2.189611736	1.568138174	2.000155803	2.291288229	2.196519233	1.912063471
Zm00001d048802_T007	0.089141501	0	0.54608299	1.233107616	0.622536835	1.019376693
Zm00001d048802_T005	4.632324003	3.793518361	4.676960376	4.519672544	4.634681489	4.608184787
Zm00001d048802_T004	1.68918553	1.292333561	1.459739536	2.972064531	2.689230715	2.632523457
Zm00001d048802_T003	1.034944628	0	0.207524324	0.755904511	0.215948164	1.096850148
Zm00001d025048_T003	3.50415891	3.018909905	3.507313873	3.879776135	3.708219688	3.916215025
Zm00001d015196_T007	2.440490355	0	2.624537498	1.663814131	2.628717846	1.630742341
Zm00001d015196_T001	2.38294387	2.268612848	2.005200548	2.774270074	2.367123885	2.70823183
Zm00001d029033_T002	1.552502004	1.258660624	1.368878956	2.757402224	2.718033208	2.831475176
Zm00001d029033_T004	2.033747891	0.961296601	1.990796609	2.368477267	2.291860486	2.223339137
Zm00001d029033_T001	2.875793822	2.385666267	2.916807949	2.96016712	2.791293982	2.564626198
Zm00001d044232_T004	4.868049816	2.855669838	4.588632313	4.788893644	4.809010958	4.682583241
Zm00001d044232_T002	0.230745306	0.059971268	0.258077456	0.84537443	0.780902175	0.807246922
Zm00001d044232_T005	1.229348582	0.745649533	0.810554155	1.676336923	1.549007671	1.285463181
Zm00001d044232_T001	1.83917637	3.491288475	2.941087338	0	1.115855598	1.256184001
Zm00001d048848_T001	2.998510731	2.787335919	2.033348288	2.677309104	1.955468672	2.114141107
Zm00001d023434_T001	0.412315453	1.155878733	0.985153559	0.442703624	0.970022393	0.887805204
Zm00001d023434_T002	4.248624015	3.525944449	4.409710059	4.452177377	4.321871973	4.455945663
Zm00001d032265_T001	1.624851416	1.914900858	1.677118059	3.011163015	2.70184178	2.99422145
Zm00001d048759_T001	2.854151482	2.257535989	2.666752275	2.008429877	2.24832625	2.356900181
Zm00001d002498_T001	3.638628096	3.247630288	3.860701441	1.525430979	1.52961958	1.560842591
Zm00001d045119_T001	1.882474807	1.479175709	2.180389457	3.635425308	3.712407811	3.585960741
Zm00001d030338_T001	4.505808569	4.507112132	4.473157429	5.022281293	4.991569191	5.233724007
Zm00001d002345_T001	2.019935288	1.458104253	1.57828712	2.157517845	2.26192581	2.173206764
Zm00001d017645_T001	4.690091307	5.362331223	5.207090832	5.64483735	5.716238851	5.714053448
Zm00001d054069_T001	4.606559724	3.679566912	4.593798822	4.015312457	3.943166597	4.005252926
Zm00001d004496_T001	3.992522644	4.299065849	3.512858449	2.296443311	3.238893224	1.764115965
Zm00001d052212_T001	3.554795725	3.600629312	3.736218987	3.976597711	4.181640002	3.917310205
Zm00001d042476_T002	3.432126411	1.54594663	3.448594676	3.416178355	3.394733493	3.405262389
Zm00001d042476_T005	2.513660777	3.094589943	3.009034662	2.951846808	3.201024037	2.645633418
Zm00001d038361_T003	4.228260125	2.218868254	3.254648461	3.555764997	3.587101153	3.437498752
Zm00001d038361_T005	0	0	3.382637513	2.39413627	1.187339562	1.301673679

Zm00001d038361_T002	3.071067235	3.925847168	3.685287593	3.891497444	3.955001947	3.744212881
Zm00001d038361_T006	3.478331184	1.448054639	2.942680957	3.537299671	3.576784049	3.660965799
Zm00001d038361_T001	1.358247708	0.76769632	1.460705349	1.543674044	1.75381716	2.175747176
Zm00001d038361_T008	2.901071741	2.698089764	2.505508616	2.511142371	2.641931852	2.538676219
Zm00001d014723_T005	0.507351173	0.285882741	0.478875536	0.477050383	0.188824998	0.333211894
Zm00001d012995_T001	1.517006029	1.312289931	1.800354497	1.426500935	1.406866967	1.342923905
Zm00001d012898_T001	3.543466435	2.96864265	3.514311408	3.240994297	3.215830778	3.118508573
Zm00001d021709_T001	8.502848229	7.871051982	8.557291027	7.79804671	7.820114697	7.759480524
Zm00001d051186_T001	2.410789909	1.971846638	2.123494705	2.814196012	2.477848524	2.291966838
Zm00001d033595_T001	5.653131433	6.078683073	5.399031809	6.451680925	6.356731177	6.431818593
Zm00001d043831_T002	0.85311819	0.449242296	0.642533382	1.238228624	1.428625353	1.270479905
Zm00001d043831_T001	1.698842992	1.227343695	1.894137912	1.636719734	1.56669876	1.423535145
Zm00001d014085_T001	3.776162011	3.153384137	4.009050074	4.338493446	4.365667064	4.128128234
Zm00001d036972_T001	4.113380041	3.229148126	4.172045006	3.337321041	3.252122991	3.364383189
Zm00001d018703_T001	1.252801352	1.195440835	1.507106548	1.054569286	1.375013289	0.919173777
Zm00001d018703_T002	3.299565286	2.642307174	3.119796555	2.768804259	2.320048482	2.585181533
Zm00001d038766_T001	0.968634172	0.819383703	1.032275085	1.333669314	1.444830302	1.845052007
Zm00001d029257_T001	3.209636771	4.061652051	3.625623521	2.796967932	3.194261861	2.918281464
Zm00001d048218_T001	3.202488718	2.658585792	3.174112512	3.789244934	3.83097122	3.835732005
Zm00001d054038_T001	1.518331687	1.142949243	2.133851181	1.863306935	2.04302107	1.817201642
Zm00001d054038_T004	3.829339149	4.005970934	2.466917848	2.489171682	0	2.626611082
Zm00001d054038_T002	4.661372918	3.67862656	4.920518835	4.885153745	5.006783449	4.656459816
Zm00001d005256_T001	5.311968378	5.00196627	5.228846236	5.475558611	5.409113844	5.347815622
Zm00001d039427_T002	3.777317974	2.706514418	3.797207677	3.99585948	3.973415383	4.029411539
Zm00001d045505_T001	2.971077402	2.386072583	2.960339147	3.373261492	3.826658408	3.401339113
Zm00001d045505_T006	2.866504679	1.358238705	2.196112843	3.203894768	2.348733252	2.521302307
Zm00001d045505_T005	1.03354916	0.323845028	0.358287099	1.853001182	1.589557852	1.427000006
Zm00001d045505_T004	2.65653321	0.85742769	3.037868193	2.190486229	1.953243631	3.239688568
Zm00001d045505_T007	3.795726018	5.532930099	4.117133146	3.524450132	4.003618696	3.611017831
Zm00001d017823_T002	5.238368471	4.540053873	5.429779873	5.193908044	5.111763578	5.113494772
Zm00001d017823_T003	2.987051768	4.237399203	0	1.815769688	2.912979942	0
Zm00001d007038_T004	1.413308656	0.59918066	0.725583247	0.824737716	1.464930124	1.108239402

Zm00001d007038_T003	5.173391237	4.702634773	5.176015289	5.088877007	5.106418928	5.130165882
Zm00001d042578_T001	1.567038663	1.317217226	1.722469959	2.097081487	2.252684202	2.152372953
Zm00001d013597_T003	5.057072479	4.269103409	5.06496638	4.613559293	4.618321244	4.486727114
Zm00001d013597_T002	1.146919757	0.760422803	1.516068617	1.242186023	1.272570294	1.395652374
Zm00001d029044_T006	0.269422203	0.166185824	0.612718611	1.19219164	0.939335669	1.092184431
Zm00001d029044_T004	0.539690866	0.294360195	0.630267533	0.930232448	0.878280401	0.964933837
Zm00001d029044_T002	3.616308877	2.97724768	3.501116812	4.484890676	4.367642384	4.256117712
Zm00001d029044_T007	4.974061003	4.067552246	5.076938574	4.628397827	4.758200398	4.683989623
Zm00001d029044_T001	0.708081265	1.044959895	0	0.727563275	0.230768665	0
Zm00001d044947_T001	5.3564282	5.015993821	5.452136152	4.956743442	4.883290356	5.083066325
Zm00001d042363_T005	3.61692871	3.529060427	4.094814299	4.293360404	4.467864723	3.788133163
Zm00001d042363_T013	2.064814232	1.52184174	1.901791382	1.482150769	2.213809492	2.350866926
Zm00001d039341_T001	0.807910459	1.112736158	0.399296897	1.669411886	1.387297464	1.081348491
Zm00001d039341_T009	0	0	0	3.668388754	3.785682366	3.86778974
Zm00001d039341_T006	0	0	0.898562065	0	1.414487367	1.345654118
Zm00001d039341_T025	0.971921295	0	0.333329841	0.810115658	0	0
Zm00001d039341_T133	0	0	1.209586867	2.680511827	3.114045131	2.262320997
Zm00001d039341_T007	1.995884619	0.492874547	0	2.206400671	1.727083122	2.082411024
Zm00001d039341_T012	0.765756225	0.208780679	0.658400706	0.460627248	0.619109649	0.17200483
Zm00001d039341_T080	1.067851389	0	1.2136459	0	0.855390177	0
Zm00001d039341_T008	0.079417026	0.323522257	0.296043849	0	0	0.425343283
Zm00001d039341_T011	4.786676544	3.844135942	4.974550879	4.266326079	4.454417946	4.50442838
Zm00001d047750_T001	5.178825408	4.474564731	5.257519961	5.510180723	5.581422627	5.492480949
Zm00001d027894_T001	8.439373096	8.119664979	8.293948338	7.539876653	7.473293019	7.57492889
Zm00001d006535_T001	2.261010509	1.979944478	2.523216672	1.593468513	1.923310464	2.089873013
Zm00001d018014_T001	0.757875807	0.604596103	0.841896656	0.700399766	0.805038156	0
Zm00001d018014_T012	1.321550636	0.405525177	0.997692007	0.857104366	0.95458197	0.809419384
Zm00001d018014_T002	0.336758685	0.078419922	0.307114887	0	0.803201407	0
Zm00001d020950_T001	4.817239204	5.059054887	4.642073938	4.464228858	4.868267762	4.793982265
Zm00001d025128_T012	2.228102628	1.664198369	2.700896647	3.350703029	3.799276544	4.032782082
Zm00001d025128_T011	4.147005835	2.921201131	4.04965943	2.828468904	0	0
Zm00001d025128_T001	1.323741288	0.988555997	1.146111014	1.721861277	1.919921239	0.986623546

Zm00001d025128_T013	0.304860284	0.229072275	0.258179995	0.991866283	0.797329298	1.235785257
Zm00001d025128_T009	0.850428202	0.348749113	0.47005159	0.836966318	0.90805121	0.989486375
Zm00001d025128_T007	0.675461874	0.976089369	0.202939223	0.546058288	1.190069282	1.05182868
Zm00001d025128_T008	1.568494277	0	1.582399923	2.560674118	2.256430103	2.418830314
Zm00001d025128_T005	1.653595252	1.669225463	1.53292234	2.088106988	1.910948283	1.558813746
Zm00001d040028_T001	6.019957432	6.313813166	5.974882463	5.15823114	5.299924773	5.265589319
Zm00001d011746_T003	1.64246453	1.086869347	1.929774715	2.634211287	2.202770006	2.520160214
Zm00001d011746_T002	1.070997879	0.518833215	0.540619506	1.769071376	2.27108857	1.518665555
Zm00001d039157_T007	1.110166104	0.406793513	0.760557359	1.040857371	1.033196029	1.110696648
Zm00001d039157_T002	2.915732748	1.971747333	2.681270239	2.545494475	2.735829096	2.521894492
Zm00001d039157_T004	1.02563056	0	0.873965136	0	1.320785028	1.398460374
Zm00001d039157_T003	0.451710676	0.375384246	0.68827079	1.390008914	1.299092818	0.809569202
Zm00001d039157_T001	6.29310218	5.49209141	6.27919718	6.390124406	6.316723429	6.31676473
Zm00001d004700_T001	11.08207952	11.06619314	11.11371411	10.67929355	10.68705613	10.70463486
Zm00001d015962_T001	4.282837122	3.187955988	4.217586537	4.508804871	4.539153599	4.380687065
Zm00001d021754_T011	0.474914656	0.259850956	0.660182029	0.363624144	0.5418528	0.460148067
Zm00001d021754_T002	3.791078161	3.019458005	3.990550486	3.977647741	4.115568742	4.107761425
Zm00001d017177_T005	3.694326181	2.861893646	3.584587111	3.416832177	3.565084221	2.681498516
Zm00001d017177_T001	0.561341779	2.451982278	1.216570959	0.21228295	1.554713102	0.272346938
Zm00001d017177_T003	4.046692206	3.124955372	4.278909783	3.872645186	4.146564481	3.924428969
Zm00001d017177_T002	5.056526305	5.412940382	4.348144444	3.372723003	0	4.145441225
Zm00001d017177_T007	3.939657441	2.98404042	4.631187477	3.654947588	3.61904918	3.273140919
Zm00001d017177_T010	1.223952573	1.10528383	0	2.119212066	2.089475778	1.763468516
Zm00001d017177_T004	0.398827541	0.458056469	0.55212914	0.685004503	1.061295104	0.679281458
Zm00001d017177_T008	1.332127016	0.598158411	1.770488678	0.825405471	0.853277912	1.066040267
Zm00001d044845_T010	3.564685821	3.324061273	3.437161276	3.766098984	3.621230788	3.303841377
Zm00001d044845_T001	0	0.010162534	0.113871159	0.578805424	0.647907585	0.706687909
Zm00001d044845_T003	1.988550544	0	1.554429717	2.584630643	3.021969308	2.807503306
Zm00001d044845_T009	3.051125657	0	3.263025088	4.09504951	4.029965557	4.279020439
Zm00001d006768_T001	1.08295732	0.750117664	0.984671007	1.56786666	1.166838181	1.775914424
Zm00001d025948_T001	0.594710008	0.5501146	0.995318803	1.233294176	1.206862612	0.913975845
Zm00001d011900_T002	3.352012695	2.170607075	3.353202732	3.057355476	3.854878255	3.629824269

Zm00001d011900_T001	3.451382576	3.052869013	3.188783229	3.208316854	2.961151922	3.204067923
Zm00001d018534_T001	3.875597159	3.469744825	3.89873618	3.021636425	3.08757367	3.245846862
Zm00001d030773_T001	0.855053635	1.082649458	0.895308826	0.977046875	0.997636373	1.03026403
Zm00001d024768_T001	2.59181942	2.125958187	2.866873171	2.213932641	2.278829889	2.26315162
Zm00001d022414_T001	4.301678215	3.492821365	4.227135719	4.486891964	4.504618994	4.454572975
Zm00001d019139_T004	5.414882435	5.619729747	5.672831898	5.042620963	5.272381802	4.519584488
Zm00001d019139_T005	3.397527126	2.044273453	3.466329807	4.620367761	4.52296098	4.227372116
Zm00001d019139_T008	0.868280605	0.443544063	0.214947902	0.94956332	1.028083222	1.010179725
Zm00001d019139_T006	5.207428858	3.345381897	4.073187502	5.166733157	5.07260654	5.689932968
Zm00001d019139_T003	3.952519576	3.368035643	5.046052833	5.08894038	5.457280072	4.900950108
Zm00001d019139_T001	0.333977805	0.152114609	0.315982343	0.710685008	0.54563236	0.390921868
Zm00001d037158_T001	3.350066354	3.418485744	3.705361446	3.335234839	3.209510604	3.145896207
Zm00001d024891_T001	4.637578891	3.878443542	4.753836731	5.637188307	5.65230989	5.640353058
Zm00001d007215_T003	4.375231523	0	4.181042265	5.440249377	5.163936305	5.563972498
Zm00001d007215_T002	7.186097239	6.198073169	7.252780314	7.232503682	7.279437457	7.258976629
Zm00001d007215_T001	4.125414214	5.261054753	3.971428502	3.455712414	3.696616731	2.911035941
Zm00001d046838_T005	3.811802278	0.889149533	3.518526704	2.531563369	2.578487363	2.323198838
Zm00001d046838_T001	2.879774763	3.050944973	3.141889038	3.44767123	3.332930299	3.513639555
Zm00001d017709_T001	3.868601137	3.61715517	4.02017965	4.664538192	4.728027482	4.853859652
Zm00001d051554_T001	0.452882153	0.463656981	0.257316027	1.7562304	2.061568158	1.705571015
Zm00001d051554_T002	2.256720541	1.581022897	1.891484904	4.555952383	4.436461637	4.539814855
Zm00001d039135_T001	5.574891534	6.13573081	5.427795916	3.720440787	3.626301286	3.819256773
Zm00001d026311_T004	2.404557117	1.040355922	2.810381816	4.051063603	3.71527279	3.803244605
Zm00001d026311_T003	0.743035785	0.718968718	0.780453731	1.812776675	1.696001383	1.533509526
Zm00001d026311_T002	4.647760317	4.054012826	4.688921718	5.56400577	5.59652812	5.54956885
Zm00001d000203_T001	1.366726742	1.331647324	1.905251918	1.658476571	1.70996109	1.83548918
Zm00001d019724_T058	1.399757898	0.559362589	1.12538396	1.338059238	1.127235081	1.168749849
Zm00001d019724_T004	0	0.197782374	0	1.021152774	1.265788525	1.623347173
Zm00001d019724_T011	0	0.316438887	0.560140696	0.54319791	0.786792053	0
Zm00001d019724_T041	1.226633681	0.828996271	1.812013891	1.581369567	0.59562205	0.595730884
Zm00001d019724_T068	4.565527415	5.800037545	4.624057777	3.991915793	3.78552199	4.015636812
Zm00001d019724_T047	0.238000499	0.067337206	0.149639177	0.83633783	0.798131826	0.932500416

Zm00001d019724_T044	0.688856219	0.143654635	0.391953543	0.737032534	0.533311166	0.893008775
Zm00001d019724_T052	0.480478149	0	0.480665298	0.766400952	0.802431475	1.076042533
Zm00001d019724_T024	0	0	1.438190102	1.919483105	2.671042634	2.506900971
Zm00001d019724_T039	1.25592064	1.08026607	1.4150485	2.184375201	1.686612577	2.01655444
Zm00001d019724_T057	1.555597281	0	1.322828056	1.092685099	1.220979331	0
Zm00001d019724_T027	0.733613785	0.514762586	0.607796636	1.508892066	1.400885992	0.925362974
Zm00001d019724_T049	0.320563237	0.107873968	0.382381729	1.014421713	0.813476257	1.203152909
Zm00001d019724_T053	0.279052839	0	0.228365575	0.716530036	0.377838949	0.854282162
Zm00001d019724_T001	0.198964289	0	0.458668625	0.754660928	1.23392793	0
Zm00001d019724_T025	0.260315973	0.130366857	0.42301658	0.601712678	0.813361325	0.314688406
Zm00001d019724_T037	0.581547932	0.376741572	0.393371167	0.12176607	0.400177211	0.187073465
Zm00001d014761_T004	2.114222748	1.772326135	2.868502269	1.640959121	1.8537917	2.485657125
Zm00001d014761_T002	0.822511535	0.612112777	0.692403093	1.227057143	0.885727411	1.05560938
Zm00001d014761_T001	3.39970363	2.28343378	3.001552946	3.832277878	4.062938956	3.828918305
Zm00001d014761_T006	0.946727866	0	1.079876418	1.676782822	1.367013143	1.23615766
Zm00001d035090_T001	2.37291422	2.742993332	2.926476378	2.530805141	3.306857746	3.289509306
Zm00001d035090_T004	0.607221878	0.416907823	0.677752674	0.476220947	0.693810308	0.717737611
Zm00001d035090_T003	2.924502796	0	2.75014018	2.614323503	0	0
Zm00001d011668_T002	3.95844581	3.479372599	4.17130492	4.168768859	4.172898375	4.102543417
Zm00001d036431_T001	2.31887472	2.616563311	2.722863804	1.780685529	1.481292741	1.263103533
Zm00001d038118_T003	0.462747577	0.382921742	0.645210994	0.568011668	0.50373035	0.497990393
Zm00001d038118_T001	3.027639057	2.581218663	3.078068552	3.057819693	3.034382658	3.115029913
Zm00001d022037_T001	3.58134498	3.554733728	3.625243366	3.566980598	3.869615606	3.730842191
Zm00001d045007_T001	3.621675527	3.014201373	3.50062298	3.76435295	3.33530245	4.047231077
Zm00001d052064_T001	2.584729246	1.891022877	2.62215774	2.628324039	2.672176385	2.807054398
Zm00001d044594_T001	3.424388569	3.010052938	3.546970496	4.046674834	4.183324212	4.058596781
Zm00001d025281_T001	2.608907863	2.15567606	2.59184359	3.160924543	3.144326069	3.286497659
Zm00001d026322_T001	0.958657108	1.182957297	0.46421657	1.426122491	0.968345898	1.126021976
Zm00001d026322_T003	4.320129593	3.657939259	4.331384558	5.104490567	5.086851518	5.118087077
Zm00001d024708_T003	4.178101425	4.643791556	3.655432892	2.916893533	2.503066021	2.700452147
Zm00001d024708_T001	4.175676687	2.822324913	4.289167987	4.697139277	4.458722097	4.435888042
Zm00001d024708_T002	1.249331256	2.45518148	1.290245088	1.105068558	1.182806692	1.543629506

Zm00001d026261_T008	0.138589071	0.6423624	0.768830507	1.499078967	1.953247729	1.893896084
Zm00001d026261_T001	1.067292456	0	0.369245351	0.701251838	0.995384657	0.483440725
Zm00001d026261_T006	2.0935835	0.826842538	1.880535469	2.598263956	2.266869578	2.260701968
Zm00001d033446_T001	8.692746125	8.012542779	8.695611691	8.525238283	8.521706316	8.547544664
Zm00001d008316_T001	1.349467191	1.086766785	1.16701295	1.352357972	1.083447585	1.509850869
Zm00001d043694_T002	4.335270289	3.502922606	4.36379653	4.743080823	4.599914686	4.472474514
Zm00001d043694_T003	2.268669434	2.246661943	2.080688018	2.361757974	2.517366662	3.239261007
Zm00001d043694_T001	1.116391374	1.329490468	1.221062262	0.582185045	0.870505184	0.968286908
Zm00001d015319_T001	3.723275128	3.154222036	3.954359	2.835388925	3.07506997	2.649980283
Zm00001d011992_T001	8.197861756	8.083016	8.306664259	7.712784558	7.707528345	7.721322529
Zm00001d033247_T003	3.109678653	0	0	1.560717889	1.825786441	0
Zm00001d033247_T001	2.375446249	2.586368924	3.250627684	2.970735694	3.056561853	3.590323202
Zm00001d033247_T007	3.036674056	4.265920214	3.568586207	1.759050984	2.215799722	0
Zm00001d033247_T006	1.576093014	1.4269324	2.928613073	2.470887182	2.488826291	2.576563979
Zm00001d033247_T002	0.870430219	0.349410573	0.553778259	1.106700742	1.171607165	0.680910319
Zm00001d004993_T006	0.680990409	0.328963958	0.641883543	1.449560318	1.570371606	1.387176126
Zm00001d004993_T003	2.505979064	1.68757674	2.378617325	3.005808304	2.857131645	2.942466086
Zm00001d010061_T002	1.598430001	2.66269771	1.621666971	1.058333122	1.196620262	0.509634029
Zm00001d010061_T001	4.496492581	3.898221996	4.438339965	3.513697913	3.430478244	3.542759253
Zm00001d012794_T009	0.902698773	0.669021321	1.051774399	0.997754863	1.097589898	0.596149912
Zm00001d012794_T004	0.71053514	0.293205663	0.770650627	0.824908756	1.014802316	1.171915829
Zm00001d012794_T006	0	0.758050694	1.174759219	0	1.197397414	0
Zm00001d012794_T010	2.327819557	1.543164248	2.135016339	1.501876282	2.069360869	2.263740252
Zm00001d012794_T013	1.17243695	0.126728423	0	1.260680894	0	0.757281884
Zm00001d012794_T002	2.099301601	1.747772783	1.649593847	1.963906128	1.202227753	1.22854967
Zm00001d012794_T005	1.480806936	1.074445837	1.704037527	0.336547334	0.484201046	1.198838599
Zm00001d039564_T013	0	1.232550248	3.316371254	1.756636484	2.062941632	2.315342476
Zm00001d039564_T005	0	0	3.004689964	3.009283386	2.458608523	0
Zm00001d039564_T019	2.540177832	0	0	3.066453134	3.420641256	2.991339165
Zm00001d013709_T004	0.562341593	0.584116834	0.645792026	1.138132271	0.883638019	0.919101298
Zm00001d013709_T001	3.34701729	2.909742196	0	3.159050442	4.177759246	3.817001537
Zm00001d013709_T003	3.332581057	2.88365444	3.506082684	4.795523795	4.722940724	4.718282378

Zm00001d039296_T006	0.896354784	0.349220321	0.89119596	1.539673001	1.384882018	1.568260777
Zm00001d039296_T003	2.103912848	1.578902977	1.980286388	1.64952717	1.733852798	1.595716087
Zm00001d039296_T005	2.43036209	1.734570465	2.823060452	2.031179199	2.376431812	2.700075447
Zm00001d039296_T007	2.998823544	2.108352494	2.515405909	3.304774733	3.099493666	2.859603065
Zm00001d006921_T004	2.747090451	2.039682373	1.918867434	2.089874707	2.238893529	2.111670699
Zm00001d047644_T001	5.52321297	5.650770274	5.091695053	6.320153228	6.430436209	6.357098223
Zm00001d021023_T001	1.579166146	1.561935105	1.804088676	2.589935546	2.276747366	2.267870141
Zm00001d021023_T004	2.545541179	1.104554054	2.256586802	0	1.494746355	0.932253971
Zm00001d042553_T001	0.823839832	0.599057799	1.194376445	1.502532249	1.472580829	1.418591525
Zm00001d009823_T001	3.88253878	3.909557788	4.102890304	3.857917515	3.343293681	3.886779166
Zm00001d022585_T002	4.216944165	3.558051704	4.206114696	3.746832022	3.66272505	3.691985551
Zm00001d022585_T001	3.231946292	3.019366729	3.414947328	2.983051601	3.225021449	2.868749378
Zm00001d037848_T005	0.720706613	0	0.494174928	1.156814672	1.453914184	1.204654085
Zm00001d037848_T002	0	0	2.31698041	3.244916887	2.922372941	3.194818776
Zm00001d032295_T001	5.835359262	6.242455885	5.785295233	5.812983167	5.866786521	5.674999895
Zm00001d043070_T001	7.642368517	7.772549352	7.614919483	7.947098545	7.981229135	7.906468347
Zm00001d045783_T049	0.132957124	0.008393661	0	0.781065059	0.555723898	0.4809682
Zm00001d045783_T014	1.652452535	0	1.370218415	2.459957713	2.497951828	2.758618079
Zm00001d045783_T012	0.059297126	0.087855201	0.081375081	0.361897466	0.488122148	0.481145983
Zm00001d045783_T056	4.364901063	3.121916058	4.331898079	4.664611858	4.472616763	4.527826338
Zm00001d045783_T046	0.241294723	0.1305461	0.348902046	0.672563834	0.922442972	0.470954321
Zm00001d045783_T013	0.55494144	0.178246785	0.479642405	1.132835419	1.337334315	0.962047
Zm00001d045783_T047	6.061287672	5.693589545	6.216887163	5.458822476	5.74950544	5.448499861
Zm00001d045783_T004	0.959275326	1.074454742	1.133059744	0	0	0
Zm00001d053643_T002	2.289404543	2.116842138	2.495720742	2.478952396	2.205275829	2.872560482
Zm00001d053643_T003	1.801022867	0.486809091	0.707695289	1.434333054	1.947546099	0.752194524
Zm00001d051632_T001	1.200537624	0.538457708	1.056771483	1.315339578	1.292700485	1.075020495
Zm00001d045154_T001	5.342013705	4.383331735	5.286251864	5.365457387	5.325450459	5.33331406
Zm00001d045154_T003	0.274531237	0.38746732	0.79526574	0.495741205	0.583096371	0.287443926
Zm00001d021240_T001	3.078267222	2.394820593	3.121743032	4.140415074	4.014060028	4.111493352
Zm00001d017049_T002	0.514978657	0.251650023	0.473261161	0.452352945	0.323660597	0.575285219
Zm00001d017049_T004	1.313593566	0.463670581	2.136364657	1.572372145	1.800390532	1.516536138

Zm00001d017049_T001	4.100972603	3.69044872	3.937982352	3.830181889	3.848686731	3.665657226
Zm00001d034675_T002	3.821577877	2.913458161	4.087380351	4.200445187	4.35020394	4.24520037
Zm00001d034675_T003	5.022139094	4.435753791	4.910678176	4.737843459	4.534391041	4.543521867
Zm00001d053432_T001	1.243273626	1.418920692	1.544509621	0.389702265	0.505090113	0.637498962
Zm00001d040130_T001	2.052918733	1.626026011	1.975585067	2.36635523	2.268481105	2.545653116
Zm00001d000120_T002	6.531665776	6.291316873	6.398640823	5.965834028	5.872258856	5.928873165
Zm00001d013643_T001	1.821451445	2.672094672	1.980350373	2.378826485	2.041978163	2.109509669
Zm00001d052478_T001	0.071853264	0.105578856	0.03468198	0.881287159	0.854753711	0.916764008
Zm00001d052461_T001	2.0898032	1.854466093	1.998962329	1.820237384	2.243824735	1.867370674
Zm00001d049668_T001	7.658068799	7.461080717	7.770907738	6.984605459	6.938244007	6.828494266
Zm00001d018057_T001	2.208089882	1.823768515	1.875666058	2.562349409	2.955448399	1.496691915
Zm00001d018057_T002	1.808883369	1.09140128	2.273193299	2.428352802	1.0943354	3.077061112
Zm00001d017469_T001	5.50478337	5.014455992	5.49532955	5.561206824	5.665940102	5.609891538
Zm00001d003124_T002	1.904894064	1.336219394	1.893374249	2.228061673	2.032619626	1.820471864
Zm00001d014199_T002	3.697793721	3.208456392	3.808871737	3.75918066	3.786293992	3.858510118
Zm00001d014199_T004	0.847547197	0.40429388	0.823975935	0.906955227	0.895777236	0.83209525
Zm00001d014199_T001	2.762277158	1.218988802	2.868848921	3.124671892	3.45785179	3.215509936
Zm00001d009415_T001	8.177383478	7.724788655	7.7870633	7.599298183	7.681148129	7.775557437
Zm00001d012476_T001	3.288031002	2.920337705	3.275331617	3.620082669	3.593433852	3.462950124
Zm00001d016543_T002	0.226538123	0.242564704	0.847210423	0.651313965	0.634111825	0.950824471
Zm00001d016543_T004	0.694589619	0.325965449	0.100638625	0.933677626	0.686064795	0.751398591
Zm00001d016543_T001	4.472330041	4.122460771	4.432196113	4.263422153	4.3176537	4.318512152
Zm00001d051193_T003	3.827684096	2.797628535	3.897685172	3.969271711	4.018459523	3.882478328
Zm00001d051193_T004	2.128563304	0	0	2.128293401	2.138130304	2.207534632
Zm00001d045564_T001	3.554117658	3.180921367	3.700325408	3.699839543	3.815646332	3.897936915
Zm00001d015140_T001	4.532723899	4.193515189	4.528786644	3.327575565	2.833885687	2.905477987
Zm00001d005624_T001	4.256612162	3.869156394	4.462099055	4.449920452	4.362760222	4.456984964
Zm00001d011401_T003	0.512992412	1.125941331	0.543228601	1.165580764	1.600333037	1.047319909
Zm00001d011401_T001	5.576268253	4.630632913	5.284262558	5.339178156	5.3221174	5.451054897
Zm00001d025814_T001	2.487167059	2.150103726	2.410606497	2.143143913	1.602129973	1.905405972
Zm00001d045031_T001	2.44594701	2.72821136	2.712859865	2.299774506	2.088935336	2.664204059
Zm00001d047739_T007	0	0	3.094437256	0	3.377242742	2.190177719

Zm00001d047739_T016	0.684633845	0	0	1.418172673	1.626738158	2.097154658
Zm00001d047739_T042	0.516770599	0.277343275	0.436427322	0.623199192	0.843823893	0.626108272
Zm00001d047739_T039	0.890870003	0.714083707	1.291469183	1.64144106	0.708468021	1.205612717
Zm00001d047739_T026	0	0	0.78565433	1.630204151	1.618097199	1.114695509
Zm00001d047739_T003	1.340119566	1.744433675	1.007174686	0	0	0
Zm00001d047739_T025	0.811253993	0.292298807	0	0.732138059	0.293270416	0.4662011
Zm00001d047739_T009	0.358860955	0.469987013	0	1.744141285	1.09506631	0
Zm00001d047739_T002	2.819193406	1.334479606	0	3.452034483	0	2.447197777
Zm00001d047739_T043	3.373678137	3.371970146	3.235098845	0	1.0955383	0
Zm00001d047739_T008	0.844409758	0	0.849128978	1.064484193	0.859289771	1.133393207
Zm00001d047739_T001	0.696537838	0.507504423	0.479375444	1.315205077	1.966060886	1.635262189
Zm00001d047739_T014	0.482463169	0	0.71486621	1.426662481	1.297288865	1.346580818
Zm00001d003784_T001	2.103741341	1.35309967	1.83308398	3.800066804	3.801997535	3.733127397
Zm00001d041913_T001	1.953642209	1.860325608	2.071790466	2.098658729	2.164601908	2.041244384
Zm00001d052411_T002	4.528374904	3.83681952	4.737768681	4.706356435	4.69338899	4.640833118
Zm00001d034809_T032	3.965876799	3.243581042	4.151900451	3.998936071	3.981851649	3.905117203
Zm00001d034809_T021	1.749742773	0.283060045	1.467958144	0	1.545405015	0.493469035
Zm00001d034809_T026	1.23451297	0.575744106	1.032178443	1.67440099	1.424430897	1.514183881
Zm00001d034809_T002	1.769782316	1.573210178	1.949194622	2.273322523	1.841177707	2.527940846
Zm00001d046882_T002	2.083872687	1.747988415	2.132422859	2.020880138	0	0
Zm00001d046882_T001	1.71250422	0.90474689	1.658997466	2.334473885	2.806364481	2.773656914
Zm00001d047662_T011	1.651274926	2.001250275	1.000570473	2.31711793	2.422152789	2.023015061
Zm00001d043387_T002	2.872169795	2.303230739	2.706264864	3.60644057	3.377411704	3.368447513
Zm00001d012755_T016	0.23629055	0	0	0.641692141	0.507929581	0.196296063
Zm00001d012755_T054	0.319562445	0	0.404635044	0.609238676	0.831834284	1.089583571
Zm00001d012755_T002	0.320931714	0.119882026	0.508905246	0.644231011	0.54754163	0.695548468
Zm00001d012755_T004	0.81653909	0.724336817	1.297487263	0	2.521573933	1.890455253
Zm00001d012755_T056	3.253959474	2.26609628	3.074787132	3.418312889	3.052976795	3.171035609
Zm00001d023596_T001	1.365344278	1.025592293	1.207503709	2.770107624	2.481352938	2.745819887
Zm00001d039693_T082	2.562645895	1.421737124	2.694436064	2.471783408	2.171824581	1.918790743
Zm00001d039693_T054	0.319955448	0	0	0.592871756	0.130148045	0.989302528
Zm00001d039693_T043	0.217158735	0.051848165	0.311897158	0.459783069	0.417469635	0.261257595

Zm00001d039693_T061	0	0	0	1.316913237	1.795473559	1.428086634
Zm00001d039693_T044	0.459548079	0.250926415	0.408465142	0.703925494	0.839066291	0.990999139
Zm00001d039693_T005	0.389346536	0	0.165717745	0.804232026	0.24316088	0
Zm00001d037743_T001	7.407467008	7.894693529	7.3416839	3.258970959	2.977929219	3.086927417
Zm00001d048481_T001	3.751088645	3.077264879	3.925650282	3.962254716	3.929607809	3.986832486
Zm00001d051906_T002	2.14323438	1.451827761	1.687305746	1.781680323	1.984141617	1.96550135
Zm00001d030523_T001	1.920561613	1.556318557	1.923324538	2.10215016	2.36271338	2.030775354
Zm00001d007530_T001	3.83474246	3.961050303	3.827150048	3.940061294	3.927450306	4.047330555
Zm00001d034702_T002	0.259520775	0.447327397	0.700308316	0.640860586	0.95854724	0.697943695
Zm00001d034702_T001	1.263941816	0.577502789	1.004690503	0	0	0.478753379
Zm00001d018214_T001	5.349053759	4.682957403	5.414872797	5.5983161	5.576133114	5.630526972
Zm00001d021845_T001	4.079857308	4.557661648	4.61973092	4.679697233	4.657815702	4.476501842
Zm00001d001866_T003	0.501710206	0	0.486470343	0.887444945	0.576209621	0.656021267
Zm00001d001866_T010	1.633582796	1.625777799	0.738641609	2.685140223	1.253808994	1.939610618
Zm00001d001866_T005	2.549168391	1.448642511	2.749806906	2.739423439	3.187380739	3.143493176
Zm00001d042973_T004	2.082777174	3.198338089	1.210171259	3.060534946	2.426051354	2.963914264
Zm00001d042973_T008	4.746011242	0	4.849955138	4.198335889	4.120031463	2.906605106
Zm00001d042973_T005	4.373083898	3.670142721	4.416113889	3.963966313	4.134511832	4.670204542
Zm00001d042973_T006	2.076265601	1.574033672	2.263719223	2.402582757	2.574165949	2.21971064
Zm00001d042973_T002	1.395527349	0.364581397	1.764800907	2.476008846	2.855986509	2.455864017
Zm00001d053897_T001	5.596385562	5.722934606	5.479626692	6.352715099	6.388579973	6.356884463
Zm00001d053897_T002	1.621175567	2.963719361	1.594365096	2.382686341	2.547190437	2.425930279
Zm00001d033575_T001	2.908685922	1.912608863	2.886764926	3.582682083	3.639535607	3.576708721
Zm00001d033575_T002	1.310729247	1.418421516	1.286372567	2.373808548	2.122377855	1.986610077
Zm00001d005072_T002	1.35625708	0.913671843	1.852800689	2.106217647	1.676752137	2.087984833
Zm00001d005072_T001	3.373781409	3.927825265	2.661138468	0	2.057511618	2.157465782
Zm00001d005072_T003	0.787351384	0.498105825	0.179903359	0.477326065	0.557030648	0.208081451
Zm00001d051403_T003	5.195656028	6.96265921	5.467095675	4.166563697	4.526625745	4.759707234
Zm00001d051403_T001	8.261075934	7.380171917	8.380514352	8.383826968	8.382022024	8.39486751
Zm00001d038960_T001	4.375669421	4.05328836	4.561910429	3.527564081	3.56007905	3.68160286
Zm00001d025062_T001	6.641921393	7.172078439	6.712017431	6.322588337	6.517370111	6.455167356
Zm00001d021775_T001	2.88827781	2.111729114	2.892150265	3.063584739	3.189763846	3.086504064

Zm00001d014764_T002	0.510066664	0.448376624	0.767559887	1.560952613	1.156626368	1.841715141
Zm00001d014764_T005	2.240684955	1.645675377	2.280281414	2.992366559	3.13492634	2.863197492
Zm00001d054070_T013	1.30137986	0.522306893	0.536281723	2.265976306	2.137044083	2.448646211
Zm00001d054070_T014	0	0.589508476	1.054364372	0.294135481	1.173413385	0
Zm00001d054070_T007	0	0.193209061	0	0.807593153	0.424563116	0.807241975
Zm00001d054070_T003	1.371640822	2.148186051	0	1.717061985	2.459255337	0
Zm00001d054070_T018	3.95137005	5.016855656	4.238961531	3.205461643	2.951099665	2.950400477
Zm00001d054070_T002	0.995197218	0.667398959	0	1.098085999	0.833431758	0.969427921
Zm00001d054070_T005	1.381936176	0.56609521	1.834832548	2.704557724	2.393843125	2.585788207
Zm00001d054070_T015	3.293360919	2.153934362	3.354457944	2.965850763	3.418842859	3.440228145
Zm00001d006244_T001	4.17269376	3.767818866	3.981295008	3.740257204	3.65771271	3.857413258
Zm00001d016797_T006	1.61299738	0.69559479	0.239951549	1.410190748	2.279395519	0.198485353
Zm00001d016797_T002	4.827492857	4.024701049	4.858183257	5.183306821	5.193028051	5.25704744
Zm00001d016797_T009	0.523456573	0.58439974	1.035049532	1.51703325	0.634969575	1.487677221
Zm00001d024872_T001	2.37947541	2.19187118	2.501956002	2.520145124	2.421387053	2.261943557
Zm00001d011958_T001	6.629950786	6.046696025	6.62767973	6.681435266	6.766921904	6.703134942
Zm00001d017459_T004	2.031436133	1.613320891	1.654232963	2.285367002	1.360539581	1.886303922
Zm00001d017459_T001	0.223020887	0.470147409	0	0.838481479	1.099089812	0
Zm00001d017459_T002	0.900768291	0.837306298	1.421235689	1.04088051	0.805749753	1.358815393
Zm00001d012284_T001	4.506386961	3.696794865	4.283996719	4.295127239	4.448123113	4.2238136
Zm00001d006943_T001	0.68974465	0.427860365	0.472458657	0.804720218	0.718547068	0.529808955
Zm00001d010800_T001	3.77758152	3.142227003	3.707173682	1.448154044	1.307283957	1.200828864
Zm00001d045846_T001	4.269748031	4.861361346	4.245547154	4.434878851	4.405924443	4.162514353
Zm00001d021664_T001	2.046681381	1.857743777	2.307556468	3.042975384	2.971146028	2.844324585
Zm00001d047701_T002	2.867791124	2.13078264	2.90844556	3.481216651	3.578806873	3.475347839
Zm00001d039287_T002	4.274412111	3.396545737	4.251208644	4.37725378	4.474687309	4.340838818
Zm00001d025338_T001	6.232149757	6.181834807	6.245501291	7.243122274	7.224137407	7.227609061
Zm00001d033477_T003	0.390479496	0.140397813	0.248159541	0.616342639	0.630319727	0.845557492
Zm00001d033477_T002	4.160187444	4.11812222	4.302662098	4.447283817	4.350959472	4.465326673
Zm00001d050017_T002	0.592422113	0.229977921	0.577120859	0.636618589	0.277734857	0.690108632
Zm00001d050017_T003	1.042884533	0.804932459	0.997894293	0.56477324	0.615720437	0.76282928
Zm00001d019944_T003	4.08272379	3.471912138	4.558021387	3.327895706	2.783495629	3.823491466

Zm00001d019944_T001	2.889063066	1.997386356	1.540333834	2.809757687	2.95956375	1.576552128
Zm00001d019944_T002	0.947610804	0.135403531	0.970473793	0	1.35759817	0.63400399
Zm00001d019944_T004	1.982078719	1.310537314	1.321509075	1.039003596	1.414230807	0.291246376
Zm00001d015423_T001	0.964751268	0	0	0.872627851	1.19641505	1.029129149
Zm00001d015423_T003	3.793612725	3.992853069	4.335130414	4.225059173	4.344456771	4.391180956
Zm00001d015423_T006	1.462076407	3.254675215	1.92244868	2.554999624	1.922147981	1.885797291
Zm00001d033002_T001	4.157831802	3.273285667	3.84511204	4.532694667	4.491134455	4.424310291
Zm00001d053393_T002	2.670837164	2.140983759	2.642634576	3.140650738	2.813521201	3.047627377
Zm00001d053393_T001	1.00286667	0.394246321	1.262993529	1.646959376	1.853253157	1.692755695
Zm00001d029297_T001	2.146003451	1.769809393	3.006455123	3.500489775	3.582208173	3.748084194
Zm00001d029297_T003	2.936583104	0	1.919997489	1.486802914	0	0
Zm00001d029297_T002	0.906193317	1.812473581	1.147218115	0.582893154	0.942930317	0.669221834
Zm00001d022215_T001	4.661615709	4.150690293	4.772170604	3.842498289	3.578957788	3.819347424
Zm00001d034628_T001	7.480951952	7.983662494	7.607635149	7.16412752	7.047615937	7.108714201
Zm00001d011841_T001	5.718874959	6.043495193	5.685528776	5.419754605	5.609204452	5.448440127
Zm00001d012229_T001	4.153046749	3.608326805	4.182357563	5.14550852	5.065349566	5.274477751
Zm00001d034514_T001	5.642595358	6.514018111	5.593987007	6.387035488	6.379830098	6.21838939
Zm00001d031272_T001	2.070986549	2.686442258	2.858275474	1.515756347	2.400872334	2.484930042
Zm00001d006226_T001	3.950315365	3.59829862	3.943442036	4.147991328	4.230762518	4.272576489
Zm00001d006226_T002	2.05067199	1.874687211	1.484907879	1.962735571	1.979364691	1.747497397
Zm00001d047106_T001	1.559535864	2.732472617	1.929929581	0.476207465	1.041060005	0.863600725
Zm00001d013101_T003	1.575967204	0.87854801	1.200289019	1.660479615	1.480857591	1.39309819
Zm00001d013101_T005	3.368810107	3.652134239	2.509858468	1.676859983	2.122382826	1.746455094
Zm00001d013101_T001	3.751480753	3.012892216	3.969441183	3.918061019	3.594988934	3.695810904
Zm00001d013101_T002	2.647739311	3.21386858	2.920407644	2.766681233	3.350030417	2.89059183
Zm00001d027988_T001	8.519530822	8.548988905	8.279816425	10.46477345	10.53635469	10.56827898
Zm00001d006903_T001	6.00077862	6.033802612	6.068116475	6.836482758	6.827795543	6.747776153
Zm00001d053060_T001	2.775924114	2.221973876	2.898151069	2.40118208	2.249857611	1.969107379
Zm00001d033223_T004	1.186117013	0.583569146	0.869467948	0.886083415	1.004131727	0.645999481
Zm00001d033223_T003	1.054262252	0	0.875684142	0.352129115	0.971358509	0.65320405
Zm00001d023677_T001	6.079001728	5.35749785	6.110031161	5.133281116	5.188694348	5.182880169
Zm00001d053698_T002	3.671274688	2.428409353	3.876767696	3.948328728	3.848055715	3.755931315

Zm00001d053698_T001	2.839130202	2.313117542	2.174230945	1.892701488	2.398312332	2.220105174
Zm00001d053698_T004	0.933451779	0.515219932	1.122211513	1.234113157	1.232107515	1.227425644
Zm00001d042024_T001	3.044639269	2.45373907	2.936573682	2.710588257	2.640435142	2.894543843
Zm00001d004667_T042	0.608053167	0.337285213	0.476253097	0.324223039	0.472089481	0.560698327
Zm00001d004667_T006	3.61870264	2.937504662	3.553918672	3.635203787	3.699401714	3.547588021
Zm00001d004667_T001	2.849878396	1.826553775	2.795236228	3.361925251	3.23341107	3.253733521
Zm00001d004667_T008	0.291302965	0.159001341	0.250043533	0.48496406	0.390196614	0.496469508
Zm00001d030834_T001	1.825048016	1.570720844	2.225259503	2.214074124	2.586706866	2.567557616
Zm00001d030231_T002	2.09275917	1.720780585	1.586561563	1.442354889	1.806188753	1.16455911
Zm00001d030231_T001	2.183074212	0.98322311	2.526980978	2.465045357	2.068446221	2.555314069
Zm00001d041604_T001	8.136156142	9.440140882	8.153375888	8.013919606	8.05506116	8.04125002
Zm00001d048129_T002	2.028663207	1.037607834	2.082114288	1.702327304	1.906067833	1.74732982
Zm00001d048129_T004	4.376703376	3.921844554	4.630294271	4.796861902	4.748669035	4.704082304
Zm00001d048129_T005	4.797130257	3.554982193	4.609938951	3.730070699	4.01065588	3.689752476
Zm00001d051856_T005	5.717083213	5.240682933	5.75774692	4.817620341	5.026432081	4.823409329
Zm00001d051856_T003	1.996847877	1.168509825	2.335302022	2.56617901	2.628443499	2.078486189
Zm00001d051856_T010	0.435436577	0	0.877150562	0.653733271	0.721687634	0.779957257
Zm00001d051856_T007	3.217155322	3.120825019	3.323623419	2.54380666	2.556932095	1.884951079
Zm00001d051856_T009	0.72981551	0.279737523	0.496599377	0.709991959	0.697143059	0.999095147
Zm00001d051856_T002	1.647160215	1.183031005	1.134936523	1.553992988	1.741167953	0
Zm00001d020183_T009	4.808682724	3.22670627	4.860047543	4.311224954	4.614037804	4.391141147
Zm00001d020183_T008	1.465094217	0	0	1.810404028	0.492321864	1.698296724
Zm00001d020183_T001	3.874614134	3.029930054	4.025684593	4.30027692	4.355104667	4.291457101
Zm00001d020183_T010	1.513240589	0.771844177	1.517888425	3.027792788	3.084520649	2.770520181
Zm00001d020183_T011	0.969758704	0.265902516	1.036074242	0.779530371	0.579542225	0.560730604
Zm00001d047253_T002	7.927296253	7.034674762	8.048674028	8.393332075	8.426389391	8.409188728
Zm00001d047253_T014	0	0.103954127	0	1.09816889	0.903649165	1.62050055
Zm00001d047253_T001	2.663166293	3.386034634	2.802056077	2.137150676	1.891121284	1.74811254
Zm00001d047253_T013	1.521678449	1.194485504	1.66526769	2.461630446	2.563291897	2.666976955
Zm00001d047253_T011	0.851543977	2.611922575	0.567370211	1.547685735	0.194329162	1.657756148
Zm00001d047253_T004	9.010511364	8.650997743	9.053297278	8.637894014	8.737901129	8.664240951
Zm00001d038290_T005	3.690310705	2.554624458	3.64174508	3.968739681	3.330081773	3.769952594

Zm00001d038290_T004	2.663200001	2.082194014	2.801712652	3.252336025	3.490562558	3.415995626
Zm00001d053442_T001	4.628566915	4.343296667	4.754022641	4.46089377	4.210764608	4.489414678
Zm00001d028866_T001	4.283438374	3.754636344	4.774425642	4.345713012	4.281442919	4.294527752
Zm00001d028866_T002	2.386983618	0.16001919	0	2.827979541	2.861411522	2.902128584
Zm00001d032825_T001	4.625748522	5.539128033	4.750458369	2.214351763	1.64884413	2.219668207
Zm00001d033279_T001	8.324113722	7.96090916	8.344929676	7.550923468	7.580332504	7.583243031
Zm00001d029829_T001	0.696481753	0.438030907	0.437401406	0.700192885	0.351721039	0.524257293
Zm00001d018345_T018	0.40935183	0.063212994	0.22312842	0.389174698	0.43233838	0.122463339
Zm00001d018345_T015	1.674079137	1.734243548	1.994588413	0	1.689088445	0.817701018
Zm00001d018345_T001	0.836334598	0.524045617	0.301893126	1.442729099	0	1.409482179
Zm00001d018345_T010	0.765748589	0.496327354	0.666596637	2.056443761	1.30250857	1.747422634
Zm00001d029028_T001	6.744230577	6.863765275	6.710530994	9.217676983	9.22041739	9.28785934
Zm00001d031593_T001	3.092293375	2.288880909	3.153994171	3.650909625	3.780313249	3.653182607
Zm00001d031593_T004	2.410565524	3.846332439	2.41653469	2.922571987	3.349737794	3.195265539
Zm00001d031593_T005	2.198313413	4.243928673	2.698082205	4.44231879	4.095393731	4.284021602
Zm00001d031593_T003	4.650517063	3.749885191	4.526243064	4.387346134	4.244172154	4.507147534
Zm00001d031593_T002	0.988559633	1.612638414	1.486997479	0.961656664	1.430341481	0.781315225
Zm00001d004372_T004	3.976973765	2.506542535	3.657370189	4.150040749	4.226216493	4.081139675
Zm00001d004372_T002	1.934274176	2.242068923	2.85603772	3.576757328	3.481471517	3.564695331
Zm00001d027318_T001	6.991406426	7.035548243	7.039148749	6.673266146	6.550532435	6.625549137
Zm00001d024311_T008	2.636581238	0	1.696083756	2.722857248	2.102983583	0
Zm00001d024311_T009	0.442700439	1.195088646	1.958236894	2.112268427	2.913523456	3.114612062
Zm00001d036563_T001	4.086590086	3.249817572	4.012054879	3.435424842	3.406265765	3.609682637
Zm00001d038222_T002	1.521684981	1.199553627	0.67675665	2.41772778	2.373403775	2.278168568
Zm00001d038222_T003	2.344420054	0.855561608	2.805018545	2.948934199	3.05174517	3.074192168
Zm00001d006597_T025	0.597108732	0	0.798934737	1.819667775	0.927144009	1.699735511
Zm00001d006597_T046	4.26548273	5.053592256	4.572520097	2.451316631	3.574070619	3.032244576
Zm00001d006597_T028	2.246237812	2.011242644	2.275714212	2.555052404	2.66779405	3.111931881
Zm00001d006597_T044	3.495418108	2.122731022	3.617367724	3.568827436	3.474403204	3.080094118
Zm00001d006597_T041	1.905594666	0	1.060675528	2.331551023	2.316591808	1.343848949
Zm00001d044605_T001	1.519805058	0.75247972	1.490224561	1.513413429	1.507807323	1.188029283
Zm00001d011850_T001	6.968309732	6.660073942	6.930501828	6.818672488	6.792857759	6.865980336

Zm00001d011850_T002	4.364538115	4.070061077	4.446161194	4.436235745	4.589502964	4.710482297
Zm00001d043782_T001	4.868624298	4.651080918	4.992836033	6.834772287	6.882064315	6.899700168
Zm00001d011330_T001	6.908336029	6.651154959	6.889539847	7.003935563	7.095735634	7.016850855
Zm00001d002968_T001	0.8897749	0.515131099	0.993236687	1.067444601	1.101418796	0.7772661
Zm00001d009182_T010	2.901196502	0	3.561006398	3.834386603	2.764255697	3.809523311
Zm00001d009182_T006	5.378098801	5.11673366	5.593054085	5.190177047	5.273859403	4.950171539
Zm00001d009182_T008	1.14597933	0.353465577	1.276249495	2.253498303	1.563249425	2.27699713
Zm00001d009182_T002	3.81530623	3.363791905	3.341323151	4.213856296	4.644892423	4.34380966
Zm00001d013156_T001	6.702310637	6.213763309	6.687698433	6.951622377	6.980330355	6.819090287
Zm00001d021470_T001	1.029259925	1.006071771	0.96036343	1.018938917	1.111739128	1.397408699
Zm00001d020046_T011	0.35121898	0.156263279	0.438630328	0.677891557	0.601356124	0.522550963
Zm00001d020046_T001	3.571826896	3.047422696	2.975670169	2.553031128	0	0
Zm00001d020046_T007	0.261850911	0.23907537	0.345983327	0	0.792073212	1.02723475
Zm00001d020046_T010	0.47547716	0.359816876	0.64054782	0.47567637	0.558397936	0.947120017
Zm00001d020046_T009	3.815752066	3.227703037	4.056346123	4.150121359	4.202983404	4.129398593
Zm00001d011125_T001	5.334306864	5.072023971	5.463852469	5.748927618	5.792150097	5.705218297
Zm00001d017806_T002	3.892213325	3.158211626	3.938543816	4.509188213	4.579141883	4.457544126
Zm00001d053623_T001	3.33634038	2.460064429	3.360642674	3.876882596	3.891379036	3.781720919
Zm00001d009971_T001	3.280102615	2.841894845	3.484185447	3.363588543	3.650244015	3.426579966
Zm00001d002042_T001	1.449148777	1.512779582	1.59251588	0.588424658	0.806193704	0.678438844
Zm00001d020315_T001	5.057235065	4.559207774	5.172210103	4.785650407	4.611112581	4.599872089
Zm00001d040343_T003	1.857140604	1.280449797	2.035473299	1.580518896	1.510790304	1.633680434
Zm00001d040343_T001	3.661760543	2.767094136	3.491071778	3.45298531	3.52111998	3.55347503
Zm00001d040343_T002	0.578055683	0.451179982	1.14594217	1.388558776	1.196547875	0.326696105
Zm00001d040340_T001	2.106055462	1.276486541	2.018857397	1.776061444	2.199704692	2.046428895
Zm00001d021537_T001	2.053706716	2.724471724	2.315361897	2.148279132	2.123154641	2.401031312
Zm00001d021537_T002	4.046293641	3.648632597	4.300185532	3.87451371	3.992142372	4.218720112
Zm00001d012846_T004	2.074256403	2.201315806	1.893537731	1.071901304	0.837672837	1.37533531
Zm00001d012846_T001	4.225006945	3.174608714	4.184621168	3.894637064	3.962261566	4.030576566
Zm00001d012846_T002	0.46891691	0.308862929	0.358410891	0.423711544	0.650136804	0.298955035
Zm00001d053293_T001	6.458536821	6.772823567	6.404440441	6.12135885	6.121550446	6.072972165
Zm00001d015769_T001	1.374270531	2.114293389	1.47182892	1.919862143	1.965933117	2.152852194

Zm00001d005910_T001	1.516680853	1.525090654	1.341528679	2.012574037	1.753068774	2.36801622
Zm00001d009032_T001	5.748781188	6.184536632	6.166020197	5.377942618	5.507242854	5.447875276
Zm00001d048040_T001	0.31071936	0.182230816	0.329898568	0.433292792	0.372101827	0.453023383
Zm00001d019718_T001	4.793366867	4.409460388	4.641674687	5.1633427	4.96414043	4.990944017
Zm00001d006051_T001	3.388375822	2.893774184	3.478252996	2.970919163	3.240361655	2.984926699
Zm00001d011132_T001	3.758556149	4.195663586	4.086502535	3.849495829	3.737729912	3.453218737
Zm00001d036322_T002	1.310901383	0.863933694	1.687736177	2.466272112	2.187962162	2.245157919
Zm00001d036322_T001	2.618098139	1.568989853	2.402389566	0.934263607	1.057945816	0
Zm00001d005594_T001	4.666896538	3.874784378	4.512393653	4.154336604	4.195560916	4.440561914
Zm00001d049685_T005	3.816583017	2.905057979	3.687711323	3.544267136	3.670445852	3.539364311
Zm00001d049685_T002	1.587040888	1.377239271	1.811692145	0	1.881653266	2.257493146
Zm00001d049685_T004	0.743444307	0	0.417235213	0.676942554	0.83117031	0
Zm00001d049685_T003	0	0.308639298	0.356933695	0	0	0.746656736
Zm00001d037612_T002	0.948882631	0.347542072	0.94441995	0.7611261	1.509885318	0.720408061
Zm00001d037612_T001	1.754902463	1.198110641	1.771108891	1.614706547	1.233989266	1.792789149
Zm00001d002787_T001	1.67179019	1.368964419	1.585973956	2.592780404	2.604315944	2.709391665
Zm00001d024521_T001	1.505466695	1.300702444	1.54703863	1.83454457	2.046246225	2.028228594
Zm00001d044399_T001	1.315709971	0.664155125	0.879370928	1.200122003	1.629596292	1.65848754
Zm00001d049805_T001	2.714087005	2.686270574	2.668128885	2.955642004	2.95716632	3.03846217
Zm00001d040160_T001	3.843938834	3.287746358	4.021152241	3.073914982	3.179604998	3.28418718
Zm00001d044251_T001	0.668019249	0.479797592	0	0.897107188	1.181555489	1.102828087
Zm00001d044251_T003	0.77597593	0.6645238	1.025884233	0.891307189	1.296673538	1.211677612
Zm00001d044251_T004	0.710564233	0.391760023	0.858900843	0.953689882	0.971222378	0.674721868
Zm00001d044251_T005	2.762516571	3.008900068	2.62673045	2.533163873	2.609572487	1.826378455
Zm00001d000439_T001	0.549236421	2.68146096	1.442205709	0.967652622	1.982003124	0
Zm00001d000439_T005	5.251168946	4.5144821	5.347205068	4.691260842	4.784747483	4.883604395
Zm00001d000439_T002	2.64129631	1.659725033	2.328237035	2.896413302	2.44631526	2.725600042
Zm00001d006613_T004	0	2.14598259	0	0.893661204	0	3.373084525
Zm00001d006613_T005	5.592202562	4.203926707	5.627000598	4.830267039	4.653686215	4.548714717
Zm00001d006613_T001	4.364778947	4.14837367	4.389190671	4.802732653	4.87982646	4.725053886
Zm00001d006613_T003	0.22428976	0.077006405	0.344377442	0.632735387	0.782521795	0.649400246
Zm00001d013497_T001	3.745650394	3.06831551	3.728912579	3.709954806	3.842907952	3.831091346

Zm00001d010956_T001	5.018529271	4.386321782	5.063607474	6.099761564	6.133700046	6.262387546
Zm00001d003185_T001	2.595605581	1.840292417	2.275603973	2.433208384	2.26210868	2.523656544
Zm00001d049793_T001	1.561896991	2.300816052	2.044793472	2.37184152	2.395753263	1.836733696
Zm00001d035558_T001	1.103714489	0.714894337	1.362440151	0.335439807	0	0
Zm00001d028922_T001	4.262123643	4.171324378	4.003230182	3.549795324	4.070192919	4.097899728
Zm00001d050470_T002	1.349876472	0.970752809	1.22391428	1.370086702	1.452133087	1.446551092
Zm00001d031979_T001	5.090851737	5.679173653	5.364172414	5.257408927	5.313440508	5.266215795
Zm00001d003025_T001	0.882362112	0.513401806	1.204672237	0.919685601	0.766632474	0.742670258
Zm00001d043256_T001	3.997882464	3.269963116	4.094034264	4.14319519	4.188742488	4.064946037
Zm00001d002696_T001	3.409659762	2.732270285	3.410715706	3.346150456	3.571350013	3.560319871
Zm00001d020536_T001	3.845897661	2.997212185	3.672157032	3.035633234	3.056289757	3.034257277
Zm00001d042062_T001	4.828894346	5.259579074	4.835669762	5.454751737	5.431049545	5.496128833
Zm00001d035751_T006	3.90977704	4.153803796	3.467377961	3.532512589	2.486173229	3.559297483
Zm00001d035751_T002	2.157705063	2.004655991	2.195636738	2.299798825	2.772124881	1.977941854
Zm00001d040117_T001	3.256594048	2.292949563	3.196148733	3.685226583	3.56474593	3.577685983
Zm00001d040117_T004	3.21759181	2.297337587	3.22291211	2.828601119	2.335794789	2.346433029
Zm00001d040117_T002	2.281756402	2.634574683	1.949814667	0.471970908	1.062762077	1.992598777
Zm00001d040117_T003	0	2.74159534	2.23634505	2.060944897	2.479908282	1.730890327
Zm00001d031540_T002	2.695345345	2.302559748	2.269714225	2.726994219	2.910493024	2.995020792
Zm00001d031540_T001	4.108078198	3.670778712	4.333054783	4.403631418	4.326830258	4.137042689
Zm00001d047438_T001	2.063794558	1.397427867	1.943472793	3.107254828	2.97993442	3.001138919
Zm00001d025193_T005	1.803850692	4.101020524	2.072022427	1.772627225	1.283911108	1.244809748
Zm00001d025193_T001	4.616410338	3.520077343	4.656692626	4.615282863	4.703884863	4.635272
Zm00001d048582_T001	1.725071015	0.751949537	1.132124058	0.536501558	0.623797581	0.440888431
Zm00001d053399_T001	4.076915193	3.33804597	4.144241137	4.212497825	4.205461408	4.222499254
Zm00001d053399_T004	2.188326857	1.090418524	0.939552323	2.627962566	2.116435958	1.857199539
Zm00001d037032_T001	1.678044403	1.351371086	1.707229684	0.718269992	0.982985911	1.25392212
Zm00001d050484_T002	1.566514653	1.110358566	2.053216319	2.029447585	1.410684097	1.853249563
Zm00001d050484_T015	2.907379875	3.463117582	2.849657054	4.208871101	4.025258473	4.011124004
Zm00001d025519_T003	2.683740689	2.532590759	2.347278713	2.881292838	3.405113	3.67738432
Zm00001d010446_T001	1.621018451	1.212634702	1.930538279	1.447344855	1.165944088	1.41915538
Zm00001d022139_T001	5.75910642	4.908451949	5.875951054	7.933124842	7.927869438	7.87456131

Zm00001d025475_T001	1.479389415	1.304281477	1.578074527	1.261954385	1.175803053	1.528732332
Zm00001d051704_T003	1.475350045	0.526417421	1.429111368	1.995928385	1.830476823	2.353761115
Zm00001d051704_T002	1.614124639	1.219449209	1.707968221	1.965354321	2.315378998	1.505688227
Zm00001d048149_T002	5.212835832	4.747113283	5.223509588	5.102992062	5.150094909	5.213239173
Zm00001d012965_T001	3.418597192	3.20693964	3.427981278	3.281002919	3.137946101	2.82862752
Zm00001d032132_T001	5.652677847	2.180821619	4.004256877	4.365728926	5.656934232	5.385362214
Zm00001d013309_T001	7.85285859	6.647966971	7.283031431	7.33387218	7.42333204	7.189242433
Zm00001d045051_T001	3.940439004	3.825455031	3.605167759	6.078999423	6.24460869	6.329636218
Zm00001d008354_T002	0.756363212	0.516916802	0.608964383	0	0	0
Zm00001d008354_T006	3.220488612	2.411404822	3.176594689	3.239558587	3.085280516	3.280830444
Zm00001d008354_T001	0	0.234175098	0.82838055	1.437629379	1.753245101	1.182904555
Zm00001d008354_T005	1.285705227	0.918370204	1.419406751	1.289345223	1.433054515	0.826447203
Zm00001d008354_T003	0.79561569	0	0	0.964790445	0	1.195946602
Zm00001d014667_T001	7.413431261	7.51955634	7.574292636	5.302406831	5.395177237	5.384266264
Zm00001d012156_T045	1.201468883	2.268464337	0.841430547	1.366096122	1.342277671	0.822735042
Zm00001d012156_T051	2.577659399	0.104771416	2.43010807	0.743514106	0.215805312	0.278743673
Zm00001d012156_T030	0	0	0	0.940479504	1.038781011	1.083744387
Zm00001d012156_T019	3.131678868	3.607616161	2.914103897	2.580745598	2.26067397	2.226566175
Zm00001d012156_T018	0.58431218	0	0.499844357	0.692202206	0	0
Zm00001d012156_T028	0	0.093389801	0	0	0.731964345	0.199955596
Zm00001d012156_T053	4.16508193	3.501181666	4.207412888	3.501520673	3.357293563	3.785077806
Zm00001d012156_T013	2.224709897	0	2.252700851	2.579415267	3.135298615	3.288598204
Zm00001d012156_T020	1.605783982	1.032983122	1.414048373	2.114347367	1.873322244	2.210307812
Zm00001d012156_T035	0	0	0.119824935	0.631751545	1.090638704	0.608335204
Zm00001d012156_T032	0	0	1.170293002	2.822294725	1.950146245	1.610286657
Zm00001d012934_T001	3.808212864	3.001026293	3.75069375	5.075492884	5.068127505	4.874014237
Zm00001d006791_T001	3.375349908	4.571851039	3.438144582	0.749448454	0.79740733	0.693634593
Zm00001d028472_T002	2.990130202	2.320888114	2.836094399	3.796941153	3.548347717	3.534626415
Zm00001d028472_T006	1.200867147	0.366947142	1.380046167	1.028429119	1.939398863	2.037975345
Zm00001d028472_T007	0	0	0	2.531953309	2.951255616	2.473807429
Zm00001d028472_T004	3.075915074	1.581428864	2.909270617	3.223074809	3.177915879	3.139428237
Zm00001d028472_T001	2.12076498	2.239764627	2.107368834	1.630076913	1.101037507	1.168254997

Zm00001d003399_T001	2.887083231	2.039566225	3.050605477	5.397209987	5.35212749	5.398006212
Zm00001d029408_T001	4.781864096	4.170358226	4.810920256	5.179160804	5.436765235	5.287198809
Zm00001d034655_T001	4.067356052	3.206434118	4.148947848	4.978968903	4.893623591	4.9775416
Zm00001d014848_T001	7.097880455	6.186921784	7.131792833	7.061147441	7.012783084	6.993893138
Zm00001d017753_T001	3.432381278	3.263698944	3.522164374	3.802251234	3.865526225	3.798212096
Zm00001d017753_T003	4.341404355	3.688737966	4.381641925	4.580115569	4.383742234	4.562319366
Zm00001d041767_T004	4.164887543	0	4.179446319	4.497017986	4.387026162	4.33094075
Zm00001d041767_T001	4.568937823	4.851925801	4.739513253	3.82207982	3.827653004	4.12539917
Zm00001d041767_T005	0.166431376	0.052689938	0.093249159	0.379111881	0.361985028	0.493010891
Zm00001d041767_T003	0.779745511	0.476457385	1.12065717	0.832460685	0.964519884	0.7162394
Zm00001d018365_T006	1.698204251	0	0	0	0.474437082	1.022346505
Zm00001d018365_T007	0.700312755	0.790053847	1.674568669	2.443596839	2.22180162	2.12145577
Zm00001d018365_T004	0	0.725230725	0	0.494402297	0.360897989	0
Zm00001d018365_T002	1.016023426	0.246371601	0.44992263	0.736222126	0.802724279	0.946444912
Zm00001d018365_T001	0.883659131	0.221966145	0.683368583	0.681097488	0.734266963	0.532128412
Zm00001d016697_T001	4.96088971	5.331954958	4.69535999	4.907808194	5.032774326	4.946848601
Zm00001d033530_T001	7.495546897	9.572455086	7.65888153	7.728618992	7.794539095	8.116612324
Zm00001d039790_T001	8.1611492	7.945352683	8.040931286	7.824382986	7.744270956	7.880812888
Zm00001d033674_T012	2.918548624	1.862488686	2.538366321	0	0	0
Zm00001d033674_T002	2.625708609	2.530625134	2.917270768	2.967778764	2.667232716	2.821734294
Zm00001d033674_T009	3.438448958	2.369659388	3.543729344	4.776886885	4.769241684	4.765550764
Zm00001d033674_T005	0.845469979	0.438602652	0.344677404	0.671693738	0.705437425	0.543207811
Zm00001d002811_T002	5.723759307	4.912247703	5.584212224	4.528632951	4.612653452	4.594999262
Zm00001d002811_T001	2.088012997	2.125751928	3.278190131	2.055552118	1.468693307	1.30714812
Zm00001d035184_T001	3.172644642	2.314592126	3.000220896	2.74660837	2.725294212	2.772591123
Zm00001d006194_T001	4.809551813	5.06637008	4.779448635	4.592466067	4.591318159	4.755462382
Zm00001d019472_T007	5.562763288	4.906976578	5.749169794	5.928162077	5.671207533	5.953055106
Zm00001d019472_T006	0.771644757	0.434656514	0.98698899	2.057487357	1.767250533	2.137569099
Zm00001d019472_T001	0.450707152	0.451177872	0.856706085	0	1.726917079	1.512506548
Zm00001d019472_T003	2.481193526	0	0	2.788483385	3.390673325	2.863475049
Zm00001d019472_T002	0	1.277295659	0	1.504526837	0.989974584	0
Zm00001d019472_T010	1.842850944	0.826347945	0	2.9230322	2.534986155	2.834317829

Zm00001d019472_T005	2.046564398	1.466580661	2.5212239	0	0	0
Zm00001d019472_T013	2.508675842	1.243028621	1.959753466	2.863452252	3.309684505	2.74274613
Zm00001d014467_T002	2.667200692	2.502047433	2.643358663	4.760255496	4.527232204	4.677144732
Zm00001d014467_T001	8.785601683	8.041804969	8.77154816	8.729163634	8.755994042	8.722724044
Zm00001d053751_T001	2.648774887	1.85511425	2.387692294	2.841045431	2.981836583	2.694265329
Zm00001d027711_T003	3.628538803	2.812544021	3.632314753	3.885467574	3.904099167	4.10890787
Zm00001d051320_T007	1.169578713	0.527375662	1.235347192	2.036077056	2.143552766	1.861501251
Zm00001d051320_T004	0.902296673	0.862813978	0.979532249	0.971460783	1.14469511	0.899584787
Zm00001d051320_T002	0.346007163	0	0.392844941	0.555549183	0.503345682	0.606097267
Zm00001d049714_T002	3.008285724	2.464199576	3.266289568	2.978838474	2.726577089	3.006553503
Zm00001d049714_T001	0.931177742	0	0.708726683	1.347017573	1.504776475	1.283710241
Zm00001d029648_T001	0.603174106	0.449585672	0.975233235	0.42187982	0.398331762	0.403075714
Zm00001d018807_T001	2.693840855	1.837098766	2.666920391	3.094605481	3.054225953	2.993340683
Zm00001d006159_T002	1.505715154	1.989003817	1.836702596	1.611704025	1.78601875	1.758537708
Zm00001d006159_T001	2.392145938	2.890836742	2.055656229	2.014300298	2.025338568	1.616102168
Zm00001d032385_T001	2.374441649	1.945146207	2.314445953	2.153059156	1.941233668	2.151368529
Zm00001d039816_T002	2.72526585	2.575579543	2.670069847	3.210042333	3.271637971	3.374026339
Zm00001d039816_T001	2.387197912	0.686928958	2.665161248	2.721256495	2.593667377	2.498470447
Zm00001d024540_T001	4.343569708	3.686247711	4.180639605	4.178661027	4.295535697	4.284217909
Zm00001d033468_T001	5.878101788	6.13989097	5.954415072	6.125568431	6.109323008	5.929579879
Zm00001d009221_T002	2.054466833	2.995945927	2.247459093	0	1.876460367	1.166438447
Zm00001d009221_T003	1.74204934	0.827039352	1.056052824	2.140976563	2.378392596	2.293491145
Zm00001d009221_T001	4.053235872	2.494915539	4.203019986	4.431070044	4.015459211	4.337715801
Zm00001d016493_T001	3.379438534	3.206190592	3.098236278	2.742169233	2.85492103	2.945890447
Zm00001d046005_T001	5.633657595	4.855955722	5.731239867	6.319626947	6.335815025	6.32256047
Zm00001d003422_T001	1.048991519	0.893611506	1.008930716	1.458959911	1.213253951	1.428982244
Zm00001d023530_T001	1.206827612	0	0.657109471	0.96954654	1.015435481	1.031139667
Zm00001d023530_T013	0.25246047	0.154496367	0.252151609	0.143058993	0.338545359	0.336053681
Zm00001d023530_T003	0	0	0.499792323	0.785879436	0	0.793281644
Zm00001d020881_T003	1.74360889	1.152378794	2.060475977	3.464048977	3.562434282	3.502478658
Zm00001d020881_T002	0	0	0	2.026598245	1.536335442	1.756524193
Zm00001d020881_T006	3.180101543	2.179885848	3.316429039	4.884826224	4.978151094	4.739786063

Zm00001d020881_T001	2.232062989	1.90748371	2.062304456	0	0	1.207383134
Zm00001d020881_T007	0.272324242	0.324144679	0.230404706	1.670449704	1.737151546	1.728161718
Zm00001d048627_T002	6.536492	5.673817832	6.520433407	6.664516717	6.708282479	6.609920193
Zm00001d048627_T001	5.467786324	4.98535087	5.510860547	5.975619709	5.922073225	5.922780994
Zm00001d020900_T003	0.922309758	0.642436341	0.912892018	0.939493649	1.181145179	0.839250155
Zm00001d015581_T001	3.257487715	2.82156451	3.310234532	3.398542461	3.575316204	3.524805274
Zm00001d025720_T005	1.086784445	0.54408274	1.041574526	1.280395751	1.422462306	1.359165232
Zm00001d025720_T002	2.891458267	2.270036099	2.90875662	2.996814622	2.95488194	2.79664468
Zm00001d012633_T001	5.280424927	4.543050509	5.347821786	5.866601806	5.853271374	5.881187321
Zm00001d006415_T005	2.198027638	0.902834581	2.080308032	1.809815709	2.364370441	1.826683522
Zm00001d006415_T008	0	0	0	0.811496514	0.740741088	0.845193754
Zm00001d006415_T001	0	0	0	2.679603504	1.739260106	2.873932664
Zm00001d006415_T004	0.501121144	0.281994918	0.406464833	0.754329125	0.802278432	0.408897688
Zm00001d006415_T018	4.329496209	3.716361237	4.513944264	4.196164317	4.266411616	4.002913644
Zm00001d006415_T002	1.359469444	1.484211876	1.323849062	0	0.389180206	0
Zm00001d006415_T006	0.257624991	0.424499696	0.791272308	0.65681485	1.045795214	0.736340772
Zm00001d006415_T016	0	0	0	0.341015657	0.750585925	0.795209208
Zm00001d025957_T001	4.070328098	4.005907088	4.282625875	2.853811058	2.888217014	3.226208862
Zm00001d027354_T002	1.239432871	0.725737667	1.006459611	1.087720127	0.920437372	0.94514246
Zm00001d027354_T001	4.351512575	3.490509161	4.255469613	4.040435359	4.212641161	4.031068806
Zm00001d039105_T004	0	0	0	1.639086265	1.797989943	1.194042913
Zm00001d039105_T003	0.266120874	0.103599691	1.332626596	1.571255952	1.400165185	1.536742244
Zm00001d039105_T001	2.324099159	1.969332502	2.029121726	2.786021051	3.150086499	3.323267592
Zm00001d039105_T005	3.266164361	2.436698885	3.133727579	3.196361224	2.91439403	2.642640582
Zm00001d028010_T001	3.714816217	2.829920577	3.786660445	3.844138051	4.01840878	3.954093177
Zm00001d053739_T001	3.253391044	2.84633926	3.490433684	3.950353535	3.690470399	3.655846954
Zm00001d016363_T001	4.279054622	5.581158242	4.184956885	3.84756704	3.720248692	3.561305605
Zm00001d016363_T002	4.805233993	4.426252676	4.975074258	4.271627588	4.351491869	4.489536706
Zm00001d045206_T003	4.145770942	2.621110612	4.190291679	5.08679578	5.052191586	5.019545362
Zm00001d045206_T001	6.151613166	7.386273008	5.943637236	6.576269024	6.585500315	6.704296597
Zm00001d045206_T005	6.118636767	6.205434527	6.144567824	7.03698636	7.056776554	7.062761451
Zm00001d045206_T002	2.657932173	0.94678475	2.733208979	3.657368245	3.642435417	3.672080412

Zm00001d046581_T022	0.397865377	0	0.86496145	0.49196902	0.352508828	0.465187753
Zm00001d046581_T009	3.888857881	2.718503669	3.852571905	3.342185918	3.293348705	3.604228517
Zm00001d046581_T010	0.787534436	0.179600221	0	0.758278443	0.462585311	0.204687885
Zm00001d046581_T001	0	0.419182893	1.196430788	1.31142348	1.102888541	1.837500478
Zm00001d046581_T005	0.214778845	0.157042416	0.40162719	0.649070926	0.450515021	0.682213702
Zm00001d046581_T012	0	0	0	1.744046967	2.510330286	2.125220278
Zm00001d046581_T003	2.055642001	1.643762469	1.595450668	2.803043071	2.772013789	2.400287076
Zm00001d031329_T004	1.249530899	0.63709363	1.451788734	0.737856328	0.681875601	0.483209557
Zm00001d031329_T005	5.183026081	4.439962763	5.244369354	4.427494279	4.485648818	4.542698593
Zm00001d012717_T004	0.273314167	0.312151659	0.191278379	1.162164266	1.00774233	0.888747534
Zm00001d023922_T005	1.934965186	0.322351608	1.278317867	3.077783566	2.588175169	2.965496363
Zm00001d023922_T006	1.050399303	1.075381337	1.431015804	1.110527617	1.165266239	1.125107509
Zm00001d023922_T004	0.648044768	0.416104699	0.676719647	1.635153046	1.594418128	0.259143505
Zm00001d023922_T001	5.079647807	4.158806699	4.940491767	5.002355935	5.24515811	5.234112237
Zm00001d014806_T001	1.05612984	0.893511327	0.558512553	1.855697941	1.70617688	1.441580683
Zm00001d045864_T005	3.377367279	0	2.739261403	1.95723041	2.730267361	2.80638098
Zm00001d045864_T002	2.586374208	2.45650076	3.107252317	3.697105017	3.497954893	3.656700919
Zm00001d045864_T006	4.423153367	5.461250038	4.392884016	3.927087673	3.785459949	3.420322049
Zm00001d034175_T001	6.559402636	6.452228801	6.588509406	5.049315187	5.017923511	5.062196203
Zm00001d043620_T001	6.455708584	6.907120411	6.29679352	6.76314884	6.916766755	6.904451655
Zm00001d016379_T001	5.701605915	6.300907254	5.456992682	7.934402486	7.889622921	8.017287662
Zm00001d050714_T001	0.731413533	0.470060964	0.619536028	1.184507553	1.100805456	1.091200177
Zm00001d018935_T003	1.325823878	1.115092521	1.50782305	1.996964625	1.895698532	2.066930273
Zm00001d018935_T001	0.315973072	0	0.082139842	0.738433222	0.837026082	0.708836137
Zm00001d025915_T006	0.847941602	1.106513821	0.786774492	1.500680209	1.174295831	0.99246536
Zm00001d025915_T002	2.753035176	2.008986633	2.598041897	1.453188836	1.85809362	1.567467567
Zm00001d025915_T004	1.193098644	0	1.371150111	0	0.39938003	0.22426753
Zm00001d025915_T007	1.644329778	1.737067156	2.086614287	2.075603823	2.436800141	2.005028037
Zm00001d025915_T005	0	0	0.22633219	2.357737221	2.034245302	2.147881708
Zm00001d049148_T001	2.533177332	1.759801386	2.086432219	1.887387623	1.845230694	1.965189171
Zm00001d044828_T001	3.23478885	2.426159535	3.003027023	3.674418731	3.595873238	3.539315917
Zm00001d045675_T001	0	3.274067947	2.773592356	2.937927191	0	2.32806868

Zm00001d045675_T002	2.617003848	3.674339518	1.935460502	0	2.534449872	0
Zm00001d045675_T003	5.923422168	4.150114614	5.963724447	6.333756531	6.300484115	6.245789241
Zm00001d045675_T004	2.76840688	1.719684189	1.696061494	3.110285727	2.97333869	3.151349531
Zm00001d037780_T001	0.583394883	0.099614346	0.415605088	0.720738128	0.697893891	0
Zm00001d006527_T001	1.333448923	0.971571143	1.37796274	1.257524223	1.24771732	1.20156737
Zm00001d001987_T003	1.965968938	1.445372871	1.614724448	1.585643773	2.170802855	1.408430902
Zm00001d053572_T012	3.757606182	4.188217886	4.109577532	4.05147434	3.638415524	3.834234081
Zm00001d053572_T007	0.508228842	0	0	0.415109632	0.457830651	1.486121215
Zm00001d053572_T004	3.990913575	0	4.066343944	3.87735251	4.213418443	3.807313289
Zm00001d053572_T009	1.386125021	0	1.657253101	1.996324023	1.781720185	1.08660104
Zm00001d053572_T010	1.827245084	1.919193994	1.46155318	1.60434987	1.898019851	1.7022577
Zm00001d053572_T008	3.253261535	1.249933135	2.890426458	2.339776762	2.66032056	2.991205632
Zm00001d035322_T001	4.087314066	3.932769774	3.990697602	2.897751572	3.24740091	2.833658032
Zm00001d028963_T001	7.454148373	7.785638194	7.4857994	7.466732969	7.385807461	7.400253094
Zm00001d009719_T002	3.168479338	1.897226095	3.035903097	3.277666278	3.361155364	3.180857733
Zm00001d009719_T003	2.177385571	1.547773572	2.167399037	2.311100535	2.604375966	2.500940963
Zm00001d009719_T001	3.095522772	3.052087703	3.184612124	3.080401161	2.424229858	3.379804753
Zm00001d052225_T001	5.185709505	4.901330038	5.283729092	5.070257547	5.125278148	5.052540577
Zm00001d007477_T001	1.757616422	1.213276973	1.859869391	2.454253028	2.546604843	2.553379919
Zm00001d025674_T001	2.650344554	1.895724897	2.623711443	2.034006129	1.853055494	1.960814896
Zm00001d034823_T002	1.00203925	0.687883954	0.889038137	1.140965115	1.259549096	1.200594747
Zm00001d030373_T006	7.661730556	5.257247338	7.467024443	7.565321984	7.284813979	7.280028004
Zm00001d030373_T004	0.363178928	0.319005121	0.294693084	0.665587422	0.461211069	0.370311612
Zm00001d030373_T007	6.010836109	0	4.860905158	0	0	3.602633132
Zm00001d030373_T003	7.598848231	6.99140011	7.650914715	7.958120248	7.962971162	7.868557612
Zm00001d030373_T005	6.995628713	8.76154841	7.113368736	4.524814612	6.101557405	6.06330404
Zm00001d040535_T012	0.413347113	0	0.410977096	0.532749828	0.37723816	0.481200761
Zm00001d040535_T009	0	0.163452359	0.080940036	0.583973435	0.519315444	0.491335942
Zm00001d040535_T011	0.236992156	0.169038594	0.287590496	0.420358433	0.758632362	0.448550011
Zm00001d040535_T002	2.117974247	1.837351515	1.666125758	2.271890264	1.521807576	1.393993822
Zm00001d040535_T004	1.908563921	0.86060054	2.233491449	1.847791709	1.661115203	1.81589836
Zm00001d026047_T002	5.297040021	4.502955566	5.426101989	5.786073264	5.801658467	5.737402169

Zm00001d026047_T001	4.739121497	6.571857864	5.188198021	4.065478737	4.134042317	4.604154727
Zm00001d023320_T004	2.361004708	1.598445723	2.201427478	2.772066802	2.657305472	2.902153866
Zm00001d023320_T001	0.418259045	0.29545738	0.672272352	1.710884661	1.817306444	1.70725045
Zm00001d023320_T005	2.169708902	1.493268679	2.420762102	2.944710984	2.687299027	2.440893479
Zm00001d015861_T002	2.919779403	2.831675412	3.268148701	2.862987702	2.806522247	3.028442734
Zm00001d015861_T004	2.650095894	0	1.488445083	1.126160781	2.173808314	1.968916125
Zm00001d015861_T003	0.9084617	0.691535952	1.120869466	0.676306229	0.492472617	0.708975591
Zm00001d015861_T001	1.81387311	2.118657073	2.496462605	2.499219319	2.494091704	1.814716934
Zm00001d012304_T001	4.29610943	3.638011493	4.185357549	2.516162436	2.517710326	2.632489955
Zm00001d009495_T001	5.022712465	4.935546684	5.13647577	4.527737215	4.626341708	4.770126658
Zm00001d018036_T002	3.824608608	3.24025662	3.930724661	4.128410871	4.096371333	4.307915826
Zm00001d018036_T003	2.551675228	1.794225366	2.56647325	2.278895589	2.229531321	2.213417587
Zm00001d018036_T001	0.705166669	0.350352405	0.557666917	0.825963054	0.949014162	0.953011884
Zm00001d021821_T002	0.87975281	1.66689154	1.416326873	1.600577102	0.818597004	1.273610773
Zm00001d021821_T001	3.791563323	2.94274119	3.815446318	3.975627068	3.979310358	4.027468766
Zm00001d048687_T002	3.345770201	2.529276242	3.387440161	3.687550539	3.597662149	3.668778242
Zm00001d047441_T001	7.370459521	7.315245823	7.469210002	7.795481416	7.803130916	7.890612069
Zm00001d008816_T001	5.338022572	4.56192692	5.448684332	6.524834228	6.555784749	6.506374774
Zm00001d007975_T001	3.430222639	2.7159621	3.391536095	3.15149826	3.040384506	3.080167132
Zm00001d004460_T006	1.701520669	0.404895495	0.684221792	1.192857589	0.672149223	0.962825875
Zm00001d004460_T009	0.581364746	1.311543222	1.589057304	1.307511296	1.119538785	1.074058036
Zm00001d019852_T004	0.554405157	0	0.308096382	0.592550324	0.82292263	0.587437964
Zm00001d019852_T005	1.474797872	1.23689116	1.420046846	1.842854161	1.903524616	1.675434323
Zm00001d013886_T001	4.617933519	5.016995213	4.640083577	5.36877226	5.17292753	5.18020703
Zm00001d013886_T002	4.839265779	4.139275311	4.751244429	6.2673105	6.226817805	6.271503498
Zm00001d005230_T001	4.432827622	5.567809054	3.918679228	4.367961653	3.91539393	3.80207066
Zm00001d015791_T001	4.588397613	4.133537634	4.598929392	3.559069589	3.606207208	3.461814948
Zm00001d039193_T001	0.461598754	0.382351845	0.643954982	0.468465499	0.225016974	0.182295661
Zm00001d015138_T002	1.956215786	1.320024791	2.344338806	4.043193035	4.015178269	4.021098926
Zm00001d015138_T004	2.111798876	1.099313385	1.812563941	2.685483167	2.724416715	2.637312079
Zm00001d015138_T001	5.91416067	5.060474118	5.900320364	6.515900367	6.487722138	6.38471348
Zm00001d015138_T005	1.394274862	0	1.216025174	4.396582657	4.075957248	4.019739004

Zm00001d002125_T013	0	0.478421017	1.136893528	0.725045704	0	0
Zm00001d002125_T001	1.444432252	0.924070993	1.279841505	0	0	0.587452366
Zm00001d002125_T016	0.339097468	0.291839273	0.582223591	1.774101781	1.815228239	1.557483614
Zm00001d002125_T006	0	0.233491755	0.248924603	2.673166075	2.559529257	2.585127199
Zm00001d002125_T018	4.448102161	3.867997647	4.66353152	3.967769728	4.314766266	3.828493885
Zm00001d002125_T024	0.722980057	0.133472779	0	0.765609425	0.975996224	0.908899736
Zm00001d019306_T001	3.905387775	3.00299355	4.128893519	3.839796066	4.153542142	3.985029474
Zm00001d019306_T002	0.913383862	1.408108031	0	0	0	1.342025015
Zm00001d044278_T005	0.363229401	0.161566546	0.511337477	0.568952411	0	0.667828559
Zm00001d044278_T003	0.569071055	0.455898727	0.836603638	0.582851737	0.683251789	0.722438919
Zm00001d044278_T001	3.016657125	2.627666366	3.160196401	3.012573322	2.537006505	2.610646444
Zm00001d003021_T002	1.538443802	2.385374433	2.169441457	2.251259781	0	2.592519946
Zm00001d003021_T004	3.225245619	0	2.909526971	3.012110407	2.490589383	2.472842813
Zm00001d021968_T001	2.813009906	2.470324187	3.041076306	3.06736526	3.046369695	3.123570687
Zm00001d034369_T001	4.468528058	4.186643964	4.029763301	2.662132565	3.548244499	2.908556043
Zm00001d011144_T001	1.037834816	2.920817492	0.918838055	1.874828829	2.04506687	2.159805799
Zm00001d011144_T002	5.818460673	5.378633588	5.856704118	7.257801837	7.300794339	7.207960506
Zm00001d022048_T005	4.376391107	3.942028364	4.50612719	4.499510123	4.566315055	4.40915316
Zm00001d022048_T001	2.491762556	0	1.858272291	2.259205285	2.240821397	1.692899389
Zm00001d010902_T002	0.672950274	0.871905139	1.423404446	0.689953928	1.617748902	0
Zm00001d010902_T004	2.484968441	1.884198561	2.457208418	2.31934856	2.686290074	2.904514926
Zm00001d010902_T003	4.190826587	3.340192504	4.268540466	4.87208994	4.869606477	4.512870771
Zm00001d010902_T001	0.60891614	0.40125145	0.911983734	1.454062685	1.391714388	1.557036041
Zm00001d010902_T007	3.907924926	4.040225359	4.318327182	4.704379439	4.560601796	5.004051615
Zm00001d012875_T001	3.392704425	2.455917396	3.416543743	3.288824225	3.257557712	3.120801468
Zm00001d043503_T001	1.961498499	1.424782379	1.869863512	0.455933436	0.850581022	0.712198682
Zm00001d024059_T002	3.367791092	2.573716496	3.70305927	3.652319787	3.512848844	3.78369237
Zm00001d011188_T001	6.218179965	5.574988021	6.244704238	6.054465096	6.017873194	6.002748442
Zm00001d002113_T015	0.334604886	0.509887344	0	0.17652831	0.139359471	0.51514927
Zm00001d002113_T026	1.653712179	1.30485386	2.157270448	1.483906044	1.420942611	1.927845646
Zm00001d002113_T011	0.583644237	0.255761754	0.259941321	1.217197208	0.739210412	0.446114336
Zm00001d002113_T025	0	0.602893908	0.583858898	0	0	0.745387081

Zm00001d002113_T019	0	0	0	0.274029022	0.647745527	0.696791528
Zm00001d002113_T024	1.303806461	0.417673778	1.108853605	1.5949918	1.5651409	1.326485001
Zm00001d002113_T001	0.539177675	0.112869577	0.387047055	1.419824706	1.505469744	1.280909411
Zm00001d009765_T009	2.150805629	1.505317802	2.324637815	1.254967668	0.76195412	1.027317567
Zm00001d009765_T008	1.3367564	0.939660638	0	0.673437928	0.781044909	0.8894751
Zm00001d041073_T002	0.641440598	0.247340597	0	0.548440571	0.640119275	0.718962583
Zm00001d041073_T001	3.987648664	3.135758775	4.013574544	4.365322229	4.315393781	4.356704929
Zm00001d010625_T002	2.726898774	1.537138493	2.896147049	3.367236156	3.559872811	3.584406595
Zm00001d010625_T003	1.778853229	1.892858053	1.959022282	1.535152676	1.567425705	0.867045616
Zm00001d036403_T001	0.565145776	0.238995913	0.22221599	0.661885473	0.683398229	0.906772097
Zm00001d036403_T002	6.130836375	5.701078136	6.247546402	6.295356758	6.223125079	6.208121826
Zm00001d044903_T009	0.59763033	0	0.657078365	0.821408177	0.275680545	0.570849541
Zm00001d044903_T005	0	0	0.693656893	2.102679629	1.884075057	2.094015425
Zm00001d044903_T001	3.042079755	2.261132402	3.002310248	2.952593936	2.939242566	2.923314458
Zm00001d032533_T001	6.797098707	9.966040384	6.163899021	6.832739624	7.075665824	6.850541231
Zm00001d007507_T006	0.146676074	0.150122903	0.580770678	0.42070552	0.290557697	0.42914137
Zm00001d007507_T003	0	1.587285773	2.116996795	0	0	1.179454997
Zm00001d007507_T005	3.080180779	2.081090407	2.732932101	2.844953018	1.946017008	2.319521404
Zm00001d007507_T007	1.027995496	0.54022074	0	0.635094988	0.453730379	0.920346662
Zm00001d007507_T001	1.240235551	0	0	1.409829177	2.031160845	1.183611643
Zm00001d015090_T001	5.05038498	4.244749403	5.150817243	5.496519968	5.617350473	5.453378505
Zm00001d015090_T005	6.518917919	7.303782227	6.608196144	6.380000888	6.442874892	6.447274542
Zm00001d015090_T002	4.738532826	5.821400803	4.712899309	4.91460657	4.738072582	5.211059205
Zm00001d015090_T003	2.1416208	1.171206187	2.143369254	0.478621893	0.816273655	0.644943457
Zm00001d052415_T001	0.989735586	2.06990457	0.916884745	2.334974942	2.738687433	2.497708925
Zm00001d005679_T001	0.31209472	0.2829059	0.251544595	0	0.444039395	0.350809514
Zm00001d046468_T005	0.650913416	0.348115684	0.630729762	1.786560817	1.93110889	1.909936658
Zm00001d046468_T003	0	0.438638844	1.193328934	2.958449615	3.009538157	2.417008587
Zm00001d046468_T004	0.732418563	0.27056841	0.624398529	1.310687954	1.191774239	1.369376583
Zm00001d046468_T002	2.04744067	1.092208115	1.322033696	0	0	1.770280612
Zm00001d038065_T004	3.574487265	2.302032677	3.502090472	3.313932928	2.823848593	3.328298656
Zm00001d038065_T001	2.091728265	1.199039697	2.058995597	2.355515594	2.227587373	1.999393219

Zm00001d038065_T003	3.691511274	2.920213445	3.94112782	4.003492142	4.230506618	3.718431773
Zm00001d038065_T002	3.020776686	3.23765289	3.394522511	2.737307761	3.174774716	3.453793318
Zm00001d016636_T001	2.841629004	3.624721483	2.966433236	2.004277375	2.628359738	2.85083882
Zm00001d054057_T001	6.866079476	6.297932754	6.9160221	9.117832024	9.188912379	9.147029715
Zm00001d016705_T002	3.00618975	3.938244007	2.834716069	1.60811185	1.723159663	1.221275137
Zm00001d016705_T001	6.36867521	6.806658254	6.463595305	3.544056273	3.94824097	3.793967131
Zm00001d038036_T001	3.401969804	4.154203886	2.361987834	2.999261692	3.063805083	2.866177447
Zm00001d035817_T001	4.724572569	3.762888476	4.809714477	5.607584827	5.604565623	5.497779521
Zm00001d044515_T001	2.805670314	2.055158163	2.442249509	2.751103109	2.68420765	2.516568158
Zm00001d012824_T001	5.440563907	5.79659521	5.483241712	5.45866226	5.431852583	5.74308753
Zm00001d045397_T001	3.601240938	2.784313007	3.530946434	3.352069635	3.424288184	3.384041377
Zm00001d031985_T001	3.031623158	3.63252462	2.442691948	2.098486934	1.198858082	1.500116228
Zm00001d026197_T001	2.364813328	1.143413665	1.957737825	1.033019078	1.003846143	0.926276541
Zm00001d038543_T004	1.279292991	1.347169556	0.926369916	1.399744229	1.907066786	2.020679272
Zm00001d038543_T001	8.139976148	8.177551129	8.199267269	8.000407566	8.108393866	8.019829518
Zm00001d051431_T006	0.220893301	0.106684663	0.449134511	0.176661064	0.348018209	0.288803854
Zm00001d051431_T005	2.167647594	1.553398844	2.606703027	3.885167756	3.946559261	4.040250964
Zm00001d051431_T010	0	0	1.770538998	2.665190361	2.798039936	3.089924185
Zm00001d051431_T008	2.233583173	0.692258457	1.338511701	2.352114987	2.351521762	1.737739969
Zm00001d051431_T002	2.897239845	2.858870018	2.300384997	0	0	0
Zm00001d018128_T001	4.52623793	3.811966846	4.634098579	4.598523736	4.666999671	4.602310541
Zm00001d009759_T001	3.201518127	2.862006364	3.21106236	3.803920314	3.687463534	3.88718298
Zm00001d037732_T002	6.828006118	6.29788352	6.852261888	6.562696473	6.479378193	6.493511803
Zm00001d037732_T004	2.541022624	5.506914867	2.04724661	1.572346919	1.397573536	1.66678431
Zm00001d037732_T003	0.717572682	0.685403774	0.828703877	0.412495396	0.745504115	0.728051108
Zm00001d043609_T001	6.306243808	5.712713139	6.31668962	6.45767085	6.43163862	6.441766656
Zm00001d043609_T004	2.996992093	2.120951059	2.960920144	3.328948883	3.436616675	3.355968676
Zm00001d014814_T002	5.617970358	4.928112463	5.66814849	5.011692466	4.939220371	5.027832721
Zm00001d014814_T004	1.274895562	1.50491729	1.063012757	0.458028112	0	0
Zm00001d016743_T001	3.879100433	3.151834469	3.963566139	2.982617079	2.680279342	2.392826536
Zm00001d042827_T003	3.064444701	2.497163713	3.1146054	3.487433225	3.614481468	3.61027189
Zm00001d042827_T001	1.307014602	1.261376157	0	1.63095756	2.102988284	1.112125609

Zm00001d016456_T007	0	0	1.26255463	1.021827922	0	1.796466103
Zm00001d016456_T001	0.868704147	0.384015535	0.988073854	2.003590724	2.245516351	2.066492227
Zm00001d016456_T003	1.735137841	1.150800755	0.962368374	0	0	0
Zm00001d005441_T001	1.266973592	0.798012347	0.900394251	0.831096516	0.922592918	0.968437326
Zm00001d004481_T001	0.898084494	0.668278911	0.942880036	1.181486794	1.304073494	1.249253576
Zm00001d003179_T001	4.330230315	3.774710957	4.087507479	4.246509559	4.222285864	4.110440672
Zm00001d011068_T001	3.331570083	2.819123698	3.326719974	3.347710722	3.446960199	3.692259796
Zm00001d021132_T002	1.049816146	0.491281548	0.935296799	1.973118232	1.760541179	1.838963462
Zm00001d021132_T005	3.066556806	1.346910382	3.022191838	3.480403309	3.402168101	3.333831295
Zm00001d021132_T001	3.641595043	4.392413118	3.643593826	3.153606263	0	0
Zm00001d021132_T004	3.689479654	2.945167749	3.820787473	4.080398518	4.3129583	4.33950122
Zm00001d048299_T002	1.859572852	1.821288568	2.068873358	2.180684793	2.336887194	2.267265211
Zm00001d007767_T001	7.132153524	6.57638604	7.216713852	7.170464739	7.217799014	7.108233819
Zm00001d003426_T002	2.344123165	1.628120794	2.258065694	1.702627847	1.967068243	2.104036682
Zm00001d003426_T003	1.196507588	0.733594697	1.103576864	1.698131336	1.633828275	1.11406446
Zm00001d039197_T001	1.874534169	1.579986731	1.937291656	0.502430411	0.623568181	0.483747171
Zm00001d015992_T006	2.661446575	1.386532863	3.283988425	3.502039665	3.227881673	3.110775479
Zm00001d015992_T003	5.123461679	5.75873122	4.893401579	5.299070246	5.09461059	5.179180235
Zm00001d015992_T001	0.324769136	0.330739658	0.315648535	0.992119435	0.727646915	0.552687908
Zm00001d015992_T002	3.319299853	2.670292878	2.994908592	4.296706352	4.481597577	4.423281064
Zm00001d027442_T001	3.60441677	3.201883959	3.576210951	2.999238056	3.286976487	3.295334973
Zm00001d000382_T001	0.729772013	0.486758644	0.627030579	1.338434114	1.190196371	1.068470642
Zm00001d020837_T001	4.067262077	4.44030357	4.440700767	6.040197736	6.23879324	6.176878239
Zm00001d028707_T003	1.794843361	1.355308383	2.281751061	2.445290757	1.996771968	2.25423786
Zm00001d028707_T002	1.621413787	1.390258259	1.615005184	1.292865364	1.595231041	1.72629719
Zm00001d028707_T001	1.475348488	1.659411306	1.395516382	1.648268929	1.17415263	1.889424089
Zm00001d009508_T001	3.095165413	1.763383103	2.826817731	3.01822039	2.983935387	2.86079876
Zm00001d038755_T001	7.10895719	7.880924804	7.022657854	6.799824154	6.846717902	6.784740993
Zm00001d021507_T002	3.096238573	2.784111743	3.048352685	3.383106081	3.263394434	3.358327895
Zm00001d004222_T001	1.955408038	1.652706754	1.939791131	2.513382855	2.466209455	2.246225344
Zm00001d054105_T001	1.322509095	0.516719172	0.653322385	0.791349	1.594902014	1.339327261
Zm00001d054105_T007	2.415096919	1.059935977	1.385946174	2.724067183	3.21917038	3.257054532

Zm00001d054105_T009	2.104247411	1.661955684	2.689536459	2.110557349	0	0
Zm00001d054105_T003	1.25312581	0.900541096	1.299004286	1.902518961	1.875877943	2.030092724
Zm00001d054105_T006	3.660122573	4.530050635	3.575867879	3.762287683	3.837953734	3.285181729
Zm00001d046602_T001	5.086442623	4.331801508	4.958659567	4.943862924	4.870451525	4.975157923
Zm00001d010214_T003	2.260552038	1.181668701	2.675965386	3.812663741	3.824891347	3.60061623
Zm00001d010214_T002	2.502960911	2.542223623	2.545286139	3.672962943	3.989694447	4.000793444
Zm00001d039824_T001	0.672390943	0.314473797	0.482115135	0.931537078	0.866418229	0.791944897
Zm00001d035588_T001	0.483131116	0.334228441	0.637623229	0.523241768	0.365788826	0.602811263
Zm00001d052068_T001	4.862134202	5.321915327	5.154570815	4.441943478	4.418853449	3.983818948
Zm00001d021697_T001	4.674032683	4.242220023	4.776377127	4.456378927	4.464833436	4.371478989
Zm00001d011619_T004	1.014620953	0	0.999499298	1.556665819	1.385818649	1.959109109
Zm00001d011619_T006	0.29236478	0.238816202	0.331908108	1.479868973	1.274114924	1.342254342
Zm00001d011619_T003	4.104414916	3.617630434	4.05652223	4.039770181	4.210782528	4.063008959
Zm00001d011619_T007	0.907314493	0.905233045	1.073793504	2.343910333	2.429581955	2.145338015
Zm00001d019895_T001	5.99200243	6.783402788	5.856849678	7.158444593	7.112271336	7.056669132
Zm00001d017925_T004	3.176367617	2.349673827	3.054310361	4.155668208	4.170351415	4.204254753
Zm00001d031882_T001	2.863650279	2.87151112	2.896265648	1.479503757	1.636973029	0.748501594
Zm00001d043565_T001	6.351947576	7.410971001	6.055394267	6.486155928	6.284170705	6.379977676
Zm00001d021224_T001	6.380936087	5.656819425	6.400561838	5.975387332	5.990608527	6.054688791
Zm00001d026321_T001	2.278685994	1.72729969	1.873992688	2.429188515	2.506240131	2.443966485
Zm00001d018374_T001	3.523961088	2.855378614	3.621232781	3.204491642	3.587286373	3.624482053
Zm00001d017454_T001	8.209455301	7.969878177	8.32821184	7.628302682	7.589914721	7.572202171
Zm00001d030916_T002	2.566017738	2.1309256	2.44206023	0	0	0.476366133
Zm00001d030916_T001	4.019295199	3.002377225	3.988467835	4.174583309	4.235792148	4.110517261
Zm00001d031995_T002	5.038995258	4.41416666	5.104006018	4.567259521	4.534557444	4.909135125
Zm00001d026460_T001	5.08822345	4.262452176	5.07778566	6.441323833	6.477117932	6.443904707
Zm00001d001902_T001	2.463442763	1.987268106	2.329658658	2.496048886	2.522310157	2.760710421
Zm00001d029597_T001	3.747497827	4.344852636	3.757225551	3.704354869	3.993892787	3.604096832
Zm00001d029597_T002	7.861719617	7.419193758	7.866241659	8.059769786	8.010347732	8.063626207
Zm00001d004545_T001	3.346204081	2.714739412	3.603126737	3.739513631	3.709701106	3.97204734
Zm00001d040276_T001	2.673515042	1.997043054	2.761124398	2.47833688	2.713568034	2.754365045
Zm00001d004414_T001	1.852161481	1.668600245	1.613690529	1.172075898	1.7000548	1.825304513

Zm00001d051674_T016	0.818745872	0	0.380022333	1.145164188	0	0.872824033
Zm00001d051674_T002	0.312114475	0	0.736631716	1.047067883	1.690842578	0.724517564
Zm00001d051674_T007	0	0.329175274	0	0.995225444	0	1.271318695
Zm00001d051674_T003	0.283853043	0.21514801	0.472971188	1.052221806	0.349387926	0
Zm00001d051674_T017	0.125488442	0.189290702	0.480820375	0.389522759	0.291641275	0.469696382
Zm00001d043171_T001	7.65607801	6.895818816	7.708869968	7.630352035	7.608059941	7.602716975
Zm00001d018904_T005	4.218329461	3.509583741	4.153484098	4.906223628	4.927551047	4.7609734
Zm00001d018904_T001	0	0	0	3.795958803	4.017953166	4.185540237
Zm00001d018904_T003	0	0	0	0.536884449	0.854547078	0.889496127
Zm00001d018904_T008	4.229177896	3.204677557	4.237576796	3.015505063	2.495724835	0
Zm00001d018904_T002	2.604323061	1.406842483	2.289607865	0	0	0
Zm00001d014790_T001	6.00750471	6.099528382	5.818975994	3.277364106	2.980467915	3.145270835
Zm00001d044573_T068	3.89151397	3.735779392	3.77947511	3.751819424	3.764593399	4.058978632
Zm00001d044573_T003	1.507811889	0	2.099175676	2.013464404	0.896409039	2.339689552
Zm00001d044573_T010	1.500634833	0.479166394	0	2.22964483	2.375534104	3.013969035
Zm00001d044573_T007	0.445815677	0	0.638771724	0	0.931654316	0
Zm00001d044573_T001	0.705149856	0.877096364	0.320171555	0	0	0.934211512
Zm00001d044573_T070	4.511838849	3.845616701	4.54955465	4.302779572	3.945033432	3.954102486
Zm00001d053046_T001	3.228450853	3.528556567	2.894013315	4.265104315	4.49144466	4.597156895
Zm00001d036396_T003	1.612234058	1.031001295	1.763789303	1.548382368	1.348743448	1.359245649
Zm00001d036396_T001	1.026456604	0.94112735	1.344039343	0.807344205	1.084254797	1.380060025
Zm00001d049351_T001	1.330245161	0.766436573	1.283769496	1.543919968	1.653565904	1.390837696
Zm00001d049351_T002	5.205025654	4.913268482	5.010749946	5.007901648	5.205795048	5.179830725
Zm00001d016765_T001	3.206373787	2.36966218	3.246870618	3.742706794	3.832572586	3.796868909
Zm00001d035561_T001	1.311361862	1.03207474	1.358430022	1.265304863	1.397257536	1.382622436
Zm00001d032356_T001	1.937973692	1.342463152	1.864420325	1.554791183	1.289182893	1.62535934
Zm00001d014774_T001	4.796349341	3.570965722	3.936077334	3.857624257	4.462154299	4.140807853
Zm00001d007632_T006	0.450315473	0.283678834	0.407011141	0.200505609	0.50320624	0
Zm00001d007632_T001	0	0.835591044	0.753248525	0.548933714	0	0.584103361
Zm00001d007632_T012	0	0	1.515636264	1.547998077	0	1.066872426
Zm00001d007632_T007	0	0	0	0.357161228	0.305559681	0.524893137
Zm00001d007632_T014	0	0.750577351	1.254017696	1.296367539	1.186019368	1.356990018

Zm00001d007632_T011	1.666144396	0.627423805	1.216325723	1.482632508	1.760581628	1.55511672
Zm00001d007632_T010	2.371221776	2.028074379	2.948477129	0	1.175405795	0
Zm00001d007632_T002	1.276400185	0.902261938	0.530724003	1.762538044	2.181968862	2.077095303
Zm00001d002537_T001	2.696573891	2.299079627	2.441061392	2.595269001	2.456399299	2.613398693
Zm00001d017878_T001	0.362822208	0.255127246	0.325899844	0.270204785	0.426330245	0.31522073
Zm00001d005808_T003	0.326009184	0.13309658	0.688901861	0.533777619	0.777258524	0.31442275
Zm00001d005808_T002	0.914359394	1.757705591	1.665325001	1.786631906	2.491351336	2.198673301
Zm00001d005808_T010	3.770183758	3.473827166	3.736993907	4.480981702	4.518572465	4.165228848
Zm00001d005808_T006	1.267909682	0.453404846	0.351429323	2.364778597	2.332634329	2.405374863
Zm00001d005808_T001	2.599861202	1.673489941	2.539918368	3.447198439	3.386899767	3.621283896
Zm00001d012918_T003	1.264234349	0.79586667	1.438981561	1.01705817	1.561428301	1.618839575
Zm00001d017139_T001	0	0.653921248	0	1.156689139	0.988441839	1.316997203
Zm00001d017139_T002	4.58265824	3.620147905	4.664603155	5.627900804	5.500507685	5.577026812
Zm00001d029934_T002	1.74728427	1.751289747	2.096672403	2.428117193	2.554400491	2.376458206
Zm00001d015208_T003	3.803902031	2.790441765	3.809511169	3.645333561	3.613633369	3.537622469
Zm00001d015208_T002	0.145831334	0.037309119	0.143894911	0.662010392	0.74738654	0.343338458
Zm00001d045393_T003	1.659299407	1.408105313	1.285835998	1.861447653	1.694964413	1.894151497
Zm00001d029137_T003	1.281850745	1.241049993	1.575000036	1.28258214	1.201658324	0.68359316
Zm00001d029137_T002	1.195806164	0	0.933011313	1.696290782	1.917715111	1.565108721
Zm00001d029137_T001	1.050957855	0.829726997	0.895102492	0.741643874	0.869854828	1.076981786
Zm00001d025964_T001	1.182614123	1.029283251	1.272087706	1.336417652	1.206295635	1.359264768
Zm00001d002184_T002	4.228204472	3.65502982	4.291787493	4.292681935	4.226935649	4.217177041
Zm00001d002184_T001	1.360041706	0.660016778	1.445775428	1.568924694	1.410398095	1.209508889
Zm00001d022315_T001	1.068494719	0.708240218	1.137626148	1.216808084	1.175180293	1.157848936
Zm00001d022110_T001	3.927803273	3.615344961	4.066923731	3.55126277	3.36073916	3.488574777
Zm00001d022110_T004	0.365735084	0.161367896	0.290715745	0.237933216	0.103198159	0.136123122
Zm00001d028232_T007	0	0.500147339	0.10144838	0.637271731	0.348073748	0.599626297
Zm00001d028232_T004	4.776946139	4.606277162	4.790983371	4.67811682	4.693006537	4.272656208
Zm00001d007089_T010	3.517984519	2.790449897	3.475755606	3.030741636	3.081545347	2.919644227
Zm00001d007089_T048	0	0	0.904071703	1.199035299	1.662883156	2.005913733
Zm00001d007089_T002	0.818776952	0.714537435	0.906797491	1.621484121	1.965892864	2.42673332
Zm00001d007089_T044	1.905891905	1.179512961	2.301980603	2.45299282	2.118655079	1.242676887

Zm00001d031445_T001	2.914481671	2.262067476	2.900146126	2.902586875	2.631585333	2.668565075
Zm00001d007435_T010	5.608324025	6.408435777	5.517874697	5.15406751	5.033277756	4.651111637
Zm00001d007435_T001	4.658701973	3.529097294	4.704647854	4.464862443	4.491256787	4.457405374
Zm00001d011487_T001	7.051581106	6.160902142	7.044342968	7.156490969	7.135725926	7.12727026
Zm00001d047194_T001	3.530963783	2.985514639	3.545321605	3.88832516	3.945251667	3.917396809
Zm00001d049174_T001	1.20324251	1.048689573	1.572840204	1.336255391	1.337484467	1.398011549
Zm00001d029251_T001	0.548326137	0.441107352	0.853091834	0.872981588	0.780969346	0.609887313
Zm00001d041959_T001	3.658167944	3.237292499	3.088618571	3.200801249	3.449977814	3.10631582
Zm00001d045497_T002	6.670339735	6.222138343	6.770936082	6.771151119	6.758593124	6.669928437
Zm00001d021632_T001	4.382763176	6.162839164	4.579875316	6.231104704	7.00757685	6.419895038
Zm00001d020781_T001	3.982480454	3.406944768	3.856403914	4.051255933	4.103406996	4.041684381
Zm00001d006931_T002	1.635477664	1.385932373	1.996516378	2.088638922	2.10748166	2.179433977
Zm00001d006931_T001	0.505012854	0.236362808	0.560759946	0.715148335	0.939680946	0.385246581
Zm00001d013090_T002	0.402594489	0.25219764	0.499368842	0.899813679	0.978721349	1.01925783
Zm00001d012530_T001	4.360603137	5.412981321	4.180961933	3.253973992	3.597143305	3.552563975
Zm00001d010341_T001	2.862383346	1.752004363	3.019018652	2.597400068	3.320698395	3.056144064
Zm00001d010341_T003	1.857435653	1.316933505	1.980355126	2.792968182	2.881988945	2.754705818
Zm00001d010341_T004	3.983931922	3.882799732	3.996597994	3.890128837	3.362794441	3.90993541
Zm00001d010341_T014	0.963852135	0.478165223	0.859342257	1.287142465	1.172626402	0.957067672
Zm00001d033422_T007	0.491777177	0	0	1.182883585	1.209949879	0.889092667
Zm00001d033422_T001	5.771700319	5.073566797	5.880715471	5.575848642	5.558666188	5.567416273
Zm00001d033422_T005	0.398362411	0.41900594	0.604746005	1.104967952	1.138199784	1.191439473
Zm00001d033422_T004	1.393746769	0.379716325	0.808759011	1.258832454	1.254155605	1.354110771
Zm00001d033422_T006	0.896138522	0.451584087	0.686701314	0.912783207	0.478835163	0.749362636
Zm00001d030551_T001	3.951677308	3.149355141	4.080161332	5.107395493	5.061400227	5.102455065
Zm00001d022322_T001	4.487715675	3.729780386	4.419866225	4.2534359	4.414168014	4.240279139
Zm00001d011766_T001	7.441422384	7.22386037	7.46897274	7.357257876	7.28195768	7.348623524
Zm00001d052040_T001	3.271717592	0	2.521330703	2.98977863	3.372348017	3.08741277
Zm00001d007080_T004	1.113080354	0.458587789	1.007262969	1.886294946	1.688288697	2.337901726
Zm00001d007080_T002	2.005646465	1.621829642	2.00842916	2.247069842	2.68181085	2.082740393
Zm00001d007080_T001	3.763750111	3.429725886	3.799554448	4.14908845	4.227976295	4.080377027
Zm00001d031159_T002	2.248234547	1.410172292	2.193305275	1.98006443	1.749311142	2.121090021

Zm00001d038585_T001	6.282114365	7.318142559	6.327101006	6.98115184	7.116476142	7.010427233
Zm00001d048132_T001	4.101362988	4.681254888	3.983722106	3.148040886	3.201075493	3.831660719
Zm00001d037577_T001	0.717703399	0.701211908	1.217648262	1.010272121	1.027573774	1.142604913
Zm00001d005950_T001	1.992601315	1.192962977	2.04659443	3.045832774	2.929207364	2.942491047
Zm00001d025716_T001	2.086488948	1.919992533	2.252676936	1.754293203	1.577881718	1.348047112
Zm00001d021915_T002	0.822931601	0.322529285	0.774789168	3.289708925	3.422647581	3.253363812
Zm00001d021915_T003	2.983009632	2.82677808	3.283148375	4.799550304	4.95446914	4.700541646
Zm00001d021915_T001	3.94417141	3.296246531	4.025902388	5.312510637	5.165405138	5.236988216
Zm00001d014358_T002	3.211631827	2.530293274	3.298245388	3.40599236	3.630962684	3.488470792
Zm00001d014358_T008	0.665229026	0.231181697	1.0257078	0.111243694	0.785270146	0.447425795
Zm00001d034383_T001	1.454202746	1.218571024	1.555935393	0	0	0
Zm00001d034383_T003	2.105658301	1.402253934	1.828223329	2.398529054	2.766088168	2.496090571
Zm00001d011742_T010	0.581928692	0	0	0.419264888	0.201885997	0.475059973
Zm00001d011742_T049	0.076483846	0.064452462	0.05070925	0.248101234	0.243728786	0.406718424
Zm00001d011742_T013	0.555144704	0.311056636	0.731463063	0	0	1.851207736
Zm00001d011742_T016	0.035500044	0.017501506	0.090587896	0.710927408	0.797510259	0.751401162
Zm00001d011742_T037	1.322956082	0	0	2.162764961	2.409053612	2.259363491
Zm00001d011742_T008	1.285012121	0.690175695	1.44827882	1.56238458	1.539004419	1.582162881
Zm00001d046533_T002	4.084859399	3.294257401	4.07003402	3.903709314	3.947565548	4.086032507
Zm00001d046533_T005	0.987982207	0.135478395	0.7180297	1.668884757	1.812768872	1.67454562
Zm00001d046533_T007	0.717287522	0.242960963	0	0	1.052694111	0
Zm00001d046533_T008	0	0	0.441821273	1.263197902	0	0.761867341
Zm00001d023446_T001	3.667277231	2.922470755	3.67520034	3.695966985	3.719770587	3.706872869
Zm00001d002418_T042	0.434112034	0	0	0.646504623	0.910990482	0
Zm00001d002418_T002	0.50860714	1.889666278	0.473010686	0	0	0
Zm00001d002418_T044	0	0	0.385120652	0.983806091	0.810221798	1.19489834
Zm00001d002418_T025	0	1.631276138	0	1.242447635	0.881808686	0.768634058
Zm00001d002418_T051	0.424897371	0.171734609	0.221338869	0.323243233	0.621336886	0.559207896
Zm00001d002418_T045	0.370141956	0.028190041	0.33471128	0	0.562324984	0.273745032
Zm00001d002418_T030	1.847421718	0	1.424763571	2.770885696	2.55805893	1.80852262
Zm00001d002418_T033	3.711566819	2.525885959	3.945219355	2.792026554	2.800230852	2.971770321
Zm00001d002418_T001	2.263142904	0	1.989365024	3.227891374	3.596197987	3.739468049

Zm00001d002418_T026	0.896550866	0.252475003	1.124727799	1.671878485	1.719696454	0
Zm00001d028565_T003	4.310555055	3.390452944	4.394009261	4.69586323	4.729830733	4.776584947
Zm00001d028565_T002	2.615515644	2.558881322	2.893109571	3.4657696	3.580356204	3.163621904
Zm00001d012957_T002	2.956783394	2.669688983	3.035871258	3.448873735	3.789962275	3.580408065
Zm00001d034338_T001	2.465418169	2.204361738	2.707949453	3.076266456	3.365569088	3.17388409
Zm00001d023220_T001	3.708142089	2.773140156	3.66434596	4.217387232	4.103409598	4.095347649
Zm00001d011225_T002	5.977609278	5.476847183	6.005430575	6.179996067	6.17352358	6.051552841
Zm00001d011225_T003	6.032404893	5.951173259	6.100271471	5.639292487	5.79173795	5.766658815
Zm00001d011225_T001	3.046609796	3.096779659	3.205297884	3.363556305	3.48922911	3.450191929
Zm00001d005661_T001	2.709196218	2.343182375	2.629274544	1.197457807	0.764884538	0.958563573
Zm00001d016855_T001	4.957559776	3.387267682	4.668278854	5.55139586	5.556312916	5.673432105
Zm00001d016855_T009	0.979154666	0.88211478	0.451122998	1.366968397	0.738462623	0.681691231
Zm00001d016855_T006	1.188119195	0.97884799	1.318474191	0.506231244	0.614663677	0.36883315
Zm00001d016855_T007	4.926471065	4.403064599	5.328505258	4.192029524	4.380647588	3.828247198
Zm00001d016855_T010	1.724233773	0.741794859	1.870839325	1.989815861	2.354685162	1.751195896
Zm00001d016855_T005	0.080650842	0.180666018	0.149535129	0.760387885	0.232466747	0.958025257
Zm00001d038975_T001	4.891093426	5.152837393	4.871372281	4.142518981	4.542233963	4.444094937
Zm00001d033670_T003	0	0.931594564	1.259158574	3.159528762	3.031938575	2.95022782
Zm00001d033670_T001	1.278789457	0.912780909	1.343898397	3.865152043	3.930950368	3.821150271
Zm00001d033670_T004	0.2076655	0.057674847	0.050664677	1.4637731	1.6018952	1.392736156
Zm00001d033989_T001	4.481136229	3.723942173	4.405971536	4.614493954	4.642355584	4.607462592
Zm00001d005849_T001	3.310783041	2.584183716	3.37619521	6.525317197	6.507977074	6.514087424
Zm00001d029810_T001	4.727593879	4.174373099	4.741504307	4.813068978	4.846931424	4.859269095
Zm00001d028218_T008	0.928531782	0.239582568	0	1.091225909	1.282827042	0
Zm00001d028218_T001	3.109327459	2.347989589	3.556962863	4.360310613	4.611531946	4.380698284
Zm00001d028218_T013	2.035867388	1.204442499	1.858131426	1.867664428	2.039679215	2.122005712
Zm00001d028218_T009	1.184239663	0	0.695184074	0.281051303	0.457040535	0.944296247
Zm00001d028218_T007	5.217453619	4.262201159	5.248777049	4.571442368	4.47059979	4.465132821
Zm00001d015656_T002	4.039494811	4.443558981	4.251050603	4.095311186	4.200396376	4.174660562
Zm00001d015656_T001	2.651003916	2.163576983	2.687441922	3.150983162	3.00320787	3.083408778
Zm00001d032598_T023	1.429874086	1.623300345	1.850345785	0.457825399	0	0
Zm00001d032598_T027	0.426644771	0.709347107	0.643396301	0.298853008	0	0.735015195

Zm00001d032598_T014	2.167987604	0.265519711	1.243770821	2.697407985	2.892986051	2.78896608
Zm00001d032598_T012	0.814372408	0	0	0.573039022	0.440489824	0.933940441
Zm00001d032598_T003	0	0	0.657944517	0.472830858	1.090530312	0
Zm00001d032598_T024	0.35281953	0.080174638	1.068697635	1.458701693	1.853965715	0.506581632
Zm00001d032598_T019	0.158956116	0	0	0.362644937	1.153460993	0.571346736
Zm00001d043798_T001	1.835162114	2.291113147	2.147989456	1.511110737	1.858955729	1.414474378
Zm00001d012983_T001	3.52940694	3.112345169	3.828666207	4.180222031	3.994266352	4.095779384
Zm00001d011573_T001	1.412919692	1.357735532	1.805444354	3.119211568	2.870777986	2.751327458
Zm00001d003224_T005	1.686048206	1.33798505	0	1.654705448	0	0.987307767
Zm00001d003224_T002	1.375372012	0.759927906	2.042103225	1.965054674	2.583270911	1.870275541
Zm00001d042367_T002	5.40817931	4.710847972	5.514520162	4.607403941	4.72956593	4.704265938
Zm00001d019222_T001	4.615976857	4.986866609	4.532127227	3.810651935	2.984697981	3.541099838
Zm00001d021371_T001	4.24495729	3.713965305	4.41208934	4.550990953	4.777356535	4.741445359
Zm00001d007294_T001	2.956044754	2.26586052	2.818372241	2.86624195	2.993454816	2.811857752
Zm00001d007294_T002	2.360827494	1.662172224	2.136152332	2.452721935	2.607748354	2.509116874
Zm00001d053878_T001	1.530216603	0.846052768	1.472788237	1.777301875	1.644750614	1.877460394
Zm00001d012380_T002	3.527384796	2.473328189	3.543452825	3.783211574	3.762415261	3.801049363
Zm00001d012380_T004	0.997788096	0	0.312141201	1.673255643	1.301180825	1.081717978
Zm00001d012380_T001	1.098635825	0.593688409	1.115106508	1.807711018	1.961092823	1.96716787
Zm00001d012380_T006	1.600688893	0.564557671	1.237110893	1.224771318	2.196085767	1.715199744
Zm00001d031948_T008	1.234038333	0.687098323	0.893957806	1.46456947	1.357390414	1.405595428
Zm00001d031948_T001	2.062652606	2.704665281	1.666924707	2.449003733	2.858811748	2.192801737
Zm00001d025916_T001	3.949132416	4.12493776	4.196253015	2.201841782	2.65363056	2.390957626
Zm00001d046910_T001	0.975288271	0.609215032	0.630614218	1.269362355	1.362843524	1.333646418
Zm00001d018246_T016	0.207997771	0.105769249	0.282279661	0.835955595	0.556798227	0.465428095
Zm00001d018246_T005	1.387915027	0.554635999	1.322596197	2.142696067	1.853520657	2.143757475
Zm00001d018246_T001	2.108042307	1.355714319	1.520301044	2.283430817	3.03513067	2.938089655
Zm00001d018246_T018	2.926408061	4.395551204	3.901142717	2.031605868	3.180034052	3.141400418
Zm00001d018246_T017	3.372139169	0	1.914666695	3.304605077	0	0
Zm00001d018246_T006	3.012104326	2.267313159	3.240659924	2.926076866	3.445168239	3.450971819
Zm00001d005182_T001	3.870201945	3.344884869	3.700184117	4.156994299	4.20818464	4.178741806
Zm00001d051499_T001	5.666943988	5.928481565	5.569152586	5.675801397	5.607749685	5.509995465

Zm00001d045107_T001	2.346551885	2.495655002	2.527563831	1.588610308	1.323662902	0.942790726
Zm00001d053215_T001	4.322978211	3.399259431	4.240583226	3.850909311	3.916670965	3.803139294
Zm00001d053215_T002	2.570321326	4.590316734	2.928792315	2.976668641	2.97220665	2.460465791
Zm00001d030617_T001	8.997621205	8.717157528	9.016655198	8.9670936	8.982686487	9.048625549
Zm00001d024128_T001	1.978680718	1.70279405	2.237088554	2.020900401	1.956711294	2.035877942
Zm00001d038855_T002	4.574793557	3.888018237	4.644329662	4.195397289	4.123778416	4.112174329
Zm00001d002501_T001	2.596948019	2.848350324	3.181972836	2.768079494	3.062391501	2.871619146
Zm00001d020909_T001	1.087590472	1.16360049	1.235171316	0.794249492	0.901331486	1.205638989
Zm00001d053965_T001	6.687510886	7.016247968	6.910859791	6.949260223	6.81889167	6.870122536
Zm00001d047873_T003	0.160378108	0.391877677	0.562811451	2.014431711	1.209341065	1.529620079
Zm00001d047873_T020	1.538484031	0.766123585	1.186565211	0.639382214	0	1.056557865
Zm00001d047873_T019	0	0	0.728727702	0.408912901	0.653241661	0
Zm00001d014603_T001	3.034834434	2.63012259	3.064916643	2.373681616	2.881284418	2.771130025
Zm00001d040851_T001	1.384436132	1.581816884	2.211810596	1.019669894	0.955675106	1.360046764
Zm00001d005586_T001	3.563686656	2.84452164	3.491376993	3.765974913	3.73907882	3.75461625
Zm00001d005279_T001	6.575414145	7.024905965	6.598769954	5.154004058	5.05378616	4.969138331
Zm00001d012430_T001	1.242780526	2.194460289	0.769553415	2.595467378	2.692634749	3.099281737
Zm00001d044119_T001	3.232944357	3.407374507	3.126064611	3.844266729	4.065433498	3.975952858
Zm00001d042464_T002	2.139937002	1.641239424	2.135043928	2.329449709	2.464128201	2.245086101
Zm00001d042382_T001	2.105082978	2.646481812	1.666805211	0.74327712	0.700408644	0.547065783
Zm00001d026454_T001	6.641094466	5.364449693	6.086657572	5.754742905	5.971986663	5.71234026
Zm00001d034604_T001	3.716563069	3.194056943	3.722526901	3.91017159	3.988468925	3.936199482
Zm00001d010304_T001	3.86892387	3.089319498	3.917532005	4.361157611	4.399024013	4.396377973
Zm00001d048311_T003	1.978667175	1.314684346	1.586563484	0.497645064	0.174532976	0.202268507
Zm00001d048311_T004	1.177396096	0.855864552	1.751576836	0	0.226464139	0
Zm00001d033860_T001	3.193040435	2.533347551	3.343796942	2.927875221	2.887499341	2.908034488
Zm00001d043775_T016	0	0	1.838407654	2.04562714	1.93347293	1.440981424
Zm00001d043775_T003	2.278854564	1.606350783	2.228473941	3.129302967	3.239632819	3.355524615
Zm00001d043775_T012	0	0	0	0.856221626	0.554950278	0.334000696
Zm00001d043775_T018	0	0.066516337	1.254881828	2.094349251	2.323586241	2.236017407
Zm00001d043775_T015	2.251769378	1.698312728	1.199466309	0	0	0
Zm00001d043775_T008	0	0	0.799008536	0.499402521	0.509921792	0.494698233

Zm00001d036356_T004	1.208126412	1.426206777	2.871447641	2.113934485	1.885301417	1.415206465
Zm00001d036356_T003	3.522679259	1.457294848	2.743054533	3.002345537	3.28043198	3.35995782
Zm00001d036356_T005	1.83245866	2.015681677	2.083147318	1.933052117	0.834852769	1.010890811
Zm00001d013138_T001	3.744766577	4.008090538	3.894476034	3.433319769	3.591471542	3.048126131
Zm00001d050811_T002	2.313151507	2.093940405	2.301055829	1.678454169	1.912725734	1.975014901
Zm00001d053076_T001	2.674676	1.903535798	2.715649827	3.368723098	3.250366036	3.112150303
Zm00001d031014_T001	5.523017989	4.581167494	5.563750923	5.496571928	5.484476783	5.451449673
Zm00001d031014_T002	0.864429042	1.869707194	1.063248205	0.675237834	1.622900373	1.956192735
Zm00001d007401_T002	0.509044139	0.336676433	0	0	0.404663379	0
Zm00001d032515_T001	1.145818946	2.100861286	1.861716417	3.327590223	2.713273501	2.905675912
Zm00001d012690_T005	1.330565857	0.778541662	1.242496415	1.661424455	1.976530077	1.42410191
Zm00001d012690_T001	1.870841297	2.413599227	2.294796876	2.635187183	1.606338464	1.422515591
Zm00001d012690_T006	1.822929563	0.68622171	1.665975735	1.264289005	1.816185986	2.176351818
Zm00001d031072_T003	0.81742188	0.407697543	0.779111766	1.341900947	1.183362645	1.0553227
Zm00001d031072_T004	0.09128008	0.143474401	0.305815303	0.719417408	0.526539614	0.731097199
Zm00001d031072_T001	3.921634935	3.387956234	4.107228795	4.106478813	4.108780857	4.000922579
Zm00001d018146_T004	2.772824187	2.33864804	2.865104818	3.546281454	3.17606549	3.356206363
Zm00001d018146_T002	4.10785146	4.666039951	3.897201114	4.219151789	4.499549028	3.961059845
Zm00001d018146_T007	2.746104625	2.280944143	2.429005552	3.06993223	2.954305951	2.75415849
Zm00001d018146_T003	3.551273108	2.734598427	3.501335701	4.086156356	4.055428824	4.008467347
Zm00001d018146_T001	0.858611265	0.59754929	0.877383826	1.33100634	1.230201783	1.483572288
Zm00001d022308_T005	0.595617277	0.170667317	0.348647149	0.395020012	0.492153659	0.473571855
Zm00001d022308_T003	5.251351897	5.801171774	5.123421575	5.289384438	5.171724042	5.031455763
Zm00001d048834_T002	4.645842106	3.780330259	4.616575485	3.895045279	4.250088191	4.150951734
Zm00001d026632_T001	7.541133633	7.991225896	7.523586858	5.142576773	5.20021348	5.183309045
Zm00001d016301_T001	0.60822921	0.627128662	0.449288788	1.199137094	1.058362218	1.133184062
Zm00001d031651_T001	3.071471287	5.037058985	3.500499463	2.780899866	2.337835521	2.21817855
Zm00001d031651_T002	5.567401426	4.913235685	5.405334525	4.707181801	4.757919051	4.823370043
Zm00001d017895_T007	1.103517782	2.425566993	2.255635479	0	0.314879787	0
Zm00001d017895_T006	3.202640104	1.819671453	3.019755459	2.771671165	2.928794588	2.162768183
Zm00001d017895_T001	2.314050576	0.713716048	2.400540115	3.211829282	3.316792058	3.629769259
Zm00001d030126_T003	2.715561315	1.737739104	2.46328162	0.596930373	2.120082488	1.542541855

Zm00001d030126_T002	0	2.177792169	0.605586307	2.606347467	2.150532057	2.387313457
Zm00001d030126_T004	0	3.32638145	2.014196731	1.589357462	0	1.88810847
Zm00001d030126_T015	3.884777929	0	3.106141583	3.865689133	3.8624991	2.22872143
Zm00001d030126_T008	3.4068063	0	3.704896304	3.750797708	3.952813546	3.817517968
Zm00001d030126_T005	0.980530612	2.636826439	0	1.295316897	1.365797223	0.784355727
Zm00001d030126_T007	3.852345182	0	3.782552827	2.917283755	3.695904198	3.665120534
Zm00001d044724_T001	3.529791718	2.411098348	3.573043871	4.077675828	3.970268545	3.826498659
Zm00001d044916_T003	1.470988149	0.881034159	1.33689062	1.491016732	1.100398436	1.844452342
Zm00001d044916_T006	2.666546647	1.989738492	2.954325868	3.319302744	3.280500873	3.110721534
Zm00001d044916_T007	4.897528306	6.312421165	5.046459541	5.040430624	4.791591869	5.041625468
Zm00001d044916_T005	0.215494715	0.124357253	0.246979579	0.75382101	1.140980161	0.800589745
Zm00001d042722_T008	1.794256563	0.573764234	0.995887513	0.955903451	0.891638491	1.936322844
Zm00001d042722_T010	0.760201355	0.35058324	0.717273481	1.153185979	1.035509897	0.830712889
Zm00001d042722_T006	2.945686914	2.683454376	2.585027901	2.958035468	2.818919463	2.387188811
Zm00001d042722_T007	1.413411568	0	2.12721923	1.036949876	0	0.671810566
Zm00001d042722_T003	0.894335071	1.090116316	0	0.969980412	1.26026478	0
Zm00001d050712_T001	3.825553842	3.292902312	4.067617981	3.093932633	2.87688348	2.972671703
Zm00001d025285_T001	1.97660156	0	2.432307108	1.581177197	0.622063537	1.209774622
Zm00001d011696_T001	5.990797925	5.04438848	5.973064559	6.325310421	6.34557647	6.336610743
Zm00001d011696_T003	4.250338595	4.261588657	4.114827414	4.906266014	4.958322933	5.238121638
Zm00001d029920_T001	3.14487878	2.424299473	3.336305242	4.51467221	4.655432091	4.590244205
Zm00001d013746_T001	1.209783354	0.54038641	1.16920219	2.388016183	2.533717825	2.541673423
Zm00001d044866_T001	4.039510165	3.898195	3.948509929	3.931817488	3.972699454	3.966714881
Zm00001d011348_T001	6.632271182	5.936142933	6.686961276	6.147744102	6.053912744	6.220429001
Zm00001d004190_T002	0.98094219	0	0.564047396	0.919086039	0.987433657	0
Zm00001d004190_T001	1.664516973	1.652589746	1.661861309	1.757623249	1.798269547	1.947112534
Zm00001d003895_T002	1.627872942	1.194491177	1.721136821	1.944530898	2.046271374	1.756847381
Zm00001d035046_T001	3.693553752	2.675763776	3.526532478	3.775950549	3.843831931	3.819276089
Zm00001d038955_T002	3.440291409	2.907920889	3.576369032	4.514450539	4.286302476	4.334195541
Zm00001d038955_T001	4.991931207	4.00486575	4.82426934	4.852895747	5.021477551	4.982243772
Zm00001d038027_T001	2.401838228	1.357720896	2.206973543	1.897233067	2.160583348	2.262927402
Zm00001d038027_T002	1.079513968	3.052636386	2.42444971	1.871573611	1.554331968	0

Zm00001d007331_T001	2.770343838	2.824841157	2.968374285	1.793596287	2.108370896	1.552018935
Zm00001d004917_T001	3.369936138	2.610469515	3.320378674	2.225937196	2.162942157	2.44340031
Zm00001d001859_T003	4.169643568	3.164548491	4.325373893	4.701626402	4.660902041	4.630175838
Zm00001d049420_T001	2.039358818	2.578884143	2.225423018	3.465320469	3.130148869	3.427951788
Zm00001d044508_T001	1.016924144	0.67172272	1.105248289	1.028361927	1.136321997	1.212550663
Zm00001d028912_T001	0.533541421	0.236435063	0.311061288	2.438887641	2.431993547	2.549204132
Zm00001d044564_T002	1.663237125	1.237874494	1.812219312	1.881358039	1.800149037	1.679348126
Zm00001d019757_T002	0	0.769383302	0.859327943	0	1.192047679	0.87761077
Zm00001d019757_T006	1.143186372	0.542275882	1.023195727	1.107130768	1.060405072	0.647665411
Zm00001d019757_T003	0.538303738	0.332737718	0.585380822	1.779843825	1.969941377	1.890970365
Zm00001d005899_T003	1.153975207	0.736919139	1.235305523	1.691256319	1.732549678	1.919580734
Zm00001d005899_T001	3.722768631	2.95483505	3.929331705	3.729835517	3.642579821	3.764606562
Zm00001d005899_T002	0.54443197	0.448228594	0.351189575	0.609621653	0.517824947	0.548582613
Zm00001d028211_T002	3.357724695	2.201115966	1.709235045	3.439104073	3.267852018	3.322249203
Zm00001d048014_T001	2.769644599	2.246986873	2.848209688	3.005346267	3.104837658	3.170394763
Zm00001d036451_T001	6.030600511	5.546143384	6.026007421	5.707231562	5.766252017	5.764246115
Zm00001d036451_T002	2.969467339	2.967556718	1.59525205	3.391496639	2.885482412	2.725235743
Zm00001d028054_T001	4.351939575	3.567871404	4.386941211	5.881575084	5.8392749	5.864811383
Zm00001d028054_T003	1.369043734	0.342866461	0.915716689	1.717339732	1.767800967	2.065975493
Zm00001d028054_T002	0.305509485	0.194460289	0.310627468	1.151899721	1.298097549	1.171909426
Zm00001d002945_T013	5.996221394	5.304910028	6.028651959	6.142648857	6.197301925	6.157114994
Zm00001d002945_T002	0.527274561	0.410203233	0.429645956	0.788567952	0.601809646	0.904747661
Zm00001d002945_T008	0.618311963	0.102709187	0.85014012	0.272162971	0.493318387	0
Zm00001d002945_T001	2.721158919	1.384669867	2.722021344	2.792122994	2.571178272	2.708801934
Zm00001d002945_T003	0.586130611	0	0.229250742	0.493328635	0.920939605	0.469153505
Zm00001d002945_T015	4.388533979	6.349645682	4.613248567	4.336621168	4.671350049	4.888451612
Zm00001d034552_T001	2.480614377	2.075229342	2.305012101	3.039826406	2.86788263	3.135299764
Zm00001d041833_T007	1.636715094	1.05322049	1.981690478	1.705507318	0	1.775042954
Zm00001d041833_T013	2.769819758	1.649209838	2.668612952	2.567291834	2.317849029	2.629114344
Zm00001d041833_T018	0.334410383	0.221075258	0	0.102546608	0.32090977	0
Zm00001d041833_T002	0.629258688	0.609323791	1.029489637	1.283492751	0.629394858	1.215016886
Zm00001d041833_T021	3.212938292	3.613131202	3.517932883	2.850238155	0	2.335887092

Zm00001d010828_T001	0.519695878	0.562322053	0.952813639	1.114099119	1.080702683	1.133300476
Zm00001d020492_T001	1.609326155	1.309559225	1.622086502	1.13959981	0.701358311	1.184042841
Zm00001d046723_T001	4.64923456	4.164155363	4.811193151	3.72229617	3.957483861	3.979791049
Zm00001d044255_T001	5.25740474	4.596278507	5.305025567	4.479497096	4.663178763	4.520759027
Zm00001d018380_T001	4.186276942	3.446204742	4.276730098	3.517140363	3.660517494	3.687525121
Zm00001d012513_T001	7.579236759	7.663911388	7.63384809	7.641211132	7.738617474	7.674256162
Zm00001d044394_T004	0.807396141	0.347716674	0.564698136	0.838949347	1.20981953	0.735325472
Zm00001d044394_T005	2.058219158	1.210587736	2.499036093	2.199438982	2.132783115	2.517406979
Zm00001d044394_T001	3.519852476	3.033882124	3.642751239	3.223605115	3.333674037	3.600364082
Zm00001d044394_T008	1.859014987	0.40867491	1.314190696	2.166953518	1.809513227	1.877360265
Zm00001d044631_T001	1.055639919	0.927294984	0.838475024	1.334051055	1.045175237	0.88864468
Zm00001d010736_T001	4.931325364	4.488021987	4.881449667	3.284182737	3.085380464	3.289950702
Zm00001d046890_T001	5.376607883	5.218684081	5.512262321	5.185429231	5.186926101	5.07422086
Zm00001d032687_T001	4.997561273	5.251750218	5.150973377	4.956087421	5.065391059	5.09390885
Zm00001d006825_T001	1.321022375	0.34070468	0.92949031	1.725794807	1.911616042	1.471716048
Zm00001d006825_T003	3.537641353	3.087561281	3.522083252	4.213765719	4.460698732	4.312076635
Zm00001d031268_T003	0.044633325	0.188015986	0	0.410897884	0	0.488330933
Zm00001d031268_T010	2.072056395	1.293853656	2.036729083	2.781747248	2.999805223	2.869002962
Zm00001d031268_T016	1.93978173	0	2.106111425	2.041198817	1.516342491	1.563563297
Zm00001d031268_T017	1.598813477	2.247212367	2.089407641	0	0	0
Zm00001d050908_T001	6.423981511	2.448709108	6.830884198	6.866441635	6.214918652	6.364785827
Zm00001d050095_T001	2.100192527	1.77169546	2.093811646	2.358901454	2.363429309	2.284259744
Zm00001d032852_T003	1.066611395	0.603060134	1.020822192	1.196056802	1.289347584	1.194661998
Zm00001d032852_T001	2.114043465	1.286852767	1.857202327	2.5308341	2.564553769	2.377834507
Zm00001d032852_T002	1.106277959	0.689895798	1.094418507	1.01715084	1.094980538	1.391235408
Zm00001d042965_T003	5.003908732	4.222372971	4.94095715	5.062521628	4.918036253	4.908417361
Zm00001d042965_T001	0.928853888	0.372245617	0.412887725	0.817616709	1.007376365	0.274483528
Zm00001d002617_T048	3.436411597	2.253464114	3.632489606	2.817516433	1.891362411	2.526455482
Zm00001d002617_T028	1.466745089	1.585257261	1.548787761	2.741476263	2.324471055	2.76484145
Zm00001d002617_T047	2.436061871	0	2.277240303	3.11288075	3.299106156	3.063622874
Zm00001d002617_T049	1.112780188	0.503193007	1.047846857	1.306830339	0.908490907	1.021445614
Zm00001d002617_T008	0.376448204	0.273716391	0.332777811	1.404031685	1.80484162	1.256783632

Zm00001d002617_T001	0.064369679	0	0.12069167	0.563377813	0.460314802	0.390440974
Zm00001d037703_T001	5.417082129	5.269935409	5.233042944	5.560021102	5.594396748	5.661669974
Zm00001d037771_T001	3.383006792	2.871568682	3.382026681	3.50493343	3.391922482	3.325760425
Zm00001d044100_T001	2.108780356	2.553718306	1.928187238	2.774968551	2.821259583	2.600766665
Zm00001d043517_T001	5.487036659	4.50710915	5.515725444	6.429358817	6.528085765	6.445383864
Zm00001d043517_T004	6.385166634	6.927942414	6.158278227	6.405327784	6.461533061	6.375841938
Zm00001d051421_T005	4.247525705	3.760935835	4.281182125	4.397445118	4.458277263	4.381825043
Zm00001d051421_T004	0.94601214	0.348455663	0.614523283	0.627370572	0.83637015	0.750309789
Zm00001d045133_T004	4.62567565	0	4.61449201	4.085603197	3.80475026	4.003546111
Zm00001d045133_T001	4.739111449	4.889967401	4.912498882	5.128305487	5.496838889	4.917727234
Zm00001d053175_T003	4.289494108	3.35440801	3.768004096	3.714012799	3.869202895	3.949399715
Zm00001d031941_T001	5.808450799	5.425752977	5.799852654	5.658370056	5.706455073	5.530330172
Zm00001d049332_T001	6.349870989	6.337683908	5.763129488	6.49540482	6.93001019	6.319005844
Zm00001d040456_T002	2.379110495	1.820090304	2.473496785	2.905408283	2.761667175	2.811483772
Zm00001d040456_T001	0.273850043	0	0.079976742	0.745900755	0.5886223	0.620177237
Zm00001d017590_T001	1.912185369	1.430712397	1.820838621	4.841422345	4.759304997	4.677472442
Zm00001d048086_T001	3.809269344	2.886606918	3.767933777	3.821828564	3.822751151	3.863105985
Zm00001d053354_T002	1.901547744	2.167843134	2.558340134	2.304677205	2.361155505	2.168679899
Zm00001d053354_T004	1.448571683	2.187043368	2.546565827	1.766689289	0	1.009935454
Zm00001d053354_T001	4.036702713	2.235802715	3.551607683	3.289963535	3.423429188	3.264246662
Zm00001d019679_T001	2.136195981	2.964432828	2.449739637	2.080057268	2.044435389	2.21418046
Zm00001d010135_T001	3.969228143	3.156189485	3.882126227	3.826698062	3.719817999	3.863302871
Zm00001d010135_T002	0.674953217	0	0	0.963465986	0	1.017348278
Zm00001d010135_T005	1.006860269	0.517597208	1.190445456	2.088070682	2.085858012	1.396144689
Zm00001d010135_T003	0.080702683	0.170264796	0	0.668489511	0.713945626	1.159536835
Zm00001d031963_T002	5.095361701	5.307524665	4.870372759	4.926110606	4.84125602	4.545909017
Zm00001d031963_T001	4.029339975	3.029285902	3.999998377	4.194572335	4.213485012	4.493509257
Zm00001d031963_T004	2.692547715	3.233214707	3.101106274	2.620763276	2.531470546	2.721654171
Zm00001d010143_T002	2.033129411	2.435018586	1.988104039	1.967041305	2.001230094	1.747279972
Zm00001d026627_T001	1.807940125	1.255477771	1.733685363	2.126124429	2.105917055	2.093051019
Zm00001d009556_T001	4.215814474	3.791612288	4.459687937	4.70149045	4.8039983	4.720353937
Zm00001d015148_T001	1.307269383	0.298194937	1.800652693	1.385252104	0	1.371871622

Zm00001d015148_T002	3.769960102	3.045528943	3.758486114	3.107960817	3.231733911	3.180233172
Zm00001d053857_T008	2.207940004	0	2.23056856	1.846232935	1.920222784	2.179860694
Zm00001d053857_T002	5.921222605	5.079382293	6.080133909	5.44499981	5.472561432	5.321072395
Zm00001d053857_T006	2.960373254	3.260477519	2.87778488	1.881709642	2.584379534	1.504681912
Zm00001d053857_T004	1.955697793	1.177461797	1.760126396	2.257238172	1.443122843	1.954582715
Zm00001d011816_T001	6.811441796	6.32127686	6.763968373	5.956119746	6.1365263	6.220286689
Zm00001d011816_T002	3.650093021	6.815490548	4.450727538	0	0	0
Zm00001d038930_T001	5.545969172	5.179707855	5.58383694	4.81111283	4.897803161	4.71055988
Zm00001d010619_T002	0.971396035	0.242306163	0.523313038	1.049252272	1.060364949	2.056368495
Zm00001d010619_T003	3.080922815	2.403656298	3.221767444	3.193796498	2.991877164	2.598261335
Zm00001d010619_T001	0	0.654967059	1.416735994	1.558183862	1.801977262	0.95957803
Zm00001d031209_T001	8.10406712	7.158044293	8.198731425	7.95343212	8.006063713	7.972609312
Zm00001d030690_T001	6.322156888	6.043214518	6.349231664	6.079553093	6.22589005	6.112995105
Zm00001d008578_T001	3.096042362	2.813958048	3.265200428	2.345046305	2.423752682	2.301181703
Zm00001d003448_T002	3.090143084	2.734669089	2.746786993	2.932003137	3.057771702	2.804712165
Zm00001d003448_T001	0	0.103523153	0.330988563	0.247220268	0.336118826	0.306448909
Zm00001d016031_T001	5.315039723	4.638882041	5.265533625	4.546550147	4.481540813	4.471130675
Zm00001d018238_T001	2.348385407	2.180061908	2.353730915	2.050918164	1.556835496	1.50387025
Zm00001d013667_T001	6.535144089	5.854841436	6.573292506	6.52766965	6.53540715	6.484017031
Zm00001d049750_T002	0.767960676	0.384522889	0.854211935	0.692622699	0.809400449	0.916237386
Zm00001d024322_T001	3.373039966	3.818363344	3.262579434	2.813224011	2.937675082	2.616304289
Zm00001d018718_T001	3.79679282	3.300183775	3.990111227	3.189408054	3.224511729	3.242706913
Zm00001d002198_T002	1.03495026	0	1.160285805	2.187461823	2.068552845	1.954898313
Zm00001d007548_T001	1.362018704	0.741278857	1.339330112	2.041876914	1.966761201	1.824347914
Zm00001d022269_T001	4.646805735	4.075195535	4.654345083	4.503880297	4.374551266	4.599951214
Zm00001d035140_T002	2.865232134	1.72357822	2.977263802	4.047751427	3.974390543	3.920592576
Zm00001d035140_T003	4.248469409	4.543024642	4.557255249	4.207258098	4.70666929	3.919569484
Zm00001d035140_T001	2.608862929	1.800388461	2.361057783	2.80098871	3.247763341	2.939265701
Zm00001d035140_T004	1.305719012	1.052500756	2.209737821	1.473763537	1.447842587	2.200606673
Zm00001d039729_T002	0.920493015	0	0	2.255624301	0	2.407596398
Zm00001d039729_T016	0.532872483	0.4866979	0.340008441	0.702964222	0.948123109	0.3860494
Zm00001d039729_T021	0.633577216	0	0.560132869	0	0.911443091	1.303562757

Zm00001d039729_T013	0.512030636	0.353910413	0.898258667	1.292703429	1.036756508	0.890810093
Zm00001d039729_T005	0.966714696	1.012665207	1.80489117	1.137975601	2.122585923	1.059375353
Zm00001d039729_T010	2.342798777	1.366107315	2.440591346	2.436158907	2.449965141	1.471677555
Zm00001d039729_T001	3.56703635	3.399048015	3.588744374	2.700059684	3.226917236	3.184836791
Zm00001d002572_T005	1.978729035	2.15829372	2.330802175	1.935806707	2.127034288	1.442098459
Zm00001d015124_T001	0.448044592	0.339315287	0.343242933	0.85876163	0.847223254	0.804853182
Zm00001d031179_T001	5.763638877	5.621466303	5.86051155	6.142865884	6.130827049	6.13713397
Zm00001d038525_T002	4.102693568	3.178438822	3.998525353	4.325908836	4.304838969	4.307147172
Zm00001d038525_T008	0.176008665	0.07593783	0.255382291	0.75380561	0.572402707	0.544840452
Zm00001d052148_T001	3.153104405	2.712165659	2.76389657	5.810536059	5.978995001	5.674030932
Zm00001d025300_T005	0.46902948	0.315024756	0.47841584	0.642701572	0.769740431	1.116666174
Zm00001d025300_T003	3.239628085	2.683414624	3.02902664	3.611327385	3.479188128	3.457520093
Zm00001d025300_T004	2.821999949	1.603334127	2.98207598	2.424412353	2.535553592	2.384177764
Zm00001d025300_T002	1.140876795	0.86933212	1.256403834	1.305782039	0.984781089	1.034323491
Zm00001d034844_T001	1.156052892	0.967291102	1.328228704	1.492229559	1.490415584	1.011297398
Zm00001d047216_T002	4.135514103	3.326953329	4.133370107	5.139537519	4.993839406	5.205452376
Zm00001d038018_T001	1.980713753	1.832683871	1.99522472	1.587787415	1.735387	1.762674103
Zm00001d013528_T001	5.026979987	4.131592118	5.166967493	4.688434627	4.688571702	4.687912557
Zm00001d017524_T001	0.980994817	0.499051405	1.070537025	0.785938845	0.994914926	1.173978724
Zm00001d048391_T001	2.436482491	2.924609776	2.481769601	1.03207474	1.22402854	1.552502988
Zm00001d023874_T001	6.461798876	5.793876197	6.348172087	6.166532104	6.174912264	6.126379249
Zm00001d017811_T001	3.08198958	2.540235869	2.963441755	3.02677386	2.693049762	2.947551709
Zm00001d052110_T019	0.606295345	0	0.438020258	1.691895463	2.019946316	1.044427694
Zm00001d052110_T007	1.8573178	1.070439478	1.995002515	2.355295132	1.719353005	2.117664792
Zm00001d052110_T029	0	0.095998662	0.280303094	0.447765373	0.805100083	0.29342699
Zm00001d052110_T020	3.538649031	3.743346173	3.322364011	3.312933186	3.154194973	2.994525237
Zm00001d019047_T001	0.990665203	0.897473562	1.321158067	0.783840484	0.62838727	0.753613949
Zm00001d051884_T005	3.887373292	2.334244176	4.225920543	3.232109511	3.928400266	3.478617363
Zm00001d051884_T003	4.037514448	3.532348382	3.457158765	3.925211701	3.521760097	3.73052584
Zm00001d051884_T001	0.815784441	0.935806707	1.466822337	1.147395277	1.209959234	2.120160143
Zm00001d051884_T008	0.721685884	0.640669973	0.947868101	1.18297636	0.585549077	1.21808243
Zm00001d051884_T007	0.146356748	0.131902879	0.244057728	0.356142683	0.721167014	0.496282353

Zm00001d033559_T001	6.72790336	6.964406638	6.623557559	6.688980041	6.777968112	6.789169277
Zm00001d013162_T001	1.702862742	0.909671738	1.526630752	0.903174047	1.198126361	0.749391815
Zm00001d042918_T001	5.389986	4.82037827	5.506093033	5.162262211	5.1683224	5.149977363
Zm00001d045181_T006	2.371783264	2.063750388	2.478956019	1.904036239	1.450149689	1.772930784
Zm00001d045181_T001	1.525575306	0.56087242	1.556544189	2.059334163	1.954981295	1.962861403
Zm00001d045181_T005	0.4232619	0.328764098	0	0.573233934	1.064391755	0.944279753
Zm00001d045181_T002	2.469774775	1.935327353	2.223772832	2.660350456	2.780519033	2.68584419
Zm00001d042284_T003	2.149746794	0.488254827	2.051028181	1.5597561	2.45482417	1.605426536
Zm00001d042284_T001	0.80721889	0.869278418	1.334115146	0.269792008	0.796240566	1.110094592
Zm00001d042284_T002	4.370265433	3.71870027	4.417269987	4.90014801	4.847834444	4.700469571
Zm00001d015952_T001	4.025260688	3.27199317	3.904461361	4.028086405	4.148255944	4.213514329
Zm00001d026669_T002	3.808489156	3.556574475	4.061475101	4.162829801	4.126519383	4.272026101
Zm00001d014377_T001	1.543523599	0	2.159311422	2.917917091	3.290677266	2.899802854
Zm00001d014377_T020	0.463945693	0.17655767	0.534493948	0.266102879	0.306811678	0.582387398
Zm00001d014377_T004	1.675493038	0	0.656082609	2.386074791	2.660186366	1.673220811
Zm00001d014377_T010	4.099004027	0	3.684138963	2.086035735	0	0
Zm00001d014377_T008	3.115146969	0	4.029008789	3.492368014	4.004744697	3.831627281
Zm00001d014377_T013	2.610625186	4.254301514	2.337155312	2.246438187	2.994546417	1.703301848
Zm00001d014377_T018	3.68551647	0	3.603948998	3.22056507	2.382733092	0
Zm00001d018669_T001	5.057872968	4.20911792	5.049605416	5.153732997	5.218127972	5.120604382
Zm00001d018669_T006	0.574142208	0.384265366	0.500250359	0.744710507	0.636522077	0.6097058
Zm00001d003607_T001	2.683309513	1.800931367	2.757421427	2.02180412	1.843173859	1.69822159
Zm00001d003607_T002	0	0.584772052	0	0	0.938564328	0.997425379
Zm00001d049222_T001	4.923258686	4.398671055	5.234882216	5.219746064	5.228947335	5.136175182
Zm00001d049222_T002	3.85951799	3.469853294	3.525179251	3.919522768	3.970320826	3.556265087
Zm00001d039615_T001	5.705600347	5.420204501	5.849563759	6.271180531	6.339792686	6.299839112
Zm00001d017721_T001	1.743070264	1.52607332	2.702894649	2.924186749	3.041489194	2.930033888
Zm00001d017721_T003	0.106667245	0.05215293	0.16782676	0.285311067	0.47208012	0.346770851
Zm00001d017721_T004	0	0.088693868	0.044352149	0.683112522	0.415887357	0.328674497
Zm00001d004143_T001	2.778379124	2.482967255	3.221773939	2.799861718	2.562098544	2.760977125
Zm00001d047077_T001	0.96530777	0.881460239	1.325228665	0.592418286	0.897778673	0.756617698
Zm00001d013265_T020	2.005230735	0	2.17058272	1.199844437	1.60973889	1.36111198

Zm00001d013265_T011	0	1.330767188	1.684408981	1.408994395	1.828926833	1.624839721
Zm00001d013265_T001	3.575013352	2.771531938	3.547235605	3.772450506	3.835053042	3.831686254
Zm00001d042500_T002	1.122515687	0	0.903617546	1.084993551	1.508514857	1.544979389
Zm00001d013596_T001	6.148573894	6.617115857	6.330651738	4.843800532	4.729354379	4.788186527
Zm00001d032566_T003	0.627860805	0	1.065528196	1.256571103	1.047289193	1.82420166
Zm00001d032566_T004	1.023005476	0.644516216	0.66931709	1.517098275	1.516725726	0.485891506
Zm00001d032566_T002	4.832360372	4.045576994	4.860744987	4.944322816	5.075562537	4.989968682
Zm00001d050216_T001	0.715060451	1.226977021	0.81717626	0.838734792	1.109133187	0.949392991
Zm00001d050216_T006	1.62634568	1.1132911	1.775845754	1.708453895	1.898447524	1.702327304
Zm00001d050216_T002	0.624697974	0.892671571	1.000530093	1.454254871	1.776492726	0.751022318
Zm00001d050216_T005	0.730065154	0.217203413	0.622012917	0.788848553	0.822801108	0.427421668
Zm00001d050216_T003	3.441452671	2.683207539	3.37417272	3.933035777	3.598174136	3.861178243
Zm00001d017846_T008	3.808499761	2.222050874	3.815545304	4.301245295	4.277677287	4.174382927
Zm00001d017846_T001	0.219699489	0.167020017	0.421331573	0.58533563	0.827931185	0.52303697
Zm00001d017846_T014	3.138819874	3.736018154	3.498867137	3.339240594	3.366050507	3.620940941
Zm00001d017846_T002	0.420820837	0.104185001	0.172255795	0.750567918	0.996641829	1.216682698
Zm00001d031487_T012	4.112632166	3.035818133	4.002068873	4.281122986	4.172829027	4.316289208
Zm00001d031487_T005	3.47208168	3.49338718	3.547966254	2.558667565	1.957147185	2.169023516
Zm00001d031487_T003	2.907329493	1.337632891	3.2539442	4.388644321	4.38740921	4.608966925
Zm00001d031487_T011	2.969466234	0.490664596	2.093434425	2.77583143	3.039428308	0.47549895
Zm00001d022084_T001	8.157171224	8.177190612	8.163777664	3.111966757	2.783684827	3.090728459
Zm00001d012520_T001	2.956323229	3.256561743	3.300034231	3.70372709	3.831214402	4.237821199
Zm00001d050434_T002	1.874478692	0.44336689	1.426459069	1.925382724	1.591341919	1.628416201
Zm00001d016156_T003	0.515199743	0.217733245	0.587012556	2.544071609	2.518907976	2.389138896
Zm00001d025271_T001	4.958568212	4.502651455	5.169094211	5.302323475	5.252277697	5.152378267
Zm00001d015088_T002	4.759503094	4.102288089	4.789512883	4.871170628	4.867006956	4.809293992
Zm00001d053776_T004	2.213318972	0	2.589985865	2.25573215	1.714323778	2.312112441
Zm00001d053776_T016	3.428795772	4.493825418	4.33420534	3.624098789	3.324493521	3.496666864
Zm00001d053776_T015	2.502929606	2.256769143	2.378055438	2.906324756	3.131407078	2.924878237
Zm00001d002341_T001	2.288279411	1.637481341	2.45812762	2.343773069	2.217920873	2.468517111
Zm00001d014258_T001	3.21071599	2.453130877	3.255091886	2.934057672	2.774845662	2.696802876
Zm00001d014258_T004	4.68741611	3.54204293	4.394759555	3.265786725	3.062404972	2.648286648

Zm00001d014258_T002	0.104070911	0.965420079	0.715840668	0.255075264	0.824604943	0.254429569
Zm00001d014258_T006	3.984775531	4.919827446	3.963821531	0	2.594775207	2.341744993
Zm00001d014258_T003	1.399298538	1.288031298	1.57439898	1.02109164	0.951911507	0.707462947
Zm00001d014258_T007	3.308974149	1.969473233	3.926423243	2.70238693	2.204787718	1.700391775
Zm00001d052783_T001	3.88255042	4.218088864	3.859713576	3.769504541	3.481923268	3.548897597
Zm00001d003668_T001	5.41130987	4.642173199	5.466054431	5.750544444	5.734443219	5.799933967
Zm00001d031236_T012	0.293439939	0.094134706	0.315645058	0.213936994	0.32287996	0.20868081
Zm00001d034773_T008	5.306121748	4.960316208	0	4.483681528	0	3.469077945
Zm00001d034773_T001	0	0.900428259	0.45966453	0.35522831	0.377962185	0
Zm00001d034773_T002	6.458110243	5.678797522	6.14081871	6.24121207	6.502355093	5.744813418
Zm00001d024926_T001	5.275573321	5.178885787	5.458040026	6.103861625	5.86850901	5.642464819
Zm00001d010743_T001	0.328229853	0.07253803	0.132991332	2.212672674	1.814908438	1.936593468
Zm00001d022274_T001	3.427009262	2.777364795	3.482618698	3.452549971	3.415337909	3.433342339
Zm00001d040985_T002	2.392319621	1.878093987	2.004820997	2.437774506	2.12960054	2.235155076
Zm00001d039456_T001	2.605313215	2.066712989	2.91104745	2.78520464	2.632992406	2.61855653
Zm00001d042761_T006	4.060796474	3.608172779	3.728419051	2.898052947	3.160369394	0
Zm00001d042761_T001	1.548930756	2.009073013	1.436456106	1.069748904	1.323311841	1.326146709
Zm00001d042761_T004	1.386680739	0.216197811	1.384008349	1.459487227	1.328642905	1.204550174
Zm00001d042761_T002	4.431124415	3.542653529	4.371780477	4.65758656	4.642874939	4.758385587
Zm00001d006505_T002	1.56281487	1.01797534	1.738879358	2.425088647	2.276035644	2.128638194
Zm00001d042796_T001	6.799628785	6.484813696	6.823236379	6.247050943	6.205525394	6.135823117
Zm00001d046735_T001	1.047572589	0.669967386	1.34214452	1.896200148	1.671151587	1.035834317
Zm00001d027296_T003	2.265429995	1.556630018	2.638297003	1.88251863	2.020885471	1.795028791
Zm00001d027296_T001	1.340822586	0.724220673	1.046812322	1.005198032	0.434595671	1.006802834
Zm00001d027392_T049	1.098148673	1.274314745	0.395467575	0.329139674	0	0.756994222
Zm00001d027392_T069	0	0	1.342859636	2.670020658	1.697090112	0
Zm00001d027392_T071	4.887303001	3.80151287	4.826088328	4.480127568	4.734694665	4.487843506
Zm00001d027392_T060	1.221560983	0	1.835178691	1.954910593	1.934178669	2.644502834
Zm00001d027392_T015	0.940383279	0.342187198	0.983551476	1.308422622	0.324013301	1.600891044
Zm00001d027392_T065	0.302300332	0.248265216	0.378411741	0.694982682	0.602644058	0.305633221
Zm00001d045632_T002	3.896074567	3.04446634	3.910854576	3.999010333	3.984910025	3.990105235
Zm00001d045632_T005	3.577506777	3.371656433	3.399090137	3.071716855	3.036284056	2.763148721

Zm00001d045632_T004	0.581765797	0.65539946	0.499196353	0.240414475	0.27833809	0.276326331
Zm00001d047396_T002	0.877882428	0.633049851	0.953128135	0.970331687	1.14681877	1.093992109
Zm00001d047396_T001	6.067526282	6.569299167	6.227198577	6.353363169	6.450522988	6.348139981
Zm00001d035637_T001	9.522233915	4.550192992	9.445236809	9.762185094	9.958640514	9.66756585
Zm00001d027869_T006	1.105432021	0	1.270739426	0.77750346	1.003974915	0.934000851
Zm00001d027869_T003	4.175178456	5.164898522	4.74941402	3.73968147	4.26943866	3.919091952
Zm00001d027869_T007	0	0	1.107080538	1.21276542	1.340344638	0.71678199
Zm00001d027869_T005	0.453459633	0.324535284	0.640321994	0.869538226	0.101413417	0.811407731
Zm00001d027869_T001	4.405637222	3.499039538	4.062399791	4.712357568	4.445485903	4.454106521
Zm00001d027763_T001	4.153528928	3.525927917	4.101375511	5.641396897	5.566609662	5.708031948
Zm00001d039174_T001	0.427995487	0.279128931	0.859311243	1.698719889	1.339466373	1.321200216
Zm00001d003878_T001	5.109299081	4.535954271	5.171895619	4.752106674	5.011025667	4.952658509
Zm00001d003878_T002	4.302380618	3.610774699	4.231346462	4.634029903	4.439096757	4.423733592
Zm00001d012641_T001	0.696354442	0.412623294	0.866593102	3.153379111	3.138005593	3.205764087
Zm00001d052118_T001	1.159425102	1.372994431	0.968185884	3.536009992	3.329463917	3.737548109
Zm00001d026370_T001	5.708274353	5.22467147	5.795511301	5.778712879	5.764693415	5.735160998
Zm00001d026370_T003	1.094768451	0.401349764	0.856665454	1.744247655	1.154690782	0.5275268
Zm00001d022209_T001	5.272325026	4.549357559	5.40589181	5.31848417	5.276563178	5.175145794
Zm00001d002889_T007	0	0	0.336615884	0.848420836	1.279864677	1.319083761
Zm00001d002889_T005	0.825987469	0.543582987	1.065596437	1.41725574	1.886145082	1.47504648
Zm00001d002889_T019	4.740327697	8.133202509	5.330005883	0	0	0
Zm00001d002889_T006	5.8892247	3.930724566	5.794832758	6.062529248	6.173105501	5.921732799
Zm00001d002889_T001	0.240877252	0	0	1.169062333	1.180278373	1.185266513
Zm00001d002889_T003	1.388113473	0.826406524	1.018725319	1.228873473	1.502830595	1.347442858
Zm00001d002889_T017	0.677735538	0.24162178	1.204977037	1.19828292	0.473255964	0.873235217
Zm00001d002889_T004	5.853310928	4.852672171	5.738485295	6.192080178	6.227246894	6.282185643
Zm00001d030968_T003	0.586863681	0.356759079	1.452756194	1.045702968	1.391879876	1.169420291
Zm00001d030968_T006	5.890324331	5.104442971	6.079863728	6.072440039	5.959163651	6.071196432
Zm00001d030968_T010	6.329723643	5.516314721	6.138473185	4.560517182	4.655324005	4.54726004
Zm00001d030968_T001	0.487858809	0.312468851	0.648975243	1.108186534	0.797679577	0.823664589
Zm00001d030968_T004	0.507719555	0	0.28833021	0.420314233	0.784179811	0.715419861
Zm00001d032939_T001	4.940220322	4.081907543	4.983055843	4.257434687	4.09260333	4.192007266

Zm00001d027361_T001	5.977543546	5.462439549	6.143885771	4.449920518	4.618279892	4.548436378
Zm00001d015274_T005	3.639633422	3.71538735	3.684924524	3.345001582	3.620795879	3.341007257
Zm00001d015274_T001	2.85643519	2.221169322	2.828291582	3.200663793	3.060702502	3.370535789
Zm00001d015274_T002	1.019267084	0.571934021	0.797141673	0.796518017	0.372798351	0.938206738
Zm00001d042066_T042	4.712795554	4.24726638	4.828675548	4.645751281	4.584815158	4.686974718
Zm00001d042066_T028	4.469689285	3.961280856	4.772990465	4.313894483	4.454160887	4.457644718
Zm00001d042066_T044	0.937650979	0.542785992	0.763212686	1.086226006	0.715923234	0.683871588
Zm00001d042066_T023	0.551160622	0.168007829	0.662647585	1.455987602	0.907576001	0.306381244
Zm00001d042066_T030	0.434845433	0.032020417	0.51230983	0.939392093	1.037567072	0.932702227
Zm00001d042066_T002	1.022310282	0.977911457	0.251559138	0	0.795352196	0.509710028
Zm00001d042066_T005	0.764853973	0	1.68040718	3.098916721	3.128252977	2.885084858
Zm00001d042066_T009	2.781201481	1.760456017	2.280113308	3.22055145	2.725712368	3.079945519
Zm00001d022386_T001	4.620847714	3.928378851	4.623237301	4.343733565	4.511772385	4.482286968
Zm00001d006102_T001	4.020748245	3.462585703	4.278901831	4.941603983	4.93903071	4.932401531
Zm00001d006102_T003	4.14280796	3.250147403	4.176184162	3.707104533	4.026708538	4.094200086
Zm00001d006102_T004	0.271130413	0.093918439	0.201095573	0.650432793	0.61686862	0.656810274
Zm00001d006102_T005	0.650896877	0.623833158	1.319486722	1.528631326	2.105778634	1.520098344
Zm00001d025681_T001	4.894284037	4.067146062	4.752180285	5.004510516	5.039377331	5.121217406
Zm00001d008424_T001	3.400015404	2.691997718	3.105064031	3.573899565	3.742921353	3.596892934
Zm00001d017030_T001	2.562057992	1.902327163	2.484923084	2.660617662	2.7304678	2.561169943
Zm00001d003164_T001	4.747764567	4.591690127	4.699106218	4.554035979	4.45597452	4.564658327
Zm00001d029371_T001	5.094658976	4.536501558	5.29613818	7.079712792	7.11332685	7.117194781
Zm00001d014292_T001	1.184631958	1.04262823	1.535963848	1.818729922	1.674410934	1.701197711
Zm00001d042567_T004	0.730921621	0.490817575	0.881761709	1.525438998	1.652084435	0.72623135
Zm00001d042567_T001	2.840173961	2.190683438	3.202017692	3.06766969	3.136312645	2.573084116
Zm00001d042567_T006	4.519448053	4.227389214	4.656817709	4.175287539	4.537132714	4.548672492
Zm00001d042567_T003	2.299100147	2.196815062	2.34043893	2.457659688	1.991597125	2.044134236
Zm00001d009085_T001	1.322357954	0.953286102	1.459671348	1.610435497	1.55006287	1.566741617
Zm00001d004090_T002	0.410112036	0	0.444251477	0.184329812	0	0.652064237
Zm00001d004090_T005	0.402565021	0.209500779	0	0.588436172	0	0
Zm00001d004090_T007	3.544581085	3.459801819	3.688050857	3.282452112	3.406199766	3.197368475
Zm00001d010478_T001	5.747673789	5.121412419	5.703017092	6.205545725	6.185468025	6.1524439

Zm00001d018179_T005	1.465396749	1.000272644	1.803353938	1.573860191	2.233290494	1.616137465
Zm00001d018179_T019	3.138195196	3.401355498	2.801259248	2.171865243	2.720563692	3.275097362
Zm00001d018179_T003	1.253835613	0.771099592	0	1.594182575	0	0
Zm00001d018179_T002	0.252146764	0	0	0.950595321	0.610430772	0.749089699
Zm00001d018179_T001	0.578926157	0.187492861	0.322994141	0.439625265	0.68780962	0.892777247
Zm00001d016410_T006	5.86634639	5.130206046	6.060297758	5.63134931	5.666934021	5.576452601
Zm00001d016410_T001	7.335717537	7.55630926	7.14147018	5.645700247	5.529753869	5.420354222
Zm00001d016410_T002	5.082890151	3.820079681	4.922333737	4.889150214	4.836147079	4.996926764
Zm00001d016410_T008	4.615678245	6.328570188	5.388633817	0	0	0
Zm00001d016410_T003	0.921030278	0.502950731	1.041786169	0.961404778	0.877449007	0.918731219
Zm00001d035766_T001	2.775561348	2.327828753	2.865510884	3.775298803	3.913238893	3.774358852
Zm00001d043634_T001	3.328236605	2.362117764	3.23042761	4.211865017	4.144728225	4.140530416
Zm00001d005751_T002	5.804803012	5.2346513	5.744974509	5.738071609	5.66201176	5.644060201
Zm00001d021877_T008	0.54319395	0.190436606	0.443474045	0.751623964	0.89364878	0.751600829
Zm00001d021877_T001	0.991255345	0.422351426	0.892230135	1.038809801	1.149752321	1.080164402
Zm00001d021877_T002	1.351586495	1.170491709	1.09779483	1.945856745	1.619808788	1.831964773
Zm00001d013003_T001	3.760555017	3.152271698	3.880079623	5.827860959	5.88979911	5.91075211
Zm00001d021835_T004	0.440109184	0.031797457	0.439429524	1.001980182	1.211956647	1.09820528
Zm00001d021835_T006	0	0	0.214351141	1.804069671	1.091404665	1.346406646
Zm00001d021835_T005	2.969670859	0	2.616791475	5.092772572	5.121153487	5.368178721
Zm00001d021835_T003	3.551627368	2.053200329	3.502245428	5.848876357	5.835394105	5.645261988
Zm00001d021835_T007	1.054130944	0	1.650509078	2.524395217	1.699103386	1.01538481
Zm00001d021835_T001	0.228555211	2.595582668	0.660353651	1.960349342	2.011705659	0
Zm00001d006731_T003	2.116897683	2.421819782	1.242879282	1.22030333	1.090827015	1.730283449
Zm00001d006731_T006	1.234685248	1.06867425	1.283188092	1.209283664	1.379103562	1.487139526
Zm00001d006731_T005	2.472491671	0	2.956096623	2.802826416	3.194234908	2.896891813
Zm00001d006731_T004	0.761148232	0	1.364810527	1.929360397	1.462973674	1.657339546
Zm00001d035578_T001	3.826446287	2.807843913	3.76682093	3.91518758	4.146751089	3.980739066
Zm00001d017379_T001	5.257387541	5.475428974	5.163123921	4.233286045	4.593691695	4.469073646
Zm00001d016648_T001	1.664532446	0.805960192	1.116134493	1.869242878	1.808167549	2.252812551
Zm00001d048418_T001	4.074428025	3.605544943	4.117316701	2.793255421	2.652036005	2.755340117
Zm00001d044457_T001	1.721111441	1.374384612	1.836259856	1.656533896	1.498485255	1.33391886

Zm00001d020804_T001	6.014031811	5.27929704	6.083258712	5.154509775	5.10395375	5.219325369
Zm00001d039636_T001	3.254660855	2.431381465	3.244879602	3.079649812	3.015364826	2.904389103
Zm00001d008238_T001	4.279439425	3.830565048	4.335154216	4.065944741	4.13950715	4.192097877
Zm00001d013627_T001	4.46045878	3.583282346	4.45616172	5.053062188	5.128165072	5.068950983
Zm00001d031002_T004	0.149991585	0.065890731	0.214286478	0.289064823	0.334887441	0.178269735
Zm00001d031002_T003	5.011967783	4.164977381	4.919813434	4.63718964	4.657802214	4.576234417
Zm00001d031002_T005	2.526292212	3.216682233	2.830798261	2.326623056	2.627230007	2.271006373
Zm00001d005864_T001	4.450104742	4.979359432	4.489172966	4.505567811	4.514783602	4.334646
Zm00001d010287_T002	2.538009906	2.132206662	2.579589047	2.645018188	2.499543097	2.531170079
Zm00001d050401_T002	3.028986165	2.390103593	2.996909867	2.893312502	2.423471948	2.106490371
Zm00001d050401_T004	0.889790472	0	1.372561013	1.416294981	0.846307163	1.077146588
Zm00001d050401_T005	0.600164117	0.64762305	1.07772564	0.825143292	0.886502544	1.005123999
Zm00001d050401_T001	3.641723234	3.020153864	3.798593783	3.962906737	4.120975022	4.178268141
Zm00001d051244_T001	2.917752913	2.077831239	2.858910587	4.024115567	4.106479567	4.057026413
Zm00001d051244_T002	1.787657293	1.357957873	1.869767197	2.102855292	1.984399773	2.095140596
Zm00001d027844_T001	2.490982857	2.331172347	2.353612366	1.540481637	1.908122708	2.265460601
Zm00001d008844_T001	2.559912691	3.114977292	2.100140036	2.770135963	2.571171232	2.234673906
Zm00001d052751_T091	3.635798612	3.008349017	3.636201755	3.382805706	3.118439633	3.194027464
Zm00001d052751_T087	0.412820539	0.175470948	0.330870424	0.381270081	0.381051858	0.368933698
Zm00001d052751_T007	1.392957563	0.362557416	0	0	0	1.744504718
Zm00001d052751_T081	0.66489875	0	1.545786539	1.152489128	1.396609439	1.817504972
Zm00001d052751_T044	0	0	0	2.127746182	2.325051896	1.280598267
Zm00001d052751_T045	0	1.266950212	1.133594431	0	0.957812102	1.365327479
Zm00001d052751_T083	1.471786269	0	0	1.52288988	1.69991981	0
Zm00001d052751_T019	0	0	0	1.227980684	0.422104875	0.935783328
Zm00001d032293_T001	5.073401114	4.204987519	5.130973483	5.121812353	5.251154514	5.162433184
Zm00001d039065_T007	0	0	0	0.876823772	0.829323519	1.429170836
Zm00001d039065_T008	0.826986492	0	0.21613944	0.747759466	0	0.809404566
Zm00001d039065_T004	3.319229898	3.172993874	3.825946981	3.476261782	3.483865038	3.677260725
Zm00001d039065_T005	1.26192972	0.288167175	0	1.314895444	1.678305422	1.243296174
Zm00001d033706_T001	4.454682327	4.168903853	4.182699606	3.833902684	3.921093898	3.55285289
Zm00001d028203_T031	0.422839021	0	0.594802671	1.412889351	1.022987728	1.361249008

Zm00001d028203_T045	1.706581386	0	1.590752425	3.514951272	3.159151868	3.184507553
Zm00001d028203_T017	0.664534722	1.008988783	0.542662195	1.493640161	2.592794514	0
Zm00001d028203_T051	3.124924116	2.252911228	3.192499877	1.908754891	2.071530666	1.676882544
Zm00001d003019_T004	1.327927602	0	1.12124953	1.400425351	1.644563745	1.210792189
Zm00001d007806_T002	3.703920623	3.447695956	4.02303627	3.882009787	3.615765628	3.853695603
Zm00001d007806_T008	0.22013428	0.049013134	0.183436968	0.414273029	0.503281561	0.472333876
Zm00001d007806_T001	2.612803049	1.729695021	2.809252878	2.628381903	2.968006861	2.713308258
Zm00001d007806_T006	3.077185734	1.96303274	1.884164559	2.883939816	3.090646494	2.788254717
Zm00001d024094_T003	0	0.75362165	1.298440722	0.774455215	0.422495676	0.482765694
Zm00001d024094_T008	1.403630396	0.245539472	0	1.177209183	1.458168314	1.195416896
Zm00001d029539_T001	3.529164402	3.527652277	3.577842935	4.107235575	3.626783473	3.47244267
Zm00001d029539_T002	6.472359645	6.010738267	6.505885421	7.046634273	7.091655379	7.135778913
Zm00001d029539_T003	4.207796669	3.250463515	4.184777613	4.856785849	5.06266629	4.96709398
Zm00001d036715_T001	4.148910032	3.388938111	4.271116814	4.412630881	4.471856617	4.456228759
Zm00001d001789_T002	4.761978207	3.983445769	4.215747624	4.840585946	5.047358913	4.842084687
Zm00001d001789_T001	4.335696263	3.774829852	4.557933681	4.49843381	4.148071157	3.900738734
Zm00001d001789_T003	2.001832141	2.265350476	2.623837605	2.597015502	2.852394625	3.740478928
Zm00001d026544_T005	0.88424155	1.435478717	1.194922287	0.89368217	1.440081536	1.302337185
Zm00001d044497_T006	3.313774356	3.984824464	3.46924795	3.608807351	3.054650375	3.570064799
Zm00001d044497_T004	2.165584623	1.273241946	3.311741268	3.655379766	3.506140205	3.238114719
Zm00001d044497_T001	3.746909179	2.550751464	2.943757456	2.161605564	3.48685529	3.344435962
Zm00001d044497_T007	2.895691941	3.240642066	2.829234609	2.781812701	2.128696257	2.805072423
Zm00001d043881_T001	3.898811817	3.829460122	4.091667088	3.814762044	3.923677279	4.181401239
Zm00001d048614_T002	0.705214453	0.534688162	0.824182104	0.888373482	0.593762019	0.152304152
Zm00001d048614_T001	1.089279154	0.28519268	0.969452235	0.646849272	0.956438842	1.328708385
Zm00001d053901_T002	2.91294757	2.419292129	3.087132511	3.222811824	3.439630584	3.241637948
Zm00001d025437_T002	2.336367947	2.241783146	1.786222049	3.045937589	3.366544215	3.043419578
Zm00001d025437_T001	1.948599352	1.309604624	1.987441298	2.875059125	2.837828023	2.941656609
Zm00001d042503_T001	5.686219104	5.059343898	5.367937618	5.324622911	5.720116208	5.674151639
Zm00001d016225_T001	3.735421987	3.080371825	3.633575473	4.222958465	4.183546936	4.252521629
Zm00001d026703_T001	3.907934056	3.664565326	3.789022752	3.484632096	3.315728517	3.238040255
Zm00001d026703_T003	3.087875563	2.42617913	2.830212319	2.71685353	2.931009768	2.29100968

Zm00001d026703_T004	2.461511534	1.625128785	2.473552633	2.416819749	2.028951345	2.504726398
Zm00001d026703_T005	2.888206296	3.660165824	3.337532849	2.753721761	2.775369817	2.776055125
Zm00001d013439_T007	0.575842835	0.069040808	0.687192407	0	0.123210606	0.763026521
Zm00001d013439_T004	4.1059938	2.738189995	4.124966535	4.492148145	4.14042489	4.180914209
Zm00001d013439_T003	2.164969017	2.433418592	2.293356063	2.34682105	2.168259812	2.644301856
Zm00001d013439_T014	2.327514345	3.244547956	2.203825093	0	2.178827121	1.830411919
Zm00001d013439_T013	0.306587728	0.338594419	0.511031774	0.579166629	0.996834141	0.797522709
Zm00001d013439_T006	0.83674662	0.682978633	0.783478447	1.371100473	2.04175043	0
Zm00001d004837_T001	6.656987101	6.011763503	6.759708606	5.643344923	5.849290919	5.6416001
Zm00001d004837_T002	4.280723193	4.070481297	4.356028418	3.713537572	3.219489325	3.45920982
Zm00001d029226_T003	0	0	0.761621437	0.908404823	1.044899062	1.128510515
Zm00001d001808_T001	0.796178257	0.862925037	1.527858046	1.118486646	1.520480081	1.410654794
Zm00001d001808_T004	4.07907802	3.464941621	4.145279314	4.180527505	4.124059342	4.290075411
Zm00001d010183_T001	5.772449371	5.127310453	5.879770916	4.843096555	4.563709959	4.486225692
Zm00001d046207_T001	3.91265111	3.320455073	3.882533205	3.92013368	3.943495392	4.043707408
Zm00001d014030_T001	5.037316807	4.538830149	4.915743693	5.142955327	5.163274582	5.048599953
Zm00001d004310_T001	7.917558451	12.38389922	8.269255249	7.860050002	8.068276627	7.707039695
Zm00001d014748_T001	2.685111061	2.491121946	3.151038221	2.852588383	2.689685617	2.768181572
Zm00001d021702_T001	2.290524669	1.605769287	2.291273197	2.368827005	2.431777249	2.272211952
Zm00001d016973_T001	2.161868339	2.649079206	2.202992491	3.606427422	3.124544358	3.634281812
Zm00001d051060_T004	3.0467376	2.21267672	3.307975043	2.866888993	2.921549822	3.201138242
Zm00001d004768_T002	4.200917903	3.37615575	4.276057388	4.002224543	4.42391904	4.328146683
Zm00001d004768_T004	4.692117936	6.222805546	4.327161274	5.056372137	4.834243892	4.750256936
Zm00001d004768_T003	0.37895768	1.334364041	2.230508308	0.823013154	1.729353509	1.819688618
Zm00001d004768_T001	4.834413303	4.115678182	4.855280729	5.082678919	5.129424023	5.133932125
Zm00001d015893_T004	0.512826599	0.447387706	0.487400938	0.727393366	0.584714336	0.527489767
Zm00001d015893_T001	3.861774569	2.967136137	3.988090856	3.901513473	3.951575673	3.783000095
Zm00001d046889_T001	0	0.277189707	0.250356485	1.937628006	1.681407434	1.38105075
Zm00001d046889_T003	2.864724584	2.370602882	2.889271629	3.214681255	3.181717832	3.129751858
Zm00001d046889_T006	0.362501309	0.042095168	0.312682597	0.619422402	0	0.559227478
Zm00001d046889_T005	0.330414984	0.294675442	0.670466927	1.085700659	0	0
Zm00001d046889_T007	1.436053606	0	0.948661395	2.002465982	2.128938378	2.581111381

Zm00001d010104_T001	4.089401115	3.975336977	3.652842679	2.969985568	3.739729421	3.14576597
Zm00001d002302_T004	3.070878387	1.990945787	3.041481309	2.823804989	3.031747181	2.847206414
Zm00001d002302_T008	2.660731055	1.617954318	2.463451656	2.305128582	2.69442024	2.681984863
Zm00001d002302_T001	1.096815747	1.008605925	0.48350367	1.659888034	1.626846538	1.525399907
Zm00001d002302_T013	0.6061475	0.216160553	0.831276534	0.755418316	0.480037582	0.759479711
Zm00001d002302_T006	2.414884024	2.069221646	1.840806419	2.26150163	1.828514602	2.235982189
Zm00001d002302_T009	1.500115718	0.986860877	1.102465983	0.896917381	0.641205661	1.4114295
Zm00001d002302_T003	0.677046326	0.913270493	0.346293168	0.325343813	0.788942074	0.294660151
Zm00001d035540_T002	4.119608253	3.9185678	4.526048023	4.463249115	4.647825062	4.451233074
Zm00001d028397_T008	2.80148752	0	3.161548972	3.485378779	2.678943345	3.150295808
Zm00001d028397_T005	0.873279322	0.753174059	0.981672214	1.87662892	1.224393494	1.455974455
Zm00001d028397_T001	4.710845107	3.989625608	4.544793231	4.780773021	4.993329858	4.837449312
Zm00001d028397_T003	0.58771062	0.246150234	0.383436116	1.211250234	0.616408515	1.297193178
Zm00001d031816_T001	1.451301331	0.590218751	1.268227167	1.621650563	1.44258845	1.516692449
Zm00001d041947_T001	1.924952325	1.239395597	1.776903251	2.645089225	2.585026699	2.525611635
Zm00001d025798_T013	3.223529899	2.331708082	3.356281874	2.473975709	2.901072706	2.15216784
Zm00001d025798_T014	1.54368691	0.758823358	1.494005402	1.401658862	1.256127225	1.497048717
Zm00001d025798_T012	3.315003881	2.536971704	3.415895873	3.467557818	3.227633578	3.472241582
Zm00001d025798_T005	0.636181449	0.697079878	0.788414266	0.746176876	0.72752581	1.07228351
Zm00001d035020_T001	2.894188951	3.821198039	2.170703846	3.166242395	3.102354949	3.368882727
Zm00001d040314_T003	5.642894862	6.015532114	5.800186631	4.728787662	4.890073616	4.792579278
Zm00001d040314_T001	5.523436405	4.625902728	5.511868855	5.287300961	5.368734027	5.255843578
Zm00001d026302_T003	3.529194643	2.897417992	3.708168692	4.068772518	3.880132141	3.727604769
Zm00001d026302_T001	0.608936951	0.263951428	0.714896095	1.775948548	1.860314879	2.29759643
Zm00001d002163_T003	2.751908844	2.242383303	3.035293284	3.522145789	3.510716387	3.592297241
Zm00001d002163_T005	0.951848113	0.68390302	1.345179472	1.653376962	1.483321017	1.432861621
Zm00001d002163_T004	3.5580653	3.048589491	3.520257288	3.253295426	2.890473542	2.695119694
Zm00001d049703_T002	2.600614684	3.76319271	0	0.631353909	0.397341883	1.784207038
Zm00001d049703_T001	4.676678525	4.007488228	4.819853464	4.555978756	4.614734576	4.417436959
Zm00001d027679_T001	2.35771217	1.873213558	2.058300215	1.573580542	1.022617089	1.747557548
Zm00001d003262_T002	3.047257954	2.085713234	2.77985832	4.495798245	4.282387538	4.424402275
Zm00001d003262_T005	2.334765331	0.76609135	2.031155904	2.324246661	2.581660724	2.755792803

Zm00001d003262_T004	2.252554629	1.266689408	1.654916664	3.187735131	4.415673092	3.2996888
Zm00001d003262_T003	3.246026764	3.109522039	3.992412516	3.607427141	0	3.227517293
Zm00001d005692_T001	4.045461756	3.512511857	4.069448006	3.67951737	3.856850474	3.503820269
Zm00001d046937_T001	5.655288738	4.998720935	5.64151499	4.273200014	4.299779267	4.3020416
Zm00001d037305_T001	1.213291906	0.77823213	1.121892691	1.83212888	1.951763086	1.985257775
Zm00001d003106_T001	6.062653016	5.015655008	6.100571749	5.843120154	5.80279101	5.889187165
Zm00001d003106_T003	3.292647631	2.612308144	3.724132686	4.509104393	4.620555034	4.353238308
Zm00001d003106_T002	1.652287774	0	0.462027183	0.943392523	0.92157191	0.214004161
Zm00001d018297_T001	5.150897606	4.557939745	5.064729747	5.144585647	5.242217736	5.151866973
Zm00001d018297_T003	1.181673153	1.929709583	2.46295745	2.824318379	2.851957874	2.908930854
Zm00001d002536_T001	1.289097293	1.013179946	1.245801693	1.366283042	1.720865494	1.464324802
Zm00001d015468_T001	3.957858895	3.25168365	4.022647269	6.784956573	6.707507646	6.72833102
Zm00001d047766_T001	5.804448857	5.469757521	5.736718268	5.340326156	5.304077036	5.344591839
Zm00001d027976_T002	4.369600975	3.68015003	4.188601347	4.631530042	4.603919038	4.537237414
Zm00001d027976_T003	1.342483064	0.981550934	1.094623214	2.227687482	2.463084092	2.329820515
Zm00001d004624_T001	0.602795113	0.424568491	0.575302648	4.502414498	4.58883808	4.502705358
Zm00001d017871_T001	1.671917877	1.187362368	2.161509147	2.343939603	2.328134473	1.514336407
Zm00001d017871_T005	2.254857646	1.598802045	1.905761774	1.815010126	0	1.505741573
Zm00001d051866_T013	1.436445445	0.71888545	1.330493578	1.438261974	1.255106091	1.612343533
Zm00001d051866_T004	0.696798648	0	1.175165599	1.641116856	2.058725172	1.589394378
Zm00001d051866_T016	0.455169641	0.074657507	0.527612871	0	0.479763453	0
Zm00001d051866_T006	1.077392047	0.812646081	1.153089971	1.042969958	0.553669163	0.865754152
Zm00001d047382_T002	2.260979507	1.560642573	2.025843492	1.898872362	2.26678774	2.110774142
Zm00001d047382_T001	4.294996352	3.538966563	4.193098092	4.725432302	4.544282532	4.672903898
Zm00001d049991_T001	3.396261713	2.962898781	3.605102316	3.484225928	3.205766276	3.368191714
Zm00001d020620_T001	1.449418749	1.596087877	1.69321038	2.074680796	2.114825749	2.100246026
Zm00001d027334_T028	3.696561651	2.998787085	3.616672576	3.394456521	3.690289806	3.114576584
Zm00001d027334_T020	0	0	0.378352918	1.874333105	0	2.811935413
Zm00001d027334_T001	1.320684533	0.643658585	1.010953095	2.037089436	1.662066921	2.335967389
Zm00001d027334_T022	2.189844164	0.887189123	2.045021424	0	0	0
Zm00001d027334_T021	0	0	0	2.899603347	3.067255966	1.802727587
Zm00001d052781_T005	4.048950293	3.20839677	4.065558526	3.693489968	3.821109647	3.828871402

Zm00001d052781_T002	0.279373825	0.245803518	0.588682739	0.378587085	0	0.459694952
Zm00001d052781_T010	0.142997587	0.589766363	0.517050892	0.688313765	0.252223077	0.341589592
Zm00001d037156_T019	0.099159177	0.208756961	0.750753125	1.037036355	0	1.278097184
Zm00001d037156_T003	0.416752205	0.458422953	0	3.100156187	3.010467997	2.909603772
Zm00001d037156_T065	0.211486989	0.062504445	0.301922383	0.522990787	0.736902691	0.529784972
Zm00001d037156_T041	0.191968115	0	0.562273201	0	0.516168488	0.722079505
Zm00001d037156_T012	2.791124955	2.190738424	2.831662239	2.833813448	2.885778748	2.874253812
Zm00001d037156_T074	0.072882348	0	0	0.316051877	0.59184694	0.420798206
Zm00001d037156_T056	2.990907314	2.251569741	2.596721942	2.483319211	0	1.957873007
Zm00001d037156_T085	1.487906645	0.521808581	0.823571662	1.768489667	2.056440639	2.122333124
Zm00001d037156_T030	0.159414768	0.052400591	0.468075482	0.448761431	0.210664409	0.501761152
Zm00001d037156_T024	2.033523788	0	1.838220455	1.569336995	0	0
Zm00001d038082_T008	0.842518705	0.623658073	0.602358056	0.885729753	0.803416352	0.900391932
Zm00001d038082_T007	0.991840168	0	1.202699807	0.939331908	1.164127842	0
Zm00001d038082_T001	4.04044211	3.353711299	3.910135412	4.385795807	4.296636246	4.313119284
Zm00001d038082_T003	0.631362291	0.510740183	0.589354106	1.165943445	0.677585815	1.597695159
Zm00001d038082_T006	0.778610624	1.075444321	0	1.075870761	0.359672966	0.164001012
Zm00001d038082_T005	0	0	0.892734512	0	0.989234942	0.835568408
Zm00001d015212_T006	1.363829884	0.053272631	0.156488522	2.45522594	2.03746727	2.128949592
Zm00001d015212_T001	1.69506244	0.822493588	1.268645782	1.51490344	1.228045968	1.308463397
Zm00001d015212_T005	2.887543207	2.053486736	3.129074924	0.861806126	1.750204295	1.531234719
Zm00001d015212_T004	3.666850421	2.959022653	3.757446069	4.021813623	4.033922374	4.029497765
Zm00001d011298_T004	3.080751623	2.375985251	3.07100097	3.059893246	3.079188812	3.022336384
Zm00001d011298_T001	1.025826133	1.136686858	1.583163782	1.983214718	1.623337808	1.968320459
Zm00001d038647_T001	4.596352999	5.188202493	3.915329676	3.810651935	3.4255537	4.053657719
Zm00001d042084_T052	4.165224345	0	3.54899744	3.692109788	2.964099156	3.682836419
Zm00001d042084_T047	0.379426889	0.517630464	0.930342222	1.047694027	0.663779981	0.306240071
Zm00001d042084_T011	2.619595654	1.229680203	2.505936648	3.472306838	3.874472495	3.656576111
Zm00001d042084_T035	5.432569293	4.737521502	5.420594875	3.928345496	4.544172962	3.593830248
Zm00001d042084_T059	0.650191962	0	0.20512472	2.648377544	2.079885632	2.610642665
Zm00001d042084_T037	3.254337497	0	2.965288928	3.644006915	3.612478596	3.754836099
Zm00001d042084_T020	2.242903055	3.20410299	3.741645492	3.897342476	3.242862518	3.685139438

Zm00001d042084_T076	0.523124314	0.280458706	1.189052807	1.074628053	0.782569601	0.795088655
Zm00001d042084_T081	0.389794762	0.223177858	0.944184531	1.699995744	2.113861827	1.320801199
Zm00001d042084_T001	1.115789028	2.814028621	0.827051551	0.844013582	0.750846577	0.728523974
Zm00001d002603_T001	2.803681889	2.038262331	2.84687277	2.987183139	2.718504327	2.705073087
Zm00001d018508_T001	5.746930886	6.316079762	5.486781455	6.080932619	6.245528729	6.141168842
Zm00001d010336_T001	2.865405369	2.833539233	3.163239475	2.941121339	3.276372937	2.804338393
Zm00001d010336_T002	0.311542641	0.094771153	0.118212193	0.226409881	0.330860101	0.299082851
Zm00001d028167_T001	6.306940695	5.584525298	6.354309354	7.589563605	7.618697282	7.573537107
Zm00001d026584_T003	2.179100158	1.303560419	2.142521922	2.594960757	2.588493021	2.614874479
Zm00001d026584_T004	5.100850313	3.751521136	5.04555061	5.06771787	5.268676694	5.015918689
Zm00001d026584_T001	2.006516342	3.194205275	1.346396434	2.773779483	2.64047008	2.406422926
Zm00001d026584_T005	0	3.234239797	3.178948194	3.445694264	0	3.447652718
Zm00001d013426_T002	3.990887624	3.146285052	3.944025283	4.554945614	4.544681289	4.497250721
Zm00001d033623_T004	3.242384523	1.722004288	2.929737416	3.415843695	3.199448563	3.358539033
Zm00001d033623_T002	1.985292026	1.769693045	2.558753513	2.200173491	2.514472253	2.801235653
Zm00001d033623_T015	3.354230972	0	2.179424422	0	3.058815031	0
Zm00001d033623_T010	0.925032489	1.703611933	1.010657401	1.843632571	2.036372509	2.237316824
Zm00001d033623_T016	3.193115207	3.273687004	4.165243483	4.916544048	4.574558652	4.855073923
Zm00001d033623_T009	0.148172707	0.142896978	0	2.270922976	1.343809163	1.818234585
Zm00001d027852_T001	2.521026859	2.060180576	2.327304511	1.983375627	1.935626827	2.06616774
Zm00001d049510_T001	4.749039483	3.93111116	4.878875046	4.713259807	4.623579125	4.663552355
Zm00001d049510_T002	4.383764833	3.028817359	4.468141709	4.25769385	4.513072784	4.714901259
Zm00001d049510_T004	5.363274999	4.801332462	5.182155954	4.720549301	4.647721585	4.573131027
Zm00001d031454_T001	3.438895224	2.70671266	3.543170436	4.574172188	4.584817142	4.590513937
Zm00001d029099_T001	1.534967486	1.56020234	1.515986898	1.948393025	1.708618986	1.663578711
Zm00001d004848_T001	3.670462281	3.434355474	3.534583215	4.528554567	4.521004489	4.614026316
Zm00001d038393_T009	0	0.061396036	1.241270313	2.708706601	2.72380274	2.673490845
Zm00001d038393_T013	2.773666197	2.200020591	2.650589955	2.158272066	2.11628357	2.216247798
Zm00001d038393_T004	0	0	0.986187368	1.182912181	0	1.490573724
Zm00001d042443_T001	4.322602758	3.64570016	4.448170038	7.7384133	7.7713583	7.792296948
Zm00001d025704_T001	3.981263128	3.996502277	4.034039947	4.181399171	4.210234861	4.305607978
Zm00001d043402_T001	3.477348604	4.074252977	3.451854262	6.021862426	5.758755654	5.812739202

Zm00001d018901_T002	3.492050059	2.857505922	3.21463339	3.110961347	3.197779544	2.865423384
Zm00001d010574_T001	4.469009155	4.744915783	4.430629578	4.210048023	4.149498386	3.718312302
Zm00001d007395_T001	2.720232929	1.846397033	2.990370405	2.711745319	2.656741642	2.553309627
Zm00001d034457_T003	2.194299847	1.422582866	2.383191397	2.00506901	1.841513073	2.259253502
Zm00001d034457_T004	1.978354537	0	1.754420207	0.781347962	2.333062729	1.251880847
Zm00001d034457_T001	3.47303888	3.118418203	3.571649266	4.285180471	3.841967686	3.877755733
Zm00001d034457_T002	0.588207797	0.526400393	0.725132981	0.285177289	0.789826888	0.693329493
Zm00001d024804_T003	0.420080272	0.182872782	0.25246047	0.985775828	1.127736279	1.070398946
Zm00001d024804_T002	4.762834009	3.942175444	4.661090285	4.832809221	4.680632275	4.813125943
Zm00001d051329_T003	1.765061974	0.931680788	1.208032743	1.815777474	2.074353349	1.794349735
Zm00001d034565_T001	2.460249002	2.146941582	2.992232577	2.056207199	1.249815449	1.784635892
Zm00001d034565_T004	2.003093786	1.04577984	0	2.345272597	2.752198593	2.259739498
Zm00001d013658_T001	4.917725993	4.157151908	4.964924598	4.703567584	4.823947412	4.780126863
Zm00001d033795_T010	0.69108476	0.296450359	0.610277679	1.202141224	0.818432577	1.202008286
Zm00001d033795_T003	0.19237599	0.135460008	0.170696797	0.14879487	0.286624514	0.292676929
Zm00001d033795_T006	3.99803073	3.307565648	3.833824679	3.992377346	3.700372576	3.767514547
Zm00001d033795_T005	0	0	0	0.524506048	0.515232045	1.058683617
Zm00001d033795_T012	0.621910736	0.138329558	0.794716124	0.191615709	1.196056802	0.93827751
Zm00001d033795_T008	1.514214691	0.412705663	1.413405068	2.328530893	2.647570096	2.395732428
Zm00001d007269_T001	2.634987226	2.857530207	3.197378698	3.890072886	3.610561171	3.688479611
Zm00001d028987_T004	3.162771566	1.631692419	2.793472685	2.660072473	2.610234205	2.007004565
Zm00001d028987_T002	3.076988454	2.038251092	3.314792219	4.312368575	4.353892281	4.383067984
Zm00001d016558_T001	2.731347925	2.461410164	2.977553414	3.502094165	3.200581565	3.298624594
Zm00001d043986_T008	0	0.110742745	0.393143782	0.57468283	0.488175632	0.384874284
Zm00001d043986_T004	4.064491868	2.981502894	3.982837094	4.002805476	4.046666803	3.914281793
Zm00001d043986_T010	4.876016447	5.182946971	5.02613578	4.344882313	3.978466659	4.246898277
Zm00001d043986_T006	0	0	0.247539908	0.25236358	0.560117213	0.289450876
Zm00001d041216_T001	6.993774545	7.157288923	7.12763883	6.703646301	6.74005669	6.753094922
Zm00001d028601_T002	7.489397601	6.592120693	7.583383464	8.593577968	8.589171748	8.507077613
Zm00001d028601_T003	3.833056041	6.816992503	4.084567674	2.309803664	3.133996631	3.4449551
Zm00001d028601_T001	0.825622018	1.015159978	0.810250595	1.945203902	1.908111176	1.784486813
Zm00001d037644_T001	2.430420703	2.892814737	2.17304522	1.337626041	1.550386028	0.819630583

Zm00001d022234_T001	4.275882906	4.182826233	3.998335162	4.067266036	4.160345028	4.098935664
Zm00001d040721_T002	6.118816663	5.293045679	6.198844805	6.16669223	6.174371841	6.139025215
Zm00001d047536_T007	0.315162753	0.032518424	0.223665965	1.716530475	1.691562304	1.573574725
Zm00001d047536_T001	0.522295843	0	0	0.34820182	0.428424362	0.37745585
Zm00001d047536_T016	0	0	0	0.376203686	0.579512297	0.71515888
Zm00001d047536_T058	4.347384388	3.25687751	4.312706845	4.394068959	4.366660307	4.489384426
Zm00001d047536_T035	1.722066386	0.534186139	1.675348052	2.022569865	1.903958755	2.13432084
Zm00001d041595_T001	2.296910482	1.925064021	2.578286153	2.436237544	2.577387254	2.372387164
Zm00001d007889_T009	0.32413085	0.47117185	0.413057846	1.190339252	1.005131187	1.120081825
Zm00001d007889_T002	3.263605059	0	3.770256505	3.791207282	4.220261379	4.703936233
Zm00001d007889_T001	3.664543141	3.362340693	1.922206223	3.980261524	3.044639794	2.86314475
Zm00001d007889_T007	3.354176939	1.978301448	4.029325485	2.657053434	3.10458022	0
Zm00001d052074_T008	2.794431047	0	2.406269984	2.749353625	2.966485287	2.702936305
Zm00001d052074_T009	3.572675552	0	3.395400669	4.390356499	4.400643054	4.370303381
Zm00001d052074_T011	0.611906999	0.182015915	0.511499413	1.698598553	0.712668849	0.716913656
Zm00001d052074_T001	0.463924774	0	0.727775851	0.509728267	0.924634287	0.948979038
Zm00001d052074_T004	4.489166478	3.861517032	4.701289714	4.739493703	4.646748028	4.396398525
Zm00001d052074_T010	3.138719629	2.83953921	2.924357799	3.527561329	3.54808935	3.25283934
Zm00001d052074_T007	3.30480656	2.027767314	3.307126548	2.770448928	3.217012589	3.224787367
Zm00001d052074_T005	1.071411218	0.673604362	1.191875916	0	1.470939748	2.141971575
Zm00001d047439_T001	4.121893271	3.411074207	3.942220212	4.377752205	4.330212384	4.25854116
Zm00001d016980_T001	0.743984516	1.004677561	1.403233905	1.736869796	1.862422031	1.557154689
Zm00001d022590_T017	0.244125943	0	0	0.306254072	0.198900188	0.328529744
Zm00001d022590_T020	0.932376443	0.368069877	1.07448283	0.598878729	0.177595103	0.251246447
Zm00001d022590_T013	1.704871964	1.055896698	1.57145596	1.539874955	1.822142347	1.678002023
Zm00001d022590_T012	0.181565666	0	0	0.516922851	0.544348875	0.434843298
Zm00001d033951_T001	1.684197549	1.313003157	1.528274248	1.756945134	1.403789616	1.425593578
Zm00001d017363_T001	3.174001263	2.460142299	2.830502176	2.804829026	3.009117993	2.585311831
Zm00001d037792_T001	1.953094602	1.420696897	1.940975937	1.161631682	1.597051507	1.428172413
Zm00001d012607_T001	7.802248655	7.672855046	7.889927538	8.282971126	8.318235199	8.236045117
Zm00001d006630_T001	3.078749521	2.412637384	3.172310528	2.80309785	2.957216663	2.764084321
Zm00001d028464_T043	4.89187579	6.101655685	4.790079607	4.187308995	3.856102877	4.301942717

Zm00001d028464_T037	3.511233222	2.669768791	3.589824597	3.825722343	3.736866225	3.76367215
Zm00001d028464_T035	0.753044806	0.346623365	0	0.67103969	1.106216307	1.084486123
Zm00001d028464_T028	0	0.525720118	0.634446436	0.45857939	0.829110776	0.693854902
Zm00001d028464_T026	0.405117767	0.124550478	0.226316158	0.560966304	0.738501535	0.759178849
Zm00001d043267_T002	2.606710843	2.140129788	2.604824508	2.933361506	4.000522792	0
Zm00001d043267_T001	3.37817921	2.597903927	3.457743071	5.152857588	4.767589706	5.347338497
Zm00001d007687_T001	6.287615778	5.959168497	6.318376392	6.277543487	6.368411559	6.229207973
Zm00001d038359_T001	3.561695532	3.295126578	3.862882995	4.010702064	4.075826617	3.976590654
Zm00001d038359_T006	0.33370423	0.152131488	0.161878655	0.467201213	0.165845067	0.510094016
Zm00001d038359_T007	3.084141161	5.726993021	3.828305497	3.131836387	3.454100466	3.672910345
Zm00001d038359_T003	3.954693536	0	2.788561061	2.575491688	2.718141739	1.670224431
Zm00001d018200_T001	7.36725732	7.272365229	7.528330086	8.260112222	8.165806873	8.158781428
Zm00001d006610_T001	1.72173881	1.232491307	1.948693162	1.783616316	2.046972577	1.91991247
Zm00001d035050_T003	0.589380954	0.549538078	0	1.327376375	0.763181235	1.330649605
Zm00001d035050_T005	0.920432036	0	0.755172166	0.761581442	0.77161518	0.666483022
Zm00001d035050_T001	1.360728346	1.038954447	1.263666649	1.624016179	1.305983941	1.739025013
Zm00001d035050_T010	0.34178313	0	0.800076954	0.805861991	1.406785896	0.37464001
Zm00001d035050_T007	3.498537846	2.650442214	3.515153182	4.303310316	4.348930011	4.215738928
Zm00001d028191_T001	4.553214076	4.166487857	4.363750843	4.967453106	4.777747934	4.981050456
Zm00001d028191_T004	0.771207796	0.407932432	0.664148752	0.866570156	1.37508949	1.206504449
Zm00001d028191_T003	1.596539718	0.490602989	1.939220935	1.671862638	1.626840465	1.682951674
Zm00001d028191_T002	1.852418397	1.414466801	2.111300791	2.284345335	2.529086297	2.147457474
Zm00001d038776_T001	1.863468309	3.690240743	2.159579782	1.906782101	1.199814291	1.60364747
Zm00001d038776_T002	4.108637443	2.00880382	4.060930548	4.324558758	4.301537971	4.090124025
Zm00001d038776_T003	5.450765407	4.2320299	5.426055381	5.151220436	5.211234073	5.224295433
Zm00001d029194_T001	1.590230729	1.089436455	1.460158029	1.447730467	1.616076283	1.690557894
Zm00001d046742_T001	2.708048589	1.611446254	2.655744509	2.684781716	2.796413564	2.639260646
Zm00001d046742_T004	3.416478347	0	2.467403917	2.212016745	1.954997296	0
Zm00001d046742_T002	3.991774151	3.665305897	4.223864713	3.89598551	3.85875636	4.12446181
Zm00001d043619_T001	7.071233884	6.62631402	6.985075153	7.268079103	7.202921392	7.178630505
Zm00001d007838_T001	1.10841004	0.500666444	0.714163735	1.392810877	1.705690882	1.322400644
Zm00001d007070_T001	4.356009256	3.865784038	4.365667064	4.149454812	4.081675717	4.098255736

Zm00001d052229_T001	8.401851745	8.381331113	8.360774673	8.03304908	8.088697684	8.096218687
Zm00001d034083_T001	1.767863667	1.274729213	1.456604841	1.077343505	1.066177386	1.543476581
Zm00001d027748_T001	1.13660287	0.799723941	1.161474319	0.805046413	1.090606864	0
Zm00001d021659_T005	3.161898644	0	2.286364287	4.126531525	4.267011584	4.328791379
Zm00001d021659_T011	0	0	0	0.780296659	0.567392603	0.534108429
Zm00001d021659_T015	3.654350296	0	4.377106739	3.536875872	4.147689467	3.986194651
Zm00001d021659_T013	4.667614627	0	4.393842987	5.384136656	5.14468104	4.967549755
Zm00001d016294_T001	5.272069337	5.313776279	5.150395374	3.828950182	3.845105515	4.13738589
Zm00001d045277_T002	4.540870228	4.851243223	4.934169562	4.562356614	4.863761273	4.680950645
Zm00001d005764_T005	1.574089395	0.884820608	1.937101411	2.644598583	2.353645109	1.791367339
Zm00001d005764_T001	2.272730623	1.43075521	2.063257525	3.093791367	3.368387165	3.42612021
Zm00001d051898_T001	6.226021037	5.523047983	6.149063648	6.744511983	6.65772094	6.643650505
Zm00001d035439_T002	1.222612313	0.525460547	1.327214244	0.842058431	0.728980147	0.513811084
Zm00001d035439_T001	3.576391165	3.048720956	3.491413296	2.633047061	3.111719434	3.200347878
Zm00001d049265_T001	4.758802042	4.284235087	4.86686971	4.664982122	4.693785	4.793282945
Zm00001d008753_T001	7.561162709	7.288278719	7.345595133	6.657866837	6.886253177	6.693398553
Zm00001d009741_T001	0.312065668	0.346902442	0.573918343	0.45728434	0.438124616	0.448746633
Zm00001d047917_T001	3.711373209	3.121867832	3.779961878	4.128117756	4.066536572	4.147682874
Zm00001d026447_T001	6.294437818	7.32388328	6.18101576	5.329215646	5.335623132	5.44969032
Zm00001d008968_T001	2.191785607	1.803006275	1.858216583	1.433615417	0.941114576	0.492411087
Zm00001d037265_T001	0.282631954	0.242576898	0.373702772	0.91299699	0.723632824	0
Zm00001d037265_T007	1.16747226	0.285985689	1.08933611	1.203999822	1.007452436	0.936238781
Zm00001d037265_T006	1.028191456	1.881459065	0	0.705124193	1.341353896	0.344270623
Zm00001d037265_T004	1.220315094	1.049448851	1.040341893	1.28068853	1.466795196	1.961060957
Zm00001d037265_T005	2.407570567	0	2.831727088	2.989502906	2.63349538	2.856653105
Zm00001d037261_T001	4.312996697	3.002308448	3.086213436	4.043043912	3.813064924	3.537086669
Zm00001d013700_T001	3.625145505	4.455401926	3.391285321	3.746599664	3.546714721	3.949361519
Zm00001d002141_T005	4.833261675	7.601608499	4.743684874	4.333637403	3.784550676	3.691464485
Zm00001d002141_T003	2.149084059	1.161908315	2.031397667	2.150450496	2.124445928	1.532704951
Zm00001d002141_T021	2.809924545	2.12181546	2.764949275	1.762714918	2.358046518	2.330629242
Zm00001d002141_T022	3.960180189	3.057522189	3.945796548	3.393668252	3.27308824	3.368830217
Zm00001d002141_T001	8.404822545	7.793206218	8.420223499	8.297718118	8.283566026	8.298506808

Zm00001d002141_T004	1.109587896	0.381285588	1.107722681	1.656388365	1.202079146	1.206510075
Zm00001d002141_T008	0.439013487	0.465485563	0.866373123	1.204931352	1.428804346	1.866983127
Zm00001d002141_T012	0.957687314	0.668510388	1.522867288	1.696678485	1.37466616	1.842673974
Zm00001d002141_T010	5.628229537	4.141638373	5.889177916	4.300207647	4.922113245	4.71103631
Zm00001d017907_T001	3.00193732	3.702986268	3.383895441	5.366344563	5.095692262	5.477764029
Zm00001d040880_T001	3.61087224	2.692258011	3.148260093	3.951230154	4.166376018	3.902319733
Zm00001d008311_T003	0.55758458	0.574464862	0.713297259	0.822231695	1.321087047	1.201280671
Zm00001d008311_T001	1.087172236	0.694921189	0.892978477	1.353483644	1.179728234	1.231249295
Zm00001d042794_T005	0.926161143	1.454803908	0.250388019	1.768561653	1.111508792	1.105759864
Zm00001d042794_T010	1.376298164	0.789164163	1.368418038	0.194945604	1.141970922	0.356165225
Zm00001d042794_T001	2.304951376	1.78848777	2.668096653	1.852841029	1.652634256	1.343624423
Zm00001d042794_T004	1.157353565	0.873183235	1.618992232	2.66240286	2.483842084	2.50836856
Zm00001d042794_T003	0.539234264	0	0.17579539	0.80941856	1.647690736	1.540222228
Zm00001d042794_T006	1.834230642	0.578033455	1.796584465	0.544859242	0	0
Zm00001d020664_T027	0.553676043	0	1.024066441	1.011059759	1.124289751	0.640646839
Zm00001d020664_T023	0.216715598	0.444168769	0	0.532568318	0	0
Zm00001d020664_T012	0.924774886	0	0.862023628	0.833600152	0.836131775	1.186149983
Zm00001d020664_T022	0	0.501782549	0.513305786	0	0.263138997	0
Zm00001d020664_T002	0.749863744	0.226922783	0.284155192	0.916817501	0.658409847	0.34721322
Zm00001d020664_T009	2.541609001	1.765788044	2.369310131	2.609683346	2.556829366	2.62292028
Zm00001d025050_T002	1.876365671	2.242002135	3.32784542	3.403614582	3.597874019	2.893075779
Zm00001d025050_T004	2.825619779	2.005895773	1.514261156	3.377049321	3.555079898	3.496621362
Zm00001d025050_T001	4.492531837	3.830645765	4.429216507	4.528845892	4.14909585	4.797277673
Zm00001d025050_T006	0.772324024	0.333324116	0.21511321	0.513635261	0.908168834	0.757301515
Zm00001d025050_T005	3.758086522	0	3.699320307	3.511872555	3.604923875	2.744330548
Zm00001d021282_T003	5.006233025	5.056120951	5.015507471	4.775195225	4.527367842	4.759697755
Zm00001d021282_T001	6.140144904	6.251140498	6.089940535	5.7663811	5.931704589	5.851033637
Zm00001d041105_T007	0	0	0	1.825733535	0.930104494	1.863623718
Zm00001d041105_T027	0.762158287	0.037425801	0.42698391	2.126669621	1.595908914	1.774119076
Zm00001d041105_T047	2.845944014	1.044579472	2.408437424	4.235198741	4.020561494	4.148198663
Zm00001d041105_T017	1.943973071	0	1.268567338	0	1.324227648	2.409177469
Zm00001d041105_T066	5.284827801	4.551161545	5.388681237	5.089653225	5.15259418	5.061287672

Zm00001d041105_T058	1.493387052	1.98131894	2.406334755	0.640746777	0	0
Zm00001d041105_T033	0.493698565	0.14583003	0.041650917	0.529860917	1.281840658	1.012728845
Zm00001d041105_T005	0.758008894	0.7851722	0.733920894	1.656668889	2.107630629	1.536323505
Zm00001d045833_T001	4.387709386	4.696370579	4.550944671	4.43472963	4.169736637	4.027406395
Zm00001d018443_T002	2.906496397	4.485788229	3.671449411	0	0	1.92328536
Zm00001d018443_T001	4.930451656	3.96982279	4.885087299	5.487428337	5.560329899	5.436769998
Zm00001d006132_T001	1.814044212	1.119205425	1.588215474	1.973145791	1.997882735	1.890738121
Zm00001d024464_T002	2.953660645	2.658841217	2.703161623	2.731984323	2.351362887	2.569446129
Zm00001d049764_T001	4.683962122	3.888150076	4.781419991	4.952783174	4.85268001	4.839841407
Zm00001d015525_T001	6.182375978	6.03371574	6.117836833	6.08239216	6.065631741	6.159072145
Zm00001d038330_T001	5.8589868	5.010408333	5.861064294	5.992326084	6.001958256	5.861492467
Zm00001d029855_T001	4.905470622	4.250902622	4.967200108	4.551649269	4.478644024	4.401893849
Zm00001d038480_T001	3.905003375	3.345186144	3.899752883	4.757131285	4.602243306	4.583725521
Zm00001d034624_T015	0	0	0.382704876	0.388683304	0.465907616	0.357231057
Zm00001d034624_T012	1.789453413	1.031241319	1.173446646	2.734095891	2.559389267	2.604529686
Zm00001d034624_T032	0.247990687	0	0.363365106	0.949924828	1.223351495	0
Zm00001d034624_T038	1.081116661	0	0.746401346	2.116685468	1.796553318	1.752433476
Zm00001d034624_T005	0	0.64250196	1.110201524	2.840321425	2.759665695	2.207742953
Zm00001d034624_T020	0	0	0	1.256142929	0.976714826	1.781129284
Zm00001d034624_T050	0.356439082	0	0	1.809286839	1.893505891	1.849300754
Zm00001d034624_T006	3.756332893	2.874088327	3.857800995	3.008394557	2.888675459	3.243535193
Zm00001d034624_T009	0	0.427908624	0	0	1.749191842	1.156940842
Zm00001d034624_T052	0.824040315	0.692774439	1.205075285	0.799491038	0.116185074	0.424933909
Zm00001d052636_T001	1.765166388	1.13437736	1.923106955	1.822825169	1.821420423	1.851636319
Zm00001d004812_T006	1.579988179	0	0	2.046075409	0	1.562744059
Zm00001d004812_T002	3.456252353	1.750892443	3.256939991	2.941922292	3.009538516	2.821250193
Zm00001d004812_T005	0	0.255323067	0.940876363	0	1.124160031	0.578002529
Zm00001d004812_T008	3.104602527	2.72179261	2.965910221	2.602863035	2.575764917	2.656354954
Zm00001d019280_T001	4.413346301	4.349360394	4.810782452	3.205211696	3.297545369	3.338680269
Zm00001d019280_T002	3.853070369	3.972146808	3.996846793	2.696782404	3.011952849	2.602153496
Zm00001d008559_T002	2.132409039	1.732331726	2.298972918	1.943063892	2.099357826	1.855029308
Zm00001d031712_T020	4.437200835	6.522699421	5.028707492	4.182298283	4.225996791	4.180100747

Zm00001d031712_T001	1.36997842	2.086370392	0	0.688749713	0.440388826	0.546003942
Zm00001d031712_T003	3.047416589	2.577978851	3.091764141	4.078027977	3.692586881	3.923713309
Zm00001d031712_T005	0.32767127	0.32804828	0.79430107	0.412612456	0.813087094	0.769572033
Zm00001d031712_T006	4.235681803	0	3.841703167	4.12028873	4.267656392	3.973830189
Zm00001d031712_T014	0.65855883	1.239592343	1.734855264	0.230044345	0.70830821	1.4748804
Zm00001d031712_T018	0.831262749	0.249650429	0.801495591	0.934769352	0.957796504	0.600040389
Zm00001d040110_T001	3.200180712	4.577048632	3.773889385	2.896035805	2.8592178	3.404391169
Zm00001d052952_T001	5.765785101	5.879333602	5.96857091	4.983022497	5.035612034	5.069811791
Zm00001d034638_T001	3.132490311	4.447226414	3.263874231	3.882394102	3.153798852	3.178868223
Zm00001d034638_T003	6.584580121	5.488751111	6.595607893	6.219662844	6.379921082	6.324637545
Zm00001d000170_T001	2.275764562	1.703376274	1.906211407	1.116833157	1.114906019	1.812694131
Zm00001d038104_T009	3.043556589	2.396793544	2.990037596	3.956510026	3.990160252	3.906105556
Zm00001d038104_T032	3.732666022	2.446958347	3.791571553	3.084368424	2.656364337	2.470870007
Zm00001d038104_T031	0	0.184097446	0.468025419	0.467146945	0.562877864	0.828113223
Zm00001d038104_T027	0.693953897	0	0	1.119328286	1.006117025	1.083935641
Zm00001d038104_T021	0	0	0	0.893865416	0.472725861	1.27228303
Zm00001d011520_T001	1.3000874	0.970266888	1.330334091	1.598089314	1.239162767	1.223276728
Zm00001d012671_T001	1.383600899	1.691298312	0.895371652	1.271084385	0.905387101	1.737806152
Zm00001d012671_T002	2.351607412	1.341335677	1.922449442	1.762523162	1.888132634	1.700716245
Zm00001d028973_T001	2.466280205	2.948684566	2.748561274	2.801163417	2.335677043	2.996188961
Zm00001d049141_T002	2.119725022	1.643118265	1.216280396	1.062507208	0.641123332	0.481565547
Zm00001d031790_T002	2.364013455	2.846101122	2.048290948	2.486549116	2.275700805	2.432950056
Zm00001d031790_T001	4.504420371	3.743330984	4.48966399	4.259857884	4.177798227	4.095036763
Zm00001d041787_T001	1.294030759	0.957282422	1.538869871	2.189452651	2.113585159	1.909899034
Zm00001d024876_T001	1.973387553	1.354774855	1.776507044	1.982119253	1.860786049	1.855641735
Zm00001d044552_T002	1.823567178	1.318948452	1.886891515	2.515234569	2.6443457	2.424491902
Zm00001d044552_T004	0.153399379	0.147512504	0.40391011	0.473395214	0.32875376	0.386307708
Zm00001d044552_T001	0.328361997	0.33518252	0.20655696	0.593753415	0.500390087	0.549271913
Zm00001d044552_T006	0.494189267	0.575975431	0.375544391	0	0.409602742	0.786289391
Zm00001d044552_T011	3.182627152	2.158541588	2.973365877	3.311036286	3.188696523	3.203659738
Zm00001d027517_T003	3.259149382	4.069108282	2.670780298	3.297990328	2.980832727	2.570867752
Zm00001d027517_T002	0.95443978	0.478831022	0.964536888	1.110724039	0.111542844	0

Zm00001d027517_T001	3.069068142	2.187639811	3.19739018	3.147050215	3.405296159	3.4294281
Zm00001d025103_T001	4.007039111	3.018850277	4.020270075	3.907449387	3.928814197	3.929625703
Zm00001d048096_T014	1.147508597	0	0.779431194	2.284155192	2.481939538	2.054530042
Zm00001d048096_T019	0.165216063	0	0.343375983	0.248653841	0.581387887	0.338355951
Zm00001d048096_T013	2.89559307	1.89016299	2.983592699	2.509936229	2.427638083	2.552132338
Zm00001d048096_T009	0	0	0	1.617706123	0.435071676	0.761355066
Zm00001d048096_T001	0.495670606	0.953222023	0.582895081	0.427300439	1.385744116	2.007296702
Zm00001d024831_T001	2.049804647	1.339424755	1.69730856	1.774989418	1.838758588	1.742941826
Zm00001d016621_T001	3.861205246	3.150240559	3.481307855	3.264133141	3.46786205	3.402926508
Zm00001d004078_T001	2.121191499	1.433082833	2.057652322	1.497226062	1.914155766	1.883374873
Zm00001d045774_T001	2.01549079	1.716705619	2.160593028	2.572514276	2.361549974	2.419039929
Zm00001d020793_T001	2.851757835	3.451711599	2.453012581	2.198189553	1.955397994	2.494897623
Zm00001d034937_T001	5.216972792	5.197357623	5.172364059	4.860154941	4.949994188	5.135939073
Zm00001d018105_T001	1.492853018	1.501121654	2.029711541	3.309530705	3.549886852	3.368504506
Zm00001d018647_T001	0.754156351	0.427643715	0.566041613	0.868821074	0.777415928	0.594790252
Zm00001d017323_T001	2.149115609	1.387230177	2.077263853	3.087421766	3.194658531	3.331900801
Zm00001d028311_T001	3.022260739	2.233740839	2.582935293	2.598289924	2.682392387	2.413635781
Zm00001d027875_T001	5.275879033	5.083600546	5.327585265	5.36369254	5.445714985	5.458416096
Zm00001d012469_T001	4.544170674	4.992838253	3.672973575	4.165983951	4.805223002	4.234605855
Zm00001d014909_T004	3.086224308	2.478477711	3.282325927	3.268654464	3.410371635	3.416593058
Zm00001d014909_T007	0.418248248	0.135821148	0.191680133	0.524147948	0.266809324	0.927109866
Zm00001d014909_T006	0.932990155	0.284552036	0.428961348	1.067114819	0.924488354	0.804916769
Zm00001d041088_T002	3.007087295	2.24378331	0.496202575	3.006506468	3.5124934	3.333086636
Zm00001d021261_T005	1.435828051	0.629391128	1.675917526	1.552606271	2.005394598	1.754145659
Zm00001d021261_T003	1.144746005	1.121408026	1.301329519	2.045641467	1.738680948	2.070239555
Zm00001d048947_T001	3.462245931	4.302853912	3.974787635	2.950075492	2.217196277	2.162089162
Zm00001d018303_T011	1.677597992	0	0	2.926180883	3.33880105	2.9134955
Zm00001d018303_T013	4.200032601	3.202437156	4.317931276	3.922277309	3.727742747	4.159606502
Zm00001d018303_T001	0.630647764	0	0	1.68370364	1.864080731	1.778509702
Zm00001d018303_T006	1.39000506	1.495604608	1.92039278	3.33311856	3.073562513	2.424681619
Zm00001d018303_T002	1.379921446	1.319251434	1.939424441	1.715021342	2.36604519	2.648838149
Zm00001d018303_T008	0.38984321	0	0	0	0.56773429	0.6025709

Zm00001d043093_T099	1.364615572	2.003414422	1.723497838	1.964812989	2.048704916	1.927375776
Zm00001d043093_T020	0.191465373	0	0	0.631808341	0.313126251	0.367155204
Zm00001d043093_T077	0.229523938	0	0.042281517	0.548379409	0.647837608	0.634839505
Zm00001d043093_T102	3.057564297	1.706725919	3.581436215	2.504078295	0	3.211048961
Zm00001d043093_T040	0.282544185	0.022458722	0	0.980203021	0.119795725	0.311611226
Zm00001d022403_T001	5.667778413	5.565208699	5.782608887	5.11235105	5.308992564	5.333295918
Zm00001d034836_T001	7.13273449	6.770888351	7.239764407	6.305750854	6.259954009	6.311383461
Zm00001d005127_T024	2.208118606	1.360113082	2.327398509	3.619438249	3.513926394	3.431829923
Zm00001d005127_T001	0.332770938	0.148780556	0.17465441	0.067326191	0.433023526	0.84442181
Zm00001d005127_T016	0.80469957	0.337405111	0.987855637	1.528167708	0	0.98517105
Zm00001d005127_T026	4.972232802	5.075301319	4.802784756	4.546965127	4.634798158	4.544539356
Zm00001d005127_T021	2.015557157	1.497253148	1.869015403	0	0	0
Zm00001d005127_T008	0.453426972	0	0.531778186	1.474081913	1.647150542	1.837655481
Zm00001d005127_T018	0.240253262	0.017817888	0.464755035	0.168540633	0.288950285	0.598234652
Zm00001d005127_T009	0	0.370425449	0	2.024719309	2.401312351	1.820587455
Zm00001d005127_T023	2.194819248	0.383262466	1.184775392	2.006132109	1.731042876	1.561131564
Zm00001d005127_T003	0	0	0	2.081608574	1.781115431	1.629662964
Zm00001d027556_T001	3.898994702	3.331465607	3.948589635	4.026593819	4.08481723	3.869478312
Zm00001d027425_T001	4.304624861	3.669342037	4.233185557	4.616635469	4.652718914	4.736583825
Zm00001d027425_T002	3.234371484	2.801248692	3.464708777	3.926866389	3.778579823	3.804502369
Zm00001d052107_T005	0.889485224	0.695888724	0.638927382	0.759702974	0.604611284	0.100641316
Zm00001d052107_T006	0.212846957	0.169051426	0.28362313	0.376817126	0.399724545	0.430775545
Zm00001d052107_T003	2.119388052	1.813971178	2.36227602	2.287443335	2.194026833	2.107410685
Zm00001d031801_T001	5.3429688	5.195909841	5.492839723	4.567911794	4.689430731	4.970616759
Zm00001d029846_T001	1.853357368	1.355337141	2.062167659	2.141518461	2.343135746	2.405472351
Zm00001d021160_T001	3.466390501	0.696443472	2.739801668	7.77746531	7.95098402	7.96631072
Zm00001d020946_T002	1.878062982	1.046911479	1.820908858	1.769579226	1.78912409	1.729690671
Zm00001d020946_T006	0.191863286	0.407294009	0.56681905	0.566788857	0.288234518	0.739029768
Zm00001d020946_T001	1.024711863	0	0.964660345	1.087571464	1.599397306	1.328439554
Zm00001d020946_T007	2.248304691	2.460059186	2.096975939	2.777788956	2.551627738	2.499161393
Zm00001d020946_T004	0.407112327	0.083586463	0.22988689	0.17344089	0.534928155	0.378320731
Zm00001d029806_T001	5.388349093	4.977330852	5.454121133	4.971263954	5.176735214	5.094336117

Zm00001d013393_T001	2.462244229	2.299693635	2.455334586	2.462297111	2.837944049	2.59216183
Zm00001d042056_T001	5.354090595	5.394610927	5.226273957	7.514706788	7.45230127	7.445524067
Zm00001d017979_T004	4.540342886	3.652947854	4.354006151	4.498129829	4.45742271	4.310810956
Zm00001d017979_T002	3.079223464	1.119842859	3.226753582	4.361883013	4.326582647	4.405341911
Zm00001d015521_T001	6.26440486	5.287198698	6.29166389	6.476296912	6.518725566	6.497684546
Zm00001d003112_T005	0	1.411392619	0	1.197281655	0	1.288041341
Zm00001d003112_T015	1.943099534	0	1.824958028	0	1.841039592	0
Zm00001d003112_T016	1.586549554	0.65525473	0.692900282	0.991534725	1.237102937	1.191157704
Zm00001d003112_T018	0	0.17708605	0	0.547457726	0.361980538	0
Zm00001d003112_T012	0.969964209	0	1.163762773	0.828308239	0.526734902	0.88044966
Zm00001d003112_T010	5.585286259	2.08844829	5.608098067	4.912406761	4.889313007	5.014989948
Zm00001d000349_T001	3.80117418	3.599172207	3.730887502	4.192633773	4.076015673	3.857704767
Zm00001d046656_T005	3.817499753	4.164355963	3.538603592	3.614001042	2.666632083	2.233007576
Zm00001d046656_T001	3.938444451	3.211453659	3.806270123	3.857838012	3.921383771	3.84789361
Zm00001d028699_T059	0.218322408	0.29225286	0.049204169	0.141967653	0.533471651	0.366867712
Zm00001d028699_T009	1.359811255	1.019665624	0	2.342401138	2.664260958	2.95019422
Zm00001d028699_T018	0.14584307	0.118277322	0.215036151	0.488889259	0.629682079	0.41774074
Zm00001d028699_T006	0	0	0.334803939	1.058099642	1.130834028	1.027205019
Zm00001d028699_T055	1.981115047	2.055773866	2.181161084	2.153448346	2.110060506	1.870060464
Zm00001d028699_T056	0.115776379	0.02638082	0.255862041	0.579093236	0.418780401	0.450758876
Zm00001d028699_T052	2.540076632	1.436701283	2.555295906	1.620436738	1.831703378	1.822308391
Zm00001d028699_T007	0.986117451	0.382586471	0.50529849	0.82207013	1.000355581	0.868112259
Zm00001d028699_T017	0.724407547	0.169216945	0.400648313	0.815808209	0.858966069	0.706580944
Zm00001d028699_T049	0	0.615201572	1.262371215	1.489243885	0	1.380876259
Zm00001d028699_T061	0.846900041	0.226000414	0.633593955	1.140230262	0.998255449	0.907056015
Zm00001d010281_T001	2.355328965	1.49898454	2.387559133	2.467339483	2.512550793	2.799384935
Zm00001d053818_T003	2.18887784	1.355264964	2.252179773	4.742757769	4.893343959	4.902394121
Zm00001d053818_T002	1.691106653	1.461537989	1.799585945	3.631100207	3.671429029	3.950226047
Zm00001d053818_T001	0.374933746	0.102416262	0	1.788079275	1.368325277	1.636975349
Zm00001d053818_T004	2.744772712	2.425481863	2.506095382	4.654458742	4.710579	4.72808317
Zm00001d022185_T010	0.153964834	0.190480856	0.176533416	1.00307975	1.315199279	0.606158873
Zm00001d022185_T004	1.161706488	1.580399261	1.529052804	0.860912754	1.251693646	0.564334265

Zm00001d022185_T007	0.095395166	0	0	0.681497842	0.822851676	1.209993534
Zm00001d022185_T008	3.008952939	0	2.365569368	3.427466589	3.482592111	3.563063412
Zm00001d022185_T009	2.48510708	1.654177501	2.793795608	2.258124202	1.78139203	1.299888187
Zm00001d045571_T001	1.129091085	1.089169304	1.171618693	1.008190689	0.84950934	0.716732832
Zm00001d052653_T005	3.988656423	2.531075733	3.896582445	6.071021356	6.272700395	6.203377547
Zm00001d052653_T006	4.940718957	5.513444255	4.832899278	6.402807568	6.303802151	6.261252724
Zm00001d052653_T002	1.303157077	0.654879097	1.109299707	3.377996461	3.482278448	3.427833956
Zm00001d052653_T004	3.687260837	2.998900608	3.771629018	6.63801975	6.566859515	6.582015584
Zm00001d007554_T001	1.446298584	1.007844927	1.503359931	1.754966154	1.561939014	1.661254329
Zm00001d018069_T001	4.029305782	4.783677337	3.833895096	3.605361342	3.361892836	3.479227974
Zm00001d045460_T001	6.279535034	2.597494943	5.940895832	11.13879932	11.0536055	11.12273727
Zm00001d044874_T001	3.987876006	3.612510561	3.875290761	0.989200784	1.102441795	1.343864864
Zm00001d034423_T001	4.245584193	3.323974981	4.198702921	4.27950168	4.276176602	4.306728648
Zm00001d029745_T005	0.86930132	1.583409325	1.671007523	1.255971987	1.724894702	1.353205273
Zm00001d024141_T001	6.104315522	5.271904378	6.147342399	6.588906033	6.633363535	6.558261863
Zm00001d013032_T001	2.565868874	1.876745599	2.350648864	3.920196482	3.932450053	3.763961776
Zm00001d039460_T002	2.402356	2.821632263	2.524861552	2.007406148	2.292265525	2.492903765
Zm00001d039460_T001	4.947957518	4.091104526	4.85483144	4.831975714	4.754117592	4.831727189
Zm00001d029234_T008	1.17898547	0.284093582	1.162965352	2.411276274	1.876261536	1.436548852
Zm00001d029234_T002	0.358556052	0.226834024	0	0.05812805	0.35486849	0.434207066
Zm00001d029234_T007	0.50123021	0	0.750665669	1.796838183	0.662004921	0
Zm00001d029234_T003	3.221996141	2.133026525	2.877846315	3.680905144	4.019831947	3.749950498
Zm00001d029234_T010	0	0.493423945	0	0	1.311085708	1.757746973
Zm00001d042646_T001	4.566463629	4.36478889	4.642668016	5.015408629	4.875981908	5.057754073
Zm00001d053117_T001	2.793243558	2.106342289	1.910581862	1.281879225	1.349642126	1.439634307
Zm00001d052911_T005	0.80160485	0.309084197	0.642288456	0.970132126	0.778303631	0.643387988
Zm00001d052911_T003	1.571881612	0.597186936	1.493509513	1.448695894	1.521477953	1.599113993
Zm00001d052911_T001	2.263547373	2.318390601	2.641357588	2.173293766	2.321403724	2.277371248
Zm00001d052153_T003	0.700828528	0.324181555	0.615313648	0.923891545	1.026866616	1.025972087
Zm00001d052153_T001	5.584279163	5.028843786	5.755919435	5.598572566	5.55394195	5.484993922
Zm00001d023655_T001	4.215162457	3.546760646	4.09619412	4.799430165	4.705920856	4.844090535
Zm00001d039104_T001	2.809302482	2.103146785	2.732288305	2.475391293	2.537895636	2.414660817

Zm00001d045282_T001	2.761972199	2.106913091	2.520900679	2.572942768	2.456823493	2.948713343
Zm00001d023669_T001	7.11623529	6.324929815	7.195595422	5.921879773	6.048347606	6.016354909
Zm00001d035820_T002	3.756914507	3.050543663	3.532579663	4.006124477	3.896003729	3.951471326
Zm00001d023261_T001	3.501651107	2.93413752	3.517392616	2.482950223	2.630025642	2.834917878
Zm00001d021788_T001	6.229423092	5.301535483	6.27234537	5.851913836	5.779408448	5.797842937
Zm00001d018289_T001	0	0.348336679	0.366031757	0.652998546	0.249123695	0.446731579
Zm00001d018289_T014	0	0	0	0.921662543	1.196980259	1.117218941
Zm00001d018289_T011	0.424666303	0.347813036	0.430111818	0.847912747	0.135756806	0.409923108
Zm00001d018289_T027	1.920003589	2.042531929	2.458090863	1.515461671	1.106622359	1.375146778
Zm00001d018289_T018	0	0.086897873	0.281624667	0.733156472	0.849528556	1.305700337
Zm00001d018289_T026	2.561323937	0.740979354	1.797008826	2.883060246	2.888567534	2.458337379
Zm00001d005636_T001	4.840204281	4.335736846	5.040453727	5.075351042	4.83838481	5.240031717
Zm00001d005636_T009	4.181490293	5.193764057	4.040913904	0	0	0
Zm00001d005636_T003	5.067981884	2.381090354	4.818034469	4.811146902	5.058549767	4.361000636
Zm00001d020471_T001	3.29566558	3.005010605	3.329123596	3.540479901	3.602585152	3.835616004
Zm00001d020471_T003	1.965445569	1.196015871	1.651884281	2.415814362	2.030296523	1.891784675
Zm00001d020471_T002	2.473408723	1.559959665	2.844942978	3.493669236	3.592824526	3.264909592
Zm00001d011565_T005	6.965053369	6.842001502	7.10258652	6.901019834	6.899265204	7.105698651
Zm00001d011565_T002	0.651937535	1.949268967	1.271071234	1.714932572	1.406704276	1.602843561
Zm00001d011565_T001	7.486699758	6.768456717	7.366393875	7.729516587	7.499864188	7.730954896
Zm00001d011565_T003	0	5.741820471	3.605583699	3.771383188	5.258118472	0
Zm00001d011565_T009	1.554584431	1.348261318	0	1.959660744	1.391513137	1.646939107
Zm00001d011565_T006	1.487605197	1.557148806	2.739622394	0	1.523736574	1.359079748
Zm00001d011565_T010	0.599512045	0.595552354	0.580272861	0.539574744	0.638313919	0.939491392
Zm00001d011565_T008	1.453993702	0.124782049	1.832864906	1.917244603	2.507812143	1.861323774
Zm00001d011565_T004	3.019475619	2.419001084	2.756811522	3.504688013	3.511309643	3.455611161
Zm00001d050258_T004	2.889407539	0	3.509879492	3.161146479	3.583787909	3.29512452
Zm00001d050258_T001	1.427381438	2.362670183	1.647866612	1.797166165	1.470337985	1.314697106
Zm00001d050258_T003	3.873215527	3.14413727	3.795508158	3.744067856	3.818675018	3.490542277
Zm00001d011869_T001	1.202351267	1.2776093	1.197612552	1.616678103	1.462507835	1.511215508
Zm00001d011350_T001	1.315635784	0.8518038	1.2960603	2.188910431	1.939046371	1.833254843
Zm00001d017719_T001	7.743838162	8.201732112	7.749491221	6.774396939	6.782923613	6.869897852

Zm00001d004554_T002	1.353718496	1.095432976	1.409485981	0.628195935	1.207169449	1.663400185
Zm00001d032837_T001	0.941452666	0.848132353	0.745459367	1.205430676	0.77038338	0.63688118
Zm00001d009260_T004	1.918995625	2.858162266	1.739637298	0	0	2.262976997
Zm00001d009260_T002	2.331514335	2.019156398	2.073916524	2.057179211	2.255493482	1.989997101
Zm00001d034677_T001	6.770074895	6.80952326	6.497603632	6.380624802	6.349096021	6.255147777
Zm00001d034150_T001	1.154303223	1.518156413	1.611475055	1.170620533	1.09670512	1.474784377
Zm00001d026505_T001	6.225353488	6.422883076	6.103862778	5.69626293	5.715956913	5.667974729
Zm00001d040678_T010	0.88942993	0.52295665	1.279236523	0.940977815	0.84319919	1.006705189
Zm00001d040678_T001	1.179035169	0.516987377	1.180748753	2.346960292	1.939806173	1.750567918
Zm00001d040678_T014	2.984080353	2.595372848	2.999663993	1.634786544	2.409018028	2.689712897
Zm00001d040678_T013	0.163046531	0	0	0.429939413	0.414441904	0.324129698
Zm00001d040678_T004	1.619132192	0	1.366866034	1.872827184	1.475485461	2.35085024
Zm00001d040678_T003	2.178021726	1.666833836	2.294036937	2.10276931	1.847096544	2.009939752
Zm00001d009820_T001	6.464470487	7.183064254	6.464113919	4.898590408	4.792586097	4.77615585
Zm00001d033645_T001	3.837812687	2.978198559	3.847224457	4.385163251	4.476381169	4.354251851
Zm00001d009684_T001	2.711260312	1.789817709	2.451212695	3.678743504	4.019548653	3.565178197
Zm00001d032591_T003	4.480222009	3.660548526	4.539934307	4.936653343	4.953755476	4.826513761
Zm00001d032591_T001	0.122032557	0.367636095	0	0	0.480432651	0.874316971
Zm00001d031670_T002	1.157341923	0.919741273	1.576517784	1.867681427	1.980367191	2.061175842
Zm00001d031670_T001	4.678511297	2.74733605	4.618340393	4.780069	5.029454211	4.67765087
Zm00001d022313_T001	1.486528008	0.993590315	1.443004519	1.250847608	1.235622912	1.57695597
Zm00001d038380_T005	4.651832975	5.907240984	4.597651245	4.45162912	3.877450381	0
Zm00001d038380_T001	6.793120004	6.334305952	6.802341693	7.332026493	7.325197772	7.424002766
Zm00001d038380_T003	6.531879985	6.218707423	6.055757492	4.762120008	5.280952566	4.124766337
Zm00001d038380_T006	1.134595532	1.959755691	0.85474334	0.534547733	0.571726315	1.330445389
Zm00001d028771_T011	1.239750576	0	1.319554352	1.104603031	0.968136475	0
Zm00001d028771_T002	0.416362006	0.909664058	0.203935318	0	0	1.420994874
Zm00001d053055_T001	7.056970756	7.825786759	6.970310483	7.416131822	7.468114332	7.435660969
Zm00001d033190_T006	3.2332773	3.252698884	3.66372534	2.869610474	2.709503495	3.062383729
Zm00001d048727_T001	3.779771876	4.102957724	3.723665695	3.837854761	3.575907199	4.02512122
Zm00001d034533_T001	3.705180606	3.064358297	3.951772689	2.91621598	3.205362013	3.169991204
Zm00001d034533_T003	1.176334263	0.580132939	0.862873475	1.196129844	1.074992418	0.702365872

Zm00001d034590_T002	3.861051164	3.637530494	3.975518027	4.113830413	3.983629361	3.932790746
Zm00001d034590_T009	0.941442149	0.371611273	0.883675552	0	1.053223967	0
Zm00001d034590_T007	0.320106842	0.125697384	0.25415621	0.631966614	0	0
Zm00001d034590_T012	3.40772174	0	3.31939712	2.607652968	3.226131931	2.987398729
Zm00001d024738_T001	3.152375711	2.892948372	3.092888193	2.779806013	2.675692879	2.651276992
Zm00001d040393_T001	4.129841108	3.332256654	4.045069492	4.081765777	4.037724931	4.228321856
Zm00001d052690_T001	2.255472332	1.425380884	2.207257551	1.850331381	1.675591946	1.752034773
Zm00001d019775_T001	3.620184511	3.990708946	3.969641392	3.937797659	3.625180932	3.954715215
Zm00001d029365_T001	1.709838932	1.107919485	2.065608155	2.675045385	2.633078224	2.78925516
Zm00001d029365_T002	6.822856251	6.025563724	6.865767388	6.376380549	6.425732	6.290408958
Zm00001d042005_T003	2.722200632	2.268987351	2.824788827	2.421063028	2.366741846	2.580203867
Zm00001d012241_T001	6.103463733	5.879228677	6.267053433	5.227835053	5.298198347	5.346423915
Zm00001d028439_T001	3.672747112	2.953318885	3.591882476	3.313941342	3.448317149	3.634758786
Zm00001d002020_T005	1.785794084	0	2.044444482	0	0	2.850321179
Zm00001d002020_T004	4.296793116	2.990015079	4.153412675	3.982374942	3.534398076	3.402672931
Zm00001d002020_T001	1.714128998	2.383565234	1.476645574	1.294499006	1.78610869	1.134144695
Zm00001d002020_T002	2.195771525	2.632158846	3.136411253	0	0	0
Zm00001d000123_T001	0.400569625	0.115213069	0.304172232	1.188871828	1.112451267	1.019631468
Zm00001d000123_T003	2.883510948	2.397514668	2.991881154	3.536087475	3.36980105	3.363022313
Zm00001d033786_T001	2.361250973	1.150564226	0	3.134908274	2.884169835	3.245806104
Zm00001d033786_T004	3.901354369	2.989432059	4.155979977	4.054938057	4.118632075	3.942148976
Zm00001d048787_T001	11.55990482	6.55442129	11.2330963	10.67556858	10.53239011	10.53189552
Zm00001d051659_T001	8.492466672	9.349741734	8.666703264	7.956752118	7.97473969	7.9370549
Zm00001d032945_T001	4.207360564	3.557437172	4.243765643	3.589629516	3.774200165	3.417842121
Zm00001d051669_T003	0.345996947	0.633131713	0.915898686	0.577333592	0.673576323	0
Zm00001d040920_T001	2.712794509	2.168864391	2.78406336	3.315955978	3.162845829	3.467000959
Zm00001d030639_T002	2.52243973	1.483358685	2.486032632	2.273862274	2.204008902	2.131681996
Zm00001d030639_T003	2.123772789	1.787399866	2.040626305	1.006995951	0.784023982	1.180182241
Zm00001d016835_T018	0.754070813	0.168955184	0	1.244459057	0.795358847	0.958542786
Zm00001d016835_T006	4.412807805	3.775550603	4.573241752	4.592540397	4.570879103	4.448791754
Zm00001d052380_T001	3.615884132	2.797522087	3.712492884	3.277738576	3.240729369	3.130614628
Zm00001d023979_T002	1.939192344	1.202281047	1.843847204	3.058946779	3.021971972	2.925820399

Zm00001d023979_T004	1.817181582	2.626062702	3.092060251	3.228517809	2.9407377	2.947774425
Zm00001d021303_T002	3.039208772	1.694529893	2.554952733	6.702628179	6.736747704	6.674997932
Zm00001d018965_T001	3.827446314	3.897798515	3.994889861	1.98497827	1.433780441	1.219548335
Zm00001d034561_T001	2.522365403	3.002210678	2.57531572	2.267345524	2.620779226	2.379923663
Zm00001d034561_T002	3.478873724	4.191489614	3.679993692	3.397291084	3.494514944	3.15288869
Zm00001d029025_T001	0.60069032	0.422780905	0.408310785	0.760430468	0.940056218	0.979887058
Zm00001d027325_T003	1.510856118	0.934451584	1.333424879	1.768674705	1.783191769	1.955348517
Zm00001d027325_T001	4.544327792	3.967157538	4.580960389	4.417074431	4.496170163	4.414430132
Zm00001d018494_T001	2.818295745	1.785329998	2.585428176	2.767136521	3.18018431	2.988341489
Zm00001d018494_T002	3.078656455	2.757536208	3.253677707	3.058786464	2.739232882	2.881607458
Zm00001d018494_T003	4.027056535	5.15174247	4.241585097	3.443935464	3.934391664	4.10025755
Zm00001d032694_T001	2.021123629	1.55673791	2.038188219	3.840553872	3.92849123	3.923994388
Zm00001d050967_T001	4.339556233	4.814392508	4.633266238	5.032176547	4.935539848	5.175311135
Zm00001d011351_T070	0	0.179545445	0.243991945	0.93025516	0.952033813	0.846917688
Zm00001d011351_T067	3.642524834	2.402854763	3.733948544	3.697193556	3.541908169	3.461606874
Zm00001d011351_T031	1.687500598	1.733906147	2.016400399	1.13921014	1.773737714	2.05935147
Zm00001d011351_T008	1.015750176	0	0.87162368	1.136115246	0	0
Zm00001d011351_T036	0.381469443	0	0	0.233710166	0.773073887	0.928322564
Zm00001d011351_T055	0.793521378	0.192171436	0.591351016	0	1.649222717	1.35960213
Zm00001d011351_T003	1.73395429	0.607816516	1.37498659	2.33425705	2.111390606	2.10189577
Zm00001d011351_T035	0	0.470798179	0.608314384	0.796226443	0	1.186880198
Zm00001d011351_T007	0.319745089	0.183550033	0.534071566	0.322696558	1.206832612	1.116027334
Zm00001d011351_T071	1.512736608	3.171643029	2.207072593	2.025114219	0	0
Zm00001d011351_T029	0.895947812	0	0.642400295	0.670938204	0.728461282	0.254671436
Zm00001d036251_T001	1.249444127	1.197780487	0.867151604	1.223907486	1.549184166	1.435305885
Zm00001d032430_T001	0.495984696	0.372248961	0.570648478	1.148541091	0.997614697	1.012208223
Zm00001d032430_T002	2.219210348	1.589188211	1.896790312	2.152171086	1.722997975	2.15439753
Zm00001d041489_T005	0.994453732	0.389315695	1.101984829	1.104182317	0.965238312	1.608669224
Zm00001d041489_T002	4.465560006	3.476974069	4.401833724	4.192469182	4.17158619	4.109776424
Zm00001d041489_T008	0.148655623	0	0.491326705	0	1.083649771	1.338640625
Zm00001d006763_T001	3.545384379	2.739826289	3.47905707	3.121521085	2.577763313	3.192845599
Zm00001d006763_T002	1.979236265	2.10689869	2.089139805	3.025596722	3.434406986	3.054927814

Zm00001d015037_T002	2.580846396	1.699392163	2.532197748	2.572276817	2.466432921	2.680949689
Zm00001d015037_T003	1.459349776	2.300343408	2.002802957	1.290112356	1.652178075	1.354136166
Zm00001d015037_T004	2.352742144	0	0.967148683	1.945116609	0.760977976	1.506715164
Zm00001d010759_T001	6.353218085	7.331796922	6.246056758	6.673109074	6.826184984	6.667535877
Zm00001d052323_T001	2.76609241	2.08751953	2.857549316	2.8020025	2.677330756	2.807222188
Zm00001d044513_T008	0	0.077233427	0.559792313	1.112643425	0.582322841	0
Zm00001d044513_T011	2.072166183	1.533236898	2.1448977	1.494426875	2.061998027	2.353618576
Zm00001d014976_T001	3.005217618	2.563898817	3.093364953	1.819339146	1.698270939	1.703807695
Zm00001d013410_T001	1.540120535	1.20304513	1.456006007	3.28767973	3.486606521	3.155318238
Zm00001d013410_T002	4.684853234	4.78382781	5.053142841	5.070358241	5.041621745	5.012649208
Zm00001d013410_T004	4.574832058	0	4.337265871	4.678140882	4.736608988	4.87299946
Zm00001d000436_T004	2.27422975	0.475051669	2.374363188	2.431132173	1.653754821	2.956289956
Zm00001d000436_T001	3.262123567	2.360067559	3.699192247	4.193956759	4.13922586	3.974915448
Zm00001d000436_T002	3.094655809	2.700801456	2.178532361	1.561780199	2.404143164	0
Zm00001d046700_T003	3.258035383	2.774218407	3.361234127	2.585721159	2.783301795	2.678174468
Zm00001d029647_T001	1.251801487	0.657583302	1.20030032	1.598551009	1.618949489	1.389045255
Zm00001d042023_T001	5.544700328	5.606017086	5.658227967	4.994706158	4.63366916	4.99327786
Zm00001d042023_T002	3.798307708	0	4.130568092	3.868614866	4.197989908	3.197047119
Zm00001d031094_T001	3.856678201	3.747118279	3.729187401	2.877651993	3.061953111	3.023861937
Zm00001d021634_T001	5.772764394	6.904304268	6.039372001	5.463658942	5.414118345	5.414941985
Zm00001d018403_T003	6.214299613	7.826277044	6.336707117	6.35869661	6.477433998	6.237831216
Zm00001d018403_T004	5.530187288	4.974878197	5.320884541	5.222823143	5.168531808	5.310986207
Zm00001d018403_T005	0.315338998	0.244859057	0.347936597	0.685171403	0.925036288	0.694224983
Zm00001d013370_T001	0.971359245	0.304319449	0.36594668	2.335520415	2.696335745	2.536325494
Zm00001d013370_T003	0	0.338855661	0	1.887702672	1.447108359	0
Zm00001d013370_T002	0.144248724	0	0.697424222	2.115251695	2.120943099	2.380948274
Zm00001d014382_T005	2.090147827	1.298825447	1.847625365	3.216253696	3.391298658	3.052591873
Zm00001d014382_T001	3.508720331	2.667630805	3.503345809	3.290333274	3.370857975	3.437976329
Zm00001d014382_T004	0.987055225	0.611342377	1.167165213	1.187100077	1.450009234	1.405960239
Zm00001d036568_T006	0.862962319	0.890992926	0.774969605	0.999795123	0.8077786	0.912695846
Zm00001d036568_T013	0.734471617	0.552543318	0.780074881	0.840010363	1.133129468	0.820980317
Zm00001d036568_T003	5.431086563	5.248519504	5.334734016	5.238182681	5.14587787	5.108547163

Zm00001d036568_T002	5.173677216	4.830828272	4.767289894	4.437215819	4.659739016	4.846963908
Zm00001d048134_T001	2.862750711	2.520837333	2.688224008	3.171457772	3.367869352	3.274026934
Zm00001d028410_T001	1.30200313	0.970704225	1.192592509	0.308831483	0.207620525	0.295408007
Zm00001d052874_T001	2.37740865	3.260356324	2.430291162	2.642794437	2.607998267	2.28791786
Zm00001d027512_T002	4.857909505	4.48904172	4.776127313	4.312562805	4.3937933	4.323065284
Zm00001d036216_T001	2.079634795	1.45577303	2.012200355	3.1510072	3.081381217	3.008721726
Zm00001d042305_T001	4.581147122	4.760295422	4.765347449	4.409408995	4.457763686	4.482316853
Zm00001d021545_T006	1.459025508	1.300272527	1.035509897	0.277046839	0.627486375	0.611537848
Zm00001d021545_T001	1.172897736	0	1.791254384	1.447035869	1.479693102	1.627539604
Zm00001d020617_T001	2.497266946	1.896106351	2.790938188	1.951053585	2.007426601	2.083211666
Zm00001d039608_T001	2.771442774	2.69717398	2.555044794	0.381345399	1.081036193	0
Zm00001d039608_T005	0	0.617661462	0	1.785670649	1.950124217	1.864879462
Zm00001d039608_T041	0.976371702	0	0	2.026882193	2.55856496	0
Zm00001d039608_T045	1.89740773	0	2.103691329	0	2.095405294	0
Zm00001d039608_T014	2.369648222	1.427803524	2.477134596	2.35755116	1.601984076	2.897052766
Zm00001d039608_T022	1.420251164	0.315072302	1.624057367	1.370316634	1.729443137	1.917593298
Zm00001d051396_T007	0.559727716	1.003819524	0.409148679	0.638059005	1.237407693	0.481452917
Zm00001d051396_T016	0.935042532	0.258872244	1.263187083	1.56843396	1.366789956	1.673895124
Zm00001d051396_T021	0.285999889	0	0	0.627565751	0.659541006	0.438549426
Zm00001d049628_T001	2.804413362	2.306234821	2.798643029	2.560011041	2.432420433	2.793179038
Zm00001d049628_T002	1.776720951	1.023699627	1.106616999	1.954993203	1.405068194	1.544409713
Zm00001d002085_T001	4.609784565	3.793011896	4.518256392	5.185732654	5.064712116	5.049281508
Zm00001d048513_T001	4.586942983	4.352501271	4.418184685	4.783600917	4.476160146	4.602801051
Zm00001d024528_T001	1.267840185	1.054051042	1.40747186	1.979873526	2.064665259	1.613929047
Zm00001d032688_T001	3.295528497	3.163249943	3.678818307	2.429806347	2.73491096	2.581790377
Zm00001d041960_T001	4.128173611	4.859298022	4.299185521	3.782573585	3.295209175	3.308527032
Zm00001d030833_T001	2.56957421	2.244343054	2.551268923	2.615656648	2.568198504	2.710509793
Zm00001d040147_T003	1.849725902	0	1.488493415	1.423802961	1.970598584	0
Zm00001d040147_T001	1.261633711	0.569802152	1.025719138	1.385593383	0.508963035	1.489523412
Zm00001d046112_T002	2.421988852	2.167166498	2.530057254	2.550528372	2.334179805	1.861871618
Zm00001d046112_T003	4.61243572	3.783750612	4.499282855	4.244729769	4.195068325	4.254921726
Zm00001d021490_T001	0	1.425670376	1.736304833	2.562891537	2.617227475	2.513725449

Zm00001d021490_T004	1.109839929	0.332957645	1.284384427	1.003192036	1.356107179	1.3492945
Zm00001d013049_T002	2.081820559	1.360508117	2.792891159	3.63814765	3.232971977	3.523999967
Zm00001d013049_T006	0.77112918	0.217950333	0.516162436	1.598392363	1.378647563	1.416885129
Zm00001d013049_T005	0.38016201	0	0	0.958454441	1.957107801	0.933709348
Zm00001d014568_T025	0	0.27164798	0	1.771098747	1.020492956	1.304604493
Zm00001d014568_T007	0	0.318607231	0.503785294	0	0.929384259	1.003009927
Zm00001d014568_T037	2.116752329	1.268421218	2.145452475	1.739177137	1.883487488	1.38746732
Zm00001d014568_T028	0.213597379	0.058816589	0	0.645909126	0.372815063	0.676312548
Zm00001d014568_T035	0.142140231	0	0.866548001	0.435927236	0.378507184	0.54071075
Zm00001d006822_T001	3.294112831	2.766795705	3.019884419	3.136639944	3.116768794	3.127978561
Zm00001d029889_T034	3.498138129	2.219396855	3.589904035	3.722636187	3.882294315	3.907851303
Zm00001d029889_T003	0.746343727	1.619100256	0.320314837	1.741612381	1.420363284	1.183179046
Zm00001d029889_T032	1.594096084	0	1.92086912	2.357217261	2.588337342	2.272213146
Zm00001d029889_T038	1.063640993	0.522009534	0.839385619	1.35233311	1.138209616	1.182905191
Zm00001d036152_T001	3.684700602	3.95465762	3.66694856	4.315729749	4.414460171	4.366800655
Zm00001d053641_T002	4.040920653	4.227652445	3.680559985	3.595317463	3.942678799	3.575657834
Zm00001d053641_T003	4.913559932	4.527476381	4.955674501	4.947866652	4.937159994	4.906963306
Zm00001d053641_T001	1.005973359	2.706731444	2.318610991	1.906914448	1.032176327	1.19352197
Zm00001d028352_T001	0.691276869	0.558912171	0.641669023	1.312018023	1.132263594	0.350283373
Zm00001d028352_T003	0	0	0.6159878	0.335389497	0.504915257	1.179325685
Zm00001d028352_T007	2.6199367	3.377640888	0	0	2.325541844	0
Zm00001d028352_T002	2.815480339	2.594704037	3.29914485	3.517970036	3.115734605	3.268531558
Zm00001d018977_T002	0	0.124717213	0.198670158	0.838005387	0.45339747	1.308063754
Zm00001d018977_T021	0.369718846	0.502029096	1.177920343	0	0.830227736	1.38260473
Zm00001d018977_T091	1.91139822	2.23794178	1.413263156	0	1.727969233	1.666453026
Zm00001d028616_T001	3.927432859	3.042871754	3.817927828	3.319314162	3.006649363	2.977125845
Zm00001d014876_T001	3.198121016	1.944680815	2.940412411	4.283023882	4.399665149	4.316525631
Zm00001d014876_T045	2.010443648	1.146306561	2.062695428	1.283714389	1.463963474	1.700986507
Zm00001d014876_T015	4.079293279	0	3.868110382	4.250344053	4.005315723	3.930457664
Zm00001d014876_T003	1.865218076	2.007182582	2.556047753	2.498520231	2.305737979	2.663879012
Zm00001d014876_T037	2.191994952	1.772112843	2.161778388	3.265836972	3.445175657	3.582379087
Zm00001d014876_T044	4.85695363	4.302838854	4.761545595	3.910396601	3.943243035	3.969432986

Zm00001d006167_T004	0.746670493	0.351769652	0.738800689	0.824173956	1.035626021	0.973955057
Zm00001d006167_T003	4.237492209	3.727937876	4.392525499	4.49288371	4.537314458	4.653629414
Zm00001d006167_T001	1.165779478	0.712503339	1.140124224	1.116195057	0	0.457383112
Zm00001d034714_T001	7.215502578	6.01884536	7.157325863	7.24891655	7.282840253	7.210170967
Zm00001d052620_T011	0	0	0.473165549	0	0.754594231	0.57195052
Zm00001d052620_T005	2.675488974	1.910822444	2.661021457	2.949071329	2.628712947	2.556262389
Zm00001d052620_T016	1.628061052	0	1.717942869	3.025774053	2.483379582	2.030449088
Zm00001d052620_T015	4.737785443	0	4.927328079	4.116648793	4.59564741	4.279640591
Zm00001d052620_T012	3.043836869	1.897209441	2.388262015	0	0	1.881842352
Zm00001d052620_T007	2.821525326	2.016576903	2.52519692	2.807938683	2.914243038	2.92680805
Zm00001d052620_T013	0	0	0	1.2456569	1.499389254	1.832263799
Zm00001d052620_T001	2.391789712	4.552007927	1.089574758	0.890045834	0	1.401314808
Zm00001d052620_T003	1.379121865	0.342683308	1.579037223	1.008420196	0	0
Zm00001d053822_T002	6.001887388	5.402384927	5.91681633	6.046215159	6.106370663	5.997061734
Zm00001d022197_T001	5.609464751	6.529037494	5.510683098	5.195958567	5.330165764	5.413073862
Zm00001d042642_T001	0.995831809	1.865531669	1.338645758	2.520687253	2.087615929	2.185666774
Zm00001d000169_T002	0	0	0.160819532	0.30143898	0.346828707	0.629546868
Zm00001d000169_T007	1.05444009	0	1.017603411	2.718670026	2.444813343	2.636934296
Zm00001d000169_T029	0	0	0	0.978128986	0.519886057	1.03434744
Zm00001d000169_T016	2.893719054	2.045909115	2.115211404	3.171497964	3.919983097	3.89257735
Zm00001d000169_T032	3.960925425	3.391623663	3.891293966	3.45433029	2.837802597	3.020946436
Zm00001d000169_T014	0	0.947800042	0	0.784266097	0.64684835	0
Zm00001d000169_T009	0.799336025	0	0.740842097	0.494488319	0.675423934	0.866888202
Zm00001d000169_T027	2.57644208	0	3.164798022	2.943867517	2.177795995	0
Zm00001d037489_T002	7.027827426	6.925460728	7.128740895	6.885753482	6.957641723	6.720761353
Zm00001d037489_T005	2.244944888	4.178700063	1.64482028	2.308336991	1.053643819	1.37980891
Zm00001d037489_T004	5.876772042	7.172599632	6.124715394	4.503403885	5.102654017	5.18887349
Zm00001d028471_T002	0.622561199	0.617827875	0.842193631	0.795281536	0.856097298	0.62645222
Zm00001d053613_T005	1.396241707	0.699650237	1.511010008	2.094903542	2.155784199	2.190582624
Zm00001d053613_T006	1.844525055	1.202319919	1.426032294	2.370634684	2.338282064	2.297836449
Zm00001d053613_T002	1.412699161	1.386654807	2.027347648	2.069658865	1.486039842	1.869092417
Zm00001d053613_T001	4.053292705	3.467604116	3.915551399	3.997948472	4.249180974	3.91087913

Zm00001d053613_T003	1.068084672	0.979110023	1.597905356	1.208154512	1.625081547	1.261701702
Zm00001d018657_T004	1.459912087	0.945277329	1.447897586	2.787616551	2.506988027	2.579456783
Zm00001d018657_T006	0.364941043	0.153176249	0.763659722	0.80278796	0.61095041	0.564479629
Zm00001d018657_T001	0.315948734	0.588055207	0.164272685	0.270332782	0.35440138	0.52901631
Zm00001d018657_T033	3.104966276	2.490947954	2.865738912	0	0	0
Zm00001d018657_T016	1.523732059	0	1.722717818	3.464429235	3.391495814	3.005094887
Zm00001d006154_T001	1.1283165	0.898556647	1.137517295	1.15604836	1.1441593	0.990881544
Zm00001d037446_T001	0.385915812	0.131144294	0.292916002	0.368767232	0.339974247	0.510231781
Zm00001d037446_T004	5.686650196	4.846751995	5.735517682	5.156635711	5.040890108	5.069651218
Zm00001d037446_T007	0.521191481	0.354922638	0.579201392	0.782048685	0.817493102	0.767798849
Zm00001d000194_T001	5.613099516	5.774124137	5.705347787	6.311200445	6.255985201	6.438771264
Zm00001d034669_T001	4.257776582	3.625713051	4.054340059	4.13812252	3.992487477	4.26820523
Zm00001d048836_T001	3.219454165	2.492829182	2.956571171	2.508077191	2.624384725	2.172789272
Zm00001d008468_T001	2.668763382	2.02954441	2.822673866	2.166525215	1.782317135	2.209948008
Zm00001d038341_T001	1.784674835	2.623574502	1.433732912	2.592995144	2.562614882	2.152429094
Zm00001d045808_T065	0.833489241	0.351447416	0.338889882	0	0.656732491	0
Zm00001d045808_T037	0	0	0.097802244	0.7392977	0.465423916	0.935775786
Zm00001d045808_T025	1.655616527	1.06298997	2.338481465	2.225498907	1.071302059	0.994505144
Zm00001d045808_T031	1.452012863	0	0.810266227	0.910907616	0	1.399381124
Zm00001d045808_T066	0.925222432	0.779544659	0.793888393	1.166736009	1.260561673	0.807497536
Zm00001d045808_T001	1.635779461	1.288336707	1.01853519	1.567486551	1.95517701	2.402643328
Zm00001d045808_T008	0	0.456810354	0	1.374306704	1.811602141	0
Zm00001d021087_T003	2.833070618	1.716234131	2.866203367	3.312831707	3.272977651	3.34451124
Zm00001d029038_T001	1.981246229	1.822452798	1.419601452	2.621286241	2.844713245	2.637844146
Zm00001d043984_T001	4.522082059	4.160096501	4.247498514	4.212949261	4.026808822	4.034838483
Zm00001d046044_T001	2.657200957	1.994487041	2.34473932	2.482546292	2.248311067	3.015504706
Zm00001d013073_T002	3.935963566	3.348069923	4.021725071	2.581050624	2.491351336	2.914929169
Zm00001d013073_T001	2.718641972	2.060659275	2.909373356	3.036731545	3.241976516	2.998167155
Zm00001d028929_T001	0.920633256	0.431066633	1.064519374	0.897028944	0.804493902	0.702216911
Zm00001d049387_T001	2.963333732	3.290813042	2.638398031	2.336863492	2.474476539	2.731170422
Zm00001d007357_T003	5.068748231	4.406227459	5.142465067	4.263342524	4.307760598	4.713026269
Zm00001d007357_T001	2.687585026	0.676396505	2.276206013	2.625139776	0.977824289	0.838884016

Zm00001d007357_T005	2.218117158	1.555593845	1.928064032	3.066952482	3.198618773	3.137459259
Zm00001d053620_T008	1.225301463	0	0	1.099431219	1.355142593	0.908278762
Zm00001d053620_T042	3.326351966	0	3.574065531	2.505984651	2.992593158	2.294803932
Zm00001d053620_T005	0	0.477087697	0.86780318	0.802543972	0	0.194559886
Zm00001d053620_T006	2.789794135	1.317675694	2.868523604	2.494235614	2.249919487	2.602447141
Zm00001d053620_T026	0.533014073	0	0	0	1.141776093	1.027509372
Zm00001d053620_T024	1.772887302	0	1.886229773	1.960224032	1.82512497	2.025971732
Zm00001d053620_T031	0.116638896	0	0	0.856745918	0.7822106	0.565472397
Zm00001d053620_T039	0	0	0.346488345	1.010686043	0.899606441	0.979712957
Zm00001d053620_T016	0	1.631924255	2.209246227	2.805273261	3.005562933	2.75119311
Zm00001d028887_T001	7.476606556	6.597377466	7.403667468	7.616214578	7.634650585	7.640869781
Zm00001d012086_T004	4.516291805	3.516612279	4.409466158	4.99948234	5.007171905	4.978412521
Zm00001d012086_T011	1.631192309	0	1.444442854	1.398707716	0	0
Zm00001d012086_T010	0	0.49449856	0	0	0.569519285	1.018814322
Zm00001d012086_T002	0.507814933	1.708340438	1.558609529	0	2.107951279	1.177055425
Zm00001d012086_T012	0.199117615	0.170032719	0.650584441	1.007406507	0.818674711	1.059915217
Zm00001d010834_T002	3.828206367	2.894278216	4.117732522	4.007655511	3.948851148	3.841189082
Zm00001d010834_T003	0.621814173	0.268840375	0.512351299	0.578809287	0.613919148	0.906394223
Zm00001d007770_T001	5.018063143	4.210096586	5.092551661	4.727982407	4.641454297	4.567943059
Zm00001d020325_T001	4.507159017	3.524062923	4.506314913	4.821517163	4.832044094	4.793171493
Zm00001d017968_T001	5.471604045	7.934041703	5.376735391	6.141362261	5.788832739	6.389473767
Zm00001d036300_T001	4.908392573	4.132938458	4.81466121	5.104779801	5.076317386	5.09605586
Zm00001d040188_T001	5.075742046	4.301926187	5.149188749	4.376039307	4.444769753	4.348146356
Zm00001d045044_T001	6.98739425	6.27335576	7.028814763	6.744565101	6.760510335	6.73490259
Zm00001d010269_T001	2.595417488	1.82834927	2.544608285	3.316132561	3.165400355	3.205441311
Zm00001d000086_T001	1.233655566	1.003879956	1.632091824	1.246535781	0.964344657	1.345192533
Zm00001d000113_T001	1.594441538	1.78494489	1.917787277	1.787823176	0.987022472	1.729707201
Zm00001d002058_T001	3.438918771	2.61964049	3.28226068	3.237790689	3.288581077	3.004962442
Zm00001d042954_T001	9.055261234	8.342414384	8.722467169	8.268158374	8.351966677	8.445347186
Zm00001d005933_T001	0.830229359	0.403514241	0.884274377	2.183666582	2.181107959	2.232741177
Zm00001d010040_T001	2.938801983	3.318439194	3.334896878	3.007468403	2.411291461	3.376615037
Zm00001d016692_T001	3.875122937	4.337847079	4.206906126	3.808945955	4.13219753	4.190712116

Zm00001d034627_T001	4.87719548	3.119223191	4.821684248	4.457957875	4.528116623	4.55953201
Zm00001d034627_T003	0.570967058	0.205916693	0.348642617	0.376609342	0.303902295	0.330962183
Zm00001d049155_T014	1.66263437	0	1.981174245	2.57687521	1.71033588	2.67127616
Zm00001d049155_T020	1.826632682	0	1.316735446	2.411215521	2.234487219	2.04086263
Zm00001d049155_T002	0.705337446	0.154937002	0.143881854	0.372435086	0.419268125	0
Zm00001d049155_T005	0.406652038	0.441569529	0	0.726224373	1.481466867	0
Zm00001d049155_T018	0.366219805	0	0	0.588479348	0.663857385	0.712117664
Zm00001d049155_T030	2.78774546	0.186993627	3.368120737	0	3.114305884	3.23528193
Zm00001d049155_T024	0.107876646	0.188203403	0.372845145	0.415664575	0.456206873	0.521505089
Zm00001d049155_T025	4.932704966	3.962163543	4.908525253	5.462518599	5.315360194	5.345044921
Zm00001d029129_T002	0.797458795	0.704132726	0.697445574	1.104522521	1.541868655	1.489847062
Zm00001d029129_T003	2.385566879	1.87346439	2.349560606	2.725110944	2.10595392	2.164318066
Zm00001d029129_T006	2.76305585	1.925530087	2.485076416	2.775572094	2.936485865	3.396158667
Zm00001d029129_T001	0.234211894	0.211230295	0.345717696	0.735133074	0.659884382	0.601124078
Zm00001d031008_T002	0.392888892	0	0.412196201	0.489328159	0.318558644	0.626286799
Zm00001d031008_T001	4.715449954	4.247417924	4.961302251	4.438635052	4.370672762	4.474724878
Zm00001d030220_T001	4.597798534	4.06852576	4.592120439	4.137328996	4.191382224	3.928439305
Zm00001d047719_T001	2.572099728	1.723399976	2.382277133	2.280654088	1.967368947	2.296658563
Zm00001d032884_T001	3.77258975	3.596759743	3.786925426	4.278523055	4.375941129	4.273404811
Zm00001d044434_T001	4.311528473	3.626902594	4.481942766	5.521842243	5.427703813	5.47600998
Zm00001d034892_T001	1.009452522	0.861009662	0.900384203	0.875099831	1.048245952	0.731442208
Zm00001d012937_T001	3.508343457	3.952643601	3.174995809	3.007156742	3.227905079	3.060287294
Zm00001d041601_T001	2.797943268	1.8460628	3.056474455	3.623812678	2.879849833	3.558464185
Zm00001d041601_T002	2.845852511	2.049430728	2.12842176	0	2.840905089	0
Zm00001d038229_T002	3.08969339	2.820021866	3.577438496	3.424463147	3.368855076	3.439507584
Zm00001d038229_T001	1.728521797	0.23393345	1.257958622	0.941687029	1.866225132	0
Zm00001d018187_T001	6.124106771	5.454297484	6.152493975	6.39594558	6.450849618	6.347436213
Zm00001d044937_T001	1.510942683	1.06085811	1.723321333	1.222009438	1.286988163	1.613867771
Zm00001d029754_T001	0.530490302	0.528340269	0.318894093	0.744065487	0.417005075	0.45623211
Zm00001d029754_T004	1.796531307	1.095939944	2.063821474	2.14457798	2.526113144	2.272770628
Zm00001d026017_T001	3.077340771	2.739337022	3.300298595	2.057863006	2.055212317	2.206660726
Zm00001d002476_T001	3.291091923	2.814894291	3.482623603	2.162076268	2.691249618	2.344777377

Zm00001d039266_T005	0.534549725	0.074047757	0.135012069	0.972273568	1.510479928	1.104673473
Zm00001d039266_T002	1.432086045	1.030509827	1.715444023	1.5247332	0.539446701	0.758135144
Zm00001d039266_T001	0.895661699	0.628223937	0.89004272	1.161169864	1.582424011	1.761342725
Zm00001d039266_T003	1.772999171	1.714135154	2.094547212	1.645948772	2.558534594	2.236285345
Zm00001d039266_T004	0.478717144	0.2177655	0.388279927	0.256106085	0.580932721	0
Zm00001d039266_T015	3.70755527	3.153270467	3.882771859	3.664807638	3.798917842	3.863071582
Zm00001d045837_T002	2.852636719	2.329490181	3.053545818	3.419506127	3.442856733	3.360606017
Zm00001d045837_T001	4.454294029	5.56081594	4.224207555	4.420196179	4.165374063	4.325123369
Zm00001d032950_T003	0.940695235	1.525886961	0.078850264	1.579014045	1.581997121	1.736102604
Zm00001d032950_T002	7.196432017	6.474566305	7.269829982	7.562433885	7.515096887	7.491820788
Zm00001d043616_T001	3.922599672	3.373890937	3.886837975	3.570781552	3.825417988	3.623903725
Zm00001d038839_T002	7.590078091	8.544981204	7.656427337	7.48639722	7.466556974	7.435579392
Zm00001d019311_T001	3.335318745	2.791490288	3.568907674	3.93477322	3.978324331	4.115766893
Zm00001d032078_T001	2.3073201	0	1.617316822	2.342242962	2.062233641	2.418762594
Zm00001d034494_T001	3.647913455	3.254960414	3.637799409	5.528645171	5.441098652	5.567989985
Zm00001d039787_T001	2.598592217	0.405248499	2.708680339	3.349301436	3.214530195	3.016114379
Zm00001d039787_T008	2.496252948	2.426228788	2.704231903	2.112699799	2.134319197	2.091702203
Zm00001d039787_T006	2.077281972	0.959799083	1.011213656	2.610076366	2.79719979	2.991769254
Zm00001d039787_T007	0.438975175	0.033325027	0	0.714970805	0.543284042	0.91009789
Zm00001d039787_T002	0	0.325430167	0.559622984	1.012826083	0.947304082	0
Zm00001d027850_T003	0.901150731	0.813046859	1.380095499	1.128110578	1.517089706	1.100964196
Zm00001d046661_T008	0	0	0	3.161368864	3.336572755	3.211359738
Zm00001d046661_T005	2.854484843	1.469676067	2.920551704	3.569420487	3.463560338	3.701586759
Zm00001d046661_T002	5.329427714	4.71972257	5.225836541	4.790125599	4.867957431	4.720173324
Zm00001d046661_T007	3.764653054	2.795085953	3.743660049	3.987713869	3.997043867	3.740064599
Zm00001d046661_T003	7.405234005	6.479341456	7.314838224	6.453377649	6.409033511	6.493745456
Zm00001d013077_T003	2.825380436	1.749822134	0	1.327839673	1.97863423	0
Zm00001d048505_T002	3.955754882	2.813160173	4.058804643	3.255678378	3.449358789	3.461585397
Zm00001d023210_T001	1.372391065	1.176641279	1.156865141	0.337609486	0.307350414	0.143431298
Zm00001d045577_T002	0.841625381	0.55680117	0.842085794	1.0958117	1.062566612	0.62814553
Zm00001d034650_T008	2.626267872	1.946958754	2.810656973	0	0	0
Zm00001d034650_T017	0	0	0	2.951953085	3.436655982	3.375048609

Zm00001d034650_T016	0	0	0	0.706761277	0.610020598	0.408719468
Zm00001d034650_T007	0.766564633	0.321292015	0.688425674	0.710416998	0.744868058	1.246138678
Zm00001d034650_T004	3.639130036	2.854400662	3.41392588	4.008160295	3.780988552	3.846492012
Zm00001d028490_T002	3.308045109	2.13883871	3.58727461	4.627357321	4.094655472	4.591400328
Zm00001d028490_T005	0.086785125	0.284227461	0.331771704	0.79530315	0.473778601	0.513459415
Zm00001d028490_T008	2.913965509	2.082559198	2.684538922	0	3.271466016	0
Zm00001d028490_T011	3.72703878	3.296472679	3.311539008	3.791975519	3.538459943	3.450559757
Zm00001d052399_T006	1.526860073	0.4806105	0.576934209	2.858130033	2.555363156	3.011672029
Zm00001d052399_T009	3.615910256	2.953581498	3.660065169	3.887748579	4.276662439	2.990748332
Zm00001d052399_T003	2.755312126	1.712279695	2.564360363	3.832617547	3.330848774	3.87685343
Zm00001d052399_T018	4.321272602	0	3.835732814	4.260750511	4.14757665	3.94026158
Zm00001d052399_T004	0.969838258	0	0	1.564807864	1.360135562	2.10898036
Zm00001d052399_T017	3.259456899	0	3.057069316	0	3.454302387	3.205841487
Zm00001d052399_T012	0.2115605	0	0.20788161	1.773708179	0.950872984	1.97343017
Zm00001d052399_T008	6.29024706	6.142633869	6.351244055	5.553342224	5.431471195	5.542565379
Zm00001d052399_T007	0.098922109	0.212665204	0.144568518	1.127246308	1.174541925	1.29396957
Zm00001d052399_T001	1.999577228	2.204222743	3.362708051	4.051974288	4.158066739	3.602981535
Zm00001d052399_T010	0	0	0.690711192	1.428243714	0	0.918579344
Zm00001d004616_T009	0.403811971	0	0.884148535	0.365065386	0.257061323	0.950183953
Zm00001d004616_T001	0	1.110141376	1.580856524	2.687030669	0	0
Zm00001d004616_T011	0.906142509	0.46295902	1.008674761	1.674645948	1.114429663	1.265186623
Zm00001d004616_T004	4.54483353	3.904370027	4.001992068	4.857836967	4.69702921	4.579161438
Zm00001d004616_T014	0	0	0.378206405	0.633277749	0.449169384	0.22522433
Zm00001d004616_T010	1.826934849	0	1.733095283	0	2.841787589	3.020931683
Zm00001d004616_T007	1.698708778	0	0	1.658243022	1.671204589	1.17847882
Zm00001d004616_T012	0.55567286	0.511356708	0	0.452930637	0.279057594	0
Zm00001d004616_T015	1.252562198	0	0.876593561	0	0.794430839	1.514038916
Zm00001d004616_T008	0	0.411677882	1.490971052	0	0.895581826	1.104711041
Zm00001d004616_T002	5.71006436	5.067833624	5.77560109	5.962217623	6.028035756	5.890855488
Zm00001d034373_T001	6.256418101	5.417312188	6.309773566	7.119403989	7.204389901	7.111406496
Zm00001d039712_T003	1.502645282	0	2.852007229	0.969549487	2.797477472	0
Zm00001d039712_T004	0	1.921747079	0.965208754	1.887324059	1.226192212	2.18913443

Zm00001d039712_T002	1.695670952	0.956911605	1.793129709	2.588562339	2.600241204	2.52066362
Zm00001d039712_T001	3.289299182	2.272156998	1.426016187	3.112531836	2.426945278	3.134085701
Zm00001d039712_T005	0.962340238	0	2.369840266	1.569135721	1.7242067	0.810112367
Zm00001d039311_T002	3.17419914	2.947024047	3.4716349	2.323359404	2.293873956	2.326193028
Zm00001d039311_T001	2.278433549	1.457396771	2.133487904	2.010328702	1.805646997	1.917623847
Zm00001d034085_T001	0.794999689	1.014420284	1.400478909	0	0	1.014900843
Zm00001d034085_T003	1.953453736	0.949504305	1.40168944	2.569367378	2.682322265	2.500385753
Zm00001d013595_T001	3.697693766	2.611943815	3.715780937	3.697458362	3.890730729	3.973316921
Zm00001d013595_T003	1.974161801	2.163767924	1.27046316	0.593368114	0.637144651	1.557931538
Zm00001d013595_T004	2.253193296	2.635750677	1.323601804	1.310089954	1.181468984	0
Zm00001d033211_T002	3.175332655	2.65964421	3.404909251	3.553587581	3.614347417	3.633996785
Zm00001d038366_T001	5.047446647	4.236221427	5.079061161	6.249185432	6.219102697	6.269657157
Zm00001d013383_T001	3.644802633	2.955033203	3.602257917	3.581845319	3.660885845	3.403875899
Zm00001d026691_T009	2.143851821	0.514298025	0	1.208375543	0	0
Zm00001d026691_T007	0.617447071	0	0	1.007273017	0.701450582	1.15412626
Zm00001d026691_T019	3.332348468	2.72028569	3.53228192	3.030156834	2.85148561	2.25509914
Zm00001d026691_T001	0	1.359796639	1.218818358	0	0	0.583083851
Zm00001d026691_T003	0.607056131	0	0.915998852	1.741680543	2.092504811	1.804090741
Zm00001d026691_T006	0	0	1.521229178	1.369553587	1.965264913	1.808970248
Zm00001d054049_T001	2.557852891	3.520913247	2.308582511	1.355143721	0.697288096	0
Zm00001d054049_T002	0	0	2.539932757	1.926626095	2.607733679	3.065460814
Zm00001d008587_T001	1.94269428	1.539282412	2.267023948	3.415617256	2.993025773	3.352774906
Zm00001d006220_T013	3.287287588	5.904131859	0	2.903401213	0	0
Zm00001d006220_T006	5.806614501	4.665828936	5.970190074	5.143070997	5.368866807	5.268445622
Zm00001d006220_T007	1.901218324	1.029519321	2.183058962	2.319245652	2.187663246	2.002598117
Zm00001d009939_T001	3.167624795	3.356285537	3.268652518	4.370585168	4.560149564	4.499716437
Zm00001d046348_T008	1.484676946	0.662486262	0.805367581	2.171963531	2.572669004	2.080922304
Zm00001d037208_T002	1.210319658	0.564996578	0.893254247	1.623561158	1.541493528	1.55016485
Zm00001d013656_T001	4.238035974	4.950045342	4.130025113	4.325788842	4.480983382	4.203029855
Zm00001d029248_T002	3.03550022	2.457595868	3.254980212	2.647252333	2.73417113	2.695097639
Zm00001d002950_T003	1.733911786	0	1.303884765	2.120723184	2.019414058	1.441423986
Zm00001d002950_T012	0	0.80326424	0.522506772	0	0.451194756	0

Zm00001d002950_T016	2.691281783	2.738444896	2.302955656	1.567856927	2.118739787	1.171005015
Zm00001d053396_T001	5.986390046	5.305288581	6.027950924	5.305504925	5.306547557	5.451505029
Zm00001d009222_T002	3.950440139	3.131757548	4.048068745	4.019694621	4.132991003	4.054900472
Zm00001d009222_T001	1.308351555	0.895940834	1.49807518	1.438133667	1.428318227	1.326796182
Zm00001d016542_T001	2.69748494	2.060476669	2.621183305	2.88325736	2.694307457	2.579598942
Zm00001d005629_T001	2.885648352	1.974155191	2.568788044	3.591752292	3.611653631	3.571667952
Zm00001d039694_T001	4.607391039	5.023661131	4.645701716	3.78269393	3.251072045	3.703909884
Zm00001d042807_T003	2.684057925	1.79306893	2.846333643	3.543035781	3.400952372	3.52667056
Zm00001d042807_T001	4.487039811	3.798132345	4.468728948	4.007708697	3.847483356	3.936939025
Zm00001d012128_T003	4.595377443	3.801062922	4.834620514	5.591520851	5.950741413	5.655306601
Zm00001d012128_T005	4.50369302	0	4.164883843	3.623488233	0	3.331305511
Zm00001d020209_T001	1.217711534	1.198108755	1.215808418	1.92506706	1.538824191	2.068607874
Zm00001d020209_T005	2.714808636	3.923276802	2.764652736	1.474342067	2.484797572	1.391814453
Zm00001d020209_T003	1.345531498	0.812409505	1.433816755	0.852967236	0.935686035	0.71397465
Zm00001d020209_T002	2.596673049	1.830609869	2.559402483	3.04842244	3.184059667	3.143070915
Zm00001d024816_T025	0.500717426	0	0.770390992	0	0.665424608	0.454724434
Zm00001d024816_T002	1.546178335	0	0	0	1.176374479	2.024002592
Zm00001d024816_T001	3.612100035	2.999275946	3.715948925	3.736627603	3.656147267	3.636581122
Zm00001d024816_T009	1.517462664	1.057721283	1.756970745	1.579029497	0	1.959142502
Zm00001d024816_T007	0	0	0	2.317354724	2.452692419	0.865090561
Zm00001d021417_T001	4.688310744	5.136704041	4.847462159	4.658801067	4.57671785	4.590935799
Zm00001d021777_T002	2.304491183	1.493908082	2.238748346	1.504318348	1.861200282	1.503430664
Zm00001d040554_T001	1.561532413	1.081260536	1.273258061	1.793254172	1.508230871	1.980278344
Zm00001d026576_T001	5.715446384	5.38573618	5.679562633	5.072107945	5.291123832	5.105267611
Zm00001d043701_T001	1.677455923	1.171890856	1.987990936	4.924403171	5.075318992	4.957898352
Zm00001d008357_T004	5.538939691	0	4.887520299	5.488040824	5.371496414	5.581677956
Zm00001d008357_T001	4.501440738	5.123946689	5.132742817	5.376152102	5.593559284	4.9949556
Zm00001d008357_T008	5.886860381	5.73689628	5.838891023	5.469853229	5.462665596	5.629333508
Zm00001d008357_T010	0.863266886	0	1.331822141	1.603462321	1.257830126	1.525258571
Zm00001d010466_T001	3.693122042	2.935725632	3.900078096	4.351873162	4.396118447	4.121643583
Zm00001d027884_T005	2.487711946	2.255095513	3.076657768	2.549668924	2.986383993	2.747057787
Zm00001d027884_T003	3.652944184	1.939194977	3.348263726	3.764315048	3.625381318	3.816192029

Zm00001d015945_T001	3.002765879	2.565714634	2.854433378	2.907598881	2.957937251	3.304589891
Zm00001d051014_T004	4.069143349	3.204068862	3.982631681	4.679951988	4.64109211	4.727776886
Zm00001d051014_T008	0	0	0.18240373	0.396852167	0.508078712	0.359729182
Zm00001d051014_T005	0.712749836	1.131727755	1.215527642	1.445227182	1.545603697	1.250786985
Zm00001d012067_T004	2.05664283	0.970536374	1.834657827	2.284406342	2.490056881	2.554179191
Zm00001d012067_T003	4.454560407	3.870701654	4.690751078	4.690169045	4.587844589	4.6427651
Zm00001d037714_T001	2.231586316	2.216105285	2.179483343	2.297033196	2.035123806	2.288729746
Zm00001d025539_T014	3.856358195	6.369444114	3.666559713	2.647287797	2.502544979	3.407054935
Zm00001d025539_T006	1.618881381	1.860959223	2.663067213	2.867299302	2.45557526	0
Zm00001d025539_T001	1.869146521	0	1.28284483	1.151825054	0	1.904141857
Zm00001d025539_T013	2.901097041	1.1626567	2.350455097	2.506567669	3.067507416	3.202436059
Zm00001d025539_T004	0.994615203	1.378186425	1.220492791	1.706403683	1.901891373	0.763222886
Zm00001d025539_T005	3.035381622	2.064906988	2.852959248	2.95689674	3.13109374	3.166624993
Zm00001d025539_T002	0.767406489	0.612871445	1.442659572	1.385661298	1.631646792	1.406583471
Zm00001d025539_T003	0	1.562042112	1.495859375	1.995786594	2.711379266	0
Zm00001d021204_T002	0.895586479	0.91248202	0	1.10731493	1.081183486	1.268260709
Zm00001d014458_T001	2.340908873	1.470028155	1.973592541	3.678678273	3.426126652	3.568185125
Zm00001d014458_T003	2.99516302	2.498270784	3.250266124	2.892039684	2.926156967	2.793388198
Zm00001d010212_T002	1.21678636	0	1.442138281	1.131141001	0.880220027	1.048453829
Zm00001d035717_T001	6.295830194	5.642436399	6.348786834	6.519119998	6.685394298	6.650932711
Zm00001d010632_T001	5.774878651	6.590545164	6.022517379	5.291943234	5.228876859	5.132781018
Zm00001d010632_T002	5.705512654	6.546289373	5.713681107	5.159989734	4.969482857	5.026539466
Zm00001d041856_T001	3.057305389	3.692202987	2.496868538	2.479401315	2.697991052	2.831463217
Zm00001d020292_T001	4.49620999	3.617552155	4.457279546	4.017152978	3.968235567	4.002832564
Zm00001d038708_T001	2.157922645	1.682301811	2.202821087	3.818327755	3.864863619	4.099136196
Zm00001d042864_T006	2.026336552	2.479061081	3.693570255	3.503597051	0	0
Zm00001d042864_T001	1.235541427	0.769077725	1.695050855	3.119549409	3.033677951	3.156088822
Zm00001d042864_T004	0	0	0	2.082434869	2.327893695	3.229142741
Zm00001d042864_T005	4.709250597	5.050608307	3.942663037	4.308425243	5.01140842	4.903872593
Zm00001d042864_T003	0	1.063070759	0.987805443	1.717662606	0	1.152879769
Zm00001d042864_T002	2.912246122	1.876377459	2.686787133	3.149052996	3.397753411	2.804118834
Zm00001d043651_T001	1.483187367	0.732109398	1.23470977	1.364993694	1.354443697	1.038258468

Zm00001d043651_T002	3.228553056	2.759892763	3.338906564	4.022814136	3.872487692	3.942018133
Zm00001d051475_T002	2.539775966	3.153680351	2.503515132	1.114728153	1.248432217	0.709383283
Zm00001d031323_T001	3.747377409	2.982666542	3.293850126	3.63729318	3.60010047	3.454056629
Zm00001d036280_T002	2.463593948	1.701977034	2.445597205	2.434262054	1.96020327	1.848968795
Zm00001d036280_T003	0	0	0	1.289312168	1.863515885	1.906923681
Zm00001d004895_T001	3.532751199	3.737459633	3.554214685	3.302840608	3.41800248	3.371232651
Zm00001d052485_T011	0	0.087939361	2.123979	1.200172863	1.243141377	0
Zm00001d052485_T001	2.626407596	0.688980614	0	2.174245648	3.019514939	3.142707502
Zm00001d052485_T002	0	1.577415776	1.730391715	2.272911831	0	0
Zm00001d052485_T015	0.646431812	0.340259172	0.755571284	1.398990575	1.161549778	1.491709461
Zm00001d019698_T001	6.532390838	6.361920375	6.696924961	7.229332352	7.188595472	7.18747277
Zm00001d045788_T001	2.921970197	2.449176781	3.050122211	2.666742733	2.464013679	2.520249492
Zm00001d052058_T001	3.036465178	3.514628283	3.217323635	2.305232501	2.332114696	2.194348076
Zm00001d011087_T002	4.082049976	2.802647775	3.298806096	4.089446117	3.369113689	3.627081606
Zm00001d011087_T003	0.613968167	0.382400544	0.415376853	0.301390982	0.733736116	0.779131942
Zm00001d011087_T004	8.421386523	7.601834941	8.455953481	8.192117895	8.23910018	8.163926903
Zm00001d053541_T002	3.607468806	2.879794168	3.744340345	3.741202585	4.001269824	3.988985645
Zm00001d044923_T001	7.723191022	9.442321929	7.62399802	7.296524401	7.590851262	7.51095624
Zm00001d013193_T002	6.156621575	6.200499371	6.455021567	3.624566039	3.719334275	3.917088084
Zm00001d046440_T001	3.524345315	3.073639114	3.381828503	3.322367473	3.492628865	3.528774082
Zm00001d026149_T001	0.294206075	0.108069413	0.37991812	0.650201155	0.640780088	0.703302734
Zm00001d043696_T006	0	0.077523306	0.324864739	0.943675326	0.759048427	0.74795706
Zm00001d043696_T001	2.698656333	2.875289778	3.060203062	3.149125204	3.262009428	3.612912363
Zm00001d043696_T007	0.298835416	0.093857608	0.427909697	0	0.387447468	0.177341237
Zm00001d043696_T004	3.328893608	0	3.24667623	3.279957956	2.912006544	2.692233457
Zm00001d040597_T001	4.051682622	3.822964936	4.159197572	3.953204605	4.203036435	4.190737792
Zm00001d048669_T001	3.006405392	2.457934111	3.35831214	2.239723696	2.472424084	2.429335833
Zm00001d048669_T002	2.868167784	2.185490762	2.67163917	1.578709312	1.350966754	1.10435008
Zm00001d045509_T005	2.421094276	3.614748472	0	2.246078769	0	0
Zm00001d045509_T007	3.346511322	5.183515416	3.103986848	3.016655878	3.200565715	3.102595149
Zm00001d045509_T004	1.521269889	0.556557922	1.524752754	1.421806051	1.298372681	1.478630176
Zm00001d045509_T006	5.023418999	3.704889887	5.012545835	5.024444331	4.957471742	5.148165547

Zm00001d045509_T002	3.599843353	0	3.775833115	3.808750775	3.784483149	3.197002755
Zm00001d047174_T001	2.876583935	3.127051132	3.051750912	3.284726139	3.246258641	3.194737474
Zm00001d007327_T001	4.338557339	4.672461211	4.428697301	4.524892009	4.337490533	4.356718167
Zm00001d008737_T001	2.116590659	2.497519632	2.258967191	2.268452659	2.126768394	2.276095812
Zm00001d037251_T001	3.045685671	3.523379161	3.038606675	3.129500147	3.441541112	2.888696498
Zm00001d027355_T001	7.047335463	6.220155199	7.027774832	7.208610673	7.301544985	7.250854978
Zm00001d026079_T002	3.113130042	3.653919185	2.642077	0	0	0
Zm00001d026079_T001	4.736875531	3.473456002	4.845879852	5.04683733	5.134582226	5.179789013
Zm00001d021704_T001	3.554236177	3.200661282	3.431172565	3.608815628	3.307549473	3.60556201
Zm00001d021704_T003	2.25349558	3.489799931	2.603549201	0.810502332	1.065465467	0.689218636
Zm00001d021704_T002	0.819403325	0.35443072	0.719383235	0.761075025	1.002862351	0.57935975
Zm00001d051902_T004	2.096525333	1.839017498	3.497689512	3.66084079	4.166040128	3.035701496
Zm00001d051902_T008	0	0	0	2.946221804	2.122364602	3.238775397
Zm00001d051902_T002	2.678144714	2.061691534	2.774902998	2.850188938	2.793455414	2.103510732
Zm00001d028187_T003	0.425186424	0.291376056	0.364585879	1.301966268	1.310578611	1.38732173
Zm00001d028187_T001	4.817193403	3.906835003	4.745141633	5.059289813	4.970802037	5.130098445
Zm00001d028187_T004	0.503391481	0	0.262882915	0.890438132	0.968295757	0.79302272
Zm00001d024421_T001	2.668092114	2.252377205	2.815811282	3.179778386	3.149806614	3.097978165
Zm00001d024421_T002	0.966087103	0.693583747	0.806925347	1.148432408	1.05720209	1.123405971
Zm00001d018017_T001	6.051283279	6.36367891	6.109844525	7.067210794	7.140622253	7.150198257
Zm00001d007442_T001	3.322821424	2.59055244	3.196873591	3.158192395	3.176306973	3.144961962
Zm00001d035999_T002	5.043504445	4.452675092	5.247260872	5.034838351	5.143439584	5.116219515
Zm00001d044683_T001	4.532074224	4.374819916	4.572194583	4.463093119	4.219078354	4.418000725
Zm00001d037315_T011	0.610657546	0.161779355	0.826421169	0.666521198	0.398629469	0.58057871
Zm00001d037315_T019	0.658349518	0.05513178	0.227933258	0.717713049	0.489358991	1.310051553
Zm00001d037315_T020	1.954712238	0.942897297	1.686186294	2.302257335	2.527893318	2.283458969
Zm00001d037315_T009	0	3.440191594	2.560392146	0	2.739519139	2.904856116
Zm00001d037315_T004	0.994942434	0.360315952	0.518091939	1.195237975	1.57847118	0.793205887
Zm00001d037315_T015	4.049312834	0	3.325444415	4.073253582	3.563574035	3.608508847
Zm00001d037315_T001	0.461173342	0.216380361	0.379061962	1.140716498	1.278559331	1.056590464
Zm00001d048613_T001	1.476427314	0.912842211	1.413577294	2.011107719	1.760010543	1.912627257
Zm00001d046913_T001	2.724157576	2.133639801	2.8328726	2.201869378	2.403698557	2.136398127

Zm00001d034641_T001	1.642589294	2.473762239	1.868114235	3.472923633	3.80557849	3.082789605
Zm00001d022212_T002	1.809218916	1.710677515	1.160951158	1.18440472	0.322953775	1.00617736
Zm00001d022212_T001	3.553989671	2.775117353	3.569347083	3.165388455	3.46541203	3.400451857
Zm00001d020396_T003	0.474764133	0.235116772	0.327649428	2.026347885	1.569993614	2.308020055
Zm00001d020396_T004	4.836618229	3.932302073	4.893817908	6.308507775	6.329741759	6.265205136
Zm00001d014192_T001	3.564979634	2.884245458	3.430394475	3.584261301	3.569867636	3.392349437
Zm00001d002396_T021	1.849947233	0	1.526236615	1.191724345	0	0.777075848
Zm00001d002396_T012	0.271600175	0.665403687	0	1.103747383	1.62265209	1.075844069
Zm00001d002396_T005	0.69588338	0.571004933	0.693588207	0.167339942	0	0.301448345
Zm00001d002396_T027	1.51690924	1.007565817	1.652168436	1.244492547	0	0.820643001
Zm00001d036654_T001	4.178578491	3.454048335	4.346089736	4.120196812	4.003196264	4.124717048
Zm00001d036654_T002	4.119130956	3.536181493	4.213473191	4.949524428	4.960245072	4.73407567
Zm00001d036654_T003	1.230815998	0.549444433	0.779285779	0.968839837	1.304168142	1.258554502
Zm00001d045149_T001	1.654482744	1.303389743	1.550926269	1.036452693	1.228095238	1.374029539
Zm00001d011179_T001	5.237418631	5.115306428	5.239356528	5.161445699	5.131503429	5.231057784
Zm00001d008870_T006	1.033800035	0.96737153	1.7081082	1.232655845	1.429655596	1.694619036
Zm00001d008870_T001	2.187307886	2.813429669	2.826715652	0	3.104376753	0
Zm00001d008870_T007	3.826019309	2.767379791	3.261931375	4.020306261	3.853379028	4.068245247
Zm00001d008870_T004	0.689241004	0.279093263	0	1.097549447	1.032947164	0.498909505
Zm00001d021572_T001	0.186451115	0.296200122	0.62307836	0.84662409	0.86558947	0.671599545
Zm00001d021036_T001	2.800954346	2.28215272	2.869734038	3.195978088	3.237066368	2.909829928
Zm00001d044090_T004	0.442680271	0	0.969969365	0.410128321	1.492823287	1.347057839
Zm00001d044090_T002	1.550607172	0.912410737	1.447531591	1.640815248	1.610272481	1.806464298
Zm00001d044090_T001	4.042076602	3.424916581	4.088927282	3.957193814	4.097989033	4.090274628
Zm00001d009022_T001	1.853921016	1.376413196	1.777662941	0.95389024	1.041692966	0.896140072
Zm00001d053736_T001	0.254716177	0	0	1.589992095	1.903472942	1.526937173
Zm00001d053026_T001	0.945063781	0.770717431	0.677889753	0.706907118	0.596989511	1.212574942
Zm00001d045383_T013	0.493014992	0.482656255	0.48516918	0.35983936	0.079810215	0.079336464
Zm00001d045383_T012	0.408520576	0	0.992907619	0	1.364135356	1.101150492
Zm00001d045383_T005	0	0.321601432	0.32903172	0.594681348	0.491025971	0.64591097
Zm00001d045383_T004	3.087574518	1.966841289	2.881205115	2.910943487	3.214597646	2.859667266
Zm00001d051484_T001	2.752178036	2.28290501	3.12131419	1.579193184	1.492288532	1.720653649

Zm00001d016928_T001	0.91477345	0.439801835	0.945654144	1.437266097	1.564061546	1.308391749
Zm00001d016928_T005	4.017478523	3.323766792	3.481077379	4.703086631	4.3687967	4.483990502
Zm00001d016928_T008	4.534901456	3.728715623	4.712681393	3.926364507	4.049966051	3.902201341
Zm00001d016928_T010	3.418069426	2.140481253	3.833176399	3.970146487	4.202981132	4.279493062
Zm00001d008256_T002	4.699519762	3.772285274	4.687561008	4.892027295	5.009276039	4.905029232
Zm00001d008256_T005	1.849326378	4.061326473	0.766574809	1.482733689	1.569725365	1.998287948
Zm00001d051166_T001	2.421433914	2.082412727	2.726093336	3.864216261	3.87938377	4.138931003
Zm00001d008308_T001	2.025060342	1.525336759	2.121581755	2.778772085	2.710112341	2.817554088
Zm00001d042269_T001	2.458380429	1.75002974	2.440093765	2.349257694	2.399547923	2.074422202
Zm00001d025210_T001	0.335727915	0.238624242	0	0.944306744	0.442197214	0.461045485
Zm00001d025210_T002	2.981069185	2.217304245	3.059399236	2.931728436	3.102981232	2.868235754
Zm00001d019404_T001	3.927904795	3.551322087	3.81414473	4.069559282	4.04634027	4.080190247
Zm00001d033552_T004	0.459498768	0.459374959	0.8092819	0	0.61015576	0
Zm00001d045500_T001	2.259546083	3.098544875	3.175741109	1.658157088	1.814777627	2.089816417
Zm00001d045500_T002	5.543510638	4.936080068	5.467811686	5.253172188	5.123614842	5.090454009
Zm00001d052651_T001	5.739408316	5.086064659	5.812165931	4.042191761	4.071255887	4.209389964
Zm00001d020953_T001	3.930911207	3.231872738	4.039467	2.748803403	2.430860898	2.732383178
Zm00001d002939_T001	4.362963631	3.259914965	4.352472598	3.777017546	3.710697239	3.693144908
Zm00001d002939_T002	4.076281338	3.596431542	4.078839934	3.446205668	3.500344702	3.517087312
Zm00001d027995_T001	3.912443121	3.366205955	3.82395821	4.043343542	4.131011237	4.095392803
Zm00001d022096_T001	3.734535132	3.009018712	3.69737718	3.823007037	3.758590472	3.751012246
Zm00001d052775_T001	2.927071929	2.323457983	2.911830189	3.597013833	3.588584765	3.606113142
Zm00001d002128_T005	5.194402765	5.877815573	5.604820743	6.194097614	6.367357383	6.404275603
Zm00001d002128_T004	4.934025439	3.681313423	4.705361557	4.573731702	4.166947575	4.446075683
Zm00001d002128_T001	3.053499769	2.704298091	2.625925048	3.677101028	3.644808862	3.544531134
Zm00001d002128_T002	5.126282597	4.583731477	5.288608391	5.953392319	6.014381339	5.970404513
Zm00001d002128_T003	1.704535591	1.206134941	1.325358781	4.008301413	4.007906177	3.993274826
Zm00001d041191_T001	1.481690049	1.180529176	1.186702115	1.55367162	0.504508532	1.362959074
Zm00001d046745_T001	3.351203995	3.698318063	3.610032768	4.655034802	4.596890847	4.798232163
Zm00001d020401_T001	6.407527757	6.452752949	6.349320069	6.400180422	6.323546252	6.232947943
Zm00001d040315_T001	4.021842932	3.971914492	4.376729974	3.84559633	3.981673492	3.916126438
Zm00001d051833_T001	3.397177019	3.457549248	3.175905703	3.497038111	3.555782786	3.627498282

Zm00001d028457_T001	1.249589755	0.828343176	1.545498429	1.788787593	2.023830896	1.767334022
Zm00001d045534_T001	0.522886366	0	0.128583759	0.706798401	0.356697113	1.094038066
Zm00001d045534_T002	0.944156038	0.134606045	0.866389741	2.110537973	2.443087559	2.258882191
Zm00001d045534_T008	0.480603263	0	0.385777795	0.97787703	1.014588105	0.644612195
Zm00001d045534_T011	3.800399852	3.506026684	3.496493412	3.198005472	0	0
Zm00001d045534_T004	2.429757616	1.877573079	2.539436278	4.0909822	3.922561617	2.770871322
Zm00001d045534_T010	3.192777124	0	3.573750785	2.060311335	4.028129824	3.685345236
Zm00001d014960_T001	3.444051856	3.27198406	3.495504325	4.595231459	4.620370752	4.569323566
Zm00001d026131_T001	2.288445092	1.673575419	2.504058204	2.450358235	2.500803328	2.409804472
Zm00001d029432_T003	1.519286756	0.875945158	1.73731728	1.658551976	1.866085437	1.476680306
Zm00001d043305_T001	2.519506176	2.199167254	2.658936705	3.203161995	3.636441684	3.286423138
Zm00001d043305_T004	2.176834966	0	1.907575232	2.162551331	0	1.697093671
Zm00001d043305_T003	0.364325892	0.116406014	0.177134539	0.710405536	0.658087149	0.516609254
Zm00001d043305_T009	1.186890337	0.629362216	0.833000161	2.096272312	2.335629885	2.430675738
Zm00001d043305_T008	0.284204951	0.163287465	0.347819838	0.76902947	0.963071593	0.831480851
Zm00001d007058_T004	0.735108805	0.221238628	0.32601724	0.547980809	1.028914933	0.54586461
Zm00001d007058_T007	0.685228826	0	0.849250705	1.038729046	0.596303557	0
Zm00001d007058_T014	2.325892075	0	1.860768572	2.703752999	0	2.372504737
Zm00001d007058_T012	1.323709588	1.373938252	1.291505136	1.128924854	2.362077916	1.946754811
Zm00001d007058_T001	4.805361132	4.218965096	4.73875449	5.207910924	5.228653791	5.090417635
Zm00001d013611_T001	4.668798094	4.47661784	4.810334512	5.779911124	5.784594332	5.737083818
Zm00001d047908_T001	4.267924434	3.49712091	4.034415851	4.862081123	4.944766213	4.840685739
Zm00001d047908_T002	1.964839599	1.625287325	2.431845965	3.039717286	2.70173756	3.233029978
Zm00001d018633_T001	2.701379605	0	2.149680144	0	3.076194442	3.887782009
Zm00001d037275_T001	10.42222149	10.68455449	10.48660154	9.945821861	9.965035734	9.874580592
Zm00001d018979_T001	6.916819686	7.04313752	7.008705515	6.359718747	6.400252983	6.373232968
Zm00001d026562_T001	3.702556817	3.389218348	3.795967943	3.437880613	3.304527542	3.217629962
Zm00001d006361_T001	5.167303529	5.247787755	5.226340244	4.892734706	4.597365442	4.750555002
Zm00001d020695_T001	1.453242578	0.90675286	1.624573993	1.19179508	1.235799959	1.544307326
Zm00001d013742_T001	3.064454531	2.375003555	2.78091435	3.466295738	3.377485558	3.29211722
Zm00001d021902_T002	3.840667866	3.011995593	3.697448243	4.157853139	4.30586629	4.372700927
Zm00001d021902_T003	4.25368822	3.783730499	4.097573549	3.596256916	3.320173288	3.46492947

Zm00001d014809_T002	4.333720543	3.649979363	4.399003636	4.088972298	3.9474308	3.910172358
Zm00001d014809_T004	0.566036741	0.194484243	0.384594722	0.912771713	0.886769413	0.898636356
Zm00001d011624_T002	2.426219661	2.164160674	2.174536172	1.514851947	1.726045584	0.766623146
Zm00001d011624_T001	3.632807031	2.97284978	3.859837693	3.825569309	3.813016065	3.824077699
Zm00001d033741_T001	0.78064102	0	0.907639833	1.521080395	1.573148118	1.400690935
Zm00001d031060_T001	1.864958281	1.372513095	1.935833103	2.086711426	1.758440489	1.736515259
Zm00001d015722_T001	2.404859263	1.979958741	2.428481714	3.444548207	2.912423767	3.46838925
Zm00001d017145_T003	1.096720635	2.564975611	0.652714096	0.754593376	1.188923087	0
Zm00001d017145_T002	5.211092233	3.991436767	5.224584849	5.446874405	5.54945694	5.567647203
Zm00001d022281_T001	2.102019442	2.596268964	2.036722754	2.323061031	2.440133907	1.884787012
Zm00001d019642_T002	1.03724726	0.648596131	1.188136923	0.91696574	0.56139555	0
Zm00001d019642_T001	1.004940696	0.091308519	0.490909971	0.51771209	1.074523932	0.98350405
Zm00001d019642_T006	1.733074018	1.560681699	1.71189396	1.742890965	1.434879051	2.131749482
Zm00001d025551_T003	7.70704758	7.049101269	7.721569232	7.35192329	7.51499877	7.399536824
Zm00001d025551_T001	6.691540921	0	5.176862964	4.938720793	4.23065855	5.092622652
Zm00001d025551_T005	0.402695985	0.198453921	0.717417386	1.752048051	1.658459662	1.673274641
Zm00001d012648_T001	4.118294351	3.493682043	4.095437787	5.102865325	5.175481807	5.103227245
Zm00001d019700_T017	0	2.384931461	2.299389448	0	0	1.721261965
Zm00001d019700_T004	0	0.289546487	0.436575502	0.552851169	0.309149406	0.423544826
Zm00001d019700_T001	0.382679425	0.414224311	2.463502402	2.327969553	2.536902348	2.395470591
Zm00001d031587_T002	0.789210915	0.214903153	0	0.573976493	0.960539882	0.797219715
Zm00001d031587_T001	0.892533248	0.663330955	1.34549346	1.690275836	1.433052912	1.442308703
Zm00001d002521_T001	7.6130408	4.235910065	7.470558901	7.648980023	7.8052195	7.696863711
Zm00001d004409_T001	3.443934933	3.418299259	3.402655197	3.508700305	3.13361876	3.433668425
Zm00001d039733_T001	8.566080787	9.888429389	8.605078778	8.035264109	8.15564783	8.178421512
Zm00001d047050_T001	2.015442618	1.640756492	1.918414859	1.645344745	1.944827343	1.70343032
Zm00001d014145_T001	1.016931987	0.844318157	0.895739242	1.01491298	1.040658215	0.871459667
Zm00001d025817_T041	0.488525292	0.121585733	0.437679443	0.51731702	0.515589338	0.123059594
Zm00001d025817_T008	5.098904639	5.271491825	5.215743353	4.548038472	4.692684178	4.518207537
Zm00001d025817_T028	0.251405225	0.048558454	0.456472898	0.408569486	0.432563948	0.548606286
Zm00001d025817_T025	0	0.019310501	0	0.676821629	0.885187772	0.409930709
Zm00001d025817_T001	1.679084592	0.871470707	0.630691559	2.386189599	2.684151982	2.642364018

Zm00001d025817_T045	1.988857345	2.460761171	2.480053873	0	0	0
Zm00001d025817_T038	2.479308955	1.368326954	2.893056358	2.481205153	3.222159253	3.023593716
Zm00001d053928_T001	1.912949868	1.387379085	1.384542782	1.965663509	2.150063096	1.353127907
Zm00001d042639_T006	3.099593981	2.000795787	2.931122888	3.403256677	3.426139403	3.818577985
Zm00001d042639_T004	0.674769767	0.790537685	1.096469673	0.497726806	0.131063934	0.281327923
Zm00001d042639_T001	3.292068911	3.356890465	3.391907502	2.418687855	2.61054936	2.433688585
Zm00001d018831_T010	1.667794959	1.169084146	1.99780977	0.435272276	0.64741675	0
Zm00001d018831_T007	0	2.969522412	2.186300557	3.139198023	3.398930944	1.944977604
Zm00001d018831_T004	1.124660975	0.990625997	0.960711123	1.061564702	0.490971565	0.873488014
Zm00001d018831_T003	3.865859938	4.429577135	3.746170532	3.671197454	3.954548099	4.096509479
Zm00001d018831_T002	3.7646055	1.770533501	3.66727144	3.557672308	3.646392295	3.619336472
Zm00001d005431_T001	2.442498215	2.10870242	2.803874864	3.708120674	2.979020001	3.309950599
Zm00001d005431_T005	0.779916087	0.454344373	0.534391352	0.524601326	0.821037481	1.217017244
Zm00001d005431_T007	2.392801537	0.983838188	2.025187234	1.716930333	2.851231327	2.336019965
Zm00001d047895_T001	0.636969318	0.402163324	0.643460953	0.910464818	0.880263135	0.528135194
Zm00001d045757_T001	3.440125533	2.567248263	3.387216527	3.172505597	2.953161108	3.175899637
Zm00001d039904_T001	2.46259578	3.312719622	3.004461661	2.450749903	2.906950418	2.410448299
Zm00001d039904_T011	3.109401014	2.659164204	3.041288548	2.874888029	3.064400206	2.778235495
Zm00001d039904_T006	6.270000487	5.300509356	6.263347594	6.417485366	6.410113376	6.40975579
Zm00001d039904_T004	1.185033664	0.331433503	0.955934687	1.743617075	1.690772417	1.68849013
Zm00001d049533_T001	0.618170044	0.305823472	0.550265345	0.826526929	0.84135003	0.866073169
Zm00001d018412_T001	5.314197912	7.969358948	5.110950242	5.935455363	5.489841861	4.928154969
Zm00001d027699_T001	5.138092818	5.019818384	5.160394163	4.864807026	4.87610198	4.736764883
Zm00001d027699_T002	0.244806704	0.13221489	0.355717701	0.339902438	0.233987426	0.038915127
Zm00001d037240_T005	1.94899249	1.585948967	2.303515122	2.022331945	2.382536118	2.018599637
Zm00001d037240_T003	1.010093053	0.598666291	0.722618155	0.810132937	0.765430359	0.601832461
Zm00001d037240_T006	0.537018685	0	0.620230736	0.649009285	0.598657717	0
Zm00001d032316_T001	5.599463187	4.801707066	5.605292233	5.677265884	5.625012061	5.637339411
Zm00001d052731_T001	2.699987529	1.777208438	2.915348417	4.70093004	4.863527779	4.871618111
Zm00001d034615_T001	4.313655452	3.996131732	4.086750568	4.359793407	4.43306814	4.511103777
Zm00001d017555_T002	6.114433848	4.48839675	6.145683018	5.876401453	5.954128316	5.799774418
Zm00001d017555_T008	5.084878273	6.269140596	4.592415954	2.914252416	0	3.895008235

Zm00001d021980_T001	0.643798019	0.444872697	0.564531332	0.698894986	0.525155818	0.926384339
Zm00001d023799_T001	0.326329097	0.491250758	1.106388524	1.667686887	1.59674436	2.088957378
Zm00001d053450_T062	0.319336997	0	0.253603286	0.596255843	0.603040188	0.779529531
Zm00001d053450_T032	1.190372126	0	0	2.460274955	2.800469434	0
Zm00001d053450_T085	1.892266278	1.27671938	1.855281328	2.240008965	0	1.448411514
Zm00001d053450_T065	0	0.882831614	1.403330447	0	0	2.198193954
Zm00001d053450_T029	0.375120637	0	0.107188363	0.393276702	0	0.634367438
Zm00001d053450_T089	3.010594568	2.041117845	3.278431319	2.419316942	2.841520118	2.907022548
Zm00001d039944_T001	1.104048092	1.127699305	1.509796156	5.923545748	6.023441378	6.159008347
Zm00001d029906_T001	9.303713428	8.820291335	9.33456515	9.039015962	9.03275419	9.056935644
Zm00001d036063_T001	1.283432299	1.664080923	1.3985151	1.883874145	1.608092921	1.994262912
Zm00001d042536_T001	6.137458152	5.671468065	6.149842639	6.996774882	7.029587111	6.967659561
Zm00001d048686_T001	2.473683283	3.203914182	3.023176383	3.066249213	2.722859433	2.685075842
Zm00001d017353_T001	6.58475113	6.190154977	6.664936882	6.903920867	6.794491105	6.786339516
Zm00001d028570_T006	4.456707273	3.324731843	4.191403071	4.245162484	4.478296492	3.761599844
Zm00001d028570_T007	2.332807594	0	2.246119215	3.196009259	3.121355472	3.129936781
Zm00001d028570_T002	0.594592499	1.112323815	0.199764674	0.155413774	0.909021133	0.55713851
Zm00001d028570_T010	0.974231198	0	0.355781964	0.787775962	0.835437436	0
Zm00001d028570_T001	0	2.405899534	0	0.523005847	1.090842593	1.877062587
Zm00001d028570_T005	2.070752017	0.559549569	1.972940372	1.635998573	1.49953927	1.369019158
Zm00001d028570_T009	1.332560148	0	1.953727498	3.589287223	3.431497404	3.758030754
Zm00001d016512_T001	3.589337925	2.680453766	3.740067406	4.123864231	4.184596018	4.163146952
Zm00001d016512_T002	0.259108545	0.575884454	0.872294519	0.325932072	0.938695297	0.891255854
Zm00001d005871_T002	1.59227236	1.106521861	1.802662663	0.509271204	1.115715797	1.278685994
Zm00001d037291_T003	2.524911186	0	2.604819528	2.643508978	2.98505298	2.361898589
Zm00001d037291_T001	3.920783676	3.037455321	3.969272172	4.5114354	4.347876093	4.45580794
Zm00001d051756_T008	1.552776425	0	1.235931657	0	0	1.284125572
Zm00001d051756_T004	0	1.116886375	1.092605951	2.536537614	2.517374473	1.993009464
Zm00001d019985_T001	1.069686358	0.803749454	1.253006568	1.035212859	0.826405711	0.540106645
Zm00001d048045_T002	2.735834078	2.645151956	2.410770104	2.450012138	2.338117159	2.008917095
Zm00001d048045_T001	1.842776941	1.028636335	1.508966584	1.387525221	1.631519681	2.00265788
Zm00001d021245_T001	3.037341629	3.462771654	3.337791287	1.24877223	1.504691572	1.377957744

Zm00001d005851_T001	0.97272647	0.535801153	0.438691002	1.033440622	1.068304846	1.135540123
Zm00001d010275_T001	2.574202045	2.718326771	2.353734584	2.295107618	2.547522382	2.887477505
Zm00001d042088_T001	3.220254258	2.485314499	3.212135064	2.428977422	2.643495817	2.383326067
Zm00001d008520_T001	0.27216775	0.094602272	0.15530755	0.937874664	0.681014705	0.764093028
Zm00001d008520_T005	3.911108059	2.952486216	3.716539035	3.976791248	3.707367084	3.981087274
Zm00001d008520_T009	0.993705516	0.131881813	1.007180428	0.68368388	0.738332909	0.505174484
Zm00001d008520_T002	1.583951779	1.332202651	2.122895317	2.608102622	3.061462226	2.112213037
Zm00001d050884_T005	2.40835318	1.988762113	2.289052129	2.429416718	2.707944154	2.322148522
Zm00001d050884_T001	3.278111907	2.777237059	3.413486986	3.520600641	3.608040271	3.512451176
Zm00001d028017_T001	2.554564049	2.693308972	3.211591959	1.340696136	0.95062294	1.248697555
Zm00001d007522_T026	3.664129633	2.388099142	3.904671082	4.954733358	4.943865361	4.733444588
Zm00001d007522_T025	0	0	0	1.080440731	0.977594992	1.197499955
Zm00001d007522_T002	1.839454556	1.536101653	1.576161721	4.130361255	4.139930782	4.51498705
Zm00001d007522_T009	1.324982219	0	1.152985523	2.881426173	2.656953704	2.463592379
Zm00001d007522_T021	0.424439497	0.435790721	0.354447647	1.786047615	1.54319692	1.461541132
Zm00001d007522_T028	0.89465947	1.179695117	0.555289033	0.931744318	1.042116887	0.992070839
Zm00001d003848_T003	3.011498859	2.065395152	3.168627596	4.709661078	4.597430879	4.739757769
Zm00001d003848_T001	3.430473293	3.01561032	3.644135699	4.92527224	4.952750238	4.857845525
Zm00001d050440_T002	1.803438671	0.672642579	1.207850382	2.091547518	1.813564091	0
Zm00001d050440_T003	4.824135759	4.000020017	4.694915118	5.069887432	5.107929986	5.064582704
Zm00001d050440_T001	3.266244435	3.698831105	3.228105708	2.751754845	2.7491618	3.253400575
Zm00001d003669_T001	4.732200428	4.417523577	4.853428784	6.00592965	6.096894496	6.100042136
Zm00001d047843_T001	1.438933138	0.857052596	1.380876813	1.242701273	2.07349739	1.096175478
Zm00001d047843_T002	3.703130608	3.071169031	3.826675388	3.956765462	3.866574013	3.961225931
Zm00001d038878_T001	3.034791129	2.625732218	3.064006762	3.457637365	3.320459839	3.45194365
Zm00001d010443_T001	2.48750127	1.860474999	2.553831821	2.447028196	2.592703396	2.259524993
Zm00001d031129_T001	2.581745313	2.309742267	2.782194242	2.782401226	2.657440621	2.712328565
Zm00001d007044_T001	3.138224363	2.352726892	3.234230445	3.673844934	3.786026699	3.791949584
Zm00001d001864_T004	0.781086887	0.554966972	1.251279174	0.63526126	0	0.610622587
Zm00001d001864_T003	0.649341378	0.239930781	0.482060393	1.002261815	1.070883195	0.990143093
Zm00001d001864_T005	2.204271267	1.531978254	2.062620834	1.760866448	2.097967044	1.470633692
Zm00001d051671_T024	4.528064099	3.564747758	4.57121948	4.229834606	3.999790343	4.02206628

Zm00001d051671_T028	1.152573494	0.495567258	1.51866203	1.806036934	1.880512352	1.649080586
Zm00001d051671_T016	1.408440685	0.54544159	0.799651008	1.155039366	0	1.175265899
Zm00001d051671_T025	3.455288156	0	2.68825736	2.878676303	3.223992719	3.226943122
Zm00001d051671_T044	0.56734295	0	0.150468693	0.944479173	1.120612053	0.542035127
Zm00001d051671_T042	0	0	0.480793496	1.256827704	1.155233707	1.021591301
Zm00001d051671_T010	2.80128305	3.00910294	3.193729014	4.071817576	3.927125989	3.844264519
Zm00001d034886_T001	5.630012271	4.798518511	5.705739174	5.465070277	5.547007711	5.51608274
Zm00001d038803_T001	3.43380821	2.720731125	3.530309506	3.436185031	3.459410765	3.325012307
Zm00001d033544_T002	1.014964382	0.388914698	0.828051463	1.902866217	1.708330726	1.67828649
Zm00001d033544_T001	0.219601613	0.314752202	0.417763419	1.258515307	1.603987797	1.615963325
Zm00001d046404_T001	6.935732962	6.748712998	6.658944885	5.635951428	5.588659595	5.790339321
Zm00001d016714_T001	2.324221023	2.010253857	1.753933512	3.314466981	3.338226432	3.448990258
Zm00001d016714_T002	3.0297179	2.844005143	2.71654562	3.815233158	3.960334699	3.630120959
Zm00001d016714_T003	3.435974693	2.651101072	3.600030038	4.853676044	4.734774262	4.740729756
Zm00001d019873_T001	4.30913929	4.469687918	4.065835598	3.961553229	3.838782082	4.019939112
Zm00001d052865_T001	4.66492929	4.29777212	4.818651655	4.236646982	4.252290529	4.269811154
Zm00001d015612_T001	5.275536559	5.265402614	5.309158066	5.511268744	5.250765387	5.470274039
Zm00001d030675_T002	5.039321001	4.30872098	5.175936513	5.340124445	5.420544113	5.305127451
Zm00001d038023_T001	4.596771548	3.970042462	4.79047883	5.607936947	5.477090029	5.552218092
Zm00001d042767_T001	6.144620212	5.687083818	6.204020428	7.377097236	7.377666419	7.463238798
Zm00001d036735_T002	1.457336879	0.735414729	1.475890076	2.23392977	1.892769866	1.861519513
Zm00001d039350_T001	7.089483702	6.809626884	7.173036173	7.064351021	7.130205552	6.963661882
Zm00001d039350_T002	6.267541697	5.739528212	6.267161181	3.865033928	3.616224526	4.452939529
Zm00001d010719_T004	1.402573753	1.911388632	1.212928489	0.715736137	0	0
Zm00001d010719_T002	0.527909077	0	0.900179366	1.841708299	2.509831872	2.405615849
Zm00001d010719_T005	2.038895467	0.883700573	2.014541689	2.088735925	0.860790419	1.403412257
Zm00001d010719_T007	0	0	0	0.276429964	0.227713971	0.327752887
Zm00001d010719_T008	1.938610244	0	1.928016642	2.853914032	2.634644378	0
Zm00001d010719_T006	3.873606522	3.102837995	4.009132866	2.724552052	3.078244504	3.77634712
Zm00001d052905_T002	4.204810015	3.191578758	4.206532814	3.955255604	3.921006467	3.971932512
Zm00001d011228_T006	2.742531653	1.696114478	1.984857632	4.159155583	3.774502972	4.044459607
Zm00001d011228_T002	2.292233126	2.006299819	2.341947901	3.910883542	4.257267896	4.126823228

Zm00001d003067_T001	1.530613649	1.104643955	0.756782493	1.768880462	1.479177261	1.96388246
Zm00001d044464_T002	4.122543931	5.368104808	3.943794488	3.421140338	3.642968371	3.815506776
Zm00001d044464_T001	2.40199983	2.394661203	2.886535906	2.489912296	2.042864577	2.237483872
Zm00001d007200_T004	0.355921753	0.615404055	1.074945165	1.113618495	0.898021013	1.068758161
Zm00001d007200_T009	0.980978736	0.341533803	0.402061791	0	0	0.721626394
Zm00001d032467_T001	6.848291802	6.661415719	6.92322356	7.09424799	7.163999312	7.049937907
Zm00001d032155_T001	3.34331543	3.242311499	3.595003739	3.894373684	4.063638663	4.054824866
Zm00001d012467_T001	1.038974107	0.873833668	1.635626249	0.842365028	0.692957398	0.502079012
Zm00001d006343_T001	1.418500317	0.769572033	1.269275571	1.594479757	1.319346825	1.52019492
Zm00001d039547_T015	4.399901419	3.355598611	4.378127177	4.248960493	4.489207205	4.002385327
Zm00001d039547_T012	3.872392241	2.873968678	3.556856336	3.526430943	3.334587725	3.414943946
Zm00001d039547_T002	0.37210963	0	2.044541004	1.129429422	0.580622122	1.129890305
Zm00001d039547_T008	2.762055356	3.741815672	2.005502033	2.208121104	3.144634752	2.391602768
Zm00001d047478_T001	2.310456462	2.632949146	2.851712068	3.163548485	2.256412893	2.549640588
Zm00001d018150_T001	4.60171553	5.461861201	4.625076695	2.531960045	1.724063471	2.567668101
Zm00001d033902_T001	3.999372562	3.718840198	4.23466563	2.600958105	2.452713239	3.19872924
Zm00001d028273_T001	4.757386916	4.334658155	4.813912913	5.230447708	5.27049265	5.345813127
Zm00001d006430_T005	0.325766324	0.47759755	0.99395255	1.162605144	0.873194262	1.181176354
Zm00001d006430_T004	0.69383974	0.316570956	0.654985383	1.239605784	1.60385965	0.522859255
Zm00001d006430_T007	0.536333452	0.062124476	0.421883051	0.929903077	1.192807733	0.640147047
Zm00001d006430_T002	0.610055571	0.300367424	0.666453026	0.880357968	0.10764904	0.393836811
Zm00001d006430_T001	1.780473049	1.191896125	1.105620423	1.954886033	1.594401406	2.098300294
Zm00001d006566_T002	4.593237885	4.203643922	4.326674104	3.844309117	3.629094521	3.98841548
Zm00001d014492_T001	1.833277516	2.799708195	1.658287358	2.200362161	2.34268729	2.306574022
Zm00001d007610_T002	2.779382654	2.210427519	2.798611511	1.975231033	2.711802575	2.838628712
Zm00001d007610_T003	3.268405198	2.184556745	3.212141758	4.076997857	3.640953801	3.841795739
Zm00001d001953_T003	2.558834803	2.737101562	2.155516427	2.985633582	3.107280275	3.012667353
Zm00001d001953_T001	3.907255647	2.356141556	4.04987442	3.906020196	3.670646619	3.586746612
Zm00001d039446_T001	2.937782784	2.402730907	3.045631334	2.975574612	2.959923132	2.926922638
Zm00001d050921_T001	2.445500007	1.562304466	2.526109637	1.928451434	2.365053904	2.034983704
Zm00001d050921_T002	7.205601516	6.94479857	7.341026415	6.756417524	6.865130547	6.717978295
Zm00001d050921_T003	3.058820398	3.606788054	2.897754669	2.789817083	2.713576832	2.343000115

Zm00001d038405_T001	0.94676529	1.089969933	1.669228184	2.068671842	2.090284029	2.28754233
Zm00001d024765_T001	5.839187584	5.166461786	5.835490822	5.789300682	5.886950905	5.699826637
Zm00001d043590_T001	5.263874493	4.817832638	5.211013089	4.706014273	4.883186378	4.899765545
Zm00001d012931_T001	3.145964987	2.367824213	3.08966763	3.254408101	3.428311392	3.200465589
Zm00001d027983_T001	2.155728188	1.640900371	1.669749747	2.710355278	2.705379031	2.913026871
Zm00001d047307_T001	0.323149829	0.261523594	0.309883971	0.998088606	1.243180992	1.14008233
Zm00001d043185_T001	4.78126061	4.331032146	4.937299755	4.454948042	4.381224874	4.369476883
Zm00001d013042_T001	3.852460845	3.977279191	3.86921484	2.517736022	2.446696383	2.508041177
Zm00001d016131_T001	1.136302964	0.769641425	0.9751393	1.36631494	1.104998806	1.371777968
Zm00001d016131_T005	0.460863111	0.119882026	0.430969253	0.421515781	0.409498472	0.431242113
Zm00001d016131_T003	3.338078783	2.544994963	3.239000803	3.611421347	3.569671296	3.403121809
Zm00001d016131_T006	2.120719204	0	2.381414898	2.043587034	2.282157762	2.254054594
Zm00001d030032_T001	3.863069996	3.624124998	4.18967625	3.075479235	2.214255388	2.515131099
Zm00001d030032_T002	5.574886025	4.890701501	5.748349753	5.338246937	5.430153912	5.279224226
Zm00001d039594_T001	4.612438846	3.810562484	4.661995291	4.272903323	4.469165816	4.240055003
Zm00001d002673_T001	3.878706118	3.300924381	4.068490076	3.070767471	3.168552346	3.330901107
Zm00001d040035_T001	3.867371959	3.193211743	3.985139438	2.89059825	2.72153453	2.961023531
Zm00001d024155_T001	1.909793453	1.517328612	1.676231295	2.268667338	1.987686496	2.228143582
Zm00001d013842_T008	4.035050148	4.427238949	4.342171763	0	2.256121185	2.764688188
Zm00001d013842_T007	4.048584086	2.436931231	3.942541811	3.359473099	3.147462521	3.155888121
Zm00001d013842_T003	3.941089592	3.193805485	4.25188274	4.410313694	4.481444521	4.402369986
Zm00001d043325_T001	0.949558091	0.590379736	0.686492459	1.027878049	1.162283523	0.98559515
Zm00001d017834_T001	1.939105815	1.469812538	2.27268793	2.296184848	2.15905884	2.247430533
Zm00001d029757_T001	3.740753283	3.564924533	3.670687404	3.660160004	3.585682466	3.548670643
Zm00001d017568_T004	3.672043854	2.792499735	0	4.523904712	0	0
Zm00001d017568_T005	0	0	3.337236823	3.714469516	5.09791443	5.032030691
Zm00001d017568_T015	0.366923649	0.379038666	0.072720487	0.867324016	0.621786984	0.497925011
Zm00001d017568_T008	0	0.39533705	0.472187244	0	0	0.660802691
Zm00001d017568_T020	0.630499598	0	0.466803547	1.050216787	0	0
Zm00001d017568_T001	1.332773802	2.282085686	1.539373741	0.972860989	0.74721895	1.003752615
Zm00001d017568_T023	0.973346038	0	0	2.047505933	0.811808031	1.959188138
Zm00001d006440_T002	4.929376826	4.244677944	4.909312482	4.957008731	4.857098542	5.008040594

Zm00001d006440_T003	1.404341844	0	0.847224858	1.168016818	1.462129819	1.484104094
Zm00001d038553_T001	2.719996028	2.230850113	2.903409697	2.142221937	2.229536551	2.40009631
Zm00001d041632_T005	3.426662347	2.549462423	3.435227997	4.153986958	4.17534559	4.074026342
Zm00001d041632_T002	1.244349449	0.84809148	0.92590602	2.115566995	2.412168012	1.92858863
Zm00001d041632_T009	3.250396963	2.396382837	3.42076318	1.863522228	0	1.894967117
Zm00001d047269_T001	5.106763546	5.069011669	5.098148505	5.092328644	5.256700919	5.080162526
Zm00001d010229_T001	2.953059577	2.260952719	2.839530947	2.395497188	2.253317364	2.433439691
Zm00001d021344_T003	2.299113631	0.565398304	2.38449526	2.728337415	2.556579992	2.511604914
Zm00001d021344_T004	0.594718606	0	0	0.624055027	0.734471617	0.753334113
Zm00001d021344_T006	1.548597408	1.336615312	2.034135407	1.462861677	1.238443873	1.329292403
Zm00001d021344_T005	1.218132662	1.037343562	1.225261971	0.782617405	1.067490035	0.450250006
Zm00001d021344_T002	3.688379233	3.394164127	3.692700467	4.001545921	4.119696971	4.206315877
Zm00001d034996_T002	3.206791049	2.486734707	3.196910878	3.268552368	2.959667976	2.871212036
Zm00001d034996_T001	0.122821122	0.072728718	0.296026223	0.660979714	1.388261739	0.734057076
Zm00001d034996_T004	0	0.38724893	0.142175529	0.808651116	0.446159871	0
Zm00001d034996_T013	0.963579178	0	0	0	1.817810284	1.218763192
Zm00001d034996_T006	2.813735024	1.982331768	2.944618226	3.279910723	3.170413511	3.269207636
Zm00001d039138_T016	2.794813216	0	2.52869158	3.733229591	3.274045875	3.522859632
Zm00001d039138_T009	3.742569485	3.172068856	3.633360295	3.840980598	4.257107273	4.016846254
Zm00001d039138_T002	0.904846292	1.019489853	1.049338714	2.823267566	2.14584796	2.14391613
Zm00001d039138_T008	4.601476471	3.767420686	4.6855149	3.483979929	3.866595674	3.605996321
Zm00001d036727_T001	3.624211462	2.963087689	3.753154801	4.330202343	4.36882505	4.43453696
Zm00001d011676_T001	5.401453361	4.319277089	5.454779073	5.807366103	5.815774093	5.791241958
Zm00001d052591_T001	3.171019431	2.383695446	3.248873459	4.021470222	3.889884	3.551375371
Zm00001d029548_T015	0.627949496	0.239472588	0.350154354	0.526990239	1.048612856	0.778642581
Zm00001d029548_T008	2.286099279	1.584604187	2.662070568	2.875287419	2.504087704	1.676086834
Zm00001d029548_T009	2.648623049	2.055241128	2.43999912	2.037856248	2.285032546	2.375763726
Zm00001d029548_T001	1.484000946	1.144052245	1.373991133	1.583869486	1.22293127	1.388140482
Zm00001d029548_T006	1.756196661	1.141196682	2.329079095	2.152139927	2.050682785	2.630352115
Zm00001d029548_T012	0.770987153	0.598636757	0.771467284	0.852872183	0.87895129	0.724177008
Zm00001d053453_T001	3.248557302	3.052903782	3.940418425	4.286532923	4.169428148	4.048031243
Zm00001d053453_T019	0.230190714	0.129107575	0.191830447	0.364795402	0.189248947	0.241597376

Zm00001d053453_T002	2.56621214	2.118118149	1.615508582	1.342710043	1.573676995	2.692344169
Zm00001d053453_T018	2.578785381	1.968586622	3.435677661	3.408381443	3.844972093	3.6538597
Zm00001d053453_T015	5.003508692	2.912110238	4.487025529	4.476357967	4.384706609	4.24829171
Zm00001d051015_T001	3.115442148	2.911574628	2.851364871	4.193354563	4.018689627	3.975645409
Zm00001d038562_T001	7.158918319	6.69284135	7.148815132	7.47465935	7.433833551	7.458448267
Zm00001d023768_T001	3.606375777	3.485079637	3.882338829	1.716796913	1.702442564	1.587848369
Zm00001d052219_T004	1.764610701	1.071092642	1.471269675	3.601176149	3.652828915	3.813690395
Zm00001d052219_T001	4.407755181	3.645574628	4.595126348	4.741966693	4.703674108	4.651096708
Zm00001d029272_T015	4.554905841	4.217602201	4.546214706	3.974502055	4.184098081	3.988129858
Zm00001d029272_T005	0.673243429	0.473876249	0.839887048	1.050564384	0.514534364	1.036245894
Zm00001d029272_T013	2.846252797	4.275639504	2.328152573	2.518872987	0	2.114903688
Zm00001d029272_T002	0.815457385	0.830867001	0.66848588	0.718983618	0.348497588	0.946916471
Zm00001d029272_T003	3.076293824	1.484695505	3.01233339	3.508394043	3.677190239	3.554575837
Zm00001d041331_T001	1.463007166	1.211896235	1.438162418	1.082458032	1.177573417	0.240074929
Zm00001d041331_T003	4.305206522	5.620160811	3.989666839	3.604854153	3.681567892	3.587367031
Zm00001d047859_T002	0	1.39781008	2.809053005	0.812029915	0.623157042	2.068773636
Zm00001d047859_T001	4.579353051	3.762498287	4.259597904	3.76121718	3.793749525	3.672584878
Zm00001d048988_T034	1.323277826	0.771494329	1.240502402	0	1.107531876	0
Zm00001d048988_T033	3.049548873	2.200313505	3.215222422	3.037912284	2.825185431	2.744663583
Zm00001d048988_T007	1.221236153	1.413840468	0.933685179	2.140863383	1.99891252	1.5585439
Zm00001d048988_T016	0	0.017160819	0.093514204	0.319394808	0.270654519	0.31233873
Zm00001d048988_T015	0.375895756	0	0.513945464	0.074504066	0	0.380573203
Zm00001d008940_T003	1.185206242	0.61660236	1.625417791	1.947535626	1.802950872	1.225895562
Zm00001d008940_T002	1.543211771	1.509220016	0	1.495978046	2.386468024	1.979697594
Zm00001d008940_T004	0.557464988	0	1.86140914	1.497984774	0	1.504187646
Zm00001d036904_T001	0.741093293	3.779762212	1.463029669	1.89845062	0.893080246	1.344241644
Zm00001d036904_T002	8.748420462	7.713442723	8.737747188	8.411085413	8.431425186	8.394350387
Zm00001d036904_T003	5.2109845	2.888853109	5.489079303	4.730971602	4.505839304	4.544002655
Zm00001d042653_T003	4.006537974	4.028451398	4.44148587	3.681929559	3.689741855	3.864767663
Zm00001d042653_T004	5.05189257	5.245259707	5.214030359	4.297629442	4.781381957	4.298260787
Zm00001d042653_T002	4.916110861	4.325468161	5.099394607	4.726384283	4.516362537	4.896151942
Zm00001d042653_T001	2.89434846	2.844705212	2.471519671	1.841937303	2.299646165	2.126352113

Zm00001d051635_T001	6.042801139	5.061048879	6.123854011	6.138026693	6.64794978	6.199223194
Zm00001d002373_T013	2.244122289	2.787011338	2.569693531	2.522290821	2.969418711	2.778811816
Zm00001d002373_T005	0.696611724	0.366360825	0.653973511	0.968153436	0.265423693	0.308078903
Zm00001d002373_T015	3.586094692	0	3.429892828	3.375345181	3.174929357	3.085988842
Zm00001d027861_T001	6.700856514	5.888876385	6.773182154	6.860941401	6.786996785	6.784352625
Zm00001d012447_T006	0.213353504	0.235193991	0.584291973	0.932192732	1.079541957	0.992140468
Zm00001d012447_T001	0.390248352	0.80000321	0.556942391	1.350210377	1.007109368	1.519528821
Zm00001d012447_T003	1.129052167	0.14090689	1.861534996	1.772282635	0	0.970735142
Zm00001d012447_T005	1.126332617	0.852695638	0.986959874	2.104650328	2.163886726	1.897443357
Zm00001d012447_T002	1.02506495	0.267880925	0	1.268060646	1.552241797	1.429382436
Zm00001d039362_T002	3.573567212	2.829910635	3.740389228	3.333776779	3.397285607	3.413476967
Zm00001d038684_T005	0.470393174	0.257676881	0.400272318	0.51918861	0.424479271	0.605025844
Zm00001d038684_T003	4.377102851	3.99746964	4.373679737	4.462008134	4.480369313	4.298192077
Zm00001d017877_T001	5.932539651	5.17628842	5.844766113	6.067324728	6.073908043	6.096532649
Zm00001d045948_T003	2.841331913	3.238411007	2.889489897	3.730994853	3.451089492	3.240671677
Zm00001d045948_T002	1.244248967	0.644592815	1.528976817	1.948498435	2.028258303	2.011188604
Zm00001d045948_T006	1.062491321	0.070297267	0.871432856	0.748320392	0	0.919284395
Zm00001d020630_T001	3.555564275	4.365904699	3.706264643	3.615210755	3.760536601	3.644556592
Zm00001d036008_T001	5.448332976	4.750187033	5.467958405	5.547435115	5.707778461	5.560496486
Zm00001d023715_T024	0.92301451	0	0.766222833	0	0.473687179	0.80144758
Zm00001d023715_T009	0.519318463	0.188606015	0.5320905	0.888152122	0.30217163	0.49328764
Zm00001d023715_T003	0.513352281	0	0.500075935	0.78261405	0.625129253	0.700790361
Zm00001d023715_T023	0.368039695	0.165721603	0.344462665	0.47015574	0.549589334	0.636874685
Zm00001d023715_T004	1.073827772	0.975523805	1.355837769	0	0.770982925	1.15143023
Zm00001d023715_T017	1.237461539	0.637212366	0.93246262	1.249958006	1.569349635	1.387242311
Zm00001d041374_T001	1.367015381	1.067568508	1.253047124	1.316602813	1.384735062	1.028831501
Zm00001d046305_T001	4.672617293	3.849973047	4.572760244	4.062951817	4.13424	3.967167224
Zm00001d022434_T001	3.724981879	2.89215046	3.568686643	4.525926038	4.511867243	4.455385486
Zm00001d042245_T001	7.386586473	7.005360035	7.531601747	8.484695525	8.487725446	8.462201269
Zm00001d038064_T001	3.070795115	2.217435161	3.098444993	3.918632865	3.883485826	3.966577203
Zm00001d025362_T002	5.500229928	4.129837318	4.781798016	5.313070729	5.251298643	5.158868083
Zm00001d025362_T001	1.436924571	3.031445309	1.436831316	1.18291091	1.515631723	1.989954971

Zm00001d037533_T001	3.584980294	3.726993566	3.739554242	2.678341033	2.417673238	2.834120387
Zm00001d051859_T001	3.338606398	3.41548435	3.409794563	3.303802808	2.89914024	2.685674004
Zm00001d003153_T007	0.994263274	0.825557708	1.073320515	0	0	1.324348061
Zm00001d003153_T008	2.835082863	0	0	2.237567695	2.925481854	0
Zm00001d003153_T001	0.172589921	1.972952132	0.462491083	1.065348273	1.174975203	0.746265464
Zm00001d003153_T010	2.710988002	0	1.460703777	1.066904104	0	0.864028814
Zm00001d033895_T002	1.888530908	1.667615138	2.005719753	3.634513349	3.582062416	3.790772768
Zm00001d033895_T010	0.557748268	0.376872679	0.38645009	1.440852827	1.825788069	1.357692186
Zm00001d033895_T006	1.703152097	0	1.751189039	3.413227811	3.642265925	2.835680978
Zm00001d033895_T008	2.466599975	1.462664876	1.70014671	2.784731153	2.77364953	2.760860275
Zm00001d033895_T009	4.373873123	3.854350291	4.460268139	4.317749631	4.276293945	4.466945965
Zm00001d048732_T003	0.971888196	0.684527028	1.632894488	1.851743845	1.422496753	1.742907344
Zm00001d048732_T001	1.071547828	0.827874293	0.383864069	0.924615286	1.3587147	1.33662331
Zm00001d048732_T002	0.170180176	0.119049343	0.165787195	0.543919474	0.468104685	0.518294418
Zm00001d048732_T004	1.990166335	0	1.60137752	1.094881253	0	0
Zm00001d048732_T005	3.573769081	2.875924522	3.425423183	3.800357707	3.924200434	3.805460141
Zm00001d036285_T001	5.008835769	4.334752891	5.024523497	5.019871835	5.162492238	5.042446873
Zm00001d022323_T001	1.405544238	1.536140454	1.148043165	0.959417783	0.820551513	0.884599484
Zm00001d011825_T001	5.965838437	6.135297753	6.033542927	5.638125808	5.487561113	5.813625141
Zm00001d034206_T001	1.788406749	1.373040105	2.298499962	2.167470012	2.192438172	2.248991071
Zm00001d015102_T001	4.498166474	3.843332475	4.48574953	4.547117744	4.364000423	4.587544656
Zm00001d011049_T001	6.866580516	7.008092085	7.041087687	6.670713814	6.715228551	6.707740147
Zm00001d033378_T007	0.560303116	0.325100841	0.31788867	1.487680822	0	1.287686822
Zm00001d033378_T010	0	0	0.109837924	0.896008283	0.517443011	0.379948054
Zm00001d033378_T018	2.264844753	0	0.483492319	2.119314008	2.401845326	3.46827298
Zm00001d033378_T006	0.158665345	0.234509904	0.562611218	0.957840327	0.353703819	1.007555054
Zm00001d033378_T012	0.581104389	0.437763589	1.092777095	1.403527875	1.472475294	1.133649007
Zm00001d033378_T019	3.636791992	3.413202893	1.491767943	0	0	0
Zm00001d033378_T022	6.098769291	6.060391691	6.105542651	5.297664251	5.37952639	5.268951506
Zm00001d033378_T004	3.778335385	3.022255056	4.385779658	4.703959204	4.800716828	4.61711014
Zm00001d033378_T016	4.188001818	0.664292592	4.6237771	5.186661236	5.17153295	4.927532132
Zm00001d033378_T020	1.10441047	0.543058307	0.596601257	1.570133551	1.631683107	1.847160302

Zm00001d010587_T001	3.419772805	2.922065944	1.969235972	3.437956893	3.227537008	3.425829062
Zm00001d020354_T001	6.863799054	9.699936718	6.718164335	6.773383168	6.660258828	6.429855177
Zm00001d052540_T001	1.242522633	0.756651854	1.270699963	1.644848423	1.601657062	1.627592364
Zm00001d044168_T001	1.868319724	1.80369284	2.417984258	1.868178651	2.079010755	2.059432118
Zm00001d035029_T002	3.859449309	0	3.489734563	3.388108099	3.258742925	2.988333308
Zm00001d035029_T008	4.158989793	0	3.401760014	0	3.493697028	0
Zm00001d035029_T007	1.269558647	0.765106117	1.104335318	2.104051448	1.807006985	1.731632493
Zm00001d035029_T001	0.609874079	0.613527881	1.680358567	0.99114503	1.191040179	2.136683578
Zm00001d035029_T009	1.68489188	2.675961096	2.195612173	2.825194184	2.628902581	1.929250927
Zm00001d035029_T005	0.375862402	0.389408217	0.456648469	0.622027916	0.257960432	0.39622636
Zm00001d035029_T010	4.897632514	4.338605828	5.242204548	3.986894271	3.080784192	3.970544104
Zm00001d033421_T001	5.144429066	4.680028416	4.952906756	4.301337348	4.432775652	4.42097137
Zm00001d054044_T001	4.003124917	3.199571215	4.060480473	2.605836596	2.48380185	2.410579906
Zm00001d019220_T001	1.067953928	1.595859278	1.811971982	1.581095227	1.101310539	1.55185312
Zm00001d014043_T001	3.432226385	3.251154627	3.635772381	4.672322483	4.621197022	4.659285819
Zm00001d045347_T001	1.262959264	2.570609622	1.514203074	1.670620109	0.877762308	0.459798803
Zm00001d012820_T001	6.715642318	6.787171953	6.825812716	6.182604927	6.162143436	6.136857179
Zm00001d038696_T001	5.841556574	6.672945227	5.989596999	4.802820524	4.514945025	5.200383663
Zm00001d025801_T007	6.808758046	0	6.508062925	6.711781751	6.887244454	6.655869703
Zm00001d025801_T001	5.646334313	4.942922719	5.915946404	5.888544959	5.796659396	5.887768802
Zm00001d025801_T006	6.237482802	7.19498684	6.625057211	4.307516687	3.910580615	4.600637399
Zm00001d025801_T005	1.389886152	4.652017242	2.426679923	2.914191364	2.000919785	1.748272726
Zm00001d037852_T008	0.139497	0.357326786	0.89501095	0.834263009	0.946568431	0.414694097
Zm00001d037852_T005	0.96108393	0.215489745	0.696570776	1.593219416	1.566100569	1.87641911
Zm00001d037852_T017	0	0.080837733	0.616952345	1.258076853	0.359493058	1.588658276
Zm00001d037852_T016	2.441792861	1.492927856	2.305202143	2.273543639	2.408640404	2.503208021
Zm00001d048007_T003	2.578299198	2.314287293	2.402032312	2.204043657	2.360310333	2.21617794
Zm00001d048007_T002	0.304199106	0.081347809	0.511826265	0.391469693	0.444867398	0.509654296
Zm00001d053322_T001	3.532221566	2.959985431	3.591598401	2.560054342	2.37778843	2.438397723
Zm00001d020936_T001	3.694996495	2.659051372	3.550333569	4.373126436	4.474853281	4.320410014
Zm00001d020936_T002	1.47238379	2.833910373	1.60755947	0.864549485	1.175735361	1.125457363
Zm00001d036415_T001	5.235226509	4.995548285	5.417074127	5.295048605	5.487385405	5.544059241

Zm00001d019078_T001	3.069912645	2.314348787	3.142125851	3.046676842	3.372930652	3.590999673
Zm00001d019078_T002	3.169067465	2.409233419	3.164241304	3.293300584	2.871156628	2.694525436
Zm00001d013982_T002	3.979740939	3.694240252	4.06471647	4.184871218	3.911947027	4.025161095
Zm00001d028197_T001	3.151533979	2.465529184	3.107780922	3.091847565	2.616208997	2.985985801
Zm00001d008545_T001	4.425581426	5.978657932	4.953121288	4.559703001	4.828324438	4.568438034
Zm00001d011434_T001	3.950244807	5.708068156	3.85959899	5.761958294	5.275152912	5.603771453
Zm00001d028593_T004	3.520373718	0	3.211331701	3.196165419	2.985953387	3.024822832
Zm00001d028593_T003	2.843573281	3.114622722	2.905219179	3.104450897	2.961949981	3.300893347
Zm00001d038690_T001	3.830702344	2.912184794	3.534853721	4.133872465	4.02782286	4.095101343
Zm00001d046538_T005	1.885628635	4.993223415	3.666302242	2.38410674	2.783289849	1.767891203
Zm00001d046538_T004	4.395626945	2.906756707	4.042227664	4.737470287	4.47978757	4.663545751
Zm00001d046538_T002	1.806207317	1.804808174	1.417766119	1.974946643	1.467109895	1.242668961
Zm00001d046538_T001	0.530550229	0.277015883	0.472943123	1.105373015	1.125227884	1.172262837
Zm00001d010974_T029	0.80192513	0.346229616	0.858945388	1.640238225	1.407982994	1.835158879
Zm00001d010974_T016	2.715020902	1.886970311	2.716465065	3.067076603	3.225672881	3.129680157
Zm00001d010974_T014	0.310305216	0.398674338	1.095212177	0	0	0.312934637
Zm00001d010974_T075	0	0	0.242067093	0.782744875	0.613509022	0.511662342
Zm00001d010974_T063	0.392642748	0	0.534870401	0.330436784	0.418817094	0
Zm00001d010974_T017	0.5893589	0.454916006	0	0	1.014553114	0
Zm00001d010974_T032	0.39043437	0	0.307937896	0.092118113	0.768827967	0.636307686
Zm00001d010974_T005	3.109808177	2.177247142	0	4.402584189	3.906333223	4.483229553
Zm00001d010974_T024	4.009566462	3.033215238	4.399473413	3.288271584	4.009221657	2.596293298
Zm00001d010974_T001	0	0.053817563	0.264573661	0.665620164	0.654361296	0.696781737
Zm00001d010974_T065	0.708130719	0.418969252	1.467425047	1.483436597	1.501309711	1.383735254
Zm00001d010974_T037	0	0	0	0.683707232	1.069184518	1.151815314
Zm00001d010974_T045	0.36610004	0.320567858	0.791580721	1.026149904	1.019682702	1.29005513
Zm00001d042935_T001	4.361223597	5.579732098	4.349973539	3.049437176	3.296812714	2.915548626
Zm00001d045972_T001	1.764825954	1.158609769	1.453954733	3.233351857	3.087011801	3.151782362
Zm00001d035514_T016	0.767520056	0.247842468	0.885017482	0	0.84896479	0.52977398
Zm00001d035514_T005	1.161599437	1.440878334	1.058286013	1.68557287	0.927007434	0.948823588
Zm00001d035514_T012	2.322006575	1.657472634	2.031919172	2.932241308	2.203027584	0
Zm00001d035514_T007	0	1.128378535	2.063526067	1.637972324	2.287274288	2.420405866

Zm00001d035514_T014	1.832715058	1.658758974	2.843867499	0	2.014424569	2.017349704
Zm00001d001944_T001	4.350572279	3.709009128	4.544593943	3.805628529	3.993801599	3.907561197
Zm00001d021342_T001	1.936917547	0.873972221	1.611367874	2.787498287	2.941166986	2.827291837
Zm00001d021342_T021	1.473454963	0	1.128624008	2.016999713	0.839104195	1.622446873
Zm00001d021342_T002	0.437531376	0.143554072	0.423259748	0.471275919	0.805587156	0.578179378
Zm00001d021342_T009	1.195065332	0.481274139	1.479837421	2.441770556	2.531554386	2.278705617
Zm00001d027714_T001	1.810899183	1.168036721	1.738568115	1.751473576	1.767308171	1.807634366
Zm00001d008613_T001	4.263248239	3.63849685	4.255825869	3.942788756	3.862388108	3.694359503
Zm00001d042792_T001	1.238783192	0.7132823	0.691754796	1.336725556	1.143089043	0.999113913
Zm00001d042792_T003	3.600698644	3.029218219	3.447999381	3.598207372	3.516178576	3.427777918
Zm00001d042792_T002	1.763874268	1.088938049	1.944653081	2.207449358	2.573045568	2.441648934
Zm00001d042792_T004	0.14583655	0	0.417343246	0.330680006	0.203761208	0.4117397
Zm00001d037900_T001	4.95507604	4.325788483	4.937142194	5.08829457	5.134555366	5.048075897
Zm00001d034069_T001	2.947619406	2.308167462	2.939539347	2.251605187	2.384785338	2.319875123
Zm00001d044427_T001	2.866630685	2.621274986	2.748951065	4.086427973	3.446372303	3.681971262
Zm00001d021225_T004	1.179446717	0.956610571	1.202702314	1.371661451	1.335749063	1.34420755
Zm00001d021225_T001	5.272326482	5.117176751	5.285054709	4.987614696	5.404461508	4.70838282
Zm00001d021225_T002	5.161416674	4.651041355	5.054851645	4.790195522	4.639388003	4.964418364
Zm00001d053887_T001	6.191179502	6.124808429	6.063226543	6.036727193	5.901435033	6.029542753
Zm00001d038672_T003	0.585290436	0.30465472	0.481296875	1.321363602	1.379693594	1.137476637
Zm00001d038672_T001	3.773745941	2.888060138	3.340336804	3.395129173	3.985763898	3.918315338
Zm00001d038672_T002	1.425985046	2.732707261	2.162518462	4.455934225	4.14608665	4.346570039
Zm00001d038672_T004	1.618461387	0.549110218	1.394447191	2.013265004	2.136510017	1.32785404
Zm00001d038672_T006	4.37523632	2.732978974	4.516219998	4.479719419	4.266955607	3.891824815
Zm00001d029635_T001	1.565087269	1.122219466	1.512560653	0.565280333	0.411270036	1.026610992
Zm00001d012895_T005	1.330865836	0.562144226	1.108588018	1.293789517	1.596024884	0.969763124
Zm00001d011687_T001	2.289521405	1.687352334	2.349112727	2.771699685	2.815001926	2.727067869
Zm00001d029031_T002	3.02585784	2.46410833	3.186519574	3.317966213	3.246842351	3.2498978
Zm00001d029031_T004	0.572937192	0.046951978	0.52761187	0.752384661	1.211568599	1.032256745
Zm00001d029031_T003	0.743676958	0.261420089	0.615481274	1.325902146	1.542834517	1.110474164
Zm00001d020984_T001	5.744039565	4.946366445	5.820041376	5.635931206	5.685681712	5.592590686
Zm00001d038508_T001	8.430187615	8.329710908	8.462770632	8.659487689	8.755211551	8.694178059

Zm00001d032326_T006	0.29325982	0	0	0.744275645	0.512117637	0.340744553
Zm00001d032326_T004	0.272017215	0.222145493	0.43376709	0.118359726	0.300195198	0.440485571
Zm00001d032326_T003	0.571846672	0.446623607	0.549693811	0.482320661	0.581049419	0.639199743
Zm00001d032326_T005	4.578935152	3.730145388	4.599379036	4.231667027	4.313230249	4.37105913
Zm00001d032326_T002	1.56007905	2.194813261	1.61448936	1.356079564	1.654370004	1.33754498
Zm00001d020011_T001	3.655691738	3.205663853	3.693852338	3.768691111	3.80934169	3.743715514
Zm00001d052344_T003	3.21927758	2.511257768	3.294531354	2.900272899	2.832239288	2.884694228
Zm00001d052344_T005	1.069451271	0.619629927	0.777007653	1.918195771	1.861077971	2.378688065
Zm00001d052344_T004	1.604261139	0.782799382	1.590996201	1.548969707	1.855851001	1.510883455
Zm00001d015639_T001	3.769485076	5.200529542	3.774560321	3.40134635	2.925164882	3.357851069
Zm00001d027896_T001	7.186506649	6.82733822	7.287151508	7.245571625	7.201929926	7.309926104
Zm00001d049625_T002	2.079101237	2.217002659	2.172448791	2.548430213	2.273191209	2.198634016
Zm00001d022551_T001	2.546437663	1.704114572	2.500928729	2.226380285	2.45469338	2.385448432
Zm00001d022551_T003	1.21126955	1.445757951	1.251898415	0.511780733	0.826443948	0.793487252
Zm00001d025891_T001	4.481688047	4.890391776	4.506272952	3.887888628	4.271936416	3.959689766
Zm00001d016496_T010	3.650933859	0.163203723	3.37473167	3.110914924	2.709531506	3.453257462
Zm00001d016496_T013	0.657474463	0.428650542	0.923922722	0.936369208	0.746672213	1.02690839
Zm00001d016496_T005	2.388012875	2.636916668	2.043255252	3.375283174	2.389730895	3.262455559
Zm00001d016496_T006	2.316852432	2.963305615	2.690141046	0	2.526905883	0
Zm00001d016496_T001	1.257778845	0.396929963	1.557396365	0.938061418	1.70785031	1.033285554
Zm00001d016496_T014	0	0	0	2.484427415	3.018348954	2.454597583
Zm00001d027484_T003	5.737634065	4.950037688	5.836849431	5.914638211	5.905204061	5.87246809
Zm00001d027484_T006	0.65964695	0.210847661	0.829126205	1.040917673	0.735529109	1.051367229
Zm00001d027484_T005	0.705511742	0.214642093	0.460803363	1.267819216	1.437105201	1.193967243
Zm00001d038866_T003	7.221053955	3.339192126	6.648385914	6.045326346	6.758720869	6.703182738
Zm00001d038866_T005	2.663802292	1.5720859	3.104267207	3.422395968	3.688613662	3.694717755
Zm00001d038866_T002	1.415759748	1.020300927	1.266696004	1.524321492	1.398710452	1.32989455
Zm00001d038866_T004	4.016926461	4.62430804	3.998174106	4.037432303	3.958771977	3.984736164
Zm00001d008405_T001	4.569262736	4.704644479	4.708479112	4.834374416	4.702237084	4.801020227
Zm00001d011790_T001	2.198093978	1.376911564	2.432795358	1.962212139	1.34640154	1.770153728
Zm00001d011790_T002	1.052838068	0.994677469	1.08545457	1.187981159	1.456899698	1.102834805
Zm00001d038530_T006	0.706550886	0.413089267	0.560330511	0.581961461	0.636176808	0.382376195

Zm00001d038530_T011	1.147886266	1.179212289	1.077485023	1.547828838	1.135308302	1.429631496
Zm00001d038530_T010	2.056248479	1.610510147	2.09694289	3.003119519	2.861708676	3.173397394
Zm00001d038530_T009	0.374910383	0.22997177	1.02170784	0.887781034	0.648491213	0.565984117
Zm00001d038530_T005	0.33075457	0	0.631477774	0	1.093422931	0.930665438
Zm00001d038530_T008	0.8035172	0.067558871	0.398743282	0.826530183	0.722727435	0.942470966
Zm00001d038530_T007	0.384354896	0.167243587	0.627604971	1.490021699	1.426556753	1.316084904
Zm00001d032510_T001	2.65168364	1.920177041	2.511672714	1.879605009	1.674176333	1.973632948
Zm00001d047760_T001	1.645263112	1.045005343	1.638529402	1.580351983	1.349283742	1.632514383
Zm00001d012522_T001	2.399704314	1.951818279	2.617383123	4.822941337	4.836850112	4.796599779
Zm00001d045559_T001	5.964319055	5.406008352	5.826767404	6.053372866	6.09050406	5.990297763
Zm00001d011370_T001	2.772143044	2.52719823	3.291677223	3.190678382	3.365959414	3.19471053
Zm00001d003227_T001	0.844208871	0.480943391	0.821988524	0.944005317	0.379707455	0.667890309
Zm00001d003227_T006	0.838551669	0.250622076	0.228422222	0.574634395	1.03690347	0.877691645
Zm00001d016916_T003	1.554254353	1.902190148	2.52841929	1.70570017	2.346536568	2.205426922
Zm00001d016916_T004	2.685978037	1.9609824	2.690634992	3.080261806	3.130775887	3.024633506
Zm00001d016916_T002	1.430216215	0	0	1.624214621	1.205418163	1.380010138
Zm00001d028100_T006	2.24692396	1.340271707	2.111304797	2.272263021	2.09087375	2.150255509
Zm00001d028100_T004	0.524624392	0.336796382	0.51802444	1.093294463	1.086192031	0.939516968
Zm00001d028100_T005	1.454952318	0	1.623003421	1.508304915	1.668158619	2.087809725
Zm00001d028100_T007	0	0.539940943	0	0.750073059	0.923946294	0
Zm00001d028100_T001	0.343839855	0.25753086	0.110591758	0.465474069	0.468903359	0.255074055
Zm00001d028100_T003	2.500234294	2.090666478	2.736381256	2.354312793	2.401783356	2.010210166
Zm00001d002625_T003	3.293641075	2.214423574	3.341286995	4.049095669	4.198130527	4.133292091
Zm00001d009292_T001	3.705500904	3.42272225	2.933819801	3.300580335	2.958153171	2.989940988
Zm00001d020639_T002	4.485293629	3.520714027	4.443621105	4.220247059	4.364030199	4.159372138
Zm00001d020639_T003	1.433461591	0.789329456	0.493980304	0	0	0
Zm00001d020639_T005	0	0.840318202	2.105616735	1.953646306	1.682020383	1.67611347
Zm00001d044442_T021	0	0.0565974	0.065131095	1.39408824	0.743909571	0.597684672
Zm00001d044442_T007	1.92691448	1.406008692	1.30751829	3.063032264	3.023710271	2.872695415
Zm00001d044442_T015	0	0.080665849	0	0.342116637	0.615641348	0.813177416
Zm00001d044442_T018	0	0	0	1.303794189	1.054540808	1.741085094
Zm00001d044442_T020	0	0.124453869	0	1.033046574	1.036463244	0.433541711

Zm00001d044442_T019	1.200774261	1.016430709	1.639645229	3.705793269	3.67703076	3.253836822
Zm00001d044442_T010	0.953089385	0	1.194317184	3.345481822	3.654584248	3.916256975
Zm00001d031570_T001	3.315944968	2.228693421	3.244340771	3.375967047	3.712078364	3.483071251
Zm00001d031570_T002	3.468799249	2.906822691	3.595989569	1.29519051	1.696751919	2.332642349
Zm00001d031570_T003	0.265240042	0.098530053	0.375756776	2.170382744	1.03748695	1.058225047
Zm00001d024212_T001	4.480505293	5.249346541	4.876004754	3.948496659	4.341893975	4.156389416
Zm00001d024212_T007	3.305291025	3.078253557	4.119516292	4.676854117	4.607472949	4.492314685
Zm00001d024212_T014	0.250875494	0.03486788	0.312779005	0.374421895	0.358611187	0
Zm00001d024212_T015	0.96193739	0.987518063	1.140184444	0.439899683	0.557228716	0.75651608
Zm00001d024212_T002	1.669930209	0.941279123	1.581495871	2.045806395	2.202712656	1.978425198
Zm00001d044546_T001	8.589043375	8.221573434	8.62421616	6.434501165	6.383204204	6.398853546
Zm00001d039920_T001	4.162477777	3.310504451	4.125593743	3.88384082	3.830591515	3.984804964
Zm00001d026348_T002	1.420873643	0.749977842	1.265305463	1.512552562	1.363822596	1.703508726
Zm00001d026348_T003	1.780435253	0.99218616	1.011139931	1.784465455	2.130139476	1.492723324
Zm00001d026348_T001	2.73324608	3.458019054	2.295361277	2.121451129	1.938290686	1.044272403
Zm00001d028089_T001	1.041587141	0.508520941	0.688391655	1.344014906	1.376381521	1.3011738
Zm00001d042362_T001	0.673941694	0.271834402	0.538582859	0	0	0.872245655
Zm00001d018506_T002	5.014148382	3.926495829	4.799800911	4.671952909	4.732275713	4.650170991
Zm00001d018506_T001	1.47298626	3.019355341	1.49151553	0.964709134	1.646229029	1.141776093
Zm00001d029051_T001	2.616834752	1.89964008	2.612794087	3.520430169	3.494994748	3.532578541
Zm00001d016552_T004	0.913265896	0.415466637	0.884323617	1.038946022	0.863489718	0.766972482
Zm00001d016552_T005	1.804381557	0.898284985	1.622660523	2.142388276	2.158313435	2.369728337
Zm00001d016552_T002	4.05750252	3.04217451	4.076090605	4.009482978	3.733012501	3.828420666
Zm00001d016552_T008	2.551185236	1.712852824	2.767094559	3.022344375	3.317043237	3.141022683
Zm00001d016552_T007	3.035535938	5.846657634	0	0	2.564451582	0
Zm00001d016552_T001	1.94926299	1.109781103	2.431422653	1.389957168	2.14901933	2.171103042
Zm00001d016552_T003	2.181592379	1.340243787	2.086502875	3.406542248	3.370476226	3.22997992
Zm00001d011713_T005	2.747998079	2.373735619	3.026943791	3.041927014	3.152658181	2.862676333
Zm00001d011713_T008	0.726778041	0	0.514942311	1.913006567	0.85756942	1.075925511
Zm00001d011713_T010	0	0.028132034	0.083391758	0.298726343	0.405870406	0.286389129
Zm00001d011713_T016	0	0	0	1.069252586	1.373488413	0.952261243
Zm00001d011713_T001	1.475168432	0	0	2.133079478	1.13972291	1.970436243

Zm00001d011713_T009	1.106535931	0	1.627805253	0	2.004184953	2.310742914
Zm00001d044315_T001	0.584931723	0.653537931	0.585945122	0.487957566	0.228387741	0.497747241
Zm00001d044315_T005	2.133275155	0	2.43090986	1.055518454	1.494448381	1.541768565
Zm00001d044315_T010	1.686122183	2.082826214	1.778576564	1.182172961	0.656892628	1.299433995
Zm00001d044315_T013	6.654657708	5.876227298	6.683933427	6.975730003	6.996452744	6.907865156
Zm00001d044315_T009	0.434601008	1.550421	0.071504581	0.527805012	0.603962168	0
Zm00001d044315_T002	5.819759524	5.051360703	5.905140917	5.687254733	5.77375989	5.613228673
Zm00001d003381_T001	3.833730582	3.621074616	3.911134143	3.083454393	3.038078624	2.903349342
Zm00001d009572_T001	4.963817694	4.718540164	4.992161229	5.321988362	5.389223752	5.330968886
Zm00001d005642_T003	6.916788808	8.198816224	6.942060959	6.127700853	6.440545187	6.413450987
Zm00001d005642_T001	7.657936395	8.166628587	7.83854933	7.104143959	7.139208697	6.995737975
Zm00001d052530_T001	1.583121891	1.621489747	1.943170817	5.964343017	6.041624833	5.950067045
Zm00001d043125_T001	4.502923243	4.509900769	4.618610031	3.879659113	3.944123233	3.82315168
Zm00001d016826_T001	2.343841845	1.766795705	2.380009861	4.511274153	4.498072307	4.23973347
Zm00001d048190_T001	4.512741411	3.960348971	4.449249362	4.120284582	4.045563452	4.159369636
Zm00001d048190_T004	2.152569276	0	0	1.277884201	1.567026003	0
Zm00001d048190_T002	3.117940513	3.076398844	4.169919231	3.253438396	3.384207334	2.704271085
Zm00001d042427_T001	4.337976347	3.768797803	4.210627787	4.113151884	4.240307536	4.234780191
Zm00001d006255_T001	1.681711918	1.38432229	1.562676175	1.210542228	1.485137229	1.443279886
Zm00001d030812_T001	2.690125398	2.063555058	2.269433274	1.956934644	2.385276405	2.117136805
Zm00001d027633_T010	0.719979825	0.436403868	0.35154579	2.151396132	2.074699975	2.028003278
Zm00001d027633_T007	0	0.191259425	0.570494019	0.710002543	0.752858181	0.387360335
Zm00001d027633_T011	1.637756293	0	1.767675983	2.170032398	2.189785981	2.274619495
Zm00001d051528_T004	2.105883205	1.744893885	2.063580252	1.360510365	1.62094528	1.730007749
Zm00001d051528_T005	3.981838409	3.999534475	3.845824316	4.722413835	4.792359328	4.767138852
Zm00001d051528_T002	0.473413918	0.334681536	0.629972039	1.104478238	0.834801811	0.731208445
Zm00001d051528_T009	4.005193091	0	4.217736967	4.425365239	4.214253756	4.496226291
Zm00001d025566_T001	2.502984326	3.123641533	2.336454216	3.438646827	3.391026114	3.291771509
Zm00001d005962_T001	3.375049304	3.810869268	3.038221236	6.596233402	6.643734234	6.729802977
Zm00001d013057_T001	3.024639179	2.540875868	3.090578581	4.445165392	4.414681854	4.349960097
Zm00001d040757_T002	5.173431415	4.954669809	4.891778309	5.258681539	5.429469947	5.271741903
Zm00001d024778_T001	9.153860485	4.35794359	9.406809135	9.872602701	9.865016833	9.846078974

Zm00001d011821_T004	1.976676705	0.134809728	1.75431544	2.144791026	2.044022643	2.292892744
Zm00001d011821_T009	0.280603612	0.290121206	0.418999465	0.468714678	0.713575292	0.505644025
Zm00001d011821_T008	0.351036884	0.487879384	1.129160341	1.427362664	1.451579867	0.602292485
Zm00001d011821_T001	4.124721762	3.210515422	3.059576442	3.784879056	4.025161449	4.134962718
Zm00001d011821_T011	5.450799877	4.874640002	5.634479806	5.340236202	5.261213244	5.098487692
Zm00001d011821_T002	0.576446676	0	1.289772508	1.739524108	1.112965631	1.533390912
Zm00001d013429_T001	1.113004985	0.711308115	1.198443234	1.066507382	0.785210709	1.083305289
Zm00001d018616_T001	1.489042427	2.754015851	1.7370849	1.501891055	1.684152656	1.465179915
Zm00001d018616_T004	5.291274118	4.492904406	5.308953042	5.199012048	5.251647638	5.133383835
Zm00001d011830_T004	1.790998431	1.20109118	2.343969724	2.190380976	1.996446598	2.649543955
Zm00001d011830_T005	0	0	1.052319201	0.721742747	1.23273688	1.00183034
Zm00001d035775_T001	3.098777964	2.160595287	2.988721401	4.986792767	5.117900296	5.046886778
Zm00001d010305_T002	0.408420576	0.205707794	0.602494888	0.798334255	0.993468584	0.945836149
Zm00001d010305_T001	2.007928864	3.408541363	2.275937941	1.412220594	1.426542262	0.784006387
Zm00001d010305_T004	3.522417759	2.456108547	3.421816416	3.955891272	4.007895326	3.891751046
Zm00001d038167_T001	3.60054951	2.876331287	3.79159041	4.729385271	4.811282358	4.641742652
Zm00001d038167_T003	1.470457733	0.292312944	1.014588819	2.780775592	2.447891769	2.789223854
Zm00001d023379_T001	6.443538924	5.578781155	6.5008728	6.527721125	6.532550989	6.567938755
Zm00001d049376_T001	4.322748106	3.381366304	4.303626317	4.446810105	4.518731828	4.327693902
Zm00001d038151_T001	1.719890921	1.085739404	1.629038551	0.885052635	0.890899568	1.310074245
Zm00001d035598_T001	5.392539443	4.668324812	5.34374234	5.893193296	5.873872686	5.848565357
Zm00001d028426_T001	7.73774245	7.796555323	7.769533057	7.108321137	7.23610121	7.221193311
Zm00001d036651_T004	0	0.711760074	1.103668168	1.503992334	1.402812206	1.562037226
Zm00001d036651_T005	2.193445642	1.918748389	2.157483245	2.807894183	1.75335123	2.394374201
Zm00001d020901_T001	3.455171878	2.86260334	3.526272428	3.577743256	3.59022474	3.694563434
Zm00001d039102_T001	5.237704662	4.808308743	5.481533515	5.456882092	5.551600916	5.39922692
Zm00001d014423_T008	4.774378515	0	4.795827821	4.122658557	4.291520165	5.022091721
Zm00001d014423_T005	3.330308416	4.0965718	3.276015538	5.153562611	5.191333185	4.329708882
Zm00001d014423_T023	3.549621491	1.684704305	3.679787579	3.608089489	3.924867408	3.768667719
Zm00001d014423_T018	2.205160387	2.672660001	2.139759904	1.460187393	1.641349957	0
Zm00001d014423_T003	1.322456025	3.366449655	0	0	1.066504627	2.192436593
Zm00001d014423_T013	1.722838454	0.963575479	1.674725935	2.420858287	2.214587699	1.943159187

Zm00001d014423_T022	1.378031015	0.083517026	1.594897716	1.209069636	0	1.219650552
Zm00001d014423_T019	5.641479182	5.765758771	5.804512333	3.951584251	4.307695108	4.001163605
Zm00001d014423_T020	0	0.072193629	0.550585502	2.682845856	2.655798079	2.230177492
Zm00001d014423_T002	0.641103905	0.202627097	1.024582802	0.8283968	0.888708575	0.474186811
Zm00001d014423_T009	1.266375183	0.10186922	1.490517761	1.333101954	1.436339366	0.684744245
Zm00001d014423_T021	4.66203746	0	4.464658466	5.009945394	5.168285128	5.102146421
Zm00001d014423_T001	1.943595816	1.400682192	1.951784343	1.331556182	2.34424619	1.689069653
Zm00001d014423_T015	1.160041141	1.459696001	1.785591143	1.580334132	0	0
Zm00001d007631_T001	6.73326798	8.608918339	6.469702698	4.083449542	3.761074493	4.262737721
Zm00001d005081_T001	2.266967897	3.050571349	2.537461079	2.470203126	2.717062424	2.432200724
Zm00001d041625_T005	0.494136005	0.220585035	1.194418683	0.707523907	0.302764733	0.802282568
Zm00001d041625_T004	0	0.330725892	0.781401682	0	0	0.700236395
Zm00001d041625_T006	1.181340488	0	0	0.712971647	1.392987777	0
Zm00001d020436_T001	0.892677788	0.365786586	0.943760081	1.02295649	0.943234969	0.816473555
Zm00001d034133_T001	2.039999892	1.550888847	2.175920869	1.68267262	1.625451926	1.61301766
Zm00001d044260_T004	0.943348259	0.736730416	1.273056302	1.263374601	1.644415616	1.040139158
Zm00001d044260_T001	1.294842447	0.516667743	1.118070626	0.537019679	0	1.216053121
Zm00001d044260_T002	1.297522479	0.884161042	1.36823307	1.294198428	1.192309703	0.881281677
Zm00001d025663_T004	2.837995299	1.463681565	2.858409163	2.864515215	2.879041111	2.643083622
Zm00001d025663_T002	3.936163951	3.318369486	4.009198272	3.367155484	3.303009741	3.283574684
Zm00001d025663_T001	1.810267872	0.273496793	1.671989412	1.793962867	1.89183016	2.09408909
Zm00001d029672_T003	1.394824159	0.745888711	1.455224362	1.282103483	1.226416046	1.284186588
Zm00001d003754_T001	0.787373117	0.488072775	0.937739855	0	0.503025047	0
Zm00001d033529_T001	1.183366457	0.932278164	1.453550765	1.112405226	1.308619496	1.275598312
Zm00001d013249_T001	5.504352007	5.058027884	5.488766501	5.953544973	5.970553307	5.953036196
Zm00001d046586_T001	3.764648065	3.165361601	3.827711123	4.564637477	4.660120348	4.699343408
Zm00001d004089_T001	3.894593995	4.393904956	4.088941355	3.798421772	4.153997575	3.893503172
Zm00001d009780_T005	2.246514195	1.393966923	2.051294831	2.446254377	2.463779377	2.13019616
Zm00001d009780_T006	3.319468946	3.884031476	3.208790978	3.332577333	3.139702285	3.311188909
Zm00001d009780_T001	0.784677347	0.672600944	1.161307264	1.500452297	1.532392771	1.540433032
Zm00001d034665_T001	4.737653884	4.232504508	4.659498478	4.955472066	5.04354994	4.929044323
Zm00001d043430_T001	4.190438502	4.520105072	4.07144726	3.538151117	3.409240616	3.393305674

Zm00001d034037_T001	7.091526309	6.662018641	7.197263586	7.051991314	7.144276097	7.110383342
Zm00001d003509_T009	4.36008168	3.704143574	4.432244562	5.647510751	5.440800083	5.45598231
Zm00001d003509_T001	2.682346089	2.743729084	2.875488743	4.158390594	4.302942792	4.556353815
Zm00001d003509_T007	2.150637333	0	1.872162898	3.596972461	3.689219195	4.216149609
Zm00001d003509_T004	1.788317787	0.545863622	1.711926109	4.352341656	4.003791106	4.027619242
Zm00001d049821_T001	1.8771647	1.487823316	1.60161713	2.190579464	1.960980176	1.732981144
Zm00001d052999_T001	1.809197509	1.67607013	1.563741437	1.638700375	1.487401967	1.373071296
Zm00001d046803_T001	5.165014255	5.31227243	5.337372283	5.040635159	4.980751356	4.869577311
Zm00001d020133_T001	4.030940224	5.538373554	4.878217857	4.200431297	3.791241775	4.264081708
Zm00001d028669_T010	1.045089943	0	0.816688989	1.617345976	1.398050416	1.177530046
Zm00001d028669_T020	0	0.649506937	0.482453875	1.013956711	0.556462769	0.478219078
Zm00001d028669_T006	0.434586064	0.056711146	0.482809054	0	0.568301642	0.831542465
Zm00001d026618_T002	0.876661135	0.63808867	0	1.18323813	1.237711162	1.029090974
Zm00001d026618_T001	0	0.399336276	0.956421742	1.252996883	1.533492582	1.459748452
Zm00001d053816_T003	0.201594971	0	1.188973078	0.169626172	0.891842212	0.810500687
Zm00001d053816_T008	0.95519524	0.886893466	1.434998044	1.058308182	0.598562443	0.714651723
Zm00001d053816_T010	3.96128595	3.313998498	3.887875472	3.539509855	3.875051259	3.988604439
Zm00001d053816_T009	3.767852652	3.582088195	3.931952285	3.678193064	3.147875523	2.515132866
Zm00001d053816_T006	0.91241457	0.924253461	1.06652047	0.498324405	1.417017501	0.389512847
Zm00001d053816_T007	0.993443222	1.124304972	1.005909422	1.790792469	0	0
Zm00001d053816_T011	0.984715478	0.380433566	1.141559005	1.104692928	1.280138555	1.70947063
Zm00001d053816_T005	0.322967615	0	0	0.991991776	0.903280499	1.286481395
Zm00001d053816_T013	2.111361558	0.56731082	2.74212201	1.82691045	1.882245104	1.54513512
Zm00001d054014_T001	4.438036231	4.08334163	4.734317479	4.334834824	4.36616622	4.24216887
Zm00001d025861_T001	1.116681476	0.882318284	0.92832029	1.294514298	1.270957651	1.066992939
Zm00001d009774_T001	5.664632791	4.937631678	5.739847725	5.805651949	5.93373708	5.816079848
Zm00001d021421_T015	6.577907149	0	6.729390723	6.22151383	6.382105073	6.163874553
Zm00001d021421_T002	5.713775645	5.010544934	5.769937232	5.873531028	5.90644839	5.609373587
Zm00001d021421_T010	3.291495705	2.404027324	2.83771098	3.503135114	3.432630222	3.299348855
Zm00001d021421_T016	5.841099113	0	5.48647414	5.396997072	4.155786222	5.210018869
Zm00001d021421_T020	4.913858932	0	4.702489884	4.515886888	4.6767364	4.346640454
Zm00001d021421_T018	0.984086188	0	0.739332269	0.984168602	0.696316157	1.04448505

Zm00001d037733_T001	4.29119848	3.837877159	4.272679645	4.380508443	4.306503447	4.258957244
Zm00001d014261_T066	0	0	1.069812822	1.364961207	1.264580267	0.831599212
Zm00001d014261_T056	1.983784571	1.103962182	1.436703415	1.739969474	0	1.804074629
Zm00001d014261_T049	0	0	0.175455618	0.717833226	1.534796711	0.463494816
Zm00001d014261_T048	0.753359788	0.579498781	0	0	0.19486746	0.68791709
Zm00001d014261_T058	0.332507446	0.24252934	0.304985243	0.272527298	0.351379567	0
Zm00001d014261_T059	0.380495629	0.518043582	0.603716301	0.99527321	1.006971543	1.312984576
Zm00001d014261_T035	0.72088694	0	0.694857912	0	0.74393886	0
Zm00001d014261_T047	0.363833807	0.281680448	0.601761163	0.547744952	0.74613817	0.612265675
Zm00001d002463_T001	4.894416226	4.931067464	4.738604538	3.387758318	0	2.92847967
Zm00001d035610_T001	8.598622764	8.150547745	8.368332306	7.971910142	8.282964243	8.225522443
Zm00001d041597_T001	5.574861751	4.960009997	5.461579372	5.063251873	5.142650001	5.078890687
Zm00001d020706_T001	5.285287391	4.828482969	5.210525163	5.169904563	5.309094568	5.209053762
Zm00001d044669_T001	1.332445576	1.377854492	1.356376539	1.365848723	0.713167023	0.770063633
Zm00001d033155_T002	2.861601692	2.188716143	2.671299488	2.054796393	2.427860365	2.454656273
Zm00001d033155_T001	2.812693515	1.72592478	3.080720759	3.590128548	3.55794979	3.419791543
Zm00001d017575_T001	5.783129785	5.354444685	5.859244392	5.905699544	5.980587321	5.935359439
Zm00001d044951_T001	0.278553384	0.252793481	0.305684579	1.573871821	1.091278726	1.317471948
Zm00001d043803_T002	4.287418511	4.397473116	3.715385044	4.369715915	3.990146453	4.271209166
Zm00001d048526_T001	6.605188979	6.824334776	6.636666103	6.202113771	6.340698272	6.152128485
Zm00001d032099_T004	5.233707789	4.341185427	5.27422975	5.336041288	5.417121972	5.241773652
Zm00001d037342_T001	4.315910633	4.182961268	4.471209185	3.588770753	3.809802849	3.893736913
Zm00001d045435_T011	2.852149893	5.316274869	3.586345262	3.230427917	2.729884014	3.31515565
Zm00001d045435_T005	0.827229634	0.599941398	1.035172029	0.58298658	1.048633779	1.192210581
Zm00001d045435_T001	5.066937719	3.996047738	4.816518047	5.055364396	5.157133471	5.144254395
Zm00001d045435_T010	0.112357181	0	0.30610822	1.090959766	0.7392977	0.811632143
Zm00001d041608_T001	3.341065914	2.597936097	2.957829186	4.633906257	4.579889974	4.641813331
Zm00001d020927_T001	3.025670775	3.008192124	3.024852966	0.924436665	0.978631302	0.849770331
Zm00001d033412_T001	1.724977187	1.395118202	1.456209502	1.148314605	1.461389731	1.340203331
Zm00001d042661_T001	3.371463656	2.628133396	3.502527539	3.564074963	3.50268843	3.417362692
Zm00001d042661_T002	0.209780235	0.142013414	0.292460199	0.365373396	0.396244996	0.478803071
Zm00001d053985_T001	1.537044039	0.968502944	1.35482675	1.379911468	1.468949223	1.173487582

Zm00001d046158_T001	4.374652181	4.797953848	3.837208087	3.397711798	2.141590066	1.549262054
Zm00001d010037_T001	1.800086482	0.989222587	1.811341963	1.799713996	2.231522113	1.884698721
Zm00001d047705_T001	7.48156185	8.695957568	7.474247557	5.81375503	5.630826487	5.742411359
Zm00001d048180_T001	3.661088517	2.814503051	3.658166459	3.486765851	3.421508645	3.38005947
Zm00001d051140_T001	5.099276644	4.477808899	5.217412166	4.667788885	4.54566757	4.601832936
Zm00001d014954_T006	8.774078615	8.268342004	8.735699921	8.552496732	8.586607013	8.620169123
Zm00001d014954_T003	2.192038207	2.055335196	2.745926993	2.256235037	2.540245046	2.325587031
Zm00001d014954_T005	4.420367664	9.058573123	5.574132094	4.487198	3.619963923	3.68026505
Zm00001d014954_T001	1.876806488	4.295644423	1.041951537	1.694266443	1.59989666	0.217539699
Zm00001d014954_T004	4.391246958	5.869296631	3.697572789	4.336438862	3.537730177	4.174593695
Zm00001d027478_T001	4.176263962	4.743420663	4.354826397	3.977373352	3.875117431	3.930844424
Zm00001d053228_T002	8.117068755	8.233390471	8.046810816	7.86940499	7.868136817	7.937414824
Zm00001d053228_T001	1.70136674	1.98987434	1.813076009	1.604178573	1.222622822	1.939924245
Zm00001d038739_T003	4.014424748	4.106754502	4.050395733	4.744306327	5.093643708	4.786855396
Zm00001d038739_T001	4.431948298	3.674689218	4.329521036	6.37708697	6.416744386	6.461672088
Zm00001d042039_T001	0.740287755	0.446928449	0.80424194	1.102416262	1.177044578	0.961522577
Zm00001d038963_T001	2.497242416	1.93882362	2.410149764	2.294152541	2.211892499	2.190771289
Zm00001d009595_T001	0.510754359	0.456257348	1.107518485	1.169782007	1.176366819	1.422864311
Zm00001d021467_T005	0.590682493	0.580552663	1.388838673	1.175639565	1.050381889	0.270221533
Zm00001d021467_T003	1.148158386	0.597667512	0.844828317	1.217121502	1.386638806	1.044288493
Zm00001d021467_T007	4.52344089	4.795271352	4.466733605	3.997893481	4.02471142	4.287071376
Zm00001d021467_T004	3.808338505	0	3.628130479	3.604835417	3.370403966	3.662788044
Zm00001d021467_T002	2.87960893	3.521289742	3.398861462	4.067068426	4.209382867	3.782797076
Zm00001d013127_T003	2.301506877	1.438224176	2.113877492	3.285075171	3.222784317	3.108647394
Zm00001d043895_T005	0.259599109	0.206215603	0.257684121	1.600007075	1.720284595	1.854780435
Zm00001d043895_T001	0.502095311	0	0	1.09686701	1.713324097	1.339938913
Zm00001d043895_T004	0.481008513	0	0.696129171	0.595867391	0.728484792	0.648496736
Zm00001d043895_T002	4.687936121	4.072692794	4.789408286	4.951708776	4.959428309	5.038523187
Zm00001d001908_T002	3.766076716	3.449284694	3.89071789	4.195296331	4.204433969	3.935485773
Zm00001d001908_T001	2.617135536	2.356300469	2.7343797	3.000173654	3.186499448	2.865790173
Zm00001d030661_T001	1.357794638	1.268012722	1.384014983	1.202240293	1.421097239	1.483584154
Zm00001d002706_T003	1.159824847	0.446538917	1.008735707	1.520384531	1.455754622	1.310016641

Zm00001d036137_T005	3.765944583	2.378749094	3.969692499	4.132599462	4.066335332	4.051590404
Zm00001d036137_T008	3.004763121	3.581083414	2.724927435	0	0	0
Zm00001d036137_T004	0.116423315	0.068394282	0.171226046	1.381062382	1.524621383	0.73204773
Zm00001d036137_T003	0.400636292	0.691200028	0.456499183	0.710202731	0	0.91833051
Zm00001d031799_T001	0.885176055	0.643285462	0.936999309	0.86980825	0.8930818	1.111614951
Zm00001d003866_T001	2.310944704	1.834003244	2.117785458	2.447465469	2.331278994	2.426907986
Zm00001d017621_T001	3.544200603	3.427931413	3.516556182	2.936126062	2.590641059	3.039932711
Zm00001d045191_T001	5.278210355	4.824908807	5.385338571	6.228375696	6.239289881	6.082858779
Zm00001d003884_T001	2.287228476	2.107747785	2.171138919	1.413571337	0.839744376	1.757729055
Zm00001d025726_T001	4.604071027	3.936887309	4.545108857	4.834045884	4.806568645	4.837620617
Zm00001d017209_T001	1.208358061	0.822453613	1.385449261	1.60355205	1.838206333	1.644732159
Zm00001d050051_T002	4.360502358	5.215101947	4.419608126	4.488382096	4.391134134	4.497881465
Zm00001d050051_T001	2.130024454	1.158965807	2.198019463	2.416935918	2.3815932	2.342160738
Zm00001d047869_T009	1.075826274	1.009372973	1.861099018	1.61676231	2.273543639	1.309229167
Zm00001d047869_T007	1.501126241	0	0	2.025861914	0	2.241348119
Zm00001d047869_T003	2.684305962	0	0	0	1.869342387	1.050246744
Zm00001d047869_T005	3.278328573	2.15749715	2.4722863	2.796460704	2.686190553	2.944800549
Zm00001d047869_T016	0.745058302	0.333253119	0.628092324	0.209919944	0.161907026	0.087731667
Zm00001d047869_T010	0.58978841	0.758164999	1.451912141	1.602510566	1.808548567	1.061483828
Zm00001d015990_T001	1.2887637	0.647929683	1.228220868	1.818288995	1.636505836	1.453235729
Zm00001d012415_T001	2.846833866	2.507756337	2.856737951	4.822039834	4.849652451	4.877032738
Zm00001d044068_T001	3.746552264	3.142733965	3.758693224	4.027040695	4.014489022	3.917340856
Zm00001d027768_T002	0.224773787	0	0.18000142	0.475454332	0.56983617	0.316586016
Zm00001d027768_T005	0	0	0	1.837214245	1.831685545	1.610512509
Zm00001d027768_T014	0.520104381	0	0.626238197	0.541749735	0.673184622	0.94624801
Zm00001d027768_T012	0.667135505	0.056534975	0.513553404	0.675030024	0.59919685	0.947675881
Zm00001d027768_T007	1.180739843	0.950409436	0.912058104	0	0	0
Zm00001d027768_T016	1.004350373	0.776548733	0.671356786	1.841812945	1.809839575	1.424129866
Zm00001d027768_T023	2.188377819	1.421109091	2.28686341	2.817168891	2.626319978	2.346731145
Zm00001d015657_T001	3.7579351	3.971635516	3.568666338	3.696717206	3.362479708	3.389791183
Zm00001d042799_T001	2.633898238	2.327175145	2.447813235	2.475041031	1.988500737	2.09809139
Zm00001d037188_T001	2.042850222	1.95191449	2.246637927	2.979451582	3.154112808	2.741576357

Zm00001d051149_T001	1.283111028	1.246887791	1.031350023	1.664156035	1.808173728	2.00751451
Zm00001d008396_T001	3.880184756	3.402791609	3.802897845	3.120047808	3.35182434	3.515368564
Zm00001d011991_T001	2.981232709	3.384245884	3.367593045	3.875873128	3.659719439	3.701986789
Zm00001d052452_T001	5.839573877	5.740864677	5.839826923	5.158368093	5.205436502	5.168043743
Zm00001d041707_T002	1.601714104	1.742773719	1.828736774	1.749243769	2.244494373	2.385912775
Zm00001d041707_T003	1.392254767	0	1.475735506	2.098133847	0.7979999	0
Zm00001d037666_T001	3.223390112	2.356977346	3.243356656	3.241029698	3.285171961	3.261157532
Zm00001d036703_T001	5.302957264	4.537407216	5.039277479	3.419612642	2.478386327	3.049657949
Zm00001d047404_T001	5.018060694	4.113990976	5.085714424	5.207680374	5.024236202	5.003189786
Zm00001d044142_T001	4.637659626	4.016778966	4.752936033	4.517108734	4.44167549	4.803138881
Zm00001d029143_T005	2.976690267	2.374064327	2.944478611	2.487690854	2.026935295	1.927290812
Zm00001d029143_T003	1.453387988	0.95757143	1.676627586	2.70736112	2.604994306	2.640717629
Zm00001d029143_T001	0.091477786	0.471359169	0.139072585	0.454088486	0.75650327	0.464229119
Zm00001d032222_T001	2.598304456	2.086248769	2.699457908	2.896633204	2.8582134	3.0805052
Zm00001d032222_T002	2.215037705	3.871168952	2.522921006	1.696329958	2.328090803	2.017831069
Zm00001d044287_T002	0.916811387	0.72030386	0.585055792	0.82936574	0.574211008	0.912785506
Zm00001d044287_T001	8.654931679	8.36816092	8.762878925	8.283471295	8.337653681	8.299982634
Zm00001d040460_T001	1.712729149	1.903440548	1.603605695	3.777443598	3.63126077	3.805756871
Zm00001d035756_T001	8.402056502	8.274111154	8.409804663	9.249443812	9.260477166	9.271603733
Zm00001d000038_T001	0.682188527	0.45883868	0.908392525	0.560167115	0.822729332	0.585410628
Zm00001d039758_T006	2.906149437	1.890353688	2.895375724	3.071436276	3.076698296	2.937353244
Zm00001d039758_T002	0.248542122	0.13281634	0.319052537	1.034045935	1.171988823	1.062981683
Zm00001d032606_T003	0.357191638	0.222100968	0.399493784	1.227867968	1.059106038	1.454468088
Zm00001d032606_T001	1.00667575	0.53020961	0.963632442	1.352423514	1.72541485	0.268237948
Zm00001d032606_T002	1.370008562	1.170003225	1.256505888	1.897005315	1.862790179	1.797265791
Zm00001d052738_T001	2.251060377	2.321744284	2.705184809	2.856584786	2.787659174	2.838451425
Zm00001d051441_T001	5.021649039	4.338604188	4.976226922	5.12657369	5.241001774	5.086530389
Zm00001d000087_T002	1.004940696	0.69694728	0	0	1.157056649	1.36334548
Zm00001d019934_T001	2.833439652	2.253454735	3.045162478	2.977280839	2.964244281	2.772335638
Zm00001d005989_T001	6.641637119	2.2663356	6.374115289	5.457090488	4.812882876	4.813227141
Zm00001d051872_T001	9.682918821	9.247254465	9.747393202	10.14083204	10.15913363	10.14036266
Zm00001d014769_T001	2.120507549	1.578776929	1.715733501	1.611097764	1.780262638	1.726012004

Zm00001d014769_T002	2.637392539	2.052098661	2.89105944	2.507428815	2.423786022	2.331769411
Zm00001d007572_T001	1.435083414	0.897824357	1.061709158	0.824085135	1.146939954	0.84008612
Zm00001d007118_T003	6.803225435	7.806229671	6.89900181	7.092565509	7.182724482	7.03488808
Zm00001d007118_T004	1.176703185	0.478834128	1.075208117	1.436050941	1.401042238	0.99385548
Zm00001d007118_T001	5.797866406	0	5.639735513	5.294252511	5.347849879	5.378696873
Zm00001d007118_T005	1.046664969	0.707067968	1.21446927	1.302187426	0.791979893	1.015412644
Zm00001d016845_T001	4.989889459	4.776012948	4.973214916	4.863423209	4.880609471	4.77279495
Zm00001d007858_T001	3.202280733	2.67656147	3.267869841	3.21678667	3.098317477	3.188887016
Zm00001d052385_T001	3.198899874	2.519387915	3.125438847	2.987224622	3.185346447	3.23675756
Zm00001d052407_T001	2.04012688	0.373651552	1.467408874	1.718672875	0.531104436	0.940724547
Zm00001d052407_T002	3.045232737	2.570931609	3.34142421	3.152622165	3.462545788	3.454046229
Zm00001d037904_T001	1.032714475	1.528319763	1.481565547	1.792872007	1.105578856	1.481556247
Zm00001d016570_T001	2.318156581	2.389255387	1.979507739	2.747156636	2.484328415	2.645421991
Zm00001d037180_T001	3.783688075	3.743939183	3.784281908	4.671980131	4.654755964	4.565522298
Zm00001d013561_T001	1.878732011	1.251221599	1.782640049	2.763358455	2.852963641	2.924479612
Zm00001d028733_T002	4.182423356	3.504349878	4.072260269	4.153509472	3.914013649	4.01053191
Zm00001d028733_T001	3.413207904	2.033948708	3.520203093	3.80503764	3.943342445	3.680767
Zm00001d028733_T003	0.948831062	1.399105453	0.297402742	0	0.677952878	0
Zm00001d009123_T001	2.515569154	1.673413504	2.379364503	2.450482293	2.50912397	2.414045666
Zm00001d022624_T001	5.931841286	4.782640311	5.782556025	5.324181123	5.692010416	5.055916344
Zm00001d039072_T013	1.249740828	0.60437217	1.032665817	1.576644998	1.257685931	1.732185393
Zm00001d039072_T001	2.095844437	0.980023837	2.401622822	2.170851555	2.479506344	2.780269779
Zm00001d039072_T017	0	0.278379724	0.177375683	1.143004119	0.829563833	0.125873241
Zm00001d039072_T015	0	0.149723711	0	1.302741926	0	0.46169304
Zm00001d025132_T001	7.063581341	6.65746	6.886416919	7.091013227	7.12858913	6.958622485
Zm00001d010530_T005	1.188054611	0.68891976	0.550726349	1.221998306	1.519618893	1.779284098
Zm00001d010530_T002	4.706423134	3.845251372	4.375558986	4.896408458	4.954943154	4.810374978
Zm00001d010530_T004	2.447480545	2.092201686	3.636720894	0	0	0
Zm00001d047955_T002	3.228873166	2.297981674	3.184262836	5.733129539	5.704709929	5.696994391
Zm00001d047955_T001	4.917482215	4.208984921	4.905051575	5.950086367	5.979683625	6.053414618
Zm00001d018133_T018	3.103337499	1.807053161	2.911573094	2.854937182	2.667393736	3.000582371
Zm00001d018133_T002	0.596152775	1.053610457	1.181717673	2.622534727	2.498942685	2.230405628

Zm00001d020176_T001	1.622193826	1.482606695	1.981617785	1.930855019	1.667316749	1.89701771
Zm00001d038117_T001	3.722045724	4.203111083	3.67265672	3.228111712	3.807553485	3.829860416
Zm00001d014035_T001	0.708124538	0.458471251	0.77919247	1.06821334	1.048620528	0.901346934
Zm00001d002669_T001	0.165079678	0.540617522	0.923734128	2.051029573	2.241883799	1.284809038
Zm00001d002669_T002	1.261265419	0.702535208	0.193178776	1.988328763	1.763362705	2.489327646
Zm00001d045845_T001	5.427797491	4.925868241	5.520944887	5.18489438	5.287152958	5.393533446
Zm00001d027805_T004	2.329860116	2.194359739	2.167439503	2.72983095	2.664773626	2.686874515
Zm00001d027805_T005	1.383614169	0.859680185	1.523036468	1.651330958	1.419918527	1.880020929
Zm00001d048147_T006	2.938215208	3.527398934	2.563508631	2.313756075	2.085904568	2.173095124
Zm00001d048147_T001	1.66917874	0.572440545	1.280195581	2.080872858	1.159551043	1.085470207
Zm00001d048147_T003	3.288884157	2.460498556	3.448036925	3.211722772	3.317878109	3.468670762
Zm00001d030587_T001	4.403296767	5.666908834	4.541501334	4.544918019	4.391949008	4.503857723
Zm00001d051337_T002	5.088953181	4.555445374	5.124214841	5.137767037	5.141922503	4.957105153
Zm00001d051337_T001	0.909972745	0.318080784	0.783247097	0.850343384	1.417627336	0.78243205
Zm00001d028170_T001	5.759438698	5.348748051	5.633142177	5.609604752	5.752726945	5.718680107
Zm00001d008859_T001	3.023438118	2.189238508	2.879053466	3.214767967	3.216689371	2.922101539
Zm00001d009480_T001	8.401274851	7.838508847	8.464861593	7.376911182	7.382036844	7.398255726
Zm00001d022537_T001	0.9493519	0.588524442	0.784586061	1.35306522	1.599302081	1.23978723
Zm00001d017120_T003	4.847392401	3.365459067	4.682220558	3.285013009	3.368676729	4.385878341
Zm00001d017120_T001	6.147894852	5.683517987	6.383488304	6.110888288	6.116767214	6.049177284
Zm00001d017120_T004	0	0	0	2.516585302	1.99088844	1.452297061
Zm00001d017920_T001	6.604017459	5.862343346	6.632750173	6.819349353	6.854164101	6.773021043
Zm00001d016531_T001	5.292068543	4.32054006	5.239137175	5.033554842	5.01608803	4.992091012
Zm00001d004877_T001	0.569341371	0.293930737	0	0.972075012	1.001185408	0
Zm00001d007718_T002	1.698794552	1.812142899	1.629557592	1.291579395	0.997408035	1.155235651
Zm00001d007718_T005	1.646238708	1.13917477	0.888455314	0.546356659	0.567129712	0.740113292
Zm00001d015599_T001	6.593461357	5.993007335	6.606076785	6.843936486	6.953139056	6.840348972
Zm00001d027305_T001	1.701226993	4.4021492	0	1.642222365	2.490320076	0
Zm00001d027305_T002	6.372000525	5.514902462	6.495217255	6.782135046	6.761927414	6.701273452
Zm00001d053372_T001	3.926941893	2.978134112	3.903675095	4.1501628	4.070960282	3.930432116
Zm00001d012604_T001	2.296185729	1.852891753	2.466070293	2.840419927	3.128994449	3.224352279
Zm00001d044717_T001	3.063255627	2.07871332	2.953128135	3.993946121	3.933668941	3.948867964

Zm00001d008782_T001	2.070643495	1.621355174	2.508875337	1.336985995	1.270998303	1.174732378
Zm00001d010056_T021	3.620315088	0	3.836823155	3.218402856	3.040880335	3.455411264
Zm00001d010056_T024	4.13779564	5.89521931	4.238556453	3.074690214	4.052591699	0
Zm00001d010056_T008	1.398665583	0	0	2.71739216	3.042771793	3.339463095
Zm00001d010056_T025	5.13663613	4.674597141	5.267274314	4.512003384	4.014273693	4.905816735
Zm00001d010056_T017	1.576340248	1.199001365	1.109327793	1.472807988	1.621987138	2.002141893
Zm00001d010056_T023	0.387937078	0.261126383	0.43496923	0.71672142	0.507357263	0.624319915
Zm00001d010056_T004	3.137988548	2.823875896	3.570229264	3.956817957	3.707551405	3.523437377
Zm00001d012922_T003	1.303679646	0.987384903	1.24451264	1.302927882	0.56587594	1.258620226
Zm00001d029455_T001	4.629945586	3.895108892	4.348748405	4.268828476	4.390276008	4.201231026
Zm00001d025919_T007	1.340517264	0	0	2.369446103	1.774539132	2.26936804
Zm00001d025919_T004	0.478774084	0.349176152	1.138005101	1.495141512	1.233199058	1.503163997
Zm00001d025919_T011	1.077682578	1.481795428	2.259566571	2.023772358	2.74268858	3.028797031
Zm00001d025919_T008	1.14673146	0.553671129	0.211129349	1.210393229	1.242748216	1.1839495
Zm00001d025919_T003	0	0.059859164	0	1.573504925	1.553802829	1.708871443
Zm00001d025919_T002	2.524759272	1.569024377	2.129088116	3.676824675	3.421275552	3.85529628
Zm00001d025919_T009	1.344750112	0.994489938	1.210591476	2.939392469	2.827133073	2.679832496
Zm00001d039297_T001	4.27440898	4.963091389	4.739218082	4.160018545	3.539248286	3.92173299
Zm00001d045053_T001	8.263707741	4.483266062	8.013201569	7.884793726	7.688447957	7.659913359
Zm00001d029699_T002	5.88711185	5.766058318	6.118673454	7.284195168	7.351021764	7.329081248
Zm00001d022120_T001	2.690256166	1.522732231	2.831805915	2.511516364	2.697818069	2.618362948
Zm00001d004992_T002	3.914454595	3.349063741	3.947033214	3.750772202	3.944565755	3.900063504
Zm00001d004992_T001	1.231617956	0.542095567	0.774517626	1.070775369	1.061301326	0.661327308
Zm00001d016767_T001	4.18523392	4.24569926	4.541618354	3.790966955	4.252504144	4.070039947
Zm00001d040689_T002	3.509678489	2.754047502	3.526392129	3.708296731	3.940206694	3.695016546
Zm00001d040689_T006	3.849061103	3.361141324	3.452566048	3.388036441	2.918378792	3.162689727
Zm00001d040689_T005	0.392586701	0.279988255	0.43048654	0.463281359	0.502436521	0.474066334
Zm00001d014039_T001	4.311705308	4.022191483	4.27668596	4.026996182	4.060157225	3.873647171
Zm00001d014039_T002	4.077350428	3.446339745	4.513216266	4.751205056	4.686038735	4.906427849
Zm00001d050319_T001	3.837528634	3.417953618	3.762851386	3.77981368	3.751019104	3.733574199
Zm00001d028752_T001	3.732647464	3.001278923	3.808965822	8.26614378	8.283810122	8.272688275
Zm00001d002256_T014	0.452410935	0.291643632	0.093527725	0.516770599	0	0.287571584

Zm00001d002256_T008	0.644041766	0	0	0	1.496173424	1.29820667
Zm00001d002256_T012	0.47994966	0.913537817	1.031544117	1.0839826	0.268212792	0.611892839
Zm00001d002639_T001	5.222528582	4.619515897	5.202887794	5.263652941	5.232781347	5.170315118
Zm00001d048471_T001	4.142724246	3.53739038	4.312785538	4.227220608	4.230664236	4.266081134
Zm00001d030732_T001	4.420531448	3.691977178	4.573077691	4.33410785	4.219911869	4.343308536
Zm00001d047076_T001	3.021772856	3.365363177	3.284866029	1.88713335	2.299913383	0
Zm00001d047076_T003	3.312871922	2.043043475	3.306435493	3.982195572	3.930507434	4.027244217
Zm00001d013037_T002	1.667628307	1.584794176	1.664371334	0.327704607	0	2.00361231
Zm00001d013037_T001	2.277331073	2.227419483	2.38190822	1.372204934	1.848111917	0.409483266
Zm00001d041536_T001	3.272487584	2.450929866	3.295885649	2.785879645	2.772090034	2.76113142
Zm00001d026213_T004	0.08987098	0.09531279	0.317552987	0.71688118	1.120975602	0.443058111
Zm00001d026213_T006	0.786893234	0.505854354	0.714491717	0.795937302	0	0.93494368
Zm00001d026213_T003	2.531333796	1.691489499	2.425043517	3.224057258	3.173936683	3.308777033
Zm00001d026213_T001	0.755706293	0.907285263	0.380321625	0.348805757	0.254217902	0.793526372
Zm00001d026213_T005	3.27268584	2.489018271	3.269728735	2.842049175	2.914078058	2.525770223
Zm00001d006705_T001	1.581042668	1.244404863	2.03830202	2.657126854	2.464942405	2.298647173
Zm00001d005648_T002	2.58081963	2.210007254	2.233819052	2.167235236	1.887990757	1.997702483
Zm00001d046530_T001	6.361531341	5.816643145	6.341078175	8.741198651	8.738509327	8.732735398
Zm00001d016760_T001	7.968828758	8.4508926	8.121967613	8.118591364	8.134518162	8.07498045
Zm00001d003658_T001	2.25809344	1.814258779	2.266653432	1.937269807	2.265641823	2.191154545
Zm00001d001913_T001	7.881585509	6.641690681	7.945032864	7.651388711	7.703412129	7.732209412
Zm00001d001913_T003	1.112615403	2.906306474	1.09705451	1.611869239	1.708024744	1.422912737
Zm00001d001913_T005	8.903783875	10.26910087	8.872557269	8.34004901	8.357409224	8.42804907
Zm00001d001913_T004	7.647120314	5.54540137	7.567583337	6.967176183	7.092626003	6.847383543
Zm00001d001913_T002	5.367971609	8.458613059	6.156196343	3.843681509	4.856920872	4.74853401
Zm00001d049683_T001	5.494548033	4.646021146	5.608191176	6.287410642	6.268097973	6.272576919
Zm00001d053284_T001	0.796067755	0	0.665331824	0.131839679	0.954232052	0.121178566
Zm00001d053284_T003	0.471780548	0.135691148	0.223284143	0.976616589	0.911104799	1.004678999
Zm00001d053284_T004	1.569763273	1.766554032	1.739345664	1.038530298	1.514659083	0.570740757
Zm00001d053284_T005	1.386915766	0	1.694361398	2.173828138	1.850969401	2.262118906
Zm00001d017822_T001	2.990475697	3.241258566	2.437411261	3.529291112	3.589026973	3.757189488
Zm00001d017900_T001	2.96022774	3.27723197	3.384637544	3.096147472	3.545265872	3.456535849

Zm00001d023465_T002	1.936217294	1.389728693	1.448051995	1.821997501	1.829877967	1.625305563
Zm00001d023465_T001	3.210421752	2.414163683	3.034677931	2.362330732	3.100807642	2.97392329
Zm00001d036108_T003	6.505619748	6.142013374	6.483826078	6.588515852	6.649950689	6.504992347
Zm00001d026366_T002	2.598130772	1.502267454	2.65742347	2.671674266	2.681659751	2.660456568
Zm00001d023615_T001	5.614712435	5.032983078	5.753036567	5.207390631	5.309146531	5.27718781
Zm00001d025425_T001	5.743217383	6.006896187	5.696841732	5.533392314	5.550150009	5.391107456
Zm00001d031580_T001	5.591275188	4.881818032	5.469372446	6.805624093	6.860069587	6.856800188
Zm00001d031580_T002	3.951607749	5.458663573	4.303590227	5.524727496	5.406933445	5.199233542
Zm00001d036971_T012	0.527741968	0.047259175	0.113200403	0.433772431	0.375505468	0.182182498
Zm00001d036971_T011	1.33054578	0.667228174	1.845811573	1.744751403	1.905325096	2.17791014
Zm00001d036971_T024	1.851198939	0	2.750625583	3.586950367	3.424623847	3.531058635
Zm00001d036971_T003	1.66711052	0	0.765798651	1.143411705	1.101590915	1.703568082
Zm00001d036971_T045	3.482065557	2.025950832	2.797397161	2.526599956	2.54231006	0.896004407
Zm00001d036971_T042	0.2057153	0.292903048	0.697858315	0.389463286	0.539134983	0.461350438
Zm00001d036971_T044	1.082088734	0.596342681	0	0	1.208319976	0.955299411
Zm00001d036971_T025	4.590593277	3.782434357	4.765807082	4.465630331	4.428529618	4.627712994
Zm00001d013166_T001	3.971711564	3.062500819	4.057665231	4.171521502	4.323028451	4.293472385
Zm00001d033419_T001	4.873739192	4.582689428	5.063548587	4.140088876	4.05288944	4.206246944
Zm00001d043224_T001	6.090542041	6.243802252	6.167779382	6.320974627	6.347124137	6.163531478
Zm00001d021269_T015	2.781628082	2.238304454	2.81860273	2.317282937	2.329845481	2.076090434
Zm00001d021269_T018	1.561729863	0.567326398	1.819509192	1.259550904	1.354637211	1.120627977
Zm00001d025144_T003	4.353232943	3.394481079	4.152803369	3.886223821	4.037663705	4.204170381
Zm00001d025144_T001	2.213380879	2.093312044	2.715740529	3.548275083	3.395611249	3.267859806
Zm00001d047911_T007	0.440127261	0.106979402	0.650764559	0.519685815	0.750362105	0.142345468
Zm00001d047911_T010	2.715518045	0	2.402293779	3.407246551	3.735992709	2.755809891
Zm00001d047911_T005	2.322903316	1.847781688	1.409311637	1.760490082	2.28060183	2.590707161
Zm00001d047911_T004	1.56134569	0.540436008	1.908381764	1.114081123	0	1.504356489
Zm00001d047911_T009	1.062174914	1.669946531	0.727590285	0.932628913	0.80193175	0
Zm00001d047911_T006	1.654567064	0.475230183	1.456787754	1.14310472	2.209453678	2.236818929
Zm00001d047911_T001	1.538795889	0	1.719333289	2.081304514	1.605142507	1.756209047
Zm00001d047911_T014	3.220427163	4.68845891	3.963927577	1.921215796	0	2.145855458
Zm00001d025667_T001	2.971231392	2.301728983	2.880786794	2.975279649	3.18262318	3.223477075

Zm00001d012042_T002	2.201318943	6.079892244	2.313982976	2.51373328	1.660052843	1.303355255
Zm00001d012042_T001	8.14912	8.269395402	8.117101689	7.968101964	8.100486866	7.972132961
Zm00001d024572_T002	3.969006319	2.981920586	4.037686456	6.200342248	6.164099757	6.288693245
Zm00001d044584_T019	3.781136734	3.121844132	3.53507589	0	3.584954205	3.31181769
Zm00001d044584_T003	0	0.238344202	0.509989671	0.153630257	0.676953383	0.415970621
Zm00001d044584_T005	1.117796094	0	0.549632702	0	0	2.056826963
Zm00001d044584_T013	2.559870609	1.644445612	2.680068205	3.28742782	0	2.6402933
Zm00001d044584_T006	0.609041947	0.157680167	0.513814116	2.430660753	2.375790689	1.349265622
Zm00001d044584_T011	0.239679098	0.061807988	0.528407287	0.722748415	0.699293097	1.002308628
Zm00001d044584_T020	1.078154139	0.390590652	1.38887889	2.345872937	2.835188193	1.96606384
Zm00001d044224_T001	3.142496926	2.730336061	3.108488493	3.298301263	3.430538994	3.422181052
Zm00001d022593_T001	2.752768712	2.305703838	2.610266338	3.951453608	3.97370586	3.963198598
Zm00001d031033_T001	1.605866457	1.853566566	1.381946694	0.860879391	0.784137923	1.3754532
Zm00001d048908_T001	2.75691504	3.956026813	3.345263796	3.451388247	3.427647201	3.657906342
Zm00001d048908_T002	5.603252249	4.521725052	5.54218644	6.578735197	6.622126472	6.453334811
Zm00001d008617_T006	0.627667533	0.643656738	1.156236092	0.483123892	0.229715883	0.697656407
Zm00001d008617_T005	1.160415544	0.187838677	0.755735344	2.224088137	1.995488861	2.123932001
Zm00001d008617_T001	5.165648613	4.288754917	5.117972294	5.181065767	5.146292385	5.136381064
Zm00001d006644_T002	3.75004754	3.162563254	3.713053827	3.973702922	3.871755744	3.90924085
Zm00001d017559_T002	5.20876765	4.415296327	5.262246269	4.471643418	4.458654321	4.612582038
Zm00001d043434_T037	3.173695763	1.169618476	3.096453649	3.664564871	3.500346487	3.527501152
Zm00001d043434_T035	0	0	1.416733832	0	2.407530867	2.255971383
Zm00001d043434_T005	2.456580924	2.43353904	2.805183063	0.738635557	1.941361014	1.413622243
Zm00001d043434_T006	0	0	0	2.07407825	1.658817916	1.51464444
Zm00001d043434_T036	0	0	0.177690769	1.227614172	0.360486767	1.305811218
Zm00001d043434_T003	1.429411897	0.883218057	0	1.853203244	0	0
Zm00001d042193_T006	3.553523442	2.301725179	3.497776104	3.259012251	3.791337747	3.128175922
Zm00001d042193_T003	3.496043644	3.064555415	3.417580216	3.14243239	2.81099293	2.959558371
Zm00001d042193_T008	0.542627531	0.310261003	0.736463738	0.913542412	0.694785718	0.937518409
Zm00001d042193_T007	2.423894104	2.22149262	2.297683874	1.690413073	1.666425756	1.576994171
Zm00001d042193_T002	1.464422047	1.307364987	1.119978278	1.480229442	0.275995134	1.69855366
Zm00001d054089_T001	6.039835024	5.241475194	6.135606465	6.09778019	6.147888158	6.189784242

Zm00001d025542_T002	5.785280243	5.553975699	5.820967582	6.200415308	6.160900388	6.149180761
Zm00001d011745_T002	1.709427398	0.980931957	1.3755038	1.676387477	2.245386437	1.771578419
Zm00001d011745_T004	0.144649434	0.082269307	0.271551174	0.809652336	0.717293664	0.87401709
Zm00001d048273_T001	6.988694933	6.556371903	6.973062917	5.43695118	5.693578256	5.823900932
Zm00001d046603_T001	2.06964065	1.556711918	1.863306142	1.624640897	1.868718763	1.576837973
Zm00001d006594_T001	5.224519639	5.428678845	5.139454187	4.516753646	4.487949015	4.319651964
Zm00001d008233_T002	4.192865636	3.597663579	4.310964765	4.376946491	4.319112599	4.291047044
Zm00001d008233_T005	0.938446145	0.337953091	0.824664407	1.199093738	1.068231917	0.941403836
Zm00001d052748_T001	2.848312061	2.254413241	2.994998353	3.250605554	3.260815759	3.291761933
Zm00001d026589_T001	5.780073069	5.731867572	5.63109421	5.365548582	5.543088135	5.088327859
Zm00001d028702_T001	2.438482625	3.102008184	2.690949375	2.7104494	3.063411477	2.748398543
Zm00001d000094_T002	1.686132495	1.016717378	1.30759989	1.15772026	1.288214433	1.069007114
Zm00001d040162_T004	2.722487009	1.920923603	2.360831145	2.835125926	2.785512679	2.704684756
Zm00001d040162_T006	1.09342834	0.960244794	0.477845157	1.490717986	1.223463328	0.778004986
Zm00001d040162_T010	1.316519404	0.564805426	1.552887064	0	0	1.105384414
Zm00001d040162_T002	0.526236115	0	0.460998321	0	0.514521235	0.774104312
Zm00001d040162_T008	0	0.224126735	0	0.795842571	0.960625876	0.705018882
Zm00001d040162_T003	0.819810403	0	1.679919625	2.433145877	2.350129454	2.020544871
Zm00001d019040_T006	0.914436703	0.411112724	0.578451857	0.195600835	1.404574556	0.571849583
Zm00001d019040_T013	2.609979952	1.327718402	2.446139751	1.713244902	1.723131696	2.31133047
Zm00001d019040_T012	1.554706718	0.822536824	1.412875264	1.675126253	1.910568815	1.702577318
Zm00001d019040_T004	0	1.767148389	2.494756594	2.058864719	2.665288386	0
Zm00001d019040_T005	0.245527303	0.137942823	0.305707924	0.385570192	0.114873379	0.302307351
Zm00001d019040_T007	2.679356234	1.681483449	1.259189916	2.791023028	2.374994377	3.104705842
Zm00001d005724_T001	3.752501664	3.886318556	3.770214105	3.785303839	3.796424155	3.48947717
Zm00001d020609_T003	1.914530082	0.613112928	1.986842314	2.4952802	2.802526188	2.371638592
Zm00001d020609_T001	3.151094252	2.504177729	3.126725615	4.388646181	4.300168176	4.380829309
Zm00001d020609_T004	4.059586478	3.585149798	4.083526046	4.689619314	4.618709041	4.667575174
Zm00001d016577_T001	1.042423014	1.174179481	0.878159529	0	0.903400827	0
Zm00001d016577_T005	2.390857226	0	1.707551295	1.552539384	0	1.822909174
Zm00001d016577_T002	2.385913604	1.9445279	2.888419075	2.106163699	2.43430049	1.922736379
Zm00001d023664_T001	3.68819558	3.177776704	3.697201787	5.216939937	5.344322045	5.404051344

Zm00001d051323_T003	0.833047943	0.456894443	0.779959778	0.69347313	0.44199545	1.328537786
Zm00001d051323_T001	1.858324813	0.688823104	2.089095387	1.330469484	1.768721278	0.983790042
Zm00001d002072_T001	1.009339289	1.937612942	1.885520872	2.720402589	2.663330728	2.746733688
Zm00001d012666_T003	2.239047255	1.53521689	2.335295732	3.046830303	3.121913406	3.012361998
Zm00001d012666_T001	0.221526957	0.606381582	0.36925317	1.125821676	1.065936903	0
Zm00001d049830_T002	8.308869226	8.292330202	8.350366002	7.84775856	7.920663539	7.905225137
Zm00001d015585_T001	2.773846775	2.629144893	2.725868736	2.811658445	2.722327869	2.625602248
Zm00001d006659_T001	3.436241676	3.017156364	3.545570832	4.067773963	4.147575429	4.296133958
Zm00001d032420_T001	2.897201308	2.831122466	3.20678558	3.037143042	2.800992643	3.084056609
Zm00001d049698_T001	3.351204136	2.642923561	3.559562417	3.981390278	3.984422925	3.795534444
Zm00001d043117_T002	4.711937174	3.802688509	4.492554012	4.516071391	4.460200501	4.490097712
Zm00001d005909_T001	3.452301147	2.725489017	3.382374258	3.426358426	3.554897248	3.416847307
Zm00001d009549_T001	0.601732643	0.506630375	0.570382293	0.56271085	0.891417631	0.924942067
Zm00001d053195_T001	1.355517003	1.311069428	0.583874298	0.640855035	1.51915388	0.811278658
Zm00001d050436_T001	4.077158383	3.681981828	4.093806323	4.310711509	3.861652803	3.889347861
Zm00001d025865_T002	1.064540757	0.148069855	1.073910699	1.615064527	1.922900753	1.200924257
Zm00001d025865_T001	3.852636819	3.287536125	3.955303503	4.429919133	4.3218675	4.45585856
Zm00001d038688_T001	2.049073794	1.86337117	2.295213143	3.253220078	2.337439081	3.091116377
Zm00001d038688_T003	2.912240373	2.21022239	2.611424534	0	2.969303397	1.498018486
Zm00001d051951_T005	1.417383757	0.733851497	1.43307375	1.837293378	2.073348967	1.677033242
Zm00001d051951_T002	3.47804184	2.468583578	3.61788581	3.841453897	3.922557906	3.755748267
Zm00001d051951_T006	3.656196474	5.11293007	3.473604713	3.12086051	2.730360845	3.048101537
Zm00001d051951_T004	3.304635739	2.582582978	3.234080345	3.575536948	3.21737684	3.252372512
Zm00001d051951_T010	3.816077006	3.840867535	4.047486477	3.933139765	3.981898307	4.000966823
Zm00001d051951_T009	0.118370358	0.673425264	0.124214303	0.749228749	0.937705209	1.305111358
Zm00001d048533_T001	6.107245746	5.444365657	5.698265243	6.903387859	6.941793294	7.25219623
Zm00001d052314_T004	4.044442646	0	4.43583145	2.716092084	3.885361361	2.925913993
Zm00001d052314_T003	1.605065207	3.370160513	1.710472103	2.868130438	3.108434965	2.56848771
Zm00001d052314_T034	3.647130966	0	3.031190141	3.812629962	3.864563153	4.043315367
Zm00001d052314_T007	0	0	2.062472324	2.601452402	0	2.3757426
Zm00001d052314_T011	2.360234751	0.807292267	1.990557021	3.119905591	2.405398827	3.022171416
Zm00001d023774_T001	2.748959435	2.683221241	2.250916413	1.148103055	1.017325471	0.317160486

Zm00001d028204_T004	0.964295117	0.586942443	1.170712178	1.043832321	1.283236107	0.613330793
Zm00001d000305_T003	4.962127998	3.380290451	5.053963665	5.026579257	4.990064425	5.054912754
Zm00001d000305_T002	3.453637283	3.854260716	3.371407337	3.602313273	3.791077327	3.681367289
Zm00001d038718_T001	2.424205936	1.97542512	2.771528557	5.132294568	5.331268351	5.32398773
Zm00001d038718_T003	1.178479457	1.996476607	1.205075285	5.462397503	5.29624554	5.262525917
Zm00001d037427_T001	1.930646516	1.719851504	1.220467407	1.478092722	1.604725134	1.779729544
Zm00001d037427_T003	2.12909867	0	2.029895609	1.576979665	1.265362479	0.904510302
Zm00001d037427_T005	1.995650216	0	1.890625681	0.910652084	1.197287317	1.555943735
Zm00001d037427_T004	1.781895143	1.829216746	1.523680378	1.673166979	2.124277507	1.626454557
Zm00001d037427_T002	1.89203813	2.402633779	2.590219709	2.741853092	2.524981622	2.681579248
Zm00001d024908_T010	0	0.187472591	0	1.2601136	0.835835166	0
Zm00001d024908_T008	3.67151327	3.101590579	3.364555904	4.240677858	3.904672334	4.481512333
Zm00001d024908_T004	4.136221661	5.012038202	4.182593466	3.944845705	3.198596302	3.354991448
Zm00001d024908_T009	0.77299706	0.245775534	0.731615985	0	1.458199293	0
Zm00001d024908_T011	0.29258977	0.124194448	0.499916793	0.381623376	0.389114109	0.381313278
Zm00001d024908_T001	3.250634202	1.150832593	3.711530368	2.849031669	3.745341254	2.467030835
Zm00001d037192_T001	2.07101745	1.429831783	1.840486599	1.901244201	1.836408932	1.676607729
Zm00001d012929_T002	4.118978271	3.469769306	4.186880752	3.96346728	4.052093182	4.067589072
Zm00001d012929_T004	1.533732774	0.995463534	0.744622684	0.762155735	0.935906254	0.39358424
Zm00001d020516_T002	0.242547632	0.243252296	0.314195338	1.184924523	0.823146074	1.024165046
Zm00001d051458_T007	3.880252848	3.400846109	3.946962028	3.908429131	3.741661778	3.818254017
Zm00001d051458_T006	1.593199812	0.749294836	1.426252945	0.828098596	0.960959425	1.569202816
Zm00001d051458_T005	0.402996066	0	0.246198889	0.757705164	0.681984414	0.168594544
Zm00001d051458_T001	3.162284168	3.09269068	3.59647436	3.596087996	3.977475201	3.867073597
Zm00001d023905_T001	4.353529658	3.405316857	4.179064478	4.996960785	4.954831701	4.890620671
Zm00001d050488_T001	1.510082873	1.300035256	1.752787976	2.744780245	2.655297326	2.531657433
Zm00001d042713_T008	2.695329308	2.437123316	2.625175554	0	2.500608576	2.098720369
Zm00001d042713_T005	0	0.338929804	0	1.221489218	1.404124903	1.477311039
Zm00001d042713_T006	0.955419197	0.841565002	1.231601983	1.49279458	1.099209684	1.65760388
Zm00001d042713_T002	0.600860605	0	0.583613431	0.729771143	1.133768671	0.68812753
Zm00001d042713_T007	1.199142749	0	1.357680927	1.926190373	0	0
Zm00001d035246_T001	6.275814034	5.587092239	6.325867958	5.874565939	5.908010726	5.884284342

Zm00001d043338_T001	7.387867677	7.788166699	7.476285513	7.238424545	7.253652759	7.15992526
Zm00001d034760_T001	10.14813972	10.98549061	9.892241357	9.835180463	9.9320886	9.94549148
Zm00001d019593_T001	4.068769767	4.215537348	4.155790512	3.706270722	3.467234216	3.7289598
Zm00001d028692_T005	4.932907646	4.316482766	4.9099273	6.5661492	6.467962577	6.519601706
Zm00001d052680_T001	1.274512154	2.769390284	1.731188456	1.892039296	1.724270014	2.422906012
Zm00001d052680_T007	3.537447784	2.422577215	4.052511016	3.003132475	3.81257082	2.629333304
Zm00001d052680_T008	3.255610857	3.920322078	0	2.883460312	0	3.162801046
Zm00001d024543_T002	1.74440052	0.694520087	1.298004263	2.833379131	2.60538742	2.967171928
Zm00001d024543_T003	0.428729857	0.284124387	0.361182181	1.178948512	1.475807088	1.160374235
Zm00001d024543_T001	1.733406841	1.617092974	2.032818837	3.552673647	3.723230778	3.374307399
Zm00001d026597_T001	4.966238027	5.188949902	5.148406863	5.437451345	5.302290716	5.706639013
Zm00001d052889_T001	2.327828753	1.790866269	2.361045427	2.868079655	2.773909423	2.899391245
Zm00001d009196_T001	1.154443871	0.814462649	0.737935048	1.081221671	0.965133379	0.985285467
Zm00001d033912_T002	2.535225601	1.575738783	2.346464233	2.484445976	2.451615999	2.49235699
Zm00001d033912_T001	1.75224463	1.083950614	1.859712384	2.093146035	1.956591615	2.078044122
Zm00001d044335_T001	2.090001447	2.337678271	2.168989511	1.929086518	1.91657677	1.804566178
Zm00001d043060_T001	1.40855427	1.42564782	1.765039053	2.592870082	2.890151508	2.611114766
Zm00001d045905_T001	5.758774199	6.504537292	5.716644935	5.569918903	5.645549237	5.45929895
Zm00001d052189_T001	0.950498276	1.044222734	1.289099064	0.447446954	0.607967977	1.201743
Zm00001d048414_T002	3.900964256	3.796735622	3.950776051	3.729426495	3.754268293	3.640055631
Zm00001d018857_T001	8.346719428	8.450300707	8.41100886	6.777481854	6.824170212	6.801762025
Zm00001d039375_T001	4.130586295	5.000297525	3.946333082	3.34540035	3.346211032	3.261606333
Zm00001d038577_T001	2.491336968	2.448346755	2.795408099	3.190469161	3.050176728	3.031238849
Zm00001d038577_T002	2.446283495	2.408983258	2.607405125	2.734944334	2.769650946	3.214054379
Zm00001d029100_T001	6.219025774	5.595198362	6.480516877	6.375761519	6.792437121	6.21050761
Zm00001d013725_T001	1.382168102	0.893442984	1.486678337	1.834298612	1.413378529	1.391771569
Zm00001d046823_T001	1.052012384	1.402169875	1.781052465	1.795652674	1.518051138	1.47538377
Zm00001d034588_T001	3.488305222	3.949438937	3.702593053	3.074910581	2.950801147	3.39192853
Zm00001d050851_T001	1.323298005	1.049963177	1.339558157	1.198198041	1.274506787	1.36819395
Zm00001d050851_T007	1.329149435	1.393579298	2.129486134	2.845351345	2.758028514	2.710906035
Zm00001d050851_T005	2.257101463	0.768678092	1.724447713	1.697146173	2.097112509	2.13300383
Zm00001d020721_T001	1.937085211	1.343833603	1.61504663	1.209505146	1.327616665	1.439280041

Zm00001d006879_T001	5.018583081	5.848278956	5.380073778	5.088867893	5.050539658	5.053443466
Zm00001d025206_T001	4.264169631	3.704562593	4.313566576	4.339787805	4.181085292	4.310807612
Zm00001d021830_T003	2.589744075	1.269720209	0	0	0	1.801431024
Zm00001d021830_T001	0	0	1.329323406	1.684983864	2.426586272	1.923494169
Zm00001d021830_T002	0	0.881999708	2.215879535	2.144051592	1.514452053	0
Zm00001d025670_T001	1.099307325	0.787711615	1.306816926	0.368475591	0.758124908	0.545902162
Zm00001d021318_T005	6.930299768	6.45672107	6.893486111	6.539186238	6.596730185	6.495038222
Zm00001d021318_T004	2.154544649	1.068829	1.910568431	1.442511485	1.1188029	1.476472939
Zm00001d021318_T006	2.844581896	1.481094304	2.950355122	2.84516404	2.883355125	2.824282122
Zm00001d021318_T007	3.387476695	2.332660106	3.428892494	3.418425699	3.586532981	4.118824158
Zm00001d021318_T002	0.322225836	0	0	0.919272952	1.120723516	1.290141263
Zm00001d024179_T001	1.929200925	1.767809016	2.099411693	1.526619736	2.089470015	1.842395201
Zm00001d030282_T015	0	0	0.516742364	0.764778406	0.782104057	0.838407251
Zm00001d030282_T035	0	0.320431533	0	0.234186137	0.487714775	0.626784875
Zm00001d030282_T008	0.659752886	0.192993266	0.782839631	1.06946502	0.922056237	0.446185285
Zm00001d033620_T001	4.601991443	4.59825252	4.53263969	4.357943098	4.584603045	4.244499092
Zm00001d021299_T002	5.606845191	4.436952879	5.666191359	5.363028203	5.401510499	5.51290104
Zm00001d021299_T001	5.086310516	4.760063785	5.194402686	5.193291751	5.29777289	5.260788178
Zm00001d039754_T001	0.84970869	0.27796333	0.90854855	1.041366356	1.169103393	1.216848429
Zm00001d052193_T007	0	0.101141749	0.385433246	0	0.670867522	0.396016967
Zm00001d052193_T014	1.894123163	1.04651411	2.300390855	2.091930308	1.511777697	1.305653648
Zm00001d052193_T004	1.044702561	0	0	1.962909883	1.747005352	2.068933533
Zm00001d052193_T005	0.885581392	0	0.749776234	0	1.023317105	0.942074545
Zm00001d022378_T120	3.136619602	1.599873339	3.087245911	3.722006693	3.29163273	3.288354428
Zm00001d022378_T127	0.63524547	0	1.098581929	1.136563498	1.363162104	1.293625332
Zm00001d022378_T044	2.523159451	0.718144589	2.86500165	2.820190198	3.019906297	0
Zm00001d022378_T064	0.16183223	0.187093741	0.215349333	0.482892673	0.380634152	0.41398503
Zm00001d022378_T130	0	0	1.616356285	1.373025623	1.025858017	2.191964642
Zm00001d022378_T124	0.183055785	0	0.063409061	0.492700255	0.884651057	0.659105277
Zm00001d022378_T002	1.306835004	1.253396339	0	3.032728931	3.298175284	3.378071257
Zm00001d022378_T065	0	0	0	0.651939372	0.597610309	0.340646578
Zm00001d022378_T131	0.5414742	0.357456291	0	0.457751869	0	0.432330897

Zm00001d022378_T132	2.081276559	0.845548661	1.914153469	1.837657903	2.459628861	0
Zm00001d022378_T128	4.224271544	3.811792621	4.209815164	3.82150655	4.16654779	4.111159713
Zm00001d039776_T001	6.563014174	7.399248756	6.43398571	7.131335922	7.346022193	7.283007458
Zm00001d011100_T002	5.389971171	4.72943884	5.348130348	5.228092929	5.215315159	5.171329182
Zm00001d019400_T001	5.743463669	4.783691479	5.140976645	6.788348225	7.324233338	6.57329793
Zm00001d033194_T001	6.413704893	5.609477399	6.414209662	5.601311875	5.738396687	5.690290728
Zm00001d040292_T001	12.56759675	13.24448135	12.45369814	12.20008316	12.22492524	12.28331872
Zm00001d039407_T002	4.576054123	3.64399272	4.327950661	5.943037651	6.043574611	5.991639094
Zm00001d039407_T003	0	1.317043526	1.926471634	2.217961497	1.362274617	1.556539284
Zm00001d032434_T001	4.485656674	4.666207411	4.215815095	3.329192356	3.984865195	4.259180272
Zm00001d007229_T001	1.191280274	0.630751192	0.618869168	0.705029502	0.761914134	0.504722077
Zm00001d007229_T002	5.31690133	4.591083117	5.442622983	5.304582956	5.29675836	5.313489776
Zm00001d006667_T001	5.887652135	5.361200257	6.047592415	6.233706159	6.253720059	6.372717589
Zm00001d006667_T005	5.414721018	4.620740759	5.214238133	5.07981094	4.563468728	4.347579135
Zm00001d006368_T003	5.29578256	4.735520174	5.272235919	5.416215655	5.443283269	5.295330895
Zm00001d031434_T001	4.221847083	3.991882968	4.271513451	2.592454645	2.857643664	2.703879438
Zm00001d027571_T003	4.278098151	3.381487578	4.299372888	4.951971729	4.987633977	4.861442541
Zm00001d027571_T006	0.753331545	0.315995092	0.683297609	0.923518128	1.229195358	0.945639913
Zm00001d027571_T004	0.761224841	0.495432179	1.021093773	0	0.689947668	0.345614381
Zm00001d019703_T001	1.941431256	1.354566691	1.976424862	2.29110961	1.975601941	2.021365658
Zm00001d031220_T001	4.69615249	5.075776012	5.106692284	4.052922296	3.842118588	4.100647539
Zm00001d013551_T001	4.003434572	4.614441949	3.925711325	3.47848237	3.899898634	3.490630455
Zm00001d053648_T008	0.344511524	0.56117361	0.650509997	0.875312979	0.630287106	0.714064359
Zm00001d053648_T002	2.800437542	2.067296931	2.553898649	2.475656139	2.70969471	2.698132003
Zm00001d053648_T007	0.681151475	0	0.244681293	0.865454861	0.851153754	0.747536925
Zm00001d020569_T001	4.33960818	3.951506203	4.614010291	4.018282092	4.20244029	4.110518263
Zm00001d042811_T001	2.70423788	2.237685161	2.694084319	5.14411887	5.002567425	5.101170793
Zm00001d043548_T001	3.239354656	2.768907556	3.446607199	3.165934122	2.941977488	3.096794668
Zm00001d042763_T001	2.54809897	2.515297909	2.609541047	2.785276215	3.037945132	2.744053428
Zm00001d047388_T003	0.80346843	0.281246011	0.85055302	1.167088762	1.500266168	0.851258517
Zm00001d047388_T002	4.330604075	3.627930825	4.390264244	4.480586396	4.322458476	4.400490727
Zm00001d052306_T003	3.962746304	2.442169606	3.999662731	4.063543323	4.148816423	4.111681965

Zm00001d024709_T004	2.850686627	2.185410835	2.499335928	2.698562772	2.267491453	2.656656763
Zm00001d024709_T001	1.016349404	0.556387231	0.761408684	0.763861098	1.113809831	0.809061239
Zm00001d025846_T001	5.305738526	4.457386527	5.218713642	4.811381527	4.771908497	4.820328586
Zm00001d025846_T002	1.503922646	0.328378083	1.363237252	0.901502178	0.992005558	1.30217046
Zm00001d040238_T005	1.373620373	1.389370217	1.656700461	1.720570915	1.647433328	1.122791972
Zm00001d040238_T001	2.725904064	2.464999631	2.899203285	3.012738319	2.910159691	2.958199025
Zm00001d040238_T004	5.415915473	4.819942852	5.456837355	5.405990794	5.269535944	5.239697426
Zm00001d002940_T003	0.719102814	0.402498444	0.778650991	0.511361769	0.48430418	0.638514111
Zm00001d002940_T001	0	0	0	0.952036795	0.838058653	0.619949138
Zm00001d040125_T001	2.141289561	1.521684479	2.262148379	1.615690779	1.642328664	1.606107219
Zm00001d048772_T001	0.807402736	0.537443187	1.007491905	0.833345125	0.863057503	1.086927078
Zm00001d048224_T001	3.682743849	4.443681436	4.087751436	3.605159697	3.474268985	3.397778597
Zm00001d023652_T002	4.760898215	3.938936129	4.727440529	4.572906399	4.553404312	4.665025512
Zm00001d005503_T001	4.880387406	5.054206326	4.87282871	4.217280356	4.33254017	4.495831304
Zm00001d007830_T001	5.848540194	5.615881249	5.919000585	6.462769119	6.43937997	6.486221025
Zm00001d053778_T001	4.958141661	4.340688232	5.043253327	5.727740624	5.752567574	5.627615096
Zm00001d031706_T001	4.213531048	3.331902807	4.361620293	4.668744664	4.630427955	4.560353074
Zm00001d049595_T001	1.681223454	1.287257737	1.713247982	1.791733653	1.864831931	2.087416674
Zm00001d037783_T001	2.152835324	1.465150653	2.239910013	1.52787956	1.381203067	1.592146039
Zm00001d028370_T001	5.761607609	4.84912127	5.793990534	5.795480676	5.705746333	5.688098764
Zm00001d034833_T001	3.012588517	3.876536788	3.224142482	2.770004625	3.291301786	3.248235913
Zm00001d034833_T004	5.408702488	4.465109372	5.610803749	5.864605617	5.818600352	5.835234639
Zm00001d034833_T003	5.352573092	3.923557395	4.783556864	4.421084309	3.662461424	3.608292027
Zm00001d034833_T002	0.480386117	0.219112128	0.533714836	0.795854205	0.835443096	0.892349056
Zm00001d024600_T002	0	0.47253872	0.316143425	0.325259756	0	0.448201101
Zm00001d024600_T003	0.469397356	0	0.29723251	0.738885409	0.98220178	0.834150531
Zm00001d049450_T005	1.651236804	0.662223733	0.833472239	1.800668429	1.926623819	1.75180518
Zm00001d049450_T002	0.813628116	0.324241476	1.054975554	0.897307814	0	0
Zm00001d049450_T008	0.301794818	0.322792299	0.304397724	0.666263947	0.721186267	0.59706486
Zm00001d037797_T001	1.812151937	0	1.409245369	2.053410274	1.732405104	1.60622948
Zm00001d037797_T003	2.767946062	2.568214559	3.203356852	2.664251854	2.882291282	3.075340259
Zm00001d006894_T001	1.631320845	1.09653714	1.571473921	1.418250947	1.57133994	1.465683024

Zm00001d029281_T005	1.08520844	0.655434266	0.819121245	0.842622487	0.807040793	0.931245077
Zm00001d029281_T001	3.299149833	0	3.339301176	3.095179932	3.061787083	3.348168528
Zm00001d029281_T004	1.53346866	0.593603322	1.449620006	0.890877005	0.895954014	1.064560761
Zm00001d029281_T007	5.610326142	4.861660097	5.624716278	5.736741887	5.68963944	5.554737534
Zm00001d049360_T002	1.151895825	0.330163684	0.888529349	1.4623424	1.484867674	2.162128488
Zm00001d049360_T001	2.144406346	1.928059482	1.035004474	2.138124077	1.699135821	1.826386185
Zm00001d049360_T003	4.664624202	4.397382	4.519210253	4.888325598	5.09903526	4.822220476
Zm00001d049360_T005	4.343996151	0	4.688116505	4.427982418	4.115573237	4.251171976
Zm00001d031717_T001	2.160039527	2.68125067	1.975298545	1.521818127	1.147647955	1.405551318
Zm00001d031619_T002	1.021567139	0.98671528	1.122640254	2.0055085	1.617096266	1.686724618
Zm00001d031619_T001	3.363525468	2.491836682	3.34859673	4.614447073	4.629361283	4.487246052
Zm00001d034752_T007	1.682105806	1.294495477	1.717180462	1.34595495	1.31610924	1.406494766
Zm00001d009487_T001	3.855659274	4.130576164	4.257893395	6.982989823	7.111892508	7.126080516
Zm00001d052425_T015	1.215071574	0	0	0	0.838757378	1.780024892
Zm00001d052425_T008	3.818101416	1.101386522	2.887706181	4.19420228	4.217685481	4.165823606
Zm00001d052425_T010	0.60164898	0	0.164104022	0.61805161	0.331786606	1.164639556
Zm00001d052756_T002	5.583015503	5.004505527	5.465439295	5.018816636	4.951692413	5.055940935
Zm00001d052756_T003	4.525093974	4.799013252	4.843347853	4.218613257	4.445510203	4.345060076
Zm00001d052756_T001	3.06094749	1.243631307	3.275797481	2.979793975	2.922424513	2.816821879
Zm00001d043446_T001	1.48757793	1.520204476	1.648950402	1.528445296	1.602859236	1.491406754
Zm00001d045136_T001	4.972753117	4.233378472	5.046921124	4.999317624	4.90973874	4.908861317
Zm00001d042993_T001	5.021168813	4.172728718	5.048044412	6.292013954	6.372214426	6.245794507
Zm00001d052605_T001	1.38785659	1.221470657	1.250301905	0.952406631	0.900382657	0.7113742
Zm00001d053006_T001	0.898772548	0.431329845	0.800342071	0.420624684	0.491994666	0.660904893
Zm00001d034875_T001	3.402255708	2.841999075	3.355807612	4.47413326	4.330417494	4.398812016
Zm00001d048085_T003	3.855048949	3.680504514	4.201470059	2.843862677	3.018463796	3.310648987
Zm00001d048085_T002	2.831371399	2.413696161	2.885896836	2.046564398	2.876010404	2.371057247
Zm00001d048085_T001	2.964603238	1.846358117	2.857791144	2.37699572	2.148942888	2.060645096
Zm00001d022206_T005	1.122621039	0.821978731	1.040675747	1.238662758	1.363484538	0.554714576
Zm00001d022206_T003	1.164504404	0.918309136	0.958673439	0	0	0
Zm00001d015758_T001	0.402407849	0	0.791033034	0.707490335	0.524470944	0.894721548
Zm00001d015758_T002	0.991420077	0.430393404	0.855022528	1.112050857	1.227463229	0.917188071

Zm00001d028140_T001	3.173488222	2.464397998	2.850717027	3.639040756	3.747420056	3.895814842
Zm00001d028140_T009	0.692754803	0.433964669	0	0.493129797	1.346594433	0
Zm00001d028140_T016	1.511294966	2.073445289	1.880571124	0.167176777	0	0
Zm00001d028140_T007	2.048465338	0.512697171	2.53989232	2.675291815	2.746464983	2.485439451
Zm00001d028140_T002	0	0	0	0.709242104	0.766848698	0.476134866
Zm00001d028140_T014	1.398817146	0.078228618	1.141644667	1.7466361	1.341288418	2.036470629
Zm00001d028140_T005	2.596487957	1.724817885	2.709872008	3.407533315	3.585240193	3.517161659
Zm00001d028140_T008	1.549110218	0.971455633	1.455464266	1.997903323	1.36817439	2.059351816
Zm00001d028140_T015	0.808985482	0.501148666	0.729164664	1.670116995	1.507452665	1.284444247
Zm00001d015609_T021	0	0	2.5197555	2.706641941	0	2.349897131
Zm00001d015609_T009	2.862849677	0	2.345715709	0	2.506872035	0
Zm00001d015609_T025	0	0	1.814377741	2.100733815	2.276710151	2.560081252
Zm00001d025584_T001	2.143895238	1.69517249	2.623598848	2.951764764	2.663264455	2.809567555
Zm00001d044443_T001	3.376533637	2.562747478	3.442410761	5.731077561	5.712787824	5.721905778
Zm00001d013432_T001	2.783577764	2.049951331	2.640249796	3.092066342	3.031055481	3.141576497
Zm00001d050174_T001	2.942230536	2.331027845	3.045373246	3.387511987	3.521258455	3.356400063
Zm00001d048346_T001	7.560298446	6.672015162	7.706529233	6.883304862	6.936227024	6.951211617
Zm00001d014820_T001	1.43430743	1.232811158	1.278644369	1.136678985	1.286322882	1.509902035
Zm00001d040720_T001	4.819906076	4.512334674	4.665391918	4.931389619	5.203544676	4.862971937
Zm00001d009731_T003	0	0	0.693105534	1.010711819	0	1.058116964
Zm00001d009731_T002	1.117970923	0.55588289	0.714115367	0.321730721	1.108996078	0
Zm00001d040111_T001	6.001298878	6.52527685	6.13525654	6.465026054	6.5499162	6.480776228
Zm00001d006020_T001	2.789066273	3.990892252	2.174098926	4.046494727	3.925714363	3.636494584
Zm00001d039345_T001	1.058857448	0.718465529	1.428282311	0.340656832	0.533625141	0.434372556
Zm00001d008173_T001	6.243031916	5.727900066	6.284894708	5.946421986	5.903048396	5.948367232
Zm00001d020412_T001	2.498804348	2.310724303	2.317096796	1.554802969	1.678056126	1.661348289
Zm00001d013153_T001	2.915827955	2.543262509	3.205776909	1.8860034	2.198539414	2.274567614
Zm00001d032044_T005	1.052906216	0.341259379	0.87998095	1.721995979	1.684442197	1.726190361
Zm00001d032044_T003	2.524707127	2.72355048	3.420906781	2.737717043	3.50811054	2.503464757
Zm00001d032044_T004	3.086567244	0	1.154736141	3.476373651	2.423127679	3.56186755
Zm00001d045043_T005	3.215389873	2.321825948	3.161414659	3.583603804	3.574287435	3.299861379
Zm00001d045043_T001	2.253376974	3.019443416	2.038027688	2.375062792	2.921455374	2.178158168

Zm00001d045043_T006	1.224591679	0	0.285088491	1.455922392	0.301213024	1.318219646
Zm00001d045043_T004	3.827841376	2.537124574	3.849151402	4.474134882	4.33437012	4.638677962
Zm00001d011770_T001	3.507327829	2.797754063	3.567695842	4.246701917	4.227210902	4.115577897
Zm00001d027532_T002	2.097346503	1.795284861	2.146176851	1.374281105	1.586651869	1.310272638
Zm00001d027532_T001	0.342199717	0.15140555	0.318154845	0.145065688	0.169549211	0.128726277
Zm00001d043815_T005	2.193779627	1.539641738	1.554734219	1.830342549	1.620525886	1.864951944
Zm00001d043815_T006	1.018835682	0.490560889	1.565041924	0.502456889	0.807210645	1.377155408
Zm00001d030665_T001	6.342174982	6.76403874	6.474143857	6.223682509	6.198775731	6.088760895
Zm00001d043158_T002	7.589627666	4.913207245	7.659498043	7.347374156	7.379215719	7.267700367
Zm00001d043158_T003	6.322320959	6.743064715	6.244055558	5.745780312	5.568127045	5.93162054
Zm00001d043158_T001	0	5.819713369	0	1.563960056	0	1.200242558
Zm00001d039534_T006	0.869352653	0	1.001689849	1.142036948	0.951393831	1.045508673
Zm00001d039534_T001	2.694576471	0	2.509907861	1.890758351	2.177339004	1.870669306
Zm00001d039534_T008	2.252794692	1.804611194	2.290820412	1.881457498	1.710578339	1.877764271
Zm00001d007328_T005	1.236993992	1.012909016	1.370578891	0	0	1.659194352
Zm00001d007328_T004	2.141516499	0.558498839	1.84885946	2.071114272	2.210184042	1.891407521
Zm00001d007328_T001	0.507400905	0.227998545	0.697579905	1.081245536	1.257618957	1.558025123
Zm00001d027982_T002	5.747598901	4.981334605	5.540853432	5.497565041	5.483205525	5.446239354
Zm00001d027982_T003	0.791493205	0.386888182	0.413795526	0.976741217	1.004380578	1.47976397
Zm00001d027982_T006	0.319999366	0.169912177	0.450317584	0.713267341	0.582815135	0.569807983
Zm00001d027982_T001	2.460963996	0	3.874520497	2.102826408	0	1.09447999
Zm00001d008427_T001	0.550098835	0.213017485	0.809989761	0.759449883	1.00554443	1.031719131
Zm00001d008772_T002	0	0.740424202	0	0	1.311741414	1.665138048
Zm00001d008772_T003	0.957496398	0	0.807181788	1.662295295	0.240228834	0.538144783
Zm00001d051535_T004	0	1.358056367	1.014466705	0.485135167	0	1.625672611
Zm00001d051535_T002	2.378474723	1.450737764	2.571538965	2.547688696	2.499752276	1.976100737
Zm00001d018498_T002	4.330095401	3.542006888	4.383029541	4.44889963	4.248133956	4.249396416
Zm00001d018498_T004	4.257008656	3.852441669	4.161342982	4.109457934	4.385016324	4.509822563
Zm00001d021363_T001	4.081366814	3.298464624	4.232238553	4.02040948	4.26439253	4.083303076
Zm00001d047183_T001	0.639581332	1.362092228	0.65868221	0	0.136665206	0.59792871
Zm00001d047183_T007	1.280093407	0.427206024	1.252964195	1.885814468	2.297446177	2.056822802
Zm00001d047183_T013	3.639630876	2.720982565	3.710517508	4.049218112	3.93567359	3.895044988

Zm00001d031473_T001	4.644620904	3.889032392	4.697371063	4.513982025	4.588718533	4.505295949
Zm00001d041789_T003	3.901172844	3.370709304	4.184484701	3.722153625	3.8162289	4.008104346
Zm00001d041789_T001	4.092801362	3.387297188	3.986723379	4.227056449	4.155017663	4.007443376
Zm00001d041789_T002	0.890324501	0.518030485	1.055521925	1.465321514	1.5203448	1.609293056
Zm00001d034410_T001	1.844911853	1.511306606	1.819115521	1.655268929	2.186529716	1.718130557
Zm00001d032396_T002	3.062673327	2.483278705	3.093533134	2.820954184	2.683177889	2.700854489
Zm00001d025576_T001	3.406889002	3.614089764	2.943179446	1.988294947	1.280268643	1.67360798
Zm00001d016159_T001	4.085924958	4.437158213	4.216655773	3.381782968	3.031135784	3.282899081
Zm00001d021365_T014	0.258682933	0.118419532	0.225626613	0.273211504	0.364458133	0.281359974
Zm00001d021365_T001	0.762773163	0.069975696	0.340010721	1.121063158	0.915772514	0.970105614
Zm00001d014666_T001	2.782925997	2.500592261	3.003062474	2.230104616	2.319307802	2.540438736
Zm00001d030891_T001	5.489733857	4.845366752	5.604880329	8.172782588	8.170735453	8.139311349
Zm00001d029196_T001	4.461902085	3.628839139	4.341257387	4.4703264	4.494641342	4.548072391
Zm00001d029196_T002	2.133823569	0.643576395	2.603836392	3.708735731	3.587131764	3.469069607
Zm00001d025018_T001	3.953767906	3.706446342	4.191329947	2.134038209	1.929398652	2.222524525
Zm00001d010740_T001	3.278379129	4.61346765	3.282702372	2.095792125	1.610722737	2.426232009
Zm00001d015194_T001	3.952622822	3.197300372	3.941984622	3.701956746	3.805576632	3.825160799
Zm00001d034358_T002	5.395205519	4.857006349	5.496658332	5.680647464	5.583084423	5.55503479
Zm00001d034358_T001	1.947027228	2.04269336	1.732378619	1.682906292	2.515015003	1.951187156
Zm00001d008849_T001	1.834621828	1.332078882	2.026229595	1.782292809	2.247546592	2.102682316
Zm00001d006562_T001	4.076059213	3.605605862	4.020792596	3.69897886	3.800232509	3.745298438
Zm00001d018384_T001	2.241082951	1.96106355	1.8578202	1.029590699	2.102416598	0.991797366
Zm00001d018384_T004	2.38696238	0.693199226	1.961177672	2.996178112	2.548469177	2.847466619
Zm00001d020064_T001	1.89429665	1.992209369	1.638880131	2.624131784	2.616201232	2.904276566
Zm00001d020064_T002	1.006253494	0.70900295	1.150727331	1.273722955	1.429697904	0.914748962
Zm00001d041782_T001	2.037457079	2.195590127	2.028671339	1.111883987	0.703240709	1.325586167
Zm00001d004451_T001	5.152744119	5.040260785	5.033195941	4.437255975	4.530667392	4.57064611
Zm00001d008602_T002	5.012134369	4.552957193	5.335572471	4.122923554	4.467446959	4.243788108
Zm00001d008602_T001	3.646797442	3.378000763	3.333658153	3.395000537	3.461994845	3.216412336
Zm00001d008602_T003	4.874377373	3.482670193	5.091981703	4.541436532	4.295082411	4.469586925
Zm00001d014553_T001	1.325711071	0.934888592	1.36470409	1.597416278	1.397936549	1.588298
Zm00001d024376_T001	3.647923698	3.458378329	3.704193938	4.451134144	4.414119428	4.59268199

Zm00001d022181_T001	0.399791251	0	0.065120063	0.254387238	0.580390577	0.570302623
Zm00001d022181_T010	0.202390137	0.258556311	0.350227919	1.014558827	0.688433731	1.462784216
Zm00001d022181_T004	2.525425467	5.956125927	2.85596997	2.887353307	3.292109267	3.441119042
Zm00001d022181_T006	0.56297943	0.237449913	0.534692146	1.325911354	1.493792826	0.759633954
Zm00001d022181_T013	0	0.137663523	0.290738153	0.768847441	0.683746751	0.678199939
Zm00001d022181_T008	0.47643872	0.59739387	0.725105926	0.775824268	1.037288734	1.034900974
Zm00001d022181_T005	7.407908213	4.366161183	7.453942629	7.53756862	7.56076405	7.550282717
Zm00001d022257_T001	4.220763163	3.585900789	4.068915309	3.891693992	3.822691404	3.70004592
Zm00001d027254_T001	0.476591142	0.263848097	0.405587259	0.364533213	0.404209939	0.376241478
Zm00001d014988_T001	2.818169536	2.253032899	2.924654998	2.734299051	2.814587336	2.610112283
Zm00001d047015_T059	2.106647817	1.183784392	2.282370708	2.345782973	2.40323745	2.412584005
Zm00001d047015_T022	0.264344259	0	0	0.788437654	0.787297885	0.753471042
Zm00001d047015_T041	0	0.046185097	0.166005812	0.849319572	0.797518559	0.689116631
Zm00001d047015_T002	0	0.129847454	0.127053444	0.456010214	0.129333139	0.547807127
Zm00001d051039_T005	0.914190216	0.992395747	0.675510653	1.382501257	1.558108904	1.128683391
Zm00001d051039_T007	0.777272835	0.801871341	0.671547917	0.735413863	0.405551318	0.868812383
Zm00001d051039_T004	1.039706234	0.057272805	0	0.989906303	0.533288238	0.91116157
Zm00001d051039_T010	1.363117799	0.780976903	2.040881562	1.713115541	1.976313406	1.839090082
Zm00001d051039_T002	0	0.604644491	0.644935153	0.495067812	0	0.21884811
Zm00001d051039_T003	0.66069592	0.35946607	0.748140885	0.102674255	0.855759326	0.756166766
Zm00001d009695_T001	5.309881607	4.966319852	5.379266517	4.771359945	4.766846313	4.766979582
Zm00001d029519_T001	2.997279941	2.501501056	3.089854543	3.102341342	3.431222854	3.243590334
Zm00001d032582_T006	4.276738509	4.430289622	4.517902342	4.608146574	4.433724768	4.522736938
Zm00001d032582_T003	0	0	0.83444425	1.125444799	0.942709666	0.94663056
Zm00001d032582_T007	0.938751746	0.459681314	0.869623502	1.564103506	1.59415916	1.601985502
Zm00001d028606_T002	6.093354515	5.098764744	6.217717757	6.486840137	6.504294208	6.511824637
Zm00001d028606_T004	0.815047437	0.26283001	0.663273565	1.10866897	1.424261575	1.550794305
Zm00001d039568_T002	4.325478595	3.689289431	4.407471384	4.214253212	3.832654203	3.961454603
Zm00001d010936_T001	0.549913583	0	0	0.593621487	0.728203522	0.728225294
Zm00001d028838_T001	0.951374433	1.33091057	0.894695941	4.032475049	3.859151292	3.891262076
Zm00001d018359_T001	4.137621394	4.269683933	4.5749662	3.949920254	3.883108745	3.762927797
Zm00001d018359_T003	7.53293468	6.822467559	7.332348262	7.056439869	6.954481133	6.678169312

Zm00001d018359_T002	7.449388876	6.703806574	7.657334508	7.830191614	7.900864695	7.924421041
Zm00001d036952_T001	2.476886348	2.318536664	2.552000239	2.264023215	3.092639945	2.376564614
Zm00001d042442_T002	0.192141129	0.148227385	0.334273071	0.699926471	0.557732586	0.485293887
Zm00001d042442_T004	3.271208867	2.860072481	3.291466384	3.899836102	4.037393644	3.982691638
Zm00001d006685_T001	3.999372562	3.839911027	3.776721898	4.397796939	4.176662181	4.117405062
Zm00001d011183_T001	2.565436813	2.448979161	2.791974686	3.309976638	3.477607522	2.954107698
Zm00001d035228_T001	2.606758212	1.935841399	2.653319404	2.780199635	2.919693418	2.451961712
Zm00001d034932_T001	1.381393024	1.016347978	1.087220448	1.28464975	1.408749354	1.095626739
Zm00001d030910_T002	4.080408496	3.86489491	4.335629527	4.199637013	4.080157749	3.855927226
Zm00001d030910_T001	1.434132856	4.336094825	1.933262539	2.014984371	1.936505653	2.16129017
Zm00001d045812_T001	4.533297272	4.995433639	4.703326989	4.072668016	4.386633564	4.330470201
Zm00001d019354_T006	0.205997993	0.114832078	0.287999394	0.95515357	1.16058722	1.064834577
Zm00001d019354_T001	0.761702275	0.537244373	0.972995496	1.068635044	0.815945892	0.85411856
Zm00001d045402_T001	0.745486904	0.813405657	0.850939406	0.580250667	0.709433575	0.451470147
Zm00001d050608_T001	3.012430118	2.117765514	3.075408722	2.736375844	2.52619955	2.799754398
Zm00001d013025_T002	3.208859633	2.577959763	3.031448838	2.460047911	2.482729301	2.543917
Zm00001d031120_T003	6.938273746	6.039365201	6.987401993	7.161500722	7.163388785	7.133981809
Zm00001d031120_T004	0.751185182	0.485226897	0.768468071	0.963463026	1.149022583	1.443567401
Zm00001d041186_T006	1.443349383	0.824404537	1.260742308	2.32946119	2.447600619	2.417641377
Zm00001d041186_T001	4.055336411	3.380378012	4.035183117	4.526922842	4.486871568	4.489756267
Zm00001d041186_T005	0	0	0	1.954719309	2.599876908	3.200737544
Zm00001d041186_T003	5.678478986	4.598414755	5.700957414	5.369874771	5.338839228	5.295334458
Zm00001d041186_T009	5.854866788	5.033807654	5.715984936	5.710099388	5.672376601	5.492175166
Zm00001d013008_T001	5.217238317	5.313964952	5.054862626	5.122598926	5.197087312	5.039040631
Zm00001d035947_T003	1.315225948	0.601244861	1.107108667	1.211551779	1.25813898	1.135385142
Zm00001d035947_T001	2.950188807	2.416512802	3.018617618	2.861351767	3.100949399	3.111560871
Zm00001d017170_T002	0.502563818	0.35408086	0.84578709	0	0.752794825	0
Zm00001d038859_T002	2.564573279	1.42150501	2.530078485	2.922845013	2.581260609	3.031288437
Zm00001d038859_T010	2.478909955	3.772824714	2.910945214	2.828336067	2.203233425	2.043613629
Zm00001d042801_T001	3.276785474	2.614666975	3.228362034	2.060353535	2.246781407	2.269267491
Zm00001d017642_T002	2.335821652	1.810698509	2.638995709	1.536665169	1.394013035	1.403899206
Zm00001d039001_T002	4.684589077	4.490971244	4.568346155	2.887985885	3.058138443	2.510056027

Zm00001d024890_T001	4.55629219	4.409895621	4.638912153	4.538138263	4.630567859	4.549097031
Zm00001d033495_T001	0.340892641	0.376391524	0.690874752	1.103380138	0.499420891	0.869445048
Zm00001d027403_T002	1.392116806	0.779188267	0.580830481	0.829165183	0.530441359	0.729571042
Zm00001d027403_T001	3.314258141	2.635594212	3.488656264	2.746552694	2.815519687	2.666751594
Zm00001d053304_T001	1.470384844	1.135471172	1.454248553	0.037834816	0.080517139	0
Zm00001d010339_T001	6.032935178	5.818792671	5.906407476	5.458798661	5.67380031	5.357674277
Zm00001d024492_T001	2.928271581	3.00560461	3.208135935	3.303123314	3.237135833	3.262001006
Zm00001d003670_T003	1.601031345	0.908299517	1.541313116	2.711275512	2.938832839	2.787487421
Zm00001d024187_T001	4.640764878	3.596668756	4.637172827	5.460590686	5.434623591	5.454838282
Zm00001d032649_T001	5.762296667	5.445812334	5.877017567	5.737663861	5.675394801	5.603943955
Zm00001d004526_T001	3.770669654	2.891255659	3.716275513	4.026684993	4.112832721	4.047850782
Zm00001d020110_T001	2.652517241	2.483432211	2.709973218	2.222535653	2.437528713	2.157384288
Zm00001d037429_T001	4.388484178	3.827266529	4.606433522	4.802139128	4.569202087	4.838729297
Zm00001d008795_T001	1.474128132	0.960108352	1.562891537	1.765682403	1.796510542	1.844322978
Zm00001d046396_T001	3.399479975	2.789392062	3.50151545	3.776720108	3.655058451	3.743874143
Zm00001d044202_T001	2.371774345	1.809013892	2.424699085	3.322557117	3.228034883	3.132453953
Zm00001d021435_T001	2.475809422	2.619400567	2.526538363	1.983993193	1.440099614	1.596792535
Zm00001d029254_T001	1.241971332	0.84789351	0.94271417	1.015796554	1.056823495	1.213135723
Zm00001d051291_T006	0.878271768	0.448505609	0.917971686	1.595729452	1.249823942	1.217073718
Zm00001d051291_T005	0.859530711	0.419396503	0.347995539	1.608837623	1.746297286	1.812768461
Zm00001d051291_T002	4.027204134	3.285663804	3.974578962	4.009680036	3.977185573	3.929692827
Zm00001d007446_T002	6.731801306	5.944604336	6.892898345	6.669260107	6.661627622	6.706985819
Zm00001d007446_T001	6.324919881	7.420026401	5.791431758	5.859799584	5.919135297	5.815716028
Zm00001d007446_T005	2.715165471	1.728628018	2.843542328	2.341179377	2.237610523	2.213608576
Zm00001d019672_T001	2.047578172	2.251004005	2.234827162	1.983321628	1.420428504	2.214344924
Zm00001d019672_T002	0.848992824	0.717443709	0.766235556	1.014283871	1.305737104	1.211022788
Zm00001d015025_T001	3.806780512	3.14437404	4.041710398	7.010984269	7.060886096	7.084627782
Zm00001d054005_T007	3.912312334	2.97761111	3.827092109	3.98244066	4.064398826	3.8282992
Zm00001d054005_T001	2.357574806	1.747181132	2.398132253	2.434504134	1.767355212	2.270978276
Zm00001d017264_T001	0.951556464	0.398391965	0.876015907	0.332435264	0.741413484	0.547208949
Zm00001d044042_T001	5.360476021	4.935439144	5.432521991	5.252348478	5.324028248	5.365458157
Zm00001d026480_T001	2.089961122	2.344854624	2.686691455	1.056017437	1.541270983	0.666713878

Zm00001d039762_T002	3.790557598	4.026847057	3.815150652	2.716064639	2.033166069	2.301214488
Zm00001d052923_T001	0.385781107	0.174934309	0.520592219	1.814250164	1.814908438	1.609600852
Zm00001d016002_T001	4.382541928	3.594341804	4.326432292	4.35880794	4.317416883	4.390449298
Zm00001d016002_T002	0.878085745	2.06977399	1.315198699	1.126329313	1.253569402	0.387854385
Zm00001d016002_T003	0.950831189	0.638100721	1.246881712	1.55528167	1.483354041	1.524546667
Zm00001d046629_T001	2.24301765	1.846442768	1.886447526	2.103559073	2.101760661	2.213867336
Zm00001d020828_T001	3.746535066	3.142059338	3.701423633	4.335025624	4.419470396	4.152702144
Zm00001d038376_T002	1.957495283	1.394631024	1.875478127	1.599494906	1.574512333	1.412447703
Zm00001d038376_T001	7.269798159	7.493015576	7.343941796	7.395795369	7.390301067	7.31857193
Zm00001d008322_T004	1.069954393	0.666652987	0.978328908	0	1.27988369	1.266886664
Zm00001d008322_T008	0.287622408	0.153370841	0.202444052	0.342652591	0.568117739	0.419049101
Zm00001d008322_T005	1.280355363	0.487390647	1.477939942	1.489221787	1.040637877	1.254629112
Zm00001d008322_T003	3.944875589	3.340306748	4.041714603	3.677991766	3.956015844	3.768438746
Zm00001d008322_T006	0	0	0.366336204	1.854137715	1.509959784	0
Zm00001d024567_T011	2.625788551	1.305615711	2.835505753	3.267929602	3.2522847	3.074075681
Zm00001d024567_T015	0	0.369821565	0	1.751836451	1.296036799	2.233048084
Zm00001d024567_T005	1.378438377	0.971544658	1.217706572	1.849580197	2.039261246	1.480788327
Zm00001d024567_T006	0.529453169	0.127915849	0.355151616	0.492914529	0.331737314	0.491794618
Zm00001d013708_T001	3.290625076	3.478646871	4.1845066	3.149867082	0	2.65641079
Zm00001d013708_T002	2.45269321	1.780834162	2.318354445	2.109149907	2.295705982	2.474912839
Zm00001d006588_T001	3.549640834	2.993960156	3.750853435	3.336344094	3.687227574	3.665554451
Zm00001d040248_T001	0.718247192	0.366415663	0.883185205	0.283848303	0.304398893	0.373034535
Zm00001d006714_T001	6.088923573	5.86166434	6.162334325	5.954868171	5.948456781	5.985218308
Zm00001d044103_T001	1.82387121	1.691192434	2.017750912	1.69290162	1.709564148	1.960306708
Zm00001d044103_T003	3.801310576	3.14294663	3.843953503	3.696317159	4.01596582	3.862411219
Zm00001d029389_T001	2.892053872	2.080513046	2.585840114	2.641506031	2.387483863	2.873614986
Zm00001d021962_T001	2.02076531	1.56472691	2.136698341	1.925981575	1.915517077	2.339396248
Zm00001d053605_T001	3.770152565	3.172800791	3.276504408	3.107169109	2.357622941	2.78113579
Zm00001d013336_T006	3.386074377	2.492890694	3.504152044	3.43974174	3.201739707	3.365786586
Zm00001d013336_T001	4.199232913	3.323909574	4.297621079	4.266913642	4.256102687	4.087445868
Zm00001d048219_T001	1.503274436	1.159215821	1.789283543	2.30722974	2.040934852	1.802866111
Zm00001d038642_T001	2.871107331	2.475347969	2.934497819	0.976715559	1.0712437	0.978451923

Zm00001d043194_T002	3.832519422	4.235615484	4.097039842	3.618518121	3.651819661	3.631876191
Zm00001d024990_T001	0.288656226	0.119986907	0.23700807	0.36783176	0.418807381	0.23567805
Zm00001d049730_T002	2.559257096	2.355379148	3.409049265	3.799398095	4.067282215	3.910954037
Zm00001d049730_T003	2.40436855	2.76658859	1.577945024	2.753707002	2.369404503	2.477159989
Zm00001d049730_T001	1.607101595	1.42254573	1.777455066	2.458614035	1.902609255	2.284272776
Zm00001d049730_T004	3.178394998	2.92832806	1.962883238	1.929071743	1.836225513	1.947212052
Zm00001d028952_T002	4.92384801	4.361982222	4.870105652	5.423106764	5.164902905	5.320213154
Zm00001d028952_T003	0.336760969	0	0	0.578260547	0.635186951	1.50792248
Zm00001d028952_T006	3.240279063	0	3.520628297	1.439147036	3.546369748	0
Zm00001d028952_T005	0.091206949	0.058582495	0.375563295	0.705126848	0.55289739	0.223638784
Zm00001d028952_T007	0	0	0	3.998017277	3.973592725	4.077001276
Zm00001d028952_T004	0.868532691	0.591835453	0	0.787643922	1.514337922	0
Zm00001d011073_T001	2.712879009	2.885573193	2.996823478	1.224140938	0.882423156	0.95233133
Zm00001d002446_T001	2.652187026	1.857355227	2.748364406	2.35379329	2.357818562	2.569454392
Zm00001d030863_T006	3.423821378	2.508673053	4.002275499	3.96484468	3.574105021	3.731176615
Zm00001d030863_T005	2.403788526	1.167411883	1.794987216	2.467578424	2.706474634	2.498040193
Zm00001d030863_T001	2.469196495	2.971924605	2.659809047	0	0	2.276898599
Zm00001d030863_T004	0.665366391	0.483578995	0.620153772	0	0	0
Zm00001d018884_T003	6.834294743	5.814238985	6.743718583	7.229422678	7.461611342	7.189841426
Zm00001d018884_T013	1.882691953	1.170619252	1.86370657	2.657281916	2.882617619	2.507794387
Zm00001d018884_T008	1.23685076	0	1.180505626	2.089621873	1.871944949	1.309948562
Zm00001d018884_T021	0	0.715657074	0.614138777	0	1.160210925	1.424534093
Zm00001d018884_T004	7.97816018	5.600311506	8.119786182	8.107994174	7.937398116	7.796734525
Zm00001d018884_T002	1.818927028	4.122705018	0	2.143048868	0	1.289259043
Zm00001d018884_T011	8.758537768	8.240632326	8.656658118	8.470867303	8.616148546	8.672056202
Zm00001d018884_T010	1.872153834	0	1.368274983	3.777713012	3.729304777	3.754788647
Zm00001d020941_T001	1.408810754	1.00989247	1.575158855	1.584516158	1.369717171	1.428112368
Zm00001d045169_T001	3.357725258	2.695146649	3.305423681	2.955420685	2.907330839	2.830892144
Zm00001d024325_T001	1.417389698	0.572341583	0.614782389	1.347124757	1.044263309	1.37817477
Zm00001d024325_T002	4.122852672	3.408121621	4.283731573	4.124154406	4.20922158	4.095890925
Zm00001d031747_T001	6.906410146	5.822686178	7.001449034	6.618283578	6.574450768	6.627711389
Zm00001d031747_T002	4.443705568	5.325850135	4.021899771	4.219321806	4.537777941	3.317754406

Zm00001d034090_T001	2.968118592	2.334287375	3.087691957	3.590563458	3.740126896	3.8974724
Zm00001d018990_T001	2.845081522	2.21285287	2.819038655	1.884857719	2.332772657	2.26334365
Zm00001d032710_T001	5.168987306	4.743123597	5.206161594	5.746963875	5.79772359	5.704778336
Zm00001d032710_T003	1.843727833	1.487036595	2.14573614	3.139278749	3.115847776	2.972207202
Zm00001d025008_T001	3.547213885	3.141015487	3.68616668	3.632673509	3.609684409	3.459244843
Zm00001d025460_T002	0.843051216	1.689150186	1.471304016	0	1.277854453	0
Zm00001d025460_T001	3.844360443	2.835141897	3.65362758	4.24692396	4.158054375	4.118647273
Zm00001d015284_T001	1.419640281	1.152107469	1.451582504	0	0.125893073	0
Zm00001d014745_T001	3.17827356	3.647770389	2.876492979	3.288960765	3.8564828	3.057889166
Zm00001d046178_T001	3.228325859	2.546712005	3.275090507	3.152136356	3.404781349	3.210199163
Zm00001d047990_T002	2.819491412	2.253678917	2.854653191	2.679937861	2.696562319	3.058397721
Zm00001d042931_T001	1.295074107	1.162262895	1.278311919	1.217014761	1.176274895	1.177488586
Zm00001d020874_T001	6.721943349	6.618846636	6.646588187	6.530929226	6.577922191	6.383632173
Zm00001d042523_T008	0.396097	0	0.337129901	0.60987597	0.531242206	0
Zm00001d042523_T042	0.877304504	0	0.706696749	0	0	0.990103873
Zm00001d042523_T018	0	0.070087017	0.140234189	0.626362502	0.526990239	0.793758576
Zm00001d042523_T019	1.379799486	1.304912254	0	1.329756808	0	1.624801362
Zm00001d042523_T030	0.560568232	0	0	0.810869166	1.162223574	1.174434543
Zm00001d042523_T001	0.436685294	0.657311646	1.250790016	0.748592615	1.704435107	1.296197772
Zm00001d049152_T001	2.450140449	1.584777824	2.603696364	3.498304361	3.379621239	3.366455531
Zm00001d009247_T001	6.035108318	8.833116838	5.605982814	5.370810872	5.466785475	5.258351844
Zm00001d031659_T001	5.375290577	4.53266961	5.388528572	5.522120391	5.515831362	5.416482198
Zm00001d024661_T001	3.99456651	3.196340606	3.924202239	4.24121989	4.034894197	4.174490712
Zm00001d026271_T001	4.828246842	5.985238895	4.969777719	3.494492032	3.808120893	3.722689406
Zm00001d042862_T001	3.017758928	3.630291883	2.724347732	2.937963149	2.767580013	3.079944837
Zm00001d049913_T001	4.6525138	3.916241972	4.546012526	4.851115865	4.83966714	4.860299979
Zm00001d006947_T001	0.79052434	0.422094108	0.819918283	1.391381703	1.608024771	1.416009548
Zm00001d004843_T003	7.72576454	7.07235214	7.776554444	7.036272954	7.104476928	7.12796605
Zm00001d003812_T002	3.794917057	2.860210375	3.85308235	3.687227126	3.637676665	3.67102893
Zm00001d048959_T001	2.828910589	1.650649231	3.08086689	2.881881692	2.886473475	2.896656452
Zm00001d048959_T002	6.165991747	6.32437611	6.207558877	6.134063907	6.269661476	6.170680615
Zm00001d051069_T001	0.551182283	0.356726406	0.729195995	0.546807062	0.885936652	0.805761307

Zm00001d041175_T006	1.548134244	0.975264056	1.728272756	1.893767972	2.325100553	1.923125215
Zm00001d041175_T005	1.704791862	1.126383503	1.529826942	1.915723954	0.85678097	1.219934862
Zm00001d023910_T001	2.380197758	1.669082113	2.312310845	2.659854248	2.757189915	2.512915321
Zm00001d025645_T001	4.413688039	5.234454032	3.925974266	2.73252732	2.022825141	2.90927465
Zm00001d010782_T009	1.308999187	1.233663542	0	2.671625811	2.495706418	2.514217974
Zm00001d042676_T001	3.728102168	3.079246338	3.773702483	4.380886577	4.271251679	4.354644686
Zm00001d042676_T002	1.570154443	0.811952671	1.344436531	1.745413758	1.716889518	1.772099327
Zm00001d053434_T001	3.982305388	2.600157693	3.660233609	2.107320956	1.697255619	2.482649802
Zm00001d041700_T006	0.968314191	0	0.665300894	0.960249243	1.067430144	1.251048254
Zm00001d041700_T005	0.569377344	0.180542543	0.312348025	0.470599335	0	1.37924
Zm00001d041700_T007	0	0	0.767112362	1.266089682	1.598598648	1.279299529
Zm00001d041700_T004	0	0.436221551	0.618443532	1.766950863	1.56530812	1.657281916
Zm00001d041700_T001	1.248786193	0.95487673	0	0.66302849	0.777739098	1.046135494
Zm00001d015290_T001	4.183093273	3.307810279	4.254114249	4.599987622	4.720898975	4.530045078
Zm00001d039305_T008	1.397563679	0.673414409	1.232561913	1.042681455	0.926072311	0.906952149
Zm00001d039305_T001	5.052798385	3.768105332	5.04255653	4.907394441	4.641915557	4.902185131
Zm00001d039305_T003	1.226245861	0	1.241206845	0.725590227	1.759739613	0
Zm00001d039305_T007	5.07513361	5.29530977	4.787874671	4.287647154	4.365936815	4.141218349
Zm00001d039211_T002	0.242532999	0.152401511	0.429269944	0.361640364	0.390952675	0.413101186
Zm00001d039211_T003	3.517825702	3.166693596	3.860138648	3.585973355	3.59417923	3.065580411
Zm00001d039211_T005	4.428184476	3.323284024	3.917741362	4.108257303	4.061286462	4.399232698
Zm00001d050716_T003	1.568367803	0.459916283	0.867156349	1.919071543	2.520322922	2.721839409
Zm00001d050716_T005	1.142512119	0.552792161	0.976342371	1.274428661	0.265003542	0.330450552
Zm00001d050716_T001	1.682596218	1.423815867	2.290225327	2.78926643	2.524704369	1.692416931
Zm00001d050716_T010	5.408512933	4.170063894	5.289085818	5.514272962	5.479302131	5.510124027
Zm00001d045578_T001	7.632152119	7.321262146	7.703013152	6.964274447	7.16163805	7.091560074
Zm00001d031668_T001	1.424859221	0.976464822	1.169721086	0.996997515	1.149786783	1.310137082
Zm00001d032338_T002	1.516885546	1.189210987	0.798214792	1.876122417	1.214619714	1.499638762
Zm00001d032338_T001	3.744235382	3.095599904	3.892250247	4.11812438	4.070513497	4.112608815
Zm00001d046865_T002	0.85895891	0.620704636	0.920632494	0.426182083	0.388888255	0.307983346
Zm00001d007503_T001	3.42095891	2.562282239	3.36789297	4.240208146	4.36463266	4.203410105
Zm00001d005598_T001	0.781868292	0.928029911	0.981390917	1.143314392	1.753100018	1.18787224

Zm00001d028214_T001	5.419051192	4.607354048	5.417946397	6.254313968	6.318401719	6.218504941
Zm00001d026026_T001	3.408662004	3.025327582	3.218394796	3.439454525	3.46518514	3.190281406
Zm00001d008410_T001	4.799163999	4.022176033	4.534584646	3.772123614	3.773900248	3.219110114
Zm00001d046449_T003	1.119701453	3.871371443	0.936899084	1.49762616	1.639964672	1.412106755
Zm00001d046449_T004	1.96154332	0.374094661	1.937135695	2.378922455	2.09497446	0
Zm00001d046449_T005	9.140709604	7.569527563	9.158961345	8.804799109	8.876098888	8.800360513
Zm00001d046449_T015	5.486552334	9.228140428	6.082671894	0	0	0
Zm00001d037828_T001	6.582372147	6.130789599	6.544393901	5.891962598	6.062726141	6.017302618
Zm00001d038972_T002	0.142336318	0.265789725	0.180184788	0.589562167	1.171184405	0.639669305
Zm00001d038972_T025	0.25220733	0.05998234	0.371662566	0.890628015	0.791280644	0.804896124
Zm00001d038972_T015	6.449963721	5.123605945	6.514946476	5.796434435	5.712218936	5.586502177
Zm00001d038972_T010	5.909860858	5.196430552	5.831031842	7.75251008	7.72779682	7.594194214
Zm00001d038972_T004	0.806931944	0.495037103	0.669513933	1.420104533	1.391432848	1.115009931
Zm00001d038972_T007	2.370145305	1.766372115	1.908266078	2.066349972	1.609914728	2.021031925
Zm00001d038972_T001	4.388676278	3.631044547	4.567000988	4.420358635	4.311137673	4.302726721
Zm00001d012917_T001	4.919927101	4.149366848	5.045279924	4.244800769	4.000535321	3.994030961
Zm00001d020538_T001	1.091210335	0.695558267	1.108706434	1.583001989	1.489282426	1.584407437
Zm00001d046363_T001	1.155693544	0.776051755	1.543816557	1.298893467	1.306314159	1.483316373
Zm00001d032350_T007	1.829969678	1.642953819	0.715983838	2.032867843	1.969770122	2.273637328
Zm00001d032350_T002	0.572109675	0.747059077	0.333043545	1.385127832	1.051699238	0.876771919
Zm00001d032350_T001	2.671249208	2.187011052	2.950807492	2.968018662	3.189185679	2.989102485
Zm00001d032350_T006	4.441819082	3.126208369	4.568644147	4.773138943	4.869068474	4.751911254
Zm00001d032350_T004	5.310744658	5.007969172	5.349075032	5.209263268	5.250449834	5.418178645
Zm00001d028022_T003	2.016238851	1.293658877	2.022564539	2.813641865	2.95954576	2.748567928
Zm00001d028022_T001	0	0	0.473265317	0.433461591	0	0.565869118
Zm00001d049193_T001	5.558983783	5.824583483	5.203673127	5.536913099	5.449652687	5.289011134
Zm00001d020675_T001	1.919083369	1.768628554	2.259617186	3.473139696	3.394784108	3.440759294
Zm00001d029910_T002	3.417019662	0	3.444033695	2.944730846	3.516853533	3.033993095
Zm00001d029910_T001	2.144007527	2.72522418	2.028548643	3.366139636	2.871739384	3.14040043
Zm00001d044412_T002	5.314724111	4.565528451	5.327904363	5.567535713	5.472950627	5.485901003
Zm00001d016342_T001	6.533195557	6.379228596	6.527507611	5.887516083	5.924983337	5.880721739
Zm00001d010822_T001	2.520572608	2.237842686	2.463265662	2.8081762	2.80676845	2.740158637

Zm00001d031971_T001	7.857884804	7.605168848	7.910987749	8.54159207	8.517672029	8.55177847
Zm00001d031971_T002	6.087291829	6.177600344	5.969365913	6.865166027	6.972777272	6.978817921
Zm00001d044814_T010	0.467906512	0.346875217	0.599294939	0.818659988	1.127569892	1.08545729
Zm00001d044814_T009	0.937036233	0.430650317	1.136343655	2.087540236	2.131368553	1.958360521
Zm00001d044814_T001	2.126861545	2.503546679	2.439456653	3.402574708	3.955035436	3.729087749
Zm00001d044814_T017	4.598958265	3.132373996	4.53960545	5.13993962	4.734304579	4.722419519
Zm00001d044814_T018	1.664715382	0.748127142	1.404224656	2.210126986	2.258950613	1.933185476
Zm00001d044814_T013	1.726277569	0.601538691	1.277267684	1.496340138	1.635961437	2.158967422
Zm00001d033088_T001	2.0301503	3.312681291	2.45777813	3.145974278	2.860812464	2.867935594
Zm00001d051405_T001	5.917569788	5.771792793	5.829454109	6.097106229	6.133520085	5.884732661
Zm00001d012078_T001	2.839260235	2.632427136	3.219625928	2.95383885	2.88225841	2.5267334
Zm00001d031988_T002	0.465470935	0.365795543	0.43520399	1.178652184	0.830510086	1.185884301
Zm00001d031988_T001	5.813528101	4.895205517	5.777373396	5.836877171	5.847355992	5.884928765
Zm00001d053172_T001	3.364161136	3.175538493	3.466531198	2.978090902	3.350615911	3.461542311
Zm00001d053172_T002	4.133532538	3.927766018	4.204649781	4.369019996	4.332631537	4.247939586
Zm00001d036825_T007	1.616385459	0	1.480653408	1.38641146	0.653905661	0.637699267
Zm00001d036825_T051	0	0	0	0.435590193	1.227514983	1.359060064
Zm00001d036825_T023	1.843643424	0	0.720130466	1.752692509	1.92425156	1.311957011
Zm00001d036825_T033	1.062510662	0	0.740924107	1.138149313	0	1.010650957
Zm00001d036825_T028	0	0	0.831478419	0.947686353	1.303386236	0.960371585
Zm00001d036825_T013	0	0.053401932	0.512844799	0.996419119	0.974625489	0.905473366
Zm00001d036825_T052	0	0	0	0.741232253	0.78778599	0.751098609
Zm00001d036825_T002	0	0	0.738620858	0.53140891	0	0.404542403
Zm00001d036825_T065	1.105714949	0	0.366909106	1.146317642	1.342442102	0.912630705
Zm00001d036825_T055	0.908928933	0.385151583	0.929100156	0.973159387	0.705671863	0.996481307
Zm00001d036825_T016	0.534140311	0.757549862	0.424097609	0.347324358	1.551264493	0.542512635
Zm00001d036825_T073	2.3254696	2.167894824	2.724894483	2.04687063	1.271180026	1.294772472
Zm00001d036825_T010	0.231991437	0.741166659	0	0	0.578159085	0.810953047
Zm00001d036825_T026	0.199711915	0.116228999	0.158692486	0	0.550448582	0.726430171
Zm00001d036707_T001	1.881125025	1.70108457	2.130000394	3.104567473	3.405916684	3.362824312
Zm00001d036707_T006	2.234708544	1.106033345	1.964522841	1.434384834	0	0
Zm00001d030343_T001	3.662458804	3.010376508	3.510712084	3.749649035	3.839312849	3.799803605

Zm00001d009599_T001	2.830298937	3.705134701	2.950813463	3.99358796	3.723918044	3.886310361
Zm00001d016491_T001	7.188287784	6.824000053	7.20823268	6.508402236	6.682201761	6.646662962
Zm00001d033186_T001	5.62310532	5.905837284	5.712481466	5.487956891	5.448401635	5.383937524
Zm00001d027794_T001	5.667205433	6.122205424	5.51788641	5.336514772	5.14437249	5.26007505
Zm00001d027794_T002	5.362214501	5.696327008	5.247845126	5.048924188	5.147490199	5.21670745
Zm00001d018324_T001	6.156445118	6.403126207	6.374551144	5.348150996	5.155068478	5.349048025
Zm00001d029988_T001	3.007938549	2.547668216	3.143205151	2.850391795	3.030971466	2.985474017
Zm00001d005997_T001	2.718818832	3.218875846	3.019196438	2.406562792	2.635665714	1.640115572
Zm00001d005997_T002	4.344645593	3.091281096	4.187182285	4.772737636	4.916146029	4.889656156
Zm00001d019856_T007	1.212145339	0.657747918	1.482476077	2.436692489	2.553323637	2.308049479
Zm00001d019856_T010	0.122769447	0.048710495	0.246741282	0.452051356	0.740551137	0.680242429
Zm00001d019856_T009	0.099525478	0.143364682	0.267541791	0.384099554	0.416422543	0.225754926
Zm00001d019856_T003	2.300220389	1.552427242	2.259969333	1.701865728	1.883231353	1.866453838
Zm00001d026463_T001	3.234862715	2.47460477	3.68964067	3.202505644	3.308697102	3.121381169
Zm00001d037659_T001	1.124175916	0.525216969	0.902477284	1.398442862	1.491382637	0.962492017
Zm00001d003212_T001	0.969086007	1.517522127	1.526744416	1.609869352	2.076124306	2.415093673
Zm00001d003212_T003	2.814544886	1.95792314	2.765889648	3.81435118	3.524881982	3.501074126
Zm00001d012749_T001	2.885145592	2.194251301	2.88461687	3.922246191	3.862046944	4.014927972
Zm00001d046200_T002	0.732062496	0.631505713	0.785550552	0.390394747	0.251397953	0
Zm00001d046200_T005	2.588051608	2.329643734	2.892926424	2.322466408	2.696154104	2.431536583
Zm00001d046200_T004	0.727707029	0.17633554	0.459207065	0.547981796	0.536736277	0.621833861
Zm00001d028096_T031	0.924026894	0.629364081	0	1.533866307	1.346735683	0.955608901
Zm00001d028096_T004	0.70173002	0.593703705	0.890446693	0	0.293688301	1.514818122
Zm00001d040748_T039	1.136428972	0.300156533	0.820881499	1.993184505	1.339969118	1.830177426
Zm00001d040748_T048	3.315144199	0	2.46998441	3.021331719	3.56248264	2.914164953
Zm00001d040748_T063	0	0.175582084	0.51568117	0.930962097	0.538390162	1.032490212
Zm00001d040748_T037	0.080416171	0.161712292	0.240474315	0.371781871	0.549361626	0.502999596
Zm00001d040748_T034	0	0	0	2.057886221	1.862328789	1.544252914
Zm00001d040748_T021	0.076557727	0	0.807429116	0.976248509	0.70328324	0.386571489
Zm00001d040748_T087	1.879636374	0	1.329832568	1.916260702	2.182184405	2.278456744
Zm00001d040748_T035	3.029606423	2.241702618	3.518650827	0	0	0
Zm00001d041518_T001	3.588505613	3.660293859	3.910966985	4.482221839	4.203532891	4.499466177

Zm00001d039498_T002	3.941449943	3.504639077	3.983373712	4.22828899	4.165605444	4.328871569
Zm00001d039498_T003	0.439568886	0.300690731	0.500743936	1.075113624	0.918451116	0.665442801
Zm00001d053548_T003	5.779004363	5.088093585	5.76437421	5.620031775	5.425184611	5.474805723
Zm00001d053548_T004	2.972768187	2.157911977	3.204852027	3.895369519	3.861151934	3.779979312
Zm00001d039316_T001	0.795875811	0.523595075	1.348166686	1.57140499	0.266981985	1.204409319
Zm00001d039316_T004	7.377960225	6.465351588	7.387552921	7.263846933	7.264403208	7.237300598
Zm00001d026066_T001	1.94777536	1.330872145	1.803585805	1.618635225	1.694964413	1.640377528
Zm00001d034064_T001	3.029042017	2.453910234	3.06343909	3.128790933	3.145316655	2.963908162
Zm00001d051840_T004	5.675724603	7.00360046	5.213689833	4.598879026	4.580569606	4.107321793
Zm00001d051840_T001	1.996614353	2.786808151	2.253384841	4.004193045	4.545471245	3.48084286
Zm00001d051840_T003	4.526530413	2.951584811	4.490060862	3.275089911	1.287224634	3.91876203
Zm00001d013271_T001	4.63009008	4.262683984	4.958679516	4.847965598	4.89971364	4.888877992
Zm00001d013935_T008	2.649594077	2.144573412	2.619539549	2.067691716	1.511861172	1.360602505
Zm00001d013935_T004	0.630563898	0.227638815	0.444170889	0.801205848	1.304342815	0.994460973
Zm00001d013935_T003	3.143564847	2.841663218	3.093287025	3.628195935	3.847449781	3.503304461
Zm00001d013935_T009	1.01931406	0.413766286	0.856756274	1.003243857	1.225790086	1.213629727
Zm00001d013935_T006	3.100200771	2.318899587	3.279083454	4.074007754	4.094930727	4.084704659
Zm00001d038998_T001	2.114710832	1.29895093	2.179124369	2.222009129	2.839843925	2.339117427
Zm00001d038998_T003	1.569032156	1.514488917	1.59341927	2.378196138	2.144458556	2.128321449
Zm00001d045204_T001	5.477114547	5.643704612	5.723331737	5.357942711	5.402767226	5.217082251
Zm00001d041083_T016	0.374372926	0.326093197	0	0.492056213	0.982129478	0
Zm00001d041083_T003	0.82597282	1.883245823	1.040622448	0.622876015	0	0
Zm00001d041083_T005	3.008460355	1.883814336	3.081922622	2.442133237	2.206224044	2.761401876
Zm00001d041083_T004	0.168018102	0.181470256	0.161990843	1.116773285	1.310291836	0.680940915
Zm00001d041083_T014	1.020656517	0.046544143	0.551242341	1.001508991	0.98458935	1.189443795
Zm00001d041083_T006	0.109174652	0.114251072	0	0.530374436	0.61331759	0.686045965
Zm00001d041083_T017	0.446961258	0.132035854	0.336087968	0.746948186	0.617779928	1.140549638
Zm00001d041083_T010	0	0	0.054455372	0.265502908	0.513943443	0.449343732
Zm00001d041083_T009	0.642187701	0.136726883	0	1.612086348	2.196055542	0
Zm00001d041083_T008	0	0	0.7647368	0.947151445	0	1.143075325
Zm00001d041083_T030	0.813770932	0.34991213	0.681816246	0.880545265	0.620130306	0.470686788
Zm00001d041083_T015	0.618975323	0.347964935	0.899316412	0.656658364	1.242062213	1.14444583

Zm00001d041083_T029	2.035670357	1.986540908	1.892136467	2.147431097	1.866215239	1.753524099
Zm00001d036404_T003	0.383042332	0.134619187	0.461234123	0.256487773	0.782224861	0.533377954
Zm00001d022582_T002	4.911687987	3.965702845	4.948761507	5.490885204	5.481443294	5.541642482
Zm00001d024083_T001	2.298258293	1.737803557	2.297545076	2.290575684	2.340355748	2.438554483
Zm00001d038977_T001	3.816851875	4.808862266	4.139298808	3.172014754	3.316795967	3.516336313
Zm00001d021752_T001	4.252202712	3.606323538	4.207134379	5.3511912	5.464967915	5.334287089
Zm00001d001811_T001	2.741038483	2.103667161	2.804804457	2.320566992	2.866859327	2.383146319
Zm00001d050489_T001	3.482087893	3.033604307	3.67164902	3.952063455	4.146894104	3.888288722
Zm00001d002066_T001	6.246472296	6.196053181	6.374760269	6.210032803	6.246697832	6.346468204
Zm00001d023239_T001	4.341049684	3.483201171	4.253360181	4.101456533	4.231078757	4.199913598
Zm00001d013797_T003	1.192502875	0.915288368	1.107076519	1.634754488	1.807641372	1.064616632
Zm00001d013797_T001	2.401402195	2.11718469	2.703106017	2.042130198	2.175518853	2.170446522
Zm00001d013797_T006	1.081808632	0.85083142	1.022554597	1.938763788	1.005540837	1.558924903
Zm00001d013797_T007	2.138116211	1.351793393	2.345041194	3.139698193	3.099518073	3.027152813
Zm00001d013797_T002	0.560655289	0.0713357	0.545783574	0.754207672	0.725914749	0.914570646
Zm00001d013797_T005	0.877040593	0.564706914	0.919946402	1.189133164	1.30291034	1.603133742
Zm00001d033925_T001	8.457390725	8.10775473	8.572549053	8.176832812	8.224615606	8.191658807
Zm00001d043920_T001	2.282964892	1.575909134	1.846674632	2.301121988	2.584067752	2.805150656
Zm00001d044734_T001	0.451643163	0.303214955	0.603429561	1.788609294	1.520775241	1.733758239
Zm00001d018811_T001	0.283967985	0.465856433	1.077031707	2.358293852	2.420067603	2.452186868
Zm00001d005451_T070	5.226216108	4.153827463	5.309334501	5.939704591	5.983850384	5.754594418
Zm00001d005451_T012	0	0	0	3.911508765	4.147054775	3.737650099
Zm00001d005451_T002	1.571465668	1.5257116	1.68315026	0	0	0
Zm00001d005451_T001	2.081211443	1.575418834	2.297732876	4.651888585	4.746981067	4.750958615
Zm00001d007940_T002	5.047695292	5.195626109	4.804924718	4.717733773	4.288704428	4.552410211
Zm00001d043508_T002	1.3650301	1.449585672	1.638440432	1.506990312	1.772034699	1.753990401
Zm00001d043508_T001	0.912004435	0.336004535	1.130452527	0.860007702	0.533784594	0.854336426
Zm00001d009627_T002	1.062727544	0.413799858	0.904156504	1.257194716	1.592119243	1.212478448
Zm00001d009627_T001	5.625630183	4.986766695	5.779454203	5.416796312	5.514214628	5.360593341
Zm00001d012564_T001	6.303188263	5.624077288	6.291737313	6.004846744	6.004515639	5.946488636
Zm00001d029333_T012	1.31052161	0.652865504	1.018931797	0.899632734	1.099777257	0.540522305
Zm00001d029333_T009	0.64558823	0.107761508	0.730966821	1.451239603	0.904299884	1.350153788

Zm00001d029333_T014	2.186105601	1.957601145	2.00734084	1.920153788	1.902166604	2.048815798
Zm00001d029333_T008	0.72336983	0.914420629	0.879232893	1.534406792	1.804005634	1.686552071
Zm00001d028107_T001	0.492202891	0.399145932	0.877492983	0.319570537	0.710355278	0.795825951
Zm00001d038995_T001	5.341704828	6.785795222	5.559163437	5.673663633	5.717769794	5.461664133
Zm00001d038995_T002	5.031017754	5.514596504	4.901924284	4.972002568	5.139219556	5.214339328
Zm00001d018529_T001	6.248029296	5.595116529	6.296073353	5.951181956	6.146583705	6.036746312
Zm00001d031860_T005	1.525253559	0.74769159	1.120954375	0.658894216	0.682605656	1.218678269
Zm00001d031860_T004	1.508920961	1.140136006	1.454519677	1.192627224	1.611243214	1.256569895
Zm00001d025508_T001	0.943892079	1.930239262	1.878300807	2.356643595	2.075496692	2.196551337
Zm00001d002496_T001	4.474261715	4.268656635	4.716110143	5.172917372	5.186886614	5.200635783
Zm00001d028994_T005	1.241356357	2.696087318	1.648082514	0.898875457	1.123047671	1.245418993
Zm00001d028994_T010	0	0.368856612	0	0.645922956	0.514819131	0.713202223
Zm00001d028994_T009	3.407467374	0	3.391409613	3.698353183	3.469604181	3.428537054
Zm00001d038185_T001	3.115233381	2.527970613	3.363760233	2.663074957	2.809227973	2.936366381
Zm00001d011534_T002	6.440329951	6.257777582	6.336327364	5.519033356	5.5400584	5.549623092
Zm00001d011534_T003	4.224022441	6.346528377	4.167160957	4.350441872	4.524934498	4.294388649
Zm00001d029048_T001	6.077153789	5.408910999	6.057816012	6.203050927	6.214844613	6.208412632
Zm00001d002241_T001	2.764035474	2.256461807	2.915611915	3.289694467	3.000136509	2.967785956
Zm00001d017651_T001	3.141378345	2.144145592	3.007755603	3.197953749	3.322237231	3.491057407
Zm00001d032517_T002	2.49210109	2.895200621	2.915379971	3.335089886	2.943159	3.456282716
Zm00001d032517_T001	4.882111453	5.560376218	4.792442522	5.415397136	5.799582215	5.50585083
Zm00001d002154_T002	0.935532918	0.30835039	0.676473235	0.311249662	0.832388578	0.826680675
Zm00001d002154_T006	1.096598533	0.527317605	1.316057092	1.131298414	0.783849701	0.87954267
Zm00001d002154_T005	0.552553155	1.526084842	1.476415907	1.741343584	1.324846886	1.503460686
Zm00001d002154_T008	2.089278476	0	1.971578132	1.088220312	1.604245955	0
Zm00001d044401_T001	1.925197741	1.725796988	1.635372717	1.915405786	2.250927324	1.958664531
Zm00001d052719_T002	3.761352087	3.094519513	3.749274666	3.181103028	2.97176885	3.010055087
Zm00001d010434_T001	5.881259208	6.425502408	5.967287597	4.732816918	5.193922706	4.955021156
Zm00001d007295_T003	0.324095126	0.166396667	0.44882168	0.844547952	0.931923549	0.951637774
Zm00001d007295_T002	1.274821035	0.622251937	2.019268507	2.572275604	3.355027826	2.441905443
Zm00001d007295_T001	3.51741164	2.56765496	3.337992041	3.017839797	2.12105752	3.152893557
Zm00001d040203_T008	0.161779355	0	0	0.881799291	0.932204829	0.79713337

Zm00001d040203_T001	1.552652991	1.596895557	1.951517672	2.478113955	2.498637662	2.338439819
Zm00001d021138_T003	0.901350796	0.659551053	0.642797671	0.541070712	0	0.136848916
Zm00001d021138_T005	0.437320435	0.104368868	0.708160744	0.988798099	0.936038973	0.883181294
Zm00001d021138_T004	3.745874731	2.905814713	3.663443339	4.120688104	3.982894122	4.250923005
Zm00001d014947_T001	6.278043439	5.343426691	6.351818352	5.319015566	5.31731192	5.423740616
Zm00001d014180_T006	0.437382229	0.319010904	0.306097718	0	0	0.29983545
Zm00001d014180_T014	1.163131571	0.581227823	1.372668544	0.662989311	1.018698973	0.887593115
Zm00001d047849_T001	3.581821824	3.265719526	3.60461757	3.761610481	3.692875739	3.430801901
Zm00001d049449_T003	2.563599659	2.595327013	2.873726988	3.728565549	3.899583531	3.754999066
Zm00001d049449_T005	4.174777112	1.251142202	3.48269936	4.165551099	4.036020332	4.15333833
Zm00001d049449_T007	0	2.967909874	2.998692867	1.209334202	1.599283035	1.078188987
Zm00001d016655_T001	9.711504789	9.296027272	9.682960231	8.155419901	8.112878916	8.187235
Zm00001d002748_T001	4.662831045	3.906980425	4.031197906	4.220642066	4.207722263	4.649261064
Zm00001d048609_T001	6.900551928	6.601397622	7.034943175	6.173887307	6.221078758	6.263756869
Zm00001d016406_T015	2.355397755	0	1.578453789	0	0	1.615287749
Zm00001d016406_T001	0	0.526794986	0	0.896843775	1.292709907	0.5323409
Zm00001d016406_T009	0	0	0.425063933	1.706290065	1.623567713	1.03358017
Zm00001d016406_T013	0.909939729	0.117766843	1.362181461	1.208142023	1.437867433	1.504168828
Zm00001d016406_T017	0.584506537	0.348760442	0.251177355	0.557643393	0.327334407	0
Zm00001d016406_T016	0.363522104	0.073536464	0.340663667	0.446015849	0.31631144	0.721135509
Zm00001d016406_T004	3.07562915	2.737796419	3.455450325	3.391923719	3.392821042	3.185270795
Zm00001d053963_T002	4.874899436	4.468105533	4.905023454	4.357503023	4.232088784	4.295244299
Zm00001d053963_T003	1.877847885	1.622064005	2.182576623	2.035749876	2.178958389	1.36671891
Zm00001d053963_T001	0.837245737	0.490476686	0.848582682	0.77054069	0.544984827	0.887747507
Zm00001d043595_T005	0.739155097	0	0	1.363871364	1.167282772	0
Zm00001d043595_T004	3.657420955	2.893369007	3.796498186	4.195851902	4.244118635	4.221954239
Zm00001d013225_T005	1.0450417	0	1.078801088	1.819431121	1.522746289	1.180265004
Zm00001d013225_T003	1.298200803	0	0	0	2.065625042	1.914913101
Zm00001d013225_T001	4.584980534	4.37117709	4.512156267	4.526224532	4.461424767	4.447588784
Zm00001d006502_T001	4.821290916	5.897502314	4.814065701	4.394041855	4.815658781	4.533561729
Zm00001d000008_T001	3.767156654	3.775293324	3.667188541	4.500271778	4.657899827	4.348250481
Zm00001d043520_T001	2.927806401	2.601788019	3.340451748	2.153261873	1.789071909	2.070566562

Zm00001d030301_T003	4.662957875	4.409211963	4.718254701	4.718391384	4.642524141	4.611892308
Zm00001d030301_T002	0.973755997	1.190717252	0.98649977	1.759895105	1.879265442	1.754053704
Zm00001d007531_T001	1.016420724	0.680177604	0.987566813	1.222789108	1.129399087	1.188983203
Zm00001d024001_T001	4.615908785	3.514511893	4.544357469	3.737843568	4.127894146	4.027266868
Zm00001d043170_T001	4.171979378	4.863053785	4.333053352	4.26652226	4.091582552	3.993251091
Zm00001d052198_T009	1.127203378	0.281129664	1.535345806	0.972036032	0.966905135	0.732853541
Zm00001d052198_T001	4.113713999	2.295491176	3.578683231	4.306057095	4.593037288	3.988029214
Zm00001d052198_T003	3.536913659	3.432885	3.995797446	3.553306063	3.331697049	3.594949415
Zm00001d052198_T008	0	0.766107467	0.535111359	0	1.358246583	1.755362338
Zm00001d045174_T001	6.381840319	7.116800706	6.600554788	5.570945297	5.792391502	5.760070254
Zm00001d020117_T002	0.350889833	0.089532048	0	0.925145698	1.423808876	1.001741003
Zm00001d020117_T001	0.791784071	0.533815486	1.039394616	2.013934207	1.829874315	2.036878509
Zm00001d028868_T001	5.974122556	5.191875759	6.065954475	5.710279916	5.830671368	5.786512699
Zm00001d028868_T002	1.282848387	0.456888136	0.580533368	1.633086596	0.910142418	1.275030894
Zm00001d020374_T001	0	0	0.105038377	0.570207401	0.336340523	0.496549272
Zm00001d020374_T002	1.716977729	1.270811175	1.467433395	1.727872981	1.598487172	1.967170821
Zm00001d014685_T009	2.20740625	1.524131395	1.976042797	3.005661015	3.116424646	3.101786373
Zm00001d014685_T005	0	0	0.313729959	0.437679443	0.563579892	0.12871704
Zm00001d014685_T002	0.378797914	0.179122458	0	0	0.386358482	0.660952341
Zm00001d005007_T001	2.666153487	2.617600345	3.116904668	0.694020781	1.78899927	0.584879784
Zm00001d032139_T002	2.86243929	3.213427542	3.326070468	2.754401179	2.644909088	2.589760851
Zm00001d032139_T005	0.515199743	0.24895374	0.626110141	0.722734428	0.648843669	0.808880075
Zm00001d037258_T002	1.693525763	0.821685728	1.816122484	1.385701603	1.355320788	1.548342908
Zm00001d037258_T001	1.605507135	1.902135725	1.789108227	1.246599623	1.074749975	1.443639536
Zm00001d012884_T001	2.568747679	1.748439335	2.564278652	2.452834985	2.107738078	2.487266893
Zm00001d012884_T007	0.649302745	0	0.734383168	1.276647925	1.293657111	1.14102857
Zm00001d003037_T001	1.792883665	1.459723276	1.623983415	3.132233812	2.971171785	2.50135481
Zm00001d003445_T001	1.635057365	2.648438061	2.497014472	2.97388381	2.177973267	3.117652659
Zm00001d003445_T002	2.020581139	1.52000629	0.942929566	2.528500057	2.731442208	2.930580667
Zm00001d018545_T001	4.056454339	2.993421483	3.97002626	3.850398497	4.056025503	3.916147271
Zm00001d018545_T003	2.392944927	4.245750972	2.113603827	1.991677297	1.798091171	1.513672145
Zm00001d020392_T002	2.687528368	1.810590348	2.777408774	2.212809924	2.601251518	2.25970395

Zm00001d010116_T001	1.763508034	1.156245155	1.857526823	1.818415806	1.670867975	1.696502229
Zm00001d004960_T004	2.437380098	1.227808219	2.193618163	1.722168274	2.329519458	2.592807906
Zm00001d004960_T001	4.286784916	3.672649593	4.340402538	4.244903875	4.182093089	3.782011455
Zm00001d017213_T001	1.314194758	1.00694211	1.502797505	1.280218748	1.397983082	1.421621879
Zm00001d029410_T001	3.360993755	2.951872912	3.476065375	3.946000159	3.78529641	3.749803045
Zm00001d029410_T002	1.756702232	0.747670969	1.864411212	1.542232787	1.800949585	1.656208032
Zm00001d007339_T002	0.904744578	0.41010335	0.451913195	0.447306236	0.787283674	0.408181417
Zm00001d007339_T001	3.277748542	2.939052955	3.470913075	3.001325228	2.769861006	2.848026762
Zm00001d003202_T001	1.163096138	1.288002939	0.812328172	1.90570479	1.850666227	2.108506726
Zm00001d035039_T001	4.508822741	3.630068757	4.460599729	5.772317213	5.81556882	5.887234729
Zm00001d045479_T001	3.476834905	2.820226558	3.684745255	4.476645833	4.481523441	4.390758676
Zm00001d043317_T001	4.280345043	3.920751097	4.23016919	3.491674704	3.878536729	3.63852917
Zm00001d033869_T001	1.123880036	0.797188166	1.30857523	1.726216524	1.141198645	1.217965838
Zm00001d036771_T001	0.658971883	0.342304414	0.488360757	0.799816761	1.314162847	1.120695651
Zm00001d033366_T002	5.356085023	4.707518165	5.380160486	5.363126737	5.28580253	5.101919464
Zm00001d038795_T001	8.621585168	3.941883147	8.879008128	8.651329889	8.777512432	8.424057547
Zm00001d009652_T002	2.717641771	0	2.592466366	2.51487315	4.080917871	3.688279184
Zm00001d009652_T006	5.530713611	7.097614842	5.598276404	5.395823922	4.590440221	5.167316135
Zm00001d009652_T004	6.325085708	5.780542943	6.086269727	5.178147848	5.490863196	5.072407002
Zm00001d009652_T005	1.70674183	2.737855246	1.942272769	1.611032118	1.72584104	1.555622311
Zm00001d051889_T002	2.233178806	1.12544546	2.482834604	2.259243558	2.125927448	2.206302513
Zm00001d051889_T001	1.804921724	1.801715342	1.552923942	1.357642645	1.691648057	1.429272086
Zm00001d021017_T001	6.208151136	6.859869217	6.266498854	5.294885997	5.312083898	5.30994918
Zm00001d018842_T001	3.740980541	3.001761717	3.681520667	4.010597253	4.16172269	3.955257836
Zm00001d052162_T001	3.498332448	3.660463642	3.368972658	2.334212706	2.455053617	2.633785505
Zm00001d031518_T015	1.907465635	1.854373931	1.733753901	0	0	0
Zm00001d031518_T006	1.647794791	0.926199104	1.756964342	2.803375019	2.724118057	2.609482895
Zm00001d031518_T012	1.675830832	0	0.978588839	2.201553256	0	2.562243646
Zm00001d038161_T001	1.34582157	0.831616236	1.458442903	0.882650874	0.97269633	0.639438711
Zm00001d018377_T003	1.039726585	0.112836223	0.703602188	1.484215485	1.738375716	1.011961434
Zm00001d018377_T010	4.011747069	3.244516141	4.236120761	4.286910785	4.090362627	3.959622448
Zm00001d018377_T002	3.793366451	2.853125378	3.719255519	3.923993058	3.73723474	3.883364705

Zm00001d042706_T002	0.293935444	0.235905931	0.37893771	0.791114741	0.779337054	0.824635897
Zm00001d038885_T001	4.846911722	3.932605482	4.718383328	4.9331145	4.873632752	4.872458387
Zm00001d038885_T002	0.379413581	0.121967597	0.369444149	0.563419793	0.290568312	0.517114405
Zm00001d048682_T001	0.869518485	0.488064546	0.773849517	0.517959961	0.390795333	0.788296485
Zm00001d045606_T004	0.994418973	1.012326237	1.476612915	1.14028066	1.368391775	1.437292201
Zm00001d025734_T017	1.015207802	0.509533704	0.905218409	1.378291873	1.270204187	1.396234582
Zm00001d025734_T008	2.566975607	2.011624445	2.045079106	2.728034559	2.01335756	3.109822716
Zm00001d025734_T009	0.541012212	0	1.822709349	2.784555911	3.010603877	2.552393303
Zm00001d025734_T003	1.726543957	0	1.730111251	1.214217458	1.287371817	1.20451324
Zm00001d025734_T002	0.587894922	0.897740731	0.529661055	0.744435827	0.982821673	1.154358317
Zm00001d025734_T015	1.288911317	0	1.298915749	3.071529122	3.457945927	3.053018167
Zm00001d025734_T014	1.71475018	1.51531633	1.544475	1.095281054	1.443843192	0
Zm00001d042142_T001	1.560195002	1.530003302	1.642765331	2.113668165	2.1817889	2.013974928
Zm00001d029378_T003	1.386406494	1.391481242	1.806163589	2.032411218	2.271828128	1.868889408
Zm00001d029378_T007	0.33858301	0.032243342	0.324680438	0.416675471	0.621715728	0.426168124
Zm00001d029378_T002	4.176805133	2.90484764	4.192386012	3.898261473	3.74452237	3.948276859
Zm00001d029378_T005	0.56124792	0.386162002	0.531868993	0.728860024	0.079320078	0.738109775
Zm00001d029378_T001	1.615959089	0.284433586	0.838526659	0	0	0
Zm00001d029378_T010	0	0.295162292	0.99775992	0.949369084	1.823755473	0.865368546
Zm00001d029378_T004	0.718341897	0	0	0.834975706	0	0.558932737
Zm00001d029378_T016	4.381690717	5.988933422	4.789409799	2.680614442	2.850146923	2.707988755
Zm00001d048574_T003	4.368706757	6.135739181	4.779883002	3.661551694	4.322538298	3.606143709
Zm00001d048574_T002	4.062653038	3.033809372	3.909493945	4.119147394	4.001336308	4.112310386
Zm00001d053979_T001	6.986436125	7.263475854	7.054677689	6.247217533	6.279932316	6.182577874
Zm00001d051005_T001	2.137486474	1.767309866	2.292826502	2.320902263	2.254663576	2.385213443
Zm00001d014863_T001	1.244737303	1.109757706	2.293102345	1.942813241	1.428035163	0.991614538
Zm00001d014863_T002	2.101180754	1.356010806	1.905216098	1.649540966	1.849384032	2.391791911
Zm00001d009600_T003	0.759285394	0	0.411227726	0.735428594	0.781130543	1.027806585
Zm00001d009600_T035	0.233450033	0.452177905	0.957581088	1.80141778	0.854232685	0.740889577
Zm00001d009600_T031	0.587192145	0	0	1.147857618	0.778423072	1.912474738
Zm00001d009600_T017	1.243475937	0	0.576269614	0	1.27059532	0
Zm00001d009600_T011	2.382321407	1.908060435	1.917217102	1.574723029	1.521153783	0.858692417

Zm00001d009600_T036	0	0.091060676	1.983748825	0.31047973	1.544261818	0.489693979
Zm00001d009600_T024	0.796692436	0.538792413	0.577254304	1.317129221	1.028264315	1.149079831
Zm00001d017397_T001	5.193884001	5.129277576	5.163856785	3.748023098	4.017194856	3.985434214
Zm00001d027859_T001	2.961660553	2.279843585	2.690794091	2.447798427	1.769163648	2.405552407
Zm00001d010880_T003	0.501056924	0.583057848	0	1.338808892	1.562498392	0.98475849
Zm00001d010880_T002	5.012998602	4.310117227	5.089814892	4.879114454	4.762592947	4.888167176
Zm00001d001860_T001	0.942325324	1.227183479	0.900511729	1.789101548	1.920077166	1.86449283
Zm00001d029052_T001	2.889091304	4.358591356	2.46675344	4.121318501	3.89570997	4.227219221
Zm00001d043091_T003	3.868726565	3.17123021	3.967718277	5.050297033	5.030902803	4.948349662
Zm00001d043091_T005	0.367080261	0.387392321	0.757071905	1.06601546	1.257234551	1.280371993
Zm00001d043091_T001	0.882897333	0.451328765	0.871240429	1.754333829	1.493919351	1.476328282
Zm00001d043091_T004	2.002369483	0	0	1.358287662	2.095267211	1.844889363
Zm00001d016704_T001	5.775290163	5.146356503	5.725538124	5.879326298	5.87089896	5.873717221
Zm00001d045482_T010	1.705948274	1.113701833	1.304189758	1.448711222	1.689724972	1.191262584
Zm00001d045482_T014	0	0.686220813	1.212061271	0.910092516	1.326189863	1.837124608
Zm00001d045482_T001	3.750283202	3.674798574	3.853614769	4.467211583	4.472910965	4.473183022
Zm00001d045482_T013	5.6916317	4.791935523	5.722246516	5.402611576	5.162136586	5.114922547
Zm00001d045482_T009	0.860426528	0	0.797283639	0.640947556	1.20187659	0.131598703
Zm00001d045482_T012	4.018981721	2.860346666	4.071665892	4.239426532	4.196396557	4.325245866
Zm00001d014065_T001	6.573038052	6.125195601	6.661514271	6.422438355	6.432519652	6.42181051
Zm00001d034145_T001	5.827748946	6.326529403	5.815522531	6.977721252	7.009856447	7.070106622
Zm00001d018183_T001	6.298329786	5.585160707	6.376035329	6.737074486	6.714078897	6.630721551
Zm00001d034915_T005	1.548946533	0.760490934	1.553513858	1.428152577	1.18390251	1.306097718
Zm00001d034915_T004	0	0.494259939	1.073005789	0.992581376	0.953301003	1.052681593
Zm00001d034915_T002	4.449640407	3.688336148	4.396078226	4.137415812	4.236765977	4.08723335
Zm00001d041850_T001	4.92206071	4.127729594	4.947734456	4.880688904	4.384864962	3.849683173
Zm00001d017612_T001	3.475407377	3.616456155	3.414265586	5.942897859	6.067012952	6.14697605
Zm00001d034188_T001	7.205763804	6.532559918	7.189593146	7.70517753	7.68120882	7.731850945
Zm00001d034188_T003	1.279603227	1.38248023	0.714302675	3.544736117	3.95117727	4.225333858
Zm00001d034188_T002	2.082884788	3.24867752	2.164744129	3.887500803	3.754520904	3.558789017
Zm00001d049054_T002	1.758276737	1.633294026	1.752500272	2.321420465	2.310779552	2.411644261
Zm00001d050988_T001	2.535794187	2.495519419	2.485130787	1.826772996	2.046041523	2.255557533

Zm00001d018082_T001	0.951384878	0.58505483	0.874194193	1.12605106	0.778802357	1.162388589
Zm00001d019631_T004	1.788501134	2.329615321	1.831861436	2.582718086	2.274201715	2.536115333
Zm00001d019631_T010	3.841959336	1.961238435	3.687018012	3.749022637	3.937940652	3.507740229
Zm00001d019631_T006	1.073239612	0.36061936	1.265198027	0.261465825	1.069289713	1.090357546
Zm00001d019631_T015	0	0	0.522236577	1.782090634	1.313150056	1.709617521
Zm00001d012321_T001	1.564687894	1.175571865	1.842545657	4.319261479	4.164820061	4.439572343
Zm00001d027463_T001	1.011791876	0.663793641	1.351193533	1.479806903	1.536330965	1.348540645
Zm00001d008177_T001	4.576593183	3.698297724	4.445175856	4.808278827	4.744940909	4.696226284
Zm00001d037899_T001	5.294998594	5.022661605	5.416598563	5.477416771	5.4600466	5.485220842
Zm00001d038300_T002	3.448125889	3.368994582	3.264052499	2.297323792	1.927860433	2.502455362
Zm00001d038300_T001	3.522368793	3.815032779	3.707307884	1.978631302	2.023775906	1.833728457
Zm00001d043997_T007	4.666669232	3.626864056	4.631772262	4.892906709	4.698483874	4.829760327
Zm00001d043997_T010	4.100243082	5.335196887	4.287048689	3.174222155	2.628104225	3.400359358
Zm00001d043997_T002	1.446115924	1.127373769	1.835232466	2.305212652	1.945902055	2.066146725
Zm00001d043997_T008	0.560878288	0.160520101	0.524193091	0.329718352	0.355988261	0.522520833
Zm00001d043997_T004	1.427001616	0.849226681	1.596403274	1.120737448	1.34512155	1.783255899
Zm00001d040222_T001	0.354375425	0.163491004	0.510152771	0.73369187	0.732026885	0.569534839
Zm00001d034366_T004	0	1.428251219	1.116791247	1.160244493	1.732802757	1.380557689
Zm00001d034366_T005	0	0.189828353	0	0.83253927	0.616428277	1.276471653
Zm00001d034366_T017	0.65332055	0.063627188	0.597788584	0.531788165	0.456276275	0
Zm00001d034366_T010	0	0	1.242566533	1.819459325	0.95633624	1.591446288
Zm00001d034366_T008	2.983482708	0	2.635750445	2.130019181	2.152270725	1.457674133
Zm00001d002836_T004	1.747759036	0	1.781845636	2.054428281	1.304667569	1.466620334
Zm00001d002836_T006	1.016677448	0.97370384	1.661841247	1.520081242	1.402200988	1.579483816
Zm00001d039685_T001	2.849762326	2.183643718	3.087675835	2.917779068	3.006536628	2.882137871
Zm00001d048546_T004	0.365156115	0	0	1.570608165	0	1.500744956
Zm00001d048546_T016	1.815334003	1.266886064	2.094465126	1.841074223	1.918987614	1.60759829
Zm00001d048546_T001	0	0.434294618	0.300294787	0	0.446050797	0.143401256
Zm00001d048546_T005	0.239390709	0.121596342	0	0.30642091	0.505683654	0.296066175
Zm00001d048546_T013	0	0	0	1.786537817	1.47343522	2.013072009
Zm00001d048546_T033	2.818842791	2.284625766	2.845931373	2.598979935	1.671921499	1.944456309
Zm00001d048546_T011	0	0.19337688	0	0.489915892	0.129403044	0.799390738

Zm00001d048546_T014	1.345650712	0	0.825948405	1.216225754	1.508480376	0
Zm00001d012091_T001	6.800679015	6.378523796	6.923438439	7.58896021	7.529737302	7.524527807
Zm00001d015662_T001	4.901964818	5.247787755	5.04171815	4.620522837	4.534508701	4.467954689
Zm00001d014957_T012	1.699415263	1.045582061	1.606890365	1.941235922	1.655131056	1.962353566
Zm00001d014957_T007	2.322343243	8.460259267	1.276928963	0	2.012695596	1.766520534
Zm00001d014957_T001	9.514568594	8.128752554	9.489169345	9.3549685	9.364622206	9.384339034
Zm00001d014957_T005	9.568851443	9.865037184	9.567308755	9.050307545	9.046961544	8.932178742
Zm00001d019344_T001	2.920068207	2.546301336	2.523071858	1.973707146	2.629192697	2.150608415
Zm00001d019344_T002	3.974680457	4.177152722	3.815835664	3.442662359	3.805749134	3.594188428
Zm00001d046135_T001	4.967175618	4.298740711	4.959220232	4.847076344	4.908018775	4.720730359
Zm00001d046135_T002	1.7253241	1.277084939	2.017213923	2.568822329	2.795243918	2.918295204
Zm00001d003679_T004	1.400892549	1.219977597	0.838774318	0	1.948977544	1.70206084
Zm00001d003679_T006	2.242522024	0.879977814	1.083295076	2.949342748	3.110733893	2.96353257
Zm00001d003679_T001	2.728156275	2.202741175	2.939371216	0	0	0
Zm00001d017419_T001	3.985088511	3.447397625	4.12113048	4.773586976	4.833422296	4.739178487
Zm00001d034030_T001	0	0.111638986	0.610273899	0.390584049	1.323018937	1.238112425
Zm00001d034030_T014	1.518109066	1.188642725	1.548724149	0	0.894383968	1.604903955
Zm00001d047005_T001	5.839419105	5.476546072	5.634906196	4.564890216	5.19289999	4.2048532
Zm00001d045118_T001	2.505789328	3.777122992	2.905502633	1.891087057	1.678011942	1.914864129
Zm00001d036796_T002	2.619237623	1.144820387	2.734618802	2.40560169	1.689926652	2.191619183
Zm00001d036796_T003	1.738710344	0.898313624	1.362169115	2.060177463	1.715448855	1.970604105
Zm00001d036796_T004	5.046592073	4.636012498	5.235953172	4.910418045	4.972155081	4.958023679
Zm00001d032530_T001	3.085070058	2.4148218	3.348409629	3.153747627	3.175760267	2.995308852
Zm00001d022360_T002	5.049782086	4.335450956	5.157293855	4.582511565	4.687186247	4.758153271
Zm00001d022360_T004	0	0	2.809752131	2.927130162	2.43325139	0
Zm00001d022360_T003	2.66756541	1.160420707	0	0	0	2.038118318
Zm00001d047424_T001	6.595268538	5.744465833	6.719157903	6.132257054	6.070465883	6.132813523
Zm00001d043200_T001	0.736990985	0.789994608	0.987122186	1.673055238	1.795326842	1.698215811
Zm00001d006097_T001	3.104706848	2.462656239	2.895950331	2.217430198	1.731645091	2.31141011
Zm00001d050628_T001	1.199733899	0.714637658	1.085685705	3.175894848	3.203828224	3.074853052
Zm00001d023694_T006	2.353483644	1.349373771	2.035434939	0.85492761	1.469362449	1.041929816
Zm00001d023694_T001	2.682153686	2.34780255	2.406007059	2.573402643	2.822247198	3.14365039

Zm00001d023694_T007	1.404567471	1.030785239	2.360025407	2.308819545	2.031428722	1.903828064
Zm00001d023694_T005	1.784640499	0	1.419901273	2.09895646	1.759604129	1.332981125
Zm00001d013208_T001	4.9558519	5.261979989	5.206816714	7.093347352	7.196857189	7.310098354
Zm00001d053841_T008	0	0	1.679792645	2.408473294	2.465303488	1.922656469
Zm00001d053841_T012	1.780507065	0.768075894	2.324556886	2.305048302	1.984454097	2.598746792
Zm00001d053841_T011	0.779595084	0.183341682	0.526722886	0.553509926	0.485693701	0.759683376
Zm00001d053841_T001	1.260956632	1.563355847	1.908152306	2.47238457	2.592880604	2.216363287
Zm00001d053841_T014	3.869645018	1.503161961	3.849717296	4.08593014	4.209141788	3.993898582
Zm00001d053841_T009	1.528813831	1.292282314	0.863282747	0	0.777638115	1.030958228
Zm00001d053841_T010	0	0	0	1.433732912	1.655771752	1.582524692
Zm00001d053841_T005	3.202825161	3.474554284	2.575994545	0	0	0
Zm00001d044704_T018	0.943711328	0.28544365	0.723964774	0.988131313	0.928399129	0.975504729
Zm00001d044704_T005	2.336193109	1.965572274	2.404256271	2.356693733	2.099203623	2.276845907
Zm00001d044704_T019	3.787542063	3.393203927	3.731806356	3.504410392	3.649716961	3.450272577
Zm00001d044704_T012	0	0.104037353	0.298986697	0.424821069	0.786255093	0.537786078
Zm00001d044704_T002	1.112318477	0	0	0.669816856	1.111970762	0
Zm00001d044704_T014	0	0.182340161	0.36350192	0	0.686273712	0.643959598
Zm00001d013503_T004	1.685918626	0.884796388	1.865083828	1.532754315	1.269324051	1.42553987
Zm00001d013503_T003	2.613130141	1.862459723	2.820696095	2.312149335	2.528337268	2.572156011
Zm00001d013503_T001	2.994379507	1.928422629	2.863993938	2.772682537	2.761000536	2.690003561
Zm00001d004340_T002	2.022252748	1.557989356	2.243558499	2.545738359	2.388423216	2.245422948
Zm00001d029361_T001	5.823664869	6.239774687	5.864546711	5.241814067	5.290745782	5.423315053
Zm00001d030666_T027	0.057992242	0.029457126	0.130995427	0.425396998	0.233295403	0.25869861
Zm00001d030666_T013	0	0	0	1.625706739	1.381461691	1.262116199
Zm00001d030666_T002	0	0	0	0.426497714	1.205069028	0.819329744
Zm00001d030666_T130	2.517734763	2.18199525	2.95295245	2.761321021	2.620522602	2.77054365
Zm00001d030666_T128	2.286283843	1.27611964	1.823181136	2.393259127	1.97164839	2.603682123
Zm00001d030666_T006	0.373693865	0.137096889	0	0.394030053	1.102174353	0.637351498
Zm00001d030666_T110	1.388846386	1.007433778	1.41328699	0.207395629	0.802014498	0.604152948
Zm00001d030666_T048	1.382448688	0.561001509	1.280509781	0	1.125499022	0
Zm00001d030666_T004	0.419368455	0	0.497152469	0.574581115	0.964240398	0.70857926
Zm00001d016029_T001	4.053981645	3.78227037	4.172636962	4.090013392	4.124822901	4.172931169

Zm00001d016029_T002	0.97817073	0.842895183	0.673683954	1.42213933	1.218935502	1.009147202
Zm00001d024396_T001	0.118018117	0.098392606	0.139706541	0.734828824	0.669735236	0.478990437
Zm00001d026182_T001	2.373150385	2.052589438	2.359704166	2.29780975	2.145232678	2.678024566
Zm00001d019123_T004	0.956930185	0.533881254	0.696531607	1.643635498	1.763700934	1.603584807
Zm00001d019123_T001	4.169228092	3.326338734	4.026476257	4.607015639	4.621996806	4.65476215
Zm00001d019123_T003	1.055514289	0.847033987	0.545329885	1.533995843	1.038134125	1.035692876
Zm00001d014906_T007	4.908597112	4.11657993	5.256836383	5.029953457	5.035157114	4.773991632
Zm00001d014906_T005	4.671933667	3.99533391	4.47575405	5.323404118	5.224594611	5.045149326
Zm00001d014906_T002	0.574364101	0	0	1.263083697	0.780268939	1.047441368
Zm00001d014906_T004	0.840873274	0.353424926	0.542055934	0.770517855	0.700585312	0.743557191
Zm00001d043681_T001	2.208355876	1.737441461	2.379488718	2.503721447	2.46949947	2.121158668
Zm00001d023792_T016	1.319906329	0.518915779	1.414562594	1.066862784	1.298025972	0.829982663
Zm00001d023792_T009	0.924867598	2.285705523	2.040744472	1.835254298	1.981710203	0.31364174
Zm00001d023792_T001	0	0	1.332040488	2.201104358	2.239224798	0
Zm00001d023792_T014	0.217296489	0.132514988	0.266346395	0.895429046	0.688168718	0.805577251
Zm00001d023792_T008	2.164253374	1.403319538	2.740711949	3.029014622	1.376690459	3.376567809
Zm00001d023792_T012	0.327701159	0.036276845	0.114151104	0.30412783	0.450602644	0.537961964
Zm00001d040109_T001	4.566556541	4.225052153	4.337813333	3.292381567	3.37337273	3.227122932
Zm00001d024919_T001	3.896168948	3.531785546	3.8104575	3.768946184	4.100805792	3.814172936
Zm00001d036386_T001	3.893651497	3.154591472	3.756527288	3.995712438	3.944878119	3.922024638
Zm00001d003103_T001	3.163207427	2.76346363	3.415829367	2.626057561	3.104723955	3.012886139
Zm00001d036293_T003	3.058897959	3.176231005	2.905679955	2.197105166	2.682878433	2.305683704
Zm00001d036293_T001	8.605946046	7.817572001	8.677843895	8.560159039	8.580610983	8.594398872
Zm00001d036293_T002	2.349032026	1.941638582	1.821509815	2.607432349	1.561842261	1.926320952
Zm00001d003549_T001	3.091417868	2.402464881	2.825459407	2.868033415	2.780306739	3.062777271
Zm00001d007822_T001	4.473791976	5.549325181	4.561763339	4.364699188	4.380467992	4.170002905
Zm00001d007075_T011	0	0	0	1.162362807	0.495858863	1.165271385
Zm00001d007075_T075	0	0.047132116	0	0.574271084	0.642960287	0.570828173
Zm00001d007075_T073	0.610009255	0.693323248	0.159521981	0.545806304	0.686159843	0.927407257
Zm00001d007075_T065	2.385113471	1.068190635	2.22935012	3.011254633	3.343262266	3.103211086
Zm00001d007075_T080	4.830150293	4.029023371	4.918909117	4.752284992	4.864601482	4.825751238
Zm00001d007075_T064	0.403301539	0.243621559	0.474659277	0	0	0

Zm00001d007075_T008	2.337332032	1.338990827	2.150791659	3.510405489	3.33572963	3.478305035
Zm00001d002610_T001	0.979202236	0.799346802	1.084551432	1.208984765	1.415470964	1.406419661
Zm00001d013885_T001	2.549034788	2.054946737	2.315186814	2.701279122	2.785013966	2.630543397
Zm00001d046260_T001	1.151052188	0.847001103	1.117841299	0	1.268680511	0
Zm00001d044970_T001	2.211678857	1.886045164	2.42293157	2.715765125	2.698294279	2.74179831
Zm00001d022000_T049	0	0	0.200668815	1.135316183	0.440766203	0.826644884
Zm00001d022000_T026	0.42678108	0.192430281	0.55322483	0.679004847	0.977096712	0.579413819
Zm00001d022000_T052	1.585142345	1.631215129	2.600439137	1.933671584	0	1.2939478
Zm00001d022000_T012	0.698193581	0	1.05356806	0.742218394	0	1.277573
Zm00001d022000_T056	0.658415331	0.542253096	0.36659359	0.330724745	0.321494067	0.520803397
Zm00001d022000_T020	0.82617138	0.94753226	0.69157079	1.402867858	2.128126089	1.300471687
Zm00001d022000_T053	1.949809813	1.362568076	1.945567628	2.379256084	2.240372036	2.21553168
Zm00001d022000_T003	0.111847273	0.410839286	0.478306071	0.554013132	1.004799069	0.85529608
Zm00001d022000_T046	0.882084251	0.702535208	1.018544448	0.459237498	0.344366079	0.502096329
Zm00001d022000_T009	1.689239662	1.181634993	1.805882209	2.462720098	2.189304304	2.886730205
Zm00001d022000_T051	2.973816232	2.123725783	2.496975881	0	0	0
Zm00001d022000_T030	0.076989992	0.029489637	0.138193229	0.364444685	0.335713054	0.566388491
Zm00001d022000_T022	0.969122855	0.600523821	1.149260016	1.509379149	1.402954059	1.711013341
Zm00001d022000_T029	1.034829153	0.660623822	1.294843623	1.298119257	1.220185059	1.195450284
Zm00001d022000_T058	2.340949594	1.493783093	2.005986289	1.76410662	1.86999179	1.942113967
Zm00001d022000_T057	0.259108545	0.86421745	0	1.188509803	0.851381663	1.034559442
Zm00001d022000_T011	1.975811016	0.589408761	1.91584516	1.182436149	1.520024903	1.637983449
Zm00001d022000_T047	2.230673381	0	1.741096746	0.913945984	1.577966287	1.018216117
Zm00001d022000_T059	3.772799805	5.157019773	3.92522889	2.489247739	2.473620947	3.036234815
Zm00001d022000_T014	0.980809153	0	0	2.267133345	1.754190993	1.723913676
Zm00001d022000_T040	0.458520597	0.300566571	0.25440538	1.051032358	0.845187329	0.871375288
Zm00001d022000_T004	1.366121867	1.588624219	1.312751129	0.773632653	0.478799965	0.711129231
Zm00001d022000_T033	0.453699831	0.190205222	0.523440514	0.314193017	0.620012033	1.444371289
Zm00001d022000_T010	0.677605659	0	0	2.05947642	2.093019909	2.112591718
Zm00001d022000_T054	0	0	0.720211036	0.442483883	0.711533672	0.149643079
Zm00001d049416_T001	4.841238204	3.77784649	4.874780149	4.809723273	4.803947636	4.594110061
Zm00001d049416_T003	2.50593614	2.6085519	2.930077047	2.903325045	2.841114893	3.033426878

Zm00001d019990_T001	2.653539995	2.426081689	2.752386374	2.611014171	2.461272634	2.485769942
Zm00001d023811_T001	2.86933982	2.683017049	2.358014719	1.691443938	1.888053123	1.956326203
Zm00001d047516_T001	2.528533813	1.901935383	2.567394307	3.189823926	2.930934477	3.155148848
Zm00001d036338_T001	3.194898182	2.60159455	3.153766594	4.984568024	4.990868387	4.994722765
Zm00001d006582_T002	2.231958884	1.536353845	2.198619873	3.159233262	3.154816651	3.071843827
Zm00001d045194_T001	7.137469608	8.024825857	7.299140709	6.864295352	6.709052249	6.940818342
Zm00001d010243_T001	4.572966832	3.786958976	4.528006945	5.016303758	4.844374856	4.918262713
Zm00001d010243_T005	2.69331321	0	2.818722765	3.60329721	3.626199875	3.458524534
Zm00001d018565_T001	5.639180666	4.353789057	5.645569699	6.071596586	6.059768462	6.019041121
Zm00001d013099_T001	2.654692618	2.516147556	3.015870398	2.516594127	2.568975509	2.851204937
Zm00001d046465_T002	3.388818151	3.984779267	3.2421383	3.285006644	2.724927216	2.820866798
Zm00001d046465_T001	3.387834538	3.406112262	3.566061712	3.003210029	3.244187	2.714709304
Zm00001d051314_T001	2.003685702	1.313454258	2.036764244	2.649420251	2.965212818	3.002514229
Zm00001d032146_T001	1.921659497	1.305357136	1.328955344	0.870435743	1.106133876	1.566876513
Zm00001d027399_T001	3.38085313	3.714268711	3.658682096	3.248296492	3.36275307	3.309785187
Zm00001d054110_T001	0.496898953	0	0	0.762248451	1.116787255	0
Zm00001d054110_T007	0.152035409	0	0	0.642560183	0.509789063	0.622385959
Zm00001d054110_T003	0.544370639	0.275306286	0.661824377	0	0	0.748015474
Zm00001d035067_T001	2.989375198	2.570228778	3.057353223	2.784643639	2.504117713	2.817556339
Zm00001d038733_T001	3.526207314	2.988475288	3.647897572	3.21836147	3.375011064	3.348845972
Zm00001d044302_T001	3.908983101	3.257122136	3.989478292	3.345057237	3.318246404	3.440336331
Zm00001d010618_T004	1.262522158	2.420394816	1.48588275	1.147466917	0.990851055	0.520192908
Zm00001d010618_T002	9.337848581	8.884314846	9.381268768	9.016439072	9.031635133	9.021265661
Zm00001d010618_T001	0.993495395	1.347801133	0.518631819	1.30018934	1.58363461	1.013330044
Zm00001d012205_T001	2.612817201	2.221235534	2.740808644	2.894335265	3.040212644	2.727346965
Zm00001d007886_T006	3.004860179	1.850445005	2.81254895	1.186928359	1.393634208	2.643673129
Zm00001d007886_T016	0.120874773	0	0	0.781114592	1.411885015	1.179960031
Zm00001d007886_T015	0	1.06815692	0	2.539401039	2.265920814	0
Zm00001d006948_T001	0.086516123	0.046141782	0.028093833	1.314738863	1.37351514	1.457589039
Zm00001d006473_T001	1.962188813	1.414175051	1.82961457	1.897706273	1.97052533	1.773180677
Zm00001d011256_T001	0.884328306	1.299808494	1.114247073	1.657235262	1.794527326	1.781057923
Zm00001d052624_T004	1.217029656	0.723417889	1.761990277	1.728517008	1.425976455	1.593047749

Zm00001d052624_T002	1.573406521	1.244630151	1.498540399	1.43630205	1.485981128	1.561213698
Zm00001d052624_T007	1.622616484	1.256413497	1.539220365	1.406156763	1.351812611	1.484733136
Zm00001d052624_T003	1.041212137	0.550491925	1.406680334	1.099489795	1.345610975	1.090233548
Zm00001d023817_T001	2.602304364	2.104257477	2.318907683	2.860577697	3.057866817	2.78102476
Zm00001d023817_T004	0.645384406	0.303106213	0.9766921	0.687344719	0	0.785922947
Zm00001d023817_T002	0.676800872	0.345018193	0.596392301	0.986429868	1.260609242	0.763174435
Zm00001d010023_T001	2.623813731	1.919390428	2.525095917	3.197553424	3.05806188	3.423367869
Zm00001d010023_T008	0.494849762	0.569424983	0.507471948	1.516627406	1.129405023	1.143189638
Zm00001d010023_T010	0.147918819	0	0.283672908	0.334211276	0	0.864001864
Zm00001d010023_T016	3.571292849	4.244507008	2.660747482	2.560110608	1.941602152	2.655425106
Zm00001d010023_T013	0	0	1.880837132	2.156077816	2.007519892	0
Zm00001d004978_T001	0.752883008	0.732468927	0.475730319	1.172134799	1.058880993	1.003374122
Zm00001d050103_T004	3.083765657	1.5212719	3.173799042	3.381693555	3.047282562	3.125849773
Zm00001d050103_T007	1.326946859	0.977230825	1.465281805	1.781524643	1.616027806	2.060152555
Zm00001d050103_T005	0.669222741	0.338669716	0.43655738	0.577924245	1.05489292	0.770637097
Zm00001d050103_T002	0.773304341	2.134210423	1.986658129	1.740085221	2.429979036	2.148596081
Zm00001d032791_T001	2.862399217	2.499453548	2.729397671	2.234726628	2.432275033	2.236324843
Zm00001d032981_T004	0.924240537	0	1.222823105	0.887536968	0	1.503429137
Zm00001d032981_T003	1.770655701	1.329850359	1.627258962	2.161649417	1.996203245	1.858160873
Zm00001d003646_T003	1.12420239	0.82430188	1.159222281	1.069961952	0.908947374	0.734533181
Zm00001d003646_T001	1.320583454	1.381349829	1.542808769	1.089812689	1.133370189	1.281507757
Zm00001d039354_T001	5.05940988	5.777101814	5.133624678	4.078605309	4.222300627	4.068576921
Zm00001d016164_T007	1.414848313	1.827669464	1.363883136	0	1.889124606	1.844809039
Zm00001d016164_T001	1.238422472	1.396616562	1.199558652	0	0.926466321	1.944841208
Zm00001d016164_T006	2.640545969	0	2.718628603	2.699623144	2.243328472	1.429330476
Zm00001d041958_T001	0.977490954	0.93462595	1.43058984	0.94751655	1.069758526	1.036904876
Zm00001d050694_T001	1.776002891	1.068100498	1.617966539	1.928487819	2.081301105	1.752903127
Zm00001d019184_T002	1.500358981	1.04685841	1.607679714	1.215372943	0.983023135	0.948458814
Zm00001d018870_T001	3.640231514	3.031349317	3.75288868	3.538161052	3.559335543	3.55023899
Zm00001d014161_T001	2.84477028	3.672164049	2.71718134	2.848911726	2.510429543	2.555078425
Zm00001d000452_T019	0	0	0.251909319	0.815670512	0.520451416	0.580882566
Zm00001d000452_T027	1.308257181	0.372518667	1.44206607	2.355306692	1.969557223	2.20122985

Zm00001d000452_T007	3.614202397	2.081549948	3.667970898	2.980894495	3.160700961	2.934535939
Zm00001d000452_T010	1.780024052	2.422755076	2.103079961	2.123997866	1.334532809	2.479629214
Zm00001d000452_T001	1.221934602	0.866457795	0.979624435	1.730185612	1.620566705	1.284245824
Zm00001d000452_T012	0	0	0.142640853	0.473551075	0.951986085	1.235644968
Zm00001d000452_T024	1.038984639	0	0	2.396924759	2.430921631	1.815603709
Zm00001d000452_T009	1.326513763	0	1.173227875	1.710053695	2.467579989	0
Zm00001d007255_T002	0	2.009853424	2.137241848	0.688823999	0.225768496	1.419364679
Zm00001d007255_T001	5.387926225	4.450800042	5.390799768	4.908397233	4.960158036	4.870329453
Zm00001d027580_T001	5.458438047	4.84774311	5.444586668	5.294839765	5.198302921	5.234861873
Zm00001d007529_T001	1.632292881	1.037217735	1.533576803	2.672227085	2.754131332	2.813020581
Zm00001d047191_T001	4.496764573	3.581009395	4.523296162	4.075498489	3.991593407	3.695682865
Zm00001d047191_T004	2.425103153	1.939791507	2.252276675	3.3520548	3.521380709	3.752115722
Zm00001d047191_T008	0.700644788	0.35034901	1.011393301	0.214869591	0.407328819	0
Zm00001d019207_T001	5.53977547	4.876135681	5.649315853	9.191164655	9.250183763	9.201987575
Zm00001d016198_T001	0	0.324963792	0.402042138	0	1.451211112	1.126717191
Zm00001d016198_T003	5.226034104	4.479011333	5.252130524	4.350957846	4.312013519	4.40150671
Zm00001d016198_T006	0	0.065568176	0	1.337284642	0.470139077	0.548616149
Zm00001d012993_T001	4.672115893	3.944470084	4.675758922	3.984017445	3.957550165	3.689315712
Zm00001d047266_T001	1.637589845	1.203266319	1.768379565	1.541874601	1.509071003	1.719188699
Zm00001d034706_T001	2.472198684	2.145742008	2.68076115	1.878910104	1.868990521	1.805365105
Zm00001d045048_T001	1.393549096	0.986804824	1.211488235	0.679588642	0.460826424	0.537311973
Zm00001d033312_T003	2.928852373	2.715562852	2.680994908	1.892095656	2.23941729	2.584379534
Zm00001d033312_T002	3.04313449	3.85798209	3.625800121	2.387553068	2.411737259	1.975714919
Zm00001d033312_T004	4.644114467	5.253591601	4.445972957	3.808185057	3.797632062	3.795854828
Zm00001d002733_T001	3.378684043	3.401635381	2.480326138	2.786544298	2.590260436	3.041835046
Zm00001d007764_T001	2.583888736	1.925101631	2.69028433	3.036683374	3.145610128	3.116185074
Zm00001d035308_T001	6.581370109	6.090471794	6.683826712	6.12517828	6.256648258	6.190656793
Zm00001d046422_T001	4.268307725	3.49092075	4.328471437	4.411538511	4.532509098	4.420537915
Zm00001d044898_T001	6.402835736	6.731410655	6.448941262	7.024947829	7.110923691	7.087450928
Zm00001d011902_T001	5.513768299	6.446201085	5.582646921	4.832289222	4.886065656	4.866712524
Zm00001d038792_T001	4.324924272	3.623298823	4.546611386	4.678393714	4.705758662	4.429184296
Zm00001d018699_T001	4.764246778	4.959667373	4.882210176	4.186088798	4.069472324	3.79403016

Zm00001d013599_T005	2.576463848	2.010795948	2.465879152	2.378602621	3.180831483	3.16248527
Zm00001d013599_T011	0.167267997	0.243937124	0.523386313	0.751054893	0.305592366	0.385280823
Zm00001d013599_T001	0.627205255	0.656705952	1.002517469	0	0.725613784	0.76774123
Zm00001d013599_T016	2.43554859	1.334219859	2.409616861	2.77319566	2.870168204	2.869868051
Zm00001d047767_T004	3.138301212	2.210487992	3.175802894	2.644299318	3.25932944	3.151596972
Zm00001d047767_T003	0.426141282	0.27796095	0.399660023	0.276389465	0.386879356	0.729041945
Zm00001d047767_T001	1.754066535	2.08087354	1.723315653	1.324380322	2.040944318	1.915032845
Zm00001d026005_T001	1.806481621	1.681858514	1.738014629	1.528244738	1.570073302	1.630754453
Zm00001d026307_T001	4.693772513	4.338250111	4.775597798	4.353520343	4.42960619	4.440609085
Zm00001d007486_T001	5.394267281	4.985360754	5.357965647	5.320052274	5.23905925	5.258076476
Zm00001d015927_T001	3.214062463	2.560828409	3.311438013	3.508980143	3.54649335	3.33723668
Zm00001d031231_T001	3.033208718	2.282895524	2.810332455	1.327293012	2.101740158	2.065430999
Zm00001d031231_T003	2.211273911	1.682430819	2.38589097	2.511243597	2.438873007	2.37793554
Zm00001d031231_T017	1.127732977	0	1.242065873	1.650730559	1.398520572	1.273996211
Zm00001d031231_T010	0.756361504	0.846699501	1.055902249	1.059833558	0.527596858	1.059030565
Zm00001d031231_T024	0.877919326	0.351650942	0.926262117	0.981423799	1.114008472	0.421358506
Zm00001d031231_T008	2.148185726	0.409730897	2.11668447	2.83093371	2.901919169	2.128301651
Zm00001d005542_T007	5.763410247	6.705439209	5.59831613	5.330989889	5.399308143	5.61894582
Zm00001d005542_T002	5.873574189	5.065407518	5.74960683	5.808642133	5.835117384	5.73534581
Zm00001d051527_T001	1.508208048	0.714349278	1.370935372	1.358701761	1.565566957	2.119733652
Zm00001d051527_T002	1.250492318	1.349670431	1.158523816	1.772333315	1.993356626	1.317815174
Zm00001d036181_T001	2.008925698	1.72962194	2.249577013	2.650254701	2.676266505	2.603201648
Zm00001d015151_T020	0.635304915	0.354112464	0.082011729	0	0.670911019	0
Zm00001d015151_T007	3.537372487	2.603793198	3.354247054	3.368454497	3.195681459	2.967097209
Zm00001d015151_T005	1.13844818	0.402156774	1.644134084	0.950359415	2.153688619	1.68660182
Zm00001d015151_T018	0	0.045194112	0.756161641	0.848389587	0	0.69850121
Zm00001d021376_T005	0.538888738	0.197890547	0.332827067	1.01323284	0.906551234	1.014448851
Zm00001d021376_T003	0	0.879170145	2.146163162	0	2.271175245	2.21824304
Zm00001d012559_T005	2.881943735	2.160196401	2.855487456	3.86536033	4.022627475	3.897825421
Zm00001d012559_T002	4.730039442	3.710418541	4.679393901	4.762824019	4.65358929	4.749216733
Zm00001d012559_T009	1.089123191	1.243243155	1.38324864	1.258965684	1.58174941	1.536574166
Zm00001d012559_T008	0.240670913	0.231875963	0.396492718	0.509448574	0.713397568	0.420663486

Zm00001d046370_T001	0.943197454	0.447371836	0.657758891	0.884860454	0.84814758	0.730586051
Zm00001d022420_T001	7.984298128	7.920860404	7.919764849	8.858917078	8.880453502	8.914973943
Zm00001d017118_T001	3.182445366	2.550349824	3.20392201	2.685522637	2.552239091	2.359010852
Zm00001d052288_T001	1.301420834	1.106087633	1.377029325	2.057099477	2.266129272	2.265043762
Zm00001d018367_T001	3.902186867	3.400136214	4.112327486	3.08057717	2.784283479	3.049441532
Zm00001d003464_T002	3.920381727	2.678675907	4.009599425	4.062205832	4.375541819	4.222722271
Zm00001d003464_T003	3.560765692	3.957001345	3.727284875	3.382769607	3.350928577	3.393294552
Zm00001d003464_T004	2.91006814	0	2.473634713	1.758243472	0	0
Zm00001d009873_T001	1.530393414	0.876951826	1.359685334	2.539511964	2.437560405	2.35230062
Zm00001d009873_T002	1.295108206	0.647763022	0.834129491	1.486521829	2.122570683	1.664503775
Zm00001d008477_T001	2.869310403	2.115280665	2.593749113	3.522038921	3.399343797	3.500902095
Zm00001d008477_T014	0.578783208	0.309913066	0.476721776	0.772992839	0.836938857	0.602363757
Zm00001d008477_T009	3.236943648	2.681951816	3.402664473	1.473285063	2.379630111	2.262672184
Zm00001d026427_T001	0.633778066	0.368043048	0.762458529	0.776890622	0.896558615	0.377850051
Zm00001d031191_T001	3.449088674	4.776247197	3.722886858	3.593295799	4.505224228	3.482784922
Zm00001d010927_T001	4.038689807	3.060058801	4.080976777	4.061676329	4.108797999	4.246079757
Zm00001d001882_T005	0	4.0320728	2.412371552	1.288809168	2.408499109	1.256482338
Zm00001d001882_T004	8.090060805	7.954192075	8.135995529	7.4991616	7.555415824	7.469494008
Zm00001d003309_T002	7.987657912	7.054971496	7.999037018	7.752608034	7.728349395	7.725907594
Zm00001d003309_T003	2.791914692	1.842863814	2.793127836	1.985248301	2.074647918	2.124524011
Zm00001d003309_T001	1.193033652	0.6001546	1.075391606	0.7059496	0.840002304	0.932468668
Zm00001d004753_T001	7.630367602	7.472037883	7.676356184	7.794832466	7.897480816	7.916395288
Zm00001d004196_T004	0.240263033	0.130270636	0.332654091	0.370714465	0.513359356	0.514431349
Zm00001d004196_T005	1.89216484	1.647022026	2.077911202	2.329786653	1.563157642	1.887627426
Zm00001d004196_T002	0.621377213	0.370982227	0.676514758	1.078840702	1.332895221	1.293549412
Zm00001d012782_T001	2.619905251	2.057172277	2.483421118	1.309622667	1.522957152	1.455724116
Zm00001d023895_T002	1.319987231	1.401825397	1.183403301	1.392297088	1.749368214	1.035003065
Zm00001d005395_T005	3.665041606	0	3.460623317	3.789048114	4.153004824	3.838066219
Zm00001d005395_T007	0.901138371	0	0.33617711	1.716098891	0.814293648	0.873862796
Zm00001d005395_T008	1.994544607	0.99979368	1.420675341	1.919949452	1.471928781	1.318100457
Zm00001d005395_T001	0	2.449106507	1.966007712	0.772004707	0.9296471	0.827802769
Zm00001d053425_T001	5.181314088	4.599387546	5.359948019	5.712705657	5.694131217	5.694839139

Zm00001d021557_T001	5.956295509	5.156685175	5.993103759	5.699131378	5.720444563	5.632878643
Zm00001d031962_T001	1.303858469	1.892451647	1.630519634	1.045189219	1.34142051	0.800869674
Zm00001d008273_T001	1.299953813	0.513519043	1.05741284	0.451385742	0.583924349	0.524400733
Zm00001d041070_T001	2.713884057	1.957877463	2.834211221	3.042879982	3.084581025	2.930472425
Zm00001d040839_T001	4.142137126	3.695897296	4.257102444	3.37956205	3.465492093	3.576933483
Zm00001d023327_T001	2.497035939	2.183959977	1.732280055	0	1.606794686	2.205619598
Zm00001d023327_T011	0	0	0	2.185986078	3.915540691	4.183172533
Zm00001d023327_T008	0.738594919	0.76629069	1.226028168	0.749856023	0	0
Zm00001d023327_T003	0	0	0	1.851892529	1.23399356	1.60493431
Zm00001d023327_T010	2.992316521	0	2.523260589	2.354936739	3.184060779	0
Zm00001d023327_T002	0	0.660883905	1.095164232	1.154929228	1.79086877	1.208228817
Zm00001d003950_T003	4.084283885	0.838064302	3.806247228	3.4963418	3.417056535	3.054832158
Zm00001d003950_T002	2.142827071	1.086565715	2.469349163	2.794910925	2.518939691	2.401515516
Zm00001d003950_T001	4.183278392	3.904808439	4.298270906	4.06792038	4.168795731	4.405255785
Zm00001d031133_T001	1.844889765	2.000893833	1.8500901	2.350305158	2.198537528	2.244924191
Zm00001d037300_T001	6.055496741	6.215415191	5.77382539	5.11672813	5.089614289	5.167086072
Zm00001d017729_T013	3.788676628	3.991985611	2.445823621	2.990176502	2.917637404	2.812907256
Zm00001d017729_T002	0	0.256055347	0.345803974	1.039946919	1.114269065	0.761160149
Zm00001d017729_T029	0.937864874	0	0.597430105	1.21109383	1.409666821	1.025574576
Zm00001d017729_T008	1.127101002	0.788209605	2.637787356	1.81808771	3.151802815	2.914991337
Zm00001d017729_T010	3.127182739	0	3.323845749	4.96195336	4.698505544	4.922895426
Zm00001d017729_T028	0.11435503	0.103575521	0.654987216	0.86363244	0.543582987	0.884371292
Zm00001d017729_T042	5.537369039	0	5.356080092	4.720498137	4.895787171	4.846879788
Zm00001d017729_T014	1.146216613	0	0.462376954	2.362590517	2.331340054	2.46751478
Zm00001d017729_T007	1.119433871	0	1.127868978	2.111452705	1.959079796	1.803597377
Zm00001d017729_T017	2.394094552	0	3.663775883	4.213002549	3.697354492	3.761898501
Zm00001d017729_T001	1.158054536	0	1.594399973	2.228623548	1.674215662	2.44850138
Zm00001d030934_T004	6.399379654	6.28436651	6.442486454	6.587322247	6.37445963	6.406322968
Zm00001d030934_T001	2.766560816	3.761176003	3.254052024	3.042643112	2.690513421	2.520694795
Zm00001d030934_T003	1.005455323	0.580693508	0.97118264	1.318760501	1.247654742	1.26430402
Zm00001d025397_T005	0.589390543	0.064474536	0.422765838	3.340562839	3.087245063	2.709226441
Zm00001d025397_T001	0	0.568900866	0	3.962889714	4.007797306	4.0821918

Zm00001d025397_T006	4.4979109	3.562046876	4.299041517	5.100567733	5.003738899	4.905110655
Zm00001d025397_T002	3.078907633	2.643687673	3.331573952	3.613919384	3.959925543	3.667878618
Zm00001d040665_T002	0.146230301	0	0.528783332	0.514955436	0.767463274	1.343426584
Zm00001d040665_T007	3.496656767	2.738516883	3.606840632	3.695088728	3.68550122	3.785567814
Zm00001d030579_T001	5.285113651	4.486475878	5.323100998	5.428859271	5.375262215	5.421288009
Zm00001d045495_T001	4.66017153	4.116278162	4.848957181	3.726135963	3.78914277	3.791833133
Zm00001d021351_T001	0.455696766	0.314269599	0.373193827	1.001793597	0.639136752	0.950786407
Zm00001d021351_T002	1.826135169	1.623958139	1.972486803	2.08124349	1.910830501	2.262357082
Zm00001d021351_T004	0.613008227	0.593518231	0.512117637	0.874519218	1.029307991	0.466471554
Zm00001d013865_T003	3.203511592	2.204053363	3.072056223	2.550938578	2.863599734	1.539205969
Zm00001d013865_T001	2.858272887	2.47923289	2.952275037	2.534092489	2.86180692	3.167871387
Zm00001d013865_T005	1.418339473	0	1.833659651	3.084404313	3.064008487	3.321902848
Zm00001d000017_T001	0.744300621	1.292554433	1.298287038	2.926882988	2.747711137	2.849151002
Zm00001d026670_T001	2.054465791	1.596648006	1.944840459	2.020843167	1.774334602	2.121774357
Zm00001d022017_T001	1.749446309	1.073676299	1.761359747	3.093378645	3.162518139	3.322113902
Zm00001d016996_T001	3.558568756	3.036861809	3.480226857	3.035984363	3.201212909	3.199660411
Zm00001d040455_T001	3.135861032	2.418981121	3.053703415	2.70742341	2.848212093	2.833912801
Zm00001d038709_T004	6.810959562	4.431467852	6.87296752	7.207598886	7.110075262	7.120161388
Zm00001d038709_T003	1.28567919	0.993220018	0.976263909	1.262519753	1.49544446	1.226104025
Zm00001d038709_T005	7.344527014	7.623494906	7.498076034	7.270824524	7.209061162	7.228702029
Zm00001d038709_T002	4.031459954	6.117248224	3.840776413	4.304674869	4.958439267	4.330240571
Zm00001d042087_T001	1.076700006	1.116241643	1.299287451	1.100506089	1.464523468	1.723868254
Zm00001d029721_T002	1.408090635	0.926832904	1.349943825	0.68656238	0.601644227	1.128273602
Zm00001d023316_T004	1.235245469	0.440392015	0.495057576	1.942301299	1.327815534	2.194875023
Zm00001d023316_T002	2.923686216	2.54809083	3.256272482	1.688790437	0	0
Zm00001d023316_T001	0.606199626	1.698218478	0.85002247	0	2.472992237	1.672016577
Zm00001d023316_T003	2.408964243	2.005477241	2.596584561	2.731450246	2.623575438	2.283626982
Zm00001d004055_T005	1.863838568	0	1.437472784	1.298760943	1.614848336	1.171366976
Zm00001d004055_T002	1.608425104	2.189086343	2.095192932	1.763035447	1.218635496	2.098677929
Zm00001d004055_T003	2.016320519	0	1.691301439	1.499209622	1.446690297	0.92759082
Zm00001d031431_T001	6.172708299	7.123638533	5.894314915	6.041158332	5.820334969	5.890843355
Zm00001d014599_T001	1.334057922	1.142362461	1.105282489	1.623001079	0.45910527	1.199649733

Zm00001d037079_T001	3.943829831	3.464698976	3.868347779	3.980819844	4.064281546	4.057781491
Zm00001d035157_T002	6.330425418	4.713070328	6.118496592	6.908489982	7.097183687	6.975055062
Zm00001d035157_T001	5.182843987	5.350385257	5.541823256	6.009877984	5.72679959	5.879373501
Zm00001d053759_T001	6.18734461	6.289846727	6.099063231	5.871604017	5.80377825	5.671343028
Zm00001d001855_T002	2.59566167	1.718370833	2.176737647	2.231164182	2.268468529	2.271186303
Zm00001d026489_T001	4.622455952	4.245545177	4.765508544	4.590361171	4.553856943	4.710359356
Zm00001d037236_T001	4.332229939	3.759433265	4.400194976	4.583684127	4.314228335	4.255986485
Zm00001d052075_T001	7.589361762	8.267898453	7.795388227	7.075813976	7.080339939	7.219495065
Zm00001d052075_T003	2.463975241	4.488220694	2.50031844	1.802915728	1.629002635	1.450557778
Zm00001d052075_T002	1.465721155	0.97350695	1.397615153	1.13696766	1.675182271	1.705888131
Zm00001d018097_T001	4.934458991	4.891459434	4.99034104	4.727037582	4.804018958	4.471708376
Zm00001d002164_T018	0	1.166705806	0.730032973	0	0	0.662079686
Zm00001d002164_T020	1.31291199	0	0.970729989	0.866525054	0.85919911	0.95263773
Zm00001d002164_T017	0	0	0	0.837230395	0.550827789	0.474365432
Zm00001d002164_T021	0.617900264	0	0.363230522	0	0.438729321	0.104519166
Zm00001d002164_T016	1.794855003	0.135444247	1.596842139	1.971163506	1.569160031	2.004980593
Zm00001d002164_T008	2.261429718	1.017889848	2.2878053	2.472786158	2.46991332	2.318543316
Zm00001d002164_T011	0.632694446	0.856777783	0.322293916	1.488675928	1.833787952	1.960863815
Zm00001d002164_T006	3.184767776	2.142074536	3.614936418	3.576488519	3.65989785	3.354282604
Zm00001d002164_T013	0.628886487	0.045479317	0.468121372	0.234268312	0.221567789	0.509733334
Zm00001d002164_T027	1.006906216	2.581270975	0.52231292	0	0	0.866164982
Zm00001d002828_T001	1.346172304	0.997727409	1.449731451	2.016391127	2.158211626	2.310236569
Zm00001d047970_T001	2.886142155	2.285591606	2.812076548	1.818345038	1.899613787	1.814126679
Zm00001d052500_T001	2.175697682	2.624399231	1.815745919	1.840305309	1.874340581	1.181107641
Zm00001d048476_T001	4.202710384	4.038214475	4.372560735	4.203481053	4.202856262	4.156311583
Zm00001d008901_T001	2.967652069	2.681900782	3.204614649	2.761834584	2.893179872	2.790971541
Zm00001d031005_T009	3.430509823	2.433943577	3.284632576	2.944142729	2.939893035	2.817981124
Zm00001d031005_T003	0.60872599	0.987894191	0.930883397	2.182847045	1.896454378	2.113418466
Zm00001d031005_T004	0	0	0	0.577915546	1.060553795	0.874671868
Zm00001d024934_T001	2.785037409	1.623662286	2.512746467	3.623200245	3.575165754	3.701589532
Zm00001d049541_T001	10.37136712	9.689317077	10.44007007	10.83117706	10.84885607	10.85224802
Zm00001d048868_T002	2.613944836	2.101265149	2.783851377	2.78132278	2.736516341	2.986301523

Zm00001d048868_T001	4.828600865	4.36914294	4.205992911	4.712693883	3.86691312	5.060346011
Zm00001d048868_T004	3.637664494	3.311343405	3.52687647	4.163666259	4.156585113	3.916393424
Zm00001d031290_T001	4.975448187	4.524523976	4.738457327	5.997047752	5.985291024	6.061325566
Zm00001d031058_T007	1.118038057	0.190683122	0.414488449	1.419996167	1.181295958	1.736741238
Zm00001d031058_T006	2.95021718	0	2.670324154	2.78661058	2.476904748	3.104862477
Zm00001d031058_T010	2.422596858	1.40744358	1.726029013	2.222818778	2.438061256	1.745943338
Zm00001d031058_T005	0	0	1.813620729	1.616973511	2.265643924	1.834341496
Zm00001d031058_T001	1.703869696	2.549633688	1.945362364	2.341576783	1.787392343	1.738105882
Zm00001d002936_T010	2.551885596	1.599546321	2.698753221	3.971040236	4.143094024	4.07372505
Zm00001d002936_T038	0.438220449	0.295906362	0.63563925	0.257877182	0.566435255	0
Zm00001d002936_T041	1.930413759	1.946344593	0	2.88102515	0	0
Zm00001d002936_T042	2.787152006	0	3.012958881	0	2.181218662	3.15989442
Zm00001d002936_T043	5.078486232	4.743170141	4.983027013	4.898529222	4.797624177	4.803234529
Zm00001d002936_T003	0.138233867	0.466143661	0.207446859	0.576597596	0.472451379	0.131029681
Zm00001d042843_T001	3.446196403	3.045688991	3.518167494	3.035920336	3.247701372	3.059070726
Zm00001d010228_T001	0.227980068	0.147449984	0.441350678	2.333466098	2.414372083	2.203960369
Zm00001d033993_T014	0	0.039257736	0.126741637	0.620004523	0.676698888	0.364920878
Zm00001d033993_T006	2.713504469	2.030821957	2.404044223	3.082803908	2.903498586	2.831918779
Zm00001d029601_T001	1.883027195	1.699497442	2.694676753	5.93581399	6.083284756	6.08698332
Zm00001d029601_T002	4.388208552	2.804063474	3.996275839	8.147006099	8.113097189	8.144601492
Zm00001d029601_T003	1.143573663	2.276182782	1.680823924	5.584928567	5.545373042	5.561256505
Zm00001d048702_T001	2.029571972	1.802906218	2.0887617	2.143396358	2.144715664	1.953883537
Zm00001d048702_T002	2.10518827	1.018139186	1.87910308	1.709212102	1.969521859	2.259373434
Zm00001d042266_T001	3.250899742	2.7676745	3.452334624	2.602997683	2.816831093	2.986202116
Zm00001d034991_T001	6.108690963	6.147015993	6.078587442	6.464759396	6.423069457	6.495966809
Zm00001d032546_T001	4.649083633	3.885201636	4.570242867	6.342454582	6.349921716	6.243562878
Zm00001d034424_T001	0.457736112	0.141262744	0.358540299	0.647672778	0.558446919	0.532173306
Zm00001d034424_T003	2.025181209	0.998903135	2.086470945	2.728153662	2.745050985	2.583078554
Zm00001d048417_T001	2.57395493	2.057558752	2.518799483	3.027605619	3.058055124	2.948697647
Zm00001d005424_T006	1.351355819	0.756549384	1.457376808	1.87561495	1.780544439	2.020578294
Zm00001d005424_T005	3.744438841	3.17034829	3.74359435	3.650894694	3.893362696	3.885261681
Zm00001d005424_T003	0.513464469	0	0	1.59471765	1.703156085	1.282872697

Zm00001d033205_T001	1.346490614	1.454400704	1.506968992	1.007664839	0.418229895	0.722487009
Zm00001d049974_T019	1.168480943	0.409245369	0.325446286	0.363002823	1.945113237	1.679058461
Zm00001d049974_T043	3.575773266	4.790776885	4.132525434	3.243611658	3.44617496	3.594054031
Zm00001d049974_T026	0.388363697	0.214271555	0.532047598	0.632113699	0	0.199976947
Zm00001d049974_T042	0.888100674	0.591330907	0.68495425	1.247795083	0.83781975	1.150104051
Zm00001d049974_T001	0	0.183196835	0	2.49460096	2.532186774	2.422823147
Zm00001d049974_T007	0	0	1.736883214	2.328542095	1.393376659	1.454829697
Zm00001d049974_T009	2.622503334	1.390437122	1.962970573	0	0	0
Zm00001d048487_T004	2.131731047	1.460679141	2.378471394	2.004500314	2.403756901	2.443560241
Zm00001d048487_T002	0	0.50895898	0.774082378	0.413702389	0.437081757	0
Zm00001d048487_T003	1.139219965	0	0.312876567	1.311597277	0.662303955	0.913983502
Zm00001d037112_T001	5.964599842	5.392456602	6.143115498	5.740083493	5.84065908	5.78987985
Zm00001d017420_T002	2.032959503	1.141233311	1.908593509	2.923034673	2.650321804	2.619212968
Zm00001d017420_T001	0	0.637132592	0	1.167955822	1.49540506	1.053881495
Zm00001d029627_T001	0.819813672	0.295563173	0.444283286	0.536353348	0.968066415	1.629366413
Zm00001d029627_T002	1.553066036	0.801755481	1.394204607	1.792669226	1.032894989	1.067636652
Zm00001d028550_T001	6.450890946	5.517333778	6.604069381	6.262932681	6.341666903	6.378801503
Zm00001d028550_T003	2.702952036	3.939793293	1.678119694	1.728636289	1.62017489	2.282150644
Zm00001d028550_T002	1.404551668	4.453056844	1.968553815	0	0	0.481243134
Zm00001d036283_T002	7.023434436	6.303574081	7.181460208	7.084654524	7.131111298	7.131727354
Zm00001d036283_T003	3.462554556	3.857621271	3.342833615	3.0240973	2.672889193	2.707476641
Zm00001d036283_T001	2.297939721	2.336625595	2.479314647	2.429855612	2.467948235	2.273298649
Zm00001d038762_T009	0.759137081	0	0.49545367	1.975631287	1.932879827	1.309850219
Zm00001d038762_T018	0	0.034349553	0.188222397	1.438983157	1.434590333	1.244008391
Zm00001d038762_T023	0	0.783661991	0	2.484739838	2.116231661	1.81766132
Zm00001d038762_T001	1.301253418	0.988638156	0	1.894076974	0.91636046	1.590065415
Zm00001d038762_T022	4.009953185	3.249621609	3.939736791	4.644613118	4.803715568	4.656332413
Zm00001d038762_T016	0.960717053	0.05120224	0.869141782	3.05901689	3.309470315	3.388988792
Zm00001d015029_T004	0	0.289019953	0	2.219233276	1.956205376	2.379690822
Zm00001d015029_T002	4.947683267	4.072575072	4.956914438	5.049456344	4.902810227	5.111382259
Zm00001d028086_T001	1.081376444	0.932473203	0.94946994	1.022756977	1.008611662	0.617022896
Zm00001d013873_T001	4.428831139	3.791666252	4.463490304	4.493406203	4.52119236	4.292275539

Zm00001d028728_T009	0.903427822	0	2.03327639	0	0.954857379	0.912700445
Zm00001d028728_T004	0.860132488	0.560090793	1.081533928	1.27503149	1.478039898	1.544796939
Zm00001d028728_T007	3.621299606	3.257032198	3.175128704	3.747404802	3.576354398	3.526916522
Zm00001d028728_T005	1.733244561	0.691114248	2.117685403	0.993441773	0	0.886344897
Zm00001d046696_T001	3.534181033	4.050940708	3.365734944	2.773080007	2.520160214	2.899694405
Zm00001d039066_T002	2.252499527	1.787063385	2.442962334	2.652350193	2.573128481	2.753004572
Zm00001d001832_T001	3.26892494	3.181925622	2.738380473	4.101353154	4.24120921	4.308730152
Zm00001d038296_T002	3.309412396	3.903149168	2.342944949	0.43314	0	0
Zm00001d038296_T001	4.386982722	3.458619678	4.647957534	2.719057678	2.948154515	2.775111873
Zm00001d036718_T001	1.464404794	0.872785429	1.878172873	2.600096782	1.98704176	2.287694208
Zm00001d036718_T002	2.611693167	2.473477563	2.624533989	2.728112511	2.564829321	3.086196108
Zm00001d003002_T001	1.14704028	0.539830795	1.10646692	1.122899954	1.273574372	1.08354698
Zm00001d034885_T002	5.181351621	4.313185762	5.084721749	5.335469321	5.297191086	5.149340791
Zm00001d032810_T003	1.952008457	0	1.831625963	2.191856655	2.577279203	2.305341375
Zm00001d032810_T013	1.404356015	0.140722386	1.196461634	0.350028719	1.231130074	2.301684798
Zm00001d032810_T007	1.60538576	1.059039567	1.380305553	4.607300365	4.886070974	4.796414706
Zm00001d032810_T014	6.330555837	7.161566435	6.445960414	7.619990567	7.706004938	7.655784157
Zm00001d032810_T004	2.383647067	1.515272422	2.279745842	3.936364684	4.305730903	3.518619986
Zm00001d032810_T010	2.231219184	0.620506649	0	1.856816021	1.815857792	2.400607604
Zm00001d032810_T006	0.621313439	0.137351377	1.161966343	3.534765342	3.168653106	3.293190651
Zm00001d032810_T009	0.595578133	0	0.344901198	3.654571303	2.533298456	3.974068898
Zm00001d032810_T002	5.544952752	4.125359657	5.493515469	6.457413435	6.449697516	6.392669174
Zm00001d032810_T005	0.891644712	2.774529855	0	1.923320354	2.101400978	2.636575206
Zm00001d032810_T011	3.28587431	0	3.563768156	3.713211793	3.365580566	3.339001092
Zm00001d032810_T008	2.120495605	2.547466364	1.040780937	3.186880831	3.66046832	3.206764329
Zm00001d028396_T003	6.061766824	5.373257663	6.114847649	6.215994064	6.174424616	6.195614142
Zm00001d045566_T001	3.447597313	2.733470836	3.524896271	3.274398543	3.243884586	3.34569201
Zm00001d044267_T003	2.150499563	1.326773751	2.014999719	2.101133679	1.581952787	2.236939517
Zm00001d015052_T001	0.640472857	0.6004791	0.749714459	1.209905599	1.017746637	1.306689793
Zm00001d023721_T001	3.522944601	2.996687198	3.752113901	3.479799661	3.423319458	3.475360941
Zm00001d053518_T001	1.821662873	1.275606059	1.706514197	2.935605142	2.917811905	2.864655062
Zm00001d003851_T003	2.385081158	1.439965621	2.358755203	2.841511262	3.229953318	3.039089601

Zm00001d003851_T002	3.924659938	3.439019872	3.977638675	4.649559302	4.634386549	4.913521541
Zm00001d003851_T004	0.907836693	0.286173817	0.946389513	0.980671717	1.119012811	1.035493006
Zm00001d003851_T001	1.40543913	0.602789413	2.257377282	2.333976089	2.295314252	1.721457097
Zm00001d003492_T001	3.789007932	3.077558853	3.71653278	4.138236325	4.30646896	4.26756561
Zm00001d051247_T002	2.61354839	1.797687462	2.387583395	3.266067487	2.729034765	2.886513275
Zm00001d051247_T001	2.592814602	2.195204267	2.692843609	2.601796812	2.548660533	2.795622131
Zm00001d047303_T001	4.19608742	4.902629995	4.243216949	4.05477009	3.779543083	4.126142357
Zm00001d031308_T001	3.553213646	5.200529542	3.736737125	3.423749321	3.731939046	3.92574645
Zm00001d007352_T001	4.515883293	4.867403492	4.613822697	4.540604505	4.453085101	4.586813733
Zm00001d029020_T002	5.132445481	4.463766824	5.247345003	5.358043176	5.387884879	5.46963511
Zm00001d029020_T004	0.968923864	0.942463459	0.860674429	2.546704598	2.662986805	2.273696998
Zm00001d029020_T005	2.046982003	0	2.619089454	1.5202085	0	0
Zm00001d028184_T001	3.01142157	2.632621167	3.38105712	3.599641054	2.877141922	2.689114618
Zm00001d028184_T002	3.666617541	3.511128579	2.986855417	2.690321658	3.506480333	3.193584575
Zm00001d006760_T001	8.88725258	8.040887966	8.985089741	9.163916933	9.206932939	9.062354156
Zm00001d006760_T002	7.618515662	8.789776907	7.233114118	5.649289408	5.354641971	6.044610573
Zm00001d037096_T008	1.604315707	2.308125805	1.948656536	1.605558336	2.201126947	2.097064289
Zm00001d037096_T007	0.319578629	0.270360294	0.418682189	0.395197737	0.511163376	0.449055253
Zm00001d037096_T001	6.00515886	5.329742997	6.067853948	6.011388962	6.025175494	5.892505393
Zm00001d013750_T001	4.706052246	5.071937119	4.835706189	4.403964493	4.520825392	4.726502324
Zm00001d007011_T001	8.671459976	4.192646397	7.055586096	7.738817243	8.535291098	8.228426763
Zm00001d048890_T002	1.309882225	1.189660754	0.993717832	0	1.073902475	1.287260692
Zm00001d048890_T003	2.843678595	1.564972685	2.763202485	2.573556549	2.884077402	2.542001933
Zm00001d048890_T004	0	1.031167904	0.699047845	1.477730165	0.185563389	0.86376167
Zm00001d038170_T002	2.114687181	1.326308386	2.126271816	2.383331874	2.282206998	2.321230816
Zm00001d038170_T003	2.58771998	1.924424883	2.651749528	2.176211374	2.02797038	2.459594765
Zm00001d014026_T001	2.180395186	1.511458425	2.333405414	1.966367339	2.139643355	2.068013452
Zm00001d007369_T013	1.247156453	0.652301084	2.25265514	2.667675762	2.79817891	1.836040455
Zm00001d007369_T020	1.676247095	1.763002292	0.907903589	0.743791546	1.645562408	1.8148498
Zm00001d007369_T021	0	0	1.348679438	2.306994777	0	2.873597861
Zm00001d007369_T018	1.624826155	3.474800078	0	1.146491655	0	0
Zm00001d007369_T010	1.471633859	1.509071003	0.876889764	0.551897897	0.826132321	0

Zm00001d007369_T006	0	0	0	0.413135856	0.606546458	0.889591913
Zm00001d007369_T009	0.572516217	0.229600227	0.768782244	0.941857519	0.944068307	0.652344226
Zm00001d007369_T004	0.481049859	0.265570117	0.56672165	0	0.285864991	0.335530133
Zm00001d007369_T019	2.385543687	0	2.32645595	1.516170506	1.857467901	0
Zm00001d007369_T014	2.821216513	1.575441586	2.652866651	2.241034123	2.332405473	2.746437895
Zm00001d044647_T003	0.611673812	0.502969055	1.145758309	1.057160491	0.662608395	0.999092981
Zm00001d031447_T004	4.662046179	4.048967899	4.825413867	4.305603528	4.416988867	4.13661058
Zm00001d031447_T012	0	0	1.414722777	0	0.593478074	2.245094013
Zm00001d031447_T001	2.204606512	1.306406327	1.954604303	3.089588147	2.550413365	2.817112392
Zm00001d031447_T014	2.101447708	0	1.390291832	1.553453403	0	0
Zm00001d031447_T007	0	0	0	2.26758134	2.755678096	2.385012664
Zm00001d031447_T003	1.341743286	0.829918548	1.052202326	0	0	0
Zm00001d029010_T006	3.404650029	3.318709461	3.431745697	3.555503046	3.504218161	3.468425615
Zm00001d029010_T001	0.997707179	1.551781277	0.684816943	0.686211847	0.778023496	1.421193671
Zm00001d029010_T005	0.799753777	0.743121119	1.111197613	1.08326716	0.812577905	1.383033482
Zm00001d029010_T002	2.146360332	0.612537454	1.830598512	2.450471471	2.533094334	2.105186258
Zm00001d039256_T001	4.466878509	4.659039438	4.44007037	2.855164894	2.992248169	2.867998635
Zm00001d049660_T002	2.129305111	1.533817978	2.498634344	4.839713895	4.976627082	4.937475236
Zm00001d049660_T001	2.46617212	3.265093585	2.096209894	4.689361396	4.226394235	3.863909121
Zm00001d030153_T001	4.105054893	5.574255036	4.730681688	3.472034746	2.818050478	2.972679238
Zm00001d002079_T001	6.72490343	7.281161238	6.589983379	6.900963942	6.810484351	6.966333651
Zm00001d018530_T001	5.356441652	5.67552917	5.474859412	5.018507283	5.072666987	5.010665412
Zm00001d013588_T001	1.182704373	1.251733633	1.287053788	0.245331365	0	0
Zm00001d047573_T001	4.349234478	3.659115669	4.461301451	2.667807445	3.089506286	2.772088767
Zm00001d013546_T006	0.924449588	0.535685713	0.935795395	0.888406995	0.99667798	0.901913764
Zm00001d013546_T002	2.364910516	1.177440747	2.255332736	2.095159842	2.398157706	2.181021427
Zm00001d013546_T004	5.461200393	5.307509948	5.373932755	5.522476892	5.407531037	5.300260335
Zm00001d002524_T001	1.847760045	1.795380875	1.174283046	2.082492777	2.543320176	2.50697686
Zm00001d007688_T002	0.12901127	0	0.727611195	0.490310319	0.66501249	0.26691604
Zm00001d007688_T001	1.38546914	0.939114468	0.584522893	1.583470464	1.362945612	1.451656349
Zm00001d008871_T001	3.88731743	3.284886012	4.025184133	3.43403208	3.564004215	3.664131681
Zm00001d031694_T001	1.996142853	2.019560662	3.165831081	0	3.205079979	3.293000345

Zm00001d031694_T006	0.381095062	0	0.668074636	0.852580593	0.628824911	0.58916136
Zm00001d031694_T005	2.279834078	1.447545344	1.971959174	2.818657125	2.696291227	2.666260538
Zm00001d031694_T002	0	1.909059163	0	1.719743759	0.818254222	1.196008315
Zm00001d031694_T009	3.260373186	0	2.785953697	3.75503497	2.777266311	2.713105421
Zm00001d031694_T015	3.5141287	0	4.115635406	3.175332016	3.795426075	3.680638857
Zm00001d002012_T004	2.954177881	0.828119724	2.764371847	2.364356152	2.715081983	2.808243761
Zm00001d002012_T005	3.421683811	4.705929866	3.987103536	3.43084538	1.575441102	3.16908158
Zm00001d002012_T001	3.121058847	2.529642066	2.908049864	2.853216222	2.849719097	2.630988536
Zm00001d044854_T001	1.025978463	0.706344001	1.179263255	1.267346423	1.08760948	0.638220301
Zm00001d044854_T003	3.215829381	2.340503592	3.250176819	3.55745347	3.581189492	3.489426174
Zm00001d039974_T001	3.237300607	3.828654124	3.594664988	2.443804743	2.791092647	2.761197603
Zm00001d000243_T001	4.466100319	4.599964421	4.353191438	4.619610561	4.464457663	4.571068663
Zm00001d013943_T001	1.853357368	2.356606131	2.276148233	2.342524879	2.125176627	2.074461251
Zm00001d027719_T001	4.266111801	3.918435373	4.383889291	4.707769821	4.900928474	4.802379307
Zm00001d039349_T002	1.346560962	0.69322421	1.609719982	1.492449031	1.670862538	1.390732061
Zm00001d046788_T001	3.109194381	2.303415755	3.285965278	3.109155759	3.227829471	2.933772034
Zm00001d009960_T001	3.613218681	2.75192405	3.665393794	4.841129993	4.718118224	4.955943659
Zm00001d038944_T003	3.291825131	2.395224339	3.343946281	3.130237024	3.342614762	3.636810781
Zm00001d038944_T002	2.572643541	2.472666343	2.600692698	2.421119866	2.565703424	3.009584735
Zm00001d038944_T004	3.21870291	2.165051692	3.536830005	3.835993176	3.90400116	3.583447116
Zm00001d038944_T009	4.451036857	3.606453244	4.02117641	3.355013162	2.888722406	2.879888836
Zm00001d018742_T001	3.440881921	2.967170637	3.649898223	1.686969285	1.861916865	1.771749963
Zm00001d018742_T005	1.762851811	0.495629677	1.088303772	0.211555517	0	0.273452631
Zm00001d032498_T001	2.143088063	3.391192507	1.766648162	1.517044339	2.041842929	1.606611836
Zm00001d051795_T001	0.653955174	0.532436659	1.707729298	2.867976106	2.918711949	3.264539433
Zm00001d028679_T002	5.296683045	4.438341628	5.494333712	5.429966018	5.376997976	5.406940688
Zm00001d028679_T001	2.61367403	1.740660794	2.255445139	2.136536594	2.890496889	2.364334578
Zm00001d009936_T001	3.694959845	3.23431783	3.872635435	3.566760976	3.311876819	3.289436858
Zm00001d010512_T001	3.048309609	2.234066239	3.116916309	2.802484622	2.854426795	2.770994127
Zm00001d017956_T001	8.941834589	8.884135778	8.849446534	8.402708582	8.495186557	8.638768148
Zm00001d035765_T001	1.853500696	1.460221475	1.305737687	1.448705408	0.671047844	1.432059847
Zm00001d035765_T002	4.177449119	3.475432282	3.587918558	2.980055105	3.104584245	3.225706501

Zm00001d020445_T001	1.138945498	0.742966825	1.184066334	1.105689475	1.201437516	1.26963584
Zm00001d049173_T001	2.143432931	2.311426677	2.620027991	1.20812329	1.083978517	1.625027759
Zm00001d029499_T001	5.830640289	5.0783364	5.90774413	5.400273548	5.296975657	5.511673189
Zm00001d029499_T002	3.09023575	2.458112917	2.92755593	3.115238709	3.073937083	2.881657964
Zm00001d022152_T001	5.01185278	4.150963672	5.036670452	5.050872641	5.081337795	5.161626483
Zm00001d002111_T001	4.318757899	4.825594951	4.715467416	4.459870459	4.638903756	4.564495177
Zm00001d028261_T001	1.136556936	0.536282718	1.333415146	2.251630938	0	0
Zm00001d028261_T013	0.348830679	0.048212466	0.462504694	0.4898974	0.762575887	0.352374358
Zm00001d028261_T010	0.405911784	0.324031741	0.993351916	0.094715762	0.49356741	0.540665129
Zm00001d028261_T012	1.998189364	0	1.460660796	2.974442308	3.375751773	3.718853127
Zm00001d028261_T007	3.714384449	3.047087432	3.779095267	3.415978596	3.463803044	3.218044291
Zm00001d045385_T001	6.921725588	6.808321114	6.825126306	5.298323371	5.822094075	5.787814637
Zm00001d030940_T001	1.09794514	0.938667449	1.551426439	0.456472898	0.568089519	0.734152483
Zm00001d023342_T001	1.915039348	1.287182072	1.650208969	2.509697615	2.498203628	2.72204802
Zm00001d024813_T002	0.990100968	0.744361767	1.151224982	1.14825212	1.306797682	1.454972315
Zm00001d019217_T003	2.25201048	1.732150218	1.260652595	0.681316122	0	0
Zm00001d019217_T002	1.411385568	1.123149673	0.739198312	1.43771193	1.195150396	1.000843009
Zm00001d019217_T005	0.294291961	0.34098946	0.54023959	0.290568312	0.477561286	0.902128391
Zm00001d019217_T004	1.607010192	0	2.748902561	2.7064932	2.764330867	2.698266271
Zm00001d015985_T006	0.098117673	0.243177945	0.596169953	0.941482715	0.519308398	0.66747617
Zm00001d015985_T005	2.508032554	1.53270894	2.751103324	1.846100922	2.050962381	1.335439807
Zm00001d039465_T001	3.764242425	2.890484049	3.772369001	3.892198557	3.956602487	3.873530438
Zm00001d005399_T001	1.802959142	1.122568695	1.693484281	1.55762869	1.26338001	1.612350611
Zm00001d024625_T002	3.429610742	2.588672426	3.258289462	3.706588569	3.75362272	3.598407966
Zm00001d024625_T004	2.863187776	3.931587662	2.910796354	1.280389217	0	1.95459872
Zm00001d050616_T003	2.225339103	0.231713793	2.268634704	2.170042977	3.20319724	3.111023798
Zm00001d050616_T001	6.247930513	5.509148521	6.310230169	6.21650492	6.221128847	6.161979279
Zm00001d050616_T002	0.196926768	2.519573355	1.335626169	0.963821809	0.341356174	1.025965002
Zm00001d037052_T003	3.614129823	4.304387356	3.237623523	4.035718737	4.123810442	4.049436827
Zm00001d037052_T002	7.410565948	7.571421935	7.264397963	7.462696339	7.45998268	7.515567443
Zm00001d046681_T001	0.944027064	0.618445411	1.27733018	1.347725744	1.414380202	1.099010341
Zm00001d039931_T005	3.17461191	2.27078965	3.262218752	3.638439621	3.584810288	3.455480705

Zm00001d039931_T001	0.525233009	0.338457504	1.099913899	0.904580435	0.808469077	0.760169839
Zm00001d039931_T003	1.152875876	1.066725042	0.715578007	1.098584624	1.179227579	1.529538126
Zm00001d052015_T001	6.540415766	7.672672227	6.697306739	4.390521524	3.832474559	3.781334532
Zm00001d001772_T001	2.656342597	3.434508404	1.724235956	1.548402098	1.711620889	2.132748903
Zm00001d028554_T001	4.054522401	0.685691719	3.74224222	3.670522899	3.773151131	3.706316039
Zm00001d028554_T002	2.569589277	1.748545817	2.181785721	1.478988367	1.932661413	2.319501173
Zm00001d028554_T003	1.70234681	2.986990264	2.782997266	2.533050216	1.460944344	0
Zm00001d004469_T001	2.520936624	1.95491357	2.258771864	2.244413388	1.997876594	2.70965347
Zm00001d017883_T001	3.423556658	2.910784652	3.035614937	3.533165992	3.645035947	3.7001345
Zm00001d017883_T031	4.346920237	2.696917911	4.423374593	4.153612099	3.908651148	4.225873203
Zm00001d034610_T003	1.254069716	1.244124725	1.528472302	1.166368385	1.486016666	2.371188593
Zm00001d034610_T002	3.545458271	2.351468335	3.608821068	3.684194856	3.642024536	3.459159842
Zm00001d043067_T025	3.580091684	2.926576381	3.7342984	3.84025958	3.721004118	3.696188831
Zm00001d043067_T002	1.104909598	0.702988151	0.878219182	0.982599739	1.313489667	1.112292451
Zm00001d043067_T011	4.164565385	3.074805795	4.150651954	5.453959835	5.476355828	5.382441079
Zm00001d049743_T001	2.199451232	1.429988138	2.114148772	1.967494007	1.998015652	1.919037972
Zm00001d012034_T002	1.569357899	0.640282193	1.418572636	2.077227957	2.141931697	1.81965674
Zm00001d012034_T003	0.878132842	0.807943422	0.746242243	0.627776777	1.178517701	1.578757127
Zm00001d012034_T004	0.853253157	0.809886073	0.625022614	1.215309567	0.956703489	1.229659287
Zm00001d022065_T001	3.265840872	2.784841689	3.308348642	2.581771339	2.715946949	2.335790216
Zm00001d024412_T002	0	0	0.440679043	0.518546217	0.731608166	0.599593927
Zm00001d024412_T005	0.782490763	0.841693809	0.772704915	1.491481154	1.373171549	1.138570068
Zm00001d040008_T010	0.687969927	0.229934866	1.688736735	2.129171224	1.402602675	3.207518389
Zm00001d040008_T002	1.916094041	0.970502507	1.829310934	2.866470654	2.767634249	2.801422953
Zm00001d040008_T006	0.061747171	0	0.245933699	0.64501911	0.467128159	0.590462138
Zm00001d040008_T009	3.980295529	0	4.391237883	2.456610098	3.047100174	1.103853444
Zm00001d040008_T004	3.990976455	4.075891121	3.605319024	5.040467799	5.030747726	4.914105285
Zm00001d040008_T008	0.880444174	0.245000279	0.836180261	0.930542821	0.631297095	1.138047055
Zm00001d020502_T001	1.356145501	1.320687421	1.406490956	1.126379538	1.576429744	1.181964411
Zm00001d010033_T003	4.497385438	4.098949219	4.720235392	4.2813783	4.672458043	4.362968889
Zm00001d010033_T001	4.399520785	3.501183068	4.295294707	4.909390206	4.69313459	4.955673293
Zm00001d003346_T001	6.914608842	2.588558022	6.942099372	6.283738906	5.103870728	5.651118958

Zm00001d028903_T001	5.04299105	4.482148187	5.077505489	5.590806398	5.580416778	5.588451974
Zm00001d038874_T002	2.905375163	2.497693343	2.910501659	2.867599989	3.008951864	2.814640448
Zm00001d030561_T009	1.501071195	1.69783608	1.651813118	2.125167698	2.03939216	1.829485492
Zm00001d030561_T008	4.602945321	0	4.65113093	4.192297471	4.025659083	2.724397942
Zm00001d030561_T003	4.304451318	4.661888551	4.336496777	4.641607758	4.678493268	4.900602439
Zm00001d030561_T006	1.531474289	1.222203004	1.636854274	2.130204399	0	2.689310344
Zm00001d003664_T002	4.260684507	4.656850308	4.597466934	4.661974889	3.992495815	4.579091124
Zm00001d003664_T001	3.219384308	2.458055156	3.307623349	4.051625204	4.086948811	4.01239132
Zm00001d025892_T012	2.174760177	1.009986317	1.984592631	2.252366002	2.23335738	0
Zm00001d025892_T005	1.592255614	0.085688424	1.563719476	2.849184637	2.241371613	2.262375425
Zm00001d025892_T001	0	0	0	2.930172069	2.76185309	2.677616708
Zm00001d025892_T016	0	0.636582398	0.700350046	0.585935511	0.275629299	1.214886994
Zm00001d025892_T002	0	1.522711142	0	0	1.623149549	1.957345578
Zm00001d025892_T018	3.618528105	3.728921283	1.994438887	3.994518449	3.837538322	3.441715854
Zm00001d025892_T008	1.506684194	0.888515322	1.295030012	2.15652347	2.274318921	2.317288437
Zm00001d025892_T017	2.10000779	0	3.691026341	3.365881889	3.536008375	4.189583984
Zm00001d008393_T003	0.338383337	0.901917625	1.161774197	0.884986234	1.099044015	1.208917363
Zm00001d008393_T002	1.272486689	0.426641551	1.010201213	0.961341058	1.107981734	0.842060845
Zm00001d050334_T001	1.138997906	0.737461796	0.948345176	1.309727427	1.078794941	1.052036737
Zm00001d012611_T001	1.588280728	1.053543733	1.725218507	1.773255383	1.711842433	1.229252588
Zm00001d016486_T001	1.449890417	0	0.440981955	2.313171248	2.087524621	1.953640347
Zm00001d016486_T011	3.11536607	2.74419081	3.03004804	0	2.810661908	1.898784541
Zm00001d016486_T006	0.144457577	0	1.044743122	0.43263129	0.852457549	0.743027165
Zm00001d016486_T004	1.057807912	0.882360547	1.161023418	0.952004729	1.581093299	0.961706295
Zm00001d016486_T005	0.776403869	0.916906141	1.164167112	1.803938287	1.566155137	1.606162664
Zm00001d016486_T003	0	0.721259777	1.577726098	2.054962708	0	2.10685783
Zm00001d024819_T004	0.716775846	0	0	1.146898909	0.746085701	1.003681385
Zm00001d024819_T007	2.04816677	0	1.445326782	1.569361302	1.643467418	1.977939291
Zm00001d024819_T003	1.248953133	1.650311463	1.493672437	1.451369387	1.483751298	1.30250974
Zm00001d012521_T001	0.156317651	0.06740605	0.258780606	0.974433864	1.137988057	0.76256143
Zm00001d028689_T001	5.922679539	5.107533089	6.018382896	6.521077976	6.586205478	6.631224575
Zm00001d045511_T001	1.498258017	0.911282776	1.411811275	1.438990607	1.018090747	0.306186399

Zm00001d009416_T001	3.039739918	4.243081877	3.005724423	2.584452899	2.876206126	2.698431199
Zm00001d038268_T001	2.450720611	2.295438865	2.976263175	2.967150528	3.027767137	2.875360363
Zm00001d017468_T001	5.331684009	4.73258837	5.336855888	4.792771037	4.840163334	4.826566079
Zm00001d019481_T002	3.283603574	3.832914312	2.611446726	2.149464893	2.393121261	2.004163016
Zm00001d019481_T001	4.343995796	4.53623391	4.522261	4.316414629	4.134637749	4.167657388
Zm00001d016684_T030	0	0.581792786	1.179189991	0	1.009922559	1.036257852
Zm00001d016684_T001	2.023579346	1.124791311	1.202377598	2.206989479	0	0
Zm00001d028827_T004	3.200888172	2.397893846	3.431582179	3.452135064	3.442139475	3.184181255
Zm00001d028827_T002	1.014922975	1.295918113	0	1.789399991	1.749092703	2.098516916
Zm00001d007313_T001	1.758077565	0.949497581	1.360170406	1.561437099	1.642399833	1.96773303
Zm00001d021711_T001	6.465301414	6.597430835	6.430456315	5.10979769	5.123799933	5.328625422
Zm00001d022164_T002	3.325050169	4.197123099	3.544171169	3.299532758	2.627482406	3.409499966
Zm00001d022164_T001	4.399354325	5.183159707	4.082105685	4.544656501	4.689222718	3.943885235
Zm00001d040155_T005	1.091953658	0.569318036	1.47156259	1.559111939	1.331888623	1.237005009
Zm00001d040155_T003	2.81806398	2.204847489	2.808500791	2.971581995	2.923830519	2.970268177
Zm00001d051607_T007	1.410278684	1.416214979	0.879896284	1.326354986	1.136907305	1.781697527
Zm00001d051607_T004	0.653066439	0	1.083215411	1.520351338	1.297345805	0.854065086
Zm00001d051607_T001	0	0	0	1.10467146	1.279737523	1.117653157
Zm00001d050741_T008	0.813907168	0.537425295	0.916259555	0.788344935	0.915064979	0.812441543
Zm00001d050741_T005	0.312007564	0.902059683	0.309762927	1.733613785	2.450201961	1.373054587
Zm00001d050741_T007	4.160911558	2.31926502	4.143278387	3.670724675	3.49896055	3.990628539
Zm00001d050741_T004	1.720571791	0.975134163	1.468754814	1.666333036	1.077287441	1.286115843
Zm00001d050741_T006	2.641109687	3.324514403	2.372305247	2.595521085	2.532381299	2.502816341
Zm00001d050741_T003	3.319477617	2.911980475	3.489445828	3.756224741	3.975382196	3.413268844
Zm00001d046652_T001	3.298479287	2.803165028	3.367390497	5.627972368	5.640040182	5.625188093
Zm00001d010159_T003	3.703835264	0	3.6444778	0	2.73545719	3.310501542
Zm00001d010159_T002	4.6551101	4.132240476	4.586122862	5.726589213	5.456604151	5.406381597
Zm00001d023499_T002	2.839045322	2.330684308	2.657744717	3.071898902	3.074404903	3.148315907
Zm00001d023499_T001	1.221652541	0	2.035061853	1.405105783	1.000194751	1.293536464
Zm00001d005484_T001	0.858233295	0	0	0.733915689	0.59913685	0
Zm00001d038514_T001	4.623793894	5.026743767	4.658480056	3.796927867	4.147924515	4.010815279
Zm00001d001767_T001	2.567755704	2.201634175	2.938940832	1.941866156	1.468230354	1.601665143

Zm00001d007605_T012	0	0	0	0.752215937	0.941898073	0.807827224
Zm00001d021403_T001	4.141307874	3.53466177	4.245152366	4.33163371	4.394396634	4.225809285
Zm00001d028482_T001	4.590395965	3.828483832	4.870574919	3.940608044	4.071677818	3.841052378
Zm00001d031266_T001	4.624671892	4.737736995	4.664687739	4.300576235	4.281071933	4.113396795
Zm00001d012221_T001	6.556104497	5.934387418	6.676862662	3.544741186	3.513171094	3.415356841
Zm00001d025862_T002	4.550118973	3.792648197	4.481356297	5.377634295	5.204590667	5.101852878
Zm00001d025862_T001	0.651274466	0.738912207	0.689449461	0.964572374	0.549501606	1.493486969
Zm00001d012119_T002	0	0.178807684	0.922106488	0	0.627804787	0.628628035
Zm00001d012119_T001	1.40592104	0.605560707	0.587816213	1.215262966	1.049601495	1.091299716
Zm00001d052543_T004	0	0	1.568773455	1.769923616	1.518722954	0
Zm00001d052543_T003	3.030691676	1.942494988	3.363551259	3.556866143	4.041264801	2.937903846
Zm00001d052543_T001	5.132427548	4.280514606	5.057129898	5.445413596	5.223617548	5.404892055
Zm00001d030533_T001	1.007867885	0.257491034	1.062304801	0.658754405	0.700863142	0.608824379
Zm00001d030533_T003	3.952755223	3.138069343	3.815261547	3.91960495	3.815646537	3.639537227
Zm00001d030533_T004	0.468853325	0.417150941	0.369524553	0.902349161	0.959873996	0.944498664
Zm00001d030751_T001	0.874046215	0.442990192	0.641952886	0.045076659	0	0
Zm00001d003434_T001	5.048088412	4.663651515	5.279531357	4.618923714	4.704660966	4.546279972
Zm00001d053704_T001	2.354666828	2.238126798	2.64031968	2.09963185	2.378327668	2.24754325
Zm00001d020725_T001	2.218116228	2.212747058	1.854887328	1.302598642	1.086062918	1.165130502
Zm00001d044551_T011	0	0.130534239	0.205371242	0.497017538	0.593391063	0.057764945
Zm00001d044551_T017	3.331513905	5.007548371	3.48452615	0	0	0.893018875
Zm00001d044551_T004	1.413471145	0	0	1.118860695	1.710255198	1.633982607
Zm00001d044551_T009	0.820161803	1.073708514	1.857349255	1.622213509	1.68126709	1.808284131
Zm00001d044551_T003	4.241722598	3.454047414	4.442535172	4.870941485	4.898494106	4.762625421
Zm00001d044551_T013	2.032275438	0.487418432	1.720934206	2.076895958	1.905184129	1.845217042
Zm00001d045973_T003	1.241355747	1.136042383	1.487760044	1.425588744	1.594955027	1.524414776
Zm00001d034550_T001	6.18387951	5.573889025	6.28550522	5.697169003	5.845843681	5.649650204
Zm00001d020038_T001	1.899815999	1.760324859	0.697948142	1.995600653	2.281398554	2.084626942
Zm00001d020038_T003	0.438190635	0.298698194	0.398155517	0.251357957	0.43184543	0.4592333
Zm00001d020038_T002	1.331135936	0.862110937	1.113952482	1.764696467	1.076446904	1.372204934
Zm00001d020038_T005	1.120213239	0	2.332295758	2.288735356	2.600368959	2.59655236
Zm00001d027555_T001	1.072580559	0.515363265	1.113219743	1.101863843	0.980353663	1.003017125

Zm00001d026171_T001	3.745813972	3.429894033	3.800525863	3.633297514	3.642069951	3.444762863
Zm00001d013521_T001	0.865830944	0.444327819	1.178852929	1.661599121	1.582472666	1.52441277
Zm00001d029886_T001	2.771674545	2.671560597	3.041531422	4.36571619	4.301427785	4.224577016
Zm00001d018613_T006	6.031696858	5.338590889	6.188410242	6.316940563	6.31336869	6.348816519
Zm00001d018613_T009	4.275394869	5.280458186	4.133681636	3.752632465	3.922396634	3.378400781
Zm00001d018613_T015	4.474686919	5.102973844	4.255391432	4.179271616	3.93498358	3.628446182
Zm00001d018613_T011	1.725328899	0	0.526698851	0.487963739	1.359119116	1.368657734
Zm00001d002423_T001	2.225157374	1.629964581	2.241963097	2.109995005	2.196613968	2.48750127
Zm00001d018891_T004	0.391300308	0	0.526235113	1.064028843	0	0.216519432
Zm00001d018891_T001	0.557422835	0.5091891	0.391250808	0.360730593	1.328675071	0.935397135
Zm00001d001899_T001	6.026414972	6.447547592	6.098649256	5.553626345	5.557290177	5.36728065
Zm00001d002452_T001	3.808513559	2.873991113	3.883003434	3.496202447	3.75248261	3.593259461
Zm00001d039477_T001	1.760443669	1.40450698	2.054098289	1.782656821	1.795363003	1.855056027
Zm00001d018490_T007	1.657842106	0.485422705	1.733613352	1.868841614	1.555985441	1.586032583
Zm00001d018490_T004	1.974635033	1.460675472	1.849645446	2.396646974	2.364993414	2.512937059
Zm00001d018490_T002	0.585728849	0.165710028	0.227891374	0.48004896	0.678921042	0.449758915
Zm00001d033870_T004	0	1.255246917	2.126170696	1.335293445	1.927206222	0
Zm00001d033870_T003	2.80691071	1.775919059	1.539083355	2.736185535	2.344467778	3.072097738
Zm00001d049848_T001	1.08762102	2.742854111	2.019304096	2.062995149	1.581870861	1.007499081
Zm00001d011975_T007	1.946902252	0.716297356	1.413889198	1.124507468	1.791145589	1.113902489
Zm00001d011975_T008	0.302033555	0.102791142	0.149734115	0.44385698	0.443169534	0.305849149
Zm00001d011975_T002	1.103425125	1.185764443	1.371204209	1.797390728	2.126693076	1.898073269
Zm00001d039714_T001	2.371460728	1.724904303	2.33193447	2.824361357	2.482581915	2.459404601
Zm00001d042166_T007	2.2340092	1.585559172	1.815505752	2.214009447	1.90658779	1.666635719
Zm00001d046714_T003	4.316070636	3.723914113	4.306715454	4.539656874	4.462023713	4.435199322
Zm00001d046714_T004	2.591001229	3.219476004	2.625405633	2.997058233	2.791026155	2.674224251
Zm00001d046714_T001	3.894980985	3.122545007	4.095836084	3.886241774	3.999609428	3.844880929
Zm00001d007381_T001	0.902230289	2.101793599	1.954876358	1.208630249	1.273945501	1.418272
Zm00001d028484_T002	1.007889407	0.356505566	0.817647814	0.679805704	0.782699591	0.744024141
Zm00001d028436_T003	3.959699502	1.093967102	3.343459844	0	0	0
Zm00001d028436_T001	4.755833546	4.309795444	5.050505441	5.607695957	5.682152028	5.603106406
Zm00001d028436_T002	0	0	0	1.737094854	1.794706975	1.806548436

Zm00001d043581_T001	3.162264829	2.279231173	3.277394163	3.15587679	3.060412855	3.071968386
Zm00001d028664_T001	0.673055238	0.689923522	1.119955045	1.419638124	1.621383309	1.15796661
Zm00001d018450_T006	0.410281398	0	0.225578493	0.584855737	0.477932173	0.372075074
Zm00001d018450_T004	0.416358763	0	1.344829065	1.645701198	2.105489013	1.097167808
Zm00001d018450_T015	2.124314568	0	2.478560028	1.366710518	1.179050461	0
Zm00001d018450_T007	0	0.206406923	0	2.397476881	1.818113486	2.295556414
Zm00001d018450_T001	3.04540872	2.277889854	2.79553673	2.499820636	2.76008998	2.967752578
Zm00001d042494_T004	2.271436135	2.514119735	1.59806358	2.254576508	2.178642943	1.6760918
Zm00001d042494_T017	2.209395033	0	2.713131822	2.389635366	2.267091685	2.044062524
Zm00001d042494_T015	0.462147623	0	0.514217216	0.369134772	1.364388652	0
Zm00001d042494_T005	0.688677214	0.8134976	0.678107972	0.646163579	0	0.846326421
Zm00001d042494_T013	0.219877881	0	0	1.133751577	0.693822795	1.288708783
Zm00001d042494_T001	1.072387793	0.290693336	1.191560756	1.198514898	1.291908213	1.43112281
Zm00001d042494_T024	0.753158652	0	0.991605832	1.308864676	1.508375913	1.680594865
Zm00001d020692_T002	0.725190581	0.56509702	0.533889226	1.434910001	1.861102593	1.64669677
Zm00001d020692_T001	2.258785731	1.696874301	2.419817965	3.747316391	3.911822042	3.630475484
Zm00001d002980_T003	4.241673639	3.610748009	4.429018411	4.34012384	4.239513526	4.304619167
Zm00001d003709_T001	5.047994045	6.329390273	5.170904259	4.135992904	4.366199449	3.902052638
Zm00001d050913_T001	2.630249824	2.232606118	2.698707222	2.799354056	2.540754635	2.848036981
Zm00001d012238_T001	1.473191531	2.219790546	1.850719827	3.078515906	3.345986732	3.017073318
Zm00001d012238_T017	0.523565971	0.152487181	0.253955391	1.420175155	1.355139773	0.870899675
Zm00001d012238_T018	0	2.238097441	0	3.527666288	1.454784961	0
Zm00001d012238_T009	4.988725536	2.909579772	4.912808399	5.82343659	5.977792836	5.929259048
Zm00001d012238_T014	0.218712983	0.030694853	0.294264901	0.651498585	0.336211394	0.761287827
Zm00001d012238_T011	4.441190436	0	4.648635473	4.512444665	4.450621844	4.370517795
Zm00001d026532_T008	4.317727205	3.177031019	4.294918005	4.403289063	4.430465799	4.547433726
Zm00001d026532_T012	0.48183107	0.16846618	0.507854503	0.857047817	0.654970724	0.621324694
Zm00001d026532_T015	4.910802493	5.406764131	4.915863417	4.049922239	4.529208889	3.615722437
Zm00001d026532_T013	0.143214459	0.172109831	0.272668225	0.343950117	0.216453623	0.159630477
Zm00001d045015_T001	1.220016615	1.200969441	1.473684062	1.936481908	1.879479151	1.935440512
Zm00001d039200_T001	5.724173023	5.259448952	5.793633298	5.571680814	5.640736135	5.678858748
Zm00001d001903_T006	4.467204278	0	5.178290932	3.877010153	4.689549877	4.720779819

Zm00001d001903_T001	5.692239205	5.711255796	5.599795399	6.160871978	5.996825963	5.897247203
Zm00001d001903_T005	0.698851436	0.591285901	0.694505823	0.662238319	0.716955787	0.683359599
Zm00001d013607_T005	3.367125563	2.626729282	3.90272636	3.21305498	3.223722488	2.071020541
Zm00001d013607_T001	3.77252071	2.563789263	3.477848265	4.404151137	4.349165393	4.422597464
Zm00001d013607_T006	3.088959073	3.612741019	3.455108605	2.617046177	2.292336506	2.281703294
Zm00001d032449_T008	0	0	0.345457693	1.157377497	0.916438427	1.019411567
Zm00001d032449_T002	3.2619031	1.957295425	3.339444851	0	0	0.537710549
Zm00001d032449_T006	3.585334067	3.246562686	3.742618849	0	0	0
Zm00001d032449_T005	1.202677869	0.282195431	0	3.02943875	1.851626726	0
Zm00001d032449_T003	0	0	0	4.245326192	4.639573866	4.66925886
Zm00001d032449_T010	0.573612047	0	1.539011866	1.752781127	0.489898428	0
Zm00001d021974_T002	4.772284905	3.846811606	4.780471737	4.689959518	4.712418652	4.627349908
Zm00001d031457_T009	1.148101102	0.288378645	1.26631161	2.254179496	2.283235514	2.479566616
Zm00001d031457_T004	0.075818746	0	0	0.923168962	0.763958802	0.576200912
Zm00001d031457_T002	0.652131256	0.707424956	0.310463442	0.312229511	0.347452496	0.286940273
Zm00001d043179_T001	5.071450778	4.381924066	5.02114922	4.802843162	4.78332338	4.786604203
Zm00001d029657_T001	4.002053477	2.956843041	3.803285322	4.042475634	3.807689176	4.048635087
Zm00001d029657_T002	0.500270758	0.214742793	0.381021947	0.704414301	0.650120257	0.61527786
Zm00001d037682_T001	1.233810157	1.164636982	1.569306855	1.044857106	0.569358871	0.950943138
Zm00001d013425_T001	1.266210847	1.340827142	1.202219602	1.040945019	1.27197002	1.148761036
Zm00001d019836_T008	1.286661771	1.246264571	1.388085913	3.41797319	3.341164714	3.470482462
Zm00001d019836_T010	3.855011462	3.276647329	3.323702528	3.570453216	3.665840019	3.16884113
Zm00001d019836_T001	3.597149146	4.129620733	3.617797203	4.037832972	4.025516198	4.280157564
Zm00001d019836_T005	1.855591108	0	0.594396629	2.627106711	2.739975305	2.853755577
Zm00001d019836_T009	1.683675796	3.251257659	3.315552609	3.54488211	3.631680314	3.028006639
Zm00001d019836_T006	2.234226612	3.230914346	1.687755434	2.034871049	2.023340882	2.223471978
Zm00001d036084_T004	4.433218001	4.315094988	4.384613647	4.673303987	4.560596539	4.412198369
Zm00001d036084_T006	0.98616552	0.815468862	1.721366097	1.294021345	1.875210342	1.872830335
Zm00001d036084_T003	0.533305185	0	0.308991036	0.29541506	0.39324924	0.835507774
Zm00001d015737_T002	4.862569123	4.527422769	4.64324886	4.178197457	3.972643032	4.336566257
Zm00001d019688_T001	5.588232777	5.793851233	5.410569934	5.219799489	4.519537502	4.407137759
Zm00001d028337_T003	2.194459658	1.247762277	2.102457249	2.123173848	2.217239093	2.207064782

Zm00001d047034_T003	3.627492562	2.775332941	3.367517005	3.573915799	3.681392141	3.287880046
Zm00001d047034_T001	6.209221892	5.726259201	6.132638016	7.07044653	7.06458084	7.086275184
Zm00001d034807_T002	2.597172631	5.318228614	3.104885115	3.256105179	3.127051462	3.178274516
Zm00001d034807_T004	5.567807867	5.445070019	5.773410771	5.459563652	5.188400094	5.255410014
Zm00001d034807_T007	0.533654043	0.170844186	1.887950998	2.402929236	1.538460191	1.972232756
Zm00001d034807_T003	5.090763513	4.087329004	5.598744857	2.692209349	3.705112689	4.684434678
Zm00001d034807_T001	2.213783369	2.695286318	0	0	1.324387812	0
Zm00001d034807_T006	6.490680687	3.511056324	6.384509316	7.056335349	7.137540503	7.007811902
Zm00001d034807_T005	1.344316647	0.758106995	1.326059247	3.367137168	3.419083764	3.397146482
Zm00001d053636_T002	6.931327492	5.481709582	6.939268311	6.352608578	6.512201898	6.557398801
Zm00001d053636_T001	7.019342463	7.299197594	7.170279871	6.737511794	6.640840158	6.607090644
Zm00001d035605_T001	11.12509012	10.53606737	11.11207766	10.15869621	10.15679456	10.16743002
Zm00001d016442_T007	4.246085611	3.713994329	4.000489712	4.555263568	4.394676429	4.621445028
Zm00001d016442_T009	0.448619783	0.287149559	0.463447733	1.401956429	0.675555816	0.758113819
Zm00001d016442_T004	0.465281805	0.343653415	0.476657497	0.492844817	0.218164908	0.243168194
Zm00001d016442_T008	0.70151712	0	1.669849049	0.310397129	2.020400501	1.324974733
Zm00001d042786_T001	5.741069314	5.09006638	5.589366721	5.529299265	5.547231101	5.561454421
Zm00001d039675_T001	3.98880455	3.215323547	3.875082526	4.19276671	4.181188999	4.082358904
Zm00001d039675_T003	0.267347621	0.354677829	0.460928097	1.098625719	1.331440956	1.633558618
Zm00001d031581_T003	3.854380813	3.411663376	3.74546808	3.591779694	3.40942719	3.742061847
Zm00001d031581_T004	0.94374658	0.633937053	0.804779681	1.000199799	1.163304213	0.910737266
Zm00001d031581_T007	2.888797595	2.014418142	2.823054539	3.777765079	3.883107376	3.812279905
Zm00001d031581_T001	1.340797524	1.120037353	1.421203367	1.284633761	1.928489335	1.885256507
Zm00001d029286_T001	0.720039383	0.336475354	0.647804459	0.481045724	0.566413822	0.573963894
Zm00001d000135_T001	3.944517311	3.48503802	3.968693792	4.03155382	4.153012041	4.001200003
Zm00001d021207_T001	0.208524751	0.236671394	0.360883385	4.407252874	4.174787337	4.138995613
Zm00001d051338_T001	2.28480519	2.797070892	2.528086422	3.226077969	3.188796678	3.355353916
Zm00001d051338_T002	1.60988495	1.481385232	1.377653519	3.040963074	3.022832597	2.940352456
Zm00001d017384_T016	1.78108185	0.368904651	1.647102177	2.045964665	2.090038721	1.003752615
Zm00001d017384_T008	0.556349952	0.55689728	0.356131412	1.089488659	0.796402558	1.013596607
Zm00001d017384_T011	3.439883065	1.216266736	3.574268662	3.64386046	3.685993618	3.939380526
Zm00001d010309_T012	4.289082829	3.800924846	4.191526093	3.880381969	4.048330624	3.97501747

Zm00001d010309_T007	0.069490455	0.069895979	0	0.347166721	0.327939095	0.708997655
Zm00001d010309_T004	2.294386076	1.386451193	2.076235838	3.004171647	3.124071504	2.761318893
Zm00001d010309_T009	0.163883829	0.127037591	0.099629157	0.792481411	0.624460295	0.749960685
Zm00001d010309_T011	0.10817248	0.305608708	0.513961629	0.731214529	0.490018618	0.878124208
Zm00001d010309_T008	0.167142085	0.105491695	0.297882805	0.719260557	0.750457297	0.571428291
Zm00001d019372_T001	3.263617077	3.163039766	3.106605777	3.017123396	3.44883476	3.234155167
Zm00001d014983_T001	4.59713496	3.926044122	4.694126397	5.806251276	5.870458405	5.780287681
Zm00001d038439_T001	0.152624761	0.532402745	0.450432671	1.430711327	1.201328981	1.233804637
Zm00001d032505_T002	2.838937652	1.968450229	2.933171498	3.214725544	3.090208307	3.283729499
Zm00001d032505_T001	2.443373521	2.338106316	2.736798376	1.975792311	2.471378159	1.975428055
Zm00001d025827_T002	1.573513651	0.970690974	1.614421983	1.751761485	1.885081538	1.975428789
Zm00001d037674_T001	1.320890713	0.339567278	0.790672797	0.888313469	0.879338773	0.910241448
Zm00001d043477_T002	5.434144635	4.036253632	5.413562638	5.374160441	5.341451467	5.507191531
Zm00001d043477_T001	5.459376697	4.552911404	5.438894958	7.061553232	7.048685127	6.988177087
Zm00001d034107_T001	1.707263704	1.532728387	1.658896043	1.259714193	1.196115991	1.156190133
Zm00001d006106_T001	2.090124787	1.670308743	2.130692707	3.951853893	4.10757506	3.919521624
Zm00001d036428_T002	3.316748043	2.044235677	3.162361679	3.487851865	3.661774336	3.525961232
Zm00001d003613_T002	3.76381458	3.192274506	3.716976303	3.81247543	3.558344053	3.615508464
Zm00001d018957_T001	7.249304905	6.491339951	7.273818486	6.446949847	6.585749877	6.502053227
Zm00001d052270_T008	3.54954313	3.796674268	4.549936186	3.594087721	3.625573844	3.268201561
Zm00001d052270_T005	4.107014231	3.184109669	3.830306747	4.021209552	3.677778941	4.007768249
Zm00001d052270_T007	0	0.628320072	0.710642694	1.209166358	0	0
Zm00001d052270_T010	0.9345867	0	0.372633444	0.790029651	1.250452297	1.31889525
Zm00001d018967_T001	5.647081967	5.076856932	5.816947539	5.354907127	5.421172661	5.370740302
Zm00001d021120_T001	2.337100493	2.068488184	2.372481613	2.811175885	2.74605538	2.837949497
Zm00001d001937_T001	3.062063671	0	2.127398864	0	2.985809702	0
Zm00001d001937_T002	4.033564665	3.450283268	4.181013075	4.290226359	3.887484621	4.267482239
Zm00001d001937_T005	3.270162616	4.032856208	3.586406877	1.845922744	2.149260992	3.529723764
Zm00001d001937_T004	0.642572197	0.523253814	0.875625958	0.879101119	0.826258445	0.949807572
Zm00001d018350_T001	4.209253558	3.594505913	4.136497959	3.827464708	3.963445085	3.929715832
Zm00001d021511_T001	7.320048735	6.980895089	7.355808669	6.865716187	6.787096893	6.882132514
Zm00001d044535_T001	3.597552627	3.452318676	3.593624117	4.329209509	4.276059994	4.411047963

Zm00001d002607_T001	3.981077316	3.33775433	3.904359525	2.419728182	2.676417267	2.177900575
Zm00001d018478_T001	5.631633232	6.250114329	5.585514887	5.356171811	5.289501707	5.279473561
Zm00001d002641_T001	1.710402009	1.169039877	1.717967866	2.037178719	2.356871455	2.28892224
Zm00001d008588_T001	3.239207533	2.479113868	3.042006539	4.433290521	4.44062317	4.346477637
Zm00001d042880_T003	1.094657667	0.41728599	1.303453457	0.972273568	0.257069774	1.125609447
Zm00001d018911_T001	6.562737054	5.517026539	6.610043017	6.04741757	6.064484258	6.053405799
Zm00001d030002_T002	1.219214376	1.320226443	1.522496729	1.184254265	1.164009382	1.28688233
Zm00001d023538_T006	3.693495321	0	3.224034098	2.971646735	3.359545487	1.215943196
Zm00001d023538_T004	0.334097979	0.189734753	0.426583589	0.954555915	1.190224822	0.889516375
Zm00001d023538_T002	2.867574885	2.720767016	3.316337654	3.775840488	3.688206213	4.181866802
Zm00001d039512_T008	0.658761716	0.313541908	0.487970939	1.352009869	1.309627323	1.388100244
Zm00001d039512_T014	2.10868603	1.48170348	2.023834444	1.087062905	0.996019167	1.585716833
Zm00001d039512_T006	3.349820861	2.81158303	3.56689307	2.810478888	3.216136025	2.531937095
Zm00001d039512_T005	0.85485901	0	0.896391213	0.839298537	0.953587085	1.14977768
Zm00001d039512_T013	1.879756338	0.839192902	1.884452179	1.917788423	2.189573152	2.077842174
Zm00001d044987_T007	0.711866656	0.264236151	0	0.685072701	0.585259665	0
Zm00001d044987_T006	3.393861242	2.373938531	3.545517827	3.138389689	3.273458151	3.158821399
Zm00001d027630_T006	0	0	0	1.617934577	2.935509157	2.101417451
Zm00001d027630_T001	2.297521305	1.94128025	2.04132605	2.195171503	2.099803846	2.561075828
Zm00001d027630_T003	2.535017271	2.417060992	3.599806466	3.825946472	3.343522238	3.540410961
Zm00001d027630_T009	2.630209744	3.407058607	2.684329081	0	0	0
Zm00001d027630_T002	1.613617929	1.087551776	0.934539902	1.854894108	2.072544203	1.858123467
Zm00001d027630_T008	4.168684953	0	3.841490027	4.683142454	4.673893541	4.69469107
Zm00001d028159_T001	2.908115597	3.02751238	3.080909687	6.4726853	6.692771203	6.560375667
Zm00001d028875_T001	0.46876993	0.346091154	0.334757038	0.870846031	0.922343253	0.851623927
Zm00001d027236_T001	4.519092911	3.827322733	3.219519682	4.116738691	3.607798884	4.254899737
Zm00001d052043_T003	0.54410154	0.327717252	0.500949883	0.844498943	0.944111049	0.760886881
Zm00001d052043_T014	0.379684169	0.241397242	0.338991397	0.869675613	0.790971333	0.86938819
Zm00001d003319_T001	0	0	0	0.252879446	0.445555095	0.530796902
Zm00001d003319_T002	0.454027404	0.153600426	0	0.444235572	0.536707437	0.335785072
Zm00001d003319_T003	0.292519096	0.138603487	0.772599356	0.290221492	0	0
Zm00001d042183_T002	2.059991335	1.219909469	1.935897205	2.483482259	2.351831547	2.3441476

Zm00001d042183_T001	3.888686757	3.363817832	3.781969504	3.600765714	3.777710592	3.644476531
Zm00001d042183_T003	0.955486155	0.38321269	0.77721896	1.342339691	1.000093772	1.094263099
Zm00001d044391_T002	6.770007573	6.58812795	6.860609329	6.572404617	6.560288364	6.486013946
Zm00001d044391_T003	1.058357368	0.675712071	1.404196857	1.376584894	1.541240746	1.724238139
Zm00001d050960_T001	0.915389341	0.420211812	0.697274749	0.644716472	0.424836115	0.461032908
Zm00001d021387_T001	1.997891043	1.596954219	2.250549922	2.297817672	2.264882876	2.450196153
Zm00001d012969_T002	0.702417296	0	0	0	0.507242569	0.508204498
Zm00001d012969_T008	1.698037074	0.764177975	2.078469452	1.990274546	2.33595653	2.309537398
Zm00001d012969_T011	2.300512101	1.16249107	2.065525439	2.123661893	2.081061766	1.870960023
Zm00001d012969_T009	0.44492781	0.188002055	0.588208757	0.400690935	0.552653483	0.563006774
Zm00001d052053_T004	2.267504637	0.840256156	2.680978935	3.053175648	3.199472748	3.470630439
Zm00001d052053_T001	1.756915254	0.4320946	2.214684052	2.896781401	2.442021203	2.592515641
Zm00001d052053_T003	2.797006335	2.487851865	2.656006618	3.267532652	3.457239287	3.110334008
Zm00001d036720_T001	1.274534815	0.732723322	1.187112749	2.037487301	2.26001903	1.989755564
Zm00001d049377_T009	0.735614022	0.276416861	0.598494794	0	0	0.586172895
Zm00001d049377_T014	0.811576249	0.334800507	0.812162208	1.372412797	1.29294132	1.357014237
Zm00001d053875_T001	3.928301243	2.405689634	3.700386448	4.853272871	4.939627683	4.877120665
Zm00001d053875_T003	0.296368126	0.941907085	0.197842751	2.552320011	1.265491506	2.109983642
Zm00001d053875_T007	2.250518695	2.667634211	3.364412606	0	0	0
Zm00001d038933_T001	4.439158342	4.3138247	4.142668215	3.754622663	4.00696598	3.641913823
Zm00001d048066_T001	2.792439347	2.265665525	2.908391948	2.774177705	2.551245295	2.384439448
Zm00001d013626_T001	6.058687859	5.886332141	6.046056327	5.784846059	5.811194539	5.794502529
Zm00001d013626_T003	0.774677015	0.297041122	0.588369007	0.969518544	1.199260244	0.877880858
Zm00001d006059_T001	6.184123657	5.888500076	6.293048439	4.282309838	4.117195997	4.051831028
Zm00001d025705_T006	0.759676559	1.040313834	1.533984384	1.961535912	0.9073368	2.181772366
Zm00001d025705_T005	1.512153549	0.422178091	0	1.457834327	0.78664236	0
Zm00001d025705_T009	1.928981574	1.711542482	0.999970424	2.132715348	2.036163588	1.482180204
Zm00001d025705_T010	3.536038598	2.263098424	3.414391162	2.740147625	3.155314189	2.662954458
Zm00001d025705_T013	0.405879117	0.134411534	0.232335346	0.668918786	0.268249928	0.645177785
Zm00001d000403_T001	4.368620441	3.774379938	4.51418546	4.347904503	4.418652103	4.280253348
Zm00001d010575_T001	8.502771156	8.399174944	8.138590018	8.043785977	8.06042582	8.099590331
Zm00001d035965_T004	1.186526546	0.301550189	1.204373633	1.004509302	0.529238255	1.278496293

Zm00001d035965_T001	2.973504562	2.268557757	2.58373617	2.046633891	2.021553282	1.697846308
Zm00001d035965_T002	1.999840573	2.072843599	2.315226528	1.646048804	2.175848073	1.461813508
Zm00001d027302_T004	3.004202575	2.54273796	3.422148617	2.920255374	2.818692093	2.910056816
Zm00001d027302_T001	0.573006049	0.374476427	0.345232849	0.25125372	0.321659151	0.441080786
Zm00001d002585_T006	4.896942173	3.998091224	4.893329832	4.984140705	5.214610273	5.30352809
Zm00001d002585_T009	0.433021388	0.215490987	0.225835115	0.571916552	0.648691839	0.575389789
Zm00001d002585_T008	5.711626643	5.519427263	5.585630344	4.579916215	4.492038198	4.228557289
Zm00001d032402_T001	5.321062904	5.361873084	5.144861572	5.731513649	5.778470409	5.812975648
Zm00001d006918_T001	3.856680392	3.528604948	4.218047547	3.842433923	3.724775656	3.820652906
Zm00001d032661_T001	4.514525401	3.867047396	4.536212085	4.179694242	4.178273082	4.192761267
Zm00001d031088_T001	3.817338067	2.957287809	3.853605887	4.201614573	4.211833564	4.073275007
Zm00001d009687_T001	4.488305029	4.775547969	4.225899572	1.381671546	1.89314045	1.817443984
Zm00001d019576_T001	4.095630198	3.59557682	4.083319162	4.040080928	3.989905122	3.947955602
Zm00001d026280_T001	2.685874458	1.94797355	2.634405543	2.958915597	2.895498264	2.753803899
Zm00001d000293_T001	0.687076818	0.598628183	1.068024806	2.030651424	2.316071288	2.028779883
Zm00001d000293_T002	1.317375854	1.097510344	1.560687567	1.200563988	1.302410304	1.203273211
Zm00001d029814_T001	5.60248889	5.199323033	5.603617088	5.946152731	5.978539831	6.010974929
Zm00001d047537_T003	0.905249992	1.073955929	0.76866539	1.038473407	0.825398144	0.837201324
Zm00001d047537_T004	1.802535286	0.44492251	1.856347139	2.546709783	2.69289805	2.412119224
Zm00001d004354_T001	2.303015296	2.199901274	2.213161547	3.197786305	3.223138153	2.79798206
Zm00001d004354_T002	3.716419737	3.158582465	3.655683381	4.075218045	3.877629124	3.637595988
Zm00001d020367_T004	0	0	0	2.428904014	2.473015363	1.234118063
Zm00001d020367_T001	0.547193152	0.514422259	0.33679524	0.567420837	1.190475166	0.634931484
Zm00001d020367_T003	1.735605357	1.038210699	1.62220976	0.9990605	0	0
Zm00001d023953_T001	3.490207101	3.830125086	4.002924533	3.441300874	3.494625535	3.0099976
Zm00001d007090_T002	2.529148032	1.987191327	2.470851009	2.331824433	2.451364376	2.351952221
Zm00001d007090_T001	0.411668121	0	1.063262703	0.921133135	0	0.705591362
Zm00001d007090_T003	0.546641141	0.271226052	1.107185684	1.219062551	1.080974817	1.294070178
Zm00001d018418_T001	4.167522438	3.694291409	4.229941939	4.326297742	4.047485517	4.205217562
Zm00001d041679_T001	3.851818389	2.85850803	3.902996223	3.290253052	3.84177471	3.57279098
Zm00001d041679_T006	3.716085827	1.530061251	3.793227532	3.362397787	2.95622601	3.370002143
Zm00001d041679_T004	0.718722405	0.300092087	0.313061221	0.758199119	0.61664282	0.637614883

Zm00001d039841_T001	5.626548503	5.416806545	5.543230829	5.016408288	4.853344542	5.156516998
Zm00001d022134_T001	7.530467696	6.806348033	7.752449687	7.001564847	6.685369992	6.636500369
Zm00001d022134_T003	9.10873057	8.659301359	9.029950764	8.013212977	8.125025919	8.030936666
Zm00001d022134_T002	8.734164334	7.927963042	8.65029365	8.190211554	8.330296171	8.290420363
Zm00001d013342_T001	1.424999997	1.325453194	1.787209273	1.947292859	1.642569887	1.932148124
Zm00001d035053_T003	3.014073154	2.671028816	2.429080294	3.555326586	3.449861107	3.680574387
Zm00001d035053_T001	1.316046083	1.544428014	1.08784502	2.172315969	1.979437863	1.691505132
Zm00001d024867_T001	1.6841338	1.177831068	2.09052015	2.991710308	3.113999807	3.136597784
Zm00001d045326_T003	1.600228356	1.118610232	1.811074749	2.586346103	2.633603022	2.38340266
Zm00001d045326_T001	4.854342162	3.984148272	4.724118166	5.531226608	5.52010743	5.57322048
Zm00001d045721_T001	3.083723626	2.0963769	3.029226348	2.227951428	1.961951092	2.243324206
Zm00001d033303_T004	3.274196794	2.360379167	3.093317284	4.252966011	4.15097228	4.306587728
Zm00001d033303_T006	0.189342579	0.192980646	0.303702439	0.751498856	0.635107993	0.591148959
Zm00001d033303_T008	4.48259024	3.770326816	4.511337604	4.778382646	4.615004477	4.440760889
Zm00001d044934_T001	3.094436918	2.614014121	3.238385478	3.307500512	3.439193456	3.563195723
Zm00001d020572_T002	1.183250836	0.926983912	1.447191958	1.407080775	1.600990445	0.731816672
Zm00001d048131_T001	2.532576796	2.034595007	2.743082547	4.764883955	4.64784388	4.607680247
Zm00001d043929_T001	8.82247781	9.025730035	8.906929154	8.686336474	8.773287333	8.696226684
Zm00001d026269_T001	6.081644958	5.7584334	6.249745113	6.31963876	6.381470914	6.241724695
Zm00001d028783_T001	1.29124166	0.525020473	1.305388658	1.324678134	1.047648663	1.165684948
Zm00001d018501_T001	4.089890042	4.120103396	4.157335778	3.512914942	3.734320839	3.260680142
Zm00001d030851_T001	2.373903183	1.741731879	2.333877653	1.714414344	1.837696248	1.692361579
Zm00001d053798_T004	2.101223793	1.614390414	2.573757449	2.114384351	2.605673297	2.593709441
Zm00001d053798_T001	4.141560394	3.147261268	3.798734362	3.566922287	3.138404763	3.202381203
Zm00001d021469_T005	1.226099092	0.738988277	1.138963841	1.019743185	0	0.623207622
Zm00001d021469_T008	0	0	0.368690137	0	0.70881054	0.89594316
Zm00001d021469_T002	0	0	0.33736743	1.058169621	1.09322752	0.87406432
Zm00001d021469_T001	0.326910054	0.250835484	0	0.408890081	0	0.163005298
Zm00001d035084_T001	3.160005794	2.633149154	3.102616815	2.1146582	1.834525558	2.147258174
Zm00001d009435_T001	2.254652693	2.259900356	1.269861411	2.78688968	2.775267825	2.580347882
Zm00001d033642_T004	4.958063177	5.428881342	4.924885836	3.871001338	4.248345227	3.956985739
Zm00001d033642_T001	4.564022512	3.601145002	4.436111523	5.014779424	5.042637465	5.027949686

Zm00001d033642_T003	0.620672735	0.218467491	0.646803201	0.535171094	0.538481547	0.321511384
Zm00001d009445_T002	0	7.315544449	0	0	3.131701419	2.483452849
Zm00001d009445_T001	6.949681495	6.442438066	6.94797279	6.956135885	7.049206598	6.9430059
Zm00001d037515_T001	4.786547958	5.504417797	4.752105764	3.096856893	3.235260636	3.321752797
Zm00001d045836_T001	7.055945597	6.265087714	7.066455825	7.095413407	7.15006721	7.152736252
Zm00001d048316_T001	6.893827383	6.925199949	6.514012649	9.145375813	9.100747745	9.26716359
Zm00001d018468_T002	0.996383684	0.310025951	0.337312619	0.726884391	0.599273988	0.873595105
Zm00001d018468_T003	6.675039074	6.07231104	6.723286938	6.560587949	6.512882841	6.607759389
Zm00001d018468_T001	1.327390748	1.478450011	1.582511686	1.474823825	0.937965785	1.455243829
Zm00001d053975_T007	2.570446415	2.105795057	2.579293367	2.031353552	0.762313093	1.885024122
Zm00001d053975_T004	0.513696902	0	0	0.6834683	1.219095396	1.538127396
Zm00001d053975_T002	1.890441245	0.558121643	1.61576704	1.611030701	1.583072776	1.815268414
Zm00001d053975_T003	1.164886015	0.768705189	1.3198757	0.985490229	0	0.269088219
Zm00001d053975_T006	1.399037621	1.578000596	1.943470543	1.755759268	1.903237688	2.341675264
Zm00001d030217_T012	1.189851752	0	1.131908146	0	0.646311989	0.711049915
Zm00001d030217_T027	4.162613039	2.363580694	3.866086328	0	2.430909057	0
Zm00001d030217_T001	0.664494673	0.915732748	0	0.940417861	1.392347101	1.356280184
Zm00001d030217_T021	3.411846519	2.961154145	3.700539372	2.727840315	2.834859239	3.180885096
Zm00001d030217_T009	0.989595365	0.934106563	0.879737913	0.893787772	0.265217234	0.795262415
Zm00001d030217_T029	2.231284015	4.168734527	3.078308217	2.731486306	0.937444586	2.141678996
Zm00001d030217_T017	3.851856759	0	0	4.578481204	4.189558209	4.133770397
Zm00001d030217_T004	1.208625255	2.665118487	4.053471009	3.056607631	3.373238937	3.559206305
Zm00001d025746_T001	4.026982996	3.214117188	3.960036232	3.969490179	3.960780893	3.822046771
Zm00001d014405_T001	3.884358201	3.380638446	4.084771573	3.958245619	4.068008807	3.913985799
Zm00001d032683_T001	3.163981375	2.307658755	3.161339838	3.151032537	2.911208366	3.093443383
Zm00001d004559_T001	0.482322726	0	0.241831643	1.427993342	1.012088053	1.751675804
Zm00001d004559_T027	0	0	0	0.352827438	0.795376303	0.688892912
Zm00001d004559_T028	0.130377401	0.21837325	0.729031501	1.092148565	1.451232216	0.998180336
Zm00001d004559_T022	0.338579587	0.308422622	0.284375543	1.565914924	1.55020229	1.398067386
Zm00001d004559_T008	3.720844048	2.88092096	3.652456436	4.962893924	4.903668588	4.932107909
Zm00001d048690_T001	1.986767696	1.527134409	1.771520106	0.724645907	1.385021778	0.783128055
Zm00001d047757_T010	0.767309866	0.69162528	0.841339562	0.550043656	0.407673617	0.516835131

Zm00001d047757_T001	8.062533365	7.294935553	8.148215424	8.087008613	8.11000737	8.104507776
Zm00001d047757_T004	0.723216031	0.343867137	1.015310584	0.657775352	0.656639146	0.548565845
Zm00001d047757_T008	3.184569915	1.784323059	3.759565995	3.666912667	2.349666468	4.010991126
Zm00001d047757_T014	6.601072912	9.101673075	6.420446981	6.102930941	6.235233002	5.738659523
Zm00001d047757_T009	4.223617084	0	5.465723766	4.173963538	4.045122365	3.957654909
Zm00001d035253_T001	1.372032156	1.13028909	0.947654189	1.681639513	1.950414662	1.580414216
Zm00001d007862_T001	2.545401555	1.772284746	2.463743548	2.328849961	2.281100279	2.308615127
Zm00001d005986_T001	2.093497302	1.611384873	2.353901383	2.457997392	2.514307873	2.506730903
Zm00001d049156_T002	6.272576825	6.595745218	6.328680869	5.802023419	5.777975092	5.811346665
Zm00001d049156_T003	5.472559937	5.51793493	5.323445697	4.108354083	3.962974828	4.309107632
Zm00001d049156_T001	3.902966616	5.313329269	3.733190754	2.773195449	2.479802248	2.585903793
Zm00001d003930_T001	4.308083783	4.460047846	4.623294726	3.576750678	3.684841286	3.529716894
Zm00001d016529_T002	1.806713398	0.882191489	1.773472726	1.65100851	1.864375949	1.503841254
Zm00001d048799_T001	4.573363134	4.174471217	4.682746152	4.236169066	4.303899738	4.36987341
Zm00001d006315_T001	1.114705502	0.545527588	1.033222112	1.222219081	0.898443654	1.308650364
Zm00001d004309_T001	7.36100856	3.900702223	7.367316902	7.642892046	7.761364388	7.794425055
Zm00001d001880_T001	0.808210393	0.400201261	0.67395345	0.379015369	0.512295669	0.59014496
Zm00001d001880_T002	4.076122681	4.046550866	4.001204418	3.367146676	3.633322626	3.634498828
Zm00001d001880_T003	5.925508107	5.890049143	6.292574678	5.471443779	5.519291537	5.624598059
Zm00001d020332_T001	0.538406055	0.340932508	0.482027339	3.33208934	3.298176017	3.431499142
Zm00001d014967_T003	2.384056716	0.924597045	0.996512401	0	0	0
Zm00001d014967_T009	1.073051048	0.305853817	1.324819818	3.413968788	3.169607894	3.368103271
Zm00001d014967_T010	0	0.371720547	1.075476497	0	0.872419035	0.935156467
Zm00001d014967_T008	1.932478494	1.334471597	2.439292011	2.309954672	2.6378562	2.273373553
Zm00001d014967_T001	1.287495345	2.380970155	2.370420427	2.447559625	2.626825517	2.256742276
Zm00001d014967_T039	1.194185414	0.563676529	1.415141009	1.548253625	0	0.768512957
Zm00001d007573_T001	2.551191882	1.825725802	2.413801753	1.044906753	2.330939818	2.252875814
Zm00001d009564_T001	2.820881295	2.031098369	2.873327954	3.123735549	3.063396289	3.154377924
Zm00001d048454_T005	0	0.202474143	1.211482629	1.04777149	1.191808343	0
Zm00001d018230_T001	5.163493459	4.456340943	5.210936318	5.623881607	5.740356709	5.586692937
Zm00001d002744_T001	2.717489553	3.715909949	2.781039242	2.129073275	2.322881401	2.396945303
Zm00001d014692_T001	5.886378799	5.732853514	5.961705137	5.97635777	5.944518248	5.881084464

Zm00001d035527_T001	2.410311794	1.777752983	2.540867439	2.786952601	2.828230845	2.771216672
Zm00001d052492_T001	2.763430484	2.465994832	2.602248295	1.852360465	1.797606955	1.848548231
Zm00001d032669_T001	4.811376697	3.940439568	4.87472001	4.725297866	4.718067193	4.657472234
Zm00001d021633_T007	2.322153138	2.066152237	2.403486155	1.875906244	2.250366491	1.997521125
Zm00001d021633_T003	0.609198954	0	0.709178569	0.589276435	0	0.626576507
Zm00001d021633_T006	1.491230231	0.959445234	1.559848099	1.765106117	1.091678172	1.458768351
Zm00001d021633_T012	1.214270933	1.02295081	1.357000156	0.841853996	0.776938614	0.973384981
Zm00001d021633_T005	0.293984867	0.182426614	0	0.254416265	0.404732037	0
Zm00001d021633_T010	1.410976011	1.142689206	1.198182951	1.735764339	1.049627283	1.441156765
Zm00001d021633_T009	0.848472116	0.060943859	0.652850823	1.420363284	1.714234526	1.485774578
Zm00001d021633_T011	1.003520206	0.801867203	0.696777287	1.343457285	1.21640023	1.009713354
Zm00001d015082_T010	0.025873605	0.187233129	0.281519033	1.029059868	0.879849245	0.733875786
Zm00001d015082_T001	0	0	0	1.932684088	1.780859772	1.586127728
Zm00001d015082_T015	0	0.062577664	0	0.359383979	0.775509096	0.687456703
Zm00001d015082_T036	3.02099549	2.0546186	2.888564611	3.347722201	3.385422892	3.361922444
Zm00001d015082_T014	0.238309958	0	0.118194913	0.651008051	0.625597806	0.872000523
Zm00001d015082_T037	0.640495068	0.423761017	0	1.157598038	1.128181202	0.559154044
Zm00001d015082_T026	0	0.324451181	0.771879664	0.762568233	1.680198312	1.460782922
Zm00001d012674_T004	4.941697357	4.343172566	4.948250456	5.224159696	5.353153637	5.332234738
Zm00001d012674_T011	3.329893402	2.3915728	3.241044194	3.383836842	3.707293857	3.682516218
Zm00001d012674_T013	2.740504293	2.862229391	2.374736538	2.202858691	1.566636906	2.48669687
Zm00001d012674_T008	0.76060164	0.771059859	0.997198453	0.958964395	1.136495252	1.385168705
Zm00001d012674_T006	2.315905853	1.252319371	2.456494714	2.988420206	2.623245555	2.927840337
Zm00001d012674_T009	5.48110351	6.288458012	5.666550255	5.769493856	5.766425177	5.688626277
Zm00001d012674_T007	1.577157123	0.741655091	2.28841113	2.799761028	2.338361086	2.169040198
Zm00001d004086_T001	5.143172287	4.566089546	5.21152908	5.442183742	5.58795755	5.418513877
Zm00001d035234_T001	1.594813656	1.343758575	1.901127556	2.362580138	2.402528459	2.076902455
Zm00001d006864_T003	1.130934164	1.87282482	0	0.590627886	1.110217563	0.670594731
Zm00001d006864_T043	1.629789305	0	1.582603209	0	1.466767533	0
Zm00001d006864_T040	0.092632334	0.053023729	0.088304451	0.218734059	0.23672037	0.210585866
Zm00001d006864_T021	0	0	2.530959914	2.536251879	1.190765917	2.253912441
Zm00001d038384_T001	3.009488711	4.131984344	3.156749966	3.177028308	3.340194641	3.271957027

Zm00001d006070_T005	1.458307978	0	1.897540552	2.092013893	2.392535871	0
Zm00001d006070_T002	3.94762511	3.593408274	4.361556992	4.14691878	4.608384295	4.300121528
Zm00001d006070_T008	2.410184777	4.11502575	2.890348823	3.167407226	0	3.599862273
Zm00001d006070_T003	2.096704108	0.900128345	1.855891654	2.429848383	1.414640523	2.516200012
Zm00001d006070_T004	3.237231609	2.558257836	2.950112456	3.608247547	3.557957385	3.570803527
Zm00001d012404_T003	2.497850177	1.946052952	2.659854248	3.107907102	3.171021994	3.138699973
Zm00001d029630_T001	6.482565007	7.929991526	6.172379921	4.760450216	5.133360612	5.266560863
Zm00001d052941_T001	4.20641927	3.554216281	4.459849874	3.985651705	3.814197756	3.947749743
Zm00001d031754_T001	1.28095572	1.149340669	1.349271284	1.707657746	1.873626207	1.893468613
Zm00001d012464_T002	1.301687725	0.807077073	1.403795614	1.822231695	1.77893605	1.826692063
Zm00001d012464_T003	0.489575817	0.357885264	0.188885751	0.714381812	0.596957081	0.86735723
Zm00001d003904_T003	0.43754629	0.319589034	0.538919521	0.466378624	0.408193376	0.362228601
Zm00001d003904_T007	2.675605269	3.755411586	2.768490514	2.520796106	3.146313568	2.692784034
Zm00001d003904_T001	5.613118911	5.104697666	5.452997826	5.043504008	5.128781119	5.049613562
Zm00001d003904_T004	2.268088201	0.730479104	2.224912668	2.76687159	2.478764508	1.787239786
Zm00001d012482_T001	0.17287406	0.207568053	0.478655024	1.153192466	1.113743166	1.46634782
Zm00001d025379_T001	3.835636214	3.244709603	3.86309042	3.740787492	3.851206836	3.906138082
Zm00001d029331_T006	4.297739405	5.395689797	4.575888991	4.076625191	4.168592458	3.041338843
Zm00001d029331_T003	0.992317427	0.738095071	1.162838419	1.009329255	1.225706193	1.533765162
Zm00001d029331_T001	4.003349293	2.862600365	4.088383496	4.254290401	4.147014874	4.309963473
Zm00001d048463_T004	4.739395516	3.959305099	4.753514258	5.14199846	5.095500028	4.973854107
Zm00001d048463_T005	0.471734774	0.283240256	0.84279866	1.378518282	0.709849076	0.635321634
Zm00001d048463_T006	0.716600272	0.205839141	0.72079853	0.454067424	0.71385591	0.880571124
Zm00001d028452_T001	0.967832611	1.084731017	1.212369495	1.022745616	1.050968996	0.849549373
Zm00001d031954_T005	1.990335911	1.444771476	1.380141503	1.789108227	1.549650937	1.210914972
Zm00001d003066_T002	4.208807049	3.601799545	4.024168504	3.765729603	3.945022004	3.742606777
Zm00001d003066_T004	3.140742997	2.785063573	3.672383814	2.850609824	2.17906639	2.780737174
Zm00001d033370_T002	2.247761062	1.194063722	2.364087998	2.547312611	2.268009727	2.344318636
Zm00001d033370_T007	2.983849495	2.862292483	2.688269895	2.593263405	2.662884751	2.803425858
Zm00001d033370_T006	3.492164428	2.823545779	3.618769348	3.459365778	3.7706353	3.455977478
Zm00001d052059_T001	3.167097114	2.590646568	2.918347877	2.133984305	2.221379088	2.393005905
Zm00001d053563_T004	3.975398706	2.149314653	3.465477596	3.407689522	3.976307998	3.499608787

Zm00001d053563_T003	0.759260676	0.516435788	0.736685396	1.047203326	1.18337408	0.905918469
Zm00001d053563_T002	3.346469339	3.319707811	3.45139748	4.056420262	3.671649359	3.883596771
Zm00001d037495_T002	2.759324813	1.842925748	2.863626295	2.859069073	2.819301128	2.753267997
Zm00001d014735_T001	3.586205808	2.718292134	3.572311626	3.540969451	3.401026259	3.555914294
Zm00001d041993_T001	0.736666348	4.227799827	1.348798393	1.605896791	0.853976491	1.399252047
Zm00001d041993_T003	6.80934744	6.482503844	6.758049321	6.569065479	6.754045604	6.747866186
Zm00001d045121_T001	6.775743427	6.709036542	6.846621972	6.309063946	6.338909237	6.238636469
Zm00001d025325_T003	1.498289679	1.331028705	1.732247054	0.22761294	1.376988777	0.73672349
Zm00001d025325_T002	3.732540127	3.1369898	3.812033613	2.709237471	2.633206825	2.455142017
Zm00001d020242_T008	0.558665361	0.2143014	0.573099147	0.467146945	0.610647153	0.530702033
Zm00001d020242_T004	2.799005634	0.975795245	2.421933395	2.875451193	2.568715824	2.449977814
Zm00001d020242_T001	2.219403051	1.845813178	2.292648662	2.35179198	2.262768986	2.436136515
Zm00001d052404_T003	4.006687598	3.454385701	4.082864781	4.218687878	4.232556541	4.088260431
Zm00001d052404_T002	0.603402024	1.108409371	0.972366954	0.748183831	1.217652604	1.078350919
Zm00001d052404_T004	1.362987679	0.495175289	0.988840262	1.317928599	1.424250286	1.509369014
Zm00001d052404_T005	1.014931542	0.965602563	0.433000016	1.433557734	0.949594694	1.247656565
Zm00001d009847_T001	4.388265873	4.610558632	4.407105323	4.195490916	3.872144671	4.034187714
Zm00001d009594_T001	1.845133925	1.067629081	2.277802391	1.899104848	1.689671305	1.757563516
Zm00001d009594_T004	8.139999281	7.254023528	8.215681984	8.167886803	8.174873715	8.12465362
Zm00001d009594_T003	2.960016394	4.08537256	2.983693745	3.114623056	3.142490227	3.569874804
Zm00001d003276_T001	0.929539545	0.667893033	1.421724738	1.520713398	1.48285809	1.634409726
Zm00001d052111_T034	0.353230685	0.818395762	0.584034068	1.152555973	1.321764773	1.676952481
Zm00001d052111_T025	0	0	0.508863677	0.374873668	0	0.899533746
Zm00001d052111_T020	0.065262095	0.010653821	0	0.511953747	0.314915741	0.35220823
Zm00001d052111_T008	1.121162648	0	1.030113565	2.073000303	1.801846515	1.852004832
Zm00001d052111_T032	3.079877271	0	2.666146214	3.389303441	3.420587094	3.466458763
Zm00001d052111_T033	0.985100355	1.689331817	1.978744042	0	0	1.47047231
Zm00001d052111_T014	0.437466393	0.189905506	0	1.002449779	1.029604127	0
Zm00001d052111_T005	2.322141021	2.420423383	2.609775525	2.822505621	3.402624775	3.043285179
Zm00001d008856_T004	3.075274874	1.641255149	2.722208502	2.708359422	2.67150376	2.600637993
Zm00001d008856_T008	3.102629244	2.279886958	2.974478652	2.311689979	2.020703804	2.170189792
Zm00001d008856_T005	1.728984934	1.701874597	2.333753169	1.390859152	1.777454224	1.482877188

Zm00001d008856_T001	3.550425679	3.124056446	3.720091056	3.140797793	2.991916699	2.963690512
Zm00001d016851_T001	1.894889145	1.321751498	1.620574681	1.520405653	1.684047149	1.802122063
Zm00001d034036_T007	0.742058828	0	1.792370625	1.584913448	2.095160855	1.744409132
Zm00001d034036_T005	1.702745299	2.811955136	3.308738014	3.214182636	2.664806386	2.813161199
Zm00001d034036_T004	2.327074807	1.265433895	2.06205918	3.168520255	3.315980171	3.372398169
Zm00001d033405_T003	3.057436582	2.539712948	3.341040145	3.10502882	2.850154926	2.734436282
Zm00001d050245_T001	5.751540578	5.529827535	5.825214414	5.656689794	5.652285766	5.52857085
Zm00001d018093_T001	0.138733225	0.071352177	0.067228427	1.135581492	1.258914443	1.104256806
Zm00001d038318_T007	4.038767581	1.873783675	3.510290783	4.566966477	2.97320054	4.16928767
Zm00001d038318_T008	3.771121889	2.843066899	3.359476613	4.041901876	4.487235052	3.642733105
Zm00001d038318_T011	3.645476061	3.679041003	4.598782814	1.609183349	3.817620188	3.104089367
Zm00001d000168_T001	4.793148442	3.88660048	4.679246885	4.459166007	4.49490914	4.482898673
Zm00001d009134_T001	3.683485592	3.469704196	4.012965226	3.708323772	3.777845859	3.736983411
Zm00001d011364_T001	2.375819875	1.714727325	2.466899321	2.490503898	2.421670078	2.496771922
Zm00001d045537_T018	4.981635776	5.884064089	5.115152339	5.673702863	5.577033883	6.059886499
Zm00001d045537_T010	2.325449452	0.94127161	1.570146669	3.317713314	2.697481827	3.407382937
Zm00001d045537_T019	1.907458329	1.147119755	2.139904268	3.018275058	2.75444287	2.691347004
Zm00001d045537_T011	1.641129344	1.464497852	1.776826208	3.304891086	3.292643214	3.240371578
Zm00001d045537_T021	0.524972348	0.168084873	0.555425495	0.486719521	0.726587115	0.823278166
Zm00001d045537_T003	1.500266678	0.639842459	1.822591887	2.10021608	2.352888723	1.652326327
Zm00001d045537_T006	0.702698319	0	1.06184738	1.876366456	2.502009995	2.136952899
Zm00001d018793_T001	3.061865521	2.668519238	2.924869878	6.145266025	6.14388016	6.16326961
Zm00001d014883_T001	7.4845426	8.307155594	7.445861288	6.957356653	7.08718691	7.090906779
Zm00001d014716_T002	2.560516876	1.760577371	2.579973702	1.900625333	1.862112921	2.133147557
Zm00001d016823_T001	2.262053942	3.254387691	2.449433804	4.164114323	3.563194014	3.956247853
Zm00001d049286_T001	0.711491383	0.250098123	0.723343616	0.091437164	0.09601216	0
Zm00001d049286_T003	1.608962491	0.967049792	2.014647373	1.134097369	0.992447232	1.33251432
Zm00001d049286_T002	1.06272271	0.669790557	0.42838684	1.027215637	1.001136396	0.899570867
Zm00001d012892_T002	1.270027126	1.809029125	1.507597786	1.648840449	0	1.389804121
Zm00001d012892_T005	4.5854965	3.260442291	4.494378166	4.78935669	4.856498835	4.752496151
Zm00001d012892_T006	3.128103647	2.635647143	2.855166289	2.690221743	2.601340196	2.334734159
Zm00001d012892_T001	0	0	0	1.35967409	2.250387715	2.138099168

Zm00001d046263_T001	5.283000094	4.585774329	5.216803701	5.324830761	5.296705104	5.172723838
Zm00001d046263_T002	0.177425438	0.10830766	0.344497889	0.662197295	0.743388293	0.604432901
Zm00001d046263_T004	0.939085123	0.421939049	1.177374408	1.947967941	1.841846752	1.877563656
Zm00001d005507_T002	0.921509454	0.62246093	0.739605329	1.937923614	1.183527802	1.572603526
Zm00001d005507_T013	2.508551365	1.596757716	2.615476094	1.401842323	1.494639358	1.110105286
Zm00001d005507_T010	0.109668123	0.051842598	0	1.991188939	1.478946962	2.009371898
Zm00001d005507_T005	2.275717489	0	2.105807124	2.246502338	2.960586586	2.743602858
Zm00001d020087_T001	0.888205127	0.637001789	0.772607801	1.599734353	1.531456822	1.582345966
Zm00001d012655_T002	2.273310586	1.449680219	2.007176481	2.31929624	2.403345991	2.292461965
Zm00001d006295_T009	0.380067785	0.094373913	0.240111574	0.67566601	0.888566754	0.326226687
Zm00001d006295_T005	0.934582926	0.860374877	0	1.441313491	0.686885941	1.117348621
Zm00001d006295_T006	2.708672394	1.924047043	2.82714832	3.025558808	3.121792589	2.658631946
Zm00001d006295_T011	1.199642195	0	0	0.964687697	0.281752842	1.120536413
Zm00001d021822_T005	0.259141094	0.352564194	0.663749018	1.422266376	1.367758528	1.299184862
Zm00001d021822_T002	3.819402814	4.093510993	3.857034177	3.633050782	3.151948739	3.479518632
Zm00001d000066_T001	3.933494364	4.22785126	4.106755088	3.415980218	3.46171098	3.804418628
Zm00001d050247_T001	1.106485011	1.010420373	1.171810173	3.353772969	3.551961738	3.498448235
Zm00001d018822_T001	6.653087411	6.307799134	6.521058151	6.027236409	5.964770279	5.897439291
Zm00001d007324_T004	1.996765823	3.748119196	2.23371446	0.484482583	1.114574918	0
Zm00001d007324_T005	3.526260532	2.738512344	3.864809551	3.706412303	3.672870529	3.848379771
Zm00001d025869_T001	0.416467943	0.081544154	0.894572557	1.075435421	1.146374345	1.384152063
Zm00001d025869_T003	3.492054931	3.126877896	3.386446779	4.739553378	4.804434633	4.558320356
Zm00001d025869_T002	2.057498101	0	1.573776835	2.988990551	2.717894408	2.648452788
Zm00001d025869_T004	0.9873638	0.635369931	0.896372611	1.595895551	1.718504984	1.775567669
Zm00001d025869_T005	0	0.816178612	0.837574349	0.954204501	1.400776173	1.968726688
Zm00001d013461_T001	4.205846881	3.645816	4.408168575	3.176417247	2.964513045	3.038132369
Zm00001d006002_T001	3.254676424	3.409307563	3.189280896	3.222974225	3.224074859	2.699466792
Zm00001d005468_T001	3.63849071	2.688339953	3.382078712	3.935825844	3.783385932	3.63137696
Zm00001d005468_T002	2.08663127	3.743388616	3.021603915	0	2.171976337	0.727812439
Zm00001d013729_T004	1.07663434	0.392015112	1.050241867	0.938193185	1.332575615	0.704264666
Zm00001d013729_T002	2.465410855	1.813871879	2.331375313	2.68795224	2.287796437	2.39175425
Zm00001d009494_T001	1.582340185	1.084799035	1.796774654	0.46701544	0.445156716	1.133545771

Zm00001d052520_T001	4.475196778	4.570118428	4.383226586	2.493862236	2.675643204	2.662565103
Zm00001d002674_T001	1.281748095	1.625089966	0.552552171	1.127911228	1.614050647	1.097038324
Zm00001d019562_T001	7.142107742	7.75623038	6.994615463	6.853573228	6.785284311	6.843422518
Zm00001d019562_T002	0.803265894	0.839750825	0.65819777	0.980103558	0.543443421	0.704640938
Zm00001d005059_T002	4.778004198	4.153431808	4.874817765	4.741158729	4.807200545	4.755595318
Zm00001d005059_T004	2.405989638	0	2.483164145	1.558544389	1.42572032	1.609759217
Zm00001d034380_T001	5.549375705	4.834015029	5.596194796	5.722578212	5.664941005	5.740075612
Zm00001d037452_T001	5.675429467	5.605152139	5.8981554	5.701490893	5.679762052	5.730692256
Zm00001d037452_T003	1.482546292	0.899695371	1.204141969	1.814675923	1.775336734	1.696751919
Zm00001d028778_T002	2.154900072	1.607442055	1.996549278	1.731914398	1.418389131	1.683115217
Zm00001d003283_T001	2.649671097	2.321354077	2.902795036	3.238717321	3.221817084	3.43577059
Zm00001d012171_T001	3.177406621	3.540028261	3.615981093	3.427258865	3.256549516	3.693557766
Zm00001d014994_T001	3.259220956	2.592673022	3.194771035	3.508400128	3.353865118	3.365111031
Zm00001d005016_T003	0.733284048	0.558073629	0.218509649	0	0.943274734	0.548129804
Zm00001d005016_T007	2.346344802	1.84505281	2.824496597	3.628193135	3.580952492	3.468241304
Zm00001d005016_T004	1.123966092	0.722147722	1.372287971	2.222545854	2.139327707	2.005281765
Zm00001d014894_T001	5.153117014	4.991467788	5.163145422	5.045093875	4.815554014	5.043486379
Zm00001d016471_T001	3.193027026	2.835909324	3.299629462	2.033922638	2.25543396	2.596635365
Zm00001d034253_T001	1.037829195	0.640802295	1.050949499	0.85995842	0.79996095	0.817700199
Zm00001d043137_T006	3.45121929	4.036466145	0	3.018956359	5.307778118	4.341283152
Zm00001d043137_T001	3.940225491	4.856717786	4.078119631	3.159123605	4.169427667	3.949707579
Zm00001d043137_T005	3.679498678	0	4.727094071	4.954030717	0	4.160866635
Zm00001d030935_T019	0.127161768	0	0.273457405	0.558622263	0.608568931	0
Zm00001d030935_T021	0	0	0	1.318292543	1.423922871	1.793554255
Zm00001d030935_T002	0.435015118	0.082376959	0.492290071	1.304401813	0	0
Zm00001d030935_T007	0.244861492	0.127927731	0	0.208708275	0.863641954	0.798961272
Zm00001d030935_T001	0.035074877	0.049733913	0.164437467	0.685590362	0.61203349	0.569894485
Zm00001d030935_T023	1.500104497	1.351374478	1.969176651	1.598769656	1.591766531	1.75959859
Zm00001d030935_T029	3.668710631	2.861005691	3.380334648	2.790076583	2.594463514	1.860383618
Zm00001d002896_T001	5.156107555	4.476931375	5.382223725	4.783649891	4.675578962	4.543053666
Zm00001d029232_T001	1.480324587	0.997256272	1.268258313	1.714929495	1.639197427	1.843655885
Zm00001d035889_T005	3.98471001	3.205781288	4.136627148	4.272939666	4.430845446	4.350396255

Zm00001d035889_T021	0.916517918	0	0.190135673	0.77187206	1.147561997	0.59697425
Zm00001d035889_T025	0.988222223	0	0	1.94342028	0	2.174783822
Zm00001d035889_T036	0.163340285	0.081241444	0.337699676	0.486360155	0.62483364	0.471269675
Zm00001d035889_T022	0.284813775	0	0.296604239	0.667549742	0	0.58111789
Zm00001d035889_T001	0	0.946858845	1.264835448	3.309014909	3.373143006	3.223879074
Zm00001d026252_T001	7.373568871	7.027607842	7.515231903	7.257013401	7.34841317	7.31674274
Zm00001d000122_T005	0	0	0.74537073	0.586439065	0.606123805	0.973279169
Zm00001d008408_T001	3.657640805	3.171427347	3.625135099	4.109124911	4.178274516	4.197223693
Zm00001d048201_T001	0.606862894	2.01881717	1.17357137	1.383526251	1.964020025	1.071693347
Zm00001d048201_T012	3.643192975	1.837104419	3.725433283	3.53854611	3.570603308	3.462279309
Zm00001d048201_T009	2.964468871	0	2.552094456	2.85299619	0	3.119117922
Zm00001d012265_T001	2.331647038	2.193901028	2.344647013	2.806835871	3.091729788	2.855283919
Zm00001d017536_T001	3.603348846	2.831434639	3.762776883	4.667621893	4.65414954	4.745695942
Zm00001d007436_T002	4.68298981	4.138921914	4.85635182	4.648351944	4.764801438	4.625218404
Zm00001d007436_T004	1.476946211	0.94705716	1.738919123	2.390485824	2.235738699	2.303469531
Zm00001d007436_T006	1.158289195	0.727834217	1.083331162	1.614886019	1.210178742	1.482362485
Zm00001d007436_T003	1.117907775	0.717042677	1.259843124	1.389333319	0.906423472	1.061745097
Zm00001d018962_T001	4.13850634	3.530294772	4.270143026	2.686912155	2.648223593	2.755050357
Zm00001d034920_T001	2.937305405	3.230971047	2.363252673	0.390595054	0.291940619	1.082621529
Zm00001d017103_T001	0.956081937	0.807271656	0.855568783	0.874582955	0.40692083	0
Zm00001d017103_T005	5.345535365	4.893761712	5.1902656	5.225428879	5.10185271	5.286432897
Zm00001d035957_T001	3.544753054	2.765392591	3.57400266	3.789159885	3.584958654	3.874668229
Zm00001d030328_T001	0.987781436	0.51121905	1.194508197	0.520211015	0.680820325	0.599824315
Zm00001d032921_T002	4.802182358	4.258876841	4.711567315	5.091737234	5.037004278	4.861015123
Zm00001d001846_T001	4.693643123	3.733128373	4.779088909	4.924311285	4.881970059	4.879855713
Zm00001d044038_T001	3.248113078	2.39582262	3.11864013	3.588531398	3.700454367	3.718206854
Zm00001d033322_T001	2.276039814	1.662360929	2.369987351	2.931149558	2.921586762	2.788707006
Zm00001d017608_T001	4.292431706	4.206961826	4.514213049	4.799925991	4.747150566	4.581290862
Zm00001d033505_T001	2.501045455	1.726927539	2.637360541	2.876381781	2.730073635	2.740886556
Zm00001d021621_T022	2.884484025	2.236793831	3.00235508	2.397098963	1.761288252	2.535660085
Zm00001d021621_T010	1.555038166	0.946834895	0	2.406758146	2.420086472	1.911704619
Zm00001d021621_T016	0.500406405	0.06711413	1.037794763	0.645321685	0.917302661	1.031275202

Zm00001d041725_T001	4.682750533	4.941984012	4.685479242	5.281747501	5.332898872	5.204275611
Zm00001d038189_T004	1.405451656	0.13976416	0.96190258	1.360404733	1.252504069	1.554306915
Zm00001d038189_T001	0.327396497	0.961924059	1.0644159	0.681550914	0.127942254	0
Zm00001d038189_T014	3.977539402	3.183424581	4.144533768	3.678153167	3.935898053	3.783188311
Zm00001d007160_T001	2.995434227	2.431980447	3.082600242	2.503312349	2.138855417	2.267648453
Zm00001d015921_T001	5.903167924	5.341269593	5.98379125	4.36992309	4.568312223	4.443506137
Zm00001d044690_T001	1.327634484	0.826434186	1.56771677	1.808444776	2.053070315	1.936584046
Zm00001d005970_T001	2.998658752	3.979896293	2.914520515	4.826696283	4.660545046	4.764833224
Zm00001d021936_T001	5.659458689	4.795861684	5.635700449	5.665832773	5.721634076	5.634030339
Zm00001d027937_T014	1.089713048	0.474177464	1.217490068	0.65180715	0.69598847	0.792097374
Zm00001d011562_T004	0.7617261	0.320727273	0.538474594	0.670940922	0.750620224	0
Zm00001d011562_T001	0.946724872	0.402269217	1.20413195	1.065655714	0.856366662	1.215404008
Zm00001d011562_T002	0	0.520561042	0.749234757	0.348733252	0.620883833	0.612302481
Zm00001d020053_T001	3.597307691	4.800726611	3.724814503	3.164312917	3.304405172	3.405090666
Zm00001d020053_T003	2.1426072	0.826334927	1.518618224	2.825981772	2.742146161	2.63562091
Zm00001d020053_T002	2.561363532	2.057035687	2.632752135	1.510973056	1.543978846	2.126385486
Zm00001d015246_T004	0.21030033	0.540086802	0.577600431	0.244050419	0.259629237	0.578937747
Zm00001d015246_T015	2.239471977	1.919499123	1.903906712	2.357360289	1.879313676	2.42251586
Zm00001d015246_T009	0.539564818	0	1.3053297	0.685751812	1.148770145	0
Zm00001d015246_T002	0.883731067	0.323975269	0.454864431	1.492614638	1.492486461	1.527478257
Zm00001d015246_T007	0.48156658	0	0	1.616095579	1.23664262	1.447111534
Zm00001d015246_T013	1.708463165	0.907142949	1.696801763	0.801892857	0.930722958	0.862767966
Zm00001d015246_T012	0.439692279	0.191417363	0.305162736	0.736083549	0.749445022	0.470258841
Zm00001d053682_T002	2.931150693	4.250695363	2.812574825	2.340996009	2.543425355	2.605364661
Zm00001d053682_T001	3.59587634	2.870925905	3.507899145	3.535611443	3.538841942	3.636665566
Zm00001d040627_T001	1.129032378	0.690730856	1.162467868	1.285634807	1.351910396	1.24199024
Zm00001d044220_T009	0.215221334	0	0	0.208302494	0.412029231	0.268740986
Zm00001d044220_T006	2.17857379	1.605229286	2.374232131	2.38089454	2.188317361	2.14040043
Zm00001d005156_T005	0	0.07220049	0.280876756	0.424065351	0.311064776	0.309928196
Zm00001d005156_T002	2.545625441	1.704569232	2.383520169	2.515316582	2.444885681	2.535903648
Zm00001d003889_T001	3.242619577	2.613578092	3.325515509	3.081827375	2.960754672	3.072547976
Zm00001d003889_T002	3.647316582	4.791068625	3.51022773	2.556345537	3.154576242	2.976109171

Zm00001d025075_T002	0.731204969	0.25977866	0.652010069	0.925650852	0.801541116	0.896011384
Zm00001d025075_T004	0.766880916	0.827503629	1.080644021	0.907526011	1.594749175	1.400353756
Zm00001d025075_T003	4.191132194	3.710866482	4.474087561	4.359282412	4.041310278	4.100201949
Zm00001d025075_T001	1.612330793	1.636667306	0.479756211	1.172989235	0.774472083	1.352103119
Zm00001d017268_T001	2.94528557	2.68527907	2.594649105	3.287042851	3.685332003	3.566026143
Zm00001d017390_T001	3.124289255	2.876887015	3.314956187	3.498107485	3.5256645	3.615130108
Zm00001d048312_T001	1.657963718	1.260273212	2.133185706	2.429547142	2.565972666	2.428100841
Zm00001d013089_T001	2.030984704	5.248504172	3.398712504	2.478795048	3.382447028	2.509060612
Zm00001d013089_T002	6.393386682	6.244278275	6.284461386	7.097359114	7.01440388	7.013471249
Zm00001d003757_T001	3.079329636	3.484408724	2.998123637	2.381389425	2.788951259	2.387653422
Zm00001d050068_T001	3.635895404	4.015146862	3.822129087	3.30316877	3.535980764	3.302747482
Zm00001d027835_T001	0.940388542	0.60373339	0.993832298	2.27311152	2.329412681	2.415965755
Zm00001d012546_T003	5.31829558	5.195285109	5.713400153	6.059932668	5.780279675	5.958347435
Zm00001d026513_T001	3.77120209	3.937694476	4.026983173	4.572689011	4.626539479	4.629478851
Zm00001d045763_T002	3.277198785	2.513212033	3.427110932	4.410383508	4.461162797	4.301149724
Zm00001d012836_T001	4.491709012	4.153814008	4.635173018	3.210488615	3.321171636	2.993999814
Zm00001d029009_T002	2.617373719	1.824769075	2.454721803	2.781223097	3.053704284	2.766187842
Zm00001d029009_T004	0	0.335322033	0	0.70087113	0	0.96847714
Zm00001d009879_T002	2.511025953	2.052088573	2.46982634	2.640567023	2.559935199	2.699073172
Zm00001d036610_T001	1.518212826	1.458569416	1.803373365	4.553923647	4.698531213	4.737946834
Zm00001d053881_T001	5.212124323	4.236276083	5.184904216	5.4460596	5.520000065	5.486821316
Zm00001d014058_T006	2.96085418	2.788666502	2.696546073	2.729253868	3.130953103	2.252633948
Zm00001d014058_T007	3.664254813	0	4.3083367	3.164991696	4.102795928	3.532233039
Zm00001d014058_T008	1.1151831	0.724784713	1.197037522	0.198662615	1.083162979	0
Zm00001d014058_T001	1.907718657	3.491885541	0.928042043	2.682674417	0.944210024	2.728121438
Zm00001d014058_T005	4.013309049	2.528591571	4.042832716	3.475401151	2.578796972	3.318439339
Zm00001d036315_T001	1.602691087	1.265264651	2.056884868	2.543474602	2.450052534	2.51761434
Zm00001d047868_T001	4.192896243	3.521108544	4.307974387	3.918786164	3.999689336	4.049852208
Zm00001d002798_T001	7.444374992	8.675064787	7.555222179	5.715386856	5.840271718	5.729843265
Zm00001d029543_T001	9.096352448	9.449235256	9.153757381	8.187062624	8.252745403	8.218381112
Zm00001d007508_T009	4.383982576	5.948629369	4.820016759	3.959899491	4.140740053	3.632292183
Zm00001d007508_T008	0.534408286	0.245848535	0.498692035	0.242190292	0	0.406075108

Zm00001d007508_T007	0.582285262	4.070834004	0	0	0	0.80538079
Zm00001d007508_T006	4.965545078	0	5.114259777	5.039936613	5.054532907	4.842034236
Zm00001d017199_T001	2.916398106	2.879571684	3.090301138	3.899714317	3.39004318	3.802289704
Zm00001d038004_T001	5.026429912	5.146550511	5.044807982	4.990927685	5.038084859	4.988188132
Zm00001d015714_T006	0.998402052	0.895012502	1.17835197	0	1.763532254	1.848910725
Zm00001d015714_T002	3.170313996	2.232947732	0	3.505665491	3.274527659	3.412893685
Zm00001d051251_T003	4.816770741	4.388269662	4.839558005	5.008843924	5.1077031	4.943705891
Zm00001d031937_T002	5.34358026	5.3125497	5.515955622	5.354961697	5.194904563	5.03850655
Zm00001d012426_T001	1.552330341	1.297491372	1.745227865	2.344266077	2.352352887	2.666273265
Zm00001d020973_T003	4.301458662	3.40188682	4.043197585	3.728187628	3.645678144	3.848087773
Zm00001d020973_T004	0.558456715	0.288141182	0.506415081	0.475545641	0.063204708	0.430913605
Zm00001d020973_T001	1.195250575	1.107335019	1.728065043	0	0	0
Zm00001d033062_T001	1.722295514	1.125045334	2.090139696	0.708540416	0.927122006	0.873819497
Zm00001d035760_T001	5.271456043	5.076177849	4.314885949	4.987059047	4.426885249	4.642180999
Zm00001d008597_T004	1.275739536	1.130808993	0.937877676	1.555046512	1.079625238	0.85600245
Zm00001d020429_T001	3.199141021	2.728322176	3.461265168	6.027792146	5.997565609	5.956138359
Zm00001d038840_T001	6.542140818	6.049881541	6.568504401	6.399807825	6.454671208	6.355056783
Zm00001d010824_T001	6.10396107	5.798330159	5.940118793	6.26023668	6.268149244	6.198772765
Zm00001d022275_T003	4.8137321	5.698940784	4.774444408	3.796293631	3.575506694	4.403683767
Zm00001d022275_T001	5.381071383	4.391388647	5.383841506	5.641280962	5.691612188	5.629750874
Zm00001d000419_T001	2.80083303	2.712561226	2.714889503	3.229781397	3.622292005	3.385811058
Zm00001d024058_T001	3.733331995	3.560816306	3.881644065	3.998086349	4.019290305	4.173822383
Zm00001d016744_T001	3.244202225	2.823945782	3.326208563	3.312956268	3.367001677	3.097745622
Zm00001d026040_T001	4.159103336	3.481157998	4.32149551	2.309432333	2.281829385	2.301329811
Zm00001d051568_T001	3.236647059	2.454495462	3.358129249	2.920323221	2.891188959	2.843830123
Zm00001d002252_T001	3.363790784	2.669918194	3.507462306	3.923993913	3.819973957	3.882665448
Zm00001d001781_T001	2.536198157	2.054229947	2.812990814	2.507131165	2.43951543	2.335319746
Zm00001d014897_T001	5.560714618	4.474884617	5.495025427	5.737628117	5.669980903	5.641662058
Zm00001d014897_T003	6.075127062	6.152557108	6.150886622	4.640314357	5.138479267	4.57126548
Zm00001d035772_T006	0	0.329890533	0	1.064472466	0.468445688	0.878316504
Zm00001d035772_T005	0.94450616	0	0.796581143	0	0.557773751	0
Zm00001d035772_T001	0.879203088	2.428872936	0.980984584	1.763175285	1.468360184	1.188457264

Zm00001d035772_T004	3.994616832	2.030203634	3.799261725	4.006201153	4.094700479	4.231944527
Zm00001d035772_T007	0.198730497	0.514155596	0.899296301	0.828206671	0.664671243	0.72422242
Zm00001d035772_T002	4.268090373	3.210711471	4.173390198	4.473261745	4.639316048	4.479990971
Zm00001d035772_T003	5.211357207	5.92082185	5.45075462	4.989551543	4.843328605	4.9085632
Zm00001d027394_T001	7.50702693	8.803586128	7.440893297	6.915969283	7.028622667	6.89286904
Zm00001d039452_T003	2.374285557	2.025373653	2.789650595	2.691464708	2.714498091	2.53760371
Zm00001d039452_T001	0.468242347	0.112658769	0.71936571	0.705248078	0.654459372	0.46171504
Zm00001d039452_T002	2.98570243	1.798900324	2.584112744	2.892432023	2.665769769	3.042028609
Zm00001d015200_T002	3.238059368	2.592595529	3.494507648	3.158087507	3.292905109	3.338497296
Zm00001d042583_T001	2.69112028	2.461271062	2.65061362	2.8518036	2.926690038	2.698647888
Zm00001d022347_T001	2.982458823	2.434912936	3.026873166	2.952247636	2.8360647	2.855608449
Zm00001d011970_T001	4.74674105	4.120644066	4.94195909	4.877913781	4.859244541	4.896506934
Zm00001d028102_T001	5.29982124	4.718700106	5.180633678	5.217215746	5.170340237	5.12753828
Zm00001d047492_T001	6.829757359	6.293776259	6.887621114	5.238983383	5.327061438	5.311171721
Zm00001d013440_T001	6.76271254	6.683310763	6.778251833	6.413358302	6.436867936	6.423339478
Zm00001d051480_T001	2.502795978	2.030431431	2.789698791	2.12306125	2.060857072	2.069546135
Zm00001d051839_T003	0.877961717	0	0.3131936	0.222532562	1.06882281	0
Zm00001d051839_T009	0	0	0.80980789	0	1.26071702	0.850055285
Zm00001d051839_T015	1.821529816	0	3.104431438	2.46759303	2.672847566	2.333576296
Zm00001d051839_T002	0	0.435175181	0	1.650551815	0.930806964	1.053655634
Zm00001d051839_T006	2.682515768	2.165615171	0	2.046521094	2.177252564	1.87350022
Zm00001d036573_T004	0.336906041	0.525293155	1.058038668	1.728065043	2.169817276	1.941309176
Zm00001d036573_T002	4.387692087	3.808183409	4.374950433	4.139938557	4.150863049	4.28097316
Zm00001d036573_T005	0.154526179	0.12853493	0.885634492	0.956383081	1.096677462	0.663446641
Zm00001d036573_T001	1.57282517	0.717635849	1.436140513	1.627876676	1.053460322	0.930257432
Zm00001d010514_T001	1.280431386	0.674976711	1.561718135	1.342522888	1.580870028	1.402149132
Zm00001d037871_T005	4.713207173	3.954566896	4.82166624	4.896136681	4.700787366	4.90302067
Zm00001d037871_T006	0.903764064	0.483892633	0.480936155	0.488531462	0.544077793	1.007153871
Zm00001d005358_T001	4.953785735	3.953427941	4.922487881	4.722776171	4.757529221	4.737772574
Zm00001d012708_T004	2.414590465	1.871748254	2.8045637	2.607245318	2.485059408	2.516400235
Zm00001d012708_T003	1.661516579	1.411593285	1.460274431	2.286217592	1.722014784	2.225612424
Zm00001d027595_T001	7.482558449	7.174203196	7.4918807	6.815485181	7.044097222	6.979260289

Zm00001d049442_T013	1.985170321	1.19655354	2.016617906	2.382514538	2.598002104	2.646539414
Zm00001d049442_T010	0.931446307	0.383008037	1.1788319	1.464292386	1.360617112	1.033662623
Zm00001d049442_T005	2.115374228	1.425978603	1.993205523	2.178470533	2.039191046	2.149582927
Zm00001d037816_T002	2.107598493	1.161309844	1.825543055	0.675542267	0.795357184	1.044741723
Zm00001d043207_T002	4.862700779	4.389108601	4.755884968	4.59860758	4.845007884	4.635907532
Zm00001d043207_T003	1.103909827	1.569506647	1.895356915	1.717244962	1.100030337	0.731841864
Zm00001d024612_T013	0.382867529	0.312580375	0.75606597	0.593740988	0.284101876	0.353931862
Zm00001d024612_T019	1.127828047	0.437668791	1.058240982	1.213358481	1.319728329	1.862932573
Zm00001d024612_T014	0.168238949	0	1.071075475	0.91118305	0.993554811	0.588072482
Zm00001d024612_T038	0.336605601	0.244473062	0.500454337	0.845929968	0.697514075	0.828945106
Zm00001d024612_T047	1.140562072	0.716398335	0	0.63207274	0.790140616	1.413104978
Zm00001d024612_T066	1.315128545	0	0	1.623342491	1.012027964	0
Zm00001d024612_T041	0.26246924	0.146192495	0.526342297	0.650006256	0.543281072	0.383477037
Zm00001d024612_T039	0	0	0.685452218	0	1.197174695	1.249723234
Zm00001d024612_T023	2.519216544	1.971559004	1.573375981	2.23380617	2.500627696	2.29858178
Zm00001d024612_T004	0	0.912113305	0.942168405	0.91592239	0.709697357	0.640872614
Zm00001d024612_T031	0	0	1.635919659	0	0.660755239	1.516431753
Zm00001d024612_T053	0.467993086	0.49306112	0.593848051	1.103549337	0.60135232	0.838583132
Zm00001d024612_T020	0.35696073	0	0.8605918	1.020178583	1.029314352	1.066823529
Zm00001d024612_T067	1.083019294	1.318020606	1.177884634	0.930536765	0.254814119	0
Zm00001d024612_T015	1.921000943	0.518340752	2.331903523	2.58194098	2.280226767	1.816060617
Zm00001d024612_T069	4.237836263	4.782506384	4.522609714	3.402943148	3.37477103	2.572552596
Zm00001d024612_T018	0.426318435	0	0	0.47497486	0.610356117	0.523718511
Zm00001d024612_T007	1.326262359	0.410467023	0.918251119	1.305219366	0.544007541	0.451055457
Zm00001d024612_T029	0.392772418	0.386414772	0.852796295	1.333978378	1.001718668	0.856543554
Zm00001d048194_T002	3.899487246	3.732664285	3.727161779	3.999389701	3.719942381	3.874889602
Zm00001d018719_T001	4.23592936	3.640771182	4.414691932	4.36884523	4.26859159	4.396351941
Zm00001d025689_T006	2.590703808	2.135013711	3.695764923	0	0	0
Zm00001d025689_T001	0	0	2.121620543	0	0.929374411	1.782378369
Zm00001d025689_T003	3.923673572	3.07195672	3.619670536	4.134631754	4.096932942	4.138030093
Zm00001d051502_T004	3.043713181	2.387629161	3.161373217	3.721258464	3.776820208	3.45807511
Zm00001d051502_T006	1.290258066	0.403864313	0	1.639361375	1.427028979	1.605553121

Zm00001d051502_T001	1.997603136	1.299445718	2.042591111	2.681780271	2.39149499	2.599476101
Zm00001d051502_T005	0.279634128	0.017718135	0.128946624	0.848496954	0.974057147	1.053634088
Zm00001d032275_T001	1.60302594	1.619686263	2.067588469	1.795576626	1.649602124	1.715149214
Zm00001d015127_T001	5.881625664	6.072631126	5.982637293	5.131053189	5.014271639	5.129082716
Zm00001d037346_T016	1.339116856	0.907855916	0	3.083730773	2.209394721	1.360045078
Zm00001d037346_T030	0	0	0.85992583	3.086224138	0	3.137442044
Zm00001d037346_T043	1.787721643	1.008074488	1.314143121	2.228622932	2.46734157	2.744746453
Zm00001d037346_T017	1.373832486	0	1.145905008	0.88814043	2.414217816	1.862602547
Zm00001d037346_T024	0	1.054215705	0	3.268197518	1.839439237	2.823971657
Zm00001d037346_T041	0.58940972	0.080759979	1.051731947	1.968598418	1.31216386	0.522846202
Zm00001d037346_T009	0.527160439	0.215935743	0.531211259	0.933268961	0.963623565	1.124472397
Zm00001d037346_T047	6.28790728	5.48225676	6.342605607	5.785053657	5.873442734	5.755577907
Zm00001d037346_T045	2.756611507	1.391426249	2.787537152	4.465704244	4.272635906	4.542470166
Zm00001d037346_T003	1.434184643	0.873602191	0.490772402	0	2.600223122	0.998644673
Zm00001d037346_T014	2.220840999	1.628200135	2.699116937	0	0	0
Zm00001d037346_T040	0.228956567	0.164062819	0.078352968	0	0.482514799	0.220088454
Zm00001d037346_T028	0.142304946	0	1.273932973	0.55705026	0.931609691	0.350260739
Zm00001d037346_T039	1.333340719	0	0	0	2.653315505	1.464520854
Zm00001d011845_T001	3.439674595	3.481177506	3.508790042	3.448886683	3.260668401	3.28378668
Zm00001d002366_T007	0.217697267	0.178207259	0.314613006	1.344819409	1.523690915	1.530528256
Zm00001d002366_T004	6.486939704	5.949172416	6.515786555	6.343353756	6.373575675	6.337763926
Zm00001d002366_T003	2.210592723	1.281643063	2.083900932	2.692878416	2.885980956	2.498458958
Zm00001d002366_T008	0.503638772	0	0.52203867	0.568167366	0.57212229	0.627791715
Zm00001d002366_T006	3.050989711	1.347923561	0	1.331847931	0	1.369991817
Zm00001d014471_T001	0.899009305	0.487064387	0.772721804	1.104088361	0.949781431	1.106015919
Zm00001d010162_T001	2.87070915	2.505042335	3.621418476	3.540442704	3.660783528	2.965582432
Zm00001d006930_T001	6.002766059	6.390217908	5.98451195	5.502664947	5.585638335	5.461233403
Zm00001d021653_T001	1.842431006	1.425639765	2.323028451	4.97918609	5.227683362	5.154770363
Zm00001d021653_T002	0.686577618	0.394977223	0.302855958	3.326289256	3.120877426	3.189559079
Zm00001d024333_T001	5.74472665	5.607586691	5.877977884	6.155874706	6.081922281	6.073928046
Zm00001d024333_T003	4.620474568	5.670741867	3.925407792	4.88645016	5.054293384	5.182607886
Zm00001d051174_T003	1.99052834	1.431668419	1.025361958	1.900265556	1.99014382	0.903913651

Zm00001d051174_T002	6.366403877	5.928723136	6.557458815	6.638790183	6.732885633	6.714732846
Zm00001d021567_T001	4.66815425	3.980389676	4.763934961	5.485175912	5.54357943	5.541833073
Zm00001d023620_T001	4.508149912	4.452462004	4.540567807	3.432781865	3.618218096	3.644704361
Zm00001d051424_T001	1.983795878	1.818079936	1.890536579	2.637831166	2.035668597	2.594809835
Zm00001d016866_T002	3.678295167	3.070314271	3.892567927	4.506569446	4.508877872	4.612653982
Zm00001d016866_T001	1.523745606	1.238162576	1.817810284	2.036539555	1.896799997	2.339613453
Zm00001d039899_T001	5.09807311	4.668504204	5.18108227	5.079334202	4.966058948	4.867257831
Zm00001d005756_T014	3.166353923	2.176518736	3.243510668	3.068332537	2.410229017	2.545198397
Zm00001d005756_T003	1.102156208	1.04744067	1.162839064	2.184457089	1.491317982	2.19256442
Zm00001d005756_T001	0.222287719	0.127194792	0	0.41065588	0.844011171	0.51699141
Zm00001d004123_T003	1.810659852	2.292054919	1.978393346	2.464010018	2.784500214	2.797130672
Zm00001d004123_T001	5.307753459	4.31078893	5.2719285	5.49822661	5.5158855	5.442074865
Zm00001d030347_T001	3.288810496	2.662740087	3.072311983	3.487058854	3.577319693	3.433060392
Zm00001d029447_T001	7.51046946	7.294198648	7.437880904	3.782983641	4.123708404	4.1747446
Zm00001d035047_T002	2.392371832	1.670587026	2.848826819	2.160440716	2.202759351	1.639923472
Zm00001d035047_T001	4.064845353	3.948969884	4.08264801	3.828263652	3.618809746	3.810179734
Zm00001d035047_T003	3.941612574	0.187801946	3.863014771	2.314927048	2.42884132	2.251854192
Zm00001d002065_T001	2.19912924	1.431850778	2.5329879	1.962441303	2.102852269	1.946300046
Zm00001d002065_T006	7.896348154	6.849361399	7.998908194	7.947339199	8.014170229	7.984938493
Zm00001d002065_T004	2.91161911	2.419711196	2.832668075	1.188962321	1.709458279	0.975055635
Zm00001d002065_T012	7.871894053	8.575822248	7.882097993	7.299863174	7.321364071	7.101814038
Zm00001d002065_T008	0.796532138	0	1.203712979	1.540187505	0	0.807021827
Zm00001d026239_T007	3.143185392	2.104358803	2.989309432	3.685579822	3.725868082	3.388543898
Zm00001d026239_T008	1.55020229	0.841188981	1.350856462	1.322614078	0.941930365	1.214260363
Zm00001d026239_T001	1.164853199	1.546208963	1.180884301	0.723216031	1.200008974	0.968809616
Zm00001d029223_T003	0.204849367	0.323228243	0.68547913	0.646836372	0.72080991	0.248579768
Zm00001d029223_T001	0	1.02215046	0	1.636448296	1.743458095	2.330054373
Zm00001d029223_T015	2.201547611	0	2.673092561	2.390754894	2.65831501	2.406204937
Zm00001d005099_T003	2.1202952	1.511671955	2.082887853	2.3293923	2.361767237	2.218471211
Zm00001d005099_T001	0.108812131	0.287439197	0.233470894	0.776192443	0.561014222	0.087981439
Zm00001d013046_T001	3.375430957	3.673418819	3.135281703	5.578004643	5.363196208	5.419859957
Zm00001d013046_T004	2.981183928	0	2.936634359	5.082519857	4.789244255	4.666154965

Zm00001d013046_T005	0	0.044574576	0	1.113299769	1.418540795	1.378216395
Zm00001d013046_T006	3.842374583	2.094010694	3.980824778	3.93946638	4.847307354	4.632722884
Zm00001d033551_T047	0.473167628	0.131440664	0.267023948	0.496398944	0.535039673	0.335307167
Zm00001d033551_T033	0	0	0.621289055	1.000979978	0.936146797	0.479260574
Zm00001d043971_T001	7.038348129	6.960764493	7.170409205	7.153128853	7.152456586	7.167583351
Zm00001d051380_T001	4.261989276	3.91698064	4.116261442	3.438656674	3.468520369	3.112650597
Zm00001d048588_T001	1.37820474	0.858841977	1.548209721	2.036347187	1.760624632	2.161488508
Zm00001d034166_T023	2.782604616	1.394431277	2.607763974	2.665407325	2.2209227	2.405730201
Zm00001d034166_T007	0.750714542	0.579210083	1.305585362	0.959783507	1.185239867	1.316677529
Zm00001d034166_T008	2.378160895	1.549390215	2.548532801	2.024611877	2.02772415	2.537507299
Zm00001d051592_T001	1.155127471	1.106407956	0.779114288	1.544198995	1.689927547	1.054920697
Zm00001d033589_T001	3.937756613	4.628582195	4.122050951	3.471233381	3.712182391	3.579248708
Zm00001d002596_T001	5.358667375	5.01858041	5.254771119	4.011211688	4.684559624	3.558833334
Zm00001d036789_T003	2.36138518	1.556226823	2.218078089	3.076853043	2.987765068	2.939501172
Zm00001d036789_T005	2.970387095	2.908890323	3.238420026	3.175882715	3.513234524	2.64691446
Zm00001d022569_T001	8.202188178	9.180898865	8.220863698	8.237561414	8.30769106	8.34439561
Zm00001d005392_T002	4.460343442	3.734308156	4.410546666	3.317566734	3.233432085	3.147296766
Zm00001d005392_T001	0.636593533	1.599610112	0.725555328	1.068356444	0.340753666	0.407012229
Zm00001d047583_T002	3.199368307	2.3522605	2.977330852	3.522188107	3.529739629	3.799720212
Zm00001d047583_T003	0.275165616	0.283986944	0.685410054	0.943427032	1.01146272	0.970308861
Zm00001d047583_T001	1.643622108	2.025166677	2.159412184	1.863421128	2.062107547	1.485380454
Zm00001d033158_T001	0.6783514	0	0.582270808	0.382838761	0.12861807	0
Zm00001d033158_T004	1.545876468	0	2.024762208	1.840530508	1.463815466	1.627806187
Zm00001d033158_T005	2.208290319	3.135516155	2.5377585	0.670039926	1.173348779	0
Zm00001d033158_T009	1.463452441	1.631750614	2.21054441	0.829552468	0.422420324	0.562528187
Zm00001d033158_T006	2.545578987	0.185068562	1.923742874	0.737444489	0.948095442	1.062657786
Zm00001d033158_T007	0.406939328	0.222790962	0.245792568	0.747577312	0	0.486998508
Zm00001d033158_T011	4.022773575	2.136943059	3.851393957	1.167307182	1.854329244	1.872866968
Zm00001d000035_T001	2.467773512	1.783197218	1.83777374	2.184840757	2.131779439	2.199386211
Zm00001d040052_T001	0.973049149	0.460563297	0.811112573	1.006798526	0.906229497	0.798061301
Zm00001d007067_T002	4.201099024	3.00070134	3.855020734	3.33433944	3.125474886	3.728801155
Zm00001d007067_T003	3.908347559	3.571568695	4.296147543	4.649507339	4.915988434	4.51638063

Zm00001d007067_T001	2.392972944	1.656797921	2.193812422	2.231934315	2.245604272	2.20197066
Zm00001d045078_T001	2.053213886	1.671002992	2.053888097	1.026371613	1.57437815	1.567009935
Zm00001d001962_T001	0.036528653	0.039774303	0.178459697	0.343290696	0.404323312	0.407821517
Zm00001d001962_T014	3.496077401	3.12777061	3.349684724	3.71133499	3.947227765	4.001294238
Zm00001d001962_T013	0.250056878	0.081661403	0.082124851	0.750356959	0.82108566	0.427083705
Zm00001d001962_T020	0.345474725	0	0.199312392	0.986142214	0	0.841964266
Zm00001d001962_T017	2.831405046	0	2.892314663	3.557750964	2.443653591	2.502303611
Zm00001d001962_T031	4.831979158	4.039466649	4.726638554	3.549241227	4.112992478	4.160688942
Zm00001d042837_T001	3.734308156	3.153867257	3.831418626	2.086418971	2.049183951	2.044807458
Zm00001d038920_T001	2.182963016	1.542888487	1.940147201	1.381982675	1.408315677	1.499253001
Zm00001d007241_T001	2.869623107	2.082815657	2.995350826	3.554307038	3.665158632	3.388531913
Zm00001d007241_T003	1.57980769	0.619273078	1.061243944	2.588335423	2.251666382	2.644119775
Zm00001d021031_T002	2.627620611	2.998972255	2.196171715	1.295747706	2.403530874	1.403708372
Zm00001d021031_T001	4.394687609	3.550645585	4.353096494	4.736860111	4.575542575	4.766768678
Zm00001d021031_T006	1.708598241	0.777638115	1.435694727	1.356959604	1.536140952	1.326238194
Zm00001d021031_T003	0.873513213	0.990059567	1.626960048	1.772704493	1.92324466	1.966427514
Zm00001d029203_T002	2.796499744	2.148237149	2.878802623	2.559472969	2.185995589	1.373530731
Zm00001d029203_T001	2.97079532	2.916681282	3.498064326	1.802523706	2.108500035	2.954763783
Zm00001d047876_T001	4.189003056	3.70417025	4.143569418	3.841791111	3.722282944	3.769435776
Zm00001d047876_T003	1.28989229	0.686148186	1.133547744	1.206631347	1.240156769	1.318647719
Zm00001d053358_T001	3.420285933	2.729371131	3.098695781	2.702474923	2.755467029	3.068471846
Zm00001d051865_T001	2.78154101	2.692852534	2.844025637	1.833244317	1.639432691	1.635580282
Zm00001d006670_T002	4.473667245	4.070049568	4.45437017	5.169426665	4.939536902	4.980682911
Zm00001d015956_T001	6.356438465	5.888287675	6.391353879	6.161473594	6.0626498	6.002953305
Zm00001d015956_T003	0.770048406	0.410509355	0.726202571	0.85717047	0.93364817	0.59644669
Zm00001d018554_T001	2.63921549	2.566943226	2.828516023	2.689737047	3.078911219	2.832863894
Zm00001d050382_T005	4.488172868	1.638120188	4.371795599	3.502121286	3.2070715	2.856779177
Zm00001d050382_T010	0.608777077	0.153257981	0.426223956	0.562482273	0.583212894	0.511670438
Zm00001d050382_T007	1.486529552	1.563440783	1.303103289	1.549266491	1.717598563	1.684463742
Zm00001d050382_T001	2.982939743	3.323987082	2.984079806	3.074509375	3.052994873	3.429366634
Zm00001d043990_T001	2.339792721	2.5231981	2.729492516	1.967029866	2.377948307	1.841684149
Zm00001d007111_T001	3.568753029	2.836435595	3.603796046	3.744813929	3.75274506	3.700223853

Zm00001d003725_T001	7.747657557	7.723597823	7.821625515	7.267160562	7.379548456	7.326077804
Zm00001d043516_T001	2.696631308	2.051599713	2.978964557	2.847127421	2.822817217	2.775797514
Zm00001d020644_T001	4.321402137	4.36254381	4.432714729	4.296587061	4.1569351	4.575953957
Zm00001d051980_T001	0	0	1.431698368	2.691963336	2.854884736	3.464184935
Zm00001d051980_T004	3.009621817	3.598728454	3.027714067	3.210179833	3.445030875	3.289296083
Zm00001d051980_T010	5.717577371	4.286327245	5.779506523	5.982539163	5.95458546	5.907143334
Zm00001d051980_T006	1.893352502	0.193684706	1.816008992	3.394198158	2.995940863	3.032336805
Zm00001d051980_T003	0.19444642	0.183335329	0	0.452549048	0.142678752	0.600755963
Zm00001d042461_T001	3.562156929	2.934255302	3.713408567	5.408231936	5.428095748	5.416178152
Zm00001d011874_T001	2.345555341	1.626028816	2.16091793	4.839208173	4.711338239	4.880947302
Zm00001d014565_T001	4.273508803	4.03717371	4.502098303	4.256355145	3.906246144	3.657097006
Zm00001d021214_T001	3.025512034	4.01600907	2.8014929	5.498423438	5.607508368	5.767720258
Zm00001d011165_T002	1.333241668	0.987323049	1.5754077	1.337124762	1.317348066	1.262240104
Zm00001d048017_T006	2.759988606	1.944076181	2.432356823	3.279100545	3.229752639	3.070994446
Zm00001d048017_T004	1.104794894	1.66877223	2.335063295	1.611346626	1.843785309	1.889101626
Zm00001d050810_T001	5.736418709	4.823938498	5.651696697	5.43583735	5.400225448	5.424916178
Zm00001d012818_T001	1.325660996	1.886522834	1.452694528	1.490494657	1.564937089	1.994922527
Zm00001d036080_T002	3.613307452	4.522383671	3.910960079	3.921287026	3.992084257	3.750968419
Zm00001d036080_T001	2.135301406	2.384740587	1.911057232	1.666138941	1.470739362	2.268201412
Zm00001d015101_T001	0.799781954	0.759911719	1.06472147	0.537977862	0.859334305	1.008818876
Zm00001d015101_T002	3.879271814	4.438158291	4.201222085	4.58881296	4.347818279	4.539087947
Zm00001d032357_T001	5.151180365	4.483474713	4.87529956	5.15375711	5.017881343	4.956999627
Zm00001d020751_T001	1.563504726	1.265424293	1.812619386	3.991597578	3.966680647	3.748231822
Zm00001d034054_T001	0.152881716	0.204900687	0.272917801	0.399081384	0.342836885	0.719314011
Zm00001d034054_T008	2.562756512	3.015331639	2.017354693	1.585886011	1.803961425	1.892324962
Zm00001d034054_T006	2.034701698	1.384190753	1.883172299	2.331994644	2.399874356	2.14465759
Zm00001d002545_T004	2.917315266	0	2.802650257	1.238810701	0.768411326	2.217487276
Zm00001d002545_T001	2.338339405	2.740326186	2.473768732	1.282178228	1.775485076	0.750737692
Zm00001d002545_T002	1.489342034	1.122160479	1.63358791	1.387305185	1.242989611	0.590771583
Zm00001d031560_T002	6.018015012	5.188666223	6.096731956	5.420602723	5.56707369	5.752793033
Zm00001d031560_T003	3.32088205	3.199654129	2.097513378	3.332196348	2.212897371	0
Zm00001d011632_T001	0.882803449	1.180485894	0.6284274	1.237603487	1.031218731	1.340067139

Zm00001d011632_T002	1.955543066	1.404716235	2.457490544	2.622648576	3.255664028	3.010171846
Zm00001d011632_T003	3.199185162	2.673889924	3.012232184	3.090297242	1.759338237	2.582135658
Zm00001d034521_T001	4.03924317	4.857655556	4.056648726	4.291720389	4.551999993	4.516798643
Zm00001d035089_T003	0.036189617	0.032770888	0.105328088	0.777641482	0.815199951	0.885458007
Zm00001d035089_T005	0.38338524	0.215769286	0.317294805	1.409727096	1.610539439	1.552192603
Zm00001d050658_T001	2.233004507	3.040531449	2.587574779	2.794148618	2.511533063	2.898393733
Zm00001d051645_T001	3.039244223	2.973655355	2.997607291	2.21105457	1.926247693	2.521504838
Zm00001d043785_T002	2.519861154	1.955793746	2.510085406	2.629472964	2.519257313	2.65735784
Zm00001d043785_T003	0.751013746	0.590178502	1.133813378	1.193140288	1.646957994	1.248438289
Zm00001d043785_T006	1.110897055	0.542434382	1.053144709	2.061618961	1.58748405	1.915749572
Zm00001d028631_T002	5.206238503	4.507675098	5.237423602	5.061356113	5.20641212	5.218538942
Zm00001d033858_T001	1.115478105	0.494643966	0.916415496	1.315875719	1.85116455	1.439342283
Zm00001d033858_T002	3.062493393	2.532236904	2.986805188	3.273400857	3.094946262	3.091975993
Zm00001d027485_T001	1.884609642	2.203913086	1.545547357	1.171854998	1.493374754	0.821278362
Zm00001d028226_T001	4.267226926	4.104641523	4.550719455	3.510672465	3.41242521	3.366705483
Zm00001d011576_T001	1.900367585	2.823996513	1.998250393	2.419038581	2.309572903	2.693132973
Zm00001d048385_T001	4.185209494	3.804747782	4.435063939	4.195424141	4.000112436	4.114210752
Zm00001d048385_T002	0.880870429	0.739719377	1.13196476	1.576798799	1.343111009	1.347196208
Zm00001d023807_T002	4.477696041	3.469264233	4.352933132	4.465764638	4.478937258	4.333327336
Zm00001d012448_T001	4.109648067	5.066327452	4.086410478	4.61081485	4.676817455	4.332566521
Zm00001d028619_T003	0.108421415	0.15309062	0.180919299	1.411562373	1.410329164	1.100992445
Zm00001d028619_T017	3.525057444	3.309121896	3.497601253	4.154354752	3.643415235	3.903863341
Zm00001d028619_T015	2.558865653	1.785248381	2.219178745	3.023927034	3.275184542	3.1393616
Zm00001d028619_T004	0	0.508405326	0	1.766052326	0	1.628635034
Zm00001d028619_T001	2.907037358	2.538630781	3.156334804	3.536046309	3.371702846	3.113280096
Zm00001d028619_T013	1.01765543	0	0.299476783	0.928417322	1.936786794	0
Zm00001d028619_T002	2.335832225	1.377454184	2.348074598	3.148688159	3.235999186	3.293368424
Zm00001d043904_T001	5.803764769	5.660200972	6.030393114	5.552188052	5.583645832	5.584724767
Zm00001d014204_T001	2.782402693	2.361236373	2.873282276	2.380978464	2.44347484	2.439163263
Zm00001d033541_T002	0.322930708	0.111277084	0.1914679	0.286734506	0.226010282	0.290668568
Zm00001d016235_T001	1.032226412	0.91194463	1.702291394	2.119324633	2.441107087	2.201589952
Zm00001d023579_T001	4.580958761	3.969697747	4.594557328	5.316453332	5.346808181	5.115633658

Zm00001d023579_T002	3.253949796	2.567495556	3.171508692	3.243271341	3.037601158	3.20304983
Zm00001d053109_T001	0.505000654	0.146650009	0.525961607	0	0.410117464	0.225878292
Zm00001d022229_T001	1.511252454	0.991524567	0.760678278	1.144565255	1.113447805	1.467428699
Zm00001d002199_T002	0.770537307	0.565563058	0.676067873	1.367355408	1.205540153	1.199178564
Zm00001d024424_T001	0.497713523	0.342148504	0.581013735	0.812518762	0.775131473	0.843798171
Zm00001d025907_T001	1.260534576	1.167612914	1.580194217	1.338160241	1.302365263	1.31895886
Zm00001d053162_T003	4.433678639	4.080662268	4.585193854	4.842799766	4.957760805	4.862819234
Zm00001d010213_T003	2.285482416	2.136699653	2.401330648	3.03198373	2.647698563	3.188230944
Zm00001d010213_T002	3.888950592	2.977857802	4.184126097	4.073202158	4.484529372	4.613347945
Zm00001d010213_T004	4.105797823	4.755887745	3.890575975	3.853958631	0	2.988424751
Zm00001d010213_T005	0	0	0	2.779089277	3.020135725	1.467363483
Zm00001d010213_T001	4.564936907	4.065326471	4.504396726	4.843894175	5.265327782	4.926973383
Zm00001d054093_T006	4.22474408	4.366983429	4.557721193	3.5489702	3.965902927	3.836910993
Zm00001d022307_T002	5.526661297	5.446284289	5.719562687	4.869984341	4.919307996	4.911768316
Zm00001d049364_T001	4.337456707	4.476381623	4.190395595	4.981351504	5.22518742	5.292057128
Zm00001d028590_T001	4.908475343	5.135153255	4.963569329	4.481354747	4.611073029	4.550723025
Zm00001d048281_T001	0.703386906	1.033313749	0.830265873	2.23037181	2.130672611	2.103488576
Zm00001d006064_T001	6.189832306	6.318220568	6.104802064	5.801717877	5.588558891	5.638568469
Zm00001d012982_T001	0.867928084	0.63844831	0.966773749	0.8772456	0.996192035	1.165963377
Zm00001d047842_T001	1.128076254	1.420088898	1.55631169	2.694594076	2.687456031	2.693250977
Zm00001d047842_T006	3.86389287	3.945652646	4.330044904	0	2.423698368	3.453929719
Zm00001d047842_T007	2.660264419	0	0	3.566378627	2.905900952	0
Zm00001d047842_T004	2.844874504	1.714448635	2.412785047	2.977765137	3.190600481	3.150333507
Zm00001d033334_T001	0	1.205229841	1.628247271	1.608786065	1.759734927	1.747100336
Zm00001d033334_T008	0.161860601	0.084363661	0.737900446	0.549455276	0.861361495	0.568939768
Zm00001d033334_T010	1.956843971	0	0	1.199620839	0.956275271	0.593488592
Zm00001d006364_T001	1.500255969	0.817937547	1.214592362	0.530223596	0.310388985	0.482096544
Zm00001d043871_T001	0.667244527	0.258826426	0.916507982	0.489613836	0.522503759	0.428833818
Zm00001d046060_T005	1.049632859	0.811749678	1.210722379	1.129525699	1.295797655	1.717548117
Zm00001d046060_T001	2.345840868	1.456608521	2.12760951	2.777685031	2.852519472	2.556347989
Zm00001d005203_T001	4.725793444	5.00022928	4.858470085	4.083779441	4.056820201	4.076274752
Zm00001d051875_T001	3.388860572	2.849782139	3.617517128	3.446600318	3.310529753	3.360345734

Zm00001d035014_T001	4.302091843	4.086378206	4.346519761	4.123883511	4.091880223	4.219401347
Zm00001d032902_T001	5.621653083	4.864444444	5.629128219	4.967634272	4.954694652	5.024817515
Zm00001d017791_T003	2.084000975	0.612671437	2.860666285	2.657902913	3.360033416	2.954020942
Zm00001d017791_T001	2.628409435	2.413634969	1.309551076	3.339021337	2.86958501	3.241654268
Zm00001d046033_T001	2.327839673	1.686032514	2.310067263	2.646683869	2.611906291	2.656134336
Zm00001d011762_T001	1.264941109	0.720742505	1.325773231	1.32547104	1.37498659	1.288965046
Zm00001d035012_T001	3.900784228	3.294681763	3.928879748	3.753052617	4.027312258	4.048299581
Zm00001d045108_T001	4.652971767	4.647623971	4.763274363	4.646055891	4.535302566	4.518453623
Zm00001d032565_T001	4.447425662	3.907396316	4.481431669	3.998627073	4.033740139	3.90344238
Zm00001d030777_T002	1.009361506	0.591560703	1.022822301	1.530743976	1.708187678	1.732498883
Zm00001d045589_T001	3.152386583	2.671950249	3.368776868	2.000947539	1.957701428	2.100743905
Zm00001d053968_T004	0	0	1.711376403	1.120996828	1.150846238	1.649228696
Zm00001d053968_T001	0	2.688629774	0.872348897	0	0.564000555	0.692242386
Zm00001d053968_T007	2.741792702	0	2.163188262	3.825363745	2.604159828	2.6807958
Zm00001d053968_T006	3.596989988	2.398070123	3.771349379	2.319459842	3.807597687	3.470999729
Zm00001d031267_T001	3.329929558	2.774978247	3.305634534	3.673569765	3.577800406	3.610541801
Zm00001d037332_T003	0	0.047429502	0	0.633685084	0.455653635	0.368569459
Zm00001d037332_T007	0.773166748	0.263735145	0.556912971	0.892666909	0.870309477	0.590928676
Zm00001d037332_T005	2.664511512	2.90169814	3.056718775	0	0	0
Zm00001d037332_T008	3.223099838	1.26380544	2.715455225	3.968425806	3.85764018	3.794351191
Zm00001d037332_T001	1.073979915	0.591160451	1.155831467	2.571015858	2.912452894	2.929357935
Zm00001d037332_T004	0.5222195	0.67675665	0.824680698	1.06334831	1.091202209	0
Zm00001d049706_T018	0.456354087	0.073033219	0.381438433	0.17672616	0	0.444590743
Zm00001d049706_T003	0.586156558	0	0	0.271970618	1.183543681	1.138755502
Zm00001d049706_T028	2.325074929	0	2.100973949	0	0.443159983	0.189694276
Zm00001d049706_T013	0	0	0	2.482878479	2.325286528	1.872070284
Zm00001d049706_T010	1.421087003	0.951276694	2.37020837	0	0	0
Zm00001d049706_T001	1.246492001	1.569037991	1.061290956	1.560371112	0.975294142	1.155771249
Zm00001d049706_T009	4.763557218	4.020505579	4.616891021	4.203796595	4.181866086	4.315141083
Zm00001d019641_T001	0.325450891	0.254038867	0.537345771	0.775538594	0.476360948	0.670527658
Zm00001d019641_T002	2.197571036	1.441923233	2.257273479	1.164243718	1.417045595	0.961808515
Zm00001d040986_T001	4.224872625	4.05212223	3.737042271	3.806427693	3.665756696	3.886833196

Zm00001d037109_T002	4.359575847	2.866086031	4.299077062	4.570083749	4.509332462	4.543627774
Zm00001d037109_T005	0.350878521	0.091905604	0.443464497	0.964308426	0.917831186	1.109011462
Zm00001d037109_T001	1.831603265	0.953220532	1.770534347	2.460107691	2.292034298	2.388374995
Zm00001d037109_T006	4.168399186	3.885108293	4.262430677	5.082462588	5.031646137	4.875664338
Zm00001d037109_T007	0.54785055	0.429291372	0.415967377	0.39350187	0.558006995	0.388467302
Zm00001d009409_T004	3.356862865	2.609714545	3.488239014	3.94911523	2.655280837	3.89037908
Zm00001d048565_T001	3.167139034	3.010512933	3.183167133	2.56778904	2.698538548	2.802064144
Zm00001d006402_T003	0.774003918	0.464160098	0.718063027	1.14715428	1.146305909	1.24317185
Zm00001d006402_T004	0.684716421	1.245441507	1.122709821	0	0	0
Zm00001d006402_T002	1.097583156	0.732810136	0.697702661	1.449443577	1.434481449	1.498194691
Zm00001d034502_T002	0	1.075735929	1.731912661	0.274328464	1.577613965	0.753409426
Zm00001d034502_T003	1.963926098	1.23225736	1.609117142	2.715981862	2.408183863	2.660010615
Zm00001d034502_T001	2.178438981	1.46991983	1.979173694	0.958002235	0.413735962	1.06525589
Zm00001d007347_T001	2.530841589	2.657427129	2.587812853	1.352683959	1.276860495	1.782450084
Zm00001d037772_T002	0.177054149	0.080635835	0.62063145	0.885723507	0.909518138	0.843004569
Zm00001d037772_T001	1.264787417	1.999502184	1.084346652	2.593928822	2.37835597	1.995749697
Zm00001d037772_T003	0.578426737	0	0	0.888703121	0	1.275325359
Zm00001d037772_T014	0	0	1.055546913	2.560251017	2.238385784	3.030340493
Zm00001d037772_T013	2.97220757	0	2.759760275	0	1.482215836	0
Zm00001d004176_T001	6.830484214	6.347197289	6.576001864	6.454388859	6.809998697	6.942463213
Zm00001d011828_T003	5.094827766	3.709396077	5.136950316	4.513597996	4.556486129	4.756537963
Zm00001d011828_T002	2.019116535	3.176927333	1.936197691	2.107722012	1.774270917	1.562891537
Zm00001d036529_T001	4.091130682	3.953289548	4.038078449	3.818436156	4.054398324	3.704037305
Zm00001d005881_T011	2.016415376	1.019459963	1.59128638	2.505549265	2.230393638	2.473879885
Zm00001d033749_T001	1.929650887	1.228056438	1.867662847	2.029353935	2.087109083	1.975222227
Zm00001d019225_T001	3.791138607	2.896419114	3.724609129	3.022434042	2.808826133	2.959581554
Zm00001d029064_T001	3.78699169	3.151643403	3.800024857	3.8853721	3.914481002	4.065962314
Zm00001d053210_T001	0.717688485	0.804174192	0.773978608	1.304039607	1.113971812	1.33077866
Zm00001d008531_T005	1.072260869	0.388436441	1.008133307	1.144728388	0.976540341	1.357312145
Zm00001d008531_T007	1.359396911	0.777516085	1.316708805	2.690763926	2.237998358	2.897844875
Zm00001d008531_T006	0.971268002	0.659431401	1.298989628	1.74309483	1.879053662	1.182007014
Zm00001d008531_T001	4.121411839	3.540803352	4.208635555	4.028131062	4.046285083	4.127843396

Zm00001d008531_T004	0	0.312652396	0	0.457825399	0	0.713262061
Zm00001d017874_T001	4.255522562	3.698493542	4.21495334	5.528601666	5.609680805	5.560490402
Zm00001d017998_T001	5.342580025	4.81504918	5.295400953	4.235629269	4.283905997	4.263484351
Zm00001d009631_T007	1.002048614	0	0.775305125	2.347688048	1.869187197	1.784758578
Zm00001d009631_T001	2.317979233	1.727355025	1.808026649	3.198777793	3.316956668	3.236275241
Zm00001d009631_T008	1.707490777	1.23962839	2.068824529	3.919698662	3.619448931	3.525688426
Zm00001d009631_T009	0.57158265	0.37028371	0.77823213	1.994198093	2.161394341	1.970330583
Zm00001d037859_T002	7.275882496	6.930351921	7.328708618	7.075561093	7.179379086	7.202340707
Zm00001d020825_T001	0.929656946	0.832097681	0.955254023	0.83761875	1.103083319	0.917476057
Zm00001d044833_T001	2.847867659	1.388343306	2.551351129	4.661474167	4.7296786	4.995632489
Zm00001d044833_T002	0	1.668522188	0	4.375371873	4.424433518	4.242159264
Zm00001d041696_T001	2.688423884	2.311577515	2.657613026	3.13788087	3.24824229	3.067229976
Zm00001d047658_T001	7.225807791	6.91664184	7.066908247	7.999262718	8.009440546	7.986810761
Zm00001d006578_T003	4.575623288	3.41282108	4.375251405	6.306755766	6.272317	6.208063283
Zm00001d006578_T004	5.108979858	5.581611921	5.274427729	6.886108114	6.907517226	6.852999447
Zm00001d006578_T001	2.229470718	2.631855592	2.380660747	3.358258822	3.661909409	3.656371087
Zm00001d006578_T002	0.351783218	0	0.16016251	1.278290507	1.460178479	1.207568053
Zm00001d021607_T001	2.56945026	1.753881326	2.516902686	2.617268857	2.744915191	2.591711966
Zm00001d039032_T001	0.734808885	0.693545389	0.974351626	1.426170809	1.434556708	1.449417164
Zm00001d026345_T003	1.861014031	2.86072746	2.664784318	1.442077221	2.127645495	1.846914078
Zm00001d026345_T002	1.831655957	1.023549895	1.869077409	2.031492243	1.984550343	1.927114419
Zm00001d034350_T003	0.285691025	0.158728673	0	0	0	0.359237776
Zm00001d034350_T010	2.685925127	2.185847838	2.756322644	3.44747671	3.693422279	3.401879995
Zm00001d034350_T002	1.850016067	1.662463475	1.671737663	1.820962758	1.860233025	1.664015368
Zm00001d034350_T009	1.886104101	0	2.030602343	2.1792967	2.171626058	2.452284144
Zm00001d047355_T001	4.056445928	3.904030457	3.78314126	4.463697911	4.645031738	4.529173525
Zm00001d053882_T001	3.166682349	2.291677223	3.025951895	3.596505131	3.626391124	3.608967576
Zm00001d012505_T001	0.311292683	0.428763083	0.415528293	2.090036349	2.62528335	2.440221632
Zm00001d013020_T001	1.547359997	1.038630729	1.383963021	1.960764123	1.885053807	2.249402257
Zm00001d012812_T001	2.41597008	1.25792303	2.277485221	1.972015439	2.106786824	2.260390952
Zm00001d016130_T001	3.664761113	2.866506064	3.991532821	5.539936199	5.56843092	5.543569315
Zm00001d016130_T002	5.70568527	5.119884391	5.623504095	7.322809276	7.302105894	7.292721022

Zm00001d022159_T001	5.710155001	5.14343791	5.776429242	5.604542288	5.638800593	5.557619776
Zm00001d022159_T002	0.723045609	0.64036457	0.634557956	0.954036954	0.873584081	0.92625908
Zm00001d022391_T001	1.769681621	1.221365475	1.922594059	2.322814215	2.197394583	2.408933818
Zm00001d022391_T002	0	0	0	0.517290813	0.743290048	0.752792257
Zm00001d013444_T004	0.257698602	0.067747466	0.200838278	0.581410063	0.42884132	0.430353793
Zm00001d013444_T002	3.590121959	2.901591378	3.796142116	4.342578781	4.323394642	4.25657065
Zm00001d050604_T011	0	0	2.114997941	3.123647161	2.070781894	2.376237032
Zm00001d050604_T009	0	0	1.018406999	1.29401958	1.183203188	1.521040181
Zm00001d050604_T015	2.624207835	1.874298083	1.890842771	0	2.293367835	1.76050456
Zm00001d050604_T002	0.469775556	0.2343885	0.362511409	0.747277394	0.621666034	0.424508295
Zm00001d050604_T006	1.10139459	0	0.967321355	0	1.026589747	0.921769925
Zm00001d050604_T010	0.908375615	0	0.479009069	0.358256712	1.251880241	0.832399111
Zm00001d016894_T002	2.649033896	1.711333668	2.647866151	3.75685912	3.67332519	3.497507752
Zm00001d007233_T001	2.504653695	1.882060376	2.422472802	2.449518588	2.568302372	2.544252667
Zm00001d047184_T002	3.737044111	3.356851881	3.740528255	3.461916422	3.386094662	3.535298148
Zm00001d047184_T001	1.01533485	0.669078484	0.841981972	0.971921295	0.315209135	0.457238105
Zm00001d037098_T005	3.673841656	4.011049469	3.83757445	2.617039828	2.884093622	3.533023669
Zm00001d037098_T001	2.319449726	0.982357326	1.65285954	2.948940925	2.961203423	2.867176716
Zm00001d037098_T004	0.69683069	0.539208451	0.885554061	1.61268842	1.671739927	1.65570765
Zm00001d037098_T006	0.153378624	0.458080624	0.52441277	1.284924497	1.562703036	1.607870003
Zm00001d031597_T001	0.786793726	2.13086598	1.08188633	1.618728243	1.587455247	0.835377606
Zm00001d026312_T001	5.839236623	6.066075582	6.27681703	5.77761726	5.778541977	5.762839987
Zm00001d026312_T004	1.630217201	1.175943537	1.344669451	1.901275486	1.792904899	1.666652987
Zm00001d026312_T005	3.925222337	3.470298544	3.68758581	3.849227481	3.685663577	3.560287951
Zm00001d026312_T002	6.976016715	6.299238832	6.932288713	7.003346662	6.960623374	6.946617952
Zm00001d026312_T003	1.227025711	1.663284041	1.864671898	0.933016603	2.435752858	1.952798733
Zm00001d026312_T007	2.466787367	1.878714357	2.592937038	3.282065071	3.37484043	2.547259547
Zm00001d020972_T005	0	0.13225438	0.258803516	0.74406118	1.004960824	0.447638437
Zm00001d020972_T003	2.729599101	1.041934721	2.840393338	3.795767675	3.434024471	3.481415458
Zm00001d020972_T009	2.915638683	2.754978763	2.746249338	3.282167847	3.769920866	3.738840564
Zm00001d021844_T026	0	0	0.083431246	0.60992229	0.812255052	1.18885664
Zm00001d021844_T021	1.554309372	0.721365659	2.260846765	2.11108775	2.274105976	2.902970956

Zm00001d021844_T049	2.226360245	1.273756965	2.204311964	1.462005188	1.657247155	0
Zm00001d021844_T009	0.255574452	0	0	0.543115734	1.043905794	0.618637104
Zm00001d021844_T027	0.077467249	0	0.195760818	0.588036972	0.72221681	0.501400418
Zm00001d021844_T036	0.526650782	0	0	1.493603274	2.026463332	0.630872315
Zm00001d021844_T071	3.25431618	1.748775071	3.507570387	2.812534575	2.324676118	1.702794493
Zm00001d021844_T030	2.040936254	1.537957492	1.119133696	2.025397042	1.648952702	0
Zm00001d021844_T077	3.899628287	4.483587894	3.898317784	2.431231948	2.37051361	2.857138812
Zm00001d021844_T024	1.015888591	0	0.978702315	1.488011572	1.101066426	2.057162918
Zm00001d021844_T061	0.299196587	0	0	0.708300263	0	1.23670078
Zm00001d021844_T054	2.582209136	0.884518214	2.266721187	2.111981442	2.287870001	2.25687419
Zm00001d021844_T032	0.749656114	0	0	2.246611783	1.903177904	2.227460764
Zm00001d021844_T057	1.09425026	0	0.678287392	0.543523599	0	1.352567584
Zm00001d021844_T075	0.681939451	0.424034168	0.526998249	0.843924367	0.913538582	0
Zm00001d021844_T025	1.04975273	1.685176787	1.167960959	1.275791374	1.146651312	0.999622686
Zm00001d021844_T058	0.201987592	0.05892462	0.428129524	1.274640366	1.493153885	0.891505513
Zm00001d021844_T044	0.254931398	0	0.135134248	0.622657712	0.317401325	0.401258004
Zm00001d021844_T048	0	0	0.59625966	1.079115919	1.046954771	1.627849135
Zm00001d025294_T001	5.100867171	4.494889881	5.032066892	4.580495078	4.601242186	4.522213912
Zm00001d033635_T002	3.528590946	2.986470098	3.378807484	3.144111485	3.104802273	3.41850369
Zm00001d017607_T001	0.489801858	0.303333041	0.523475643	0.423509329	0.232105673	0.422877763
Zm00001d014280_T001	4.723027364	3.443892908	4.871477015	4.970676343	4.905968274	5.009051508
Zm00001d014280_T003	0.154132094	0.126803739	0.236206041	0.832601649	0.441137106	0.314386784
Zm00001d007039_T001	3.988860071	3.096897194	4.004856764	3.547874604	3.610440221	3.56140276
Zm00001d023294_T001	3.060909283	2.548656342	3.217245764	2.296374	2.082756059	2.388289572
Zm00001d010204_T001	2.007486164	1.870362747	2.124673216	2.140516264	1.987178227	2.316434252
Zm00001d006184_T001	2.835163933	2.123845281	2.659407652	3.511043037	3.354312934	3.356439082
Zm00001d043159_T001	3.567963983	3.096155401	3.537079336	4.333836875	4.274161676	4.24434374
Zm00001d050831_T001	1.12143654	2.02981506	1.420609594	2.182372263	1.800408757	2.405139012
Zm00001d016492_T001	2.507232926	4.369781441	3.158392452	3.277432848	3.381600814	2.739295108
Zm00001d048336_T005	0	0.200390098	0.379885968	0.617852319	0.178729936	0.576325734
Zm00001d048336_T004	4.996095884	4.482841444	5.110247928	4.885154038	5.015746029	4.860148185
Zm00001d039653_T003	1.389113007	1.530596171	2.112315474	1.418218559	2.179978179	1.48260876

Zm00001d039653_T009	3.998487895	2.961765368	4.328629622	3.916121946	3.807252075	3.902215236
Zm00001d039653_T005	1.276784283	2.861802156	0.598652001	1.887266342	1.008427367	1.648998704
Zm00001d039653_T002	6.382540008	5.499251789	6.292913592	6.277643052	6.40006703	6.358357156
Zm00001d039653_T015	0.440209138	0	0.273951467	1.192942152	1.127594983	1.168995607
Zm00001d039653_T007	0.596347452	1.409658132	0.827112541	0.320952503	1.218640456	0.981488829
Zm00001d008299_T002	5.243232757	4.676281402	5.265582944	5.000790741	4.950927092	5.167770733
Zm00001d021418_T001	5.500071472	5.808062081	5.589140351	5.42460816	5.390384979	5.527748004
Zm00001d019219_T001	8.115262876	7.665294647	8.106634182	7.687563059	7.585046633	7.655637191
Zm00001d019219_T003	5.999438615	8.349861216	6.181885919	5.087566881	5.235638995	4.175467036
Zm00001d015272_T021	1.192889774	0.784387558	1.085969133	2.105697184	1.901697561	1.961033907
Zm00001d015272_T011	1.978179152	0.193746516	0	2.253879775	0	0
Zm00001d015272_T024	4.120680806	3.343918432	4.105769919	4.04558774	3.615335661	3.934583115
Zm00001d015272_T013	0.956655172	0.444286467	0.479896903	1.014183159	1.116304199	0.948023654
Zm00001d015272_T012	0	0	2.219661393	0	2.357298068	2.438182649
Zm00001d015272_T023	0.959428912	1.08205875	0.972150025	0	2.096021609	1.529202766
Zm00001d015272_T008	0	0	0.210421284	1.147674002	1.118821501	0
Zm00001d015272_T004	0.892968377	0.58461044	0.720370409	2.791208741	2.693480044	2.47009273
Zm00001d032644_T002	0.59627302	0.463367164	0.753778231	1.044441684	1.124232835	1.03560702
Zm00001d048542_T001	1.4065198	1.083263755	1.702027141	1.54062149	1.282284407	1.721265903
Zm00001d047906_T001	0.745223561	0.488786453	0.787471749	0.697711556	0.621957609	1.099429872
Zm00001d047906_T006	1.079688038	0.835355775	0.903302869	1.519211763	1.445750536	0
Zm00001d047906_T009	1.14931075	1.247458485	1.315104193	2.172929409	3.079248045	2.879594228
Zm00001d047906_T012	2.309219561	0.89245631	3.249014744	3.844820184	3.733453699	2.127031976
Zm00001d047906_T010	0.230082476	0	0.654690327	0.785350506	0.75485245	1.004073464
Zm00001d047906_T011	2.434130454	1.649529929	2.045757827	1.060082672	2.558538512	2.250580239
Zm00001d047906_T007	0.938887965	0.554892338	0.801623059	1.543667611	1.002099036	1.437770512
Zm00001d047906_T008	0.967705736	0.221522008	0.441702311	0.762664324	0.802913661	0
Zm00001d047906_T013	3.026037442	2.352079949	2.313022609	1.88459167	1.313404336	3.706492647
Zm00001d033979_T001	6.321124741	5.712297967	6.40225419	5.963906151	6.018565968	5.997003365
Zm00001d048159_T001	7.403066133	6.74659425	7.358790651	6.663258078	6.687415382	6.747864039
Zm00001d012752_T008	1.102764944	0.619836483	0.962526073	1.492613613	1.257945351	1.333617225
Zm00001d023575_T001	3.237253793	3.444763261	3.27360958	3.612993135	3.49191901	3.459574307

Zm00001d011119_T008	0.891951058	0.339853423	1.491697149	1.075534685	0.756790177	1.598889207
Zm00001d011119_T016	0.993757679	0.441267803	1.109064969	2.300133977	1.651610172	0
Zm00001d011119_T017	2.405343273	1.197929538	1.835277748	3.340096059	3.096850822	0
Zm00001d011119_T019	2.633998179	1.361954719	1.367574035	2.351264217	2.321679641	2.651739886
Zm00001d011119_T023	1.221713783	1.305984525	2.054291778	0	1.900171636	2.830370135
Zm00001d011119_T013	2.155143018	1.115570657	1.621176973	1.292010732	2.010229505	1.778052101
Zm00001d011119_T022	0	0	2.245349013	2.503216164	0	3.146526521
Zm00001d011119_T011	1.309311252	0.088455074	1.069766774	1.57077621	1.965817893	1.59940302
Zm00001d011119_T018	4.365620457	0	4.473532567	4.137741631	3.57914309	4.493791609
Zm00001d011119_T015	1.17063976	0	1.84575779	0	2.750706611	0
Zm00001d011119_T003	1.77345669	0	0	2.884652424	2.151104159	2.317312173
Zm00001d025296_T002	4.170413912	4.426171144	4.344865629	3.844785643	3.924904073	3.716800424
Zm00001d025296_T001	4.081371331	3.134499264	4.274649459	3.251609883	3.070998909	2.997096542
Zm00001d042152_T002	1.277680113	0	0.925587812	0.980600801	0.347563615	0.484783654
Zm00001d042152_T015	0.645390862	0	0.400882168	0	0.569274283	0.136218953
Zm00001d042152_T011	0	0	0	1.760548843	1.19505714	1.441143482
Zm00001d042152_T012	0.856342756	0.920582954	0.544013478	1.340410728	1.150858582	1.545614569
Zm00001d042152_T006	0.184733506	0.221022035	0.316390227	0.332144206	0.428298929	0.270259814
Zm00001d042152_T008	1.319576895	0	1.294590167	0.728678079	0.839858837	0.801027007
Zm00001d042152_T005	3.187901687	2.131024411	3.356782597	4.041017938	4.356167691	4.108136508
Zm00001d001978_T090	0	0	0	0.780037074	0.708812305	0.865267179
Zm00001d001978_T133	2.946159095	1.550142681	2.932410083	0	0	0
Zm00001d001978_T115	0.202696046	0.14019099	0.242500073	1.407424	1.279428504	1.055978579
Zm00001d001978_T137	0.309854875	0.094292831	0.463217525	0.656669346	0	0.434360812
Zm00001d001978_T134	2.107394949	2.578798421	2.504435563	0	0	0
Zm00001d001978_T121	2.605216957	0	0	2.842901819	2.458025486	3.921699195
Zm00001d001978_T014	0.347318688	0.397984725	0	2.770607499	2.783256319	2.755823775
Zm00001d001978_T131	0.070370092	0.112792195	0.175424958	0.831533547	0.872208612	0.670625547
Zm00001d001978_T001	0.738018954	1.477933726	1.102222066	2.78735828	2.388827103	2.632022704
Zm00001d001978_T122	0	0	2.449475273	2.674383362	2.915068613	0
Zm00001d001978_T009	0.164494106	0.057687323	0.04101303	0.633683224	0.436851566	0.522900423
Zm00001d047275_T003	0.496628008	0.403971171	0.620192255	1.228144506	1.370912502	0.843447657

Zm00001d031217_T002	3.947946721	3.069099429	3.956228148	4.395268012	4.417423389	4.400828694
Zm00001d031217_T005	0	0	0.743665757	0.884886236	0	1.044957098
Zm00001d042899_T001	3.759338876	2.590303079	3.642199024	4.76184304	4.967296129	4.66827318
Zm00001d042899_T004	2.044039435	1.533993352	1.867634779	2.442217389	2.280653197	2.722448319
Zm00001d042899_T002	2.509298318	3.015858091	2.684032557	3.625787382	3.582179143	3.699147928
Zm00001d042899_T003	0.768595103	0.448931601	0.516749423	1.289357618	0.818301676	1.369248143
Zm00001d027800_T001	1.26517942	0.763966449	1.10571562	1.539675979	1.682810132	2.017392823
Zm00001d045417_T003	1.495095962	0.549968767	0.752358112	1.67259144	0.916323004	1.450167641
Zm00001d045417_T001	4.311518737	3.424007554	4.089258727	4.015128213	4.066627323	3.970398784
Zm00001d045417_T002	0.713687018	0.785569801	1.571770964	2.464685255	1.969794062	2.125470258
Zm00001d036091_T001	6.426659898	6.171041776	6.411325786	5.826160547	5.905073267	5.921343065
Zm00001d038012_T001	5.303711461	4.7477556	5.30243308	5.686584453	5.717238326	5.679481085
Zm00001d051404_T015	2.681135729	2.17660522	2.707280051	2.302257335	2.170551636	2.000464112
Zm00001d051404_T001	1.340326405	0.983645594	1.975940114	0.991532549	0.526861074	1.716998355
Zm00001d051404_T014	1.052999393	0	0	2.512367482	2.726347986	2.427973236
Zm00001d006243_T025	0	1.800177621	0	1.449280864	1.538877815	1.510471826
Zm00001d006243_T016	1.06790438	0	0.533314157	0.835068711	0.956339214	0.744102525
Zm00001d006243_T002	0	0	0	0.97905806	0.571720491	0.713162623
Zm00001d013122_T003	0.46172761	0.190968775	0.378722463	0.53627476	0.463211246	0.715402288
Zm00001d013122_T012	2.417400771	1.174242132	1.568538541	2.639539658	2.730971167	2.938813272
Zm00001d013122_T015	2.234829614	1.906886364	2.558894544	2.557595853	2.389576723	2.176233399
Zm00001d013122_T014	0.506165219	0.198463979	0.969415395	0.993328726	0.553602325	0.443830464
Zm00001d002051_T001	5.959704486	5.123597875	5.87636125	6.000452034	5.980744686	5.96734778
Zm00001d042972_T001	1.914793347	1.318748934	2.377736799	2.698177131	3.089738128	3.173147906
Zm00001d042972_T007	2.310840323	1.270008581	0	1.894436745	0	0
Zm00001d042972_T004	0.480418174	0.273811858	0.555403898	0.945979191	0.770544918	0.650389593
Zm00001d042972_T002	2.33231753	1.968898804	2.777400146	2.464165065	2.418573986	2.282231911
Zm00001d003525_T003	0.61886635	0.229251973	0.511154266	0.706354611	0.678386559	0.744907659
Zm00001d031894_T003	1.611621411	1.14131376	1.403972262	1.190906846	2.057038114	1.059663998
Zm00001d031894_T006	4.48842773	3.920618013	4.694949429	4.703466589	4.438976772	4.569019696
Zm00001d042141_T001	2.804877544	2.380048939	3.094388947	3.877731197	4.100335354	3.962741585
Zm00001d028597_T002	4.318981919	3.596230076	4.458416982	4.131085918	4.117483609	4.107180159

Zm00001d028597_T007	0.367288304	0.113032331	0.609200846	0.793843458	1.011015376	0.881659921
Zm00001d012595_T001	1.434845966	1.393253085	1.545442579	2.127014472	1.991939912	1.653765825
Zm00001d010583_T001	6.940843848	6.49992776	6.916811638	6.853861798	6.896760492	6.866272679
Zm00001d040118_T001	4.877617886	5.504967996	4.781175719	4.190947211	4.025973504	4.347904291
Zm00001d025339_T006	2.003051677	1.626573704	2.24911277	2.475790489	2.594734129	2.662931223
Zm00001d025339_T004	1.678727731	2.588768595	0.878960704	1.653667243	1.724239886	1.36403111
Zm00001d025339_T002	6.859097793	7.865307673	6.912533072	7.152280998	7.044003807	6.93703263
Zm00001d025339_T001	5.808832798	5.130126088	5.66913451	5.930516612	5.869627622	5.736909103
Zm00001d025339_T003	3.038063344	3.778795104	2.898737147	2.755539026	3.419322066	2.766962732
Zm00001d030998_T015	1.055914046	0.729486643	1.513062674	1.905314697	1.945048045	1.875954198
Zm00001d030998_T020	0.651259769	0.878955997	0.598930166	0	0.92646708	0
Zm00001d030998_T019	2.645150572	1.787042901	2.860960216	2.600597083	2.511390108	2.690887264
Zm00001d030998_T016	1.007115828	0.649943735	0	1.744273062	1.843867299	1.023983437
Zm00001d037616_T001	2.790410693	2.123652624	2.599685325	3.263998585	3.252632132	3.069059031
Zm00001d040731_T002	1.659575256	1.923915498	2.239551102	2.32674787	1.624144421	1.805443941
Zm00001d040731_T004	2.930989149	1.87581898	2.824142787	2.971270945	3.295783405	3.460893508
Zm00001d040731_T003	0.690544932	0.159399266	0.291037684	1.963258813	1.910559989	1.888144327
Zm00001d012052_T001	8.867579095	9.085708633	8.965163033	8.162444514	8.188126724	8.208595955
Zm00001d005257_T001	4.648309028	6.320478865	4.297832782	5.222687075	5.217523763	4.782125712
Zm00001d005257_T002	1.585784603	0.806173902	1.656485387	2.250599642	2.336777819	2.304714582
Zm00001d005257_T006	2.551919301	2.227262973	2.727470917	3.536892031	3.922900373	3.899924342
Zm00001d038336_T001	1.41969313	1.034805916	1.436320708	1.894340504	1.845559499	1.635762748
Zm00001d032739_T007	1.679545406	2.399053212	2.216187255	1.288507992	1.422314823	1.741022946
Zm00001d032739_T004	1.530098217	1.225258269	1.93430664	1.47207596	1.604222229	1.226178645
Zm00001d032739_T002	0.621041433	0.7491824	1.482896802	1.17488319	0.584371837	0.367059007
Zm00001d032739_T005	0.890497279	0.26933243	0.634877598	0.793820157	0.886328507	0.583506568
Zm00001d032739_T006	2.131817295	0.794064794	2.225783918	0.397580655	1.432796427	1.384000611
Zm00001d032739_T001	0.424339519	0.055724592	0.290650876	0.522874317	0.395050731	0.38549399
Zm00001d032739_T009	4.372508011	3.355760684	4.29870963	4.493992277	4.312729132	4.309443903
Zm00001d051561_T001	2.879102296	2.073246468	2.933489926	3.28173326	3.09155835	3.159618853
Zm00001d003390_T001	3.672303358	3.469231276	3.730450953	3.390133316	3.269977922	3.306755548
Zm00001d030748_T001	6.436555281	3.191426207	6.971682816	6.548315779	6.424081564	6.53120399

Zm00001d010493_T001	6.54219305	6.602589591	6.633657188	6.278909523	6.419640922	6.33351025
Zm00001d039328_T001	2.06206298	0.066289001	1.193286033	0.717106744	0.684690393	0.722413562
Zm00001d039328_T002	2.950043568	1.754063969	3.351362887	1.96011725	1.797716094	1.691060186
Zm00001d039328_T004	4.365942412	4.922861754	4.554064966	0.741385007	1.577738665	0.793309948
Zm00001d039328_T006	5.589276076	3.472591096	5.761725223	3.744406225	3.243547988	3.324110107
Zm00001d027319_T006	1.809033654	0.809558501	1.458634506	1.870253837	2.062198059	1.996090052
Zm00001d027319_T001	2.708631125	1.956602765	2.748552901	2.857809851	3.154437228	2.888985747
Zm00001d029706_T001	2.516132676	2.223493293	2.199062633	0.708468904	0.6780692	0.961174338
Zm00001d005030_T005	0.796591109	0.850272165	0.609753071	1.486426578	1.948711101	1.953021199
Zm00001d005030_T006	2.827283299	2.999245454	2.736782144	2.361786042	2.509448067	1.61758248
Zm00001d005030_T001	1.382995314	0.841615721	1.101033471	1.454864431	0.876731063	1.551981542
Zm00001d005030_T008	0.608617186	0	0.318906816	0.858366193	0.543883849	0.877674371
Zm00001d005030_T004	0.973845614	0.884010956	0.554095673	0.657891483	0.838082057	0
Zm00001d016100_T001	1.843606444	1.221961816	1.696107354	1.946369299	1.875738786	1.474028423
Zm00001d013644_T002	1.564830784	1.10104356	0	0.825659462	2.084211249	0
Zm00001d013644_T004	7.755104765	6.728777977	7.782326441	8.284537989	8.353871154	8.313117905
Zm00001d013644_T003	0.369900832	0.932158709	0.511259539	0.750923734	0.572312475	0.266354792
Zm00001d011643_T001	1.605149146	0.992030945	1.521210581	1.740634028	1.846608847	1.703653568
Zm00001d008679_T008	4.924686966	0	4.669839754	4.537463129	4.469586729	4.976037022
Zm00001d008679_T002	0	3.712058659	3.463146755	0	1.676506634	0
Zm00001d008679_T009	1.535835485	0	1.219819658	0.841812945	1.238147898	0.998330558
Zm00001d008679_T005	5.023918609	4.460962293	4.800661027	5.169212014	5.111368945	4.952602555
Zm00001d008679_T010	1.893797866	0.298232482	0.503911455	2.783519516	2.898478482	2.576117207
Zm00001d048777_T014	0.115150465	0.059027103	0.190987732	0.752249341	0.515600439	0.333304649
Zm00001d048777_T006	4.678495297	3.048021126	4.553054113	4.045181354	4.093026334	4.393496035
Zm00001d048777_T017	2.400492571	0	2.102585239	2.081067903	2.996373199	2.62226131
Zm00001d048777_T026	4.577013756	4.040916271	4.179847003	3.652236483	3.607215604	4.197354556
Zm00001d048777_T018	0.985248301	0.421906742	1.069312401	1.118124463	1.152595559	0.975520137
Zm00001d048777_T013	1.152271049	2.935973369	2.531113422	1.950590842	1.220521889	1.219452926
Zm00001d048777_T008	3.790448646	1.397320523	4.031231613	4.60319405	4.329615249	4.014739127
Zm00001d024510_T002	5.202383907	4.540032292	5.219554608	5.446219368	5.437833385	5.269832356
Zm00001d024510_T004	1.468667244	0.804729302	1.265538313	1.779562308	1.888438948	1.829322302

Zm00001d024510_T001	3.885403925	3.918057679	3.938983914	3.082910498	2.701175749	3.645080922
Zm00001d044656_T001	1.487660759	0.804255159	1.463555499	1.668686013	1.745694275	1.395776291
Zm00001d037962_T012	4.45550247	5.185229558	3.971361912	0	5.688198322	0
Zm00001d037962_T011	6.030213192	0	6.374800724	6.890352192	6.414004013	6.890072862
Zm00001d037962_T010	0.436379347	0.244772613	0.803287389	1.950881567	2.306019242	1.835419245
Zm00001d037962_T017	0	0	0	1.083446904	1.229595921	0.806427177
Zm00001d037962_T008	2.29384483	2.921859975	2.398661753	1.464106238	1.559261257	1.077485707
Zm00001d037962_T013	5.773019407	5.46975993	5.34155725	4.486281113	3.139716035	4.277820241
Zm00001d045560_T001	2.243174288	2.324474511	2.514612378	2.754068246	2.606444834	2.671121235
Zm00001d042395_T001	4.65826199	4.893553069	4.691630193	4.850389195	4.985574066	4.777745304
Zm00001d044481_T002	1.684059272	2.529821946	1.947371791	1.87796525	2.517211684	1.555444638
Zm00001d044481_T006	2.610176553	0	2.276092535	3.191922174	2.712723207	2.584836981
Zm00001d044481_T005	0.8827166	0.210952366	1.311258964	0	1.137089673	2.158870829
Zm00001d044481_T004	4.318846528	4.061104281	4.88118666	4.462593621	4.078140814	4.608934704
Zm00001d018182_T001	4.038037436	3.096060415	3.869413855	4.060872805	3.95735468	4.151168632
Zm00001d011365_T001	2.139658415	1.396255958	2.466257231	1.692378988	1.596598395	1.379785072
Zm00001d010679_T001	1.579842921	1.004761683	1.692195063	1.324320983	1.362583785	1.384785891
Zm00001d018698_T001	2.450056494	1.753109434	2.472462297	2.954069161	3.200550335	2.894277245
Zm00001d048031_T024	1.579449059	0.850879415	1.737601106	2.084872492	2.172589601	2.14941579
Zm00001d048031_T009	3.23557972	0.900328552	1.619389063	3.282396805	3.403430391	3.378312684
Zm00001d048031_T040	3.05269046	2.587337385	3.912243056	3.357772122	3.450819437	3.52046688
Zm00001d048031_T007	0	0	0	1.992194501	2.410421704	0.870573045
Zm00001d048031_T048	0.353404599	0.130122998	0	0	0.901537704	0.680140688
Zm00001d048031_T018	0.617978289	0.237035	0.33805239	0.998428049	1.093167336	1.186799081
Zm00001d048031_T082	0	0	0	0.796500574	1.189961154	0.966521279
Zm00001d048031_T020	2.386589146	1.267037136	2.209306749	2.886807839	2.697440458	3.080845406
Zm00001d048031_T065	0.792546379	0	0.80687092	1.045710655	0.839299343	0.919655858
Zm00001d048031_T025	0	0	0.306763858	1.754628007	1.400095763	1.232872541
Zm00001d048031_T066	0.347037423	0.207144455	0.121633472	0	0	0.818113486
Zm00001d048031_T015	0.655068758	0.255547865	0.272305129	1.301893711	1.03029511	0.861104181
Zm00001d008693_T003	1.864614852	2.444280901	1.621972608	2.582539384	0	2.233431625
Zm00001d008693_T002	4.64742786	3.944554886	4.774253678	5.039961522	4.905872896	5.146841697

Zm00001d012856_T001	3.668303762	2.934854443	3.791293982	3.430779158	3.424453338	3.384249062
Zm00001d012856_T003	0.477230722	0.135253789	0.382272152	0.512027601	0.813851362	0.456095401
Zm00001d029357_T001	2.212033559	2.32640964	2.201536946	2.274503507	2.496343462	2.799062846
Zm00001d043414_T001	4.670109798	3.857413855	4.692290488	4.515731688	4.641169527	4.674020364
Zm00001d043414_T003	0.278257198	0.133852825	0.321237744	0.430800162	0.539030731	0.521963317
Zm00001d012291_T001	2.61756908	3.19194254	2.017828931	0.412300276	0.781909407	1.131148905
Zm00001d023305_T001	3.097319194	3.222561928	2.926391171	2.835026455	2.691592006	3.216722271
Zm00001d009411_T002	2.208888342	1.407810642	2.157926848	2.089428997	2.141918621	2.284552924
Zm00001d050121_T001	4.552954734	4.281313159	4.668107322	4.469868724	4.308627941	4.50873947
Zm00001d029585_T001	2.319810107	1.905448334	2.144899331	0.202822654	0.113187064	0.239092483
Zm00001d009749_T009	0	0	0	3.685666941	3.506429173	3.337831383
Zm00001d009749_T021	0.76696909	0.327800016	0.337202991	0.618303505	1.219382914	0.390021574
Zm00001d009749_T012	0.513739343	0.303382144	1.165605203	0.875982891	0.761184834	0.445182147
Zm00001d009749_T017	0.70080545	0.068706577	0.439477398	0	0	0.600728374
Zm00001d009749_T024	2.480300284	2.744256913	0	1.443602939	2.183602434	2.19180708
Zm00001d009749_T005	5.561738202	0	5.585498543	4.945558732	5.088977639	5.108037706
Zm00001d009749_T001	0.581823631	4.096802172	2.004737957	2.798756236	1.995229424	0
Zm00001d009749_T023	1.413468437	1.465280237	0.933991035	0.491578125	0	1.91379169
Zm00001d009749_T006	1.772823976	3.017217665	0	0	0	2.04662551
Zm00001d026689_T007	3.298746135	2.59671908	3.404541858	3.175171509	3.090962814	2.70275239
Zm00001d026689_T006	1.625706272	0.352096338	0.444884356	0.734867834	0.775673432	0.835811726
Zm00001d015164_T001	1.293625332	0.902048875	1.074836958	4.450719687	4.376731572	4.412748539
Zm00001d030213_T001	2.726484667	2.027567404	2.728166508	3.196740009	3.439565163	3.231355448
Zm00001d006640_T001	3.86846187	3.096116092	3.810325051	4.077154964	4.22810717	4.012691128
Zm00001d006640_T002	1.647238516	0.310007332	1.946748075	2.151514983	1.283216546	1.670744727
Zm00001d006476_T005	0.474899085	0.131955544	0.534913219	0	0.290516413	0.453136146
Zm00001d050838_T001	4.88927528	3.899864131	4.960621289	4.344644456	4.459727669	4.521886454
Zm00001d050838_T002	4.430477575	4.94831228	4.70034089	3.326186555	3.56573096	2.230676147
Zm00001d006886_T002	2.328139357	1.941855266	2.4593141	1.972612522	1.867334692	1.800724333
Zm00001d006886_T003	0.893234829	0.529286238	0.960662199	0.747733691	0	0
Zm00001d011446_T001	2.746118817	2.324339131	2.954686185	2.226375044	2.292983418	2.079876418
Zm00001d012102_T004	0.811318944	0.871408409	1.491380071	1.132930811	1.735257442	1.370064935

Zm00001d012102_T005	2.71152354	2.466119118	2.542765442	2.924990888	2.523422447	2.590247979
Zm00001d012102_T001	2.26160182	1.44319447	2.36619798	2.868988151	2.564660095	2.735864831
Zm00001d005324_T002	1.603451401	0	1.831352345	1.280321507	1.341476303	0.620503834
Zm00001d005324_T003	2.543374375	1.690469394	2.577976193	2.600687941	2.065717401	2.214672552
Zm00001d005324_T004	0	0	0	0.865566509	1.247142474	2.029015682
Zm00001d027585_T001	3.838843989	3.179939177	3.795008939	3.092570942	3.140951049	3.213952231
Zm00001d030947_T002	3.090709831	2.409440632	3.10368579	3.013985466	2.944146478	3.134164085
Zm00001d030947_T004	1.031529296	0.579066196	1.298458318	1.482943771	1.176956526	0.99402571
Zm00001d042453_T002	2.584305925	2.189188526	2.820489428	2.737873413	2.575803635	2.808562776
Zm00001d042453_T001	9.601186345	8.909628131	9.654849526	9.70799295	9.742635565	9.745458504
Zm00001d043943_T002	4.700678686	4.040624376	4.590035884	4.636657099	4.57239258	4.535405532
Zm00001d043943_T003	0.691853934	1.591828274	1.416154434	1.415971162	0.821394298	0.736924332
Zm00001d031013_T008	3.407189028	2.51410837	3.389880784	3.431668018	2.859851902	3.258536412
Zm00001d031013_T005	1.017289834	0	1.684135147	1.397468941	1.753284472	1.678901216
Zm00001d031013_T007	0	0.434768588	0	1.056189508	0.660762539	0.944745274
Zm00001d031013_T006	1.492453133	0	1.167329665	1.82659567	1.561438077	1.453710365
Zm00001d031013_T002	0	1.16397397	0	0.515734652	1.814390867	1.799822976
Zm00001d037267_T003	0.998238115	0.478802036	1.088068307	0.757626662	0.757804139	0.751796184
Zm00001d037267_T002	2.545627418	2.626208754	2.737083385	2.73545719	2.560831588	2.772721592
Zm00001d037267_T001	4.344267569	3.952035211	4.316852359	4.088059654	4.174985745	4.080758359
Zm00001d045213_T004	2.506791571	2.544369897	2.494104252	2.576905193	1.509526103	1.786659086
Zm00001d045213_T002	3.548082936	2.498813792	3.615822234	3.455704524	3.77436465	3.593108002
Zm00001d005027_T002	5.588955879	4.862787502	5.476790166	5.998752296	5.988318991	5.934286303
Zm00001d005027_T001	1.335764496	1.769654119	1.16321403	1.65742873	2.187932717	1.420722225
Zm00001d005027_T004	1.412837336	0.492091089	1.640260441	1.963602111	1.664760885	1.254851601
Zm00001d036626_T001	7.128814199	7.350613436	7.045490064	7.09159625	7.180653995	7.162070808
Zm00001d033967_T001	1.135704936	1.125828287	1.223808041	1.917764368	2.076289548	2.359955713
Zm00001d005271_T001	0.252599739	0.098105543	0.448403056	0.431297748	0.40070077	0.092540329
Zm00001d005271_T002	5.604398147	4.863489817	5.604005891	6.051760959	6.046384343	5.975174526
Zm00001d033594_T001	2.342008511	1.559872566	2.361104399	2.446308113	2.121587059	2.494834405
Zm00001d028189_T001	5.446019853	4.775117669	5.456532267	5.473223358	5.422542333	5.406045099
Zm00001d028189_T003	0.794380929	0.5151099	0.427314386	0.50650039	0.847815757	1.23251955

Zm00001d042449_T001	2.567633545	2.651448988	2.550028383	4.971960277	4.895419108	4.88495728
Zm00001d039095_T001	0.912274294	0.592609641	1.224419426	0.718914375	0.869343177	0.74009429
Zm00001d031490_T006	0.37560444	0.101086602	0.397544513	1.404452482	1.432227722	1.021571403
Zm00001d031490_T004	1.023269548	0.840633225	0.796184903	1.625741334	1.620871637	2.229550086
Zm00001d031490_T003	5.829517357	5.047091316	5.79304593	5.885022364	5.854837846	5.757373927
Zm00001d028359_T003	1.76604045	4.797784976	2.420119358	1.871151304	1.784794168	1.136636991
Zm00001d028359_T001	6.475802598	5.462675214	6.529648125	6.680783594	6.79275308	6.713551978
Zm00001d020606_T002	2.172347976	3.042449105	2.31268463	2.549676809	2.244753132	3.059433848
Zm00001d018471_T001	4.778830053	4.784112319	4.831406769	4.06452265	3.90037058	4.107625524
Zm00001d018471_T003	1.208476681	0.714449514	1.243851843	1.190626869	0.947227017	0.835757569
Zm00001d005396_T001	3.292694449	3.672221879	2.696762823	3.329381679	0	0
Zm00001d005396_T002	4.594316541	4.078318466	4.77550504	4.700310591	5.178515829	5.238379287
Zm00001d046434_T006	3.46994574	2.793408175	3.392090009	3.467674407	3.851887134	3.906630599
Zm00001d046434_T001	1.043895298	0.424729716	0.934468191	0.904110249	0.871752984	0.710899204
Zm00001d046434_T002	2.924990318	2.076029531	2.892973815	3.277583712	3.054030544	2.989655487
Zm00001d015851_T009	0.955183335	0.511815135	0.875137587	1.228899941	1.507236479	1.47797516
Zm00001d015851_T010	1.703137032	2.836798519	2.784484928	2.139860404	2.231646216	1.029367364
Zm00001d015851_T003	4.268224696	3.248644734	4.281515547	4.748098635	4.694736752	4.806090309
Zm00001d038746_T001	1.307207003	0.441729928	0.973016075	0.741511858	0.921293882	0.896447016
Zm00001d042910_T005	3.734606447	1.990106778	3.732733523	3.557132872	3.742924693	3.725887381
Zm00001d042910_T008	1.490738521	0	1.701748204	1.670599716	1.038275327	1.861573505
Zm00001d042910_T010	0.873688015	0.908819055	0.827505255	0.567670048	0.737422856	0.209160118
Zm00001d012320_T001	1.458444478	0.820386491	1.235287139	1.523367744	1.767923398	1.641533082
Zm00001d042695_T001	4.802103912	4.156786283	4.819854639	5.147848585	5.113242834	5.084127934
Zm00001d042695_T009	0.823769737	0.608492288	1.235106966	2.692937318	2.322873039	2.28525809
Zm00001d006884_T003	0.107551292	0.131815978	0.293411687	0.331522934	0.602707712	0.359504303
Zm00001d006884_T004	0.936576508	0.358156543	0.847661842	1.592448904	1.443706364	1.462898836
Zm00001d006884_T002	3.263489985	2.46970654	3.382960465	3.463949615	3.47545057	3.497182622
Zm00001d051068_T001	0.58562022	0.314154725	0.49322102	1.305517073	1.138377401	1.308020637
Zm00001d008500_T001	6.463770665	6.717675148	6.404781519	5.759377842	5.789722679	5.709306822
Zm00001d039919_T001	2.316032176	2.023999754	2.355026416	3.589682243	3.876855394	3.71087188
Zm00001d039919_T002	0	0.375523261	1.594026314	3.476906692	3.508851764	3.538121559

Zm00001d020067_T001	1.771274152	1.617953378	1.789926187	1.300915598	1.368744327	1.113167057
Zm00001d039925_T001	2.795187801	3.499256956	2.618545254	2.64236656	2.601759024	2.356202419
Zm00001d030688_T006	2.409240209	3.055575544	2.478421794	0	2.130814593	2.873175949
Zm00001d030688_T002	2.717766121	2.381238513	2.988971652	2.752600247	2.686463549	1.89395276
Zm00001d047293_T001	4.540166732	4.027055296	4.409203408	4.346212522	4.458839861	4.543091229
Zm00001d028417_T005	1.039424798	0.370942066	1.202218348	1.333756317	1.248282828	1.303250616
Zm00001d028417_T002	2.934211135	1.454160097	3.241017949	4.040258242	4.20684269	4.172823908
Zm00001d028417_T001	2.950691054	2.739499913	2.876132635	3.832510713	3.609280998	3.66713153
Zm00001d016378_T002	0.347901457	0.136291151	0.724187487	1.126278425	1.069745467	0.721754119
Zm00001d016378_T004	0.240355854	0.108896415	0.284752194	0.287524304	0.462281666	0.337906292
Zm00001d016378_T005	1.127821445	0.371794135	0.930181722	0.730166044	0.663429334	1.028757256
Zm00001d016378_T003	5.071564688	3.057790414	4.924061869	5.001820074	5.174769843	5.112128279
Zm00001d025307_T001	2.401656404	2.066029931	2.492139811	4.070812371	4.091862541	3.927148656
Zm00001d025307_T002	0	0.063754184	0.054690136	0.56020234	0.502966001	0.608115636
Zm00001d028711_T003	2.145951623	1.613368515	2.151254213	3.103664644	3.03915419	2.826521235
Zm00001d047361_T001	0.530578194	0	1.012786045	1.057568109	0.833298973	0.661691234
Zm00001d047361_T004	0.930919721	0.634307954	0.777912436	0.567358528	0.878473461	1.163766636
Zm00001d047361_T002	1.344018316	1.109424083	0	0.605455457	0.808950073	0
Zm00001d031104_T005	1.012284755	0.408424924	0.775496454	1.037554421	0.792080711	1.003191316
Zm00001d002283_T004	0.288028934	0.586621607	0.468229833	1.145501387	1.370090051	0.817886807
Zm00001d002283_T002	4.63452938	3.748569431	4.546990122	4.979345162	5.181137822	4.999042996
Zm00001d002283_T001	2.218741187	2.521383725	2.487152134	2.69924867	2.111823909	2.699768154
Zm00001d024292_T001	4.789220046	4.060029134	5.03425983	5.180810602	4.99391384	5.216426966
Zm00001d024292_T002	1.707841036	2.515283778	1.892353719	1.737981327	2.311470564	1.201308905
Zm00001d033029_T001	1.212940314	1.008075923	1.368305719	2.180017656	2.01273671	2.296322015
Zm00001d026487_T002	4.79972995	3.345814475	4.729439275	3.888930045	3.991127792	3.957178302
Zm00001d026487_T001	2.321934443	2.861206238	2.49239955	2.608593059	2.466529502	2.663351907
Zm00001d052509_T001	4.647583392	3.966828095	4.740177963	3.966148952	3.947782091	4.001366306
Zm00001d039563_T013	0	0	0	1.375970799	1.237698926	0.747667532
Zm00001d039563_T004	0	0	0.730659083	1.860685551	1.821188547	2.17588958
Zm00001d039563_T039	0	0	0.248211772	0.417051538	0.720626947	0.657220168
Zm00001d045520_T001	7.024131007	5.785355974	6.609784122	6.652983135	6.721530784	6.916026186

Zm00001d034440_T001	5.195994815	4.442058371	5.290738853	4.263772772	4.311285707	4.398288592
Zm00001d052186_T001	5.640325378	4.809175639	5.69936973	5.52406703	5.490662959	5.445614055
Zm00001d002439_T001	0.839918482	0.604084612	0.783428995	0.920720138	0.78819958	0.99263793
Zm00001d016285_T001	3.438176127	2.569830824	3.511067586	2.436341499	2.549457248	2.547835993
Zm00001d038127_T001	4.617896034	3.99191951	4.521424306	4.979682597	4.844860347	4.844215049
Zm00001d020417_T001	5.083766636	4.616232997	5.260031868	4.349281264	4.312356593	4.144789884
Zm00001d042845_T001	3.600242274	2.937174875	3.544063446	3.534968357	3.483651349	3.552305746
Zm00001d047220_T003	2.927093174	3.019254805	2.901110367	2.495657816	2.755617001	2.366849253
Zm00001d047220_T001	3.562367727	2.243080734	3.450451279	3.387790984	3.570182623	3.644646685
Zm00001d038388_T001	2.10704772	2.131545037	2.060597371	1.676809897	1.45538062	1.748750172
Zm00001d003729_T001	4.365969209	4.632016769	4.51756899	4.449139003	4.579339956	4.301985357
Zm00001d034948_T003	1.946215065	1.944955871	2.643848803	2.660307324	2.636902519	2.915322026
Zm00001d006536_T001	3.717086888	2.831760321	3.696476857	2.395125607	2.293126777	2.569142528
Zm00001d017158_T001	0.992519019	0.853164511	1.054383823	1.079479834	1.083157532	0.796876798
Zm00001d010793_T001	3.3584763	2.440155706	3.582233227	3.049694886	3.292746565	2.550617022
Zm00001d010793_T005	1.998630954	3.97491976	2.999944816	2.886525371	3.117225592	2.82080881
Zm00001d031777_T001	4.388061728	3.591148959	4.364836015	5.473619908	5.328417364	5.26751194
Zm00001d019004_T002	1.481417783	0.946456142	1.659848313	0.515950577	0.483440725	0.77345458
Zm00001d033906_T001	3.861276622	3.143129216	3.890932049	4.046463383	3.939608832	3.950752633
Zm00001d011638_T012	0	0.396978173	0	0.660975152	0	1.626493339
Zm00001d011638_T004	3.666410076	3.101428041	3.727545523	5.803029918	5.923223833	5.924379678
Zm00001d011638_T011	0.363901073	0.052610652	0.311347329	2.42106653	2.669047635	2.331280714
Zm00001d011638_T005	0.195363978	0	0	2.982900874	2.794122827	2.919063722
Zm00001d011638_T010	0.780693927	0	0.693630133	1.559873055	1.723377694	0.772546997
Zm00001d011638_T008	0	0.013628049	0.062353851	1.779603488	1.486559928	0.899000021
Zm00001d011638_T002	1.531104436	1.49515533	1.670397582	0	0	0
Zm00001d003510_T001	2.598700826	2.229430725	2.543716601	1.809537099	2.157421802	1.836714309
Zm00001d003013_T001	1.723929836	1.264828844	1.766863111	1.630953368	2.000714318	2.107209123
Zm00001d013209_T001	2.383395194	2.095555178	2.280965516	3.395561348	3.338735741	2.894140629
Zm00001d005936_T001	1.869132304	1.3661325	1.640251184	3.756564755	4.099169531	3.986225421
Zm00001d006409_T002	4.128671102	3.904338231	4.275045126	4.156133328	4.355476762	4.429758352
Zm00001d006409_T001	1.456094875	0.921058469	1.381322139	1.419091182	1.307687313	0.987451121

Zm00001d006409_T004	0.692675363	0.311045007	0.811909116	1.207975289	0.771024352	1.133084082
Zm00001d009374_T001	1.533962464	0.836299853	1.546840639	1.690850621	1.74905193	1.567285019
Zm00001d052971_T004	3.451903835	2.144692174	3.633776207	3.528963069	3.378585837	3.506913661
Zm00001d052971_T005	1.963109705	2.209418429	1.818378991	1.416421462	1.47897077	1.146223131
Zm00001d052971_T001	0.644933308	0.480066544	0.805171091	0.561818805	0.747342711	0.601190653
Zm00001d052971_T003	0.624712009	0.573862129	1.137291694	1.030400354	0.82914407	0.129476902
Zm00001d012634_T008	0.325807763	0	0.696253831	1.294266078	1.464257877	1.083522472
Zm00001d012634_T001	3.36587895	2.845253982	2.769055079	4.283756534	4.299248761	4.257920316
Zm00001d022106_T001	5.061368816	4.204087022	5.130056383	4.326926659	4.194284952	4.186150538
Zm00001d042768_T001	5.524203938	5.575502065	5.556771808	6.045725681	5.986027478	6.087046988
Zm00001d045030_T001	2.003718079	2.822225779	0.422585018	1.12067243	1.398309322	2.152429094
Zm00001d017789_T001	4.220211918	4.538991325	4.19053261	2.570665477	2.79938307	2.686904089
Zm00001d016616_T002	2.0744085	2.104367862	2.05289961	2.004146113	2.217503717	1.887347067
Zm00001d025082_T002	3.397408973	2.89148782	3.628801119	2.848401005	2.864807769	2.673746133
Zm00001d021264_T001	1.879308971	1.089125226	1.952522558	2.655879346	1.430117707	1.871779396
Zm00001d010832_T001	3.400748034	3.932614552	3.067232558	3.534569769	3.594743444	3.353033029
Zm00001d052797_T003	2.627505986	1.829269524	2.670115635	2.633571404	2.870816447	2.683539714
Zm00001d052797_T002	1.068587582	0.929317595	1.2754976	1.475294526	1.641062738	1.226817998
Zm00001d052797_T001	0.607396129	0.154311001	0.320521648	0.455104395	0.678880489	1.418654127
Zm00001d052797_T005	2.999283524	4.352611297	3.171247987	2.350576169	0	2.330831138
Zm00001d016815_T001	1.15854385	0.677791454	0.783428995	0.684579989	0.953911094	0.839554931
Zm00001d019915_T001	1.378041006	1.029045022	1.118833458	1.213932641	1.425908264	0.640826352
Zm00001d050011_T004	1.924434005	1.241451549	2.269031051	2.725540714	2.811567412	2.705855184
Zm00001d050011_T003	2.826933222	1.180643742	2.359806195	3.38730808	3.695999602	3.188895876
Zm00001d050011_T005	1.471358128	1.30690323	1.675763551	2.774164841	3.042800504	3.149235136
Zm00001d050011_T001	5.983508063	5.628211249	6.204500837	6.773903306	6.794266845	6.779672624
Zm00001d022607_T001	6.444329326	6.28546995	6.446555575	6.612984778	6.604421722	6.68465226
Zm00001d032386_T003	6.440611926	5.469723436	6.422596573	6.284063887	6.228001124	6.211625832
Zm00001d032386_T004	0.701367184	0.482335119	0.930928045	0.535689693	0.330000718	0.453097154
Zm00001d032386_T001	3.088674875	2.95200939	3.420708618	1.718565918	2.186363318	1.88087748
Zm00001d008562_T001	4.844608157	5.597553252	5.125717921	3.627222652	3.859926624	3.684350739
Zm00001d038540_T001	2.638315077	2.278731781	2.622780453	2.837324667	2.97781495	3.056989923

Zm00001d047116_T001	7.047493936	7.696993717	6.971807181	5.698686667	5.468781104	5.483037802
Zm00001d015406_T001	0	0	2.063660319	3.753814486	4.005809202	3.610844844
Zm00001d015406_T003	3.605026318	3.878513286	3.0848645	0	0	0
Zm00001d027676_T001	3.904876246	3.409514086	4.029091416	4.535198099	4.590456928	4.53494614
Zm00001d022451_T001	4.984577138	4.486916027	5.069079789	7.752574726	7.74718682	7.775145245
Zm00001d013620_T025	0.775262135	0.802259404	1.928806903	1.199216892	1.351985002	1.203466174
Zm00001d013620_T032	1.047371565	0.259629237	0.756607451	0.305181418	0.785460995	0.170460943
Zm00001d013620_T012	0.658835735	0.102314128	1.09006278	0.768148747	0.562318145	0.648531709
Zm00001d013620_T029	0.260912084	0.22530208	0	0.221973567	0.230565794	0.577525991
Zm00001d013620_T006	1.29948792	0.442168544	1.325209665	0.938522928	1.074293058	1.207529946
Zm00001d013620_T009	0.296167224	0.617334221	0	1.970419676	1.892130637	1.857977413
Zm00001d013620_T002	2.640403443	3.299958061	3.238226025	3.097541188	2.912900064	3.265312815
Zm00001d013620_T001	3.867617483	1.003371963	3.401957657	4.064558002	4.22152657	3.858645576
Zm00001d033456_T001	3.563548899	3.377820906	3.630538854	5.260526333	5.227665228	5.268847859
Zm00001d028768_T001	1.667275414	2.072601138	1.585493315	1.785380222	2.314173871	2.224822243
Zm00001d032131_T001	4.165359751	3.83522913	4.244411713	4.090355937	4.067565066	4.117586836
Zm00001d028114_T001	1.597302327	0.853321035	1.59978814	1.224940443	1.319238715	0.680430586
Zm00001d007076_T001	5.629897456	5.00401304	5.566163999	4.008083634	3.980576674	3.972766533
Zm00001d045263_T001	4.313599153	4.460451244	4.520949946	4.391046605	4.155091193	4.089182951
Zm00001d045263_T002	0	0	0	1.830133607	2.462542909	2.70168589
Zm00001d045263_T003	5.261764726	5.118970466	5.361958543	5.820659262	5.886295873	5.846236521
Zm00001d002644_T018	0	0.622801065	1.414685439	1.593403493	1.645858876	0
Zm00001d002644_T052	1.573184966	0.653493907	0.962759258	0.798007368	1.149825795	0.522591135
Zm00001d002644_T023	0.927590062	0	0	0	1.579143453	2.497317027
Zm00001d002644_T055	3.529061802	4.053433647	3.364424654	3.715688261	3.373310638	3.648034986
Zm00001d029649_T002	4.416844672	3.669115684	4.385669649	4.37921948	4.263559992	4.233663925
Zm00001d023791_T002	3.728875692	2.904926424	3.823690368	3.873214641	3.962788496	3.684856992
Zm00001d034689_T003	2.560303605	1.984656061	2.459019998	2.095397529	2.514331357	2.281856975
Zm00001d002387_T003	3.422448043	3.603788808	3.676731267	2.780546539	3.479294726	2.998627705
Zm00001d050705_T001	3.784206409	3.991946984	3.797634033	3.751185825	3.074756996	3.512693253
Zm00001d033472_T002	0.882182096	0.440399458	0.680546708	1.137790067	1.150343256	1.28326041
Zm00001d033472_T001	3.783357642	3.269391531	3.841174285	3.736166372	3.815764873	3.502844467

Zm00001d043052_T004	0.462158095	1.784849853	0.533487599	1.288459562	2.339736863	2.254871248
Zm00001d043052_T009	2.708861955	0	2.158391967	3.762650931	3.379361315	3.869153136
Zm00001d043052_T008	3.871798022	3.050303346	3.831545403	3.948783976	3.78520464	3.701880473
Zm00001d043052_T003	0.937759437	0.119487657	0.635205529	1.742013543	1.199775352	1.467211649
Zm00001d043052_T001	1.409541919	0	1.941197979	1.795526756	0	0
Zm00001d053369_T001	4.601796634	4.055466568	4.678959622	3.283910811	3.417802028	3.189380536
Zm00001d008581_T001	3.891135189	3.115418843	3.902436668	4.13668284	3.98999928	4.027196436
Zm00001d045728_T003	1.295677774	1.760117026	0.957742282	1.249925856	1.56780291	0.95647304
Zm00001d045728_T008	1.246660423	1.027847624	1.381593477	0.987320138	1.398254589	0.911195324
Zm00001d045728_T004	3.518302098	1.935362811	3.680655846	3.466828731	3.539322493	3.667617749
Zm00001d045728_T005	1.33908036	0.373755105	0.888832462	0.843953302	1.119743943	1.194163977
Zm00001d012263_T003	2.186071679	1.804173779	2.186811122	2.610845316	2.851885135	2.930723715
Zm00001d012263_T006	1.060051535	0.69516447	1.129054146	1.092513944	1.541446941	0
Zm00001d012263_T005	0.281079798	0.051533591	0.025522133	0.500892792	0.441449483	0.648541833
Zm00001d038668_T001	1.911864887	2.368939563	1.832333482	1.534435678	1.708449922	1.811929661
Zm00001d017863_T006	0.878263134	0.568134282	0.808547332	1.043521593	0.758369702	0.726169431
Zm00001d017863_T004	4.520471029	3.694730791	4.572186213	3.874334285	3.850116111	3.99808671
Zm00001d051313_T001	2.537875514	2.094220528	2.306487698	2.284626358	2.472400427	2.555849768
Zm00001d028304_T001	4.903568961	4.294418794	4.987962204	3.792943202	4.014297798	4.012618461
Zm00001d040888_T002	0.213521483	0.08000131	0.053957933	0.223735151	0	0.215678597
Zm00001d040888_T001	2.156159708	1.180391049	2.408049047	2.396416816	2.117022736	2.325961133
Zm00001d040888_T004	0.411896943	0.340171421	0.673015425	0.855744179	0.938330211	0.964933837
Zm00001d038205_T002	3.098027365	2.560411468	3.28817323	3.524230083	3.580169851	3.459219396
Zm00001d038205_T003	1.104129299	0	0.606123805	2.132318878	0.38340736	1.657892398
Zm00001d015289_T001	0	0	0	1.577662299	2.366426294	1.945191164
Zm00001d015289_T002	3.526156348	2.193132086	3.608755673	4.363162034	4.114881789	4.113355034
Zm00001d015289_T009	0.581405242	0	0.830659352	0.339501149	0.87060145	0.667074631
Zm00001d015289_T007	1.356312866	0.865266387	1.251945059	3.13265646	3.0088273	3.154532498
Zm00001d015289_T004	0	0.211424689	0	0.424334144	0.468338286	0
Zm00001d023331_T001	0.574618896	0.529389198	0.577143101	0.698072645	0.8317127	0.669679007
Zm00001d028691_T002	4.875777655	5.063207902	4.884395715	5.158024878	5.171192734	5.242717696
Zm00001d022193_T002	0.288782596	0.400066791	0.666901081	1.343042198	1.481870326	0.95000623

Zm00001d029703_T001	3.153291061	2.503541336	3.138304325	1.920878645	2.227005989	2.183703419
Zm00001d039280_T012	5.470861711	6.065026028	5.429150444	4.204193079	4.681982053	4.53603866
Zm00001d039280_T005	0.54060562	1.078880998	1.261282271	0.626827853	0.792562204	0.656934718
Zm00001d039280_T010	1.537082816	0	1.871644969	1.135783725	1.002975374	0.84334715
Zm00001d039280_T008	2.904315493	2.318421261	2.573238055	2.659013455	2.161782902	2.342497571
Zm00001d039280_T009	0.416300387	1.331757375	0.542565132	1.074669152	2.268272987	1.493575607
Zm00001d039280_T007	1.660206678	0	1.915102468	1.678684019	1.773422508	1.788728302
Zm00001d039280_T004	2.583604045	2.294946522	3.046330752	2.796974575	2.655473422	3.154775506
Zm00001d039280_T001	4.012505208	2.543054346	4.134879533	4.51395683	3.97063751	3.895480523
Zm00001d039280_T006	1.504866456	0.609055189	0.849595008	1.638906999	1.30988688	1.488811641
Zm00001d045050_T001	2.173988955	1.871629593	1.419860833	2.585441156	2.130866309	2.345417666
Zm00001d045050_T002	3.390517466	2.887280967	3.759307873	3.775181632	4.015242777	3.928331851
Zm00001d024839_T001	9.219236284	8.823342583	9.24518767	9.453112378	9.49380572	9.501093866
Zm00001d035367_T001	7.293236964	6.85936338	7.489067297	6.727577488	6.634548401	6.781017269
Zm00001d035750_T001	2.360937027	2.222736875	2.135427829	2.880901962	2.516274153	2.888121916
Zm00001d040235_T005	4.822685694	3.884215854	4.887763832	5.146481635	5.148619263	5.175668384
Zm00001d040235_T001	0.752528532	1.548146577	1.033964913	1.019503375	1.036762134	1.286108153
Zm00001d040235_T002	5.468698976	6.08486059	5.323761316	5.072037009	4.923281509	5.329033658
Zm00001d040235_T003	0.584715298	0.490176801	1.055203291	1.272336187	1.515981854	1.286560053
Zm00001d032442_T003	6.228301767	6.360148365	6.174322899	5.8453663	5.97198055	6.009630125
Zm00001d032442_T007	4.682121879	5.894491774	4.777823456	4.569027233	4.906658447	4.292930575
Zm00001d032442_T008	3.054090646	3.218254201	1.87952581	2.541869399	2.035065373	2.702108284
Zm00001d032442_T005	3.995398859	0	3.953995529	4.878800515	4.636246425	4.980185789
Zm00001d030138_T001	5.297110547	6.717329038	5.343570454	5.723360819	5.617646036	5.311621216
Zm00001d028285_T001	2.842584676	2.246812107	3.254489132	3.575637869	3.746023984	3.753798979
Zm00001d028285_T002	2.376103643	1.551204436	1.014693073	1.733668879	1.67241629	1.544432465
Zm00001d027742_T001	7.704797041	8.304263153	7.677036428	4.768709741	5.083601695	4.876842971
Zm00001d032266_T004	0.52127391	0.360681157	0.467205387	0.72157915	0.660520688	0.729064575
Zm00001d032266_T001	4.103792024	4.076041506	4.154197566	4.033510045	3.9132816	4.116602303
Zm00001d029550_T008	1.825314691	1.116766632	1.580631287	2.212926933	1.310864166	2.179123095
Zm00001d029550_T002	0.312384041	0.282304574	0.405075276	0.669859477	1.184251726	0.920719375
Zm00001d029550_T004	0.441619456	0	0.342791383	0	1.082212753	0

Zm00001d029550_T005	1.06551441	1.034667191	1.271315109	1.432178003	1.723185007	0.628110993
Zm00001d029550_T017	1.483289025	3.40137202	1.872397758	0	0	0
Zm00001d029550_T007	1.089161166	0	1.26362639	1.117735597	1.318870961	0.963175196
Zm00001d029550_T016	1.209717861	0	1.040872096	0.792149029	0	0.970170421
Zm00001d029550_T006	0	0	0.358684322	1.102070857	0	1.24820084
Zm00001d012600_T002	1.37236766	1.139946822	1.334459583	0.899319506	1.196851879	1.038141151
Zm00001d023477_T001	6.19039765	6.296612314	6.519545427	5.372625609	5.40931975	5.668065102
Zm00001d041221_T001	1.616815466	1.664941062	1.900972658	2.101701168	1.824541403	1.953124409
Zm00001d010198_T001	5.170123559	4.433809545	5.206502456	5.345078817	5.37724118	5.257424466
Zm00001d006608_T001	3.016736274	2.640475402	2.962380406	2.921425667	3.176101244	3.157931859
Zm00001d022598_T001	1.50585791	0.942672138	1.488626574	1.092080215	1.206581964	1.357146586
Zm00001d034414_T002	8.04581585	8.211485486	8.118632075	9.010167721	9.023209433	8.996668301
Zm00001d048401_T001	1.355400856	1.696820009	1.294908304	0.413925474	0.124129585	0.482534418
Zm00001d010693_T001	2.847595902	2.352278582	2.779514402	3.223737622	3.397516174	3.104716408
Zm00001d014349_T001	3.582342834	3.921146563	3.761427302	2.097388982	2.570595293	2.307239651
Zm00001d053300_T005	0	0	1.726621989	0.909368363	0	0.860346269
Zm00001d053300_T003	3.611037667	2.56453938	3.458603405	2.454118763	2.546101516	2.590502379
Zm00001d053300_T001	1.302422588	1.219337683	0.767858159	2.190324712	1.479389932	1.140629471
Zm00001d053300_T007	0	0	0	1.526722385	2.083721244	2.506629868
Zm00001d035187_T001	2.23031155	1.847823773	3.126828018	2.329094886	2.43272642	2.753177055
Zm00001d035187_T002	3.318068778	3.24202455	2.509897476	3.554794375	3.434031946	3.159490334
Zm00001d014958_T001	3.942769148	3.445009547	3.89620073	3.785187688	3.859432909	3.83981259
Zm00001d033046_T002	3.802278018	2.70382231	3.825250978	3.541953628	3.645310616	3.57697906
Zm00001d033046_T001	0.857676107	0.103974263	1.008828196	1.035789986	0.919378223	1.006646311
Zm00001d018649_T001	0.670616483	0.644972058	0.113837829	0.752381235	0.339396248	1.00922963
Zm00001d018649_T002	1.104691586	0.609020192	1.736436461	0.852108334	1.607156529	0.515677133
Zm00001d039532_T001	3.118314869	2.626425119	3.122815987	3.241496549	2.989411168	3.172717279
Zm00001d009339_T001	4.25008766	3.24553141	4.119332851	4.509555809	4.091963134	4.522400244
Zm00001d009339_T003	3.694954609	4.810817354	3.686648655	3.686587591	3.780270094	2.433194227
Zm00001d012659_T006	1.011999349	0	0.681104687	1.2082719	0.63400306	0.599654859
Zm00001d012659_T008	1.296240069	0.737016088	1.605427484	1.845784682	2.088661986	1.380501724
Zm00001d012659_T007	4.013788728	3.368257939	4.09796755	3.851329287	3.817598496	3.942122696

Zm00001d012659_T005	1.853119787	1.274306992	1.837517433	2.711293576	2.863961636	2.679087971
Zm00001d026586_T003	3.244863623	2.803477936	3.368631894	4.211573816	3.875213389	4.033763922
Zm00001d026586_T002	1.047526523	0.698262047	0.760360631	1.327280938	1.202463486	1.21034148
Zm00001d026586_T004	3.630876041	2.557173563	3.464233433	3.734357045	3.925189289	3.772168703
Zm00001d021324_T001	1.109147232	0.448187354	0.755676387	1.38321269	1.35543807	1.255404047
Zm00001d018309_T015	1.183872664	0	0.727932649	1.515915265	0	1.583814621
Zm00001d018309_T003	6.324482468	5.477115584	6.316347231	6.662625327	6.66996784	6.63497226
Zm00001d040659_T001	1.019036449	0.629730565	0.979279807	1.046053051	0.870662205	1.180335667
Zm00001d050283_T002	0.40941592	0.512586946	0.424717893	0.940849308	0.231227172	0.889630848
Zm00001d010445_T001	6.775362903	6.248204465	6.725212399	6.021886672	5.952911717	6.008027346
Zm00001d010445_T002	2.998775354	2.226608405	2.926838216	2.826934849	2.883403614	2.812916084
Zm00001d003796_T003	5.212426074	5.618617783	5.103066403	4.922153691	5.201751193	5.102819439
Zm00001d053650_T001	4.158811626	3.44786731	4.029103169	3.574647352	3.311420574	3.256117561
Zm00001d009476_T001	4.453313046	4.062566699	4.533824766	3.454956396	4.067700491	3.670296617
Zm00001d009476_T002	6.02235656	5.780102264	6.050481215	5.9644164	5.83104981	5.913547295
Zm00001d052888_T009	0	0.416509019	0.42795152	1.610039975	1.802236652	1.471102119
Zm00001d052888_T003	3.408653581	2.542519073	3.23524746	3.54109141	3.569569835	3.574574213
Zm00001d052888_T020	0.256680994	0	0.782065465	0.47311878	0	0.642071222
Zm00001d016306_T001	3.261154974	2.936818636	3.457086383	2.398023866	2.020463442	2.149037545
Zm00001d031804_T001	3.531156227	3.071016077	3.294304755	2.573395129	2.668110726	2.141230368
Zm00001d040344_T001	4.951360491	4.129574329	4.96202668	5.188469529	5.195738065	5.155453848
Zm00001d008727_T004	0	0.157740952	3.073915667	2.322838148	2.235408161	3.01130545
Zm00001d008727_T003	1.376242589	0.801789413	1.134658611	1.268505658	1.175956307	1.293818939
Zm00001d008727_T001	3.338162096	3.795607482	2.886186257	0	1.452915354	0.616954226
Zm00001d008727_T002	4.58187044	2.128429019	4.16077033	3.62526897	3.7165195	3.122843646
Zm00001d008727_T006	0	0	2.335064439	1.285522364	0.418439328	0.502471147
Zm00001d050219_T017	4.17891936	3.17373829	4.02716874	3.7044257	3.702428046	3.399965383
Zm00001d050219_T006	1.264102807	0	0.60083492	1.152169138	1.519207233	1.934129592
Zm00001d050219_T018	0.443076151	0.453391149	0.207051971	0.590798405	0.675302882	0.563911748
Zm00001d050219_T009	1.655209842	1.219998035	2.234216494	2.791043456	2.815711905	2.779692359
Zm00001d018409_T004	6.006704404	5.147207653	5.934787913	6.251291049	6.21282774	6.294778187
Zm00001d007261_T001	3.933926565	3.719483341	4.229694045	3.990283624	3.592626742	3.845102002

Zm00001d047470_T002	3.381187559	4.056162276	3.554116307	3.193052739	3.480989649	3.329455593
Zm00001d047470_T003	7.696181645	7.498454187	7.698751235	8.401051252	8.439791311	8.404879771
Zm00001d047470_T001	3.858509323	4.048680855	3.980404484	4.218996781	3.864847379	4.222130883
Zm00001d047470_T004	2.195877329	2.285965574	1.852058782	0.595893164	1.456658456	0.391254108
Zm00001d047149_T001	3.031035007	2.459114716	3.322923644	3.082303205	3.030324071	3.044794871
Zm00001d008462_T001	0.930651814	1.422425168	0	0.673603458	0.900769064	0.40317063
Zm00001d008462_T002	4.034243452	3.150332207	4.274575367	3.11929973	3.022602887	3.175986637
Zm00001d028755_T002	7.50775208	6.982305022	7.5608366	7.264712282	7.266175795	7.226718233
Zm00001d028755_T003	1.541105911	0.631490812	1.51668539	3.014438853	3.015743933	3.07179304
Zm00001d020511_T003	7.323997247	7.158684055	7.311576998	7.494578288	7.516036026	7.514049547
Zm00001d038093_T002	0.953742767	0	0.5628847	1.880990682	1.233394196	0
Zm00001d038093_T007	2.850790624	2.370974418	3.283538089	3.022116895	3.348657345	2.91434772
Zm00001d046997_T001	1.869775092	1.231181697	1.858141772	2.661423315	2.675938521	2.586290371
Zm00001d052354_T006	0.609573432	0.109919475	0.333597763	0.394968445	0.87970655	0.848308655
Zm00001d052354_T001	3.70839485	5.477941333	4.071036592	2.722956239	3.070626838	2.699907376
Zm00001d052354_T005	3.33200281	2.437082823	3.359875476	3.744583507	3.616600361	3.619683564
Zm00001d047938_T001	3.547553104	2.791152259	3.659529475	4.74374653	4.788878509	4.689533551
Zm00001d014278_T001	4.719692566	4.196263875	4.797349222	4.655357725	4.753562072	4.568512822
Zm00001d035007_T001	4.096021271	3.560547445	4.201399483	4.785533656	4.639666817	4.75522126
Zm00001d048163_T001	6.153370902	6.498743773	6.26092529	5.819241698	5.998812692	5.788637794
Zm00001d014486_T002	4.169448834	3.643902471	4.453009155	4.012893109	4.168045068	3.895172598
Zm00001d043019_T001	3.25057645	2.860867277	3.222089372	3.894010695	3.935141094	4.380290936
Zm00001d043019_T005	1.370742359	2.767046662	0	2.411481566	2.778911877	2.820300501
Zm00001d043019_T002	7.096799537	5.714588728	7.107238798	7.85538087	7.878993725	7.824173943
Zm00001d018806_T001	3.294451512	3.059785807	3.332860142	3.713874601	3.733617581	3.883684446
Zm00001d015515_T001	4.368949897	3.529950971	4.396090354	7.188454584	7.25736674	7.272572337
Zm00001d043333_T001	1.975897939	1.924642647	1.833699316	1.850575422	1.491272824	1.686771672
Zm00001d052008_T005	0.424072878	0.333053853	0.549223604	0.834885121	1.136615337	1.86744224
Zm00001d052008_T001	4.355465838	3.306194275	4.303652763	5.141228201	5.220263353	5.167483098
Zm00001d052008_T006	0.694367639	0.122840996	0	2.651658156	3.035667894	2.837976333
Zm00001d052008_T004	1.397090472	1.914954419	1.906993697	1.675393221	0	0
Zm00001d033278_T014	0.508805884	0	0	1.390527371	0	0.393958688

Zm00001d033278_T010	0	0	0	2.690632981	3.698096544	2.741799388
Zm00001d033278_T001	0	0.039969372	0	0.407820429	0.241906064	0.252735361
Zm00001d033278_T011	2.730230618	0	2.787415956	4.132953753	3.3488767	4.214639917
Zm00001d033278_T007	4.607147827	3.618366472	4.647135975	4.166039164	4.513068929	4.409857547
Zm00001d033278_T012	0	1.757148303	0	1.617011138	0.568078815	0
Zm00001d033278_T016	0	1.714589745	0	1.305534583	1.22151149	0
Zm00001d033278_T002	0.313380536	0.3456246	0	1.69212854	2.308421457	2.129248062
Zm00001d033053_T001	1.586490948	0.991102933	0.879761434	0	0.076992728	0.240161655
Zm00001d012718_T001	3.746739707	3.149579676	3.802685304	3.582582496	3.520113812	3.390544567
Zm00001d050310_T001	6.275287828	5.648264901	6.369685314	5.948907247	5.946557436	5.94831728
Zm00001d030184_T002	3.486706136	2.570823803	3.432347869	3.615956853	3.669349748	3.710232382
Zm00001d022102_T001	7.643381003	7.325150612	7.78565892	7.560541591	7.550458202	7.551909429
Zm00001d003184_T001	3.012763522	2.933789404	2.879263285	1.418863498	1.490359102	1.658190913
Zm00001d043443_T001	4.827056633	4.057456857	4.874878539	4.214400881	4.172380542	4.179053886
Zm00001d017770_T002	3.672387549	3.073946335	3.776337117	3.604838619	3.528510184	3.3343074
Zm00001d032874_T004	0.380678477	0	0	0.362077074	0.346744759	0.514799947
Zm00001d051542_T046	1.372693615	1.851319292	0	0	0	0.950620701
Zm00001d051542_T050	0.506330783	0	0.343599979	0.214241709	0.531870989	0.576252197
Zm00001d051542_T043	2.071410532	0	2.751692512	2.162791381	3.260674121	0
Zm00001d051542_T071	1.006896165	0	1.292062578	2.49031263	2.512807388	2.101891401
Zm00001d051542_T076	3.258124655	2.718517697	3.040134071	1.032875951	1.598116	1.766285177
Zm00001d048229_T001	4.034472373	3.223516616	3.919064676	2.23485536	2.288950285	1.932269848
Zm00001d014582_T001	3.259479497	3.302191959	3.212807901	2.70167325	2.666072797	2.857442023
Zm00001d020233_T001	3.660418346	2.746451224	3.577658432	3.702674718	3.740669751	3.629228025
Zm00001d041476_T001	2.220667679	1.493139535	1.824180882	2.483345785	2.610026978	2.524235224
Zm00001d012667_T018	0.523318057	0.121522079	0.915838278	0.823932742	1.024522522	0.503525817
Zm00001d012667_T006	0.659703572	0	0.956179351	0.496847835	0.156689139	0.707459413
Zm00001d012667_T029	2.945018258	2.089536794	3.083509537	3.437483837	3.913128289	3.574179516
Zm00001d012667_T024	1.43757878	0.639221048	1.404325493	0	1.636306294	1.347863482
Zm00001d012667_T005	0	0.216313305	0.553620018	1.639227532	0.297883979	0.600369673
Zm00001d012667_T031	1.024851548	0.402975336	0.527542813	0.956725045	0.867735189	1.229873973
Zm00001d012667_T012	0.670369933	0.313350349	0.40380979	1.492919655	1.163942422	0.901148414

Zm00001d012667_T009	0.212763553	0	0	0.50657757	0.445778606	1.273523051
Zm00001d012667_T001	1.855924336	2.282064626	1.969202075	2.42549744	2.262863677	2.519457363
Zm00001d012667_T004	2.744122121	1.024836659	2.866330191	2.47645557	3.12470762	2.85186715
Zm00001d012667_T030	2.856049078	3.550644847	3.202675362	2.772672404	0	0
Zm00001d050134_T001	2.918711186	2.163249137	2.962195663	3.385134875	3.485235657	3.512188574
Zm00001d035475_T001	1.206068032	0.677356692	1.08162323	0.825812487	0.679882253	0.815301619
Zm00001d035475_T002	3.281324659	2.832033052	3.38176276	1.987136742	2.156250333	2.356074773
Zm00001d030372_T001	3.655146745	3.165325418	3.812849666	2.963981567	3.077260092	2.867051153
Zm00001d004231_T001	5.850447156	6.009016674	6.030196526	5.921041659	6.033521498	6.029720396
Zm00001d002941_T001	5.406330163	4.719185084	5.11989302	5.350213949	5.313647907	5.381809784
Zm00001d002941_T002	3.757275376	2.60176045	4.309293787	3.403894845	3.637202279	3.342123039
Zm00001d036968_T017	0	2.450668886	2.208253793	2.075785893	0	1.543428573
Zm00001d036968_T011	1.082157559	0.148396613	0	0.686271919	0	0.57646022
Zm00001d036968_T019	0.136789869	0.152047095	0.419103051	0.764363142	0.653832306	0.59460874
Zm00001d036968_T010	2.204543914	1.172060532	2.304199982	3.373245203	2.990845066	3.372019058
Zm00001d036968_T020	2.795435946	0.686165223	2.333036387	0	0	0
Zm00001d036968_T014	0	0	0	3.274356347	3.404709424	3.033312163
Zm00001d016000_T009	0.444174071	0.152310643	0.172519518	1.148399216	1.010181873	1.139267124
Zm00001d016000_T002	1.097923571	1.343700596	1.134015859	1.346914919	0.799152806	0.241625441
Zm00001d016000_T010	2.304564485	2.297308823	2.163702885	1.917210226	1.613065766	2.334680678
Zm00001d016000_T003	1.492927856	0.517859205	2.130362903	1.789351994	2.047878956	1.928513211
Zm00001d016000_T008	1.270675447	1.635881594	2.506591026	1.880502557	2.125714574	0
Zm00001d016000_T004	2.297287691	0	0	2.516649339	1.864242811	3.04290519
Zm00001d016000_T001	1.162190697	0.647604631	0.774083222	1.205911064	1.175837537	1.566878461
Zm00001d003142_T001	1.590009347	1.164342205	1.497990393	0.768517192	0.591463044	0.550472224
Zm00001d030062_T003	0.680700624	0.595033825	0.89090657	0.965652058	1.076053482	0.98147933
Zm00001d008715_T001	3.514002799	2.624625692	3.583298354	3.172843025	3.203709378	3.108535664
Zm00001d050300_T002	0.721298281	0.40311499	0.813467227	0.803912257	0.779143712	0.47039005
Zm00001d050300_T001	6.541326266	6.382319314	6.675633833	5.958249726	5.945270281	5.975927668
Zm00001d042063_T001	0.224266295	0.262486078	0	3.116888204	2.779796769	2.8046273
Zm00001d008764_T001	0.792139032	0.664634839	0.879085431	0.415353054	0.694667171	0.539726594
Zm00001d027446_T001	5.402862845	4.621356522	5.450146389	5.568263453	5.515916179	5.495284198

Zm00001d008196_T001	3.864840645	4.659661676	3.977859084	2.549749743	2.254998497	1.997063293
Zm00001d018725_T001	6.292806794	6.488839367	6.23266398	5.707508833	5.717588227	5.525429664
Zm00001d045646_T001	5.256036621	4.735162596	5.385463066	5.383149984	5.318352782	5.255303765
Zm00001d019065_T001	1.239074759	0.502568909	1.033604131	1.148036004	1.638413555	1.835897402
Zm00001d020961_T001	6.339216948	5.935331055	6.300145054	7.80933174	7.831083084	7.78098585
Zm00001d020961_T002	1.039580612	0.123933656	0.356763585	1.155857366	1.356469507	1.314154725
Zm00001d034729_T002	3.547556312	3.052405634	3.550469023	2.839730672	2.595862141	2.71890824
Zm00001d034729_T001	0.732883924	0	1.731203231	0.813583791	0.567966902	0.583506568
Zm00001d023919_T001	3.219313983	2.591339764	3.245156702	3.089200836	2.996657554	3.03393902
Zm00001d009161_T002	6.66014136	5.623838688	6.638031541	6.066741636	6.127578063	6.07267809
Zm00001d016105_T001	7.002041006	6.388609797	7.023319955	6.375604701	6.378586669	6.394069491
Zm00001d053713_T001	1.54469359	1.823740802	1.753965593	1.923026304	2.30979057	1.956780421
Zm00001d053262_T031	0.298224269	0	0	0.546243048	0.284242862	0
Zm00001d053262_T030	2.29678306	0.681504138	1.861853757	2.221695225	2.99700655	2.771675601
Zm00001d053262_T016	0	0	0	1.112837558	0.627820659	1.068888144
Zm00001d053262_T037	3.770695128	4.636355428	3.446929639	2.481663961	1.998482208	0
Zm00001d053262_T033	0	0	1.253899133	1.982930437	1.462260199	2.279915476
Zm00001d053262_T036	1.128508535	0.612627092	1.35501288	0.850587423	0.929122129	0.950613983
Zm00001d053262_T014	0.360403609	0	0.463559684	0.063966739	0.208824369	0
Zm00001d009004_T011	2.566284973	2.257327493	2.542616388	1.907466405	2.095246954	1.607499817
Zm00001d009004_T001	1.843394992	1.050345666	2.058140521	2.607057789	2.64918523	2.853487721
Zm00001d020688_T001	3.52149039	3.137381217	3.795477403	3.401743087	3.391716312	3.403853545
Zm00001d037656_T001	4.978100973	5.073993106	5.123181382	1.938253041	1.648556098	1.875128934
Zm00001d017107_T001	3.05208892	3.244503202	3.19420228	2.730589094	1.874940933	2.598670817
Zm00001d024605_T001	3.516092955	2.972423203	3.727092382	3.88702531	4.182118839	4.132802113
Zm00001d027576_T003	1.053807139	0.173995988	0.824857446	0.470234889	0.705972595	0.279120608
Zm00001d027576_T001	3.049573442	3.326417262	2.632198406	2.567489472	2.503435752	1.97499692
Zm00001d027576_T005	6.098630246	5.543524465	5.964751292	5.324164738	5.290377107	5.47113396
Zm00001d027576_T002	4.126260664	3.627564934	4.442546518	4.244799247	4.1301941	3.970652233
Zm00001d027576_T004	1.636603741	1.066556978	1.739965155	1.208925477	1.349205598	1.441330491
Zm00001d049753_T003	1.0970943	0.72398399	0.828645391	1.17313703	0.707885199	1.176491928
Zm00001d049753_T002	0.539176682	0	0.896652383	0.267746719	0.853666763	0

Zm00001d018869_T001	2.228065369	1.599314937	2.233207649	2.987038303	2.954857006	3.011931387
Zm00001d004956_T002	3.470983856	2.816079874	3.273504253	3.441148264	3.411823881	3.239764932
Zm00001d004956_T003	2.87420895	2.026506534	2.686707588	2.452798621	2.739154449	3.076856975
Zm00001d004956_T001	2.086784785	1.424596975	2.346281252	2.005925586	1.857136224	2.104715401
Zm00001d011785_T001	4.085068528	5.151384441	4.069731635	3.547911487	3.712898594	3.603038169
Zm00001d020184_T001	7.247867004	7.781473603	7.44793186	6.475263538	6.47542758	6.536160461
Zm00001d026406_T001	5.346101688	6.454372046	5.450614884	2.681864585	2.926419447	2.915548626
Zm00001d006682_T002	1.889409681	0.95074237	1.869391349	2.855107072	3.166601696	2.882407308
Zm00001d006682_T001	2.664136233	2.118036063	2.725400453	3.412361931	3.428848286	3.371726958
Zm00001d049188_T002	1.312057534	0.531501737	1.625537492	1.690768395	2.192059044	2.002185109
Zm00001d049188_T007	0	0.1425742	0.967787616	0.887730353	0.821543694	0
Zm00001d049188_T005	2.334629483	0.684716421	2.475677667	1.93487048	1.791659064	2.074979406
Zm00001d017677_T003	0.543123655	0.660778053	1.171153014	1.274778106	1.773483698	1.861065262
Zm00001d017677_T002	2.355955853	1.771598278	2.178984514	2.229942554	2.235762897	2.476095194
Zm00001d036535_T001	4.671640982	4.133520537	4.704186079	2.516269866	2.341776869	2.286735984
Zm00001d034783_T001	1.612378451	2.006452789	2.234227225	5.817586908	5.688666165	5.616190026
Zm00001d017284_T001	2.649476586	1.923243518	2.732076393	5.319694192	5.369620237	5.32588891
Zm00001d025037_T001	1.691372914	1.534103946	1.989815135	1.319249121	1.230538742	1.160082459
Zm00001d030171_T001	3.289246794	2.712111059	3.438997258	3.428109821	3.296122575	3.242771687
Zm00001d049581_T001	3.684949875	3.179171356	3.71026611	3.440097354	3.442649355	3.307594644
Zm00001d047634_T002	3.33335217	3.060595815	3.426353058	3.470834614	3.153300142	3.579446887
Zm00001d047634_T001	3.754104387	3.368162653	3.356509791	3.176766525	3.553680835	3.397613236
Zm00001d024999_T002	1.202087298	1.865823028	1.730814264	1.197340794	1.507025844	0.82805065
Zm00001d009424_T013	1.476984046	0	1.416736534	1.493428556	0	0
Zm00001d009424_T010	2.027046801	0	1.64603267	2.947157618	3.058072446	2.952238129
Zm00001d009424_T011	0	0.66329725	0	1.08512004	2.148920767	0
Zm00001d025507_T017	1.880692577	0.873554159	1.577164375	1.778431062	2.047416938	1.766406889
Zm00001d025507_T020	0.275163232	0.137595329	0.187760147	0.484567137	0.401199013	0.461701421
Zm00001d025507_T008	1.046664271	0.380764906	1.064076455	1.45269769	0	0
Zm00001d025507_T019	2.12857815	1.694941687	2.512198563	2.681128756	1.831949779	1.423307623
Zm00001d025507_T014	4.084357112	4.759862091	4.065213834	3.624646746	3.168699473	3.456623239
Zm00001d025507_T015	1.145537906	0	0	1.039145414	2.177433731	2.010437203

Zm00001d025507_T004	0.170243	0.836231978	1.053581961	0.875121855	0.916595877	0.208250046
Zm00001d025507_T013	0.412717585	0.189652532	0.46706867	0.651467357	0.498986072	0.792378955
Zm00001d025507_T018	1.14514201	1.090159686	1.005444544	0.701236753	2.04241636	1.847321888
Zm00001d045472_T002	1.618115528	1.403860496	2.296569294	2.302662975	1.899567	1.98327237
Zm00001d045472_T001	3.986043525	3.431128695	3.857348956	3.544123925	3.663761426	3.594348135
Zm00001d047603_T005	0.562080711	0.303435921	1.298075842	0.951913744	1.265883318	0.407165637
Zm00001d047603_T003	2.922271504	2.822473805	3.056369189	2.238074506	2.348377758	2.866121253
Zm00001d047603_T001	2.577201363	1.966598058	2.530407149	2.6470826	2.799126482	2.50109617
Zm00001d042093_T002	2.301738054	2.19480948	2.948061792	2.635346247	2.650351218	2.595990524
Zm00001d042093_T001	1.24908485	0.964228567	1.23971331	1.412833001	0.571585562	1.138492087
Zm00001d012832_T001	2.419001354	2.221791414	2.806009497	2.784655992	1.948897952	2.260612253
Zm00001d012832_T004	4.636989033	4.983688684	4.753962545	3.57629973	3.84271128	3.849747716
Zm00001d049886_T001	3.891948629	4.434859908	3.998493671	4.10048725	4.275516	4.502938068
Zm00001d047252_T001	2.517357087	2.282268095	2.335550141	3.498035085	3.561340435	3.37158451
Zm00001d028534_T005	1.853697499	1.27041711	1.833905719	3.399583605	3.492244559	3.435599927
Zm00001d028534_T006	0.10266888	0.10390043	0	0.576149625	1.078980707	1.220118179
Zm00001d028534_T003	0.150363405	0	0	1.75947545	1.718809189	1.341247991
Zm00001d028534_T001	2.227654832	2.411600877	0	3.563721062	0	0
Zm00001d047263_T001	2.57436071	2.771300566	2.694285167	0.725970574	0.668809891	0.9373685
Zm00001d038752_T001	0	0.235714808	0.338117444	1.03787276	1.0505811	1.37865755
Zm00001d038752_T005	0.407126471	0.184501205	0	0.562880794	0.489565542	0.290281661
Zm00001d038752_T006	1.069001612	0.493109297	1.0035641	0.716075178	0.989899038	0.740357707
Zm00001d012787_T002	1.364017658	0.486556839	1.339358049	1.530021786	1.443869707	1.86285523
Zm00001d015884_T001	2.071366251	1.572849418	1.869484925	2.372605587	2.420859364	2.416993729
Zm00001d015485_T001	1.163154119	0.711881629	1.351010868	1.091983438	1.287578676	1.138296132
Zm00001d050917_T003	4.163581733	0	3.974049252	4.424007285	4.389252358	4.100996396
Zm00001d050917_T002	4.74007329	4.299412528	4.82189457	6.183019369	6.204976216	6.171609806
Zm00001d050917_T001	0.226515928	0.060365641	0	2.038535565	2.376635177	1.599176374
Zm00001d027728_T001	5.120471261	4.351777919	5.14509806	5.331318626	5.326094419	5.349481982
Zm00001d052996_T001	0.87457666	0.395543252	1.144603756	1.711216473	1.720820414	2.155306579
Zm00001d052996_T002	0.651893463	0.541379038	0	1.196115991	1.885267832	1.17759
Zm00001d049657_T001	4.645057743	5.816829992	4.858947824	3.58418107	3.83209991	3.574777151

Zm00001d017837_T001	6.581739123	6.310914394	6.330448796	7.176464183	7.057706718	7.234467493
Zm00001d048634_T001	2.759636084	2.338975999	2.870922355	2.997602956	3.267845278	3.323963167
Zm00001d017360_T004	0	1.67354557	2.705570573	3.851608038	3.420271918	3.777062483
Zm00001d017360_T006	2.685633193	1.569966403	2.324681014	2.355076046	2.339873371	1.839251768
Zm00001d017360_T016	4.246320444	3.493781172	3.864640503	1.671677436	3.065253305	2.541358717
Zm00001d017360_T009	0.322126596	0	0.32371247	0.206463188	0.327133171	0.100304906
Zm00001d017360_T008	0.307426194	0.266240836	0.1765832	1.044294789	0.655422359	0.591100117
Zm00001d017360_T003	0.157277886	0	0.458449202	0.870243972	1.01067387	0.905009635
Zm00001d027955_T001	2.65666866	2.10778862	2.313315517	2.088980098	2.653604194	1.305437107
Zm00001d022425_T002	3.387389977	2.58074391	3.618554181	4.1497446	3.936741385	3.954080518
Zm00001d022425_T009	0.842763266	0.478903486	0.639798941	0.997787374	0.946972599	0.914308872
Zm00001d022425_T001	3.863284333	3.319312861	3.724150262	2.625945382	3.353917751	3.167573256
Zm00001d014097_T001	3.373138132	3.456427293	3.248157871	3.669195977	3.766251036	3.758755361
Zm00001d037736_T001	4.19392649	4.726448972	4.038616419	3.911396302	3.752066039	4.047444684
Zm00001d014062_T001	3.370001584	2.747280402	3.248676913	3.373600469	3.223479083	3.514991025
Zm00001d052375_T002	5.441775236	5.524875872	5.610437446	5.69028704	5.427444129	5.488661244
Zm00001d052375_T001	4.329858394	5.43636029	4.6837687	3.896540403	4.333948118	4.26517942
Zm00001d034916_T001	4.59024145	4.129751363	4.613657412	3.475950889	3.286223507	3.466439186
Zm00001d010366_T001	1.393448053	0.955998646	1.244476715	1.051674879	1.225115102	1.636801387
Zm00001d017186_T002	2.757815231	1.765901738	2.922085359	5.326139517	5.356720386	5.3577343
Zm00001d017186_T003	7.345763212	6.006424086	7.404278823	8.662532669	8.679511645	8.661695164
Zm00001d017186_T001	4.731384151	5.644516677	4.955989071	6.091553103	6.018290349	6.068423713
Zm00001d006142_T003	0.530754961	0.535318431	1.041571021	2.224538276	2.333229644	2.027325706
Zm00001d006142_T006	4.48992334	0	4.12338387	3.157248778	0	2.418452011
Zm00001d006142_T002	4.277729427	4.414669407	4.45631689	6.093461364	6.228363997	6.076465375
Zm00001d006142_T011	4.104876144	2.762379009	4.399223467	3.756589733	3.121101628	3.822763386
Zm00001d025573_T001	0.946626069	0.833793618	0.980801112	0.190267181	0.326444157	0.223711678
Zm00001d006342_T002	1.387982281	1.106391204	0.906678985	0.93379469	0.553673094	0.778592963
Zm00001d006342_T003	4.197468737	3.798734155	4.392235874	4.092743697	4.129653783	3.997914882
Zm00001d032555_T011	0.511374927	0.598273724	1.400440654	1.659269262	1.99786034	1.396291036
Zm00001d032555_T008	3.349334277	0	3.528613449	3.065092999	2.98120366	3.201880353
Zm00001d032555_T005	0.569464843	2.157258158	1.275404628	0.651608794	1.107430103	1.312893408

Zm00001d032555_T014	3.881576528	2.182388473	4.094491813	2.907868988	2.874433837	1.88320124
Zm00001d032555_T010	1.61466933	0.81479239	1.175633178	1.676340534	1.266190453	1.483138341
Zm00001d032555_T002	2.034818239	0.714195393	0.886253579	2.919092524	2.817723322	3.156678462
Zm00001d008890_T001	1.697235154	0.854673136	1.219478328	1.816133136	1.781882976	1.933269716
Zm00001d049922_T002	1.810322172	0.721796109	1.267490854	1.956026534	1.738843914	1.478634317
Zm00001d041702_T001	2.129495366	1.520388554	2.288932573	1.94457625	1.951503871	2.207459041
Zm00001d021617_T001	2.499843336	1.555208525	2.469403869	2.724629755	2.553880959	2.536275008
Zm00001d021617_T002	0.636813449	0.544473516	0.735260476	1.327158471	0	1.622153522
Zm00001d053281_T008	4.744781321	5.732416528	2.87072059	4.223018029	2.756891136	3.174192268
Zm00001d053281_T005	3.328713554	5.843388132	3.234257274	3.80063002	2.24301125	0.927109108
Zm00001d053281_T007	1.652430048	1.053552768	1.936604397	1.410671617	1.667437111	1.589233283
Zm00001d053281_T006	1.899469554	1.180489076	1.407635008	1.244306212	1.568786585	1.049032657
Zm00001d053281_T001	7.063142299	1.774067193	7.152444133	6.803898209	7.032522666	6.980767964
Zm00001d051740_T006	0.515444009	0.163478122	0.731303171	0.940823004	1.221862855	1.104723116
Zm00001d051740_T007	0.568523462	0	0.182164696	0.423662068	0.624925324	0.717935852
Zm00001d051740_T002	2.405077183	1.730629958	2.423019271	2.238958017	2.258427566	2.348582851
Zm00001d004468_T003	0.93738432	0.663273565	0.309304266	0.684277457	0.732289173	0.837822171
Zm00001d029212_T010	0.559399791	0	0	1.10409373	0.693292912	0.761345704
Zm00001d029212_T009	4.760555496	0	4.678678611	4.496877611	4.450375855	4.403294517
Zm00001d029212_T004	4.070224525	1.928620463	2.846129813	4.25969913	4.475133404	4.307426631
Zm00001d040644_T001	3.708997655	3.293977954	3.782545593	3.413291594	3.471275008	3.514331736
Zm00001d006752_T001	2.248227259	2.074000473	2.533493579	2.236903709	1.682960211	1.595835414
Zm00001d006752_T002	1.220037052	0.615677127	1.251312506	1.624326934	1.88404887	1.716787696
Zm00001d035720_T001	7.608441323	6.855269889	7.556990726	7.111245207	7.191942481	7.100251735
Zm00001d035720_T002	5.722089153	5.194567017	5.885938604	6.050344338	5.996120725	6.000140701
Zm00001d039046_T003	0.763032472	0	0.37598803	0.617041709	0	0
Zm00001d039046_T006	0	0	1.548526883	0	1.989749026	2.651011956
Zm00001d039046_T001	2.569294944	2.108004826	0	1.145551601	1.44377584	0
Zm00001d039046_T024	3.552038738	3.84427135	3.302260845	2.807285259	2.524601576	2.166720265
Zm00001d039046_T011	1.834856408	0.527572838	2.872468681	2.985762168	2.749370145	2.217791242
Zm00001d048857_T004	1.14996428	0.743184901	1.020516425	1.383404042	1.468319516	1.330857807
Zm00001d048857_T003	0.958297781	0.705980555	1.008435256	2.131448895	1.877623333	2.328850535

Zm00001d048857_T006	3.925618003	2.635193453	3.957533729	4.279619569	4.470192061	4.301406859
Zm00001d048857_T002	1.006264986	2.139026075	0.6825715	0.695462946	0.742853033	1.42366153
Zm00001d026475_T001	4.440194651	3.876573818	4.502094483	4.515103906	4.642642084	4.614452668
Zm00001d023737_T001	2.815855129	3.107458894	2.723883759	2.706698074	2.704881479	2.386561281
Zm00001d047030_T029	0	0	0	2.303457548	1.885396701	1.949018645
Zm00001d047030_T001	1.158271742	2.03885123	1.496936267	2.229781089	2.748900415	2.291226923
Zm00001d047030_T074	4.68643559	3.390177763	4.988534411	4.433949451	4.558769551	4.67501856
Zm00001d047030_T011	2.339573549	0	2.538652383	3.162369575	2.766994736	3.106327379
Zm00001d047030_T085	4.320525835	5.031306216	3.933363961	4.285671127	4.249396416	4.10784075
Zm00001d047030_T047	0.313891292	0.091980054	0.234742874	0.318186089	0.891503957	0.832941849
Zm00001d047030_T023	0.246214701	0.040843347	0.083821981	1.041273827	1.459889012	0.536108617
Zm00001d047030_T018	3.572793162	2.969703088	3.421838897	2.672212599	2.617932227	2.235113401
Zm00001d029628_T001	7.88810079	8.634261916	7.973700379	6.626034921	6.741116679	6.628059229
Zm00001d039411_T009	4.961612633	4.040005242	5.011974579	4.891025746	4.728502696	4.901453184
Zm00001d039411_T002	4.437038399	6.340185382	4.441223309	3.938931143	3.724414751	3.06103479
Zm00001d039411_T001	1.449967517	0.555640469	1.014168159	1.479266266	1.26424456	1.481963296
Zm00001d039411_T003	2.548778639	0	2.415840857	4.072193715	4.284633761	3.893762925
Zm00001d026675_T004	1.767579802	3.466453151	2.729311739	2.071718743	2.072262927	2.845961472
Zm00001d026675_T002	4.675559994	3.687233846	4.576020675	4.422197337	4.475465875	4.448468408
Zm00001d029783_T003	3.441046648	3.30613287	3.460513628	4.35072806	4.397367692	4.406408912
Zm00001d029783_T004	1.882132783	1.341535511	1.844152216	2.218316207	1.824719389	2.207275046
Zm00001d029783_T001	2.168364444	1.376092527	2.583422564	1.003498618	0.302213752	1.986576585
Zm00001d006725_T001	6.715317317	6.03661863	6.535156021	7.161191928	7.1527566	7.119644449
Zm00001d025922_T001	3.79741698	3.123885497	3.802525774	4.781991371	4.816181377	4.723509579
Zm00001d016364_T001	4.467905991	3.505696483	4.530675131	3.140350691	3.078553308	3.15230253
Zm00001d016364_T003	0	4.486572671	2.774553469	1.724952311	1.422158172	2.072173731
Zm00001d016084_T004	0.341301514	0.114077791	0.0732115	0.708793769	1.010254932	0.634861803
Zm00001d016084_T005	0.540789097	0	0.691493072	0.524156976	0	0.29371184
Zm00001d016084_T002	3.199597912	2.302663853	3.164615583	3.263342749	3.262181912	3.319645967
Zm00001d040466_T001	3.282101555	2.949234597	3.114550765	3.717172235	3.830367396	3.728580894
Zm00001d040466_T002	2.679701455	0.853622853	2.993500467	0	0	0
Zm00001d027499_T001	0.726403139	0.460188966	0.620432515	1.025821173	1.023921003	0.996252787

Zm00001d049479_T002	5.030718333	4.328466554	5.090531751	5.126265909	4.974067751	5.011459768
Zm00001d049479_T004	1.078859826	0.453839927	1.078777866	1.870031258	1.763162959	1.743165939
Zm00001d049479_T001	0.585501006	0	0.816295777	2.289867213	2.171029683	2.223585655
Zm00001d019185_T005	0.163498732	0.223129656	0.181465167	0.548417882	0.567936733	0.558487083
Zm00001d030361_T001	3.339046857	2.970780782	3.600010407	2.243168194	2.787450435	2.642012055
Zm00001d029118_T001	3.514213176	2.8641376	3.685624559	3.668730257	3.643776666	3.636114498
Zm00001d029118_T003	1.08533763	0	0.881531502	1.636492844	0.932424068	1.670345004
Zm00001d049738_T001	6.200199058	5.942315329	6.118666976	5.800729639	5.858815104	5.752537095
Zm00001d005298_T001	3.233815832	2.293650932	3.34831104	3.249798311	2.965422296	3.061005921
Zm00001d021390_T004	2.611874667	2.328192506	2.459246417	2.619844229	2.212724027	2.34818397
Zm00001d021390_T005	0.505262916	0.276800359	0.648227966	0.581899777	0.900439852	0.818088119
Zm00001d021390_T001	3.154229977	2.158808153	3.281483128	3.17973524	3.297223704	3.174935427
Zm00001d021390_T003	0.470976179	0.425713876	0.935900975	1.204930101	1.499805077	1.129675377
Zm00001d000042_T002	2.312527519	1.272439509	2.166944523	2.747705982	2.768204655	2.608552373
Zm00001d000042_T001	0.452592272	0.65763086	0.840350432	0.769330825	0.254883037	0.880955822
Zm00001d039637_T001	2.49657637	1.906994466	2.919460795	2.40056498	2.373613135	2.562989928
Zm00001d011570_T004	1.188408521	0	0.786480942	0.567190084	0.848520189	1.080796128
Zm00001d011570_T001	0	1.401502136	0.484645499	1.369334143	1.475680002	0
Zm00001d011570_T006	1.093168013	1.170393	1.493694467	2.521872387	2.483037189	2.497206642
Zm00001d016878_T001	1.524214158	1.23645833	1.857129454	1.418205603	1.511638562	1.73462292
Zm00001d045894_T005	0.49114504	2.504179	0	2.259849148	2.23456815	0.849144194
Zm00001d045894_T001	3.912043921	3.56700957	4.144398188	4.162187071	4.050489769	4.175194747
Zm00001d045894_T004	3.505049197	0	3.241977279	3.659202574	2.063746247	0
Zm00001d045894_T002	2.930346953	2.21315688	2.988609437	3.370104562	3.946535026	4.040172306
Zm00001d045894_T003	1.410648282	0.612071246	1.399347762	1.530068244	1.683001099	1.742361989
Zm00001d046460_T001	3.66889576	3.220321441	3.700975413	3.383114654	3.396512035	3.411836759
Zm00001d042874_T002	2.913529583	4.766682346	2.698308949	2.274778106	2.401287226	0
Zm00001d042874_T005	5.066892139	4.257673864	5.050534521	5.216183995	5.273278877	5.187402277
Zm00001d042874_T004	5.112696505	0.275716298	5.234577845	4.864232112	4.895995443	4.941735427
Zm00001d043449_T001	2.684335589	2.03827041	2.845595025	2.411858989	2.613722821	2.784252484
Zm00001d006256_T001	5.668103066	6.260079605	5.898140667	4.916781298	4.886822371	4.746199507
Zm00001d051334_T001	3.282014941	2.6967626	3.182461257	4.167932627	4.290295744	4.218291018

Zm00001d045231_T002	0.407485457	0.70051518	1.03122579	1.536484152	1.851336884	1.675564849
Zm00001d050139_T006	1.284318089	0.695128827	1.028051386	0.61862301	0.887229683	0.90747525
Zm00001d050139_T004	1.319508109	0.528788332	1.211286996	1.683767858	1.565806254	1.639916066
Zm00001d050139_T007	5.056475496	4.148789258	5.15655684	5.102214758	5.095037987	5.019229047
Zm00001d024393_T001	3.439248387	3.510624618	3.338406871	4.035703784	3.498312404	4.1705542
Zm00001d024393_T003	4.293392704	2.815725633	4.277532607	4.153408216	4.555963423	3.916766492
Zm00001d024393_T002	1.891781176	3.19485848	2.387451328	2.834026916	2.712226421	2.394722108
Zm00001d027683_T001	2.546148942	1.860464669	2.550904604	2.57132756	2.62777631	2.517574785
Zm00001d002158_T001	9.713427821	6.224029351	9.622103955	6.857132901	6.79603983	6.905730166
Zm00001d014901_T001	5.660878972	4.577274006	5.635263437	5.827576184	6.001782047	5.835330558
Zm00001d014901_T006	2.046324465	0.952166547	2.003307551	2.304039899	2.307710625	2.059779924
Zm00001d014901_T002	0	2.650974286	1.676985868	0	1.313949321	0
Zm00001d052795_T001	3.947792656	3.464025968	3.964084828	5.081699447	5.076366047	4.902781147
Zm00001d005841_T001	1.566772786	1.790746186	2.109516355	3.903541775	3.871721349	4.204738272
Zm00001d015060_T001	1.458606161	1.248094553	1.946103498	2.197996825	2.448566133	1.912850257
Zm00001d015060_T002	1.857776018	1.446566971	1.595211941	1.952736123	1.763448544	2.323502947
Zm00001d024347_T001	5.273339831	5.075857201	5.173710272	5.159501435	5.143640718	5.003990336
Zm00001d037574_T001	5.50919987	5.59801509	5.312582009	4.59605656	4.715259774	4.848247453
Zm00001d031505_T001	4.296923693	3.580288179	4.14533239	4.10516245	4.01042843	4.217435316
Zm00001d031505_T002	1.059655692	0.685413642	1.008979464	1.544727216	1.413036718	1.40119082
Zm00001d045128_T001	4.044903782	3.364427595	4.155273706	4.683201531	4.752737461	4.680595484
Zm00001d033317_T001	4.244400143	4.739534639	4.587498392	4.35060891	4.348382078	4.404994377
Zm00001d048430_T014	0.079583598	0.106242442	0.089992975	1.13285384	0.859636458	1.156461986
Zm00001d048430_T001	0	0	0	0.845054818	0.642776419	0.43612985
Zm00001d048430_T002	0.728207005	0.922200132	2.010465132	2.140479617	2.658058578	1.414591818
Zm00001d048430_T016	3.968692134	3.115284161	3.798099053	3.747109576	4.265922014	3.724503594
Zm00001d048430_T005	1.43176789	0.765042449	0.355178685	3.214085317	2.668354712	3.419265563
Zm00001d012492_T001	0.899703877	0.733599036	1.14255917	0.699532088	0.535557324	0.67902377
Zm00001d043422_T001	6.316729148	7.449822703	6.397503236	5.215453051	5.053462321	5.185278805
Zm00001d022602_T001	3.862897273	3.732490852	3.78792982	3.675005062	3.85917207	3.848279307
Zm00001d045391_T001	8.699045662	8.517388687	8.833883433	2.454415445	2.624899597	2.745638993
Zm00001d044982_T007	0.453457526	0.177829793	0.240045612	1.774054538	1.395565735	1.611658706

Zm00001d044982_T015	1.425765966	0.666604817	1.177706075	2.643170689	2.508934648	2.885513063
Zm00001d044982_T011	0	0	1.095339124	1.599073039	1.386587491	0
Zm00001d044982_T016	3.304556162	3.314131372	3.691414791	1.475001848	2.539783161	1.860797171
Zm00001d044982_T010	1.709088115	0.826227525	1.239890466	2.828818405	3.06237993	2.84432338
Zm00001d044982_T009	1.856228001	0.933961585	1.600112723	3.674339292	3.827796063	3.718615562
Zm00001d042333_T001	3.242295338	2.521928151	3.180791869	3.225748294	3.417790419	3.447602338
Zm00001d009667_T001	3.225547801	2.813402784	3.130480204	3.45966689	3.472877766	3.277846123
Zm00001d023282_T001	1.83300421	1.807365639	1.962487945	1.704810449	1.394460362	1.605272437
Zm00001d028366_T001	6.268588091	5.495704084	6.330569819	5.081381899	5.092722051	5.089507473
Zm00001d034800_T001	0.446123866	0.377660182	0.58268124	1.516544206	1.609111467	1.579037706
Zm00001d008731_T005	0.376956004	0.4376624	0.104052119	0.957590002	1.114460314	1.19511133
Zm00001d008731_T003	4.814774142	4.433835645	4.959588277	4.473531593	4.311709376	4.398421655
Zm00001d008731_T006	0.645806779	0	0.725692303	0.831287885	0.726578396	0
Zm00001d028725_T001	4.716896705	3.910014013	4.618593705	6.035074371	6.001921157	6.095364022
Zm00001d007125_T001	0.777210542	0.441010649	0.903302869	0.576301545	0.612159025	1.033863452
Zm00001d034404_T001	6.071440781	6.15055464	6.00458112	5.823494166	5.900926398	5.739655362
Zm00001d035425_T001	1.514320751	0.635284481	0.58036935	2.538314417	2.461380039	2.945553208
Zm00001d008323_T007	0	0	0	1.518047612	2.351767391	1.414786629
Zm00001d008323_T008	0.409946996	0.42145869	0.580547839	1.385796566	1.289741823	1.023412212
Zm00001d008323_T004	3.323440545	2.535829763	3.171979378	3.427180274	2.813649663	3.190317916
Zm00001d008323_T011	0.571347707	0.296619509	0.301544336	1.094288101	1.752983599	1.351545225
Zm00001d008323_T017	0.587263207	0	0.426157387	1.003635336	1.212596107	0.96887448
Zm00001d049046_T002	2.419846005	1.64377909	2.439008432	2.885002053	2.996931915	2.969975257
Zm00001d009849_T005	0.584116834	0.24177552	0.717347191	0.777489153	0.47178991	0.771038724
Zm00001d009849_T001	3.634586183	3.112582211	3.692285354	4.150579578	4.039613247	3.922106392
Zm00001d010924_T001	4.586916209	3.989555584	4.669573117	3.71563577	3.713480604	3.712396915
Zm00001d005833_T003	3.429289229	3.216860687	2.966260088	2.316724732	2.33297053	2.280359223
Zm00001d005833_T004	4.313022246	3.201340274	4.448237251	3.976878111	4.018638447	4.20045578
Zm00001d005833_T001	1.200220583	0.643629034	1.130086745	1.380435783	1.57237263	1.3548448
Zm00001d044171_T001	2.157800765	1.782504182	2.217177351	4.698695889	4.805258916	4.733509991
Zm00001d007105_T002	2.735986971	2.220121895	3.09360057	2.492098013	1.957218149	2.347277579
Zm00001d018789_T001	3.299091645	2.438754068	3.308913736	3.740740116	3.781579301	3.75815519

Zm00001d003050_T001	1.204243405	1.043996755	1.230626658	1.44258208	1.67679997	1.42372929
Zm00001d043834_T002	6.115857324	5.205484164	6.082859396	6.164332388	6.203308845	6.164305937
Zm00001d032188_T001	3.245865873	2.103705091	3.51782079	3.773565561	3.691964899	3.636275664
Zm00001d032188_T002	2.193045325	2.226195604	1.71865052	3.132526175	3.177926401	3.248528764
Zm00001d017403_T001	3.342112226	2.417762069	3.301622762	3.919509134	3.96655487	4.081228319
Zm00001d010814_T001	6.49596056	6.304801979	6.477857135	7.741183712	7.858230628	7.726302804
Zm00001d032707_T001	1.528219729	0.86051155	1.550765744	1.843102285	1.94676267	1.887022579
Zm00001d036245_T009	1.879626965	2.619015246	1.843675983	1.485533995	1.931678519	0.783233685
Zm00001d036245_T005	3.864857578	2.364897072	3.636423122	3.958448872	3.857949355	3.822975028
Zm00001d036245_T001	1.032951394	0.851276909	0.731135441	1.276574084	1.212573696	0.927939677
Zm00001d023923_T001	3.837354038	3.116039315	3.761562721	3.95814426	4.019811491	3.960449898
Zm00001d052614_T001	1.964743507	2.961621662	1.268481105	1.56073794	1.391020888	1.562646383
Zm00001d053177_T001	6.116429429	5.103786779	6.067586512	5.77000711	5.680962288	5.576315544
Zm00001d053177_T002	0	0	1.196328801	1.394316574	0	0.862244271
Zm00001d030796_T007	1.345311776	0.795604054	1.15337603	1.384567094	1.686179569	1.355210265
Zm00001d030796_T003	1.475462114	1.097181295	1.703293873	1.459269503	1.18757774	1.232942515
Zm00001d030796_T006	3.145608661	3.108652077	3.281593512	2.886368702	2.588886819	3.131629156
Zm00001d015450_T002	5.113990517	4.206797612	5.150666047	5.069033545	4.999829797	4.938992662
Zm00001d028807_T001	3.390879782	2.972617852	3.397088829	2.988030032	2.711157871	0
Zm00001d028807_T002	9.250914833	8.912173537	9.281757602	8.480243889	8.540468215	8.466423682
Zm00001d029560_T001	2.544486375	1.664847791	2.312741546	1.636932672	1.398084903	1.455637855
Zm00001d014188_T005	0	0	0.704902942	2.106625038	1.702693887	1.52730309
Zm00001d014188_T034	1.338470055	1.237326309	1.608721259	2.188462012	2.234926157	0
Zm00001d014188_T033	1.980991527	1.052392939	1.928478343	0	2.189971904	2.820693032
Zm00001d014188_T035	1.392949322	1.242238471	1.824077801	1.551619863	1.245557118	0.98103794
Zm00001d025793_T004	0.30069893	0.112830887	0.183071035	0.27155715	0.493520273	0.425537722
Zm00001d025793_T002	3.507472201	2.502239698	3.237655949	3.243325273	3.624230649	3.380029123
Zm00001d032911_T001	6.410033352	5.931191148	6.506508864	5.729611092	5.74725153	5.66301935
Zm00001d043386_T001	1.036716425	1.933193787	1.247960315	2.33470556	2.427299903	2.383294267
Zm00001d020851_T002	1.377949973	0.792528887	1.429821609	1.199383384	1.058886533	0
Zm00001d020851_T001	0.74243917	0.339773627	0.484587759	0.925830082	1.142050676	1.346164359
Zm00001d048422_T001	3.076293482	2.566641046	3.040380998	2.858681676	2.95585362	3.209752635

Zm00001d003256_T001	4.386480785	3.447092618	4.289299182	4.631930655	4.464835396	4.473461976
Zm00001d031539_T003	4.438274152	3.859511032	4.402612429	3.771462741	3.983350999	4.002214369
Zm00001d031539_T001	2.534401811	2.185371822	2.295092332	2.017543552	2.050465477	1.653498952
Zm00001d013243_T002	2.596866702	1.725379074	2.581773749	1.98543303	1.693230902	1.842155406
Zm00001d010171_T001	0.41771374	0.415891682	0.768543445	1.909856419	1.604140609	1.180282192
Zm00001d010171_T003	1.79055812	1.55394631	2.011506373	3.457951704	3.333888384	3.051611719
Zm00001d010171_T002	7.746843856	7.434836544	7.810088428	7.471609987	7.575581669	7.494465126
Zm00001d003555_T002	0.880192594	0.571437028	0.94175688	1.174812259	1.093001649	1.332299482
Zm00001d045315_T002	7.251171702	6.784635762	7.268315633	7.250166736	7.302768662	7.234108576
Zm00001d045315_T004	5.459115995	0	5.489522738	4.621668847	4.888400614	4.755708322
Zm00001d037239_T001	7.48556411	6.82837447	7.576883976	7.480741942	7.447567816	7.442770876
Zm00001d040733_T001	3.07007671	2.637531189	3.162708093	2.752731039	2.483049833	2.426112022
Zm00001d040733_T002	1.906369592	1.135750897	1.824898168	1.914292414	1.986592603	2.744081207
Zm00001d039854_T001	3.373614249	2.924132579	3.42846215	3.535717061	3.605018967	3.467431177
Zm00001d034245_T003	0.900225747	0.648079752	1.180219166	1.694442082	1.511006971	1.545046133
Zm00001d034245_T009	3.383132492	2.293903965	3.559254893	4.320792463	4.354873637	4.135768624
Zm00001d034245_T001	2.351869983	3.886136398	2.061693262	0	0	0
Zm00001d034245_T014	0.833341887	0.481893052	0.439210348	1.02135393	0.984462482	1.543152367
Zm00001d045607_T001	7.20292055	7.243108218	7.349156598	6.674438985	6.81910231	6.825948913
Zm00001d007881_T004	0.543783899	1.81994893	0	1.743262469	1.184871218	1.482219451
Zm00001d007881_T010	2.912929372	3.305301095	2.735664923	0	2.993859645	3.068212136
Zm00001d007881_T005	3.814034775	2.914031546	3.340280396	3.650523209	3.502889646	3.225001236
Zm00001d021947_T004	0.093910329	0.110226919	0.248212987	1.391007686	1.440969733	1.244609451
Zm00001d021947_T005	0.230415774	0.026254741	0.133186035	2.016231005	2.08250504	2.102729005
Zm00001d021947_T002	4.866082568	3.968046871	4.885835744	5.615339252	5.707737441	5.68374605
Zm00001d006658_T003	2.283803567	1.578876899	2.452869241	2.288882385	2.407434334	2.688352487
Zm00001d006658_T005	0.41310552	0.270389002	0.346058239	1.226882102	1.072144912	1.146820725
Zm00001d006658_T007	0.286864592	0.080660392	0.36543387	0.785018152	0.744409993	0
Zm00001d006658_T002	0	0.412155002	0.130145408	1.120286241	1.01584293	0.495575445
Zm00001d005301_T015	0.4818445	0.119272502	0.219523554	0.456976424	0.686975558	0.411516276
Zm00001d005301_T016	0.761355917	0.443387048	0.762108098	1.305878326	1.121600983	0.974466904
Zm00001d005301_T012	0	0	0.43142506	0.499972902	0	0.547335317

Zm00001d040834_T001	5.114717411	5.793206797	5.393992484	4.719447582	4.95852534	4.740725385
Zm00001d037356_T001	1.827309318	1.394772586	1.871523145	1.46822149	1.651527056	1.810860119
Zm00001d017504_T001	2.432201793	2.627742931	2.314642007	2.623846031	2.434285543	2.694076516
Zm00001d050061_T005	1.425388941	1.42371961	0.885743027	0	0	0
Zm00001d050061_T006	1.706042462	0	2.339703802	1.320196399	1.651416381	1.667611959
Zm00001d050061_T002	1.099085771	0.98040558	1.396110156	1.744838357	1.661335518	1.794807187
Zm00001d026284_T001	4.52535643	4.990749738	4.656347917	4.782410138	4.69808265	4.631834527
Zm00001d049435_T001	1.207903469	0.835014526	0.828550347	2.393511755	2.801706859	2.164403675
Zm00001d027642_T001	1.764102798	1.544250935	1.856843902	1.820693645	2.077256674	1.672105764
Zm00001d028408_T001	0.901485187	0.617697191	0.490957193	2.052560922	1.645241896	1.324493233
Zm00001d017830_T011	3.156761451	0.854751318	2.2843634	3.285508456	3.631241559	3.481902608
Zm00001d017830_T012	3.705533196	0	3.52421002	3.665651769	3.697180765	3.536009121
Zm00001d017830_T001	0	2.946844063	0.435800321	1.126191185	0.739943991	1.529677544
Zm00001d017830_T003	4.507101853	3.974056229	4.890298719	4.446556319	4.571188771	4.41521201
Zm00001d017830_T014	0.185341372	0.185081252	0.809932157	1.372060025	0.909216268	1.086289878
Zm00001d017830_T009	2.222561001	0.148028192	2.190557025	2.277898183	2.869895681	2.885138757
Zm00001d030421_T001	4.249184464	3.642394288	4.278559331	4.687916531	4.716814359	4.412203993
Zm00001d037553_T002	0.696182598	1.945301679	1.434812881	0.757205066	0.497901514	0.795042925
Zm00001d037553_T003	5.175427433	4.354939841	5.182502216	5.088017069	5.070564974	5.070625378
Zm00001d037553_T001	0.258003865	0.205926699	0.492547475	0.753182619	0.328733083	0.485074354
Zm00001d031700_T003	1.1132911	0.763592591	1.285203927	0.853160518	1.017376074	0.912584722
Zm00001d031700_T002	3.564104116	2.261448674	3.903980054	3.919313193	3.710081034	3.96758964
Zm00001d031700_T004	0.582080004	0.190168551	0.613046901	0.561422924	0.734046668	0.516016156
Zm00001d023235_T001	1.378912193	0.834944164	0.952074827	1.813622781	1.837590495	2.069363963
Zm00001d023235_T002	2.974937284	2.688611424	3.345082224	2.427864387	2.538430889	2.473619129
Zm00001d017415_T001	3.236432766	3.252893975	3.248570812	2.420291593	3.467051188	2.807535042
Zm00001d038831_T001	1.42263776	0.744409132	1.354448211	0.868117002	1.249793609	0.986046802
Zm00001d024751_T001	6.388375563	5.942337547	6.509946772	7.54982944	7.61343477	7.62099346
Zm00001d017237_T003	3.920309214	3.042497959	3.82832002	3.614171294	3.734427719	3.604287593
Zm00001d041710_T004	0	1.223764184	0	1.326133475	0.857527221	1.166358101
Zm00001d041710_T006	1.00568742	0.635059688	0.864486887	1.184864238	1.407910141	1.234868539
Zm00001d041710_T007	0.822394874	0.058611586	0.812746286	0.442207832	0.806057562	0.651872344

Zm00001d041710_T005	1.055351857	1.83771441	0.982463934	1.490066383	1.142152651	0.559653327
Zm00001d041710_T002	5.0378916	3.670669391	4.974915585	5.121155186	5.234804711	5.139461596
Zm00001d039535_T001	8.032890444	6.919903296	8.132775421	8.244124939	8.284806443	8.289632291
Zm00001d049073_T001	0.726709171	0.60561001	1.149731514	1.296006834	1.397240557	1.429664165
Zm00001d010798_T001	0.877694	1.000549562	1.602063917	3.444923703	2.988334399	3.62389682
Zm00001d032213_T001	1.861156998	1.366693175	1.634884566	3.510063372	3.515096019	3.804138974
Zm00001d027924_T001	7.374729418	6.833687199	7.503908712	4.9176964	4.980788045	4.920695941
Zm00001d002402_T002	2.484676946	2.982512855	2.984116455	1.898304722	2.619091098	2.705686459
Zm00001d002402_T001	5.918847046	5.147706478	5.794959439	3.830369729	3.779583003	3.654798689
Zm00001d022021_T001	1.930841775	1.310597223	1.858104763	1.659537353	1.689010145	1.686508595
Zm00001d002890_T001	3.364635041	3.323097935	3.426775177	2.897305103	2.786568971	2.879306422
Zm00001d019173_T001	4.813050194	4.000007213	4.895599856	5.11480514	5.072180378	5.070735018
Zm00001d005001_T003	1.014572395	0.355627504	0.571333143	1.241660827	1.173026985	0.752397507
Zm00001d005001_T001	5.147724751	4.433360167	5.035692876	5.222207487	5.220379218	5.153720433
Zm00001d005001_T002	3.005258227	2.148149924	3.262752752	3.277684576	3.77809364	3.499166369
Zm00001d005654_T001	3.047888376	4.844841169	3.185476172	4.332890998	3.728120349	3.728083552
Zm00001d032539_T001	3.557371734	2.644250165	3.465067828	4.103105062	4.13691936	4.192090537
Zm00001d044132_T001	0.559516286	0.219819658	0.274372599	1.251228266	0.946464377	1.13183112
Zm00001d044132_T002	1.094933936	0.73742026	0.884467418	1.756426411	1.800079854	1.853363357
Zm00001d044801_T001	1.121408689	0.740245437	1.122729697	1.781488973	1.61330533	1.96764377
Zm00001d009825_T001	3.314150808	2.578693857	3.210666435	3.515875411	4.018350022	4.066610448
Zm00001d036524_T002	2.944129607	1.247601883	3.147046632	2.513668103	2.212004601	2.193861298
Zm00001d036524_T001	3.43947168	2.815740797	3.391054856	3.865302817	3.774834384	3.850029374
Zm00001d039483_T001	5.717716475	5.143822971	5.649158524	5.715782996	5.664729204	5.658550919
Zm00001d000432_T001	5.331902843	5.060750224	5.471518338	4.387072222	4.402802317	4.045322217
Zm00001d000432_T003	5.179795103	3.558673442	5.005930121	5.34417683	5.359075882	5.37802654
Zm00001d009288_T012	1.787679858	0	1.29383365	2.049029171	1.429059398	1.749825137
Zm00001d009288_T016	2.519961015	1.42920673	2.581465501	2.02984721	1.679485955	0
Zm00001d009288_T003	0.750908303	1.544058004	0	1.445271155	1.681566655	1.895980761
Zm00001d009288_T006	0.585263512	0.219414513	0.232456923	0.451893156	0.794443316	0.689302739
Zm00001d033388_T002	3.564223647	2.959192962	3.435167978	4.073506901	3.910125624	3.852216524
Zm00001d033388_T003	3.443997903	1.973316645	3.54991543	3.14421462	3.328441852	3.233693449

Zm00001d052216_T001	3.229547164	3.334875145	3.128439412	3.060366506	2.749481493	3.538082811
Zm00001d007208_T001	2.771089236	2.025506542	2.745974306	3.795607898	3.682491385	3.783427947
Zm00001d040076_T016	0.848808595	0	0.627527464	1.386941693	0.57694388	1.348902046
Zm00001d040076_T001	0.709514743	0.957885633	0.837797956	0.894567125	0.987368894	0.679136402
Zm00001d040076_T011	0.786660759	0.411957667	0.859698471	1.637230918	0.629602819	1.654316381
Zm00001d040076_T002	0.977468973	0.309637217	0.346036674	0.862708465	1.900908917	0.94645689
Zm00001d040076_T010	0	0.176841028	0.1744371	0.739584592	1.151815314	0.515392535
Zm00001d040076_T008	3.294764533	2.664154214	2.964759954	3.645327681	3.015101623	3.47504142
Zm00001d049220_T001	0	3.611757722	2.720434986	3.547557299	3.652540645	3.668318514
Zm00001d003840_T001	4.696001327	4.544280862	4.866688739	3.616951639	3.570109136	3.729428562
Zm00001d048268_T001	0.613608972	0.250497169	0.767780208	2.672072263	2.337517864	2.621199719
Zm00001d033172_T003	1.063932926	0.705171094	0.938366347	1.479008551	1.688672291	1.433403903
Zm00001d033172_T005	2.131564133	0.891706142	2.330218194	2.082074083	1.305761031	2.746656951
Zm00001d033172_T009	1.60770149	1.079281843	1.460669707	2.62857298	3.065277435	1.05020982
Zm00001d033172_T007	1.208698912	0.534621435	0.846916886	0.321301252	0	0.851392858
Zm00001d011379_T016	2.134480864	0	2.096365767	2.223452825	2.361256589	2.01996588
Zm00001d011379_T023	0.202686018	0.131892346	0.179425696	0.944775254	0.796992219	0.533055949
Zm00001d011379_T004	0	0	0	1.607408913	1.425874436	0.740055421
Zm00001d011379_T005	0.622325979	0	0.618922716	0	0	1.417106643
Zm00001d011379_T007	0.946084029	0.589313833	0.445778606	0.155984914	1.059139965	0.193663261
Zm00001d035612_T001	0.942732934	0.830973247	0.998738533	1.165562756	1.174192268	1.100423331
Zm00001d025905_T003	3.400290355	3.852310422	3.636344116	3.6270168	3.67005704	3.342940825
Zm00001d025905_T004	0.640142418	0.515350143	0.512302749	1.634560279	1.45657961	1.566023585
Zm00001d028074_T001	2.176120079	1.792446427	2.345522982	6.395617931	6.404500815	6.31756129
Zm00001d035649_T001	2.454733645	2.440295795	2.532766531	2.321500417	2.186313236	2.221687802
Zm00001d053665_T001	4.388446016	4.031935753	4.541462119	5.23424087	5.10397485	5.155678003
Zm00001d053665_T002	2.129818776	0.288841644	1.839030402	1.669962852	2.011591171	1.776660319
Zm00001d012175_T001	2.41859989	1.67375495	2.470091949	2.687801783	2.355477819	2.320437598
Zm00001d012175_T003	2.34988949	1.864251133	2.0782457	2.303935309	2.374290287	2.659442819
Zm00001d012175_T004	4.372572087	4.0366512	4.373085987	4.725478874	4.778328235	4.720705628
Zm00001d012175_T002	0.463693596	0	0.708637525	0.422758304	0.826094889	0.458713765
Zm00001d016902_T004	3.320829064	2.595583861	3.243915348	3.098310065	3.13273706	2.783273713

Zm00001d016902_T003	4.350217733	3.49885463	4.386559763	4.966443896	4.979041409	4.947372399
Zm00001d016902_T002	2.090584847	0	1.017404582	0	1.871827093	2.038375073
Zm00001d030340_T039	0.041879366	0	0.197989908	0.36302638	0.416959691	0.166682028
Zm00001d030340_T060	0.416550093	0.290885568	0.726875674	0.925081877	0.864376741	1.028255827
Zm00001d030340_T024	2.554487184	1.769884274	2.558989781	2.216985282	2.414901068	2.106459885
Zm00001d030340_T003	1.512416536	0.608425104	1.187584074	1.75083586	1.724180501	1.819413135
Zm00001d030340_T019	0	0.244966192	0.115616597	0.762859888	0.697758699	0.283057674
Zm00001d044618_T001	1.600518112	0.821526551	1.646068625	0.974709179	0.771820518	1.141991188
Zm00001d047960_T002	3.223005127	2.110786835	3.330518675	3.492368271	3.09785145	3.277665683
Zm00001d047960_T004	1.138433107	0.614129352	0.193234299	1.55308226	2.089304581	1.571872392
Zm00001d047960_T001	1.520043012	1.405519187	1.049988261	0.589535322	0.892473408	1.355656818
Zm00001d028514_T001	2.356678241	1.86507868	2.594661047	3.595872999	3.654891009	3.475693619
Zm00001d028514_T002	0.265770526	0	0.317128063	1.104518495	1.044325568	0.686480806
Zm00001d052845_T002	2.494593792	1.804686355	2.521490516	2.417379706	2.348013108	2.519312424
Zm00001d033574_T001	8.483489578	9.224672552	8.551940435	8.164196041	8.259957002	8.223911578
Zm00001d045944_T008	0	0.675093274	0	1.617324346	0.572753879	0.660081603
Zm00001d045944_T007	2.373295741	0.656446945	2.819405573	3.245685495	4.164761343	3.548795528
Zm00001d045944_T005	0.417327042	0.323406965	0	1.13002742	0.894632311	0.754777212
Zm00001d045944_T011	0.420095368	0.120807107	0.247231207	1.223572681	1.431264046	1.919039879
Zm00001d045944_T006	0.947344483	0	1.218426571	1.685266958	2.592144604	2.551624047
Zm00001d045944_T002	3.400003651	2.96651242	3.016461554	3.287476285	0	2.447652189
Zm00001d045747_T005	2.695853322	2.770867729	3.306548942	2.570668148	1.76003397	1.866187934
Zm00001d045747_T004	2.114720825	1.283992276	2.111535833	2.786181266	2.566525856	2.792091334
Zm00001d046766_T003	0.688034403	0.866640575	1.51587642	0	0	0.854886132
Zm00001d046766_T001	0	0.643728765	0	1.639025123	0	0.72824184
Zm00001d046766_T005	0.822925893	0	0.588126226	0	0.969563485	0
Zm00001d016992_T001	5.162733627	1.605694865	4.255761981	5.525259699	6.476981487	6.4748005
Zm00001d052813_T001	0.832796059	0.789982092	0.839684724	0.267141437	0.469621371	0.240757604
Zm00001d049359_T001	2.048533345	1.752260047	2.245723215	2.23083075	2.393575728	2.281965847
Zm00001d001945_T017	0	0	0.546865328	0	0.704666609	0.537755271
Zm00001d042117_T002	3.678306549	2.684360278	3.703361987	3.240396461	3.334825958	3.018791716
Zm00001d042117_T004	0.527434718	0.278407083	0.270873354	0.330126961	0.080222402	0.318043752

Zm00001d042117_T001	3.881645729	4.519057675	3.978605678	3.685910218	2.385318931	3.370318727
Zm00001d012536_T006	1.708936316	0.683799741	1.804633082	1.63227985	2.224902484	1.756767978
Zm00001d012536_T002	2.935754292	2.498359385	2.855363065	3.08736796	3.008371608	3.318966089
Zm00001d012536_T009	0.756814083	0.46675344	1.080862286	1.097084184	1.162680544	1.1576834
Zm00001d028238_T001	2.054116354	1.254549903	1.559401749	2.679709336	2.331606626	2.398303575
Zm00001d028238_T009	3.817097141	3.063061092	3.845229088	3.492212122	3.253938302	3.19669658
Zm00001d028238_T007	0	0.069047685	1.076583721	0.341422218	1.464918626	0.743821699
Zm00001d028238_T012	0.053287925	0.104256135	0.093666988	0.254115081	0.411328616	0.423651312
Zm00001d020554_T001	2.97583559	2.755764608	2.667256337	3.877583287	3.993002125	4.02108382
Zm00001d012396_T001	0.632087635	0.135076444	0.861733089	1.503873811	0.412098624	0.830958649
Zm00001d012396_T005	0	2.119120412	3.617057583	1.721510907	2.499466305	2.553399089
Zm00001d012396_T006	0.111840598	0.089994331	0.869482162	0.639496131	0.653390262	0.695774721
Zm00001d012396_T007	3.688127418	2.082523774	0	4.188932816	4.088631121	3.985354423
Zm00001d002761_T001	1.364171223	1.419222812	1.40496632	0.849175428	0.824243215	0.803783338
Zm00001d040857_T001	2.881430872	2.214756468	2.771539332	2.561886974	2.606447677	2.60659786
Zm00001d011558_T001	2.728027156	2.815060147	2.207919707	2.076921263	1.733533961	2.010351262
Zm00001d010416_T002	3.524267199	2.809933186	3.604927788	3.623622491	3.723925905	3.586067903
Zm00001d005402_T003	0.341500784	0.864570878	1.118051351	1.765392167	0	1.896264873
Zm00001d005402_T005	1.309368301	0.222760056	0.84844247	1.196134881	0.593558387	1.063211613
Zm00001d005402_T008	1.968253726	3.804532932	2.226468763	1.781096962	0.956114657	1.810692341
Zm00001d005402_T007	0.334548826	0.283732165	0.328657265	0.747836789	0.554648768	0.805909855
Zm00001d005402_T004	1.541610983	0	1.137583526	1.457174003	2.10599179	0
Zm00001d033383_T001	2.990240958	2.387314561	3.112845562	3.085870585	3.259886801	3.139150862
Zm00001d033383_T002	1.519082907	0.853266732	0.872950866	1.292443118	0.570971913	0.443460253
Zm00001d034059_T005	8.9736188	7.766206617	9.019339489	8.455766066	8.484029309	8.433150393
Zm00001d028999_T001	3.977815316	3.050902671	3.909130996	3.638910126	3.834204546	3.634849144
Zm00001d044074_T001	2.530368692	2.048432903	2.472594345	2.729557121	2.527591854	2.490796529
Zm00001d013794_T001	2.217042999	1.695680305	1.912341751	0.190343045	0.753790209	0.813422897
Zm00001d040766_T001	6.724759043	6.108245781	6.763803588	6.787039374	6.879392458	6.848950849
Zm00001d029214_T001	4.666121666	4.241189375	4.73950958	4.912830526	5.026977066	4.938933871
Zm00001d029214_T006	0.151540636	0.223106173	0.215226305	0.802525774	0.665719296	0.495405571
Zm00001d029214_T005	0.8539725	0.227405928	0.697430449	0.912880524	1.11304367	1.099148402

Zm00001d044500_T001	2.698550549	0.935360925	2.433075353	2.54472326	2.728089431	1.879960176
Zm00001d044500_T002	0.654219212	1.513792897	0	1.051945585	0.234811531	1.86584084
Zm00001d006195_T001	3.751184003	3.027211213	3.699623477	3.164083742	3.280281266	3.606561737
Zm00001d012438_T001	1.677717049	1.451799809	1.901790996	2.204858755	2.091126704	2.261315373
Zm00001d021279_T001	1.25684159	1.512475702	1.220022808	0.116276915	0.12535883	0.486806002
Zm00001d026381_T003	4.233777722	3.334326853	4.084061798	4.334304468	4.453353612	4.234934355
Zm00001d026381_T002	3.999489827	3.185286814	3.855853492	3.833729975	3.809840193	3.973932655
Zm00001d031969_T001	1.630645901	1.849141391	2.24801225	1.937250595	2.08266206	2.028116117
Zm00001d031969_T002	2.019684125	2.345497718	2.136406002	1.370375786	1.481191976	2.538070764
Zm00001d000150_T001	4.585299271	3.586550875	4.399457006	4.814016722	4.737974407	4.67942042
Zm00001d028670_T001	2.674732261	2.670812469	2.916460975	1.060345579	1.420534682	0.874556988
Zm00001d048111_T001	2.218400221	1.4673447	2.152726316	2.944740964	2.852652697	3.011531956
Zm00001d050612_T008	1.931701965	1.089773376	1.931275717	1.425888934	2.002643839	1.969536226
Zm00001d050612_T001	0.531478781	0.467431829	0	1.526930665	1.116766632	0.883744359
Zm00001d027283_T001	8.515398378	8.609154569	8.605034992	7.62265739	7.701340379	7.729090706
Zm00001d020358_T001	4.081722494	3.530596421	4.205203092	3.370139724	3.517218614	3.509479865
Zm00001d022614_T002	3.535563296	2.876510857	3.417836316	3.445139496	3.587676661	3.401315491
Zm00001d022614_T001	1.433505925	0.925252821	1.640040124	1.645180092	1.301211853	1.273592871
Zm00001d014755_T001	3.474388926	3.176848526	3.48002698	6.202557968	6.224970357	6.236855543
Zm00001d017166_T002	1.53672683	0.834739527	1.409779766	1.932214279	2.104417516	1.60482522
Zm00001d017166_T001	2.514646712	1.908852865	2.784610558	3.208177459	3.042497959	3.161033257
Zm00001d032035_T001	5.423250166	4.722720386	5.575635237	6.524836939	6.441555304	6.50014541
Zm00001d025513_T001	2.769684583	2.441815431	3.024625882	3.084049293	3.053462929	3.193433183
Zm00001d039499_T002	5.417821872	4.722462636	5.52905221	6.251786739	6.269606126	6.264659678
Zm00001d034740_T001	5.484089718	4.975485607	5.530026032	5.227172117	5.255636008	5.347321133
Zm00001d034740_T002	0.908251089	1.009266184	2.016929848	1.062230186	1.079440921	1.086497101
Zm00001d048065_T002	5.131785323	4.223174459	5.176943285	5.735392524	6.033943049	5.909768425
Zm00001d048065_T001	1.3652446	0.927194841	1.485751912	1.676496705	1.665852523	1.734347614
Zm00001d048065_T003	4.459874786	3.914438904	4.198485903	3.781652525	0	0
Zm00001d008622_T002	6.026361254	5.395700763	6.046566493	6.307089871	6.29902008	6.261890485
Zm00001d008622_T001	2.293566774	1.894097545	2.162033073	2.194042282	2.269735765	1.988448747
Zm00001d046055_T001	5.661687814	7.647484013	5.260476654	1.395891974	1.857576188	0

Zm00001d014124_T004	0	0	4.895538397	5.291087391	6.350340717	0
Zm00001d014124_T003	6.391805725	0	5.762189308	6.119830329	4.709460153	6.716586636
Zm00001d014124_T001	1.681730805	5.076127984	1.468207411	1.767784444	2.220611965	1.242953039
Zm00001d014124_T006	0.878661002	0.472591746	0.828477231	1.601248189	1.676954285	1.808772601
Zm00001d014674_T005	0.936847839	0.456330954	0.5608333	1.200404538	1.12570135	1.357539619
Zm00001d014674_T001	2.544997682	2.033374368	2.72995382	3.098755903	3.05953543	3.123922734
Zm00001d014674_T004	0.390990083	0.094206337	0.252071658	0.794207063	0.523159451	0.547118114
Zm00001d020018_T001	0.243971235	0.245076971	0.274898543	0.743170248	1.252923032	1.028943921
Zm00001d013026_T003	1.277023027	0.72531101	1.035438811	1.267675417	1.29310264	1.268182244
Zm00001d013026_T002	1.452853168	1.498338703	2.140717806	2.089789982	2.324363904	1.701931803
Zm00001d013026_T001	0.147199877	0.093143668	1.072754096	1.285312843	0.940378769	1.089668986
Zm00001d013026_T004	1.261371945	1.518482264	1.233406468	1.208403013	1.270265795	1.02719794
Zm00001d005144_T006	1.161005999	0.836404085	0.506442503	0.248142535	0.909912855	1.599608208
Zm00001d005144_T010	0.276179804	0.384621244	0.527084352	0.532380801	0.470367142	0.40060897
Zm00001d005144_T001	4.165842173	3.741535695	4.267669125	4.579181778	4.648844647	4.288552287
Zm00001d005144_T012	1.096820469	0	0.986554378	1.06790782	0.374230461	0
Zm00001d005144_T005	1.668474534	1.013885269	1.892724799	2.753247241	2.658215597	2.803847179
Zm00001d026248_T001	2.910957874	2.347828907	3.063131521	3.423636792	3.562484105	3.505811935
Zm00001d048717_T003	1.120891357	0.69257271	0.931664905	1.328530319	1.46236596	1.368246482
Zm00001d002847_T001	0.887808323	0.827554845	1.272063811	2.352566454	2.008746462	2.184878833
Zm00001d053100_T004	1.178065083	1.607911654	0	1.518959576	1.048667255	0.378514953
Zm00001d053100_T005	2.152099029	1.296308798	2.37497324	0	1.820524963	1.926846563
Zm00001d053100_T003	2.303903171	1.582394624	2.492955023	2.865200455	2.745733852	2.474087106
Zm00001d053100_T001	0.912345583	0.313010125	0.898558195	1.395423702	1.261976643	1.342331156
Zm00001d052234_T001	5.159511082	5.296375689	5.247029422	5.09498649	5.16946567	5.041867673
Zm00001d044700_T001	4.359970748	4.92658288	3.899610307	5.302100656	4.837137479	4.951155071
Zm00001d038903_T002	2.454286987	2.516602446	2.413735421	3.197001025	2.956866082	3.168253874
Zm00001d038903_T001	2.905014642	2.748219475	2.934508576	3.501192114	3.746013016	3.34276536
Zm00001d024652_T001	3.535656851	3.657073562	3.630192616	4.763436965	4.947020165	4.645348088
Zm00001d051429_T001	2.91370171	2.479883714	2.698587885	4.109301379	3.931006458	4.019099005
Zm00001d047801_T002	0	3.783663981	3.730241489	1.91529334	3.049806738	2.272602242
Zm00001d047801_T001	3.723641233	3.104285661	3.705265554	4.266365437	3.880177211	3.876356731

Zm00001d044839_T001	1.89214735	1.614435176	1.889553755	0.90828568	0.814524174	0.608177156
Zm00001d032807_T001	4.233062967	3.165836547	4.118436311	3.674812695	3.60680783	3.590639981
Zm00001d032807_T002	0.793160097	2.307046962	1.148841066	1.112206363	1.504349369	1.608380628
Zm00001d051995_T001	5.514057606	4.90950369	5.53896616	5.201483586	5.270270056	5.159431359
Zm00001d049163_T003	1.178205346	0.719986832	1.711316045	1.32709637	1.447479752	1.033872612
Zm00001d049163_T006	4.121355555	2.90573925	4.083648409	3.839982961	4.077219495	3.74929977
Zm00001d049163_T001	4.896026066	4.640988495	4.861893994	5.070258449	4.902772466	4.844668409
Zm00001d014770_T001	4.795010706	3.911625437	4.690877991	4.468483941	4.179526815	4.281473855
Zm00001d023987_T007	7.233893542	7.472953209	7.403444316	6.111126174	6.28960709	6.213021997
Zm00001d023987_T008	0.202219602	0.042994464	0.327731047	0.419710387	0.340798094	0.446690297
Zm00001d023987_T002	6.479248737	5.613361619	6.447567923	6.378415903	6.437186134	6.395121151
Zm00001d023987_T004	0.692106663	0.255865666	0.61220716	0.746736697	0.774312671	1.003135174
Zm00001d023987_T006	3.698653333	1.846840283	2.806306114	1.904752669	1.933728607	1.956065576
Zm00001d032160_T001	5.718062589	5.13720852	5.711998	5.864638151	5.927686669	5.749205414
Zm00001d006775_T001	3.993734858	5.231433061	4.270918044	4.328379663	4.681767905	4.851176097
Zm00001d044729_T001	3.579655174	2.860152357	3.558885117	3.590371352	3.595769785	3.71327966
Zm00001d043580_T001	2.229685125	1.672680139	2.479493927	2.186873227	1.796700325	2.221001302
Zm00001d043237_T001	0.581508404	0.284033155	0.207596788	1.32280441	1.746019037	1.614395126
Zm00001d024004_T001	3.176008505	2.566523664	3.099467577	3.199192859	3.075013818	3.201905125
Zm00001d011811_T002	0.630840637	1.004307941	0.555022945	0.977931966	0.130435394	1.090173917
Zm00001d044451_T001	3.999305175	3.501711353	4.136958558	4.223029308	4.349132548	4.310623251
Zm00001d044451_T002	3.862069366	3.046289886	3.802676516	4.320294761	4.286582083	4.182179239
Zm00001d043942_T002	8.334744253	7.506918714	8.345420306	7.366966317	7.489583058	7.367157485
Zm00001d043942_T003	3.40054517	7.880765573	3.83860804	1.359180416	0	0
Zm00001d051359_T001	2.598665815	2.610050845	2.857287539	1.983393505	2.469853945	2.237080292
Zm00001d017952_T002	3.729207744	2.192303389	4.093219068	5.014859612	4.907643198	4.935984774
Zm00001d014179_T001	2.888370559	2.092267324	2.659353302	2.518127703	2.60358718	2.284725251
Zm00001d015130_T001	4.432624677	3.687802791	4.44991801	4.347105121	4.29718129	4.308288421
Zm00001d040562_T007	0.241659607	0.171578344	0.062784868	0.612075966	0.69012741	0.929667549
Zm00001d040562_T006	1.807108819	4.222403808	2.741932026	3.672966336	3.104364004	3.58549704
Zm00001d040562_T025	0.398325194	0.501733641	0.089100813	1.886873961	2.099024484	0.990205553
Zm00001d040562_T017	1.616070165	0.77805799	1.042724174	2.797428497	2.431504758	2.556647183

Zm00001d040562_T009	0	0.120635939	0.136767562	0.908654606	0.826037111	0.797206431
Zm00001d040562_T005	0.178714641	0.149333515	0.279957362	0.402335806	0.457862162	0.396983651
Zm00001d040562_T020	1.847096143	0.659499905	1.664864171	4.606125168	4.397012206	4.700241778
Zm00001d040562_T019	0.273149425	0.128596955	1.318677217	0.883970305	2.329685348	1.943703078
Zm00001d040562_T013	4.092260303	2.897960625	4.042484652	4.978601376	5.184698639	4.932000491
Zm00001d002791_T011	0.806357881	1.011661654	1.30600553	1.992986993	1.205459453	1.718377848
Zm00001d002791_T007	3.401981405	3.48482399	3.562544061	2.882887358	2.728670896	2.513781782
Zm00001d002791_T009	0.088828173	0.183200647	0.529870909	0.324342876	0.274787646	0.435190119
Zm00001d002791_T006	0.514469728	0.375164019	0.636363375	0.818305767	0.491343126	0.10614996
Zm00001d002791_T013	0.559111939	0	0	1.033974776	1.967901945	1.573767627
Zm00001d002791_T001	1.530462834	1.303609514	1.444484731	1.753577582	1.909541179	2.1861503
Zm00001d049361_T002	4.333876366	3.664196321	4.368238379	4.286922684	4.342156043	4.23446162
Zm00001d049361_T003	5.032017859	2.872254719	5.15218492	5.380559871	5.236192836	5.264244222
Zm00001d051806_T001	5.34248278	4.606942939	5.333524705	4.893750211	4.836523911	4.877197787
Zm00001d051806_T002	2.363824558	1.467595117	2.727592245	3.02207001	3.246685046	2.961867029
Zm00001d005082_T001	1.4707175	1.200916726	1.475397779	0.856212859	0.982855982	0.82954435
Zm00001d041855_T003	1.861841452	1.768111897	1.370408709	2.060118652	2.090773843	2.267023648
Zm00001d041855_T006	1.065833531	0.651285489	1.259943128	2.539069206	2.413807709	2.528930823
Zm00001d041855_T001	1.322396029	0.545878445	1.21007897	1.014168159	1.291419673	0.731684621
Zm00001d041855_T005	1.06646054	0.409716779	1.217372186	1.14618011	1.050432042	1.185315362
Zm00001d045305_T001	8.429817237	7.709626019	8.472702178	7.744942644	7.806723953	7.706308371
Zm00001d047449_T032	0	0	1.865878046	0	1.345411705	1.759711069
Zm00001d047449_T094	2.769381609	1.664369059	3.055747147	2.096668018	2.024933793	2.463287114
Zm00001d047449_T007	1.559546143	1.722150346	0.868124115	2.031646799	2.008913869	1.730365628
Zm00001d051881_T001	6.47374776	5.694302804	6.484149235	6.21359701	6.200362416	6.253410522
Zm00001d013416_T001	1.083118718	0.762037492	1.384569305	0.728148655	0.648318175	0.583627872
Zm00001d000093_T001	0.34070468	0.496443942	0.600464828	1.247947559	1.184588798	1.193173098
Zm00001d012251_T003	0.952352951	0.801522078	0.83076318	0.537291097	0.573075873	0.967067505
Zm00001d016477_T001	3.225745673	2.587991143	3.298645707	3.732258995	3.602489899	3.627341736
Zm00001d047089_T001	4.465395638	3.716143021	4.527087105	4.249176347	4.118103693	4.372897369
Zm00001d025528_T002	1.043145692	0	0	0.372337011	2.755275588	0
Zm00001d025528_T001	3.370746682	2.423208643	3.426923144	2.635152117	0	2.831464636

Zm00001d025528_T003	1.255872311	1.961534801	2.107597154	0.930272573	0.855017743	0.757054832
Zm00001d039590_T004	0.776495674	0.371401621	1.135078415	0.682744972	0.855823897	1.461107319
Zm00001d039590_T001	1.249205024	1.166630616	1.417174711	1.552693809	1.680493149	1.803093095
Zm00001d005816_T001	2.940508821	2.368657454	2.944550576	3.805153958	3.854807857	3.961161925
Zm00001d043578_T014	0.998425883	0.662537304	0.848032172	1.046090081	1.020486556	0.851380064
Zm00001d043578_T013	0.1630388	0.140090185	0	0.423013352	0.095649016	0.538350427
Zm00001d042905_T001	0.406488779	0.194748976	0.128065033	0.94345704	1.36604519	1.431379593
Zm00001d043105_T001	1.175541846	1.413432691	1.241079289	2.875156661	2.541168125	2.763413699
Zm00001d011797_T002	2.155677355	2.112608398	1.820725908	1.029621087	0.942478473	1.656919621
Zm00001d037946_T001	2.47079922	1.664675794	2.376380966	3.540567931	3.613540375	3.443316492
Zm00001d021689_T001	1.34462003	0.954102486	1.237354457	0.84230307	1.038624408	1.090570282
Zm00001d031201_T001	3.595847465	2.721281435	3.499708019	3.555949868	3.651289623	3.566643968
Zm00001d045625_T001	4.045616133	4.149713226	4.105573492	4.210147174	4.479437339	4.256491396
Zm00001d032334_T002	1.783734061	1.052138319	1.775858814	2.876225972	2.817417991	2.752273751
Zm00001d043734_T001	3.822188041	3.152995741	3.921770687	4.008457666	4.050567344	3.875743209
Zm00001d043860_T009	3.195515168	1.671366751	3.298970719	2.746555059	3.147571277	3.244306212
Zm00001d043860_T004	1.740147409	0.493009866	1.881158706	2.587101393	2.238649614	2.42154675
Zm00001d043860_T005	0.155767363	0.526006694	0	1.141605433	0.530591178	0.723872185
Zm00001d036750_T001	5.07074751	4.441373122	4.982038	6.094025859	6.100907256	6.16525074
Zm00001d050781_T002	3.059623681	2.17095664	3.028709172	3.407123615	3.3218196	3.287756994
Zm00001d010292_T001	2.30552291	1.802088967	2.379497036	3.094842753	3.236457718	3.067863088
Zm00001d035037_T001	8.268356551	4.519606188	8.306918578	8.250053722	8.219241873	8.244911984
Zm00001d035037_T008	2.977122913	1.627733827	2.815820707	3.022473814	2.442717953	2.286232676
Zm00001d035037_T003	3.54099424	2.44333002	3.538558028	3.372982595	3.356370904	3.834977829
Zm00001d035037_T006	7.009957953	7.199500301	7.008936933	6.677770232	7.14329644	6.752294815
Zm00001d020986_T002	1.836445694	1.447103068	1.79782481	1.503704912	1.83932192	1.626283528
Zm00001d029105_T001	4.975120999	4.226925171	4.968340736	4.968906174	4.919077313	5.067115723
Zm00001d005168_T006	1.131900904	1.035066429	2.373079373	1.581016146	1.197611294	1.160015317
Zm00001d005168_T003	1.557078206	0.632003852	0.692962753	2.192607026	2.371549663	2.481257603
Zm00001d018393_T001	5.803907867	6.769571715	6.062325267	5.823886771	5.773279519	5.752960539
Zm00001d003775_T001	3.040953257	3.659626173	2.744542606	2.828837492	3.055232103	2.826623734
Zm00001d022541_T003	1.474484846	1.249940415	1.439004973	1.083973753	1.30067082	1.528179713

Zm00001d022541_T004	2.983165639	2.271854417	2.309152608	3.19489771	2.862579537	2.838548643
Zm00001d022541_T002	1.52151514	0.197957208	1.881722169	0.901128329	1.3173938	1.148586645
Zm00001d013573_T001	4.674783493	4.05671574	4.928566222	4.086761606	4.143461258	4.017994041
Zm00001d037018_T001	8.450437517	9.396698284	8.488771184	8.234448092	8.174276978	8.347502848
Zm00001d048492_T002	2.796106598	2.86319888	3.146058374	2.414704379	2.344014053	1.964162754
Zm00001d048492_T003	1.224123029	0.944708548	1.382844294	1.3108694	1.396940372	1.28267465
Zm00001d050642_T001	1.307446595	1.314196498	1.674922945	2.452890058	2.4662789	2.50492034
Zm00001d049831_T005	3.169080136	2.731427436	2.865040066	0	2.473999599	1.806043947
Zm00001d049831_T002	2.857595694	1.924672288	3.172492156	3.148858633	2.32947669	2.869910877
Zm00001d049428_T001	2.955881139	2.682423627	2.705934123	4.156504944	4.188371409	4.135959998
Zm00001d012425_T003	2.276293274	2.8023715	2.202836129	2.689547417	2.276060367	2.251417951
Zm00001d012425_T002	5.777440231	5.750771559	5.822160451	5.593287641	5.362015337	5.584812332
Zm00001d025402_T006	4.718977483	4.14455669	4.863717769	4.935256012	5.154222117	5.19189648
Zm00001d025402_T002	2.92762571	2.570681505	2.599038274	3.21973263	3.0639833	3.2836135
Zm00001d014944_T002	7.414606125	6.646734652	7.494276023	6.520486807	6.519697419	6.525464885
Zm00001d014944_T001	3.189970007	2.216217371	3.095367483	0.731450898	2.039421289	1.290770586
Zm00001d028951_T001	4.853041517	4.252124505	4.868133452	4.846532429	4.826139746	4.698807162
Zm00001d022560_T002	1.515375881	1.257430086	1.749569021	1.271632444	0.723446724	1.682667676
Zm00001d022560_T001	1.783005231	0	1.54098544	2.633119852	2.743188348	0
Zm00001d022560_T003	0.282642628	0.249094561	0.415950076	0.705659479	0.951965949	0.605151991
Zm00001d052026_T001	2.975578831	3.401713193	3.13439149	3.748817569	3.488602925	3.622821325
Zm00001d037803_T002	1.760493915	1.043956173	1.76911582	2.143262793	2.070732442	2.11826403
Zm00001d037803_T001	0.192550218	0.093209939	0.329110964	0.38844085	0.70776419	0.595107366
Zm00001d035004_T002	0.716921556	0.214070081	0.774948527	2.268638596	1.294651918	2.18324353
Zm00001d035004_T039	2.854427393	1.315914545	2.730810352	3.342446795	3.214283213	3.504171753
Zm00001d035004_T034	0	0	0	2.409939124	2.572227574	1.903673842
Zm00001d035004_T015	0.70693982	0	0	1.275873597	2.494550529	2.005811358
Zm00001d035004_T026	0	0.017263461	0.23124315	0	0.47630806	0.802135301
Zm00001d026352_T001	2.981104267	3.995648136	2.922520991	2.568796069	2.809586693	2.757790275
Zm00001d009628_T001	1.430621952	1.790802059	1.966854575	1.274855616	1.749997572	1.907058323
Zm00001d050872_T001	4.2107985	3.511870404	4.267747243	6.01189385	6.062573649	6.071181798
Zm00001d004126_T001	4.330950427	3.682564533	4.217327901	4.567201099	4.767435941	4.71524143

Zm00001d010703_T007	2.408145268	3.125182249	2.510658162	4.022537464	4.213601034	3.802827967
Zm00001d010703_T009	5.101516456	2.713390529	4.705791003	4.204127019	4.084811619	4.743298451
Zm00001d010703_T010	3.375024691	0	2.09132003	2.813168178	2.035709061	0
Zm00001d010703_T005	2.63538154	0	3.437406067	2.317270779	1.56747925	3.13561613
Zm00001d038729_T001	2.757109678	3.185487431	3.07408476	1.375882414	1.107034324	1.711161396
Zm00001d033112_T001	1.365429951	1.087186496	1.06289053	2.293404623	2.138865899	1.979934603
Zm00001d033112_T006	2.921775637	1.853305064	2.929886417	2.473694971	2.340907449	0
Zm00001d024756_T005	0.780805612	0.415228644	0.930696469	0.603715352	0.655833563	0.813928505
Zm00001d038915_T001	0.271332442	0.044070919	0.140104586	2.906173494	2.991118175	2.945868728
Zm00001d017833_T001	6.558935445	5.708358346	6.44702006	6.615165149	6.672022236	6.674407007
Zm00001d017833_T002	3.179985024	4.965854779	2.743686005	3.38118119	2.478766061	2.877718733
Zm00001d038552_T001	2.993867976	2.572821775	3.288794701	3.67977407	3.847529658	3.792798414
Zm00001d008623_T001	1.437277817	1.106241102	1.448442175	1.540252983	1.560445952	1.726132365
Zm00001d013038_T001	4.817792227	4.899287261	4.862262921	5.914671789	5.947172209	5.843600489
Zm00001d017876_T007	1.76630723	1.518894132	2.066489128	2.921750506	2.612136137	0
Zm00001d017876_T010	1.200385704	0.191198773	0	1.638266184	1.371433405	2.104422883
Zm00001d017876_T012	0.383300074	0	1.005462509	1.102273809	0.541665494	1.946603987
Zm00001d017876_T011	0.259879873	0.181919264	0.12023514	0.769776816	0.392797692	0.225468675
Zm00001d017876_T006	1.282055431	0.996559401	1.347866316	2.004613568	1.914184857	2.124033941
Zm00001d017876_T009	2.939054084	2.324507346	2.678901216	2.880080113	2.688718834	3.158552737
Zm00001d017876_T018	2.124935858	0	2.615532829	3.033489078	3.448151533	3.066501699
Zm00001d021699_T001	4.444829043	4.057200703	4.496103677	4.339733514	4.329470447	4.223496845
Zm00001d003119_T002	2.403895117	1.557007604	2.318616775	0	0.37085393	1.27268375
Zm00001d003119_T001	2.233835921	1.640109092	2.389947809	3.55557814	3.348508352	2.803617421
Zm00001d003119_T006	0	0	0	1.345579185	1.799652665	2.280006969
Zm00001d003446_T003	0.484160822	0.072481779	0.142342854	0.408686865	0.347577221	0.318545919
Zm00001d003446_T002	4.218391541	3.469953031	4.360872291	3.119778797	3.390354985	3.247747849
Zm00001d013554_T001	2.730588224	2.343480595	2.740104007	2.619866525	2.579795383	2.139234047
Zm00001d046313_T003	4.240940735	5.884134074	3.416959691	4.024836216	4.315952573	4.335735131
Zm00001d046313_T001	4.915349278	4.255899949	5.195707007	5.071892854	4.960143112	4.918184128
Zm00001d034630_T001	5.135138107	4.269212125	5.092898296	5.26420657	5.224965285	5.228870665
Zm00001d028649_T002	3.647521515	3.067408287	3.746774097	4.493222494	4.766001849	4.57330502

Zm00001d028649_T001	1.851826583	1.172205221	2.06023212	3.523330479	3.396596836	3.652800813
Zm00001d034270_T001	3.539221358	4.64962069	3.704646249	3.887819434	3.988286493	3.872005943
Zm00001d034270_T002	1.362698233	2.172940927	1.66903221	2.261942955	1.97906794	1.560006148
Zm00001d040290_T003	3.493746849	1.620180053	3.661955114	3.897664748	3.818804351	3.635725607
Zm00001d040290_T005	4.013480305	4.748999889	4.157081718	4.290633406	4.205158901	4.310240423
Zm00001d040290_T004	0.188969281	0.290076369	0.263432294	1.586575494	1.020764599	1.276147041
Zm00001d013185_T001	3.929809079	3.348842574	4.037675827	5.795513716	5.727852211	5.727886047
Zm00001d006516_T001	5.046362317	6.077198341	5.292628013	5.533346585	5.536948368	5.661633123
Zm00001d017522_T011	0	0.302593963	0.869374765	0	0.846893625	0.873313187
Zm00001d017522_T007	1.564577181	0.182365589	1.423681966	1.775437456	2.109508332	1.994018466
Zm00001d017522_T018	4.72304408	3.562467131	4.7700943	5.261206323	5.37273526	5.084651814
Zm00001d017522_T016	1.036620784	0.603650793	1.18083021	1.329936444	1.362508603	1.240614134
Zm00001d017522_T020	0	0	1.09624026	0.300586484	0.863441348	1.594504599
Zm00001d017522_T023	6.514107123	7.319167437	6.647548396	6.275730896	6.130788096	6.286299537
Zm00001d017522_T012	2.920883979	1.515576218	3.011553603	4.620774007	4.53763949	4.641268156
Zm00001d017522_T009	0	0.568949494	0	1.621072388	1.047796613	0
Zm00001d011276_T001	2.627350492	1.858688041	2.702221123	3.964208879	4.120369028	3.898829903
Zm00001d053864_T003	6.037287284	4.963132969	6.067624327	5.685724597	5.65542608	5.506738296
Zm00001d028623_T001	4.000786415	3.240802473	4.053531308	4.103150562	3.801677271	4.042556005
Zm00001d028623_T002	0.112837558	0.081973565	0.495754506	0.521654833	1.044728436	0.597303281
Zm00001d041734_T001	3.834533345	3.148332666	4.117773907	3.891001386	3.806907102	3.986300066
Zm00001d013962_T002	1.605894421	1.899003502	1.287236456	1.469832851	1.68040808	1.777136041
Zm00001d013962_T004	4.144383829	0	4.079974182	3.814375177	3.778443889	3.775011549
Zm00001d013962_T001	0	3.266918138	0	0	1.012607288	1.509382189
Zm00001d036025_T005	1.647147318	1.486912553	2.176737009	2.081932674	2.351174023	2.243865245
Zm00001d036025_T001	3.500346742	2.600050145	4.023405559	3.883496481	3.880268327	3.776592421
Zm00001d036025_T006	2.925504072	3.16823285	1.907827851	0	0	1.169949367
Zm00001d036025_T003	1.177335495	0.842774528	1.453134039	0.871638661	0.312159793	0.428057682
Zm00001d036025_T007	1.159363742	0.352573234	0.244018746	0.817353929	0.488708315	0.780202575
Zm00001d008941_T008	1.442058106	0.873373828	0.8858578	1.760633573	0.690769289	1.428980101
Zm00001d008941_T004	0.397069109	0.299168447	0	0.442015628	0	0.78572379
Zm00001d012420_T004	1.93103209	0	2.397128269	2.547237333	0	0

Zm00001d012420_T002	0	2.845348936	2.255333038	2.409376815	0	1.266961003
Zm00001d012420_T003	2.665178761	1.781624516	2.007659817	2.127679828	3.402724769	2.949114483
Zm00001d043420_T001	3.002010977	2.409681483	3.112663106	2.331043328	2.496006945	2.349800342
Zm00001d022059_T001	3.165932997	2.378465567	3.181442586	3.184451217	3.15233547	3.035148974
Zm00001d027298_T001	3.046244129	2.352970621	3.104043897	2.226008741	2.504779779	2.43653073
Zm00001d033976_T001	8.048292028	6.971235647	8.063738666	7.506876897	7.4876855	7.516337597
Zm00001d033976_T004	1.011916364	3.805000275	1.89274267	2.269933801	1.516862355	1.865554632
Zm00001d028603_T005	3.549190575	2.826908824	3.594226535	2.680321431	3.141565708	3.084673709
Zm00001d028603_T001	1.530875789	1.416194978	1.359932106	1.50020803	1.864623568	1.611079818
Zm00001d028603_T006	2.888998407	1.41390111	2.769816373	3.271615114	2.922571226	2.95468879
Zm00001d004790_T006	1.673398126	1.665474637	1.825340745	2.953762692	2.852798292	2.47294936
Zm00001d004790_T002	4.919627782	4.085820799	5.077506985	4.930949327	4.893481816	4.975029764
Zm00001d008604_T011	4.978789292	4.272490944	5.114234827	4.913008099	4.960674616	5.307731933
Zm00001d008604_T003	2.381581849	1.232994073	1.808746657	3.569151158	3.61942921	3.980211065
Zm00001d008604_T007	0.33640794	0.285988056	1.6590733	2.812595976	2.031447073	0.755747306
Zm00001d008604_T005	0.724004079	0.397654031	0.602209806	1.113168391	0.853246768	0.639207153
Zm00001d008604_T001	2.941982933	2.035251224	3.18083546	4.146922282	4.482940416	4.034119292
Zm00001d008604_T004	2.111240354	1.614656611	1.741616695	2.458198768	2.343956937	2.748351094
Zm00001d008604_T010	0	0.544190585	0	1.291460342	1.724369999	1.21088007
Zm00001d012532_T001	7.7147805	6.922284945	7.796673405	7.841894751	7.843889962	7.768550901
Zm00001d002382_T002	2.678323002	2.165781729	2.798625197	3.551361835	3.391729645	3.568137201
Zm00001d002382_T001	2.282079457	1.127239704	2.109745004	2.732693804	2.855057224	2.565926375
Zm00001d015131_T002	1.748876377	1.403345173	1.977397166	2.428566934	2.524983878	2.453621876
Zm00001d015131_T005	0.461698278	0.104529901	0.565786273	0.341632856	0.660680406	0.578287603
Zm00001d015131_T001	1.60317838	0.758594842	1.477496008	1.485128983	1.668624297	1.625877837
Zm00001d043842_T008	2.661989421	0	0	2.028103737	1.621666503	1.841189786
Zm00001d043842_T007	0.7284012	0.730422584	0.955936919	1.352164717	1.365841446	0.981483714
Zm00001d043842_T004	0.112286445	0.025795665	0.387198188	0.555399971	0	0.484422774
Zm00001d043842_T013	4.004783522	3.572722233	4.038468403	3.609948167	3.319441922	3.610418841
Zm00001d043842_T003	1.973031877	1.973092143	2.921043992	3.458013277	3.645479174	3.377971066
Zm00001d023264_T004	1.965142985	0.612942196	2.245270509	1.484572293	1.549275856	0.283665797
Zm00001d023264_T002	2.808953984	2.134273191	2.002117044	2.635480683	2.496521917	2.910248549

Zm00001d023264_T007	2.39015726	2.281232655	2.41196336	1.364573553	1.914036332	1.717624883
Zm00001d033420_T001	5.177473238	4.848842939	5.220768269	4.397359409	4.495759366	4.426323602
Zm00001d034163_T001	3.128585078	2.927449737	3.182692933	1.709485629	1.945240241	1.891575503
Zm00001d052488_T002	0.930536765	0.607785275	0.979172962	0.881261313	0.901059573	0.68946288
Zm00001d018984_T001	5.04182033	5.50092309	5.034651698	4.866752724	5.070441324	5.008523282
Zm00001d015213_T001	4.929094852	4.324195311	5.071532897	3.949871616	3.985747052	3.982342356
Zm00001d036370_T001	3.797223036	4.116770208	3.636297592	2.713889334	2.705291657	2.167238769
Zm00001d037786_T001	1.112790861	0.833868083	1.267641861	1.905764854	1.633497705	1.857749349
Zm00001d011555_T001	2.519730092	2.788942283	2.637188249	2.890034157	2.376612675	2.802395284
Zm00001d017767_T002	3.308439078	2.546788298	3.555879946	3.961777868	4.096791801	4.091055851
Zm00001d017767_T003	2.837671223	4.442274793	2.510117822	3.795166496	3.886514835	3.914963888
Zm00001d044880_T001	5.178345197	5.618480328	5.136751036	5.170231221	5.078273841	5.035899535
Zm00001d004810_T002	0.884074275	0.696429228	1.570887419	0.835726044	1.557443421	0.789132438
Zm00001d004810_T001	2.249529989	1.560109874	2.184718274	2.002127488	1.646289867	1.943842956
Zm00001d000011_T007	0.780130328	0.552578729	0.432235741	0.532259101	0.104061515	0.448732891
Zm00001d000011_T001	1.942470966	1.713322337	1.382327495	1.161062773	1.62369693	1.423735743
Zm00001d000011_T024	1.331809532	0.302039406	0.950380319	1.635489738	1.39010084	1.713508428
Zm00001d000011_T002	1.317433164	0.832758799	1.404957059	1.598855391	1.80846537	1.565646889
Zm00001d000011_T019	0.649262272	0.911599553	1.009660331	1.277822323	0.844667656	0.745604791
Zm00001d000011_T022	0.445695985	0.1696685	0.951372941	0.853249962	0.73758034	1.094747511
Zm00001d013116_T001	1.213504686	0.903659961	0.161926369	0.1568742	0.610781312	1.16338022
Zm00001d013116_T004	3.042910966	2.143541991	3.139276457	3.778360935	0	3.68525922
Zm00001d045462_T001	1.665092102	0.973633315	1.525021977	1.973592541	1.513027796	1.745626732
Zm00001d045462_T006	1.392709233	0	0	1.166039887	0	1.624390107
Zm00001d052146_T001	1.41633444	0.746084841	0.859935368	0.926461007	1.148509854	1.185212587
Zm00001d051422_T001	8.590913163	7.438251527	8.124202168	8.097134173	8.043526826	8.136896419
Zm00001d003496_T001	0.893380074	0.989514713	1.759691897	1.640829127	1.86498957	1.691129886
Zm00001d038022_T001	4.399042203	3.598058814	4.381386794	4.38792054	4.443769211	4.369290935
Zm00001d052493_T001	3.245575979	3.150227071	3.401859658	2.281354039	2.161988587	1.6349928
Zm00001d052493_T003	8.656909449	8.277097087	8.696804791	7.059482066	7.038940076	7.114210398
Zm00001d052493_T002	6.398698944	5.727902108	6.317658258	4.255219494	4.079598275	4.163013593
Zm00001d048372_T002	2.671618339	2.443411715	3.143355702	3.117338148	3.04203982	3.09804354

Zm00001d044059_T001	2.964153325	2.272754208	2.952350715	3.343113426	3.212442027	3.167004598
Zm00001d041416_T003	7.002657497	5.984104262	7.118780468	6.678579295	6.627280663	6.601496513
Zm00001d041416_T002	7.509998646	8.392428287	7.479489578	7.040292648	6.950132512	6.916895264
Zm00001d041416_T004	1.072722545	0.746074519	1.06329239	1.109750352	0.681637264	1.066886888
Zm00001d041416_T001	4.299282835	4.159390466	4.133430774	3.851581854	4.096968267	3.553766588
Zm00001d019279_T001	4.464227485	3.989008905	4.524750686	4.312672506	4.337368643	4.042037543
Zm00001d040320_T004	0.330116633	0.114265733	0.21336968	0.943341507	1.087743206	1.385035036
Zm00001d040320_T002	1.790276183	0.93301358	1.40265888	1.801247657	2.010221268	1.697887665
Zm00001d052749_T001	5.91061906	5.961114568	6.137923463	5.607597668	5.529471691	5.623516707
Zm00001d038123_T002	4.229845678	3.5319119	4.358456537	4.673464783	4.552988476	4.487611242
Zm00001d038123_T004	2.150487215	0.252187949	1.611261159	1.763718354	2.185583052	2.509187326
Zm00001d043095_T001	1.142128465	0	0.890291033	0.942144377	0	1.09991861
Zm00001d043095_T002	0	0.772760647	0.414279525	0	1.227921555	0
Zm00001d016132_T001	4.55646749	4.688511279	4.531724048	5.191917517	5.398539144	5.154778786
Zm00001d045421_T001	3.709210668	3.250531126	3.827101258	3.876729394	4.058461624	4.157461578
Zm00001d028810_T001	5.047312579	4.307612275	5.081091131	4.857196702	4.953048492	4.854933643
Zm00001d034345_T003	2.968491701	1.940278026	2.903004903	7.603908922	7.592557963	7.55958916
Zm00001d034345_T002	6.362549403	5.161489395	6.355852512	9.524529858	9.572370877	9.528010353
Zm00001d034345_T001	3.984184829	4.501052782	4.031805307	7.017462532	6.995791929	6.93752919
Zm00001d053309_T001	0.570472646	0.380075545	0.40835318	1.371261651	1.428644647	1.570705789
Zm00001d017643_T001	2.714259367	2.081865202	2.714485562	3.438978634	3.284835239	3.257781409
Zm00001d025750_T019	0.762227186	0.278966041	0.465613027	0.882573407	0.744288564	0.742663361
Zm00001d025750_T011	3.77673095	2.977543157	4.048020602	4.311621688	4.277507389	4.292916591
Zm00001d025750_T012	0.565666385	0.493206671	0.560993686	0.670350897	0.346913785	0.350209811
Zm00001d025750_T024	2.971495548	1.749759075	2.57328387	1.684441299	1.452227466	2.150512561
Zm00001d041740_T001	3.776080189	2.980830534	3.781478167	4.024302124	4.142185742	4.122928025
Zm00001d015613_T001	5.45733839	5.141741072	5.627878398	5.571652906	5.423253293	5.568115763
Zm00001d015613_T002	1.309091184	1.140353309	1.171051146	1.170664113	0.650481506	1.319753758
Zm00001d053685_T005	0	1.962523482	3.794783798	2.614889316	3.648249828	3.231364204
Zm00001d053685_T007	3.456577902	0	0	2.060132144	0	1.467871047
Zm00001d042766_T001	3.204739211	3.927749049	2.986260195	2.299853908	2.602159198	2.336936595
Zm00001d029919_T002	1.934045402	1.338143693	2.10523253	2.046084841	2.419856789	2.056022294

Zm00001d007433_T001	1.009170856	0.798110254	0.935609101	0.286948551	0.414591277	0.274271205
Zm00001d048737_T001	2.591476449	1.995331467	2.586747933	2.007893353	2.048950031	1.631912617
Zm00001d022364_T001	4.127359323	6.560448887	4.613535955	4.855377468	4.133565252	3.45530052
Zm00001d022364_T003	5.519839552	4.388947889	5.474373317	5.926929918	5.989153452	6.099838617
Zm00001d025710_T002	1.724327212	1.1313972	1.612036322	1.537582838	1.620350399	1.193798232
Zm00001d038845_T004	0	0.040119514	0.074719153	0.808357041	0.716024239	0.88924612
Zm00001d038845_T007	1.422222773	0.325001798	1.01860213	1.275020163	1.368686785	1.081690032
Zm00001d038845_T011	0.70450195	0.312440969	1.429779304	0	0	0
Zm00001d038845_T006	0	1.026358864	0.654683912	0.667495244	0	0.946161903
Zm00001d038845_T012	1.223464564	0	0.510845487	0.864566124	1.199705006	0
Zm00001d043358_T001	2.51541146	1.944853574	2.075878631	2.227023246	1.943463041	2.181550401
Zm00001d012887_T001	4.363331601	3.581196243	4.425717098	5.093359797	5.127405344	4.959985477
Zm00001d012887_T002	1.097142182	1.453839927	1.573769565	2.021803054	1.859129918	2.216376326
Zm00001d040743_T001	2.284054482	1.701477641	2.392834777	2.299182224	2.459272913	2.457842205
Zm00001d016850_T002	5.092385226	5.734012434	4.993986323	4.882568957	4.841799663	4.751375773
Zm00001d010975_T005	0.594685169	0.331156005	0.34331344	1.047488832	1.193588833	1.045407322
Zm00001d010975_T004	2.227856265	1.317384537	2.779634373	3.255050028	3.095108687	2.534145541
Zm00001d010975_T006	0.815522147	0.673013615	0.664391816	0.683525791	0.986164063	0.581070636
Zm00001d010975_T003	4.997672273	4.022463605	5.022168175	4.813059073	5.127234792	4.910059263
Zm00001d010975_T001	4.460257325	3.518758324	4.157162259	5.088454481	4.809073385	5.228882514
Zm00001d052083_T019	0	0	0.280272207	0.686841131	0.886557951	1.005622753
Zm00001d052083_T006	0.727343697	0	1.108159764	0.726252279	0.296631254	0
Zm00001d024213_T001	4.115591713	3.855547156	4.038130261	2.594901537	2.594247797	2.848649178
Zm00001d041911_T033	1.402573753	0.343607938	1.144531973	1.47618776	1.249402257	1.447243278
Zm00001d041911_T027	1.013941709	0.322235068	0.767187806	1.588013458	1.823490141	1.641857192
Zm00001d041911_T026	1.498572055	1.673723749	1.295657206	0.29709631	0	1.867841522
Zm00001d041911_T011	3.049826773	1.947505703	3.030562089	3.397840877	3.264598881	3.387079875
Zm00001d041911_T030	0.299758098	0.105834942	0.408665129	0.320553995	0.345399782	0.423953519
Zm00001d041911_T025	0.950928958	0.450925644	0.828882569	1.025771573	0.731814065	1.480709238
Zm00001d048893_T001	5.348551055	4.558790057	5.41113561	5.446014591	5.523207856	5.459260649
Zm00001d034279_T001	6.446104772	6.158322342	6.563097117	6.19505716	6.090029742	6.125273684
Zm00001d018217_T004	2.546185992	1.325145754	2.451135397	2.784304002	2.810857241	2.630984111

Zm00001d018217_T009	0	0	0	1.387959128	1.332381984	1.193736425
Zm00001d018217_T002	0	1.149193018	0.642391977	1.728247501	1.782535215	0.97928932
Zm00001d018217_T003	1.116859766	0	0.317625918	2.820457567	2.44692739	2.705200959
Zm00001d030967_T005	5.301356409	5.89492561	5.208020136	4.688031996	4.441741745	4.173942278
Zm00001d030967_T002	4.307897252	3.3375965	4.21652401	4.991097081	5.012644292	5.08075725
Zm00001d030967_T006	0.673259714	0.628149264	0.800262541	1.079234731	0.774224945	0.998057547
Zm00001d030967_T007	0.819114704	0.484145351	0.767875105	0.727710514	0	0.746099464
Zm00001d030967_T010	1.044751514	1.087008575	1.488915987	1.2173362	1.016017719	0.499552536
Zm00001d020963_T002	3.135513364	0	3.749248275	0	2.525318966	0
Zm00001d020963_T001	5.288894379	4.599259347	5.338025283	5.445448591	5.488402406	5.558396283
Zm00001d012736_T001	4.69061907	5.105551283	4.677078076	3.18669039	3.591421513	3.567526951
Zm00001d000041_T001	4.217304245	4.013001864	4.103106909	4.168028696	4.13700948	4.028927218
Zm00001d018625_T021	0	0.629863893	0.828137602	0	1.105397154	1.370860624
Zm00001d029170_T002	1.933383791	3.163258961	1.844627086	2.091677157	2.102094715	2.334101126
Zm00001d029170_T005	3.352180682	4.00713153	3.339218641	4.158823176	3.617776285	4.125170178
Zm00001d029170_T003	7.468672912	7.161391792	7.492949705	7.620833502	7.744215882	7.61910631
Zm00001d019304_T001	4.127690062	5.139532075	4.29492315	3.724973696	3.761314425	3.732101147
Zm00001d051890_T001	1.785004338	1.141831668	1.346322673	2.225601628	1.886088099	1.890344348
Zm00001d010711_T002	3.402093726	2.920455285	3.602412933	2.841328491	3.063806636	3.333961352
Zm00001d010711_T001	1.188443338	1.559706182	0	2.906095355	2.90444691	2.053724786
Zm00001d053259_T004	4.559792007	3.716170245	4.59410821	4.174387881	4.664522321	4.056966
Zm00001d053259_T001	0.459966628	0.800334615	0	0	2.421755971	1.478479005
Zm00001d053259_T003	3.816381596	3.33382271	4.018067328	4.386713292	3.94430515	4.401529442
Zm00001d021956_T006	1.545138581	0.531908907	1.074871202	2.131958835	1.918694969	1.967433877
Zm00001d021956_T007	0.256509512	0.433968941	0.258883095	0.958582874	1.135604475	0.656090849
Zm00001d021956_T002	2.693316779	2.041210034	2.98255721	2.756897752	2.777331336	2.756069173
Zm00001d021956_T008	0.785312838	0	0	0.585793254	0.90959494	1.135974112
Zm00001d011817_T002	1.330361629	0.2718583	0	0.567183268	1.045038904	1.055312287
Zm00001d011817_T001	0.25283707	2.151806549	1.274434625	0.863706966	1.227336917	0.789346151
Zm00001d011817_T003	4.811205073	4.325164683	4.887630106	4.980181309	4.852426986	4.797002547
Zm00001d030656_T001	4.391096249	3.466193398	4.404478914	4.853773289	4.690663986	4.785314774
Zm00001d046093_T001	2.325112357	1.963310239	2.487863438	2.673297937	2.51523785	2.672225274

Zm00001d033572_T004	0.579579876	0.625905416	0	1.135374634	0	0
Zm00001d033572_T001	1.777341858	0.835982266	1.638096549	1.891240297	1.965326243	0
Zm00001d033572_T006	0	0.31066818	0.74109243	0.699182916	1.009580077	0.955512193
Zm00001d029217_T001	2.795695684	3.439621675	3.025652882	3.211171567	3.257475345	2.836993777
Zm00001d007151_T011	0	0.205346216	0	0	0.509027918	0.650046709
Zm00001d007151_T009	1.377514154	0.824884323	1.671705059	1.86499155	1.531346024	1.547587034
Zm00001d007151_T004	0.667378066	0.535541399	0.388975296	0	0	0.779739628
Zm00001d027962_T001	4.641290818	3.88235859	4.714631614	5.120372927	5.010792682	5.072785988
Zm00001d004919_T001	1.960511339	0	2.02905598	0	1.786220794	0
Zm00001d012269_T004	2.408113195	1.412012425	1.546777435	2.910342583	2.653444608	3.064688537
Zm00001d012269_T002	1.890137302	1.67929272	1.957987381	2.43106476	2.474621641	2.449445954
Zm00001d012269_T003	0	2.687158806	2.665425063	0	2.453983697	0
Zm00001d012269_T006	3.735898074	1.377167071	2.905974097	4.11604231	3.683129145	3.958070836
Zm00001d023635_T001	6.650956772	5.974012415	6.692035936	6.632108434	6.751376831	6.676806428
Zm00001d005064_T002	3.027742194	2.789334673	3.191371563	3.047552522	2.71846794	2.837498662
Zm00001d035962_T001	3.046275391	2.531264668	2.890722948	4.700352543	4.650876203	4.73800333
Zm00001d028210_T001	3.427235395	2.700039925	3.387736402	3.249120205	3.434737902	3.307266905
Zm00001d007514_T001	5.47034241	5.776722003	5.59055217	4.596270932	4.84472409	4.953620048
Zm00001d033363_T001	4.871745642	5.408463953	4.899425421	4.183067858	4.300753173	4.376899135
Zm00001d044415_T002	2.79754512	3.041515828	2.774654457	2.465161626	1.077054958	2.656095198
Zm00001d044415_T001	1.394460911	1.116845797	1.16320179	1.795214197	2.029871586	1.933394745
Zm00001d003226_T001	1.501350988	1.132270176	1.301943448	2.031054245	2.266379681	1.915328146
Zm00001d043738_T001	0.106340278	0.130821511	0.099886302	2.426938839	2.226893197	2.7307069
Zm00001d019259_T001	5.025965755	4.456789397	5.18150035	4.849532259	4.854879102	4.995500529
Zm00001d024088_T001	3.402164962	2.548295554	3.238312098	3.33203003	3.307293867	3.441731521
Zm00001d003534_T001	0.810253063	0.284039079	0.777543017	1.633853378	1.655410909	1.627989172
Zm00001d044130_T002	3.617804489	4.258368014	3.912214596	3.706128465	3.553573205	3.036128765
Zm00001d029684_T001	2.342381225	1.605671163	2.256623936	3.343274633	3.462932592	3.398071492
Zm00001d028957_T001	4.49076868	4.375221651	4.410249644	3.832756369	3.692117825	3.890294535
Zm00001d045350_T002	4.280986963	3.884684558	4.255243442	4.132824152	4.320998772	4.167089565
Zm00001d045350_T001	1.186816192	0	2.076329231	2.080831936	0	1.877862801
Zm00001d045350_T003	0.36470577	1.912267778	0	1.210969192	2.516334421	1.845944817

Zm00001d000126_T001	6.674769386	6.291323854	6.689888015	6.346421238	6.296610718	6.319312102
Zm00001d035143_T001	0.559314616	0.204699152	0.478147616	0.541035018	0.838688005	0.724846692
Zm00001d042041_T001	1.144428209	1.067881669	0.953027533	2.533770892	2.508294519	2.843228542
Zm00001d012844_T005	3.216540695	4.681308081	3.598130176	2.302980799	2.41590547	2.907223916
Zm00001d012844_T002	6.317293503	5.251160082	6.327732681	6.385650738	6.426881459	6.32110114
Zm00001d021205_T001	1.605961724	1.767359026	0.620670858	6.68204934	6.628170339	6.738166643
Zm00001d021420_T001	3.796558405	3.447860435	3.63784264	3.68854496	3.528238235	3.645761366
Zm00001d021420_T002	0.960029743	1.264323839	0.802251958	1.15527905	1.29775723	0
Zm00001d021420_T003	3.138374616	2.410885134	3.54039633	3.363327816	3.493128516	3.613145586
Zm00001d047306_T005	2.787217214	1.956004595	2.829345645	3.031014003	3.070166552	3.15830891
Zm00001d047306_T012	0.370678759	0.122420929	0.579320162	0.739809227	0.152305451	0
Zm00001d013022_T001	3.577360786	3.377583422	3.643922898	3.933119648	3.92002732	3.9376941
Zm00001d006746_T001	2.296903729	2.307288912	2.682968299	2.929057915	3.049437001	3.088852251
Zm00001d018482_T005	1.169636434	1.330840601	1.966863063	0	0.633191245	0
Zm00001d018482_T004	2.653119192	0	1.985795861	1.323926286	1.614405963	1.801494763
Zm00001d018482_T001	2.258000547	2.373279035	2.515813862	3.27842894	3.287552229	3.261116602
Zm00001d018482_T003	0.062819399	0.29857269	0.059374661	0.724857167	0.640564478	0
Zm00001d005372_T003	2.523660307	0	2.341918306	1.627853336	0	2.536176768
Zm00001d005372_T002	2.870836564	2.790084718	2.989800787	3.991727176	4.100703375	3.659116925
Zm00001d005372_T016	0.591156621	0.134481193	0.467883564	0.606839211	0.507454695	0.465159536
Zm00001d005372_T009	0.916241208	0.233776419	0.737854597	1.049926943	1.384740035	1.044561987
Zm00001d005372_T006	1.115946794	0.062936798	1.121388795	1.223356438	0.991103659	0.798131826
Zm00001d009660_T001	5.609605875	4.954283568	5.731872051	4.533620407	4.554872513	4.578750668
Zm00001d052445_T020	0.148930201	0.15849861	0.489081487	0.515701352	0.709946097	0.634789332
Zm00001d052445_T001	0.69961737	0	0.771187509	1.3475296	1.014183159	1.446142928
Zm00001d052445_T012	0	0	0.072022084	1.245914233	0.565620571	0.547616645
Zm00001d052445_T008	3.551817937	2.514240702	3.438313613	3.565869971	3.586981822	3.631084022
Zm00001d004438_T001	0.690299977	0.623359348	0.796594432	0.638254597	1.097629673	0.876028484
Zm00001d011304_T001	1.14507743	1.156332539	1.321439227	0.984202149	0.905521887	0.638149852
Zm00001d025098_T007	0.406088173	0.165374304	0.322864965	0.644891788	0.783060985	0.504490226
Zm00001d025098_T003	1.100249054	0	1.886948467	2.087253041	2.344589921	1.185326147
Zm00001d025098_T008	0.785650146	0.370904135	0.629972039	1.021744789	0.94710131	1.118830137

Zm00001d025098_T005	5.125305139	4.393704971	4.990419467	5.188705701	5.113760999	5.263205641
Zm00001d042720_T006	0.425285269	0	0.363094804	1.013599466	0.910371175	1.172323651
Zm00001d042720_T007	0.411213622	0.316020587	0.70234282	1.306360244	0.927533175	0.619238327
Zm00001d042720_T001	0.484254677	0.323898048	0.572398826	0.760732773	1.130244273	0.875350729
Zm00001d042720_T002	2.455167537	3.150040018	3.136543484	2.470270557	1.148168802	0
Zm00001d042720_T005	2.704402348	1.368758293	2.403155084	3.018448663	2.990731815	3.137104596
Zm00001d034995_T001	4.161181395	4.355024372	4.296819321	5.64051942	5.6412226	5.774451129
Zm00001d047201_T002	1.031229319	0.787708272	1.346114421	2.309900846	2.125749945	2.212379768
Zm00001d047201_T003	1.025331481	0.952023372	1.088891929	0.797803234	1.207281919	0.948334709
Zm00001d013953_T001	1.242651891	1.465406675	2.323462595	1.89188925	2.904325899	2.397445392
Zm00001d011652_T007	3.502987889	2.730541925	3.490389911	2.700891322	2.834565402	3.286105343
Zm00001d011652_T010	0.649540046	0	0.923816261	1.279496257	0.784195728	1.110452783
Zm00001d011652_T011	1.991743681	1.060563479	1.862535104	1.962793677	2.267599317	1.844376413
Zm00001d011652_T003	0.496571768	0.177670361	0.48106743	0.976060766	0.949593947	1.079556293
Zm00001d011652_T012	0.272243012	0.103465412	0.309902592	0.464936918	0.487105559	0.357720896
Zm00001d011652_T005	0	0.464981862	0.514502046	1.716643728	1.919343134	1.337332032
Zm00001d037759_T001	1.271518904	0.85550739	1.391670401	1.866638993	1.484332025	1.649074606
Zm00001d000226_T009	0.864417156	0.497727828	1.051106162	1.551191636	1.450593671	1.38604388
Zm00001d000226_T001	1.556808525	1.375539386	1.689250846	2.1043172	1.462092117	1.898283824
Zm00001d027588_T003	0.866304273	0.278628313	0.660991575	1.30815581	1.252880052	1.261897234
Zm00001d027588_T001	1.258854761	0.828353739	1.215291548	1.089811334	1.253982007	1.239002636
Zm00001d027588_T002	4.298648126	4.074724205	4.599093099	4.962608756	4.922698898	5.002059284
Zm00001d013010_T005	0.79954906	0.504040656	1.065953442	0.763202485	0.661848998	0.763263685
Zm00001d013010_T003	1.165485578	1.075154024	1.308507665	0.7696135	0.781080171	0.937296929
Zm00001d013010_T001	5.417820218	5.056836671	5.509555936	5.183650228	4.960911806	5.055042603
Zm00001d037818_T001	0.482100675	0.652448864	0.587855568	0	0.256738955	0
Zm00001d037818_T002	2.40407775	1.690431399	2.560452556	2.049201729	2.143477992	2.412907231
Zm00001d009365_T002	1.054142755	0.434593536	0.915629505	1.342696391	1.034722824	1.423977176
Zm00001d009365_T001	3.501112097	2.87396179	3.384892791	3.811019141	3.839205199	3.607653205
Zm00001d031533_T001	2.349622879	1.783891179	2.361825056	2.06713582	2.103348914	2.002637359
Zm00001d020461_T001	4.166041334	3.357210784	4.231569651	4.390955357	4.340563978	4.351915412
Zm00001d011227_T001	1.070681959	0.887197703	1.272783465	1.425348117	1.219960875	1.613349654

Zm00001d033704_T008	2.200389471	0	2.491412398	3.008865656	2.722415748	2.602424573
Zm00001d033704_T007	3.120651696	2.497611855	3.103499654	2.588397553	3.119591571	2.823787261
Zm00001d033704_T001	0.929403955	0.694213391	0.90590307	1.377667955	1.194538454	1.030652483
Zm00001d002420_T001	1.557517924	1.274831767	1.399012457	2.057102597	1.801092833	2.144053877
Zm00001d003807_T010	1.813733178	2.285704635	1.014812312	2.208325283	2.577165342	2.422007158
Zm00001d003807_T001	3.857568524	3.115877399	3.969947453	3.106720002	3.159620145	3.080438514
Zm00001d003807_T004	2.354442287	1.364133674	2.03078065	2.228416373	2.524802141	2.251482183
Zm00001d040780_T005	2.176072514	3.647209614	2.244170403	1.391266208	2.338294616	2.536681082
Zm00001d040780_T002	3.164564902	2.61729378	3.039964986	3.025577411	3.077795186	2.855704518
Zm00001d040780_T003	3.326938089	2.496495329	3.413229843	3.361215033	3.147249543	3.227384824
Zm00001d040780_T004	2.250196985	0.592908104	1.786347946	1.700220856	1.847836598	1.996527224
Zm00001d047245_T001	2.473700166	1.778216989	2.585387793	2.688052088	2.628180767	2.595514879
Zm00001d043914_T001	2.837640345	2.044748017	2.870725718	3.020358894	2.954387478	2.889995427
Zm00001d033294_T007	1.256462411	0.569545532	1.085255357	0.61147836	0.142281416	0.414529581
Zm00001d033294_T008	1.001843308	0.756850796	0.844734332	1.304485926	1.736786256	1.284094767
Zm00001d033294_T003	3.384142667	2.867436112	3.354859607	3.405948262	3.120677572	3.257770097
Zm00001d033294_T005	0.631033485	0.264502803	0.963258813	0.870784495	0.735587162	0.580363561
Zm00001d031427_T001	1.564690333	0.839486403	1.429704866	0.880906467	0.788361642	0.949483387
Zm00001d031611_T002	5.785468188	7.36503662	5.764859094	5.222566487	4.90120582	4.567893911
Zm00001d031611_T001	7.733190245	7.330174721	7.76011457	7.082633727	7.172828827	7.1574102
Zm00001d022334_T002	0.23361937	0.053503418	0.149509116	1.571023384	1.361499443	1.204665352
Zm00001d022334_T001	3.011629454	2.353773251	3.043872556	4.389584364	4.433659145	4.515089583
Zm00001d003897_T001	1.767635308	1.548768037	1.900612582	2.483599115	2.705598661	2.773803742
Zm00001d018552_T001	2.817230505	2.178688512	2.585976359	2.323377275	2.482301297	2.456279693
Zm00001d036278_T003	2.14479755	1.969527385	1.735948857	2.028872157	1.995994573	1.837676874
Zm00001d020902_T002	6.179374716	5.584318735	6.262535707	6.524055947	6.583535706	6.462756835
Zm00001d032079_T001	2.769775547	2.003082989	2.84568795	3.900739604	3.900744144	3.897372392
Zm00001d020835_T012	1.128729575	0.580240053	0.775723993	0.799971722	1.512806377	0.745744177
Zm00001d020835_T027	0.112426579	0.681499641	0.663870134	0.222193728	0.279210963	0.728950552
Zm00001d020835_T004	0.090910324	0.186830134	0.642364249	1.54253096	1.553566447	1.751613253
Zm00001d020835_T020	2.006273964	1.393428832	1.842511061	1.481042107	0	0
Zm00001d020835_T011	0.262326109	0	0.06852361	1.198259029	0	0.816341657

Zm00001d020835_T003	4.74864172	4.417084831	4.870181769	4.343503833	4.209099043	4.339747693
Zm00001d020835_T014	1.16146787	0.852784313	1.441733114	1.04878372	0.976059299	1.217514263
Zm00001d007035_T001	3.674257363	4.076743183	3.767968619	2.962359304	2.848539819	3.131412511
Zm00001d016898_T001	2.369465925	1.872124277	2.406678157	1.640471931	1.582500606	1.606488659
Zm00001d022573_T001	6.515614141	5.811132692	6.504608492	6.555948381	6.515957072	6.515396178
Zm00001d048027_T001	1.868319724	1.981859593	1.831002443	4.972205639	4.999888232	4.793474142
Zm00001d034031_T001	5.092466755	4.622865124	5.268354179	7.598698102	7.724244791	7.783681691
Zm00001d047600_T004	3.752269469	0	3.566463873	4.092527475	4.28311088	3.495814615
Zm00001d047600_T003	4.214720105	4.010565746	4.404810976	5.171273731	5.114684475	5.289770442
Zm00001d044276_T001	1.673332992	1.371293996	1.788923704	1.159463854	1.115105176	1.00467109
Zm00001d043523_T001	6.314242186	6.718225776	6.470361951	3.751327886	4.013371407	3.823506445
Zm00001d022394_T001	0.302622036	0.272681362	0	0.640825426	0.496273148	0
Zm00001d022394_T002	0.820154449	0.293339876	0.953577401	0	0.133779191	0.709766161
Zm00001d012785_T001	3.470624973	2.63677935	3.351363311	3.192529861	2.882261541	3.260731621
Zm00001d024432_T001	2.692452418	2.3004424	3.012651979	3.351652214	3.390785429	3.385200049
Zm00001d018003_T001	2.137208725	2.860442818	2.13084951	2.606719606	1.799122544	2.482777308
Zm00001d027769_T003	1.387191569	0.90802507	0.73376301	0.356253135	0.922471896	0.629906779
Zm00001d027769_T002	3.251057346	2.101015985	3.120353268	3.357818984	3.208332931	3.235713889
Zm00001d035599_T001	3.178789682	2.376894344	3.134490393	3.50881615	3.466189612	3.373967197
Zm00001d020442_T001	5.845564793	6.348842592	5.901685857	5.554222238	5.876718934	5.413258484
Zm00001d053817_T001	2.047499302	1.654400252	1.982374486	2.141860762	2.336958297	2.35965076
Zm00001d048870_T001	3.463087101	3.133109406	3.362451232	1.089816078	0.550557922	1.188921189
Zm00001d002772_T001	2.786504152	2.935810101	2.414825588	3.17674722	3.106658536	3.040179146
Zm00001d033156_T001	4.197298956	4.651394165	4.5424259	2.904113333	3.123874905	3.988109312
Zm00001d037636_T008	5.044359844	3.950351389	5.052991614	5.20443753	5.188552925	5.06268127
Zm00001d037636_T006	5.478545371	0	5.526989582	6.331274927	6.119860887	6.130156366
Zm00001d037636_T001	2.836647665	4.747586817	2.599794328	4.009715324	3.871178712	4.156191751
Zm00001d037636_T005	3.139011172	0	3.187120194	3.81799074	4.813980975	5.114276687
Zm00001d011149_T001	3.800701247	3.587981545	3.733317676	3.697645399	3.645918346	3.828587309
Zm00001d011149_T002	3.671386001	4.896659019	3.824540792	3.812556959	3.148769657	3.782094619
Zm00001d038136_T004	1.039313192	0.467946149	1.130386627	1.484956844	1.518998338	1.301969193
Zm00001d038136_T002	7.016992295	6.454432373	7.054954386	7.268398723	7.243206208	7.184021382

Zm00001d011168_T010	0.341822972	0.261677635	0.303960727	0.641112231	1.103266656	0.49273409
Zm00001d011168_T006	0	0	0	1.589027573	1.700595077	1.562453943
Zm00001d011168_T011	0.603047786	0.518627791	0.453404846	1.20528365	0.827507694	0.843296491
Zm00001d011168_T017	1.918746863	2.245615832	2.173810553	2.129160341	2.64856185	2.240833911
Zm00001d011168_T007	2.120916233	1.646743306	2.352146069	2.211815268	2.680619168	2.122776072
Zm00001d011168_T002	0	0	0.228290452	0.813539465	0.866698332	0.827863727
Zm00001d011168_T008	1.186059949	0.172336453	0.92547084	0	0.551814246	0.708493624
Zm00001d011168_T005	1.068088801	0.341744424	0.86835173	1.731810156	1.101249347	2.140031925
Zm00001d011168_T018	0.202831428	0.193335242	0.669951064	2.102528468	2.014993651	2.083093862
Zm00001d011168_T016	3.894034276	5.479042385	3.93332836	4.289429111	3.986776614	3.761612502
Zm00001d025777_T007	0.405396649	0.485790547	0.928294514	2.301938767	1.182514969	1.711520897
Zm00001d025777_T009	4.282972673	2.914311743	4.386815705	4.53105539	4.717374447	4.615855654
Zm00001d025777_T004	2.709851942	2.091253333	2.719587382	3.519732608	3.665805577	3.287825095
Zm00001d025966_T001	4.664722151	4.146476748	4.557565405	4.903712833	4.736730307	4.95243827
Zm00001d039103_T001	4.055292328	5.497333507	3.700774828	4.91116296	5.054405792	5.024910884
Zm00001d025133_T001	1.043581785	0.860091954	1.001648059	1.205728435	1.331480512	1.026119442
Zm00001d040953_T001	2.482612374	1.713473236	2.199362652	2.901906043	2.705121095	3.103237276
Zm00001d028270_T002	0.048269671	0.025059988	0.054648465	1.713391409	1.275645984	1.685950013
Zm00001d036650_T001	4.656069963	5.68703711	4.689142692	4.990129203	4.89316856	4.988758978
Zm00001d042279_T001	5.666903836	4.82909829	5.735421987	5.541222033	5.482845089	5.4733454
Zm00001d034492_T024	4.461630184	0	4.322329685	4.297845178	4.591682827	2.806709687
Zm00001d034492_T007	0	0.057401748	0.934511218	1.343549953	0.969399922	0.437534572
Zm00001d034492_T006	3.072323989	3.805099051	3.393189097	3.461790332	3.639909237	3.005433045
Zm00001d034492_T019	0	0	0	2.985475474	2.6516616	3.935388177
Zm00001d033300_T001	1.28713478	0.663022112	1.237074171	2.060970137	1.824050094	2.021460894
Zm00001d053930_T003	1.160838244	1.577278478	1.84036977	1.7014701	1.66406499	2.095760062
Zm00001d053930_T001	3.949819242	3.193565021	4.010103797	3.506770756	3.483234714	3.606819435
Zm00001d053930_T002	0.419596059	0.292287026	0.522653399	0.441485604	0.470932462	0.340223844
Zm00001d015421_T001	5.135037524	4.747020019	5.253710303	5.29019325	5.368326814	5.46851975
Zm00001d015421_T002	4.221118964	2.928948801	4.085240738	4.301432834	4.336377374	4.448660284
Zm00001d009618_T001	2.478409109	2.009676812	2.766521806	2.342448075	2.901301748	2.575076784
Zm00001d041179_T006	1.326479248	1.576060111	1.313605755	1.948642334	2.030205753	1.960735585

Zm00001d021591_T001	4.724346204	4.367705349	4.650163522	5.09847906	5.232884089	5.226044126
Zm00001d053316_T003	3.24069396	2.444890185	3.321991284	3.086784106	3.536746222	3.445334993
Zm00001d053316_T012	2.263417871	0.379747373	1.830228142	1.705928374	1.903172504	1.845280481
Zm00001d053316_T008	0.388376924	0.411959836	0.545409958	0.702196516	1.389620774	0.441681066
Zm00001d053316_T001	0.895467048	0.747084864	1.098250429	1.397727951	1.047467195	0.9159935
Zm00001d053316_T011	1.589990178	0	0.913661121	2.057759054	1.032930948	1.042155419
Zm00001d053316_T007	1.8012311	2.548166803	2.490686928	1.139526468	1.849663459	2.163564425
Zm00001d053316_T006	0.692420056	0.429428502	0.367416918	1.696239139	1.298221922	1.022480028
Zm00001d045547_T001	3.638179979	3.292667212	3.539117112	3.553861427	3.423896389	3.272667031
Zm00001d016876_T001	5.002434341	4.626311391	5.029075465	4.422699379	4.655141248	4.602040215
Zm00001d004193_T004	0	0	0.243073115	0.68188999	0.288336117	0.263730338
Zm00001d004193_T001	4.761853994	4.157140911	4.566611519	4.667464361	4.495492621	4.740364562
Zm00001d004193_T003	0.442177039	0	0.296659444	0	0.526000683	0
Zm00001d004193_T005	0.697434898	0.113529819	0.901956229	0.716114699	0.396574917	0.799894658
Zm00001d004033_T001	4.231276564	3.496555791	4.133567142	4.483874	4.273282682	4.263620907
Zm00001d002868_T001	5.253984162	5.941471564	5.200160266	5.229866053	5.000954298	4.410788689
Zm00001d002726_T008	2.139361109	1.36190757	2.491455242	3.005297037	3.297629149	3.34627345
Zm00001d002726_T001	0.721111879	0.237929546	0.077565689	1.82821886	1.114531609	1.466608328
Zm00001d002726_T009	1.368789577	0.823451009	1.880978148	0	0	0
Zm00001d019897_T003	0.660161944	0	0.761994105	1.695629531	1.929820533	1.815817634
Zm00001d019897_T001	0	0.409483266	0.11424574	0.995330382	1.180196884	0.85272999
Zm00001d026621_T006	0	2.10073516	1.010902982	0	1.714757652	0
Zm00001d026621_T019	0.827095464	0.268465535	0.531563619	0.461163911	1.447400931	1.352594702
Zm00001d026621_T016	0	0	0	0.72907676	0.82152002	1.01810072
Zm00001d026621_T009	0.745484323	0.596607935	0.917845695	1.357807585	0.565834031	0.724784713
Zm00001d026621_T018	0	0	0.663304538	0.724789078	1.248345986	1.386302741
Zm00001d026621_T007	2.787016146	0	2.901620724	3.389459845	2.935221542	1.610328712
Zm00001d024712_T002	0.579808655	0.606375896	1.038816121	0.508329258	0.939657629	0.959776831
Zm00001d024712_T001	0.620470052	0.130741133	0.587305458	0.772801177	0.488155062	0
Zm00001d049079_T001	3.841489121	3.37252605	3.726017238	3.457157451	3.086731464	3.009397877
Zm00001d007254_T001	2.240357381	1.52455469	2.494257123	1.975610011	2.11894539	1.925405893
Zm00001d002319_T001	1.327733346	1.312227769	1.664923773	2.408068618	2.289442317	1.644096696

Zm00001d021641_T018	0.337495314	1.542803818	1.121011421	0.662140771	0.502743032	0.443014601
Zm00001d021641_T016	1.14433488	0.29442372	1.047150966	1.076524891	1.427651223	1.125121399
Zm00001d021641_T002	1.718309014	1.196274028	0.700415747	1.076068536	1.77960769	1.070644182
Zm00001d021641_T008	0	0	1.596703339	0.625599676	0	1.65853004
Zm00001d021641_T004	1.847570848	0.578915533	1.798576054	2.109849288	1.941421115	1.815446318
Zm00001d045483_T004	1.176057829	0.83454457	1.855562006	2.385579579	2.117748893	2.305222576
Zm00001d045483_T001	3.853688618	2.859235097	3.734742348	4.02614707	4.105335464	4.070954702
Zm00001d045483_T003	0.452719827	0.195633589	0.366912462	0.894806125	1.075051995	0.719115083
Zm00001d048573_T001	2.077427939	1.741633089	1.845756185	1.912667491	1.831880078	2.144368492
Zm00001d048606_T001	3.351534483	2.799286078	3.444752926	4.353566143	4.24540553	4.371222334
Zm00001d024963_T001	8.54372482	8.625538237	8.544624179	8.752836262	8.759383448	8.786464772
Zm00001d024963_T002	2.400432729	5.671463649	2.079872323	2.990279812	3.103067872	3.271209764
Zm00001d009028_T002	2.202594188	1.719432303	1.975893171	2.06235696	2.100055239	2.199005135
Zm00001d013997_T002	4.420196381	3.835838096	4.480255944	3.183705324	3.171781997	3.160478795
Zm00001d036598_T001	0.73608788	0.875959308	1.651009888	1.673954806	0.621440983	0.583721249
Zm00001d036598_T008	0.379244992	0	0.982566884	1.589391022	1.691833839	1.198627416
Zm00001d036598_T004	2.682938419	1.156434803	2.121233613	2.717298052	2.991666414	2.968112692
Zm00001d032906_T001	2.400301013	2.022760173	2.654012707	1.593143388	1.312671562	1.605698184
Zm00001d036122_T001	4.143009591	3.617812128	4.387133724	6.606508113	6.646123795	6.592179206
Zm00001d028235_T001	2.071187742	1.425755763	2.043192247	1.293471134	1.239517803	1.339502289
Zm00001d022144_T002	3.107317106	2.29342699	3.266287619	3.596456708	3.674476583	3.573592659
Zm00001d049881_T001	6.426157941	6.180391188	6.474517732	6.680652921	6.640137978	6.747757372
Zm00001d014879_T004	3.412130743	1.222228357	3.417353644	3.421931645	3.122874781	3.211946527
Zm00001d021730_T001	6.839306978	7.07335833	6.739675409	6.441572682	6.519797653	6.468464016
Zm00001d021946_T001	2.438169871	2.04864424	2.52221297	5.120103811	5.344170082	5.312049036
Zm00001d021927_T001	4.607153687	3.87379204	4.828434122	4.339655066	4.18153283	4.3717887
Zm00001d009907_T001	5.254086123	4.967707212	5.120775844	4.668816868	4.949210267	4.75312008
Zm00001d020976_T001	0.883968741	0.767453103	1.068936971	0.937738349	0.272979889	0.486850271
Zm00001d005012_T004	1.509516476	0.851922904	1.466673578	1.819724582	1.765637004	1.714983549
Zm00001d005012_T014	1.769759047	0	1.649837077	2.086429502	1.915323556	1.628830975
Zm00001d005012_T013	2.150473567	1.694866824	2.336336238	1.335724485	1.861404376	1.456894443
Zm00001d005012_T015	0	1.483296765	0	3.155673146	3.111659683	3.561804511

Zm00001d005012_T006	0.420180546	0.060997796	0.308886222	0.461418546	0.431625099	0.338432402
Zm00001d005012_T002	1.049540161	0.639837829	0.825018699	0.8025936	1.045638672	0.971858039
Zm00001d002658_T015	0.222856482	0.138645424	0.392996566	0.671255323	1.118170322	1.295666608
Zm00001d002658_T007	0.139034591	0.074383494	0.376931562	0.37777233	0.510148719	0.489086627
Zm00001d002658_T002	1.252723253	0.621362208	0.700480557	1.534745923	1.913072074	1.713478954
Zm00001d002658_T014	1.141570776	1.34785668	2.02874276	1.030249196	0.483082605	0
Zm00001d002658_T016	1.467073888	0.518592544	0	2.32922494	2.044614092	1.836012573
Zm00001d037725_T001	5.846334721	5.158125285	5.871490273	5.818157979	5.761177599	5.743542677
Zm00001d006458_T001	6.975152602	2.308813139	6.834229036	6.599172029	6.349765	6.222401528
Zm00001d036704_T002	0.470582677	0.083153446	0	1.450322336	0.766021786	1.103118914
Zm00001d036704_T001	2.618518709	1.804400144	2.754706673	3.174387242	3.278132876	3.11735045
Zm00001d018019_T001	0.99960104	0.703724433	1.134581076	1.51527545	1.22558713	1.384624007
Zm00001d013159_T003	1.630718115	1.087281559	1.217215203	1.455721487	1.432535086	1.592987971
Zm00001d013159_T001	8.546990674	7.796237366	8.560852757	8.091828111	8.149547587	8.084170262
Zm00001d013159_T004	0	0	0.93306194	5.498165069	5.2671789	5.423051989
Zm00001d012515_T002	3.975109211	2.141597913	3.963038198	3.297754442	3.310024787	3.408931237
Zm00001d012515_T001	0	2.058344899	1.148013869	1.150903413	1.483437113	0.741947553
Zm00001d045503_T001	4.295966367	4.702832717	4.076266285	4.470758556	4.326203528	4.418783571
Zm00001d039372_T003	5.376346651	5.289415942	5.375161204	4.872581363	4.945791446	5.000137365
Zm00001d039372_T001	2.369109639	2.755247169	2.518633833	1.720919764	2.544203694	2.388659611
Zm00001d032849_T002	4.336798809	3.262925598	4.293380123	2.485732079	2.393866458	2.55579579
Zm00001d032849_T001	4.429607663	4.999539075	4.758238888	3.127672235	2.908501091	2.9769712
Zm00001d006118_T001	1.104667435	0	1.766880916	1.474244969	1.467123463	0.908171909
Zm00001d006118_T002	4.209204343	3.309052464	4.077647461	4.225832802	4.211861124	4.220168339
Zm00001d011613_T001	1.070695009	0.869013033	1.53175224	1.098201237	1.025483154	1.237071111
Zm00001d051854_T002	0.385304019	0.047506282	0.152015933	2.12861708	1.72163558	2.214396529
Zm00001d051854_T001	3.355218019	2.757762758	3.297148267	3.526242125	4.276270492	3.77089563
Zm00001d051854_T003	3.132214561	2.153465209	3.390014005	4.060115452	3.180231422	3.447013909
Zm00001d014924_T001	0.598516709	1.443814554	1.690013397	1.133256411	0.241426531	1.202123667
Zm00001d014924_T004	3.03691472	1.976924106	2.893647615	3.376885872	3.361717836	3.279257624
Zm00001d014924_T005	0.281756402	0.601724087	0.674482346	0.258211358	0.298551576	0.660402942
Zm00001d014924_T002	4.12845591	3.653688909	4.079260677	3.585966027	3.72724359	3.772034804

Zm00001d012158_T001	1.349205598	0.952026355	1.565234019	1.552349525	1.405253402	1.533832925
Zm00001d048236_T005	0.703797066	0	1.096726032	0	1.116240978	1.964298074
Zm00001d048236_T008	2.385328319	1.881872885	2.339466658	1.637562952	1.19232296	2.00267624
Zm00001d048236_T009	0.48306609	0.409819947	0.72244504	0.659408566	0.495123087	0.663249879
Zm00001d048236_T001	1.430144476	1.423894911	1.584827843	1.408091178	1.187414313	1.037196645
Zm00001d048236_T013	0	0	0.118917823	2.267792854	1.658443667	1.199191759
Zm00001d048236_T015	0.762159988	0.063536076	0.428022295	0.734714389	0.533670986	0
Zm00001d048997_T001	5.500017884	6.17389518	5.655551359	5.470621329	5.534747666	5.562688507
Zm00001d006581_T001	4.75041608	4.278414963	4.850024922	4.254390262	4.045170343	4.165901651
Zm00001d010519_T001	3.263146361	2.703425225	3.368335895	3.271570148	3.333353887	3.110339688
Zm00001d043563_T004	4.854245603	3.787248041	5.033624128	5.151157508	5.172323411	5.064030008
Zm00001d043563_T003	1.248036243	1.484899116	1.610063605	1.941515768	1.965312204	2.341960422
Zm00001d043563_T005	5.659277169	5.758032646	5.832779403	5.341830727	5.282414784	5.400034675
Zm00001d043563_T002	1.667068726	1.568106062	1.015493288	1.993035921	1.916892768	1.581959052
Zm00001d001900_T002	3.642084743	3.208279236	3.632258559	4.072553121	3.75522687	3.859719042
Zm00001d001900_T001	3.906345058	3.920443375	3.956177399	4.070700848	3.430905044	4.262424738
Zm00001d001900_T003	2.330816513	0	1.869643241	2.80035926	3.307731605	2.995372898
Zm00001d021662_T002	2.892473408	2.292343869	2.56181343	2.268131929	2.64134672	2.46456555
Zm00001d021662_T001	0.517924697	0.068205772	1.691637785	0.957370097	0.701185288	0.579557672
Zm00001d008495_T001	2.510011959	1.821823667	2.604226025	3.672404069	3.569871888	3.497837151
Zm00001d045519_T003	1.122706508	0.682478013	1.007326128	1.101827545	1.070803528	1.170340438
Zm00001d045519_T005	2.5908281	1.862628729	2.416553876	3.492815854	3.652571505	3.137939217
Zm00001d045519_T001	4.132770861	3.222424676	4.260036499	5.464240851	5.601159625	5.539781145
Zm00001d045519_T004	1.167166498	0.905578108	1.04548421	1.128053152	1.094891384	0.965259002
Zm00001d029847_T001	2.036771276	1.672022915	2.378606228	3.172728478	3.193127827	3.01785369
Zm00001d003773_T006	4.933477179	5.75274704	5.025085376	4.498199925	4.270326577	4.287622703
Zm00001d003773_T007	0.506209913	0	0.264721372	0.704916218	0.639316453	0.376551556
Zm00001d003773_T002	3.576492631	1.121978866	3.585725244	4.493266629	4.823934015	4.915803485
Zm00001d003773_T001	2.079492805	3.098565087	2.74267888	3.292760992	2.40270608	2.541686307
Zm00001d003773_T008	3.633984467	0	3.208962454	3.329543711	3.841104524	2.44560462
Zm00001d040628_T003	2.902897465	2.981404983	3.080598999	2.99950038	2.96876631	2.999542413
Zm00001d040628_T002	2.172819988	3.332980695	2.861091672	2.247508615	1.90401735	1.408053668

Zm00001d025117_T001	4.17419906	3.604203604	4.051020435	3.825570733	3.876795391	3.720535457
Zm00001d026360_T001	1.729702851	1.454570211	1.349157465	0.868556395	1.149096744	0.34361817
Zm00001d043390_T001	4.270827093	4.325765101	4.042587084	7.667739129	7.741711374	7.781874585
Zm00001d018372_T001	1.000293559	1.012705249	1.097836621	3.371926389	3.004180278	3.154113132
Zm00001d008293_T001	0.408940066	0.739722833	0.745127162	0.384854396	0.488607552	0.298472982
Zm00001d008293_T004	0.907600612	0.575297807	0.32620137	1.716016335	0.827214998	1.475242637
Zm00001d008293_T006	4.251899399	2.983434915	4.247859022	3.974332259	4.114519116	4.100865322
Zm00001d008293_T007	1.372714229	0	1.373730609	1.430831734	0	0
Zm00001d053452_T004	2.597396254	1.630726967	2.379154589	2.490084615	2.547675125	2.483439693
Zm00001d053452_T003	4.59857864	3.430256098	4.697882718	4.925513946	4.845499033	4.978434442
Zm00001d023462_T002	1.995281894	1.391507088	1.993962691	2.316048401	2.164346389	2.168867279
Zm00001d035757_T009	3.166839948	2.583512104	3.18543954	1.906340343	1.952564305	1.903123518
Zm00001d035757_T002	1.755790453	1.157668527	2.287195373	3.022066458	2.85662761	2.715741188
Zm00001d035757_T006	0.593243802	0.392973494	0.352055647	0.483180658	0.726388316	0.096655875
Zm00001d035757_T003	1.276120831	1.187195749	1.284293803	0.968954083	0.660976977	1.006968672
Zm00001d035757_T007	0.651231292	0.140507757	0.529320226	0.584759547	0	0.566871643
Zm00001d024770_T001	2.169739042	1.887161822	2.009774614	1.058304026	1.225167557	1.011394733
Zm00001d042361_T002	5.563789293	5.059652967	5.619952218	5.787308334	5.677679055	5.724630519
Zm00001d046810_T002	0.402719994	0	0.48265419	0.916843482	0.620805027	0.690715661
Zm00001d046810_T005	2.883010963	0	3.623878096	3.173734293	3.357242742	3.113868993
Zm00001d046810_T003	3.117833987	2.963990627	1.651031479	2.62275539	2.570045849	2.707563884
Zm00001d043314_T001	5.560073271	5.41033242	5.680105291	5.619929541	5.519140384	5.563045133
Zm00001d014378_T001	3.506336496	2.836594953	3.772318744	3.472438511	3.594059168	3.683099718
Zm00001d011769_T001	2.179390024	1.620658192	2.337536417	1.242304333	1.422580175	1.288530435
Zm00001d021335_T003	3.798757169	3.321787858	3.6078248	3.120010637	2.804280563	2.470975919
Zm00001d021335_T004	0.233582559	0.390025978	0.480237199	0.483086734	0.567636952	0.279416618
Zm00001d021335_T005	0.4465368	0.061219054	0.647890091	0.425089721	0.036413305	0.296249468
Zm00001d005892_T001	0	0.334897735	0.445217115	3.536271402	3.640920379	3.933949691
Zm00001d038740_T001	2.817398548	4.042501812	2.709471513	2.300424828	2.546874463	2.630613985
Zm00001d038740_T002	5.364185829	5.416498108	5.667839031	4.852365509	4.859076232	4.793299126
Zm00001d032410_T001	2.464695448	2.943453664	2.840011169	1.594781655	1.594581511	1.523434993
Zm00001d025735_T001	1.409396911	0.87999898	1.709888767	3.343643893	3.31909865	3.156174273

Zm00001d012302_T001	5.227515637	4.714167088	5.122682119	4.365656987	4.129735458	4.440132911
Zm00001d046286_T001	2.557133117	1.849802751	2.67664293	3.293019921	3.009414898	3.190414639
Zm00001d017945_T001	3.164914487	2.641843323	3.437278483	3.339949741	3.316155157	3.391220008
Zm00001d029797_T002	4.3064099	5.306601471	4.471734969	4.319228452	4.42825993	4.581832547
Zm00001d036475_T002	1.799517563	1.224192194	1.959022653	3.293555445	3.488227058	3.348983462
Zm00001d036475_T003	1.238284274	1.926651142	0	1.412060133	2.079855261	1.540701824
Zm00001d036475_T009	4.463888687	3.442820115	4.752901789	4.834281822	4.932921343	4.703933576
Zm00001d036475_T006	0.54477716	0.063404919	0.455101238	0.945210645	0.9202933	1.148610722
Zm00001d036475_T001	0	0.340792398	0	0.808867722	0.960439796	0.684008986
Zm00001d036475_T008	2.66089554	0	1.98800221	3.895230097	3.627287682	3.912977643
Zm00001d048393_T001	2.535893698	2.308096673	2.408919692	3.86497749	3.615243131	3.776890095
Zm00001d005478_T026	4.539724795	2.772803922	4.490654906	5.280935349	5.216577283	5.092374612
Zm00001d005478_T025	4.48927536	3.312034873	4.163299543	4.494401977	4.476082683	4.866167356
Zm00001d005478_T009	0.399570344	0.054712361	0.061825957	0.887343558	0.986370157	0.876284722
Zm00001d005478_T021	2.795420152	2.318114054	2.427221045	3.593570338	3.639604715	3.1964865
Zm00001d005478_T001	2.397975418	2.211670137	2.749782025	0	1.933111053	2.767044967
Zm00001d005478_T007	0	1.678555127	2.983748825	2.379776479	2.409306748	2.571063443
Zm00001d005478_T003	0.206601968	0	0.0874384	1.088466608	1.330381136	0.59106085
Zm00001d005478_T008	2.267337732	0	0	4.624681541	4.371906252	4.312939355
Zm00001d005478_T005	0	0	0.198538157	0.433068407	0.873028064	0.481983956
Zm00001d013144_T001	2.732043822	2.652940285	2.884954399	2.179744474	2.686509267	2.822682023
Zm00001d036077_T003	4.338689895	2.714316744	4.052686809	3.815724506	3.731763679	3.920038186
Zm00001d036077_T001	3.883391296	3.77973217	4.123078968	3.646561878	3.911114772	3.561340435
Zm00001d036077_T002	1.810375235	3.363882996	2.45293696	2.33286859	1.948385175	0
Zm00001d018507_T002	6.21406085	6.656629536	6.172310648	6.223616911	6.234693007	6.156926062
Zm00001d018507_T001	0.420169764	0.272194032	0.322132366	1.038757837	0.908078887	0.817782048
Zm00001d044459_T001	5.655860833	5.529444985	5.691698357	5.455666752	5.742982207	5.178185901
Zm00001d044459_T005	5.288136234	0	5.687769485	5.575767156	5.349998264	5.848912151
Zm00001d044459_T004	0.414825047	0.26276147	0.246654951	0.415044713	0.514138423	0.395586024
Zm00001d044459_T008	6.171457472	7.629670292	5.645289978	5.733118094	5.583826833	5.438073536
Zm00001d033402_T001	5.995176025	6.194404085	5.965152477	5.282384758	5.408468878	5.534698401
Zm00001d033402_T002	4.360165488	4.997764571	4.709833144	4.084571416	4.297486897	4.231050486

Zm00001d038059_T001	3.456596562	2.69504885	3.553428213	3.684530619	3.617831166	3.556971075
Zm00001d041698_T001	3.230049265	2.465707574	3.207633018	3.516539038	3.40545152	3.569802516
Zm00001d018208_T001	6.06278776	5.827509777	6.092573606	5.58086204	5.626744874	5.660703278
Zm00001d041750_T001	2.160704348	2.354123469	2.241549175	2.049886527	1.417315159	1.626516234
Zm00001d041750_T004	2.829998083	1.731704603	2.874804243	2.682900451	2.406007875	1.637967224
Zm00001d041750_T002	6.532132496	5.53683187	6.576883734	6.038086374	6.147003677	6.019069641
Zm00001d041750_T008	0.59965962	0.207298164	0.495375893	0.34052467	0.486212882	0.283703722
Zm00001d041750_T006	5.396545189	7.306964384	4.523467801	3.346716255	0	3.522625908
Zm00001d023769_T001	3.188350439	2.689177477	3.233980526	0.547294841	0.608405231	0.652765485
Zm00001d023769_T002	0	0	0	1.059168352	1.023393759	1.099813606
Zm00001d041299_T001	2.914450672	2.583934455	2.87698698	2.49408018	2.860553265	2.744989654
Zm00001d020163_T001	2.432247769	1.933091408	1.999354249	1.309239064	1.156409561	1.316125463
Zm00001d004758_T001	8.503455077	8.622649239	8.424574369	8.226172714	8.197488086	8.249719134
Zm00001d048491_T001	7.896761649	8.565806711	8.016273751	7.924384524	7.947398028	7.998641125
Zm00001d003857_T001	7.309878161	6.50751546	7.246629444	7.906742147	7.925312587	7.869217315
Zm00001d003857_T002	7.022686203	6.921577325	7.161970695	6.325663316	6.160177176	6.318573828
Zm00001d034931_T001	1.409171494	0.046010431	0	3.131272564	1.392139342	0
Zm00001d034931_T008	1.067209837	1.416456593	0.673075144	0	1.165093188	0
Zm00001d034931_T015	0.21848981	0.077377006	0.340709237	0.552719382	0.645635263	0.485000139
Zm00001d034931_T011	0.764797086	0.411099704	0.616295584	0.75411786	1.043238101	1.220236455
Zm00001d034931_T006	0	0	0	1.131722488	0.611253603	0.678048461
Zm00001d034931_T034	1.007250768	1.274921199	0.836819318	1.325556235	0.941482715	1.313925529
Zm00001d034931_T032	3.915182511	2.559752185	3.739845079	4.178229891	4.25232361	3.992984094
Zm00001d044768_T001	0.590546452	0.492893001	0.381478303	0.40271672	0.60162236	0.649519813
Zm00001d015917_T001	7.040950475	6.629859669	7.025208755	7.679060158	7.641122869	7.675383037
Zm00001d031653_T002	3.416980087	2.829012919	3.396312137	2.858497885	2.328264901	2.590354344
Zm00001d005004_T001	4.692284238	3.857167086	4.642314337	4.607502835	4.498623431	4.584139449
Zm00001d014246_T003	3.09263521	2.073687609	3.082803057	3.083347333	3.132442273	3.05602958
Zm00001d014246_T010	3.83991153	4.307472607	3.850710227	3.343639488	2.877391875	3.429056585
Zm00001d014246_T009	0.258652786	0.294300196	0.631201157	0.817103384	0.778029385	0.64825282
Zm00001d021729_T003	2.150593144	0.894439849	2.053463798	2.728190894	2.914901623	3.130787746
Zm00001d021729_T001	4.895113498	4.097403984	5.006288553	5.091453455	5.019019319	5.106560889

Zm00001d045954_T004	2.29691606	1.643419853	2.340287661	2.941186709	2.855411706	2.910578792
Zm00001d051628_T002	3.893117437	2.998261407	3.995772758	4.37256561	4.384279321	4.401464181
Zm00001d051628_T001	1.767848416	1.595781479	1.352117248	1.683651994	1.932500038	1.907865528
Zm00001d046616_T001	4.207851007	2.831733167	3.842762462	3.941328991	3.952781031	3.908992705
Zm00001d038528_T001	3.152631899	2.90849475	2.908670552	2.442564034	2.815409222	2.241645879
Zm00001d033018_T001	3.269193271	2.618214219	3.278599621	2.90578141	2.867555711	2.772384204
Zm00001d049980_T001	3.485555376	2.583003916	3.304222766	3.895794779	4.039669566	4.103178684
Zm00001d026371_T001	2.154352808	1.833149575	2.547320509	2.01013245	1.894307129	2.046538556
Zm00001d011486_T003	1.753668284	1.241631542	2.372925639	2.109438459	1.05123148	0.700699824
Zm00001d011486_T001	2.649989247	2.325628762	3.294367253	2.888459211	3.005977669	2.960849177
Zm00001d034129_T002	4.545922543	4.082967281	4.320827765	4.507874731	4.627356913	4.579726246
Zm00001d034129_T001	4.868907182	3.926240196	4.928717334	5.362868206	5.242077233	5.36654376
Zm00001d034336_T005	1.406103416	0.977754698	0.550631796	1.263081293	0.919370595	1.133504344
Zm00001d026022_T001	3.573094056	3.551755319	3.696155662	2.951156563	2.630142165	2.52472969
Zm00001d026022_T005	3.270112968	2.33742595	3.196703661	3.286430679	3.475502062	3.447453964
Zm00001d019905_T004	0	0.277461118	1.12393829	0.303494372	0	0.745481741
Zm00001d019905_T005	0.904265196	0.274429854	0	0.662389642	0.870126372	0
Zm00001d009596_T001	1.695914997	1.19556997	1.301114084	1.269078642	1.111502783	1.353462754
Zm00001d042662_T006	0.609078836	0	0.817559409	0.656315136	0	0
Zm00001d042662_T005	0.276130958	0	0	0.712543838	0.540424105	0
Zm00001d042662_T001	1.248474115	1.077597817	1.414803944	2.166868061	2.387242035	2.327034268
Zm00001d045603_T009	0	0	0	1.146985558	1.082226381	0.75868523
Zm00001d045603_T061	0.090548604	0	0.037855896	0.388530122	0.261411664	0.289005784
Zm00001d045603_T051	0.297618732	0.119139674	0.144225227	0.937179396	0.727743618	0.63349259
Zm00001d045603_T010	0.69165297	0	0	1.485316045	0.938709598	0
Zm00001d045603_T029	0	0.586340097	0.734000697	0.200980109	0.633932405	0.546111643
Zm00001d045603_T056	0	0.522558997	1.246579559	2.24303685	2.470725308	2.514197013
Zm00001d045603_T069	3.982314698	3.095145323	3.759101918	3.349407318	3.545357812	3.583730033
Zm00001d045603_T068	0	0.816158947	1.746246973	0.526160981	0.394801665	0.507956974
Zm00001d043378_T001	1.129856024	0.698850547	1.010310081	1.482421864	1.441908364	1.426420423
Zm00001d015379_T001	4.716034284	4.491783268	4.849756272	4.59362806	4.447235011	4.505556823
Zm00001d038584_T001	0.670153261	0.959270132	0.616631529	3.315009245	3.341903935	3.399411204

Zm00001d014986_T002	0.371002307	0.170064776	0	1.382484657	1.21189499	1.378142579
Zm00001d014986_T007	1.016669604	0.139802134	0.53303202	0	0.583210005	0.352235354
Zm00001d014986_T001	0	0	0.506558276	0.281205647	0.863571388	0.49333376
Zm00001d014986_T005	2.34775692	0	2.640492755	2.004536628	0	0
Zm00001d014986_T009	0.420693664	0.273237767	0	0.455264349	0	0
Zm00001d014986_T004	3.864909367	3.24899623	3.998474084	3.733449577	3.40409642	3.318925031
Zm00001d014986_T006	0	1.256383906	0	0	2.522686037	2.746830195
Zm00001d027838_T001	1.451953801	0.976329905	1.520309092	1.441827115	1.307884287	1.249729301
Zm00001d016501_T001	6.823929762	5.96117098	6.863759403	6.651669363	6.738218887	6.639238531
Zm00001d017242_T001	6.685236789	7.00361846	6.63443948	5.939615272	6.17919021	6.025778615
Zm00001d034832_T008	0.367173101	0	0.5966232	0.519953469	0.518868456	0.531808123
Zm00001d034832_T006	0.432515844	0.370095074	0.504387509	0.965968938	1.492094167	0.843383333
Zm00001d034832_T002	2.425436477	1.653496659	2.351225766	2.342501269	1.800032211	2.346061361
Zm00001d039612_T001	0.588133903	0.398495946	0	1.207419995	1.359995619	1.366747441
Zm00001d039612_T013	0	0	0.875929435	1.415464474	2.238632801	0
Zm00001d039612_T028	0	0	0	1.087348779	0.616115816	1.663524063
Zm00001d039612_T033	0.37048348	0.208697039	0.299156722	1.278666371	1.473697049	1.051056032
Zm00001d039612_T025	2.285532721	1.294069589	0	1.244164313	1.050565777	1.604094577
Zm00001d039612_T009	0.036515994	0.085673469	0.288702287	0.294360195	0.608405231	0.50291408
Zm00001d028160_T001	4.048262434	4.276437483	4.525863727	5.089344119	5.239724383	5.212718464
Zm00001d037080_T001	1.841172875	1.302892797	1.919735935	1.799873941	1.879924114	1.659440992
Zm00001d014272_T002	1.379864348	1.844996188	1.940820374	1.845053212	1.783366129	2.011246939
Zm00001d014272_T001	3.196186354	2.969196548	3.153970183	3.350404176	3.338397315	2.890136134
Zm00001d007072_T001	4.419197593	3.996721269	4.306062565	4.622610686	4.814548937	4.712995135
Zm00001d039404_T001	3.059405813	2.344748976	2.980608112	3.852376146	3.831060733	3.719220575
Zm00001d012562_T001	5.365744601	4.815288757	5.145458385	5.040895455	5.092543458	5.119866592
Zm00001d008397_T001	9.110199861	10.0266512	9.202433847	8.128253431	8.140257133	8.190062569
Zm00001d011838_T001	1.17997404	0.614490302	0.94169454	1.302164024	1.076416802	1.147496874
Zm00001d032600_T001	4.335382779	3.728327183	4.471011829	3.930111214	3.987445664	3.954859984
Zm00001d034422_T003	6.555888641	6.876588146	6.597923259	5.787139622	6.093935125	5.708018148
Zm00001d034422_T002	7.672440384	8.034221747	7.902673657	7.11426112	7.162868331	7.191244134
Zm00001d034422_T001	4.888091758	6.507186202	3.38752012	3.193812422	3.679896549	2.611708038

Zm00001d043272_T002	5.38003567	4.841551617	5.388801726	6.343596178	6.316391439	6.301569412
Zm00001d019550_T003	5.672636639	5.457134069	5.941552317	6.130894843	6.167747294	6.087499142
Zm00001d019550_T002	1.205841018	2.357282301	2.019311569	2.331451275	1.689949457	1.97322773
Zm00001d019550_T001	1.035672468	1.066479828	1.722020469	1.169859598	1.665093922	1.687846788
Zm00001d005043_T001	1.908176906	1.544566001	1.64806226	2.024181006	2.485985763	1.882389504
Zm00001d011117_T001	1.979070502	1.654498783	2.115120495	1.995386467	2.037939863	1.763915474
Zm00001d011060_T001	6.383245961	5.783609638	6.5226928	5.927063204	6.05146031	5.982312485
Zm00001d032031_T002	0.547817983	0.217610421	0.551860501	0.641169585	0.670993478	0.389369667
Zm00001d032031_T001	5.302500272	4.761595909	5.293462857	5.217457341	5.374697872	5.27278354
Zm00001d017072_T001	0.886428406	0.69650757	1.803197686	0.76146145	0.990158345	1.477124491
Zm00001d017072_T002	6.030246834	5.700203513	5.783948758	5.808353282	5.827350276	5.826698571
Zm00001d017072_T006	3.859088361	2.829810195	3.853382322	3.872579984	3.82891394	3.860193089
Zm00001d017072_T008	5.542218422	0.278989822	5.880822075	5.391491415	5.286475665	5.202027922
Zm00001d034838_T002	1.310681557	0	0.774945155	0.11004246	0	0.722067261
Zm00001d030235_T002	2.832727411	2.067434275	2.966446895	2.722112521	2.46315395	2.747392771
Zm00001d030235_T001	1.579479954	1.266601864	0.867333505	1.178713367	2.340244072	2.148841392
Zm00001d006547_T001	7.487401179	7.67631125	7.521277931	7.118949345	7.108074569	7.137313614
Zm00001d045475_T001	9.610702994	9.675203347	9.611188958	8.612973738	8.672863155	8.638988065
Zm00001d034867_T001	6.208313459	5.497901546	6.361504636	6.223938156	6.311062669	6.177092889
Zm00001d015527_T002	3.485220327	2.597910122	3.413904629	4.179030789	4.361692644	4.223974885
Zm00001d015527_T004	0.65401477	0	0.495610236	1.184235854	1.050122733	1.063438054
Zm00001d009738_T001	3.72169846	4.604832097	3.569606168	4.444517529	4.209712014	4.163789416
Zm00001d025575_T002	0.779368995	0.424594288	0.611375429	0.972951397	0.784420225	0.827339401
Zm00001d039575_T001	4.020036399	3.55123508	4.181720375	5.424235234	5.511131774	5.45994021
Zm00001d042833_T001	6.292367338	5.39675769	6.259062038	5.143594029	5.267074601	5.212453545
Zm00001d035413_T003	3.768565887	3.324537589	3.955203425	3.994127295	3.750595679	3.875091179
Zm00001d035413_T001	2.52350224	1.70283305	2.382353505	2.765831529	3.052638298	3.02614158
Zm00001d035413_T004	3.290539266	2.367911695	3.100400454	3.061968832	3.042114785	3.173237791
Zm00001d013418_T001	1.655449837	2.146090806	1.921559343	1.429553837	0.867349322	1.332623159
Zm00001d011178_T001	3.106993635	2.555253443	3.068162425	3.587761138	3.537061315	3.37958007
Zm00001d034935_T001	0	0	0.562607311	0.517403703	0.467677015	0
Zm00001d034935_T007	4.093071815	3.266837802	4.260865352	4.11564947	4.094511406	4.108975762

Zm00001d034935_T011	0.812680579	0.397641984	0	1.433286915	1.3764282	0.755972855
Zm00001d034935_T003	0.381008653	0.411544477	0.084416729	0	0.223701794	0
Zm00001d017090_T001	5.881278862	5.627557697	5.909609244	5.437155882	5.721727766	5.623456774
Zm00001d025989_T001	1.120412328	1.192126606	1.390697398	0	0.747634022	0.743260745
Zm00001d002084_T009	1.57107194	0.714589305	1.133341252	1.777779069	0.677960994	0.890321388
Zm00001d002084_T004	2.938944406	2.32255755	2.753772028	2.645388095	3.035595935	3.092131647
Zm00001d002084_T011	1.252060755	1.33788118	1.45741726	0	0	1.665972552
Zm00001d013477_T001	1.906676291	1.241060369	1.580102058	3.078914292	2.857560463	3.209998835
Zm00001d013477_T008	2.709798357	1.690458219	2.674886121	2.662739175	2.369599649	2.586592066
Zm00001d013477_T012	0.456247884	0.528556317	0.447670172	1.853044312	1.432076956	1.868064834
Zm00001d013477_T010	0.142533685	0.127361222	0.320031725	1.66789939	1.677464493	1.365423231
Zm00001d009431_T003	6.603970785	5.363569306	6.60761193	6.493291996	6.466873143	6.353986486
Zm00001d009431_T002	2.689425307	1.853394499	2.654794337	3.457097681	3.285439506	3.412116513
Zm00001d052987_T001	5.905053453	5.02852199	5.926366002	6.064523491	6.008138709	5.9919359
Zm00001d044254_T007	4.110445098	4.417688359	4.103838005	3.633703681	2.596370831	2.407501228
Zm00001d044254_T001	1.990041046	0	2.152434287	2.924357039	3.384944995	3.084206996
Zm00001d044254_T003	4.591494341	3.919808096	4.188741538	3.085035715	3.626750071	3.33227284
Zm00001d044254_T006	0.236463228	0.183139654	0.416954288	2.247188664	1.479541525	2.627346756
Zm00001d016059_T001	5.640001357	5.706098812	5.631200575	5.34681279	5.228319162	5.3864862
Zm00001d052757_T001	3.374610105	3.950567514	3.515161005	3.12803764	3.121309051	3.722689406
Zm00001d005151_T001	0	0.230180875	0	1.882349981	1.743342188	0.707798638
Zm00001d005151_T004	3.036924036	2.529937607	3.290557844	3.4284856	3.555456295	3.585715511
Zm00001d005151_T006	0.737024743	0	0.410425775	0	0.634733582	1.106066186
Zm00001d005151_T007	2.830424697	0	2.533196772	2.224197752	2.347544624	2.391061319
Zm00001d049347_T001	2.913325838	3.2427662	2.792955484	2.643994913	2.883088602	2.61800226
Zm00001d043249_T001	6.799509611	6.769735341	6.92348535	7.345075445	7.393345724	7.371031738
Zm00001d012249_T001	1.879129749	1.634432495	1.768588329	0.734251353	1.283988129	1.242291527
Zm00001d008176_T015	3.02258176	0	3.200210694	3.575802183	3.957388857	3.666593001
Zm00001d008176_T008	1.278293481	0	0	2.084339167	1.949782551	2.284201989
Zm00001d008176_T006	2.344374033	1.340810055	2.983980428	3.797871489	3.612859535	3.847421918
Zm00001d051511_T001	4.591275308	4.064217383	4.619245606	4.463821873	4.650699832	4.448722453
Zm00001d032990_T001	2.878642759	2.367498834	2.73195783	2.66718979	2.795610911	2.981009252

Zm00001d046660_T001	2.756899246	2.402308789	3.004675943	2.793856148	2.925047875	2.917930072
Zm00001d043857_T001	1.379164018	0.982466855	1.392181666	1.754054987	1.328554445	1.365641591
Zm00001d007231_T002	1.055036657	0.808974777	1.351258563	0.953055851	1.360542952	1.355602698
Zm00001d006397_T001	0.350782363	0.571802995	0.952458818	1.612344949	1.588193882	1.481276722
Zm00001d019678_T001	3.148672053	2.718547726	1.181261604	3.186807953	3.350497671	2.878323371
Zm00001d022400_T003	3.882894008	2.472828519	3.250560382	3.119471223	3.200417251	3.372529532
Zm00001d022400_T004	1.430652993	0.727073535	1.143540358	1.510683478	1.397212623	1.367001957
Zm00001d022400_T005	3.057495329	3.166904847	3.738749465	3.005636225	3.122264864	3.188015986
Zm00001d022400_T006	3.290481615	3.827761722	2.756458863	2.79179428	1.746022048	2.850695227
Zm00001d010795_T001	1.55484569	1.11570914	1.557911448	2.225850844	2.314262637	2.325020225
Zm00001d050645_T003	0	1.323474412	1.596780611	1.962789606	1.959120611	1.783266378
Zm00001d050645_T002	3.314405782	2.152109741	3.099039133	3.327929182	3.473126055	3.340483082
Zm00001d042133_T018	4.898010755	6.686730906	5.141358316	4.737168369	4.693707122	4.34531866
Zm00001d042133_T004	5.111148986	4.138644523	5.022540393	5.191849945	5.111369154	5.24131673
Zm00001d042133_T001	1.90299468	0.805456736	0.888297101	1.987688679	1.632295208	1.706276359
Zm00001d025382_T001	5.905496519	7.344815637	5.504156812	6.309345089	6.74487021	6.476881068
Zm00001d012423_T001	8.754951894	8.757315334	8.601876103	8.214123635	8.267104559	8.280004496
Zm00001d028566_T001	3.229149664	2.866213854	3.505208155	2.924743158	3.609971917	2.71867945
Zm00001d028566_T002	3.162665561	1.580290712	2.56127994	4.412484489	4.383241588	4.613800247
Zm00001d003108_T003	3.923091072	4.317865232	3.384674977	3.271713858	3.490235089	3.552000116
Zm00001d003108_T007	1.26404514	0.535512535	1.102109165	1.417194158	1.912580507	1.493676024
Zm00001d003108_T001	3.684031547	3.29972279	3.891825884	3.837300847	3.837305692	4.044192653
Zm00001d003108_T004	1.293853068	0	1.346068455	1.249599463	1.069606625	1.086777653
Zm00001d003108_T005	0	0	2.452512676	3.218234669	3.243805546	2.96414593
Zm00001d038682_T001	5.462945086	4.858412645	5.631973102	5.009350084	4.974882188	4.84101533
Zm00001d010465_T001	4.354006504	4.323064779	4.516586563	4.847724672	4.756685102	4.770010335
Zm00001d049305_T006	4.058277267	6.668318103	4.763062226	3.994646427	3.389382333	2.864176436
Zm00001d049305_T005	1.448780457	0.811639541	1.044693469	0.533049966	1.04297976	0.862734646
Zm00001d049305_T001	6.595991493	6.229030213	6.562835487	6.205028998	6.357431429	6.396891322
Zm00001d049305_T003	7.368252839	6.649785871	7.438599824	6.598766902	6.46316417	6.676465393
Zm00001d037343_T001	2.940562756	2.760590996	3.444209858	3.391365205	3.283868892	3.154379382
Zm00001d037343_T003	4.04116508	3.582856794	3.999219381	4.381320132	4.382574159	4.336043681

Zm00001d025108_T001	1.612510561	1.441327303	1.352117813	2.274556284	2.305301387	2.244417955
Zm00001d050184_T001	0.757819497	1.00989247	0.266262429	1.340471119	0.749433865	1.009597992
Zm00001d050184_T003	2.916804701	2.279585102	2.980579964	3.98299321	3.903237688	3.70264336
Zm00001d050184_T011	0	0.524257293	0.892194379	0.84009982	0	0.953510355
Zm00001d050184_T010	4.432688342	3.854051019	4.478901609	4.145438209	4.20673464	4.051896354
Zm00001d050184_T002	1.257802374	0	1.304008639	1.926651521	2.124290744	2.35473424
Zm00001d006631_T001	2.868987164	2.280499388	2.9715579	3.231667257	3.22339073	3.29201191
Zm00001d003982_T001	3.94746194	3.511745697	3.859533296	3.527102873	3.457230355	3.514505833
Zm00001d043599_T001	4.514870752	3.673755177	4.64806134	3.766657596	3.828436	3.774603332
Zm00001d024803_T001	1.996143576	2.47901942	2.166440054	2.585006262	2.291311807	2.441540581
Zm00001d029720_T001	3.134765545	4.274324661	2.69854766	2.387338275	2.383519339	2.468341414
Zm00001d053658_T002	5.833000389	5.064503982	5.874496939	6.097776777	6.110200605	6.030211691
Zm00001d053658_T001	0	1.346883158	1.129096361	1.512364448	1.047907565	1.191792554
Zm00001d010178_T001	3.404299058	2.713352474	3.507262996	2.993032115	2.871254822	2.84992162
Zm00001d002388_T007	1.147716327	0.364091641	0.65579785	2.130750686	1.287695686	0.955821638
Zm00001d002388_T003	4.635084886	3.662839303	4.629822404	5.651340345	5.635232292	5.614953197
Zm00001d002388_T002	4.456927814	3.934101372	4.551231757	0	0	0
Zm00001d017659_T001	2.487307802	2.719198557	3.028893369	1.960799331	2.412865782	2.363133782
Zm00001d013128_T001	2.362156768	2.179344159	2.261683652	2.356951719	1.959187396	1.85259817
Zm00001d013128_T005	0.610435497	1.021887604	0	1.135391709	0.543387987	1.324581367
Zm00001d013128_T004	1.991326828	0	1.489216134	0.595781479	1.52538387	0.975691077
Zm00001d017138_T001	5.769495549	5.194780095	5.756821127	5.657645521	5.646532012	5.656621872
Zm00001d052820_T001	5.917208125	5.110947403	5.882673321	5.773829372	5.642672752	5.693436692
Zm00001d048740_T001	2.007318592	1.614654726	2.15777684	1.2275501	1.251545201	1.647238055
Zm00001d038335_T001	2.752228785	3.610094797	3.444831626	2.794734628	3.301665486	3.116133661
Zm00001d022111_T002	4.780958746	4.494269541	4.977057777	4.121554487	4.090753861	4.256594123
Zm00001d022111_T001	2.843002759	1.768239384	2.879070135	2.799036106	2.489297071	2.539006157
Zm00001d008570_T001	2.164022902	1.947118147	2.498677485	5.008265014	4.923060638	4.819593544
Zm00001d019579_T008	2.410207304	2.221229037	3.183884888	2.896040263	2.963705307	3.001204508
Zm00001d019579_T002	1.395390797	1.039801671	1.746069788	1.316562268	1.787640997	1.730516928
Zm00001d019579_T007	3.018481779	1.666306674	2.641661625	2.950921122	2.625314214	2.974332902
Zm00001d005228_T001	4.922171916	4.342431505	5.129022154	4.666875978	4.803916079	4.795408723

Zm00001d004702_T006	0.67855062	0.61258935	0.794096411	0.973153508	1.009323521	1.124086563
Zm00001d004702_T003	4.875412807	3.579498781	4.325314955	5.087429532	5.255822773	5.460335184
Zm00001d004702_T002	0.956191992	3.537950537	1.055755128	0	1.191594865	1.550346622
Zm00001d004702_T004	3.180249407	0	3.949314263	4.113676999	3.269086124	0
Zm00001d012127_T001	6.321222336	6.44234739	6.213727057	6.459038168	6.49054239	6.445732877
Zm00001d054001_T002	1.476065375	0.455840876	1.378933272	1.55968416	1.305409088	1.231014532
Zm00001d054001_T011	2.197332301	0	0	2.805979584	2.638527085	3.305777371
Zm00001d054001_T016	0	2.16935294	2.418377527	2.659689416	3.09954029	0
Zm00001d054001_T022	0.274531237	0	0.355342216	0	0.523793772	0
Zm00001d054001_T001	0	0.23177768	0	0.798607986	0.970328005	0.851149755
Zm00001d054001_T028	0.945389709	0	0.803717221	1.861767623	2.01342081	1.339434447
Zm00001d054001_T004	1.903593639	0	1.806105832	1.17338652	0	0
Zm00001d042441_T003	4.475478327	4.049021414	3.794246996	2.915858542	2.106557706	3.326937658
Zm00001d042441_T001	5.211093946	4.429088129	5.434346299	5.131014942	5.310822514	5.115263766
Zm00001d029704_T001	3.478981509	3.765796105	3.674492741	2.751060679	2.712306112	2.83963696
Zm00001d002958_T001	4.125660854	4.546501376	4.353855451	5.5649608	5.600449187	5.476501551
Zm00001d036558_T001	2.72064555	2.2264925	2.950168833	2.730425193	2.507513049	2.623742809
Zm00001d029077_T001	0.512684026	0.29398016	0.634340484	0.305109023	0.683141275	0.649594307
Zm00001d029077_T002	2.460917616	1.567877366	2.056316813	1.488735047	1.845935587	1.711732326
Zm00001d042373_T002	1.715827493	0.378511623	1.079682577	2.045324139	1.487572785	1.872480501
Zm00001d042373_T001	5.026969588	4.193401798	5.170071107	5.434205998	5.473771102	5.5053266
Zm00001d042373_T003	0	0.741680112	0.183611008	0.892122086	0.699332191	0.536070809
Zm00001d011347_T002	1.401406564	0.499066717	2.236507007	1.656839556	1.597736152	1.543165733
Zm00001d011347_T001	3.502778287	2.612752104	3.236499966	4.257660062	4.255509042	4.240341809
Zm00001d033210_T002	10.25551792	9.433852802	10.31831956	9.980380668	9.986863406	9.969440876
Zm00001d033210_T005	8.123246396	6.452844225	8.065092784	0	0	0
Zm00001d033210_T004	2.894539575	0	2.446018232	0.715152729	1.572390094	1.919841554
Zm00001d033210_T001	3.875241114	1.939013638	1.536951569	3.117230247	2.782175157	2.087742188
Zm00001d048884_T001	5.540562227	5.8615577	5.484056776	5.287318362	5.429095195	5.334572459
Zm00001d017678_T001	3.904313277	3.942863431	4.289878129	3.985246479	4.026401183	3.774923549
Zm00001d016982_T001	1.63420478	1.185017166	1.669297586	1.854155672	1.900406617	2.084450066
Zm00001d024913_T001	5.180533194	4.6847038	5.150334238	4.410583637	4.058365508	4.204197306

Zm00001d045870_T001	0.593452258	0.802203972	1.303429491	1.017915496	1.061238413	1.58124277
Zm00001d045870_T002	0.609030597	0.221849866	0.292999609	2.251773316	2.230623892	1.595872642
Zm00001d045870_T003	3.76665855	3.028891424	3.838184848	4.546474274	4.558642527	4.493847445
Zm00001d053208_T001	0.717083048	0.407748656	0.681221655	0.532341898	0.746143331	0.638975558
Zm00001d001995_T001	2.185131693	1.080952311	1.901293638	0	0	1.597552627
Zm00001d001995_T002	0	0.689219531	0	2.425630903	1.871941402	1.342493304
Zm00001d029121_T004	0.130039945	0.155786789	0	0	0.434518812	0.785253403
Zm00001d029121_T001	3.26129491	3.169908491	2.724277436	2.589676971	1.86030733	1.866697937
Zm00001d029121_T003	0.417047216	0	0.399226886	1.054541503	0.469810975	0.506532888
Zm00001d029121_T005	3.7507677	3.114074291	3.86229159	3.72577387	4.146399356	4.05675007
Zm00001d029121_T002	0	0	0.738919987	1.10181679	0.824791474	0
Zm00001d034532_T001	1.7281922	1.112353177	1.71513691	2.350507997	2.295865818	2.264068568
Zm00001d034532_T002	4.891266986	3.945165314	4.814243344	4.675400052	4.442900646	4.747993516
Zm00001d036131_T001	5.031550601	4.225188384	5.156710655	5.242384332	5.32178656	5.326701749
Zm00001d036131_T004	3.32573582	3.96606504	3.01240473	0	2.650321575	0
Zm00001d028265_T004	3.505938807	3.136944699	3.545331862	0	2.061453067	2.36064127
Zm00001d028265_T002	0	1.537443187	0	3.389850505	2.084688504	1.940130283
Zm00001d028265_T005	3.399815308	0	3.685347704	3.662098832	4.017764628	3.698587663
Zm00001d028265_T001	1.291185658	0.447330571	0.347090731	0.26389736	1.034463657	1.290206155
Zm00001d028265_T007	0.587048091	0.290347725	0.308873411	0.579310506	0.488298023	0.206571963
Zm00001d054010_T001	2.523834662	2.022451975	2.33765344	2.841991027	2.888850966	2.976905599
Zm00001d036532_T001	2.706647024	2.292324725	2.95941259	4.262611228	4.195595245	4.145772002
Zm00001d008380_T001	2.084160555	2.691473419	3.183521926	3.251802093	2.402221184	3.19253065
Zm00001d008380_T002	3.298536766	0	1.738273237	0	2.330613468	0
Zm00001d010442_T001	1.868608545	1.096889943	1.818400262	2.211579812	2.142021913	2.06672883
Zm00001d013326_T001	8.965343676	10.07612479	9.042798748	7.465070367	7.482212673	7.434191358
Zm00001d014181_T001	1.966843872	1.295206383	1.862374022	2.188837655	2.143637657	1.977998985
Zm00001d052578_T001	1.485812181	1.536984381	1.557083109	0	0.245701313	0.692223635
Zm00001d023216_T001	2.871145783	2.24637525	2.886654712	2.105541981	2.531053019	1.796797076
Zm00001d036201_T001	5.171384471	4.588655697	5.112278604	4.704110698	4.855694253	4.784762661
Zm00001d046016_T001	4.546543541	3.692856439	4.53129761	5.241281946	5.214180382	5.088923594
Zm00001d031489_T003	1.386677429	2.107831797	2.540268608	2.62181722	2.678864043	2.125497038

Zm00001d031489_T002	2.485250859	0.802277604	1.69120807	1.19489771	1.801853549	2.261220279
Zm00001d031489_T004	1.570331771	0	1.392773517	2.391713013	1.764513891	2.107982069
Zm00001d049556_T009	0	0	0	0.730167783	1.507977264	0.960137273
Zm00001d049556_T001	1.387956923	0.833055231	0.99335409	1.258356104	0.718951188	1.217933587
Zm00001d021817_T001	3.60833343	2.901108436	3.865970664	5.719143949	5.833301427	5.687456003
Zm00001d047272_T001	7.339641955	7.917028931	7.343308314	7.172612602	7.073681322	7.145835195
Zm00001d051576_T001	0.21920756	0.195554223	0.340999711	0.460862063	0.458319003	0.382147069
Zm00001d043045_T001	2.059925251	2.89058366	3.051628075	1.481150634	2.334146618	2.256786047
Zm00001d032868_T001	0.360795756	0.292938376	0.305840979	1.495149189	1.109700213	1.368260453
Zm00001d008720_T002	2.263028996	1.834844276	2.091968886	2.106989784	2.084088423	2.230215313
Zm00001d020669_T001	5.741111311	4.877036322	5.874220461	5.736695406	5.797496796	5.765905291
Zm00001d002483_T006	1.687517171	1.621618213	1.969765334	0.630498666	0.982333958	1.12879885
Zm00001d002483_T004	1.310982791	0.322643494	1.31239624	1.663173353	0.801344104	1.914299686
Zm00001d002483_T001	0	0	0.310899638	1.162636078	1.176883144	1.191140645
Zm00001d045269_T014	0.259988308	0.028057046	0	0.425369066	0.381830441	1.494231261
Zm00001d045269_T031	0	0	0	2.494524672	2.64214148	2.338946914
Zm00001d045269_T039	1.133860057	0.365538014	0.783454978	1.54796058	1.845975718	1.312431094
Zm00001d045269_T004	0	0.067604301	0.433887777	0.466916283	1.404654661	0.77761371
Zm00001d045269_T011	0	0.916669246	0.934835765	2.831996178	0	1.963876913
Zm00001d045269_T012	0.38568504	0.205401272	0.514617174	0	1.510624239	1.399495971
Zm00001d045269_T042	3.50591671	2.581432238	3.555240925	3.665803758	3.758157855	3.991451915
Zm00001d053011_T006	0.34673001	0.09732088	0	0.516786732	0.673332992	0.448517238
Zm00001d053011_T003	1.954071582	1.34155202	1.99257485	2.23125544	2.315091726	2.243721171
Zm00001d053011_T004	0	0	0.475423202	1.409975228	0.754319717	1.172934848
Zm00001d022525_T001	5.264260213	4.209724177	5.139158927	4.653917753	4.624838025	4.704190174
Zm00001d016551_T001	1.766487035	1.323194225	1.41271325	1.169996814	0.955364885	0
Zm00001d016551_T002	2.506454944	2.008168813	2.649748802	2.104253115	2.281462356	2.173673379
Zm00001d033021_T001	2.825972616	1.531542659	2.198744639	3.543770538	3.384843485	3.379398192
Zm00001d010191_T001	4.244660745	3.39578383	3.865526621	3.669045593	3.946779604	4.275827943
Zm00001d023424_T001	2.69402368	2.2437099	3.066175319	2.620521429	2.51666497	2.512657987
Zm00001d035207_T001	1.859046007	1.77017403	2.602305315	0	1.36877617	0
Zm00001d035207_T002	1.752121289	1.685141793	0.563087825	1.863102317	1.792694211	2.118107515

Zm00001d035207_T003	2.763550313	0.727741876	1.951415094	2.454502042	2.879057192	2.881523083
Zm00001d029039_T001	4.458390601	3.904229063	4.333208888	4.87605467	4.857082812	4.71200367
Zm00001d008815_T002	2.698641222	2.002422774	2.848736299	2.630644736	2.748002374	2.581745554
Zm00001d042747_T004	0	0.065995477	0.697461587	2.641163803	2.515356199	2.276020751
Zm00001d042747_T001	4.360741899	3.444938408	3.977225146	4.105502758	4.438057463	4.215186148
Zm00001d042747_T006	0.750567061	0.347817571	0.447441665	1.282635512	1.183622441	1.470919971
Zm00001d042747_T002	2.760924767	1.950780436	2.579011872	3.213633615	2.126994985	2.878104388
Zm00001d042747_T008	0	0.147707862	0.501352517	1.651841125	1.881399546	0
Zm00001d042747_T005	1.475141967	0.893507444	2.638198981	3.077251375	3.316078821	3.468989612
Zm00001d048841_T001	0.939829863	1.111871972	1.430670119	1.327507447	1.319267044	1.403542599
Zm00001d014294_T007	0.162237111	0	0	0	0.650430955	0.342931299
Zm00001d014294_T027	0	0	0.682294619	2.138935344	2.384010837	2.216926943
Zm00001d014294_T021	4.874030866	3.93072116	4.942089657	5.713783506	5.639638978	5.69985925
Zm00001d014294_T008	0	0	0.711514289	2.624967889	2.704577421	2.667314932
Zm00001d014294_T011	1.668521734	0.914465789	1.092053822	3.377213863	3.269655885	3.228165598
Zm00001d014294_T009	0.414254625	0.304312439	0.341514447	0	0	0
Zm00001d008457_T001	1.654433708	1.394144229	1.937605033	1.804158907	1.896760093	1.402386018
Zm00001d014036_T014	0	0	0.247673577	1.336231393	1.917388216	2.113614162
Zm00001d014036_T007	0.190277296	0.829181423	1.154483405	0.981401878	1.809849863	1.869482556
Zm00001d014036_T002	0.412480221	0.597098241	0.168277465	0.267805435	0.294230783	0.75796368
Zm00001d014036_T005	3.447626404	1.488795192	3.440396135	4.065861528	3.917561315	4.001239194
Zm00001d014036_T009	2.621702601	3.414214432	1.86059617	0	0	0.714445997
Zm00001d014036_T018	0.153200899	0.075387499	0.247127887	0.51166133	0.541593142	0.709891413
Zm00001d014036_T003	0.526238118	0.323620249	0.547090468	0.951958491	1.41417451	1.413315698
Zm00001d041920_T001	4.455441974	3.961308179	4.603867659	5.134365161	5.024765266	5.06186997
Zm00001d017121_T002	3.840814876	7.847841189	5.542815299	4.406250797	4.209222672	3.932561359
Zm00001d017121_T001	8.307879215	7.110180577	8.269575098	7.15449658	7.152067007	7.176684548
Zm00001d009243_T001	3.07628493	2.724663804	3.0638546	4.37642299	4.268639719	4.224513118
Zm00001d009243_T002	3.510565377	1.855494632	3.31244126	3.781703296	4.24460321	4.086125862
Zm00001d009243_T003	0	0	1.586684051	2.704943655	1.981644087	2.095828237
Zm00001d015970_T002	4.199725969	4.569027172	4.118703662	4.576744027	4.500726858	4.531520888
Zm00001d015970_T003	4.447451253	5.476881651	4.496364684	4.141714795	4.398243574	4.401060402

Zm00001d040768_T001	4.677670769	3.782605978	4.735368964	4.248335435	4.239128084	4.111567465
Zm00001d012677_T015	1.591297392	0.692043262	1.344324034	0.99136275	2.42628569	1.646019763
Zm00001d012677_T007	1.244559524	0	1.458359954	1.619866995	1.789485548	1.697609261
Zm00001d012677_T001	1.33601825	0.907302955	1.242051235	1.886633644	1.907628682	2.033713714
Zm00001d012677_T011	3.8600849	3.445551917	3.791525087	3.989429516	3.847585179	4.062967009
Zm00001d036678_T001	2.10590633	1.371937398	1.909424435	2.059262856	2.102216354	2.244916278
Zm00001d036678_T007	0.152093836	0	0.033226341	0.407242879	0.332698769	0.57272672
Zm00001d052621_T004	0.662499934	0.18567375	0.589903436	1.101733435	0.128467623	0.738447923
Zm00001d052621_T008	0.717600757	0.542146097	0.788786758	0.833459285	1.107165593	1.054313661
Zm00001d052621_T012	0.575852514	0.432523327	0.400072257	1.30702568	1.549147686	1.759379147
Zm00001d052621_T001	1.408152063	0.970207241	1.805323002	1.813007443	1.908611954	1.608915667
Zm00001d052621_T014	4.32725061	6.401180442	4.672801299	2.800553922	0	0
Zm00001d052621_T006	0.70341703	0.045133988	0.282073228	0.867949428	0.455319066	0.738242103
Zm00001d052621_T005	1.528930823	1.874653771	1.505467203	1.51798666	1.501864057	1.232119798
Zm00001d052621_T007	5.603058739	2.14591381	5.488313932	5.395825224	5.671881909	5.417151043
Zm00001d052621_T002	2.191705713	4.080718201	2.391967286	2.724688903	0.931008255	2.928516433
Zm00001d053197_T015	1.136633054	0.411918631	1.005916606	0.965984448	0.815254066	0
Zm00001d053197_T006	1.125711929	0	1.307282791	2.912656187	2.853156325	2.931634273
Zm00001d053197_T002	4.325859272	2.966847009	4.202942043	3.968485895	4.017624433	4.026004322
Zm00001d053197_T014	0.582239972	1.017433803	0.770754637	1.694869052	1.682613746	1.498669571
Zm00001d053197_T011	0	0	0.198993196	0.722692466	0.590625012	0.874826075
Zm00001d053197_T004	4.239009513	0	3.401773255	3.289785489	3.854321663	4.088345246
Zm00001d053197_T018	6.025768451	6.495544235	6.020281567	5.425607541	5.39370782	5.627392315
Zm00001d053197_T005	3.756930514	4.327778033	4.786231879	3.505887628	2.966632386	0
Zm00001d026645_T020	0.439899683	0.069824505	0.281213957	0.176269149	0.534576616	0.065434446
Zm00001d026645_T016	0	0	0	0.482976288	0.461023475	0.477456633
Zm00001d026645_T004	0.349587214	0.349056096	0.226395083	0	0	0.608284101
Zm00001d026645_T015	2.959075343	1.695805894	2.834394087	2.806402223	2.563257236	2.93302227
Zm00001d026645_T007	1.00978142	0.080705411	0.945472866	1.926189614	1.759698287	1.730360845
Zm00001d014641_T001	4.289647921	3.486428766	4.34799058	4.07630289	4.05486931	3.970305547
Zm00001d009773_T001	6.258941985	5.623382966	6.289379218	5.042392413	5.059560305	5.015626911
Zm00001d022225_T001	3.280082863	2.619048828	3.425404249	4.985252856	4.981724631	4.807776591

Zm00001d016816_T002	3.918802285	3.241007725	3.814070471	3.859563311	3.735708351	3.788048989
Zm00001d046728_T001	6.37767493	6.507121791	6.540329262	5.573152724	5.49428196	5.531161156
Zm00001d020593_T006	3.75865805	2.761192708	3.854977962	3.972343884	3.745687822	3.697795055
Zm00001d020593_T002	2.258790554	0	1.375237988	2.729181418	3.46451066	3.14664447
Zm00001d052322_T001	5.932811293	6.390175854	5.625297525	6.074496831	5.968220773	5.938453673
Zm00001d041322_T002	6.870854216	6.832972903	6.974952319	6.259162812	6.394915745	6.36515433
Zm00001d010835_T003	1.815058917	1.669853583	1.857942788	0	0	2.374853086
Zm00001d010835_T005	0	1.78691372	1.864212299	1.332919847	0	2.007324692
Zm00001d010835_T006	3.425688233	2.234604629	3.496723739	3.797706031	3.762513276	3.021378096
Zm00001d051047_T001	3.095873967	3.417824977	3.19956776	4.058111334	4.240650385	3.949478531
Zm00001d032670_T021	1.875784387	1.610823352	1.528738332	1.644692941	1.070722483	1.24543664
Zm00001d032670_T009	2.474240296	1.7526741	2.014020649	1.925961832	1.628433466	1.930108659
Zm00001d032670_T013	0.984405607	0	1.214931121	1.073362336	0	1.044675286
Zm00001d032670_T011	0.653232488	0.50652781	0.81890372	0.753304157	0.69589763	0.641386029
Zm00001d032670_T001	0.364720336	0.022158984	0.503309041	0	0	0.275967729
Zm00001d032670_T020	0.858374946	0.953367314	0.762598847	1.645129816	1.379691376	1.624599258
Zm00001d032670_T003	2.802557206	2.37731841	3.129452173	2.511915807	2.798152776	2.59414148
Zm00001d032670_T006	0.10777088	0	0.349135379	0.264079981	1.0010153	0.347667924
Zm00001d034893_T002	1.301302006	1.251377955	1.061584056	1.307159197	1.26832779	1.150185957
Zm00001d034893_T001	5.801471586	5.790854594	6.084952044	6.031899769	6.387795102	6.271964961
Zm00001d034893_T004	4.602502786	5.223128034	4.114975044	5.145707042	4.738106261	4.472083045
Zm00001d031671_T001	3.36481935	3.394923323	3.607692851	4.186683815	4.014375291	4.267013457
Zm00001d022542_T002	2.945333146	2.856955621	3.695512835	3.998499177	3.907025722	3.89104077
Zm00001d022542_T004	1.516016156	1.237363024	1.741662425	2.664331964	2.425915513	2.728892014
Zm00001d022542_T007	1.295999784	1.715491029	1.237841462	1.579770528	2.088759326	0.54046279
Zm00001d022542_T005	4.429020085	2.990675549	4.444295281	4.857145184	4.296542792	4.419397042
Zm00001d022542_T003	3.355782246	2.089944178	3.751983696	3.674318725	3.164660793	3.323844596
Zm00001d022542_T001	3.470117987	4.164619606	0	2.84154407	3.753751066	3.811517476
Zm00001d022542_T006	5.265905253	3.347871134	5.101201811	4.652543456	5.126202668	4.649263479
Zm00001d029753_T003	1.126292965	0.930557959	1.463068393	1.505414863	1.446979778	1.670856195
Zm00001d029753_T002	4.213644812	3.925875645	4.252876268	4.482892286	4.546883165	4.47835785
Zm00001d013136_T001	0.60792538	0.426705951	0.451785569	0.771654053	0.653478315	0.565128224

Zm00001d008307_T001	2.757133365	2.233268709	2.722300324	3.182833541	3.19678438	3.292818405
Zm00001d041816_T005	1.016683152	0.658845786	1.205700914	1.221709453	1.517669262	1.391008786
Zm00001d041816_T004	1.360093412	0	1.091888684	0.881753097	0	0
Zm00001d041816_T002	0.903241159	0.672663395	0	0.798239681	0	0
Zm00001d041816_T003	0	0	1.15494089	1.717970497	2.280264189	1.786840972
Zm00001d049665_T001	7.240884978	6.78700141	7.039948092	6.844211344	6.859098303	6.969371255
Zm00001d013362_T001	4.511365691	4.352511865	4.554381027	5.811900513	5.820595445	5.823880888
Zm00001d034049_T002	5.516411424	3.813565117	5.790193932	5.918955543	5.97977683	6.040585212
Zm00001d034049_T001	4.979043101	4.409919307	4.874198817	5.279791632	5.32472342	5.154669722
Zm00001d034049_T003	5.934826001	5.295758614	5.658921028	5.774318892	5.787031091	5.598955289
Zm00001d027756_T002	8.984632315	8.505528472	9.058383057	9.316220489	9.339619993	9.276384595
Zm00001d027756_T001	2.211208175	4.087990432	2.332280861	2.281570368	2.200339874	2.259102518
Zm00001d052709_T002	0.335835369	0.236188893	0.159972705	0.759261528	0.638818053	1.158843691
Zm00001d052709_T010	0	0.636414421	0	0.966277625	0.917348495	0.88369119
Zm00001d052709_T013	5.367729283	4.80580644	5.44619915	5.018193679	4.912355644	4.399639176
Zm00001d052709_T004	4.634151971	3.421312989	4.71078291	5.293050721	5.14230274	5.365243725
Zm00001d052709_T005	1.370952106	1.265658924	0.960146913	1.527079847	0.942708165	1.65438467
Zm00001d052709_T003	1.988020031	0	1.76471812	2.280501764	2.642747081	1.76146145
Zm00001d052709_T008	0.424867281	0.424323393	0.489353852	0.790482636	0.968060515	1.031967502
Zm00001d041124_T017	0	0	0.586054688	0	0.966459262	0.620094637
Zm00001d041124_T007	0.609220707	0.292420147	0	0.836517195	0	0
Zm00001d041124_T005	0.623566308	0.799294575	1.255509193	1.280635085	1.403925376	0.969526648
Zm00001d041124_T010	0	0	0.423106966	1.011120604	0.313728798	0.834157814
Zm00001d014032_T001	5.771637891	6.820945634	5.787064796	4.799940286	4.685106631	4.731792999
Zm00001d001802_T001	2.944478986	4.394111569	3.281395438	3.763109618	4.256480149	4.135400001
Zm00001d046336_T001	0.614462033	3.46888603	1.660405224	1.313002576	1.705657267	0.948071513
Zm00001d046336_T002	7.51895879	6.463469476	7.479934531	7.492142216	7.499533474	7.473906414
Zm00001d006808_T002	5.436116355	5.175296722	5.400269449	5.880298502	5.857552401	5.727951513
Zm00001d006808_T001	2.708886227	4.336954728	2.43781924	2.455175955	2.998286143	2.906580287
Zm00001d006944_T003	3.45983945	2.720803344	3.569937733	3.343758291	3.773116836	3.50442463
Zm00001d006944_T002	1.659276113	2.005757114	2.185536114	3.473599778	3.23160444	3.162875147
Zm00001d006944_T004	0.565282283	0.495128205	0.645222064	0.90125965	1.395420412	1.0903257

Zm00001d020273_T001	2.315868475	2.210826158	2.482770855	1.583402585	1.697027374	1.871814873
Zm00001d048705_T001	2.6596401	1.789581532	2.412230622	1.952047236	2.03566156	2.036848625
Zm00001d027502_T001	1.635661536	2.653711262	1.771212023	1.742452105	1.535201459	1.299340795
Zm00001d027502_T002	5.245316988	4.733560262	5.379543544	5.338096508	5.384902631	5.41234418
Zm00001d052776_T001	6.394944873	7.881273998	6.471976288	5.776139844	5.601617725	5.836544386
Zm00001d018295_T001	0	0	0	2.303563634	2.561390662	2.367500791
Zm00001d018295_T002	1.928790231	1.965097537	2.381286418	0	0	0
Zm00001d018295_T007	3.028442204	1.67819498	2.881649155	3.082889385	2.835980447	2.697810287
Zm00001d032185_T001	1.126955681	0.854009216	1.179252425	0.84655349	0.857479447	1.156414092
Zm00001d016106_T001	2.510147199	2.355253968	2.200822588	2.871963474	2.373694421	2.851414247
Zm00001d016106_T003	4.333027224	3.542263251	4.496920869	4.643265256	4.783767528	4.871898093
Zm00001d050211_T001	1.896397026	1.8710322	2.287386003	3.044975627	2.931474863	2.94856534
Zm00001d028894_T003	0.499879045	0.318872118	0.420549233	1.332239895	1.114690179	0.919218025
Zm00001d028894_T001	5.733717328	5.131830009	5.717269971	5.843846776	5.70287587	5.804256992
Zm00001d037590_T001	6.323935902	5.632741406	6.406973127	6.644632093	6.627106813	6.546912142
Zm00001d037590_T004	5.652397607	5.788897715	5.445164994	5.062790349	4.26098327	4.916859815
Zm00001d052793_T001	3.819732347	3.104173257	4.019096247	4.470236451	4.330557181	4.468913978
Zm00001d034768_T002	3.573510136	2.163406951	3.463615264	3.641394931	3.022382551	3.288083287
Zm00001d034768_T005	1.61213071	2.84154407	1.799880156	2.92510638	2.87753205	2.912626107
Zm00001d034768_T003	7.087153806	6.554573181	7.222855381	6.945569477	6.81718304	6.793730202
Zm00001d034768_T001	4.647545	4.13456243	4.764851797	4.825463071	4.984574677	4.873738257
Zm00001d005959_T001	1.788034159	1.385177542	2.138067704	2.633942864	2.591222459	2.893180455
Zm00001d024516_T001	4.522204306	3.536436034	4.402138692	4.822187021	4.886510934	4.831907432
Zm00001d048954_T001	5.462867205	4.711667194	5.511304392	5.514268953	5.552767943	5.515352162
Zm00001d027802_T002	0.918923519	0.786816304	0.622125402	1.565891046	1.65232495	1.425289566
Zm00001d027802_T004	2.419102242	1.47924298	2.668522188	3.240790111	3.06997415	3.073651281
Zm00001d027802_T001	3.625267216	2.751332814	3.737811991	4.637723492	4.489111103	4.347488355
Zm00001d027802_T003	4.499671031	3.728616265	4.507006682	4.669512969	4.894269289	5.0016563
Zm00001d023724_T006	2.051155594	0.659985735	1.530539742	1.834955891	1.700791249	2.022195212
Zm00001d023724_T015	1.990823469	0	1.530237082	2.277182564	1.986857965	1.697796945
Zm00001d023724_T012	3.486258584	2.704822841	2.858385092	2.961432376	0	3.477068651
Zm00001d023724_T001	0.298840107	1.046401653	0.935746372	1.578365868	1.439430056	1.496768088

Zm00001d023724_T007	0.962103283	0.927122006	1.003620945	1.246618471	0.70077616	0.831873189
Zm00001d023724_T008	0.89883058	0	0.959448944	1.03554931	2.178759889	0
Zm00001d023724_T017	1.741107535	1.99940657	2.160735968	2.289007555	2.110825246	2.405571739
Zm00001d023724_T011	2.789844203	3.095072894	2.737626846	1.57536752	1.570074274	2.387374122
Zm00001d023724_T003	2.990958671	1.782304553	2.636235055	2.784635474	2.659535069	2.117049675
Zm00001d006027_T002	3.272579848	2.359692361	3.226756202	3.205035862	3.37306322	3.239972469
Zm00001d029777_T001	3.017616951	3.217246229	3.075722924	4.963687738	5.0732109	4.97894699
Zm00001d035797_T001	1.249827582	0.229985301	1.36026594	1.529562113	1.439591757	1.055140806
Zm00001d028399_T001	4.74837557	4.128909929	4.938574867	5.193921287	5.236523424	5.05921885
Zm00001d021898_T001	2.481222464	1.834922326	2.246820618	2.160648851	2.263564799	2.275027913
Zm00001d020395_T002	2.103827601	1.704903385	2.38260224	2.555433594	2.390366405	2.464858784
Zm00001d046438_T014	2.858655422	2.312199591	2.942477159	2.763059463	3.012614975	1.868875583
Zm00001d046438_T009	0.93152422	0.666307583	0	2.326038243	2.405356073	2.116275251
Zm00001d046438_T013	1.160207698	0	1.410355759	1.758082683	0.690675439	2.258046693
Zm00001d041857_T002	1.961975903	1.156337069	1.779412703	2.459318035	2.689096051	2.264925201
Zm00001d041857_T001	2.426320851	1.697364613	2.415445544	2.548041494	2.746211495	2.681673917
Zm00001d011297_T002	5.848103452	5.307519966	5.950170606	3.602299613	3.769216025	3.36673835
Zm00001d011297_T001	5.042811271	4.831964064	5.1266237	2.483275867	2.445457365	2.803788297
Zm00001d028547_T001	4.24377798	3.930580761	4.195284597	3.216287537	3.318438471	4.030690793
Zm00001d040666_T002	0.107366491	0.145610943	0.127559327	0.287484116	0.258720315	0.148278155
Zm00001d040666_T003	2.277895506	1.573221329	2.057652322	2.291763849	2.662086524	2.271927902
Zm00001d035864_T001	0.853986867	0.774687978	1.20692386	1.44741257	1.787586673	1.495683908
Zm00001d040735_T003	2.467275045	1.618122107	2.396158667	3.125077579	3.353972922	3.431526287
Zm00001d040735_T004	2.155000495	1.596824015	2.297708229	3.559076076	3.108223508	3.167981505
Zm00001d025141_T003	0	0	0	2.54059843	2.031998193	2.068706921
Zm00001d025141_T002	0	0	0	2.408960711	2.815897745	2.875404599
Zm00001d037479_T001	8.600649358	9.537847566	8.715385521	7.953665819	7.942836079	7.87374482
Zm00001d042262_T004	4.293436849	3.418613517	4.315787197	4.220475223	4.26355203	4.219535325
Zm00001d027337_T004	2.524472448	1.65671007	1.895582601	0.825251589	0.621668847	0
Zm00001d027337_T012	0.311671672	0	0	2.022947971	2.184990833	1.75885533
Zm00001d053749_T001	4.932674451	4.339534143	4.953976585	4.87743924	4.914867764	4.829100524
Zm00001d005315_T001	7.654624415	7.579266451	7.661602299	8.621393423	8.67722611	8.696638377

Zm00001d050897_T002	0.409417006	0.676224073	0.597311863	0.874571152	1.058458507	1.172962362
Zm00001d050897_T001	2.922013217	2.193388235	3.085059687	3.146593968	3.152390153	2.803374192
Zm00001d025875_T001	2.591620779	2.221467873	2.669674472	2.631278699	2.620277899	2.546478163
Zm00001d037250_T001	3.93547078	3.135048527	3.765999092	3.280896942	3.251513996	3.314809762
Zm00001d013431_T001	3.531964784	3.453972506	3.585969151	4.189763925	4.084841801	4.052474498
Zm00001d013431_T003	2.910336442	2.891091918	2.705348064	2.799379547	2.696817339	2.879059545
Zm00001d015399_T003	0.974240745	0.614535532	0.99501482	1.569256781	2.051702022	1.581330034
Zm00001d015399_T005	6.571915096	6.151087289	6.789021957	5.739469076	6.183987897	5.808682698
Zm00001d015399_T002	5.629881695	5.124687523	5.742598425	5.611039497	5.55147249	5.590713717
Zm00001d015399_T004	2.939603096	1.236138675	2.682835296	1.878258033	0.820214914	3.18079171
Zm00001d017087_T001	1.924088861	1.523199104	1.902081685	1.794449971	2.181369433	1.578457171
Zm00001d003012_T001	3.819674007	3.449251145	3.90408211	5.262483785	5.15653795	5.218266486
Zm00001d018732_T004	3.353086258	2.989095217	3.083280948	3.384638096	3.332410199	3.440459791
Zm00001d018732_T005	1.458539495	2.738567034	0.919801518	1.047037162	1.272416815	1.86218951
Zm00001d018732_T007	3.603763057	2.103275721	3.821817036	3.330112903	3.255122713	3.805572402
Zm00001d018732_T001	0.849354004	0.435209325	0.645620508	0.590929633	1.057402442	1.009765658
Zm00001d018732_T002	0.644680483	0.403855589	0.465202383	0	0.578101103	0.736694919
Zm00001d009747_T001	2.29604003	1.73679751	2.639125404	2.76809919	2.832863489	2.761027991
Zm00001d035683_T001	5.410636039	1.986499042	5.906538391	5.973087274	6.394335921	6.239112957
Zm00001d026551_T002	2.444913237	2.382250844	2.667143228	2.476858878	2.352835911	2.275450221
Zm00001d039415_T001	0.752832496	0.687981569	1.200567755	0.392744947	0.42016653	0.456184789
Zm00001d052929_T001	3.392027618	2.59005032	3.198352706	3.440827318	3.473371574	3.270378984
Zm00001d052929_T004	1.538937395	1.026093231	1.474012843	1.861451623	1.589341161	0.763119181
Zm00001d038097_T004	1.858335158	1.748089777	1.605699132	1.18538895	1.890568096	1.742053653
Zm00001d038097_T002	0.65288569	0.340652274	0.885654013	1.568804093	1.64927515	1.551907738
Zm00001d035948_T005	8.486261405	8.310920259	8.545870508	8.275377883	8.363009571	8.321795086
Zm00001d035948_T001	2.624204091	4.932654847	2.866764987	1.968878534	2.507361576	2.580503942
Zm00001d035948_T004	2.900715552	5.304485816	2.507912334	2.839110243	3.05521249	2.986641384
Zm00001d035948_T002	1.652002265	2.911028844	1.904398545	1.926278439	1.979053302	2.191028489
Zm00001d037926_T001	5.79747301	5.502590389	5.788134234	5.52613005	5.578860298	5.593851636
Zm00001d031759_T001	1.626676023	1.361947422	2.094018805	1.074036793	1.35391888	1.351402749
Zm00001d013234_T001	2.081848844	2.315228267	2.339119993	3.113960481	3.273778296	3.294834362

Zm00001d052618_T001	4.025686453	3.634195485	4.139022554	3.884916119	3.808380211	3.691419147
Zm00001d020584_T001	9.360158657	9.350348179	9.450649448	8.797399295	8.772000172	8.71691774
Zm00001d038546_T005	4.544393577	3.639032882	4.662992272	4.953690767	4.86241236	4.885102971
Zm00001d038546_T002	0	4.202092236	4.335848871	2.91094061	0	2.636429271
Zm00001d026684_T001	2.695183183	2.331542424	2.811071254	2.359552093	2.274148925	2.065299325
Zm00001d045800_T001	1.720832669	1.150843639	1.61688179	2.922010933	2.824362986	2.905748106
Zm00001d036467_T001	4.778388639	5.433469503	4.932921768	4.659570176	4.324136973	4.583404391
Zm00001d028181_T001	5.885679122	6.329129464	5.990011061	5.912807633	5.901032171	5.957125542
Zm00001d039967_T001	2.232103216	1.896129219	2.344727959	1.677088734	1.657787239	1.960432011
Zm00001d028205_T003	3.388440299	2.509742453	3.317325778	2.944671258	2.963361293	3.067306053
Zm00001d044021_T001	3.623843925	2.864235282	3.601501728	3.97543952	4.194226081	4.108596548
Zm00001d030992_T001	5.082336718	4.889886822	4.946044107	5.10039074	4.838146668	5.059939134
Zm00001d013810_T002	0.860845233	0.223187745	0.653266429	0.717770068	0.739766031	0.570470703
Zm00001d013810_T001	4.222489053	3.345033101	4.134617544	5.317604357	5.260312322	5.338636311
Zm00001d013810_T003	0	1.794938573	2.261671919	1.797684557	0.86857931	0.476494714
Zm00001d012979_T006	3.955332799	4.310248931	4.339692901	3.322985635	3.098661256	3.225140095
Zm00001d012979_T003	0.791761571	0.407308151	0.581864114	0.453183568	0.895266166	0.624615633
Zm00001d012979_T020	0.444064847	0.439652922	0.632092289	0.878920695	0.856801682	0.655647665
Zm00001d012979_T009	0	0	0.527686928	0.482542678	0.836660186	0.566840477
Zm00001d012979_T011	0.62124216	0.139536291	0	0	0.804732606	0
Zm00001d012979_T035	0.9149219	1.027282883	1.122219466	0.627827194	0.251199174	0.391176006
Zm00001d012979_T004	3.609177911	3.48544499	3.582859202	3.409551151	3.228363727	3.36574782
Zm00001d012979_T024	0.707151921	0.099941495	0.531863006	0	0	0.622537772
Zm00001d029130_T001	1.730699075	1.255619165	1.867722935	2.044947309	2.146815187	2.147263385
Zm00001d050253_T003	0.596328368	0.287731141	0.235746663	0.542830556	0.125488442	0.953922265
Zm00001d050253_T006	0	0	0.37361926	0.406283047	0.652477317	0
Zm00001d050253_T004	3.008908492	2.202676302	3.000315735	2.925870899	3.398417277	3.241550548
Zm00001d050253_T001	2.153741791	1.812286272	1.981739425	2.221334847	1.206584465	2.127213285
Zm00001d032561_T001	1.652071582	2.336227964	1.730092117	1.925531986	2.31342001	2.127577816
Zm00001d007441_T001	5.017365695	3.877835323	5.077058377	5.469097389	5.479617315	5.556404001
Zm00001d007441_T002	0.394893835	0.187929867	0.339796426	0.606473498	0.628168866	0.715107909
Zm00001d023762_T001	4.241861839	3.511746456	4.037110791	4.255349809	4.092715625	4.133167536

Zm00001d043228_T001	3.107840332	2.372825648	3.101614445	2.868567062	3.050393208	2.917283373
Zm00001d051901_T001	0.956226196	0.643490507	1.569714673	2.609840047	2.71579587	2.634079522
Zm00001d036050_T001	3.426294279	2.634295638	3.274927906	3.386811636	3.468029722	3.208383659
Zm00001d026235_T001	5.618496215	5.658747294	5.183722194	5.58807983	5.717496873	5.693320738
Zm00001d019414_T001	3.445039618	3.111987449	3.420533334	1.708915575	1.816016367	2.270711024
Zm00001d027402_T002	1.030312769	0.675525105	0.549886975	1.087449263	1.327879902	1.166842037
Zm00001d038576_T001	3.038463926	2.056549888	3.060732933	4.045541349	3.983346347	3.934738975
Zm00001d038576_T002	0.322621576	0.209191317	0.282697184	1.995288769	1.864903623	1.868770907
Zm00001d052185_T001	4.4973245	3.503030391	4.580469994	4.7217077	4.70281632	4.685985659
Zm00001d037290_T002	2.930327081	2.247192007	3.060442081	2.582791536	2.676012341	2.563286039
Zm00001d038478_T001	2.461429548	2.83566865	2.623432396	4.787174213	4.481011226	4.814136474
Zm00001d029705_T069	1.290726949	2.057559792	1.508137041	0.60029069	1.089744229	1.181234885
Zm00001d029705_T040	2.685636781	1.26329527	2.732902153	3.033171356	3.061722463	2.537426537
Zm00001d029705_T042	0	0.571653513	0	1.012311218	1.005336026	0.876795489
Zm00001d029705_T043	0.75304395	0.423194118	0.784017279	0.976648847	0.617987689	0.76138145
Zm00001d029705_T029	1.438737304	0	1.062588716	1.593609059	1.817912586	1.455206999
Zm00001d029705_T039	0.746236222	0.093387096	0.808041482	0.417460994	0.97864448	0.577937775
Zm00001d029705_T028	0.78347677	0.223103701	0.42771665	0.526246132	0.639746162	0.62285353
Zm00001d029705_T046	0.553748774	0.503283596	0.1433895	0	0	0.495434226
Zm00001d029705_T008	0.683626395	0.225648822	0.173393556	0.731281446	0.874327202	0.699158924
Zm00001d029705_T004	0	0.236928499	0.751250322	0.387696704	0.267233744	0.3346884
Zm00001d053726_T001	2.50022027	1.763304909	2.54221223	2.7747742	2.746481107	2.783743698
Zm00001d005638_T001	2.777409615	3.087346912	3.222650486	3.312736029	3.09925194	3.190139151
Zm00001d005638_T014	3.454766409	0	3.068897428	2.558408957	2.848891701	2.841098383
Zm00001d006109_T001	4.265503733	5.301392921	4.315749671	4.088998494	4.536767106	4.332234953
Zm00001d004523_T001	5.50404091	6.877193246	5.444013247	5.361956578	5.448752215	5.432495733
Zm00001d051307_T001	4.848829722	4.130368834	4.992644183	5.526057885	5.587460767	5.585607031
Zm00001d038535_T006	7.996933106	8.108221177	8.150220347	7.372239722	7.200013575	7.017606495
Zm00001d038535_T003	6.583173773	7.443265256	6.933674133	6.549072473	5.732216061	5.777532049
Zm00001d038535_T004	8.244541686	8.045908089	8.09602589	7.297268576	7.453543481	7.569819829
Zm00001d038535_T005	0.842071307	0.805977521	0.995141488	0.944197278	0.877072013	1.158322807
Zm00001d025079_T001	3.965378888	3.576626618	3.668525932	4.024291751	4.100892311	4.018120666

Zm00001d043157_T002	3.889533897	3.839613783	4.096662705	4.191718582	3.899422763	4.163874332
Zm00001d017847_T001	6.693261419	6.328103965	6.824358786	6.796281027	6.766346418	6.592645054
Zm00001d017847_T002	4.542526812	5.253029381	4.483211879	4.12279661	4.563935475	4.697755474
Zm00001d024298_T001	3.034197048	2.856144719	2.47143461	2.380774878	2.051956023	2.210557813
Zm00001d028826_T001	3.041510571	2.407831576	3.104325588	4.662075412	4.89179522	4.718181257
Zm00001d024034_T001	2.305761906	1.762102569	2.235960751	3.042373632	2.941015388	2.989565757
Zm00001d047124_T001	3.129108234	2.217613833	3.324257464	3.372299117	3.477104021	3.409912249
Zm00001d018475_T001	1.087699084	0.498798222	0.822152546	2.428950899	2.080180097	2.474787491
Zm00001d011984_T004	0.984496023	0.454684433	1.152892097	0.986099971	1.090724734	0.749539416
Zm00001d011984_T001	1.291019997	1.381442309	2.064243428	2.31646814	2.762221657	2.657384139
Zm00001d011984_T003	2.165436696	1.512898386	1.094319183	0	0	0.646695388
Zm00001d011334_T003	2.316022326	1.612101922	2.125158108	2.685842396	3.006512033	2.572913915
Zm00001d011334_T002	2.542336065	2.18343411	2.798894727	3.181372613	3.376577254	3.434243502
Zm00001d045336_T001	5.88606229	5.242503198	5.983077876	5.970629872	5.918684475	5.868802804
Zm00001d032440_T005	2.195507294	0	2.395246553	3.711592478	3.780165192	3.953980821
Zm00001d032440_T004	0.385465275	1.649962124	0	1.104790199	1.243993773	0.716672259
Zm00001d032440_T006	2.992659318	2.396740945	2.916691408	2.253301327	2.310885682	0
Zm00001d032440_T009	0.81953903	0.286301588	0.124639141	1.32089822	1.522824613	1.343545405
Zm00001d052726_T015	0.373189371	0	0	0.54454968	0.50728926	0.290095248
Zm00001d052726_T045	6.15250229	5.173963498	6.229398787	5.712691407	5.81101837	5.438628698
Zm00001d052726_T028	0	0	0.698933202	3.422934529	3.395547913	4.402130709
Zm00001d052726_T004	0	0	0	5.481380485	5.411496447	5.259401985
Zm00001d052726_T007	1.622981875	0.309812975	0.515262328	2.687581443	1.313417107	2.632541604
Zm00001d052726_T003	0	0	0	1.510389284	1.110709342	0.819372258
Zm00001d052726_T012	0	0	0	0.411770065	0.884420528	0.637830007
Zm00001d052726_T011	3.014808921	2.29408871	2.763157221	2.739558239	2.702977959	2.192318541
Zm00001d052726_T009	0	0	0.405977116	1.037138999	1.807122011	1.357439963
Zm00001d052726_T016	0.404047494	0.180883664	0.162054026	0.756105264	0.642677546	1.251396741
Zm00001d052726_T024	1.048681204	0.677066177	0.996239769	1.075755778	0.947803033	0.817709203
Zm00001d052726_T029	0.272756597	0.19106735	0.338444953	0.552098638	0.475134702	0.177000552
Zm00001d052726_T018	0.299677226	0	0.13661665	0.821686544	0.143990227	0.811908294
Zm00001d052726_T032	0.226640463	0.029683275	0.196235622	0.57046876	0.812194252	0.678729985

Zm00001d052726_T008	0.177401199	0	0.392443825	3.133743523	2.371816155	3.292914677
Zm00001d052726_T034	0.38648651	0.153998547	0.521486998	0.866828079	0.864301457	1.068508477
Zm00001d052726_T039	0.827466232	0.419350115	0.709626784	1.001692731	1.010749051	1.150936548
Zm00001d052726_T006	1.53187997	0.273966978	1.076011054	1.958742843	1.932991667	2.564819812
Zm00001d052726_T010	1.48019583	1.963862859	2.173545147	3.458778192	3.524479971	3.168503407
Zm00001d052726_T001	0	0.297290039	1.091870409	0.548433665	2.5831503	0
Zm00001d052726_T030	0	0	0.441301803	0.453182514	0.760103397	0.173558578
Zm00001d003396_T001	1.367144578	1.199419822	1.635985575	1.625549648	1.603767092	1.617574957
Zm00001d006499_T002	1.664169236	1.132536045	1.596584084	1.174959229	1.543435997	1.533667498
Zm00001d030143_T001	0.940490028	0.585546193	1.138699154	1.202148122	0.963334287	1.014962954
Zm00001d020626_T001	3.632923097	4.188826343	3.375143163	3.607780424	3.553307907	4.089705761
Zm00001d001865_T001	3.617329401	3.989190791	3.61971537	4.134588632	4.338219656	4.302991396
Zm00001d035937_T001	4.907738507	3.891767861	4.833602327	4.522782941	4.484843448	4.460703253
Zm00001d002161_T001	9.13170708	8.232810409	8.94395778	3.785100202	3.480190141	3.892885149
Zm00001d038574_T004	2.956552772	2.141505382	3.010429683	2.232988549	2.595343485	2.296641238
Zm00001d038574_T002	0.86048374	0.828629145	1.63549856	1.119755229	1.772307975	0.779341257
Zm00001d038574_T001	2.81484611	2.500099907	2.840830785	2.373344747	2.437129975	2.817821742
Zm00001d034022_T001	3.773848884	3.432419498	3.644334277	3.558933349	3.990500839	3.78618388
Zm00001d025003_T004	3.984359667	3.200623778	4.106979319	3.692679491	3.940980633	3.663481481
Zm00001d025003_T001	2.323912455	1.472513246	2.192690973	3.224408312	3.199114945	3.20185605
Zm00001d039619_T005	2.479524711	2.67562514	2.55676954	2.205033359	2.457784432	2.615225589
Zm00001d039619_T001	0.449130284	0.243753154	0	0	0.608479041	0
Zm00001d039619_T003	0	0	0.595242023	0.416242007	0	0.722752786
Zm00001d039619_T009	2.338257529	2.521690508	1.907616377	2.582377281	0	0
Zm00001d015747_T012	0.459563816	0.359247898	0.584826881	0.211032136	0.685591259	0.586587022
Zm00001d015747_T024	1.455589989	0.41123532	1.025266979	1.618920837	1.604188063	1.478192669
Zm00001d015747_T008	1.26324839	0.604364579	1.373339178	1.42630931	1.70009787	1.92425042
Zm00001d015747_T022	0.624209473	0.069387337	0.214174553	0.840232787	0.896133871	0
Zm00001d009658_T001	0.553536467	0.425547389	0.4836863	1.916439191	1.599217801	1.759590921
Zm00001d037307_T001	3.21999339	2.287094284	3.135328498	3.879184459	3.952105215	3.815560367
Zm00001d044671_T001	2.040110394	3.190829431	1.86949677	2.561597174	2.29142763	1.572179057
Zm00001d010863_T001	4.272337979	3.372335061	4.300141374	4.045172527	4.255412809	4.112033587

Zm00001d010863_T002	2.369291702	2.429163603	2.329856385	2.493300964	0	0
Zm00001d045403_T002	5.90684087	5.371724519	5.751143584	5.695104545	5.621433305	5.619162249
Zm00001d012232_T003	1.272229871	1.043686763	1.00623482	0.888106131	1.788939569	1.310426796
Zm00001d012232_T001	1.667947062	1.117883179	1.712462399	1.930825505	1.745301881	1.970490727
Zm00001d012232_T002	3.40356414	3.749004718	3.870711023	3.770415097	3.20127424	3.770025141
Zm00001d050173_T001	1.487236274	1.362729649	1.765859316	1.432522792	1.594453481	1.689741072
Zm00001d024982_T001	3.830495686	4.841794532	4.009523736	3.122923636	2.96161518	3.05199725
Zm00001d024982_T002	3.875207294	3.795837585	3.89125284	3.035719265	2.781628922	3.06825273
Zm00001d007199_T001	3.253051966	3.096957897	3.176581605	4.20453648	4.372950636	4.52640033
Zm00001d007267_T002	2.106450169	1.330164832	2.244610973	1.14191666	0	1.330287618
Zm00001d050026_T005	1.486077954	0.753649032	1.236335865	1.999483423	1.54746661	1.569460954
Zm00001d050026_T003	0.444282226	0.270659302	0.545095571	0.945157446	0.936693337	1.237990712
Zm00001d050026_T001	1.51196083	1.386611217	0.916756366	1.519695878	1.683789413	1.971925708
Zm00001d012647_T001	3.675731151	3.073156304	3.56357611	4.513054966	4.412248714	4.354756099
Zm00001d012647_T002	0.580235228	0.586862721	0.909428275	1.657160246	1.947674385	1.970941612
Zm00001d039002_T004	3.840634031	3.029193654	3.800078404	3.970212948	3.849354004	4.126704721
Zm00001d039002_T021	0.82531754	0.710807536	1.359499243	1.217169284	1.166004526	0.696862731
Zm00001d039002_T009	0	0	0.739128303	2.16575247	1.305068153	2.009058318
Zm00001d039002_T016	0	0	0	1.295518506	1.385916916	1.615782575
Zm00001d039002_T027	0.670959951	0	0	0.757438922	0.944828467	0.755167037
Zm00001d039002_T012	0.251834201	0	0	0.715078028	0.432625946	0.421401597
Zm00001d008239_T001	3.428241167	3.087106876	3.537374724	3.483975287	3.406359792	3.547951699
Zm00001d030672_T001	3.096886571	2.142258867	3.019708141	3.82203912	3.863618961	3.846953381
Zm00001d041504_T004	1.61800414	2.967215836	2.524970593	0	0	0
Zm00001d041504_T002	3.986330834	1.732327818	3.853830716	3.944668916	3.840751039	3.743536403
Zm00001d010103_T003	3.706916951	2.553182798	3.881750063	4.090803223	4.032306651	3.8094732
Zm00001d045738_T001	3.528526062	4.493175922	3.500767897	4.115097933	4.026140517	4.253645338
Zm00001d016286_T011	0	0	1.499732635	1.806686181	0.796320318	0.947130493
Zm00001d016286_T008	0	0	0.950481852	1.29860436	1.548518498	1.897769768
Zm00001d016286_T001	1.536018574	1.04076551	1.602057739	1.492430572	1.289747134	0
Zm00001d007932_T001	2.006118821	1.395806446	1.823771367	1.987532615	1.999910189	1.840648531
Zm00001d007932_T002	0.611889063	0.385400112	0.503394534	0.844927918	1.097140833	0.867515384

Zm00001d016431_T001	1.476319468	0.963640579	1.345968571	0.854823114	0.6826497	1.002570035
Zm00001d009918_T001	2.052399199	1.820154041	2.00787183	1.811726665	1.922179957	2.113920153
Zm00001d046047_T012	0.980251286	0	0	1.696803098	1.096005411	0
Zm00001d046047_T009	0.161157607	0.408340136	0.601484494	1.089814723	1.432733902	1.190380976
Zm00001d046047_T007	2.019920704	1.4637731	2.424974744	2.01763049	1.492774588	2.343077173
Zm00001d046047_T021	0.965630635	0	0	2.236172971	2.65779867	2.28901464
Zm00001d046047_T001	0.532399753	0.504255287	0.40942461	0.59994235	0.902114495	0.95916699
Zm00001d046047_T022	0.905546532	0.345226035	0.818547923	1.21988903	0.840598584	0.911191489
Zm00001d006180_T001	2.106826012	1.597204579	2.156274931	2.724379386	2.746038822	2.680171527
Zm00001d028050_T001	3.33591624	2.475586106	3.275817739	3.417201856	3.458323991	3.395238189
Zm00001d041961_T001	3.061694471	2.93719616	3.101652597	3.346184504	2.949804024	2.962071439
Zm00001d053595_T002	2.920904744	1.875849641	2.049864925	2.854418217	2.689600865	2.82073183
Zm00001d053595_T005	0	0	0.294802462	0.268382889	0.358134032	0.518712381
Zm00001d053595_T003	0.3143659	0.188229994	0.199015818	0.381328785	0.255442722	0
Zm00001d053595_T004	5.547743255	4.710930053	5.729740504	6.039459235	6.001294216	5.97824854
Zm00001d036620_T001	2.740467594	2.262346557	2.78312596	2.903410661	2.819682079	2.954742942
Zm00001d027652_T001	10.65472135	10.29690178	10.73557974	10.13434313	10.14606157	10.17395378
Zm00001d010536_T001	3.311117687	3.395875252	3.353234779	3.124888559	3.432233068	3.057816402
Zm00001d043767_T001	1.131689567	0.496366218	0.942048264	0.990469162	0.604553407	0.988665784
Zm00001d043767_T003	2.14236867	1.469028959	1.603937963	1.421064914	2.060268788	2.218102585
Zm00001d043767_T006	3.342524168	2.26374626	3.322881978	3.548923236	2.652391957	2.761353364
Zm00001d043767_T004	1.33818649	2.627714219	2.042650991	1.284243454	1.846166729	1.351467769
Zm00001d029572_T001	3.392095643	3.494940748	3.336709134	3.794741697	3.913425416	3.864641197
Zm00001d006503_T001	2.975304966	2.128282841	3.00199549	4.682231684	4.707446713	4.693271889
Zm00001d030363_T001	6.861944946	7.03548952	6.830803318	6.367604873	6.416939278	6.457962027
Zm00001d002021_T001	7.027084244	8.088431137	6.992494059	6.798930332	6.805105657	7.03009034
Zm00001d022622_T007	1.132899891	0.862281571	0.849996058	0.865544338	0.83091242	0.770470494
Zm00001d022622_T001	0	3.258784224	0	4.690950046	4.277998133	3.888590522
Zm00001d022622_T003	5.168404843	0	5.275092668	4.774483311	4.989370202	5.241766102
Zm00001d022622_T004	4.255740835	4.484302891	4.173638844	4.609018064	4.682661553	4.550108257
Zm00001d047330_T001	1.156147408	0.640164635	0.76735055	2.435105822	2.147443146	2.109456179
Zm00001d004287_T001	1.048636569	1.354954224	1.58331689	3.362720112	3.616413926	3.618505201

Zm00001d006880_T001	2.104607391	1.539190581	2.153704182	2.291822774	2.146216939	1.900727723
Zm00001d012138_T001	3.011234234	2.292705785	3.026045058	2.271428963	1.973068992	2.155660195
Zm00001d047367_T015	0.760374258	0.593273446	0.713144142	1.822613096	1.681700225	1.590761525
Zm00001d047367_T001	1.645301854	1.540141867	1.437766252	2.851865951	2.642409538	2.789517056
Zm00001d047367_T016	1.754066963	1.008440993	1.697185325	1.770684452	1.983438746	1.952155362
Zm00001d047367_T019	2.905794693	3.280493746	3.429225882	1.989860901	2.068768133	2.036380598
Zm00001d047367_T010	0.255048668	0	0	0.718442733	0.782186272	0.794474925
Zm00001d047367_T006	2.699985753	1.989220407	2.528163706	3.190692444	3.122473444	3.215601102
Zm00001d047367_T014	0.924768807	0.186352224	0.843492682	0.88299434	0.933033226	1.303785423
Zm00001d047367_T012	0.499708657	0.165385882	0.467069714	1.523151921	1.426960302	1.603714402
Zm00001d047367_T003	3.477541081	0.614475225	3.384080346	3.736913079	3.580928742	3.33040437
Zm00001d047367_T008	0.80491264	0.049509493	0.5967539	0.916638676	0.966714696	0.839022741
Zm00001d047367_T013	1.524358104	1.481516466	1.152002949	2.291698438	2.378991517	2.200413641
Zm00001d047367_T002	3.101218917	1.998826973	3.073907615	2.796185942	2.969411343	2.715720544
Zm00001d047367_T020	0.950637123	2.691583073	1.868791448	0.341407415	0	0
Zm00001d012413_T001	1.537035091	1.085571502	1.806021256	1.91266251	2.038970596	2.205510439
Zm00001d038381_T001	8.883552913	8.828362257	8.956466486	8.587120679	8.55490115	8.545187093
Zm00001d053735_T001	0.904482556	0.510758409	0.968008152	1.323768953	1.480075853	1.318131702
Zm00001d053217_T001	6.034468852	5.540083608	6.093067546	5.9598962	5.92555997	5.802741492
Zm00001d029047_T001	6.357563529	5.436791581	6.415201039	7.032540011	7.007784683	7.06462395
Zm00001d005682_T001	4.6603225	4.086140897	4.657406892	5.292419484	5.303896305	5.399226954
Zm00001d051534_T001	1.635744642	1.599925693	1.630887221	2.337251526	1.999121131	2.124738715
Zm00001d035171_T001	4.539333785	5.930961364	4.774973135	4.139974563	4.286681581	4.097848754
Zm00001d044654_T001	0.693812984	0.472664523	0.870692976	0.141186871	0.256226883	0.204764247
Zm00001d022272_T004	3.194286134	4.786413818	3.493130822	2.624265631	2.271755833	1.57491384
Zm00001d022272_T003	2.378908032	1.415712162	2.440767798	2.479931816	2.672161899	2.50667607
Zm00001d022272_T001	1.664435963	1.703689887	1.641867825	2.016397546	1.42005709	1.416137676
Zm00001d022272_T002	1.984580966	1.35608689	2.236125507	2.828874853	3.002554193	2.823576553
Zm00001d044316_T001	1.567947438	2.227553489	2.320417668	2.180221713	2.310396257	2.656832236
Zm00001d048776_T001	3.401985227	2.594494567	3.417524454	2.90011617	2.875570326	2.840336936
Zm00001d047932_T001	4.177297778	4.192821932	4.053033464	4.397432659	4.278001777	4.408576415
Zm00001d011352_T001	3.24848292	2.375619174	3.418402625	2.967893094	3.299934038	3.141492793

Zm00001d031213_T001	3.027371006	2.407540384	3.164568281	1.88877052	2.1124506	2.185499325
Zm00001d025617_T009	1.098915374	0	1.042387291	0.213757866	0.671139356	1.225339103
Zm00001d025617_T005	0	0.303565679	0.904474078	1.297301779	0.906591253	0
Zm00001d025617_T002	0.957147185	0.247518034	0.450055702	1.013702361	1.357378026	1.55191069
Zm00001d025617_T006	0.716572178	2.827730428	0	0.243084086	1.333221055	1.285780969
Zm00001d025617_T003	7.712777062	5.064356572	7.834844149	7.768929986	7.854295841	7.761797753
Zm00001d025617_T001	7.527861806	7.437491594	7.341081023	7.244609926	7.172721828	7.221210821
Zm00001d034797_T001	3.039223865	2.12412065	2.967551369	2.785120713	2.662348167	2.594167762
Zm00001d036708_T001	6.510646311	7.324709381	6.37744936	6.485858396	6.543926385	6.486319846
Zm00001d009364_T001	3.323275087	2.711830982	3.306310222	3.604465282	3.549168638	3.532764288
Zm00001d027734_T006	2.689417703	0	2.318145877	2.195270737	3.159278318	2.154328178
Zm00001d027734_T001	0	1.640982713	0	3.299702572	2.280269831	3.091901036
Zm00001d027734_T007	1.970827892	2.195085495	2.26313479	2.480538381	2.067497263	2.224550624
Zm00001d027734_T002	3.158466134	2.264805729	3.096025827	2.992545485	2.889706964	2.875973851
Zm00001d027734_T005	4.652120239	3.845584689	4.694254908	3.67197832	4.123386353	4.203921228
Zm00001d050043_T006	1.418865117	0.961083189	1.633962619	1.038501502	0.476523746	0.959290166
Zm00001d050043_T005	4.886987279	2.296509975	4.783386823	4.693324084	4.471169053	4.38247857
Zm00001d050043_T004	4.977754011	4.995369868	5.340537595	4.899925308	5.052885876	5.06636741
Zm00001d050043_T002	1.238490343	0.78969587	0	0	1.170836497	0
Zm00001d039480_T001	0.765508438	0.286939091	0.626156878	0.75699849	0.979056596	0.7602712
Zm00001d023994_T004	5.105347199	6.654589246	4.976354607	2.542760491	2.816665031	2.828979825
Zm00001d023994_T003	3.69056985	3.125620522	3.912040758	3.187244533	3.187230753	3.217781162
Zm00001d023994_T002	8.943005281	7.914716229	9.009044026	8.404447913	8.431319869	8.391187475
Zm00001d005208_T001	3.311635492	2.903508805	3.467560296	6.738541567	6.813074919	6.774952492
Zm00001d032806_T001	2.99020755	2.341149482	2.958877559	2.777468743	2.967726022	2.509549158
Zm00001d014765_T001	3.716009199	3.411830523	3.698436867	3.569319008	3.628268155	3.598578997
Zm00001d011912_T004	3.351199047	2.757401157	3.244528167	2.285287982	2.207669248	2.151382168
Zm00001d042772_T002	6.65338066	6.177276926	6.45603672	6.306226325	6.158737841	6.266284452
Zm00001d042772_T004	7.244681845	6.53786803	7.414995032	6.356718995	6.744943989	6.363032672
Zm00001d042772_T003	0.898640999	1.076695902	0.438985818	0.551086775	0.826643257	0.527064328
Zm00001d036726_T001	1.174044581	0.730409541	0.870209245	5.0156142	5.249057422	5.085617473
Zm00001d025901_T001	4.495972356	3.854475667	4.664802121	4.156989608	4.335142279	4.312143671

Zm00001d033483_T001	10.75641718	10.19767032	10.55132281	11.00516456	11.0322328	10.97479154
Zm00001d041708_T002	0	0.495997994	0	0.41645281	0.913316454	1.858375344
Zm00001d041708_T001	2.731958916	1.99552902	2.868274084	2.744965981	2.939308397	2.185212587
Zm00001d036454_T004	1.859229331	1.588243303	3.086572169	1.134808414	2.373964136	1.704102174
Zm00001d036454_T002	0.81865917	0	0.901887513	0.636668698	1.42854764	0.330809631
Zm00001d036454_T005	3.792295965	3.343689227	3.18916654	2.688964506	2.909497785	2.46268372
Zm00001d036454_T003	5.679116832	5.111761742	5.765745433	4.875822567	4.709535972	4.937867227
Zm00001d036454_T001	0.973631111	0.552681023	1.041043879	0.985340851	0.782247511	0.899655933
Zm00001d008369_T006	3.818766728	2.493887336	3.348404104	4.013437068	3.81236812	3.535867079
Zm00001d008369_T008	4.622023873	3.952348198	4.538462302	3.564819568	3.670782224	3.853907746
Zm00001d008369_T004	1.400812781	1.170261591	1.890838492	1.284143344	1.226375352	1.864812918
Zm00001d053883_T001	4.096153714	3.775370133	4.242300827	4.006296228	3.803995098	4.012571623
Zm00001d052933_T001	2.514129584	2.180729979	2.672711816	2.408155324	2.259504505	2.51241249
Zm00001d042050_T001	2.203941581	1.899331495	2.463447994	2.24321634	2.261663796	2.097131729
Zm00001d033682_T004	3.545147974	2.241561378	3.625089732	3.497916455	3.290515086	3.221498962
Zm00001d033682_T002	2.722835613	2.325308406	2.71863737	2.977890581	3.04573197	3.015715392
Zm00001d033855_T003	5.343478783	5.390978737	5.249612129	5.837590041	5.857018894	5.951895099
Zm00001d033855_T002	4.04605768	3.569215456	4.181379293	5.161947605	5.052529535	4.884724115
Zm00001d033855_T004	6.390896407	5.137941061	6.32371274	7.481412721	7.455229517	7.458352571
Zm00001d050008_T001	2.856846691	2.429111636	3.16662676	4.698572606	4.768739166	4.575206666
Zm00001d011283_T005	1.725861103	0.978223458	1.624263759	1.792695877	1.973020118	1.683546451
Zm00001d011283_T002	3.971523781	3.354239859	4.041749116	3.610980283	3.568531244	3.169923238
Zm00001d011283_T006	1.087269337	0.911225243	0.959469717	1.139679041	1.799720626	1.946638046
Zm00001d043814_T001	1.162564542	0.958762513	1.220942815	1.881803597	1.817399367	1.584541172
Zm00001d043814_T002	3.021926148	2.592182406	2.959122837	3.17771022	3.42580624	2.939462431
Zm00001d043814_T003	3.001379999	3.593287671	3.310981919	2.416112808	0	3.087357096
Zm00001d052666_T009	0	0	0	1.173465196	2.201684982	0.903894375
Zm00001d052666_T103	2.830238082	3.222718176	2.786976431	2.705370626	2.777785801	1.983896909
Zm00001d052666_T100	1.574862992	0	1.937320661	0	0	1.730737762
Zm00001d052666_T089	0.904414731	1.131432103	0	1.517538756	0	0.387505921
Zm00001d052666_T017	0	0	0	0.875036903	0.99094615	0.551399859
Zm00001d004733_T001	5.635776124	6.746774836	5.655757014	4.916489973	4.802322278	4.5904816

Zm00001d028890_T014	1.674219278	0	2.012873985	2.412634945	2.016402895	2.166018349
Zm00001d028890_T008	0.429740211	0.185970542	0.513553404	0.361434873	0.581353176	0.521200528
Zm00001d028890_T018	2.270083956	0.175934597	2.084628303	2.319337287	2.616614592	2.557915123
Zm00001d028890_T005	0.483727569	1.24699052	0.583541227	0	0.949365348	0
Zm00001d022321_T010	1.314213903	0.831860221	0.848695643	1.369427955	1.355251994	1.544369649
Zm00001d022321_T012	1.330937524	0.573584904	1.492553114	1.091718788	1.658647025	1.5847961
Zm00001d022321_T006	2.445936154	1.775233896	2.274095537	2.02157922	1.756534867	2.522936318
Zm00001d022321_T001	1.542647339	1.25870886	1.800926813	2.167823228	1.996748833	1.615130461
Zm00001d021774_T001	5.906261804	5.620924935	5.893314055	5.885164632	5.935007397	5.978326803
Zm00001d003047_T001	1.205544532	0.737113896	1.39653053	1.566018225	1.369038148	1.588375724
Zm00001d013534_T002	2.282048015	2.452484738	2.833233183	2.037540716	2.018195816	2.053630266
Zm00001d013534_T001	1.200998308	1.206188091	1.014982229	1.223843249	1.370727296	0.982502633
Zm00001d011971_T001	4.723697964	4.258639069	4.862648961	4.542477162	4.742048747	4.609933515
Zm00001d014671_T001	4.748854593	4.08034624	4.721090273	4.987582592	4.951792734	4.88562151
Zm00001d005264_T001	4.240649011	3.176673669	4.155604341	4.30765504	4.320741207	4.248068207
Zm00001d017593_T002	1.171515577	1.885874585	0.570982596	2.424049222	2.603238924	2.458922389
Zm00001d017593_T001	0.746734118	0.39442524	0	1.422466612	1.768872419	1.766910591
Zm00001d019312_T001	4.016896874	3.95855188	4.159121102	3.393330526	2.564162785	3.136765757
Zm00001d019312_T002	5.934481542	5.345235298	6.062581463	4.908366535	4.936889288	4.897406713
Zm00001d039634_T001	3.852021317	3.261052796	4.116350034	3.799139022	3.697808063	3.73655628
Zm00001d032668_T001	6.640064252	6.302789861	6.662244501	5.942967417	5.999543405	5.972024656
Zm00001d030603_T001	2.50262848	2.916052946	2.365540254	1.785967293	1.788933307	2.130147056
Zm00001d040244_T003	0.502579093	1.183964105	1.84781215	0.927924511	1.862997618	1.84333549
Zm00001d040244_T001	6.051668855	5.924118489	6.044456722	6.127753424	6.253983954	6.07521522
Zm00001d037810_T020	0.549001755	0.295219903	0.656479896	0.381577973	0.178096325	0.605666897
Zm00001d037810_T005	0.296054424	0	0.414568547	0.41887429	0	0.360446313
Zm00001d037810_T024	0.935492185	0.945713317	1.244117417	1.003256092	1.296755754	1.066226305
Zm00001d037810_T026	2.915477686	2.139450503	2.970963876	2.044879832	2.325364538	2.087100594
Zm00001d037810_T016	1.290857855	0.653068274	1.13238798	0.657616227	0.620309574	0.514463668
Zm00001d037810_T001	1.513598375	0.809214388	1.229031655	0.681999701	0	0.395463188
Zm00001d042078_T001	3.171795285	3.865882005	3.613302383	3.300949559	3.612733117	2.823914609
Zm00001d053731_T001	5.055579882	5.148552561	4.950735419	4.832634356	4.869414744	4.978493841

Zm00001d007502_T001	1.125116108	0.885330706	1.24250739	1.316060569	1.275075008	1.316857065
Zm00001d036016_T001	2.939779662	3.068289194	2.588057606	4.538979099	4.439472278	4.46323865
Zm00001d031640_T001	6.4635196	5.800657507	6.453898944	5.967850291	5.929796323	5.949649013
Zm00001d014497_T001	6.699696927	6.053618233	6.647963562	6.772019188	6.621675981	6.717219198
Zm00001d013009_T001	7.385014002	7.20228716	7.088136497	6.963973836	7.123744187	7.097967508
Zm00001d044037_T001	5.740873445	5.161285655	5.748086932	5.590889045	5.669417499	5.705754598
Zm00001d040191_T006	0	0	0	0.959399977	1.083194302	1.056113882
Zm00001d040191_T001	4.727762566	3.96045138	4.76743859	5.114615144	5.125188367	4.941155527
Zm00001d040191_T007	1.018094309	0.36438529	0.995992403	0	0	0
Zm00001d040191_T008	4.751679766	4.644109562	4.853300472	5.089611493	4.71748034	5.030155554
Zm00001d020505_T001	2.196365001	1.520126513	2.234053053	3.157869465	3.287624181	3.245052625
Zm00001d002462_T021	1.025758819	0.35537943	0.404819217	1.565548923	0.816172057	1.517920163
Zm00001d002462_T067	0	0	0.735092337	0	1.084181309	0.968384977
Zm00001d002462_T074	0.818651809	0.534932138	1.027998326	1.737943266	1.009739148	1.694412216
Zm00001d002462_T060	0	0.161816755	0	0.309318238	0.260810943	0
Zm00001d002462_T191	2.404587089	0	3.268137619	3.406304411	4.005517483	3.633103806
Zm00001d002462_T179	0.384291894	0	0.79950513	0.916489638	1.14555747	1.113671832
Zm00001d002462_T165	0	0	0	1.224472524	1.354669367	0.932597923
Zm00001d002462_T011	0	0.188264182	0.648116577	0.929114552	0	0
Zm00001d002462_T185	0.857586936	0.40834231	0.531735275	0	0	1.303461055
Zm00001d002462_T181	0.575362679	1.118119811	0	0	1.429149942	0
Zm00001d002462_T189	0	4.47295787	1.965697859	3.379021748	2.90668668	2.649284118
Zm00001d002462_T149	0.606897944	0.22179296	0.415195105	1.094930559	1.123492718	0.860919109
Zm00001d002462_T176	2.32140026	0	1.863827073	2.817494944	2.930639893	2.856310681
Zm00001d002462_T187	1.20464094	0	1.071350804	0	0.756804692	1.421995044
Zm00001d002462_T073	1.138313173	0.510088951	1.802085657	0.850350586	0	1.390990083
Zm00001d002462_T079	0.178506865	0	0	1.064440045	0.743517553	0.914074613
Zm00001d002462_T171	2.855002788	2.128502596	2.401563572	2.595861664	2.310976104	2.5509275
Zm00001d002462_T116	0	0.097488095	0	0.707003454	0.733037564	0.645886076
Zm00001d002462_T001	0	0.030727336	0.066016149	0.530838843	0.614084109	0.482689294
Zm00001d002462_T190	0.535401053	0.061220437	0.757089831	0.925928041	0.995624892	0.859176046
Zm00001d002462_T087	0.112521328	0	0.423704015	1.456882355	1.348595597	1.600530005

Zm00001d042372_T002	6.477414277	6.533987576	6.454105896	6.522170166	6.612005054	6.533029184
Zm00001d037050_T001	1.949859106	1.763790153	1.225455101	1.349755341	1.284633168	1.573018172
Zm00001d037050_T002	2.638072678	1.615651705	2.906194279	2.324979916	2.599466817	2.415621312
Zm00001d006755_T001	2.029326015	1.539219868	2.292163613	2.40633666	2.477088474	2.42592491
Zm00001d018282_T001	4.957221261	3.939693168	4.867157388	5.170518108	5.213800746	5.246878217
Zm00001d045978_T005	0.975847326	0.449669127	1.123983965	0.936234258	1.167226884	1.094443506
Zm00001d051474_T001	2.440105196	1.911081783	2.554009202	2.128923205	1.778211941	1.861172882
Zm00001d014491_T001	6.97079892	6.794115027	6.801136185	9.653957346	9.629186684	9.655303267
Zm00001d012435_T001	1.767468782	0.970233015	1.259716603	0.974184933	1.125510263	1.052036737
Zm00001d008663_T002	4.099657348	3.374921925	4.191857523	4.434791068	4.276781603	4.372850298
Zm00001d005008_T003	0.477239013	0.221237391	1.430884175	0.421825974	0	0.207740455
Zm00001d005008_T004	1.872430856	1.229786011	0.98899364	0	1.94190746	1.735181609
Zm00001d029120_T005	3.213189082	3.656262958	3.623921044	2.463140345	2.836072781	2.76806361
Zm00001d029120_T002	2.204839666	0	1.700652777	3.59394065	3.388085086	3.046141258
Zm00001d029120_T001	0.476226133	0.310717034	0.126963608	1.071531353	1.100350662	1.632569288
Zm00001d029120_T004	0	0.451308717	0.710332353	0	0.704873735	0.280838756
Zm00001d029120_T014	1.736266295	0.34361476	1.598203203	0.868900862	1.063265465	0.809897594
Zm00001d029120_T010	3.474363225	0	3.446716363	4.270385039	4.022160583	4.132896026
Zm00001d029120_T008	0.401245988	0	0	0.466686628	1.208073333	0.565404154
Zm00001d052213_T004	0	1.604707583	1.843001754	1.730453453	0	0
Zm00001d052213_T001	3.33218847	2.34963505	3.415443651	3.414928662	3.365971168	3.342650884
Zm00001d052213_T003	2.158234251	0.696972198	1.582848366	2.217057893	2.638817589	2.850312176
Zm00001d047560_T001	6.212120469	6.425739703	6.285849551	4.904763795	4.855609146	4.820072378
Zm00001d012821_T007	1.756072804	1.220824597	0.475416976	2.649634542	2.619346568	2.036575071
Zm00001d012821_T002	1.355166843	0	0	2.552006881	2.708689388	2.627409096
Zm00001d012821_T009	0	0	1.938853724	0.957924996	0	1.311222338
Zm00001d012821_T003	0.386354067	0.753387174	1.015621737	0.287514848	0.289443793	0.544633753
Zm00001d012821_T004	4.68006708	3.348180286	4.635233511	4.279697785	4.451553427	4.34300246
Zm00001d012821_T008	2.841637658	3.202976667	3.060245611	2.958067958	2.365960114	2.924895145
Zm00001d012821_T005	2.220290946	0.964485136	2.396018063	2.213390212	2.92839098	2.539442234
Zm00001d038865_T001	6.968160027	6.620982481	7.008577433	6.582773204	6.352319531	6.293275824
Zm00001d038865_T012	1.909162877	7.982614969	1.851744645	1.404070935	2.260132574	1.906382293

Zm00001d038865_T002	2.02010318	5.477708279	1.862603737	1.950534855	1.956617261	1.344986958
Zm00001d038865_T006	6.491822623	6.166934624	6.757879579	6.067698319	6.073385346	6.100606627
Zm00001d038865_T009	3.179175656	2.839004997	2.797368522	1.756805972	2.693429416	2.741879619
Zm00001d038865_T013	9.506912646	10.06105676	9.494400545	8.632784232	8.744511549	8.556386952
Zm00001d009011_T002	1.80484575	1.296588382	1.901507584	1.469335876	1.479866904	1.494819048
Zm00001d038531_T001	2.87871063	2.460300122	3.160924866	1.617407574	2.029083904	2.698703889
Zm00001d038531_T004	0.880296053	0.340679617	0.751631676	0.919983002	0.835151197	0.845528589
Zm00001d038531_T003	4.403640484	4.003561312	4.61674908	4.867260204	4.793391735	4.80774996
Zm00001d044301_T002	4.316095624	4.251430297	4.195527058	4.6720318	4.63979761	5.168169456
Zm00001d044301_T001	4.100339055	3.240758977	4.13732252	5.340664166	5.420090549	5.246903216
Zm00001d027895_T004	0.625095578	0	0	2.177217796	1.511408326	1.205976105
Zm00001d027895_T014	2.074196793	0	1.317318542	2.871587409	2.702915255	2.374760182
Zm00001d027895_T006	0	0	1.094280668	0.888656368	1.080848646	1.607586454
Zm00001d027895_T019	3.19263827	2.116835486	2.570880863	2.664144655	3.031225437	2.732462849
Zm00001d027895_T002	4.571128385	3.597673828	4.673556085	4.622823199	4.675166403	4.617919124
Zm00001d027895_T032	2.282573244	2.029023459	2.752359825	2.060261523	2.56153559	1.972705151
Zm00001d023867_T013	0.645697048	0.146652616	0.62737244	1.044875986	0.839196934	0.987504238
Zm00001d023867_T017	3.443388772	0.137254309	2.862944273	1.955431473	0.79343065	1.471417961
Zm00001d023867_T003	2.840444099	2.170479851	3.32650082	3.713430123	3.941588068	3.721239212
Zm00001d023867_T001	0.932950106	0.114580248	0.425114435	0.858253987	1.419100353	0.271075418
Zm00001d028596_T001	3.860901633	3.367758668	3.842573111	4.20808434	4.13738589	4.082288896
Zm00001d029980_T001	0.320396872	0.181577115	0.290214413	0.554647786	0.350843452	0.535302504
Zm00001d029980_T003	3.68823755	3.095100583	3.699962551	3.506221467	3.317273095	3.471398841
Zm00001d002956_T003	0	1.044571778	0.680296448	1.116577685	1.396515186	1.760931153
Zm00001d002956_T004	0.971627055	1.16753456	1.0798225	1.423195194	1.232202091	1.194893298
Zm00001d002956_T005	3.50126168	0	3.873911507	4.379796229	4.398767767	4.494492672
Zm00001d002956_T001	3.96414667	3.75620232	3.647756462	3.614235031	3.420042803	3.670419111
Zm00001d049192_T002	3.43052655	2.667997911	3.315129849	3.856026761	3.951065992	3.797215356
Zm00001d049192_T001	0.488253799	0.068746468	0.613393977	0.658708713	0.911951531	0.749801973
Zm00001d026273_T003	1.065300014	0.873386428	1.203494365	1.432268886	1.462362818	1.03785168
Zm00001d010910_T002	1.386043328	0.53074098	0.187736081	1.394824159	0.923533341	1.432019212
Zm00001d010910_T001	3.777831765	3.209744683	3.699812786	3.385132251	3.823736421	3.655989222

Zm00001d010910_T004	3.037487653	2.754171106	2.631650982	2.875720506	0.865984516	1.370145305
Zm00001d019479_T002	2.28054482	1.548692588	0	1.193381927	0	0
Zm00001d019479_T006	0	0	1.8765472	0	1.254669622	1.249431384
Zm00001d007204_T003	1.35715447	0.937933404	1.600146034	1.210393852	1.245009409	1.493320436
Zm00001d042102_T001	7.513496154	7.784597152	7.537849786	7.734935266	7.742812548	7.715083384
Zm00001d033734_T002	4.315125645	3.347957425	4.310409127	4.510249887	4.616708094	4.411261018
Zm00001d043280_T001	2.700758851	1.669171935	2.037505926	2.074197478	2.651914811	2.691662572
Zm00001d043280_T006	1.650637743	0	2.410799948	2.143127256	1.605685859	1.501131847
Zm00001d043280_T008	0.180422877	0.572482262	1.136423722	0.703746578	0.855989697	0.89512654
Zm00001d047793_T001	5.734050788	5.002247095	5.776933457	5.225405703	5.265360153	5.233139146
Zm00001d049260_T001	3.884682507	2.923208523	3.826633496	3.879579819	3.908210731	3.922743609
Zm00001d025652_T005	1.547848082	1.447645313	1.363007871	2.413933596	1.762859888	2.34421238
Zm00001d025652_T004	0.994349454	0.117188348	1.212343346	0.706238779	1.078238867	0
Zm00001d005482_T001	3.090813807	2.359343493	2.803147665	2.439553461	2.488135005	3.064045749
Zm00001d005482_T002	2.076687694	1.454034249	1.776317533	1.643458182	1.510443975	1.164660793
Zm00001d019492_T001	5.174484919	4.488211245	5.043992601	4.859642967	4.890418481	4.724257351
Zm00001d012891_T001	0.536573172	0.235292042	0.257579133	0.379007603	0.290486923	0.415633209
Zm00001d013043_T003	2.605488408	1.616668224	2.577710632	1.404468287	1.888235526	0.965753999
Zm00001d013043_T001	4.78459674	4.2291002	4.52344089	3.880893051	4.057998132	3.878934424
Zm00001d013043_T004	2.448956967	1.522423409	2.48996931	2.603065358	2.450961529	2.263275135
Zm00001d024082_T001	3.668449572	3.640692182	3.792201203	3.503368582	3.468679233	3.36526028
Zm00001d048090_T002	3.89352715	3.110377777	3.852649602	3.631458799	3.643962599	3.775980564
Zm00001d021227_T003	4.208340268	2.892404821	3.925795908	4.574486842	4.747524895	4.451660701
Zm00001d021227_T004	3.597714941	4.236798575	3.98638645	2.627412598	2.412815933	2.636343188
Zm00001d021227_T001	1.510173537	0.700797462	2.218541267	2.940500928	2.53823991	2.594281961
Zm00001d052239_T001	6.292285425	5.796397288	6.335557322	6.435513054	6.486847745	6.463354003
Zm00001d003457_T001	6.153485963	5.967653752	6.301673002	5.698962419	5.968186483	5.869874071
Zm00001d002751_T001	0.776204236	0.562820241	0.604679595	0.971229737	0.403150993	1.326095498
Zm00001d002751_T004	1.10818118	0.413244197	0.977381046	0.997525099	0.886185672	1.525105692
Zm00001d002751_T002	3.218030337	2.882954442	3.120051126	3.320826754	3.284717553	3.092152964
Zm00001d039546_T001	0.977729058	0.44615034	1.006145756	1.049034052	1.14339995	1.040451316
Zm00001d039546_T003	3.385675101	2.748593689	3.329307761	2.857569022	2.987241725	2.869322249

Zm00001d039546_T011	0.585311591	0	0.404769089	0	0.406353805	0.562331823
Zm00001d039546_T010	1.599821459	0.870345779	1.709161802	2.524355847	2.007420501	2.307603387
Zm00001d014533_T002	3.238490954	2.344403578	3.402060153	3.33463363	3.524365	3.428150835
Zm00001d014533_T003	0.7437545	0.384462104	0.559635708	0.840415697	0.697102125	0.64701051
Zm00001d014533_T001	3.621591959	3.406564288	3.386102528	3.439266608	3.513323601	3.722256381
Zm00001d011241_T001	2.768538575	2.629468301	2.654033794	3.254295617	3.178438981	3.280574216
Zm00001d014911_T017	1.369445265	0.366911343	0.783183386	1.917776586	0	1.928859572
Zm00001d014911_T002	0.796541274	0.637214222	1.09864054	0.604116882	0.469903684	1.138443592
Zm00001d014911_T011	0.575173864	0.869831934	0	1.399948709	1.200184165	1.414558806
Zm00001d014911_T019	0.193955892	0.260900044	0.330957596	0.448084779	0.209160118	0.319804039
Zm00001d014911_T008	2.346713844	2.241319743	2.648128084	1.106655856	0.983855695	1.561508463
Zm00001d014911_T015	2.009740223	0	0	1.136356781	1.912757153	0
Zm00001d020426_T001	3.264300266	2.766151367	3.230807392	3.378600124	3.506708056	3.303667812
Zm00001d039361_T001	2.63231708	1.917796441	2.763305971	3.431108766	3.366778628	3.28749106
Zm00001d011843_T001	3.716316455	3.31374737	3.639603673	3.92685216	4.013308066	3.887057684
Zm00001d040429_T007	3.716792743	4.684258547	4.204580612	3.028546522	3.238074965	2.732932752
Zm00001d040429_T012	0.313804244	0.017025377	0.090973989	0.900121388	0.305830475	0.269961919
Zm00001d040429_T003	2.943939325	2.180820347	2.992885147	3.132616323	3.104521514	3.383947129
Zm00001d017288_T001	8.224083635	7.706309082	8.308186288	8.431627573	8.498339393	8.499612977
Zm00001d036905_T004	5.251060339	4.862249876	4.841554888	4.817174469	4.795917048	4.849264769
Zm00001d036905_T006	4.244966496	0	4.134161538	2.784197613	3.474759202	2.112623743
Zm00001d036905_T001	0	0.178718465	3.327163214	3.587073062	2.939371028	3.778177871
Zm00001d036905_T003	0.788937064	0.249958613	0.464447664	0.38945007	0.747807578	0.685995748
Zm00001d015186_T001	6.018281157	5.31721415	6.088924272	6.687066695	6.694868648	6.636329599
Zm00001d027373_T004	4.779836683	4.121882084	4.901447101	4.582448821	4.795498495	4.799174829
Zm00001d014341_T001	2.770520393	2.142566032	2.755379003	1.621641186	1.743884158	1.79574534
Zm00001d040789_T004	0.993804769	0.437812583	1.354911922	0.957741539	0.96967178	0.989450044
Zm00001d040789_T002	4.240580173	3.762384218	4.443391292	4.685198994	4.525532398	4.525876753
Zm00001d037972_T001	7.700912976	8.595038683	7.618945211	7.300077889	7.151316903	7.141129401
Zm00001d032263_T002	3.604854983	4.184847262	3.87904219	4.428220193	4.001375134	4.35656761
Zm00001d032263_T001	3.814963588	4.163222807	3.956208443	4.068528684	4.390761909	3.933448569
Zm00001d039110_T001	3.891419187	4.367067047	3.823730206	5.000045895	5.077406148	5.36980105

Zm00001d046579_T029	1.930173015	0	1.756317946	2.83681326	2.462850162	2.575660617
Zm00001d046579_T020	0.032397112	0	0.070901737	0.833794427	0.537495868	0.833475478
Zm00001d046579_T003	0.416840823	0.144097287	0.912380843	1.248699984	1.116466567	0.561027912
Zm00001d046579_T034	4.885423791	4.023240269	4.746101453	3.786756931	3.712722437	3.861380652
Zm00001d046579_T031	0.412997168	0.138291545	0	0.523224702	0.645294013	0.475867255
Zm00001d046579_T016	1.508475812	0.929869757	1.118116487	2.800228988	2.960633474	2.955479459
Zm00001d046579_T027	0	0	0.647412144	1.097523154	1.406979043	1.33748618
Zm00001d017071_T001	1.519524292	0.891604275	1.031431193	1.053151662	1.180895119	1.050397214
Zm00001d028923_T002	3.537908927	2.926214098	3.722328087	4.187233683	3.882816162	4.303247693
Zm00001d028923_T005	2.59393145	0	2.821966286	0	2.857445407	0
Zm00001d028923_T004	0.466369226	0.46677745	0.64624009	0.904986521	1.29583585	0.828121349
Zm00001d028923_T008	0.820090713	0.555662063	0.458665475	1.158595552	0.577767663	0.971383527
Zm00001d028923_T009	3.042641536	3.521911573	3.052990875	0	0	0
Zm00001d017649_T001	5.856335758	5.330969818	5.957799452	6.597223682	6.635059688	6.636053766
Zm00001d017169_T001	6.565026214	7.101064282	6.58029626	6.408060481	6.54019338	6.382685269
Zm00001d034152_T002	4.324543565	3.379121033	4.363154253	4.987494779	5.013739561	4.939285733
Zm00001d034152_T005	0.285354868	0.231539316	0.456799842	1.262521557	1.427928464	1.070471078
Zm00001d042654_T003	1.662312159	1.435208791	1.621625246	2.133412278	2.401476196	1.963707526
Zm00001d042654_T011	0.605545536	0	0	1.078689071	1.051900355	0.888646238
Zm00001d042654_T009	1.984895173	0	1.740381456	1.268308025	1.192639217	1.725807892
Zm00001d042654_T006	1.435089283	1.588198681	2.057024939	0	0	0
Zm00001d026308_T001	4.906815718	4.600369732	3.886174549	4.25056258	5.318033734	4.965912436
Zm00001d035617_T001	4.742099261	9.63011142	5.090322905	3.347741049	3.398353928	3.942974781
Zm00001d031265_T034	0.355413261	0.10215688	0.538435855	0.282807475	0.356232849	0.242354948
Zm00001d031265_T024	0.188651586	0.230805548	0.33776018	0.141659053	0.528441295	0.098329269
Zm00001d031265_T023	2.492764335	1.750106084	2.397175102	2.177361012	1.753858654	2.483664375
Zm00001d031265_T028	0.651083387	0.497681848	0.770941498	0.834176426	0.719070387	0.482973191
Zm00001d013967_T019	1.680247831	0.768292748	1.541825548	1.136161193	1.297145625	1.195523987
Zm00001d013967_T015	0.502269491	0	0.455955523	1.41829791	1.288086832	1.008387923
Zm00001d021016_T001	6.242937038	5.610329745	6.381478424	6.382888153	6.492901458	6.483364748
Zm00001d020989_T011	2.073601242	0.796490607	2.011009649	2.570320112	1.929894368	1.664886465
Zm00001d020989_T014	1.225273696	1.646118408	1.921235985	0.587201748	1.118751081	1.731692006

Zm00001d020989_T008	1.384120005	0.493041644	0	1.074153968	1.655155334	1.231438553
Zm00001d025407_T001	4.296728447	4.323789341	4.501360543	4.050813014	4.215074448	4.070365025
Zm00001d016800_T001	4.054015779	3.280791998	4.165174007	4.141388891	4.257449849	4.199682159
Zm00001d034108_T008	1.878422844	0	1.027595712	1.859145824	1.206188091	2.172464793
Zm00001d034108_T005	1.400528093	2.116890034	1.278029961	1.479656373	0.905743678	1.931273447
Zm00001d034108_T001	0.827010076	0.357846988	0.330076465	0.85666625	0.92807768	0
Zm00001d034108_T006	2.515423067	3.251841925	4.003373582	3.995521965	3.640305449	0
Zm00001d034108_T007	3.424775931	0	0	2.567759354	3.528330141	4.34657089
Zm00001d034108_T009	2.901774201	0	3.471173801	2.221786775	2.023528604	0
Zm00001d007360_T001	5.340774563	4.705716756	5.392976652	4.558307314	4.432201727	4.446344377
Zm00001d018158_T001	0.211494465	0.730386063	0.194305205	4.00007619	4.13337545	3.887724018
Zm00001d014153_T001	2.418560493	2.956289212	2.48475556	1.775508253	1.999167325	1.685387626
Zm00001d024016_T001	0.582899897	0.428465098	0.739317577	0.807447252	0.57909227	0.760065063
Zm00001d036086_T001	1.487994085	1.026205865	1.376445981	0.983112179	1.289573635	1.303786008
Zm00001d005350_T011	2.844777309	1.849670263	2.643092398	2.879190342	2.264651422	1.919108546
Zm00001d005350_T006	0.307186015	0	0.093082804	0.544928463	0.792350633	0.105255666
Zm00001d005350_T001	1.106106398	0.905518036	1.544877042	2.226079973	0.257989387	2.676785079
Zm00001d040446_T001	6.809187565	5.935774254	6.818300858	7.12169479	7.147136702	7.061720659
Zm00001d051588_T001	5.641164266	5.967748267	5.500171182	5.579202962	5.4967755	5.32911545
Zm00001d052457_T001	4.788179896	3.909005574	4.797185831	5.687004626	5.692258987	5.660472427
Zm00001d052457_T002	6.678773541	7.255739863	6.683493859	6.902254484	6.997920593	7.000937672
Zm00001d050609_T001	1.357521602	2.555681694	0	2.053622273	1.605205576	1.952734632
Zm00001d015129_T001	0.957165763	0.93902944	1.359458761	1.642684477	1.430956947	1.640801369
Zm00001d005195_T003	0.912262029	0.860576704	0.584309293	0.538437842	0	0
Zm00001d005195_T008	0	0	1.365818495	1.216233205	1.235290203	2.154156403
Zm00001d005195_T013	0	1.481503033	1.74485988	1.308788389	0.873917116	1.308995694
Zm00001d005195_T014	0.79678462	0.897904881	1.209017216	0	0.670823118	1.505199388
Zm00001d005195_T007	2.923486943	1.917708237	2.785349669	2.621011181	2.568724821	0
Zm00001d005195_T001	0.412584276	0.740994892	0.323146369	0.592553194	0.552126189	0.911369456
Zm00001d005195_T004	0	0	0	0.954258112	1.349716849	1.458299052
Zm00001d005195_T006	0.356453731	0.230446517	0.208501029	0.310480893	0.56799999	0.662623889
Zm00001d005195_T002	0	0	1.015763019	1.057132064	1.580380446	1.592522577

Zm00001d008530_T001	3.601262454	3.456270229	3.796076583	3.458936427	3.170151487	3.352585662
Zm00001d022432_T003	0	0.807682995	0.974418445	1.657880511	0	0
Zm00001d022432_T001	3.722100277	2.137023748	3.037303495	3.591642559	3.779188267	3.893706921
Zm00001d022432_T004	1.107821756	1.595109754	2.414758491	1.944719416	1.345388994	0
Zm00001d022432_T002	1.799612882	1.743377521	1.653564528	0	1.545972322	1.261878585
Zm00001d032754_T002	2.102754196	1.492182891	2.066323103	2.060138717	1.897743054	2.342139399
Zm00001d032754_T003	1.062454709	0.341928835	1.059034028	1.305882994	1.066682339	1.044465465
Zm00001d032754_T001	2.854712427	2.548023486	2.644572512	2.042648539	2.139790349	1.669380137
Zm00001d037943_T001	7.370253409	7.076823687	7.184141722	6.52186069	6.615931334	6.58240724
Zm00001d006769_T001	0	1.105434033	0	1.283140669	1.403376807	2.004637655
Zm00001d006769_T008	2.272135494	1.826096109	2.25794897	0	0	0.79910223
Zm00001d006769_T003	3.237299384	1.745302741	2.889866969	3.163406468	3.159658085	2.646476513
Zm00001d006769_T009	3.189935227	2.275036557	3.525410933	3.044111848	2.944864067	3.120426263
Zm00001d017041_T001	7.106558994	6.665211385	7.245092092	5.857521001	5.819863705	6.00269181
Zm00001d044694_T001	1.024572873	1.107281447	1.149803688	1.590344762	1.505031661	1.452399337
Zm00001d044694_T002	7.112650711	6.452602699	7.109225551	7.332779324	7.32214323	7.255486807
Zm00001d016948_T001	2.037705167	2.516904451	1.837378563	5.220033336	4.959064119	4.536777859
Zm00001d029826_T003	2.395039212	3.424119517	2.543711653	2.271627663	2.466877922	2.206519452
Zm00001d029826_T006	3.337238678	4.114194674	3.110840443	3.049656555	3.172767835	3.107606192
Zm00001d029826_T002	1.806182565	2.171385552	1.792829954	2.349140193	2.154413409	1.525472575
Zm00001d007799_T002	0.5074892	0.485552542	0.833515957	1.056589077	1.40793352	0.791049709
Zm00001d007799_T001	1.148597057	0.277786028	0.914763502	0.883638019	0	0.810409375
Zm00001d033504_T001	3.642080467	2.925899755	3.738378311	3.501842406	3.599441346	3.708242647
Zm00001d013641_T001	3.5657323	3.807410877	3.689599076	3.911717656	3.768129051	3.766707202
Zm00001d036035_T001	4.542849557	5.342934711	4.58042308	3.890981159	3.941378857	3.955018877
Zm00001d021189_T001	3.617651354	2.957042405	3.645569901	3.591012962	3.716022263	3.713980807
Zm00001d035747_T021	2.067289358	2.241078984	2.632184211	3.154375817	3.279743168	3.677615693
Zm00001d035747_T009	0.787289525	0.066680273	0.64400853	0.977364926	1.21263408	1.562717199
Zm00001d035747_T013	0.599625345	0.028417803	0	0.659192068	0.467452698	0.143877937
Zm00001d035747_T096	4.680758732	3.530645982	4.492292891	4.478668419	4.630777689	4.243578148
Zm00001d035747_T027	0.305354217	0.151014509	0.10424674	0.418568859	0.590696863	0.437631509
Zm00001d004009_T004	6.669625156	6.196454474	6.766331801	6.105138789	6.294409438	5.983658156

Zm00001d004009_T007	5.659383446	8.336871367	5.850568396	4.462583282	4.190495552	5.130989375
Zm00001d004009_T005	2.916638103	4.224919149	2.975014534	2.00264996	2.481783806	2.248812298
Zm00001d004009_T006	1.384993054	0.847397262	1.344746136	1.388697629	0.955236165	1.041433647
Zm00001d000039_T001	1.974813418	1.275778266	1.430696878	2.770803046	2.544342445	2.427319482
Zm00001d000039_T002	0	0.592522577	1.793495575	0	0.458674923	0.715691335
Zm00001d046590_T001	3.856575623	3.113021493	3.990497118	4.14169003	4.123260857	4.081066199
Zm00001d046590_T003	1.066275222	0.587940034	0.990191754	1.986825207	2.320574789	1.498799243
Zm00001d029978_T003	0.628389136	0	0.522514807	0.736404854	0.936812418	0.623187015
Zm00001d029978_T004	0.368967212	0.222222173	0.240879694	0.473687179	0.281628228	0.333069887
Zm00001d029978_T002	3.984646857	3.202946743	4.075123724	4.049480741	3.971544474	3.708275649
Zm00001d029978_T001	4.037140493	3.678277475	4.093541923	3.91836114	4.192529861	3.921110278
Zm00001d022496_T001	5.764588543	5.298436176	5.857615425	6.456141509	6.373582078	6.325735874
Zm00001d031459_T001	3.915664685	3.4037355	3.887703257	3.79422651	3.970121356	3.824585698
Zm00001d039163_T002	3.20590825	2.485326093	3.380805764	3.583762521	3.705321851	3.533739501
Zm00001d045042_T020	7.897578627	7.387047365	8.132854451	6.571574019	6.867111068	6.652342146
Zm00001d045042_T002	1.473763018	4.050056892	1.514604551	2.13541174	0.966249565	2.159603031
Zm00001d045042_T007	6.812123615	3.676544432	6.219036309	6.234180675	6.390662527	6.188667666
Zm00001d045042_T013	0	0.716195492	0	1.152962167	0.770099164	0
Zm00001d045042_T015	3.792063214	4.478500038	4.213288095	2.175478613	2.689270306	1.612371845
Zm00001d045042_T010	0.118666706	0	0	0.656878903	0.722814852	0.557417933
Zm00001d045042_T005	3.638989918	3.459729308	5.538600985	3.956278524	3.978791076	4.53165681
Zm00001d045042_T019	4.956698565	0	0	4.749268498	0	3.77687936
Zm00001d019059_T001	4.278737875	3.881649546	3.800411552	3.829716038	3.658988329	3.602676717
Zm00001d033273_T006	0.73091119	0.54121844	0.83174107	0	0.837890779	0.986764056
Zm00001d033273_T001	0.52369242	0	0	1.711027001	1.960661087	1.437259704
Zm00001d033273_T010	3.395555179	2.874715339	3.712599632	0	2.732089204	2.615906372
Zm00001d033273_T009	1.888707796	0.81038305	2.092713511	3.868777918	2.620174186	3.042617724
Zm00001d010954_T001	6.424105304	5.863029172	6.345891277	5.910589776	5.9955356	5.912088915
Zm00001d023492_T001	1.272229871	0.913450501	1.279406514	2.106133206	2.100626852	2.277016776
Zm00001d023492_T002	2.51888658	1.803744495	2.754430469	2.377999376	2.196378222	2.197016127
Zm00001d024767_T005	3.384080623	2.860906598	3.32550342	2.837387849	3.267987113	2.714764025
Zm00001d024767_T007	5.552150264	0.65636365	5.748195426	5.584340174	5.506170806	4.643576799

Zm00001d024767_T008	6.512742485	7.785935361	6.512472667	5.53854164	5.833882171	5.965577053
Zm00001d024767_T002	2.788121049	0	2.714303114	2.737198931	2.934575378	2.672062303
Zm00001d024767_T003	6.240756097	5.31989376	6.255634139	6.241600102	6.229887889	6.283800234
Zm00001d026236_T001	5.450600599	4.889654111	5.385749672	5.205227612	5.00624106	5.121075139
Zm00001d052718_T001	9.263039473	9.058458845	9.233979081	8.639589232	8.614287154	8.579066694
Zm00001d034814_T002	0.738810198	0.275014201	1.777693236	1.646963522	2.122084589	1.747496967
Zm00001d034814_T001	4.288148049	3.498908995	4.217920175	4.815733574	4.520396349	4.591661228
Zm00001d012339_T001	3.197278981	2.877428589	3.427847764	2.789274778	2.482605921	2.720712522
Zm00001d005164_T010	1.057241608	0.589911104	0.90350572	1.063921884	1.404845914	2.595302423
Zm00001d005164_T020	0.421649345	0.134216996	0.399649087	1.379302115	1.440776301	1.453331617
Zm00001d005164_T015	0.708984417	0.408577095	0.926685674	3.594289247	3.295630025	3.497536621
Zm00001d005164_T016	0.138911432	0.037484841	0	1.640359479	1.537196156	1.680386925
Zm00001d005164_T011	0.29678629	0	0	0.567710929	0.637178973	0.460738371
Zm00001d005164_T001	4.915605796	4.073614009	4.861825426	5.916908815	5.855291171	5.863998991
Zm00001d053352_T001	2.907598689	3.445547547	3.316407026	3.164533688	3.234935964	3.105420119
Zm00001d051427_T008	0	5.852454128	1.351916613	2.423790324	3.441560499	3.785648367
Zm00001d051427_T004	3.622519968	6.937075439	4.752210959	5.423383468	5.709450641	5.711149142
Zm00001d051427_T006	3.968861121	5.249113718	4.155492545	4.056000263	3.643286039	4.050985272
Zm00001d051427_T003	6.638954076	5.35737039	6.645156193	6.719872719	6.675718887	6.583789549
Zm00001d002919_T001	5.152347518	4.330703523	4.950954519	5.098940842	5.013956086	5.124011725
Zm00001d024406_T002	2.192981277	1.413688851	2.001365225	2.162816511	1.943073647	2.089920457
Zm00001d037729_T001	3.310687664	2.70719014	3.174330351	2.095531211	2.21480091	2.34132856
Zm00001d005038_T001	4.165923834	3.687856751	4.142257641	3.344564073	3.325930777	3.434712952
Zm00001d023223_T001	4.497646277	4.242315615	4.578204382	3.838360453	3.526869711	3.486559799
Zm00001d023223_T003	1.230247905	1.062465071	1.444733847	0.79110557	0.934295315	0.135533556
Zm00001d032520_T001	1.920609629	0.98688781	1.480054132	2.627283479	2.626257124	2.543274637
Zm00001d032520_T002	2.554086584	1.883615734	2.880566618	2.999166964	2.738730661	2.987974388
Zm00001d024222_T001	5.796636375	5.127548143	5.869971636	5.797704968	5.738935898	5.834630803
Zm00001d002837_T003	1.085710176	1.334923471	1.257734199	1.236096416	1.793132623	1.868578125
Zm00001d002837_T002	3.974122878	3.729582244	4.232933615	4.282750033	4.160107314	4.402233193
Zm00001d016950_T001	2.777516085	2.824840139	2.765078528	3.691685015	3.653321812	3.490946799
Zm00001d016950_T002	4.017108693	3.643904894	4.078852227	5.24467433	5.249800851	5.078880912

Zm00001d050575_T004	0.358948702	2.008495136	2.087173255	1.255614331	2.096132626	0.433741457
Zm00001d050575_T001	3.921226462	2.770324173	3.589005154	4.221835794	4.081297952	4.276010772
Zm00001d053254_T001	1.778566472	1.252557354	1.879726544	1.60183056	1.716565593	1.536162342
Zm00001d017618_T001	0.885012795	0.762449174	1.154078288	1.53451337	1.549422746	1.31233873
Zm00001d006016_T001	8.611286396	9.177223438	8.425919532	9.264984734	9.30676071	9.396888534
Zm00001d027291_T001	10.14802126	9.706538311	9.967492568	9.258038344	9.303910612	9.317111946
Zm00001d046210_T003	1.948498435	1.03835962	1.823259413	2.69888121	3.514648606	3.148429968
Zm00001d046210_T006	0.630683173	0.756326487	0.935747127	1.611109098	1.773503953	1.630500996
Zm00001d046210_T013	4.370423496	4.18728595	4.22833625	4.202929979	4.45835536	4.600609035
Zm00001d046210_T009	1.064343464	0.369447499	1.073755807	1.192973704	1.444882236	1.698589219
Zm00001d046210_T012	4.100620124	3.468375434	4.239874584	5.311897884	5.158763689	5.28508764
Zm00001d046210_T002	2.372939008	1.611319712	2.07563531	2.893475603	2.377602994	2.57662033
Zm00001d046210_T004	2.550662329	2.804969	1.331425477	3.19599084	3.624656921	3.561885019
Zm00001d046210_T011	1.644734005	0	1.813550547	0	0.734892973	0.356739926
Zm00001d046210_T008	0	0.58179857	0	2.758272472	1.917597498	0
Zm00001d046210_T005	1.581779292	0.812017589	1.876269003	2.428718067	2.752029848	2.490623525
Zm00001d046210_T010	1.797290281	0	1.58629926	0	1.939593318	2.713730782
Zm00001d046210_T014	2.182662109	0.598993984	2.906250089	2.744920141	2.239631445	0
Zm00001d030391_T001	2.925407033	2.399125146	2.730617351	3.181623704	3.236565325	3.061676674
Zm00001d038980_T001	3.757196424	3.220648644	3.862116691	3.744127827	3.76185511	4.043919876
Zm00001d014953_T001	0.640365496	2.377132358	0	1.409734698	1.127477447	1.400747761
Zm00001d014953_T002	5.07608898	4.121573301	5.004848676	5.059092104	5.160999305	5.075177904
Zm00001d043617_T001	1.207245055	0.873410053	1.006663544	0.519620403	0.082130302	0.331330308
Zm00001d037751_T001	6.645079985	6.705305455	6.482498406	7.263037402	7.227846082	7.331831015
Zm00001d032980_T046	1.234430196	1.193662631	0	0	1.154358965	0
Zm00001d032980_T052	2.168007187	0	2.147848503	1.227356018	2.221529741	1.144494776
Zm00001d032980_T056	0	1.977362361	1.682280684	1.581464055	1.299594591	1.982548266
Zm00001d032980_T031	0.320575945	0.120517834	0.163864512	0.205587695	0.177393544	0.453926296
Zm00001d032980_T053	1.415067435	0.376145884	1.194126776	1.374435807	0.78845603	0.531353012
Zm00001d032980_T049	2.221192528	0	2.255255076	3.223864404	2.363795689	2.961192493
Zm00001d032980_T020	1.802646121	0.660386512	1.271160899	1.799109278	2.300522644	1.641347645
Zm00001d032980_T006	0	1.263647421	1.307576576	1.95587147	0.98004066	1.45395684

Zm00001d032980_T054	0.289657437	0.08577407	0.255350866	0.599747205	0.383172868	0.586877129
Zm00001d029965_T001	5.713916683	5.227274642	5.842378455	5.967166832	5.974644026	5.936330759
Zm00001d006787_T001	1.072153146	0.829419325	0.886415919	0.775924535	0.710536022	1.123982641
Zm00001d052401_T001	3.654983322	3.26328385	3.603765549	3.896260803	4.276388199	4.207553763
Zm00001d019890_T001	5.69670875	4.84199158	5.714489546	5.551290644	5.611139057	5.532515207
Zm00001d044753_T001	3.737397436	4.075198702	4.043283079	4.170043217	4.531649325	4.032748588
Zm00001d010731_T002	3.548660533	3.038352596	3.543289858	3.088698108	3.089673223	3.509065808
Zm00001d010731_T001	1.392295439	0.654554691	1.375791245	1.291499831	1.439956581	1.492133657
Zm00001d012165_T001	4.748682719	6.307653164	5.272329244	4.85555513	4.307481642	4.37246225
Zm00001d052307_T015	0.908018919	0.357669104	0.985399147	0.538174588	0.292585058	1.052339375
Zm00001d052307_T001	1.48749664	0	0	2.106758689	2.758886022	1.463903331
Zm00001d052307_T002	2.783152787	2.301177313	3.117931705	2.786754004	1.452153123	2.747013733
Zm00001d045072_T029	0.769204704	0	0.480184453	0.228183303	0.484898086	0.207541816
Zm00001d045072_T012	5.419108918	4.798500781	5.57962727	5.04834196	4.994691452	4.93845499
Zm00001d045072_T037	0	0.458962535	0	0	1.997304694	1.825483627
Zm00001d045072_T010	0.280488401	0	0.470867925	1.206168082	0.544610015	0.448481293
Zm00001d045072_T004	0.522398298	0	0.581678076	1.028999775	0	0.844236193
Zm00001d045072_T033	0	0.754984961	0.516342995	0.635375504	1.659381163	1.350082483
Zm00001d045072_T003	2.322929843	1.773447828	1.551168006	2.188961688	2.185702608	1.945905799
Zm00001d045072_T020	0	0	0	2.053452328	3.354217428	3.239191185
Zm00001d045072_T035	0	0	0.119033402	0.379170673	0.811295924	0.909499705
Zm00001d045072_T028	1.512608183	0	2.364629579	2.674315109	1.668031053	2.612034198
Zm00001d045072_T021	2.134525221	0	1.514959475	0	0	0.899032514
Zm00001d045072_T026	0.913274323	0.714234965	1.121365585	1.50854021	0.967671065	1.065109031
Zm00001d045072_T001	2.099239987	0.91786784	2.148947442	1.773354563	2.429426092	2.514048261
Zm00001d045072_T002	0	0	0.261701702	0.839290473	1.426664091	0.980232273
Zm00001d045072_T044	0.469904726	0	0	1.703625221	1.000492596	1.613375588
Zm00001d051567_T001	1.481193526	1.549093456	1.73908379	1.108361862	0.700191997	0.755484975
Zm00001d007117_T002	2.111367568	1.394048717	2.048330711	1.45498284	1.604135389	1.779636684
Zm00001d040874_T001	8.728550295	9.258636968	8.803344509	7.903137096	8.032144323	7.979407783
Zm00001d008641_T001	1.996563739	2.486087482	2.355384505	1.977705982	2.49719591	2.594608501
Zm00001d021480_T001	0	3.236969815	2.445520665	2.538801847	2.724982643	1.914961688

Zm00001d021480_T003	3.021928101	2.314881527	3.030400354	3.120341986	2.515172488	3.077232401
Zm00001d021480_T004	2.030128753	2.188289822	2.147847527	2.404563384	2.562957946	2.776292263
Zm00001d005450_T005	4.065193408	3.056122555	4.1493901	4.382999394	4.623294492	4.485020175
Zm00001d005450_T011	3.295962181	3.521000342	2.791443819	0	1.548894762	2.687690722
Zm00001d005450_T002	1.39725918	0	1.186736973	2.031374374	1.998121832	1.872134523
Zm00001d029350_T001	0.539758351	0.533852357	0.933151854	1.408154237	1.139883318	1.539805986
Zm00001d009522_T002	2.200619227	0	0	1.639136752	2.015238138	2.247111781
Zm00001d009522_T003	3.079446041	2.749906853	3.146413938	3.216353819	2.382139044	2.77483196
Zm00001d009522_T004	3.942878816	3.343672458	3.915822124	3.299553271	3.639725212	3.633864535
Zm00001d023732_T002	1.35053685	0.551105483	1.110004363	1.748727849	1.859506063	1.638369067
Zm00001d023732_T005	2.879610498	3.406742774	2.371259978	1.454368064	0	1.220911869
Zm00001d023732_T003	1.178086123	0.840119968	1.342520043	0	0.216603861	0.161148575
Zm00001d019432_T008	0.871998947	0.166840109	0.915204984	2.064877679	2.457044476	1.161832875
Zm00001d019432_T010	0.463951969	0.247939662	0.340474537	1.082711444	1.068239485	1.174018366
Zm00001d019432_T002	2.613966753	2.331945932	2.881566153	3.91738306	3.770864769	4.173112239
Zm00001d019432_T011	4.045316188	1.47945978	3.884470739	4.075857073	4.325936603	3.932253782
Zm00001d019432_T007	0.200651239	0	0.317365434	0.59254267	0.539520153	0.749178967
Zm00001d019432_T001	2.558010669	3.590720812	1.834272314	0	0	0
Zm00001d039343_T001	2.04947255	1.3322525	2.180108387	1.670065767	2.197846524	1.865997973
Zm00001d039343_T002	1.676269665	0.910427976	1.219076805	1.21324524	1.350101725	1.249098203
Zm00001d020214_T001	4.05407536	4.152745944	4.009493996	3.591668168	3.555494457	3.49813034
Zm00001d031933_T007	0.157817253	0	0	1.251656082	1.036375323	1.016577616
Zm00001d031933_T005	2.325528892	2.558772367	2.90891299	2.41018722	2.716195273	2.948682324
Zm00001d031933_T002	0.601269587	0.394309988	0.288997518	2.024493799	1.261584972	1.511527244
Zm00001d031933_T001	3.968405529	2.523336878	3.706568899	3.799846594	3.619004443	3.900335798
Zm00001d031933_T003	0	1.53437143	1.541608505	2.200046651	1.800946272	1.790629429
Zm00001d031933_T010	1.857813036	0.914510948	0.543265232	1.668382399	1.805779051	2.061383939
Zm00001d048364_T001	3.699101497	3.296712151	3.790936938	3.649121065	3.486589918	3.637024634
Zm00001d048364_T002	2.513455373	1.763602788	2.389104194	2.888722016	3.032949808	3.033976538
Zm00001d032152_T001	7.534225693	6.918121442	7.601271823	8.482847985	8.503424045	8.489392089
Zm00001d017957_T001	1.451157294	0.734478554	1.603998713	1.892448538	1.997386356	1.843992283
Zm00001d017957_T003	0	0.457748717	0	0.516279449	0	0.653208637

Zm00001d017957_T002	4.314734224	3.618545254	4.529633571	4.659164375	4.68121097	4.482092154
Zm00001d029053_T001	0.518499889	0.270104294	0.375434293	0	0	0
Zm00001d029053_T010	1.808634231	0.523039982	0	2.79310161	2.427266107	2.202176965
Zm00001d029053_T011	0	0	0.374657814	0.667459819	0.501738736	0.782988043
Zm00001d029053_T003	0	1.434717889	1.963521843	0	0	1.32552918
Zm00001d029053_T004	0.506832438	0.444325698	1.027044323	0.670489588	0.622287553	1.07819377
Zm00001d014862_T001	4.206308843	3.299798386	4.266570158	4.627135142	4.592331031	4.544729998
Zm00001d014862_T011	2.687680197	1.145483127	4.177958841	3.929908662	4.01339928	3.961033258
Zm00001d025820_T001	0.663763589	0.33331839	0.523601096	0.464259445	0.539826826	0.442611273
Zm00001d028406_T001	3.700508189	3.221973721	3.536941128	5.231805246	5.197530424	5.285127119
Zm00001d023583_T005	0.57661114	0.486690693	1.503965375	1.67236288	1.540547597	1.389888354
Zm00001d023583_T002	1.267569356	0.578384225	0	0.159683431	0.957907172	0.33391142
Zm00001d013487_T001	5.705622325	1.763955829	5.76802742	7.39129217	6.795247866	7.334987335
Zm00001d016827_T002	0.853870329	0	0.638888469	0	1.010355201	0.377125971
Zm00001d016827_T003	2.70242727	2.183621805	2.670901504	2.060059147	2.460309297	2.334276504
Zm00001d016827_T004	2.022101445	0	1.990511277	2.755854532	2.44864674	2.516524288
Zm00001d022457_T001	5.503693624	4.865371466	5.458783933	3.169646374	3.081289344	3.299155549
Zm00001d025629_T003	1.41887375	0.61755427	0.843680002	1.336667294	0.91799612	1.316539678
Zm00001d025629_T004	2.969185494	1.251754232	3.250693013	2.517221008	2.513042455	2.386103218
Zm00001d025629_T007	0	0	0	2.638078241	2.500092766	2.739334429
Zm00001d025629_T001	1.094961627	1.396619302	0.838844493	0.380194156	0.913768334	0.396558477
Zm00001d023896_T003	4.755533738	4.150492658	4.88473144	3.983391681	4.178685086	4.18715227
Zm00001d000400_T001	2.258229754	2.377732913	2.471519671	3.996447863	3.638660298	3.587674861
Zm00001d029921_T001	2.207202566	2.112810541	2.419752179	3.47286971	3.413326531	3.448727606
Zm00001d027466_T001	3.684283293	2.928140611	3.688625746	4.406461159	4.294201957	4.348443765
Zm00001d007700_T001	5.407576039	5.019576229	5.405523101	3.896953698	3.870904902	3.931786008
Zm00001d043451_T001	1.951647098	1.366329489	1.824433867	1.921760406	1.95898332	1.732890868
Zm00001d039522_T002	0.36903759	1.198027009	1.383637944	1.651229914	2.318986328	2.839371509
Zm00001d039522_T018	3.223963536	0	2.596864794	3.68358968	3.594739622	2.983601089
Zm00001d039522_T023	0.504674286	0	0	0.709266811	0.551781769	0.671056905
Zm00001d039522_T006	3.780524912	3.28873698	3.621147428	2.05751647	0	2.405472351
Zm00001d039522_T014	1.728002767	1.276228052	2.352510523	2.987631749	3.096134313	3.57165327

Zm00001d039522_T024	0.889943072	0.271800943	1.309345016	0.810119772	1.290863162	1.12815084
Zm00001d042730_T001	8.540125341	8.374961228	8.517363831	7.976392525	8.115989748	8.05108831
Zm00001d017537_T001	2.611323961	1.858202258	2.378767956	1.487172977	1.331090636	1.857403005
Zm00001d045175_T001	1.615060759	2.261500727	2.831413964	3.402491895	3.398605255	3.473905075
Zm00001d010588_T001	3.448925656	2.414350162	3.397006385	0.224384849	0.099666857	0.616822522
Zm00001d010588_T003	2.581151642	5.211439447	2.878925206	0	0.545036245	0
Zm00001d026004_T001	2.39929006	1.888658316	2.349433504	1.748082476	1.404466107	1.134427306
Zm00001d044261_T004	3.944825095	3.523610129	3.660260083	2.956766485	2.788103295	2.829969475
Zm00001d044261_T002	1.962246571	1.067646977	2.192079882	1.745782462	1.692323188	1.446678123
Zm00001d044261_T001	4.573046417	3.725798841	4.680020256	4.379858666	4.485482666	4.439357178
Zm00001d034217_T001	4.647273692	3.979323347	4.806016562	3.97481039	3.891330717	3.96580035
Zm00001d011719_T008	1.25467325	1.032683447	1.101980124	0.685944632	1.055079706	0
Zm00001d011719_T007	2.227547636	0.200957518	2.112156977	1.613610386	1.238018848	0.947884555
Zm00001d011719_T006	0.972838938	1.43239825	1.54517368	1.393578749	1.492065445	1.849621828
Zm00001d011719_T001	0.376948227	0.470636816	0.619993259	0.560094707	0.355071535	0.286008171
Zm00001d011719_T003	0.271851131	0.145770046	0.221677904	0.799094768	0.842975615	0.568303588
Zm00001d014005_T001	5.601570156	6.151062887	5.646666072	5.311390273	5.654791244	5.491506551
Zm00001d053416_T001	2.650966017	2.290199371	2.544989524	2.80199257	2.761749712	2.779425311
Zm00001d048545_T006	0.827484118	0	1.286102237	0.960103902	1.168699152	1.671559692
Zm00001d048545_T001	6.353117865	5.33270933	6.29611055	6.663642081	6.701201385	6.661219563
Zm00001d048545_T008	1.412040616	0.745292414	1.43296956	1.26515301	0.74620956	1.170853157
Zm00001d048545_T007	2.904474464	3.34334528	2.75817225	2.69417817	2.646780625	2.769014231
Zm00001d021558_T001	4.511013615	4.573159573	4.712600622	5.010255155	5.067903992	4.92524095
Zm00001d021558_T003	3.749498978	3.681166208	3.682616443	4.106763294	4.156052245	4.310951609
Zm00001d021558_T002	2.975464742	0	2.889615469	2.871555278	2.296100838	1.736742969
Zm00001d014417_T002	7.632230264	7.426461082	7.68002091	7.674855649	7.750497488	7.78830198
Zm00001d014417_T003	0.461935013	0.259022951	0.436750313	0.551611497	0.62923537	0.475912898
Zm00001d015473_T002	7.137368429	7.706955218	7.223900006	7.087879762	7.216825968	7.13001056
Zm00001d015473_T001	3.641797092	2.972277429	3.7878378	3.803849555	3.875720998	3.882015365
Zm00001d049543_T001	0.636277984	0.041080337	1.16439048	0.936069134	0	0
Zm00001d049543_T011	1.398742734	0.321275849	0	0.75117661	0.644286395	0.907759798
Zm00001d049543_T009	0	0	0	0.644411924	1.479866904	1.578469731

Zm00001d049543_T007	0.394902613	0.232803178	0.146944514	0.183497948	0.073260866	0.594881
Zm00001d049543_T013	2.359755322	2.241835608	2.822061969	1.494370549	0	0
Zm00001d049543_T016	1.982035992	0	1.264149659	1.05048707	1.756399509	0
Zm00001d049543_T017	1.40254101	1.908991937	2.08699703	0	0	0
Zm00001d037738_T001	2.689933136	3.144629205	2.596728858	3.929591241	3.492561061	3.82078686
Zm00001d036917_T009	0	0.121162648	0	0.441167922	0.672006164	0.408108574
Zm00001d032285_T001	1.469025832	2.303102997	1.644397618	1.93136423	2.151676358	1.866181603
Zm00001d043703_T008	0.094810331	0.214618471	0.117145783	0.784364104	0.959670016	0.693255439
Zm00001d043703_T011	2.223796305	1.248400033	2.142363114	1.935969976	1.699880288	2.373186865
Zm00001d043703_T003	1.11194206	0.863160612	1.265981705	1.763654199	0.995964192	1.632556726
Zm00001d043703_T007	1.731190194	0.724271324	1.505423502	2.876858733	2.222636731	2.442915637
Zm00001d043703_T004	0.172966201	0	0.227091655	0.809765094	1.628939198	1.094071857
Zm00001d011945_T002	2.856211863	2.075864259	2.867762267	2.835280578	2.915857395	2.977416217
Zm00001d014436_T013	4.649665694	3.925036573	4.650476738	4.965032364	4.847814304	4.867042551
Zm00001d014436_T010	5.25211947	4.291054192	5.025790306	4.577494692	4.184645683	4.454300741
Zm00001d014436_T001	2.071139678	2.486968141	2.241513786	1.735633949	1.807646317	1.073153904
Zm00001d014436_T002	2.764299653	2.487550919	3.276925837	3.360599837	2.911093291	3.073766945
Zm00001d014436_T011	0.781362232	0.256426178	0.756816645	0.559962601	0.598696777	0.855532904
Zm00001d033890_T002	5.019286746	4.309793625	4.988174996	4.850814522	4.915592986	4.946008115
Zm00001d033890_T001	1.68255487	0.963255114	2.117043023	2.221573666	1.765942886	1.752534526
Zm00001d003420_T001	6.842635196	1.591750736	6.09481565	9.377179305	9.123689618	9.409061557
Zm00001d044423_T001	5.594347	5.793686198	5.339084708	5.263236407	4.932139051	5.240082105
Zm00001d049375_T001	4.50851885	3.87272437	4.629299669	3.819332708	3.915554554	3.750457726
Zm00001d016679_T003	1.963271763	0.957259388	1.848086671	1.577633782	1.588213075	1.353834778
Zm00001d016679_T005	0	0	1.065639172	0.86393528	0.924723208	1.186989191
Zm00001d016679_T006	1.937035856	1.362155085	1.756542553	1.600718385	1.729441831	1.487677221
Zm00001d016679_T008	1.835288664	1.285380319	2.228144198	1.598043088	2.048705264	1.579564912
Zm00001d016679_T001	0.511144143	0	0.270296896	1.654036316	1.381584618	1.026664811
Zm00001d047786_T001	5.620974097	5.171162864	5.475105317	8.902872151	8.895022921	8.893228894
Zm00001d037438_T002	1.910471725	1.566388979	1.837086652	1.599622013	2.509252199	2.280140634
Zm00001d037438_T007	0.903656876	0.215715867	0.421951971	1.273693717	1.011982895	0.120716881
Zm00001d037438_T006	2.023680468	1.536642792	2.3849519	2.475412047	2.065348273	2.17839468

Zm00001d037438_T001	0.313631293	0	0.40322845	0.563530107	0.617330459	0.665503743
Zm00001d018830_T001	0.854008418	0.774139744	1.163878034	1.348554241	1.071286268	1.2995647
Zm00001d043242_T001	3.141083029	2.978353073	3.473782107	3.604200283	3.630037645	3.659888034
Zm00001d021707_T001	7.079862342	7.370971629	7.169857484	6.870917955	6.82192988	6.891353333
Zm00001d033604_T001	1.173877056	0.785682784	1.572429873	2.237393926	1.979694668	1.370263061
Zm00001d007187_T003	2.140679201	1.994563071	2.582188177	1.838763025	1.952899722	2.178076878
Zm00001d007187_T002	3.550054988	2.749192271	3.445843083	3.994461335	4.112424326	4.061211881
Zm00001d042828_T001	0.607763501	0.283281749	1.068732025	3.331858104	3.386480578	3.448617801
Zm00001d028119_T008	0	0.211729935	0	0.820132387	0.576969026	0
Zm00001d028119_T019	0	0.032397112	0.570980654	1.159774481	0.93048605	0.763352931
Zm00001d028119_T018	0.93064803	0.479065998	0.892861161	0	0.436785486	0.510594365
Zm00001d010640_T001	4.573702683	4.35467254	4.550634505	8.165228004	8.131405622	8.173772443
Zm00001d045144_T001	3.452255805	4.31754293	3.983361489	2.854380913	3.130988181	3.510526767
Zm00001d027991_T003	3.667909378	3.534386247	3.629597457	4.776834102	4.771115126	4.552445749
Zm00001d027991_T001	4.601957758	3.89279473	4.8146547	5.799578848	5.927980458	5.92748252
Zm00001d012015_T001	4.154489724	3.351125531	3.93671331	5.018354563	5.093807928	5.042604418
Zm00001d044579_T001	1.184982901	1.061342114	1.264957318	0	0	0
Zm00001d051836_T001	3.936929793	3.722131652	4.072300748	3.771745527	3.786776583	3.749859776
Zm00001d019719_T001	1.873780132	1.614772026	2.041306773	1.469191545	1.1649999	1.451807193
Zm00001d042470_T004	0.836985701	0.02615132	0.883214928	1.651680426	1.174150712	1.046709666
Zm00001d042470_T008	0	0.21406635	0	0.974140871	1.378080415	1.463585317
Zm00001d042470_T012	1.304695602	0.936854621	1.203898369	1.9493971	2.150269484	1.531304598
Zm00001d042470_T014	0	0.178987381	0	2.25605444	2.088599916	2.018942832
Zm00001d042470_T007	3.049346905	2.150687369	2.964442809	3.149169926	3.172711039	3.098786552
Zm00001d042470_T006	0.902976549	0	0.843115555	0.958937677	1.369427955	1.348994931
Zm00001d009513_T001	3.744106832	4.443692707	4.064620857	3.877843959	3.714357631	3.721414113
Zm00001d009513_T004	1.945387836	1.503735438	1.944730284	2.423870176	2.399824603	2.771233789
Zm00001d009513_T002	1.343744933	0.62288913	1.354944071	1.893421238	1.703328872	1.561654112
Zm00001d009513_T003	0.646920219	0.41821586	0.784455404	0.577119892	0.646705525	0.898352325
Zm00001d039607_T001	2.364640784	2.039235272	2.544538306	2.537808437	2.625398152	2.397664435
Zm00001d013014_T001	0.626391474	0.123140401	0.778113515	1.128893189	0.853724243	1.016549805
Zm00001d013014_T003	2.120667786	1.609967664	2.078432561	2.814127704	3.098714306	2.84390367

Zm00001d018344_T001	3.465002113	3.924933519	3.699450023	2.353166873	2.588958996	2.740666837
Zm00001d026289_T001	0.841889412	0.246336331	0.365109071	0.364302357	0.243689795	0.272483105
Zm00001d026289_T004	1.747103344	1.183274342	1.632264027	1.968171873	1.963352045	2.207984657
Zm00001d026289_T003	1.625022147	0.695404147	1.672733084	0	0	0
Zm00001d027536_T005	0	0.047122341	0	0.532018663	0.373451106	0.331362414
Zm00001d027536_T003	0.564521577	0.807362342	0.306436076	0.152999796	0.961689257	0
Zm00001d027536_T004	0.652971939	0.561963446	0.169052709	0	0.864341081	0
Zm00001d027536_T008	2.520785548	1.877785469	2.774687135	2.993628716	3.033307229	3.184001569
Zm00001d032663_T001	7.642197414	7.660676712	7.594171345	6.895898628	6.723641956	7.059825708
Zm00001d002257_T001	2.069471894	1.918165615	2.129213106	2.463911438	2.587677261	2.584588313
Zm00001d002257_T005	7.260373835	6.946707739	7.309701083	7.730410988	7.691277916	7.573724591
Zm00001d002599_T001	2.770098318	1.992969597	2.963435467	2.138523542	1.912959446	2.139952059
Zm00001d002599_T002	1.568270507	1.128160741	1.627412598	1.938038828	2.103919896	1.841771892
Zm00001d048825_T003	2.960532468	4.13452136	3.718333786	3.057830089	2.854699662	2.468198547
Zm00001d048825_T001	5.50501781	5.522689207	5.449230573	4.825116013	4.999978495	5.031379668
Zm00001d021930_T001	5.608751682	5.041717844	5.675664937	6.469890257	6.450236164	6.48648492
Zm00001d021930_T005	1.549853962	1.066376495	1.497534194	3.126438848	3.13837478	3.219419934
Zm00001d021930_T002	2.312786264	0.916056195	2.745191931	3.985335203	3.902184552	3.885401094
Zm00001d011422_T001	2.125835229	1.776433769	2.387079875	1.28489134	1.597725666	1.219268286
Zm00001d039147_T001	2.974584927	2.547466857	2.766229194	3.757383448	3.844453749	3.690968477
Zm00001d013741_T007	0	0	0	3.008218125	2.843059661	2.754547841
Zm00001d013741_T001	2.287999099	1.451496523	2.514510631	1.898532269	2.003933191	2.413323281
Zm00001d013741_T003	1.186832669	0.590848216	0	0.987555171	0.66366159	0.332571605
Zm00001d013741_T008	1.254286848	0.323891132	0.714852146	0.717581456	1.019072755	0.785520421
Zm00001d013741_T002	3.588984173	3.009178024	3.775867451	2.941969979	2.730139083	3.199100492
Zm00001d041670_T001	3.355465697	3.314783665	3.397478251	1.370809861	1.440887368	1.783879448
Zm00001d002440_T001	4.875517052	4.044780011	4.96891313	5.046381789	5.090751617	5.08470198
Zm00001d002440_T003	1.441887123	0.146042566	0.257104783	2.353000554	1.666459843	2.408653174
Zm00001d002440_T004	1.154434797	1.001230815	2.295120552	2.815133331	2.99360861	2.418762324
Zm00001d002440_T002	2.717665019	2.574565373	2.988044579	3.366590793	3.123726776	3.433284645
Zm00001d050715_T002	1.87040615	1.248187479	1.968099602	2.355794365	2.532042859	2.558572552
Zm00001d028363_T001	3.83783438	2.971592294	3.840759397	3.955307874	4.029013296	3.875579074

Zm00001d037857_T001	2.199662923	2.840527487	2.247414734	3.033297889	3.068647253	3.108293774
Zm00001d047972_T001	4.05642876	3.123689204	3.820566727	3.179976746	3.130408375	3.390959964
Zm00001d047972_T002	2.473531853	2.670447665	2.62934776	2.691397705	2.619540253	2.354234922
Zm00001d000444_T001	2.030909158	1.678933658	2.086304486	2.38554562	1.957419876	2.297677712
Zm00001d029066_T021	1.218414171	0	1.531968775	2.152796394	1.587650621	1.40033408
Zm00001d029066_T017	1.059135811	0	0	0	1.268804452	1.58028637
Zm00001d029066_T010	1.220801076	0.506930921	0.864566124	1.711009375	1.451257014	1.713317498
Zm00001d029066_T004	0	0	0	0.65377637	0.708188119	0.40995134
Zm00001d041726_T001	3.376674347	2.809350232	3.516219178	2.80430225	2.262682105	2.619053759
Zm00001d020424_T002	0	1.014686646	1.399795078	1.647683829	1.715357476	1.674313753
Zm00001d020424_T011	0.374060152	0.116286231	0.136730819	0.599539656	0.56506289	0.767817489
Zm00001d017084_T001	0.71021684	0.496706741	1.141304604	3.349072519	3.459557783	3.462326693
Zm00001d008327_T001	5.470896128	4.904419211	5.628613135	4.610247437	4.635937125	4.620155356
Zm00001d050074_T004	0.90767367	0	0.171019752	1.942589195	2.104449387	1.484038596
Zm00001d050074_T007	0.340732021	0.193079084	0.717177829	0.505124676	0.816094214	0.683915593
Zm00001d050074_T002	2.138579897	1.342740759	0	2.088576851	0	2.445933242
Zm00001d050074_T005	1.503417942	3.77105542	1.735165142	0	0	0
Zm00001d050074_T003	0	0	2.194403238	1.140024724	2.340554863	0
Zm00001d010671_T002	6.119040377	5.911760312	6.265522749	6.773294	6.834973444	6.815299095
Zm00001d043025_T001	2.003357569	1.67620105	2.558711641	3.965831927	3.932790557	3.861265305
Zm00001d025036_T003	0	0	0.722015658	0.978200756	1.111484755	1.008129003
Zm00001d025036_T002	1.461943393	0	1.680916618	0	1.178687237	0
Zm00001d042302_T005	3.963464413	2.88799524	4.045917413	3.443285722	3.101310875	2.987991663
Zm00001d042302_T001	2.971941154	2.620339372	2.972141384	3.999054996	4.159586967	4.042379148
Zm00001d042302_T004	0.320504318	0.128208922	0.169473528	0.815121236	0.600895801	0.571437999
Zm00001d042302_T006	0.101615118	0.049903948	0.144655959	0.355111012	0.3904872	0.373132563
Zm00001d018943_T002	1.230828292	0.8172827	0.967352346	1.678115636	1.286988755	1.394503715
Zm00001d018943_T001	0.737251508	1.072442676	1.698651889	0	1.309795517	0
Zm00001d038035_T004	2.841439002	2.086719917	2.752710918	2.557675983	2.996775583	2.430274031
Zm00001d038035_T002	1.257951383	0.844101184	1.098195172	1.302500382	1.08104574	1.175847116
Zm00001d038035_T005	3.026567085	3.034335994	2.585863903	2.006207168	2.452554583	2.583598509
Zm00001d006472_T003	1.21608976	0.857746165	1.069874675	2.745562198	2.68710594	2.634479196

Zm00001d006472_T030	4.424551292	3.663205239	4.36481928	3.629555611	4.006525318	3.866356702
Zm00001d006472_T029	1.77149771	0.566718728	1.30103563	2.513062927	2.181696049	2.423404714
Zm00001d042338_T001	0.065561283	0.215956859	0.228727586	0.657883254	0.657308902	0.488534546
Zm00001d042338_T002	3.70844937	2.81177454	3.546077185	4.119851904	3.996691355	3.97773034
Zm00001d039519_T001	3.454764435	2.790752649	3.270492463	3.638235943	3.732848224	3.555143231
Zm00001d016723_T001	8.994224601	8.904542753	8.844030949	8.637641757	8.493835087	8.676096202
Zm00001d013692_T001	4.203356696	3.466557039	4.278955488	4.266894982	4.293752445	4.139991174
Zm00001d007341_T001	2.731186718	2.63358977	3.128990326	1.974984075	1.539526109	2.228439462
Zm00001d034137_T001	3.382762138	2.857186	3.433612747	3.061194683	3.195143937	3.243595665
Zm00001d002312_T004	0.096283446	0.294007225	0	0.568385315	0.435349095	0.566460585
Zm00001d020908_T006	0.26904033	0.360571043	0.745415479	0.552622992	0.767947121	0.340517834
Zm00001d020908_T004	4.83414517	3.974071652	4.799270015	4.855347117	4.911444529	4.934399355
Zm00001d054017_T006	3.611347807	4.924863609	0	4.244477629	3.044481902	0
Zm00001d054017_T004	4.195643588	3.424752555	4.117412875	4.136986766	3.964798668	4.055630116
Zm00001d054017_T007	1.843878552	2.466607806	2.521229429	2.817450328	2.550869644	3.051348608
Zm00001d054017_T005	5.097291042	4.3156978	5.243784985	5.070705226	5.303486043	5.513044951
Zm00001d054017_T002	1.748249536	1.900444876	1.41612362	1.707296398	1.799018486	1.831590295
Zm00001d014658_T005	0.512543461	0.160521392	0.507103503	2.643126348	2.564359632	2.937592416
Zm00001d014658_T001	5.767307694	4.401939436	5.739822537	6.668715013	6.82048085	6.654302115
Zm00001d014658_T002	2.370917522	1.310171409	2.200353058	4.073802928	4.156811841	4.01850074
Zm00001d014658_T003	6.162184432	5.648964548	6.110735939	5.874352632	5.610666256	5.854328272
Zm00001d031766_T001	1.782920966	0.982663987	1.762689834	1.869115323	1.715900397	1.47626969
Zm00001d026594_T001	4.961426912	4.717725823	5.172383142	3.331503156	3.157205762	3.4749227
Zm00001d048728_T001	7.820816403	7.6331122	7.8302057	8.251163657	8.271871272	8.314621317
Zm00001d007422_T008	4.046702595	2.931029442	3.935961209	4.725373948	4.771391218	4.731675662
Zm00001d007422_T006	0	0	0.940286297	1.820138515	1.292742884	1.322021578
Zm00001d007422_T001	0	1.513294162	0.645594686	0.662054157	0	0.760042062
Zm00001d007422_T012	4.655754353	7.163985157	5.173437372	3.019054424	0	4.330430617
Zm00001d007422_T011	4.096968351	2.943930701	4.02329794	4.001283878	4.491565234	3.066966943
Zm00001d007422_T010	4.496738565	2.180863938	4.644342931	5.106133834	4.754957765	4.663538522
Zm00001d007422_T007	0.814938371	0.4568072	0.840672699	0.25155429	0.804115529	0.514494977
Zm00001d038734_T004	2.737306463	1.413368779	2.618423328	2.502144969	2.65244611	2.29366329

Zm00001d038734_T001	3.086618533	2.337861205	3.063645824	2.461668422	2.545036492	2.800199162
Zm00001d038734_T006	0.923213087	1.05610625	0.6582901	0.666608452	0.679390467	1.4107465
Zm00001d042669_T003	4.329204055	3.680308489	4.330436999	3.38032107	3.933823672	3.862376205
Zm00001d042669_T006	0.528048153	0.163002721	0.288281774	0.699432588	0.876823772	0.69713594
Zm00001d042669_T005	3.110637022	2.901527664	3.600665703	4.413198289	4.011079802	4.301606083
Zm00001d042669_T001	1.322140444	0.800802596	0.944891421	1.98098568	2.232882669	2.182826392
Zm00001d042669_T004	2.782024774	0.249353103	2.297854346	3.62385118	3.25380612	2.882954442
Zm00001d037337_T001	4.063053756	4.097893072	4.022234369	3.801541737	3.707350076	3.760782477
Zm00001d048094_T001	2.857990348	3.653845602	2.879182891	1.775716831	2.857725068	2.938319106
Zm00001d049613_T001	2.332037623	1.709841137	2.201971914	2.241979871	2.312218763	2.127549753
Zm00001d028454_T001	1.403808154	0.955736101	1.546196119	1.255784108	1.14280159	1.275330127
Zm00001d043902_T001	3.913678544	3.478881229	3.991538807	5.619716074	5.707377825	5.723301343
Zm00001d013219_T001	3.5225423	3.018754156	3.6618539	2.93112024	3.044988562	2.848589492
Zm00001d012407_T004	2.552136274	0	2.639229616	2.718342116	2.554287266	2.313726767
Zm00001d012407_T006	3.941386839	4.863660883	4.005847818	3.962729834	3.818034725	3.612524126
Zm00001d012407_T001	1.305848565	1.081062107	1.321563335	0.791921566	1.151882191	1.399816948
Zm00001d012407_T007	6.65626732	7.28820092	6.543741003	6.270332764	6.305701121	6.310893114
Zm00001d001786_T004	3.910648246	2.700799459	3.834206164	3.729663048	3.888953709	3.54737172
Zm00001d012022_T001	2.178071459	1.391933752	2.015001504	0.683372176	0.930996905	0.958034168
Zm00001d006604_T001	3.05493771	4.17464043	3.391943235	3.261884751	3.121904126	3.087197361
Zm00001d048449_T001	6.897975081	6.44070771	7.005207241	7.038147627	7.024482795	7.055120877
Zm00001d035748_T001	1.82705277	1.095233786	1.900131438	2.623250004	2.881100742	2.459045449
Zm00001d038946_T002	3.128517444	2.217018485	2.666638672	3.139962043	3.228546746	3.300089158
Zm00001d028841_T001	1.805925947	2.221026676	1.863083678	1.976623188	2.195830096	1.616987151
Zm00001d040966_T001	1.211944814	0.919340845	1.366934836	1.387734196	1.291368394	1.143365988
Zm00001d015265_T003	0.964788227	0.071725606	1.304869043	1.584472382	1.748535513	1.582715437
Zm00001d015265_T001	3.819680444	3.188508854	3.788440891	3.873909637	3.730535078	3.628855116
Zm00001d034734_T007	1.474041925	0.523596079	1.516334926	0.54361169	1.25094157	0
Zm00001d034734_T004	0	0.188626269	0.296057949	0.142768921	0.808074441	0.604344651
Zm00001d034734_T003	0	0	0	1.717971374	0.97929371	1.140618347
Zm00001d034734_T001	0	0.619131253	0.865949687	1.48595486	1.233310744	1.577246085
Zm00001d036426_T001	2.157918443	2.19936705	2.299239968	2.278632773	1.653115063	2.228323088

Zm00001d017692_T001	0.780667054	0.254797191	0.799947692	0.705674517	0.874002921	0.684441748
Zm00001d035913_T004	1.228417912	0.489272662	1.174214643	1.131405103	1.719155835	0
Zm00001d035913_T010	0.442377716	0.513432127	0.281742161	0.357028317	0.24987854	0.523493709
Zm00001d035913_T007	0.258674492	0	0.590280081	0.468215233	0	0.850983398
Zm00001d035913_T003	1.01631089	0.510727019	0.898829033	0.8005972	0.655971825	0.909798445
Zm00001d032096_T012	1.900400821	0.832130096	1.876393176	2.114628885	1.722386455	2.024716827
Zm00001d032096_T010	0.55501018	0.359605503	0.638962588	0.774423165	1.098251103	1.057560485
Zm00001d032096_T005	0	0	0	0.963511116	1.206735734	1.001986665
Zm00001d039053_T001	4.880901375	4.25898618	4.794935091	4.332922496	4.384677602	4.338124006
Zm00001d012992_T002	8.030708712	8.438266907	8.134676157	7.303187249	7.407550377	7.394331616
Zm00001d023629_T001	3.367690604	2.44162105	3.152951624	3.523173003	3.674570025	3.243630089
Zm00001d048222_T001	5.031657825	4.657537287	5.262891933	5.073998246	5.096026924	5.112797282
Zm00001d052317_T002	0.220945291	0.185789177	0.350580978	0.457955643	0.157096759	0.513683766
Zm00001d030638_T002	1.200391354	0.831687571	1.675425741	1.816933054	1.339285068	1.285751975
Zm00001d028129_T002	4.936940202	4.129725568	5.11855006	5.669857267	5.735937759	5.758932993
Zm00001d032603_T001	3.975775622	2.979350788	3.8174491	3.492340581	3.428191043	3.599943183
Zm00001d032603_T002	0.784619561	0.371344742	0.820241878	0.771601659	0.615568846	0.516521515
Zm00001d047912_T002	5.183391589	4.713282685	5.278783511	5.085915952	5.098499104	4.890206629
Zm00001d029869_T001	1.788740828	2.289923856	2.205663071	1.139364056	1.678968352	1.219548335
Zm00001d037004_T001	5.789874113	5.199082545	5.890662132	6.055941499	6.08422692	5.942849641
Zm00001d037004_T004	0.546882116	0.283416889	0.568881415	1.562952086	1.61914769	1.424020189
Zm00001d031221_T001	4.651607244	3.996843991	4.809429056	4.988275676	4.952307611	4.831679262
Zm00001d042179_T001	5.021694206	4.690084824	5.109439588	4.285770984	4.209252232	4.229811077
Zm00001d035603_T001	2.247960315	2.074672919	2.447402783	1.74964024	2.127161438	1.451702237
Zm00001d006521_T001	2.283344875	2.355467388	2.497101616	2.532285287	2.340935356	2.128072624
Zm00001d043056_T006	3.450338966	2.580138006	3.319430216	3.570178857	3.654856304	3.50110356
Zm00001d053207_T001	0.516790765	0.31733533	0.518499889	0.952599713	0.600390607	0.877899699
Zm00001d032120_T001	0.663453928	0.40059039	0.147994339	3.747777294	3.771415728	3.714292013
Zm00001d044581_T002	2.115803673	0.407078599	2.225428571	2.466241567	2.565950739	2.196950367
Zm00001d044581_T015	1.80525613	1.691595355	1.926824556	2.035552829	1.722708639	1.438463729
Zm00001d012081_T001	9.752270561	9.968367635	9.552110309	9.252348087	9.172997778	9.103049825
Zm00001d042192_T001	4.991942814	4.533810317	5.055360144	5.141709033	5.177796075	5.006282986

Zm00001d037163_T002	7.287231875	7.26636331	7.283220427	7.468325007	7.376526587	7.371497076
Zm00001d042962_T002	2.860651587	2.143619047	2.966200275	3.564777263	3.493528214	3.465428095
Zm00001d042962_T007	1.117095236	0.834649737	1.445866517	1.73208421	1.749449741	1.186477738
Zm00001d021073_T002	1.639564663	1.390347418	1.953608688	1.746937441	2.004097921	1.988682142
Zm00001d021073_T004	1.236181544	0.875036903	1.408860198	1.07827303	1.484890354	1.422347658
Zm00001d011525_T001	1.94695389	1.031590695	2.535047141	2.982542243	2.918499966	2.943190513
Zm00001d011525_T008	1.755085405	0.987510059	1.379264957	1.268691888	1.197148897	1.262826402
Zm00001d011525_T007	5.137167566	4.405010653	5.092145309	4.946403597	4.990252487	5.024805106
Zm00001d011525_T004	0	0.11180722	0.945753014	0.199365165	1.295273398	0.910771798
Zm00001d031134_T001	0.844497336	0.617822235	1.221354957	0.860324813	0.618088269	0.976762476
Zm00001d017977_T002	1.604788222	0.928892536	1.750976885	2.231609969	1.639587351	2.239570043
Zm00001d017977_T014	1.379142942	2.232907836	2.30930543	0	1.094033335	0
Zm00001d017977_T016	0.277259946	0.146232908	0.226704575	0.838443558	1.234416093	0.942535524
Zm00001d017977_T006	0.330877307	0.277238517	0.564696185	1.67991107	1.348528748	0.750872296
Zm00001d017977_T013	2.87026094	1.597645584	2.731161297	3.723260927	3.662898989	3.760777687
Zm00001d040010_T004	0.193534586	0.03363232	0.09216413	0.424020189	0.407630114	0.574485207
Zm00001d040010_T003	5.223399419	4.257005714	5.21309718	5.390243811	5.39517854	5.373678241
Zm00001d027457_T001	4.089621534	4.228763521	3.618189077	3.546863723	2.594925416	3.434522949
Zm00001d001914_T002	0.816755333	0.910150862	0.209618057	1.003428815	0.50521311	0.744144731
Zm00001d001914_T003	3.117847282	2.278871509	3.086589152	3.540251619	3.540467006	3.552994499
Zm00001d001914_T001	1.388758786	1.192652472	2.01042646	1.199792309	1.673368273	1.594731025
Zm00001d017840_T001	8.474843735	8.022058499	8.442787117	8.293419108	8.355442474	8.372786182
Zm00001d024597_T001	3.006518137	2.378080137	3.223703647	3.471794721	3.31144833	3.220986913
Zm00001d017435_T001	4.207459744	3.857442521	4.447195992	3.739601009	3.665350017	3.795928785
Zm00001d027359_T001	5.213163258	4.439487903	5.176522566	4.910085174	4.893841444	4.914726483
Zm00001d051685_T002	2.284222722	4.475302114	0	0	1.039848685	0.423997608
Zm00001d051685_T001	7.853955383	6.896589832	7.934375056	7.745623781	7.759194271	7.700209021
Zm00001d021051_T004	5.17753184	3.942990633	5.323863218	4.792938362	5.067642287	5.145708265
Zm00001d021051_T002	5.931090163	5.676607503	5.783701378	6.008941447	6.026075808	5.832187377
Zm00001d021051_T003	0.637027765	0.620379961	0.989697081	1.533776622	0.172680801	0.688311975
Zm00001d021051_T001	1.221243579	0	0.765152806	0	1.61178805	1.2173635
Zm00001d053223_T001	6.675869805	6.027224685	6.67762219	6.084220414	6.077071818	6.091927876

Zm00001d030007_T001	2.62110217	2.159947202	2.449201349	1.868641335	2.449674409	2.107756153
Zm00001d007868_T001	1.89212986	1.631120584	1.731890945	3.261004038	3.199969882	3.130053293
Zm00001d027525_T001	2.456371436	1.670438373	2.484453195	2.796823651	2.214122629	2.205373118
Zm00001d008956_T001	2.670021338	2.589326059	2.518150621	2.777805575	2.763995971	3.186336851
Zm00001d018844_T001	1.329609294	1.037947943	1.083160937	4.217041525	4.95202752	4.620655787
Zm00001d020170_T003	1.144603103	0.72755979	0.7378892	0.849372421	1.807236204	0.185390853
Zm00001d020170_T001	4.940837002	4.095962216	5.083001545	5.304190123	5.199811818	5.184263947
Zm00001d044189_T001	3.402126751	2.718040663	3.237877706	3.488172032	3.559612339	3.372202705
Zm00001d023271_T002	1.896596976	1.754299618	2.344544758	1.886072486	1.759575582	2.030529602
Zm00001d007390_T002	3.537073867	4.148882707	3.349131415	2.747105708	3.327831483	3.402137805
Zm00001d053632_T002	6.339072769	5.0956083	6.471573466	6.102049161	6.20893673	6.296836313
Zm00001d053632_T001	6.502545551	6.631889837	6.549563028	6.257507346	6.008549725	5.895258215
Zm00001d016665_T001	3.824317564	3.272553424	3.90178839	3.622190077	3.57326872	3.861744203
Zm00001d016665_T002	4.830834963	4.584469736	4.869438977	4.663723405	4.703766231	4.638474665
Zm00001d044922_T001	1.238212724	1.262592513	1.711790464	1.940009596	1.745909357	2.123748293
Zm00001d044922_T002	1.587891563	0.715549015	0.889035021	1.339914406	1.856705288	1.336657012
Zm00001d036836_T001	3.0086943	3.568933568	3.206277503	3.226384601	2.381513742	2.948069644
Zm00001d021802_T003	1.068325485	0.495835333	1.027955169	1.852979217	1.498921756	1.537147938
Zm00001d021802_T002	6.54743931	5.741479202	6.573548127	6.329622101	6.309631088	6.256770577
Zm00001d021802_T004	1.070634565	0.296023873	1.971462255	1.316149798	1.456995868	1.609615035
Zm00001d021802_T005	1.582690873	0.633648819	1.647208117	2.846805791	2.89000185	3.100721538
Zm00001d047241_T005	1.359861283	0.679036385	0.80210386	1.28809333	1.173613581	0.792756255
Zm00001d047241_T003	1.013107036	2.466135567	0.742641806	0.215714625	0.604048544	1.133628623
Zm00001d047241_T006	0.491135802	0.329898568	0.701453244	0.828485355	0.97783894	0.930586722
Zm00001d047241_T004	4.150890338	2.39475448	4.198911893	4.143587784	4.154097088	3.932969375
Zm00001d048451_T001	0.914539267	0.608339936	1.012479284	0.500625656	0.493101097	0.662315806
Zm00001d032636_T001	2.39588704	2.065423416	2.651752513	2.055264733	1.957190284	1.589580383
Zm00001d015568_T003	0.667390784	0.515604475	0.493132872	0.887622747	1.137221515	0.862423618
Zm00001d015568_T002	6.604261955	6.568977089	6.641393631	6.441928526	6.429514068	6.415428236
Zm00001d047236_T003	1.382306465	0.406440886	1.321608358	1.613418967	1.469872956	1.289839186
Zm00001d043473_T024	0	0	0.492503381	0	1.039946217	1.526962204
Zm00001d043473_T016	0.105525219	0	0.392725167	0.711452616	0	0.631775754

Zm00001d043473_T009	0.651448988	0.22055532	0.633905443	1.092444259	0.545151928	0.348456796
Zm00001d043473_T025	1.182265781	0.810979362	0.685176787	1.230721331	1.460010679	1.317422744
Zm00001d043473_T010	0.784409336	0	0.706806357	1.784998895	1.691515405	1.39471278
Zm00001d043473_T001	0.589581341	0.402936055	0	0.407769318	0.969531805	0.919010504
Zm00001d043473_T008	0	1.731689834	0.730481713	0	1.579371336	1.785413704
Zm00001d043473_T012	3.075317837	1.029004017	3.098945515	3.543229963	3.640895282	3.629576476
Zm00001d013828_T003	1.279035598	0.39256582	0.570743671	1.853303866	1.181058013	1.833534982
Zm00001d013828_T002	1.968525062	1.534656293	2.273107939	2.166659535	2.527720953	1.869853644
Zm00001d013828_T001	0.824452605	0.299163757	0.496943935	0.569788544	0.481668869	0.959687077
Zm00001d033932_T001	3.836112784	3.251451885	4.162275062	4.207516515	4.359237706	4.059730094
Zm00001d033932_T004	0.784897582	0.055465006	0.3373857	0.889581011	0.250823359	0.793329927
Zm00001d022092_T001	3.853439614	3.59033494	3.617001731	3.642900578	3.756239796	3.735270117
Zm00001d030021_T001	1.985803874	1.328232726	2.217078062	2.454533627	0	2.264895484
Zm00001d030021_T005	1.947224772	1.45588032	1.850450606	0.972897741	1.560706641	0.652692989
Zm00001d044621_T001	5.088296987	5.09228923	5.381394028	4.426729563	4.698462649	4.761372513
Zm00001d053828_T001	1.649677994	1.888275277	0.394798373	1.723308225	1.481746872	0.461039196
Zm00001d017708_T002	2.62424036	1.836696134	2.769459051	2.415518828	2.57948985	2.720413096
Zm00001d017708_T001	6.589153583	6.742151174	6.666168105	6.772952023	6.674185332	6.658382296
Zm00001d017206_T001	3.737269899	4.086465509	3.932876238	4.146781143	3.917545944	4.064151664
Zm00001d050583_T001	2.387889392	2.307097687	2.426683411	3.214607126	3.153351707	3.106033512
Zm00001d049716_T001	5.791671097	5.167498393	5.868401467	5.436532029	5.495530388	5.501237759
Zm00001d032547_T012	3.426517439	0	3.778805615	3.367459554	3.641175597	3.827791491
Zm00001d032547_T007	0.87828825	0.624947776	0.683994618	2.176470226	2.187971661	1.5920221
Zm00001d032547_T011	1.401756601	0	1.315830517	1.97791695	2.539951114	1.985459262
Zm00001d032547_T002	0.302582266	1.231347614	0.280041723	0.444872697	0.524887121	0.877824332
Zm00001d032547_T004	3.552808635	2.555056577	3.16540936	4.09678084	3.748946022	3.687203607
Zm00001d032547_T006	1.873306493	2.131825524	1.454074796	1.271064061	1.77549814	0.774494855
Zm00001d017457_T001	3.700451926	3.229153357	3.924606261	6.212690064	6.165892046	6.191261104
Zm00001d009426_T006	2.47693144	1.704414301	2.430621952	2.348534696	2.363473885	2.075917641
Zm00001d019752_T001	1.86724928	1.381301648	1.916601609	2.052452413	2.166338817	2.066698869
Zm00001d013468_T001	0	0	1.714710623	0	2.41308087	0.790219038
Zm00001d013468_T003	0.309570866	0.092382013	0.304662896	0.894875955	1.174501018	1.57380349

Zm00001d013468_T002	3.583836639	2.855670834	3.274269564	5.703879328	5.455559085	5.581592821
Zm00001d034618_T002	5.685914001	4.460021822	5.725901611	5.189803017	5.206714794	5.129429381
Zm00001d031569_T001	5.738524828	5.142875541	5.815182707	6.208622446	6.191006452	6.264135449
Zm00001d012963_T001	1.942166152	1.241225764	1.692192384	1.497965875	1.358994822	1.20507904
Zm00001d047712_T001	0.81659643	0.401303885	0.679147214	0.728368981	0.460155408	0.499025884
Zm00001d012108_T001	0.176569159	0.345874356	0.44900981	0.720677723	0.812299418	0.293615327
Zm00001d012108_T012	0.500151419	0.173025066	0.398365695	0.386273491	0.286081531	0.368678963
Zm00001d012108_T011	4.361507654	3.965031671	4.467792485	3.96293847	3.967209287	4.000530363
Zm00001d012108_T006	0.061270215	0	0.150971631	0.933325622	0.645089225	1.021122918
Zm00001d012108_T009	0.533908158	0.265650524	0.969290133	0.728200038	1.377308691	1.19390229
Zm00001d012108_T002	3.605329218	3.180244632	3.44060583	3.999951669	3.751232751	3.748509107
Zm00001d006798_T012	0.109767065	0.292427215	0.265340873	0.607834503	0.33003974	1.070811083
Zm00001d006798_T002	0	0.550817941	0.568464119	0.666637536	1.929123265	0.837764861
Zm00001d006798_T003	0.844194406	0.375893532	0.688889332	1.089952311	1.413809604	1.247409265
Zm00001d006798_T001	3.64089216	3.125307743	3.523388195	2.532656333	2.850300773	2.620707216
Zm00001d006798_T013	1.503182319	0.557807074	1.638548863	1.933780719	0	2.089154724
Zm00001d006798_T007	0	0	0.864943231	1.73589515	1.484307274	0
Zm00001d006798_T020	0.265747726	0	0.220679132	0.482964933	1.320088352	1.408383074
Zm00001d005785_T014	0.676895629	0.268732604	0	0	1.133631253	0
Zm00001d005785_T011	1.548696533	0.409094355	0.944581872	1.454232757	1.104212516	1.22413044
Zm00001d005785_T002	0.28870583	0.169599236	0.659188414	0.585408705	0.714570843	0.573198057
Zm00001d005785_T005	0.699387277	0.841147911	0.908214959	0.615937909	0.264911093	0.92857726
Zm00001d028543_T001	4.159691826	3.517690928	4.321989841	4.173821743	4.228851853	4.279375162
Zm00001d010261_T001	3.607874263	4.541015931	3.897579661	3.238956641	3.096971723	3.592897463
Zm00001d013100_T001	4.621576956	3.890521403	4.803640667	4.479198607	4.593817104	4.624031098
Zm00001d013100_T002	5.967312985	6.495003754	6.051371275	5.45971908	5.609585369	5.596942594
Zm00001d027344_T005	3.138556307	3.69500006	3.264946512	3.433148148	3.654992942	3.301070466
Zm00001d027344_T006	4.967480128	2.645520907	4.906796192	5.364484012	5.288807839	5.398589249
Zm00001d027344_T001	3.876602302	2.361741975	3.225269685	2.80677979	3.008114657	2.429537501
Zm00001d027344_T003	0	0	0	4.086890911	3.649150848	4.282743436
Zm00001d027344_T009	1.67139121	4.725450789	2.697614154	1.233400332	1.741749565	1.123790002
Zm00001d027344_T008	0.924906353	0.323480753	1.356434574	1.113713166	1.101257416	1.563781943

Zm00001d002450_T002	8.097013049	8.163953075	8.206955288	7.47821212	7.469896482	7.499231144
Zm00001d045465_T001	5.504710288	5.056768492	5.55870855	5.815149525	5.761339799	5.782626054
Zm00001d050265_T002	3.150002149	1.37646154	3.408454	2.88382704	1.495961678	2.536251879
Zm00001d050265_T001	4.287155322	3.946273654	4.558210317	4.646805159	4.807547818	4.596977826
Zm00001d018776_T019	0.591596127	0.263777203	0.842650644	0.799222449	0.583691408	0.802917795
Zm00001d018776_T007	0	0	0.988159677	0.608021931	0.929185772	0
Zm00001d018776_T005	0.445685392	0.230508001	0.46064507	1.23393529	0.793393192	1.017768725
Zm00001d018776_T002	1.410941289	0.609478876	0.334704416	0.862613258	1.014188874	0.161090514
Zm00001d030542_T001	2.196912609	1.494448381	1.603388729	1.028761499	1.029102992	1.16118083
Zm00001d026547_T001	6.507981449	6.153118474	6.513916764	6.264189396	6.247941447	6.230166231
Zm00001d013076_T001	5.29424468	4.549148117	5.258942023	4.567525126	4.55827737	4.709213646
Zm00001d002711_T002	1.32032177	0.346605212	0.980818656	1.514884761	1.68980368	1.238268986
Zm00001d002711_T003	2.794799702	1.008256676	2.380193602	2.263393232	2.415440406	1.994288622
Zm00001d002711_T001	2.42315915	2.179533662	2.876383942	3.075950148	2.661384776	3.232119338
Zm00001d022516_T001	7.692545421	7.963705023	7.77070846	8.734025265	8.751201514	8.771350571
Zm00001d004709_T001	2.269194767	1.45121375	2.120738443	2.558333276	2.524703366	2.499848948
Zm00001d039695_T001	5.509371991	4.722791141	5.572189064	6.429833892	6.45535032	6.394153492
Zm00001d037426_T001	4.08426866	4.42608236	4.27499274	4.522902619	4.932878269	5.001707276
Zm00001d011788_T001	1.506375472	0.897738408	1.392355345	0.88140542	0.908664596	0.999914878
Zm00001d020636_T003	0.919147837	0.456422431	0.92134416	1.156386908	1.028122838	0.905553463
Zm00001d020636_T001	8.435577	7.734228872	8.474909741	8.732353677	8.794000548	8.744357713
Zm00001d002119_T020	1.567994638	0.315707653	2.02401146	1.313383438	1.44558052	0
Zm00001d002119_T009	0	0	0	1.518371473	1.331683435	1.741125229
Zm00001d002119_T006	1.231481562	0.870902831	0.458909006	0	0.81064299	0.802915314
Zm00001d045667_T001	5.331026949	4.486068297	5.450810432	4.830945419	4.888130929	4.885010305
Zm00001d034575_T002	0.601820103	0.220203636	0.381699783	0.698040631	0.403537146	0.325691501
Zm00001d034575_T001	4.53133442	3.675352343	4.544279069	4.682348168	4.660327806	4.633314197
Zm00001d041539_T001	4.317005237	4.706677522	4.366697791	2.776041224	2.689932689	2.984304246
Zm00001d041539_T002	3.50724333	4.531655313	3.606120132	2.03342864	2.599456343	1.884387313
Zm00001d016072_T002	3.054856289	2.815711495	3.345174504	2.987005913	2.723286597	2.995350283
Zm00001d003643_T003	2.551518232	3.578326132	4.036086905	3.289336222	3.357680082	3.330449979
Zm00001d003643_T005	3.365232699	0	2.871592929	3.830257657	3.83394649	3.826673863

Zm00001d003643_T006	1.575976882	1.875210342	1.665944819	1.80128781	1.79283495	1.563710203
Zm00001d028643_T001	5.689335591	5.040556215	5.67743323	6.29171573	6.219166139	6.26486511
Zm00001d036970_T001	2.0638301	1.407600204	1.870995915	2.115903526	2.184348222	1.950580765
Zm00001d006626_T001	4.246244882	3.682904832	4.168391403	7.270361855	7.348792924	7.294924501
Zm00001d053716_T001	3.802038184	3.170203575	3.734478879	3.416130378	3.569049418	3.46376447
Zm00001d045127_T002	3.133296366	2.730967473	3.164048494	3.421955067	3.559811031	3.347764147
Zm00001d015767_T002	5.152844733	5.00610652	5.233718333	5.257778619	5.066663872	5.144945163
Zm00001d016118_T001	1.14675622	1.185409884	1.805748927	0.90049859	0.979841706	0.856851868
Zm00001d037518_T003	2.594984636	1.769819123	1.811888571	0	2.416718432	1.439902874
Zm00001d037518_T005	0	0.311488003	0	2.030585041	0.87495824	2.099017413
Zm00001d037518_T008	0.764369937	0	1.971446804	1.770207444	0	0.56892129
Zm00001d029246_T001	4.401945987	3.461881463	4.264463394	3.287099605	3.294531501	3.283489936
Zm00001d017808_T009	1.909547323	1.254682924	1.995790211	2.547189202	1.538525748	1.855794402
Zm00001d017808_T012	0.368267718	0.175509272	0.410218432	0.498461256	0.631854894	0.440470688
Zm00001d017808_T001	0.513555425	0	0	1.337193855	2.211925196	2.155490198
Zm00001d017808_T008	1.052042999	0.962472028	1.241440566	2.383157105	1.470973577	1.537319428
Zm00001d017808_T013	3.203013171	2.755813736	3.304140829	0	2.175905863	1.873808081
Zm00001d017808_T014	0.453306855	0.240019959	0.584442075	0.792498069	0.883535581	0.583952261
Zm00001d016170_T001	4.92261694	4.912634825	4.763472606	4.440730131	4.655473995	4.676215326
Zm00001d019376_T007	1.022946551	0	0.948433395	0.361879504	0.510848524	0.487876298
Zm00001d003349_T001	2.493257661	1.990550123	2.373955786	3.084203594	3.221392081	2.990312673
Zm00001d040941_T001	1.870874037	3.811019244	2.299520449	0	2.60451403	0
Zm00001d040941_T002	3.920597911	2.365921212	3.74544732	4.029323188	3.671671209	4.053979039
Zm00001d040941_T006	1.683700945	1.027078304	0	1.945107992	1.987697046	2.227903385
Zm00001d011473_T002	0	0	0	1.111453373	2.103512411	1.604630734
Zm00001d011473_T006	1.932523848	1.243545399	1.918793414	3.471125146	3.124842417	3.047901111
Zm00001d028687_T001	4.891916121	4.105982071	5.089504888	5.461463666	5.510583162	5.489328224
Zm00001d044475_T001	5.447771488	4.750980099	5.402856298	5.544804078	5.36961598	5.451314389
Zm00001d031913_T001	5.501623754	4.80072159	5.216928184	4.489687493	4.206887768	3.502227348
Zm00001d031913_T004	0	0	3.630034964	0	2.549943639	3.823499924
Zm00001d017768_T008	2.450437422	1.632585574	1.524040099	3.386186011	4.144290497	3.94159126
Zm00001d017768_T001	1.526094359	0.851416846	1.475139372	2.548962065	2.531236965	2.489892521

Zm00001d017768_T013	0.244013873	0.266889662	0.368360482	1.114411671	0.940927466	1.218536307
Zm00001d017768_T003	0	1.005310872	1.829126611	2.468748038	0.792561371	2.349927128
Zm00001d042096_T001	8.56067609	9.111517024	8.558536629	7.462354965	7.410189518	7.457958285
Zm00001d036195_T002	3.597877832	2.51383685	3.701436054	4.854196174	4.863283243	4.79757111
Zm00001d036195_T003	2.688923564	2.66856394	3.575309789	0	0	0
Zm00001d036195_T001	0.221731103	0.122675369	0.2244256	0.483160016	0.584820148	0.610546998
Zm00001d053649_T003	5.268259698	0	5.286758491	5.537634769	5.278013527	5.698335345
Zm00001d053649_T002	3.299282469	4.770714207	3.377873089	2.53652717	3.762133831	0
Zm00001d046187_T001	2.302486052	1.752155552	2.425943971	0.922021212	0.403171721	0.99655217
Zm00001d010965_T001	1.988567994	2.802584915	1.785260937	1.64699669	1.725953565	2.072260526
Zm00001d020381_T011	0	1.053899563	1.635361108	0	2.159664704	2.52711714
Zm00001d020381_T004	3.681354694	0	4.196467851	4.216691466	4.560239398	4.432900964
Zm00001d020381_T010	4.172668241	0	2.269041827	3.369633846	2.2593656	2.683036369
Zm00001d020381_T001	2.449457048	3.880278516	2.97546676	2.804491424	3.332383989	2.903181375
Zm00001d020381_T005	1.550143667	0.408330352	1.259541263	0.5693929	1.282910643	0.797027093
Zm00001d041395_T010	3.583331334	2.698488541	3.534009045	3.190334195	3.176458258	3.031143197
Zm00001d041395_T002	1.316226276	0.729364822	1.451983332	2.349788738	2.619280356	2.59603252
Zm00001d041395_T003	0.291312397	0.244765308	0	0.794049817	0.858351074	0.415055533
Zm00001d042045_T001	2.329701994	1.801347415	2.401928585	2.845082928	2.852159283	2.65320749
Zm00001d002370_T001	6.425322266	5.801560154	6.433431562	7.819345527	7.857451448	7.773928948
Zm00001d031000_T001	2.671237204	3.246743562	2.54727559	2.628244471	2.905776212	2.174734935
Zm00001d045220_T001	6.855588292	6.216462374	6.935919958	6.493676136	6.63667763	6.509109303
Zm00001d042626_T001	1.102615807	0.873266721	1.124301001	1.3406443	1.667615592	1.406440342
Zm00001d007387_T005	1.977277359	1.885092865	1.834559132	2.166491794	2.530596421	2.419200425
Zm00001d007387_T004	0.053524271	0	0.314032878	0	0.335283154	0.352250046
Zm00001d032029_T003	2.35419796	1.735204576	2.38406252	2.138333818	2.204599313	2.078894315
Zm00001d009571_T007	3.737125256	2.250040501	2.939380056	4.55403518	4.605501801	4.310962367
Zm00001d009571_T004	0	0	0	2.078073506	1.105722994	2.26648523
Zm00001d009571_T005	1.014726632	0.252045006	0.776519256	2.22589217	2.298321061	2.232336574
Zm00001d009571_T008	1.07779399	0	1.089677799	1.392216843	1.61454684	1.012195348
Zm00001d009571_T001	1.53464235	1.927375018	2.987727056	0	1.370082237	1.24417893
Zm00001d018512_T001	0.3986623	1.358593184	1.172495836	1.510337629	1.68033336	1.246746754

Zm00001d018512_T002	4.946824604	4.052896307	4.931365648	4.831226007	4.910524348	4.872471488
Zm00001d023658_T009	0.998950057	0.38012432	0.953471616	1.267689198	1.248257321	1.088784084
Zm00001d023658_T008	0.31086591	0.276971828	0.087423464	0.117377216	0.571807848	0.79951259
Zm00001d023658_T007	0	0.436711942	0	0.536537365	1.599203516	0.843814247
Zm00001d023658_T003	0	1.65980996	0	0.836495382	0	2.386171661
Zm00001d023658_T001	4.707680824	2.309234406	4.264003391	5.636070867	5.54792191	5.554234458
Zm00001d023658_T002	3.794767061	3.821078209	4.024144031	1.926834801	2.262376627	0
Zm00001d023658_T004	2.776166749	2.388074613	3.511013552	3.801610333	3.939417294	3.772509203
Zm00001d004640_T001	3.335010398	2.514079073	3.092599863	3.822663772	3.571154118	3.58070231
Zm00001d004640_T002	1.532386287	1.927544932	2.271162692	2.218894595	2.178733123	1.96129549
Zm00001d025353_T020	0.440108121	0.255404047	0.726995091	0	0.962781464	0.733531358
Zm00001d025353_T001	2.504507515	1.228125415	2.539229548	3.055162155	2.640786565	2.603787502
Zm00001d025353_T019	2.298936565	2.171761505	1.914918074	1.715210289	1.608835731	1.186322428
Zm00001d025353_T010	0	0.431797303	0.555246816	0.409209519	0.70342589	0
Zm00001d007130_T001	2.48873736	2.39236139	2.890746291	2.632738178	2.824487432	2.109650741
Zm00001d051516_T001	1.041018639	0.749680139	0.816217121	0.49879618	0.482083116	0.819132693
Zm00001d024672_T001	3.39948435	3.130311004	3.173235712	3.690973728	3.813556395	3.946748543
Zm00001d041497_T003	0	0.990931632	0	0.624545452	0.559542717	0
Zm00001d041497_T008	2.83836832	1.946657882	2.858689234	2.308691715	0	1.714504025
Zm00001d041497_T005	1.619192305	0	1.400778359	1.643591171	2.525143286	2.597873185
Zm00001d041694_T001	0	0.072990702	0.070477261	0.449782153	0.376339287	0.289176989
Zm00001d043927_T011	0	0	0	2.856869194	1.569625245	1.766828772
Zm00001d043927_T002	2.163575695	1.705720957	1.995488499	1.302696895	2.828716874	2.611231173
Zm00001d043927_T009	3.447273831	2.236298205	3.621711274	2.982803605	3.291162372	3.378940484
Zm00001d043927_T010	1.094489448	0.425191796	0.541857755	1.132995937	1.734622053	1.316197308
Zm00001d040348_T003	0.188587026	0.064735264	0.156035412	1.13502455	0.551065113	0.711819091
Zm00001d040348_T005	0.323630624	0.183865043	0.412010798	0.324933846	0.73100159	1.048932251
Zm00001d040348_T001	3.252133439	2.818847494	3.099214229	3.482579075	3.632470062	3.540320814
Zm00001d013499_T028	0.733002844	0.355087326	0.890364195	1.791959896	1.334696408	1.417082332
Zm00001d013499_T029	1.15332025	0.473572894	0.857576586	1.806748038	1.316909184	1.46858436
Zm00001d013499_T002	2.591578415	2.135103376	2.77520945	2.968622193	3.0251805	2.965559345
Zm00001d013499_T027	0.927937402	0.160982127	0.598306124	0.350680543	1.307495558	0.819355089

Zm00001d013499_T025	0.031238496	0.092982712	0.223018415	0.371091549	0.687328593	0.791017192
Zm00001d016521_T001	2.068688006	1.225729017	1.96366684	2.409108751	2.628060585	2.473919098
Zm00001d048415_T009	1.092901551	0.581907487	1.031042243	0.714467099	0.448024499	1.221892544
Zm00001d048415_T015	0.309577851	0	0.284088843	0.849855183	0.806699377	0.119122405
Zm00001d048415_T007	0.657546719	0	0.917360717	0	0	0.806836283
Zm00001d048415_T022	0.830156328	0.477058675	0.685337386	0.384223362	0.706830222	1.212255554
Zm00001d048415_T010	0	0	0	1.898249378	1.809389747	2.134936523
Zm00001d048415_T012	2.37726343	1.721525343	2.634494995	0.82404276	0.703079431	0.496355991
Zm00001d048415_T016	2.980051448	1.545225585	0	1.649727652	2.468488177	0
Zm00001d048415_T019	2.114478638	0.851376866	0	2.872461589	2.816465158	2.751788687
Zm00001d048415_T008	1.728110333	1.314716245	2.192381356	1.937030958	0.940441165	1.635629035
Zm00001d048415_T018	0.76088603	0.424059975	0.54252254	1.497533172	1.615988271	1.291090155
Zm00001d034160_T001	4.03681918	3.423819093	4.056281259	7.668803248	7.707042705	7.625725754
Zm00001d035316_T002	0.685536541	0.458024961	0.89870987	1.152135383	1.020847788	1.185020339
Zm00001d041138_T003	2.795573717	2.31642035	3.044381878	3.14913301	2.50849356	2.168513034
Zm00001d041138_T001	0.258789047	0.229960699	0.317735886	0.661979391	0.64825374	0.758532591
Zm00001d041138_T006	2.960202899	2.170593295	2.969851517	3.971721403	3.91535119	4.077664037
Zm00001d041138_T005	5.056195969	6.502596149	5.143662144	4.872083979	4.652053507	4.519806756
Zm00001d041138_T004	4.152964762	2.355165997	4.250432135	3.568425569	3.812890626	4.102533843
Zm00001d039395_T001	5.238993888	4.858530806	5.314011228	4.842376293	4.784201069	4.698916205
Zm00001d020703_T001	3.519927939	2.72637153	3.594827149	4.490765921	4.437361387	4.461930562
Zm00001d011890_T001	2.37212217	1.794345991	2.340743698	0.38148827	0.258966287	0.589017502
Zm00001d049476_T024	2.21912855	2.569788787	1.774907635	1.769255492	2.181432726	1.648409299
Zm00001d049476_T166	2.588566176	1.474128651	2.272417711	2.390531773	0	2.467181123
Zm00001d019726_T001	1.318761658	0.985089422	1.955268904	3.383957908	3.069591503	2.889030931
Zm00001d009077_T002	3.222414938	2.543891271	3.6431551	3.725666675	3.557585561	3.508778001
Zm00001d009077_T003	0.067759854	0.078559285	0.53089576	0.351961829	0.807897273	0.867475056
Zm00001d009077_T004	0.764081985	0.130658115	0.130208683	0.708068901	0.712752477	0.660723298
Zm00001d036177_T003	1.534759367	1.555635071	1.805960605	1.74461106	2.326924718	2.341732471
Zm00001d012393_T001	3.235529173	3.17196129	3.24743585	4.073189216	3.373086892	3.922493304
Zm00001d012393_T002	1.950728188	0.778292696	1.651318558	1.202353775	2.061347645	1.766706672
Zm00001d012393_T004	2.316234966	1.866671432	2.757623035	0	2.474145009	0

Zm00001d013683_T002	4.032101637	3.343077315	3.975156913	4.094736618	4.029211592	3.643354275
Zm00001d013683_T009	1.34218037	1.385143298	1.5723736	1.759653552	1.64632812	1.289139798
Zm00001d013683_T001	2.914836582	2.100116818	2.879369948	3.72113518	3.689540708	3.717635301
Zm00001d024828_T002	0	1.710808858	0	0	0.47394273	2.060013132
Zm00001d024828_T004	2.42119825	1.575579543	2.158706703	2.123738363	2.016548379	1.116351448
Zm00001d024828_T001	4.521823968	4.084856679	5.112424201	4.671383227	4.680698543	4.174123781
Zm00001d024828_T005	3.79610909	0	2.173759394	2.181631495	2.402017299	3.041255951
Zm00001d038891_T002	2.620708624	2.002921023	2.829009265	4.837211166	4.670437014	4.587575919
Zm00001d038891_T004	3.514738226	2.466565783	3.114545768	3.490190796	3.59675867	3.367742316
Zm00001d049760_T001	2.171909746	1.673905975	2.09046053	2.131448566	2.080718712	2.304471031
Zm00001d017967_T001	0	0.094346886	0.459726423	0.787062967	0.726316811	0.416872162
Zm00001d017967_T003	0	0.050693928	0	0.325304663	0.256761898	0.626298949
Zm00001d017967_T006	1.751362168	0	0	2.006329984	1.377645191	1.218985703
Zm00001d017967_T002	0.813846438	1.502377452	0.746444345	1.303528857	1.600544753	1.746904773
Zm00001d042306_T004	4.624837908	3.914952124	4.727303339	4.321834822	4.325750353	4.213104764
Zm00001d037191_T001	3.471925531	3.991600934	3.450499185	4.022020725	4.022775616	4.218684391
Zm00001d037191_T002	3.087561111	2.956490511	3.265046613	3.707963143	3.594278497	3.458998614
Zm00001d016260_T001	4.048090897	4.243663826	3.933386719	2.245816294	1.887978674	1.484971791
Zm00001d053626_T001	1.441636984	1.407682861	1.258078662	0.931608935	1.556295502	1.262186573
Zm00001d053626_T002	2.347903157	1.883383475	2.596966619	2.271623182	2.164110138	2.181381519
Zm00001d052723_T004	0.438510038	0.221805331	0.219045199	0	0	0
Zm00001d052723_T010	3.536523441	0	3.743836991	2.680203264	3.518287996	3.29392853
Zm00001d052723_T002	3.998743316	3.457731253	3.957613402	4.766907518	4.557423815	4.635772381
Zm00001d052723_T009	0.656726085	0.540820831	1.029623207	1.599413018	1.308708604	1.377599663
Zm00001d037711_T001	3.522664446	3.220286921	3.630019233	3.649921324	3.644995008	3.429411361
Zm00001d009726_T001	1.151888034	0.456578033	1.094117136	0.817375215	0.763346981	1.36657177
Zm00001d009726_T003	1.92842604	2.181790808	2.151802653	1.275213302	0	0
Zm00001d012936_T001	5.290423777	5.781012166	5.501029527	4.878951045	4.843241107	5.000089129
Zm00001d024967_T006	0.501848774	0.240625735	0.800085239	1.139769398	1.805688265	1.038540131
Zm00001d024967_T003	2.700417967	2.19112864	2.642428716	3.350903125	3.0318091	3.625212383
Zm00001d024967_T005	0.356016442	0.166750146	0.168032227	0.553480436	0.527022278	0.505461105
Zm00001d024967_T002	1.186463159	0.415387671	0.765553416	0.882568712	1.361803725	0.912337151

Zm00001d051457_T002	0.626595196	1.313219146	0.577291048	1.220372678	0.911354882	1.251348867
Zm00001d051457_T001	4.20404037	3.401583914	4.224755191	4.654074535	4.675196275	4.693944087
Zm00001d019950_T002	3.977405868	3.900846043	3.501442457	3.165388134	3.443723601	3.343914169
Zm00001d019950_T001	1.692373185	1.104492328	1.864112435	2.351446002	2.238364687	2.124143816
Zm00001d032480_T001	5.14945782	4.202634619	4.918210658	5.603481757	5.52125745	5.458301119
Zm00001d032480_T002	4.135610713	3.855505197	4.441693282	4.779593509	5.010974437	5.069568819
Zm00001d009309_T001	3.03747465	3.502058257	2.687434083	5.559787511	5.682440314	5.67046163
Zm00001d013154_T001	4.740762995	3.667475488	4.72485804	5.510758314	5.552144453	5.543042124
Zm00001d031480_T001	4.212802844	3.807566261	4.159358897	3.987181957	3.924231509	3.729781474
Zm00001d043510_T001	4.176360675	3.350914437	4.25219128	2.956810152	3.126153015	3.032247222
Zm00001d043510_T002	1.584309293	1.508407354	1.194822715	2.781084578	2.140975582	2.731194974
Zm00001d020646_T001	2.08533457	1.867241371	1.728280158	1.55367162	1.506915184	1.46313956
Zm00001d020727_T002	0.224836748	0.104073596	0.199647848	1.050833209	0.962112169	1.019457828
Zm00001d020727_T022	5.102660315	5.252071431	5.231205779	5.145585633	5.33886992	5.098175671
Zm00001d020727_T020	0.501957785	0.414022928	0.26270856	1.122791972	0.787628878	0.43232662
Zm00001d020727_T001	0	0.211045846	0.739288194	0	0	0.40781608
Zm00001d020727_T005	0	0.135893365	0.398386492	1.308830318	1.327528716	1.232533057
Zm00001d020727_T016	0	2.531580835	3.571433388	4.607837225	4.504256813	4.422271221
Zm00001d015730_T001	3.351721886	4.267755332	3.409792391	3.240556132	2.940615373	2.966022667
Zm00001d044017_T001	4.40597909	3.672372046	4.439915702	4.366433988	4.496162364	4.35127482
Zm00001d047639_T001	7.969220388	7.323372907	7.90502904	8.400666619	8.493553332	8.400177104
Zm00001d047639_T005	5.521683474	5.232021148	4.920323316	5.617269445	5.652383982	5.816836032
Zm00001d047639_T002	7.523683341	6.858643674	7.525587684	7.757998877	7.783808491	7.744082983
Zm00001d047639_T003	0.347028349	1.835430564	0.838002159	0.588495659	1.353112095	0.80726506
Zm00001d047639_T004	4.836830121	3.05020686	5.055888067	5.040088601	5.059786196	5.139969039
Zm00001d053610_T004	2.855869534	1.987119275	2.994743172	3.708199047	3.343166026	3.39410361
Zm00001d053610_T005	1.350023626	0.481958131	0.877611555	1.294117243	1.251147051	1.225943055
Zm00001d053610_T003	4.766474579	3.937279695	4.748274497	4.092808971	4.310040352	4.457897612
Zm00001d010610_T001	3.2070251	2.536009867	3.227939417	3.445649641	3.387409003	3.642638734
Zm00001d052174_T001	4.604076959	4.081431497	4.711844966	4.89489637	4.84585517	4.871215092
Zm00001d032979_T001	2.556512799	1.723900137	2.673442451	3.310251549	3.17681662	3.084275039
Zm00001d032979_T010	5.012914646	4.626527857	4.881849202	4.603210611	4.409430448	4.024254955

Zm00001d032979_T006	0	0	1.326847943	0.801200053	1.554392877	1.577386287
Zm00001d032979_T003	3.341051392	2.565758252	2.932014858	4.37996212	4.242359902	4.410474621
Zm00001d003183_T001	2.733447842	2.319125392	2.772619202	1.753235686	1.437768914	1.528726332
Zm00001d002962_T001	3.517144144	3.072588619	3.576888872	4.393368902	4.32775806	4.286190751
Zm00001d018413_T012	0	0	0.582446171	0.680742025	0.909915927	0
Zm00001d018413_T017	0	0	0	1.59363057	2.168155172	2.816109783
Zm00001d018413_T002	1.620411869	0	0.552613156	0	1.575113825	1.346419128
Zm00001d018413_T015	5.096779617	4.34490631	5.151732973	5.264447329	5.209811266	5.073277707
Zm00001d018413_T008	1.812454276	1.519265617	1.296259455	1.718060396	2.330170283	1.815655349
Zm00001d018413_T001	0	0	1.723441481	2.363056666	0	1.408897688
Zm00001d029497_T001	4.933000781	4.500653698	4.995943756	5.806514359	5.845428302	5.83438008
Zm00001d003962_T001	9.59445506	5.141922993	9.309751672	8.854261214	8.851765354	8.742332169
Zm00001d053964_T001	5.585894661	5.164893254	5.603640408	5.258981056	5.353289376	5.169970365
Zm00001d039989_T001	6.697853076	5.979079239	6.816763037	7.324064748	7.336391854	7.253083668
Zm00001d039183_T001	3.671748305	2.773492559	3.602982485	3.990719201	4.002783878	4.082142568
Zm00001d048270_T002	2.165576905	1.692950259	2.57338301	1.858550399	0	2.169447871
Zm00001d048270_T001	1.710963985	1.523112267	1.640986876	2.200640883	2.673624035	2.001379999
Zm00001d044095_T001	5.597511747	5.352084858	5.73304996	7.53821217	7.571555142	7.569790017
Zm00001d050735_T001	2.165987809	2.217470525	2.316400075	2.661031721	2.743319781	2.57367748
Zm00001d050735_T003	1.397880707	0	1.419171564	1.577140683	1.37752637	1.477147811
Zm00001d029710_T001	0.551767006	0.524187072	0.522509785	1.310842068	1.576476668	1.851494805
Zm00001d042315_T001	5.436382345	1.373293514	4.773008458	6.685008794	5.453602881	6.327208674
Zm00001d039703_T001	3.964351774	3.347073293	4.133918484	3.831540235	3.629088458	3.934593211
Zm00001d022211_T001	1.649851789	2.073780482	2.280035486	2.555164096	2.432280647	3.175481168
Zm00001d042312_T008	3.019966058	1.363070127	2.675873277	2.666521198	2.782306231	2.061663196
Zm00001d042312_T013	0	1.201245536	2.304413204	2.649255143	3.022777214	2.336318812
Zm00001d042312_T002	3.458913073	2.454192742	3.399941328	3.76288008	3.773948762	3.805633069
Zm00001d042312_T003	1.613873899	0.856159463	1.355124547	1.188493345	1.511501943	1.667064183
Zm00001d012707_T006	3.514955941	3.904570224	3.812766614	4.173791608	4.431092983	4.51746657
Zm00001d012707_T001	0.930165823	0.590268581	1.213322394	1.452521638	1.319933489	1.522619757
Zm00001d034804_T007	0.594129056	0.474575179	0.639731347	0.748122847	0.672620856	0.757804139
Zm00001d034804_T001	2.870338282	1.856792521	2.897728148	2.443379356	2.595223401	2.574252917

Zm00001d006512_T002	4.357637016	2.756905649	4.195080061	4.353877183	4.555858601	4.358035473
Zm00001d006512_T004	2.836426101	3.042390968	3.088084933	3.719046066	3.280473405	3.360886193
Zm00001d040202_T002	4.187649312	3.975978896	4.161862052	3.611011691	3.365150235	3.523317555
Zm00001d040202_T005	0.989567028	0.86451779	1.482245271	1.316538519	1.594919685	1.379650902
Zm00001d029059_T001	2.477316479	1.762918977	2.332872313	1.923032771	2.005351119	2.054884587
Zm00001d001835_T001	1.722589304	1.317434901	1.664094125	1.74761383	1.57640217	1.355996715
Zm00001d025238_T001	3.324934998	2.624227257	3.470253374	3.674177011	3.677884793	3.582003747
Zm00001d016473_T001	0.661155802	0.30442109	0.478847586	0.750933164	0.762861588	0.623910863
Zm00001d022355_T001	3.390776764	2.682506554	3.067435479	2.980226422	2.9311558	2.808922897
Zm00001d045388_T001	2.81659172	2.814545502	2.871490224	1.172295485	0.613769254	1.55949475
Zm00001d043367_T001	6.996809674	6.744632505	6.709421746	6.764734996	6.925759301	6.678812998
Zm00001d006813_T001	2.037083812	2.160432325	2.05779717	1.305936678	1.135273493	0.919230994
Zm00001d036318_T001	4.242296177	3.668613406	4.074578991	4.418903629	4.341290269	4.252279476
Zm00001d007577_T001	4.698711444	4.04818395	4.551482242	4.596806248	4.70633958	4.524016647
Zm00001d047834_T002	2.142704562	1.250697864	2.042349815	1.918513705	1.224091533	2.454656799
Zm00001d047834_T007	2.615171433	1.639280793	2.547996597	2.905860528	2.777950719	2.775902207
Zm00001d047834_T018	2.356254544	5.46190382	2.847542787	2.580423382	1.959667049	0
Zm00001d047834_T016	2.317673089	0	0	1.687823502	0	2.304165805
Zm00001d047834_T012	2.098050954	1.462737109	2.227445361	2.56063646	1.822693443	2.387515019
Zm00001d029390_T003	0.609595179	0.533439755	0.742500395	0.402196076	0.390201018	0.441264615
Zm00001d029390_T007	0.540068942	0	0.695443347	0.65702164	0	0
Zm00001d029390_T008	2.227514058	1.878617062	1.927304467	1.496511947	1.893971393	1.69694372
Zm00001d007624_T001	5.19420094	5.845790702	5.180210811	4.136984962	4.268103176	4.166228011
Zm00001d014442_T009	0.250875494	0.093560176	0.102581544	0.527684927	0.434602075	0.444531377
Zm00001d014442_T014	3.33290553	3.184360283	3.430966712	2.189513694	2.435580059	0
Zm00001d014442_T001	0.959801308	0.546236132	0.776354174	1.849060102	1.332089769	1.567229032
Zm00001d014442_T010	0.3900513	0.155276459	0.464602403	0.728678079	0.694244599	0.702911932
Zm00001d014442_T011	2.767314104	0	2.676104893	3.513527381	3.429401854	3.750522792
Zm00001d014442_T008	2.357338892	1.885322505	2.649541885	3.779445483	3.696908788	3.544618423
Zm00001d011778_T001	4.852120172	4.30078282	4.961073045	4.559198411	4.723311229	4.70384329
Zm00001d034517_T001	5.230372156	4.671672568	5.199794154	5.297964402	5.312559392	5.298370628
Zm00001d046613_T004	0.229990222	0	0.42900957	0.539084348	0.711116012	0.448198986

Zm00001d046613_T005	0.383983474	0.809839164	0.49459072	0.481237967	0.7933241	1.073671501
Zm00001d046613_T008	2.827197922	1.744202869	2.553398598	2.512474437	2.246963775	2.395712415
Zm00001d020485_T001	5.928790681	5.56399534	5.823122961	6.235152203	6.129753774	6.065693816
Zm00001d029177_T003	1.637272658	1.193504938	1.687829771	1.991463253	1.562294696	1.619291392
Zm00001d029177_T001	5.906006554	5.118821459	5.903494513	5.429346948	5.33779992	5.318245898
Zm00001d043551_T001	0.903560477	0.736247242	1.034732682	1.364233429	1.347385026	1.287646637
Zm00001d043551_T003	6.981888058	6.427878697	7.013979103	7.239468998	7.226287003	7.269760073
Zm00001d029675_T001	1.11633348	0.898285759	1.414504145	1.816903982	1.594708575	1.822719544
Zm00001d008601_T001	8.448004537	9.133085143	8.493954001	7.95883269	7.963582761	7.977321091
Zm00001d012797_T011	2.174411852	1.330491857	2.076791317	2.160459756	2.304653844	2.051918447
Zm00001d012797_T012	1.041763745	0.401638111	0.836433171	2.103882978	0.673344753	1.668057838
Zm00001d012797_T006	0.935112705	0.928933456	1.390349069	0.781797807	0.678217069	0.487729179
Zm00001d012797_T014	0	0.159285585	0.439948605	0	0	0.628084856
Zm00001d012797_T013	3.089371706	0	2.762563131	3.728697776	3.583069044	3.557359479
Zm00001d012797_T010	1.493987475	0.724148188	1.902017221	1.658514503	2.845866357	3.020715181
Zm00001d012797_T007	0	1.016543387	0	2.870565548	2.407553164	1.162016633
Zm00001d012797_T009	0	0.06637994	0.31721491	0.366036235	0.641988018	0.483946275
Zm00001d012797_T015	2.116062278	0	0.697750694	0.432480567	0	0.516135198
Zm00001d012797_T001	3.672644162	3.617534641	3.962107356	0	0	0
Zm00001d016373_T010	4.11178736	1.509310734	4.123765672	3.405205468	3.678480986	3.666898469
Zm00001d016373_T003	4.335044495	3.588975301	4.318504054	4.982562276	4.799150578	4.955299225
Zm00001d016373_T007	6.217560346	6.66785881	6.36216901	5.995831741	5.881048626	5.829880351
Zm00001d048354_T001	3.439182151	3.463895356	3.361117877	3.781611717	4.090197973	3.678966324
Zm00001d023560_T002	2.864062897	2.228958414	2.950809731	2.922931571	2.912306106	2.803170196
Zm00001d023560_T001	3.717843533	3.147700374	3.660201543	3.598163057	3.525855021	3.614508913
Zm00001d025047_T001	3.86633672	3.567495678	3.873312006	4.619767126	4.556777325	4.52895582
Zm00001d011403_T001	0.828231048	1.007897299	1.075760569	2.989133738	3.035348364	2.987192237
Zm00001d016453_T001	1.114361028	0.791495706	1.145112004	1.707935984	1.7403223	1.496433715
Zm00001d022489_T002	1.663130078	0.325450891	1.398634393	0.716768823	1.50709995	1.220476694
Zm00001d022489_T001	2.434816349	2.029136218	2.499085602	2.606715817	2.560890023	2.489301182
Zm00001d022489_T003	7.089193737	7.364701569	7.202307448	7.141204746	7.006645549	7.029608245
Zm00001d038737_T001	7.311092622	7.074812248	7.229269876	6.440606611	6.397007686	6.531405651

Zm00001d018358_T001	2.27757538	1.713951343	2.551228557	1.259691297	1.536596545	1.910884214
Zm00001d025023_T002	3.516359259	2.198968688	3.349321679	3.631776219	3.566347207	3.259857282
Zm00001d025023_T001	6.092320673	5.621615781	6.081465415	5.739952764	5.769003569	5.811774823
Zm00001d014989_T001	3.174030355	2.858204446	3.191061031	2.552121515	2.243973367	2.813498831
Zm00001d052519_T007	1.490217885	1.452482629	2.004132087	1.651800722	1.566791292	1.183081201
Zm00001d052519_T004	0.182520689	0.097989623	0.689814413	0.748932608	1.042543485	0
Zm00001d009473_T001	3.063457038	2.274371704	2.954500454	2.76474147	2.978324148	2.713586069
Zm00001d006591_T001	1.621393156	1.244772004	1.114550266	1.863184802	1.883508602	2.20169
Zm00001d017866_T001	3.243596731	3.204966711	3.395364746	3.696717539	4.307311429	3.92920405
Zm00001d017866_T002	2.777847437	1.8184653	3.105407882	3.249177409	2.775643516	2.827218454
Zm00001d017866_T003	4.766740113	3.603931556	4.532517451	4.932612899	4.808683033	4.988583899
Zm00001d037596_T001	1.372827319	0.478255326	1.742444775	1.149029088	1.588507172	0.992519744
Zm00001d037596_T002	4.628069103	3.735727194	4.592653948	4.845327607	4.895168187	4.887816364
Zm00001d037596_T003	2.194670192	0.877116787	1.541749735	2.495982906	2.380658531	2.398479255
Zm00001d005140_T004	0.279229984	0.17383741	0.262321298	0.208132656	0.19113559	0.159799664
Zm00001d005140_T002	2.785686759	2.817213924	2.596638704	2.926034347	2.876948291	2.528614824
Zm00001d011541_T002	3.649907187	2.743045698	3.727609343	3.775988357	3.71022169	4.183256713
Zm00001d011541_T009	0.895263063	0.431527757	0.744164541	0.843126009	0.921084374	1.040970962
Zm00001d011541_T007	2.180958433	1.996556147	2.790543524	1.647049204	0	0
Zm00001d011541_T003	0	1.567305952	0.947470917	0	0.702044877	0
Zm00001d011541_T008	2.841579292	0	1.680885121	3.41216083	3.741207116	3.07479244
Zm00001d008296_T001	2.990745791	2.515563856	2.722639356	4.602131517	4.765327768	4.890835137
Zm00001d012282_T001	2.573191754	3.473873393	2.469175652	1.745920541	1.272566711	2.475765332
Zm00001d046083_T001	1.422496215	0.945179925	1.571194294	1.443652796	1.499084071	1.386049952
Zm00001d022180_T007	5.169433039	5.880293555	5.568001055	5.180297789	4.062453414	4.45729892
Zm00001d022180_T009	9.029721309	8.495989619	9.056838064	8.13601471	8.240333828	8.122656875
Zm00001d022180_T001	6.47916282	5.672064001	6.487633541	5.854213482	5.908547229	5.880611846
Zm00001d050768_T003	6.650043563	6.18775132	6.731483781	5.185019268	5.331029063	5.390187361
Zm00001d051629_T008	4.966382014	4.034034751	5.00233622	5.345408653	5.470588728	5.36115217
Zm00001d051629_T001	5.001521376	5.36646263	4.983988406	3.504488192	3.347483253	3.549667323
Zm00001d015168_T001	6.20874988	5.355130257	6.323299428	5.812163671	5.808978071	5.786016658
Zm00001d008708_T001	5.056952912	4.196329667	5.07797305	4.981122903	4.907996142	4.964627634

Zm00001d049049_T004	0.115059885	0.061243944	0.295346876	1.491616603	1.445996786	1.365771472
Zm00001d049049_T003	0.243828695	0.23727613	0.249121267	1.03286608	1.145159622	1.341373254
Zm00001d049049_T002	4.041319304	3.20029467	4.095811784	4.819267964	4.868945643	4.842090119
Zm00001d038512_T001	2.036268756	1.547324458	2.028690432	2.141904565	2.440114501	2.219103452
Zm00001d053984_T002	2.986656673	1.963960119	3.06788339	2.727766486	2.738674896	2.858748106
Zm00001d053984_T003	1.256449729	1.037623295	0.888417126	1.345439525	1.931596833	1.492375706
Zm00001d048888_T001	1.018732439	0.501202689	0.744616657	0.856695728	0.838634763	0.707703239
Zm00001d023590_T005	3.882642463	0	0	3.490073188	0	2.690557223
Zm00001d023590_T007	0.833598532	0.488308307	1.013158503	1.891648211	1.931122509	1.723112469
Zm00001d023590_T002	5.089108272	4.683464201	5.551306243	5.242277082	5.470183209	5.372542766
Zm00001d023590_T004	0.395213095	0.194767884	0.648610856	0.17935053	0.49475557	0
Zm00001d023590_T006	0	0.12327551	0.083971712	0.355108756	0.515163403	0.65899107
Zm00001d037059_T027	2.157871567	1.759720015	2.484834685	1.75682689	2.494035877	2.425932964
Zm00001d037059_T016	0	0.192671407	0	0.068120453	0.470251552	0.502723686
Zm00001d037059_T005	0.47427197	0	0	1.399524408	0.526532604	0.47721414
Zm00001d037059_T004	0.297961431	0	0.176881868	0	0.696490656	1.195079194
Zm00001d037059_T026	2.128572212	1.021654546	1.574836841	1.74369979	0.864088261	1.40388285
Zm00001d047749_T001	2.26613347	1.703295202	2.208120167	2.182116694	1.994155361	1.882993557
Zm00001d018636_T002	3.292825913	3.762957872	3.63174817	2.739840112	2.267271205	2.248563981
Zm00001d018636_T003	3.937939805	2.966034669	3.703943097	3.423151753	3.338858798	3.385113747
Zm00001d014726_T010	4.763166465	3.674392967	4.613348652	5.858757976	5.911151597	5.961647959
Zm00001d014726_T011	0	0	2.773428416	1.616795709	2.640116498	2.470291645
Zm00001d014726_T012	1.432104223	1.017977477	1.620653031	4.072514706	4.147454624	3.54066922
Zm00001d014726_T005	0.916138001	0.37291422	1.237815159	2.916938997	2.897428254	2.925367911
Zm00001d014726_T008	3.88360635	4.496913329	3.573196481	0	0	0
Zm00001d032718_T001	2.312126967	1.716507209	2.326295441	2.040295947	1.931008255	1.778315828
Zm00001d001933_T001	6.342499296	5.87050728	6.481906627	5.593129969	5.711010917	5.697248585
Zm00001d029762_T001	5.847046895	5.321120356	5.996367458	5.256664164	5.302002654	4.950084967
Zm00001d029762_T002	0.994201714	0.640185	0.871119754	0.602091956	1.113245085	0.930544335
Zm00001d029762_T004	3.195789159	1.948243867	3.153784749	4.08187321	3.648332557	4.083740813
Zm00001d029762_T008	5.800045235	4.597829515	5.715850687	6.22549748	6.290300371	6.20728309
Zm00001d037966_T008	4.042458561	4.65255097	4.176236272	4.164846362	4.394432854	4.231782825

Zm00001d037966_T005	3.339197971	2.9111959	3.289932412	3.406294341	3.422733012	3.037985357
Zm00001d037966_T002	0.784522409	0.991830011	0.426450481	0.736109534	1.214455592	1.435722993
Zm00001d037966_T004	0.773648687	0.530539242	1.09580495	1.244423131	1.281194369	1.101529734
Zm00001d037966_T006	2.985444324	2.513418483	2.798278465	2.969364371	2.706925679	3.117694544
Zm00001d037966_T001	3.945111176	2.815957365	3.822129801	4.733810073	4.53505131	4.715389108
Zm00001d040268_T012	0.152676675	0.066127777	0.472097801	0.254399333	0.608342775	0.849538164
Zm00001d040268_T008	0.631880032	0.831285453	0	1.668015617	0.746168275	1.609220707
Zm00001d040268_T002	2.324777775	1.409810716	2.231103648	1.729408331	2.536189452	1.864320476
Zm00001d033726_T013	0	0	0	2.164890841	1.849293547	2.959819851
Zm00001d033726_T036	3.35094611	1.885759424	2.993359162	3.537561096	3.626634325	3.563409421
Zm00001d033726_T011	0	1.089334076	2.068426271	2.519597508	2.348246302	0
Zm00001d033726_T038	5.12840852	5.966495047	5.082990435	4.564979146	4.836404387	4.723034684
Zm00001d033726_T007	2.455549222	0.99180317	1.752485286	2.394625262	2.779478892	2.612729461
Zm00001d006127_T002	2.236724962	1.565916873	2.575243583	2.580439301	2.520178824	2.272667628
Zm00001d006127_T001	3.987941837	4.726661985	4.173466235	3.480477632	2.88559252	3.610983708
Zm00001d006127_T003	3.403236905	2.300669648	3.223612528	3.472860744	3.513016296	3.498767719
Zm00001d019643_T009	0	0	0.626834393	0.557015939	0	0.999595267
Zm00001d019643_T001	0.991526744	0.544309304	0.513033862	0.925156335	0.756021549	0.863896437
Zm00001d019643_T002	1.454823381	1.065597126	1.304816487	1.083905015	1.08407107	0.895702022
Zm00001d028339_T001	3.91781515	4.070277686	4.16700275	4.470575519	4.077206418	4.03557447
Zm00001d028339_T023	0.565574755	0.382330815	0.865774736	0.812719182	0.513944454	0.864244396
Zm00001d028339_T004	1.034188242	0	0	0.529873907	2.431607717	2.18573527
Zm00001d028339_T018	0.663046713	0	0.357768182	0.834862474	0.542494806	0.871325604
Zm00001d028339_T007	4.156150645	0	3.768099931	0	0	1.451930071
Zm00001d005502_T003	3.807141181	1.895869892	3.519903036	2.37601082	3.687523665	2.975887119
Zm00001d005502_T002	0	2.414235679	2.443266093	4.861543582	4.679180832	4.903778956
Zm00001d005502_T015	3.855616122	3.40715911	3.874681211	3.591527194	2.884359373	1.945894191
Zm00001d005502_T018	1.927549483	1.14356452	1.845222262	1.245394044	1.70430274	1.882485371
Zm00001d005502_T009	0	0	0	1.070947062	0.705620555	1.066273155
Zm00001d044204_T001	0.601832461	0.654393378	0.863106679	0.778898213	0.917223213	0.986720376
Zm00001d046506_T001	3.896511729	3.166380598	3.95380524	4.033928803	4.034101063	4.192030472
Zm00001d046506_T003	0	0	0	1.646551625	1.8504162	1.294780117

Zm00001d046506_T004	0.736559851	0.627355629	0.656175075	1.310065517	1.732267028	1.523151921
Zm00001d037537_T003	4.212986057	0	4.238256679	3.663583037	3.480820375	3.85255243
Zm00001d037537_T002	0	3.672211807	0	2.552963769	2.703522901	1.207852256
Zm00001d003911_T008	4.838162	0	4.974456901	5.607684241	5.407410369	5.394878165
Zm00001d003911_T001	4.774853936	4.412300615	4.438105848	3.854695474	4.302768754	4.33050964
Zm00001d003911_T011	2.86873496	1.625922244	2.452933271	3.040094258	2.768140911	2.797199997
Zm00001d003911_T010	2.778235706	2.12783762	2.693019867	2.229276587	2.432396112	1.961396998
Zm00001d035198_T001	3.694974104	2.892220418	3.729441396	3.731461759	3.795019644	3.696686497
Zm00001d032045_T001	3.274496947	2.672248361	3.215732483	3.154422969	3.326268831	3.324874962
Zm00001d041882_T003	6.680556835	7.169313099	6.587923762	6.549243984	6.49947002	6.391730229
Zm00001d041882_T001	5.779436815	6.508658366	5.858429579	5.339551316	5.427829699	5.627112636
Zm00001d041882_T002	5.549980776	5.21210747	5.64153944	5.50036118	5.387990481	5.363133642
Zm00001d047737_T001	2.108302976	1.738566818	2.466419608	1.892908943	2.187983375	2.073451459
Zm00001d044691_T001	5.981147227	5.808066639	5.999166716	6.221028475	6.259234366	6.155636922
Zm00001d039626_T003	0.952546041	0.613712686	1.400226405	1.28122761	1.219589223	1.154228681
Zm00001d039626_T001	2.441090616	1.942546033	2.161705521	2.146301021	2.291767679	2.213872934
Zm00001d043299_T001	3.155763964	3.783646696	2.754333401	1.448112274	1.52217329	1.533985879
Zm00001d021893_T003	0.485602001	0.285911141	0.557272836	0.770389301	0.658137432	0.509136388
Zm00001d021893_T005	0	0.321975415	0	0.884459603	0	0.86948453
Zm00001d021893_T002	1.240625735	2.275715702	1.284592307	4.231116477	4.272526028	4.228292222
Zm00001d021893_T004	5.464963604	4.816362549	5.406538507	3.435159442	4.651231522	5.36558501
Zm00001d007890_T005	3.19190023	0	3.071711021	2.989761376	2.92006592	3.432965285
Zm00001d007890_T006	3.152417412	1.935070073	2.963248639	2.732508869	0	0
Zm00001d007890_T003	1.032609401	0.767212389	1.191501379	1.357711889	1.739084222	0.825560965
Zm00001d007890_T001	2.773077686	2.987058865	2.86687772	3.104338505	3.246007604	2.893922481
Zm00001d037613_T004	1.69347536	2.330757151	1.33599882	1.668011985	1.716918922	1.78370473
Zm00001d037613_T005	1.013843831	0.502229767	0.856576221	1.638308821	0.908855938	1.098408103
Zm00001d037613_T001	5.564372162	4.869279948	5.588530648	5.891462107	5.915887432	5.845435805
Zm00001d006561_T001	6.600872109	6.880685942	6.453821873	5.822512478	5.896555079	6.050934289
Zm00001d045581_T001	2.515725066	2.957526486	2.546619411	2.489633872	2.041708033	2.404363373
Zm00001d018243_T002	3.2390034	4.752467677	4.098986684	2.520737281	3.198869394	2.043873956
Zm00001d018243_T001	4.138348155	3.078792551	3.998311781	3.867892116	3.586586422	3.615420182

Zm00001d019100_T001	6.521375244	5.98158605	6.649458134	6.532213507	6.6307158	6.608030154
Zm00001d050357_T004	2.72874359	2.071993262	2.661741392	2.525834355	2.850940006	2.525981646
Zm00001d050357_T007	1.141049504	0.494516992	1.259741908	1.215218849	0.794461616	1.164541733
Zm00001d050357_T001	1.590146877	1.344698421	1.587757178	1.26780903	1.272226884	1.622923325
Zm00001d050357_T006	4.996887954	4.655531124	5.209728699	5.034724188	5.026730313	5.106576173
Zm00001d050357_T005	1.441143482	0	1.218212656	1.00442948	0.671325986	1.341158593
Zm00001d013831_T012	1.448455391	1.827662148	2.792097374	2.628423667	2.627762071	3.147274295
Zm00001d013831_T013	0.757144462	0.423495345	0.94434423	1.55012199	1.444499043	1.347208116
Zm00001d013831_T002	2.228559829	2.865881015	2.375775122	2.27648803	1.73875703	2.234695057
Zm00001d013831_T011	2.37255962	0	0	1.980978005	1.433220672	2.154657733
Zm00001d013831_T010	3.034357477	2.702619204	3.34081632	3.613651519	3.809454782	3.312841434
Zm00001d013831_T014	0.888942315	0.642137783	0.27746945	0	1.2976604	0.264198912
Zm00001d013831_T015	2.925883808	0	2.465160581	1.701567243	0	0
Zm00001d013831_T006	3.347502671	2.285981252	2.982124913	3.17575915	3.828263144	3.614223721
Zm00001d040898_T001	3.530599916	3.275741919	3.572675794	3.835282195	4.107640002	4.087583853
Zm00001d052010_T002	1.544766776	1.798177873	2.105363627	1.978863728	1.760401512	1.792698375
Zm00001d029074_T003	3.613122006	2.637910438	3.511137816	3.666170989	3.631161337	3.687253893
Zm00001d029074_T002	1.166067533	0.483841052	1.232312626	1.11544348	0.991233573	0.88857143
Zm00001d048483_T001	0.867195104	2.28734049	1.122762822	2.413869706	1.473396253	0
Zm00001d048483_T002	5.497147231	4.647338746	5.281340833	5.005122426	5.111385389	5.290945893
Zm00001d048483_T003	1.394414264	1.636639468	1.611238964	1.696570331	1.897240038	0.7698132
Zm00001d001824_T001	5.1059393	5.426165071	5.036460738	4.811049978	5.098931707	4.862157714
Zm00001d049708_T001	2.186888436	2.115095519	3.127173987	2.416581168	1.195839543	2.136948307
Zm00001d049708_T008	1.66253457	0.572305683	0	2.104593973	2.23372857	1.589946568
Zm00001d049708_T006	2.046925794	0.971492421	0.94824125	1.899905689	2.490547797	1.708977356
Zm00001d017076_T001	1.883465591	2.166350066	1.808279187	1.744619671	1.511148192	1.702866287
Zm00001d005437_T001	2.763603	2.35410682	2.004147551	2.43317072	2.772958857	2.012211084
Zm00001d013543_T007	2.006112715	1.160889218	1.399209383	1.541453879	0.492947334	1.424911879
Zm00001d013543_T009	4.813667772	4.22994686	4.969557867	4.612908177	4.732087196	4.735094775
Zm00001d013543_T005	0.467215823	0	0.948148533	1.478449494	1.979732343	1.381992085
Zm00001d013543_T002	1.28026211	0.907359107	1.196321876	2.028689372	2.204911013	2.25990638
Zm00001d005733_T002	4.688880998	4.903942661	4.551985971	4.763960183	4.776534574	4.73063224

Zm00001d005733_T001	2.603421727	2.535872055	2.834789274	1.919720301	1.767975503	1.957327746
Zm00001d009957_T001	6.914203446	6.627269703	7.013819383	6.998646715	6.746259645	6.956209756
Zm00001d016732_T005	0.718577754	0.243778741	0.427916131	0.923467927	1.158854676	1.378438932
Zm00001d016732_T003	3.488551513	2.978026634	3.759249915	3.138903897	3.043858911	3.195426188
Zm00001d040051_T039	1.799150734	2.19908117	1.300514444	1.179447354	1.291126706	1.910893421
Zm00001d021026_T001	5.245921647	4.762589865	5.239690744	4.768764992	4.87460902	4.766158683
Zm00001d002235_T027	0.343803479	0.055424745	0.846870363	1.174752189	1.097161064	0.177068185
Zm00001d002235_T009	3.324983227	2.879850029	3.631033951	3.330652903	3.230263432	2.943679638
Zm00001d002235_T019	0	0.60397261	1.017698184	1.257673864	1.225565538	1.45544638
Zm00001d002235_T026	0.97127536	0.50415357	1.058256916	1.121931139	0.65661352	1.215022479
Zm00001d002235_T017	0.178961894	0.069159078	0.21683105	1.397902606	0.725369476	0.733739586
Zm00001d002235_T006	0.633109387	0	0	0	1.103818539	1.10883153
Zm00001d002235_T010	0	0.027212098	0.367036635	0.352395829	0.433337664	0
Zm00001d002235_T008	2.466410993	1.252394464	1.549687897	1.680047045	2.652321049	3.207129302
Zm00001d035899_T001	8.598755552	7.853199944	8.643236488	7.800548234	7.875536919	7.863320714
Zm00001d000027_T029	0.288404633	0.037064478	0.27664197	0.920206393	1.081593236	0.663361927
Zm00001d000027_T020	0.890230321	1.539112644	1.573753572	1.70888027	1.928658359	0.826272277
Zm00001d000027_T031	0.134908274	0	0.110740072	1.291093692	1.135651752	0.886127909
Zm00001d000027_T035	2.689951693	3.33736586	2.630295028	2.092460835	2.528189467	2.228816228
Zm00001d000027_T007	1.673164718	0	1.162707609	1.122889355	0.634658316	1.835340817
Zm00001d000027_T030	0.538632519	0.296295288	0.488554084	0.333824427	0.072789075	0.576092531
Zm00001d021823_T002	4.558732394	3.76281355	4.511727926	5.428379637	5.356283459	5.364809302
Zm00001d021823_T004	0.154377114	0.049303162	0.074417749	0.277542055	0.422408482	0.296658269
Zm00001d021823_T003	0.532625167	0.200973834	0.366792753	0.648727727	0.707806588	0.621389404
Zm00001d037279_T009	0	0.026288741	0.197074019	0.70613355	0.938124668	0.774623044
Zm00001d037279_T011	2.293130604	0	0	3.559334075	2.822182942	3.386316677
Zm00001d037279_T002	2.618607741	2.125110486	2.01141656	1.812636225	2.156215702	2.712043247
Zm00001d037279_T005	0	0.675077913	2.061596152	3.432721877	3.489664825	3.159716526
Zm00001d037279_T013	4.895695625	4.134545838	5.026286218	5.01179125	5.107836106	4.83408574
Zm00001d022631_T002	4.27813875	3.836292682	4.355071957	4.654171542	4.499476382	4.484442238
Zm00001d022631_T001	0.249133406	0.117181698	0.197906898	0.328201125	0.417547407	0.564434753
Zm00001d022631_T003	1.131573681	0.757833148	1.037709032	1.30537815	1.12716441	1.384775394

Zm00001d042863_T001	1.234615358	1.928111041	0.823837386	1.216128262	1.537032605	1.404388716
Zm00001d053168_T001	1.212403738	0.746392747	1.378572104	1.669701227	1.675589236	1.844980928
Zm00001d008489_T001	2.254617927	1.571579252	2.401585143	2.930224498	3.107334851	2.853500696
Zm00001d018099_T002	2.371198074	1.273010336	1.895810578	2.432706112	2.528106181	2.574550842
Zm00001d018099_T001	5.117217029	4.534113411	5.157064331	4.978710734	4.854677573	4.929769225
Zm00001d016567_T001	9.510446151	5.193715651	9.371580435	10.04967535	9.960918773	10.09505755
Zm00001d038200_T001	0.587087467	0.182282946	0	0.576711744	0	0
Zm00001d038200_T005	5.200530601	1.694328855	5.103934327	5.499052426	5.123706708	5.298403512
Zm00001d038200_T010	0	2.324945652	1.798306049	4.409748001	4.014643535	3.718836911
Zm00001d038200_T004	0	0.10132735	0.626412969	0.914826251	1.301697088	1.077519204
Zm00001d038200_T008	5.386982136	6.300097049	5.418956977	5.448083324	5.649746359	5.283475749
Zm00001d038200_T003	4.43876072	4.014802138	4.431853452	4.179665582	4.589323841	4.445284334
Zm00001d038200_T009	0.743613198	0.361650469	0.582323804	1.087547703	1.218145064	1.437055649
Zm00001d012005_T024	2.834907566	1.974559414	3.036131227	3.546688672	3.502075574	3.487101957
Zm00001d012005_T004	3.282504006	2.442526347	3.433746663	4.37297967	4.43194001	4.364925639
Zm00001d012005_T025	2.555212698	0.962140309	1.869454129	2.966583662	2.8129167	2.759197601
Zm00001d012005_T002	0.702911045	0.074860244	0.677308878	0.577817926	0.285886291	0.905271561
Zm00001d012005_T015	1.910405333	1.642052271	1.630904457	0	0	0
Zm00001d041382_T001	9.360636321	9.59266946	9.033272116	6.540175909	6.006897332	6.225474324
Zm00001d043806_T001	3.386815912	2.898114686	3.032254452	3.0236496	2.838826748	2.945553208
Zm00001d033641_T001	5.039294721	4.8988727	5.186104214	5.319163155	5.160970393	5.088209328
Zm00001d033641_T003	3.593088635	2.80608211	3.654659742	3.532651596	3.882896061	3.877547559
Zm00001d033641_T005	2.719035987	1.814726366	2.350681674	2.231989594	2.270887404	2.217428026
Zm00001d033641_T004	1.970358195	0	1.415177795	0.631415377	2.147749209	0.922688814
Zm00001d033232_T001	4.153177708	3.660937513	4.149339206	3.988446838	4.233965039	3.908654894
Zm00001d041111_T001	1.756139005	1.770766052	1.752711774	2.92485107	2.72625664	2.777429605
Zm00001d041111_T002	5.938062218	5.061254098	5.888380155	7.288167507	7.243012526	7.230037099
Zm00001d028575_T001	5.178547977	4.889622769	5.304190854	5.461274107	5.488903137	5.421332549
Zm00001d013755_T003	3.54790754	3.345656247	3.342986892	4.131516064	4.041300465	4.019548653
Zm00001d013755_T002	4.778180395	4.609960042	4.93992871	5.516851705	5.601257967	5.598747834
Zm00001d017821_T002	1.538967184	0.674350366	1.459569586	2.710670242	2.713024454	2.424860564
Zm00001d017821_T001	1.28859658	1.160556888	1.537742351	2.512920377	2.660151217	2.684356014

Zm00001d013335_T001	2.684532414	1.933351307	2.393073746	2.996365967	2.719812305	2.908698986
Zm00001d045009_T001	1.957126007	3.006604845	1.849706689	1.903417796	2.748331771	2.626716668
Zm00001d006910_T009	2.255030837	2.168413869	2.868395195	2.412514907	2.631646559	2.956052376
Zm00001d006910_T004	1.350620012	1.061736113	1.69741399	1.407733975	0	1.074284836
Zm00001d006910_T015	3.892326808	5.168564781	4.233186477	4.118864681	0	4.666626857
Zm00001d006910_T008	1.12761017	0.574258488	1.163379576	2.503419978	2.37034342	1.934288521
Zm00001d006910_T013	0	0	0	1.021749053	2.257105084	1.076400383
Zm00001d006910_T001	0.45323731	0	0.111669697	1.162253226	1.808964895	1.742037695
Zm00001d006910_T010	4.864028071	2.746375117	4.596080957	4.94672955	5.384545821	4.827480256
Zm00001d006910_T005	1.8934092	1.606798949	1.433700335	1.520609315	0	1.257215841
Zm00001d006910_T011	1.043327005	3.275046244	2.567732343	3.216496772	2.022326263	2.115794021
Zm00001d049826_T001	8.674549387	8.844524091	8.793759209	8.21072328	8.279678854	8.160298226
Zm00001d007595_T017	1.52822423	0.640743075	1.33505729	1.222651259	1.23926177	1.069576381
Zm00001d007595_T013	0.249592182	0.461045485	0.148591851	0.799468658	0.405471807	0.666937429
Zm00001d007595_T025	0	0	0	1.477942013	0.952434962	1.421444148
Zm00001d007595_T012	0.483475809	0.242856118	0.248927031	0.43877296	0.162595477	0.375021632
Zm00001d007595_T014	2.472263161	1.252758973	2.310717034	2.647627885	2.443186511	2.437587835
Zm00001d007595_T027	1.668008807	0	1.73955003	1.294028406	2.049033006	1.610048482
Zm00001d007595_T022	0.1696685	0.455834565	0.424485721	0.952629531	0.614869062	0.24951573
Zm00001d007595_T010	0.949430346	0	0.707166944	0	0.688357635	0.545358553
Zm00001d043389_T001	4.12098323	3.364692045	4.277817266	5.921025897	5.783369299	5.795969813
Zm00001d043389_T003	0.4760187	0.840477736	0.123584095	1.107049059	1.889502357	1.679437761
Zm00001d034183_T003	0.236735063	0.556094853	0	0.705240999	0	0.923562244
Zm00001d034183_T006	2.28947596	1.661000243	2.646324663	2.677778828	2.617334691	2.742308089
Zm00001d034183_T002	2.663678894	2.251603672	2.284303873	2.914336621	2.303018512	2.878106939
Zm00001d034183_T004	0.857229404	0	1.094592814	0.299688946	0.974734873	0
Zm00001d024493_T008	3.695980789	2.271967033	3.542393025	3.438522547	3.861383928	2.948429097
Zm00001d024493_T002	2.168842253	2.504597004	2.568110441	4.170993562	4.101446363	4.196982618
Zm00001d024493_T009	4.951616561	5.268262693	5.027476861	4.66182261	4.648366211	4.857852838
Zm00001d024493_T003	0.879566194	0.454316997	1.023468281	2.098714979	2.092714187	2.010801318
Zm00001d024493_T006	2.722416622	1.639649396	2.967579403	2.777444545	2.233665996	2.780129698
Zm00001d053753_T007	0.816275294	0.289336369	0.930553418	0.384407949	0.338316012	0.687616153

Zm00001d053753_T001	2.329763121	2.58804441	1.823671518	3.020709314	2.192441328	3.437472252
Zm00001d053753_T003	4.785606365	3.506456213	4.716918264	5.316259082	5.122014826	4.996392498
Zm00001d053753_T013	5.969374548	5.365376196	5.913150363	4.753829779	5.08988318	5.211048845
Zm00001d053753_T008	0.843660708	0.365732845	0.584757623	0.664466456	0.638730027	0.414812061
Zm00001d021093_T001	3.271745824	4.355242549	3.004040016	2.658869773	2.194705173	2.669948117
Zm00001d005532_T001	3.876509383	4.762411859	4.4105641	4.05677521	4.451718719	4.42101885
Zm00001d005532_T003	2.241832863	1.545088155	2.406784808	3.160683376	2.818173219	2.882340394
Zm00001d005532_T002	6.480727492	6.419936454	6.324663573	6.446028789	6.467499961	6.409829466
Zm00001d042932_T001	2.356745559	1.604298625	2.26506147	2.031155198	2.425720051	2.131901892
Zm00001d037120_T001	2.555593845	1.876823379	2.646314293	2.517135323	2.440591612	2.523405133
Zm00001d042148_T001	1.898623975	1.228814997	1.782160266	2.378198913	2.163980087	2.163861937
Zm00001d019759_T001	3.725029884	2.671586185	3.448054771	4.293517483	4.331630701	4.21395091
Zm00001d042281_T001	4.410043633	3.943863768	4.613396806	4.110359234	4.177116515	4.114071709
Zm00001d036118_T001	3.875571702	3.429436403	3.989039342	1.526687835	1.733133038	1.767375977
Zm00001d045557_T003	2.40055569	1.040945019	2.585740144	4.984943738	4.826199351	5.378237624
Zm00001d045557_T004	4.148799832	3.392723244	4.025676178	6.473709305	6.521712255	6.290609318
Zm00001d045557_T001	3.624550014	2.674717575	3.54736555	6.453177047	6.548152697	6.42532047
Zm00001d037449_T002	2.32168801	1.82892196	2.36485366	2.624707331	2.399493237	2.169656315
Zm00001d024420_T005	0.728455187	0.666274856	0.686251298	1.023484604	0.325659269	0.780356298
Zm00001d024420_T001	3.03482264	2.355909635	3.037287152	2.818745667	2.820890278	2.752944434
Zm00001d024420_T006	1.071302059	0.513364409	1.463032285	1.115141141	1.29303258	1.317139643
Zm00001d006192_T002	0.762687283	0.353479128	0.577257205	0.480342684	0.733673651	1.022776149
Zm00001d006192_T001	4.21303351	3.375918269	4.265397362	4.216762385	4.304593688	4.199983619
Zm00001d041444_T002	3.174227908	2.664131226	3.117770666	2.119229002	2.540898428	2.282393247
Zm00001d039929_T001	1.155693544	1.894081631	1.259144711	2.215970212	1.961612587	2.171400284
Zm00001d028130_T001	4.072860745	3.916395717	4.367374281	4.103951275	3.916115161	4.12071713
Zm00001d028130_T002	0.314599086	0.097056533	0.389385087	0.25326562	0.32708487	0.37197363
Zm00001d033187_T002	3.055623435	1.987859275	2.189280264	3.488713584	3.424307536	3.010840696
Zm00001d033187_T003	2.075205378	0	3.029045905	0	2.080654935	2.544962579
Zm00001d033187_T001	1.579652278	2.515372853	2.385066797	3.267685603	2.685545511	3.220797517
Zm00001d009638_T001	6.865262613	6.013942892	6.962905639	6.780283849	6.871384283	6.748107869
Zm00001d038038_T001	3.274708194	3.453941568	3.292062578	3.360053228	3.518290892	3.50903134

Zm00001d002789_T001	0.872236985	0.456239471	0.705463968	0.614595837	0.453040245	0.581070636
Zm00001d051967_T001	2.382158416	2.218978266	2.318118683	2.247891977	2.155363254	2.20765738
Zm00001d002969_T001	5.366126169	6.086229085	5.666037167	4.574739375	4.74891619	4.722364759
Zm00001d045094_T001	3.125646969	2.829067533	3.188479578	2.893616165	3.016754099	3.036446539
Zm00001d020526_T001	0.568174178	0.440892682	0.619725698	0.10158957	0	0.221137143
Zm00001d052922_T005	0.872259053	0.520843617	0.962474989	1.110342528	1.024638824	1.196743628
Zm00001d052922_T009	3.935653509	3.087194475	3.5279481	3.509866701	3.427801647	3.712434445
Zm00001d052922_T007	2.243099933	1.086825878	2.765515227	2.637785733	2.652224664	1.98179604
Zm00001d052922_T010	1.00311574	0.444682967	0.515712452	1.15390389	0	0.590576152
Zm00001d052673_T001	3.767782432	3.170371847	3.627174899	6.560142241	6.625469781	6.555676909
Zm00001d052673_T002	2.212612603	1.313238304	2.918839963	2.346912651	3.169651184	2.814959078
Zm00001d016248_T001	6.473146793	5.623362509	6.501372821	6.273521783	6.288883529	6.25335237
Zm00001d028414_T003	2.225598544	1.168081663	2.401474831	2.370538719	2.198958005	2.365296119
Zm00001d028414_T001	0.32886518	0.211567976	0.222955375	0.360495757	0.517015607	0.411204943
Zm00001d015026_T004	3.7562162	2.627828128	3.610296344	3.739656307	3.938364089	3.985615459
Zm00001d015026_T001	2.811227273	2.611527461	2.73371768	3.096157089	2.917099067	2.950003803
Zm00001d015026_T002	0.881890891	0.395885395	0.910800191	0.886631303	0.725907771	0.817508656
Zm00001d026483_T004	5.92711283	5.115213319	5.664946579	5.431931822	5.573609321	5.469281428
Zm00001d026483_T001	4.389452686	3.585296807	4.706552267	5.528425542	5.370833813	5.396207587
Zm00001d026483_T002	1.38479694	0.762354769	1.633594885	1.793060188	1.812465777	1.973838269
Zm00001d042800_T001	1.435757657	0.732373408	1.202950503	1.430102716	1.376587672	1.6844992
Zm00001d013177_T004	0	0.43397962	1.225389082	0	0.601070817	0
Zm00001d039860_T007	2.624406718	2.288725317	1.612114193	1.409962741	0	2.381821583
Zm00001d039860_T011	0	0.536742244	1.179009045	1.22591345	1.187921635	1.361745905
Zm00001d039860_T008	4.821274331	3.51987109	4.946826101	5.159479395	5.180118773	5.028018445
Zm00001d039860_T002	1.834907364	1.331424331	1.26302058	1.996688825	1.683791658	2.921830086
Zm00001d039860_T009	1.01657619	0.249514516	0.859774791	2.913028978	2.835361233	2.897226289
Zm00001d039860_T013	0.644834582	0.115139809	0.642268121	0.800987261	0.962512007	0.709842019
Zm00001d052480_T001	4.431994015	4.142347429	4.736332816	3.149239201	2.949928749	3.088063726
Zm00001d040310_T001	2.590847976	2.145990739	2.86751657	3.534150772	3.348018917	3.623410623
Zm00001d037609_T003	1.655555166	0.970755754	1.5455266	0.598659622	0	0.666622994
Zm00001d037609_T006	1.705234363	0	1.678268008	0	2.194827126	2.197566948

Zm00001d037609_T002	3.986667594	3.417458968	4.240142035	4.256439614	3.943202987	3.747490308
Zm00001d037609_T001	1.032202428	1.904293717	1.416245791	0.948370596	0	1.23827877
Zm00001d015058_T003	5.96470985	5.74834001	5.906941474	5.633393864	5.554487952	5.469834511
Zm00001d015058_T001	2.967734505	3.745293705	2.985042776	2.821926705	3.262252885	3.234099664
Zm00001d045692_T019	0.183570358	0.069610062	0.245798652	1.399141556	1.246554022	1.010742607
Zm00001d045692_T028	0.472649968	0	0.710609195	1.908360626	1.852332896	1.17745478
Zm00001d045692_T029	0.2592978	0	0.039829037	0.378100952	0.321683393	0.282537069
Zm00001d045692_T031	2.531414151	1.739443746	2.494902742	2.317623892	2.50460819	2.394974754
Zm00001d024757_T001	2.772788301	2.704317792	2.955832236	2.202182922	2.010627685	2.270841069
Zm00001d048698_T006	0.590944959	0.250049599	0.825006484	1.18438758	1.038676377	0.908237252
Zm00001d048698_T004	2.153382192	1.571027269	2.217311071	0.95983246	1.221759558	1.578126229
Zm00001d048698_T003	2.596170669	2.507883926	2.434041557	2.284789794	1.664305337	1.593672157
Zm00001d048698_T005	2.937315387	2.325877112	3.226659882	2.594077447	2.494651133	2.677054672
Zm00001d027971_T001	0.909683257	0.772197319	0.954744989	1.390194413	1.609349324	1.47780113
Zm00001d027971_T003	2.124143485	1.702718264	2.568690047	0.996951975	0.85622322	1.529306231
Zm00001d027971_T004	3.461658339	4.961652636	3.997547047	3.315497108	3.219974965	3.474117486
Zm00001d027971_T002	3.496816397	2.621414022	3.391222895	4.232504277	4.138031159	3.998442221
Zm00001d037235_T001	0.177964978	0.140397813	0.16967876	1.357018179	1.094538768	1.380951044
Zm00001d039461_T001	3.496325181	3.61437404	3.394167283	4.815411836	4.91621082	4.874032883
Zm00001d017991_T001	5.907432707	8.257818701	5.345521385	7.341380905	7.383712508	7.331931471
Zm00001d016998_T001	1.857731039	1.576225103	1.771355719	2.094404994	1.763413274	1.89539143
Zm00001d015008_T001	3.728884724	2.822948116	3.723105259	3.726101077	3.815390982	3.600646556
Zm00001d046836_T003	3.314249728	2.547723486	3.415287875	3.398046721	3.262492091	3.344023004
Zm00001d052123_T001	2.198663558	1.977723198	2.066928552	1.762639239	1.591384529	1.825589863
Zm00001d002052_T003	1.873323426	0.733540035	1.508464656	2.265416192	2.369978141	2.269138202
Zm00001d002052_T001	5.959693545	4.890149222	6.234046709	7.682826252	7.763498507	7.694671175
Zm00001d002052_T002	3.68436342	5.806964904	0	0	0	2.445720877
Zm00001d007445_T001	4.774837388	4.030196747	4.88130997	3.812762302	3.835823547	3.801939818
Zm00001d040259_T002	0.774156616	0.293478785	1.206275627	0.356158463	0.648711163	0.975946349
Zm00001d040259_T004	2.736150674	2.193954	2.700296773	2.666136668	2.75079385	2.366970075
Zm00001d028392_T001	7.035677702	6.787926217	7.017412856	6.801938383	6.783196157	6.777627793
Zm00001d044102_T006	0	0.180953661	0	2.179071487	2.439548674	2.750233242

Zm00001d044102_T001	5.12531787	4.230387566	5.099426127	5.082896196	5.035237189	4.822562368
Zm00001d029534_T001	1.354432413	0.986953323	1.319210963	0.943958073	1.433597792	1.435023655
Zm00001d029534_T002	4.983062914	4.232815071	4.951263684	5.12538644	5.147840121	5.023460119
Zm00001d049452_T001	1.330389167	0.998833109	1.612046705	1.769915579	1.907907818	1.783904166
Zm00001d021943_T004	0.945885579	0.515448046	1.009269051	0.77156532	0.715676401	0.778282602
Zm00001d021943_T003	3.843233568	3.961586196	3.642063827	3.572987987	3.189986606	3.275546765
Zm00001d007016_T001	1.744427647	1.159106001	1.873064298	1.965995526	2.010758359	2.138177826
Zm00001d007951_T001	5.938498651	5.406342137	5.980027883	5.837233498	5.864592222	5.922060805
Zm00001d026297_T001	4.633873542	3.865252824	4.896881256	4.97565701	5.005741397	5.199557435
Zm00001d052473_T003	5.94689416	4.969075228	5.997738337	5.013558511	4.95095811	5.107837403
Zm00001d052473_T001	0	0	0	0.456697877	0.450041973	0.491666367
Zm00001d052473_T002	4.332480092	4.464464982	3.790108496	2.718278761	3.533389915	3.146345668
Zm00001d014882_T006	0.667726847	1.302634318	2.121021702	1.41154285	0	1.347913359
Zm00001d014882_T005	4.14256693	3.827064359	3.508119036	3.839273641	4.237401345	4.101843089
Zm00001d029134_T002	0.768300371	0.333665308	0.887213303	0.88421263	0.904485639	0
Zm00001d029134_T005	0	0	0.080710868	0.468003516	0.542289752	0.971957335
Zm00001d047761_T005	1.71830726	3.150260384	1.829594681	2.758137917	2.938545322	2.289164888
Zm00001d047761_T009	3.275239231	0	2.895442812	3.376227861	3.868591359	3.290863899
Zm00001d047761_T001	1.12014886	0	0.760076989	1.92584489	1.509116621	1.655295036
Zm00001d047761_T014	5.120309925	2.653536326	5.005377889	4.772838752	4.618127277	4.628347372
Zm00001d047761_T015	0.591153747	2.041146589	0	1.133053824	0.5905522	1.206089293
Zm00001d047761_T012	1.286972791	1.203535084	1.815832386	1.901711847	1.42946439	2.394570663
Zm00001d047761_T006	0	0	0	2.387642394	1.989879062	2.592194607
Zm00001d047761_T008	0	0	0	0.755930995	1.380730557	1.820865165
Zm00001d002694_T005	2.659517031	1.829732679	2.510755118	3.080381717	3.028490474	3.04535245
Zm00001d024239_T001	7.741036797	8.867612229	7.740424047	7.691760287	7.736174093	7.601319119
Zm00001d042034_T001	0	0	1.550590427	2.20759179	2.485730276	2.163828132
Zm00001d042034_T004	1.252329061	0.727445648	1.000054821	0	1.427354618	2.046290934
Zm00001d042034_T003	2.320322925	1.652035776	2.022645139	2.30440415	1.24033326	1.547551007
Zm00001d051001_T004	7.541258994	6.539488685	7.544457999	6.455105974	6.65429186	6.681276818
Zm00001d051001_T002	7.142922202	6.530002443	7.267117751	6.784237471	6.729291166	6.659732624
Zm00001d008987_T001	3.00120685	2.251091289	2.979962947	3.238026646	3.070824132	3.37093858

Zm00001d049557_T002	7.169781596	8.099454269	7.136506757	6.907873037	6.870491868	6.906132609
Zm00001d021883_T001	1.365162836	0.953295788	1.629398122	1.424566341	1.528147199	1.514463163
Zm00001d004248_T001	3.78417646	2.676095412	3.764138157	3.460787508	3.478450659	3.310836107
Zm00001d010388_T001	4.498012995	1.007631833	4.699581004	6.744674668	6.108372443	6.376811328
Zm00001d027936_T003	0	0	0.417112045	2.326837878	0.690443021	2.428237013
Zm00001d027936_T009	4.003666545	2.956214485	4.167216847	2.829525475	2.614677339	2.473834438
Zm00001d027936_T007	4.457350802	4.978890171	4.350793747	4.651714977	4.960948678	4.605717975
Zm00001d027936_T004	1.141293485	0.966366231	1.594416217	1.135588715	1.369383842	1.198731754
Zm00001d027936_T005	3.575697038	2.298455679	3.378294509	4.261078454	4.515521532	4.647431026
Zm00001d045064_T001	4.69154427	4.634307779	4.748165525	4.743225948	4.84894812	4.594500539
Zm00001d049832_T001	2.1348682	1.40326445	2.042428267	2.114420334	2.132845616	2.249669844
Zm00001d028230_T001	4.700202708	3.820474212	4.58804333	5.308231293	5.431018579	5.246039309
Zm00001d002860_T001	1.265333071	1.001574563	1.419313436	1.445176849	1.045479317	1.545136603
Zm00001d012170_T002	5.479072305	4.918194101	5.52062484	6.259586719	6.182354742	6.210034089
Zm00001d012170_T001	3.531862757	2.566356341	3.455050328	2.995327668	2.999927322	3.013339693
Zm00001d036613_T001	0.330434489	0.338867068	0.48913802	0.619028867	0.430027224	0.543552303
Zm00001d022031_T005	1.741248219	1.007501234	1.522396289	2.157733839	2.072609369	2.239183852
Zm00001d022031_T001	1.560775595	1.126339226	1.433882971	1.159224865	1.351479077	1.152404107
Zm00001d022031_T002	3.319637731	3.041999708	3.533900311	3.531473541	3.413278458	3.374284444
Zm00001d020454_T003	3.393964452	2.484345173	3.58914182	4.108023734	4.09692097	4.327203319
Zm00001d020454_T002	3.45234016	2.012088768	2.805384711	3.945454699	3.795230408	3.910805179
Zm00001d020454_T004	0.276807504	1.616831459	0.827421518	1.425960347	2.115294982	0.358353498
Zm00001d024752_T001	8.104591437	8.118918835	8.145287595	8.717041707	8.783656861	8.783844179
Zm00001d028806_T001	2.866722265	2.095323256	2.8786151	4.038477358	4.022272903	4.115519471
Zm00001d053661_T003	4.914836486	4.580383642	4.850873466	4.039070909	4.451112577	4.209473797
Zm00001d008730_T001	4.884424191	4.742588509	4.699831216	4.729378364	4.518037915	4.510460179
Zm00001d011233_T050	4.109096738	3.500665169	4.209530567	3.810026379	4.18545857	4.100143149
Zm00001d011233_T026	0.96491462	0.374757957	0	0.613414724	1.555485377	0
Zm00001d011233_T019	2.473854954	1.513459415	2.776573367	2.03414844	1.78929523	1.843754361
Zm00001d011233_T018	0.224887362	0	0.481877557	1.59089994	1.515103338	1.94307102
Zm00001d011233_T041	0.500763309	0.07334314	0.169445306	0.432599223	0.439295469	0.412527914
Zm00001d011233_T001	0	0.213465492	0.096781346	0.632526947	0.651929272	0.47238379

Zm00001d011233_T007	2.008017101	0	1.57939644	3.489068895	2.874482233	2.890340262
Zm00001d011233_T006	0	1.513674671	0.506933967	0	0	0.852363261
Zm00001d032899_T001	2.367937967	1.769715469	2.525229751	2.203408539	2.411278172	2.310263039
Zm00001d029662_T001	6.119243779	5.609496576	6.263049603	6.95999863	7.071009103	6.92240038
Zm00001d041831_T003	2.551827778	3.19543548	2.672349754	2.591885467	3.159849868	3.300587217
Zm00001d041831_T001	0.636254779	1.577820825	0.875615736	1.434171296	1.737733048	1.379982977
Zm00001d041831_T002	4.364861713	2.478788319	4.443336519	5.221308241	5.08991118	5.040741054
Zm00001d038824_T001	4.76936484	3.861394549	4.723125688	3.710539769	3.73559366	3.419347283
Zm00001d038824_T002	0.747712211	0.721462784	0.929531213	0.559316574	0.783193446	0.86412472
Zm00001d003841_T002	4.86793095	4.519495797	4.774994371	6.345451253	6.385420631	6.23235277
Zm00001d051111_T018	0.461704564	0.328785923	0.620843492	0.070165349	0.049080069	0
Zm00001d034125_T002	2.36736128	2.620250681	3.695033032	3.502270382	3.023321009	2.753089104
Zm00001d034125_T001	6.049590518	5.161108054	5.928508521	6.355692034	6.328541	6.304184427
Zm00001d050116_T003	2.833105441	1.754103318	2.364199244	2.911367155	0	1.655140676
Zm00001d050116_T001	0.480295113	1.068544935	0.755279862	1.209943019	2.008514139	1.52066714
Zm00001d050116_T002	2.360028498	1.399521674	2.729263441	2.908986174	3.560425041	3.294341225
Zm00001d009740_T003	6.836982168	6.174299647	6.954760817	6.325895852	6.27045789	6.270694039
Zm00001d009740_T002	2.684066231	0	2.607811783	2.581644577	2.71018818	0
Zm00001d018894_T003	0	2.708695567	2.918118853	2.292715502	0	2.533815985
Zm00001d018894_T007	1.606488185	0.558017774	1.258241515	1.250800928	1.41735783	1.120089789
Zm00001d018894_T009	3.394479021	3.799257061	4.043047413	3.217116537	3.271504413	3.294824072
Zm00001d018894_T001	3.547644035	0	2.147172519	3.409290182	3.652351455	3.043067454
Zm00001d018894_T008	0.541067738	0.282731577	0.708302912	0.338471196	0.428603379	0.432346933
Zm00001d018902_T001	4.10640025	3.534102078	4.072673761	4.376852472	4.345777155	4.421249291
Zm00001d041323_T012	1.216431894	0.517340204	1.588689933	1.073690008	1.217572579	1.319359544
Zm00001d041323_T003	0.581456342	0.548207748	0.184540559	1.325742727	1.013292164	1.080532147
Zm00001d048202_T002	1.667879412	0.950763269	1.592820102	1.295918701	1.668755441	1.631492675
Zm00001d048202_T001	2.44961578	3.183760735	2.829298755	2.913216486	2.373392916	3.036865149
Zm00001d052112_T003	2.023316395	1.569822077	2.509439959	1.672614068	1.263753771	1.322902451
Zm00001d052112_T002	2.691653863	2.228531816	2.771794958	1.92921532	2.11801712	2.330902541
Zm00001d017503_T003	1.982375946	1.838068337	2.26319099	1.155487607	1.173724861	1.145933694
Zm00001d017503_T004	2.589629996	2.282412819	2.071123885	1.254854019	1.950603905	1.480970784

Zm00001d009167_T001	0.883766251	0.340573662	0.564245478	0.524601326	0.738780805	0.633189385
Zm00001d040381_T001	1.840347209	1.56213592	1.897971464	1.77582511	2.06906075	1.818339311
Zm00001d017616_T001	4.374271296	3.921244841	4.2265802	4.159012324	4.399441283	4.35702191
Zm00001d039213_T002	4.260531188	4.229753485	4.046920382	3.608021813	3.670425343	3.973642498
Zm00001d039213_T001	3.712884401	3.637296658	3.568551187	2.957550258	3.380919191	3.237587427
Zm00001d047115_T004	4.412022183	4.788134364	4.37844788	3.410833317	3.392310965	3.560964104
Zm00001d018258_T004	0.884154788	0.107107997	0.542277863	1.294667797	0.650875743	1.72778674
Zm00001d018258_T009	1.340391927	1.277230184	2.128788954	1.780969766	2.334105704	1.165459207
Zm00001d018258_T012	0.544176734	0	0	1.082089416	1.180549544	0.725313628
Zm00001d018258_T013	3.29295339	2.78896775	3.178683572	4.004967474	4.012250513	3.854035954
Zm00001d018258_T011	2.283490381	0	1.654954689	2.261110131	2.095753649	1.762037492
Zm00001d018258_T001	0.326949159	0.086445467	0.388110166	0.843524841	0.678093545	0.906716692
Zm00001d029078_T001	3.96670593	3.632596857	3.88068484	3.682306644	3.648779718	3.760450801
Zm00001d038648_T003	7.044111706	6.31117312	7.129409989	7.486716094	7.447556113	7.370387251
Zm00001d038648_T005	1.627534468	1.60391091	0.920528839	1.088933301	1.330992577	0.85182938
Zm00001d038648_T001	1.119744606	0	1.200362475	2.25034375	1.972158115	2.376274267
Zm00001d038648_T004	1.134337271	0.870493348	1.256542119	1.659225869	1.936694845	1.741662856
Zm00001d038648_T002	1.369016924	1.529240254	0.905410208	2.501448317	2.695723282	2.288172491
Zm00001d002416_T002	1.668272556	0.894796814	1.608804513	1.800112996	1.781465053	1.599343505
Zm00001d002416_T001	4.685618281	4.101173105	4.77233701	5.243710966	5.142624272	5.279580459
Zm00001d050365_T001	4.39302781	3.468183816	4.38270785	3.677417021	3.728150288	3.569802152
Zm00001d050365_T003	1.862606514	1.084688844	2.04092188	1.267530405	1.340623223	1.574623739
Zm00001d050365_T002	2.01931584	1.299058813	2.024227114	1.17747264	1.171247827	0.977187588
Zm00001d050365_T004	4.273810516	4.876911025	3.832805171	2.281966737	3.033457009	2.970136545
Zm00001d023447_T001	1.172416467	1.050859671	0.5697866	1.307213999	0.786336235	1.188571202
Zm00001d035235_T001	5.267858271	4.830087605	5.248880251	5.26511133	5.32341554	5.233525807
Zm00001d039537_T053	0	0.260986731	0	0.677617385	0.715585914	0
Zm00001d039537_T068	0.290022092	0	0	0	0.351144335	0.278404704
Zm00001d039537_T069	0.164867355	0.148408328	0.281841845	0.623901501	0.422701262	0.468579147
Zm00001d039537_T048	0	0	0	0.411550985	0.56459669	0.467562252
Zm00001d039537_T002	2.195849619	1.703562767	1.942350474	1.699890502	1.438345022	0.352116682
Zm00001d039537_T019	0.146755569	0	0.299892875	0	0.735581097	0.727077892

Zm00001d039537_T032	1.420443596	1.16824922	1.401296238	2.175341279	2.325544147	1.756438367
Zm00001d039537_T018	1.481030737	1.023737235	1.966669297	2.765039478	2.766572689	2.534636872
Zm00001d039537_T067	0.059414812	0.025814087	0	0.37344331	0.414818554	0.568262723
Zm00001d039537_T024	0	0.42933744	0.878077111	1.103599019	0.610812488	1.193866974
Zm00001d039537_T066	0.349598536	0.131099504	0.299273969	0.371125013	0.599375881	0.32074691
Zm00001d039537_T036	1.756676617	0	1.652868715	0	0	1.395363924
Zm00001d039537_T039	0.660789916	0.580859418	0	1.046526682	0.930631378	0.979265171
Zm00001d048437_T002	3.365957595	2.583065794	3.282852241	3.206185121	3.180324049	3.128954045
Zm00001d048437_T001	2.330996305	3.421282285	2.149820268	2.131842971	0.911778237	1.276335861
Zm00001d048496_T004	1.223076509	4.02544382	0	1.828030333	0	2.143177227
Zm00001d048496_T001	4.703756764	3.740251375	4.584147328	5.310326014	5.299755021	5.182421608
Zm00001d025431_T001	0.818556103	0.429129584	1.07677798	1.534268825	1.057263793	1.535683722
Zm00001d025431_T002	4.456435113	3.671580977	4.55145111	5.145428793	5.173160262	5.081658719
Zm00001d025431_T003	1.545585411	0.850029674	1.690180166	2.741695642	2.792494738	2.607699833
Zm00001d044004_T001	4.361394515	4.503382767	4.334889014	3.005145383	3.122325006	3.181015223
Zm00001d038791_T001	3.582950342	2.841985192	3.734631157	6.534698961	6.580693176	6.554093738
Zm00001d016736_T001	4.550426664	4.271417832	4.579003603	4.548858705	4.759188225	4.728950661
Zm00001d037227_T004	5.681041756	5.168863188	5.336563365	5.01174689	5.288562437	4.927226326
Zm00001d037227_T008	2.966558562	4.493323639	1.766665122	3.715961112	4.304393928	3.505262281
Zm00001d037227_T002	2.772155504	3.271048219	4.065758668	3.740391711	3.594345626	2.868303721
Zm00001d037227_T007	1.069553697	0.474560643	0.994482697	1.543672065	1.485463925	1.329880203
Zm00001d037227_T005	3.64874153	0	2.651090737	2.745207639	2.92313054	3.840010665
Zm00001d037227_T003	1.436841974	1.361680785	0.738685704	2.015917842	1.651137131	0.857129853
Zm00001d038101_T003	1.091316644	0.221174273	0.870423906	0.984113903	1.119694814	0.79639342
Zm00001d038101_T002	4.952162959	4.002814116	5.003196579	5.674198117	5.735399294	5.671484653
Zm00001d038101_T001	0.561944878	0.970990557	0.578539774	1.534490461	0.861668781	1.062174914
Zm00001d010576_T002	2.000932401	1.199601995	1.866332367	1.796296642	1.496836589	1.979130515
Zm00001d010576_T003	1.09898138	1.304507537	0.853102217	0.83847099	1.210008501	0.31986761
Zm00001d024470_T002	0	0	0	1.009177307	1.40503115	0.467564339
Zm00001d024470_T001	0.671739022	0.477402749	0.795257427	0.786838045	0	0.793155935
Zm00001d023888_T001	3.345213543	3.063302401	3.638929582	2.602917893	2.748854486	3.110338853
Zm00001d009591_T002	3.460360546	2.824982053	3.673668127	2.413194362	2.426648796	2.65691802

Zm00001d009591_T003	2.89472601	1.338327424	2.793134288	3.623788452	3.531334919	3.446882674
Zm00001d037321_T015	0	0.082411024	0.136037788	0.937256999	0.588507172	0.914546155
Zm00001d037321_T031	2.461141641	0	2.112785191	2.592777534	2.531129146	2.567458075
Zm00001d037321_T013	0.233126028	0.216638624	0.350789151	1.057379565	0.771964998	0.912107172
Zm00001d037321_T024	0.49108448	0.745316511	0.49601027	0.913317986	1.331591722	0
Zm00001d037321_T033	1.289272619	1.901299045	1.653473271	1.876008439	1.732995466	2.281351368
Zm00001d037321_T002	0.322301993	0.141980728	0.336466213	0.673205431	0.563837575	0.62783373
Zm00001d032724_T001	0.290309975	0.138379367	0.261773902	1.413422942	1.452988604	1.246920617
Zm00001d016158_T001	6.65373072	7.045585709	6.740913546	6.048833602	6.114405591	6.139396679
Zm00001d009848_T001	8.549488384	8.47659569	8.605908481	7.969073685	7.959763636	7.889656855
Zm00001d009848_T002	1.299056468	0.920468624	1.546474212	1.465967675	1.213138834	1.156232856
Zm00001d026672_T001	3.025227993	2.568624145	3.003749197	3.42166779	3.184269026	3.466319887
Zm00001d005928_T002	2.031057069	1.633860816	2.02610917	2.495841215	2.540608348	2.56084528
Zm00001d007121_T052	0.285039946	0.256486565	0.08727409	0	0.59951014	0.377265929
Zm00001d007121_T057	0	0	0	1.950429967	1.891234463	1.664228412
Zm00001d007121_T066	0.345188558	0.394713877	0.585384668	0.618854137	0.622943466	0.76679952
Zm00001d007121_T022	1.230220232	0.54171604	0.758487394	1.511815135	2.060465601	0
Zm00001d007121_T049	1.321039121	0.23038257	1.078441784	0	0.518894635	0.277323038
Zm00001d007121_T063	3.138984313	2.270045372	3.026012646	2.744246363	2.922149694	2.881417754
Zm00001d007121_T044	0.319543948	0.057509885	0.573714799	1.195219703	1.35730201	0.859011408
Zm00001d007121_T026	0	0	0.548564859	0.964495486	0	0.585216393
Zm00001d007121_T004	0.612890313	0.403470613	0.611909831	0.748431177	0.667739562	0.835171413
Zm00001d007121_T033	0.240267918	0.425402369	0.292393052	0.512072114	0.361696504	0.243923723
Zm00001d007121_T065	0.418520286	0	0.360710369	1.231540545	1.196568647	1.244849926
Zm00001d007121_T001	1.112732822	0.731665508	1.170259668	1.788523685	1.163061993	1.743968149
Zm00001d007121_T046	0.351068554	0.442005008	0.378975431	1.272719577	0.874237481	1.119909242
Zm00001d017456_T009	0	0.290302896	1.037751898	1.468789737	1.432298822	1.756013861
Zm00001d017456_T015	2.609925362	0	0	5.049454602	4.730868215	5.030104955
Zm00001d017456_T005	0.151408148	0	0	0.503324308	0.662913682	0.535484665
Zm00001d017456_T014	4.046512975	4.235690303	3.510307116	0	2.531300106	3.065431344
Zm00001d017456_T004	5.512139386	5.141239852	5.511141612	5.813276275	5.930442548	5.838260121
Zm00001d017456_T010	1.580395884	1.715393501	1.692637427	2.532071793	2.337643165	2.295864643

Zm00001d017456_T012	4.033171444	2.831411127	4.219708859	4.972877574	5.169282418	4.769692939
Zm00001d017456_T011	0.449496931	0.144807337	1.015042195	2.788274348	2.90392059	2.430808189
Zm00001d017456_T001	1.543840307	1.696007171	1.999674996	2.418646572	2.55320959	1.154938298
Zm00001d047618_T001	7.334330831	7.42253484	7.302867471	7.858298409	7.93865039	7.961534263
Zm00001d035725_T001	2.274852635	1.830532396	2.084044189	0.934465926	0.343424879	0.375201838
Zm00001d053080_T002	3.090016696	1.767743349	3.0005346	3.468826222	3.46687466	3.492293276
Zm00001d005969_T008	0	0	0.123449014	1.256111521	0.632063431	1.491507834
Zm00001d005969_T012	2.850687027	0.88741297	3.105715452	3.778084281	3.70673962	3.021364236
Zm00001d005969_T003	0.950997616	2.475669108	0.989383191	2.350297802	1.682406996	1.284922721
Zm00001d005969_T009	2.140980161	0.077229324	1.942943825	3.061800559	2.978518003	3.532471445
Zm00001d005969_T013	1.095672644	0.417815257	0.466614592	2.321195597	1.990433581	2.105539634
Zm00001d005969_T005	0.094956224	0	0	0.707338368	0.958103975	0.757042027
Zm00001d005969_T004	0	0	0.201730456	0.552770524	0.7022976	0.677018353
Zm00001d005969_T015	3.718911746	2.624183967	3.62465926	3.950557437	3.776886201	3.770048088
Zm00001d003575_T001	2.508929325	2.187213172	2.490079736	1.645905899	1.835534454	1.92794271
Zm00001d030766_T001	1.729000601	0.994227787	1.716415457	1.908908188	1.849697082	1.813997849
Zm00001d015134_T001	7.804403481	7.316727175	7.762062089	7.664840227	7.628663049	7.588165025
Zm00001d048875_T001	2.345682785	1.794096827	2.231586009	2.55485772	2.707912358	2.975984122
Zm00001d029636_T001	4.402767056	3.752592969	4.451942463	5.572423839	5.582861881	5.526056977
Zm00001d045862_T001	1.611824869	1.004978796	1.322928401	1.726363464	2.159539095	1.850063289
Zm00001d038523_T001	5.618053725	6.2215663	5.656982698	4.763912181	4.776892516	4.753103603
Zm00001d003076_T002	3.040109692	2.426201141	2.448307369	1.103980304	1.636754994	1.831420045
Zm00001d003076_T001	3.196034447	2.105575168	3.412020828	3.31071747	3.191314074	3.231438092
Zm00001d021973_T001	3.741471625	3.238903004	3.783574098	6.75177202	6.745143193	6.729605124
Zm00001d047058_T003	4.344265367	5.059029743	3.997578208	3.236984352	3.484114022	3.778063143
Zm00001d047058_T001	2.939704826	2.585869911	3.168299772	2.947400594	3.061123814	2.924468211
Zm00001d033475_T001	1.13353525	0.469794308	0.683559027	1.143158284	1.378115938	1.415045795
Zm00001d038623_T002	6.336653317	5.677539249	6.411798922	6.500852011	6.508003007	6.442334484
Zm00001d039451_T004	1.497679805	0.743028027	1.340451748	1.099642621	0.867621338	1.101687723
Zm00001d039451_T007	4.092213275	3.473773017	3.761282082	3.829914084	4.020596517	3.746479924
Zm00001d039451_T006	2.795540886	2.331167473	3.071611837	1.83164704	1.970088676	1.727078328
Zm00001d049097_T005	3.497971366	2.360077114	3.578864947	3.516876723	3.211031357	3.208069587

Zm00001d049097_T003	2.42532878	1.64790068	1.917787659	1.460858918	0.554784308	2.177666215
Zm00001d049097_T001	1.618633346	1.826546454	1.815591413	1.800265026	1.922277404	1.741705564
Zm00001d049097_T006	5.298638889	6.567705987	5.241011807	4.910216307	4.801110945	4.405250678
Zm00001d049097_T007	3.037196997	2.067850012	3.117539795	3.796904512	3.999385642	4.021244826
Zm00001d049097_T002	0.63908117	0	0.326596021	0.498726751	0.666466661	0.756001901
Zm00001d011204_T001	4.991759959	5.148898809	4.873828107	4.583426114	4.70557284	4.788953451
Zm00001d048856_T005	3.831604988	3.61234082	3.130664869	1.353175344	2.994741181	2.999674636
Zm00001d048856_T003	3.384047458	2.701710949	3.528036397	3.149614304	3.172401905	3.043981362
Zm00001d024497_T001	4.518242542	3.709816991	4.496151816	4.210816186	4.138383463	4.273210236
Zm00001d011413_T001	1.873345477	1.433463728	1.973194294	1.016607566	1.109974953	0.711764478
Zm00001d036898_T001	3.242759494	4.116946575	3.428360039	2.546869525	2.705605738	3.088444219
Zm00001d026195_T001	2.574883574	1.910259488	1.944084054	2.408439598	2.112191013	1.796510127
Zm00001d026195_T005	0	0	2.104479916	0	0.924809844	1.510289517
Zm00001d035876_T007	0	0	1.218634256	1.222373048	2.389441534	0
Zm00001d035876_T004	2.047169816	1.946568431	2.097623606	1.221744093	1.472014074	0.890512065
Zm00001d035876_T002	1.548014359	1.199468194	1.702579534	1.297590561	0.601474034	1.538266484
Zm00001d035876_T009	2.148021356	0.380296132	1.732180616	1.655456248	0.539698805	1.990535601
Zm00001d043972_T001	4.033192328	4.081324116	3.428793762	3.690977972	3.37512606	3.562107583
Zm00001d015092_T001	5.20579407	4.87937034	5.129510697	4.994940986	5.083358779	5.109169803
Zm00001d017927_T001	4.573197754	4.024887442	4.5290718	4.068604951	3.979311456	4.218437266
Zm00001d021843_T001	5.135983549	4.655479605	5.317027385	3.494930895	3.243181449	3.104205805
Zm00001d052353_T006	2.181947242	0.835020187	1.50893262	2.164468039	1.501412138	1.733861473
Zm00001d052353_T004	0.152385934	0.208683307	0.32997432	0.611834309	0.287573948	0.642379962
Zm00001d052353_T008	1.981737233	3.330394187	2.785912069	0	0	1.630419917
Zm00001d052353_T001	2.646515452	1.758260531	2.845177692	2.957452379	3.145631321	2.911033256
Zm00001d040416_T004	2.449383882	1.596389915	2.371334147	2.994736475	3.142619289	2.922789841
Zm00001d040416_T003	3.145310133	3.659765671	3.507139793	2.488038059	1.986006742	3.275378404
Zm00001d010825_T001	5.919826278	5.061533165	6.035426163	6.140089163	6.18462573	6.182865176
Zm00001d010825_T002	1.815863939	0.502317361	1.066929585	1.55609289	0.67939407	1.354779368
Zm00001d011765_T002	2.536961761	1.154583857	2.195124247	2.009671079	1.842234269	1.679696276
Zm00001d048924_T001	4.435660728	3.551898512	4.585649721	5.074375787	5.057480989	5.116630455
Zm00001d051977_T001	5.786019482	4.854517158	5.842812384	6.131704053	6.162786508	6.115707018

Zm00001d027998_T001	2.725327154	4.189976646	3.874484496	2.195560207	2.152446293	2.852208432
Zm00001d048233_T001	2.815019351	2.010038253	2.839485395	4.15411297	4.359888896	4.339472074
Zm00001d021806_T028	0	0	1.10778293	2.23364207	1.756277377	0
Zm00001d021806_T016	4.512946348	4.266584174	4.630549687	4.205724135	4.131939909	4.357925931
Zm00001d021806_T014	0.58860695	1.605513298	0.915081046	0.391577474	0	1.26485586
Zm00001d021806_T027	0	0	0	1.798878764	1.162456911	1.747399001
Zm00001d021806_T022	1.122816484	0.043967368	0.496822275	1.245083666	2.217338682	2.209899362
Zm00001d021806_T032	0	0.015040767	0	0.511850548	1.039575699	0.736761583
Zm00001d021806_T068	0.152642931	0	0.070093889	0.278166783	0.359235527	0.504711908
Zm00001d021806_T034	3.587586899	2.076673672	3.557459597	4.040656198	4.136590156	4.165841209
Zm00001d021806_T049	0.349486441	0	0	0.539583677	0.411879593	0
Zm00001d024753_T001	4.319297468	4.27735875	4.312897255	4.76833081	4.90298764	4.839829517
Zm00001d012710_T002	4.491230872	3.991463797	4.505582864	4.471698687	4.572169899	4.358475526
Zm00001d011195_T001	0.685335592	0.374537634	0.706656969	0.773923767	0.983639028	0.761917537
Zm00001d011195_T002	2.500661855	1.702675715	2.296617453	2.957430277	3.120888372	3.088522239
Zm00001d025717_T001	3.33942333	2.526535859	3.206014252	3.526522463	3.467711833	3.626987373
Zm00001d027479_T002	3.070401522	2.383312241	3.374764633	3.119344888	3.281955168	2.99108134
Zm00001d002876_T001	3.162692143	2.243511582	3.049689659	3.388326635	3.314198094	3.317439387
Zm00001d009617_T003	2.750415276	1.370819344	2.594843985	2.824187808	3.03188407	2.763732374
Zm00001d009617_T001	0.488906735	1.204848741	0.913536285	0.39232182	0.388925716	0.288150634
Zm00001d021721_T005	4.84152983	3.997093018	4.599140541	3.65590487	4.445723989	3.873139226
Zm00001d021721_T003	4.510811567	3.735720697	4.321717302	5.404896652	5.114551723	5.346936862
Zm00001d021721_T001	2.820535175	2.576310737	3.375799028	2.833178322	2.883322081	2.907090251
Zm00001d013732_T001	2.174812898	2.266027296	2.308809063	2.609505352	2.339785027	2.427756066
Zm00001d032181_T002	0.589596681	0.638639216	0.62015471	0.806442026	0.630812686	0.999909829
Zm00001d032181_T001	6.406996402	6.670003501	6.47654173	5.882493344	6.053086328	5.983365959
Zm00001d031471_T001	1.736900094	1.290085809	1.925065541	1.617527942	1.69343343	1.581726275
Zm00001d027534_T001	3.430912669	2.951095374	3.520494161	2.654533153	2.532285287	2.673762186
Zm00001d006268_T001	1.594925416	1.970744712	1.996793295	1.751878003	1.990476786	2.067927433
Zm00001d050466_T001	3.414657975	3.529160028	3.475933384	1.956934644	2.299448063	1.960960536
Zm00001d014275_T002	3.366904351	2.370104562	3.219473991	3.333558694	3.44084645	3.38315351
Zm00001d014275_T003	0.443384926	0.099058159	0.120671766	0.685901586	0.726467664	0.788760871

Zm00001d039799_T001	1.420803594	1.505466695	1.613818278	1.696479083	2.291358079	1.915828719
Zm00001d039506_T002	3.763378322	3.07298213	3.789436093	4.363814819	4.359582734	4.383704154
Zm00001d006274_T002	0	0.501683714	1.135517796	1.162728875	1.135145414	1.419018888
Zm00001d006274_T001	0.82404276	0.339681287	0.385458648	0.568893086	0.866918262	0.19288725
Zm00001d006274_T005	1.597635574	0.884198561	0.57620188	0.785929641	0.362560782	0
Zm00001d007977_T001	3.222402108	3.734441376	3.137825307	2.390156434	2.399275566	1.848236134
Zm00001d027680_T002	8.666401089	8.488563487	8.734549076	8.258427943	8.270033926	8.326320386
Zm00001d043560_T001	4.026396048	3.650760079	4.385404599	3.885181914	3.847611736	3.932973909
Zm00001d043560_T007	1.04152967	0.297665681	1.192590615	1.405136288	1.052977838	0.57776863
Zm00001d043560_T005	0.555915276	0.128051832	0.093798127	0.576418618	0.240529267	0.762254405
Zm00001d052796_T004	7.509660914	6.366279999	7.572504833	7.002823846	7.004013423	6.911625557
Zm00001d052796_T002	4.253272126	6.462303508	3.897679461	3.958691811	3.934543581	4.458618103
Zm00001d052796_T001	3.17064729	5.828057786	3.787494944	2.779729334	3.077635755	2.672094672
Zm00001d042619_T002	2.830707414	4.004322595	2.951950847	3.835562749	3.915939881	3.812787454
Zm00001d042619_T001	6.220739486	5.167415015	6.188673897	6.800530303	6.796015526	6.742449397
Zm00001d042619_T003	2.703486799	0.617138593	3.662326745	1.999456723	2.239574625	2.630606997
Zm00001d011122_T001	3.515313807	3.216906151	3.635852463	3.828292496	3.408011536	3.55974778
Zm00001d050054_T001	6.284434864	6.347540194	6.241397509	6.174990897	6.10818348	6.070104883
Zm00001d051501_T001	3.349287564	3.855598084	3.540344994	3.448484729	3.329166661	3.766906564
Zm00001d003756_T001	2.998779505	3.35585482	3.298982153	2.95071102	2.926115968	2.683010759
Zm00001d029102_T001	7.438952103	7.234226718	7.620566449	6.356503277	6.234317753	6.293468688
Zm00001d010048_T004	0.709899351	1.475644727	0	0.917769331	1.240833301	0.88375296
Zm00001d010048_T003	2.594115437	2.61688767	1.655986933	1.790845005	1.751327029	2.06633516
Zm00001d010048_T001	3.909999617	2.542241208	4.090433599	4.417896048	4.404214027	4.540620436
Zm00001d010048_T002	0.619888118	1.879080332	2.480729399	2.448229915	2.514551532	2.421056563
Zm00001d004775_T006	0.64830897	0.340907449	0.516326857	0.822422612	0.758973408	0.589468208
Zm00001d011461_T001	2.008431311	3.020862186	2.266836603	3.181573139	2.934242279	2.823155655
Zm00001d031880_T001	0.659287075	0.851850963	1.23174758	3.060499323	2.893218905	3.01948327
Zm00001d002032_T045	0.361734677	0.205587695	0.413692642	0.91795107	1.059956045	1.13478739
Zm00001d002032_T026	0.638363969	0	0.525094664	0.956017238	0.422635607	1.178603109
Zm00001d002032_T044	1.504907123	0.243849407	0.68430888	2.269506883	1.614560503	2.466633645
Zm00001d002032_T024	0.508969118	0	0.472675959	0.875207589	0.825172606	1.000984304

Zm00001d002032_T022	0.424641582	0.407400614	0.540267368	1.383127514	1.5957137	1.122761497
Zm00001d002032_T013	0	0	0.323945303	0.799246491	1.417130413	0.533125739
Zm00001d002032_T002	0.588526361	0.441469669	0.280396938	0.155823049	0.273637626	1.317643282
Zm00001d002032_T033	2.047583058	1.653243955	2.359255209	2.043402254	1.853893478	1.488946313
Zm00001d002032_T001	0.871590563	0.350903408	0.261550071	0.820994199	0	0.515575209
Zm00001d002032_T015	0.923922722	0.523918195	0.893548603	1.593422139	1.792011972	1.681768579
Zm00001d002032_T034	0	0	0.191843077	0.553936483	0.7008019	0.573182542
Zm00001d002032_T003	1.081545517	0.162448532	0.49867774	0.532812653	1.072987273	0.706361685
Zm00001d002032_T010	0.36590302	0.127730997	0.180003966	1.172445271	1.22917505	0.955358189
Zm00001d002032_T020	0.436448644	0.027142725	0.593208418	0	0.5972165	0.752545658
Zm00001d002032_T043	0.116802556	0.019653527	0.04369866	0.477634849	0.366194062	0.4177483
Zm00001d045657_T001	6.181297429	6.547526793	6.139803894	8.135472685	8.017486902	8.268222572
Zm00001d046400_T002	1.313934233	0.560641595	0.689817096	1.095037269	1.735496616	1.439195451
Zm00001d046400_T001	5.503234645	4.866161123	5.678149645	5.276219378	5.258029765	5.367933111
Zm00001d018421_T001	2.092133677	1.237917924	2.072796276	2.378534096	2.325731791	2.193973864
Zm00001d018421_T006	0	0	1.434597272	1.424452397	0	0.82744916
Zm00001d018421_T003	3.430033515	1.665258592	3.660564498	3.604136101	3.02487211	2.559432832
Zm00001d018421_T002	3.177690131	2.73155017	3.246697661	4.081173087	4.414273909	4.182539917
Zm00001d018421_T005	3.880601488	4.062643368	3.392335834	4.313676056	4.163559675	4.400634174
Zm00001d018421_T004	3.182022274	0	4.045841247	3.504344792	3.60777001	4.066653411
Zm00001d029801_T001	5.197798058	5.03805061	5.403212529	5.050787204	5.054016778	4.919224128
Zm00001d048142_T001	2.095713822	1.478947997	2.205979544	3.016252938	3.073076591	2.984072877
Zm00001d002897_T001	8.607624967	8.03043904	8.680987336	7.833450386	7.863920534	7.835791373
Zm00001d013182_T002	3.603043512	4.880908181	4.298369821	1.942928816	2.689621886	2.632780281
Zm00001d013182_T004	5.184548335	3.976759177	5.147040728	4.628305081	4.821876972	4.651211369
Zm00001d013182_T003	1.588793536	0.970115925	1.993092819	1.257443965	0.817967826	1.410145693
Zm00001d012027_T006	4.120978255	3.197637555	4.025038631	0	2.27770927	0
Zm00001d012027_T014	0	0	0.900517138	2.136951587	2.585641129	2.029708007
Zm00001d012027_T001	0	0	0	4.405388888	3.924312188	4.305699097
Zm00001d045098_T001	2.98253914	2.254077277	2.915059432	2.927434376	2.890601169	2.944136168
Zm00001d007296_T001	3.684175888	3.296427158	3.959302409	3.941666186	3.760853783	3.852785711
Zm00001d003890_T001	3.284082622	2.642591373	3.521546044	2.666324627	2.957529271	2.692599267

Zm00001d028775_T001	4.604657774	4.574125795	4.579425405	3.963074091	4.099933839	4.201688353
Zm00001d025931_T003	2.342475384	1.845472789	2.514843869	2.993891338	2.99759844	2.899893318
Zm00001d025931_T001	2.204751416	2.671887541	2.396196762	2.372475763	2.207490277	2.414468965
Zm00001d002377_T002	5.354767169	4.599055537	5.337744305	4.827694409	4.838297819	4.828996474
Zm00001d018394_T003	0.976960388	1.382393903	1.024973496	0.981133683	1.259658761	1.351989523
Zm00001d018394_T001	5.009869007	4.011920567	5.097537902	5.054574148	4.985045646	5.063409967
Zm00001d046568_T001	5.369398465	4.963713906	5.49783399	5.44507651	5.572690739	5.528305446
Zm00001d046568_T003	3.916718825	3.332988426	3.840923916	4.126227783	3.84557104	3.820474927
Zm00001d018557_T001	5.50073562	6.021152241	5.485334112	5.17882951	4.814919714	5.058405297
Zm00001d042978_T001	1.006746832	0.310252858	1.020835701	1.222154151	1.100781912	1.204197697
Zm00001d037886_T003	1.447522069	0.97795101	1.791050126	0	1.715345174	1.205632108
Zm00001d037886_T004	8.993790922	8.250490944	9.025726135	8.572527449	8.732919158	8.615252869
Zm00001d037886_T002	1.329594369	0.525898483	1.15215226	0.998487263	1.196547245	1.367158001
Zm00001d026678_T001	4.385649358	4.073946506	4.636696478	3.577317034	3.732820119	3.995656547
Zm00001d036024_T003	1.02628945	1.824202067	2.506068208	3.293942357	3.095733735	3.151223521
Zm00001d036024_T001	5.335721234	4.614436413	5.443754228	5.471741894	5.54829577	5.655509085
Zm00001d036024_T005	2.424729985	0	1.91092373	2.53283833	2.691960434	2.117997512
Zm00001d036024_T004	1.336075968	2.160582703	0.603327005	2.274536008	1.104755987	0.410774174
Zm00001d009117_T002	5.051147849	5.20850813	5.517386285	4.764642652	5.02199031	4.987816764
Zm00001d022122_T001	3.988622706	3.173468234	4.007410095	3.598753818	3.76756762	3.590529326
Zm00001d009264_T001	2.933308435	2.587284092	3.124960003	2.24579926	2.078132273	2.560601002
Zm00001d018382_T020	1.431349102	0	0.739015937	1.932293284	1.641493777	1.839488015
Zm00001d018382_T033	3.608763833	2.411260001	3.727727175	4.162043388	4.119943761	4.055200167
Zm00001d018382_T001	1.161532365	1.042137204	1.400599134	1.269601732	1.702043991	1.47742451
Zm00001d018382_T040	0.031262496	0.071293134	0.212017679	0.504989472	0.309608116	0.585201007
Zm00001d018382_T019	0	0	0	0.587288173	0.371518717	0.217166181
Zm00001d018382_T028	0.355872152	0.334738735	0.194105968	0	0	0.427451705
Zm00001d018382_T003	2.074512629	1.375710632	1.994480524	2.939246328	2.598085263	2.367496317
Zm00001d007947_T003	0.834148912	0.344831905	1.27883643	1.132037171	1.280828662	0.85211233
Zm00001d007947_T001	3.899995761	3.539609606	3.774547144	4.7289147	4.645706557	4.622608285
Zm00001d037565_T001	0.546491992	0.214998863	0.451364639	0.431818693	0.5667947	0.570132579
Zm00001d037565_T002	2.415588594	2.023933065	2.5236021	2.134530149	2.608545513	2.698302503

Zm00001d034568_T005	0	0.45719502	1.585879283	1.605882098	2.517723426	1.53369889
Zm00001d034568_T003	0	0	0	1.064444873	0.990402357	0.707704123
Zm00001d034568_T004	0	0.35908706	1.022698041	1.079098164	0.747558407	0
Zm00001d034568_T002	1.011874154	0.814593898	1.366185668	0	0	0
Zm00001d034568_T006	0.780828284	0	0	1.222313071	1.138150624	1.528728332
Zm00001d034568_T001	3.538847155	2.717712829	3.373907497	3.142880645	3.293218614	3.181831985
Zm00001d012830_T002	3.362452214	2.811710227	3.214911544	2.898528013	3.015679179	2.965446678
Zm00001d028895_T001	3.360249081	3.227984071	3.393762966	3.406115528	3.409172716	3.519650342
Zm00001d008229_T001	1.498875304	1.135803421	1.47346951	0.935102141	0.71168784	0.872084866
Zm00001d032162_T001	6.169866731	6.294258798	6.251839615	6.172287983	6.250934921	6.227022803
Zm00001d044816_T001	3.815410452	3.161603306	3.692049847	4.30604951	4.265973456	4.318808214
Zm00001d034992_T001	2.547992897	2.692656618	2.551842048	1.850232153	2.047910705	2.165925763
Zm00001d010884_T001	1.350381827	0.945727548	1.600252148	0.918632006	1.156300175	0.998307448
Zm00001d016969_T001	1.621596178	1.048845784	1.217193485	1.47160889	1.337462202	0.960604378
Zm00001d020490_T001	2.434819551	2.469022184	2.680305226	1.31575054	1.924988039	1.320871655
Zm00001d009488_T004	4.377986123	0	5.240323527	5.294236298	5.854955354	5.514897603
Zm00001d009488_T001	5.565294288	5.157423985	4.875782618	5.852048666	5.480813914	5.793970538
Zm00001d009488_T003	7.149899631	5.698314923	6.981402745	6.749410963	6.682724497	6.578601587
Zm00001d009488_T002	5.738718557	5.617538021	6.044421399	6.071904929	6.088726725	6.143319188
Zm00001d003958_T017	1.263601153	0	1.540464774	3.631175775	2.808467841	3.260830058
Zm00001d003958_T011	0	0.596947542	0.741882854	0	0.716511599	0.196430788
Zm00001d003958_T013	1.692170954	0.785143736	0	2.300433322	3.027951278	3.127211964
Zm00001d003958_T007	3.731549952	2.731459152	3.81995414	4.04937993	4.175116645	3.977810097
Zm00001d052025_T001	8.615135995	9.05424648	8.604707412	7.830000556	7.746910986	7.688036341
Zm00001d015837_T003	0.6397832	0.282290338	0.435897374	0.47527377	0.630201355	0.549158531
Zm00001d015837_T001	2.582837289	1.87720358	2.979685523	3.192064885	3.389831236	3.245426599
Zm00001d038932_T001	4.660909455	4.812418336	4.486143488	4.102215682	4.061033667	4.045499325
Zm00001d011551_T001	2.357969692	1.482532352	2.155075321	0.974174652	1.111539506	1.012555802
Zm00001d035044_T010	0	0	0	2.991119445	2.581719286	2.62445585
Zm00001d035044_T004	0.712148487	0	0.359875335	0	0.58684351	0.144132537
Zm00001d035044_T001	0	0	2.473810026	2.507581803	2.346928815	2.02295365
Zm00001d035044_T023	1.96014061	2.643369977	2.220376393	1.465748838	1.865599764	1.948469653

Zm00001d035044_T011	3.205933422	0.081327355	2.307070284	1.346921158	1.896585351	1.058335893
Zm00001d035044_T031	2.516074165	2.589309278	2.068006227	0	0	1.069597689
Zm00001d035044_T024	4.217909942	1.776203815	4.055678265	0	0	0
Zm00001d035044_T002	2.330216185	0.473083443	0.874015516	2.288958551	2.455432439	2.592094359
Zm00001d035044_T003	0	2.896541178	2.788898028	3.551434068	3.719153096	3.519683676
Zm00001d033159_T001	0.286956828	0.056561333	0.176883144	0.746989449	1.165583337	1.265069574
Zm00001d033159_T002	5.853906149	5.124459494	5.859061741	5.749571702	5.604207845	5.653234609
Zm00001d002790_T001	2.343730438	1.590259957	2.395749425	2.293064372	2.307043755	2.373537413
Zm00001d002790_T002	1.304215463	1.91512236	1.841463558	2.766145641	2.557272591	2.287776937
Zm00001d009928_T001	1.307207003	1.031990079	1.22491452	0.445522254	0.87809752	0.745774289
Zm00001d004659_T001	3.268592488	2.677321057	3.405688954	2.213738584	2.359685053	2.276665491
Zm00001d004659_T011	0	0.039433221	1.116350782	1.47802954	1.454272772	1.088459145
Zm00001d004659_T012	1.542706763	0.838759798	1.910844313	2.342484486	2.042192899	2.649577063
Zm00001d004659_T007	1.123051646	0	0	1.523579519	2.616468036	1.854041541
Zm00001d004659_T004	0.48518464	0.157037241	0.322964155	0.942590321	0.946380529	0.896201311
Zm00001d002611_T001	0.738860339	0.31686633	0.634436213	1.164927195	1.278129902	1.062935417
Zm00001d002611_T002	4.491458642	4.08845677	4.406200515	4.42146926	4.492342952	4.602121122
Zm00001d017254_T002	2.671031082	3.682030274	2.512604138	2.746821598	2.356322164	2.053831461
Zm00001d002253_T001	8.087351352	7.749813421	7.980440187	7.327773183	7.415309089	7.489615232
Zm00001d035753_T001	2.722966291	2.94417797	2.639468348	3.767452997	3.603878872	3.746505507
Zm00001d043735_T001	1.212044457	1.488541744	1.404387626	2.112376199	1.81379145	2.401064363
Zm00001d038149_T004	0.2517821	0.103708447	0.340915423	0.549965811	0.717121664	0.886524395
Zm00001d038149_T003	0.372488579	1.426246503	1.257360686	1.052322679	1.347943398	1.793486419
Zm00001d038149_T005	2.106976723	1.163188906	2.062777616	1.713452559	1.537435732	0
Zm00001d012913_T008	4.520645645	2.66197506	4.617166398	4.788735609	4.890422761	4.733076568
Zm00001d012913_T009	0.396472989	0.273046751	0.360450808	0.34573586	0.335642175	0.268037882
Zm00001d012913_T005	0.579563464	0	0	0.600912923	0	0.843628552
Zm00001d044726_T001	2.532455611	1.675785677	2.431285445	2.759204207	2.925284728	2.649678913
Zm00001d026625_T001	3.494145736	4.77682084	3.626611315	3.601347922	2.83342346	2.871001042
Zm00001d026625_T002	5.882137871	5.750655835	5.982494806	5.09717021	5.089162607	5.229487907
Zm00001d043935_T001	5.670616625	5.109383671	5.721975151	7.094486471	7.018916602	7.13943497
Zm00001d032257_T001	5.281705482	4.191582942	5.192928585	5.522312512	5.48437537	5.547541938

Zm00001d032257_T003	5.212884729	5.880644021	5.274681995	4.664107896	4.969430776	4.700021997
Zm00001d049440_T006	2.170412229	2.111602266	2.480268225	2.174043302	2.026535925	2.238760879
Zm00001d049440_T003	1.446903577	1.491773586	1.498160984	2.421418563	2.314303827	1.743399065
Zm00001d049440_T004	0	1.32731371	0.840410863	1.800391361	2.587955391	0.896022238
Zm00001d049440_T002	1.165454705	1.144342059	0	0.826254376	0.544021394	0
Zm00001d049440_T005	3.020147284	0	3.060183343	2.855606256	2.144264063	3.32911283
Zm00001d010655_T001	5.092499356	4.637306513	5.160621989	4.6484009	4.766798672	4.699412931
Zm00001d012459_T001	3.361120966	3.11536041	3.317900822	3.415394567	3.253759385	3.034666135
Zm00001d011694_T001	5.353328055	5.076412184	4.121213551	4.3219097	4.8419452	4.571403534
Zm00001d048362_T001	4.118305815	3.135823774	4.147453078	4.120425102	4.09130471	4.409473558
Zm00001d012378_T001	4.075309621	5.018833012	4.374784382	4.749517481	4.909393951	5.027101533
Zm00001d037142_T012	5.273173713	5.903911579	5.361802076	5.095915182	4.783197166	4.797036434
Zm00001d037142_T007	0.070337115	0.243527729	0.071007493	0.721221272	0.549949058	0.838554089
Zm00001d037142_T002	3.538542882	0	3.298064254	2.474766469	3.410691287	2.747972094
Zm00001d037142_T022	0	0	2.285153314	3.123021176	3.659207256	3.964549271
Zm00001d037142_T001	2.151476666	2.664204514	0.705261351	1.241949983	1.693677409	1.175179015
Zm00001d007257_T001	3.95289758	3.615270325	3.923751525	3.644819588	3.415252445	3.401170609
Zm00001d018713_T002	0.762041745	0.417864934	1.034336874	0.698958975	0.667803132	0.654507032
Zm00001d025326_T001	0.890694157	0.666871094	0.764760575	1.106846107	0.540322922	1.841176097
Zm00001d025326_T002	3.418622017	2.886083805	3.505770785	4.344961756	4.293537935	4.099488954
Zm00001d005344_T001	5.114131818	6.249756052	4.611530058	4.954188259	4.823722819	4.449376685
Zm00001d005344_T002	6.847333164	6.175410846	6.951787734	6.812107977	6.815648804	6.934072573
Zm00001d027368_T001	4.958388781	5.620070056	4.964702851	3.9876965	4.010778407	3.768853898
Zm00001d031674_T004	3.984690417	3.155579894	4.090539712	3.849290444	3.742393465	3.624965667
Zm00001d045937_T001	7.048620746	6.185459006	6.108786021	6.900574171	6.482120284	6.458230665
Zm00001d021867_T001	3.785422388	3.095093661	3.708137894	3.491677526	3.814918792	3.717303207
Zm00001d004705_T001	0.822966669	0.088768483	0.53210347	1.474226276	1.573981339	1.344930164
Zm00001d040611_T004	0	0.133780505	0	0.550983383	0	0.465605714
Zm00001d040611_T005	0.441321991	0	0.343485143	0	0.42230944	0
Zm00001d040611_T002	3.138141447	2.450914562	3.196027678	2.499703555	2.599824315	2.449392335
Zm00001d034407_T001	5.712838823	6.43182755	5.702160077	4.869111818	4.936113716	4.525132509
Zm00001d044133_T002	0.17108254	0.222121994	0.121974226	0.145923915	0.667073723	0.694707282

Zm00001d044133_T008	1.026350364	0.451542943	1.315063605	0.922813622	1.265785525	1.12992063
Zm00001d044133_T011	3.700020277	1.829459513	3.383884523	3.584545742	3.38542731	3.324713845
Zm00001d044133_T009	2.941583186	2.050707857	2.778174086	2.723913021	3.108686531	3.068123549
Zm00001d044133_T001	1.632529738	1.444671307	1.889169006	1.857727457	1.388716362	1.510185186
Zm00001d044133_T014	0.208527248	0.327111321	0.476242726	0.547913707	1.498447469	0.45429699
Zm00001d044133_T010	2.323421089	2.329279485	2.609033434	2.539492113	2.385112367	2.458103466
Zm00001d042758_T001	0.869173376	0.590990933	0.875584284	1.945133094	1.62896532	1.715924991
Zm00001d038408_T001	2.131138366	2.647421125	2.503691176	1.973339792	2.102448514	1.425719246
Zm00001d027721_T001	4.922619271	4.248301579	4.963864154	4.874980954	5.082245118	4.978756533
Zm00001d012612_T001	0.762484892	0.55403082	0.791447361	1.226145344	0.983760862	0.937772993
Zm00001d025337_T001	2.70242328	2.59162772	2.780407535	2.932688622	3.131998662	3.187837569
Zm00001d047597_T001	7.788224584	8.198992754	7.72620349	7.902769578	7.859146744	7.901553217
Zm00001d033489_T001	3.722903139	3.316049559	3.669159914	3.678058943	3.682335638	3.643030501
Zm00001d015126_T001	6.348585241	4.109259333	5.754282191	3.785100202	4.492916516	3.765060913
Zm00001d033368_T071	3.963957253	4.895700616	4.303547268	0	0	0
Zm00001d033368_T064	1.456179006	0.726493822	1.623781664	1.30122766	0.990525073	2.199201812
Zm00001d033368_T062	0	1.498499551	0	2.832201203	2.813505399	0
Zm00001d033368_T030	1.588149258	1.019603004	0.853978087	0	1.471468427	0.744830179
Zm00001d033368_T065	0.311455451	0.120683709	0.462036609	0	0.933312779	0
Zm00001d033368_T006	0.481654404	0.58771734	0.479962073	0.656728831	0.928360468	0
Zm00001d033368_T015	0.195962345	0	0	0.550715515	1.108056028	1.549683462
Zm00001d033368_T010	0	0	0	1.39103134	1.585705778	2.207211314
Zm00001d033368_T066	5.095684921	3.512041385	4.956713292	4.747483594	4.790698285	4.885949533
Zm00001d033368_T045	1.397177567	0	2.161785481	2.804510629	3.057200703	3.1522162
Zm00001d053020_T001	2.027096711	1.758443474	1.824622456	0.551794563	0.84121314	1.131475566
Zm00001d013273_T003	3.207313159	2.389810728	3.209482531	3.222058141	3.457675971	3.158171062
Zm00001d013273_T001	0.311712355	0.344570606	0.377025992	0.613609915	0.463291823	0.612995021
Zm00001d050498_T001	2.570001389	2.179816118	2.869112361	2.333162937	2.542371975	2.417986688
Zm00001d050498_T002	0.616952345	0.243158442	0.698005059	0.633926826	0.819158041	0.821272647
Zm00001d010500_T001	5.699399882	4.948852643	5.694636223	5.713747248	5.615122131	5.53805111
Zm00001d032570_T001	6.105359017	5.392544561	6.135604536	6.315068407	6.299654333	6.229880739
Zm00001d032570_T002	0.671478171	1.672695072	1.403734	1.374708443	1.496544159	2.055893575

Zm00001d019256_T001	2.498709138	1.812016357	2.73214023	1.427987444	1.689921286	1.923953898
Zm00001d043861_T001	3.463575901	2.60051716	3.454783908	4.120592148	4.078096398	4.036681264
Zm00001d043932_T001	5.908716229	6.625470717	5.945867417	7.322968867	7.502618735	7.323363701
Zm00001d043506_T005	0.244447488	0.110295083	0.111618957	0	0.535310468	0.286261365
Zm00001d043506_T004	0.706290065	0.156950547	0.600626577	0.984759219	1.196208548	0.706062803
Zm00001d008690_T001	4.472610135	3.648793636	4.364284145	4.212633535	4.222223796	4.067626327
Zm00001d002797_T001	0.823908293	0	0.185591297	0.667552467	0	0.502599459
Zm00001d002797_T008	3.032069273	2.356103234	3.269224246	2.80893772	2.829954664	1.944875308
Zm00001d002797_T003	3.386836876	2.480638933	3.245357228	3.752215402	3.912036637	3.993902567
Zm00001d002797_T005	1.567768844	0.825749	1.14974777	2.236915339	1.959475281	1.919912089
Zm00001d004467_T001	3.571620266	2.848242745	3.557090218	2.778998898	2.728542041	2.810404028
Zm00001d042389_T001	1.317031366	0.873052483	1.156255512	1.615299521	1.219485762	1.103614461
Zm00001d015658_T002	0.753060214	0.455363258	1.295705982	1.348686802	0.823120795	1.283789643
Zm00001d015658_T001	0.129647025	0.539953843	0.411509768	0	0.988854801	0.610889953
Zm00001d015410_T002	2.52291197	1.718296299	2.310718488	1.647599566	1.823059636	1.516123597
Zm00001d015410_T001	1.580664086	0.790651947	1.769186081	1.178798763	1.362804819	1.009463271
Zm00001d015410_T003	3.071050068	3.632909839	3.469490874	2.112854234	1.418842994	2.257606588
Zm00001d003223_T001	3.919401107	4.507959383	4.183028066	4.410647536	4.578161803	4.776358807
Zm00001d031187_T001	0.772080741	0.604821899	1.199596341	1.012108797	0.466332678	0.912192268
Zm00001d031187_T002	1.81469971	1.629513297	1.470238013	2.097815726	2.145667674	1.853303866
Zm00001d016853_T001	1.933763726	1.082746864	1.64568506	1.357286243	1.550447105	1.447853164
Zm00001d039062_T014	1.282010343	0	0.617503493	0	0.544268743	1.025933828
Zm00001d039062_T006	0	0	0.488553055	0.864551862	1.161085353	0
Zm00001d031793_T002	2.070839241	1.650516891	2.215824256	2.15179194	2.590219709	2.110940473
Zm00001d031793_T001	1.001862038	0.4021797	0.437207494	1.344466642	0.606132335	1.379479014
Zm00001d007156_T001	1.246378899	0.610715179	1.085656475	0.909622588	1.238075118	1.142407556
Zm00001d018709_T002	3.05548288	2.235900112	3.13173796	2.224182931	2.333175534	2.312927379
Zm00001d012518_T004	5.189088992	4.946709575	4.855935396	4.96785626	4.850295172	4.882066981
Zm00001d012518_T008	5.473235764	0	5.533134712	5.324503746	5.547665378	5.405295376
Zm00001d012518_T001	0	2.543951386	1.757237501	0.350277714	0	1.538301751
Zm00001d053211_T003	1.026795103	0.539878428	0.703053731	0.96226027	0.620004523	1.283776014
Zm00001d053211_T002	0.561251831	0	0	0.80124476	1.016882793	0.966417177

Zm00001d053211_T008	5.406803103	4.673462126	5.157566026	5.633714112	5.377015022	5.294122538
Zm00001d053211_T005	0	0	2.835961455	0	3.061402258	3.13670605
Zm00001d026426_T005	2.09838789	1.747564422	2.324920601	2.377242048	2.536185224	2.05038781
Zm00001d026426_T001	4.889151431	3.841453695	4.915459951	4.227924404	4.156249281	4.140200726
Zm00001d012551_T001	2.216815533	2.515698577	2.51672497	2.138806279	1.438646827	1.800093525
Zm00001d045109_T031	2.050514235	0	2.14254414	1.40757954	1.815364337	0
Zm00001d045109_T003	0	0	0	1.242258596	1.095441078	0.687470141
Zm00001d045109_T037	5.739401538	7.401960694	5.682595544	4.900360822	5.193337214	4.797164505
Zm00001d045109_T034	4.318373246	3.496902658	4.403845367	4.762174236	4.540442394	3.998120297
Zm00001d045109_T010	2.287713118	0.695288323	2.349282609	3.128007441	3.70478301	3.755621914
Zm00001d045109_T008	0	0.198286692	0	0.944802236	0	1.156202432
Zm00001d045109_T022	0.424359946	0.078880315	0.067112753	0.829165995	0.649571315	0.374043454
Zm00001d045109_T036	5.092642989	2.004205092	4.654895533	3.849390037	3.934689633	4.353224473
Zm00001d052794_T001	0.909645627	0.74255903	0.557785512	1.317728362	1.123186763	1.281180716
Zm00001d003471_T001	3.367194912	2.742245131	3.669117045	2.295939266	2.615485276	2.338252679
Zm00001d034486_T002	2.34790004	2.070036511	2.519304875	2.433892316	2.569110195	2.24069686
Zm00001d000046_T002	1.765328087	2.375983028	2.745024731	3.880968062	3.574019499	3.671258041
Zm00001d000046_T001	3.288466945	2.968046503	3.163749089	3.614238212	3.733777758	3.608142493
Zm00001d022251_T002	2.876044598	2.547166741	3.147144346	3.136570881	3.163155085	3.406242088
Zm00001d052785_T001	5.591234968	4.943327018	5.626165787	5.411528343	5.524834166	5.40422234
Zm00001d033650_T001	3.091789524	2.309764091	3.093774638	3.190857238	2.946042094	3.089921982
Zm00001d015097_T003	1.44835072	1.033015553	1.690516326	1.904637463	2.127057407	1.883308785
Zm00001d015097_T001	2.492189814	1.804360491	2.062383902	2.599697463	2.598074302	2.119937454
Zm00001d015097_T005	2.033925456	1.452795722	1.687147158	0	0	0
Zm00001d015097_T004	0	0	2.25665473	2.811561248	2.794763943	3.042976959
Zm00001d013958_T003	1.422611928	1.324268555	1.824530405	1.811609128	1.84719238	1.899715476
Zm00001d028917_T001	5.600263895	2.33412001	6.034377882	5.606362392	5.415367692	5.510001638
Zm00001d038667_T001	0.531148365	0.557292444	0.937998918	0.990908403	0.929212289	1.049105866
Zm00001d053967_T001	3.720700594	3.262984963	3.970606498	3.248521478	3.303236293	3.391636722
Zm00001d007468_T001	0.772049483	0.543429562	0.71457436	0.525656984	0.701788562	0.49429681
Zm00001d047538_T003	1.023424278	0.861228078	1.036696032	1.280602424	1.1716885	0.848262178
Zm00001d029932_T001	4.007980516	3.1670621	3.87577908	5.394383188	5.433651334	5.384530107

Zm00001d013021_T001	0.849143393	0.763949457	0.221854814	1.341235465	1.312695955	1.597184552
Zm00001d032319_T020	0	0.468210019	0	0.522266713	0	0.582536735
Zm00001d032319_T012	0.410737276	0.245114707	0.262938223	0.362809867	0.158214212	0.435836583
Zm00001d032319_T014	0.771849247	0	1.280186077	1.540705792	1.712304791	1.791324826
Zm00001d032319_T040	0.793699488	0.436587228	0.622373775	1.275471377	0.640160932	1.376129766
Zm00001d032319_T029	3.731426024	0	3.906669173	3.047616035	3.143407134	0
Zm00001d032319_T017	1.575918329	0	0	2.612078561	3.118546945	2.79998933
Zm00001d032319_T002	1.841734058	3.183106933	1.860569156	1.974322989	1.965640239	3.02405509
Zm00001d014756_T004	2.498483467	2.181191623	2.463449825	3.039773426	3.352325058	3.256558271
Zm00001d014756_T002	0.323808144	0	1.062570066	1.084452787	1.263483375	1.335486686
Zm00001d014756_T001	0.622836666	0.051211987	0.794343496	1.112601392	0.742308089	1.156628309
Zm00001d046929_T002	7.020483222	6.972279554	7.087801644	6.96300294	6.858474325	6.890327031
Zm00001d046929_T001	1.479904145	1.028641992	1.478432408	1.358186932	1.262674288	1.509161731
Zm00001d013445_T003	3.49532229	3.269652145	3.57693977	2.926003974	2.640815711	2.869578298
Zm00001d013673_T002	5.713241465	5.542041474	5.947464745	5.962163821	6.017021323	5.88861838
Zm00001d030694_T003	5.556923054	4.190599611	5.663141922	5.568562405	5.614274673	5.548472507
Zm00001d026600_T002	3.715935421	3.007091243	3.780957067	3.52154127	3.547001848	3.399904426
Zm00001d048914_T001	2.793782293	2.538133606	3.07912821	3.24784915	3.448551333	3.693216625
Zm00001d038049_T001	6.836800677	6.146250711	6.914352708	6.079714274	6.087750333	5.927873017
Zm00001d042064_T001	0	0.047383432	0.468052537	1.063197804	1.076177336	1.022710823
Zm00001d042064_T007	2.256529136	1.872489957	2.258028596	2.210691836	1.766639682	2.000040756
Zm00001d042064_T013	0.725440155	0.920784152	0.390445377	1.574791802	1.27157448	0.772995372
Zm00001d042064_T002	0.918464855	1.030171492	0.889837967	0.798712488	0.895594233	0.999632066
Zm00001d042064_T008	1.019214411	0.828584466	1.333375643	0.984979363	1.139392217	0.998324058
Zm00001d042064_T003	1.187863375	0	1.299430479	0.411383941	0.975823853	0
Zm00001d042064_T005	0.751669377	0	0.489228468	0.304089269	0.715915329	0.643523755
Zm00001d040297_T001	11.92985971	11.44593464	11.89735935	11.62379442	11.69761989	11.74177601
Zm00001d040681_T001	4.917007573	4.942477441	4.7653971	4.711369244	4.833196187	4.556958695
Zm00001d036409_T001	4.471120072	4.491151969	5.082535909	2.597494466	2.402024396	2.653361369
Zm00001d026649_T001	4.188799447	3.821602978	4.265773076	4.956871796	5.034704471	5.129522773
Zm00001d026649_T002	3.931167528	2.79804284	4.253073227	4.448990853	4.399442104	4.103950352
Zm00001d046949_T001	5.639209816	5.006403551	5.71293149	5.295267556	5.470934284	5.456578296

Zm00001d040649_T001	3.50051629	2.812817329	3.560035628	3.702317329	3.933838209	3.772178735
Zm00001d017862_T001	1.883950761	1.248558517	1.915349182	1.867813062	1.989994922	1.751772194
Zm00001d044806_T001	1.27709744	0.799535798	0.915261594	1.840326259	2.157947861	2.259739498
Zm00001d012923_T008	1.336366233	0.819700061	1.777600666	1.706600835	1.779703073	1.264487189
Zm00001d012923_T004	3.286486866	3.821223453	2.818902494	0	1.691189306	1.327401096
Zm00001d012923_T006	0.445336848	0	0	0.361275401	0	0.944126795
Zm00001d012923_T002	1.590112376	1.172539999	1.439795985	2.171935358	2.193722549	2.132628167
Zm00001d006341_T001	1.552359855	1.492156223	1.650184608	1.677618287	1.199620839	1.3317184
Zm00001d049438_T001	3.114564424	3.117261338	3.249981815	4.64852889	4.744067694	4.673379807
Zm00001d025247_T001	4.134168768	4.071125945	4.211786694	2.696643325	1.500340623	2.042681455
Zm00001d053761_T001	2.022620285	0.755792589	1.761129079	1.920560851	2.292005724	1.788742499
Zm00001d053761_T009	0	1.641375393	1.163417578	1.631971734	0	0
Zm00001d053761_T012	2.299272796	1.155856071	1.899121093	1.254980967	1.222345842	1.982343086
Zm00001d039190_T001	1.675889983	1.626585852	1.682498689	1.051221037	1.054310882	1.59090904
Zm00001d006860_T002	5.450435673	4.896286092	5.46120655	6.62758701	6.68904071	6.532314465
Zm00001d024861_T002	0.362592201	0.20025072	0.176578094	0.387612895	0.602725763	0.616946701
Zm00001d024861_T004	0.808501203	0.371439539	2.174978397	0.951596746	1.111309138	0
Zm00001d024861_T001	0.490796015	0.438926221	0.394566822	0.827464606	1.259753355	0.926412426
Zm00001d024861_T005	2.526474262	1.712806612	1.146851999	2.633300536	2.295677774	2.918498058
Zm00001d028998_T001	7.903738545	7.376541347	7.984297102	6.498698129	6.460408091	6.456049883
Zm00001d051448_T001	2.820079477	2.109337489	2.964592704	2.945396639	3.184814263	2.783708502
Zm00001d022228_T001	8.303612148	8.298412453	8.373574957	8.446374238	8.429341776	8.538086836
Zm00001d037701_T001	1.86693606	1.664014002	1.827788951	1.171634063	0.903214932	0.71244347
Zm00001d033147_T001	7.706699007	7.16472036	7.679659201	7.188138407	7.231192115	7.02059135
Zm00001d050558_T001	2.671346142	2.870911311	2.713248202	1.822414454	1.566205319	1.293261586
Zm00001d034023_T001	1.716605978	2.039009915	1.758989605	1.133856769	1.315843846	1.366447557
Zm00001d034023_T002	3.443955216	2.259497274	3.240146234	2.88767928	3.08065016	3.079300961
Zm00001d044054_T002	6.474468752	6.24010875	6.55334098	6.265321086	6.311956992	6.258743848
Zm00001d003250_T001	2.277539079	1.742116619	2.159915565	2.722071415	2.928164114	2.727488997
Zm00001d013417_T006	0.914543093	0.553502062	1.188128059	0.803069944	1.516634465	1.058612278
Zm00001d013417_T003	1.090878491	0.0700719	0.777110364	0.140074475	0.377752344	0.693033253
Zm00001d013417_T002	1.71227133	0	1.530394413	2.272454738	2.074873599	1.924688628

Zm00001d013417_T005	1.03527199	1.491917215	1.20824068	1.019798684	0.988945666	0.660012213
Zm00001d018362_T003	0.453698777	0.220057489	0	0	0	0.506728872
Zm00001d028505_T001	5.451055655	4.827950385	5.33053108	6.666736059	6.727813473	6.742989426
Zm00001d028505_T002	3.903957502	5.137883165	3.828020988	4.611753355	4.893402792	4.736002833
Zm00001d022306_T001	1.603594301	1.636576366	1.778666549	1.351415753	1.361532008	1.060526818
Zm00001d022306_T002	3.223165808	2.685764369	3.131325087	2.925837866	2.777983532	2.990629809
Zm00001d014232_T008	0.461038148	0.26407037	0.470879375	0	0	0.605367269
Zm00001d014232_T021	2.171980179	1.876071717	2.225789469	0	1.444901843	1.449471047
Zm00001d014232_T023	0.693424955	0	0	1.021274324	1.771283027	0
Zm00001d014232_T002	1.948519367	1.902030732	2.638001295	2.999178151	2.932815969	3.049907606
Zm00001d014232_T007	1.929759947	0	0	1.811475141	1.258589475	1.499245346
Zm00001d014232_T018	0.135683269	0.039221232	0.028540864	0.463216478	0.485315529	0.310867073
Zm00001d014232_T005	0.224626867	0.075695546	0.129751199	0.343092811	0.312738352	0.398113915
Zm00001d050150_T001	0.780153851	0	0	0.788543731	0.929468343	0.877791358
Zm00001d038270_T001	4.828280308	4.045725855	4.812337928	7.468491419	7.472479567	7.446419732
Zm00001d006065_T001	1.081790229	2.583926274	1.14587567	1.423313003	0.876128312	1.424176638
Zm00001d006065_T004	2.073926461	4.779860841	2.97001779	2.071438679	3.019531663	0
Zm00001d006065_T002	3.652552003	0	3.425832552	3.432194042	3.559515552	3.644006915
Zm00001d046519_T001	2.603078417	1.950476626	2.561021556	2.176451716	2.23828458	2.001643736
Zm00001d023578_T001	4.315670487	3.770014565	4.433612546	4.052828506	4.210491654	4.196130709
Zm00001d029559_T001	5.048905971	5.663874658	5.046786266	4.518017514	4.72155427	4.680689317
Zm00001d051065_T007	4.778387693	4.46544625	4.924411628	4.520120728	4.475245426	4.561968149
Zm00001d051065_T008	1.019543939	0.355709809	0.999323939	1.395705561	1.808815427	1.555454455
Zm00001d051065_T004	0.30372114	0.385517182	0.564216207	0.580233298	0.325131934	0.223418843
Zm00001d051065_T003	1.039249312	0.642968602	1.411175108	1.2716916	1.634119727	0.723361092
Zm00001d033909_T001	5.410203131	7.02424784	5.968582291	2.746757332	2.683499965	2.746539581
Zm00001d044212_T002	1.228034267	1.165127285	1.200054186	1.304953712	1.339832904	1.80419526
Zm00001d044212_T004	2.096250719	0	1.56185741	1.805133523	1.886874742	1.728942718
Zm00001d044212_T001	5.425057519	5.152145444	5.620984827	5.155911227	4.970437761	5.046603161
Zm00001d044646_T041	0	0	0	0.79757998	1.137206429	1.153058183
Zm00001d044646_T055	0.245305806	0	0	0.55021904	0.527248534	0.287893044
Zm00001d044646_T052	1.081661403	1.719480493	1.785835506	1.084392236	1.220190013	1.367211689

Zm00001d044646_T038	0.749215016	0.58672824	0.883896036	0	0	0.333854188
Zm00001d044646_T053	4.290156012	5.100576227	4.468445427	3.757939579	4.118477509	4.461171902
Zm00001d044646_T059	4.243917404	4.04326444	4.521430839	4.694668508	4.475263522	4.485436681
Zm00001d044646_T037	1.332133892	0	0	1.64893568	1.244023619	0
Zm00001d044646_T057	3.009097205	2.187979259	2.765741164	3.801287706	3.828255018	3.740060604
Zm00001d044646_T003	0.981688286	0.427944013	1.251172507	0	1.204891925	1.73968957
Zm00001d030179_T002	3.366631075	2.056788474	3.137857269	1.712094767	2.17764485	2.136551687
Zm00001d030179_T004	2.337286355	2.994748602	2.590526332	2.292519685	2.262310773	0
Zm00001d049905_T001	3.379771905	2.55023702	3.382374534	2.95213411	3.022252215	3.061793475
Zm00001d004019_T001	4.782213851	3.794187201	5.011209406	4.574605273	4.668677391	4.52704443
Zm00001d004019_T002	3.116328323	4.353326609	0	0.537049508	0	2.306777271
Zm00001d053662_T002	0.249673484	0.237053362	0.323206333	0.29610965	0.5621667	0
Zm00001d027757_T001	2.905471825	2.237906914	3.072500643	2.915475965	2.836887974	2.865486535
Zm00001d045076_T001	0.405326933	1.433944912	0.357171364	0.805417112	0.342323759	0.942996355
Zm00001d045076_T005	1.607865743	0.702514818	2.047925708	1.020017103	1.539475983	1.167633465
Zm00001d045076_T009	3.776748634	4.420228861	3.979471155	3.946741339	4.259982284	4.095887719
Zm00001d045076_T004	4.897071359	3.601380135	4.89955221	4.566659128	4.791517533	4.737977813
Zm00001d045076_T006	1.22655292	1.009651016	1.000371446	0	0.552869854	1.159215821
Zm00001d045076_T003	1.284700677	0.345237392	1.591238021	0	0.390442075	0.987328143
Zm00001d045076_T002	4.636985322	4.092889715	4.556577479	4.541368692	4.432311385	4.283862967
Zm00001d011097_T001	2.561477181	2.353980683	2.555104446	2.337976489	2.446815993	2.20907026
Zm00001d008791_T005	3.083457116	5.134803199	2.983831805	0	0	0
Zm00001d008791_T002	4.92853993	3.722217138	4.815184014	5.542806355	5.565846123	5.510824066
Zm00001d002150_T001	2.764821497	2.232255824	2.950459082	2.084577963	1.790560622	2.439327388
Zm00001d035921_T001	0.976714093	0.83498622	1.253230518	0	1.011879877	0.50043904
Zm00001d035921_T004	1.706598182	1.791704901	2.145866868	1.820003683	0.851886135	1.424416385
Zm00001d008236_T013	3.423731844	1.927715963	3.641003414	4.197691647	4.089359163	3.870673152
Zm00001d008236_T027	0.432155549	0.221950064	0.444790025	0.236476699	0.369818215	0.070905858
Zm00001d008236_T019	0	0	1.712824217	1.869383452	1.952421915	0
Zm00001d008236_T016	0.851845368	0.419735193	0	0	0	2.29414107
Zm00001d008236_T007	0.781142296	1.000864637	0.734890372	0.604119729	1.715658831	1.642574045
Zm00001d008236_T028	4.138972849	3.485762601	3.986863425	3.896319324	4.129290189	4.189125651

Zm00001d008236_T009	0.298159737	0.322885726	0.948645697	1.873125737	1.644115621	1.798965418
Zm00001d008236_T012	1.102300016	0.524117851	0.307152201	1.333835873	1.517210172	1.22636487
Zm00001d008236_T011	0.212175851	0.088995018	0.295188159	0.280099942	0	0.420120167
Zm00001d038067_T001	3.220700952	2.890433073	3.568291912	2.719161532	2.945370605	2.545541179
Zm00001d036371_T001	4.531371853	3.608407242	4.662223847	4.478839498	4.424857206	4.34240569
Zm00001d000102_T001	4.548345621	4.187296324	4.415155142	4.439672534	4.319515696	4.370495337
Zm00001d024041_T004	0.513811084	0.229128894	0	0.63890422	0.96207292	0.152741564
Zm00001d024041_T012	2.252738994	0	0	2.246096407	1.598500511	1.365457947
Zm00001d024041_T016	3.504851078	3.193000681	3.791466949	4.392672626	4.455661131	4.454645022
Zm00001d024041_T002	0.847394857	1.380712827	1.041738517	1.324742067	1.867835988	1.686471841
Zm00001d038357_T001	4.233918883	4.321820899	4.315937942	4.715673216	4.420828246	4.751460026
Zm00001d038357_T004	7.114713986	6.462485537	7.114603922	7.69050159	7.723491742	7.733884346
Zm00001d038357_T003	3.468815146	2.589525255	3.448205991	5.184640923	5.384414822	5.181314963
Zm00001d038357_T002	0	1.46240889	1.464124017	3.669781828	3.933965455	3.458908744
Zm00001d018574_T001	1.714211662	2.113879158	1.948766785	0.788516168	0.905948497	1.51267897
Zm00001d014797_T002	1.098228192	0	1.650407057	1.269714225	2.12937073	1.722355414
Zm00001d014797_T007	2.749451672	1.213426919	2.900168157	3.920592004	3.814004003	3.819964764
Zm00001d014797_T003	4.766110543	4.101888293	4.650421419	4.353234355	4.598435123	4.3147074
Zm00001d014797_T001	3.068776387	2.816520044	3.144951034	3.952284357	3.828109769	3.967115566
Zm00001d006500_T001	2.830242747	2.49723935	2.75260089	2.035466612	1.732645185	1.999604647
Zm00001d044091_T005	2.336349093	1.092375247	2.442416733	2.60169818	2.226656183	2.386672187
Zm00001d044091_T001	3.147541158	2.555287806	3.526934545	3.476037759	3.603076874	3.272559694
Zm00001d044091_T002	1.411736988	1.451549273	0	1.738237346	1.21559225	0
Zm00001d044091_T004	0.654876348	0.276114278	0.288788501	1.028628556	0.772312198	0.748529931
Zm00001d037095_T001	1.590317931	2.203382539	2.054734585	3.38445644	3.341700737	3.559706794
Zm00001d002455_T002	1.341248561	0.63460442	0.948275644	0.522734741	1.156478166	1.38331943
Zm00001d002455_T004	1.427900581	0.995170438	1.422779291	1.000846614	1.103610432	1.10528316
Zm00001d015550_T001	6.897516109	6.466007822	6.648637156	6.121778915	5.887080111	6.287742535
Zm00001d041976_T001	0.076416802	0	0.041220549	0.211297588	0.300773889	0.254265076
Zm00001d051063_T001	3.703511827	4.096688424	3.746656306	3.066249213	3.478622669	3.686255108
Zm00001d053852_T001	4.227000211	3.393753906	4.288907701	4.60522567	4.755808983	4.662171084
Zm00001d043082_T001	2.483747945	1.918483556	2.377598552	2.617153642	2.546279849	2.545737371

Zm00001d007184_T001	2.280287651	1.458048067	2.136936827	2.481881431	2.459065652	2.615835061
Zm00001d047667_T001	1.302850695	1.182966193	1.671599092	1.128380514	1.062158332	1.202664079
Zm00001d047667_T002	4.780735337	3.926122897	4.72056506	3.965267038	4.089623313	3.755468951
Zm00001d029848_T003	4.94145708	4.142688063	4.830181737	4.712506696	4.760842449	4.769646661
Zm00001d029848_T005	1.50627492	1.158241361	1.42095231	1.040358026	1.026198782	0
Zm00001d029848_T007	1.011937826	0.460049484	1.004074903	0.790298289	0.764341059	0
Zm00001d002191_T001	2.667107567	1.93878298	2.960097784	2.452851323	2.534756629	2.487697027
Zm00001d053009_T006	1.000585615	0.409564728	1.047704495	1.452671863	1.805012558	1.785866466
Zm00001d053009_T002	3.46964351	2.678998314	3.314684346	3.773657125	3.880626856	3.767159409
Zm00001d042505_T016	0.481061229	0	0	1.909359146	2.088107667	2.317611158
Zm00001d042505_T031	4.6603229	3.36291939	4.664874067	4.691022654	4.470301212	4.69062812
Zm00001d042505_T026	0	2.251565197	1.679181902	0	0	1.306350327
Zm00001d042505_T001	1.326467743	0	0.874387013	2.86505373	3.331097374	2.517850137
Zm00001d042505_T035	0.056859556	0	0	0.718267362	0.479645509	0.468581232
Zm00001d042505_T020	0.134251174	0.088706078	0.488310364	0.640129458	0.351849917	0.841618136
Zm00001d053800_T002	6.284534621	6.20045735	6.363319843	6.674818371	6.861423198	6.768992021
Zm00001d018276_T003	3.515442243	3.462261115	0	2.411777385	0.99502423	0
Zm00001d018276_T004	0	0	2.993951101	1.891265965	2.953179923	2.540536935
Zm00001d018276_T006	1.568522489	0.520023897	1.33758494	1.198894532	0.189634822	0.623985755
Zm00001d004557_T001	0.516330891	0.637103835	0.993467135	0.758118937	0	0
Zm00001d026029_T001	7.603848622	7.279567961	7.435845924	7.125280225	7.021328154	6.667058064
Zm00001d021731_T001	2.650307327	1.891290077	2.761378258	2.240832996	2.245273552	2.267005064
Zm00001d007311_T002	3.034849045	2.479221247	2.220821811	1.995965277	2.283326204	2.464888575
Zm00001d007311_T003	4.049458958	3.421957624	4.354838313	4.540177956	4.777554063	4.899840596
Zm00001d007311_T001	3.243612115	2.666493702	3.16913082	3.405802211	3.681158336	3.355124406
Zm00001d047262_T001	5.190041816	4.689868298	5.265746376	5.018380069	4.859267554	4.839491139
Zm00001d004868_T002	2.106914431	1.278304782	1.769454819	0	0	1.272365452
Zm00001d004868_T001	0.995373802	0.869019353	0	0.777978905	1.81112984	1.876152678
Zm00001d004868_T004	0.676196987	0.293107938	0	1.386338062	1.589012227	0
Zm00001d026344_T002	0.449149306	1.001014579	1.136087677	0.999157942	1.419659695	1.923135486
Zm00001d026344_T001	4.022613716	2.936498114	3.885077047	3.920346471	3.869777164	3.707951551
Zm00001d013920_T008	0	0	0	1.643448022	1.781895143	2.099844906

Zm00001d013920_T016	1.132852524	0.093449299	0.412211379	0.262237097	0.517962983	1.187580907
Zm00001d013920_T002	0.508987367	0.294387252	0.264359874	0.912271994	0.304123156	1.444176191
Zm00001d013920_T018	0.552427242	0.359016195	0.530319496	0.839034838	1.576180591	1.617056289
Zm00001d013920_T012	0.428888475	0.147416118	0.260482186	0.520204979	0.545912044	0.734409183
Zm00001d013920_T014	0	0	1.293425224	1.197237613	1.286950325	0
Zm00001d013920_T005	1.017774425	0.738413333	0.406185069	1.575852998	0.879838269	1.53122823
Zm00001d013920_T007	1.087758818	0.956173402	1.910636735	2.233856777	1.570407068	1.218060105
Zm00001d013920_T003	4.009274068	3.214277462	3.962932734	4.495035055	4.563209698	4.441546224
Zm00001d052076_T004	0.978202221	0.310694933	0.821709399	1.040817402	1.125795232	0.900975362
Zm00001d052076_T013	0.284872986	0.182299475	0.950691613	0.715949584	0.360451931	0.816927322
Zm00001d052076_T018	0.691477885	0	0	0.880384614	1.022603597	0.700674082
Zm00001d052076_T022	0	0	0.312031968	0.783836294	0.622619295	0
Zm00001d052076_T010	0.40533238	0.53194383	0.680773525	0	0	0.950114509
Zm00001d052076_T003	0.734749067	0	0.349960804	1.109761717	1.050049576	0.767299695
Zm00001d052076_T002	2.870240421	2.64120358	2.931119861	2.611706857	2.667389876	2.473014844
Zm00001d029654_T004	3.826946337	2.932359434	4.043281504	4.838465796	4.747788355	4.650187366
Zm00001d029654_T005	1.610199709	0	1.598785374	2.496401501	2.87518419	3.150836491
Zm00001d029654_T006	0.966311591	0.829783004	0	1.484868705	1.74834315	0.817815604
Zm00001d042482_T001	5.703546959	5.013289439	5.81003952	6.265399932	6.34010231	6.367998404
Zm00001d038091_T007	0.821622875	0.442513608	0.518834222	0.73883354	0.843932404	0.847573654
Zm00001d038091_T005	0.66874455	0	0.503633684	1.444192097	1.305066402	0.684658978
Zm00001d038091_T006	3.655613665	2.971380951	3.683449659	3.53978068	3.751762556	3.740774219
Zm00001d051823_T002	4.028708553	3.191258478	4.226307679	3.914391638	4.148343324	3.96197868
Zm00001d011108_T003	3.618878798	2.901546393	3.792641847	3.510568415	3.406806572	3.478208074
Zm00001d011108_T005	1.752042054	0.625878304	1.24587591	1.198573358	1.720436956	1.328444724
Zm00001d052838_T001	5.090366396	4.532770146	5.285319983	4.498684313	4.330118641	4.240881983
Zm00001d038747_T004	0.824864776	0.4049685	0.921931363	0.849640642	0	0.254430778
Zm00001d038747_T001	4.252888224	4.260383123	4.738619399	4.00197631	4.238002257	3.786704769
Zm00001d038747_T008	4.051281604	0	3.861956555	2.736843393	2.244494069	2.582923013
Zm00001d038747_T007	0.903242702	0	0	0	0.971167186	1.358059181
Zm00001d040567_T001	6.573779606	5.619036587	6.538776167	8.73843254	8.720841063	8.703586329
Zm00001d023230_T009	2.138758778	1.646698153	2.258650977	0	0	2.420715489

Zm00001d023230_T003	4.350292144	4.036973606	4.403013251	3.925083491	3.611479304	3.517999253
Zm00001d023230_T006	0.273728325	0.11989663	0.06611124	0.330622646	0.273821404	0.385309542
Zm00001d023230_T012	0.584381459	0.348247152	0	1.374148649	0	0
Zm00001d023230_T013	0	0	0.774819523	0	1.659670238	1.061138852
Zm00001d023230_T016	1.472268361	0.821362457	1.416123079	1.649494061	2.060842895	1.978132282
Zm00001d023230_T002	1.248675698	0.754920844	1.188499675	2.565306658	2.485807803	2.226770534
Zm00001d035390_T001	2.669453613	3.574360467	2.783264492	1.85238124	1.879003065	2.313043222
Zm00001d025977_T001	4.448541025	3.611037431	4.353131366	3.909120528	3.973733041	4.052682027
Zm00001d006046_T015	1.130922306	0	0.639445194	1.561941946	1.060225893	1.066468117
Zm00001d006046_T004	0	0.332407765	0.402439503	0.335231694	0.792780405	0.088345161
Zm00001d006046_T008	1.256036621	0.424914566	1.233989879	2.14593402	2.058740063	1.523733062
Zm00001d006046_T006	1.850513816	0.990966474	1.970776733	1.82150655	1.664136461	1.099698497
Zm00001d006046_T014	0.922240481	0.672907744	0.648441513	0.793204222	0.544008531	0.889194711
Zm00001d006046_T010	2.72177752	2.130888049	3.468005993	2.462326955	2.832480129	3.093398084
Zm00001d006046_T005	3.364805066	2.310562325	1.45693123	3.055401491	1.147707862	2.289748314
Zm00001d006046_T007	4.740199771	4.271708704	4.871132522	4.370426146	4.845006027	4.655290685
Zm00001d006046_T012	0.836929973	0.408810754	0.192715588	1.716548034	0.816835597	1.79905414
Zm00001d010784_T002	1.513381086	1.284993767	1.840882939	1.827567039	1.581813992	1.689683381
Zm00001d010790_T028	0.113484479	0	0	1.273487841	1.129326544	1.011031841
Zm00001d010790_T026	1.550465821	0.751973523	1.173449845	1.210605814	1.443936524	0
Zm00001d010790_T002	2.629363848	2.531639719	2.591207137	1.38479915	1.671996656	1.762185081
Zm00001d010790_T039	0	0	2.196365631	0	2.69070203	2.524007993
Zm00001d010790_T077	0	0	0.03439745	0.248743696	0.311625176	0.289285606
Zm00001d010790_T027	0.279761291	0	0.57193208	0	0	0.679873247
Zm00001d010790_T011	2.085249918	1.212182078	0	2.646997151	0	0
Zm00001d013836_T007	0.983036273	0.262843236	0	1.114492964	1.688431941	1.740016119
Zm00001d013836_T005	0.742131282	0.090369742	0.91633447	1.346908681	1.994750231	1.000726214
Zm00001d013836_T006	0.938519164	0.529211263	0.380585393	0.985499702	1.407018758	0
Zm00001d013836_T001	2.683426527	2.050131791	2.849705088	3.23959219	2.746951195	3.076461783
Zm00001d013836_T008	0	0	0.680349564	0.545850775	0	1.30903587
Zm00001d033385_T001	3.054061985	2.814374254	2.997655881	4.645648404	4.675938239	4.740563442
Zm00001d006881_T011	0.258387463	0	0.36543947	0.122921815	0	0.370927563

Zm00001d006881_T018	2.586644904	2.381291957	2.778539769	2.432401457	3.21741236	2.870356236
Zm00001d006881_T014	1.460434861	0.874691538	1.616497913	1.77808365	1.270354313	1.434087474
Zm00001d006881_T016	1.175380245	0.494654205	1.119092517	0.834059894	0.497285343	0.641340708
Zm00001d022028_T001	2.634211519	2.582358492	2.317902269	1.335038419	1.541824061	0
Zm00001d022028_T011	1.744550355	1.263745359	1.771831925	2.279424641	1.923690779	1.752444181
Zm00001d045179_T008	0.463956153	0.198766951	0	0.50658671	0	0.223767273
Zm00001d045179_T021	1.556195426	0.831185717	1.493815365	0	1.150965134	0.694126007
Zm00001d008904_T006	0.442884062	1.557090954	1.344533112	1.59621767	0.983165456	1.201981948
Zm00001d008904_T009	1.481614109	0	0.64176704	1.241740137	1.35356946	1.765170632
Zm00001d008904_T010	1.288927259	0.95063787	1.420801977	1.186099894	1.55750469	1.405625923
Zm00001d008904_T005	2.550401297	1.585990776	2.615436308	2.756541272	2.678111128	2.695808344
Zm00001d008904_T008	1.869917587	1.754247872	2.119095505	1.333715106	1.485667943	1.636990192
Zm00001d042842_T006	2.127018435	1.142658495	1.908901657	2.729154003	2.507661716	2.659595349
Zm00001d042842_T002	0	0.478913837	0.260844657	0.137482539	1.280446828	1.236206653
Zm00001d042842_T007	2.998206879	2.417058831	3.337378991	2.149870008	2.673547379	1.92067746
Zm00001d027846_T001	5.429875358	4.660972643	5.437293799	3.501806619	3.61731212	3.338622085
Zm00001d042451_T006	0.120343977	0.212585524	0.604539175	0.247216621	0.668015617	0.753260507
Zm00001d042451_T004	1.489956469	2.254572275	1.712498497	0.562143249	1.371041351	1.111855283
Zm00001d042451_T002	4.011632674	2.455657843	3.809562925	4.441718509	4.218203044	4.34418688
Zm00001d018034_T001	1.875759621	1.45780229	2.278826322	1.608972424	1.685001811	1.874547546
Zm00001d002942_T003	3.63083528	3.267506584	3.722524278	3.322854873	3.194681694	3.183079295
Zm00001d002942_T001	3.911456034	3.855538386	3.828145626	4.095084882	4.195499105	4.308650873
Zm00001d002942_T002	5.407090533	4.668407249	5.235160208	5.211279363	5.176542512	5.227700034
Zm00001d048616_T001	5.575523939	4.921181752	5.583632775	6.61621207	6.679691365	6.638788736
Zm00001d008222_T002	1.867245721	1.00964815	1.955892295	0.87441377	0.641233411	0
Zm00001d008222_T001	1.43034041	1.645651401	1.814239498	0	0.262445185	1.084149327
Zm00001d053913_T001	4.385818304	3.785967293	4.465700849	4.113727083	4.136317485	4.168731239
Zm00001d025706_T003	1.377363669	1.233967185	1.452222721	0.57682298	0.990636162	1.647883185
Zm00001d025706_T001	0.602380862	0.330230243	0.440614201	0.758340705	0.382447028	0
Zm00001d025706_T002	0.998382554	0	0.744755277	1.033348993	0	0.922345536
Zm00001d010141_T001	0.078009953	0.27111248	0.775393628	0.892040462	0.672813629	1.000602199
Zm00001d010141_T012	2.923579737	2.631673097	2.768114438	2.762541871	2.643747233	2.471119552

Zm00001d010141_T006	1.687428036	1.022393382	1.731234517	2.225659618	2.634761457	2.55035278
Zm00001d020068_T002	2.953900853	3.777763712	3.161368864	3.343670326	2.81920751	3.018781391
Zm00001d011594_T001	3.764365902	3.738478728	3.643823988	2.161954738	2.592044113	2.813463533
Zm00001d041889_T027	0.775944756	0.244586314	0.7800732	1.001429723	0.471054242	1.458289076
Zm00001d041889_T153	1.840716199	1.912134388	0.989274186	1.827269477	0	0
Zm00001d041889_T170	0	0	2.760424719	0	2.218529798	2.251359472
Zm00001d038193_T005	4.813878086	4.318258771	4.861365515	4.437781296	4.625856626	4.251278492
Zm00001d038193_T007	0	0	0.56537978	0	0.689263372	0.944684562
Zm00001d038193_T006	1.278657452	0.91165017	0.901517624	1.550649525	1.549952015	1.363829323
Zm00001d038193_T009	1.760601214	0.680205515	1.957159818	1.28542471	0	0.812646081
Zm00001d038193_T003	0	0	1.01912045	0.154454889	0.715590307	0.736507899
Zm00001d038193_T001	0.480236164	0.132043753	0.228661101	0.442271541	0.48773741	0.414033756
Zm00001d046626_T001	3.358943358	2.723335096	3.510704109	3.884374809	3.91193658	3.817201642
Zm00001d007240_T001	1.339253708	1.025427164	1.638158658	1.936902099	1.998740338	1.700085438
Zm00001d015225_T001	1.236809135	0.940245697	1.339565568	1.120639256	1.054983886	1.08307173
Zm00001d038897_T002	1.875611804	1.248695734	1.731754992	2.10587248	1.825442921	1.962420202
Zm00001d015986_T001	1.694866824	1.010115258	1.687761256	1.69048325	1.676089995	1.614619864
Zm00001d015986_T002	5.05114737	4.701609658	5.01811884	5.109536456	4.996514028	5.003471903
Zm00001d021048_T001	2.891841434	2.577078551	2.775958237	2.771245624	2.582980079	2.781119419
Zm00001d033621_T001	4.581945016	4.143829296	4.607024104	3.99706519	3.815787924	3.903772643
Zm00001d012537_T005	2.782029388	4.903314294	2.95155721	2.982141711	2.885223116	3.341693195
Zm00001d012537_T004	0.461083214	0.351903048	0.466644868	0.490837081	0.570863138	0.288842825
Zm00001d012537_T001	8.287982533	7.746710333	8.305639586	8.180522995	8.234782399	8.142802202
Zm00001d019216_T001	5.487481108	5.893786341	5.425369805	5.233361867	5.362845172	5.429481563
Zm00001d033632_T002	0.953140802	0.365993697	1.030874207	1.563172777	1.380975417	1.925914753
Zm00001d033632_T007	0	0.519921273	0.819019029	1.317166855	1.717143604	0.395616732
Zm00001d033632_T009	2.533922856	1.984537584	2.64038123	2.11862651	2.181456898	2.649180861
Zm00001d033632_T008	1.014630236	0	0	1.812494529	1.793269157	1.594406184
Zm00001d043225_T001	4.337514795	4.016215848	4.383778101	4.101491831	4.123166894	4.165661877
Zm00001d012439_T001	9.13120508	8.914414407	8.938142365	8.775189361	8.832566349	8.836631553
Zm00001d048416_T001	2.792530345	2.243610592	2.605604559	4.830425153	4.964236609	5.151801395
Zm00001d046531_T002	1.374316721	0.700635024	1.095300636	1.502196664	1.4335118	1.449251277

Zm00001d046531_T005	2.583066516	2.772895534	2.476085341	2.281144505	2.443535045	2.856143523
Zm00001d046531_T001	1.521152778	2.64949774	1.648986284	0.772067224	0.594158683	1.813156067
Zm00001d046531_T006	4.593328072	2.82012442	4.757445109	5.198526527	5.104669364	5.070431835
Zm00001d049588_T001	2.153347491	1.47563487	2.263956534	1.880741939	1.668583908	1.703128614
Zm00001d040670_T005	1.102634618	0.726407499	0	0	1.300054591	0.927254018
Zm00001d040670_T004	3.489637083	2.640632495	3.531490509	3.728339918	3.700918946	3.586637939
Zm00001d040670_T007	0.677370224	0	1.343476615	1.117028058	0	0
Zm00001d053349_T001	6.340791206	6.891880005	6.301434736	6.24304104	6.303936934	6.177045136
Zm00001d008392_T001	1.65291964	1.863578128	2.173822383	1.842587492	2.069593565	2.072395683
Zm00001d036635_T001	0.73508367	0.47728254	0.689258899	1.072544889	0.843979022	1.103149807
Zm00001d011038_T001	3.02344415	2.181518916	2.912887995	2.952636799	2.933483883	2.792949655
Zm00001d010635_T001	4.36212078	4.251725303	4.304179534	4.696516473	4.711908603	4.519581972
Zm00001d012019_T001	1.083670873	0.890790642	0.953737554	0.748021488	0.451263345	0.800743796
Zm00001d030744_T001	4.501037428	3.832516586	4.446252524	4.602686396	4.767129422	4.64508738
Zm00001d010492_T007	1.005642872	1.815884018	1.192980015	2.206296573	1.817345742	1.925296883
Zm00001d010492_T011	0	0.064438665	0.516422676	0.700403317	0.151217188	0.321780355
Zm00001d010492_T002	1.011826934	0.580230403	1.049384722	1.799427211	1.806910504	1.903610606
Zm00001d010492_T010	1.355332066	1.884765135	1.889775679	2.019719348	2.179790645	2.134697706
Zm00001d010492_T001	4.706770337	2.760025877	4.670241146	3.980536553	4.027299783	3.558206765
Zm00001d010492_T003	2.841512872	2.872506308	2.670216272	2.785134736	2.714043032	2.823613847
Zm00001d010492_T006	3.663433547	2.240080731	3.935638895	2.83570826	2.763719203	3.085943648
Zm00001d024573_T001	6.711039725	6.057574174	6.751414177	6.284498716	6.547028725	6.520760221
Zm00001d028288_T001	1.678048461	1.903728593	1.592619208	1.912896999	1.891016265	2.059273587
Zm00001d018755_T001	5.626063549	5.206450216	5.468233744	4.980048476	5.048727799	5.110407638
Zm00001d029747_T001	6.95431591	6.274679125	6.952434379	6.19473517	6.252794579	6.261157494
Zm00001d021162_T001	4.069808698	3.502953403	4.107146673	3.85230283	3.619679339	3.657270024
Zm00001d017467_T001	5.912475169	5.437275919	6.075426201	5.480426899	5.516328275	5.447147646
Zm00001d014773_T002	4.549302598	3.603959677	4.527418703	4.929867343	5.080842294	4.957807971
Zm00001d014773_T001	3.781370311	3.212582256	3.669888949	3.895402773	3.909551356	3.956648668
Zm00001d011189_T001	6.465413761	6.562825247	6.084871748	6.200311916	6.079382933	6.284637055
Zm00001d024203_T002	3.091961272	1.009011723	3.198538942	2.516988386	2.714461823	2.896176506
Zm00001d024203_T001	1.348842008	2.496650504	1.458873319	0.95121178	1.818151535	1.520369443

Zm00001d041785_T001	1.414893764	0	1.580913431	1.255892851	1.210964207	1.255385313
Zm00001d041785_T002	4.392640894	4.368568271	4.103281513	5.012574439	4.855598084	5.126148719
Zm00001d041785_T011	5.337313262	2.08286061	4.786525741	5.729809855	5.586851975	5.393601399
Zm00001d041785_T009	4.621873705	3.539198896	5.264999715	0	3.440864783	0
Zm00001d039956_T001	0.526760939	0.397459083	0.808440245	0.78610618	0.605816688	1.037421584
Zm00001d017377_T001	3.039538327	2.535870064	3.120496435	2.496804383	2.284192511	2.262793335
Zm00001d040154_T001	2.238183063	1.755428999	2.433753205	3.470694987	3.302348153	3.159577845
Zm00001d053939_T001	4.78705654	5.201782749	4.934294513	4.702845403	4.911365573	4.733581737
Zm00001d049100_T001	5.014945552	4.194618664	5.082295412	5.662806355	5.663357885	5.618436425
Zm00001d012766_T001	3.006824193	2.547649463	3.003241698	2.758468419	2.741199241	2.720535019
Zm00001d006028_T001	1.895001253	1.660317137	2.204902251	1.500129998	1.182917264	0.950150352
Zm00001d040534_T001	1.245446984	0.607434007	1.242115886	1.863762066	1.907887057	1.785262612
Zm00001d030152_T001	3.967628278	3.32231483	3.823515922	3.928821113	3.883273003	3.955919999
Zm00001d040518_T001	4.209434026	4.531406602	3.558696705	3.003219566	3.208668014	3.374320616
Zm00001d006019_T001	4.812754191	5.771942372	4.775144698	4.607388257	4.627127845	4.786941261
Zm00001d047780_T012	3.721183861	2.819574998	3.754334363	4.191687239	4.042124944	3.926053234
Zm00001d047780_T010	0.133680567	0	0	0.811928017	0.503731368	0.462510976
Zm00001d047780_T011	0	0	0	0.592564675	0.595156073	0.950819993
Zm00001d047780_T002	1.024675699	0.683650647	0.997095819	3.003342816	3.008594094	2.76596855
Zm00001d044665_T001	5.636432867	4.831852419	5.740098231	6.064518554	5.988344193	5.854856667
Zm00001d044665_T002	0.985368542	3.286392529	1.052143189	0.658780906	0	1.737229439
Zm00001d015202_T001	2.970792007	2.111226664	2.983742806	3.733753793	3.824836677	3.755637722
Zm00001d043491_T001	3.598029864	3.506569066	3.92538168	3.303757957	3.375117022	3.397332709
Zm00001d025274_T001	3.867551065	3.234787471	3.94795233	3.186840907	3.411143238	3.60860595
Zm00001d051020_T001	3.590138491	3.213928598	3.416388221	3.609768316	3.762538682	3.285450308
Zm00001d020791_T001	4.54533631	4.208020877	4.674814841	3.385238711	3.313992696	3.284400271
Zm00001d045528_T001	6.388647627	6.000428732	6.428516704	5.685750103	5.790331083	5.89772781
Zm00001d027799_T002	3.156673446	2.696214876	3.386321368	2.432942843	2.528720831	2.077992185
Zm00001d027799_T001	3.009415614	1.388876135	2.710620435	2.986661041	2.671024286	3.059120232
Zm00001d031279_T001	2.385931821	1.537534633	2.329443681	2.140151716	1.849598211	1.939097162
Zm00001d047479_T007	0	7.22286023	0	1.809345704	1.762607776	0.405895451
Zm00001d047479_T008	4.81329231	4.515992888	4.869331034	4.183880998	4.351810704	3.989588371

Zm00001d047479_T012	5.15240504	7.496686986	4.65007791	4.605394413	4.321713262	4.127888452
Zm00001d047479_T016	2.115307302	2.189238508	2.34435017	0	1.463029146	1.575211145
Zm00001d047479_T017	2.650373968	3.797650633	3.268004036	3.034417171	3.430876015	2.754267544
Zm00001d047479_T013	1.217499374	1.157751298	1.181446721	1.576783323	1.32775921	1.800470055
Zm00001d047479_T003	8.682726368	7.995186695	8.637340585	8.373914121	8.375918	8.404079428
Zm00001d008858_T002	3.875460532	4.150120953	3.847334217	3.225012037	3.759109803	3.588376563
Zm00001d008858_T001	2.817886602	2.72542314	2.653633082	2.521922625	2.292028701	2.458065658
Zm00001d054078_T002	5.680017161	5.068297063	5.568855277	5.645342064	5.628610743	5.660054384
Zm00001d028045_T010	3.932343369	3.258338916	4.021177121	4.33716195	4.434155613	4.22098699
Zm00001d028045_T001	1.534419741	0.727000321	1.508323172	2.032959855	1.450575197	1.208718886
Zm00001d028045_T012	0.556799208	0.311594952	0.605096981	0.683295812	0.574066621	0.660038691
Zm00001d028045_T013	4.417198412	3.642315839	4.53913126	3.852858903	3.429913309	3.85975591
Zm00001d054084_T001	6.794502711	6.25555015	6.80862714	5.898401666	5.88409714	5.844751252
Zm00001d013861_T001	1.697436677	1.309280398	1.550311649	1.566005069	1.807976796	1.206546333
Zm00001d032049_T002	4.497256853	3.82677442	4.603056453	4.473404047	4.574449179	4.427392702
Zm00001d032049_T006	2.748523705	2.67921231	2.244653896	1.883210626	0	0
Zm00001d027700_T001	8.424199657	7.80649143	8.465552933	7.497168321	7.579374067	7.5754109
Zm00001d034682_T001	4.76210342	4.269822896	4.965481633	4.950054536	5.102929304	4.87421018
Zm00001d034682_T002	3.403788389	3.662140885	3.327606318	3.0097121	3.051677663	3.128529486
Zm00001d050195_T001	6.99038612	7.337009017	7.098431959	5.932271998	5.962459118	6.081819239
Zm00001d016708_T002	4.892353671	4.103098011	4.850978049	5.305385812	5.341148877	5.334574175
Zm00001d016708_T001	0.672243381	0.332567022	0.905814521	1.710811061	2.07973377	1.89524755
Zm00001d020826_T001	4.147912145	3.132921601	4.036606543	4.514236661	4.484189701	4.557980169
Zm00001d042712_T001	2.219466866	1.623492799	2.199602937	2.766630142	2.684038619	2.785342764
Zm00001d029448_T001	6.064586153	5.507436966	6.235155612	6.25179611	6.1501525	6.208392107
Zm00001d042267_T005	3.11041219	0	2.99288424	4.092448572	3.831149226	4.643756063
Zm00001d042267_T004	3.871356657	2.915805587	3.768289042	5.331649545	5.54905343	5.224728185
Zm00001d042267_T006	5.874653919	5.408863866	5.948823285	6.325498977	6.177980142	6.134551814
Zm00001d042267_T011	1.804921724	1.537175775	2.272656581	3.794868827	4.091435556	4.118551845
Zm00001d051759_T001	3.204564571	2.357935922	3.3544681	3.866792479	3.678247836	4.013369174
Zm00001d051759_T003	0.40330263	0.190833539	0.477502225	1.031635861	1.666078934	1.339679007
Zm00001d051759_T002	0.708228739	0.806611949	0.255277136	1.081015735	1.019644277	0

Zm00001d018389_T001	6.143379928	5.321722244	6.115217377	6.16617817	6.207174311	6.229096508
Zm00001d051676_T006	1.932586586	0.188837655	2.279252572	0	1.278785295	2.141653495
Zm00001d051676_T003	3.663517687	2.178140954	3.683863393	3.569235997	3.761715677	3.760500728
Zm00001d051676_T002	4.873461191	4.320919154	4.641475222	4.611753591	4.591813078	4.553980766
Zm00001d051676_T001	0.136430284	0.068479585	0.39103409	0.669898471	0.975312487	0.726445866
Zm00001d051676_T005	1.378639241	0.934349671	1.237080292	1.444514945	1.616750079	1.102942947
Zm00001d006688_T001	4.483566355	3.749066845	4.698007505	5.575073032	5.596495888	5.641335707
Zm00001d033104_T016	0	0	1.16092535	2.094304318	2.076981102	1.938323435
Zm00001d033104_T001	3.116814364	2.341409692	2.699564069	2.740755549	2.524377914	2.479296019
Zm00001d033104_T007	0.539997502	0	0	1.525316711	2.314879787	2.269483544
Zm00001d033104_T010	0.480173076	0	0.618787433	0.946944161	0	0
Zm00001d046682_T001	6.80030707	7.474274656	6.799525619	6.590830121	6.708941598	6.590177918
Zm00001d027801_T016	0.645665694	0.89303985	1.392937786	0.871972145	0	0.93551406
Zm00001d027801_T019	0.25004596	0.133491192	0.206319394	0.429403862	0.509256	0.342501269
Zm00001d002166_T037	4.276693925	0	3.969200969	0	0	3.26104482
Zm00001d002166_T012	3.776053967	3.877259541	4.004956781	4.608153199	4.881268215	4.821868454
Zm00001d002166_T013	0	0	0.108652914	0.71520985	1.13668489	0.776939456
Zm00001d002166_T034	0.355822549	0.265068374	1.031164375	0.469525518	0.99154053	0.396490525
Zm00001d002166_T026	0	0	1.758044296	3.328407526	0	1.526655789
Zm00001d002166_T010	0	0	0.498406108	1.029244373	0.435396037	0.766177875
Zm00001d038248_T005	5.188174793	0	4.784001831	4.867096683	5.316613927	5.228555827
Zm00001d038248_T003	2.779523858	2.70036514	2.728989286	2.676352044	2.292121197	2.675971481
Zm00001d038248_T009	1.928999381	2.893442013	1.555866698	2.169814711	2.357957028	1.997449226
Zm00001d038248_T004	0.604927195	1.084175865	0.332997732	1.195820649	1.06799384	1.294401957
Zm00001d038248_T001	3.368420133	2.787349503	3.35717066	3.036883431	2.674407092	2.575094458
Zm00001d038248_T006	5.702122999	6.267860892	5.604270985	4.810553693	4.685290341	4.618834878
Zm00001d038248_T002	3.759424316	3.865512863	3.945278078	4.064424696	4.086984466	3.837617741
Zm00001d038248_T008	5.133672595	4.480967877	4.953638345	4.399350223	4.471074408	4.671528273
Zm00001d023524_T001	2.759014541	2.017698184	2.588185485	2.726419271	2.816307861	2.823722259
Zm00001d041267_T002	5.510540281	5.071267387	5.613370402	5.800071931	5.780944105	5.756243265
Zm00001d041267_T001	0.203742418	0.325098538	0.460523457	0.537070388	0.335167653	0.723088435
Zm00001d025759_T001	2.983779282	2.678864719	3.249568215	2.327539638	2.17227404	2.043615029

Zm00001d030010_T001	3.413536948	3.24706498	3.593729038	3.671226785	3.819631605	3.647738275
Zm00001d003602_T001	6.160411187	5.24490456	6.186636557	6.218356083	6.159463834	6.203392015
Zm00001d026531_T002	0.78339882	0.82104483	1.151154178	2.048756174	2.425865039	2.337664286
Zm00001d026531_T001	2.453841507	1.821723275	2.62327178	3.497593845	3.29171759	3.478074467
Zm00001d036241_T001	2.23671486	1.533175587	2.024326417	1.940889515	1.66540778	2.017450551
Zm00001d028336_T001	0.86690956	0.546522613	0.73156733	0.833594485	0.976899551	0.975168655
Zm00001d008788_T008	4.800017296	3.206450998	4.85708545	3.984523001	3.626988658	4.034646593
Zm00001d008788_T003	2.696819564	3.070763693	2.617708473	2.243214816	2.646662213	1.845438264
Zm00001d008788_T004	0	0	0	2.454999942	2.704844305	2.610796664
Zm00001d032887_T001	3.37448227	4.39079045	3.009336601	3.578949579	3.908264445	4.148858308
Zm00001d014021_T001	3.178360735	2.644610349	3.05526803	3.142452977	3.148368789	3.051332597
Zm00001d049636_T001	3.537628184	2.773530749	3.531604539	3.412477105	3.243650347	3.303536309
Zm00001d021529_T001	3.839284627	3.48457139	3.816286969	2.923465645	2.840025676	3.178866152
Zm00001d040726_T001	1.112948955	0.897958302	1.355067587	1.298159737	1.968902858	1.584230388
Zm00001d011392_T004	0.953122173	0.227670849	0.354528892	1.629489517	1.239119375	0.957696228
Zm00001d011392_T001	1.066157405	3.679179312	1.893182008	3.340927668	1.04477599	2.038282352
Zm00001d011392_T002	5.848124164	3.486387445	5.781862943	6.042318469	6.21685933	6.080614582
Zm00001d010754_T001	0.986067196	0.477601694	0.926495924	0.8856056	0.803602336	0.654507949
Zm00001d014109_T007	2.390815689	0.707386964	1.479479958	1.289967812	1.932581295	1.973910987
Zm00001d014109_T011	0.76498472	0.794963103	2.16985543	1.375335866	1.268258912	0
Zm00001d014109_T002	2.167737822	1.842063662	2.28541672	2.357453194	2.078344087	2.328875518
Zm00001d014109_T006	1.212501482	0.688877698	1.322580623	0.917036798	0.704787879	0.828096971
Zm00001d014109_T010	0.153383813	0.376254816	0.413504182	0.462168568	0.445610181	0.844273961
Zm00001d017365_T001	1.696329067	1.344803505	1.808413884	2.398849973	2.251147051	2.393876614
Zm00001d029287_T001	4.091460437	3.717673572	4.053695162	3.593467557	3.560941611	3.472165144
Zm00001d013097_T001	2.496127906	1.933711991	2.678684019	3.86984121	3.797515861	4.092428275
Zm00001d036875_T001	1.382638482	1.042147713	1.318999339	1.427265571	1.504420562	1.377082092
Zm00001d035243_T001	7.98098564	7.188200495	7.989445496	6.381585518	6.366668629	6.340211931
Zm00001d002364_T001	4.110047974	4.891665027	4.064374164	6.642613971	6.641908897	6.632618274
Zm00001d005793_T001	4.825867832	3.980949956	4.158946909	4.800496251	4.41086655	4.499607065
Zm00001d028469_T001	5.376524399	6.685796516	5.054932241	4.628785783	4.804660596	4.666766248
Zm00001d010343_T007	1.11481076	2.680832699	1.233570289	1.939859945	2.049396572	2.215486328

Zm00001d010343_T006	3.044424372	2.925155575	3.022910519	3.041037219	3.139599482	2.991348055
Zm00001d010343_T003	0.987374716	0.657690304	1.172961722	1.781132642	1.498955446	1.705440522
Zm00001d030862_T001	2.206714169	1.782513827	2.345859882	2.119020118	1.935888155	1.90868035
Zm00001d034425_T001	2.375743156	2.889464781	2.718966527	1.669046727	1.892547237	2.093551049
Zm00001d005110_T001	4.62819856	4.293833429	4.604673488	4.729802406	4.826845588	4.847041957
Zm00001d038106_T009	0.79082374	0	0.817252407	0	1.851174547	2.112657435
Zm00001d038106_T010	0.849886401	0.339439578	0.698673667	0.865575218	1.081361445	0.999496412
Zm00001d038106_T011	0.184786815	0.143633739	0.074406787	0.295123491	0.385668476	0.449094354
Zm00001d038106_T004	2.923877667	2.146955589	3.025292319	2.738114532	2.600373241	2.482417733
Zm00001d039660_T009	0.562531118	0.877747391	0.656259296	0.522931549	0.658983761	0.659920906
Zm00001d039660_T010	2.61709462	1.620527763	2.47354562	2.771546093	2.729440309	2.611298699
Zm00001d038332_T001	3.738721583	3.081153654	3.892122184	3.648118418	3.50654774	3.662513834
Zm00001d024583_T003	0.813521406	0	0.654326464	2.583999661	2.356047158	1.839180402
Zm00001d024583_T001	0.639333125	0.874359469	0.889219637	0.81633838	0.822640423	1.142710116
Zm00001d024583_T011	4.44044597	3.487071463	4.510932179	4.205861266	4.223182879	4.105140654
Zm00001d050944_T001	5.683646043	6.447620288	5.76984924	5.638633396	5.608470997	5.50582092
Zm00001d014966_T001	3.724102991	3.024008267	3.580109778	3.7356033	3.411610503	3.52042602
Zm00001d043988_T001	4.385765027	4.565454605	4.31262989	3.299381534	3.148592014	3.17045886
Zm00001d043822_T003	0.878837536	0.612872389	0.878752802	0.74265905	0.892647482	0.310229587
Zm00001d006084_T008	0.593823198	0.401143297	0.953826933	0.537978856	0.839435609	0.539835757
Zm00001d006084_T010	0.410246659	0	0	0.324280654	0	0.562488135
Zm00001d006084_T007	5.440353907	4.537857255	5.406981118	5.060736997	5.043109593	5.076561361
Zm00001d003745_T001	1.549018025	1.578006878	1.981493213	1.245249209	1.442799683	1.205326821
Zm00001d046231_T009	1.304511626	0.540860498	1.294849504	2.016795097	1.891736856	2.129242456
Zm00001d046231_T008	0.309528958	0.085079238	0.381103924	0.750553341	1.041190405	0.691051697
Zm00001d046231_T012	0	0.604369323	0.487006743	1.106415327	1.362923176	0.944336733
Zm00001d046231_T004	0.95402206	0.718702243	0.62427499	1.26655689	1.362748161	1.663012545
Zm00001d046231_T011	0.454498094	0.067918143	0.303240677	0.964000796	0.750794279	0.885160433
Zm00001d046231_T005	0.844763251	0.381711964	0.092571449	1.116748005	0.804918421	1.185066658
Zm00001d046231_T010	2.855809349	1.95146284	2.987808171	2.370754074	2.61731259	2.540749676
Zm00001d046231_T001	1.294671325	2.020618117	1.142798323	0	0	0
Zm00001d027877_T001	2.841080062	2.843748131	2.609731327	1.273923427	1.80436834	2.026667998

Zm00001d033291_T007	1.011744654	0.441452671	1.322633689	0.681701125	0.74484137	1.143006079
Zm00001d033291_T008	4.105082642	2.635146312	3.991473684	3.971088073	3.718906706	4.056033136
Zm00001d033291_T004	1.386912456	2.345284521	0.622348472	1.127472164	1.58772406	0.971461519
Zm00001d033291_T005	5.576951587	5.207197411	5.586338026	5.507780752	5.53515137	5.51267818
Zm00001d033291_T006	0.802786306	0.732645185	0.905642798	1.662622522	1.457809117	1.436876612
Zm00001d006878_T001	3.899224267	4.822808296	4.020743712	3.32348205	4.015267045	3.791282728
Zm00001d010319_T001	4.083028402	3.285861293	4.184034904	3.670058853	3.789868403	3.884211164
Zm00001d023279_T001	1.623279272	1.247391035	1.733271899	1.55134473	1.794845855	1.5356046
Zm00001d016977_T003	2.450311513	1.563124443	2.464607631	1.387163991	0	1.941403836
Zm00001d016977_T002	3.076958363	2.967814171	3.606844539	2.692141488	2.72280327	2.827326799
Zm00001d016660_T001	11.49289329	11.94548061	11.438952	11.31563336	11.30369721	11.36182057
Zm00001d048639_T001	1.099146382	0.662037745	1.122104805	1.396743136	1.25062147	1.386139923
Zm00001d021620_T001	2.794201864	2.65431936	2.66128717	2.628302339	2.492907353	2.631955908
Zm00001d034890_T001	4.614133358	4.739866514	4.654183632	4.696557089	4.608017908	4.778609205
Zm00001d009186_T001	2.874395473	2.09654726	2.938288239	5.188752535	5.138792602	5.085310858
Zm00001d048098_T004	1.625318189	0	2.122305953	1.885995984	2.624812589	0
Zm00001d048098_T001	0.895653945	1.687966793	0	0.752111439	0.634793977	2.593853309
Zm00001d048098_T002	1.222962173	1.05903195	1.555923617	1.691460465	1.571989825	1.716193297
Zm00001d048098_T003	1.348177453	0	1.259821435	0.90579065	0	1.461326337
Zm00001d052108_T003	1.10305444	3.026539466	0.667004669	0.724958421	1.189634822	1.324990857
Zm00001d052108_T007	5.370743788	6.926456358	5.449925007	5.043669967	5.715686202	5.497460361
Zm00001d052108_T005	5.691263271	4.255627549	5.813745489	6.31642283	6.253173285	6.025370286
Zm00001d052108_T001	4.576906825	3.877956909	4.392572826	4.536485457	4.482358613	4.686246198
Zm00001d039789_T001	0.149470095	0.113759166	0.142693127	1.510656643	1.265308464	1.534959022
Zm00001d043915_T002	1.340575371	0.263524839	0.875102978	2.436612278	2.107627951	2.251655476
Zm00001d043915_T001	0.83426139	0.930480752	1.482211189	1.722050643	2.060365987	1.801829964
Zm00001d026654_T001	1.675898111	1.176472141	1.573250418	2.039679215	2.051338688	1.97766569
Zm00001d032016_T007	1.365355477	1.67601189	1.314326452	1.773073042	1.959091298	1.490351913
Zm00001d032016_T014	0.714314106	0	1.209840112	1.19813202	1.551168499	1.521417145
Zm00001d032016_T017	1.612695496	1.058768804	1.662088347	2.34270663	2.2839668	1.948727171
Zm00001d032016_T023	1.282744622	0.525623912	1.173816308	1.821647772	1.861069234	2.24656983
Zm00001d032016_T011	0	0	0.517195051	1.509357358	0.84912177	0.409761305

Zm00001d032016_T003	0	0	0.145087867	0.597356682	0.375914656	0.961752961
Zm00001d032016_T006	0.846760468	0	0.618171924	0.795528418	0.939359743	0.927523314
Zm00001d027515_T002	1.803968036	0.638070129	1.671176503	1.88764809	1.624117276	1.870440478
Zm00001d027515_T011	0.973275495	0.809304128	1.168061118	0.626140053	0.921855215	0.692848518
Zm00001d031620_T002	1.638963051	0.750810569	1.444000157	1.379687495	0.872932748	1.049489976
Zm00001d045611_T006	0.367894367	0.033262997	0.22720135	0.560183749	0.245984794	0.419553993
Zm00001d045611_T004	1.997099795	2.805061276	1.860347461	1.882827311	2.19213292	1.734695315
Zm00001d045611_T001	3.748509107	1.360892372	3.566708378	4.458657536	4.566427948	4.461253968
Zm00001d003435_T001	4.348546523	4.96934245	4.428868314	4.291893114	4.296037974	4.16991354
Zm00001d051397_T001	3.536177763	2.697680201	3.554662028	4.525330932	4.650264641	4.602005284
Zm00001d032552_T007	0.749505092	0	1.394705098	0	1.761625267	0
Zm00001d032552_T004	1.091751279	0.840161067	0	2.511619841	1.460570636	2.336463642
Zm00001d032552_T009	2.004480538	1.557524295	2.074435218	2.05116917	2.174230625	1.609140788
Zm00001d032552_T008	1.167837034	1.145197455	1.56386783	1.327131446	1.161545264	1.233400332
Zm00001d032552_T002	2.876759937	1.85685147	2.450665983	2.710963104	2.949512149	2.934428182
Zm00001d032552_T003	2.95976589	3.495548456	3.636314763	2.434701345	2.591797403	2.351522045
Zm00001d032552_T001	3.751032712	3.537854646	3.842234068	2.249598856	2.390294033	2.139662344
Zm00001d046960_T005	3.453350715	3.994426396	2.78867903	2.8412071	2.457657062	0
Zm00001d046960_T003	1.063986751	0	0.263309694	0.987379082	0.723629329	0.969631999
Zm00001d046960_T004	2.266710694	2.064899058	2.676018888	2.556338179	2.329368475	2.984917223
Zm00001d021762_T002	2.562535513	2.340367998	2.761736736	2.394160422	2.847131631	2.565019495
Zm00001d021762_T001	1.839987796	1.104283648	1.564497189	1.911668959	1.772107774	1.521212591
Zm00001d027541_T001	6.300661468	5.822724923	6.246983131	6.526806736	6.57855656	6.518784882
Zm00001d021781_T001	2.116638896	1.648539992	2.086418291	1.231868592	1.269286943	1.491069083
Zm00001d037799_T001	3.293674766	2.816507142	2.908113867	2.457727971	2.201637625	2.520177315
Zm00001d020019_T003	0.882427852	0.378747984	0.707455879	0.666392123	0.597096334	0.31189832
Zm00001d020019_T001	4.986007652	4.294264828	4.878827582	5.192493604	5.122272444	4.977739635
Zm00001d020019_T005	0.506360237	0.228132805	0.562142272	0.312227188	0.194857377	0.232047942
Zm00001d033788_T003	3.020918708	5.276415409	3.908310951	0	0	0
Zm00001d033788_T001	5.510632245	4.996996069	5.596554924	6.164176547	6.076519526	6.104852855
Zm00001d033788_T002	1.164108528	1.199887771	1.619251006	1.250568719	1.728159541	1.368309072
Zm00001d033788_T004	0.770902606	0.517164809	0.839466247	0.932016559	1.017224971	0.734894707

Zm00001d036543_T001	6.456109105	5.627579087	6.547738706	6.9608933	6.944166922	6.924073821
Zm00001d008370_T001	2.299499056	2.380856593	2.881692807	3.815570101	3.836048133	3.79977781
Zm00001d027950_T003	0.504797318	0.298287624	0.478780296	0.365677981	0.540198914	0.763861098
Zm00001d027950_T002	2.148160339	1.935929632	2.152887879	2.267963	2.073598158	2.438616224
Zm00001d032310_T001	3.020064766	2.455855865	3.253148205	2.940236487	3.05795569	2.916599507
Zm00001d032699_T020	0.914497936	0	0.640138715	1.708277747	1.800582705	1.602205529
Zm00001d032699_T006	1.341484843	0	1.604288186	1.979231874	0	1.266349394
Zm00001d032699_T018	0	0	0	0.885372881	1.3315258	1.630576478
Zm00001d032699_T022	3.6853172	2.598868012	3.556713512	3.089521371	3.385620853	3.308771792
Zm00001d032699_T005	0.641404527	1.182516875	0.364548901	1.29866594	1.529745	0.777493361
Zm00001d018605_T001	6.148924651	6.564750852	6.135899991	5.55137291	5.688336679	5.730626507
Zm00001d004698_T001	3.218710968	2.397132377	3.23022592	3.671272649	3.777530813	3.7321297
Zm00001d033397_T001	0.568191693	0.176675105	0.435175181	0	0.729974696	0.813303856
Zm00001d033397_T004	2.65730753	1.799405244	2.649481644	2.608363121	2.512941103	2.592973863
Zm00001d045084_T003	4.914920418	4.119980601	4.862946703	4.552565389	4.664698944	4.66417743
Zm00001d045084_T005	1.083049258	0	1.484853757	1.477690796	0.888416347	0.642911319
Zm00001d045084_T004	0.452563808	0.121682535	0.477609983	1.021725604	0.825687138	1.118913173
Zm00001d031318_T001	3.387723721	2.447817731	3.491373401	4.160489687	4.111846355	4.047531584
Zm00001d031318_T003	3.772661742	3.449857542	3.952289856	3.094212757	3.050135797	3.162245974
Zm00001d031318_T002	0.718867043	1.298883499	0.743939722	1.515344087	1.949526342	1.595021409
Zm00001d051211_T025	4.536077712	5.407437325	4.583053816	4.559790233	4.101229761	0
Zm00001d051211_T002	3.275004812	2.600264757	3.00469248	2.990167242	3.218987562	3.161885587
Zm00001d051211_T016	4.657868166	0	2.831656768	4.853697799	4.455073678	5.392684233
Zm00001d051211_T030	4.721736186	5.48326632	5.505158411	4.905274497	5.06693475	4.939570141
Zm00001d051211_T005	4.424398983	0	4.531143934	4.363910041	4.085673894	4.23745152
Zm00001d051211_T022	0.367164153	0.083590548	0.414138781	0.355540683	0.235312876	0.131892346
Zm00001d051211_T019	2.792374374	4.820309182	3.699389831	2.405938734	4.025780342	2.073239954
Zm00001d051211_T010	0.563793655	0.322941088	0.72496715	0.334237596	0	0.603117121
Zm00001d051211_T027	6.014900977	4.743847921	6.007743496	6.213008345	6.155918167	6.160204813
Zm00001d034551_T001	2.995691818	1.96841705	3.021717077	3.627908535	3.624618791	3.527752976
Zm00001d026051_T001	0	0.753855232	0.47715092	0.881402287	1.107296849	0.387875334
Zm00001d026051_T002	3.333208029	2.244216385	3.163823624	5.039481783	5.031739066	5.069592663

Zm00001d038218_T001	5.677450764	4.157331816	5.470714377	6.013875088	6.090327945	5.925818026
Zm00001d029808_T001	2.8352733	2.068871983	2.874153658	3.342391893	3.379111188	3.438519486
Zm00001d008548_T001	3.983044302	4.090373468	4.153100756	1.297034076	0.537442193	0.814157448
Zm00001d049762_T013	0	0	0.880028768	1.028806046	0	0.450591032
Zm00001d049762_T006	1.181945971	0.744793159	0	1.02076531	0.367009787	1.804998108
Zm00001d049762_T008	1.470831491	0.953226493	1.617573077	0.909968906	1.918729311	0
Zm00001d049762_T010	0.473010686	0	0.352526907	1.004978796	0.260519521	0.931239024
Zm00001d020607_T001	4.047389714	3.996671563	4.25754572	4.174891257	4.02233301	4.156257534
Zm00001d043462_T009	0.516042386	0.095875823	0.501425897	1.647445302	1.538672743	1.505394029
Zm00001d043462_T010	1.970547417	1.401634289	1.61104298	3.152613242	2.957834385	3.026090752
Zm00001d043462_T008	4.056122208	3.10121337	4.099031388	4.741615886	4.799610862	4.653842622
Zm00001d002286_T001	2.669535475	1.844944383	2.751226966	1.904380432	1.430475835	1.929438043
Zm00001d037530_T001	2.647320266	2.220744438	1.919441153	0	0	2.09851961
Zm00001d037530_T002	3.645517218	3.050871335	3.897982593	4.098716832	3.911629367	3.826364014
Zm00001d040596_T001	4.133629856	4.411326582	4.396729507	4.042016611	4.203800353	3.982921586
Zm00001d040596_T002	1.211091337	0.682338672	0.924944347	1.468387295	1.253138521	1.227688098
Zm00001d042493_T001	4.118674347	3.482461233	4.26701548	4.611539382	4.623241399	4.511297623
Zm00001d012217_T001	2.390892436	2.942512441	2.652200796	2.374917336	2.568548998	2.759163932
Zm00001d037650_T005	0.845909099	0.60343146	0.816850339	2.333582021	2.619984341	2.62493234
Zm00001d037650_T001	3.151668079	2.187427616	3.106058813	4.308217129	4.172928049	4.119181433
Zm00001d037650_T002	1.292667507	0.501023283	0.965050609	2.659621835	2.744376838	2.805536599
Zm00001d030094_T004	4.083129698	1.872629427	4.076291258	4.621430844	4.775100756	4.63394298
Zm00001d030094_T001	1.695811684	1.692231225	2.192675194	2.542590141	2.632824942	2.74201764
Zm00001d030094_T005	3.467861659	3.278908817	3.46204394	2.279663543	0	0
Zm00001d005631_T001	4.881783974	5.080894043	4.871717062	5.133113888	5.296938079	5.219531685
Zm00001d023208_T002	4.278521865	3.481239	4.329196949	4.120976763	4.137697291	4.125340644
Zm00001d044465_T001	4.916691456	4.123813918	4.837055863	5.005258137	4.937659782	4.967639759
Zm00001d003187_T002	2.45084754	2.4759505	2.534992378	0.537935135	0.835894169	0.690106844
Zm00001d003187_T001	2.384447737	1.861071219	1.911598019	0.431445386	0.303300306	0
Zm00001d050256_T001	6.278452042	6.119938554	6.167771736	6.404357582	6.391876818	6.374159762
Zm00001d034763_T001	3.628894301	4.74355746	3.751174575	3.502055073	3.841256724	3.414028477
Zm00001d019169_T005	2.774617984	2.004607815	2.917997456	3.489344089	3.385022331	3.282935989

Zm00001d019169_T007	1.572420171	0.333324116	0.879958216	0.741636109	1.010857162	0
Zm00001d019169_T002	0.925459446	0.765064521	0.390598355	1.598371876	1.832262989	2.30034165
Zm00001d026331_T009	0.435962429	0.140985395	0	0.50354617	0.60224782	0
Zm00001d026331_T018	0.464470667	0.059045106	0.50218495	0	0.226956066	1.129888327
Zm00001d026331_T006	3.420270975	0	3.552201581	4.62696034	4.649149468	4.554784124
Zm00001d026331_T031	5.201222164	4.625774234	5.186068984	5.350653071	5.225003358	5.287537417
Zm00001d013030_T002	3.29741859	2.153232035	3.32763707	3.65849131	3.681358068	3.731430152
Zm00001d013030_T001	4.11719475	3.36450786	4.249398692	4.010189842	4.044915144	3.778880556
Zm00001d041985_T001	7.094279728	6.528775363	7.154753374	7.479845285	7.516373183	7.486274282
Zm00001d022599_T001	3.368997514	2.47526443	3.353327244	2.86778559	2.823332386	2.921354634
Zm00001d046665_T001	2.719005311	3.044769871	2.541041711	2.021531251	1.48919712	2.005033428
Zm00001d026445_T001	3.764178294	2.985343401	3.816183733	4.270685089	4.230754373	4.132646015
Zm00001d026445_T002	1.255136312	0.727526681	1.080029289	1.306082548	1.353113224	1.190796884
Zm00001d041530_T001	3.705901398	3.561586666	3.634311904	3.793821822	3.653433143	3.638915569
Zm00001d009005_T001	0	0.714719413	1.21005652	0	1.079878466	0.625104932
Zm00001d009005_T002	3.70702135	2.706231263	3.648293322	3.71480479	3.968855869	3.602917062
Zm00001d009005_T003	0.98421163	0	0	1.25485946	0	0.933422318
Zm00001d028715_T002	0.236294225	0.27272913	0.356658806	0.488467707	0.32921317	0.45702372
Zm00001d028715_T001	2.586236079	1.988488376	2.715536276	2.842497585	2.548502716	2.751879502
Zm00001d046811_T001	1.840734324	1.929620212	1.8947231	1.772697315	1.721002477	1.664146476
Zm00001d009436_T003	3.943524273	3.263999186	3.896109936	4.452640041	4.361776078	4.461350045
Zm00001d049526_T001	5.148488663	4.349850577	5.212741728	5.064046051	5.029962554	5.119316498
Zm00001d049526_T003	1.023112673	0.494041767	1.05086176	1.496258829	1.371327455	1.226262511
Zm00001d053427_T009	3.333151342	2.18874494	3.404580005	3.121863357	3.387661141	3.031161375
Zm00001d053427_T001	0.39023184	0	0.787112294	0.977011695	0.907211415	0.21008084
Zm00001d053427_T011	1.141339268	0	0.503875846	1.847456195	1.197304304	2.528414789
Zm00001d053427_T005	0.449853978	0	0.241878004	0.54902049	0.497137137	0.597730432
Zm00001d053427_T012	0.901889057	0.890066075	0.941176941	1.205262377	1.358869393	0.90372705
Zm00001d009446_T001	5.627293111	5.562752239	5.73990906	5.179502889	5.270188972	5.211389527
Zm00001d013323_T001	4.306279595	4.691160546	4.389646447	3.885449026	4.084698452	3.919132579
Zm00001d027707_T001	6.071911684	5.212236523	6.126245091	5.93582245	5.970526412	5.892515277
Zm00001d023337_T003	0.93150304	0.322957235	0.78576898	1.572767943	1.328945581	1.373587524

Zm00001d023337_T027	1.525210453	0.668378768	0.681431274	1.424197066	1.734168528	0.501610348
Zm00001d023337_T024	0	0.504661067	1.398916172	0.263968249	0	0.913069009
Zm00001d022388_T012	0	0.159381181	0.084884734	1.556222408	1.571343338	1.819178074
Zm00001d022388_T004	0	0	0.43389632	0.290079909	0	0.690169436
Zm00001d022388_T018	3.519931712	4.773505061	3.597088346	3.178449339	2.739145374	3.627532951
Zm00001d022388_T003	0.950113762	0.543841297	0.646430891	2.817167867	2.999524012	2.605932575
Zm00001d022388_T017	5.740926346	4.369079759	5.736432185	6.169506078	6.269609006	5.962750352
Zm00001d022388_T014	2.834257345	1.806010941	2.584309774	4.319700586	4.127257206	4.108166122
Zm00001d022388_T016	0	0	0	3.083589697	3.510076162	3.74977795
Zm00001d022388_T011	3.052726798	2.267361406	3.205084515	1.609361618	2.375000774	2.511972717
Zm00001d022388_T019	0	0	0.423445862	0.772518282	0	0.908629245
Zm00001d013300_T001	2.924820483	3.406726994	3.056059242	2.775898416	2.655570964	2.362697392
Zm00001d013300_T003	0.984283098	0.602026373	0.752478007	0.579783559	0.463737533	0.615446432
Zm00001d013300_T002	6.663045901	6.501390593	6.601464526	6.182276231	6.191066797	6.258348226
Zm00001d026319_T004	3.750260259	4.470234238	3.573163027	4.178317467	4.026098013	3.789485443
Zm00001d026319_T003	3.071375519	2.846313983	2.885290678	3.574046391	3.303760002	3.251212962
Zm00001d034460_T007	3.502940168	2.498905166	3.335009111	3.289045488	3.275107943	2.896403807
Zm00001d034460_T011	0.98217768	0.27423661	1.145104176	0.774426538	0.64476261	1.386900871
Zm00001d034460_T014	0.659993039	0.307972858	0.485451559	1.423425423	1.607523962	0.814058983
Zm00001d034460_T016	1.993900031	1.378467787	2.546290963	2.730501277	2.52068323	2.342852811
Zm00001d034460_T010	1.631718025	0.572461888	0.754962734	1.762201668	0.627270637	1.495080607
Zm00001d034460_T015	0	1.711967505	0.254111452	0	1.34236757	2.06877845
Zm00001d007465_T001	2.136419456	2.00337808	2.306960958	0.56422694	1.575777017	1.476839959
Zm00001d007465_T002	1.555579612	0.707067968	1.403866494	1.104338673	1.218789846	1.218375111
Zm00001d042558_T003	0.228838388	0.317019206	0.418127326	0.338715349	0.550667254	0.277652741
Zm00001d042558_T002	0	0	0	0.807794259	0.459422176	0.432985055
Zm00001d026573_T001	7.164281828	6.41099027	7.175266198	7.230187111	7.229378348	7.237721849
Zm00001d053976_T001	3.894369222	3.599787426	3.827106238	3.510727399	3.144961146	3.370544577
Zm00001d027423_T001	1.02678873	1.055176909	1.084105776	0.699229122	1.063503633	1.056838752
Zm00001d027423_T002	1.338889882	0.882597665	1.550405238	1.837076154	1.433910204	1.312044751
Zm00001d044195_T004	1.215945059	0	1.079370601	1.506235307	0.521802552	1.304923932
Zm00001d044195_T015	1.074235505	0.736716564	1.27454555	0	1.214843486	1.380453515

Zm00001d044195_T003	4.115933398	3.900422075	4.209981607	4.138188477	4.266630571	4.171530949
Zm00001d044195_T020	4.178074727	2.499648456	4.428787733	2.058963398	1.560364753	0
Zm00001d044195_T007	1.169353581	0.316743555	0.95608268	2.077648743	1.92278166	1.579204772
Zm00001d044195_T011	0.668904267	0.053275412	0.280564418	0	0.943913827	0.551537673
Zm00001d044195_T017	1.828800942	2.032557213	0.839300956	2.187126688	1.648421264	1.976697232
Zm00001d039683_T004	0.599579645	0.317364276	0.586761862	0.570799035	0.999373734	0.945670623
Zm00001d039683_T006	1.506174869	1.528993314	1.143409746	1.460865731	0.790116422	1.621010946
Zm00001d039683_T001	0.715840668	0.310835671	0.589404926	0.823361329	0.95469214	0.480918582
Zm00001d039683_T009	3.034546236	2.28745811	3.358212537	3.045021774	3.084911599	3.050136145
Zm00001d008569_T002	6.430479749	5.701056366	6.396733924	5.898109702	5.907681288	5.867673224
Zm00001d008569_T004	1.819228769	1.262459016	1.864750332	1.908638083	1.929381608	1.824784958
Zm00001d053580_T001	2.436458771	3.319294072	1.885297903	3.264806029	2.655038753	1.477911973
Zm00001d053580_T005	2.997098168	2.725285049	2.933054761	0.619944444	0	0.947722255
Zm00001d053580_T003	4.496568125	3.011130088	4.539142802	4.849078422	4.973192227	5.079086001
Zm00001d003614_T001	3.437043794	2.837931337	3.384517225	4.117299577	4.056238853	4.137323012
Zm00001d020770_T001	1.477554033	1.565068741	1.543224146	0	0	2.242167117
Zm00001d020770_T003	4.260227664	3.752873912	4.120519742	4.382526434	4.53874009	4.221720587
Zm00001d027443_T001	2.054906809	2.224392567	2.438996992	3.058657818	3.197201278	3.094580823
Zm00001d051339_T001	2.652328163	2.479512553	2.994442689	3.646607957	3.717586829	3.556698677
Zm00001d020331_T002	3.464141927	3.556161943	3.584080985	1.525223485	2.295472074	2.747162008
Zm00001d015449_T001	4.774663786	4.019031644	4.544583681	4.290206302	4.120567101	4.216030609
Zm00001d035111_T001	2.741920811	3.281676294	3.056672827	3.35726048	2.877299399	3.24130586
Zm00001d034000_T008	0	0	0.187746214	0.662932817	0.718647013	1.071069982
Zm00001d034000_T002	2.988470016	2.232810851	2.985686766	3.677186066	3.629187684	3.443542471
Zm00001d034000_T005	0.704749817	0	0.474348817	1.228848237	1.645591919	1.75642086
Zm00001d034000_T007	2.880758001	2.363463232	2.961200644	1.586495751	2.281555234	2.493993621
Zm00001d007679_T001	1.697004681	1.253089494	1.633002872	3.450304914	3.577073957	3.36475927
Zm00001d041067_T001	4.116756071	3.590342486	4.371651067	2.127834649	2.034507677	2.205878543
Zm00001d015692_T001	4.701677463	3.994216289	4.616851627	4.999516977	5.043740168	5.066537562
Zm00001d034782_T001	6.044411432	5.275425935	6.101631399	6.708915257	6.75043634	6.720199569
Zm00001d028007_T002	2.154521643	1.385709885	1.959274955	1.500123368	2.314010249	2.100934938
Zm00001d028007_T004	0.766888547	0.518504925	1.295110557	2.175195626	0.982298176	1.023508733

Zm00001d007836_T001	5.085855718	5.335538458	4.79476597	4.185035805	4.252051745	4.166682349
Zm00001d008582_T001	3.132610236	3.366192383	3.030787534	3.425109465	3.298663447	3.640807499
Zm00001d043277_T012	1.689740178	0	1.286747515	1.403540963	1.720458846	1.802146884
Zm00001d043277_T008	0	0	0	1.491695097	1.320294039	0.756035217
Zm00001d043277_T016	0	1.082249547	0.392593295	0	0	0.441405925
Zm00001d052640_T001	3.495560735	3.715757768	3.410821652	3.734687296	3.683871139	3.571510448
Zm00001d035052_T001	5.495643491	5.198518709	5.527193538	5.219854112	5.299879434	5.322374071
Zm00001d006496_T002	0.628856632	0.280818568	0.460428046	0.436436917	0.449156703	0.568409638
Zm00001d006496_T003	3.999164257	3.445826799	4.097455564	3.831075938	3.968388202	4.077609523
Zm00001d006496_T001	1.273132712	0	1.979294808	0.965045436	0.303025528	0
Zm00001d047090_T002	5.151263103	4.581107704	5.15797942	5.43365574	5.565804122	5.454157135
Zm00001d052881_T001	1.479583947	1.13344779	1.664602529	1.21346176	1.264678141	1.815008896
Zm00001d041692_T001	0.683997312	0.4169035	0.983232597	1.046632145	0.725643447	0.46986306
Zm00001d041692_T002	3.282535883	2.869526184	3.415572775	3.735000027	3.725381801	3.856845894
Zm00001d028946_T001	4.507958179	3.80241979	4.513774646	4.339286636	4.434327782	4.325175622
Zm00001d012605_T002	2.503165524	3.470552092	3.100456973	2.160351644	2.478605586	2.716258939
Zm00001d012605_T001	2.92069194	2.156234798	2.927020523	2.851798005	2.991270767	3.021173033
Zm00001d041610_T001	1.99051164	1.906560084	2.724449241	2.26430402	2.460988626	2.36652701
Zm00001d041082_T001	1.264396508	1.013430815	1.279588371	0.901225662	1.008260262	1.095248642
Zm00001d013702_T001	1.283249148	1.163602422	1.414369377	1.950639736	1.915528167	1.953164647
Zm00001d043012_T001	0.351378436	0.13922455	0.418102493	0.4105506	0	0
Zm00001d043012_T008	0.561044536	0.308567076	0.421456536	0.346525789	0.418727518	0.443816676
Zm00001d043012_T018	1.916280577	0.764505823	1.357913407	2.423399067	2.352385658	2.391143003
Zm00001d043012_T015	0.887501096	0.103586263	1.125702012	0.619445879	0.965060955	1.088727785
Zm00001d043012_T016	0	0.426884108	0.907664442	0.419601452	0.809511581	0.485112492
Zm00001d006388_T004	0	0	4.46514386	0	4.960067101	3.975066369
Zm00001d006388_T001	7.839801841	7.218130859	7.696640251	7.171622856	7.022251017	7.046014798
Zm00001d006388_T006	4.893852314	8.520244231	5.628080305	4.608345377	0	3.953696586
Zm00001d052972_T001	2.16168295	1.694777251	2.182388156	2.23880795	2.280230925	2.160472019
Zm00001d028608_T010	0.061463781	0.4566327	0.301525606	0.978126056	0.815926223	0.743290048
Zm00001d028608_T002	4.231938845	3.553690418	4.548026261	5.244584716	5.210671928	4.919327877
Zm00001d028608_T005	0.101781835	0.320677602	0.108666294	0.56506679	0.442655857	0.887195363

Zm00001d002668_T003	4.08041438	3.12780395	3.940986457	4.919274049	4.75913804	4.726500743
Zm00001d002668_T008	0.736050635	0.954377241	1.29887529	1.211495088	1.155790026	1.473035632
Zm00001d036671_T001	5.090955448	4.922869792	5.330042143	4.947667232	4.470066042	5.258654331
Zm00001d037808_T001	4.555784442	3.605697828	4.467717505	4.969420645	5.209606712	5.179941207
Zm00001d037808_T007	3.906935222	5.734186308	3.290059555	0	0	0
Zm00001d037808_T004	3.977283313	2.752227929	4.302907635	4.818210601	4.75690789	4.793773867
Zm00001d037808_T003	0.366895681	0.139121058	0.201316433	0.496711853	0.490389397	0.5862786
Zm00001d019364_T001	7.795961542	8.220280797	7.708761806	5.364013245	5.338283205	5.019011666
Zm00001d013721_T002	1.857633513	3.361354016	2.036510367	1.835196482	2.392952618	1.011537144
Zm00001d013721_T007	1.053251775	0.291116684	0.988912228	1.117909104	0.851771025	0.930629865
Zm00001d013721_T001	4.824263687	4.310966801	4.758394169	4.315323056	4.258636205	4.44997326
Zm00001d013721_T009	2.248218149	1.97252577	2.53617826	1.904282925	2.187050971	1.87770617
Zm00001d013721_T003	3.0181342	4.272412858	3.108212634	2.942118472	2.7841729	3.034805212
Zm00001d013721_T004	4.34171945	4.638122042	4.421489323	3.976083502	3.313564835	3.414011559
Zm00001d052327_T008	0	0.584597934	0	1.811655158	0	0.731649869
Zm00001d052327_T018	0.869035151	0.446478573	0.565691728	0.908041215	1.133962612	1.630936133
Zm00001d052327_T022	0.533612185	0.110669257	0	0.192256039	0.483572804	0.357449534
Zm00001d052327_T029	2.95867381	2.455654161	3.043089858	1.544576386	1.747564422	1.999012498
Zm00001d052327_T026	0.586220944	0	0.536806884	0	0.742871999	1.091864317
Zm00001d052327_T034	0.507373502	0.115346259	0.279892008	0.626253151	0.818657534	0.601924668
Zm00001d009481_T001	1.243413784	0.961856659	1.348096416	2.104472535	2.289092865	2.207875364
Zm00001d046998_T001	6.742163168	7.0918381	6.706041412	5.954498709	5.830459076	6.204853044
Zm00001d033128_T002	4.364844698	2.122139933	3.064389341	3.235111409	3.575652268	3.40137161
Zm00001d033128_T001	9.501648934	9.401614021	9.596374262	9.096389854	9.13731622	9.105688652
Zm00001d036107_T001	1.190604747	0.975387332	1.521294517	1.548973651	1.434816616	0.980852281
Zm00001d046893_T001	3.830659251	3.084517758	3.880369332	3.362230425	3.279353617	3.159719271
Zm00001d018445_T001	6.308909114	6.341708421	6.510257864	5.439784418	5.448205132	5.244363112
Zm00001d018445_T004	7.328141952	5.921575909	7.313467446	7.220037623	7.313459573	7.270544743
Zm00001d018445_T003	1.081143937	1.601941778	0.700849829	0.614826668	0	0
Zm00001d018445_T002	2.321479347	0	2.457533358	1.503966901	1.424538393	1.621204643
Zm00001d023929_T002	0.555816155	0.284877722	0.475710607	0.212554398	0.133017645	0.495613305
Zm00001d023929_T001	4.433375124	3.652929389	4.504476179	3.940371251	4.017191203	4.03275705

Zm00001d003275_T001	3.076319994	2.443603204	3.050071001	3.037901569	2.907980669	2.691485479
Zm00001d020414_T001	0.64249087	0.147096955	0.034894637	1.587310739	1.326179506	1.594172062
Zm00001d020414_T004	1.457307983	1.04377144	1.775224624	2.500245769	2.527306344	2.216165832
Zm00001d007077_T001	4.772619466	4.878323077	4.784898105	4.699095444	4.846507056	4.764203246
Zm00001d024364_T004	5.225543135	3.905507157	5.144585484	5.50687781	5.519113454	5.390673497
Zm00001d024364_T001	1.951066271	2.965318484	2.212141291	2.763176772	2.845705812	3.123849253
Zm00001d024364_T002	0.480942358	0.278719888	0.681998802	1.019994337	1.537944575	1.118438138
Zm00001d024364_T003	0.1219729	0.126346496	0.177930545	0.702617653	0.586889615	0.471667152
Zm00001d025067_T001	1.124041553	0.879452488	1.155481778	0	0.570406582	0.964434861
Zm00001d041430_T001	6.066269861	6.557557685	5.858919263	6.312393644	6.037383825	6.140211117
Zm00001d010055_T001	3.017277895	3.19092454	3.472640481	3.497097655	3.466903104	3.744395891
Zm00001d010055_T003	4.955368047	3.791474243	4.918466764	5.416573771	5.396940543	5.287572508
Zm00001d010055_T002	3.296885967	6.62522387	3.367138706	2.778942776	3.389435339	0
Zm00001d010055_T005	4.093280771	3.581073288	4.022444429	4.533563286	4.656564213	4.882904667
Zm00001d025044_T005	1.622733138	1.119414614	1.800153594	3.081529327	2.911869871	3.089717115
Zm00001d025044_T040	3.464951551	2.989036707	3.251199629	3.383430863	3.37095615	2.9066432
Zm00001d035672_T001	1.154749101	1.173905831	0.975985222	1.568137201	1.510112757	1.397575726
Zm00001d032103_T001	1.299686602	0.98205206	1.601775423	1.624552471	1.670676302	1.71779463
Zm00001d019669_T001	5.842658563	5.425399079	5.997021956	7.469628013	7.537410571	7.460511809
Zm00001d053564_T006	3.133384	1.23179058	3.020345202	3.323747052	3.294812752	3.501562708
Zm00001d053564_T002	4.847754734	4.091481425	4.882710781	4.419579745	4.460760515	4.372097647
Zm00001d019978_T001	4.954419259	4.137955279	4.796715897	5.073772814	5.067245381	5.049500649
Zm00001d019978_T006	0	0	0	1.568194612	1.090032283	1.548649191
Zm00001d019978_T009	2.66650211	0.684120331	2.471711367	3.047676579	2.564324752	2.108313682
Zm00001d019978_T010	0.405524088	0	0.514566681	1.348800659	1.96858183	1.221685946
Zm00001d019978_T003	0.439800771	0.373137018	0.481051926	0.952857613	1.005961146	1.07932554
Zm00001d049399_T002	7.02107489	5.977691285	6.992857736	5.656868409	5.912221328	5.744590314
Zm00001d049399_T001	5.400437204	4.686657282	5.362195702	5.261835306	5.171286143	5.116216479
Zm00001d048665_T002	2.613192627	1.933063073	2.711664055	3.427107177	3.534972091	3.407769862
Zm00001d016619_T002	3.45439465	1.518608153	2.061462745	3.172489596	2.334228441	2.791405059
Zm00001d016619_T001	6.363265238	5.841308184	6.52513583	6.135112182	6.345594796	6.256741144
Zm00001d020666_T020	2.519141799	1.9748637	2.740980001	2.073337655	2.060779616	1.159728634

Zm00001d020666_T013	0.710102199	0.823812935	1.406329312	1.992210819	1.479893283	1.290088168
Zm00001d020666_T015	1.082720299	0	0	0	1.254869133	1.354340442
Zm00001d025938_T001	2.861346009	2.271274167	2.432567689	2.589641739	2.366809256	2.20489568
Zm00001d052609_T001	3.119182346	3.229261511	2.476549927	2.792985668	2.263918687	2.571146471
Zm00001d037619_T001	3.31198098	2.518545462	3.408918741	4.271302931	4.559667826	4.605728285
Zm00001d037619_T002	3.878598033	4.003442938	3.931616026	5.163911757	4.818129544	4.928069718
Zm00001d029428_T001	0.851797405	0.513847459	0.5750441	1.02213057	0.81858228	0.608355077
Zm00001d026488_T001	1.246159965	0.638059932	1.022446649	1.77969467	1.146794011	1.27104254
Zm00001d007302_T008	3.155573903	2.364202046	2.786866267	2.463434915	2.606258859	2.137186424
Zm00001d007302_T009	0.172990515	0.985936088	0.399322056	1.201769343	0.791742403	0.592238389
Zm00001d007302_T013	2.882295	1.735691998	2.636958881	3.410131986	3.719958913	3.695591673
Zm00001d007302_T002	0.946955387	0.954300553	1.04074307	0.851290504	0.652475481	0.488653822
Zm00001d013625_T001	2.983857336	2.424440035	2.736281234	2.097739218	1.511257009	2.110801532
Zm00001d008913_T001	2.398314795	2.142458205	2.113808831	2.718467502	2.481675585	2.668973911
Zm00001d048603_T001	2.544903989	1.799473631	2.506848176	2.940563132	2.868415938	2.907134102
Zm00001d004664_T001	1.542577513	0.614081281	1.673105002	4.131855973	4.190761019	3.863027957
Zm00001d051584_T001	4.02535744	3.403194768	4.039282042	3.956633429	4.010718263	4.054544368
Zm00001d013204_T002	4.131899176	3.679318058	4.281851932	3.924326061	4.120848569	3.818647412
Zm00001d020811_T001	3.209109729	2.394870519	3.228048432	4.178143186	4.25627988	4.192010186
Zm00001d049496_T002	5.2284527	4.554186007	5.298810018	5.605779864	5.619766686	5.507281108
Zm00001d049496_T001	6.082733284	5.960981774	5.94466559	6.129157332	6.04666115	5.813742283
Zm00001d010862_T006	2.263991677	0	2.4733643	0	2.129080861	1.611293741
Zm00001d010862_T009	0.956973291	0.378600402	0.865393887	0.759234253	0.66971347	0.670277466
Zm00001d010862_T001	3.659304203	2.832547979	3.539465437	3.89538125	3.564133634	3.980433549
Zm00001d010862_T007	4.206511794	4.800540566	4.606424935	4.071446659	3.801573914	3.292031058
Zm00001d049965_T001	3.757051524	3.592247001	3.868458215	3.79389297	3.528692705	3.368332961
Zm00001d016545_T001	3.500139434	3.394538699	3.306842147	2.609420012	2.804599011	2.649443475
Zm00001d013566_T001	2.45030333	2.250609343	2.681234251	3.256013365	3.04806578	3.03879207
Zm00001d002156_T001	6.126807786	6.008106341	6.044034887	5.587383175	5.618799265	5.509490284
Zm00001d028296_T001	4.344986958	3.919687508	4.472302744	4.352498376	4.198040529	4.514872204
Zm00001d045582_T002	1.395230647	0.729069797	1.375792356	1.719946979	1.518992801	1.449366977
Zm00001d045582_T001	5.053956152	4.505051738	5.217215126	5.609114423	5.681117762	5.621052689

Zm00001d049294_T001	1.355758289	0.943355762	1.411505429	1.069734471	1.454349638	1.225140404
Zm00001d037412_T001	4.556541675	3.855372933	4.400345148	4.290236166	4.340767977	4.340145209
Zm00001d039731_T002	1.703258873	1.222482484	1.496575859	2.388927369	2.569381233	2.591783523
Zm00001d000165_T001	2.327919556	3.071972504	2.14556986	2.122432526	1.75456302	1.592811015
Zm00001d010529_T001	2.496635933	1.930914802	2.297705294	3.170393961	2.402873041	3.160138786
Zm00001d042582_T001	4.51325651	3.874409245	4.32063912	4.353166944	4.215282849	4.082373296
Zm00001d042582_T002	2.643300703	2.670253442	2.71523797	2.801258213	2.660781247	2.885117666
Zm00001d038847_T001	4.169098261	3.748508356	3.783490076	4.368002246	4.477995877	4.470384128
Zm00001d011425_T001	1.082103726	0.912770947	1.09591902	3.016633594	3.144698373	3.047690363
Zm00001d038921_T001	1.220336766	1.158903781	1.069839625	3.345520427	3.416720593	3.270300186
Zm00001d013040_T007	3.333902692	3.924540515	3.433893784	4.113473477	3.747194025	3.709666702
Zm00001d013040_T008	3.969450853	3.239335713	3.910659182	3.615152948	3.69436775	3.723108099
Zm00001d013040_T002	1.169113658	0.590386443	0.986157508	1.294509005	1.694955946	1.389279896
Zm00001d013040_T005	2.305724556	1.781090665	2.32782473	3.188132175	3.091602184	2.895154854
Zm00001d033169_T001	5.698621915	5.100596536	5.814133833	5.054568678	4.925747589	5.036279439
Zm00001d037125_T003	0.747725099	0	1.041601859	1.796831955	0	1.559151106
Zm00001d037125_T007	2.649521192	1.97337249	2.981286604	2.417547948	2.856509094	2.417682959
Zm00001d037125_T010	1.450154441	0.585458702	0	1.504981339	1.188906634	1.889401114
Zm00001d037125_T001	0.373248405	0.763807571	0.417859534	1.006601793	0.880520972	0.895524438
Zm00001d036337_T008	0.214916826	0.521266874	0.588885141	1.507096397	1.934670104	1.727927859
Zm00001d036337_T005	0.62046536	0.105320041	0	1.016245984	0.893314832	0.896264098
Zm00001d036337_T007	4.689649726	3.4319104	4.610854763	4.889709202	4.616157113	4.686381801
Zm00001d036337_T010	1.394293523	0.828053901	1.249681978	1.221882029	1.574499739	1.511310655
Zm00001d036337_T001	1.967494007	0.829577634	1.591362027	3.780977532	3.678071567	3.682678013
Zm00001d036337_T003	2.124729453	2.317883751	2.165204483	2.493211283	3.090856817	2.728696361
Zm00001d009160_T001	6.076986445	5.448887541	6.03970608	2.985923522	2.880367177	2.931698751
Zm00001d030034_T001	2.248025006	2.06620529	2.298223976	2.4395016	2.298111923	2.150861181
Zm00001d030034_T003	3.733781662	5.485973177	4.020137237	2.678976912	3.456550304	3.243903469
Zm00001d030034_T002	2.168483831	0.983082985	1.310921154	1.380408075	1.596869325	1.492361861
Zm00001d043478_T001	2.538901151	2.255429428	2.309753034	1.298165604	1.506030621	1.249044183
Zm00001d039514_T003	0	1.421870666	2.033419125	1.410337848	0	0
Zm00001d039514_T001	2.11622068	0	0	1.286073841	0	0.728062431

Zm00001d039514_T002	2.313257753	2.193044694	2.677650081	2.121670932	2.618124692	2.629012431
Zm00001d016083_T001	5.924425643	5.337624792	5.511501816	4.97442363	5.471976401	5.314745279
Zm00001d017274_T003	0.962250644	1.427127698	0.674697466	0.550925284	0	0.591394105
Zm00001d017274_T005	2.222301941	1.109748347	2.654467621	2.45678171	2.519641536	2.427751776
Zm00001d017274_T002	9.6244564	8.810068043	9.718100801	9.457289947	9.442947219	9.35127304
Zm00001d017274_T004	1.267791655	1.434632497	1.129425466	1.480713891	1.403003704	0
Zm00001d017274_T001	9.672690192	8.774893911	9.765000182	9.595495013	9.632982187	9.681619862
Zm00001d019949_T001	4.913182874	3.956808015	4.889028497	5.110549582	5.120559719	5.116646672
Zm00001d015251_T001	6.148216482	5.389885119	6.19434977	5.706442612	5.908835912	5.785093295
Zm00001d015201_T001	0.852075566	0.468709466	0.848670808	0.258360928	0.320721497	0.116652202
Zm00001d048898_T001	4.981648561	4.521253429	5.164185942	5.067223607	5.069888677	5.16381308
Zm00001d045314_T001	5.162873214	4.513158837	5.200013369	5.23146962	5.238555727	5.140937556
Zm00001d009097_T001	2.98882227	2.480704327	2.809372459	1.914908127	2.013937065	2.091686973
Zm00001d012485_T005	1.446768627	1.199537294	1.215418298	0.507829138	0.557172828	0.582648487
Zm00001d016877_T003	1.053194072	1.045669423	1.752501129	2.07855826	2.186933111	2.139020507
Zm00001d016877_T004	3.586494791	3.269202548	3.429029662	3.572226846	3.211316592	3.352252307
Zm00001d016877_T002	2.896241231	2.217498443	2.541560682	2.538226001	2.659200975	2.700928154
Zm00001d039638_T001	2.052042999	1.390405754	2.040663825	2.619432027	2.58672848	2.606296056
Zm00001d006257_T009	0.670182273	0	0.533009087	0	0.551390999	0
Zm00001d047708_T001	1.768330016	1.639240963	2.046438325	2.986304072	2.893259102	3.045101653
Zm00001d010403_T001	5.150649599	4.628674982	5.246408999	4.837544832	4.906519487	4.866605069
Zm00001d010403_T002	1.040440093	0.753126126	1.110180138	0.772176201	0.735269142	1.219242261
Zm00001d017931_T001	0.924070993	0.653934085	1.213974931	0.788957939	1.124197095	0.857342486
Zm00001d014000_T001	1.294330784	1.036297245	1.252999304	1.283422816	1.140143207	1.446501864
Zm00001d032245_T003	2.197152043	1.129780867	1.879150535	1.825437222	2.025893798	1.875578386
Zm00001d032245_T001	1.331459874	1.170242359	1.527433717	0.997538828	1.276665789	1.084445304
Zm00001d017960_T001	4.703030745	3.865786611	4.624802122	4.230632576	4.267358409	4.287355489
Zm00001d017988_T001	2.520057097	1.870240815	2.504649373	2.593712786	2.577260589	1.795989651
Zm00001d017988_T005	1.509240289	0.564095212	1.417770439	1.963574369	1.662359561	1.795291096
Zm00001d012463_T001	3.448053317	2.803826106	3.588682978	3.015889126	2.796257181	3.132113198
Zm00001d033377_T001	2.438428331	2.10803896	2.405138467	3.184609426	3.312160229	3.092981529
Zm00001d033214_T001	5.593467586	5.403275836	5.756022964	5.51604185	5.679299252	5.584801571

Zm00001d011878_T001	1.884512744	1.520148645	1.960903839	2.682748343	2.693673841	2.659761105
Zm00001d012831_T001	3.489212666	2.721443097	3.523912802	3.363213418	3.263341547	2.991676935
Zm00001d000110_T001	2.081698552	1.650928117	0	2.207358143	2.534643844	1.779224414
Zm00001d000110_T005	0.877159202	0.630731625	1.007346941	1.647946716	1.741520055	1.601261503
Zm00001d000110_T003	2.685443471	1.413554549	2.974773412	3.07017411	2.557428471	3.481759786
Zm00001d000110_T006	2.722708858	2.903994606	3.110364747	2.663083157	2.294648684	0
Zm00001d000110_T002	0.636813449	0.305209441	1.533083865	0.736440358	1.205782851	1.229536858
Zm00001d000110_T004	0	0.209045298	0.366828555	1.46987608	1.687703485	1.334578572
Zm00001d039710_T004	3.991911077	5.273330692	4.168156135	3.799235817	3.823609975	4.008369994
Zm00001d039710_T003	4.99285257	4.651116575	5.12521197	4.411837504	4.416041176	4.348498863
Zm00001d039710_T005	1.70332267	3.744530658	1.441726741	1.778690516	2.329706012	2.574590803
Zm00001d039710_T002	1.27108319	1.956558534	0	1.678474	2.257234249	1.268482303
Zm00001d039710_T001	4.825540918	4.864147953	5.236356723	5.200183616	5.267065497	5.18488276
Zm00001d006054_T001	1.136873191	1.648370641	1.022911762	1.546943341	1.579339473	1.846831861
Zm00001d036668_T002	1.55056334	0.791695734	1.782845082	2.123381469	2.518171272	2.61236689
Zm00001d036668_T005	3.330131121	2.761972199	3.340857044	3.13119995	2.882591209	2.754878312
Zm00001d036668_T006	1.71863737	0.810785279	1.402194438	1.95691049	1.357756923	1.710071774
Zm00001d036668_T004	0.98424226	0.93377581	1.378198635	2.127002582	2.232368505	1.771094097
Zm00001d019936_T001	4.909982294	4.687185183	4.952354023	5.687255965	5.723542425	5.603591661
Zm00001d001766_T001	5.238711972	5.458819655	5.3066322	5.622988637	5.770874123	5.908308044
Zm00001d052439_T003	3.21860667	2.028436192	3.165913546	3.529094045	3.295550831	3.488410248
Zm00001d053552_T001	3.580974555	2.819961597	3.575640289	3.921863116	3.994575561	3.895228449
Zm00001d037735_T001	1.400012671	1.127080526	1.664697636	1.590867373	1.890974255	1.741716349
Zm00001d048126_T005	1.185407981	1.073208757	1.180305747	2.032530413	1.906648972	2.522850469
Zm00001d048126_T008	3.376708099	1.786686684	2.961263814	3.795544522	3.910646136	3.236556295
Zm00001d048126_T004	0.672107575	0.517924697	0.689512082	1.024116099	1.57272187	1.186627961
Zm00001d048126_T006	2.781972755	0	2.622226397	3.184475656	3.268904735	3.201244281
Zm00001d048126_T010	3.629982876	6.038446256	3.876010601	3.274290739	0	3.668449459
Zm00001d049823_T001	3.549993279	3.465433581	3.659831762	3.547668339	3.921339589	3.374209034
Zm00001d044783_T001	4.35252839	4.829047688	4.692339928	3.922386547	4.082050999	3.946422734
Zm00001d015249_T002	3.613982425	2.783089281	3.296338756	4.151852162	4.086697671	4.179517261
Zm00001d010726_T001	5.775021824	5.036971233	5.485089848	6.211209791	6.049105169	6.267515255

Zm00001d005445_T001	3.281716794	2.511947424	3.192016422	3.633229963	3.50003105	3.227492648
Zm00001d015426_T001	5.29927613	4.658862748	5.426356447	6.01112548	5.959744194	6.038561969
Zm00001d037858_T001	3.645054399	2.751081037	3.583073137	3.770167157	4.00093159	3.906834329
Zm00001d023646_T003	0.608542438	0.251223417	0.156164887	0.510936609	0.56254577	0.312076127
Zm00001d023646_T001	0.532407733	0.625983944	0.987860729	0.932999979	0.767813253	1.1185903
Zm00001d022396_T002	0	0.267519019	0	1.315181306	1.891508235	1.218350929
Zm00001d022396_T001	1.941525909	1.205513879	1.881141474	1.579912415	0	1.924728148
Zm00001d046633_T001	1.708115707	1.287607634	1.616277231	2.189729061	2.195330273	1.97508279
Zm00001d046633_T002	2.245365747	2.381340968	2.554304214	1.546036056	2.36657177	2.273741153
Zm00001d049921_T001	2.147153303	1.807200339	1.716239839	2.091150067	1.521715631	1.490512113
Zm00001d041792_T001	4.993142377	6.307777899	4.750964187	4.008047588	4.049885395	3.687709083
Zm00001d018749_T001	2.967413956	2.321408631	2.867073102	3.483253291	3.616378636	3.546124241
Zm00001d033863_T001	1.364603247	1.218874762	1.592894713	0.904232821	0.915756455	1.110767462
Zm00001d014319_T001	6.588758553	6.826953758	6.546021481	6.009975818	5.988402459	6.201017389
Zm00001d013080_T001	5.241122012	4.555091436	5.26939082	5.361242375	5.32861677	5.387649183
Zm00001d012239_T002	2.172537119	1.272793615	2.232244157	2.329923245	2.6855565	2.590677463
Zm00001d012239_T003	1.295650153	1.195394217	1.766448871	0.962251384	0.720773144	1.685038603
Zm00001d034968_T004	1.065115926	0.493455713	0.820177328	1.349525505	1.347348739	1.092984741
Zm00001d034968_T001	0.963196656	0.585291398	0.782329718	1.464792668	0.810485058	1.119756557
Zm00001d009715_T001	0.845297344	0.9614233	0.486191253	0.685052063	0.665824787	0.782043651
Zm00001d013400_T002	1.524370141	1.033167126	1.365196438	0.399386593	0.395566284	0
Zm00001d049059_T001	8.708147946	7.95964228	8.754923335	8.341768967	8.365345074	8.34968131
Zm00001d028667_T001	7.061342104	7.341933584	7.263207988	5.943607068	6.260695157	6.336395567
Zm00001d012719_T001	2.458000017	2.299555909	2.597262752	2.172574561	2.275156377	2.286642551
Zm00001d018349_T003	3.327054106	2.791345458	3.021539246	3.725866337	3.397345032	3.763250298
Zm00001d018349_T006	0	0	3.13990656	0	2.513995474	2.039902714
Zm00001d018349_T004	0.635572389	0	2.05300878	0.658452807	3.487731237	2.259948248
Zm00001d018349_T008	4.325752439	5.375186787	4.08479572	3.042691084	0	3.253150324
Zm00001d018349_T001	1.308085312	0.669597379	1.218975787	2.06490354	1.997075219	2.127615122
Zm00001d043600_T001	3.877090571	3.27246295	3.911716985	4.380449498	4.295573605	4.374374039
Zm00001d014736_T001	1.054137197	0.918757165	1.170489145	0.962149936	0.69982078	0.909285404
Zm00001d019249_T004	2.495443948	2.455854813	3.200827922	2.817924248	2.849712493	3.134833873

Zm00001d019249_T007	2.329952513	2.909394287	2.27529824	1.780170233	2.245962592	1.863799327
Zm00001d019249_T003	2.246422681	1.04583225	1.029240132	1.515801249	1.133543799	1.198971201
Zm00001d015914_T005	3.659710421	2.97850519	3.680083062	3.542381633	3.615074888	3.557819325
Zm00001d045729_T002	6.733152336	6.713683678	6.723464445	6.816328165	6.844599684	6.833121675
Zm00001d045729_T001	0.501468699	0.123892614	0.633157758	0.517397656	0.455560006	0.457737162
Zm00001d032882_T001	4.4027247	4.56045867	4.396571697	4.271845156	4.169792027	4.283332206
Zm00001d023439_T002	2.101929714	1.720001284	0.806847829	1.561081695	1.749743202	1.332357348
Zm00001d023439_T001	3.707747406	3.189399514	3.924237591	3.586789599	3.639194185	3.612346836
Zm00001d037693_T002	1.350543639	1.373473379	1.696859616	0	0	0.759802664
Zm00001d037693_T003	2.321149118	1.572014087	2.063556093	1.657527512	1.662836227	1.443970462
Zm00001d012573_T001	0.480596025	0.314971409	0.403388805	0.389660419	0.223510283	0.690288354
Zm00001d034252_T003	1.154231273	0.831035694	0.837039004	0.558489043	1.303017342	0.854487237
Zm00001d034252_T001	4.444738219	4.634260493	4.865394431	4.31417039	4.303028378	4.131778122
Zm00001d036178_T001	3.145707613	2.498920735	3.350912599	2.790736804	2.770195389	2.390139371
Zm00001d036178_T002	1.318033337	0.931385033	1.153507689	0.759067181	0.483998883	0.660648464
Zm00001d034157_T006	1.377895572	1.051773007	0.945994917	0	1.95351855	2.388357084
Zm00001d034157_T016	0.622194764	0.501030419	1.244173449	1.191023118	1.567632571	1.438462132
Zm00001d034157_T017	0.25051536	0.084994911	0.0936751	0.628648564	0.619682508	0.642544472
Zm00001d034157_T022	4.391209076	3.8666833	4.339594997	4.045189044	4.18645785	4.349998582
Zm00001d034157_T019	2.074833533	0.445199102	2.493169003	1.881946084	1.653282482	1.822057889
Zm00001d034157_T010	0	0	0.286636342	0.222867608	0.339078079	0.415799762
Zm00001d034157_T013	1.475561205	0.722435422	1.164261098	1.616471094	1.737581638	1.407896549
Zm00001d034157_T003	0.849137787	1.421041747	1.2900628	0	0	0.51837802
Zm00001d034157_T001	0.312396821	0.22518607	0.394880668	0.79704453	1.122933739	0.679068824
Zm00001d034157_T018	0	0.135266925	0.298532807	0.713334216	0.41863254	0.557612026
Zm00001d034157_T008	0.114852062	0.067501049	0.160145725	1.186326866	1.036631333	0.998373166
Zm00001d017700_T025	0.214230516	0.212296648	0.56341296	2.48389418	2.633201477	2.656719908
Zm00001d017700_T024	2.367728061	1.524285882	1.58886212	2.802677343	2.076793369	2.031641153
Zm00001d017700_T013	1.162906719	0	1.633819907	2.824566045	3.105766735	3.421230033
Zm00001d017700_T005	0	0	0	1.153327384	1.315946416	0.478563911
Zm00001d053401_T012	4.016358854	3.010711103	3.819201889	3.778967264	3.988228132	3.324298802
Zm00001d053401_T003	0	0.213222839	0.297023508	0	0.554240107	0.700403317

Zm00001d053401_T005	1.809960137	1.343755733	1.661814802	3.468743998	3.296569882	3.713816987
Zm00001d053401_T007	0.619834606	0.179453724	0.402213543	0.536076779	0.452977011	0.516527566
Zm00001d033539_T002	3.721447035	3.218442381	3.626941364	5.804624977	5.858321058	5.880483088
Zm00001d018522_T001	5.034221395	4.220037439	5.286562789	5.342608895	5.118617458	5.258442718
Zm00001d018522_T004	0.990739983	1.010720411	1.378438932	1.03917841	1.5148055	1.070725917
Zm00001d018522_T003	3.995464077	0	3.133480013	1.372908649	2.899322213	2.689145936
Zm00001d018522_T002	2.431267791	3.121358456	1.924700028	0	0	0
Zm00001d016824_T002	1.277464093	0.571587503	1.141177059	1.286478438	1.1380405	1.268334378
Zm00001d016824_T001	3.091557504	2.70536576	3.198446849	2.919165766	2.835837793	2.933595109
Zm00001d008212_T002	1.63985218	1.676008278	2.529122038	2.614839622	2.49330122	2.646242165
Zm00001d004818_T001	0.788034994	0.607971764	0.999861495	1.249641328	0.761390812	0.808202154
Zm00001d036255_T001	3.376045977	2.777530182	3.247504058	3.811185752	3.85186715	3.826169346
Zm00001d037512_T003	3.281898507	2.678019156	3.375405795	3.391683872	3.437448282	3.439451733
Zm00001d037512_T002	1.069436147	1.14294271	1.383998952	1.159585271	0.825251589	0
Zm00001d030061_T030	0.919694753	0	1.448588598	2.066549057	1.929918222	1.672523555
Zm00001d030061_T028	1.504841038	1.608353184	0.950892388	0.692899389	0.727990138	0.934247752
Zm00001d030061_T014	1.863015465	0	1.631271481	0	2.414104946	0
Zm00001d030061_T001	0	0.792574697	0.699650237	2.44842288	1.041674044	2.657923715
Zm00001d030061_T002	0.346230751	0	0.492891976	0.307556759	0.757481592	0.29057303
Zm00001d030061_T026	0	0	0	0.485146505	0.32717802	0.516759507
Zm00001d048313_T001	2.578386158	3.055403053	2.60719607	2.70917901	2.809005245	3.320718754
Zm00001d031152_T001	7.975058364	8.20312431	7.898430322	8.008805987	8.251874657	8.156457127
Zm00001d002891_T001	2.032117069	1.321375725	1.961492202	2.082782964	2.231218569	2.22689104
Zm00001d038719_T001	4.609930679	4.195985488	4.166640497	4.517581714	4.316511801	4.528722894
Zm00001d033744_T001	4.762061471	4.771512605	4.868871831	5.031189435	5.067781448	4.857064394
Zm00001d014088_T002	1.809233736	1.536019569	2.269309686	1.874004102	1.880344253	1.978666077
Zm00001d017336_T006	0.965292253	0	0.999388168	0.860151563	1.122043825	0.851234526
Zm00001d017336_T004	4.502299474	3.317247911	4.094705038	4.868278579	4.536750387	4.44195297
Zm00001d017336_T005	3.347079531	2.653994141	3.768727205	3.777186867	4.43515424	4.240828417
Zm00001d017336_T002	1.085706097	1.55662168	1.137754662	0.791810738	1.339851143	1.178504316
Zm00001d049433_T001	2.152239568	2.472209083	1.964848838	3.997704289	3.910735156	4.463755644
Zm00001d034662_T001	3.540233389	2.937572644	3.629497793	3.536805144	3.543659693	3.650034987

Zm00001d043789_T006	1.695954183	0	1.457439324	0	0.723231761	0
Zm00001d043789_T001	0.346526923	0.238910339	0.493033443	0.299569389	0.196078211	0.205187293
Zm00001d043789_T012	0.446526214	0.185087597	0.490050461	0.060744691	0	0.236125201
Zm00001d043789_T005	0	1.024400536	0	0.671539765	0	0.555151577
Zm00001d009024_T001	4.090137578	5.072909094	4.688094735	4.177129515	4.165872234	3.823768515
Zm00001d011658_T001	3.806032498	3.170804297	3.717895614	4.056099659	3.895828993	3.980358416
Zm00001d025616_T001	3.279712418	3.931236566	3.074889522	2.939793763	2.923862458	3.237627806
Zm00001d002530_T001	3.798916287	3.823380896	4.189767324	4.860306138	4.868150544	4.820706918
Zm00001d018871_T001	1.7721441	1.235466677	1.733564763	2.822671012	3.016524311	2.767380215
Zm00001d016822_T008	0.579532571	0.36239919	0.493007816	0.956284194	1.125043349	1.280388029
Zm00001d016822_T021	0.131185131	0.288879433	0.655693455	1.038251443	1.279821304	0.459041251
Zm00001d016822_T001	0	1.145187671	0.465903438	1.575175317	2.480718026	0.612459134
Zm00001d016822_T006	4.417856902	3.639792806	4.328448673	3.238026188	3.783756792	3.448023838
Zm00001d016822_T003	3.32929025	1.15309062	2.735615971	3.681650194	3.967281417	3.534796338
Zm00001d025620_T005	0.674312397	0.736395328	0.936144535	1.532237154	1.754541641	1.756479786
Zm00001d025620_T001	1.553970878	0.78149317	1.459231201	1.693897266	1.752578198	1.537935632
Zm00001d025620_T006	2.996446236	3.102710699	2.666559599	2.644020071	2.527729209	1.363106582
Zm00001d017042_T002	0.472419144	0	0.945491594	2.560425408	1.826113198	2.71539394
Zm00001d017042_T003	4.337152742	4.032599968	4.643583321	6.320204434	6.210597086	6.267879502
Zm00001d017042_T001	4.968472301	3.691480789	4.81978384	0	3.293436113	0
Zm00001d033545_T001	1.435501653	4.306901189	0	2.489360275	0.939559093	0
Zm00001d023291_T001	3.262548767	2.542429925	3.268253521	3.926102397	3.935080071	3.960758656
Zm00001d044027_T001	4.048883962	1.409475119	4.065077485	5.451117721	5.635610115	4.563934133
Zm00001d041945_T005	4.5695558	3.914807312	4.594611487	4.989393909	4.852486264	4.857456654
Zm00001d041945_T002	5.742269468	5.074880918	5.666005968	5.378498063	5.469256711	5.382705498
Zm00001d041945_T001	1.494489343	1.282255342	1.119394693	3.50910623	3.731249183	3.589680685
Zm00001d041945_T006	0	0.103976948	0.538359368	1.978221993	1.995264163	1.726649016
Zm00001d045232_T001	0.744930902	0.468374782	0.965611428	2.906471191	2.914035757	2.77800099
Zm00001d026096_T001	4.656860602	4.36132945	4.67080454	4.554065273	4.851013942	4.79068072
Zm00001d005671_T002	1.828051463	1.296301749	1.541380525	2.021006332	2.45035058	1.840767754
Zm00001d005671_T001	0.486639212	0	1.229664208	0.934118644	0	1.152348289
Zm00001d048730_T001	1.156554534	0.854622076	1.190299424	1.321211763	1.223361381	1.138330213

Zm00001d006580_T001	0.766392471	0.589797996	1.192006003	1.186702749	1.511202348	1.358583057
Zm00001d006580_T010	4.04852637	2.965126728	3.884569204	4.207780351	4.190791907	3.988741757
Zm00001d027638_T001	1.680712324	0.964605641	1.99557352	1.348451697	1.154226088	1.194515761
Zm00001d038312_T001	0.72438921	0.301992598	0.56238458	2.14970778	2.457346861	2.328280414
Zm00001d007032_T001	4.916606433	5.153430227	4.955896199	4.532212775	5.097102604	4.490990941
Zm00001d007032_T002	2.749039054	3.592085028	2.594168239	2.516964441	2.259676837	3.126559277
Zm00001d006355_T018	2.614519278	1.434849168	2.816871428	3.179242869	3.280252606	3.288205425
Zm00001d006355_T001	0.271154324	1.011944264	0.165257233	0.546060264	0.543165238	0.505139923
Zm00001d006355_T021	0.249725661	0.185269051	0.176509162	0.913429055	0.511149204	1.113801831
Zm00001d006355_T012	0.787627207	0.250725146	0.646605077	0.686545348	0	0.522267717
Zm00001d015704_T001	4.009417585	4.444694561	3.884445927	4.036164467	3.993584247	4.079069484
Zm00001d015965_T001	3.966323959	3.376187985	3.999484866	3.955915444	3.741653365	3.733197371
Zm00001d029604_T001	2.973326382	2.902366528	3.393583691	2.267234943	2.003755492	2.061705703
Zm00001d014489_T010	1.743939722	2.572570543	2.198005	2.339236602	2.785052689	2.948654293
Zm00001d014489_T006	0.279722074	0.170573748	0.43543231	0.759950056	1.014833731	0.542402684
Zm00001d014489_T005	1.685942839	1.301337129	2.28302655	2.785522932	2.269561938	0.693305403
Zm00001d014489_T001	2.891124007	1.421744124	2.646112646	3.367818763	3.410836844	3.393639425
Zm00001d013428_T003	5.589705011	3.174771521	5.418815306	5.385670822	5.167126747	5.123557816
Zm00001d013428_T002	5.45269601	5.161439209	5.495885271	5.706993539	5.772495163	5.78819509
Zm00001d032282_T012	3.940621574	3.254259935	4.034495968	4.201820302	4.251789294	4.227749623
Zm00001d032282_T005	0.766241494	0	0.626549408	1.643598097	1.44433365	1.432221307
Zm00001d032282_T006	0	0	0	0.91468621	0.837693019	0.645694281
Zm00001d032282_T003	0.465860611	0	0	1.554616355	0	1.791653647
Zm00001d043206_T001	1.650407517	0.965277475	1.64142395	1.833581532	1.643432783	1.908610801
Zm00001d037573_T001	6.780945102	6.972084779	7.176337166	7.522444077	7.517407002	7.468532603
Zm00001d037573_T002	7.688216901	7.785709727	7.513814384	7.49838996	7.577571557	7.65506626
Zm00001d010066_T002	4.447361057	3.95471782	4.52149328	4.84511184	5.074985527	4.998718949
Zm00001d010066_T001	2.79519071	1.604756915	1.474711187	2.5449784	2.260804019	2.007143104
Zm00001d010066_T003	3.13597083	2.310731864	3.938656347	4.256511851	4.281011379	4.110363076
Zm00001d043835_T006	1.051589273	0.584342008	1.042293426	1.751274749	1.310466933	2.010095918
Zm00001d043835_T010	2.324997191	2.669499193	2.726630272	1.394859821	1.472813186	0.828878508
Zm00001d043835_T009	1.527632887	0.581608668	1.452508986	1.965566364	1.80980501	1.72254078

Zm00001d043835_T008	0.789774315	0.484279428	0.712332528	0.732465453	1.329214892	1.222661768
Zm00001d039144_T002	5.319499692	4.312952784	5.278276269	5.939162271	5.946994278	5.865881559
Zm00001d022636_T001	3.618483235	2.851633921	3.639756811	3.442974538	3.645511223	3.57009711
Zm00001d023398_T001	1.547219809	1.066694737	1.720366469	0.908360242	0.805829806	1.069122635
Zm00001d025825_T001	2.883110896	2.186542076	2.800728474	3.014114228	3.148066112	3.019560662
Zm00001d023566_T002	1.50468598	1.199762162	1.638438579	1.385812576	1.608919924	1.045526846
Zm00001d023566_T003	1.47012033	0	1.08498267	1.978015465	1.487130262	1.744529259
Zm00001d042769_T001	4.572647481	4.96382583	4.443764504	5.7329742	5.648590638	5.708803809
Zm00001d043128_T001	1.50558153	1.269921238	1.073184074	2.737836431	2.603394664	2.776967872
Zm00001d040285_T001	2.008061218	1.639395181	2.178384799	2.96266932	2.858180372	2.247730686
Zm00001d020219_T001	2.705763195	3.510554364	3.215956238	3.131422883	3.007344429	2.384229718
Zm00001d020219_T004	3.072108888	4.489056368	0	0	2.859442053	3.579477661
Zm00001d020219_T002	5.985686561	3.824728145	6.174112972	5.958687983	5.8324858	5.694611347
Zm00001d018504_T001	5.115787821	4.321800122	5.042417717	4.92133121	4.792245097	4.741643389
Zm00001d050929_T004	1.332232446	0.378054327	1.398093662	1.412478053	1.466818162	1.890171552
Zm00001d045650_T001	4.38956633	4.497495361	4.11084729	4.086975807	4.338550422	4.174056809
Zm00001d015546_T001	3.677164638	3.048611984	3.689082404	4.41864846	4.48933105	4.388360735
Zm00001d053445_T001	5.012352789	5.068077877	4.768830719	2.360608685	2.105857733	1.86628251
Zm00001d044596_T001	2.716790988	1.977289083	2.598545293	3.696534945	3.831609446	3.822697113
Zm00001d005159_T006	5.075135835	0	4.941243483	4.706157481	4.727648767	4.816163095
Zm00001d005159_T001	3.172461273	4.408122436	3.773473781	1.60718873	2.881471595	2.693972848
Zm00001d051016_T002	4.062181909	3.549419542	4.060916544	3.88274692	4.022423389	3.968771009
Zm00001d038267_T001	6.958677023	6.712925274	7.033469266	6.245395907	6.20714549	6.125799054
Zm00001d021629_T003	7.066439798	6.058804492	7.118336291	5.968077063	5.981554587	6.042814991
Zm00001d021629_T001	1.116777942	0.679174244	1.084487484	1.412372365	1.45813891	1.475093186
Zm00001d021598_T007	0.831835094	0.403074623	0	0.15244824	1.191234786	0
Zm00001d021598_T010	0.895041206	0.703129057	1.065395152	1.557369405	1.412313285	1.54125413
Zm00001d021598_T002	1.349057795	0	1.828608836	2.527724456	2.55982828	2.446593437
Zm00001d021598_T008	0.107998467	0	0.94750084	1.155137836	0.393260225	1.446326113
Zm00001d021598_T005	3.067420679	2.545832987	3.37694781	3.270859155	3.329423158	3.478864408
Zm00001d021598_T001	1.469100874	0.950714752	0	0	1.438785733	1.597735199
Zm00001d021598_T003	1.502450779	0.936413686	1.202752457	1.473274151	1.757493113	1.155336698

Zm00001d011716_T001	2.43942713	1.608169585	2.3800021	2.455287761	2.486799311	2.311649296
Zm00001d009030_T001	1.941058972	1.505293917	1.754594659	1.718184053	1.774576661	1.482680002
Zm00001d021646_T005	3.050164884	2.401072284	3.20598658	2.835873357	2.89417556	2.745976457
Zm00001d021646_T004	1.09338304	0.61916037	0.441558906	0.943083401	0.33379352	0.208873052
Zm00001d021646_T014	0	0.346077535	0	2.619915812	2.801247037	2.874187306
Zm00001d033730_T001	6.764701004	7.770468697	6.724425952	6.530281926	6.413581	6.61028109
Zm00001d024841_T002	1.702360553	1.172975159	1.736402256	1.968352165	1.88372403	1.772860283
Zm00001d024841_T001	1.38881939	1.098402039	1.60814687	1.43213256	1.516586059	1.658064292
Zm00001d025178_T003	3.208047574	1.87162644	2.853412265	2.488908534	1.06982588	2.160788881
Zm00001d025178_T002	1.425752541	1.054693609	0.914275956	0.931441012	1.465508027	1.58658462
Zm00001d025178_T004	0.884779199	0.721174015	1.237988266	1.169382446	1.040159503	1.134975939
Zm00001d025178_T001	5.4131882	4.928144923	5.537271183	5.329139782	5.38282825	5.300989558
Zm00001d040040_T001	3.573876546	2.99221372	3.702792166	3.182228908	3.078083415	3.102722623
Zm00001d034547_T001	3.864625846	2.885520677	3.902141225	3.331375743	3.193591987	3.127692538
Zm00001d005834_T001	3.963250396	3.713850302	4.062192618	3.375567882	3.428216507	3.012361282
Zm00001d053591_T002	3.845433847	3.183913781	3.924397898	3.525578688	3.755182102	3.578534098
Zm00001d017270_T001	4.834635833	4.328889516	4.833983669	3.803674864	3.706271607	3.748592615
Zm00001d029151_T002	4.538730469	3.95709043	4.683785371	5.685549996	5.748502747	5.818377073
Zm00001d029151_T001	1.716711325	1.983999393	1.261079432	2.17162862	2.273682081	1.950880447
Zm00001d029151_T003	0.478699543	0.248605269	0.17839468	1.146264195	0.846982656	1.075392291
Zm00001d031196_T001	3.304795026	3.784953054	2.850233753	1.627105776	2.288871757	2.85148481
Zm00001d021487_T001	1.899648587	1.330236554	1.849775134	2.127618754	2.214189476	2.068013452
Zm00001d008248_T001	0.822703231	0.081405079	0.621613525	0.515770978	0.945808438	1.908005464
Zm00001d008248_T005	4.47734854	3.803008342	4.529695033	5.046615296	5.072941627	5.126275658
Zm00001d008248_T003	1.776002891	0.709958445	1.466626076	3.361763026	3.153082673	3.567683918
Zm00001d008248_T002	2.290105277	1.555867188	2.184812517	4.644075232	4.708361685	4.646485384
Zm00001d050244_T005	0.73214327	0.449026719	0.642450205	0.576044143	0.652590205	1.16197408
Zm00001d050244_T017	4.231388168	3.932418304	4.351723088	3.792151841	3.940684524	4.064148645
Zm00001d050244_T002	3.073729077	1.852442368	3.084403462	3.961663145	3.927019764	3.845940603
Zm00001d050244_T011	1.040700292	0.366144813	0.591295477	0.772320645	1.332749746	1.015383382
Zm00001d050244_T006	0.234696283	0.04690729	0.278954151	0.638849555	1.046478488	0.566275473
Zm00001d027596_T005	2.867470312	2.409694787	2.634690839	4.7407467	4.894568628	5.128099934

Zm00001d027596_T007	0.564932213	0.189236294	0	2.257868435	2.571503773	2.276624701
Zm00001d027596_T008	0	0	0.675840315	1.43780779	0.940124644	1.183392502
Zm00001d027596_T003	3.790542481	2.827280657	3.864180894	5.007449431	4.913902483	4.317444669
Zm00001d027596_T002	2.148720043	1.253179678	2.111709086	3.288488207	3.232853359	3.215035064
Zm00001d023838_T008	0	3.019312993	3.353006061	2.885566751	0	3.532519695
Zm00001d023838_T013	2.670664746	2.216161174	2.237075395	1.531064001	0	0
Zm00001d023838_T017	0.252886711	0.146914545	0.518951018	1.063340716	0.985167406	0.329874464
Zm00001d023838_T019	4.395067874	0	3.827915641	3.938741397	4.337939465	3.877137503
Zm00001d023838_T005	0.621593834	0.202545607	0.351202015	0.424285765	0.823583889	1.267792854
Zm00001d023838_T001	2.434424068	2.2836895	2.348437816	3.215758572	3.598708686	3.300733482
Zm00001d017851_T001	7.788434085	8.535064222	7.577873887	8.20247239	8.141372439	8.234253686
Zm00001d006312_T001	4.529796839	4.780115207	4.288414969	4.067477213	3.989834204	3.897778963
Zm00001d011796_T008	1.046109644	0.629569249	1.159012324	1.528164707	1.409850896	1.217638336
Zm00001d049326_T008	3.3622091	1.49124306	3.813806634	3.058765514	3.173611183	3.282853131
Zm00001d049326_T003	2.446842189	2.591673912	1.739589345	3.292653962	3.595646753	3.348264576
Zm00001d049326_T002	0.70254496	0.416333899	0.767860701	0.882514717	1.201456963	1.01101824
Zm00001d039926_T001	2.813210053	2.145127985	2.714297619	3.182659408	3.199799218	3.019044992
Zm00001d012338_T001	6.934876517	6.155675797	7.014128738	6.175477076	6.100085861	6.118597151
Zm00001d048110_T001	5.282318995	4.3816721	5.24433902	5.278060561	5.664214216	5.157572251
Zm00001d020937_T001	0.851110567	0.511301039	0.934896893	1.184847103	1.320692042	1.238761796
Zm00001d039039_T002	3.041112938	2.407002165	2.907665019	1.970113715	1.891644712	1.950564715
Zm00001d026012_T001	3.443253227	3.538203648	3.553475767	3.084860079	3.065303806	3.38135869
Zm00001d053513_T001	2.515957135	2.092135369	3.110985226	2.170088498	1.742953033	2.148121608
Zm00001d032604_T003	2.293341347	2.168426385	2.431836607	2.792169441	2.839964826	2.493405242
Zm00001d032604_T006	1.048599604	0	0.956452967	0	1.583257671	1.707128061
Zm00001d025694_T002	4.34100747	4.258508674	4.172854703	3.338636346	3.045487355	3.368499757
Zm00001d025694_T001	4.61414944	4.250212981	4.797208299	4.518101825	4.445266784	4.340035511
Zm00001d035115_T001	3.768308312	2.871917358	3.740195776	0	0	0
Zm00001d035115_T008	0.770894151	0.390760121	0.863722823	0.730090377	0.372574385	0.638206397
Zm00001d035115_T006	3.488231944	1.922885915	3.445458821	2.828173965	3.039276865	2.488862273
Zm00001d035115_T004	0.875010158	0.783356071	0	0.584571958	0.147100864	0.931552206
Zm00001d035115_T005	0	2.434759515	2.052502843	3.922092878	3.937358989	4.125582585

Zm00001d035115_T007	1.589551621	0.130403762	0.757085563	1.209878157	0	1.42368089
Zm00001d049369_T001	2.899528912	2.662139403	3.390643613	2.243909409	2.140459003	2.48214767
Zm00001d049369_T002	5.085771308	4.434873848	5.072203749	4.05407041	4.094296885	3.973298459
Zm00001d019034_T001	8.600033618	9.089889436	8.632532926	8.641216494	8.642972525	8.655090352
Zm00001d025830_T003	0.480249609	0.285314619	0.48105296	1.444074921	1.523011871	1.359678588
Zm00001d025830_T002	0	0	0	1.598619132	1.722473457	1.791253968
Zm00001d025830_T009	3.661523534	2.705676286	3.716677527	4.000951594	4.026185588	3.994892123
Zm00001d025830_T059	0.170821118	0	0	0.31129152	0.469271268	0.551783737
Zm00001d025830_T065	0.629611211	0.179597673	0.324746098	1.291938262	1.08518736	0.952625058
Zm00001d034733_T003	0.821553489	0.307912257	0.836604446	0.830058948	1.309158721	1.081860431
Zm00001d034733_T001	4.687290516	3.918128778	4.875863939	5.106781256	5.033664649	4.99946421
Zm00001d017361_T001	2.809084501	2.301424054	2.492983726	2.758647498	2.654938426	2.418661952
Zm00001d049709_T002	0.263802436	0.177248099	0.305896999	0.578125263	0.677003916	0.254814119
Zm00001d049709_T001	4.181391458	3.372614223	4.144920045	4.678804225	4.754700795	4.739003026
Zm00001d049709_T004	0.433855737	0.147072201	0.515899121	0.429926562	0.416812725	0.491001334
Zm00001d012988_T002	4.480482092	4.591480398	4.494812457	4.04606065	4.121787865	4.124051978
Zm00001d030952_T002	0.495098521	0.322250068	0.586171934	1.29915848	0.862407748	0.738853423
Zm00001d030952_T003	3.560495844	2.62384252	3.38512148	3.065010944	3.338462924	3.075708208
Zm00001d044838_T001	0.553677026	0.333388238	0.537675765	0.850297772	0.674234648	0.474427736
Zm00001d044838_T002	3.466028385	3.050466174	3.106685668	3.325064277	3.287870592	3.2665401
Zm00001d019981_T001	6.548029206	5.590454563	6.346604733	6.571848552	6.541558483	6.592992081
Zm00001d019981_T003	2.181151541	1.508966584	2.07286486	2.61503933	2.393431028	2.831474973
Zm00001d019981_T002	5.646628721	5.881380359	5.770173052	5.815196851	5.847689324	5.626298861
Zm00001d016911_T001	3.954687581	3.15748761	3.868284951	4.332579911	4.518252993	4.372266655
Zm00001d008846_T007	2.187911819	0	0	2.256529136	0	2.425333614
Zm00001d008846_T003	1.714701392	3.111320323	0	1.24296523	2.513332066	1.557314014
Zm00001d008846_T001	4.948922288	3.573957958	5.114923088	5.752676027	5.743961581	5.756939692
Zm00001d009448_T001	0.448824851	0.126123107	0.137012924	1.94010735	2.195572805	1.981813207
Zm00001d002756_T001	1.749845298	1.576147206	1.814704221	2.08351192	1.651781439	1.7488459
Zm00001d046919_T001	3.703115875	2.944543267	3.91357276	4.885644838	4.991076622	4.988181632
Zm00001d006328_T001	5.036918455	4.199709167	5.071254428	5.488991223	5.46292376	5.461802706
Zm00001d053101_T001	1.997793876	1.344070031	1.797704475	1.585818727	1.757161961	1.568990339

Zm00001d038299_T001	0.739870564	0.738172048	1.201748645	0.83458502	0.734510637	0.997053173
Zm00001d038299_T005	2.355066741	2.254949533	2.121265778	2.46542914	2.848057819	2.828305801
Zm00001d038299_T002	2.276434728	0	1.64804799	1.654052818	1.690333057	1.518408237
Zm00001d034371_T001	0	0.04949834	0.259676234	0.694085879	0.850545819	0.654787464
Zm00001d034371_T002	3.712485951	2.781025179	3.792992225	3.714871264	3.733520187	3.670344438
Zm00001d040061_T002	4.876688488	4.241164963	4.991131194	5.539435968	5.59081906	5.543764384
Zm00001d039115_T001	4.738205671	4.440857144	4.673099065	3.723774786	4.066999911	3.831030524
Zm00001d044205_T001	2.514135898	2.680430361	2.240647714	2.036016903	1.819376754	1.808231814
Zm00001d035918_T001	3.115213569	2.596167806	2.784529109	2.544446561	2.674744914	2.721518781
Zm00001d020363_T003	1.157956266	0	0	1.846733996	1.791253968	0
Zm00001d020363_T002	2.639891993	1.935475212	2.752509692	3.238675138	3.106912589	3.177804285
Zm00001d020363_T001	2.669138593	2.425690246	2.629986022	1.408804234	1.842517498	1.823981232
Zm00001d033928_T001	3.663375819	2.537877253	3.632037948	3.653395995	3.524624768	3.597658693
Zm00001d033928_T008	0.357059856	0.229315969	0.526233109	0.561753321	0.598038323	0.553193367
Zm00001d033928_T006	0.650657042	0.290338288	0.496861126	0.316506081	0.63405326	0.533732774
Zm00001d033928_T010	0.404923825	0.501799869	0.096662621	0.829656376	0.16707784	0.89292487
Zm00001d007793_T001	3.172568161	2.556089456	2.691027123	2.332286877	2.768705613	2.765573783
Zm00001d040638_T001	4.691533104	5.499561146	4.794593034	3.582510843	3.561283851	3.732699339
Zm00001d036423_T001	1.3626354	0.873759662	1.585022131	0.800264198	0.716106795	1.123814497
Zm00001d036423_T002	0.826237289	0.383725855	0.770560986	1.019742473	0.650114741	0.270339959
Zm00001d028255_T001	0.608766671	0.450842262	0.423722299	0.176728713	0.434083203	0.824065577
Zm00001d028255_T006	0	0.578341712	0.903892062	0	1.380784296	1.172056051
Zm00001d034883_T003	3.885660553	3.240545753	4.025420697	3.774095665	3.811430339	3.938925123
Zm00001d034883_T004	0.239681542	0.113944483	0.29376951	0.188990796	0.353857356	0.342361311
Zm00001d006861_T005	1.128245882	0.960067565	1.073469283	0	1.402734181	0
Zm00001d006861_T014	0	0.297122142	0	0.533830434	0.563383671	0.474291702
Zm00001d006861_T011	0	0.433836513	0	1.182818131	1.478043006	1.086620741
Zm00001d006861_T019	0	0.020619539	0.455097028	0.800596372	0.719816028	0.70016447
Zm00001d006861_T009	1.22498735	0.831857789	1.440010818	2.158092033	1.774350628	1.782515924
Zm00001d006861_T035	0.748241377	0.51898827	0.87614639	0.576540519	0.802600217	0.876539342
Zm00001d006861_T007	0.277231375	0.300838304	0.308606682	0.548620094	0.803573407	1.069174204
Zm00001d006861_T028	0.544806829	0.158119831	0.391041791	0.346319269	0.618472663	0.5002116

Zm00001d006861_T008	0	0	0.522176304	1.725721092	0.900586693	1.607065129
Zm00001d006861_T015	0	0	0.072805535	0.476882461	1.10992549	0.391198007
Zm00001d006861_T004	0.742729749	0.404308051	0.749558295	0.799973379	1.400540115	0.82029662
Zm00001d006861_T001	0.768245314	0.313229596	0.562862238	1.029512253	0	1.182488272
Zm00001d006861_T013	1.136289182	0	0.742546096	1.210689966	0	0.711053441
Zm00001d006861_T034	0.315205656	0	0.171652636	0.855650903	0.926871606	0.759942389
Zm00001d038171_T001	3.706514197	3.086114904	3.977114485	4.160550353	3.943320407	4.071444771
Zm00001d014610_T001	1.431679115	1.398373904	1.840184034	0.954753176	1.215606538	0.605116899
Zm00001d016841_T002	3.739593341	3.667154244	3.894182353	4.091676226	4.073233698	4.35599904
Zm00001d016841_T001	2.015128213	1.397136485	2.35733298	2.447502762	1.985112745	2.604373119
Zm00001d026032_T001	2.283261299	2.051020173	2.20907806	2.978738003	2.676586066	2.744378775
Zm00001d043941_T001	3.686423208	3.3310925	3.779534258	3.198714627	3.192744305	3.349947079
Zm00001d011846_T001	5.027954329	4.788617332	5.122275095	3.741743957	3.806115218	3.726888314
Zm00001d025904_T001	2.635091969	1.958532393	2.72337835	2.846220898	2.739431216	2.487650727
Zm00001d010877_T003	0.96626138	0.553135357	0.517867266	0.992070113	0.911343376	1.193339659
Zm00001d046456_T007	1.795302734	0.6399406	1.281265006	2.369299241	2.935907951	2.137419255
Zm00001d046456_T006	0.414668122	0.337045387	0.544916597	0.731542133	0.704262895	0.556277351
Zm00001d046456_T002	0.581807245	0	0.745811284	1.460422279	1.174561099	1.657611654
Zm00001d046456_T008	3.049703946	1.943963697	3.355372664	3.360236016	3.146049574	3.45614956
Zm00001d046456_T001	4.210603943	3.612936536	4.131311421	4.727682472	4.674649338	4.640378569
Zm00001d022338_T005	3.002087151	1.802290014	2.684666607	3.652649857	3.356651904	3.42710208
Zm00001d022338_T004	4.908377488	4.076348387	4.985114704	4.34203405	4.605129466	4.147451368
Zm00001d022338_T001	2.276272427	1.600427242	2.293071142	3.111375581	2.798516748	3.598479192
Zm00001d022338_T006	0.090834465	0.027890077	0	0.276849182	0.31633693	0.250189101
Zm00001d022338_T002	1.096249032	0.566177062	0.997361785	3.073432435	3.082553748	3.127638067
Zm00001d009826_T001	3.720044966	3.812452839	4.101841157	2.981420511	2.748789237	3.000951504
Zm00001d049956_T002	4.350501915	3.77769534	4.230704041	4.041016974	4.108734448	4.125995783
Zm00001d013029_T002	1.069334401	0.972916117	1.053128718	1.314618227	0.668916064	0.994173468
Zm00001d025656_T001	1.077575259	0.426663018	0.847814154	0.766275423	0.852699633	1.013687356
Zm00001d011997_T001	1.223465181	0	1.213128255	1.094919077	0.444176191	0
Zm00001d011997_T003	0	0.613221393	0	0	0.969758704	1.098825116
Zm00001d033004_T006	0.429334226	0	0.212893014	1.224447827	1.279665623	0.64804661

Zm00001d033004_T004	2.006053454	1.987494416	0.987750881	2.774178549	2.36987655	2.694695249
Zm00001d033004_T001	1.794668303	1.243242546	1.845200178	3.348917622	3.256730805	3.163773076
Zm00001d033004_T005	4.002766419	0	3.874164284	4.382083971	4.829703152	4.752800069
Zm00001d033004_T002	2.76007635	3.161758559	3.216086034	2.578597013	1.863875034	0
Zm00001d052898_T001	3.995690552	5.022000212	3.918801427	4.775818159	5.115168364	5.078313896
Zm00001d015183_T003	0.120554992	0	0.162688277	0.677884343	0.700559568	0.575512745
Zm00001d015183_T001	0.745560047	1.038912319	1.358659569	1.430981025	1.736565479	2.232840622
Zm00001d015183_T009	2.549208568	1.428520305	2.61919207	3.082381046	2.394699063	2.804112844
Zm00001d005698_T001	6.390899502	6.908404427	6.454335242	6.826216477	6.970144347	7.054353355
Zm00001d041375_T001	6.010215113	6.051688668	5.785749654	5.305233413	5.158620028	5.221790525
Zm00001d046444_T003	5.07121276	4.155957075	5.21945858	4.323463027	4.474950273	4.52682196
Zm00001d046444_T001	0.928670486	1.372057795	1.280682591	1.507020768	0.97478112	0.881821213
Zm00001d006553_T001	4.472880365	3.576911359	4.560231876	3.956382338	3.954117751	3.84173104
Zm00001d052136_T001	2.45101509	2.170954398	2.842250766	2.357414907	2.555793582	2.392025007
Zm00001d045323_T003	0.394727046	0	0	0.50871767	0.506005731	0.352411649
Zm00001d045323_T009	9.41284824	8.775545451	9.487537812	9.274497608	9.277994668	9.231496856
Zm00001d045323_T001	4.491833283	3.831222004	4.453102226	4.113031998	4.297678299	4.265120372
Zm00001d045323_T007	4.258479879	6.704105467	4.145279396	4.346432887	3.985420825	3.98065874
Zm00001d045323_T002	0.038490967	0.118023434	0.418259045	0.489432982	0.890727616	0.721782112
Zm00001d026398_T006	4.207409062	3.534207932	3.924502986	4.31108738	4.864477676	4.35515641
Zm00001d026398_T007	6.255268089	4.403039437	6.366389766	6.34565073	6.335361018	6.438371439
Zm00001d026398_T009	2.216387191	0.915205749	2.215615856	2.521771904	1.700584868	2.579412129
Zm00001d026398_T004	1.65025677	3.911097127	3.117922233	2.45815545	1.711742897	1.915947241
Zm00001d026398_T010	1.511908219	2.533887731	0.871864934	1.822884299	2.311648424	1.338964593
Zm00001d026398_T002	0	0	2.064833887	1.210477394	0.343231561	1.052883964
Zm00001d018199_T073	3.105241248	2.741669327	3.236638182	2.098013212	2.240147608	1.953635877
Zm00001d018199_T002	0	0.231024365	0.867183241	1.14633915	1.382423786	0
Zm00001d018199_T062	0.105845667	0	0	0.678639854	0.370332818	0.638538206
Zm00001d018199_T018	0.82233042	0	0	0.943949824	0	1.94636855
Zm00001d018199_T011	0.0945185	0.142457878	0.080634471	0.743935415	0.344773969	0.574708984
Zm00001d018199_T017	0.370139724	0.409787368	0	0.375187378	0.162201011	0.480567074
Zm00001d018199_T050	1.280117763	0	1.455054406	2.877955437	1.858804986	2.361518813

Zm00001d018199_T069	1.382823271	0	1.125172328	0.185624279	1.188762344	1.005570298
Zm00001d018199_T013	0	0.680703324	0.471209315	0	1.837434274	0
Zm00001d018199_T070	0.79510279	0.380181963	0.397696741	0	0.452132559	0
Zm00001d032679_T001	4.680379273	3.756956552	4.615647997	5.118716576	5.029909387	5.097277767
Zm00001d023243_T004	4.547481294	3.744090466	4.642003677	3.965657784	3.999002934	3.861211301
Zm00001d050225_T001	6.430745611	6.274631999	6.34985461	6.286704274	6.342019644	6.345716596
Zm00001d011447_T005	0.718870549	1.864333948	1.124888559	2.22650144	1.525050551	2.149490907
Zm00001d011447_T002	2.621666971	0	2.351254322	2.56217354	2.525161081	2.518731513
Zm00001d011447_T001	0	0.048511025	0	0.189185679	1.542268947	0.505603378
Zm00001d013241_T003	0	0.562021102	0	0.988364759	1.787810642	1.157349684
Zm00001d013241_T004	2.119651327	0	2.697518079	3.115884056	3.149777517	3.932569295
Zm00001d013241_T006	0.398430275	0	1.15370094	1.36438529	0	1.115846278
Zm00001d013241_T011	0.63234826	0.03644988	0.062794536	1.890710887	1.812542584	1.767969572
Zm00001d013241_T002	3.667599357	3.155893624	3.257911116	4.643065376	4.656292308	4.267376539
Zm00001d038698_T009	0.619341637	0.396098096	0.223240889	0.886035797	1.045178732	1.231552218
Zm00001d038698_T007	0	0.464197746	1.162992412	1.780801834	2.33373056	1.632850761
Zm00001d038698_T002	1.257232136	1.280216966	1.837890779	2.575773629	2.871447839	2.474398402
Zm00001d038698_T006	0.352077123	0.218755134	0.615230769	2.896887358	2.764897486	2.914359968
Zm00001d038698_T008	0	0	0	1.532238151	0.946551214	1.164636338
Zm00001d029391_T003	0.21514801	0	0.21113932	0.727291411	0.66090398	0.659694439
Zm00001d029391_T002	0.639889679	0.328283861	0.796098497	0.711575079	1.115281664	0.960737067
Zm00001d007910_T001	2.006847705	2.51952857	2.314799758	1.106133206	1.708266267	1.396594096
Zm00001d033374_T001	4.86033723	5.704158545	4.854852828	5.474448374	5.122996585	4.999982462
Zm00001d021518_T002	1.678179652	1.108619462	2.065554734	1.374312825	1.797251262	1.218703685
Zm00001d053706_T002	1.574458564	1.001470078	1.735497916	2.523109506	2.548240306	2.470511876
Zm00001d023801_T004	0.836928358	1.573039992	2.305898457	2.388851344	2.313667278	2.281748688
Zm00001d010497_T001	2.972040077	2.262452401	2.7325347	2.991442935	2.861063872	2.624057601
Zm00001d048282_T003	4.02871863	3.116669168	3.897099054	3.273064809	3.128201662	2.727944409
Zm00001d048282_T002	2.19260892	1.726085269	2.583324834	3.062857209	3.117240555	3.301851726
Zm00001d046472_T001	6.265584237	6.292333414	6.280255223	6.192590043	6.250479509	6.155462612
Zm00001d046472_T002	2.508150736	1.781107036	2.463618795	3.271128172	3.233482245	3.180403621
Zm00001d032157_T001	4.365302489	3.831957681	4.130326656	4.484499147	4.572210411	4.644340335

Zm00001d018863_T002	2.954782391	2.120834638	3.007790401	2.749428287	2.666916075	2.532270573
Zm00001d018863_T004	0.393524934	0.098707906	0.171382349	0.241515617	0.428975279	1.063878408
Zm00001d027967_T001	4.517208849	4.308019472	4.728044956	3.855600177	3.921542777	3.831598705
Zm00001d046397_T001	2.967074885	2.298993146	2.97419191	2.620960524	2.403711371	2.675162845
Zm00001d009236_T001	2.992016438	2.004239616	2.392975142	3.029294914	3.084638677	3.40621664
Zm00001d009236_T002	1.620131714	0.532550365	0.447665941	1.038964979	0.729352639	0.184497397
Zm00001d009236_T004	0.160383272	0.492422368	1.580575816	1.303203847	1.199596969	0.416136054
Zm00001d009236_T003	1.753443657	3.668688396	3.147723652	2.34768578	2.88166599	2.562678129
Zm00001d019180_T001	3.153144787	3.430772335	3.162002287	2.946075604	3.065237965	3.029587343
Zm00001d021347_T002	3.744574788	2.018413765	3.731197256	3.900074134	3.826646104	3.861308884
Zm00001d021347_T005	0.318064583	0.273808278	0.49553349	0.277252803	1.164070544	0
Zm00001d021347_T001	0	1.854708637	0	0.982934816	0.737352762	0.872340229
Zm00001d021347_T016	0.614597722	0.111669697	0.575012141	0.993759128	0.41095865	1.192836763
Zm00001d051442_T002	2.619193948	2.25646543	2.982496426	2.435882177	2.384645556	2.584702071
Zm00001d030936_T001	3.899362627	4.527472252	3.793470084	3.545138828	3.132012815	3.297847892
Zm00001d007027_T001	3.306902356	3.873249591	3.746254176	2.451362793	3.067671583	2.360843222
Zm00001d045183_T001	7.00381668	6.216051277	7.057749893	6.087526722	6.055628944	6.053579962
Zm00001d051864_T015	0.905822221	0.829613352	0.247690588	1.065174533	0.546977902	0.843458109
Zm00001d051864_T006	0.729429214	0.197734574	1.244574137	0.721562527	0.916105127	0.443882434
Zm00001d051864_T001	0.270790846	0	0.551560313	0.486565077	1.035139646	0.598125054
Zm00001d038907_T001	2.484527181	2.255335758	2.320924785	1.831201123	1.938531584	2.168707494
Zm00001d021032_T004	1.536174778	3.849063105	2.121599326	2.610336036	2.339060971	2.357036765
Zm00001d021032_T001	3.760232445	2.511311667	3.734151399	3.780304534	4.142337053	3.621210505
Zm00001d021032_T002	3.955880953	4.524073019	3.945028936	4.387218044	3.203492486	4.184039666
Zm00001d045199_T024	5.907998497	4.695596182	5.776421925	4.703735782	5.067729055	5.048258466
Zm00001d045199_T004	0.413925474	0	0	2.676899916	2.670475765	2.781493589
Zm00001d045199_T022	4.496369158	3.145182289	4.404361397	4.943519913	4.946102889	4.861270964
Zm00001d045199_T017	3.279705436	1.810021854	3.515588076	2.69596064	2.786550989	1.751953392
Zm00001d045199_T005	0.41181995	0.415040385	0.79694572	0.939985533	0.95267351	1.34116087
Zm00001d026512_T001	3.184433761	3.190030395	3.357269349	3.437745349	3.118053511	3.015556444
Zm00001d052801_T023	1.093844766	0.598647237	1.255719462	0.627944828	0	0
Zm00001d052801_T016	0	0	0.07961773	0.494567169	0.528341269	0.797865473

Zm00001d052801_T024	0.064082665	0.056888682	0	0.921529257	0.797163259	0.750783133
Zm00001d052801_T009	0.628103525	0.768852522	0.090862913	1.419078234	0.73357908	1.281931436
Zm00001d052801_T001	3.00674055	2.208228192	3.29771395	3.592930104	3.682192686	3.455086899
Zm00001d034878_T001	2.098635151	1.558932737	1.899264587	2.233429785	1.924345066	2.111237683
Zm00001d013847_T002	1.370727854	0.872610517	1.433678438	1.66158316	1.758318529	1.533408356
Zm00001d013847_T003	2.212237808	1.774198373	1.687950673	1.292454309	0	1.796115529
Zm00001d013847_T001	0.542767176	0.23400828	0.554534826	1.018399165	0.843729039	0.73300024
Zm00001d047979_T005	1.025504416	0.913429821	1.157550829	1.17031031	1.237449913	1.19324187
Zm00001d007675_T006	1.480525714	0.497662434	1.349096304	1.751456437	2.360860636	2.235932576
Zm00001d007675_T002	1.205861031	1.255291641	1.049976416	1.506552183	1.954685441	1.520478069
Zm00001d007675_T007	2.953340864	2.301555456	3.246563598	2.705366866	1.595759046	2.066362028
Zm00001d050140_T001	4.059475468	3.6526806	4.235091338	3.230729015	3.404728768	3.31899876
Zm00001d035519_T001	7.663750491	7.514388463	7.727368103	7.951649277	7.867152111	7.878414377
Zm00001d036382_T001	2.307102643	1.370482922	1.895689227	1.252841913	1.713836559	1.709376665
Zm00001d020748_T001	3.237658855	3.070210869	3.606820975	3.395569985	3.227215832	3.475997047
Zm00001d020748_T002	4.9017784	3.962586871	4.981912095	4.335906738	4.141170355	3.99677748
Zm00001d044469_T008	1.25257673	0.943021119	0	2.03986342	0	0
Zm00001d044469_T002	3.795637922	3.243094904	4.120496186	3.215919906	3.677729901	3.786742401
Zm00001d044469_T006	2.18986029	0	0	0	2.29006457	2.0841119
Zm00001d011891_T001	5.051403383	4.326032561	5.093829938	5.075927307	4.976643852	4.960374273
Zm00001d044228_T001	7.541248437	6.907276934	7.597024921	8.099977787	8.141306597	8.094940595
Zm00001d024339_T002	5.404944869	4.2800966	5.367056875	5.567297828	5.481768568	5.4635452
Zm00001d024339_T003	2.244595143	1.946222178	2.502145988	2.961794997	2.617687084	3.130084932
Zm00001d024339_T001	0.620976706	0.260381015	0.620498204	0.821531449	0.95843291	0.854341214
Zm00001d033488_T001	5.091574048	4.469033128	5.005337733	4.603671146	4.589701117	4.596498333
Zm00001d053313_T002	0.855610243	0.996240492	1.118929116	1.414487367	1.914014128	0.46912224
Zm00001d053313_T006	0.829704268	0.446017967	1.326614428	0.80672412	0.943409027	0
Zm00001d053313_T003	0.906829809	0	0	0.854945956	0	1.618219858
Zm00001d053313_T005	0	0	0	1.405780023	1.157160156	1.715587671
Zm00001d039013_T001	3.009216191	2.503092488	2.964962846	2.208668014	2.633443301	2.660478474
Zm00001d039013_T002	0	0.552264917	1.321473863	1.413725132	0	0
Zm00001d031476_T001	2.807344205	2.67083875	2.66816906	2.162142026	2.709556649	2.313244981

Zm00001d012933_T001	3.138758614	2.732091593	3.332277137	2.547932703	2.545728229	2.786825502
Zm00001d025765_T001	1.632324061	1.643151059	2.563932486	2.706543151	2.853125977	2.652970104
Zm00001d036987_T001	3.089062158	2.354960992	3.109210765	4.069559797	4.05013989	3.949465831
Zm00001d017117_T001	6.902591481	6.220896144	6.900135279	6.801819839	6.776514426	6.833260954
Zm00001d052396_T001	0.6970692	0.240543921	0.685709658	0.864171482	1.088219633	0.91805033
Zm00001d052167_T001	0.581550824	1.625261136	1.326252002	5.448013857	5.338671962	5.298344305
Zm00001d051394_T001	2.597509483	2.496788281	2.077785446	1.867569943	1.899627321	1.776446824
Zm00001d007062_T001	4.672644615	4.120044074	4.768981584	4.980220252	4.922230204	4.984317464
Zm00001d050637_T002	2.515075576	1.767893321	2.03652162	1.416740857	1.660721929	1.31716396
Zm00001d048400_T001	1.411066612	0.956335497	1.410663477	2.489471262	2.48830008	2.281627634
Zm00001d027901_T001	4.43734221	4.66415632	4.497200446	8.923302994	8.965043239	8.986446271
Zm00001d014100_T001	7.67973606	8.24455863	7.846853719	6.793316205	6.773692462	6.757445042
Zm00001d038142_T002	0.780756069	0	0.55485993	0.984734433	0.793524707	0.454663379
Zm00001d038142_T011	3.701114741	3.256360504	3.681799945	3.577218164	3.838844594	3.771232521
Zm00001d038142_T017	0.392716376	0.192172698	0.420645163	0.77009578	0	0.768645067
Zm00001d038142_T020	0.348243752	0	0.430775545	0.39334151	0.429446714	0.588648203
Zm00001d038142_T027	0.71672142	0.336164539	0.737234199	0.748539377	0.813401552	0.474801506
Zm00001d038142_T004	1.279925279	0.981014551	1.176687231	1.807503306	1.68489547	2.054100026
Zm00001d038142_T023	1.859739017	0.657562267	1.217528532	1.92310277	1.690983334	1.055201903
Zm00001d038142_T013	0.629599089	0.380839142	0.710787262	0	0	0
Zm00001d038142_T016	1.382245587	1.26827149	1.957587774	1.874381898	1.56707372	1.751349741
Zm00001d029444_T001	6.686323933	2.398889638	7.153073287	7.692505473	7.30129478	7.360544119
Zm00001d009813_T004	0	1.315178987	1.420740008	0.98346246	0	1.420147661
Zm00001d009813_T005	3.135271523	3.148207857	2.906145973	2.819937489	2.143021758	1.919331691
Zm00001d009813_T001	5.314264341	4.51060753	5.278755119	5.534070507	5.52788178	5.446147991
Zm00001d009813_T003	0.230927254	0.622373775	0.907006776	0.861639405	0.803794082	1.208592171
Zm00001d016669_T001	3.744940586	3.018476259	3.603765312	5.034326044	5.046178416	5.058857318
Zm00001d041255_T001	3.742277472	3.030816132	3.765018785	3.929360586	4.112026662	3.903751244
Zm00001d034520_T002	1.722016533	6.000028561	2.843577702	2.931383709	3.105005345	2.081307241
Zm00001d034520_T003	5.876909969	4.981635959	5.793052773	6.128531053	6.098194498	6.070534814
Zm00001d040190_T001	7.203711893	6.65926428	7.262062119	7.054259321	6.969909418	7.019867221
Zm00001d013609_T006	1.535552845	0.456006007	1.046782294	1.085173081	0.837024467	0.478142438

Zm00001d013609_T003	0.615691249	0.296641826	1.019965169	0.750732548	0.849124173	1.098578561
Zm00001d013609_T001	0.114749472	0	0	0.387855488	0.55048503	0.370562711
Zm00001d013609_T007	4.327249388	3.681324894	3.976729213	4.290296039	4.467463719	4.23780713
Zm00001d013047_T001	4.085459499	0	3.752389907	2.993104598	3.681474341	3.769251683
Zm00001d013047_T005	2.735862015	2.533192037	0.96647772	2.316795388	2.5745661	1.598631517
Zm00001d013047_T004	4.947784475	4.284504065	5.043802449	5.474185286	5.46792343	5.56252028
Zm00001d013047_T002	0	2.862446234	2.278831376	3.342754553	2.250939448	1.954407022
Zm00001d047802_T001	1.748538948	1.42843133	1.896821693	1.922313564	1.867025446	1.986687979
Zm00001d011373_T007	0.292031353	0	0.376833792	0	0.335650177	0
Zm00001d011373_T013	2.134739757	2.549051304	2.240168373	0	0	0
Zm00001d011373_T005	2.270794733	0.825136778	2.273753982	3.824807967	4.020434372	3.935790681
Zm00001d011373_T011	0	0.072422781	0	0.781750814	0.723139999	0.784485557
Zm00001d011373_T001	0.379943619	0.976781535	0.399759539	0.518302476	0.689407415	0.361605556
Zm00001d011373_T006	2.695509494	0	2.732646921	3.764184877	3.294486361	3.66415467
Zm00001d011286_T001	4.976976697	5.479603444	5.021015841	4.814167961	5.296901344	5.236607302
Zm00001d011286_T002	2.310949938	2.662744643	2.453723005	2.880823814	2.137516311	2.62255745
Zm00001d022546_T001	6.652843984	5.888222811	6.623853857	6.715648852	6.791140222	6.78474306
Zm00001d022546_T004	0.918534313	0	1.474503017	3.251568681	2.776335854	3.440336597
Zm00001d051492_T001	4.485365447	4.73908433	3.465794149	3.62232879	3.876200821	3.729513508
Zm00001d049082_T002	6.447839942	5.787175362	6.368989031	6.507572178	6.52554278	6.432662121
Zm00001d049082_T004	0.128829197	0.215771771	0	0.347510324	0.335104753	0
Zm00001d049082_T006	0.083872346	0	0.238983688	0.456446613	0.334934337	0.435224263
Zm00001d027790_T001	2.678856157	2.402853672	2.186348103	3.101410223	3.289908811	2.833627675
Zm00001d033528_T003	5.767943017	5.26915283	6.098587845	6.56511928	6.400139784	6.482492033
Zm00001d033528_T007	1.04246574	0.366565616	1.06244642	2.325689775	2.273237469	2.281764412
Zm00001d033528_T006	5.261860499	3.837626722	4.917499974	4.855857328	5.290955769	5.100799149
Zm00001d033528_T002	1.067779125	0.595735657	0.974717989	1.908857091	1.830061789	1.695879818
Zm00001d006776_T001	2.588408587	2.785818979	1.473609258	2.241908504	1.899272323	1.235200732
Zm00001d003612_T001	3.423241458	3.114792607	3.507865537	3.696139634	3.84664184	3.907700778
Zm00001d004478_T002	1.410858276	0.761400174	1.279417212	0.96493088	0.652056892	0.775769497
Zm00001d022439_T001	0.569869215	0.258228246	0.414914865	2.903668829	2.624002605	2.744582645
Zm00001d013005_T004	6.016266245	3.674433985	5.96536545	5.512812981	5.347684044	5.501819323

Zm00001d013005_T001	0.384048701	1.115119829	0.437462131	0.967655573	0.437090282	0.414746045
Zm00001d013005_T003	5.54359505	5.505983921	5.472265534	5.436018316	5.654728415	5.469815923
Zm00001d007758_T006	0.566129315	0.098446509	0	0.68615267	0.445423727	0
Zm00001d007758_T003	5.201585365	4.086364278	5.210549358	5.535945129	5.713756897	5.668197715
Zm00001d007758_T008	2.626626032	1.808278363	2.990860855	3.839936617	3.60530148	3.453142338
Zm00001d007758_T001	1.547677839	1.950850221	1.591296913	2.163409205	1.928443475	2.005779027
Zm00001d007758_T013	0.290222672	0.678704749	0.472052038	0	1.321625097	1.17960659
Zm00001d024409_T001	1.379649239	0.886111517	1.373834156	2.013432959	1.842136092	2.231681848
Zm00001d032168_T003	2.252500738	3.384312342	3.081617776	5.006233878	4.365729206	4.599622668
Zm00001d017571_T001	1.677708481	0.99154706	1.733196829	3.313102591	3.434238297	3.330755431
Zm00001d038810_T001	5.301071271	6.238335546	5.074718597	6.957949041	7.195964293	7.041726483
Zm00001d039706_T001	4.401862524	3.618681382	4.445255592	4.525341896	4.488307729	4.413914646
Zm00001d049109_T001	2.933883049	2.199183905	2.80767805	2.856829564	2.968837073	2.604293168
Zm00001d017772_T001	3.639875328	3.255816732	3.647251066	3.284795716	3.515620874	3.212997104
Zm00001d017772_T003	0.614761662	0.730375628	1.473804052	0.844947194	0.918584687	0.642785659
Zm00001d005391_T001	7.395937074	6.731767494	7.421549249	6.353805338	6.497859483	6.365224597
Zm00001d042601_T001	2.555850014	2.380747177	2.647102638	2.621020092	2.876273915	2.863292462
Zm00001d049383_T002	3.470003549	2.758661994	3.517987919	3.321358118	3.445636665	3.484968956
Zm00001d049383_T001	0.983904568	0.466369226	0.514374789	1.263863717	1.67674221	1.630645901
Zm00001d020071_T001	2.344299317	1.677937548	2.245406823	2.677932814	2.395894441	2.592207048
Zm00001d040527_T001	3.02054096	2.678373487	3.12430398	4.302657346	4.476299181	4.481094692
Zm00001d029035_T001	0.866869216	0.119143659	0.588888978	0.844708626	0.858299348	0.940167502
Zm00001d011518_T001	2.802183289	2.17045197	2.866530989	2.862315894	3.054292646	3.107119885
Zm00001d051383_T001	3.684447134	3.105836451	3.725855106	3.364024384	3.612882176	3.213776682
Zm00001d016664_T001	3.551019694	2.5998543	3.557595853	5.45395266	5.447683263	5.40930298
Zm00001d027544_T002	1.890798033	1.377570235	2.062308601	2.055993845	2.41465297	2.175208084
Zm00001d018177_T001	7.628308566	7.221571558	7.673935053	8.082935415	8.008578778	7.9435753
Zm00001d016926_T001	1.615431364	0.756845673	1.443469801	2.035341677	1.973559113	1.824000384
Zm00001d016926_T002	0.556075228	0.967030604	0.382662826	0.508772424	0.743714868	0.323320489
Zm00001d031562_T001	5.515173624	6.277743857	5.531425068	5.319875267	5.39972082	5.454023816
Zm00001d024425_T004	5.034532415	4.154094089	4.924414336	5.279547849	5.357418144	5.47095045
Zm00001d024425_T007	0.721986795	0.643157064	0	2.967384442	2.670677661	1.695282977

Zm00001d024425_T005	2.030194097	0.937042261	1.552969178	3.64873808	3.575190326	3.488572721
Zm00001d048442_T013	1.668753626	0.758946127	0.876423039	0	1.474072566	0
Zm00001d048442_T001	0.528827331	0.293045532	0	0.48263767	0.638698522	0.713228623
Zm00001d048442_T010	3.156662445	2.775313343	3.250161201	2.362140493	2.562303734	2.891156678
Zm00001d048442_T002	1.099304631	0.135611041	0.26388174	0	0.867615803	1.005931693
Zm00001d048442_T004	1.210721756	0.942290788	1.815905326	3.339432166	3.215024188	3.299254184
Zm00001d048442_T005	0.884690125	0.894941127	1.974696332	1.29074405	0	0
Zm00001d041036_T001	4.642511895	4.045878457	4.641141544	4.3323499	4.418890409	4.269838451
Zm00001d032272_T001	3.561045881	2.92179734	3.415687828	3.233611394	3.175952795	3.053417399
Zm00001d044088_T003	1.954525021	0.967530158	0	2.273076002	2.045368877	1.924336324
Zm00001d044088_T004	0	0	1.166118322	1.511210953	1.019999317	0
Zm00001d044088_T002	0.548715765	0.998532033	0.956957684	1.763420073	1.744668749	1.538717434
Zm00001d044088_T001	4.435866246	3.668566776	4.508052336	4.431631985	4.570934887	4.418748294
Zm00001d001806_T003	1.063354523	0	0.994002531	1.503684562	1.423437256	1.290075189
Zm00001d016681_T001	4.132638448	3.655400719	4.147386077	4.912925397	4.856005937	4.795003586
Zm00001d037648_T004	1.344201868	0	1.040818103	2.446032529	2.744363705	2.528997813
Zm00001d037648_T012	0.553902088	0	0.181187806	0	0.498009802	0.413546425
Zm00001d037648_T011	1.482578301	0	1.767594208	2.858382506	2.25630902	2.371577541
Zm00001d037648_T010	1.082507765	0.219110889	1.070517104	1.256673145	1.8429169	0.523091184
Zm00001d037648_T013	3.336304671	2.685120931	3.120655346	1.243625824	1.823260228	2.908233024
Zm00001d041571_T001	8.601745169	8.01749924	8.638282216	7.80494453	7.79372071	7.870541254
Zm00001d002595_T003	1.811391701	1.393868105	1.754519835	1.466453804	1.691222813	1.955889692
Zm00001d026435_T001	2.897651293	2.484884942	2.725415505	1.837527928	1.611844222	2.18033503
Zm00001d010603_T001	5.772382198	5.594130758	5.875773036	5.697250114	5.813007032	5.580560561
Zm00001d010603_T002	3.95453228	4.086570216	3.896808618	3.943886641	4.018896022	4.283532014
Zm00001d034900_T003	0.398595542	0.116323498	0.380076653	0.636519293	0.579613664	0.715499815
Zm00001d034900_T001	2.107769207	1.830435853	2.233276994	2.54770498	2.684030762	2.765887739
Zm00001d034900_T002	1.984786192	1.670349083	1.91466019	2.516023218	1.978508668	1.518618727
Zm00001d034900_T004	2.057078676	1.791924483	1.804712784	2.378253302	2.258876765	2.04816084
Zm00001d045785_T001	4.067109999	3.628968002	4.215174885	4.202304715	4.233850106	4.149965499
Zm00001d010667_T001	1.920716327	1.583050144	2.08498845	1.912042771	1.618591062	1.991052489
Zm00001d032040_T001	0.731754123	0.554179191	0.809800483	0.77361915	0.786141318	0.80645275

Zm00001d047882_T002	1.958005205	1.048401514	1.303865481	2.037229334	2.576943397	2.619378263
Zm00001d047882_T005	0.448916805	0.235376605	0.44632823	0.819805499	0.601729791	1.052395026
Zm00001d047882_T007	2.621336651	2.315365955	2.728185015	2.576951376	1.633324254	1.062389774
Zm00001d047882_T003	0.597412941	0	0.663631536	1.309940415	1.57082186	0.998474265
Zm00001d047882_T001	0.419140815	0.346102504	0.211094453	0.2842322	0.306383577	0.541898384
Zm00001d033850_T001	6.141383802	4.647565376	6.350472159	6.078478909	5.9164942	6.233367543
Zm00001d033850_T019	8.187160501	7.987760504	8.153540561	7.36272389	7.273640833	7.142597449
Zm00001d033850_T013	7.190377588	0	6.929196663	5.97726284	6.096100739	6.226106415
Zm00001d033850_T015	7.274634253	7.069388519	7.548581257	6.545756272	6.495261106	6.455996574
Zm00001d033850_T017	0.344625142	3.482715234	1.682173242	1.282842458	0.921119421	1.292397765
Zm00001d033850_T024	8.557538207	6.728084041	8.633582349	6.953783629	7.008569557	6.564585732
Zm00001d033850_T003	5.900330533	6.716434005	5.840072017	5.109709823	5.289922381	4.619712788
Zm00001d033850_T018	8.053355993	0	8.12854556	8.10395043	8.089031238	8.265301848
Zm00001d025773_T002	6.401997117	5.222461077	6.421687378	6.374592787	6.389866487	6.403177824
Zm00001d002859_T001	5.160061437	4.672869115	5.285338924	4.72351362	4.753148916	4.921770782
Zm00001d018151_T003	0.953850022	0.470043258	1.021878368	0.909884446	0.612103338	0.723799682
Zm00001d002473_T001	2.130814264	1.965437442	2.336712704	2.836261674	3.001435849	3.062285457
Zm00001d007043_T002	3.458491331	0	3.304435108	3.981435033	3.981904242	3.867092283
Zm00001d007043_T004	4.857643664	4.684501444	4.940075722	4.318505645	4.246265635	4.272581043
Zm00001d023825_T003	2.513399785	2.466786584	2.884930767	3.289288557	1.998601351	2.308341069
Zm00001d023825_T006	4.842258863	3.612198902	4.887590532	4.701082074	4.859323122	4.84953371
Zm00001d023825_T001	0.429683445	0.464010541	0	0.794617154	0.717550749	0.177542802
Zm00001d041716_T005	2.542769404	1.517616356	1.630882563	3.116811038	3.308106433	2.681831984
Zm00001d041716_T008	0.354876387	0.28542471	0.731963476	1.448750861	1.240977359	1.271271478
Zm00001d041716_T001	1.036435813	0.515069519	1.205088426	1.739993661	1.837192442	2.191384514
Zm00001d041716_T009	0	6.351811852	4.395873265	2.052417285	0	3.235676978
Zm00001d041716_T006	5.714148043	4.672074187	5.692884217	5.576561802	5.583338194	5.482033117
Zm00001d027651_T001	4.755959934	5.633777921	4.670040436	5.315641435	5.109722985	5.361692714
Zm00001d033028_T002	1.357321718	0.885321334	1.65000028	2.942175725	2.100555202	2.991161178
Zm00001d033028_T001	2.040912765	2.679304657	3.066556117	3.861485072	3.862739406	3.712279695
Zm00001d033028_T003	4.036823926	2.450876566	3.605985658	3.377896543	3.951644487	3.585824734
Zm00001d030159_T002	6.022264446	4.584311518	5.951701597	6.131090303	6.20779337	6.198815561

Zm00001d030159_T001	6.503502141	6.113966834	6.654877021	6.207268681	6.158655107	6.054047372
Zm00001d014721_T001	3.222634104	2.70745853	3.387249895	2.951064779	2.955956627	3.083972732
Zm00001d052885_T001	2.510961666	2.283476453	2.312262626	3.640846592	3.674986425	3.565726818
Zm00001d052885_T002	9.00878405	8.767501229	8.971022943	8.163841483	8.116150352	8.197225492
Zm00001d052885_T004	3.489414356	7.174674961	3.291301786	3.144667704	3.641067016	3.02734234
Zm00001d022626_T001	1.672323954	1.20615495	2.011302408	1.729572347	1.795487274	1.665615162
Zm00001d028064_T001	6.664723061	7.279952386	6.72898696	5.90990225	6.016968212	5.897154539
Zm00001d047940_T012	1.032354086	0.941161914	1.359675215	1.230119376	0.662404227	1.161325325
Zm00001d047940_T008	1.254893918	0.3161666	1.533235902	1.476095453	0.711423541	0.868237137
Zm00001d047940_T011	0.512493906	0.259835292	0.640053548	0.743019408	0.7554431	0.349378866
Zm00001d014224_T001	6.180266337	5.993237615	6.159185864	5.270479718	5.074571927	5.222852812
Zm00001d051606_T001	3.677573413	3.117487599	3.958576842	2.715921038	2.824892467	3.245395869
Zm00001d011720_T002	0.064725609	0.132983438	0.376990443	1.531248196	1.163227557	0.811212884
Zm00001d011720_T001	2.558015814	1.683228429	2.316899629	2.080450282	2.31855662	2.356141274
Zm00001d011720_T004	0.473569777	0.242940238	1.024577128	0.934736144	0.755875463	0.680764525
Zm00001d036792_T001	6.060722019	6.273004926	6.185292226	5.572374813	5.352105556	5.536126619
Zm00001d021710_T014	3.615488218	3.687647504	3.460268532	3.583084814	4.126644183	3.910113916
Zm00001d021710_T013	0.739020259	0.547917654	1.016195341	1.243121265	0.587871886	0.564854192
Zm00001d021710_T005	0.965582616	0.473945846	0.74485084	1.192933949	0.994471835	0.955216819
Zm00001d021710_T001	0.816620185	0.983337682	1.269441952	1.301260443	1.166010955	1.34953513
Zm00001d021710_T011	4.025616653	3.149173829	4.060483327	4.362028246	4.022364351	4.001808727
Zm00001d044506_T012	3.391740917	2.183341047	3.13802067	2.991744588	3.668175982	3.266470687
Zm00001d044506_T013	1.57980576	0.771238227	1.458739484	2.503663448	2.162307373	2.63213092
Zm00001d044506_T009	1.72131972	1.159602708	1.68823811	2.073490192	1.997651185	1.912308023
Zm00001d044506_T011	0.314108308	0.347932063	0.805332083	0.527700939	0.654978053	0.659297124
Zm00001d044506_T014	4.323661029	4.233355922	4.380517655	3.2128801	2.973628173	2.642288456
Zm00001d044506_T004	2.803812883	3.854552464	2.469657054	2.57917194	3.073575194	2.594533979
Zm00001d044506_T002	0	0.473255964	0.95208303	2.62871598	0	2.343747491
Zm00001d044506_T003	0.606561619	0	0.621620089	1.020121676	0.939386826	0
Zm00001d044506_T006	0.480546394	0	0.635910375	0.188790824	0.311922726	0.484429993
Zm00001d037097_T001	6.370055691	5.992213811	6.472972163	5.43161818	5.518920971	5.403638848
Zm00001d002028_T001	7.244124078	7.788662868	7.097366131	6.768470016	6.74038124	6.723284986

Zm00001d017425_T001	6.530035664	7.601063178	6.416187342	6.33683788	6.29649553	6.390596429
Zm00001d023304_T002	1.339843733	0.522597161	0	0.90372011	0.843464541	1.333586886
Zm00001d023304_T006	0.548206762	0.567889045	1.373206636	1.108360524	0	0
Zm00001d023304_T003	0.70438951	0.209067764	0.97680426	0.683624598	0.687520306	0.698673667
Zm00001d023304_T004	0	0	0	1.306490906	1.709114148	1.085935832
Zm00001d047570_T001	1.395707206	0.787808553	1.281131445	1.27389121	1.46011241	1.32381506
Zm00001d049746_T003	0	0	0	2.743005616	1.749742773	2.291020882
Zm00001d049746_T002	3.175732009	2.300682533	3.546128317	2.573508076	3.833571919	3.285239888
Zm00001d049746_T006	2.185048892	1.598998747	1.918566369	2.012175678	2.097282445	1.671584147
Zm00001d049746_T004	4.061729892	2.826152461	3.674755307	3.676541047	3.104393192	3.455216338
Zm00001d017147_T001	7.996504548	7.685406557	8.079706488	6.984632104	7.037454476	6.905701938
Zm00001d017147_T002	2.121219685	2.895876289	1.833536197	1.219792404	1.296926629	0.934565566
Zm00001d035443_T001	5.039223382	4.331713814	5.14222349	5.07526807	5.093553923	5.040872359
Zm00001d039468_T001	7.568277607	8.057418781	7.594766199	6.574560908	6.37504701	6.180229273
Zm00001d044213_T001	3.547855977	2.923948195	3.682649138	3.558844842	3.634526824	3.430158664
Zm00001d044213_T002	2.301552823	2.258537316	2.863393572	3.191027226	2.844892375	3.13094668
Zm00001d028555_T001	6.536832258	7.492281433	6.361653434	5.866642603	5.828697455	6.043320005
Zm00001d007259_T001	3.561454941	3.408115913	3.645955341	4.060974545	4.032591505	3.951906846
Zm00001d005485_T001	6.803060008	6.167585057	6.926571602	6.191689667	6.260752976	6.145269734
Zm00001d012693_T003	3.031507594	2.341743286	3.208394585	2.478310731	2.28935024	2.559621515
Zm00001d043592_T003	2.024903661	2.146246596	2.326431213	1.977061533	1.902681024	2.198549157
Zm00001d043592_T005	1.355482611	0.649676154	1.498595541	2.182998917	1.88557905	2.114271064
Zm00001d043592_T001	0.474657201	0.312924185	0.501112989	0.951927914	0.829105091	0.666513017
Zm00001d043592_T004	1.560821563	1.044359844	1.261927314	2.549990199	2.386320954	2.219847221
Zm00001d043592_T002	0.33510361	0.134560048	0.297758403	0.150780613	0.31304148	0.124977863
Zm00001d033636_T001	6.942149422	2.224259503	7.061065592	7.127974363	7.572766026	8.037446206
Zm00001d006702_T002	0.194401031	0.099028525	0.189744872	0.611241326	0.705923067	0.523872039
Zm00001d006702_T001	4.325958256	3.606374948	4.522444501	4.378872807	4.424279919	4.487530918
Zm00001d013307_T003	3.385766201	2.803585599	3.469049413	3.011172141	3.059967981	2.946140001
Zm00001d013307_T002	1.288573548	0.830705589	1.313772905	1.205820378	1.099173993	1.408573834
Zm00001d014360_T002	1.321217537	0.510971031	1.215609023	2.235879285	2.091550903	2.020500068
Zm00001d014360_T001	3.163389238	2.651735524	3.421991948	3.503825484	3.806237637	3.565827696

Zm00001d020486_T001	6.429582005	6.652311009	6.373675179	6.251985058	6.311750767	6.17608648
Zm00001d027718_T001	1.845425818	1.677694501	1.876136565	0.216771463	0.433690188	0
Zm00001d022280_T001	5.663080481	4.967987824	5.557340668	5.718456871	5.800429776	5.776601527
Zm00001d002523_T001	6.50728964	6.774937593	6.543496796	7.18248131	7.159381837	7.1056089
Zm00001d032368_T001	0.817836065	0.561988854	0.748263708	1.129691201	1.299192483	1.511439195
Zm00001d051908_T002	1.419645674	1.904595848	2.366903512	0.691796774	1.192046416	0.605041969
Zm00001d051908_T001	3.723236895	3.315178262	3.691139603	2.478761402	2.015150699	2.295468841
Zm00001d048958_T003	0.525640949	0	0.292789993	0.446967608	0.653662658	0.255368996
Zm00001d048958_T002	1.421225993	1.014913694	1.491458064	1.639302559	1.765132009	1.623171561
Zm00001d017053_T001	1.910436035	1.488127805	1.769857621	1.736186184	2.166700986	1.847391248
Zm00001d017053_T006	1.693784889	0	1.442257207	1.936809027	1.740889145	1.572866876
Zm00001d013511_T002	4.427286023	4.9061057	4.311820523	2.714287067	2.766895542	3.005449035
Zm00001d013511_T001	2.529409439	1.962434269	2.662983388	2.071966499	2.399485854	2.273720568
Zm00001d030212_T001	1.719560661	1.100815546	1.780704424	1.981077042	1.932114477	1.94515295
Zm00001d005269_T003	3.595820737	2.651046638	3.029043784	3.320429222	3.309216359	3.472504408
Zm00001d005269_T005	1.965371684	1.342875562	2.221739145	2.594586288	2.647200517	2.626510861
Zm00001d005269_T004	0	0	2.288285909	1.30699536	1.485997094	0
Zm00001d020102_T001	1.770161764	3.504617342	2.466002404	2.076921263	2.594165372	2.905021962
Zm00001d021201_T001	3.941374349	2.915755308	3.985305781	4.260095304	4.380802025	4.278724348
Zm00001d021201_T003	3.254740059	2.972846288	3.331334608	3.508537548	3.244432721	3.5486593
Zm00001d038080_T003	0.922350104	0.839088872	1.541226371	1.221162515	1.604869331	0.921841508
Zm00001d038080_T001	1.486552206	0.771687009	0	1.062612891	0.899070422	1.796339009
Zm00001d016402_T082	4.750008241	3.72035421	4.60734002	5.279353617	5.331190409	5.320435288
Zm00001d016402_T020	0.378390654	0.110309784	0.17901924	0.667433477	0.502702304	0.419219576
Zm00001d016402_T081	0.411570508	0.637152072	0.584621985	1.220303949	1.604439069	1.09217225
Zm00001d016402_T025	0.857794726	0.529670049	0	1.321818446	1.02973274	1.203850771
Zm00001d016402_T024	2.94024363	2.717595273	3.611528642	0	0	0
Zm00001d016402_T005	2.950059064	1.819771988	2.708589633	4.369277741	4.455808532	4.335459031
Zm00001d016402_T040	0.870229766	0.116307527	0.582594539	0.77177996	0.229521477	1.274181733
Zm00001d004187_T001	2.958303721	2.626906097	2.983217454	2.985613182	3.272492661	3.051164819
Zm00001d039973_T003	1.610892314	1.630344892	1.828340739	1.190676169	1.896236193	1.480322518
Zm00001d039973_T007	2.108010515	0.869267361	1.1223593	2.472320357	1.996814261	1.877936204

Zm00001d039973_T004	2.415473939	0.841981167	2.949793941	2.973310767	2.957816745	2.900842662
Zm00001d013508_T001	2.311785001	2.992827515	1.855508187	2.18719765	1.809516108	1.84811392
Zm00001d006595_T002	2.630422014	1.666452572	2.745874731	2.224002601	2.511906702	2.433063598
Zm00001d006595_T001	3.382148592	3.185961823	3.803742119	3.777840916	3.890914739	3.593363692
Zm00001d018096_T001	1.456817712	2.199568389	1.656602538	1.939046747	1.710114986	1.864936894
Zm00001d003334_T003	0.673023569	0.663909289	0.700027714	1.221553559	1.432198853	1.44635523
Zm00001d003334_T001	0.138184053	0.164580348	0.282851352	1.154360262	1.422924575	1.225012654
Zm00001d003334_T002	3.351696731	3.055660394	3.515380423	3.796892887	3.763854514	3.913651835
Zm00001d024598_T001	1.933238363	1.373197725	1.654931325	1.649081046	1.498647618	1.291704331
Zm00001d041854_T004	3.82965252	3.065559215	3.876729885	4.239195845	4.340347344	4.144839634
Zm00001d041854_T005	0.184871853	0.023702465	0.22561921	0.211194155	0.099015055	0.256079508
Zm00001d041854_T001	0.656472573	0.247762277	0.532065557	0.608618132	0.88248498	0.835943472
Zm00001d010805_T001	4.008758023	4.045433447	3.994932209	4.984413537	4.779082446	4.618127982
Zm00001d034512_T002	3.038524328	3.382962124	2.938995578	2.846250389	2.461446312	2.759715116
Zm00001d030849_T001	3.021055918	2.379776756	3.07228008	2.681335464	2.759489725	2.657851021
Zm00001d005699_T001	4.505039603	3.878131861	4.576124284	4.018211219	4.179873749	4.187587636
Zm00001d033610_T001	3.305155146	2.808222133	3.515665907	3.387061948	3.378489289	3.486898398
Zm00001d014446_T001	1.11269146	1.093846794	1.168384664	2.860784461	2.772396663	2.497071461
Zm00001d005612_T001	2.199473848	3.52528689	2.361188639	2.396521214	2.491013139	2.811269615
Zm00001d053727_T005	1.571075339	1.183421723	1.770727578	2.277105477	2.570744885	2.604391623
Zm00001d053727_T009	1.205018966	1.000143541	1.346179681	0.9530171	0	0
Zm00001d046553_T004	0.722781634	0.386303293	0.875934938	0.449786378	0.58566348	0
Zm00001d046553_T001	3.609465047	3.396394757	3.80772029	3.55460555	3.80202691	3.734733028
Zm00001d038973_T001	4.175370982	3.766373281	4.419571925	4.064837249	4.168519292	3.913992977
Zm00001d023969_T002	1.565532351	0.720046389	1.437776903	1.180914209	1.000419763	0.936301357
Zm00001d014079_T001	3.132935909	2.327908637	3.011211688	2.52819822	2.557898218	2.745958607
Zm00001d036481_T001	2.937803495	0	2.439643083	2.338170227	0	1.261102306
Zm00001d036481_T003	1.800138681	1.630444614	2.400301833	1.897628061	2.2804495	2.023948673
Zm00001d036481_T006	4.352024564	4.178319858	4.291562304	4.623986165	4.725902428	4.932347432
Zm00001d036481_T004	0.932025633	0.472359873	1.247692411	1.951188649	2.155350947	1.818637085
Zm00001d036481_T002	6.688727142	6.342730894	6.79855121	7.158478201	7.121229074	7.032251498
Zm00001d036481_T007	1.668194027	0.972447099	1.358795705	2.107802343	2.053814435	2.135588387

Zm00001d036481_T005	5.281591398	3.044326792	4.964778526	5.108972543	5.370001026	5.195751567
Zm00001d017351_T001	4.301157554	4.355013374	4.324105353	3.83119788	4.174381329	3.820191627
Zm00001d047053_T011	2.709716322	1.572483717	2.504045742	1.222347697	1.269487434	2.014949747
Zm00001d047053_T008	2.591920165	1.458489625	2.451035142	2.425958468	2.735555537	2.299684551
Zm00001d038880_T001	4.48561971	3.521784967	4.670834672	5.133681965	5.136546478	5.328795256
Zm00001d038880_T004	0.916185401	0.370332818	0.829093722	1.6234235	2.07154165	2.047715312
Zm00001d038880_T009	3.570963294	4.750663043	3.337159452	3.150654716	3.207909402	2.81722375
Zm00001d038880_T006	6.019316084	4.962590573	5.948569078	5.546118776	5.543867212	5.434276567
Zm00001d038880_T003	2.431757997	1.421916434	2.158142457	2.416239305	2.007866091	2.203020691
Zm00001d038880_T007	6.675449552	5.241723322	6.770097115	6.357995864	6.409754263	6.353922619
Zm00001d038880_T002	2.653696589	2.938893422	2.493558956	2.29721754	2.145383344	1.980205946
Zm00001d008187_T001	4.225729557	3.831061746	4.137119355	4.026131131	4.292931163	4.324731123
Zm00001d048054_T004	1.846851112	1.125635894	2.118639798	1.7967078	1.597689439	1.607841603
Zm00001d048054_T002	4.712374792	3.936595635	4.771352021	4.28817419	4.332343813	4.433635913
Zm00001d048054_T003	4.754278931	4.382165542	4.876957914	4.049871632	3.995982095	3.816514924
Zm00001d017790_T001	4.23590149	3.305833976	4.0968762	4.006801398	4.129851904	4.046904584
Zm00001d032497_T001	6.855362342	6.999090252	7.04668412	6.615753904	6.672408001	6.657651065
Zm00001d005361_T001	2.489828056	1.428018006	2.108439482	3.597074755	3.378417706	3.127377402
Zm00001d005361_T010	0.948017672	0.665138958	0.707690872	0.957908657	1.899810586	1.825665974
Zm00001d005361_T021	2.370902182	0	2.828624271	1.699438807	2.609614326	2.281734744
Zm00001d005361_T018	0.71131164	0	0	0.469423406	0	0.607810836
Zm00001d005361_T004	0.390250553	0.350252817	0.610691559	1.156368138	1.082490052	0.756761147
Zm00001d005361_T007	1.72590559	2.045975845	1.376019714	0	0.989419524	1.342895468
Zm00001d005361_T011	0.843589159	0.247801158	1.137577624	1.241739527	1.975667968	2.171494121
Zm00001d005361_T012	0.757004466	0	0.91809843	1.62396282	1.496623918	1.124163341
Zm00001d025319_T001	2.409836236	1.919411405	2.570983325	2.263633301	2.236437818	2.172663201
Zm00001d021294_T001	2.120594471	1.418411261	1.74800173	1.439851291	1.706343117	1.641214911
Zm00001d006242_T009	2.259291773	1.63370926	2.193101483	2.037093303	1.669529806	1.726048201
Zm00001d006242_T003	4.886686363	3.115332774	4.9115776	4.951197183	4.835816525	4.84040628
Zm00001d006242_T001	2.628382837	3.623242745	2.609717618	2.351912939	3.155209416	3.019404628
Zm00001d006242_T004	0.821204877	0.444178312	0.360383381	0.304467819	0.702256813	0.5255728
Zm00001d013176_T003	0.730821653	0.384689757	0.564764462	0.599448249	0.428311794	0.655138386

Zm00001d013176_T001	1.624215557	1.436312179	1.880351698	1.936190151	1.959250469	1.631872118
Zm00001d003493_T002	6.675863032	7.062175454	6.806367691	6.697308296	6.752066441	6.659040294
Zm00001d018738_T001	6.490402074	7.214718415	6.46581922	3.518967757	3.11801712	3.27518484
Zm00001d003174_T001	5.739227184	5.277052309	5.76361819	5.29360547	5.132879744	5.177332705
Zm00001d051188_T001	4.564237917	4.300778574	4.415479144	4.604804408	4.493322999	4.540634383
Zm00001d036710_T002	4.503396506	4.065174878	4.626454849	4.719424362	4.587451286	4.847130178
Zm00001d036710_T001	2.612858238	1.483765741	2.810625511	2.650365465	2.828053494	2.943904265
Zm00001d032722_T001	1.832645798	1.806121096	2.025388537	1.603425763	1.623377142	2.067802869
Zm00001d028229_T001	0.768681479	0.745024731	1.22636487	0.915182798	0.78651858	0.766249976
Zm00001d053765_T006	0.362835671	0.751820173	0.581131391	1.529179773	1.779335373	0.714103055
Zm00001d053765_T003	5.715411376	5.014141596	5.7051808	6.185775086	6.277266307	6.190615769
Zm00001d053765_T005	5.166328171	0	3.879613635	4.841344595	4.795692619	4.587897922
Zm00001d053765_T004	6.61622385	6.507903996	6.95261899	5.954840865	6.131130935	6.094717789
Zm00001d053765_T007	4.578236088	0	4.831539425	4.590710873	4.029298183	4.632571789
Zm00001d038180_T001	2.787112712	2.448697744	2.412343096	3.04365842	2.917992684	3.458903627
Zm00001d043607_T001	4.929706033	4.100076607	4.918365815	5.551288768	5.502020246	5.429136984
Zm00001d024531_T001	4.020708515	3.374946679	4.224621466	4.964994303	4.766781979	4.831355436
Zm00001d024531_T006	5.114745266	4.325385191	5.185777484	4.899400768	4.754960009	4.723429139
Zm00001d024531_T005	2.762201455	0	2.773010569	1.926024855	0	1.956426203
Zm00001d024531_T002	3.686328626	2.686774585	3.396528064	3.665151809	4.46853451	4.068781459
Zm00001d039991_T001	7.189855774	6.921419751	7.099886155	7.537448033	7.51107525	7.621694521
Zm00001d048323_T001	2.409373284	1.739791517	2.531390195	2.996437739	2.976534659	2.863391589
Zm00001d018796_T001	2.457291959	2.98062712	2.070759229	2.018594297	1.700912845	2.034805916
Zm00001d042260_T001	2.262888025	1.772089189	1.8345203	1.644704937	1.373322472	1.62246749
Zm00001d022062_T001	7.73704121	7.791678265	7.744670303	6.34765749	6.470617116	6.204639238
Zm00001d035064_T001	2.975106642	2.579920619	3.251020523	2.716262671	2.675852958	2.992709344
Zm00001d052002_T001	2.447243013	3.466382278	3.212588326	2.151787719	2.286249831	2.216449588
Zm00001d025946_T001	1.42434812	0	1.828621021	0.920795583	1.839096533	0.71404589
Zm00001d025946_T003	1.608598736	2.214104284	1.666465752	3.590122319	3.201809326	3.405007316
Zm00001d025946_T007	0.264446354	0.111845938	0.264475179	0.464260491	0.609619762	0.610072585
Zm00001d025946_T004	3.637488181	2.598938501	3.556029477	3.225473148	3.786365303	3.152248168
Zm00001d015964_T001	1.109494291	0.493734426	0.757590823	1.268997228	0.917383633	0.682742276

Zm00001d038700_T001	3.516706567	3.13492207	3.696007617	3.808795661	4.112253746	3.973563246
Zm00001d040696_T003	0.8122797	0.691179477	0.840584888	1.268149899	1.130223183	1.295500874
Zm00001d018479_T001	6.752673806	6.01817162	6.781571722	6.785294749	6.764667025	6.735380162
Zm00001d044184_T001	5.75472356	5.086953098	5.875365524	5.654025485	5.739784578	5.574786293
Zm00001d011366_T006	2.267829101	2.288736242	2.26660786	2.490337021	2.304294037	2.560888312
Zm00001d011366_T002	1.533697894	1.380527767	1.725252542	2.598448105	3.103612782	2.855954227
Zm00001d011366_T003	2.127887792	0	1.383868491	1.621479901	0	0
Zm00001d037975_T048	0	0	0	1.940388542	1.674719156	0.732927326
Zm00001d037975_T018	0.935919828	0	0.260526747	0.849618226	0.915170558	0.790556868
Zm00001d037975_T004	0.79864448	0.961412187	1.243230966	0	0.855924336	0.674867369
Zm00001d037975_T008	0.91023761	0.29950609	0	0	1.017783688	1.242437269
Zm00001d037975_T001	1.231050179	1.389450621	1.535903151	1.18184995	1.314771921	1.267206173
Zm00001d037975_T012	0.668841653	0	0.683574297	0.723720187	0.45539272	0.616159113
Zm00001d037975_T037	0.098914027	0	0.229240896	0.392538344	0.391291508	0.624339569
Zm00001d011706_T003	3.297908623	2.600414396	3.466336855	3.600158645	3.612659289	3.648748892
Zm00001d044030_T001	4.051737776	3.516405404	4.264497999	3.399971533	3.335422228	3.446203551
Zm00001d041663_T001	1.8444656	2.0455569	2.235163656	0	0.57312533	0.404543493
Zm00001d028347_T001	0.10208161	0.339513691	0.921603138	1.765070463	1.724828797	1.653527842
Zm00001d042469_T006	1.885873805	2.497022139	1.793823486	1.928311193	1.884959282	2.085531733
Zm00001d015777_T001	3.071714968	3.723619391	3.34570393	3.822816809	3.789430041	3.968544231
Zm00001d023377_T001	3.652206419	2.974460847	3.62400752	2.767990964	2.723598751	2.724567549
Zm00001d007012_T001	3.68838315	3.047437355	3.505645676	3.87695595	3.620512397	3.781364435
Zm00001d014835_T001	1.094979187	0.590405607	1.347817571	1.145923263	0.924141702	1.049538767
Zm00001d048050_T004	1.811360872	1.456821391	1.867180077	1.228615545	0.921493459	0.815207331
Zm00001d048050_T001	2.260706182	1.698058417	2.285536272	1.954286778	2.002544472	2.211956958
Zm00001d048050_T003	4.535426498	3.876532957	4.816338687	4.090006276	4.036842736	3.866813542
Zm00001d048050_T005	3.469947954	5.908970592	2.720686697	0	3.091423453	2.856650515
Zm00001d017175_T002	5.031993651	4.204462454	4.947098551	4.608747099	4.563228738	4.473645037
Zm00001d017175_T001	0.569406511	0.29661481	0.447371836	0.463824357	0.409556039	0.493313262
Zm00001d021021_T002	6.32851418	6.597776057	6.377778871	6.253116448	6.405571552	6.275455733
Zm00001d021021_T003	0.327870132	0.199086197	0.462931807	0.443131332	0.133275484	0.72869375
Zm00001d004676_T003	1.41173265	0	1.222568419	1.076756777	1.652346062	0.531420888

Zm00001d004676_T002	6.475283872	5.666921953	6.475982866	7.219434049	7.169102511	7.09912098
Zm00001d042479_T002	3.97978474	3.331441529	4.001125945	3.48304493	3.245105273	3.56259852
Zm00001d017380_T001	1.774695146	1.294779528	2.042152617	1.571588474	1.810737577	1.619881546
Zm00001d002503_T002	5.042470556	3.836628479	4.911469505	4.959639231	4.724437617	4.82046446
Zm00001d002503_T003	3.477261036	0.102272465	3.612918495	4.962665248	5.031155021	4.883498338
Zm00001d002503_T001	3.237206824	3.40430955	3.202993744	4.071931757	4.056544426	4.202907888
Zm00001d045295_T001	4.52357977	3.789150806	4.590980278	5.305649015	5.249569505	5.190164362
Zm00001d045295_T002	2.390668236	0.721768116	2.423640288	3.808199476	3.797226875	3.917377522
Zm00001d007815_T003	8.367922731	8.882859499	8.397356645	7.841954915	7.772835157	7.769492772
Zm00001d043036_T001	2.65746806	2.410931794	2.667289268	2.596528269	2.408519217	2.527037046
Zm00001d041649_T010	2.45994906	2.433277567	2.903644923	1.714954105	0	1.087147111
Zm00001d041649_T006	1.570659163	0.724769	1.396662591	2.047384828	3.03068073	2.35302484
Zm00001d041649_T003	2.73149543	3.412031129	2.869353048	1.698374521	2.290051886	1.102232818
Zm00001d041649_T001	0.971629998	0	1.27716054	1.977766419	0	2.168756587
Zm00001d041649_T005	0.477969463	0.257430689	0.115550016	0.37723816	0.826536691	0.477582008
Zm00001d041649_T004	2.762684732	1.600431524	2.36928891	1.593668811	2.460117392	2.538155215
Zm00001d041649_T008	2.086347631	1.20782415	1.962387625	3.207582889	2.085076518	2.646168649
Zm00001d052248_T004	0.833868892	0.987895646	0.706760393	1.815338102	2.180381819	1.983537249
Zm00001d052248_T009	0.902471882	0.785686132	0.891327411	1.840202971	0.592158002	2.011181088
Zm00001d052248_T018	0.126493199	0	1.082115311	0.605882098	0.922765679	0.566476172
Zm00001d052248_T001	3.118298422	3.011646091	3.122671725	2.767391022	2.116491187	2.110076881
Zm00001d052248_T016	2.543995667	1.187480826	2.199459085	1.94199307	3.00235724	0
Zm00001d006113_T011	4.319122573	3.837186688	4.225900343	3.792923946	3.164946658	3.563770353
Zm00001d006113_T001	2.486923105	1.668986841	2.682092319	2.790840627	3.058468897	3.00733151
Zm00001d013814_T001	1.615247722	1.236624254	1.333728843	1.475919121	1.491418555	1.244602144
Zm00001d048081_T001	4.323595536	3.369517574	4.381619431	4.257510797	4.234186827	4.14766106
Zm00001d048503_T001	5.663606234	4.934383407	5.731668576	6.369943517	6.460130091	6.416511907
Zm00001d003485_T001	1.684466883	0	2.897014612	6.052859299	5.591180981	6.189755011
Zm00001d016839_T001	2.852459547	3.108665959	2.944569691	2.636572886	1.975110312	2.380620577
Zm00001d047854_T005	0.380053373	2.104990757	0.879308186	1.168187592	1.018212555	0.784072576
Zm00001d047854_T003	1.572067462	0.664568398	1.869391349	2.751196324	2.810777672	2.627582793
Zm00001d047854_T007	0.547886076	0	0.373046789	0.143211846	0.466844258	0.556776651

Zm00001d047854_T006	0.110158752	0.21932653	0.188994593	1.594751086	1.355901462	1.327047493
Zm00001d047854_T002	3.806820511	2.641682894	3.602931192	3.990400814	4.12615632	4.290057122
Zm00001d047854_T004	0.318940356	0.288311308	0.307037925	1.597619366	1.314722044	1.216204021
Zm00001d024885_T001	1.313626069	0.770498404	1.212942181	0.626385867	0.44248176	0.62099453
Zm00001d021453_T004	1.304523892	0.805038981	0.189365353	0.513342173	0.869372396	0.14330982
Zm00001d021453_T001	1.81745258	1.482341831	2.147605302	1.917352314	2.107334349	2.254772101
Zm00001d008451_T001	3.698706222	4.320880751	4.501261234	3.909768113	3.096730754	3.495508035
Zm00001d030698_T001	7.994950301	8.799293607	8.011324265	6.832184592	6.938422432	6.950884027
Zm00001d024511_T002	8.364066888	7.955583167	8.468788605	7.86362427	7.883931998	7.904197993
Zm00001d007318_T005	1.122639591	0.265501708	0.591929258	0	0.894018362	0
Zm00001d007318_T007	2.362281351	3.401056578	2.043236001	1.595242023	2.075267343	1.647885487
Zm00001d007318_T001	0	0.560450844	0.693057347	1.378008812	0.636853346	1.120563617
Zm00001d007318_T009	3.065344999	0	3.765883709	4.296887362	4.303431026	4.447337052
Zm00001d039884_T002	0.900451445	1.270833894	0.766122737	0.215440043	1.212754216	0.762615004
Zm00001d039884_T001	2.125876546	1.088827494	1.849863988	1.81299184	1.472706109	2.159475803
Zm00001d053375_T001	5.352123676	4.880838308	5.368810282	4.856384342	4.861331269	4.816335615
Zm00001d002090_T001	1.901569368	1.377363669	1.939089637	2.179918323	2.513627429	2.404294425
Zm00001d026126_T001	1.996435389	1.496088525	1.913901573	5.63342534	5.681716752	5.664327498
Zm00001d027506_T003	2.564083746	1.883626681	2.701167319	3.247562845	3.017789388	3.386551486
Zm00001d038868_T002	2.822195181	2.048682599	2.829615788	3.196626085	3.127450538	3.214119986
Zm00001d038868_T004	1.365744601	0.429294586	1.233859231	1.091823029	1.458830808	0.721403285
Zm00001d009723_T001	1.364995374	1.50431072	1.809618589	1.904804683	1.841726411	2.01938986
Zm00001d028180_T001	2.614474754	2.364044561	2.654117911	2.821212838	2.355317123	2.717237064
Zm00001d028180_T011	3.234016713	2.060058801	3.508492419	2.361289159	3.073342968	2.843213464
Zm00001d028574_T001	0.587896842	0.641765191	0.400760874	3.453367046	3.790577094	3.611070254
Zm00001d003935_T005	1.881353731	1.624153781	0	0	2.176239145	1.492635146
Zm00001d003935_T012	2.216185082	1.270219739	1.810823111	2.439242269	1.996650866	2.114700172
Zm00001d003935_T009	3.439478595	2.533000862	3.405205877	1.772028785	2.793941649	1.757019405
Zm00001d003935_T011	0.969486126	0.344902334	0.902910196	0.822731779	1.007945364	1.389374072
Zm00001d003935_T007	1.834368196	1.298042986	1.637254107	0.896446241	0.799424725	1.079313933
Zm00001d003935_T010	1.966440804	1.583613913	2.601150946	2.610053208	2.4385622	2.807945276
Zm00001d042314_T009	4.71155008	4.656988874	4.78504327	4.472696687	4.462419688	4.148111599

Zm00001d042314_T004	3.190274451	0	3.048418081	3.851553271	3.846318999	4.317888887
Zm00001d042314_T010	1.893795148	2.244911713	2.216919495	3.098548412	3.029093448	2.469024008
Zm00001d042314_T011	0	0	0	1.365921492	1.967648565	1.882928235
Zm00001d015400_T004	1.223755536	0.582889302	1.022865612	1.499694372	1.630780542	1.026402777
Zm00001d015400_T002	0.869565862	0.345646171	0.857605249	0.93357717	0.709484747	0.818390035
Zm00001d015400_T003	0.455272767	0.216124536	0.312019185	0.594614473	0.739883522	0.603853005
Zm00001d015464_T008	0.319439898	0.247497374	0.736939048	0.657717739	0.550827789	0.076134912
Zm00001d015464_T003	4.478978792	4.478837169	4.461258422	4.46045393	4.564462069	4.589397854
Zm00001d015464_T006	5.458284351	3.654342734	5.48320746	4.839810777	4.721448292	4.793141937
Zm00001d015464_T005	1.189507685	0.78723268	1.742069179	0.765093384	1.278227457	1.08760337
Zm00001d046385_T001	2.263322014	2.106760364	2.551301412	2.459029182	2.693191419	2.729365039
Zm00001d046385_T002	3.122124026	1.948810135	2.942320444	2.942299047	3.466571003	3.009415794
Zm00001d021535_T001	3.838946121	2.936380139	3.84361187	3.37121369	3.497087433	3.45206533
Zm00001d021535_T003	0.642747774	0.335169941	0.60595888	0.441076535	0.560953591	0.212665204
Zm00001d040281_T001	7.120057059	7.490409383	7.267374488	7.089393318	7.12583464	7.139664052
Zm00001d038720_T010	1.309188996	1.023918165	1.483953496	2.093617301	2.083984982	2.005265953
Zm00001d038720_T006	2.388825726	2.17602782	2.571265905	2.459151971	2.031503183	2.674882506
Zm00001d038720_T005	1.8463196	0.663017556	0.98056132	1.101364332	1.602738113	1.041483411
Zm00001d038720_T007	0.893859204	0	1.036083388	1.714220016	1.130264046	1.359986064
Zm00001d038720_T001	0.939357486	0.810039134	0.746617184	1.691508705	1.654416751	1.608138352
Zm00001d038720_T004	0.166535499	0.138077864	0.210436247	0.922031872	1.026921134	1.022019038
Zm00001d038720_T008	0.959169958	0.568901839	0.697729346	0.490889439	0.844627488	1.005946779
Zm00001d038720_T009	0.068256686	0	0.328606717	1.036956907	1.07012	0.685711451
Zm00001d038720_T003	0.887451184	0.541865682	1.148352355	1.435868579	1.564329387	1.414789875
Zm00001d033153_T002	3.156760642	2.462769561	3.237013425	3.676528636	3.645392822	3.522143403
Zm00001d033153_T006	0.684304391	0.522763862	0.761190793	1.287968672	1.155941536	1.206672603
Zm00001d023311_T033	0.328074713	0.147883661	0.333237087	1.088395369	1.272308116	1.125212673
Zm00001d023311_T030	1.628780593	0.574215853	1.526289707	0.838487126	0.802569613	0.463531436
Zm00001d023311_T007	2.056032008	0.681223454	1.969223814	4.132808363	3.731884212	3.929552043
Zm00001d023311_T040	1.147425887	0.797443854	0.348486257	0	0.863858387	0.322561587
Zm00001d023311_T010	0.869452944	0	0.924242057	1.727091402	2.153657169	1.749617073
Zm00001d023311_T037	1.517448554	0.485108369	1.757239208	1.834516659	1.506278475	1.85341526

Zm00001d023311_T001	1.948926352	1.420322318	1.679019715	3.157139294	3.186731269	2.944512907
Zm00001d023311_T034	1.553511401	0	2.156938901	1.297849359	1.45785586	1.405334014
Zm00001d023311_T013	0.209704139	0.199319931	0	0.243525292	0.315908171	0.29515171
Zm00001d023311_T035	3.494391672	3.132563847	3.318290374	3.088648419	2.841619344	3.174275215
Zm00001d023311_T029	3.725352678	2.453435927	3.616862858	0	0	0
Zm00001d023311_T022	2.593227784	2.214073813	2.71452095	3.612117614	3.71212515	3.840645007
Zm00001d023311_T041	0.843331872	0.358532423	0.956139196	0.71495059	0.485159904	0.541619903
Zm00001d005028_T001	2.184471054	1.906853277	2.23591175	3.542347333	3.442170137	3.451338393
Zm00001d048794_T003	3.377903344	1.075410091	2.416157678	3.29400649	1.750174275	3.176926695
Zm00001d048794_T004	3.830116363	3.765004883	3.985768998	4.084601433	4.418109309	4.128415903
Zm00001d048794_T001	1.87184759	1.52415497	2.393481002	2.451026171	2.502626953	2.186794645
Zm00001d023934_T003	3.877859563	3.579133796	3.906436364	3.768104908	3.826922955	3.796211281
Zm00001d023934_T001	0.704115015	0.977049074	1.062807658	0.912241331	1.113867826	0
Zm00001d023934_T002	1.237107833	0.923605597	1.331756802	1.944478049	1.664902845	1.959663711
Zm00001d015997_T002	5.71357444	5.587444655	5.696832748	5.077511727	5.278173996	5.211522344
Zm00001d021778_T001	2.361314148	2.997273979	1.604895418	2.177423525	1.584704716	2.074089557
Zm00001d046537_T002	0.648807784	0.143243198	0.328796261	0.842703738	0.469057621	1.027918378
Zm00001d011750_T004	3.253010654	0.961961091	2.876624795	3.327616952	2.692476076	2.568510572
Zm00001d011750_T003	3.624995603	3.107081375	3.857172163	4.181869186	4.29942535	4.306535454
Zm00001d011750_T001	3.822236998	3.785490604	2.871153473	1.84581077	2.431538187	0
Zm00001d011750_T002	3.200814899	2.099337289	3.193305433	3.219227079	3.158721888	3.319514902
Zm00001d005980_T003	1.696002719	0.248317444	2.079881878	1.088007229	1.605305629	1.693027898
Zm00001d005980_T002	1.598439053	1.239798225	1.520317641	1.922632875	1.831816047	1.447625743
Zm00001d031327_T004	1.964994441	0.801673546	2.263520632	2.41006942	2.938777523	2.466231124
Zm00001d031327_T025	0	0.177680565	0.494388984	0.572498754	0.482401211	0.652762732
Zm00001d031327_T011	0.447713542	0.320837005	0.538527238	0.381373088	0.425374438	0.526533605
Zm00001d031327_T008	3.40313681	2.859039644	3.321816426	3.887942422	3.761709295	3.815673689
Zm00001d043558_T001	4.754843579	4.674609286	4.866281026	4.168821639	4.019289682	3.94062101
Zm00001d014271_T001	5.547136473	5.50328172	5.712218716	5.997245205	5.924664593	6.002006768
Zm00001d034495_T001	3.940295976	3.559733833	4.236453432	3.447811517	3.252996883	3.296020788
Zm00001d043610_T001	4.314980759	3.907784693	4.148227141	5.09906864	4.800397004	5.207453028
Zm00001d037273_T001	4.59309515	4.331313323	4.71356281	4.92259249	4.770520974	4.786558569

Zm00001d018930_T006	0.501153763	0.459935162	0.838930797	0.956119862	0.361111419	0.552477412
Zm00001d028071_T001	2.993657517	2.612858002	3.009434606	2.948806585	2.920168465	3.345536607
Zm00001d037190_T001	1.146844181	0.87681513	1.033584398	1.317584244	0.953495456	0.754884937
Zm00001d039294_T001	1.406979043	1.357808148	1.682460035	1.499081008	1.675596011	1.246157532
Zm00001d039294_T002	3.283067011	2.799357372	3.444509909	2.577245118	2.444494537	2.782715944
Zm00001d034415_T001	6.643217598	6.463067837	6.585404093	6.999516109	7.05320563	7.047132159
Zm00001d037160_T002	2.742655601	1.971334595	2.85878311	2.136180884	2.120681387	2.155823049
Zm00001d027899_T001	1.25634586	0.785859354	0.926171772	4.01350755	4.204015869	4.235147951
Zm00001d049548_T009	4.23213377	4.726526901	4.113763999	4.282775975	4.327750373	4.01283368
Zm00001d049548_T006	0.694031483	1.285522956	1.576225587	1.130167818	1.169563962	1.18472589
Zm00001d049548_T001	0.491326705	0.609678382	0.570364805	1.331487965	1.520399618	1.361584783
Zm00001d049548_T007	1.081934719	0	0.815899999	2.145181474	1.892008977	1.998574996
Zm00001d049548_T008	0.490224047	0.220421591	0.734122995	1.215231898	0.696519143	0.609694455
Zm00001d049548_T005	1.755642208	0.440446236	1.525648966	1.980450549	2.452694791	1.860464272
Zm00001d033876_T003	0.561043558	0.259971441	0.793470604	1.765571238	1.360081048	1.493754917
Zm00001d033876_T004	4.22396593	3.163787242	4.687138702	4.87738839	5.008586878	4.827923515
Zm00001d033876_T005	3.64774426	3.01742525	2.222910874	2.864508084	2.58513946	2.96865463
Zm00001d034586_T002	3.835109247	3.390703037	3.747982509	3.450870761	3.265917664	3.583466853
Zm00001d036262_T001	1.776680951	0.937327818	1.555689055	1.956129529	1.621040495	1.74112825
Zm00001d025031_T003	0	0	0.363021893	0.932256996	1.709827465	1.312493829
Zm00001d025031_T002	2.198178235	1.308556592	2.392728464	1.787835293	1.379283813	1.828111598
Zm00001d025031_T005	1.136335124	0	0	0.902572212	0.174553429	1.01387098
Zm00001d025031_T004	0.688953767	0	0	1.510641454	1.687088467	0.687492536
Zm00001d031893_T001	2.859032287	2.0142778	2.999768068	2.204314781	2.322503904	2.359984097
Zm00001d039598_T001	2.319447413	1.715696167	2.528157203	2.600926953	2.628042616	2.393084183
Zm00001d017345_T002	3.468757681	2.312135682	3.496582633	3.749207828	3.907620991	3.427899777
Zm00001d017345_T001	1.104913622	1.890143529	0.774865898	1.781808715	0	1.95232872
Zm00001d035186_T001	4.561144826	3.81183433	4.566045633	4.495929837	4.373721145	4.344974463
Zm00001d035186_T002	1.01950551	1.071984341	1.455630491	1.914702664	1.531649948	1.340585056
Zm00001d027453_T001	1.458137334	2.640490441	2.07170124	2.608990632	2.705326386	1.877247563
Zm00001d018954_T002	3.263463093	2.952748608	3.347160625	3.675758132	4.005632184	4.052450239
Zm00001d022192_T001	3.645802515	3.137898899	3.728319345	2.565432914	2.400398298	2.468684706

Zm00001d022192_T002	2.941558397	2.428387644	3.13154619	1.72226928	1.598655335	1.303760295
Zm00001d020561_T007	1.421091313	0.997918855	1.599971382	1.385194112	1.346368065	1.431641144
Zm00001d036758_T001	6.312999382	4.954295993	6.099937519	6.180248253	6.144593233	6.091776578
Zm00001d035195_T001	3.892521785	3.664678183	3.974420739	3.730372041	3.755184667	3.726497092
Zm00001d035254_T002	2.687077714	1.831871568	2.652466073	2.489268037	2.80184217	3.055642175
Zm00001d035254_T001	0	0.515591356	0.301848654	1.830076802	1.274691647	0
Zm00001d053301_T001	3.39653601	2.89843398	3.510080214	4.214622511	4.139675767	4.222800389
Zm00001d053301_T003	5.036287177	5.044238825	5.156615286	5.404103337	5.428778556	5.272881681
Zm00001d022324_T001	6.633908813	6.15324896	6.74526217	6.574095557	6.636949792	6.612995582
Zm00001d007470_T001	1.948595615	2.54192613	1.867015163	0.600596131	0.740203117	0.319045598
Zm00001d008356_T005	2.731433302	2.657647322	2.839638169	2.593297831	3.342861769	2.436713541
Zm00001d008356_T004	1.846571541	2.416285251	1.054116354	1.46430232	0	1.80656947
Zm00001d008356_T003	0.435976292	0.132551838	0.317577298	0.592419243	0.587349628	0.55925881
Zm00001d047349_T001	5.61959407	4.781376659	5.505762085	5.877959043	5.883346791	5.822453613
Zm00001d020886_T001	3.892617273	3.062326563	4.006997207	4.881096777	4.929343399	4.927230499
Zm00001d047717_T011	0.884025027	0.282532324	0.560627901	1.618943853	0.901023262	1.282325927
Zm00001d047717_T018	1.359547595	0.311976186	0.261584972	1.349891188	1.740209163	1.159164788
Zm00001d047717_T007	0	0.136557593	0.046007636	0.514210145	0.56390394	0.337103634
Zm00001d047717_T019	0.324865891	0.055691279	0.474217968	1.062015302	1.115717129	1.157308935
Zm00001d047717_T026	0.106848117	0.036182582	0.157380084	0.355713192	0.258786635	0.235489348
Zm00001d047717_T014	2.464485566	2.470203647	2.488553055	3.418963047	3.490156516	3.24458053
Zm00001d047717_T017	2.607335049	0	2.85556978	1.548079483	1.908460163	2.640579747
Zm00001d047717_T003	0.579983353	0.488903651	1.020268919	0.938088525	1.033575236	1.061405714
Zm00001d019348_T007	1.462093164	1.143384928	0.619256172	1.522692564	1.47783169	1.461812461
Zm00001d019348_T001	3.249951637	2.318065161	3.462031896	3.33803798	3.661367103	3.797474151
Zm00001d019348_T004	0.982620182	0.662797954	1.081979698	1.104364842	1.14035789	1.103443254
Zm00001d019348_T005	2.88164798	2.107769207	2.425491263	3.377889049	3.461882249	2.961952203
Zm00001d026549_T006	3.323448471	2.653943025	2.937808014	3.168850435	3.151934131	3.192921485
Zm00001d026549_T008	1.242823199	0.611739902	1.346730578	1.442550233	1.380734989	1.610720375
Zm00001d049176_T001	6.653824598	7.964358492	6.889128281	5.615151536	5.821502213	5.885986591
Zm00001d027278_T009	0	0	0	0.917959469	1.324031741	1.999478371
Zm00001d027278_T074	0.461326337	0	0	1.579938957	0	1.55261414

Zm00001d027278_T114	2.232906915	2.123307299	2.029346866	2.272515354	2.220994494	1.82875302
Zm00001d027278_T054	0.706621609	0.213565031	0.711418255	1.543139496	0	0
Zm00001d027278_T116	3.034218885	0.701211021	3.425040562	3.255109113	3.08019272	3.413397079
Zm00001d027278_T122	2.659835757	2.972617117	1.948008324	0	0.374907045	0
Zm00001d027278_T008	0	0.175048042	0.10224693	0.565413903	0.540385418	0
Zm00001d010522_T003	0.286620966	0.191563914	0.415688369	0.655775873	0.499370883	0.354579667
Zm00001d010522_T001	4.677018466	3.369505988	4.670645883	5.117063102	5.060670557	5.064741773
Zm00001d004401_T001	3.860491785	3.297547277	3.63278726	3.453346105	3.616306759	3.173860111
Zm00001d021430_T013	0.674918879	0.338351387	0.71032089	1.052391548	1.182764113	1.030242132
Zm00001d021430_T019	2.072704712	1.255594996	1.904684472	2.042033164	1.546340359	0
Zm00001d043149_T003	2.042855824	1.319260684	2.123845943	2.384122492	2.279386901	2.078008928
Zm00001d043149_T001	7.69778614	7.22515937	7.753553461	7.459959794	7.465914683	7.416784196
Zm00001d031077_T001	7.368111725	6.459875769	7.41807377	6.186086005	6.184819399	6.195679175
Zm00001d029740_T001	6.44955492	6.303443867	6.404495859	6.41846795	6.470461214	6.267307016
Zm00001d042180_T001	4.908642646	4.114086039	4.967025207	5.265920926	5.281065885	5.119239586
Zm00001d044111_T001	5.032758416	4.430289689	5.089358739	5.966229835	6.072549048	5.959474099
Zm00001d014952_T001	4.719674279	4.252858334	4.986312947	4.586004051	4.535594336	4.323789269
Zm00001d013252_T003	6.696601265	8.084370013	6.906412515	6.21175703	6.290225972	6.092813664
Zm00001d013252_T001	5.065564471	3.313819623	5.095020005	4.593524386	4.590433214	4.682488969
Zm00001d052930_T002	1.096735476	1.244527862	0	0	0.406342919	1.005931693
Zm00001d052930_T004	4.047405769	3.350129171	4.374841334	4.733253511	4.788261818	4.813117373
Zm00001d052930_T003	5.41928987	4.512776485	5.307764714	5.409369788	5.508770682	5.350448096
Zm00001d052930_T006	0.450415778	0	0.440725813	0	0.956655916	0.537516742
Zm00001d038513_T002	3.782863949	3.154456833	3.882767947	3.988916408	3.687650639	3.920575904
Zm00001d038513_T001	0.998779685	0.495096473	0.761806082	1.333170667	1.076654177	1.475899412
Zm00001d016237_T001	0.958214618	0.733481032	0.736867199	1.052220415	1.14962227	0.970084257
Zm00001d002822_T001	2.406394081	1.907401027	2.464744843	2.861510779	2.656340766	2.726820321
Zm00001d006788_T001	1.63548463	1.565449487	1.676909165	1.359921426	1.54059471	1.842712587
Zm00001d016832_T002	4.375911946	3.702702308	3.861513955	4.811368785	4.665063101	3.956739075
Zm00001d016832_T001	2.081169508	2.24430408	3.489513778	0	0	3.760440475
Zm00001d006797_T010	1.053116203	0.507885955	1.203511905	1.000199799	1.066366161	1.353369028
Zm00001d006797_T009	2.548887859	2.208914243	2.694937677	2.246289809	2.069962639	1.398752583

Zm00001d006797_T007	0.402328165	0.518472696	0.933549978	1.278665777	1.289953062	1.31674645
Zm00001d006797_T001	2.45650076	1.532632148	1.827297528	2.52667732	2.720195929	2.722919309
Zm00001d018337_T001	6.222459376	6.387092217	6.093007736	5.702742474	5.774087203	5.967521859
Zm00001d041640_T001	3.201619121	2.42988426	2.860597759	3.356557821	3.303788491	3.218955025
Zm00001d017296_T006	0.932817103	0	0	1.398561067	1.427973504	0
Zm00001d017296_T005	0	0.576366372	0.983836729	0.274279555	0	1.600380616
Zm00001d017296_T002	3.057173491	2.478512916	3.042406553	3.874531218	3.88106138	3.745896561
Zm00001d028113_T001	3.088997053	2.670834219	3.126235301	3.370194139	3.306485802	3.233554951
Zm00001d035491_T002	1.136661925	0.550229877	1.193810215	1.657835705	1.115419508	1.025800625
Zm00001d035491_T001	1.750007008	1.317804756	1.514586878	1.668288897	1.800695346	1.694160783
Zm00001d017699_T001	4.082072635	3.578922777	4.096108753	6.329620989	6.258290631	6.250476761
Zm00001d006524_T001	3.032059749	2.350019947	2.859594916	3.637775533	3.766574597	3.627979019
Zm00001d033597_T001	0.371195286	0.475850657	0.537141973	0.711408563	0.843028697	0.809610358
Zm00001d033597_T006	3.017356653	1.662451626	2.755263623	3.092809056	3.62822732	3.2700023
Zm00001d033597_T004	2.706596856	2.379599338	3.286064227	3.743163569	3.378736195	3.506142871
Zm00001d033597_T010	0.802601871	0.248251856	0.502559744	0.763294284	0.903329094	0.768103002
Zm00001d033597_T007	0	0	0	0.672978326	0.349379999	0.768633211
Zm00001d032931_T003	0.420305608	0.117446372	0.715984716	0.802074073	0.821167317	0.721489908
Zm00001d032931_T001	2.967037246	2.485153204	2.99348525	2.566882356	2.976869499	2.988605075
Zm00001d032931_T004	1.199582522	0.945187418	1.178708268	0.947140221	1.233285585	1.275752049
Zm00001d031090_T001	4.941065171	4.039844475	4.974805114	5.156941812	5.100488722	5.128601863
Zm00001d031090_T002	0.587865167	0.819183389	0.998325502	1.779441701	2.322636861	1.643769395
Zm00001d003198_T021	0.103813169	0	0	1.584774457	0.959690044	1.207821652
Zm00001d003198_T025	0.320770012	0	0.184937848	0.329511708	0.437797672	0.340510998
Zm00001d003198_T017	1.768958355	1.485812696	1.688929157	1.246213485	0.906998313	1.240243491
Zm00001d003198_T024	3.022530096	2.013402585	2.949493099	2.223934044	2.606846552	2.295825273
Zm00001d003198_T004	0.386366208	0.343805752	0.654608764	0.68375214	0.555412733	0.625705337
Zm00001d003198_T001	0	0.166754002	0.28936234	0	0.371661451	0.529656058
Zm00001d021913_T002	5.177696868	5.586714641	5.009033945	5.099937709	5.22063665	5.040047734
Zm00001d004669_T005	1.741838426	0.227971445	0.746479602	2.465616945	2.476513637	2.498555461
Zm00001d004669_T011	0.369965582	0.199185476	0.078012687	0.933802243	0.662686774	0.840824945
Zm00001d004669_T014	0.845584789	0.30878606	0.921051612	0.889937622	1.613575027	0

Zm00001d004669_T006	0	0	0	1.64630185	0.943708328	1.689297371
Zm00001d004669_T010	2.176959716	3.079836833	2.742352287	3.161882847	3.351291077	3.799275922
Zm00001d004669_T020	3.406957554	0	3.220174996	2.670679021	2.744326027	0
Zm00001d018439_T005	0.954200033	2.379021193	1.555771009	1.150286708	1.152610484	0.62093543
Zm00001d018439_T011	4.012145546	1.843764007	4.011370847	4.060708381	3.70260635	4.006536987
Zm00001d018439_T006	0.321345128	0.039215616	0.256177357	0.252356313	0.626887646	0.780186614
Zm00001d018439_T008	0.820602976	0.119034731	0	1.255298288	1.997500892	1.700810776
Zm00001d018439_T009	0.934827464	0.499976983	0.416917548	0.966763415	1.47103811	1.139398767
Zm00001d018439_T001	3.33366359	2.639161995	3.159330961	3.7298826	3.480644491	3.430723101
Zm00001d011642_T002	4.809573317	3.877445768	4.717280447	5.451417724	5.262770451	5.317440798
Zm00001d011642_T001	4.413015453	3.953141734	4.62294856	3.456202404	3.450342265	3.445274334
Zm00001d011642_T004	0.6988701	0.321627983	0.286186832	0.869452944	0.700823202	1.219840718
Zm00001d004611_T001	5.30035084	5.062997479	5.290268352	5.549034233	5.795755443	5.705363879
Zm00001d008757_T001	6.269527641	5.504809233	6.314291662	5.915185619	5.882999987	5.92261675
Zm00001d022450_T001	6.39126815	5.825043079	6.400547851	6.961807103	7.018334831	6.987899215
Zm00001d050196_T001	6.540929121	5.995485469	6.451838902	6.747828963	6.87842732	6.866904727
Zm00001d050196_T002	1.466122252	0.97911661	1.185585589	2.564461825	2.490344724	2.410439343
Zm00001d009394_T002	3.116413001	3.360435918	3.444645999	2.670503638	2.351794523	2.498399981
Zm00001d037741_T001	0.641561751	1.047275232	0.785069224	0.86988009	1.206334399	1.096570872
Zm00001d003089_T002	0.943944574	0.991655894	0.924293753	1.839115485	0.813523868	1.122583272
Zm00001d047452_T001	2.321896067	1.946567308	2.09366158	3.229678204	3.308949839	3.360663039
Zm00001d008749_T004	4.451788074	4.211836833	4.456154687	4.284257004	4.307269601	4.237235587
Zm00001d008749_T001	1.30792799	1.749518822	0.925435898	1.381234636	0.883743577	1.138542546
Zm00001d008749_T003	3.347553978	2.862227407	3.398188633	3.059677667	3.129603672	3.1487503
Zm00001d034312_T001	1.830833747	1.412544172	2.110705667	3.359914119	3.494351987	3.700209645
Zm00001d050580_T003	0.424497546	0.136721634	0.538245125	0.590579026	0.705639133	0.744072378
Zm00001d022445_T001	0.837936785	3.461146881	0.535582206	0.182434242	0.86717375	0
Zm00001d022445_T002	5.522847175	4.145885775	5.521451036	5.006842949	5.055347171	4.846896131
Zm00001d027239_T003	3.56295819	2.98648193	3.254392227	3.138318744	3.815385243	3.836739855
Zm00001d027239_T001	1.020343602	1.409054698	0.643701986	1.908260313	1.965737379	0.799844108
Zm00001d027239_T004	1.267810828	0.763583243	2.724508396	2.280472663	0.643387064	1.423545364
Zm00001d027239_T002	4.154779799	3.153391434	4.026742085	4.428133277	3.894771596	4.093774469

Zm00001d032479_T001	3.548449819	4.115176773	3.444077837	2.024918904	1.851989246	2.100006108
Zm00001d014158_T003	4.145823429	3.137887754	3.661557167	4.115858926	4.21106049	4.108962886
Zm00001d014158_T007	1.579689444	0.552787244	1.188826897	1.682208757	1.526328273	1.421920203
Zm00001d014158_T002	1.113649164	0.651855816	2.259700335	1.609166325	1.065117994	1.197855958
Zm00001d014158_T004	1.054954028	0	0	1.686278643	2.018739204	1.890112392
Zm00001d019044_T001	2.862081173	2.207062907	2.922012075	3.978921006	4.01813331	4.063732789
Zm00001d024729_T001	5.545083335	4.710214856	5.523263538	5.271893512	5.321130966	5.237779869
Zm00001d020743_T001	0.659851509	0.374538747	0.801907752	1.017474425	1.209180086	1.119380747
Zm00001d007827_T001	4.896057027	4.701535805	5.04394817	5.608161895	5.471122089	5.589988232
Zm00001d000196_T001	1.720218479	1.169879476	1.417888691	1.553118641	1.887758812	1.567727477
Zm00001d008528_T001	2.233948478	1.646751599	1.946662747	1.414523087	1.533484608	1.529290237
Zm00001d025789_T007	1.566168779	3.208724504	2.596090501	1.457445103	0	0
Zm00001d025789_T003	2.975150309	1.535221868	2.826126421	2.105857398	2.3134084	1.923189503
Zm00001d025789_T002	1.520145627	1.093272149	1.319856052	2.479623782	2.318906237	2.698144897
Zm00001d034984_T001	1.655269845	1.195016181	1.521398048	1.723079695	1.893187057	1.937103295
Zm00001d022502_T009	1.605900109	0	1.65875075	3.550039715	3.490978622	3.531161967
Zm00001d022502_T015	1.659409022	0	1.417447491	2.091774971	1.836178645	2.280071427
Zm00001d022502_T013	0	0.147023994	0.321618748	0	0.443889859	0
Zm00001d022502_T017	0.20557018	0.038839293	0.316350834	0.316409922	0.223656081	0.395922675
Zm00001d022502_T018	0.440026238	0	0.089926557	0.866574112	0.91513154	0.511797934
Zm00001d022502_T019	3.937582813	3.538259282	4.098611404	3.808914971	3.97521287	3.842797755
Zm00001d029715_T001	2.700698936	2.273391457	2.75971256	3.142092186	3.05982387	3.224349655
Zm00001d039300_T004	4.486962158	3.990050216	4.663154221	4.897402066	4.933768493	4.793341581
Zm00001d039300_T006	1.339278796	0.900847105	1.264301017	1.064906988	1.285942499	0.948983522
Zm00001d039300_T003	1.27203215	0.348022743	0.826899471	0.635550101	1.505095704	1.410925555
Zm00001d032438_T001	5.0184293	4.407557651	5.000842513	6.896362487	6.842941633	6.905961971
Zm00001d032855_T019	2.640416863	1.441539253	2.470775017	3.013102211	3.389475264	2.779525118
Zm00001d032855_T001	2.959383654	2.317660646	3.163694996	3.556448422	3.234695057	3.656072195
Zm00001d032855_T014	0	0	0.032598822	0.505378784	0.847470227	0.979620045
Zm00001d032855_T005	0.537621103	0.198450149	0.441856322	1.794064378	1.930644245	1.556656011
Zm00001d032855_T006	0	0	0	0.331759094	0.452682932	0.354209528
Zm00001d032855_T003	0.181509693	0	0.077065215	1.098352852	1.059335202	0.681997003

Zm00001d053062_T001	3.965164139	3.120274793	3.969253566	3.32847833	3.437158746	3.317332725
Zm00001d050375_T002	7.862766658	6.820985586	7.948694529	7.988340705	7.985316188	7.913139918
Zm00001d053825_T002	4.864679128	4.35888451	4.940463483	5.878687361	5.795225706	5.802357954
Zm00001d053825_T004	3.124357584	0	3.774613873	3.448140826	3.584219682	3.526993118
Zm00001d030172_T003	0	1.567635978	1.334203838	1.948245363	0	0
Zm00001d030172_T006	0.864866391	0.578606432	0	0	0.902627777	0.539356369
Zm00001d030172_T001	0.393841203	0.189398249	0.66060922	0.795856698	0.866381036	0.701443485
Zm00001d030172_T004	2.745888066	1.188968649	1.743763546	0	2.391918084	2.163546714
Zm00001d030172_T005	1.940614058	0	2.166165895	1.745848707	1.715160638	2.112256081
Zm00001d001999_T006	0.74247625	0.2860626	0.450617424	0.829381166	0.591336652	0.906274914
Zm00001d001999_T004	6.447023136	7.311523886	6.326064516	5.582040942	5.407016174	5.51190961
Zm00001d001999_T007	3.827105019	3.560058379	3.801568017	3.48453427	3.48080293	3.455867961
Zm00001d001999_T008	4.835737158	3.140630289	4.374733617	4.25525213	2.50140373	4.262935443
Zm00001d001999_T001	4.791869955	3.953765485	4.947736233	5.056972327	5.445223109	4.703914644
Zm00001d001999_T003	2.099920966	1.972746318	2.1449861	1.132605795	2.655998149	2.369091209
Zm00001d001999_T002	0.53998758	0.463149502	0.746256004	0.83152544	0.959563193	0.807695358
Zm00001d038436_T001	6.355874865	6.065629457	6.18285616	3.899533842	4.31212871	4.328513085
Zm00001d010962_T001	2.42516789	1.484909425	2.189454865	1.972520255	0	0
Zm00001d009707_T001	4.61405536	4.137893736	4.427259402	4.398462837	4.582994044	4.543837895
Zm00001d020618_T003	3.689524941	2.95539632	3.870645142	3.946850331	3.775186901	3.824263585
Zm00001d050330_T003	4.885172052	4.549038978	4.798061146	4.591850948	4.478057895	4.628343813
Zm00001d050330_T005	2.32782904	1.984140158	3.035575878	1.936465323	2.738210535	2.550462127
Zm00001d050330_T004	1.867809504	0	1.779754754	1.68797933	2.192659415	1.388592388
Zm00001d050330_T006	0	1.191984533	0	1.477198595	0	1.780558297
Zm00001d028453_T003	1.15970797	0	1.323997166	0	0.319820221	0
Zm00001d028453_T002	0	0.819324021	0	1.019287013	0.700717577	0.891353856
Zm00001d028453_T004	5.61478233	5.9910465	5.362514635	5.480625428	5.388645733	5.234432035
Zm00001d029975_T003	4.657352295	4.104986565	4.718734843	5.558843648	5.645792689	5.589880641
Zm00001d036927_T002	1.480154977	0.269333627	1.962171411	2.166594948	1.761244418	2.360918491
Zm00001d036927_T001	2.483928738	1.888592081	2.189616163	2.020696337	2.425844634	1.806338082
Zm00001d042394_T002	2.024301947	1.595637803	2.273472623	3.702841802	3.468429004	3.705472704
Zm00001d042394_T007	3.256528231	2.182997646	3.042513543	1.345729616	2.152133435	0

Zm00001d042394_T006	1.406192145	0.926105718	1.511398205	1.307169108	1.324384931	1.293355769
Zm00001d020185_T006	0.908766802	0.636425558	0.80833974	2.062545545	1.983409558	2.154612695
Zm00001d020185_T003	5.409664717	4.616693216	5.474577742	5.818833718	5.859686272	5.84164352
Zm00001d020185_T007	0.459202868	0	0	1.155438385	1.048735601	1.463317984
Zm00001d020185_T004	5.661431268	5.332894756	5.873542569	5.120067388	4.876017135	4.595502944
Zm00001d020185_T005	4.804180027	2.702180999	4.742863486	6.372030589	6.440097304	6.415717553
Zm00001d020185_T008	0.316602234	0.274043339	0.85216268	1.611318767	0.741823329	1.284925089
Zm00001d020185_T001	2.65111646	1.827412981	2.865035116	4.162200447	4.33118919	4.24376709
Zm00001d044332_T001	5.513377486	5.417884439	5.572186881	5.598489762	5.547204074	5.530538618
Zm00001d018632_T001	6.619735497	5.938481832	6.648803873	6.020992269	6.025655473	6.006665362
Zm00001d000238_T001	1.981271075	1.207016976	2.030130519	1.290885568	1.583787669	1.677022865
Zm00001d050728_T001	0.507910305	0.244736086	0.457714052	0.627063274	0.686214537	0.750722259
Zm00001d011581_T001	8.974003033	8.886076646	9.071593591	9.197190186	9.19986358	9.182769783
Zm00001d044953_T001	3.468286928	3.760156742	3.795711994	3.632235405	3.505982619	3.544270103
Zm00001d012224_T001	2.784460848	2.707069515	2.863086454	3.639385919	3.997779788	3.848054413
Zm00001d012224_T008	0.520734013	0	0	1.123633754	1.217908779	0.859458354
Zm00001d012224_T009	3.962761849	0	4.015667227	3.254547182	2.460426735	0
Zm00001d002271_T001	1.440367031	2.46584442	1.476228207	0.789351994	1.059460499	0.869663769
Zm00001d034700_T001	3.511798566	3.045761671	3.463316414	3.354475716	3.448684795	3.411836759
Zm00001d024048_T004	5.399144359	4.714591558	5.617191058	5.477997301	5.467678938	5.22646514
Zm00001d024048_T006	5.687854036	5.062269092	5.543935739	4.358513153	4.400573382	4.851390809
Zm00001d009177_T001	2.051985247	1.268980466	2.09227815	2.288258737	2.115949456	2.251013704
Zm00001d038075_T001	7.761196126	3.803979708	7.872724006	3.8797582	3.674570251	3.76109737
Zm00001d020655_T001	4.95173987	4.366107175	4.475399335	3.721004556	3.346689027	3.597504835
Zm00001d047239_T002	0.140607223	0.716298235	0.761269101	1.379467924	0.70012895	1.335023551
Zm00001d047239_T010	0	0	1.062853238	1.129425466	1.006795654	0.88825813
Zm00001d047239_T003	1.514003051	0	1.386944452	2.187752547	2.109982974	2.271761808
Zm00001d047239_T011	1.730036452	0.518025448	1.194062461	0.840269049	1.127103644	1.17512535
Zm00001d028533_T001	3.367292219	2.451626284	3.372184592	3.518685066	3.208745101	3.308284853
Zm00001d024344_T001	2.712103794	2.513010104	2.566701438	2.185790762	2.057650936	1.85908856
Zm00001d052273_T002	2.35583805	2.346872665	2.873363393	3.760294091	3.528169834	3.731434714
Zm00001d052273_T001	0.183317542	0.316106343	0.655345417	1.003926717	1.035136126	0.971858039

Zm00001d012924_T001	5.111855367	4.300839768	5.087882604	4.726867284	4.87703421	4.685177123
Zm00001d029820_T001	6.36460435	5.830249847	6.399433712	6.160153755	6.437464029	6.212771411
Zm00001d027892_T001	5.265088295	4.494767408	5.221223159	6.402900081	6.36281113	6.423447627
Zm00001d037610_T001	5.022790527	4.896517784	5.274990318	2.716451895	2.656809359	3.035426493
Zm00001d050816_T001	1.354204449	1.345254995	1.5300048	1.818979367	2.091532286	1.983173849
Zm00001d050816_T002	5.386530242	4.93248114	5.471643808	4.942014613	4.894096866	4.723931255
Zm00001d038489_T001	2.78863414	2.129288623	2.966773934	2.929814853	2.95387609	3.09386893
Zm00001d001936_T001	1.724649835	1.095350603	1.852109533	1.555588446	1.895330932	1.636801387
Zm00001d039717_T001	0.561919469	0.800685822	0.680438688	0.516448899	0.372994431	0.662531835
Zm00001d039717_T003	4.157596098	3.4439523	4.165546437	4.770856526	4.231641071	4.303273342
Zm00001d039717_T002	0.772639047	0	1.177885271	0.976681837	0.895345281	0
Zm00001d039717_T006	4.027026359	3.297895861	3.737837944	3.070414231	4.213533381	4.150516135
Zm00001d052997_T001	3.019195192	2.206242802	2.890983785	3.163586644	3.176273458	3.177781646
Zm00001d029579_T001	3.584059572	3.407135039	3.743570439	4.710725227	4.616547138	4.670891536
Zm00001d013645_T001	4.418718412	4.805586486	4.357948234	4.234513047	4.229603149	4.304923494
Zm00001d029241_T001	3.034739724	2.256455466	3.018481067	2.024907915	2.095027139	2.11147741
Zm00001d052918_T001	1.685517255	1.326754196	1.832031634	1.794083514	1.728128623	2.042975909
Zm00001d040593_T001	1.215075924	0.979576148	1.437809388	0.881043559	1.154291556	0.968401935
Zm00001d004929_T001	2.582305978	1.942542655	2.645534971	1.705321077	1.9468682	1.757427827
Zm00001d004525_T001	1.474234065	0.824322249	1.842282149	1.890694935	1.904884818	1.829072202
Zm00001d004525_T003	4.524115155	3.634719412	4.386856875	5.050754735	5.091318972	5.007645511
Zm00001d040606_T007	2.901264092	1.296110238	2.75274902	2.77427872	2.291284397	3.413550487
Zm00001d040606_T008	1.538956261	2.786250492	1.523241768	1.904564251	2.24766902	1.874351206
Zm00001d040606_T002	0.141567506	0.042819411	0.203925298	0.783546335	0.9159935	0.651232211
Zm00001d040606_T001	1.773722103	0.943396274	2.040541093	2.670054886	2.845074696	2.501527042
Zm00001d049357_T001	3.638737324	2.760666356	3.694716307	4.065987725	4.037239439	4.00265617
Zm00001d003999_T001	3.745890754	4.794723453	3.60870423	3.693342876	3.633617203	3.428144669
Zm00001d010482_T001	2.126768064	2.64614906	2.490572184	3.29311721	3.159770445	3.606298899
Zm00001d039606_T001	1.460332628	0.926824556	2.042113034	2.596667802	2.172783193	2.298637789
Zm00001d039606_T002	0.998538531	1.170018613	0.577695166	0.308874576	1.050610353	0.910427208
Zm00001d047255_T002	0.471975069	0.391260708	0.547123051	0.603293767	1.051936539	0.585913404
Zm00001d047255_T001	4.174233742	3.254511506	4.151008987	4.465723505	4.497569704	4.324151954

Zm00001d051288_T001	3.595834459	3.1314647	3.646663711	2.566274742	2.777753404	2.713237202
Zm00001d021505_T001	3.513360746	3.10440829	3.567361814	3.242068313	3.247510894	3.279400571
Zm00001d045512_T001	0.796478978	0.464473804	0.57111661	0.766066748	0.789765136	0.772502236
Zm00001d050963_T001	1.586202205	1.235837938	0.983382924	1.025891318	1.566269627	1.881551078
Zm00001d002598_T001	4.567709469	3.922770245	4.470091363	4.585129603	4.65396142	4.597930676
Zm00001d022327_T001	3.819146185	3.508317847	3.835062443	6.506826886	6.500208556	6.581452018
Zm00001d019218_T001	1.13201084	0.553447996	1.68375214	1.173317434	1.236613233	1.031971736
Zm00001d009074_T011	0.459124161	0.185425108	0.323093322	0.571947608	0.883676334	0
Zm00001d009074_T002	6.190990378	4.996371255	6.082820936	5.7715523	5.918624589	5.820771495
Zm00001d009074_T010	0.686801697	0.259242352	0.563064389	0.509790076	0.703305392	0.781296758
Zm00001d009074_T009	0	5.293350986	2.814676538	2.028035822	2.310114973	2.647091583
Zm00001d050411_T001	6.027342428	6.308801856	6.113503919	5.473022379	5.368260768	5.439803164
Zm00001d022468_T007	0.19809052	0.040446406	0.229992682	0.713358853	0.826365031	0.77645272
Zm00001d022468_T002	0	0	0	0.729432695	0.230739158	0.399834991
Zm00001d022468_T005	2.965459977	2.272007657	2.709231294	3.311754054	3.304733708	3.344218915
Zm00001d022468_T006	0	0.030581863	0.047401581	0.447426853	0.807143861	0.69419645
Zm00001d002510_T020	0.582364273	0	0.96357252	0	0.816050784	0
Zm00001d002510_T001	0.082280209	0	0	0.390927369	0.577166309	0.460555958
Zm00001d040234_T004	0.2302436	0.422497829	0.37103912	0.378230825	0	0.117712328
Zm00001d040234_T016	1.251300385	1.021240206	1.424345432	0.869816145	1.157969196	0.884094599
Zm00001d040234_T008	2.416529826	1.500687856	1.817294572	1.824895318	2.714018846	2.751276249
Zm00001d040234_T011	0	0	0	2.388962075	1.443954023	1.071045948
Zm00001d040234_T015	0	0	0.909702455	0.313394468	0.993775791	1.40579418
Zm00001d012662_T001	1.90463284	2.897662909	2.161155026	1.845228686	2.544315735	2.068998518
Zm00001d012662_T002	4.779653702	3.652734629	4.844587821	5.247210468	5.211918423	5.1887627
Zm00001d041923_T001	3.552167021	3.042871754	3.288947038	2.303158539	2.378617047	2.372381313
Zm00001d041923_T002	2.99231634	2.223448809	2.835069924	3.478384515	3.601317135	3.331053076
Zm00001d041923_T003	0.734977056	0.48315692	0.55415954	0.686056725	0.479261609	1.172422228
Zm00001d003006_T001	7.938941725	7.533892908	8.020042602	8.679260645	8.706367056	8.710896408
Zm00001d032328_T004	0.349084412	0.263596947	0.274508576	0.614919932	0.728331537	0.679679614
Zm00001d032328_T001	2.050940795	1.905979294	2.613176122	0	1.838930797	1.734208857
Zm00001d032328_T002	0	1.364933761	1.061493505	1.130924282	0	1.368733713

Zm00001d032328_T005	3.147150697	2.317105192	3.185393232	2.628705249	3.024537237	2.648023133
Zm00001d011139_T002	1.013349341	0.653662658	1.145884797	0.73819367	0.550541177	1.013523718
Zm00001d011139_T004	7.124038554	6.61300752	7.202938861	6.706326649	6.718984837	6.627047321
Zm00001d045459_T001	5.527352608	5.275021616	5.371798908	7.767795486	7.808387458	7.984631136
Zm00001d028924_T001	4.639633712	4.466570024	4.823955307	4.630986032	4.843100777	4.855426558
Zm00001d006673_T003	1.573862614	1.234186751	1.759173734	1.774328276	1.609476039	2.221096918
Zm00001d030087_T001	2.633281934	1.798456196	2.562893978	2.954958411	2.831768224	2.923641154
Zm00001d013526_T002	2.665309536	3.578006395	2.941118333	2.312811817	2.143557337	2.304528856
Zm00001d013526_T001	0.707541577	0.409627722	1.275727619	1.226005965	1.442231723	1.328410829
Zm00001d013526_T004	0.92562275	0.629880675	0.93130787	0.827840157	0.733793374	0.223983455
Zm00001d013526_T005	5.235791306	4.117086506	5.415123765	5.234831377	5.105612545	5.190540788
Zm00001d033040_T001	1.52588646	0.661134819	0.861783897	1.101174029	1.234761264	0.98407233
Zm00001d017207_T002	4.831954743	3.850310876	4.860384313	2.877863783	3.000269939	2.946281889
Zm00001d048957_T001	3.621607079	5.605761614	3.66886502	2.938845633	3.084944925	2.730966821
Zm00001d048957_T003	7.364696999	7.223294156	7.389044394	7.386401881	7.417248633	7.352068063
Zm00001d042632_T001	4.119031244	3.417292067	4.289038108	4.253861626	4.188402586	4.325039588
Zm00001d036835_T004	2.693643513	3.11771532	2.809859532	3.73634088	3.445722864	3.36986413
Zm00001d036835_T001	4.007361563	3.518093451	4.115032743	4.905995655	4.879738942	4.890352666
Zm00001d034582_T009	1.725409615	0	0	1.739328812	1.361552781	1.017347565
Zm00001d034582_T021	1.297169108	1.048969904	2.29982666	0	1.777622547	1.59605829
Zm00001d034582_T016	0.712704941	0	0.352043214	1.132039804	1.341329983	1.406952929
Zm00001d034582_T015	0.49419951	0	0	0.833738575	0.767759872	0.317451109
Zm00001d034582_T001	0	1.138695877	1.144393619	0.210685603	0.554917871	0.236488945
Zm00001d034582_T012	0	0.567949385	0.760838352	0.816747143	0.610966468	1.038327308
Zm00001d029359_T001	6.967654709	6.275625947	6.921616038	7.49476896	7.510758963	7.478401019
Zm00001d029359_T002	1.607461941	1.287833958	1.262086725	0.703641166	0.842953094	0.675319143
Zm00001d034776_T002	6.864421489	5.060110565	6.992721409	6.454520302	6.299821148	6.863801767
Zm00001d034776_T008	5.306487953	6.301833275	6.649399599	0	3.300929212	0
Zm00001d031701_T002	1.739059156	4.558648343	2.215111035	2.095013969	0.897969141	1.70561569
Zm00001d031701_T001	7.305552203	8.077590528	7.383659922	6.442987041	6.630934546	6.54979634
Zm00001d045893_T001	3.965169219	3.169356307	4.086100294	4.230253209	4.21014733	4.086268135
Zm00001d049761_T037	0.129618013	0.039030275	0.099291164	1.144471282	0.897527774	1.060403689

Zm00001d049761_T019	0.265777725	0.135460008	0.485156812	1.464500988	1.081177349	1.466468943
Zm00001d049761_T036	0	0	0	0.946502556	0.632710264	0.771616025
Zm00001d049761_T006	3.644915201	2.639311359	3.817602691	3.908714644	3.906935511	3.897065452
Zm00001d051543_T001	8.427209247	9.139760055	8.399712909	8.005808321	7.86045064	8.061317756
Zm00001d021465_T001	3.605741083	3.429770067	3.488935262	2.652787049	2.943309809	2.833524863
Zm00001d049678_T001	1.673950285	1.220590607	1.322150253	1.103837334	1.138603487	0.877376758
Zm00001d046405_T001	3.028641816	2.44186535	2.875328315	2.809416502	2.503310314	2.372825926
Zm00001d017448_T006	2.378111775	2.73207509	3.173117037	2.642759325	2.572694467	2.716952276
Zm00001d017448_T007	1.251557926	1.155371674	1.987691225	1.517226805	2.01151997	1.278416004
Zm00001d017448_T008	3.996283252	2.413181361	3.764880612	4.235437726	4.226876632	4.134509039
Zm00001d017448_T010	0	4.526792295	3.610270709	0	0	3.041266116
Zm00001d017448_T009	1.015533965	1.121791263	0.663426601	1.548064189	1.672985565	1.304839261
Zm00001d017448_T005	2.09150859	1.996358012	1.854778441	1.714951469	2.337963363	1.439571013
Zm00001d007195_T019	0	0	0.173003312	0.319568225	0.396387496	0.296371651
Zm00001d007195_T012	1.478681943	0	1.748741587	3.062675571	2.886659784	2.93652054
Zm00001d007195_T009	0.47153814	0.22356465	0.650662556	0.882431765	0.657166193	1.047874769
Zm00001d007195_T001	0.847514325	0.574511364	0.712203966	0.409773251	0	0.718233162
Zm00001d028696_T001	1.242557388	0.544676282	1.480723712	1.112550685	1.117367906	1.146425832
Zm00001d028696_T003	1.207925328	1.443299516	2.11860392	1.445470872	1.444351675	1.57281935
Zm00001d029107_T001	4.746001403	5.874580324	4.356204461	3.329909615	2.770396913	2.924930669
Zm00001d032166_T008	0.458030212	0.027084676	0.158471466	0.516662701	0.231492622	0.45857834
Zm00001d032166_T013	3.915429116	3.499196736	3.825173726	3.791010626	3.647666332	3.669290442
Zm00001d032166_T014	0.506085985	0	0	0	0.968749908	0.785427515
Zm00001d032166_T003	3.014280835	2.461408854	2.98765303	3.102147639	3.099443675	3.270176074
Zm00001d032166_T010	3.629495812	2.043343455	3.790084509	4.058683011	4.119475207	4.091653126
Zm00001d032166_T001	0.18731042	0.344803505	0.494839524	0.333723692	0.462973674	0.37153656
Zm00001d032166_T005	0.970489254	0.861993465	0.925233068	1.647010049	1.550621454	1.681769029
Zm00001d019069_T002	5.859848822	5.266466189	6.007923932	5.594636834	5.757858477	5.648245801
Zm00001d025867_T002	3.435211326	3.683038615	3.527696435	3.181274964	3.544782847	3.104103461
Zm00001d023833_T003	4.285725346	3.824664	3.996668129	4.333228785	4.218998563	4.329886659
Zm00001d039331_T001	1.169070031	0.410384526	1.159896518	1.086486231	1.101783852	1.043364805
Zm00001d012694_T005	1.141955232	0.313837903	0.70848303	1.100740206	0.972826442	0.857233386

Zm00001d012694_T004	3.28108529	2.636815073	3.428583549	3.615545658	3.686698065	3.654539454
Zm00001d012694_T001	0.753897153	0.129242126	0.57329793	0.875388477	0.869522433	0.190340517
Zm00001d033110_T001	3.319233077	3.149788896	3.497108644	3.552498069	3.806433467	3.55912932
Zm00001d048412_T002	1.493618644	1.559251466	1.26913491	1.415853834	1.463526727	1.798396471
Zm00001d048412_T003	2.417306516	1.2189386	2.644494528	2.565317139	2.291648936	1.742055809
Zm00001d006175_T001	3.876399463	3.491087816	3.922485883	3.633113573	3.627864423	3.490511985
Zm00001d034880_T001	5.359142912	4.869333256	5.338469735	5.100533085	5.136145194	5.187064436
Zm00001d011787_T001	4.872385346	4.713207338	5.01520526	6.146575803	6.068565141	6.174782245
Zm00001d028328_T001	2.774432021	3.925604712	2.831967812	3.316744858	3.347791072	3.51558404
Zm00001d010235_T016	0.841878948	0.522031637	2.124255337	0.925675155	1.532950758	1.482150769
Zm00001d010235_T008	0.615152595	0.399420501	0.099190155	0.349539659	0.289847446	0.082021269
Zm00001d010235_T019	0	0.27521211	0.97528607	0.832288108	0	0.798287798
Zm00001d010235_T010	0.316810735	0.152462519	0.260576124	0.90121562	0.802011188	0.231850164
Zm00001d010235_T013	0	1.122787335	1.014434568	0.61603769	0.815122876	0.75380561
Zm00001d010235_T015	1.732196249	1.050302475	1.702712059	1.614619393	1.26785037	1.530850326
Zm00001d010235_T014	1.262742231	0.196092064	0.98885989	1.234531977	1.653709886	2.113330111
Zm00001d010235_T001	4.381451239	3.674686846	4.110520852	3.681308137	3.421262758	3.16695914
Zm00001d010235_T009	0	0	0	0.651598692	1.401375433	1.091172414
Zm00001d010235_T020	1.141574045	0	1.582797797	0	1.878821845	1.815422954
Zm00001d012661_T013	2.511342538	0.732547942	2.512176815	2.355606363	2.075302945	2.197744009
Zm00001d012661_T005	0.571687488	0.570604765	0.951166263	1.941630695	1.969120275	2.058216387
Zm00001d012661_T008	1.701262929	1.510355354	2.022066636	0.771677714	1.07562915	0.942512253
Zm00001d012661_T001	1.562660058	1.431831527	1.034948148	0.369036473	0	0.187159635
Zm00001d017215_T002	1.674880473	0.909333798	0.821968122	0.551909706	1.903334494	0.516758498
Zm00001d017215_T001	0.593045836	0.96264304	1.503812766	1.432319136	0	1.632463199
Zm00001d022119_T001	1.902011431	1.484135037	1.875025104	1.822163154	1.641431811	1.575306521
Zm00001d020519_T002	4.383246635	3.829102351	4.464853362	4.375391267	4.458320381	4.51450476
Zm00001d035700_T001	3.458163195	2.015271328	2.74568352	3.454497699	3.112769514	2.886568486
Zm00001d032435_T001	4.004600445	3.28718089	3.906577689	4.131423377	4.062942581	4.087966254
Zm00001d050182_T001	5.298634344	4.945933556	5.26775829	4.553005992	4.529198392	4.631726638
Zm00001d040504_T001	3.620661241	3.375698957	3.560419539	3.050247789	2.389953589	2.601329022
Zm00001d030276_T001	1.574804393	1.828051463	2.204210533	1.441267803	1.336146254	1.543816062

Zm00001d049940_T001	1.192688448	0.775852074	1.127604888	2.602072709	2.648422645	2.4677044
Zm00001d049940_T002	3.782574528	2.585214229	3.8850209	4.197956657	3.900379083	4.254016638
Zm00001d010109_T008	3.977151678	0	4.078588744	3.161263884	2.863747796	3.76538665
Zm00001d010109_T002	3.587652301	3.459031674	3.610073766	4.003559153	3.963980735	3.468567417
Zm00001d010109_T004	2.579352025	0.611167658	2.009137884	2.224796626	2.947981402	2.688836753
Zm00001d047821_T001	2.82341473	3.724416169	3.088536825	2.982693919	3.172165049	3.646788112
Zm00001d023682_T006	4.705500517	3.744361875	4.648532284	4.414265519	4.312145922	4.387857693
Zm00001d052164_T006	3.517579572	2.397075408	3.535658095	8.262752499	8.27171227	8.249856075
Zm00001d052164_T001	0.495361565	1.159058194	0.54961299	2.854742941	3.355269193	3.138645096
Zm00001d052164_T002	0	1.087327052	1.903296698	4.077053248	4.124342612	3.973298734
Zm00001d052164_T005	3.942390358	2.594890313	4.098603993	6.949428279	6.741135439	6.931696234
Zm00001d016175_T001	2.824241586	2.181096824	2.787073418	2.854743141	2.86218832	2.771770243
Zm00001d017654_T001	0	0	0.155545894	0.513171347	0.452224303	0.744401381
Zm00001d017654_T002	2.750186497	2.328131025	0.720391424	1.488351501	1.859521568	2.194081692
Zm00001d017654_T008	0.424623309	0.252582785	0.652758143	0.764950761	0.796728978	0.770825664
Zm00001d017654_T005	3.738437006	2.790608579	3.956444325	4.19464624	4.153520659	3.954915059
Zm00001d038465_T001	2.331122748	1.485846693	2.330181185	0.681706521	0.811968284	1.066841435
Zm00001d015700_T001	4.127684863	4.489087269	4.227269135	4.345010455	4.192987745	4.225700255
Zm00001d048021_T001	6.294876074	5.705469386	6.40758598	7.408970092	7.502120268	7.478567729
Zm00001d036196_T001	3.833219012	3.698279052	4.2732888	3.888327011	3.958959571	3.707830437
Zm00001d024698_T002	3.948016269	2.924053696	3.789113862	3.684399891	3.806842777	3.836665033
Zm00001d032556_T001	4.943608521	5.069593222	5.009091068	3.997189148	3.802363952	3.8770076
Zm00001d029247_T001	3.708201475	2.86253927	3.560049327	2.63878238	2.671287257	2.652630126
Zm00001d039883_T001	6.290949228	6.651290742	6.308542195	6.168556959	5.948215034	6.098007926
Zm00001d032876_T001	4.281089297	4.849887251	4.17067677	4.068993017	4.144652533	4.138232147
Zm00001d045319_T001	2.291788303	1.67071346	1.982174028	2.443871298	2.470947815	2.412030316
Zm00001d031817_T007	2.320711679	1.566410412	2.547516459	2.471235593	2.343152521	2.278034423
Zm00001d031817_T001	1.750053759	1.146107102	2.464998585	1.686019063	1.801218267	2.3728081
Zm00001d031817_T006	3.564789089	1.095687495	3.069216662	4.161548488	3.468892284	3.817344412
Zm00001d031817_T008	5.487298926	6.472218037	5.594986755	4.819317531	4.975402191	4.714300806
Zm00001d031817_T003	4.478717661	3.812267068	4.535182046	4.416513883	4.922888958	4.482195309
Zm00001d005752_T001	5.89175525	5.598016162	6.006060099	4.258254557	4.439462504	4.407978305

Zm00001d035054_T002	4.54297668	4.77668574	4.492973859	4.229862978	4.630192442	4.859342356
Zm00001d035054_T004	6.461374194	6.373251729	6.547875005	6.596202163	6.600526586	6.593997551
Zm00001d014626_T003	0.452563808	0.185299502	0.578056649	0.257312406	0.29515171	0.252586418
Zm00001d014626_T002	5.094006893	4.446579609	4.996521304	5.039837677	5.202309653	5.039172618
Zm00001d019591_T001	2.878494845	4.091403819	3.081781198	0	1.801822102	0
Zm00001d019591_T011	0.721775114	0.238775856	0	1.195259396	0.26299954	0.741140766
Zm00001d019591_T006	0	0.853432028	0	0	1.275262775	0.882537411
Zm00001d019591_T014	2.656080549	1.61285871	2.551387307	3.142625824	2.141274844	2.504877894
Zm00001d019591_T009	0.998268449	0	0	1.128702525	1.783098713	0.947870345
Zm00001d019591_T035	0	0.268758948	0	0.769652426	0.698254045	0.3321889
Zm00001d042178_T001	3.541005643	3.476078729	3.377986054	2.226603473	2.913313007	2.786257812
Zm00001d024027_T001	6.117810117	5.937272444	6.151761036	5.2338996	5.158121285	5.256555705
Zm00001d032301_T002	0.241648625	0.698807884	1.087539557	0	0.279358372	1.006175924
Zm00001d032301_T005	1.314719725	0.315958006	0	0.961255848	0.879855517	0
Zm00001d022104_T001	3.976919525	3.497949785	3.941210564	4.037603969	3.921650358	4.221362614
Zm00001d010360_T001	3.356658946	3.218777914	3.391575137	1.635830064	1.622756561	1.832630407
Zm00001d030856_T001	1.457444053	0.901820338	1.235820786	2.487026816	2.58163566	2.384972339
Zm00001d030173_T001	0.405810514	0.377005996	0.622890067	0.579025634	0.633441441	0.529354212
Zm00001d030173_T017	0	0.272100848	0	0.822950359	0.723366335	0.937457393
Zm00001d030173_T003	0.110728047	0	0.360798003	0.215540688	0.368939284	0.283163195
Zm00001d030173_T013	0.415412551	0.347263119	0.529053304	1.058242367	0.970829364	1.032015478
Zm00001d030173_T006	0.298099896	0	0.705866462	1.034812253	0.474650971	0.606397691
Zm00001d030173_T005	4.512309577	3.620548525	4.488789023	5.030975349	5.114918259	5.087926211
Zm00001d030173_T009	0.349563436	0	0.376683792	0.66326901	0.866482325	0
Zm00001d015434_T001	5.527813705	6.113809935	5.361746115	4.319168973	4.313292731	4.075359429
Zm00001d052901_T002	1.04281661	1.172839506	1.523266361	1.865957603	1.786529871	1.114546268
Zm00001d052901_T004	0.517616356	0	0.508662913	0.989893953	0.607686815	0.790775374
Zm00001d052901_T003	3.485254982	3.310344774	3.734818313	2.237110281	2.767481919	3.066068863
Zm00001d052901_T001	3.426841181	2.057599646	2.800417869	3.727202631	3.697351489	3.704072397
Zm00001d040124_T001	4.203481288	4.240571625	4.340594882	4.54893242	4.504378228	4.354150627
Zm00001d038407_T001	0.672500473	0.3670646	0.591027328	0.849342794	1.129005993	0.879055623
Zm00001d038407_T002	0.276190526	0.176446609	0	0	0	0.535385126

Zm00001d024545_T001	4.176995528	5.229941401	4.515081886	5.229200204	5.091687692	5.285405141
Zm00001d019510_T001	1.263117358	0.820169157	1.483229166	1.978365887	1.912948336	1.826974699
Zm00001d037770_T001	4.853018754	4.2451583	4.969741624	4.644807708	4.532768713	4.521800731
Zm00001d006022_T001	4.353318986	3.535434772	4.417472403	3.007217931	2.918963197	2.929234449
Zm00001d010812_T001	2.104666764	1.708744338	1.936402378	5.007188593	5.220305961	5.016557293
Zm00001d038850_T001	3.187182761	2.752842128	3.520079607	2.784314682	2.827921229	2.963206091
Zm00001d049169_T001	4.071272193	3.266087133	4.044846617	2.940958089	3.136750995	2.979694302
Zm00001d027313_T001	4.980908703	4.581605293	5.118726583	4.78459339	4.606166988	4.537639427
Zm00001d031269_T002	1.09873081	1.893416189	1.556095834	1.045268914	1.126531525	0.642745926
Zm00001d031269_T005	4.584794297	3.125069311	4.655651042	4.641953926	4.618229668	4.688057685
Zm00001d031269_T003	2.390790106	2.332942755	2.776707266	2.198770094	2.698494542	2.69263341
Zm00001d031269_T010	2.451984651	5.807598768	3.807913755	0	0	0
Zm00001d025208_T002	1.329691375	0.823624647	1.516040873	1.518821635	1.109988321	1.607827876
Zm00001d025208_T001	2.116301538	1.795961815	2.139710797	1.630368658	1.915175913	1.602786563
Zm00001d003175_T001	3.82052384	3.601342335	3.985267249	3.405121444	3.527470125	3.512319944
Zm00001d045036_T001	3.549907547	3.59101368	3.869131909	3.106996147	3.288112678	3.148958664
Zm00001d052660_T019	4.937386345	5.617303773	4.956561182	4.187627461	4.364149857	4.857458894
Zm00001d052660_T008	0.745451623	0.381544751	0	1.660418004	1.213179282	1.558953792
Zm00001d052660_T015	0	0	2.274332042	2.239032586	1.728056769	2.92912819
Zm00001d052660_T001	0	0.713536582	1.310325577	0	2.014099585	2.006594612
Zm00001d052660_T003	1.744410423	2.096130601	2.866562639	3.12357466	3.514865073	2.965308509
Zm00001d052660_T007	1.203913399	0.602657359	1.3693777	1.59259242	0.899746407	1.273571388
Zm00001d052660_T014	1.940624957	1.001440533	0	2.594682303	0	0
Zm00001d052660_T005	0.260261769	0.018034469	0.152565058	0.457593241	0.475691933	0.386042776
Zm00001d052660_T006	1.719898805	0.785169689	1.784171853	2.483440209	2.510668795	1.857971443
Zm00001d031222_T001	5.755242896	5.150527061	5.806948772	6.131305494	6.053560132	6.133474834
Zm00001d045296_T001	7.244978556	8.54547011	7.2375665	7.011071414	6.727018965	7.044544928
Zm00001d026664_T001	5.653917122	5.895715228	5.691808329	5.692453367	5.663301805	5.747527258
Zm00001d008388_T004	3.526715124	4.396213	1.898869267	0	0.928052658	1.25656748
Zm00001d008388_T001	3.304025584	3.071120452	3.674467657	4.300122773	4.3777436	3.917557401
Zm00001d008388_T003	4.145450763	0	4.214973227	4.13597879	3.74034756	4.417666961
Zm00001d031081_T001	1.154428316	0.978998043	0.748230212	0.92401853	0.675168266	0.297774834

Zm00001d031081_T004	3.669809487	2.877116394	3.695300351	3.620104376	3.651712567	3.684397535
Zm00001d031081_T002	4.181813225	4.040423084	3.717754827	3.505090749	3.693885672	3.489240801
Zm00001d017490_T001	1.73325541	5.993990375	1.773286615	1.961824069	2.098120032	1.852649701
Zm00001d005716_T002	3.604067053	4.012965941	3.589190369	3.321882938	3.305370708	2.984890982
Zm00001d005716_T001	3.212918843	3.789125029	3.42723325	3.310911559	3.551662925	3.294569436
Zm00001d038115_T002	2.787929716	2.079752199	2.655441822	2.868945692	2.757710497	3.088147535
Zm00001d026440_T001	1.900367585	3.260814856	2.060509871	1.984311903	2.605977364	1.633550248
Zm00001d010054_T002	1.52303948	2.087679738	1.852392028	1.933664787	2.249312747	1.907537163
Zm00001d010054_T006	5.042543879	5.672402457	5.422592519	5.510365957	5.139383458	5.032958137
Zm00001d010054_T001	5.558270941	4.736869147	5.435559491	6.002566457	5.966618544	6.09633321
Zm00001d010054_T004	1.055210928	0.718498847	1.262913575	1.049329652	1.286536988	1.432779326
Zm00001d028194_T001	2.184789354	3.004745686	2.576918492	2.42942877	2.260651692	1.868035588
Zm00001d028194_T004	5.052875706	0	5.127212171	5.614943218	5.705337833	5.543375891
Zm00001d028194_T005	4.092591406	2.636177504	3.880811376	3.516752826	0	3.257338809
Zm00001d028194_T003	0	4.201660676	0.851688684	1.019141093	2.662722088	2.735048566
Zm00001d047726_T001	3.370753098	2.666368262	3.489237204	3.848061025	3.574227723	3.532522438
Zm00001d028522_T001	3.979169029	3.427041588	4.246159357	3.762235692	3.783747679	3.764051509
Zm00001d006605_T001	3.563985307	2.898971589	3.630530001	3.487238204	3.485126277	3.535123182
Zm00001d015741_T001	4.548895008	4.496979906	4.661634861	4.005220044	4.232837169	4.019234608
Zm00001d021267_T001	1.064447633	0.593797388	0.962295071	1.23804209	1.376394303	1.462598921
Zm00001d021267_T002	5.467328037	5.587550237	5.406761615	5.626437151	5.635278125	5.529912033
Zm00001d012394_T001	4.486654785	3.794254899	4.629825318	5.486032825	5.543722292	5.40925036
Zm00001d008271_T003	4.105855387	3.298335139	3.915233863	3.643395839	3.674862964	3.605225492
Zm00001d025286_T001	6.934254099	6.288714504	6.947656655	6.514118361	6.51459622	6.561675448
Zm00001d003257_T001	5.87152524	5.135565692	5.797675661	5.9272291	5.932670648	5.866942364
Zm00001d031311_T001	3.341907777	3.364980531	3.545956017	2.81427888	3.081859068	3.260169316
Zm00001d008957_T001	5.50138357	4.52469478	5.527551352	4.940457375	4.840971386	4.972356798
Zm00001d008564_T001	4.214365053	4.049051308	3.965718081	4.389351149	4.260939324	4.343667839
Zm00001d044192_T001	4.169699442	3.502578711	4.234323885	4.182407862	4.311291448	4.129806909
Zm00001d044192_T002	4.723623924	5.781326977	4.631783377	4.470107962	4.616716326	4.778162308
Zm00001d028294_T001	2.999718467	2.315606514	2.799171254	2.449772646	2.207294727	2.223304535
Zm00001d011931_T001	1.77636723	1.173548984	1.691005675	2.553757129	2.21848578	2.447810591

Zm00001d011931_T002	0.823277351	0.89345075	1.519579645	0.460344162	0.686237849	1.488540202
Zm00001d031981_T008	0.785469365	0.135241967	0.932645541	1.572596735	1.067953928	0.808273008
Zm00001d031981_T006	0.829096971	0.647183705	0.948783228	1.606029971	1.834448295	1.726786759
Zm00001d031981_T003	3.951019911	3.131100821	3.858693511	4.262127628	4.007902769	4.217001728
Zm00001d031981_T007	0	0.852759548	1.328043683	1.483991147	2.089082503	0
Zm00001d031981_T001	0.632295208	0.308052101	0.302622036	0	0.443638475	1.778357465
Zm00001d002848_T002	0.740993165	0.390443175	0.920338277	1.126780616	1.173948033	0.90700216
Zm00001d002848_T001	3.853667561	3.457183066	3.988799189	3.483272512	3.419247087	3.652511276
Zm00001d043016_T001	1.680722225	0	1.796435784	0.989135373	1.654372754	0.826482999
Zm00001d013898_T001	1.627793116	0.949309313	0.990813306	0.608116583	0.599390165	0.48231653
Zm00001d053575_T012	0.341132968	0.690610187	0	1.042446128	0.896006733	1.321184627
Zm00001d053575_T007	1.506585694	0	1.067790825	2.797439288	2.839732083	2.444687472
Zm00001d053575_T011	0	0	0.520426272	0.571985458	0.84520339	0.671688304
Zm00001d053575_T010	3.194952536	2.527094364	3.204711358	3.709261407	3.951218962	3.682938195
Zm00001d053575_T003	0	0.260524339	0.503289703	1.268773917	0.950581884	1.278304187
Zm00001d053575_T005	0	0.618440713	0	2.212336808	1.748552257	2.378692226
Zm00001d017458_T002	3.529610835	3.135764356	3.633292049	4.040029273	3.872996949	3.867029302
Zm00001d045202_T004	0.828991398	0.779456409	0.782779256	1.311435106	1.560147546	1.104294385
Zm00001d045202_T002	2.90002108	3.178480095	2.852778321	4.302943158	4.106150296	3.918010531
Zm00001d045202_T005	3.525211706	0	3.550332215	0	0	1.809528044
Zm00001d045202_T001	1.515403133	1.759612224	1.656362735	1.086262019	1.567918728	1.294414897
Zm00001d006625_T002	2.728263394	1.818416215	2.525867421	2.123104966	3.112949122	2.244037628
Zm00001d006625_T006	0.221770692	0.456182686	0.556511818	2.273968768	1.061258462	2.202188565
Zm00001d018502_T001	2.126290652	1.744633878	2.282004707	0.902420942	1.21800491	0.889183806
Zm00001d037655_T001	2.925530277	3.365586305	2.6333682	2.302135942	2.616236762	2.515832024
Zm00001d048432_T002	0.471656748	0.401914391	0.853148539	1.379704683	0.616883672	1.057747619
Zm00001d048432_T001	2.623843222	1.750148543	2.457057876	2.417816607	2.590465971	2.32108647
Zm00001d033665_T002	3.738265994	4.205275047	3.670759567	3.913627233	3.633598139	3.704684424
Zm00001d051695_T001	2.000998	1.527250536	1.918446918	3.294330343	3.415102058	3.336098968
Zm00001d005766_T001	4.525870177	4.631348146	4.418714837	9.024748434	9.057512484	9.054066936
Zm00001d020365_T002	6.058170855	5.208868215	5.987100693	6.204620322	6.24417598	6.178628901
Zm00001d020365_T005	0.340369707	0.09317748	1.459476735	0.656628163	1.031251908	1.180380864

Zm00001d020365_T003	1.615016016	0.921355586	0.477511551	2.537289605	2.456231585	2.248471079
Zm00001d002403_T001	3.532385289	4.6890767	3.021184051	3.122510054	3.136755096	3.148577209
Zm00001d052321_T002	2.264843853	2.432040064	2.064364161	2.512024313	2.040250701	1.823803562
Zm00001d052321_T001	7.463306947	7.658072863	7.418818873	7.289068143	7.323863461	7.314159611
Zm00001d047969_T001	4.640222489	3.763327433	4.601084073	5.080467253	5.016995481	4.868761476
Zm00001d010199_T001	1.011811909	0.529006312	1.326998615	1.372684144	1.286648169	1.460509303
Zm00001d010199_T003	3.907561581	3.200820077	3.869797592	4.289138396	4.462335397	4.425454672
Zm00001d010199_T004	0.976804993	0.438287528	0	1.766598978	1.371922348	1.549828832
Zm00001d007901_T001	5.446361946	4.959727041	5.485591987	7.191975426	7.163688235	7.147494381
Zm00001d002451_T001	7.596447957	7.519005842	7.649632609	8.484922367	8.444701227	8.46181769
Zm00001d016581_T002	0.519383889	0.16708555	0	0.812557369	1.0846766	0.916930592
Zm00001d016581_T018	1.187248968	0.760920936	1.358034417	0.917622702	1.20915575	0
Zm00001d017352_T007	0.29998311	0.419655381	0.07605006	0.66139481	0.757872394	1.117170391
Zm00001d017352_T003	3.26423495	3.891798768	3.119593563	3.364239313	2.747056283	2.785756846
Zm00001d017352_T008	2.998498997	2.660031843	3.605568885	2.655788006	2.370393921	3.368194229
Zm00001d017352_T012	4.27359399	0	4.195404376	4.574826792	4.827793218	4.619392409
Zm00001d017352_T002	1.730360845	1.773995481	1.341918021	1.615573083	2.299303865	2.013560163
Zm00001d017352_T006	2.171426546	1.030427899	2.038137638	2.43602588	2.093689972	1.581845319
Zm00001d022142_T004	2.460581382	1.573268356	2.722494222	2.883303897	2.756691559	2.727526028
Zm00001d040116_T001	2.764939724	2.320471681	2.97496224	2.551103514	2.674149661	2.427519018
Zm00001d053589_T002	4.615612512	3.510463217	4.673254003	4.757203946	4.874762496	4.56391803
Zm00001d053589_T005	3.658442867	6.071241898	3.807238265	2.955726059	3.442417662	3.30126615
Zm00001d053589_T003	0.150913159	0.033071249	0.150063096	0.223745035	0.429793763	0.156814672
Zm00001d006646_T001	4.422148617	4.831873999	3.282202549	4.49626113	4.92660048	4.985529575
Zm00001d021516_T001	1.654777384	2.818359969	2.477916635	0.252155243	0	0.302902738
Zm00001d021516_T007	4.041418138	0.193967243	3.709502833	4.013931439	4.105059588	3.974333911
Zm00001d021516_T004	0	1.660932723	0.29812219	2.165234396	1.805274706	2.173415304
Zm00001d021516_T011	0.50219921	0.285988056	0.241513176	0.511365817	0.312101692	0.329905454
Zm00001d021516_T010	0.239594787	0.21835837	0.524408758	0.698693222	0.631045595	0.748902561
Zm00001d047110_T003	0.625899807	0.603037338	1.120996165	1.729486643	0	1.278056137
Zm00001d047110_T005	4.020956211	3.357686838	4.010081592	5.138089663	5.228717506	5.148873679
Zm00001d047110_T004	1.309339195	0.770141462	1.639201132	2.715637088	3.226689472	3.176062138

Zm00001d043489_T001	2.07780185	1.360481148	1.64697596	1.949142691	1.781865355	1.862008943
Zm00001d039166_T003	2.698425865	2.595570017	2.327367752	1.841165628	2.111599596	1.502928842
Zm00001d039166_T004	3.232431596	3.039624125	2.839788106	0.963482262	0.846685863	0
Zm00001d039166_T001	6.694153552	5.821056416	6.779245653	5.668635103	5.645921833	5.798325804
Zm00001d051896_T001	6.758414371	6.144957742	6.901925466	6.052281244	6.115114438	6.077474918
Zm00001d014649_T001	0.455891364	0.184219348	0.437833884	0.379135177	0.451975422	0.537479965
Zm00001d045261_T011	2.597129476	1.550179137	2.574648926	2.724783622	2.47364744	2.635283785
Zm00001d049999_T002	2.650736073	3.302886365	2.923240856	1.962490907	1.438780943	1.884636601
Zm00001d007820_T002	4.985045372	4.052679854	5.163864794	2.726199736	2.730041018	2.897058189
Zm00001d040760_T002	4.027071314	3.199374432	3.99001063	4.319771097	4.302413375	4.275718234
Zm00001d046330_T001	3.787846365	3.023138406	3.828319715	2.543217711	2.565481414	2.459393583
Zm00001d047392_T002	2.183332471	1.29016604	2.039780619	2.317009073	2.221274827	2.005753162
Zm00001d047392_T006	0.307342252	0.815182733	0.931947747	0.683592262	1.279906267	1.107117373
Zm00001d047392_T001	1.047025293	0.931027929	0.955382742	1.484419681	1.679142709	1.209030945
Zm00001d049500_T002	8.58408344	8.221189356	8.586101892	7.933390726	7.873774221	7.854346719
Zm00001d049500_T003	1.039332846	0.559069834	1.013618045	1.029422494	1.01156863	0.855157317
Zm00001d017865_T006	0.542324425	0	0.692884217	2.077885231	1.558030513	2.067207771
Zm00001d017865_T002	1.488407034	0.48749664	1.505489053	1.567209558	1.283674686	0.714960258
Zm00001d017865_T001	2.191053765	1.423529767	2.407653225	3.736404529	3.918916938	3.958175913
Zm00001d017865_T005	0.163939202	0	0	1.204072463	1.074025143	0.801379701
Zm00001d029613_T001	0.52316447	0.224562663	0.505157204	2.022902887	2.3007938	2.146150777
Zm00001d036490_T001	4.264613143	3.54412442	4.204590002	4.173225396	4.101837124	4.194978687
Zm00001d019670_T010	0	0	0	0.748256837	0.952727178	1.721810542
Zm00001d019670_T025	0.099903802	0.023597442	0.026253325	0.30216929	0.344677404	0.268942152
Zm00001d019670_T012	3.67112837	2.936929982	3.580806608	2.8855361	2.637813781	3.076641694
Zm00001d019670_T001	1.370991709	0.950372853	1.774517204	4.005135859	3.976657553	4.161008257
Zm00001d019670_T013	3.457095842	0	3.624246795	4.648530501	4.468582144	4.425207912
Zm00001d019670_T005	2.139507805	2.868170352	1.850657826	1.069654053	2.036704471	1.100467065
Zm00001d019670_T009	1.867845079	0	1.11770701	2.821175278	2.783002506	1.569762787
Zm00001d010988_T001	6.043076359	6.669210578	6.08251017	5.48880695	5.47561399	5.49078469
Zm00001d028754_T001	2.643310171	2.324937877	2.403515332	3.689542273	3.945619595	3.931892072
Zm00001d049006_T001	6.108546306	6.468120264	6.064279174	6.07575642	6.123188171	5.934119281

Zm00001d000298_T002	1.908284143	1.914074996	2.015829374	1.482915384	1.603507423	1.208048979
Zm00001d045746_T001	1.268990045	0.718547945	1.160272249	2.079788715	2.036782878	2.117595314
Zm00001d036139_T001	6.473974881	7.310390803	6.256203743	5.381520422	4.998854361	5.39623304
Zm00001d031531_T002	2.226137636	1.698304282	2.178728024	1.53732092	1.64566385	1.72260067
Zm00001d037005_T001	0.877726192	0.622580877	0.683061304	1.086616665	0.706181302	0.761361024
Zm00001d026164_T003	5.615436367	4.85702452	5.493836592	5.90585894	5.854449785	5.812017974
Zm00001d026164_T007	3.876790284	5.434649543	3.441511499	2.680564711	2.863772174	3.325279907
Zm00001d026164_T005	2.530664333	2.089609671	3.008988066	2.914979287	2.514356608	2.69142205
Zm00001d026164_T004	2.966307346	1.995683136	2.765967066	2.233609247	2.262651139	2.317597556
Zm00001d026164_T006	2.912702552	2.179476973	3.01224488	3.025458172	3.346437993	3.094844104
Zm00001d025463_T001	0.385656329	0.346349908	0.506542028	0.642145178	0.880678473	0.972049271
Zm00001d030091_T001	4.370663556	3.777055221	4.350124432	4.295856122	4.315644913	4.20283605
Zm00001d034597_T003	3.559654428	2.550506208	3.582645115	3.427918276	3.564789699	3.449870349
Zm00001d032978_T001	1.655778162	1.473944807	1.94831041	1.757690232	1.703730633	1.821166092
Zm00001d023459_T001	5.223396252	4.988345352	5.065833445	5.585878502	5.592499794	5.579337753
Zm00001d052177_T001	5.360094325	6.9834016	5.271862445	4.788259364	4.822388245	4.913719228
Zm00001d021846_T018	0.615470915	0	0.342610495	0.656083525	1.503599595	0.791589056
Zm00001d021846_T010	4.116074758	2.978668639	4.205250723	4.459977444	3.924226947	4.148317534
Zm00001d021846_T012	1.016318022	0.722494004	0.645231288	2.212841978	2.049144212	2.345203322
Zm00001d021846_T015	0.744614074	0	0.834189373	1.854628857	2.3507241	2.502761614
Zm00001d021846_T001	6.026873498	5.301107058	6.075373785	5.701866919	5.587285743	5.728084722
Zm00001d021846_T002	3.168687921	2.310462279	3.027496456	4.668993023	4.73134559	4.316933288
Zm00001d021846_T020	0.185286814	0.175893731	0.104927037	0.216921667	0.507568357	0.39403664
Zm00001d002340_T001	1.721671012	0.844904625	1.581962907	1.162157175	0.656011196	1.965935702
Zm00001d002340_T003	4.213548312	3.618587304	4.291635308	4.297036425	4.259695967	4.222185998
Zm00001d021504_T001	3.972564919	5.381641267	3.75368668	4.586641301	4.584726481	4.759296634
Zm00001d007839_T001	3.732714749	3.326207124	3.812860137	3.521016303	3.762206346	3.684828834
Zm00001d002296_T003	4.090814992	3.154651253	4.063888242	3.809126597	3.691997495	3.764244336
Zm00001d012862_T001	0.737061963	0.464964094	0.485470107	1.655553792	1.255204006	1.441083974
Zm00001d023237_T001	3.441108681	2.925315685	3.416223628	3.312840563	3.312825319	3.266211596
Zm00001d017976_T001	1.731372257	1.26782341	1.297378676	2.19440765	2.24765778	2.340920833
Zm00001d043432_T001	3.660721815	3.103223846	3.548081579	3.737826266	3.671622754	3.692810698

Zm00001d052282_T097	2.505746399	0.724252985	3.123266238	0	1.11986543	1.997224111
Zm00001d052282_T064	0	0	0	0.539232278	0.585510621	0.380015681
Zm00001d052282_T082	1.00389938	0	0.606791844	1.176622771	1.816577999	1.437158479
Zm00001d052282_T095	4.358324941	3.529350589	4.222138382	4.511300913	4.581354923	4.383828342
Zm00001d052282_T017	0	0	0	2.577871328	1.602921455	2.017098803
Zm00001d052282_T006	0.49098183	0.158564531	0.330288766	0.540602645	0.320995235	0.450024019
Zm00001d052282_T045	0.860871447	1.028820188	1.045691087	0.598752032	1.470005762	1.622786074
Zm00001d052282_T088	0.259127834	0.130163864	0.242584215	0.168142653	0.145747877	0.26559892
Zm00001d047458_T001	3.349336684	4.336089111	3.296622445	2.976535759	2.887109559	3.019998072
Zm00001d044836_T001	2.461572824	2.385859502	2.565767755	0.97603803	0.785720443	1.22172368
Zm00001d047992_T002	6.313775299	5.599824612	6.222375502	5.918967584	6.072315328	5.874808374
Zm00001d047992_T004	1.808746246	0.922938419	1.576040272	1.365478664	0.445638783	2.066259031
Zm00001d047992_T003	1.219563203	0	0.86709782	1.400324242	1.587465808	0.749367785
Zm00001d047992_T005	0.716442237	0.318522781	1.29627649	1.464370811	1.313530299	1.231856307
Zm00001d047992_T001	1.416474429	1.080379329	1.652016037	1.331007487	1.125638539	1.776776527
Zm00001d028782_T002	5.650960964	4.852714312	5.673127707	5.60497489	5.518908448	5.447661346
Zm00001d040777_T001	3.673386705	3.055282436	3.599979948	5.415699995	5.290827046	5.259126364
Zm00001d012044_T002	3.792558976	6.152255248	3.92719541	3.766349851	4.155098076	3.589991257
Zm00001d012044_T001	8.128802159	8.430430844	8.142599257	8.11927839	8.198897395	8.123907312
Zm00001d007069_T005	5.388885776	5.675124276	5.300650249	5.150234384	5.347102995	5.240033625
Zm00001d007069_T004	1.991679837	3.485708383	2.367269011	2.487775477	2.951698753	2.33290868
Zm00001d007069_T002	1.48257572	4.032200488	1.743146977	1.587520054	2.21754063	1.894771984
Zm00001d023217_T002	4.456953433	3.687655006	4.432999882	4.89365009	4.934384823	4.779622398
Zm00001d023217_T004	1.569073972	0.803104671	1.64751668	2.195221593	2.124110722	2.097772248
Zm00001d042625_T001	6.799437392	7.646024452	6.55868564	6.297612056	6.267151421	6.386901629
Zm00001d046039_T024	0	0	0	2.002827075	1.546757683	1.976502949
Zm00001d046039_T023	1.010393159	0	1.275415356	0	1.743028027	1.932722633
Zm00001d046039_T007	0.070570684	0.027526358	0.184742391	0.298709923	0.368485648	0.187127956
Zm00001d046039_T016	3.104553048	2.320420268	2.935444661	2.374535408	2.596298785	2.255725504
Zm00001d046039_T036	0	0	0	0.456897596	0.914944855	0.847505505
Zm00001d046039_T018	0.290197896	0.315316969	0.063910153	0.324339419	0.707750056	0.782816153
Zm00001d046039_T038	0.390106345	0.119459768	0.385060997	1.256082528	1.133327441	1.017573482

Zm00001d046039_T012	0	0	0	1.346847992	1.048559849	1.073456258
Zm00001d046039_T015	0.285597524	0.088107667	0.071829929	0.399296897	0.465772865	0.35022226
Zm00001d029763_T001	5.285045126	5.855817595	5.50365429	4.516188979	4.707834743	4.793006952
Zm00001d016203_T004	3.890500975	3.174954596	3.820987564	3.837527928	3.899621231	3.939761987
Zm00001d016203_T001	0.132068767	0.219413274	0.408136842	0.944167286	0.813987591	1.074136838
Zm00001d016203_T003	4.277849023	4.458468889	4.519391123	4.387248102	4.590292478	4.360141393
Zm00001d052921_T002	4.271129218	3.345642197	4.130775228	3.659252019	3.504401366	3.649338274
Zm00001d052921_T006	2.790578345	3.829480824	2.329661528	2.153855586	2.506532634	0
Zm00001d052921_T001	1.326331399	0.927930578	1.202473516	0.961483312	1.555395553	1.414179382
Zm00001d024092_T001	2.705459323	3.777600771	2.592493396	3.073837196	2.95027206	3.043133965
Zm00001d011174_T003	0.507161364	0.342977935	0.53914094	1.138513712	1.23156082	1.047629819
Zm00001d011174_T001	5.502659092	4.227961667	5.564157967	5.900131752	5.924546097	5.759520004
Zm00001d011174_T004	0.283128813	0	0	0.515301694	0.53246758	0.562947203
Zm00001d011174_T002	3.83086842	4.212482261	4.042637334	3.286789646	3.435755124	3.887090447
Zm00001d031481_T001	4.414029966	4.156497582	4.816328856	4.406791881	4.343699885	4.381491247
Zm00001d038217_T004	2.283950507	1.527275562	2.103421433	2.427941601	2.492968606	2.491558115
Zm00001d002444_T001	3.43064443	4.19906067	3.387788227	3.470259753	3.246442748	3.424997311
Zm00001d009792_T001	3.960859646	3.700876456	4.111134251	4.099121633	4.232228881	3.84224473
Zm00001d013979_T001	5.565246207	4.848668405	5.767641478	6.373462347	6.311680735	6.383720844
Zm00001d036090_T003	0.79645572	0.776061865	0.944429696	2.214371348	2.240184252	2.826045249
Zm00001d036090_T008	3.066040784	2.391674525	3.097158872	2.799661161	3.136029583	2.801511732
Zm00001d036090_T005	0.401768068	0.342246376	0	0.32544168	1.119759876	0
Zm00001d036090_T006	1.312147011	0	0.924175915	1.840641281	1.630357474	0
Zm00001d029387_T004	6.016442409	4.935520283	5.943823902	7.019231617	7.099792234	7.442832819
Zm00001d029387_T002	0	0	4.39679957	5.770597034	6.219207386	5.080335494
Zm00001d029387_T003	6.883378343	7.473579452	6.977171202	7.827108411	7.771964754	7.851051633
Zm00001d029387_T001	7.014578208	6.065702841	6.911622285	7.277496854	7.232666944	7.373523075
Zm00001d029387_T005	8.520488288	8.705214277	8.609969281	9.268373751	9.368215056	9.304413815
Zm00001d018488_T001	1.781220369	1.574053539	1.841853996	3.244013721	3.29683635	3.26106017
Zm00001d018488_T003	1.881518189	1.032342095	1.896545828	3.220637037	3.245180286	3.478248335
Zm00001d038722_T001	1.381639433	1.057929878	0.991769798	0.791623227	1.587998581	1.267037735
Zm00001d023429_T001	4.447202804	3.689051309	4.509810278	4.403529784	4.486093919	4.296426204

Zm00001d041870_T001	2.007671297	1.387720413	1.695266494	1.861346803	2.022947616	1.940664794
Zm00001d014186_T017	4.07567253	0	4.06230981	3.80354799	3.669122035	3.839244208
Zm00001d014186_T020	1.140392577	0.638500209	0.737892661	0.85435877	0.994662989	0.922352387
Zm00001d014186_T019	1.418212081	0	1.511699277	1.804877957	2.154266276	2.058858487
Zm00001d014186_T014	1.110776815	0.697361943	1.049235541	0.941551825	1.36618231	0.907161412
Zm00001d014186_T007	2.85136807	0	1.77443666	3.416201195	2.962561238	2.887948659
Zm00001d014186_T013	2.201795058	3.804651253	2.702347475	1.693447705	2.146229975	1.698018843
Zm00001d014186_T016	2.11236352	0	0	2.732741336	2.441307381	1.94093949
Zm00001d014186_T002	1.002845793	0	0.478302964	2.323095052	1.122363939	1.91067242
Zm00001d014186_T011	0.900112884	0.266125672	0.548199855	0.903025926	0.647680146	1.513490746
Zm00001d014186_T022	2.412915358	0	3.290629793	2.541072943	2.884677819	3.246545965
Zm00001d014186_T001	0.702109614	0.92964407	0.875209162	1.443133985	2.714294101	2.218950686
Zm00001d014186_T021	2.425995516	0	1.274366635	1.962647112	1.686340953	2.1062086
Zm00001d007060_T001	0	0	0.772715048	1.355201806	0.643506207	0
Zm00001d007060_T004	4.134468871	3.338199756	3.988000119	4.217878232	4.217719443	3.943343008
Zm00001d013178_T001	4.028488264	4.363849783	3.887749846	6.265904691	6.182171332	6.175734583
Zm00001d016008_T006	1.016707396	0	1.749481064	1.742120069	1.5628471	1.579479471
Zm00001d016008_T024	0	0	1.441640701	1.570356061	1.406847379	1.333186127
Zm00001d016008_T014	0	0.222620349	0.420312077	0.960517641	0.91593386	0.679065219
Zm00001d016008_T027	2.701095662	2.722840858	2.355612846	2.024714345	1.637064409	1.801496832
Zm00001d016008_T015	0	0	0	1.286532257	1.011038999	1.442509362
Zm00001d016008_T004	0.273232992	0.279511708	0.210997237	0.698614998	1.173932687	1.229642061
Zm00001d016008_T005	2.603742883	0.528721331	2.383030993	3.365429391	3.758767298	3.537789183
Zm00001d039006_T007	0	1.611861215	0	0	0.637408074	1.850701427
Zm00001d039006_T004	2.475897597	1.506337893	0	2.22411531	1.546971484	1.930108659
Zm00001d039006_T003	2.646930123	0.652677388	2.62327576	3.518829565	3.5094411	2.998109192
Zm00001d039006_T008	2.289637962	2.639046662	3.195508869	0	1.490055084	0
Zm00001d048324_T002	4.422752385	3.801115809	4.319466273	4.280671754	4.339207237	4.289394952
Zm00001d002243_T005	0.512857943	0.303377468	0.790359185	1.041683154	0.773443608	0.782919289
Zm00001d002243_T004	3.448693648	2.635786657	3.800169025	3.897458847	3.962888974	3.887407218
Zm00001d002243_T001	0.970281615	1.686207364	0.655071506	0	0.207211939	0.659037668
Zm00001d014943_T041	3.030313829	2.370614599	2.759391718	2.98622797	2.92835971	3.098866541

Zm00001d014943_T001	1.051188316	0	1.465992743	2.690672981	2.759760062	2.697007351
Zm00001d014943_T042	1.740382751	1.395481285	2.458242085	1.667969763	1.324008115	0.424514745
Zm00001d039471_T002	2.757736735	1.641961207	2.456049653	2.271755534	1.434692806	1.953964344
Zm00001d039471_T001	0.351763999	1.050416718	0.826321909	0.349233912	1.738169021	0.649533608
Zm00001d039076_T003	3.211226868	3.882247256	2.986529441	2.895434087	2.473370535	3.255014667
Zm00001d039076_T001	1.454449663	1.451930071	1.709514302	1.334708992	1.973642498	1.332352192
Zm00001d017820_T001	2.370982506	1.760626335	2.263630897	2.511001403	2.376541833	2.54404712
Zm00001d046054_T001	1.088495102	1.121890039	1.268022307	1.574728841	1.5030337	1.394018525
Zm00001d027384_T001	2.869750815	2.17598504	2.7329959	2.889025089	2.593656143	2.637883088
Zm00001d042454_T001	4.495662164	5.226293535	4.449405278	4.579863975	4.178466709	4.11386591
Zm00001d008628_T001	4.142811145	3.670719011	4.48910031	4.606511992	4.518788721	4.645701774
Zm00001d008628_T002	6.422967602	6.177734395	6.484752741	6.678050645	6.743095436	6.722378231
Zm00001d038909_T001	3.682567455	3.225466361	3.678907412	4.943749908	4.951516507	4.999131958
Zm00001d034439_T001	1.329683339	1.202738041	1.518988774	0.966902182	0.938973755	1.401583505
Zm00001d040711_T001	3.959951501	3.806395108	3.804156015	3.751029283	3.664654747	3.707002459
Zm00001d030868_T004	1.424723267	0	1.812985271	1.722276276	2.099000912	2.665819558
Zm00001d030868_T008	0.100477158	0.049880256	0.592103451	0.31901553	0.687308882	0.687077714
Zm00001d030868_T014	0.994045267	0.794614659	1.156148056	0.673973343	0.503493252	0.67130062
Zm00001d030868_T006	1.710789906	0.974466904	1.580147414	1.740040305	2.53315515	2.212009894
Zm00001d030868_T010	2.346972486	3.417753835	3.38103621	2.788606997	0	3.500714367
Zm00001d030868_T007	4.982069817	3.224972383	4.52438224	4.772740803	4.480455788	3.978811024
Zm00001d030868_T005	0.871004592	1.080943446	1.192585566	1.874222134	2.109999015	1.68185042
Zm00001d030868_T001	0.427143793	0.504683437	0.433337664	0.562819265	0.588730704	0.498093567
Zm00001d030868_T003	1.52857932	0.667636935	2.141898028	2.381394409	2.407059831	2.430121455
Zm00001d030868_T009	1.36410285	0	1.002079588	0	2.67864053	1.148036004
Zm00001d030868_T012	0	0	0.682952573	0	0.688595759	1.039955339
Zm00001d018320_T001	4.179638276	4.654826122	3.817477037	2.81870518	3.155978764	3.547746556
Zm00001d003846_T002	0.922959725	0.356105488	0.803355178	1.121636787	1.263300679	1.238270209
Zm00001d003846_T001	4.572888335	3.994407568	4.527198168	4.434902264	4.31319556	4.343289914
Zm00001d036031_T006	3.978419431	3.227526843	4.817223545	4.584099752	3.587549697	0
Zm00001d036031_T016	6.152585845	5.721699717	6.092509568	5.375315081	5.637832673	5.187676705
Zm00001d036031_T009	3.907029184	0	0	3.681784318	4.640415417	5.360721078

Zm00001d036031_T012	1.137813014	0.580326896	1.090889328	1.191203827	0.781244713	1.270086947
Zm00001d036031_T003	0	2.175781022	1.309470751	0	0	0.758501891
Zm00001d036031_T005	4.495440814	3.825338506	4.401051047	4.540699283	4.389894822	4.285374622
Zm00001d003626_T001	3.002031327	2.313311744	2.838045135	1.929304716	1.807422521	2.182121144
Zm00001d029193_T001	1.373952168	0.949866575	1.432061986	1.311695501	1.286237113	0.944206275
Zm00001d031967_T002	5.124561603	4.131080731	5.034353735	5.435865246	5.259908564	5.108726211
Zm00001d031967_T004	4.528129629	3.177830431	4.670359848	4.636542667	4.678202643	5.083825001
Zm00001d039454_T001	4.192876522	3.629342747	4.228630089	3.775858498	3.687034478	3.950855818
Zm00001d016647_T001	3.429318825	4.033467141	3.531420639	3.030133698	3.692078646	3.579850401
Zm00001d031599_T001	1.638442749	1.954175089	2.031437192	2.096822155	2.590434112	2.534366698
Zm00001d029716_T001	2.826686166	2.887654913	2.723454369	2.607495556	2.510783217	1.95350812
Zm00001d003672_T001	7.348356493	7.353213391	7.32667104	6.744328517	6.85464348	6.771095035
Zm00001d014834_T001	4.51833452	4.258536713	4.39834394	4.409025566	4.657383281	4.339166823
Zm00001d047904_T001	3.595121573	3.12996282	3.692468376	3.710776905	3.767387102	3.774920809
Zm00001d048253_T001	6.837173932	6.156835054	6.926030337	6.574500586	6.559020138	6.512689113
Zm00001d048253_T002	0.893439101	0.804426992	1.025332898	1.038757837	0.661794284	0.728766007
Zm00001d005776_T001	0.556252823	0	0.429946909	0	0	1.141092678
Zm00001d005776_T004	0.528157203	0.501710206	0.654742562	0.949680596	0.677802276	0
Zm00001d005776_T003	0.633319604	0.313611559	0.871210458	0	0	0.505316785
Zm00001d022301_T001	6.754605668	7.475937031	6.712240854	5.797353268	5.919247515	6.020575072
Zm00001d030893_T027	5.136579739	4.512065033	5.231367853	5.214577131	5.107652345	4.995767287
Zm00001d030893_T020	1.1123772	0.356563033	1.332129308	3.114589409	2.696822679	2.723603119
Zm00001d030893_T018	1.333304649	0	0	0.7988543	1.334363469	1.385701051
Zm00001d030893_T026	1.470441073	0.962393548	1.400923143	2.524986635	2.446113277	2.51626331
Zm00001d030893_T004	3.048734032	2.352355994	3.232028288	4.38208743	4.77831914	4.733294299
Zm00001d030893_T001	0.345893653	0.091224555	0.183778676	1.290717514	1.402498444	1.480758346
Zm00001d030893_T025	0	0	0	2.279065323	1.946290312	1.19144579
Zm00001d024588_T002	1.650216782	1.072420037	1.367307876	1.292749951	1.353002531	1.346176843
Zm00001d024588_T001	5.203394873	4.584535219	5.23951639	5.330068216	5.316682633	5.281669841
Zm00001d002830_T001	3.146924155	2.389848165	3.149126993	4.219013127	4.199066953	4.202878119
Zm00001d035926_T001	3.883890759	2.96988632	3.874253025	4.06071668	4.112035673	3.983669122
Zm00001d019397_T006	2.884620777	2.125153478	3.028874455	3.033914007	2.783437167	2.803591798

Zm00001d026094_T001	1.698807884	1.357018179	1.785748477	1.292210447	1.23448354	0.703487907
Zm00001d053234_T001	1.691358172	0.960646632	1.603684971	1.811713103	1.829979417	1.828139633
Zm00001d026127_T001	0.553251375	0.286635159	0.360489014	0.980943652	1.150893667	1.042451031
Zm00001d035898_T001	3.866401215	3.506885615	4.01186217	3.438901211	3.599275298	3.624422277
Zm00001d002943_T008	1.822103179	0.965643932	1.270883518	1.812271484	1.883219621	1.878331415
Zm00001d002943_T012	5.947790061	4.445407504	5.698641166	6.004094796	5.882651461	6.005090597
Zm00001d002943_T013	4.699464848	4.80038054	5.249127337	4.338332273	4.951204645	4.542286903
Zm00001d002943_T011	2.791710943	2.642055506	2.678259443	2.534051141	0	1.563195723
Zm00001d002943_T002	3.727784236	2.70502043	3.891342675	3.977950186	4.047396869	4.058870692
Zm00001d053374_T001	2.06174959	1.904852071	2.563968595	2.404302873	2.496033031	2.206306264
Zm00001d019684_T002	5.876877465	5.664444639	5.994731611	5.674550252	5.656740098	5.661256439
Zm00001d053859_T001	5.121378226	5.111135002	5.665938824	7.217679297	7.224861824	7.244459285
Zm00001d010284_T001	2.332270835	2.127640873	2.211324692	1.813463533	2.03833363	1.871291298
Zm00001d012593_T001	1.300154775	0.740564089	0.708040641	1.270332782	1.264311227	1.26674577
Zm00001d020681_T001	2.43230791	3.101130821	2.699156036	2.122652511	2.110090248	1.790932143
Zm00001d032704_T001	0.27739446	0.349692508	0.302947178	0.223002346	0.825244261	0.131747507
Zm00001d032704_T055	0.828136789	0.856643146	1.072859716	1.667819932	1.282525801	1.246600839
Zm00001d032704_T057	0.323057571	0.027551835	0.051033751	0.700930595	1.121624189	0.67659419
Zm00001d032704_T052	0.988273131	0	0	2.07239534	0	2.468971893
Zm00001d032704_T003	0.898532656	0.901668989	1.946593508	2.44838799	1.754828083	1.218999957
Zm00001d032704_T004	0	0	0	0.558822071	0.546410003	0.66849405
Zm00001d032704_T013	4.090551991	3.08988911	4.089349755	2.954353788	3.393372403	3.599099587
Zm00001d003313_T001	2.224586431	2.207250991	1.994380231	1.963751539	1.913922247	1.984843053
Zm00001d020560_T003	1.726257512	0.665150786	1.603455674	1.418572097	1.577281379	1.588995922
Zm00001d020560_T001	0.829362492	0.80313609	0.974749554	0.945428666	0.774048632	1.046608399
Zm00001d042044_T001	1.421933126	0.999831195	1.454140089	1.443685149	1.674880924	1.125461992
Zm00001d042044_T002	1.156513115	1.146366524	1.75468829	1.318063425	1.410277599	1.348039745
Zm00001d042709_T001	3.394407815	2.989695446	3.539753637	3.754487232	3.679159715	3.821992197
Zm00001d020419_T005	4.63971601	3.813981641	4.676675704	4.776313532	4.761969327	4.741977314
Zm00001d020419_T007	2.53461521	1.30667113	2.604414398	2.851225729	2.627137302	2.691422944
Zm00001d020419_T004	2.281137678	1.919341227	0	1.380828617	2.01655551	0
Zm00001d020419_T006	0.435786455	0.187394042	0.487960653	0.897898687	1.169665934	1.213692554

Zm00001d012602_T001	3.99056646	3.861519612	4.142452323	4.659601514	4.648390373	4.535147263
Zm00001d045858_T001	4.655422874	4.322085124	3.942936883	4.638877988	3.910857645	3.504226171
Zm00001d049362_T008	2.181102233	1.192363996	1.924627067	1.045674315	1.489363101	1.391252458
Zm00001d049362_T002	3.2714197	1.509813887	3.446419814	3.521911447	3.221153078	3.422862024
Zm00001d049362_T009	2.308599984	4.311025529	2.014334581	2.03980974	2.461626255	2.431595683
Zm00001d049362_T001	5.056328043	5.734983151	4.873189585	3.939002444	3.491592105	4.099907084
Zm00001d049362_T003	6.23413284	6.092406582	6.298707926	6.620028152	6.543614814	6.495449177
Zm00001d049362_T004	0.810743334	0.548345868	0.938328705	1.118076608	1.055998702	1.231162646
Zm00001d027454_T001	6.625827848	6.008073255	6.704510471	6.452226593	6.47850562	6.509570155
Zm00001d020794_T001	6.192507965	6.435207708	6.195618964	5.975492141	6.078521286	5.945183554
Zm00001d003945_T002	5.145613959	4.293057013	5.16005603	3.79227566	3.725771253	3.747825405
Zm00001d002708_T001	2.648117498	2.052001947	2.725969047	3.06092588	3.024217893	2.908465159
Zm00001d006774_T001	7.160683628	7.788873036	7.186623633	7.666587072	7.768779479	7.73788843
Zm00001d006638_T001	1.568282183	1.256542119	1.658927111	1.397176472	1.642867432	1.5868608
Zm00001d047640_T002	2.845757991	3.878390962	2.786282489	3.040915745	3.069558337	3.07512749
Zm00001d047640_T004	2.263132085	1.15476854	2.180063818	2.167026121	2.253561839	2.726200608
Zm00001d047640_T003	2.892290374	3.915769551	3.147624838	2.933599264	3.310826076	3.420780154
Zm00001d023836_T001	4.311381481	4.717715187	4.588267353	4.29156945	4.311083164	4.193089337
Zm00001d010356_T001	2.674237586	1.928685644	2.578884143	0.772456629	1.139302492	1.392727914
Zm00001d047614_T001	3.786670481	3.223082071	3.684804602	3.834643266	3.356670636	3.792503692
Zm00001d053981_T001	2.213005972	1.402330348	1.993521118	1.460639305	1.661879546	1.372389393
Zm00001d053981_T005	0.667554284	0	0.647954542	0.900740473	0.647098031	0
Zm00001d053981_T004	4.889348202	4.478805789	4.978568385	4.980031746	4.702829615	4.822418278
Zm00001d050317_T001	3.24420862	3.009758851	3.516349929	2.720089742	3.046472725	2.895370682
Zm00001d027503_T013	1.587013516	0	0.958166352	1.728368546	1.798211059	1.397015972
Zm00001d027503_T029	1.173413385	0.230913731	0.760484973	1.063992271	0.841820994	0.776673372
Zm00001d027503_T019	0	0	1.105153061	0.706872648	0.493090846	1.00272988
Zm00001d027503_T042	0.780080762	0.882982605	0.424737239	0	1.097078115	0
Zm00001d027503_T043	0.923224499	0	0.939007618	0	0.556888454	0.392132746
Zm00001d027503_T033	0	0.932575248	1.448512481	1.507241554	1.548014852	1.770374076
Zm00001d027503_T021	2.526709617	1.475814868	2.447250685	1.95025638	2.637283557	2.540061749
Zm00001d027503_T001	4.81817373	4.524310019	4.605164973	4.365639002	4.371386635	4.029959816

Zm00001d000072_T001	1.688324509	1.317142538	2.054903684	1.771034074	2.062235023	2.038731504
Zm00001d023273_T001	0.681554512	0.301085396	0.503197079	0.52512875	0.619237388	0.493030368
Zm00001d014250_T001	2.109571515	2.356264687	1.970697967	2.201968151	2.252501041	2.047711125
Zm00001d004591_T003	2.733079226	2.309314745	2.723408714	2.8542265	2.835461893	2.84803678
Zm00001d004591_T001	5.618153507	4.731702214	5.53356531	5.050776019	5.062768077	5.109005024
Zm00001d004591_T004	5.989574794	5.732533778	6.130371429	5.692686967	5.789520082	5.657139605
Zm00001d004591_T002	0.972091927	0.705317096	1.080607867	1.357527232	1.391336056	1.49688924
Zm00001d004591_T005	3.46698191	2.743444738	3.193703943	2.243554538	2.079613293	2.163206621
Zm00001d043922_T001	2.374054865	1.574730294	2.648052824	2.431201187	2.577807777	2.392749892
Zm00001d015754_T052	3.48730613	2.928059293	3.527387423	2.467293046	1.972512903	1.535567775
Zm00001d015754_T053	0.217452843	0.256364581	0.686059415	1.122284414	0.505970174	0.718375217
Zm00001d015754_T021	0	0	0	0.740030372	1.072444734	1.40654701
Zm00001d015754_T050	1.972750361	0.569078834	1.868507405	2.441332616	2.762616279	2.313236563
Zm00001d015754_T044	1.125126029	0.787400701	0.969383712	1.802807395	0	1.978562848
Zm00001d018386_T005	3.64985041	3.470695898	3.145287141	3.010326554	2.32129519	3.0669635
Zm00001d018386_T003	1.625954497	0.974521236	1.6188485	1.412414099	1.546446553	2.046530523
Zm00001d018386_T001	4.144972155	2.850157727	4.377667886	4.01428762	4.263073779	3.745945273
Zm00001d023729_T003	0.674995687	0.28227373	0.569799236	1.140567961	0.971874221	0.831530304
Zm00001d023729_T002	3.645537277	3.413571202	3.610634161	4.422795233	4.551044188	4.353338466
Zm00001d023729_T001	2.861315237	1.880085208	2.836712088	3.161711002	2.829962171	2.80366226
Zm00001d041951_T001	5.967077837	5.178249893	5.940987326	5.848193285	5.818702062	5.805124854
Zm00001d013151_T001	1.999079628	1.496211269	2.161558162	0.313148314	0.836866166	0.366171676
Zm00001d050565_T001	1.403988072	1.126349139	1.397765183	1.198446377	1.45384572	1.582911455
Zm00001d033637_T001	5.205688678	5.926215688	5.305316638	5.488944996	5.247188247	5.373762585
Zm00001d049707_T020	0.306390577	0.107717325	0.300530258	0.934437996	0.821279995	0.660925879
Zm00001d049707_T018	1.479280237	0.896770166	1.563601856	2.398814957	2.202199538	2.052831809
Zm00001d049707_T022	3.800416523	4.310064644	3.838221867	0	1.763539478	0
Zm00001d049707_T021	3.427212864	2.400263026	3.214284457	3.587242441	2.996458348	3.519687073
Zm00001d049707_T006	0.418940116	0.479173639	0.43809693	0.21383375	1.352091251	0.588218353
Zm00001d049707_T002	2.177034688	0.648750732	2.247147336	2.971882312	3.027902282	2.762306502
Zm00001d008600_T003	2.082862653	2.664721525	2.790523923	2.126655415	2.460515593	2.363986831
Zm00001d008600_T001	2.916143735	1.666261674	2.463995114	3.165934122	3.033097687	3.151438023

Zm00001d044203_T003	1.575048458	0.500856089	0.520006793	1.756430254	1.812045117	1.579137658
Zm00001d044203_T001	4.226424987	3.521360732	4.143558398	3.989662117	3.917509283	3.833705084
Zm00001d008906_T008	0	1.225583429	0	2.735395449	2.190815528	2.480749042
Zm00001d008906_T010	2.573297445	3.139174934	3.034240547	0.937433287	1.616231585	2.00245482
Zm00001d008906_T003	2.652089025	1.506915692	2.083485369	0.757294687	1.283380734	1.258160091
Zm00001d008906_T001	1.966282425	0	2.521472174	3.522779552	3.542318481	3.551913396
Zm00001d008906_T009	0.905477987	0.439308237	0.725813562	0.888814542	1.47545952	0.923351543
Zm00001d033109_T008	2.213934817	3.481072598	2.434529754	0	0.936386547	0
Zm00001d033109_T004	2.844618852	2.003468754	2.845323441	3.176272341	3.204166703	3.224790608
Zm00001d016439_T003	5.980353115	5.326396408	5.951334051	6.259741513	6.263955427	6.204310399
Zm00001d022384_T001	1.612451113	2.426521062	2.348399572	3.073734389	2.911393618	2.702811777
Zm00001d022384_T003	2.649350347	2.039111716	2.93762537	3.254504854	3.073017789	3.307431294
Zm00001d022384_T002	1.871765599	1.006089729	1.012078753	0	0.450027188	1.414014807
Zm00001d047785_T001	0.293721256	0	0.200126398	1.262946639	1.597495896	1.362361595
Zm00001d012750_T002	3.612515633	2.431837677	3.350742484	4.106293539	4.251327656	4.160051632
Zm00001d035222_T001	3.551253171	3.000941232	2.633111248	2.730067111	3.058743872	3.174960666
Zm00001d053994_T008	0	0.926735005	1.702086558	2.330259791	1.061089069	2.967799418
Zm00001d053994_T001	0	0	0.694481753	0.915126185	1.868095266	1.030534546
Zm00001d053994_T006	2.348132402	0.931960603	1.660299337	2.226454274	2.358945608	2.612134486
Zm00001d053994_T007	4.175535379	3.21808088	4.164880063	4.578083648	4.626034424	4.179782207
Zm00001d013442_T088	2.109036544	2.25581069	2.045760273	2.39542096	2.620134999	2.075296441
Zm00001d013442_T008	0	0.082124851	0	0.954691396	1.688513852	0.976920075
Zm00001d013442_T012	0	0.399167812	0	0.92012253	0.170600666	0.564718619
Zm00001d013442_T078	2.794382385	0	2.864122937	2.790365858	3.314894285	3.211776806
Zm00001d045668_T001	5.439921319	8.224194259	5.915723906	5.513305344	5.554735416	5.908378761
Zm00001d014889_T001	3.428195332	4.07195063	3.647288142	3.292434136	3.455720961	3.445271552
Zm00001d005313_T001	1.753187755	0.911755232	1.170065417	1.329893402	1.248145572	1.526100871
Zm00001d023521_T002	1.034267139	1.565380268	1.087007896	0.507910305	0.407173253	0.783354394
Zm00001d023521_T007	1.276195887	0.353074821	0.941582622	0.839950722	0.699896275	0.37451649
Zm00001d023521_T001	2.148683929	0	2.188183776	3.936124837	3.934303431	3.77429443
Zm00001d023521_T004	1.037510143	0.25782168	1.111359889	1.710759055	1.815433202	1.575695223
Zm00001d023521_T005	3.609921581	3.0085534	3.738095287	3.411637753	3.108330579	3.563153492

Zm00001d039347_T001	2.884607493	2.919359534	2.966428621	3.704356751	4.026279533	4.046356336
Zm00001d038274_T002	4.129375924	3.136564975	4.191584364	2.579327886	2.931474863	2.685131475
Zm00001d038274_T001	3.727632758	3.239911082	3.816130882	3.211607844	2.749615571	3.221079745
Zm00001d021297_T008	2.458115018	2.381764006	2.470390831	3.32844458	3.270742715	3.553133513
Zm00001d021297_T015	0.837249775	0.721617645	0.86048215	1.516162436	2.219889339	0.826797804
Zm00001d021297_T014	3.124607217	1.238908505	3.746681133	4.428958936	4.265266676	4.22182497
Zm00001d021297_T013	2.504128903	0	0	0	1.85122373	2.396515734
Zm00001d021297_T001	1.399220869	0.734749067	1.245142095	1.967051268	2.901413745	2.417484218
Zm00001d021297_T011	1.442220043	0.348258485	0.592898539	2.071944882	1.13861135	1.609614089
Zm00001d021297_T016	2.284951732	3.074472383	2.790202144	2.109563826	0	0
Zm00001d040331_T001	4.667070883	4.194703754	4.861411771	4.546828974	4.660503803	4.637944221
Zm00001d002053_T001	3.00221572	2.401467185	3.112324149	3.011636788	2.889422337	2.929430089
Zm00001d047828_T001	2.791088687	0	2.763450669	3.123235939	3.116225005	2.864394967
Zm00001d047828_T005	2.272338277	2.222964028	2.380823908	1.69425619	0	0
Zm00001d047828_T011	4.475562826	5.592557768	4.730967853	2.94087843	3.127507327	3.370970095
Zm00001d047828_T003	0.557232637	1.50783624	0	0.944671071	0.457312712	0.709063846
Zm00001d046827_T002	1.633482826	0.558375404	1.797188582	0.918174776	0.822957699	0.850702627
Zm00001d038784_T002	2.604513081	2.030699443	2.580757173	3.333361473	2.839892287	3.257696942
Zm00001d028971_T001	4.031144167	3.21123824	3.877193369	3.822932927	3.96989433	3.917828704
Zm00001d038471_T001	3.629414094	2.97083102	3.925126229	5.66984596	5.750880066	5.741947526
Zm00001d028391_T001	4.952920544	4.055581791	4.919830925	5.825978771	5.892894084	5.915078249
Zm00001d051254_T006	1.435874445	1.188215432	0.786279352	0.858412346	1.766764332	1.346341397
Zm00001d023968_T001	4.136932071	4.106938879	3.939133844	4.384959289	4.364156792	4.282207443
Zm00001d023968_T003	2.826744528	2.869617975	3.017031615	2.576698201	2.868828579	2.247207505
Zm00001d017099_T001	5.334152697	4.683518548	5.328279983	4.935123646	5.201250673	4.961729582
Zm00001d044831_T003	1.455245408	0.318741416	0.909566524	2.007325051	1.965613275	1.929527805
Zm00001d044831_T002	4.819544599	4.068494633	4.596764751	4.473077661	4.375566006	4.400822069
Zm00001d044831_T001	3.777326812	3.15201009	3.955729127	3.93584055	3.811667384	4.04777088
Zm00001d041351_T001	2.017578826	1.473088639	2.235859684	1.399751337	1.573252842	1.598668197
Zm00001d022472_T001	3.832225715	3.6507386	3.501158604	2.55037864	2.097404827	2.800912114
Zm00001d020251_T001	2.625720998	3.538283745	2.29797698	3.650993236	3.483496963	3.488737746
Zm00001d032280_T002	4.467176818	3.195427133	4.518290515	4.101869892	4.00326077	4.084858974

Zm00001d035479_T001	1.513863625	0.876452116	1.244387203	2.355646389	2.46556967	2.35390618
Zm00001d040933_T001	3.780555147	4.014382522	3.745574137	4.069073041	4.184982108	4.025732778
Zm00001d048271_T001	5.906091747	5.472480006	5.951268743	5.832815549	5.789058315	5.761154004
Zm00001d054107_T006	5.952490129	0	5.885371001	4.768723924	4.75659982	4.444388982
Zm00001d054107_T004	1.350242066	0.628629902	1.44402879	1.235177445	1.044761304	1.320311371
Zm00001d054107_T002	6.110633953	6.000708527	6.121692065	6.150727677	6.195984759	6.190550921
Zm00001d042848_T003	2.875817604	2.392314675	2.971416637	3.299541549	3.300710204	3.149090239
Zm00001d042848_T001	3.186811122	2.423846517	3.169658559	4.725024047	4.69618833	4.607603497
Zm00001d042848_T002	1.018367828	0.225466207	1.013120618	0.889058392	1.01183695	1.137531722
Zm00001d024645_T001	1.473905335	0.988653424	1.519843797	1.554574609	1.460937531	1.496866237
Zm00001d025185_T001	6.210008423	5.481049536	6.122083035	5.994668012	5.976397411	5.862632647
Zm00001d002029_T001	1.201074238	0.606280182	1.22740716	1.736549461	1.964752377	1.85561981
Zm00001d002029_T003	6.798782269	6.374704652	6.836759254	6.990395855	6.857198083	6.801278871
Zm00001d002029_T002	4.961103939	5.406480514	5.535720731	4.229999755	4.747765534	5.070753047
Zm00001d046510_T001	5.7150089	5.537334619	5.613452827	4.985595787	4.851703424	4.955621036
Zm00001d043611_T001	4.082939102	3.450273369	3.930270586	4.397882417	4.174884468	4.204941522
Zm00001d048662_T001	4.130361667	4.157368523	4.12267628	1.727430835	1.887707741	2.100370512
Zm00001d046687_T001	3.578186142	5.018589178	3.343096508	2.066621727	2.092042656	1.74721938
Zm00001d052886_T003	0.251809968	0.097803593	0.166982753	2.236414244	2.259876861	2.308516985
Zm00001d052886_T004	0.474426698	0.210160659	0.333461518	2.446771273	2.450903216	2.523237752
Zm00001d052886_T001	1.748952782	1.993970297	1.893891425	3.89912032	3.977210031	3.92758371
Zm00001d052886_T002	1.50305355	0.339588941	1.45817199	2.82009153	3.397753	2.756503484
Zm00001d032371_T001	4.828898762	4.117440055	4.972841832	5.317225078	5.297884602	5.236684212
Zm00001d032371_T002	0.972211063	0.712753357	0.871234908	1.088505957	1.212573074	0.778127817
Zm00001d027874_T009	5.232912784	4.402464812	4.941712519	4.616986915	4.54474168	4.326732197
Zm00001d027874_T005	2.265415291	1.917369501	3.32708717	2.041919656	2.10051248	2.334255906
Zm00001d027874_T004	2.701770379	2.564458655	2.85445173	0.950703556	1.218742117	1.205043997
Zm00001d027874_T008	0.618144666	0	0.973552501	0.816978914	1.117214286	0.980238123
Zm00001d027874_T007	3.181726576	2.182165649	3.06752583	1.993987319	2.121260472	1.784134153
Zm00001d027874_T003	2.662574217	3.487313591	2.256310832	0	2.071312357	2.381124141
Zm00001d027874_T006	0.60544313	0.256100045	0.54964453	1.105680089	0.579304713	0.807259289
Zm00001d027874_T010	4.150385178	2.927628364	4.190430443	3.006598203	3.08703608	3.100899626

Zm00001d035337_T001	3.044103453	2.923141382	3.463096258	3.547848082	3.644835274	3.2208704
Zm00001d035337_T002	3.6991296	2.324676982	3.725926198	4.076328461	4.279111394	4.14844876
Zm00001d022387_T036	3.253948132	2.677117382	3.201239105	2.868717578	2.72425495	2.323555691
Zm00001d022387_T013	1.129581088	0.444608764	1.143717319	1.507679981	1.445198572	1.137103448
Zm00001d022387_T019	0.588293202	0.284824433	0.493211796	0.75058421	0.830721812	0.769560185
Zm00001d043827_T002	5.098457078	4.346931793	4.896915589	4.926594977	5.026523974	4.87542539
Zm00001d043827_T004	0.76752853	1.044130388	1.255819148	1.202422737	1.303938523	0.989272732
Zm00001d043827_T003	0.383940357	0.181416824	0.238958017	0.560825475	0.478509033	0.84124374
Zm00001d006683_T001	3.840773594	3.089232536	3.685928042	3.705891337	3.574207132	3.529764237
Zm00001d039591_T001	3.554472695	4.93330631	3.355685287	2.6239895	1.926776746	1.916058871
Zm00001d043813_T001	3.331755512	2.556084795	3.586333251	3.995086479	4.06815133	3.860845829
Zm00001d043813_T004	0.536253869	0	0.975989623	0.704204453	0.983004158	1.879445823
Zm00001d043813_T002	2.676360846	2.415642943	2.406870503	2.185855131	2.60112289	2.335606448
Zm00001d043813_T003	1.242278111	0.640853184	0.602854011	1.661790637	1.455007046	1.453734593
Zm00001d038203_T001	6.48404601	7.109547665	6.540963099	6.269531755	6.299844624	6.122716964
Zm00001d008842_T001	2.846598418	2.577248261	2.981207497	2.495680326	2.718493805	2.7274243
Zm00001d005945_T001	9.289106856	9.437965636	9.272197731	8.409810678	8.413015999	8.418063977
Zm00001d048355_T001	3.752992373	3.047770444	3.713952442	3.508259906	3.437200302	3.504097114
Zm00001d047443_T003	6.492839979	6.6497063	6.634913962	6.522053473	6.577432726	6.483804623
Zm00001d047443_T001	0.957204403	0.456167964	1.206264998	1.187948231	1.148773399	1.105591594
Zm00001d009701_T001	2.839930371	3.596550929	2.578385916	0.610501642	0.513722165	0.212118562
Zm00001d025149_T001	2.347186284	1.528592822	2.224512963	2.919602471	2.872460407	2.604905378
Zm00001d035990_T005	0.723745521	0	0.469320246	1.285783928	0.998509648	1.461468839
Zm00001d035990_T017	4.392267546	3.54613795	4.530014355	3.863193427	3.595749857	3.801653165
Zm00001d035990_T006	2.450564904	2.294552821	1.906720155	2.549186631	1.983957087	3.035603325
Zm00001d035990_T007	0.156428978	0.148077667	0.23811793	0.827773508	1.061747171	0.6319061
Zm00001d035990_T013	0	0	0.464700675	0.627293987	0.581210466	1.619393289
Zm00001d035990_T009	2.60206368	1.536463264	2.856898667	3.148475362	3.557331048	1.76929062
Zm00001d035990_T002	0.136280649	0.239422483	0.297104529	0.571412757	0.491044448	0.413673147
Zm00001d029755_T001	3.110772305	3.283301013	2.310055335	3.738925282	3.773531593	3.769669351
Zm00001d009474_T001	0.742207182	0.430775545	0.693724683	2.501253143	2.456320439	2.567522814
Zm00001d025011_T002	2.315212034	0.467200169	2.366095562	3.156474283	3.423086791	3.271943883

Zm00001d025011_T003	1.384973168	0.670251176	0.51823398	2.725350715	2.318762815	1.95749937
Zm00001d025011_T001	3.026071801	2.847815356	3.224466967	5.515494693	5.532411598	5.52726624
Zm00001d004763_T001	2.187595791	1.470822122	1.908701484	1.781040712	1.916621862	2.26463641
Zm00001d024854_T001	3.085430945	2.490391194	3.045690389	2.649933162	3.017001317	2.880595415
Zm00001d043407_T003	5.33251475	5.078896834	5.544311376	5.696839395	5.620724457	5.51174263
Zm00001d043407_T004	4.295916057	4.903117539	3.146367339	0	1.93207516	2.505659521
Zm00001d009783_T002	0.788432643	2.388379679	0.849309963	1.126861875	0.743661449	0.971036924
Zm00001d009783_T004	5.417646203	4.43994136	5.400390032	5.684430975	5.696791277	5.684955007
Zm00001d009783_T001	0.471117731	0.50958032	0.486928509	0.552229498	0.756364066	0.667222723
Zm00001d009783_T006	2.193284456	1.46384737	2.247519249	2.324407975	2.351989806	2.115959774
Zm00001d002169_T011	0.669692611	0.104646638	0.634230807	0.542741426	0.385767857	0.696245817
Zm00001d002169_T001	1.78771454	0.113476477	2.113138045	2.373759558	1.811901309	2.06640164
Zm00001d002169_T005	1.84093489	2.906588367	2.037935998	1.570861196	0.655271219	1.662959698
Zm00001d002169_T008	5.631166489	4.625177015	5.691151945	5.317398178	5.521239136	5.251176749
Zm00001d002169_T004	0.370085028	0.253763015	0	1.064450392	1.511598589	1.158703472
Zm00001d019597_T001	7.318838612	7.100361439	7.319170292	7.421472896	7.429663043	7.424863973
Zm00001d021890_T015	1.784426507	0	0	0.712494535	3.364188316	3.056949878
Zm00001d021890_T031	4.188881242	1.845356765	4.657189121	3.303945535	4.130829828	3.763224373
Zm00001d021890_T037	0	1.648463602	1.963470795	2.658987187	3.61594744	0
Zm00001d021890_T024	2.610745884	2.035312817	0	4.351485508	2.541056831	4.113219243
Zm00001d021890_T053	5.906243209	7.779214727	5.842043567	5.006159269	5.141600529	5.381744767
Zm00001d021890_T017	0.253464114	0	0	2.351546073	1.26787074	0.791459031
Zm00001d021890_T011	4.689187879	3.76471865	4.993042354	4.328710036	3.375674495	4.58071672
Zm00001d021890_T052	0.497527551	0.646723955	0.598267053	1.074604764	0.673003662	0.943816331
Zm00001d021890_T018	5.672157372	3.856121905	5.330543736	4.318879274	5.205210874	4.294335269
Zm00001d021890_T036	3.179542101	0	2.016830747	4.043685801	1.825975261	3.794233581
Zm00001d021890_T029	0.936059332	0.342687859	1.520797866	0	1.398604845	1.69136666
Zm00001d021890_T044	1.169964114	0	1.203591461	1.764687551	1.99072147	1.996816068
Zm00001d021890_T032	0.135450814	0.061317227	0.380002378	0.852169073	0.175875851	0.435442978
Zm00001d023828_T001	2.993708051	4.670935146	3.184446457	3.545410081	3.283237885	3.625862878
Zm00001d028443_T001	2.077211205	2.17280751	1.899693051	0	1.635256152	0
Zm00001d028443_T002	3.177425917	1.927642775	3.238479031	3.646029789	3.58237716	3.603598455

Zm00001d052420_T001	3.648976278	2.715003105	3.698557105	5.516909114	5.458590512	5.415673295
Zm00001d005248_T001	5.224030817	4.322887529	5.277362172	5.404664333	5.410953564	5.315800635
Zm00001d005248_T005	1.507737312	1.960694074	1.258825823	1.363659464	0.791054712	1.21932653
Zm00001d005196_T001	4.535245822	5.727839171	4.482452649	4.945544687	4.818014011	4.910174661
Zm00001d037728_T001	1.626260629	1.061532215	1.660742006	1.065655714	0.966434897	1.247644414
Zm00001d044445_T001	6.215933278	5.883766031	6.266662595	6.043732798	6.018744388	5.964518613
Zm00001d050606_T009	2.00681468	2.6725446	2.399067982	2.528450798	2.647449677	2.243862504
Zm00001d050606_T021	5.478291508	4.54521471	5.375767339	5.563320058	5.579481433	5.413503607
Zm00001d050606_T022	0.33782753	0	0	1.462470142	0.295403304	0.733112208
Zm00001d050606_T035	1.845955251	0	0	3.828950893	3.869987646	4.174002542
Zm00001d050606_T016	3.272147889	0	3.50500129	3.86067274	3.871080908	3.794658635
Zm00001d017317_T001	4.347811548	3.676753942	4.336899401	3.837780198	3.778553541	3.84586425
Zm00001d004448_T001	1.970830468	1.226178028	1.836362474	1.593707051	1.653835057	1.748401549
Zm00001d043773_T007	0	0	1.311840209	1.530706527	1.27854149	1.556024202
Zm00001d043773_T001	2.74825061	1.409854154	2.156759672	0	0	3.050043306
Zm00001d043773_T006	1.066049225	0.68824393	0.404153249	1.283841785	1.515054376	1.333201015
Zm00001d043773_T004	2.501608564	2.279534886	2.691904171	3.825724378	3.984685496	3.218772645
Zm00001d012801_T002	2.059691337	1.258920472	1.967450109	1.895570193	1.904611647	1.716470772
Zm00001d027242_T005	0.736756388	0.568412556	1.058784733	1.939607986	1.830663408	2.228350181
Zm00001d027242_T008	2.150205783	1.948304055	2.214272487	1.357794076	1.407366892	0.673683954
Zm00001d027242_T004	3.105943698	2.241764539	2.788357674	2.822908358	2.634911045	2.701551053
Zm00001d027242_T009	1.646826697	0.821188547	1.328761801	2.228756826	2.023826284	1.372431743
Zm00001d027242_T006	1.982685524	1.2182449	2.090277592	2.322702614	2.292978119	2.036493135
Zm00001d027242_T007	0.60368782	0.163511613	0.92419112	0.499315771	1.143923637	1.700503195
Zm00001d027242_T011	1.609005057	0.958233182	1.358992573	0.606717001	1.589556894	0.905304683
Zm00001d027242_T010	0.988790829	0.876473334	0.922656088	1.710069128	1.855104679	1.936971051
Zm00001d051887_T001	1.745681369	0.559646476	1.436964535	2.235064371	2.054629712	2.182253702
Zm00001d051887_T002	5.122109569	4.505242841	5.219363087	5.709950782	5.727542065	5.706030523
Zm00001d021306_T001	5.306617692	4.642224272	5.137243897	5.267329529	5.256047419	5.221440611
Zm00001d044300_T001	3.771878502	3.445691351	4.104655192	6.18995321	6.257055664	6.236042136
Zm00001d043864_T002	3.07779997	2.751591617	3.496787003	3.108429445	2.534029221	3.117211293
Zm00001d043864_T001	4.682609252	4.05404088	4.741467418	5.398040734	5.41138828	5.475831887

Zm00001d043864_T003	2.974855809	2.37916069	3.009989362	3.448467285	3.521390509	3.200159991
Zm00001d038797_T001	0.73856725	0.569369566	1.130345109	1.363658903	1.383191673	1.034043822
Zm00001d038797_T003	0.579695718	0.405569833	0.303923331	1.094319859	1.164866712	1.159377952
Zm00001d009010_T001	2.563908088	0.768967668	2.961337539	2.920346852	2.422647447	2.285127859
Zm00001d009010_T002	4.606037257	4.504037667	4.411381635	4.218116538	4.319884369	4.397084104
Zm00001d002630_T002	3.581290501	2.87107302	3.729896083	4.68988339	5.061733521	4.946940606
Zm00001d002630_T001	3.152084098	4.160085929	3.29675546	4.328185971	4.359285715	4.633268738
Zm00001d028256_T002	1.393361281	0.441976334	1.140988011	1.018257429	1.666013924	1.139837489
Zm00001d028256_T001	1.161631682	0.538635499	0.860478972	0.901764742	0.859896418	0.641675496
Zm00001d014945_T001	5.503105053	5.250804528	5.593284951	3.422273509	2.972251692	3.251475821
Zm00001d003004_T001	0.555354811	0.192364627	0.559173627	0.614593953	0.317165118	0.536202137
Zm00001d025221_T001	3.513386898	3.861805431	3.689861143	2.754214301	2.891558394	2.779041566
Zm00001d040829_T006	0.987168765	0.487956538	0.959654438	0.968625326	0.941360263	0.740889577
Zm00001d040829_T008	5.902092397	5.579244997	5.834431533	5.230851304	5.009207635	5.011061862
Zm00001d040829_T003	4.204137899	3.270410382	4.374444223	4.900806926	4.873575321	4.864517295
Zm00001d022166_T001	1.431264046	1.21379643	1.433793792	1.852169872	1.986375982	2.077869512
Zm00001d022166_T002	2.306277116	1.462961114	2.436606948	2.674976937	2.557348818	2.38222843
Zm00001d020152_T002	3.998225928	4.037309383	4.361748291	4.502596086	4.487722363	4.354604561
Zm00001d020152_T001	2.704437542	0.800115896	1.875666844	2.094088414	2.063800079	2.216918564
Zm00001d039111_T011	1.526725389	0.57731232	1.684650002	2.156517645	2.192875891	1.754810129
Zm00001d039111_T008	1.473553672	1.495016118	1.85290693	1.314456975	1.381402992	1.287351721
Zm00001d039111_T009	1.369350896	1.719659221	1.671127125	1.656650128	2.331332028	2.219131029
Zm00001d039111_T007	3.272810333	2.211134334	3.21540494	1.860140833	2.622409388	2.817787368
Zm00001d039111_T010	2.832700275	3.464258138	2.990783905	2.597594578	2.369667205	1.727290976
Zm00001d039111_T003	3.012078217	1.377831732	2.86226927	2.561776534	1.892485454	2.37587352
Zm00001d031208_T001	2.433615684	2.724680828	2.127649126	2.915462006	3.281147325	3.231386018
Zm00001d031635_T001	5.227147544	4.592254298	5.338823187	7.002550154	7.037210101	7.011588052
Zm00001d009147_T002	3.252609881	3.26011616	3.338316868	3.022091322	3.16442041	3.516595387
Zm00001d009147_T001	0.474892857	0.56170836	0.821258767	0.819188295	0.766618906	0.500963137
Zm00001d047847_T006	0.9502631	0.601507314	0.721630768	0.934304375	0.864543938	1.122991369
Zm00001d047847_T007	1.616451333	0.634857158	0.901983251	0.837476661	1.093964399	1.395799318
Zm00001d047847_T005	2.863634224	2.640981788	3.423202322	2.365200078	2.027842671	1.476422129

Zm00001d047847_T010	3.700570886	3.042934073	3.813358965	2.746227191	3.117702854	2.544655018
Zm00001d047847_T011	1.113267092	0.649574074	1.463976026	0.640808772	1.073851074	2.150756247
Zm00001d047847_T009	3.34550666	2.521572426	3.450211992	3.444457432	3.559281084	3.346881598
Zm00001d047847_T002	2.624315469	1.60445947	2.368235585	1.465465188	1.448258201	1.922633256
Zm00001d018277_T004	0.949603658	0.426886255	0.85288017	2.046192782	2.025251385	2.207856628
Zm00001d018277_T001	4.630004201	3.783535649	4.556679796	5.275984485	5.39293006	5.341514127
Zm00001d018277_T002	1.646375589	1.537490899	1.629954326	2.056601215	2.367935172	2.167496024
Zm00001d037233_T001	0	1.032620684	0.463768916	0.411451194	0.573262054	0.551862469
Zm00001d037233_T002	0	0.243976108	0.186246986	1.660995681	1.162581299	0.921712808
Zm00001d037233_T003	1.999572177	0	1.656024471	2.200805642	2.287599065	2.200283682
Zm00001d037233_T004	0.110985893	0.137089017	0.272184475	0.318164103	0.518208796	0.395333759
Zm00001d019062_T007	3.319057307	1.679292269	4.156770591	3.909038035	3.969652166	3.725653044
Zm00001d019062_T008	6.065723238	5.287124841	5.908225312	5.564462039	5.411673984	5.337217837
Zm00001d019062_T001	2.999337471	2.406900971	3.635406385	4.242651052	4.070566305	4.014426801
Zm00001d019062_T003	3.90720728	0.407047047	3.137429584	4.635251914	4.728981452	4.913085146
Zm00001d022563_T001	1.139095512	0.598892064	0.899135405	1.201251183	1.503748156	1.277770558
Zm00001d018813_T001	5.03056381	4.635405688	5.125818329	4.328568088	4.027961183	4.15972145
Zm00001d021554_T001	6.719227586	6.482856139	6.735558204	6.56232906	6.62591589	6.645221098
Zm00001d036831_T007	2.267833295	1.439153953	2.005145562	2.940132163	2.601127407	2.482369714
Zm00001d036831_T001	1.152348938	1.040143367	1.402371282	2.873442144	3.016526807	3.331183385
Zm00001d036831_T012	4.660192756	4.106262881	4.67062651	5.49450928	5.17254668	5.268981364
Zm00001d036831_T008	2.175304867	0	1.869121247	4.351252272	4.347837834	4.071629341
Zm00001d036831_T010	1.832581801	1.615219467	1.738430196	0	2.923920821	2.160915672
Zm00001d048611_T001	14.05116282	13.70842338	14.02774461	14.30328709	14.31482505	14.29279885
Zm00001d006416_T001	1.271193177	1.030274626	1.305118365	1.320522225	1.238906672	1.433083367
Zm00001d004506_T001	2.449102016	1.975060039	2.26075736	1.356845263	1.724383097	1.52185832
Zm00001d053042_T001	5.88000312	7.503194534	5.463607549	5.205285801	5.565506852	5.151120683
Zm00001d002781_T003	3.698351627	2.630814783	3.717120896	4.233798196	4.297362387	4.159964958
Zm00001d002781_T014	2.097232882	1.386490373	2.602018057	3.000009558	3.172520958	2.967099976
Zm00001d002781_T011	0.804517031	0.286227057	0	0	0.309143584	1.660228132
Zm00001d002781_T027	4.520864859	4.361681557	4.532986529	3.3797446	3.432598822	3.034403789
Zm00001d002781_T023	2.473257264	1.898032625	2.089039439	2.597343808	2.571348192	2.225822162

Zm00001d003144_T001	1.780834582	1.707077247	1.994881627	1.105128918	1.111649669	1.324820394
Zm00001d042968_T003	2.294349607	2.85246494	2.63502578	2.641876147	2.634282858	1.994866425
Zm00001d042968_T001	5.108454453	4.853650746	5.109427803	5.463361605	5.368202123	5.357147782
Zm00001d042968_T002	4.57502231	5.09839488	4.760799239	4.592353997	4.686952314	4.659847628
Zm00001d020869_T001	6.328480359	6.169867533	5.7948826	6.257074037	6.508241236	6.480675492
Zm00001d053345_T001	1.823153005	2.18882215	1.644018224	1.977009496	1.486717977	2.172629922
Zm00001d039852_T001	2.941156278	2.111202622	2.766855904	3.027244483	3.182757281	3.063931545
Zm00001d049011_T001	2.954948736	2.308871664	3.187453588	1.994895019	2.203935632	2.450148897
Zm00001d024940_T001	5.102907306	4.184831	5.273428236	4.112729904	4.06323992	4.143417746
Zm00001d048536_T011	1.624457955	1.057963139	1.175229485	1.494345458	1.395192801	1.391370704
Zm00001d048536_T004	1.09705451	1.349989669	2.000829672	1.981859958	1.181292141	2.022230373
Zm00001d048536_T001	1.903272785	0.620438145	1.552664794	1.910148559	1.925282069	1.516230526
Zm00001d048536_T006	0.190910638	0.277984747	0.282700742	0.443120721	0.673490393	0.717659536
Zm00001d048536_T012	2.22409246	1.948918504	2.612482725	1.85146762	2.662004921	1.723814532
Zm00001d011242_T003	4.559437604	3.686081272	4.481233058	4.305151132	4.049613866	4.276211299
Zm00001d005846_T003	1.95447142	1.381378072	1.702640258	2.098384184	2.005294701	1.537248846
Zm00001d030303_T001	4.054942744	3.312909813	4.189613238	3.384776775	3.379151816	3.443741633
Zm00001d033272_T008	0.849105753	0.427908624	0.762656671	1.205576435	1.064887679	1.1116717
Zm00001d033272_T007	0.78068217	0.068537368	0.504425139	0.963888377	0.705287011	1.096612025
Zm00001d033272_T006	0.876041847	0.831262749	1.193422932	1.180774209	1.368906327	1.321499839
Zm00001d033272_T001	1.271734621	0.413438107	1.489908187	2.630436226	2.28814384	2.555316523
Zm00001d033272_T003	3.210200722	2.519648581	3.147903031	3.820677206	3.915699864	3.77762181
Zm00001d033272_T009	0	0.215330693	0	1.209806432	0.685000017	1.201480802
Zm00001d013712_T005	2.442533779	1.6105881	2.499082029	2.264148157	2.075230712	2.504370982
Zm00001d013712_T001	3.777593618	4.425798454	3.760046002	2.059923867	2.023900073	0
Zm00001d013712_T002	2.503782242	1.52781802	2.727029302	2.560733049	2.633733436	2.969230998
Zm00001d052720_T008	0.827210933	0.40142841	0.6006085	1.178130116	0	1.092000357
Zm00001d052720_T001	2.419767278	1.571826289	2.758452642	2.106639442	1.898991124	0
Zm00001d052720_T003	1.345003995	1.281362349	0	0	1.574710921	2.206509762
Zm00001d043217_T001	8.040540731	8.660503218	7.978384004	8.042116274	7.945424499	7.983609679
Zm00001d041714_T001	1.393612244	1.096990439	1.651327743	1.540435016	1.638918117	1.091433778
Zm00001d024494_T001	1.168788993	1.271266098	1.667796321	4.251081136	4.232639039	4.251725

Zm00001d045022_T001	2.893839794	4.447431217	2.654519176	5.467118929	5.511205923	5.440528959
Zm00001d051595_T004	7.834562583	7.39182538	7.833124313	8.058293022	8.095005738	8.045917555
Zm00001d051595_T001	1.23400828	0.70232686	0	0	1.38897805	1.344580832
Zm00001d043525_T001	5.251093183	5.304427478	5.303159635	6.264315074	6.186084023	6.278761567
Zm00001d038794_T002	1.062338653	0.492221353	1.283957913	1.525228498	1.288163039	1.144409283
Zm00001d036230_T001	2.099667866	1.687148502	2.108605078	2.341727348	2.254772706	2.069612812
Zm00001d004132_T001	3.051884176	2.290518772	2.964143527	3.46860065	3.409767956	3.393948806
Zm00001d011710_T001	6.335329858	6.415564595	6.399656093	6.003071854	5.86912332	5.795036715
Zm00001d042340_T001	4.293242528	4.323171739	4.223337592	4.434211604	4.411829507	4.257049477
Zm00001d004489_T001	1.67610083	1.217853578	1.289610225	1.644068539	1.495925362	1.912685118
Zm00001d039218_T002	1.921301119	1.411613892	2.033629149	1.537614146	1.397933264	1.436068002
Zm00001d011644_T002	0.182258788	0	0.035950414	0.553582666	0.49095514	0.520122489
Zm00001d049987_T001	4.117840884	3.325751648	4.107446758	3.059888056	2.983031894	2.776803683
Zm00001d033557_T004	4.683238368	3.834929707	4.452507405	4.439780629	4.675677186	4.663209851
Zm00001d033557_T003	0.294163719	0.16690822	0.450247894	0.456041765	0.419711465	0.298831898
Zm00001d033557_T006	3.185550703	3.21789994	3.710223233	1.513661029	0	0
Zm00001d003039_T002	2.228101705	1.455774082	2.585213027	3.434342929	3.231209812	2.885399239
Zm00001d003039_T001	2.698983193	2.407594223	3.076024227	3.537081697	3.357817155	3.75603842
Zm00001d035008_T002	0.772490412	0.207100713	0.435526188	0.70681166	1.293991928	0.987196421
Zm00001d035008_T004	0.287300887	0.069600439	0.611484025	0.622668956	0.816987104	0.658206912
Zm00001d035008_T003	2.368493191	0	1.326659868	1.018048717	0	1.579741087
Zm00001d035008_T001	2.66358964	2.477515436	3.173390997	2.874350812	2.714718974	2.47539285
Zm00001d000019_T001	7.63793563	2.676343243	7.961038909	8.30926467	8.017388513	8.025405952
Zm00001d009402_T001	2.382969039	2.305307809	2.624393616	1.482703232	1.742014837	1.770108047
Zm00001d016947_T001	3.013709685	4.822930531	1.417974	6.106760992	6.397406269	6.048093579
Zm00001d042058_T001	4.976417621	4.064200479	5.008396888	4.969819383	4.964235731	4.905590719
Zm00001d047214_T001	3.727993295	3.570990487	3.970422714	4.582305135	4.758535203	4.80682484
Zm00001d037842_T001	4.200474064	3.884318439	4.042922345	4.204187287	4.41424867	4.359302866
Zm00001d011454_T001	3.615019195	4.353845362	3.610213767	5.68509791	5.612056527	5.482960126
Zm00001d031625_T001	5.977235382	1.202914782	4.623265575	5.849747816	5.656934232	5.872759454
Zm00001d042908_T001	1.410519124	1.325405988	1.497136115	2.112821214	2.214313836	2.118542128
Zm00001d042908_T004	0.841731643	0.922960486	1.542587912	1.365629274	1.253248069	1.150797507

Zm00001d027841_T002	4.920255755	4.305872709	4.923958746	4.959360283	4.981211881	4.922435931
Zm00001d022127_T001	7.374249	7.094774457	7.2514778	8.392343211	8.444780564	8.306929407
Zm00001d047209_T011	1.263858911	1.005674487	0	5.429300244	5.522859287	5.502765878
Zm00001d047209_T020	0	0.307992669	0	3.557572328	3.296673832	3.900749746
Zm00001d047209_T013	1.587088428	0	2.066215281	3.530289028	2.765619184	3.131706851
Zm00001d047209_T021	0	0	0.088468643	1.217900096	1.235791995	1.356195093
Zm00001d047209_T006	1.503526835	1.114648206	1.587903561	2.742650428	2.539707737	2.65505616
Zm00001d047209_T022	5.208394819	4.343629966	5.197660867	6.301558639	6.375706341	6.210929598
Zm00001d047209_T014	0.59116524	0	0.203885217	2.268863126	2.521753063	1.088750847
Zm00001d002428_T001	5.150415197	5.541555354	5.376938888	4.926426137	5.05898058	4.959773029
Zm00001d002428_T002	2.026859536	2.168871129	1.646799054	2.078974561	1.699562293	1.85665908
Zm00001d003477_T001	3.233848956	2.297542435	3.182585363	2.620669216	2.598010205	2.847392451
Zm00001d003477_T002	0.419640281	0.396060821	0	0.649798457	1.272788839	0.567575625
Zm00001d043312_T005	0.919733647	0	0.504854255	1.161056322	0.52837928	0.7040238
Zm00001d043312_T009	2.215465515	1.929640283	1.945652272	2.535762342	2.580894141	2.198655701
Zm00001d043312_T011	3.89390113	2.67317761	3.891528551	3.726613489	3.783463045	3.883373504
Zm00001d043312_T001	1.259302621	1.443118068	1.758821226	3.050381366	3.21088677	2.777149089
Zm00001d037382_T001	0.35499032	0.463735441	0.839684724	1.012286185	1.435028457	0.977173664
Zm00001d021061_T001	8.581470891	8.141643063	8.619155351	8.034781518	8.011086636	8.035449588
Zm00001d041108_T002	4.114095703	3.112934948	3.9908859	4.216984661	4.287942823	3.961491091
Zm00001d041108_T021	0.502934442	0.114087122	0.378822324	1.011884885	0.988889694	0.680657421
Zm00001d041108_T012	0.331834749	0.179268988	0.294726016	0.69742867	0.660019517	0.273272386
Zm00001d041108_T006	1.097621583	3.557447465	2.463775977	1.451246989	2.457172426	0
Zm00001d041108_T003	0.359910185	0.358370379	0.459669775	0	0.428724498	0.536675612
Zm00001d013028_T001	3.869769664	4.54167014	3.653451946	4.0777511	4.229656749	4.206305326
Zm00001d019039_T002	5.275834869	4.701902202	5.326617627	5.009163285	5.042976522	4.941720734
Zm00001d013523_T001	3.334657655	2.717700109	3.282815035	2.655902009	3.280107665	3.346008866
Zm00001d013523_T002	0	0	2.066076788	2.863952719	2.276964089	2.33600625
Zm00001d026695_T001	5.195185916	4.45386988	5.342573127	4.611102605	4.575584686	4.472588302
Zm00001d026695_T002	0.943445787	0.769730277	0.686268333	0.550865212	1.006656364	0.71344772
Zm00001d008501_T001	3.545219406	3.5599878	3.754306139	4.007555592	3.411685609	4.12151578
Zm00001d015306_T001	3.71309563	2.87139382	3.717108938	3.758339532	3.722921604	3.807194053

Zm00001d040298_T001	10.0881681	8.737392045	9.775782415	9.373880956	9.308179392	9.363831988
Zm00001d032239_T001	7.527253031	7.157722391	7.655982347	7.194816895	7.291678742	7.27403605
Zm00001d032667_T001	0.422318053	0.143894911	0.41090331	0.843751547	0.825046388	0.735939762
Zm00001d032667_T005	2.523327342	2.60294734	2.638237449	3.354662456	3.394101277	3.588669068
Zm00001d032667_T010	3.97027416	1.945897561	3.814990035	3.800581048	3.68167684	3.587499652
Zm00001d032667_T007	0.558120663	0.300162391	0.579110619	0	0	0.32718032
Zm00001d010727_T001	4.125848946	3.391112338	4.053242911	3.278989376	3.108483141	3.114694176
Zm00001d010997_T001	2.67024075	1.856631195	2.729818119	3.152170599	3.033478682	2.949401396
Zm00001d010210_T002	1.380094944	0.681425876	1.130014237	1.063401467	1.219619578	1.18004599
Zm00001d010210_T001	0.456521261	0.33137044	0.7091971	0.501539016	0.294860088	0.494212825
Zm00001d016025_T001	2.031098722	2.49693729	2.456686051	2.581231681	2.908291253	2.757924863
Zm00001d052210_T001	3.217845204	2.997638902	3.590478786	2.943917952	2.755465533	3.089488659
Zm00001d037826_T001	1.225883843	1.930536008	1.860324416	2.040345049	1.962119574	2.25737879
Zm00001d007501_T001	1.865447734	1.558859287	2.076987599	2.038220885	1.98445592	1.845334282
Zm00001d034017_T001	5.136797455	4.371338748	5.275074933	5.639631165	5.746879896	5.762869718
Zm00001d034017_T002	3.315370303	2.774006027	3.010744934	3.554215053	3.729765815	3.501866986
Zm00001d027256_T001	1.517006029	1.161003418	1.36322884	0.643991912	1.030773941	0.253482268
Zm00001d029922_T001	2.667054415	2.374158667	2.859138269	2.448874792	2.666360989	2.434345065
Zm00001d029922_T002	3.621115184	4.851458675	3.850053084	3.292191448	3.158076679	3.283582388
Zm00001d049817_T001	0.288487321	0.203768724	0.396100289	0.564059106	0.291465647	0
Zm00001d049817_T007	0.528425292	0	0	0	0.929642555	1.586839668
Zm00001d049817_T006	2.949179491	3.239066049	3.008292896	3.324066891	3.141840167	2.658948811
Zm00001d049817_T004	1.426133765	0.891339856	1.522020083	1.787262775	1.719596143	1.237875106
Zm00001d011514_T001	3.264307173	3.523476019	3.542687946	3.620977058	3.754963055	3.825423891
Zm00001d011514_T002	5.176361433	5.095274597	5.190591711	5.619073896	5.81541678	5.457606275
Zm00001d005547_T003	0.446442577	0.193217894	0.438766574	0.318029865	0.249243866	0.540160221
Zm00001d005547_T001	3.330743242	2.441401675	3.329171111	3.619443883	3.556815145	3.655214995
Zm00001d005547_T002	1.903983427	2.642083703	2.269573308	2.403955634	2.751480218	2.284971862
Zm00001d023384_T001	6.242266791	5.999878313	6.31335298	4.528120875	4.347471772	4.343473701
Zm00001d041362_T001	1.326467167	0	1.085531393	1.907676746	1.989021625	2.046759249
Zm00001d041362_T003	1.716614318	1.449227502	1.849293547	1.678595238	1.753744863	1.497574047
Zm00001d028241_T003	3.417693894	2.459920216	3.215558393	3.766541099	3.869762065	3.913662079

Zm00001d028241_T001	0.534311661	0.30234479	0.96858994	1.243238889	0.564449387	0.942253248
Zm00001d018414_T001	3.277547708	3.064051614	3.33348685	2.25643735	2.246495042	2.580986249
Zm00001d003463_T001	5.865436129	6.701792969	5.896351079	9.826157016	9.811777296	9.815184903
Zm00001d002542_T001	4.419938948	3.597598272	4.495424696	4.962218294	4.924100313	4.872506899
Zm00001d038970_T002	3.914410775	2.654370233	3.757784515	3.786573049	3.966436835	3.720124336
Zm00001d041142_T031	3.413612495	2.983259599	3.187915144	4.076968365	3.792371562	2.660772349
Zm00001d041142_T005	1.005851231	0	1.070729352	1.525119727	0	1.954110305
Zm00001d041142_T003	2.869395495	2.753855018	2.972615096	3.56177519	4.139225614	3.900182555
Zm00001d041142_T006	2.55397358	0.559470277	2.074733194	1.855932706	0.939111458	1.823758734
Zm00001d041142_T001	0.485768911	0.344122881	0.29727595	0.401008914	0.411415398	0.511648175
Zm00001d045951_T001	3.979677384	4.53235711	3.816719602	4.082774365	3.782678626	3.857254988
Zm00001d022190_T002	4.579695235	4.016016738	5.096161559	5.228698538	5.267613736	5.168868442
Zm00001d022190_T012	1.668220355	0.833267394	1.390170745	1.92613457	1.444958015	1.575844771
Zm00001d022190_T006	3.247191551	2.440591612	3.46057614	2.299790035	2.634751468	2.522108744
Zm00001d022190_T001	2.591110651	1.813321918	2.336219965	2.753844965	2.687516275	2.507544763
Zm00001d022190_T007	0	0.264078779	0.957963616	0.904501054	1.088473392	1.158354479
Zm00001d022190_T014	4.989537193	5.726433767	5.187136509	4.816620133	4.948204519	4.862655209
Zm00001d044545_T001	3.209803157	3.930951219	3.191452581	3.342132286	2.701823598	3.028551472
Zm00001d010385_T001	0.628212736	0.390445377	0.783289849	2.440209404	2.463608333	2.223670289
Zm00001d033148_T001	1.642019914	0.993556261	1.660128165	1.836126118	1.994051786	1.83879287
Zm00001d013908_T003	3.033365909	2.410513425	2.760810468	3.176386607	2.159343718	0
Zm00001d013908_T001	0.489719665	0	0	0	1.208848087	2.119496621
Zm00001d013908_T002	2.194571547	1.264161071	2.345619774	3.467999865	3.580249823	3.392107736
Zm00001d007851_T001	1.940738452	1.199845065	1.688871433	2.382546355	2.483822999	2.317265279
Zm00001d011758_T001	4.335474824	3.598960528	4.298759623	3.755402613	3.906359586	3.762741596
Zm00001d047942_T001	4.660852766	4.305921725	4.687545612	4.670209359	4.859059032	4.858128341
Zm00001d047942_T002	2.515661997	2.212106731	2.545386974	3.315501745	3.347994973	3.45660379
Zm00001d036756_T001	1.994786067	1.574325345	2.157807877	2.301910095	2.26284534	2.261466126
Zm00001d012996_T001	2.410364444	1.483887475	2.396680399	2.191849393	2.353437629	2.454197218
Zm00001d020971_T001	2.206165893	2.030116744	2.335793931	2.667974303	2.282049201	2.739314552
Zm00001d009539_T002	5.242244379	4.452370144	5.290081974	5.487910246	5.45236194	5.48644772
Zm00001d009539_T001	2.851511396	1.658090348	2.680472445	2.568470686	2.605828775	2.656145093

Zm00001d033165_T001	1.104924354	2.040159152	1.848728087	1.507314634	1.752045909	1.4103677
Zm00001d044575_T001	6.539344333	6.158995486	6.545629024	6.215885261	6.177727998	6.278533072
Zm00001d038143_T002	4.530802644	3.895455706	4.525776674	5.038469632	5.1317487	4.916364425
Zm00001d038143_T001	1.426665701	0.792834534	1.952647421	1.264686548	1.522498235	1.933579436
Zm00001d038541_T002	3.650216897	2.528032896	3.056854182	3.954920548	4.142087283	3.939271344
Zm00001d038541_T001	3.139303474	2.856486186	4.126832312	3.70277876	3.531241458	3.382773757
Zm00001d009578_T001	6.198057213	6.27090547	6.268287979	5.889088919	5.93324528	6.006232082
Zm00001d018144_T001	2.130725979	2.049941924	1.554335897	1.833983011	1.91421012	1.687318289
Zm00001d018144_T004	3.173641402	2.267331739	3.39623006	3.486239531	3.290072977	3.375339064
Zm00001d012757_T001	2.262767182	1.840688408	2.15999272	2.250354059	2.233817825	2.117293426
Zm00001d003214_T009	0.87589799	0	0	2.295773268	2.489849116	2.235192766
Zm00001d003214_T014	1.366326131	0	0.337690543	1.44124974	1.658899241	1.187974193
Zm00001d003214_T003	1.41259674	4.041206354	1.253402996	0.73103375	0.613325135	0.232458151
Zm00001d003214_T004	0.831177608	0.623297536	1.039191046	1.166843964	0.358885705	1.85784647
Zm00001d003214_T015	0	0.540448903	1.07265807	0.443453887	0	0
Zm00001d003214_T001	3.512017105	2.696554752	3.819232551	3.864384468	3.790710534	3.880455048
Zm00001d003214_T002	1.258351882	0.563431509	0.533526471	0.957327003	0.544524952	1.359639797
Zm00001d003214_T010	0.679289567	0.325889486	0	0.998160112	0.576688528	0.598144115
Zm00001d011377_T002	1.716371994	1.545039212	3.082420903	3.929192117	4.11000194	3.77777381
Zm00001d007092_T001	1.956394605	1.048062466	0	1.414849937	1.973833861	1.793177164
Zm00001d007092_T002	0.836646453	0.530602164	0.910699664	0.886984744	0.788233832	1.224671315
Zm00001d025153_T012	0.472553276	0.155948658	0.223551058	0.778772085	0	0
Zm00001d025153_T005	2.854370939	2.237264196	2.908543746	2.368944032	2.792957357	2.600112485
Zm00001d025153_T002	0.342035828	0.138999216	0.526371345	0.206601968	0.624025072	0.52388408
Zm00001d025153_T007	0.041209333	0.018135625	0.04975482	0.3485837	0.814938371	1.023656341
Zm00001d025153_T006	1.201390464	0.759704678	0.876032414	1.137658933	0.782073015	1.234454722
Zm00001d021600_T003	0.290213234	0.099237293	0.600048955	1.53987942	0	1.179944749
Zm00001d021600_T002	0	0.645146421	0.702004083	0.61438099	0.383749076	0.695329308
Zm00001d021600_T004	2.795916944	1.461212117	2.634573754	3.028762029	3.44627198	3.276877463
Zm00001d021600_T008	2.671516667	3.302600981	2.510304204	2.470477777	2.108435801	2.626161318
Zm00001d041917_T001	5.794374586	6.437693523	5.839212936	5.551880429	5.667175026	5.527623223
Zm00001d015389_T001	0.150948242	0	0.662439776	0.964781575	1.459369713	0.843738686

Zm00001d015389_T002	3.276450659	2.556629037	3.25557702	3.308034913	3.279715538	3.507667551
Zm00001d011445_T006	0.733230242	0.59748064	0.958366832	1.279474267	1.002567875	1.14452349
Zm00001d011445_T002	0.701125834	0.337173299	0.586232475	0.315034033	0.2542808	0
Zm00001d011445_T003	1.190039564	0	0.840451148	0.615609334	0.663437532	1.341784268
Zm00001d014126_T001	3.097748992	2.579297954	2.830085931	2.990823832	2.953981286	2.983358114
Zm00001d034062_T001	3.422703684	2.622134307	3.348611035	2.826757339	2.575493382	2.45774399
Zm00001d031940_T001	4.460001895	2.911988909	4.044709729	4.623066183	4.464848593	4.420745127
Zm00001d052835_T001	4.456996131	3.487062199	4.439075607	4.161830941	4.086154997	4.150325707
Zm00001d035618_T001	3.022148507	3.116560386	2.940960343	2.310095773	1.988301492	1.987476225
Zm00001d036698_T001	1.043651071	0.722308631	1.026984146	1.076756093	0.925057563	0.866233046
Zm00001d027621_T001	3.581469117	2.917558738	3.597445122	2.916684339	2.944251449	3.117777979
Zm00001d031216_T005	1.160519456	0.643021263	1.136734756	0.243476546	1.031682436	1.318336511
Zm00001d031216_T002	1.934291163	1.389402159	1.548083923	1.917666616	1.736603577	1.788970881
Zm00001d031216_T004	0	0.668501311	0	1.144662484	1.48342473	0.267840185
Zm00001d004931_T001	4.375023927	3.629792918	4.603477217	4.730529807	4.859189119	4.660022599
Zm00001d011605_T002	2.538760634	3.804365345	2.829582505	3.086190842	2.849725301	2.979380606
Zm00001d047535_T001	4.670486699	5.301794123	4.703669956	5.459153381	5.60967203	5.580819087
Zm00001d053515_T007	1.253368502	0.556991424	0	0.873744703	0.175021207	0.933928358
Zm00001d053515_T006	0	0	0	0.967095548	1.748160211	2.17384988
Zm00001d053515_T005	1.792659232	0.903214932	2.423749993	2.2624518	2.360311737	1.349392455
Zm00001d053515_T009	2.158895382	1.093186947	1.672132021	3.762734794	3.762816313	3.770573143
Zm00001d053515_T014	0.349746851	0.098255146	0	0.486888361	0	0.497424337
Zm00001d053515_T011	3.928656844	3.255600283	4.056185172	3.423241996	3.257307879	3.62139972
Zm00001d036013_T004	6.640826973	5.742003165	6.708024303	7.227134901	7.275265262	7.191444754
Zm00001d041982_T001	2.444636856	1.965229444	2.510132258	2.445060018	2.248581286	2.178179845
Zm00001d022448_T001	1.999233365	3.546277132	2.811869669	2.687226902	2.785335858	3.344638208
Zm00001d051746_T001	1.350143602	1.121488925	1.407968315	1.092227739	1.159291398	1.134578447
Zm00001d002003_T002	1.639453529	1.052145972	1.497340533	2.208805957	2.201807601	2.333589462
Zm00001d002003_T008	3.477249377	2.729271708	3.548304927	3.155412478	3.297127719	2.867887571
Zm00001d007129_T001	1.543335025	1.210092689	1.48357074	1.703191973	1.347573253	1.347996673
Zm00001d006904_T001	7.090764201	7.030383093	6.754296437	6.367836738	6.732963947	6.751657274
Zm00001d026326_T001	4.831309474	4.157748146	4.771500404	5.281626188	5.396677522	5.389124712

Zm00001d016724_T001	4.922049527	3.41239662	4.111704663	3.675429242	3.05806673	3.198833414
Zm00001d006130_T001	3.457222736	2.711852783	3.456141147	5.709541707	5.775534827	5.687568007
Zm00001d037289_T003	4.57118865	3.500178067	4.684693983	4.252332013	4.192113663	4.245508365
Zm00001d015338_T001	2.126265868	1.937848306	2.291970962	1.642422477	1.572448791	1.90215734
Zm00001d005925_T003	0.608162013	0.250727571	0.434169695	1.947755165	2.237734409	1.802950872
Zm00001d005925_T004	1.092371187	0.772010621	1.129861957	3.384222809	3.664669764	3.856593849
Zm00001d005925_T002	5.3368188	4.721086225	5.632754956	6.333957597	6.27762864	6.215044696
Zm00001d049464_T001	2.808356423	3.319627616	3.389409731	2.280356551	2.397197834	2.190759281
Zm00001d017528_T001	4.477642749	3.67358582	4.413789163	5.053792936	4.951440692	5.126774381
Zm00001d047679_T005	2.508576464	1.753266927	2.192354526	2.702997678	2.731729581	2.529445172
Zm00001d047679_T004	0.266454345	0.043446701	0.052143189	1.414969511	1.37614644	1.247694841
Zm00001d053938_T001	0.906732083	1.406667274	1.080926396	3.547252759	3.603519174	3.314161397
Zm00001d026477_T006	0.707650237	0.316193252	1.119575968	3.190798306	3.309839453	2.813944713
Zm00001d026477_T003	7.163990761	6.791332146	7.229177483	7.444798073	7.526098594	7.523342203
Zm00001d026477_T007	1.305351298	0.622789822	0.744029309	2.707861572	2.501669448	2.549972955
Zm00001d026477_T002	5.497792579	5.398476587	5.635154207	5.966020036	5.931463327	5.803336475
Zm00001d023507_T001	3.886188306	3.12482224	3.937995341	3.806574625	3.821468997	3.613501832
Zm00001d013261_T001	3.204005614	3.260714461	3.66991536	5.410305485	5.456234214	5.474094604
Zm00001d027341_T001	1.950139151	1.07544569	1.675558526	2.337746196	2.428200023	2.342926465
Zm00001d006751_T005	3.415951833	2.141597586	3.34723817	4.426476111	4.446660459	4.333992112
Zm00001d006751_T004	4.509618322	3.625781422	4.607945555	4.437014755	4.45554771	4.466642911
Zm00001d006751_T003	1.945230501	1.191237313	1.538983071	2.5818605	2.361711942	2.37100147
Zm00001d006751_T008	2.270278654	4.919152463	2.954497849	2.553953189	2.279345296	2.689568886
Zm00001d006751_T001	1.69487128	1.256383906	1.650111983	1.555998198	1.534620439	1.550981414
Zm00001d006751_T007	0.053788375	0.044605349	0.107213811	0.425587133	0.697323685	0.422355732
Zm00001d042438_T001	0.888508308	0.663062202	1.288328438	2.784743086	2.484771798	2.610477074
Zm00001d016479_T036	5.787762748	5.328577817	5.791545637	5.121740629	5.219835956	5.204208224
Zm00001d016479_T035	0.394208998	0.215677354	0.571040865	0.62370957	0.794430007	0.735800292
Zm00001d016479_T034	3.593762497	2.686920892	3.194887469	3.502320671	3.382234241	3.574278594
Zm00001d016479_T016	3.278712009	2.256453051	3.067715289	4.083828191	3.982024397	3.927444332
Zm00001d051130_T001	0.870433375	0.452364543	0.682272143	0.325161876	0.467081194	0.443564219
Zm00001d024906_T001	5.227739844	5.015815015	5.322207482	4.738830028	4.658694833	4.602340772

Zm00001d041220_T001	3.298852128	3.828263042	3.701999316	3.420721551	3.486738439	3.180191791
Zm00001d047664_T001	2.9801862	3.469188809	3.027371006	5.186509155	5.051415173	5.540601808
Zm00001d012021_T001	4.046204658	3.69198343	3.846332239	3.553752092	3.364805906	3.268218782
Zm00001d008479_T001	2.57532516	3.708193307	1.435392303	2.465270832	2.056268945	0
Zm00001d032209_T002	2.345840301	2.898786863	2.128486099	2.960073683	2.638009175	2.95596518
Zm00001d052977_T001	1.338879616	1.239159711	1.521306579	1.440448362	1.381147127	1.375075029
Zm00001d053171_T001	2.088568031	1.503448982	2.138500278	2.255550585	2.308673951	2.414727377
Zm00001d018107_T001	4.154368445	4.337769955	4.134139272	6.368220985	6.386425981	6.41886161
Zm00001d035065_T011	0.893192106	0.301673094	0.375465433	1.049388207	1.297807696	0.755695185
Zm00001d035065_T001	4.168112961	3.372778713	4.332042565	4.532439652	4.557228777	4.600674265
Zm00001d002275_T045	5.676296354	4.806087628	5.704775818	4.933620365	5.480843538	5.087230082
Zm00001d002275_T027	0	0	0	1.20861277	0.326937658	1.133878464
Zm00001d002275_T012	0	0	0.075262892	1.1160899	0.411480481	1.346369767
Zm00001d002275_T037	0	0	0	1.822167234	2.843614885	2.775538804
Zm00001d002275_T031	2.948997347	1.563738509	3.623139246	4.394393958	3.366727721	3.914195479
Zm00001d002275_T042	2.90314724	0	1.957632345	4.935242667	3.796594847	4.619494241
Zm00001d002275_T002	1.066924764	0.810265404	1.278785295	3.229353504	3.559307885	3.116045472
Zm00001d002275_T014	1.948795187	0.246080897	0	3.402830075	3.236695117	3.566181081
Zm00001d002275_T028	0	0	0	0.408879215	0.578168748	0.382689384
Zm00001d045147_T019	1.152335957	0.226013983	1.100919804	2.584981256	2.370869829	1.73849332
Zm00001d045147_T021	0.645997637	0.185157389	0.680510702	1.118446112	0.958446274	1.49243211
Zm00001d045147_T015	3.212542259	2.173761312	3.366803382	3.175238589	3.281747056	3.034299717
Zm00001d045147_T011	0	0.41392764	1.137778921	0.3041956	0.256860908	0.787827772
Zm00001d045147_T023	0.337741915	0.068457572	0.475599595	0.815324575	1.068330301	0.538178562
Zm00001d045147_T001	1.592040285	1.623312521	1.814007285	1.727384652	2.059688222	1.793624585
Zm00001d044329_T002	4.005305931	5.071899803	4.285141178	3.661119879	3.796902125	3.739769919
Zm00001d044329_T001	1.353982094	0.506011827	0.937011366	1.380327166	1.24215248	1.413246364
Zm00001d044329_T004	0.273481277	0.181105096	0.491940297	0.574893985	0.480502966	0.435810986
Zm00001d004839_T005	2.45480996	4.716620573	2.207106962	2.079769263	1.935388083	2.09031452
Zm00001d004839_T007	6.911432461	4.15635495	6.587132034	6.506554468	6.494882699	6.331446259
Zm00001d004839_T003	1.566862391	5.179233671	2.924206136	1.521166349	0	0
Zm00001d017958_T003	8.474034748	0	8.726459054	8.530418312	8.566457856	8.536953033

Zm00001d017958_T001	9.386367087	9.118518581	9.473927972	9.653533428	9.671901662	9.659886279
Zm00001d017958_T002	1.45180772	4.384901008	1.64866009	2.088748134	1.270981564	1.532083017
Zm00001d017958_T004	1.448081606	5.103675175	1.060454187	1.15679979	0.960606602	1.088187062
Zm00001d022072_T002	2.577367433	1.937027944	2.590377101	2.04262893	2.008503741	2.169395596
Zm00001d022072_T001	1.520149651	0.783877353	1.508352079	1.886314849	1.845819199	1.736052801
Zm00001d030498_T001	7.650589632	4.610981877	7.724162373	5.613572759	5.947085269	6.141526778
Zm00001d047514_T001	2.909483384	2.574581115	2.634085797	3.53260522	3.601181737	3.615110799
Zm00001d031926_T001	5.108337814	4.357897155	5.125616389	5.291260192	5.266535303	5.286669865
Zm00001d029768_T001	2.086051365	1.15101126	1.916312302	2.237120991	2.211939209	0
Zm00001d015412_T001	2.440485306	2.865879432	1.977006198	2.399144838	1.996485284	2.446289054
Zm00001d038522_T001	4.299587411	3.763826157	4.292219283	3.061824747	2.891670373	3.0046044
Zm00001d038522_T002	4.590723506	3.536793211	4.728978623	7.182480495	7.253686603	7.229685201
Zm00001d003352_T004	0.394023465	0.168240233	0.243007288	0.286180916	0.453312124	0.399986977
Zm00001d003352_T001	3.658197884	2.906240852	3.575253145	3.57495137	3.553418013	3.77984351
Zm00001d003352_T003	1.927236189	1.824249326	2.152029891	0.880346212	0.800800939	0.796199858
Zm00001d040088_T001	6.204308912	5.823656336	6.175741448	5.921135016	6.101777172	6.090769631
Zm00001d032284_T002	0.310463442	0.538434862	0.840044212	1.136822673	1.052472237	0.760697862
Zm00001d013223_T004	5.021721074	5.311817654	4.707121158	4.091412197	4.184590782	4.122748661
Zm00001d013223_T006	0.295712447	0.282232208	0.455135966	0.511149204	0.875593719	0.164003587
Zm00001d013223_T001	4.266916865	3.510667402	4.263559767	3.850807223	3.951996619	3.802166224
Zm00001d047787_T001	8.880381874	8.830291112	8.958992692	8.73059176	8.759325845	8.764303488
Zm00001d024538_T001	3.852984009	3.668290372	3.904146	4.023508024	3.972198377	3.826843555
Zm00001d011799_T001	1.883987113	2.619086871	2.546722869	2.333225922	2.356661622	1.685387626
Zm00001d014351_T001	1.256274596	1.565380755	1.635967936	1.782418211	1.896792637	1.661131626
Zm00001d021627_T001	4.224483174	4.31270895	4.446128897	4.317301463	4.428564188	4.397728293
Zm00001d034005_T004	1.947038453	1.391276658	1.906882132	1.875484418	1.16409179	1.735806356
Zm00001d034005_T006	4.664481247	3.797219818	4.954114074	2.999529424	3.875865757	2.903890134
Zm00001d034005_T001	3.516898023	3.296997821	3.627407462	3.099205138	3.410311794	3.422085763
Zm00001d034005_T003	3.632307075	1.474908427	3.321278159	3.899852919	3.371567087	3.777204229
Zm00001d006353_T001	4.133200587	3.630635767	4.113988393	3.326054212	3.13202022	3.408969132
Zm00001d002650_T006	1.065684662	0.108001144	1.194986564	1.389625179	0.823570031	1.986255462
Zm00001d002650_T004	0.77945809	0.921345683	0.216605102	0.898840639	1.29976513	1.392291591

Zm00001d002650_T009	0.35350284	0.027258817	0.087776466	0.451726499	0.369476534	0.5427236
Zm00001d002650_T016	3.601684751	0	3.397644996	3.949961609	4.147260861	3.575184153
Zm00001d002650_T064	1.289043569	1.060756448	1.542841944	1.954618819	1.025162069	0.888772468
Zm00001d002650_T011	3.067633382	3.036924564	3.085585438	3.181137385	2.852810075	3.626897105
Zm00001d002650_T015	0.783857243	0.189359027	0.421664424	0.683745853	0.069145326	0.333108825
Zm00001d002650_T063	4.343594792	3.729384292	4.489676512	1.646095361	1.420049542	0
Zm00001d022082_T001	3.060864161	2.54375891	3.122707171	5.029374697	4.991601252	5.06025668
Zm00001d025166_T002	4.908273525	3.892207981	4.998735736	5.662004295	5.751725017	5.755913508
Zm00001d025166_T001	0.223450971	2.595717519	0.187929867	3.061253623	1.681872004	2.272999888
Zm00001d027290_T001	7.77508676	8.249432337	8.053103477	6.298886523	6.099115761	6.13947895
Zm00001d048765_T001	2.439307971	1.571416155	2.952132432	3.811598648	3.797043284	4.207880439
Zm00001d034013_T020	0.875803649	0.364106212	0.144953481	0.577246569	1.136051573	0.629189666
Zm00001d034013_T017	0	0.030748521	0	0.700587087	0.649860984	0.6448475
Zm00001d034013_T010	2.162216483	1.587620861	2.345581455	1.125991569	2.003763766	0
Zm00001d017106_T001	4.867330935	4.001893646	4.739735631	5.00609166	4.991886595	4.850269964
Zm00001d017106_T003	0.486495056	0.960271488	1.094706305	0.997294576	1.637105226	1.797935592
Zm00001d017106_T005	1.330854366	1.013078443	1.361791375	2.331306801	2.383774784	2.07150664
Zm00001d017106_T002	0.58727473	0.14230756	0.261959197	0.845557492	0.970094568	0.91468774
Zm00001d010290_T001	6.286757032	6.228726759	6.262260534	6.383261982	6.424008378	6.391637255
Zm00001d025235_T002	2.289138617	1.072853544	0	2.687333968	2.403578864	3.015787635
Zm00001d025235_T020	1.912545251	2.129089106	2.510819668	0	0	0
Zm00001d025235_T007	0	0	0	3.379503135	3.548258311	2.932377388
Zm00001d025235_T021	0.135253789	0.124959341	0.349678922	0.641344408	0.586020089	0.413180277
Zm00001d033600_T002	4.020271675	3.855504599	4.229237204	3.651526941	4.086546948	3.963785842
Zm00001d033600_T001	1.032692615	1.273222248	0.643414773	1.642393364	1.539000944	0.596228168
Zm00001d027514_T004	0.513463458	0.593419749	0.663307271	0.808182379	0.961086894	0.912508079
Zm00001d027514_T003	3.43242324	3.598827408	3.351702101	2.264479082	2.219550194	1.616163819
Zm00001d027514_T002	4.318361459	3.266331402	4.27087029	4.674598383	4.614045404	4.676520117
Zm00001d017165_T001	1.490936662	1.8323428	2.563781455	0	0	0
Zm00001d017165_T004	0.532543384	0.129246083	0	0.452425696	0.640157229	0.424163198
Zm00001d017165_T002	2.348130702	1.71525906	2.603254356	2.907668672	3.001774325	2.969401027
Zm00001d005470_T001	5.809556211	6.873548536	5.62795323	6.728369784	6.651590627	6.743359679

Zm00001d042433_T001	2.001209193	1.851901322	2.138962858	2.030610818	1.543920958	1.727967056
Zm00001d012451_T001	4.546458222	3.61987826	4.480068871	4.387967328	4.549388613	4.345222131
Zm00001d035467_T001	2.404575918	1.759478007	2.57803128	3.375214491	3.498330533	3.518721569
Zm00001d010908_T001	2.958334906	2.242075632	3.005486942	3.42927289	3.524388822	3.496999521
Zm00001d015181_T001	2.758025102	2.083136763	2.79741148	2.39603862	2.748318674	3.103646685
Zm00001d002534_T003	2.336788386	1.564464508	2.109972948	1.735374001	2.089223552	1.850402998
Zm00001d002534_T004	0.341875336	0.088018087	0.159078868	0.398483906	0	0.329210873
Zm00001d002258_T001	7.349729975	6.461177518	7.420485316	7.52875923	7.553447612	7.501290887
Zm00001d002258_T003	2.897916688	2.027877695	2.879258971	2.130891673	2.239549575	2.20734721
Zm00001d044910_T001	1.01881361	0.699857196	1.098674224	1.976924473	2.171816897	1.915458182
Zm00001d043370_T002	5.112545097	4.981564222	4.849654452	4.591388958	4.64625795	4.739057698
Zm00001d052028_T038	1.180927572	0	1.994964512	2.159267175	1.977215436	2.164089859
Zm00001d052028_T044	0.959833202	1.169000739	0.918404554	0.975140768	1.148231941	1.288723546
Zm00001d052028_T002	1.908941227	1.360354724	1.443299516	2.175621363	2.307462333	2.242579032
Zm00001d052028_T042	2.91632071	1.310718778	2.445486499	2.529784473	2.86321831	3.04054232
Zm00001d052028_T028	0.52766291	0.168938502	0.360895742	0.683241007	0.744653681	0.716073422
Zm00001d052028_T013	0.423963197	0	0.610831382	0.435415241	1.02628945	0.621813235
Zm00001d026286_T001	8.524178725	8.902619857	8.49214385	7.923092754	7.983821461	7.857659618
Zm00001d004466_T006	0.538134848	0.89284096	0	0.166421092	0.528304258	1.095497117
Zm00001d004466_T013	0.253888849	0.049772939	0.383352059	0.383517957	0.473048104	0.501697979
Zm00001d004466_T023	3.557252001	2.767538488	3.850412799	4.034995937	3.849502335	3.733716054
Zm00001d004466_T022	0.779226936	0.096195722	0.888572989	1.381190882	0.869604552	1.199612673
Zm00001d004466_T015	0.577722232	0	0.602788463	0.496360082	0.686010992	0
Zm00001d004466_T001	2.369357318	2.925896148	3.676466126	3.750548732	4.047174092	4.026445361
Zm00001d028773_T001	6.569239446	6.516614658	6.493614449	6.494991421	6.475262874	6.542202121
Zm00001d017130_T001	2.143592927	0.649835238	1.606234219	1.289120317	2.054203895	1.361497759
Zm00001d017130_T002	0.240957826	1.319183789	1.182245438	1.475582475	0	1.666225764
Zm00001d004198_T001	2.562446372	2.110717024	2.240494464	1.929394107	1.565743875	2.050995803
Zm00001d052247_T001	4.846743973	4.329472241	4.969206865	4.934106374	4.989125652	4.973034541
Zm00001d038034_T001	1.196738593	1.216868911	1.225756159	1.65933686	1.568525408	1.558943999
Zm00001d044510_T031	5.171296312	4.19911683	5.257667114	0	0	0
Zm00001d044510_T022	0.177032455	0.314438992	0	0.696172803	0.741234842	0

Zm00001d044510_T006	0.378943257	0.224064978	0.619900322	0.839174354	0.569920726	0.667959321
Zm00001d044510_T030	1.601865257	0.479986898	2.115868911	2.14026757	1.990714936	1.901032147
Zm00001d044510_T002	5.460154916	4.662081167	5.36941773	5.236475474	5.285750347	5.22731897
Zm00001d044510_T015	0.275033278	0.044252816	0	1.617016782	1.897951334	1.293305735
Zm00001d044510_T005	0.526266167	0.094341481	0	0.453997915	0.465208654	0.306733533
Zm00001d016305_T001	4.728315481	4.517747988	4.548648143	4.804517547	4.823637995	4.740548871
Zm00001d016180_T001	2.52091928	2.173752679	2.618096729	2.47187027	2.444523161	2.220610108
Zm00001d052157_T019	2.351730366	3.265663125	1.924367111	1.756343996	1.25477724	2.099506964
Zm00001d052157_T004	0	0	0.277868137	0	0.413279948	0.4127566
Zm00001d052157_T006	0.522740766	0	1.559189782	0.883652876	1.293510568	2.037672489
Zm00001d052157_T009	0.269574205	0.713109821	0	1.194830277	0	1.519385902
Zm00001d052157_T015	1.816832321	0	1.565362231	2.182614123	1.972681995	0
Zm00001d032766_T002	0	0	0	0.606311456	0.573300839	0.902808346
Zm00001d025081_T001	4.178830148	4.838309872	4.643336611	1.420690969	1.563283598	0.696367797
Zm00001d025081_T004	4.509088427	4.365952067	3.067214829	0	0	0
Zm00001d009316_T002	2.357200085	4.670947662	2.812208425	1.422869154	2.181088235	2.915048912
Zm00001d009316_T001	3.589780023	4.040223254	3.069243304	3.915050729	3.848962988	3.517932631
Zm00001d045082_T001	2.224492589	0	1.760829838	3.090453755	2.844523849	2.634858784
Zm00001d045082_T002	2.476448312	2.405114499	2.721744497	3.296196451	3.363289124	3.526836542
Zm00001d045374_T001	4.08766718	3.939430459	4.090477129	3.114979291	3.098087178	3.210639008
Zm00001d005802_T001	3.15981274	3.602624938	1.892183885	1.771621518	2.880319173	2.794529614
Zm00001d039518_T009	4.465246144	1.93560269	4.541676025	4.278177937	4.172453992	4.545979362
Zm00001d039518_T013	6.863935342	6.359129468	6.990008417	5.950742883	5.800798947	5.845614594
Zm00001d039518_T002	3.261064685	3.483383065	3.292045934	4.417402054	4.217091559	4.179633499
Zm00001d039518_T012	4.383545259	3.756550025	3.927783555	0	0	0
Zm00001d039518_T005	1.714489079	0.134081593	1.435415241	2.547398251	3.441093804	2.295379499
Zm00001d043736_T003	0.449821234	0.295735953	0.612678042	0.822559665	0.83467077	0.857814628
Zm00001d043736_T004	2.049897328	1.802644467	1.613042185	1.332552701	1.481926625	1.645920651
Zm00001d043736_T002	3.991409192	3.482297166	4.088944068	4.064716556	4.192862638	4.040882176
Zm00001d053838_T004	4.000332773	8.949629817	3.960385951	3.897170419	4.072741321	3.997300899
Zm00001d053838_T001	3.895088528	2.039742725	3.388168179	3.71652817	3.831512874	3.873734173
Zm00001d053838_T002	3.745112207	1.931874773	4.074336992	3.212408719	3.681315785	3.501679256

Zm00001d053838_T005	3.297157073	4.154866461	3.294758212	3.555996113	3.62363244	3.718460488
Zm00001d053838_T003	9.323372184	8.443423697	9.318864207	9.071174119	9.080412245	9.111678694
Zm00001d044101_T001	1.935658505	0.93823836	1.182938869	0	0	0
Zm00001d044101_T002	1.627904684	1.502605059	1.87008888	2.901265251	2.92275883	3.072971329
Zm00001d002576_T001	4.501470483	5.57164599	4.522647437	5.721148034	6.035222912	5.782635279
Zm00001d029139_T001	0.887196923	0.538784469	1.089544929	0.89734964	0.949732881	0.816503865
Zm00001d003957_T002	4.060260053	3.883465493	4.100783594	3.993193293	4.186216396	3.954991714
Zm00001d005347_T002	0.70487462	0.482811118	0.792229008	1.163903145	1.169129055	1.091938092
Zm00001d005347_T001	7.319085378	6.595303647	7.348697317	7.542593399	7.619640299	7.545424446
Zm00001d042916_T002	0	0	0.108785373	0.265343274	0.468405022	0.400953191
Zm00001d042916_T003	2.228216557	1.602081976	2.094863354	2.69100076	2.503879152	2.696899443
Zm00001d005026_T001	1.166013527	2.170371847	1.328278116	0.627284647	1.924917751	1.734064015
Zm00001d005026_T002	1.410447485	1.062168005	1.626360166	0.97027867	1.407758987	1.471252504
Zm00001d005026_T004	3.561851424	2.379535019	3.775492135	3.55051766	3.266839451	3.268079216
Zm00001d035658_T001	2.444792144	2.044699064	2.478223739	1.950142885	2.086603758	2.391143553
Zm00001d046169_T001	5.701597072	6.33466173	5.961320312	5.263379822	5.212541403	5.156651808
Zm00001d026509_T001	5.939482201	5.744770479	5.863843349	5.509582157	5.386011346	5.750132379
Zm00001d012472_T001	4.484068058	4.072746037	4.657311132	3.903655141	3.793930311	4.116138486
Zm00001d043202_T004	2.239181713	1.335115618	2.091358962	2.01598419	1.75316336	2.06657041
Zm00001d043202_T002	3.392103476	3.19095977	3.597387434	2.959916828	3.041021093	2.988107857
Zm00001d048968_T001	1.971453058	1.463851554	2.191849708	2.077864386	2.257964655	2.321165573
Zm00001d021356_T001	4.509676336	1.923817021	4.796592511	5.318316986	5.236811698	5.413331101
Zm00001d037734_T001	3.73401696	3.186680566	4.139946576	4.35422286	4.920719328	4.647585867
Zm00001d037734_T006	4.725050504	3.875960585	4.560965754	4.703477109	4.083035042	4.433319368
Zm00001d037734_T011	0	0	0.198932867	0.537835765	0.517192027	0.596013431
Zm00001d037734_T007	0.292463732	0.161748403	0.253316457	0.363184537	0.328464256	0.284145713
Zm00001d009383_T007	1.665845248	1.330660504	1.626106402	2.1120305	1.756139859	1.810305306
Zm00001d009383_T002	1.32664894	1.370913618	1.36169089	1.301344153	0.583232153	1.070000435
Zm00001d036570_T001	8.857906579	8.499556008	8.646311669	8.302321039	8.358595457	8.438203741
Zm00001d022466_T006	5.445492657	4.793236117	5.367952605	4.984750699	5.025266979	4.817633387
Zm00001d025571_T001	5.279286675	4.578468523	5.196591623	5.112689208	5.096275855	4.980809335
Zm00001d012177_T001	0.771509542	0.517904546	1.114710832	1.032794158	1.161861891	0.938109609

Zm00001d050033_T002	1.970893402	1.338021573	1.943955823	2.479633352	2.672419684	2.519228121
Zm00001d009693_T003	4.248229005	3.35816808	4.176243773	4.884630008	5.33382668	4.978896164
Zm00001d009693_T002	2.427230165	2.167315212	3.087345893	4.52368389	3.795946442	4.220592464
Zm00001d017193_T002	3.85613117	3.23335784	4.089154639	4.077424435	3.946205612	4.001343335
Zm00001d017193_T003	0.732957708	0.449976494	0.597565498	0.524149954	0.588029294	0.559050249
Zm00001d050063_T001	0.727142387	0.929993945	1.093602767	0.675769873	0.765117153	0.962095877
Zm00001d018944_T009	0	0.455404294	0	0.641578397	0.73502473	1.613277037
Zm00001d018944_T010	4.63793379	0	3.665591628	3.328411547	3.479381007	1.757133792
Zm00001d018944_T001	3.921192084	4.213167147	3.953638205	4.383322887	4.262313555	4.062298324
Zm00001d025474_T002	1.051157683	0.763978342	1.01746516	1.416189572	0.238490954	1.214754603
Zm00001d025474_T003	2.585526965	2.067486248	2.687812979	1.278744862	2.486467253	2.429981178
Zm00001d025474_T001	3.827693647	2.821186301	3.978468123	2.94576556	2.968929207	2.283002539
Zm00001d004772_T004	2.208842781	1.09714353	2.078267222	1.670354069	2.505841908	2.281281032
Zm00001d004772_T003	1.096724683	1.518477229	1.677509595	1.477858106	0.833753955	1.709869362
Zm00001d040201_T001	3.478329242	2.861347995	3.505723156	3.555441815	3.657781181	3.746600524
Zm00001d009562_T058	0.205726559	0.093351937	0.405004456	1.075338205	1.022913892	1.086469246
Zm00001d009562_T020	0.688166032	1.270274766	0.652727861	2.282788204	2.036449528	1.78136391
Zm00001d009562_T029	1.032774414	0	1.113696499	0.702398677	0	1.251794823
Zm00001d009562_T039	0.776579051	0.271435238	0.824965765	1.773436856	1.395371602	1.764995757
Zm00001d009562_T067	2.814690072	3.320108864	2.958617208	3.079988002	3.929773579	3.315088393
Zm00001d009562_T087	4.738138371	3.023766327	4.802873864	4.87697068	0	4.517320422
Zm00001d009562_T052	1.496472576	0	1.480439373	2.588325348	0	0
Zm00001d009562_T015	0	0	0	1.206096171	2.604245006	2.166032172
Zm00001d032267_T010	0.785932988	0.710294437	0.433098327	0.655721844	0.214285234	0.652018332
Zm00001d032267_T011	3.181410303	2.458719014	3.139070128	3.489897144	3.629907828	3.696678708
Zm00001d032267_T009	0.437450412	0.373045675	0.287668502	0.453044461	0.555842653	0.793290802
Zm00001d053977_T003	3.636515581	2.824002829	3.445147444	3.95032871	4.000078625	4.205918413
Zm00001d053977_T002	2.330823396	1.134382618	2.301834903	2.901167726	2.591750258	2.549030843
Zm00001d053977_T004	3.140214553	3.60004598	3.756780678	2.53267603	2.926776177	0
Zm00001d036360_T002	2.470025291	1.825384303	2.187697131	2.507274796	2.571116125	2.729532976
Zm00001d043439_T011	0	2.881151069	3.047210657	0	1.481605327	0
Zm00001d043439_T006	3.140682309	1.901938857	3.076052969	3.233039645	3.297938548	2.90935012

Zm00001d043439_T004	0.513636271	0.156337069	0.346072995	0.597664652	0.286372567	0.296218921
Zm00001d044075_T001	2.998173294	2.927623434	2.999606632	3.095579482	3.265239142	3.840450141
Zm00001d005694_T001	4.973542353	5.758964643	5.112417028	4.912761224	4.590872402	4.547473582
Zm00001d040684_T001	2.964135947	2.717285766	2.975438144	2.771799394	2.84725473	2.954368865
Zm00001d040684_T002	1.366578484	0	1.175122794	1.390000656	0.884708097	0.815051537
Zm00001d013035_T008	1.177644212	1.181551037	1.459616796	0.630268465	0	0.986649029
Zm00001d002186_T002	4.473504773	3.209987766	4.368090626	5.036935946	5.090273273	4.953577122
Zm00001d002186_T001	2.605129466	2.510827515	2.386423601	4.049425762	3.911830285	3.90824196
Zm00001d025690_T001	0.645954304	0.436157574	0.540596694	1.146388683	1.121888713	0.966592891
Zm00001d025690_T003	4.185784579	3.654395211	4.28381327	4.772237655	4.775108712	4.714847861
Zm00001d025690_T004	1.269022371	0	1.375567187	1.161859312	1.454598636	0.876222631
Zm00001d022427_T001	0.141126693	0.47713641	0.356824421	0.541224388	0.803004621	0.427942941
Zm00001d022427_T005	3.317769453	2.510618416	3.303254562	3.875719327	3.893138216	3.867587141
Zm00001d022427_T008	1.135768624	0.533235403	0	1.458834482	0.896550091	0
Zm00001d022427_T007	0.368038577	0	1.044014947	0	0.840174767	0.835681584
Zm00001d049189_T001	6.892930515	7.032251774	6.886953648	6.462667035	6.430478411	6.580666015
Zm00001d041758_T001	4.495205292	4.012544628	4.638023603	4.414767217	4.37483139	4.492279173
Zm00001d000023_T006	0	0	1.289048882	1.240291735	1.016601149	1.52965256
Zm00001d000023_T001	1.347692299	1.06360303	1.127470183	1.899036382	1.814873994	1.469702112
Zm00001d000023_T004	0.573855345	0.136293776	0	0.803328724	0.698903873	0.338436966
Zm00001d048226_T001	2.563435902	2.227099667	2.64702502	4.614162989	4.950834407	4.831754597
Zm00001d008412_T001	3.031646623	2.274600114	2.991672581	3.539799536	3.557874452	3.454091251
Zm00001d006148_T001	2.156336422	1.482726462	2.195896222	2.278745159	2.331325721	2.063765572
Zm00001d003071_T001	4.133059168	3.631249825	4.338242194	4.135254857	4.249779656	4.040021029
Zm00001d046500_T001	2.736458109	2.029204435	2.855853791	2.389238038	2.657033763	2.463908561
Zm00001d003511_T001	2.589575349	2.41756685	3.014177623	2.396855454	2.733677772	2.886250262
Zm00001d042030_T001	6.867261538	3.124524011	7.246097139	3.60401212	2.982769478	3.435933368
Zm00001d010152_T006	6.443232951	5.952817762	6.60200821	6.220805235	6.289115686	6.200361769
Zm00001d010152_T003	2.711431691	1.577895732	2.841917382	3.072163439	3.551393583	3.315023451
Zm00001d010152_T007	2.34460526	1.669502141	2.562654686	5.043916683	4.815000183	4.821089794
Zm00001d010152_T005	2.974194847	0	0	2.938927474	1.929461147	3.455446774
Zm00001d010152_T001	2.88549764	2.756366842	2.843293476	3.208301089	2.796470464	2.950774651

Zm00001d034918_T005	1.625667936	0.88004915	1.626318576	0.857668145	0.977208108	0.99252627
Zm00001d034918_T003	1.295921051	0.957763823	1.074938317	1.213720545	1.144601798	1.3924125
Zm00001d034918_T002	2.674653405	1.851474816	2.877209078	2.395151388	2.64772711	2.209794581
Zm00001d026064_T004	0.460956397	0.386676325	0.735525643	1.170286592	1.306072046	1.282697184
Zm00001d026064_T002	2.659124691	2.170475685	2.615936497	2.837939408	2.88926053	2.917877767
Zm00001d028705_T001	3.552015737	3.174968014	3.561458484	3.502894227	3.48428523	3.33810489
Zm00001d053969_T002	1.700605286	0.708483913	1.459510834	1.93447272	2.077888648	2.027028748
Zm00001d053969_T003	0.268191229	0.131208842	0.258704639	0.350749555	0.423154309	0.245788918
Zm00001d053969_T001	1.536430439	0.774218196	0.708801713	1.824722647	1.793274152	1.47622354
Zm00001d053969_T004	1.449195802	2.205965786	2.359907093	0	0	0
Zm00001d007153_T001	6.677396597	6.923212969	6.737490513	6.161786348	6.139764017	6.150645456
Zm00001d013164_T002	2.96083287	2.211757964	3.029473029	2.855936093	2.977675397	3.045385129
Zm00001d014614_T001	4.057330346	1.168754982	4.367458785	4.282255045	4.110139371	3.910575434
Zm00001d007972_T001	4.90870816	4.216090537	4.886319922	4.96622169	4.899455343	5.035261255
Zm00001d007972_T005	5.132708851	5.275721064	5.190250862	4.400015609	4.488114112	4.252039025
Zm00001d043011_T001	3.4527081	4.34069343	3.533884368	2.698717667	3.268840076	2.85083862
Zm00001d018659_T003	3.09040599	0	3.318662468	4.025536041	0	0
Zm00001d018659_T004	2.047099651	1.592661302	1.711783417	2.204537967	2.368924202	2.482147412
Zm00001d018659_T001	3.445893393	3.763154459	3.532905638	3.311946261	4.062843742	4.210335791
Zm00001d018659_T002	1.540607108	1.118157695	1.810156387	2.040965352	2.005512452	1.834380737
Zm00001d027685_T001	4.873936009	5.225981025	4.959335009	5.214258769	5.136072661	5.176126743
Zm00001d013240_T002	2.513962892	1.449496402	2.331945932	2.184567218	2.126414563	2.203635309
Zm00001d034498_T001	0.190001624	0.098600119	0.211037121	0.373985566	0.778526523	0.473293376
Zm00001d034498_T007	3.397347633	2.217325652	3.193772216	3.589903077	3.462563848	3.577444781
Zm00001d034498_T004	0.528669329	0	0.449377543	0.534019756	0.917498208	0.942193933
Zm00001d023810_T001	2.753164858	2.040954484	2.829234	2.61983132	2.520917018	2.549611018
Zm00001d001817_T001	3.005557544	2.42062657	3.139275802	2.660730143	2.938718818	2.803550056
Zm00001d039040_T001	1.193946433	1.311069428	1.301649684	1.236586295	1.414826129	1.020707715
Zm00001d018031_T001	2.703449811	2.268118152	2.648417813	1.997331794	2.170268001	2.284705118
Zm00001d042099_T001	3.190725942	3.152106819	3.108171476	2.592964298	2.294964749	2.549779555
Zm00001d017852_T001	8.819226741	4.856624473	9.123412601	9.816170615	10.04439529	9.952367775
Zm00001d038645_T001	4.551897036	3.915528549	4.423254503	4.812605166	4.883967764	5.029983175

Zm00001d030790_T002	3.569899101	2.796325095	3.701390585	4.990461628	5.159973472	5.039000305
Zm00001d044972_T001	1.634672254	1.287665548	1.55235346	1.709608699	1.06520487	2.011591171
Zm00001d007354_T001	3.558191823	2.671135958	3.60423172	3.921688628	3.963725834	3.832873005
Zm00001d018524_T003	2.621606024	1.743509367	2.64232566	3.454368327	3.618470901	3.494346866
Zm00001d018524_T009	4.100043545	3.339363038	3.978730774	3.904508375	3.529234631	3.843519515
Zm00001d018524_T005	0.417137976	0	0.441182798	0.629386465	0	1.280689717
Zm00001d018524_T002	0.829929099	0.441804279	0.871376076	1.049752033	1.13076551	0.831157335
Zm00001d040468_T001	2.103028918	1.733884461	2.155521284	2.298510226	2.696508461	2.506183249
Zm00001d012499_T001	2.696796645	2.068255654	2.879365438	2.655010121	2.562683012	2.837523488
Zm00001d036880_T001	4.68353483	3.860941847	4.634187875	2.196866983	1.912536054	2.382767947
Zm00001d036880_T002	5.420312481	4.444278846	5.332507446	2.957073989	3.227463075	2.942393642
Zm00001d048680_T005	6.189186509	4.98757227	6.269295883	6.624222708	6.648329192	6.587112977
Zm00001d048680_T004	1.166521359	0.362620252	1.009274068	1.1154821	0.818614182	0.901034851
Zm00001d048680_T002	4.141135114	4.232023067	4.367256708	4.359893252	4.484765677	4.38737557
Zm00001d048680_T001	2.434493725	1.79852131	2.52743722	2.729885536	2.505525892	2.390029006
Zm00001d048680_T009	2.30548497	2.28011806	2.611218895	0	2.598509325	0
Zm00001d048680_T007	1.031684553	1.038044199	1.131540099	1.531995715	1.534945082	1.647728951
Zm00001d026194_T001	4.045988684	3.607660897	4.258938331	4.800055411	4.607834917	4.728728083
Zm00001d044695_T001	2.560938677	2.001314778	2.561880377	2.551224865	2.56786374	2.195553908
Zm00001d040060_T001	2.70464293	2.053781426	2.500962882	2.497092672	2.724311712	2.587350828
Zm00001d026379_T001	2.476227947	1.731007239	2.22507869	2.141436061	2.111298787	2.184791892
Zm00001d019114_T001	0	0.549658329	0	0.771992034	1.096026333	0.634264269
Zm00001d019114_T002	2.181643897	1.258011103	2.478996389	1.578904909	1.836754294	2.304704362
Zm00001d019114_T008	1.664484661	1.536570685	1.876607312	2.232932081	1.834921922	1.670503638
Zm00001d014623_T001	4.661502328	4.03032822	4.86912564	3.324747394	3.536234345	3.545423674
Zm00001d052932_T001	2.598428572	1.737556545	2.394767373	3.756808854	3.77832466	3.756776089
Zm00001d010241_T002	4.806846231	4.921979477	4.910721295	4.635115599	4.765661825	5.225548263
Zm00001d010241_T001	2.485777926	1.688325404	2.515382442	2.05729187	2.146552262	2.134383603
Zm00001d010043_T001	4.259979724	4.502353772	4.293004687	4.118517959	3.681651768	4.138886454
Zm00001d026176_T002	4.469173372	3.665051046	4.384817313	4.276113906	4.275013903	4.302020683
Zm00001d026176_T001	0.790090558	0.198660101	0.544622873	0.853942169	0.760836649	0.736059297
Zm00001d049459_T002	3.502751051	5.0163971	2.059646349	0	2.467548428	0.632136971

Zm00001d049459_T001	5.658941016	5.03684111	5.843414641	5.546111026	5.527735307	5.542515576
Zm00001d052777_T001	1.884089909	2.017513265	1.800526795	2.293655934	2.307017517	1.777923795
Zm00001d052777_T003	3.775472434	3.371375413	3.923912361	4.30442328	4.425932158	4.609004585
Zm00001d052777_T002	0.318960017	0.097786067	0.317166276	0.81326691	0.921501837	0.878146971
Zm00001d008585_T001	1.877382255	1.183677061	1.425454471	2.083778079	1.865726442	2.257293093
Zm00001d008585_T005	0.557722785	0.111124817	0	1.103801086	0.813481182	0.310750765
Zm00001d008585_T002	0	0.112126276	0.195701614	1.156329949	1.618292227	0.965121555
Zm00001d042290_T001	2.214016599	1.431891952	2.039268968	1.875021958	1.817960052	1.723245743
Zm00001d030267_T001	4.308168554	4.426630012	4.31223641	2.857041644	2.520970054	3.013853834
Zm00001d048428_T003	1.210413803	0.71192743	1.187550503	1.645392707	1.717262951	1.43699917
Zm00001d048428_T001	4.169902159	3.357729199	4.120232817	3.964228567	4.109158434	4.06032102
Zm00001d040582_T002	2.254514227	2.878951879	1.809883193	2.834545379	3.138341847	1.983170565
Zm00001d008215_T001	4.715948541	4.343593158	4.775067034	4.985101448	4.917076862	4.76740739
Zm00001d008215_T002	1.140079057	1.175360442	1.269194169	1.286502686	1.427609927	1.639544289
Zm00001d034351_T001	5.2910728	4.503563215	5.219090129	5.234632218	5.182884022	5.159297494
Zm00001d049171_T002	3.43670941	2.782827263	3.392602361	3.365044382	3.547266581	3.380677923
Zm00001d049171_T001	0.635151653	0	0.837597761	1.721210771	0.856188951	1.930704037
Zm00001d020580_T001	8.590012327	8.585630273	8.650040065	8.071067654	8.282465326	8.16916617
Zm00001d018440_T003	0.898962885	0.527222506	1.431335729	2.240927001	2.723317619	2.287544694
Zm00001d018440_T002	4.385605738	3.432973433	4.39361046	5.146292426	5.192403254	5.189824677
Zm00001d018440_T001	3.674829753	3.208889747	3.937646272	4.056745302	3.830328958	3.999937422
Zm00001d039976_T002	3.555537403	3.371535027	3.381602751	2.955701326	2.980901622	2.541269744
Zm00001d014580_T003	5.1212264	5.724516773	4.962040798	4.343614262	4.664612256	4.667059072
Zm00001d014580_T001	1.879058368	1.640634809	1.974565655	2.207091652	1.777117099	1.580668427
Zm00001d035436_T002	0.70869667	0.312933476	0.651087981	0.679116579	1.171338791	1.044254914
Zm00001d035436_T003	1.548944561	1.300512101	1.551322087	1.594729114	1.180782482	1.232351312
Zm00001d015570_T002	3.281213067	2.868771894	3.25909875	4.357125326	4.392187987	4.40070077
Zm00001d015570_T007	4.852739926	3.724788423	4.810149188	3.560103024	3.713967064	3.856198315
Zm00001d015570_T004	1.014061725	0	1.681478501	2.784984452	2.454077692	1.876015514
Zm00001d015570_T003	0.737537942	0.678409997	1.335727343	1.075055419	0.88985276	0.55264168
Zm00001d016149_T001	4.24510999	3.578872432	4.423190622	3.811995197	3.76876097	3.881699561
Zm00001d018744_T001	11.51687921	7.560992532	11.52200344	11.95522682	11.89921486	11.97166861

Zm00001d018580_T001	0.657967375	0.324855525	0.833979774	2.422099491	2.497003994	2.486879611
Zm00001d007479_T001	3.681978007	3.09669888	3.805346014	3.648057427	3.348276617	3.397956531
Zm00001d038194_T003	0.231238234	0.091197469	0.208322473	0.331081461	0.466579095	0.402286683
Zm00001d038194_T001	4.598619489	3.741517682	4.510732778	3.76671547	3.84330604	3.85386524
Zm00001d017703_T004	0.603060134	0.204297251	0.819164583	0.627359364	0.927414843	0.537001782
Zm00001d017703_T003	2.363228279	2.365795543	1.984052273	1.809482358	1.61529905	1.606245591
Zm00001d017703_T001	3.138845098	2.446104011	3.576384634	2.972580175	3.048925278	2.723249239
Zm00001d052325_T001	4.585315136	4.332898586	4.326685967	4.402587122	3.998941575	4.180088331
Zm00001d039643_T001	1.568439797	1.087234708	1.691806152	1.173529797	1.266248634	1.5978739
Zm00001d023213_T001	2.522191121	2.137800557	2.315522758	1.049281551	1.452256992	1.374436363
Zm00001d042596_T005	2.321262282	1.592971711	2.321650782	2.39477286	1.900027845	2.058626477
Zm00001d037174_T003	4.783555083	3.984793027	4.75949308	4.44940475	4.211241589	4.290307246
Zm00001d027366_T001	1.365455708	1.417161746	1.010152506	1.217990025	0.72165089	1.432981315
Zm00001d027366_T002	3.438514829	2.950097521	3.569592923	2.99228987	3.109396835	2.93859933
Zm00001d028936_T004	3.090459684	2.139570343	3.048049035	3.557411561	3.829251661	3.644701015
Zm00001d028936_T013	1.201501504	0.559486919	1.712885831	1.924197583	1.944748272	2.223888956
Zm00001d028936_T010	0.528630326	0.365708212	0.402105462	1.132097729	0.988470198	0.975702814
Zm00001d028936_T011	1.206697605	0.528058158	1.166666605	1.72548291	1.6810381	1.927340122
Zm00001d028936_T009	5.287987578	0	5.355251571	4.756946575	5.052094487	4.965940641
Zm00001d028936_T003	4.702381222	4.241706278	4.677821271	5.402055377	5.189627272	5.013849547
Zm00001d041305_T001	4.641746582	4.350971137	4.586884333	3.61146691	3.938689464	3.81310752
Zm00001d018233_T005	1.238342979	0.788288132	0.879317598	2.06729521	2.005305841	2.228325859
Zm00001d018233_T014	2.504816382	2.173844125	2.469082896	1.448878756	2.171994265	1.943323501
Zm00001d053873_T001	3.472713516	2.798411195	3.322263194	3.615638052	3.577060176	3.525195291
Zm00001d051617_T001	4.611464549	4.491533616	4.457518124	4.312681871	4.148547681	4.18546071
Zm00001d036060_T010	2.994230503	2.150609065	2.501621557	2.386196498	1.731599477	2.293728906
Zm00001d041962_T002	2.415120452	1.898008238	2.608546696	2.767105156	3.055198605	2.776960084
Zm00001d010823_T003	1.957915713	1.016560502	1.540443944	2.648156392	2.627330412	2.169919872
Zm00001d010823_T001	2.175531946	1.894773148	2.300565693	2.52239177	1.904972653	2.538407545
Zm00001d044784_T001	3.797757071	3.201283181	4.004931889	2.890012554	3.112546015	3.129873824
Zm00001d035854_T001	5.20288188	4.304522285	5.234054893	5.080109386	4.929415176	4.821068614
Zm00001d046149_T001	5.586260162	5.8445239	5.304189466	5.268926998	5.417300135	5.405300823

Zm00001d011263_T001	4.678701256	4.047853137	4.65010187	4.157338203	4.059906134	4.329648972
Zm00001d043076_T002	1.857158126	1.640491829	1.7504633	1.788674854	1.881618813	2.063885654
Zm00001d043076_T001	4.006921202	3.417833076	4.233888904	3.832227539	4.06532423	4.020267674
Zm00001d016694_T003	1.205156632	0.759352726	0.513145064	1.627454623	1.42557854	1.473793145
Zm00001d016694_T007	1.181930709	0.952266463	1.489193523	0	1.026204449	0.968272898
Zm00001d032971_T001	2.604037866	2.00001623	2.570136709	3.173464236	3.491334787	3.342321341
Zm00001d028438_T019	1.096372514	0	2.186853265	2.211154586	2.182265464	3.067767769
Zm00001d028438_T021	4.961803839	4.003739214	4.718011939	4.813647098	4.878298354	4.647795884
Zm00001d028438_T001	0	0	0.047772886	0.267798246	0.267822211	0.347599897
Zm00001d028438_T002	0	1.411908871	1.774485999	3.148345033	2.392883672	3.407563089
Zm00001d002480_T002	2.224701564	1.467503303	1.646117947	2.203338057	2.288601009	1.859604655
Zm00001d003983_T002	2.188198655	2.305606665	2.776710214	1.242662865	2.391704216	1.719686379
Zm00001d003983_T001	3.153349437	2.395933639	2.771116077	3.013693251	2.836724003	3.154931657
Zm00001d003983_T003	1.250435318	0.887590775	1.288673942	1.222556673	1.219739753	1.280687342
Zm00001d002731_T001	1.220245124	1.617914365	1.714491277	1.806614836	1.138214859	0.734975323
Zm00001d045575_T001	2.5631884	2.031443544	2.100222137	1.534602014	1.820076004	1.705341428
Zm00001d004744_T001	2.70701207	2.619336237	3.147365317	3.517289931	3.636820988	3.483890957
Zm00001d017480_T001	3.784699854	4.050016393	3.528683705	2.296551381	2.290031827	2.092720613
Zm00001d040186_T001	0.882015365	0.862496621	0.878797523	0.547972915	0.662139859	0.588207797
Zm00001d013703_T001	0.902238008	0.22480712	0.943493048	0.68326257	0.286120576	0.618891715
Zm00001d013703_T002	0.206945733	0.537511772	0.503610279	0.280332792	0.82253764	0.824956806
Zm00001d039313_T001	1.09691355	0.988616345	0.775466956	1.585146192	1.480227374	1.105818184
Zm00001d014150_T002	1.084008461	1.3380016	1.490845294	0.941179195	0.56271378	0
Zm00001d014150_T007	0.077152742	0.058111421	0.179842227	0.430322745	0.405788735	0.310177226
Zm00001d014150_T010	1.24991615	0	1.387770034	2.337707953	2.232253061	2.385353999
Zm00001d014150_T005	2.474221343	1.636878397	2.059693759	2.971286582	2.779394212	2.659431173
Zm00001d048453_T001	1.472259002	1.149631374	1.527753977	3.771908814	3.895108795	3.863026668
Zm00001d019374_T001	2.900852321	2.305174704	3.143411705	2.151734799	1.961159888	2.263539261
Zm00001d006160_T001	5.985512225	5.000664524	5.981342644	5.432369351	5.423080066	5.35460516
Zm00001d003091_T001	2.701033099	2.239185991	2.454078482	1.907372568	1.525508156	1.773918282
Zm00001d016475_T001	1.946947155	1.292812369	1.984022733	2.595363538	2.078460571	2.186549365
Zm00001d041766_T001	1.521334724	1.885481045	1.738258103	1.707071503	1.652085812	2.385012664

Zm00001d048318_T002	0.31954048	0.161235018	0.129045571	0.950351202	0.931373686	1.201956236
Zm00001d048318_T001	3.656117742	3.049516811	3.698359629	4.034897893	3.888410405	3.796917176
Zm00001d040462_T003	4.472170409	3.412928768	4.495135754	4.323905468	4.40374511	4.188513285
Zm00001d040462_T001	1.728540081	0	2.024991929	1.906615111	1.745851288	1.690194025
Zm00001d027429_T002	4.177092111	3.407327052	4.122630398	3.410524958	3.497452951	3.4082336
Zm00001d027429_T001	2.016329434	2.248011339	2.257514266	1.715573615	2.03944761	2.03659617
Zm00001d047402_T001	2.528303508	2.002405131	2.653318486	3.803024983	3.91019961	3.879094942
Zm00001d006790_T009	4.084897062	0	2.980110141	2.317476001	0	2.282838307
Zm00001d006790_T011	0.533722808	1.387211425	0	1.603059659	0	0.728779936
Zm00001d006790_T005	2.018999786	0.992091148	1.573845652	2.278033233	2.159229386	2.40958455
Zm00001d006790_T002	1.876005295	1.490825788	1.385263702	0	1.554629615	0.990422689
Zm00001d006790_T012	0.778998267	0.350447459	0.560121127	0.881855661	1.104786174	0.667583349
Zm00001d006790_T006	2.532097483	3.491992486	4.05869946	4.533299702	4.649119915	4.344678824
Zm00001d005657_T002	1.862749719	1.798162939	2.350470373	2.485456454	2.375664487	2.321198484
Zm00001d053725_T001	5.528895326	4.542946973	5.39456768	5.565328047	5.711335293	5.542058659
Zm00001d053725_T003	4.081220563	4.591153209	4.624912168	3.369963071	3.27697153	4.153098404
Zm00001d053834_T005	4.383383443	7.123115781	3.080065286	3.842180554	3.363367209	3.732208082
Zm00001d053834_T004	1.913331775	0.468360184	1.782669401	1.491916189	2.06618152	1.669629577
Zm00001d053834_T001	9.501441482	8.129964056	9.528821585	9.40303875	9.448084799	9.419915892
Zm00001d053834_T002	3.586204727	7.613453645	3.562668117	3.081479049	3.476507934	3.967190008
Zm00001d029861_T003	0.81396051	0	0	0.650756289	0.882554627	0.962492017
Zm00001d029861_T004	0	0.399207195	0.426737076	0.660312573	0.934571604	0.811826112
Zm00001d034112_T001	4.414317821	3.68268026	4.351698639	5.090220716	5.248606256	5.258954758
Zm00001d010887_T006	0	0	1.333910275	2.111847607	2.612280775	0
Zm00001d010887_T009	2.462522754	1.213098386	1.711222201	2.444631821	2.207599286	3.037626809
Zm00001d010887_T005	1.273317748	0.725572778	1.506858329	2.485875024	2.173906151	2.524249017
Zm00001d010887_T007	4.395001566	3.546478163	4.153640551	4.278420836	4.27242428	4.194161613
Zm00001d011308_T001	4.140397158	3.606924467	4.239604333	4.330086292	4.428779896	4.490079287
Zm00001d015948_T005	1.527434718	0.546195625	1.668559402	2.222482484	2.450131737	2.037021943
Zm00001d015948_T013	0	0.257458448	0.280823319	0.725810945	0.827871042	0.535924544
Zm00001d015948_T010	2.879592071	1.777466849	2.705690661	3.492662702	3.456921245	3.587827371
Zm00001d015948_T007	4.173867145	5.048668781	4.628115777	4.995088198	4.68067711	4.742836815

Zm00001d031814_T001	2.025280801	1.902954946	1.719992525	2.601363256	2.786332053	2.545940454
Zm00001d028169_T001	0.748085053	0.218257922	0.626364372	0.606691421	0.432493395	0.405878028
Zm00001d037916_T001	1.99533581	1.846442768	1.585527926	2.131668169	1.868747601	2.116333813
Zm00001d026035_T001	5.379245442	5.334478855	5.447282164	5.729841362	5.806343367	5.69922579
Zm00001d035211_T001	2.781846056	2.016057668	2.507575461	2.198990682	2.556975979	2.438495666
Zm00001d006871_T001	7.329856448	6.885401179	7.325744211	6.64267333	6.725360218	6.789111879
Zm00001d002853_T001	4.867783168	3.886720354	4.849890853	5.073369835	5.079684667	5.00264744
Zm00001d021745_T001	2.929002601	2.339655636	2.947597901	2.95862927	3.552739298	3.063356422
Zm00001d020549_T008	0.406963266	0.221409405	0	0.303184555	0.413325448	0
Zm00001d020549_T001	2.169165303	1.57733891	1.82583487	1.953989292	2.757899908	1.495099544
Zm00001d020549_T010	0	0	0.331809532	0.524563215	0.730023405	0.644428538
Zm00001d020549_T016	2.580365249	0	2.388683579	1.651312587	2.829921591	2.185448262
Zm00001d020549_T015	0	1.729516227	1.638146144	2.681026853	0	2.600279984
Zm00001d020178_T002	1.701903865	1.646960298	0	2.554439786	2.72342903	3.446392552
Zm00001d020178_T005	3.656915046	0	3.056018304	3.497230406	3.349410715	3.214260829
Zm00001d020178_T001	2.223803408	2.850795223	3.219409556	0	0	0
Zm00001d020178_T004	0	0	1.986725836	2.253396944	2.42776116	0
Zm00001d013325_T001	5.639285279	6.255989562	5.780439689	5.636298665	5.504348702	5.66420881
Zm00001d003400_T001	2.740431542	2.755840648	2.65071218	4.192659494	4.208187216	4.058010605
Zm00001d036667_T001	1.307086318	0.781448686	0.92592956	1.65440346	1.358046799	1.26587072
Zm00001d041984_T001	8.463010731	7.977571147	8.516370311	8.287499173	8.350442407	8.365965295
Zm00001d011839_T001	1.585028863	1.369402269	1.556740852	2.229852751	2.303576492	1.838414513
Zm00001d053010_T001	8.19675623	7.89948747	8.129111888	8.041630089	8.041007547	8.097695738
Zm00001d032408_T002	1.456345149	1.001223608	1.360298532	1.892245292	2.175198181	1.6335177
Zm00001d001939_T002	0	0	1.442622419	0	1.633867789	2.358553802
Zm00001d001939_T003	3.855447888	3.199929691	3.888538019	3.337105775	3.688361551	3.281109629
Zm00001d001939_T005	4.066694048	4.14102211	3.89191238	2.722374869	2.731276883	3.381473181
Zm00001d001939_T001	1.344335965	1.235403566	1.079954903	1.468657861	1.374660596	0.218391851
Zm00001d014198_T001	4.640007722	3.861903668	4.561550009	1.567854007	1.904854768	1.916959628
Zm00001d050234_T001	3.88427809	3.539068958	3.809115481	3.602119518	3.407408908	3.555228407
Zm00001d025988_T001	3.293315153	2.696767941	3.316252782	3.781483518	3.887967857	3.797804064
Zm00001d034674_T001	3.045431612	2.425327437	3.175546477	3.372252862	3.306177939	3.417946464

Zm00001d053624_T001	4.939896044	4.316248509	4.854099655	5.13730063	5.144949281	5.193558753
Zm00001d040357_T001	1.248842044	2.211978133	0.994765072	0.657641834	1.056187426	0.905207624
Zm00001d040357_T004	0.477368553	0.41773642	0.826176262	0.73303062	0.627589096	0.848417631
Zm00001d040357_T003	4.599369275	4.061092181	4.722841787	4.452495281	4.576376168	4.651074717
Zm00001d040357_T002	0.732491503	0.437060445	1.308979972	1.43020979	1.24124102	1.275819378
Zm00001d025418_T001	2.474635657	2.518556792	2.565603756	2.457028712	2.77443434	2.374818317
Zm00001d002817_T022	0.713562975	0.222138072	0	0.791842404	0.269389885	1.50663596
Zm00001d002817_T039	0.748486136	0.100556548	0.71890298	0.698339402	1.106563401	0.660759801
Zm00001d002817_T004	0	0	0	1.210970439	0.969980412	0.923199393
Zm00001d002817_T001	0.521530215	0.567386762	0.689961977	0.714317623	0.49768287	0.280377932
Zm00001d002817_T005	0.33917616	0	0.193752823	0.611833365	0	0.345194236
Zm00001d052125_T001	2.071824782	1.698588774	1.958621476	0.934329288	0.988999455	0.591402722
Zm00001d022367_T001	4.128886592	3.178550526	3.988446111	5.107080286	5.037302573	5.093141682
Zm00001d022367_T002	1.886655102	1.76784545	1.988641064	1.92160428	2.552299351	2.140170044
Zm00001d023939_T001	6.415765358	5.662119175	6.475285364	5.861561943	5.947075564	5.943017742
Zm00001d052852_T001	2.393601262	1.786877765	2.984364225	2.411766541	1.848968795	2.427523041
Zm00001d052852_T003	1.546755214	0.701863954	0	0	1.045208793	0.995980106
Zm00001d052852_T002	1.257681708	0.488901595	0.534902265	0.37281952	0.711442925	0.712476927
Zm00001d014286_T050	4.232570816	0	4.399396301	3.699237341	3.916443873	2.838507901
Zm00001d014286_T012	4.06046612	0	4.223243824	5.056834591	5.098485165	5.177213051
Zm00001d014286_T030	1.138193229	0.647686592	1.518328665	1.15719056	1.034871401	1.653814425
Zm00001d014286_T008	0.032148815	0.016502025	0.327102121	0.254721014	0.60266686	0.407639902
Zm00001d014286_T004	0.808488023	0	0.90260231	1.149646981	0.35344751	1.047109774
Zm00001d014286_T005	4.090113775	3.228613236	3.782611639	3.938172292	3.991519216	3.62616716
Zm00001d014286_T042	2.780726258	2.484032923	2.574336972	1.789233872	1.777244635	1.199402231
Zm00001d014286_T053	0	0	0	0.751724214	0.447543228	0.781702982
Zm00001d051325_T001	2.250389535	3.134170494	1.534002817	2.026669768	2.616015569	1.724594396
Zm00001d034417_T001	3.242537114	2.605924992	3.411788366	4.687785718	4.811666305	4.969510716
Zm00001d043291_T001	3.069722443	2.600120813	3.035878998	3.655453959	3.71399312	3.749815164
Zm00001d053634_T002	3.548972788	3.186206569	3.594729592	3.956884663	3.935493693	4.014285746
Zm00001d053634_T001	2.530151167	2.381360905	2.261387291	2.52064577	2.426275491	2.388062762
Zm00001d027880_T002	1.021050408	0.637547186	1.349907036	1.552340179	1.421146802	1.202082281

Zm00001d027880_T004	2.228583839	1.431082149	2.145723099	1.995616933	2.278350283	2.178968584
Zm00001d017800_T001	0.880164376	0.957745253	1.066935782	1.305287669	1.2634359	1.461066446
Zm00001d048361_T001	3.383388282	2.677106103	3.414253272	3.839773295	3.836592631	3.867146661
Zm00001d013840_T003	0.502471147	0	0.588805526	0.683565315	1.309426512	1.281479864
Zm00001d013840_T002	4.064751558	3.189675143	4.048997882	4.434392774	4.437381497	4.352727315
Zm00001d018432_T001	3.584519044	2.928277836	3.699644685	2.943753518	2.788062566	3.02766064
Zm00001d020883_T001	3.127023389	3.460916437	3.140413847	3.653410327	3.264613593	3.566443901
Zm00001d045617_T002	1.113082355	0.451804556	1.218564825	1.78342187	1.418508412	1.295392724
Zm00001d045617_T001	3.496755052	2.863285126	3.562049075	3.126236127	3.26254997	3.333614077
Zm00001d025107_T001	5.134079457	4.822155096	5.146368357	5.135583544	5.192164332	5.08458821
Zm00001d031275_T001	7.437798995	6.854507259	7.606756436	7.764471461	7.803909939	7.798119517
Zm00001d050824_T001	2.00271188	1.995509121	1.848568662	1.187025309	1.053351881	2.142999873
Zm00001d048819_T001	4.008595797	3.228088618	3.949936404	4.249907809	4.333397971	4.305224182
Zm00001d013059_T022	0.677147382	0.302726136	0.484118534	0.994225614	0.893006445	1.044803262
Zm00001d013059_T012	1.062370431	0.744138701	1.561189254	2.338500577	2.251942333	2.368381716
Zm00001d013059_T002	3.472825401	3.320458972	3.432372993	4.215882408	4.558126358	4.361095553
Zm00001d013059_T017	0.474987316	0	0.496965403	0.431076263	1.137149364	0.373574718
Zm00001d013059_T018	3.269204344	0	3.12666929	1.96229322	0	1.575985593
Zm00001d013059_T003	1.318648876	0	1.792583442	2.409273886	2.255612519	2.303743055
Zm00001d019303_T001	4.075375603	5.23264092	4.001680212	2.703793745	2.594967682	3.051626857
Zm00001d012656_T003	3.047870757	2.269561639	3.350117146	3.7437727	3.767716551	3.726249336
Zm00001d012656_T001	3.762182742	4.447118876	3.93347548	4.170884235	4.110967859	3.813640736
Zm00001d042017_T002	4.432969159	4.440352212	4.384416929	4.678730717	4.795174446	4.89378093
Zm00001d028354_T001	3.266796733	2.61554201	3.327021042	3.695891841	3.679460169	3.782613212
Zm00001d052872_T001	8.520834968	7.625217169	7.99883748	8.261663429	8.268230494	8.330585643
Zm00001d032991_T001	4.454784172	3.782848541	4.506645925	4.666458082	4.673204639	4.577722051
Zm00001d045086_T001	1.695309261	1.191635921	1.875542608	1.890517901	1.851589949	1.762301186
Zm00001d037897_T001	3.642785313	3.728920304	3.841071908	3.785875148	3.307979122	3.5519348
Zm00001d027447_T002	4.832303705	4.760887094	5.120750178	4.527350513	4.43401793	4.376253079
Zm00001d027447_T010	0.051596233	0.255057131	0.600098447	0.217529773	0.330900247	0.520145627
Zm00001d027447_T005	0.308605517	0.194132451	0.700840954	0.658126461	0.616160995	0.493489531
Zm00001d027447_T011	0.738001654	0.27524549	0.271926409	0.607247449	0.569720505	0.724371746

Zm00001d027447_T007	2.248077242	3.764094515	2.980149451	0	0	1.540154268
Zm00001d027447_T004	0.85869003	0.482771888	0.8123216	1.004586963	1.116614944	0.842471236
Zm00001d010646_T001	4.149797593	3.399300862	4.251512708	3.962869545	4.208623772	4.099096294
Zm00001d019865_T001	4.165497316	4.201519539	4.341541773	4.33739669	4.309471697	4.222201612
Zm00001d002343_T001	4.293908304	3.869813381	4.243238508	4.919858377	4.85197316	4.931575985
Zm00001d017473_T002	3.483267997	2.687180311	3.389728968	3.42285893	3.485449627	3.325424122
Zm00001d017473_T001	1.735290374	1.900293383	1.954483332	1.211540566	1.145637027	1.891750074
Zm00001d007602_T001	0.376271488	0.169376032	0.360873274	0.879906475	0.648210475	0.483971032
Zm00001d032069_T001	2.301112328	1.707988093	2.251988068	4.012150106	3.987906285	4.041426637
Zm00001d049638_T015	0.111394612	0.080543062	0.208683307	0.607856276	1.000015148	0.822825577
Zm00001d049638_T013	0.162317042	0.12640465	0.158864368	0.396154008	0.244309866	0.391584073
Zm00001d049638_T008	2.118009144	1.523130338	2.507329352	2.896906146	2.796293527	2.959124136
Zm00001d049638_T028	2.560782686	4.016233591	3.040509882	1.841689784	0.94008404	0
Zm00001d004517_T007	0.311526366	0.065457884	0.116583008	0.31724849	0.661249768	0.413666649
Zm00001d004517_T005	2.48121523	0	2.673112467	2.32790835	2.689779755	2.636869582
Zm00001d004517_T008	0.504744446	0.239851371	0.631980579	0.652907712	0.401716742	0.614016242
Zm00001d004517_T010	3.527425334	3.91096804	3.113696499	2.90548049	2.437344407	3.05471184
Zm00001d004517_T006	3.344613923	1.803389899	3.32820055	3.241449413	3.240384096	3.264226691
Zm00001d022188_T001	1.165284893	0.831155713	1.14885473	1.221173654	1.226809985	1.290414967
Zm00001d022188_T002	2.792368543	2.069254305	2.104386986	1.991702328	1.587409159	1.341811589
Zm00001d022188_T003	3.434949621	4.222496549	3.597464908	3.678576873	3.634485237	3.900067756
Zm00001d032439_T002	1.830232199	1.339091195	1.701586316	1.766669786	1.969650784	1.970824211
Zm00001d032439_T001	2.313765651	2.191249317	2.198653816	1.648509621	2.006825808	1.82551212
Zm00001d032439_T003	1.648339346	0.65459685	1.374787998	1.866085041	1.903968007	1.703597759
Zm00001d018452_T001	5.61697989	5.090988846	5.864816186	6.0195074	6.024556319	6.03381153
Zm00001d032897_T001	7.048189335	6.662577137	7.192705282	6.338921838	6.433765171	6.356685547
Zm00001d043740_T001	1.978669005	1.763690738	2.185107266	5.126198496	4.929490452	4.846823488
Zm00001d021301_T009	2.786352756	0	3.277481947	3.761440174	3.775129787	3.863862945
Zm00001d021301_T020	0.555161396	0	0.920573045	1.850997795	0.957768279	1.217415618
Zm00001d021301_T001	3.318953367	3.010004047	3.381462383	4.033267579	4.268440532	4.155604584
Zm00001d021301_T008	5.964609684	5.391793148	5.955855386	5.617778606	5.670054036	5.552683882
Zm00001d021301_T006	0.870779761	0.722156468	0.498154855	0.552606271	0	0

Zm00001d021301_T016	0.25662303	0.034869288	0.077621742	0.450939365	0.899284699	0.374185938
Zm00001d033267_T001	1.181525594	0.712510383	1.18668627	1.569538727	1.870535564	1.370631337
Zm00001d009440_T003	1.393017441	1.768755995	0.881903417	0.89975182	1.464267288	1.101984829
Zm00001d009440_T004	1.03318475	0.540352682	0.746120968	1.167595574	0.986493217	0.936532789
Zm00001d009440_T001	4.357979823	3.933759572	4.641276886	4.624537791	4.530782672	4.633381221
Zm00001d037199_T001	3.175185244	2.626500114	3.003572375	3.624379578	3.727103277	3.521653326
Zm00001d028481_T026	1.937570761	0.778271667	2.134859331	3.116694117	2.90527002	2.703354124
Zm00001d028481_T002	2.905711336	2.233320255	3.064875438	4.182260854	4.015141687	4.093528824
Zm00001d028481_T001	0.399297991	0.171343915	0.118139085	0.948336205	0.333515331	0.952349969
Zm00001d028481_T040	5.466046369	4.695043448	5.21145288	5.371273118	5.444280603	5.18995483
Zm00001d028481_T041	3.980454936	2.284122314	4.147821646	3.753672241	3.156059366	3.61493995
Zm00001d004716_T003	1.841867277	1.314722624	2.056063926	2.278242625	2.653875173	2.414915677
Zm00001d004716_T015	0.806344682	0.215330693	0.71890298	0.743017684	0.862469642	0.289938312
Zm00001d004716_T012	0	0	0	1.145855458	0.866314561	0.854369941
Zm00001d004716_T019	0.358673071	0.173251545	0	0.295906362	0.269704651	0.532944277
Zm00001d004716_T017	0	0	0.477095988	0	0.600315432	1.283408591
Zm00001d004716_T007	0.580511178	0.383409572	0.067944294	0.582741928	0.262570264	0.323657138
Zm00001d052627_T001	2.035500396	1.613385962	2.264308524	1.787037048	1.698692333	1.68736846
Zm00001d033718_T001	2.477566207	1.864785983	2.389833576	2.987831631	2.869376739	2.804992327
Zm00001d018959_T001	4.029846327	3.280782349	3.907912047	3.828431735	3.741173672	3.963900673
Zm00001d028662_T004	0.375909097	0.327689663	0.569829366	1.222677841	0.733182509	1.032827299
Zm00001d028662_T007	0.705171094	0.307232655	0.838584745	0.719987708	0.76172525	0.730158216
Zm00001d028662_T001	4.672071188	3.7696135	4.552427119	4.700825865	4.624091885	4.557784838
Zm00001d028662_T005	1.165216063	0	1.726461561	1.261838878	1.41564078	1.555336158
Zm00001d030902_T004	1.049357536	1.180119847	1.377161517	0.313226113	1.269693881	0
Zm00001d030902_T003	4.88948206	3.742339674	4.797651151	3.650007405	3.721901733	3.543955221
Zm00001d030902_T002	1.862462897	0	1.460938579	0.614828552	0.327840247	1.167598143
Zm00001d030902_T001	2.024015007	2.237860731	2.328326377	1.328546977	0.856341959	1.02356054
Zm00001d033647_T002	0.1875296	0.200455388	0.123148349	0.396357901	0.428443658	0.460634587
Zm00001d033647_T001	5.554362945	4.770250531	5.560937974	5.55970554	5.44695375	5.416991467
Zm00001d033647_T006	3.065761166	1.487969911	2.84540434	2.062824923	2.470469447	2.193862559
Zm00001d033647_T007	0.791777404	0.32890653	0.660848318	0.847371603	0.945553021	1.000094493

Zm00001d033647_T003	0.303819317	0	0	0.421064375	0.612679929	0
Zm00001d000291_T001	4.757772837	4.150492739	4.66037784	4.010124929	4.175750848	4.27610847
Zm00001d049007_T001	5.823000386	6.456742586	5.621183774	6.080208819	6.044094729	5.746144272
Zm00001d039673_T023	2.286075615	0	2.469188939	1.854979457	1.935161749	0
Zm00001d039673_T005	0.176290854	0	0.266064488	1.098286817	0.746885431	1.236826887
Zm00001d039673_T007	0.608786538	0.229541165	0.391591771	0.929075153	1.374033992	0.90785361
Zm00001d039673_T026	4.711496448	3.160140722	4.478332608	4.718558795	4.607362689	4.712227136
Zm00001d039673_T013	0.617804372	0	0.107339706	0.927189531	1.377032102	0.72203665
Zm00001d039673_T027	3.619125617	6.852694677	4.64857111	3.285588943	4.197256488	2.675188595
Zm00001d039673_T003	3.715396137	2.988535457	3.692299417	4.393806958	4.323018504	4.515364968
Zm00001d031229_T001	1.662114331	1.692476745	1.632282642	2.112218042	1.804613259	1.594688991
Zm00001d002087_T005	3.910285491	2.880651833	3.675892805	3.881037684	4.178794302	3.985411534
Zm00001d002087_T001	2.399317407	2.284456685	2.299204794	3.508549844	3.235170397	3.47333936
Zm00001d002087_T004	0.43963803	0.325938977	0.782911743	0.650389593	0.54753472	0.71944457
Zm00001d016036_T001	2.647011431	2.274068096	2.560708352	2.983923169	2.584702311	2.514415694
Zm00001d016837_T001	4.067461811	3.352116682	4.09067359	4.220970744	4.063618215	4.213471325
Zm00001d016615_T003	1.7469383	0.865111946	1.823336055	1.360876644	1.359851165	1.444919861
Zm00001d016615_T002	2.857541354	2.569773722	2.736766561	3.692031987	3.729236354	3.706244416
Zm00001d019125_T002	4.679685806	3.77453365	4.718805628	4.05840586	4.071497287	4.186349688
Zm00001d019125_T004	1.649185	2.223878765	1.374092435	0.384054229	1.203548239	0.988286948
Zm00001d052227_T001	2.133937631	1.520009308	2.075504222	2.202163484	2.207586793	2.617126365
Zm00001d039387_T001	2.660034354	2.674621093	3.085462389	3.390363516	3.511716732	3.479111669
Zm00001d039387_T002	4.462940049	4.210597554	4.46178169	4.205051976	4.422010927	4.388513315
Zm00001d015975_T002	3.675787709	2.462656239	3.598083238	3.545935389	3.970430997	3.751198896
Zm00001d017025_T001	2.739629306	2.142533358	2.605598396	4.058039101	4.129979712	4.203325762
Zm00001d053762_T001	3.550637583	2.860537373	3.727335745	3.578734669	3.763444826	3.452699535
Zm00001d015475_T001	3.029029998	3.295216523	3.319111081	2.663259217	2.646302772	2.789016595
Zm00001d011649_T001	6.254355168	5.850655426	6.389519232	7.205438427	7.274451576	7.304300582
Zm00001d018400_T001	6.237119079	7.388379843	6.316306081	4.84477274	5.133883231	4.741153928
Zm00001d044320_T001	1.873513213	1.152447591	1.438519619	1.652745296	1.25176756	1.388429829
Zm00001d047772_T002	0.446732638	1.53380004	0.510260143	0	2.207649884	1.563803415
Zm00001d047772_T001	4.358475315	3.987594006	4.241350255	5.004667585	4.677358891	4.835061382

Zm00001d047772_T003	3.551630198	0	3.997047752	1.686749266	2.618950194	2.759933018
Zm00001d037249_T001	3.576108982	3.001376756	3.515012731	3.268950533	3.064035399	3.155127147
Zm00001d043692_T002	5.94229602	5.509941484	5.881633323	5.879951552	6.019830857	5.846706169
Zm00001d043692_T001	1.804098591	1.720523199	1.506240385	1.48447485	1.203493738	1.17196001
Zm00001d016588_T005	0.269422203	0.489845007	0.883447213	0.376690459	0.799859853	0
Zm00001d016588_T006	2.864328797	1.880729011	2.542619112	1.633113573	2.253592092	1.483369521
Zm00001d016588_T001	4.903275823	4.266175833	4.881129137	3.458453665	3.304803202	3.581572275
Zm00001d037245_T001	4.487324719	3.924902174	4.375684641	3.959976346	3.763853239	3.962996013
Zm00001d038227_T001	0.192662571	0.044133886	0.477204813	0.896970064	0.834073652	0.743607167
Zm00001d038227_T002	6.067791492	5.195103217	6.072353019	7.065371023	7.065280473	7.049357993
Zm00001d011353_T005	4.633425863	0	3.618193424	5.426638834	5.207638015	5.267843855
Zm00001d011353_T001	0.171173514	1.1897961	0.17269616	1.160232228	0.167339942	1.470315596
Zm00001d011353_T004	4.46968629	4.227234858	4.595957355	4.483053833	4.782752684	4.985162623
Zm00001d011353_T007	1.067012909	1.980367191	1.752874019	1.611623299	2.512390998	2.098297262
Zm00001d011353_T002	2.934353635	3.055821924	3.298872065	4.036778132	4.126186228	3.936511494
Zm00001d016788_T001	4.034362408	3.911123115	4.257199695	5.255498203	5.267058529	5.365174002
Zm00001d020187_T001	2.601672749	2.823125076	3.032738804	2.936915098	2.67806672	3.00126523
Zm00001d033957_T003	0.184068239	0.240054163	0.23388806	1.129106255	1.268530211	1.270089339
Zm00001d033957_T002	1.909883678	1.749344613	1.81246167	4.009659615	4.168143456	3.709870355
Zm00001d033957_T001	1.652475022	0.775074147	1.836655339	4.125184812	4.150619625	4.22188288
Zm00001d033192_T001	3.976777228	3.695897074	3.708807451	2.381151835	2.885496664	2.834869349
Zm00001d029023_T002	1.37917622	1.286307503	1.12448894	1.547539655	1.982981166	1.666655713
Zm00001d029023_T004	1.531318074	0	1.453814648	2.303498464	2.133321522	1.646661292
Zm00001d017241_T004	1.982298907	1.081624593	2.029963084	2.30897939	2.129944362	2.256313248
Zm00001d017241_T007	0.984978634	0.482220484	0.854144898	1.213323016	0.823134658	0.994516729
Zm00001d017241_T002	4.142601891	3.588484745	4.365995516	4.616226056	4.613144171	4.593863824
Zm00001d017241_T005	1.668670585	1.236962164	1.400114896	1.177931821	1.634169458	0.99593381
Zm00001d017241_T003	1.40796016	0	1.03407482	1.219334585	1.265347475	0
Zm00001d012223_T001	3.131839679	2.075823537	2.825180138	3.514396125	3.733816797	3.753576833
Zm00001d022406_T001	2.555025155	1.740794183	2.799977937	2.662538216	2.896128057	2.935381105
Zm00001d022406_T003	0.901551606	0.458709566	0.789857763	1.523012875	1.802331792	1.52369242
Zm00001d051660_T001	4.922467282	5.999482182	4.901007232	4.887584732	4.824101229	4.920590289

Zm00001d045017_T001	7.370276106	8.153289708	7.310078672	8.262080127	8.355796968	8.358190489
Zm00001d016544_T006	1.345699531	1.25184874	1.764828926	0.450846484	0.683592262	1.537334837
Zm00001d016544_T001	1.006801398	0.507065943	1.3301918	1.290823066	0.76267878	1.150520034
Zm00001d016544_T007	2.412277239	1.537730923	2.505889406	2.690993835	1.62372502	2.498307553
Zm00001d016544_T010	1.045819672	0.326659293	0.777299771	0.859285795	1.215405251	0.605653624
Zm00001d016544_T004	0.988760296	0	0	1.394188141	1.058796506	1.409879672
Zm00001d016544_T002	1.064801474	1.145837854	1.515121509	1.320003988	1.981925334	1.014853722
Zm00001d016544_T005	2.042899238	1.450482293	1.738686568	0.648280435	1.440440389	1.419393266
Zm00001d016544_T003	1.073017447	0.424210506	1.461257701	1.431197175	0.878903436	1.41732002
Zm00001d047471_T018	4.292687677	3.975149483	4.395882654	4.133444913	4.414810708	4.075432255
Zm00001d047471_T009	0	0.077262144	0.517519608	2.854480455	0	2.456838734
Zm00001d047471_T001	1.679725548	0.961345504	1.444280636	1.718896407	3.034874217	2.210012555
Zm00001d047471_T012	1.845590007	1.033511102	1.885036621	2.13788841	0	0
Zm00001d006169_T001	0.333478692	0.329814776	0	3.776620423	3.773012679	3.694426369
Zm00001d036982_T006	1.289355848	0.184418685	1.141127347	2.690817106	2.79812104	2.272498334
Zm00001d036982_T004	2.677040235	1.697091002	2.534507643	3.789772125	3.928645475	3.781183956
Zm00001d036982_T001	2.338055528	2.218633946	2.607868109	2.820340535	2.628147631	3.111283428
Zm00001d012277_T003	0.610014926	0.497150425	0.514048514	0.97235666	1.421597106	1.812049226
Zm00001d012277_T001	2.368178581	1.349123487	2.088659951	3.6985121	3.587977226	3.612422214
Zm00001d012277_T002	7.608110401	6.737348921	7.628636142	8.025036349	8.038823379	7.990362894
Zm00001d022637_T001	4.749068776	4.157272469	4.761484162	4.206796674	4.315971044	4.320704315
Zm00001d011610_T033	4.856089178	3.667605601	4.772121713	5.254220472	5.246661525	5.029385962
Zm00001d011610_T031	0.19280647	0.020386276	0	0.361778464	0.329962843	0
Zm00001d011610_T020	4.111358386	3.14419667	4.214499655	3.425234907	3.683433602	3.600080245
Zm00001d011610_T002	1.34866471	2.082704973	2.229810308	0	0	0
Zm00001d011610_T043	0.564892228	0.127857755	0.509977514	0.660888468	0.931524976	0
Zm00001d011610_T001	0.906321869	0.580022923	0.729608454	2.293992516	2.000772356	2.131047796
Zm00001d011610_T074	1.147631024	0.714560293	1.023030324	0.421827051	0.425328242	0.418345411
Zm00001d011610_T016	0.081575512	0	0.095809675	0.764827653	0.728490016	1.45623842
Zm00001d021668_T004	8.050930709	9.046051479	8.172656996	7.575898526	7.58842726	7.447381102
Zm00001d021668_T009	9.843556766	8.981171198	9.906961843	9.563695503	9.556923044	9.556139301
Zm00001d021668_T007	9.234339218	9.472048737	9.061484859	9.147684479	9.121381402	9.126138606

Zm00001d021668_T001	7.283792735	8.122954538	7.273442392	7.328227618	7.429518604	7.40167427
Zm00001d021668_T002	2.177037559	0	2.052606826	2.234216494	1.385263702	2.121110583
Zm00001d021668_T006	2.661781973	3.517171488	2.954359931	4.138309323	3.805113294	3.551124191
Zm00001d021668_T003	0	1.527375662	2.163285855	2.195803645	0	2.677390522
Zm00001d021668_T005	2.416459296	4.501938238	2.354288811	1.12659628	1.246149626	1.642523216
Zm00001d006676_T001	4.779500272	4.194680355	4.8330075	4.641572733	4.727326486	4.844701145
Zm00001d003281_T001	0.93542203	0.436056272	0.6937006	1.207624273	1.272039318	1.380660193
Zm00001d022492_T003	1.367388959	1.170653218	1.761961352	2.649625115	1.755849406	1.453206224
Zm00001d022492_T002	4.183811382	3.469536588	4.261164679	4.56993743	4.743416193	4.737561088
Zm00001d027694_T002	1.325439378	0.894322652	1.109620656	1.314845572	1.050469657	1.073803099
Zm00001d041119_T005	4.335644032	3.639431186	4.374897588	4.067193657	3.873299011	4.123821614
Zm00001d041119_T001	2.950718671	2.153955109	2.077434776	3.34510281	3.32840968	3.32895721
Zm00001d014513_T005	3.993169013	4.918434801	4.380118293	4.501225623	4.522472498	4.443838485
Zm00001d014513_T002	4.149387254	3.624134592	3.96330284	3.400851572	3.253611454	3.917821259
Zm00001d014513_T003	3.068443641	3.069902508	3.095932013	3.364492171	3.143632107	2.609912364
Zm00001d022283_T001	4.892234313	4.674518559	4.942251512	4.929783093	4.710557401	5.020067123
Zm00001d039268_T001	4.837294186	5.941236533	4.604077196	5.117201317	5.072610355	5.300562618
Zm00001d050335_T001	2.293531461	1.565249132	1.878523684	2.426343396	2.179463278	2.043494647
Zm00001d031973_T001	1.426561584	0.908477072	1.152711715	1.877228713	2.094941703	1.999517338
Zm00001d031973_T005	2.498880409	1.278370802	2.876208484	3.328275387	2.904410299	3.080460857
Zm00001d011622_T001	4.361645908	4.988283085	4.652386564	4.162455541	4.160295003	4.074693296
Zm00001d011622_T012	3.612453708	2.419080122	3.337893308	3.557365729	3.70338746	3.753004786
Zm00001d011622_T026	0.889197827	0.367699828	1.090438173	1.442293307	1.614024254	1.399383859
Zm00001d011622_T013	0.625576299	0	0	0.900078869	0	0.937946959
Zm00001d011622_T008	0.220050057	0.174130254	0.735612289	0.867494034	1.193772374	0.907629066
Zm00001d011622_T006	0	0	0	2.020800506	1.936189397	1.701823598
Zm00001d011622_T009	0	0	0.457535459	0.504382423	0.46221674	0.231472961
Zm00001d011622_T030	0.505877722	0.251200386	0	0	0	0.726723992
Zm00001d022505_T008	1.123345047	3.260908923	0.716693328	0.749271663	1.719864644	1.412627088
Zm00001d022505_T003	7.031943161	6.701468728	7.139134558	7.332910076	7.255150592	7.125819579
Zm00001d022505_T005	5.441492576	6.930003541	5.632038559	5.315595936	5.410931421	5.557827563
Zm00001d000257_T005	5.333312343	4.613959448	5.354861722	5.310619579	5.266259017	5.340984441

[truncated: 490,837 more chars]
