# Supplementary material for: High-resolution profile of transcriptomes reveals a role of alternative splicing for modulating response to nitrogen in maize
Source: BMC Genomics. 2020 May 11;21:353. doi: 10.1186/s12864-020-6769-8 (PMC7216474; doi:10.1186/s12864-020-6769-8)
Supplement: Supplementary file 15 — Additional file 15: Table S9. The profile of high-confidence isoforms identified in the untreated sample. [file 12864_2020_6769_MOESM15_ESM.pdf]

Supplemental Table S9: The profile of high-confidence isoforms identified in the untreated sample.

Isoform	Chr	Length	Exons	Structural Category	Associated Gene	Associated Transcript	Iso Exp	Gene Exp	Coding	ORF Length	CDS Start	CDS End
PB.10565.1	9	1230	1	novel_in_catalog	gene: Zm00001d047639	novel	687.75	1341.95	coding	327	121	1101
PB.1521.2	1	1316	1	novel_in_catalog	gene: Zm00001d034558	novel	145.43	512.66	coding	309	115	1041
PB.1591.1	1	3586	1	full-splice_match	gene: Zm00001d034781	Zm00001d034781_T001	16.90	43.63	coding	852	148	2703
PB.5575.1	4	1999	5	full-splice_match	gene: Zm00001d052696	Zm00001d052696_T001	13.49	13.49	coding	420	409	1668
PB.2110.2	10	1592	4	full-splice_match	gene: Zm00001d025689	Zm00001d025689_T003	5.47	33.81	coding	458	140	1513
PB.2110.1	10	1735	4	full-splice_match	gene: Zm00001d025689	Zm00001d025689_T003	28.34	33.81	coding	458	123	1496
PB.3189.1	2	1258	5	full-splice_match	gene: Zm00001d005795	Zm00001d005795_T001	14.47	14.47	coding	271	332	1144
PB.3742.3	3	1453	3	full-splice_match	gene: Zm00001d039613	Zm00001d039613_T004	4.53	97.39	coding	324	322	1293
PB.3742.2	3	2542	2	novel_in_catalog	gene: Zm00001d039613	novel	2.61	97.39	coding	233	1529	2227
PB.3742.1	3	1598	3	full-splice_match	gene: Zm00001d039613	Zm00001d039613_T002	66.59	97.39	coding	324	309	1280
PB.6488.1	5	2175	5	incomplete-splice_match	gene: Zm00001d015141	Zm00001d015141_T001	4.01	4.01	coding	588	16	1779
PB.3742.5	3	1326	3	full-splice_match	gene: Zm00001d039613	Zm00001d039613_T004	23.66	97.39	coding	324	263	1234
PB.8351.2	7	1273	2	antisense	novelGene_gene: Zm00001d019641_A	novel	7.09	12.07	coding	127	73	453
PB.8351.1	7	2030	1	antisense	novelGene_gene: Zm00001d019641_A	novel	4.98	12.07	coding	77	101	331
PB.926.13	1	2420	13	novel_in_catalog	gene: Zm00001d031927	novel	3.82	74.67	coding	247	1045	1785
PB.10650.1	9	1700	6	novel_in_catalog	gene: Zm00001d047911	novel	26.27	29.49	coding	332	458	1453

PB.10650.2	9	1699	6	novel_in_catalog	gene: Zm00001d047911	novel	3.22	29.49	coding	260	654	1433
PB.240.2	1	2134	7	full-splice_match	gene: Zm00001d028303	Zm00001d028303_T002	2.05	2.05	coding	403	750	1958
PB.792.3	1	1261	3	novel_in_catalog	gene: Zm00001d031204	novel	13.46	13.46	coding	120	41	400
PB.4689.4	3	1276	4	novel_in_catalog	gene: Zm00001d044287	novel	3.94	786.59	coding	184	66	617
PB.4086.3	3	2418	4	full-splice_match	gene: Zm00001d041715	Zm00001d041715_T009	8.03	21.06	coding	697	273	2363
PB.4086.2	3	2878	3	full-splice_match	gene: Zm00001d041715	Zm00001d041715_T010	13.03	21.06	coding	505	1231	2745
PB.7847.1	6	935	6	full-splice_match	gene: Zm00001d038139	Zm00001d038139_T001	84.68	84.68	coding	177	79	609
PB.4689.1	3	968	5	full-splice_match	gene: Zm00001d044287	Zm00001d044287_T001	782.65	786.59	coding	207	102	722
PB.7144.5	5	1018	6	incomplete-splice_match	gene: Zm00001d018409	Zm00001d018409_T004	59.81	146.45	coding	234	81	782
PB.4689.3	3	847	5	full-splice_match	gene: Zm00001d044287	Zm00001d044287_T001	0.00	786.59	coding	207	70	690
PB.2476.2	2	1160	2	genic	gene: Zm00001d002105	novel	152.86	228.85	coding	269	64	870
PB.2476.3	2	1189	1	full-splice_match	gene: Zm00001d002105	Zm00001d002105_T001	15.27	228.85	coding	269	13	819
PB.2476.1	2	1295	2	genic	gene: Zm00001d002105	novel	60.73	228.85	coding	269	80	886
PB.2476.4	2	924	1	full-splice_match	gene: Zm00001d002105	Zm00001d002105_T001	0.00	228.85	coding	211	39	671
PB.5267.1	4	1273	1	full-splice_match	gene: Zm00001d051344	Zm00001d051344_T001	10.23	10.23	coding	293	62	940
PB.6810.22	5	1954	9	novel_in_catalog	gene: Zm00001d017096	novel	0.15	118.92	coding	171	106	618
PB.250.2	1	659	4	intergenic	novelGene_86	novel	335.74	612.46	coding	129	72	458

PB.250.1	1	759	4	intergenic	novelGene_86	novel	276.72	612.46	coding	129	70	456
PB.3372.1	2	1021	4	full-splice_match	gene: Zm00001d006630	Zm00001d006630_T001	14.19	14.19	coding	274	55	876
PB.5794.1	4	2447	2	full-splice_match	gene: Zm00001d053732	Zm00001d053732_T001	10.92	10.92	coding	655	155	2119
PB.10418.1	9	1917	7	incomplete-splice_match	gene: Zm00001d046928	Zm00001d046928_T005	10.26	10.26	coding	359	450	1526
PB.8045.2	6	1625	11	full-splice_match	gene: Zm00001d038866	Zm00001d038866_T005	23.05	244.12	coding	407	151	1371
PB.9473.2	8	2620	7	incomplete-splice_match	gene: Zm00001d010315	Zm00001d010315_T010	32.76	42.22	coding	652	114	2069
PB.9473.3	8	1488	6	incomplete-splice_match	gene: Zm00001d010315	Zm00001d010315_T008	9.45	42.22	coding	324	51	1022
PB.10446.1	9	2236	12	full-splice_match	gene: Zm00001d047054	Zm00001d047054_T001	87.94	99.42	coding	561	162	1844
PB.767.2	1	2738	1	genic	gene: Zm00001d031078	novel	0.61	77.46	coding	625	300	2174
PB.6291.1	5	2362	8	full-splice_match	gene: Zm00001d014382	Zm00001d014382_T005	16.89	23.02	coding	693	117	2195
PB.3561.2	2	532	3	novel_not_in_catalog	gene: Zm00001d007633	novel	109.82	255.13	coding	68	117	320
PB.6291.2	5	2403	7	novel_in_catalog	gene: Zm00001d014382	novel	6.13	23.02	coding	506	83	1600
PB.6006.2	5	1681	4	full-splice_match	gene: Zm00001d013159	Zm00001d013159_T004	101.04	673.44	coding	450	153	1502
PB.6006.1	5	1730	4	full-splice_match	gene: Zm00001d013159	Zm00001d013159_T001	572.39	673.44	coding	450	155	1504
PB.10739.1	9	1398	5	full-splice_match	gene: Zm00001d048253	Zm00001d048253_T001	202.46	202.46	coding	308	149	1072
PB.10739.4	9	1229	5	full-splice_match	gene: Zm00001d048253	Zm00001d048253_T001	0.00	202.46	coding	308	122	1045
PB.10739.5	9	1360	5	full-splice_match	gene: Zm00001d048256	Zm00001d048256_T002	143.18	143.18	coding	308	126	1049

PB.1521.1	1	1684	2	full-splice_match	gene: Zm00001d034558	Zm00001d034558_T001	47.10	512.66	coding	260	595	1374
PB.9508.1	8	2155	2	full-splice_match	gene: Zm00001d010497	Zm00001d010497_T001	10.14	10.14	coding	242	120	845
PB.9508.2	8	2033	2	full-splice_match	gene: Zm00001d010497	Zm00001d010497_T001	0.00	10.14	coding	242	142	867
PB.3082.4	2	1882	13	full-splice_match	gene: Zm00001d005240	Zm00001d005240_T003	4.32	16.27	coding	581	109	1851
PB.9084.1	8	2760	2	full-splice_match	gene: Zm00001d008293	Zm00001d008293_T001	34.18	34.18	coding	806	82	2499
PB.6903.1	5	951	1	genic	gene: Zm00001d017473	novel	11.57	25.83	coding	161	101	583
PB.1875.15	10	4863	1	antisense	novelGene_gene: Zm00001d024500_A	novel	3.23	3.23	coding	123	540	908
PB.8059.1	6	1014	5	full-splice_match	gene: Zm00001d038910	Zm00001d038910_T001	29.69	29.69	coding	227	32	712
PB.10293.2	9	775	3	full-splice_match	gene: Zm00001d046169	Zm00001d046169_T001	29.24	76.61	coding	181	96	638
PB.10293.1	9	970	3	full-splice_match	gene: Zm00001d046169	Zm00001d046169_T001	47.37	76.61	coding	181	234	776
PB.3082.3	2	1545	8	novel_in_catalog	gene: Zm00001d005240	novel	1.33	16.27	coding	186	509	1066
PB.5549.1	4	744	1	full-splice_match	gene: Zm00001d052545	Zm00001d052545_T001	87.84	87.84	non_coding	NA	NA	NA
PB.2969.1	2	1664	4	full-splice_match	gene: Zm00001d004545	Zm00001d004545_T001	20.95	20.95	coding	387	165	1325
PB.3925.1	3	2218	17	novel_not_in_catalog	gene: Zm00001d040603	novel	41.73	46.38	coding	608	137	1960
PB.3925.2	3	2283	16	novel_not_in_catalog	gene: Zm00001d040603	novel	4.65	46.38	coding	500	101	1600
PB.9521.1	8	2968	1	antisense	novelGene_gene: Zm00001d010564_A	novel	4.73	23.35	non_coding	NA	NA	NA
PB.6882.1	5	874	2	full-splice_match	gene: Zm00001d017379	Zm00001d017379_T001	91.28	91.28	coding	173	91	609

PB.4647.1	3	1922	16	incomplete-splice_match	gene: Zm00001d044129	Zm00001d044129_T011	13.62	13.62	coding	516	86	1633
PB.7791.2	6	2746	19	full-splice_match	gene: Zm00001d037950	Zm00001d037950_T010	23.90	29.82	coding	788	100	2463
PB.7791.1	6	3832	28	incomplete-splice_match	gene: Zm00001d037950	Zm00001d037950_T015	5.92	29.82	coding	1140	17	3436
PB.8492.3	7	1151	4	full-splice_match	gene: Zm00001d020505	Zm00001d020505_T001	28.90	28.90	coding	223	339	1007
PB.7217.8	6	942	5	full-splice_match	gene: Zm00001d035028	Zm00001d035028_T001	11.37	234.37	coding	233	39	737
PB.7217.9	6	1763	4	novel_in_catalog	gene: Zm00001d035028	novel	0.00	234.37	coding	125	1192	1566
PB.5238.1	4	2072	1	novel_in_catalog	gene: Zm00001d051163	novel	156.63	156.63	coding	557	77	1747
PB.7217.4	6	1894	4	novel_in_catalog	gene: Zm00001d035028	novel	0.00	234.37	coding	125	1194	1568
PB.7217.5	6	2184	2	novel_in_catalog	gene: Zm00001d035028	novel	8.16	234.37	non_coding	NA	NA	NA
PB.7217.7	6	1845	4	novel_not_in_catalog	gene: Zm00001d035028	novel	3.13	234.37	non_coding	NA	NA	NA
PB.8402.1	7	738	3	incomplete-splice_match	gene: Zm00001d020046	Zm00001d020046_T006	47.31	47.31	coding	69	398	604
PB.7217.1	6	1069	5	full-splice_match	gene: Zm00001d035028	Zm00001d035028_T001	173.06	234.37	coding	233	34	732
PB.7217.2	6	1074	5	novel_not_in_catalog	gene: Zm00001d035028	novel	36.77	234.37	coding	194	34	615
PB.7217.3	6	1995	3	novel_in_catalog	gene: Zm00001d035028	novel	1.88	234.37	coding	128	1187	1570
PB.8333.2	7	1516	15	novel_in_catalog	gene: Zm00001d019552	novel	22.51	22.97	coding	418	46	1299
PB.8333.1	7	1638	15	novel_in_catalog	gene: Zm00001d019552	novel	0.46	22.97	coding	418	42	1295
PB.9964.1	8	792	1	full-splice_match	gene: Zm00001d012545	Zm00001d012545_T001	50.21	50.21	coding	131	92	484

PB.4992.3	4	1473	6	novel_in_catalog	gene: Zm00001d049598	novel	6.66	461.83	coding	186	225	782
PB.10689.2	9	1942	3	full-splice_match	gene: Zm00001d048055	Zm00001d048055_T001	16.44	31.01	coding	501	256	1758
PB.4992.1	4	1482	7	full-splice_match	gene: Zm00001d049598	Zm00001d049598_T001	428.97	461.83	coding	228	325	1008
PB.9878.2	8	4054	20	full-splice_match	gene: Zm00001d012155	Zm00001d012155_T019	11.37	22.90	coding	983	839	3787
PB.4534.3	3	1420	11	novel_in_catalog	gene: Zm00001d043669	novel	2.93	102.56	coding	294	419	1300
PB.4534.2	3	1451	12	full-splice_match	gene: Zm00001d043669	Zm00001d043669_T004	33.84	102.56	coding	391	117	1289
PB.4992.5	4	1355	7	full-splice_match	gene: Zm00001d049598	Zm00001d049598_T001	0.00	461.83	coding	228	305	988
PB.4992.4	4	567	1	novel_in_catalog	gene: Zm00001d049598	novel	26.20	461.83	non_coding	NA	NA	NA
PB.2567.1	2	1175	3	novel_in_catalog	gene: Zm00001d002496	novel	6.46	38.15	coding	191	527	1099
PB.6038.1	5	811	3	full-splice_match	gene: Zm00001d013257	Zm00001d013257_T001	64.63	64.63	coding	135	204	608
PB.8271.3	7	1678	13	full-splice_match	gene: Zm00001d019164	Zm00001d019164_T001	133.67	133.67	coding	440	66	1385
PB.2567.2	2	1040	4	full-splice_match	gene: Zm00001d002496	Zm00001d002496_T001	31.69	38.15	coding	306	49	966
PB.8271.5	7	1129	3	incomplete-splice_match	gene: Zm00001d019165	Zm00001d019165_T002	3.72	11.66	coding	293	1	879
PB.8271.4	7	2131	4	full-splice_match	gene: Zm00001d019165	Zm00001d019165_T002	7.94	11.66	coding	487	160	1620
PB.8934.1	7	2196	2	genic	gene: Zm00001d022247	novel	7.52	65.62	coding	581	111	1853
PB.9961.1	8	3079	5	full-splice_match	gene: Zm00001d012529	Zm00001d012529_T001	9.26	9.26	coding	903	93	2801
PB.6285.2	5	710	4	incomplete-splice_match	gene: Zm00001d014344	Zm00001d014344_T001	2.13	46.65	coding	163	50	538

PB.6285.3	5	2608	5	incomplete-splice_match	gene: Zm00001d014344	Zm00001d014344_T029	6.45	46.65	coding	508	69	1592
PB.5058.1	4	907	2	incomplete-splice_match	gene: Zm00001d049926	Zm00001d049926_T001	12.67	12.67	coding	185	194	748
PB.6285.1	5	3451	8	incomplete-splice_match	gene: Zm00001d014344	Zm00001d014344_T001	4.04	46.65	coding	300	115	1014
PB.6285.4	5	1784	2	full-splice_match	gene: Zm00001d014344	Zm00001d014344_T039	20.66	46.65	coding	300	56	955
PB.6285.5	5	5254	8	full-splice_match	gene: Zm00001d014344	Zm00001d014344_T034	13.37	46.65	coding	1399	433	4629
PB.8150.2	7	1210	7	full-splice_match	gene: Zm00001d018616	Zm00001d018616_T004	67.99	67.99	coding	307	94	1014
PB.3806.1	3	995	1	full-splice_match	gene: Zm00001d039991	Zm00001d039991_T001	53.78	144.87	coding	185	186	740
PB.3806.2	3	884	1	full-splice_match	gene: Zm00001d039991	Zm00001d039991_T001	91.09	144.87	coding	185	189	743
PB.5591.1	4	2020	14	full-splice_match	gene: Zm00001d052758	Zm00001d052758_T001	62.19	62.19	coding	552	128	1783
PB.10336.4	9	1699	7	full-splice_match	gene: Zm00001d046445	Zm00001d046445_T002	0.00	149.37	coding	340	340	1359
PB.9059.10	8	1091	1	novel_in_catalog	gene: Zm00001d008181	novel	0.00	133.77	non_coding	NA	NA	NA
PB.9059.11	8	884	2	full-splice_match	gene: Zm00001d008181	Zm00001d008181_T002	9.19	133.77	non_coding	NA	NA	NA
PB.9059.12	8	859	1	genic	gene: Zm00001d008181	novel	0.00	133.77	non_coding	NA	NA	NA
PB.10336.2	9	1838	7	full-splice_match	gene: Zm00001d046445	Zm00001d046445_T002	144.80	149.37	coding	340	337	1356
PB.6609.3	5	1202	4	novel_in_catalog	gene: Zm00001d016041	novel	16.59	126.61	coding	152	105	560
PB.7009.5	5	2146	5	antisense	novelGene_gene: Zm00001d017932_A	novel	5.06	81.66	non_coding	NA	NA	NA
PB.10398.10	9	837	3	full-splice_match	gene: Zm00001d046767	Zm00001d046767_T001	0.00	1894.58	coding	174	120	641

PB.9926.1	8	2207	1	full-splice_match	gene: Zm00001d012340	Zm00001d012340_T001	3.47	3.59	coding	628	211	2094
PB.9926.3	8	1862	1	full-splice_match	gene: Zm00001d012340	Zm00001d012340_T001	0.12	3.59	coding	594	79	1860
PB.9926.2	8	2077	1	full-splice_match	gene: Zm00001d012340	Zm00001d012340_T001	0.00	3.59	coding	683	27	2075
PB.4693.1	3	1988	11	full-splice_match	gene: Zm00001d044297	Zm00001d044297_T001	24.57	24.57	coding	513	157	1695
PB.2070.1	10	936	3	full-splice_match	gene: Zm00001d025445	Zm00001d025445_T001	80.07	85.21	coding	154	147	608
PB.3094.1	2	1624	3	novel_not_in_catalog	gene: Zm00001d005295	novel	9.28	110.79	coding	328	160	1143
PB.2070.2	10	1453	1	novel_in_catalog	gene: Zm00001d025445	novel	5.14	85.21	non_coding	NA	NA	NA
PB.2084.1	10	1628	8	full-splice_match	gene: Zm00001d025542	Zm00001d025542_T002	8.74	92.14	coding	187	170	730
PB.947.2	1	1503	2	incomplete-splice_match	gene: Zm00001d031992	Zm00001d031992_T001	76.41	76.41	coding	339	178	1194
PB.8478.3	7	1822	10	full-splice_match	gene: Zm00001d020441	Zm00001d020441_T002	0.00	45.18	coding	511	56	1588
PB.3712.2	3	1895	2	intergenic	novelGene_27	novel	39.64	44.08	coding	183	1171	1719
PB.8478.1	7	1932	10	full-splice_match	gene: Zm00001d020441	Zm00001d020441_T002	45.18	45.18	coding	511	67	1599
PB.4740.1	3	3608	2	novel_in_catalog	gene: Zm00001d044500	novel	4.46	11.76	coding	595	1654	3438
PB.9902.1	8	1052	2	full-splice_match	gene: Zm00001d012255	Zm00001d012255_T001	25.38	25.38	coding	251	150	902
PB.1280.1	1	1921	2	full-splice_match	gene: Zm00001d033528	Zm00001d033528_T006	244.93	244.93	coding	465	115	1509
PB.8418.2	7	1689	5	full-splice_match	gene: Zm00001d020140	Zm00001d020140_T018	8.33	38.65	coding	156	538	1005
PB.8418.5	7	2672	2	novel_in_catalog	gene: Zm00001d020140	novel	0.96	38.65	coding	165	712	1206

PB.8418.6	7	1449	3	full-splice_match	gene: Zm00001d020140	Zm00001d020140_T003	5.22	38.65	coding	134	44	445
PB.8418.7	7	1832	4	full-splice_match	gene: Zm00001d020140	Zm00001d020140_T013	16.90	38.65	coding	156	781	1248
PB.788.1	1	923	2	full-splice_match	gene: Zm00001d031168	Zm00001d031168_T001	7023.07	14591.48	coding	155	100	564
PB.8265.3	7	1480	14	full-splice_match	gene: Zm00001d019139	Zm00001d019139_T003	9.67	142.61	coding	344	200	1231
PB.2577.1	2	2064	7	full-splice_match	gene: Zm00001d002541	Zm00001d002541_T001	4.84	4.84	coding	352	624	1679
PB.7551.1	6	2090	4	full-splice_match	gene: Zm00001d036895	Zm00001d036895_T001	75.31	75.31	coding	441	227	1549
PB.788.7	1	704	2	full-splice_match	gene: Zm00001d031168	Zm00001d031168_T001	2415.95	14591.48	coding	155	100	564
PB.2071.2	10	1642	10	novel_in_catalog	gene: Zm00001d025446	novel	26.83	26.83	coding	517	23	1573
PB.5171.1	4	549	2	novel_in_catalog	gene: Zm00001d050696	novel	75.58	162.87	non_coding	NA	NA	NA
PB.7275.1	6	1337	2	novel_not_in_catalog	gene: Zm00001d035308	novel	145.32	155.22	coding	342	100	1125
PB.7275.2	6	1620	1	genic	gene: Zm00001d035308	novel	9.90	155.22	coding	259	283	1059
PB.5171.2	4	744	5	full-splice_match	gene: Zm00001d050696	Zm00001d050696_T002	87.30	162.87	non_coding	NA	NA	NA
PB.6211.1	5	1615	8	full-splice_match	gene: Zm00001d013996	Zm00001d013996_T003	18.22	18.22	coding	320	124	1083
PB.2694.1	2	1076	7	full-splice_match	gene: Zm00001d003013	Zm00001d003013_T001	5.58	5.58	coding	249	96	842
PB.3479.3	2	1505	4	full-splice_match	gene: Zm00001d007172	Zm00001d007172_T001	23.67	23.67	coding	362	66	1151
PB.6211.2	5	1474	8	full-splice_match	gene: Zm00001d013996	Zm00001d013996_T003	0.00	18.22	coding	320	118	1077
PB.1531.4	1	2488	7	incomplete-splice_match	gene: Zm00001d034590	Zm00001d034590_T017	1.92	90.00	coding	252	120	875

PB.3391.1	2	1970	2	novel_in_catalog	gene: Zm00001d006749	novel	0.79	13.57	coding	307	146	1066
PB.1082.4	1	1729	12	full-splice_match	gene: Zm00001d032566	Zm00001d032566_T002	0.00	48.45	coding	461	71	1453
PB.3094.3	2	1225	3	novel_not_in_catalog	gene: Zm00001d005295	novel	9.59	110.79	coding	328	85	1068
PB.8756.2	7	1804	1	full-splice_match	gene: Zm00001d021638	Zm00001d021638_T001	2.60	6.30	coding	521	123	1685
PB.5143.1	4	1796	9	full-splice_match	gene: Zm00001d050428	Zm00001d050428_T002	7.29	7.29	coding	498	154	1647
PB.7012.3	5	2936	14	incomplete-splice_match	gene: Zm00001d017946	Zm00001d017946_T010	2.72	30.15	coding	871	68	2680
PB.7484.2	6	3501	1	genic	gene: Zm00001d036534	novel	1.45	24.35	coding	1067	194	3394
PB.7012.1	5	4231	18	incomplete-splice_match	gene: Zm00001d017946	Zm00001d017946_T040	7.38	30.15	coding	1136	323	3730
PB.6828.4	5	1287	1	genic	gene: Zm00001d017147	novel	21.58	375.74	coding	140	371	790
PB.7956.3	6	2522	2	genic	gene: Zm00001d038538	novel	6.01	6.01	coding	287	1506	2366
PB.6828.3	5	1190	2	novel_in_catalog	gene: Zm00001d017147	novel	29.15	375.74	coding	175	142	666
PB.8909.1	7	2409	14	novel_in_catalog	gene: Zm00001d022152	novel	48.72	48.72	coding	473	656	2074
PB.9954.1	8	2491	4	intergenic	novelGene_288	novel	5.23	5.23	coding	451	1139	2491
PB.8744.1	7	2916	10	novel_not_in_catalog	gene: Zm00001d021592	novel	10.38	10.38	coding	833	160	2658
PB.6828.2	5	1254	3	full-splice_match	gene: Zm00001d017147	Zm00001d017147_T001	325.02	375.74	coding	258	295	1068
PB.1164.7	1	2307	3	novel_in_catalog	gene: Zm00001d032923	novel	8.25	29.12	coding	227	868	1548
PB.1164.6	1	1973	5	full-splice_match	gene: Zm00001d032923	Zm00001d032923_T003	10.81	29.12	coding	227	752	1432

PB.1164.5	1	2310	4	full-splice_match	gene: Zm00001d032923	Zm00001d032923_T001	8.87	29.12	coding	227	858	1538
PB.7484.1	6	3584	1	genic	gene: Zm00001d036534	novel	22.91	24.35	coding	1067	55	3255
PB.1164.2	1	2169	4	novel_in_catalog	gene: Zm00001d032923	novel	1.20	29.12	coding	227	1012	1692
PB.654.1	1	1952	2	full-splice_match	gene: Zm00001d030300	Zm00001d030300_T001	7.65	7.65	coding	246	874	1611
PB.3776.1	3	1442	11	full-splice_match	gene: Zm00001d039851	Zm00001d039851_T002	10.39	10.39	coding	360	164	1243
PB.4768.2	3	1102	7	full-splice_match	gene: Zm00001d044615	Zm00001d044615_T002	2.95	105.81	coding	116	95	442
PB.4768.3	3	975	8	full-splice_match	gene: Zm00001d044615	Zm00001d044615_T003	46.82	105.81	coding	231	80	772
PB.957.1	1	1041	2	full-splice_match	gene: Zm00001d032058	Zm00001d032058_T001	17.28	17.28	coding	221	90	752
PB.4768.1	3	1092	8	full-splice_match	gene: Zm00001d044615	Zm00001d044615_T003	56.03	105.81	coding	231	91	783
PB.1533.3	1	1587	5	incomplete-splice_match	gene: Zm00001d034597	Zm00001d034597_T004	0.00	27.67	coding	419	330	1586
PB.1917.1	10	1752	1	full-splice_match	gene: Zm00001d024717	Zm00001d024717_T001	3.16	3.16	coding	501	19	1521
PB.10842.2	B73V4 ctg103	1864	12	full-splice_match	gene: Zm00001d000299	Zm00001d000299_T003	43.28	50.88	coding	425	367	1641
PB.4486.1	3	1841	4	full-splice_match	gene: Zm00001d043485	Zm00001d043485_T001	11.70	11.70	coding	413	396	1634
PB.2109.2	10	1815	1	full-splice_match	gene: Zm00001d025675	Zm00001d025675_T001	20.46	43.81	coding	497	67	1557
PB.10842.5	B73V4 ctg103	1888	11	novel_not_in_catalog	gene: Zm00001d000299	novel	7.61	50.88	coding	289	818	1684
PB.5038.1	4	1679	3	full-splice_match	gene: Zm00001d049786	Zm00001d049786_T001	3.72	28.55	coding	397	130	1320
PB.5038.2	4	1557	3	full-splice_match	gene: Zm00001d049786	Zm00001d049786_T001	24.83	28.55	coding	397	131	1321

PB.2255.2	10	2326	9	full-splice_match	gene: Zm00001d026302	Zm00001d026302_T001	1.09	29.94	coding	121	60	422
PB.8747.1	7	2242	2	full-splice_match	gene: Zm00001d021613	Zm00001d021613_T001	4.69	4.69	coding	615	114	1958
PB.7286.10	6	412	1	novel_in_catalog	gene: Zm00001d035356	novel	0.00	28.23	non_coding	NA	NA	NA
PB.1465.1	1	1374	1	incomplete-splice_match	gene: Zm00001d034350	Zm00001d034350_T002	5.04	23.85	coding	322	115	1080
PB.2135.1	10	2922	9	novel_in_catalog	gene: Zm00001d025777	novel	35.53	78.90	coding	785	142	2496
PB.5385.2	4	1250	4	full-splice_match	gene: Zm00001d051912	Zm00001d051912_T001	86.20	183.74	coding	329	84	1070
PB.2227.1	10	1351	5	full-splice_match	gene: Zm00001d026212	Zm00001d026212_T003	51.11	51.11	non_coding	NA	NA	NA
PB.1233.3	1	1097	2	full-splice_match	gene: Zm00001d033319	Zm00001d033319_T001	0.00	79.95	coding	226	167	844
PB.341.1	1	1067	10	full-splice_match	gene: Zm00001d028692	Zm00001d028692_T005	66.01	66.01	coding	242	59	784
PB.8775.1	7	2204	4	full-splice_match	gene: Zm00001d021701	Zm00001d021701_T004	6.97	67.00	coding	193	1399	1977
PB.4455.1	3	1189	1	full-splice_match	gene: Zm00001d043390	Zm00001d043390_T001	25.34	25.34	coding	297	86	976
PB.6104.1	5	2659	10	full-splice_match	gene: Zm00001d013470	Zm00001d013470_T009	27.63	27.63	coding	799	106	2502
PB.6392.4	5	1508	13	full-splice_match	gene: Zm00001d014786	Zm00001d014786_T002	0.00	24.88	coding	393	28	1206
PB.6392.3	5	2275	12	novel_in_catalog	gene: Zm00001d014786	novel	1.13	24.88	coding	311	29	961
PB.6392.1	5	1672	13	full-splice_match	gene: Zm00001d014786	Zm00001d014786_T002	23.75	24.88	coding	393	101	1279
PB.10287.1	9	1559	8	full-splice_match	gene: Zm00001d046135	Zm00001d046135_T001	47.54	52.81	coding	395	141	1325
PB.2883.2	2	337	1	incomplete-splice_match	gene: Zm00001d003879	Zm00001d003879_T005	124.96	529.65	non_coding	NA	NA	NA

PB.4241.4	3	1453	4	novel_in_catalog	gene: Zm00001d042455	novel	7.99	132.32	coding	130	745	1134
PB.2883.1	2	1223	2	full-splice_match	gene: Zm00001d003879	Zm00001d003879_T007	345.26	529.65	coding	273	136	954
PB.10287.2	9	1923	8	novel_in_catalog	gene: Zm00001d046135	novel	5.27	52.81	coding	206	1082	1699
PB.5813.1	4	1749	8	full-splice_match	gene: Zm00001d053808	Zm00001d053808_T001	11.54	11.54	coding	437	145	1455
PB.2716.2	2	1925	1	full-splice_match	gene: Zm00001d003104	Zm00001d003104_T001	74.82	87.80	coding	442	252	1577
PB.1144.1	1	940	1	full-splice_match	gene: Zm00001d032821	Zm00001d032821_T001	251.46	251.46	coding	196	31	618
PB.8065.1	6	780	4	intergenic	novelGene_274	novel	198.64	198.64	coding	111	159	491
PB.3637.1	3	1082	2	full-splice_match	gene: Zm00001d039256	Zm00001d039256_T001	27.66	27.66	coding	236	224	931
PB.2716.1	2	2082	1	full-splice_match	gene: Zm00001d003104	Zm00001d003104_T001	2.30	87.80	coding	442	193	1518
PB.373.1	1	1094	1	full-splice_match	gene: Zm00001d028870	Zm00001d028870_T001	11.95	11.95	coding	214	168	809
PB.6563.1	5	1668	1	full-splice_match	gene: Zm00001d015585	Zm00001d015585_T001	8.73	8.73	coding	395	92	1276
PB.836.1	1	1308	1	incomplete-splice_match	gene: Zm00001d031444	Zm00001d031444_T001	9.56	9.56	coding	331	173	1165
PB.4178.1	3	474	1	intergenic	novelGene_251	novel	27.29	27.29	non_coding	NA	NA	NA
PB.7839.1	6	2248	1	full-splice_match	gene: Zm00001d038125	Zm00001d038125_T001	14.09	14.09	coding	586	164	1921
PB.4023.1	3	1733	2	novel_in_catalog	gene: Zm00001d041410	novel	66.79	66.79	coding	165	458	952
PB.2132.2	10	1549	10	full-splice_match	gene: Zm00001d025767	Zm00001d025767_T002	3.47	41.22	coding	448	93	1436
PB.7452.1	6	1161	1	genic	gene: Zm00001d036370	novel	33.58	33.58	coding	261	46	828

PB.1200.1	1	878	3	intergenic	novelGene_368	novel	19.24	61.74	coding	149	77	523
PB.7908.5	6	1406	5	full-splice_match	gene: Zm00001d038346	Zm00001d038346_T002	0.00	78.74	coding	362	58	1143
PB.7908.4	6	1729	5	full-splice_match	gene: Zm00001d038346	Zm00001d038346_T002	0.00	78.74	coding	362	276	1361
PB.6432.1	5	852	1	full-splice_match	gene: Zm00001d014949	Zm00001d014949_T001	165.70	165.70	coding	203	49	657
PB.1907.2	10	1283	7	full-splice_match	gene: Zm00001d024661	Zm00001d024661_T001	19.83	26.43	coding	229	285	971
PB.7329.1	6	341	1	genic	gene: Zm00001d035617	novel	274.51	274.51	non_coding	NA	NA	NA
PB.4158.1	3	1944	5	full-splice_match	gene: Zm00001d042043	Zm00001d042043_T001	35.67	35.67	coding	394	410	1591
PB.7908.2	6	2281	4	novel_in_catalog	gene: Zm00001d038346	novel	36.12	78.74	coding	236	754	1461
PB.1381.3	1	3283	5	incomplete-splice_match	gene: Zm00001d033984	Zm00001d033984_T008	17.37	104.75	coding	866	114	2711
PB.5160.2	4	2894	2	genic_intron	novelGene_166	novel	1.09	46.27	non_coding	NA	NA	NA
PB.1804.1	10	1874	10	full-splice_match	gene: Zm00001d023987	Zm00001d023987_T002	95.11	341.43	coding	490	211	1680
PB.2559.2	2	2639	11	novel_in_catalog	gene: Zm00001d002440	novel	52.88	52.88	coding	598	479	2272
PB.428.1	1	2508	1	full-splice_match	gene: Zm00001d029100	Zm00001d029100_T001	8.14	8.14	coding	106	1956	2273
PB.1804.2	10	1776	10	full-splice_match	gene: Zm00001d023987	Zm00001d023987_T006	27.29	341.43	coding	264	164	955
PB.7725.1	6	644	1	incomplete-splice_match	gene: Zm00001d037712	Zm00001d037712_T001	17.95	17.95	coding	121	49	411
PB.3160.7	2	2201	4	novel_in_catalog	gene: Zm00001d005682	novel	3.40	38.30	coding	165	1094	1588
PB.10446.2	9	2229	12	full-splice_match	gene: Zm00001d047054	Zm00001d047054_T001	11.48	99.42	coding	561	322	2004

PB.5399.10	4	2060	3	full-splice_match	gene: Zm00001d051981	Zm00001d051981_T003	0.81	138.16	coding	405	498	1712
PB.9056.1	8	1380	2	full-splice_match	gene: Zm00001d008173	Zm00001d008173_T001	367.01	367.01	coding	348	96	1139
PB.3228.4	2	2144	5	novel_in_catalog	gene: Zm00001d006034	novel	6.43	61.89	coding	295	347	1231
PB.5160.1	4	1366	4	genic_intron	novelGene_166	novel	9.25	46.27	coding	184	158	709
PB.3228.2	2	1876	7	full-splice_match	gene: Zm00001d006034	Zm00001d006034_T004	48.16	61.89	coding	377	385	1515
PB.1043.1	1	1893	8	full-splice_match	gene: Zm00001d032435	Zm00001d032435_T001	25.72	25.72	coding	456	190	1557
PB.3228.1	2	2043	6	novel_in_catalog	gene: Zm00001d006034	novel	7.31	61.89	coding	377	549	1679
PB.1376.2	1	1481	10	novel_in_catalog	gene: Zm00001d033969	novel	11.60	73.48	coding	374	119	1240
PB.10892.5	B73V4 ctg167	1620	1	intergenic	novelGene_7	novel	0.00	5924.92	coding	103	96	404
PB.1519.1	1	1238	5	novel_not_in_catalog	gene: Zm00001d034534	novel	25.62	25.62	coding	206	239	856
PB.8813.5	7	1528	5	full-splice_match	gene: Zm00001d021817	Zm00001d021817_T001	7.69	32.50	coding	431	181	1473
PB.3683.2	3	1632	8	novel_not_in_catalog	gene: Zm00001d039385	novel	2.17	3.74	coding	395	326	1510
PB.3683.1	3	2099	7	novel_not_in_catalog	gene: Zm00001d039385	novel	1.56	3.74	coding	280	1085	1924
PB.10892.3	B73V4 ctg167	3768	1	intergenic	novelGene_7	novel	5405.41	5924.92	coding	103	831	1139
PB.9415.2	8	1575	12	fusion	gene: Zm00001d010043_g	novel	0.00	40.59	coding	399	118	1314
PB.4186.9	3	3040	3	full-splice_match	gene: Zm00001d042185	Zm00001d042185_T005	8.83	93.73	coding	309	1592	2518
PB.9415.1	8	1680	12	fusion	gene: Zm00001d010043_g	novel	40.59	40.59	coding	399	118	1314

PB.1372.8	1	1994	8	novel_in_catalog	gene: Zm00001d033931	novel	6.61	1100.09	coding	247	854	1594
PB.4186.5	3	2868	4	full-splice_match	gene: Zm00001d042185	Zm00001d042185_T012	1.04	93.73	coding	309	1423	2349
PB.4186.6	3	2234	5	full-splice_match	gene: Zm00001d042185	Zm00001d042185_T013	70.21	93.73	coding	491	207	1679
PB.4186.7	3	2421	4	novel_in_catalog	gene: Zm00001d042185	novel	13.64	93.73	coding	363	785	1873
PB.4878.2	4	1227	2	full-splice_match	gene: Zm00001d049020	Zm00001d049020_T001	0.00	43.14	coding	364	38	1129
PB.1745.11	10	1290	8	novel_not_in_catalog	gene: Zm00001d023636	novel	27.92	66.48	coding	340	54	1073
PB.1745.12	10	1788	9	novel_not_in_catalog	gene: Zm00001d023636	novel	2.95	66.48	coding	501	71	1573
PB.4878.1	4	1293	2	full-splice_match	gene: Zm00001d049020	Zm00001d049020_T001	43.14	43.14	coding	364	42	1133
PB.9820.2	8	913	3	full-splice_match	gene: Zm00001d011880	Zm00001d011880_T002	36.45	48.74	coding	251	23	775
PB.3593.1	2	708	2	novel_not_in_catalog	gene: Zm00001d007822	novel	98.03	98.03	coding	76	285	512
PB.7933.1	6	3157	1	genic_intron	novelGene_256	novel	8.05	15.51	non_coding	NA	NA	NA
PB.9820.1	8	1022	3	full-splice_match	gene: Zm00001d011880	Zm00001d011880_T002	12.29	48.74	coding	251	12	764
PB.3252.1	2	1917	10	full-splice_match	gene: Zm00001d006128	Zm00001d006128_T002	21.41	21.41	coding	572	80	1795
PB.7795.1	6	718	3	full-splice_match	gene: Zm00001d037968	Zm00001d037968_T002	28.50	28.50	coding	178	45	578
PB.3552.1	2	980	9	full-splice_match	gene: Zm00001d007594	Zm00001d007594_T001	68.98	68.98	coding	229	73	759
PB.9840.1	8	1520	2	full-splice_match	gene: Zm00001d011971	Zm00001d011971_T001	22.65	51.90	coding	288	156	1019
PB.737.1	1	2108	1	full-splice_match	gene: Zm00001d030950	Zm00001d030950_T001	9.15	9.15	coding	530	360	1949

PB.9840.3	8	1409	2	full-splice_match	gene: Zm00001d011971	Zm00001d011971_T001	8.77	51.90	coding	288	320	1183
PB.4162.2	3	1178	6	incomplete-splice_match	gene: Zm00001d042055	Zm00001d042055_T002	22.26	22.26	coding	183	14	562
PB.10548.1	9	1651	2	full-splice_match	gene: Zm00001d047579	Zm00001d047579_T001	12.38	12.38	coding	347	164	1204
PB.7009.3	5	1977	5	antisense	novelGene_gene: Zm00001d017932_A	novel	29.00	81.66	non_coding	NA	NA	NA
PB.1487.2	1	2002	9	full-splice_match	gene: Zm00001d034424	Zm00001d034424_T003	5.50	5.50	coding	564	102	1793
PB.10312.3	9	1933	5	novel_in_catalog	gene: Zm00001d046263	novel	25.06	60.33	coding	372	554	1669
PB.2677.10	2	1760	10	novel_not_in_catalog	gene: Zm00001d002942	novel	6.72	67.49	coding	246	832	1569
PB.10312.1	9	1933	6	full-splice_match	gene: Zm00001d046263	Zm00001d046263_T001	24.24	60.33	coding	486	230	1687
PB.9409.1	8	405	1	full-splice_match	gene: Zm00001d010001	Zm00001d010001_T001	38.21	144.19	non_coding	NA	NA	NA
PB.10312.5	9	1577	6	full-splice_match	gene: Zm00001d046263	Zm00001d046263_T001	0.00	60.33	coding	486	108	1565
PB.10312.4	9	1645	6	full-splice_match	gene: Zm00001d046263	Zm00001d046263_T001	0.00	60.33	coding	486	107	1564
PB.2210.1	10	946	3	full-splice_match	gene: Zm00001d026137	Zm00001d026137_T001	193.56	215.63	coding	147	273	713
PB.2210.2	10	1693	1	genic	gene: Zm00001d026137	novel	13.86	215.63	coding	110	1125	1454
PB.2210.3	10	965	3	full-splice_match	gene: Zm00001d026137	Zm00001d026137_T001	8.21	215.63	coding	147	347	787
PB.4591.1	3	1071	4	novel_in_catalog	gene: Zm00001d043874	novel	19.66	96.12	coding	175	25	549
PB.4943.1	4	1291	9	full-splice_match	gene: Zm00001d049333	Zm00001d049333_T002	31.27	31.27	coding	262	61	846
PB.6866.1	5	2434	6	incomplete-splice_match	gene: Zm00001d017303	Zm00001d017303_T018	23.27	23.27	coding	613	36	1874

PB.4474.1	3	1978	3	genic	gene: Zm00001d043446	novel	3.21	3.21	coding	384	57	1208
PB.9867.1	8	1971	2	full-splice_match	gene: Zm00001d012098	Zm00001d012098_T003	64.33	100.63	coding	241	1010	1732
PB.4581.2	3	2300	2	novel_in_catalog	gene: Zm00001d043850	novel	0.98	227.52	coding	426	78	1355
PB.9867.3	8	1134	1	novel_in_catalog	gene: Zm00001d012098	novel	0.00	100.63	coding	32	272	367
PB.1921.1	10	1175	2	full-splice_match	gene: Zm00001d024738	Zm00001d024738_T001	11.52	11.52	coding	336	4	1011
PB.9024.2	7	1526	8	full-splice_match	gene: Zm00001d022542	Zm00001d022542_T006	76.43	144.00	coding	324	110	1081
PB.9024.3	7	1771	10	novel_in_catalog	gene: Zm00001d022542	novel	3.13	144.00	coding	312	393	1328
PB.9024.1	7	1773	10	full-splice_match	gene: Zm00001d022542	Zm00001d022542_T002	38.51	144.00	coding	333	324	1322
PB.9024.6	7	1745	10	full-splice_match	gene: Zm00001d022542	Zm00001d022542_T002	12.04	144.00	coding	333	385	1383
PB.9024.7	7	1489	9	full-splice_match	gene: Zm00001d022542	Zm00001d022542_T005	11.95	144.00	coding	326	177	1154
PB.9825.1	8	2233	5	incomplete-splice_match	gene: Zm00001d011898	Zm00001d011898_T001	25.77	30.64	coding	528	162	1745
PB.1814.3	10	1884	7	full-splice_match	gene: Zm00001d024059	Zm00001d024059_T002	0.00	16.55	coding	430	443	1732
PB.1814.1	10	2035	7	full-splice_match	gene: Zm00001d024059	Zm00001d024059_T002	16.55	16.55	coding	430	524	1813
PB.1375.10	1	2278	12	novel_not_in_catalog	gene: Zm00001d033948	novel	1.24	50.33	coding	115	153	497
PB.892.4	1	1565	2	novel_in_catalog	gene: Zm00001d031732	novel	20.71	218.99	coding	147	70	510
PB.9883.2	8	2852	1	intergenic	novelGene_278	novel	1.91	31.02	non_coding	NA	NA	NA
PB.9883.1	8	1755	5	intergenic	novelGene_278	novel	29.11	31.02	non_coding	NA	NA	NA

PB.3619.1	2	825	1	full-splice_match	gene: Zm00001d007910	Zm00001d007910_T001	5.17	5.17	coding	207	148	768
PB.9547.1	8	1598	1	intergenic	novelGene_197	novel	7.87	7.87	non_coding	NA	NA	NA
PB.5026.1	4	1680	2	novel_in_catalog	gene: Zm00001d049734	novel	38.12	38.12	coding	219	129	785
PB.5172.1	4	2084	1	full-splice_match	gene: Zm00001d050698	Zm00001d050698_T001	22.18	22.18	coding	398	738	1931
PB.1557.1	1	1616	1	genic	gene: Zm00001d034669	novel	24.65	30.88	coding	400	58	1257
PB.10798.1	9	1220	8	novel_in_catalog	gene: Zm00001d048445	novel	32.39	32.39	coding	305	107	1021
PB.2579.2	2	2047	6	full-splice_match	gene: Zm00001d002545	Zm00001d002545_T004	7.81	21.46	coding	257	889	1659
PB.2579.3	2	1947	6	full-splice_match	gene: Zm00001d002545	Zm00001d002545_T002	13.65	21.46	coding	440	241	1560
PB.8600.1	7	1548	5	novel_not_in_catalog	gene: Zm00001d020975	novel	22.03	65.37	coding	297	285	1175
PB.10506.8	9	2204	1	novel_in_catalog	gene: Zm00001d047424	novel	5.49	173.54	coding	194	1452	2033
PB.8600.3	7	935	4	incomplete-splice_match	gene: Zm00001d020975	Zm00001d020975_T005	39.35	65.37	non_coding	NA	NA	NA
PB.8600.2	7	2312	4	novel_not_in_catalog	gene: Zm00001d020975	novel	3.99	65.37	coding	238	285	998
PB.1039.1	1	2081	10	full-splice_match	gene: Zm00001d032427	Zm00001d032427_T001	13.12	15.13	coding	467	73	1473
PB.1039.2	1	1986	10	full-splice_match	gene: Zm00001d032427	Zm00001d032427_T001	2.00	15.13	coding	467	100	1500
PB.9525.1	8	2186	7	novel_in_catalog	gene: Zm00001d010579	novel	33.34	43.30	coding	541	165	1787
PB.9330.2	8	3374	14	full-splice_match	gene: Zm00001d009564	Zm00001d009564_T001	1.49	9.88	coding	1049	135	3281
PB.9330.1	8	3410	14	full-splice_match	gene: Zm00001d009564	Zm00001d009564_T001	8.39	9.88	coding	1049	58	3204

PB.7701.8	6	1168	6	incomplete-splice_match	gene: Zm00001d037609	Zm00001d037609_T006	10.05	34.44	coding	32	764	859
PB.287.1	1	477	1	full-splice_match	gene: Zm00001d028458	Zm00001d028458_T001	78.46	78.46	non_coding	NA	NA	NA
PB.2114.1	10	1165	1	full-splice_match	gene: Zm00001d025704	Zm00001d025704_T001	6.27	25.93	coding	218	310	963
PB.6555.2	5	2440	6	full-splice_match	gene: Zm00001d015521	Zm00001d015521_T001	110.11	133.04	coding	631	269	2161
PB.6555.1	5	3163	5	novel_in_catalog	gene: Zm00001d015521	novel	22.93	133.04	coding	595	1076	2860
PB.5387.1	4	2135	3	incomplete-splice_match	gene: Zm00001d051917	Zm00001d051917_T005	12.25	17.70	coding	563	177	1865
PB.5387.2	4	3528	5	novel_in_catalog	gene: Zm00001d051917	novel	1.11	17.70	coding	926	598	3375
PB.5387.3	4	3370	6	novel_not_in_catalog	gene: Zm00001d051917	novel	4.34	17.70	coding	926	439	3216
PB.5569.2	4	1393	5	novel_in_catalog	gene: Zm00001d052667	novel	1.59	5.64	coding	176	436	963
PB.6555.8	5	3031	5	novel_in_catalog	gene: Zm00001d015521	novel	0.00	133.04	coding	595	1012	2796
PB.6555.9	5	2361	6	full-splice_match	gene: Zm00001d015521	Zm00001d015521_T001	0.00	133.04	coding	631	267	2159
PB.1506.1	1	682	5	incomplete-splice_match	gene: Zm00001d034503	Zm00001d034503_T001	32.43	32.43	coding	104	78	389
PB.6842.1	5	338	1	incomplete-splice_match	gene: Zm00001d017206	Zm00001d017206_T001	69.47	69.47	non_coding	NA	NA	NA
PB.5400.1	4	1120	4	full-splice_match	gene: Zm00001d051995	Zm00001d051995_T001	150.88	150.88	coding	263	100	888
PB.2730.8	2	1908	3	full-splice_match	gene: Zm00001d003157	Zm00001d003157_T001	0.00	787.59	coding	400	577	1776
PB.2730.3	2	2918	2	novel_in_catalog	gene: Zm00001d003157	novel	2.08	787.59	coding	400	1429	2628
PB.2730.2	2	2166	2	novel_in_catalog	gene: Zm00001d003157	novel	8.54	787.59	coding	400	674	1873

PB.10107.1	9	385	1	incomplete-splice_match	gene: Zm00001d045113	Zm00001d045113_T002	41.36	41.36	non_coding	NA	NA	NA
PB.5569.1	4	1240	6	full-splice_match	gene: Zm00001d052667	Zm00001d052667_T002	4.05	5.64	coding	222	378	1043
PB.3354.3	2	927	5	full-splice_match	gene: Zm00001d006570	Zm00001d006570_T002	44.98	57.89	coding	201	100	702
PB.2730.4	2	3008	1	novel_in_catalog	gene: Zm00001d003157	novel	1.14	787.59	coding	400	1522	2721
PB.297.1	1	2223	9	full-splice_match	gene: Zm00001d028514	Zm00001d028514_T001	15.48	15.48	coding	600	112	1911
PB.5417.3	4	3064	3	full-splice_match	gene: Zm00001d052060	Zm00001d052060_T002	35.98	58.94	coding	855	269	2833
PB.959.1	1	1045	3	novel_not_in_catalog	gene: Zm00001d032073	novel	113.25	113.25	coding	194	264	845
PB.982.1	1	1793	2	genic	gene: Zm00001d032187	novel	43.42	43.42	coding	394	256	1437
PB.2248.2	10	1241	2	full-splice_match	gene: Zm00001d026285	Zm00001d026285_T001	13.79	15.10	coding	289	128	994
PB.10542.8	9	2426	9	full-splice_match	gene: Zm00001d047539	Zm00001d047539_T005	8.39	24.22	coding	684	120	2171
PB.1823.5	10	2087	7	novel_not_in_catalog	gene: Zm00001d024160	novel	0.68	115.49	coding	247	851	1591
PB.7902.2	6	1516	3	full-splice_match	gene: Zm00001d038325	Zm00001d038325_T001	61.47	104.15	coding	383	77	1225
PB.1223.1	1	1100	2	incomplete-splice_match	gene: Zm00001d033280	Zm00001d033280_T001	25.26	25.26	coding	264	2	793
PB.5770.1	4	1010	5	full-splice_match	gene: Zm00001d053636	Zm00001d053636_T001	235.95	826.59	coding	221	74	736
PB.414.5	1	956	1	full-splice_match	gene: Zm00001d029028	Zm00001d029028_T001	136.37	145.43	coding	188	93	656
PB.414.4	1	1085	1	full-splice_match	gene: Zm00001d029028	Zm00001d029028_T001	4.99	145.43	coding	188	89	652
PB.4800.5	4	451	2	full-splice_match	gene: Zm00001d048611	Zm00001d048611_T001	#######	31115.73	non_coding	NA	NA	NA

PB.4800.4	4	580	2	full-splice_match	gene: Zm00001d048611	Zm00001d048611_T001	4673.38	31115.73	non_coding	NA	NA	NA
PB.4800.3	4	517	2	novel_not_in_catalog	gene: Zm00001d048611	novel	85.57	31115.73	non_coding	NA	NA	NA
PB.4800.2	4	785	1	genic	gene: Zm00001d048611	novel	291.61	31115.73	non_coding	NA	NA	NA
PB.4800.1	4	643	2	full-splice_match	gene: Zm00001d048611	Zm00001d048611_T001	#######	31115.73	non_coding	NA	NA	NA
PB.4742.1	3	2665	14	novel_in_catalog	gene: Zm00001d044503	novel	1.86	26.50	coding	502	292	1797
PB.5920.3	5	1185	4	full-splice_match	gene: Zm00001d012852	Zm00001d012852_T006	17.71	23.34	coding	131	543	935
PB.4568.1	3	1473	1	antisense	novelGene_gene: Zm00001d043782_A	novel	42.12	42.12	non_coding	NA	NA	NA
PB.5920.1	5	2002	3	novel_in_catalog	gene: Zm00001d012852	novel	3.79	23.34	coding	265	376	1170
PB.7183.3	5	1423	4	incomplete-splice_match	gene: Zm00001d018515	Zm00001d018515_T003	0.00	48.94	non_coding	NA	NA	NA
PB.9777.1	8	2313	1	full-splice_match	gene: Zm00001d011721	Zm00001d011721_T001	36.02	36.02	coding	607	136	1956
PB.864.1	1	1649	2	antisense	novelGene_gene: Zm00001d031601_A	novel	1.54	1.87	coding	172	339	854
PB.7640.1	6	2229	11	full-splice_match	gene: Zm00001d037297	Zm00001d037297_T003	11.81	39.59	coding	614	104	1945
PB.5920.4	5	1095	1	novel_in_catalog	gene: Zm00001d012852	novel	1.84	23.34	non_coding	NA	NA	NA
PB.3157.3	2	1090	2	incomplete-splice_match	gene: Zm00001d005672	Zm00001d005672_T001	1.60	19.99	non_coding	NA	NA	NA
PB.9006.1	7	1096	1	full-splice_match	gene: Zm00001d022478	Zm00001d022478_T001	20.33	20.33	coding	314	35	976
PB.10211.1	9	2437	6	full-splice_match	gene: Zm00001d045550	Zm00001d045550_T001	2.71	2.71	coding	253	1590	2348
PB.3082.2	2	2147	13	full-splice_match	gene: Zm00001d005240	Zm00001d005240_T003	5.64	16.27	coding	581	91	1833

PB.7717.1	6	1068	2	intergenic	novelGene_213	novel	5.33	8.07	non_coding	NA	NA	NA
PB.1591.4	1	3101	1	full-splice_match	gene: Zm00001d034781	Zm00001d034781_T001	10.03	43.63	coding	852	312	2867
PB.7717.4	6	1175	4	intergenic	novelGene_213	novel	2.75	8.07	non_coding	NA	NA	NA
PB.9162.1	8	1314	3	incomplete-splice_match	gene: Zm00001d008646	Zm00001d008646_T001	21.28	66.58	coding	255	442	1206
PB.6051.1	5	1060	4	incomplete-splice_match	gene: Zm00001d013296	Zm00001d013296_T001	518.16	518.16	coding	205	166	780
PB.9162.2	8	1205	2	novel_in_catalog	gene: Zm00001d008646	novel	11.53	66.58	coding	296	197	1084
PB.3639.4	3	1375	5	full-splice_match	gene: Zm00001d039264	Zm00001d039264_T001	25.83	25.83	coding	340	250	1269
PB.10392.1	9	1160	1	full-splice_match	gene: Zm00001d046743	Zm00001d046743_T001	20.22	20.22	coding	327	57	1037
PB.3639.1	3	2329	6	fusion	gene: Zm00001d039263_g	novel	104.82	104.82	coding	340	250	1269
PB.3639.2	3	1465	5	full-splice_match	gene: Zm00001d039264	Zm00001d039264_T001	0.00	25.83	coding	340	212	1231
PB.4458.1	3	1835	3	full-splice_match	gene: Zm00001d043400	Zm00001d043400_T001	8.47	14.16	coding	524	62	1633
PB.10123.1	9	2155	6	novel_not_in_catalog	gene: Zm00001d045191	novel	57.85	57.85	coding	212	82	717
PB.5899.3	4	2173	15	full-splice_match	gene: Zm00001d054089	Zm00001d054089_T001	49.64	110.50	coding	605	186	2000
PB.1045.2	1	2006	14	novel_not_in_catalog	gene: Zm00001d032441	novel	7.20	55.74	coding	487	220	1680
PB.872.1	1	615	3	full-splice_match	gene: Zm00001d031636	Zm00001d031636_T001	48.24	48.24	coding	134	2	403
PB.2238.2	10	811	2	genic	gene: Zm00001d026259	novel	59.84	93.30	coding	148	61	504
PB.9488.1	8	1211	5	full-splice_match	gene: Zm00001d010411	Zm00001d010411_T003	166.49	263.77	coding	246	138	875

PB.9488.2	8	1622	4	novel_in_catalog	gene: Zm00001d010411	novel	4.74	263.77	coding	214	137	778
PB.9488.3	8	2175	3	novel_in_catalog	gene: Zm00001d010411	novel	3.71	263.77	coding	214	196	837
PB.9488.4	8	1146	5	full-splice_match	gene: Zm00001d010411	Zm00001d010411_T003	84.57	263.77	coding	246	177	914
PB.9488.5	8	967	4	novel_in_catalog	gene: Zm00001d010411	novel	4.26	263.77	coding	219	62	718
PB.515.1	1	1221	1	full-splice_match	gene: Zm00001d029501	Zm00001d029501_T001	0.11	0.11	coding	307	58	978
PB.3121.1	2	1612	2	full-splice_match	gene: Zm00001d005456	Zm00001d005456_T001	5.76	5.76	coding	470	62	1471
PB.8761.1	7	1531	7	full-splice_match	gene: Zm00001d021656	Zm00001d021656_T001	23.63	23.63	coding	381	78	1220
PB.5082.1	4	731	1	full-splice_match	gene: Zm00001d050100	Zm00001d050100_T001	1199.54	1199.54	coding	150	80	529
PB.9383.9	8	2139	2	novel_in_catalog	gene: Zm00001d009858	novel	9.73	534.95	coding	312	63	998
PB.6037.1	5	2974	1	antisense	novelGene_gene: Zm00001d013256_A	novel	17.70	17.70	non_coding	NA	NA	NA
PB.6604.1	5	2271	11	full-splice_match	gene: Zm00001d015971	Zm00001d015971_T004	13.33	13.33	coding	618	230	2083
PB.3490.1	2	503	1	full-splice_match	gene: Zm00001d007206	Zm00001d007206_T001	9.74	9.74	coding	82	153	398
PB.2899.3	2	2569	6	full-splice_match	gene: Zm00001d003941	Zm00001d003941_T005	10.46	84.92	coding	250	127	876
PB.5346.1	4	2026	2	full-splice_match	gene: Zm00001d051742	Zm00001d051742_T001	82.36	125.51	coding	535	60	1664
PB.5828.2	4	549	3	novel_in_catalog	gene: Zm00001d053863	novel	1.17	2468.45	coding	102	95	400
PB.5584.1	4	4589	2	intergenic	novelGene_317	novel	7.02	9.42	coding	215	1841	2485
PB.5584.2	4	1036	2	intergenic	novelGene_317	novel	2.40	9.42	coding	200	171	770

PB.10394.1	9	1144	1	full-splice_match	gene: Zm00001d046746	Zm00001d046746_T001	17.69	17.69	coding	312	75	1010
PB.7984.1	6	1440	6	full-splice_match	gene: Zm00001d038646	Zm00001d038646_T002	82.45	87.75	coding	309	278	1204
PB.9027.2	7	1606	1	genic	gene: Zm00001d022549	novel	2.46	4.66	coding	406	89	1306
PB.4279.1	3	2028	3	incomplete-splice_match	gene: Zm00001d042627	Zm00001d042627_T002	17.93	54.98	coding	371	282	1394
PB.2899.1	2	2345	7	full-splice_match	gene: Zm00001d003941	Zm00001d003941_T004	74.46	84.92	coding	668	183	2186
PB.9713.1	8	1268	4	novel_not_in_catalog	gene: Zm00001d011446	novel	24.82	24.82	coding	285	135	989
PB.4279.2	3	2552	5	full-splice_match	gene: Zm00001d042627	Zm00001d042627_T007	37.05	54.98	coding	634	377	2278
PB.5469.1	4	1092	11	full-splice_match	gene: Zm00001d052270	Zm00001d052270_T005	36.32	36.32	coding	246	121	858
PB.3141.1	2	3071	15	full-splice_match	gene: Zm00001d005573	Zm00001d005573_T016	23.98	23.98	coding	885	127	2781
PB.5372.1	4	1646	8	full-splice_match	gene: Zm00001d051881	Zm00001d051881_T001	159.47	159.47	coding	344	182	1213
PB.5453.1	4	1912	9	full-splice_match	gene: Zm00001d052227	Zm00001d052227_T001	6.32	6.32	coding	356	179	1246
PB.3470.1	2	890	1	full-splice_match	gene: Zm00001d007097	Zm00001d007097_T001	540.31	540.31	coding	159	101	577
PB.2296.1	10	4284	9	full-splice_match	gene: Zm00001d026426	Zm00001d026426_T013	53.32	53.32	coding	1266	252	4049
PB.1157.12	1	1961	2	novel_not_in_catalog	gene: Zm00001d032897	novel	5.08	297.96	coding	186	295	852
PB.9464.1	8	1768	3	novel_not_in_catalog	gene: Zm00001d010265	novel	18.98	18.98	coding	498	133	1626
PB.8680.1	7	1385	4	full-splice_match	gene: Zm00001d021363	Zm00001d021363_T001	47.01	47.01	coding	292	216	1091
PB.6122.1	5	1904	2	genic	gene: Zm00001d013552	novel	10.93	10.93	coding	180	962	1501

PB.8680.3	7	1183	4	full-splice_match	gene: Zm00001d021363	Zm00001d021363_T001	0.00	47.01	coding	292	149	1024
PB.5366.2	4	2956	7	full-splice_match	gene: Zm00001d051871	Zm00001d051871_T003	5.66	13.24	coding	760	302	2581
PB.9971.2	8	828	3	full-splice_match	gene: Zm00001d012561	Zm00001d012561_T001	320.59	320.59	coding	134	99	500
PB.2596.1	2	1440	6	novel_in_catalog	gene: Zm00001d002600	novel	22.81	22.81	coding	303	104	1012
PB.7002.2	5	1817	5	full-splice_match	gene: Zm00001d017890	Zm00001d017890_T003	66.13	66.13	coding	446	93	1430
PB.9694.1	8	1741	12	full-splice_match	gene: Zm00001d011355	Zm00001d011355_T005	42.66	42.66	coding	444	94	1425
PB.9694.3	8	1418	12	full-splice_match	gene: Zm00001d011355	Zm00001d011355_T005	0.00	42.66	coding	444	14	1345
PB.9694.2	8	1694	12	full-splice_match	gene: Zm00001d011355	Zm00001d011355_T005	0.00	42.66	coding	444	191	1522
PB.3162.2	2	1419	3	full-splice_match	gene: Zm00001d005692	Zm00001d005692_T001	22.47	25.58	coding	283	444	1292
PB.3162.1	2	1333	4	novel_not_in_catalog	gene: Zm00001d005692	novel	3.12	25.58	coding	283	357	1205
PB.2750.1	2	3048	4	novel_not_in_catalog	gene: Zm00001d003208	novel	3.98	3.98	coding	239	2174	2890
PB.5859.2	4	1621	4	novel_not_in_catalog	gene: Zm00001d053972	novel	18.46	20.51	coding	338	215	1228
PB.3587.3	2	2748	1	full-splice_match	gene: Zm00001d007789	Zm00001d007789_T001	1.61	67.38	coding	866	27	2624
PB.2127.1	10	1182	2	full-splice_match	gene: Zm00001d025753	Zm00001d025753_T001	59.51	103.28	coding	279	60	896
PB.678.2	1	1443	6	novel_not_in_catalog	gene: Zm00001d030507	novel	9.53	52.08	coding	290	394	1263
PB.3501.3	2	1908	3	novel_in_catalog	gene: Zm00001d007270	novel	3.04	101.91	non_coding	NA	NA	NA
PB.9847.2	8	1224	9	full-splice_match	gene: Zm00001d012003	Zm00001d012003_T001	0.00	52.90	coding	281	210	1052

PB.5859.1	4	2088	3	novel_not_in_catalog	gene: Zm00001d053972	novel	2.05	20.51	coding	338	667	1680
PB.4205.1	3	1342	6	full-splice_match	gene: Zm00001d042284	Zm00001d042284_T002	39.05	39.05	coding	225	322	996
PB.5238.2	4	1758	1	incomplete-splice_match	gene: Zm00001d051163	Zm00001d051163_T001	0.00	156.63	coding	557	55	1725
PB.4330.1	3	2453	5	novel_in_catalog	gene: Zm00001d042848	novel	4.42	38.75	coding	362	1108	2193
PB.4330.2	3	2303	6	full-splice_match	gene: Zm00001d042848	Zm00001d042848_T003	12.45	38.75	coding	641	123	2045
PB.5346.2	4	1876	2	full-splice_match	gene: Zm00001d051742	Zm00001d051742_T001	43.15	125.51	coding	535	79	1683
PB.4293.1	3	988	2	full-splice_match	gene: Zm00001d042665	Zm00001d042665_T001	29.72	29.72	coding	262	83	868
PB.448.2	1	1732	5	full-splice_match	gene: Zm00001d029189	Zm00001d029189_T001	5.47	12.06	coding	328	210	1193
PB.4265.2	3	2803	4	novel_in_catalog	gene: Zm00001d042569	novel	1.41	53.18	coding	540	1067	2686
PB.2499.2	2	3316	2	genic	gene: Zm00001d002175	novel	39.09	52.70	coding	897	384	3074
PB.2499.1	2	3498	2	genic	gene: Zm00001d002175	novel	13.61	52.70	coding	897	455	3145
PB.9564.2	8	2031	5	full-splice_match	gene: Zm00001d010733	Zm00001d010733_T005	10.05	568.49	coding	114	1290	1631
PB.6734.1	5	741	2	genic	gene: Zm00001d016743	novel	11.92	11.92	coding	150	127	576
PB.4745.7	3	2055	3	novel_in_catalog	gene: Zm00001d044516	novel	4.10	381.23	coding	330	123	1112
PB.4273.2	3	2749	5	full-splice_match	gene: Zm00001d042608	Zm00001d042608_T003	0.00	7.13	coding	800	119	2518
PB.2952.1	2	1241	1	full-splice_match	gene: Zm00001d004451	Zm00001d004451_T001	58.18	58.18	coding	337	67	1077
PB.9232.1	8	1504	5	full-splice_match	gene: Zm00001d008976	Zm00001d008976_T002	2.07	10.03	coding	352	99	1154

PB.6047.4	5	2946	21	full-splice_match	gene: Zm00001d013274	Zm00001d013274_T028	16.19	16.19	coding	821	179	2641
PB.448.1	1	1939	5	full-splice_match	gene: Zm00001d029189	Zm00001d029189_T001	6.60	12.06	coding	328	227	1210
PB.1461.2	1	2846	5	full-splice_match	gene: Zm00001d034344	Zm00001d034344_T001	21.73	23.36	coding	770	102	2411
PB.10566.1	9	1046	1	novel_in_catalog	gene: Zm00001d047639	novel	56.11	1341.95	coding	148	219	662
PB.10566.2	9	1290	1	novel_in_catalog	gene: Zm00001d047639	novel	598.09	1341.95	coding	327	121	1101
PB.1461.1	1	3027	4	novel_in_catalog	gene: Zm00001d034344	novel	1.62	23.36	coding	769	103	2409
PB.9106.1	8	918	4	full-splice_match	gene: Zm00001d008395	Zm00001d008395_T001	171.70	171.70	coding	221	126	788
PB.9564.6	8	1719	6	novel_in_catalog	gene: Zm00001d010733	novel	1.12	568.49	coding	114	1125	1466
PB.3699.2	3	815	5	intergenic	novelGene_22	novel	23.27	163.89	coding	182	79	624
PB.3699.1	3	947	5	intergenic	novelGene_22	novel	140.61	163.89	coding	182	104	649
PB.3338.13	2	2360	4	full-splice_match	gene: Zm00001d006480	Zm00001d006480_T001	0.00	96.23	coding	658	186	2159
PB.10913.1	B73V4 ctg44	1634	1	full-splice_match	gene: Zm00001d000237	Zm00001d000237_T001	59.43	59.43	coding	462	70	1455
PB.10265.3	9	1978	10	full-splice_match	gene: Zm00001d045938	Zm00001d045938_T005	11.95	12.67	coding	560	50	1729
PB.10265.4	9	1876	10	full-splice_match	gene: Zm00001d045938	Zm00001d045938_T005	0.71	12.67	coding	560	67	1746
PB.3338.10	2	2659	4	full-splice_match	gene: Zm00001d006480	Zm00001d006480_T001	0.00	96.23	coding	658	182	2155
PB.3839.1	3	2890	1	genic	gene: Zm00001d040188	novel	28.88	28.88	coding	316	1807	2754
PB.1491.1	1	2173	1	intergenic	novelGene_379	novel	3.47	3.47	non_coding	NA	NA	NA

PB.5746.8	4	1997	6	full-splice_match	gene: Zm00001d053564	Zm00001d053564_T003	0.00	79.10	coding	80	518	757
PB.3338.11	2	2371	4	full-splice_match	gene: Zm00001d006480	Zm00001d006480_T001	0.00	96.23	coding	658	59	2032
PB.8695.7	7	2373	10	novel_in_catalog	gene: Zm00001d021413	novel	11.10	54.32	non_coding	NA	NA	NA
PB.7617.1	6	1678	5	full-splice_match	gene: Zm00001d037228	Zm00001d037228_T001	7.48	7.48	coding	294	402	1283
PB.5746.3	4	1670	1	incomplete-splice_match	gene: Zm00001d053564	Zm00001d053564_T006	27.84	79.10	coding	379	206	1342
PB.5746.2	4	2464	7	full-splice_match	gene: Zm00001d053564	Zm00001d053564_T002	0.00	79.10	coding	692	32	2107
PB.3956.2	3	1272	7	novel_not_in_catalog	gene: Zm00001d040798	novel	21.80	23.32	coding	259	156	932
PB.8695.2	7	1061	6	novel_not_in_catalog	gene: Zm00001d021413	novel	43.22	54.32	coding	118	226	579
PB.5746.7	4	2802	2	novel_in_catalog	gene: Zm00001d053564	novel	0.87	79.10	coding	379	1499	2635
PB.3956.1	3	2007	4	novel_in_catalog	gene: Zm00001d040798	novel	1.52	23.32	coding	172	1127	1642
PB.4625.1	3	2487	21	full-splice_match	gene: Zm00001d044024	Zm00001d044024_T002	3.21	40.56	coding	619	169	2025
PB.7975.1	6	2131	2	novel_in_catalog	gene: Zm00001d038595	novel	28.76	28.76	coding	507	346	1866
PB.2993.1	2	2254	4	full-splice_match	gene: Zm00001d004706	Zm00001d004706_T001	34.42	34.42	coding	647	138	2078
PB.10078.4	9	2378	5	full-splice_match	gene: Zm00001d044976	Zm00001d044976_T001	13.68	13.68	coding	724	169	2340
PB.4740.2	3	2638	3	novel_in_catalog	gene: Zm00001d044500	novel	7.30	11.76	coding	742	302	2527
PB.5354.3	4	2295	16	full-splice_match	gene: Zm00001d051809	Zm00001d051809_T001	0.00	31.98	coding	671	63	2075
PB.289.5	1	2261	3	incomplete-splice_match	gene: Zm00001d028472	Zm00001d028472_T006	10.09	35.72	coding	344	201	1232

PB.5285.1	4	2543	6	full-splice_match	gene: Zm00001d051442	Zm00001d051442_T002	11.87	11.87	coding	647	75	2015
PB.8510.2	7	1960	4	novel_in_catalog	gene: Zm00001d020560	novel	0.89	9.33	coding	296	183	1070
PB.289.1	1	3054	5	full-splice_match	gene: Zm00001d028472	Zm00001d028472_T007	4.91	35.72	coding	666	683	2680
PB.289.3	1	2734	6	full-splice_match	gene: Zm00001d028472	Zm00001d028472_T004	19.91	35.72	coding	666	359	2356
PB.306.3	1	1929	14	novel_in_catalog	gene: Zm00001d028569	novel	2.37	50.54	coding	392	218	1393
PB.306.2	1	1862	15	full-splice_match	gene: Zm00001d028569	Zm00001d028569_T005	48.17	50.54	coding	441	206	1528
PB.9945.3	8	3078	7	novel_in_catalog	gene: Zm00001d012452	novel	1.15	15.31	coding	540	255	1874
PB.289.8	1	2904	4	novel_in_catalog	gene: Zm00001d028472	novel	0.82	35.72	coding	532	489	2084
PB.6186.2	5	1926	14	full-splice_match	gene: Zm00001d013865	Zm00001d013865_T005	8.20	29.53	coding	477	78	1508
PB.10422.1	9	2401	4	full-splice_match	gene: Zm00001d046938	Zm00001d046938_T001	10.44	10.44	coding	660	143	2122
PB.6186.1	5	1961	14	full-splice_match	gene: Zm00001d013865	Zm00001d013865_T005	21.33	29.53	coding	437	119	1429
PB.8981.1	7	2494	5	incomplete-splice_match	gene: Zm00001d022417	Zm00001d022417_T016	25.46	25.46	coding	591	148	1920
PB.943.1	1	818	2	full-splice_match	gene: Zm00001d031979	Zm00001d031979_T001	6.86	98.33	coding	135	29	433
PB.8314.1	7	911	3	full-splice_match	gene: Zm00001d019459	Zm00001d019459_T001	50.73	50.73	coding	166	85	582
PB.7321.1	6	2918	20	full-splice_match	gene: Zm00001d035574	Zm00001d035574_T002	19.78	19.78	coding	818	217	2670
PB.5841.3	4	2371	13	novel_not_in_catalog	gene: Zm00001d053911	novel	4.51	4.51	coding	679	243	2279
PB.10445.1	9	2105	10	full-splice_match	gene: Zm00001d047053	Zm00001d047053_T012	19.42	19.42	coding	505	225	1739

PB.9761.1	8	1162	1	full-splice_match	gene: Zm00001d011650	Zm00001d011650_T001	172.60	172.60	coding	240	168	887
PB.9761.2	8	936	1	full-splice_match	gene: Zm00001d011650	Zm00001d011650_T001	0.00	172.60	coding	240	68	787
PB.4534.4	3	1448	10	full-splice_match	gene: Zm00001d043669	Zm00001d043669_T008	3.63	102.56	coding	189	881	1447
PB.602.2	1	978	1	genic	gene: Zm00001d029988	novel	6.85	16.97	coding	269	8	814
PB.5299.1	4	1972	6	full-splice_match	gene: Zm00001d051529	Zm00001d051529_T003	23.56	23.56	coding	575	69	1793
PB.602.1	1	1573	3	novel_in_catalog	gene: Zm00001d029988	novel	10.12	16.97	coding	326	277	1254
PB.5940.1	5	2738	10	full-splice_match	gene: Zm00001d012913	Zm00001d012913_T008	36.64	38.12	coding	721	238	2400
PB.5940.3	5	2504	10	full-splice_match	gene: Zm00001d012913	Zm00001d012913_T008	0.00	38.12	coding	721	242	2404
PB.5940.2	5	2641	10	full-splice_match	gene: Zm00001d012913	Zm00001d012913_T008	1.47	38.12	coding	721	269	2431
PB.5940.5	5	2392	10	full-splice_match	gene: Zm00001d012913	Zm00001d012913_T008	0.00	38.12	coding	711	258	2390
PB.334.1	1	1863	1	full-splice_match	gene: Zm00001d028667	Zm00001d028667_T001	35.21	170.64	coding	182	133	678
PB.10948.6	B73V4 ctg98	3011	11	novel_in_catalog	gene: Zm00001d000228	novel	23.48	46.40	coding	313	389	1327
PB.334.3	1	839	1	full-splice_match	gene: Zm00001d028667	Zm00001d028667_T001	0.00	170.64	coding	182	133	678
PB.334.4	1	707	1	full-splice_match	gene: Zm00001d028667	Zm00001d028667_T001	11.36	170.64	coding	182	135	680
PB.4534.1	3	1614	12	full-splice_match	gene: Zm00001d043669	Zm00001d043669_T004	60.66	102.56	coding	391	1	1173
PB.9087.9	8	1063	4	incomplete-splice_match	gene: Zm00001d008303	Zm00001d008303_T005	2.43	56.61	non_coding	NA	NA	NA
PB.9087.8	8	1010	6	incomplete-splice_match	gene: Zm00001d008303	Zm00001d008303_T005	0.00	56.61	coding	31	210	302

PB.7633.2	6	943	5	full-splice_match	gene: Zm00001d037267	Zm00001d037267_T002	5.25	43.54	coding	182	187	732
PB.9087.6	8	1001	4	incomplete-splice_match	gene: Zm00001d008303	Zm00001d008303_T005	21.52	56.61	non_coding	NA	NA	NA
PB.9087.4	8	1710	9	novel_in_catalog	gene: Zm00001d008303	novel	23.14	56.61	coding	117	668	1018
PB.10948.9	B73V4 ctg98	2594	13	full-splice_match	gene: Zm00001d000228	Zm00001d000228_T077	22.92	46.40	coding	313	309	1247
PB.9087.1	8	3761	10	novel_in_catalog	gene: Zm00001d008303	novel	9.50	56.61	non_coding	NA	NA	NA
PB.10288.2	9	1557	4	novel_in_catalog	gene: Zm00001d046136	novel	2.48	44.56	coding	338	101	1114
PB.9352.2	8	2276	2	intergenic	novelGene_138	novel	4.45	6.76	coding	108	1080	1403
PB.9352.3	8	2013	1	intergenic	novelGene_138	novel	1.02	6.76	coding	108	864	1187
PB.9352.1	8	2216	2	intergenic	novelGene_138	novel	1.29	6.76	coding	108	900	1223
PB.7876.1	6	2208	1	intergenic	novelGene_249	novel	4.47	4.47	coding	121	135	497
PB.4497.1	3	1657	2	full-splice_match	gene: Zm00001d043520	Zm00001d043520_T001	10.75	10.75	coding	447	91	1431
PB.4390.1	3	2281	4	full-splice_match	gene: Zm00001d043131	Zm00001d043131_T001	9.85	9.85	coding	559	310	1986
PB.1778.1	10	1337	4	novel_not_in_catalog	gene: Zm00001d023853	novel	25.90	25.90	coding	303	29	937
PB.990.1	1	1754	1	genic	gene: Zm00001d032222	novel	6.49	9.39	coding	496	88	1575
PB.2665.1	2	3487	1	full-splice_match	gene: Zm00001d002882	Zm00001d002882_T001	3.16	38.17	coding	229	408	1094
PB.2665.2	2	1490	1	full-splice_match	gene: Zm00001d002882	Zm00001d002882_T001	35.00	38.17	coding	409	56	1282
PB.8934.4	7	2040	1	full-splice_match	gene: Zm00001d022247	Zm00001d022247_T001	0.00	65.62	coding	581	106	1848

PB.5956.1	5	1015	9	intergenic	novelGene_17	novel	304.81	304.81	coding	214	236	877
PB.261.1	1	1724	1	full-splice_match	gene: Zm00001d028374	Zm00001d028374_T001	1.00	1.00	coding	447	139	1479
PB.8334.1	7	498	3	novel_not_in_catalog	gene: Zm00001d019553	novel	837.89	837.89	non_coding	NA	NA	NA
PB.1718.1	10	2260	1	full-splice_match	gene: Zm00001d023524	Zm00001d023524_T001	11.46	11.46	coding	584	289	2040
PB.6693.4	5	1364	3	full-splice_match	gene: Zm00001d016531	Zm00001d016531_T001	0.00	62.84	coding	274	518	1339
PB.8691.1	7	3015	4	full-splice_match	gene: Zm00001d021400	Zm00001d021400_T001	34.77	38.07	coding	687	407	2467
PB.5291.1	4	1826	5	novel_not_in_catalog	gene: Zm00001d051480	novel	10.01	13.00	coding	449	169	1515
PB.5291.2	4	1904	5	novel_not_in_catalog	gene: Zm00001d051480	novel	2.98	13.00	coding	348	589	1632
PB.8934.2	7	2133	1	full-splice_match	gene: Zm00001d022247	Zm00001d022247_T001	58.10	65.62	coding	581	71	1813
PB.1784.3	10	1345	12	full-splice_match	gene: Zm00001d023874	Zm00001d023874_T001	89.97	153.89	coding	293	97	975
PB.2298.1	10	1430	1	full-splice_match	gene: Zm00001d026435	Zm00001d026435_T001	11.41	11.41	coding	441	15	1337
PB.1784.1	10	1477	12	full-splice_match	gene: Zm00001d023874	Zm00001d023874_T001	63.93	153.89	coding	293	100	978
PB.3000.1	2	1569	8	full-splice_match	gene: Zm00001d004734	Zm00001d004734_T001	38.57	38.57	coding	415	82	1326
PB.8297.1	7	2277	2	genic	gene: Zm00001d019297	novel	3.40	4.41	coding	486	48	1505
PB.831.3	1	2225	5	novel_in_catalog	gene: Zm00001d031430	novel	13.32	58.80	coding	354	909	1970
PB.8297.3	7	2317	1	full-splice_match	gene: Zm00001d019297	Zm00001d019297_T001	0.00	4.41	coding	751	65	2317
PB.8297.2	7	2999	1	full-splice_match	gene: Zm00001d019297	Zm00001d019297_T001	1.01	4.41	coding	879	65	2701

PB.4727.1	3	1625	13	full-splice_match	gene: Zm00001d044468	Zm00001d044468_T004	6.09	6.09	coding	435	134	1438
PB.2038.1	10	2045	5	novel_in_catalog	gene: Zm00001d025263	novel	2.62	2.62	coding	174	204	725
PB.831.4	1	2698	7	novel_in_catalog	gene: Zm00001d031430	novel	4.33	58.80	coding	548	257	1900
PB.142.1	1	1198	7	novel_not_in_catalog	gene: Zm00001d027850	novel	3.43	3.43	coding	158	271	744
PB.3060.2	2	4287	1	genic	gene: Zm00001d005110	novel	1.93	20.53	coding	841	104	2626
PB.3060.3	2	2399	2	novel_in_catalog	gene: Zm00001d005110	novel	14.22	20.53	coding	266	37	834
PB.3060.4	2	893	1	genic	gene: Zm00001d005110	novel	4.38	20.53	coding	85	173	427
PB.1350.1	1	1350	2	full-splice_match	gene: Zm00001d033840	Zm00001d033840_T001	21.37	21.37	non_coding	NA	NA	NA
PB.5486.1	4	595	2	full-splice_match	gene: Zm00001d052333	Zm00001d052333_T002	190.49	190.49	coding	109	82	408
PB.1297.2	1	2877	23	novel_in_catalog	gene: Zm00001d033597	novel	21.44	23.44	coding	798	98	2491
PB.2399.1	2	2721	15	full-splice_match	gene: Zm00001d001804	Zm00001d001804_T005	5.24	5.24	coding	594	799	2580
PB.6714.3	5	1595	1	full-splice_match	gene: Zm00001d016653	Zm00001d016653_T001	0.00	39.54	coding	416	88	1335
PB.6714.2	5	1739	1	full-splice_match	gene: Zm00001d016653	Zm00001d016653_T001	39.54	39.54	coding	416	148	1395
PB.3827.1	3	2067	2	genic	gene: Zm00001d040118	novel	17.90	17.90	coding	247	1101	1841
PB.848.2	1	921	2	full-splice_match	gene: Zm00001d031510	Zm00001d031510_T001	0.00	21.39	coding	252	130	885
PB.7837.1	6	1746	1	novel_in_catalog	gene: Zm00001d038121	novel	6.70	6.70	coding	485	48	1502
PB.10467.4	9	1710	6	novel_in_catalog	gene: Zm00001d047208	novel	12.03	54.45	coding	346	68	1105

PB.613.1	1	671	3	full-splice_match	gene: Zm00001d030079	Zm00001d030079_T001	9.81	65.90	coding	91	77	349
PB.613.2	1	540	3	full-splice_match	gene: Zm00001d030079	Zm00001d030079_T001	56.09	65.90	coding	91	67	339
PB.10467.7	9	1381	7	full-splice_match	gene: Zm00001d047208	Zm00001d047208_T001	0.00	54.45	coding	388	34	1197
PB.3966.3	3	1115	10	full-splice_match	gene: Zm00001d040941	Zm00001d040941_T002	16.20	31.27	coding	294	34	915
PB.3966.2	3	1160	10	full-splice_match	gene: Zm00001d040941	Zm00001d040941_T002	15.07	31.27	coding	152	404	859
PB.10467.2	9	854	4	full-splice_match	gene: Zm00001d047207	Zm00001d047207_T001	15.74	15.74	coding	180	73	612
PB.9529.1	8	2452	1	full-splice_match	gene: Zm00001d010602	Zm00001d010602_T001	44.48	44.48	coding	662	231	2216
PB.10467.8	9	1530	6	novel_in_catalog	gene: Zm00001d047208	novel	0.00	54.45	coding	346	68	1105
PB.10467.9	9	1277	6	novel_in_catalog	gene: Zm00001d047208	novel	0.00	54.45	coding	346	61	1098
PB.8038.1	6	1585	11	full-splice_match	gene: Zm00001d038851	Zm00001d038851_T001	72.23	231.18	coding	370	159	1268
PB.9840.4	8	1125	2	full-splice_match	gene: Zm00001d011971	Zm00001d011971_T001	20.47	51.90	coding	288	154	1017
PB.5865.4	4	2322	5	novel_not_in_catalog	gene: Zm00001d053982	novel	0.00	27.39	coding	645	131	2065
PB.7160.5	5	2127	14	full-splice_match	gene: Zm00001d018440	Zm00001d018440_T002	0.00	67.73	coding	468	453	1856
PB.3711.1	3	1599	1	antisense	novelGene_gene: Zm00001d039492_A	novel	78.78	78.78	coding	24	311	382
PB.10490.4	9	922	2	full-splice_match	gene: Zm00001d047340	Zm00001d047340_T003	0.00	159.64	coding	238	67	780
PB.7160.1	5	2205	14	full-splice_match	gene: Zm00001d018440	Zm00001d018440_T002	27.05	67.73	coding	468	459	1862
PB.5559.1	4	1895	8	full-splice_match	gene: Zm00001d052622	Zm00001d052622_T001	5.62	5.62	coding	546	82	1719

PB.7160.3	5	2331	13	novel_in_catalog	gene: Zm00001d018440	novel	3.11	67.73	coding	389	829	1995
PB.3377.2	2	3220	4	novel_not_in_catalog	gene: Zm00001d006679	novel	5.20	147.77	coding	243	114	842
PB.6543.13	5	1960	6	full-splice_match	gene: Zm00001d015459	Zm00001d015459_T002	0.00	229.13	coding	555	150	1814
PB.6543.11	5	2240	5	novel_in_catalog	gene: Zm00001d015459	novel	5.70	229.13	coding	483	151	1599
PB.6543.10	5	2502	5	novel_in_catalog	gene: Zm00001d015459	novel	2.47	229.13	coding	330	1243	2232
PB.5865.1	4	2731	5	novel_not_in_catalog	gene: Zm00001d053982	novel	1.45	27.39	coding	645	131	2065
PB.8691.6	7	2339	3	novel_in_catalog	gene: Zm00001d021400	novel	1.31	38.07	coding	687	111	2171
PB.10351.1	9	1418	3	full-splice_match	gene: Zm00001d046500	Zm00001d046500_T001	11.98	11.98	coding	305	195	1109
PB.4743.5	3	567	2	full-splice_match	gene: Zm00001d044504	Zm00001d044504_T001	50.12	105.91	coding	153	30	488
PB.4743.4	3	1598	2	full-splice_match	gene: Zm00001d044504	Zm00001d044504_T001	6.63	105.91	coding	381	249	1391
PB.9243.1	8	2578	9	full-splice_match	gene: Zm00001d009014	Zm00001d009014_T001	47.75	47.75	coding	705	145	2259
PB.4743.2	3	2019	2	full-splice_match	gene: Zm00001d044504	Zm00001d044504_T001	40.70	105.91	coding	482	249	1694
PB.9535.2	8	1333	2	genic	gene: Zm00001d010635	novel	1.36	26.13	coding	312	169	1104
PB.3092.1	2	2266	2	genic	gene: Zm00001d005275	novel	12.12	12.12	coding	578	140	1873
PB.10869.1	B73V4 ctg150	823	2	incomplete-splice_match	gene: Zm00001d000023	Zm00001d000023_T006	25.76	25.76	coding	126	56	433
PB.8103.1	6	1111	8	full-splice_match	gene: Zm00001d039091	Zm00001d039091_T001	208.79	583.87	coding	221	95	757
PB.7276.2	6	585	4	full-splice_match	gene: Zm00001d035309	Zm00001d035309_T001	0.00	43.57	non_coding	NA	NA	NA

PB.7276.1	6	786	4	full-splice_match	gene: Zm00001d035309	Zm00001d035309_T001	43.57	43.57	coding	116	412	759
PB.8103.2	6	982	8	full-splice_match	gene: Zm00001d039091	Zm00001d039091_T001	375.09	583.87	coding	221	98	760
PB.3837.1	3	1038	3	full-splice_match	gene: Zm00001d040178	Zm00001d040178_T001	188.00	188.00	coding	201	213	815
PB.3525.3	2	1785	3	full-splice_match	gene: Zm00001d007434	Zm00001d007434_T010	10.26	67.30	coding	248	879	1622
PB.3525.4	2	1203	4	full-splice_match	gene: Zm00001d007434	Zm00001d007434_T014	41.56	67.30	coding	280	206	1045
PB.1418.1	1	1729	10	full-splice_match	gene: Zm00001d034125	Zm00001d034125_T001	53.35	182.98	coding	383	118	1266
PB.3525.6	2	1359	2	novel_in_catalog	gene: Zm00001d007434	novel	0.00	67.30	coding	160	879	1358
PB.5689.2	4	1783	8	full-splice_match	gene: Zm00001d053232	Zm00001d053232_T002	0.00	132.90	coding	491	130	1602
PB.3390.1	2	765	2	intergenic	novelGene_334	novel	444.37	444.37	coding	15	404	448
PB.7355.1	6	2017	13	incomplete-splice_match	gene: Zm00001d035757	Zm00001d035757_T002	9.96	22.10	coding	580	93	1832
PB.4670.3	3	1936	12	novel_in_catalog	gene: Zm00001d044213	novel	25.57	33.65	coding	400	439	1638
PB.8371.2	7	2314	9	full-splice_match	gene: Zm00001d019824	Zm00001d019824_T013	254.37	453.10	coding	656	148	2115
PB.5689.1	4	1901	8	full-splice_match	gene: Zm00001d053232	Zm00001d053232_T002	132.90	132.90	coding	491	129	1601
PB.4595.1	3	2697	13	full-splice_match	gene: Zm00001d043890	Zm00001d043890_T002	9.96	18.96	coding	660	390	2369
PB.3958.1	3	1823	8	novel_in_catalog	gene: Zm00001d040804	novel	1.65	14.17	coding	337	2	1012
PB.3958.2	3	1492	9	novel_in_catalog	gene: Zm00001d040804	novel	8.33	14.17	coding	450	2	1351
PB.3958.3	3	1675	8	novel_in_catalog	gene: Zm00001d040804	novel	2.51	14.17	coding	337	60	1070

PB.3958.4	3	1725	7	novel_in_catalog	gene: Zm00001d040804	novel	1.68	14.17	coding	337	2	1012
PB.4595.2	3	2143	11	novel_in_catalog	gene: Zm00001d043890	novel	9.00	18.96	coding	540	285	1904
PB.43.1	1	2627	1	full-splice_match	gene: Zm00001d027387	Zm00001d027387_T001	76.29	76.29	coding	748	93	2336
PB.10814.2	9	1303	1	full-splice_match	gene: Zm00001d048508	Zm00001d048508_T001	14.21	14.21	coding	171	143	655
PB.3725.1	3	1309	4	full-splice_match	gene: Zm00001d039529	Zm00001d039529_T001	6.30	6.30	coding	263	322	1110
PB.9868.17	8	2441	9	novel_in_catalog	gene: Zm00001d012100	novel	2.04	79.52	coding	138	623	1036
PB.1972.1	10	537	2	incomplete-splice_match	gene: Zm00001d024934	Zm00001d024934_T001	19.76	19.76	non_coding	NA	NA	NA
PB.8691.4	7	3015	4	full-splice_match	gene: Zm00001d021400	Zm00001d021400_T001	0.00	38.07	coding	687	606	2666
PB.5471.1	4	1714	3	novel_in_catalog	gene: Zm00001d052294	novel	37.29	37.29	coding	196	913	1500
PB.6023.1	5	2087	8	full-splice_match	gene: Zm00001d013223	Zm00001d013223_T001	16.23	51.19	coding	377	47	1177
PB.6023.2	5	1421	8	full-splice_match	gene: Zm00001d013223	Zm00001d013223_T001	34.96	51.19	coding	377	79	1209
PB.10735.1	9	1643	3	full-splice_match	gene: Zm00001d048229	Zm00001d048229_T001	15.39	67.55	coding	223	394	1062
PB.9626.2	8	1555	2	full-splice_match	gene: Zm00001d010982	Zm00001d010982_T001	100.31	117.96	coding	294	212	1093
PB.3859.1	3	621	3	novel_not_in_catalog	gene: Zm00001d040276	novel	49.62	49.62	coding	106	91	408
PB.7108.1	5	1293	6	full-splice_match	gene: Zm00001d018297	Zm00001d018297_T001	94.77	103.10	coding	260	208	987
PB.8506.1	7	2450	12	full-splice_match	gene: Zm00001d020549	Zm00001d020549_T006	8.83	8.83	coding	342	17	1042
PB.10004.1	8	2152	7	novel_in_catalog	gene: Zm00001d012672	novel	7.76	7.76	coding	191	44	616

PB.4731.1	3	1194	7	novel_in_catalog	gene: Zm00001d044480	novel	87.77	87.77	coding	247	127	867
PB.7884.8	6	989	4	full-splice_match	gene: Zm00001d038248	Zm00001d038248_T008	92.65	251.94	coding	137	84	494
PB.7884.9	6	1108	4	novel_in_catalog	gene: Zm00001d038248	novel	116.66	251.94	coding	190	72	641
PB.7884.6	6	2210	1	novel_in_catalog	gene: Zm00001d038248	novel	0.00	251.94	non_coding	NA	NA	NA
PB.7884.7	6	1588	3	full-splice_match	gene: Zm00001d038248	Zm00001d038248_T002	0.00	251.94	coding	183	72	620
PB.7884.4	6	2193	2	novel_in_catalog	gene: Zm00001d038248	novel	5.72	251.94	coding	92	67	342
PB.7578.1	6	1734	5	full-splice_match	gene: Zm00001d037034	Zm00001d037034_T002	44.88	44.88	coding	412	238	1473
PB.7884.2	6	1918	4	novel_in_catalog	gene: Zm00001d038248	novel	8.05	251.94	coding	190	62	631
PB.7884.3	6	1949	3	full-splice_match	gene: Zm00001d038248	Zm00001d038248_T002	7.29	251.94	coding	183	145	693
PB.7884.1	6	3502	1	genic	gene: Zm00001d038248	novel	1.50	251.94	non_coding	NA	NA	NA
PB.10185.1	9	1904	14	novel_not_in_catalog	gene: Zm00001d045472	novel	18.43	18.69	non_coding	NA	NA	NA
PB.7520.4	6	1725	7	novel_in_catalog	gene: Zm00001d036673	novel	0.00	14.22	coding	446	273	1610
PB.10185.3	9	1740	14	novel_not_in_catalog	gene: Zm00001d045472	novel	0.27	18.69	non_coding	NA	NA	NA
PB.6720.3	5	2126	5	incomplete-splice_match	gene: Zm00001d016675	Zm00001d016675_T003	5.35	24.19	coding	160	1350	1829
PB.7520.1	6	1990	8	novel_in_catalog	gene: Zm00001d036673	novel	6.58	14.22	coding	418	274	1527
PB.7520.2	6	723	5	novel_in_catalog	gene: Zm00001d036673	novel	7.64	14.22	coding	118	33	386
PB.3337.3	2	2173	9	full-splice_match	gene: Zm00001d006479	Zm00001d006479_T002	0.00	100.07	coding	531	421	2013

PB.215.3	1	1169	8	incomplete-splice_match	gene: Zm00001d028199	Zm00001d028199_T005	0.00	20.26	coding	260	288	1067
PB.3337.1	2	2270	9	full-splice_match	gene: Zm00001d006479	Zm00001d006479_T002	75.58	100.07	coding	531	437	2029
PB.215.1	1	2013	13	full-splice_match	gene: Zm00001d028199	Zm00001d028199_T004	20.26	20.26	coding	523	140	1708
PB.989.1	1	2659	2	intergenic	novelGene_340	novel	1.71	12.51	coding	288	1530	2393
PB.4160.4	3	2841	2	full-splice_match	gene: Zm00001d042051	Zm00001d042051_T001	5.93	20.14	coding	679	549	2585
PB.2383.5	10	1159	5	full-splice_match	gene: Zm00001d026706	Zm00001d026706_T005	8.79	269.39	coding	185	111	665
PB.8455.4	7	1786	6	full-splice_match	gene: Zm00001d020353	Zm00001d020353_T003	0.00	8.54	coding	325	167	1141
PB.8455.1	7	2205	6	full-splice_match	gene: Zm00001d020353	Zm00001d020353_T002	8.54	8.54	coding	325	330	1304
PB.1325.1	1	1775	14	full-splice_match	gene: Zm00001d033708	Zm00001d033708_T001	27.92	36.24	coding	367	278	1378
PB.8455.3	7	2085	6	full-splice_match	gene: Zm00001d020353	Zm00001d020353_T003	0.00	8.54	coding	325	220	1194
PB.2383.2	10	2229	1	incomplete-splice_match	gene: Zm00001d026706	Zm00001d026706_T002	71.32	269.39	coding	653	111	2069
PB.10718.5	9	986	6	full-splice_match	gene: Zm00001d048159	Zm00001d048159_T001	125.88	336.56	coding	260	72	851
PB.10718.4	9	1096	6	full-splice_match	gene: Zm00001d048159	Zm00001d048159_T001	210.68	336.56	coding	260	77	856
PB.2532.3	2	1110	5	full-splice_match	gene: Zm00001d002332	Zm00001d002332_T026	10.51	59.67	coding	297	104	994
PB.2532.2	2	1469	5	full-splice_match	gene: Zm00001d002332	Zm00001d002332_T026	32.06	59.67	coding	297	355	1245
PB.2532.1	2	2072	6	full-splice_match	gene: Zm00001d002332	Zm00001d002332_T025	4.31	59.67	coding	285	90	944
PB.3488.1	2	3125	20	full-splice_match	gene: Zm00001d007202	Zm00001d007202_T013	18.61	18.61	coding	864	352	2943

PB.6794.1	5	1621	1	antisense	novelGene_gene: Zm00001d017006_A	novel	1.62	1.62	coding	243	252	980
PB.2556.1	2	1454	3	full-splice_match	gene: Zm00001d002419	Zm00001d002419_T001	9.75	9.75	coding	328	164	1147
PB.5205.1	4	638	2	full-splice_match	gene: Zm00001d050944	Zm00001d050944_T001	114.94	114.94	coding	96	78	365
PB.6107.2	5	656	2	intergenic	novelGene_46	novel	17.06	29.75	non_coding	NA	NA	NA
PB.6107.1	5	1051	1	intergenic	novelGene_46	novel	12.69	29.75	coding	56	167	334
PB.7311.6	6	2342	5	full-splice_match	gene: Zm00001d035492	Zm00001d035492_T009	94.41	99.56	coding	476	635	2062
PB.5106.1	4	1098	7	full-splice_match	gene: Zm00001d050196	Zm00001d050196_T001	414.58	414.58	coding	264	81	872
PB.308.1	1	2025	5	full-splice_match	gene: Zm00001d028571	Zm00001d028571_T003	17.58	17.58	coding	464	220	1611
PB.308.3	1	548	2	novel_in_catalog	gene: Zm00001d028571	novel	0.00	17.58	coding	157	78	548
PB.4414.1	3	2710	4	novel_in_catalog	gene: Zm00001d043202	novel	1.49	27.24	coding	244	1685	2416
PB.4414.2	3	1982	3	novel_in_catalog	gene: Zm00001d043202	novel	6.55	27.24	coding	244	970	1701
PB.4414.3	3	1872	6	novel_in_catalog	gene: Zm00001d043202	novel	1.52	27.24	coding	244	960	1691
PB.4414.4	3	1701	7	novel_in_catalog	gene: Zm00001d043202	novel	17.68	27.24	coding	434	233	1534
PB.5493.1	4	4126	3	full-splice_match	gene: Zm00001d052361	Zm00001d052361_T001	12.91	12.91	coding	1095	535	3819
PB.4853.2	4	1149	1	full-splice_match	gene: Zm00001d048887	Zm00001d048887_T001	20.40	41.13	coding	330	54	1043
PB.4853.1	4	949	1	full-splice_match	gene: Zm00001d048887	Zm00001d048887_T001	20.72	41.13	coding	81	469	711
PB.1070.2	1	3006	2	novel_in_catalog	gene: Zm00001d032536	novel	55.11	87.72	coding	177	100	630

PB.1185.1	1	2628	2	full-splice_match	gene: Zm00001d033039	Zm00001d033039_T001	29.59	29.59	coding	773	86	2404
PB.5698.1	4	2231	12	full-splice_match	gene: Zm00001d053284	Zm00001d053284_T005	8.10	8.10	coding	665	111	2105
PB.10746.1	9	1476	9	full-splice_match	gene: Zm00001d048286	Zm00001d048286_T002	15.85	16.87	coding	304	218	1129
PB.10746.2	9	1498	8	novel_in_catalog	gene: Zm00001d048286	novel	1.02	16.87	coding	262	183	968
PB.3604.1	2	702	3	novel_not_in_catalog	gene: Zm00001d007851	novel	15.24	15.24	non_coding	NA	NA	NA
PB.8973.2	7	2067	12	novel_in_catalog	gene: Zm00001d022388	novel	55.24	93.42	coding	233	389	1087
PB.5138.5	4	1164	11	novel_in_catalog	gene: Zm00001d050395	novel	6.16	206.22	coding	172	137	652
PB.5138.6	4	1021	11	incomplete-splice_match	gene: Zm00001d050395	Zm00001d050395_T005	8.89	206.22	coding	232	143	838
PB.5245.4	4	2559	1	genic	gene: Zm00001d051219	novel	0.00	30.37	coding	253	1545	2303
PB.5138.2	4	1284	12	full-splice_match	gene: Zm00001d050395	Zm00001d050395_T006	191.16	206.22	coding	231	141	833
PB.5138.3	4	1377	11	novel_in_catalog	gene: Zm00001d050395	novel	0.00	206.22	coding	172	134	649
PB.6926.1	5	3180	6	novel_in_catalog	gene: Zm00001d017568	novel	8.00	30.85	coding	581	1186	2928
PB.1206.2	1	1078	3	full-splice_match	gene: Zm00001d033169	Zm00001d033169_T001	93.95	113.95	coding	253	149	907
PB.6739.1	5	2466	3	novel_in_catalog	gene: Zm00001d016768	novel	39.78	42.03	coding	415	818	2062
PB.912.3	1	1436	11	full-splice_match	gene: Zm00001d031849	Zm00001d031849_T002	0.00	219.38	coding	365	176	1270
PB.6926.7	5	2421	7	full-splice_match	gene: Zm00001d017568	Zm00001d017568_T017	22.85	30.85	coding	296	1476	2363
PB.5483.2	4	852	1	genic	gene: Zm00001d052325	novel	9.74	32.40	non_coding	NA	NA	NA

PB.5483.1	4	947	3	novel_in_catalog	gene: Zm00001d052325	novel	22.66	32.40	coding	100	473	772
PB.9827.1	8	3207	2	novel_in_catalog	gene: Zm00001d011901	novel	7.04	7.75	coding	182	2280	2825
PB.9176.4	8	2457	12	full-splice_match	gene: Zm00001d008725	Zm00001d008725_T013	25.63	49.22	coding	665	179	2173
PB.718.1	1	2380	6	full-splice_match	gene: Zm00001d030790	Zm00001d030790_T002	19.44	19.44	coding	538	416	2029
PB.976.1	1	1748	1	full-splice_match	gene: Zm00001d032155	Zm00001d032155_T001	15.14	15.14	coding	448	297	1640
PB.5711.1	4	1731	3	genic	gene: Zm00001d053358	novel	12.66	13.31	coding	405	341	1555
PB.5330.1	4	2354	3	intergenic	novelGene_272	novel	7.32	7.32	coding	583	383	2131
PB.2277.1	10	1326	2	novel_in_catalog	gene: Zm00001d026368	novel	14.36	82.08	coding	100	139	438
PB.7499.1	6	300	2	incomplete-splice_match	gene: Zm00001d036568	Zm00001d036568_T005	133.83	133.83	non_coding	NA	NA	NA
PB.1271.1	1	1569	3	full-splice_match	gene: Zm00001d033504	Zm00001d033504_T001	21.25	21.25	coding	399	72	1268
PB.10875.4	B73V4 ctg173	2424	4	novel_not_in_catalog	gene: Zm00001d000396	novel	45.92	48.92	coding	602	469	2274
PB.10875.6	B73V4 ctg173	1451	3	novel_not_in_catalog	gene: Zm00001d000396	novel	2.99	48.92	coding	381	308	1450
PB.3188.1	2	3381	16	full-splice_match	gene: Zm00001d005794	Zm00001d005794_T018	20.14	21.39	coding	768	828	3131
PB.8629.4	7	3564	9	intergenic	novelGene_231	novel	27.58	54.18	coding	287	2533	3393
PB.8036.1	6	1843	4	full-splice_match	gene: Zm00001d038846	Zm00001d038846_T001	27.35	27.35	coding	370	421	1530
PB.5590.1	4	1703	11	full-splice_match	gene: Zm00001d052747	Zm00001d052747_T001	22.61	24.18	coding	481	86	1528
PB.320.1	1	1539	6	full-splice_match	gene: Zm00001d028622	Zm00001d028622_T002	314.49	314.49	coding	389	165	1331

PB.4709.1	3	2255	1	genic	gene: Zm00001d044388	novel	17.63	17.63	coding	665	21	2015
PB.5130.7	4	2067	6	novel_in_catalog	gene: Zm00001d050357	novel	2.87	42.70	coding	223	1216	1884
PB.4473.1	3	1541	1	novel_in_catalog	gene: Zm00001d043444	novel	12.02	12.02	coding	101	206	508
PB.5130.1	4	2150	6	novel_in_catalog	gene: Zm00001d050357	novel	17.17	42.70	coding	223	1140	1808
PB.5130.3	4	2122	6	novel_in_catalog	gene: Zm00001d050357	novel	4.51	42.70	coding	226	1138	1815
PB.3843.1	3	1310	4	full-splice_match	gene: Zm00001d040193	Zm00001d040193_T062	7.82	12.64	non_coding	NA	NA	NA
PB.3843.2	3	2087	7	incomplete-splice_match	gene: Zm00001d040193	Zm00001d040193_T056	4.82	12.64	non_coding	NA	NA	NA
PB.710.4	1	1186	8	full-splice_match	gene: Zm00001d030739	Zm00001d030739_T006	0.00	171.06	non_coding	NA	NA	NA
PB.710.2	1	1212	8	full-splice_match	gene: Zm00001d030739	Zm00001d030739_T006	155.20	171.06	non_coding	NA	NA	NA
PB.4843.1	4	1126	2	full-splice_match	gene: Zm00001d048837	Zm00001d048837_T001	19.95	19.95	coding	216	74	721
PB.3672.1	3	620	3	incomplete-splice_match	gene: Zm00001d039361	Zm00001d039361_T001	24.73	24.73	coding	130	13	402
PB.710.1	1	1385	7	novel_in_catalog	gene: Zm00001d030739	novel	9.62	171.06	non_coding	NA	NA	NA
PB.9729.4	8	1212	2	genic	gene: Zm00001d011531	novel	0.00	28.94	coding	378	2	1135
PB.2798.1	2	1777	12	novel_in_catalog	gene: Zm00001d003426	novel	3.53	9.29	coding	391	96	1268
PB.2798.2	2	1670	13	full-splice_match	gene: Zm00001d003426	Zm00001d003426_T002	5.76	9.29	coding	446	93	1430
PB.3616.1	2	909	2	genic	gene: Zm00001d007898	novel	160.05	160.05	coding	182	16	561
PB.730.1	1	1170	6	novel_in_catalog	gene: Zm00001d030895	novel	459.22	459.22	coding	180	282	821

PB.7041.1	5	878	3	full-splice_match	gene: Zm00001d018029	Zm00001d018029_T001	36.73	36.73	coding	202	47	652
PB.8261.1	7	3400	9	incomplete-splice_match	gene: Zm00001d019115	Zm00001d019115_T003	12.02	12.02	coding	991	36	3008
PB.7998.2	6	2091	7	full-splice_match	gene: Zm00001d038730	Zm00001d038730_T002	1.45	59.77	coding	164	212	703
PB.6942.4	5	1326	3	incomplete-splice_match	gene: Zm00001d017660	Zm00001d017660_T036	4.58	22.57	coding	286	90	947
PB.7804.4	6	3466	19	full-splice_match	gene: Zm00001d038006	Zm00001d038006_T001	21.19	21.19	coding	1018	124	3177
PB.3978.1	3	2444	7	fusion	gene: Zm00001d041076_g	novel	6.97	6.97	coding	715	59	2203
PB.1163.3	1	1433	1	novel_in_catalog	gene: Zm00001d032911	novel	3.59	138.85	coding	256	636	1403
PB.3465.1	2	1114	6	novel_in_catalog	gene: Zm00001d007085	novel	20.63	20.63	coding	272	104	919
PB.7772.1	6	1746	8	full-splice_match	gene: Zm00001d037898	Zm00001d037898_T006	6.49	6.49	coding	381	297	1439
PB.7998.4	6	1844	5	novel_in_catalog	gene: Zm00001d038730	novel	12.40	59.77	coding	148	209	652
PB.6873.1	5	679	2	novel_not_in_catalog	gene: Zm00001d017347	novel	33.96	114.88	coding	102	131	436
PB.8542.2	7	1491	1	full-splice_match	gene: Zm00001d020736	Zm00001d020736_T001	16.31	16.31	coding	346	96	1133
PB.7998.5	6	1702	5	novel_in_catalog	gene: Zm00001d038730	novel	0.00	59.77	coding	148	212	655
PB.5157.2	4	1532	13	full-splice_match	gene: Zm00001d050501	Zm00001d050501_T010	32.67	38.12	coding	199	497	1093
PB.7998.6	6	2060	6	novel_in_catalog	gene: Zm00001d038730	novel	0.00	59.77	coding	148	168	611
PB.7132.1	5	2175	10	full-splice_match	gene: Zm00001d018375	Zm00001d018375_T001	7.49	9.82	coding	520	139	1698
PB.1926.1	10	1254	1	full-splice_match	gene: Zm00001d024752	Zm00001d024752_T001	483.79	483.79	coding	340	95	1114

PB.5157.5	4	1541	10	novel_in_catalog	gene: Zm00001d050501	novel	5.44	38.12	coding	160	475	954
PB.7998.7	6	1185	8	full-splice_match	gene: Zm00001d038730	Zm00001d038730_T003	0.00	59.77	coding	211	212	844
PB.4016.1	3	668	5	novel_not_in_catalog	gene: Zm00001d041375	novel	44.51	44.51	coding	13	330	368
PB.881.1	1	1565	5	novel_in_catalog	gene: Zm00001d031667	novel	6.59	21.70	coding	192	511	1086
PB.6588.1	5	1390	8	full-splice_match	gene: Zm00001d015839	Zm00001d015839_T001	17.61	27.83	coding	284	58	909
PB.6588.2	5	1221	8	full-splice_match	gene: Zm00001d015839	Zm00001d015839_T001	10.22	27.83	coding	284	60	911
PB.7748.1	6	1793	3	intergenic	novelGene_218	novel	10.51	10.51	coding	363	462	1550
PB.1534.10	1	2404	11	novel_in_catalog	gene: Zm00001d034602	novel	0.00	213.93	coding	418	63	1316
PB.181.2	1	1768	1	novel_in_catalog	gene: Zm00001d028055	novel	2.10	26.44	coding	87	78	338
PB.8469.1	7	1414	4	novel_not_in_catalog	gene: Zm00001d020402	novel	82.82	92.61	coding	359	159	1235
PB.8469.2	7	1705	3	full-splice_match	gene: Zm00001d020402	Zm00001d020402_T001	9.79	92.61	coding	187	955	1515
PB.181.1	1	1178	2	full-splice_match	gene: Zm00001d028055	Zm00001d028055_T001	24.33	26.44	coding	272	79	894
PB.8469.4	7	1259	4	novel_not_in_catalog	gene: Zm00001d020402	novel	0.00	92.61	coding	359	97	1173
PB.9878.4	8	3015	16	incomplete-splice_match	gene: Zm00001d012155	Zm00001d012155_T021	0.00	22.90	coding	745	781	3015
PB.1383.1	1	1063	4	full-splice_match	gene: Zm00001d033986	Zm00001d033986_T003	256.06	256.06	coding	213	200	838
PB.7174.2	5	2624	7	full-splice_match	gene: Zm00001d018497	Zm00001d018497_T006	0.00	6.93	coding	683	346	2394
PB.7174.1	5	2774	7	full-splice_match	gene: Zm00001d018497	Zm00001d018497_T006	6.93	6.93	coding	683	457	2505

PB.9898.2	8	1091	1	incomplete-splice_match	gene: Zm00001d012239	Zm00001d012239_T002	16.71	18.60	coding	239	248	964
PB.3799.2	3	2009	12	full-splice_match	gene: Zm00001d039971	Zm00001d039971_T004	17.00	30.32	coding	484	38	1489
PB.3799.1	3	1973	11	novel_in_catalog	gene: Zm00001d039971	novel	5.68	30.32	coding	296	72	959
PB.3799.4	3	1801	12	full-splice_match	gene: Zm00001d039971	Zm00001d039971_T004	7.64	30.32	coding	484	106	1557
PB.7695.1	6	1622	10	full-splice_match	gene: Zm00001d037586	Zm00001d037586_T002	46.18	46.18	coding	350	305	1354
PB.8847.8	7	1707	4	novel_in_catalog	gene: Zm00001d021930	novel	0.00	101.07	coding	243	126	854
PB.8847.9	7	1249	5	full-splice_match	gene: Zm00001d021930	Zm00001d021930_T001	58.87	101.07	coding	344	128	1159
PB.6552.1	5	1055	5	full-splice_match	gene: Zm00001d015509	Zm00001d015509_T002	59.93	62.51	coding	253	38	796
PB.8847.5	7	2857	4	full-splice_match	gene: Zm00001d021930	Zm00001d021930_T003	2.84	101.07	coding	219	126	782
PB.8847.6	7	3299	3	novel_in_catalog	gene: Zm00001d021930	novel	0.28	101.07	coding	219	126	782
PB.6552.2	5	2279	4	novel_in_catalog	gene: Zm00001d015509	novel	2.59	62.51	coding	140	1620	2039
PB.8847.1	7	1989	4	novel_in_catalog	gene: Zm00001d021930	novel	7.02	101.07	coding	243	126	854
PB.8847.2	7	1664	4	novel_in_catalog	gene: Zm00001d021930	novel	27.84	101.07	coding	304	400	1311
PB.10716.1	9	2086	2	full-splice_match	gene: Zm00001d048154	Zm00001d048154_T001	11.62	11.62	coding	467	477	1877
PB.4563.4	3	1672	11	full-splice_match	gene: Zm00001d043768	Zm00001d043768_T001	0.00	15.83	coding	391	124	1296
PB.5536.2	4	1599	1	genic	gene: Zm00001d052480	novel	13.64	33.51	coding	351	471	1523
PB.4563.1	3	1868	11	full-splice_match	gene: Zm00001d043768	Zm00001d043768_T001	14.52	15.83	coding	391	124	1296

PB.5536.1	4	1724	1	genic	gene: Zm00001d052480	novel	19.87	33.51	coding	351	469	1521
PB.5981.1	5	1988	14	full-splice_match	gene: Zm00001d013048	Zm00001d013048_T005	3.65	44.29	coding	401	136	1338
PB.5981.2	5	1885	14	full-splice_match	gene: Zm00001d013048	Zm00001d013048_T005	40.63	44.29	coding	401	136	1338
PB.5981.3	5	1598	14	full-splice_match	gene: Zm00001d013048	Zm00001d013048_T005	0.00	44.29	coding	401	124	1326
PB.1131.1	1	2032	19	full-splice_match	gene: Zm00001d032763	Zm00001d032763_T002	9.27	69.78	coding	526	166	1743
PB.1131.2	1	2240	18	full-splice_match	gene: Zm00001d032763	Zm00001d032763_T003	60.51	69.78	coding	526	224	1801
PB.6434.1	5	1991	9	full-splice_match	gene: Zm00001d014953	Zm00001d014953_T002	73.25	73.25	coding	547	109	1749
PB.987.2	1	2046	3	incomplete-splice_match	gene: Zm00001d032220	Zm00001d032220_T005	21.15	21.15	non_coding	NA	NA	NA
PB.8863.1	7	2002	4	novel_not_in_catalog	gene: Zm00001d021976	novel	2.81	2.81	coding	562	71	1756
PB.2884.1	2	2331	9	full-splice_match	gene: Zm00001d003879	Zm00001d003879_T010	59.44	529.65	coding	637	245	2155
PB.9057.6	8	2174	8	novel_in_catalog	gene: Zm00001d008176	novel	18.87	35.58	coding	448	625	1968
PB.9837.1	8	1495	8	full-splice_match	gene: Zm00001d011956	Zm00001d011956_T007	15.49	15.49	coding	122	842	1207
PB.3682.1	3	2007	1	antisense	novelGene_gene: Zm00001d039384_A	novel	31.95	31.95	coding	185	52	606
PB.7576.1	6	2092	1	full-splice_match	gene: Zm00001d037017	Zm00001d037017_T001	4.17	8.16	coding	591	61	1833
PB.7576.2	6	1970	1	full-splice_match	gene: Zm00001d037017	Zm00001d037017_T001	3.99	8.16	coding	591	61	1833
PB.8560.3	7	1060	8	novel_in_catalog	gene: Zm00001d020827	novel	0.00	28.12	coding	321	97	1059
PB.8669.3	7	1454	2	novel_not_in_catalog	gene: Zm00001d021318	novel	68.50	248.77	coding	333	196	1194

PB.417.1	1	1156	7	full-splice_match	gene: Zm00001d029036	Zm00001d029036_T002	99.73	99.73	coding	258	99	872
PB.2267.2	10	1714	1	incomplete-splice_match	gene: Zm00001d026344	Zm00001d026344_T002	0.00	29.26	coding	499	3	1499
PB.8669.4	7	1402	1	incomplete-splice_match	gene: Zm00001d021318	Zm00001d021318_T006	0.00	248.77	coding	333	236	1234
PB.9057.1	8	3237	12	full-splice_match	gene: Zm00001d008176	Zm00001d008176_T006	16.71	35.58	coding	877	189	2819
PB.2312.3	10	433	1	intergenic	novelGene_239	novel	10.55	13.46	non_coding	NA	NA	NA
PB.5468.2	4	1148	5	full-splice_match	gene: Zm00001d052268	Zm00001d052268_T001	56.29	124.56	coding	230	299	988
PB.8161.1	7	1737	2	novel_in_catalog	gene: Zm00001d018652	novel	5.46	5.46	coding	292	14	889
PB.8418.9	7	1808	5	novel_in_catalog	gene: Zm00001d020140	novel	1.13	38.65	coding	133	740	1138
PB.9001.1	7	1194	3	novel_in_catalog	gene: Zm00001d022469	novel	879.27	922.79	coding	259	146	922
PB.9001.2	7	1876	2	novel_in_catalog	gene: Zm00001d022469	novel	10.10	922.79	coding	106	1305	1622
PB.6652.2	5	3206	2	full-splice_match	gene: Zm00001d016285	Zm00001d016285_T001	0.00	16.04	coding	1032	35	3130
PB.7117.1	5	1312	1	novel_in_catalog	gene: Zm00001d018334	novel	3.52	23.66	coding	103	916	1224
PB.7180.1	5	1228	7	full-splice_match	gene: Zm00001d018506	Zm00001d018506_T002	56.13	56.13	coding	312	70	1005
PB.6652.1	5	3287	2	full-splice_match	gene: Zm00001d016285	Zm00001d016285_T001	16.04	16.04	coding	1032	36	3131
PB.4954.1	4	1844	3	full-splice_match	gene: Zm00001d049375	Zm00001d049375_T001	3.51	35.19	coding	386	51	1208
PB.8265.1	7	1599	14	full-splice_match	gene: Zm00001d019139	Zm00001d019139_T003	132.94	142.61	coding	344	196	1227
PB.788.5	1	820	2	full-splice_match	gene: Zm00001d031168	Zm00001d031168_T001	5065.00	14591.48	coding	155	100	564

PB.5007.1	4	2414	1	intergenic	novelGene_97	novel	2.07	2.07	coding	71	136	348
PB.9180.2	8	1561	9	novel_not_in_catalog	gene: Zm00001d008737	novel	99.00	99.00	coding	5	290	304
PB.1235.3	1	2164	11	full-splice_match	gene: Zm00001d033321	Zm00001d033321_T003	5.66	33.48	coding	523	240	1808
PB.548.2	1	1386	4	full-splice_match	gene: Zm00001d029632	Zm00001d029632_T001	0.00	23.86	coding	279	267	1103
PB.548.1	1	1445	4	full-splice_match	gene: Zm00001d029632	Zm00001d029632_T001	23.86	23.86	coding	279	267	1103
PB.6173.1	5	958	4	novel_not_in_catalog	gene: Zm00001d013777	novel	1621.72	1621.72	coding	220	64	723
PB.4837.1	4	932	2	genic	gene: Zm00001d048801	novel	34.98	34.98	coding	129	354	740
PB.4573.1	3	1542	7	incomplete-splice_match	gene: Zm00001d043807	Zm00001d043807_T003	35.00	49.41	coding	408	1	1224
PB.4573.2	3	1415	7	incomplete-splice_match	gene: Zm00001d043807	Zm00001d043807_T003	14.40	49.41	coding	408	41	1264
PB.7108.2	5	1210	6	full-splice_match	gene: Zm00001d018297	Zm00001d018297_T001	8.33	103.10	coding	260	212	991
PB.4650.2	3	1569	5	novel_in_catalog	gene: Zm00001d044138	novel	11.01	72.12	coding	354	25	1086
PB.61.2	1	1831	9	novel_not_in_catalog	gene: Zm00001d027460	novel	9.81	11.69	coding	440	297	1616
PB.5186.2	4	2022	1	antisense	novelGene_gene: Zm00001d050848_A	novel	69.81	69.81	coding	432	100	1395
PB.1119.1	1	2492	2	full-splice_match	gene: Zm00001d032707	Zm00001d032707_T001	3.10	3.10	coding	582	324	2069
PB.61.5	1	1745	8	novel_not_in_catalog	gene: Zm00001d027460	novel	1.88	11.69	coding	440	224	1543
PB.1372.7	1	1590	10	novel_in_catalog	gene: Zm00001d033931	novel	971.01	1100.09	coding	379	197	1333
PB.4026.1	3	1558	1	genic	gene: Zm00001d041422	novel	32.97	32.97	coding	363	134	1222

PB.4680.1	3	2153	16	novel_not_in_catalog	gene: Zm00001d044251	novel	7.90	7.90	coding	506	227	1744
PB.1518.1	1	2255	7	novel_not_in_catalog	gene: Zm00001d034532	novel	13.21	125.80	coding	222	172	837
PB.6449.1	5	594	1	genic	gene: Zm00001d014992	novel	32.36	32.36	non_coding	NA	NA	NA
PB.9196.4	8	1453	11	full-splice_match	gene: Zm00001d008820	Zm00001d008820_T002	117.88	318.56	coding	334	137	1138
PB.9196.5	8	1743	9	novel_in_catalog	gene: Zm00001d008820	novel	8.73	318.56	coding	205	139	753
PB.9196.6	8	1465	10	novel_in_catalog	gene: Zm00001d008820	novel	6.93	318.56	coding	262	137	922
PB.9196.7	8	1560	10	full-splice_match	gene: Zm00001d008820	Zm00001d008820_T004	40.51	318.56	coding	205	185	799
PB.7978.1	6	1870	1	genic	gene: Zm00001d038605	novel	27.62	50.07	coding	493	11	1489
PB.9196.1	8	1338	1	genic	gene: Zm00001d008820	novel	19.68	318.56	non_coding	NA	NA	NA
PB.9196.2	8	1688	9	novel_in_catalog	gene: Zm00001d008820	novel	15.95	318.56	coding	205	134	748
PB.9196.3	8	1483	11	full-splice_match	gene: Zm00001d008820	Zm00001d008820_T005	40.11	318.56	coding	329	137	1123
PB.8208.1	7	1337	1	full-splice_match	gene: Zm00001d018855	Zm00001d018855_T001	30.54	30.54	coding	368	63	1166
PB.4222.8	3	2502	5	full-splice_match	gene: Zm00001d042347	Zm00001d042347_T007	22.94	1197.41	non_coding	NA	NA	NA
PB.8208.2	7	1228	1	full-splice_match	gene: Zm00001d018855	Zm00001d018855_T001	0.00	30.54	coding	368	63	1166
PB.9196.8	8	1374	11	full-splice_match	gene: Zm00001d008820	Zm00001d008820_T005	68.76	318.56	coding	329	136	1122
PB.8561.1	7	2873	3	genic	gene: Zm00001d020828	novel	16.23	19.21	coding	647	456	2396
PB.9327.1	8	1476	8	full-splice_match	gene: Zm00001d009554	Zm00001d009554_T001	60.71	106.27	coding	298	91	984

PB.251.2	1	628	4	intergenic	novelGene_87	novel	637.27	859.24	coding	129	74	460
PB.5073.2	4	2102	4	full-splice_match	gene: Zm00001d050023	Zm00001d050023_T001	35.10	35.10	coding	566	83	1780
PB.2632.1	2	1317	1	full-splice_match	gene: Zm00001d002771	Zm00001d002771_T001	0.71	0.71	coding	426	31	1308
PB.6967.1	5	420	2	incomplete-splice_match	gene: Zm00001d017777	Zm00001d017777_T003	69.97	69.97	non_coding	NA	NA	NA
PB.7415.1	6	1167	8	full-splice_match	gene: Zm00001d036108	Zm00001d036108_T003	42.25	270.16	coding	244	132	863
PB.7415.2	6	1038	8	full-splice_match	gene: Zm00001d036108	Zm00001d036108_T003	227.91	270.16	coding	244	152	883
PB.3794.3	3	2215	4	full-splice_match	gene: Zm00001d039920	Zm00001d039920_T001	29.37	29.37	coding	680	38	2077
PB.8088.12	6	515	5	incomplete-splice_match	gene: Zm00001d039039	Zm00001d039039_T002	25.79	25.79	coding	69	208	414
PB.8088.11	6	1967	1	full-splice_match	gene: Zm00001d039038	Zm00001d039038_T001	0.00	42.20	coding	582	81	1826
PB.4966.1	4	2256	6	novel_not_in_catalog	gene: Zm00001d049452	novel	4.05	4.05	coding	124	251	622
PB.5450.3	4	1812	6	full-splice_match	gene: Zm00001d052219	Zm00001d052219_T004	3.56	42.95	coding	212	119	754
PB.1247.3	1	2284	6	novel_not_in_catalog	gene: Zm00001d033383	novel	10.18	20.71	coding	643	71	1999
PB.5450.1	4	2665	4	novel_in_catalog	gene: Zm00001d052219	novel	2.83	42.95	coding	200	920	1519
PB.83.2	1	906	3	full-splice_match	gene: Zm00001d027541	Zm00001d027541_T001	63.18	138.26	coding	226	61	738
PB.83.1	1	1010	3	full-splice_match	gene: Zm00001d027541	Zm00001d027541_T001	75.08	138.26	coding	226	64	741
PB.2477.1	2	1052	8	full-splice_match	gene: Zm00001d002115	Zm00001d002115_T001	14.00	14.00	coding	266	83	880
PB.7023.1	5	1822	1	full-splice_match	gene: Zm00001d017984	Zm00001d017984_T001	86.24	86.24	coding	365	248	1342

PB.3219.2	2	1061	4	novel_in_catalog	gene: Zm00001d005998	novel	12.86	98.74	coding	101	600	902
PB.2228.4	10	2324	9	novel_in_catalog	gene: Zm00001d026213	novel	4.12	20.33	coding	381	929	2071
PB.8958.1	7	2162	3	novel_in_catalog	gene: Zm00001d022342	novel	9.66	9.66	coding	169	1416	1922
PB.4018.1	3	1501	1	full-splice_match	gene: Zm00001d041381	Zm00001d041381_T001	0.77	0.77	coding	282	309	1154
PB.3504.1	2	1800	1	full-splice_match	gene: Zm00001d007274	Zm00001d007274_T001	49.83	338.75	coding	373	83	1201
PB.8958.2	7	1935	3	novel_in_catalog	gene: Zm00001d022342	novel	0.00	9.66	coding	169	1358	1864
PB.1693.2	10	1324	2	full-splice_match	gene: Zm00001d023396	Zm00001d023396_T001	0.37	0.37	coding	279	103	939
PB.8649.1	7	1115	3	full-splice_match	gene: Zm00001d021242	Zm00001d021242_T001	2.40	2.40	coding	308	88	1011
PB.1693.1	10	3069	3	fusion	gene: Zm00001d023396_g	novel	51.49	51.49	coding	279	57	893
PB.3239.1	2	2444	2	incomplete-splice_match	gene: Zm00001d006079	Zm00001d006079_T001	10.61	10.61	coding	460	495	1874
PB.9950.1	8	558	2	genic	gene: Zm00001d012469	novel	1088.22	1088.22	coding	71	100	312
PB.4002.5	3	2124	4	intergenic	novelGene_182	novel	8.86	61.78	coding	121	1365	1727
PB.473.1	1	549	1	intergenic	novelGene_124	novel	32.72	32.72	coding	60	188	367
PB.7328.6	6	1953	1	genic	gene: Zm00001d035605	novel	614.09	3577.33	coding	250	879	1628
PB.10492.1	9	984	5	full-splice_match	gene: Zm00001d047354	Zm00001d047354_T001	53.85	124.11	coding	160	139	618
PB.3931.1	3	2149	1	genic	gene: Zm00001d040644	novel	28.60	28.60	coding	475	453	1877
PB.10601.1	9	3277	17	full-splice_match	gene: Zm00001d047762	Zm00001d047762_T009	26.02	26.02	coding	878	104	2737

PB.4119.1	3	2275	11	incomplete-splice_match	gene: Zm00001d041912	Zm00001d041912_T006	34.41	34.41	coding	545	277	1911
PB.954.1	1	1177	6	full-splice_match	gene: Zm00001d032031	Zm00001d032031_T001	137.81	137.81	coding	273	137	955
PB.93.4	1	2059	3	full-splice_match	gene: Zm00001d027593	Zm00001d027593_T001	0.00	68.25	coding	612	82	1917
PB.4002.4	3	2952	8	intergenic	novelGene_182	novel	9.05	61.78	coding	196	1968	2555
PB.93.1	1	2175	3	full-splice_match	gene: Zm00001d027593	Zm00001d027593_T001	68.25	68.25	coding	612	88	1923
PB.2974.1	2	3189	20	full-splice_match	gene: Zm00001d004564	Zm00001d004564_T006	43.96	52.51	coding	905	106	2820
PB.3253.3	2	2751	5	full-splice_match	gene: Zm00001d006130	Zm00001d006130_T001	8.66	32.62	coding	761	283	2565
PB.3383.4	2	2118	6	full-splice_match	gene: Zm00001d006702	Zm00001d006702_T001	15.94	30.36	coding	610	40	1869
PB.5277.1	4	861	5	full-splice_match	gene: Zm00001d051405	Zm00001d051405_T001	104.83	104.83	coding	174	83	604
PB.8641.1	7	2317	1	genic	gene: Zm00001d021219	novel	0.18	7.40	coding	497	209	1699
PB.8641.2	7	1942	1	genic	gene: Zm00001d021219	novel	7.22	7.40	coding	497	210	1700
PB.8641.3	7	1829	1	genic	gene: Zm00001d021219	novel	0.00	7.40	coding	497	252	1742
PB.6749.3	5	2033	9	novel_not_in_catalog	gene: Zm00001d016811	novel	5.74	15.92	coding	460	419	1798
PB.6749.2	5	1982	9	full-splice_match	gene: Zm00001d016811	Zm00001d016811_T001	10.18	15.92	coding	460	337	1716
PB.3260.1	2	1787	12	full-splice_match	gene: Zm00001d006166	Zm00001d006166_T001	68.68	68.68	coding	470	168	1577
PB.530.2	1	3430	16	full-splice_match	gene: Zm00001d029561	Zm00001d029561_T001	36.12	36.12	coding	1031	77	3169
PB.5198.3	4	514	4	full-splice_match	gene: Zm00001d050916	Zm00001d050916_T002	0.00	182.18	coding	114	59	400

PB.6169.1	5	1457	4	full-splice_match	gene: Zm00001d013751	Zm00001d013751_T005	439.64	439.64	coding	364	122	1213
PB.5198.1	4	736	4	full-splice_match	gene: Zm00001d050916	Zm00001d050916_T002	10.74	182.18	coding	114	59	400
PB.9493.1	8	1934	8	novel_not_in_catalog	gene: Zm00001d010431	novel	2.14	149.31	coding	229	228	914
PB.9493.2	8	1286	8	novel_not_in_catalog	gene: Zm00001d010431	novel	54.59	149.31	coding	229	230	916
PB.9493.3	8	1739	7	novel_not_in_catalog	gene: Zm00001d010431	novel	7.07	149.31	coding	229	769	1455
PB.9493.5	8	1248	8	novel_not_in_catalog	gene: Zm00001d010431	novel	69.03	149.31	coding	229	279	965
PB.9493.6	8	1072	8	novel_not_in_catalog	gene: Zm00001d010431	novel	16.49	149.31	coding	229	228	914
PB.7323.1	6	710	3	incomplete-splice_match	gene: Zm00001d035579	Zm00001d035579_T003	46.32	46.32	non_coding	NA	NA	NA
PB.9967.1	8	1387	6	full-splice_match	gene: Zm00001d012550	Zm00001d012550_T001	247.54	247.54	coding	364	66	1157
PB.9746.4	8	2644	7	incomplete-splice_match	gene: Zm00001d011610	Zm00001d011610_T033	61.91	133.97	coding	811	102	2534
PB.9930.3	8	1601	6	novel_not_in_catalog	gene: Zm00001d012394	novel	0.00	34.14	coding	144	172	603
PB.9746.1	8	3632	9	full-splice_match	gene: Zm00001d011610	Zm00001d011610_T033	39.33	133.97	coding	1133	2	3400
PB.2610.1	2	901	7	full-splice_match	gene: Zm00001d002659	Zm00001d002659_T001	147.40	147.40	coding	123	151	519
PB.2610.2	2	717	7	full-splice_match	gene: Zm00001d002659	Zm00001d002659_T001	0.00	147.40	coding	123	93	461
PB.9746.2	8	1672	2	incomplete-splice_match	gene: Zm00001d011610	Zm00001d011610_T074	3.30	133.97	coding	163	40	528
PB.2835.20	2	2598	5	novel_not_in_catalog	gene: Zm00001d003616	novel	0.26	253.94	coding	155	172	636
PB.6752.7	5	2156	11	novel_in_catalog	gene: Zm00001d016825	novel	0.69	35.33	coding	203	72	680

PB.2835.22	2	1418	9	full-splice_match	gene: Zm00001d003616	Zm00001d003616_T022	61.95	253.94	coding	334	4	1005
PB.2032.1	10	673	2	full-splice_match	gene: Zm00001d025233	Zm00001d025233_T002	9.66	9.66	coding	103	190	498
PB.3572.1	2	3641	9	novel_in_catalog	gene: Zm00001d007709	novel	21.64	40.73	coding	1052	75	3230
PB.3572.2	2	3973	8	novel_in_catalog	gene: Zm00001d007709	novel	2.11	40.73	coding	848	92	2635
PB.3572.3	2	3572	9	novel_in_catalog	gene: Zm00001d007709	novel	16.98	40.73	coding	1052	248	3403
PB.9669.1	8	649	3	full-splice_match	gene: Zm00001d011224	Zm00001d011224_T001	180.46	180.46	coding	139	51	467
PB.953.1	1	797	1	full-splice_match	gene: Zm00001d032030	Zm00001d032030_T001	272.45	272.45	coding	138	113	526
PB.5432.1	4	1574	12	incomplete-splice_match	gene: Zm00001d052110	Zm00001d052110_T007	17.48	17.48	coding	203	637	1245
PB.6060.2	5	2490	6	full-splice_match	gene: Zm00001d013319	Zm00001d013319_T001	258.65	260.13	coding	621	286	2148
PB.7614.5	6	2275	4	novel_not_in_catalog	gene: Zm00001d037213	novel	0.20	104.93	coding	596	301	2088
PB.7614.4	6	6833	8	fusion	gene: Zm00001d037213_g	novel	3.86	26.15	coding	2144	216	6647
PB.7614.2	6	3522	4	novel_not_in_catalog	gene: Zm00001d037213	novel	104.72	104.93	coding	983	291	3239
PB.6060.5	5	2792	5	novel_in_catalog	gene: Zm00001d013319	novel	1.48	260.13	coding	193	171	749
PB.6060.4	5	2350	6	full-splice_match	gene: Zm00001d013319	Zm00001d013319_T001	0.00	260.13	coding	621	263	2125
PB.1753.2	10	780	3	full-splice_match	gene: Zm00001d023733	Zm00001d023733_T001	0.00	102.22	coding	187	32	592
PB.1753.1	10	889	3	full-splice_match	gene: Zm00001d023733	Zm00001d023733_T001	102.22	102.22	coding	187	114	674
PB.1291.3	1	792	2	novel_not_in_catalog	gene: Zm00001d033574	novel	0.00	582.10	coding	170	68	577

PB.9335.3	8	2026	1	full-splice_match	gene: Zm00001d009608	Zm00001d009608_T001	0.00	67.84	coding	505	247	1761
PB.9335.2	8	2166	1	full-splice_match	gene: Zm00001d009608	Zm00001d009608_T001	1.22	67.84	coding	505	309	1823
PB.9335.1	8	2246	1	full-splice_match	gene: Zm00001d009608	Zm00001d009608_T001	66.62	67.84	coding	505	292	1806
PB.4528.26	3	2588	9	novel_in_catalog	gene: Zm00001d043649	novel	3.28	96.43	coding	125	1164	1538
PB.6500.1	5	2377	1	full-splice_match	gene: Zm00001d015213	Zm00001d015213_T001	46.20	46.20	coding	656	231	2198
PB.5707.2	4	2181	15	novel_not_in_catalog	gene: Zm00001d053334	novel	24.07	30.13	coding	466	393	1790
PB.5707.3	4	2050	15	novel_not_in_catalog	gene: Zm00001d053334	novel	4.24	30.13	coding	306	235	1152
PB.4588.1	3	2852	1	full-splice_match	gene: Zm00001d043870	Zm00001d043870_T001	10.68	10.68	coding	819	152	2608
PB.4520.16	3	2428	12	novel_not_in_catalog	gene: Zm00001d043613	novel	0.12	284.34	coding	300	1226	2125
PB.5707.6	4	1938	15	novel_not_in_catalog	gene: Zm00001d053334	novel	0.00	30.13	coding	306	235	1152
PB.5707.7	4	2065	14	novel_not_in_catalog	gene: Zm00001d053334	novel	1.82	30.13	coding	485	346	1800
PB.6181.9	5	2255	7	full-splice_match	gene: Zm00001d013831	Zm00001d013831_T010	23.50	56.47	coding	179	367	903
PB.7528.10	6	1851	4	intergenic	novelGene_172	novel	0.00	72.68	coding	126	286	663
PB.6181.1	5	1447	8	full-splice_match	gene: Zm00001d013831	Zm00001d013831_T002	4.80	56.47	coding	74	125	346
PB.6181.3	5	1842	9	full-splice_match	gene: Zm00001d013831	Zm00001d013831_T006	10.57	56.47	coding	173	214	732
PB.109.1	1	782	6	full-splice_match	gene: Zm00001d027675	Zm00001d027675_T002	77.16	77.16	coding	183	82	630
PB.6181.5	5	2135	8	novel_in_catalog	gene: Zm00001d013831	novel	3.25	56.47	coding	176	292	819

PB.2618.1	2	1920	6	novel_in_catalog	gene: Zm00001d002703	novel	53.28	53.28	coding	377	287	1417
PB.6181.6	5	1823	9	full-splice_match	gene: Zm00001d013831	Zm00001d013831_T003	14.35	56.47	coding	173	209	727
PB.2765.1	2	1440	8	full-splice_match	gene: Zm00001d003257	Zm00001d003257_T001	99.62	99.62	coding	388	44	1207
PB.10556.1	9	2387	6	full-splice_match	gene: Zm00001d047608	Zm00001d047608_T003	105.27	127.39	coding	149	658	1104
PB.6520.1	5	1948	4	full-splice_match	gene: Zm00001d015328	Zm00001d015328_T010	33.39	61.20	coding	357	580	1650
PB.7855.2	6	1410	1	full-splice_match	gene: Zm00001d038158	Zm00001d038158_T001	0.00	28.01	coding	403	51	1259
PB.5643.2	4	1036	2	full-splice_match	gene: Zm00001d052967	Zm00001d052967_T001	10.52	13.15	coding	127	325	705
PB.9984.1	8	1702	1	full-splice_match	gene: Zm00001d012606	Zm00001d012606_T001	17.83	17.83	coding	453	52	1410
PB.6755.1	5	952	1	genic	gene: Zm00001d016836	novel	27.07	27.07	coding	246	53	790
PB.6990.1	5	1962	2	full-splice_match	gene: Zm00001d017864	Zm00001d017864_T001	24.55	24.55	coding	478	37	1470
PB.5847.4	4	2492	5	full-splice_match	gene: Zm00001d053927	Zm00001d053927_T008	3.92	49.24	coding	270	1317	2126
PB.2326.2	10	1168	5	novel_not_in_catalog	gene: Zm00001d026549	novel	0.54	19.84	coding	155	35	499
PB.169.1	1	1394	1	full-splice_match	gene: Zm00001d027978	Zm00001d027978_T001	2.90	2.90	coding	376	141	1268
PB.4619.1	3	1151	6	full-splice_match	gene: Zm00001d043991	Zm00001d043991_T001	326.28	453.38	coding	230	126	815
PB.2326.1	10	1187	5	full-splice_match	gene: Zm00001d026549	Zm00001d026549_T006	19.30	19.84	coding	294	34	915
PB.6636.4	5	1375	3	full-splice_match	gene: Zm00001d016182	Zm00001d016182_T001	0.00	20.05	coding	322	315	1280
PB.5847.3	4	1943	6	full-splice_match	gene: Zm00001d053927	Zm00001d053927_T009	13.05	49.24	coding	270	747	1556

PB.5379.1	4	3514	3	novel_not_in_catalog	gene: Zm00001d051889	novel	10.23	10.23	coding	754	742	3003
PB.1562.2	1	2111	14	full-splice_match	gene: Zm00001d034678	Zm00001d034678_T004	3.36	12.01	coding	529	349	1935
PB.6636.1	5	1586	3	full-splice_match	gene: Zm00001d016182	Zm00001d016182_T001	5.03	20.05	coding	322	120	1085
PB.1937.1	10	2302	3	novel_not_in_catalog	gene: Zm00001d024784	novel	11.01	26.61	coding	430	147	1436
PB.10556.4	9	1511	5	full-splice_match	gene: Zm00001d047608	Zm00001d047608_T007	0.00	127.39	coding	149	663	1109
PB.5975.1	5	1108	2	full-splice_match	gene: Zm00001d013034	Zm00001d013034_T001	551.99	556.94	coding	274	87	908
PB.5975.2	5	1871	1	genic	gene: Zm00001d013034	novel	4.95	556.94	coding	248	87	830
PB.3927.2	3	1407	6	full-splice_match	gene: Zm00001d040614	Zm00001d040614_T004	5.87	64.14	coding	114	115	456
PB.4829.1	4	922	1	full-splice_match	gene: Zm00001d048760	Zm00001d048760_T001	22.53	22.53	coding	238	43	756
PB.8667.2	7	560	5	incomplete-splice_match	gene: Zm00001d021310	Zm00001d021310_T001	27.45	81.16	coding	152	104	559
PB.3927.1	3	1078	7	full-splice_match	gene: Zm00001d040614	Zm00001d040614_T006	58.27	64.14	coding	229	124	810
PB.4520.14	3	2987	12	full-splice_match	gene: Zm00001d043613	Zm00001d043613_T005	0.00	284.34	coding	300	1783	2682
PB.10244.3	9	2439	8	full-splice_match	gene: Zm00001d045763	Zm00001d045763_T002	5.72	25.02	coding	499	618	2114
PB.10244.2	9	2371	9	novel_not_in_catalog	gene: Zm00001d045763	novel	2.38	25.02	coding	513	484	2022
PB.10244.1	9	2460	8	full-splice_match	gene: Zm00001d045763	Zm00001d045763_T002	16.92	25.02	coding	499	482	1978
PB.9329.1	8	1543	3	full-splice_match	gene: Zm00001d009556	Zm00001d009556_T001	48.88	48.88	coding	351	123	1175
PB.9329.2	8	1460	3	full-splice_match	gene: Zm00001d009556	Zm00001d009556_T001	0.00	48.88	coding	351	119	1171

PB.1541.2	1	1168	4	full-splice_match	gene: Zm00001d034618	Zm00001d034618_T002	46.30	123.85	coding	318	98	1051
PB.5723.2	4	1231	1	novel_in_catalog	gene: Zm00001d053425	novel	6.60	74.10	non_coding	NA	NA	NA
PB.4590.1	3	1606	8	novel_in_catalog	gene: Zm00001d043872	novel	3.89	17.96	coding	205	148	762
PB.6528.1	5	1892	6	novel_in_catalog	gene: Zm00001d015374	novel	6.78	6.78	coding	164	50	541
PB.1743.1	10	2702	2	intergenic	novelGene_47	novel	11.24	39.07	coding	126	1197	1574
PB.5969.3	5	1567	1	novel_in_catalog	gene: Zm00001d013015	novel	19.23	238.62	coding	244	680	1411
PB.5969.1	5	1126	2	full-splice_match	gene: Zm00001d013015	Zm00001d013015_T001	219.39	238.62	coding	283	107	955
PB.3882.1	3	1323	6	full-splice_match	gene: Zm00001d040334	Zm00001d040334_T001	64.88	154.30	coding	307	70	990
PB.3882.2	3	1186	6	full-splice_match	gene: Zm00001d040334	Zm00001d040334_T001	89.41	154.30	coding	307	73	993
PB.4324.2	3	2227	2	full-splice_match	gene: Zm00001d042812	Zm00001d042812_T001	19.12	21.81	coding	523	94	1662
PB.4262.2	3	2822	3	full-splice_match	gene: Zm00001d042543	Zm00001d042543_T003	0.19	10.71	coding	570	817	2526
PB.4347.1	3	2699	8	novel_in_catalog	gene: Zm00001d042908	novel	7.06	7.06	coding	290	1296	2165
PB.8786.1	7	2950	9	full-splice_match	gene: Zm00001d021729	Zm00001d021729_T001	48.64	53.48	coding	863	96	2684
PB.10908.1	B73V4 ctg35	1979	8	fusion	gene: Zm00001d000350_g	novel	11.46	11.46	coding	553	88	1746
PB.7922.2	6	2424	11	incomplete-splice_match	gene: Zm00001d038393	Zm00001d038393_T008	22.42	22.42	coding	571	69	1781
PB.1983.1	10	979	3	full-splice_match	gene: Zm00001d024966	Zm00001d024966_T001	25.77	25.77	coding	267	28	828
PB.4363.1	3	2311	5	full-splice_match	gene: Zm00001d042993	Zm00001d042993_T001	45.20	53.22	coding	541	341	1963

PB.4363.3	3	2208	5	full-splice_match	gene: Zm00001d042993	Zm00001d042993_T001	5.04	53.22	coding	541	345	1967
PB.2771.1	2	2054	7	full-splice_match	gene: Zm00001d003284	Zm00001d003284_T002	20.76	20.76	coding	557	92	1762
PB.4528.6	3	2433	5	full-splice_match	gene: Zm00001d043649	Zm00001d043649_T015	46.47	96.43	coding	125	420	794
PB.8288.1	7	2791	1	novel_in_catalog	gene: Zm00001d019241	novel	2.94	48.33	non_coding	NA	NA	NA
PB.3083.1	2	4286	12	full-splice_match	gene: Zm00001d005241	Zm00001d005241_T004	6.98	33.55	coding	1331	119	4111
PB.8288.3	7	783	2	novel_not_in_catalog	gene: Zm00001d019241	novel	26.80	48.33	non_coding	NA	NA	NA
PB.8288.2	7	1013	3	novel_not_in_catalog	gene: Zm00001d019241	novel	18.59	48.33	non_coding	NA	NA	NA
PB.4520.12	3	2999	13	full-splice_match	gene: Zm00001d043613	Zm00001d043613_T008	0.00	284.34	coding	300	1671	2570
PB.3804.1	3	1085	3	intergenic	novelGene_64	novel	1.05	1.05	non_coding	NA	NA	NA
PB.6839.1	5	1150	8	full-splice_match	gene: Zm00001d017193	Zm00001d017193_T002	26.04	26.04	coding	243	51	779
PB.10292.1	9	1567	1	full-splice_match	gene: Zm00001d046157	Zm00001d046157_T001	1.12	19.62	coding	175	102	626
PB.5961.1	5	1463	7	full-splice_match	gene: Zm00001d012996	Zm00001d012996_T001	17.22	17.22	coding	279	205	1041
PB.5.1	1	2473	7	full-splice_match	gene: Zm00001d027257	Zm00001d027257_T005	17.85	17.85	coding	186	1735	2292
PB.3558.1	2	2250	1	full-splice_match	gene: Zm00001d007625	Zm00001d007625_T001	0.57	0.57	coding	644	105	2036
PB.4010.2	3	1544	7	full-splice_match	gene: Zm00001d041346	Zm00001d041346_T001	0.00	20.51	coding	460	9	1388
PB.8728.2	7	2096	19	full-splice_match	gene: Zm00001d021546	Zm00001d021546_T003	0.00	39.53	coding	619	58	1914
PB.3425.3	2	3317	1	novel_in_catalog	gene: Zm00001d006903	novel	0.82	101.68	coding	84	266	517

PB.8728.1	7	2435	19	full-splice_match	gene: Zm00001d021546	Zm00001d021546_T003	39.53	39.53	coding	619	222	2078
PB.3425.4	2	1071	1	genic_intron	novelGene_343	novel	0.53	0.53	non_coding	NA	NA	NA
PB.8003.4	6	900	2	full-splice_match	gene: Zm00001d038741	Zm00001d038741_T002	36.77	118.37	coding	200	110	709
PB.8003.1	6	1086	2	full-splice_match	gene: Zm00001d038741	Zm00001d038741_T002	77.29	118.37	coding	200	110	709
PB.8003.2	6	1152	1	novel_in_catalog	gene: Zm00001d038741	novel	4.31	118.37	coding	116	111	458
PB.8003.3	6	1040	1	novel_in_catalog	gene: Zm00001d038741	novel	0.00	118.37	coding	116	110	457
PB.3876.1	3	3920	22	full-splice_match	gene: Zm00001d040311	Zm00001d040311_T004	5.48	20.61	coding	767	22	2322
PB.9641.1	8	1632	1	full-splice_match	gene: Zm00001d011076	Zm00001d011076_T001	45.14	45.14	coding	387	152	1312
PB.3876.2	3	3715	23	full-splice_match	gene: Zm00001d040311	Zm00001d040311_T009	15.13	20.61	coding	1066	54	3251
PB.4968.1	4	3627	16	full-splice_match	gene: Zm00001d049457	Zm00001d049457_T028	45.48	45.48	coding	1028	211	3294
PB.8234.2	7	2182	3	novel_not_in_catalog	gene: Zm00001d018941	novel	13.18	13.18	coding	281	797	1639
PB.7096.4	5	3427	23	full-splice_match	gene: Zm00001d018246	Zm00001d018246_T004	0.00	45.36	coding	994	174	3155
PB.3058.1	2	2919	3	incomplete-splice_match	gene: Zm00001d005100	Zm00001d005100_T020	8.18	62.42	coding	738	9	2222
PB.3058.3	2	2812	3	incomplete-splice_match	gene: Zm00001d005100	Zm00001d005100_T030	40.17	62.42	coding	586	90	1847
PB.3058.2	2	4209	14	full-splice_match	gene: Zm00001d005100	Zm00001d005100_T041	14.06	62.42	coding	1167	171	3671
PB.3058.4	2	1992	2	incomplete-splice_match	gene: Zm00001d005100	Zm00001d005100_T026	0.00	62.42	coding	268	121	924
PB.9327.2	8	1353	8	full-splice_match	gene: Zm00001d009554	Zm00001d009554_T001	45.57	106.27	coding	298	77	970

PB.1168.3	1	1200	2	full-splice_match	gene: Zm00001d032955	Zm00001d032955_T001	70.18	116.99	coding	300	208	1107
PB.7917.3	6	1144	9	full-splice_match	gene: Zm00001d038380	Zm00001d038380_T001	96.30	294.87	coding	215	203	847
PB.7528.6	6	2028	4	intergenic	novelGene_172	novel	2.83	72.68	coding	126	331	708
PB.7978.2	6	1769	1	genic	gene: Zm00001d038605	novel	0.00	50.07	coding	493	11	1489
PB.7978.3	6	1736	1	genic	gene: Zm00001d038605	novel	22.45	50.07	coding	493	79	1557
PB.7553.9	6	1416	3	full-splice_match	gene: Zm00001d036904	Zm00001d036904_T002	0.00	1538.54	coding	432	120	1415
PB.7553.8	6	1614	3	full-splice_match	gene: Zm00001d036904	Zm00001d036904_T002	8.35	1538.54	coding	447	142	1482
PB.7553.7	6	1687	3	full-splice_match	gene: Zm00001d036904	Zm00001d036904_T002	1255.86	1538.54	coding	447	121	1461
PB.7528.1	6	2099	4	intergenic	novelGene_172	novel	21.13	72.68	coding	126	291	668
PB.7553.4	6	1687	3	full-splice_match	gene: Zm00001d036904	Zm00001d036904_T003	274.33	1538.54	coding	447	89	1429
PB.5610.1	4	2465	2	genic	gene: Zm00001d052816	novel	26.76	37.07	coding	603	145	1953
PB.1253.4	1	608	1	novel_in_catalog	gene: Zm00001d033402	novel	0.00	85.10	coding	80	119	358
PB.8581.1	7	664	1	full-splice_match	gene: Zm00001d020930	Zm00001d020930_T001	272.68	272.68	coding	112	85	420
PB.5610.2	4	2310	2	genic	gene: Zm00001d052816	novel	0.00	37.07	coding	603	131	1939
PB.10346.1	9	2807	7	full-splice_match	gene: Zm00001d046488	Zm00001d046488_T002	47.82	47.82	coding	765	237	2531
PB.6747.1	5	717	3	novel_not_in_catalog	gene: Zm00001d016800	novel	149.92	149.92	non_coding	NA	NA	NA
PB.2812.2	2	799	3	full-splice_match	gene: Zm00001d003502	Zm00001d003502_T005	161.80	161.80	coding	146	120	557

PB.8720.2	7	1288	9	novel_in_catalog	gene: Zm00001d021522	novel	12.41	47.06	coding	246	55	792
PB.8720.3	7	1146	9	novel_in_catalog	gene: Zm00001d021522	novel	8.62	47.06	coding	246	84	821
PB.7883.6	6	2375	5	full-splice_match	gene: Zm00001d038229	Zm00001d038229_T002	15.60	15.60	coding	632	282	2177
PB.8720.4	7	1146	9	full-splice_match	gene: Zm00001d021522	Zm00001d021522_T003	15.79	47.06	coding	260	84	863
PB.723.1	1	1873	2	full-splice_match	gene: Zm00001d030851	Zm00001d030851_T001	6.99	6.99	coding	518	106	1659
PB.10045.1	9	2567	6	full-splice_match	gene: Zm00001d044812	Zm00001d044812_T001	11.00	65.55	coding	601	160	1962
PB.1837.2	10	1252	1	novel_in_catalog	gene: Zm00001d024265	novel	9.43	43.94	coding	166	584	1081
PB.5670.1	4	1539	9	full-splice_match	gene: Zm00001d053101	Zm00001d053101_T001	5.20	5.20	coding	331	187	1179
PB.10045.7	9	2212	6	full-splice_match	gene: Zm00001d044812	Zm00001d044812_T001	54.55	65.55	coding	601	160	1962
PB.8666.1	7	2738	1	novel_in_catalog	gene: Zm00001d021305	novel	1.83	18.68	coding	342	122	1147
PB.10158.2	9	1106	10	full-splice_match	gene: Zm00001d045352	Zm00001d045352_T006	0.00	196.68	coding	332	110	1105
PB.10158.1	9	1542	12	full-splice_match	gene: Zm00001d045352	Zm00001d045352_T002	196.68	196.68	coding	380	109	1248
PB.6973.1	5	2820	3	full-splice_match	gene: Zm00001d017793	Zm00001d017793_T001	11.71	16.16	coding	713	452	2590
PB.2422.1	2	760	4	full-splice_match	gene: Zm00001d001882	Zm00001d001882_T004	353.53	624.91	coding	146	96	533
PB.2422.3	2	479	4	full-splice_match	gene: Zm00001d001882	Zm00001d001882_T004	53.30	624.91	coding	135	73	477
PB.2422.2	2	622	4	full-splice_match	gene: Zm00001d001882	Zm00001d001882_T004	218.07	624.91	coding	146	80	517
PB.1369.1	1	2517	6	novel_in_catalog	gene: Zm00001d033917	novel	48.81	105.08	coding	621	173	2035

PB.40.3	1	1804	4	novel_not_in_catalog	gene: Zm00001d027374	novel	1.60	68.29	coding	291	814	1686
PB.40.2	1	1498	5	novel_not_in_catalog	gene: Zm00001d027374	novel	66.69	68.29	coding	349	163	1209
PB.6996.11	5	2486	14	novel_in_catalog	gene: Zm00001d017876	novel	0.80	25.93	coding	273	1387	2205
PB.6996.10	5	2680	14	full-splice_match	gene: Zm00001d017876	Zm00001d017876_T020	0.00	25.93	coding	415	316	1560
PB.8887.1	7	2184	10	full-splice_match	gene: Zm00001d022071	Zm00001d022071_T002	30.33	36.73	coding	586	124	1881
PB.8887.2	7	2027	9	novel_in_catalog	gene: Zm00001d022071	novel	2.48	36.73	coding	500	117	1616
PB.175.4	1	2412	7	full-splice_match	gene: Zm00001d028006	Zm00001d028006_T002	1.59	21.95	coding	195	107	691
PB.3017.2	2	1768	3	genic	gene: Zm00001d004851	novel	12.08	12.08	coding	436	256	1563
PB.175.1	1	1816	8	full-splice_match	gene: Zm00001d028006	Zm00001d028006_T001	20.36	21.95	coding	466	141	1538
PB.268.2	1	1541	11	full-splice_match	gene: Zm00001d028396	Zm00001d028396_T003	162.14	240.34	coding	400	107	1306
PB.5525.2	4	861	6	incomplete-splice_match	gene: Zm00001d052445	Zm00001d052445_T008	68.72	68.72	coding	193	147	725
PB.7721.1	6	2275	5	full-splice_match	gene: Zm00001d037695	Zm00001d037695_T003	10.60	10.60	coding	627	154	2034
PB.6042.3	5	1503	11	full-splice_match	gene: Zm00001d013265	Zm00001d013265_T012	0.00	27.15	coding	418	250	1503
PB.9673.1	8	1214	2	full-splice_match	gene: Zm00001d011237	Zm00001d011237_T001	41.31	62.67	coding	298	18	911
PB.6042.1	5	2360	12	full-splice_match	gene: Zm00001d013265	Zm00001d013265_T001	20.50	27.15	coding	565	278	1972
PB.4459.1	3	984	1	intergenic	novelGene_316	novel	0.61	0.61	non_coding	NA	NA	NA
PB.10503.1	9	825	1	full-splice_match	gene: Zm00001d047420	Zm00001d047420_T001	35.03	35.03	coding	188	43	606

PB.5812.1	4	827	4	full-splice_match	gene: Zm00001d053805	Zm00001d053805_T003	18.02	18.02	coding	209	16	642
PB.1654.1	10	1243	5	novel_not_in_catalog	gene: Zm00001d023242	novel	102.55	165.03	coding	235	51	755
PB.1654.3	10	972	5	novel_not_in_catalog	gene: Zm00001d023242	novel	44.58	165.03	coding	203	244	852
PB.1654.2	10	2068	3	novel_not_in_catalog	gene: Zm00001d023242	novel	8.15	165.03	coding	118	47	400
PB.1654.5	10	984	4	novel_not_in_catalog	gene: Zm00001d023242	novel	0.52	165.03	coding	154	51	512
PB.1654.4	10	2044	1	genic	gene: Zm00001d023242	novel	9.22	165.03	coding	118	51	404
PB.5903.4	5	1824	7	novel_in_catalog	gene: Zm00001d012794	novel	7.64	16.40	coding	131	1031	1423
PB.1654.6	10	816	4	novel_not_in_catalog	gene: Zm00001d023242	novel	0.00	165.03	coding	154	60	521
PB.10182.2	9	2179	3	full-splice_match	gene: Zm00001d045463	Zm00001d045463_T002	16.61	16.88	coding	435	241	1545
PB.10182.3	9	685	1	novel_in_catalog	gene: Zm00001d045463	novel	0.27	16.88	non_coding	NA	NA	NA
PB.7141.4	5	1198	1	intergenic	novelGene_345	novel	147.21	272.48	non_coding	NA	NA	NA
PB.7141.5	5	851	1	intergenic	novelGene_345	novel	0.00	272.48	non_coding	NA	NA	NA
PB.7141.2	5	1464	1	intergenic	novelGene_345	novel	41.35	272.48	non_coding	NA	NA	NA
PB.7141.3	5	1321	1	intergenic	novelGene_345	novel	19.27	272.48	non_coding	NA	NA	NA
PB.5678.1	4	2427	11	incomplete-splice_match	gene: Zm00001d053155	Zm00001d053155_T012	24.26	24.26	coding	644	115	2046
PB.7141.1	5	2205	1	intergenic	novelGene_345	novel	3.51	272.48	non_coding	NA	NA	NA
PB.10527.1	9	3132	13	full-splice_match	gene: Zm00001d047491	Zm00001d047491_T002	10.61	11.64	coding	895	123	2807

PB.10527.2	9	2687	12	full-splice_match	gene: Zm00001d047491	Zm00001d047491_T011	1.03	11.64	coding	573	294	2012
PB.5836.1	4	1365	7	full-splice_match	gene: Zm00001d053899	Zm00001d053899_T003	109.36	109.36	coding	197	388	978
PB.9141.1	8	575	2	full-splice_match	gene: Zm00001d008548	Zm00001d008548_T001	24.72	24.72	coding	102	60	365
PB.1326.1	1	817	2	full-splice_match	gene: Zm00001d033711	Zm00001d033711_T001	146.78	158.71	coding	130	96	485
PB.1101.1	1	1386	1	full-splice_match	gene: Zm00001d032643	Zm00001d032643_T001	35.69	35.69	coding	320	74	1033
PB.10370.1	9	1766	2	full-splice_match	gene: Zm00001d046603	Zm00001d046603_T001	5.84	5.84	coding	509	7	1533
PB.6818.2	5	1755	3	full-splice_match	gene: Zm00001d017120	Zm00001d017120_T001	140.14	144.27	coding	348	434	1477
PB.5606.1	4	1846	1	full-splice_match	gene: Zm00001d052799	Zm00001d052799_T001	2.21	23.83	coding	307	46	966
PB.6818.1	5	2180	3	full-splice_match	gene: Zm00001d017120	Zm00001d017120_T001	4.14	144.27	coding	348	428	1471
PB.7829.1	6	806	4	full-splice_match	gene: Zm00001d038088	Zm00001d038088_T001	162.00	162.00	non_coding	NA	NA	NA
PB.10723.4	9	1651	11	full-splice_match	gene: Zm00001d048174	Zm00001d048174_T001	0.00	46.35	coding	458	69	1442
PB.10051.3	9	2720	8	full-splice_match	gene: Zm00001d044823	Zm00001d044823_T011	0.00	12.44	coding	637	653	2563
PB.10051.2	9	2894	8	full-splice_match	gene: Zm00001d044823	Zm00001d044823_T004	4.10	12.44	coding	637	542	2452
PB.10051.1	9	2922	8	full-splice_match	gene: Zm00001d044823	Zm00001d044823_T011	8.33	12.44	coding	637	535	2445
PB.5853.5	4	732	2	intergenic	novelGene_364	novel	70.61	224.73	non_coding	NA	NA	NA
PB.7374.5	6	1982	9	incomplete-splice_match	gene: Zm00001d035889	Zm00001d035889_T035	23.36	37.35	coding	597	92	1882
PB.14.4	1	2246	10	full-splice_match	gene: Zm00001d027293	Zm00001d027293_T002	261.37	309.60	coding	625	163	2037

PB.8067.1	6	910	6	full-splice_match	gene: Zm00001d038946	Zm00001d038946_T002	16.52	16.52	coding	83	301	549
PB.14.2	1	2566	9	novel_in_catalog	gene: Zm00001d027293	novel	1.91	309.60	coding	516	105	1652
PB.14.3	1	2441	10	full-splice_match	gene: Zm00001d027293	Zm00001d027293_T002	41.15	309.60	coding	625	105	1979
PB.7374.2	6	4340	13	full-splice_match	gene: Zm00001d035889	Zm00001d035889_T001	13.99	37.35	coding	1289	187	4053
PB.8341.2	7	466	4	novel_in_catalog	gene: Zm00001d019577	novel	148.21	162.48	non_coding	NA	NA	NA
PB.4908.2	4	1341	10	full-splice_match	gene: Zm00001d049163	Zm00001d049163_T001	51.82	84.16	coding	333	116	1114
PB.6074.1	5	1789	9	full-splice_match	gene: Zm00001d013375	Zm00001d013375_T004	8.21	8.21	coding	311	250	1182
PB.4908.1	4	1229	9	full-splice_match	gene: Zm00001d049163	Zm00001d049163_T006	28.98	84.16	coding	294	96	977
PB.9657.2	8	1060	10	full-splice_match	gene: Zm00001d011159	Zm00001d011159_T002	16.55	143.23	coding	204	131	742
PB.9657.3	8	1106	11	full-splice_match	gene: Zm00001d011159	Zm00001d011159_T001	5.99	143.23	coding	246	158	895
PB.9657.1	8	1229	11	full-splice_match	gene: Zm00001d011159	Zm00001d011159_T001	120.69	143.23	coding	246	174	911
PB.2845.1	2	2754	1	full-splice_match	gene: Zm00001d003668	Zm00001d003668_T001	63.79	63.79	coding	771	194	2506
PB.2517.2	2	1762	2	novel_in_catalog	gene: Zm00001d002285	novel	25.64	46.50	coding	196	910	1497
PB.2517.3	2	2535	1	genic	gene: Zm00001d002285	novel	0.75	46.50	coding	109	1948	2274
PB.2517.4	2	2158	2	novel_in_catalog	gene: Zm00001d002285	novel	20.11	46.50	coding	216	176	823
PB.4417.1	3	1943	3	full-splice_match	gene: Zm00001d043218	Zm00001d043218_T001	17.83	17.83	coding	516	157	1704
PB.8114.1	6	2074	1	full-splice_match	gene: Zm00001d039146	Zm00001d039146_T001	9.57	9.57	coding	453	65	1423

PB.6355.1	5	1003	1	full-splice_match	gene: Zm00001d014667	Zm00001d014667_T001	25.25	243.95	coding	93	255	533
PB.6355.2	5	1069	1	full-splice_match	gene: Zm00001d014667	Zm00001d014667_T001	13.56	243.95	coding	205	126	740
PB.6355.3	5	945	1	full-splice_match	gene: Zm00001d014667	Zm00001d014667_T001	191.54	243.95	coding	205	126	740
PB.6355.4	5	863	1	full-splice_match	gene: Zm00001d014667	Zm00001d014667_T001	13.60	243.95	coding	205	126	740
PB.5868.3	4	1465	1	full-splice_match	gene: Zm00001d053988	Zm00001d053988_T001	12.75	36.42	coding	162	702	1187
PB.8361.1	7	1439	1	full-splice_match	gene: Zm00001d019718	Zm00001d019718_T001	39.10	39.10	coding	371	106	1218
PB.5868.1	4	1894	1	full-splice_match	gene: Zm00001d053988	Zm00001d053988_T001	23.66	36.42	coding	162	956	1441
PB.1842.2	10	2081	1	genic	gene: Zm00001d024294	novel	9.83	309.46	coding	194	78	659
PB.1842.3	10	1016	3	novel_not_in_catalog	gene: Zm00001d024294	novel	6.96	309.46	coding	252	142	897
PB.1666.1	10	1566	5	antisense	novelGene_gene: Zm00001d023277_A	novel	36.02	56.67	coding	153	476	934
PB.6810.6	5	1808	11	novel_in_catalog	gene: Zm00001d017096	novel	2.22	118.92	coding	310	623	1552
PB.8887.3	7	2132	10	full-splice_match	gene: Zm00001d022071	Zm00001d022071_T002	3.92	36.73	coding	586	184	1941
PB.1666.4	10	1061	2	antisense	novelGene_gene: Zm00001d023277_A	novel	9.57	56.67	coding	153	301	759
PB.6810.5	5	2099	9	full-splice_match	gene: Zm00001d017096	Zm00001d017096_T012	1.08	118.92	coding	171	158	670
PB.8077.1	6	1178	5	full-splice_match	gene: Zm00001d038973	Zm00001d038973_T001	29.87	29.87	coding	250	66	815
PB.5808.1	4	1756	14	novel_in_catalog	gene: Zm00001d053798	novel	90.82	90.82	coding	412	206	1441
PB.3453.1	2	2124	8	novel_in_catalog	gene: Zm00001d007037	novel	9.88	31.18	coding	420	412	1671

PB.3453.4	2	1800	9	novel_in_catalog	gene: Zm00001d007037	novel	0.00	31.18	coding	441	339	1661
PB.22.1	1	1339	2	full-splice_match	gene: Zm00001d027313	Zm00001d027313_T001	45.08	45.08	coding	301	256	1158
PB.3943.1	3	1938	2	full-splice_match	gene: Zm00001d040721	Zm00001d040721_T002	106.39	111.89	coding	503	120	1628
PB.3943.2	3	1864	1	novel_in_catalog	gene: Zm00001d040721	novel	5.49	111.89	coding	409	354	1580
PB.9129.1	8	1503	6	full-splice_match	gene: Zm00001d008491	Zm00001d008491_T002	176.28	176.28	coding	387	102	1262
PB.3943.4	3	1701	2	full-splice_match	gene: Zm00001d040721	Zm00001d040721_T002	0.00	111.89	coding	503	98	1606
PB.6925.2	5	1324	7	full-splice_match	gene: Zm00001d017564	Zm00001d017564_T003	39.84	43.92	coding	353	58	1116
PB.1205.1	1	3230	14	novel_not_in_catalog	gene: Zm00001d033168	novel	22.87	34.71	coding	921	43	2805
PB.1086.1	1	1539	6	full-splice_match	gene: Zm00001d032572	Zm00001d032572_T001	9.57	9.57	coding	408	122	1345
PB.1476.2	1	1275	1	genic	gene: Zm00001d034400	novel	8.33	91.19	non_coding	NA	NA	NA
PB.7626.1	6	1719	1	novel_in_catalog	gene: Zm00001d037249	novel	5.67	20.50	coding	385	329	1483
PB.234.1	1	1635	3	full-splice_match	gene: Zm00001d028266	Zm00001d028266_T001	37.77	37.77	coding	484	33	1484
PB.2093.1	10	568	1	antisense	novelGene_gene: Zm00001d025612_A	novel	513.97	513.97	non_coding	NA	NA	NA
PB.4071.1	3	2024	1	intergenic	novelGene_205	novel	7.02	7.02	coding	43	290	418
PB.4280.1	3	2105	4	full-splice_match	gene: Zm00001d042632	Zm00001d042632_T001	3.24	26.97	coding	475	300	1724
PB.8601.1	7	2027	3	full-splice_match	gene: Zm00001d020982	Zm00001d020982_T001	35.81	57.70	coding	376	440	1567
PB.937.3	1	1207	6	full-splice_match	gene: Zm00001d031963	Zm00001d031963_T001	13.34	87.37	coding	274	30	851

PB.6691.2	5	2391	12	full-splice_match	gene: Zm00001d016512	Zm00001d016512_T001	19.13	19.13	coding	677	180	2210
PB.8601.2	7	1728	3	full-splice_match	gene: Zm00001d020982	Zm00001d020982_T001	21.89	57.70	coding	376	353	1480
PB.8980.2	7	730	1	full-splice_match	gene: Zm00001d022416	Zm00001d022416_T001	47.40	97.60	coding	113	148	486
PB.1476.1	1	1917	5	novel_not_in_catalog	gene: Zm00001d034400	novel	19.89	91.19	coding	427	155	1435
PB.3606.1	2	1095	3	full-splice_match	gene: Zm00001d007857	Zm00001d007857_T001	2.20	2.20	coding	258	174	947
PB.7626.2	6	1580	2	full-splice_match	gene: Zm00001d037249	Zm00001d037249_T001	14.82	20.50	coding	454	53	1414
PB.942.2	1	2731	8	novel_in_catalog	gene: Zm00001d031977	novel	3.70	18.53	coding	624	396	2267
PB.10143.1	9	826	1	intergenic	novelGene_59	novel	42.08	42.08	coding	91	94	366
PB.1892.1	10	609	4	full-splice_match	gene: Zm00001d024571	Zm00001d024571_T001	530.27	530.27	coding	119	76	432
PB.8375.1	7	2000	6	full-splice_match	gene: Zm00001d019850	Zm00001d019850_T001	39.17	49.36	coding	496	100	1587
PB.8375.3	7	2012	5	novel_in_catalog	gene: Zm00001d019850	novel	3.75	49.36	coding	378	533	1666
PB.8375.4	7	2074	4	novel_in_catalog	gene: Zm00001d019850	novel	3.25	49.36	coding	436	489	1796
PB.8375.6	7	1834	5	novel_in_catalog	gene: Zm00001d019850	novel	2.04	49.36	coding	325	705	1679
PB.8375.7	7	1962	4	novel_in_catalog	gene: Zm00001d019850	novel	1.15	49.36	coding	436	545	1852
PB.8126.1	6	2421	1	full-splice_match	gene: Zm00001d039177	Zm00001d039177_T001	4.55	4.55	coding	430	664	1953
PB.8990.1	7	1956	7	novel_not_in_catalog	gene: Zm00001d022434	novel	24.04	24.04	coding	486	278	1735
PB.2971.8	2	2875	7	full-splice_match	gene: Zm00001d004552	Zm00001d004552_T012	0.98	108.54	coding	107	1086	1406

PB.6917.1	5	1056	3	full-splice_match	gene: Zm00001d017523	Zm00001d017523_T002	41.37	41.37	coding	224	32	703
PB.1589.2	1	2961	3	full-splice_match	gene: Zm00001d034771	Zm00001d034771_T002	208.18	336.20	coding	843	173	2701
PB.1237.4	1	2121	7	intergenic	novelGene_370	novel	15.69	16.76	coding	73	104	322
PB.1237.6	1	395	3	intergenic	novelGene_370	novel	1.07	16.76	non_coding	NA	NA	NA
PB.10310.1	9	1474	5	novel_in_catalog	gene: Zm00001d046243	novel	22.52	39.81	coding	245	279	1013
PB.8396.1	7	2114	2	incomplete-splice_match	gene: Zm00001d020014	Zm00001d020014_T012	23.24	23.24	coding	291	402	1274
PB.10310.3	9	2174	8	full-splice_match	gene: Zm00001d046243	Zm00001d046243_T003	10.16	39.81	coding	510	345	1874
PB.10310.2	9	2077	7	novel_in_catalog	gene: Zm00001d046243	novel	3.44	39.81	coding	403	94	1302
PB.10310.4	9	2099	8	full-splice_match	gene: Zm00001d046243	Zm00001d046243_T002	0.00	39.81	coding	511	317	1849
PB.9282.2	8	1558	13	fusion	gene: Zm00001d009317_g	novel	18.93	18.93	coding	436	27	1334
PB.2971.7	2	2124	8	full-splice_match	gene: Zm00001d004552	Zm00001d004552_T001	3.20	108.54	coding	191	82	654
PB.7056.3	5	1619	3	novel_in_catalog	gene: Zm00001d018099	novel	17.27	138.11	coding	183	168	716
PB.7056.2	5	1413	4	full-splice_match	gene: Zm00001d018099	Zm00001d018099_T001	54.33	138.11	coding	321	161	1123
PB.7056.1	5	1632	4	full-splice_match	gene: Zm00001d018099	Zm00001d018099_T001	13.78	138.11	coding	321	168	1130
PB.10348.2	9	1537	3	novel_in_catalog	gene: Zm00001d046491	novel	7.15	50.03	coding	228	642	1325
PB.10348.1	9	1369	4	full-splice_match	gene: Zm00001d046491	Zm00001d046491_T001	42.88	50.03	coding	344	126	1157
PB.3657.1	3	1546	8	novel_in_catalog	gene: Zm00001d039312	novel	18.86	18.86	coding	367	18	1118

PB.4710.2	3	1783	1	full-splice_match	gene: Zm00001d044390	Zm00001d044390_T001	1.69	62.28	coding	485	192	1646
PB.3305.4	2	2652	5	full-splice_match	gene: Zm00001d006355	Zm00001d006355_T018	0.00	15.81	coding	734	238	2439
PB.6854.2	5	1751	8	full-splice_match	gene: Zm00001d017252	Zm00001d017252_T002	32.73	40.00	coding	457	124	1494
PB.599.2	1	1037	1	full-splice_match	gene: Zm00001d029969	Zm00001d029969_T001	30.28	30.28	coding	210	94	723
PB.2911.1	2	1564	12	full-splice_match	gene: Zm00001d004020	Zm00001d004020_T004	6.31	6.31	coding	411	50	1282
PB.5458.1	4	831	2	full-splice_match	gene: Zm00001d052234	Zm00001d052234_T001	61.94	71.81	coding	157	108	578
PB.7648.1	6	1393	3	novel_not_in_catalog	gene: Zm00001d037342	novel	30.58	30.58	coding	324	89	1060
PB.10297.1	9	534	4	intergenic	novelGene_129	novel	339.97	339.97	coding	76	105	332
PB.5163.1	4	1038	2	intergenic	novelGene_173	novel	105.82	105.82	coding	188	274	837
PB.10423.1	9	2246	6	full-splice_match	gene: Zm00001d046939	Zm00001d046939_T002	16.41	25.76	coding	465	432	1826
PB.6054.2	5	2689	2	incomplete-splice_match	gene: Zm00001d013301	Zm00001d013301_T001	10.63	18.46	coding	753	15	2273
PB.9542.1	8	1704	2	genic	gene: Zm00001d010654	novel	216.96	216.96	coding	357	276	1346
PB.10674.1	9	1969	1	full-splice_match	gene: Zm00001d047982	Zm00001d047982_T001	0.85	0.85	coding	627	19	1899
PB.1076.19	1	1634	3	novel_in_catalog	gene: Zm00001d032555	novel	4.73	39.90	coding	434	55	1356
PB.6907.2	5	2061	3	full-splice_match	gene: Zm00001d017492	Zm00001d017492_T001	4.29	9.06	coding	502	231	1736
PB.6907.1	5	2105	2	novel_in_catalog	gene: Zm00001d017492	novel	4.77	9.06	coding	481	160	1602
PB.4797.1	4	1556	1	novel_in_catalog	gene: Zm00001d048603	novel	12.85	22.75	coding	417	23	1273

PB.870.2	1	736	2	full-splice_match	gene: Zm00001d031627	Zm00001d031627_T001	613.61	721.59	coding	124	143	514
PB.870.1	1	884	2	full-splice_match	gene: Zm00001d031627	Zm00001d031627_T001	107.99	721.59	coding	124	85	456
PB.4222.9	3	822	6	full-splice_match	gene: Zm00001d042347	Zm00001d042347_T011	226.60	1197.41	non_coding	NA	NA	NA
PB.7780.1	6	730	7	incomplete-splice_match	gene: Zm00001d037925	Zm00001d037925_T011	28.29	67.95	coding	118	30	383
PB.2932.1	2	2503	21	novel_in_catalog	gene: Zm00001d004159	novel	8.80	8.80	coding	667	224	2224
PB.8269.2	7	1796	16	full-splice_match	gene: Zm00001d019148	Zm00001d019148_T004	22.11	22.90	coding	473	101	1519
PB.8269.3	7	2559	13	novel_in_catalog	gene: Zm00001d019148	novel	0.78	22.90	coding	171	1163	1675
PB.5857.1	4	2028	11	full-splice_match	gene: Zm00001d053968	Zm00001d053968_T004	0.89	27.09	coding	300	145	1044
PB.7213.1	6	2381	3	incomplete-splice_match	gene: Zm00001d035008	Zm00001d035008_T004	14.74	14.74	coding	324	1008	1979
PB.4520.4	3	3237	12	full-splice_match	gene: Zm00001d043613	Zm00001d043613_T005	0.00	284.34	coding	300	1780	2679
PB.9476.1	8	1388	1	full-splice_match	gene: Zm00001d010329	Zm00001d010329_T001	16.76	16.76	coding	384	46	1197
PB.8602.1	7	2091	3	intergenic	novelGene_223	novel	1.82	1.82	non_coding	NA	NA	NA
PB.196.2	1	1676	1	full-splice_match	gene: Zm00001d028125	Zm00001d028125_T001	9.34	9.34	coding	382	346	1491
PB.4081.1	3	406	2	incomplete-splice_match	gene: Zm00001d041700	Zm00001d041700_T007	21.66	21.66	non_coding	NA	NA	NA
PB.7692.1	6	1758	2	novel_in_catalog	gene: Zm00001d037565	novel	6.33	7.18	coding	278	116	949
PB.3531.1	2	715	1	full-splice_match	gene: Zm00001d007477	Zm00001d007477_T001	9.66	9.66	coding	58	317	490
PB.7692.2	6	2156	1	incomplete-splice_match	gene: Zm00001d037565	Zm00001d037565_T001	0.85	7.18	coding	134	963	1364

PB.2260.1	10	1825	7	full-splice_match	gene: Zm00001d026322	Zm00001d026322_T003	31.54	31.54	coding	374	488	1609
PB.7237.2	6	815	1	full-splice_match	gene: Zm00001d035095	Zm00001d035095_T001	212.50	654.81	coding	148	107	550
PB.7237.1	6	907	1	full-splice_match	gene: Zm00001d035095	Zm00001d035095_T001	442.31	654.81	coding	148	81	524
PB.9790.1	8	1043	2	full-splice_match	gene: Zm00001d011778	Zm00001d011778_T001	64.51	64.51	coding	254	38	799
PB.7060.4	5	907	6	full-splice_match	gene: Zm00001d018112	Zm00001d018112_T003	30.11	201.70	coding	212	43	678
PB.7060.5	5	2462	3	incomplete-splice_match	gene: Zm00001d018112	Zm00001d018112_T009	0.00	201.70	non_coding	NA	NA	NA
PB.7060.6	5	405	4	incomplete-splice_match	gene: Zm00001d018112	Zm00001d018112_T002	68.66	201.70	coding	93	46	324
PB.10324.15	9	1196	2	genic	gene: Zm00001d046378	novel	0.00	784.46	coding	138	201	614
PB.2563.1	2	2500	7	full-splice_match	gene: Zm00001d002478	Zm00001d002478_T001	15.04	15.04	coding	645	149	2083
PB.7060.1	5	1186	6	full-splice_match	gene: Zm00001d018112	Zm00001d018112_T003	88.11	201.70	coding	212	52	687
PB.7060.2	5	3562	3	incomplete-splice_match	gene: Zm00001d018112	Zm00001d018112_T001	14.82	201.70	non_coding	NA	NA	NA
PB.2563.2	2	2339	7	full-splice_match	gene: Zm00001d002478	Zm00001d002478_T001	0.00	15.04	coding	645	149	2083
PB.4518.1	3	522	1	full-splice_match	gene: Zm00001d043610	Zm00001d043610_T001	17.73	17.73	coding	108	42	365
PB.3750.2	3	2201	1	full-splice_match	gene: Zm00001d039679	Zm00001d039679_T001	0.59	0.59	coding	580	93	1832
PB.5982.4	5	2230	4	incomplete-splice_match	gene: Zm00001d013049	Zm00001d013049_T006	2.92	16.92	coding	489	186	1652
PB.5982.3	5	2705	7	full-splice_match	gene: Zm00001d013049	Zm00001d013049_T002	8.18	16.92	coding	775	252	2576
PB.7121.1	5	1970	4	novel_not_in_catalog	gene: Zm00001d018338	novel	23.78	23.78	non_coding	NA	NA	NA

PB.5982.1	5	2833	7	full-splice_match	gene: Zm00001d013049	Zm00001d013049_T002	5.83	16.92	coding	775	230	2554
PB.4236.3	3	1675	15	full-splice_match	gene: Zm00001d042421	Zm00001d042421_T002	0.00	50.83	coding	401	225	1427
PB.6466.1	5	1140	6	full-splice_match	gene: Zm00001d015061	Zm00001d015061_T001	28.80	28.80	coding	231	237	929
PB.9938.1	8	760	1	full-splice_match	gene: Zm00001d012427	Zm00001d012427_T001	12.78	12.78	coding	169	38	544
PB.8563.8	7	1839	6	novel_not_in_catalog	gene: Zm00001d020835	novel	7.68	44.58	coding	288	109	972
PB.9512.1	8	1656	9	full-splice_match	gene: Zm00001d010523	Zm00001d010523_T006	27.49	38.31	coding	423	170	1438
PB.9512.2	8	1693	8	full-splice_match	gene: Zm00001d010523	Zm00001d010523_T005	10.82	38.31	coding	348	409	1452
PB.8563.3	7	2171	7	novel_in_catalog	gene: Zm00001d020835	novel	2.46	44.58	coding	527	269	1849
PB.2055.2	10	2971	3	full-splice_match	gene: Zm00001d025342	Zm00001d025342_T003	22.45	32.56	coding	791	396	2768
PB.7575.1	6	2063	5	novel_not_in_catalog	gene: Zm00001d037015	novel	5.31	5.31	coding	316	183	1130
PB.8563.7	7	1980	8	incomplete-splice_match	gene: Zm00001d020835	Zm00001d020835_T007	0.00	44.58	coding	497	269	1759
PB.8563.5	7	2293	6	novel_in_catalog	gene: Zm00001d020835	novel	0.00	44.58	coding	206	267	884
PB.8563.4	7	2177	7	incomplete-splice_match	gene: Zm00001d020835	Zm00001d020835_T014	6.34	44.58	coding	225	1183	1857
PB.2069.2	10	2068	11	full-splice_match	gene: Zm00001d025433	Zm00001d025433_T003	0.00	25.16	coding	626	16	1893
PB.9300.2	8	589	3	genic	gene: Zm00001d009418	novel	104.97	144.20	non_coding	NA	NA	NA
PB.9300.1	8	675	3	genic	gene: Zm00001d009418	novel	31.09	144.20	non_coding	NA	NA	NA
PB.7549.1	6	1006	2	full-splice_match	gene: Zm00001d036877	Zm00001d036877_T001	25.34	25.34	coding	274	10	831

PB.5214.1	4	1262	3	full-splice_match	gene: Zm00001d050992	Zm00001d050992_T001	69.97	70.92	coding	326	31	1008
PB.1852.4	10	1122	1	full-splice_match	gene: Zm00001d024379	Zm00001d024379_T001	0.00	311.40	coding	286	129	986
PB.8930.3	7	2413	20	full-splice_match	gene: Zm00001d022240	Zm00001d022240_T008	2.16	11.19	coding	701	48	2150
PB.8930.2	7	3923	16	novel_in_catalog	gene: Zm00001d022240	novel	0.31	11.19	coding	383	18	1166
PB.8422.1	7	1417	7	novel_not_in_catalog	gene: Zm00001d020176	novel	2.86	2.86	coding	199	725	1321
PB.7769.1	6	1697	3	full-splice_match	gene: Zm00001d037873	Zm00001d037873_T002	702.42	702.42	coding	447	83	1423
PB.7403.1	6	622	1	antisense	novelGene_gene: Zm00001d036025_A	novel	14.05	14.05	non_coding	NA	NA	NA
PB.7120.2	5	874	1	full-splice_match	gene: Zm00001d018337	Zm00001d018337_T001	101.33	102.56	coding	188	101	664
PB.3300.2	2	419	2	full-splice_match	gene: Zm00001d006336	Zm00001d006336_T001	605.21	667.26	non_coding	NA	NA	NA
PB.4043.1	3	1652	6	full-splice_match	gene: Zm00001d041514	Zm00001d041514_T016	4.76	4.76	coding	391	139	1311
PB.1852.1	10	1457	1	full-splice_match	gene: Zm00001d024379	Zm00001d024379_T001	11.40	311.40	coding	286	125	982
PB.5506.4	4	1318	9	novel_not_in_catalog	gene: Zm00001d052391	novel	74.11	242.70	coding	318	143	1096
PB.7466.2	6	951	7	full-splice_match	gene: Zm00001d036442	Zm00001d036442_T002	431.37	485.98	coding	215	135	779
PB.5054.2	4	1428	9	novel_not_in_catalog	gene: Zm00001d049892	novel	34.58	34.58	coding	336	71	1078
PB.5506.1	4	1507	9	novel_not_in_catalog	gene: Zm00001d052391	novel	155.16	242.70	coding	318	138	1091
PB.7466.1	6	1090	7	full-splice_match	gene: Zm00001d036442	Zm00001d036442_T002	54.60	485.98	coding	215	90	734
PB.1579.3	1	2907	3	novel_in_catalog	gene: Zm00001d034738	novel	2.03	19.79	coding	315	130	1074

PB.852.1	1	476	1	intergenic	novelGene_310	novel	222.59	222.59	coding	23	232	300
PB.8910.1	7	1286	1	full-splice_match	gene: Zm00001d022154	Zm00001d022154_T001	70.62	70.62	coding	271	225	1037
PB.6492.1	5	1236	1	incomplete-splice_match	gene: Zm00001d015164	Zm00001d015164_T001	4.78	4.78	coding	243	22	750
PB.9906.1	8	1433	2	full-splice_match	gene: Zm00001d012267	Zm00001d012267_T001	73.90	73.90	coding	417	136	1386
PB.9588.4	8	2109	18	full-splice_match	gene: Zm00001d010835	Zm00001d010835_T008	24.28	25.74	coding	604	139	1950
PB.1967.1	10	1359	3	novel_not_in_catalog	gene: Zm00001d024925	novel	124.50	124.50	coding	321	197	1159
PB.7583.3	6	3133	1	novel_in_catalog	gene: Zm00001d037058	novel	1.74	40.68	coding	298	2001	2894
PB.573.1	1	2102	15	full-splice_match	gene: Zm00001d029768	Zm00001d029768_T001	5.70	5.70	coding	565	171	1865
PB.2132.1	10	1632	10	full-splice_match	gene: Zm00001d025767	Zm00001d025767_T002	37.76	41.22	coding	448	38	1381
PB.2447.3	2	2026	6	novel_in_catalog	gene: Zm00001d001984	novel	25.16	116.65	coding	346	821	1858
PB.3980.1	3	2156	2	incomplete-splice_match	gene: Zm00001d041098	Zm00001d041098_T001	26.18	26.18	coding	543	190	1818
PB.9427.2	8	2980	15	full-splice_match	gene: Zm00001d010109	Zm00001d010109_T002	48.95	48.95	coding	763	274	2562
PB.1043.2	1	1718	8	full-splice_match	gene: Zm00001d032435	Zm00001d032435_T001	0.00	25.72	coding	456	162	1529
PB.8069.1	6	2370	16	novel_in_catalog	gene: Zm00001d038955	novel	62.30	62.30	coding	609	254	2080
PB.1265.1	1	1550	1	full-splice_match	gene: Zm00001d033475	Zm00001d033475_T001	2.04	2.04	coding	260	481	1260
PB.8619.2	7	1058	7	full-splice_match	gene: Zm00001d021061	Zm00001d021061_T001	553.65	693.39	coding	258	72	845
PB.1232.1	1	2138	7	incomplete-splice_match	gene: Zm00001d033309	Zm00001d033309_T005	12.19	49.16	coding	150	92	541

PB.393.1	1	1468	13	novel_not_in_catalog	gene: Zm00001d028958	novel	75.18	75.18	coding	387	114	1274
PB.8098.1	6	1442	13	full-splice_match	gene: Zm00001d039079	Zm00001d039079_T003	504.65	504.65	coding	323	125	1093
PB.1474.2	1	3600	9	full-splice_match	gene: Zm00001d034387	Zm00001d034387_T011	31.49	31.49	coding	1126	36	3413
PB.9576.1	8	1843	3	full-splice_match	gene: Zm00001d010795	Zm00001d010795_T001	3.50	3.50	coding	326	592	1569
PB.6414.1	5	653	2	incomplete-splice_match	gene: Zm00001d014880	Zm00001d014880_T001	73.19	73.19	coding	63	180	368
PB.1150.1	1	2468	1	full-splice_match	gene: Zm00001d032870	Zm00001d032870_T001	4.45	4.45	coding	688	38	2101
PB.4896.2	4	1466	2	full-splice_match	gene: Zm00001d049107	Zm00001d049107_T001	0.00	15.17	coding	320	109	1068
PB.1108.1	1	783	1	full-splice_match	gene: Zm00001d032672	Zm00001d032672_T001	4.07	119.54	coding	166	6	503
PB.8619.1	7	1176	7	full-splice_match	gene: Zm00001d021061	Zm00001d021061_T001	139.73	693.39	coding	258	76	849
PB.9127.4	8	2387	2	novel_in_catalog	gene: Zm00001d008477	novel	6.18	30.09	coding	428	946	2229
PB.3772.3	3	726	1	novel_in_catalog	gene: Zm00001d039826	novel	0.00	246.16	non_coding	NA	NA	NA
PB.9127.7	8	2399	3	incomplete-splice_match	gene: Zm00001d008488	Zm00001d008488_T006	38.22	38.22	coding	683	82	2130
PB.4067.2	3	2987	1	full-splice_match	gene: Zm00001d041626	Zm00001d041626_T001	3.80	14.07	coding	941	73	2895
PB.4067.3	3	2005	1	full-splice_match	gene: Zm00001d041626	Zm00001d041626_T001	0.00	14.07	coding	529	417	2003
PB.3772.4	3	849	2	full-splice_match	gene: Zm00001d039826	Zm00001d039826_T001	16.10	246.16	non_coding	NA	NA	NA
PB.9127.3	8	1965	4	novel_not_in_catalog	gene: Zm00001d008477	novel	23.91	30.09	coding	464	416	1807
PB.4308.4	3	1815	4	novel_in_catalog	gene: Zm00001d042731	novel	21.18	56.15	coding	375	155	1279

PB.10701.2	9	2631	5	incomplete-splice_match	gene: Zm00001d048096	Zm00001d048096_T013	25.21	37.11	coding	652	215	2170
PB.10701.1	9	5476	7	full-splice_match	gene: Zm00001d048096	Zm00001d048096_T007	11.90	37.11	coding	1553	121	4779
PB.1612.2	1	2130	5	full-splice_match	gene: Zm00001d034839	Zm00001d034839_T002	4.50	14.21	coding	545	353	1987
PB.7096.2	5	3539	23	full-splice_match	gene: Zm00001d018246	Zm00001d018246_T004	13.39	45.36	coding	994	162	3143
PB.6120.1	5	2055	8	novel_not_in_catalog	gene: Zm00001d013534	novel	11.07	11.07	coding	305	947	1861
PB.4806.1	4	3566	21	full-splice_match	gene: Zm00001d048628	Zm00001d048628_T001	31.25	31.25	coding	1092	61	3336
PB.9696.2	8	1371	7	full-splice_match	gene: Zm00001d011363	Zm00001d011363_T003	67.35	108.94	coding	345	63	1097
PB.9696.1	8	1433	7	full-splice_match	gene: Zm00001d011363	Zm00001d011363_T003	41.59	108.94	coding	345	26	1060
PB.1196.4	1	2512	17	full-splice_match	gene: Zm00001d033108	Zm00001d033108_T006	26.82	82.41	coding	605	127	1941
PB.8081.2	6	2232	4	full-splice_match	gene: Zm00001d038999	Zm00001d038999_T003	35.25	872.48	coding	151	1564	2016
PB.8081.1	6	1317	5	full-splice_match	gene: Zm00001d038999	Zm00001d038999_T001	837.23	872.48	coding	229	438	1124
PB.1317.2	1	1931	14	full-splice_match	gene: Zm00001d033674	Zm00001d033674_T009	60.16	60.16	coding	473	133	1551
PB.1612.1	1	1936	5	full-splice_match	gene: Zm00001d034839	Zm00001d034839_T002	9.71	14.21	coding	545	65	1699
PB.1907.1	10	1301	7	full-splice_match	gene: Zm00001d024661	Zm00001d024661_T001	6.60	26.43	coding	229	210	896
PB.742.1	1	2122	1	intergenic	novelGene_283	novel	6.43	6.43	coding	170	1294	1803
PB.5654.3	4	729	3	full-splice_match	gene: Zm00001d053010	Zm00001d053010_T001	154.35	918.66	coding	165	127	621
PB.9997.2	8	1489	6	novel_in_catalog	gene: Zm00001d012656	novel	4.10	22.69	coding	395	57	1241

PB.658.1	1	1302	1	full-splice_match	gene: Zm00001d030338	Zm00001d030338_T001	35.12	35.12	coding	320	93	1052
PB.4743.3	3	2306	1	novel_in_catalog	gene: Zm00001d044504	novel	6.48	105.91	non_coding	NA	NA	NA
PB.8905.1	7	1035	8	full-splice_match	gene: Zm00001d022134	Zm00001d022134_T001	1318.48	1318.48	coding	229	116	802
PB.4694.8	3	2468	19	incomplete-splice_match	gene: Zm00001d044303	Zm00001d044303_T003	0.93	42.18	coding	697	377	2467
PB.8128.2	6	2329	8	novel_not_in_catalog	gene: Zm00001d039180	novel	53.25	53.25	coding	513	400	1938
PB.9620.1	8	1638	3	novel_not_in_catalog	gene: Zm00001d010969	novel	0.00	60.34	coding	279	47	883
PB.4144.3	3	1729	13	full-splice_match	gene: Zm00001d041995	Zm00001d041995_T003	2.79	19.82	coding	488	61	1524
PB.9797.1	8	1214	2	full-splice_match	gene: Zm00001d011793	Zm00001d011793_T001	6.30	6.30	coding	332	106	1101
PB.4144.1	3	1818	13	full-splice_match	gene: Zm00001d041995	Zm00001d041995_T003	17.03	19.82	coding	488	46	1509
PB.9620.2	8	1828	2	full-splice_match	gene: Zm00001d010969	Zm00001d010969_T001	11.54	60.34	coding	318	47	1000
PB.3399.1	2	1977	6	full-splice_match	gene: Zm00001d006760	Zm00001d006760_T001	0.82	2189.04	coding	160	85	564
PB.4522.1	3	965	2	novel_not_in_catalog	gene: Zm00001d043619	novel	187.61	187.61	coding	202	28	633
PB.6591.2	5	696	4	novel_not_in_catalog	gene: Zm00001d015861	novel	0.00	24.72	coding	144	133	564
PB.8743.3	7	2348	20	novel_in_catalog	gene: Zm00001d021584	novel	0.63	15.33	coding	682	210	2255
PB.7948.4	6	737	3	full-splice_match	gene: Zm00001d038519	Zm00001d038519_T004	32.24	63.23	non_coding	NA	NA	NA
PB.6591.1	5	1243	6	full-splice_match	gene: Zm00001d015861	Zm00001d015861_T002	24.72	24.72	coding	217	325	975
PB.7777.2	6	2114	1	full-splice_match	gene: Zm00001d037914	Zm00001d037914_T001	3.31	3.31	coding	161	53	535

PB.10648.1	9	1307	1	full-splice_match	gene: Zm00001d047901	Zm00001d047901_T001	66.60	66.60	coding	336	51	1058
PB.9479.1	8	1160	7	full-splice_match	gene: Zm00001d010368	Zm00001d010368_T002	78.83	83.80	coding	243	122	850
PB.6567.4	5	2766	1	genic	gene: Zm00001d015638	novel	2.00	46.52	coding	194	1888	2469
PB.9479.2	8	1736	4	novel_in_catalog	gene: Zm00001d010368	novel	4.98	83.80	coding	162	1008	1493
PB.6567.3	5	1521	2	full-splice_match	gene: Zm00001d015638	Zm00001d015638_T004	41.96	46.52	coding	355	255	1319
PB.6567.2	5	1622	2	full-splice_match	gene: Zm00001d015638	Zm00001d015638_T004	2.55	46.52	coding	355	254	1318
PB.2361.1	10	1117	5	novel_not_in_catalog	gene: Zm00001d026640	novel	14.25	14.25	coding	143	467	895
PB.6436.3	5	1093	1	novel_in_catalog	gene: Zm00001d014957	novel	11.23	3442.44	non_coding	NA	NA	NA
PB.6436.6	5	636	5	full-splice_match	gene: Zm00001d014957	Zm00001d014957_T001	2207.29	3442.44	coding	167	75	575
PB.198.1	1	736	2	incomplete-splice_match	gene: Zm00001d028130	Zm00001d028130_T001	40.78	40.78	coding	126	51	428
PB.7456.1	6	1157	8	full-splice_match	gene: Zm00001d036403	Zm00001d036403_T002	262.93	262.93	coding	258	174	947
PB.6436.5	5	708	5	novel_in_catalog	gene: Zm00001d014957	novel	1223.92	3442.44	coding	156	75	542
PB.4445.2	3	2145	1	novel_in_catalog	gene: Zm00001d043347	novel	1.69	12.29	non_coding	NA	NA	NA
PB.4445.3	3	3185	7	novel_not_in_catalog	gene: Zm00001d043347	novel	0.51	12.29	coding	897	101	2791
PB.6772.1	5	738	3	incomplete-splice_match	gene: Zm00001d016892	Zm00001d016892_T001	49.59	156.87	coding	145	27	461
PB.5368.3	4	2196	15	novel_in_catalog	gene: Zm00001d051873	novel	7.75	35.79	coding	405	665	1879
PB.8483.1	7	3842	5	full-splice_match	gene: Zm00001d020454	Zm00001d020454_T003	34.33	35.14	coding	1130	202	3591

PB.4679.1	3	898	1	full-splice_match	gene: Zm00001d044247	Zm00001d044247_T001	65.14	1129.73	coding	148	81	524
PB.2341.2	10	2342	2	novel_in_catalog	gene: Zm00001d026588	novel	2.33	163.02	coding	170	24	533
PB.2341.1	10	567	3	full-splice_match	gene: Zm00001d026588	Zm00001d026588_T002	160.69	163.02	coding	96	52	339
PB.6793.1	5	976	4	full-splice_match	gene: Zm00001d017000	Zm00001d017000_T001	44.82	44.82	coding	238	101	814
PB.1600.10	1	2578	11	novel_in_catalog	gene: Zm00001d034809	novel	0.00	80.83	coding	255	506	1270
PB.2572.1	2	1667	10	full-splice_match	gene: Zm00001d002504	Zm00001d002504_T002	22.68	31.55	coding	319	368	1324
PB.1600.12	1	2207	13	full-splice_match	gene: Zm00001d034809	Zm00001d034809_T002	0.00	80.83	coding	321	201	1163
PB.10925.10	B73V4 ctg52	1628	3	full-splice_match	gene: Zm00001d000112	Zm00001d000112_T001	0.00	64.88	coding	359	131	1207
PB.684.1	1	1177	2	full-splice_match	gene: Zm00001d030544	Zm00001d030544_T001	21.46	21.46	coding	320	84	1043
PB.7383.9	6	1904	2	full-splice_match	gene: Zm00001d035934	Zm00001d035934_T002	0.00	156.37	coding	593	29	1807
PB.9554.1	8	571	1	intergenic	novelGene_199	novel	3.23	3.23	non_coding	NA	NA	NA
PB.8488.1	7	1082	5	full-splice_match	gene: Zm00001d020485	Zm00001d020485_T001	184.89	184.89	coding	218	207	860
PB.5792.2	4	998	5	full-splice_match	gene: Zm00001d053724	Zm00001d053724_T001	22.79	32.66	coding	178	185	718
PB.4854.1	4	2034	5	novel_in_catalog	gene: Zm00001d048891	novel	7.57	7.57	coding	609	38	1864
PB.7383.3	6	2506	2	full-splice_match	gene: Zm00001d035934	Zm00001d035934_T002	135.32	156.37	coding	593	318	2096
PB.4893.12	4	798	5	incomplete-splice_match	gene: Zm00001d049097	Zm00001d049097_T007	0.00	54.78	coding	194	74	655
PB.1558.1	1	1786	1	full-splice_match	gene: Zm00001d034671	Zm00001d034671_T001	3.00	4.68	coding	478	85	1518

PB.8748.4	7	1780	10	novel_not_in_catalog	gene: Zm00001d021615	novel	0.00	543.59	coding	158	99	572
PB.1128.4	1	2099	4	full-splice_match	gene: Zm00001d032754	Zm00001d032754_T002	12.44	12.44	coding	528	241	1824
PB.8748.5	7	1664	11	novel_not_in_catalog	gene: Zm00001d021615	novel	6.62	543.59	coding	274	104	925
PB.5877.1	4	1905	5	full-splice_match	gene: Zm00001d054008	Zm00001d054008_T002	33.37	33.37	coding	405	410	1624
PB.6388.2	5	731	1	full-splice_match	gene: Zm00001d014778	Zm00001d014778_T001	11.96	29.62	coding	187	15	575
PB.8748.6	7	1449	11	novel_not_in_catalog	gene: Zm00001d021615	novel	13.96	543.59	coding	192	683	1258
PB.8795.1	7	1661	9	novel_not_in_catalog	gene: Zm00001d021752	novel	37.21	37.21	coding	417	142	1392
PB.6388.1	5	952	1	full-splice_match	gene: Zm00001d014778	Zm00001d014778_T001	17.66	29.62	coding	187	135	695
PB.9411.3	8	910	7	full-splice_match	gene: Zm00001d010009	Zm00001d010009_T001	67.91	219.48	coding	171	118	630
PB.6095.1	5	1600	2	full-splice_match	gene: Zm00001d013443	Zm00001d013443_T001	0.31	0.31	coding	413	73	1311
PB.9411.1	8	1080	7	full-splice_match	gene: Zm00001d010009	Zm00001d010009_T001	151.58	219.48	coding	171	179	691
PB.10660.1	9	1766	1	full-splice_match	gene: Zm00001d047937	Zm00001d047937_T001	12.84	12.84	coding	411	284	1516
PB.10723.1	9	2029	11	full-splice_match	gene: Zm00001d048174	Zm00001d048174_T001	23.86	46.35	coding	458	70	1443
PB.7946.1	6	1209	7	full-splice_match	gene: Zm00001d038506	Zm00001d038506_T002	25.10	25.10	coding	284	122	973
PB.5704.3	4	707	3	incomplete-splice_match	gene: Zm00001d053313	Zm00001d053313_T003	15.04	15.04	coding	124	110	481
PB.6503.2	5	2401	22	fusion	gene: Zm00001d015228_g	novel	3.67	31.82	coding	653	191	2149
PB.6503.1	5	2486	22	fusion	gene: Zm00001d015228_g	novel	28.15	31.82	coding	653	141	2099

PB.8748.1	7	1467	12	novel_not_in_catalog	gene: Zm00001d021615	novel	55.16	543.59	coding	347	106	1146
PB.9221.1	8	607	6	full-splice_match	gene: Zm00001d008916	Zm00001d008916_T001	287.94	287.94	non_coding	NA	NA	NA
PB.1414.2	1	2080	1	antisense	novelGene_gene: Zm00001d034103_A	novel	0.71	0.71	coding	203	1240	1848
PB.6374.1	5	1786	1	full-splice_match	gene: Zm00001d014748	Zm00001d014748_T001	11.88	11.88	coding	448	103	1446
PB.4089.1	3	628	4	incomplete-splice_match	gene: Zm00001d041734	Zm00001d041734_T001	113.19	113.19	coding	98	153	446
PB.9249.13	8	2609	1	novel_in_catalog	gene: Zm00001d009054	novel	0.00	23.92	non_coding	NA	NA	NA
PB.9249.12	8	1550	3	novel_in_catalog	gene: Zm00001d009054	novel	1.17	23.92	non_coding	NA	NA	NA
PB.9076.1	8	500	3	full-splice_match	gene: Zm00001d008259	Zm00001d008259_T001	603.85	603.85	coding	75	75	299
PB.9249.10	8	2011	3	novel_not_in_catalog	gene: Zm00001d009054	novel	0.22	23.92	non_coding	NA	NA	NA
PB.8507.1	7	969	1	full-splice_match	gene: Zm00001d020550	Zm00001d020550_T001	81.21	81.21	coding	234	178	879
PB.6229.1	5	1116	5	full-splice_match	gene: Zm00001d014065	Zm00001d014065_T001	87.79	215.45	coding	216	83	730
PB.1748.1	10	826	2	novel_not_in_catalog	gene: Zm00001d023671	novel	35.53	35.53	coding	75	70	294
PB.2375.1	10	2239	16	full-splice_match	gene: Zm00001d026677	Zm00001d026677_T001	217.73	258.36	coding	562	188	1873
PB.971.1	1	348	1	intergenic	novelGene_332	novel	7.63	7.63	non_coding	NA	NA	NA
PB.2375.5	10	2458	14	novel_in_catalog	gene: Zm00001d026677	novel	0.00	258.36	coding	313	774	1712
PB.9360.1	8	1106	9	full-splice_match	gene: Zm00001d009726	Zm00001d009726_T002	7.67	7.67	coding	254	109	870
PB.3590.1	2	1540	11	full-splice_match	gene: Zm00001d007806	Zm00001d007806_T002	23.28	46.08	coding	323	137	1105

PB.10781.1	9	1695	1	full-splice_match	gene: Zm00001d048398	Zm00001d048398_T001	5.27	5.27	coding	429	95	1381
PB.3590.3	2	2077	10	full-splice_match	gene: Zm00001d007806	Zm00001d007806_T001	8.45	46.08	coding	323	803	1771
PB.7709.8	6	2158	4	full-splice_match	gene: Zm00001d037625	Zm00001d037625_T004	0.00	62.70	coding	335	974	1978
PB.3590.5	2	1977	10	full-splice_match	gene: Zm00001d007806	Zm00001d007806_T001	0.00	46.08	coding	323	846	1814
PB.7878.1	6	759	3	intergenic	novelGene_250	novel	43.53	99.35	coding	104	52	363
PB.7709.4	6	1835	5	full-splice_match	gene: Zm00001d037625	Zm00001d037625_T003	45.30	62.70	coding	422	292	1557
PB.7709.5	6	2335	3	full-splice_match	gene: Zm00001d037625	Zm00001d037625_T006	1.54	62.70	coding	287	1197	2057
PB.1520.1	1	3721	14	full-splice_match	gene: Zm00001d034553	Zm00001d034553_T002	40.17	40.17	coding	1076	276	3503
PB.7709.2	6	2259	4	full-splice_match	gene: Zm00001d037625	Zm00001d037625_T004	6.50	62.70	coding	335	975	1979
PB.7709.3	6	2171	5	full-splice_match	gene: Zm00001d037625	Zm00001d037625_T002	9.36	62.70	coding	310	959	1888
PB.2595.2	2	1122	4	intergenic	novelGene_55	novel	22.31	22.31	coding	66	257	454
PB.9897.1	8	2240	6	full-splice_match	gene: Zm00001d012238	Zm00001d012238_T009	99.01	136.30	coding	256	168	935
PB.9897.2	8	2224	6	full-splice_match	gene: Zm00001d012238	Zm00001d012238_T012	5.06	136.30	coding	284	237	1088
PB.9897.3	8	2461	6	novel_in_catalog	gene: Zm00001d012238	novel	1.43	136.30	coding	205	168	782
PB.9897.4	8	2130	6	full-splice_match	gene: Zm00001d012238	Zm00001d012238_T009	0.00	136.30	coding	256	168	935
PB.9897.5	8	2100	6	full-splice_match	gene: Zm00001d012238	Zm00001d012238_T012	30.80	136.30	coding	284	168	1019
PB.7676.2	6	2166	16	full-splice_match	gene: Zm00001d037484	Zm00001d037484_T003	0.00	109.49	coding	551	274	1926

PB.144.6	1	1942	12	full-splice_match	gene: Zm00001d027854	Zm00001d027854_T002	0.00	20.93	coding	520	279	1838
PB.8196.3	7	930	3	incomplete-splice_match	gene: Zm00001d018798	Zm00001d018798_T002	79.62	151.62	coding	241	45	767
PB.8196.2	7	1537	3	incomplete-splice_match	gene: Zm00001d018798	Zm00001d018798_T002	34.70	151.62	coding	352	207	1262
PB.8196.1	7	3258	7	full-splice_match	gene: Zm00001d018798	Zm00001d018798_T002	37.31	151.62	coding	941	44	2866
PB.432.2	1	1454	10	full-splice_match	gene: Zm00001d029121	Zm00001d029121_T005	0.00	29.52	coding	339	68	1084
PB.6753.2	5	2643	6	novel_not_in_catalog	gene: Zm00001d016827	novel	16.33	16.33	coding	433	6	1304
PB.4645.2	3	1862	1	full-splice_match	gene: Zm00001d044120	Zm00001d044120_T001	6.16	32.96	coding	494	251	1732
PB.4645.3	3	1649	1	full-splice_match	gene: Zm00001d044120	Zm00001d044120_T001	10.86	32.96	coding	494	121	1602
PB.5639.2	4	2718	6	novel_in_catalog	gene: Zm00001d052944	novel	6.75	37.40	coding	212	1586	2221
PB.9976.1	8	1043	1	full-splice_match	gene: Zm00001d012585	Zm00001d012585_T001	26.41	26.41	coding	241	55	777
PB.632.1	1	1188	2	novel_in_catalog	gene: Zm00001d030149	novel	50.03	50.03	coding	289	107	973
PB.9028.1	7	945	3	full-splice_match	gene: Zm00001d022550	Zm00001d022550_T001	13.18	13.18	coding	98	146	439
PB.6081.4	5	2985	9	full-splice_match	gene: Zm00001d013391	Zm00001d013391_T021	3.59	20.28	coding	879	146	2782
PB.6081.5	5	1978	5	incomplete-splice_match	gene: Zm00001d013391	Zm00001d013391_T022	0.00	20.28	coding	629	92	1978
PB.3881.1	3	1939	1	full-splice_match	gene: Zm00001d040324	Zm00001d040324_T001	5.73	39.42	coding	398	113	1306
PB.5338.4	4	1382	8	full-splice_match	gene: Zm00001d051676	Zm00001d051676_T003	16.57	76.62	coding	300	194	1093
PB.6081.1	5	3032	9	full-splice_match	gene: Zm00001d013391	Zm00001d013391_T013	4.98	20.28	coding	509	1210	2736

PB.6081.2	5	3062	8	full-splice_match	gene: Zm00001d013391	Zm00001d013391_T017	10.68	20.28	coding	901	146	2848
PB.6081.3	5	2812	10	novel_not_in_catalog	gene: Zm00001d013391	novel	1.04	20.28	coding	565	914	2608
PB.4659.3	3	1527	10	full-splice_match	gene: Zm00001d044172	Zm00001d044172_T002	407.55	408.12	coding	361	93	1175
PB.9048.1	7	2763	12	full-splice_match	gene: Zm00001d022621	Zm00001d022621_T017	14.47	17.65	coding	561	750	2432
PB.9048.3	7	2728	11	novel_in_catalog	gene: Zm00001d022621	novel	3.18	17.65	coding	356	657	1724
PB.9048.4	7	2511	12	full-splice_match	gene: Zm00001d022621	Zm00001d022621_T017	0.00	17.65	coding	561	658	2340
PB.4659.5	3	1376	10	full-splice_match	gene: Zm00001d044172	Zm00001d044172_T002	0.00	408.12	coding	361	50	1132
PB.4659.4	3	1486	9	novel_in_catalog	gene: Zm00001d044172	novel	0.57	408.12	coding	95	169	453
PB.3900.2	3	1937	3	full-splice_match	gene: Zm00001d040446	Zm00001d040446_T001	157.02	184.98	coding	509	158	1684
PB.3900.1	3	2052	3	full-splice_match	gene: Zm00001d040446	Zm00001d040446_T001	27.96	184.98	coding	509	158	1684
PB.9235.1	8	1014	3	full-splice_match	gene: Zm00001d008992	Zm00001d008992_T001	82.52	82.52	coding	225	111	785
PB.6302.1	5	834	5	full-splice_match	gene: Zm00001d014428	Zm00001d014428_T001	128.61	280.13	coding	159	66	542
PB.6302.2	5	736	5	full-splice_match	gene: Zm00001d014428	Zm00001d014428_T001	151.52	280.13	coding	159	71	547
PB.1462.3	1	1417	6	full-splice_match	gene: Zm00001d034345	Zm00001d034345_T002	87.91	177.64	coding	381	69	1211
PB.2677.9	2	1878	8	novel_not_in_catalog	gene: Zm00001d002942	novel	16.26	67.49	coding	8	421	444
PB.2677.8	2	1446	9	novel_not_in_catalog	gene: Zm00001d002942	novel	13.88	67.49	coding	246	501	1238
PB.1041.1	1	1286	8	full-splice_match	gene: Zm00001d032430	Zm00001d032430_T002	7.93	8.41	coding	306	164	1081

PB.8585.3	7	2794	2	genic	gene: Zm00001d020939	novel	13.02	33.46	coding	609	541	2367
PB.1041.2	1	1320	7	novel_in_catalog	gene: Zm00001d032430	novel	0.48	8.41	coding	151	166	618
PB.2677.1	2	1777	5	full-splice_match	gene: Zm00001d002941	Zm00001d002941_T001	13.45	97.91	coding	344	143	1174
PB.4741.1	3	1507	3	genic	gene: Zm00001d044501	novel	16.30	36.47	coding	188	490	1053
PB.2677.2	2	1607	5	full-splice_match	gene: Zm00001d002941	Zm00001d002941_T001	84.45	97.91	coding	344	177	1208
PB.2677.7	2	1933	10	novel_not_in_catalog	gene: Zm00001d002942	novel	30.62	67.49	coding	246	832	1569
PB.9620.5	8	1467	2	full-splice_match	gene: Zm00001d010969	Zm00001d010969_T001	0.00	60.34	coding	318	46	999
PB.2.1	1	1948	9	full-splice_match	gene: Zm00001d027231	Zm00001d027231_T005	72.92	72.92	coding	517	174	1724
PB.1810.3	10	1059	1	full-splice_match	gene: Zm00001d024027	Zm00001d024027_T001	0.32	111.22	non_coding	NA	NA	NA
PB.1810.2	10	1445	1	full-splice_match	gene: Zm00001d024027	Zm00001d024027_T001	110.91	111.22	coding	378	115	1248
PB.4438.5	3	1906	6	full-splice_match	gene: Zm00001d043309	Zm00001d043309_T016	22.08	44.80	coding	472	213	1628
PB.1073.2	1	1714	2	full-splice_match	gene: Zm00001d032543	Zm00001d032543_T001	0.00	28.49	coding	517	45	1595
PB.4498.1	3	689	3	full-splice_match	gene: Zm00001d043523	Zm00001d043523_T001	185.46	185.46	coding	132	106	501
PB.491.2	1	1667	4	novel_not_in_catalog	gene: Zm00001d029390	novel	14.55	21.43	coding	83	136	384
PB.491.3	1	1833	2	novel_not_in_catalog	gene: Zm00001d029390	novel	6.89	21.43	coding	78	133	366
PB.6242.11	5	2141	14	full-splice_match	gene: Zm00001d014138	Zm00001d014138_T019	27.14	71.54	coding	314	971	1912
PB.6753.1	5	2297	6	fusion	gene: Zm00001d016827_g	novel	5.65	5.65	coding	231	1417	2109

PB.10794.1	9	1105	3	incomplete-splice_match	gene: Zm00001d048437	Zm00001d048437_T002	19.93	29.88	coding	189	58	624	
PB.10794.3	9	1475	5	novel_not_in_catalog	gene: Zm00001d048437	novel	9.95	29.88	coding	396	153	1340	
PB.10794.2	9	1021	3	incomplete-splice_match	gene: Zm00001d048437	Zm00001d048437_T002	0.00	29.88	coding	235	31	735	
PB.5209.1	4	1564	4	fusion	gene: Zm00001d050967_g	novel	63.67	80.55	coding	373	185	1303	
PB.1284.1	1	2450	4	full-splice_match	gene: Zm00001d033544	Zm00001d033544_T002	1.76	1.92	coding	730	73	2262	
PB.10892.4	B73V4 ctg167	2740	1	intergenic	novelGene_7	novel	519.50	5924.92	coding	103	831	1139	
PB.3030.1	2	518	1	novel_in_catalog	gene: Zm00001d004924	novel	18.07	18.07	coding	92	55	330	
PB.5785.1	4	1239	5	novel_not_in_catalog	gene: Zm00001d053686	novel	45.55	45.55	coding	267	124	924	
PB.6822.1	5	1428	2	genic	gene: Zm00001d017131	novel	16.59	26.21	coding	311	116	1048	
PB.2734.2	2	1199	1	genic	gene: Zm00001d003175	novel	10.18	18.58	coding	239	308	1024	
PB.2734.1	2	1577	2	full-splice_match	gene: Zm00001d003175	Zm00001d003175_T001	8.40	18.58	coding	257	167	937	
PB.6822.2	5	1262	1	full-splice_match	gene: Zm00001d017131	Zm00001d017131_T001	9.62	26.21	coding	311	135	1067	
PB.551.1	1	4152	2	genic	gene: Zm00001d029652	novel	6.16	19.38	coding	1256	155	3922	
PB.6617.2	5	3030	6	full-splice_match	gene: Zm00001d016070	Zm00001d016070_T001	68.19	84.03	coding	923	44	2812	
PB.6617.1	5	4376	7	full-splice_match	gene: Zm00001d016070	Zm00001d016070_T002	15.84	84.03	coding	1286	296	4153	
PB.551.2	1	2520	1	full-splice_match	gene: Zm00001d029652	Zm00001d029652_T001	13.22	19.38	coding	780	26	2365	
PB.6239.1	5	1069	1	intergenic	novelGene_91	novel	5.10	5.10	non_coding	NA	NA	NA	

PB.7298.1	6	2283	4	full-splice_match	gene: Zm00001d035428	Zm00001d035428_T003	14.45	14.45	coding	598	181	1974
PB.6617.4	5	1470	2	full-splice_match	gene: Zm00001d016070	Zm00001d016070_T008	0.00	84.03	coding	391	298	1470
PB.3387.2	2	2142	2	genic	gene: Zm00001d006725	novel	4.20	196.31	coding	437	156	1466
PB.7097.2	5	1116	5	novel_not_in_catalog	gene: Zm00001d018255	novel	29.03	109.30	coding	271	57	869
PB.3277.2	2	1415	1	full-splice_match	gene: Zm00001d006244	Zm00001d006244_T001	26.63	26.63	coding	402	30	1235
PB.3387.1	2	1987	3	genic	gene: Zm00001d006725	novel	192.11	196.31	coding	437	152	1462
PB.8419.3	7	2686	11	incomplete-splice_match	gene: Zm00001d020157	Zm00001d020157_T036	28.22	28.22	coding	646	469	2406
PB.3387.4	2	1883	3	genic	gene: Zm00001d006725	novel	0.00	196.31	coding	437	157	1467
PB.1728.1	10	2075	1	full-splice_match	gene: Zm00001d023565	Zm00001d023565_T001	34.73	34.73	coding	405	374	1588
PB.1728.2	10	1919	1	full-splice_match	gene: Zm00001d023565	Zm00001d023565_T001	0.00	34.73	coding	405	332	1546
PB.9241.1	8	1829	11	full-splice_match	gene: Zm00001d009011	Zm00001d009011_T002	6.60	6.60	coding	502	75	1580
PB.10892.1	B73V4 ctg167	1829	1	intergenic	novelGene_7	novel	0.00	5924.92	coding	103	831	1139
PB.10215.1	9	1567	5	full-splice_match	gene: Zm00001d045558	Zm00001d045558_T004	15.49	62.37	coding	219	165	821
PB.7844.2	6	2831	1	novel_in_catalog	gene: Zm00001d038133	novel	0.00	20.63	coding	135	1245	1649
PB.10215.3	9	1222	6	novel_not_in_catalog	gene: Zm00001d045558	novel	0.00	62.37	coding	333	76	1074
PB.10215.2	9	1316	6	novel_not_in_catalog	gene: Zm00001d045558	novel	40.84	62.37	coding	333	28	1026
PB.10215.5	9	1383	4	novel_in_catalog	gene: Zm00001d045558	novel	6.04	62.37	coding	219	41	697

PB.10215.4	9	1321	5	full-splice_match	gene: Zm00001d045558	Zm00001d045558_T004	0.00	62.37	coding	219	91	747
PB.5924.1	5	3622	12	full-splice_match	gene: Zm00001d012863	Zm00001d012863_T009	16.88	18.16	coding	654	48	2009
PB.1431.1	1	841	1	full-splice_match	gene: Zm00001d034195	Zm00001d034195_T001	1.93	1.93	coding	185	89	643
PB.10508.4	9	2280	19	novel_in_catalog	gene: Zm00001d047426	novel	27.73	42.05	coding	327	1112	2092
PB.6571.1	5	963	2	full-splice_match	gene: Zm00001d015657	Zm00001d015657_T001	16.56	16.56	coding	131	65	457
PB.10508.6	9	2755	19	full-splice_match	gene: Zm00001d047426	Zm00001d047426_T005	9.41	42.05	coding	412	258	1493
PB.7713.1	6	3144	4	novel_not_in_catalog	gene: Zm00001d037650	novel	28.59	45.14	coding	822	406	2871
PB.10888.8	B73V4 ctg190	3039	7	novel_in_catalog	gene: Zm00001d000435	novel	4.65	31.98	coding	466	884	2281
PB.10508.3	9	2730	18	novel_in_catalog	gene: Zm00001d047426	novel	3.39	42.05	coding	278	258	1091
PB.10888.5	B73V4 ctg190	3604	6	full-splice_match	gene: Zm00001d000435	Zm00001d000435_T014	4.42	31.98	coding	466	1547	2944
PB.10888.4	B73V4 ctg190	2289	1	incomplete-splice_match	gene: Zm00001d000435	Zm00001d000435_T009	10.97	31.98	coding	263	187	975
PB.10888.7	B73V4 ctg190	3451	4	full-splice_match	gene: Zm00001d000435	Zm00001d000435_T004	0.00	31.98	coding	466	1340	2737
PB.9166.1	8	2010	6	full-splice_match	gene: Zm00001d008651	Zm00001d008651_T001	5.71	5.71	coding	593	46	1824
PB.10888.3	B73V4 ctg190	3522	4	full-splice_match	gene: Zm00001d000435	Zm00001d000435_T004	11.94	31.98	coding	466	1309	2706
PB.2576.2	2	2530	7	full-splice_match	gene: Zm00001d002540	Zm00001d002540_T002	0.99	80.90	coding	547	592	2232
PB.6963.7	5	2066	9	full-splice_match	gene: Zm00001d017755	Zm00001d017755_T006	0.00	16.31	coding	541	270	1892
PB.2576.1	2	2327	7	full-splice_match	gene: Zm00001d002540	Zm00001d002540_T002	72.82	80.90	coding	547	126	1766

PB.2714.1	2	610	1	intergenic	novelGene_88	novel	289.63	4129.20	non_coding	NA	NA	NA
PB.4247.1	3	1354	7	full-splice_match	gene: Zm00001d042478	Zm00001d042478_T001	3.89	3.89	coding	358	120	1193
PB.1531.1	1	2485	14	full-splice_match	gene: Zm00001d034590	Zm00001d034590_T002	49.82	90.00	coding	647	79	2019
PB.9844.1	8	2863	2	genic	gene: Zm00001d011985	novel	0.54	66.55	coding	315	65	1009
PB.7313.1	6	1351	8	full-splice_match	gene: Zm00001d035503	Zm00001d035503_T004	31.91	47.43	coding	299	71	967
PB.9951.3	8	2102	4	novel_in_catalog	gene: Zm00001d012477	novel	14.32	53.45	coding	327	980	1960
PB.5348.1	4	1924	1	antisense	novelGene_gene: Zm00001d051749_A	novel	53.02	53.02	non_coding	NA	NA	NA
PB.7313.2	6	1241	8	full-splice_match	gene: Zm00001d035503	Zm00001d035503_T004	15.52	47.43	coding	299	51	947
PB.7877.1	6	1693	10	incomplete-splice_match	gene: Zm00001d038209	Zm00001d038209_T001	30.95	55.08	coding	273	39	857
PB.5473.1	4	1788	3	full-splice_match	gene: Zm00001d052298	Zm00001d052298_T002	9.11	9.11	coding	531	99	1691
PB.6021.2	5	1329	3	full-splice_match	gene: Zm00001d013212	Zm00001d013212_T001	43.80	308.81	coding	328	35	1018
PB.6021.3	5	1230	3	full-splice_match	gene: Zm00001d013212	Zm00001d013212_T001	50.10	308.81	coding	328	26	1009
PB.9670.1	8	2230	7	full-splice_match	gene: Zm00001d011226	Zm00001d011226_T002	42.89	45.59	coding	340	957	1976
PB.4784.3	3	849	2	full-splice_match	gene: Zm00001d044691	Zm00001d044691_T001	2.59	108.71	non_coding	NA	NA	NA
PB.3159.1	2	743	5	full-splice_match	gene: Zm00001d005675	Zm00001d005675_T003	193.56	387.55	non_coding	NA	NA	NA
PB.6021.4	5	1122	3	full-splice_match	gene: Zm00001d013212	Zm00001d013212_T001	214.91	308.81	coding	328	33	1016
PB.3159.2	2	1169	1	incomplete-splice_match	gene: Zm00001d005675	Zm00001d005675_T001	6.03	387.55	non_coding	NA	NA	NA

PB.8765.6	7	577	5	full-splice_match	gene: Zm00001d021668	Zm00001d021668_T001	714.27	2691.00	coding	115	157	501
PB.8765.5	7	788	4	novel_in_catalog	gene: Zm00001d021668	novel	2.40	2691.00	non_coding	NA	NA	NA
PB.8765.3	7	906	5	full-splice_match	gene: Zm00001d021668	Zm00001d021668_T001	1791.98	2691.00	coding	115	299	643
PB.8765.2	7	1012	5	full-splice_match	gene: Zm00001d021668	Zm00001d021668_T001	128.67	2691.00	coding	115	255	599
PB.8765.1	7	991	5	full-splice_match	gene: Zm00001d021668	Zm00001d021668_T001	53.68	2691.00	coding	115	131	475
PB.3070.3	2	1296	9	full-splice_match	gene: Zm00001d005159	Zm00001d005159_T006	0.00	66.30	coding	318	110	1063
PB.2825.2	2	2571	8	novel_in_catalog	gene: Zm00001d003572	novel	4.53	25.52	coding	409	1114	2340
PB.5368.4	4	2113	14	full-splice_match	gene: Zm00001d051873	Zm00001d051873_T003	0.00	35.79	coding	545	162	1796
PB.5629.1	4	1900	4	incomplete-splice_match	gene: Zm00001d052905	Zm00001d052905_T002	75.48	75.48	coding	444	3	1334
PB.2825.1	2	2455	9	full-splice_match	gene: Zm00001d003572	Zm00001d003572_T004	20.99	25.52	coding	652	259	2214
PB.10500.1	9	699	3	full-splice_match	gene: Zm00001d047396	Zm00001d047396_T001	109.71	109.71	coding	131	53	445
PB.2825.4	2	2367	9	full-splice_match	gene: Zm00001d003572	Zm00001d003572_T004	0.00	25.52	coding	652	205	2160
PB.5368.2	4	2274	14	full-splice_match	gene: Zm00001d051873	Zm00001d051873_T003	28.04	35.79	coding	545	161	1795
PB.2125.3	10	1948	6	novel_not_in_catalog	gene: Zm00001d025749	novel	0.00	24.75	coding	207	1164	1784
PB.1745.10	10	2100	10	novel_not_in_catalog	gene: Zm00001d023636	novel	1.75	66.48	coding	230	47	736
PB.1825.2	10	1960	2	full-splice_match	gene: Zm00001d024170	Zm00001d024170_T001	0.00	10.59	coding	490	210	1679
PB.1825.1	10	2086	2	full-splice_match	gene: Zm00001d024170	Zm00001d024170_T001	10.59	10.59	coding	490	255	1724

PB.4186.1	3	1915	2	genic	gene: Zm00001d042184	novel	249.96	251.53	coding	480	199	1638
PB.6035.1	5	1481	1	intergenic	novelGene_33	novel	8.23	8.23	non_coding	NA	NA	NA
PB.9733.1	8	1017	1	full-splice_match	gene: Zm00001d011543	Zm00001d011543_T001	109.61	109.61	coding	159	137	613
PB.4186.2	3	2404	1	full-splice_match	gene: Zm00001d042184	Zm00001d042184_T001	1.57	251.53	coding	480	732	2171
PB.5719.2	4	1124	3	full-splice_match	gene: Zm00001d053396	Zm00001d053396_T001	40.19	153.18	coding	217	258	908
PB.9733.2	8	871	1	full-splice_match	gene: Zm00001d011543	Zm00001d011543_T001	0.00	109.61	coding	159	137	613
PB.1669.1	10	1441	2	novel_not_in_catalog	gene: Zm00001d023292	novel	0.79	5.58	non_coding	NA	NA	NA
PB.1531.3	1	1664	11	incomplete-splice_match	gene: Zm00001d034590	Zm00001d034590_T021	38.25	90.00	coding	397	153	1343
PB.1669.2	10	2191	1	novel_in_catalog	gene: Zm00001d023292	novel	4.79	5.58	non_coding	NA	NA	NA
PB.9794.1	8	1537	1	full-splice_match	gene: Zm00001d011787	Zm00001d011787_T001	39.14	42.64	coding	381	84	1226
PB.3371.1	2	1100	1	intergenic	novelGene_330	novel	23.32	23.32	non_coding	NA	NA	NA
PB.253.1	1	2037	5	full-splice_match	gene: Zm00001d028354	Zm00001d028354_T001	13.48	14.93	coding	592	141	1916
PB.253.2	1	1969	5	full-splice_match	gene: Zm00001d028354	Zm00001d028354_T001	1.46	14.93	coding	592	181	1956
PB.1443.1	1	2509	5	full-splice_match	gene: Zm00001d034257	Zm00001d034257_T002	8.64	8.64	coding	586	523	2280
PB.2475.1	2	1801	8	full-splice_match	gene: Zm00001d002103	Zm00001d002103_T003	31.95	42.71	coding	401	181	1383
PB.1069.2	1	623	1	full-splice_match	gene: Zm00001d032533	Zm00001d032533_T001	35.09	83.17	non_coding	NA	NA	NA
PB.7487.3	6	2022	4	novel_in_catalog	gene: Zm00001d036541	novel	1.74	174.28	coding	328	814	1797

PB.2475.2	2	1688	8	full-splice_match	gene: Zm00001d002103	Zm00001d002103_T003	10.76	42.71	coding	401	182	1384
PB.122.2	1	2828	7	novel_in_catalog	gene: Zm00001d027723	novel	5.49	5.49	coding	479	1116	2552
PB.8585.4	7	988	1	full-splice_match	gene: Zm00001d020939	Zm00001d020939_T001	12.50	33.46	coding	138	77	490
PB.2125.1	10	2287	6	novel_not_in_catalog	gene: Zm00001d025749	novel	24.75	24.75	coding	509	427	1953
PB.8779.1	7	849	2	full-splice_match	gene: Zm00001d021711	Zm00001d021711_T001	11.03	261.74	coding	114	64	405
PB.8779.3	7	2277	1	novel_in_catalog	gene: Zm00001d021711	novel	0.00	261.74	coding	96	64	351
PB.8779.4	7	714	2	full-splice_match	gene: Zm00001d021711	Zm00001d021711_T001	47.24	261.74	coding	114	64	405
PB.8779.5	7	2140	1	novel_in_catalog	gene: Zm00001d021711	novel	10.84	261.74	coding	96	64	351
PB.8779.6	7	602	2	full-splice_match	gene: Zm00001d021711	Zm00001d021711_T001	192.63	261.74	coding	114	64	405
PB.3145.1	2	2376	2	genic	gene: Zm00001d005591	novel	1.73	2.23	coding	679	108	2144
PB.10683.4	9	2466	6	full-splice_match	gene: Zm00001d048046	Zm00001d048046_T001	56.87	66.01	coding	686	220	2277
PB.2162.1	10	1336	2	full-splice_match	gene: Zm00001d025901	Zm00001d025901_T001	1.49	52.05	coding	223	195	863
PB.4334.1	3	465	1	full-splice_match	gene: Zm00001d042857	Zm00001d042857_T001	126.30	126.30	non_coding	NA	NA	NA
PB.10683.3	9	2448	6	full-splice_match	gene: Zm00001d048046	Zm00001d048046_T002	9.14	66.01	coding	686	194	2251
PB.2236.4	10	2096	3	incomplete-splice_match	gene: Zm00001d026257	Zm00001d026257_T010	29.14	55.22	coding	128	1301	1684
PB.4593.1	3	1264	5	full-splice_match	gene: Zm00001d043878	Zm00001d043878_T001	211.29	211.29	coding	269	100	906
PB.5352.3	4	2012	9	full-splice_match	gene: Zm00001d051761	Zm00001d051761_T002	0.00	225.19	coding	557	135	1805

PB.3302.1	2	3720	6	full-splice_match	gene: Zm00001d006344	Zm00001d006344_T002	14.21	84.46	coding	359	136	1212
PB.5352.1	4	2154	9	full-splice_match	gene: Zm00001d051761	Zm00001d051761_T002	225.19	225.19	coding	557	149	1819
PB.6633.7	5	3197	10	full-splice_match	gene: Zm00001d016166	Zm00001d016166_T001	0.00	142.61	coding	960	179	3058
PB.6633.6	5	2449	1	novel_in_catalog	gene: Zm00001d016166	novel	4.91	142.61	coding	380	1	1140
PB.6633.1	5	3443	10	full-splice_match	gene: Zm00001d016166	Zm00001d016166_T001	39.14	142.61	coding	960	179	3058
PB.118.1	1	2764	9	full-splice_match	gene: Zm00001d027707	Zm00001d027707_T001	110.17	110.17	coding	807	118	2538
PB.118.2	1	2665	9	full-splice_match	gene: Zm00001d027707	Zm00001d027707_T001	0.00	110.17	coding	807	118	2538
PB.6858.1	5	2523	2	full-splice_match	gene: Zm00001d017274	Zm00001d017274_T001	264.42	2528.25	coding	703	102	2210
PB.7639.4	6	2380	11	full-splice_match	gene: Zm00001d037291	Zm00001d037291_T001	0.31	31.14	coding	606	232	2049
PB.3922.3	3	822	4	intergenic	novelGene_114	novel	7.40	23.60	coding	33	371	469
PB.3922.2	3	2721	1	intergenic	novelGene_114	novel	3.89	23.60	coding	53	367	525
PB.3922.1	3	556	4	intergenic	novelGene_114	novel	12.31	23.60	non_coding	NA	NA	NA
PB.2956.5	2	1671	2	full-splice_match	gene: Zm00001d004467	Zm00001d004467_T001	7.79	29.42	coding	421	192	1454
PB.2956.4	2	1795	2	full-splice_match	gene: Zm00001d004467	Zm00001d004467_T001	0.00	29.42	coding	421	192	1454
PB.7920.4	6	2414	8	novel_not_in_catalog	gene: Zm00001d038383	novel	6.11	8.27	coding	98	333	626
PB.2956.3	2	1933	2	full-splice_match	gene: Zm00001d004467	Zm00001d004467_T001	18.79	29.42	coding	421	192	1454
PB.2956.2	2	2051	1	genic	gene: Zm00001d004467	novel	2.84	29.42	coding	353	175	1233

PB.1604.2	1	1541	3	novel_in_catalog	gene: Zm00001d034824	novel	35.82	35.82	coding	282	590	1435
PB.8388.3	7	1740	2	intergenic	novelGene_144	novel	22.09	22.09	coding	18	400	453
PB.10562.7	9	2860	4	full-splice_match	gene: Zm00001d047635	Zm00001d047635_T004	15.60	242.87	coding	188	2091	2654
PB.2984.2	2	1529	8	novel_not_in_catalog	gene: Zm00001d004644	novel	4.83	18.53	coding	408	109	1332
PB.4653.9	3	1871	4	novel_not_in_catalog	gene: Zm00001d044146	novel	3.98	46.01	coding	350	673	1722
PB.2984.3	2	1387	8	novel_not_in_catalog	gene: Zm00001d004644	novel	0.00	18.53	coding	408	74	1297
PB.1637.2	1	988	6	full-splice_match	gene: Zm00001d034937	Zm00001d034937_T001	0.00	59.47	coding	249	106	852
PB.4269.8	3	1574	7	full-splice_match	gene: Zm00001d042590	Zm00001d042590_T001	0.00	119.12	coding	293	445	1323
PB.7827.1	6	1397	2	novel_in_catalog	gene: Zm00001d038080	novel	3.64	3.64	coding	164	397	888
PB.3261.1	2	1215	7	full-splice_match	gene: Zm00001d006167	Zm00001d006167_T003	35.27	35.27	coding	272	221	1036
PB.1841.1	10	1136	6	full-splice_match	gene: Zm00001d024292	Zm00001d024292_T001	62.57	62.57	coding	264	88	879
PB.592.2	1	1426	4	full-splice_match	gene: Zm00001d029899	Zm00001d029899_T001	131.10	197.61	coding	282	103	948
PB.592.3	1	1298	4	full-splice_match	gene: Zm00001d029899	Zm00001d029899_T001	61.42	197.61	coding	282	107	952
PB.592.1	1	2337	4	full-splice_match	gene: Zm00001d029899	Zm00001d029899_T001	5.09	197.61	coding	282	107	952
PB.4269.6	3	1217	7	full-splice_match	gene: Zm00001d042590	Zm00001d042590_T001	60.22	119.12	coding	266	37	834
PB.1736.1	10	2417	6	full-splice_match	gene: Zm00001d023590	Zm00001d023590_T002	76.28	76.28	coding	208	1578	2201
PB.4269.4	3	1810	7	full-splice_match	gene: Zm00001d042590	Zm00001d042590_T001	29.73	119.12	coding	293	445	1323

PB.1736.3	10	2231	6	full-splice_match	gene: Zm00001d023590	Zm00001d023590_T002	0.00	76.28	coding	208	1514	2137
PB.3178.2	2	1438	10	full-splice_match	gene: Zm00001d005765	Zm00001d005765_T003	31.48	39.79	coding	279	337	1173
PB.3178.1	2	1599	9	full-splice_match	gene: Zm00001d005765	Zm00001d005765_T002	8.31	39.79	coding	279	246	1082
PB.10074.1	9	1681	10	novel_not_in_catalog	gene: Zm00001d044945	novel	11.50	35.76	coding	365	24	1118
PB.10074.2	9	1569	10	novel_not_in_catalog	gene: Zm00001d044945	novel	11.28	35.76	coding	365	49	1143
PB.10074.3	9	521	3	incomplete-splice_match	gene: Zm00001d044945	Zm00001d044945_T001	12.98	35.76	non_coding	NA	NA	NA
PB.5631.2	4	1352	3	novel_not_in_catalog	gene: Zm00001d052916	novel	7.66	14.39	coding	343	221	1249
PB.5631.1	4	1835	2	genic	gene: Zm00001d052916	novel	6.73	14.39	coding	336	723	1730
PB.8409.3	7	2152	12	novel_in_catalog	gene: Zm00001d020069	novel	0.31	96.74	coding	302	131	1036
PB.3809.1	3	3439	15	full-splice_match	gene: Zm00001d040008	Zm00001d040008_T004	52.37	56.05	coding	822	670	3135
PB.4894.1	4	1852	8	full-splice_match	gene: Zm00001d049099	Zm00001d049099_T004	74.25	291.86	coding	331	222	1214
PB.4894.2	4	2004	10	full-splice_match	gene: Zm00001d049099	Zm00001d049099_T008	217.60	291.86	coding	514	164	1705
PB.358.1	1	1503	10	full-splice_match	gene: Zm00001d028787	Zm00001d028787_T001	67.96	67.96	coding	368	201	1304
PB.7325.2	6	1936	3	incomplete-splice_match	gene: Zm00001d035593	Zm00001d035593_T001	0.00	4.77	coding	509	302	1828
PB.3914.1	3	1996	3	novel_not_in_catalog	gene: Zm00001d040527	novel	11.34	12.92	coding	427	298	1578
PB.3914.2	3	1803	3	novel_not_in_catalog	gene: Zm00001d040527	novel	1.57	12.92	coding	427	309	1589
PB.7325.1	6	2427	3	incomplete-splice_match	gene: Zm00001d035593	Zm00001d035593_T001	4.77	4.77	coding	707	25	2145

PB.10312.2	9	1911	5	novel_in_catalog	gene: Zm00001d046263	novel	11.03	60.33	coding	221	986	1648
PB.849.17	1	1881	7	novel_not_in_catalog	gene: Zm00001d031529	novel	4.39	121.67	coding	238	857	1570
PB.8409.6	7	2047	12	novel_in_catalog	gene: Zm00001d020069	novel	0.00	96.74	coding	302	131	1036
PB.849.15	1	2731	3	novel_in_catalog	gene: Zm00001d031529	novel	0.51	121.67	coding	121	1590	1952
PB.849.14	1	1884	7	novel_not_in_catalog	gene: Zm00001d031529	novel	0.00	121.67	coding	233	858	1556
PB.10644.1	9	490	1	full-splice_match	gene: Zm00001d047888	Zm00001d047888_T001	110.80	110.80	coding	91	63	335
PB.849.10	1	3924	9	novel_not_in_catalog	gene: Zm00001d031529	novel	0.24	121.67	coding	935	319	3123
PB.7218.7	6	2022	5	full-splice_match	gene: Zm00001d035031	Zm00001d035031_T006	4.31	96.90	coding	381	581	1723
PB.7218.6	6	1740	5	novel_not_in_catalog	gene: Zm00001d035031	novel	41.68	96.90	coding	423	158	1426
PB.7218.5	6	2155	4	novel_in_catalog	gene: Zm00001d035031	novel	9.70	96.90	coding	381	708	1850
PB.7218.4	6	2690	4	full-splice_match	gene: Zm00001d035031	Zm00001d035031_T003	0.00	96.90	coding	393	1198	2376
PB.7218.3	6	2042	5	full-splice_match	gene: Zm00001d035031	Zm00001d035031_T001	8.08	96.90	coding	381	600	1742
PB.7218.2	6	1973	5	full-splice_match	gene: Zm00001d035031	Zm00001d035031_T006	10.18	96.90	coding	381	416	1558
PB.4093.1	3	1354	5	full-splice_match	gene: Zm00001d041758	Zm00001d041758_T001	38.50	38.50	coding	363	62	1150
PB.5442.1	4	2818	2	novel_in_catalog	gene: Zm00001d052188	novel	2.28	27.63	coding	421	46	1308
PB.3170.1	2	642	2	intergenic	novelGene_304	novel	27.41	27.41	non_coding	NA	NA	NA
PB.7218.8	6	1668	5	full-splice_match	gene: Zm00001d035031	Zm00001d035031_T001	22.95	96.90	coding	381	331	1473

PB.10429.4	9	1661	1	full-splice_match	gene: Zm00001d046968	Zm00001d046968_T001	11.56	90.09	coding	364	469	1560
PB.10429.2	9	1357	2	genic	gene: Zm00001d046968	novel	0.73	90.09	coding	336	229	1236
PB.10429.3	9	1448	2	genic	gene: Zm00001d046968	novel	17.83	90.09	coding	364	274	1365
PB.10429.1	9	1484	2	genic	gene: Zm00001d046968	novel	59.96	90.09	coding	364	236	1327
PB.6360.1	5	533	2	full-splice_match	gene: Zm00001d014679	Zm00001d014679_T001	180.70	1770.95	non_coding	NA	NA	NA
PB.6360.2	5	602	2	full-splice_match	gene: Zm00001d014679	Zm00001d014679_T001	1590.25	1770.95	coding	120	84	443
PB.9409.2	8	1044	1	full-splice_match	gene: Zm00001d010001	Zm00001d010001_T001	105.98	144.19	coding	231	186	878
PB.1400.4	1	2636	6	full-splice_match	gene: Zm00001d034035	Zm00001d034035_T001	15.81	61.04	coding	748	201	2444
PB.10294.1	9	1970	6	novel_in_catalog	gene: Zm00001d046174	novel	9.51	9.51	coding	531	169	1761
PB.4817.1	4	831	1	intergenic	novelGene_19	novel	14.54	14.54	coding	121	64	426
PB.10557.1	9	524	2	incomplete-splice_match	gene: Zm00001d047612	Zm00001d047612_T001	40.54	84.25	non_coding	NA	NA	NA
PB.10141.1	9	2653	1	full-splice_match	gene: Zm00001d045313	Zm00001d045313_T001	8.04	23.14	coding	385	410	1564
PB.10141.3	9	2223	1	full-splice_match	gene: Zm00001d045313	Zm00001d045313_T001	0.00	23.14	coding	385	884	2038
PB.10141.2	9	2414	1	full-splice_match	gene: Zm00001d045313	Zm00001d045313_T001	15.10	23.14	coding	705	4	2118
PB.8685.2	7	2160	11	novel_in_catalog	gene: Zm00001d021384	novel	1.61	26.30	coding	395	101	1285
PB.5758.1	4	3335	4	incomplete-splice_match	gene: Zm00001d053611	Zm00001d053611_T001	13.14	13.14	coding	975	127	3051
PB.4608.2	3	2134	1	full-splice_match	gene: Zm00001d043955	Zm00001d043955_T001	16.67	85.26	coding	580	122	1861

PB.7949.5	6	3185	19	full-splice_match	gene: Zm00001d038522	Zm00001d038522_T002	30.27	66.10	coding	974	195	3116
PB.7949.3	6	3314	18	novel_in_catalog	gene: Zm00001d038522	novel	15.17	66.10	coding	252	2444	3199
PB.518.1	1	2402	2	full-splice_match	gene: Zm00001d029516	Zm00001d029516_T001	27.65	29.09	coding	543	386	2014
PB.265.3	1	1291	1	full-splice_match	gene: Zm00001d028389	Zm00001d028389_T001	0.00	35.95	coding	256	409	1176
PB.265.2	1	1632	1	full-splice_match	gene: Zm00001d028389	Zm00001d028389_T001	29.50	35.95	coding	256	409	1176
PB.265.1	1	1740	1	full-splice_match	gene: Zm00001d028389	Zm00001d028389_T001	6.45	35.95	coding	256	408	1175
PB.5269.2	4	1554	2	antisense	novelGene_gene: Zm00001d051349_A	novel	21.20	103.06	coding	102	963	1268
PB.8491.1	7	1395	3	novel_not_in_catalog	gene: Zm00001d020500	novel	25.62	25.62	non_coding	NA	NA	NA
PB.7834.6	6	2283	4	full-splice_match	gene: Zm00001d038105	Zm00001d038105_T002	13.23	60.46	coding	555	367	2031
PB.7834.4	6	2414	4	full-splice_match	gene: Zm00001d038105	Zm00001d038105_T002	11.50	60.46	coding	555	247	1911
PB.606.2	1	1259	4	novel_in_catalog	gene: Zm00001d030009	novel	7.84	85.64	coding	169	432	938
PB.606.3	1	1147	4	novel_in_catalog	gene: Zm00001d030009	novel	0.00	85.64	coding	169	434	940
PB.7834.8	6	2234	1	incomplete-splice_match	gene: Zm00001d038105	Zm00001d038105_T001	2.10	60.46	non_coding	NA	NA	NA
PB.1493.12	1	1001	3	genic	gene: Zm00001d034434	novel	0.00	39.47	non_coding	NA	NA	NA
PB.606.7	1	982	5	full-splice_match	gene: Zm00001d030009	Zm00001d030009_T002	21.58	85.64	coding	243	109	837
PB.7210.1	6	543	1	genic	gene: Zm00001d034984	novel	7.16	7.16	coding	97	14	304
PB.606.5	1	1506	3	novel_in_catalog	gene: Zm00001d030009	novel	6.96	85.64	coding	129	702	1088

PB.8330.4	7	3304	2	novel_in_catalog	gene: Zm00001d019542	novel	13.28	78.97	coding	555	528	2192
PB.8330.7	7	2475	9	full-splice_match	gene: Zm00001d019542	Zm00001d019542_T023	0.00	78.97	coding	731	282	2474
PB.8330.6	7	2639	9	full-splice_match	gene: Zm00001d019542	Zm00001d019542_T023	9.22	78.97	coding	786	281	2638
PB.1714.1	10	1629	9	full-splice_match	gene: Zm00001d023514	Zm00001d023514_T003	52.07	54.18	coding	355	271	1335
PB.8330.3	7	3839	10	novel_not_in_catalog	gene: Zm00001d019542	novel	56.47	78.97	coding	635	1620	3524
PB.1714.3	10	1404	9	full-splice_match	gene: Zm00001d023514	Zm00001d023514_T003	0.00	54.18	coding	355	279	1343
PB.9610.1	8	1542	2	full-splice_match	gene: Zm00001d010924	Zm00001d010924_T001	37.44	37.44	coding	336	204	1211
PB.1276.1	1	1243	4	intergenic	novelGene_371	novel	27.69	35.58	coding	145	26	460
PB.2253.1	10	1555	6	incomplete-splice_match	gene: Zm00001d026295	Zm00001d026295_T005	11.80	11.80	coding	337	36	1046
PB.4490.1	3	851	8	full-splice_match	gene: Zm00001d043497	Zm00001d043497_T001	281.88	281.88	coding	163	146	634
PB.835.2	1	3127	5	antisense	novelGene_gene: Zm00001d031443_A	novel	11.53	11.53	non_coding	NA	NA	NA
PB.5275.5	4	1419	4	antisense	novelGene_gene: Zm00001d051399_A	novel	13.36	13.36	non_coding	NA	NA	NA
PB.8320.4	7	1552	4	genic	gene: Zm00001d019493	novel	6.65	27.26	coding	324	402	1373
PB.8320.5	7	1394	4	genic	gene: Zm00001d019493	novel	3.32	27.26	coding	324	267	1238
PB.8320.2	7	3432	1	full-splice_match	gene: Zm00001d019493	Zm00001d019493_T001	0.49	27.26	coding	423	1978	3246
PB.8320.3	7	1634	3	genic	gene: Zm00001d019493	novel	13.30	27.26	coding	423	190	1458
PB.8320.1	7	2398	2	genic	gene: Zm00001d019493	novel	3.50	27.26	coding	423	941	2209

PB.3709.1	3	823	1	genic	gene: Zm00001d039480	novel	3.99	3.99	coding	115	143	487
PB.4232.1	3	2352	19	full-splice_match	gene: Zm00001d042398	Zm00001d042398_T002	75.12	111.35	coding	711	1	2133
PB.1386.1	1	1594	1	novel_in_catalog	gene: Zm00001d033995	novel	16.63	183.77	coding	258	573	1346
PB.6947.2	5	319	1	incomplete-splice_match	gene: Zm00001d017682	Zm00001d017682_T001	0.00	78.80	non_coding	NA	NA	NA
PB.1792.1	10	3031	1	full-splice_match	gene: Zm00001d023919	Zm00001d023919_T001	30.38	30.38	coding	956	37	2904
PB.5570.1	4	1970	3	full-splice_match	gene: Zm00001d052668	Zm00001d052668_T001	2.07	2.07	coding	477	86	1516
PB.9230.1	8	974	2	full-splice_match	gene: Zm00001d008970	Zm00001d008970_T001	17.05	17.05	coding	247	68	808
PB.9606.5	8	1560	8	full-splice_match	gene: Zm00001d010915	Zm00001d010915_T001	2.41	40.25	coding	421	54	1316
PB.10718.1	9	1124	6	full-splice_match	gene: Zm00001d048158	Zm00001d048158_T001	196.20	196.20	coding	260	75	854
PB.9606.1	8	2007	8	full-splice_match	gene: Zm00001d010915	Zm00001d010915_T001	0.83	40.25	coding	421	50	1312
PB.9606.2	8	1854	8	full-splice_match	gene: Zm00001d010915	Zm00001d010915_T001	24.17	40.25	coding	421	31	1293
PB.9606.3	8	1666	8	full-splice_match	gene: Zm00001d010915	Zm00001d010915_T001	12.84	40.25	coding	421	46	1308
PB.8598.1	7	2460	4	incomplete-splice_match	gene: Zm00001d020972	Zm00001d020972_T003	24.01	24.01	coding	709	42	2168
PB.5547.11	4	2390	10	novel_in_catalog	gene: Zm00001d052532	novel	1.73	938.93	coding	102	92	397
PB.6294.2	5	471	1	intergenic	novelGene_105	novel	8.25	10.31	non_coding	NA	NA	NA
PB.8598.2	7	2554	3	incomplete-splice_match	gene: Zm00001d020972	Zm00001d020972_T005	0.00	24.01	coding	638	349	2262
PB.5547.14	4	1778	13	novel_in_catalog	gene: Zm00001d052532	novel	25.90	938.93	coding	102	103	408

PB.5547.15	4	2446	2	novel_in_catalog	gene: Zm00001d052532	novel	28.06	938.93	non_coding	NA	NA	NA
PB.5783.4	4	1199	3	full-splice_match	gene: Zm00001d053682	Zm00001d053682_T001	13.86	25.60	coding	288	209	1072
PB.4581.3	3	1583	3	full-splice_match	gene: Zm00001d043850	Zm00001d043850_T001	20.85	227.52	coding	447	76	1416
PB.5783.1	4	1393	3	full-splice_match	gene: Zm00001d053682	Zm00001d053682_T001	10.15	25.60	coding	358	59	1132
PB.5783.3	4	2583	1	novel_in_catalog	gene: Zm00001d053682	novel	1.59	25.60	coding	328	1449	2432
PB.1343.1	1	2522	5	full-splice_match	gene: Zm00001d033800	Zm00001d033800_T002	5.83	45.77	coding	587	422	2182
PB.1343.2	1	2402	5	full-splice_match	gene: Zm00001d033800	Zm00001d033800_T002	39.93	45.77	coding	587	375	2135
PB.1252.1	1	320	1	full-splice_match	gene: Zm00001d033395	Zm00001d033395_T001	3.83	3.83	non_coding	NA	NA	NA
PB.9114.2	8	2155	1	full-splice_match	gene: Zm00001d008424	Zm00001d008424_T001	6.66	15.36	coding	643	164	2092
PB.7842.1	6	2198	13	full-splice_match	gene: Zm00001d038128	Zm00001d038128_T004	13.44	13.44	coding	524	85	1656
PB.9114.1	8	2265	1	full-splice_match	gene: Zm00001d008424	Zm00001d008424_T001	8.70	15.36	coding	643	164	2092
PB.5546.3	4	2141	9	novel_not_in_catalog	gene: Zm00001d052525	novel	0.47	42.92	coding	426	618	1895
PB.5546.2	4	2058	8	novel_in_catalog	gene: Zm00001d052525	novel	10.44	42.92	coding	183	226	774
PB.5546.1	4	2134	9	full-splice_match	gene: Zm00001d052525	Zm00001d052525_T001	19.06	42.92	coding	468	135	1538
PB.9867.2	8	2378	1	novel_in_catalog	gene: Zm00001d012098	novel	36.30	100.63	coding	241	1425	2147
PB.5546.5	4	2038	8	novel_in_catalog	gene: Zm00001d052525	novel	0.00	42.92	coding	183	333	881
PB.855.1	1	1199	1	antisense	novelGene_gene: Zm00001d031554_A	novel	3.66	3.66	coding	232	10	705

PB.5546.9	4	1670	9	full-splice_match	gene: Zm00001d052525	Zm00001d052525_T001	0.00	42.92	coding	468	141	1544
PB.5546.8	4	1888	9	full-splice_match	gene: Zm00001d052525	Zm00001d052525_T001	12.95	42.92	coding	468	289	1692
PB.1951.3	10	2167	5	full-splice_match	gene: Zm00001d024861	Zm00001d024861_T005	8.60	10.21	coding	289	328	1194
PB.10054.1	9	1575	1	full-splice_match	gene: Zm00001d044841	Zm00001d044841_T001	6.73	6.73	coding	437	246	1556
PB.370.1	1	2446	3	full-splice_match	gene: Zm00001d028866	Zm00001d028866_T001	15.36	59.89	coding	628	145	2028
PB.370.3	1	2308	3	full-splice_match	gene: Zm00001d028866	Zm00001d028866_T001	44.53	59.89	coding	628	145	2028
PB.2880.1	2	2169	5	novel_in_catalog	gene: Zm00001d003857	novel	50.38	364.78	coding	403	568	1776
PB.6564.2	5	2272	3	full-splice_match	gene: Zm00001d015618	Zm00001d015618_T003	13.42	718.92	coding	229	1236	1922
PB.2552.2	2	1546	4	full-splice_match	gene: Zm00001d002402	Zm00001d002402_T001	105.44	105.44	coding	363	214	1302
PB.63.2	1	2297	20	novel_not_in_catalog	gene: Zm00001d027466	novel	10.64	20.34	coding	584	87	1838
PB.63.3	1	2360	20	novel_not_in_catalog	gene: Zm00001d027466	novel	9.70	20.34	coding	587	174	1934
PB.2880.9	2	1876	6	full-splice_match	gene: Zm00001d003857	Zm00001d003857_T001	0.00	364.78	coding	534	72	1673
PB.9978.1	8	1735	3	novel_not_in_catalog	gene: Zm00001d012591	novel	5.78	7.25	coding	402	211	1416
PB.9978.2	8	1732	2	novel_in_catalog	gene: Zm00001d012591	novel	1.47	7.25	coding	213	167	805
PB.9978.3	8	1557	3	novel_not_in_catalog	gene: Zm00001d012591	novel	0.00	7.25	coding	402	167	1372
PB.6003.1	5	1064	3	full-splice_match	gene: Zm00001d013151	Zm00001d013151_T001	7.97	7.97	coding	281	9	851
PB.1529.2	1	849	4	full-splice_match	gene: Zm00001d034586	Zm00001d034586_T002	23.05	39.57	coding	182	113	658

PB.10843.2	B73V4 ctg104	1241	4	novel_not_in_catalog	gene: Zm00001d000288	novel	1.89	8.42	coding	194	206	787
PB.1553.1	1	2338	4	novel_in_catalog	gene: Zm00001d034655	novel	23.49	38.32	coding	615	35	1879
PB.2890.1	2	3880	4	full-splice_match	gene: Zm00001d003911	Zm00001d003911_T011	80.87	80.87	coding	440	2138	3457
PB.5140.1	4	811	4	full-splice_match	gene: Zm00001d050411	Zm00001d050411_T001	7.85	232.09	coding	119	77	433
PB.4997.3	4	1695	4	full-splice_match	gene: Zm00001d049624	Zm00001d049624_T001	14.99	14.99	coding	474	14	1435
PB.5140.2	4	725	4	full-splice_match	gene: Zm00001d050411	Zm00001d050411_T001	224.24	232.09	coding	119	119	475
PB.2054.1	10	1244	4	full-splice_match	gene: Zm00001d025340	Zm00001d025340_T004	13.73	13.73	coding	316	46	993
PB.7643.1	6	2105	2	full-splice_match	gene: Zm00001d037318	Zm00001d037318_T001	26.67	31.89	coding	554	235	1896
PB.10843.1	B73V4 ctg104	1254	4	full-splice_match	gene: Zm00001d000288	Zm00001d000288_T001	6.53	8.42	coding	197	207	797
PB.673.1	1	998	7	fusion	gene: Zm00001d030411_g	novel	34.68	34.68	coding	250	64	813
PB.4697.1	3	1514	10	full-splice_match	gene: Zm00001d044315	Zm00001d044315_T013	174.07	275.21	coding	351	202	1254
PB.5655.1	4	3270	5	full-splice_match	gene: Zm00001d053011	Zm00001d053011_T004	4.77	4.77	coding	936	341	3148
PB.3775.1	3	944	2	genic	gene: Zm00001d039841	novel	59.09	59.09	coding	168	280	783
PB.657.1	1	791	2	full-splice_match	gene: Zm00001d030317	Zm00001d030317_T001	15.56	15.56	coding	185	69	623
PB.5879.2	4	3207	3	novel_in_catalog	gene: Zm00001d054014	novel	5.13	44.48	coding	167	111	611
PB.6966.1	5	1050	5	full-splice_match	gene: Zm00001d017771	Zm00001d017771_T001	15.97	15.97	coding	310	19	948
PB.10435.1	9	2796	14	novel_not_in_catalog	gene: Zm00001d046997	novel	5.05	9.44	coding	705	195	2309

PB.10435.3	9	1518	4	novel_not_in_catalog	gene: Zm00001d046997	novel	4.39	9.44	coding	155	599	1063
PB.8681.5	7	1620	7	novel_not_in_catalog	gene: Zm00001d021371	novel	8.07	35.05	coding	267	536	1336
PB.2177.4	10	3137	14	full-splice_match	gene: Zm00001d025951	Zm00001d025951_T031	53.88	53.88	coding	939	194	3010
PB.4328.1	3	1143	2	full-splice_match	gene: Zm00001d042843	Zm00001d042843_T001	19.42	19.42	coding	262	22	807
PB.8037.6	6	3394	2	novel_not_in_catalog	gene: Zm00001d038847	novel	17.47	17.47	coding	109	1114	1440
PB.972.1	1	917	1	full-splice_match	gene: Zm00001d032115	Zm00001d032115_T001	200.76	200.76	coding	178	117	650
PB.972.2	1	873	1	full-splice_match	gene: Zm00001d032115	Zm00001d032115_T001	0.00	200.76	coding	178	119	652
PB.7417.1	6	1801	3	full-splice_match	gene: Zm00001d036122	Zm00001d036122_T001	27.97	27.97	coding	496	22	1509
PB.562.1	1	334	1	intergenic	novelGene_148	novel	1.71	1.71	non_coding	NA	NA	NA
PB.8407.1	7	2019	7	novel_in_catalog	gene: Zm00001d020063	novel	9.02	26.94	coding	353	836	1894
PB.8407.2	7	867	6	incomplete-splice_match	gene: Zm00001d020063	Zm00001d020063_T002	17.92	26.94	non_coding	NA	NA	NA
PB.7720.1	6	1756	3	full-splice_match	gene: Zm00001d037688	Zm00001d037688_T001	10.50	10.50	coding	462	128	1513
PB.9695.1	8	2583	21	full-splice_match	gene: Zm00001d011357	Zm00001d011357_T003	25.91	25.91	coding	696	85	2172
PB.9923.1	8	1389	1	full-splice_match	gene: Zm00001d012336	Zm00001d012336_T001	118.34	118.34	coding	327	62	1042
PB.10457.1	9	699	4	novel_not_in_catalog	gene: Zm00001d047119	novel	345.01	345.01	coding	123	121	489
PB.7244.1	6	1145	1	full-splice_match	gene: Zm00001d035133	Zm00001d035133_T001	29.03	40.91	coding	231	6	698
PB.7244.2	6	3297	1	full-splice_match	gene: Zm00001d035133	Zm00001d035133_T001	11.88	40.91	coding	982	107	3052

PB.7986.4	6	2757	5	full-splice_match	gene: Zm00001d038671	Zm00001d038671_T003	6.39	32.27	coding	797	131	2521
PB.7860.1	6	2239	2	novel_not_in_catalog	gene: Zm00001d038171	novel	37.68	37.68	coding	617	219	2069
PB.4386.4	3	2105	7	novel_in_catalog	gene: Zm00001d043109	novel	1.65	42.88	coding	380	413	1552
PB.4386.2	3	2968	12	novel_in_catalog	gene: Zm00001d043109	novel	5.84	42.88	coding	727	229	2409
PB.4386.3	3	2943	13	full-splice_match	gene: Zm00001d043109	Zm00001d043109_T001	0.00	42.88	coding	765	207	2501
PB.8301.1	7	1514	8	full-splice_match	gene: Zm00001d019314	Zm00001d019314_T008	19.28	19.28	coding	382	164	1309
PB.4386.1	3	3097	13	full-splice_match	gene: Zm00001d043109	Zm00001d043109_T001	35.39	42.88	coding	765	249	2543
PB.8417.3	7	1842	2	genic	gene: Zm00001d020139	novel	19.56	40.24	coding	451	186	1538
PB.8417.2	7	1983	2	genic	gene: Zm00001d020139	novel	20.67	40.24	coding	451	114	1466
PB.8417.1	7	1926	2	genic	gene: Zm00001d020139	novel	0.00	40.24	coding	397	56	1246
PB.4470.3	3	2858	4	incomplete-splice_match	gene: Zm00001d043434	Zm00001d043434_T040	4.37	40.57	coding	856	290	2857
PB.5684.1	4	1576	9	full-splice_match	gene: Zm00001d053189	Zm00001d053189_T005	29.06	29.06	coding	356	291	1358
PB.7651.3	6	1286	4	full-splice_match	gene: Zm00001d037359	Zm00001d037359_T001	63.66	198.34	coding	345	90	1124
PB.1195.1	1	1788	7	incomplete-splice_match	gene: Zm00001d033104	Zm00001d033104_T001	15.17	22.73	coding	427	124	1404
PB.7651.1	6	1353	4	full-splice_match	gene: Zm00001d037359	Zm00001d037359_T001	134.68	198.34	coding	345	90	1124
PB.3612.1	2	1192	4	novel_not_in_catalog	gene: Zm00001d007877	novel	9.45	9.45	coding	321	41	1003
PB.734.1	1	1832	12	full-splice_match	gene: Zm00001d030939	Zm00001d030939_T002	23.87	23.87	coding	422	258	1523

PB.1689.9	10	1034	5	full-splice_match	gene: Zm00001d023376	Zm00001d023376_T002	0.00	1404.98	coding	238	106	819
PB.1689.8	10	1856	4	novel_in_catalog	gene: Zm00001d023376	novel	0.00	1404.98	coding	114	1295	1636
PB.1689.7	10	1504	2	novel_in_catalog	gene: Zm00001d023376	novel	2.91	1404.98	non_coding	NA	NA	NA
PB.5497.1	4	1210	2	full-splice_match	gene: Zm00001d052375	Zm00001d052375_T002	52.94	52.94	coding	135	527	931
PB.1689.5	10	2270	1	novel_in_catalog	gene: Zm00001d023376	novel	16.85	1404.98	non_coding	NA	NA	NA
PB.1689.4	10	1171	5	full-splice_match	gene: Zm00001d023376	Zm00001d023376_T002	949.31	1404.98	coding	238	169	882
PB.1689.3	10	2154	3	novel_in_catalog	gene: Zm00001d023376	novel	9.48	1404.98	non_coding	NA	NA	NA
PB.1689.2	10	1999	4	novel_in_catalog	gene: Zm00001d023376	novel	14.26	1404.98	coding	114	1295	1636
PB.1689.1	10	1270	5	full-splice_match	gene: Zm00001d023376	Zm00001d023376_T002	412.18	1404.98	coding	238	106	819
PB.7754.5	6	2861	1	incomplete-splice_match	gene: Zm00001d037810	Zm00001d037810_T024	13.90	32.81	coding	881	34	2676
PB.10060.1	9	1111	4	novel_not_in_catalog	gene: Zm00001d044880	novel	62.45	62.45	coding	284	8	859
PB.6431.1	5	765	4	full-splice_match	gene: Zm00001d014926	Zm00001d014926_T001	109.21	109.21	coding	132	163	558
PB.8784.5	7	2952	6	full-splice_match	gene: Zm00001d021726	Zm00001d021726_T003	0.00	4.67	coding	848	217	2760
PB.8784.1	7	3229	6	full-splice_match	gene: Zm00001d021726	Zm00001d021726_T003	4.67	4.67	coding	848	162	2705
PB.4195.1	3	1318	2	full-splice_match	gene: Zm00001d042245	Zm00001d042245_T001	252.22	264.55	coding	144	259	690
PB.8784.3	7	3061	6	full-splice_match	gene: Zm00001d021726	Zm00001d021726_T003	0.00	4.67	coding	848	71	2614
PB.10733.1	9	1066	1	full-splice_match	gene: Zm00001d048214	Zm00001d048214_T001	9.16	9.16	coding	178	98	631

PB.6633.3	5	3367	10	full-splice_match	gene: Zm00001d016166	Zm00001d016166_T001	98.55	142.61	coding	960	240	3119
PB.8870.3	7	1715	4	full-splice_match	gene: Zm00001d022016	Zm00001d022016_T003	16.28	43.68	coding	171	195	707
PB.6790.1	5	1802	6	full-splice_match	gene: Zm00001d016993	Zm00001d016993_T006	14.42	45.23	coding	311	135	1067
PB.6790.2	5	1610	6	full-splice_match	gene: Zm00001d016993	Zm00001d016993_T006	29.17	45.23	coding	311	209	1141
PB.10790.14	9	2548	12	full-splice_match	gene: Zm00001d048415	Zm00001d048415_T010	0.00	31.23	coding	396	72	1259
PB.7726.1	6	1557	2	full-splice_match	gene: Zm00001d037713	Zm00001d037713_T002	10.60	21.50	coding	284	250	1101
PB.7726.3	6	1414	3	novel_not_in_catalog	gene: Zm00001d037713	novel	10.90	21.50	coding	284	105	956
PB.6896.1	5	2309	9	novel_in_catalog	gene: Zm00001d017445	novel	14.39	50.46	coding	266	302	1099
PB.4662.1	3	433	1	incomplete-splice_match	gene: Zm00001d044179	Zm00001d044179_T002	157.37	157.37	non_coding	NA	NA	NA
PB.8465.1	7	1477	4	full-splice_match	gene: Zm00001d020392	Zm00001d020392_T002	6.26	9.37	coding	342	89	1114
PB.8465.3	7	1287	4	full-splice_match	gene: Zm00001d020392	Zm00001d020392_T002	3.11	9.37	coding	342	86	1111
PB.7025.1	5	2428	5	full-splice_match	gene: Zm00001d017987	Zm00001d017987_T002	35.36	35.95	coding	612	21	1856
PB.3163.2	2	689	3	full-splice_match	gene: Zm00001d005695	Zm00001d005695_T001	380.00	380.00	coding	90	95	364
PB.2528.4	2	2385	6	full-splice_match	gene: Zm00001d002323	Zm00001d002323_T006	0.00	37.89	coding	582	425	2170
PB.7025.2	5	2326	5	full-splice_match	gene: Zm00001d017987	Zm00001d017987_T002	0.00	35.95	coding	612	21	1856
PB.2528.2	2	3172	5	full-splice_match	gene: Zm00001d002323	Zm00001d002323_T016	4.68	37.89	coding	327	1887	2867
PB.10451.1	9	1395	4	full-splice_match	gene: Zm00001d047087	Zm00001d047087_T001	29.93	29.93	coding	286	26	883

PB.2528.1	2	2492	6	full-splice_match	gene: Zm00001d002323	Zm00001d002323_T005	33.21	37.89	coding	582	425	2170
PB.324.3	1	1414	9	full-splice_match	gene: Zm00001d028631	Zm00001d028631_T002	0.00	61.24	coding	308	239	1162
PB.324.2	1	1637	8	novel_in_catalog	gene: Zm00001d028631	novel	3.55	61.24	coding	254	242	1003
PB.324.1	1	1520	9	full-splice_match	gene: Zm00001d028631	Zm00001d028631_T002	57.69	61.24	coding	308	226	1149
PB.9416.1	8	666	3	incomplete-splice_match	gene: Zm00001d010047	Zm00001d010047_T007	54.80	54.80	coding	118	57	410
PB.2548.2	2	2694	17	incomplete-splice_match	gene: Zm00001d002390	Zm00001d002390_T011	25.16	25.16	coding	567	765	2465
PB.6137.2	5	1841	2	novel_in_catalog	gene: Zm00001d013612	novel	5.58	239.82	coding	213	931	1569
PB.6137.3	5	333	1	incomplete-splice_match	gene: Zm00001d013612	Zm00001d013612_T001	0.00	239.82	non_coding	NA	NA	NA
PB.6137.1	5	1763	3	full-splice_match	gene: Zm00001d013612	Zm00001d013612_T003	234.24	239.82	coding	447	127	1467
PB.8750.1	7	1298	1	full-splice_match	gene: Zm00001d021620	Zm00001d021620_T001	9.97	9.97	coding	359	96	1172
PB.10851.4	B73V4 ctg11	1221	6	novel_in_catalog	gene: Zm00001d000241	novel	0.00	44.63	coding	151	204	656
PB.439.2	1	1226	12	full-splice_match	gene: Zm00001d029141	Zm00001d029141_T003	48.46	63.05	coding	256	120	887
PB.10851.2	B73V4 ctg11	1408	5	novel_in_catalog	gene: Zm00001d000241	novel	3.34	44.63	coding	151	250	702
PB.10851.1	B73V4 ctg11	1200	7	full-splice_match	gene: Zm00001d000241	Zm00001d000241_T002	29.11	44.63	coding	206	254	871
PB.439.1	1	2055	9	novel_in_catalog	gene: Zm00001d029141	novel	5.10	63.05	coding	77	117	347
PB.9823.1	8	900	2	incomplete-splice_match	gene: Zm00001d011890	Zm00001d011890_T001	13.68	13.68	coding	71	298	510
PB.1347.1	1	1851	10	full-splice_match	gene: Zm00001d033830	Zm00001d033830_T002	56.54	56.54	coding	488	71	1534

PB.4181.1	3	1303	1	antisense	novelGene_gene: Zm00001d042141_A	novel	10.12	10.12	coding	105	148	462
PB.6885.1	5	650	1	intergenic	novelGene_312	novel	29.17	29.17	non_coding	NA	NA	NA
PB.4450.1	3	2107	2	novel_not_in_catalog	gene: Zm00001d043365	novel	28.34	28.34	coding	606	175	1992
PB.5114.1	4	717	5	incomplete-splice_match	gene: Zm00001d050248	Zm00001d050248_T002	30.34	30.34	coding	79	73	309
PB.661.2	1	2039	1	full-splice_match	gene: Zm00001d030343	Zm00001d030343_T001	11.24	18.86	coding	535	71	1675
PB.4161.1	3	2130	11	full-splice_match	gene: Zm00001d042052	Zm00001d042052_T009	22.47	22.47	coding	565	205	1899
PB.4661.1	3	1493	2	full-splice_match	gene: Zm00001d044176	Zm00001d044176_T001	6.69	6.69	coding	290	131	1000
PB.7124.1	5	2184	1	full-splice_match	gene: Zm00001d018343	Zm00001d018343_T001	15.12	27.24	coding	430	208	1497
PB.1325.2	1	1530	14	full-splice_match	gene: Zm00001d033708	Zm00001d033708_T001	8.32	36.24	coding	367	284	1384
PB.7124.2	5	1819	1	full-splice_match	gene: Zm00001d018343	Zm00001d018343_T001	12.12	27.24	coding	430	380	1669
PB.8518.1	7	1495	2	incomplete-splice_match	gene: Zm00001d020615	Zm00001d020615_T001	11.26	11.26	coding	183	947	1495
PB.1227.2	1	1524	9	full-splice_match	gene: Zm00001d033294	Zm00001d033294_T003	8.31	21.40	coding	272	543	1358
PB.5489.2	4	2184	4	incomplete-splice_match	gene: Zm00001d052344	Zm00001d052344_T003	9.23	21.85	coding	403	453	1661
PB.4761.3	3	1460	5	novel_in_catalog	gene: Zm00001d044594	novel	4.44	19.56	coding	354	13	1074
PB.4761.2	3	1619	6	novel_in_catalog	gene: Zm00001d044594	novel	7.58	19.56	coding	421	224	1486
PB.4761.1	3	1489	6	incomplete-splice_match	gene: Zm00001d044594	Zm00001d044594_T001	7.55	19.56	coding	365	70	1164
PB.8546.1	7	2000	9	full-splice_match	gene: Zm00001d020764	Zm00001d020764_T004	19.39	19.39	coding	304	532	1443

PB.1347.2	1	1721	10	full-splice_match	gene: Zm00001d033830	Zm00001d033830_T002	0.00	56.54	coding	488	43	1506
PB.4641.1	3	936	7	full-splice_match	gene: Zm00001d044096	Zm00001d044096_T003	209.13	272.02	coding	206	75	692
PB.9013.1	7	2296	7	full-splice_match	gene: Zm00001d022499	Zm00001d022499_T001	41.69	41.69	coding	571	216	1928
PB.4477.1	3	2767	1	full-splice_match	gene: Zm00001d043454	Zm00001d043454_T001	1.85	14.93	coding	372	89	1204
PB.4477.2	3	1627	1	full-splice_match	gene: Zm00001d043454	Zm00001d043454_T001	13.08	14.93	coding	372	286	1401
PB.9857.1	8	879	5	full-splice_match	gene: Zm00001d012052	Zm00001d012052_T001	523.46	893.24	coding	156	169	636
PB.9585.2	8	2607	11	full-splice_match	gene: Zm00001d010823	Zm00001d010823_T003	11.52	11.52	coding	642	8	1933
PB.9857.3	8	696	5	full-splice_match	gene: Zm00001d012052	Zm00001d012052_T001	369.78	893.24	coding	156	120	587
PB.8639.1	7	1318	7	novel_in_catalog	gene: Zm00001d021203	novel	15.04	20.53	coding	363	53	1141
PB.1958.1	10	1739	3	full-splice_match	gene: Zm00001d024883	Zm00001d024883_T001	42.17	42.17	coding	499	28	1524
PB.5208.2	4	392	1	full-splice_match	gene: Zm00001d050964	Zm00001d050964_T001	68.99	102.10	non_coding	NA	NA	NA
PB.10859.1	B73V4 ctg134	662	1	intergenic	novelGene_8	novel	250.84	250.84	non_coding	NA	NA	NA
PB.431.1	1	2241	16	novel_in_catalog	gene: Zm00001d029120	novel	69.26	69.26	coding	442	420	1745
PB.1922.4	10	2326	4	novel_in_catalog	gene: Zm00001d024744	novel	3.90	36.88	coding	307	105	1025
PB.1922.7	10	1968	8	incomplete-splice_match	gene: Zm00001d024744	Zm00001d024744_T005	10.89	36.88	coding	470	16	1425
PB.1922.6	10	2031	7	novel_in_catalog	gene: Zm00001d024744	novel	0.00	36.88	coding	357	112	1182
PB.1922.1	10	3574	6	novel_in_catalog	gene: Zm00001d024744	novel	0.00	36.88	coding	734	145	2346

PB.1922.3	10	3091	7	full-splice_match	gene: Zm00001d024744	Zm00001d024744_T002	13.06	36.88	coding	857	307	2877
PB.1922.2	10	3233	9	full-splice_match	gene: Zm00001d024744	Zm00001d024744_T006	4.09	36.88	coding	831	198	2690
PB.8393.1	7	1877	2	genic	gene: Zm00001d019993	novel	9.75	9.75	coding	499	79	1575
PB.1226.1	1	406	3	novel_not_in_catalog	gene: Zm00001d022965	novel	71.42	71.42	non_coding	NA	NA	NA
PB.4863.1	4	727	2	full-splice_match	gene: Zm00001d048949	Zm00001d048949_T001	48.59	48.59	coding	149	95	541
PB.5021.3	4	1651	8	full-splice_match	gene: Zm00001d049716	Zm00001d049716_T001	73.52	100.67	coding	417	83	1333
PB.5021.2	4	1768	7	novel_in_catalog	gene: Zm00001d049716	novel	1.45	100.67	coding	454	83	1444
PB.5021.1	4	1780	8	full-splice_match	gene: Zm00001d049716	Zm00001d049716_T001	19.63	100.67	coding	417	81	1331
PB.2882.2	2	1575	4	full-splice_match	gene: Zm00001d003878	Zm00001d003878_T002	85.75	85.75	coding	427	85	1365
PB.372.4	1	3621	2	novel_in_catalog	gene: Zm00001d028868	novel	7.98	112.10	coding	238	2681	3394
PB.1321.1	1	2602	1	incomplete-splice_match	gene: Zm00001d033704	Zm00001d033704_T001	28.04	30.95	coding	758	58	2331
PB.9333.1	8	1142	1	full-splice_match	gene: Zm00001d009582	Zm00001d009582_T001	21.45	21.45	coding	317	2	952
PB.706.1	1	2095	1	full-splice_match	gene: Zm00001d030682	Zm00001d030682_T001	1.93	1.93	coding	505	383	1897
PB.3668.1	3	914	2	full-splice_match	gene: Zm00001d039348	Zm00001d039348_T001	65.46	65.46	coding	164	143	634
PB.10474.1	9	3129	15	full-splice_match	gene: Zm00001d047253	Zm00001d047253_T002	460.00	821.31	coding	816	163	2610
PB.3247.1	2	957	1	full-splice_match	gene: Zm00001d006111	Zm00001d006111_T001	308.58	308.58	coding	220	84	743
PB.2782.1	2	1083	6	novel_not_in_catalog	gene: Zm00001d003347	novel	92.59	118.60	coding	209	137	763

PB.2782.2	2	933	6	novel_not_in_catalog	gene: Zm00001d003347	novel	26.01	118.60	coding	209	133	759
PB.2016.1	10	3036	17	full-splice_match	gene: Zm00001d025111	Zm00001d025111_T024	8.09	36.59	coding	791	173	2545
PB.3608.1	2	1045	4	full-splice_match	gene: Zm00001d007864	Zm00001d007864_T001	6.52	6.52	coding	177	227	757
PB.8522.1	7	585	3	incomplete-splice_match	gene: Zm00001d020641	Zm00001d020641_T001	48.49	48.49	coding	103	31	339
PB.1522.1	1	981	2	novel_in_catalog	gene: Zm00001d034564	novel	10.64	10.64	coding	82	491	736
PB.3694.2	3	2668	4	full-splice_match	gene: Zm00001d039427	Zm00001d039427_T002	22.40	22.40	coding	574	732	2453
PB.4388.2	3	2178	1	full-splice_match	gene: Zm00001d043125	Zm00001d043125_T001	4.31	27.68	coding	350	821	1870
PB.4388.3	3	1691	2	genic	gene: Zm00001d043125	novel	18.57	27.68	coding	350	516	1565
PB.6556.1	5	585	1	novel_in_catalog	gene: Zm00001d015525	novel	115.24	115.24	non_coding	NA	NA	NA
PB.5380.1	4	1293	1	full-splice_match	gene: Zm00001d051894	Zm00001d051894_T001	9.71	9.71	coding	310	61	990
PB.3789.1	3	1697	1	full-splice_match	gene: Zm00001d039907	Zm00001d039907_T001	115.36	115.36	coding	378	285	1418
PB.3789.2	3	1550	1	full-splice_match	gene: Zm00001d039907	Zm00001d039907_T001	0.00	115.36	coding	378	285	1418
PB.4467.1	3	1194	1	full-splice_match	gene: Zm00001d043430	Zm00001d043430_T001	28.98	28.98	coding	279	52	888
PB.5752.3	4	1788	1	full-splice_match	gene: Zm00001d053586	Zm00001d053586_T001	0.00	13.88	coding	509	47	1573
PB.3323.3	2	2106	3	novel_in_catalog	gene: Zm00001d006430	novel	6.47	6.47	coding	465	612	2006
PB.8867.1	7	871	1	genic	gene: Zm00001d022006	novel	22.42	22.42	coding	151	142	594
PB.10754.8	9	2209	3	full-splice_match	gene: Zm00001d048310	Zm00001d048310_T003	0.00	111.41	coding	428	756	2039

PB.6881.2	5	1713	3	genic	gene: Zm00001d017378	novel	3.60	22.37	coding	255	140	904
PB.6881.3	5	1587	2	genic	gene: Zm00001d017378	novel	9.08	22.37	coding	255	335	1099
PB.10108.1	9	1996	1	antisense	novelGene_gene: Zm00001d045130_A	novel	5.53	5.53	coding	428	466	1749
PB.10754.6	9	1837	2	full-splice_match	gene: Zm00001d048310	Zm00001d048310_T004	0.00	111.41	coding	428	306	1589
PB.10754.1	9	1882	1	incomplete-splice_match	gene: Zm00001d048310	Zm00001d048310_T003	78.48	111.41	coding	428	55	1338
PB.2194.1	10	2208	1	full-splice_match	gene: Zm00001d026035	Zm00001d026035_T001	65.96	65.96	coding	230	220	909
PB.10754.2	9	2342	3	full-splice_match	gene: Zm00001d048310	Zm00001d048310_T003	25.16	111.41	coding	428	753	2036
PB.5013.1	4	2525	2	full-splice_match	gene: Zm00001d049690	Zm00001d049690_T001	2.15	19.60	coding	516	20	1567
PB.5013.2	4	1869	2	full-splice_match	gene: Zm00001d049690	Zm00001d049690_T001	17.46	19.60	coding	516	179	1726
PB.3167.1	2	2351	3	full-splice_match	gene: Zm00001d005708	Zm00001d005708_T001	7.13	7.13	coding	619	191	2047
PB.6125.3	5	2476	3	full-splice_match	gene: Zm00001d013561	Zm00001d013561_T001	0.00	4.82	coding	748	72	2315
PB.6125.2	5	2594	3	full-splice_match	gene: Zm00001d013561	Zm00001d013561_T001	4.82	4.82	coding	748	72	2315
PB.8236.1	7	1206	1	full-splice_match	gene: Zm00001d018965	Zm00001d018965_T001	19.40	19.40	coding	307	158	1078
PB.5853.6	4	1160	2	intergenic	novelGene_364	novel	4.81	224.73	non_coding	NA	NA	NA
PB.1213.1	1	4046	25	full-splice_match	gene: Zm00001d033218	Zm00001d033218_T002	15.39	15.39	coding	1227	30	3710
PB.9590.2	8	1804	4	full-splice_match	gene: Zm00001d010840	Zm00001d010840_T001	43.09	60.90	coding	534	98	1699
PB.10699.4	9	1386	9	full-splice_match	gene: Zm00001d048091	Zm00001d048091_T002	168.32	433.40	coding	330	89	1078

PB.9590.1	8	1950	4	full-splice_match	gene: Zm00001d010840	Zm00001d010840_T001	17.81	60.90	coding	534	97	1698
PB.7110.1	5	1636	10	full-splice_match	gene: Zm00001d018309	Zm00001d018309_T003	137.22	137.22	coding	426	96	1373
PB.7110.5	5	1503	10	full-splice_match	gene: Zm00001d018309	Zm00001d018309_T003	0.00	137.22	coding	426	94	1371
PB.1381.1	1	5098	5	incomplete-splice_match	gene: Zm00001d033984	Zm00001d033984_T008	12.29	104.75	coding	1364	307	4398
PB.9782.2	8	1777	1	incomplete-splice_match	gene: Zm00001d011745	Zm00001d011745_T002	18.76	18.76	coding	156	44	511
PB.4625.2	3	2444	21	full-splice_match	gene: Zm00001d044024	Zm00001d044024_T001	20.22	40.56	coding	603	173	1981
PB.10212.1	9	647	6	full-splice_match	gene: Zm00001d045555	Zm00001d045555_T003	304.53	304.53	coding	114	108	449
PB.5388.3	4	1502	2	full-splice_match	gene: Zm00001d051928	Zm00001d051928_T001	0.00	6.95	coding	387	61	1221
PB.5388.1	4	1579	2	full-splice_match	gene: Zm00001d051928	Zm00001d051928_T001	6.95	6.95	coding	387	9	1169
PB.9005.5	7	2451	2	incomplete-splice_match	gene: Zm00001d022475	Zm00001d022475_T012	3.14	25.62	non_coding	NA	NA	NA
PB.1450.1	1	1539	11	full-splice_match	gene: Zm00001d034318	Zm00001d034318_T001	6.60	6.60	coding	431	110	1402
PB.4133.2	3	677	3	full-splice_match	gene: Zm00001d041963	Zm00001d041963_T001	400.80	478.47	coding	135	126	530
PB.9005.1	7	2158	1	genic	gene: Zm00001d022475	novel	10.62	25.62	non_coding	NA	NA	NA
PB.2207.1	10	2397	5	novel_in_catalog	gene: Zm00001d026122	novel	5.90	5.90	coding	148	1919	2362
PB.9005.3	7	2439	6	novel_not_in_catalog	gene: Zm00001d022475	novel	8.03	25.62	non_coding	NA	NA	NA
PB.9005.2	7	2424	3	incomplete-splice_match	gene: Zm00001d022475	Zm00001d022475_T010	3.83	25.62	coding	126	949	1326
PB.4950.4	4	2750	3	novel_in_catalog	gene: Zm00001d049362	novel	0.61	288.48	coding	115	937	1281

PB.693.1	1	2234	14	full-splice_match	gene: Zm00001d030612	Zm00001d030612_T001	6.67	6.67	coding	545	148	1782
PB.4849.1	4	1643	1	full-splice_match	gene: Zm00001d048880	Zm00001d048880_T001	1.52	1.52	coding	539	27	1643
PB.4950.1	4	787	6	full-splice_match	gene: Zm00001d049362	Zm00001d049362_T003	276.79	288.48	coding	110	225	554
PB.3257.1	2	999	4	full-splice_match	gene: Zm00001d006159	Zm00001d006159_T002	6.51	6.51	coding	164	43	534
PB.4950.3	4	1923	3	novel_in_catalog	gene: Zm00001d049362	novel	3.15	288.48	coding	94	22	303
PB.4950.2	4	2697	4	full-splice_match	gene: Zm00001d049362	Zm00001d049362_T008	7.49	288.48	coding	115	959	1303
PB.4581.1	3	1696	3	full-splice_match	gene: Zm00001d043850	Zm00001d043850_T001	205.69	227.52	coding	447	78	1418
PB.2498.1	2	2467	1	genic_intron	novelGene_34	novel	2.30	2.30	coding	320	1008	1967
PB.5003.2	4	1314	11	incomplete-splice_match	gene: Zm00001d049641	Zm00001d049641_T005	1708.71	1807.46	coding	337	78	1088
PB.5003.1	4	1396	11	incomplete-splice_match	gene: Zm00001d049641	Zm00001d049641_T001	98.75	1807.46	coding	337	56	1066
PB.6323.2	5	1235	10	novel_in_catalog	gene: Zm00001d014533	novel	0.00	39.54	coding	306	80	997
PB.6323.1	5	1387	10	novel_in_catalog	gene: Zm00001d014533	novel	39.54	39.54	coding	306	92	1009
PB.1115.7	1	1178	7	full-splice_match	gene: Zm00001d032695	Zm00001d032695_T001	89.93	802.50	coding	332	96	1091
PB.8786.4	7	2985	8	full-splice_match	gene: Zm00001d021729	Zm00001d021729_T003	4.37	53.48	coding	654	121	2082
PB.1036.1	1	2814	6	full-splice_match	gene: Zm00001d032408	Zm00001d032408_T002	2.90	2.90	coding	878	74	2707
PB.9153.4	8	2696	7	full-splice_match	gene: Zm00001d008618	Zm00001d008618_T005	17.92	36.12	coding	691	300	2372
PB.8885.1	7	2427	1	intergenic	novelGene_279	novel	10.50	10.50	coding	673	230	2248

PB.6863.1	5	2042	2	incomplete-splice_match	gene: Zm00001d017294	Zm00001d017294_T001	0.35	0.35	coding	180	22	561
PB.8786.3	7	3067	9	full-splice_match	gene: Zm00001d021729	Zm00001d021729_T001	0.47	53.48	coding	863	274	2862
PB.6139.1	5	1521	10	full-splice_match	gene: Zm00001d013624	Zm00001d013624_T003	6.53	6.53	coding	342	76	1101
PB.10381.4	9	930	1	full-splice_match	gene: Zm00001d046682	Zm00001d046682_T001	13.56	146.54	coding	233	39	737
PB.10381.3	9	1144	1	full-splice_match	gene: Zm00001d046682	Zm00001d046682_T001	51.46	146.54	coding	233	206	904
PB.10381.2	9	1190	1	full-splice_match	gene: Zm00001d046682	Zm00001d046682_T001	76.07	146.54	coding	233	96	794
PB.10381.1	9	1321	1	full-splice_match	gene: Zm00001d046682	Zm00001d046682_T001	5.45	146.54	coding	233	77	775
PB.1413.1	1	532	3	intergenic	novelGene_376	novel	2.81	2.81	non_coding	NA	NA	NA
PB.7587.1	6	1985	6	full-splice_match	gene: Zm00001d037085	Zm00001d037085_T001	61.22	65.08	coding	535	179	1783
PB.7587.2	6	2204	4	novel_in_catalog	gene: Zm00001d037085	novel	3.87	65.08	coding	215	106	750
PB.10112.6	9	3508	15	full-splice_match	gene: Zm00001d045147	Zm00001d045147_T024	0.00	37.67	coding	428	50	1333
PB.10112.1	9	3797	15	full-splice_match	gene: Zm00001d045147	Zm00001d045147_T010	7.11	37.67	coding	428	68	1351
PB.7513.1	6	1492	9	full-splice_match	gene: Zm00001d036632	Zm00001d036632_T004	25.31	25.31	coding	333	146	1144
PB.10112.3	9	2437	16	full-splice_match	gene: Zm00001d045147	Zm00001d045147_T015	1.77	37.67	coding	706	69	2186
PB.10112.2	9	1503	10	novel_not_in_catalog	gene: Zm00001d045147	novel	28.79	37.67	coding	387	89	1249
PB.2668.1	2	1464	3	full-splice_match	gene: Zm00001d002897	Zm00001d002897_T001	334.09	635.04	coding	357	51	1121
PB.350.6	1	2076	6	full-splice_match	gene: Zm00001d028759	Zm00001d028759_T001	0.00	340.10	coding	606	157	1974

PB.2668.3	2	1358	2	novel_in_catalog	gene: Zm00001d002897	novel	23.77	635.04	coding	133	834	1232
PB.2668.2	2	1455	3	full-splice_match	gene: Zm00001d002897	Zm00001d002897_T001	277.18	635.04	coding	357	174	1244
PB.2668.4	2	1331	3	full-splice_match	gene: Zm00001d002897	Zm00001d002897_T001	0.00	635.04	coding	357	150	1220
PB.7653.1	6	2453	14	incomplete-splice_match	gene: Zm00001d037377	Zm00001d037377_T005	33.15	33.15	coding	656	78	2045
PB.2636.1	2	2009	2	full-splice_match	gene: Zm00001d002789	Zm00001d002789_T001	1.43	1.43	coding	513	121	1659
PB.5870.1	4	991	1	full-splice_match	gene: Zm00001d053995	Zm00001d053995_T001	2.25	2.25	coding	269	66	872
PB.8891.1	7	1423	2	novel_not_in_catalog	gene: Zm00001d022083	novel	54.73	100.93	coding	220	210	869
PB.6873.2	5	580	2	novel_not_in_catalog	gene: Zm00001d017347	novel	80.92	114.88	coding	102	143	448
PB.2295.3	10	1919	3	novel_not_in_catalog	gene: Zm00001d026452	novel	91.38	91.38	coding	482	4	1449
PB.8891.2	7	1100	2	novel_not_in_catalog	gene: Zm00001d022083	novel	46.20	100.93	coding	220	114	773
PB.9623.2	8	3610	17	full-splice_match	gene: Zm00001d010974	Zm00001d010974_T005	37.57	48.61	coding	876	276	2903
PB.3142.2	2	1493	8	full-splice_match	gene: Zm00001d005579	Zm00001d005579_T012	37.28	89.25	coding	310	238	1167
PB.1938.1	10	1566	7	full-splice_match	gene: Zm00001d024788	Zm00001d024788_T002	15.43	15.43	coding	344	196	1227
PB.2730.1	2	2063	3	full-splice_match	gene: Zm00001d003157	Zm00001d003157_T001	775.83	787.59	coding	400	577	1776
PB.4938.1	4	1397	4	full-splice_match	gene: Zm00001d049286	Zm00001d049286_T003	9.12	12.29	coding	238	80	793
PB.6216.2	5	2289	2	incomplete-splice_match	gene: Zm00001d014017	Zm00001d014017_T002	9.82	109.11	coding	9	429	455
PB.4508.5	3	1906	14	novel_not_in_catalog	gene: Zm00001d043563	novel	1.30	91.14	coding	320	98	1057

PB.7834.1	6	1548	1	genic	gene: Zm00001d038105	novel	19.64	60.46	coding	180	264	803
PB.6216.1	5	835	3	full-splice_match	gene: Zm00001d014017	Zm00001d014017_T004	99.29	109.11	non_coding	NA	NA	NA
PB.87.1	1	1244	1	full-splice_match	gene: Zm00001d027555	Zm00001d027555_T001	3.59	3.59	coding	245	92	826
PB.1142.2	1	1285	10	intergenic	novelGene_359	novel	5.51	46.12	coding	374	60	1181
PB.10029.1	8	2173	8	full-splice_match	gene: Zm00001d012774	Zm00001d012774_T001	7.44	7.44	non_coding	NA	NA	NA
PB.10389.3	9	1002	3	full-splice_match	gene: Zm00001d046718	Zm00001d046718_T001	0.00	136.97	coding	229	180	866
PB.10389.2	9	1619	2	novel_in_catalog	gene: Zm00001d046718	novel	5.51	136.97	coding	165	9	503
PB.10389.1	9	1187	3	full-splice_match	gene: Zm00001d046718	Zm00001d046718_T001	131.46	136.97	coding	300	33	932
PB.1697.1	10	1695	1	full-splice_match	gene: Zm00001d023417	Zm00001d023417_T001	9.68	9.68	coding	456	56	1423
PB.2974.6	2	3114	20	full-splice_match	gene: Zm00001d004564	Zm00001d004564_T006	1.90	52.51	coding	905	106	2820
PB.4387.1	3	1038	7	novel_not_in_catalog	gene: Zm00001d043113	novel	34.33	97.17	coding	181	131	673
PB.1000.1	1	396	2	incomplete-splice_match	gene: Zm00001d032266	Zm00001d032266_T001	33.48	95.51	non_coding	NA	NA	NA
PB.1000.2	1	489	5	incomplete-splice_match	gene: Zm00001d032266	Zm00001d032266_T003	62.04	95.51	coding	118	15	368
PB.4590.2	3	1472	9	full-splice_match	gene: Zm00001d043872	Zm00001d043872_T001	14.08	17.96	coding	342	99	1124
PB.3235.1	2	1269	1	novel_in_catalog	gene: Zm00001d006059	novel	22.52	133.32	coding	113	143	481
PB.3235.2	2	1056	2	full-splice_match	gene: Zm00001d006059	Zm00001d006059_T001	110.80	133.32	coding	262	145	930
PB.1142.1	1	1398	10	intergenic	novelGene_359	novel	40.61	46.12	coding	374	51	1172

PB.4202.2	3	1004	6	full-splice_match	gene: Zm00001d042274	Zm00001d042274_T001	0.00	33.67	coding	251	101	853
PB.4202.1	3	1115	6	full-splice_match	gene: Zm00001d042274	Zm00001d042274_T001	33.67	33.67	coding	251	104	856
PB.4625.3	3	2080	16	novel_in_catalog	gene: Zm00001d044024	novel	17.14	40.56	coding	447	338	1678
PB.1020.7	1	995	7	full-splice_match	gene: Zm00001d032350	Zm00001d032350_T004	24.04	135.32	coding	196	152	739
PB.1020.4	1	2440	7	full-splice_match	gene: Zm00001d032350	Zm00001d032350_T007	0.00	135.32	coding	114	163	504
PB.5383.3	4	2707	10	full-splice_match	gene: Zm00001d051902	Zm00001d051902_T008	0.00	37.10	coding	832	108	2603
PB.6191.3	5	1562	17	full-splice_match	gene: Zm00001d013892	Zm00001d013892_T004	0.00	111.16	coding	390	171	1340
PB.1020.3	1	2815	6	full-splice_match	gene: Zm00001d032350	Zm00001d032350_T001	0.00	135.32	coding	114	229	570
PB.1020.1	1	2479	7	full-splice_match	gene: Zm00001d032350	Zm00001d032350_T006	39.53	135.32	coding	114	211	552
PB.2652.2	2	2415	13	full-splice_match	gene: Zm00001d002835	Zm00001d002835_T001	30.28	36.30	coding	653	281	2239
PB.2652.3	2	2486	12	full-splice_match	gene: Zm00001d002835	Zm00001d002835_T006	6.01	36.30	coding	351	1261	2313
PB.2300.1	10	835	1	full-splice_match	gene: Zm00001d026457	Zm00001d026457_T001	366.82	366.82	non_coding	NA	NA	NA
PB.2652.6	2	2365	13	full-splice_match	gene: Zm00001d002835	Zm00001d002835_T001	0.00	36.30	coding	653	255	2213
PB.2652.4	2	1203	9	incomplete-splice_match	gene: Zm00001d002835	Zm00001d002835_T001	0.00	36.30	coding	343	173	1201
PB.4766.1	3	949	2	full-splice_match	gene: Zm00001d044606	Zm00001d044606_T001	31.79	31.79	coding	289	37	903
PB.9487.2	8	1313	3	full-splice_match	gene: Zm00001d010410	Zm00001d010410_T001	61.33	63.20	coding	257	168	938
PB.9487.1	8	1890	1	novel_in_catalog	gene: Zm00001d010410	novel	1.87	63.20	coding	125	1140	1514

PB.9455.1	8	2159	1	full-splice_match	gene: Zm00001d010234	Zm00001d010234_T001	17.29	17.29	coding	650	70	2019
PB.1191.2	1	3019	9	novel_not_in_catalog	gene: Zm00001d033071	novel	2.23	19.96	coding	689	672	2738
PB.3527.3	2	1965	4	full-splice_match	gene: Zm00001d007446	Zm00001d007446_T005	3.38	490.59	coding	230	141	830
PB.6044.1	5	2543	7	novel_not_in_catalog	gene: Zm00001d013268	novel	119.44	123.10	coding	626	426	2303
PB.8645.1	7	2149	10	full-splice_match	gene: Zm00001d021227	Zm00001d021227_T003	38.78	40.66	coding	548	220	1863
PB.8645.2	7	3218	9	novel_in_catalog	gene: Zm00001d021227	novel	1.88	40.66	coding	535	146	1750
PB.6044.2	5	2600	6	novel_not_in_catalog	gene: Zm00001d013268	novel	3.67	123.10	coding	374	1270	2391
PB.9772.1	8	2706	17	novel_in_catalog	gene: Zm00001d011713	novel	14.30	48.17	coding	655	283	2247
PB.9772.2	8	3536	23	incomplete-splice_match	gene: Zm00001d011713	Zm00001d011713_T008	24.15	48.17	coding	935	324	3128
PB.9772.3	8	2106	15	incomplete-splice_match	gene: Zm00001d011713	Zm00001d011713_T001	9.72	48.17	coding	567	209	1909
PB.7138.1	5	2595	1	full-splice_match	gene: Zm00001d018389	Zm00001d018389_T001	81.40	111.84	coding	661	190	2172
PB.1476.3	1	1735	5	novel_not_in_catalog	gene: Zm00001d034400	novel	0.00	91.19	coding	427	114	1394
PB.6962.4	5	4289	17	novel_in_catalog	gene: Zm00001d017754	novel	0.00	44.58	coding	883	1453	4101
PB.3249.1	2	856	1	full-splice_match	gene: Zm00001d006119	Zm00001d006119_T001	134.65	134.65	coding	159	36	512
PB.9621.3	8	2645	1	genic	gene: Zm00001d010971	novel	53.02	53.02	coding	757	180	2450
PB.3249.2	2	409	1	full-splice_match	gene: Zm00001d006119	Zm00001d006119_T001	0.00	134.65	non_coding	NA	NA	NA
PB.5499.2	4	1964	3	genic	gene: Zm00001d052377	novel	1.08	2.21	coding	522	118	1683

PB.3495.2	2	1094	8	novel_not_in_catalog	gene: Zm00001d007234	novel	84.67	419.80	coding	168	84	587
PB.2494.1	2	3199	4	full-splice_match	gene: Zm00001d002166	Zm00001d002166_T008	152.71	152.71	coding	930	180	2969
PB.3495.4	2	1030	8	full-splice_match	gene: Zm00001d007234	Zm00001d007234_T004	144.34	419.80	coding	227	79	759
PB.9020.4	7	1570	7	full-splice_match	gene: Zm00001d022526	Zm00001d022526_T001	0.00	16.57	coding	367	294	1394
PB.5446.1	4	2551	10	full-splice_match	gene: Zm00001d052197	Zm00001d052197_T013	22.27	22.27	coding	727	172	2352
PB.7981.2	6	2585	3	full-splice_match	gene: Zm00001d038622	Zm00001d038622_T001	0.00	18.82	coding	751	233	2485
PB.2334.1	10	1718	5	full-splice_match	gene: Zm00001d026577	Zm00001d026577_T001	76.69	78.80	coding	470	99	1508
PB.1441.1	1	2459	17	full-splice_match	gene: Zm00001d034245	Zm00001d034245_T009	8.47	24.20	coding	570	481	2190
PB.8482.4	7	647	4	full-splice_match	gene: Zm00001d020450	Zm00001d020450_T001	62.39	522.38	coding	120	77	436
PB.3576.1	2	2168	8	full-splice_match	gene: Zm00001d007754	Zm00001d007754_T002	14.76	37.31	coding	472	87	1502
PB.3576.2	2	2481	7	novel_in_catalog	gene: Zm00001d007754	novel	2.01	37.31	coding	402	27	1232
PB.5499.1	4	2129	3	genic	gene: Zm00001d052377	novel	1.13	2.21	coding	522	82	1647
PB.7841.1	6	1374	3	full-splice_match	gene: Zm00001d038127	Zm00001d038127_T001	47.59	47.59	coding	336	193	1200
PB.9020.3	7	1674	7	full-splice_match	gene: Zm00001d022526	Zm00001d022526_T001	11.34	16.57	coding	367	297	1397
PB.3955.1	3	1981	9	full-splice_match	gene: Zm00001d040796	Zm00001d040796_T002	10.85	16.42	coding	493	43	1521
PB.7130.7	5	1959	10	full-splice_match	gene: Zm00001d018370	Zm00001d018370_T001	150.67	150.67	coding	535	178	1782
PB.3955.2	3	1906	8	novel_in_catalog	gene: Zm00001d040796	novel	5.57	16.42	coding	460	54	1433

PB.9704.6	8	1896	3	incomplete-splice_match	gene: Zm00001d011393	Zm00001d011393_T001	8.04	70.73	coding	566	67	1764
PB.9704.5	8	2351	4	novel_not_in_catalog	gene: Zm00001d011393	novel	62.68	70.73	coding	634	107	2008
PB.2378.2	10	1288	1	full-splice_match	gene: Zm00001d026683	Zm00001d026683_T001	0.00	73.54	coding	375	56	1180
PB.7618.1	6	654	2	incomplete-splice_match	gene: Zm00001d037233	Zm00001d037233_T008	14.41	14.41	coding	114	140	481
PB.9020.1	7	1978	7	full-splice_match	gene: Zm00001d022526	Zm00001d022526_T001	5.24	16.57	coding	367	300	1400
PB.6307.1	5	1212	6	full-splice_match	gene: Zm00001d014455	Zm00001d014455_T001	6.76	6.76	coding	347	68	1108
PB.1173.1	1	2543	10	full-splice_match	gene: Zm00001d032981	Zm00001d032981_T002	5.38	5.38	coding	656	319	2286
PB.7196.1	5	491	2	novel_in_catalog	gene: Zm00001d018535	novel	231.43	301.71	non_coding	NA	NA	NA
PB.2482.1	2	2141	7	novel_not_in_catalog	gene: Zm00001d002140	novel	11.60	11.60	coding	526	234	1811
PB.8782.1	7	1287	9	full-splice_match	gene: Zm00001d021719	Zm00001d021719_T001	56.13	56.13	coding	301	159	1061
PB.4367.1	3	2214	1	genic	gene: Zm00001d043015	novel	13.01	13.01	coding	647	94	2034
PB.1029.2	1	2973	6	full-splice_match	gene: Zm00001d032376	Zm00001d032376_T009	15.17	18.26	coding	778	56	2389
PB.2240.1	10	555	2	intergenic	novelGene_228	novel	55.28	55.28	non_coding	NA	NA	NA
PB.3322.4	2	2390	6	novel_in_catalog	gene: Zm00001d006422	novel	2.80	31.73	coding	325	824	1798
PB.414.7	1	534	1	full-splice_match	gene: Zm00001d029028	Zm00001d029028_T001	0.00	145.43	coding	85	150	404
PB.8992.1	7	1771	15	full-splice_match	gene: Zm00001d022445	Zm00001d022445_T002	74.63	74.63	coding	425	161	1435
PB.10009.1	8	2168	14	full-splice_match	gene: Zm00001d012693	Zm00001d012693_T003	14.09	14.09	coding	524	410	1981

PB.9781.1	8	3428	14	full-splice_match	gene: Zm00001d011742	Zm00001d011742_T027	11.59	11.59	coding	972	353	3268
PB.6279.11	5	2054	8	novel_in_catalog	gene: Zm00001d014330	novel	4.58	82.37	non_coding	NA	NA	NA
PB.7322.1	6	2860	21	full-splice_match	gene: Zm00001d035578	Zm00001d035578_T001	21.90	21.90	coding	785	246	2600
PB.414.1	1	2300	1	full-splice_match	gene: Zm00001d029028	Zm00001d029028_T001	4.07	145.43	coding	188	93	656
PB.8169.2	7	1354	1	incomplete-splice_match	gene: Zm00001d018693	Zm00001d018693_T001	12.82	18.43	coding	329	64	1050
PB.7490.1	6	745	2	intergenic	novelGene_158	novel	1.65	1.65	non_coding	NA	NA	NA
PB.7989.4	6	1320	11	novel_not_in_catalog	gene: Zm00001d038689	novel	11.16	115.37	coding	309	139	1065
PB.10188.1	9	2575	4	full-splice_match	gene: Zm00001d045479	Zm00001d045479_T001	17.35	17.35	coding	552	303	1958
PB.633.1	1	1208	6	full-splice_match	gene: Zm00001d030157	Zm00001d030157_T001	81.64	81.64	coding	194	194	775
PB.5753.2	4	759	6	novel_not_in_catalog	gene: Zm00001d053591	novel	6.00	27.03	coding	101	21	323
PB.9779.1	8	965	2	full-splice_match	gene: Zm00001d011732	Zm00001d011732_T001	266.51	375.30	coding	200	81	680
PB.6751.1	5	1746	6	full-splice_match	gene: Zm00001d016824	Zm00001d016824_T001	13.72	16.99	coding	472	121	1536
PB.6751.2	5	1903	1	incomplete-splice_match	gene: Zm00001d016824	Zm00001d016824_T002	3.26	16.99	coding	178	1160	1693
PB.2761.1	2	1141	5	full-splice_match	gene: Zm00001d003248	Zm00001d003248_T001	8.21	38.56	coding	156	202	669
PB.2761.2	2	955	5	full-splice_match	gene: Zm00001d003248	Zm00001d003248_T001	30.34	38.56	coding	156	161	628
PB.2322.2	10	1759	4	full-splice_match	gene: Zm00001d026536	Zm00001d026536_T004	0.00	20.56	coding	458	281	1654
PB.2322.1	10	1924	4	full-splice_match	gene: Zm00001d026536	Zm00001d026536_T004	20.56	20.56	coding	458	281	1654

PB.4088.1	3	509	3	full-splice_match	gene: Zm00001d041727	Zm00001d041727_T001	53.07	1395.62	non_coding	NA	NA	NA
PB.7183.1	5	2150	4	novel_in_catalog	gene: Zm00001d018515	novel	7.85	48.94	coding	327	585	1565
PB.8212.1	7	2522	16	novel_in_catalog	gene: Zm00001d018864	novel	15.97	18.56	coding	618	317	2170
PB.8212.2	7	2441	16	novel_in_catalog	gene: Zm00001d018864	novel	2.59	18.56	coding	618	373	2226
PB.3512.1	2	1167	8	full-splice_match	gene: Zm00001d007366	Zm00001d007366_T003	10.32	10.32	coding	304	3	914
PB.7183.2	5	1948	5	full-splice_match	gene: Zm00001d018515	Zm00001d018515_T002	41.09	48.94	coding	388	540	1703
PB.504.1	1	2073	3	novel_in_catalog	gene: Zm00001d029457	novel	105.03	105.03	coding	388	745	1908
PB.3138.1	2	1788	1	novel_in_catalog	gene: Zm00001d005554	novel	3.01	3.01	non_coding	NA	NA	NA
PB.9613.2	8	2225	4	full-splice_match	gene: Zm00001d010945	Zm00001d010945_T002	11.19	11.19	coding	670	50	2059
PB.9750.1	8	2021	4	incomplete-splice_match	gene: Zm00001d011619	Zm00001d011619_T007	15.25	15.25	coding	100	153	452
PB.4411.1	3	1445	5	full-splice_match	gene: Zm00001d043196	Zm00001d043196_T001	49.19	49.19	coding	377	117	1247
PB.10912.4	B73V4 ctg41	1139	2	novel_in_catalog	gene: Zm00001d000391	novel	13.14	25.42	coding	121	414	776
PB.10240.1	9	780	1	full-splice_match	gene: Zm00001d045738	Zm00001d045738_T001	13.53	13.53	coding	60	383	562
PB.5971.1	5	1396	1	full-splice_match	gene: Zm00001d013028	Zm00001d013028_T001	26.24	40.95	coding	256	128	895
PB.8076.3	6	4407	11	full-splice_match	gene: Zm00001d038972	Zm00001d038972_T010	0.00	254.63	coding	871	1471	4083
PB.3159.5	2	471	5	full-splice_match	gene: Zm00001d005675	Zm00001d005675_T003	182.80	387.55	coding	78	74	307
PB.5971.2	5	977	1	full-splice_match	gene: Zm00001d013028	Zm00001d013028_T001	14.71	40.95	coding	256	82	849

PB.9090.3	8	1225	3	incomplete-splice_match	gene: Zm00001d008314	Zm00001d008314_T007	5.33	178.84	non_coding	NA	NA	NA
PB.9090.2	8	1200	5	full-splice_match	gene: Zm00001d008314	Zm00001d008314_T008	168.95	178.84	coding	306	59	976
PB.9090.1	8	2121	3	incomplete-splice_match	gene: Zm00001d008314	Zm00001d008314_T007	4.56	178.84	coding	129	1411	1797
PB.9351.1	8	2999	1	full-splice_match	gene: Zm00001d009694	Zm00001d009694_T001	33.11	33.11	coding	881	187	2829
PB.9351.3	8	2909	1	full-splice_match	gene: Zm00001d009694	Zm00001d009694_T001	0.00	33.11	coding	881	187	2829
PB.10264.1	9	966	1	full-splice_match	gene: Zm00001d045931	Zm00001d045931_T001	16.02	16.02	coding	162	173	658
PB.6986.1	5	585	1	full-splice_match	gene: Zm00001d017851	Zm00001d017851_T001	263.25	263.25	coding	58	134	307
PB.6972.1	5	1903	7	full-splice_match	gene: Zm00001d017787	Zm00001d017787_T001	12.78	48.05	coding	478	102	1535
PB.7602.1	6	454	2	genic	gene: Zm00001d037158	novel	26.42	26.42	non_coding	NA	NA	NA
PB.10260.1	9	1858	7	novel_not_in_catalog	gene: Zm00001d045887	novel	11.44	17.36	coding	369	163	1269
PB.1203.2	1	2407	12	novel_not_in_catalog	gene: Zm00001d033159	novel	86.37	228.23	coding	643	141	2069
PB.10260.3	9	1950	7	novel_not_in_catalog	gene: Zm00001d045887	novel	5.92	17.36	coding	369	152	1258
PB.5951.2	5	1358	6	full-splice_match	gene: Zm00001d012955	Zm00001d012955_T001	0.00	19.24	coding	285	213	1067
PB.5951.1	5	1564	6	full-splice_match	gene: Zm00001d012955	Zm00001d012955_T001	19.24	19.24	coding	285	237	1091
PB.6126.1	5	818	2	full-splice_match	gene: Zm00001d013568	Zm00001d013568_T001	3700.06	3770.33	coding	159	89	565
PB.1717.3	10	2118	2	full-splice_match	gene: Zm00001d023523	Zm00001d023523_T013	4.83	115.79	coding	446	474	1811
PB.1717.2	10	1710	2	novel_in_catalog	gene: Zm00001d023523	novel	1.20	115.79	coding	293	831	1709

PB.1717.1	10	3594	3	full-splice_match	gene: Zm00001d023523	Zm00001d023523_T015	6.70	115.79	coding	446	280	1617
PB.5296.2	4	1128	4	full-splice_match	gene: Zm00001d051511	Zm00001d051511_T001	8.63	44.43	coding	251	100	852
PB.5296.1	4	1244	4	full-splice_match	gene: Zm00001d051511	Zm00001d051511_T001	35.80	44.43	coding	251	98	850
PB.984.12	1	1530	8	full-splice_match	gene: Zm00001d032198	Zm00001d032198_T011	35.47	199.16	coding	276	648	1475
PB.1987.2	10	1623	2	novel_not_in_catalog	gene: Zm00001d024995	novel	4.72	15.69	coding	504	34	1545
PB.6739.8	5	1328	2	novel_in_catalog	gene: Zm00001d016768	novel	0.00	42.03	coding	137	209	619
PB.1987.1	10	1721	2	novel_not_in_catalog	gene: Zm00001d024995	novel	10.96	15.69	coding	504	24	1535
PB.8698.1	7	515	2	full-splice_match	gene: Zm00001d001250	Zm00001d001250_T001	7.64	7.64	non_coding	NA	NA	NA
PB.5272.1	4	2495	2	full-splice_match	gene: Zm00001d051389	Zm00001d051389_T001	20.29	22.52	coding	776	30	2357
PB.5272.2	4	2537	1	genic	gene: Zm00001d051389	novel	2.23	22.52	coding	364	17	1108
PB.6739.6	5	2251	3	novel_in_catalog	gene: Zm00001d016768	novel	0.00	42.03	coding	415	817	2061
PB.2749.2	2	2438	14	novel_in_catalog	gene: Zm00001d003207	novel	22.14	58.90	coding	260	256	1035
PB.2749.1	2	2432	15	full-splice_match	gene: Zm00001d003207	Zm00001d003207_T001	36.77	58.90	coding	589	351	2117
PB.6739.5	5	2478	3	novel_in_catalog	gene: Zm00001d016768	novel	2.25	42.03	coding	415	948	2192
PB.9625.1	8	2352	3	full-splice_match	gene: Zm00001d010976	Zm00001d010976_T001	6.93	136.56	coding	365	274	1368
PB.10931.1	B73V4 ctg61	607	2	novel_not_in_catalog	gene: Zm00001d000345	novel	149.08	149.08	coding	80	94	333
PB.9625.3	8	2424	2	novel_in_catalog	gene: Zm00001d010976	novel	6.07	136.56	coding	338	197	1210

PB.9625.2	8	1819	3	full-splice_match	gene: Zm00001d010976	Zm00001d010976_T001	89.71	136.56	coding	365	354	1448
PB.9625.4	8	1242	3	full-splice_match	gene: Zm00001d010976	Zm00001d010976_T001	33.85	136.56	coding	194	390	971
PB.5476.1	4	1969	7	incomplete-splice_match	gene: Zm00001d052308	Zm00001d052308_T189	183.33	183.33	coding	530	28	1617
PB.5919.3	5	2396	3	full-splice_match	gene: Zm00001d012850	Zm00001d012850_T001	0.00	36.94	coding	366	109	1206
PB.4629.3	3	1595	5	full-splice_match	gene: Zm00001d044036	Zm00001d044036_T004	0.00	36.60	coding	117	815	1165
PB.6311.1	5	652	1	full-splice_match	gene: Zm00001d014493	Zm00001d014493_T001	123.89	302.39	coding	103	122	430
PB.6311.2	5	525	1	full-splice_match	gene: Zm00001d014493	Zm00001d014493_T001	178.50	302.39	coding	103	122	430
PB.5760.2	4	1435	6	novel_in_catalog	gene: Zm00001d053613	novel	18.26	26.90	non_coding	NA	NA	NA
PB.151.1	1	1382	6	incomplete-splice_match	gene: Zm00001d027884	Zm00001d027884_T004	43.36	43.36	coding	300	159	1058
PB.3077.1	2	2169	8	full-splice_match	gene: Zm00001d005199	Zm00001d005199_T001	11.47	11.47	coding	508	237	1760
PB.623.2	1	1893	9	full-splice_match	gene: Zm00001d030106	Zm00001d030106_T082	9.39	80.51	coding	104	904	1215
PB.10268.1	9	880	4	novel_not_in_catalog	gene: Zm00001d045951	novel	25.94	26.85	coding	101	262	564
PB.10268.2	9	2794	3	novel_in_catalog	gene: Zm00001d045951	novel	0.91	26.85	coding	101	2244	2546
PB.1203.4	1	1648	9	novel_not_in_catalog	gene: Zm00001d033159	novel	0.00	228.23	coding	514	107	1648
PB.5959.1	5	638	5	full-splice_match	gene: Zm00001d012992	Zm00001d012992_T002	953.55	953.55	coding	132	89	484
PB.6272.1	5	2151	1	antisense	novelGene_gene: Zm00001d014315_A	novel	1.32	1.32	non_coding	NA	NA	NA
PB.311.9	1	2349	12	full-splice_match	gene: Zm00001d028579	Zm00001d028579_T018	0.00	74.00	coding	743	120	2348

PB.311.8	1	2577	12	full-splice_match	gene: Zm00001d028579	Zm00001d028579_T018	0.00	74.00	coding	806	160	2577
PB.311.7	1	3806	9	incomplete-splice_match	gene: Zm00001d028579	Zm00001d028579_T014	1.00	74.00	coding	809	1154	3580
PB.311.6	1	3117	12	full-splice_match	gene: Zm00001d028579	Zm00001d028579_T018	35.37	74.00	coding	901	154	2856
PB.311.5	1	2515	7	incomplete-splice_match	gene: Zm00001d028579	Zm00001d028579_T002	27.19	74.00	coding	191	1517	2089
PB.311.4	1	3301	14	full-splice_match	gene: Zm00001d028579	Zm00001d028579_T009	2.88	74.00	coding	901	150	2852
PB.3421.1	2	1315	2	novel_in_catalog	gene: Zm00001d006880	novel	11.01	11.01	coding	103	844	1152
PB.311.1	1	1730	2	incomplete-splice_match	gene: Zm00001d028579	Zm00001d028579_T011	7.55	74.00	non_coding	NA	NA	NA
PB.5945.1	5	2099	3	full-splice_match	gene: Zm00001d012924	Zm00001d012924_T001	154.62	263.84	coding	542	222	1847
PB.5945.2	5	2230	2	novel_in_catalog	gene: Zm00001d012924	novel	3.16	263.84	coding	442	176	1501
PB.5945.3	5	1847	3	full-splice_match	gene: Zm00001d012924	Zm00001d012924_T001	106.07	263.84	coding	542	77	1702
PB.1049.3	1	2104	2	genic	gene: Zm00001d032460	novel	51.10	51.10	coding	439	196	1512
PB.3680.1	3	2176	7	genic	gene: Zm00001d039379	novel	66.08	424.27	coding	575	71	1795
PB.10891.1	B73V4 ctg205	2479	1	full-splice_match	gene: ENSRNA049479077	ENSRNA049479077-T1	8523.18	8523.18	non_coding	NA	NA	NA
PB.1616.3	1	1817	11	novel_in_catalog	gene: Zm00001d034856	novel	3.09	128.98	coding	307	626	1546
PB.3680.5	3	1953	7	genic	gene: Zm00001d039379	novel	358.20	424.27	coding	575	64	1788
PB.1340.1	1	716	1	incomplete-splice_match	gene: Zm00001d033797	Zm00001d033797_T002	7.35	7.35	non_coding	NA	NA	NA
PB.3903.1	3	1623	12	full-splice_match	gene: Zm00001d040462	Zm00001d040462_T003	53.16	68.52	coding	438	77	1390

PB.8028.1	6	2161	9	full-splice_match	gene: Zm00001d038830	Zm00001d038830_T005	0.23	15.67	coding	495	337	1821
PB.779.2	1	1423	4	novel_in_catalog	gene: Zm00001d031127	novel	17.82	174.58	coding	280	120	959
PB.6490.2	5	818	1	intergenic	novelGene_141	novel	0.00	0.00	non_coding	NA	NA	NA
PB.3271.2	2	3097	17	full-splice_match	gene: Zm00001d006214	Zm00001d006214_T011	0.00	11.01	coding	885	74	2728
PB.7905.1	6	1601	9	novel_in_catalog	gene: Zm00001d038337	novel	15.78	19.20	coding	375	58	1182
PB.6490.1	5	1904	1	full-splice_match	gene: Zm00001d015153	Zm00001d015153_T001	49.90	49.90	coding	170	969	1478
PB.159.5	1	1128	2	full-splice_match	gene: Zm00001d027925	Zm00001d027925_T001	2.71	771.22	coding	340	43	1062
PB.159.4	1	1235	2	full-splice_match	gene: Zm00001d027925	Zm00001d027925_T001	0.00	771.22	coding	340	40	1059
PB.1965.1	10	1862	2	full-splice_match	gene: Zm00001d024916	Zm00001d024916_T001	28.50	28.50	coding	470	183	1592
PB.159.1	1	1345	2	full-splice_match	gene: Zm00001d027925	Zm00001d027925_T001	653.15	771.22	coding	340	37	1056
PB.159.3	1	1366	1	novel_in_catalog	gene: Zm00001d027925	novel	0.00	771.22	coding	114	890	1231
PB.159.2	1	1516	1	genic	gene: Zm00001d027925	novel	115.37	771.22	coding	114	890	1231
PB.5448.1	4	376	1	full-splice_match	gene: Zm00001d052205	Zm00001d052205_T001	98.36	98.36	non_coding	NA	NA	NA
PB.4754.1	3	2016	7	incomplete-splice_match	gene: Zm00001d044553	Zm00001d044553_T024	27.95	27.95	coding	198	1090	1683
PB.8311.1	7	2787	6	full-splice_match	gene: Zm00001d019450	Zm00001d019450_T002	66.12	66.12	coding	815	74	2518
PB.9631.2	8	1406	8	novel_in_catalog	gene: Zm00001d011004	novel	10.50	214.67	non_coding	NA	NA	NA
PB.9631.3	8	2268	6	novel_in_catalog	gene: Zm00001d011004	novel	6.32	214.67	coding	314	1136	2077

PB.9631.1	8	1307	7	novel_in_catalog	gene: Zm00001d011004	novel	118.22	214.67	coding	325	75	1049
PB.9631.6	8	1222	7	novel_in_catalog	gene: Zm00001d011004	novel	51.04	214.67	non_coding	NA	NA	NA
PB.9631.4	8	2354	5	novel_in_catalog	gene: Zm00001d011004	novel	3.24	214.67	coding	180	1113	1652
PB.9631.5	8	1497	7	novel_in_catalog	gene: Zm00001d011004	novel	25.35	214.67	coding	314	365	1306
PB.8302.11	7	1709	6	novel_not_in_catalog	gene: Zm00001d019327	novel	13.60	173.25	coding	261	188	970
PB.2301.26	10	1741	5	full-splice_match	gene: Zm00001d026458	Zm00001d026458_T001	90.47	1555.47	coding	469	81	1487
PB.9118.3	8	2847	8	full-splice_match	gene: Zm00001d008433	Zm00001d008433_T007	1.00	20.91	coding	212	2092	2727
PB.7979.1	6	403	1	full-splice_match	gene: Zm00001d038607	Zm00001d038607_T001	175.92	175.92	coding	101	38	340
PB.5329.2	4	2059	9	novel_in_catalog	gene: Zm00001d051644	novel	7.64	65.93	coding	231	292	984
PB.5329.3	4	3129	20	incomplete-splice_match	gene: Zm00001d051644	Zm00001d051644_T049	45.15	65.93	coding	939	72	2888
PB.1971.1	10	1458	8	full-splice_match	gene: Zm00001d024933	Zm00001d024933_T003	452.10	597.21	coding	319	61	1017
PB.8292.1	7	562	3	full-splice_match	gene: Zm00001d019260	Zm00001d019260_T001	149.14	250.57	non_coding	NA	NA	NA
PB.8292.2	7	479	3	full-splice_match	gene: Zm00001d019260	Zm00001d019260_T001	101.43	250.57	non_coding	NA	NA	NA
PB.1971.2	10	732	3	incomplete-splice_match	gene: Zm00001d024933	Zm00001d024933_T003	145.11	597.21	coding	106	51	368
PB.8776.3	7	2190	2	antisense	novelGene_gene: Zm00001d021702_A	novel	7.97	17.87	coding	179	1182	1718
PB.6684.4	5	916	9	full-splice_match	gene: Zm00001d016469	Zm00001d016469_T001	3.53	712.60	coding	218	234	887
PB.6893.1	5	2500	7	full-splice_match	gene: Zm00001d017429	Zm00001d017429_T001	182.47	210.43	coding	682	74	2119

PB.8023.1	6	3071	3	full-splice_match	gene: Zm00001d038809	Zm00001d038809_T002	23.43	24.08	coding	593	1183	2961
PB.3409.3	2	728	1	incomplete-splice_match	gene: Zm00001d006828	Zm00001d006828_T002	33.23	44.28	coding	207	24	644
PB.5340.1	4	1073	10	novel_not_in_catalog	gene: Zm00001d051682	novel	37.66	37.66	coding	219	148	804
PB.8023.4	6	2343	1	novel_in_catalog	gene: Zm00001d038809	novel	0.66	24.08	coding	348	1145	2188
PB.10720.1	9	1703	1	full-splice_match	gene: Zm00001d048169	Zm00001d048169_T001	5.31	5.31	coding	372	445	1560
PB.2301.27	10	2429	4	novel_in_catalog	gene: Zm00001d026458	novel	0.00	1555.47	coding	298	74	967
PB.1314.2	1	1736	10	incomplete-splice_match	gene: Zm00001d033657	Zm00001d033657_T019	42.15	56.34	coding	135	740	1144
PB.1314.3	1	2325	10	incomplete-splice_match	gene: Zm00001d033657	Zm00001d033657_T059	4.16	56.34	coding	135	1334	1738
PB.1314.4	1	2377	6	incomplete-splice_match	gene: Zm00001d033657	Zm00001d033657_T046	3.94	56.34	coding	124	747	1118
PB.9125.1	8	2186	3	full-splice_match	gene: Zm00001d008468	Zm00001d008468_T001	11.44	11.44	coding	607	79	1899
PB.3400.1	2	616	1	intergenic	novelGene_336	novel	11.85	11.85	non_coding	NA	NA	NA
PB.10703.2	9	1296	3	novel_in_catalog	gene: Zm00001d048102	novel	38.38	157.56	coding	99	244	540
PB.10703.4	9	1992	9	full-splice_match	gene: Zm00001d048102	Zm00001d048102_T002	119.19	157.56	coding	530	116	1705
PB.7887.1	6	1737	8	full-splice_match	gene: Zm00001d038268	Zm00001d038268_T001	7.17	7.17	coding	522	58	1623
PB.7179.1	5	2300	4	novel_in_catalog	gene: Zm00001d018504	novel	35.18	35.18	coding	326	1023	2000
PB.826.1	1	2376	3	novel_in_catalog	gene: Zm00001d031332	novel	50.92	98.73	coding	351	120	1172
PB.10007.1	8	1413	5	incomplete-splice_match	gene: Zm00001d012688	Zm00001d012688_T001	11.59	11.59	coding	346	28	1065

PB.616.1	1	1509	4	novel_not_in_catalog	gene: Zm00001d030087	novel	19.75	22.26	coding	203	534	1142
PB.3734.1	3	3272	1	full-splice_match	gene: Zm00001d039567	Zm00001d039567_T001	1.00	4.88	coding	997	79	3069
PB.3734.2	3	2383	1	full-splice_match	gene: Zm00001d039567	Zm00001d039567_T001	3.88	4.88	coding	777	1	2331
PB.616.2	1	3969	3	full-splice_match	gene: Zm00001d030087	Zm00001d030087_T001	2.51	22.26	coding	167	481	981
PB.7698.2	6	2862	5	novel_in_catalog	gene: Zm00001d037604	novel	0.29	5.93	coding	565	433	2127
PB.7698.1	6	2882	6	full-splice_match	gene: Zm00001d037604	Zm00001d037604_T001	5.64	5.93	coding	629	528	2414
PB.2872.1	2	1620	1	full-splice_match	gene: Zm00001d003830	Zm00001d003830_T001	42.54	42.54	coding	426	128	1405
PB.10186.2	9	1558	3	novel_not_in_catalog	gene: Zm00001d045477	novel	6.10	15.19	coding	115	922	1266
PB.10186.3	9	2348	2	novel_in_catalog	gene: Zm00001d045477	novel	0.41	15.19	coding	330	1079	2068
PB.10186.1	9	1859	3	novel_not_in_catalog	gene: Zm00001d045477	novel	8.68	15.19	coding	330	266	1255
PB.7863.3	6	2386	2	full-splice_match	gene: Zm00001d038189	Zm00001d038189_T014	0.00	25.81	coding	654	79	2040
PB.7863.2	6	2496	2	full-splice_match	gene: Zm00001d038189	Zm00001d038189_T014	0.00	25.81	coding	654	81	2042
PB.9139.7	8	1830	8	novel_in_catalog	gene: Zm00001d008531	novel	10.69	63.33	coding	243	732	1460
PB.9139.1	8	2604	9	full-splice_match	gene: Zm00001d008531	Zm00001d008531_T007	52.64	63.33	coding	421	942	2204
PB.4722.1	3	1194	5	full-splice_match	gene: Zm00001d044447	Zm00001d044447_T001	36.75	59.95	coding	291	220	1092
PB.2402.8	2	2110	17	novel_not_in_catalog	gene: Zm00001d001809	novel	3.09	48.07	coding	440	670	1989
PB.1322.2	1	1640	2	novel_not_in_catalog	gene: Zm00001d033705	novel	14.51	40.25	coding	322	133	1098

PB.1322.1	1	1635	2	novel_not_in_catalog	gene: Zm00001d033705	novel	25.75	40.25	coding	309	28	954
PB.915.1	1	794	5	full-splice_match	gene: Zm00001d031856	Zm00001d031856_T002	43.64	43.64	coding	167	82	582
PB.2402.3	2	3216	17	novel_in_catalog	gene: Zm00001d001809	novel	1.59	48.07	coding	451	1506	2858
PB.2402.2	2	3177	16	novel_in_catalog	gene: Zm00001d001809	novel	6.42	48.07	coding	150	1537	1986
PB.2402.1	2	2270	18	full-splice_match	gene: Zm00001d001809	Zm00001d001809_T003	14.18	48.07	coding	451	559	1911
PB.2402.7	2	2309	16	novel_in_catalog	gene: Zm00001d001809	novel	5.77	48.07	coding	451	766	2118
PB.2402.6	2	3107	17	novel_in_catalog	gene: Zm00001d001809	novel	3.75	48.07	coding	451	1564	2916
PB.2402.5	2	2258	16	full-splice_match	gene: Zm00001d001809	Zm00001d001809_T010	13.27	48.07	coding	451	674	2026
PB.5833.1	4	953	1	full-splice_match	gene: Zm00001d053887	Zm00001d053887_T001	87.08	87.08	coding	189	127	693
PB.2855.2	2	703	3	full-splice_match	gene: Zm00001d003729	Zm00001d003729_T001	40.85	44.04	non_coding	NA	NA	NA
PB.2855.1	2	858	3	full-splice_match	gene: Zm00001d003729	Zm00001d003729_T001	3.19	44.04	non_coding	NA	NA	NA
PB.10326.1	9	2100	4	full-splice_match	gene: Zm00001d046383	Zm00001d046383_T001	18.25	18.25	coding	592	123	1898
PB.7370.1	6	1306	11	full-splice_match	gene: Zm00001d035874	Zm00001d035874_T003	68.71	68.71	coding	334	43	1044
PB.5223.2	4	2018	2	genic	gene: Zm00001d051044	novel	23.36	81.69	coding	451	261	1613
PB.5563.1	4	3163	8	full-splice_match	gene: Zm00001d052653	Zm00001d052653_T004	48.29	48.29	coding	932	163	2958
PB.6110.2	5	2603	1	full-splice_match	gene: Zm00001d013497	Zm00001d013497_T001	0.00	21.85	coding	747	30	2270
PB.6110.1	5	3017	2	genic	gene: Zm00001d013497	novel	21.85	21.85	coding	747	182	2422

PB.56.3	1	588	4	full-splice_match	gene: Zm00001d027447	Zm00001d027447_T002	97.55	101.03	coding	105	91	405
PB.10462.1	9	806	1	antisense	novelGene_gene: Zm00001d047183_A	novel	16.36	16.36	coding	93	98	376
PB.10462.2	9	387	1	intergenic	novelGene_195	novel	11.11	11.11	non_coding	NA	NA	NA
PB.5832.1	4	4415	3	genic	gene: Zm00001d053882	novel	41.56	41.56	coding	1280	79	3918
PB.5284.1	4	1538	5	full-splice_match	gene: Zm00001d051441	Zm00001d051441_T001	50.08	50.08	coding	375	104	1228
PB.9975.1	8	2072	7	novel_not_in_catalog	gene: Zm00001d012581	novel	7.73	30.03	coding	463	383	1771
PB.5494.1	4	2122	1	incomplete-splice_match	gene: Zm00001d052362	Zm00001d052362_T001	21.76	21.76	coding	142	1695	2120
PB.7350.2	6	1737	5	full-splice_match	gene: Zm00001d035720	Zm00001d035720_T002	658.53	791.54	coding	377	188	1318
PB.6810.9	5	2637	3	full-splice_match	gene: Zm00001d017096	Zm00001d017096_T010	6.66	118.92	coding	129	108	494
PB.466.1	1	2262	13	full-splice_match	gene: Zm00001d029260	Zm00001d029260_T005	24.67	24.67	coding	494	270	1751
PB.10523.1	9	877	4	full-splice_match	gene: Zm00001d047477	Zm00001d047477_T001	701.91	701.91	coding	203	67	675
PB.2849.2	2	2243	7	full-splice_match	gene: Zm00001d003673	Zm00001d003673_T001	17.78	17.78	coding	501	439	1941
PB.5223.1	4	2370	1	full-splice_match	gene: Zm00001d051044	Zm00001d051044_T001	12.46	81.69	coding	451	589	1941
PB.2988.1	2	952	3	full-splice_match	gene: Zm00001d004672	Zm00001d004672_T001	7.79	7.79	coding	239	59	775
PB.8110.1	6	731	2	full-splice_match	gene: Zm00001d039118	Zm00001d039118_T001	11.72	11.72	non_coding	NA	NA	NA
PB.7165.1	5	1254	3	full-splice_match	gene: Zm00001d018461	Zm00001d018461_T001	97.13	97.13	coding	264	250	1041
PB.1638.1	1	591	1	incomplete-splice_match	gene: Zm00001d034944	Zm00001d034944_T002	893.95	893.95	coding	135	2	406

PB.2680.1	2	1289	6	full-splice_match	gene: Zm00001d002953	Zm00001d002953_T002	19.81	19.81	coding	335	49	1053
PB.4920.3	4	1868	1	full-splice_match	gene: Zm00001d049201	Zm00001d049201_T001	17.79	30.20	coding	518	220	1773
PB.4437.1	3	2773	19	novel_not_in_catalog	gene: Zm00001d043305	novel	18.87	19.62	coding	801	16	2418
PB.6796.1	5	777	1	full-splice_match	gene: Zm00001d017041	Zm00001d017041_T001	102.84	102.84	coding	109	307	633
PB.5649.1	4	1558	5	novel_in_catalog	gene: Zm00001d052990	novel	13.59	22.37	coding	274	184	1005
PB.4437.2	3	2742	20	novel_not_in_catalog	gene: Zm00001d043305	novel	0.75	19.62	coding	814	98	2539
PB.6929.1	5	2478	2	genic	gene: Zm00001d017603	novel	1.54	1.54	coding	501	29	1531
PB.7666.1	6	1657	5	full-splice_match	gene: Zm00001d037427	Zm00001d037427_T003	25.04	25.04	coding	107	1127	1447
PB.10458.1	9	1356	4	full-splice_match	gene: Zm00001d047124	Zm00001d047124_T001	23.97	23.97	coding	348	154	1197
PB.7505.2	6	1874	7	novel_in_catalog	gene: Zm00001d036602	novel	29.64	33.15	coding	381	527	1669
PB.7505.3	6	2725	6	full-splice_match	gene: Zm00001d036602	Zm00001d036602_T001	0.42	33.15	coding	381	1441	2583
PB.2757.1	2	1908	2	novel_in_catalog	gene: Zm00001d003228	novel	10.38	10.38	coding	455	307	1671
PB.4323.2	3	2408	8	full-splice_match	gene: Zm00001d042809	Zm00001d042809_T003	158.28	172.86	coding	490	446	1915
PB.923.4	1	2180	3	novel_in_catalog	gene: Zm00001d031909	novel	346.11	1145.30	coding	363	59	1147
PB.923.5	1	4488	1	novel_in_catalog	gene: Zm00001d031909	novel	18.82	1145.30	coding	310	2529	3458
PB.230.1	1	2016	2	full-splice_match	gene: Zm00001d028241	Zm00001d028241_T003	23.44	23.60	coding	494	203	1684
PB.5887.2	4	1864	1	full-splice_match	gene: Zm00001d054060	Zm00001d054060_T001	182.50	250.97	coding	524	82	1653

PB.230.3	1	1830	2	full-splice_match	gene: Zm00001d028241	Zm00001d028241_T003	0.00	23.60	coding	494	207	1688
PB.230.2	1	2032	1	novel_in_catalog	gene: Zm00001d028241	novel	0.16	23.60	coding	239	207	923
PB.8466.6	7	1986	5	full-splice_match	gene: Zm00001d020399	Zm00001d020399_T002	17.60	17.60	coding	426	273	1550
PB.3589.1	2	1861	7	full-splice_match	gene: Zm00001d007802	Zm00001d007802_T001	46.32	46.32	coding	6	469	486
PB.6000.1	5	952	4	intergenic	novelGene_24	novel	2.70	2.70	non_coding	NA	NA	NA
PB.1306.2	1	2024	4	full-splice_match	gene: Zm00001d033641	Zm00001d033641_T005	7.76	81.41	coding	147	237	677
PB.10876.1	B73V4 ctg178	795	1	full-splice_match	gene: Zm00001d000197	Zm00001d000197_T001	131.34	131.34	coding	172	78	593
PB.5333.1	4	1410	7	full-splice_match	gene: Zm00001d051664	Zm00001d051664_T001	78.52	78.52	coding	356	105	1172
PB.7358.2	6	1093	7	full-splice_match	gene: Zm00001d035762	Zm00001d035762_T001	115.11	149.98	coding	228	168	851
PB.2841.5	2	1307	11	novel_in_catalog	gene: Zm00001d003652	novel	6.02	43.99	coding	121	718	1080
PB.8365.1	7	1357	8	full-splice_match	gene: Zm00001d019754	Zm00001d019754_T002	65.38	80.81	coding	316	118	1065
PB.2841.7	2	1184	11	full-splice_match	gene: Zm00001d003652	Zm00001d003652_T004	0.00	43.99	coding	318	25	978
PB.2513.1	2	1972	6	novel_not_in_catalog	gene: Zm00001d002274	novel	7.24	7.24	coding	540	121	1740
PB.2841.2	2	1460	11	full-splice_match	gene: Zm00001d003652	Zm00001d003652_T003	17.94	43.99	coding	318	60	1013
PB.2841.3	2	1236	11	full-splice_match	gene: Zm00001d003652	Zm00001d003652_T004	20.03	43.99	coding	318	56	1009
PB.3332.2	2	3149	10	incomplete-splice_match	gene: Zm00001d006459	Zm00001d006459_T006	19.83	22.95	coding	930	182	2971
PB.10857.1	B73V4 ctg134	587	1	incomplete-splice_match	gene: Zm00001d000270	Zm00001d000270_T009	149.26	149.26	non_coding	NA	NA	NA

PB.210.1	1	1633	3	novel_in_catalog	gene: Zm00001d028188	novel	58.53	58.53	coding	288	206	1069
PB.10444.2	9	1353	7	novel_not_in_catalog	gene: Zm00001d047046	novel	0.00	15.68	coding	252	347	1102
PB.7172.2	5	1920	13	full-splice_match	gene: Zm00001d018487	Zm00001d018487_T003	9.09	147.71	coding	328	244	1227
PB.4700.1	3	761	4	incomplete-splice_match	gene: Zm00001d044327	Zm00001d044327_T003	25.50	25.50	coding	123	19	387
PB.3881.2	3	1528	1	full-splice_match	gene: Zm00001d040324	Zm00001d040324_T001	33.69	39.42	coding	398	113	1306
PB.10852.1	B73V4 ctg115	1771	1	antisense	novelGene_gene: Zm00001d000358_A	novel	0.00	0.00	coding	154	1087	1548
PB.5220.2	4	1281	8	full-splice_match	gene: Zm00001d051021	Zm00001d051021_T003	91.88	91.88	coding	213	324	962
PB.5137.2	4	1600	2	full-splice_match	gene: Zm00001d050394	Zm00001d050394_T001	0.00	18.73	coding	370	201	1310
PB.5137.1	4	1715	2	full-splice_match	gene: Zm00001d050394	Zm00001d050394_T001	18.73	18.73	coding	370	140	1249
PB.8752.2	7	2751	6	novel_in_catalog	gene: Zm00001d021624	novel	2.88	15.36	coding	384	84	1235
PB.6737.1	5	582	2	full-splice_match	gene: Zm00001d016760	Zm00001d016760_T001	586.19	622.35	coding	106	177	494
PB.6737.2	5	619	1	genic	gene: Zm00001d016760	novel	36.16	622.35	non_coding	NA	NA	NA
PB.4479.3	3	1646	12	full-splice_match	gene: Zm00001d043459	Zm00001d043459_T004	0.00	24.56	coding	471	115	1527
PB.4844.4	4	2012	2	full-splice_match	gene: Zm00001d048839	Zm00001d048839_T002	0.00	13.43	non_coding	NA	NA	NA
PB.450.1	1	2328	7	full-splice_match	gene: Zm00001d029193	Zm00001d029193_T001	2.50	2.50	coding	586	269	2026
PB.4844.2	4	2296	1	novel_in_catalog	gene: Zm00001d048839	novel	0.00	13.43	coding	66	118	315
PB.4844.1	4	2260	2	full-splice_match	gene: Zm00001d048839	Zm00001d048839_T002	9.79	13.43	coding	66	118	315

PB.4479.2	3	2671	11	novel_in_catalog	gene: Zm00001d043459	novel	1.12	24.56	coding	275	156	980
PB.9834.1	8	2054	15	full-splice_match	gene: Zm00001d011941	Zm00001d011941_T004	15.60	15.60	coding	487	240	1700
PB.3315.2	2	1992	14	full-splice_match	gene: Zm00001d006384	Zm00001d006384_T005	11.88	12.28	coding	534	126	1727
PB.3643.1	3	1476	1	full-splice_match	gene: Zm00001d039270	Zm00001d039270_T001	6.44	6.44	coding	397	34	1224
PB.761.1	1	1274	1	genic	gene: Zm00001d031066	novel	10.22	10.22	coding	216	74	721
PB.4479.1	3	1842	12	full-splice_match	gene: Zm00001d043459	Zm00001d043459_T004	23.44	24.56	coding	471	165	1577
PB.5211.18	4	2014	9	novel_in_catalog	gene: Zm00001d050970	novel	0.00	68.24	coding	220	1159	1818
PB.5211.19	4	1705	11	novel_in_catalog	gene: Zm00001d050970	novel	0.30	68.24	coding	171	90	602
PB.7807.1	6	3155	3	novel_not_in_catalog	gene: Zm00001d038021	novel	30.70	42.10	coding	870	214	2823
PB.9286.1	8	1171	5	full-splice_match	gene: Zm00001d009339	Zm00001d009339_T001	40.68	40.68	coding	310	74	1003
PB.5211.14	4	2380	5	novel_in_catalog	gene: Zm00001d050970	novel	0.00	68.24	coding	123	90	458
PB.5211.15	4	1895	10	novel_in_catalog	gene: Zm00001d050970	novel	1.67	68.24	coding	211	87	719
PB.7826.1	6	1459	4	full-splice_match	gene: Zm00001d038076	Zm00001d038076_T002	10.86	10.86	coding	353	15	1073
PB.5211.11	4	2177	7	novel_in_catalog	gene: Zm00001d050970	novel	4.00	68.24	coding	123	86	454
PB.1459.1	1	1439	9	full-splice_match	gene: Zm00001d034341	Zm00001d034341_T002	10.55	22.38	coding	237	293	1003
PB.729.1	1	1785	5	novel_in_catalog	gene: Zm00001d030894	novel	14.83	15.46	coding	345	155	1189
PB.701.1	1	789	5	incomplete-splice_match	gene: Zm00001d030657	Zm00001d030657_T003	110.91	110.91	non_coding	NA	NA	NA

PB.4708.2	3	912	2	full-splice_match	gene: Zm00001d044376	Zm00001d044376_T001	1.13	1.28	coding	197	129	719
PB.4317.1	3	1809	1	full-splice_match	gene: Zm00001d042786	Zm00001d042786_T001	70.08	70.08	coding	391	220	1392
PB.4015.1	3	2881	5	full-splice_match	gene: Zm00001d041363	Zm00001d041363_T002	52.98	52.98	coding	647	496	2436
PB.4015.3	3	2521	5	full-splice_match	gene: Zm00001d041363	Zm00001d041363_T002	0.00	52.98	coding	647	386	2326
PB.4015.2	3	2660	5	full-splice_match	gene: Zm00001d041363	Zm00001d041363_T002	0.00	52.98	coding	647	403	2343
PB.6806.1	5	1957	2	full-splice_match	gene: Zm00001d017085	Zm00001d017085_T002	37.23	88.85	coding	388	308	1471
PB.4876.1	4	1763	2	intergenic	novelGene_45	novel	4.01	6.41	non_coding	NA	NA	NA
PB.10804.1	9	1734	7	full-splice_match	gene: Zm00001d048467	Zm00001d048467_T001	68.12	68.12	coding	526	68	1645
PB.4022.7	3	2713	3	full-splice_match	gene: Zm00001d041397	Zm00001d041397_T003	0.00	31.28	coding	343	378	1406
PB.818.2	1	2532	13	full-splice_match	gene: Zm00001d031300	Zm00001d031300_T003	26.41	29.79	coding	747	45	2285
PB.7032.4	5	2011	9	full-splice_match	gene: Zm00001d018005	Zm00001d018005_T002	0.00	58.12	coding	563	26	1714
PB.4876.2	4	1216	2	intergenic	novelGene_45	novel	2.40	6.41	non_coding	NA	NA	NA
PB.7752.1	6	2008	15	full-splice_match	gene: Zm00001d037808	Zm00001d037808_T004	6.54	63.65	coding	437	166	1476
PB.5280.2	4	1234	5	full-splice_match	gene: Zm00001d051419	Zm00001d051419_T002	0.00	40.82	coding	281	195	1037
PB.8848.1	7	2240	1	antisense	novelGene_gene: Zm00001d021935_A	novel	203.12	203.12	coding	202	485	1090
PB.6810.2	5	2561	4	full-splice_match	gene: Zm00001d017096	Zm00001d017096_T017	6.48	118.92	coding	129	106	492
PB.6587.3	5	3360	8	novel_in_catalog	gene: Zm00001d015829	novel	1.26	59.66	coding	841	233	2755

PB.6587.1	5	3392	9	full-splice_match	gene: Zm00001d015829	Zm00001d015829_T003	58.39	59.66	coding	974	311	3232
PB.4022.4	3	2277	3	full-splice_match	gene: Zm00001d041397	Zm00001d041397_T001	2.27	31.28	coding	367	469	1569
PB.3417.2	2	1834	5	incomplete-splice_match	gene: Zm00001d006861	Zm00001d006861_T028	11.34	26.09	non_coding	NA	NA	NA
PB.3417.3	2	897	5	incomplete-splice_match	gene: Zm00001d006861	Zm00001d006861_T028	0.00	26.09	non_coding	NA	NA	NA
PB.10129.3	9	4001	10	full-splice_match	gene: Zm00001d045217	Zm00001d045217_T011	5.86	5.86	coding	1040	129	3248
PB.3417.1	2	2041	6	incomplete-splice_match	gene: Zm00001d006861	Zm00001d006861_T034	14.76	26.09	non_coding	NA	NA	NA
PB.5078.1	4	2371	6	novel_not_in_catalog	gene: Zm00001d050048	novel	0.78	0.78	coding	587	160	1920
PB.9941.3	8	600	1	full-splice_match	gene: Zm00001d012439	Zm00001d012439_T001	1963.47	1963.47	coding	103	55	363
PB.66.1	1	1823	14	full-splice_match	gene: Zm00001d027479	Zm00001d027479_T002	12.64	12.64	coding	435	238	1542
PB.822.2	1	1439	9	full-splice_match	gene: Zm00001d031323	Zm00001d031323_T001	11.63	30.60	coding	332	155	1150
PB.6703.3	5	1391	7	full-splice_match	gene: Zm00001d016588	Zm00001d016588_T001	67.30	67.30	coding	376	137	1264
PB.4475.1	3	2756	8	novel_in_catalog	gene: Zm00001d043451	novel	6.96	6.96	coding	823	8	2476
PB.7177.1	5	951	2	full-splice_match	gene: Zm00001d018501	Zm00001d018501_T001	28.66	28.66	coding	187	82	642
PB.7984.2	6	1191	6	full-splice_match	gene: Zm00001d038646	Zm00001d038646_T002	5.30	87.75	coding	295	307	1191
PB.9806.3	8	2141	6	full-splice_match	gene: Zm00001d011831	Zm00001d011831_T001	0.00	27.19	coding	582	240	1985
PB.9451.1	8	987	1	incomplete-splice_match	gene: Zm00001d010228	Zm00001d010228_T001	0.15	0.15	coding	272	99	914
PB.9597.1	8	2832	16	full-splice_match	gene: Zm00001d010866	Zm00001d010866_T004	3.92	3.92	coding	868	119	2722

PB.3653.1	3	2570	10	full-splice_match	gene: Zm00001d039304	Zm00001d039304_T002	29.38	36.21	coding	673	346	2364
PB.3653.3	3	2374	10	full-splice_match	gene: Zm00001d039304	Zm00001d039304_T002	6.83	36.21	coding	673	184	2202
PB.846.8	1	1525	9	full-splice_match	gene: Zm00001d031502	Zm00001d031502_T006	2.60	54.70	coding	354	186	1247
PB.2915.2	2	2021	9	full-splice_match	gene: Zm00001d004055	Zm00001d004055_T002	13.36	13.36	coding	475	212	1636
PB.9615.1	8	1833	2	genic	gene: Zm00001d010954	novel	101.28	127.28	coding	414	216	1457
PB.5535.1	4	1913	7	full-splice_match	gene: Zm00001d052475	Zm00001d052475_T001	21.90	21.90	coding	503	125	1633
PB.7064.1	5	971	5	full-splice_match	gene: Zm00001d018131	Zm00001d018131_T002	31.79	31.79	coding	132	116	511
PB.5573.1	4	1275	2	novel_not_in_catalog	gene: Zm00001d052684	novel	48.85	48.85	coding	332	70	1065
PB.8840.1	7	1256	9	full-splice_match	gene: Zm00001d021898	Zm00001d021898_T001	8.81	8.81	coding	340	37	1056
PB.1035.1	1	2201	2	full-splice_match	gene: Zm00001d032407	Zm00001d032407_T001	5.73	24.77	coding	614	54	1895
PB.9615.3	8	1153	1	full-splice_match	gene: Zm00001d010954	Zm00001d010954_T001	0.00	127.28	coding	253	254	1012
PB.984.2	1	1724	8	full-splice_match	gene: Zm00001d032198	Zm00001d032198_T006	45.86	199.16	coding	126	677	1054
PB.8860.1	7	1240	2	full-splice_match	gene: Zm00001d021971	Zm00001d021971_T001	16.06	16.06	coding	271	147	959
PB.9105.1	8	1338	10	full-splice_match	gene: Zm00001d008379	Zm00001d008379_T001	248.48	248.48	coding	305	147	1061
PB.9457.2	8	669	3	full-splice_match	gene: Zm00001d010236	Zm00001d010236_T001	270.25	376.64	coding	112	90	425
PB.4001.1	3	1653	3	genic	gene: Zm00001d041258	novel	9.81	9.81	coding	424	56	1327
PB.4427.2	3	1243	12	full-splice_match	gene: Zm00001d043272	Zm00001d043272_T002	7.79	74.11	coding	287	122	982

PB.4427.1	3	1327	12	full-splice_match	gene: Zm00001d043272	Zm00001d043272_T002	66.32	74.11	coding	287	119	979
PB.2172.3	10	990	9	full-splice_match	gene: Zm00001d025939	Zm00001d025939_T001	0.00	66.55	coding	250	124	873
PB.2172.2	10	1188	9	full-splice_match	gene: Zm00001d025939	Zm00001d025939_T001	17.22	66.55	coding	250	170	919
PB.7412.1	6	1136	4	full-splice_match	gene: Zm00001d036077	Zm00001d036077_T001	62.82	62.82	coding	153	296	754
PB.984.7	1	1757	11	novel_in_catalog	gene: Zm00001d032198	novel	23.99	199.16	coding	296	116	1003
PB.553.1	1	2180	11	full-splice_match	gene: Zm00001d029654	Zm00001d029654_T004	26.24	26.24	coding	598	129	1922
PB.6614.3	5	1075	2	genic	gene: Zm00001d016062	novel	21.48	36.28	coding	151	337	789
PB.6462.3	5	2188	11	novel_in_catalog	gene: Zm00001d015037	novel	2.97	22.16	coding	364	20	1111
PB.6462.2	5	1933	12	full-splice_match	gene: Zm00001d015037	Zm00001d015037_T002	19.19	22.16	coding	563	41	1729
PB.2264.1	10	2412	11	full-splice_match	gene: Zm00001d026334	Zm00001d026334_T001	2.78	35.08	coding	482	573	2018
PB.6810.1	5	1444	9	full-splice_match	gene: Zm00001d017096	Zm00001d017096_T004	26.34	118.92	coding	193	106	684
PB.2264.2	10	2195	11	full-splice_match	gene: Zm00001d026334	Zm00001d026334_T001	32.30	35.08	coding	482	459	1904
PB.7571.3	6	2977	9	novel_not_in_catalog	gene: Zm00001d036987	novel	3.24	23.69	coding	284	73	924
PB.7571.2	6	2995	13	novel_not_in_catalog	gene: Zm00001d036987	novel	9.93	23.69	coding	494	1378	2859
PB.7571.1	6	2357	12	novel_not_in_catalog	gene: Zm00001d036987	novel	10.52	23.69	coding	690	144	2213
PB.846.1	1	1628	11	novel_in_catalog	gene: Zm00001d031502	novel	5.35	54.70	coding	335	183	1187
PB.984.4	1	1789	10	full-splice_match	gene: Zm00001d032198	Zm00001d032198_T002	78.41	199.16	coding	464	157	1548

PB.58.2	1	1713	14	full-splice_match	gene: Zm00001d027455	Zm00001d027455_T001	9.50	15.90	coding	445	149	1483
PB.5706.1	4	2318	1	full-splice_match	gene: Zm00001d053322	Zm00001d053322_T001	17.27	17.27	coding	683	84	2132
PB.58.1	1	1488	12	incomplete-splice_match	gene: Zm00001d027455	Zm00001d027455_T001	6.40	15.90	coding	363	4	1092
PB.6651.1	5	1622	2	novel_not_in_catalog	gene: Zm00001d016278	novel	39.62	39.62	coding	100	1058	1357
PB.7233.2	6	1997	8	novel_in_catalog	gene: Zm00001d035086	novel	66.62	66.62	coding	228	777	1460
PB.468.1	1	1995	2	full-splice_match	gene: Zm00001d029272	Zm00001d029272_T014	15.19	15.19	coding	55	346	510
PB.6446.1	5	1795	1	full-splice_match	gene: Zm00001d014984	Zm00001d014984_T001	25.14	31.22	coding	515	35	1579
PB.2204.1	10	1255	7	full-splice_match	gene: Zm00001d026089	Zm00001d026089_T001	19.67	19.67	coding	331	103	1095
PB.6170.1	5	1187	8	full-splice_match	gene: Zm00001d013755	Zm00001d013755_T002	39.30	62.22	coding	217	176	826
PB.2928.3	2	3160	9	full-splice_match	gene: Zm00001d004140	Zm00001d004140_T001	12.44	17.07	coding	772	381	2696
PB.6170.3	5	1636	6	novel_in_catalog	gene: Zm00001d013755	novel	1.59	62.22	coding	122	337	702
PB.6170.2	5	1278	7	full-splice_match	gene: Zm00001d013755	Zm00001d013755_T003	18.50	62.22	coding	197	329	919
PB.6170.4	5	1062	8	full-splice_match	gene: Zm00001d013755	Zm00001d013755_T002	2.83	62.22	coding	217	177	827
PB.2928.4	2	3141	9	full-splice_match	gene: Zm00001d004140	Zm00001d004140_T001	4.62	17.07	coding	911	66	2798
PB.3470.2	2	776	1	full-splice_match	gene: Zm00001d007097	Zm00001d007097_T001	0.00	540.31	coding	159	101	577
PB.4763.1	3	2677	7	full-splice_match	gene: Zm00001d044601	Zm00001d044601_T003	7.74	9.46	coding	724	128	2299
PB.2583.1	2	2392	1	full-splice_match	gene: Zm00001d002573	Zm00001d002573_T001	79.11	79.11	coding	623	226	2094

PB.1225.3	1	2434	6	full-splice_match	gene: Zm00001d033292	Zm00001d033292_T004	63.31	84.80	coding	559	181	1857
PB.10630.2	9	1874	11	novel_not_in_catalog	gene: Zm00001d047849	novel	0.47	34.33	coding	414	496	1737
PB.664.2	1	1491	10	novel_not_in_catalog	gene: Zm00001d030348	novel	1.70	69.37	coding	379	106	1242
PB.7759.8	6	2026	8	incomplete-splice_match	gene: Zm00001d037829	Zm00001d037829_T006	0.00	52.49	coding	469	440	1846
PB.4148.2	3	1363	3	novel_in_catalog	gene: Zm00001d042017	novel	1.27	36.58	non_coding	NA	NA	NA
PB.1920.1	10	1877	13	full-splice_match	gene: Zm00001d024729	Zm00001d024729_T001	74.94	129.97	coding	378	331	1464
PB.664.3	1	1180	6	novel_in_catalog	gene: Zm00001d030348	novel	0.00	69.37	coding	142	548	973
PB.7759.2	6	2305	1	novel_in_catalog	gene: Zm00001d037829	novel	5.92	52.49	coding	109	1571	1897
PB.7759.1	6	2810	9	full-splice_match	gene: Zm00001d037829	Zm00001d037829_T014	46.57	52.49	coding	515	838	2382
PB.9423.1	8	1073	3	incomplete-splice_match	gene: Zm00001d010062	Zm00001d010062_T002	7.07	7.07	coding	279	60	896
PB.7759.6	6	2591	9	full-splice_match	gene: Zm00001d037829	Zm00001d037829_T014	0.00	52.49	coding	515	793	2337
PB.6338.3	5	2463	2	full-splice_match	gene: Zm00001d014594	Zm00001d014594_T002	0.00	43.08	coding	629	209	2095
PB.6338.2	5	2529	2	full-splice_match	gene: Zm00001d014594	Zm00001d014594_T002	26.17	43.08	coding	629	217	2103
PB.2412.2	2	4496	6	full-splice_match	gene: Zm00001d001848	Zm00001d001848_T008	28.23	28.23	coding	1386	105	4262
PB.10765.1	9	1652	5	full-splice_match	gene: Zm00001d048346	Zm00001d048346_T001	10.31	404.71	coding	310	112	1041
PB.4502.1	3	1455	2	novel_in_catalog	gene: Zm00001d043539	novel	11.77	20.87	coding	68	377	580
PB.10765.3	9	1791	4	novel_in_catalog	gene: Zm00001d048346	novel	0.00	404.71	coding	152	935	1390

PB.10765.2	9	1482	5	full-splice_match	gene: Zm00001d048346	Zm00001d048346_T001	95.84	404.71	coding	310	112	1041
PB.7877.4	6	2051	14	full-splice_match	gene: Zm00001d038209	Zm00001d038209_T001	22.59	55.08	coding	507	94	1614
PB.1225.2	1	2827	5	full-splice_match	gene: Zm00001d033292	Zm00001d033292_T003	8.31	84.80	coding	487	765	2225
PB.8892.1	7	1236	1	full-splice_match	gene: Zm00001d022092	Zm00001d022092_T001	22.50	22.50	coding	343	66	1094
PB.1377.1	1	1280	5	full-splice_match	gene: Zm00001d033976	Zm00001d033976_T001	521.01	521.01	coding	237	257	967
PB.5998.1	5	963	8	incomplete-splice_match	gene: Zm00001d013116	Zm00001d013116_T007	105.48	105.48	coding	237	63	773
PB.9324.1	8	1082	5	full-splice_match	gene: Zm00001d009525	Zm00001d009525_T002	8.84	8.84	coding	201	119	721
PB.7565.1	6	2589	12	full-splice_match	gene: Zm00001d036971	Zm00001d036971_T045	91.25	111.61	coding	640	444	2363
PB.6257.1	5	1777	2	full-splice_match	gene: Zm00001d014201	Zm00001d014201_T001	10.90	10.90	coding	348	163	1206
PB.9191.4	8	1925	3	full-splice_match	gene: Zm00001d008794	Zm00001d008794_T001	38.13	69.38	coding	540	77	1696
PB.9191.2	8	2297	1	genic	gene: Zm00001d008794	novel	3.55	69.38	coding	263	1180	1968
PB.9191.1	8	2172	2	novel_in_catalog	gene: Zm00001d008794	novel	27.69	69.38	coding	471	434	1846
PB.6418.1	5	2671	7	full-splice_match	gene: Zm00001d014895	Zm00001d014895_T003	16.30	90.33	coding	762	256	2541
PB.2256.2	10	3425	24	full-splice_match	gene: Zm00001d026306	Zm00001d026306_T009	69.77	152.36	coding	1028	83	3166
PB.2920.1	2	1738	13	full-splice_match	gene: Zm00001d004091	Zm00001d004091_T003	30.28	30.28	coding	474	64	1485
PB.4132.1	3	1023	1	novel_in_catalog	gene: Zm00001d041955	novel	9.01	9.01	coding	155	145	609
PB.2256.7	10	3641	23	novel_in_catalog	gene: Zm00001d026306	novel	6.83	152.36	coding	635	44	1948

PB.2256.5	10	1609	4	novel_in_catalog	gene: Zm00001d026306	novel	73.19	152.36	coding	157	878	1348
PB.4063.2	3	2499	10	incomplete-splice_match	gene: Zm00001d041599	Zm00001d041599_T008	4.50	22.85	coding	259	1335	2111
PB.6478.1	5	1423	4	full-splice_match	gene: Zm00001d015123	Zm00001d015123_T001	60.77	60.77	coding	244	427	1158
PB.4063.1	3	2910	13	full-splice_match	gene: Zm00001d041599	Zm00001d041599_T002	18.35	22.85	coding	752	246	2501
PB.10352.1	9	921	2	full-splice_match	gene: Zm00001d046510	Zm00001d046510_T001	84.91	84.91	coding	147	130	570
PB.4393.1	3	785	1	full-splice_match	gene: Zm00001d043136	Zm00001d043136_T001	30.27	30.27	coding	129	80	466
PB.2995.1	2	1589	9	incomplete-splice_match	gene: Zm00001d004709	Zm00001d004709_T001	11.41	11.41	coding	397	11	1201
PB.2635.1	2	1045	2	full-splice_match	gene: Zm00001d002788	Zm00001d002788_T001	50.73	50.73	coding	207	96	716
PB.6645.5	5	2641	2	incomplete-splice_match	gene: Zm00001d016230	Zm00001d016230_T001	0.07	6.26	coding	250	1480	2229
PB.2174.2	10	683	6	full-splice_match	gene: Zm00001d025941	Zm00001d025941_T002	16.86	20.00	coding	153	8	466
PB.6122.2	5	353	2	intergenic	novelGene_48	novel	106.95	106.95	non_coding	NA	NA	NA
PB.2714.2	2	479	1	intergenic	novelGene_88	novel	3839.56	4129.20	non_coding	NA	NA	NA
PB.9619.1	8	3342	1	full-splice_match	gene: Zm00001d010961	Zm00001d010961_T001	4.50	6.99	coding	814	86	2527
PB.84.1	1	1040	2	full-splice_match	gene: Zm00001d027542	Zm00001d027542_T002	31.32	31.32	coding	224	49	720
PB.5421.2	4	1840	15	incomplete-splice_match	gene: Zm00001d052070	Zm00001d052070_T048	32.14	32.14	coding	481	108	1550
PB.9851.1	8	1825	3	full-splice_match	gene: Zm00001d012016	Zm00001d012016_T001	71.96	71.96	coding	382	121	1266
PB.8075.2	6	2366	6	novel_in_catalog	gene: Zm00001d038972	novel	4.47	254.63	coding	261	152	934

PB.9619.2	8	1448	1	full-splice_match	gene: Zm00001d010961	Zm00001d010961_T001	2.49	6.99	coding	361	172	1254
PB.1690.1	10	2670	2	full-splice_match	gene: Zm00001d023377	Zm00001d023377_T001	1.07	18.51	coding	494	99	1580
PB.3522.3	2	2244	5	incomplete-splice_match	gene: Zm00001d007407	Zm00001d007407_T025	10.22	28.05	coding	109	266	592
PB.1690.3	10	1619	2	full-splice_match	gene: Zm00001d023377	Zm00001d023377_T001	0.00	18.51	coding	494	104	1585
PB.1690.2	10	1767	2	full-splice_match	gene: Zm00001d023377	Zm00001d023377_T001	17.44	18.51	coding	494	132	1613
PB.4971.2	4	2546	2	incomplete-splice_match	gene: Zm00001d049487	Zm00001d049487_T001	54.27	57.97	coding	739	178	2394
PB.9199.1	8	938	5	full-splice_match	gene: Zm00001d008839	Zm00001d008839_T002	53.74	53.74	coding	109	414	740
PB.4971.1	4	2678	2	incomplete-splice_match	gene: Zm00001d049487	Zm00001d049487_T001	3.71	57.97	coding	739	158	2374
PB.854.2	1	1637	1	genic	gene: Zm00001d031545	novel	36.06	181.18	coding	126	209	586
PB.2921.5	2	2110	7	intergenic	novelGene_146	novel	0.00	44.65	coding	150	1037	1486
PB.1211.3	1	1796	6	full-splice_match	gene: Zm00001d033210	Zm00001d033210_T002	2352.12	2362.83	coding	419	159	1415
PB.8043.6	6	680	4	full-splice_match	gene: Zm00001d038865	Zm00001d038865_T006	208.94	1550.98	coding	143	91	519
PB.1134.1	1	1290	3	full-splice_match	gene: Zm00001d032788	Zm00001d032788_T001	3.42	3.42	coding	390	7	1176
PB.8.2	1	2212	5	full-splice_match	gene: Zm00001d027271	Zm00001d027271_T001	11.10	33.41	non_coding	NA	NA	NA
PB.8.3	1	2075	4	full-splice_match	gene: Zm00001d027271	Zm00001d027271_T006	22.31	33.41	non_coding	NA	NA	NA
PB.9185.1	8	652	1	full-splice_match	gene: Zm00001d008753	Zm00001d008753_T001	231.65	231.65	non_coding	NA	NA	NA
PB.9992.1	8	3938	6	full-splice_match	gene: Zm00001d012634	Zm00001d012634_T001	20.19	20.19	coding	1068	90	3293

PB.10584.1	9	1499	2	genic	gene: Zm00001d047709	novel	26.36	32.94	coding	333	140	1138
PB.10584.2	9	956	1	full-splice_match	gene: Zm00001d047709	Zm00001d047709_T001	6.58	32.94	coding	269	37	843
PB.9316.1	8	681	4	full-splice_match	gene: Zm00001d009496	Zm00001d009496_T001	103.20	103.20	coding	111	114	446
PB.6247.1	5	1866	2	incomplete-splice_match	gene: Zm00001d014159	Zm00001d014159_T023	127.21	127.21	coding	461	37	1419
PB.2359.1	10	1728	16	full-splice_match	gene: Zm00001d026635	Zm00001d026635_T001	80.85	80.85	coding	506	65	1582
PB.896.1	1	2023	7	novel_in_catalog	gene: Zm00001d031749	novel	52.17	52.17	coding	497	229	1719
PB.4197.1	3	888	1	intergenic	novelGene_258	novel	13.09	13.09	non_coding	NA	NA	NA
PB.3103.4	2	1464	3	novel_in_catalog	gene: Zm00001d005338	novel	13.49	57.07	coding	135	96	500
PB.3103.5	2	1805	4	novel_in_catalog	gene: Zm00001d005338	novel	0.00	57.07	coding	267	184	984
PB.6631.1	5	2807	12	incomplete-splice_match	gene: Zm00001d016155	Zm00001d016155_T003	12.22	12.22	coding	776	100	2427
PB.3618.2	2	3189	27	full-splice_match	gene: Zm00001d007905	Zm00001d007905_T015	2.46	15.01	coding	966	161	3058
PB.5028.1	4	2095	2	novel_not_in_catalog	gene: Zm00001d049737	novel	9.40	9.40	coding	553	95	1753
PB.3618.1	2	1781	13	incomplete-splice_match	gene: Zm00001d007905	Zm00001d007905_T004	12.55	15.01	coding	403	177	1385
PB.1452.2	1	1888	4	full-splice_match	gene: Zm00001d034320	Zm00001d034320_T003	56.31	56.31	coding	501	120	1622
PB.5375.2	4	1461	2	full-splice_match	gene: Zm00001d051885	Zm00001d051885_T003	8.74	423.03	coding	277	279	1109
PB.3996.2	3	3235	2	full-splice_match	gene: Zm00001d041232	Zm00001d041232_T001	15.08	15.08	coding	1021	4	3066
PB.3098.1	2	1586	1	full-splice_match	gene: Zm00001d005312	Zm00001d005312_T001	45.70	45.70	coding	454	58	1419

PB.6776.1	5	791	4	intergenic	novelGene_299	novel	11.08	28.40	non_coding	NA	NA	NA
PB.6776.3	5	1254	5	intergenic	novelGene_299	novel	6.97	28.40	non_coding	NA	NA	NA
PB.6776.2	5	2049	3	intergenic	novelGene_299	novel	10.35	28.40	non_coding	NA	NA	NA
PB.7502.1	6	1976	4	incomplete-splice_match	gene: Zm00001d036593	Zm00001d036593_T001	83.25	83.25	coding	508	93	1616
PB.2617.3	2	1904	7	full-splice_match	gene: Zm00001d002696	Zm00001d002696_T001	0.00	15.46	coding	462	352	1737
PB.2617.2	2	1846	7	full-splice_match	gene: Zm00001d002696	Zm00001d002696_T001	10.51	15.46	coding	462	226	1611
PB.2617.1	2	2077	7	full-splice_match	gene: Zm00001d002696	Zm00001d002696_T001	4.95	15.46	coding	462	203	1588
PB.8007.1	6	835	4	full-splice_match	gene: Zm00001d038753	Zm00001d038753_T001	127.76	129.15	coding	113	176	514
PB.7943.1	6	1488	1	antisense	novelGene_gene: Zm00001d038478_A	novel	3.18	3.18	coding	125	420	794
PB.3575.1	2	1321	1	full-splice_match	gene: Zm00001d007753	Zm00001d007753_T001	34.16	34.16	coding	380	78	1217
PB.5375.1	4	1355	3	full-splice_match	gene: Zm00001d051885	Zm00001d051885_T002	54.22	423.03	coding	277	65	895
PB.9852.2	8	884	6	full-splice_match	gene: Zm00001d012017	Zm00001d012017_T002	0.00	117.79	coding	202	39	644
PB.1211.6	1	1794	5	novel_in_catalog	gene: Zm00001d033210	novel	0.00	2362.83	coding	258	700	1473
PB.8652.3	7	1304	2	full-splice_match	gene: Zm00001d021268	Zm00001d021268_T001	0.00	112.84	coding	239	396	1112
PB.966.1	1	871	1	full-splice_match	gene: Zm00001d032094	Zm00001d032094_T001	14.40	14.40	coding	133	187	585
PB.9239.1	8	702	4	full-splice_match	gene: Zm00001d009000	Zm00001d009000_T001	537.19	537.19	coding	130	93	482
PB.8975.2	7	810	6	full-splice_match	gene: Zm00001d022393	Zm00001d022393_T001	8.73	73.18	coding	153	101	559

PB.10227.1	9	1150	1	full-splice_match	gene: Zm00001d045625	Zm00001d045625_T001	23.25	23.25	coding	310	5	934
PB.4491.1	3	2182	3	novel_in_catalog	gene: Zm00001d043500	novel	9.78	9.78	coding	454	525	1886
PB.5708.1	4	1243	2	intergenic	novelGene_349	novel	35.25	35.25	non_coding	NA	NA	NA
PB.4782.1	3	660	2	full-splice_match	gene: Zm00001d044685	Zm00001d044685_T002	53.69	53.69	coding	123	76	444
PB.2021.1	10	2020	1	full-splice_match	gene: Zm00001d025152	Zm00001d025152_T001	0.83	0.83	coding	632	83	1978
PB.2991.1	2	1934	1	novel_in_catalog	gene: Zm00001d000919	novel	38.38	38.38	non_coding	NA	NA	NA
PB.1484.1	1	1638	10	full-splice_match	gene: Zm00001d034420	Zm00001d034420_T007	194.52	251.69	coding	411	148	1380
PB.1484.3	1	1803	10	full-splice_match	gene: Zm00001d034420	Zm00001d034420_T009	16.91	251.69	coding	411	316	1548
PB.9225.5	8	2242	6	full-splice_match	gene: Zm00001d008952	Zm00001d008952_T001	0.00	19.34	coding	645	127	2061
PB.2101.1	10	2535	7	full-splice_match	gene: Zm00001d025653	Zm00001d025653_T007	7.09	35.52	coding	220	22	681
PB.10450.1	9	1562	5	full-splice_match	gene: Zm00001d047078	Zm00001d047078_T002	45.49	45.49	coding	351	213	1265
PB.1484.8	1	1725	9	full-splice_match	gene: Zm00001d034420	Zm00001d034420_T005	12.64	251.69	coding	367	134	1234
PB.9225.2	8	2358	6	full-splice_match	gene: Zm00001d008952	Zm00001d008952_T001	19.34	19.34	coding	645	124	2058
PB.5728.1	4	816	1	full-splice_match	gene: Zm00001d053434	Zm00001d053434_T001	8.12	8.12	coding	146	106	543
PB.6752.8	5	2359	10	novel_in_catalog	gene: Zm00001d016825	novel	6.13	35.33	coding	303	66	974
PB.2325.1	10	2751	15	incomplete-splice_match	gene: Zm00001d026548	Zm00001d026548_T001	18.02	18.02	coding	793	79	2457
PB.6752.4	5	2854	9	full-splice_match	gene: Zm00001d016825	Zm00001d016825_T009	0.00	35.33	coding	203	69	677

PB.6752.3	5	1877	10	novel_in_catalog	gene: Zm00001d016825	novel	22.22	35.33	coding	441	216	1538		
PB.6752.1	5	2296	10	full-splice_match	gene: Zm00001d016825	Zm00001d016825_T012	6.29	35.33	coding	203	111	719		
PB.3899.1	3	692	5	full-splice_match	gene: Zm00001d040435	Zm00001d040435_T002	102.70	147.82	coding	121	111	473		
PB.3899.2	3	721	5	full-splice_match	gene: Zm00001d040435	Zm00001d040435_T003	45.12	147.82	coding	121	142	504		
PB.4726.3	3	1519	2	genic	gene: Zm00001d044461	novel	67.89	86.13	coding	311	231	1163		
PB.1332.1	1	3228	2	incomplete-splice_match	gene: Zm00001d033775	Zm00001d033775_T001	39.43	40.09	coding	914	291	3032		
PB.8233.2	7	1171	4	novel_not_in_catalog	gene: Zm00001d018938	novel	181.01	238.97	coding	249	189	935		
PB.8233.1	7	1275	4	novel_not_in_catalog	gene: Zm00001d018938	novel	57.95	238.97	coding	249	191	937		
PB.6362.2	5	829	5	full-splice_match	gene: Zm00001d014692	Zm00001d014692_T001	47.24	104.20	coding	183	108	656		
PB.9753.2	8	2443	5	full-splice_match	gene: Zm00001d011625	Zm00001d011625_T001	50.66	50.66	coding	718	74	2227		
PB.4641.2	3	825	7	full-splice_match	gene: Zm00001d044096	Zm00001d044096_T003	62.89	272.02	coding	206	76	693		
PB.6362.1	5	973	5	full-splice_match	gene: Zm00001d014692	Zm00001d014692_T001	56.97	104.20	coding	183	102	650		
PB.9872.1	8	633	2	full-splice_match	gene: Zm00001d012127	Zm00001d012127_T001	211.09	211.09	non_coding	NA	NA	NA		
PB.6336.5	5	2536	6	novel_in_catalog	gene: Zm00001d014580	novel	2.78	21.76	non_coding	NA	NA	NA		
PB.5996.1	5	1358	2	full-splice_match	gene: Zm00001d013108	Zm00001d013108_T001	187.30	187.30	coding	311	6	938		
PB.6336.7	5	2485	5	novel_not_in_catalog	gene: Zm00001d014580	novel	0.53	21.76	coding	52	149	304		
PB.6336.6	5	2590	4	novel_in_catalog	gene: Zm00001d014580	novel	5.44	21.76	coding	52	172	327		

PB.8192.3	7	1264	1	novel_in_catalog	gene: Zm00001d018790	novel	14.80	79.25	coding	139	35	451
PB.8192.2	7	1252	2	full-splice_match	gene: Zm00001d018790	Zm00001d018790_T001	56.37	79.25	coding	313	49	987
PB.8192.1	7	1337	2	full-splice_match	gene: Zm00001d018790	Zm00001d018790_T001	8.07	79.25	coding	313	30	968
PB.4002.7	3	2974	7	intergenic	novelGene_182	novel	10.46	61.78	coding	198	2072	2665
PB.4637.1	3	1729	12	full-splice_match	gene: Zm00001d044088	Zm00001d044088_T001	23.61	38.74	coding	404	38	1249
PB.9072.1	8	1440	1	novel_in_catalog	gene: Zm00001d008242	novel	14.00	14.00	coding	224	185	856
PB.6336.9	5	1572	1	genic	gene: Zm00001d014580	novel	0.00	21.76	coding	52	183	338
PB.10243.1	9	3138	2	novel_not_in_catalog	gene: Zm00001d045757	novel	1.31	16.30	coding	642	683	2608
PB.10243.2	9	3016	2	novel_not_in_catalog	gene: Zm00001d045757	novel	14.99	16.30	coding	642	686	2611
PB.6079.1	5	1018	1	full-splice_match	gene: Zm00001d013385	Zm00001d013385_T001	5.33	5.33	coding	168	419	922
PB.4818.9	4	1399	3	full-splice_match	gene: Zm00001d048680	Zm00001d048680_T005	0.00	159.29	coding	194	97	678
PB.4905.1	4	1666	2	full-splice_match	gene: Zm00001d049148	Zm00001d049148_T001	7.54	7.54	coding	447	38	1378
PB.4528.22	3	2922	7	novel_in_catalog	gene: Zm00001d043649	novel	1.44	96.43	coding	125	1051	1425
PB.6259.1	5	1668	15	full-splice_match	gene: Zm00001d014258	Zm00001d014258_T001	13.03	77.62	coding	486	92	1549
PB.1952.2	10	1601	1	full-splice_match	gene: Zm00001d024866	Zm00001d024866_T001	5.88	7.72	coding	366	209	1306
PB.5413.2	4	2429	1	novel_in_catalog	gene: Zm00001d052053	novel	9.94	40.67	coding	440	371	1690
PB.6259.2	5	1732	14	novel_in_catalog	gene: Zm00001d014258	novel	3.36	77.62	coding	496	76	1563

PB.6085.1	5	1542	5	full-splice_match	gene: Zm00001d013410	Zm00001d013410_T002	199.69	199.69	coding	377	93	1223
PB.1744.3	10	1071	8	full-splice_match	gene: Zm00001d023635	Zm00001d023635_T001	0.00	195.35	coding	275	68	892
PB.1744.2	10	1279	8	full-splice_match	gene: Zm00001d023635	Zm00001d023635_T001	195.35	195.35	coding	275	143	967
PB.4818.6	4	1541	3	full-splice_match	gene: Zm00001d048680	Zm00001d048680_T005	122.08	159.29	coding	194	173	754
PB.4818.7	4	1620	3	novel_not_in_catalog	gene: Zm00001d048680	novel	26.89	159.29	coding	194	61	642
PB.6358.1	5	1837	5	full-splice_match	gene: Zm00001d014673	Zm00001d014673_T001	433.87	929.57	coding	414	141	1382
PB.6321.1	5	1759	1	antisense	novelGene_gene: Zm00001d014530_A	novel	6.99	6.99	coding	153	548	1006
PB.6301.18	5	3524	2	full-splice_match	gene: Zm00001d014423	Zm00001d014423_T001	0.00	169.16	coding	410	469	1698
PB.3885.3	3	1172	7	full-splice_match	gene: Zm00001d040357	Zm00001d040357_T003	44.65	44.65	coding	254	112	873
PB.7042.1	5	2016	10	full-splice_match	gene: Zm00001d018032	Zm00001d018032_T002	5.37	9.58	coding	537	118	1728
PB.8463.12	7	1553	8	full-splice_match	gene: Zm00001d020378	Zm00001d020378_T001	0.00	49.96	coding	390	57	1226
PB.6358.6	5	1168	2	incomplete-splice_match	gene: Zm00001d014673	Zm00001d014673_T002	313.22	929.57	coding	286	155	1012
PB.1980.1	10	1254	1	genic	gene: Zm00001d024961	novel	11.18	11.18	coding	218	32	685
PB.6301.12	5	2526	7	full-splice_match	gene: Zm00001d014423	Zm00001d014423_T021	0.00	169.16	coding	593	480	2258
PB.6301.10	5	2150	3	incomplete-splice_match	gene: Zm00001d014423	Zm00001d014423_T025	23.88	169.16	coding	113	1540	1878
PB.5938.1	5	2316	2	full-splice_match	gene: Zm00001d012909	Zm00001d012909_T002	57.35	57.35	coding	232	1395	2090
PB.6301.15	5	2236	3	novel_in_catalog	gene: Zm00001d014423	novel	5.59	169.16	coding	288	21	884

PB.6301.14	5	2685	7	novel_in_catalog	gene: Zm00001d014423	novel	3.85	169.16	coding	489	425	1891
PB.3014.1	2	1332	9	full-splice_match	gene: Zm00001d004837	Zm00001d004837_T001	213.87	218.64	coding	276	177	1004
PB.4350.2	3	1150	3	full-splice_match	gene: Zm00001d042918	Zm00001d042918_T001	24.07	84.10	coding	269	117	923
PB.4350.1	3	1418	3	full-splice_match	gene: Zm00001d042918	Zm00001d042918_T001	60.03	84.10	coding	269	317	1123
PB.1117.2	1	2320	8	full-splice_match	gene: Zm00001d032703	Zm00001d032703_T007	9.50	50.11	coding	609	228	2054
PB.2710.1	2	2654	8	novel_in_catalog	gene: Zm00001d003072	novel	5.39	8.35	coding	726	177	2354
PB.830.1	1	3385	2	full-splice_match	gene: Zm00001d031427	Zm00001d031427_T001	3.25	3.25	coding	1088	72	3335
PB.2710.3	2	2596	8	novel_in_catalog	gene: Zm00001d003072	novel	2.96	8.35	coding	726	222	2399
PB.3014.6	2	1942	8	novel_in_catalog	gene: Zm00001d004837	novel	4.77	218.64	coding	224	982	1653
PB.8888.1	7	3423	5	full-splice_match	gene: Zm00001d022072	Zm00001d022072_T001	9.14	9.14	coding	951	446	3298
PB.7258.1	6	2640	5	full-splice_match	gene: Zm00001d035199	Zm00001d035199_T001	17.60	17.60	coding	657	267	2237
PB.8223.1	7	1446	3	full-splice_match	gene: Zm00001d018902	Zm00001d018902_T001	27.27	27.27	coding	354	100	1161
PB.769.1	1	2424	8	novel_in_catalog	gene: Zm00001d031081	novel	11.10	29.83	coding	598	91	1884
PB.535.2	1	866	2	full-splice_match	gene: Zm00001d029590	Zm00001d029590_T001	105.82	169.85	coding	125	280	654
PB.535.1	1	927	1	incomplete-splice_match	gene: Zm00001d029590	Zm00001d029590_T001	64.03	169.85	coding	125	57	431
PB.1600.3	1	2359	13	full-splice_match	gene: Zm00001d034809	Zm00001d034809_T002	10.60	80.83	coding	321	201	1163
PB.4629.2	3	1771	4	novel_in_catalog	gene: Zm00001d044036	novel	10.08	36.60	coding	119	821	1177

PB.10898.2	B73V4 ctg24	1294	2	full-splice_match	gene: Zm00001d000380	Zm00001d000380_T001	12.96	29.19	coding	252	105	860
PB.4629.1	3	1693	5	full-splice_match	gene: Zm00001d044036	Zm00001d044036_T004	17.67	36.60	coding	117	814	1164
PB.2700.2	2	2207	3	novel_in_catalog	gene: Zm00001d003038	novel	1.04	4.37	coding	307	1118	2038
PB.640.2	1	1411	1	full-splice_match	gene: Zm00001d030181	Zm00001d030181_T001	10.13	18.31	coding	368	55	1158
PB.4629.4	3	1471	5	full-splice_match	gene: Zm00001d044036	Zm00001d044036_T004	8.85	36.60	coding	117	820	1170
PB.2700.1	2	1814	5	full-splice_match	gene: Zm00001d003038	Zm00001d003038_T001	3.33	4.37	coding	517	66	1616
PB.5306.1	4	2257	3	full-splice_match	gene: Zm00001d051550	Zm00001d051550_T001	4.95	12.34	coding	466	459	1856
PB.5306.2	4	1812	3	full-splice_match	gene: Zm00001d051550	Zm00001d051550_T001	7.39	12.34	coding	466	267	1664
PB.9684.1	8	1793	4	intergenic	novelGene_237	novel	11.21	11.21	non_coding	NA	NA	NA
PB.6.2	1	3813	25	incomplete-splice_match	gene: Zm00001d027259	Zm00001d027259_T064	2.63	16.33	coding	953	745	3603
PB.2017.1	10	1537	1	intergenic	novelGene_178	novel	0.91	0.91	non_coding	NA	NA	NA
PB.6.1	1	3021	20	full-splice_match	gene: Zm00001d027259	Zm00001d027259_T004	13.70	16.33	coding	774	276	2597
PB.4253.1	3	1896	13	full-splice_match	gene: Zm00001d042507	Zm00001d042507_T001	27.01	27.01	coding	480	219	1658
PB.4253.3	3	1899	13	full-splice_match	gene: Zm00001d042507	Zm00001d042507_T001	0.00	27.01	coding	480	312	1751
PB.7629.7	6	1501	9	novel_not_in_catalog	gene: Zm00001d037257	novel	8.03	107.46	coding	206	184	801
PB.9030.3	7	1402	1	full-splice_match	gene: Zm00001d022557	Zm00001d022557_T001	14.89	31.90	coding	363	69	1157
PB.182.1	1	1131	3	full-splice_match	gene: Zm00001d028062	Zm00001d028062_T001	8.06	8.06	coding	181	325	867

PB.8953.4	7	1116	4	novel_not_in_catalog	gene: Zm00001d022330	novel	0.00	52.68	coding	318	14	967
PB.5437.1	4	2019	2	full-splice_match	gene: Zm00001d052168	Zm00001d052168_T001	4.97	4.97	coding	589	19	1785
PB.7294.1	6	2312	7	full-splice_match	gene: Zm00001d035401	Zm00001d035401_T001	9.39	9.39	coding	706	46	2163
PB.7947.1	6	1137	1	full-splice_match	gene: Zm00001d038514	Zm00001d038514_T001	31.40	31.40	coding	226	134	811
PB.4911.1	4	2036	4	novel_in_catalog	gene: Zm00001d049171	novel	15.64	16.77	coding	169	964	1470
PB.4911.2	4	2098	3	novel_in_catalog	gene: Zm00001d049171	novel	1.13	16.77	coding	169	1048	1554
PB.907.4	1	1821	4	full-splice_match	gene: Zm00001d031825	Zm00001d031825_T003	0.29	651.03	coding	75	108	332
PB.2835.9	2	1503	5	novel_in_catalog	gene: Zm00001d003616	novel	13.74	253.94	coding	161	199	681
PB.1515.4	1	591	2	novel_not_in_catalog	gene: Zm00001d034521	novel	0.00	55.00	non_coding	NA	NA	NA
PB.2835.7	2	1724	3	novel_in_catalog	gene: Zm00001d003616	novel	13.16	253.94	non_coding	NA	NA	NA
PB.2835.1	2	1761	2	novel_in_catalog	gene: Zm00001d003616	novel	13.52	253.94	coding	122	691	1056
PB.2414.4	2	1117	7	intergenic	novelGene_15	novel	38.37	307.17	coding	278	71	904
PB.2835.3	2	2388	8	novel_in_catalog	gene: Zm00001d003616	novel	12.74	253.94	coding	269	1099	1905
PB.8743.1	7	2585	20	novel_in_catalog	gene: Zm00001d021584	novel	4.06	15.33	coding	682	190	2235
PB.991.1	1	859	3	full-splice_match	gene: Zm00001d032223	Zm00001d032223_T001	56.47	56.47	coding	178	127	660
PB.495.1	1	623	4	full-splice_match	gene: Zm00001d029418	Zm00001d029418_T002	127.47	1007.97	non_coding	NA	NA	NA
PB.495.2	1	504	4	full-splice_match	gene: Zm00001d029418	Zm00001d029418_T002	880.50	1007.97	non_coding	NA	NA	NA

PB.9805.1	8	2403	16	full-splice_match	gene: Zm00001d011829	Zm00001d011829_T004	3.19	3.19	coding	724	96	2267
PB.9859.3	8	1872	4	novel_not_in_catalog	gene: Zm00001d012074	novel	0.52	31.39	coding	340	240	1259
PB.3026.2	2	2041	2	genic	gene: Zm00001d004899	novel	1.97	30.93	coding	226	1240	1917
PB.104.2	1	3321	2	full-splice_match	gene: Zm00001d027645	Zm00001d027645_T001	0.00	23.73	coding	1040	94	3213
PB.104.1	1	3426	2	full-splice_match	gene: Zm00001d027645	Zm00001d027645_T001	23.73	23.73	coding	1040	94	3213
PB.3026.1	2	1991	3	novel_not_in_catalog	gene: Zm00001d004899	novel	28.96	30.93	coding	583	91	1839
PB.9383.12	8	1702	1	novel_in_catalog	gene: Zm00001d009858	novel	0.00	534.95	coding	262	63	848
PB.9383.10	8	3025	1	novel_in_catalog	gene: Zm00001d009858	novel	3.48	534.95	coding	262	63	848
PB.10063.1	9	3254	17	incomplete-splice_match	gene: Zm00001d044903	Zm00001d044903_T042	9.17	10.50	coding	952	87	2942
PB.4580.3	3	1571	12	full-splice_match	gene: Zm00001d043843	Zm00001d043843_T001	0.00	85.24	coding	479	134	1570
PB.10469.2	9	1464	3	novel_in_catalog	gene: Zm00001d047210	novel	2.67	12.39	coding	222	550	1215
PB.9859.1	8	2195	3	full-splice_match	gene: Zm00001d012074	Zm00001d012074_T001	19.26	31.39	coding	500	292	1791
PB.2815.3	2	1061	11	incomplete-splice_match	gene: Zm00001d003512	Zm00001d003512_T001	8.15	18.34	coding	292	49	924
PB.2815.2	2	1867	13	novel_in_catalog	gene: Zm00001d003512	novel	3.25	18.34	coding	367	333	1433
PB.2815.1	2	2801	16	full-splice_match	gene: Zm00001d003512	Zm00001d003512_T001	6.94	18.34	coding	664	373	2364
PB.1834.1	10	1867	3	incomplete-splice_match	gene: Zm00001d024229	Zm00001d024229_T004	12.09	12.09	coding	409	100	1326
PB.2142.1	10	1919	1	full-splice_match	gene: Zm00001d025807	Zm00001d025807_T001	17.86	17.86	coding	542	65	1690

PB.9717.1	8	1119	2	incomplete-splice_match	gene: Zm00001d011470	Zm00001d011470_T001	15.35	15.35	coding	141	541	963
PB.489.1	1	1419	4	full-splice_match	gene: Zm00001d029388	Zm00001d029388_T001	8.05	8.05	coding	349	87	1133
PB.9318.1	8	1767	2	full-splice_match	gene: Zm00001d009510	Zm00001d009510_T001	39.40	39.40	coding	469	240	1646
PB.1551.3	1	1940	7	full-splice_match	gene: Zm00001d034650	Zm00001d034650_T017	17.12	48.82	coding	474	287	1708
PB.10157.1	9	639	2	full-splice_match	gene: Zm00001d045351	Zm00001d045351_T001	68.75	68.75	coding	119	54	410
PB.5374.1	4	2061	4	novel_in_catalog	gene: Zm00001d051884	novel	24.29	51.48	coding	430	364	1653
PB.4269.5	3	2343	6	novel_in_catalog	gene: Zm00001d042590	novel	0.00	119.12	coding	221	1200	1862
PB.5712.3	4	1002	3	full-splice_match	gene: Zm00001d053371	Zm00001d053371_T001	146.39	264.95	coding	188	244	807
PB.5374.4	4	1976	4	full-splice_match	gene: Zm00001d051884	Zm00001d051884_T003	27.19	51.48	coding	125	1235	1609
PB.5712.1	4	1120	3	full-splice_match	gene: Zm00001d053371	Zm00001d053371_T001	114.72	264.95	coding	188	250	813
PB.3450.7	2	2251	11	full-splice_match	gene: Zm00001d007029	Zm00001d007029_T005	5.51	45.54	coding	587	339	2099
PB.1287.3	1	1800	13	novel_not_in_catalog	gene: Zm00001d033558	novel	0.00	36.09	coding	473	216	1634
PB.3450.3	2	2593	11	full-splice_match	gene: Zm00001d007029	Zm00001d007029_T002	20.26	45.54	coding	592	645	2420
PB.3450.2	2	2799	11	full-splice_match	gene: Zm00001d007029	Zm00001d007029_T002	19.77	45.54	coding	592	511	2286
PB.4265.1	3	2342	6	novel_not_in_catalog	gene: Zm00001d042569	novel	51.77	53.18	coding	666	121	2118
PB.7317.1	6	1209	3	full-splice_match	gene: Zm00001d035542	Zm00001d035542_T002	26.35	26.35	coding	175	374	898
PB.6019.1	5	884	2	full-splice_match	gene: Zm00001d013210	Zm00001d013210_T001	36.16	36.16	coding	94	262	543

PB.1593.1	1	1143	5	novel_not_in_catalog	gene: Zm00001d034790	novel	779.78	1199.41	coding	265	96	890
PB.10590.1	9	1552	3	full-splice_match	gene: Zm00001d047732	Zm00001d047732_T001	20.48	20.48	coding	388	110	1273
PB.5881.1	4	1517	12	full-splice_match	gene: Zm00001d054017	Zm00001d054017_T004	90.94	160.46	coding	346	232	1269
PB.6834.1	5	894	4	incomplete-splice_match	gene: Zm00001d017175	Zm00001d017175_T002	43.94	84.08	coding	85	234	488
PB.9674.1	8	2836	1	full-splice_match	gene: Zm00001d011239	Zm00001d011239_T001	8.47	8.47	coding	867	74	2674
PB.6834.3	5	1689	10	full-splice_match	gene: Zm00001d017175	Zm00001d017175_T002	2.68	84.08	coding	467	104	1504
PB.1529.1	1	627	1	genic	gene: Zm00001d034586	novel	16.52	39.57	non_coding	NA	NA	NA
PB.31.1	1	1888	7	full-splice_match	gene: Zm00001d027344	Zm00001d027344_T006	80.79	80.79	coding	248	83	826
PB.3440.3	2	1327	11	full-splice_match	gene: Zm00001d006944	Zm00001d006944_T003	39.93	39.93	coding	365	2	1096
PB.2829.6	2	3071	14	full-splice_match	gene: Zm00001d003601	Zm00001d003601_T001	0.00	59.22	coding	661	588	2570
PB.10231.2	9	1097	3	intergenic	novelGene_89	novel	12.64	16.10	coding	64	169	360
PB.2829.4	2	3208	13	novel_in_catalog	gene: Zm00001d003601	novel	1.89	59.22	coding	411	545	1777
PB.7849.10	6	997	1	incomplete-splice_match	gene: Zm00001d038146	Zm00001d038146_T009	2.67	116.48	non_coding	NA	NA	NA
PB.2829.2	2	2589	11	novel_in_catalog	gene: Zm00001d003601	novel	5.67	59.22	coding	293	937	1815
PB.2829.1	2	3138	14	full-splice_match	gene: Zm00001d003601	Zm00001d003601_T001	51.66	59.22	coding	661	596	2578
PB.2133.1	10	535	3	full-splice_match	gene: Zm00001d025772	Zm00001d025772_T001	349.05	897.44	non_coding	NA	NA	NA
PB.1412.1	1	2336	9	full-splice_match	gene: Zm00001d034085	Zm00001d034085_T003	8.78	8.78	coding	544	441	2072

PB.3600.1	2	1145	3	full-splice_match	gene: Zm00001d007832	Zm00001d007832_T001	78.13	133.93	coding	191	110	682
PB.2809.1	2	954	5	full-splice_match	gene: Zm00001d003493	Zm00001d003493_T002	38.88	190.65	coding	136	319	726
PB.2809.2	2	689	5	full-splice_match	gene: Zm00001d003493	Zm00001d003493_T002	151.77	190.65	coding	136	124	531
PB.10377.8	9	2293	5	novel_in_catalog	gene: Zm00001d046661	novel	15.41	391.13	coding	333	976	1974
PB.10377.9	9	1903	5	novel_in_catalog	gene: Zm00001d046661	novel	25.55	391.13	coding	200	110	709
PB.10524.1	9	561	1	full-splice_match	gene: Zm00001d047478	Zm00001d047478_T001	12.86	12.86	non_coding	NA	NA	NA
PB.7898.1	6	602	4	incomplete-splice_match	gene: Zm00001d038306	Zm00001d038306_T001	72.33	72.33	coding	101	95	397
PB.5910.7	5	2130	13	full-splice_match	gene: Zm00001d012817	Zm00001d012817_T005	1.28	62.97	coding	132	1544	1939
PB.10377.6	9	1934	5	novel_in_catalog	gene: Zm00001d046661	novel	61.23	391.13	coding	416	92	1339
PB.5910.5	5	2715	12	full-splice_match	gene: Zm00001d012817	Zm00001d012817_T003	5.66	62.97	coding	445	1178	2512
PB.5910.2	5	2197	13	full-splice_match	gene: Zm00001d012817	Zm00001d012817_T002	31.77	62.97	coding	496	496	1983
PB.10377.1	9	2408	5	novel_in_catalog	gene: Zm00001d046661	novel	1.73	391.13	coding	459	714	2090
PB.5910.1	5	2244	13	full-splice_match	gene: Zm00001d012817	Zm00001d012817_T002	24.26	62.97	coding	496	424	1911
PB.8691.3	7	3164	4	full-splice_match	gene: Zm00001d021400	Zm00001d021400_T001	2.00	38.07	coding	687	630	2690
PB.5839.4	4	1176	1	full-splice_match	gene: Zm00001d053908	Zm00001d053908_T001	21.98	715.10	coding	249	90	836
PB.257.8	1	1863	4	novel_in_catalog	gene: Zm00001d028366	novel	0.00	133.55	coding	230	172	861
PB.2778.1	2	694	4	full-splice_match	gene: Zm00001d003310	Zm00001d003310_T002	134.42	134.42	coding	122	66	431

PB.257.4	1	3343	4	novel_in_catalog	gene: Zm00001d028366	novel	2.13	133.55	coding	291	172	1044
PB.257.5	1	1795	4	novel_in_catalog	gene: Zm00001d028366	novel	1.62	133.55	coding	380	170	1309
PB.257.6	1	1869	5	full-splice_match	gene: Zm00001d028366	Zm00001d028366_T001	0.00	133.55	coding	455	170	1534
PB.3375.6	2	1379	2	full-splice_match	gene: Zm00001d006677	Zm00001d006677_T001	0.00	69.89	coding	324	152	1123
PB.3375.1	2	1416	2	full-splice_match	gene: Zm00001d006677	Zm00001d006677_T001	67.18	69.89	coding	324	153	1124
PB.257.1	1	3459	2	novel_in_catalog	gene: Zm00001d028366	novel	6.83	133.55	coding	117	444	794
PB.257.2	1	2061	5	full-splice_match	gene: Zm00001d028366	Zm00001d028366_T001	98.01	133.55	coding	455	164	1528
PB.257.3	1	2087	4	novel_in_catalog	gene: Zm00001d028366	novel	21.50	133.55	coding	230	162	851
PB.126.3	1	1047	1	full-splice_match	gene: Zm00001d027742	Zm00001d027742_T001	17.85	181.51	coding	162	404	889
PB.126.2	1	935	1	full-splice_match	gene: Zm00001d027742	Zm00001d027742_T001	159.08	181.51	coding	162	148	633
PB.126.1	1	1282	1	full-splice_match	gene: Zm00001d027742	Zm00001d027742_T001	4.59	181.51	coding	162	151	636
PB.7917.4	6	1021	9	full-splice_match	gene: Zm00001d038380	Zm00001d038380_T001	0.00	294.87	coding	215	190	834
PB.9654.1	8	1804	7	full-splice_match	gene: Zm00001d011152	Zm00001d011152_T042	12.09	25.02	coding	104	806	1117
PB.4312.1	3	1508	2	genic	gene: Zm00001d042767	novel	52.30	113.55	coding	403	98	1306
PB.9654.3	8	2230	11	novel_in_catalog	gene: Zm00001d011152	novel	5.19	25.02	coding	104	1151	1462
PB.9654.2	8	2321	9	novel_in_catalog	gene: Zm00001d011152	novel	7.74	25.02	coding	104	1053	1364
PB.6487.2	5	4484	4	incomplete-splice_match	gene: Zm00001d015138	Zm00001d015138_T002	6.06	109.75	coding	1453	126	4484

PB.7373.2	6	2512	15	full-splice_match	gene: Zm00001d035878	Zm00001d035878_T002	9.31	9.31	coding	730	91	2280
PB.6487.1	5	4015	6	full-splice_match	gene: Zm00001d015138	Zm00001d015138_T002	103.68	109.75	coding	1203	103	3711
PB.1307.2	1	2392	6	novel_in_catalog	gene: Zm00001d033642	novel	13.85	65.09	coding	634	246	2147
PB.2064.2	10	816	1	novel_in_catalog	gene: Zm00001d025401	novel	54.77	439.87	coding	181	76	618
PB.7544.1	6	2182	10	full-splice_match	gene: Zm00001d036827	Zm00001d036827_T001	20.74	86.18	coding	582	270	2015
PB.2113.1	10	1243	1	full-splice_match	gene: Zm00001d025699	Zm00001d025699_T001	12.52	12.52	coding	109	821	1147
PB.8701.1	7	3579	5	full-splice_match	gene: Zm00001d021442	Zm00001d021442_T004	71.38	76.11	coding	851	244	2796
PB.10135.2	9	604	1	full-splice_match	gene: Zm00001d045296	Zm00001d045296_T001	56.44	108.95	coding	117	63	413
PB.7709.10	6	2059	5	full-splice_match	gene: Zm00001d037625	Zm00001d037625_T002	0.00	62.70	coding	310	960	1889
PB.7544.4	6	1726	3	novel_in_catalog	gene: Zm00001d036827	novel	65.44	86.18	coding	499	64	1560
PB.9737.1	8	1120	8	full-splice_match	gene: Zm00001d011565	Zm00001d011565_T001	25.62	970.93	coding	153	149	607
PB.993.2	1	1203	6	full-splice_match	gene: Zm00001d032231	Zm00001d032231_T002	9.59	432.32	coding	256	217	984
PB.2518.1	2	1227	1	full-splice_match	gene: Zm00001d002286	Zm00001d002286_T001	8.20	8.20	coding	344	30	1061
PB.2237.1	10	2054	5	full-splice_match	gene: Zm00001d026258	Zm00001d026258_T002	32.01	32.01	coding	482	344	1789
PB.5247.1	4	1166	3	full-splice_match	gene: Zm00001d051225	Zm00001d051225_T001	30.56	47.12	coding	239	135	851
PB.8701.8	7	3498	5	full-splice_match	gene: Zm00001d021442	Zm00001d021442_T004	0.00	76.11	coding	851	233	2785
PB.9737.7	8	2619	5	novel_in_catalog	gene: Zm00001d011565	novel	3.99	970.93	coding	109	1082	1408

PB.5247.2	4	1031	3	full-splice_match	gene: Zm00001d051225	Zm00001d051225_T001	16.56	47.12	coding	239	136	852
PB.5356.2	4	566	5	incomplete-splice_match	gene: Zm00001d051830	Zm00001d051830_T002	17.37	34.04	coding	101	137	439
PB.5356.1	4	1492	9	novel_in_catalog	gene: Zm00001d051830	novel	16.67	34.04	coding	197	75	665
PB.8733.8	7	2170	15	novel_not_in_catalog	gene: Zm00001d021567	novel	0.00	64.74	coding	569	294	2000
PB.8733.7	7	2320	15	novel_not_in_catalog	gene: Zm00001d021567	novel	0.00	64.74	coding	569	326	2032
PB.8733.5	7	2432	15	novel_not_in_catalog	gene: Zm00001d021567	novel	0.00	64.74	coding	569	326	2032
PB.8733.3	7	3113	13	novel_not_in_catalog	gene: Zm00001d021567	novel	6.33	64.74	coding	309	322	1248
PB.8733.1	7	2513	15	novel_not_in_catalog	gene: Zm00001d021567	novel	58.42	64.74	coding	569	353	2059
PB.3194.1	2	1849	14	full-splice_match	gene: Zm00001d005818	Zm00001d005818_T003	92.32	105.84	coding	509	58	1584
PB.3194.4	2	869	1	incomplete-splice_match	gene: Zm00001d005818	Zm00001d005818_T004	10.06	105.84	non_coding	NA	NA	NA
PB.3194.5	2	1457	7	incomplete-splice_match	gene: Zm00001d005818	Zm00001d005818_T002	3.46	105.84	coding	188	58	621
PB.1235.1	1	2287	11	full-splice_match	gene: Zm00001d033321	Zm00001d033321_T002	27.82	33.48	coding	568	248	1951
PB.8824.1	7	1918	7	genic	gene: Zm00001d021845	novel	0.00	43.34	coding	151	135	587
PB.6803.2	5	2997	7	full-splice_match	gene: Zm00001d017072	Zm00001d017072_T006	189.11	189.11	coding	809	195	2621
PB.5615.1	4	1094	2	full-splice_match	gene: Zm00001d052842	Zm00001d052842_T001	71.86	77.62	coding	246	84	821
PB.5615.2	4	993	2	full-splice_match	gene: Zm00001d052842	Zm00001d052842_T001	5.76	77.62	coding	246	131	868
PB.1671.1	10	1804	3	full-splice_match	gene: Zm00001d023294	Zm00001d023294_T001	12.06	12.06	coding	475	156	1580

PB.9378.1	8	1092	6	full-splice_match	gene: Zm00001d009824	Zm00001d009824_T004	13.80	13.80	coding	187	228	788
PB.9454.1	8	656	6	incomplete-splice_match	gene: Zm00001d010232	Zm00001d010232_T001	2.09	2.09	coding	112	112	447
PB.1085.2	1	1487	2	genic	gene: Zm00001d032571	novel	64.70	64.70	coding	390	143	1312
PB.1651.1	10	3208	14	incomplete-splice_match	gene: Zm00001d023230	Zm00001d023230_T016	28.87	28.87	coding	857	100	2670
PB.233.4	1	2639	20	full-splice_match	gene: Zm00001d028261	Zm00001d028261_T012	0.00	47.08	coding	711	310	2442
PB.233.2	1	2918	19	novel_in_catalog	gene: Zm00001d028261	novel	2.30	47.08	coding	340	336	1355
PB.233.1	1	2808	20	full-splice_match	gene: Zm00001d028261	Zm00001d028261_T012	44.78	47.08	coding	711	340	2472
PB.3369.1	2	1378	1	full-splice_match	gene: Zm00001d006623	Zm00001d006623_T001	132.12	132.12	coding	331	53	1045
PB.938.2	1	1874	7	novel_in_catalog	gene: Zm00001d031967	novel	1.61	94.25	coding	263	129	917
PB.7907.5	6	2550	9	novel_in_catalog	gene: Zm00001d038343	novel	1.88	97.25	coding	281	1415	2257
PB.7907.6	6	1864	11	novel_in_catalog	gene: Zm00001d038343	novel	12.37	97.25	non_coding	NA	NA	NA
PB.938.1	1	1362	8	full-splice_match	gene: Zm00001d031967	Zm00001d031967_T002	40.94	94.25	coding	304	135	1046
PB.2427.1	2	1249	5	full-splice_match	gene: Zm00001d001900	Zm00001d001900_T002	40.03	40.03	coding	280	238	1077
PB.9507.1	8	1688	8	full-splice_match	gene: Zm00001d010495	Zm00001d010495_T001	10.88	10.88	coding	362	262	1347
PB.5609.2	4	1548	6	full-splice_match	gene: Zm00001d052810	Zm00001d052810_T002	23.11	28.60	coding	398	196	1389
PB.5609.1	4	1704	6	full-splice_match	gene: Zm00001d052810	Zm00001d052810_T002	5.49	28.60	coding	398	177	1370
PB.7161.1	5	2222	5	full-splice_match	gene: Zm00001d018441	Zm00001d018441_T003	11.11	11.11	coding	632	61	1956

PB.7532.7	6	3007	12	novel_in_catalog	gene: Zm00001d036742	novel	2.42	19.37	coding	428	200	1483
PB.5223.3	4	1928	2	genic	gene: Zm00001d051044	novel	45.87	81.69	coding	451	321	1673
PB.7532.1	6	2798	13	full-splice_match	gene: Zm00001d036742	Zm00001d036742_T005	16.95	19.37	coding	638	74	1987
PB.2012.2	10	2526	5	full-splice_match	gene: Zm00001d025103	Zm00001d025103_T001	0.00	23.68	coding	744	50	2281
PB.2012.3	10	2375	5	full-splice_match	gene: Zm00001d025103	Zm00001d025103_T001	0.00	23.68	coding	744	45	2276
PB.8118.2	6	743	2	full-splice_match	gene: Zm00001d039158	Zm00001d039158_T001	65.28	156.37	coding	154	45	506
PB.3438.2	2	1320	2	full-splice_match	gene: Zm00001d006933	Zm00001d006933_T001	199.48	208.62	coding	362	48	1133
PB.3438.1	2	1423	2	full-splice_match	gene: Zm00001d006933	Zm00001d006933_T001	9.15	208.62	coding	362	47	1132
PB.1131.4	1	2124	18	full-splice_match	gene: Zm00001d032763	Zm00001d032763_T008	0.00	69.78	coding	526	228	1805
PB.5237.27	4	1700	2	novel_in_catalog	gene: Zm00001d051161	novel	2.44	110.85	coding	520	123	1682
PB.2178.1	10	2880	9	full-splice_match	gene: Zm00001d025963	Zm00001d025963_T001	37.83	37.83	coding	751	377	2629
PB.4890.2	4	1774	9	full-splice_match	gene: Zm00001d049092	Zm00001d049092_T002	41.08	41.08	coding	375	143	1267
PB.6611.2	5	1769	1	intergenic	novelGene_226	novel	0.00	8.00	coding	110	227	556
PB.5237.23	4	2559	2	novel_in_catalog	gene: Zm00001d051161	novel	0.00	110.85	coding	520	123	1682
PB.5817.1	4	1175	9	full-splice_match	gene: Zm00001d053822	Zm00001d053822_T002	109.97	125.34	coding	208	137	760
PB.7126.1	5	1808	4	full-splice_match	gene: Zm00001d018360	Zm00001d018360_T001	19.93	19.93	coding	499	125	1621
PB.4395.3	3	778	3	full-splice_match	gene: Zm00001d043144	Zm00001d043144_T001	0.00	203.70	coding	183	97	645

PB.5521.2	4	378	3	novel_not_in_catalog	gene: Zm00001d052436	novel	686.40	920.63	non_coding	NA	NA	NA
PB.6344.1	5	1679	1	antisense	novelGene_gene: Zm00001d014623_A	novel	36.02	36.02	non_coding	NA	NA	NA
PB.9780.3	8	695	5	full-splice_match	gene: Zm00001d011741	Zm00001d011741_T001	772.53	1159.02	coding	137	100	510
PB.4395.1	3	1057	3	full-splice_match	gene: Zm00001d043144	Zm00001d043144_T001	55.41	203.70	coding	183	97	645
PB.6840.2	5	2866	15	full-splice_match	gene: Zm00001d017197	Zm00001d017197_T006	0.32	59.93	coding	762	294	2579
PB.1641.1	1	1386	7	fusion	gene: Zm00001d034957_g	novel	10.66	57.51	coding	120	485	844
PB.8131.1	6	555	1	incomplete-splice_match	gene: Zm00001d039183	Zm00001d039183_T001	47.63	47.63	non_coding	NA	NA	NA
PB.1235.4	1	2091	11	full-splice_match	gene: Zm00001d033321	Zm00001d033321_T002	0.00	33.48	coding	568	244	1947
PB.4208.3	3	1787	10	full-splice_match	gene: Zm00001d042303	Zm00001d042303_T001	6.13	66.16	coding	499	185	1681
PB.3658.2	3	2196	4	novel_in_catalog	gene: Zm00001d039316	novel	5.67	306.55	coding	144	58	489
PB.3658.1	3	1243	5	full-splice_match	gene: Zm00001d039316	Zm00001d039316_T004	300.88	306.55	coding	283	64	912
PB.4208.2	3	1889	9	novel_in_catalog	gene: Zm00001d042303	novel	4.48	66.16	coding	373	120	1238
PB.1379.1	1	2186	7	incomplete-splice_match	gene: Zm00001d033982	Zm00001d033982_T001	20.06	35.72	coding	529	39	1625
PB.6807.1	5	837	1	full-splice_match	gene: Zm00001d017090	Zm00001d017090_T001	70.91	70.91	coding	200	24	623
PB.4400.3	3	2063	13	novel_not_in_catalog	gene: Zm00001d043152	novel	40.52	93.99	coding	435	387	1691
PB.1202.1	1	2062	12	incomplete-splice_match	gene: Zm00001d033155	Zm00001d033155_T001	26.89	38.05	coding	423	27	1295
PB.2912.1	2	2182	4	full-splice_match	gene: Zm00001d004022	Zm00001d004022_T004	0.45	118.23	coding	232	872	1567

PB.6063.1	5	2967	11	novel_in_catalog	gene: Zm00001d013330	novel	25.04	25.04	coding	815	202	2646
PB.2912.3	2	1916	4	full-splice_match	gene: Zm00001d004022	Zm00001d004022_T004	81.37	118.23	coding	232	976	1671
PB.2912.2	2	2057	4	full-splice_match	gene: Zm00001d004022	Zm00001d004022_T004	36.41	118.23	coding	232	871	1566
PB.3089.3	2	2186	2	intergenic	novelGene_273	novel	5.49	15.99	coding	123	84	452
PB.10298.1	9	1259	1	full-splice_match	gene: Zm00001d046188	Zm00001d046188_T001	31.26	31.26	coding	263	114	902
PB.7647.1	6	1417	6	full-splice_match	gene: Zm00001d037336	Zm00001d037336_T001	54.11	60.46	coding	377	93	1223
PB.10516.1	9	908	6	full-splice_match	gene: Zm00001d047448	Zm00001d047448_T002	136.49	139.67	coding	170	117	626
PB.10516.2	9	1887	5	full-splice_match	gene: Zm00001d047448	Zm00001d047448_T003	3.17	139.67	non_coding	NA	NA	NA
PB.5251.1	4	1978	2	full-splice_match	gene: Zm00001d051235	Zm00001d051235_T001	10.80	10.80	coding	390	650	1819
PB.851.1	1	3198	5	full-splice_match	gene: Zm00001d031534	Zm00001d031534_T001	24.13	36.64	coding	837	296	2806
PB.851.2	1	2270	1	genic	gene: Zm00001d031534	novel	12.51	36.64	coding	462	145	1530
PB.851.3	1	1774	4	incomplete-splice_match	gene: Zm00001d031534	Zm00001d031534_T001	0.00	36.64	coding	555	109	1773
PB.2578.1	2	609	1	full-splice_match	gene: Zm00001d002543	Zm00001d002543_T001	768.97	768.97	coding	103	67	375
PB.574.4	1	2279	7	novel_in_catalog	gene: Zm00001d029782	novel	0.99	74.83	coding	489	99	1565
PB.4545.1	3	571	1	intergenic	novelGene_325	novel	210.66	379.86	non_coding	NA	NA	NA
PB.5831.2	4	3318	24	novel_in_catalog	gene: Zm00001d053881	novel	1.39	92.51	coding	740	162	2381
PB.574.3	1	3049	6	full-splice_match	gene: Zm00001d029782	Zm00001d029782_T010	4.71	74.83	coding	489	102	1568

PB.574.1	1	2211	8	full-splice_match	gene: Zm00001d029782	Zm00001d029782_T003	69.12	74.83	coding	578	154	1887
PB.6696.6	5	1994	3	novel_in_catalog	gene: Zm00001d016553	novel	10.53	140.34	coding	364	629	1720
PB.7280.2	6	2023	4	full-splice_match	gene: Zm00001d035323	Zm00001d035323_T001	20.63	20.63	coding	496	164	1651
PB.8632.1	7	2735	8	incomplete-splice_match	gene: Zm00001d021170	Zm00001d021170_T007	7.09	20.72	coding	350	1529	2578
PB.2293.1	10	487	1	intergenic	novelGene_234	novel	75.98	75.98	non_coding	NA	NA	NA
PB.6118.2	5	2533	3	novel_in_catalog	gene: Zm00001d013527	novel	3.25	74.65	coding	509	138	1664
PB.4097.3	3	1892	13	full-splice_match	gene: Zm00001d041769	Zm00001d041769_T001	16.68	41.54	coding	517	178	1728
PB.4097.2	3	2065	13	full-splice_match	gene: Zm00001d041769	Zm00001d041769_T001	0.64	41.54	coding	517	316	1866
PB.4097.1	3	1935	13	full-splice_match	gene: Zm00001d041769	Zm00001d041769_T001	19.25	41.54	coding	295	624	1508
PB.6696.4	5	2620	1	novel_in_catalog	gene: Zm00001d016553	novel	0.00	140.34	coding	202	1564	2169
PB.863.1	1	2388	9	full-splice_match	gene: Zm00001d031589	Zm00001d031589_T001	3.12	24.96	coding	283	133	981
PB.4097.4	3	2081	12	novel_in_catalog	gene: Zm00001d041769	novel	4.96	41.54	coding	480	129	1568
PB.2445.5	2	1781	3	novel_in_catalog	gene: Zm00001d001979	novel	120.25	123.22	coding	349	522	1568
PB.2445.6	2	1444	3	novel_in_catalog	gene: Zm00001d001979	novel	0.00	123.22	coding	349	301	1347
PB.6696.5	5	2314	3	novel_in_catalog	gene: Zm00001d016553	novel	1.44	140.34	coding	202	1259	1864
PB.2445.1	2	2414	2	novel_in_catalog	gene: Zm00001d001979	novel	2.98	123.22	coding	349	1141	2187
PB.2197.2	10	2228	1	full-splice_match	gene: Zm00001d026040	Zm00001d026040_T001	1.21	27.22	coding	407	158	1378

PB.2197.3	10	1432	1	full-splice_match	gene: Zm00001d026040	Zm00001d026040_T001	25.62	27.22	coding	407	157	1377
PB.6696.2	5	2570	2	novel_in_catalog	gene: Zm00001d016553	novel	1.55	140.34	coding	202	1498	2103
PB.1202.3	1	2089	13	incomplete-splice_match	gene: Zm00001d033155	Zm00001d033155_T001	4.54	38.05	coding	470	44	1453
PB.1945.1	10	2714	4	novel_not_in_catalog	gene: Zm00001d024824	novel	18.34	18.34	coding	598	554	2347
PB.6948.3	5	2311	3	incomplete-splice_match	gene: Zm00001d017685	Zm00001d017685_T002	0.00	17.88	non_coding	NA	NA	NA
PB.8808.2	7	1809	15	novel_in_catalog	gene: Zm00001d021810	novel	0.00	69.29	coding	140	322	741
PB.8808.1	7	2100	16	full-splice_match	gene: Zm00001d021810	Zm00001d021810_T005	12.71	69.29	coding	140	662	1081
PB.7234.1	6	3563	17	fusion	gene: Zm00001d035088_g	novel	14.55	14.55	coding	1029	44	3130
PB.1945.2	10	2292	4	novel_not_in_catalog	gene: Zm00001d024824	novel	0.00	18.34	coding	588	529	2292
PB.27.1	1	3203	3	full-splice_match	gene: Zm00001d027335	Zm00001d027335_T001	10.55	10.55	coding	809	235	2661
PB.6696.1	5	2104	4	full-splice_match	gene: Zm00001d016553	Zm00001d016553_T003	99.30	140.34	coding	364	532	1623
PB.3341.2	2	2994	6	full-splice_match	gene: Zm00001d006504	Zm00001d006504_T007	0.00	9.96	coding	920	36	2795
PB.7067.2	5	1560	8	full-splice_match	gene: Zm00001d018144	Zm00001d018144_T004	0.00	19.71	coding	460	8	1387
PB.7067.1	5	1723	8	full-splice_match	gene: Zm00001d018144	Zm00001d018144_T004	19.71	19.71	coding	460	49	1428
PB.1586.3	1	1613	5	full-splice_match	gene: Zm00001d034761	Zm00001d034761_T001	0.00	254.48	coding	377	212	1342
PB.6918.2	5	351	2	incomplete-splice_match	gene: Zm00001d017526	Zm00001d017526_T003	617.39	1736.15	non_coding	NA	NA	NA
PB.7834.3	6	2569	4	full-splice_match	gene: Zm00001d038105	Zm00001d038105_T002	13.99	60.46	coding	555	278	1942

PB.3771.1	3	2691	2	full-splice_match	gene: Zm00001d039822	Zm00001d039822_T001	3.86	3.86	coding	786	150	2507
PB.5875.1	4	1924	4	full-splice_match	gene: Zm00001d054002	Zm00001d054002_T001	18.74	18.74	coding	565	30	1724
PB.9120.1	8	810	1	full-splice_match	gene: Zm00001d008449	Zm00001d008449_T001	4.10	4.10	coding	184	60	611
PB.6883.1	5	1016	8	full-splice_match	gene: Zm00001d017390	Zm00001d017390_T001	18.09	18.09	coding	225	166	840
PB.4767.1	3	2046	7	incomplete-splice_match	gene: Zm00001d044614	Zm00001d044614_T190	115.43	115.43	coding	538	21	1634
PB.2111.3	10	1543	4	full-splice_match	gene: Zm00001d025690	Zm00001d025690_T003	23.43	32.52	coding	377	122	1252
PB.958.2	1	2828	5	novel_in_catalog	gene: Zm00001d032060	novel	6.78	11.82	coding	236	375	1082
PB.7471.1	6	1606	1	full-splice_match	gene: Zm00001d036450	Zm00001d036450_T001	0.70	0.70	coding	460	62	1441
PB.5204.4	4	2491	4	full-splice_match	gene: Zm00001d050943	Zm00001d050943_T002	0.00	19.42	coding	608	337	2160
PB.5746.4	4	2410	7	full-splice_match	gene: Zm00001d053564	Zm00001d053564_T002	21.30	79.10	coding	692	147	2222
PB.9592.4	8	2027	3	novel_not_in_catalog	gene: Zm00001d010851	novel	3.11	15.56	coding	264	219	1010
PB.6489.3	5	2116	6	novel_in_catalog	gene: Zm00001d015148	novel	21.39	21.39	coding	445	573	1907
PB.7919.1	6	863	1	full-splice_match	gene: Zm00001d038382	Zm00001d038382_T001	433.76	433.76	coding	188	40	603
PB.2491.1	2	955	1	intergenic	novelGene_31	novel	20.23	817.66	coding	88	50	313
PB.10411.1	9	1309	3	full-splice_match	gene: Zm00001d046898	Zm00001d046898_T001	24.14	24.14	coding	380	98	1237
PB.7919.2	6	757	1	full-splice_match	gene: Zm00001d038382	Zm00001d038382_T001	0.00	433.76	coding	188	40	603
PB.778.8	1	2335	13	novel_in_catalog	gene: Zm00001d031120	novel	1.62	204.87	coding	313	84	1022

PB.10282.1	9	2838	13	incomplete-splice_match	gene: Zm00001d046039	Zm00001d046039_T016	19.85	19.85	coding	858	56	2629
PB.7026.2	5	2097	13	full-splice_match	gene: Zm00001d017988	Zm00001d017988_T001	9.12	11.21	coding	588	129	1892
PB.3743.1	3	1375	5	full-splice_match	gene: Zm00001d039614	Zm00001d039614_T001	62.70	62.70	coding	343	53	1081
PB.241.1	1	1731	3	full-splice_match	gene: Zm00001d028304	Zm00001d028304_T001	13.10	68.83	coding	292	193	1068
PB.847.3	1	2336	4	full-splice_match	gene: Zm00001d031505	Zm00001d031505_T002	2.28	86.51	coding	321	1069	2031
PB.241.3	1	1839	1	genic	gene: Zm00001d028304	novel	5.44	68.83	non_coding	NA	NA	NA
PB.241.2	1	1558	3	full-splice_match	gene: Zm00001d028304	Zm00001d028304_T001	9.99	68.83	coding	292	193	1068
PB.241.5	1	1201	3	full-splice_match	gene: Zm00001d028304	Zm00001d028304_T001	40.30	68.83	coding	292	196	1071
PB.6298.1	5	980	1	incomplete-splice_match	gene: Zm00001d014414	Zm00001d014414_T001	15.23	15.23	coding	211	82	714
PB.1069.1	1	623	1	full-splice_match	gene: Zm00001d032533	Zm00001d032533_T001	48.08	83.17	coding	85	57	311
PB.4614.1	3	1357	10	full-splice_match	gene: Zm00001d043975	Zm00001d043975_T001	33.18	33.18	coding	239	457	1173
PB.10651.1	9	1186	6	full-splice_match	gene: Zm00001d047912	Zm00001d047912_T002	76.72	76.72	coding	295	98	982
PB.4612.1	3	1678	3	full-splice_match	gene: Zm00001d043965	Zm00001d043965_T001	2.15	2.15	coding	426	100	1377
PB.10651.3	9	452	3	incomplete-splice_match	gene: Zm00001d047912	Zm00001d047912_T002	0.00	76.72	coding	118	97	450
PB.7465.1	6	1670	1	full-splice_match	gene: Zm00001d036441	Zm00001d036441_T001	4.36	552.52	coding	108	87	410
PB.2105.3	10	1744	7	full-splice_match	gene: Zm00001d025665	Zm00001d025665_T001	13.08	36.43	coding	468	85	1488
PB.7465.2	6	783	1	full-splice_match	gene: Zm00001d036441	Zm00001d036441_T001	548.16	552.52	coding	108	82	405

PB.4044.2	3	1696	1	full-splice_match	gene: Zm00001d041518	Zm00001d041518_T001	6.59	18.85	coding	341	473	1495
PB.2105.7	10	2242	6	novel_in_catalog	gene: Zm00001d025665	novel	0.00	36.43	coding	171	1055	1567
PB.2105.6	10	2096	7	full-splice_match	gene: Zm00001d025665	Zm00001d025665_T003	13.15	36.43	coding	302	999	1904
PB.1384.2	1	1213	1	novel_in_catalog	gene: Zm00001d033989	novel	18.49	47.15	coding	299	121	1017
PB.9592.3	8	2522	2	novel_in_catalog	gene: Zm00001d010851	novel	1.20	15.56	coding	3	538	546
PB.2259.5	10	1306	8	novel_in_catalog	gene: Zm00001d026319	novel	19.68	19.68	non_coding	NA	NA	NA
PB.2259.4	10	484	2	intergenic	novelGene_230	novel	184.27	184.27	non_coding	NA	NA	NA
PB.847.2	1	1485	5	full-splice_match	gene: Zm00001d031505	Zm00001d031505_T001	10.33	86.51	coding	321	221	1183
PB.4521.1	3	720	1	full-splice_match	gene: Zm00001d043617	Zm00001d043617_T001	2.76	2.76	coding	165	93	587
PB.3476.1	2	1423	10	full-splice_match	gene: Zm00001d007146	Zm00001d007146_T001	19.32	19.32	coding	306	155	1072
PB.5688.3	4	1275	9	novel_not_in_catalog	gene: Zm00001d053223	novel	143.15	189.89	coding	289	106	972
PB.5688.1	4	1412	9	novel_not_in_catalog	gene: Zm00001d053223	novel	46.74	189.89	coding	289	94	960
PB.3638.1	3	1618	3	full-splice_match	gene: Zm00001d039259	Zm00001d039259_T001	28.20	28.20	coding	336	223	1230
PB.6948.6	5	2273	7	full-splice_match	gene: Zm00001d017685	Zm00001d017685_T002	4.65	17.88	coding	121	182	544
PB.5893.1	4	1182	1	incomplete-splice_match	gene: Zm00001d054076	Zm00001d054076_T002	9.23	36.67	coding	133	6	404
PB.374.1	1	2207	1	full-splice_match	gene: Zm00001d028887	Zm00001d028887_T001	263.43	263.43	coding	601	258	2060
PB.8246.3	7	1306	3	full-splice_match	gene: Zm00001d019041	Zm00001d019041_T001	85.16	98.90	coding	293	105	983

PB.9156.1	8	1656	2	incomplete-splice_match	gene: Zm00001d008621	Zm00001d008621_T001	7.85	7.85	coding	265	640	1434
PB.8246.1	7	942	1	genic	gene: Zm00001d019041	novel	13.74	98.90	non_coding	NA	NA	NA
PB.6007.1	5	1720	1	full-splice_match	gene: Zm00001d013163	Zm00001d013163_T001	22.48	22.48	coding	437	135	1445
PB.9575.1	8	2150	2	novel_in_catalog	gene: Zm00001d010791	novel	0.55	0.55	non_coding	NA	NA	NA
PB.5045.1	4	881	7	full-splice_match	gene: Zm00001d049815	Zm00001d049815_T002	361.47	361.47	coding	171	130	642
PB.6290.1	5	2609	2	novel_in_catalog	gene: Zm00001d014378	novel	5.74	19.87	coding	255	1564	2328
PB.6290.2	5	2138	7	full-splice_match	gene: Zm00001d014378	Zm00001d014378_T001	14.13	19.87	coding	640	61	1980
PB.5513.2	4	2338	8	full-splice_match	gene: Zm00001d052407	Zm00001d052407_T002	0.00	21.96	coding	577	316	2046
PB.1153.1	1	2041	13	full-splice_match	gene: Zm00001d032875	Zm00001d032875_T009	15.79	56.66	coding	537	67	1677
PB.9509.1	8	1335	1	full-splice_match	gene: Zm00001d010507	Zm00001d010507_T001	22.38	22.38	coding	316	200	1147
PB.8633.1	7	2686	4	full-splice_match	gene: Zm00001d021173	Zm00001d021173_T001	5.27	5.27	coding	762	89	2374
PB.1153.2	1	2270	14	full-splice_match	gene: Zm00001d032875	Zm00001d032875_T016	11.57	56.66	coding	314	1013	1954
PB.1153.5	1	1936	13	full-splice_match	gene: Zm00001d032875	Zm00001d032875_T009	22.03	56.66	coding	537	82	1692
PB.394.1	1	3055	6	full-splice_match	gene: Zm00001d028960	Zm00001d028960_T004	15.00	15.00	coding	843	330	2858
PB.1396.2	1	1648	6	full-splice_match	gene: Zm00001d034028	Zm00001d034028_T001	26.88	26.88	coding	247	157	897
PB.5144.1	4	2073	1	antisense	novelGene_gene: Zm00001d050433_A	novel	1.25	1.25	coding	154	280	741
PB.1541.1	1	1246	4	full-splice_match	gene: Zm00001d034618	Zm00001d034618_T002	77.55	123.85	coding	318	61	1014

PB.1020.8	1	2823	6	full-splice_match	gene: Zm00001d032350	Zm00001d032350_T001	9.17	135.32	coding	114	248	589
PB.9985.2	8	2527	2	novel_in_catalog	gene: Zm00001d012607	novel	2.77	406.73	coding	152	1629	2084
PB.6902.2	5	1486	9	novel_in_catalog	gene: Zm00001d017470	novel	1.63	33.49	coding	257	89	859
PB.6902.1	5	1410	10	full-splice_match	gene: Zm00001d017470	Zm00001d017470_T002	31.87	33.49	coding	345	86	1120
PB.4064.1	3	1440	1	full-splice_match	gene: Zm00001d041604	Zm00001d041604_T001	51.01	501.67	coding	152	639	1094
PB.8462.3	7	548	1	incomplete-splice_match	gene: Zm00001d020371	Zm00001d020371_T002	0.00	15.20	coding	110	39	368
PB.4064.2	3	1333	1	full-splice_match	gene: Zm00001d041604	Zm00001d041604_T001	450.66	501.67	coding	152	638	1093
PB.7663.1	6	2292	10	full-splice_match	gene: Zm00001d037413	Zm00001d037413_T003	18.31	23.74	coding	584	242	1993
PB.4565.3	3	1965	15	full-splice_match	gene: Zm00001d043773	Zm00001d043773_T004	0.00	17.82	coding	560	152	1831
PB.7663.3	6	1472	7	incomplete-splice_match	gene: Zm00001d037413	Zm00001d037413_T004	5.42	23.74	coding	445	42	1376
PB.2968.1	2	2886	14	full-splice_match	gene: Zm00001d004541	Zm00001d004541_T009	16.42	16.42	coding	853	54	2612
PB.8929.1	7	1844	11	full-splice_match	gene: Zm00001d022239	Zm00001d022239_T001	41.31	122.71	coding	440	87	1406
PB.6601.2	5	1948	15	full-splice_match	gene: Zm00001d015948	Zm00001d015948_T010	23.37	23.37	coding	559	26	1702
PB.8929.3	7	2514	10	novel_in_catalog	gene: Zm00001d022239	novel	0.00	122.71	coding	344	1177	2208
PB.5239.1	4	1273	1	full-splice_match	gene: Zm00001d051166	Zm00001d051166_T001	18.74	18.74	coding	396	18	1205
PB.8929.5	7	2345	10	novel_in_catalog	gene: Zm00001d022239	novel	1.54	122.71	coding	307	85	1005
PB.8929.4	7	1817	11	full-splice_match	gene: Zm00001d022239	Zm00001d022239_T001	65.91	122.71	coding	440	197	1516

PB.8929.7	7	2275	10	novel_in_catalog	gene: Zm00001d022239	novel	4.52	122.71	coding	344	1068	2099
PB.10767.1	9	1029	9	incomplete-splice_match	gene: Zm00001d048349	Zm00001d048349_T004	20.55	20.55	coding	10	735	764
PB.6123.1	5	1474	2	antisense	novelGene_gene: Zm00001d013555_A	novel	5.15	5.15	non_coding	NA	NA	NA
PB.3325.3	2	1384	5	full-splice_match	gene: Zm00001d006440	Zm00001d006440_T002	116.04	133.58	coding	275	198	1022
PB.8403.3	7	1380	2	novel_in_catalog	gene: Zm00001d020052	novel	60.93	63.09	coding	334	127	1128
PB.8403.5	7	2114	1	genic	gene: Zm00001d020052	novel	2.16	63.09	coding	334	945	1946
PB.590.1	1	1027	4	full-splice_match	gene: Zm00001d029887	Zm00001d029887_T002	44.20	44.20	coding	187	325	885
PB.4746.2	3	1032	3	full-splice_match	gene: Zm00001d044527	Zm00001d044527_T001	33.04	103.91	coding	240	104	823
PB.5562.2	4	1194	3	full-splice_match	gene: Zm00001d052651	Zm00001d052651_T001	39.15	92.26	coding	297	74	964
PB.449.3	1	2019	7	full-splice_match	gene: Zm00001d029191	Zm00001d029191_T002	0.00	7.99	coding	494	79	1560
PB.449.2	1	2161	6	novel_in_catalog	gene: Zm00001d029191	novel	0.66	7.99	coding	459	128	1504
PB.449.1	1	2198	7	full-splice_match	gene: Zm00001d029191	Zm00001d029191_T002	7.33	7.99	coding	494	112	1593
PB.8082.1	6	540	3	full-splice_match	gene: Zm00001d039001	Zm00001d039001_T002	65.76	65.76	coding	96	64	351
PB.4535.1	3	1346	1	full-splice_match	gene: Zm00001d043674	Zm00001d043674_T001	18.91	18.91	coding	316	152	1099
PB.8427.2	7	578	4	full-splice_match	gene: Zm00001d020201	Zm00001d020201_T001	954.68	1100.02	coding	112	44	379
PB.8427.3	7	1832	3	full-splice_match	gene: Zm00001d020201	Zm00001d020201_T002	4.18	1100.02	coding	112	44	379
PB.8427.1	7	712	4	full-splice_match	gene: Zm00001d020201	Zm00001d020201_T001	141.16	1100.02	coding	112	60	395

PB.8810.7	7	3100	19	full-splice_match	gene: Zm00001d021810	Zm00001d021810_T023	0.00	69.29	coding	773	460	2778
PB.2671.1	2	1590	1	full-splice_match	gene: Zm00001d002901	Zm00001d002901_T001	242.78	242.78	coding	344	345	1376
PB.1175.1	1	2399	9	full-splice_match	gene: Zm00001d032994	Zm00001d032994_T002	5.92	5.92	coding	514	307	1848
PB.4993.1	4	726	3	full-splice_match	gene: Zm00001d049606	Zm00001d049606_T001	66.26	66.26	coding	164	83	574
PB.8270.5	7	3501	14	full-splice_match	gene: Zm00001d019149	Zm00001d019149_T006	12.00	202.09	coding	1077	164	3394
PB.8270.6	7	2717	10	incomplete-splice_match	gene: Zm00001d019149	Zm00001d019149_T008	91.27	202.09	coding	870	14	2623
PB.8270.1	7	3612	14	full-splice_match	gene: Zm00001d019149	Zm00001d019149_T006	98.83	202.09	coding	1077	133	3363
PB.1207.4	1	2346	3	novel_in_catalog	gene: Zm00001d033174	novel	0.00	548.07	coding	161	1593	2075
PB.3410.1	2	2242	6	full-splice_match	gene: Zm00001d006835	Zm00001d006835_T002	9.10	9.10	coding	293	1086	1964
PB.2201.1	10	1396	1	full-splice_match	gene: Zm00001d026061	Zm00001d026061_T001	18.22	18.22	coding	383	32	1180
PB.5059.2	4	2542	8	full-splice_match	gene: Zm00001d049928	Zm00001d049928_T007	23.22	23.22	coding	604	327	2138
PB.455.4	1	3038	22	full-splice_match	gene: Zm00001d029212	Zm00001d029212_T004	23.54	64.28	coding	871	159	2771
PB.1127.1	1	2048	1	full-splice_match	gene: Zm00001d032753	Zm00001d032753_T001	9.60	9.60	coding	642	7	1932
PB.3999.3	3	679	2	full-splice_match	gene: Zm00001d041246	Zm00001d041246_T001	567.31	1474.17	coding	145	87	521
PB.3999.2	3	835	1	genic	gene: Zm00001d041246	novel	9.08	1474.17	coding	100	81	380
PB.8151.1	7	1980	1	full-splice_match	gene: Zm00001d018622	Zm00001d018622_T001	1.95	1.95	coding	200	1232	1831
PB.8151.2	7	979	1	genic	gene: Zm00001d018624	novel	1.65	1.65	coding	145	455	889

PB.7206.1	6	1629	4	antisense	novelGene_gene: Zm00001d034967_A	novel	2.46	2.46	non_coding	NA	NA	NA
PB.3759.3	3	610	1	full-splice_match	gene: Zm00001d039733	Zm00001d039733_T001	1415.33	1487.16	coding	103	77	385
PB.3759.2	3	746	1	full-splice_match	gene: Zm00001d039733	Zm00001d039733_T001	68.68	1487.16	coding	103	77	385
PB.3759.1	3	1080	1	full-splice_match	gene: Zm00001d039733	Zm00001d039733_T001	3.15	1487.16	coding	103	77	385
PB.9018.2	7	1278	5	full-splice_match	gene: Zm00001d022513	Zm00001d022513_T002	14.06	1516.94	coding	151	582	1034
PB.10790.3	9	2343	13	full-splice_match	gene: Zm00001d048415	Zm00001d048415_T011	6.86	31.23	coding	411	73	1305
PB.9018.1	7	1008	6	full-splice_match	gene: Zm00001d022513	Zm00001d022513_T001	1502.89	1516.94	coding	219	93	749
PB.4692.1	3	2224	1	antisense	novelGene_gene: Zm00001d044293_A	novel	1.00	1.00	coding	113	622	960
PB.6592.1	5	532	1	full-splice_match	gene: Zm00001d015862	Zm00001d015862_T001	63.94	63.94	coding	95	62	346
PB.10319.1	9	1648	8	full-splice_match	gene: Zm00001d046342	Zm00001d046342_T001	4.69	4.69	coding	505	73	1587
PB.8627.1	7	1127	6	full-splice_match	gene: Zm00001d021147	Zm00001d021147_T003	196.89	262.93	coding	263	168	956
PB.1889.1	10	1616	1	full-splice_match	gene: Zm00001d024545	Zm00001d024545_T001	2.94	20.09	coding	249	116	862
PB.1889.2	10	1223	1	full-splice_match	gene: Zm00001d024545	Zm00001d024545_T001	17.15	20.09	coding	249	245	991
PB.8972.1	7	1769	5	full-splice_match	gene: Zm00001d022386	Zm00001d022386_T001	34.66	38.72	coding	414	20	1261
PB.8627.2	7	1000	7	full-splice_match	gene: Zm00001d021147	Zm00001d021147_T004	66.04	262.93	coding	263	45	833
PB.8972.3	7	1529	5	full-splice_match	gene: Zm00001d022386	Zm00001d022386_T001	0.00	38.72	coding	414	21	1262
PB.8972.2	7	1682	5	full-splice_match	gene: Zm00001d022386	Zm00001d022386_T001	4.07	38.72	coding	414	39	1280

PB.10218.1	9	1470	5	novel_not_in_catalog	gene: Zm00001d045576	novel	10.47	16.50	coding	366	55	1152
PB.10218.2	9	1809	5	novel_not_in_catalog	gene: Zm00001d045576	novel	6.02	16.50	coding	366	506	1603
PB.10218.3	9	1419	5	novel_not_in_catalog	gene: Zm00001d045576	novel	0.00	16.50	coding	366	241	1338
PB.6435.1	5	426	2	incomplete-splice_match	gene: Zm00001d014954	Zm00001d014954_T005	947.49	1078.41	non_coding	NA	NA	NA
PB.7009.11	5	1845	5	antisense	novelGene_gene: Zm00001d017932_A	novel	0.00	81.66	non_coding	NA	NA	NA
PB.1875.14	10	1829	3	novel_not_in_catalog	gene: Zm00001d024499	novel	84.39	276.67	coding	438	399	1712
PB.6435.2	5	1376	4	full-splice_match	gene: Zm00001d014954	Zm00001d014954_T003	130.92	1078.41	coding	129	76	462
PB.1908.1	10	1939	13	full-splice_match	gene: Zm00001d024698	Zm00001d024698_T002	23.22	23.22	coding	379	498	1634
PB.5666.1	4	2719	1	intergenic	novelGene_309	novel	34.21	34.21	coding	101	353	655
PB.946.1	1	1909	4	novel_not_in_catalog	gene: Zm00001d031991	novel	12.15	12.15	non_coding	NA	NA	NA
PB.738.1	1	2097	1	antisense	novelGene_gene: Zm00001d030950_A	novel	8.45	8.45	coding	222	226	891
PB.9903.4	8	2084	2	genic	gene: Zm00001d012260	novel	3.82	51.34	coding	519	348	1904
PB.9903.2	8	2662	1	full-splice_match	gene: Zm00001d012260	Zm00001d012260_T001	3.43	51.34	coding	519	926	2482
PB.9903.1	8	2110	2	genic	gene: Zm00001d012260	novel	44.09	51.34	coding	519	242	1798
PB.7550.1	6	2893	2	full-splice_match	gene: Zm00001d036880	Zm00001d036880_T002	57.47	112.91	coding	626	195	2072
PB.7550.2	6	2431	1	novel_in_catalog	gene: Zm00001d036880	novel	19.46	112.91	coding	583	107	1855
PB.7550.3	6	2624	2	full-splice_match	gene: Zm00001d036880	Zm00001d036880_T002	35.98	112.91	coding	626	195	2072

PB.7550.4	6	2509	2	full-splice_match	gene: Zm00001d036880	Zm00001d036880_T002	0.00	112.91	coding	626	195	2072
PB.7550.5	6	2401	2	full-splice_match	gene: Zm00001d036880	Zm00001d036880_T002	0.00	112.91	coding	626	195	2072
PB.7550.6	6	2253	2	full-splice_match	gene: Zm00001d036880	Zm00001d036880_T002	0.00	112.91	coding	626	195	2072
PB.5754.1	4	606	2	novel_not_in_catalog	gene: Zm00001d027020	novel	1408.41	1408.41	non_coding	NA	NA	NA
PB.5170.1	4	1798	13	novel_not_in_catalog	gene: Zm00001d050664	novel	8.15	11.16	coding	362	28	1113
PB.5170.2	4	2298	13	novel_not_in_catalog	gene: Zm00001d050664	novel	3.01	11.16	coding	441	190	1512
PB.6305.6	5	1555	6	full-splice_match	gene: Zm00001d014447	Zm00001d014447_T001	0.00	11.47	coding	496	56	1543
PB.6305.5	5	1785	6	full-splice_match	gene: Zm00001d014447	Zm00001d014447_T001	0.00	11.47	coding	496	103	1590
PB.4666.1	3	849	1	genic	gene: Zm00001d044202	novel	19.55	19.55	coding	188	57	620
PB.8824.2	7	1922	7	genic	gene: Zm00001d021845	novel	4.28	43.34	coding	151	95	547
PB.6305.1	5	1951	6	full-splice_match	gene: Zm00001d014447	Zm00001d014447_T001	11.47	11.47	coding	496	65	1552
PB.1179.1	1	2170	2	genic	gene: Zm00001d033012	novel	53.02	53.02	coding	446	312	1649
PB.6718.1	5	1897	1	full-splice_match	gene: Zm00001d016669	Zm00001d016669_T001	20.61	20.61	coding	468	327	1730
PB.5428.5	4	2552	20	incomplete-splice_match	gene: Zm00001d052079	Zm00001d052079_T050	15.64	15.64	coding	782	111	2456
PB.271.1	1	2165	1	full-splice_match	gene: Zm00001d028406	Zm00001d028406_T001	20.98	20.98	coding	547	124	1764
PB.6286.7	5	1098	5	novel_in_catalog	gene: Zm00001d014347	novel	1.74	26.00	non_coding	NA	NA	NA
PB.6222.2	5	2408	13	novel_in_catalog	gene: Zm00001d014037	novel	0.00	30.58	coding	540	237	1856

PB.1210.1	1	1901	2	incomplete-splice_match	gene: Zm00001d033193	Zm00001d033193_T007	5.71	5.71	coding	104	1146	1457
PB.9412.1	8	1532	9	full-splice_match	gene: Zm00001d010026	Zm00001d010026_T001	3.75	50.59	coding	391	78	1250
PB.2544.1	2	2164	6	incomplete-splice_match	gene: Zm00001d002373	Zm00001d002373_T015	33.63	33.63	coding	617	20	1870
PB.9412.3	8	1393	8	full-splice_match	gene: Zm00001d010026	Zm00001d010026_T002	0.00	50.59	coding	395	80	1264
PB.9412.2	8	1526	8	full-splice_match	gene: Zm00001d010026	Zm00001d010026_T002	46.84	50.59	coding	395	80	1264
PB.7637.2	6	2044	16	full-splice_match	gene: Zm00001d037278	Zm00001d037278_T001	62.07	109.54	coding	564	61	1752
PB.3626.1	2	788	6	full-splice_match	gene: Zm00001d007940	Zm00001d007940_T002	62.02	62.02	coding	160	76	555
PB.7013.1	5	2934	1	antisense	novelGene_gene: Zm00001d017951_A	novel	14.22	14.22	non_coding	NA	NA	NA
PB.1165.3	1	2733	4	novel_in_catalog	gene: Zm00001d032931	novel	6.21	6.21	coding	129	11	397
PB.1234.3	1	2024	1	novel_in_catalog	gene: Zm00001d033320	novel	7.15	21.30	coding	163	77	565
PB.1622.1	1	1114	1	full-splice_match	gene: Zm00001d034874	Zm00001d034874_T001	61.12	61.12	coding	215	83	727
PB.8908.2	7	2261	13	full-splice_match	gene: Zm00001d022144	Zm00001d022144_T002	0.00	12.51	coding	496	367	1854
PB.6286.3	5	1410	5	novel_in_catalog	gene: Zm00001d014347	novel	11.86	26.00	coding	105	445	759
PB.8908.1	7	2370	13	full-splice_match	gene: Zm00001d022144	Zm00001d022144_T002	12.51	12.51	coding	496	363	1850
PB.7972.1	6	3313	8	full-splice_match	gene: Zm00001d038587	Zm00001d038587_T007	23.03	25.48	coding	593	189	1967
PB.6226.1	5	1003	3	full-splice_match	gene: Zm00001d014046	Zm00001d014046_T001	54.75	54.75	coding	192	86	661
PB.7637.1	6	2197	16	full-splice_match	gene: Zm00001d037278	Zm00001d037278_T001	47.47	109.54	coding	564	66	1757

PB.10635.1	9	659	4	full-splice_match	gene: Zm00001d047857	Zm00001d047857_T001	269.62	269.62	coding	111	44	376
PB.4487.1	3	1471	2	full-splice_match	gene: Zm00001d043489	Zm00001d043489_T001	9.17	9.17	coding	409	159	1385
PB.9450.2	8	1656	5	incomplete-splice_match	gene: Zm00001d010227	Zm00001d010227_T001	380.26	380.26	coding	417	33	1283
PB.3120.1	2	3274	22	novel_in_catalog	gene: Zm00001d005453	novel	9.94	27.13	coding	951	169	3021
PB.8210.1	7	4109	32	full-splice_match	gene: Zm00001d018859	Zm00001d018859_T001	31.95	31.95	coding	1117	203	3553
PB.1916.1	10	1619	6	full-splice_match	gene: Zm00001d024715	Zm00001d024715_T001	16.39	32.09	coding	303	295	1203
PB.1916.2	10	1468	6	full-splice_match	gene: Zm00001d024715	Zm00001d024715_T001	15.69	32.09	coding	303	295	1203
PB.6886.1	5	2208	3	novel_in_catalog	gene: Zm00001d017403	novel	16.24	16.24	coding	533	238	1836
PB.8248.1	7	2093	1	genic	gene: Zm00001d019044	novel	5.23	10.91	coding	335	661	1665
PB.3587.1	2	2851	1	full-splice_match	gene: Zm00001d007789	Zm00001d007789_T001	65.78	67.38	coding	866	58	2655
PB.8248.3	7	1566	1	genic	gene: Zm00001d019044	novel	0.00	10.91	coding	335	500	1504
PB.8248.2	7	1697	1	genic	gene: Zm00001d019044	novel	5.68	10.91	coding	335	502	1506
PB.2364.1	10	1278	7	full-splice_match	gene: Zm00001d026647	Zm00001d026647_T009	26.19	164.86	coding	202	142	747
PB.2364.2	10	1163	7	full-splice_match	gene: Zm00001d026647	Zm00001d026647_T009	21.19	164.86	coding	202	142	747
PB.2364.3	10	1052	7	full-splice_match	gene: Zm00001d026647	Zm00001d026647_T009	117.48	164.86	coding	202	136	741
PB.8174.1	7	2649	1	full-splice_match	gene: Zm00001d018711	Zm00001d018711_T001	12.57	12.57	coding	746	178	2415
PB.8287.2	7	1127	8	intergenic	novelGene_71	novel	72.89	141.76	coding	214	231	872

PB.5730.3	4	1893	3	full-splice_match	gene: Zm00001d053438	Zm00001d053438_T002	8.28	22.12	coding	490	174	1643
PB.964.1	1	899	6	novel_not_in_catalog	gene: Zm00001d032084	novel	71.30	75.38	coding	157	87	557
PB.964.2	1	1056	5	novel_not_in_catalog	gene: Zm00001d032084	novel	4.08	75.38	non_coding	NA	NA	NA
PB.5763.1	4	2066	1	antisense	novelGene_gene: Zm00001d053623_A	novel	20.71	20.71	non_coding	NA	NA	NA
PB.10401.4	9	1584	9	novel_not_in_catalog	gene: Zm00001d046801	novel	0.00	31.05	coding	495	98	1582
PB.5410.1	4	1705	1	full-splice_match	gene: Zm00001d052042	Zm00001d052042_T001	1.89	1.89	coding	471	31	1443
PB.6393.1	5	1688	13	full-splice_match	gene: Zm00001d014787	Zm00001d014787_T003	22.64	22.64	coding	455	103	1467
PB.8774.1	7	2386	6	novel_not_in_catalog	gene: Zm00001d021699	novel	0.00	96.59	coding	559	10	1686
PB.8774.2	7	2315	7	novel_not_in_catalog	gene: Zm00001d021699	novel	70.38	96.59	coding	702	11	2116
PB.8774.5	7	2438	5	novel_not_in_catalog	gene: Zm00001d021699	novel	6.86	96.59	coding	447	96	1436
PB.10642.1	9	3421	4	full-splice_match	gene: Zm00001d047881	Zm00001d047881_T001	30.62	30.62	coding	870	354	2963
PB.8774.7	7	2495	5	novel_not_in_catalog	gene: Zm00001d021699	novel	11.74	96.59	coding	559	17	1693
PB.8774.6	7	2418	5	novel_not_in_catalog	gene: Zm00001d021699	novel	6.92	96.59	coding	330	59	1048
PB.8774.9	7	2277	7	novel_not_in_catalog	gene: Zm00001d021699	novel	0.68	96.59	coding	734	32	2233
PB.6086.3	5	1698	3	full-splice_match	gene: Zm00001d013411	Zm00001d013411_T002	1.90	13.87	coding	416	250	1497
PB.6086.1	5	1714	3	full-splice_match	gene: Zm00001d013411	Zm00001d013411_T002	11.97	13.87	coding	416	138	1385
PB.9589.1	8	1547	2	full-splice_match	gene: Zm00001d010838	Zm00001d010838_T001	13.36	13.36	coding	238	438	1151

PB.2587.1	2	336	1	incomplete-splice_match	gene: Zm00001d002589	Zm00001d002589_T001	23.54	23.54	non_coding	NA	NA	NA
PB.6562.1	5	1737	4	full-splice_match	gene: Zm00001d015581	Zm00001d015581_T001	14.97	14.97	coding	370	223	1332
PB.2133.2	10	425	3	full-splice_match	gene: Zm00001d025772	Zm00001d025772_T001	548.39	897.44	non_coding	NA	NA	NA
PB.7453.1	6	1780	3	novel_in_catalog	gene: Zm00001d036387	novel	18.75	18.75	coding	161	75	557
PB.4159.1	3	2204	15	novel_not_in_catalog	gene: Zm00001d042045	novel	12.76	12.76	coding	552	258	1913
PB.8450.1	7	2166	1	antisense	novelGene_gene: Zm00001d020322_A	novel	1.91	1.91	coding	182	134	679
PB.1070.1	1	859	2	full-splice_match	gene: Zm00001d032536	Zm00001d032536_T001	32.61	87.72	coding	173	51	569
PB.1990.2	10	865	4	incomplete-splice_match	gene: Zm00001d025010	Zm00001d025010_T015	50.37	50.37	coding	138	68	481
PB.158.10	1	1254	2	novel_in_catalog	gene: Zm00001d027924	novel	24.10	289.31	coding	338	85	1098
PB.158.11	1	1211	2	novel_in_catalog	gene: Zm00001d027924	novel	0.00	289.31	coding	341	77	1099
PB.726.1	1	3379	2	genic	gene: Zm00001d030867	novel	1.81	1.81	non_coding	NA	NA	NA
PB.5245.1	4	1845	2	full-splice_match	gene: Zm00001d051219	Zm00001d051219_T001	19.56	30.37	coding	417	99	1349
PB.2461.7	2	1173	8	full-splice_match	gene: Zm00001d002035	Zm00001d002035_T001	123.06	196.16	coding	291	93	965
PB.334.2	1	973	1	full-splice_match	gene: Zm00001d028667	Zm00001d028667_T001	124.06	170.64	coding	182	133	678
PB.6514.3	5	1150	2	full-splice_match	gene: Zm00001d015307	Zm00001d015307_T001	0.00	47.70	coding	320	96	1055
PB.6514.2	5	1361	2	full-splice_match	gene: Zm00001d015307	Zm00001d015307_T001	44.05	47.70	coding	320	157	1116
PB.6514.1	5	1308	2	full-splice_match	gene: Zm00001d015307	Zm00001d015307_T001	3.66	47.70	coding	301	55	957

PB.1774.1	10	3470	8	full-splice_match	gene: Zm00001d023838	Zm00001d023838_T001	34.47	69.36	coding	884	343	2994
PB.8983.1	7	978	1	full-splice_match	gene: Zm00001d022420	Zm00001d022420_T001	633.08	633.08	coding	205	97	711
PB.6409.1	5	1670	5	incomplete-splice_match	gene: Zm00001d014863	Zm00001d014863_T001	9.68	9.68	coding	287	361	1221
PB.5750.5	4	1507	8	novel_not_in_catalog	gene: Zm00001d053576	novel	3.09	74.01	coding	414	83	1324
PB.10893.8	B73V4 ctg205	2663	11	novel_not_in_catalog	gene: Zm00001d000452	novel	4.59	58.01	coding	125	263	637
PB.9898.1	8	1585	6	novel_not_in_catalog	gene: Zm00001d012239	novel	1.89	18.60	coding	239	741	1457
PB.5158.1	4	2315	7	full-splice_match	gene: Zm00001d050518	Zm00001d050518_T001	53.06	53.06	coding	612	209	2044
PB.1934.1	10	1279	3	incomplete-splice_match	gene: Zm00001d024768	Zm00001d024768_T001	11.43	11.43	coding	367	1	1101
PB.10893.1	B73V4 ctg205	2342	9	novel_not_in_catalog	gene: Zm00001d000452	novel	9.71	58.01	non_coding	NA	NA	NA
PB.10893.3	B73V4 ctg205	2079	10	novel_not_in_catalog	gene: Zm00001d000452	novel	7.52	58.01	non_coding	NA	NA	NA
PB.9057.7	8	2947	12	full-splice_match	gene: Zm00001d008176	Zm00001d008176_T006	0.00	35.58	coding	877	189	2819
PB.10893.5	B73V4 ctg205	2454	9	novel_not_in_catalog	gene: Zm00001d000452	novel	0.00	58.01	non_coding	NA	NA	NA
PB.10893.4	B73V4 ctg205	2287	9	novel_not_in_catalog	gene: Zm00001d000452	novel	26.32	58.01	non_coding	NA	NA	NA
PB.10893.7	B73V4 ctg205	402	4	novel_not_in_catalog	gene: Zm00001d000452	novel	9.86	58.01	non_coding	NA	NA	NA
PB.10893.6	B73V4 ctg205	2430	12	novel_not_in_catalog	gene: Zm00001d000452	novel	0.00	58.01	coding	125	212	586
PB.7009.7	5	2019	6	antisense	novelGene_gene: Zm00001d017932_A	novel	0.00	81.66	non_coding	NA	NA	NA
PB.7009.6	5	3750	5	antisense	novelGene_gene: Zm00001d017932_A	novel	7.06	81.66	non_coding	NA	NA	NA

PB.6238.4	5	2623	9	full-splice_match	gene: Zm00001d014124	Zm00001d014124_T004	0.00	136.17	coding	811	100	2532
PB.6238.5	5	3192	8	novel_in_catalog	gene: Zm00001d014124	novel	3.68	136.17	coding	718	73	2226
PB.6238.2	5	3449	8	novel_in_catalog	gene: Zm00001d014124	novel	2.67	136.17	coding	720	1077	3236
PB.7009.2	5	2015	7	antisense	novelGene_gene: Zm00001d017932_A	novel	19.38	81.66	non_coding	NA	NA	NA
PB.7009.1	5	1994	5	antisense	novelGene_gene: Zm00001d017932_A	novel	1.37	81.66	non_coding	NA	NA	NA
PB.6238.1	5	2825	9	full-splice_match	gene: Zm00001d014124	Zm00001d014124_T003	129.83	136.17	coding	811	180	2612
PB.985.1	1	863	2	incomplete-splice_match	gene: Zm00001d032208	Zm00001d032208_T001	3.88	3.88	coding	210	128	757
PB.7009.9	5	1814	6	antisense	novelGene_gene: Zm00001d017932_A	novel	0.00	81.66	non_coding	NA	NA	NA
PB.7009.8	5	1773	6	antisense	novelGene_gene: Zm00001d017932_A	novel	19.79	81.66	non_coding	NA	NA	NA
PB.7328.2	6	1783	2	novel_not_in_catalog	gene: Zm00001d035605	novel	173.98	3577.33	non_coding	NA	NA	NA
PB.7328.1	6	2097	1	genic	gene: Zm00001d035605	novel	88.72	3577.33	coding	250	961	1710
PB.7633.1	6	1043	5	full-splice_match	gene: Zm00001d037267	Zm00001d037267_T001	38.29	43.54	coding	182	249	794
PB.7328.7	6	1843	1	genic	gene: Zm00001d035605	novel	2660.77	3577.33	coding	250	879	1628
PB.10216.3	9	1786	6	novel_in_catalog	gene: Zm00001d045563	novel	12.96	12.96	coding	514	129	1670
PB.3253.1	2	1888	4	incomplete-splice_match	gene: Zm00001d006130	Zm00001d006130_T001	23.97	32.62	coding	319	626	1582
PB.6443.1	5	895	1	full-splice_match	gene: Zm00001d001575	Zm00001d001575_T001	23.87	23.87	non_coding	NA	NA	NA
PB.5839.1	4	1492	1	full-splice_match	gene: Zm00001d053908	Zm00001d053908_T001	18.42	715.10	coding	249	86	832

PB.9047.2	7	1417	9	full-splice_match	gene: Zm00001d022619	Zm00001d022619_T001	5.55	17.79	coding	414	59	1300
PB.9047.1	7	1539	9	full-splice_match	gene: Zm00001d022619	Zm00001d022619_T001	12.24	17.79	coding	414	59	1300
PB.10559.1	9	889	1	full-splice_match	gene: Zm00001d047618	Zm00001d047618_T001	149.17	149.17	coding	173	139	657
PB.6242.8	5	2244	13	full-splice_match	gene: Zm00001d014138	Zm00001d014138_T008	0.00	71.54	coding	314	1044	1985
PB.4964.1	4	1938	6	novel_in_catalog	gene: Zm00001d049449	novel	52.93	52.93	coding	354	106	1167
PB.6242.5	5	2017	13	full-splice_match	gene: Zm00001d014138	Zm00001d014138_T005	22.60	71.54	coding	536	82	1689
PB.9408.1	8	1159	4	novel_not_in_catalog	gene: Zm00001d010000	novel	21.56	21.56	coding	221	215	877
PB.6242.7	5	2112	15	novel_not_in_catalog	gene: Zm00001d014138	novel	3.24	71.54	coding	326	827	1804
PB.1562.1	1	2437	14	full-splice_match	gene: Zm00001d034678	Zm00001d034678_T004	8.65	12.01	coding	529	559	2145
PB.6242.2	5	2374	13	full-splice_match	gene: Zm00001d014138	Zm00001d014138_T008	18.56	71.54	coding	314	1056	1997
PB.8693.1	7	3399	2	genic	gene: Zm00001d021411	novel	0.33	0.33	coding	639	50	1966
PB.6540.5	5	2974	16	full-splice_match	gene: Zm00001d015435	Zm00001d015435_T001	1.76	29.02	coding	585	1001	2755
PB.6540.6	5	2789	17	full-splice_match	gene: Zm00001d015435	Zm00001d015435_T002	0.00	29.02	coding	787	321	2681
PB.4262.1	3	2752	4	full-splice_match	gene: Zm00001d042543	Zm00001d042543_T002	10.51	10.71	coding	771	38	2350
PB.6540.1	5	3136	17	full-splice_match	gene: Zm00001d015435	Zm00001d015435_T002	20.47	29.02	coding	787	39	2399
PB.6540.2	5	3218	17	full-splice_match	gene: Zm00001d015435	Zm00001d015435_T002	0.00	29.02	coding	787	195	2555
PB.6540.3	5	3112	17	full-splice_match	gene: Zm00001d015435	Zm00001d015435_T002	6.80	29.02	coding	787	327	2687

PB.5470.1	4	669	2	full-splice_match	gene: Zm00001d052286	Zm00001d052286_T004	40.49	40.49	non_coding	NA	NA	NA
PB.5452.1	4	960	3	full-splice_match	gene: Zm00001d052225	Zm00001d052225_T001	93.29	93.29	coding	248	30	773
PB.3580.5	2	1181	5	full-splice_match	gene: Zm00001d007767	Zm00001d007767_T001	319.67	519.93	coding	222	222	887
PB.3384.1	2	2090	4	full-splice_match	gene: Zm00001d006704	Zm00001d006704_T003	57.61	57.61	coding	470	277	1686
PB.5231.1	4	445	1	incomplete-splice_match	gene: Zm00001d051129	Zm00001d051129_T007	363.44	363.44	non_coding	NA	NA	NA
PB.6141.2	5	883	4	full-splice_match	gene: Zm00001d013626	Zm00001d013626_T001	119.12	119.12	coding	199	105	701
PB.4580.2	3	1862	12	full-splice_match	gene: Zm00001d043843	Zm00001d043843_T001	39.03	85.24	coding	504	182	1693
PB.9866.1	8	1588	9	full-splice_match	gene: Zm00001d012087	Zm00001d012087_T002	33.22	33.22	coding	332	274	1269
PB.4580.1	3	1961	12	full-splice_match	gene: Zm00001d043843	Zm00001d043843_T001	46.21	85.24	coding	504	179	1690
PB.1815.2	10	809	3	full-splice_match	gene: Zm00001d024066	Zm00001d024066_T001	0.00	55.86	coding	199	75	671
PB.1815.1	10	991	3	full-splice_match	gene: Zm00001d024066	Zm00001d024066_T001	55.86	55.86	coding	199	77	673
PB.5461.1	4	2208	18	incomplete-splice_match	gene: Zm00001d052248	Zm00001d052248_T020	12.76	19.85	coding	491	423	1895
PB.7198.1	5	1121	7	incomplete-splice_match	gene: Zm00001d018542	Zm00001d018542_T006	175.44	175.44	coding	242	66	791
PB.1546.3	1	1035	3	full-splice_match	gene: Zm00001d034634	Zm00001d034634_T001	8.27	30.45	coding	210	94	723
PB.8228.1	7	1387	2	genic	gene: Zm00001d018917	novel	6.50	6.50	coding	242	480	1205
PB.4934.1	4	436	1	full-splice_match	gene: Zm00001d049258	Zm00001d049258_T001	135.41	135.41	non_coding	NA	NA	NA
PB.1550.1	1	1225	4	full-splice_match	gene: Zm00001d034649	Zm00001d034649_T002	48.36	54.79	non_coding	NA	NA	NA

PB.9035.1	7	2193	3	full-splice_match	gene: Zm00001d022582	Zm00001d022582_T002	32.98	50.82	coding	529	309	1895
PB.9035.2	7	2161	3	full-splice_match	gene: Zm00001d022582	Zm00001d022582_T002	17.84	50.82	coding	529	368	1954
PB.2382.1	10	857	3	full-splice_match	gene: Zm00001d026704	Zm00001d026704_T002	31.45	117.45	coding	166	24	521
PB.1306.1	1	1242	6	full-splice_match	gene: Zm00001d033641	Zm00001d033641_T001	71.43	81.41	coding	276	202	1029
PB.9737.5	8	2338	7	full-splice_match	gene: Zm00001d011565	Zm00001d011565_T004	0.00	970.93	coding	112	1719	2054
PB.3336.4	2	1833	3	novel_in_catalog	gene: Zm00001d006474	novel	0.71	531.17	non_coding	NA	NA	NA
PB.1051.5	1	2209	14	novel_in_catalog	gene: Zm00001d032472	novel	0.00	37.15	coding	509	177	1703
PB.9173.1	8	2278	6	novel_in_catalog	gene: Zm00001d008716	novel	22.10	63.69	coding	321	905	1867
PB.9173.5	8	2014	7	full-splice_match	gene: Zm00001d008716	Zm00001d008716_T001	41.58	63.69	coding	444	354	1685
PB.7583.4	6	1680	2	full-splice_match	gene: Zm00001d037058	Zm00001d037058_T001	0.00	40.68	coding	481	22	1464
PB.121.1	1	1491	2	full-splice_match	gene: Zm00001d027721	Zm00001d027721_T001	40.70	40.70	coding	356	301	1368
PB.6843.1	5	1805	2	full-splice_match	gene: Zm00001d017210	Zm00001d017210_T001	11.17	11.17	coding	499	12	1508
PB.1676.1	10	2006	2	full-splice_match	gene: Zm00001d023320	Zm00001d023320_T005	40.77	40.77	coding	535	156	1760
PB.7583.1	6	1703	2	full-splice_match	gene: Zm00001d037058	Zm00001d037058_T001	38.94	40.68	coding	481	22	1464
PB.1852.3	10	1248	1	full-splice_match	gene: Zm00001d024379	Zm00001d024379_T001	214.18	311.40	coding	286	128	985
PB.1852.2	10	1328	1	full-splice_match	gene: Zm00001d024379	Zm00001d024379_T001	85.82	311.40	coding	286	129	986
PB.9252.2	8	1102	6	full-splice_match	gene: Zm00001d009071	Zm00001d009071_T001	6.02	71.70	coding	239	232	948

PB.9252.1	8	1162	6	full-splice_match	gene: Zm00001d009071	Zm00001d009071_T001	65.69	71.70	coding	239	171	887
PB.3686.2	3	2069	2	antisense	novelGene_gene: Zm00001d039397_A	novel	7.89	12.53	coding	176	252	779
PB.9582.1	8	1151	7	full-splice_match	gene: Zm00001d010806	Zm00001d010806_T001	127.64	127.64	coding	273	141	959
PB.811.1	1	1192	2	full-splice_match	gene: Zm00001d031270	Zm00001d031270_T001	12.33	12.33	coding	314	103	1044
PB.3146.1	2	1649	1	full-splice_match	gene: Zm00001d005600	Zm00001d005600_T001	2.12	2.12	coding	473	68	1486
PB.1370.11	1	905	6	novel_not_in_catalog	gene: Zm00001d033925	novel	0.71	1241.38	coding	205	135	749
PB.1024.1	1	1332	8	full-splice_match	gene: Zm00001d032363	Zm00001d032363_T001	6.66	6.66	coding	375	117	1241
PB.8893.1	7	683	1	full-splice_match	gene: Zm00001d022099	Zm00001d022099_T001	19.90	19.90	coding	124	26	397
PB.2731.1	2	1845	1	full-splice_match	gene: Zm00001d003161	Zm00001d003161_T001	3.49	3.49	coding	470	312	1721
PB.3392.1	2	2395	12	full-splice_match	gene: Zm00001d006751	Zm00001d006751_T005	36.93	58.08	coding	631	68	1960
PB.296.3	1	3760	20	full-splice_match	gene: Zm00001d028511	Zm00001d028511_T008	24.73	24.73	coding	1047	202	3342
PB.3064.1	2	1382	12	full-splice_match	gene: Zm00001d005144	Zm00001d005144_T001	30.99	38.64	coding	364	88	1179
PB.9728.1	8	1867	3	full-splice_match	gene: Zm00001d011527	Zm00001d011527_T001	4.93	13.90	coding	414	215	1456
PB.10694.1	9	3137	6	full-splice_match	gene: Zm00001d048080	Zm00001d048080_T002	23.23	54.74	coding	915	74	2818
PB.10694.2	9	1199	5	incomplete-splice_match	gene: Zm00001d048080	Zm00001d048080_T006	31.50	54.74	coding	319	57	1013
PB.4801.1	4	1405	2	genic	gene: Zm00001d048613	novel	8.71	8.71	coding	183	255	803
PB.9728.4	8	2525	2	novel_in_catalog	gene: Zm00001d011527	novel	1.83	13.90	coding	225	1737	2411

PB.8748.2	7	1337	12	novel_not_in_catalog	gene: Zm00001d021615	novel	467.86	543.59	coding	347	106	1146
PB.9776.1	8	2081	5	full-splice_match	gene: Zm00001d011719	Zm00001d011719_T007	4.79	14.32	coding	223	471	1139
PB.9776.2	8	1555	6	full-splice_match	gene: Zm00001d011719	Zm00001d011719_T006	9.53	14.32	coding	239	538	1254
PB.5851.1	4	1416	6	full-splice_match	gene: Zm00001d053932	Zm00001d053932_T003	45.30	151.82	coding	311	112	1044
PB.8748.8	7	1208	12	novel_not_in_catalog	gene: Zm00001d021615	novel	0.00	543.59	coding	347	91	1131
PB.3979.1	3	1914	11	novel_in_catalog	gene: Zm00001d041083	novel	21.95	21.95	coding	515	65	1609
PB.5921.2	5	904	8	full-splice_match	gene: Zm00001d012854	Zm00001d012854_T004	34.64	44.81	coding	173	144	662
PB.3154.1	2	353	1	antisense	novelGene_gene: Zm00001d005648_A	novel	44.07	44.07	non_coding	NA	NA	NA
PB.5921.1	5	1174	7	full-splice_match	gene: Zm00001d012854	Zm00001d012854_T006	10.17	44.81	coding	146	128	565
PB.9728.2	8	1432	3	full-splice_match	gene: Zm00001d011527	Zm00001d011527_T001	7.14	13.90	coding	414	40	1281
PB.3392.2	2	2210	11	novel_not_in_catalog	gene: Zm00001d006751	novel	1.65	58.08	coding	588	77	1840
PB.1378.2	1	954	4	incomplete-splice_match	gene: Zm00001d033981	Zm00001d033981_T001	0.00	162.01	coding	205	230	844
PB.3499.1	2	2300	12	fusion	gene: Zm00001d007259_g	novel	14.60	14.60	coding	423	96	1364
PB.3499.2	2	958	6	novel_not_in_catalog	gene: Zm00001d007261	novel	29.76	29.76	coding	137	102	512
PB.6795.1	5	762	2	full-splice_match	gene: Zm00001d017019	Zm00001d017019_T001	28.28	28.28	coding	122	179	544
PB.6693.3	5	1881	3	full-splice_match	gene: Zm00001d016531	Zm00001d016531_T001	0.00	62.84	coding	521	126	1688
PB.6050.2	5	894	3	novel_in_catalog	gene: Zm00001d013282	novel	5.26	822.89	non_coding	NA	NA	NA

PB.9163.2	8	1079	5	incomplete-splice_match	gene: Zm00001d008646	Zm00001d008646_T001	33.77	66.58	coding	241	63	785
PB.6050.1	5	619	4	novel_in_catalog	gene: Zm00001d013282	novel	817.63	822.89	non_coding	NA	NA	NA
PB.8621.1	7	1912	5	full-splice_match	gene: Zm00001d021065	Zm00001d021065_T001	40.03	43.74	coding	443	295	1623
PB.1828.4	10	1230	1	full-splice_match	gene: Zm00001d024200	Zm00001d024200_T001	2.30	107.89	coding	278	96	929
PB.1828.5	10	760	1	full-splice_match	gene: Zm00001d024200	Zm00001d024200_T001	0.00	107.89	non_coding	NA	NA	NA
PB.1828.2	10	1446	1	full-splice_match	gene: Zm00001d024200	Zm00001d024200_T001	20.72	107.89	coding	278	93	926
PB.1828.3	10	1339	1	full-splice_match	gene: Zm00001d024200	Zm00001d024200_T001	77.65	107.89	coding	278	93	926
PB.9794.2	8	1480	1	full-splice_match	gene: Zm00001d011787	Zm00001d011787_T001	3.51	42.64	coding	381	174	1316
PB.1828.1	10	1539	1	full-splice_match	gene: Zm00001d024200	Zm00001d024200_T001	7.22	107.89	coding	278	84	917
PB.1731.2	10	541	1	intergenic	novelGene_42	novel	145.84	168.94	coding	58	121	294
PB.3263.4	2	1618	1	incomplete-splice_match	gene: Zm00001d006177	Zm00001d006177_T029	12.39	22.79	non_coding	NA	NA	NA
PB.8323.1	7	4248	14	full-splice_match	gene: Zm00001d019507	Zm00001d019507_T001	28.18	328.94	coding	1086	188	3445
PB.8323.3	7	4063	14	full-splice_match	gene: Zm00001d019507	Zm00001d019507_T001	69.62	328.94	coding	1086	188	3445
PB.8323.2	7	3929	12	novel_in_catalog	gene: Zm00001d019507	novel	0.71	328.94	coding	430	283	1572
PB.5276.1	4	3963	13	full-splice_match	gene: Zm00001d051404	Zm00001d051404_T014	9.09	9.09	coding	895	673	3357
PB.7175.4	5	1509	11	novel_in_catalog	gene: Zm00001d018498	novel	13.01	70.12	coding	280	112	951
PB.8323.7	7	4008	14	full-splice_match	gene: Zm00001d019507	Zm00001d019507_T001	0.00	328.94	coding	1086	188	3445

PB.8323.6	7	1880	3	novel_in_catalog	gene: Zm00001d019507	novel	85.02	328.94	coding	106	279	596
PB.8323.9	7	3859	14	full-splice_match	gene: Zm00001d019507	Zm00001d019507_T001	0.00	328.94	coding	1086	188	3445
PB.8323.8	7	4089	13	novel_in_catalog	gene: Zm00001d019507	novel	2.04	328.94	coding	791	189	2561
PB.4218.2	3	3046	22	full-splice_match	gene: Zm00001d042335	Zm00001d042335_T004	3.73	18.83	coding	869	73	2679
PB.4218.1	3	3043	22	full-splice_match	gene: Zm00001d042335	Zm00001d042335_T004	15.10	18.83	coding	869	53	2659
PB.3068.4	2	2916	14	full-splice_match	gene: Zm00001d005157	Zm00001d005157_T002	0.20	26.18	coding	732	382	2577
PB.4325.1	3	1998	1	full-splice_match	gene: Zm00001d042814	Zm00001d042814_T001	74.50	74.50	coding	547	119	1759
PB.4893.8	4	2057	5	novel_in_catalog	gene: Zm00001d049097	novel	9.51	54.78	coding	194	362	943
PB.1421.1	1	1882	7	full-splice_match	gene: Zm00001d034139	Zm00001d034139_T002	76.64	76.64	coding	475	144	1568
PB.4893.6	4	1881	6	full-splice_match	gene: Zm00001d049097	Zm00001d049097_T007	8.88	54.78	coding	194	245	826
PB.4893.4	4	1413	6	novel_in_catalog	gene: Zm00001d049097	novel	26.80	54.78	coding	260	285	1064
PB.4893.2	4	2215	4	novel_in_catalog	gene: Zm00001d049097	novel	9.60	54.78	coding	194	396	977
PB.1049.4	1	1927	2	genic	gene: Zm00001d032460	novel	0.00	51.10	coding	439	199	1515
PB.3455.2	2	832	2	full-splice_match	gene: Zm00001d007045	Zm00001d007045_T001	141.69	142.89	coding	197	27	617
PB.7326.1	6	1461	10	novel_in_catalog	gene: Zm00001d035601	novel	17.53	17.53	coding	221	511	1173
PB.4520.9	3	3144	12	full-splice_match	gene: Zm00001d043613	Zm00001d043613_T005	0.00	284.34	coding	300	1779	2678
PB.6962.1	5	3710	18	full-splice_match	gene: Zm00001d017754	Zm00001d017754_T019	44.58	44.58	coding	940	484	3303

PB.6729.1	5	1235	8	full-splice_match	gene: Zm00001d016714	Zm00001d016714_T003	35.37	35.37	coding	314	87	1028
PB.7500.1	6	699	1	full-splice_match	gene: Zm00001d036570	Zm00001d036570_T001	484.59	484.59	coding	137	62	472
PB.8529.8	7	2531	11	novel_in_catalog	gene: Zm00001d020685	novel	11.76	208.26	coding	497	374	1864
PB.8681.1	7	2198	7	novel_not_in_catalog	gene: Zm00001d021371	novel	9.23	35.05	coding	368	572	1675
PB.8681.2	7	1841	8	novel_not_in_catalog	gene: Zm00001d021371	novel	13.36	35.05	coding	359	259	1335
PB.8681.3	7	2234	6	novel_not_in_catalog	gene: Zm00001d021371	novel	4.38	35.05	coding	240	1021	1740
PB.3392.5	2	2216	12	full-splice_match	gene: Zm00001d006751	Zm00001d006751_T005	0.00	58.08	coding	631	26	1918
PB.2990.1	2	1469	2	full-splice_match	gene: Zm00001d004689	Zm00001d004689_T001	329.46	336.02	coding	363	41	1129
PB.8681.7	7	1386	8	novel_not_in_catalog	gene: Zm00001d021371	novel	0.00	35.05	coding	240	577	1296
PB.8681.8	7	482	1	genic	gene: Zm00001d021371	novel	0.00	35.05	non_coding	NA	NA	NA
PB.4914.4	4	1533	4	full-splice_match	gene: Zm00001d049178	Zm00001d049178_T001	10.40	22.47	coding	333	355	1353
PB.10820.1	9	1230	6	novel_not_in_catalog	gene: Zm00001d048515	novel	10.98	14.09	coding	121	743	1105
PB.4914.1	4	2420	1	genic	gene: Zm00001d049178	novel	1.27	22.47	coding	178	1503	2036
PB.4914.3	4	1664	3	novel_in_catalog	gene: Zm00001d049178	novel	0.00	22.47	coding	284	600	1451
PB.4914.2	4	1652	4	full-splice_match	gene: Zm00001d049178	Zm00001d049178_T001	10.80	22.47	coding	333	356	1354
PB.1703.1	10	2089	5	full-splice_match	gene: Zm00001d023434	Zm00001d023434_T002	36.44	36.44	coding	501	283	1785
PB.1703.2	10	1984	5	full-splice_match	gene: Zm00001d023434	Zm00001d023434_T002	0.00	36.44	coding	501	292	1794

PB.1998.1	10	3639	12	novel_in_catalog	gene: Zm00001d025031	novel	8.67	8.67	coding	856	926	3493
PB.10543.2	9	2858	8	incomplete-splice_match	gene: Zm00001d047540	Zm00001d047540_T005	10.32	26.38	coding	860	15	2594
PB.10543.1	9	2828	8	incomplete-splice_match	gene: Zm00001d047540	Zm00001d047540_T007	16.06	26.38	coding	796	71	2458
PB.10928.2	B73V4 ctg56	980	1	novel_in_catalog	gene: Zm00001d000126	novel	13.25	263.78	coding	108	234	557
PB.10928.3	B73V4 ctg56	772	2	full-splice_match	gene: Zm00001d000126	Zm00001d000126_T001	125.86	263.78	coding	108	78	401
PB.10928.1	B73V4 ctg56	904	2	full-splice_match	gene: Zm00001d000126	Zm00001d000126_T001	124.66	263.78	coding	108	80	403
PB.7494.1	6	1286	1	intergenic	novelGene_159	novel	11.13	11.13	non_coding	NA	NA	NA
PB.3392.6	2	2137	12	full-splice_match	gene: Zm00001d006751	Zm00001d006751_T005	0.00	58.08	coding	631	75	1967
PB.10142.2	9	1984	12	full-splice_match	gene: Zm00001d045316	Zm00001d045316_T002	0.00	8.44	coding	431	246	1538
PB.10142.1	9	2132	12	full-splice_match	gene: Zm00001d045316	Zm00001d045316_T002	8.44	8.44	coding	431	246	1538
PB.5757.3	4	2579	14	full-splice_match	gene: Zm00001d053610	Zm00001d053610_T004	0.00	54.49	coding	692	166	2241
PB.5757.1	4	2676	14	full-splice_match	gene: Zm00001d053610	Zm00001d053610_T004	54.49	54.49	coding	692	212	2287
PB.4776.2	3	944	1	full-splice_match	gene: Zm00001d044660	Zm00001d044660_T001	100.83	124.96	coding	227	141	821
PB.266.4	1	2725	1	full-splice_match	gene: Zm00001d028391	Zm00001d028391_T001	1.22	49.52	coding	794	96	2477
PB.634.1	1	2315	3	full-splice_match	gene: Zm00001d030158	Zm00001d030158_T001	16.53	16.53	coding	596	312	2099
PB.768.2	1	2020	1	antisense	novelGene_gene: Zm00001d031077_A	novel	0.00	206.01	coding	289	1135	2001
PB.266.1	1	2908	1	full-splice_match	gene: Zm00001d028391	Zm00001d028391_T001	48.29	49.52	coding	794	94	2475

PB.1076.20	1	1528	4	full-splice_match	gene: Zm00001d032555	Zm00001d032555_T014	4.04	39.90	coding	432	122	1417
PB.266.3	1	2858	1	full-splice_match	gene: Zm00001d028391	Zm00001d028391_T001	0.00	49.52	coding	794	90	2471
PB.2446.2	2	1460	16	full-splice_match	gene: Zm00001d001983	Zm00001d001983_T001	17.74	37.06	coding	378	139	1272
PB.2446.1	2	1701	16	full-splice_match	gene: Zm00001d001983	Zm00001d001983_T001	19.33	37.06	coding	378	144	1277
PB.6715.7	5	844	2	genic	gene: Zm00001d016655	novel	368.50	807.42	coding	119	97	453
PB.4331.2	3	2050	5	novel_not_in_catalog	gene: Zm00001d042848	novel	21.34	38.75	coding	569	141	1847
PB.4331.3	3	2145	4	novel_not_in_catalog	gene: Zm00001d042848	novel	0.54	38.75	coding	358	883	1956
PB.3392.7	2	1337	1	incomplete-splice_match	gene: Zm00001d006751	Zm00001d006751_T006	0.00	58.08	coding	134	686	1087
PB.6799.4	5	896	4	full-splice_match	gene: Zm00001d017047	Zm00001d017047_T001	0.00	389.64	coding	219	64	720
PB.4364.1	3	746	1	full-splice_match	gene: Zm00001d043006	Zm00001d043006_T001	28.04	28.04	coding	115	230	574
PB.3148.1	2	1457	5	full-splice_match	gene: Zm00001d005614	Zm00001d005614_T002	20.43	20.43	coding	387	11	1171
PB.1713.1	10	1103	9	full-splice_match	gene: Zm00001d023506	Zm00001d023506_T002	65.02	65.02	coding	223	176	844
PB.4292.5	3	1741	1	novel_in_catalog	gene: Zm00001d042664	novel	4.63	169.79	coding	32	231	326
PB.2745.1	2	1435	9	novel_in_catalog	gene: Zm00001d003198	novel	21.19	21.19	coding	194	297	878
PB.10692.1	9	2231	3	full-splice_match	gene: Zm00001d048074	Zm00001d048074_T001	23.31	23.31	coding	575	168	1892
PB.4292.1	3	2296	4	full-splice_match	gene: Zm00001d042664	Zm00001d042664_T002	162.42	169.79	coding	571	205	1917
PB.4292.2	3	2247	3	novel_in_catalog	gene: Zm00001d042664	novel	2.75	169.79	coding	451	66	1418

PB.4292.3	3	2320	4	full-splice_match	gene: Zm00001d042664	Zm00001d042664_T002	0.00	169.79	coding	571	258	1970
PB.10436.2	9	2221	1	full-splice_match	gene: Zm00001d046998	Zm00001d046998_T001	15.05	158.71	coding	277	1067	1897
PB.10272.10	9	3544	3	novel_in_catalog	gene: Zm00001d045965	novel	1.79	472.17	coding	255	2239	3003
PB.10436.1	9	1519	2	genic	gene: Zm00001d046998	novel	143.66	158.71	coding	277	343	1173
PB.5876.1	4	2812	11	incomplete-splice_match	gene: Zm00001d054003	Zm00001d054003_T021	23.50	40.22	coding	731	52	2244
PB.9107.1	8	848	4	full-splice_match	gene: Zm00001d008405	Zm00001d008405_T001	57.26	57.26	coding	130	248	637
PB.2953.1	2	2421	14	novel_in_catalog	gene: Zm00001d004463	novel	39.48	49.56	coding	608	200	2023
PB.2953.2	2	2366	14	novel_not_in_catalog	gene: Zm00001d004463	novel	10.08	49.56	coding	511	86	1618
PB.6799.1	5	1116	4	full-splice_match	gene: Zm00001d017047	Zm00001d017047_T001	174.97	389.64	coding	219	56	712
PB.10639.5	9	3213	8	full-splice_match	gene: Zm00001d047869	Zm00001d047869_T007	0.00	44.65	coding	305	2251	3165
PB.1297.6	1	2618	23	novel_in_catalog	gene: Zm00001d033597	novel	0.00	23.44	coding	798	82	2475
PB.6028.1	5	976	5	novel_not_in_catalog	gene: Zm00001d013242	novel	24.70	24.70	coding	244	42	773
PB.5955.2	5	2421	14	full-splice_match	gene: Zm00001d012969	Zm00001d012969_T012	10.02	30.31	coding	522	155	1720
PB.5955.1	5	2526	14	full-splice_match	gene: Zm00001d012969	Zm00001d012969_T008	10.51	30.31	coding	530	225	1814
PB.5955.5	5	2459	14	novel_in_catalog	gene: Zm00001d012969	novel	9.79	30.31	coding	296	1300	2187
PB.329.2	1	1734	3	full-splice_match	gene: Zm00001d028656	Zm00001d028656_T001	236.69	251.38	coding	454	123	1484
PB.329.3	1	2133	2	novel_in_catalog	gene: Zm00001d028656	novel	6.90	251.38	coding	309	97	1023

PB.329.1	1	2181	3	full-splice_match	gene: Zm00001d028656	Zm00001d028656_T001	7.79	251.38	coding	454	96	1457
PB.329.6	1	1650	3	full-splice_match	gene: Zm00001d028656	Zm00001d028656_T001	0.00	251.38	coding	454	98	1459
PB.9977.2	8	1172	4	full-splice_match	gene: Zm00001d012590	Zm00001d012590_T001	0.00	40.78	coding	282	31	876
PB.10912.3	B73V4 ctg41	1923	4	novel_in_catalog	gene: Zm00001d000391	novel	1.06	25.42	coding	372	446	1561
PB.10912.2	B73V4 ctg41	1620	5	full-splice_match	gene: Zm00001d000391	Zm00001d000391_T002	11.22	25.42	coding	372	152	1267
PB.8694.1	7	1855	1	full-splice_match	gene: Zm00001d021412	Zm00001d021412_T001	11.40	11.40	coding	509	127	1653
PB.3430.1	2	679	2	genic	gene: Zm00001d006915	novel	43.35	45.07	coding	160	91	570
PB.10639.4	9	3342	8	full-splice_match	gene: Zm00001d047869	Zm00001d047869_T007	4.45	44.65	coding	305	2253	3167
PB.8995.1	7	1378	3	novel_in_catalog	gene: Zm00001d022456	novel	65.94	65.94	coding	321	102	1064
PB.9117.1	8	1006	3	incomplete-splice_match	gene: Zm00001d008430	Zm00001d008430_T001	3.01	33.09	coding	126	4	381
PB.2943.2	2	3630	12	novel_in_catalog	gene: Zm00001d004335	novel	6.58	22.82	coding	862	934	3519
PB.9117.3	8	837	4	full-splice_match	gene: Zm00001d008430	Zm00001d008430_T006	16.79	33.09	coding	126	129	506
PB.9117.2	8	997	5	full-splice_match	gene: Zm00001d008430	Zm00001d008430_T003	10.89	33.09	coding	126	295	672
PB.9117.4	8	1829	3	novel_in_catalog	gene: Zm00001d008430	novel	2.40	33.09	coding	143	1076	1504
PB.3903.2	3	1568	12	full-splice_match	gene: Zm00001d040462	Zm00001d040462_T003	15.36	68.52	coding	438	113	1426
PB.7594.1	6	1837	7	full-splice_match	gene: Zm00001d037108	Zm00001d037108_T002	80.46	80.46	coding	461	133	1515
PB.7845.3	6	2689	19	full-splice_match	gene: Zm00001d038134	Zm00001d038134_T009	0.00	17.18	coding	739	31	2247

PB.7845.2	6	2717	19	full-splice_match	gene: Zm00001d038134	Zm00001d038134_T004	3.90	17.18	coding	784	70	2421
PB.7845.1	6	2752	19	full-splice_match	gene: Zm00001d038134	Zm00001d038134_T009	13.28	17.18	coding	739	36	2252
PB.2670.2	2	1209	1	full-splice_match	gene: Zm00001d002899	Zm00001d002899_T001	119.28	224.05	coding	337	79	1089
PB.826.3	1	838	3	incomplete-splice_match	gene: Zm00001d031332	Zm00001d031332_T001	0.00	98.73	coding	241	115	837
PB.2670.1	2	1379	1	full-splice_match	gene: Zm00001d002899	Zm00001d002899_T001	104.77	224.05	coding	337	176	1186
PB.60.2	1	656	3	full-splice_match	gene: Zm00001d027458	Zm00001d027458_T001	14.92	14.92	coding	114	68	409
PB.10858.1	B73V4 ctg134	1994	1	genic_intron	novelGene_6	novel	12.74	12.74	non_coding	NA	NA	NA
PB.2306.1	10	2287	11	full-splice_match	gene: Zm00001d026487	Zm00001d026487_T002	46.98	46.98	coding	579	105	1841
PB.4920.1	4	1966	1	full-splice_match	gene: Zm00001d049201	Zm00001d049201_T001	12.41	30.20	coding	518	220	1773
PB.8315.1	7	403	2	incomplete-splice_match	gene: Zm00001d019461	Zm00001d019461_T001	49.30	51.51	coding	70	92	301
PB.10444.1	9	1583	7	novel_not_in_catalog	gene: Zm00001d047046	novel	15.68	15.68	coding	252	380	1135
PB.5150.1	4	2197	9	full-splice_match	gene: Zm00001d050457	Zm00001d050457_T002	12.88	20.16	coding	483	176	1624
PB.9609.3	8	2688	7	intergenic	novelGene_212	novel	13.41	22.22	non_coding	NA	NA	NA
PB.9609.2	8	2679	7	intergenic	novelGene_212	novel	8.81	22.22	coding	102	157	462
PB.2893.1	2	2429	1	intergenic	novelGene_126	novel	36.89	36.89	coding	68	113	316
PB.603.1	1	993	2	intergenic	novelGene_167	novel	5.08	5.08	coding	134	40	441
PB.818.1	1	2587	12	novel_in_catalog	gene: Zm00001d031300	novel	3.38	29.79	coding	490	128	1597

PB.8408.2	7	891	6	novel_not_in_catalog	gene: Zm00001d020064	novel	10.73	10.73	coding	151	405	857
PB.818.3	1	2275	13	full-splice_match	gene: Zm00001d031300	Zm00001d031300_T003	0.00	29.79	coding	672	133	2148
PB.4317.3	3	1632	1	full-splice_match	gene: Zm00001d042786	Zm00001d042786_T001	0.00	70.08	coding	391	216	1388
PB.9858.2	8	2131	7	full-splice_match	gene: Zm00001d012067	Zm00001d012067_T004	51.64	52.05	coding	424	157	1428
PB.7261.3	6	2087	4	novel_in_catalog	gene: Zm00001d035207	novel	2.38	18.30	coding	294	1014	1895
PB.7261.2	6	1685	7	full-splice_match	gene: Zm00001d035207	Zm00001d035207_T003	15.92	18.30	coding	294	580	1461
PB.9615.2	8	1854	2	genic	gene: Zm00001d010954	novel	26.00	127.28	coding	414	241	1482
PB.10949.2	B73V4 ctg98	2624	1	genic	gene: Zm00001d000233	novel	125.24	125.24	coding	739	191	2407
PB.4994.1	4	1636	5	full-splice_match	gene: Zm00001d049610	Zm00001d049610_T001	23.79	23.79	coding	427	139	1419
PB.6614.1	5	1726	2	genic	gene: Zm00001d016062	novel	14.80	36.28	coding	301	85	987
PB.6614.2	5	938	2	genic	gene: Zm00001d016062	novel	0.00	36.28	coding	42	289	414
PB.9648.1	8	1031	6	novel_in_catalog	gene: Zm00001d011122	novel	27.28	27.28	coding	238	100	813
PB.9086.2	8	1161	4	full-splice_match	gene: Zm00001d008299	Zm00001d008299_T002	0.00	66.97	coding	341	61	1083
PB.3405.1	2	2104	19	full-splice_match	gene: Zm00001d006797	Zm00001d006797_T001	14.04	14.04	coding	524	229	1800
PB.9086.1	8	1273	4	full-splice_match	gene: Zm00001d008299	Zm00001d008299_T002	66.97	66.97	coding	341	71	1093
PB.335.1	1	2099	3	incomplete-splice_match	gene: Zm00001d028671	Zm00001d028671_T019	46.58	54.80	coding	537	220	1830
PB.335.3	1	1795	3	incomplete-splice_match	gene: Zm00001d028671	Zm00001d028671_T019	0.00	54.80	coding	537	137	1747

PB.335.2	1	3684	8	incomplete-splice_match	gene: Zm00001d028671	Zm00001d028671_T008	8.22	54.80	coding	1021	466	3528
PB.3706.3	3	690	1	full-splice_match	gene: Zm00001d039468	Zm00001d039468_T001	69.16	254.99	coding	120	97	456
PB.3706.2	3	803	1	full-splice_match	gene: Zm00001d039468	Zm00001d039468_T001	185.83	254.99	coding	120	97	456
PB.7877.2	6	2184	13	novel_in_catalog	gene: Zm00001d038209	novel	1.53	55.08	coding	394	528	1709
PB.664.1	1	1551	10	full-splice_match	gene: Zm00001d030348	Zm00001d030348_T002	67.67	69.37	coding	398	104	1297
PB.10350.1	9	1416	1	full-splice_match	gene: Zm00001d046496	Zm00001d046496_T001	26.70	43.64	coding	209	138	764
PB.7877.5	6	1943	14	full-splice_match	gene: Zm00001d038209	Zm00001d038209_T001	0.00	55.08	coding	507	94	1614
PB.10350.3	9	933	1	full-splice_match	gene: Zm00001d046496	Zm00001d046496_T001	0.00	43.64	coding	209	139	765
PB.10350.2	9	1304	1	full-splice_match	gene: Zm00001d046496	Zm00001d046496_T001	16.95	43.64	coding	209	133	759
PB.7414.1	6	1573	1	full-splice_match	gene: Zm00001d036103	Zm00001d036103_T001	2.96	5.59	coding	382	108	1253
PB.4368.2	3	2160	4	full-splice_match	gene: Zm00001d043019	Zm00001d043019_T001	59.81	238.63	coding	492	474	1949
PB.2174.3	10	1985	5	novel_in_catalog	gene: Zm00001d025941	novel	3.14	20.00	coding	81	201	443
PB.7414.2	6	1472	1	full-splice_match	gene: Zm00001d036103	Zm00001d036103_T001	2.63	5.59	coding	382	110	1255
PB.3293.2	2	1802	3	full-splice_match	gene: Zm00001d006308	Zm00001d006308_T001	24.16	24.16	coding	424	78	1349
PB.801.2	1	2908	1	full-splice_match	gene: Zm00001d031230	Zm00001d031230_T001	10.76	30.76	coding	900	113	2812
PB.1187.1	1	727	3	full-splice_match	gene: Zm00001d033054	Zm00001d033054_T001	116.38	164.37	coding	119	160	516
PB.1225.5	1	2432	5	novel_not_in_catalog	gene: Zm00001d033292	novel	13.18	84.80	coding	487	839	2299

PB.8827.2	7	2818	3	novel_not_in_catalog	gene: Zm00001d021858	novel	0.00	140.53	coding	736	113	2320
PB.876.1	1	616	2	full-splice_match	gene: Zm00001d031651	Zm00001d031651_T002	82.62	82.62	non_coding	NA	NA	NA
PB.6692.1	5	1979	15	novel_not_in_catalog	gene: Zm00001d016516	novel	58.69	58.69	coding	501	81	1583
PB.8803.2	7	2205	1	genic	gene: Zm00001d021777	novel	6.32	6.32	coding	569	192	1898
PB.8400.1	7	1553	9	full-splice_match	gene: Zm00001d020040	Zm00001d020040_T003	17.13	17.13	coding	309	351	1277
PB.3098.2	2	1500	1	full-splice_match	gene: Zm00001d005312	Zm00001d005312_T001	0.00	45.70	coding	454	49	1410
PB.4778.1	3	2863	1	intergenic	novelGene_355	novel	4.43	4.43	coding	42	170	295
PB.3047.1	2	2021	8	full-splice_match	gene: Zm00001d005059	Zm00001d005059_T002	64.56	64.56	coding	115	1407	1751
PB.4778.2	3	1779	1	intergenic	novelGene_355	novel	0.00	4.43	non_coding	NA	NA	NA
PB.10467.5	9	1530	7	full-splice_match	gene: Zm00001d047208	Zm00001d047208_T001	30.24	54.45	coding	388	68	1231
PB.10941.1	B73V4 ctg77	1690	10	incomplete-splice_match	gene: Zm00001d000257	Zm00001d000257_T005	156.03	156.03	coding	315	421	1365
PB.4726.1	3	798	1	full-splice_match	gene: Zm00001d044461	Zm00001d044461_T001	8.25	86.13	non_coding	NA	NA	NA
PB.4726.2	3	2128	1	full-splice_match	gene: Zm00001d044461	Zm00001d044461_T001	9.99	86.13	coding	311	840	1772
PB.8827.1	7	2313	3	novel_not_in_catalog	gene: Zm00001d021858	novel	101.00	140.53	coding	520	27	1586
PB.1785.2	10	1787	5	full-splice_match	gene: Zm00001d023880	Zm00001d023880_T001	0.00	21.43	non_coding	NA	NA	NA
PB.5475.2	4	4112	14	full-splice_match	gene: Zm00001d052307	Zm00001d052307_T054	7.30	21.79	coding	1174	227	3748
PB.1785.1	10	2066	5	full-splice_match	gene: Zm00001d023880	Zm00001d023880_T001	21.43	21.43	non_coding	NA	NA	NA

PB.4385.1	3	2031	6	full-splice_match	gene: Zm00001d043108	Zm00001d043108_T001	13.65	13.65	coding	562	69	1754
PB.1586.1	1	1838	5	full-splice_match	gene: Zm00001d034761	Zm00001d034761_T001	28.97	254.48	coding	377	206	1336
PB.3283.1	2	2259	1	full-splice_match	gene: Zm00001d006255	Zm00001d006255_T001	3.11	3.11	coding	535	134	1738
PB.5475.3	4	2494	3	incomplete-splice_match	gene: Zm00001d052307	Zm00001d052307_T015	1.72	21.79	coding	712	36	2171
PB.7867.1	6	2357	3	genic	gene: Zm00001d038195	novel	23.31	23.31	coding	606	234	2051
PB.913.2	1	2712	9	full-splice_match	gene: Zm00001d031851	Zm00001d031851_T005	9.37	9.37	coding	720	238	2397
PB.6715.2	5	1228	1	full-splice_match	gene: Zm00001d016655	Zm00001d016655_T001	63.17	807.42	non_coding	NA	NA	NA
PB.6715.3	5	922	2	genic	gene: Zm00001d016655	novel	344.67	807.42	coding	119	94	450
PB.7537.1	6	3741	9	full-splice_match	gene: Zm00001d036765	Zm00001d036765_T003	63.33	63.33	coding	1085	133	3387
PB.6715.6	5	915	1	full-splice_match	gene: Zm00001d016655	Zm00001d016655_T001	31.08	807.42	coding	119	186	542
PB.9529.3	8	2288	1	full-splice_match	gene: Zm00001d010602	Zm00001d010602_T001	0.00	44.48	coding	662	174	2159
PB.3826.2	3	1333	4	full-splice_match	gene: Zm00001d040117	Zm00001d040117_T004	11.00	26.89	coding	181	462	1004
PB.1511.1	1	4222	18	full-splice_match	gene: Zm00001d034515	Zm00001d034515_T001	5.49	5.49	coding	1146	183	3620
PB.3826.1	3	1561	4	full-splice_match	gene: Zm00001d040117	Zm00001d040117_T001	15.89	26.89	coding	182	581	1126
PB.5117.1	4	2243	1	antisense	novelGene_gene: Zm00001d050294_A	novel	3.09	3.09	coding	118	198	551
PB.7253.1	6	3414	3	full-splice_match	gene: Zm00001d035183	Zm00001d035183_T004	12.65	37.40	coding	905	259	2973
PB.977.3	1	1236	1	novel_in_catalog	gene: Zm00001d032158	novel	50.45	56.45	coding	286	292	1149

PB.10467.3	9	1644	7	full-splice_match	gene: Zm00001d047208	Zm00001d047208_T001	12.18	54.45	coding	388	69	1232
PB.10466.1	9	1281	2	full-splice_match	gene: Zm00001d047194	Zm00001d047194_T001	19.45	19.45	coding	362	47	1132
PB.4556.1	3	1447	4	full-splice_match	gene: Zm00001d043736	Zm00001d043736_T002	29.01	29.01	coding	286	99	956
PB.7652.1	6	3713	21	full-splice_match	gene: Zm00001d037362	Zm00001d037362_T048	9.56	13.28	coding	1116	48	3395
PB.1259.2	1	2422	13	full-splice_match	gene: Zm00001d033451	Zm00001d033451_T001	8.37	8.37	coding	559	182	1858
PB.7652.2	6	2381	20	full-splice_match	gene: Zm00001d037362	Zm00001d037362_T028	3.73	13.28	coding	759	75	2351
PB.6905.3	5	1021	2	full-splice_match	gene: Zm00001d017485	Zm00001d017485_T002	85.30	674.17	coding	250	75	824
PB.5498.1	4	1701	6	novel_not_in_catalog	gene: Zm00001d052376	novel	51.48	51.48	coding	224	894	1565
PB.612.1	1	2145	5	incomplete-splice_match	gene: Zm00001d030061	Zm00001d030061_T030	39.62	39.62	coding	599	69	1865
PB.3046.1	2	2511	5	novel_not_in_catalog	gene: Zm00001d005036	novel	17.86	17.86	coding	226	1507	2184
PB.3730.3	3	2108	8	incomplete-splice_match	gene: Zm00001d039537	Zm00001d039537_T036	17.63	17.63	coding	226	103	780
PB.9622.1	8	552	1	genic	gene: Zm00001d010972	novel	2.15	2.15	coding	146	91	528
PB.9826.1	8	1872	9	full-splice_match	gene: Zm00001d011900	Zm00001d011900_T002	29.09	29.09	coding	455	168	1532
PB.9939.1	8	1590	4	full-splice_match	gene: Zm00001d012428	Zm00001d012428_T001	137.97	168.43	coding	370	113	1222
PB.8038.2	6	1438	11	full-splice_match	gene: Zm00001d038851	Zm00001d038851_T001	158.95	231.18	coding	370	154	1263
PB.3089.2	2	1588	5	intergenic	novelGene_273	novel	4.04	15.99	coding	123	144	512
PB.6996.5	5	2428	15	full-splice_match	gene: Zm00001d017876	Zm00001d017876_T018	15.39	25.93	coding	617	238	2088

PB.3710.3	3	963	6	novel_not_in_catalog	gene: Zm00001d039483	novel	0.00	196.57	coding	267	2	802
PB.3710.1	3	1221	6	novel_not_in_catalog	gene: Zm00001d039483	novel	196.57	196.57	coding	267	149	949
PB.4742.2	3	2797	13	novel_in_catalog	gene: Zm00001d044503	novel	2.31	26.50	coding	484	283	1734
PB.9242.1	8	2413	1	full-splice_match	gene: Zm00001d009013	Zm00001d009013_T001	26.14	26.14	coding	611	226	2058
PB.9534.2	8	1320	5	novel_in_catalog	gene: Zm00001d010622	novel	4.24	21.16	coding	170	456	965
PB.9534.3	8	889	5	incomplete-splice_match	gene: Zm00001d010622	Zm00001d010622_T005	16.92	21.16	coding	182	3	548
PB.4742.6	3	2447	14	novel_in_catalog	gene: Zm00001d044503	novel	1.34	26.50	coding	433	158	1456
PB.6823.1	5	2547	1	novel_in_catalog	gene: Zm00001d017138	novel	7.63	7.63	coding	131	218	610
PB.7525.1	6	3896	13	full-splice_match	gene: Zm00001d036704	Zm00001d036704_T002	7.07	16.94	coding	965	584	3478
PB.10868.2	B73V4 ctg150	2190	5	novel_in_catalog	gene: Zm00001d000021	novel	8.81	212.95	coding	313	293	1231
PB.10868.1	B73V4 ctg150	1740	6	full-splice_match	gene: Zm00001d000021	Zm00001d000021_T003	204.15	212.95	coding	387	290	1450
PB.5204.2	4	2635	4	full-splice_match	gene: Zm00001d050943	Zm00001d050943_T002	13.36	19.42	coding	608	357	2180
PB.10190.2	9	1949	12	novel_in_catalog	gene: Zm00001d045483	novel	9.25	37.99	coding	408	196	1419
PB.10190.1	9	1919	13	full-splice_match	gene: Zm00001d045483	Zm00001d045483_T001	28.74	37.99	coding	466	295	1692
PB.5204.1	4	2901	4	full-splice_match	gene: Zm00001d050943	Zm00001d050943_T002	6.05	19.42	coding	608	346	2169
PB.778.4	1	2434	14	full-splice_match	gene: Zm00001d031120	Zm00001d031120_T003	94.30	204.87	coding	624	197	2068
PB.4232.2	3	1522	6	novel_in_catalog	gene: Zm00001d042398	novel	36.23	111.35	coding	35	390	494

PB.778.1	1	2503	14	full-splice_match	gene: Zm00001d031120	Zm00001d031120_T003	105.44	204.87	coding	624	186	2057
PB.3448.2	2	1714	1	full-splice_match	gene: Zm00001d007018	Zm00001d007018_T001	0.00	95.81	coding	125	152	526
PB.868.1	1	2074	5	intergenic	novelGene_313	novel	6.12	89.57	coding	395	194	1378
PB.7277.2	6	326	1	full-splice_match	gene: Zm00001d001307	Zm00001d001307_T001	17.74	17.74	non_coding	NA	NA	NA
PB.8462.2	7	1588	1	novel_in_catalog	gene: Zm00001d020371	novel	4.59	15.20	coding	238	531	1244
PB.8462.1	7	2188	4	full-splice_match	gene: Zm00001d020371	Zm00001d020371_T001	10.61	15.20	coding	539	167	1783
PB.7277.1	6	566	3	full-splice_match	gene: Zm00001d035312	Zm00001d035312_T001	354.17	354.17	non_coding	NA	NA	NA
PB.2696.1	2	1978	1	antisense	novelGene_gene: Zm00001d003016_A	novel	55.04	55.04	coding	212	1005	1640
PB.1613.1	1	1193	3	full-splice_match	gene: Zm00001d034841	Zm00001d034841_T002	35.23	35.23	coding	217	249	899
PB.6134.1	5	1627	5	incomplete-splice_match	gene: Zm00001d013599	Zm00001d013599_T005	19.32	23.55	coding	446	29	1366
PB.6134.3	5	2292	12	full-splice_match	gene: Zm00001d013599	Zm00001d013599_T006	4.22	23.55	coding	723	19	2187
PB.7354.2	6	1423	1	full-splice_match	gene: Zm00001d035756	Zm00001d035756_T001	13.54	537.68	coding	350	105	1154
PB.327.1	1	1354	13	full-splice_match	gene: Zm00001d028653	Zm00001d028653_T003	594.24	594.24	coding	350	115	1164
PB.6781.1	5	1676	6	full-splice_match	gene: Zm00001d016918	Zm00001d016918_T001	19.09	21.48	coding	417	127	1377
PB.7354.1	6	1448	1	full-splice_match	gene: Zm00001d035756	Zm00001d035756_T001	524.14	537.68	coding	350	94	1143
PB.4433.1	3	1827	6	full-splice_match	gene: Zm00001d043288	Zm00001d043288_T001	27.42	34.87	coding	317	99	1049
PB.2547.1	2	2249	14	full-splice_match	gene: Zm00001d002388	Zm00001d002388_T003	60.15	71.96	coding	565	157	1851

PB.4433.2	3	3872	6	full-splice_match	gene: Zm00001d043288	Zm00001d043288_T001	7.45	34.87	coding	1074	45	3266
PB.1186.1	1	1361	4	full-splice_match	gene: Zm00001d033044	Zm00001d033044_T001	37.38	37.38	coding	386	30	1187
PB.2547.4	2	1760	12	incomplete-splice_match	gene: Zm00001d002388	Zm00001d002388_T005	11.81	71.96	coding	455	118	1482
PB.868.2	1	685	4	intergenic	novelGene_313	novel	47.12	89.57	coding	63	171	359
PB.9485.2	8	1285	3	full-splice_match	gene: Zm00001d010403	Zm00001d010403_T001	19.55	125.38	coding	349	220	1266
PB.10856.1	B73V4 ctg129	2471	12	full-splice_match	gene: Zm00001d000213	Zm00001d000213_T003	3.18	5.51	coding	546	315	1952
PB.10856.2	B73V4 ctg129	2477	11	novel_in_catalog	gene: Zm00001d000213	novel	2.33	5.51	coding	384	251	1402
PB.7109.2	5	1511	3	novel_not_in_catalog	gene: Zm00001d018305	novel	130.74	150.59	coding	363	94	1182
PB.7109.3	5	1345	4	novel_not_in_catalog	gene: Zm00001d018305	novel	5.22	150.59	coding	310	100	1029
PB.4848.1	4	2584	11	novel_in_catalog	gene: Zm00001d048877	novel	37.83	37.83	coding	369	1243	2349
PB.7109.1	5	1648	3	novel_not_in_catalog	gene: Zm00001d018305	novel	14.62	150.59	coding	363	101	1189
PB.805.1	1	2071	4	full-splice_match	gene: Zm00001d031253	Zm00001d031253_T001	25.56	25.56	coding	551	117	1769
PB.3634.1	2	562	2	incomplete-splice_match	gene: Zm00001d007972	Zm00001d007972_T005	154.11	154.11	coding	109	87	413
PB.4192.3	3	3883	13	full-splice_match	gene: Zm00001d042212	Zm00001d042212_T005	2.90	24.00	coding	1175	165	3689
PB.4192.2	3	3078	6	incomplete-splice_match	gene: Zm00001d042212	Zm00001d042212_T013	1.52	24.00	coding	670	875	2884
PB.4192.1	3	2348	3	incomplete-splice_match	gene: Zm00001d042212	Zm00001d042212_T010	16.60	24.00	coding	670	18	2027
PB.10757.1	9	1772	1	incomplete-splice_match	gene: Zm00001d048320	Zm00001d048320_T002	24.79	24.79	coding	119	1262	1618

PB.8864.1	7	2866	2	genic	gene: Zm00001d021980	novel	0.99	0.99	coding	598	583	2376
PB.9109.1	8	1385	1	full-splice_match	gene: Zm00001d008408	Zm00001d008408_T001	21.62	21.62	coding	345	97	1131
PB.4192.5	3	1563	10	incomplete-splice_match	gene: Zm00001d042212	Zm00001d042212_T018	1.67	24.00	coding	459	186	1562
PB.4192.4	3	2622	12	novel_in_catalog	gene: Zm00001d042212	novel	1.31	24.00	coding	822	155	2620
PB.868.3	1	1509	4	intergenic	novelGene_313	novel	35.19	89.57	coding	395	207	1391
PB.9830.2	8	1604	8	novel_not_in_catalog	gene: Zm00001d011918	novel	18.79	18.79	coding	333	168	1166
PB.7521.1	6	2098	7	novel_in_catalog	gene: Zm00001d036678	novel	6.22	6.22	coding	135	1151	1555
PB.4447.1	3	736	6	full-splice_match	gene: Zm00001d043352	Zm00001d043352_T002	14.48	14.48	coding	174	85	606
PB.8549.1	7	1628	2	full-splice_match	gene: Zm00001d020781	Zm00001d020781_T001	23.52	23.52	coding	367	202	1302
PB.7160.2	5	2013	15	full-splice_match	gene: Zm00001d018440	Zm00001d018440_T001	37.57	67.73	coding	468	256	1659
PB.6789.1	5	1028	2	novel_in_catalog	gene: Zm00001d016977	novel	22.14	22.14	coding	197	134	724
PB.1361.1	1	1673	1	full-splice_match	gene: Zm00001d033882	Zm00001d033882_T001	2.16	2.16	coding	299	582	1478
PB.5502.4	4	2419	9	full-splice_match	gene: Zm00001d052385	Zm00001d052385_T001	16.39	18.21	coding	633	68	1966
PB.8454.1	7	1751	3	full-splice_match	gene: Zm00001d020340	Zm00001d020340_T001	1.97	1.97	coding	519	33	1589
PB.2382.2	10	783	3	full-splice_match	gene: Zm00001d026704	Zm00001d026704_T002	86.00	117.45	coding	166	56	553
PB.3336.2	2	679	6	full-splice_match	gene: Zm00001d006474	Zm00001d006474_T001	522.31	531.17	coding	119	111	467
PB.9850.2	8	2778	1	novel_in_catalog	gene: Zm00001d012015	novel	11.08	11.08	coding	208	1994	2617

PB.214.1	1	1249	6	full-splice_match	gene: Zm00001d028197	Zm00001d028197_T001	13.42	13.42	coding	239	232	948
PB.3336.1	2	1747	4	novel_in_catalog	gene: Zm00001d006474	novel	6.50	531.17	non_coding	NA	NA	NA
PB.967.1	1	926	2	full-splice_match	gene: Zm00001d032098	Zm00001d032098_T001	37.47	37.47	coding	186	147	704
PB.4011.1	3	1298	4	novel_not_in_catalog	gene: Zm00001d041351	novel	6.47	6.47	coding	335	49	1053
PB.1937.3	10	1866	3	novel_not_in_catalog	gene: Zm00001d024784	novel	0.00	26.61	coding	430	231	1520
PB.8529.1	7	1094	1	genic	gene: Zm00001d020685	novel	5.17	208.26	non_coding	NA	NA	NA
PB.10077.1	9	892	2	intergenic	novelGene_38	novel	1.58	1.58	non_coding	NA	NA	NA
PB.8529.2	7	2033	10	incomplete-splice_match	gene: Zm00001d020685	Zm00001d020685_T003	140.84	208.26	coding	341	269	1291
PB.6106.1	5	1660	10	full-splice_match	gene: Zm00001d013485	Zm00001d013485_T006	31.04	31.04	coding	267	67	867
PB.2990.3	2	1370	2	full-splice_match	gene: Zm00001d004689	Zm00001d004689_T001	0.00	336.02	coding	363	39	1127
PB.8394.2	7	1601	8	novel_in_catalog	gene: Zm00001d020004	novel	1.21	101.12	coding	394	138	1319
PB.8394.1	7	1581	9	full-splice_match	gene: Zm00001d020004	Zm00001d020004_T005	99.91	101.12	coding	397	147	1337
PB.7040.4	5	2106	6	full-splice_match	gene: Zm00001d018024	Zm00001d018024_T001	0.00	65.32	coding	585	28	1782
PB.8041.1	6	2027	16	full-splice_match	gene: Zm00001d038857	Zm00001d038857_T001	32.75	32.75	coding	580	69	1808
PB.8920.1	7	2043	2	incomplete-splice_match	gene: Zm00001d022201	Zm00001d022201_T002	21.48	21.48	coding	561	146	1828
PB.7040.1	5	2537	6	full-splice_match	gene: Zm00001d018024	Zm00001d018024_T001	65.32	65.32	coding	595	221	2005
PB.6859.17	5	914	1	novel_in_catalog	gene: Zm00001d017275	novel	8.20	88.65	coding	138	164	577

PB.5107.2	4	1189	5	full-splice_match	gene: Zm00001d050202	Zm00001d050202_T002	0.00	37.30	coding	310	92	1021
PB.10478.2	9	2540	2	full-splice_match	gene: Zm00001d047273	Zm00001d047273_T001	20.57	20.57	coding	543	434	2062
PB.5107.1	4	1363	5	full-splice_match	gene: Zm00001d050202	Zm00001d050202_T002	37.30	37.30	coding	310	114	1043
PB.705.1	1	904	2	incomplete-splice_match	gene: Zm00001d030678	Zm00001d030678_T003	22.15	151.68	coding	177	28	558
PB.705.2	1	1113	3	full-splice_match	gene: Zm00001d030678	Zm00001d030678_T004	52.92	151.68	coding	211	242	874
PB.705.3	1	1298	2	novel_in_catalog	gene: Zm00001d030678	novel	49.82	151.68	coding	177	538	1068
PB.705.4	1	1157	3	full-splice_match	gene: Zm00001d030678	Zm00001d030678_T001	19.25	151.68	coding	242	209	934
PB.705.6	1	1347	1	novel_in_catalog	gene: Zm00001d030678	novel	7.54	151.68	coding	135	748	1152
PB.4743.6	3	1542	1	genic	gene: Zm00001d044504	novel	1.97	105.91	coding	393	126	1304
PB.10747.1	9	1518	10	full-splice_match	gene: Zm00001d048292	Zm00001d048292_T002	30.81	45.30	coding	311	33	965
PB.5699.1	4	1810	14	full-splice_match	gene: Zm00001d053286	Zm00001d053286_T002	54.88	54.88	coding	409	129	1355
PB.6859.14	5	2480	2	full-splice_match	gene: Zm00001d017275	Zm00001d017275_T001	0.00	88.65	coding	719	160	2316
PB.10747.4	9	1402	10	full-splice_match	gene: Zm00001d048292	Zm00001d048292_T002	14.50	45.30	coding	311	41	973
PB.9858.1	8	2271	6	novel_in_catalog	gene: Zm00001d012067	novel	0.41	52.05	coding	342	158	1183
PB.4852.1	4	755	1	full-splice_match	gene: Zm00001d048886	Zm00001d048886_T001	69.64	69.64	coding	220	43	702
PB.3254.3	2	2067	17	novel_not_in_catalog	gene: Zm00001d006131	novel	3.06	88.89	coding	335	923	1927
PB.5191.1	4	1129	10	full-splice_match	gene: Zm00001d050874	Zm00001d050874_T001	224.75	224.75	coding	251	127	879

PB.1607.1	1	1405	4	full-splice_match	gene: Zm00001d034829	Zm00001d034829_T001	47.31	47.31	coding	379	30	1166
PB.725.2	1	2124	2	novel_in_catalog	gene: Zm00001d030864	novel	4.08	11.55	coding	363	208	1296
PB.9535.1	8	1414	2	genic	gene: Zm00001d010635	novel	24.78	26.13	coding	312	144	1079
PB.3607.1	2	3576	3	incomplete-splice_match	gene: Zm00001d007862	Zm00001d007862_T001	8.74	8.74	coding	1079	168	3404
PB.725.1	1	2471	5	full-splice_match	gene: Zm00001d030864	Zm00001d030864_T001	7.47	11.55	coding	641	457	2379
PB.6893.11	5	2130	6	novel_in_catalog	gene: Zm00001d017429	novel	0.00	210.43	coding	304	941	1852
PB.6979.1	5	1465	2	full-splice_match	gene: Zm00001d017823	Zm00001d017823_T002	18.07	70.16	coding	331	92	1084
PB.6979.2	5	1351	2	full-splice_match	gene: Zm00001d017823	Zm00001d017823_T002	52.08	70.16	coding	331	116	1108
PB.6893.12	5	2172	7	full-splice_match	gene: Zm00001d017429	Zm00001d017429_T001	0.00	210.43	coding	668	169	2172
PB.5282.1	4	2502	7	full-splice_match	gene: Zm00001d051431	Zm00001d051431_T005	17.71	21.35	coding	565	559	2253
PB.719.2	1	2500	3	full-splice_match	gene: Zm00001d030795	Zm00001d030795_T001	4.81	20.11	coding	720	195	2354
PB.719.1	1	2462	3	full-splice_match	gene: Zm00001d030795	Zm00001d030795_T001	15.31	20.11	coding	652	201	2156
PB.9088.3	8	1607	6	novel_not_in_catalog	gene: Zm00001d008311	novel	15.66	15.66	coding	96	150	437
PB.7141.6	5	508	1	intergenic	novelGene_345	novel	0.00	272.48	non_coding	NA	NA	NA
PB.3786.1	3	1496	2	novel_not_in_catalog	gene: Zm00001d039880	novel	12.74	12.74	coding	437	18	1328
PB.4039.4	3	3831	14	full-splice_match	gene: Zm00001d041497	Zm00001d041497_T005	0.00	10.97	coding	888	861	3524
PB.4039.1	3	3942	14	full-splice_match	gene: Zm00001d041497	Zm00001d041497_T005	10.97	10.97	coding	888	866	3529

PB.5482.1	4	1446	4	full-splice_match	gene: Zm00001d052324	Zm00001d052324_T001	10.63	10.63	coding	401	242	1444
PB.2276.2	10	2606	5	novel_in_catalog	gene: Zm00001d026366	novel	5.09	17.17	coding	400	1065	2264
PB.2276.3	10	1546	6	incomplete-splice_match	gene: Zm00001d026366	Zm00001d026366_T002	12.09	17.17	coding	421	109	1371
PB.180.2	1	1825	2	genic	gene: Zm00001d028051	novel	21.70	29.90	coding	491	135	1607
PB.180.1	1	1659	2	genic	gene: Zm00001d028051	novel	8.20	29.90	coding	424	26	1297
PB.6649.1	5	1256	1	full-splice_match	gene: Zm00001d016271	Zm00001d016271_T001	41.45	41.45	coding	315	86	1030
PB.4969.1	4	1987	2	novel_in_catalog	gene: Zm00001d049459	novel	10.31	125.93	coding	185	102	656
PB.1937.2	10	2176	3	novel_not_in_catalog	gene: Zm00001d024784	novel	15.60	26.61	coding	430	359	1648
PB.4969.3	4	1337	3	novel_in_catalog	gene: Zm00001d049459	novel	8.16	125.93	coding	205	123	737
PB.4969.2	4	1211	4	full-splice_match	gene: Zm00001d049459	Zm00001d049459_T001	106.28	125.93	coding	314	90	1031
PB.4969.5	4	1046	4	full-splice_match	gene: Zm00001d049459	Zm00001d049459_T001	0.00	125.93	coding	314	30	971
PB.4969.4	4	1842	3	novel_in_catalog	gene: Zm00001d049459	novel	1.19	125.93	coding	185	60	614
PB.5282.2	4	2400	7	full-splice_match	gene: Zm00001d051431	Zm00001d051431_T008	3.63	21.35	coding	546	559	2196
PB.5444.1	4	2363	11	incomplete-splice_match	gene: Zm00001d052193	Zm00001d052193_T014	19.97	24.55	coding	540	59	1678
PB.9593.1	8	1874	2	novel_in_catalog	gene: Zm00001d010855	novel	211.80	211.80	coding	113	96	434
PB.2330.1	10	961	5	full-splice_match	gene: Zm00001d026562	Zm00001d026562_T001	26.12	32.01	coding	207	97	717
PB.4472.1	3	1827	5	full-splice_match	gene: Zm00001d043443	Zm00001d043443_T001	45.20	47.55	coding	453	142	1500

PB.6624.1	5	1692	5	novel_in_catalog	gene: Zm00001d016130	novel	102.96	102.96	coding	378	232	1365
PB.4472.2	3	1683	5	full-splice_match	gene: Zm00001d043443	Zm00001d043443_T001	2.35	47.55	coding	453	143	1501
PB.4236.5	3	1766	13	novel_in_catalog	gene: Zm00001d042421	novel	1.71	50.83	coding	268	219	1022
PB.6810.10	5	2260	7	novel_in_catalog	gene: Zm00001d017096	novel	0.00	118.92	coding	171	106	618
PB.10473.2	9	3092	3	full-splice_match	gene: Zm00001d047240	Zm00001d047240_T001	19.84	84.90	coding	785	507	2861
PB.7407.1	6	2111	9	intergenic	novelGene_127	novel	17.74	17.74	coding	613	51	1889
PB.3491.1	2	2160	1	full-splice_match	gene: Zm00001d007208	Zm00001d007208_T001	8.96	8.96	coding	654	163	2124
PB.3673.3	3	2711	7	full-splice_match	gene: Zm00001d039362	Zm00001d039362_T002	0.00	18.36	coding	670	412	2421
PB.711.1	1	2410	1	full-splice_match	gene: Zm00001d030759	Zm00001d030759_T001	30.65	30.65	coding	690	92	2161
PB.3673.1	3	2675	7	full-splice_match	gene: Zm00001d039362	Zm00001d039362_T002	18.36	18.36	coding	670	272	2281
PB.6655.1	5	1402	1	full-splice_match	gene: Zm00001d016306	Zm00001d016306_T001	15.15	15.15	coding	333	284	1282
PB.422.2	1	972	1	incomplete-splice_match	gene: Zm00001d029055	Zm00001d029055_T012	16.15	46.69	coding	157	64	534
PB.422.3	1	2667	12	incomplete-splice_match	gene: Zm00001d029055	Zm00001d029055_T029	9.30	46.69	coding	874	44	2665
PB.2799.2	2	1409	5	full-splice_match	gene: Zm00001d003432	Zm00001d003432_T002	38.57	51.53	coding	361	209	1291
PB.422.1	1	2295	4	incomplete-splice_match	gene: Zm00001d029055	Zm00001d029055_T012	21.24	46.69	coding	503	211	1719
PB.2799.4	2	895	3	incomplete-splice_match	gene: Zm00001d003432	Zm00001d003432_T002	12.97	51.53	coding	184	295	846
PB.2480.2	2	5111	1	intergenic	novelGene_28	novel	7.44	7.44	coding	1627	92	4972

PB.3250.4	2	1168	6	full-splice_match	gene: Zm00001d006125	Zm00001d006125_T002	154.41	154.41	coding	263	214	1002
PB.8154.1	7	743	1	full-splice_match	gene: Zm00001d018629	Zm00001d018629_T001	18.27	18.27	coding	163	105	593
PB.2229.1	10	1248	12	full-splice_match	gene: Zm00001d026214	Zm00001d026214_T003	44.11	100.48	coding	270	150	959
PB.7680.4	6	1326	8	novel_in_catalog	gene: Zm00001d037507	novel	56.77	157.79	coding	1	383	385
PB.7680.5	6	1547	7	full-splice_match	gene: Zm00001d037507	Zm00001d037507_T008	34.25	157.79	non_coding	NA	NA	NA
PB.4429.5	3	3433	5	novel_in_catalog	gene: Zm00001d043275	novel	1.95	52.72	coding	149	2591	3037
PB.7680.7	6	2018	3	novel_in_catalog	gene: Zm00001d037507	novel	2.18	157.79	non_coding	NA	NA	NA
PB.7680.1	6	1475	7	full-splice_match	gene: Zm00001d037507	Zm00001d037507_T005	0.00	157.79	coding	1	383	385
PB.7680.2	6	1309	5	full-splice_match	gene: Zm00001d037507	Zm00001d037507_T001	24.62	157.79	non_coding	NA	NA	NA
PB.7680.3	6	1909	4	full-splice_match	gene: Zm00001d037507	Zm00001d037507_T023	6.35	157.79	non_coding	NA	NA	NA
PB.6543.2	5	3169	5	full-splice_match	gene: Zm00001d015459	Zm00001d015459_T004	4.69	229.13	coding	445	46	1380
PB.6543.1	5	2226	6	full-splice_match	gene: Zm00001d015459	Zm00001d015459_T002	207.02	229.13	coding	555	148	1812
PB.7680.8	6	1763	4	novel_in_catalog	gene: Zm00001d037507	novel	29.89	157.79	non_coding	NA	NA	NA
PB.6543.6	5	2103	1	novel_in_catalog	gene: Zm00001d015459	novel	6.88	229.13	non_coding	NA	NA	NA
PB.5653.1	4	1863	6	full-splice_match	gene: Zm00001d053004	Zm00001d053004_T001	25.14	25.14	coding	485	86	1540
PB.6543.4	5	2295	5	novel_in_catalog	gene: Zm00001d015459	novel	2.36	229.13	coding	552	148	1803
PB.10093.1	9	1909	7	novel_not_in_catalog	gene: Zm00001d045050	novel	20.82	20.82	coding	331	420	1412

PB.10093.2	9	1768	7	novel_not_in_catalog	gene: Zm00001d045050	novel	0.00	20.82	coding	331	356	1348
PB.9939.3	8	1373	4	full-splice_match	gene: Zm00001d012428	Zm00001d012428_T001	25.85	168.43	coding	370	58	1167
PB.8770.4	7	1203	2	novel_in_catalog	gene: Zm00001d021692	novel	0.20	10.91	coding	387	15	1175
PB.6442.2	5	3563	1	genic	gene: Zm00001d014971	novel	2.63	28.30	coding	895	212	2896
PB.6442.1	5	3488	2	full-splice_match	gene: Zm00001d014971	Zm00001d014971_T001	25.67	28.30	coding	1033	215	3313
PB.10818.1	9	2098	9	incomplete-splice_match	gene: Zm00001d048512	Zm00001d048512_T010	9.50	9.50	coding	615	29	1873
PB.1071.3	1	616	2	incomplete-splice_match	gene: Zm00001d032539	Zm00001d032539_T001	10.06	38.95	non_coding	NA	NA	NA
PB.6864.1	5	1312	4	incomplete-splice_match	gene: Zm00001d017296	Zm00001d017296_T004	48.44	48.44	coding	275	29	853
PB.4679.2	3	734	1	full-splice_match	gene: Zm00001d044247	Zm00001d044247_T001	1064.59	1129.73	coding	148	81	524
PB.10111.2	9	1371	12	full-splice_match	gene: Zm00001d045146	Zm00001d045146_T002	59.49	59.49	coding	353	133	1191
PB.10386.1	9	1798	2	full-splice_match	gene: Zm00001d046690	Zm00001d046690_T001	5.51	5.51	coding	501	21	1523
PB.3525.1	2	1939	3	full-splice_match	gene: Zm00001d007434	Zm00001d007434_T010	12.33	67.30	coding	248	877	1620
PB.9320.5	8	1793	9	novel_not_in_catalog	gene: Zm00001d009513	novel	0.00	17.90	coding	520	125	1684
PB.9320.4	8	1935	8	novel_not_in_catalog	gene: Zm00001d009513	novel	0.00	17.90	coding	504	121	1632
PB.7588.1	6	1237	9	full-splice_match	gene: Zm00001d037096	Zm00001d037096_T001	211.10	211.10	coding	239	352	1068
PB.9320.1	8	2057	8	novel_not_in_catalog	gene: Zm00001d009513	novel	8.90	17.90	coding	504	122	1633
PB.2208.2	10	982	4	novel_in_catalog	gene: Zm00001d026124	novel	30.06	30.06	coding	213	94	732

PB.9320.3	8	2117	9	novel_not_in_catalog	gene: Zm00001d009513	novel	6.83	17.90	coding	520	140	1699
PB.9320.2	8	2784	1	novel_in_catalog	gene: Zm00001d009513	novel	2.17	17.90	non_coding	NA	NA	NA
PB.711.2	1	2215	1	full-splice_match	gene: Zm00001d030759	Zm00001d030759_T001	0.00	30.65	coding	690	54	2123
PB.2639.1	2	1288	9	full-splice_match	gene: Zm00001d002796	Zm00001d002796_T002	19.11	19.11	coding	267	211	1011
PB.8535.1	7	2830	1	full-splice_match	gene: Zm00001d020703	Zm00001d020703_T001	18.07	18.07	coding	430	1281	2570
PB.7195.1	5	1527	1	full-splice_match	gene: Zm00001d018534	Zm00001d018534_T001	20.66	20.66	coding	419	65	1321
PB.3204.1	2	756	1	full-splice_match	gene: Zm00001d005879	Zm00001d005879_T001	82.34	82.34	coding	91	183	455
PB.3204.2	2	448	1	full-splice_match	gene: Zm00001d005879	Zm00001d005879_T001	0.00	82.34	non_coding	NA	NA	NA
PB.472.1	1	1024	4	intergenic	novelGene_123	novel	57.63	57.63	coding	59	295	471
PB.1985.1	10	760	1	full-splice_match	gene: Zm00001d024969	Zm00001d024969_T001	0.23	0.23	coding	180	30	569
PB.8490.1	7	1876	1	full-splice_match	gene: Zm00001d020496	Zm00001d020496_T001	72.00	87.76	coding	464	209	1600
PB.9036.1	7	958	8	novel_in_catalog	gene: Zm00001d022585	novel	67.88	67.88	coding	211	92	724
PB.8796.6	7	1282	8	incomplete-splice_match	gene: Zm00001d021753	Zm00001d021753_T001	20.43	113.38	coding	381	56	1198
PB.4949.3	4	1744	3	full-splice_match	gene: Zm00001d049361	Zm00001d049361_T002	7.80	132.43	coding	423	269	1537
PB.4949.2	4	1996	3	full-splice_match	gene: Zm00001d049361	Zm00001d049361_T003	72.84	132.43	coding	423	414	1682
PB.3798.4	3	1528	2	full-splice_match	gene: Zm00001d039970	Zm00001d039970_T001	3.67	10.54	coding	369	160	1266
PB.3798.5	3	1417	2	full-splice_match	gene: Zm00001d039970	Zm00001d039970_T001	6.87	10.54	coding	369	160	1266

PB.3798.2	3	1551	2	full-splice_match	gene: Zm00001d039969	Zm00001d039969_T001	13.21	13.93	coding	403	120	1328
PB.8120.10	6	1996	14	full-splice_match	gene: Zm00001d039162	Zm00001d039162_T001	0.00	103.90	coding	474	265	1686
PB.3798.1	3	1751	2	full-splice_match	gene: Zm00001d039969	Zm00001d039969_T001	0.72	13.93	coding	403	72	1280
PB.9195.3	8	2235	10	novel_in_catalog	gene: Zm00001d008816	novel	6.28	73.53	coding	223	1419	2087
PB.9033.2	7	1805	4	novel_in_catalog	gene: Zm00001d022573	novel	9.78	125.41	coding	492	42	1517
PB.9195.6	8	1878	11	full-splice_match	gene: Zm00001d008816	Zm00001d008816_T001	67.25	73.53	coding	531	151	1743
PB.7189.3	5	1943	21	full-splice_match	gene: Zm00001d018525	Zm00001d018525_T001	0.00	44.59	coding	586	126	1883
PB.7189.1	5	2099	21	full-splice_match	gene: Zm00001d018525	Zm00001d018525_T001	44.59	44.59	coding	586	126	1883
PB.5062.1	4	1261	12	full-splice_match	gene: Zm00001d049939	Zm00001d049939_T005	51.25	57.74	coding	340	148	1167
PB.5062.2	4	1330	11	novel_in_catalog	gene: Zm00001d049939	novel	6.49	57.74	coding	266	75	872
PB.1130.1	1	2596	4	full-splice_match	gene: Zm00001d032761	Zm00001d032761_T002	24.54	26.05	coding	569	556	2262
PB.1130.3	1	2629	2	novel_in_catalog	gene: Zm00001d032761	novel	1.51	26.05	coding	401	1124	2326
PB.1007.2	1	823	2	full-splice_match	gene: Zm00001d032292	Zm00001d032292_T001	138.23	170.43	coding	189	97	663
PB.1007.1	1	581	2	full-splice_match	gene: Zm00001d032292	Zm00001d032292_T001	32.19	170.43	coding	7	299	319
PB.8019.1	6	2034	10	full-splice_match	gene: Zm00001d038796	Zm00001d038796_T001	14.61	14.61	coding	564	86	1777
PB.3232.1	2	806	4	incomplete-splice_match	gene: Zm00001d006051	Zm00001d006051_T001	26.66	26.66	coding	113	303	641
PB.7107.3	5	2065	7	novel_in_catalog	gene: Zm00001d018291	novel	1.90	1.90	coding	224	138	809

PB.10105.1	9	2191	1	antisense	novelGene_gene: Zm00001d045098_A	novel	10.72	10.72	coding	80	468	707
PB.1023.2	1	1243	1	novel_in_catalog	gene: Zm00001d032359	novel	22.95	400.26	non_coding	NA	NA	NA
PB.9348.2	8	1113	4	full-splice_match	gene: Zm00001d009686	Zm00001d009686_T002	45.47	55.54	coding	245	68	802
PB.9348.1	8	1420	2	novel_in_catalog	gene: Zm00001d009686	novel	10.08	55.54	coding	180	111	650
PB.507.1	1	2090	1	full-splice_match	gene: Zm00001d029474	Zm00001d029474_T001	15.19	25.36	coding	526	13	1590
PB.507.2	1	1968	1	full-splice_match	gene: Zm00001d029474	Zm00001d029474_T001	10.17	25.36	coding	526	89	1666
PB.9495.5	8	1844	2	novel_in_catalog	gene: Zm00001d010445	novel	0.00	239.50	coding	171	169	681
PB.2321.1	10	1680	2	full-splice_match	gene: Zm00001d026531	Zm00001d026531_T001	19.85	27.53	coding	475	14	1438
PB.2321.2	10	1531	2	full-splice_match	gene: Zm00001d026531	Zm00001d026531_T001	7.68	27.53	non_coding	NA	NA	NA
PB.9495.2	8	1963	2	novel_in_catalog	gene: Zm00001d010445	novel	18.35	239.50	coding	171	185	697
PB.3107.2	2	1630	1	full-splice_match	gene: Zm00001d005351	Zm00001d005351_T001	22.57	30.42	coding	422	213	1478
PB.3107.3	2	1452	1	full-splice_match	gene: Zm00001d005351	Zm00001d005351_T001	0.00	30.42	coding	422	111	1376
PB.10477.1	9	2904	3	genic	gene: Zm00001d047268	novel	2.10	2.10	coding	790	269	2638
PB.416.7	1	1755	5	novel_in_catalog	gene: Zm00001d029033	novel	0.00	20.35	coding	216	869	1516
PB.8160.1	7	1820	11	full-splice_match	gene: Zm00001d018642	Zm00001d018642_T007	7.60	21.36	coding	336	581	1588
PB.8160.2	7	1640	10	full-splice_match	gene: Zm00001d018642	Zm00001d018642_T008	13.76	21.36	coding	302	504	1409
PB.416.3	1	2112	6	full-splice_match	gene: Zm00001d029033	Zm00001d029033_T004	10.51	20.35	coding	216	766	1413

PB.416.2	1	2063	6	novel_not_in_catalog	gene: Zm00001d029033	novel	6.32	20.35	coding	216	715	1362
PB.2166.1	10	2333	7	full-splice_match	gene: Zm00001d025911	Zm00001d025911_T002	12.29	12.29	coding	475	278	1702
PB.7181.2	5	1117	6	novel_in_catalog	gene: Zm00001d018511	novel	20.36	33.02	coding	174	256	777
PB.9775.4	8	592	2	full-splice_match	gene: Zm00001d011718	Zm00001d011718_T003	0.00	83.02	coding	65	254	448
PB.9775.3	8	912	2	full-splice_match	gene: Zm00001d011718	Zm00001d011718_T003	80.10	83.02	coding	207	37	657
PB.416.9	1	1505	6	full-splice_match	gene: Zm00001d029033	Zm00001d029033_T004	0.00	20.35	coding	216	733	1380
PB.416.8	1	1487	7	full-splice_match	gene: Zm00001d029033	Zm00001d029033_T003	0.24	20.35	coding	161	790	1272
PB.5445.1	4	1034	2	full-splice_match	gene: Zm00001d052194	Zm00001d052194_T001	7.46	7.46	coding	218	93	746
PB.5445.2	4	926	2	full-splice_match	gene: Zm00001d052194	Zm00001d052194_T001	0.00	7.46	coding	218	93	746
PB.8626.1	7	958	1	full-splice_match	gene: Zm00001d021141	Zm00001d021141_T001	77.81	309.09	coding	165	87	581
PB.4955.1	4	2445	1	genic	gene: Zm00001d049376	novel	35.32	44.15	coding	476	304	1731
PB.4955.2	4	3473	9	novel_not_in_catalog	gene: Zm00001d049376	novel	8.39	44.15	coding	912	160	2895
PB.4955.3	4	3321	9	novel_not_in_catalog	gene: Zm00001d049376	novel	0.43	44.15	coding	912	207	2942
PB.4790.3	3	1818	9	novel_in_catalog	gene: Zm00001d044713	novel	146.93	146.93	coding	178	136	669
PB.5096.2	4	2505	13	full-splice_match	gene: Zm00001d050153	Zm00001d050153_T002	30.60	30.60	coding	652	156	2111
PB.9498.3	8	1411	4	novel_in_catalog	gene: Zm00001d010451	novel	72.45	194.40	coding	368	157	1260
PB.6108.1	5	2537	9	novel_in_catalog	gene: Zm00001d013492	novel	3.04	3.04	coding	410	733	1962

PB.9498.1	8	1722	4	novel_in_catalog	gene: Zm00001d010451	novel	14.91	194.40	coding	368	134	1237
PB.5006.1	4	709	1	full-splice_match	gene: Zm00001d049657	Zm00001d049657_T001	65.01	65.01	coding	120	256	615
PB.8626.2	7	870	1	full-splice_match	gene: Zm00001d021141	Zm00001d021141_T001	231.29	309.09	coding	165	133	627
PB.4836.1	4	1332	3	full-splice_match	gene: Zm00001d048799	Zm00001d048799_T001	37.55	37.55	coding	327	83	1063
PB.4027.1	3	2649	2	incomplete-splice_match	gene: Zm00001d041443	Zm00001d041443_T019	14.22	14.22	coding	720	217	2376
PB.1768.1	10	906	2	full-splice_match	gene: Zm00001d023811	Zm00001d023811_T001	9.09	11.90	coding	161	54	536
PB.1768.2	10	748	2	full-splice_match	gene: Zm00001d023811	Zm00001d023811_T001	2.82	11.90	coding	161	56	538
PB.9878.3	8	1871	8	novel_in_catalog	gene: Zm00001d012155	novel	11.52	22.90	coding	424	17	1288
PB.7636.4	6	536	3	full-splice_match	gene: Zm00001d037275	Zm00001d037275_T001	3048.14	3319.79	coding	87	104	364
PB.10171.2	9	1115	3	full-splice_match	gene: Zm00001d045404	Zm00001d045404_T001	4.42	4.42	coding	299	96	992
PB.3123.2	2	1413	2	genic	gene: Zm00001d005461	novel	5.56	51.74	coding	305	209	1123
PB.517.2	1	1238	1	full-splice_match	gene: Zm00001d029506	Zm00001d029506_T001	0.00	81.90	coding	313	125	1063
PB.517.1	1	1341	1	full-splice_match	gene: Zm00001d029506	Zm00001d029506_T001	81.90	81.90	coding	313	124	1062
PB.3123.1	2	1621	2	genic	gene: Zm00001d005461	novel	46.18	51.74	coding	305	235	1149
PB.4223.1	3	1595	5	novel_not_in_catalog	gene: Zm00001d042349	novel	35.25	35.25	coding	404	201	1412
PB.4551.2	3	1429	11	full-splice_match	gene: Zm00001d043727	Zm00001d043727_T001	21.91	26.21	coding	355	196	1260
PB.9618.1	8	1787	11	intergenic	novelGene_214	novel	25.98	25.98	non_coding	NA	NA	NA

PB.8951.1	7	1318	5	full-splice_match	gene: Zm00001d022324	Zm00001d022324_T001	165.33	165.33	coding	273	180	998
PB.8951.2	7	1196	5	full-splice_match	gene: Zm00001d022324	Zm00001d022324_T001	0.00	165.33	coding	273	178	996
PB.4187.1	3	2684	15	novel_not_in_catalog	gene: Zm00001d042191	novel	18.17	18.17	coding	815	44	2488
PB.9490.1	8	1005	7	full-splice_match	gene: Zm00001d010424	Zm00001d010424_T001	5.86	5.86	coding	273	18	836
PB.10036.1	9	1317	3	full-splice_match	gene: Zm00001d044729	Zm00001d044729_T001	17.13	17.13	coding	317	54	1004
PB.3274.3	2	1085	2	incomplete-splice_match	gene: Zm00001d006236	Zm00001d006236_T006	49.83	132.37	coding	273	158	976
PB.5451.2	4	985	1	novel_in_catalog	gene: Zm00001d052224	novel	35.52	265.54	coding	142	254	679
PB.5451.3	4	799	2	full-splice_match	gene: Zm00001d052224	Zm00001d052224_T001	39.38	265.54	coding	187	47	607
PB.5451.1	4	932	2	full-splice_match	gene: Zm00001d052224	Zm00001d052224_T001	190.64	265.54	coding	187	44	604
PB.7022.1	5	3040	2	full-splice_match	gene: Zm00001d017983	Zm00001d017983_T001	9.83	9.83	coding	921	77	2839
PB.1623.1	1	2412	5	novel_in_catalog	gene: Zm00001d034876	novel	22.13	22.13	coding	697	47	2137
PB.6061.1	5	1665	9	novel_not_in_catalog	gene: Zm00001d013320	novel	16.75	16.75	coding	318	108	1061
PB.10540.1	9	2897	1	full-splice_match	gene: Zm00001d047533	Zm00001d047533_T001	116.16	141.27	coding	684	517	2568
PB.10540.2	9	3146	1	full-splice_match	gene: Zm00001d047533	Zm00001d047533_T001	25.12	141.27	coding	888	233	2896
PB.4639.1	3	1678	6	full-splice_match	gene: Zm00001d044091	Zm00001d044091_T001	17.63	28.51	coding	382	213	1358
PB.7171.4	5	1658	14	novel_in_catalog	gene: Zm00001d018484	novel	19.76	40.17	coding	105	1128	1442
PB.70.1	1	771	2	genic	gene: Zm00001d027493	novel	70.99	368.73	coding	108	74	397

PB.7935.3	6	2338	1	full-splice_match	gene: Zm00001d038447	Zm00001d038447_T001	2.55	10.96	coding	581	350	2092
PB.70.2	1	707	2	genic	gene: Zm00001d027493	novel	297.74	368.73	coding	108	94	417
PB.9757.1	8	3770	2	full-splice_match	gene: Zm00001d011643	Zm00001d011643_T001	2.67	4.21	coding	1099	38	3334
PB.7423.2	6	2222	15	incomplete-splice_match	gene: Zm00001d036161	Zm00001d036161_T018	12.70	34.34	coding	423	356	1624
PB.1476.4	1	1660	4	full-splice_match	gene: Zm00001d034400	Zm00001d034400_T003	62.98	91.19	coding	428	242	1525
PB.8856.1	7	1505	5	full-splice_match	gene: Zm00001d021961	Zm00001d021961_T001	15.36	15.36	coding	396	96	1283
PB.5705.1	4	2217	15	full-splice_match	gene: Zm00001d053316	Zm00001d053316_T003	29.53	29.53	coding	414	142	1383
PB.7935.1	6	2411	1	full-splice_match	gene: Zm00001d038447	Zm00001d038447_T001	8.41	10.96	coding	581	298	2040
PB.3505.1	2	1836	12	full-splice_match	gene: Zm00001d007288	Zm00001d007288_T005	35.88	35.88	coding	476	93	1520
PB.7601.1	6	2576	11	incomplete-splice_match	gene: Zm00001d037156	Zm00001d037156_T012	26.44	51.56	coding	586	13	1770
PB.7082.1	5	3015	4	novel_in_catalog	gene: Zm00001d018206	novel	60.18	60.18	coding	890	89	2758
PB.7601.3	6	2735	11	incomplete-splice_match	gene: Zm00001d037156	Zm00001d037156_T104	0.73	51.56	coding	377	151	1281
PB.7601.2	6	3603	18	full-splice_match	gene: Zm00001d037156	Zm00001d037156_T106	0.00	51.56	coding	965	6	2900
PB.7601.5	6	624	1	incomplete-splice_match	gene: Zm00001d037156	Zm00001d037156_T056	20.56	51.56	non_coding	NA	NA	NA
PB.7601.4	6	3705	18	full-splice_match	gene: Zm00001d037156	Zm00001d037156_T106	3.84	51.56	coding	1027	9	3089
PB.7096.1	5	2633	3	incomplete-splice_match	gene: Zm00001d018246	Zm00001d018246_T005	31.97	45.36	coding	232	1537	2232
PB.6527.1	5	810	4	full-splice_match	gene: Zm00001d015367	Zm00001d015367_T001	42.22	42.22	coding	160	87	566

PB.10493.1	9	1935	1	full-splice_match	gene: Zm00001d047364	Zm00001d047364_T001	6.64	25.67	coding	357	437	1507
PB.7103.2	5	2713	1	full-splice_match	gene: Zm00001d018282	Zm00001d018282_T001	20.32	51.05	coding	783	27	2375
PB.10493.2	9	1815	1	full-splice_match	gene: Zm00001d047364	Zm00001d047364_T001	19.03	25.67	coding	357	466	1536
PB.1218.1	1	3196	18	full-splice_match	gene: Zm00001d033246	Zm00001d033246_T004	93.37	93.37	coding	185	2396	2950
PB.10761.2	9	2148	13	full-splice_match	gene: Zm00001d048335	Zm00001d048335_T003	8.42	8.42	coding	457	509	1879
PB.6726.2	5	2327	12	novel_in_catalog	gene: Zm00001d016702	novel	5.64	20.56	coding	431	127	1419
PB.10263.1	9	2447	4	novel_in_catalog	gene: Zm00001d045897	novel	38.14	38.14	coding	305	1335	2249
PB.92.8	1	2438	4	novel_in_catalog	gene: Zm00001d027571	novel	3.32	85.29	coding	241	462	1184
PB.92.9	1	1829	5	full-splice_match	gene: Zm00001d027571	Zm00001d027571_T003	11.35	85.29	coding	399	580	1776
PB.9201.1	8	2397	16	full-splice_match	gene: Zm00001d008847	Zm00001d008847_T001	52.28	78.06	coding	639	136	2052
PB.9626.1	8	1888	1	novel_in_catalog	gene: Zm00001d010982	novel	7.17	117.96	coding	102	1060	1365
PB.10037.1	9	2116	5	full-splice_match	gene: Zm00001d044744	Zm00001d044744_T001	43.51	43.51	coding	542	326	1951
PB.7082.2	5	2922	4	novel_in_catalog	gene: Zm00001d018206	novel	0.00	60.18	coding	890	91	2760
PB.5799.3	4	1141	6	novel_not_in_catalog	gene: Zm00001d053751	novel	7.63	12.82	coding	162	44	529
PB.2309.2	10	1761	2	full-splice_match	gene: Zm00001d026495	Zm00001d026495_T002	3.87	23.17	coding	499	59	1555
PB.2309.1	10	1892	1	novel_in_catalog	gene: Zm00001d026495	novel	19.30	23.17	coding	561	39	1721
PB.172.1	1	1032	2	full-splice_match	gene: Zm00001d028002	Zm00001d028002_T001	92.45	92.45	coding	217	163	813

PB.6168.2	5	1534	1	full-splice_match	gene: Zm00001d013747	Zm00001d013747_T001	21.71	23.69	coding	449	29	1375
PB.531.2	1	2190	9	full-splice_match	gene: Zm00001d029566	Zm00001d029566_T007	4.36	4.36	coding	548	305	1948
PB.7508.1	6	3120	2	novel_in_catalog	gene: Zm00001d036613	novel	1.69	1.69	coding	246	2144	2881
PB.6168.1	5	2343	1	full-splice_match	gene: Zm00001d013747	Zm00001d013747_T001	1.98	23.69	coding	449	33	1379
PB.4947.1	4	987	8	full-splice_match	gene: Zm00001d049348	Zm00001d049348_T006	236.06	242.94	coding	182	145	690
PB.5732.1	4	633	1	full-splice_match	gene: Zm00001d053445	Zm00001d053445_T001	54.46	54.46	coding	189	40	606
PB.2288.1	10	2410	12	full-splice_match	gene: Zm00001d026396	Zm00001d026396_T004	44.45	44.45	coding	623	255	2123
PB.4947.2	4	2578	7	full-splice_match	gene: Zm00001d049348	Zm00001d049348_T008	6.88	242.94	coding	54	179	340
PB.5283.4	4	1112	1	incomplete-splice_match	gene: Zm00001d051439	Zm00001d051439_T001	8.47	70.62	coding	216	300	947
PB.5283.3	4	1624	2	full-splice_match	gene: Zm00001d051439	Zm00001d051439_T001	52.38	70.62	coding	367	219	1319
PB.5283.2	4	2612	1	genic	gene: Zm00001d051439	novel	9.76	70.62	coding	351	1242	2294
PB.624.7	1	2692	6	novel_in_catalog	gene: Zm00001d030110	novel	1.01	158.92	coding	451	477	1829
PB.624.4	1	2030	6	full-splice_match	gene: Zm00001d030110	Zm00001d030110_T020	44.33	158.92	coding	362	54	1139
PB.624.5	1	1430	1	novel_in_catalog	gene: Zm00001d030110	novel	15.97	158.92	coding	112	326	661
PB.156.2	1	958	8	full-splice_match	gene: Zm00001d027896	Zm00001d027896_T001	0.00	331.93	coding	246	70	807
PB.624.3	1	2896	6	full-splice_match	gene: Zm00001d030110	Zm00001d030110_T005	0.65	158.92	coding	489	472	1938
PB.3070.1	2	1524	9	full-splice_match	gene: Zm00001d005159	Zm00001d005159_T001	7.47	66.30	coding	318	101	1054

PB.156.1	1	1120	8	full-splice_match	gene: Zm00001d027896	Zm00001d027896_T001	331.93	331.93	coding	246	131	868
PB.5302.1	4	873	2	genic	gene: Zm00001d051543	novel	292.61	568.72	coding	166	56	553
PB.624.8	1	2683	6	novel_in_catalog	gene: Zm00001d030110	novel	3.39	158.92	coding	572	475	2190
PB.5302.2	4	764	2	genic	gene: Zm00001d051543	novel	276.10	568.72	coding	166	55	552
PB.312.1	1	892	1	full-splice_match	gene: Zm00001d028584	Zm00001d028584_T001	10.19	10.19	coding	158	123	596
PB.1103.1	1	1293	3	incomplete-splice_match	gene: Zm00001d032652	Zm00001d032652_T001	74.39	92.27	coding	55	162	326
PB.1384.1	1	1666	2	full-splice_match	gene: Zm00001d033989	Zm00001d033989_T001	28.67	47.15	coding	451	116	1468
PB.891.1	1	2581	1	full-splice_match	gene: Zm00001d031726	Zm00001d031726_T001	12.63	12.63	coding	692	305	2380
PB.7615.3	6	2417	7	full-splice_match	gene: Zm00001d037215	Zm00001d037215_T010	3.55	12.46	coding	713	102	2240
PB.7615.1	6	2767	9	novel_not_in_catalog	gene: Zm00001d037215	novel	8.91	12.46	coding	713	239	2377
PB.10897.2	B73V4 ctg24	801	6	full-splice_match	gene: Zm00001d000379	Zm00001d000379_T002	83.18	92.04	coding	184	36	587
PB.4036.1	3	1793	7	incomplete-splice_match	gene: Zm00001d041488	Zm00001d041488_T008	19.00	26.75	coding	330	25	1014
PB.9334.1	8	1977	9	full-splice_match	gene: Zm00001d009596	Zm00001d009596_T001	3.70	3.70	coding	416	26	1273
PB.6261.1	5	1044	7	full-splice_match	gene: Zm00001d014272	Zm00001d014272_T001	5.42	16.05	coding	226	51	728
PB.6261.2	5	934	7	full-splice_match	gene: Zm00001d014272	Zm00001d014272_T001	10.63	16.05	coding	226	51	728
PB.7684.1	6	2000	4	novel_in_catalog	gene: Zm00001d037530	novel	4.48	27.35	coding	306	832	1749
PB.1752.6	10	1798	6	full-splice_match	gene: Zm00001d023724	Zm00001d023724_T008	9.05	57.44	coding	122	1228	1593

PB.1752.7	10	2379	5	full-splice_match	gene: Zm00001d023724	Zm00001d023724_T003	0.00	57.44	coding	141	1198	1620
PB.1752.4	10	1706	7	novel_in_catalog	gene: Zm00001d023724	novel	28.83	57.44	coding	124	1134	1505
PB.7083.2	5	2774	12	novel_in_catalog	gene: Zm00001d018207	novel	2.59	16.70	coding	487	327	1787
PB.1752.2	10	1873	7	novel_not_in_catalog	gene: Zm00001d023724	novel	7.06	57.44	coding	116	1206	1553
PB.1752.3	10	2552	4	novel_in_catalog	gene: Zm00001d023724	novel	12.50	57.44	coding	141	1356	1778
PB.1167.1	1	2351	4	novel_in_catalog	gene: Zm00001d032939	novel	56.74	60.10	coding	190	463	1032
PB.4374.1	3	1274	3	novel_not_in_catalog	gene: Zm00001d043060	novel	2.20	2.62	coding	270	173	982
PB.1331.2	1	1271	5	full-splice_match	gene: Zm00001d033766	Zm00001d033766_T002	26.30	45.07	coding	283	164	1012
PB.4533.2	3	849	1	full-splice_match	gene: Zm00001d043665	Zm00001d043665_T001	16.04	24.69	coding	168	48	551
PB.4374.2	3	1539	1	genic	gene: Zm00001d043060	novel	0.42	2.62	coding	126	178	555
PB.8312.1	7	864	1	intergenic	novelGene_92	novel	1.85	1.85	non_coding	NA	NA	NA
PB.8312.2	7	968	2	genic	gene: Zm00001d019452	novel	2.48	2.48	non_coding	NA	NA	NA
PB.3078.1	2	1331	7	full-splice_match	gene: Zm00001d005205	Zm00001d005205_T001	33.31	33.31	coding	281	12	854
PB.3597.1	2	1912	8	incomplete-splice_match	gene: Zm00001d007826	Zm00001d007826_T001	36.23	65.94	coding	393	383	1561
PB.108.4	1	2099	4	incomplete-splice_match	gene: Zm00001d027671	Zm00001d027671_T001	0.00	19.43	coding	679	63	2099
PB.108.3	1	3093	7	novel_not_in_catalog	gene: Zm00001d027671	novel	10.89	19.43	coding	808	466	2889
PB.108.1	1	3179	7	novel_not_in_catalog	gene: Zm00001d027671	novel	8.54	19.43	coding	808	456	2879

PB.7639.1	6	2402	10	novel_in_catalog	gene: Zm00001d037291	novel	10.19	31.14	coding	204	76	687
PB.4314.1	3	1225	2	full-splice_match	gene: Zm00001d042769	Zm00001d042769_T001	38.23	38.23	coding	299	116	1012
PB.4497.2	3	1543	2	full-splice_match	gene: Zm00001d043520	Zm00001d043520_T001	0.00	10.75	coding	447	91	1431
PB.7639.2	6	2504	11	full-splice_match	gene: Zm00001d037291	Zm00001d037291_T001	20.64	31.14	coding	606	228	2045
PB.168.1	1	2499	3	full-splice_match	gene: Zm00001d027975	Zm00001d027975_T001	18.01	18.89	coding	598	115	1908
PB.10783.2	9	497	2	novel_in_catalog	gene: Zm00001d048402	novel	96.56	274.26	non_coding	NA	NA	NA
PB.3006.2	2	2533	3	intergenic	novelGene_220	novel	8.22	20.19	non_coding	NA	NA	NA
PB.168.2	1	2349	3	full-splice_match	gene: Zm00001d027975	Zm00001d027975_T001	0.00	18.89	coding	598	114	1907
PB.168.4	1	2243	2	novel_in_catalog	gene: Zm00001d027975	novel	0.88	18.89	coding	346	110	1147
PB.7588.2	6	1074	9	full-splice_match	gene: Zm00001d037096	Zm00001d037096_T001	0.00	211.10	coding	139	616	1032
PB.2000.1	10	2650	1	full-splice_match	gene: Zm00001d025035	Zm00001d025035_T001	23.72	23.72	coding	800	20	2419
PB.6991.4	5	1639	2	incomplete-splice_match	gene: Zm00001d017866	Zm00001d017866_T003	0.00	84.19	coding	335	120	1124
PB.6991.2	5	2475	3	incomplete-splice_match	gene: Zm00001d017866	Zm00001d017866_T002	8.11	84.19	coding	467	85	1485
PB.293.1	1	1815	2	full-splice_match	gene: Zm00001d028482	Zm00001d028482_T001	38.41	38.41	coding	454	6	1367
PB.3533.1	2	1806	1	full-splice_match	gene: Zm00001d007486	Zm00001d007486_T001	52.17	66.66	coding	476	72	1499
PB.6991.1	5	3028	6	full-splice_match	gene: Zm00001d017866	Zm00001d017866_T003	76.07	84.19	coding	467	89	1489
PB.8852.1	7	2005	1	antisense	novelGene_gene: Zm00001d021949_A	novel	18.09	18.09	coding	372	249	1364

PB.4226.2	3	1976	7	full-splice_match	gene: Zm00001d042355	Zm00001d042355_T001	6.14	11.11	coding	593	23	1801
PB.740.1	1	1738	12	full-splice_match	gene: Zm00001d030967	Zm00001d030967_T002	60.57	60.57	coding	469	101	1507
PB.9126.1	8	2618	3	intergenic	novelGene_40	novel	24.59	24.59	non_coding	NA	NA	NA
PB.2000.2	10	2479	1	full-splice_match	gene: Zm00001d025035	Zm00001d025035_T001	0.00	23.72	coding	800	23	2422
PB.5974.1	5	1024	5	full-splice_match	gene: Zm00001d013033	Zm00001d013033_T001	20.45	32.95	coding	194	117	698
PB.5974.2	5	634	5	full-splice_match	gene: Zm00001d013033	Zm00001d013033_T001	12.50	32.95	coding	110	57	386
PB.4102.1	3	824	1	full-splice_match	gene: Zm00001d041790	Zm00001d041790_T001	48.95	78.27	coding	163	44	532
PB.4102.2	3	653	1	full-splice_match	gene: Zm00001d041790	Zm00001d041790_T001	29.33	78.27	coding	163	58	546
PB.8281.1	7	1574	8	full-splice_match	gene: Zm00001d019218	Zm00001d019218_T001	1.92	1.92	coding	427	22	1302
PB.3337.2	2	2223	9	novel_not_in_catalog	gene: Zm00001d006479	novel	24.49	100.07	coding	531	378	1970
PB.2811.3	2	1382	2	full-splice_match	gene: Zm00001d003500	Zm00001d003500_T001	9.17	30.37	coding	423	40	1308
PB.2811.2	2	1520	2	full-splice_match	gene: Zm00001d003500	Zm00001d003500_T001	15.30	30.37	coding	423	35	1303
PB.2811.1	2	413	1	genic	gene: Zm00001d003500	novel	5.89	30.37	non_coding	NA	NA	NA
PB.6856.1	5	1449	13	full-splice_match	gene: Zm00001d017271	Zm00001d017271_T005	233.24	479.93	coding	342	39	1064
PB.4110.2	3	1909	8	incomplete-splice_match	gene: Zm00001d041847	Zm00001d041847_T007	0.00	7.42	coding	548	152	1795
PB.3867.2	3	810	7	full-splice_match	gene: Zm00001d040294	Zm00001d040294_T001	137.33	151.01	coding	146	152	589
PB.3867.1	3	988	7	full-splice_match	gene: Zm00001d040294	Zm00001d040294_T001	13.68	151.01	coding	146	18	455

PB.743.1	1	1092	5	incomplete-splice_match	gene: Zm00001d030985	Zm00001d030985_T001	12.76	12.76	coding	69	693	899
PB.619.2	1	1351	1	full-splice_match	gene: Zm00001d030098	Zm00001d030098_T001	14.09	32.49	coding	308	155	1078
PB.619.1	1	1444	1	full-splice_match	gene: Zm00001d030098	Zm00001d030098_T001	18.40	32.49	coding	308	92	1015
PB.6320.4	5	1970	16	full-splice_match	gene: Zm00001d014526	Zm00001d014526_T001	22.94	59.72	coding	544	94	1725
PB.6980.4	5	1586	1	full-splice_match	gene: Zm00001d017825	Zm00001d017825_T001	0.00	93.25	coding	324	544	1515
PB.2871.1	2	1664	14	novel_not_in_catalog	gene: Zm00001d003813	novel	18.55	18.55	coding	430	112	1401
PB.6320.1	5	2174	16	full-splice_match	gene: Zm00001d014526	Zm00001d014526_T001	7.94	59.72	coding	544	92	1723
PB.1116.2	1	2167	13	full-splice_match	gene: Zm00001d032699	Zm00001d032699_T011	7.34	35.84	coding	289	207	1073
PB.6320.3	5	2087	16	full-splice_match	gene: Zm00001d014526	Zm00001d014526_T001	28.85	59.72	coding	544	106	1737
PB.6856.2	5	2452	12	full-splice_match	gene: Zm00001d017271	Zm00001d017271_T004	15.29	479.93	coding	133	109	507
PB.5847.2	4	1693	7	novel_not_in_catalog	gene: Zm00001d053927	novel	4.39	49.24	coding	262	516	1301
PB.10909.1	B73V4 ctg4	2007	8	full-splice_match	gene: Zm00001d000399	Zm00001d000399_T002	3.46	3.46	coding	572	67	1782
PB.4725.1	3	1404	6	full-splice_match	gene: Zm00001d044452	Zm00001d044452_T003	8.07	8.07	coding	262	141	926
PB.4638.3	3	1156	7	novel_in_catalog	gene: Zm00001d044090	novel	0.64	32.80	coding	100	549	848
PB.2383.4	10	935	6	full-splice_match	gene: Zm00001d026706	Zm00001d026706_T001	160.14	269.39	coding	215	112	756
PB.7250.2	6	2612	10	full-splice_match	gene: Zm00001d035167	Zm00001d035167_T001	14.24	16.15	coding	691	24	2096
PB.7250.1	6	3369	9	novel_in_catalog	gene: Zm00001d035167	novel	1.91	16.15	coding	635	518	2422

PB.8129.2	6	1650	3	novel_in_catalog	gene: Zm00001d039181	novel	3.56	10.68	coding	157	1001	1471
PB.8129.5	6	1658	6	incomplete-splice_match	gene: Zm00001d039181	Zm00001d039181_T001	0.00	10.68	coding	350	609	1658
PB.8129.4	6	2326	7	full-splice_match	gene: Zm00001d039181	Zm00001d039181_T001	7.12	10.68	coding	527	608	2188
PB.457.1	1	1574	2	genic	gene: Zm00001d029217	novel	6.90	6.90	coding	265	656	1450
PB.10487.1	9	1080	3	full-splice_match	gene: Zm00001d047310	Zm00001d047310_T001	50.92	50.92	coding	177	291	821
PB.2383.1	10	3589	5	full-splice_match	gene: Zm00001d026706	Zm00001d026706_T008	29.13	269.39	coding	653	1353	3311
PB.1102.1	1	1305	2	genic	gene: Zm00001d032646	novel	13.27	13.27	coding	234	342	1043
PB.10461.4	9	2750	10	novel_in_catalog	gene: Zm00001d047166	novel	113.50	330.25	coding	257	148	918
PB.1637.1	1	1116	6	full-splice_match	gene: Zm00001d034937	Zm00001d034937_T001	59.47	59.47	coding	249	127	873
PB.9650.4	8	2042	7	full-splice_match	gene: Zm00001d011138	Zm00001d011138_T002	0.00	33.90	coding	578	207	1940
PB.2383.3	10	2056	1	incomplete-splice_match	gene: Zm00001d026706	Zm00001d026706_T007	0.00	269.39	coding	653	27	1985
PB.9650.3	8	2233	7	full-splice_match	gene: Zm00001d011138	Zm00001d011138_T002	33.90	33.90	coding	578	250	1983
PB.10832.2	9	970	4	full-splice_match	gene: Zm00001d048551	Zm00001d048551_T002	100.33	100.33	coding	239	75	791
PB.10520.3	9	1294	1	novel_in_catalog	gene: Zm00001d047470	novel	4.30	514.72	coding	103	449	757
PB.3424.6	2	1918	5	full-splice_match	gene: Zm00001d006900	Zm00001d006900_T004	0.00	180.26	coding	537	87	1697
PB.10520.1	9	1192	2	full-splice_match	gene: Zm00001d047470	Zm00001d047470_T002	112.14	514.72	coding	138	444	857
PB.55.2	1	1070	3	full-splice_match	gene: Zm00001d027442	Zm00001d027442_T001	11.54	22.20	coding	283	40	888

PB.55.1	1	1149	3	full-splice_match	gene: Zm00001d027442	Zm00001d027442_T001	10.66	22.20	coding	283	6	854
PB.10520.5	9	822	3	full-splice_match	gene: Zm00001d047470	Zm00001d047470_T003	364.39	514.72	coding	138	216	629
PB.5290.2	4	502	1	full-splice_match	gene: Zm00001d051478	Zm00001d051478_T001	365.57	683.73	coding	103	77	385
PB.7353.1	6	2629	7	full-splice_match	gene: Zm00001d035736	Zm00001d035736_T001	36.15	36.63	coding	610	520	2349
PB.2595.3	2	831	4	intergenic	novelGene_55	novel	0.00	22.31	coding	66	214	411
PB.7353.2	6	2572	7	full-splice_match	gene: Zm00001d035736	Zm00001d035736_T001	0.48	36.63	coding	610	540	2369
PB.7162.6	5	1942	11	novel_in_catalog	gene: Zm00001d018445	novel	163.41	433.77	coding	243	288	1016
PB.9640.2	8	1293	8	full-splice_match	gene: Zm00001d011067	Zm00001d011067_T001	18.45	53.43	coding	273	205	1023
PB.7162.2	5	3068	14	novel_in_catalog	gene: Zm00001d018445	novel	51.46	433.77	coding	701	597	2699
PB.7162.1	5	2949	15	full-splice_match	gene: Zm00001d018445	Zm00001d018445_T004	0.00	433.77	coding	805	203	2617
PB.9640.1	8	674	2	incomplete-splice_match	gene: Zm00001d011067	Zm00001d011067_T001	34.98	53.43	non_coding	NA	NA	NA
PB.1649.3	10	1735	4	novel_in_catalog	gene: Zm00001d023225	novel	1.51	98.20	coding	318	73	1026
PB.1649.2	10	1672	5	full-splice_match	gene: Zm00001d023225	Zm00001d023225_T001	40.49	98.20	coding	439	79	1395
PB.1649.1	10	1973	2	novel_in_catalog	gene: Zm00001d023225	novel	5.38	98.20	coding	202	74	679
PB.9443.1	8	2535	12	full-splice_match	gene: Zm00001d010199	Zm00001d010199_T003	18.90	30.45	coding	649	202	2148
PB.1649.7	10	1688	4	novel_in_catalog	gene: Zm00001d023225	novel	6.60	98.20	coding	202	71	676
PB.1649.5	10	1654	4	novel_in_catalog	gene: Zm00001d023225	novel	4.19	98.20	coding	265	7	801

PB.1649.4	10	1706	4	novel_in_catalog	gene: Zm00001d023225	novel	0.00	98.20	coding	196	5	592
PB.10193.1	9	2126	3	full-splice_match	gene: Zm00001d045492	Zm00001d045492_T002	20.66	38.42	coding	536	95	1702
PB.10193.2	9	2171	2	full-splice_match	gene: Zm00001d045492	Zm00001d045492_T004	7.11	38.42	coding	532	7	1602
PB.10193.3	9	1813	1	incomplete-splice_match	gene: Zm00001d045492	Zm00001d045492_T004	10.64	38.42	coding	455	1	1365
PB.10193.4	9	1846	2	full-splice_match	gene: Zm00001d045492	Zm00001d045492_T004	0.00	38.42	coding	536	94	1701
PB.7882.9	6	2681	5	full-splice_match	gene: Zm00001d038227	Zm00001d038227_T002	122.89	122.89	coding	452	918	2273
PB.2754.1	2	2201	17	full-splice_match	gene: Zm00001d003214	Zm00001d003214_T001	21.10	25.72	coding	511	250	1782
PB.10718.3	9	1024	6	full-splice_match	gene: Zm00001d048158	Zm00001d048158_T001	0.00	196.20	coding	260	77	856
PB.2754.3	2	3698	14	full-splice_match	gene: Zm00001d003214	Zm00001d003214_T012	4.63	25.72	coding	203	2788	3396
PB.2754.2	2	2935	15	novel_in_catalog	gene: Zm00001d003214	novel	0.00	25.72	coding	266	1812	2609
PB.3351.1	2	516	3	novel_not_in_catalog	gene: Zm00001d006555	novel	391.64	391.64	coding	69	93	299
PB.10347.1	9	1787	4	incomplete-splice_match	gene: Zm00001d046489	Zm00001d046489_T004	0.14	39.21	coding	225	182	856
PB.3001.1	2	2903	4	incomplete-splice_match	gene: Zm00001d004737	Zm00001d004737_T009	17.44	17.44	coding	797	72	2462
PB.10347.3	9	1333	6	full-splice_match	gene: Zm00001d046489	Zm00001d046489_T003	10.58	39.21	coding	346	105	1142
PB.10347.2	9	1475	6	full-splice_match	gene: Zm00001d046489	Zm00001d046489_T003	28.49	39.21	coding	346	104	1141
PB.7429.1	6	314	1	incomplete-splice_match	gene: Zm00001d036213	Zm00001d036213_T001	3793.09	3793.09	non_coding	NA	NA	NA
PB.5671.3	4	1239	8	full-splice_match	gene: Zm00001d053104	Zm00001d053104_T004	0.00	19.94	coding	266	85	882

PB.2149.3	10	2729	10	full-splice_match	gene: Zm00001d025830	Zm00001d025830_T009	6.27	36.86	coding	682	297	2342
PB.2149.2	10	2320	7	incomplete-splice_match	gene: Zm00001d025830	Zm00001d025830_T009	11.63	36.86	coding	436	489	1796
PB.8933.1	7	2236	5	novel_in_catalog	gene: Zm00001d022246	novel	3.96	15.26	coding	417	309	1559
PB.1303.1	1	1165	7	incomplete-splice_match	gene: Zm00001d033635	Zm00001d033635_T002	24.98	24.98	coding	183	262	810
PB.1871.2	10	629	3	full-splice_match	gene: Zm00001d024471	Zm00001d024471_T001	174.88	174.88	coding	71	108	320
PB.10017.1	8	596	3	full-splice_match	gene: Zm00001d012729	Zm00001d012729_T010	38.31	71.44	coding	96	46	333
PB.6851.1	5	2888	2	full-splice_match	gene: Zm00001d017247	Zm00001d017247_T002	15.35	15.35	coding	696	249	2336
PB.5187.1	4	1694	2	full-splice_match	gene: Zm00001d050860	Zm00001d050860_T001	94.14	94.14	coding	490	2	1471
PB.10044.1	9	1831	1	full-splice_match	gene: Zm00001d044808	Zm00001d044808_T001	12.43	19.16	coding	411	316	1548
PB.2846.2	2	1291	2	full-splice_match	gene: Zm00001d003669	Zm00001d003669_T001	0.00	44.40	coding	336	83	1090
PB.2846.1	2	1434	2	full-splice_match	gene: Zm00001d003669	Zm00001d003669_T001	44.40	44.40	coding	336	83	1090
PB.10044.2	9	1310	1	full-splice_match	gene: Zm00001d044808	Zm00001d044808_T001	6.74	19.16	coding	195	588	1172
PB.41.4	1	2057	8	incomplete-splice_match	gene: Zm00001d027376	Zm00001d027376_T008	0.00	34.08	coding	490	325	1794
PB.213.1	1	1864	9	full-splice_match	gene: Zm00001d028195	Zm00001d028195_T003	8.40	8.40	coding	503	93	1601
PB.41.2	1	2044	7	incomplete-splice_match	gene: Zm00001d027376	Zm00001d027376_T001	26.12	34.08	coding	490	66	1535
PB.41.3	1	2210	8	incomplete-splice_match	gene: Zm00001d027376	Zm00001d027376_T008	4.53	34.08	coding	490	329	1798
PB.10017.2	8	630	3	full-splice_match	gene: Zm00001d012729	Zm00001d012729_T017	33.13	71.44	non_coding	NA	NA	NA

PB.4297.1	3	2168	14	full-splice_match	gene: Zm00001d042690	Zm00001d042690_T001	43.10	51.27	coding	571	140	1852
PB.4297.2	3	2201	14	full-splice_match	gene: Zm00001d042690	Zm00001d042690_T001	8.17	51.27	coding	571	249	1961
PB.4424.1	3	3174	6	full-splice_match	gene: Zm00001d043253	Zm00001d043253_T002	13.26	14.05	coding	852	130	2685
PB.2039.1	10	2891	5	full-splice_match	gene: Zm00001d025268	Zm00001d025268_T001	18.14	18.14	coding	573	663	2381
PB.5120.1	4	2810	11	full-splice_match	gene: Zm00001d050315	Zm00001d050315_T001	19.14	19.14	coding	840	96	2615
PB.6043.1	5	1439	4	full-splice_match	gene: Zm00001d013265	Zm00001d013265_T020	6.65	27.15	coding	380	98	1237
PB.4424.5	3	1564	1	genic	gene: Zm00001d043253	novel	0.79	14.05	coding	441	142	1464
PB.5365.1	4	1370	3	full-splice_match	gene: Zm00001d051870	Zm00001d051870_T001	19.44	19.44	coding	374	81	1202
PB.4424.7	3	1090	3	novel_in_catalog	gene: Zm00001d043254	novel	8.83	13.93	coding	121	82	444
PB.4424.6	3	780	1	novel_in_catalog	gene: Zm00001d043254	novel	5.09	13.93	non_coding	NA	NA	NA
PB.4424.9	3	3100	4	novel_in_catalog	gene: Zm00001d043256	novel	3.85	34.21	coding	566	99	1796
PB.4424.8	3	1712	7	full-splice_match	gene: Zm00001d043256	Zm00001d043256_T001	13.48	34.21	coding	510	3	1532
PB.9458.1	8	1356	6	full-splice_match	gene: Zm00001d010240	Zm00001d010240_T001	1.16	1.16	coding	387	32	1192
PB.7519.3	6	2148	15	full-splice_match	gene: Zm00001d036672	Zm00001d036672_T005	3.85	3.85	coding	575	281	2005
PB.10828.1	9	2383	3	incomplete-splice_match	gene: Zm00001d048541	Zm00001d048541_T018	28.67	28.67	coding	623	57	1925
PB.6340.2	5	2162	6	full-splice_match	gene: Zm00001d014609	Zm00001d014609_T001	7.54	34.25	coding	585	177	1931
PB.9273.1	8	1871	8	novel_in_catalog	gene: Zm00001d009221	novel	38.46	38.46	coding	453	10	1368

PB.8109.1	6	820	1	full-splice_match	gene: Zm00001d039110	Zm00001d039110_T001	17.88	17.88	coding	172	67	582
PB.6340.1	5	2266	6	full-splice_match	gene: Zm00001d014609	Zm00001d014609_T001	26.71	34.25	coding	585	177	1931
PB.1105.1	1	888	1	full-splice_match	gene: Zm00001d032663	Zm00001d032663_T001	216.87	216.87	coding	180	92	631
PB.1879.4	10	1895	2	full-splice_match	gene: Zm00001d024516	Zm00001d024516_T001	0.00	39.50	coding	476	205	1632
PB.9289.1	8	1943	10	novel_not_in_catalog	gene: Zm00001d009364	novel	14.00	14.00	coding	551	45	1697
PB.5679.1	4	1164	2	full-splice_match	gene: Zm00001d053156	Zm00001d053156_T001	10.60	10.60	coding	288	44	907
PB.5100.1	4	2240	2	novel_in_catalog	gene: Zm00001d050164	novel	20.47	20.47	coding	266	61	858
PB.1655.1	10	2475	12	full-splice_match	gene: Zm00001d023243	Zm00001d023243_T004	39.59	39.59	coding	626	323	2200
PB.1879.3	10	2120	2	full-splice_match	gene: Zm00001d024516	Zm00001d024516_T001	30.05	39.50	coding	476	242	1669
PB.8088.3	6	1061	2	full-splice_match	gene: Zm00001d039016	Zm00001d039016_T001	100.77	105.65	coding	216	133	780
PB.8088.1	6	2243	5	full-splice_match	gene: Zm00001d039012	Zm00001d039012_T003	9.10	9.10	coding	462	92	1477
PB.6956.2	5	1466	4	full-splice_match	gene: Zm00001d017727	Zm00001d017727_T001	27.32	41.79	coding	347	140	1180
PB.8088.5	6	895	2	full-splice_match	gene: Zm00001d039016	Zm00001d039016_T001	2.27	105.65	coding	216	129	776
PB.8088.4	6	1561	1	novel_in_catalog	gene: Zm00001d039016	novel	2.60	105.65	coding	110	980	1309
PB.8088.9	6	2897	9	novel_not_in_catalog	gene: Zm00001d039037	novel	1.06	41.63	coding	368	1342	2445
PB.8088.8	6	2416	10	novel_not_in_catalog	gene: Zm00001d039037	novel	40.57	41.63	coding	589	198	1964
PB.5295.2	4	4165	6	incomplete-splice_match	gene: Zm00001d051507	Zm00001d051507_T058	8.44	20.94	coding	1228	92	3775

PB.4949.1	4	1909	3	full-splice_match	gene: Zm00001d049361	Zm00001d049361_T002	51.79	132.43	coding	423	246	1514
PB.8015.5	6	1775	13	novel_in_catalog	gene: Zm00001d038776	novel	0.00	85.48	coding	499	78	1574
PB.6963.6	5	2582	8	novel_in_catalog	gene: Zm00001d017755	novel	1.19	16.31	coding	541	760	2382
PB.1327.2	1	1171	1	intergenic	novelGene_373	novel	0.35	0.35	non_coding	NA	NA	NA
PB.8015.4	6	1878	13	novel_in_catalog	gene: Zm00001d038776	novel	61.96	85.48	coding	499	94	1590
PB.88.1	1	1287	4	full-splice_match	gene: Zm00001d027556	Zm00001d027556_T001	30.14	30.14	coding	297	88	978
PB.5270.1	4	1344	2	full-splice_match	gene: Zm00001d051362	Zm00001d051362_T001	1169.23	2366.20	coding	249	364	1110
PB.2036.2	10	927	3	novel_not_in_catalog	gene: Zm00001d025252	novel	63.38	130.70	coding	138	185	598
PB.1329.2	1	2450	2	full-splice_match	gene: Zm00001d033745	Zm00001d033745_T001	0.00	35.54	coding	694	124	2205
PB.1243.3	1	813	4	full-splice_match	gene: Zm00001d033371	Zm00001d033371_T005	8.88	125.13	coding	128	245	628
PB.8340.1	7	833	3	full-splice_match	gene: Zm00001d019576	Zm00001d019576_T001	34.85	34.85	coding	162	167	652
PB.8120.11	6	2033	13	novel_in_catalog	gene: Zm00001d039162	novel	0.15	103.90	coding	197	210	800
PB.7516.1	6	1748	8	full-splice_match	gene: Zm00001d036654	Zm00001d036654_T002	13.44	102.24	coding	349	236	1282
PB.7600.2	6	3520	22	novel_not_in_catalog	gene: Zm00001d037142	novel	1.25	89.93	coding	723	77	2245
PB.4416.2	3	2305	11	novel_not_in_catalog	gene: Zm00001d043211	novel	20.37	20.37	coding	593	180	1958
PB.10873.1	B73V4 ctg205	1554	1	full-splice_match	gene: ENSRNA049479077	ENSRNA049479077-T1	0.00	8523.18	non_coding	NA	NA	NA
PB.8015.2	6	2136	12	novel_in_catalog	gene: Zm00001d038776	novel	0.96	85.48	coding	382	687	1832

PB.8360.2	7	2327	10	full-splice_match	gene: Zm00001d019703	Zm00001d019703_T001	4.99	4.99	coding	570	92	1801
PB.1378.1	1	1844	5	full-splice_match	gene: Zm00001d033981	Zm00001d033981_T001	162.01	162.01	coding	487	54	1514
PB.1883.1	10	4385	25	full-splice_match	gene: Zm00001d024523	Zm00001d024523_T002	31.77	79.72	coding	1141	490	3912
PB.8816.1	7	2715	5	intergenic	novelGene_267	novel	16.98	21.78	coding	105	554	868
PB.3869.1	3	985	1	full-splice_match	gene: Zm00001d040298	Zm00001d040298_T001	400.93	400.93	coding	108	159	482
PB.8040.1	6	1534	17	full-splice_match	gene: Zm00001d038855	Zm00001d038855_T002	37.68	37.68	coding	379	103	1239
PB.1883.5	10	4376	25	full-splice_match	gene: Zm00001d024523	Zm00001d024523_T002	0.00	79.72	coding	1141	491	3913
PB.3869.2	3	441	1	full-splice_match	gene: Zm00001d040298	Zm00001d040298_T001	0.00	400.93	coding	17	265	315
PB.2916.1	2	2281	2	full-splice_match	gene: Zm00001d004072	Zm00001d004072_T001	3.85	3.85	coding	653	96	2054
PB.3306.3	2	1187	4	full-splice_match	gene: Zm00001d006360	Zm00001d006360_T001	0.00	212.80	coding	272	106	921
PB.10262.1	9	1154	2	genic	gene: Zm00001d045896	novel	8.68	8.68	coding	151	491	943
PB.3306.1	2	1288	4	full-splice_match	gene: Zm00001d006360	Zm00001d006360_T001	198.64	212.80	coding	272	106	921
PB.5853.3	4	843	3	intergenic	novelGene_364	novel	114.52	224.73	non_coding	NA	NA	NA
PB.3057.1	2	2334	7	full-splice_match	gene: Zm00001d005099	Zm00001d005099_T003	8.99	8.99	coding	551	338	1990
PB.8076.9	6	2943	7	incomplete-splice_match	gene: Zm00001d038972	Zm00001d038972_T010	0.00	254.63	coding	565	1249	2943
PB.958.1	1	2774	5	novel_in_catalog	gene: Zm00001d032060	novel	5.03	11.82	coding	515	380	1924
PB.8076.7	6	3910	11	full-splice_match	gene: Zm00001d038972	Zm00001d038972_T010	48.82	254.63	coding	871	1129	3741

PB.330.1	1	939	2	novel_in_catalog	gene: Zm00001d028661	novel	23.37	23.37	non_coding	NA	NA	NA
PB.1407.1	1	1432	6	full-splice_match	gene: Zm00001d034063	Zm00001d034063_T002	87.06	87.06	coding	303	230	1138
PB.635.1	1	2072	5	novel_in_catalog	gene: Zm00001d030159	novel	147.69	324.18	coding	566	105	1802
PB.2655.1	2	1802	3	genic	gene: Zm00001d002845	novel	6.11	6.11	coding	187	1056	1616
PB.6519.10	5	1813	2	full-splice_match	gene: Zm00001d015327	Zm00001d015327_T001	0.47	1235.58	coding	533	67	1665
PB.10355.1	9	1388	11	full-splice_match	gene: Zm00001d046538	Zm00001d046538_T004	39.31	39.31	coding	327	93	1073
PB.899.1	1	1305	1	full-splice_match	gene: Zm00001d031801	Zm00001d031801_T001	60.04	60.04	coding	355	38	1102
PB.2129.4	10	2077	2	novel_in_catalog	gene: Zm00001d025761	novel	0.00	127.05	coding	102	102	407
PB.2129.3	10	2566	1	genic	gene: Zm00001d025761	novel	3.40	127.05	coding	133	1866	2264
PB.2129.2	10	2509	3	novel_in_catalog	gene: Zm00001d025761	novel	11.85	127.05	coding	161	1746	2228
PB.7449.1	6	774	1	full-splice_match	gene: Zm00001d036348	Zm00001d036348_T001	10.17	10.17	coding	188	76	639
PB.8740.1	7	1840	12	full-splice_match	gene: Zm00001d021580	Zm00001d021580_T002	22.29	22.29	coding	475	154	1578
PB.2904.4	2	2261	7	incomplete-splice_match	gene: Zm00001d003958	Zm00001d003958_T017	0.00	33.69	coding	728	78	2261
PB.4281.1	3	1211	3	full-splice_match	gene: Zm00001d042634	Zm00001d042634_T001	399.21	399.21	coding	265	136	930
PB.2307.2	10	1886	2	genic	gene: Zm00001d026489	novel	19.35	32.42	coding	426	330	1607
PB.2904.1	2	3562	12	novel_in_catalog	gene: Zm00001d003958	novel	33.69	33.69	coding	996	291	3278
PB.2904.2	2	3396	12	novel_in_catalog	gene: Zm00001d003958	novel	0.00	33.69	coding	996	266	3253

PB.2904.3	2	2842	11	full-splice_match	gene: Zm00001d003958	Zm00001d003958_T013	0.00	33.69	coding	868	238	2841
PB.8368.1	7	1816	1	full-splice_match	gene: Zm00001d019764	Zm00001d019764_T001	2.96	2.96	coding	456	39	1406
PB.4070.2	3	2924	7	full-splice_match	gene: Zm00001d041635	Zm00001d041635_T002	101.43	106.27	coding	663	401	2389
PB.8851.2	7	1765	5	incomplete-splice_match	gene: Zm00001d021947	Zm00001d021947_T002	0.00	47.01	coding	466	114	1511
PB.10669.1	9	715	5	full-splice_match	gene: Zm00001d047962	Zm00001d047962_T001	148.80	148.80	coding	132	99	494
PB.9854.3	8	1323	3	novel_not_in_catalog	gene: Zm00001d012021	novel	18.21	32.26	coding	269	328	1134
PB.3907.13	3	1591	5	full-splice_match	gene: Zm00001d040470	Zm00001d040470_T002	0.00	183.26	coding	436	141	1448
PB.3907.10	3	1870	5	full-splice_match	gene: Zm00001d040470	Zm00001d040470_T002	0.00	183.26	coding	436	279	1586
PB.2397.1	2	1501	6	fusion	gene: Zm00001d001802_g	novel	26.57	26.57	coding	255	411	1175
PB.1538.3	1	2421	10	novel_in_catalog	gene: Zm00001d034610	novel	13.09	19.55	coding	548	387	2030
PB.8991.1	7	3438	12	novel_in_catalog	gene: Zm00001d022440	novel	18.24	69.63	coding	533	1597	3195
PB.8991.2	7	2618	9	incomplete-splice_match	gene: Zm00001d022440	Zm00001d022440_T009	51.39	69.63	coding	753	23	2281
PB.1893.1	10	2152	3	full-splice_match	gene: Zm00001d024572	Zm00001d024572_T002	23.88	23.88	coding	464	413	1804
PB.6450.9	5	2287	8	full-splice_match	gene: Zm00001d014993	Zm00001d014993_T003	0.00	190.83	coding	663	124	2112
PB.6837.1	5	1544	2	novel_in_catalog	gene: Zm00001d017187	novel	27.14	27.14	coding	254	464	1225
PB.7683.1	6	894	3	incomplete-splice_match	gene: Zm00001d037529	Zm00001d037529_T004	2.80	2.80	coding	200	47	646
PB.1560.1	1	2218	4	full-splice_match	gene: Zm00001d034674	Zm00001d034674_T001	13.46	13.46	coding	588	187	1950

PB.9947.6	8	1865	7	full-splice_match	gene: Zm00001d012457	Zm00001d012457_T003	0.00	46.11	coding	536	79	1686
PB.10349.3	9	1436	4	novel_in_catalog	gene: Zm00001d046492	novel	0.00	250.90	coding	219	188	844
PB.10349.1	9	2766	4	novel_in_catalog	gene: Zm00001d046492	novel	14.88	250.90	coding	219	187	843
PB.6686.2	5	1231	5	incomplete-splice_match	gene: Zm00001d016475	Zm00001d016475_T001	8.39	8.39	coding	227	150	830
PB.10349.7	9	1324	3	novel_in_catalog	gene: Zm00001d046492	novel	9.62	250.90	coding	164	187	678
PB.7226.1	6	610	1	incomplete-splice_match	gene: Zm00001d035047	Zm00001d035047_T002	45.21	98.06	coding	70	91	300
PB.7226.2	6	2728	9	full-splice_match	gene: Zm00001d035047	Zm00001d035047_T001	52.85	98.06	coding	771	220	2532
PB.10349.4	9	1267	4	novel_in_catalog	gene: Zm00001d046492	novel	57.86	250.90	coding	219	187	843
PB.10094.2	9	1405	1	full-splice_match	gene: Zm00001d045051	Zm00001d045051_T001	20.11	20.11	coding	331	146	1138
PB.10094.1	9	1517	1	full-splice_match	gene: Zm00001d045051	Zm00001d045051_T001	0.00	20.11	coding	331	143	1135
PB.7057.2	5	2495	1	full-splice_match	gene: Zm00001d018102	Zm00001d018102_T001	0.00	4.63	coding	814	52	2493
PB.9854.1	8	1753	2	novel_not_in_catalog	gene: Zm00001d012021	novel	10.82	32.26	coding	292	547	1422
PB.7057.1	5	2734	1	full-splice_match	gene: Zm00001d018102	Zm00001d018102_T001	4.63	4.63	coding	849	44	2590
PB.8882.1	7	543	1	full-splice_match	gene: Zm00001d022057	Zm00001d022057_T001	189.88	189.88	coding	105	52	366
PB.9571.1	8	1243	3	novel_not_in_catalog	gene: Zm00001d010783	novel	28.82	31.45	coding	322	74	1039
PB.4505.2	3	1720	12	full-splice_match	gene: Zm00001d043555	Zm00001d043555_T004	1.78	33.52	coding	247	137	877
PB.4505.1	3	1731	13	full-splice_match	gene: Zm00001d043555	Zm00001d043555_T001	31.08	33.52	coding	452	140	1495

PB.9571.2	8	1827	2	novel_in_catalog	gene: Zm00001d010783	novel	2.62	31.45	coding	135	1222	1626
PB.1157.5	1	2477	1	genic	gene: Zm00001d032897	novel	8.15	297.96	coding	186	1708	2265
PB.1157.7	1	1639	4	novel_not_in_catalog	gene: Zm00001d032897	novel	8.16	297.96	coding	262	760	1545
PB.1473.5	1	1769	2	full-splice_match	gene: Zm00001d034382	Zm00001d034382_T001	0.00	12.26	coding	529	130	1716
PB.1157.1	1	909	1	genic	gene: Zm00001d032897	novel	45.13	297.96	coding	163	1	489
PB.1380.1	1	1172	4	full-splice_match	gene: Zm00001d033983	Zm00001d033983_T002	615.36	621.61	coding	255	106	870
PB.1157.3	1	1727	4	novel_not_in_catalog	gene: Zm00001d032897	novel	36.49	297.96	coding	430	214	1503
PB.2856.1	2	647	1	novel_in_catalog	gene: Zm00001d003730	novel	772.95	772.95	coding	136	73	480
PB.958.5	1	2609	6	full-splice_match	gene: Zm00001d032060	Zm00001d032060_T001	0.00	11.82	coding	713	375	2513
PB.5530.1	4	1183	5	full-splice_match	gene: Zm00001d052458	Zm00001d052458_T001	80.39	80.39	coding	296	105	992
PB.6906.1	5	2115	2	novel_not_in_catalog	gene: Zm00001d017487	novel	9.53	9.53	non_coding	NA	NA	NA
PB.5705.5	4	2104	15	full-splice_match	gene: Zm00001d053316	Zm00001d053316_T003	0.00	29.53	coding	414	139	1380
PB.3137.1	2	1991	10	full-splice_match	gene: Zm00001d005546	Zm00001d005546_T002	13.00	13.00	coding	510	159	1688
PB.9543.2	8	1664	5	novel_not_in_catalog	gene: Zm00001d010655	novel	0.00	69.98	coding	392	141	1316
PB.9543.3	8	1525	5	novel_not_in_catalog	gene: Zm00001d010655	novel	55.77	69.98	coding	392	142	1317
PB.10675.1	9	1220	5	full-splice_match	gene: Zm00001d047983	Zm00001d047983_T002	92.59	92.59	coding	226	243	920
PB.9543.1	8	1799	5	novel_not_in_catalog	gene: Zm00001d010655	novel	14.22	69.98	coding	392	141	1316

PB.4434.2	3	3398	6	full-splice_match	gene: Zm00001d043295	Zm00001d043295_T002	9.12	34.21	coding	969	285	3191
PB.5201.2	4	1281	8	full-splice_match	gene: Zm00001d050923	Zm00001d050923_T003	39.26	60.79	coding	322	132	1097
PB.5201.1	4	1402	8	full-splice_match	gene: Zm00001d050923	Zm00001d050923_T001	21.53	60.79	coding	322	105	1070
PB.4434.3	3	3294	6	full-splice_match	gene: Zm00001d043295	Zm00001d043295_T001	25.08	34.21	coding	980	150	3089
PB.471.2	1	1536	8	novel_in_catalog	gene: Zm00001d029281	novel	62.99	62.99	coding	183	240	788
PB.2241.4	10	3264	13	full-splice_match	gene: Zm00001d026265	Zm00001d026265_T010	0.00	37.17	coding	1010	114	3143
PB.8603.1	7	1541	2	full-splice_match	gene: Zm00001d020984	Zm00001d020984_T001	85.56	85.56	coding	415	89	1333
PB.2933.1	2	1721	2	novel_not_in_catalog	gene: Zm00001d004167	novel	11.20	11.20	coding	515	78	1622
PB.5041.1	4	1847	1	full-splice_match	gene: Zm00001d049800	Zm00001d049800_T001	5.18	23.63	coding	348	413	1456
PB.1023.1	1	1091	3	novel_in_catalog	gene: Zm00001d032359	novel	377.31	400.26	non_coding	NA	NA	NA
PB.5041.3	4	1675	1	full-splice_match	gene: Zm00001d049800	Zm00001d049800_T001	0.00	23.63	coding	348	416	1459
PB.5041.2	4	1537	2	genic	gene: Zm00001d049800	novel	18.45	23.63	coding	348	234	1277
PB.8107.1	6	802	4	full-splice_match	gene: Zm00001d039106	Zm00001d039106_T001	229.25	229.25	coding	140	165	584
PB.2261.1	10	2375	2	genic	gene: Zm00001d026326	novel	15.84	42.28	coding	564	272	1963
PB.2261.2	10	2131	2	genic	gene: Zm00001d026326	novel	26.18	42.28	coding	564	176	1867
PB.390.1	1	1157	3	full-splice_match	gene: Zm00001d028951	Zm00001d028951_T001	47.19	47.19	coding	249	72	818
PB.2261.4	10	2005	2	genic	gene: Zm00001d026326	novel	0.26	42.28	coding	564	208	1899

PB.2261.5	10	1957	2	genic	gene: Zm00001d026326	novel	0.00	42.28	coding	564	176	1867
PB.197.1	1	2260	2	novel_in_catalog	gene: Zm00001d028128	novel	0.57	0.57	coding	127	87	467
PB.281.3	1	1995	2	full-splice_match	gene: Zm00001d028450	Zm00001d028450_T001	28.16	141.69	coding	182	70	615
PB.2289.2	10	2005	2	genic	gene: Zm00001d026397	novel	22.68	34.44	coding	521	65	1627
PB.4539.5	3	2169	14	novel_not_in_catalog	gene: Zm00001d043693	novel	81.68	192.92	coding	470	307	1716
PB.8086.1	6	2650	1	full-splice_match	gene: Zm00001d039014	Zm00001d039014_T001	137.48	137.48	coding	307	1563	2483
PB.773.5	1	2040	6	full-splice_match	gene: Zm00001d031092	Zm00001d031092_T001	8.25	81.05	coding	330	550	1539
PB.8039.2	6	1481	3	full-splice_match	gene: Zm00001d038852	Zm00001d038852_T001	17.28	63.78	coding	233	218	916
PB.9438.1	8	1146	8	full-splice_match	gene: Zm00001d010174	Zm00001d010174_T003	21.17	21.17	coding	263	11	799
PB.5061.2	4	658	4	novel_in_catalog	gene: Zm00001d049933	novel	292.40	327.98	coding	108	67	390
PB.5061.1	4	825	4	novel_in_catalog	gene: Zm00001d049933	novel	35.58	327.98	coding	44	392	523
PB.4789.1	3	812	4	incomplete-splice_match	gene: Zm00001d044705	Zm00001d044705_T001	21.27	21.27	coding	173	91	609
PB.5126.1	4	596	3	intergenic	novelGene_145	novel	22.73	22.73	non_coding	NA	NA	NA
PB.8039.1	6	1651	3	full-splice_match	gene: Zm00001d038852	Zm00001d038852_T001	0.95	63.78	coding	233	217	915
PB.4539.8	3	2076	13	novel_not_in_catalog	gene: Zm00001d043693	novel	0.46	192.92	coding	504	309	1820
PB.4539.9	3	1928	14	novel_not_in_catalog	gene: Zm00001d043693	novel	0.00	192.92	coding	470	278	1687
PB.4519.1	3	1268	3	novel_not_in_catalog	gene: Zm00001d043611	novel	13.25	24.71	coding	313	100	1038

PB.4519.2	3	1435	2	novel_in_catalog	gene: Zm00001d043611	novel	3.42	24.71	coding	132	813	1208
PB.9343.3	8	1579	14	full-splice_match	gene: Zm00001d009661	Zm00001d009661_T007	0.00	13.20	coding	422	139	1404
PB.4519.4	3	1689	2	novel_not_in_catalog	gene: Zm00001d043611	novel	3.94	24.71	coding	318	511	1464
PB.1206.1	1	1122	3	full-splice_match	gene: Zm00001d033169	Zm00001d033169_T001	20.00	113.95	coding	253	51	809
PB.5429.4	4	2534	1	full-splice_match	gene: Zm00001d052104	Zm00001d052104_T001	125.18	505.95	coding	780	80	2419
PB.1133.1	1	1074	2	full-splice_match	gene: Zm00001d032787	Zm00001d032787_T001	47.72	47.72	coding	294	47	928
PB.9989.1	8	2070	14	fusion	gene: Zm00001d012628_g	novel	14.48	14.48	coding	593	15	1793
PB.1525.2	1	1595	2	full-splice_match	gene: Zm00001d034569	Zm00001d034569_T001	25.57	30.52	coding	469	55	1461
PB.8039.5	6	2338	2	novel_in_catalog	gene: Zm00001d038852	novel	5.44	63.78	coding	233	1377	2075
PB.1525.3	1	1478	2	full-splice_match	gene: Zm00001d034569	Zm00001d034569_T001	0.00	30.52	coding	469	55	1461
PB.6467.4	5	1861	5	incomplete-splice_match	gene: Zm00001d015065	Zm00001d015065_T008	13.56	133.78	coding	238	830	1543
PB.6467.5	5	1292	6	full-splice_match	gene: Zm00001d015065	Zm00001d015065_T007	107.14	133.78	coding	279	200	1036
PB.6467.6	5	2398	5	full-splice_match	gene: Zm00001d015065	Zm00001d015065_T006	0.00	133.78	coding	238	1446	2159
PB.8039.4	6	1372	3	full-splice_match	gene: Zm00001d038852	Zm00001d038852_T001	0.00	63.78	coding	233	215	913
PB.4233.1	3	908	4	novel_in_catalog	gene: Zm00001d042400	novel	71.08	71.08	coding	207	84	704
PB.5102.2	4	1959	1	novel_in_catalog	gene: Zm00001d050179	novel	2.76	534.87	non_coding	NA	NA	NA
PB.5215.1	4	1410	3	full-splice_match	gene: Zm00001d050993	Zm00001d050993_T001	45.36	45.36	coding	348	46	1089

PB.4127.1	3	1843	11	full-splice_match	gene: Zm00001d041945	Zm00001d041945_T005	115.94	124.21	coding	397	360	1550
PB.6977.1	5	3813	24	novel_not_in_catalog	gene: Zm00001d017818	novel	30.22	30.22	coding	1092	231	3506
PB.5215.4	4	1407	2	novel_in_catalog	gene: Zm00001d050993	novel	0.00	45.36	coding	258	483	1256
PB.10822.1	9	1112	1	novel_in_catalog	gene: Zm00001d048522	novel	2.79	2.79	coding	119	583	939
PB.895.2	1	1449	3	full-splice_match	gene: Zm00001d031740	Zm00001d031740_T001	38.22	78.71	coding	348	102	1145
PB.7574.1	6	1678	12	full-splice_match	gene: Zm00001d037010	Zm00001d037010_T003	47.10	47.10	coding	403	114	1322
PB.912.1	1	1633	11	full-splice_match	gene: Zm00001d031849	Zm00001d031849_T002	219.38	219.38	coding	365	220	1314
PB.9301.2	8	1618	12	full-splice_match	gene: Zm00001d009418	Zm00001d009418_T005	5.66	144.20	coding	376	136	1263
PB.7509.4	6	2467	1	genic	gene: Zm00001d036626	novel	2.17	250.91	coding	235	1564	2268
PB.3104.1	2	983	6	full-splice_match	gene: Zm00001d005344	Zm00001d005344_T002	239.87	239.87	coding	145	291	725
PB.9301.1	8	2021	11	novel_in_catalog	gene: Zm00001d009418	novel	2.48	144.20	coding	399	467	1663
PB.8423.4	7	2227	5	full-splice_match	gene: Zm00001d020178	Zm00001d020178_T002	0.00	28.19	coding	698	134	2227
PB.9623.4	8	3315	17	full-splice_match	gene: Zm00001d010974	Zm00001d010974_T005	0.00	48.61	coding	876	261	2888
PB.10810.1	9	1354	8	full-splice_match	gene: Zm00001d048497	Zm00001d048497_T001	87.33	87.33	coding	294	253	1134
PB.8423.1	7	3520	5	full-splice_match	gene: Zm00001d020178	Zm00001d020178_T002	28.19	28.19	coding	982	170	3115
PB.942.1	1	2710	9	full-splice_match	gene: Zm00001d031977	Zm00001d031977_T001	14.82	18.53	coding	682	209	2254
PB.9623.3	8	3676	18	full-splice_match	gene: Zm00001d010974	Zm00001d010974_T016	11.04	48.61	coding	855	293	2857

PB.4228.1	3	943	3	incomplete-splice_match	gene: Zm00001d042367	Zm00001d042367_T002	24.63	82.90	coding	168	87	590
PB.5507.1	4	1814	3	novel_not_in_catalog	gene: Zm00001d052393	novel	3.70	3.70	coding	424	484	1755
PB.1765.1	10	2125	16	full-splice_match	gene: Zm00001d023801	Zm00001d023801_T004	5.74	5.74	coding	626	10	1887
PB.6477.2	5	1733	12	full-splice_match	gene: Zm00001d015120	Zm00001d015120_T004	0.00	27.56	coding	455	94	1458
PB.6477.1	5	1896	12	full-splice_match	gene: Zm00001d015120	Zm00001d015120_T002	27.56	27.56	coding	455	93	1457
PB.9907.1	8	1210	3	full-splice_match	gene: Zm00001d012270	Zm00001d012270_T003	44.16	44.16	coding	276	143	970
PB.5503.1	4	1234	1	full-splice_match	gene: Zm00001d052387	Zm00001d052387_T001	1.52	23.23	coding	98	49	342
PB.2401.1	2	1255	6	incomplete-splice_match	gene: Zm00001d001808	Zm00001d001808_T002	12.37	37.05	coding	252	1	756
PB.2401.2	2	1754	10	full-splice_match	gene: Zm00001d001808	Zm00001d001808_T004	24.68	37.05	coding	461	143	1525
PB.2153.3	10	1296	3	incomplete-splice_match	gene: Zm00001d025855	Zm00001d025855_T019	19.56	31.60	non_coding	NA	NA	NA
PB.2029.1	10	1974	3	full-splice_match	gene: Zm00001d025208	Zm00001d025208_T002	9.55	9.55	coding	586	98	1855
PB.6996.2	5	2796	14	full-splice_match	gene: Zm00001d017876	Zm00001d017876_T020	7.13	25.93	coding	415	328	1572
PB.8587.2	7	2076	3	full-splice_match	gene: Zm00001d020947	Zm00001d020947_T001	16.70	38.02	coding	574	112	1833
PB.1346.1	1	1764	6	incomplete-splice_match	gene: Zm00001d033825	Zm00001d033825_T014	12.25	12.25	coding	511	75	1607
PB.1141.2	1	2338	1	full-splice_match	gene: Zm00001d032819	Zm00001d032819_T001	36.71	46.62	coding	637	137	2047
PB.1141.3	1	1728	1	full-splice_match	gene: Zm00001d032819	Zm00001d032819_T001	9.91	46.62	coding	458	222	1595
PB.1493.4	1	2299	2	genic	gene: Zm00001d034434	novel	0.00	39.47	non_coding	NA	NA	NA

PB.7045.1	5	2232	5	full-splice_match	gene: Zm00001d018046	Zm00001d018046_T021	22.66	22.66	non_coding	NA	NA	NA
PB.625.2	1	2563	12	full-splice_match	gene: Zm00001d030117	Zm00001d030117_T007	9.95	9.95	coding	304	109	1020
PB.5558.2	4	1232	5	full-splice_match	gene: Zm00001d052620	Zm00001d052620_T012	64.10	77.09	non_coding	NA	NA	NA
PB.9827.2	8	2983	1	incomplete-splice_match	gene: Zm00001d011901	Zm00001d011901_T005	0.72	7.75	coding	917	93	2843
PB.3981.2	3	2607	1	genic	gene: Zm00001d041111	novel	2.99	2.99	non_coding	NA	NA	NA
PB.3981.3	3	2055	1	intergenic	novelGene_168	novel	0.00	0.00	non_coding	NA	NA	NA
PB.8976.1	7	4662	18	full-splice_match	gene: Zm00001d022396	Zm00001d022396_T002	4.35	4.35	coding	1388	83	4246
PB.9757.2	8	2043	1	genic	gene: Zm00001d011643	novel	1.54	4.21	coding	346	47	1084
PB.7187.3	5	2557	10	full-splice_match	gene: Zm00001d018523	Zm00001d018523_T012	6.07	145.84	coding	231	81	773
PB.4377.1	3	2185	5	novel_in_catalog	gene: Zm00001d043074	novel	10.41	10.41	coding	553	274	1932
PB.1547.1	1	1085	4	full-splice_match	gene: Zm00001d034635	Zm00001d034635_T001	204.17	204.17	coding	231	109	801
PB.10667.2	9	755	5	full-splice_match	gene: Zm00001d047958	Zm00001d047958_T001	602.29	735.30	coding	127	155	535
PB.3071.4	2	2520	11	novel_in_catalog	gene: Zm00001d005164	novel	0.84	53.65	coding	351	1252	2304
PB.416.6	1	2786	3	full-splice_match	gene: Zm00001d029033	Zm00001d029033_T002	0.00	20.35	coding	216	1440	2087
PB.3622.1	2	1120	10	full-splice_match	gene: Zm00001d007930	Zm00001d007930_T001	77.14	77.14	coding	293	46	924
PB.8952.1	7	1265	1	full-splice_match	gene: Zm00001d022327	Zm00001d022327_T001	21.17	21.17	coding	357	44	1114
PB.3071.5	2	2683	11	novel_in_catalog	gene: Zm00001d005164	novel	0.49	53.65	coding	425	1275	2549

PB.4137.1	3	2377	11	novel_in_catalog	gene: Zm00001d041983	novel	5.17	12.74	coding	516	320	1867
PB.4137.2	3	1811	8	novel_in_catalog	gene: Zm00001d041983	novel	7.57	12.74	coding	404	9	1220
PB.5166.7	4	1868	9	full-splice_match	gene: Zm00001d050610	Zm00001d050610_T004	0.00	57.17	coding	477	103	1533
PB.1483.1	1	1057	4	full-splice_match	gene: Zm00001d034415	Zm00001d034415_T001	98.43	164.15	coding	201	115	717
PB.2608.3	2	2536	4	full-splice_match	gene: Zm00001d002654	Zm00001d002654_T002	24.54	50.22	coding	588	308	2071
PB.2356.2	10	1045	1	full-splice_match	gene: Zm00001d026632	Zm00001d026632_T001	0.00	287.06	coding	265	88	882
PB.2608.1	2	2570	4	full-splice_match	gene: Zm00001d002654	Zm00001d002654_T002	18.87	50.22	coding	588	271	2034
PB.4623.1	3	2571	19	full-splice_match	gene: Zm00001d044016	Zm00001d044016_T001	51.26	66.64	coding	655	191	2155
PB.2608.5	2	2658	4	full-splice_match	gene: Zm00001d002654	Zm00001d002654_T002	1.23	50.22	coding	588	515	2278
PB.185.1	1	1395	8	full-splice_match	gene: Zm00001d028075	Zm00001d028075_T035	23.75	75.10	coding	237	250	960
PB.9697.2	8	3094	17	full-splice_match	gene: Zm00001d011366	Zm00001d011366_T002	9.05	17.26	coding	768	343	2646
PB.1912.1	10	1488	9	full-splice_match	gene: Zm00001d024704	Zm00001d024704_T005	16.79	16.79	coding	373	133	1251
PB.9697.1	8	3507	16	novel_in_catalog	gene: Zm00001d011366	novel	0.52	17.26	coding	533	1442	3040
PB.4484.4	3	2595	8	novel_not_in_catalog	gene: Zm00001d043480	novel	0.00	133.65	coding	705	232	2346
PB.4916.1	4	2991	11	full-splice_match	gene: Zm00001d049188	Zm00001d049188_T002	25.15	25.15	coding	562	907	2592
PB.804.3	1	932	1	novel_in_catalog	gene: Zm00001d031244	novel	0.00	16.90	non_coding	NA	NA	NA
PB.6763.3	5	2307	3	novel_in_catalog	gene: Zm00001d016858	novel	15.64	58.43	coding	589	311	2077

PB.6763.2	5	2180	4	full-splice_match	gene: Zm00001d016858	Zm00001d016858_T011	7.60	58.43	coding	589	186	1952
PB.6763.1	5	1237	2	incomplete-splice_match	gene: Zm00001d016858	Zm00001d016858_T004	22.32	58.43	coding	237	98	808
PB.1349.1	1	3374	4	full-splice_match	gene: Zm00001d033839	Zm00001d033839_T001	16.20	16.20	coding	796	606	2993
PB.5766.1	4	1321	9	full-splice_match	gene: Zm00001d053628	Zm00001d053628_T003	24.20	24.20	coding	291	198	1070
PB.6763.5	5	2179	3	novel_in_catalog	gene: Zm00001d016858	novel	0.00	58.43	coding	589	288	2054
PB.6763.4	5	2194	4	full-splice_match	gene: Zm00001d016858	Zm00001d016858_T002	12.87	58.43	coding	589	205	1971
PB.1368.1	1	1421	8	full-splice_match	gene: Zm00001d033913	Zm00001d033913_T001	109.86	109.86	coding	318	149	1102
PB.804.1	1	1535	1	genic	gene: Zm00001d031244	novel	1.08	16.90	non_coding	NA	NA	NA
PB.2628.1	2	1136	1	full-splice_match	gene: Zm00001d002744	Zm00001d002744_T001	12.07	12.07	coding	220	83	742
PB.6622.1	5	1481	8	full-splice_match	gene: Zm00001d016106	Zm00001d016106_T003	61.05	61.05	coding	365	175	1269
PB.7181.1	5	1890	5	novel_in_catalog	gene: Zm00001d018511	novel	12.65	33.02	coding	174	1029	1550
PB.5805.1	4	830	3	incomplete-splice_match	gene: Zm00001d053782	Zm00001d053782_T006	4.89	14.62	coding	111	288	620
PB.3333.3	2	2531	10	novel_not_in_catalog	gene: Zm00001d006461	novel	0.07	44.45	coding	348	1113	2156
PB.2277.2	10	646	3	full-splice_match	gene: Zm00001d026368	Zm00001d026368_T001	67.72	82.08	coding	78	100	333
PB.1013.1	1	760	1	full-splice_match	gene: Zm00001d032318	Zm00001d032318_T001	46.30	46.30	non_coding	NA	NA	NA
PB.9687.1	8	1564	12	full-splice_match	gene: Zm00001d011329	Zm00001d011329_T002	12.22	13.49	coding	357	23	1093
PB.8181.1	7	632	2	full-splice_match	gene: Zm00001d018727	Zm00001d018727_T001	30.21	30.21	coding	98	102	395

PB.4622.1	3	528	1	intergenic	novelGene_337	novel	17.72	17.72	non_coding	NA	NA	NA
PB.9775.1	8	1423	8	novel_not_in_catalog	gene: Zm00001d011718	novel	2.92	83.02	coding	388	218	1381
PB.5414.1	4	1173	2	novel_not_in_catalog	gene: Zm00001d052054	novel	10.37	10.37	coding	236	120	827
PB.9817.1	8	1872	6	full-splice_match	gene: Zm00001d011855	Zm00001d011855_T001	49.12	71.85	coding	476	135	1562
PB.10589.1	9	3286	6	novel_not_in_catalog	gene: Zm00001d047726	novel	1.18	13.99	coding	406	200	1417
PB.3398.3	2	3301	9	full-splice_match	gene: Zm00001d006759	Zm00001d006759_T017	3.26	36.59	coding	705	733	2847
PB.10589.3	9	2042	7	novel_not_in_catalog	gene: Zm00001d047726	novel	0.00	13.99	coding	583	141	1889
PB.10589.2	9	2285	7	novel_not_in_catalog	gene: Zm00001d047726	novel	12.81	13.99	coding	583	284	2032
PB.3398.6	2	1857	4	incomplete-splice_match	gene: Zm00001d006759	Zm00001d006759_T009	0.00	36.59	coding	374	734	1855
PB.3398.4	2	2147	8	incomplete-splice_match	gene: Zm00001d006759	Zm00001d006759_T007	12.82	36.59	coding	539	90	1706
PB.6672.1	5	1678	15	full-splice_match	gene: Zm00001d016397	Zm00001d016397_T005	5.26	5.26	coding	532	39	1634
PB.6538.1	5	1738	2	full-splice_match	gene: Zm00001d015433	Zm00001d015433_T001	11.54	22.82	coding	440	93	1412
PB.7800.3	6	1653	11	novel_in_catalog	gene: Zm00001d037982	novel	1.88	13.32	coding	357	120	1190
PB.6538.2	5	1615	2	full-splice_match	gene: Zm00001d015433	Zm00001d015433_T001	11.27	22.82	coding	440	57	1376
PB.7800.6	6	2830	19	full-splice_match	gene: Zm00001d037982	Zm00001d037982_T005	1.96	13.32	coding	869	112	2718
PB.9404.1	8	1693	3	full-splice_match	gene: Zm00001d009967	Zm00001d009967_T001	21.53	21.53	coding	470	157	1566
PB.763.3	1	1699	9	full-splice_match	gene: Zm00001d031072	Zm00001d031072_T003	1.86	28.59	coding	265	745	1539

PB.7800.5	6	3057	20	full-splice_match	gene: Zm00001d037982	Zm00001d037982_T001	0.00	13.32	coding	869	112	2718
PB.4248.1	3	1693	6	full-splice_match	gene: Zm00001d042482	Zm00001d042482_T001	27.81	94.32	coding	362	153	1238
PB.6566.1	5	1554	3	full-splice_match	gene: Zm00001d015635	Zm00001d015635_T002	0.55	36.60	coding	115	361	705
PB.6566.2	5	921	4	full-splice_match	gene: Zm00001d015635	Zm00001d015635_T001	7.45	36.60	coding	115	307	651
PB.6566.3	5	955	3	full-splice_match	gene: Zm00001d015635	Zm00001d015635_T002	28.60	36.60	coding	115	362	706
PB.4248.5	3	3117	1	genic	gene: Zm00001d042482	novel	3.58	94.32	coding	153	1542	2000
PB.4248.6	3	1150	4	novel_in_catalog	gene: Zm00001d042482	novel	5.91	94.32	coding	183	1	549
PB.199.1	1	2254	3	novel_in_catalog	gene: Zm00001d028139	novel	23.98	25.04	coding	238	1330	2043
PB.9216.2	8	1335	4	novel_in_catalog	gene: Zm00001d008902	novel	7.76	17.21	coding	267	237	1037
PB.9216.1	8	2051	6	full-splice_match	gene: Zm00001d008902	Zm00001d008902_T001	9.45	17.21	coding	507	244	1764
PB.7457.1	6	1145	2	full-splice_match	gene: Zm00001d036409	Zm00001d036409_T001	34.27	34.27	coding	131	392	784
PB.199.4	1	2144	3	novel_in_catalog	gene: Zm00001d028139	novel	0.00	25.04	coding	238	1324	2037
PB.1755.4	10	2313	17	novel_in_catalog	gene: Zm00001d023741	novel	41.55	89.23	coding	350	30	1079
PB.536.2	1	1009	2	full-splice_match	gene: Zm00001d029594	Zm00001d029594_T001	0.00	276.32	coding	238	23	736
PB.1755.6	10	2428	18	incomplete-splice_match	gene: Zm00001d023741	Zm00001d023741_T004	0.00	89.23	coding	769	122	2428
PB.1755.1	10	3470	22	full-splice_match	gene: Zm00001d023741	Zm00001d023741_T004	15.35	89.23	coding	1002	20	3025
PB.2360.1	10	1451	6	full-splice_match	gene: Zm00001d026639	Zm00001d026639_T002	90.97	90.97	coding	328	80	1063

PB.1755.3	10	3268	22	full-splice_match	gene: Zm00001d023741	Zm00001d023741_T004	0.00	89.23	coding	1002	18	3023
PB.1755.2	10	3349	22	full-splice_match	gene: Zm00001d023741	Zm00001d023741_T004	32.32	89.23	coding	954	122	2983
PB.2703.1	2	3211	1	full-splice_match	gene: Zm00001d003047	Zm00001d003047_T001	2.40	2.40	coding	821	595	3057
PB.9375.2	8	1238	2	novel_not_in_catalog	gene: Zm00001d009797	novel	49.97	67.69	coding	121	653	1015
PB.9375.1	8	2420	1	genic	gene: Zm00001d009797	novel	3.22	67.69	coding	121	1759	2121
PB.4520.6	3	2546	13	full-splice_match	gene: Zm00001d043613	Zm00001d043613_T008	5.89	284.34	coding	300	1093	1992
PB.4155.1	3	3743	4	genic	gene: Zm00001d042033	novel	6.52	6.52	coding	821	410	2872
PB.4155.2	3	2008	2	genic	gene: Zm00001d042033	novel	0.00	6.52	coding	535	403	2007
PB.9375.4	8	2238	2	genic	gene: Zm00001d009797	novel	14.50	67.69	coding	121	1655	2017
PB.1074.1	1	1852	3	full-splice_match	gene: Zm00001d032546	Zm00001d032546_T001	41.29	41.29	coding	499	127	1623
PB.6773.1	5	2107	9	full-splice_match	gene: Zm00001d016894	Zm00001d016894_T002	13.45	13.45	coding	446	174	1511
PB.2612.6	2	1833	7	novel_in_catalog	gene: Zm00001d002678	novel	7.85	49.62	coding	17	352	402
PB.2612.5	2	3140	4	novel_in_catalog	gene: Zm00001d002678	novel	3.54	49.62	non_coding	NA	NA	NA
PB.2340.2	10	2480	3	full-splice_match	gene: Zm00001d026587	Zm00001d026587_T002	0.00	20.15	coding	679	218	2254
PB.313.3	1	1638	5	incomplete-splice_match	gene: Zm00001d028599	Zm00001d028599_T001	2.06	4.90	coding	438	174	1487
PB.2612.1	2	1633	3	full-splice_match	gene: Zm00001d002678	Zm00001d002678_T002	32.93	49.62	non_coding	NA	NA	NA
PB.5434.1	4	2215	9	full-splice_match	gene: Zm00001d052147	Zm00001d052147_T001	25.22	65.34	coding	525	108	1682

PB.5434.3	4	547	3	incomplete-splice_match	gene: Zm00001d052147	Zm00001d052147_T002	37.24	65.34	non_coding	NA	NA	NA
PB.5434.2	4	2325	9	full-splice_match	gene: Zm00001d052147	Zm00001d052147_T001	2.88	65.34	coding	525	347	1921
PB.685.1	1	1456	2	full-splice_match	gene: Zm00001d030549	Zm00001d030549_T001	12.22	15.67	coding	332	70	1065
PB.2612.8	2	2033	3	full-splice_match	gene: Zm00001d002678	Zm00001d002678_T002	5.29	49.62	non_coding	NA	NA	NA
PB.685.2	1	1207	2	full-splice_match	gene: Zm00001d030549	Zm00001d030549_T001	2.23	15.67	coding	332	98	1093
PB.5913.1	5	1884	2	novel_not_in_catalog	gene: Zm00001d012823	novel	43.60	63.65	coding	497	228	1718
PB.8947.2	7	2329	10	novel_in_catalog	gene: Zm00001d022305	novel	12.92	56.69	coding	284	14	865
PB.3896.1	3	1522	8	full-splice_match	gene: Zm00001d040411	Zm00001d040411_T002	41.32	41.32	coding	292	264	1139
PB.5913.2	5	1751	2	novel_not_in_catalog	gene: Zm00001d012823	novel	20.05	63.65	coding	497	225	1715
PB.3273.2	2	1329	9	full-splice_match	gene: Zm00001d006221	Zm00001d006221_T001	48.05	53.69	coding	298	117	1010
PB.3901.1	3	2179	16	fusion	gene: Zm00001d040448_g	novel	48.79	48.79	coding	602	51	1856
PB.6940.1	5	1875	13	novel_in_catalog	gene: Zm00001d017654	novel	29.88	29.88	coding	350	455	1504
PB.9053.1	7	1045	3	full-splice_match	gene: Zm00001d022629	Zm00001d022629_T001	268.49	268.49	coding	249	87	833
PB.9053.2	7	849	3	full-splice_match	gene: Zm00001d022629	Zm00001d022629_T001	0.00	268.49	coding	80	504	743
PB.10222.3	9	3163	14	full-splice_match	gene: Zm00001d045590	Zm00001d045590_T005	0.00	13.86	coding	1000	67	3066
PB.10222.2	9	3296	14	full-splice_match	gene: Zm00001d045590	Zm00001d045590_T005	0.00	13.86	coding	1000	67	3066
PB.10222.1	9	3475	14	full-splice_match	gene: Zm00001d045590	Zm00001d045590_T005	13.86	13.86	coding	1000	69	3068

PB.4541.2	3	1518	2	genic	gene: Zm00001d043695	novel	0.00	27.30	coding	398	38	1231
PB.4129.5	3	1711	2	novel_in_catalog	gene: Zm00001d041951	novel	2.18	103.95	coding	352	348	1403
PB.4129.4	3	1770	3	novel_in_catalog	gene: Zm00001d041951	novel	25.11	103.95	coding	359	291	1367
PB.4129.6	3	1561	4	full-splice_match	gene: Zm00001d041951	Zm00001d041951_T001	0.73	103.95	coding	419	43	1299
PB.4129.1	3	1761	3	novel_in_catalog	gene: Zm00001d041951	novel	8.66	103.95	coding	440	39	1358
PB.4129.3	3	1878	1	novel_in_catalog	gene: Zm00001d041951	novel	4.08	103.95	coding	377	345	1475
PB.4129.2	3	1737	4	full-splice_match	gene: Zm00001d041951	Zm00001d041951_T001	63.20	103.95	coding	419	39	1295
PB.1320.1	1	1062	1	intergenic	novelGene_372	novel	11.53	11.53	coding	235	117	821
PB.6502.1	5	963	6	full-splice_match	gene: Zm00001d015228	Zm00001d015228_T001	26.32	26.32	coding	197	130	720
PB.6094.2	5	3944	3	novel_in_catalog	gene: Zm00001d013441	novel	6.57	38.05	coding	1217	165	3815
PB.3568.3	2	1580	3	full-splice_match	gene: Zm00001d007682	Zm00001d007682_T003	0.41	16.08	coding	114	89	430
PB.6094.1	5	2919	1	genic	gene: Zm00001d013441	novel	31.48	38.05	coding	848	90	2633
PB.9077.1	8	1057	7	novel_in_catalog	gene: Zm00001d008262	novel	8.86	8.86	coding	152	70	525
PB.1632.3	1	1828	1	full-splice_match	gene: Zm00001d034916	Zm00001d034916_T001	3.63	46.44	coding	486	233	1690
PB.1632.2	1	1910	1	full-splice_match	gene: Zm00001d034916	Zm00001d034916_T001	27.02	46.44	coding	486	175	1632
PB.6375.2	5	927	3	novel_in_catalog	gene: Zm00001d014749	novel	3.48	409.45	non_coding	NA	NA	NA
PB.554.1	1	2223	10	full-splice_match	gene: Zm00001d029663	Zm00001d029663_T001	43.82	81.74	coding	520	225	1784

PB.6375.1	5	719	5	full-splice_match	gene: Zm00001d014749	Zm00001d014749_T001	405.97	409.45	coding	112	75	410
PB.7920.1	6	2997	8	novel_not_in_catalog	gene: Zm00001d038383	novel	2.16	8.27	coding	238	490	1203
PB.1614.3	1	809	2	novel_in_catalog	gene: Zm00001d034853	novel	9.27	30.52	coding	214	74	715
PB.1749.1	10	1398	8	full-splice_match	gene: Zm00001d023695	Zm00001d023695_T001	39.68	39.68	coding	354	53	1114
PB.9361.4	8	1028	1	incomplete-splice_match	gene: Zm00001d009750	Zm00001d009750_T008	10.03	19.32	coding	196	148	735
PB.7982.1	6	1352	12	full-splice_match	gene: Zm00001d038623	Zm00001d038623_T002	114.68	141.19	coding	282	112	957
PB.4349.3	3	2114	10	novel_in_catalog	gene: Zm00001d042910	novel	0.27	27.97	coding	560	165	1844
PB.6946.1	5	2203	1	full-splice_match	gene: Zm00001d017678	Zm00001d017678_T001	19.01	19.01	coding	405	475	1689
PB.9361.1	8	1910	3	incomplete-splice_match	gene: Zm00001d009750	Zm00001d009750_T003	6.66	19.32	coding	453	16	1374
PB.9361.2	8	1257	1	incomplete-splice_match	gene: Zm00001d009750	Zm00001d009750_T001	2.63	19.32	non_coding	NA	NA	NA
PB.9361.3	8	4266	17	fusion	gene: Zm00001d009751_g	novel	4.85	4.85	coding	1210	2	3631
PB.8730.1	7	1359	13	novel_in_catalog	gene: Zm00001d021558	novel	64.82	64.82	coding	311	82	1014
PB.6616.5	5	1679	3	genic	gene: Zm00001d016066	novel	4.98	28.23	coding	415	313	1557
PB.6616.2	5	2000	3	genic	gene: Zm00001d016066	novel	0.00	28.23	coding	415	424	1668
PB.6616.3	5	1937	3	genic	gene: Zm00001d016066	novel	8.81	28.23	coding	415	473	1717
PB.6616.1	5	2006	3	genic	gene: Zm00001d016066	novel	14.43	28.23	coding	415	312	1556
PB.7982.2	6	1209	12	full-splice_match	gene: Zm00001d038623	Zm00001d038623_T002	26.51	141.19	coding	282	74	919

PB.2152.4	10	2063	11	novel_in_catalog	gene: Zm00001d025846	novel	8.86	66.24	coding	365	779	1873
PB.4213.1	3	2204	14	full-splice_match	gene: Zm00001d042314	Zm00001d042314_T004	34.96	34.96	coding	550	226	1875
PB.8665.5	7	1678	6	full-splice_match	gene: Zm00001d021304	Zm00001d021304_T001	0.00	138.20	coding	500	40	1539
PB.8197.2	7	1894	14	novel_in_catalog	gene: Zm00001d018799	novel	50.88	54.17	coding	449	340	1686
PB.8665.3	7	2123	6	full-splice_match	gene: Zm00001d021304	Zm00001d021304_T001	0.00	138.20	coding	526	220	1797
PB.8197.1	7	2006	14	novel_in_catalog	gene: Zm00001d018799	novel	3.30	54.17	coding	449	341	1687
PB.975.1	1	606	3	full-splice_match	gene: Zm00001d032153	Zm00001d032153_T002	57.11	57.11	coding	117	26	376
PB.7920.6	6	1622	4	fusion	gene: Zm00001d038384_g	novel	10.98	10.98	coding	254	179	940
PB.2002.1	10	702	6	novel_in_catalog	gene: Zm00001d025047	novel	30.74	30.74	coding	112	151	486
PB.4028.4	3	1099	5	novel_in_catalog	gene: Zm00001d041445	novel	97.02	157.23	coding	133	317	715
PB.3443.1	2	958	4	novel_in_catalog	gene: Zm00001d006951	novel	205.21	205.21	coding	229	79	765
PB.4213.2	3	1991	14	full-splice_match	gene: Zm00001d042314	Zm00001d042314_T004	0.00	34.96	coding	550	143	1792
PB.4028.7	3	1774	7	incomplete-splice_match	gene: Zm00001d041445	Zm00001d041445_T022	0.00	157.23	coding	557	102	1772
PB.6833.1	5	1148	6	full-splice_match	gene: Zm00001d017171	Zm00001d017171_T001	10.70	26.27	coding	184	384	935
PB.10339.1	9	1720	2	full-splice_match	gene: Zm00001d046455	Zm00001d046455_T001	6.08	53.51	coding	425	69	1343
PB.9886.2	8	1828	13	novel_in_catalog	gene: Zm00001d012170	novel	1.07	84.78	coding	232	75	770
PB.10871.1	B73V4 ctg165	1260	2	intergenic	novelGene_3	novel	24.60	24.60	coding	157	342	812

PB.1591.5	1	2838	1	full-splice_match	gene: Zm00001d034781	Zm00001d034781_T001	0.00	43.63	coding	852	190	2745
PB.9886.1	8	1832	14	full-splice_match	gene: Zm00001d012170	Zm00001d012170_T002	70.97	84.78	coding	415	104	1348
PB.2580.1	2	1200	3	full-splice_match	gene: Zm00001d002548	Zm00001d002548_T001	63.74	63.74	coding	258	145	918
PB.9886.4	8	1668	14	full-splice_match	gene: Zm00001d012170	Zm00001d012170_T002	12.73	84.78	coding	415	73	1317
PB.807.1	1	1101	2	full-splice_match	gene: Zm00001d031258	Zm00001d031258_T004	54.49	62.40	coding	192	169	744
PB.3836.1	3	3029	1	full-splice_match	gene: Zm00001d040169	Zm00001d040169_T001	6.10	6.10	coding	912	39	2774
PB.4658.4	3	2540	11	novel_in_catalog	gene: Zm00001d044170	novel	0.00	14.40	coding	456	884	2251
PB.4833.1	4	439	3	novel_not_in_catalog	gene: Zm00001d048787	novel	1887.65	1887.65	non_coding	NA	NA	NA
PB.4658.3	3	2657	12	full-splice_match	gene: Zm00001d044170	Zm00001d044170_T001	0.00	14.40	coding	702	167	2272
PB.5903.1	5	1820	1	novel_in_catalog	gene: Zm00001d012794	novel	8.77	16.40	coding	108	766	1089
PB.4658.1	3	2716	12	full-splice_match	gene: Zm00001d044170	Zm00001d044170_T001	14.40	14.40	coding	702	212	2317
PB.5799.2	4	2445	14	novel_not_in_catalog	gene: Zm00001d053751	novel	1.04	12.82	coding	351	446	1498
PB.1040.1	1	1681	6	full-splice_match	gene: Zm00001d032428	Zm00001d032428_T004	108.89	108.89	coding	415	142	1386
PB.1040.4	1	1622	6	full-splice_match	gene: Zm00001d032428	Zm00001d032428_T004	0.00	108.89	coding	415	136	1380
PB.10200.1	9	2762	1	intergenic	novelGene_79	novel	4.74	4.74	non_coding	NA	NA	NA
PB.6717.1	5	1096	6	full-splice_match	gene: Zm00001d016665	Zm00001d016665_T002	65.67	65.67	coding	271	16	828
PB.5636.1	4	1895	7	full-splice_match	gene: Zm00001d052941	Zm00001d052941_T001	23.92	30.53	coding	515	58	1602

PB.2015.2	10	1498	2	genic	gene: Zm00001d025107	novel	0.00	44.13	coding	375	295	1419
PB.7535.1	6	2407	1	genic	gene: Zm00001d036763	novel	2.12	2.12	coding	324	35	1006
PB.710.3	1	2815	5	incomplete-splice_match	gene: Zm00001d030739	Zm00001d030739_T004	6.24	171.06	coding	160	2038	2517
PB.2838.5	2	2080	11	novel_not_in_catalog	gene: Zm00001d003632	novel	8.37	67.67	coding	417	354	1604
PB.2838.6	2	1999	11	full-splice_match	gene: Zm00001d003632	Zm00001d003632_T003	13.71	67.67	coding	443	354	1682
PB.2838.1	2	2219	11	full-splice_match	gene: Zm00001d003632	Zm00001d003632_T003	40.17	67.67	coding	443	549	1877
PB.2838.3	2	1991	10	novel_in_catalog	gene: Zm00001d003632	novel	5.41	67.67	coding	384	210	1361
PB.490.1	1	1455	5	full-splice_match	gene: Zm00001d029389	Zm00001d029389_T001	11.43	11.43	coding	378	52	1185
PB.6484.1	5	760	1	incomplete-splice_match	gene: Zm00001d015129	Zm00001d015129_T001	1.37	1.37	non_coding	NA	NA	NA
PB.7963.1	6	1607	2	full-splice_match	gene: Zm00001d038553	Zm00001d038553_T001	8.75	8.75	coding	457	66	1436
PB.1811.2	10	2700	15	fusion	gene: Zm00001d024043_g	novel	0.00	43.58	coding	376	108	1235
PB.1811.3	10	1685	13	fusion	gene: Zm00001d024043_g	novel	43.58	43.58	coding	376	189	1316
PB.125.2	1	2762	10	novel_not_in_catalog	gene: Zm00001d027732	novel	9.93	12.04	coding	695	335	2419
PB.1811.1	10	2795	11	full-splice_match	gene: Zm00001d024041	Zm00001d024041_T012	8.96	8.96	coding	312	3	938
PB.4495.1	3	2414	2	full-splice_match	gene: Zm00001d043512	Zm00001d043512_T001	4.99	4.99	coding	652	88	2043
PB.4256.1	3	2453	5	full-splice_match	gene: Zm00001d042520	Zm00001d042520_T002	14.00	14.00	coding	511	472	2004
PB.4375.1	3	1942	3	full-splice_match	gene: Zm00001d043069	Zm00001d043069_T001	8.73	12.73	coding	446	242	1579

PB.2735.2	2	2713	5	novel_not_in_catalog	gene: Zm00001d003176	novel	0.00	45.55	coding	837	6	2516
PB.2735.3	2	2724	5	novel_not_in_catalog	gene: Zm00001d003176	novel	24.47	45.55	coding	846	8	2545
PB.1831.2	10	2478	11	full-splice_match	gene: Zm00001d024212	Zm00001d024212_T002	5.64	44.91	coding	255	1399	2163
PB.2735.1	2	3138	5	novel_not_in_catalog	gene: Zm00001d003176	novel	21.08	45.55	coding	686	186	2243
PB.1831.5	10	2078	12	novel_in_catalog	gene: Zm00001d024212	novel	0.18	44.91	coding	255	1098	1862
PB.1831.6	10	1935	13	full-splice_match	gene: Zm00001d024212	Zm00001d024212_T009	0.00	44.91	coding	500	220	1719
PB.7299.2	6	1217	1	full-splice_match	gene: Zm00001d035440	Zm00001d035440_T001	286.22	316.60	coding	135	569	973
PB.8484.2	7	1631	1	full-splice_match	gene: Zm00001d020470	Zm00001d020470_T001	17.56	17.56	coding	421	170	1432
PB.7299.1	6	1317	1	full-splice_match	gene: Zm00001d035440	Zm00001d035440_T001	30.38	316.60	coding	135	566	970
PB.6071.3	5	2294	1	full-splice_match	gene: Zm00001d013362	Zm00001d013362_T001	1.36	36.81	coding	406	791	2008
PB.3031.1	2	1399	7	full-splice_match	gene: Zm00001d004930	Zm00001d004930_T001	37.46	37.46	coding	374	108	1229
PB.8290.2	7	1725	2	antisense	novelGene_gene: Zm00001d019254_A	novel	3.53	4.53	coding	195	1140	1724
PB.10497.2	9	1779	4	full-splice_match	gene: Zm00001d047388	Zm00001d047388_T002	22.68	37.55	coding	469	60	1466
PB.10515.6	9	2322	4	novel_in_catalog	gene: Zm00001d047447	novel	37.07	37.07	coding	318	1179	2132
PB.9699.1	8	2245	5	full-splice_match	gene: Zm00001d011377	Zm00001d011377_T002	10.52	18.90	coding	583	196	1944
PB.9699.2	8	950	2	incomplete-splice_match	gene: Zm00001d011377	Zm00001d011377_T003	8.38	18.90	coding	81	525	767
PB.8290.3	7	1338	2	antisense	novelGene_gene: Zm00001d019254_A	novel	0.00	4.53	non_coding	NA	NA	NA

PB.9536.2	8	2052	1	full-splice_match	gene: Zm00001d010640	Zm00001d010640_T001	2.47	26.35	coding	354	699	1760
PB.9536.3	8	1762	2	genic	gene: Zm00001d010640	novel	0.00	26.35	coding	354	531	1592
PB.9240.1	8	942	5	full-splice_match	gene: Zm00001d009010	Zm00001d009010_T002	672.95	672.95	coding	194	92	673
PB.1856.1	10	1472	10	full-splice_match	gene: Zm00001d024393	Zm00001d024393_T003	21.52	50.09	coding	374	93	1214
PB.9218.4	8	1760	1	full-splice_match	gene: Zm00001d008905	Zm00001d008905_T001	0.00	65.15	coding	313	708	1646
PB.6800.3	5	2757	7	novel_in_catalog	gene: Zm00001d017049	novel	1.22	29.94	coding	279	168	1004
PB.1856.2	10	1327	10	full-splice_match	gene: Zm00001d024393	Zm00001d024393_T001	28.57	50.09	coding	383	9	1157
PB.9218.1	8	2230	1	full-splice_match	gene: Zm00001d008905	Zm00001d008905_T001	2.94	65.15	coding	313	805	1743
PB.9218.3	8	2031	1	full-splice_match	gene: Zm00001d008905	Zm00001d008905_T001	39.04	65.15	coding	587	54	1814
PB.9218.2	8	2164	1	full-splice_match	gene: Zm00001d008905	Zm00001d008905_T001	23.17	65.15	coding	587	54	1814
PB.10476.2	9	801	3	full-splice_match	gene: Zm00001d047262	Zm00001d047262_T001	0.00	69.82	coding	129	121	507
PB.10168.1	9	859	3	full-splice_match	gene: Zm00001d045391	Zm00001d045391_T001	940.15	940.15	coding	115	273	617
PB.5612.1	4	1182	1	intergenic	novelGene_319	novel	1.05	1.05	non_coding	NA	NA	NA
PB.10509.1	9	2627	11	full-splice_match	gene: Zm00001d047428	Zm00001d047428_T009	15.78	15.78	coding	595	515	2299
PB.7712.1	6	1152	1	full-splice_match	gene: Zm00001d037644	Zm00001d037644_T001	4.35	4.35	coding	251	274	1026
PB.2083.9	10	2032	4	novel_in_catalog	gene: Zm00001d025539	novel	8.99	41.72	coding	194	322	903
PB.2251.4	10	2405	8	incomplete-splice_match	gene: Zm00001d026289	Zm00001d026289_T004	6.21	11.85	coding	601	520	2322

PB.7137.2	5	2293	18	incomplete-splice_match	gene: Zm00001d018384	Zm00001d018384_T004	4.68	18.80	coding	613	157	1995
PB.5818.1	4	1809	11	full-splice_match	gene: Zm00001d053825	Zm00001d053825_T002	54.78	54.78	coding	402	222	1427
PB.1300.3	1	2090	14	novel_not_in_catalog	gene: Zm00001d033616	novel	27.87	27.87	coding	263	864	1652
PB.2083.3	10	1047	5	incomplete-splice_match	gene: Zm00001d025539	Zm00001d025539_T005	20.19	41.72	coding	105	229	543
PB.1872.1	10	740	2	genic	gene: Zm00001d023122	novel	22.23	22.23	non_coding	NA	NA	NA
PB.9167.3	8	970	1	novel_in_catalog	gene: Zm00001d008666	novel	2.63	2.63	coding	304	38	949
PB.7079.1	5	1676	5	full-splice_match	gene: Zm00001d018195	Zm00001d018195_T001	336.37	340.37	coding	394	112	1293
PB.2083.7	10	2953	3	full-splice_match	gene: Zm00001d025539	Zm00001d025539_T012	3.26	41.72	coding	113	1449	1787
PB.2083.4	10	1981	5	novel_in_catalog	gene: Zm00001d025539	novel	9.28	41.72	coding	194	196	777
PB.730.5	1	770	6	incomplete-splice_match	gene: Zm00001d030895	Zm00001d030895_T001	0.00	459.22	coding	180	107	646
PB.9504.1	8	1642	5	full-splice_match	gene: Zm00001d010478	Zm00001d010478_T001	8.69	86.63	coding	335	156	1160
PB.2866.1	2	1850	3	intergenic	novelGene_117	novel	1.29	16.32	non_coding	NA	NA	NA
PB.2806.1	2	1327	2	novel_in_catalog	gene: Zm00001d003471	novel	21.46	21.46	coding	350	169	1218
PB.9504.2	8	1509	5	full-splice_match	gene: Zm00001d010478	Zm00001d010478_T001	68.64	86.63	coding	335	155	1159
PB.9504.5	8	1585	4	novel_in_catalog	gene: Zm00001d010478	novel	5.39	86.63	coding	241	141	863
PB.3396.1	2	1117	7	incomplete-splice_match	gene: Zm00001d006758	Zm00001d006758_T005	34.93	34.93	non_coding	NA	NA	NA
PB.5773.2	4	994	5	full-splice_match	gene: Zm00001d053641	Zm00001d053641_T002	83.72	83.72	coding	109	464	790

PB.7960.1	6	847	2	incomplete-splice_match	gene: Zm00001d038543	Zm00001d038543_T001	242.80	651.67	coding	149	73	519
PB.7407.2	6	1995	9	intergenic	novelGene_127	novel	0.00	17.74	coding	613	46	1884
PB.7900.2	6	1304	8	novel_not_in_catalog	gene: Zm00001d038319	novel	29.01	29.01	coding	258	160	933
PB.2420.1	2	1715	5	full-splice_match	gene: Zm00001d001868	Zm00001d001868_T001	51.26	51.26	coding	404	223	1434
PB.2866.2	2	823	4	intergenic	novelGene_117	novel	15.03	16.32	non_coding	NA	NA	NA
PB.2379.1	10	1944	6	full-splice_match	gene: Zm00001d026685	Zm00001d026685_T002	43.30	43.30	coding	545	113	1747
PB.8327.4	7	2406	2	full-splice_match	gene: Zm00001d019527	Zm00001d019527_T001	4.76	13.03	coding	377	205	1335
PB.5349.2	4	2773	16	novel_in_catalog	gene: Zm00001d051753	novel	3.12	26.14	coding	344	1582	2613
PB.7312.1	6	628	1	genic	gene: Zm00001d035501	novel	18.46	80.59	coding	41	271	393
PB.7312.2	6	1334	2	full-splice_match	gene: Zm00001d035501	Zm00001d035501_T001	62.13	80.59	coding	294	96	977
PB.8327.2	7	2074	2	full-splice_match	gene: Zm00001d019527	Zm00001d019527_T005	8.27	13.03	coding	430	205	1494
PB.5234.1	4	2334	4	novel_in_catalog	gene: Zm00001d051139	novel	4.89	4.89	coding	183	183	731
PB.7804.5	6	3284	19	full-splice_match	gene: Zm00001d038006	Zm00001d038006_T001	0.00	21.19	coding	1018	83	3136
PB.3724.1	3	825	4	intergenic	novelGene_32	novel	54.22	59.17	non_coding	NA	NA	NA
PB.9671.1	8	3058	19	full-splice_match	gene: Zm00001d011233	Zm00001d011233_T007	52.07	52.07	coding	775	231	2555
PB.10501.1	9	1895	5	full-splice_match	gene: Zm00001d047399	Zm00001d047399_T001	40.54	40.54	coding	429	307	1593
PB.5369.1	4	2916	6	novel_in_catalog	gene: Zm00001d051875	novel	16.27	16.27	coding	498	1238	2731

PB.1427.2	1	1063	7	full-splice_match	gene: Zm00001d034180	Zm00001d034180_T002	116.08	122.73	coding	227	122	802
PB.1978.1	10	3258	20	novel_not_in_catalog	gene: Zm00001d024952	novel	72.18	124.67	coding	905	86	2800
PB.5545.1	4	1371	1	antisense	novelGene_gene: Zm00001d052519_A	novel	2.58	2.58	coding	215	395	1039
PB.1483.2	1	846	4	full-splice_match	gene: Zm00001d034415	Zm00001d034415_T001	65.72	164.15	coding	201	114	716
PB.2239.1	10	1644	12	full-splice_match	gene: Zm00001d026261	Zm00001d026261_T001	19.82	19.82	coding	383	198	1346
PB.4160.1	3	3599	2	full-splice_match	gene: Zm00001d042051	Zm00001d042051_T001	14.21	20.14	coding	1043	19	3147
PB.4551.1	3	1566	11	full-splice_match	gene: Zm00001d043727	Zm00001d043727_T001	4.30	26.21	coding	355	194	1258
PB.9732.1	8	2279	6	full-splice_match	gene: Zm00001d011541	Zm00001d011541_T002	24.83	39.66	coding	520	354	1913
PB.7364.1	6	661	1	antisense	novelGene_gene: Zm00001d035815_A	novel	24.41	24.41	non_coding	NA	NA	NA
PB.9732.3	8	2096	6	full-splice_match	gene: Zm00001d011541	Zm00001d011541_T002	11.30	39.66	coding	520	363	1922
PB.9732.5	8	2660	5	novel_in_catalog	gene: Zm00001d011541	novel	3.53	39.66	coding	518	357	1910
PB.6034.4	5	2263	17	full-splice_match	gene: Zm00001d013254	Zm00001d013254_T001	25.36	45.92	coding	648	163	2106
PB.4315.2	3	998	6	full-splice_match	gene: Zm00001d042772	Zm00001d042772_T002	107.60	265.94	coding	216	206	853
PB.3816.1	3	2215	6	full-splice_match	gene: Zm00001d040038	Zm00001d040038_T001	56.94	56.94	coding	610	82	1911
PB.9143.1	8	942	1	full-splice_match	gene: Zm00001d008573	Zm00001d008573_T001	16.96	16.96	coding	205	141	755
PB.6034.2	5	2384	16	novel_in_catalog	gene: Zm00001d013254	novel	20.56	45.92	coding	450	855	2204
PB.7889.2	6	2243	3	incomplete-splice_match	gene: Zm00001d038273	Zm00001d038273_T001	47.87	168.88	coding	503	97	1605

PB.10075.5	9	3001	2	novel_in_catalog	gene: Zm00001d044947	novel	0.00	76.83	coding	371	1837	2949
PB.10075.4	9	4172	1	genic	gene: Zm00001d044947	novel	0.89	76.83	coding	304	3144	4055
PB.2474.2	2	2605	9	full-splice_match	gene: Zm00001d002099	Zm00001d002099_T003	34.84	46.36	coding	723	258	2426
PB.1068.1	1	1767	4	novel_in_catalog	gene: Zm00001d032528	novel	28.21	28.21	coding	244	561	1292
PB.10075.1	9	1744	3	novel_not_in_catalog	gene: Zm00001d044947	novel	70.46	76.83	coding	474	205	1626
PB.10075.2	9	1838	2	genic	gene: Zm00001d044947	novel	5.48	76.83	coding	304	807	1718
PB.252.5	1	1217	4	full-splice_match	gene: Zm00001d028349	Zm00001d028349_T001	235.81	392.06	coding	334	56	1057
PB.252.4	1	1400	3	novel_in_catalog	gene: Zm00001d028349	novel	0.00	392.06	non_coding	NA	NA	NA
PB.4315.1	3	1284	6	full-splice_match	gene: Zm00001d042772	Zm00001d042772_T002	158.34	265.94	coding	216	102	749
PB.252.1	1	1590	3	novel_in_catalog	gene: Zm00001d028349	novel	156.25	392.06	non_coding	NA	NA	NA
PB.3370.1	2	3083	4	incomplete-splice_match	gene: Zm00001d006628	Zm00001d006628_T003	39.47	71.39	coding	857	29	2599
PB.3370.3	2	3672	8	incomplete-splice_match	gene: Zm00001d006628	Zm00001d006628_T003	31.92	71.39	coding	1058	23	3196
PB.6928.1	5	1738	2	full-splice_match	gene: Zm00001d017575	Zm00001d017575_T001	65.60	79.67	coding	374	212	1333
PB.4818.10	4	1698	1	novel_in_catalog	gene: Zm00001d048680	novel	2.72	159.29	coding	162	63	548
PB.1914.1	10	2162	4	novel_in_catalog	gene: Zm00001d024712	novel	2.89	2.89	coding	401	826	2028
PB.6804.2	5	1797	2	full-splice_match	gene: Zm00001d017077	Zm00001d017077_T001	15.85	17.70	coding	515	55	1599
PB.9821.2	8	597	3	intergenic	novelGene_268	novel	0.00	24.32	non_coding	NA	NA	NA

PB.581.4	1	1861	3	full-splice_match	gene: Zm00001d029842	Zm00001d029842_T001	136.74	226.49	coding	499	63	1559
PB.1334.1	1	1446	1	intergenic	novelGene_374	novel	12.50	12.50	non_coding	NA	NA	NA
PB.4335.1	3	1515	6	novel_not_in_catalog	gene: Zm00001d042868	novel	3.95	17.50	coding	173	618	1136
PB.4335.2	3	1393	6	novel_not_in_catalog	gene: Zm00001d042868	novel	3.44	17.50	coding	98	26	319
PB.4335.3	3	1721	6	novel_not_in_catalog	gene: Zm00001d042868	novel	7.02	17.50	coding	173	593	1111
PB.4335.4	3	1574	4	full-splice_match	gene: Zm00001d042868	Zm00001d042868_T001	3.08	17.50	coding	250	603	1352
PB.5242.1	4	2250	5	incomplete-splice_match	gene: Zm00001d051183	Zm00001d051183_T001	10.16	26.43	coding	566	10	1707
PB.5242.2	4	3268	12	incomplete-splice_match	gene: Zm00001d051183	Zm00001d051183_T019	16.27	26.43	coding	918	88	2841
PB.224.2	1	1402	1	genic	gene: Zm00001d028226	novel	1.10	30.87	coding	293	57	935
PB.224.3	1	1255	2	novel_not_in_catalog	gene: Zm00001d028226	novel	9.15	30.87	coding	345	61	1095
PB.5798.1	4	858	1	full-splice_match	gene: Zm00001d053750	Zm00001d053750_T001	42.94	42.94	coding	102	193	498
PB.224.1	1	1378	2	novel_not_in_catalog	gene: Zm00001d028226	novel	20.63	30.87	coding	345	127	1161
PB.24.1	1	908	6	full-splice_match	gene: Zm00001d027318	Zm00001d027318_T001	316.52	316.52	coding	169	104	610
PB.119.1	1	1287	3	full-splice_match	gene: Zm00001d027708	Zm00001d027708_T001	2.84	9.45	coding	182	170	715
PB.6632.1	5	1873	8	full-splice_match	gene: Zm00001d016164	Zm00001d016164_T002	20.58	20.58	coding	427	153	1433
PB.119.2	1	1243	3	full-splice_match	gene: Zm00001d027708	Zm00001d027708_T001	6.61	9.45	coding	182	275	820
PB.7330.4	6	1687	4	full-splice_match	gene: Zm00001d035628	Zm00001d035628_T001	93.15	350.98	coding	50	163	312

PB.7330.5	6	1678	4	novel_in_catalog	gene: Zm00001d035628	novel	2.80	350.98	coding	50	155	304
PB.7330.6	6	1844	3	novel_in_catalog	gene: Zm00001d035628	novel	6.75	350.98	coding	323	598	1566
PB.7330.7	6	1922	3	novel_in_catalog	gene: Zm00001d035628	novel	2.11	350.98	coding	293	805	1683
PB.8685.1	7	2090	12	full-splice_match	gene: Zm00001d021384	Zm00001d021384_T004	17.88	26.30	coding	542	38	1663
PB.7330.2	6	1791	4	full-splice_match	gene: Zm00001d035628	Zm00001d035628_T001	246.16	350.98	coding	293	610	1488
PB.6880.1	5	2196	1	full-splice_match	gene: Zm00001d017366	Zm00001d017366_T001	7.02	7.02	coding	449	228	1574
PB.2957.1	2	1819	2	genic	gene: Zm00001d004469	novel	8.40	8.40	coding	385	151	1305
PB.10432.1	9	2720	10	full-splice_match	gene: Zm00001d046973	Zm00001d046973_T002	41.28	41.28	coding	820	77	2536
PB.1341.1	1	2725	7	full-splice_match	gene: Zm00001d033798	Zm00001d033798_T006	28.57	32.54	coding	779	11	2347
PB.7070.1	5	1289	10	full-splice_match	gene: Zm00001d018148	Zm00001d018148_T005	14.42	14.42	coding	364	69	1160
PB.5512.1	4	837	1	genic	gene: Zm00001d052405	novel	11.99	19.26	coding	86	99	356
PB.5872.1	4	2216	11	full-splice_match	gene: Zm00001d053997	Zm00001d053997_T001	26.41	26.41	coding	491	452	1924
PB.7973.1	6	2953	14	full-splice_match	gene: Zm00001d038589	Zm00001d038589_T001	2.87	16.75	coding	720	76	2235
PB.3740.1	3	1409	8	full-splice_match	gene: Zm00001d039611	Zm00001d039611_T003	98.21	133.34	coding	330	59	1048
PB.4468.3	3	1429	4	novel_in_catalog	gene: Zm00001d043432	novel	1.27	18.68	coding	240	109	828
PB.3740.2	3	795	5	incomplete-splice_match	gene: Zm00001d039611	Zm00001d039611_T005	35.13	133.34	coding	109	238	564
PB.9874.1	8	918	1	intergenic	novelGene_276	novel	32.25	61.28	coding	129	316	702

PB.2198.3	10	1034	4	full-splice_match	gene: Zm00001d026047	Zm00001d026047_T002	12.59	133.34	coding	253	125	883
PB.2198.2	10	1498	4	full-splice_match	gene: Zm00001d026047	Zm00001d026047_T002	120.75	133.34	coding	253	143	901
PB.10652.2	9	1947	1	full-splice_match	gene: Zm00001d047915	Zm00001d047915_T001	17.08	20.07	coding	479	157	1593
PB.10652.3	9	1654	1	full-splice_match	gene: Zm00001d047915	Zm00001d047915_T001	0.00	20.07	coding	479	55	1491
PB.7849.9	6	2378	4	novel_in_catalog	gene: Zm00001d038146	novel	25.67	116.48	coding	324	1284	2255
PB.10300.1	9	2291	10	full-splice_match	gene: Zm00001d046194	Zm00001d046194_T017	12.19	12.19	coding	599	174	1970
PB.5557.1	4	1614	2	full-splice_match	gene: Zm00001d052618	Zm00001d052618_T001	24.79	24.79	coding	404	74	1285
PB.593.2	1	2008	5	novel_not_in_catalog	gene: Zm00001d029920	novel	0.00	17.66	coding	486	286	1743
PB.593.1	1	2108	5	novel_not_in_catalog	gene: Zm00001d029920	novel	17.66	17.66	coding	486	282	1739
PB.7660.3	6	953	6	incomplete-splice_match	gene: Zm00001d037408	Zm00001d037408_T004	20.87	35.80	non_coding	NA	NA	NA
PB.7849.1	6	1529	3	incomplete-splice_match	gene: Zm00001d038146	Zm00001d038146_T005	88.14	116.48	coding	198	178	771
PB.9874.2	8	742	1	intergenic	novelGene_276	novel	29.03	61.28	coding	129	253	639
PB.5803.1	4	1961	20	full-splice_match	gene: Zm00001d053776	Zm00001d053776_T015	27.02	27.02	coding	525	130	1704
PB.4858.1	4	1538	6	novel_not_in_catalog	gene: Zm00001d048899	novel	36.39	36.39	coding	311	258	1190
PB.7205.1	6	1670	9	full-splice_match	gene: Zm00001d034965	Zm00001d034965_T001	22.87	22.87	coding	341	279	1301
PB.8590.1	7	1748	11	full-splice_match	gene: Zm00001d020953	Zm00001d020953_T001	23.06	23.06	coding	418	286	1539
PB.270.1	1	924	1	full-splice_match	gene: Zm00001d028404	Zm00001d028404_T001	54.58	100.78	coding	154	83	544

PB.6719.1	5	1783	2	full-splice_match	gene: Zm00001d016674	Zm00001d016674_T001	3.39	6.38	coding	457	46	1416
PB.7884.5	6	956	4	full-splice_match	gene: Zm00001d038248	Zm00001d038248_T001	20.06	251.94	coding	123	62	430
PB.270.2	1	763	1	full-splice_match	gene: Zm00001d028404	Zm00001d028404_T001	46.20	100.78	coding	154	76	537
PB.359.3	1	872	1	full-splice_match	gene: Zm00001d028793	Zm00001d028793_T001	5.09	1066.00	coding	178	21	554
PB.359.2	1	1148	1	full-splice_match	gene: Zm00001d028793	Zm00001d028793_T001	965.84	1066.00	coding	220	102	761
PB.359.1	1	1801	1	full-splice_match	gene: Zm00001d028793	Zm00001d028793_T001	10.03	1066.00	non_coding	NA	NA	NA
PB.8624.4	7	1829	9	full-splice_match	gene: Zm00001d021119	Zm00001d021119_T001	20.31	57.68	coding	533	71	1669
PB.8624.3	7	2041	8	full-splice_match	gene: Zm00001d021119	Zm00001d021119_T008	12.57	57.68	coding	571	73	1785
PB.8624.2	7	1976	9	full-splice_match	gene: Zm00001d021119	Zm00001d021119_T001	24.80	57.68	coding	533	124	1722
PB.8156.1	7	1321	8	full-splice_match	gene: Zm00001d018634	Zm00001d018634_T001	36.54	36.54	coding	322	92	1057
PB.359.4	1	987	1	full-splice_match	gene: Zm00001d028793	Zm00001d028793_T001	85.03	1066.00	coding	220	102	761
PB.3808.2	3	1253	2	full-splice_match	gene: Zm00001d040004	Zm00001d040004_T001	54.29	60.91	coding	298	154	1047
PB.2059.1	10	1229	1	full-splice_match	gene: Zm00001d025360	Zm00001d025360_T001	29.64	29.64	coding	308	42	965
PB.3808.1	3	1359	2	full-splice_match	gene: Zm00001d040004	Zm00001d040004_T001	6.62	60.91	coding	298	159	1052
PB.9973.3	8	1789	12	full-splice_match	gene: Zm00001d012571	Zm00001d012571_T001	0.00	7.53	coding	530	3	1592
PB.9605.1	8	1264	2	full-splice_match	gene: Zm00001d010914	Zm00001d010914_T001	26.19	26.19	coding	327	26	1006
PB.7973.3	6	2594	14	full-splice_match	gene: Zm00001d038589	Zm00001d038589_T001	0.87	16.75	coding	720	77	2236

PB.2934.1	2	1033	4	novel_not_in_catalog	gene: Zm00001d004176	novel	104.64	104.64	coding	210	116	745
PB.7105.1	5	885	2	full-splice_match	gene: Zm00001d001747	Zm00001d001747_T001	11.56	11.56	non_coding	NA	NA	NA
PB.7219.2	6	1226	7	full-splice_match	gene: Zm00001d035034	Zm00001d035034_T001	13.33	40.93	coding	286	32	889
PB.10488.1	9	384	1	full-splice_match	gene: Zm00001d047334	Zm00001d047334_T001	0.00	0.00	non_coding	NA	NA	NA
PB.1049.1	1	1948	1	full-splice_match	gene: Zm00001d032459	Zm00001d032459_T001	18.96	18.96	coding	531	173	1765
PB.7219.1	6	1366	7	full-splice_match	gene: Zm00001d035034	Zm00001d035034_T001	27.60	40.93	coding	286	30	887
PB.6004.1	5	1218	1	antisense	novelGene_gene: Zm00001d013153_A	novel	6.49	6.49	coding	17	741	791
PB.5260.1	4	1445	2	novel_in_catalog	gene: Zm00001d051309	novel	6.97	6.97	coding	235	182	886
PB.10448.2	9	1233	6	full-splice_match	gene: Zm00001d047059	Zm00001d047059_T009	130.36	130.36	coding	279	181	1017
PB.7155.3	5	2991	2	full-splice_match	gene: Zm00001d018428	Zm00001d018428_T001	0.00	56.36	coding	739	422	2638
PB.2897.1	2	1349	7	full-splice_match	gene: Zm00001d003935	Zm00001d003935_T010	46.77	46.77	coding	212	470	1105
PB.928.5	1	3311	18	novel_in_catalog	gene: Zm00001d031938	novel	23.55	23.55	coding	911	356	3088
PB.10428.3	9	728	7	full-splice_match	gene: Zm00001d046959	Zm00001d046959_T002	73.17	186.68	coding	177	6	536
PB.10428.2	9	832	7	full-splice_match	gene: Zm00001d046959	Zm00001d046959_T002	83.65	186.68	coding	177	1	531
PB.10428.1	9	955	7	full-splice_match	gene: Zm00001d046959	Zm00001d046959_T002	29.86	186.68	coding	177	5	535
PB.7297.1	6	593	3	intergenic	novelGene_81	novel	11.37	11.37	non_coding	NA	NA	NA
PB.10295.1	9	2470	10	full-splice_match	gene: Zm00001d046182	Zm00001d046182_T001	20.30	52.32	coding	617	282	2132

PB.10295.3	9	984	3	incomplete-splice_match	gene: Zm00001d046182	Zm00001d046182_T001	19.89	52.32	coding	38	561	674
PB.10295.2	9	1697	5	novel_in_catalog	gene: Zm00001d046182	novel	5.40	52.32	coding	199	153	749
PB.3872.1	3	443	1	antisense	novelGene_gene: Zm00001d040304_A	novel	32.15	32.15	non_coding	NA	NA	NA
PB.10295.4	9	1509	8	incomplete-splice_match	gene: Zm00001d046182	Zm00001d046182_T001	6.73	52.32	coding	381	275	1417
PB.2874.1	2	1346	1	antisense	novelGene_gene: Zm00001d003841_A	novel	33.12	33.12	coding	184	325	876
PB.9573.1	8	2140	1	intergenic	novelGene_202	novel	9.57	11.26	coding	689	48	2114
PB.9972.3	8	1468	2	full-splice_match	gene: Zm00001d012564	Zm00001d012564_T001	76.75	127.84	coding	308	238	1161
PB.5777.1	4	1772	2	genic	gene: Zm00001d053650	novel	26.19	26.54	coding	427	176	1456
PB.9538.1	8	1036	2	genic	gene: Zm00001d010649	novel	13.76	89.69	coding	152	49	504
PB.3340.2	2	2502	8	full-splice_match	gene: Zm00001d006497	Zm00001d006497_T001	11.08	18.06	coding	746	34	2271
PB.9538.3	8	694	2	genic	gene: Zm00001d010649	novel	75.93	89.69	coding	152	49	504
PB.3297.1	2	2171	3	novel_in_catalog	gene: Zm00001d006324	novel	1.28	20.19	coding	235	9	713
PB.3297.2	2	1024	4	full-splice_match	gene: Zm00001d006324	Zm00001d006324_T007	18.91	20.19	coding	235	23	727
PB.6824.2	5	2604	20	full-splice_match	gene: Zm00001d017139	Zm00001d017139_T002	41.64	42.16	coding	746	203	2440
PB.8298.2	7	1631	5	incomplete-splice_match	gene: Zm00001d019299	Zm00001d019299_T008	20.25	20.25	coding	100	250	549
PB.8490.2	7	1565	1	full-splice_match	gene: Zm00001d020496	Zm00001d020496_T001	15.76	87.76	coding	464	69	1460
PB.786.1	1	837	1	full-splice_match	gene: Zm00001d031157	Zm00001d031157_T001	93.42	93.42	coding	177	86	616

PB.4378.1	3	1647	3	full-splice_match	gene: Zm00001d043076	Zm00001d043076_T001	27.62	27.62	coding	273	535	1353
PB.7211.1	6	1673	5	full-splice_match	gene: Zm00001d034990	Zm00001d034990_T001	19.47	19.47	coding	493	74	1552
PB.7835.2	6	2598	4	full-splice_match	gene: Zm00001d038106	Zm00001d038106_T011	19.26	19.26	coding	628	391	2274
PB.5268.1	4	2209	13	full-splice_match	gene: Zm00001d051345	Zm00001d051345_T005	17.68	18.45	coding	606	113	1930
PB.5268.2	4	2269	12	novel_in_catalog	gene: Zm00001d051345	novel	0.77	18.45	coding	213	1375	2013
PB.10604.2	9	1043	1	full-splice_match	gene: Zm00001d047775	Zm00001d047775_T001	9.42	60.58	coding	305	72	986
PB.8331.1	7	2195	9	full-splice_match	gene: Zm00001d019547	Zm00001d019547_T002	14.88	63.78	coding	517	304	1854
PB.8331.2	7	2129	9	full-splice_match	gene: Zm00001d019547	Zm00001d019547_T002	37.98	63.78	coding	517	351	1901
PB.8331.3	7	2184	8	novel_in_catalog	gene: Zm00001d019547	novel	10.92	63.78	coding	379	818	1954
PB.607.1	1	2515	11	full-splice_match	gene: Zm00001d030011	Zm00001d030011_T001	117.04	117.04	coding	720	191	2350
PB.4990.1	4	1425	3	genic	gene: Zm00001d049584	novel	17.74	17.74	coding	314	293	1234
PB.5839.3	4	1279	1	full-splice_match	gene: Zm00001d053908	Zm00001d053908_T001	674.70	715.10	coding	249	86	832
PB.1270.1	1	921	6	full-splice_match	gene: Zm00001d033484	Zm00001d033484_T001	28.75	28.75	coding	165	75	569
PB.7400.1	6	989	6	incomplete-splice_match	gene: Zm00001d036013	Zm00001d036013_T004	333.46	333.46	coding	112	144	479
PB.8424.5	7	1783	11	novel_not_in_catalog	gene: Zm00001d020183	novel	49.15	177.20	coding	323	664	1632
PB.8424.4	7	2175	10	novel_in_catalog	gene: Zm00001d020183	novel	20.46	177.20	coding	421	78	1340
PB.8424.7	7	1213	2	genic	gene: Zm00001d020183	novel	95.82	177.20	coding	81	429	671

PB.8936.1	7	1184	7	full-splice_match	gene: Zm00001d022254	Zm00001d022254_T002	19.68	22.75	coding	264	125	916
PB.10154.2	9	1389	6	novel_in_catalog	gene: Zm00001d045340	novel	56.63	103.02	coding	275	79	903
PB.10154.1	9	1347	6	novel_in_catalog	gene: Zm00001d045340	novel	46.39	103.02	coding	314	83	1024
PB.8424.2	7	2446	12	novel_not_in_catalog	gene: Zm00001d020183	novel	0.00	177.20	coding	612	459	2294
PB.5508.1	4	1848	5	genic	gene: Zm00001d052394	novel	2.17	2.17	coding	266	324	1121
PB.7930.1	6	1634	1	full-splice_match	gene: Zm00001d001456	Zm00001d001456_T001	1.61	1.61	non_coding	NA	NA	NA
PB.8424.9	7	2543	1	intergenic	novelGene_170	novel	2.22	2.22	non_coding	NA	NA	NA
PB.8424.8	7	3665	1	intergenic	novelGene_169	novel	7.97	7.97	non_coding	NA	NA	NA
PB.2561.1	2	2225	3	full-splice_match	gene: Zm00001d002452	Zm00001d002452_T001	19.53	19.53	coding	505	439	1953
PB.9972.1	8	1566	2	full-splice_match	gene: Zm00001d012564	Zm00001d012564_T001	46.93	127.84	coding	308	234	1157
PB.6398.4	5	1666	7	full-splice_match	gene: Zm00001d014809	Zm00001d014809_T002	0.48	36.20	coding	474	142	1563
PB.10486.1	9	2719	1	full-splice_match	gene: Zm00001d047307	Zm00001d047307_T001	0.67	0.67	coding	825	27	2501
PB.6398.1	5	2211	6	full-splice_match	gene: Zm00001d014809	Zm00001d014809_T005	2.19	36.20	coding	310	1075	2004
PB.6398.3	5	1772	6	novel_in_catalog	gene: Zm00001d014809	novel	1.06	36.20	coding	500	135	1634
PB.6398.2	5	1762	7	full-splice_match	gene: Zm00001d014809	Zm00001d014809_T002	32.46	36.20	coding	474	137	1558
PB.5633.1	4	798	2	incomplete-splice_match	gene: Zm00001d052930	Zm00001d052930_T002	118.50	118.50	non_coding	NA	NA	NA
PB.648.1	1	2092	4	full-splice_match	gene: Zm00001d030235	Zm00001d030235_T002	13.05	13.05	coding	583	193	1941

PB.4986.1	4	2308	20	full-splice_match	gene: Zm00001d049563	Zm00001d049563_T003	14.92	14.92	coding	600	82	1881
PB.379.3	1	1515	8	full-splice_match	gene: Zm00001d028903	Zm00001d028903_T001	0.00	52.29	coding	368	225	1328
PB.4986.3	4	2167	20	full-splice_match	gene: Zm00001d049563	Zm00001d049563_T003	0.00	14.92	coding	600	78	1877
PB.3986.4	3	2677	24	novel_not_in_catalog	gene: Zm00001d041138	novel	0.00	38.72	coding	806	148	2565
PB.4639.2	3	1446	6	full-splice_match	gene: Zm00001d044091	Zm00001d044091_T001	10.88	28.51	coding	382	186	1331
PB.3986.1	3	2794	24	novel_not_in_catalog	gene: Zm00001d041138	novel	38.72	38.72	coding	806	90	2507
PB.6648.1	5	1324	2	full-splice_match	gene: Zm00001d016255	Zm00001d016255_T001	33.64	33.64	coding	251	226	978
PB.4012.1	3	1128	6	full-splice_match	gene: Zm00001d041352	Zm00001d041352_T001	39.97	604.71	coding	180	116	655
PB.7119.1	5	1835	12	full-splice_match	gene: Zm00001d018336	Zm00001d018336_T003	7.29	9.41	coding	526	16	1593
PB.9920.1	8	1900	1	novel_in_catalog	gene: Zm00001d012322	novel	0.64	0.64	coding	336	106	1113
PB.4438.3	3	2040	5	full-splice_match	gene: Zm00001d043309	Zm00001d043309_T017	13.84	44.80	coding	459	201	1577
PB.3714.2	3	2008	13	full-splice_match	gene: Zm00001d039505	Zm00001d039505_T003	70.17	128.20	coding	505	149	1663
PB.3714.3	3	1814	13	full-splice_match	gene: Zm00001d039505	Zm00001d039505_T003	0.00	128.20	coding	505	124	1638
PB.3714.1	3	2069	13	full-splice_match	gene: Zm00001d039505	Zm00001d039505_T001	58.03	128.20	coding	505	109	1623
PB.4041.1	3	2357	6	full-splice_match	gene: Zm00001d041510	Zm00001d041510_T002	27.86	27.86	coding	512	423	1958
PB.9900.1	8	2468	4	full-splice_match	gene: Zm00001d012247	Zm00001d012247_T001	20.90	20.90	coding	585	320	2074
PB.5547.7	4	1781	12	novel_in_catalog	gene: Zm00001d052532	novel	2.60	938.93	coding	102	94	399

PB.10055.1	9	1079	3	full-splice_match	gene: Zm00001d044844	Zm00001d044844_T002	21.05	21.05	coding	118	265	618
PB.9900.2	8	2282	4	full-splice_match	gene: Zm00001d012247	Zm00001d012247_T001	0.00	20.90	coding	585	283	2037
PB.9298.1	8	574	1	genic	gene: Zm00001d009415	novel	85.10	755.96	non_coding	NA	NA	NA
PB.5547.3	4	2517	10	novel_in_catalog	gene: Zm00001d052532	novel	0.37	938.93	coding	140	92	511
PB.9298.2	8	494	1	genic	gene: Zm00001d009415	novel	670.86	755.96	non_coding	NA	NA	NA
PB.2553.1	2	1191	3	full-splice_match	gene: Zm00001d002405	Zm00001d002405_T001	304.29	328.22	coding	298	82	975
PB.5816.1	4	3770	14	full-splice_match	gene: Zm00001d053819	Zm00001d053819_T009	9.13	14.72	coding	847	922	3462
PB.2553.4	2	1342	1	genic	gene: Zm00001d002405	novel	9.00	328.22	coding	204	82	693
PB.2553.5	2	992	2	novel_in_catalog	gene: Zm00001d002405	novel	14.93	328.22	coding	78	331	564
PB.2553.6	2	1088	3	full-splice_match	gene: Zm00001d002405	Zm00001d002405_T001	0.00	328.22	coding	298	82	975
PB.847.1	1	1460	5	novel_not_in_catalog	gene: Zm00001d031505	novel	73.90	86.51	coding	319	274	1230
PB.371.4	1	768	6	novel_in_catalog	gene: Zm00001d028867	novel	2.00	70.84	non_coding	NA	NA	NA
PB.371.1	1	935	7	full-splice_match	gene: Zm00001d028867	Zm00001d028867_T002	2.52	70.84	non_coding	NA	NA	NA
PB.1339.4	1	2895	19	full-splice_match	gene: Zm00001d033795	Zm00001d033795_T008	2.18	30.20	coding	548	963	2606
PB.371.3	1	769	4	novel_in_catalog	gene: Zm00001d028867	novel	7.70	70.84	coding	91	34	306
PB.371.2	1	666	7	full-splice_match	gene: Zm00001d028867	Zm00001d028867_T002	58.62	70.84	non_coding	NA	NA	NA
PB.7273.2	6	1658	4	full-splice_match	gene: Zm00001d035298	Zm00001d035298_T004	1.02	19.48	coding	172	815	1330

PB.7273.1	6	1275	5	full-splice_match	gene: Zm00001d035298	Zm00001d035298_T002	18.46	19.48	coding	172	234	749
PB.1262.1	1	585	1	full-splice_match	gene: Zm00001d033463	Zm00001d033463_T001	0.38	0.38	coding	103	121	429
PB.1919.3	10	2389	19	novel_in_catalog	gene: Zm00001d024723	novel	1.51	39.76	coding	440	942	2261
PB.1919.2	10	2288	20	full-splice_match	gene: Zm00001d024723	Zm00001d024723_T002	7.59	39.76	coding	670	103	2112
PB.1919.1	10	1879	14	novel_in_catalog	gene: Zm00001d024723	novel	30.66	39.76	coding	469	185	1591
PB.7836.1	6	1506	5	full-splice_match	gene: Zm00001d038114	Zm00001d038114_T001	35.58	35.58	coding	335	230	1234
PB.672.1	1	3547	19	full-splice_match	gene: Zm00001d030398	Zm00001d030398_T003	2.93	2.93	coding	1089	43	3309
PB.6130.1	5	448	1	full-splice_match	gene: Zm00001d013593	Zm00001d013593_T001	2.97	2.97	non_coding	NA	NA	NA
PB.5141.1	4	1081	5	full-splice_match	gene: Zm00001d050412	Zm00001d050412_T001	8.41	8.41	coding	284	104	955
PB.8959.1	7	1586	1	incomplete-splice_match	gene: Zm00001d022343	Zm00001d022343_T006	27.40	27.40	coding	452	72	1427
PB.365.3	1	1720	1	full-splice_match	gene: Zm00001d028811	Zm00001d028811_T001	43.89	55.33	coding	447	202	1542
PB.365.1	1	2024	1	full-splice_match	gene: Zm00001d028811	Zm00001d028811_T001	11.44	55.33	coding	447	500	1840
PB.2543.3	2	631	1	genic	gene: Zm00001d002370	novel	6.21	137.31	coding	160	100	579
PB.2543.2	2	3687	1	genic	gene: Zm00001d002370	novel	10.23	137.31	coding	294	2625	3506
PB.2543.1	2	1714	2	full-splice_match	gene: Zm00001d002370	Zm00001d002370_T001	120.86	137.31	coding	460	106	1485
PB.3621.7	2	2963	6	intergenic	novelGene_363	novel	2.49	35.51	coding	279	1838	2674
PB.3621.5	2	2441	6	intergenic	novelGene_363	novel	8.60	35.51	coding	279	1277	2113

PB.3621.4	2	1998	8	intergenic	novelGene_363	novel	1.09	35.51	coding	279	828	1664
PB.8073.1	6	1382	1	genic	gene: Zm00001d038969	novel	19.60	19.60	coding	157	765	1235
PB.3621.2	2	1850	7	intergenic	novelGene_363	novel	7.10	35.51	coding	279	673	1509
PB.3621.1	2	2234	7	intergenic	novelGene_363	novel	16.24	35.51	coding	279	1060	1896
PB.973.1	1	599	3	full-splice_match	gene: Zm00001d032123	Zm00001d032123_T001	13.92	211.54	non_coding	NA	NA	NA
PB.973.2	1	651	5	novel_not_in_catalog	gene: Zm00001d032123	novel	197.62	211.54	non_coding	NA	NA	NA
PB.6406.2	5	1521	6	full-splice_match	gene: Zm00001d014850	Zm00001d014850_T002	14.55	16.75	coding	413	108	1346
PB.6406.1	5	1609	6	full-splice_match	gene: Zm00001d014850	Zm00001d014850_T002	2.20	16.75	coding	388	66	1229
PB.10753.2	9	1814	2	incomplete-splice_match	gene: Zm00001d048308	Zm00001d048308_T002	3.48	25.22	coding	497	324	1814
PB.10600.1	9	929	1	intergenic	novelGene_225	novel	3.31	3.31	coding	127	403	783
PB.10753.1	9	2574	5	incomplete-splice_match	gene: Zm00001d048308	Zm00001d048308_T002	21.74	25.22	coding	790	6	2375
PB.4724.2	3	1228	6	novel_in_catalog	gene: Zm00001d044451	novel	0.00	47.57	coding	310	68	997
PB.9635.4	8	1805	6	full-splice_match	gene: Zm00001d011051	Zm00001d011051_T003	14.45	27.71	coding	441	92	1414
PB.7478.3	6	1594	9	novel_in_catalog	gene: Zm00001d036496	novel	9.70	22.98	coding	112	79	414
PB.7478.1	6	1450	10	full-splice_match	gene: Zm00001d036496	Zm00001d036496_T003	13.27	22.98	coding	408	62	1285
PB.6785.3	5	3156	5	full-splice_match	gene: Zm00001d016956	Zm00001d016956_T008	1.91	12.39	coding	107	144	464
PB.1911.6	10	797	3	full-splice_match	gene: Zm00001d024703	Zm00001d024703_T012	15.79	44.13	non_coding	NA	NA	NA

PB.6785.1	5	1745	1	incomplete-splice_match	gene: Zm00001d016956	Zm00001d016956_T001	5.14	12.39	non_coding	NA	NA	NA
PB.1911.3	10	2428	2	incomplete-splice_match	gene: Zm00001d024703	Zm00001d024703_T010	28.34	44.13	coding	705	57	2171
PB.7861.3	6	1963	3	novel_not_in_catalog	gene: Zm00001d038186	novel	0.51	27.54	coding	487	401	1861
PB.8950.2	7	1940	8	novel_in_catalog	gene: Zm00001d022321	novel	7.31	26.63	coding	204	269	880
PB.7737.2	6	2046	7	novel_not_in_catalog	gene: Zm00001d037745	novel	0.88	34.48	coding	400	508	1707
PB.7737.1	6	2013	7	full-splice_match	gene: Zm00001d037745	Zm00001d037745_T001	17.57	34.48	coding	522	154	1719
PB.4724.1	3	1409	6	novel_in_catalog	gene: Zm00001d044451	novel	47.57	47.57	coding	310	93	1022
PB.3580.3	2	2032	4	novel_in_catalog	gene: Zm00001d007767	novel	9.09	519.93	coding	176	195	722
PB.7737.6	6	1875	7	novel_not_in_catalog	gene: Zm00001d037745	novel	3.89	34.48	coding	400	533	1732
PB.7737.5	6	1941	6	novel_in_catalog	gene: Zm00001d037745	novel	4.84	34.48	coding	386	641	1798
PB.7737.4	6	2096	6	novel_in_catalog	gene: Zm00001d037745	novel	7.29	34.48	coding	386	732	1889
PB.1988.1	10	3739	2	full-splice_match	gene: Zm00001d025002	Zm00001d025002_T001	12.41	12.41	coding	1147	106	3546
PB.6559.1	5	459	1	full-splice_match	gene: Zm00001d015550	Zm00001d015550_T001	72.02	72.02	non_coding	NA	NA	NA
PB.963.6	1	1645	1	novel_in_catalog	gene: Zm00001d032081	novel	0.00	115.28	coding	109	228	554
PB.963.4	1	1935	1	novel_in_catalog	gene: Zm00001d032081	novel	5.28	115.28	coding	109	232	558
PB.963.5	1	1072	3	full-splice_match	gene: Zm00001d032081	Zm00001d032081_T001	95.90	115.28	coding	131	230	622
PB.963.2	1	2169	3	full-splice_match	gene: Zm00001d032081	Zm00001d032081_T001	3.48	115.28	coding	131	227	619

PB.963.3	1	1973	2	novel_in_catalog	gene: Zm00001d032081	novel	9.59	115.28	coding	109	225	551
PB.4681.2	3	2186	4	full-splice_match	gene: Zm00001d044253	Zm00001d044253_T001	42.99	42.99	coding	562	167	1852
PB.963.1	1	2379	2	novel_in_catalog	gene: Zm00001d032081	novel	1.03	115.28	coding	150	232	681
PB.8398.1	7	2000	2	full-splice_match	gene: Zm00001d020030	Zm00001d020030_T001	0.60	0.60	coding	168	44	547
PB.6158.3	5	1417	1	novel_in_catalog	gene: Zm00001d013707	novel	5.33	57.86	coding	69	114	320
PB.6158.1	5	1138	3	full-splice_match	gene: Zm00001d013707	Zm00001d013707_T001	52.53	57.86	coding	224	118	789
PB.1896.2	10	1896	6	novel_in_catalog	gene: Zm00001d024588	novel	3.47	64.22	coding	144	315	746
PB.9677.12	8	2282	9	novel_not_in_catalog	gene: Zm00001d011253	novel	6.02	141.59	coding	207	827	1447
PB.357.4	1	1088	1	novel_in_catalog	gene: Zm00001d028785	novel	15.98	88.51	coding	143	544	972
PB.9677.10	8	2272	5	novel_not_in_catalog	gene: Zm00001d011253	novel	0.33	141.59	non_coding	NA	NA	NA
PB.735.2	1	1345	4	full-splice_match	gene: Zm00001d030944	Zm00001d030944_T001	10.89	10.89	coding	293	123	1001
PB.357.1	1	1019	2	full-splice_match	gene: Zm00001d028785	Zm00001d028785_T001	5.52	88.51	coding	170	110	619
PB.9677.17	8	1599	5	novel_in_catalog	gene: Zm00001d011253	novel	1.49	141.59	coding	149	1052	1498
PB.357.3	1	882	2	full-splice_match	gene: Zm00001d028785	Zm00001d028785_T001	27.30	88.51	coding	170	178	687
PB.357.2	1	990	2	full-splice_match	gene: Zm00001d028785	Zm00001d028785_T001	39.71	88.51	coding	170	182	691
PB.9407.1	8	1451	7	full-splice_match	gene: Zm00001d009997	Zm00001d009997_T001	22.76	22.76	coding	375	29	1153
PB.1194.2	1	924	6	full-splice_match	gene: Zm00001d033099	Zm00001d033099_T001	64.51	321.05	coding	142	106	531

PB.5492.3	4	2350	1	novel_in_catalog	gene: Zm00001d052357	novel	4.14	79.45	coding	154	1362	1823
PB.1194.1	1	1063	6	full-splice_match	gene: Zm00001d033099	Zm00001d033099_T001	49.00	321.05	coding	142	130	555
PB.8847.4	7	2382	5	full-splice_match	gene: Zm00001d021930	Zm00001d021930_T002	4.23	101.07	coding	219	126	782
PB.5492.2	4	1411	2	novel_in_catalog	gene: Zm00001d052357	novel	9.38	79.45	coding	154	402	863
PB.8988.1	7	3316	9	incomplete-splice_match	gene: Zm00001d022429	Zm00001d022429_T026	26.35	26.35	coding	962	118	3003
PB.7450.1	6	1990	8	full-splice_match	gene: Zm00001d036359	Zm00001d036359_T003	16.55	16.55	coding	458	474	1847
PB.9854.2	8	1818	1	genic	gene: Zm00001d012021	novel	3.23	32.26	coding	150	1080	1529
PB.9586.1	8	1738	5	full-splice_match	gene: Zm00001d010825	Zm00001d010825_T001	99.25	99.25	coding	440	92	1411
PB.3588.2	2	2128	3	novel_in_catalog	gene: Zm00001d007801	novel	0.86	20.84	coding	132	1439	1834
PB.3588.1	2	2054	4	full-splice_match	gene: Zm00001d007801	Zm00001d007801_T001	19.98	20.84	coding	429	445	1731
PB.2064.1	10	743	2	full-splice_match	gene: Zm00001d025401	Zm00001d025401_T001	385.11	439.87	coding	104	136	447
PB.5167.4	4	1049	6	full-splice_match	gene: Zm00001d050616	Zm00001d050616_T001	45.07	229.36	coding	205	339	953
PB.9058.6	8	1470	9	full-splice_match	gene: Zm00001d008177	Zm00001d008177_T001	0.00	37.95	coding	357	166	1236
PB.9058.3	8	1672	9	full-splice_match	gene: Zm00001d008177	Zm00001d008177_T001	10.89	37.95	coding	357	168	1238
PB.5167.1	4	1736	6	full-splice_match	gene: Zm00001d050616	Zm00001d050616_T001	21.07	229.36	coding	420	58	1317
PB.9058.1	8	1756	9	full-splice_match	gene: Zm00001d008177	Zm00001d008177_T001	27.06	37.95	coding	357	135	1205
PB.5167.3	4	1577	6	full-splice_match	gene: Zm00001d050616	Zm00001d050616_T001	0.00	229.36	coding	420	154	1413

PB.7989.1	6	1424	10	full-splice_match	gene: Zm00001d038689	Zm00001d038689_T001	24.26	115.37	coding	369	1	1107
PB.4156.6	3	1667	12	full-splice_match	gene: Zm00001d042040	Zm00001d042040_T001	66.66	89.47	non_coding	NA	NA	NA
PB.8341.1	7	308	1	incomplete-splice_match	gene: Zm00001d019577	Zm00001d019577_T001	14.27	162.48	non_coding	NA	NA	NA
PB.4156.4	3	2022	11	novel_in_catalog	gene: Zm00001d042040	novel	22.81	89.47	coding	405	131	1345
PB.4188.2	3	1481	8	novel_in_catalog	gene: Zm00001d042193	novel	7.37	43.02	coding	219	123	779
PB.4188.3	3	1300	9	full-splice_match	gene: Zm00001d042193	Zm00001d042193_T006	0.00	43.02	coding	286	120	977
PB.2331.1	10	1091	1	full-splice_match	gene: Zm00001d026563	Zm00001d026563_T001	41.45	41.45	coding	178	287	820
PB.721.2	1	1819	1	genic	gene: Zm00001d030845	novel	6.23	20.33	coding	324	43	1014
PB.7644.1	6	1877	4	full-splice_match	gene: Zm00001d037322	Zm00001d037322_T001	8.22	10.74	coding	531	34	1626
PB.7644.3	6	2102	2	novel_in_catalog	gene: Zm00001d037322	novel	2.52	10.74	coding	292	962	1837
PB.4035.2	3	2007	4	full-splice_match	gene: Zm00001d041484	Zm00001d041484_T003	0.00	6.55	coding	452	153	1508
PB.4035.1	3	2870	5	full-splice_match	gene: Zm00001d041484	Zm00001d041484_T001	6.55	6.55	coding	574	520	2241
PB.2037.1	10	2795	6	full-splice_match	gene: Zm00001d025253	Zm00001d025253_T007	9.10	9.10	coding	384	1221	2372
PB.7727.1	6	1122	4	incomplete-splice_match	gene: Zm00001d037717	Zm00001d037717_T001	12.05	34.29	coding	66	426	623
PB.7727.2	6	1911	9	full-splice_match	gene: Zm00001d037717	Zm00001d037717_T001	22.25	34.29	coding	481	78	1520
PB.5423.10	4	887	8	incomplete-splice_match	gene: Zm00001d052074	Zm00001d052074_T007	19.97	84.05	coding	132	491	886
PB.4488.1	3	1561	15	full-splice_match	gene: Zm00001d043490	Zm00001d043490_T001	35.92	35.92	coding	384	192	1343

PB.8743.2	7	2474	20	novel_in_catalog	gene: Zm00001d021584	novel	10.64	15.33	coding	682	207	2252
PB.92.7	1	2027	5	full-splice_match	gene: Zm00001d027571	Zm00001d027571_T003	70.63	85.29	coding	399	582	1778
PB.9748.1	8	612	1	full-splice_match	gene: Zm00001d008121	Zm00001d008121_T001	1.37	1.37	non_coding	NA	NA	NA
PB.9201.2	8	2353	16	full-splice_match	gene: Zm00001d008847	Zm00001d008847_T001	25.78	78.06	coding	639	136	2052
PB.2986.6	2	3245	13	full-splice_match	gene: Zm00001d004659	Zm00001d004659_T005	15.44	15.44	coding	907	192	2912
PB.1989.1	10	1830	2	novel_in_catalog	gene: Zm00001d025008	novel	24.63	24.63	coding	375	190	1314
PB.2781.1	2	4404	4	full-splice_match	gene: Zm00001d003333	Zm00001d003333_T002	17.12	17.12	coding	1238	373	4086
PB.8382.2	7	1442	9	novel_in_catalog	gene: Zm00001d019907	novel	13.48	23.19	coding	302	98	1003
PB.8757.2	7	3280	7	full-splice_match	gene: Zm00001d021641	Zm00001d021641_T004	9.28	9.28	coding	864	260	2851
PB.9050.1	7	2838	1	intergenic	novelGene_302	novel	12.54	12.54	coding	848	97	2640
PB.8382.1	7	1815	12	incomplete-splice_match	gene: Zm00001d019907	Zm00001d019907_T012	5.74	23.19	coding	302	186	1091
PB.5588.1	4	1482	6	full-splice_match	gene: Zm00001d052742	Zm00001d052742_T004	9.69	43.89	non_coding	NA	NA	NA
PB.2857.1	2	1607	2	intergenic	novelGene_116	novel	203.54	203.54	coding	371	80	1192
PB.438.1	1	1052	4	novel_in_catalog	gene: Zm00001d029140	novel	5.35	5.35	coding	220	155	814
PB.5588.2	4	1284	6	novel_in_catalog	gene: Zm00001d052742	novel	1.88	43.89	coding	189	480	1046
PB.1633.2	1	2171	11	novel_in_catalog	gene: Zm00001d034918	novel	4.91	10.94	coding	587	240	2000
PB.6451.1	5	1360	5	full-splice_match	gene: Zm00001d014995	Zm00001d014995_T002	47.95	47.95	coding	235	347	1051

PB.1321.2	1	2463	1	incomplete-splice_match	gene: Zm00001d033704	Zm00001d033704_T001	2.91	30.95	coding	758	70	2343
PB.4180.2	3	2138	15	full-splice_match	gene: Zm00001d042128	Zm00001d042128_T005	0.00	7.35	coding	588	132	1895
PB.8203.7	7	1894	1	full-splice_match	gene: Zm00001d018813	Zm00001d018813_T001	17.96	41.55	coding	410	476	1705
PB.8203.4	7	1978	1	full-splice_match	gene: Zm00001d018813	Zm00001d018813_T001	13.24	41.55	coding	410	484	1713
PB.4180.1	3	2314	15	full-splice_match	gene: Zm00001d042128	Zm00001d042128_T005	7.35	7.35	coding	588	140	1903
PB.8751.2	7	3099	12	novel_in_catalog	gene: Zm00001d021621	novel	8.47	8.47	coding	579	1187	2923
PB.3935.10	3	1677	4	incomplete-splice_match	gene: Zm00001d040667	Zm00001d040667_T003	0.00	252.28	coding	205	67	681
PB.4673.3	3	2345	4	novel_in_catalog	gene: Zm00001d044225	novel	2.42	93.87	coding	321	55	1017
PB.4673.2	3	1672	5	full-splice_match	gene: Zm00001d044225	Zm00001d044225_T001	88.89	93.87	coding	450	101	1450
PB.9869.3	8	1356	2	full-splice_match	gene: Zm00001d012103	Zm00001d012103_T001	72.55	2175.90	coding	355	172	1236
PB.9869.2	8	2140	2	novel_not_in_catalog	gene: Zm00001d012103	novel	7.83	2175.90	coding	128	1530	1913
PB.9869.1	8	1365	2	full-splice_match	gene: Zm00001d012103	Zm00001d012103_T001	2095.52	2175.90	coding	355	78	1142
PB.4673.4	3	1579	5	full-splice_match	gene: Zm00001d044225	Zm00001d044225_T001	2.56	93.87	coding	450	149	1498
PB.7125.1	5	1775	6	full-splice_match	gene: Zm00001d018359	Zm00001d018359_T002	524.29	524.29	coding	413	292	1530
PB.7125.2	5	1594	6	full-splice_match	gene: Zm00001d018359	Zm00001d018359_T002	0.00	524.29	coding	413	157	1395
PB.5662.2	4	682	1	full-splice_match	gene: Zm00001d053055	Zm00001d053055_T001	407.85	649.95	coding	171	25	537
PB.4747.1	3	2007	11	full-splice_match	gene: Zm00001d044533	Zm00001d044533_T001	183.07	183.07	coding	462	312	1697

PB.2217.1	10	826	1	novel_in_catalog	gene: Zm00001d026163	novel	239.08	4617.38	coding	122	121	486
PB.5664.1	4	759	3	intergenic	novelGene_328	novel	37.51	37.51	non_coding	NA	NA	NA
PB.4760.3	3	2976	11	full-splice_match	gene: Zm00001d044593	Zm00001d044593_T001	0.00	65.85	coding	921	135	2897
PB.3036.1	2	837	1	full-splice_match	gene: Zm00001d004972	Zm00001d004972_T001	57.89	57.89	coding	187	96	656
PB.2374.3	10	1524	4	novel_in_catalog	gene: Zm00001d026676	novel	3.96	11.70	coding	135	12	416
PB.10109.1	9	1053	6	full-splice_match	gene: Zm00001d045133	Zm00001d045133_T001	75.12	75.12	coding	153	300	758
PB.8444.1	7	1029	2	full-splice_match	gene: Zm00001d020292	Zm00001d020292_T001	36.91	61.24	coding	177	140	670
PB.1027.2	1	1994	5	novel_in_catalog	gene: Zm00001d032373	novel	9.80	9.80	coding	240	1094	1813
PB.5039.2	4	2010	1	intergenic	novelGene_108	novel	3.60	4.13	non_coding	NA	NA	NA
PB.8519.1	7	2049	5	novel_not_in_catalog	gene: Zm00001d020618	novel	27.89	27.89	coding	518	135	1688
PB.291.1	1	1373	5	intergenic	novelGene_95	novel	20.14	20.14	non_coding	NA	NA	NA
PB.6547.1	5	1426	9	full-splice_match	gene: Zm00001d015485	Zm00001d015485_T001	2.82	2.82	coding	402	82	1287
PB.430.2	1	2224	8	full-splice_match	gene: Zm00001d029118	Zm00001d029118_T003	2.26	19.08	coding	249	1	747
PB.430.1	1	1744	9	full-splice_match	gene: Zm00001d029118	Zm00001d029118_T001	16.83	19.08	coding	413	173	1411
PB.4476.1	3	3013	7	incomplete-splice_match	gene: Zm00001d043452	Zm00001d043452_T003	44.58	56.98	coding	904	15	2726
PB.4476.2	3	2592	6	incomplete-splice_match	gene: Zm00001d043452	Zm00001d043452_T003	12.40	56.98	coding	717	293	2443
PB.9743.1	8	1381	2	novel_in_catalog	gene: Zm00001d011581	novel	87.34	1136.33	non_coding	NA	NA	NA

PB.1923.1	10	1268	1	full-splice_match	gene: Zm00001d024745	Zm00001d024745_T001	116.60	116.60	coding	338	105	1118
PB.1149.1	1	830	1	full-splice_match	gene: Zm00001d032869	Zm00001d032869_T001	40.28	50.91	coding	65	165	359
PB.4583.4	3	2634	10	full-splice_match	gene: Zm00001d043860	Zm00001d043860_T004	22.90	22.90	coding	624	289	2160
PB.5702.2	4	2426	16	novel_not_in_catalog	gene: Zm00001d053301	novel	18.35	24.99	coding	496	734	2221
PB.10115.2	9	1379	3	novel_not_in_catalog	gene: Zm00001d045159	novel	35.69	35.69	coding	303	243	1151
PB.9861.2	8	563	1	full-splice_match	gene: Zm00001d012081	Zm00001d012081_T001	630.29	905.23	coding	100	95	394
PB.9861.1	8	669	1	full-splice_match	gene: Zm00001d012081	Zm00001d012081_T001	274.93	905.23	coding	100	90	389
PB.1816.4	10	1123	2	full-splice_match	gene: Zm00001d024071	Zm00001d024071_T001	0.00	51.73	coding	287	109	969
PB.1816.1	10	1719	2	full-splice_match	gene: Zm00001d024071	Zm00001d024071_T001	1.31	51.73	coding	287	34	894
PB.1816.3	10	1361	1	novel_in_catalog	gene: Zm00001d024071	novel	4.98	51.73	coding	181	521	1063
PB.1816.2	10	1272	2	full-splice_match	gene: Zm00001d024071	Zm00001d024071_T001	45.44	51.73	coding	287	114	974
PB.5020.2	4	1597	7	full-splice_match	gene: Zm00001d049714	Zm00001d049714_T001	17.05	17.78	coding	386	190	1347
PB.9881.3	8	948	5	full-splice_match	gene: Zm00001d012160	Zm00001d012160_T002	37.11	96.04	coding	196	140	727
PB.5020.1	4	2163	3	novel_in_catalog	gene: Zm00001d049714	novel	0.73	17.78	coding	226	1235	1912
PB.1149.2	1	2363	1	full-splice_match	gene: Zm00001d032869	Zm00001d032869_T001	4.04	50.91	coding	667	11	2011
PB.1442.4	1	2060	23	novel_in_catalog	gene: Zm00001d034256	novel	107.61	178.19	coding	506	330	1847
PB.1442.5	1	2296	22	novel_in_catalog	gene: Zm00001d034256	novel	70.58	178.19	coding	209	1364	1990

PB.4186.12	3	2111	5	full-splice_match	gene: Zm00001d042185	Zm00001d042185_T013	0.00	93.73	non_coding	NA	NA	NA
PB.4186.13	3	2151	4	full-splice_match	gene: Zm00001d042185	Zm00001d042185_T012	0.00	93.73	coding	268	1347	2150
PB.9793.1	8	746	3	full-splice_match	gene: Zm00001d011786	Zm00001d011786_T001	82.85	257.23	coding	135	91	495
PB.6322.1	5	1406	2	full-splice_match	gene: Zm00001d014532	Zm00001d014532_T001	49.06	49.06	coding	321	317	1279
PB.10203.1	9	1701	4	full-splice_match	gene: Zm00001d045513	Zm00001d045513_T001	74.94	159.29	coding	322	69	1034
PB.10203.2	9	2073	3	novel_in_catalog	gene: Zm00001d045513	novel	11.01	159.29	coding	190	255	824
PB.10203.3	9	1438	4	novel_not_in_catalog	gene: Zm00001d045513	novel	27.87	159.29	coding	118	443	796
PB.10203.4	9	1614	4	full-splice_match	gene: Zm00001d045513	Zm00001d045513_T001	2.50	159.29	coding	322	257	1222
PB.10203.5	9	1254	4	novel_not_in_catalog	gene: Zm00001d045513	novel	0.00	159.29	coding	154	2	463
PB.7191.3	5	1074	1	full-splice_match	gene: Zm00001d018530	Zm00001d018530_T001	48.42	54.38	coding	207	45	665
PB.10203.7	9	1472	4	full-splice_match	gene: Zm00001d045513	Zm00001d045513_T001	42.97	159.29	coding	322	249	1214
PB.6962.3	5	3608	18	full-splice_match	gene: Zm00001d017754	Zm00001d017754_T035	0.00	44.58	coding	940	483	3302
PB.7039.2	5	3206	2	genic	gene: Zm00001d018019	novel	2.51	2.51	coding	745	107	2341
PB.6696.7	5	2236	2	novel_in_catalog	gene: Zm00001d016553	novel	0.97	140.34	coding	364	865	1956
PB.9032.2	7	1385	3	incomplete-splice_match	gene: Zm00001d022564	Zm00001d022564_T001	6.06	7.85	coding	343	63	1091
PB.9032.1	7	2109	3	incomplete-splice_match	gene: Zm00001d022564	Zm00001d022564_T001	1.79	7.85	coding	388	543	1706
PB.7955.1	6	1955	2	genic	gene: Zm00001d038537	novel	8.19	30.39	coding	354	540	1601

PB.10382.1	9	2042	13	incomplete-splice_match	gene: Zm00001d046683	Zm00001d046683_T012	57.29	57.29	coding	472	75	1490
PB.3525.7	2	2143	1	incomplete-splice_match	gene: Zm00001d007434	Zm00001d007434_T005	3.14	67.30	non_coding	NA	NA	NA
PB.8224.1	7	946	4	full-splice_match	gene: Zm00001d018903	Zm00001d018903_T001	1313.82	1313.82	coding	204	63	674
PB.1149.3	1	2242	1	full-splice_match	gene: Zm00001d032869	Zm00001d032869_T001	6.59	50.91	coding	667	114	2114
PB.3246.1	2	2045	5	full-splice_match	gene: Zm00001d006102	Zm00001d006102_T001	46.34	46.34	coding	431	419	1711
PB.5381.2	4	1315	2	full-splice_match	gene: Zm00001d051896	Zm00001d051896_T001	53.81	226.58	coding	350	24	1073
PB.6557.1	5	919	7	full-splice_match	gene: Zm00001d015526	Zm00001d015526_T001	73.79	73.79	coding	204	116	727
PB.5381.1	4	1430	2	full-splice_match	gene: Zm00001d051896	Zm00001d051896_T001	47.29	226.58	coding	350	21	1070
PB.7739.1	6	1738	4	full-splice_match	gene: Zm00001d037756	Zm00001d037756_T001	70.19	134.17	coding	387	218	1378
PB.4435.1	3	2556	18	full-splice_match	gene: Zm00001d043301	Zm00001d043301_T006	16.78	16.78	coding	715	131	2275
PB.7739.3	6	844	1	novel_in_catalog	gene: Zm00001d037756	novel	14.20	134.17	coding	111	275	607
PB.2028.2	10	1461	9	full-splice_match	gene: Zm00001d025180	Zm00001d025180_T005	7.22	38.15	coding	440	31	1350
PB.789.2	1	971	7	full-splice_match	gene: Zm00001d031179	Zm00001d031179_T001	0.00	89.32	coding	204	164	775
PB.4577.2	3	1061	2	full-splice_match	gene: Zm00001d043837	Zm00001d043837_T001	19.76	25.46	coding	201	255	857
PB.772.1	1	2291	7	full-splice_match	gene: Zm00001d031090	Zm00001d031090_T001	50.61	50.61	coding	596	167	1954
PB.3695.2	3	517	2	incomplete-splice_match	gene: Zm00001d039435	Zm00001d039435_T001	12.28	21.31	coding	116	46	393
PB.2028.1	10	1646	9	full-splice_match	gene: Zm00001d025180	Zm00001d025180_T002	29.23	38.15	coding	440	114	1433

PB.6318.3	5	2679	9	full-splice_match	gene: Zm00001d014524	Zm00001d014524_T006	15.11	16.33	coding	737	320	2530
PB.3695.1	3	3000	4	full-splice_match	gene: Zm00001d039435	Zm00001d039435_T001	9.03	21.31	coding	813	66	2504
PB.2793.1	2	528	1	intergenic	novelGene_104	novel	13.89	13.89	non_coding	NA	NA	NA
PB.10813.8	9	1673	8	full-splice_match	gene: Zm00001d048507	Zm00001d048507_T007	0.00	86.16	coding	458	68	1441
PB.2732.1	2	1443	2	full-splice_match	gene: Zm00001d003162	Zm00001d003162_T001	30.62	30.62	coding	320	308	1267
PB.9344.2	8	1608	6	novel_not_in_catalog	gene: Zm00001d009667	novel	0.00	14.95	coding	496	51	1538
PB.9344.1	8	1901	6	novel_not_in_catalog	gene: Zm00001d009667	novel	14.95	14.95	coding	496	240	1727
PB.5527.3	4	1020	6	full-splice_match	gene: Zm00001d052450	Zm00001d052450_T002	79.77	139.39	coding	246	140	877
PB.6124.1	5	1945	3	novel_in_catalog	gene: Zm00001d013555	novel	4.94	4.94	coding	250	955	1704
PB.9342.3	8	2386	5	novel_in_catalog	gene: Zm00001d009660	novel	1.43	93.43	coding	233	1501	2199
PB.503.1	1	1322	5	full-splice_match	gene: Zm00001d029448	Zm00001d029448_T001	53.10	124.70	coding	228	140	823
PB.503.2	1	1222	5	full-splice_match	gene: Zm00001d029448	Zm00001d029448_T001	71.60	124.70	coding	228	171	854
PB.9783.1	8	685	2	full-splice_match	gene: Zm00001d011747	Zm00001d011747_T001	62.68	62.68	coding	174	49	570
PB.412.1	1	1703	6	full-splice_match	gene: Zm00001d029014	Zm00001d029014_T002	12.80	12.80	coding	519	45	1601
PB.8219.4	7	1785	4	full-splice_match	gene: Zm00001d018887	Zm00001d018887_T002	0.00	26.60	coding	432	64	1359
PB.4802.1	4	1508	1	full-splice_match	gene: Zm00001d048615	Zm00001d048615_T001	7.45	7.45	coding	265	511	1305
PB.8219.2	7	2177	3	novel_in_catalog	gene: Zm00001d018887	novel	3.49	26.60	coding	292	118	993

PB.8219.1	7	2060	4	full-splice_match	gene: Zm00001d018887	Zm00001d018887_T002	23.11	26.60	coding	432	73	1368
PB.5922.1	5	2583	6	full-splice_match	gene: Zm00001d012855	Zm00001d012855_T001	9.77	9.77	coding	725	137	2311
PB.9779.2	8	900	2	full-splice_match	gene: Zm00001d011732	Zm00001d011732_T001	108.79	375.30	coding	200	86	685
PB.8718.1	7	1314	7	full-splice_match	gene: Zm00001d021517	Zm00001d021517_T002	25.99	25.99	coding	310	78	1007
PB.5922.2	5	2455	6	full-splice_match	gene: Zm00001d012855	Zm00001d012855_T001	0.00	9.77	coding	725	133	2307
PB.6810.25	5	2364	6	novel_in_catalog	gene: Zm00001d017096	novel	2.89	118.92	coding	171	106	618
PB.6810.24	5	1520	10	novel_in_catalog	gene: Zm00001d017096	novel	0.00	118.92	coding	180	106	645
PB.2056.1	10	1599	5	full-splice_match	gene: Zm00001d025343	Zm00001d025343_T001	4.95	4.95	coding	444	92	1423
PB.9004.1	7	1204	3	full-splice_match	gene: Zm00001d022474	Zm00001d022474_T001	25.48	25.48	coding	58	810	983
PB.416.10	1	2291	2	novel_in_catalog	gene: Zm00001d029033	novel	3.29	20.35	coding	196	1442	2029
PB.4566.2	3	2590	5	full-splice_match	gene: Zm00001d043776	Zm00001d043776_T006	15.64	15.64	coding	396	51	1238
PB.3153.2	2	630	4	full-splice_match	gene: Zm00001d005642	Zm00001d005642_T001	693.67	693.67	coding	112	38	373
PB.2107.1	10	3827	2	incomplete-splice_match	gene: Zm00001d025668	Zm00001d025668_T005	9.58	9.58	coding	993	533	3511
PB.3598.1	2	2676	2	novel_in_catalog	gene: Zm00001d007828	novel	4.61	4.61	coding	350	1426	2475
PB.4951.2	4	1549	9	novel_not_in_catalog	gene: Zm00001d049363	novel	8.92	8.92	coding	141	229	651
PB.5441.1	4	1102	8	novel_not_in_catalog	gene: Zm00001d052180	novel	48.92	48.92	coding	228	135	818
PB.3573.1	2	4498	8	full-splice_match	gene: Zm00001d007719	Zm00001d007719_T003	19.65	19.65	coding	1353	274	4332

PB.6053.1	5	2014	1	intergenic	novelGene_35	novel	3.21	3.21	coding	505	149	1663
PB.7955.2	6	1781	2	genic	gene: Zm00001d038537	novel	22.20	30.39	coding	354	543	1604
PB.5002.1	4	2172	9	full-splice_match	gene: Zm00001d049636	Zm00001d049636_T001	40.18	40.18	coding	534	154	1755
PB.9719.1	8	1788	4	incomplete-splice_match	gene: Zm00001d011473	Zm00001d011473_T006	11.30	11.30	coding	413	23	1261
PB.6748.1	5	1551	1	antisense	novelGene_gene: Zm00001d016806_A	novel	12.58	12.58	coding	117	849	1199
PB.9164.2	8	1743	7	incomplete-splice_match	gene: Zm00001d008648	Zm00001d008648_T004	0.00	12.24	non_coding	NA	NA	NA
PB.9164.1	8	3039	13	full-splice_match	gene: Zm00001d008648	Zm00001d008648_T001	12.24	12.24	coding	908	85	2808
PB.8669.1	7	1688	1	novel_in_catalog	gene: Zm00001d021318	novel	174.58	248.77	coding	333	408	1406
PB.2172.1	10	1285	9	full-splice_match	gene: Zm00001d025939	Zm00001d025939_T001	49.33	66.55	coding	250	177	926
PB.1686.1	10	1310	6	novel_not_in_catalog	gene: Zm00001d023371	novel	80.46	249.35	coding	241	307	1029
PB.1686.2	10	1241	6	full-splice_match	gene: Zm00001d023371	Zm00001d023371_T001	160.00	249.35	coding	239	315	1031
PB.1686.3	10	1505	5	novel_in_catalog	gene: Zm00001d023371	novel	8.90	249.35	coding	156	841	1308
PB.6736.1	5	1522	1	full-splice_match	gene: Zm00001d016748	Zm00001d016748_T001	16.36	26.87	coding	287	149	1009
PB.3106.1	2	2913	17	novel_not_in_catalog	gene: Zm00001d005348	novel	22.15	22.15	coding	769	211	2517
PB.6736.2	5	1156	1	full-splice_match	gene: Zm00001d016748	Zm00001d016748_T001	10.51	26.87	coding	287	239	1099
PB.4295.1	3	1638	10	full-splice_match	gene: Zm00001d042685	Zm00001d042685_T003	37.41	37.41	coding	416	225	1472
PB.1037.1	1	1912	3	full-splice_match	gene: Zm00001d032409	Zm00001d032409_T001	27.73	27.73	coding	536	219	1826

PB.1722.3	10	811	3	full-splice_match	gene: Zm00001d023542	Zm00001d023542_T001	231.81	389.81	coding	165	129	623
PB.9150.11	8	1368	3	full-splice_match	gene: Zm00001d008602	Zm00001d008602_T002	0.00	155.50	coding	299	303	1199
PB.1722.1	10	1108	3	full-splice_match	gene: Zm00001d023542	Zm00001d023542_T001	158.00	389.81	coding	165	196	690
PB.8763.1	7	880	1	full-splice_match	gene: Zm00001d021665	Zm00001d021665_T001	44.13	44.13	coding	124	91	462
PB.624.2	1	2666	7	full-splice_match	gene: Zm00001d030110	Zm00001d030110_T002	83.14	158.92	coding	592	541	2316
PB.2267.1	10	2245	3	full-splice_match	gene: Zm00001d026344	Zm00001d026344_T001	29.26	29.26	coding	519	368	1924
PB.2880.11	2	1415	4	novel_in_catalog	gene: Zm00001d003857	novel	0.00	364.78	coding	448	71	1414
PB.3070.2	2	1406	9	full-splice_match	gene: Zm00001d005159	Zm00001d005159_T006	58.83	66.30	coding	318	102	1055
PB.5637.1	4	1966	17	incomplete-splice_match	gene: Zm00001d052943	Zm00001d052943_T024	29.02	29.02	coding	409	291	1517
PB.6724.1	5	581	2	full-splice_match	gene: Zm00001d016692	Zm00001d016692_T001	26.46	26.46	coding	106	58	375
PB.2171.1	10	3555	5	novel_not_in_catalog	gene: Zm00001d025931	novel	7.31	25.04	coding	12	291	326
PB.2669.4	2	1374	3	full-splice_match	gene: Zm00001d002898	Zm00001d002898_T001	195.09	231.62	coding	351	86	1138
PB.2669.5	2	1417	2	novel_in_catalog	gene: Zm00001d002898	novel	2.98	231.62	coding	211	570	1202
PB.7986.3	6	2948	5	full-splice_match	gene: Zm00001d038671	Zm00001d038671_T003	5.23	32.27	coding	797	280	2670
PB.2669.7	2	1316	3	full-splice_match	gene: Zm00001d002898	Zm00001d002898_T001	0.00	231.62	coding	351	142	1194
PB.2669.1	2	2560	3	full-splice_match	gene: Zm00001d002898	Zm00001d002898_T001	0.34	231.62	coding	355	43	1107
PB.2669.2	2	1567	3	full-splice_match	gene: Zm00001d002898	Zm00001d002898_T001	0.00	231.62	coding	351	61	1113

PB.2669.3	2	1453	3	full-splice_match	gene: Zm00001d002898	Zm00001d002898_T001	33.20	231.62	coding	351	57	1109
PB.3143.1	2	701	1	antisense	novelGene_gene: Zm00001d005583_A	novel	14.22	14.22	coding	100	175	474
PB.4939.8	4	1423	3	incomplete-splice_match	gene: Zm00001d049295	Zm00001d049295_T009	28.24	91.57	coding	379	69	1205
PB.1012.2	1	1494	3	full-splice_match	gene: Zm00001d032316	Zm00001d032316_T001	42.35	82.53	coding	270	481	1290
PB.2238.1	10	964	2	genic	gene: Zm00001d026259	novel	33.46	93.30	coding	148	93	536
PB.2339.3	10	2003	10	novel_in_catalog	gene: Zm00001d026586	novel	0.00	38.46	coding	144	1520	1951
PB.4939.2	4	3077	15	full-splice_match	gene: Zm00001d049295	Zm00001d049295_T006	47.49	91.57	coding	849	210	2756
PB.4939.3	4	3184	14	novel_in_catalog	gene: Zm00001d049295	novel	3.53	91.57	coding	671	851	2863
PB.4939.6	4	2958	15	full-splice_match	gene: Zm00001d049295	Zm00001d049295_T006	0.00	91.57	coding	124	2388	2759
PB.4939.7	4	2504	11	novel_in_catalog	gene: Zm00001d049295	novel	7.05	91.57	coding	671	318	2330
PB.4939.4	4	1777	4	incomplete-splice_match	gene: Zm00001d049295	Zm00001d049295_T009	5.26	91.57	coding	379	221	1357
PB.8890.3	7	1614	6	full-splice_match	gene: Zm00001d022082	Zm00001d022082_T001	0.00	31.16	coding	447	99	1439
PB.10083.2	9	1280	3	full-splice_match	gene: Zm00001d045021	Zm00001d045021_T007	6.32	64.96	coding	220	222	881
PB.3276.5	2	1563	7	novel_in_catalog	gene: Zm00001d006242	novel	0.00	142.91	coding	363	63	1151
PB.3276.4	2	1494	8	full-splice_match	gene: Zm00001d006242	Zm00001d006242_T003	0.00	142.91	coding	440	74	1393
PB.3276.3	2	1867	6	full-splice_match	gene: Zm00001d006242	Zm00001d006242_T009	3.05	142.91	coding	345	64	1098
PB.3276.2	2	1613	8	full-splice_match	gene: Zm00001d006242	Zm00001d006242_T003	127.16	142.91	coding	440	74	1393

PB.3904.1	3	1048	3	novel_in_catalog	gene: Zm00001d040463	novel	3.73	3.73	coding	116	118	465
PB.7619.1	6	993	5	full-splice_match	gene: Zm00001d037237	Zm00001d037237_T002	17.02	17.02	non_coding	NA	NA	NA
PB.9265.1	8	953	3	full-splice_match	gene: Zm00001d009181	Zm00001d009181_T002	14.60	14.60	coding	160	41	520
PB.10544.4	9	1778	1	full-splice_match	gene: Zm00001d047560	Zm00001d047560_T001	18.98	97.71	coding	309	558	1484
PB.10544.5	9	1552	2	genic	gene: Zm00001d047560	novel	27.13	97.71	coding	309	339	1265
PB.10083.1	9	1405	3	full-splice_match	gene: Zm00001d045021	Zm00001d045021_T002	36.06	64.96	coding	220	346	1005
PB.10544.1	9	1633	2	genic	gene: Zm00001d047560	novel	29.06	97.71	coding	309	221	1147
PB.10544.2	9	1898	1	full-splice_match	gene: Zm00001d047560	Zm00001d047560_T001	4.27	97.71	coding	309	597	1523
PB.10544.3	9	1489	2	genic	gene: Zm00001d047560	novel	18.28	97.71	coding	309	189	1115
PB.5596.1	4	1878	2	genic	gene: Zm00001d052775	novel	9.99	9.99	coding	548	161	1804
PB.10812.4	9	3007	3	novel_in_catalog	gene: Zm00001d048503	novel	0.00	120.19	coding	345	1955	2989
PB.4203.2	3	1202	6	full-splice_match	gene: Zm00001d042275	Zm00001d042275_T001	9.95	17.97	coding	251	221	973
PB.4203.3	3	1230	5	novel_in_catalog	gene: Zm00001d042275	novel	0.00	17.97	coding	148	149	592
PB.3180.1	2	1466	1	novel_in_catalog	gene: Zm00001d005767	novel	0.35	66.38	non_coding	NA	NA	NA
PB.4203.1	3	1406	5	novel_in_catalog	gene: Zm00001d042275	novel	8.03	17.97	coding	148	212	655
PB.7605.1	6	1201	1	full-splice_match	gene: Zm00001d037167	Zm00001d037167_T001	4.45	4.45	coding	340	76	1095
PB.6225.1	5	1198	5	novel_in_catalog	gene: Zm00001d014043	novel	19.12	19.12	coding	320	132	1091

PB.1505.2	1	2649	11	novel_in_catalog	gene: Zm00001d034502	novel	8.33	8.33	coding	394	136	1317
PB.4336.4	3	1210	7	full-splice_match	gene: Zm00001d042869	Zm00001d042869_T001	0.00	231.40	coding	310	80	1009
PB.9364.1	8	2019	14	full-splice_match	gene: Zm00001d009756	Zm00001d009756_T002	46.52	46.52	coding	475	191	1615
PB.4336.2	3	1458	7	full-splice_match	gene: Zm00001d042869	Zm00001d042869_T001	224.63	231.40	coding	310	227	1156
PB.591.1	1	643	2	intergenic	novelGene_162	novel	20.22	26.45	non_coding	NA	NA	NA
PB.9364.2	8	1859	14	full-splice_match	gene: Zm00001d009756	Zm00001d009756_T002	0.00	46.52	coding	475	157	1581
PB.4745.2	3	1709	4	full-splice_match	gene: Zm00001d044516	Zm00001d044516_T001	78.98	381.23	coding	365	285	1379
PB.6613.2	5	2399	1	novel_in_catalog	gene: Zm00001d016059	novel	0.59	179.61	coding	75	130	354
PB.6613.1	5	588	2	full-splice_match	gene: Zm00001d016059	Zm00001d016059_T001	179.02	179.61	coding	73	155	373
PB.8564.1	7	2044	6	incomplete-splice_match	gene: Zm00001d020852	Zm00001d020852_T001	16.39	16.39	coding	608	94	1917
PB.9544.1	8	1804	9	full-splice_match	gene: Zm00001d010656	Zm00001d010656_T001	12.54	12.54	coding	446	179	1516
PB.7333.1	6	1930	7	novel_not_in_catalog	gene: Zm00001d035657	novel	323.12	323.12	coding	408	469	1692
PB.9486.2	8	1927	13	incomplete-splice_match	gene: Zm00001d010406	Zm00001d010406_T002	19.52	19.52	coding	489	177	1643
PB.5804.1	4	635	1	intergenic	novelGene_361	novel	18.78	18.78	non_coding	NA	NA	NA
PB.332.1	1	613	4	incomplete-splice_match	gene: Zm00001d028664	Zm00001d028664_T001	4.10	4.10	non_coding	NA	NA	NA
PB.4954.2	4	1446	3	full-splice_match	gene: Zm00001d049375	Zm00001d049375_T001	31.68	35.19	coding	386	65	1222
PB.7647.2	6	785	4	incomplete-splice_match	gene: Zm00001d037336	Zm00001d037336_T001	6.35	60.46	coding	199	63	659

PB.9668.5	8	2630	7	full-splice_match	gene: Zm00001d011220	Zm00001d011220_T005	0.00	53.00	coding	704	255	2366
PB.8918.3	7	1264	5	full-splice_match	gene: Zm00001d022190	Zm00001d022190_T002	72.58	98.23	coding	289	118	984
PB.7092.2	5	1410	10	full-splice_match	gene: Zm00001d018230	Zm00001d018230_T001	63.17	64.71	coding	395	59	1243
PB.7092.1	5	2224	11	novel_not_in_catalog	gene: Zm00001d018230	novel	1.53	64.71	coding	506	64	1581
PB.10249.13	9	2491	4	full-splice_match	gene: Zm00001d045804	Zm00001d045804_T014	0.00	37.21	coding	305	204	1118
PB.1371.3	1	927	1	novel_in_catalog	gene: Zm00001d033927	novel	0.36	13.97	non_coding	NA	NA	NA
PB.2954.1	2	652	3	full-splice_match	gene: Zm00001d004465	Zm00001d004465_T001	58.60	58.60	coding	102	272	577
PB.5760.1	4	2304	6	novel_in_catalog	gene: Zm00001d053613	novel	8.64	26.90	coding	225	149	823
PB.3375.2	2	1586	1	genic	gene: Zm00001d006677	novel	2.71	69.89	coding	238	581	1294
PB.9763.1	8	1448	2	genic	gene: Zm00001d011669	novel	2.56	18.16	coding	139	740	1156
PB.2027.1	10	1032	6	full-splice_match	gene: Zm00001d025178	Zm00001d025178_T001	96.85	96.85	coding	212	98	733
PB.10267.2	9	1236	1	novel_in_catalog	gene: Zm00001d045946	novel	9.88	9.88	coding	249	87	833
PB.10911.1	B73V4 ctg41	783	1	intergenic	novelGene_1	novel	2.39	2.39	non_coding	NA	NA	NA
PB.3529.1	2	1589	4	novel_in_catalog	gene: Zm00001d007465	novel	9.11	9.11	coding	369	206	1312
PB.4884.2	4	2002	15	novel_in_catalog	gene: Zm00001d049049	novel	2.07	26.46	coding	350	112	1161
PB.4884.1	4	1938	16	full-splice_match	gene: Zm00001d049049	Zm00001d049049_T002	24.38	26.46	coding	527	116	1696
PB.7824.1	6	2603	1	full-splice_match	gene: Zm00001d038068	Zm00001d038068_T001	31.50	31.50	coding	694	222	2303

PB.2263.3	10	1782	3	novel_not_in_catalog	gene: Zm00001d026333	novel	2.94	48.08	coding	431	127	1419
PB.3954.4	3	3321	9	fusion	gene: Zm00001d040795_g	novel	7.76	13.45	coding	962	120	3005
PB.6306.1	5	1730	5	full-splice_match	gene: Zm00001d014449	Zm00001d014449_T002	8.05	31.89	coding	380	174	1313
PB.6306.2	5	1569	5	full-splice_match	gene: Zm00001d014449	Zm00001d014449_T002	23.84	31.89	coding	380	196	1335
PB.9668.3	8	2732	7	full-splice_match	gene: Zm00001d011220	Zm00001d011220_T003	53.00	53.00	coding	704	251	2362
PB.4424.12	3	3094	5	novel_in_catalog	gene: Zm00001d043256	novel	1.87	34.21	coding	711	166	2298
PB.1466.5	1	3196	7	full-splice_match	gene: Zm00001d034350	Zm00001d034350_T002	7.78	23.85	coding	958	103	2976
PB.3954.2	3	874	4	fusion	gene: Zm00001d040795_g	novel	5.69	13.45	non_coding	NA	NA	NA
PB.9705.1	8	1024	4	full-splice_match	gene: Zm00001d011395	Zm00001d011395_T005	14.85	14.85	coding	101	1	303
PB.3998.3	3	1382	3	intergenic	novelGene_180	novel	3.45	100.18	non_coding	NA	NA	NA
PB.9980.1	8	1716	2	novel_in_catalog	gene: Zm00001d012600	novel	3.82	3.82	coding	352	77	1132
PB.2263.1	10	1875	3	novel_not_in_catalog	gene: Zm00001d026333	novel	26.29	48.08	coding	447	110	1450
PB.2483.9	2	2001	2	full-splice_match	gene: Zm00001d002141	Zm00001d002141_T022	20.86	691.17	coding	521	53	1615
PB.2483.6	2	2883	2	novel_in_catalog	gene: Zm00001d002141	novel	11.26	691.17	coding	134	2252	2653
PB.2483.4	2	870	2	novel_in_catalog	gene: Zm00001d002141	novel	533.12	691.17	coding	134	180	581
PB.2483.5	2	1843	2	full-splice_match	gene: Zm00001d002141	Zm00001d002141_T002	25.06	691.17	coding	171	1146	1658
PB.2483.2	2	3399	1	genic	gene: Zm00001d002141	novel	0.66	691.17	coding	134	2661	3062

PB.1295.1	1	676	2	novel_not_in_catalog	gene: Zm00001d033589	novel	123.15	123.15	coding	110	68	397
PB.273.4	1	2996	10	novel_in_catalog	gene: Zm00001d028417	novel	0.00	23.30	coding	887	204	2864
PB.2483.1	2	1547	2	full-splice_match	gene: Zm00001d002141	Zm00001d002141_T002	82.45	691.17	coding	171	689	1201
PB.5287.1	4	2389	1	full-splice_match	gene: Zm00001d051461	Zm00001d051461_T001	6.41	21.78	coding	607	176	1996
PB.6603.1	5	1537	1	full-splice_match	gene: Zm00001d015965	Zm00001d015965_T001	34.45	34.45	coding	485	42	1496
PB.10925.5	B73V4 ctg52	2625	1	genic	gene: Zm00001d000112	novel	4.12	64.88	coding	148	1478	1921
PB.5287.2	4	2245	1	full-splice_match	gene: Zm00001d051461	Zm00001d051461_T001	15.37	21.78	coding	607	176	1996
PB.2673.1	2	1506	4	novel_not_in_catalog	gene: Zm00001d002934	novel	5.38	5.60	coding	320	131	1090
PB.10925.2	B73V4 ctg52	2524	2	novel_in_catalog	gene: Zm00001d000112	novel	3.54	64.88	coding	148	1372	1815
PB.10925.1	B73V4 ctg52	1919	3	full-splice_match	gene: Zm00001d000112	Zm00001d000112_T001	57.22	64.88	coding	359	131	1207
PB.2673.2	2	1502	3	novel_not_in_catalog	gene: Zm00001d002934	novel	0.22	5.60	coding	196	570	1157
PB.152.2	1	961	8	incomplete-splice_match	gene: Zm00001d027886	Zm00001d027886_T001	14.73	39.10	coding	212	149	784
PB.7491.1	6	803	3	genic	gene: Zm00001d036550	novel	103.04	103.04	coding	149	66	512
PB.3501.1	2	868	6	full-splice_match	gene: Zm00001d007270	Zm00001d007270_T001	26.20	101.91	coding	142	110	535
PB.152.1	1	1665	14	full-splice_match	gene: Zm00001d027886	Zm00001d027886_T001	24.38	39.10	coding	380	240	1379
PB.10008.1	8	964	5	full-splice_match	gene: Zm00001d012689	Zm00001d012689_T001	25.59	25.59	non_coding	NA	NA	NA
PB.10925.8	B73V4 ctg52	1813	3	full-splice_match	gene: Zm00001d000112	Zm00001d000112_T001	0.00	64.88	coding	359	131	1207

PB.5752.2	4	1920	1	full-splice_match	gene: Zm00001d053586	Zm00001d053586_T001	7.31	13.88	coding	509	50	1576
PB.6184.1	5	2213	5	full-splice_match	gene: Zm00001d013840	Zm00001d013840_T002	13.41	35.05	coding	559	166	1842
PB.6184.2	5	1913	4	incomplete-splice_match	gene: Zm00001d013840	Zm00001d013840_T003	15.77	35.05	coding	412	276	1511
PB.6184.3	5	2075	5	full-splice_match	gene: Zm00001d013840	Zm00001d013840_T002	0.00	35.05	coding	559	120	1796
PB.6184.4	5	2049	5	full-splice_match	gene: Zm00001d013840	Zm00001d013840_T003	0.00	35.05	coding	554	156	1817
PB.6184.5	5	484	1	incomplete-splice_match	gene: Zm00001d013840	Zm00001d013840_T001	5.87	35.05	coding	121	10	372
PB.1772.2	10	2760	7	novel_in_catalog	gene: Zm00001d023825	novel	26.58	52.78	coding	660	423	2402
PB.1772.3	10	2726	7	novel_not_in_catalog	gene: Zm00001d023825	novel	3.66	52.78	coding	644	437	2368
PB.9988.1	8	2348	1	full-splice_match	gene: Zm00001d012625	Zm00001d012625_T001	12.04	19.91	coding	679	122	2158
PB.2760.1	2	1672	7	full-splice_match	gene: Zm00001d003246	Zm00001d003246_T005	4.76	4.76	coding	416	95	1342
PB.5263.1	4	2511	3	full-splice_match	gene: Zm00001d051323	Zm00001d051323_T001	5.37	5.37	coding	269	1372	2178
PB.9988.2	8	1012	1	full-splice_match	gene: Zm00001d012625	Zm00001d012625_T001	7.87	19.91	coding	255	110	874
PB.173.3	1	458	1	intergenic	novelGene_62	novel	0.00	4.81	non_coding	NA	NA	NA
PB.8459.1	7	2470	4	novel_not_in_catalog	gene: Zm00001d020361	novel	13.63	14.43	coding	162	93	578
PB.6493.1	5	1952	10	full-splice_match	gene: Zm00001d015168	Zm00001d015168_T001	124.03	128.96	coding	546	55	1692
PB.4332.5	3	1881	12	full-splice_match	gene: Zm00001d042851	Zm00001d042851_T009	5.27	28.44	coding	324	549	1520
PB.8459.2	7	2563	3	novel_not_in_catalog	gene: Zm00001d020361	novel	0.80	14.43	coding	146	69	506

PB.600.1	1	1385	9	full-splice_match	gene: Zm00001d029975	Zm00001d029975_T003	43.13	43.13	coding	333	151	1149
PB.6699.2	5	1051	2	full-splice_match	gene: Zm00001d016566	Zm00001d016566_T001	57.61	57.61	coding	262	53	838
PB.4591.3	3	870	5	full-splice_match	gene: Zm00001d043874	Zm00001d043874_T001	41.19	96.12	coding	195	8	592
PB.5322.2	4	3296	2	novel_in_catalog	gene: Zm00001d051615	novel	13.98	70.39	coding	757	99	2369
PB.4294.11	3	2172	4	novel_in_catalog	gene: Zm00001d042684	novel	12.26	417.27	coding	542	454	2079
PB.5322.7	4	2712	2	novel_in_catalog	gene: Zm00001d051615	novel	0.00	70.39	coding	757	118	2388
PB.6493.3	5	1942	9	novel_in_catalog	gene: Zm00001d015168	novel	2.85	128.96	coding	495	24	1508
PB.5322.5	4	2789	2	novel_in_catalog	gene: Zm00001d051615	novel	17.55	70.39	coding	757	133	2403
PB.5322.4	4	2571	2	incomplete-splice_match	gene: Zm00001d051615	Zm00001d051615_T001	38.86	70.39	coding	459	661	2037
PB.5775.2	4	1596	7	full-splice_match	gene: Zm00001d053643	Zm00001d053643_T002	3.69	11.25	coding	397	259	1449
PB.5775.1	4	1506	8	full-splice_match	gene: Zm00001d053643	Zm00001d053643_T003	7.56	11.25	coding	397	122	1312
PB.5970.1	5	648	2	incomplete-splice_match	gene: Zm00001d013022	Zm00001d013022_T001	30.88	30.88	coding	76	265	492
PB.6493.4	5	1831	10	full-splice_match	gene: Zm00001d015168	Zm00001d015168_T001	2.07	128.96	coding	546	58	1695
PB.7286.8	6	1171	6	novel_not_in_catalog	gene: Zm00001d035356	novel	5.25	28.23	non_coding	NA	NA	NA
PB.5496.1	4	964	1	full-splice_match	gene: Zm00001d052371	Zm00001d052371_T001	14.67	14.67	coding	220	111	770
PB.4380.1	3	2367	11	full-splice_match	gene: Zm00001d043083	Zm00001d043083_T002	10.67	58.95	coding	432	363	1658
PB.8723.2	7	1473	5	novel_in_catalog	gene: Zm00001d021532	novel	0.00	3.39	coding	409	9	1235

PB.4380.2	3	2224	11	full-splice_match	gene: Zm00001d043083	Zm00001d043083_T002	44.67	58.95	coding	432	380	1675
PB.3294.1	2	1493	6	novel_in_catalog	gene: Zm00001d006310	novel	29.31	29.31	coding	184	1	552
PB.9091.1	8	1830	1	full-splice_match	gene: Zm00001d008316	Zm00001d008316_T001	2.66	2.66	coding	505	22	1536
PB.5769.4	4	2814	2	novel_in_catalog	gene: Zm00001d053634	novel	5.46	41.21	non_coding	NA	NA	NA
PB.1331.3	1	2869	4	full-splice_match	gene: Zm00001d033766	Zm00001d033766_T004	0.98	45.07	coding	249	1960	2706
PB.5769.6	4	2906	8	novel_not_in_catalog	gene: Zm00001d053634	novel	2.50	41.21	coding	305	341	1255
PB.5950.1	5	1111	3	novel_not_in_catalog	gene: Zm00001d012954	novel	112.92	112.92	coding	215	106	750
PB.5769.2	4	2734	1	genic	gene: Zm00001d053634	novel	14.11	41.21	non_coding	NA	NA	NA
PB.1112.1	1	1751	8	full-splice_match	gene: Zm00001d032686	Zm00001d032686_T002	119.32	119.32	coding	356	347	1414
PB.5297.2	4	1338	3	full-splice_match	gene: Zm00001d051520	Zm00001d051520_T001	8.30	8.30	coding	340	65	1084
PB.4380.4	3	3124	10	full-splice_match	gene: Zm00001d043083	Zm00001d043083_T003	3.61	58.95	coding	432	1299	2594
PB.7035.2	5	1061	1	novel_in_catalog	gene: Zm00001d018011	novel	7.46	7.46	non_coding	NA	NA	NA
PB.817.2	1	932	1	full-splice_match	gene: Zm00001d031292	Zm00001d031292_T001	33.77	49.10	coding	250	37	786
PB.4318.2	3	1977	4	full-splice_match	gene: Zm00001d042787	Zm00001d042787_T002	0.87	25.72	coding	281	899	1741
PB.4318.1	3	1634	5	full-splice_match	gene: Zm00001d042787	Zm00001d042787_T001	24.85	25.72	coding	398	9	1202
PB.817.1	1	945	1	full-splice_match	gene: Zm00001d031292	Zm00001d031292_T001	10.39	49.10	coding	183	50	598
PB.1786.1	10	842	2	novel_in_catalog	gene: Zm00001d023882	novel	1.16	1.16	coding	180	80	619

PB.2515.2	2	1039	2	genic	gene: Zm00001d002278	novel	11.49	206.32	coding	144	333	764
PB.5560.1	4	1700	15	novel_not_in_catalog	gene: Zm00001d052624	novel	13.12	13.12	coding	418	94	1347
PB.1716.5	10	1816	5	full-splice_match	gene: Zm00001d023523	Zm00001d023523_T004	0.00	115.79	coding	436	187	1494
PB.6656.4	5	1579	11	novel_in_catalog	gene: Zm00001d016308	novel	25.33	29.98	coding	348	228	1271
PB.3049.1	2	2035	4	full-splice_match	gene: Zm00001d005065	Zm00001d005065_T002	33.46	33.46	coding	434	98	1399
PB.1716.1	10	2002	5	full-splice_match	gene: Zm00001d023523	Zm00001d023523_T004	103.06	115.79	coding	436	261	1568
PB.833.1	1	1415	1	full-splice_match	gene: Zm00001d031434	Zm00001d031434_T001	26.26	26.26	coding	381	58	1200
PB.4729.1	3	1578	2	full-splice_match	gene: Zm00001d044475	Zm00001d044475_T001	71.24	71.24	coding	428	96	1379
PB.2748.1	2	2181	12	incomplete-splice_match	gene: Zm00001d003203	Zm00001d003203_T013	22.15	22.15	coding	599	11	1807
PB.5477.2	4	1494	4	novel_in_catalog	gene: Zm00001d052310	novel	6.09	29.95	coding	193	747	1325
PB.9548.2	8	1810	1	genic	gene: Zm00001d010673	novel	11.07	52.94	coding	274	812	1633
PB.2538.1	2	1645	2	novel_not_in_catalog	gene: Zm00001d002353	novel	11.00	11.00	coding	426	79	1356
PB.1112.6	1	1533	8	full-splice_match	gene: Zm00001d032686	Zm00001d032686_T002	0.00	119.32	coding	356	332	1399
PB.2538.2	2	1542	2	novel_not_in_catalog	gene: Zm00001d002353	novel	0.00	11.00	coding	426	79	1356
PB.10930.4	B73V4 ctg58	2573	2	full-splice_match	gene: Zm00001d000408	Zm00001d000408_T001	0.00	141.27	coding	626	251	2128
PB.9624.6	8	1752	6	full-splice_match	gene: Zm00001d010975	Zm00001d010975_T004	6.75	81.63	coding	467	192	1592
PB.10930.6	B73V4 ctg58	2465	2	full-splice_match	gene: Zm00001d000408	Zm00001d000408_T001	0.00	141.27	coding	626	253	2130

PB.10930.1	B73V4 ctg58	2826	1	genic	gene: Zm00001d000408	novel	1.44	141.27	coding	512	42	1577
PB.9624.1	8	2145	6	full-splice_match	gene: Zm00001d010975	Zm00001d010975_T001	74.89	81.63	coding	467	467	1867
PB.10930.2	B73V4 ctg58	2898	2	full-splice_match	gene: Zm00001d000408	Zm00001d000408_T001	139.83	141.27	coding	626	251	2128
PB.3825.1	3	1818	1	full-splice_match	gene: Zm00001d040111	Zm00001d040111_T001	0.48	72.45	coding	172	149	664
PB.7254.2	6	1834	9	novel_in_catalog	gene: Zm00001d035183	novel	4.60	37.40	coding	404	293	1504
PB.7254.1	6	1912	10	full-splice_match	gene: Zm00001d035183	Zm00001d035183_T009	20.15	37.40	coding	435	278	1582
PB.3825.2	3	1079	1	full-splice_match	gene: Zm00001d040111	Zm00001d040111_T001	27.59	72.45	coding	172	158	673
PB.10950.1	Mt	792	1	intergenic	novelGene_10	novel	503.54	503.54	non_coding	NA	NA	NA
PB.5958.1	5	1452	4	full-splice_match	gene: Zm00001d012991	Zm00001d012991_T001	17.14	17.14	coding	345	222	1256
PB.5761.1	4	2971	13	novel_in_catalog	gene: Zm00001d053620	novel	26.90	50.91	coding	445	132	1466
PB.5761.3	4	2389	6	incomplete-splice_match	gene: Zm00001d053620	Zm00001d053620_T006	24.01	50.91	coding	260	151	930
PB.744.1	1	1807	2	novel_in_catalog	gene: Zm00001d030989	novel	7.61	139.59	coding	226	251	928
PB.1946.1	10	3130	7	full-splice_match	gene: Zm00001d024825	Zm00001d024825_T012	2.97	2.97	coding	938	30	2843
PB.10465.3	9	2739	6	full-splice_match	gene: Zm00001d047191	Zm00001d047191_T001	5.49	44.26	coding	732	89	2284
PB.3964.2	3	2657	7	incomplete-splice_match	gene: Zm00001d040864	Zm00001d040864_T007	24.49	24.49	coding	696	334	2421
PB.2875.1	2	2455	11	incomplete-splice_match	gene: Zm00001d003847	Zm00001d003847_T001	10.65	17.83	coding	670	137	2146
PB.3420.1	2	1740	4	novel_in_catalog	gene: Zm00001d006871	novel	161.67	161.67	coding	149	1080	1526

PB.2875.2	2	1786	8	incomplete-splice_match	gene: Zm00001d003847	Zm00001d003847_T001	7.18	17.83	coding	433	326	1624
PB.744.3	1	2121	1	full-splice_match	gene: Zm00001d022856	Zm00001d022856_T001	2.06	2.06	coding	226	615	1292
PB.3737.1	3	2178	1	antisense	novelGene_gene: Zm00001d039579_A	novel	5.90	5.90	coding	148	500	943
PB.615.1	1	1309	2	full-splice_match	gene: Zm00001d030086	Zm00001d030086_T001	54.65	54.65	coding	249	70	816
PB.4429.3	3	2563	6	full-splice_match	gene: Zm00001d043275	Zm00001d043275_T010	7.88	52.72	coding	345	1133	2167
PB.5781.1	4	1350	8	full-splice_match	gene: Zm00001d053667	Zm00001d053667_T001	19.23	19.23	coding	286	152	1009
PB.3713.1	3	1975	11	full-splice_match	gene: Zm00001d039499	Zm00001d039499_T002	54.18	72.09	coding	528	250	1833
PB.9633.2	8	1196	2	novel_not_in_catalog	gene: Zm00001d011041	novel	14.46	14.46	coding	100	506	805
PB.3713.3	3	2152	10	novel_in_catalog	gene: Zm00001d039499	novel	5.65	72.09	coding	362	249	1334
PB.3713.2	3	1769	9	novel_in_catalog	gene: Zm00001d039499	novel	12.27	72.09	coding	379	341	1477
PB.744.4	1	1061	3	full-splice_match	gene: Zm00001d030989	Zm00001d030989_T001	0.00	139.59	coding	159	251	727
PB.2313.1	10	441	1	full-splice_match	gene: Zm00001d026510	Zm00001d026510_T001	65.14	65.14	non_coding	NA	NA	NA
PB.10070.5	9	1277	1	full-splice_match	gene: Zm00001d044921	Zm00001d044921_T001	108.01	132.83	coding	330	141	1130
PB.8794.1	7	1898	7	novel_in_catalog	gene: Zm00001d021748	novel	30.47	36.95	coding	374	271	1392
PB.2023.1	10	1459	4	full-splice_match	gene: Zm00001d025166	Zm00001d025166_T002	48.55	48.55	coding	339	145	1161
PB.2403.2	2	1023	3	full-splice_match	gene: Zm00001d001814	Zm00001d001814_T001	34.47	197.17	coding	207	213	833
PB.10070.1	9	1293	1	full-splice_match	gene: Zm00001d044921	Zm00001d044921_T001	24.82	132.83	coding	330	6	995

PB.8794.5	7	1789	7	novel_not_in_catalog	gene: Zm00001d021748	novel	1.25	36.95	coding	277	641	1471
PB.2403.1	2	1291	3	full-splice_match	gene: Zm00001d001814	Zm00001d001814_T001	162.70	197.17	coding	299	82	978
PB.5207.1	4	1997	2	full-splice_match	gene: Zm00001d050961	Zm00001d050961_T001	25.70	42.31	coding	388	369	1532
PB.3935.3	3	2589	3	novel_in_catalog	gene: Zm00001d040667	novel	14.14	252.28	coding	205	64	678
PB.5207.2	4	1892	2	full-splice_match	gene: Zm00001d050961	Zm00001d050961_T001	16.61	42.31	coding	388	376	1539
PB.5669.1	4	2107	2	full-splice_match	gene: Zm00001d053091	Zm00001d053091_T001	24.31	24.31	coding	618	109	1962
PB.1493.11	1	2133	3	genic	gene: Zm00001d034434	novel	0.00	39.47	non_coding	NA	NA	NA
PB.10197.1	9	1875	7	novel_not_in_catalog	gene: Zm00001d045499	novel	82.57	82.57	coding	408	344	1567
PB.880.3	1	1230	5	full-splice_match	gene: Zm00001d031666	Zm00001d031666_T002	0.00	45.30	coding	346	54	1091
PB.7274.1	6	947	5	full-splice_match	gene: Zm00001d035304	Zm00001d035304_T001	88.68	88.68	coding	195	131	715
PB.7858.4	6	1762	8	full-splice_match	gene: Zm00001d038167	Zm00001d038167_T003	2.23	24.08	coding	220	124	783
PB.7274.3	6	724	5	full-splice_match	gene: Zm00001d035304	Zm00001d035304_T001	0.00	88.68	coding	195	20	604
PB.7858.1	6	2203	7	full-splice_match	gene: Zm00001d038167	Zm00001d038167_T002	0.00	24.08	coding	380	295	1434
PB.4732.1	3	1776	12	novel_not_in_catalog	gene: Zm00001d044481	novel	23.62	23.62	coding	462	46	1431
PB.7858.3	6	1843	8	full-splice_match	gene: Zm00001d038167	Zm00001d038167_T001	20.03	24.08	coding	476	295	1722
PB.7858.2	6	2294	7	novel_in_catalog	gene: Zm00001d038167	novel	1.82	24.08	coding	142	295	720
PB.4222.1	3	1190	5	full-splice_match	gene: Zm00001d042347	Zm00001d042347_T001	126.21	1197.41	coding	109	576	902

PB.880.1	1	1348	5	full-splice_match	gene: Zm00001d031666	Zm00001d031666_T002	45.30	45.30	coding	346	54	1091
PB.1268.1	1	2085	1	full-splice_match	gene: Zm00001d033482	Zm00001d033482_T001	18.86	18.86	coding	551	126	1778
PB.9403.1	8	343	2	intergenic	novelGene_161	novel	3870.15	3870.15	non_coding	NA	NA	NA
PB.5918.1	5	849	2	full-splice_match	gene: Zm00001d012845	Zm00001d012845_T001	52.40	52.40	coding	172	122	637
PB.4222.3	3	3180	4	full-splice_match	gene: Zm00001d042347	Zm00001d042347_T012	5.66	1197.41	coding	109	2568	2894
PB.2049.1	10	2469	6	full-splice_match	gene: Zm00001d025323	Zm00001d025323_T004	51.81	51.81	coding	521	560	2122
PB.79.1	1	786	5	incomplete-splice_match	gene: Zm00001d027536	Zm00001d027536_T008	18.10	18.10	coding	97	60	350
PB.8471.1	7	2261	3	incomplete-splice_match	gene: Zm00001d020414	Zm00001d020414_T004	6.99	6.99	coding	549	243	1889
PB.4222.2	3	3075	5	full-splice_match	gene: Zm00001d042347	Zm00001d042347_T007	0.00	1197.41	coding	109	2463	2789
PB.7794.2	6	1265	8	full-splice_match	gene: Zm00001d037967	Zm00001d037967_T002	2.77	11.95	coding	313	123	1061
PB.1263.1	1	686	1	full-splice_match	gene: Zm00001d033469	Zm00001d033469_T001	13.16	13.16	coding	150	66	515
PB.3408.2	2	2264	4	novel_in_catalog	gene: Zm00001d006823	novel	1.26	38.36	coding	565	563	2257
PB.3408.1	2	1240	2	incomplete-splice_match	gene: Zm00001d006823	Zm00001d006823_T001	14.36	38.36	coding	298	137	1030
PB.2011.1	10	2194	17	full-splice_match	gene: Zm00001d025100	Zm00001d025100_T008	33.96	88.80	coding	537	180	1790
PB.4243.1	3	2133	4	full-splice_match	gene: Zm00001d042463	Zm00001d042463_T001	10.73	115.55	coding	615	67	1911
PB.4222.4	3	1050	5	full-splice_match	gene: Zm00001d042347	Zm00001d042347_T001	0.00	1197.41	coding	109	575	901
PB.5481.1	4	2475	5	full-splice_match	gene: Zm00001d052318	Zm00001d052318_T002	32.77	32.77	coding	446	4	1341

PB.6928.4	5	1534	1	novel_in_catalog	gene: Zm00001d017575	novel	1.75	79.67	coding	359	341	1417
PB.6928.5	5	1449	2	full-splice_match	gene: Zm00001d017575	Zm00001d017575_T001	0.00	79.67	coding	374	213	1334
PB.6928.2	5	1624	2	full-splice_match	gene: Zm00001d017575	Zm00001d017575_T001	8.87	79.67	coding	374	214	1335
PB.4222.7	3	659	5	full-splice_match	gene: Zm00001d042347	Zm00001d042347_T001	801.11	1197.41	non_coding	NA	NA	NA
PB.3678.2	3	2120	5	full-splice_match	gene: Zm00001d039369	Zm00001d039369_T008	23.30	31.87	coding	490	192	1661
PB.4222.6	3	1074	4	novel_in_catalog	gene: Zm00001d042347	novel	14.88	1197.41	non_coding	NA	NA	NA
PB.4420.1	3	956	2	full-splice_match	gene: Zm00001d043232	Zm00001d043232_T001	42.56	42.56	coding	214	85	726
PB.10006.3	8	1339	8	full-splice_match	gene: Zm00001d012681	Zm00001d012681_T010	0.00	24.17	coding	328	141	1124
PB.10377.16	9	1848	3	novel_in_catalog	gene: Zm00001d046661	novel	0.48	391.13	coding	232	990	1685
PB.3122.6	2	1156	10	novel_in_catalog	gene: Zm00001d005460	novel	3.06	82.41	coding	195	5	589
PB.7178.4	5	1549	5	novel_not_in_catalog	gene: Zm00001d018503	novel	46.42	148.93	coding	317	303	1253
PB.7178.2	5	1803	4	novel_not_in_catalog	gene: Zm00001d018503	novel	61.57	148.93	coding	317	389	1339
PB.10637.3	9	1879	5	full-splice_match	gene: Zm00001d047863	Zm00001d047863_T007	0.00	17.20	coding	483	43	1491
PB.1193.2	1	1365	9	incomplete-splice_match	gene: Zm00001d033094	Zm00001d033094_T001	9.40	23.26	coding	290	131	1000
PB.10187.2	9	1135	1	full-splice_match	gene: Zm00001d045478	Zm00001d045478_T001	20.29	722.68	coding	274	78	899
PB.10187.1	9	1051	2	genic	gene: Zm00001d045478	novel	702.38	722.68	coding	235	75	779
PB.3122.4	2	1705	13	novel_in_catalog	gene: Zm00001d005460	novel	79.36	82.41	coding	405	184	1398

PB.1646.1	10	1728	1	intergenic	novelGene_12	novel	43.85	108.48	coding	395	9	1193
PB.3401.1	2	3211	6	incomplete-splice_match	gene: Zm00001d006762	Zm00001d006762_T001	22.26	25.35	coding	994	30	3011
PB.4618.3	3	2008	2	novel_not_in_catalog	gene: Zm00001d043989	novel	6.75	9.44	coding	402	569	1774
PB.9138.1	8	855	6	novel_not_in_catalog	gene: Zm00001d008530	novel	41.30	41.30	coding	235	56	760
PB.217.2	1	315	1	incomplete-splice_match	gene: Zm00001d028205	Zm00001d028205_T002	85.80	85.80	non_coding	NA	NA	NA
PB.3335.2	2	2522	10	novel_not_in_catalog	gene: Zm00001d006473	novel	5.16	5.16	coding	740	127	2346
PB.7699.2	6	1753	2	novel_in_catalog	gene: Zm00001d037605	novel	12.47	108.33	coding	106	929	1246
PB.7699.3	6	854	3	full-splice_match	gene: Zm00001d037605	Zm00001d037605_T001	0.00	108.33	coding	142	1	426
PB.7699.1	6	1126	3	full-splice_match	gene: Zm00001d037605	Zm00001d037605_T001	95.85	108.33	coding	143	143	571
PB.1631.2	1	2545	20	incomplete-splice_match	gene: Zm00001d034914	Zm00001d034914_T032	32.94	63.90	coding	695	7	2091
PB.1631.3	1	2114	17	full-splice_match	gene: Zm00001d034914	Zm00001d034914_T034	0.00	63.90	coding	595	4	1788
PB.4618.1	3	2662	1	genic	gene: Zm00001d043989	novel	2.69	9.44	coding	402	1123	2328
PB.3122.1	2	1515	1	genic	gene: Zm00001d005460	novel	0.00	82.41	coding	224	844	1515
PB.7862.1	6	1456	6	novel_in_catalog	gene: Zm00001d038189	novel	1.68	25.81	coding	438	49	1362
PB.516.1	1	1765	5	novel_in_catalog	gene: Zm00001d029504	novel	18.09	18.09	coding	404	335	1546
PB.10867.1	B73V4 ctg150	1297	1	full-splice_match	gene: Zm00001d000020	Zm00001d000020_T001	1.44	1.44	coding	302	82	987
PB.1646.2	10	1942	2	intergenic	novelGene_12	novel	20.71	108.48	coding	422	250	1515

PB.2391.1	2	2215	12	full-splice_match	gene: Zm00001d001780	Zm00001d001780_T001	51.88	54.84	coding	490	218	1687
PB.2391.2	2	2732	11	full-splice_match	gene: Zm00001d001780	Zm00001d001780_T010	2.97	54.84	coding	490	199	1668
PB.2391.3	2	2789	11	novel_in_catalog	gene: Zm00001d001780	novel	0.00	54.84	coding	481	817	2259
PB.7043.1	5	1674	10	full-splice_match	gene: Zm00001d018036	Zm00001d018036_T002	28.71	28.71	coding	394	57	1238
PB.7043.3	5	1639	10	full-splice_match	gene: Zm00001d018036	Zm00001d018036_T002	0.00	28.71	coding	394	56	1237
PB.354.1	1	1154	4	novel_not_in_catalog	gene: Zm00001d028779	novel	27.59	27.59	coding	170	199	708
PB.8828.1	7	877	1	intergenic	novelGene_270	novel	15.93	73.17	non_coding	NA	NA	NA
PB.2502.1	2	2564	6	novel_not_in_catalog	gene: Zm00001d002198	novel	2.87	2.87	coding	588	568	2331
PB.5104.1	4	1615	5	full-splice_match	gene: Zm00001d050187	Zm00001d050187_T001	18.48	18.48	coding	369	87	1193
PB.7972.5	6	2998	7	incomplete-splice_match	gene: Zm00001d038587	Zm00001d038587_T017	0.00	25.48	coding	593	15	1793
PB.775.6	1	3257	4	novel_in_catalog	gene: Zm00001d031098	novel	1.35	45.68	coding	324	2136	3107
PB.8042.1	6	1890	1	genic	gene: Zm00001d038860	novel	13.83	13.83	coding	486	251	1708
PB.931.1	1	805	1	full-splice_match	gene: Zm00001d031944	Zm00001d031944_T001	29.40	29.40	coding	190	87	656
PB.775.3	1	1740	7	novel_in_catalog	gene: Zm00001d031098	novel	19.18	45.68	coding	223	903	1571
PB.775.2	1	3068	5	novel_in_catalog	gene: Zm00001d031098	novel	0.00	45.68	coding	255	2131	2895
PB.7051.4	5	864	3	novel_not_in_catalog	gene: Zm00001d018072	novel	233.83	349.46	coding	118	247	600
PB.6111.4	5	904	5	novel_not_in_catalog	gene: Zm00001d013498	novel	0.00	59.53	coding	129	235	621

PB.5495.1	4	2613	4	full-splice_match	gene: Zm00001d052368	Zm00001d052368_T001	6.01	6.01	coding	731	161	2353
PB.4448.3	3	2135	6	novel_not_in_catalog	gene: Zm00001d043354	novel	8.26	17.01	coding	526	171	1748
PB.6111.3	5	1025	5	novel_not_in_catalog	gene: Zm00001d013498	novel	52.73	59.53	coding	129	241	627
PB.4448.1	3	2554	4	novel_not_in_catalog	gene: Zm00001d043354	novel	5.44	17.01	coding	658	141	2114
PB.6111.1	5	699	3	novel_not_in_catalog	gene: Zm00001d013498	novel	6.80	59.53	non_coding	NA	NA	NA
PB.4851.1	4	859	1	full-splice_match	gene: Zm00001d048884	Zm00001d048884_T001	60.91	60.91	coding	215	174	818
PB.5833.2	4	832	1	full-splice_match	gene: Zm00001d053887	Zm00001d053887_T001	0.00	87.08	coding	189	135	701
PB.763.1	1	1301	10	full-splice_match	gene: Zm00001d031072	Zm00001d031072_T001	26.73	28.59	coding	174	431	952
PB.5164.3	4	3062	13	novel_in_catalog	gene: Zm00001d050606	novel	36.44	69.27	coding	766	429	2726
PB.8111.1	6	1153	1	full-splice_match	gene: Zm00001d039140	Zm00001d039140_T001	13.15	13.15	coding	280	117	956
PB.5164.1	4	3629	17	full-splice_match	gene: Zm00001d050606	Zm00001d050606_T035	23.11	69.27	coding	986	316	3273
PB.9809.2	8	2135	11	full-splice_match	gene: Zm00001d011835	Zm00001d011835_T003	11.62	20.28	coding	569	244	1950
PB.8418.10	7	1567	5	novel_in_catalog	gene: Zm00001d020140	novel	6.10	38.65	coding	133	586	984
PB.467.1	1	514	3	full-splice_match	gene: Zm00001d029263	Zm00001d029263_T001	90.50	90.50	coding	97	69	359
PB.5164.4	4	3468	17	full-splice_match	gene: Zm00001d050606	Zm00001d050606_T035	0.00	69.27	coding	986	246	3203
PB.5648.2	4	2989	2	full-splice_match	gene: Zm00001d052988	Zm00001d052988_T010	4.90	4.90	coding	910	106	2835
PB.4436.1	3	1904	9	full-splice_match	gene: Zm00001d043303	Zm00001d043303_T001	85.98	88.18	coding	494	193	1674

PB.8418.11	7	1552	3	full-splice_match	gene: Zm00001d020140	Zm00001d020140_T011	0.00	38.65	coding	156	739	1206
PB.10459.3	9	2388	5	incomplete-splice_match	gene: Zm00001d047126	Zm00001d047126_T004	7.05	41.20	coding	632	492	2387
PB.3641.5	3	2696	12	novel_in_catalog	gene: Zm00001d039267	novel	0.00	84.86	coding	266	103	900
PB.10459.1	9	3532	6	novel_in_catalog	gene: Zm00001d047126	novel	0.00	41.20	coding	637	376	2286
PB.2681.1	2	1067	4	full-splice_match	gene: Zm00001d002954	Zm00001d002954_T002	20.58	20.58	coding	149	213	659
PB.8536.1	7	1153	1	full-splice_match	gene: Zm00001d020707	Zm00001d020707_T001	13.63	13.63	coding	315	54	998
PB.6586.2	5	2533	7	novel_not_in_catalog	gene: Zm00001d015818	novel	46.49	46.49	coding	215	469	1113
PB.2036.1	10	1080	3	novel_not_in_catalog	gene: Zm00001d025252	novel	67.32	130.70	coding	138	205	618
PB.183.1	1	2251	6	full-splice_match	gene: Zm00001d028073	Zm00001d028073_T005	25.85	25.85	coding	549	210	1856
PB.8980.1	7	863	1	full-splice_match	gene: Zm00001d022416	Zm00001d022416_T001	50.20	97.60	coding	113	145	483
PB.922.1	1	897	8	full-splice_match	gene: Zm00001d031908	Zm00001d031908_T001	109.21	109.21	coding	206	47	664
PB.5860.2	4	1308	1	genic	gene: Zm00001d027041	novel	7.96	9.07	non_coding	NA	NA	NA
PB.2907.3	2	2635	2	genic	gene: Zm00001d003984	novel	33.31	33.31	coding	750	68	2317
PB.8467.2	7	1406	4	full-splice_match	gene: Zm00001d020400	Zm00001d020400_T001	34.70	36.97	coding	365	65	1159
PB.8467.3	7	1286	4	full-splice_match	gene: Zm00001d020400	Zm00001d020400_T001	0.00	36.97	coding	365	62	1156
PB.4398.1	3	1043	6	incomplete-splice_match	gene: Zm00001d043149	Zm00001d043149_T001	424.72	438.61	coding	216	147	794
PB.5101.1	4	955	6	novel_not_in_catalog	gene: Zm00001d050165	novel	43.20	43.20	coding	202	26	631

PB.10877.2	B73V4 ctg181	990	8	novel_not_in_catalog	gene: Zm00001d000178	novel	10.91	129.62	coding	185	117	671
PB.10877.1	B73V4 ctg181	1337	10	novel_not_in_catalog	gene: Zm00001d000178	novel	118.70	129.62	coding	311	170	1102
PB.5552.1	4	2149	8	fusion	gene: Zm00001d052565_g	novel	45.58	45.58	coding	504	273	1784
PB.1211.13	1	1662	6	full-splice_match	gene: Zm00001d033210	Zm00001d033210_T002	0.00	2362.83	coding	419	166	1422
PB.1211.11	1	1739	6	full-splice_match	gene: Zm00001d033210	Zm00001d033210_T002	10.71	2362.83	coding	419	166	1422
PB.8364.2	7	1350	1	full-splice_match	gene: Zm00001d019753	Zm00001d019753_T001	0.38	5.07	coding	422	61	1326
PB.8364.1	7	2209	1	full-splice_match	gene: Zm00001d019753	Zm00001d019753_T001	4.69	5.07	coding	422	50	1315
PB.5136.2	4	1596	6	full-splice_match	gene: Zm00001d050383	Zm00001d050383_T001	0.00	50.84	coding	472	43	1458
PB.5136.3	4	1687	5	novel_in_catalog	gene: Zm00001d050383	novel	0.14	50.84	coding	423	44	1312
PB.6964.2	5	1242	7	novel_not_in_catalog	gene: Zm00001d017756	novel	9.13	46.65	coding	291	158	1030
PB.5136.1	4	1711	6	full-splice_match	gene: Zm00001d050383	Zm00001d050383_T001	50.71	50.84	coding	472	46	1461
PB.3231.3	2	1597	6	full-splice_match	gene: Zm00001d006046	Zm00001d006046_T005	15.31	79.51	coding	301	217	1119
PB.10853.1	B73V4 ctg115	1944	3	incomplete-splice_match	gene: Zm00001d000358	Zm00001d000358_T001	13.25	23.92	coding	387	222	1382
PB.2746.2	2	2324	6	novel_not_in_catalog	gene: Zm00001d003199	novel	6.17	6.17	coding	107	1542	1862
PB.10853.2	B73V4 ctg115	2582	3	novel_in_catalog	gene: Zm00001d000358	novel	10.67	23.92	coding	691	324	2396
PB.8179.3	7	1351	14	incomplete-splice_match	gene: Zm00001d018724	Zm00001d018724_T009	15.76	37.93	coding	267	390	1190
PB.4845.1	4	1695	10	full-splice_match	gene: Zm00001d048856	Zm00001d048856_T003	20.72	20.72	coding	512	70	1605

PB.1597.1	1	1215	2	full-splice_match	gene: Zm00001d034803	Zm00001d034803_T001	40.12	40.12	coding	234	301	1002
PB.10737.1	9	2490	17	incomplete-splice_match	gene: Zm00001d048235	Zm00001d048235_T001	25.39	30.30	coding	618	136	1989
PB.9319.1	8	3107	1	full-splice_match	gene: Zm00001d009511	Zm00001d009511_T001	20.94	20.94	coding	926	105	2882
PB.2908.1	2	1420	3	full-splice_match	gene: Zm00001d004007	Zm00001d004007_T001	52.55	52.55	coding	358	81	1154
PB.9319.2	8	2633	1	full-splice_match	gene: Zm00001d009511	Zm00001d009511_T001	0.00	20.94	coding	730	318	2507
PB.8722.1	7	934	4	full-splice_match	gene: Zm00001d021526	Zm00001d021526_T002	399.67	1033.27	coding	149	124	570
PB.9835.1	8	1802	11	full-splice_match	gene: Zm00001d011945	Zm00001d011945_T002	14.66	14.66	coding	400	364	1563
PB.760.1	1	3947	14	full-splice_match	gene: Zm00001d031064	Zm00001d031064_T003	10.19	21.64	coding	897	887	3577
PB.3642.1	3	1182	2	genic	gene: Zm00001d039268	novel	37.31	37.31	coding	219	144	800
PB.8855.1	7	1473	5	full-splice_match	gene: Zm00001d021960	Zm00001d021960_T001	6.36	18.48	coding	334	14	1015
PB.8855.2	7	1290	5	full-splice_match	gene: Zm00001d021960	Zm00001d021960_T001	12.12	18.48	coding	334	88	1089
PB.10331.2	9	2671	5	full-splice_match	gene: Zm00001d046422	Zm00001d046422_T001	12.47	24.92	coding	565	597	2291
PB.7661.1	6	1384	2	full-splice_match	gene: Zm00001d037410	Zm00001d037410_T001	39.14	39.14	coding	338	154	1167
PB.7687.1	6	1331	3	full-splice_match	gene: Zm00001d037550	Zm00001d037550_T001	177.89	177.89	coding	357	46	1116
PB.10331.1	9	2666	5	full-splice_match	gene: Zm00001d046422	Zm00001d046422_T001	12.46	24.92	coding	565	154	1848
PB.700.1	1	2715	5	full-splice_match	gene: Zm00001d030652	Zm00001d030652_T001	18.81	23.57	coding	714	141	2282
PB.1538.5	1	2071	11	full-splice_match	gene: Zm00001d034610	Zm00001d034610_T002	6.46	19.55	coding	548	148	1791

PB.700.3	1	2460	5	full-splice_match	gene: Zm00001d030652	Zm00001d030652_T001	0.00	23.57	coding	714	127	2268
PB.8544.1	7	2746	5	novel_in_catalog	gene: Zm00001d020748	novel	36.41	36.41	coding	278	70	903
PB.5450.5	4	1454	8	full-splice_match	gene: Zm00001d052219	Zm00001d052219_T001	36.56	42.95	coding	363	139	1227
PB.10597.3	9	1575	3	full-splice_match	gene: Zm00001d047750	Zm00001d047750_T001	14.99	68.56	coding	352	215	1270
PB.10737.2	9	2670	20	full-splice_match	gene: Zm00001d048235	Zm00001d048235_T001	4.92	30.30	coding	746	118	2355
PB.10762.3	9	3353	13	full-splice_match	gene: Zm00001d048338	Zm00001d048338_T026	0.00	33.66	coding	1054	71	3232
PB.10762.1	9	3634	13	full-splice_match	gene: Zm00001d048338	Zm00001d048338_T026	18.26	33.66	coding	1054	187	3348
PB.8373.1	7	1238	4	intergenic	novelGene_128	novel	14.67	14.67	coding	309	25	951
PB.4014.1	3	4563	3	novel_in_catalog	gene: Zm00001d041362	novel	3.14	13.23	coding	1261	6	3788
PB.4014.2	3	3718	3	novel_in_catalog	gene: Zm00001d041362	novel	5.22	13.23	coding	1011	175	3207
PB.4014.3	3	1749	4	incomplete-splice_match	gene: Zm00001d041362	Zm00001d041362_T003	4.87	13.23	coding	414	126	1367
PB.9829.3	8	2766	10	full-splice_match	gene: Zm00001d011913	Zm00001d011913_T001	0.00	47.73	coding	782	143	2488
PB.9829.2	8	3456	8	novel_in_catalog	gene: Zm00001d011913	novel	1.76	47.73	coding	357	2079	3149
PB.9829.1	8	2764	10	full-splice_match	gene: Zm00001d011913	Zm00001d011913_T001	45.97	47.73	coding	782	94	2439
PB.8849.1	7	1973	2	full-splice_match	gene: Zm00001d021938	Zm00001d021938_T001	55.53	72.90	coding	513	64	1602
PB.4109.1	3	2477	6	incomplete-splice_match	gene: Zm00001d041839	Zm00001d041839_T001	17.34	17.34	coding	457	105	1475
PB.8849.4	7	1676	2	full-splice_match	gene: Zm00001d021938	Zm00001d021938_T001	0.00	72.90	coding	513	62	1600

PB.7746.2	6	603	4	novel_not_in_catalog	gene: Zm00001d037778	novel	1.32	2.88	coding	148	15	458
PB.7746.1	6	1308	2	novel_in_catalog	gene: Zm00001d037778	novel	1.55	2.88	coding	131	85	477
PB.10080.1	9	728	1	full-splice_match	gene: Zm00001d045000	Zm00001d045000_T001	1379.71	1379.71	coding	142	101	526
PB.935.1	1	2038	5	novel_in_catalog	gene: Zm00001d031959	novel	9.66	54.85	coding	373	671	1789
PB.10489.1	9	1190	2	full-splice_match	gene: Zm00001d047335	Zm00001d047335_T001	71.92	71.92	coding	271	198	1010
PB.2658.1	2	527	1	intergenic	novelGene_78	novel	318.51	394.66	coding	20	368	427
PB.2279.2	10	1352	5	novel_in_catalog	gene: Zm00001d026370	novel	7.03	89.40	coding	198	610	1203
PB.2279.1	10	1497	6	full-splice_match	gene: Zm00001d026370	Zm00001d026370_T001	82.37	89.40	coding	343	258	1286
PB.8688.3	7	335	2	genic	gene: Zm00001d021389	novel	32.22	88.17	non_coding	NA	NA	NA
PB.8250.1	7	2214	1	full-splice_match	gene: Zm00001d019049	Zm00001d019049_T001	11.62	11.62	coding	625	61	1935
PB.5184.1	4	2776	3	full-splice_match	gene: Zm00001d026882	Zm00001d026882_T001	0.59	0.59	non_coding	NA	NA	NA
PB.7050.1	5	1888	3	full-splice_match	gene: Zm00001d018070	Zm00001d018070_T001	11.88	11.88	coding	331	619	1611
PB.5184.3	4	2762	2	full-splice_match	gene: Zm00001d026881	Zm00001d026881_T001	0.00	2.83	non_coding	NA	NA	NA
PB.5184.2	4	2895	2	full-splice_match	gene: Zm00001d026881	Zm00001d026881_T001	2.83	2.83	non_coding	NA	NA	NA
PB.6779.4	5	1809	3	full-splice_match	gene: Zm00001d016911	Zm00001d016911_T001	6.50	35.18	coding	388	16	1179
PB.4831.1	4	3043	8	full-splice_match	gene: Zm00001d048777	Zm00001d048777_T018	57.19	57.19	coding	301	1416	2318
PB.8089.3	6	973	4	novel_not_in_catalog	gene: Zm00001d001483	novel	5.72	8.32	non_coding	NA	NA	NA

PB.778.9	1	2317	14	full-splice_match	gene: Zm00001d031120	Zm00001d031120_T003	0.00	204.87	coding	624	176	2047
PB.6666.1	5	1087	2	full-splice_match	gene: Zm00001d016364	Zm00001d016364_T001	46.16	46.16	coding	270	64	873
PB.7176.4	5	1875	5	novel_not_in_catalog	gene: Zm00001d018499	novel	43.00	43.00	coding	165	1079	1573
PB.6957.2	5	1232	1	incomplete-splice_match	gene: Zm00001d017728	Zm00001d017728_T001	0.00	5.32	coding	392	17	1192
PB.475.1	1	1592	1	antisense	novelGene_gene: Zm00001d029297_A	novel	3.24	3.24	coding	138	60	473
PB.4501.3	3	2433	5	novel_in_catalog	gene: Zm00001d043527	novel	3.23	30.27	coding	156	247	714
PB.9596.1	8	2307	3	full-splice_match	gene: Zm00001d010863	Zm00001d010863_T001	32.63	32.63	coding	590	174	1943
PB.4501.1	3	1996	6	full-splice_match	gene: Zm00001d043527	Zm00001d043527_T002	1.68	30.27	coding	340	80	1099
PB.8089.1	6	1762	2	novel_not_in_catalog	gene: Zm00001d001482	novel	6.57	6.57	non_coding	NA	NA	NA
PB.2219.1	10	560	2	full-splice_match	gene: Zm00001d026187	Zm00001d026187_T001	60.76	60.76	coding	98	33	326
PB.2658.2	2	397	1	intergenic	novelGene_78	novel	76.15	394.66	coding	11	364	396
PB.4501.4	3	1456	6	full-splice_match	gene: Zm00001d043527	Zm00001d043527_T002	22.05	30.27	coding	340	152	1171
PB.7697.1	6	2709	5	full-splice_match	gene: Zm00001d037596	Zm00001d037596_T003	5.32	59.20	coding	281	234	1076
PB.2147.1	10	1575	3	full-splice_match	gene: Zm00001d025823	Zm00001d025823_T001	6.41	6.41	coding	278	160	993
PB.7697.3	6	1613	6	full-splice_match	gene: Zm00001d037596	Zm00001d037596_T002	0.00	59.20	non_coding	NA	NA	NA
PB.4248.3	3	1597	6	full-splice_match	gene: Zm00001d042482	Zm00001d042482_T001	55.03	94.32	coding	362	164	1249
PB.5772.1	4	1927	4	full-splice_match	gene: Zm00001d053640	Zm00001d053640_T001	9.43	12.91	coding	529	105	1691

PB.4439.2	3	1691	1	genic	gene: Zm00001d043314	novel	1.35	77.47	coding	99	1108	1404
PB.8841.2	7	525	1	full-splice_match	gene: Zm00001d021901	Zm00001d021901_T001	4.63	4.99	coding	99	53	349
PB.1072.4	1	1944	2	incomplete-splice_match	gene: Zm00001d032540	Zm00001d032540_T008	28.45	28.45	coding	226	791	1468
PB.8841.1	7	723	1	full-splice_match	gene: Zm00001d021901	Zm00001d021901_T001	0.37	4.99	coding	99	18	314
PB.4976.1	4	720	3	full-splice_match	gene: Zm00001d049507	Zm00001d049507_T001	416.29	416.29	coding	110	50	379
PB.3406.4	2	3384	5	incomplete-splice_match	gene: Zm00001d006798	Zm00001d006798_T010	1.66	28.79	coding	541	1512	3134
PB.2796.1	2	1432	2	novel_in_catalog	gene: Zm00001d003422	novel	2.17	2.17	coding	343	147	1175
PB.5065.2	4	2435	14	full-splice_match	gene: Zm00001d049958	Zm00001d049958_T001	0.00	18.64	coding	646	19	1956
PB.5065.1	4	2534	14	full-splice_match	gene: Zm00001d049958	Zm00001d049958_T001	18.64	18.64	coding	646	19	1956
PB.4561.2	3	3000	6	novel_in_catalog	gene: Zm00001d043766	novel	3.75	3.75	coding	711	742	2874
PB.816.1	1	377	1	full-splice_match	gene: Zm00001d031290	Zm00001d031290_T001	43.92	43.92	non_coding	NA	NA	NA
PB.7503.1	6	2364	7	full-splice_match	gene: Zm00001d036594	Zm00001d036594_T001	10.10	10.10	coding	647	118	2058
PB.2173.2	10	2224	2	genic	gene: Zm00001d025940	novel	7.82	7.82	coding	266	714	1511
PB.4248.2	3	2130	3	novel_in_catalog	gene: Zm00001d042482	novel	2.00	94.32	coding	284	164	1015
PB.6463.1	5	1563	1	full-splice_match	gene: Zm00001d015052	Zm00001d015052_T001	3.08	3.08	coding	223	81	749
PB.9339.3	8	2306	9	novel_in_catalog	gene: Zm00001d009638	novel	2.28	195.22	coding	239	116	832
PB.9339.1	8	2058	10	full-splice_match	gene: Zm00001d009638	Zm00001d009638_T001	187.71	195.22	coding	583	116	1864

PB.624.13	1	2409	7	full-splice_match	gene: Zm00001d030110	Zm00001d030110_T002	0.00	158.92	coding	592	412	2187
PB.9339.7	8	1999	10	full-splice_match	gene: Zm00001d009638	Zm00001d009638_T001	0.00	195.22	coding	583	116	1864
PB.6825.1	5	1941	16	full-splice_match	gene: Zm00001d017144	Zm00001d017144_T002	35.89	35.89	coding	405	359	1573
PB.9339.5	8	2082	9	novel_in_catalog	gene: Zm00001d009638	novel	5.22	195.22	coding	474	67	1488
PB.9339.4	8	2398	8	novel_in_catalog	gene: Zm00001d009638	novel	0.00	195.22	coding	239	116	832
PB.415.1	1	1921	5	full-splice_match	gene: Zm00001d029031	Zm00001d029031_T002	14.51	14.51	coding	524	220	1791
PB.3108.1	2	2092	13	fusion	gene: Zm00001d005356_g	novel	58.68	58.68	coding	444	359	1690
PB.3219.1	2	999	5	full-splice_match	gene: Zm00001d005998	Zm00001d005998_T003	85.88	98.74	coding	248	99	842
PB.1030.1	1	1200	6	full-splice_match	gene: Zm00001d032384	Zm00001d032384_T001	25.13	25.25	coding	267	129	929
PB.2265.1	10	855	6	full-splice_match	gene: Zm00001d026335	Zm00001d026335_T003	303.70	478.90	coding	120	289	648
PB.2265.3	10	747	7	full-splice_match	gene: Zm00001d026335	Zm00001d026335_T006	175.19	478.90	coding	120	184	543
PB.469.1	1	1269	2	full-splice_match	gene: Zm00001d029275	Zm00001d029275_T001	67.92	67.92	coding	295	85	969
PB.6447.3	5	1691	3	full-splice_match	gene: Zm00001d014986	Zm00001d014986_T004	0.00	33.41	coding	203	905	1513
PB.2646.3	2	1784	3	full-splice_match	gene: Zm00001d002811	Zm00001d002811_T002	24.79	120.79	coding	387	118	1278
PB.6447.1	5	1840	3	full-splice_match	gene: Zm00001d014986	Zm00001d014986_T004	33.41	33.41	coding	203	908	1516
PB.8719.1	7	1094	8	full-splice_match	gene: Zm00001d021520	Zm00001d021520_T002	100.71	100.71	coding	273	75	893
PB.2646.4	2	1573	3	full-splice_match	gene: Zm00001d002811	Zm00001d002811_T002	51.19	120.79	coding	387	144	1304

PB.3504.2	2	1610	1	full-splice_match	gene: Zm00001d007274	Zm00001d007274_T001	288.92	338.75	coding	373	89	1207
PB.3366.1	2	1397	1	full-splice_match	gene: Zm00001d006611	Zm00001d006611_T001	14.82	20.00	coding	334	144	1145
PB.6650.6	5	2793	7	full-splice_match	gene: Zm00001d016274	Zm00001d016274_T006	20.42	83.51	coding	745	363	2597
PB.6650.1	5	3065	6	full-splice_match	gene: Zm00001d016274	Zm00001d016274_T011	49.85	83.51	coding	745	493	2727
PB.998.2	1	697	2	intergenic	novelGene_344	novel	11.02	32.76	non_coding	NA	NA	NA
PB.998.3	1	650	1	intergenic	novelGene_344	novel	15.97	32.76	coding	107	101	421
PB.4346.2	3	2787	2	genic	gene: Zm00001d042907	novel	0.00	22.18	coding	809	197	2623
PB.978.1	1	870	2	full-splice_match	gene: Zm00001d032162	Zm00001d032162_T001	128.76	128.76	coding	181	87	629
PB.3128.1	2	2023	1	novel_in_catalog	gene: Zm00001d005495	novel	1.66	90.47	coding	120	1281	1640
PB.4013.1	3	1695	11	novel_in_catalog	gene: Zm00001d041353	novel	6.36	6.36	coding	390	238	1407
PB.2205.1	10	1130	2	full-splice_match	gene: Zm00001d026094	Zm00001d026094_T001	3.85	3.85	coding	318	79	1032
PB.5005.1	4	1640	11	full-splice_match	gene: Zm00001d049652	Zm00001d049652_T007	42.78	55.75	coding	424	106	1377
PB.4645.1	3	1998	1	full-splice_match	gene: Zm00001d044120	Zm00001d044120_T001	15.95	32.96	coding	494	125	1606
PB.5005.2	4	1563	11	full-splice_match	gene: Zm00001d049652	Zm00001d049652_T007	12.97	55.75	coding	424	131	1402
PB.6171.1	5	1991	3	novel_in_catalog	gene: Zm00001d013765	novel	11.66	13.71	coding	417	498	1748
PB.4952.1	4	1066	8	novel_not_in_catalog	gene: Zm00001d049365	novel	3.23	3.23	coding	79	149	385
PB.6171.3	5	1800	3	novel_in_catalog	gene: Zm00001d013765	novel	0.00	13.71	coding	417	497	1747

PB.10585.4	9	1357	4	incomplete-splice_match	gene: Zm00001d047710	Zm00001d047710_T001	14.00	26.28	non_coding	NA	NA	NA
PB.4346.1	3	3086	2	genic	gene: Zm00001d042907	novel	22.18	22.18	coding	809	283	2709
PB.10585.1	9	1125	1	genic	gene: Zm00001d047710	novel	5.51	26.28	non_coding	NA	NA	NA
PB.8090.1	6	1531	1	full-splice_match	gene: Zm00001d039050	Zm00001d039050_T001	7.72	7.72	coding	291	104	976
PB.10585.3	9	1528	3	full-splice_match	gene: Zm00001d047710	Zm00001d047710_T022	6.77	26.28	non_coding	NA	NA	NA
PB.3394.1	2	2494	1	genic	gene: Zm00001d006753	novel	1.11	12.53	coding	454	758	2119
PB.1685.2	10	2172	14	novel_not_in_catalog	gene: Zm00001d023368	novel	8.00	12.95	coding	319	733	1689
PB.1685.1	10	3258	16	novel_not_in_catalog	gene: Zm00001d023368	novel	1.75	12.95	coding	255	2186	2950
PB.1685.7	10	2050	14	novel_not_in_catalog	gene: Zm00001d023368	novel	0.00	12.95	coding	319	736	1692
PB.1685.5	10	2128	13	novel_not_in_catalog	gene: Zm00001d023368	novel	3.21	12.95	coding	156	19	486
PB.4024.5	3	1449	5	full-splice_match	gene: Zm00001d041416	Zm00001d041416_T003	185.49	332.91	coding	271	179	991
PB.10405.11	9	2170	9	novel_in_catalog	gene: Zm00001d046828	novel	1.55	85.56	coding	193	150	728
PB.4024.1	3	1576	5	full-splice_match	gene: Zm00001d041416	Zm00001d041416_T003	141.94	332.91	coding	271	205	1017
PB.4024.2	3	1715	3	novel_in_catalog	gene: Zm00001d041416	novel	5.49	332.91	coding	239	203	919
PB.7175.3	5	1400	12	full-splice_match	gene: Zm00001d018498	Zm00001d018498_T004	23.48	70.12	coding	280	108	947
PB.1459.4	1	1427	8	full-splice_match	gene: Zm00001d034341	Zm00001d034341_T006	3.92	22.38	coding	108	294	617
PB.1459.5	1	1282	9	full-splice_match	gene: Zm00001d034341	Zm00001d034341_T002	4.73	22.38	coding	237	243	953

PB.1459.2	1	2103	8	novel_in_catalog	gene: Zm00001d034341	novel	0.00	22.38	coding	173	1203	1721
PB.1459.3	1	2192	7	full-splice_match	gene: Zm00001d034341	Zm00001d034341_T001	3.18	22.38	non_coding	NA	NA	NA
PB.6339.1	5	1525	4	novel_not_in_catalog	gene: Zm00001d014596	novel	1.59	1.59	coding	386	253	1410
PB.9599.4	8	754	2	full-splice_match	gene: Zm00001d010869	Zm00001d010869_T003	12.38	39.73	coding	104	442	753
PB.9190.1	8	3978	22	full-splice_match	gene: Zm00001d008789	Zm00001d008789_T035	18.15	18.15	coding	1181	35	3577
PB.3213.1	2	4195	4	full-splice_match	gene: Zm00001d005958	Zm00001d005958_T002	12.22	22.36	coding	1176	126	3653
PB.3213.2	2	3234	3	incomplete-splice_match	gene: Zm00001d005958	Zm00001d005958_T002	10.14	22.36	coding	814	322	2763
PB.3213.3	2	1638	2	incomplete-splice_match	gene: Zm00001d005958	Zm00001d005958_T002	0.00	22.36	coding	498	76	1569
PB.4133.1	3	750	3	full-splice_match	gene: Zm00001d041963	Zm00001d041963_T001	70.54	478.47	coding	135	94	498
PB.10599.4	9	1467	4	novel_in_catalog	gene: Zm00001d047758	novel	5.29	168.10	coding	404	149	1360
PB.4133.3	3	1971	2	novel_in_catalog	gene: Zm00001d041963	novel	7.13	478.47	coding	78	120	353
PB.6256.2	5	2368	2	full-splice_match	gene: Zm00001d014200	Zm00001d014200_T001	1.67	28.88	coding	121	90	452
PB.9325.1	8	1224	5	full-splice_match	gene: Zm00001d009532	Zm00001d009532_T001	98.28	98.89	coding	281	121	963
PB.9325.2	8	1204	5	full-splice_match	gene: Zm00001d009532	Zm00001d009532_T001	0.61	98.89	coding	281	123	965
PB.6256.1	5	1188	3	novel_not_in_catalog	gene: Zm00001d014200	novel	27.21	28.88	coding	120	187	546
PB.8253.1	7	1889	2	full-splice_match	gene: Zm00001d019078	Zm00001d019078_T002	14.31	29.54	coding	512	105	1640
PB.10625.2	9	1517	4	full-splice_match	gene: Zm00001d047835	Zm00001d047835_T004	197.41	314.58	coding	323	124	1092

PB.10625.3	9	1453	4	full-splice_match	gene: Zm00001d047835	Zm00001d047835_T004	2.90	314.58	coding	323	149	1117
PB.8956.2	7	2060	7	incomplete-splice_match	gene: Zm00001d022338	Zm00001d022338_T005	25.36	71.06	coding	623	8	1876
PB.8956.1	7	3045	10	incomplete-splice_match	gene: Zm00001d022338	Zm00001d022338_T002	45.71	71.06	coding	946	8	2845
PB.2634.2	2	2900	2	genic	gene: Zm00001d002787	novel	3.19	15.11	coding	886	189	2846
PB.39.1	1	1080	7	novel_in_catalog	gene: Zm00001d027366	novel	17.85	17.85	coding	237	144	854
PB.2634.1	2	1654	1	novel_in_catalog	gene: Zm00001d002787	novel	11.91	15.11	coding	360	410	1489
PB.1951.1	10	2691	4	full-splice_match	gene: Zm00001d024861	Zm00001d024861_T001	0.73	10.21	coding	289	672	1538
PB.1440.1	1	1073	8	full-splice_match	gene: Zm00001d034244	Zm00001d034244_T001	273.42	291.44	coding	223	174	842
PB.8657.1	7	2715	9	full-splice_match	gene: Zm00001d021287	Zm00001d021287_T005	17.85	17.85	coding	683	206	2254
PB.3366.2	2	1169	1	full-splice_match	gene: Zm00001d006611	Zm00001d006611_T001	5.18	20.00	coding	334	25	1026
PB.5430.1	4	2028	11	incomplete-splice_match	gene: Zm00001d052107	Zm00001d052107_T003	14.90	14.90	coding	180	1159	1698
PB.1440.2	1	868	8	full-splice_match	gene: Zm00001d034244	Zm00001d034244_T001	18.02	291.44	coding	223	69	737
PB.3382.5	2	1876	1	novel_in_catalog	gene: Zm00001d006697	novel	2.99	35.13	coding	474	297	1718
PB.6062.1	5	400	1	genic	gene: Zm00001d013325	novel	294.11	294.11	non_coding	NA	NA	NA
PB.2871.2	2	1448	14	novel_not_in_catalog	gene: Zm00001d003813	novel	0.00	18.55	coding	430	15	1304
PB.10492.2	9	828	5	full-splice_match	gene: Zm00001d047354	Zm00001d047354_T001	70.26	124.11	coding	160	64	543
PB.7654.1	6	1705	1	full-splice_match	gene: Zm00001d037382	Zm00001d037382_T001	0.36	0.36	coding	475	90	1514

PB.9198.9	8	3418	9	incomplete-splice_match	gene: Zm00001d008826	Zm00001d008826_T068	50.31	277.29	coding	868	248	2851
PB.7966.1	6	1692	1	full-splice_match	gene: Zm00001d038574	Zm00001d038574_T005	0.29	17.72	non_coding	NA	NA	NA
PB.85.1	1	2097	8	full-splice_match	gene: Zm00001d027544	Zm00001d027544_T002	2.89	4.51	coding	431	65	1357
PB.85.2	1	1865	8	full-splice_match	gene: Zm00001d027544	Zm00001d027544_T002	1.63	4.51	coding	431	81	1373
PB.9198.2	8	4242	15	novel_in_catalog	gene: Zm00001d008826	novel	89.74	277.29	coding	866	1379	3976
PB.3303.1	2	1163	7	full-splice_match	gene: Zm00001d006345	Zm00001d006345_T002	18.84	18.84	coding	293	72	950
PB.9198.6	8	3153	7	incomplete-splice_match	gene: Zm00001d008826	Zm00001d008826_T042	128.83	277.29	coding	607	275	2095
PB.9198.5	8	3689	8	novel_in_catalog	gene: Zm00001d008826	novel	0.54	277.29	coding	751	283	2535
PB.3506.1	2	4276	9	novel_in_catalog	gene: Zm00001d007295	novel	2.57	23.93	coding	402	453	1658
PB.3506.3	2	3544	10	full-splice_match	gene: Zm00001d007295	Zm00001d007295_T002	0.16	23.93	coding	906	554	3271
PB.3506.2	2	3859	10	full-splice_match	gene: Zm00001d007295	Zm00001d007295_T002	21.19	23.93	coding	906	530	3247
PB.5817.3	4	1123	9	full-splice_match	gene: Zm00001d053822	Zm00001d053822_T002	15.37	125.34	coding	208	283	906
PB.3933.1	3	1408	5	novel_in_catalog	gene: Zm00001d040652	novel	3.97	3.97	coding	370	104	1213
PB.10490.3	9	1047	1	novel_in_catalog	gene: Zm00001d047340	novel	0.00	159.64	coding	123	74	442
PB.3374.2	2	821	4	full-splice_match	gene: Zm00001d006670	Zm00001d006670_T002	0.00	61.58	coding	121	166	528
PB.10490.1	9	1097	2	full-splice_match	gene: Zm00001d047340	Zm00001d047340_T003	130.86	159.64	coding	238	74	787
PB.8177.1	7	1449	2	full-splice_match	gene: Zm00001d018718	Zm00001d018718_T001	19.56	28.67	coding	322	173	1138

PB.8177.2	7	474	1	incomplete-splice_match	gene: Zm00001d018718	Zm00001d018718_T001	9.11	28.67	coding	94	24	305
PB.3237.1	2	1658	8	full-splice_match	gene: Zm00001d006065	Zm00001d006065_T002	21.79	21.79	coding	350	130	1179
PB.5894.1	4	995	8	full-splice_match	gene: Zm00001d054077	Zm00001d054077_T001	11.76	11.76	coding	219	59	715
PB.1204.2	1	1384	4	full-splice_match	gene: Zm00001d033160	Zm00001d033160_T001	69.04	74.31	coding	335	132	1136
PB.10599.3	9	637	2	full-splice_match	gene: Zm00001d047758	Zm00001d047758_T006	10.55	168.10	coding	115	76	420
PB.1550.2	1	1489	3	novel_in_catalog	gene: Zm00001d034649	novel	6.42	54.79	coding	254	494	1255
PB.7629.3	6	2104	11	novel_not_in_catalog	gene: Zm00001d037257	novel	36.05	107.46	coding	441	249	1571
PB.10617.3	9	1599	8	incomplete-splice_match	gene: Zm00001d047808	Zm00001d047808_T017	0.00	27.10	coding	498	1	1494
PB.10617.2	9	2739	10	full-splice_match	gene: Zm00001d047808	Zm00001d047808_T008	4.58	27.10	coding	680	340	2379
PB.10617.1	9	3226	10	full-splice_match	gene: Zm00001d047808	Zm00001d047808_T008	22.52	27.10	coding	680	347	2386
PB.1307.1	1	2428	6	novel_in_catalog	gene: Zm00001d033642	novel	51.25	65.09	coding	634	176	2077
PB.6630.1	5	1342	4	full-splice_match	gene: Zm00001d016154	Zm00001d016154_T001	42.40	42.40	coding	252	304	1059
PB.5715.1	4	772	5	full-splice_match	gene: Zm00001d053377	Zm00001d053377_T001	20.44	20.44	coding	206	7	624
PB.1659.2	10	2261	5	incomplete-splice_match	gene: Zm00001d023262	Zm00001d023262_T033	50.01	97.36	coding	459	56	1432
PB.3374.3	2	694	4	full-splice_match	gene: Zm00001d006670	Zm00001d006670_T002	44.62	61.58	coding	121	210	572
PB.10023.1	8	1759	2	full-splice_match	gene: Zm00001d012749	Zm00001d012749_T001	11.53	11.53	coding	433	267	1565
PB.6668.7	5	1224	10	incomplete-splice_match	gene: Zm00001d016373	Zm00001d016373_T003	0.00	73.43	coding	393	46	1224

PB.6668.1	5	3008	19	full-splice_match	gene: Zm00001d016373	Zm00001d016373_T003	57.66	73.43	coding	879	91	2727
PB.6668.3	5	2838	19	full-splice_match	gene: Zm00001d016373	Zm00001d016373_T003	15.77	73.43	coding	879	92	2728
PB.7225.2	6	3029	15	novel_in_catalog	gene: Zm00001d035046	novel	4.04	20.63	coding	872	356	2971
PB.1659.4	10	3033	22	incomplete-splice_match	gene: Zm00001d023262	Zm00001d023262_T068	0.00	97.36	coding	944	200	3031
PB.8647.1	7	1431	2	incomplete-splice_match	gene: Zm00001d021236	Zm00001d021236_T003	5.43	7.17	coding	266	16	813
PB.2484.1	2	2269	10	novel_in_catalog	gene: Zm00001d002143	novel	63.24	63.24	coding	401	197	1399
PB.1875.5	10	2659	2	novel_in_catalog	gene: Zm00001d024499	novel	16.39	276.67	coding	438	1165	2478
PB.10162.1	9	2725	11	full-splice_match	gene: Zm00001d045383	Zm00001d045383_T004	13.46	15.02	coding	722	197	2362
PB.10162.2	9	3208	9	novel_in_catalog	gene: Zm00001d045383	novel	1.32	15.02	coding	414	218	1459
PB.10162.3	9	1934	7	novel_in_catalog	gene: Zm00001d045383	novel	0.25	15.02	coding	200	1334	1933
PB.5735.3	4	2792	17	full-splice_match	gene: Zm00001d053515	Zm00001d053515_T009	0.00	22.41	coding	787	295	2655
PB.532.1	1	832	3	full-splice_match	gene: Zm00001d029567	Zm00001d029567_T001	63.87	63.87	coding	117	226	576
PB.5735.1	4	2908	17	full-splice_match	gene: Zm00001d053515	Zm00001d053515_T009	20.13	22.41	coding	787	304	2664
PB.4626.1	3	1723	6	incomplete-splice_match	gene: Zm00001d044026	Zm00001d044026_T055	55.98	55.98	coding	494	99	1580
PB.6777.1	5	2305	8	full-splice_match	gene: Zm00001d016902	Zm00001d016902_T003	71.06	76.14	coding	554	205	1866
PB.6777.3	5	2182	8	full-splice_match	gene: Zm00001d016902	Zm00001d016902_T003	0.00	76.14	coding	554	208	1869
PB.2707.1	2	1389	9	full-splice_match	gene: Zm00001d003058	Zm00001d003058_T001	14.87	22.73	coding	370	28	1137

PB.2707.2	2	1380	9	full-splice_match	gene: Zm00001d003058	Zm00001d003058_T001	7.86	22.73	coding	370	139	1248
PB.2616.2	2	1390	4	full-splice_match	gene: Zm00001d002690	Zm00001d002690_T001	1213.15	1263.20	coding	288	322	1185
PB.10736.1	9	1143	2	full-splice_match	gene: Zm00001d048234	Zm00001d048234_T001	10.01	10.01	coding	182	315	860
PB.2616.1	2	2592	3	novel_in_catalog	gene: Zm00001d002690	novel	46.18	1263.20	coding	286	80	937
PB.820.1	1	733	1	genic	gene: Zm00001d031315	novel	44.48	44.48	coding	72	129	344
PB.560.3	1	1843	5	full-splice_match	gene: Zm00001d029688	Zm00001d029688_T003	42.39	43.53	coding	526	57	1634
PB.2616.4	2	1498	3	novel_in_catalog	gene: Zm00001d002690	novel	0.00	1263.20	coding	286	82	939
PB.2616.5	2	1199	2	novel_in_catalog	gene: Zm00001d002690	novel	3.87	1263.20	coding	177	78	608
PB.10599.1	9	1625	5	full-splice_match	gene: Zm00001d047758	Zm00001d047758_T003	138.81	168.10	coding	374	258	1379
PB.3574.1	2	2550	3	full-splice_match	gene: Zm00001d007745	Zm00001d007745_T001	8.95	10.54	coding	731	70	2262
PB.3574.6	2	2118	2	novel_in_catalog	gene: Zm00001d007745	novel	1.00	10.54	coding	385	70	1224
PB.7285.14	6	1317	9	novel_in_catalog	gene: Zm00001d035346	novel	14.08	65.77	non_coding	NA	NA	NA
PB.3112.1	2	1257	1	genic	gene: Zm00001d005375	novel	51.78	51.78	coding	184	100	651
PB.5461.6	4	1087	10	incomplete-splice_match	gene: Zm00001d052248	Zm00001d052248_T030	0.00	19.85	coding	344	55	1086
PB.10278.1	9	1421	10	novel_in_catalog	gene: Zm00001d045993	novel	6.12	6.12	coding	347	96	1136
PB.6327.3	5	2352	1	genic	gene: Zm00001d014557	novel	9.15	72.82	coding	722	9	2174
PB.6327.2	5	3230	1	genic	gene: Zm00001d014557	novel	9.71	72.82	coding	882	247	2892

PB.6327.1	5	1836	1	genic	gene: Zm00001d014557	novel	53.96	72.82	coding	394	87	1268
PB.10497.1	9	2440	3	novel_in_catalog	gene: Zm00001d047388	novel	1.83	37.55	coding	201	117	719
PB.2153.2	10	2073	5	novel_in_catalog	gene: Zm00001d025855	novel	12.04	31.60	non_coding	NA	NA	NA
PB.1751.1	10	747	1	intergenic	novelGene_56	novel	5.64	5.64	non_coding	NA	NA	NA
PB.6266.5	5	3364	13	full-splice_match	gene: Zm00001d014286	Zm00001d014286_T012	0.00	90.62	coding	1020	95	3154
PB.6266.4	5	3458	13	full-splice_match	gene: Zm00001d014286	Zm00001d014286_T005	5.80	90.62	coding	810	813	3242
PB.6266.3	5	3655	12	novel_in_catalog	gene: Zm00001d014286	novel	10.28	90.62	coding	1008	178	3201
PB.6266.2	5	3578	13	full-splice_match	gene: Zm00001d014286	Zm00001d014286_T012	74.55	90.62	coding	1020	256	3315
PB.4634.2	3	1619	2	full-splice_match	gene: Zm00001d044061	Zm00001d044061_T001	2.09	38.94	coding	431	68	1360
PB.7756.1	6	1231	3	incomplete-splice_match	gene: Zm00001d037815	Zm00001d037815_T001	5.08	5.08	coding	260	161	940
PB.288.1	1	1141	1	full-splice_match	gene: Zm00001d028469	Zm00001d028469_T001	6.69	50.32	coding	211	89	721
PB.288.2	1	1030	1	full-splice_match	gene: Zm00001d028469	Zm00001d028469_T001	43.62	50.32	coding	211	155	787
PB.6722.4	5	1696	2	novel_in_catalog	gene: Zm00001d016682	novel	4.81	39.88	coding	108	220	543
PB.5709.2	4	1865	6	full-splice_match	gene: Zm00001d053354	Zm00001d053354_T001	0.00	30.76	coding	537	169	1779
PB.1542.1	1	3818	5	incomplete-splice_match	gene: Zm00001d034624	Zm00001d034624_T005	21.77	35.15	coding	699	39	2135
PB.5709.1	4	1973	6	full-splice_match	gene: Zm00001d053354	Zm00001d053354_T001	30.76	30.76	coding	537	165	1775
PB.1995.3	10	2299	8	full-splice_match	gene: Zm00001d025021	Zm00001d025021_T009	1.15	5.28	coding	723	58	2226

PB.9224.1	8	2390	21	incomplete-splice_match	gene: Zm00001d008941	Zm00001d008941_T001	6.85	6.85	coding	577	348	2078
PB.1771.1	10	1134	5	full-splice_match	gene: Zm00001d023824	Zm00001d023824_T004	52.97	52.97	coding	145	82	516
PB.10033.2	9	1376	5	novel_in_catalog	gene: Zm00001d044717	novel	11.31	22.43	coding	236	282	989
PB.10033.1	9	2221	10	incomplete-splice_match	gene: Zm00001d044717	Zm00001d044717_T001	11.12	22.43	coding	411	740	1972
PB.5729.1	4	722	1	antisense	novelGene_gene: Zm00001d053434_A	novel	3.47	3.47	non_coding	NA	NA	NA
PB.9399.2	8	2962	10	full-splice_match	gene: Zm00001d009948	Zm00001d009948_T004	121.06	132.51	coding	833	143	2641
PB.9399.5	8	2753	10	full-splice_match	gene: Zm00001d009948	Zm00001d009948_T004	0.00	132.51	coding	833	175	2673
PB.9399.4	8	2872	10	full-splice_match	gene: Zm00001d009948	Zm00001d009948_T004	11.45	132.51	coding	833	191	2689
PB.3785.2	3	1090	8	full-splice_match	gene: Zm00001d039879	Zm00001d039879_T001	128.92	128.92	coding	218	113	766
PB.2763.8	2	1022	8	full-splice_match	gene: Zm00001d003254	Zm00001d003254_T004	18.49	54.71	coding	241	1	723
PB.2763.7	2	1924	6	full-splice_match	gene: Zm00001d003254	Zm00001d003254_T007	2.98	54.71	coding	221	4	666
PB.1090.1	1	776	4	full-splice_match	gene: Zm00001d032588	Zm00001d032588_T003	240.66	240.66	coding	118	204	557
PB.2763.4	2	2042	6	full-splice_match	gene: Zm00001d003254	Zm00001d003254_T007	0.00	54.71	coding	178	111	644
PB.2763.3	2	1178	9	full-splice_match	gene: Zm00001d003254	Zm00001d003254_T006	4.45	54.71	coding	241	61	783
PB.2763.2	2	1670	7	full-splice_match	gene: Zm00001d003254	Zm00001d003254_T001	4.72	54.71	coding	178	112	645
PB.2763.1	2	1368	8	full-splice_match	gene: Zm00001d003254	Zm00001d003254_T004	24.08	54.71	coding	284	50	901
PB.8232.1	7	642	2	genic	gene: Zm00001d018936	novel	22.42	136.59	coding	110	111	440

PB.10336.1	9	648	1	genic	gene: Zm00001d046445	novel	4.57	149.37	coding	133	60	458
PB.5458.2	4	1511	1	novel_in_catalog	gene: Zm00001d052234	novel	9.87	71.81	coding	151	144	596
PB.10497.3	9	1656	4	full-splice_match	gene: Zm00001d047388	Zm00001d047388_T002	13.04	37.55	coding	469	91	1497
PB.3870.7	3	2324	6	full-splice_match	gene: Zm00001d040301	Zm00001d040301_T003	0.00	38.04	coding	242	283	1008
PB.3870.6	3	1882	7	full-splice_match	gene: Zm00001d040301	Zm00001d040301_T001	0.00	38.04	coding	451	138	1490
PB.3870.5	3	2389	6	full-splice_match	gene: Zm00001d040301	Zm00001d040301_T003	0.00	38.04	coding	242	173	898
PB.3870.4	3	2114	7	full-splice_match	gene: Zm00001d040301	Zm00001d040301_T001	23.24	38.04	coding	451	236	1588
PB.8734.1	7	1895	5	full-splice_match	gene: Zm00001d021569	Zm00001d021569_T001	9.96	9.96	coding	483	235	1683
PB.3870.2	3	2581	6	full-splice_match	gene: Zm00001d040301	Zm00001d040301_T003	14.79	38.04	coding	242	293	1018
PB.8734.3	7	1740	5	full-splice_match	gene: Zm00001d021569	Zm00001d021569_T001	0.00	9.96	coding	483	203	1651
PB.1882.3	10	2609	2	novel_in_catalog	gene: Zm00001d024521	novel	2.41	6.28	coding	514	43	1584
PB.5317.3	4	2343	15	full-splice_match	gene: Zm00001d051599	Zm00001d051599_T001	14.93	48.00	coding	671	132	2144
PB.6337.1	5	2375	2	novel_in_catalog	gene: Zm00001d014584	novel	0.34	0.34	non_coding	NA	NA	NA
PB.5317.1	4	2430	15	full-splice_match	gene: Zm00001d051599	Zm00001d051599_T001	33.07	48.00	coding	671	57	2069
PB.8193.1	7	2127	2	genic	gene: Zm00001d018792	novel	4.45	4.45	coding	615	281	2125
PB.9752.1	8	709	5	novel_not_in_catalog	gene: Zm00001d011623	novel	61.92	61.92	coding	140	19	438
PB.10242.1	9	1741	6	incomplete-splice_match	gene: Zm00001d045744	Zm00001d045744_T003	17.37	17.37	coding	432	28	1323

PB.1882.1	10	1573	1	genic	gene: Zm00001d024521	novel	0.71	6.28	non_coding	NA	NA	NA
PB.5601.1	4	2008	10	fusion	gene: Zm00001d052785_g	novel	54.08	56.39	coding	520	139	1698
PB.5973.1	5	629	4	full-splice_match	gene: Zm00001d013031	Zm00001d013031_T002	135.37	135.37	coding	83	110	358
PB.10937.1	B73V4 ctg73	1703	2	full-splice_match	gene: Zm00001d000294	Zm00001d000294_T001	6.91	6.91	coding	473	75	1493
PB.8011.1	6	1813	1	full-splice_match	gene: Zm00001d038765	Zm00001d038765_T001	8.64	63.74	coding	478	48	1481
PB.1578.1	1	1952	9	full-splice_match	gene: Zm00001d034736	Zm00001d034736_T002	6.86	17.79	coding	400	586	1785
PB.6258.2	5	3780	18	novel_not_in_catalog	gene: Zm00001d014232	novel	24.86	24.86	coding	798	960	3353
PB.1745.9	10	2733	9	novel_not_in_catalog	gene: Zm00001d023636	novel	4.35	66.48	coding	238	58	771
PB.9073.3	8	1440	8	full-splice_match	gene: Zm00001d008244	Zm00001d008244_T005	6.27	97.68	coding	365	132	1226
PB.8286.1	7	1243	5	full-splice_match	gene: Zm00001d019234	Zm00001d019234_T002	79.94	79.94	coding	249	149	895
PB.9073.1	8	1562	8	full-splice_match	gene: Zm00001d008244	Zm00001d008244_T005	91.40	97.68	coding	365	128	1222
PB.1745.3	10	1955	9	novel_not_in_catalog	gene: Zm00001d023636	novel	0.24	66.48	coding	573	47	1765
PB.1745.1	10	2051	10	novel_not_in_catalog	gene: Zm00001d023636	novel	8.00	66.48	coding	596	57	1844
PB.1745.6	10	1862	10	novel_not_in_catalog	gene: Zm00001d023636	novel	16.15	66.48	coding	524	67	1638
PB.6078.1	5	1765	2	full-splice_match	gene: Zm00001d013383	Zm00001d013383_T001	19.14	19.14	coding	478	50	1483
PB.1745.4	10	1973	11	novel_not_in_catalog	gene: Zm00001d023636	novel	5.12	66.48	coding	544	118	1749
PB.7289.2	6	2337	2	antisense	novelGene_gene: Zm00001d035361_A	novel	0.51	0.51	coding	463	769	2157

PB.3884.1	3	1492	1	full-splice_match	gene: Zm00001d040356	Zm00001d040356_T001	16.90	16.90	coding	445	69	1403
PB.1273.2	1	3364	2	novel_in_catalog	gene: Zm00001d033511	novel	0.00	27.66	coding	259	2325	3101
PB.9548.1	8	1578	2	full-splice_match	gene: Zm00001d010673	Zm00001d010673_T001	41.87	52.94	coding	408	192	1415
PB.1439.1	1	1724	8	full-splice_match	gene: Zm00001d034241	Zm00001d034241_T001	10.96	10.96	coding	358	166	1239
PB.6359.2	5	776	4	incomplete-splice_match	gene: Zm00001d014674	Zm00001d014674_T002	24.88	24.88	coding	148	96	539
PB.9768.2	8	2111	5	novel_in_catalog	gene: Zm00001d011697	novel	5.28	5.28	coding	131	1508	1900
PB.2711.1	2	653	4	full-splice_match	gene: Zm00001d003081	Zm00001d003081_T003	641.38	641.38	coding	113	52	390
PB.177.5	1	2136	12	full-splice_match	gene: Zm00001d028010	Zm00001d028010_T001	0.00	29.10	coding	589	99	1865
PB.177.7	1	1906	12	full-splice_match	gene: Zm00001d028010	Zm00001d028010_T001	0.00	29.10	coding	589	25	1791
PB.7076.2	5	1458	6	full-splice_match	gene: Zm00001d018183	Zm00001d018183_T001	153.98	153.98	coding	366	54	1151
PB.177.1	1	2210	12	full-splice_match	gene: Zm00001d028010	Zm00001d028010_T001	29.10	29.10	coding	589	42	1808
PB.3015.1	2	1590	5	novel_in_catalog	gene: Zm00001d004839	novel	201.88	201.88	coding	389	206	1372
PB.4351.1	3	1191	8	fusion	gene: Zm00001d042924_g	novel	25.51	25.51	coding	196	222	809
PB.754.1	1	835	7	incomplete-splice_match	gene: Zm00001d031040	Zm00001d031040_T016	18.14	18.14	coding	132	62	457
PB.4371.1	3	1068	3	full-splice_match	gene: Zm00001d043036	Zm00001d043036_T001	8.60	8.60	coding	237	104	814
PB.4371.2	3	865	3	full-splice_match	gene: Zm00001d043036	Zm00001d043036_T001	0.00	8.60	coding	237	53	763
PB.8966.1	7	1938	4	full-splice_match	gene: Zm00001d022366	Zm00001d022366_T001	36.98	36.98	coding	422	235	1500

PB.8902.1	7	736	1	full-splice_match	gene: Zm00001d022124	Zm00001d022124_T001	1244.31	1648.96	coding	145	64	498
PB.10812.1	9	1452	3	novel_in_catalog	gene: Zm00001d048503	novel	108.82	120.19	coding	362	163	1248
PB.7033.1	5	1954	6	full-splice_match	gene: Zm00001d018006	Zm00001d018006_T002	11.40	11.40	coding	232	885	1580
PB.8775.4	7	1685	3	full-splice_match	gene: Zm00001d021701	Zm00001d021701_T006	14.67	67.00	coding	109	98	424
PB.4252.2	3	2502	1	full-splice_match	gene: Zm00001d042503	Zm00001d042503_T001	1.83	54.02	coding	149	10	456
PB.6480.1	5	1358	4	full-splice_match	gene: Zm00001d015125	Zm00001d015125_T001	6.97	6.97	coding	256	75	842
PB.7.1	1	1974	2	full-splice_match	gene: Zm00001d027268	Zm00001d027268_T001	89.00	89.00	coding	525	195	1769
PB.6726.6	5	2141	12	novel_in_catalog	gene: Zm00001d016702	novel	0.00	20.56	coding	431	85	1377
PB.167.2	1	891	6	novel_not_in_catalog	gene: Zm00001d027966	novel	23.54	112.96	coding	189	35	601
PB.167.3	1	691	6	novel_not_in_catalog	gene: Zm00001d027966	novel	0.00	112.96	coding	113	194	532
PB.3009.1	2	1470	6	incomplete-splice_match	gene: Zm00001d004782	Zm00001d004782_T001	37.77	37.77	coding	192	278	853
PB.167.1	1	954	6	novel_not_in_catalog	gene: Zm00001d027966	novel	89.42	112.96	coding	113	225	563
PB.799.4	1	1797	1	full-splice_match	gene: Zm00001d031221	Zm00001d031221_T001	0.00	42.01	coding	529	85	1671
PB.5198.2	4	615	4	full-splice_match	gene: Zm00001d050916	Zm00001d050916_T002	171.44	182.18	coding	114	59	400
PB.528.2	1	1235	3	genic	gene: Zm00001d029559	novel	45.26	45.26	coding	250	232	981
PB.2291.1	10	2066	3	novel_in_catalog	gene: Zm00001d026405	novel	13.59	13.59	coding	531	243	1835
PB.7870.1	6	370	1	full-splice_match	gene: Zm00001d038203	Zm00001d038203_T001	155.74	155.74	non_coding	NA	NA	NA

PB.3423.1	2	2484	10	novel_in_catalog	gene: Zm00001d006885	novel	17.13	29.30	coding	434	178	1479
PB.1848.1	10	2243	9	full-splice_match	gene: Zm00001d024339	Zm00001d024339_T001	71.80	126.96	coding	592	50	1825
PB.1848.2	10	2153	9	full-splice_match	gene: Zm00001d024339	Zm00001d024339_T001	55.16	126.96	coding	592	175	1950
PB.746.1	1	1860	1	antisense	novelGene_gene: Zm00001d030998_A	novel	4.96	4.96	coding	166	197	694
PB.10057.1	9	1757	10	full-splice_match	gene: Zm00001d044854	Zm00001d044854_T003	11.58	15.42	coding	384	160	1311
PB.2834.1	2	3831	6	full-splice_match	gene: Zm00001d003615	Zm00001d003615_T002	43.19	43.19	coding	1124	183	3554
PB.10057.2	9	1983	8	novel_in_catalog	gene: Zm00001d044854	novel	1.73	15.42	coding	209	155	781
PB.10057.4	9	1885	9	novel_in_catalog	gene: Zm00001d044854	novel	2.11	15.42	coding	209	160	786
PB.10511.1	9	1726	2	full-splice_match	gene: Zm00001d047436	Zm00001d047436_T001	7.01	7.01	coding	512	73	1608
PB.10421.2	9	1481	6	full-splice_match	gene: Zm00001d046937	Zm00001d046937_T001	16.99	99.27	coding	282	143	988
PB.7153.1	5	3317	3	full-splice_match	gene: Zm00001d018422	Zm00001d018422_T001	15.64	15.64	coding	932	280	3075
PB.494.8	1	1273	10	novel_in_catalog	gene: Zm00001d029410	novel	4.36	21.99	coding	316	210	1157
PB.494.5	1	1795	12	novel_in_catalog	gene: Zm00001d029410	novel	8.53	21.99	coding	287	83	943
PB.494.7	1	2076	8	novel_in_catalog	gene: Zm00001d029410	novel	0.00	21.99	coding	235	1122	1826
PB.494.6	1	2354	12	novel_in_catalog	gene: Zm00001d029410	novel	3.38	21.99	coding	235	86	790
PB.494.1	1	1817	12	novel_in_catalog	gene: Zm00001d029410	novel	5.71	21.99	coding	367	457	1557
PB.6994.5	5	1654	10	full-splice_match	gene: Zm00001d017872	Zm00001d017872_T025	6.07	162.08	coding	313	63	1001

PB.10344.2	9	665	1	novel_in_catalog	gene: Zm00001d046474	novel	5.72	199.88	non_coding	NA	NA	NA
PB.2814.1	2	479	1	incomplete-splice_match	gene: Zm00001d003511	Zm00001d003511_T001	17.24	17.24	non_coding	NA	NA	NA
PB.10827.1	9	2346	4	incomplete-splice_match	gene: Zm00001d048535	Zm00001d048535_T033	22.76	38.39	coding	536	85	1692
PB.10827.2	9	2936	4	incomplete-splice_match	gene: Zm00001d048535	Zm00001d048535_T033	15.63	38.39	coding	786	190	2547
PB.4733.1	3	2548	21	full-splice_match	gene: Zm00001d044488	Zm00001d044488_T002	10.10	10.10	coding	608	261	2084
PB.8816.2	7	3016	4	intergenic	novelGene_267	novel	4.80	21.78	coding	105	901	1215
PB.8014.3	6	2156	14	full-splice_match	gene: Zm00001d038775	Zm00001d038775_T002	69.49	69.49	coding	575	233	1957
PB.10047.1	9	870	3	full-splice_match	gene: Zm00001d044816	Zm00001d044816_T001	34.15	34.15	coding	186	87	644
PB.5672.1	4	1678	7	full-splice_match	gene: Zm00001d053107	Zm00001d053107_T002	61.28	78.86	coding	430	100	1389
PB.1876.2	10	973	7	full-splice_match	gene: Zm00001d024510	Zm00001d024510_T002	0.00	84.70	coding	215	110	754
PB.1876.1	10	1142	7	full-splice_match	gene: Zm00001d024510	Zm00001d024510_T002	84.70	84.70	coding	215	125	769
PB.5672.2	4	1540	7	full-splice_match	gene: Zm00001d053107	Zm00001d053107_T002	17.58	78.86	coding	430	100	1389
PB.9493.7	8	1592	7	novel_not_in_catalog	gene: Zm00001d010431	novel	0.00	149.31	coding	229	773	1459
PB.1861.2	10	2036	11	novel_not_in_catalog	gene: Zm00001d024420	novel	13.65	13.65	coding	539	58	1674
PB.115.5	1	1806	1	full-splice_match	gene: Zm00001d027700	Zm00001d027700_T001	0.00	535.38	coding	524	134	1705
PB.3451.1	2	434	5	incomplete-splice_match	gene: Zm00001d007032	Zm00001d007032_T001	113.67	113.67	non_coding	NA	NA	NA
PB.4309.1	3	965	1	full-splice_match	gene: Zm00001d042735	Zm00001d042735_T001	186.94	186.94	coding	224	84	755

PB.7348.2	6	1460	6	full-splice_match	gene: Zm00001d035701	Zm00001d035701_T001	0.00	18.97	coding	382	146	1291
PB.115.1	1	2114	1	full-splice_match	gene: Zm00001d027700	Zm00001d027700_T001	260.89	535.38	coding	524	258	1829
PB.10704.4	9	2180	3	full-splice_match	gene: Zm00001d048105	Zm00001d048105_T006	11.91	39.41	coding	315	948	1892
PB.115.2	1	2039	1	full-splice_match	gene: Zm00001d027700	Zm00001d027700_T001	274.49	535.38	coding	524	206	1777
PB.20.3	1	1691	14	novel_in_catalog	gene: Zm00001d027309	novel	17.20	27.63	coding	373	118	1236
PB.7316.1	6	2273	1	full-splice_match	gene: Zm00001d035527	Zm00001d035527_T001	12.13	12.13	coding	694	50	2131
PB.10704.5	9	1948	4	full-splice_match	gene: Zm00001d048105	Zm00001d048105_T002	15.60	39.41	coding	534	165	1766
PB.20.7	1	2362	13	full-splice_match	gene: Zm00001d027309	Zm00001d027309_T008	0.00	27.63	coding	169	1433	1939
PB.20.6	1	2335	14	novel_not_in_catalog	gene: Zm00001d027309	novel	10.43	27.63	coding	169	1514	2020
PB.8071.1	6	1025	3	full-splice_match	gene: Zm00001d038960	Zm00001d038960_T001	29.36	29.36	coding	263	86	874
PB.30.1	1	1726	3	full-splice_match	gene: Zm00001d027342	Zm00001d027342_T002	19.27	291.13	non_coding	NA	NA	NA
PB.30.2	1	644	3	full-splice_match	gene: Zm00001d027342	Zm00001d027342_T002	271.86	291.13	non_coding	NA	NA	NA
PB.9675.1	8	998	2	full-splice_match	gene: Zm00001d011241	Zm00001d011241_T001	9.04	9.04	coding	110	508	837
PB.8668.1	7	1343	5	full-splice_match	gene: Zm00001d021313	Zm00001d021313_T001	28.40	28.40	coding	276	140	967
PB.10539.4	9	2300	3	novel_not_in_catalog	gene: Zm00001d047528	novel	5.98	12.25	coding	381	1016	2158
PB.2505.1	2	1930	10	full-splice_match	gene: Zm00001d002243	Zm00001d002243_T004	25.86	25.86	coding	454	122	1483
PB.10539.1	9	2711	2	novel_in_catalog	gene: Zm00001d047528	novel	6.27	12.25	coding	381	1309	2451

PB.2853.3	2	1443	5	incomplete-splice_match	gene: Zm00001d003683	Zm00001d003683_T001	0.00	490.97	coding	389	133	1299
PB.10904.1	B73V4 ctg3	1871	1	full-splice_match	gene: Zm00001d000434	Zm00001d000434_T001	8.75	8.75	coding	573	25	1743
PB.440.2	1	2889	15	full-splice_match	gene: Zm00001d029142	Zm00001d029142_T007	13.93	29.55	coding	872	125	2740
PB.5901.2	4	1867	15	full-splice_match	gene: Zm00001d054105	Zm00001d054105_T007	20.32	20.32	coding	480	145	1584
PB.440.1	1	1480	4	incomplete-splice_match	gene: Zm00001d029142	Zm00001d029142_T013	15.63	29.55	coding	302	67	972
PB.6343.1	5	1388	2	full-splice_match	gene: Zm00001d014617	Zm00001d014617_T001	22.69	22.69	coding	298	131	1024
PB.9746.3	8	2220	6	novel_in_catalog	gene: Zm00001d011610	novel	29.43	133.97	coding	398	1	1194
PB.1423.2	1	2080	11	full-splice_match	gene: Zm00001d034152	Zm00001d034152_T002	7.69	35.02	coding	582	10	1755
PB.10317.3	9	1822	3	genic	gene: Zm00001d046332	novel	1.47	23.89	coding	264	699	1490
PB.10723.2	9	1798	11	full-splice_match	gene: Zm00001d048174	Zm00001d048174_T001	22.00	46.35	coding	458	75	1448
PB.8136.1	6	3030	7	full-splice_match	gene: Zm00001d039196	Zm00001d039196_T001	11.11	11.11	coding	862	157	2742
PB.1652.1	10	1253	10	novel_not_in_catalog	gene: Zm00001d023237	novel	21.87	21.87	coding	318	59	1012
PB.7143.3	5	1476	1	full-splice_match	gene: Zm00001d018407	Zm00001d018407_T001	0.00	9.95	coding	403	53	1261
PB.10525.3	9	1260	6	full-splice_match	gene: Zm00001d047479	Zm00001d047479_T016	6.93	1033.31	non_coding	NA	NA	NA
PB.3572.4	2	2865	9	novel_in_catalog	gene: Zm00001d007709	novel	0.00	40.73	coding	706	653	2770
PB.10525.1	9	956	8	full-splice_match	gene: Zm00001d047479	Zm00001d047479_T003	305.41	1033.31	coding	152	132	587
PB.3348.2	2	2170	7	full-splice_match	gene: Zm00001d006536	Zm00001d006536_T001	17.97	22.28	coding	657	9	1979

PB.5834.1	4	1525	6	full-splice_match	gene: Zm00001d053888	Zm00001d053888_T003	41.51	41.51	coding	10	485	514
PB.10525.5	9	936	7	full-splice_match	gene: Zm00001d047479	Zm00001d047479_T008	41.62	1033.31	non_coding	NA	NA	NA
PB.10525.4	9	857	8	full-splice_match	gene: Zm00001d047479	Zm00001d047479_T003	679.34	1033.31	coding	152	136	591
PB.3951.5	3	2409	7	novel_not_in_catalog	gene: Zm00001d040784	novel	1.13	21.69	coding	461	856	2238
PB.3951.4	3	3285	6	novel_in_catalog	gene: Zm00001d040784	novel	0.00	21.69	coding	576	84	1811
PB.5503.2	4	1664	1	full-splice_match	gene: Zm00001d052387	Zm00001d052387_T001	21.71	23.23	coding	501	50	1552
PB.10376.2	9	1697	5	full-splice_match	gene: Zm00001d046660	Zm00001d046660_T001	1.26	12.58	coding	421	229	1491
PB.3951.1	3	2614	6	full-splice_match	gene: Zm00001d040784	Zm00001d040784_T013	8.67	21.69	coding	500	906	2405
PB.3951.3	3	2608	7	full-splice_match	gene: Zm00001d040784	Zm00001d040784_T004	7.87	21.69	coding	732	226	2421
PB.3951.2	3	3409	5	novel_in_catalog	gene: Zm00001d040784	novel	4.03	21.69	coding	344	887	1918
PB.9383.4	8	1724	3	full-splice_match	gene: Zm00001d009858	Zm00001d009858_T002	384.16	534.95	coding	501	63	1565
PB.208.1	1	2284	14	full-splice_match	gene: Zm00001d028183	Zm00001d028183_T002	61.97	61.97	coding	558	248	1921
PB.208.2	1	2175	14	full-splice_match	gene: Zm00001d028183	Zm00001d028183_T002	0.00	61.97	coding	558	259	1932
PB.2779.1	2	1430	4	novel_in_catalog	gene: Zm00001d003322	novel	10.14	10.14	coding	227	308	988
PB.9383.1	8	1844	3	full-splice_match	gene: Zm00001d009858	Zm00001d009858_T002	121.88	534.95	coding	501	63	1565
PB.4717.1	3	2400	15	incomplete-splice_match	gene: Zm00001d044432	Zm00001d044432_T020	35.01	35.01	coding	702	2	2107
PB.256.1	1	2420	12	full-splice_match	gene: Zm00001d028363	Zm00001d028363_T001	7.87	25.19	coding	611	255	2087

PB.2392.2	2	2192	1	full-splice_match	gene: Zm00001d001781	Zm00001d001781_T001	5.03	9.56	coding	380	957	2096
PB.2392.1	2	1660	1	full-splice_match	gene: Zm00001d001781	Zm00001d001781_T001	4.52	9.56	coding	380	221	1360
PB.256.2	1	2344	12	full-splice_match	gene: Zm00001d028363	Zm00001d028363_T001	17.33	25.19	coding	611	212	2044
PB.2842.1	2	1496	9	full-splice_match	gene: Zm00001d003659	Zm00001d003659_T002	88.87	88.87	coding	356	196	1263
PB.1894.1	10	835	2	genic	gene: Zm00001d024573	novel	57.08	77.24	coding	146	128	565
PB.7372.2	6	2994	6	novel_in_catalog	gene: Zm00001d035876	novel	0.67	13.28	coding	620	903	2762
PB.925.5	1	1921	2	novel_in_catalog	gene: Zm00001d031926	novel	0.00	54.93	coding	495	230	1714
PB.309.2	1	1817	4	full-splice_match	gene: Zm00001d028572	Zm00001d028572_T002	20.09	261.42	coding	361	436	1518
PB.309.3	1	2735	3	full-splice_match	gene: Zm00001d028572	Zm00001d028572_T001	6.75	261.42	coding	361	1385	2467
PB.4738.1	3	1088	10	full-splice_match	gene: Zm00001d044497	Zm00001d044497_T004	23.66	44.09	coding	204	276	887
PB.309.5	1	1695	4	full-splice_match	gene: Zm00001d028572	Zm00001d028572_T004	73.95	261.42	coding	361	342	1424
PB.925.2	1	2534	1	genic	gene: Zm00001d031926	novel	4.44	54.93	coding	430	230	1519
PB.2436.1	2	600	3	full-splice_match	gene: Zm00001d001940	Zm00001d001940_T001	44.01	44.01	non_coding	NA	NA	NA
PB.5218.1	4	1236	6	full-splice_match	gene: Zm00001d051016	Zm00001d051016_T002	27.45	27.97	coding	242	287	1012
PB.8700.1	7	810	1	full-splice_match	gene: Zm00001d021435	Zm00001d021435_T001	19.89	19.89	coding	160	116	595
PB.5218.2	4	2043	5	full-splice_match	gene: Zm00001d051016	Zm00001d051016_T001	0.52	27.97	coding	242	1097	1822
PB.9263.1	8	2266	1	intergenic	novelGene_101	novel	0.21	0.21	non_coding	NA	NA	NA

PB.5246.1	4	1058	2	full-splice_match	gene: Zm00001d051223	Zm00001d051223_T001	24.10	24.10	coding	229	97	783
PB.8139.1	6	1574	1	full-splice_match	gene: Zm00001d039216	Zm00001d039216_T001	80.18	80.18	coding	391	178	1350
PB.7545.2	6	1683	8	novel_in_catalog	gene: Zm00001d036830	novel	4.14	14.28	coding	318	514	1467
PB.8593.3	7	1382	4	novel_in_catalog	gene: Zm00001d020961	novel	105.79	105.79	coding	245	584	1318
PB.864.2	1	1731	1	antisense	novelGene_gene: Zm00001d031601_A	novel	0.33	1.87	coding	172	309	824
PB.7545.1	6	1637	8	novel_in_catalog	gene: Zm00001d036830	novel	10.14	14.28	coding	317	399	1349
PB.8363.1	7	1507	9	full-splice_match	gene: Zm00001d019721	Zm00001d019721_T002	26.93	26.93	coding	412	26	1261
PB.2519.1	2	1643	1	antisense	novelGene_gene: Zm00001d002287_A	novel	3.80	3.80	coding	271	539	1351
PB.8112.4	6	2268	10	novel_not_in_catalog	gene: Zm00001d039141	novel	0.00	29.94	coding	601	220	2022
PB.8112.2	6	2310	10	novel_not_in_catalog	gene: Zm00001d039141	novel	0.00	29.94	coding	601	141	1943
PB.8112.1	6	2443	10	novel_not_in_catalog	gene: Zm00001d039141	novel	29.94	29.94	coding	601	218	2020
PB.9540.1	8	963	1	full-splice_match	gene: Zm00001d010651	Zm00001d010651_T001	15.94	15.94	coding	165	300	794
PB.1649.8	10	1062	4	incomplete-splice_match	gene: Zm00001d023225	Zm00001d023225_T001	40.03	98.20	coding	299	3	899
PB.10299.1	9	1920	6	full-splice_match	gene: Zm00001d046190	Zm00001d046190_T003	44.30	44.30	coding	401	341	1543
PB.3131.2	2	1760	2	incomplete-splice_match	gene: Zm00001d005507	Zm00001d005507_T019	18.93	29.78	coding	444	73	1404
PB.8061.1	6	2619	9	full-splice_match	gene: Zm00001d038922	Zm00001d038922_T001	20.04	20.04	coding	795	117	2501
PB.7646.1	6	3537	32	full-splice_match	gene: Zm00001d037333	Zm00001d037333_T033	45.59	45.59	coding	1043	165	3293

PB.1616.2	1	1706	12	full-splice_match	gene: Zm00001d034856	Zm00001d034856_T003	63.11	128.98	coding	452	80	1435
PB.10672.1	9	2286	7	full-splice_match	gene: Zm00001d047977	Zm00001d047977_T001	177.08	204.44	coding	593	171	1949
PB.10672.2	9	2244	7	full-splice_match	gene: Zm00001d047977	Zm00001d047977_T004	20.54	204.44	coding	593	245	2023
PB.10672.3	9	1378	1	novel_in_catalog	gene: Zm00001d047977	novel	6.82	204.44	coding	73	277	495
PB.1310.1	1	1858	11	full-splice_match	gene: Zm00001d033650	Zm00001d033650_T001	12.17	12.17	coding	506	191	1708
PB.1084.1	1	1300	5	full-splice_match	gene: Zm00001d032570	Zm00001d032570_T001	73.67	138.86	coding	317	57	1007
PB.232.9	1	1538	10	novel_in_catalog	gene: Zm00001d028260	novel	0.00	1186.23	coding	164	803	1294
PB.7614.6	6	3565	2	incomplete-splice_match	gene: Zm00001d037214	Zm00001d037214_T009	0.00	0.00	coding	988	602	3565
PB.8565.1	7	1408	3	novel_in_catalog	gene: Zm00001d020853	novel	5.47	45.37	coding	138	138	551
PB.232.1	1	1512	11	novel_in_catalog	gene: Zm00001d028260	novel	837.14	1186.23	coding	357	83	1153
PB.3645.1	3	1391	1	novel_in_catalog	gene: Zm00001d039276	novel	3.65	10.38	coding	256	366	1133
PB.232.6	1	1545	10	novel_in_catalog	gene: Zm00001d028260	novel	18.39	1186.23	coding	267	496	1296
PB.232.5	1	1634	9	novel_in_catalog	gene: Zm00001d028260	novel	22.40	1186.23	coding	196	795	1382
PB.3645.2	3	1352	2	novel_in_catalog	gene: Zm00001d039276	novel	6.74	10.38	coding	300	200	1099
PB.2903.7	2	1993	3	full-splice_match	gene: Zm00001d003950	Zm00001d003950_T001	31.88	58.16	coding	571	108	1820
PB.2903.6	2	2703	2	novel_in_catalog	gene: Zm00001d003950	novel	8.48	58.16	coding	481	1086	2528
PB.2903.4	2	2563	3	full-splice_match	gene: Zm00001d003950	Zm00001d003950_T001	13.95	58.16	coding	481	153	1595

PB.4073.5	3	746	3	full-splice_match	gene: Zm00001d041649	Zm00001d041649_T003	45.01	45.01	coding	126	104	481
PB.2903.2	2	3535	2	novel_in_catalog	gene: Zm00001d003950	novel	0.00	58.16	coding	481	1057	2499
PB.2903.1	2	2890	3	full-splice_match	gene: Zm00001d003950	Zm00001d003950_T001	3.85	58.16	coding	571	106	1818
PB.10471.4	9	1312	5	full-splice_match	gene: Zm00001d047217	Zm00001d047217_T005	0.00	192.52	coding	198	625	1218
PB.6935.3	5	1805	6	full-splice_match	gene: Zm00001d017642	Zm00001d017642_T002	0.00	8.13	coding	441	203	1525
PB.10285.2	9	1337	8	full-splice_match	gene: Zm00001d046060	Zm00001d046060_T001	15.12	41.07	coding	322	137	1102
PB.3744.2	3	3661	2	genic	gene: Zm00001d039636	novel	13.55	13.55	coding	1097	215	3505
PB.9202.4	8	1591	1	full-splice_match	gene: Zm00001d008850	Zm00001d008850_T001	17.98	78.35	coding	282	285	1130
PB.9202.5	8	1020	1	full-splice_match	gene: Zm00001d008850	Zm00001d008850_T001	0.00	78.35	non_coding	NA	NA	NA
PB.9202.6	8	1184	1	full-splice_match	gene: Zm00001d008850	Zm00001d008850_T001	0.00	78.35	coding	282	132	977
PB.7614.1	6	4735	5	fusion	gene: Zm00001d037213_g	novel	22.30	26.15	coding	1444	81	4412
PB.9202.3	8	1718	1	full-splice_match	gene: Zm00001d008850	Zm00001d008850_T001	60.36	78.35	coding	282	302	1147
PB.2026.1	10	2657	16	fusion	gene: Zm00001d025175_g	novel	15.71	15.71	coding	730	35	2224
PB.10172.1	9	2235	6	full-splice_match	gene: Zm00001d045417	Zm00001d045417_T001	33.75	33.75	coding	615	123	1967
PB.5222.1	4	1753	12	full-splice_match	gene: Zm00001d051043	Zm00001d051043_T001	42.24	42.24	coding	483	90	1538
PB.10820.2	9	596	3	novel_in_catalog	gene: Zm00001d048515	novel	3.11	14.09	coding	178	21	554
PB.2154.1	10	373	3	incomplete-splice_match	gene: Zm00001d025857	Zm00001d025857_T001	534.61	534.61	non_coding	NA	NA	NA

PB.3439.1	2	672	6	full-splice_match	gene: Zm00001d006942	Zm00001d006942_T001	71.82	71.82	coding	130	87	476
PB.10536.1	9	646	1	incomplete-splice_match	gene: Zm00001d047519	Zm00001d047519_T001	36.91	36.91	non_coding	NA	NA	NA
PB.3554.1	2	2623	5	full-splice_match	gene: Zm00001d007599	Zm00001d007599_T005	14.73	14.73	coding	256	1642	2409
PB.48.1	1	1643	2	novel_in_catalog	gene: Zm00001d027411	novel	28.77	155.62	coding	274	554	1375
PB.10668.1	9	2626	14	full-splice_match	gene: Zm00001d047960	Zm00001d047960_T002	27.49	28.69	coding	737	201	2411
PB.48.3	1	1379	3	full-splice_match	gene: Zm00001d027411	Zm00001d027411_T001	126.85	155.62	coding	329	188	1174
PB.2026.2	10	2502	16	fusion	gene: Zm00001d025175_g	novel	0.00	15.71	coding	730	14	2203
PB.5517.3	4	1180	11	incomplete-splice_match	gene: Zm00001d052416	Zm00001d052416_T003	0.00	38.13	coding	300	279	1178
PB.8752.1	7	2596	8	incomplete-splice_match	gene: Zm00001d021624	Zm00001d021624_T024	12.48	15.36	coding	704	183	2294
PB.4408.2	3	448	2	incomplete-splice_match	gene: Zm00001d043188	Zm00001d043188_T001	0.00	19.84	non_coding	NA	NA	NA
PB.9284.2	8	2102	9	novel_not_in_catalog	gene: Zm00001d009330	novel	7.67	30.17	coding	571	135	1847
PB.9284.3	8	2202	8	novel_not_in_catalog	gene: Zm00001d009330	novel	22.50	30.17	coding	571	197	1909
PB.6953.1	5	861	3	full-splice_match	gene: Zm00001d017708	Zm00001d017708_T001	153.09	163.14	coding	157	104	574
PB.7221.7	6	2182	2	novel_in_catalog	gene: Zm00001d035037	novel	0.00	643.97	coding	256	1183	1950
PB.7221.6	6	1454	3	full-splice_match	gene: Zm00001d035037	Zm00001d035037_T001	0.00	643.97	coding	335	119	1123
PB.7221.4	6	1569	2	novel_in_catalog	gene: Zm00001d035037	novel	0.00	643.97	coding	305	119	1033
PB.7221.3	6	2380	2	novel_in_catalog	gene: Zm00001d035037	novel	0.00	643.97	coding	256	1186	1953

PB.7221.2	6	1552	3	full-splice_match	gene: Zm00001d035037	Zm00001d035037_T001	630.11	643.97	coding	335	117	1121
PB.7221.1	6	2470	1	full-splice_match	gene: Zm00001d035038	Zm00001d035038_T005	24.58	24.58	coding	226	1180	1857
PB.1629.1	1	780	5	full-splice_match	gene: Zm00001d034893	Zm00001d034893_T001	134.86	134.86	coding	147	78	518
PB.5517.2	4	1882	15	full-splice_match	gene: Zm00001d052416	Zm00001d052416_T003	26.75	38.13	coding	440	289	1608
PB.2585.1	2	2330	8	full-splice_match	gene: Zm00001d002580	Zm00001d002580_T004	27.99	31.64	coding	531	179	1771
PB.7221.8	6	1418	3	full-splice_match	gene: Zm00001d035037	Zm00001d035037_T001	13.86	643.97	coding	335	196	1200
PB.8689.2	7	2431	7	full-splice_match	gene: Zm00001d021392	Zm00001d021392_T002	159.26	174.94	coding	639	166	2082
PB.2913.1	2	1033	6	full-splice_match	gene: Zm00001d004023	Zm00001d004023_T001	87.53	92.31	coding	213	6	644
PB.2913.2	2	888	6	full-splice_match	gene: Zm00001d004023	Zm00001d004023_T001	0.00	92.31	coding	114	281	622
PB.2913.3	2	2881	3	novel_in_catalog	gene: Zm00001d004023	novel	4.78	92.31	coding	126	760	1137
PB.3659.2	3	1720	9	full-splice_match	gene: Zm00001d039319	Zm00001d039319_T006	0.00	17.83	coding	419	34	1290
PB.3659.1	3	2114	9	full-splice_match	gene: Zm00001d039319	Zm00001d039319_T006	17.83	17.83	coding	446	154	1491
PB.6712.2	5	3481	16	full-splice_match	gene: Zm00001d016640	Zm00001d016640_T021	0.00	34.20	coding	681	901	2943
PB.4401.2	3	1748	4	full-splice_match	gene: Zm00001d043158	Zm00001d043158_T002	332.87	345.56	coding	446	85	1422
PB.4401.3	3	1758	4	full-splice_match	gene: Zm00001d043158	Zm00001d043158_T002	11.86	345.56	coding	496	50	1537
PB.850.1	1	2131	5	full-splice_match	gene: Zm00001d031531	Zm00001d031531_T002	6.21	7.59	coding	446	512	1849
PB.2149.1	10	1463	4	incomplete-splice_match	gene: Zm00001d025830	Zm00001d025830_T009	18.97	36.86	coding	275	4	828

PB.850.2	1	1131	2	novel_in_catalog	gene: Zm00001d031531	novel	1.38	7.59	non_coding	NA	NA	NA
PB.10059.2	9	831	6	full-splice_match	gene: Zm00001d044869	Zm00001d044869_T002	95.75	200.62	coding	199	79	675
PB.10059.1	9	956	6	full-splice_match	gene: Zm00001d044869	Zm00001d044869_T002	104.87	200.62	coding	199	75	671
PB.575.1	1	1011	3	full-splice_match	gene: Zm00001d029801	Zm00001d029801_T001	61.67	61.67	coding	246	113	850
PB.770.3	1	1339	7	intergenic	novelGene_292	novel	2.35	8.37	non_coding	NA	NA	NA
PB.2242.1	10	1972	3	full-splice_match	gene: Zm00001d026267	Zm00001d026267_T002	109.61	109.61	coding	414	393	1634
PB.782.2	1	631	3	full-splice_match	gene: Zm00001d031132	Zm00001d031132_T002	376.71	1446.26	coding	84	105	356
PB.1969.4	10	1892	6	full-splice_match	gene: Zm00001d024927	Zm00001d024927_T002	0.00	33.42	coding	517	240	1790
PB.5671.1	4	1480	8	full-splice_match	gene: Zm00001d053104	Zm00001d053104_T004	17.41	19.94	coding	266	168	965
PB.1969.2	10	2339	3	novel_in_catalog	gene: Zm00001d024927	novel	4.04	33.42	coding	370	241	1350
PB.1969.3	10	2074	5	novel_in_catalog	gene: Zm00001d024927	novel	2.03	33.42	coding	399	242	1438
PB.8589.1	7	809	2	full-splice_match	gene: Zm00001d020950	Zm00001d020950_T001	45.59	45.59	coding	117	221	571
PB.1969.1	10	2002	6	full-splice_match	gene: Zm00001d024927	Zm00001d024927_T002	27.35	33.42	coding	517	241	1791
PB.7229.2	6	834	4	novel_not_in_catalog	gene: Zm00001d035054	novel	163.89	253.90	coding	141	173	595
PB.7229.1	6	882	4	novel_not_in_catalog	gene: Zm00001d035054	novel	90.01	253.90	coding	141	116	538
PB.194.1	1	1911	3	full-splice_match	gene: Zm00001d028111	Zm00001d028111_T004	19.21	19.21	coding	467	280	1680
PB.4096.4	3	1034	5	full-splice_match	gene: Zm00001d041767	Zm00001d041767_T001	17.26	71.31	coding	257	124	894

PB.3496.2	2	1632	1	incomplete-splice_match	gene: Zm00001d007241	Zm00001d007241_T001	13.66	22.24	coding	531	39	1631
PB.4096.2	3	1149	5	full-splice_match	gene: Zm00001d041767	Zm00001d041767_T001	52.35	71.31	coding	257	121	891
PB.4096.3	3	2546	4	full-splice_match	gene: Zm00001d041767	Zm00001d041767_T003	1.70	71.31	coding	133	124	522
PB.3236.1	2	2476	6	novel_in_catalog	gene: Zm00001d006061	novel	12.17	12.17	non_coding	NA	NA	NA
PB.7931.1	6	2846	7	full-splice_match	gene: Zm00001d038432	Zm00001d038432_T004	11.29	22.10	coding	234	1827	2528
PB.2971.6	2	2015	8	full-splice_match	gene: Zm00001d004552	Zm00001d004552_T015	0.00	108.54	coding	107	1093	1413
PB.7235.1	6	1731	11	full-splice_match	gene: Zm00001d035090	Zm00001d035090_T001	6.47	17.55	coding	394	173	1354
PB.7235.2	6	1483	11	full-splice_match	gene: Zm00001d035090	Zm00001d035090_T001	11.08	17.55	coding	394	32	1213
PB.1544.1	1	716	4	full-splice_match	gene: Zm00001d034628	Zm00001d034628_T001	420.65	420.65	coding	142	143	568
PB.7066.2	5	656	3	novel_in_catalog	gene: Zm00001d018135	novel	13.31	120.20	coding	144	39	470
PB.1113.1	1	670	4	full-splice_match	gene: Zm00001d032687	Zm00001d032687_T001	65.06	65.06	coding	116	91	438
PB.7066.1	5	856	4	full-splice_match	gene: Zm00001d018135	Zm00001d018135_T001	106.90	120.20	coding	162	79	564
PB.1975.1	10	1802	2	full-splice_match	gene: Zm00001d024943	Zm00001d024943_T001	46.14	46.14	coding	532	39	1634
PB.1975.2	10	1683	2	full-splice_match	gene: Zm00001d024943	Zm00001d024943_T001	0.00	46.14	coding	532	9	1604
PB.6016.4	5	1598	4	novel_in_catalog	gene: Zm00001d013202	novel	7.14	25.56	coding	132	152	547
PB.6448.1	5	598	1	genic	gene: Zm00001d014989	novel	18.02	18.02	coding	56	189	356
PB.9121.1	8	1042	1	full-splice_match	gene: Zm00001d008452	Zm00001d008452_T001	147.66	147.66	coding	165	175	669

PB.8673.1	7	1085	7	full-splice_match	gene: Zm00001d021335	Zm00001d021335_T003	26.01	26.01	coding	202	256	861
PB.6016.2	5	1689	4	novel_in_catalog	gene: Zm00001d013202	novel	9.96	25.56	coding	299	472	1368
PB.6016.3	5	1088	4	incomplete-splice_match	gene: Zm00001d013202	Zm00001d013202_T001	8.47	25.56	coding	264	81	872
PB.10732.1	9	647	1	full-splice_match	gene: Zm00001d048208	Zm00001d048208_T001	20.55	20.55	coding	35	256	360
PB.6919.4	5	3539	12	full-splice_match	gene: Zm00001d017525	Zm00001d017525_T005	0.00	9.34	coding	893	717	3395
PB.6919.2	5	3929	13	novel_in_catalog	gene: Zm00001d017525	novel	9.34	9.34	coding	893	693	3371
PB.6919.3	5	3734	13	novel_in_catalog	gene: Zm00001d017525	novel	0.00	9.34	coding	893	680	3358
PB.1239.1	1	2102	3	genic	gene: Zm00001d033335	novel	4.09	8.89	coding	281	201	1043
PB.4528.4	3	3838	6	full-splice_match	gene: Zm00001d043649	Zm00001d043649_T034	12.45	96.43	coding	125	1671	2045
PB.2438.1	2	393	1	incomplete-splice_match	gene: Zm00001d001949	Zm00001d001949_T001	24.90	24.90	non_coding	NA	NA	NA
PB.7918.2	6	849	2	full-splice_match	gene: Zm00001d038381	Zm00001d038381_T001	0.00	822.27	coding	160	77	556
PB.7426.1	6	1856	4	full-splice_match	gene: Zm00001d036178	Zm00001d036178_T001	8.01	17.99	coding	439	416	1732
PB.5919.2	5	2887	3	full-splice_match	gene: Zm00001d012850	Zm00001d012850_T001	3.22	36.94	coding	366	115	1212
PB.7577.1	6	485	1	full-splice_match	gene: Zm00001d037033	Zm00001d037033_T001	9.53	9.53	non_coding	NA	NA	NA
PB.8170.3	7	1999	9	full-splice_match	gene: Zm00001d018694	Zm00001d018694_T001	8.66	86.25	coding	535	121	1725
PB.8561.3	7	2831	3	genic	gene: Zm00001d020828	novel	1.72	19.21	coding	647	449	2389
PB.8561.5	7	2878	3	genic	gene: Zm00001d020828	novel	1.26	19.21	coding	647	506	2446

PB.8561.7	7	2567	3	genic	gene: Zm00001d020828	novel	0.00	19.21	coding	647	447	2387
PB.8561.6	7	2718	3	genic	gene: Zm00001d020828	novel	0.00	19.21	coding	647	447	2387
PB.9302.1	8	2012	11	full-splice_match	gene: Zm00001d009431	Zm00001d009431_T002	113.42	113.42	coding	305	873	1787
PB.7568.1	6	1112	3	full-splice_match	gene: Zm00001d036978	Zm00001d036978_T001	117.64	117.64	coding	275	28	852
PB.6231.1	5	1194	4	full-splice_match	gene: Zm00001d014091	Zm00001d014091_T001	14.84	14.84	coding	243	51	779
PB.9429.1	8	480	4	full-splice_match	gene: Zm00001d010120	Zm00001d010120_T007	125.64	125.64	non_coding	NA	NA	NA
PB.10824.2	9	2013	12	novel_not_in_catalog	gene: Zm00001d048529	novel	44.71	137.46	coding	399	214	1410
PB.8420.1	7	2422	7	novel_not_in_catalog	gene: Zm00001d020158	novel	13.12	13.12	coding	631	219	2111
PB.10031.1	8	1630	1	full-splice_match	gene: Zm00001d012782	Zm00001d012782_T001	7.91	7.91	coding	474	43	1464
PB.8932.3	7	1550	2	novel_not_in_catalog	gene: Zm00001d022244	novel	5.92	104.80	coding	126	1051	1428
PB.7939.1	6	1482	8	full-splice_match	gene: Zm00001d038460	Zm00001d038460_T009	77.43	77.43	coding	329	100	1086
PB.7819.1	6	685	4	full-splice_match	gene: Zm00001d038056	Zm00001d038056_T001	84.45	98.06	coding	145	82	516
PB.7819.2	6	715	3	novel_in_catalog	gene: Zm00001d038056	novel	13.62	98.06	non_coding	NA	NA	NA
PB.6181.4	5	1938	8	full-splice_match	gene: Zm00001d013831	Zm00001d013831_T018	0.00	56.47	coding	179	378	914
PB.4045.1	3	2709	1	full-splice_match	gene: Zm00001d041536	Zm00001d041536_T001	27.41	27.41	coding	710	178	2307
PB.9647.12	8	3026	9	full-splice_match	gene: Zm00001d011119	Zm00001d011119_T013	1.57	60.56	coding	306	82	999
PB.5823.1	4	609	1	full-splice_match	gene: Zm00001d053843	Zm00001d053843_T001	102.99	102.99	coding	98	90	383

PB.7464.1	6	1428	5	novel_in_catalog	gene: Zm00001d036432	novel	95.17	95.17	coding	353	13	1071
PB.41.1	1	2302	8	incomplete-splice_match	gene: Zm00001d027376	Zm00001d027376_T008	3.43	34.08	coding	490	316	1785
PB.389.1	1	1994	15	full-splice_match	gene: Zm00001d028949	Zm00001d028949_T002	67.12	68.82	coding	449	198	1544
PB.389.2	1	2769	14	novel_in_catalog	gene: Zm00001d028949	novel	1.70	68.82	coding	387	1207	2367
PB.10586.1	9	2823	4	full-splice_match	gene: Zm00001d047713	Zm00001d047713_T004	3.67	97.32	coding	144	2116	2547
PB.4520.8	3	3313	11	full-splice_match	gene: Zm00001d043613	Zm00001d043613_T002	0.00	284.34	coding	300	1948	2847
PB.4428.3	3	2880	25	incomplete-splice_match	gene: Zm00001d043274	Zm00001d043274_T019	14.08	14.08	coding	759	482	2758
PB.375.1	1	2103	1	full-splice_match	gene: Zm00001d028888	Zm00001d028888_T001	262.10	262.10	coding	601	133	1935
PB.4520.5	3	1643	12	full-splice_match	gene: Zm00001d043613	Zm00001d043613_T006	184.75	284.34	coding	300	190	1089
PB.9795.3	8	521	1	novel_in_catalog	gene: Zm00001d011789	novel	336.17	421.90	non_coding	NA	NA	NA
PB.9795.2	8	616	2	novel_not_in_catalog	gene: Zm00001d011789	novel	42.51	421.90	non_coding	NA	NA	NA
PB.9795.4	8	429	2	novel_not_in_catalog	gene: Zm00001d011789	novel	10.75	421.90	non_coding	NA	NA	NA
PB.4520.2	3	3447	12	full-splice_match	gene: Zm00001d043613	Zm00001d043613_T005	35.22	284.34	coding	300	1863	2762
PB.4520.3	3	3402	11	full-splice_match	gene: Zm00001d043613	Zm00001d043613_T002	46.91	284.34	coding	300	1943	2842
PB.3982.1	3	1193	6	full-splice_match	gene: Zm00001d041112	Zm00001d041112_T002	50.15	131.62	coding	241	48	770
PB.10024.3	8	1724	6	full-splice_match	gene: Zm00001d012750	Zm00001d012750_T002	0.00	113.43	coding	558	29	1702
PB.8436.6	7	1875	2	full-splice_match	gene: Zm00001d020260	Zm00001d020260_T001	0.00	36.60	coding	569	130	1836

PB.3982.2	3	1076	6	full-splice_match	gene: Zm00001d041112	Zm00001d041112_T002	81.46	131.62	coding	241	93	815
PB.8436.1	7	2123	2	full-splice_match	gene: Zm00001d020260	Zm00001d020260_T001	1.91	36.60	coding	569	108	1814
PB.8436.2	7	2004	2	full-splice_match	gene: Zm00001d020260	Zm00001d020260_T001	33.66	36.60	coding	569	130	1836
PB.8436.3	7	3428	1	genic	gene: Zm00001d020260	novel	1.04	36.60	coding	316	2324	3271
PB.6264.1	5	2488	12	full-splice_match	gene: Zm00001d014280	Zm00001d014280_T001	42.18	42.18	coding	701	109	2211
PB.1056.1	1	2685	2	novel_in_catalog	gene: Zm00001d032485	novel	1.91	130.84	coding	143	1006	1434
PB.8247.2	7	1377	1	full-splice_match	gene: Zm00001d019043	Zm00001d019043_T001	0.00	15.95	coding	345	204	1238
PB.9421.1	8	2162	4	novel_not_in_catalog	gene: Zm00001d010060	novel	19.44	19.44	coding	549	199	1845
PB.8247.1	7	1776	1	full-splice_match	gene: Zm00001d019043	Zm00001d019043_T001	15.95	15.95	coding	448	231	1574
PB.1269.5	1	510	2	genic	gene: Zm00001d033483	novel	0.00	876.45	coding	100	95	394
PB.395.1	1	923	1	full-splice_match	gene: Zm00001d028968	Zm00001d028968_T001	27.16	27.16	coding	165	137	631
PB.1625.2	1	3024	1	full-splice_match	gene: Zm00001d034879	Zm00001d034879_T001	4.16	12.47	coding	762	414	2699
PB.6949.4	5	1296	5	novel_not_in_catalog	gene: Zm00001d017691	novel	6.21	29.32	coding	243	347	1075
PB.3210.3	2	677	1	full-splice_match	gene: Zm00001d005945	Zm00001d005945_T001	953.74	1458.67	coding	146	71	508
PB.5145.1	4	1219	1	full-splice_match	gene: Zm00001d050433	Zm00001d050433_T001	17.57	17.57	coding	280	295	1134
PB.3210.1	2	497	1	full-splice_match	gene: Zm00001d005945	Zm00001d005945_T001	504.93	1458.67	non_coding	NA	NA	NA
PB.8144.2	7	2033	1	novel_in_catalog	gene: Zm00001d018601	novel	10.60	35.65	coding	571	193	1905

PB.8144.3	7	1864	2	novel_not_in_catalog	gene: Zm00001d018601	novel	21.66	35.65	coding	585	1	1755
PB.5577.1	4	516	3	full-splice_match	gene: Zm00001d052718	Zm00001d052718_T001	1173.03	1173.03	non_coding	NA	NA	NA
PB.8144.1	7	2032	1	genic	gene: Zm00001d018601	novel	3.38	35.65	coding	479	48	1484
PB.4516.3	3	2450	19	full-splice_match	gene: Zm00001d043601	Zm00001d043601_T002	16.69	194.87	coding	593	111	1889
PB.8575.1	7	1956	6	full-splice_match	gene: Zm00001d020901	Zm00001d020901_T001	16.66	16.66	coding	535	243	1847
PB.4516.6	3	2152	18	novel_in_catalog	gene: Zm00001d043601	novel	0.38	194.87	coding	445	113	1447
PB.4516.5	3	2157	19	full-splice_match	gene: Zm00001d043601	Zm00001d043601_T002	122.02	194.87	coding	593	197	1975
PB.4516.4	3	2201	19	full-splice_match	gene: Zm00001d043601	Zm00001d043601_T002	55.79	194.87	coding	593	200	1978
PB.7662.2	6	1367	5	full-splice_match	gene: Zm00001d037412	Zm00001d037412_T001	37.93	37.93	coding	275	194	1018
PB.4130.1	3	4644	17	novel_in_catalog	gene: Zm00001d041953	novel	8.26	8.26	coding	798	58	2451
PB.8097.2	6	3939	9	full-splice_match	gene: Zm00001d039072	Zm00001d039072_T025	1.90	11.70	coding	1120	33	3392
PB.878.2	1	2127	5	full-splice_match	gene: Zm00001d031657	Zm00001d031657_T001	0.00	53.36	coding	626	51	1928
PB.878.1	1	2241	5	full-splice_match	gene: Zm00001d031657	Zm00001d031657_T001	53.36	53.36	coding	626	46	1923
PB.6949.3	5	1342	5	full-splice_match	gene: Zm00001d017691	Zm00001d017691_T001	21.08	29.32	coding	254	341	1102
PB.6583.1	5	1197	9	incomplete-splice_match	gene: Zm00001d015785	Zm00001d015785_T002	21.56	21.56	coding	298	4	897
PB.749.1	1	4051	3	full-splice_match	gene: Zm00001d031005	Zm00001d031005_T024	12.28	34.82	coding	1265	64	3858
PB.7864.1	6	1346	1	full-splice_match	gene: Zm00001d038191	Zm00001d038191_T001	52.71	52.71	coding	99	149	445

PB.6949.2	5	1476	5	full-splice_match	gene: Zm00001d017691	Zm00001d017691_T001	2.03	29.32	coding	254	341	1102
PB.682.2	1	1961	12	novel_in_catalog	gene: Zm00001d030533	novel	2.88	26.79	coding	272	741	1556
PB.8928.1	7	514	3	novel_not_in_catalog	gene: Zm00001d022233	novel	62.45	62.45	coding	89	59	325
PB.682.1	1	2164	13	full-splice_match	gene: Zm00001d030533	Zm00001d030533_T003	23.91	26.79	coding	551	107	1759
PB.8916.3	7	891	2	novel_in_catalog	gene: Zm00001d022184	novel	37.59	131.46	coding	112	234	569
PB.1326.2	1	937	1	novel_in_catalog	gene: Zm00001d033711	novel	11.93	158.71	non_coding	NA	NA	NA
PB.5646.1	4	1452	2	incomplete-splice_match	gene: Zm00001d052981	Zm00001d052981_T001	23.27	23.27	coding	397	186	1376
PB.1757.1	10	1260	1	intergenic	novelGene_58	novel	81.18	81.18	coding	134	283	684
PB.8916.2	7	1271	5	full-splice_match	gene: Zm00001d022182	Zm00001d022182_T002	8.88	8.88	coding	330	56	1045
PB.6978.1	5	2230	2	full-splice_match	gene: Zm00001d017820	Zm00001d017820_T001	6.69	6.69	coding	523	295	1863
PB.4694.1	3	4087	32	incomplete-splice_match	gene: Zm00001d044303	Zm00001d044303_T008	22.83	42.18	coding	1224	24	3695
PB.2250.1	10	1977	5	full-splice_match	gene: Zm00001d026288	Zm00001d026288_T001	27.19	33.43	coding	432	235	1530
PB.2385.3	2	2215	12	novel_not_in_catalog	gene: Zm00001d001765	novel	6.14	80.07	coding	631	277	2169
PB.3341.1	2	3351	6	full-splice_match	gene: Zm00001d006504	Zm00001d006504_T001	9.96	9.96	coding	920	271	3030
PB.454.1	1	659	5	novel_not_in_catalog	gene: Zm00001d029205	novel	8.73	12.39	non_coding	NA	NA	NA
PB.6154.2	5	1614	3	novel_not_in_catalog	gene: Zm00001d013667	novel	170.09	178.26	coding	400	43	1242
PB.6154.1	5	3970	1	genic	gene: Zm00001d013667	novel	8.17	178.26	coding	247	2872	3612

PB.454.2	1	2064	3	full-splice_match	gene: Zm00001d029205	Zm00001d029205_T002	3.66	12.39	non_coding	NA	NA	NA
PB.6731.4	5	538	5	full-splice_match	gene: Zm00001d016722	Zm00001d016722_T004	166.53	331.89	coding	69	100	306
PB.8453.1	7	2149	1	antisense	novelGene_gene: Zm00001d020339_A	novel	35.17	35.17	coding	279	888	1724
PB.4619.2	3	1049	6	full-splice_match	gene: Zm00001d043991	Zm00001d043991_T001	127.10	453.38	coding	230	136	825
PB.4164.1	3	1395	4	novel_not_in_catalog	gene: Zm00001d042059	novel	37.79	97.94	coding	283	132	980
PB.4164.2	3	2149	3	full-splice_match	gene: Zm00001d042059	Zm00001d042059_T001	4.17	97.94	coding	120	1409	1768
PB.3758.1	3	1574	9	novel_in_catalog	gene: Zm00001d039732	novel	44.98	44.98	coding	335	263	1267
PB.6227.2	5	1984	3	full-splice_match	gene: Zm00001d014060	Zm00001d014060_T001	13.84	18.09	coding	610	86	1915
PB.7129.1	5	1633	2	full-splice_match	gene: Zm00001d018367	Zm00001d018367_T001	21.90	21.90	coding	442	51	1376
PB.6227.1	5	2261	2	novel_in_catalog	gene: Zm00001d014060	novel	4.24	18.09	coding	493	705	2183
PB.10614.1	9	2052	1	full-splice_match	gene: Zm00001d047804	Zm00001d047804_T001	105.55	105.55	coding	544	184	1815
PB.10000.9	8	627	1	incomplete-splice_match	gene: Zm00001d012660	Zm00001d012660_T001	0.00	59.00	coding	86	370	627
PB.9366.3	8	2189	3	full-splice_match	gene: Zm00001d009760	Zm00001d009760_T001	0.00	15.55	coding	678	71	2104
PB.9366.2	8	2635	3	full-splice_match	gene: Zm00001d009760	Zm00001d009760_T001	5.70	15.55	coding	734	212	2413
PB.9366.1	8	1482	1	novel_in_catalog	gene: Zm00001d009760	novel	9.85	15.55	coding	333	113	1111
PB.10318.1	9	2011	15	full-splice_match	gene: Zm00001d046336	Zm00001d046336_T002	282.13	635.63	coding	487	131	1591
PB.1997.1	10	971	4	full-splice_match	gene: Zm00001d025025	Zm00001d025025_T001	63.46	64.93	coding	255	45	809

PB.1108.2	1	645	1	full-splice_match	gene: Zm00001d032672	Zm00001d032672_T001	115.47	119.54	coding	166	10	507
PB.7775.1	6	1944	1	genic	gene: Zm00001d037903	novel	1.80	182.38	coding	229	1143	1829
PB.1887.1	10	1379	2	genic	gene: Zm00001d024538	novel	17.66	17.66	coding	317	40	990
PB.10318.7	9	1938	15	full-splice_match	gene: Zm00001d046336	Zm00001d046336_T002	114.70	635.63	coding	487	160	1620
PB.10318.6	9	886	6	novel_in_catalog	gene: Zm00001d046336	novel	238.80	635.63	coding	124	192	563
PB.10318.9	9	1801	15	full-splice_match	gene: Zm00001d046336	Zm00001d046336_T002	0.00	635.63	coding	487	130	1590
PB.6569.2	5	1586	1	full-splice_match	gene: Zm00001d015651	Zm00001d015651_T001	5.89	14.01	coding	490	50	1519
PB.6569.1	5	1865	1	full-splice_match	gene: Zm00001d015651	Zm00001d015651_T001	8.13	14.01	coding	490	109	1578
PB.8973.3	7	3570	24	incomplete-splice_match	gene: Zm00001d022388	Zm00001d022388_T014	17.19	93.42	coding	1057	32	3202
PB.1170.4	1	915	4	intergenic	novelGene_366	novel	19.09	72.75	coding	188	161	724
PB.5928.2	5	1858	2	full-splice_match	gene: Zm00001d012883	Zm00001d012883_T001	0.00	50.34	coding	505	106	1620
PB.5928.1	5	2102	2	full-splice_match	gene: Zm00001d012883	Zm00001d012883_T001	27.02	50.34	coding	505	110	1624
PB.7454.1	6	1363	3	full-splice_match	gene: Zm00001d036388	Zm00001d036388_T002	8.39	8.39	coding	382	110	1255
PB.4504.4	3	1526	10	full-splice_match	gene: Zm00001d043548	Zm00001d043548_T001	0.00	18.57	coding	428	56	1339
PB.1170.2	1	1038	4	intergenic	novelGene_366	novel	53.66	72.75	coding	188	155	718
PB.5928.4	5	1765	2	full-splice_match	gene: Zm00001d012883	Zm00001d012883_T001	23.32	50.34	coding	505	107	1621
PB.10219.1	9	1517	4	full-splice_match	gene: Zm00001d045579	Zm00001d045579_T001	5.54	5.54	coding	443	25	1353

PB.2367.1	10	1880	5	full-splice_match	gene: Zm00001d026652	Zm00001d026652_T001	25.01	25.01	coding	397	586	1776
PB.6643.4	5	2421	1	full-splice_match	gene: Zm00001d016225	Zm00001d016225_T001	0.00	34.07	coding	729	216	2402
PB.1420.1	1	1634	2	genic	gene: Zm00001d034131	novel	19.21	19.21	coding	306	204	1121
PB.6509.1	5	548	2	incomplete-splice_match	gene: Zm00001d015289	Zm00001d015289_T002	65.26	65.26	non_coding	NA	NA	NA
PB.6636.2	5	1424	2	novel_in_catalog	gene: Zm00001d016182	novel	13.67	20.05	coding	151	239	691
PB.381.1	1	1696	1	incomplete-splice_match	gene: Zm00001d028915	Zm00001d028915_T016	20.02	20.02	coding	501	12	1514
PB.2611.1	2	1227	4	novel_not_in_catalog	gene: Zm00001d002677	novel	53.42	53.42	coding	236	176	883
PB.6636.3	5	1258	1	novel_in_catalog	gene: Zm00001d016182	novel	1.35	20.05	coding	125	782	1156
PB.6774.2	5	1608	2	novel_not_in_catalog	gene: Zm00001d016896	novel	2.83	25.80	coding	157	1017	1487
PB.6774.1	5	1663	2	full-splice_match	gene: Zm00001d016896	Zm00001d016896_T001	22.96	25.80	coding	488	41	1504
PB.9885.5	8	2195	6	novel_not_in_catalog	gene: Zm00001d012167	novel	66.91	113.70	coding	560	327	2006
PB.6335.2	5	2639	16	full-splice_match	gene: Zm00001d014575	Zm00001d014575_T008	48.44	48.44	coding	620	478	2337
PB.1731.1	10	663	1	intergenic	novelGene_42	novel	23.10	168.94	coding	58	121	294
PB.9885.1	8	2405	6	novel_not_in_catalog	gene: Zm00001d012167	novel	8.02	113.70	coding	560	332	2011
PB.8255.1	7	2051	4	full-splice_match	gene: Zm00001d019086	Zm00001d019086_T003	30.70	30.70	coding	469	212	1618
PB.3270.1	2	767	1	full-splice_match	gene: Zm00001d006213	Zm00001d006213_T001	1447.54	1447.54	coding	157	77	547
PB.4667.1	3	2181	10	full-splice_match	gene: Zm00001d044203	Zm00001d044203_T001	31.48	33.04	coding	560	85	1764

PB.4667.2	3	2002	11	novel_not_in_catalog	gene: Zm00001d044203	novel	1.57	33.04	coding	560	35	1714
PB.5324.2	4	1392	8	novel_in_catalog	gene: Zm00001d051628	novel	10.67	32.01	coding	184	559	1110
PB.3902.5	3	1617	2	incomplete-splice_match	gene: Zm00001d040456	Zm00001d040456_T005	2.02	22.29	coding	538	3	1616
PB.3902.4	3	1153	4	incomplete-splice_match	gene: Zm00001d040456	Zm00001d040456_T003	20.27	22.29	coding	301	65	967
PB.6643.1	5	2748	1	full-splice_match	gene: Zm00001d016225	Zm00001d016225_T001	22.44	34.07	coding	729	108	2294
PB.1107.1	1	2792	20	full-splice_match	gene: Zm00001d032667	Zm00001d032667_T005	15.29	28.39	coding	698	145	2238
PB.6643.2	5	2856	1	full-splice_match	gene: Zm00001d016225	Zm00001d016225_T001	11.63	34.07	coding	729	338	2524
PB.4534.5	3	1051	10	incomplete-splice_match	gene: Zm00001d043669	Zm00001d043669_T004	1.49	102.56	coding	310	121	1050
PB.1106.3	1	1858	3	full-splice_match	gene: Zm00001d032664	Zm00001d032664_T001	11.33	51.41	coding	542	148	1773
PB.3145.2	2	2396	2	genic	gene: Zm00001d005591	novel	0.50	2.23	coding	679	226	2262
PB.8800.1	7	1371	5	full-splice_match	gene: Zm00001d021764	Zm00001d021764_T001	30.15	31.17	coding	284	133	984
PB.1178.2	1	2298	1	novel_in_catalog	gene: Zm00001d033005	novel	2.35	32.14	coding	224	152	823
PB.8667.1	7	1089	9	full-splice_match	gene: Zm00001d021310	Zm00001d021310_T001	53.71	81.16	coding	256	108	875
PB.6097.1	5	1785	6	full-splice_match	gene: Zm00001d013449	Zm00001d013449_T001	25.29	28.15	coding	516	53	1600
PB.8769.2	7	1518	1	full-splice_match	gene: Zm00001d021677	Zm00001d021677_T001	0.00	2.27	coding	444	68	1399
PB.8769.1	7	1646	1	full-splice_match	gene: Zm00001d021677	Zm00001d021677_T001	2.27	2.27	coding	444	67	1398
PB.6097.2	5	1818	5	novel_in_catalog	gene: Zm00001d013449	novel	2.85	28.15	coding	350	21	1070

PB.1493.6	1	2152	4	genic	gene: Zm00001d034434	novel	3.50	39.47	non_coding	NA	NA	NA
PB.14.19	1	2194	10	full-splice_match	gene: Zm00001d027293	Zm00001d027293_T006	5.18	309.60	coding	625	213	2087
PB.8825.1	7	3247	26	full-splice_match	gene: Zm00001d021846	Zm00001d021846_T002	51.96	51.96	coding	992	48	3023
PB.1879.1	10	2236	2	full-splice_match	gene: Zm00001d024516	Zm00001d024516_T001	9.46	39.50	coding	493	173	1651
PB.6376.1	5	1190	1	full-splice_match	gene: Zm00001d014752	Zm00001d014752_T001	24.51	26.72	coding	276	90	917
PB.1416.3	1	1736	4	full-splice_match	gene: Zm00001d034109	Zm00001d034109_T001	6.33	48.70	coding	376	286	1413
PB.6376.2	5	1131	1	full-splice_match	gene: Zm00001d014752	Zm00001d014752_T001	2.21	26.72	coding	276	130	957
PB.9074.1	8	1504	4	full-splice_match	gene: Zm00001d008250	Zm00001d008250_T001	24.46	24.46	coding	330	123	1112
PB.7068.1	5	1674	7	incomplete-splice_match	gene: Zm00001d018146	Zm00001d018146_T001	1.62	49.87	coding	330	247	1236
PB.7068.2	5	1507	7	incomplete-splice_match	gene: Zm00001d018146	Zm00001d018146_T001	35.94	49.87	coding	330	303	1292
PB.7068.3	5	1449	7	incomplete-splice_match	gene: Zm00001d018146	Zm00001d018146_T001	0.00	49.87	coding	330	300	1289
PB.3214.3	2	2651	2	genic	gene: Zm00001d005966	novel	3.45	150.85	coding	650	423	2372
PB.7439.1	6	635	1	genic	gene: Zm00001d036301	novel	9.61	163.84	non_coding	NA	NA	NA
PB.5752.1	4	2030	1	full-splice_match	gene: Zm00001d053586	Zm00001d053586_T001	6.57	13.88	coding	509	46	1572
PB.9227.1	8	1673	3	novel_in_catalog	gene: Zm00001d008957	novel	93.66	93.66	coding	470	68	1477
PB.2736.5	2	1885	3	full-splice_match	gene: Zm00001d003181	Zm00001d003181_T005	46.98	139.72	coding	415	254	1498
PB.6956.1	5	1479	4	full-splice_match	gene: Zm00001d017727	Zm00001d017727_T001	14.47	41.79	coding	347	46	1086

PB.2736.7	2	2251	3	novel_in_catalog	gene: Zm00001d003181	novel	7.07	139.72	coding	318	44	997
PB.2736.6	2	1655	4	novel_in_catalog	gene: Zm00001d003181	novel	1.63	139.72	coding	415	254	1498
PB.2736.1	2	2509	4	full-splice_match	gene: Zm00001d003181	Zm00001d003181_T002	8.49	139.72	coding	378	58	1191
PB.2736.3	2	2280	4	novel_not_in_catalog	gene: Zm00001d003181	novel	0.00	139.72	coding	415	263	1507
PB.2736.2	2	2073	5	novel_not_in_catalog	gene: Zm00001d003181	novel	0.50	139.72	coding	378	149	1282
PB.6615.1	5	2050	6	full-splice_match	gene: Zm00001d016063	Zm00001d016063_T001	31.92	44.25	coding	539	147	1763
PB.2976.3	2	1458	1	intergenic	novelGene_189	novel	1.86	1.86	non_coding	NA	NA	NA
PB.6615.2	5	1974	6	full-splice_match	gene: Zm00001d016063	Zm00001d016063_T001	12.33	44.25	coding	539	172	1788
PB.755.1	1	1491	7	full-splice_match	gene: Zm00001d031049	Zm00001d031049_T002	31.13	31.13	coding	377	194	1324
PB.2597.1	2	884	2	full-splice_match	gene: Zm00001d002601	Zm00001d002601_T001	844.57	857.67	coding	196	95	682
PB.2597.2	2	876	1	novel_in_catalog	gene: Zm00001d002601	novel	13.11	857.67	coding	155	308	772
PB.5837.1	4	1638	5	full-splice_match	gene: Zm00001d053900	Zm00001d053900_T001	23.94	23.94	coding	369	161	1267
PB.4828.1	4	1868	1	full-splice_match	gene: Zm00001d048758	Zm00001d048758_T001	1.30	1.30	coding	524	186	1757
PB.4356.4	3	2516	14	novel_in_catalog	gene: Zm00001d042961	novel	0.00	30.59	coding	785	1	2355
PB.923.12	1	1249	3	novel_in_catalog	gene: Zm00001d031909	novel	1.13	1145.30	coding	363	67	1155
PB.8666.2	7	1583	2	full-splice_match	gene: Zm00001d021305	Zm00001d021305_T001	16.84	18.68	coding	462	124	1509
PB.4356.1	3	2651	14	novel_in_catalog	gene: Zm00001d042961	novel	30.59	30.59	coding	804	47	2458

PB.2301.11	10	1817	4	novel_not_in_catalog	gene: Zm00001d026458	novel	1.54	1555.47	coding	141	1055	1477
PB.4713.1	3	2958	9	novel_in_catalog	gene: Zm00001d044405	novel	29.91	29.91	coding	802	334	2739
PB.555.1	1	2311	12	full-splice_match	gene: Zm00001d029664	Zm00001d029664_T015	2.92	45.04	coding	310	336	1265
PB.8007.2	6	1979	3	novel_in_catalog	gene: Zm00001d038753	novel	1.39	129.15	coding	105	1358	1672
PB.5411.6	4	2737	21	full-splice_match	gene: Zm00001d052050	Zm00001d052050_T002	0.00	60.81	coding	775	79	2403
PB.5411.7	4	1691	15	incomplete-splice_match	gene: Zm00001d052050	Zm00001d052050_T002	0.00	60.81	coding	537	79	1689
PB.4496.1	3	1380	7	novel_not_in_catalog	gene: Zm00001d043515	novel	25.47	78.68	coding	357	42	1112
PB.4496.6	3	1203	6	full-splice_match	gene: Zm00001d043515	Zm00001d043515_T002	0.00	78.68	coding	230	372	1061
PB.5411.3	4	2769	20	novel_in_catalog	gene: Zm00001d052050	novel	1.96	60.81	coding	753	79	2337
PB.4496.4	3	1326	7	novel_not_in_catalog	gene: Zm00001d043515	novel	0.00	78.68	coding	357	89	1159
PB.5411.1	4	2839	21	full-splice_match	gene: Zm00001d052050	Zm00001d052050_T002	58.85	60.81	coding	775	67	2391
PB.6087.2	5	1638	4	incomplete-splice_match	gene: Zm00001d013412	Zm00001d013412_T010	24.00	42.32	coding	345	47	1081
PB.3883.3	3	2820	5	full-splice_match	gene: Zm00001d040343	Zm00001d040343_T002	1.17	35.37	coding	803	10	2418
PB.3883.2	3	1475	3	full-splice_match	gene: Zm00001d040343	Zm00001d040343_T003	6.90	35.37	coding	242	12	737
PB.3883.5	3	2042	2	incomplete-splice_match	gene: Zm00001d040343	Zm00001d040343_T004	0.00	35.37	coding	581	95	1837
PB.3883.4	3	1806	4	full-splice_match	gene: Zm00001d040343	Zm00001d040343_T001	13.30	35.37	coding	403	68	1276
PB.8775.2	7	1149	5	full-splice_match	gene: Zm00001d021701	Zm00001d021701_T002	35.19	67.00	coding	252	169	924

PB.3883.6	3	1465	2	incomplete-splice_match	gene: Zm00001d040343	Zm00001d040343_T004	0.00	35.37	coding	461	16	1398
PB.3111.1	2	2842	9	full-splice_match	gene: Zm00001d005373	Zm00001d005373_T008	36.07	45.50	coding	709	220	2346
PB.5741.1	4	1349	2	genic	gene: Zm00001d053552	novel	60.05	74.47	coding	306	52	969
PB.5399.9	4	1948	3	full-splice_match	gene: Zm00001d051981	Zm00001d051981_T005	0.00	138.16	coding	405	386	1600
PB.3391.2	2	1902	3	full-splice_match	gene: Zm00001d006749	Zm00001d006749_T002	12.78	13.57	coding	505	247	1761
PB.10580.2	9	2508	1	genic	gene: Zm00001d047702	novel	19.70	19.70	coding	219	44	700
PB.5399.5	4	3291	2	novel_in_catalog	gene: Zm00001d051981	novel	7.24	138.16	coding	301	1602	2504
PB.5399.7	4	2485	3	full-splice_match	gene: Zm00001d051981	Zm00001d051981_T003	0.00	138.16	coding	405	496	1710
PB.5399.6	4	2446	3	full-splice_match	gene: Zm00001d051981	Zm00001d051981_T005	7.99	138.16	coding	405	440	1654
PB.5399.1	4	2067	1	genic	gene: Zm00001d051981	novel	62.03	138.16	coding	301	176	1078
PB.861.3	1	1049	1	full-splice_match	gene: Zm00001d031586	Zm00001d031586_T001	59.12	62.25	coding	250	165	914
PB.8584.3	7	3112	10	novel_in_catalog	gene: Zm00001d020938	novel	0.28	11.79	coding	454	1368	2729
PB.1157.15	1	2464	2	novel_in_catalog	gene: Zm00001d032897	novel	2.71	297.96	coding	186	1781	2338
PB.1157.14	1	1881	3	novel_not_in_catalog	gene: Zm00001d032897	novel	172.20	297.96	coding	245	1034	1768
PB.873.2	1	855	4	full-splice_match	gene: Zm00001d031637	Zm00001d031637_T004	14.04	71.62	coding	213	33	671
PB.1157.13	1	1879	3	novel_not_in_catalog	gene: Zm00001d032897	novel	9.11	297.96	coding	186	1112	1669
PB.5723.3	4	1385	5	full-splice_match	gene: Zm00001d053425	Zm00001d053425_T001	0.00	74.10	coding	382	106	1251

PB.2347.1	10	1314	1	full-splice_match	gene: Zm00001d026597	Zm00001d026597_T001	72.75	78.95	coding	309	66	992
PB.8584.1	7	2239	11	full-splice_match	gene: Zm00001d020938	Zm00001d020938_T002	3.66	11.79	coding	486	164	1621
PB.1157.19	1	2374	1	genic	gene: Zm00001d032897	novel	0.00	297.96	coding	186	1702	2259
PB.5723.1	4	1725	5	full-splice_match	gene: Zm00001d053425	Zm00001d053425_T001	67.50	74.10	coding	382	256	1401
PB.1071.8	1	3083	7	novel_in_catalog	gene: Zm00001d032539	novel	0.00	38.95	coding	380	1832	2971
PB.1812.1	10	1328	5	intergenic	novelGene_98	novel	50.36	58.71	non_coding	NA	NA	NA
PB.1812.2	10	1244	5	intergenic	novelGene_98	novel	8.35	58.71	non_coding	NA	NA	NA
PB.1071.1	1	1831	6	incomplete-splice_match	gene: Zm00001d032539	Zm00001d032539_T001	13.81	38.95	coding	346	12	1049
PB.1071.2	1	3674	9	novel_not_in_catalog	gene: Zm00001d032539	novel	15.08	38.95	coding	823	746	3214
PB.5408.1	4	369	1	incomplete-splice_match	gene: Zm00001d052025	Zm00001d052025_T001	1602.86	1602.86	non_coding	NA	NA	NA
PB.806.2	1	1020	1	genic	gene: Zm00001d031257	novel	31.69	343.85	coding	126	370	747
PB.837.3	1	3132	11	full-splice_match	gene: Zm00001d031447	Zm00001d031447_T001	16.71	69.53	coding	898	140	2833
PB.1512.1	1	1854	8	novel_in_catalog	gene: Zm00001d034518	novel	43.53	43.53	coding	419	344	1600
PB.10131.1	9	1262	3	full-splice_match	gene: Zm00001d045233	Zm00001d045233_T001	18.78	18.78	coding	345	72	1106
PB.6515.2	5	772	2	full-splice_match	gene: Zm00001d015308	Zm00001d015308_T001	0.00	59.80	coding	172	140	655
PB.6515.1	5	952	2	full-splice_match	gene: Zm00001d015308	Zm00001d015308_T001	59.80	59.80	coding	172	140	655
PB.4935.2	4	2217	12	full-splice_match	gene: Zm00001d049260	Zm00001d049260_T001	0.00	34.48	coding	488	309	1772

PB.4935.3	4	2063	12	full-splice_match	gene: Zm00001d049260	Zm00001d049260_T001	2.23	34.48	coding	488	315	1778
PB.4935.1	4	2370	12	full-splice_match	gene: Zm00001d049260	Zm00001d049260_T001	32.25	34.48	coding	488	314	1777
PB.4604.1	3	823	3	novel_not_in_catalog	gene: Zm00001d043944	novel	7.79	7.79	coding	188	229	792
PB.9899.2	8	1199	3	full-splice_match	gene: Zm00001d012242	Zm00001d012242_T001	18.22	28.53	coding	316	67	1014
PB.895.1	1	1496	3	full-splice_match	gene: Zm00001d031740	Zm00001d031740_T001	40.49	78.71	coding	348	98	1141
PB.6033.1	5	2389	7	full-splice_match	gene: Zm00001d013253	Zm00001d013253_T003	24.01	24.01	coding	647	185	2125
PB.9899.1	8	716	1	genic	gene: Zm00001d012242	novel	10.32	28.53	coding	42	237	362
PB.1800.4	10	1020	4	full-splice_match	gene: Zm00001d023962	Zm00001d023962_T004	0.00	70.63	coding	245	105	839
PB.3385.1	2	837	5	full-splice_match	gene: Zm00001d006714	Zm00001d006714_T001	150.78	150.78	coding	157	56	526
PB.7008.1	5	1572	1	full-splice_match	gene: Zm00001d017931	Zm00001d017931_T001	1.77	1.77	coding	488	47	1510
PB.5243.1	4	2581	1	intergenic	novelGene_240	novel	13.21	13.21	non_coding	NA	NA	NA
PB.1800.1	10	1345	5	full-splice_match	gene: Zm00001d023962	Zm00001d023962_T003	64.68	70.63	coding	256	260	1027
PB.1800.3	10	1156	4	full-splice_match	gene: Zm00001d023962	Zm00001d023962_T004	5.95	70.63	coding	245	116	850
PB.5578.1	4	1976	10	full-splice_match	gene: Zm00001d052720	Zm00001d052720_T003	10.62	10.62	coding	491	246	1718
PB.2837.2	2	312	1	incomplete-splice_match	gene: Zm00001d003626	Zm00001d003626_T001	5.16	16.93	non_coding	NA	NA	NA
PB.2837.1	2	1016	3	full-splice_match	gene: Zm00001d003626	Zm00001d003626_T001	11.76	16.93	coding	211	50	682
PB.3142.8	2	1403	9	full-splice_match	gene: Zm00001d005579	Zm00001d005579_T005	0.00	89.25	coding	310	254	1183

PB.10217.1	9	2794	10	full-splice_match	gene: Zm00001d045566	Zm00001d045566_T001	18.18	18.18	coding	768	146	2449
PB.8805.1	7	2100	11	full-splice_match	gene: Zm00001d021802	Zm00001d021802_T002	96.24	168.02	coding	468	211	1614
PB.5926.1	5	2290	1	full-splice_match	gene: Zm00001d012881	Zm00001d012881_T001	18.23	18.23	coding	689	57	2123
PB.5385.1	4	1302	4	full-splice_match	gene: Zm00001d051912	Zm00001d051912_T001	97.54	183.74	coding	329	79	1065
PB.4929.5	4	2319	1	novel_in_catalog	gene: Zm00001d049234	novel	8.17	609.53	coding	110	179	508
PB.4929.1	4	1578	4	full-splice_match	gene: Zm00001d049234	Zm00001d049234_T001	252.55	609.53	coding	347	125	1165
PB.3918.1	3	2299	6	incomplete-splice_match	gene: Zm00001d040562	Zm00001d040562_T022	159.46	159.46	coding	704	37	2148
PB.4929.3	4	2754	3	novel_in_catalog	gene: Zm00001d049234	novel	4.05	609.53	coding	344	118	1149
PB.4929.2	4	2001	4	full-splice_match	gene: Zm00001d049234	Zm00001d049234_T001	344.76	609.53	coding	526	144	1721
PB.5849.1	4	2412	14	full-splice_match	gene: Zm00001d053928	Zm00001d053928_T001	6.54	6.54	coding	579	297	2033
PB.6243.1	5	581	3	full-splice_match	gene: Zm00001d014140	Zm00001d014140_T001	27.61	27.61	non_coding	NA	NA	NA
PB.9046.2	7	1577	5	full-splice_match	gene: Zm00001d022614	Zm00001d022614_T002	16.51	20.69	coding	403	98	1306
PB.10558.1	9	2766	1	full-splice_match	gene: Zm00001d047617	Zm00001d047617_T001	16.51	16.51	coding	828	101	2584
PB.9046.1	7	2993	6	full-splice_match	gene: Zm00001d022614	Zm00001d022614_T001	4.18	20.69	coding	403	1559	2767
PB.4263.1	3	1482	5	full-splice_match	gene: Zm00001d042549	Zm00001d042549_T001	14.36	14.36	coding	260	554	1333
PB.4591.2	3	862	4	novel_in_catalog	gene: Zm00001d043874	novel	35.27	96.12	coding	160	103	582
PB.7701.7	6	1521	6	novel_in_catalog	gene: Zm00001d037609	novel	15.55	34.44	non_coding	NA	NA	NA

PB.843.2	1	1854	5	novel_not_in_catalog	gene: Zm00001d031481	novel	34.11	34.11	coding	376	557	1684
PB.7701.3	6	2739	5	novel_in_catalog	gene: Zm00001d037609	novel	8.84	34.44	coding	120	1024	1383
PB.116.2	1	2221	14	full-splice_match	gene: Zm00001d027701	Zm00001d027701_T001	34.60	46.24	coding	579	163	1899
PB.116.1	1	2276	14	full-splice_match	gene: Zm00001d027701	Zm00001d027701_T001	11.64	46.24	coding	579	57	1793
PB.7592.2	6	1373	7	full-splice_match	gene: Zm00001d037104	Zm00001d037104_T005	11.70	41.44	coding	58	153	326
PB.7592.3	6	1004	8	full-splice_match	gene: Zm00001d037104	Zm00001d037104_T002	29.74	41.44	coding	183	154	702
PB.6942.3	5	1998	4	full-splice_match	gene: Zm00001d017660	Zm00001d017660_T012	6.87	22.57	coding	401	153	1355
PB.6140.1	5	1328	2	full-splice_match	gene: Zm00001d013625	Zm00001d013625_T001	9.18	9.18	coding	199	404	1000
PB.2451.1	2	2367	1	full-splice_match	gene: Zm00001d001997	Zm00001d001997_T001	4.67	4.67	coding	411	715	1947
PB.4611.1	3	894	7	full-splice_match	gene: Zm00001d043963	Zm00001d043963_T001	21.35	21.35	coding	145	203	637
PB.9672.1	8	852	1	full-splice_match	gene: Zm00001d011236	Zm00001d011236_T001	442.41	1075.86	coding	150	114	563
PB.3157.2	2	3293	2	novel_in_catalog	gene: Zm00001d005672	novel	15.42	19.99	coding	162	1090	1575
PB.3157.1	2	1422	2	novel_in_catalog	gene: Zm00001d005672	novel	2.97	19.99	non_coding	NA	NA	NA
PB.9672.2	8	744	1	full-splice_match	gene: Zm00001d011236	Zm00001d011236_T001	633.45	1075.86	coding	150	117	566
PB.7315.1	6	1008	4	full-splice_match	gene: Zm00001d035519	Zm00001d035519_T001	332.39	332.39	coding	196	153	740
PB.3238.6	2	914	5	full-splice_match	gene: Zm00001d006070	Zm00001d006070_T002	8.69	52.81	coding	198	210	803
PB.8767.1	7	1782	4	novel_in_catalog	gene: Zm00001d021673	novel	12.49	92.15	coding	165	187	681

PB.1057.1	1	1047	6	fusion	gene: Zm00001d032496_g	novel	53.40	150.50	coding	170	59	568
PB.1057.2	1	877	6	fusion	gene: Zm00001d032496_g	novel	97.09	150.50	coding	170	118	627
PB.8767.2	7	1094	5	full-splice_match	gene: Zm00001d021673	Zm00001d021673_T001	79.67	92.15	coding	194	200	781
PB.5366.3	4	2753	7	full-splice_match	gene: Zm00001d051871	Zm00001d051871_T003	0.00	13.24	coding	760	302	2581
PB.7358.3	6	1173	6	novel_in_catalog	gene: Zm00001d035762	novel	4.27	149.98	coding	120	574	933
PB.5366.1	4	2068	7	full-splice_match	gene: Zm00001d051871	Zm00001d051871_T003	7.58	13.24	coding	432	156	1451
PB.269.2	1	1705	3	full-splice_match	gene: Zm00001d028399	Zm00001d028399_T001	22.34	53.52	coding	335	269	1273
PB.269.1	1	1658	3	full-splice_match	gene: Zm00001d028399	Zm00001d028399_T001	31.18	53.52	coding	335	61	1065
PB.5516.1	4	1629	6	full-splice_match	gene: Zm00001d052411	Zm00001d052411_T002	67.03	67.03	coding	332	285	1280
PB.7810.1	6	780	3	full-splice_match	gene: Zm00001d038027	Zm00001d038027_T001	18.34	18.34	coding	104	70	381
PB.4235.3	3	1819	3	novel_not_in_catalog	gene: Zm00001d042416	novel	16.23	113.72	non_coding	NA	NA	NA
PB.4273.1	3	2776	5	full-splice_match	gene: Zm00001d042608	Zm00001d042608_T003	7.13	7.13	coding	800	126	2525
PB.3338.12	2	982	3	incomplete-splice_match	gene: Zm00001d006480	Zm00001d006480_T011	43.43	96.23	non_coding	NA	NA	NA
PB.5465.2	4	1719	2	full-splice_match	gene: Zm00001d052260	Zm00001d052260_T001	19.87	59.77	coding	490	91	1560
PB.5465.1	4	1798	2	full-splice_match	gene: Zm00001d052260	Zm00001d052260_T001	39.89	59.77	coding	490	39	1508
PB.4543.2	3	1064	5	full-splice_match	gene: Zm00001d043700	Zm00001d043700_T002	222.98	228.89	coding	221	188	850
PB.3338.14	2	2242	4	full-splice_match	gene: Zm00001d006480	Zm00001d006480_T001	0.00	96.23	coding	658	187	2160

PB.5423.1	4	2043	7	novel_in_catalog	gene: Zm00001d052074	novel	7.83	84.05	coding	212	1141	1776
PB.1106.1	1	2165	3	full-splice_match	gene: Zm00001d032664	Zm00001d032664_T001	40.09	51.41	coding	542	217	1842
PB.4650.1	3	1500	6	full-splice_match	gene: Zm00001d044138	Zm00001d044138_T001	61.11	72.12	coding	379	25	1161
PB.9380.1	8	3167	12	full-splice_match	gene: Zm00001d009840	Zm00001d009840_T002	89.43	89.43	coding	797	268	2658
PB.10076.1	9	1448	2	genic	gene: Zm00001d044953	novel	18.36	18.36	coding	310	214	1143
PB.5423.3	4	1665	9	full-splice_match	gene: Zm00001d052074	Zm00001d052074_T010	44.15	84.05	coding	212	806	1441
PB.9198.13	8	3682	16	novel_not_in_catalog	gene: Zm00001d008826	novel	7.87	277.29	coding	900	65	2764
PB.1151.2	1	621	3	incomplete-splice_match	gene: Zm00001d032873	Zm00001d032873_T001	19.69	223.13	coding	155	156	620
PB.3065.3	2	665	2	genic	gene: Zm00001d005146	novel	20.85	5365.41	coding	115	50	394
PB.3377.7	2	936	5	novel_not_in_catalog	gene: Zm00001d006679	novel	0.00	147.77	coding	274	115	936
PB.1853.1	10	1236	2	full-splice_match	gene: Zm00001d024382	Zm00001d024382_T001	90.13	90.13	coding	281	83	925
PB.1677.2	10	2191	2	full-splice_match	gene: Zm00001d023323	Zm00001d023323_T021	50.70	50.70	coding	540	272	1891
PB.9172.2	8	2192	3	full-splice_match	gene: Zm00001d008708	Zm00001d008708_T001	73.59	88.51	coding	563	178	1866
PB.9172.3	8	2109	3	full-splice_match	gene: Zm00001d008708	Zm00001d008708_T001	0.00	88.51	coding	563	146	1834
PB.3377.1	2	1706	5	novel_not_in_catalog	gene: Zm00001d006679	novel	110.44	147.77	coding	344	393	1424
PB.9172.1	8	429	1	genic	gene: Zm00001d008708	novel	14.92	88.51	non_coding	NA	NA	NA
PB.4363.2	3	2445	1	genic	gene: Zm00001d042993	novel	2.98	53.22	coding	108	1772	2095

PB.9521.6	8	2723	1	antisense	novelGene_gene: Zm00001d010564_A	novel	0.00	23.35	non_coding	NA	NA	NA
PB.9253.4	8	2903	6	novel_in_catalog	gene: Zm00001d009077	novel	0.29	14.88	coding	518	1149	2702
PB.3584.1	2	3376	2	incomplete-splice_match	gene: Zm00001d007776	Zm00001d007776_T012	3.03	3.03	coding	825	67	2541
PB.9253.2	8	2488	6	novel_in_catalog	gene: Zm00001d009077	novel	2.22	14.88	coding	346	1200	2237
PB.9521.3	8	556	4	antisense	novelGene_gene: Zm00001d010564_A	novel	14.55	23.35	non_coding	NA	NA	NA
PB.9253.1	8	2318	7	full-splice_match	gene: Zm00001d009077	Zm00001d009077_T002	12.36	14.88	coding	633	220	2118
PB.4146.1	3	1308	7	full-splice_match	gene: Zm00001d042005	Zm00001d042005_T003	15.36	15.36	coding	342	96	1121
PB.8489.2	7	1383	3	full-splice_match	gene: Zm00001d020492	Zm00001d020492_T001	0.00	3.99	coding	302	121	1026
PB.8489.3	7	1225	3	full-splice_match	gene: Zm00001d020492	Zm00001d020492_T001	2.15	3.99	coding	302	121	1026
PB.8489.1	7	1667	3	full-splice_match	gene: Zm00001d020492	Zm00001d020492_T001	1.84	3.99	coding	302	121	1026
PB.5245.3	4	1691	2	full-splice_match	gene: Zm00001d051219	Zm00001d051219_T001	10.18	30.37	coding	417	100	1350
PB.5245.2	4	2671	1	novel_in_catalog	gene: Zm00001d051219	novel	0.63	30.37	coding	253	1522	2280
PB.8489.4	7	1084	3	full-splice_match	gene: Zm00001d020492	Zm00001d020492_T001	0.00	3.99	coding	302	121	1026
PB.5590.2	4	1791	10	full-splice_match	gene: Zm00001d052747	Zm00001d052747_T002	1.58	24.18	coding	377	500	1630
PB.3538.1	2	1440	2	novel_not_in_catalog	gene: Zm00001d007501	novel	5.15	6.90	coding	331	205	1197
PB.7290.4	6	1496	3	fusion	gene: Zm00001d035383_g	novel	15.99	15.99	coding	152	80	535
PB.7290.3	6	550	3	intergenic	novelGene_74	novel	8.63	19.49	non_coding	NA	NA	NA

PB.7290.1	6	2525	8	intergenic	novelGene_74	novel	10.86	19.49	coding	123	1749	2117
PB.1306.4	1	1200	6	full-splice_match	gene: Zm00001d033641	Zm00001d033641_T001	0.00	81.41	coding	276	202	1029
PB.9089.1	8	887	1	genic	gene: Zm00001d008312	novel	11.51	763.11	non_coding	NA	NA	NA
PB.9729.5	8	1259	1	genic	gene: Zm00001d011531	novel	4.84	28.94	coding	328	239	1222
PB.9089.3	8	2382	2	novel_in_catalog	gene: Zm00001d008312	novel	12.67	763.11	non_coding	NA	NA	NA
PB.9089.2	8	751	3	full-splice_match	gene: Zm00001d008312	Zm00001d008312_T001	506.75	763.11	coding	100	148	447
PB.9089.5	8	2285	1	novel_in_catalog	gene: Zm00001d008312	novel	0.44	763.11	non_coding	NA	NA	NA
PB.9089.4	8	732	3	full-splice_match	gene: Zm00001d008312	Zm00001d008312_T002	37.34	763.11	non_coding	NA	NA	NA
PB.9729.2	8	1433	2	genic	gene: Zm00001d011531	novel	24.09	28.94	coding	378	8	1141
PB.9089.6	8	602	3	full-splice_match	gene: Zm00001d008312	Zm00001d008312_T001	194.40	763.11	coding	100	152	451
PB.3329.2	2	1317	12	full-splice_match	gene: Zm00001d006455	Zm00001d006455_T002	0.00	57.82	coding	411	84	1316
PB.4117.1	3	2015	12	incomplete-splice_match	gene: Zm00001d041880	Zm00001d041880_T010	146.12	146.12	coding	591	33	1805
PB.4973.2	4	1139	7	full-splice_match	gene: Zm00001d049496	Zm00001d049496_T002	82.11	153.62	coding	215	196	840
PB.7337.1	6	561	2	full-splice_match	gene: Zm00001d035663	Zm00001d035663_T001	45.22	45.22	non_coding	NA	NA	NA
PB.6964.1	5	1504	6	novel_not_in_catalog	gene: Zm00001d017756	novel	15.74	46.65	coding	171	662	1174
PB.1874.1	10	1417	1	genic	gene: Zm00001d024497	novel	8.75	56.38	coding	189	154	720
PB.2958.1	2	1685	3	full-splice_match	gene: Zm00001d004473	Zm00001d004473_T001	134.74	134.74	coding	428	211	1494

PB.1565.1	1	2039	10	full-splice_match	gene: Zm00001d034689	Zm00001d034689_T003	8.83	8.83	coding	491	213	1685
PB.6920.4	5	759	1	intergenic	novelGene_315	novel	0.00	0.00	non_coding	NA	NA	NA
PB.5077.2	4	1312	11	full-splice_match	gene: Zm00001d050043	Zm00001d050043_T004	92.46	97.16	coding	320	114	1073
PB.3329.1	2	1629	12	full-splice_match	gene: Zm00001d006455	Zm00001d006455_T002	57.82	57.82	coding	461	85	1467
PB.6964.4	5	2121	5	novel_not_in_catalog	gene: Zm00001d017756	novel	1.32	46.65	coding	120	1434	1793
PB.1306.3	1	1787	5	novel_in_catalog	gene: Zm00001d033641	novel	2.22	81.41	coding	232	203	898
PB.10560.1	9	728	2	incomplete-splice_match	gene: Zm00001d047632	Zm00001d047632_T001	49.20	49.20	coding	111	92	424
PB.6920.2	5	2754	1	full-splice_match	gene: Zm00001d017528	Zm00001d017528_T001	11.76	32.46	coding	534	976	2577
PB.1874.2	10	2792	3	full-splice_match	gene: Zm00001d024497	Zm00001d024497_T001	8.63	56.38	coding	727	238	2418
PB.6920.3	5	2686	1	full-splice_match	gene: Zm00001d017528	Zm00001d017528_T001	0.00	32.46	coding	534	959	2560
PB.1372.12	1	1316	10	incomplete-splice_match	gene: Zm00001d033931	Zm00001d033931_T001	77.19	1100.09	coding	379	104	1240
PB.5861.2	4	1065	4	full-splice_match	gene: Zm00001d053976	Zm00001d053976_T001	21.53	22.01	coding	248	81	824
PB.5861.1	4	1860	4	full-splice_match	gene: Zm00001d053976	Zm00001d053976_T001	0.48	22.01	coding	248	84	827
PB.1372.11	1	2396	8	novel_in_catalog	gene: Zm00001d033931	novel	1.66	1100.09	coding	268	1391	2194
PB.2978.1	2	439	1	incomplete-splice_match	gene: Zm00001d004591	Zm00001d004591_T001	160.44	160.44	non_coding	NA	NA	NA
PB.6089.1	5	2399	16	full-splice_match	gene: Zm00001d013426	Zm00001d013426_T002	46.77	53.89	coding	611	191	2023
PB.6089.2	5	2298	16	full-splice_match	gene: Zm00001d013426	Zm00001d013426_T002	7.13	53.89	coding	611	215	2047

PB.1730.3	10	2218	12	full-splice_match	gene: Zm00001d023576	Zm00001d023576_T003	94.52	199.87	coding	596	111	1898
PB.7621.1	6	1563	1	incomplete-splice_match	gene: Zm00001d037240	Zm00001d037240_T003	6.20	13.98	non_coding	NA	NA	NA
PB.712.3	1	2174	2	genic	gene: Zm00001d030760	novel	18.05	53.70	coding	361	346	1428
PB.10570.1	9	2058	3	full-splice_match	gene: Zm00001d047663	Zm00001d047663_T001	43.22	43.22	coding	582	128	1873
PB.1730.4	10	2113	12	full-splice_match	gene: Zm00001d023576	Zm00001d023576_T009	102.13	199.87	coding	596	112	1899
PB.1730.5	10	2001	12	full-splice_match	gene: Zm00001d023576	Zm00001d023576_T009	3.22	199.87	coding	596	131	1918
PB.7489.3	6	2045	3	full-splice_match	gene: Zm00001d036543	Zm00001d036543_T001	77.35	168.24	coding	531	202	1794
PB.7489.2	6	2060	3	full-splice_match	gene: Zm00001d036543	Zm00001d036543_T001	57.96	168.24	coding	531	89	1681
PB.7489.1	6	1800	3	full-splice_match	gene: Zm00001d036543	Zm00001d036543_T001	32.93	168.24	coding	358	220	1293
PB.2744.2	2	957	1	full-splice_match	gene: Zm00001d003197	Zm00001d003197_T001	34.42	48.26	coding	222	64	729
PB.10153.1	9	827	4	full-splice_match	gene: Zm00001d045339	Zm00001d045339_T002	109.94	109.94	coding	143	209	637
PB.2744.1	2	1161	1	full-splice_match	gene: Zm00001d003197	Zm00001d003197_T001	13.84	48.26	coding	222	2	667
PB.1291.1	1	470	2	novel_not_in_catalog	gene: Zm00001d033574	novel	32.56	582.10	non_coding	NA	NA	NA
PB.1291.2	1	883	2	novel_not_in_catalog	gene: Zm00001d033574	novel	549.54	582.10	coding	170	69	578
PB.3282.2	2	1933	2	full-splice_match	gene: Zm00001d006254	Zm00001d006254_T001	17.87	18.20	coding	463	318	1706
PB.8322.1	7	1567	6	full-splice_match	gene: Zm00001d019505	Zm00001d019505_T001	24.61	59.26	coding	284	267	1118
PB.8322.2	7	1390	6	full-splice_match	gene: Zm00001d019505	Zm00001d019505_T001	34.65	59.26	coding	284	262	1113

PB.1874.8	10	1316	2	novel_not_in_catalog	gene: Zm00001d024497	novel	8.11	56.38	coding	331	225	1217
PB.3234.1	2	1698	6	full-splice_match	gene: Zm00001d006057	Zm00001d006057_T001	57.74	57.74	coding	421	214	1476
PB.277.2	1	1223	1	full-splice_match	gene: Zm00001d028440	Zm00001d028440_T001	0.00	14.42	coding	331	130	1122
PB.2800.3	2	1217	4	novel_in_catalog	gene: Zm00001d003434	novel	11.25	55.77	coding	325	108	1082
PB.277.1	1	1603	1	full-splice_match	gene: Zm00001d028440	Zm00001d028440_T001	14.42	14.42	coding	331	130	1122
PB.1883.7	10	2111	12	full-splice_match	gene: Zm00001d024523	Zm00001d024523_T004	47.95	79.72	coding	596	52	1839
PB.4324.4	3	1832	2	full-splice_match	gene: Zm00001d042812	Zm00001d042812_T001	2.69	21.81	coding	523	211	1779
PB.4892.1	4	1533	1	full-splice_match	gene: Zm00001d049096	Zm00001d049096_T001	9.70	9.70	coding	346	228	1265
PB.2189.1	10	2239	15	full-splice_match	gene: Zm00001d026022	Zm00001d026022_T005	0.00	24.31	coding	593	75	1853
PB.2189.2	10	2110	15	full-splice_match	gene: Zm00001d026022	Zm00001d026022_T005	24.31	24.31	coding	593	75	1853
PB.7327.2	6	1486	1	full-splice_match	gene: Zm00001d035604	Zm00001d035604_T001	236.16	407.17	coding	368	152	1255
PB.7327.3	6	1237	2	genic	gene: Zm00001d035604	novel	171.01	407.17	coding	145	577	1011
PB.1370.10	1	1963	1	novel_in_catalog	gene: Zm00001d033925	novel	27.62	1241.38	non_coding	NA	NA	NA
PB.2463.1	2	2147	5	full-splice_match	gene: Zm00001d002051	Zm00001d002051_T001	116.48	116.48	coding	626	60	1937
PB.1238.1	1	1678	5	incomplete-splice_match	gene: Zm00001d033334	Zm00001d033334_T001	9.33	9.33	coding	394	159	1340
PB.3617.1	2	1277	1	genic	gene: Zm00001d007901	novel	63.26	63.26	coding	233	345	1043
PB.7528.5	6	2175	3	intergenic	novelGene_172	novel	9.47	72.68	coding	126	288	665

PB.7528.4	6	2281	1	intergenic	novelGene_172	novel	15.57	72.68	non_coding	NA	NA	NA
PB.7528.7	6	1926	4	intergenic	novelGene_172	novel	0.00	72.68	coding	126	274	651
PB.8926.1	7	1570	2	genic	gene: Zm00001d022229	novel	2.09	2.09	coding	384	207	1358
PB.2008.1	10	1355	4	fusion	gene: Zm00001d025079_g	novel	26.22	26.22	coding	276	48	875
PB.7528.3	6	1964	4	intergenic	novelGene_172	novel	17.15	72.68	coding	126	291	668
PB.2008.2	10	1231	4	fusion	gene: Zm00001d025079_g	novel	0.00	26.22	coding	276	28	855
PB.4091.5	3	1590	2	novel_not_in_catalog	gene: Zm00001d041741	novel	3.48	27.85	coding	286	5	862
PB.8354.2	7	1147	2	genic	gene: Zm00001d019656	novel	3.39	12.29	coding	318	155	1108
PB.8354.1	7	1746	2	genic	gene: Zm00001d019656	novel	8.90	12.29	coding	318	606	1559
PB.4091.1	3	1858	2	novel_not_in_catalog	gene: Zm00001d041741	novel	0.00	27.85	coding	212	194	829
PB.5267.2	4	1104	1	full-splice_match	gene: Zm00001d051344	Zm00001d051344_T001	0.00	10.23	coding	293	53	931
PB.4091.3	3	1675	2	novel_not_in_catalog	gene: Zm00001d041741	novel	15.67	27.85	coding	212	194	829
PB.4091.2	3	1972	1	genic	gene: Zm00001d041741	novel	8.70	27.85	coding	286	5	862
PB.4915.1	4	1908	1	full-splice_match	gene: Zm00001d049187	Zm00001d049187_T001	131.53	131.53	coding	507	142	1662
PB.609.1	1	907	1	full-splice_match	gene: Zm00001d030038	Zm00001d030038_T001	3.94	3.94	coding	232	9	704
PB.4915.3	4	1814	1	full-splice_match	gene: Zm00001d049187	Zm00001d049187_T001	0.00	131.53	coding	507	123	1643
PB.4915.4	4	1471	1	full-splice_match	gene: Zm00001d049187	Zm00001d049187_T001	0.00	131.53	coding	365	340	1434

PB.5544.1	4	1053	2	full-splice_match	gene: Zm00001d052518	Zm00001d052518_T001	37.70	37.70	coding	285	37	891
PB.8640.1	7	923	1	full-splice_match	gene: Zm00001d021208	Zm00001d021208_T001	26.49	26.49	coding	231	98	790
PB.6280.1	5	2343	9	novel_not_in_catalog	gene: Zm00001d014337	novel	35.66	35.66	coding	295	76	960
PB.3717.1	3	1261	3	incomplete-splice_match	gene: Zm00001d039514	Zm00001d039514_T002	21.37	21.37	coding	325	134	1108
PB.5853.1	4	1425	1	intergenic	novelGene_364	novel	25.13	224.73	coding	43	288	416
PB.5853.2	4	1096	3	intergenic	novelGene_364	novel	9.67	224.73	coding	75	115	339
PB.8967.2	7	1088	6	incomplete-splice_match	gene: Zm00001d022374	Zm00001d022374_T001	6.08	15.85	coding	311	5	937
PB.10728.1	9	1862	2	novel_in_catalog	gene: Zm00001d048198	novel	7.43	7.43	coding	156	113	580
PB.8967.1	7	1910	7	full-splice_match	gene: Zm00001d022374	Zm00001d022374_T001	9.77	15.85	coding	524	52	1623
PB.8115.1	6	1553	1	antisense	novelGene_gene: Zm00001d039146_A	novel	8.57	8.57	non_coding	NA	NA	NA
PB.9249.6	8	2022	2	novel_in_catalog	gene: Zm00001d009054	novel	1.00	23.92	non_coding	NA	NA	NA
PB.9249.5	8	1449	4	novel_in_catalog	gene: Zm00001d009054	novel	5.35	23.92	non_coding	NA	NA	NA
PB.9249.4	8	1676	3	novel_in_catalog	gene: Zm00001d009054	novel	0.00	23.92	non_coding	NA	NA	NA
PB.9249.3	8	2223	3	novel_not_in_catalog	gene: Zm00001d009054	novel	2.11	23.92	non_coding	NA	NA	NA
PB.9249.1	8	2106	3	novel_not_in_catalog	gene: Zm00001d009054	novel	4.66	23.92	non_coding	NA	NA	NA
PB.7017.1	5	2204	8	full-splice_match	gene: Zm00001d017965	Zm00001d017965_T001	7.15	7.15	coding	620	168	2027
PB.785.1	1	776	1	full-splice_match	gene: Zm00001d031152	Zm00001d031152_T001	232.94	232.94	coding	171	26	538

PB.3298.1	2	1281	2	full-splice_match	gene: Zm00001d006327	Zm00001d006327_T001	5.75	5.75	coding	376	89	1216
PB.1580.1	1	1317	8	full-splice_match	gene: Zm00001d034739	Zm00001d034739_T001	61.41	61.41	coding	138	203	616
PB.7495.1	6	2436	1	intergenic	novelGene_160	novel	12.64	12.64	non_coding	NA	NA	NA
PB.9249.9	8	2208	3	novel_in_catalog	gene: Zm00001d009054	novel	5.25	23.92	non_coding	NA	NA	NA
PB.9249.8	8	2510	1	novel_in_catalog	gene: Zm00001d009054	novel	0.00	23.92	non_coding	NA	NA	NA
PB.267.1	1	1052	3	full-splice_match	gene: Zm00001d028392	Zm00001d028392_T001	111.24	267.72	coding	143	151	579
PB.267.3	1	899	3	full-splice_match	gene: Zm00001d028392	Zm00001d028392_T001	116.21	267.72	coding	143	111	539
PB.267.2	1	2459	2	novel_in_catalog	gene: Zm00001d028392	novel	12.61	267.72	non_coding	NA	NA	NA
PB.267.4	1	777	3	full-splice_match	gene: Zm00001d028392	Zm00001d028392_T001	27.66	267.72	coding	143	144	572
PB.604.1	1	1509	1	full-splice_match	gene: Zm00001d029999	Zm00001d029999_T001	0.76	0.76	coding	463	120	1508
PB.5405.2	4	1660	4	novel_in_catalog	gene: Zm00001d052009	novel	15.57	41.71	coding	432	95	1390
PB.1351.1	1	1574	3	incomplete-splice_match	gene: Zm00001d033848	Zm00001d033848_T003	6.73	6.73	coding	223	194	862
PB.4737.4	3	1831	9	full-splice_match	gene: Zm00001d044495	Zm00001d044495_T001	696.32	807.82	coding	481	92	1534
PB.4601.1	3	1850	2	incomplete-splice_match	gene: Zm00001d043927	Zm00001d043927_T009	14.45	70.75	coding	374	90	1211
PB.2447.6	2	2082	5	novel_in_catalog	gene: Zm00001d001984	novel	0.00	116.65	coding	244	1183	1914
PB.2447.7	2	2004	4	novel_in_catalog	gene: Zm00001d001984	novel	0.30	116.65	coding	282	991	1836
PB.2447.4	2	1731	7	full-splice_match	gene: Zm00001d001984	Zm00001d001984_T002	66.01	116.65	coding	474	142	1563

PB.1712.1	10	1069	9	full-splice_match	gene: Zm00001d023504	Zm00001d023504_T002	48.42	48.42	coding	223	203	871
PB.2447.2	2	2010	5	novel_in_catalog	gene: Zm00001d001984	novel	15.37	116.65	coding	244	979	1710
PB.171.5	1	1510	7	novel_in_catalog	gene: Zm00001d027996	novel	101.64	101.64	coding	140	330	749
PB.2447.1	2	1970	7	full-splice_match	gene: Zm00001d001984	Zm00001d001984_T002	9.80	116.65	coding	474	235	1656
PB.3054.2	2	3907	18	full-splice_match	gene: Zm00001d005090	Zm00001d005090_T154	48.49	72.35	coding	1163	115	3603
PB.2447.8	2	1962	6	novel_in_catalog	gene: Zm00001d001984	novel	0.00	116.65	coding	346	819	1856
PB.3425.1	2	911	2	full-splice_match	gene: Zm00001d006903	Zm00001d006903_T001	100.86	101.68	coding	112	266	601
PB.3404.5	2	341	1	incomplete-splice_match	gene: Zm00001d006789	Zm00001d006789_T024	74.28	74.28	coding	31	249	341
PB.8300.1	7	1184	4	full-splice_match	gene: Zm00001d019312	Zm00001d019312_T002	139.69	139.69	coding	306	50	967
PB.7106.1	5	1278	6	full-splice_match	gene: Zm00001d018290	Zm00001d018290_T001	81.66	96.25	coding	258	117	890
PB.6015.2	5	3641	1	novel_in_catalog	gene: Zm00001d013196	novel	2.87	44.58	coding	206	80	697
PB.6015.1	5	1586	2	full-splice_match	gene: Zm00001d013196	Zm00001d013196_T001	41.71	44.58	coding	432	79	1374
PB.9620.3	8	979	3	novel_not_in_catalog	gene: Zm00001d010969	novel	14.92	60.34	coding	148	14	457
PB.9620.4	8	1472	3	novel_not_in_catalog	gene: Zm00001d010969	novel	7.26	60.34	coding	279	175	1011
PB.10437.2	9	2258	3	novel_not_in_catalog	gene: Zm00001d046999	novel	4.77	4.77	coding	99	198	494
PB.9620.6	8	1188	3	novel_not_in_catalog	gene: Zm00001d010969	novel	26.63	60.34	coding	279	50	886
PB.9620.7	8	1357	2	full-splice_match	gene: Zm00001d010969	Zm00001d010969_T001	0.00	60.34	coding	318	48	1001

PB.2572.3	2	1364	10	full-splice_match	gene: Zm00001d002504	Zm00001d002504_T002	5.80	31.55	coding	319	118	1074
PB.2572.2	2	1694	9	novel_in_catalog	gene: Zm00001d002504	novel	3.07	31.55	coding	158	893	1366
PB.8260.1	7	1833	7	novel_in_catalog	gene: Zm00001d019114	novel	41.78	41.78	coding	376	531	1658
PB.6029.1	5	944	2	incomplete-splice_match	gene: Zm00001d013243	Zm00001d013243_T002	19.97	19.97	coding	256	13	780
PB.5799.5	4	2657	14	novel_not_in_catalog	gene: Zm00001d053751	novel	1.41	12.82	coding	480	734	2173
PB.5799.4	4	2373	15	novel_not_in_catalog	gene: Zm00001d053751	novel	2.74	12.82	coding	480	446	1885
PB.5799.6	4	2429	13	novel_not_in_catalog	gene: Zm00001d053751	novel	0.00	12.82	coding	351	691	1743
PB.9513.1	8	2180	2	full-splice_match	gene: Zm00001d010529	Zm00001d010529_T002	13.19	13.19	coding	649	115	2061
PB.778.5	1	2536	13	novel_in_catalog	gene: Zm00001d031120	novel	3.52	204.87	coding	484	197	1648
PB.5576.1	4	1722	11	novel_not_in_catalog	gene: Zm00001d052701	novel	11.61	15.56	coding	341	352	1374
PB.9600.3	8	924	4	full-splice_match	gene: Zm00001d010870	Zm00001d010870_T001	29.62	99.18	coding	234	62	763
PB.9600.1	8	1064	4	full-splice_match	gene: Zm00001d010870	Zm00001d010870_T001	69.56	99.18	coding	234	193	894
PB.5954.2	5	1885	1	novel_in_catalog	gene: Zm00001d012966	novel	4.73	2659.90	non_coding	NA	NA	NA
PB.5954.3	5	1754	2	novel_in_catalog	gene: Zm00001d012966	novel	0.65	2659.90	coding	125	1159	1533
PB.4981.1	4	2862	7	incomplete-splice_match	gene: Zm00001d049543	Zm00001d049543_T003	24.23	24.23	coding	720	447	2606
PB.5954.1	5	874	3	full-splice_match	gene: Zm00001d012966	Zm00001d012966_T001	2654.52	2659.90	coding	139	196	612
PB.328.7	1	935	5	incomplete-splice_match	gene: Zm00001d028655	Zm00001d028655_T002	0.00	197.93	coding	253	9	767

PB.328.6	1	1135	5	incomplete-splice_match	gene: Zm00001d028655	Zm00001d028655_T001	127.02	197.93	coding	253	80	838
PB.328.5	1	1514	6	full-splice_match	gene: Zm00001d028655	Zm00001d028655_T001	39.93	197.93	coding	253	207	965
PB.328.4	1	1608	6	full-splice_match	gene: Zm00001d028655	Zm00001d028655_T001	16.75	197.93	coding	253	85	843
PB.328.3	1	562	2	incomplete-splice_match	gene: Zm00001d028655	Zm00001d028655_T001	0.00	197.93	non_coding	NA	NA	NA
PB.328.2	1	1965	6	full-splice_match	gene: Zm00001d028655	Zm00001d028655_T001	0.00	197.93	coding	253	85	843
PB.328.1	1	1975	6	full-splice_match	gene: Zm00001d028655	Zm00001d028655_T001	14.24	197.93	coding	253	80	838
PB.8076.1	6	4484	11	full-splice_match	gene: Zm00001d038972	Zm00001d038972_T010	166.70	254.63	coding	871	1457	4069
PB.5785.2	4	1107	5	novel_not_in_catalog	gene: Zm00001d053686	novel	0.00	45.55	coding	267	99	899
PB.10427.1	9	3129	9	novel_in_catalog	gene: Zm00001d046953	novel	16.03	18.86	coding	1002	53	3058
PB.5651.1	4	1348	1	incomplete-splice_match	gene: Zm00001d052993	Zm00001d052993_T001	11.45	11.45	coding	153	300	758
PB.10427.3	9	3217	8	novel_in_catalog	gene: Zm00001d046953	novel	2.83	18.86	coding	451	53	1405
PB.10427.4	9	3094	9	novel_in_catalog	gene: Zm00001d046953	novel	0.00	18.86	coding	1002	53	3058
PB.9116.4	8	2318	10	incomplete-splice_match	gene: Zm00001d008429	Zm00001d008429_T017	50.28	50.28	coding	349	924	1970
PB.7844.3	6	1966	2	full-splice_match	gene: Zm00001d038133	Zm00001d038133_T002	8.83	20.63	coding	234	1195	1896
PB.7844.1	6	1789	2	novel_in_catalog	gene: Zm00001d038133	novel	11.80	20.63	coding	135	180	584
PB.1379.2	1	2566	8	incomplete-splice_match	gene: Zm00001d033982	Zm00001d033982_T001	15.66	35.72	coding	627	134	2014
PB.6899.1	5	861	2	full-splice_match	gene: Zm00001d017455	Zm00001d017455_T001	329.93	329.93	coding	212	49	684

PB.2942.1	2	2275	21	fusion	gene: Zm00001d004259_g	novel	23.51	23.51	coding	590	122	1891
PB.8415.1	7	1845	6	full-splice_match	gene: Zm00001d020129	Zm00001d020129_T003	7.52	7.69	coding	491	68	1540
PB.9951.1	8	2183	5	full-splice_match	gene: Zm00001d012477	Zm00001d012477_T002	39.14	53.45	coding	571	337	2049
PB.8415.2	7	2108	5	novel_in_catalog	gene: Zm00001d020129	novel	0.17	7.69	coding	377	677	1807
PB.10835.1	9	906	2	full-splice_match	gene: Zm00001d048560	Zm00001d048560_T001	7.62	7.62	coding	241	29	751
PB.4686.1	3	1467	8	full-splice_match	gene: Zm00001d044278	Zm00001d044278_T001	13.27	13.27	coding	294	262	1143
PB.6109.1	5	3055	9	full-splice_match	gene: Zm00001d013493	Zm00001d013493_T001	13.61	13.61	coding	887	194	2854
PB.1453.1	1	2518	1	genic	gene: Zm00001d034322	novel	24.30	28.13	coding	333	1323	2321
PB.952.1	1	2043	4	full-splice_match	gene: Zm00001d032029	Zm00001d032029_T003	7.41	7.41	coding	576	65	1792
PB.10646.2	9	3423	1	intergenic	novelGene_232	novel	4.57	4.57	coding	975	300	3224
PB.9931.8	8	1501	5	full-splice_match	gene: Zm00001d012395	Zm00001d012395_T006	0.00	56.49	coding	361	228	1310
PB.3990.5	3	1811	11	incomplete-splice_match	gene: Zm00001d041183	Zm00001d041183_T047	12.57	33.97	coding	371	162	1274
PB.8068.1	6	1831	1	antisense	novelGene_gene: Zm00001d038948_A	novel	2.73	2.73	non_coding	NA	NA	NA
PB.4060.1	3	1642	2	incomplete-splice_match	gene: Zm00001d041592	Zm00001d041592_T054	65.86	65.86	coding	333	163	1161
PB.9608.1	8	1591	2	full-splice_match	gene: Zm00001d010920	Zm00001d010920_T002	1580.37	1709.65	coding	396	150	1337
PB.9608.7	8	2736	1	novel_in_catalog	gene: Zm00001d010920	novel	9.14	1709.65	coding	396	1323	2510
PB.9931.2	8	1750	5	full-splice_match	gene: Zm00001d012395	Zm00001d012395_T006	56.49	56.49	coding	361	232	1314

PB.8409.2	7	826	4	incomplete-splice_match	gene: Zm00001d020069	Zm00001d020069_T001	13.49	96.74	coding	5	526	540
PB.5110.1	4	3555	3	full-splice_match	gene: Zm00001d050227	Zm00001d050227_T001	28.74	28.74	coding	1028	217	3300
PB.8409.1	7	3371	12	novel_in_catalog	gene: Zm00001d020069	novel	1.39	96.74	coding	581	125	1867
PB.7270.1	6	884	1	novel_in_catalog	gene: Zm00001d035263	novel	4.99	4.99	coding	183	90	638
PB.8409.7	7	2047	12	novel_in_catalog	gene: Zm00001d020069	novel	4.47	96.74	coding	272	1195	2010
PB.8409.4	7	1008	8	novel_not_in_catalog	gene: Zm00001d020069	novel	1.43	96.74	coding	85	164	418
PB.8409.5	7	2119	13	novel_in_catalog	gene: Zm00001d020069	novel	31.23	96.74	coding	587	263	2023
PB.2892.4	2	1596	12	novel_in_catalog	gene: Zm00001d003923	novel	0.74	140.05	coding	409	98	1324
PB.2892.5	2	1625	13	novel_not_in_catalog	gene: Zm00001d003923	novel	0.17	140.05	coding	401	135	1337
PB.2892.6	2	1585	13	full-splice_match	gene: Zm00001d003923	Zm00001d003923_T001	0.00	140.05	coding	446	76	1413
PB.8409.9	7	764	5	novel_not_in_catalog	gene: Zm00001d020069	novel	44.41	96.74	coding	189	164	730
PB.828.2	1	1193	1	novel_in_catalog	gene: Zm00001d031344	novel	9.82	1026.24	non_coding	NA	NA	NA
PB.2892.1	2	1796	13	full-splice_match	gene: Zm00001d003923	Zm00001d003923_T001	31.21	140.05	coding	446	78	1415
PB.2892.2	2	1740	13	full-splice_match	gene: Zm00001d003923	Zm00001d003923_T001	107.93	140.05	coding	446	124	1461
PB.4995.1	4	1693	7	full-splice_match	gene: Zm00001d049617	Zm00001d049617_T007	10.22	15.22	coding	400	11	1210
PB.4995.2	4	2463	7	full-splice_match	gene: Zm00001d049617	Zm00001d049617_T007	4.99	15.22	coding	641	185	2107
PB.75.1	1	1195	9	full-splice_match	gene: Zm00001d027512	Zm00001d027512_T002	509.66	509.66	coding	301	47	949

PB.8487.1	7	1248	5	full-splice_match	gene: Zm00001d020476	Zm00001d020476_T001	21.65	21.65	coding	184	434	985
PB.5146.1	4	2236	8	full-splice_match	gene: Zm00001d050434	Zm00001d050434_T002	4.51	4.51	coding	597	133	1923
PB.7260.1	6	1326	4	full-splice_match	gene: Zm00001d035201	Zm00001d035201_T001	613.40	613.40	coding	319	171	1127
PB.5741.2	4	3419	2	genic	gene: Zm00001d053552	novel	14.42	74.47	coding	1038	48	3161
PB.3976.1	3	488	2	genic	gene: Zm00001d041065	novel	74.98	74.98	coding	79	60	296
PB.10455.1	9	730	1	full-splice_match	gene: Zm00001d047116	Zm00001d047116_T001	95.34	527.62	coding	126	89	466
PB.665.4	1	2037	8	full-splice_match	gene: Zm00001d030357	Zm00001d030357_T013	7.85	32.05	coding	613	28	1866
PB.5485.1	4	1239	2	full-splice_match	gene: Zm00001d052331	Zm00001d052331_T001	17.58	17.58	coding	131	69	461
PB.665.1	1	3505	13	full-splice_match	gene: Zm00001d030357	Zm00001d030357_T009	24.20	32.05	coding	935	125	2929
PB.3707.1	3	3226	16	full-splice_match	gene: Zm00001d039475	Zm00001d039475_T014	6.05	6.05	coding	879	267	2903
PB.1309.2	1	1941	11	full-splice_match	gene: Zm00001d033649	Zm00001d033649_T001	137.25	170.16	coding	502	163	1668
PB.3632.2	2	1341	5	full-splice_match	gene: Zm00001d007962	Zm00001d007962_T001	24.00	29.02	coding	345	70	1104
PB.3632.1	2	1529	4	novel_in_catalog	gene: Zm00001d007962	novel	5.02	29.02	coding	281	70	912
PB.2129.1	10	1284	4	full-splice_match	gene: Zm00001d025761	Zm00001d025761_T002	111.80	127.05	coding	292	90	965
PB.8401.1	7	1914	5	full-splice_match	gene: Zm00001d020043	Zm00001d020043_T003	7.32	7.32	coding	283	814	1662
PB.3791.2	3	416	1	incomplete-splice_match	gene: Zm00001d039914	Zm00001d039914_T001	0.00	47.15	non_coding	NA	NA	NA
PB.3791.1	3	744	2	full-splice_match	gene: Zm00001d039914	Zm00001d039914_T001	47.15	47.15	non_coding	NA	NA	NA

PB.5829.1	4	2919	4	novel_not_in_catalog	gene: Zm00001d053875	novel	27.23	27.23	coding	758	97	2370
PB.4779.1	3	923	2	intergenic	novelGene_356	novel	5.83	5.83	non_coding	NA	NA	NA
PB.7866.2	6	1926	4	full-splice_match	gene: Zm00001d038194	Zm00001d038194_T001	0.00	39.73	coding	424	403	1674
PB.7866.1	6	2051	4	full-splice_match	gene: Zm00001d038194	Zm00001d038194_T001	38.54	39.73	coding	424	425	1696
PB.7866.4	6	789	1	incomplete-splice_match	gene: Zm00001d038194	Zm00001d038194_T003	1.19	39.73	coding	122	422	787
PB.7536.1	6	1180	5	novel_in_catalog	gene: Zm00001d036764	novel	5.26	5.26	coding	126	168	545
PB.2016.3	10	2932	17	full-splice_match	gene: Zm00001d025111	Zm00001d025111_T046	20.33	36.59	coding	791	172	2544
PB.2016.4	10	2960	16	full-splice_match	gene: Zm00001d025111	Zm00001d025111_T031	8.17	36.59	coding	828	172	2655
PB.4384.1	3	2161	3	full-splice_match	gene: Zm00001d043104	Zm00001d043104_T001	128.39	128.39	coding	321	635	1597
PB.4598.3	3	2118	13	full-splice_match	gene: Zm00001d043907	Zm00001d043907_T003	0.33	18.25	coding	558	172	1845
PB.3282.1	2	2277	2	full-splice_match	gene: Zm00001d006254	Zm00001d006254_T001	0.33	18.20	coding	463	76	1464
PB.895.3	1	1333	3	full-splice_match	gene: Zm00001d031740	Zm00001d031740_T001	0.00	78.71	coding	348	100	1143
PB.5116.1	4	1233	7	full-splice_match	gene: Zm00001d050258	Zm00001d050258_T003	38.80	38.80	coding	256	128	895
PB.8175.1	7	363	1	full-splice_match	gene: Zm00001d018712	Zm00001d018712_T001	299.86	299.86	non_coding	NA	NA	NA
PB.6129.3	5	4502	2	full-splice_match	gene: Zm00001d013590	Zm00001d013590_T001	0.00	21.18	coding	1415	97	4341
PB.6129.2	5	4296	1	genic	gene: Zm00001d013590	novel	12.78	21.18	coding	1169	602	4108
PB.6129.1	5	4622	2	full-splice_match	gene: Zm00001d013590	Zm00001d013590_T001	8.40	21.18	coding	1415	188	4432

PB.7653.3	6	1482	12	incomplete-splice_match	gene: Zm00001d037377	Zm00001d037377_T052	0.00	33.15	coding	394	301	1482
PB.350.2	1	2378	6	full-splice_match	gene: Zm00001d028759	Zm00001d028759_T001	43.72	340.10	coding	606	157	1974
PB.350.3	1	2185	6	full-splice_match	gene: Zm00001d028759	Zm00001d028759_T001	294.99	340.10	coding	606	157	1974
PB.350.1	1	1789	3	incomplete-splice_match	gene: Zm00001d028759	Zm00001d028759_T001	1.39	340.10	coding	111	103	435
PB.4444.2	3	2799	5	incomplete-splice_match	gene: Zm00001d043330	Zm00001d043330_T016	5.60	5.60	coding	863	139	2727
PB.10649.1	9	2958	18	full-splice_match	gene: Zm00001d047910	Zm00001d047910_T001	122.53	122.53	coding	820	165	2624
PB.4598.2	3	2781	11	novel_in_catalog	gene: Zm00001d043907	novel	1.30	18.25	coding	390	103	1272
PB.1401.2	1	3787	4	full-splice_match	gene: Zm00001d034038	Zm00001d034038_T002	7.29	31.79	coding	1135	145	3549
PB.3105.1	2	1680	10	full-splice_match	gene: Zm00001d005347	Zm00001d005347_T001	268.08	268.08	coding	435	162	1466
PB.4070.5	3	2758	7	full-splice_match	gene: Zm00001d041635	Zm00001d041635_T002	4.85	106.27	coding	663	414	2402
PB.9478.1	8	2777	19	full-splice_match	gene: Zm00001d010341	Zm00001d010341_T003	21.02	21.63	coding	748	52	2295
PB.2522.1	2	616	1	genic	gene: Zm00001d002302	novel	9.82	40.83	coding	100	114	413
PB.9478.2	8	2658	19	full-splice_match	gene: Zm00001d010341	Zm00001d010341_T003	0.62	21.63	coding	748	58	2301
PB.6381.3	5	1594	3	full-splice_match	gene: Zm00001d014763	Zm00001d014763_T001	3.09	6.98	coding	391	161	1333
PB.6381.2	5	1916	2	incomplete-splice_match	gene: Zm00001d014763	Zm00001d014763_T001	0.29	6.98	coding	228	88	771
PB.6381.1	5	2315	4	novel_not_in_catalog	gene: Zm00001d014763	novel	3.60	6.98	coding	420	26	1285
PB.10748.1	9	1685	1	full-splice_match	gene: Zm00001d048293	Zm00001d048293_T001	58.56	58.56	coding	323	400	1368

PB.4855.1	4	1141	8	full-splice_match	gene: Zm00001d048892	Zm00001d048892_T001	79.17	89.90	coding	229	128	814
PB.8169.3	7	1971	2	full-splice_match	gene: Zm00001d018693	Zm00001d018693_T001	5.61	18.43	coding	571	202	1914
PB.4855.2	4	1269	7	novel_in_catalog	gene: Zm00001d048892	novel	10.73	89.90	coding	162	126	611
PB.9009.1	7	749	4	novel_in_catalog	gene: Zm00001d022489	novel	447.53	447.53	coding	130	131	520
PB.5160.3	4	952	4	genic_intron	novelGene_166	novel	35.93	46.27	coding	184	119	670
PB.3815.1	3	3098	7	full-splice_match	gene: Zm00001d040036	Zm00001d040036_T024	38.20	38.20	coding	949	103	2949
PB.5692.1	4	1951	3	novel_in_catalog	gene: Zm00001d053239	novel	291.01	291.01	non_coding	NA	NA	NA
PB.10470.1	9	1293	5	full-splice_match	gene: Zm00001d047212	Zm00001d047212_T001	6.20	6.20	coding	381	82	1224
PB.6972.2	5	1808	7	full-splice_match	gene: Zm00001d017787	Zm00001d017787_T001	35.27	48.05	coding	478	122	1555
PB.6200.1	5	1952	10	incomplete-splice_match	gene: Zm00001d013935	Zm00001d013935_T006	30.50	44.60	coding	336	534	1541
PB.6067.2	5	368	1	intergenic	novelGene_39	novel	107.16	107.16	non_coding	NA	NA	NA
PB.2966.4	2	2238	14	novel_in_catalog	gene: Zm00001d004525	novel	0.94	46.19	coding	449	163	1509
PB.1422.1	1	1351	1	full-splice_match	gene: Zm00001d034145	Zm00001d034145_T001	92.99	92.99	coding	278	116	949
PB.779.1	1	1508	5	full-splice_match	gene: Zm00001d031127	Zm00001d031127_T001	156.76	174.58	coding	309	298	1224
PB.8532.4	7	1539	6	full-splice_match	gene: Zm00001d020694	Zm00001d020694_T002	13.13	98.12	coding	396	251	1438
PB.10434.10	9	2618	1	genic	gene: Zm00001d046996	novel	0.00	801.09	coding	234	1718	2419
PB.578.1	1	2133	4	full-splice_match	gene: Zm00001d029808	Zm00001d029808_T001	12.15	12.15	coding	476	219	1646

PB.8532.1	7	1743	6	full-splice_match	gene: Zm00001d020694	Zm00001d020694_T002	42.47	98.12	coding	396	242	1429
PB.8532.3	7	1646	6	full-splice_match	gene: Zm00001d020694	Zm00001d020694_T002	41.46	98.12	coding	396	248	1435
PB.8532.2	7	1753	5	novel_in_catalog	gene: Zm00001d020694	novel	1.05	98.12	coding	302	246	1151
PB.8463.2	7	2300	5	novel_in_catalog	gene: Zm00001d020378	novel	4.77	49.96	coding	198	66	659
PB.8463.3	7	2042	7	full-splice_match	gene: Zm00001d020378	Zm00001d020378_T002	15.40	49.96	coding	256	103	870
PB.8463.1	7	2249	7	full-splice_match	gene: Zm00001d020378	Zm00001d020378_T002	3.16	49.96	coding	256	184	951
PB.8463.6	7	2143	6	novel_in_catalog	gene: Zm00001d020378	novel	0.00	49.96	coding	256	50	817
PB.5610.3	4	2268	1	full-splice_match	gene: Zm00001d052816	Zm00001d052816_T001	10.31	37.07	coding	603	138	1946
PB.8463.5	7	1393	1	genic	gene: Zm00001d020378	novel	6.07	49.96	non_coding	NA	NA	NA
PB.828.1	1	1149	2	full-splice_match	gene: Zm00001d031344	Zm00001d031344_T001	246.37	1026.24	coding	167	139	639
PB.8463.8	7	1709	8	full-splice_match	gene: Zm00001d020378	Zm00001d020378_T001	20.55	49.96	coding	390	62	1231
PB.4664.1	3	2935	4	novel_in_catalog	gene: Zm00001d044186	novel	43.24	43.24	non_coding	NA	NA	NA
PB.326.1	1	1183	2	full-splice_match	gene: Zm00001d028651	Zm00001d028651_T001	33.08	125.69	coding	268	57	860
PB.326.2	1	1075	2	full-splice_match	gene: Zm00001d028651	Zm00001d028651_T001	92.60	125.69	coding	268	55	858
PB.902.1	1	1197	6	full-splice_match	gene: Zm00001d031809	Zm00001d031809_T002	31.07	31.07	coding	306	2	919
PB.10549.1	9	1460	7	full-splice_match	gene: Zm00001d047581	Zm00001d047581_T002	12.43	12.43	coding	401	44	1246
PB.1187.2	1	484	3	full-splice_match	gene: Zm00001d033054	Zm00001d033054_T001	47.99	164.37	coding	115	138	482

PB.2960.1	2	1929	15	full-splice_match	gene: Zm00001d004478	Zm00001d004478_T002	2.87	2.87	coding	532	174	1769
PB.6135.2	5	1962	10	novel_in_catalog	gene: Zm00001d013603	novel	5.88	5.88	coding	346	442	1479
PB.8200.1	7	1148	2	full-splice_match	gene: Zm00001d018809	Zm00001d018809_T001	77.85	170.79	coding	222	98	763
PB.1902.2	10	1293	3	full-splice_match	gene: Zm00001d024630	Zm00001d024630_T003	0.00	19.33	coding	351	76	1128
PB.1902.1	10	1641	3	full-splice_match	gene: Zm00001d024630	Zm00001d024630_T003	19.33	19.33	coding	351	214	1266
PB.8200.2	7	1082	2	full-splice_match	gene: Zm00001d018809	Zm00001d018809_T001	92.95	170.79	coding	222	97	762
PB.2546.2	2	1351	5	full-splice_match	gene: Zm00001d002386	Zm00001d002386_T003	52.51	54.36	coding	248	187	930
PB.2546.3	2	1300	5	full-splice_match	gene: Zm00001d002386	Zm00001d002386_T003	0.00	54.36	coding	248	248	991
PB.8625.1	7	1501	14	novel_not_in_catalog	gene: Zm00001d021132	novel	40.63	45.44	coding	344	217	1248
PB.2546.1	2	2862	3	incomplete-splice_match	gene: Zm00001d002386	Zm00001d002386_T002	1.85	54.36	coding	133	581	979
PB.1501.1	1	807	3	full-splice_match	gene: Zm00001d034491	Zm00001d034491_T001	125.72	410.14	coding	133	177	575
PB.1367.2	1	1720	10	full-splice_match	gene: Zm00001d033906	Zm00001d033906_T001	22.38	32.73	coding	473	108	1526
PB.7942.2	6	2848	5	intergenic	novelGene_260	novel	7.40	54.59	non_coding	NA	NA	NA
PB.1367.1	1	1869	10	full-splice_match	gene: Zm00001d033906	Zm00001d033906_T001	10.35	32.73	coding	473	103	1521
PB.3787.2	3	2597	5	full-splice_match	gene: Zm00001d039901	Zm00001d039901_T003	0.00	17.32	coding	600	391	2190
PB.3787.3	3	2437	5	full-splice_match	gene: Zm00001d039901	Zm00001d039901_T003	11.88	17.32	coding	600	376	2175
PB.3787.1	3	1888	2	incomplete-splice_match	gene: Zm00001d039901	Zm00001d039901_T003	5.44	17.32	coding	258	515	1288

PB.9831.1	8	2857	12	novel_in_catalog	gene: Zm00001d011923	novel	0.96	7.10	coding	723	178	2346
PB.9831.2	8	2706	13	full-splice_match	gene: Zm00001d011923	Zm00001d011923_T004	6.14	7.10	coding	797	139	2529
PB.4193.2	3	1347	5	novel_not_in_catalog	gene: Zm00001d042233	novel	27.82	58.44	coding	247	355	1095
PB.984.6	1	2999	6	full-splice_match	gene: Zm00001d032198	Zm00001d032198_T018	3.28	199.16	coding	120	632	991
PB.10756.1	9	1870	9	full-splice_match	gene: Zm00001d048319	Zm00001d048319_T001	7.14	7.14	coding	539	79	1695
PB.4193.1	3	652	3	incomplete-splice_match	gene: Zm00001d042233	Zm00001d042233_T002	30.61	58.44	coding	50	148	297
PB.9966.1	8	2384	3	intergenic	novelGene_291	novel	20.76	20.76	coding	64	128	319
PB.828.3	1	1020	3	novel_not_in_catalog	gene: Zm00001d031344	novel	33.82	1026.24	coding	151	134	586
PB.4763.2	3	2690	7	full-splice_match	gene: Zm00001d044601	Zm00001d044601_T003	1.73	9.46	coding	724	295	2466
PB.3555.1	2	503	2	novel_not_in_catalog	gene: Zm00001d007605	novel	21.70	24.30	non_coding	NA	NA	NA
PB.5822.4	4	2489	10	full-splice_match	gene: Zm00001d053841	Zm00001d053841_T008	0.00	46.13	coding	320	1335	2294
PB.9583.2	8	1973	11	full-splice_match	gene: Zm00001d010811	Zm00001d010811_T005	66.89	70.26	coding	567	1	1701
PB.9583.3	8	1743	11	full-splice_match	gene: Zm00001d010811	Zm00001d010811_T005	0.00	70.26	coding	503	121	1629
PB.9583.1	8	1764	10	novel_in_catalog	gene: Zm00001d010811	novel	3.37	70.26	coding	492	5	1480
PB.10447.1	9	1080	6	full-splice_match	gene: Zm00001d047057	Zm00001d047057_T002	65.86	65.86	coding	174	78	599
PB.8548.1	7	1888	3	full-splice_match	gene: Zm00001d020776	Zm00001d020776_T002	30.72	30.72	coding	447	259	1599
PB.777.4	1	2139	1	genic	gene: Zm00001d031115	novel	10.06	10.06	coding	383	750	1898

PB.4010.1	3	1972	7	full-splice_match	gene: Zm00001d041346	Zm00001d041346_T001	20.51	20.51	coding	460	37	1416
PB.10100.10	9	2053	5	novel_in_catalog	gene: Zm00001d045076	novel	3.21	95.12	coding	138	136	549
PB.10100.11	9	1455	7	novel_in_catalog	gene: Zm00001d045076	novel	39.51	95.12	coding	184	128	679
PB.4471.1	3	2122	4	full-splice_match	gene: Zm00001d043442	Zm00001d043442_T001	18.82	18.82	coding	558	179	1852
PB.8528.1	7	1441	4	full-splice_match	gene: Zm00001d020670	Zm00001d020670_T001	8.43	8.43	coding	333	123	1121
PB.3907.11	3	2724	3	incomplete-splice_match	gene: Zm00001d040470	Zm00001d040470_T004	3.51	183.26	coding	427	1158	2438
PB.8871.1	7	643	1	incomplete-splice_match	gene: Zm00001d022017	Zm00001d022017_T003	10.25	10.25	coding	89	256	522
PB.8395.2	7	1964	3	full-splice_match	gene: Zm00001d020013	Zm00001d020013_T001	5.73	17.74	coding	526	194	1771
PB.4716.1	3	1172	1	full-splice_match	gene: Zm00001d044425	Zm00001d044425_T001	19.70	19.70	coding	324	39	1010
PB.8395.1	7	2081	3	full-splice_match	gene: Zm00001d020013	Zm00001d020013_T001	12.01	17.74	coding	526	126	1703
PB.7461.1	6	3445	4	full-splice_match	gene: Zm00001d036422	Zm00001d036422_T008	5.79	5.79	coding	857	329	2899
PB.1494.1	1	1542	2	full-splice_match	gene: Zm00001d034440	Zm00001d034440_T001	130.98	130.98	coding	404	86	1297
PB.2101.2	10	1803	10	full-splice_match	gene: Zm00001d025653	Zm00001d025653_T001	28.44	35.52	coding	481	53	1495
PB.5001.5	4	1948	1	full-splice_match	gene: Zm00001d049632	Zm00001d049632_T001	0.00	62.77	coding	479	406	1842
PB.5001.7	4	1866	2	genic	gene: Zm00001d049632	novel	0.00	62.77	coding	479	298	1734
PB.3075.4	2	3078	4	novel_in_catalog	gene: Zm00001d005190	novel	7.02	76.54	coding	309	47	973
PB.5001.1	4	2098	2	genic	gene: Zm00001d049632	novel	12.53	62.77	coding	479	298	1734

PB.1952.1	10	1737	1	full-splice_match	gene: Zm00001d024866	Zm00001d024866_T001	1.83	7.72	coding	366	171	1268
PB.5001.3	4	1997	2	genic	gene: Zm00001d049632	novel	43.51	62.77	coding	479	298	1734
PB.5001.2	4	2195	1	full-splice_match	gene: Zm00001d049632	Zm00001d049632_T001	6.74	62.77	coding	479	406	1842
PB.132.12	1	2429	5	full-splice_match	gene: Zm00001d027802	Zm00001d027802_T003	0.00	63.41	coding	587	390	2150
PB.132.13	1	2592	3	novel_in_catalog	gene: Zm00001d027802	novel	0.00	63.41	coding	360	321	1400
PB.132.10	1	2477	4	novel_in_catalog	gene: Zm00001d027802	novel	0.00	63.41	coding	382	262	1407
PB.132.11	1	2506	4	novel_in_catalog	gene: Zm00001d027802	novel	4.88	63.41	coding	343	1174	2202
PB.3394.3	2	2241	1	novel_in_catalog	gene: Zm00001d006753	novel	0.00	12.53	coding	454	757	2118
PB.1441.4	1	2206	17	full-splice_match	gene: Zm00001d034245	Zm00001d034245_T009	14.53	24.20	coding	570	281	1990
PB.132.14	1	2225	5	full-splice_match	gene: Zm00001d027802	Zm00001d027802_T001	0.00	63.41	coding	587	188	1948
PB.7428.1	6	2667	3	intergenic	novelGene_137	novel	7.11	7.11	non_coding	NA	NA	NA
PB.9859.2	8	2045	3	full-splice_match	gene: Zm00001d012074	Zm00001d012074_T001	11.61	31.39	coding	500	251	1750
PB.704.4	1	1899	10	full-splice_match	gene: Zm00001d030675	Zm00001d030675_T002	5.43	5.43	coding	429	253	1539
PB.704.6	1	1732	10	fusion	gene: Zm00001d030675_g	novel	0.00	54.84	coding	444	109	1440
PB.704.1	1	1818	10	fusion	gene: Zm00001d030675_g	novel	54.84	54.84	coding	444	124	1455
PB.1885.1	10	384	3	intergenic	novelGene_139	novel	67.13	67.13	non_coding	NA	NA	NA
PB.9859.4	8	1958	3	full-splice_match	gene: Zm00001d012074	Zm00001d012074_T001	0.00	31.39	coding	500	265	1764

PB.5033.1	4	2045	14	full-splice_match	gene: Zm00001d049764	Zm00001d049764_T001	64.56	64.56	coding	456	119	1486
PB.3245.1	2	711	1	full-splice_match	gene: Zm00001d006101	Zm00001d006101_T001	9.87	43.71	non_coding	NA	NA	NA
PB.8653.1	7	613	1	novel_in_catalog	gene: Zm00001d021269	novel	151.38	151.38	non_coding	NA	NA	NA
PB.3245.2	2	835	1	full-splice_match	gene: Zm00001d006101	Zm00001d006101_T001	33.84	43.71	coding	151	86	538
PB.1837.1	10	1140	2	full-splice_match	gene: Zm00001d024265	Zm00001d024265_T001	34.52	43.94	coding	300	70	969
PB.5190.2	4	2947	10	full-splice_match	gene: Zm00001d050868	Zm00001d050868_T001	4.88	94.38	coding	364	1458	2549
PB.5190.3	4	2497	10	novel_in_catalog	gene: Zm00001d050868	novel	9.76	94.38	coding	411	216	1448
PB.8879.1	7	3717	2	full-splice_match	gene: Zm00001d022044	Zm00001d022044_T001	6.04	6.04	coding	1101	177	3479
PB.5190.1	4	2511	11	full-splice_match	gene: Zm00001d050868	Zm00001d050868_T004	79.75	94.38	coding	623	244	2112
PB.9021.3	7	2294	3	incomplete-splice_match	gene: Zm00001d022527	Zm00001d022527_T020	28.72	35.63	coding	644	161	2092
PB.7902.5	6	1414	3	full-splice_match	gene: Zm00001d038325	Zm00001d038325_T001	0.00	104.15	coding	383	74	1222
PB.3696.3	3	1323	2	full-splice_match	gene: Zm00001d039439	Zm00001d039439_T001	0.00	73.64	coding	345	159	1193
PB.3696.2	3	1527	2	full-splice_match	gene: Zm00001d039439	Zm00001d039439_T001	73.64	73.64	coding	345	194	1228
PB.5382.1	4	1723	1	full-splice_match	gene: Zm00001d051899	Zm00001d051899_T001	8.61	8.61	coding	429	159	1445
PB.9021.5	7	839	2	novel_in_catalog	gene: Zm00001d022527	novel	2.13	35.63	coding	161	133	615
PB.8054.3	6	1922	10	full-splice_match	gene: Zm00001d038894	Zm00001d038894_T001	29.62	29.62	coding	562	26	1711
PB.4038.1	3	1476	6	full-splice_match	gene: Zm00001d041491	Zm00001d041491_T002	22.18	22.18	coding	215	348	992

PB.7970.1	6	976	1	full-splice_match	gene: Zm00001d038585	Zm00001d038585_T001	99.72	99.72	coding	211	135	767
PB.4044.1	3	1704	1	full-splice_match	gene: Zm00001d041518	Zm00001d041518_T001	12.26	18.85	coding	341	103	1125
PB.6871.1	5	1747	2	full-splice_match	gene: Zm00001d017329	Zm00001d017329_T001	21.26	26.53	coding	513	46	1584
PB.6628.1	5	464	2	intergenic	novelGene_238	novel	182.81	182.81	non_coding	NA	NA	NA
PB.6871.2	5	1948	1	genic	gene: Zm00001d017329	novel	5.27	26.53	coding	175	47	571
PB.4208.1	3	1887	10	full-splice_match	gene: Zm00001d042303	Zm00001d042303_T001	55.55	66.16	coding	499	159	1655
PB.10774.2	9	2892	9	novel_not_in_catalog	gene: Zm00001d048364	novel	6.82	18.51	coding	376	1445	2572
PB.10774.1	9	3664	10	novel_not_in_catalog	gene: Zm00001d048364	novel	11.69	18.51	coding	970	415	3324
PB.2638.1	2	2211	18	full-splice_match	gene: Zm00001d002791	Zm00001d002791_T011	11.75	11.75	coding	262	963	1748
PB.7902.1	6	1571	3	full-splice_match	gene: Zm00001d038325	Zm00001d038325_T001	42.68	104.15	coding	383	25	1173
PB.10461.38	9	2547	10	novel_not_in_catalog	gene: Zm00001d047166	novel	0.00	330.25	coding	162	92	577
PB.8897.3	7	842	6	full-splice_match	gene: Zm00001d022111	Zm00001d022111_T001	87.04	98.54	coding	232	36	731
PB.8897.2	7	1845	1	novel_in_catalog	gene: Zm00001d022111	novel	5.45	98.54	coding	106	1346	1663
PB.8897.1	7	2382	6	full-splice_match	gene: Zm00001d022111	Zm00001d022111_T001	6.05	98.54	coding	232	31	726
PB.10126.1	9	916	1	full-splice_match	gene: Zm00001d045204	Zm00001d045204_T001	53.77	53.77	coding	137	94	504
PB.6127.1	5	1825	3	full-splice_match	gene: Zm00001d013573	Zm00001d013573_T001	39.94	39.94	coding	463	101	1489
PB.1936.1	10	462	1	intergenic	novelGene_155	novel	383.46	383.46	non_coding	NA	NA	NA

PB.7595.5	6	2916	5	full-splice_match	gene: Zm00001d037109	Zm00001d037109_T005	3.34	71.30	coding	528	1114	2697
PB.9592.5	8	1387	4	novel_not_in_catalog	gene: Zm00001d010851	novel	0.42	15.56	coding	405	171	1385
PB.7595.6	6	2399	6	novel_in_catalog	gene: Zm00001d037109	novel	16.39	71.30	coding	375	379	1503
PB.9592.1	8	2036	4	novel_not_in_catalog	gene: Zm00001d010851	novel	10.83	15.56	coding	422	183	1448
PB.9592.2	8	2048	3	novel_not_in_catalog	gene: Zm00001d010851	novel	0.00	15.56	coding	264	135	926
PB.7595.2	6	2331	6	novel_in_catalog	gene: Zm00001d037109	novel	0.00	71.30	coding	528	551	2134
PB.282.2	1	1780	1	full-splice_match	gene: Zm00001d028451	Zm00001d028451_T001	8.95	36.26	coding	330	531	1520
PB.282.3	1	1435	1	full-splice_match	gene: Zm00001d028451	Zm00001d028451_T001	13.30	36.26	coding	330	283	1272
PB.282.1	1	1544	1	full-splice_match	gene: Zm00001d028451	Zm00001d028451_T001	14.01	36.26	coding	330	183	1172
PB.7427.1	6	2403	15	novel_not_in_catalog	gene: Zm00001d036181	novel	8.11	8.11	coding	330	152	1141
PB.282.4	1	1096	1	full-splice_match	gene: Zm00001d028451	Zm00001d028451_T001	0.00	36.26	coding	330	71	1060
PB.4565.1	3	2229	15	full-splice_match	gene: Zm00001d043773	Zm00001d043773_T004	15.50	17.82	coding	560	213	1892
PB.6654.1	5	1415	7	full-splice_match	gene: Zm00001d016299	Zm00001d016299_T001	24.79	24.79	coding	335	95	1099
PB.6654.2	5	1323	7	full-splice_match	gene: Zm00001d016299	Zm00001d016299_T001	0.00	24.79	coding	335	115	1119
PB.4565.2	3	2696	14	full-splice_match	gene: Zm00001d043773	Zm00001d043773_T007	2.32	17.82	coding	347	127	1167
PB.2642.1	2	1298	2	full-splice_match	gene: Zm00001d002799	Zm00001d002799_T001	4.83	4.83	coding	261	89	871
PB.2314.2	10	2139	6	incomplete-splice_match	gene: Zm00001d026511	Zm00001d026511_T021	50.09	50.09	coding	603	45	1853

PB.6189.1	5	1989	3	novel_not_in_catalog	gene: Zm00001d013886	novel	16.63	70.28	coding	143	195	623
PB.2348.1	10	2111	2	incomplete-splice_match	gene: Zm00001d026603	Zm00001d026603_T002	1.29	1.29	coding	581	130	1872
PB.7106.2	5	1679	4	novel_in_catalog	gene: Zm00001d018290	novel	5.11	96.25	coding	211	118	750
PB.6189.2	5	1865	3	novel_not_in_catalog	gene: Zm00001d013886	novel	40.21	70.28	coding	143	189	617
PB.6189.5	5	1991	1	novel_in_catalog	gene: Zm00001d013886	novel	10.04	70.28	non_coding	NA	NA	NA
PB.6189.4	5	2629	2	novel_in_catalog	gene: Zm00001d013886	novel	3.41	70.28	coding	143	1059	1487
PB.6189.7	5	851	2	genic	gene: Zm00001d013886	novel	0.00	70.28	coding	143	192	620
PB.6189.6	5	1776	3	novel_not_in_catalog	gene: Zm00001d013886	novel	0.00	70.28	coding	143	202	630
PB.3251.2	2	3411	3	full-splice_match	gene: Zm00001d006127	Zm00001d006127_T002	6.73	34.65	coding	230	200	889
PB.8155.1	7	1177	1	full-splice_match	gene: Zm00001d018631	Zm00001d018631_T001	0.00	287.63	coding	250	46	795
PB.8155.2	7	1087	1	full-splice_match	gene: Zm00001d018631	Zm00001d018631_T001	68.80	287.63	coding	250	65	814
PB.8155.3	7	1028	1	full-splice_match	gene: Zm00001d018631	Zm00001d018631_T001	218.83	287.63	coding	250	105	854
PB.95.2	1	2417	2	full-splice_match	gene: Zm00001d027596	Zm00001d027596_T005	13.21	40.01	coding	552	274	1929
PB.95.1	1	3415	3	full-splice_match	gene: Zm00001d027596	Zm00001d027596_T004	1.74	40.01	coding	580	1356	3095
PB.6222.3	5	2292	13	novel_in_catalog	gene: Zm00001d014037	novel	0.00	30.58	coding	540	256	1875
PB.95.6	1	2887	3	full-splice_match	gene: Zm00001d027596	Zm00001d027596_T004	12.83	40.01	coding	580	952	2691
PB.6222.1	5	2496	13	novel_in_catalog	gene: Zm00001d014037	novel	30.58	30.58	coding	540	256	1875

PB.95.4	1	2600	3	full-splice_match	gene: Zm00001d027596	Zm00001d027596_T002	12.23	40.01	coding	537	648	2258
PB.7702.1	6	1525	1	full-splice_match	gene: Zm00001d037611	Zm00001d037611_T001	7.01	7.01	coding	445	52	1386
PB.10825.1	9	2222	8	full-splice_match	gene: Zm00001d048531	Zm00001d048531_T001	13.88	15.52	coding	655	44	2008
PB.10666.1	9	2950	2	novel_in_catalog	gene: Zm00001d047955	novel	23.23	55.91	coding	268	1881	2684
PB.1034.1	1	1459	4	full-splice_match	gene: Zm00001d032405	Zm00001d032405_T001	28.06	50.38	coding	351	169	1221
PB.10342.4	9	2043	7	novel_not_in_catalog	gene: Zm00001d046468	novel	17.22	17.22	coding	218	1071	1724
PB.6865.9	5	1839	9	full-splice_match	gene: Zm00001d017298	Zm00001d017298_T007	0.00	57.49	coding	295	558	1442
PB.6542.2	5	2051	3	full-splice_match	gene: Zm00001d015450	Zm00001d015450_T002	27.60	60.72	coding	460	334	1713
PB.6542.3	5	1920	3	full-splice_match	gene: Zm00001d015450	Zm00001d015450_T002	0.00	60.72	coding	460	306	1685
PB.1681.1	10	837	2	full-splice_match	gene: Zm00001d023344	Zm00001d023344_T004	11.07	11.07	coding	129	91	477
PB.6542.1	5	2231	3	full-splice_match	gene: Zm00001d015450	Zm00001d015450_T002	33.12	60.72	coding	460	390	1769
PB.6865.1	5	1724	9	full-splice_match	gene: Zm00001d017298	Zm00001d017298_T004	10.48	57.49	coding	352	555	1610
PB.6865.6	5	2247	8	novel_in_catalog	gene: Zm00001d017298	novel	4.26	57.49	coding	298	427	1320
PB.9151.1	8	2527	6	full-splice_match	gene: Zm00001d008611	Zm00001d008611_T006	2.57	49.10	coding	216	359	1006
PB.6865.5	5	1976	9	full-splice_match	gene: Zm00001d017298	Zm00001d017298_T007	42.75	57.49	coding	295	558	1442
PB.10387.1	9	930	1	full-splice_match	gene: Zm00001d046696	Zm00001d046696_T001	5.85	18.70	coding	29	484	570
PB.1634.2	1	2003	7	full-splice_match	gene: Zm00001d034919	Zm00001d034919_T001	38.19	111.65	coding	584	111	1862

PB.10387.2	9	1159	1	full-splice_match	gene: Zm00001d046696	Zm00001d046696_T001	12.85	18.70	coding	271	99	911
PB.10110.8	9	2524	8	full-splice_match	gene: Zm00001d045139	Zm00001d045139_T002	0.00	353.77	coding	762	95	2380
PB.10110.7	9	2675	8	full-splice_match	gene: Zm00001d045139	Zm00001d045139_T002	111.54	353.77	coding	762	105	2390
PB.10110.6	9	2839	7	novel_in_catalog	gene: Zm00001d045139	novel	2.70	353.77	coding	702	101	2206
PB.1054.1	1	928	1	full-splice_match	gene: Zm00001d032479	Zm00001d032479_T001	19.17	19.17	coding	275	48	872
PB.9916.1	8	1879	1	antisense	novelGene_gene: Zm00001d012304_A	novel	43.43	43.43	coding	268	575	1378
PB.518.4	1	2171	1	incomplete-splice_match	gene: Zm00001d029516	Zm00001d029516_T002	1.44	29.09	coding	496	469	1956
PB.10110.1	9	2803	8	full-splice_match	gene: Zm00001d045139	Zm00001d045139_T002	239.53	353.77	coding	762	20	2305
PB.8502.1	7	1899	8	full-splice_match	gene: Zm00001d020537	Zm00001d020537_T001	17.45	17.45	coding	529	145	1731
PB.7589.1	6	709	3	full-splice_match	gene: Zm00001d037097	Zm00001d037097_T001	282.61	282.61	coding	139	71	487
PB.8365.2	7	1256	8	full-splice_match	gene: Zm00001d019754	Zm00001d019754_T002	15.43	80.81	coding	316	172	1119
PB.9151.4	8	1467	7	full-splice_match	gene: Zm00001d008611	Zm00001d008611_T003	30.74	49.10	coding	260	362	1141
PB.6578.1	5	924	4	full-splice_match	gene: Zm00001d015737	Zm00001d015737_T002	55.89	55.89	coding	166	59	556
PB.4653.3	3	1917	4	novel_not_in_catalog	gene: Zm00001d044146	novel	5.52	46.01	coding	275	885	1709
PB.8746.1	7	2127	1	antisense	novelGene_gene: Zm00001d021598_A	novel	24.32	24.32	coding	188	871	1434
PB.10349.6	9	1198	4	incomplete-splice_match	gene: Zm00001d046492	Zm00001d046492_T002	0.00	250.90	coding	285	229	1083
PB.9706.1	8	603	1	full-splice_match	gene: Zm00001d011412	Zm00001d011412_T001	406.79	406.79	non_coding	NA	NA	NA

PB.3578.2	2	2775	7	full-splice_match	gene: Zm00001d007760	Zm00001d007760_T001	20.52	20.52	coding	778	177	2510
PB.10349.5	9	1273	4	novel_in_catalog	gene: Zm00001d046492	novel	167.82	250.90	coding	285	254	1108
PB.5029.1	4	2012	2	novel_in_catalog	gene: Zm00001d049743	novel	12.13	12.13	coding	334	870	1871
PB.2048.1	10	726	1	genic	gene: Zm00001d025322	novel	59.10	59.10	coding	56	291	458
PB.6309.1	5	849	4	full-splice_match	gene: Zm00001d014488	Zm00001d014488_T002	12.53	12.53	coding	192	88	663
PB.3953.4	3	1668	6	full-splice_match	gene: Zm00001d040789	Zm00001d040789_T002	27.51	31.97	non_coding	NA	NA	NA
PB.3953.3	3	2194	5	full-splice_match	gene: Zm00001d040789	Zm00001d040789_T003	4.46	31.97	coding	181	164	706
PB.7194.1	5	1236	10	incomplete-splice_match	gene: Zm00001d018533	Zm00001d018533_T008	7.46	7.46	coding	326	22	999
PB.6214.1	5	1162	6	full-splice_match	gene: Zm00001d014016	Zm00001d014016_T001	7.04	7.04	coding	207	14	634
PB.6214.2	5	529	1	incomplete-splice_match	gene: Zm00001d014016	Zm00001d014016_T001	0.00	7.04	non_coding	NA	NA	NA
PB.10829.1	9	3451	25	incomplete-splice_match	gene: Zm00001d048546	Zm00001d048546_T047	14.00	41.83	coding	1000	118	3117
PB.9037.2	7	1873	2	genic	gene: Zm00001d022587	novel	15.30	22.60	coding	506	12	1529
PB.3132.1	2	498	3	incomplete-splice_match	gene: Zm00001d005513	Zm00001d005513_T002	53.30	53.30	coding	91	32	304
PB.9037.1	7	2109	1	full-splice_match	gene: Zm00001d022587	Zm00001d022587_T001	1.57	22.60	coding	506	86	1603
PB.1534.8	1	2186	11	novel_in_catalog	gene: Zm00001d034602	novel	0.00	213.93	coding	313	63	1001
PB.4948.2	4	3278	11	full-splice_match	gene: Zm00001d049357	Zm00001d049357_T001	13.60	62.26	coding	988	197	3160
PB.4948.1	4	2676	8	incomplete-splice_match	gene: Zm00001d049357	Zm00001d049357_T001	48.66	62.26	coding	766	153	2450

PB.1695.1	10	1697	1	full-splice_match	gene: Zm00001d023399	Zm00001d023399_T001	12.65	12.65	coding	456	84	1451
PB.5087.1	4	962	3	full-splice_match	gene: Zm00001d050121	Zm00001d050121_T001	40.85	40.85	coding	218	110	763
PB.10349.8	9	2666	1	novel_in_catalog	gene: Zm00001d046492	novel	0.71	250.90	coding	144	190	621
PB.9194.2	8	543	2	intergenic	novelGene_63	novel	22.50	79.82	non_coding	NA	NA	NA
PB.9673.2	8	1231	1	genic	gene: Zm00001d011237	novel	21.36	62.67	coding	276	153	980
PB.7993.1	6	1002	2	intergenic	novelGene_266	novel	45.30	45.30	coding	220	205	864
PB.9194.1	8	659	1	intergenic	novelGene_63	novel	57.32	79.82	non_coding	NA	NA	NA
PB.89.1	1	1039	1	novel_in_catalog	gene: Zm00001d027557	novel	10.87	52.57	coding	114	70	411
PB.89.2	1	947	2	full-splice_match	gene: Zm00001d027557	Zm00001d027557_T001	41.70	52.57	coding	254	1	762
PB.1006.1	1	976	1	full-splice_match	gene: Zm00001d032285	Zm00001d032285_T001	2.60	2.60	coding	257	92	862
PB.3502.1	2	1566	1	full-splice_match	gene: Zm00001d007272	Zm00001d007272_T001	6.61	6.61	coding	395	189	1373
PB.6637.3	5	1490	3	novel_not_in_catalog	gene: Zm00001d016185	novel	11.14	189.77	coding	312	44	979
PB.3233.2	2	2177	1	genic	gene: Zm00001d006053	novel	5.47	27.30	coding	236	1110	1817
PB.3233.3	2	1973	2	novel_in_catalog	gene: Zm00001d006053	novel	0.00	27.30	coding	236	913	1620
PB.6637.2	5	1639	2	novel_in_catalog	gene: Zm00001d016185	novel	20.96	189.77	coding	357	46	1116
PB.7749.1	6	1815	3	full-splice_match	gene: Zm00001d037792	Zm00001d037792_T001	4.57	4.57	coding	485	139	1593
PB.890.1	1	1486	1	full-splice_match	gene: Zm00001d031725	Zm00001d031725_T001	36.06	36.06	coding	350	137	1186

PB.9349.1	8	1248	1	full-splice_match	gene: Zm00001d009687	Zm00001d009687_T001	33.58	33.58	coding	291	76	948
PB.5269.5	4	955	1	antisense	novelGene_gene: Zm00001d051349_A	novel	19.00	103.06	non_coding	NA	NA	NA
PB.8953.3	7	1275	4	novel_not_in_catalog	gene: Zm00001d022330	novel	23.05	52.68	coding	318	14	967
PB.2320.1	10	1121	1	full-splice_match	gene: Zm00001d026530	Zm00001d026530_T001	40.53	40.53	coding	149	176	622
PB.8890.1	7	1733	6	full-splice_match	gene: Zm00001d022082	Zm00001d022082_T001	30.77	31.16	coding	447	99	1439
PB.6996.8	5	2859	13	novel_in_catalog	gene: Zm00001d017876	novel	0.73	25.93	coding	170	1096	1605
PB.506.5	1	1870	3	full-splice_match	gene: Zm00001d029468	Zm00001d029468_T002	0.77	31.85	coding	472	336	1751
PB.8935.1	7	2091	3	novel_in_catalog	gene: Zm00001d022252	novel	7.21	7.21	coding	232	98	793
PB.6996.7	5	2897	12	novel_in_catalog	gene: Zm00001d017876	novel	1.88	25.93	coding	301	740	1642
PB.506.1	1	1800	3	full-splice_match	gene: Zm00001d029468	Zm00001d029468_T002	27.98	31.85	coding	472	155	1570
PB.506.2	1	2935	2	novel_in_catalog	gene: Zm00001d029468	novel	3.10	31.85	coding	268	155	958
PB.9485.1	8	1329	3	full-splice_match	gene: Zm00001d010403	Zm00001d010403_T001	105.83	125.38	coding	349	95	1141
PB.8266.1	7	1311	3	genic	gene: Zm00001d019142	novel	44.69	115.31	coding	253	87	845
PB.3340.3	2	2645	7	novel_in_catalog	gene: Zm00001d006497	novel	1.39	18.06	coding	444	72	1403
PB.4505.3	3	1544	13	full-splice_match	gene: Zm00001d043555	Zm00001d043555_T001	0.66	33.52	coding	386	385	1542
PB.9774.1	8	966	7	novel_not_in_catalog	gene: Zm00001d011717	novel	59.58	59.58	coding	206	97	714
PB.5269.4	4	1369	2	antisense	novelGene_gene: Zm00001d051349_A	novel	0.00	103.06	coding	103	942	1250

PB.4676.1	3	3083	4	full-splice_match	gene: Zm00001d044232	Zm00001d044232_T004	41.05	70.69	coding	756	257	2524
PB.2158.3	10	1283	3	full-splice_match	gene: Zm00001d025864	Zm00001d025864_T001	19.22	43.22	coding	277	175	1005
PB.2158.2	10	1395	3	full-splice_match	gene: Zm00001d025864	Zm00001d025864_T001	18.96	43.22	coding	277	173	1003
PB.4603.3	3	1043	2	full-splice_match	gene: Zm00001d043935	Zm00001d043935_T001	0.00	79.60	coding	220	268	927
PB.6810.21	5	1538	8	novel_in_catalog	gene: Zm00001d017096	novel	0.00	118.92	coding	193	105	683
PB.5550.1	4	1465	8	full-splice_match	gene: Zm00001d052561	Zm00001d052561_T002	47.06	47.06	coding	367	102	1202
PB.4719.1	3	1045	2	novel_in_catalog	gene: Zm00001d044434	novel	66.68	66.68	coding	262	19	804
PB.7438.1	6	3216	16	full-splice_match	gene: Zm00001d036296	Zm00001d036296_T001	10.31	10.31	coding	931	97	2889
PB.5444.2	4	1328	5	incomplete-splice_match	gene: Zm00001d052193	Zm00001d052193_T015	4.58	24.55	coding	193	520	1098
PB.7983.1	6	2521	8	full-splice_match	gene: Zm00001d038625	Zm00001d038625_T001	14.33	192.70	coding	635	102	2006
PB.7983.2	6	2362	8	full-splice_match	gene: Zm00001d038625	Zm00001d038625_T001	99.11	192.70	coding	635	100	2004
PB.6341.4	5	1171	3	novel_not_in_catalog	gene: Zm00001d014611	novel	22.34	165.59	coding	348	79	1122
PB.7983.4	6	359	3	incomplete-splice_match	gene: Zm00001d038625	Zm00001d038625_T001	79.27	192.70	non_coding	NA	NA	NA
PB.10249.8	9	2917	4	full-splice_match	gene: Zm00001d045804	Zm00001d045804_T014	0.00	37.21	coding	305	333	1247
PB.2229.2	10	1147	12	full-splice_match	gene: Zm00001d026214	Zm00001d026214_T003	56.37	100.48	coding	270	150	959
PB.6341.3	5	1368	2	novel_in_catalog	gene: Zm00001d014611	novel	11.53	165.59	coding	104	837	1148
PB.8770.1	7	2238	4	full-splice_match	gene: Zm00001d021692	Zm00001d021692_T001	3.07	10.91	coding	694	68	2149

PB.5097.1	4	753	4	full-splice_match	gene: Zm00001d050155	Zm00001d050155_T001	59.82	59.82	coding	197	58	648
PB.8770.3	7	2066	5	full-splice_match	gene: Zm00001d021692	Zm00001d021692_T003	7.64	10.91	coding	651	30	1982
PB.4791.1	4	722	1	genic	gene: Zm00001d048583	novel	167.78	269.54	coding	77	490	720
PB.7049.5	5	1973	21	incomplete-splice_match	gene: Zm00001d018060	Zm00001d018060_T003	2.73	24.41	coding	621	111	1973
PB.1769.1	10	1950	6	novel_not_in_catalog	gene: Zm00001d023813	novel	27.39	27.39	coding	449	216	1562
PB.6341.1	5	1235	4	novel_not_in_catalog	gene: Zm00001d014611	novel	126.08	165.59	coding	262	257	1042
PB.10170.1	9	1093	2	full-splice_match	gene: Zm00001d045400	Zm00001d045400_T001	56.17	56.17	coding	272	117	932
PB.2158.4	10	1903	2	novel_in_catalog	gene: Zm00001d025864	novel	0.00	43.22	coding	131	176	568
PB.2480.1	2	2507	2	full-splice_match	gene: Zm00001d002131	Zm00001d002131_T001	18.77	18.77	coding	195	1471	2055
PB.7636.2	6	1371	1	novel_in_catalog	gene: Zm00001d037275	novel	271.66	3319.79	coding	102	83	388
PB.5994.1	5	967	5	full-splice_match	gene: Zm00001d013092	Zm00001d013092_T001	66.57	261.79	coding	152	127	582
PB.10128.2	9	2500	2	full-splice_match	gene: Zm00001d045210	Zm00001d045210_T001	1.31	36.55	coding	154	64	525
PB.10128.3	9	703	3	novel_not_in_catalog	gene: Zm00001d045210	novel	34.59	36.55	coding	154	63	524
PB.10128.1	9	2620	2	full-splice_match	gene: Zm00001d045210	Zm00001d045210_T001	0.65	36.55	coding	154	66	527
PB.6897.1	5	1430	7	novel_in_catalog	gene: Zm00001d017448	novel	5.89	38.54	coding	153	655	1113
PB.3165.1	2	794	1	full-splice_match	gene: Zm00001d005698	Zm00001d005698_T001	124.14	124.14	coding	180	81	620
PB.5731.1	4	2975	4	intergenic	novelGene_352	novel	2.73	2.73	coding	730	164	2353

PB.1157.2	1	1939	3	novel_not_in_catalog	gene: Zm00001d032897	novel	0.00	297.96	coding	245	1032	1766
PB.1292.2	1	2158	9	full-splice_match	gene: Zm00001d033575	Zm00001d033575_T001	7.90	23.02	coding	529	218	1804
PB.1292.1	1	2066	9	full-splice_match	gene: Zm00001d033575	Zm00001d033575_T001	15.12	23.02	coding	475	96	1520
PB.7446.1	6	598	1	intergenic	novelGene_150	novel	18.38	18.38	non_coding	NA	NA	NA
PB.10922.4	B73V4 ctg51	2810	9	fusion	gene: Zm00001d000312_g	novel	9.61	9.61	coding	644	311	2242
PB.1004.1	1	1321	7	full-splice_match	gene: Zm00001d032280	Zm00001d032280_T002	37.06	37.06	coding	256	268	1035
PB.2126.2	10	1306	6	full-splice_match	gene: Zm00001d025752	Zm00001d025752_T004	49.69	53.67	coding	159	478	954
PB.6810.23	5	2424	5	novel_in_catalog	gene: Zm00001d017096	novel	3.62	118.92	coding	129	108	494
PB.10922.3	B73V4 ctg51	2730	9	fusion	gene: Zm00001d000312_g	novel	0.00	9.61	coding	645	332	2266
PB.2460.1	2	1196	9	novel_in_catalog	gene: Zm00001d002034	novel	98.81	98.81	coding	214	326	967
PB.9491.1	8	1185	6	full-splice_match	gene: Zm00001d010425	Zm00001d010425_T005	169.52	169.52	coding	215	227	871
PB.554.2	1	2098	10	full-splice_match	gene: Zm00001d029663	Zm00001d029663_T001	37.92	81.74	coding	520	202	1761
PB.4917.2	4	2222	13	full-splice_match	gene: Zm00001d049190	Zm00001d049190_T008	18.86	26.75	coding	606	60	1877
PB.1991.4	10	1852	5	full-splice_match	gene: Zm00001d025011	Zm00001d025011_T003	0.00	28.58	coding	222	313	978
PB.1991.3	10	2319	4	novel_in_catalog	gene: Zm00001d025011	novel	1.24	28.58	coding	222	550	1215
PB.1991.2	10	1778	6	full-splice_match	gene: Zm00001d025011	Zm00001d025011_T001	21.39	28.58	coding	419	223	1479
PB.1775.1	10	1390	6	incomplete-splice_match	gene: Zm00001d023839	Zm00001d023839_T002	5.36	5.36	coding	375	24	1148

PB.4153.3	3	3053	1	incomplete-splice_match	gene: Zm00001d042028	Zm00001d042028_T013	0.23	31.09	coding	986	95	3052
PB.1578.2	1	1863	9	full-splice_match	gene: Zm00001d034736	Zm00001d034736_T003	10.93	17.79	coding	564	62	1753
PB.524.4	1	464	2	full-splice_match	gene: Zm00001d029546	Zm00001d029546_T001	27.14	12712.93	coding	79	79	315
PB.527.1	1	822	1	full-splice_match	gene: Zm00001d029558	Zm00001d029558_T001	781.98	921.69	coding	160	102	581
PB.524.1	1	691	2	full-splice_match	gene: Zm00001d029546	Zm00001d029546_T001	1296.91	12712.93	non_coding	NA	NA	NA
PB.6193.1	5	1603	7	novel_not_in_catalog	gene: Zm00001d013910	novel	50.31	50.31	coding	362	47	1132
PB.524.3	1	583	2	full-splice_match	gene: Zm00001d029546	Zm00001d029546_T001	#######	12712.93	coding	79	82	318
PB.524.2	1	727	1	novel_in_catalog	gene: Zm00001d029546	novel	93.49	12712.93	non_coding	NA	NA	NA
PB.9647.5	8	3047	9	novel_in_catalog	gene: Zm00001d011119	novel	0.00	60.56	coding	314	79	1020
PB.9647.4	8	1819	11	full-splice_match	gene: Zm00001d011119	Zm00001d011119_T014	35.98	60.56	coding	484	80	1531
PB.9647.7	8	1836	11	novel_in_catalog	gene: Zm00001d011119	novel	17.01	60.56	coding	492	80	1555
PB.9647.6	8	3235	7	novel_in_catalog	gene: Zm00001d011119	novel	2.22	60.56	coding	158	2516	2989
PB.4638.4	3	2298	2	novel_in_catalog	gene: Zm00001d044090	novel	4.46	32.80	non_coding	NA	NA	NA
PB.2077.1	10	1969	5	novel_not_in_catalog	gene: Zm00001d025475	novel	3.48	3.48	coding	498	49	1542
PB.9756.2	8	2061	11	novel_in_catalog	gene: Zm00001d011642	novel	26.63	72.25	coding	449	241	1587
PB.9756.3	8	1803	12	full-splice_match	gene: Zm00001d011642	Zm00001d011642_T002	45.63	72.25	coding	502	242	1747
PB.7318.5	6	852	3	full-splice_match	gene: Zm00001d035543	Zm00001d035543_T001	41.50	389.67	coding	199	115	711

PB.10541.1	9	2363	12	full-splice_match	gene: Zm00001d047534	Zm00001d047534_T002	77.23	101.76	coding	604	119	1930
PB.10541.3	9	2223	12	full-splice_match	gene: Zm00001d047534	Zm00001d047534_T002	5.72	101.76	coding	604	149	1960
PB.10541.2	9	1938	11	novel_not_in_catalog	gene: Zm00001d047534	novel	18.80	101.76	coding	109	410	736
PB.1578.3	1	1414	7	novel_in_catalog	gene: Zm00001d034736	novel	0.00	17.79	coding	452	58	1413
PB.4787.2	3	1406	5	full-splice_match	gene: Zm00001d044699	Zm00001d044699_T001	27.08	81.61	coding	350	163	1212
PB.9677.5	8	1585	9	novel_not_in_catalog	gene: Zm00001d011253	novel	52.58	141.59	coding	154	225	686
PB.9092.1	8	1349	3	novel_in_catalog	gene: Zm00001d008323	novel	3.71	3.71	coding	160	870	1349
PB.7097.1	5	1293	5	novel_not_in_catalog	gene: Zm00001d018255	novel	80.27	109.30	coding	271	97	909
PB.8585.5	7	2548	2	genic	gene: Zm00001d020939	novel	0.20	33.46	coding	609	392	2218
PB.7600.4	6	1651	16	incomplete-splice_match	gene: Zm00001d037142	Zm00001d037142_T002	0.00	89.93	coding	508	126	1649
PB.7600.5	6	1819	11	full-splice_match	gene: Zm00001d037142	Zm00001d037142_T026	0.00	89.93	coding	581	77	1819
PB.7318.1	6	1053	3	full-splice_match	gene: Zm00001d035543	Zm00001d035543_T001	348.17	389.67	coding	199	214	810
PB.8015.1	6	2056	12	full-splice_match	gene: Zm00001d038776	Zm00001d038776_T001	22.56	85.48	coding	528	69	1652
PB.7600.1	6	3359	22	full-splice_match	gene: Zm00001d037142	Zm00001d037142_T023	14.62	89.93	coding	1040	150	3269
PB.1999.1	10	2800	9	full-splice_match	gene: Zm00001d025034	Zm00001d025034_T002	7.48	7.48	coding	776	142	2469
PB.7600.3	6	1871	16	incomplete-splice_match	gene: Zm00001d037142	Zm00001d037142_T002	74.06	89.93	coding	542	163	1788
PB.2318.1	10	815	4	full-splice_match	gene: Zm00001d026521	Zm00001d026521_T005	66.55	66.55	coding	94	64	345

PB.10914.1	B73V4 ctg48	2141	11	full-splice_match	gene: Zm00001d000357	Zm00001d000357_T003	7.62	7.62	coding	426	522	1799
PB.5953.1	5	2110	2	novel_not_in_catalog	gene: Zm00001d012963	novel	6.52	6.52	coding	426	316	1593
PB.9677.9	8	2804	7	novel_not_in_catalog	gene: Zm00001d011253	novel	0.00	141.59	coding	137	1539	1949
PB.1715.1	10	682	3	novel_not_in_catalog	gene: Zm00001d023516	novel	27.16	27.16	coding	151	62	514
PB.7741.4	6	323	3	incomplete-splice_match	gene: Zm00001d037760	Zm00001d037760_T005	6.84	24.11	non_coding	NA	NA	NA
PB.9811.1	8	548	3	incomplete-splice_match	gene: Zm00001d011840	Zm00001d011840_T001	21.46	21.46	coding	107	73	393
PB.1634.1	1	2038	7	full-splice_match	gene: Zm00001d034919	Zm00001d034919_T001	73.46	111.65	coding	584	30	1781
PB.4627.1	3	1746	1	full-splice_match	gene: Zm00001d044030	Zm00001d044030_T001	26.02	26.02	coding	501	100	1602
PB.800.2	1	1802	1	full-splice_match	gene: Zm00001d031222	Zm00001d031222_T001	0.00	83.29	coding	527	39	1619
PB.800.3	1	1690	1	full-splice_match	gene: Zm00001d031222	Zm00001d031222_T001	0.00	83.29	coding	527	39	1619
PB.639.2	1	2407	8	full-splice_match	gene: Zm00001d030173	Zm00001d030173_T005	39.12	39.12	coding	527	311	1891
PB.2308.1	10	2352	1	full-splice_match	gene: Zm00001d026492	Zm00001d026492_T001	4.45	4.45	coding	421	640	1902
PB.639.4	1	2091	8	full-splice_match	gene: Zm00001d030173	Zm00001d030173_T005	0.00	39.12	coding	527	203	1783
PB.800.4	1	1542	1	full-splice_match	gene: Zm00001d031222	Zm00001d031222_T001	0.00	83.29	coding	502	36	1541
PB.8303.1	7	3138	17	full-splice_match	gene: Zm00001d019335	Zm00001d019335_T017	6.22	6.22	coding	297	816	1706
PB.173.2	1	1993	1	intergenic	novelGene_62	novel	4.81	4.81	non_coding	NA	NA	NA
PB.1977.1	10	2382	5	antisense	novelGene_gene: Zm00001d024947_A	novel	8.34	8.34	non_coding	NA	NA	NA

PB.10933.2	B73V4 ctg68	2235	7	full-splice_match	gene: Zm00001d000428	Zm00001d000428_T001	3.48	66.09	coding	640	119	2038
PB.10933.1	B73V4 ctg68	2339	7	full-splice_match	gene: Zm00001d000428	Zm00001d000428_T001	62.61	66.09	coding	640	97	2016
PB.9991.9	8	2215	9	novel_in_catalog	gene: Zm00001d012633	novel	2.16	111.96	coding	231	287	979
PB.9991.8	8	1987	10	full-splice_match	gene: Zm00001d012633	Zm00001d012633_T001	42.77	111.96	coding	509	288	1814
PB.8282.2	7	717	5	full-splice_match	gene: Zm00001d019219	Zm00001d019219_T001	876.54	900.08	coding	140	90	509
PB.8282.1	7	355	1	genic	gene: Zm00001d019219	novel	23.53	900.08	non_coding	NA	NA	NA
PB.7509.5	6	1613	3	novel_not_in_catalog	gene: Zm00001d036626	novel	0.00	250.91	coding	445	94	1428
PB.7509.2	6	1569	1	genic	gene: Zm00001d036626	novel	0.00	250.91	coding	235	560	1264
PB.7509.3	6	1720	3	novel_not_in_catalog	gene: Zm00001d036626	novel	46.08	250.91	coding	445	94	1428
PB.9991.1	8	784	4	incomplete-splice_match	gene: Zm00001d012633	Zm00001d012633_T001	65.20	111.96	coding	118	43	396
PB.7509.1	6	1817	3	novel_not_in_catalog	gene: Zm00001d036626	novel	202.67	250.91	coding	445	83	1417
PB.157.2	1	985	8	full-splice_match	gene: Zm00001d027922	Zm00001d027922_T001	194.93	194.93	coding	226	115	792
PB.3071.1	2	2123	12	full-splice_match	gene: Zm00001d005164	Zm00001d005164_T001	31.53	53.65	coding	512	202	1737
PB.1278.1	1	1961	11	novel_not_in_catalog	gene: Zm00001d033525	novel	4.42	5.79	coding	509	148	1674
PB.5089.3	4	3600	14	full-splice_match	gene: Zm00001d050125	Zm00001d050125_T002	33.66	33.66	coding	1059	112	3288
PB.442.1	1	2209	10	novel_in_catalog	gene: Zm00001d029144	novel	136.19	138.20	coding	564	256	1947
PB.5358.2	4	2861	9	novel_in_catalog	gene: Zm00001d051837	novel	0.97	4.40	coding	453	25	1383

PB.5303.1	4	2820	7	full-splice_match	gene: Zm00001d051544	Zm00001d051544_T002	14.62	14.62	coding	820	91	2550
PB.4288.1	3	777	3	full-splice_match	gene: Zm00001d042658	Zm00001d042658_T002	32.52	32.52	coding	152	45	500
PB.7378.2	6	1342	2	genic	gene: Zm00001d035918	novel	0.00	14.32	coding	373	9	1127
PB.6897.4	5	1070	8	novel_in_catalog	gene: Zm00001d017448	novel	0.00	38.54	coding	153	425	883
PB.107.16	1	2581	5	full-splice_match	gene: Zm00001d027667	Zm00001d027667_T013	1.18	139.20	coding	535	741	2345
PB.107.17	1	2487	5	novel_not_in_catalog	gene: Zm00001d027667	novel	0.00	139.20	coding	535	578	2182
PB.107.14	1	2437	5	full-splice_match	gene: Zm00001d027667	Zm00001d027667_T014	0.00	139.20	coding	535	593	2197
PB.5606.3	4	1173	1	full-splice_match	gene: Zm00001d052799	Zm00001d052799_T001	21.62	23.83	coding	307	49	969
PB.107.12	1	2219	5	novel_in_catalog	gene: Zm00001d027667	novel	2.04	139.20	coding	535	362	1966
PB.107.13	1	2373	5	novel_in_catalog	gene: Zm00001d027667	novel	0.00	139.20	coding	535	516	2120
PB.5782.1	4	3256	12	full-splice_match	gene: Zm00001d053672	Zm00001d053672_T007	25.62	25.62	coding	879	376	3012
PB.1409.1	1	1415	4	full-splice_match	gene: Zm00001d034073	Zm00001d034073_T001	3.31	3.31	coding	331	215	1207
PB.5782.3	4	3127	12	full-splice_match	gene: Zm00001d053672	Zm00001d053672_T007	0.00	25.62	coding	879	376	3012
PB.313.1	1	2130	6	full-splice_match	gene: Zm00001d028599	Zm00001d028599_T001	2.46	4.90	coding	576	102	1829
PB.107.18	1	2418	4	full-splice_match	gene: Zm00001d027667	Zm00001d027667_T004	0.00	139.20	coding	535	597	2201
PB.313.2	1	2663	4	novel_in_catalog	gene: Zm00001d028599	novel	0.37	4.90	coding	283	1525	2373
PB.3720.3	3	2064	6	novel_in_catalog	gene: Zm00001d039521	novel	3.16	22.35	coding	243	1168	1896

PB.3720.2	3	2278	4	novel_in_catalog	gene: Zm00001d039521	novel	5.11	22.35	coding	191	68	640
PB.3720.1	3	2190	5	full-splice_match	gene: Zm00001d039521	Zm00001d039521_T003	14.08	22.35	coding	258	1262	2035
PB.9856.1	8	1726	3	full-splice_match	gene: Zm00001d012033	Zm00001d012033_T002	76.91	76.91	coding	392	148	1323
PB.1632.1	1	1149	1	full-splice_match	gene: Zm00001d034916	Zm00001d034916_T001	15.79	46.44	coding	189	162	728
PB.4400.5	3	1947	13	full-splice_match	gene: Zm00001d043152	Zm00001d043152_T005	47.39	93.99	coding	538	108	1721
PB.5358.3	4	2707	11	full-splice_match	gene: Zm00001d051837	Zm00001d051837_T001	3.44	4.40	coding	849	32	2578
PB.2122.1	10	2827	3	novel_in_catalog	gene: Zm00001d025727	novel	26.13	114.82	coding	309	661	1587
PB.2152.1	10	2077	12	full-splice_match	gene: Zm00001d025846	Zm00001d025846_T001	13.18	66.24	coding	519	125	1681
PB.1598.2	1	2186	4	full-splice_match	gene: Zm00001d034805	Zm00001d034805_T006	5.90	83.94	coding	228	98	781
PB.2152.3	10	1907	12	full-splice_match	gene: Zm00001d025846	Zm00001d025846_T001	11.35	66.24	coding	519	127	1683
PB.7920.5	6	2890	9	fusion	gene: Zm00001d038384_g	novel	0.00	10.98	coding	254	128	889
PB.2400.4	2	2133	2	novel_in_catalog	gene: Zm00001d001806	novel	4.49	4.49	coding	161	640	1122
PB.2152.7	10	1873	11	novel_in_catalog	gene: Zm00001d025846	novel	1.34	66.24	coding	365	606	1700
PB.2152.6	10	1862	12	full-splice_match	gene: Zm00001d025846	Zm00001d025846_T001	31.50	66.24	coding	519	125	1681
PB.8009.1	6	1735	1	antisense	novelGene_gene: Zm00001d038764_A	novel	14.99	14.99	coding	236	272	979
PB.2122.3	10	4487	10	novel_not_in_catalog	gene: Zm00001d025727	novel	30.30	114.82	coding	298	3509	4402
PB.8138.2	6	3033	23	full-splice_match	gene: Zm00001d039214	Zm00001d039214_T024	15.18	23.75	coding	910	179	2908

PB.3079.2	2	1211	2	full-splice_match	gene: Zm00001d005208	Zm00001d005208_T001	50.18	57.37	coding	301	109	1011
PB.8190.1	7	1707	7	full-splice_match	gene: Zm00001d018787	Zm00001d018787_T002	10.00	10.00	coding	484	87	1538
PB.2241.1	10	3372	13	full-splice_match	gene: Zm00001d026265	Zm00001d026265_T001	37.17	37.17	coding	1010	113	3142
PB.3700.1	3	1538	11	full-splice_match	gene: Zm00001d039448	Zm00001d039448_T006	38.67	38.67	coding	387	49	1209
PB.662.1	1	1055	2	full-splice_match	gene: Zm00001d030345	Zm00001d030345_T001	17.74	94.13	coding	221	34	696
PB.662.2	1	953	2	full-splice_match	gene: Zm00001d030345	Zm00001d030345_T001	76.39	94.13	coding	221	34	696
PB.662.3	1	744	2	full-splice_match	gene: Zm00001d030345	Zm00001d030345_T001	0.00	94.13	coding	60	442	621
PB.4756.1	3	2691	7	incomplete-splice_match	gene: Zm00001d044564	Zm00001d044564_T002	5.98	5.98	coding	841	48	2570
PB.5517.1	4	1966	15	full-splice_match	gene: Zm00001d052416	Zm00001d052416_T005	11.38	38.13	coding	244	332	1063
PB.7145.1	5	526	2	full-splice_match	gene: Zm00001d018410	Zm00001d018410_T001	818.44	818.44	coding	4	312	323
PB.10787.3	9	1323	7	full-splice_match	gene: Zm00001d048410	Zm00001d048410_T005	3.18	98.77	coding	157	195	665
PB.7437.1	6	1459	3	full-splice_match	gene: Zm00001d036285	Zm00001d036285_T001	76.84	76.84	coding	324	201	1172
PB.10723.3	9	1760	11	novel_not_in_catalog	gene: Zm00001d048174	novel	0.49	46.35	coding	446	70	1407
PB.10340.4	9	1429	10	full-splice_match	gene: Zm00001d046456	Zm00001d046456_T001	29.67	57.19	coding	427	39	1319
PB.10787.2	9	1092	8	full-splice_match	gene: Zm00001d048410	Zm00001d048410_T001	95.59	98.77	coding	200	192	791
PB.10340.3	9	1670	10	full-splice_match	gene: Zm00001d046456	Zm00001d046456_T001	0.00	57.19	coding	380	137	1276
PB.10340.1	9	1232	6	incomplete-splice_match	gene: Zm00001d046456	Zm00001d046456_T001	27.53	57.19	coding	195	102	686

PB.7889.6	6	2145	2	novel_in_catalog	gene: Zm00001d038273	novel	0.00	168.88	coding	262	187	972
PB.7889.5	6	2401	3	novel_in_catalog	gene: Zm00001d038273	novel	102.86	168.88	coding	599	221	2017
PB.9561.1	8	1829	3	full-splice_match	gene: Zm00001d010724	Zm00001d010724_T001	5.66	31.57	coding	427	29	1309
PB.7889.3	6	2487	3	novel_in_catalog	gene: Zm00001d038273	novel	0.00	168.88	coding	599	218	2014
PB.9347.1	8	2779	4	full-splice_match	gene: Zm00001d009676	Zm00001d009676_T009	4.95	13.64	coding	143	434	862
PB.9347.2	8	2696	5	full-splice_match	gene: Zm00001d009676	Zm00001d009676_T003	5.79	13.64	coding	143	434	862
PB.9347.3	8	2581	5	novel_in_catalog	gene: Zm00001d009676	novel	2.90	13.64	coding	254	1636	2397
PB.292.1	1	2499	17	incomplete-splice_match	gene: Zm00001d028481	Zm00001d028481_T009	158.08	158.08	coding	578	407	2140
PB.10710.1	9	459	1	intergenic	novelGene_246	novel	24.92	24.92	coding	108	44	367
PB.1489.1	1	1432	1	full-splice_match	gene: Zm00001d034427	Zm00001d034427_T001	153.18	153.18	coding	392	97	1272
PB.7889.8	6	2297	2	novel_in_catalog	gene: Zm00001d038273	novel	18.15	168.88	coding	358	275	1348
PB.8582.1	7	1598	3	full-splice_match	gene: Zm00001d020931	Zm00001d020931_T003	14.82	14.82	coding	457	51	1421
PB.7660.2	6	3406	9	full-splice_match	gene: Zm00001d037408	Zm00001d037408_T027	12.83	35.80	coding	677	847	2877
PB.10456.1	9	757	1	full-splice_match	gene: Zm00001d047117	Zm00001d047117_T001	1778.40	1778.40	coding	129	211	597
PB.3975.1	3	2924	3	full-splice_match	gene: Zm00001d041063	Zm00001d041063_T001	7.60	7.60	coding	790	336	2705
PB.7660.5	6	3565	8	full-splice_match	gene: Zm00001d037408	Zm00001d037408_T029	2.10	35.80	coding	677	1067	3097
PB.7638.5	6	2223	8	novel_in_catalog	gene: Zm00001d037289	novel	10.83	40.04	coding	379	911	2047

PB.7798.2	6	1877	1	incomplete-splice_match	gene: Zm00001d037975	Zm00001d037975_T048	0.00	10.96	coding	523	295	1863
PB.3949.1	3	1549	2	full-splice_match	gene: Zm00001d040777	Zm00001d040777_T001	19.24	19.24	coding	399	171	1367
PB.7638.1	6	2859	11	full-splice_match	gene: Zm00001d037289	Zm00001d037289_T003	23.57	40.04	coding	842	116	2641
PB.7638.2	6	2058	8	novel_in_catalog	gene: Zm00001d037289	novel	5.64	40.04	coding	148	1421	1864
PB.10823.1	9	3291	19	full-splice_match	gene: Zm00001d048527	Zm00001d048527_T030	4.83	48.39	coding	842	476	3001
PB.10535.2	9	2349	11	incomplete-splice_match	gene: Zm00001d047518	Zm00001d047518_T002	20.01	20.01	coding	630	39	1928
PB.10823.4	9	3267	19	full-splice_match	gene: Zm00001d048527	Zm00001d048527_T030	36.32	48.39	coding	842	476	3001
PB.10823.5	9	3136	19	full-splice_match	gene: Zm00001d048527	Zm00001d048527_T030	0.00	48.39	coding	842	472	2997
PB.10823.6	9	1053	5	incomplete-splice_match	gene: Zm00001d048527	Zm00001d048527_T030	0.00	48.39	coding	191	481	1053
PB.3160.1	2	1896	6	full-splice_match	gene: Zm00001d005682	Zm00001d005682_T001	8.31	38.30	coding	377	458	1588
PB.2810.7	2	1784	6	novel_in_catalog	gene: Zm00001d003495	novel	5.22	67.99	coding	126	164	541
PB.2261.3	10	2125	2	genic	gene: Zm00001d026326	novel	0.00	42.28	coding	564	219	1910
PB.2810.2	2	1026	6	novel_not_in_catalog	gene: Zm00001d003495	novel	62.77	67.99	coding	214	173	814
PB.6535.1	5	974	4	full-splice_match	gene: Zm00001d015423	Zm00001d015423_T006	44.87	89.33	coding	127	257	637
PB.8918.8	7	1171	5	full-splice_match	gene: Zm00001d022190	Zm00001d022190_T002	0.00	98.23	coding	289	130	996
PB.7147.1	5	1903	11	full-splice_match	gene: Zm00001d018413	Zm00001d018413_T008	11.94	77.88	coding	488	84	1547
PB.5676.2	4	1590	2	full-splice_match	gene: Zm00001d053144	Zm00001d053144_T001	0.26	2.61	coding	468	74	1477

PB.3866.2	3	2010	1	full-splice_match	gene: Zm00001d040293	Zm00001d040293_T001	79.49	79.49	coding	579	96	1832
PB.7881.2	6	1811	3	incomplete-splice_match	gene: Zm00001d038225	Zm00001d038225_T003	99.64	99.64	coding	446	157	1494
PB.2870.1	2	1296	8	novel_not_in_catalog	gene: Zm00001d003807	novel	16.19	75.53	coding	179	237	773
PB.4349.1	3	2336	9	full-splice_match	gene: Zm00001d042910	Zm00001d042910_T005	27.70	27.97	coding	560	247	1926
PB.5415.1	4	2025	17	full-splice_match	gene: Zm00001d052056	Zm00001d052056_T005	174.89	176.75	coding	545	127	1761
PB.618.1	1	1251	7	full-splice_match	gene: Zm00001d030091	Zm00001d030091_T001	33.36	33.36	coding	328	99	1082
PB.7861.1	6	2196	3	full-splice_match	gene: Zm00001d038186	Zm00001d038186_T002	6.74	27.54	coding	597	47	1837
PB.9137.1	8	1302	3	full-splice_match	gene: Zm00001d008516	Zm00001d008516_T001	4.30	4.30	coding	310	218	1147
PB.7861.2	6	2049	3	full-splice_match	gene: Zm00001d038186	Zm00001d038186_T002	20.29	27.54	coding	597	149	1939
PB.10328.1	9	1696	4	full-splice_match	gene: Zm00001d046397	Zm00001d046397_T001	10.22	13.37	coding	147	365	805
PB.10328.2	9	1112	5	novel_not_in_catalog	gene: Zm00001d046397	novel	3.15	13.37	coding	142	366	791
PB.6731.3	5	728	5	full-splice_match	gene: Zm00001d016722	Zm00001d016722_T004	165.36	331.89	coding	69	100	306
PB.1435.2	1	1569	1	full-splice_match	gene: Zm00001d034221	Zm00001d034221_T001	0.00	31.20	coding	435	134	1438
PB.7517.3	6	1534	4	intergenic	novelGene_165	novel	4.05	14.86	non_coding	NA	NA	NA
PB.7517.2	6	997	3	intergenic	novelGene_165	novel	5.72	14.86	non_coding	NA	NA	NA
PB.2741.1	2	1104	2	incomplete-splice_match	gene: Zm00001d003186	Zm00001d003186_T001	28.97	28.97	coding	299	28	924
PB.4539.7	3	2441	13	novel_not_in_catalog	gene: Zm00001d043693	novel	3.97	192.92	coding	267	1243	2043

PB.456.2	1	1048	5	full-splice_match	gene: Zm00001d029214	Zm00001d029214_T001	0.00	68.10	coding	210	159	788
PB.456.1	1	1119	5	full-splice_match	gene: Zm00001d029214	Zm00001d029214_T001	68.10	68.10	coding	210	159	788
PB.4623.7	3	1848	17	incomplete-splice_match	gene: Zm00001d044016	Zm00001d044016_T001	0.00	66.64	coding	585	92	1846
PB.1564.1	1	470	1	full-splice_match	gene: Zm00001d034680	Zm00001d034680_T001	91.42	91.42	coding	96	29	316
PB.2501.1	2	2612	17	full-splice_match	gene: Zm00001d002186	Zm00001d002186_T001	42.26	42.26	coding	777	1	2331
PB.8444.5	7	1378	1	incomplete-splice_match	gene: Zm00001d020292	Zm00001d020292_T001	0.00	61.24	coding	418	125	1378
PB.8444.4	7	1871	1	novel_in_catalog	gene: Zm00001d020292	novel	0.91	61.24	coding	481	121	1563
PB.7251.2	6	1246	10	full-splice_match	gene: Zm00001d035169	Zm00001d035169_T001	0.00	26.17	coding	291	65	937
PB.8444.2	7	2303	2	full-splice_match	gene: Zm00001d020292	Zm00001d020292_T001	23.42	61.24	coding	641	147	2069
PB.2396.1	2	1099	7	full-splice_match	gene: Zm00001d001799	Zm00001d001799_T003	25.67	25.67	coding	197	151	741
PB.7251.1	6	1407	10	full-splice_match	gene: Zm00001d035169	Zm00001d035169_T001	26.17	26.17	coding	291	113	985
PB.4539.2	3	2263	13	novel_not_in_catalog	gene: Zm00001d043693	novel	3.67	192.92	coding	504	252	1763
PB.6814.1	5	1380	3	full-splice_match	gene: Zm00001d017111	Zm00001d017111_T001	26.72	89.41	coding	326	88	1065
PB.9999.2	8	728	3	intergenic	novelGene_297	novel	7.63	7.63	coding	128	37	420
PB.6112.1	5	789	4	novel_not_in_catalog	gene: Zm00001d013501	novel	24.41	24.41	non_coding	NA	NA	NA
PB.9651.1	8	1185	3	full-splice_match	gene: Zm00001d011145	Zm00001d011145_T001	25.68	25.68	coding	284	93	944
PB.54.1	1	1557	1	genic	gene: Zm00001d027436	novel	25.03	25.03	coding	196	210	797

PB.772.2	1	2181	7	full-splice_match	gene: Zm00001d031090	Zm00001d031090_T001	0.00	50.61	coding	596	126	1913
PB.4908.3	4	2137	9	full-splice_match	gene: Zm00001d049163	Zm00001d049163_T003	3.36	84.16	coding	211	111	743
PB.4419.1	3	1657	2	genic	gene: Zm00001d043225	novel	32.04	32.04	coding	393	66	1244
PB.2521.2	2	1294	8	full-splice_match	gene: Zm00001d002296	Zm00001d002296_T003	25.77	26.21	coding	266	88	885
PB.2521.1	2	1353	8	full-splice_match	gene: Zm00001d002296	Zm00001d002296_T003	0.45	26.21	coding	266	25	822
PB.4539.1	3	2183	14	novel_not_in_catalog	gene: Zm00001d043693	novel	103.14	192.92	coding	470	254	1663
PB.5677.1	4	2514	4	incomplete-splice_match	gene: Zm00001d053151	Zm00001d053151_T003	65.22	108.01	coding	671	4	2016
PB.1201.1	1	2171	3	full-splice_match	gene: Zm00001d033153	Zm00001d033153_T002	14.27	15.76	coding	425	548	1822
PB.464.1	1	1985	1	full-splice_match	gene: Zm00001d029255	Zm00001d029255_T001	6.86	6.86	coding	546	7	1644
PB.9385.1	8	1555	11	full-splice_match	gene: Zm00001d009878	Zm00001d009878_T001	69.36	69.36	coding	434	1	1302
PB.10878.1	B73V4 ctg181	659	1	genic	gene: Zm00001d000184	novel	93.50	93.50	non_coding	NA	NA	NA
PB.8274.1	7	2626	4	full-splice_match	gene: Zm00001d019173	Zm00001d019173_T001	46.76	46.76	coding	742	116	2341
PB.1188.1	1	1706	5	full-splice_match	gene: Zm00001d033056	Zm00001d033056_T001	181.86	198.24	coding	460	30	1409
PB.1188.2	1	1785	4	novel_in_catalog	gene: Zm00001d033056	novel	3.18	198.24	coding	357	26	1096
PB.1188.4	1	2383	4	novel_in_catalog	gene: Zm00001d033056	novel	3.71	198.24	coding	294	74	955
PB.1188.7	1	1591	5	full-splice_match	gene: Zm00001d033056	Zm00001d033056_T001	9.48	198.24	coding	460	62	1441
PB.3352.1	2	1244	3	full-splice_match	gene: Zm00001d006562	Zm00001d006562_T001	26.11	26.11	coding	254	70	831

PB.9262.2	8	1726	2	full-splice_match	gene: Zm00001d009163	Zm00001d009163_T001	0.00	67.92	coding	499	84	1580
PB.8918.5	7	2070	3	full-splice_match	gene: Zm00001d022190	Zm00001d022190_T014	0.00	98.23	coding	212	1156	1791
PB.9262.1	8	1965	2	full-splice_match	gene: Zm00001d009163	Zm00001d009163_T001	67.92	67.92	coding	499	156	1652
PB.10083.6	9	1243	3	incomplete-splice_match	gene: Zm00001d045021	Zm00001d045021_T010	15.36	64.96	coding	220	417	1076
PB.1348.1	1	1894	1	full-splice_match	gene: Zm00001d033834	Zm00001d033834_T001	3.26	3.26	coding	551	154	1806
PB.10083.5	9	1343	4	novel_not_in_catalog	gene: Zm00001d045021	novel	7.22	64.96	coding	220	369	1028
PB.6585.3	5	1923	12	fusion	gene: Zm00001d015811_g	novel	18.94	18.94	coding	442	265	1590
PB.1648.2	10	1038	8	full-splice_match	gene: Zm00001d023223	Zm00001d023223_T001	32.70	38.43	coding	252	94	849
PB.1648.1	10	1130	7	novel_in_catalog	gene: Zm00001d023223	novel	5.73	38.43	coding	124	57	428
PB.5826.4	4	1902	15	full-splice_match	gene: Zm00001d053857	Zm00001d053857_T007	1.95	117.03	coding	560	59	1738
PB.2755.1	2	1402	14	full-splice_match	gene: Zm00001d003220	Zm00001d003220_T002	41.09	41.09	coding	348	132	1175
PB.6947.1	5	1279	4	full-splice_match	gene: Zm00001d017682	Zm00001d017682_T001	78.80	78.80	coding	252	133	888
PB.10192.1	9	2762	11	full-splice_match	gene: Zm00001d045491	Zm00001d045491_T004	24.80	47.32	coding	698	301	2394
PB.5826.1	4	2086	14	full-splice_match	gene: Zm00001d053857	Zm00001d053857_T002	109.13	117.03	coding	560	144	1823
PB.5826.2	4	1980	15	full-splice_match	gene: Zm00001d053857	Zm00001d053857_T004	5.95	117.03	coding	560	134	1813
PB.10192.2	9	2491	11	full-splice_match	gene: Zm00001d045491	Zm00001d045491_T004	22.51	47.32	coding	698	207	2300
PB.8464.1	7	1590	7	full-splice_match	gene: Zm00001d020380	Zm00001d020380_T001	23.20	23.20	coding	403	89	1297

PB.1628.1	1	1370	11	full-splice_match	gene: Zm00001d034887	Zm00001d034887_T001	50.74	50.74	coding	306	132	1049
PB.4194.1	3	2813	13	full-splice_match	gene: Zm00001d042234	Zm00001d042234_T005	10.03	17.42	coding	800	135	2534
PB.2845.2	2	2626	1	full-splice_match	gene: Zm00001d003668	Zm00001d003668_T001	0.00	63.79	coding	771	194	2506
PB.4194.2	3	2039	10	novel_in_catalog	gene: Zm00001d042234	novel	7.39	17.42	coding	597	87	1877
PB.3350.2	2	913	3	full-splice_match	gene: Zm00001d006541	Zm00001d006541_T001	19.66	23.21	coding	207	102	722
PB.3350.1	2	2978	2	novel_in_catalog	gene: Zm00001d006541	novel	3.56	23.21	coding	155	2277	2741
PB.8367.1	7	1586	4	full-splice_match	gene: Zm00001d019759	Zm00001d019759_T001	27.72	27.72	coding	418	203	1456
PB.2515.3	2	871	2	genic	gene: Zm00001d002278	novel	116.68	206.32	coding	144	170	601
PB.2515.1	2	855	2	genic	gene: Zm00001d002278	novel	78.15	206.32	coding	144	139	570
PB.4077.1	3	1201	1	full-splice_match	gene: Zm00001d041685	Zm00001d041685_T001	29.71	165.58	coding	124	154	525
PB.2148.1	10	2634	2	novel_not_in_catalog	gene: Zm00001d025825	novel	11.17	11.17	coding	703	246	2354
PB.4077.2	3	685	1	full-splice_match	gene: Zm00001d041685	Zm00001d041685_T001	135.87	165.58	coding	124	112	483
PB.212.1	1	1879	9	full-splice_match	gene: Zm00001d028194	Zm00001d028194_T004	19.57	77.94	coding	386	129	1286
PB.3330.1	2	2761	18	novel_in_catalog	gene: Zm00001d006456	novel	1.37	10.02	coding	617	168	2018
PB.212.3	1	2095	9	full-splice_match	gene: Zm00001d028194	Zm00001d028194_T004	58.37	77.94	coding	530	145	1734
PB.744.2	1	1672	3	full-splice_match	gene: Zm00001d030989	Zm00001d030989_T001	131.99	139.59	coding	159	241	717
PB.212.4	1	2031	9	full-splice_match	gene: Zm00001d028194	Zm00001d028194_T004	0.00	77.94	coding	530	156	1745

PB.2049.4	10	2284	6	full-splice_match	gene: Zm00001d025323	Zm00001d025323_T004	0.00	51.81	coding	521	538	2100
PB.4296.1	3	2092	2	genic	gene: Zm00001d042686	novel	18.37	61.71	coding	514	377	1918
PB.4296.2	3	3442	1	full-splice_match	gene: Zm00001d042686	Zm00001d042686_T001	7.60	61.71	coding	514	1687	3228
PB.4296.3	3	1941	2	genic	gene: Zm00001d042686	novel	35.74	61.71	coding	514	226	1767
PB.4296.4	3	1968	2	genic	gene: Zm00001d042686	novel	0.00	61.71	coding	514	302	1843
PB.7569.1	6	1977	16	full-splice_match	gene: Zm00001d036982	Zm00001d036982_T004	15.17	15.17	coding	494	144	1625
PB.9982.1	8	1049	3	full-splice_match	gene: Zm00001d012603	Zm00001d012603_T001	129.93	129.93	coding	245	83	817
PB.6928.3	5	1668	1	genic	gene: Zm00001d017575	novel	3.45	79.67	coding	359	334	1410
PB.9962.3	8	942	7	full-splice_match	gene: Zm00001d012537	Zm00001d012537_T001	582.79	582.79	coding	181	150	692
PB.3401.3	2	2124	4	incomplete-splice_match	gene: Zm00001d006762	Zm00001d006762_T009	0.00	25.35	coding	552	468	2123
PB.3401.2	2	2307	4	incomplete-splice_match	gene: Zm00001d006762	Zm00001d006762_T009	3.09	25.35	coding	611	474	2306
PB.918.5	1	1021	1	incomplete-splice_match	gene: Zm00001d031871	Zm00001d031871_T002	22.54	59.27	coding	290	43	912
PB.1435.1	1	1759	1	full-splice_match	gene: Zm00001d034221	Zm00001d034221_T001	31.20	31.20	coding	435	195	1499
PB.5419.2	4	3152	3	novel_not_in_catalog	gene: Zm00001d052064	novel	1.62	10.58	coding	420	662	1921
PB.5121.2	4	1230	3	incomplete-splice_match	gene: Zm00001d050316	Zm00001d050316_T033	0.00	106.47	coding	288	193	1056
PB.6810.8	5	2182	8	full-splice_match	gene: Zm00001d017096	Zm00001d017096_T003	4.28	118.92	coding	171	106	618
PB.5121.1	4	1446	3	incomplete-splice_match	gene: Zm00001d050316	Zm00001d050316_T016	76.37	106.47	coding	288	280	1143

PB.3641.1	3	2646	14	full-splice_match	gene: Zm00001d039267	Zm00001d039267_T004	46.64	84.86	coding	686	112	2169
PB.8825.5	7	3150	26	full-splice_match	gene: Zm00001d021846	Zm00001d021846_T002	0.00	51.96	coding	992	46	3021
PB.3641.3	3	2732	14	full-splice_match	gene: Zm00001d039267	Zm00001d039267_T004	10.02	84.86	coding	686	310	2367
PB.3641.2	3	2757	13	novel_in_catalog	gene: Zm00001d039267	novel	13.44	84.86	coding	610	98	1927
PB.3856.3	3	3229	19	full-splice_match	gene: Zm00001d040268	Zm00001d040268_T015	2.13	7.75	coding	534	826	2427
PB.3641.4	3	2751	13	novel_in_catalog	gene: Zm00001d039267	novel	0.00	84.86	coding	610	262	2091
PB.3856.1	3	3102	20	full-splice_match	gene: Zm00001d040268	Zm00001d040268_T008	5.61	7.75	coding	699	833	2929
PB.9459.1	8	2136	13	novel_in_catalog	gene: Zm00001d010243	novel	16.83	48.05	coding	210	288	917
PB.3641.9	3	2783	13	novel_in_catalog	gene: Zm00001d039267	novel	1.67	84.86	coding	652	260	2215
PB.3641.8	3	2634	13	novel_in_catalog	gene: Zm00001d039267	novel	1.53	84.86	coding	400	1054	2253
PB.2139.6	10	2258	9	full-splice_match	gene: Zm00001d025801	Zm00001d025801_T011	0.00	381.01	coding	652	177	2132
PB.9832.1	8	1798	1	full-splice_match	gene: Zm00001d011932	Zm00001d011932_T001	3.47	3.47	coding	523	67	1635
PB.8108.1	6	1249	1	full-splice_match	gene: Zm00001d039108	Zm00001d039108_T001	8.12	8.12	coding	305	153	1067
PB.9700.2	8	2597	2	genic	gene: Zm00001d011378	novel	0.68	4.17	coding	711	158	2290
PB.5304.3	4	1439	4	incomplete-splice_match	gene: Zm00001d051545	Zm00001d051545_T005	9.75	39.00	coding	193	5	583
PB.9288.4	8	2273	8	full-splice_match	gene: Zm00001d009352	Zm00001d009352_T002	7.00	37.07	coding	638	77	1990
PB.9288.2	8	2230	3	novel_in_catalog	gene: Zm00001d009352	novel	7.52	37.07	coding	125	1553	1927

PB.8817.1	7	2678	7	full-splice_match	gene: Zm00001d021828	Zm00001d021828_T001	12.50	12.50	coding	776	196	2523
PB.9288.1	8	2352	8	full-splice_match	gene: Zm00001d009352	Zm00001d009352_T002	22.55	37.07	coding	638	41	1954
PB.3661.3	3	2203	10	novel_in_catalog	gene: Zm00001d039321	novel	1.20	36.25	coding	534	157	1758
PB.3661.2	3	2286	10	novel_in_catalog	gene: Zm00001d039321	novel	5.95	36.25	coding	269	122	928
PB.3661.1	3	2250	11	novel_in_catalog	gene: Zm00001d039321	novel	25.62	36.25	coding	595	125	1909
PB.8089.2	6	2297	1	genic	gene: Zm00001d001483	novel	2.60	8.32	non_coding	NA	NA	NA
PB.5304.1	4	1382	5	incomplete-splice_match	gene: Zm00001d051545	Zm00001d051545_T001	17.78	39.00	coding	290	28	897
PB.3661.5	3	2303	9	novel_in_catalog	gene: Zm00001d039321	novel	3.47	36.25	coding	573	65	1783
PB.3661.4	3	2085	11	novel_in_catalog	gene: Zm00001d039321	novel	0.00	36.25	coding	595	125	1909
PB.1666.2	10	1636	5	antisense	novelGene_gene: Zm00001d023277_A	novel	11.09	56.67	coding	153	572	1030
PB.4439.1	3	974	2	full-splice_match	gene: Zm00001d043314	Zm00001d043314_T001	76.12	77.47	coding	187	100	660
PB.6810.3	5	1643	9	novel_in_catalog	gene: Zm00001d017096	novel	12.33	118.92	coding	180	103	642
PB.5185.1	4	1408	1	full-splice_match	gene: Zm00001d050840	Zm00001d050840_T001	7.30	7.30	coding	331	153	1145
PB.8918.1	7	2193	2	novel_in_catalog	gene: Zm00001d022190	novel	8.73	98.23	coding	136	1156	1563
PB.8963.1	7	2363	4	novel_not_in_catalog	gene: Zm00001d022355	novel	15.16	15.16	coding	392	965	2140
PB.9445.1	8	1896	3	full-splice_match	gene: Zm00001d010214	Zm00001d010214_T002	5.14	15.42	coding	352	538	1593
PB.9445.3	8	1718	3	full-splice_match	gene: Zm00001d010214	Zm00001d010214_T002	5.25	15.42	coding	352	536	1591

PB.9113.2	8	1901	19	full-splice_match	gene: Zm00001d008417	Zm00001d008417_T011	21.82	21.82	coding	488	98	1561
PB.10405.12	9	1587	11	novel_in_catalog	gene: Zm00001d046831	novel	15.81	33.24	coding	246	147	884
PB.10405.13	9	1437	12	full-splice_match	gene: Zm00001d046831	Zm00001d046831_T004	17.43	33.24	coding	366	192	1289
PB.1842.1	10	1543	2	full-splice_match	gene: Zm00001d024294	Zm00001d024294_T001	292.68	309.46	coding	419	83	1339
PB.1365.1	1	2136	1	novel_in_catalog	gene: Zm00001d033903	novel	29.61	29.61	coding	600	80	1879
PB.9066.2	8	1420	4	novel_in_catalog	gene: Zm00001d008215	novel	9.54	180.60	coding	199	156	752
PB.2581.1	2	2232	1	full-splice_match	gene: Zm00001d002550	Zm00001d002550_T001	4.63	4.63	coding	603	45	1853
PB.1951.2	10	2370	5	full-splice_match	gene: Zm00001d024861	Zm00001d024861_T004	0.88	10.21	coding	289	487	1353
PB.8253.3	7	1917	2	full-splice_match	gene: Zm00001d019078	Zm00001d019078_T002	15.24	29.54	coding	512	256	1791
PB.2581.2	2	1059	1	full-splice_match	gene: Zm00001d002550	Zm00001d002550_T001	0.00	4.63	coding	281	80	922
PB.6176.2	5	2447	11	novel_in_catalog	gene: Zm00001d013787	novel	4.60	58.55	coding	284	1439	2290
PB.6176.1	5	1268	13	novel_not_in_catalog	gene: Zm00001d013787	novel	53.95	58.55	coding	315	86	1030
PB.7914.1	6	2257	5	novel_in_catalog	gene: Zm00001d038371	novel	28.09	28.09	coding	587	174	1934
PB.3415.1	2	663	1	incomplete-splice_match	gene: Zm00001d006844	Zm00001d006844_T002	964.35	964.35	coding	145	65	499
PB.4698.1	3	2556	2	genic	gene: Zm00001d044320	novel	3.75	3.75	coding	562	436	2121
PB.7171.1	5	1917	14	novel_in_catalog	gene: Zm00001d018484	novel	20.41	40.17	coding	105	1194	1508
PB.6810.4	5	1877	10	novel_in_catalog	gene: Zm00001d017096	novel	0.28	118.92	coding	171	106	618

PB.1754.1	10	1410	11	full-splice_match	gene: Zm00001d023738	Zm00001d023738_T001	63.96	69.44	coding	304	137	1048
PB.64.2	1	2497	10	novel_in_catalog	gene: Zm00001d027472	novel	49.52	49.52	coding	597	507	2297
PB.7225.1	6	2816	16	incomplete-splice_match	gene: Zm00001d035046	Zm00001d035046_T001	16.59	20.63	coding	913	5	2743
PB.1754.2	10	1314	11	full-splice_match	gene: Zm00001d023738	Zm00001d023738_T001	4.38	69.44	coding	304	119	1030
PB.3868.1	3	805	1	full-splice_match	gene: Zm00001d040297	Zm00001d040297_T001	2108.37	2108.37	coding	138	114	527
PB.3304.1	2	1357	3	full-splice_match	gene: Zm00001d006348	Zm00001d006348_T001	58.87	58.87	coding	273	166	984
PB.1754.4	10	1253	11	full-splice_match	gene: Zm00001d023738	Zm00001d023738_T001	1.11	69.44	coding	304	167	1078
PB.9599.1	8	3326	2	full-splice_match	gene: Zm00001d010869	Zm00001d010869_T001	27.35	39.73	coding	985	44	2998
PB.3655.1	3	1670	2	full-splice_match	gene: Zm00001d039310	Zm00001d039310_T001	11.02	11.02	coding	515	7	1551
PB.1882.2	10	2174	1	genic	gene: Zm00001d024521	novel	3.16	6.28	coding	308	52	975
PB.190.1	1	2370	2	novel_in_catalog	gene: Zm00001d028097	novel	13.51	13.51	coding	123	1861	2229
PB.170.3	1	1494	10	novel_not_in_catalog	gene: Zm00001d027995	novel	18.74	25.87	coding	382	125	1270
PB.331.8	1	1570	16	full-splice_match	gene: Zm00001d028662	Zm00001d028662_T001	0.00	45.53	coding	464	160	1551
PB.5321.2	4	1228	2	full-splice_match	gene: Zm00001d051611	Zm00001d051611_T001	24.21	31.66	coding	271	102	914
PB.5321.3	4	1124	2	full-splice_match	gene: Zm00001d051611	Zm00001d051611_T001	7.45	31.66	coding	271	107	919
PB.331.1	1	2156	16	full-splice_match	gene: Zm00001d028662	Zm00001d028662_T001	28.16	45.53	coding	464	139	1530
PB.331.3	1	2602	15	full-splice_match	gene: Zm00001d028662	Zm00001d028662_T005	3.00	45.53	coding	331	1117	2109

PB.331.2	1	2057	16	full-splice_match	gene: Zm00001d028662	Zm00001d028662_T001	14.37	45.53	coding	464	158	1549
PB.331.7	1	1609	16	full-splice_match	gene: Zm00001d028662	Zm00001d028662_T001	0.00	45.53	coding	464	42	1433
PB.331.6	1	1836	16	full-splice_match	gene: Zm00001d028662	Zm00001d028662_T001	0.00	45.53	coding	464	160	1551
PB.10354.3	9	1980	8	full-splice_match	gene: Zm00001d046531	Zm00001d046531_T005	53.94	72.84	coding	385	524	1678
PB.10354.4	9	1848	9	full-splice_match	gene: Zm00001d046531	Zm00001d046531_T006	18.90	72.84	coding	385	483	1637
PB.10777.1	9	3420	2	full-splice_match	gene: Zm00001d048390	Zm00001d048390_T001	9.31	9.31	coding	1047	109	3249
PB.7448.1	6	1705	2	genic	gene: Zm00001d036339	novel	1.09	1.09	coding	449	153	1499
PB.5655.3	4	3165	5	full-splice_match	gene: Zm00001d053011	Zm00001d053011_T004	0.00	4.77	coding	936	273	3080
PB.232.11	1	1328	11	novel_in_catalog	gene: Zm00001d028260	novel	290.98	1186.23	coding	357	84	1154
PB.8830.1	7	1645	2	full-splice_match	gene: Zm00001d021864	Zm00001d021864_T001	29.00	29.00	coding	440	125	1444
PB.10482.3	9	625	4	novel_not_in_catalog	gene: Zm00001d027176	novel	0.00	70.65	non_coding	NA	NA	NA
PB.9868.30	8	932	6	novel_in_catalog	gene: Zm00001d012100	novel	0.00	79.52	non_coding	NA	NA	NA
PB.8002.2	6	621	2	full-splice_match	gene: Zm00001d038740	Zm00001d038740_T002	0.00	66.23	coding	141	49	471
PB.7410.1	6	1056	4	full-splice_match	gene: Zm00001d036057	Zm00001d036057_T002	66.63	66.63	coding	250	97	846
PB.10482.2	9	570	1	intergenic	novelGene_200	novel	21.58	21.58	non_coding	NA	NA	NA
PB.8850.2	7	2504	7	novel_not_in_catalog	gene: Zm00001d021945	novel	2.76	2.76	coding	439	162	1478
PB.6467.3	5	2518	5	full-splice_match	gene: Zm00001d015065	Zm00001d015065_T006	13.07	133.78	coding	238	1457	2170

PB.10482.1	9	887	4	novel_not_in_catalog	gene: Zm00001d027176	novel	70.65	70.65	non_coding	NA	NA	NA
PB.7077.1	5	1499	6	full-splice_match	gene: Zm00001d018187	Zm00001d018187_T001	128.54	128.54	coding	366	83	1180
PB.8915.4	7	2223	5	full-splice_match	gene: Zm00001d022181	Zm00001d022181_T004	53.01	288.80	coding	538	92	1705
PB.3943.3	3	1805	2	full-splice_match	gene: Zm00001d040721	Zm00001d040721_T002	0.00	111.89	coding	503	98	1606
PB.2053.1	10	531	3	full-splice_match	gene: Zm00001d025339	Zm00001d025339_T002	442.84	442.84	coding	80	74	313
PB.6935.1	5	1915	6	full-splice_match	gene: Zm00001d017642	Zm00001d017642_T002	8.13	8.13	coding	441	200	1522
PB.8565.3	7	1386	6	full-splice_match	gene: Zm00001d020853	Zm00001d020853_T004	0.00	45.37	coding	377	63	1193
PB.8565.2	7	1531	6	full-splice_match	gene: Zm00001d020853	Zm00001d020853_T004	39.90	45.37	coding	377	106	1236
PB.1205.3	1	2377	9	incomplete-splice_match	gene: Zm00001d033168	Zm00001d033168_T003	8.96	34.71	coding	635	171	2075
PB.9712.1	8	1318	7	incomplete-splice_match	gene: Zm00001d011445	Zm00001d011445_T009	5.94	5.94	coding	264	217	1008
PB.9306.1	8	546	5	full-splice_match	gene: Zm00001d009450	Zm00001d009450_T003	66.71	66.71	coding	103	161	469
PB.10668.2	9	2889	13	full-splice_match	gene: Zm00001d047960	Zm00001d047960_T004	1.20	28.69	coding	451	1328	2680
PB.6444.4	5	3552	6	incomplete-splice_match	gene: Zm00001d014977	Zm00001d014977_T007	26.26	88.44	coding	1115	40	3384
PB.8915.7	7	2080	5	full-splice_match	gene: Zm00001d022181	Zm00001d022181_T004	149.52	288.80	coding	538	166	1779
PB.6444.1	5	2315	5	incomplete-splice_match	gene: Zm00001d014977	Zm00001d014977_T005	62.18	88.44	coding	697	44	2134
PB.2206.1	10	3188	10	full-splice_match	gene: Zm00001d026097	Zm00001d026097_T003	24.85	43.03	coding	844	86	2617
PB.2206.3	10	3004	9	novel_in_catalog	gene: Zm00001d026097	novel	1.91	43.03	coding	749	132	2378

PB.2206.2	10	2889	10	full-splice_match	gene: Zm00001d026097	Zm00001d026097_T003	16.26	43.03	coding	844	133	2664
PB.6657.3	5	1708	16	full-splice_match	gene: Zm00001d016311	Zm00001d016311_T002	5.27	48.71	coding	509	52	1578
PB.2206.4	10	2862	10	full-splice_match	gene: Zm00001d026097	Zm00001d026097_T003	0.00	43.03	coding	844	132	2663
PB.6657.1	5	1774	16	full-splice_match	gene: Zm00001d016311	Zm00001d016311_T002	43.44	48.71	coding	509	11	1537
PB.8915.1	7	2746	5	full-splice_match	gene: Zm00001d022181	Zm00001d022181_T004	3.55	288.80	coding	538	93	1706
PB.458.5	1	1689	8	incomplete-splice_match	gene: Zm00001d029223	Zm00001d029223_T015	4.40	51.50	coding	439	334	1650
PB.458.4	1	2499	9	incomplete-splice_match	gene: Zm00001d029223	Zm00001d029223_T015	20.41	51.50	coding	689	37	2103
PB.5076.1	4	1608	6	full-splice_match	gene: Zm00001d050033	Zm00001d050033_T002	4.84	4.84	coding	347	153	1193
PB.458.1	1	1408	5	incomplete-splice_match	gene: Zm00001d029223	Zm00001d029223_T015	26.68	51.50	coding	295	72	956
PB.8170.1	7	2073	9	full-splice_match	gene: Zm00001d018694	Zm00001d018694_T001	77.59	86.25	coding	535	89	1693
PB.10586.2	9	1390	5	full-splice_match	gene: Zm00001d047713	Zm00001d047713_T002	93.65	97.32	coding	339	103	1119
PB.4524.1	3	1545	6	full-splice_match	gene: Zm00001d043625	Zm00001d043625_T002	395.40	433.32	coding	378	204	1337
PB.4524.2	3	1599	5	novel_in_catalog	gene: Zm00001d043625	novel	36.16	433.32	coding	307	202	1122
PB.6241.1	5	1912	1	genic	gene: Zm00001d014134	novel	37.20	37.20	coding	305	58	972
PB.4524.4	3	976	3	novel_in_catalog	gene: Zm00001d043625	novel	1.75	433.32	coding	258	91	864
PB.1561.7	1	686	2	genic	gene: Zm00001d034677	novel	112.96	140.30	coding	123	70	438
PB.9623.5	8	3447	18	full-splice_match	gene: Zm00001d010974	Zm00001d010974_T016	0.00	48.61	coding	855	299	2863

PB.1561.5	1	1025	2	genic	gene: Zm00001d034675	novel	13.86	13.86	coding	123	70	438
PB.4963.3	4	1724	6	full-splice_match	gene: Zm00001d049441	Zm00001d049441_T001	0.00	37.30	coding	387	266	1426
PB.4963.2	4	1799	7	novel_in_catalog	gene: Zm00001d049441	novel	7.84	37.30	coding	387	99	1259
PB.4963.1	4	2045	6	full-splice_match	gene: Zm00001d049441	Zm00001d049441_T001	29.46	37.30	coding	387	272	1432
PB.8097.4	6	2806	8	full-splice_match	gene: Zm00001d039072	Zm00001d039072_T001	1.76	11.70	coding	813	23	2461
PB.7682.3	6	2414	11	full-splice_match	gene: Zm00001d037518	Zm00001d037518_T008	0.00	9.24	coding	733	92	2290
PB.8097.1	6	2461	7	full-splice_match	gene: Zm00001d039072	Zm00001d039072_T017	8.04	11.70	coding	560	128	1807
PB.7682.1	6	2619	11	full-splice_match	gene: Zm00001d037518	Zm00001d037518_T008	9.24	9.24	coding	733	134	2332
PB.4504.2	3	1875	9	novel_in_catalog	gene: Zm00001d043548	novel	1.75	18.57	coding	262	143	928
PB.8883.1	7	1735	14	full-splice_match	gene: Zm00001d022058	Zm00001d022058_T001	22.38	22.38	coding	434	130	1431
PB.4822.1	4	1877	2	full-splice_match	gene: Zm00001d048703	Zm00001d048703_T001	11.19	11.19	coding	530	135	1724
PB.4504.1	3	1840	10	full-splice_match	gene: Zm00001d043548	Zm00001d043548_T001	11.09	18.57	coding	428	87	1370
PB.5048.1	4	1302	4	full-splice_match	gene: Zm00001d049835	Zm00001d049835_T001	4.30	207.87	coding	213	488	1126
PB.5048.2	4	1018	5	full-splice_match	gene: Zm00001d049835	Zm00001d049835_T003	203.57	207.87	coding	213	193	831
PB.3545.3	2	583	2	novel_in_catalog	gene: Zm00001d007560	novel	0.00	91.97	coding	159	79	555
PB.1156.1	1	1017	2	full-splice_match	gene: Zm00001d032893	Zm00001d032893_T001	15.43	15.43	coding	200	146	745
PB.1575.1	1	1406	2	full-splice_match	gene: Zm00001d034731	Zm00001d034731_T001	21.66	21.66	coding	262	448	1233

PB.9193.1	8	2521	10	full-splice_match	gene: Zm00001d008800	Zm00001d008800_T006	7.54	7.54	coding	604	284	2095
PB.3214.2	2	3509	1	full-splice_match	gene: Zm00001d005966	Zm00001d005966_T001	2.06	150.85	coding	650	1128	3077
PB.3214.1	2	2875	2	genic	gene: Zm00001d005966	novel	145.34	150.85	coding	650	410	2359
PB.1009.1	1	1458	1	full-splice_match	gene: Zm00001d032297	Zm00001d032297_T001	14.51	14.51	coding	114	532	873
PB.9322.3	8	1641	1	novel_in_catalog	gene: Zm00001d009517	novel	17.74	77.09	coding	413	58	1296
PB.9322.2	8	1786	1	genic	gene: Zm00001d009517	novel	19.41	77.09	coding	413	85	1323
PB.9322.1	8	1929	1	genic	gene: Zm00001d009517	novel	2.56	77.09	coding	413	84	1322
PB.5200.2	4	825	4	full-splice_match	gene: Zm00001d050920	Zm00001d050920_T002	430.64	430.64	coding	219	14	670
PB.5200.3	4	676	4	full-splice_match	gene: Zm00001d050921	Zm00001d050921_T002	333.38	333.38	coding	131	127	519
PB.2615.1	2	823	3	full-splice_match	gene: Zm00001d002685	Zm00001d002685_T001	45.71	45.71	non_coding	NA	NA	NA
PB.2347.2	10	1157	1	full-splice_match	gene: Zm00001d026597	Zm00001d026597_T001	6.20	78.95	coding	309	65	991
PB.5211.2	4	1847	10	full-splice_match	gene: Zm00001d050970	Zm00001d050970_T020	0.76	68.24	coding	171	90	602
PB.4636.1	3	1013	1	full-splice_match	gene: Zm00001d044076	Zm00001d044076_T001	85.91	207.04	coding	136	284	691
PB.6396.1	5	498	2	intergenic	novelGene_120	novel	40.15	40.73	coding	94	69	350
PB.7131.2	5	2043	15	full-splice_match	gene: Zm00001d018371	Zm00001d018371_T005	8.88	17.94	coding	365	471	1565
PB.10870.1	B73V4 ctg140	2123	1	intergenic	novelGene_9	novel	0.00	0.00	non_coding	NA	NA	NA
PB.8106.2	6	1504	6	incomplete-splice_match	gene: Zm00001d039104	Zm00001d039104_T001	13.59	13.59	coding	441	21	1343

PB.4235.9	3	2130	10	full-splice_match	gene: Zm00001d042416	Zm00001d042416_T002	0.00	113.72	coding	571	105	1817
PB.470.1	1	1251	2	full-splice_match	gene: Zm00001d029280	Zm00001d029280_T001	137.53	137.53	coding	337	61	1071
PB.4235.6	3	2281	10	full-splice_match	gene: Zm00001d042416	Zm00001d042416_T004	1.89	113.72	coding	473	104	1522
PB.4235.1	3	3560	9	full-splice_match	gene: Zm00001d042416	Zm00001d042416_T003	35.92	113.72	coding	473	245	1663
PB.5040.1	4	983	4	full-splice_match	gene: Zm00001d049798	Zm00001d049798_T001	21.52	21.52	coding	247	19	759
PB.7511.1	6	2460	5	novel_not_in_catalog	gene: Zm00001d036630	novel	19.78	19.78	coding	677	210	2240
PB.4235.2	3	2220	10	full-splice_match	gene: Zm00001d042416	Zm00001d042416_T002	57.25	113.72	coding	571	101	1813
PB.391.7	1	2272	18	full-splice_match	gene: Zm00001d028952	Zm00001d028952_T006	12.77	65.35	coding	618	227	2080
PB.391.6	1	2523	18	full-splice_match	gene: Zm00001d028952	Zm00001d028952_T002	34.68	65.35	coding	670	308	2317
PB.391.5	1	3555	16	full-splice_match	gene: Zm00001d028952	Zm00001d028952_T004	0.00	65.35	coding	697	1115	3205
PB.391.4	1	2614	16	novel_in_catalog	gene: Zm00001d028952	novel	12.20	65.35	coding	494	163	1644
PB.391.3	1	3542	17	full-splice_match	gene: Zm00001d028952	Zm00001d028952_T007	0.00	65.35	coding	670	1178	3187
PB.391.2	1	3525	17	full-splice_match	gene: Zm00001d028952	Zm00001d028952_T007	3.59	65.35	coding	670	1112	3121
PB.4280.4	3	1908	4	full-splice_match	gene: Zm00001d042632	Zm00001d042632_T001	5.27	26.97	coding	475	347	1771
PB.4973.4	4	1047	7	full-splice_match	gene: Zm00001d049496	Zm00001d049496_T002	18.11	153.62	coding	215	177	821
PB.5423.8	4	1555	9	full-splice_match	gene: Zm00001d052074	Zm00001d052074_T007	0.00	84.05	coding	212	802	1437
PB.8931.1	7	1078	5	full-splice_match	gene: Zm00001d022242	Zm00001d022242_T002	55.29	55.29	coding	210	177	806

PB.4973.1	4	1279	7	full-splice_match	gene: Zm00001d049496	Zm00001d049496_T002	53.41	153.62	coding	215	209	853
PB.391.9	1	2487	16	novel_in_catalog	gene: Zm00001d028952	novel	0.69	65.35	coding	334	163	1164
PB.1404.1	1	2567	5	full-splice_match	gene: Zm00001d034044	Zm00001d034044_T001	12.31	12.31	coding	592	523	2298
PB.5060.4	4	2036	14	full-splice_match	gene: Zm00001d049929	Zm00001d049929_T006	0.00	37.78	coding	591	199	1971
PB.5060.2	4	2165	14	full-splice_match	gene: Zm00001d049929	Zm00001d049929_T009	20.47	37.78	coding	304	200	1111
PB.5060.3	4	2255	13	novel_in_catalog	gene: Zm00001d049929	novel	0.00	37.78	coding	381	184	1326
PB.5060.1	4	2112	14	full-splice_match	gene: Zm00001d049929	Zm00001d049929_T006	17.31	37.78	coding	591	197	1969
PB.8884.1	7	1589	4	novel_in_catalog	gene: Zm00001d022065	novel	2.36	13.83	coding	308	355	1278
PB.7391.1	6	1507	17	full-splice_match	gene: Zm00001d035974	Zm00001d035974_T001	35.01	35.01	coding	429	61	1347
PB.1132.1	1	3484	8	full-splice_match	gene: Zm00001d032776	Zm00001d032776_T001	118.99	118.99	coding	1078	49	3282
PB.7056.4	5	1230	4	full-splice_match	gene: Zm00001d018099	Zm00001d018099_T001	52.73	138.11	coding	321	124	1086
PB.4009.2	3	1929	4	novel_in_catalog	gene: Zm00001d041345	novel	5.25	24.05	coding	214	691	1332
PB.3782.6	3	2167	10	novel_in_catalog	gene: Zm00001d039871	novel	0.12	87.77	coding	512	68	1603
PB.3782.5	3	2099	11	full-splice_match	gene: Zm00001d039871	Zm00001d039871_T001	32.54	87.77	coding	510	128	1657
PB.4009.1	3	1871	5	full-splice_match	gene: Zm00001d041345	Zm00001d041345_T001	18.80	24.05	coding	349	230	1276
PB.3782.2	3	2162	11	full-splice_match	gene: Zm00001d039871	Zm00001d039871_T001	55.00	87.77	coding	510	73	1602
PB.3782.1	3	2290	10	novel_in_catalog	gene: Zm00001d039871	novel	0.11	87.77	coding	428	71	1354

PB.2096.1	10	1933	15	full-splice_match	gene: Zm00001d025626	Zm00001d025626_T002	34.36	34.36	coding	506	121	1638
PB.989.2	1	886	1	intergenic	novelGene_340	novel	7.27	12.51	coding	185	78	632
PB.4280.2	3	1988	4	full-splice_match	gene: Zm00001d042632	Zm00001d042632_T001	18.46	26.97	coding	475	324	1748
PB.993.1	1	1310	6	full-splice_match	gene: Zm00001d032231	Zm00001d032231_T002	418.03	432.32	coding	256	82	849
PB.989.4	1	3233	3	intergenic	novelGene_340	novel	3.53	12.51	coding	288	2239	3102
PB.989.5	1	2003	1	genic	gene: Zm00001d032221	novel	1.02	1.02	non_coding	NA	NA	NA
PB.894.1	1	1027	4	novel_in_catalog	gene: Zm00001d031738	novel	1.14	1.14	coding	149	74	520
PB.1331.1	1	1396	5	full-splice_match	gene: Zm00001d033766	Zm00001d033766_T002	17.79	45.07	coding	283	246	1094
PB.2961.2	2	1593	6	novel_in_catalog	gene: Zm00001d004498	novel	8.96	30.04	coding	159	193	669
PB.8607.1	7	1836	2	full-splice_match	gene: Zm00001d021016	Zm00001d021016_T001	6.77	125.44	coding	403	151	1359
PB.2961.1	2	1345	7	full-splice_match	gene: Zm00001d004498	Zm00001d004498_T002	14.34	30.04	coding	297	182	1072
PB.937.2	1	1463	1	antisense	novelGene_gene: Zm00001d031962_A	novel	1.85	1.85	coding	339	81	1097
PB.8911.1	7	900	5	novel_not_in_catalog	gene: Zm00001d022164	novel	34.12	34.12	coding	152	234	689
PB.5778.1	4	1562	16	full-splice_match	gene: Zm00001d053658	Zm00001d053658_T002	90.67	90.67	coding	408	63	1286
PB.937.4	1	927	6	full-splice_match	gene: Zm00001d031963	Zm00001d031963_T001	74.03	87.37	coding	186	130	687
PB.9024.8	7	2413	8	novel_in_catalog	gene: Zm00001d022542	novel	1.94	144.00	coding	323	1104	2072
PB.9546.1	8	2016	1	intergenic	novelGene_196	novel	18.02	18.02	non_coding	NA	NA	NA

PB.4229.1	3	731	1	full-splice_match	gene: Zm00001d042383	Zm00001d042383_T001	46.65	46.65	coding	106	101	418
PB.6778.1	5	2024	1	antisense	novelGene_gene: Zm00001d016909_A	novel	57.24	57.24	coding	232	876	1571
PB.8122.1	6	3545	19	full-splice_match	gene: Zm00001d039165	Zm00001d039165_T048	52.85	72.08	coding	1089	81	3347
PB.10745.1	9	370	2	incomplete-splice_match	gene: Zm00001d048285	Zm00001d048285_T004	50.19	50.19	non_coding	NA	NA	NA
PB.7921.1	6	3724	11	full-splice_match	gene: Zm00001d038389	Zm00001d038389_T002	16.53	16.53	coding	969	373	3279
PB.7044.1	5	2553	2	full-splice_match	gene: Zm00001d018043	Zm00001d018043_T001	19.32	19.32	coding	736	95	2302
PB.3887.1	3	925	1	antisense	novelGene_gene: Zm00001d040363_A	novel	0.33	0.33	coding	168	340	843
PB.7433.2	6	1702	2	genic	gene: Zm00001d036255	novel	17.75	17.75	coding	507	51	1571
PB.3555.3	2	2792	8	novel_not_in_catalog	gene: Zm00001d007605	novel	2.60	24.30	non_coding	NA	NA	NA
PB.4816.1	4	1209	1	full-splice_match	gene: Zm00001d048671	Zm00001d048671_T001	26.08	26.08	coding	322	120	1085
PB.7797.1	6	2850	1	full-splice_match	gene: Zm00001d037973	Zm00001d037973_T001	5.96	5.96	coding	874	15	2636
PB.1594.1	1	757	3	full-splice_match	gene: Zm00001d034793	Zm00001d034793_T001	269.38	269.38	coding	116	115	462
PB.10229.1	9	626	2	incomplete-splice_match	gene: Zm00001d045655	Zm00001d045655_T009	16.38	16.38	coding	143	70	498
PB.3549.1	2	1827	1	genic	gene: Zm00001d007575	novel	4.60	4.60	coding	312	603	1538
PB.8977.1	7	1215	1	full-splice_match	gene: Zm00001d022403	Zm00001d022403_T001	11.40	75.33	coding	203	147	755
PB.8977.2	7	1099	1	full-splice_match	gene: Zm00001d022403	Zm00001d022403_T001	63.93	75.33	coding	203	143	751
PB.10666.3	9	2446	2	novel_in_catalog	gene: Zm00001d047955	novel	0.00	55.91	coding	268	1483	2286

PB.10666.2	9	1751	3	full-splice_match	gene: Zm00001d047955	Zm00001d047955_T001	32.68	55.91	coding	448	198	1541
PB.9678.1	8	1969	9	novel_in_catalog	gene: Zm00001d011258	novel	49.03	49.03	coding	214	997	1638
PB.8896.1	7	1523	5	full-splice_match	gene: Zm00001d022104	Zm00001d022104_T001	24.74	24.74	coding	366	180	1277
PB.4633.1	3	2434	7	full-splice_match	gene: Zm00001d044059	Zm00001d044059_T001	11.89	11.89	coding	652	281	2236
PB.10666.4	9	1284	1	incomplete-splice_match	gene: Zm00001d047955	Zm00001d047955_T002	0.00	55.91	non_coding	NA	NA	NA
PB.591.2	1	575	2	intergenic	novelGene_162	novel	6.23	26.45	non_coding	NA	NA	NA
PB.4786.2	3	1188	6	full-splice_match	gene: Zm00001d044694	Zm00001d044694_T002	105.12	277.83	coding	240	83	802
PB.1493.3	1	2145	3	genic	gene: Zm00001d034434	novel	4.94	39.47	coding	98	458	751
PB.4123.2	3	1382	4	full-splice_match	gene: Zm00001d041920	Zm00001d041920_T001	36.79	45.02	coding	321	167	1129
PB.1482.2	1	1070	1	novel_in_catalog	gene: Zm00001d034414	novel	15.36	521.97	coding	151	107	559
PB.10249.7	9	1997	5	full-splice_match	gene: Zm00001d045804	Zm00001d045804_T013	0.00	37.21	coding	407	318	1538
PB.10249.4	9	2147	5	full-splice_match	gene: Zm00001d045804	Zm00001d045804_T013	0.00	37.21	coding	407	333	1553
PB.10249.5	9	3080	4	full-splice_match	gene: Zm00001d045804	Zm00001d045804_T014	0.00	37.21	coding	305	329	1243
PB.10249.2	9	3280	4	full-splice_match	gene: Zm00001d045804	Zm00001d045804_T014	6.53	37.21	coding	305	379	1293
PB.10249.3	9	2277	5	full-splice_match	gene: Zm00001d045804	Zm00001d045804_T013	30.68	37.21	coding	407	328	1548
PB.8953.1	7	1382	4	novel_not_in_catalog	gene: Zm00001d022330	novel	29.63	52.68	coding	318	16	969
PB.10034.1	9	2404	11	full-splice_match	gene: Zm00001d044724	Zm00001d044724_T001	17.67	17.67	coding	606	233	2050

PB.10034.2	9	2376	11	full-splice_match	gene: Zm00001d044724	Zm00001d044724_T001	0.00	17.67	coding	606	243	2060
PB.10249.9	9	1509	4	incomplete-splice_match	gene: Zm00001d045804	Zm00001d045804_T009	0.00	37.21	coding	394	36	1217
PB.184.1	1	2982	5	full-splice_match	gene: Zm00001d028075	Zm00001d028075_T001	51.35	75.10	coding	613	877	2715
PB.10461.57	9	573	6	incomplete-splice_match	gene: Zm00001d047166	Zm00001d047166_T001	0.00	330.25	coding	95	289	573
PB.10461.50	9	1828	10	novel_in_catalog	gene: Zm00001d047166	novel	6.69	330.25	coding	470	87	1496
PB.2126.1	10	1447	5	novel_in_catalog	gene: Zm00001d025752	novel	3.98	53.67	coding	208	472	1095
PB.10461.52	9	1778	11	full-splice_match	gene: Zm00001d047166	Zm00001d047166_T001	210.06	330.25	coding	509	149	1675
PB.1493.1	1	2342	2	genic	gene: Zm00001d034434	novel	11.43	39.47	coding	98	499	792
PB.7296.1	6	672	3	novel_not_in_catalog	gene: Zm00001d035411	novel	126.66	126.66	non_coding	NA	NA	NA
PB.4917.3	4	3189	18	full-splice_match	gene: Zm00001d049190	Zm00001d049190_T001	0.00	26.75	coding	957	88	2958
PB.4917.1	4	3511	18	full-splice_match	gene: Zm00001d049190	Zm00001d049190_T001	7.89	26.75	coding	957	93	2963
PB.2609.2	2	3483	12	full-splice_match	gene: Zm00001d002656	Zm00001d002656_T003	5.07	13.03	coding	973	158	3076
PB.1493.2	1	2187	3	genic	gene: Zm00001d034434	novel	0.00	39.47	coding	98	497	790
PB.2609.1	2	2550	12	full-splice_match	gene: Zm00001d002656	Zm00001d002656_T003	7.96	13.03	coding	609	129	1955
PB.5409.1	4	1345	1	full-splice_match	gene: Zm00001d052031	Zm00001d052031_T001	8.98	45.06	coding	287	78	938
PB.5409.2	4	1196	1	full-splice_match	gene: Zm00001d052031	Zm00001d052031_T001	36.08	45.06	coding	287	141	1001
PB.8231.1	7	1100	2	novel_in_catalog	gene: Zm00001d018936	novel	114.16	136.59	coding	274	72	893

PB.6531.1	5	825	4	full-splice_match	gene: Zm00001d015379	Zm00001d015379_T001	53.30	55.26	coding	157	104	574
PB.6531.2	5	887	3	novel_in_catalog	gene: Zm00001d015379	novel	1.96	55.26	coding	225	1	675
PB.4344.1	3	1471	1	antisense	novelGene_gene: Zm00001d042887_A	novel	20.35	20.35	coding	206	45	662
PB.9751.1	8	1595	8	full-splice_match	gene: Zm00001d011622	Zm00001d011622_T012	57.45	73.11	coding	384	38	1189
PB.2629.1	2	1284	1	full-splice_match	gene: Zm00001d002748	Zm00001d002748_T001	21.90	21.90	coding	150	506	955
PB.9751.2	8	4138	14	full-splice_match	gene: Zm00001d011622	Zm00001d011622_T006	15.66	73.11	coding	1232	209	3904
PB.1099.4	1	2385	3	incomplete-splice_match	gene: Zm00001d032633	Zm00001d032633_T005	2.82	9.87	coding	128	1586	1969
PB.178.2	1	917	3	full-splice_match	gene: Zm00001d028025	Zm00001d028025_T001	42.91	42.91	coding	172	132	647
PB.178.3	1	814	3	full-splice_match	gene: Zm00001d028025	Zm00001d028025_T001	0.00	42.91	coding	172	130	645
PB.8662.2	7	1938	3	full-splice_match	gene: Zm00001d021299	Zm00001d021299_T002	86.20	113.27	coding	251	74	826
PB.9070.1	8	2918	4	novel_in_catalog	gene: Zm00001d008238	novel	10.69	10.69	coding	259	1946	2722
PB.1493.7	1	2300	2	genic	gene: Zm00001d034434	novel	0.00	39.47	coding	98	499	792
PB.4262.3	3	2651	4	full-splice_match	gene: Zm00001d042543	Zm00001d042543_T002	0.00	10.71	coding	771	45	2357
PB.698.1	1	2331	14	full-splice_match	gene: Zm00001d030623	Zm00001d030623_T002	5.80	5.80	coding	588	246	2009
PB.6623.1	5	1580	2	genic	gene: Zm00001d016129	novel	32.00	32.00	coding	380	104	1243
PB.4907.1	4	915	2	full-splice_match	gene: Zm00001d049157	Zm00001d049157_T001	11.65	11.65	coding	228	98	781
PB.5415.3	4	2243	16	novel_in_catalog	gene: Zm00001d052056	novel	1.87	176.75	coding	429	105	1391

PB.8807.3	7	3106	13	novel_in_catalog	gene: Zm00001d021806	novel	36.25	73.05	coding	678	698	2731
PB.4623.4	3	2268	19	full-splice_match	gene: Zm00001d044016	Zm00001d044016_T001	0.00	66.64	coding	655	87	2051
PB.6824.5	5	2231	17	full-splice_match	gene: Zm00001d017139	Zm00001d017139_T004	0.52	42.16	coding	676	203	2230
PB.7485.1	6	1282	1	full-splice_match	gene: Zm00001d036536	Zm00001d036536_T001	32.18	32.18	coding	197	445	1035
PB.10808.1	9	774	1	full-splice_match	gene: Zm00001d048491	Zm00001d048491_T001	751.36	751.36	coding	149	97	543
PB.1099.1	1	1648	13	full-splice_match	gene: Zm00001d032633	Zm00001d032633_T004	7.04	9.87	coding	403	22	1230
PB.10634.2	9	1244	4	full-splice_match	gene: Zm00001d047856	Zm00001d047856_T001	125.15	125.15	coding	329	104	1090
PB.9686.1	8	1169	5	full-splice_match	gene: Zm00001d011325	Zm00001d011325_T001	38.12	38.12	coding	291	90	962
PB.8180.1	7	842	1	full-splice_match	gene: Zm00001d018725	Zm00001d018725_T001	66.58	66.58	coding	136	193	600
PB.1217.1	1	1763	1	full-splice_match	gene: Zm00001d033234	Zm00001d033234_T001	12.68	12.68	coding	471	93	1505
PB.4116.4	3	1550	3	incomplete-splice_match	gene: Zm00001d041873	Zm00001d041873_T001	3.63	6.74	coding	328	384	1367
PB.4116.2	3	3054	4	novel_in_catalog	gene: Zm00001d041873	novel	3.11	6.74	coding	887	69	2729
PB.4786.1	3	1418	6	full-splice_match	gene: Zm00001d044694	Zm00001d044694_T002	75.14	277.83	coding	240	217	936
PB.10155.1	9	1069	2	intergenic	novelGene_61	novel	39.32	39.32	coding	271	57	869
PB.7823.1	6	2241	4	full-splice_match	gene: Zm00001d038065	Zm00001d038065_T001	1.60	63.52	coding	324	84	1055
PB.9994.3	8	906	2	genic	gene: Zm00001d012644	novel	45.43	45.43	coding	104	354	665
PB.4352.1	3	2376	6	full-splice_match	gene: Zm00001d042943	Zm00001d042943_T001	112.54	112.54	coding	231	83	775

PB.4600.2	3	5710	18	fusion	gene: Zm00001d043915_g	novel	8.42	19.80	coding	1783	91	5439
PB.9066.1	8	1173	5	full-splice_match	gene: Zm00001d008215	Zm00001d008215_T001	171.06	180.60	coding	263	133	921
PB.9217.8	8	2730	4	full-splice_match	gene: Zm00001d008904	Zm00001d008904_T005	0.00	20.42	coding	106	1620	1937
PB.10839.4	9	3491	8	novel_in_catalog	gene: Zm00001d048575	novel	14.53	14.53	coding	145	2462	2896
PB.9217.6	8	3236	2	novel_in_catalog	gene: Zm00001d008904	novel	0.00	20.42	coding	114	2677	3018
PB.9217.7	8	1674	6	novel_in_catalog	gene: Zm00001d008904	novel	7.77	20.42	coding	103	493	801
PB.9217.4	8	2393	5	full-splice_match	gene: Zm00001d008904	Zm00001d008904_T009	5.92	20.42	coding	106	1600	1917
PB.170.2	1	1732	7	novel_not_in_catalog	gene: Zm00001d027995	novel	7.13	25.87	coding	270	529	1338
PB.9217.3	8	3434	1	novel_in_catalog	gene: Zm00001d008904	novel	2.09	20.42	coding	114	2875	3216
PB.3012.1	2	525	1	antisense	novelGene_gene: Zm00001d004810_A	novel	90.81	90.81	coding	154	54	515
PB.9217.1	8	1443	4	novel_not_in_catalog	gene: Zm00001d008904	novel	4.65	20.42	coding	101	676	978
PB.1089.2	1	798	3	full-splice_match	gene: Zm00001d032582	Zm00001d032582_T006	39.08	39.08	coding	182	95	640
PB.1075.1	1	746	2	full-splice_match	gene: Zm00001d032548	Zm00001d032548_T001	27.10	27.10	coding	162	47	532
PB.2702.1	2	2568	14	full-splice_match	gene: Zm00001d003040	Zm00001d003040_T002	6.62	11.70	coding	631	355	2247
PB.2702.2	2	2301	14	full-splice_match	gene: Zm00001d003040	Zm00001d003040_T002	5.08	11.70	coding	631	221	2113
PB.5726.1	4	1007	5	full-splice_match	gene: Zm00001d053432	Zm00001d053432_T001	5.27	5.27	coding	227	66	746
PB.7745.1	6	1747	6	full-splice_match	gene: Zm00001d037777	Zm00001d037777_T011	3.93	3.93	coding	476	152	1579

PB.4245.1	3	2046	5	novel_not_in_catalog	gene: Zm00001d042473	novel	34.02	34.02	coding	552	301	1956
PB.5183.2	4	1980	3	novel_in_catalog	gene: Zm00001d050834	novel	3.99	44.16	coding	208	40	663
PB.4327.1	3	2352	3	full-splice_match	gene: Zm00001d042833	Zm00001d042833_T001	133.71	133.71	coding	598	290	2083
PB.5183.1	4	1266	4	full-splice_match	gene: Zm00001d050834	Zm00001d050834_T001	40.17	44.16	coding	331	40	1032
PB.1589.1	1	3065	3	full-splice_match	gene: Zm00001d034771	Zm00001d034771_T002	128.02	336.20	coding	843	173	2701
PB.7287.1	6	556	2	intergenic	novelGene_68	novel	8.47	8.47	non_coding	NA	NA	NA
PB.5912.1	5	893	3	full-splice_match	gene: Zm00001d012820	Zm00001d012820_T001	203.90	203.90	coding	223	106	774
PB.3897.1	3	2623	21	full-splice_match	gene: Zm00001d040416	Zm00001d040416_T004	10.74	10.74	coding	726	116	2293
PB.3434.2	2	2509	14	full-splice_match	gene: Zm00001d006922	Zm00001d006922_T001	0.00	25.04	coding	655	193	2157
PB.3434.1	2	2613	14	full-splice_match	gene: Zm00001d006922	Zm00001d006922_T001	25.04	25.04	coding	655	195	2159
PB.1543.2	1	1091	5	full-splice_match	gene: Zm00001d034627	Zm00001d034627_T001	21.30	71.31	coding	250	149	898
PB.4543.1	3	1558	6	novel_not_in_catalog	gene: Zm00001d043700	novel	5.91	228.89	coding	221	159	821
PB.8739.2	7	1364	1	antisense	novelGene_gene: Zm00001d021577_A	novel	35.34	35.34	non_coding	NA	NA	NA
PB.6263.1	5	720	3	incomplete-splice_match	gene: Zm00001d014275	Zm00001d014275_T002	37.89	37.89	coding	150	6	455
PB.3569.1	2	2266	3	antisense	novelGene_gene: Zm00001d007688_A	novel	2.02	2.02	non_coding	NA	NA	NA
PB.3801.1	3	3048	3	full-splice_match	gene: Zm00001d039975	Zm00001d039975_T001	12.33	12.33	coding	853	283	2841
PB.5464.2	4	1722	6	novel_not_in_catalog	gene: Zm00001d052259	novel	19.60	82.76	coding	473	41	1459

PB.493.1	1	1608	1	full-splice_match	gene: Zm00001d029409	Zm00001d029409_T001	42.27	42.27	coding	396	55	1242
PB.8379.4	7	1888	3	novel_in_catalog	gene: Zm00001d019875	novel	6.42	20.40	coding	269	167	973
PB.5964.1	5	973	1	full-splice_match	gene: Zm00001d013004	Zm00001d013004_T001	0.92	0.92	coding	219	79	735
PB.2817.1	2	3015	6	full-splice_match	gene: Zm00001d003518	Zm00001d003518_T004	17.17	17.17	coding	632	684	2579
PB.1230.2	1	2310	14	full-splice_match	gene: Zm00001d033297	Zm00001d033297_T011	24.41	24.41	coding	503	483	1991
PB.10255.1	9	1346	2	full-splice_match	gene: Zm00001d045845	Zm00001d045845_T001	43.60	69.79	coding	320	152	1111
PB.10255.2	9	1225	2	full-splice_match	gene: Zm00001d045845	Zm00001d045845_T001	26.19	69.79	coding	320	152	1111
PB.5354.1	4	2490	16	full-splice_match	gene: Zm00001d051809	Zm00001d051809_T001	31.98	31.98	coding	671	63	2075
PB.2971.3	2	1339	9	full-splice_match	gene: Zm00001d004552	Zm00001d004552_T009	104.35	108.54	coding	191	126	698
PB.1488.1	1	795	2	novel_not_in_catalog	gene: Zm00001d034425	novel	30.67	30.67	coding	138	19	432
PB.8485.1	7	2140	7	novel_not_in_catalog	gene: Zm00001d020471	novel	36.70	36.70	non_coding	NA	NA	NA
PB.8731.1	7	3128	2	novel_in_catalog	gene: Zm00001d021565	novel	168.95	168.95	coding	722	647	2812
PB.6826.1	5	1148	5	full-splice_match	gene: Zm00001d017145	Zm00001d017145_T002	71.97	71.97	coding	245	120	854
PB.6499.3	5	2611	4	novel_in_catalog	gene: Zm00001d015212	novel	4.40	67.45	coding	205	1598	2212
PB.6499.2	5	2195	5	full-splice_match	gene: Zm00001d015212	Zm00001d015212_T004	4.67	67.45	coding	205	1180	1794
PB.6499.1	5	1976	4	novel_in_catalog	gene: Zm00001d015212	novel	37.44	67.45	coding	205	813	1427
PB.10310.7	9	1810	8	full-splice_match	gene: Zm00001d046243	Zm00001d046243_T003	3.68	39.81	coding	510	90	1619

PB.6499.5	5	3190	4	novel_in_catalog	gene: Zm00001d015212	novel	20.93	67.45	coding	205	2440	3054
PB.112.1	1	1230	1	full-splice_match	gene: Zm00001d027684	Zm00001d027684_T001	6.41	6.41	coding	313	66	1004
PB.7319.3	6	1445	7	full-splice_match	gene: Zm00001d035551	Zm00001d035551_T002	21.94	293.64	coding	222	559	1224
PB.7319.1	6	1359	7	novel_in_catalog	gene: Zm00001d035551	novel	266.97	293.64	coding	377	8	1138
PB.567.1	1	1133	3	full-splice_match	gene: Zm00001d029738	Zm00001d029738_T001	14.46	14.46	coding	216	60	707
PB.7319.4	6	1815	6	full-splice_match	gene: Zm00001d035551	Zm00001d035551_T001	4.73	293.64	coding	222	964	1629
PB.2092.5	10	3847	10	novel_in_catalog	gene: Zm00001d025590	novel	2.01	34.73	coding	1198	25	3618
PB.9676.1	8	1183	6	full-splice_match	gene: Zm00001d011242	Zm00001d011242_T003	45.89	45.89	coding	275	99	923
PB.2092.1	10	2593	3	incomplete-splice_match	gene: Zm00001d025590	Zm00001d025590_T011	20.87	34.73	coding	597	256	2046
PB.2720.2	2	534	3	incomplete-splice_match	gene: Zm00001d003114	Zm00001d003114_T003	65.22	65.22	coding	95	12	296
PB.8886.1	7	2792	1	genic	gene: Zm00001d022069	novel	0.17	9.16	coding	192	486	1061
PB.33.5	1	1672	3	novel_in_catalog	gene: Zm00001d027347	novel	0.90	40.62	coding	133	85	483
PB.33.4	1	3481	6	novel_in_catalog	gene: Zm00001d027347	novel	2.19	40.62	coding	962	468	3353
PB.8499.1	7	1680	2	full-splice_match	gene: Zm00001d020530	Zm00001d020530_T001	27.66	61.04	coding	434	68	1369
PB.5677.13	4	2276	4	incomplete-splice_match	gene: Zm00001d053151	Zm00001d053151_T003	0.00	108.01	coding	671	87	2099
PB.33.1	1	3450	7	novel_in_catalog	gene: Zm00001d027347	novel	37.53	40.62	coding	1061	62	3244
PB.8499.2	7	1555	2	full-splice_match	gene: Zm00001d020530	Zm00001d020530_T001	33.38	61.04	coding	434	64	1365

PB.4832.8	4	1090	7	full-splice_match	gene: Zm00001d048785	Zm00001d048785_T003	22.35	169.67	coding	275	136	960
PB.4832.9	4	1200	6	novel_in_catalog	gene: Zm00001d048785	novel	0.00	169.67	coding	115	133	477
PB.8725.1	7	1509	6	full-splice_match	gene: Zm00001d021534	Zm00001d021534_T001	8.73	8.73	coding	408	19	1242
PB.7379.1	6	1323	5	full-splice_match	gene: Zm00001d035920	Zm00001d035920_T001	125.49	125.49	coding	270	167	976
PB.5362.2	4	1441	7	incomplete-splice_match	gene: Zm00001d051856	Zm00001d051856_T002	56.17	99.21	coding	308	108	1031
PB.5362.1	4	1535	7	incomplete-splice_match	gene: Zm00001d051856	Zm00001d051856_T002	43.04	99.21	coding	308	91	1014
PB.4832.1	4	1817	6	novel_in_catalog	gene: Zm00001d048785	novel	19.64	169.67	coding	115	136	480
PB.4832.2	4	1579	7	full-splice_match	gene: Zm00001d048785	Zm00001d048785_T003	39.23	169.67	coding	275	136	960
PB.4832.3	4	1189	7	full-splice_match	gene: Zm00001d048785	Zm00001d048785_T003	0.00	169.67	coding	275	121	945
PB.4832.4	4	1285	5	novel_in_catalog	gene: Zm00001d048785	novel	10.04	169.67	coding	115	136	480
PB.4832.5	4	1460	5	novel_in_catalog	gene: Zm00001d048785	novel	10.75	169.67	coding	115	136	480
PB.4832.6	4	1313	6	novel_in_catalog	gene: Zm00001d048785	novel	49.33	169.67	coding	115	131	475
PB.4832.7	4	1621	3	novel_in_catalog	gene: Zm00001d048785	novel	13.20	169.67	coding	115	136	480
PB.6815.2	5	1796	6	incomplete-splice_match	gene: Zm00001d017112	Zm00001d017112_T001	9.50	27.52	coding	441	53	1375
PB.1847.1	10	3717	30	novel_in_catalog	gene: Zm00001d024327	novel	24.39	63.22	coding	1052	106	3261
PB.5930.1	5	1543	5	full-splice_match	gene: Zm00001d012887	Zm00001d012887_T001	33.62	33.62	coding	408	125	1348
PB.9205.1	8	2159	5	novel_in_catalog	gene: Zm00001d008859	novel	7.81	7.81	coding	178	17	550

PB.594.3	1	1552	6	novel_not_in_catalog	gene: Zm00001d029922	novel	6.44	19.61	coding	133	230	628
PB.2014.2	10	1658	5	novel_in_catalog	gene: Zm00001d025106	novel	11.09	43.95	coding	105	239	553
PB.2014.3	10	3083	3	full-splice_match	gene: Zm00001d025106	Zm00001d025106_T006	1.57	43.95	coding	40	213	332
PB.2014.1	10	1543	6	novel_in_catalog	gene: Zm00001d025106	novel	22.46	43.95	coding	105	160	474
PB.2014.4	10	1721	5	novel_in_catalog	gene: Zm00001d025106	novel	8.83	43.95	coding	109	169	495
PB.9384.4	8	1619	3	full-splice_match	gene: Zm00001d009868	Zm00001d009868_T003	0.00	569.77	coding	447	95	1435
PB.9384.3	8	1555	4	novel_in_catalog	gene: Zm00001d009868	novel	1.33	569.77	coding	354	95	1156
PB.9384.2	8	1650	3	full-splice_match	gene: Zm00001d009868	Zm00001d009868_T007	153.01	569.77	coding	447	67	1407
PB.9384.1	8	1712	3	full-splice_match	gene: Zm00001d009868	Zm00001d009868_T003	415.44	569.77	coding	447	91	1431
PB.3305.1	2	2975	5	full-splice_match	gene: Zm00001d006355	Zm00001d006355_T018	15.81	15.81	coding	734	267	2468
PB.259.1	1	2003	15	full-splice_match	gene: Zm00001d028372	Zm00001d028372_T013	17.18	24.23	coding	381	434	1576
PB.10361.1	9	721	1	full-splice_match	gene: Zm00001d046574	Zm00001d046574_T001	81.21	81.21	coding	108	151	474
PB.2728.1	2	1866	2	novel_in_catalog	gene: Zm00001d003142	novel	3.58	3.58	coding	319	64	1020
PB.259.4	1	1225	7	incomplete-splice_match	gene: Zm00001d028372	Zm00001d028372_T014	7.04	24.23	coding	146	275	712
PB.7915.1	6	1648	1	genic	gene: Zm00001d038373	novel	27.33	27.33	coding	430	93	1382
PB.9480.1	8	2027	1	full-splice_match	gene: Zm00001d010380	Zm00001d010380_T001	49.89	49.89	coding	397	66	1256
PB.2435.3	2	1776	5	novel_in_catalog	gene: Zm00001d001937	novel	40.32	40.32	coding	293	168	1046

PB.6781.2	5	1819	5	novel_in_catalog	gene: Zm00001d016918	novel	2.39	21.48	coding	220	89	748
PB.124.4	1	2871	8	novel_in_catalog	gene: Zm00001d027731	novel	3.06	562.10	coding	577	309	2039
PB.6809.2	5	1222	1	genic	gene: Zm00001d017095	novel	38.56	2390.54	coding	158	497	970
PB.3100.2	2	2148	9	novel_in_catalog	gene: Zm00001d005324	novel	3.72	11.40	coding	411	643	1875
PB.124.8	1	1952	6	novel_not_in_catalog	gene: Zm00001d027731	novel	420.02	562.10	coding	390	366	1535
PB.6485.1	5	1584	5	full-splice_match	gene: Zm00001d015130	Zm00001d015130_T001	28.74	34.37	coding	356	191	1258
PB.6485.3	5	1497	5	full-splice_match	gene: Zm00001d015130	Zm00001d015130_T001	5.63	34.37	coding	356	193	1260
PB.7546.1	6	2393	11	full-splice_match	gene: Zm00001d036831	Zm00001d036831_T011	30.28	30.28	coding	448	831	2174
PB.3357.1	2	1361	2	full-splice_match	gene: Zm00001d006581	Zm00001d006581_T001	46.92	51.93	coding	345	65	1099
PB.239.1	1	2313	1	antisense	novelGene_gene: Zm00001d028286_A	novel	19.53	19.53	coding	76	82	309
PB.3357.2	2	2272	1	novel_in_catalog	gene: Zm00001d006581	novel	5.00	51.93	coding	222	58	723
PB.2848.1	2	869	5	full-splice_match	gene: Zm00001d003672	Zm00001d003672_T001	681.92	681.92	coding	181	82	624
PB.5249.1	4	3056	10	full-splice_match	gene: Zm00001d051229	Zm00001d051229_T002	5.55	5.55	coding	731	185	2377
PB.2848.2	2	548	3	incomplete-splice_match	gene: Zm00001d003672	Zm00001d003672_T001	0.00	681.92	coding	136	8	415
PB.6706.2	5	1373	8	incomplete-splice_match	gene: Zm00001d016595	Zm00001d016595_T004	16.04	16.04	coding	305	253	1167
PB.5211.1	4	1427	9	full-splice_match	gene: Zm00001d050970	Zm00001d050970_T005	15.00	68.24	coding	193	90	668
PB.4402.1	3	1684	2	full-splice_match	gene: Zm00001d043165	Zm00001d043165_T001	4.28	4.28	coding	470	64	1473

PB.6065.1	5	688	1	full-splice_match	gene: Zm00001d013332	Zm00001d013332_T001	40.35	40.35	coding	70	267	476
PB.4919.1	4	831	6	novel_not_in_catalog	gene: Zm00001d049193	novel	76.50	77.48	coding	185	20	574
PB.4919.2	4	737	6	novel_not_in_catalog	gene: Zm00001d049193	novel	0.97	77.48	coding	185	30	584
PB.3817.1	3	1885	3	full-splice_match	gene: Zm00001d040040	Zm00001d040040_T001	21.52	21.52	coding	462	156	1541
PB.7645.1	6	1712	12	full-splice_match	gene: Zm00001d037326	Zm00001d037326_T001	85.02	196.84	coding	402	114	1319
PB.7645.3	6	1908	11	novel_in_catalog	gene: Zm00001d037326	novel	1.95	196.84	coding	272	110	925
PB.7645.2	6	1564	12	full-splice_match	gene: Zm00001d037326	Zm00001d037326_T001	109.87	196.84	coding	402	171	1376
PB.10514.1	9	1081	1	full-splice_match	gene: Zm00001d047442	Zm00001d047442_T001	13.34	13.34	coding	215	213	857
PB.3307.1	2	1229	2	genic	gene: Zm00001d006361	novel	43.66	43.66	coding	287	170	1030
PB.8123.3	6	1504	4	full-splice_match	gene: Zm00001d039166	Zm00001d039166_T004	4.23	225.80	coding	179	202	738
PB.9219.1	8	1728	10	full-splice_match	gene: Zm00001d008907	Zm00001d008907_T001	41.30	60.15	coding	302	382	1287
PB.9219.2	8	1524	10	full-splice_match	gene: Zm00001d008907	Zm00001d008907_T001	18.85	60.15	coding	302	383	1288
PB.3712.1	3	2749	1	intergenic	novelGene_27	novel	4.43	44.08	coding	439	599	1915
PB.8123.6	6	1128	5	full-splice_match	gene: Zm00001d039166	Zm00001d039166_T001	11.38	225.80	coding	276	143	970
PB.8123.5	6	1282	5	full-splice_match	gene: Zm00001d039166	Zm00001d039166_T001	4.57	225.80	coding	276	213	1040
PB.1555.1	1	1213	3	full-splice_match	gene: Zm00001d034663	Zm00001d034663_T001	28.03	28.03	coding	268	152	955
PB.5613.1	4	1586	4	full-splice_match	gene: Zm00001d052837	Zm00001d052837_T001	303.90	303.90	coding	332	276	1271

PB.5654.1	4	984	3	full-splice_match	gene: Zm00001d053010	Zm00001d053010_T001	764.31	918.66	coding	165	169	663
PB.1857.1	10	1371	9	full-splice_match	gene: Zm00001d024408	Zm00001d024408_T001	53.25	53.25	coding	332	98	1093
PB.6801.2	5	2168	2	full-splice_match	gene: Zm00001d017067	Zm00001d017067_T003	12.90	12.90	coding	503	92	1600
PB.8062.3	6	1979	12	full-splice_match	gene: Zm00001d038927	Zm00001d038927_T003	5.89	56.53	coding	515	105	1649
PB.8062.2	6	2252	9	novel_in_catalog	gene: Zm00001d038927	novel	3.28	56.53	coding	224	108	779
PB.5819.1	4	1212	2	full-splice_match	gene: Zm00001d053830	Zm00001d053830_T001	14.65	14.65	coding	275	89	913
PB.8062.7	6	1782	11	full-splice_match	gene: Zm00001d038927	Zm00001d038927_T002	0.00	56.53	coding	515	96	1640
PB.8062.6	6	1950	12	full-splice_match	gene: Zm00001d038927	Zm00001d038927_T005	38.79	56.53	coding	511	108	1640
PB.8062.4	6	2066	11	full-splice_match	gene: Zm00001d038927	Zm00001d038927_T002	8.56	56.53	coding	515	102	1646
PB.7063.1	5	849	1	genic	gene: Zm00001d018128	novel	6.35	72.50	non_coding	NA	NA	NA
PB.10838.1	9	2322	10	full-splice_match	gene: Zm00001d048574	Zm00001d048574_T002	30.72	30.72	coding	597	250	2040
PB.10838.2	9	2060	10	full-splice_match	gene: Zm00001d048574	Zm00001d048574_T002	0.00	30.72	coding	597	117	1907
PB.5897.1	4	1470	5	full-splice_match	gene: Zm00001d054081	Zm00001d054081_T001	8.20	8.20	coding	339	229	1245
PB.1352.1	1	1321	2	full-splice_match	gene: Zm00001d033849	Zm00001d033849_T002	16.77	16.77	coding	319	66	1022
PB.3086.1	2	2186	8	incomplete-splice_match	gene: Zm00001d005250	Zm00001d005250_T003	171.56	171.56	coding	624	49	1920
PB.4701.1	3	1685	8	novel_not_in_catalog	gene: Zm00001d044328	novel	5.67	5.67	coding	422	17	1282
PB.2421.1	2	1172	8	full-splice_match	gene: Zm00001d001870	Zm00001d001870_T001	22.16	22.16	coding	250	225	974

PB.9505.1	8	1189	2	genic	gene: Zm00001d010481	novel	28.57	28.57	coding	263	118	906
PB.2807.1	2	2710	5	full-splice_match	gene: Zm00001d003477	Zm00001d003477_T001	15.34	15.34	coding	688	296	2359
PB.8378.1	7	3296	1	genic_intron	novelGene_131	novel	2.20	2.20	coding	904	365	3076
PB.6639.1	5	2113	1	full-splice_match	gene: Zm00001d016199	Zm00001d016199_T001	17.79	17.79	coding	561	112	1794
PB.10003.14	8	1960	9	novel_in_catalog	gene: Zm00001d012667	novel	0.00	43.63	coding	379	561	1697
PB.8326.3	7	1420	2	incomplete-splice_match	gene: Zm00001d019522	Zm00001d019522_T002	37.38	37.38	coding	445	85	1419
PB.2356.1	10	1175	1	full-splice_match	gene: Zm00001d026632	Zm00001d026632_T001	287.06	287.06	coding	265	88	882
PB.875.2	1	2024	5	full-splice_match	gene: Zm00001d031648	Zm00001d031648_T001	109.58	114.31	coding	435	318	1622
PB.875.3	1	1777	3	novel_in_catalog	gene: Zm00001d031648	novel	0.00	114.31	coding	243	648	1376
PB.875.1	1	1219	2	genic	gene: Zm00001d031648	novel	4.74	114.31	non_coding	NA	NA	NA
PB.875.4	1	1758	5	full-splice_match	gene: Zm00001d031648	Zm00001d031648_T001	0.00	114.31	coding	435	252	1556
PB.875.5	1	1545	5	full-splice_match	gene: Zm00001d031648	Zm00001d031648_T001	0.00	114.31	coding	435	220	1524
PB.2263.4	10	1793	3	novel_not_in_catalog	gene: Zm00001d026333	novel	0.97	48.08	coding	447	130	1470
PB.4099.1	3	1058	2	genic	gene: Zm00001d041774	novel	78.23	100.82	coding	117	597	947
PB.3468.1	2	1741	11	novel_in_catalog	gene: Zm00001d007095	novel	20.61	20.61	coding	422	181	1446
PB.5235.1	4	1302	13	full-splice_match	gene: Zm00001d051157	Zm00001d051157_T001	95.55	95.55	coding	342	101	1126
PB.2608.4	2	2461	4	full-splice_match	gene: Zm00001d002654	Zm00001d002654_T013	5.58	50.22	coding	588	265	2028

PB.9810.1	8	811	1	genic	gene: Zm00001d011839	novel	14.52	14.52	non_coding	NA	NA	NA
PB.8133.2	6	813	2	intergenic	novelGene_284	novel	79.23	111.14	non_coding	NA	NA	NA
PB.10640.1	9	1247	4	full-splice_match	gene: Zm00001d047876	Zm00001d047876_T001	43.16	43.16	coding	225	133	807
PB.5597.1	4	1047	1	full-splice_match	gene: Zm00001d052776	Zm00001d052776_T001	121.51	121.51	coding	213	112	750
PB.3560.1	2	2220	11	full-splice_match	gene: Zm00001d007632	Zm00001d007632_T011	7.63	17.91	coding	299	796	1692
PB.2263.5	10	746	3	novel_not_in_catalog	gene: Zm00001d026333	novel	7.84	48.08	coding	179	3	539
PB.3308.1	2	1307	4	full-splice_match	gene: Zm00001d006365	Zm00001d006365_T001	18.87	18.87	coding	350	79	1128
PB.1595.3	1	1075	5	full-splice_match	gene: Zm00001d034795	Zm00001d034795_T001	0.00	151.38	coding	289	53	919
PB.7873.1	6	1888	14	full-splice_match	gene: Zm00001d038205	Zm00001d038205_T002	15.26	15.26	coding	492	179	1654
PB.316.1	1	2598	9	novel_in_catalog	gene: Zm00001d028608	novel	31.64	31.64	coding	398	371	1564
PB.7365.1	6	1046	1	genic	gene: Zm00001d035835	novel	8.31	8.31	coding	120	420	779
PB.2066.1	10	1043	4	novel_not_in_catalog	gene: Zm00001d025425	novel	65.70	65.70	coding	150	345	794
PB.4046.1	3	1587	1	full-splice_match	gene: Zm00001d041537	Zm00001d041537_T001	2.78	2.78	coding	346	1	1038
PB.8962.1	7	786	6	novel_not_in_catalog	gene: Zm00001d022352	novel	28.01	28.01	coding	123	80	448
PB.8645.3	7	1923	10	full-splice_match	gene: Zm00001d021227	Zm00001d021227_T003	0.00	40.66	coding	548	101	1744
PB.7848.1	6	2969	9	full-splice_match	gene: Zm00001d038142	Zm00001d038142_T026	14.58	14.58	coding	333	1621	2619
PB.5540.4	4	1835	2	novel_in_catalog	gene: Zm00001d052493	novel	15.69	1238.52	coding	140	925	1344

PB.5540.7	4	926	4	full-splice_match	gene: Zm00001d052493	Zm00001d052493_T003	449.02	1238.52	coding	235	102	806
PB.7463.4	6	1977	13	full-splice_match	gene: Zm00001d036429	Zm00001d036429_T001	7.79	50.83	coding	549	118	1764
PB.2103.3	10	1700	7	full-splice_match	gene: Zm00001d025658	Zm00001d025658_T001	2.03	63.14	coding	346	311	1348
PB.2103.2	10	1804	7	full-splice_match	gene: Zm00001d025658	Zm00001d025658_T001	50.54	63.14	coding	346	303	1340
PB.7463.1	6	2108	13	full-splice_match	gene: Zm00001d036429	Zm00001d036429_T001	43.04	50.83	coding	549	94	1740
PB.5540.2	4	1600	3	full-splice_match	gene: Zm00001d052493	Zm00001d052493_T002	34.13	1238.52	coding	111	928	1260
PB.2962.4	2	1644	2	full-splice_match	gene: Zm00001d004506	Zm00001d004506_T001	0.00	7.21	coding	383	435	1583
PB.3755.2	3	918	3	full-splice_match	gene: Zm00001d039716	Zm00001d039716_T001	0.00	3.71	coding	213	19	657
PB.3755.1	3	1079	3	full-splice_match	gene: Zm00001d039716	Zm00001d039716_T001	3.71	3.71	coding	213	71	709
PB.6497.1	5	1366	6	full-splice_match	gene: Zm00001d015195	Zm00001d015195_T001	42.48	42.48	coding	334	41	1042
PB.2962.1	2	1883	2	full-splice_match	gene: Zm00001d004506	Zm00001d004506_T001	3.38	7.21	coding	383	436	1584
PB.2962.2	2	2215	1	genic	gene: Zm00001d004506	novel	0.30	7.21	coding	200	437	1036
PB.2962.3	2	1768	2	full-splice_match	gene: Zm00001d004506	Zm00001d004506_T001	3.53	7.21	coding	383	436	1584
PB.8342.2	7	1457	7	full-splice_match	gene: Zm00001d019579	Zm00001d019579_T007	17.79	21.04	coding	352	75	1130
PB.8342.1	7	1643	10	novel_not_in_catalog	gene: Zm00001d019579	novel	3.25	21.04	coding	352	56	1111
PB.8837.1	7	2049	1	novel_in_catalog	gene: Zm00001d021883	novel	2.79	2.79	coding	628	39	1922
PB.770.2	1	2342	5	intergenic	novelGene_292	novel	4.93	8.37	non_coding	NA	NA	NA

PB.10526.1	9	1760	1	intergenic	novelGene_209	novel	7.19	7.19	coding	445	76	1410
PB.10315.1	9	1581	2	full-splice_match	gene: Zm00001d046305	Zm00001d046305_T001	48.96	48.96	coding	355	319	1383
PB.5520.4	4	1788	16	full-splice_match	gene: Zm00001d052434	Zm00001d052434_T003	7.15	17.33	coding	484	142	1593
PB.2092.2	10	4351	10	novel_in_catalog	gene: Zm00001d025590	novel	11.85	34.73	coding	1264	166	3957
PB.5520.1	4	1927	16	full-splice_match	gene: Zm00001d052434	Zm00001d052434_T003	6.51	17.33	coding	484	156	1607
PB.4095.1	3	929	3	full-splice_match	gene: Zm00001d041760	Zm00001d041760_T001	847.37	1277.61	coding	194	90	671
PB.10068.1	9	1387	1	full-splice_match	gene: Zm00001d044918	Zm00001d044918_T001	10.55	10.55	coding	393	78	1256
PB.4095.2	3	830	3	full-splice_match	gene: Zm00001d041760	Zm00001d041760_T001	430.24	1277.61	coding	194	84	665
PB.7784.1	6	1550	2	full-splice_match	gene: Zm00001d037935	Zm00001d037935_T001	3.19	3.19	coding	451	78	1430
PB.7832.3	6	2018	3	full-splice_match	gene: Zm00001d038101	Zm00001d038101_T002	0.00	57.66	coding	443	484	1812
PB.7832.2	6	1919	3	full-splice_match	gene: Zm00001d038101	Zm00001d038101_T002	17.70	57.66	coding	443	289	1617
PB.6439.1	5	2322	3	full-splice_match	gene: Zm00001d014961	Zm00001d014961_T001	62.57	62.57	coding	612	169	2004
PB.6439.3	5	2169	3	full-splice_match	gene: Zm00001d014961	Zm00001d014961_T001	0.00	62.57	coding	612	114	1949
PB.2443.1	2	679	3	full-splice_match	gene: Zm00001d001972	Zm00001d001972_T001	36.61	36.61	coding	67	219	419
PB.1093.1	1	1763	7	full-splice_match	gene: Zm00001d032604	Zm00001d032604_T003	10.10	10.10	coding	416	55	1302
PB.225.6	1	2324	12	novel_in_catalog	gene: Zm00001d028228	novel	1.91	106.06	coding	437	272	1582
PB.225.5	1	2223	11	novel_in_catalog	gene: Zm00001d028228	novel	5.64	106.06	coding	247	174	914

PB.225.4	1	2218	13	full-splice_match	gene: Zm00001d028228	Zm00001d028228_T001	22.44	106.06	coding	553	272	1930
PB.225.3	1	2339	12	novel_in_catalog	gene: Zm00001d028228	novel	4.47	106.06	coding	202	267	872
PB.225.1	1	2240	13	full-splice_match	gene: Zm00001d028228	Zm00001d028228_T001	71.60	106.06	coding	553	272	1930
PB.4784.1	3	882	2	full-splice_match	gene: Zm00001d044691	Zm00001d044691_T001	9.97	108.71	coding	86	133	390
PB.4047.1	3	802	1	full-splice_match	gene: Zm00001d041538	Zm00001d041538_T001	20.00	20.00	coding	140	60	479
PB.3559.1	2	2081	2	incomplete-splice_match	gene: Zm00001d007630	Zm00001d007630_T004	6.24	6.24	coding	461	138	1520
PB.10433.1	9	1488	9	novel_in_catalog	gene: Zm00001d046974	novel	20.74	20.74	coding	310	87	1016
PB.9948.1	8	1259	4	full-splice_match	gene: Zm00001d012460	Zm00001d012460_T001	27.51	27.51	coding	241	389	1111
PB.10324.6	9	1744	3	genic	gene: Zm00001d046378	novel	5.16	784.46	coding	138	194	607
PB.3442.1	2	770	1	full-splice_match	gene: Zm00001d006950	Zm00001d006950_T001	40.16	40.16	coding	156	85	552
PB.5873.2	4	2072	10	novel_not_in_catalog	gene: Zm00001d053998	novel	0.00	10.09	coding	530	239	1828
PB.1584.3	1	691	1	full-splice_match	gene: Zm00001d034756	Zm00001d034756_T001	0.00	304.16	non_coding	NA	NA	NA
PB.5873.1	4	2104	10	novel_not_in_catalog	gene: Zm00001d053998	novel	10.09	10.09	coding	530	162	1751
PB.9122.1	8	1846	1	novel_in_catalog	gene: Zm00001d008455	novel	17.46	17.46	coding	280	700	1539
PB.5677.10	4	2574	5	novel_not_in_catalog	gene: Zm00001d053151	novel	0.00	108.01	coding	671	247	2259
PB.6011.1	5	949	4	full-splice_match	gene: Zm00001d013179	Zm00001d013179_T001	155.75	155.75	coding	214	39	680
PB.3597.2	2	2234	14	full-splice_match	gene: Zm00001d007826	Zm00001d007826_T009	29.70	65.94	coding	644	139	2070

PB.784.1	1	587	1	full-splice_match	gene: Zm00001d031146	Zm00001d031146_T001	21.38	21.38	coding	111	22	354
PB.1169.2	1	2611	18	full-splice_match	gene: Zm00001d032956	Zm00001d032956_T014	0.00	43.59	coding	774	148	2469
PB.8960.2	7	1434	1	novel_in_catalog	gene: Zm00001d022347	novel	7.94	7.94	coding	325	69	1043
PB.5528.1	4	890	5	full-splice_match	gene: Zm00001d052452	Zm00001d052452_T001	99.17	99.17	coding	159	157	633
PB.7071.1	5	1088	1	full-splice_match	gene: Zm00001d018150	Zm00001d018150_T001	40.57	40.57	coding	209	166	792
PB.10417.1	9	806	1	full-splice_match	gene: Zm00001d046927	Zm00001d046927_T001	148.57	148.57	coding	184	123	674
PB.9517.1	8	464	1	antisense	novelGene_gene: Zm00001d010540_A	novel	30.90	30.90	non_coding	NA	NA	NA
PB.4302.3	3	2235	14	full-splice_match	gene: Zm00001d042720	Zm00001d042720_T005	3.79	15.50	coding	620	231	2090
PB.6936.3	5	1555	5	full-splice_match	gene: Zm00001d017643	Zm00001d017643_T001	9.37	9.37	coding	387	209	1369
PB.1216.1	1	826	2	full-splice_match	gene: Zm00001d033232	Zm00001d033232_T001	28.98	28.98	coding	190	15	584
PB.2199.1	10	2114	2	full-splice_match	gene: Zm00001d026051	Zm00001d026051_T002	14.87	14.87	coding	606	52	1869
PB.10280.2	9	2265	10	full-splice_match	gene: Zm00001d046020	Zm00001d046020_T001	10.52	18.65	coding	672	116	2131
PB.3741.2	3	1674	7	incomplete-splice_match	gene: Zm00001d039612	Zm00001d039612_T025	12.25	12.25	coding	494	14	1495
PB.10805.1	9	1656	6	full-splice_match	gene: Zm00001d048471	Zm00001d048471_T001	39.58	68.53	coding	318	167	1120
PB.5556.1	4	2201	14	full-splice_match	gene: Zm00001d052615	Zm00001d052615_T005	7.54	7.54	coding	668	71	2074
PB.10722.1	9	2504	3	novel_in_catalog	gene: Zm00001d048173	novel	6.21	6.21	coding	253	1473	2231
PB.1375.3	1	3102	9	novel_not_in_catalog	gene: Zm00001d033948	novel	1.24	50.33	coding	115	192	536

PB.6895.2	5	955	2	full-splice_match	gene: Zm00001d017441	Zm00001d017441_T001	132.23	136.90	coding	209	80	706
PB.6895.1	5	2486	1	genic	gene: Zm00001d017441	novel	4.68	136.90	coding	124	80	451
PB.8101.3	6	1411	7	full-splice_match	gene: Zm00001d039089	Zm00001d039089_T001	186.07	302.45	coding	340	166	1185
PB.10301.3	9	1903	6	novel_in_catalog	gene: Zm00001d046200	novel	4.33	12.91	coding	81	67	309
PB.10301.2	9	1185	7	full-splice_match	gene: Zm00001d046200	Zm00001d046200_T005	8.58	12.91	non_coding	NA	NA	NA
PB.1375.5	1	2486	11	novel_not_in_catalog	gene: Zm00001d033948	novel	21.61	50.33	coding	115	193	537
PB.1375.4	1	2484	8	novel_not_in_catalog	gene: Zm00001d033948	novel	11.34	50.33	coding	67	117	317
PB.5541.3	4	1967	16	full-splice_match	gene: Zm00001d052494	Zm00001d052494_T001	115.00	128.72	coding	527	159	1739
PB.8157.2	7	1777	4	novel_in_catalog	gene: Zm00001d018638	novel	0.52	5.58	coding	369	537	1643
PB.8625.3	7	2549	12	novel_not_in_catalog	gene: Zm00001d021132	novel	2.12	45.44	coding	221	1642	2304
PB.1157.9	1	1701	4	novel_not_in_catalog	gene: Zm00001d032897	novel	10.93	297.96	coding	245	755	1489
PB.8157.1	7	1719	5	full-splice_match	gene: Zm00001d018638	Zm00001d018638_T001	5.07	5.58	coding	500	40	1539
PB.8411.1	7	1307	2	novel_not_in_catalog	gene: Zm00001d020102	novel	12.43	12.43	coding	216	133	780
PB.1497.7	1	1162	8	full-splice_match	gene: Zm00001d034460	Zm00001d034460_T007	51.40	56.18	coding	274	132	953
PB.8678.1	7	655	3	full-splice_match	gene: Zm00001d021359	Zm00001d021359_T001	34.60	34.60	coding	101	174	476
PB.2247.1	10	1273	7	full-splice_match	gene: Zm00001d026283	Zm00001d026283_T002	277.30	277.30	coding	287	193	1053
PB.2935.2	2	1656	1	full-splice_match	gene: Zm00001d004187	Zm00001d004187_T001	11.53	11.53	coding	483	32	1480

PB.2935.3	2	1489	1	full-splice_match	gene: Zm00001d004187	Zm00001d004187_T001	0.00	11.53	coding	468	9	1412
PB.4985.6	4	1617	3	incomplete-splice_match	gene: Zm00001d049558	Zm00001d049558_T003	22.48	22.48	coding	460	236	1615
PB.4527.1	3	1357	6	full-splice_match	gene: Zm00001d043632	Zm00001d043632_T001	304.99	304.99	coding	378	47	1180
PB.8244.1	7	759	3	full-splice_match	gene: Zm00001d019034	Zm00001d019034_T001	184.12	905.92	coding	122	71	436
PB.6005.1	5	3455	5	novel_not_in_catalog	gene: Zm00001d013154	novel	32.31	40.66	coding	949	480	3326
PB.6005.2	5	3502	3	novel_not_in_catalog	gene: Zm00001d013154	novel	8.35	40.66	coding	629	726	2612
PB.8244.2	7	611	3	full-splice_match	gene: Zm00001d019034	Zm00001d019034_T001	721.80	905.92	coding	122	72	437
PB.6005.4	5	3488	4	novel_not_in_catalog	gene: Zm00001d013154	novel	0.00	40.66	coding	757	352	2622
PB.8431.2	7	829	1	incomplete-splice_match	gene: Zm00001d020227	Zm00001d020227_T002	9.07	43.17	non_coding	NA	NA	NA
PB.6005.6	5	3339	5	novel_not_in_catalog	gene: Zm00001d013154	novel	0.00	40.66	coding	949	446	3292
PB.6005.7	5	2442	4	novel_not_in_catalog	gene: Zm00001d013154	novel	0.00	40.66	coding	722	276	2441
PB.6296.2	5	1560	6	incomplete-splice_match	gene: Zm00001d014413	Zm00001d014413_T006	17.91	22.31	coding	354	36	1097
PB.5092.1	4	3171	19	fusion	gene: Zm00001d050134_g	novel	22.75	25.28	coding	920	54	2813
PB.2896.4	2	964	1	incomplete-splice_match	gene: Zm00001d003931	Zm00001d003931_T001	0.00	59.92	coding	229	208	894
PB.6296.4	5	4137	7	full-splice_match	gene: Zm00001d014413	Zm00001d014413_T032	4.40	22.31	coding	415	2388	3632
PB.7144.2	5	1801	9	full-splice_match	gene: Zm00001d018409	Zm00001d018409_T004	86.64	146.45	coding	428	245	1528
PB.8631.1	7	1759	2	full-splice_match	gene: Zm00001d021168	Zm00001d021168_T001	2.48	6.25	coding	472	185	1600

PB.8631.2	7	1560	2	full-splice_match	gene: Zm00001d021168	Zm00001d021168_T001	3.78	6.25	coding	472	95	1510
PB.396.1	1	1260	2	genic	gene: Zm00001d028972	novel	13.05	13.05	coding	165	213	707
PB.1169.1	1	2732	18	full-splice_match	gene: Zm00001d032956	Zm00001d032956_T014	43.59	43.59	coding	774	152	2473
PB.7436.1	6	2230	3	novel_in_catalog	gene: Zm00001d036283	novel	143.34	143.34	coding	211	186	818
PB.5092.2	4	2839	17	fusion	gene: Zm00001d050134_g	novel	2.53	25.28	coding	822	52	2517
PB.1155.3	1	1064	2	full-splice_match	gene: Zm00001d032883	Zm00001d032883_T001	35.66	60.62	coding	250	81	830
PB.1155.2	1	1121	2	full-splice_match	gene: Zm00001d032883	Zm00001d032883_T001	18.78	60.62	coding	250	81	830
PB.1155.1	1	619	1	genic	gene: Zm00001d032883	novel	6.18	60.62	non_coding	NA	NA	NA
PB.9560.2	8	1126	9	full-splice_match	gene: Zm00001d010723	Zm00001d010723_T001	11.58	56.22	coding	280	174	1013
PB.6629.4	5	1889	3	full-splice_match	gene: Zm00001d016149	Zm00001d016149_T001	0.00	29.79	coding	471	201	1613
PB.8904.1	7	2610	13	full-splice_match	gene: Zm00001d022132	Zm00001d022132_T004	13.75	13.75	coding	750	194	2443
PB.9560.1	8	1230	9	full-splice_match	gene: Zm00001d010723	Zm00001d010723_T001	44.64	56.22	coding	280	148	987
PB.2352.3	10	2447	7	novel_in_catalog	gene: Zm00001d026612	novel	3.33	23.34	coding	113	269	607
PB.1663.1	10	895	1	full-splice_match	gene: Zm00001d023269	Zm00001d023269_T001	25.62	25.62	coding	115	263	607
PB.7901.1	6	1722	5	full-splice_match	gene: Zm00001d038321	Zm00001d038321_T001	24.05	24.05	coding	432	187	1482
PB.4141.3	3	2136	8	novel_not_in_catalog	gene: Zm00001d041989	novel	1.44	7.57	coding	243	1311	2039
PB.4141.2	3	1855	9	novel_not_in_catalog	gene: Zm00001d041989	novel	4.05	7.57	coding	524	153	1724

PB.4141.1	3	2216	9	novel_not_in_catalog	gene: Zm00001d041989	novel	2.08	7.57	coding	524	126	1697
PB.6901.2	5	1144	2	full-splice_match	gene: Zm00001d017467	Zm00001d017467_T001	134.18	134.18	coding	249	49	795
PB.7375.5	6	1614	2	full-splice_match	gene: Zm00001d035899	Zm00001d035899_T001	0.00	613.12	coding	486	60	1517
PB.5845.1	4	2599	7	full-splice_match	gene: Zm00001d053923	Zm00001d053923_T005	13.42	73.38	coding	761	34	2316
PB.8439.1	7	2548	1	genic	gene: Zm00001d020279	novel	8.17	8.17	coding	387	457	1617
PB.1847.6	10	1858	16	incomplete-splice_match	gene: Zm00001d024327	Zm00001d024327_T003	38.83	63.22	coding	486	136	1593
PB.8773.10	7	2413	6	novel_not_in_catalog	gene: Zm00001d021698	novel	0.00	192.02	coding	535	134	1738
PB.641.1	1	1487	3	full-splice_match	gene: Zm00001d030191	Zm00001d030191_T001	35.69	35.69	coding	262	177	962
PB.3767.1	3	1433	1	full-splice_match	gene: Zm00001d039788	Zm00001d039788_T001	38.97	38.97	coding	362	106	1191
PB.9915.1	8	1704	1	full-splice_match	gene: Zm00001d012302	Zm00001d012302_T001	55.81	55.81	coding	394	185	1366
PB.6629.3	5	1988	3	full-splice_match	gene: Zm00001d016149	Zm00001d016149_T001	0.00	29.79	coding	471	177	1589
PB.9687.3	8	1492	12	full-splice_match	gene: Zm00001d011329	Zm00001d011329_T002	1.27	13.49	coding	357	93	1163
PB.6411.2	5	3000	15	incomplete-splice_match	gene: Zm00001d014876	Zm00001d014876_T003	6.88	109.60	coding	852	74	2629
PB.6411.1	5	2901	16	full-splice_match	gene: Zm00001d014876	Zm00001d014876_T001	11.25	109.60	coding	857	74	2644
PB.9929.1	8	1801	12	full-splice_match	gene: Zm00001d012380	Zm00001d012380_T002	19.52	27.73	coding	324	436	1407
PB.8937.4	7	2727	3	full-splice_match	gene: Zm00001d022273	Zm00001d022273_T009	27.15	31.12	coding	650	32	1981
PB.8937.5	7	2639	3	full-splice_match	gene: Zm00001d022273	Zm00001d022273_T009	3.97	31.12	coding	650	93	2042

PB.9929.4	8	1338	6	novel_in_catalog	gene: Zm00001d012380	novel	3.29	27.73	coding	56	157	324
PB.8937.7	7	2341	3	full-splice_match	gene: Zm00001d022273	Zm00001d022273_T014	0.00	31.12	coding	650	75	2024
PB.2560.2	2	734	2	full-splice_match	gene: Zm00001d002450	Zm00001d002450_T002	277.87	507.90	coding	166	77	574
PB.2560.1	2	834	2	full-splice_match	gene: Zm00001d002450	Zm00001d002450_T002	230.03	507.90	coding	166	77	574
PB.9656.1	8	1792	8	novel_not_in_catalog	gene: Zm00001d011156	novel	4.44	4.44	coding	427	148	1428
PB.3088.2	2	2232	15	fusion	gene: Zm00001d005256_g	novel	0.30	32.71	coding	450	715	2064
PB.6399.1	5	999	4	full-splice_match	gene: Zm00001d014811	Zm00001d014811_T001	43.00	43.00	coding	208	76	699
PB.1167.2	1	2241	4	novel_in_catalog	gene: Zm00001d032939	novel	2.93	60.10	coding	190	463	1032
PB.1167.3	1	2101	4	incomplete-splice_match	gene: Zm00001d032939	Zm00001d032939_T001	0.43	60.10	coding	190	467	1036
PB.3207.1	2	2188	14	full-splice_match	gene: Zm00001d005925	Zm00001d005925_T002	87.38	87.38	coding	616	129	1976
PB.6224.1	5	2712	15	novel_not_in_catalog	gene: Zm00001d014040	novel	9.63	9.63	coding	625	461	2335
PB.8722.3	7	1573	3	novel_in_catalog	gene: Zm00001d021526	novel	6.78	1033.27	coding	115	985	1329
PB.2336.1	10	1784	3	full-splice_match	gene: Zm00001d026580	Zm00001d026580_T002	18.00	18.00	coding	458	124	1497
PB.7386.1	6	1063	1	full-splice_match	gene: Zm00001d035963	Zm00001d035963_T001	50.99	50.99	coding	246	109	846
PB.5521.1	4	611	3	novel_not_in_catalog	gene: Zm00001d052436	novel	234.23	920.63	non_coding	NA	NA	NA
PB.4817.2	4	679	1	intergenic	novelGene_19	novel	0.00	14.54	coding	121	64	426
PB.3987.1	3	1601	2	genic	gene: Zm00001d041161	novel	2.35	2.35	coding	371	109	1221

PB.10369.1	9	1123	2	genic	gene: Zm00001d046599	novel	389.44	400.31	coding	204	289	900
PB.5154.2	4	1355	1	full-splice_match	gene: Zm00001d050493	Zm00001d050493_T001	69.79	88.70	coding	327	56	1036
PB.5154.3	4	1250	1	full-splice_match	gene: Zm00001d050493	Zm00001d050493_T001	0.00	88.70	coding	327	56	1036
PB.5154.1	4	517	1	full-splice_match	gene: Zm00001d050493	Zm00001d050493_T001	18.91	88.70	non_coding	NA	NA	NA
PB.7005.3	5	696	4	full-splice_match	gene: Zm00001d017914	Zm00001d017914_T001	134.35	210.66	coding	150	65	514
PB.7005.2	5	933	4	full-splice_match	gene: Zm00001d017914	Zm00001d017914_T001	59.77	210.66	coding	150	65	514
PB.7005.1	5	1085	4	full-splice_match	gene: Zm00001d017914	Zm00001d017914_T001	16.54	210.66	coding	150	64	513
PB.2336.2	10	1657	3	full-splice_match	gene: Zm00001d026580	Zm00001d026580_T002	0.00	18.00	coding	458	122	1495
PB.1233.2	1	1356	1	novel_in_catalog	gene: Zm00001d033319	novel	4.51	79.95	coding	162	500	985
PB.9868.20	8	2142	10	full-splice_match	gene: Zm00001d012100	Zm00001d012100_T036	9.90	79.52	coding	78	124	357
PB.2170.1	10	1276	4	novel_in_catalog	gene: Zm00001d025922	novel	18.83	18.83	coding	188	498	1061
PB.7118.1	5	3173	3	full-splice_match	gene: Zm00001d018335	Zm00001d018335_T001	20.19	20.65	coding	953	48	2906
PB.2079.2	10	1631	1	full-splice_match	gene: Zm00001d025490	Zm00001d025490_T001	7.91	152.24	coding	146	552	989
PB.7118.6	5	3018	3	full-splice_match	gene: Zm00001d018335	Zm00001d018335_T001	0.45	20.65	coding	953	51	2909
PB.9432.2	8	1247	2	novel_in_catalog	gene: Zm00001d010137	novel	2.24	32.17	coding	384	29	1180
PB.9432.1	8	1544	3	full-splice_match	gene: Zm00001d010137	Zm00001d010137_T001	29.93	32.17	coding	403	144	1352
PB.5893.3	4	1438	3	novel_in_catalog	gene: Zm00001d054076	novel	1.89	36.67	coding	146	368	805

PB.1177.2	1	1857	6	full-splice_match	gene: Zm00001d033004	Zm00001d033004_T005	40.20	45.33	coding	183	1170	1718
PB.6385.1	5	1127	5	full-splice_match	gene: Zm00001d014770	Zm00001d014770_T001	55.96	55.96	coding	195	111	695
PB.7785.1	6	852	1	full-splice_match	gene: Zm00001d037937	Zm00001d037937_T001	78.16	78.16	coding	173	52	570
PB.9299.1	8	758	4	full-splice_match	gene: Zm00001d009417	Zm00001d009417_T001	259.49	449.67	coding	146	60	497
PB.9299.2	8	655	4	full-splice_match	gene: Zm00001d009417	Zm00001d009417_T001	190.18	449.67	coding	146	61	498
PB.8703.2	7	1194	7	novel_in_catalog	gene: Zm00001d021446	novel	14.26	14.26	coding	210	325	954
PB.6423.4	5	2088	9	full-splice_match	gene: Zm00001d014903	Zm00001d014903_T001	0.00	126.79	coding	586	178	1935
PB.7780.2	6	1115	14	full-splice_match	gene: Zm00001d037925	Zm00001d037925_T001	39.65	67.95	coding	276	66	893
PB.2370.1	10	880	1	full-splice_match	gene: Zm00001d026665	Zm00001d026665_T001	22.15	48.52	coding	100	158	457
PB.8399.2	7	1899	13	novel_not_in_catalog	gene: Zm00001d020038	novel	11.15	17.43	coding	342	430	1455
PB.6423.1	5	2373	9	full-splice_match	gene: Zm00001d014903	Zm00001d014903_T001	105.75	126.79	coding	586	243	2000
PB.6423.2	5	1009	3	novel_in_catalog	gene: Zm00001d014903	novel	21.04	126.79	coding	130	338	727
PB.6423.3	5	2242	9	full-splice_match	gene: Zm00001d014903	Zm00001d014903_T002	0.00	126.79	coding	586	271	2028
PB.8275.1	7	1080	4	novel_not_in_catalog	gene: Zm00001d019180	novel	19.97	19.97	coding	180	229	768
PB.8772.5	7	2168	4	novel_not_in_catalog	gene: Zm00001d021697	novel	0.00	56.72	coding	660	83	2062
PB.8772.4	7	2341	3	novel_not_in_catalog	gene: Zm00001d021697	novel	0.00	56.72	coding	568	116	1819
PB.8772.3	7	2322	4	novel_not_in_catalog	gene: Zm00001d021697	novel	26.39	56.72	coding	660	107	2086

PB.8772.2	7	2465	3	novel_not_in_catalog	gene: Zm00001d021697	novel	20.59	56.72	coding	568	118	1821
PB.8772.1	7	2334	5	novel_not_in_catalog	gene: Zm00001d021697	novel	9.74	56.72	coding	667	91	2091
PB.7148.2	5	1862	7	novel_in_catalog	gene: Zm00001d018413	novel	0.88	77.88	coding	228	729	1412
PB.7776.1	6	849	1	full-splice_match	gene: Zm00001d037909	Zm00001d037909_T001	0.57	0.57	coding	173	123	641
PB.5583.2	4	2460	3	full-splice_match	gene: Zm00001d052733	Zm00001d052733_T001	5.40	60.17	coding	309	1049	1975
PB.3088.1	2	2591	17	fusion	gene: Zm00001d005256_g	novel	32.41	32.71	coding	695	208	2292
PB.7060.3	5	404	1	incomplete-splice_match	gene: Zm00001d018112	Zm00001d018112_T003	0.00	201.70	non_coding	NA	NA	NA
PB.2350.1	10	2669	6	full-splice_match	gene: Zm00001d026607	Zm00001d026607_T002	68.01	68.01	coding	720	182	2341
PB.5869.1	4	2184	1	intergenic	novelGene_365	novel	6.58	6.58	non_coding	NA	NA	NA
PB.5593.5	4	1046	5	full-splice_match	gene: Zm00001d052766	Zm00001d052766_T002	0.00	58.65	coding	281	204	1046
PB.5711.4	4	2079	2	genic	gene: Zm00001d053358	novel	0.65	13.31	coding	405	679	1893
PB.2138.1	10	1458	8	incomplete-splice_match	gene: Zm00001d025789	Zm00001d025789_T003	40.11	40.11	coding	306	115	1032
PB.5619.3	4	2389	3	full-splice_match	gene: Zm00001d052855	Zm00001d052855_T001	175.34	585.42	coding	699	96	2192
PB.2844.1	2	1702	1	incomplete-splice_match	gene: Zm00001d003667	Zm00001d003667_T002	2.33	2.33	non_coding	NA	NA	NA
PB.6131.2	5	791	2	full-splice_match	gene: Zm00001d013596	Zm00001d013596_T001	123.43	123.43	coding	191	115	687
PB.5583.1	4	1787	2	novel_in_catalog	gene: Zm00001d052733	novel	17.23	60.17	coding	343	274	1302
PB.8385.1	7	1666	13	full-splice_match	gene: Zm00001d019926	Zm00001d019926_T004	38.93	39.94	coding	374	240	1361

PB.8385.2	7	1595	13	full-splice_match	gene: Zm00001d019926	Zm00001d019926_T004	1.02	39.94	coding	374	294	1415
PB.2542.2	2	3143	4	full-splice_match	gene: Zm00001d002366	Zm00001d002366_T003	8.02	179.76	coding	279	2075	2911
PB.2542.3	2	1188	5	full-splice_match	gene: Zm00001d002366	Zm00001d002366_T004	122.08	179.76	coding	279	125	961
PB.2542.1	2	1372	5	full-splice_match	gene: Zm00001d002366	Zm00001d002366_T004	49.66	179.76	coding	279	202	1038
PB.7159.5	5	2830	11	full-splice_match	gene: Zm00001d018439	Zm00001d018439_T010	34.14	34.14	coding	649	589	2535
PB.4674.1	3	900	5	full-splice_match	gene: Zm00001d044226	Zm00001d044226_T002	43.52	43.52	coding	176	114	641
PB.1485.1	1	1063	4	novel_in_catalog	gene: Zm00001d034422	novel	7.60	1248.88	coding	152	83	538
PB.8072.1	6	2179	3	novel_in_catalog	gene: Zm00001d038964	novel	4.34	4.34	coding	425	632	1906
PB.4871.1	4	741	1	full-splice_match	gene: Zm00001d048985	Zm00001d048985_T001	88.46	88.46	coding	168	9	512
PB.1485.2	1	803	4	novel_in_catalog	gene: Zm00001d034422	novel	263.12	1248.88	coding	152	87	542
PB.5593.4	4	1526	5	novel_not_in_catalog	gene: Zm00001d052766	novel	0.00	58.65	coding	285	393	1247
PB.7800.8	6	1211	1	novel_in_catalog	gene: Zm00001d037982	novel	0.13	13.32	coding	188	112	675
PB.364.1	1	2288	2	genic	gene: Zm00001d028809	novel	13.23	13.23	coding	564	440	2131
PB.6516.1	5	1379	7	full-splice_match	gene: Zm00001d015313	Zm00001d015313_T001	25.82	25.82	coding	307	111	1031
PB.5350.4	4	1902	9	full-splice_match	gene: Zm00001d051754	Zm00001d051754_T002	244.24	287.92	coding	550	128	1777
PB.3911.2	3	1695	4	novel_not_in_catalog	gene: Zm00001d040509	novel	625.11	832.21	coding	445	78	1412
PB.9462.1	8	1801	13	novel_not_in_catalog	gene: Zm00001d010255	novel	24.12	24.12	coding	445	166	1500

PB.4034.1	3	2271	7	full-splice_match	gene: Zm00001d041480	Zm00001d041480_T001	35.77	35.77	coding	599	91	1887
PB.7445.3	6	1085	6	incomplete-splice_match	gene: Zm00001d036337	Zm00001d036337_T007	0.00	62.85	coding	316	136	1083
PB.7445.2	6	1809	10	full-splice_match	gene: Zm00001d036337	Zm00001d036337_T010	2.05	62.85	coding	340	135	1154
PB.7445.1	6	1811	11	full-splice_match	gene: Zm00001d036337	Zm00001d036337_T007	48.07	62.85	coding	394	168	1349
PB.9849.1	8	874	1	full-splice_match	gene: Zm00001d012010	Zm00001d012010_T001	57.66	57.66	coding	119	182	538
PB.124.3	1	2870	8	novel_in_catalog	gene: Zm00001d027731	novel	12.10	562.10	coding	617	294	2144
PB.2119.2	10	1809	2	novel_in_catalog	gene: Zm00001d025717	novel	0.00	18.13	coding	473	272	1690
PB.9868.15	8	2033	9	novel_in_catalog	gene: Zm00001d012100	novel	2.02	79.52	coding	138	214	627
PB.6407.4	5	1198	7	full-splice_match	gene: Zm00001d014858	Zm00001d014858_T001	638.39	2470.46	coding	157	160	630
PB.6407.5	5	1189	6	novel_in_catalog	gene: Zm00001d014858	novel	21.20	2470.46	coding	106	521	838
PB.3534.1	2	1664	5	full-splice_match	gene: Zm00001d007489	Zm00001d007489_T001	15.78	15.78	coding	410	173	1402
PB.6407.3	5	1261	2	full-splice_match	gene: Zm00001d014853	Zm00001d014853_T001	53.52	53.52	coding	335	189	1193
PB.8512.3	7	1352	3	full-splice_match	gene: Zm00001d020569	Zm00001d020569_T001	14.56	36.06	coding	369	89	1195
PB.8512.2	7	1463	4	novel_not_in_catalog	gene: Zm00001d020569	novel	11.17	36.06	coding	342	169	1194
PB.8512.1	7	1493	3	full-splice_match	gene: Zm00001d020569	Zm00001d020569_T001	10.33	36.06	coding	369	110	1216
PB.1910.1	10	1731	5	full-splice_match	gene: Zm00001d024702	Zm00001d024702_T001	26.98	26.98	coding	479	79	1515
PB.9868.19	8	1684	7	full-splice_match	gene: Zm00001d012100	Zm00001d012100_T045	13.34	79.52	non_coding	NA	NA	NA

PB.6407.8	5	1297	5	novel_in_catalog	gene: Zm00001d014858	novel	2.31	2470.46	non_coding	NA	NA	NA
PB.6407.9	5	1088	6	novel_in_catalog	gene: Zm00001d014858	novel	32.60	2470.46	non_coding	NA	NA	NA
PB.6784.4	5	2457	11	full-splice_match	gene: Zm00001d016928	Zm00001d016928_T010	0.00	87.77	coding	626	391	2268
PB.6784.5	5	2247	11	full-splice_match	gene: Zm00001d016928	Zm00001d016928_T010	5.40	87.77	coding	626	268	2145
PB.6784.2	5	2440	11	full-splice_match	gene: Zm00001d016928	Zm00001d016928_T010	45.24	87.77	coding	626	242	2119
PB.4173.1	3	986	1	full-splice_match	gene: Zm00001d042096	Zm00001d042096_T001	205.55	532.50	coding	235	44	748
PB.4173.2	3	902	1	full-splice_match	gene: Zm00001d042096	Zm00001d042096_T001	326.96	532.50	coding	235	73	777
PB.6784.1	5	2023	8	incomplete-splice_match	gene: Zm00001d016928	Zm00001d016928_T005	37.13	87.77	coding	499	7	1503
PB.9983.1	8	1313	1	genic_intron	novelGene_294	novel	3.93	3.93	coding	341	96	1118
PB.7736.4	6	1869	13	full-splice_match	gene: Zm00001d037734	Zm00001d037734_T001	12.33	63.55	coding	483	187	1635
PB.7736.5	6	1742	13	full-splice_match	gene: Zm00001d037734	Zm00001d037734_T006	0.00	63.55	coding	483	124	1572
PB.199.3	1	2801	2	novel_in_catalog	gene: Zm00001d028139	novel	1.06	25.04	coding	306	1710	2627
PB.7736.1	6	1926	13	full-splice_match	gene: Zm00001d037734	Zm00001d037734_T006	51.22	63.55	coding	483	124	1572
PB.962.2	1	1867	8	novel_not_in_catalog	gene: Zm00001d032079	novel	0.56	15.19	coding	355	221	1285
PB.8027.7	6	3644	8	full-splice_match	gene: Zm00001d038819	Zm00001d038819_T054	9.34	28.86	coding	253	2594	3352
PB.2137.1	10	1802	1	full-splice_match	gene: Zm00001d025788	Zm00001d025788_T001	19.14	19.14	coding	446	360	1697
PB.3560.2	2	2055	10	full-splice_match	gene: Zm00001d007632	Zm00001d007632_T012	7.85	17.91	coding	538	10	1623

PB.6159.2	5	2033	8	full-splice_match	gene: Zm00001d013712	Zm00001d013712_T005	10.39	23.91	coding	359	803	1879
PB.6159.3	5	1913	6	novel_in_catalog	gene: Zm00001d013712	novel	4.54	23.91	coding	315	98	1042
PB.1897.2	10	814	4	fusion	gene: Zm00001d024591_g	novel	0.00	85.99	non_coding	NA	NA	NA
PB.6159.1	5	1911	7	full-splice_match	gene: Zm00001d013712	Zm00001d013712_T002	8.98	23.91	coding	527	129	1709
PB.8159.4	7	1972	1	genic	gene: Zm00001d018639	novel	4.39	75.50	coding	633	38	1936
PB.10791.1	9	1426	3	full-splice_match	gene: Zm00001d048422	Zm00001d048422_T001	13.00	13.00	coding	355	171	1235
PB.9045.2	7	2916	6	novel_not_in_catalog	gene: Zm00001d022615	novel	10.40	10.40	coding	224	2088	2759
PB.8159.1	7	2429	1	genic	gene: Zm00001d018639	novel	4.34	75.50	coding	633	7	1905
PB.8159.2	7	2088	1	novel_in_catalog	gene: Zm00001d018639	novel	1.93	75.50	coding	521	334	1896
PB.1076.18	1	2311	5	full-splice_match	gene: Zm00001d032555	Zm00001d032555_T013	26.14	39.90	coding	517	527	2077
PB.8828.2	7	728	1	intergenic	novelGene_270	novel	57.24	73.17	non_coding	NA	NA	NA
PB.1147.4	1	3587	16	novel_not_in_catalog	gene: Zm00001d032855	novel	2.93	25.91	coding	992	80	3055
PB.6564.8	5	1743	4	novel_not_in_catalog	gene: Zm00001d015618	novel	1.09	718.92	coding	229	794	1480
PB.6811.1	5	957	6	full-splice_match	gene: Zm00001d017100	Zm00001d017100_T006	50.61	53.50	coding	229	98	784
PB.536.1	1	1151	2	full-splice_match	gene: Zm00001d029594	Zm00001d029594_T001	276.32	276.32	coding	238	56	769
PB.3890.5	3	2787	9	full-splice_match	gene: Zm00001d040368	Zm00001d040368_T008	40.64	40.64	non_coding	NA	NA	NA
PB.6564.1	5	1652	4	full-splice_match	gene: Zm00001d015618	Zm00001d015618_T001	215.82	718.92	coding	367	148	1248

PB.5431.1	4	1159	10	full-splice_match	gene: Zm00001d052108	Zm00001d052108_T005	193.59	200.76	coding	212	123	758
PB.6564.5	5	1540	4	full-splice_match	gene: Zm00001d015618	Zm00001d015618_T001	278.19	718.92	coding	367	148	1248
PB.9059.2	8	1452	1	genic	gene: Zm00001d008181	novel	9.24	133.77	non_coding	NA	NA	NA
PB.9059.3	8	1869	1	genic	gene: Zm00001d008181	novel	9.87	133.77	non_coding	NA	NA	NA
PB.9855.4	8	2116	1	genic	gene: Zm00001d012031	novel	0.00	88.09	coding	118	1580	1933
PB.9855.5	8	1084	3	full-splice_match	gene: Zm00001d012031	Zm00001d012031_T001	9.19	88.09	coding	228	260	943
PB.9855.2	8	1194	3	full-splice_match	gene: Zm00001d012031	Zm00001d012031_T001	65.99	88.09	coding	228	255	938
PB.9059.7	8	1507	1	novel_in_catalog	gene: Zm00001d008181	novel	0.00	133.77	non_coding	NA	NA	NA
PB.9059.4	8	1633	1	novel_in_catalog	gene: Zm00001d008181	novel	97.75	133.77	non_coding	NA	NA	NA
PB.9059.5	8	1531	2	novel_not_in_catalog	gene: Zm00001d008181	novel	5.46	133.77	non_coding	NA	NA	NA
PB.1076.13	1	2413	2	novel_in_catalog	gene: Zm00001d032552	novel	7.06	48.82	coding	225	1506	2180
PB.9059.8	8	1266	1	novel_in_catalog	gene: Zm00001d008181	novel	0.00	133.77	non_coding	NA	NA	NA
PB.9059.9	8	1141	2	full-splice_match	gene: Zm00001d008181	Zm00001d008181_T001	2.27	133.77	non_coding	NA	NA	NA
PB.3241.1	2	1939	14	full-splice_match	gene: Zm00001d006084	Zm00001d006084_T007	53.40	70.54	coding	490	79	1548
PB.3241.2	2	1807	14	full-splice_match	gene: Zm00001d006084	Zm00001d006084_T007	17.14	70.54	coding	490	75	1544
PB.3602.2	2	1627	1	full-splice_match	gene: Zm00001d007843	Zm00001d007843_T001	5.42	28.53	coding	481	134	1576
PB.720.1	1	1875	2	incomplete-splice_match	gene: Zm00001d030796	Zm00001d030796_T003	14.68	14.68	coding	572	31	1746

PB.3602.1	2	2324	1	full-splice_match	gene: Zm00001d007843	Zm00001d007843_T001	23.11	28.53	coding	701	77	2179
PB.4157.2	3	2635	6	novel_not_in_catalog	gene: Zm00001d042042	novel	6.00	32.48	coding	324	436	1407
PB.2360.2	10	1317	6	full-splice_match	gene: Zm00001d026639	Zm00001d026639_T002	0.00	90.97	coding	328	79	1062
PB.6970.2	5	1867	2	novel_in_catalog	gene: Zm00001d017785	novel	20.63	110.51	coding	279	123	959
PB.8563.2	7	2081	8	incomplete-splice_match	gene: Zm00001d020835	Zm00001d020835_T007	21.39	44.58	coding	497	269	1759
PB.10671.2	9	1681	2	full-splice_match	gene: Zm00001d047969	Zm00001d047969_T001	33.96	123.55	coding	510	43	1572
PB.10671.1	9	2604	2	full-splice_match	gene: Zm00001d047969	Zm00001d047969_T001	89.59	123.55	coding	735	145	2349
PB.1076.3	1	2319	4	full-splice_match	gene: Zm00001d032547	Zm00001d032547_T011	3.86	33.58	coding	509	520	2046
PB.2055.1	10	3208	3	full-splice_match	gene: Zm00001d025342	Zm00001d025342_T003	10.10	32.56	coding	803	479	2887
PB.1076.1	1	2328	5	full-splice_match	gene: Zm00001d032547	Zm00001d032547_T004	24.03	33.58	coding	517	593	2143
PB.6809.4	5	1063	1	novel_in_catalog	gene: Zm00001d017095	novel	0.00	2390.54	coding	158	493	966
PB.1277.1	1	1258	8	novel_not_in_catalog	gene: Zm00001d033523	novel	34.76	34.76	coding	299	126	1022
PB.6875.6	5	2632	5	full-splice_match	gene: Zm00001d017352	Zm00001d017352_T006	0.00	64.36	coding	193	1905	2483
PB.1395.1	1	1738	3	novel_not_in_catalog	gene: Zm00001d034022	novel	36.86	37.65	coding	445	114	1448
PB.6875.3	5	2618	5	novel_in_catalog	gene: Zm00001d017352	novel	5.84	64.36	coding	193	1889	2467
PB.6875.2	5	1802	6	full-splice_match	gene: Zm00001d017352	Zm00001d017352_T012	58.53	64.36	coding	351	568	1620
PB.1867.1	10	1103	1	genic	gene: Zm00001d024463	novel	24.52	24.52	coding	277	45	875

PB.4242.1	3	2143	1	genic	gene: Zm00001d042461	novel	16.55	16.55	coding	335	890	1894
PB.1932.1	10	1622	2	full-splice_match	gene: Zm00001d024763	Zm00001d024763_T001	506.38	506.38	coding	435	89	1393
PB.7388.1	6	1638	4	full-splice_match	gene: Zm00001d035965	Zm00001d035965_T001	19.00	19.00	coding	374	215	1336
PB.8623.1	7	3213	19	full-splice_match	gene: Zm00001d021087	Zm00001d021087_T003	9.28	9.28	coding	837	382	2892
PB.7487.1	6	1922	5	novel_not_in_catalog	gene: Zm00001d036541	novel	168.38	174.28	coding	484	81	1532
PB.9051.1	7	2295	4	novel_not_in_catalog	gene: Zm00001d022626	novel	4.02	4.02	coding	642	172	2097
PB.1311.3	1	1900	11	full-splice_match	gene: Zm00001d033651	Zm00001d033651_T008	0.00	26.12	coding	564	83	1774
PB.1311.2	1	2049	10	full-splice_match	gene: Zm00001d033651	Zm00001d033651_T010	1.01	26.12	coding	392	49	1224
PB.2780.3	2	2779	9	full-splice_match	gene: Zm00001d003329	Zm00001d003329_T004	15.59	15.59	coding	765	244	2538
PB.1807.1	10	779	5	full-splice_match	gene: Zm00001d024005	Zm00001d024005_T001	31.93	31.93	non_coding	NA	NA	NA
PB.728.3	1	1660	5	novel_not_in_catalog	gene: Zm00001d030891	novel	1.18	94.88	coding	360	341	1420
PB.728.2	1	1973	2	novel_in_catalog	gene: Zm00001d030891	novel	11.22	94.88	coding	151	168	620
PB.728.1	1	1586	5	novel_not_in_catalog	gene: Zm00001d030891	novel	82.49	94.88	coding	360	140	1219
PB.5386.5	4	1537	5	full-splice_match	gene: Zm00001d051916	Zm00001d051916_T007	29.48	31.97	non_coding	NA	NA	NA
PB.5386.4	4	1719	5	novel_in_catalog	gene: Zm00001d051916	novel	2.48	31.97	coding	112	1116	1451
PB.728.4	1	1730	3	novel_not_in_catalog	gene: Zm00001d030891	novel	0.00	94.88	coding	293	134	1012
PB.9123.3	8	1597	3	novel_not_in_catalog	gene: Zm00001d008456	novel	5.31	23.41	coding	66	125	322

PB.3942.3	3	1785	2	genic	gene: Zm00001d040697	novel	390.96	1614.59	coding	394	88	1269
PB.9868.4	8	2182	10	novel_in_catalog	gene: Zm00001d012100	novel	16.58	79.52	coding	78	124	357
PB.9868.5	8	2018	9	novel_in_catalog	gene: Zm00001d012100	novel	10.18	79.52	coding	114	179	520
PB.8079.3	6	4395	17	novel_in_catalog	gene: Zm00001d038982	novel	0.00	28.25	coding	572	501	2216
PB.9868.8	8	2303	8	full-splice_match	gene: Zm00001d012100	Zm00001d012100_T023	0.00	79.52	coding	114	118	459
PB.10365.6	9	2397	13	full-splice_match	gene: Zm00001d046590	Zm00001d046590_T008	0.00	25.26	coding	639	123	2039
PB.3269.1	2	2593	8	full-splice_match	gene: Zm00001d006212	Zm00001d006212_T003	21.47	21.47	coding	631	267	2159
PB.4672.1	3	1286	6	novel_not_in_catalog	gene: Zm00001d044224	novel	19.15	19.15	coding	212	416	1051
PB.1026.1	1	893	6	full-splice_match	gene: Zm00001d032370	Zm00001d032370_T001	370.66	370.66	coding	205	82	696
PB.9345.3	8	2023	18	full-splice_match	gene: Zm00001d009669	Zm00001d009669_T003	0.00	56.33	coding	506	294	1811
PB.9345.1	8	2166	18	full-splice_match	gene: Zm00001d009669	Zm00001d009669_T003	56.33	56.33	coding	506	315	1832
PB.290.1	1	3300	16	novel_in_catalog	gene: Zm00001d028474	novel	18.19	18.19	coding	235	494	1198
PB.5214.2	4	1736	2	novel_in_catalog	gene: Zm00001d050992	novel	0.95	70.92	coding	284	36	887
PB.7591.1	6	3245	16	full-splice_match	gene: Zm00001d037099	Zm00001d037099_T010	12.71	47.76	coding	765	304	2598
PB.6546.1	5	859	1	full-splice_match	gene: Zm00001d015475	Zm00001d015475_T001	7.83	7.83	coding	180	53	592
PB.5394.1	4	992	2	incomplete-splice_match	gene: Zm00001d051946	Zm00001d051946_T001	34.39	41.17	coding	263	105	893
PB.7706.1	6	984	1	genic	gene: Zm00001d037619	novel	42.37	42.37	coding	5	302	316

PB.8930.1	7	1960	18	incomplete-splice_match	gene: Zm00001d022240	Zm00001d022240_T008	7.14	11.19	coding	500	69	1568
PB.8717.1	7	3282	13	full-splice_match	gene: Zm00001d021516	Zm00001d021516_T007	29.07	29.07	coding	913	132	2870
PB.1389.1	1	1241	8	incomplete-splice_match	gene: Zm00001d034012	Zm00001d034012_T003	16.70	16.70	coding	297	51	941
PB.9860.2	8	638	1	full-splice_match	gene: Zm00001d012080	Zm00001d012080_T001	0.00	85.64	coding	107	91	411
PB.10114.2	9	1577	12	full-splice_match	gene: Zm00001d045153	Zm00001d045153_T002	29.48	43.37	coding	137	1064	1474
PB.10114.1	9	1822	12	full-splice_match	gene: Zm00001d045153	Zm00001d045153_T002	13.89	43.37	coding	137	1091	1501
PB.9860.1	8	804	1	full-splice_match	gene: Zm00001d012080	Zm00001d012080_T001	26.73	85.64	coding	107	92	412
PB.9359.1	8	2624	7	full-splice_match	gene: Zm00001d009724	Zm00001d009724_T011	10.74	11.41	coding	678	120	2153
PB.4582.1	3	1670	1	full-splice_match	gene: Zm00001d043858	Zm00001d043858_T001	204.46	204.46	coding	396	88	1275
PB.9359.3	8	2581	7	novel_not_in_catalog	gene: Zm00001d009724	novel	0.66	11.41	coding	658	124	2097
PB.9359.2	8	2470	7	full-splice_match	gene: Zm00001d009724	Zm00001d009724_T011	0.00	11.41	coding	678	77	2110
PB.6849.1	5	1773	5	novel_in_catalog	gene: Zm00001d017241	novel	48.59	48.59	coding	159	187	663
PB.7996.1	6	1919	4	novel_not_in_catalog	gene: Zm00001d038720	novel	2.77	23.97	coding	111	1556	1888
PB.1050.2	1	2601	3	incomplete-splice_match	gene: Zm00001d032470	Zm00001d032470_T002	11.86	28.80	coding	709	309	2435
PB.1050.1	1	3295	4	full-splice_match	gene: Zm00001d032470	Zm00001d032470_T002	16.94	28.80	coding	989	59	3025
PB.1168.2	1	1382	1	novel_in_catalog	gene: Zm00001d032955	novel	8.58	116.99	coding	108	89	412
PB.1405.5	1	2047	5	novel_in_catalog	gene: Zm00001d034049	novel	1.50	239.03	coding	259	285	1061

PB.8930.4	7	2773	18	novel_in_catalog	gene: Zm00001d022240	novel	1.58	11.19	coding	505	744	2258
PB.9747.1	8	2506	6	full-splice_match	gene: Zm00001d011611	Zm00001d011611_T002	18.68	18.68	coding	264	1539	2330
PB.1619.1	1	2015	14	novel_not_in_catalog	gene: Zm00001d034868	novel	9.31	9.31	coding	608	25	1848
PB.6584.1	5	1384	9	full-splice_match	gene: Zm00001d015804	Zm00001d015804_T007	38.12	38.12	coding	322	197	1162
PB.10573.1	9	1644	3	incomplete-splice_match	gene: Zm00001d047676	Zm00001d047676_T001	50.80	75.13	coding	303	84	992
PB.4577.1	3	793	1	genic	gene: Zm00001d043837	novel	5.70	25.46	coding	138	14	427
PB.4813.1	4	2697	1	genic	gene: Zm00001d048663	novel	9.31	9.31	coding	548	182	1825
PB.8606.2	7	1259	1	novel_in_catalog	gene: Zm00001d021006	novel	8.03	169.98	coding	110	120	449
PB.5933.1	5	2262	18	full-splice_match	gene: Zm00001d012892	Zm00001d012892_T005	47.12	47.12	coding	658	236	2209
PB.2340.1	10	2513	3	full-splice_match	gene: Zm00001d026587	Zm00001d026587_T002	20.15	20.15	coding	679	219	2255
PB.9702.1	8	1449	3	novel_not_in_catalog	gene: Zm00001d011384	novel	63.22	63.22	non_coding	NA	NA	NA
PB.9516.2	8	1109	9	full-splice_match	gene: Zm00001d010538	Zm00001d010538_T005	80.92	235.70	coding	215	182	826
PB.4879.3	4	1803	3	incomplete-splice_match	gene: Zm00001d049026	Zm00001d049026_T008	1.64	12.60	coding	105	37	351
PB.9033.3	7	1780	6	novel_not_in_catalog	gene: Zm00001d022573	novel	93.89	125.41	coding	447	294	1634
PB.9516.3	8	999	9	full-splice_match	gene: Zm00001d010538	Zm00001d010538_T005	0.00	235.70	coding	215	145	789
PB.10202.1	9	1242	7	full-splice_match	gene: Zm00001d045509	Zm00001d045509_T006	61.67	78.38	coding	262	131	916
PB.1929.1	10	447	2	intergenic	novelGene_152	novel	50.73	50.73	non_coding	NA	NA	NA

PB.10202.2	9	1351	6	novel_in_catalog	gene: Zm00001d045509	novel	16.71	78.38	coding	197	133	723
PB.5634.1	4	2073	2	novel_in_catalog	gene: Zm00001d052933	novel	9.29	9.29	coding	509	86	1612
PB.6716.1	5	1960	14	novel_in_catalog	gene: Zm00001d016662	novel	20.72	20.72	coding	441	295	1617
PB.9171.1	8	2466	5	full-splice_match	gene: Zm00001d008700	Zm00001d008700_T002	16.83	16.83	coding	530	628	2217
PB.7738.1	6	1533	3	novel_in_catalog	gene: Zm00001d037748	novel	6.47	6.47	coding	178	235	768
PB.6024.1	5	1823	2	genic	gene: Zm00001d013230	novel	15.99	15.99	coding	521	119	1681
PB.10383.1	9	1760	2	genic	gene: Zm00001d046684	novel	4.89	4.89	coding	396	235	1422
PB.9726.1	8	981	3	full-splice_match	gene: Zm00001d011514	Zm00001d011514_T002	219.34	219.34	coding	180	105	644
PB.8123.2	6	2153	2	novel_in_catalog	gene: Zm00001d039166	novel	7.79	225.80	coding	125	1395	1769
PB.6319.1	5	2030	3	full-splice_match	gene: Zm00001d014525	Zm00001d014525_T001	205.16	205.16	coding	529	219	1805
PB.1678.1	10	1242	4	genic	gene: Zm00001d023330	novel	10.79	10.79	coding	132	167	562
PB.6319.3	5	1618	3	full-splice_match	gene: Zm00001d014525	Zm00001d014525_T001	0.00	205.16	coding	497	55	1545
PB.8123.1	6	1369	5	full-splice_match	gene: Zm00001d039166	Zm00001d039166_T001	197.82	225.80	coding	276	133	960
PB.1689.11	10	921	5	full-splice_match	gene: Zm00001d023376	Zm00001d023376_T002	0.00	1404.98	coding	238	106	819
PB.1689.10	10	1750	4	novel_in_catalog	gene: Zm00001d023376	novel	0.00	1404.98	coding	114	1292	1633
PB.9250.1	8	2850	3	full-splice_match	gene: Zm00001d009060	Zm00001d009060_T001	9.70	9.70	coding	830	58	2547
PB.1257.1	1	1808	3	full-splice_match	gene: Zm00001d033423	Zm00001d033423_T001	14.33	14.33	coding	461	75	1457

PB.2820.1	2	999	4	full-splice_match	gene: Zm00001d003540	Zm00001d003540_T001	27.82	27.82	coding	196	116	703
PB.5805.2	4	2234	14	novel_in_catalog	gene: Zm00001d053782	novel	9.73	14.62	coding	572	320	2035
PB.685.3	1	1903	1	novel_in_catalog	gene: Zm00001d030549	novel	1.22	15.67	coding	202	1196	1801
PB.9233.1	8	1820	1	intergenic	novelGene_83	novel	15.86	38.92	coding	146	224	661
PB.8000.3	6	2428	8	novel_not_in_catalog	gene: Zm00001d038734	novel	5.88	22.67	coding	98	125	418
PB.1160.3	1	522	1	incomplete-splice_match	gene: Zm00001d032905	Zm00001d032905_T014	0.00	44.34	non_coding	NA	NA	NA
PB.10238.1	9	664	5	novel_not_in_catalog	gene: Zm00001d045724	novel	66.35	66.35	coding	95	41	325
PB.1129.2	1	702	2	full-splice_match	gene: Zm00001d032760	Zm00001d032760_T001	703.93	1444.84	coding	164	76	567
PB.3264.1	2	1345	1	full-splice_match	gene: Zm00001d006182	Zm00001d006182_T001	18.73	18.73	coding	350	29	1078
PB.103.1	1	1884	1	full-splice_match	gene: Zm00001d027643	Zm00001d027643_T001	8.55	8.55	coding	503	191	1699
PB.502.1	1	1729	1	intergenic	novelGene_132	novel	6.48	6.48	coding	354	86	1147
PB.2106.2	10	1960	2	full-splice_match	gene: Zm00001d025667	Zm00001d025667_T001	12.24	16.54	coding	488	104	1567
PB.6780.1	5	1585	7	novel_not_in_catalog	gene: Zm00001d016916	novel	21.38	21.38	coding	327	214	1194
PB.2106.3	10	2183	2	genic	gene: Zm00001d025667	novel	4.30	16.54	coding	182	1370	1915
PB.8218.2	7	979	5	full-splice_match	gene: Zm00001d018884	Zm00001d018884_T004	598.38	1203.47	coding	152	133	588
PB.8218.3	7	955	5	full-splice_match	gene: Zm00001d018884	Zm00001d018884_T005	572.19	1203.47	coding	152	248	703
PB.8218.1	7	4084	3	full-splice_match	gene: Zm00001d018884	Zm00001d018884_T011	7.37	1203.47	non_coding	NA	NA	NA

PB.8218.6	7	5485	4	full-splice_match	gene: Zm00001d018884	Zm00001d018884_T010	3.50	1203.47	non_coding	NA	NA	NA
PB.8218.7	7	2141	2	incomplete-splice_match	gene: Zm00001d018884	Zm00001d018884_T009	0.00	1203.47	non_coding	NA	NA	NA
PB.8218.4	7	875	5	novel_in_catalog	gene: Zm00001d018884	novel	22.02	1203.47	non_coding	NA	NA	NA
PB.5593.2	4	1756	5	full-splice_match	gene: Zm00001d052766	Zm00001d052766_T002	20.06	58.65	coding	409	241	1467
PB.10706.1	9	418	1	intergenic	novelGene_245	novel	62.45	62.45	non_coding	NA	NA	NA
PB.7997.1	6	2244	15	incomplete-splice_match	gene: Zm00001d038728	Zm00001d038728_T017	45.73	45.73	coding	576	328	2055
PB.745.1	1	3226	15	full-splice_match	gene: Zm00001d030996	Zm00001d030996_T002	49.06	56.99	coding	799	493	2889
PB.2139.5	10	2391	9	full-splice_match	gene: Zm00001d025801	Zm00001d025801_T007	0.00	381.01	coding	652	181	2136
PB.3152.2	2	2039	4	incomplete-splice_match	gene: Zm00001d005638	Zm00001d005638_T014	16.73	32.09	coding	571	30	1742
PB.1513.1	1	2666	14	full-splice_match	gene: Zm00001d034519	Zm00001d034519_T001	29.28	31.00	coding	729	169	2355
PB.3152.1	2	3477	5	full-splice_match	gene: Zm00001d005638	Zm00001d005638_T001	15.36	32.09	coding	982	86	3031
PB.9778.1	8	1420	2	genic	gene: Zm00001d011730	novel	13.89	13.89	coding	350	112	1161
PB.7905.2	6	1494	9	novel_in_catalog	gene: Zm00001d038337	novel	3.42	19.20	coding	375	68	1192
PB.5440.2	4	1051	4	full-splice_match	gene: Zm00001d052174	Zm00001d052174_T001	0.00	45.29	coding	263	33	821
PB.3273.1	2	1415	9	full-splice_match	gene: Zm00001d006221	Zm00001d006221_T001	5.63	53.69	coding	298	58	951
PB.8319.1	7	1484	7	full-splice_match	gene: Zm00001d019483	Zm00001d019483_T001	10.62	10.62	coding	332	159	1154
PB.4899.4	4	1569	3	novel_in_catalog	gene: Zm00001d049120	novel	0.00	53.07	coding	139	982	1398

PB.9718.3	8	2264	5	full-splice_match	gene: Zm00001d011471	Zm00001d011471_T001	0.00	77.55	coding	634	3	1904
PB.9718.4	8	2249	5	full-splice_match	gene: Zm00001d011471	Zm00001d011471_T001	48.09	77.55	coding	634	143	2044
PB.9718.5	8	2147	5	full-splice_match	gene: Zm00001d011471	Zm00001d011471_T001	19.55	77.55	coding	634	143	2044
PB.4899.3	4	1775	2	novel_in_catalog	gene: Zm00001d049120	novel	1.19	53.07	coding	115	1208	1552
PB.4899.2	4	1797	3	novel_in_catalog	gene: Zm00001d049120	novel	51.88	53.07	coding	139	1041	1457
PB.10412.1	9	740	4	full-splice_match	gene: Zm00001d046900	Zm00001d046900_T003	382.63	382.63	coding	135	57	461
PB.3522.2	2	2297	10	incomplete-splice_match	gene: Zm00001d007407	Zm00001d007407_T012	17.83	28.05	coding	307	77	997
PB.3830.1	3	2017	3	novel_not_in_catalog	gene: Zm00001d040130	novel	7.35	7.35	coding	478	218	1651
PB.9165.1	8	1989	7	full-splice_match	gene: Zm00001d008649	Zm00001d008649_T004	11.81	11.81	coding	514	278	1819
PB.9683.1	8	1298	1	full-splice_match	gene: Zm00001d011302	Zm00001d011302_T001	23.54	23.54	coding	329	80	1066
PB.6052.1	5	857	2	full-splice_match	gene: Zm00001d013300	Zm00001d013300_T001	312.67	312.67	coding	137	206	616
PB.5225.1	4	766	4	full-splice_match	gene: Zm00001d051055	Zm00001d051055_T001	216.76	275.73	coding	77	131	361
PB.3175.1	2	1509	1	full-splice_match	gene: Zm00001d005752	Zm00001d005752_T001	90.52	90.52	coding	421	102	1364
PB.1711.1	10	2268	3	novel_in_catalog	gene: Zm00001d023492	novel	10.65	18.00	coding	230	1359	2048
PB.3577.2	2	3197	17	novel_in_catalog	gene: Zm00001d007758	novel	24.89	125.91	coding	619	1084	2940
PB.1711.2	10	2188	4	incomplete-splice_match	gene: Zm00001d023492	Zm00001d023492_T004	7.35	18.00	coding	669	2	2008
PB.2747.1	2	1890	8	full-splice_match	gene: Zm00001d003200	Zm00001d003200_T003	28.71	28.71	coding	346	559	1596

PB.4294.3	3	2006	3	novel_in_catalog	gene: Zm00001d042684	novel	164.00	417.27	coding	504	261	1772
PB.2031.2	10	962	2	novel_not_in_catalog	gene: Zm00001d025221	novel	0.00	34.08	coding	180	250	789
PB.4294.6	3	2290	2	novel_in_catalog	gene: Zm00001d042684	novel	7.56	417.27	coding	368	54	1157
PB.5225.3	4	550	4	full-splice_match	gene: Zm00001d051055	Zm00001d051055_T001	0.00	275.73	coding	77	89	319
PB.4294.4	3	2407	1	genic	gene: Zm00001d042684	novel	6.08	417.27	coding	330	261	1250
PB.1542.6	1	3109	8	incomplete-splice_match	gene: Zm00001d034624	Zm00001d034624_T012	4.43	35.15	coding	993	3	2981
PB.1542.5	1	1919	5	incomplete-splice_match	gene: Zm00001d034624	Zm00001d034624_T039	0.00	35.15	coding	515	46	1590
PB.1723.8	10	2178	14	novel_not_in_catalog	gene: Zm00001d023543	novel	0.00	54.88	coding	460	119	1498
PB.745.3	1	3141	15	full-splice_match	gene: Zm00001d030996	Zm00001d030996_T002	7.30	56.99	coding	799	486	2882
PB.1723.4	10	2392	14	novel_not_in_catalog	gene: Zm00001d023543	novel	3.15	54.88	coding	391	228	1400
PB.1723.5	10	2183	13	novel_not_in_catalog	gene: Zm00001d023543	novel	10.55	54.88	coding	341	102	1124
PB.1723.2	10	2419	15	novel_not_in_catalog	gene: Zm00001d023543	novel	32.75	54.88	coding	610	373	2202
PB.1723.3	10	2321	14	novel_not_in_catalog	gene: Zm00001d023543	novel	8.43	54.88	coding	534	181	1782
PB.8325.1	7	1676	8	full-splice_match	gene: Zm00001d019515	Zm00001d019515_T001	27.11	63.67	coding	368	165	1268
PB.8815.1	7	3486	4	novel_in_catalog	gene: Zm00001d021824	novel	11.63	11.63	coding	116	896	1243
PB.7285.7	6	1242	7	novel_in_catalog	gene: Zm00001d035346	novel	7.76	65.77	non_coding	NA	NA	NA
PB.4327.3	3	2165	3	full-splice_match	gene: Zm00001d042833	Zm00001d042833_T001	0.00	133.71	coding	598	256	2049

PB.8256.1	7	2370	5	novel_not_in_catalog	gene: Zm00001d019089	novel	155.45	155.45	coding	487	770	2230
PB.3497.1	2	3771	2	full-splice_match	gene: Zm00001d007254	Zm00001d007254_T001	9.01	9.01	coding	1035	281	3385
PB.3330.2	2	2519	19	novel_in_catalog	gene: Zm00001d006456	novel	8.65	10.02	coding	732	25	2220
PB.3360.1	2	2156	2	full-splice_match	gene: Zm00001d006590	Zm00001d006590_T001	7.83	7.83	coding	603	188	1996
PB.2464.2	2	3108	1	novel_in_catalog	gene: Zm00001d002052	novel	3.96	111.39	coding	182	2336	2881
PB.2464.1	2	1422	3	full-splice_match	gene: Zm00001d002052	Zm00001d002052_T001	107.42	111.39	coding	337	142	1152
PB.4276.1	3	1602	5	full-splice_match	gene: Zm00001d042619	Zm00001d042619_T003	148.39	148.39	coding	447	33	1373
PB.681.1	1	663	1	full-splice_match	gene: Zm00001d030530	Zm00001d030530_T001	210.24	210.24	coding	127	62	442
PB.5468.3	4	1499	4	novel_in_catalog	gene: Zm00001d052268	novel	16.01	124.56	coding	231	238	930
PB.6727.1	5	1298	9	full-splice_match	gene: Zm00001d016704	Zm00001d016704_T001	100.50	100.81	coding	300	186	1085
PB.5468.1	4	1294	4	novel_in_catalog	gene: Zm00001d052268	novel	14.70	124.56	coding	153	31	489
PB.5468.6	4	518	3	novel_in_catalog	gene: Zm00001d052268	novel	8.85	124.56	coding	126	109	486
PB.5468.7	4	1139	3	novel_in_catalog	gene: Zm00001d052268	novel	3.23	124.56	coding	153	30	488
PB.5468.5	4	1252	4	novel_in_catalog	gene: Zm00001d052268	novel	25.48	124.56	coding	153	30	488
PB.2941.1	2	1934	1	genic	gene: Zm00001d004248	novel	11.77	19.51	coding	489	150	1616
PB.2941.3	2	1694	1	genic	gene: Zm00001d004248	novel	0.00	19.51	coding	489	149	1615
PB.3905.1	3	2922	9	full-splice_match	gene: Zm00001d040466	Zm00001d040466_T001	19.25	19.25	coding	782	319	2664

PB.5786.1	4	1836	6	full-splice_match	gene: Zm00001d053687	Zm00001d053687_T002	15.86	19.02	coding	488	73	1536
PB.5786.2	4	1654	6	full-splice_match	gene: Zm00001d053687	Zm00001d053687_T002	3.15	19.02	coding	488	65	1528
PB.1618.1	1	1619	6	full-splice_match	gene: Zm00001d034867	Zm00001d034867_T001	157.50	163.97	coding	322	140	1105
PB.1618.2	1	1545	6	full-splice_match	gene: Zm00001d034867	Zm00001d034867_T001	6.47	163.97	coding	322	190	1155
PB.9111.2	8	2792	1	full-splice_match	gene: Zm00001d008413	Zm00001d008413_T001	9.92	26.23	coding	727	270	2450
PB.9111.1	8	1461	1	full-splice_match	gene: Zm00001d008413	Zm00001d008413_T001	16.31	26.23	coding	270	135	944
PB.10545.2	9	3084	1	full-splice_match	gene: Zm00001d047563	Zm00001d047563_T001	34.31	217.27	coding	641	904	2826
PB.10545.5	9	2334	2	genic	gene: Zm00001d047563	novel	182.95	217.27	coding	641	210	2132
PB.5854.1	4	1420	11	full-splice_match	gene: Zm00001d053954	Zm00001d053954_T003	29.97	29.97	coding	291	321	1193
PB.2139.1	10	2496	9	full-splice_match	gene: Zm00001d025801	Zm00001d025801_T007	381.01	381.01	coding	652	181	2136
PB.1492.1	1	1271	4	incomplete-splice_match	gene: Zm00001d034434	Zm00001d034434_T001	19.61	39.47	coding	99	556	852
PB.3161.6	2	3210	4	full-splice_match	gene: Zm00001d005687	Zm00001d005687_T003	27.32	61.25	coding	864	185	2776
PB.3161.7	2	2825	3	novel_in_catalog	gene: Zm00001d005687	novel	0.59	61.25	coding	663	216	2204
PB.3161.2	2	3554	3	novel_in_catalog	gene: Zm00001d005687	novel	33.34	61.25	coding	756	218	2485
PB.5403.1	4	2073	16	full-splice_match	gene: Zm00001d052008	Zm00001d052008_T001	37.09	37.09	coding	553	188	1846
PB.4345.6	3	2064	6	full-splice_match	gene: Zm00001d042901	Zm00001d042901_T001	0.00	32.97	coding	582	65	1810
PB.10438.1	9	2453	8	incomplete-splice_match	gene: Zm00001d047015	Zm00001d047015_T060	40.31	40.31	coding	600	245	2044

PB.5755.1	4	2119	7	full-splice_match	gene: Zm00001d053595	Zm00001d053595_T004	29.71	92.93	coding	517	126	1676
PB.3568.1	2	1473	4	full-splice_match	gene: Zm00001d007682	Zm00001d007682_T004	4.12	16.08	coding	162	57	542
PB.2034.1	10	670	3	full-splice_match	gene: Zm00001d025239	Zm00001d025239_T001	220.71	220.71	non_coding	NA	NA	NA
PB.5755.2	4	1953	7	full-splice_match	gene: Zm00001d053595	Zm00001d053595_T004	63.22	92.93	coding	517	91	1641
PB.9096.1	8	1220	1	full-splice_match	gene: Zm00001d008343	Zm00001d008343_T001	2.12	2.12	coding	299	60	956
PB.7492.2	6	2103	3	novel_not_in_catalog	gene: Zm00001d036551	novel	2.16	22.99	coding	364	211	1302
PB.7492.1	6	2111	4	novel_not_in_catalog	gene: Zm00001d036551	novel	20.83	22.99	coding	388	397	1560
PB.5865.7	4	2079	5	novel_not_in_catalog	gene: Zm00001d053982	novel	0.00	27.39	coding	645	133	2067
PB.7141.7	5	560	1	intergenic	novelGene_345	novel	61.13	272.48	non_coding	NA	NA	NA
PB.3181.2	2	2232	5	full-splice_match	gene: Zm00001d005769	Zm00001d005769_T001	18.58	24.75	coding	625	156	2030
PB.3181.3	2	1423	1	novel_in_catalog	gene: Zm00001d005769	novel	6.17	24.75	coding	181	44	586
PB.2444.1	2	2104	9	incomplete-splice_match	gene: Zm00001d001974	Zm00001d001974_T001	16.07	16.07	coding	526	87	1664
PB.9365.1	8	1649	2	full-splice_match	gene: Zm00001d009759	Zm00001d009759_T001	10.48	12.92	coding	467	39	1439
PB.9365.2	8	1659	1	genic	gene: Zm00001d009759	novel	2.44	12.92	coding	498	37	1530
PB.7964.2	6	625	3	full-splice_match	gene: Zm00001d038558	Zm00001d038558_T001	250.00	262.03	coding	110	106	435
PB.8410.2	7	2020	5	full-splice_match	gene: Zm00001d020097	Zm00001d020097_T002	6.05	22.86	coding	414	545	1786
PB.4337.5	3	1409	5	incomplete-splice_match	gene: Zm00001d042874	Zm00001d042874_T005	0.00	130.44	coding	361	195	1277

PB.7263.1	6	1884	7	novel_in_catalog	gene: Zm00001d035211	novel	9.08	9.08	coding	139	332	748
PB.6612.1	5	1860	3	full-splice_match	gene: Zm00001d016052	Zm00001d016052_T001	32.97	32.97	coding	383	85	1233
PB.5323.2	4	1642	2	genic	gene: Zm00001d051620	novel	0.00	47.99	coding	317	285	1235
PB.5323.3	4	1859	2	genic	gene: Zm00001d051620	novel	0.00	47.99	coding	314	730	1671
PB.8480.1	7	957	3	incomplete-splice_match	gene: Zm00001d020444	Zm00001d020444_T003	29.81	29.81	coding	159	392	868
PB.5323.1	4	1964	2	genic	gene: Zm00001d051620	novel	47.99	47.99	coding	314	566	1507
PB.3407.1	2	1871	2	genic	gene: Zm00001d006822	novel	8.22	13.49	coding	484	37	1488
PB.333.1	1	1079	1	full-splice_match	gene: Zm00001d028665	Zm00001d028665_T001	55.74	55.74	coding	267	109	909
PB.3407.2	2	1579	1	full-splice_match	gene: Zm00001d006822	Zm00001d006822_T001	5.27	13.49	coding	216	763	1410
PB.7332.2	6	2019	6	full-splice_match	gene: Zm00001d035634	Zm00001d035634_T001	39.26	70.50	coding	443	128	1456
PB.7332.3	6	1613	4	novel_in_catalog	gene: Zm00001d035634	novel	28.52	70.50	coding	209	436	1062
PB.8307.1	7	1722	3	novel_not_in_catalog	gene: Zm00001d019414	novel	13.20	13.20	coding	525	68	1642
PB.7332.1	6	2195	6	full-splice_match	gene: Zm00001d035634	Zm00001d035634_T001	2.71	70.50	coding	443	125	1453
PB.2955.2	2	3642	12	full-splice_match	gene: Zm00001d004466	Zm00001d004466_T006	0.54	47.29	coding	1005	251	3265
PB.2955.3	2	3459	13	full-splice_match	gene: Zm00001d004466	Zm00001d004466_T001	1.88	47.29	coding	1005	110	3124
PB.2955.1	2	3567	13	full-splice_match	gene: Zm00001d004466	Zm00001d004466_T001	38.72	47.29	coding	1005	108	3122
PB.5258.1	4	2132	3	full-splice_match	gene: Zm00001d051307	Zm00001d051307_T001	42.54	45.19	coding	536	249	1856

PB.10430.1	9	2443	5	novel_not_in_catalog	gene: Zm00001d046971	novel	15.10	18.81	coding	677	149	2179
PB.10430.2	9	2362	5	novel_not_in_catalog	gene: Zm00001d046971	novel	3.71	18.81	coding	677	144	2174
PB.5258.2	4	2084	3	full-splice_match	gene: Zm00001d051307	Zm00001d051307_T001	2.65	45.19	coding	536	303	1910
PB.9664.2	8	781	1	full-splice_match	gene: Zm00001d011189	Zm00001d011189_T001	66.66	74.11	coding	119	158	514
PB.7093.1	5	2460	5	full-splice_match	gene: Zm00001d018237	Zm00001d018237_T001	18.00	18.00	coding	710	128	2257
PB.5957.1	5	1815	5	full-splice_match	gene: Zm00001d012983	Zm00001d012983_T001	16.34	16.34	coding	471	198	1610
PB.745.4	1	3008	15	full-splice_match	gene: Zm00001d030996	Zm00001d030996_T008	0.63	56.99	coding	799	384	2780
PB.8915.12	7	1905	5	full-splice_match	gene: Zm00001d022181	Zm00001d022181_T004	0.00	288.80	coding	538	89	1702
PB.8915.13	7	630	2	incomplete-splice_match	gene: Zm00001d022181	Zm00001d022181_T014	0.91	288.80	coding	146	67	504
PB.9603.1	8	868	1	genic	gene: Zm00001d010907	novel	15.40	15.40	coding	107	5	325
PB.4739.2	3	3703	20	full-splice_match	gene: Zm00001d044498	Zm00001d044498_T012	0.65	17.19	coding	908	87	2810
PB.9975.2	8	1967	7	novel_not_in_catalog	gene: Zm00001d012581	novel	22.30	30.03	coding	463	383	1771
PB.6299.1	5	1413	3	full-splice_match	gene: Zm00001d014419	Zm00001d014419_T001	21.75	25.17	coding	381	114	1256
PB.7308.4	6	1988	5	novel_not_in_catalog	gene: Zm00001d035481	novel	7.28	57.76	coding	109	663	989
PB.8031.1	6	2239	7	novel_not_in_catalog	gene: Zm00001d038838	novel	61.88	61.88	coding	582	227	1972
PB.7308.6	6	2076	6	novel_not_in_catalog	gene: Zm00001d035481	novel	0.00	57.76	coding	147	261	701
PB.7308.1	6	1128	7	novel_not_in_catalog	gene: Zm00001d035481	novel	10.85	57.76	coding	83	171	419

PB.7308.2	6	3161	2	genic	gene: Zm00001d035481	novel	7.96	57.76	coding	92	108	383
PB.3281.1	2	1085	4	full-splice_match	gene: Zm00001d006251	Zm00001d006251_T001	171.19	171.19	coding	238	53	766
PB.4728.4	3	1992	1	novel_in_catalog	gene: Zm00001d044469	novel	5.35	31.20	coding	447	95	1435
PB.4728.5	3	1546	1	novel_in_catalog	gene: Zm00001d044469	novel	0.00	31.20	coding	447	94	1434
PB.7865.1	6	1211	2	full-splice_match	gene: Zm00001d038192	Zm00001d038192_T001	42.37	42.37	coding	267	37	837
PB.1233.1	1	1199	2	full-splice_match	gene: Zm00001d033319	Zm00001d033319_T001	75.44	79.95	coding	226	154	831
PB.1739.1	10	3118	2	full-splice_match	gene: Zm00001d023608	Zm00001d023608_T001	10.39	10.39	coding	923	136	2904
PB.8789.2	7	2670	2	novel_in_catalog	gene: Zm00001d021736	novel	15.79	15.79	coding	749	357	2603
PB.7539.1	6	722	1	full-splice_match	gene: Zm00001d036780	Zm00001d036780_T001	2.25	2.25	coding	124	108	479
PB.4383.3	3	3239	18	novel_in_catalog	gene: Zm00001d043091	novel	0.00	28.15	coding	514	1604	3145
PB.4383.2	3	3220	19	full-splice_match	gene: Zm00001d043091	Zm00001d043091_T003	8.90	28.15	coding	954	265	3126
PB.4383.1	3	3527	18	novel_in_catalog	gene: Zm00001d043091	novel	19.25	28.15	coding	514	1540	3081
PB.9220.1	8	2314	2	genic_intron	novelGene_80	novel	15.74	15.74	coding	594	309	2090
PB.2672.1	2	1812	10	novel_not_in_catalog	gene: Zm00001d002919	novel	66.49	66.49	coding	307	355	1275
PB.272.1	1	906	1	incomplete-splice_match	gene: Zm00001d028415	Zm00001d028415_T004	24.69	24.69	coding	188	174	737
PB.1588.1	1	2127	14	fusion	gene: Zm00001d034766_g	novel	12.69	12.69	coding	583	121	1869
PB.1415.1	1	2054	17	novel_in_catalog	gene: Zm00001d034108	novel	37.12	37.12	coding	498	411	1904

PB.5286.1	4	1826	9	full-splice_match	gene: Zm00001d051458	Zm00001d051458_T007	31.05	31.05	coding	284	273	1124
PB.8317.1	7	1491	9	full-splice_match	gene: Zm00001d019467	Zm00001d019467_T001	20.09	41.95	coding	333	52	1050
PB.8317.2	7	1382	9	full-splice_match	gene: Zm00001d019467	Zm00001d019467_T001	21.86	41.95	coding	333	114	1112
PB.8317.3	7	1264	9	full-splice_match	gene: Zm00001d019467	Zm00001d019467_T001	0.00	41.95	coding	333	110	1108
PB.3075.3	2	3049	5	full-splice_match	gene: Zm00001d005190	Zm00001d005190_T001	0.00	76.54	coding	898	136	2829
PB.153.1	1	1168	4	full-splice_match	gene: Zm00001d027887	Zm00001d027887_T001	33.43	33.43	coding	327	82	1062
PB.3075.1	2	3240	5	full-splice_match	gene: Zm00001d005190	Zm00001d005190_T001	68.97	76.54	coding	898	159	2852
PB.844.2	1	3015	3	incomplete-splice_match	gene: Zm00001d031489	Zm00001d031489_T003	8.82	24.38	coding	809	193	2619
PB.844.3	1	2076	2	novel_in_catalog	gene: Zm00001d031489	novel	15.56	24.38	coding	216	356	1003
PB.4752.1	3	1948	1	full-splice_match	gene: Zm00001d044546	Zm00001d044546_T001	0.71	168.48	coding	141	987	1409
PB.844.4	1	2515	3	incomplete-splice_match	gene: Zm00001d031489	Zm00001d031489_T003	0.00	24.38	coding	397	1017	2207
PB.4752.2	3	1725	1	full-splice_match	gene: Zm00001d044546	Zm00001d044546_T001	167.77	168.48	coding	141	1030	1452
PB.601.1	1	1733	4	full-splice_match	gene: Zm00001d029983	Zm00001d029983_T001	11.24	11.24	coding	461	109	1491
PB.6698.1	5	1563	1	intergenic	novelGene_275	novel	12.54	12.54	non_coding	NA	NA	NA
PB.6698.2	5	2021	3	novel_not_in_catalog	gene: Zm00001d016558	novel	9.10	9.10	coding	544	82	1713
PB.4311.1	3	1582	3	full-splice_match	gene: Zm00001d042764	Zm00001d042764_T001	29.87	29.87	coding	401	162	1364
PB.7686.2	6	1507	1	full-splice_match	gene: Zm00001d037544	Zm00001d037544_T001	37.83	60.44	coding	447	93	1433

PB.8753.1	7	467	1	full-splice_match	gene: Zm00001d021627	Zm00001d021627_T001	40.91	40.91	non_coding	NA	NA	NA
PB.4554.1	3	2559	11	novel_not_in_catalog	gene: Zm00001d043731	novel	5.08	5.08	coding	791	53	2425
PB.248.2	1	3955	8	full-splice_match	gene: Zm00001d028339	Zm00001d028339_T001	88.70	88.70	coding	1168	337	3840
PB.248.3	1	1290	7	incomplete-splice_match	gene: Zm00001d028339	Zm00001d028339_T043	0.00	88.70	coding	353	230	1288
PB.3812.2	3	691	1	novel_in_catalog	gene: Zm00001d040029	novel	7.61	972.07	non_coding	NA	NA	NA
PB.3812.1	3	549	2	full-splice_match	gene: Zm00001d040029	Zm00001d040029_T001	964.45	972.07	non_coding	NA	NA	NA
PB.2686.1	2	1897	1	genic	gene: Zm00001d002968	novel	1.92	1.92	coding	545	35	1669
PB.1376.1	1	1605	11	full-splice_match	gene: Zm00001d033969	Zm00001d033969_T006	56.81	73.48	coding	384	246	1397
PB.5774.1	4	2981	2	full-splice_match	gene: Zm00001d053642	Zm00001d053642_T001	85.55	85.55	coding	651	249	2201
PB.3704.1	3	2454	3	full-splice_match	gene: Zm00001d039456	Zm00001d039456_T001	8.30	8.30	coding	641	201	2123
PB.666.1	1	717	1	full-splice_match	gene: Zm00001d030363	Zm00001d030363_T001	132.01	132.01	coding	166	50	547
PB.5681.1	4	5150	5	full-splice_match	gene: Zm00001d053171	Zm00001d053171_T001	5.35	5.35	coding	1687	16	5076
PB.3704.2	3	2342	3	full-splice_match	gene: Zm00001d039456	Zm00001d039456_T001	0.00	8.30	coding	641	207	2129
PB.4312.3	3	1378	2	genic	gene: Zm00001d042767	novel	61.26	113.55	coding	403	88	1296
PB.10452.1	9	1795	5	full-splice_match	gene: Zm00001d047104	Zm00001d047104_T001	8.96	53.18	coding	431	1	1293
PB.3971.1	3	3975	2	genic	gene: Zm00001d040986	novel	9.77	9.77	coding	1207	68	3688
PB.10452.3	9	1913	5	full-splice_match	gene: Zm00001d047104	Zm00001d047104_T001	27.94	53.18	coding	488	183	1646

PB.2970.2	2	1131	2	full-splice_match	gene: Zm00001d004546	Zm00001d004546_T001	7.16	15.20	coding	312	112	1047
PB.9809.1	8	2240	11	full-splice_match	gene: Zm00001d011835	Zm00001d011835_T003	8.66	20.28	coding	569	245	1951
PB.8644.2	7	970	7	full-splice_match	gene: Zm00001d021225	Zm00001d021225_T002	109.53	133.22	coding	175	141	665
PB.3577.6	2	2974	18	full-splice_match	gene: Zm00001d007758	Zm00001d007758_T003	0.00	125.91	coding	888	169	2832
PB.1097.1	1	2468	3	novel_not_in_catalog	gene: Zm00001d032613	novel	12.72	17.95	coding	647	181	2121
PB.642.2	1	2800	4	incomplete-splice_match	gene: Zm00001d030217	Zm00001d030217_T021	76.34	76.34	coding	848	42	2585
PB.3564.5	2	2267	9	novel_in_catalog	gene: Zm00001d007640	novel	4.61	33.97	coding	623	80	1948
PB.3764.1	3	1694	3	incomplete-splice_match	gene: Zm00001d039772	Zm00001d039772_T003	12.63	12.63	non_coding	NA	NA	NA
PB.4319.1	3	2451	5	full-splice_match	gene: Zm00001d042795	Zm00001d042795_T007	22.33	22.33	coding	113	194	532
PB.1354.1	1	2211	12	full-splice_match	gene: Zm00001d033855	Zm00001d033855_T004	163.88	178.17	coding	479	260	1696
PB.3615.3	2	2562	17	full-splice_match	gene: Zm00001d007889	Zm00001d007889_T002	37.47	77.98	coding	704	218	2329
PB.4349.4	3	1582	8	full-splice_match	gene: Zm00001d042910	Zm00001d042910_T010	0.00	27.97	coding	444	251	1582
PB.10324.9	9	1661	2	genic	gene: Zm00001d046378	novel	0.00	784.46	coding	138	197	610
PB.3048.1	2	954	7	incomplete-splice_match	gene: Zm00001d005062	Zm00001d005062_T001	35.69	35.69	coding	76	390	617
PB.3523.3	2	1569	8	intergenic	novelGene_351	novel	18.11	18.11	coding	360	261	1340
PB.9500.5	8	1943	7	novel_not_in_catalog	gene: Zm00001d010456	novel	10.41	10.41	coding	302	19	924
PB.10324.1	9	2386	2	genic	gene: Zm00001d046378	novel	2.15	784.46	coding	138	166	579

PB.10324.2	9	1901	2	genic	gene: Zm00001d046378	novel	364.74	784.46	coding	138	251	664
PB.10324.3	9	1929	1	full-splice_match	gene: Zm00001d046378	Zm00001d046378_T001	9.94	784.46	coding	138	292	705
PB.10324.4	9	1182	2	genic	gene: Zm00001d046378	novel	395.43	784.46	coding	94	220	501
PB.1787.1	10	2384	1	novel_in_catalog	gene: Zm00001d023885	novel	3.38	15.29	coding	650	265	2214
PB.1787.2	10	2260	2	full-splice_match	gene: Zm00001d023885	Zm00001d023885_T003	11.91	15.29	coding	650	158	2107
PB.5561.1	4	969	1	full-splice_match	gene: Zm00001d052628	Zm00001d052628_T001	10.56	10.56	coding	255	15	779
PB.2539.1	2	2652	15	full-splice_match	gene: Zm00001d002357	Zm00001d002357_T007	21.23	24.37	coding	762	115	2400
PB.2539.2	2	2332	13	novel_in_catalog	gene: Zm00001d002357	novel	3.13	24.37	coding	332	201	1196
PB.1371.2	1	1801	2	full-splice_match	gene: Zm00001d033927	Zm00001d033927_T028	0.77	13.97	coding	379	334	1470
PB.8176.1	7	1125	1	intergenic	novelGene_18	novel	4.12	7.34	non_coding	NA	NA	NA
PB.1376.3	1	1608	11	full-splice_match	gene: Zm00001d033969	Zm00001d033969_T006	5.07	73.48	coding	384	304	1455
PB.8176.2	7	726	2	intergenic	novelGene_18	novel	3.22	7.34	non_coding	NA	NA	NA
PB.408.1	1	2374	6	incomplete-splice_match	gene: Zm00001d029004	Zm00001d029004_T114	23.89	23.89	coding	698	22	2115
PB.7255.2	6	1804	6	novel_in_catalog	gene: Zm00001d035185	novel	0.00	13.04	coding	411	250	1482
PB.3824.1	3	1893	8	incomplete-splice_match	gene: Zm00001d040082	Zm00001d040082_T012	14.55	35.10	coding	418	164	1417
PB.3824.2	3	464	1	incomplete-splice_match	gene: Zm00001d040082	Zm00001d040082_T005	12.42	35.10	non_coding	NA	NA	NA
PB.3824.3	3	2319	11	incomplete-splice_match	gene: Zm00001d040082	Zm00001d040082_T012	8.12	35.10	coding	688	115	2178

PB.5355.1	4	2098	2	full-splice_match	gene: Zm00001d051824	Zm00001d051824_T001	37.88	37.88	coding	628	80	1963
PB.6707.1	5	2641	9	full-splice_match	gene: Zm00001d016602	Zm00001d016602_T002	33.50	33.50	coding	743	74	2302
PB.3772.2	3	1004	1	novel_in_catalog	gene: Zm00001d039826	novel	52.99	246.16	non_coding	NA	NA	NA
PB.10464.1	9	858	6	full-splice_match	gene: Zm00001d047187	Zm00001d047187_T001	70.79	70.79	coding	176	122	649
PB.1371.1	1	2055	2	full-splice_match	gene: Zm00001d033927	Zm00001d033927_T028	12.85	13.97	coding	379	103	1239
PB.614.2	1	1442	1	full-splice_match	gene: Zm00001d030083	Zm00001d030083_T001	31.23	35.78	coding	342	65	1090
PB.614.1	1	983	1	full-splice_match	gene: Zm00001d030083	Zm00001d030083_T001	4.55	35.78	non_coding	NA	NA	NA
PB.3736.1	3	1540	3	novel_not_in_catalog	gene: Zm00001d039576	novel	31.20	31.20	coding	349	172	1218
PB.6116.1	5	2148	8	full-splice_match	gene: Zm00001d013524	Zm00001d013524_T009	35.92	35.92	coding	590	128	1897
PB.893.1	1	1437	2	full-splice_match	gene: Zm00001d031737	Zm00001d031737_T001	9.54	9.54	coding	290	271	1140
PB.3040.4	2	2065	8	full-splice_match	gene: Zm00001d004986	Zm00001d004986_T014	14.04	81.46	coding	506	417	1934
PB.1947.1	10	1973	1	full-splice_match	gene: Zm00001d024831	Zm00001d024831_T001	4.81	4.81	coding	611	42	1874
PB.5701.1	4	2365	5	incomplete-splice_match	gene: Zm00001d053300	Zm00001d053300_T003	25.17	30.94	coding	632	73	1968
PB.5466.5	4	1437	5	novel_not_in_catalog	gene: Zm00001d052261	novel	203.22	413.82	coding	303	158	1066
PB.5466.6	4	1847	4	full-splice_match	gene: Zm00001d052261	Zm00001d052261_T001	55.06	413.82	coding	211	158	790
PB.3772.1	3	945	2	full-splice_match	gene: Zm00001d039826	Zm00001d039826_T001	177.06	246.16	coding	10	582	611
PB.2525.3	2	2371	13	incomplete-splice_match	gene: Zm00001d002313	Zm00001d002313_T007	52.18	52.18	coding	310	1247	2176

PB.5466.1	4	1913	2	incomplete-splice_match	gene: Zm00001d052261	Zm00001d052261_T001	1.21	413.82	non_coding	NA	NA	NA
PB.5466.2	4	1552	5	novel_not_in_catalog	gene: Zm00001d052261	novel	154.34	413.82	coding	303	153	1061
PB.5466.3	4	2019	4	full-splice_match	gene: Zm00001d052261	Zm00001d052261_T001	0.00	413.82	coding	211	156	788
PB.3304.2	2	1325	3	full-splice_match	gene: Zm00001d006348	Zm00001d006348_T001	0.00	58.87	coding	273	166	984
PB.9969.2	8	1534	4	full-splice_match	gene: Zm00001d012556	Zm00001d012556_T001	0.00	119.24	coding	448	89	1432
PB.1571.2	1	1589	6	novel_in_catalog	gene: Zm00001d034719	novel	0.51	46.56	coding	403	223	1431
PB.9969.1	8	1678	4	full-splice_match	gene: Zm00001d012556	Zm00001d012556_T001	119.24	119.24	coding	448	86	1429
PB.10071.1	9	717	1	full-splice_match	gene: Zm00001d044923	Zm00001d044923_T001	118.31	118.31	coding	112	83	418
PB.10087.8	9	1667	2	genic	gene: Zm00001d045028	novel	0.00	111.95	coding	508	144	1667
PB.6420.3	5	2002	11	novel_in_catalog	gene: Zm00001d014896	novel	5.57	24.30	coding	353	196	1254
PB.10087.7	9	2280	3	genic	gene: Zm00001d045028	novel	111.95	111.95	coding	670	140	2149
PB.6420.1	5	1959	11	novel_in_catalog	gene: Zm00001d014896	novel	8.74	24.30	coding	303	121	1029
PB.4067.1	3	2124	1	full-splice_match	gene: Zm00001d041626	Zm00001d041626_T001	10.27	14.07	coding	603	109	1917
PB.7879.1	6	1949	9	full-splice_match	gene: Zm00001d038222	Zm00001d038222_T003	17.47	17.47	coding	361	491	1573
PB.10196.1	9	586	3	full-splice_match	gene: Zm00001d045497	Zm00001d045497_T002	176.39	347.25	non_coding	NA	NA	NA
PB.5641.2	4	2075	13	full-splice_match	gene: Zm00001d052948	Zm00001d052948_T001	63.38	94.36	coding	542	219	1844
PB.10196.2	9	466	3	full-splice_match	gene: Zm00001d045497	Zm00001d045497_T002	170.86	347.25	non_coding	NA	NA	NA

PB.5488.1	4	3427	4	novel_in_catalog	gene: Zm00001d052343	novel	25.19	46.11	coding	667	1055	3055
PB.5488.3	4	2444	2	novel_in_catalog	gene: Zm00001d052343	novel	20.93	46.11	coding	634	300	2201
PB.776.2	1	1797	3	full-splice_match	gene: Zm00001d031101	Zm00001d031101_T002	23.22	157.98	coding	428	256	1539
PB.7859.1	6	2188	7	novel_in_catalog	gene: Zm00001d038170	novel	11.52	11.52	coding	639	117	2033
PB.776.1	1	1714	3	full-splice_match	gene: Zm00001d031101	Zm00001d031101_T002	134.76	157.98	coding	428	126	1409
PB.8460.1	7	1989	9	incomplete-splice_match	gene: Zm00001d020363	Zm00001d020363_T002	20.21	20.21	coding	540	27	1646
PB.1624.1	1	1013	1	genic	gene: Zm00001d034878	novel	15.23	15.23	non_coding	NA	NA	NA
PB.7162.20	5	1463	7	novel_in_catalog	gene: Zm00001d018445	novel	0.76	433.77	coding	422	52	1317
PB.1542.3	1	1951	4	incomplete-splice_match	gene: Zm00001d034624	Zm00001d034624_T012	5.62	35.15	coding	440	33	1352
PB.5641.1	4	2225	13	full-splice_match	gene: Zm00001d052948	Zm00001d052948_T001	30.98	94.36	coding	542	214	1839
PB.78.1	1	2088	2	full-splice_match	gene: Zm00001d027534	Zm00001d027534_T001	24.69	24.69	coding	559	171	1847
PB.78.2	1	1748	2	full-splice_match	gene: Zm00001d027534	Zm00001d027534_T001	0.00	24.69	coding	483	187	1635
PB.905.1	1	438	1	intergenic	novelGene_320	novel	41.12	41.12	non_coding	NA	NA	NA
PB.8665.2	7	2224	6	full-splice_match	gene: Zm00001d021304	Zm00001d021304_T001	107.28	138.20	coding	526	220	1797
PB.8470.4	7	1508	7	full-splice_match	gene: Zm00001d020409	Zm00001d020409_T001	0.00	60.04	coding	404	67	1278
PB.6132.3	5	1570	15	incomplete-splice_match	gene: Zm00001d013607	Zm00001d013607_T005	54.79	54.79	coding	359	276	1352
PB.8470.3	7	1720	6	novel_in_catalog	gene: Zm00001d020409	novel	2.35	60.04	coding	201	51	653

PB.8470.1	7	1809	7	full-splice_match	gene: Zm00001d020409	Zm00001d020409_T001	18.93	60.04	coding	404	105	1316
PB.5119.3	4	4132	2	novel_in_catalog	gene: Zm00001d050310	novel	1.97	135.00	coding	186	500	1057
PB.5119.2	4	1637	3	full-splice_match	gene: Zm00001d050310	Zm00001d050310_T001	40.15	135.00	coding	272	502	1317
PB.5119.1	4	1341	3	full-splice_match	gene: Zm00001d050310	Zm00001d050310_T001	4.93	135.00	coding	272	81	896
PB.3600.2	2	933	3	full-splice_match	gene: Zm00001d007832	Zm00001d007832_T001	0.00	133.93	coding	191	89	661
PB.8053.3	6	2111	15	full-splice_match	gene: Zm00001d038892	Zm00001d038892_T001	68.05	68.05	coding	563	203	1891
PB.5119.4	4	1523	3	full-splice_match	gene: Zm00001d050310	Zm00001d050310_T001	87.95	135.00	coding	272	497	1312
PB.3784.1	3	941	5	full-splice_match	gene: Zm00001d039874	Zm00001d039874_T001	49.83	49.83	coding	191	117	689
PB.3087.1	2	656	4	full-splice_match	gene: Zm00001d005253	Zm00001d005253_T002	334.03	334.03	coding	110	111	440
PB.8665.1	7	2360	6	full-splice_match	gene: Zm00001d021304	Zm00001d021304_T001	30.92	138.20	coding	526	220	1797
PB.5480.1	4	1166	8	novel_in_catalog	gene: Zm00001d052316	novel	53.70	53.70	coding	254	105	866
PB.8133.1	6	749	2	intergenic	novelGene_284	novel	31.90	111.14	non_coding	NA	NA	NA
PB.6884.4	5	2869	7	novel_in_catalog	gene: Zm00001d017392	novel	5.28	33.52	coding	486	1136	2593
PB.4866.1	4	1076	3	novel_in_catalog	gene: Zm00001d048959	novel	180.70	180.70	coding	243	199	927
PB.6884.6	5	2318	7	full-splice_match	gene: Zm00001d017392	Zm00001d017392_T005	4.32	33.52	coding	605	266	2080
PB.6884.1	5	2398	7	full-splice_match	gene: Zm00001d017392	Zm00001d017392_T005	23.91	33.52	coding	605	249	2063
PB.1253.3	1	1581	1	novel_in_catalog	gene: Zm00001d033402	novel	46.16	85.10	coding	80	101	340

PB.1192.1	1	1675	12	novel_in_catalog	gene: Zm00001d033090	novel	19.01	64.61	coding	123	139	507
PB.1192.2	1	1560	13	full-splice_match	gene: Zm00001d033090	Zm00001d033090_T001	35.80	64.61	coding	313	152	1090
PB.1192.3	1	1803	13	full-splice_match	gene: Zm00001d033090	Zm00001d033090_T001	0.46	64.61	coding	313	561	1499
PB.1192.4	1	1348	13	full-splice_match	gene: Zm00001d033090	Zm00001d033090_T001	9.35	64.61	coding	313	314	1252
PB.10751.1	9	1632	7	full-splice_match	gene: Zm00001d048299	Zm00001d048299_T002	5.69	5.69	coding	386	166	1323
PB.1539.1	1	952	3	novel_not_in_catalog	gene: Zm00001d034615	novel	33.71	33.71	coding	200	119	718
PB.1799.4	10	1203	1	full-splice_match	gene: Zm00001d023950	Zm00001d023950_T001	0.00	112.81	coding	309	114	1040
PB.6219.3	5	2110	4	intergenic	novelGene_84	novel	5.87	5.87	coding	339	882	1898
PB.6219.2	5	487	3	novel_not_in_catalog	gene: Zm00001d014030	novel	21.17	71.87	non_coding	NA	NA	NA
PB.1799.1	10	1624	1	full-splice_match	gene: Zm00001d023950	Zm00001d023950_T001	83.36	112.81	coding	309	127	1053
PB.6219.4	5	1364	7	novel_not_in_catalog	gene: Zm00001d014030	novel	13.80	71.87	coding	328	100	1083
PB.1799.3	10	1504	2	genic	gene: Zm00001d023950	novel	20.83	112.81	coding	309	127	1053
PB.1799.2	10	1283	2	genic	gene: Zm00001d023950	novel	8.63	112.81	coding	320	127	1086
PB.6720.1	5	3105	16	full-splice_match	gene: Zm00001d016675	Zm00001d016675_T001	16.73	24.19	coding	800	406	2805
PB.4170.2	3	3042	7	fusion	gene: Zm00001d042080_g	novel	0.85	7.52	coding	908	150	2873
PB.4170.1	3	2399	7	fusion	gene: Zm00001d042080_g	novel	6.67	7.52	coding	684	36	2087
PB.6953.3	5	673	3	full-splice_match	gene: Zm00001d017708	Zm00001d017708_T001	0.00	163.14	coding	157	73	543

PB.1698.1	10	3171	2	genic	gene: Zm00001d023422	novel	4.52	4.52	coding	716	315	2462
PB.9580.4	8	349	1	incomplete-splice_match	gene: Zm00001d010805	Zm00001d010805_T001	118.13	133.54	non_coding	NA	NA	NA
PB.1698.3	10	2706	2	genic	gene: Zm00001d023422	novel	0.00	4.52	coding	716	315	2462
PB.1698.2	10	2992	2	genic	gene: Zm00001d023422	novel	0.00	4.52	coding	716	315	2462
PB.9580.1	8	2061	2	full-splice_match	gene: Zm00001d010805	Zm00001d010805_T001	4.08	133.54	coding	229	37	723
PB.7622.1	6	2514	5	full-splice_match	gene: Zm00001d037242	Zm00001d037242_T001	43.32	43.32	coding	731	88	2280
PB.9580.3	8	1935	1	genic	gene: Zm00001d010805	novel	4.99	133.54	coding	258	36	809
PB.7622.3	6	2385	5	full-splice_match	gene: Zm00001d037242	Zm00001d037242_T001	0.00	43.32	coding	731	93	2285
PB.965.1	1	1061	1	full-splice_match	gene: Zm00001d032088	Zm00001d032088_T001	69.38	69.38	coding	137	143	553
PB.10304.4	9	2083	14	full-splice_match	gene: Zm00001d046214	Zm00001d046214_T003	0.00	56.57	coding	511	302	1834
PB.10304.2	9	2146	13	novel_in_catalog	gene: Zm00001d046214	novel	0.69	56.57	coding	450	297	1646
PB.10304.3	9	2181	14	full-splice_match	gene: Zm00001d046214	Zm00001d046214_T003	0.00	56.57	coding	511	292	1824
PB.10304.1	9	2325	14	full-splice_match	gene: Zm00001d046214	Zm00001d046214_T003	55.88	56.57	coding	511	288	1820
PB.420.1	1	2053	10	full-splice_match	gene: Zm00001d029041	Zm00001d029041_T003	8.69	19.64	coding	446	282	1619
PB.420.2	1	1920	10	full-splice_match	gene: Zm00001d029041	Zm00001d029041_T003	10.95	19.64	coding	446	286	1623
PB.420.3	1	1740	10	full-splice_match	gene: Zm00001d029041	Zm00001d029041_T003	0.00	19.64	coding	446	209	1546
PB.2202.2	10	2145	4	novel_not_in_catalog	gene: Zm00001d026067	novel	4.52	4.52	coding	512	294	1829

PB.8829.1	7	1904	8	full-splice_match	gene: Zm00001d021861	Zm00001d021861_T001	12.19	12.19	coding	414	254	1495
PB.4624.14	3	2214	1	genic_intron	novelGene_338	novel	4.67	4.67	coding	185	1075	1629
PB.8043.1	6	1351	3	full-splice_match	gene: Zm00001d038865	Zm00001d038865_T002	7.58	1550.98	coding	143	577	1005
PB.8043.2	6	837	4	full-splice_match	gene: Zm00001d038865	Zm00001d038865_T001	292.65	1550.98	coding	143	72	500
PB.8043.3	6	857	4	full-splice_match	gene: Zm00001d038865	Zm00001d038865_T006	0.00	1550.98	coding	143	92	520
PB.8043.4	6	805	4	full-splice_match	gene: Zm00001d038865	Zm00001d038865_T009	12.13	1550.98	coding	143	66	494
PB.8043.5	6	728	4	full-splice_match	gene: Zm00001d038865	Zm00001d038865_T008	458.49	1550.98	coding	143	74	502
PB.355.1	1	993	4	full-splice_match	gene: Zm00001d028780	Zm00001d028780_T001	103.86	103.86	coding	202	133	738
PB.8043.7	6	612	4	full-splice_match	gene: Zm00001d038865	Zm00001d038865_T013	571.19	1550.98	coding	143	68	496
PB.4449.2	3	1702	7	full-splice_match	gene: Zm00001d043358	Zm00001d043358_T001	0.00	8.43	coding	379	52	1188
PB.5668.1	4	2352	1	intergenic	novelGene_331	novel	9.01	9.01	coding	111	835	1167
PB.4449.1	3	1787	7	full-splice_match	gene: Zm00001d043358	Zm00001d043358_T001	8.43	8.43	coding	379	52	1188
PB.871.4	1	1290	3	full-splice_match	gene: Zm00001d031635	Zm00001d031635_T001	0.00	58.73	coding	337	175	1185
PB.5105.2	4	1184	1	full-splice_match	gene: Zm00001d050195	Zm00001d050195_T001	218.25	218.25	coding	306	120	1037
PB.6833.2	5	1307	6	full-splice_match	gene: Zm00001d017171	Zm00001d017171_T001	15.57	26.27	coding	373	94	1212
PB.1542.7	1	3133	7	incomplete-splice_match	gene: Zm00001d034624	Zm00001d034624_T038	3.34	35.15	coding	521	285	1847
PB.5030.2	4	1595	8	full-splice_match	gene: Zm00001d049760	Zm00001d049760_T001	7.02	7.02	coding	478	29	1462

PB.5617.2	4	2066	4	full-splice_match	gene: Zm00001d052850	Zm00001d052850_T001	8.47	8.47	coding	604	33	1844
PB.4850.1	4	1001	2	novel_not_in_catalog	gene: Zm00001d048883	novel	126.22	135.92	coding	229	49	735
PB.4850.2	4	951	1	genic	gene: Zm00001d048883	novel	9.70	135.92	coding	84	340	591
PB.703.1	1	857	6	full-splice_match	gene: Zm00001d030670	Zm00001d030670_T001	6.55	6.55	coding	217	78	728
PB.2787.1	2	1879	1	novel_in_catalog	gene: Zm00001d003382	novel	0.68	17.45	non_coding	NA	NA	NA
PB.2787.3	2	1487	6	full-splice_match	gene: Zm00001d003382	Zm00001d003382_T011	5.75	17.45	coding	252	519	1274
PB.2787.2	2	1577	7	full-splice_match	gene: Zm00001d003382	Zm00001d003382_T010	11.01	17.45	coding	347	145	1185
PB.349.4	1	573	4	full-splice_match	gene: Zm00001d028755	Zm00001d028755_T002	0.00	357.34	coding	164	81	572
PB.727.1	1	2087	3	full-splice_match	gene: Zm00001d030877	Zm00001d030877_T001	5.49	8.02	coding	584	39	1790
PB.727.2	1	1893	3	full-splice_match	gene: Zm00001d030877	Zm00001d030877_T001	2.52	8.02	coding	584	40	1791
PB.2245.1	10	3191	14	full-splice_match	gene: Zm00001d026280	Zm00001d026280_T001	9.00	9.00	coding	881	360	3002
PB.349.1	1	1111	4	full-splice_match	gene: Zm00001d028755	Zm00001d028755_T002	354.18	357.34	coding	209	137	763
PB.349.2	1	2287	3	full-splice_match	gene: Zm00001d028755	Zm00001d028755_T003	3.16	357.34	coding	163	75	563
PB.349.3	1	923	4	full-splice_match	gene: Zm00001d028755	Zm00001d028755_T002	0.00	357.34	coding	209	80	706
PB.6720.4	5	3071	16	full-splice_match	gene: Zm00001d016675	Zm00001d016675_T001	2.12	24.19	coding	800	470	2869
PB.1172.1	1	1182	7	full-splice_match	gene: Zm00001d032974	Zm00001d032974_T008	52.73	52.73	coding	299	88	984
PB.7747.1	6	500	4	full-splice_match	gene: Zm00001d037785	Zm00001d037785_T003	72.55	72.55	non_coding	NA	NA	NA

PB.9981.1	8	1608	2	full-splice_match	gene: Zm00001d012602	Zm00001d012602_T001	13.11	13.11	coding	208	788	1411
PB.8537.1	7	1854	5	incomplete-splice_match	gene: Zm00001d020708	Zm00001d020708_T010	27.03	27.03	coding	287	194	1054
PB.6797.2	5	1991	13	full-splice_match	gene: Zm00001d017042	Zm00001d017042_T003	76.80	80.03	coding	533	183	1781
PB.4399.2	3	1799	5	novel_in_catalog	gene: Zm00001d043153	novel	3.02	108.47	coding	88	286	549
PB.4399.1	3	1307	9	novel_not_in_catalog	gene: Zm00001d043153	novel	105.44	108.47	coding	312	115	1050
PB.10773.1	9	1667	4	full-splice_match	gene: Zm00001d048359	Zm00001d048359_T009	54.04	54.04	coding	399	30	1226
PB.5751.1	4	2117	18	full-splice_match	gene: Zm00001d053580	Zm00001d053580_T003	49.02	49.02	coding	539	180	1796
PB.1935.1	10	1046	2	intergenic	novelGene_154	novel	6.09	6.09	non_coding	NA	NA	NA
PB.1949.1	10	1127	3	full-splice_match	gene: Zm00001d024839	Zm00001d024839_T001	656.44	1120.31	coding	223	242	910
PB.1949.2	10	1167	2	novel_in_catalog	gene: Zm00001d024839	novel	7.44	1120.31	coding	128	568	951
PB.1949.3	10	1264	1	novel_in_catalog	gene: Zm00001d024839	novel	17.23	1120.31	coding	128	665	1048
PB.1949.6	10	856	3	full-splice_match	gene: Zm00001d024839	Zm00001d024839_T001	439.21	1120.31	coding	223	114	782
PB.8961.1	7	1934	2	full-splice_match	gene: Zm00001d022351	Zm00001d022351_T001	19.38	20.51	coding	526	160	1737
PB.9595.3	8	1981	11	novel_in_catalog	gene: Zm00001d010862	novel	87.13	87.13	coding	341	77	1099
PB.9934.1	8	1741	5	incomplete-splice_match	gene: Zm00001d012408	Zm00001d012408_T001	17.12	17.12	coding	357	438	1508
PB.8527.3	7	2407	1	full-splice_match	gene: Zm00001d020659	Zm00001d020659_T001	2.28	91.80	coding	139	1781	2197
PB.8527.2	7	765	2	genic	gene: Zm00001d020659	novel	79.14	91.80	coding	123	195	563

PB.8527.1	7	2551	1	full-splice_match	gene: Zm00001d020659	Zm00001d020659_T001	4.25	91.80	coding	139	1710	2126
PB.924.2	1	1550	4	full-splice_match	gene: Zm00001d031913	Zm00001d031913_T004	0.00	74.61	coding	393	131	1309
PB.8527.4	7	627	2	genic	gene: Zm00001d020659	novel	6.13	91.80	non_coding	NA	NA	NA
PB.2005.1	10	642	2	full-splice_match	gene: Zm00001d025062	Zm00001d025062_T001	184.36	184.36	coding	124	37	408
PB.8961.2	7	3167	1	genic	gene: Zm00001d022351	novel	1.13	20.51	coding	423	1741	3009
PB.1542.4	1	2405	5	incomplete-splice_match	gene: Zm00001d034624	Zm00001d034624_T012	0.00	35.15	coding	605	114	1928
PB.10529.4	9	1269	14	full-splice_match	gene: Zm00001d047498	Zm00001d047498_T010	19.30	170.79	coding	285	157	1011
PB.5540.6	4	1070	4	full-splice_match	gene: Zm00001d052493	Zm00001d052493_T003	277.26	1238.52	coding	235	102	806
PB.3230.1	2	1700	1	full-splice_match	gene: Zm00001d006043	Zm00001d006043_T001	17.63	17.63	coding	129	589	975
PB.6279.9	5	3083	6	full-splice_match	gene: Zm00001d014330	Zm00001d014330_T061	0.00	82.37	non_coding	NA	NA	NA
PB.6279.8	5	2897	7	incomplete-splice_match	gene: Zm00001d014330	Zm00001d014330_T019	0.00	82.37	coding	69	390	596
PB.4110.1	3	2732	9	full-splice_match	gene: Zm00001d041847	Zm00001d041847_T002	7.08	7.42	coding	773	110	2428
PB.6279.6	5	3473	8	full-splice_match	gene: Zm00001d014330	Zm00001d014330_T019	8.92	82.37	coding	101	428	730
PB.6279.5	5	3923	7	full-splice_match	gene: Zm00001d014330	Zm00001d014330_T050	14.36	82.37	non_coding	NA	NA	NA
PB.6279.4	5	3603	8	full-splice_match	gene: Zm00001d014330	Zm00001d014330_T028	5.20	82.37	coding	101	467	769
PB.6279.3	5	1511	7	full-splice_match	gene: Zm00001d014330	Zm00001d014330_T001	40.52	82.37	coding	339	238	1254
PB.1559.2	1	926	2	full-splice_match	gene: Zm00001d034673	Zm00001d034673_T001	27.45	83.84	coding	127	128	508

PB.6279.1	5	1642	6	novel_in_catalog	gene: Zm00001d014330	novel	7.86	82.37	coding	234	145	846
PB.3585.3	2	2005	3	full-splice_match	gene: Zm00001d007778	Zm00001d007778_T001	0.00	15.15	coding	568	197	1900
PB.10330.3	9	671	1	full-splice_match	gene: Zm00001d046404	Zm00001d046404_T001	33.70	118.23	coding	135	127	531
PB.10330.2	9	859	1	full-splice_match	gene: Zm00001d046404	Zm00001d046404_T001	83.87	118.23	coding	135	128	532
PB.10330.1	9	1444	1	full-splice_match	gene: Zm00001d046404	Zm00001d046404_T001	0.65	118.23	coding	135	127	531
PB.7686.1	6	3628	2	genic	gene: Zm00001d037544	novel	22.60	60.44	coding	836	982	3489
PB.2103.1	10	1009	2	incomplete-splice_match	gene: Zm00001d025658	Zm00001d025658_T006	10.57	63.14	coding	119	67	423
PB.9014.1	7	2778	20	novel_in_catalog	gene: Zm00001d022502	novel	10.15	10.15	coding	893	99	2777
PB.5253.1	4	1599	7	incomplete-splice_match	gene: Zm00001d051268	Zm00001d051268_T007	55.89	55.89	coding	275	19	843
PB.6541.1	5	1845	13	incomplete-splice_match	gene: Zm00001d015446	Zm00001d015446_T017	12.46	12.46	coding	400	79	1278
PB.3220.7	2	2375	6	novel_in_catalog	gene: Zm00001d006001	novel	0.00	41.26	coding	332	392	1387
PB.3220.4	2	2557	5	novel_in_catalog	gene: Zm00001d006001	novel	2.20	41.26	coding	332	529	1524
PB.4099.2	3	1155	1	full-splice_match	gene: Zm00001d041774	Zm00001d041774_T001	22.60	100.82	coding	117	693	1043
PB.3220.2	2	2369	7	novel_not_in_catalog	gene: Zm00001d006001	novel	0.21	41.26	coding	412	443	1678
PB.8854.1	7	783	5	full-splice_match	gene: Zm00001d021958	Zm00001d021958_T001	229.30	229.30	coding	157	108	578
PB.3220.1	2	2409	7	full-splice_match	gene: Zm00001d006001	Zm00001d006001_T001	28.89	41.26	coding	567	448	2148
PB.10091.3	9	1076	1	genic	gene: Zm00001d027055	novel	79.98	79.98	coding	172	235	750

PB.10091.2	9	1609	3	fusion	gene: Zm00001d027055_g	novel	15.98	15.98	coding	363	187	1275
PB.10091.1	9	1701	2	full-splice_match	gene: Zm00001d045044	Zm00001d045044_T001	167.58	167.58	coding	363	188	1276
PB.1207.14	1	860	4	full-splice_match	gene: Zm00001d033174	Zm00001d033174_T001	0.00	548.07	coding	161	295	777
PB.6440.1	5	2630	1	genic	gene: Zm00001d014966	novel	21.22	21.22	coding	748	68	2311
PB.2072.3	10	2717	11	full-splice_match	gene: Zm00001d025449	Zm00001d025449_T003	15.95	27.86	coding	727	236	2416
PB.1056.2	1	732	4	novel_not_in_catalog	gene: Zm00001d032485	novel	63.29	130.84	non_coding	NA	NA	NA
PB.8117.4	6	1793	10	full-splice_match	gene: Zm00001d039157	Zm00001d039157_T002	10.44	155.40	coding	163	149	637
PB.10384.1	9	898	1	full-splice_match	gene: Zm00001d046687	Zm00001d046687_T001	20.68	20.68	coding	209	49	675
PB.1962.5	10	1471	11	incomplete-splice_match	gene: Zm00001d024891	Zm00001d024891_T001	75.78	75.78	coding	469	64	1470
PB.9828.1	8	969	1	full-splice_match	gene: Zm00001d011902	Zm00001d011902_T001	68.59	68.59	coding	154	78	539
PB.4792.1	4	1423	9	full-splice_match	gene: Zm00001d048583	Zm00001d048583_T001	89.11	269.54	coding	310	72	1001
PB.3306.2	2	1355	3	novel_in_catalog	gene: Zm00001d006360	novel	14.15	212.80	coding	158	72	545
PB.4792.2	4	1317	9	full-splice_match	gene: Zm00001d048583	Zm00001d048583_T001	12.64	269.54	coding	310	82	1011
PB.3520.1	2	1779	13	full-splice_match	gene: Zm00001d007397	Zm00001d007397_T003	33.08	33.08	coding	413	328	1566
PB.3115.4	2	1471	1	incomplete-splice_match	gene: Zm00001d005393	Zm00001d005393_T001	6.24	71.47	non_coding	NA	NA	NA
PB.10231.3	9	3178	2	intergenic	novelGene_89	novel	2.02	16.10	coding	64	143	334
PB.1207.12	1	1432	4	full-splice_match	gene: Zm00001d033174	Zm00001d033174_T001	59.46	548.07	coding	364	120	1211

PB.6871.4	5	1639	2	full-splice_match	gene: Zm00001d017329	Zm00001d017329_T001	0.00	26.53	coding	513	39	1577
PB.3115.3	2	1547	6	full-splice_match	gene: Zm00001d005393	Zm00001d005393_T004	4.12	71.47	coding	170	58	567
PB.3115.2	2	1463	5	full-splice_match	gene: Zm00001d005393	Zm00001d005393_T007	61.11	71.47	coding	354	58	1119
PB.2657.2	2	1549	1	full-splice_match	gene: Zm00001d002851	Zm00001d002851_T001	13.94	21.65	coding	342	83	1108
PB.474.1	1	1537	1	full-splice_match	gene: Zm00001d029287	Zm00001d029287_T001	33.07	33.07	coding	383	95	1243
PB.1324.1	1	992	4	full-splice_match	gene: Zm00001d033707	Zm00001d033707_T001	110.44	416.69	coding	148	61	504
PB.6456.1	5	1268	1	antisense	novelGene_gene: Zm00001d015012_A	novel	7.21	7.21	coding	127	28	408
PB.9034.1	7	2897	18	novel_in_catalog	gene: Zm00001d022581	novel	65.36	65.36	coding	750	60	2309
PB.4926.2	4	1024	3	full-splice_match	gene: Zm00001d049228	Zm00001d049228_T001	4.84	9.10	coding	333	10	1008
PB.6667.5	5	2045	18	full-splice_match	gene: Zm00001d016365	Zm00001d016365_T001	78.31	79.99	coding	542	179	1804
PB.7193.2	5	600	6	incomplete-splice_match	gene: Zm00001d018531	Zm00001d018531_T009	0.00	25.71	coding	115	104	448
PB.7193.1	5	773	6	incomplete-splice_match	gene: Zm00001d018531	Zm00001d018531_T009	25.71	25.71	coding	115	111	455
PB.2284.3	10	1132	1	full-splice_match	gene: Zm00001d026390	Zm00001d026390_T001	0.00	77.39	coding	27	553	633
PB.2284.1	10	1689	1	full-splice_match	gene: Zm00001d026390	Zm00001d026390_T001	14.60	77.39	coding	27	557	637
PB.4341.8	3	2723	7	full-splice_match	gene: Zm00001d042885	Zm00001d042885_T008	0.00	39.75	coding	234	210	911
PB.509.1	1	1522	7	full-splice_match	gene: Zm00001d029478	Zm00001d029478_T001	8.03	8.03	coding	216	401	1048
PB.9197.1	8	638	1	full-splice_match	gene: Zm00001d008825	Zm00001d008825_T001	21.13	21.13	coding	142	85	510

PB.4977.1	4	1218	1	full-splice_match	gene: Zm00001d049521	Zm00001d049521_T001	80.03	80.03	coding	49	749	895
PB.4926.1	4	1036	3	full-splice_match	gene: Zm00001d049228	Zm00001d049228_T001	4.26	9.10	coding	273	20	838
PB.5427.1	4	1608	10	full-splice_match	gene: Zm00001d052078	Zm00001d052078_T002	212.94	212.94	coding	426	35	1312
PB.6164.1	5	2046	3	genic	gene: Zm00001d013732	novel	5.65	5.65	coding	299	312	1208
PB.2072.1	10	2295	9	novel_in_catalog	gene: Zm00001d025449	novel	11.91	27.86	coding	239	1180	1896
PB.5064.3	4	1600	9	full-splice_match	gene: Zm00001d049954	Zm00001d049954_T001	0.00	255.02	coding	433	213	1511
PB.4560.2	3	1619	5	full-splice_match	gene: Zm00001d043764	Zm00001d043764_T003	4.72	6.89	coding	310	124	1053
PB.5064.1	4	1792	9	full-splice_match	gene: Zm00001d049954	Zm00001d049954_T001	255.02	255.02	coding	433	289	1587
PB.76.1	1	2094	8	full-splice_match	gene: Zm00001d027514	Zm00001d027514_T002	38.90	38.90	coding	536	234	1841
PB.2797.2	2	1330	11	novel_not_in_catalog	gene: Zm00001d003423	novel	10.17	29.76	coding	374	9	1130
PB.3553.1	2	1322	3	full-splice_match	gene: Zm00001d007595	Zm00001d007595_T033	40.38	40.38	non_coding	NA	NA	NA
PB.2797.1	2	1440	11	novel_not_in_catalog	gene: Zm00001d003423	novel	19.60	29.76	coding	374	4	1125
PB.900.1	1	1368	1	antisense	novelGene_gene: Zm00001d031805_A	novel	19.43	19.43	non_coding	NA	NA	NA
PB.4031.2	3	1527	2	genic	gene: Zm00001d041456	novel	0.00	129.86	coding	426	101	1378
PB.4031.1	3	1645	2	genic	gene: Zm00001d041456	novel	129.86	129.86	coding	426	145	1422
PB.10643.1	9	1570	1	full-splice_match	gene: Zm00001d047883	Zm00001d047883_T001	8.93	8.93	coding	365	167	1261
PB.9914.1	8	887	4	novel_in_catalog	gene: Zm00001d012301	novel	47.14	47.14	coding	145	144	578

PB.4231.1	3	763	4	full-splice_match	gene: Zm00001d042395	Zm00001d042395_T001	44.65	44.65	coding	113	204	542
PB.7781.1	6	1488	14	intergenic	novelGene_227	novel	40.11	40.11	coding	353	201	1259
PB.2921.6	2	2861	6	intergenic	novelGene_146	novel	0.00	44.65	coding	453	999	2357
PB.2015.1	10	1678	2	genic	gene: Zm00001d025107	novel	44.13	44.13	coding	375	296	1420
PB.3399.6	2	612	6	full-splice_match	gene: Zm00001d006760	Zm00001d006760_T001	527.67	2189.04	coding	160	82	561
PB.98.2	1	2760	1	antisense	novelGene_gene: Zm00001d027621_A	novel	11.89	16.13	coding	335	1439	2443
PB.1031.1	1	2337	19	full-splice_match	gene: Zm00001d032386	Zm00001d032386_T003	92.19	154.28	coding	618	259	2112
PB.1031.2	1	2283	18	novel_in_catalog	gene: Zm00001d032386	novel	53.27	154.28	coding	448	664	2007
PB.98.1	1	2872	1	antisense	novelGene_gene: Zm00001d027621_A	novel	4.24	16.13	coding	335	1449	2453
PB.1031.4	1	2359	18	novel_in_catalog	gene: Zm00001d032386	novel	8.81	154.28	coding	645	194	2128
PB.3399.5	2	754	6	full-splice_match	gene: Zm00001d006760	Zm00001d006760_T001	337.67	2189.04	coding	160	81	560
PB.501.1	1	544	2	genic	gene: Zm00001d029444	novel	46.89	46.89	non_coding	NA	NA	NA
PB.3399.4	2	1341	5	novel_in_catalog	gene: Zm00001d006760	novel	9.05	2189.04	coding	168	76	579
PB.3109.1	2	2508	3	full-splice_match	gene: Zm00001d005370	Zm00001d005370_T001	10.39	10.39	coding	245	1576	2310
PB.8648.1	7	2407	5	full-splice_match	gene: Zm00001d021240	Zm00001d021240_T001	13.33	32.93	coding	568	170	1873
PB.8648.3	7	1940	5	full-splice_match	gene: Zm00001d021240	Zm00001d021240_T001	0.00	32.93	coding	568	187	1890
PB.8648.2	7	2251	5	full-splice_match	gene: Zm00001d021240	Zm00001d021240_T001	19.60	32.93	coding	568	188	1891

PB.2647.1	2	1055	1	novel_in_catalog	gene: Zm00001d002815	novel	3.69	3.69	coding	172	89	604
PB.10588.1	9	792	2	full-splice_match	gene: Zm00001d047720	Zm00001d047720_T001	238.13	238.13	coding	110	116	445
PB.3129.3	2	924	6	incomplete-splice_match	gene: Zm00001d005497	Zm00001d005497_T005	15.08	15.08	coding	127	120	500
PB.3498.1	2	1348	1	full-splice_match	gene: Zm00001d007257	Zm00001d007257_T001	21.03	21.03	coding	346	59	1096
PB.10787.4	9	835	8	full-splice_match	gene: Zm00001d048410	Zm00001d048410_T001	0.00	98.77	coding	200	92	691
PB.5443.2	4	817	2	full-splice_match	gene: Zm00001d052191	Zm00001d052191_T001	18.20	19.02	coding	205	71	685
PB.5443.1	4	1413	2	full-splice_match	gene: Zm00001d052191	Zm00001d052191_T001	0.82	19.02	coding	205	78	692
PB.9689.2	8	1745	2	full-splice_match	gene: Zm00001d011336	Zm00001d011336_T001	50.28	60.31	coding	448	55	1398
PB.6827.2	5	869	4	incomplete-splice_match	gene: Zm00001d017146	Zm00001d017146_T001	0.00	23.91	coding	142	193	618
PB.90.4	1	3010	20	full-splice_match	gene: Zm00001d027558	Zm00001d027558_T002	66.72	66.72	coding	795	461	2845
PB.5895.1	4	1091	9	full-splice_match	gene: Zm00001d054078	Zm00001d054078_T002	89.51	89.51	coding	278	52	885
PB.7832.1	6	2795	3	full-splice_match	gene: Zm00001d038101	Zm00001d038101_T002	39.96	57.66	coding	778	45	2378
PB.10583.4	9	2395	3	antisense	novelGene_gene: Zm00001d047708_A	novel	3.01	20.54	non_coding	NA	NA	NA
PB.8117.1	6	1634	11	full-splice_match	gene: Zm00001d039157	Zm00001d039157_T001	143.00	155.40	coding	377	148	1278
PB.10894.1	B73V4 ctg23	1359	3	intergenic	novelGene_4	novel	18.58	18.58	non_coding	NA	NA	NA
PB.9498.2	8	1584	4	novel_in_catalog	gene: Zm00001d010451	novel	107.04	194.40	coding	368	213	1316
PB.3517.3	2	1706	15	full-splice_match	gene: Zm00001d007391	Zm00001d007391_T005	0.00	4.73	coding	491	37	1509

PB.4025.2	3	1702	7	incomplete-splice_match	gene: Zm00001d041420	Zm00001d041420_T015	46.83	46.83	coding	417	75	1325
PB.1684.2	10	2917	3	novel_in_catalog	gene: Zm00001d023367	novel	16.45	22.33	coding	795	371	2755
PB.1684.3	10	2972	3	novel_in_catalog	gene: Zm00001d023367	novel	2.32	22.33	coding	795	462	2846
PB.1684.1	10	3118	3	novel_in_catalog	gene: Zm00001d023367	novel	3.57	22.33	coding	795	379	2763
PB.3944.2	3	1957	7	full-splice_match	gene: Zm00001d040724	Zm00001d040724_T001	0.00	26.30	coding	592	6	1781
PB.3944.1	3	2102	7	full-splice_match	gene: Zm00001d040724	Zm00001d040724_T001	26.30	26.30	coding	592	37	1812
PB.9677.1	8	4223	6	novel_not_in_catalog	gene: Zm00001d011253	novel	6.75	141.59	coding	129	2581	2967
PB.7801.1	6	908	3	full-splice_match	gene: Zm00001d038002	Zm00001d038002_T001	45.72	45.72	coding	189	46	612
PB.4557.2	3	886	4	full-splice_match	gene: Zm00001d043737	Zm00001d043737_T001	62.06	79.59	coding	157	134	604
PB.8814.1	7	2434	5	full-splice_match	gene: Zm00001d021823	Zm00001d021823_T002	28.05	38.84	coding	614	79	1920
PB.10598.1	9	1625	4	full-splice_match	gene: Zm00001d047754	Zm00001d047754_T001	186.48	186.48	coding	324	245	1216
PB.4557.1	3	1027	4	full-splice_match	gene: Zm00001d043737	Zm00001d043737_T001	17.53	79.59	coding	152	145	600
PB.5889.1	4	2334	3	full-splice_match	gene: Zm00001d054066	Zm00001d054066_T001	30.71	98.73	coding	571	190	1902
PB.10598.6	9	1163	9	incomplete-splice_match	gene: Zm00001d047755	Zm00001d047755_T008	22.86	38.56	coding	189	137	703
PB.10598.5	9	1700	12	full-splice_match	gene: Zm00001d047755	Zm00001d047755_T008	15.70	38.56	coding	343	68	1096
PB.5889.2	4	2199	3	full-splice_match	gene: Zm00001d054066	Zm00001d054066_T001	65.06	98.73	coding	571	184	1896
PB.8957.2	7	2150	6	full-splice_match	gene: Zm00001d022340	Zm00001d022340_T001	24.84	24.84	coding	94	325	606

PB.1121.1	1	1352	2	full-splice_match	gene: Zm00001d032718	Zm00001d032718_T001	7.08	7.08	coding	314	119	1060
PB.1183.1	1	2826	14	full-splice_match	gene: Zm00001d033028	Zm00001d033028_T001	5.40	29.98	coding	724	498	2669
PB.2191.1	10	964	1	full-splice_match	gene: Zm00001d026028	Zm00001d026028_T001	117.41	117.41	non_coding	NA	NA	NA
PB.6203.1	5	741	3	incomplete-splice_match	gene: Zm00001d013957	Zm00001d013957_T001	50.55	50.55	coding	122	141	506
PB.8957.4	7	1568	6	full-splice_match	gene: Zm00001d022340	Zm00001d022340_T001	0.00	24.84	non_coding	NA	NA	NA
PB.10038.1	9	1239	1	genic	gene: Zm00001d044747	novel	133.29	2226.37	coding	99	74	370
PB.10038.2	9	572	3	novel_not_in_catalog	gene: Zm00001d044747	novel	2093.07	2226.37	non_coding	NA	NA	NA
PB.8701.9	7	3126	5	full-splice_match	gene: Zm00001d021442	Zm00001d021442_T017	0.00	76.11	coding	851	243	2795
PB.9677.2	8	1734	9	novel_not_in_catalog	gene: Zm00001d011253	novel	24.62	141.59	coding	190	260	829
PB.5431.3	4	2016	8	novel_in_catalog	gene: Zm00001d052108	novel	2.10	200.76	coding	176	891	1418
PB.6675.2	5	1311	9	full-splice_match	gene: Zm00001d016410	Zm00001d016410_T006	154.08	222.35	coding	329	127	1113
PB.6675.1	5	1321	9	full-splice_match	gene: Zm00001d016410	Zm00001d016410_T002	68.27	222.35	coding	327	146	1126
PB.10204.1	9	2936	10	full-splice_match	gene: Zm00001d045516	Zm00001d045516_T001	19.13	19.13	coding	766	380	2677
PB.8656.2	7	1610	3	novel_not_in_catalog	gene: Zm00001d021286	novel	4.82	16.76	coding	234	681	1382
PB.8656.3	7	1153	3	novel_not_in_catalog	gene: Zm00001d021286	novel	0.00	16.76	coding	343	22	1050
PB.7020.1	5	1775	1	full-splice_match	gene: Zm00001d017976	Zm00001d017976_T001	3.04	3.04	coding	452	159	1514
PB.523.4	1	1292	3	full-splice_match	gene: Zm00001d029539	Zm00001d029539_T003	20.34	189.73	coding	118	152	505

PB.6194.4	5	2365	18	novel_in_catalog	gene: Zm00001d013911	novel	2.36	36.28	coding	552	169	1824
PB.6194.3	5	2393	17	novel_in_catalog	gene: Zm00001d013911	novel	0.84	36.28	coding	311	80	1012
PB.523.3	1	1188	4	full-splice_match	gene: Zm00001d029539	Zm00001d029539_T002	152.87	189.73	coding	190	150	719
PB.6194.1	5	2317	19	full-splice_match	gene: Zm00001d013911	Zm00001d013911_T003	33.09	36.28	coding	613	189	2027
PB.1978.16	10	3614	17	novel_not_in_catalog	gene: Zm00001d024952	novel	0.00	124.67	coding	882	86	2731
PB.5935.1	5	3379	6	full-splice_match	gene: Zm00001d012895	Zm00001d012895_T005	3.81	3.81	coding	899	470	3166
PB.1978.14	10	3154	15	incomplete-splice_match	gene: Zm00001d024952	Zm00001d024952_T007	5.19	124.67	coding	708	39	2162
PB.9480.3	8	1378	1	full-splice_match	gene: Zm00001d010380	Zm00001d010380_T001	0.00	49.89	coding	397	59	1249
PB.1362.1	1	1613	2	full-splice_match	gene: Zm00001d033892	Zm00001d033892_T001	10.45	10.45	coding	365	351	1445
PB.1978.10	10	3149	20	novel_not_in_catalog	gene: Zm00001d024952	novel	0.00	124.67	coding	905	89	2803
PB.125.1	1	2984	10	novel_not_in_catalog	gene: Zm00001d027732	novel	2.11	12.04	coding	695	334	2418
PB.8130.3	6	1426	8	novel_in_catalog	gene: Zm00001d039182	novel	3.98	104.02	coding	307	149	1069
PB.9755.1	8	1225	5	full-splice_match	gene: Zm00001d011636	Zm00001d011636_T002	22.96	22.96	coding	179	345	881
PB.2625.1	2	826	2	incomplete-splice_match	gene: Zm00001d002738	Zm00001d002738_T001	755.41	755.41	coding	176	90	617
PB.2337.2	10	1672	2	novel_in_catalog	gene: Zm00001d026584	novel	3.45	65.39	coding	152	13	468
PB.125.3	1	2629	10	novel_not_in_catalog	gene: Zm00001d027732	novel	0.00	12.04	coding	695	334	2418
PB.3507.1	2	2284	9	novel_in_catalog	gene: Zm00001d007301	novel	62.71	62.71	coding	274	48	869

PB.941.1	1	497	2	full-splice_match	gene: Zm00001d031975	Zm00001d031975_T001	452.86	453.94	non_coding	NA	NA	NA
PB.3641.11	3	1132	2	incomplete-splice_match	gene: Zm00001d039267	Zm00001d039267_T012	10.88	84.86	coding	231	40	732
PB.3641.10	3	2510	14	full-splice_match	gene: Zm00001d039267	Zm00001d039267_T004	0.00	84.86	coding	686	142	2199
PB.3641.12	3	2259	12	full-splice_match	gene: Zm00001d039267	Zm00001d039267_T013	0.69	84.86	coding	610	203	2032
PB.7094.1	5	1213	6	full-splice_match	gene: Zm00001d018241	Zm00001d018241_T001	64.84	64.84	coding	263	161	949
PB.6073.2	5	2114	10	novel_in_catalog	gene: Zm00001d013371	novel	0.18	96.45	coding	395	721	1905
PB.6073.1	5	1966	12	novel_in_catalog	gene: Zm00001d013371	novel	73.38	96.45	coding	515	211	1755
PB.3932.1	3	1998	2	full-splice_match	gene: Zm00001d040649	Zm00001d040649_T001	86.71	86.71	coding	551	81	1733
PB.7607.1	6	1282	1	full-splice_match	gene: Zm00001d037180	Zm00001d037180_T001	158.82	158.82	coding	325	64	1038
PB.6073.4	5	1787	12	novel_in_catalog	gene: Zm00001d013371	novel	22.88	96.45	coding	515	173	1717
PB.1091.6	1	2040	7	full-splice_match	gene: Zm00001d032597	Zm00001d032597_T020	17.26	62.22	coding	541	176	1798
PB.1091.7	1	2710	8	full-splice_match	gene: Zm00001d032597	Zm00001d032597_T003	0.00	62.22	coding	770	166	2475
PB.1766.1	10	519	1	full-splice_match	gene: Zm00001d023804	Zm00001d023804_T001	128.41	128.41	non_coding	NA	NA	NA
PB.9340.2	8	1474	7	novel_in_catalog	gene: Zm00001d009640	novel	89.32	108.77	coding	340	208	1227
PB.9340.1	8	1615	7	novel_in_catalog	gene: Zm00001d009640	novel	19.45	108.77	coding	340	207	1226
PB.2163.1	10	551	1	incomplete-splice_match	gene: Zm00001d025903	Zm00001d025903_T006	98.81	98.81	non_coding	NA	NA	NA
PB.5714.1	4	1839	7	full-splice_match	gene: Zm00001d053376	Zm00001d053376_T006	11.40	25.69	coding	485	132	1586

PB.5714.2	4	1770	4	novel_in_catalog	gene: Zm00001d053376	novel	4.20	25.69	coding	172	390	905
PB.5714.3	4	1907	6	full-splice_match	gene: Zm00001d053376	Zm00001d053376_T005	0.98	25.69	coding	327	703	1683
PB.5714.4	4	2268	4	full-splice_match	gene: Zm00001d053376	Zm00001d053376_T002	2.32	25.69	coding	227	1414	2094
PB.6993.1	5	1988	3	full-splice_match	gene: Zm00001d017869	Zm00001d017869_T001	95.91	95.91	coding	465	90	1484
PB.6669.1	5	1737	13	full-splice_match	gene: Zm00001d016374	Zm00001d016374_T002	7.66	7.66	coding	498	78	1571
PB.10903.4	B73V4 ctg28	1715	1	full-splice_match	gene: Zm00001d000403	Zm00001d000403_T001	8.31	33.47	coding	537	32	1642
PB.2659.1	2	1303	2	full-splice_match	gene: Zm00001d002859	Zm00001d002859_T001	63.55	63.55	coding	325	210	1184
PB.10903.2	B73V4 ctg28	2484	1	full-splice_match	gene: Zm00001d000403	Zm00001d000403_T001	8.91	33.47	coding	679	237	2273
PB.10271.1	9	850	1	full-splice_match	gene: Zm00001d045957	Zm00001d045957_T001	41.69	41.69	coding	116	111	458
PB.4298.1	3	1612	9	full-splice_match	gene: Zm00001d042695	Zm00001d042695_T001	55.65	57.58	coding	361	347	1429
PB.2485.1	2	1980	3	incomplete-splice_match	gene: Zm00001d002146	Zm00001d002146_T001	3.70	3.70	coding	503	247	1755
PB.6676.2	5	2282	4	full-splice_match	gene: Zm00001d016411	Zm00001d016411_T001	1.43	36.25	coding	655	110	2074
PB.1758.4	10	1852	5	novel_in_catalog	gene: Zm00001d023762	novel	2.46	55.30	coding	63	516	704
PB.1758.5	10	3448	6	novel_in_catalog	gene: Zm00001d023762	novel	0.75	55.30	coding	155	2209	2673
PB.1758.6	10	1794	8	full-splice_match	gene: Zm00001d023762	Zm00001d023762_T001	13.89	55.30	coding	483	213	1661
PB.5016.1	4	3356	7	novel_in_catalog	gene: Zm00001d049698	novel	5.65	28.06	coding	556	447	2114
PB.2156.1	10	436	1	antisense	novelGene_gene: Zm00001d025859_A	novel	17.63	17.63	coding	107	114	434

PB.1758.1	10	1962	8	full-splice_match	gene: Zm00001d023762	Zm00001d023762_T001	31.72	55.30	coding	483	190	1638
PB.1758.2	10	2083	7	novel_in_catalog	gene: Zm00001d023762	novel	6.49	55.30	coding	263	164	952
PB.1831.1	10	2043	13	full-splice_match	gene: Zm00001d024212	Zm00001d024212_T007	39.09	44.91	coding	500	274	1773
PB.7884.10	6	1171	3	full-splice_match	gene: Zm00001d038248	Zm00001d038248_T002	0.00	251.94	coding	183	63	611
PB.2706.1	2	3695	3	genic	gene: Zm00001d003050	novel	2.41	2.41	coding	643	57	1985
PB.2706.2	2	1724	1	full-splice_match	gene: Zm00001d003050	Zm00001d003050_T001	0.00	2.41	coding	567	24	1724
PB.8056.1	6	1875	1	full-splice_match	gene: Zm00001d038904	Zm00001d038904_T001	86.94	86.94	coding	525	65	1639
PB.10163.1	9	552	2	full-splice_match	gene: Zm00001d045385	Zm00001d045385_T001	205.41	205.41	non_coding	NA	NA	NA
PB.3271.1	2	3238	17	full-splice_match	gene: Zm00001d006214	Zm00001d006214_T011	11.01	11.01	coding	885	105	2759
PB.6983.2	5	1829	2	genic	gene: Zm00001d017840	novel	556.58	566.72	coding	387	209	1369
PB.6707.2	5	2478	9	full-splice_match	gene: Zm00001d016602	Zm00001d016602_T002	0.00	33.50	coding	743	73	2301
PB.3072.1	2	1162	9	incomplete-splice_match	gene: Zm00001d005170	Zm00001d005170_T001	41.90	41.90	coding	269	247	1053
PB.154.1	1	1723	3	novel_in_catalog	gene: Zm00001d027888	novel	7.68	19.70	coding	314	98	1039
PB.154.2	1	1414	4	full-splice_match	gene: Zm00001d027888	Zm00001d027888_T002	12.03	19.70	coding	362	24	1109
PB.3072.2	2	924	9	incomplete-splice_match	gene: Zm00001d005170	Zm00001d005170_T001	0.00	41.90	coding	243	195	923
PB.9677.6	8	2434	10	novel_not_in_catalog	gene: Zm00001d011253	novel	11.32	141.59	coding	187	1007	1567
PB.6983.8	5	2956	1	full-splice_match	gene: Zm00001d017840	Zm00001d017840_T001	10.14	566.72	coding	387	1579	2739

PB.8674.4	7	2127	6	novel_in_catalog	gene: Zm00001d021344	novel	2.25	42.25	coding	396	230	1417
PB.4306.1	3	685	1	full-splice_match	gene: Zm00001d042730	Zm00001d042730_T001	645.08	645.08	coding	136	72	479
PB.6326.1	5	1027	1	full-splice_match	gene: Zm00001d014556	Zm00001d014556_T001	3.55	3.55	coding	103	173	481
PB.8674.1	7	2091	7	full-splice_match	gene: Zm00001d021344	Zm00001d021344_T002	4.74	42.25	coding	532	216	1811
PB.8674.2	7	2426	5	novel_in_catalog	gene: Zm00001d021344	novel	1.15	42.25	coding	396	80	1267
PB.8674.3	7	1989	7	full-splice_match	gene: Zm00001d021344	Zm00001d021344_T002	34.11	42.25	coding	532	230	1825
PB.5916.1	5	2241	7	fusion	gene: Zm00001d012839_g	novel	16.54	16.54	coding	545	49	1683
PB.10379.1	9	1585	1	intergenic	novelGene_174	novel	0.72	0.72	non_coding	NA	NA	NA
PB.10279.1	9	2637	5	full-splice_match	gene: Zm00001d046016	Zm00001d046016_T001	35.07	35.07	coding	568	741	2444
PB.8026.3	6	845	7	incomplete-splice_match	gene: Zm00001d038815	Zm00001d038815_T001	26.02	26.02	coding	104	24	335
PB.6267.3	5	4217	10	novel_in_catalog	gene: Zm00001d014294	novel	20.78	94.58	coding	1302	120	4025
PB.9336.2	8	3956	7	full-splice_match	gene: Zm00001d009617	Zm00001d009617_T003	0.42	11.88	coding	1121	316	3678
PB.6267.1	5	1881	2	incomplete-splice_match	gene: Zm00001d014294	Zm00001d014294_T021	73.80	94.58	coding	503	79	1587
PB.1570.1	1	1996	11	full-splice_match	gene: Zm00001d034713	Zm00001d034713_T001	3.90	3.90	coding	577	51	1781
PB.8690.1	7	733	3	intergenic	novelGene_247	novel	270.79	270.79	coding	172	58	573
PB.1750.1	10	1518	9	full-splice_match	gene: Zm00001d023698	Zm00001d023698_T003	6.42	25.68	coding	355	150	1214
PB.1750.3	10	1552	8	full-splice_match	gene: Zm00001d023698	Zm00001d023698_T002	19.26	25.68	coding	414	31	1272

PB.3565.4	2	702	9	incomplete-splice_match	gene: Zm00001d007645	Zm00001d007645_T001	0.00	39.62	coding	177	171	701
PB.3565.5	2	1053	6	incomplete-splice_match	gene: Zm00001d007645	Zm00001d007645_T008	0.55	39.62	coding	115	171	515
PB.1333.3	1	3430	13	full-splice_match	gene: Zm00001d033776	Zm00001d033776_T004	3.87	22.12	coding	995	107	3091
PB.5857.2	4	1902	12	full-splice_match	gene: Zm00001d053968	Zm00001d053968_T007	26.21	27.09	coding	455	145	1509
PB.1333.5	1	1896	8	incomplete-splice_match	gene: Zm00001d033776	Zm00001d033776_T008	5.77	22.12	coding	493	106	1584
PB.3565.1	2	1311	11	full-splice_match	gene: Zm00001d007645	Zm00001d007645_T001	17.78	39.62	coding	262	175	960
PB.3565.2	2	970	9	incomplete-splice_match	gene: Zm00001d007645	Zm00001d007645_T006	19.63	39.62	coding	95	74	358
PB.3565.3	2	2119	9	novel_in_catalog	gene: Zm00001d007645	novel	1.66	39.62	coding	154	1349	1810
PB.1333.8	1	2322	9	incomplete-splice_match	gene: Zm00001d033776	Zm00001d033776_T011	0.00	22.12	coding	738	107	2320
PB.4372.5	3	2352	2	genic	gene: Zm00001d043038	novel	9.68	10.82	coding	513	585	2123
PB.4372.4	3	2292	3	novel_not_in_catalog	gene: Zm00001d043038	novel	1.14	10.82	coding	513	484	2022
PB.791.1	1	1916	2	full-splice_match	gene: Zm00001d031201	Zm00001d031201_T001	26.98	26.98	coding	229	804	1490
PB.10364.1	9	3313	19	full-splice_match	gene: Zm00001d046587	Zm00001d046587_T028	16.22	16.22	coding	904	220	2931
PB.624.14	1	2226	6	novel_in_catalog	gene: Zm00001d030110	novel	2.35	158.92	coding	368	475	1578
PB.624.15	1	3188	4	novel_in_catalog	gene: Zm00001d030110	novel	3.54	158.92	coding	424	475	1746
PB.624.16	1	2685	7	full-splice_match	gene: Zm00001d030110	Zm00001d030110_T015	0.00	158.92	coding	489	475	1941
PB.624.17	1	1756	2	novel_in_catalog	gene: Zm00001d030110	novel	0.00	158.92	coding	424	475	1746

PB.1770.2	10	2129	10	novel_in_catalog	gene: Zm00001d023817	novel	2.14	12.28	coding	369	17	1123
PB.1770.3	10	2175	10	full-splice_match	gene: Zm00001d023817	Zm00001d023817_T001	8.03	12.28	coding	633	13	1911
PB.1770.1	10	2293	9	novel_in_catalog	gene: Zm00001d023817	novel	2.11	12.28	coding	397	26	1216
PB.9398.4	8	1127	12	full-splice_match	gene: Zm00001d009941	Zm00001d009941_T002	0.00	74.78	coding	298	74	967
PB.2762.1	2	1874	16	full-splice_match	gene: Zm00001d003252	Zm00001d003252_T006	25.72	39.48	coding	507	88	1608
PB.9398.1	8	1460	11	novel_in_catalog	gene: Zm00001d009941	novel	5.48	74.78	coding	262	66	851
PB.9398.2	8	1245	12	full-splice_match	gene: Zm00001d009941	Zm00001d009941_T002	63.01	74.78	coding	298	81	974
PB.2762.5	2	1812	16	full-splice_match	gene: Zm00001d003252	Zm00001d003252_T002	13.75	39.48	coding	507	148	1668
PB.4313.2	3	1440	1	full-splice_match	gene: Zm00001d042768	Zm00001d042768_T001	0.77	62.31	coding	301	354	1256
PB.1366.1	1	1220	4	full-splice_match	gene: Zm00001d033905	Zm00001d033905_T001	13.07	13.07	coding	227	260	940
PB.5459.1	4	1719	10	full-splice_match	gene: Zm00001d052237	Zm00001d052237_T002	24.71	26.36	coding	485	88	1542
PB.5899.1	4	2216	15	full-splice_match	gene: Zm00001d054089	Zm00001d054089_T001	60.86	110.50	coding	605	67	1881
PB.3871.2	3	2081	8	full-splice_match	gene: Zm00001d040302	Zm00001d040302_T005	37.06	37.06	coding	471	120	1532
PB.8735.1	7	2061	9	full-splice_match	gene: Zm00001d021570	Zm00001d021570_T002	9.73	12.63	coding	446	237	1574
PB.8735.2	7	1947	10	novel_in_catalog	gene: Zm00001d021570	novel	2.90	12.63	coding	267	698	1498
PB.1299.5	1	1230	3	full-splice_match	gene: Zm00001d033611	Zm00001d033611_T001	0.00	86.53	coding	257	255	1025
PB.9642.1	8	721	3	full-splice_match	gene: Zm00001d011081	Zm00001d011081_T001	131.54	430.15	coding	133	36	434

PB.9642.2	8	607	3	full-splice_match	gene: Zm00001d011081	Zm00001d011081_T001	298.61	430.15	coding	133	36	434
PB.3519.1	2	2309	4	novel_not_in_catalog	gene: Zm00001d007395	novel	9.25	9.25	coding	660	119	2098
PB.5972.1	5	2096	8	full-splice_match	gene: Zm00001d013030	Zm00001d013030_T002	49.75	49.75	coding	534	201	1802
PB.4313.3	3	1068	1	full-splice_match	gene: Zm00001d042768	Zm00001d042768_T001	41.29	62.31	coding	301	34	936
PB.7716.1	6	3882	2	full-splice_match	gene: Zm00001d037672	Zm00001d037672_T001	16.83	16.83	coding	1098	306	3599
PB.3418.1	2	2299	13	full-splice_match	gene: Zm00001d006866	Zm00001d006866_T002	43.96	43.96	coding	505	344	1858
PB.1332.2	1	2019	1	incomplete-splice_match	gene: Zm00001d033775	Zm00001d033775_T001	0.65	40.09	coding	564	327	2018
PB.37.1	1	1679	6	full-splice_match	gene: Zm00001d027359	Zm00001d027359_T001	66.81	66.81	coding	445	136	1470
PB.8287.1	7	1288	8	intergenic	novelGene_71	novel	68.87	141.76	coding	214	254	895
PB.2813.1	2	1307	4	novel_in_catalog	gene: Zm00001d003504	novel	5.12	62.59	coding	107	129	449
PB.3446.1	2	1104	4	full-splice_match	gene: Zm00001d007012	Zm00001d007012_T001	29.74	29.74	coding	268	82	885
PB.2813.2	2	984	6	full-splice_match	gene: Zm00001d003504	Zm00001d003504_T002	57.47	62.59	coding	239	132	848
PB.1229.1	1	2013	6	full-splice_match	gene: Zm00001d033296	Zm00001d033296_T002	22.40	22.40	coding	495	223	1707
PB.5864.1	4	1250	7	full-splice_match	gene: Zm00001d053981	Zm00001d053981_T004	52.08	58.74	coding	255	202	966
PB.9634.2	8	777	3	full-splice_match	gene: Zm00001d011049	Zm00001d011049_T001	51.99	211.98	coding	127	90	470
PB.9634.1	8	874	3	full-splice_match	gene: Zm00001d011049	Zm00001d011049_T001	159.99	211.98	coding	127	32	412
PB.1176.1	1	1479	1	antisense	novelGene_gene: Zm00001d032999_A	novel	71.99	71.99	coding	155	627	1091

PB.3739.1	3	1943	3	incomplete-splice_match	gene: Zm00001d039608	Zm00001d039608_T014	25.65	42.65	coding	440	57	1376
PB.9769.1	8	2359	7	incomplete-splice_match	gene: Zm00001d011700	Zm00001d011700_T013	18.77	24.19	coding	615	234	2078
PB.9769.2	8	3337	13	full-splice_match	gene: Zm00001d011700	Zm00001d011700_T002	5.42	24.19	coding	987	200	3160
PB.3739.2	3	3203	4	full-splice_match	gene: Zm00001d039608	Zm00001d039608_T041	0.00	42.65	coding	925	85	2859
PB.3739.5	3	3979	4	full-splice_match	gene: Zm00001d039608	Zm00001d039608_T041	4.26	42.65	coding	1196	185	3772
PB.3739.7	3	2870	4	full-splice_match	gene: Zm00001d039608	Zm00001d039608_T005	12.75	42.65	coding	897	77	2767
PB.6057.2	5	1803	4	novel_in_catalog	gene: Zm00001d013307	novel	32.15	32.15	coding	395	171	1355
PB.7892.1	6	2694	14	full-splice_match	gene: Zm00001d038290	Zm00001d038290_T004	29.44	29.44	coding	747	163	2403
PB.9536.1	8	1974	2	genic	gene: Zm00001d010640	novel	23.88	26.35	coding	354	531	1592
PB.4705.2	3	2161	9	novel_not_in_catalog	gene: Zm00001d044358	novel	7.05	7.05	coding	377	900	2030
PB.4299.2	3	1126	3	full-splice_match	gene: Zm00001d042699	Zm00001d042699_T001	3.22	13.37	coding	278	33	866
PB.4299.1	3	1253	3	full-splice_match	gene: Zm00001d042699	Zm00001d042699_T001	10.15	13.37	coding	278	32	865
PB.7467.1	6	1148	3	full-splice_match	gene: Zm00001d036443	Zm00001d036443_T002	70.94	85.62	coding	187	57	617
PB.2867.4	2	1663	1	novel_in_catalog	gene: Zm00001d003800	novel	50.62	69.70	coding	145	1085	1519
PB.10359.1	9	1768	10	full-splice_match	gene: Zm00001d046561	Zm00001d046561_T002	36.09	42.18	coding	465	52	1446
PB.10359.2	9	2473	8	novel_in_catalog	gene: Zm00001d046561	novel	6.09	42.18	coding	277	883	1713
PB.6732.1	5	2828	7	full-splice_match	gene: Zm00001d016731	Zm00001d016731_T001	33.37	36.11	coding	792	180	2555

PB.10179.1	9	2465	4	full-splice_match	gene: Zm00001d045445	Zm00001d045445_T001	4.64	4.64	coding	682	101	2146
PB.6732.3	5	2680	7	full-splice_match	gene: Zm00001d016731	Zm00001d016731_T001	0.00	36.11	coding	792	173	2548
PB.6732.2	5	2866	6	novel_in_catalog	gene: Zm00001d016731	novel	2.74	36.11	coding	625	155	2029
PB.7252.1	6	975	1	full-splice_match	gene: Zm00001d035171	Zm00001d035171_T001	26.08	42.88	coding	218	109	762
PB.926.15	1	2926	11	novel_in_catalog	gene: Zm00001d031927	novel	2.94	74.67	coding	102	1474	1779
PB.7252.2	6	822	1	full-splice_match	gene: Zm00001d035171	Zm00001d035171_T001	16.80	42.88	coding	218	111	764
PB.8792.1	7	1940	4	novel_in_catalog	gene: Zm00001d021745	novel	2.38	11.03	coding	233	151	849
PB.926.12	1	2574	12	novel_in_catalog	gene: Zm00001d031927	novel	0.00	74.67	coding	217	1289	1939
PB.926.11	1	2576	11	full-splice_match	gene: Zm00001d031927	Zm00001d031927_T021	18.97	74.67	coding	217	1219	1869
PB.8792.2	7	1420	6	full-splice_match	gene: Zm00001d021745	Zm00001d021745_T001	8.65	11.03	coding	307	148	1068
PB.6836.6	5	2838	2	full-splice_match	gene: Zm00001d017186	Zm00001d017186_T002	0.00	256.78	coding	166	77	574
PB.6800.1	5	1471	8	full-splice_match	gene: Zm00001d017049	Zm00001d017049_T001	24.57	29.94	coding	322	132	1097
PB.6836.4	5	1894	3	full-splice_match	gene: Zm00001d017186	Zm00001d017186_T001	146.54	256.78	coding	446	314	1651
PB.6836.2	5	4633	2	full-splice_match	gene: Zm00001d017186	Zm00001d017186_T002	11.08	256.78	coding	286	3532	4389
PB.7911.8	6	2058	6	full-splice_match	gene: Zm00001d038361	Zm00001d038361_T001	0.00	74.57	coding	117	149	499
PB.5663.1	4	991	1	full-splice_match	gene: Zm00001d053068	Zm00001d053068_T001	160.29	160.29	coding	211	79	711
PB.6836.1	5	2290	3	full-splice_match	gene: Zm00001d017186	Zm00001d017186_T003	99.17	256.78	coding	446	701	2038

PB.7911.5	6	1155	7	full-splice_match	gene: Zm00001d038361	Zm00001d038361_T003	16.11	74.57	coding	268	16	819
PB.6481.1	5	839	1	full-splice_match	gene: Zm00001d015126	Zm00001d015126_T001	13.25	31.69	coding	106	181	498
PB.6481.2	5	693	1	full-splice_match	gene: Zm00001d015126	Zm00001d015126_T001	18.44	31.69	coding	106	181	498
PB.4085.1	3	409	1	antisense	novelGene_gene: Zm00001d041710_A	novel	24.10	24.10	non_coding	NA	NA	NA
PB.7911.1	6	1707	5	novel_in_catalog	gene: Zm00001d038361	novel	0.26	74.57	coding	167	477	977
PB.2424.1	2	1827	10	full-splice_match	gene: Zm00001d001895	Zm00001d001895_T004	85.75	150.50	coding	461	117	1499
PB.7911.3	6	1589	7	full-splice_match	gene: Zm00001d038361	Zm00001d038361_T002	0.00	74.57	coding	270	130	939
PB.2431.2	2	2079	11	full-splice_match	gene: Zm00001d001914	Zm00001d001914_T003	14.82	14.82	coding	115	95	439
PB.53.1	1	2297	2	full-splice_match	gene: Zm00001d027428	Zm00001d027428_T001	18.63	18.63	coding	669	65	2071
PB.9917.1	8	880	4	full-splice_match	gene: Zm00001d012306	Zm00001d012306_T001	61.29	61.29	coding	145	187	621
PB.5359.2	4	2315	3	full-splice_match	gene: Zm00001d051838	Zm00001d051838_T001	0.00	45.52	coding	649	150	2096
PB.10388.3	9	2980	5	incomplete-splice_match	gene: Zm00001d046717	Zm00001d046717_T011	5.15	18.10	coding	738	214	2427
PB.2290.1	10	1621	9	incomplete-splice_match	gene: Zm00001d026398	Zm00001d026398_T007	173.81	173.81	coding	377	67	1197
PB.3422.1	2	2077	8	full-splice_match	gene: Zm00001d006884	Zm00001d006884_T002	16.37	16.37	coding	529	101	1687
PB.3008.2	2	1875	10	full-splice_match	gene: Zm00001d004779	Zm00001d004779_T001	0.00	10.02	coding	491	88	1560
PB.3008.1	2	2068	10	full-splice_match	gene: Zm00001d004779	Zm00001d004779_T001	10.02	10.02	coding	491	105	1577
PB.9660.4	8	2305	23	full-splice_match	gene: Zm00001d011177	Zm00001d011177_T002	1.58	30.95	coding	601	335	2137

PB.9660.1	8	2442	23	full-splice_match	gene: Zm00001d011177	Zm00001d011177_T002	29.38	30.95	coding	601	321	2123
PB.6733.1	5	1097	7	novel_in_catalog	gene: Zm00001d016736	novel	106.57	106.57	coding	260	97	876
PB.8273.2	7	1313	9	incomplete-splice_match	gene: Zm00001d019172	Zm00001d019172_T001	0.00	47.72	coding	343	43	1071
PB.10510.2	9	1956	9	full-splice_match	gene: Zm00001d047434	Zm00001d047434_T006	6.95	15.60	coding	451	383	1735
PB.10510.1	9	1960	8	novel_in_catalog	gene: Zm00001d047434	novel	8.65	15.60	coding	279	383	1219
PB.4406.1	3	1490	11	full-splice_match	gene: Zm00001d043182	Zm00001d043182_T001	24.94	24.94	coding	404	26	1237
PB.4494.2	3	1791	4	novel_not_in_catalog	gene: Zm00001d043510	novel	19.39	31.56	coding	414	306	1547
PB.1319.1	1	1374	9	full-splice_match	gene: Zm00001d033684	Zm00001d033684_T002	47.89	47.89	coding	282	141	986
PB.9661.1	8	1189	6	full-splice_match	gene: Zm00001d011178	Zm00001d011178_T001	11.34	11.34	coding	279	126	962
PB.8773.9	7	2487	5	novel_not_in_catalog	gene: Zm00001d021698	novel	6.54	192.02	coding	462	137	1522
PB.9261.1	8	2134	1	full-splice_match	gene: Zm00001d009160	Zm00001d009160_T001	19.86	117.37	coding	533	51	1649
PB.10280.1	9	2457	9	novel_in_catalog	gene: Zm00001d046020	novel	8.14	18.65	coding	313	145	1083
PB.9261.3	8	1890	1	full-splice_match	gene: Zm00001d009160	Zm00001d009160_T001	64.31	117.37	coding	533	134	1732
PB.9261.2	8	1793	1	full-splice_match	gene: Zm00001d009160	Zm00001d009160_T001	33.20	117.37	coding	426	178	1455
PB.10191.2	9	2475	1	novel_in_catalog	gene: Zm00001d045490	novel	1.29	12.88	coding	604	433	2244
PB.10191.3	9	2274	2	full-splice_match	gene: Zm00001d045490	Zm00001d045490_T001	0.00	12.88	coding	694	83	2164
PB.10191.1	9	2493	2	full-splice_match	gene: Zm00001d045490	Zm00001d045490_T001	11.58	12.88	coding	694	69	2150

PB.9261.8	8	1560	1	full-splice_match	gene: Zm00001d009160	Zm00001d009160_T001	0.00	117.37	coding	500	12	1511
PB.10530.1	9	635	1	full-splice_match	gene: Zm00001d047499	Zm00001d047499_T001	54.75	54.75	coding	134	104	505
PB.10826.2	9	948	1	full-splice_match	gene: Zm00001d048533	Zm00001d048533_T001	44.64	44.64	coding	136	233	640
PB.5821.1	4	1794	2	full-splice_match	gene: Zm00001d053838	Zm00001d053838_T003	1005.69	1005.69	coding	533	83	1681
PB.572.1	1	1727	1	genic	gene: Zm00001d029764	novel	1.29	1.29	coding	124	706	1077
PB.1627.3	1	1651	7	full-splice_match	gene: Zm00001d034885	Zm00001d034885_T002	58.10	117.75	coding	416	203	1450
PB.1627.2	1	1775	7	full-splice_match	gene: Zm00001d034885	Zm00001d034885_T002	39.63	117.75	coding	416	288	1535
PB.1627.1	1	2180	7	full-splice_match	gene: Zm00001d034885	Zm00001d034885_T002	20.02	117.75	coding	416	204	1451
PB.3354.2	2	1207	3	novel_in_catalog	gene: Zm00001d006570	novel	0.56	57.89	coding	99	682	978
PB.1877.2	10	2783	10	full-splice_match	gene: Zm00001d024512	Zm00001d024512_T011	7.01	24.73	coding	407	1438	2658
PB.8773.2	7	2512	6	novel_not_in_catalog	gene: Zm00001d021698	novel	9.73	192.02	coding	535	130	1734
PB.5673.2	4	2312	2	full-splice_match	gene: Zm00001d053119	Zm00001d053119_T001	34.24	48.27	coding	679	176	2212
PB.1877.1	10	1519	7	incomplete-splice_match	gene: Zm00001d024512	Zm00001d024512_T006	17.72	24.73	coding	417	135	1385
PB.5640.1	4	2130	1	full-splice_match	gene: Zm00001d052947	Zm00001d052947_T001	3.72	3.72	coding	623	15	1883
PB.2419.2	2	775	1	full-splice_match	gene: Zm00001d001865	Zm00001d001865_T001	24.13	28.76	coding	156	73	540
PB.2419.1	2	972	1	full-splice_match	gene: Zm00001d001865	Zm00001d001865_T001	4.63	28.76	coding	156	73	540
PB.2011.3	10	1549	14	incomplete-splice_match	gene: Zm00001d025100	Zm00001d025100_T008	0.00	88.80	coding	462	164	1549

PB.8773.1	7	2422	7	novel_not_in_catalog	gene: Zm00001d021698	novel	168.98	192.02	coding	677	134	2164
PB.7069.1	5	1253	10	full-splice_match	gene: Zm00001d018146	Zm00001d018146_T007	11.96	49.87	coding	348	41	1084
PB.9052.1	7	1850	4	full-splice_match	gene: Zm00001d022627	Zm00001d022627_T003	11.50	11.50	coding	485	104	1558
PB.7349.1	6	913	3	novel_not_in_catalog	gene: Zm00001d035702	novel	312.50	312.50	coding	166	121	618
PB.7349.2	6	776	3	novel_not_in_catalog	gene: Zm00001d035702	novel	0.00	312.50	coding	166	95	592
PB.8773.7	7	1095	1	intergenic	novelGene_262	novel	30.27	30.27	non_coding	NA	NA	NA
PB.5122.1	4	739	3	incomplete-splice_match	gene: Zm00001d050316	Zm00001d050316_T041	30.10	106.47	coding	122	372	737
PB.6041.1	5	1062	3	full-splice_match	gene: Zm00001d013263	Zm00001d013263_T001	124.07	124.07	coding	217	113	763
PB.201.1	1	1893	2	full-splice_match	gene: Zm00001d028144	Zm00001d028144_T001	1.73	9.82	coding	525	70	1644
PB.3327.1	2	2680	3	incomplete-splice_match	gene: Zm00001d006453	Zm00001d006453_T004	5.92	5.92	coding	114	209	550
PB.8028.3	6	1994	9	novel_in_catalog	gene: Zm00001d038830	novel	1.17	15.67	coding	454	144	1505
PB.201.2	1	1748	2	full-splice_match	gene: Zm00001d028144	Zm00001d028144_T001	8.09	9.82	coding	525	73	1647
PB.9108.1	8	1498	8	full-splice_match	gene: Zm00001d008406	Zm00001d008406_T001	74.23	95.60	coding	372	95	1210
PB.2852.1	2	1424	6	full-splice_match	gene: Zm00001d003680	Zm00001d003680_T002	236.87	236.87	coding	389	60	1226
PB.9108.3	8	1427	7	novel_in_catalog	gene: Zm00001d008406	novel	3.62	95.60	coding	213	650	1288
PB.9108.2	8	1575	7	novel_in_catalog	gene: Zm00001d008406	novel	1.46	95.60	coding	178	754	1287
PB.10861.2	B73V4 ctg142	760	6	full-splice_match	gene: Zm00001d000120	Zm00001d000120_T002	93.44	174.32	coding	151	42	494

PB.10861.3	B73V4 ctg142	633	6	full-splice_match	gene: Zm00001d000120	Zm00001d000120_T002	80.88	174.32	coding	151	39	491
PB.4422.1	3	828	1	antisense	novelGene_gene: Zm00001d043240_A	novel	82.66	82.66	coding	108	394	717
PB.6342.1	5	1483	1	full-splice_match	gene: Zm00001d014612	Zm00001d014612_T001	7.26	7.26	coding	397	128	1318
PB.1422.2	1	1259	1	full-splice_match	gene: Zm00001d034145	Zm00001d034145_T001	0.00	92.99	coding	278	114	947
PB.3359.2	2	1312	5	full-splice_match	gene: Zm00001d006588	Zm00001d006588_T001	17.84	17.84	coding	284	126	977
PB.8822.1	7	1381	1	full-splice_match	gene: Zm00001d021836	Zm00001d021836_T001	81.38	81.38	coding	332	52	1047
PB.7767.1	6	1736	9	full-splice_match	gene: Zm00001d037871	Zm00001d037871_T005	49.55	49.55	coding	456	95	1462
PB.1375.7	1	3107	10	novel_not_in_catalog	gene: Zm00001d033948	novel	0.00	50.33	coding	115	193	537
PB.10574.1	9	832	1	genic	gene: Zm00001d047676	novel	24.32	75.13	coding	187	54	614
PB.7815.2	6	643	3	full-splice_match	gene: Zm00001d038038	Zm00001d038038_T001	10.48	16.32	coding	152	85	540
PB.441.1	1	2146	7	full-splice_match	gene: Zm00001d029143	Zm00001d029143_T003	18.72	18.72	coding	547	161	1801
PB.1375.6	1	2321	10	novel_not_in_catalog	gene: Zm00001d033948	novel	6.61	50.33	coding	115	152	496
PB.832.1	1	697	1	full-splice_match	gene: Zm00001d031431	Zm00001d031431_T001	60.94	60.94	coding	120	122	481
PB.5835.1	4	2380	5	full-splice_match	gene: Zm00001d053890	Zm00001d053890_T003	5.92	12.65	coding	482	630	2075
PB.5835.2	4	2094	6	full-splice_match	gene: Zm00001d053890	Zm00001d053890_T008	5.60	12.65	coding	482	500	1945
PB.5835.4	4	2423	5	full-splice_match	gene: Zm00001d053890	Zm00001d053890_T003	1.14	12.65	coding	482	825	2270
PB.5381.3	4	1330	1	novel_in_catalog	gene: Zm00001d051896	novel	26.72	226.58	coding	275	352	1176

PB.3950.1	3	979	1	full-splice_match	gene: Zm00001d040783	Zm00001d040783_T001	39.65	39.65	coding	167	67	567
PB.6950.2	5	1212	1	full-splice_match	gene: Zm00001d017696	Zm00001d017696_T001	0.00	544.08	coding	337	176	1186
PB.6950.1	5	1414	1	full-splice_match	gene: Zm00001d017696	Zm00001d017696_T001	544.08	544.08	coding	337	174	1184
PB.10455.2	9	620	1	full-splice_match	gene: Zm00001d047116	Zm00001d047116_T001	432.28	527.62	coding	126	89	466
PB.2083.1	10	2185	4	novel_in_catalog	gene: Zm00001d025539	novel	0.00	41.72	coding	194	423	1004
PB.3594.2	2	2423	1	full-splice_match	gene: Zm00001d007823	Zm00001d007823_T001	26.81	55.81	coding	709	115	2241
PB.10824.3	9	1877	12	novel_in_catalog	gene: Zm00001d048529	novel	0.00	137.46	coding	399	150	1346
PB.1241.1	1	1694	7	full-splice_match	gene: Zm00001d033365	Zm00001d033365_T002	7.20	23.35	coding	372	124	1239
PB.1895.1	10	1292	1	full-splice_match	gene: Zm00001d024574	Zm00001d024574_T001	4.65	4.65	coding	351	76	1128
PB.209.1	1	1470	6	novel_not_in_catalog	gene: Zm00001d028185	novel	9.03	10.75	coding	283	457	1305
PB.3091.2	2	1417	6	full-splice_match	gene: Zm00001d005271	Zm00001d005271_T002	0.00	79.82	coding	357	120	1190
PB.209.2	1	1238	6	novel_not_in_catalog	gene: Zm00001d028185	novel	1.72	10.75	coding	272	302	1117
PB.4004.1	3	914	8	incomplete-splice_match	gene: Zm00001d041268	Zm00001d041268_T004	32.14	32.14	coding	114	383	724
PB.5318.3	4	1614	3	incomplete-splice_match	gene: Zm00001d051600	Zm00001d051600_T029	114.40	171.13	coding	499	16	1512
PB.8441.2	7	1782	3	full-splice_match	gene: Zm00001d020281	Zm00001d020281_T001	7.89	11.56	coding	509	210	1736
PB.9549.1	8	933	1	full-splice_match	gene: Zm00001d010689	Zm00001d010689_T001	2.68	17.76	non_coding	NA	NA	NA
PB.9549.2	8	1898	1	full-splice_match	gene: Zm00001d010689	Zm00001d010689_T001	15.08	17.76	coding	480	9	1448

PB.2393.1	2	3585	3	genic	gene: Zm00001d001785	novel	15.89	15.89	coding	1012	320	3355
PB.924.1	1	1691	4	full-splice_match	gene: Zm00001d031913	Zm00001d031913_T001	74.61	74.61	coding	393	131	1309
PB.4739.1	3	3607	21	full-splice_match	gene: Zm00001d044498	Zm00001d044498_T007	16.54	17.19	coding	1024	67	3138
PB.924.3	1	1419	4	full-splice_match	gene: Zm00001d031913	Zm00001d031913_T004	0.00	74.61	coding	393	135	1313
PB.4224.2	3	1633	4	full-splice_match	gene: Zm00001d042350	Zm00001d042350_T002	17.57	46.82	coding	234	714	1415
PB.8337.11	7	1230	3	full-splice_match	gene: Zm00001d019563	Zm00001d019563_T006	1171.29	2290.28	coding	290	94	963
PB.8337.13	7	2965	2	novel_in_catalog	gene: Zm00001d019563	novel	2.56	2290.28	coding	247	92	832
PB.586.1	1	2596	1	full-splice_match	gene: Zm00001d029855	Zm00001d029855_T001	47.73	47.73	coding	645	276	2210
PB.8337.16	7	3023	1	novel_in_catalog	gene: Zm00001d019563	novel	0.00	2290.28	coding	247	92	832
PB.8337.17	7	2346	1	novel_in_catalog	gene: Zm00001d019563	novel	0.00	2290.28	coding	247	91	831
PB.8337.18	7	1347	1	novel_in_catalog	gene: Zm00001d019563	novel	0.00	2290.28	coding	247	91	831
PB.865.2	1	780	7	full-splice_match	gene: Zm00001d031611	Zm00001d031611_T001	220.40	509.56	coding	171	74	586
PB.9504.3	8	1423	5	novel_in_catalog	gene: Zm00001d010478	novel	3.91	86.63	coding	162	155	640
PB.8097.5	6	1292	3	incomplete-splice_match	gene: Zm00001d039072	Zm00001d039072_T034	0.00	11.70	coding	267	490	1290
PB.1324.2	1	870	4	full-splice_match	gene: Zm00001d033707	Zm00001d033707_T001	60.42	416.69	coding	148	59	502
PB.1881.1	10	997	8	full-splice_match	gene: Zm00001d024519	Zm00001d024519_T015	65.29	82.59	coding	169	251	757
PB.8625.2	7	1916	13	novel_not_in_catalog	gene: Zm00001d021132	novel	2.69	45.44	coding	221	1008	1670

PB.8814.2	7	2398	5	full-splice_match	gene: Zm00001d021823	Zm00001d021823_T002	10.37	38.84	coding	614	78	1919
PB.8814.5	7	2311	5	full-splice_match	gene: Zm00001d021823	Zm00001d021823_T002	0.42	38.84	coding	614	109	1950
PB.8814.6	7	1879	5	full-splice_match	gene: Zm00001d021823	Zm00001d021823_T002	0.00	38.84	coding	600	80	1879
PB.1314.1	1	3220	10	incomplete-splice_match	gene: Zm00001d033657	Zm00001d033657_T001	6.09	56.34	coding	135	2041	2445
PB.8113.1	6	1624	6	full-splice_match	gene: Zm00001d039142	Zm00001d039142_T001	25.98	25.98	coding	407	117	1337
PB.6904.1	5	1360	5	full-splice_match	gene: Zm00001d017482	Zm00001d017482_T001	47.03	65.68	coding	303	124	1032
PB.6904.2	5	1104	4	novel_in_catalog	gene: Zm00001d017482	novel	18.65	65.68	coding	294	11	892
PB.7621.2	6	1159	7	novel_in_catalog	gene: Zm00001d037240	novel	7.79	13.98	coding	262	122	907
PB.3081.3	2	1198	6	full-splice_match	gene: Zm00001d005231	Zm00001d005231_T012	19.02	1988.67	coding	181	361	903
PB.815.1	1	886	4	novel_not_in_catalog	gene: Zm00001d022873	novel	3.76	6.24	non_coding	NA	NA	NA
PB.9541.4	8	508	2	novel_not_in_catalog	gene: Zm00001d010653	novel	10.06	27.04	non_coding	NA	NA	NA
PB.7160.6	5	1997	14	full-splice_match	gene: Zm00001d018440	Zm00001d018440_T002	0.00	67.73	coding	468	432	1835
PB.10673.2	9	1759	5	full-splice_match	gene: Zm00001d047981	Zm00001d047981_T001	14.80	26.65	coding	490	101	1570
PB.9541.1	8	1950	2	novel_not_in_catalog	gene: Zm00001d010653	novel	2.63	27.04	non_coding	NA	NA	NA
PB.9541.2	8	1174	3	novel_not_in_catalog	gene: Zm00001d010653	novel	4.54	27.04	non_coding	NA	NA	NA
PB.9541.3	8	962	2	novel_not_in_catalog	gene: Zm00001d010653	novel	9.82	27.04	non_coding	NA	NA	NA
PB.7049.3	5	3034	25	novel_not_in_catalog	gene: Zm00001d018060	novel	21.68	24.41	coding	541	1180	2802

PB.10357.2	9	928	7	incomplete-splice_match	gene: Zm00001d046549	Zm00001d046549_T001	14.15	37.98	coding	302	10	915
PB.7669.2	6	2104	6	incomplete-splice_match	gene: Zm00001d037448	Zm00001d037448_T013	19.98	30.98	coding	100	1602	1901
PB.7669.1	6	2755	9	full-splice_match	gene: Zm00001d037448	Zm00001d037448_T009	11.00	30.98	coding	100	2302	2601
PB.10357.1	9	3374	10	novel_in_catalog	gene: Zm00001d046549	novel	23.82	37.98	coding	995	42	3026
PB.766.1	1	1427	1	antisense	novelGene_gene: Zm00001d031075_A	novel	11.44	11.44	coding	286	92	949
PB.3644.1	3	782	2	full-splice_match	gene: Zm00001d039273	Zm00001d039273_T001	129.53	129.53	coding	227	55	735
PB.5524.1	4	1727	9	full-splice_match	gene: Zm00001d052444	Zm00001d052444_T003	78.68	78.68	coding	394	340	1521
PB.1370.9	1	2072	5	novel_not_in_catalog	gene: Zm00001d033925	novel	0.00	1241.38	coding	150	190	639
PB.2902.2	2	2875	2	genic	gene: Zm00001d003948	novel	0.00	39.04	coding	736	207	2414
PB.5750.1	4	1601	8	full-splice_match	gene: Zm00001d053576	Zm00001d053576_T002	70.92	74.01	coding	414	78	1319
PB.2902.1	2	3038	2	genic	gene: Zm00001d003948	novel	39.04	39.04	coding	736	262	2469
PB.4283.10	3	1524	5	novel_not_in_catalog	gene: Zm00001d042639	novel	0.62	58.00	coding	186	84	641
PB.9249.11	8	1698	4	novel_not_in_catalog	gene: Zm00001d009054	novel	4.16	23.92	non_coding	NA	NA	NA
PB.10708.1	9	1258	2	genic	gene: Zm00001d048112	novel	44.59	44.59	coding	261	291	1073
PB.3745.1	3	1786	4	novel_not_in_catalog	gene: Zm00001d039637	novel	9.33	9.33	coding	499	238	1734
PB.10284.1	9	597	1	full-splice_match	gene: Zm00001d046055	Zm00001d046055_T001	60.41	60.41	non_coding	NA	NA	NA
PB.4669.1	3	1334	5	full-splice_match	gene: Zm00001d044212	Zm00001d044212_T002	68.11	68.11	coding	174	74	595

PB.4072.1	3	1205	3	intergenic	novelGene_208	novel	5.95	16.68	coding	105	891	1205
PB.1432.2	1	2546	5	full-splice_match	gene: Zm00001d034205	Zm00001d034205_T001	22.09	22.09	coding	709	225	2351
PB.9555.3	8	2562	9	full-splice_match	gene: Zm00001d010710	Zm00001d010710_T004	0.00	21.66	coding	658	313	2286
PB.2375.2	10	2369	15	novel_in_catalog	gene: Zm00001d026677	novel	11.14	258.36	coding	431	769	2061
PB.9555.1	8	2747	9	full-splice_match	gene: Zm00001d010710	Zm00001d010710_T004	20.03	21.66	coding	658	335	2308
PB.9555.6	8	1345	2	incomplete-splice_match	gene: Zm00001d010710	Zm00001d010710_T012	1.62	21.66	coding	38	204	317
PB.6932.1	5	576	2	full-splice_match	gene: Zm00001d017617	Zm00001d017617_T001	126.00	126.00	coding	87	86	346
PB.2980.1	2	807	1	full-splice_match	gene: Zm00001d004611	Zm00001d004611_T001	51.55	51.55	coding	195	25	609
PB.7812.1	6	1676	1	intergenic	novelGene_235	novel	46.42	46.42	non_coding	NA	NA	NA
PB.6229.2	5	1024	5	full-splice_match	gene: Zm00001d014065	Zm00001d014065_T001	127.66	215.45	coding	216	132	779
PB.3081.1	2	1029	7	full-splice_match	gene: Zm00001d005231	Zm00001d005231_T004	457.68	1988.67	coding	181	90	632
PB.2925.1	2	1215	5	full-splice_match	gene: Zm00001d004133	Zm00001d004133_T001	10.33	10.33	coding	323	70	1038
PB.1599.1	1	2919	2	full-splice_match	gene: Zm00001d034807	Zm00001d034807_T005	130.70	130.70	coding	319	127	1083
PB.7200.1	5	1900	8	full-splice_match	gene: Zm00001d018545	Zm00001d018545_T001	26.28	26.28	coding	512	93	1628
PB.7200.2	5	1782	8	full-splice_match	gene: Zm00001d018545	Zm00001d018545_T001	0.00	26.28	coding	512	26	1561
PB.5381.4	4	1187	2	full-splice_match	gene: Zm00001d051896	Zm00001d051896_T001	98.76	226.58	coding	350	32	1081
PB.1318.2	1	2302	13	full-splice_match	gene: Zm00001d033682	Zm00001d033682_T002	21.38	21.38	coding	588	219	1982

PB.8572.1	7	1435	4	full-splice_match	gene: Zm00001d020882	Zm00001d020882_T001	11.82	11.82	coding	396	112	1299
PB.6640.1	5	940	3	novel_in_catalog	gene: Zm00001d016200	novel	406.33	406.33	coding	172	212	727
PB.189.1	1	3424	9	incomplete-splice_match	gene: Zm00001d028096	Zm00001d028096_T016	5.06	5.06	coding	947	80	2920
PB.3853.2	3	1022	4	full-splice_match	gene: Zm00001d040244	Zm00001d040244_T001	103.70	110.63	coding	231	176	868
PB.3100.3	2	2097	10	full-splice_match	gene: Zm00001d005324	Zm00001d005324_T003	5.34	11.40	coding	601	75	1877
PB.3853.1	3	1047	3	novel_in_catalog	gene: Zm00001d040244	novel	6.92	110.63	coding	140	468	887
PB.7681.1	6	967	9	full-splice_match	gene: Zm00001d037509	Zm00001d037509_T001	56.67	59.90	coding	227	52	732
PB.10360.2	9	4169	9	full-splice_match	gene: Zm00001d046569	Zm00001d046569_T003	34.96	34.96	coding	1114	293	3634
PB.7681.2	6	977	8	novel_in_catalog	gene: Zm00001d037509	novel	3.22	59.90	coding	155	367	831
PB.7220.1	6	2484	1	full-splice_match	gene: Zm00001d035035	Zm00001d035035_T001	18.12	18.12	coding	749	39	2285
PB.2939.1	2	1806	1	full-splice_match	gene: Zm00001d004222	Zm00001d004222_T001	7.51	7.51	coding	392	79	1254
PB.7055.1	5	1340	3	novel_not_in_catalog	gene: Zm00001d018097	novel	53.10	56.34	coding	322	106	1071
PB.7055.2	5	1550	2	novel_in_catalog	gene: Zm00001d018097	novel	3.23	56.34	coding	204	673	1284
PB.821.3	1	2414	15	full-splice_match	gene: Zm00001d031316	Zm00001d031316_T009	3.50	15.93	coding	608	252	2075
PB.5519.3	4	1260	2	full-splice_match	gene: Zm00001d052433	Zm00001d052433_T001	15.49	64.61	coding	349	87	1133
PB.5519.2	4	1309	1	novel_in_catalog	gene: Zm00001d052433	novel	10.08	64.61	coding	193	593	1171
PB.5519.1	4	1362	2	full-splice_match	gene: Zm00001d052433	Zm00001d052433_T001	39.05	64.61	coding	349	86	1132

PB.3158.1	2	1655	7	full-splice_match	gene: Zm00001d005674	Zm00001d005674_T001	10.69	10.69	coding	476	122	1549
PB.3590.4	2	1496	11	full-splice_match	gene: Zm00001d007806	Zm00001d007806_T002	14.35	46.08	coding	323	372	1340
PB.1159.1	1	773	1	full-splice_match	gene: Zm00001d032900	Zm00001d032900_T001	27.71	27.71	non_coding	NA	NA	NA
PB.7136.1	5	4152	9	incomplete-splice_match	gene: Zm00001d018382	Zm00001d018382_T036	25.43	25.43	coding	1258	238	4011
PB.2407.2	2	1179	2	novel_in_catalog	gene: Zm00001d001826	novel	2.66	91.92	coding	110	89	418
PB.2407.3	2	808	3	full-splice_match	gene: Zm00001d001826	Zm00001d001826_T002	21.60	91.92	coding	115	279	623
PB.2407.1	2	1017	3	full-splice_match	gene: Zm00001d001826	Zm00001d001826_T002	67.67	91.92	coding	115	361	705
PB.9295.1	8	1055	3	full-splice_match	gene: Zm00001d009398	Zm00001d009398_T003	73.82	73.82	coding	261	112	894
PB.8431.6	7	2764	5	full-splice_match	gene: Zm00001d020227	Zm00001d020227_T032	15.24	43.17	coding	76	86	313
PB.8556.1	7	1398	3	full-splice_match	gene: Zm00001d020808	Zm00001d020808_T002	34.97	34.97	coding	347	158	1198
PB.8431.5	7	3764	4	full-splice_match	gene: Zm00001d020227	Zm00001d020227_T029	5.34	43.17	coding	201	597	1199
PB.3100.1	2	2938	10	full-splice_match	gene: Zm00001d005324	Zm00001d005324_T003	2.35	11.40	coding	601	776	2578
PB.9313.1	8	1232	7	full-splice_match	gene: Zm00001d009482	Zm00001d009482_T001	30.97	30.97	coding	243	310	1038
PB.8431.4	7	4155	3	full-splice_match	gene: Zm00001d020227	Zm00001d020227_T017	3.26	43.17	coding	169	541	1047
PB.5203.1	4	1486	4	full-splice_match	gene: Zm00001d050942	Zm00001d050942_T001	2.09	33.28	coding	295	57	941
PB.5203.3	4	1143	4	full-splice_match	gene: Zm00001d050942	Zm00001d050942_T001	0.00	33.28	coding	295	69	953
PB.5203.2	4	1286	4	full-splice_match	gene: Zm00001d050942	Zm00001d050942_T001	31.20	33.28	coding	295	94	978

PB.7228.2	6	3407	7	novel_in_catalog	gene: Zm00001d035050	novel	0.00	23.24	coding	550	410	2059
PB.9972.2	8	1640	1	genic	gene: Zm00001d012564	novel	4.17	127.84	coding	308	321	1244
PB.8923.1	7	1773	1	intergenic	novelGene_285	novel	50.19	50.19	coding	500	240	1739
PB.7228.1	6	3120	8	full-splice_match	gene: Zm00001d035050	Zm00001d035050_T007	20.31	23.24	coding	753	484	2742
PB.1016.1	1	1015	3	incomplete-splice_match	gene: Zm00001d032333	Zm00001d032333_T005	13.07	13.07	coding	14	270	311
PB.2931.1	2	2801	13	fusion	gene: Zm00001d004153_g	novel	13.52	13.52	coding	832	59	2554
PB.7228.4	6	2985	4	incomplete-splice_match	gene: Zm00001d035050	Zm00001d035050_T010	2.92	23.24	coding	221	70	732
PB.2243.3	10	1623	5	novel_not_in_catalog	gene: Zm00001d026269	novel	96.28	96.28	coding	318	331	1284
PB.1096.5	1	1656	11	novel_not_in_catalog	gene: Zm00001d032608	novel	2.27	78.90	coding	224	846	1517
PB.4050.1	3	403	3	full-splice_match	gene: Zm00001d000603	Zm00001d000603_T001	137.37	137.37	non_coding	NA	NA	NA
PB.1096.4	1	1523	12	novel_not_in_catalog	gene: Zm00001d032608	novel	6.55	78.90	coding	224	714	1385
PB.392.1	1	1034	1	full-splice_match	gene: Zm00001d028953	Zm00001d028953_T001	33.92	33.92	coding	201	139	741
PB.9604.1	8	645	6	incomplete-splice_match	gene: Zm00001d010908	Zm00001d010908_T001	21.91	21.91	coding	143	99	527
PB.195.1	1	1028	6	full-splice_match	gene: Zm00001d028117	Zm00001d028117_T003	643.17	643.17	coding	260	76	855
PB.5047.2	4	657	2	full-splice_match	gene: Zm00001d049826	Zm00001d049826_T001	348.49	922.03	coding	124	78	449
PB.5047.1	4	760	2	full-splice_match	gene: Zm00001d049826	Zm00001d049826_T001	573.54	922.03	coding	124	78	449
PB.6005.8	5	1927	3	novel_not_in_catalog	gene: Zm00001d013154	novel	0.00	40.66	coding	513	388	1926

PB.8764.1	7	1474	2	novel_not_in_catalog	gene: Zm00001d021666	novel	0.17	0.17	coding	357	16	1086
PB.9911.1	8	1028	1	full-splice_match	gene: Zm00001d012279	Zm00001d012279_T001	59.62	59.62	coding	219	74	730
PB.2896.1	2	1819	4	full-splice_match	gene: Zm00001d003931	Zm00001d003931_T001	59.92	59.92	coding	451	186	1538
PB.5063.1	4	944	6	full-splice_match	gene: Zm00001d049945	Zm00001d049945_T004	1338.79	1338.79	coding	215	92	736
PB.1974.1	10	2686	4	full-splice_match	gene: Zm00001d024937	Zm00001d024937_T001	5.87	5.87	coding	713	413	2551
PB.560.1	1	2515	5	full-splice_match	gene: Zm00001d029688	Zm00001d029688_T003	1.14	43.53	coding	526	63	1640
PB.4974.1	4	1210	9	full-splice_match	gene: Zm00001d049499	Zm00001d049499_T001	18.86	18.86	coding	328	71	1054
PB.9188.1	8	1314	8	full-splice_match	gene: Zm00001d008762	Zm00001d008762_T007	113.21	113.21	coding	326	49	1026
PB.3993.2	3	2535	1	genic	gene: Zm00001d041203	novel	5.54	5.54	coding	185	46	600
PB.1481.1	1	670	2	full-splice_match	gene: Zm00001d034407	Zm00001d034407_T001	90.30	90.30	coding	104	105	416
PB.1096.3	1	1786	10	novel_not_in_catalog	gene: Zm00001d032608	novel	3.92	78.90	coding	142	52	477
PB.5717.2	4	2847	6	full-splice_match	gene: Zm00001d053379	Zm00001d053379_T001	11.05	38.70	coding	671	532	2544
PB.8807.1	7	4110	18	full-splice_match	gene: Zm00001d021806	Zm00001d021806_T055	14.74	73.05	coding	1176	224	3751
PB.2220.1	10	1264	4	full-splice_match	gene: Zm00001d026190	Zm00001d026190_T001	41.35	41.35	coding	208	195	818
PB.244.11	1	1479	1	intergenic	novelGene_85	novel	0.00	94.41	non_coding	NA	NA	NA
PB.6920.1	5	3015	1	full-splice_match	gene: Zm00001d017528	Zm00001d017528_T001	20.70	32.46	coding	534	980	2581
PB.8484.3	7	1404	1	full-splice_match	gene: Zm00001d020470	Zm00001d020470_T001	0.00	17.56	coding	421	73	1335

PB.8138.1	6	3115	22	full-splice_match	gene: Zm00001d039214	Zm00001d039214_T011	8.58	23.75	coding	560	115	1794
PB.1020.2	1	1096	7	full-splice_match	gene: Zm00001d032350	Zm00001d032350_T004	58.05	135.32	coding	196	166	753
PB.380.1	1	1635	8	incomplete-splice_match	gene: Zm00001d028907	Zm00001d028907_T019	14.53	14.53	coding	520	34	1593
PB.8431.9	7	3501	5	full-splice_match	gene: Zm00001d020227	Zm00001d020227_T020	0.00	43.17	coding	201	543	1145
PB.3081.4	2	1077	6	full-splice_match	gene: Zm00001d005231	Zm00001d005231_T012	0.00	1988.67	coding	181	360	902
PB.1664.1	10	1462	6	full-splice_match	gene: Zm00001d023271	Zm00001d023271_T002	7.39	7.39	coding	337	99	1109
PB.6461.2	5	1127	7	full-splice_match	gene: Zm00001d015036	Zm00001d015036_T005	21.81	72.77	coding	197	251	841
PB.6461.1	5	1376	7	full-splice_match	gene: Zm00001d015036	Zm00001d015036_T005	50.96	72.77	coding	197	396	986
PB.1125.2	1	2288	3	full-splice_match	gene: Zm00001d032744	Zm00001d032744_T001	0.94	8.58	coding	596	383	2170
PB.4058.1	3	3311	10	incomplete-splice_match	gene: Zm00001d041582	Zm00001d041582_T004	25.10	25.10	coding	905	324	3038
PB.1125.1	1	2184	3	full-splice_match	gene: Zm00001d032744	Zm00001d032744_T001	7.64	8.58	coding	596	85	1872
PB.9428.2	8	618	1	incomplete-splice_match	gene: Zm00001d010115	Zm00001d010115_T003	1.01	18.46	non_coding	NA	NA	NA
PB.9428.1	8	2030	8	full-splice_match	gene: Zm00001d010115	Zm00001d010115_T001	17.45	18.46	coding	516	360	1907
PB.6230.4	5	2108	17	full-splice_match	gene: Zm00001d014090	Zm00001d014090_T002	0.00	83.70	coding	577	163	1893
PB.6230.5	5	1473	13	incomplete-splice_match	gene: Zm00001d014090	Zm00001d014090_T001	0.00	83.70	coding	426	195	1472
PB.6230.2	5	2335	17	full-splice_match	gene: Zm00001d014090	Zm00001d014090_T002	2.34	83.70	coding	577	171	1901
PB.6230.3	5	2258	17	full-splice_match	gene: Zm00001d014090	Zm00001d014090_T002	81.36	83.70	coding	577	198	1928

PB.9919.1	8	1943	17	full-splice_match	gene: Zm00001d012320	Zm00001d012320_T002	3.78	3.78	coding	490	78	1547
PB.1096.8	1	1567	12	novel_not_in_catalog	gene: Zm00001d032608	novel	1.97	78.90	coding	224	760	1431
PB.432.1	1	1611	10	full-splice_match	gene: Zm00001d029121	Zm00001d029121_T005	29.52	29.52	coding	339	119	1135
PB.9134.1	8	1482	1	full-splice_match	gene: zma-MIR159a	zma-mir159a	22.93	22.93	coding	176	728	1255
PB.8978.1	7	2316	9	incomplete-splice_match	gene: Zm00001d022405	Zm00001d022405_T030	14.56	28.61	coding	363	778	1866
PB.4558.1	3	2983	3	novel_not_in_catalog	gene: Zm00001d043743	novel	6.73	8.42	coding	700	431	2530
PB.4558.3	3	2395	3	novel_not_in_catalog	gene: Zm00001d043743	novel	1.69	8.42	coding	700	154	2253
PB.9147.1	8	1858	8	full-splice_match	gene: Zm00001d008596	Zm00001d008596_T002	181.83	181.83	coding	435	278	1582
PB.8664.1	7	3192	17	full-splice_match	gene: Zm00001d021301	Zm00001d021301_T001	60.17	60.17	coding	885	111	2765
PB.8183.1	7	2425	11	novel_in_catalog	gene: Zm00001d018733	novel	23.88	23.88	coding	102	786	1091
PB.5416.4	4	2279	11	full-splice_match	gene: Zm00001d052057	Zm00001d052057_T005	0.00	30.97	coding	201	1557	2159
PB.6155.5	5	1680	6	full-splice_match	gene: Zm00001d013669	Zm00001d013669_T005	379.35	1235.68	coding	419	128	1384
PB.5416.1	4	2395	11	full-splice_match	gene: Zm00001d052057	Zm00001d052057_T005	28.86	30.97	coding	497	654	2144
PB.6155.1	5	1775	6	full-splice_match	gene: Zm00001d013669	Zm00001d013669_T005	856.32	1235.68	coding	419	164	1420
PB.6088.1	5	885	1	incomplete-splice_match	gene: Zm00001d013419	Zm00001d013419_T001	38.33	38.33	coding	174	58	579
PB.9766.2	8	1569	3	full-splice_match	gene: Zm00001d011687	Zm00001d011687_T001	5.88	11.06	coding	443	106	1434
PB.9681.2	8	1860	14	full-splice_match	gene: Zm00001d011282	Zm00001d011282_T001	44.60	54.97	coding	343	407	1435

PB.9681.1	8	2231	13	novel_in_catalog	gene: Zm00001d011282	novel	10.37	54.97	coding	238	99	812
PB.388.2	1	812	1	full-splice_match	gene: Zm00001d022729	Zm00001d022729_T001	0.41	0.41	coding	100	341	640
PB.7047.1	5	1211	2	full-splice_match	gene: Zm00001d018056	Zm00001d018056_T001	21.02	21.02	non_coding	NA	NA	NA
PB.9420.1	8	1160	8	full-splice_match	gene: Zm00001d010055	Zm00001d010055_T003	101.56	101.56	coding	209	208	834
PB.3983.1	3	2498	8	full-splice_match	gene: Zm00001d041119	Zm00001d041119_T001	68.23	68.23	coding	703	116	2224
PB.8807.4	7	2727	13	incomplete-splice_match	gene: Zm00001d021806	Zm00001d021806_T070	22.06	73.05	coding	833	2	2500
PB.4765.7	3	1722	1	novel_in_catalog	gene: Zm00001d044604	novel	5.94	337.61	coding	221	818	1480
PB.6149.1	5	2016	7	novel_not_in_catalog	gene: Zm00001d013648	novel	40.85	46.46	coding	86	351	608
PB.6149.2	5	1314	6	novel_not_in_catalog	gene: Zm00001d013648	novel	5.60	46.46	coding	86	384	641
PB.3354.1	2	1304	3	novel_in_catalog	gene: Zm00001d006570	novel	12.35	57.89	coding	99	678	974
PB.6981.4	5	2406	17	full-splice_match	gene: Zm00001d017830	Zm00001d017830_T015	42.81	42.81	coding	480	217	1656
PB.7704.1	6	1431	4	full-splice_match	gene: Zm00001d037616	Zm00001d037616_T001	9.68	9.68	coding	371	14	1126
PB.9499.1	8	1039	3	novel_in_catalog	gene: Zm00001d010452	novel	17.75	17.75	coding	195	7	591
PB.4131.2	3	1010	4	full-splice_match	gene: Zm00001d041954	Zm00001d041954_T005	5.78	43.53	coding	240	77	796
PB.8950.3	7	1482	7	incomplete-splice_match	gene: Zm00001d022321	Zm00001d022321_T013	9.00	26.63	coding	152	28	483
PB.4131.1	3	1650	6	full-splice_match	gene: Zm00001d041954	Zm00001d041954_T004	37.75	43.53	coding	362	256	1341
PB.4517.2	3	1764	9	full-splice_match	gene: Zm00001d043607	Zm00001d043607_T001	0.00	48.03	coding	504	91	1602

PB.7972.6	6	3023	7	full-splice_match	gene: Zm00001d038587	Zm00001d038587_T025	2.45	25.48	coding	593	186	1964
PB.4517.1	3	1884	9	full-splice_match	gene: Zm00001d043607	Zm00001d043607_T001	48.03	48.03	coding	504	106	1617
PB.6582.2	5	1710	7	full-splice_match	gene: Zm00001d015780	Zm00001d015780_T006	15.18	27.49	coding	388	349	1512
PB.6582.3	5	1582	1	novel_in_catalog	gene: Zm00001d015780	novel	1.54	27.49	non_coding	NA	NA	NA
PB.6582.1	5	1943	7	full-splice_match	gene: Zm00001d015780	Zm00001d015780_T006	10.77	27.49	coding	388	317	1480
PB.7742.1	6	2060	1	genic	gene: Zm00001d037768	novel	11.44	11.44	coding	206	610	1227
PB.683.1	1	1756	2	full-splice_match	gene: Zm00001d030542	Zm00001d030542_T001	5.95	5.95	coding	441	129	1451
PB.2124.1	10	1235	8	full-splice_match	gene: Zm00001d025745	Zm00001d025745_T001	38.89	38.89	coding	259	211	987
PB.3599.1	2	1041	1	full-splice_match	gene: Zm00001d007831	Zm00001d007831_T001	112.85	670.00	coding	181	69	611
PB.3599.3	2	826	1	full-splice_match	gene: Zm00001d007831	Zm00001d007831_T001	542.75	670.00	coding	181	74	616
PB.10788.1	9	1361	4	full-splice_match	gene: Zm00001d048411	Zm00001d048411_T001	34.20	34.20	coding	350	89	1138
PB.2354.1	10	2142	3	full-splice_match	gene: Zm00001d026619	Zm00001d026619_T001	31.69	170.74	coding	515	113	1657
PB.6403.1	5	2929	14	novel_not_in_catalog	gene: Zm00001d014844	novel	11.72	28.87	coding	821	175	2637
PB.764.2	1	3001	3	novel_not_in_catalog	gene: Zm00001d031073	novel	18.12	18.12	coding	735	446	2650
PB.187.2	1	2312	5	incomplete-splice_match	gene: Zm00001d028093	Zm00001d028093_T001	10.05	10.05	coding	696	19	2106
PB.9532.1	8	1577	4	full-splice_match	gene: Zm00001d010615	Zm00001d010615_T001	31.95	31.95	coding	357	200	1270
PB.1517.1	1	1025	11	novel_not_in_catalog	gene: Zm00001d034524	novel	33.51	33.51	coding	246	33	770

PB.6761.1	5	3366	6	novel_in_catalog	gene: Zm00001d016855	novel	53.53	87.40	coding	463	1819	3207
PB.6761.3	5	4091	6	novel_in_catalog	gene: Zm00001d016855	novel	33.87	87.40	coding	309	243	1169
PB.10231.4	9	944	2	intergenic	novelGene_90	novel	3.75	3.75	coding	64	142	333
PB.558.1	1	2335	10	full-splice_match	gene: Zm00001d029684	Zm00001d029684_T001	9.89	9.89	coding	656	33	2000
PB.6536.1	5	2286	19	fusion	gene: Zm00001d015426_g	novel	118.22	118.22	coding	621	115	1977
PB.2334.2	10	1888	4	novel_in_catalog	gene: Zm00001d026577	novel	2.12	78.80	coding	494	101	1582
PB.2626.1	2	1642	6	full-splice_match	gene: Zm00001d002739	Zm00001d002739_T004	41.98	41.98	coding	438	98	1411
PB.2334.4	10	1574	5	full-splice_match	gene: Zm00001d026577	Zm00001d026577_T001	0.00	78.80	coding	470	99	1508
PB.6620.1	5	1052	3	full-splice_match	gene: Zm00001d016098	Zm00001d016098_T002	6.59	6.59	coding	301	63	965
PB.246.11	1	2349	9	incomplete-splice_match	gene: Zm00001d028330	Zm00001d028330_T171	4.49	86.01	coding	123	364	732
PB.246.12	1	2197	3	full-splice_match	gene: Zm00001d028330	Zm00001d028330_T240	10.28	86.01	coding	123	411	779
PB.3475.1	2	1257	9	full-splice_match	gene: Zm00001d007145	Zm00001d007145_T001	134.83	134.83	coding	260	210	989
PB.883.1	1	1647	9	incomplete-splice_match	gene: Zm00001d031678	Zm00001d031678_T004	10.30	10.30	coding	407	121	1341
PB.8080.2	6	1965	7	full-splice_match	gene: Zm00001d038991	Zm00001d038991_T001	71.15	78.74	coding	473	317	1735
PB.10256.1	9	2225	10	full-splice_match	gene: Zm00001d045864	Zm00001d045864_T002	30.67	30.67	coding	592	190	1965
PB.9367.2	8	1239	4	novel_in_catalog	gene: Zm00001d009762	novel	2.65	140.31	coding	299	36	932
PB.9367.3	8	1182	5	full-splice_match	gene: Zm00001d009762	Zm00001d009762_T001	0.00	140.31	coding	297	98	988

PB.9367.1	8	1282	5	full-splice_match	gene: Zm00001d009762	Zm00001d009762_T001	137.66	140.31	coding	297	105	995
PB.9041.1	7	2472	4	full-splice_match	gene: Zm00001d022598	Zm00001d022598_T001	2.99	2.99	coding	723	128	2296
PB.5696.1	4	1423	10	full-splice_match	gene: Zm00001d053275	Zm00001d053275_T001	12.65	26.87	coding	381	4	1146
PB.4652.4	3	1700	6	novel_not_in_catalog	gene: Zm00001d044144	novel	33.24	50.50	coding	397	228	1418
PB.6068.2	5	1626	3	novel_in_catalog	gene: Zm00001d013346	novel	22.71	22.71	coding	472	61	1476
PB.6248.5	5	2661	19	novel_not_in_catalog	gene: Zm00001d014178	novel	0.49	21.81	coding	759	278	2554
PB.6248.4	5	2564	16	incomplete-splice_match	gene: Zm00001d014178	Zm00001d014178_T011	13.94	21.81	coding	783	99	2447
PB.6248.3	5	2335	16	novel_in_catalog	gene: Zm00001d014178	novel	0.00	21.81	coding	653	255	2213
PB.6248.2	5	2451	16	novel_in_catalog	gene: Zm00001d014178	novel	7.38	21.81	coding	653	231	2189
PB.4652.2	3	1919	7	novel_not_in_catalog	gene: Zm00001d044144	novel	15.49	50.50	coding	460	255	1634
PB.9855.1	8	2242	1	genic	gene: Zm00001d012031	novel	10.05	88.09	coding	118	1585	1938
PB.8080.4	6	2034	5	novel_in_catalog	gene: Zm00001d038991	novel	7.58	78.74	coding	427	275	1555
PB.5717.3	4	2919	6	novel_in_catalog	gene: Zm00001d053379	novel	2.49	38.70	coding	277	883	1713
PB.3752.1	3	1931	8	full-splice_match	gene: Zm00001d039702	Zm00001d039702_T001	79.74	79.74	coding	388	221	1384
PB.5717.1	4	897	1	incomplete-splice_match	gene: Zm00001d053379	Zm00001d053379_T001	23.26	38.70	coding	79	169	405
PB.4012.3	3	801	6	full-splice_match	gene: Zm00001d041352	Zm00001d041352_T001	0.00	604.71	coding	223	1	669
PB.6367.3	5	2078	13	full-splice_match	gene: Zm00001d014716	Zm00001d014716_T002	0.00	9.03	coding	633	69	1967

PB.6367.2	5	2284	12	novel_in_catalog	gene: Zm00001d014716	novel	0.00	9.03	coding	612	83	1918
PB.6367.1	5	2236	13	full-splice_match	gene: Zm00001d014716	Zm00001d014716_T002	9.03	9.03	coding	633	120	2018
PB.9214.2	8	1844	12	full-splice_match	gene: Zm00001d008893	Zm00001d008893_T001	34.80	79.50	coding	406	214	1431
PB.2135.4	10	1027	3	incomplete-splice_match	gene: Zm00001d025777	Zm00001d025777_T008	0.00	78.90	coding	337	15	1025
PB.9214.4	8	1729	12	full-splice_match	gene: Zm00001d008893	Zm00001d008893_T001	0.00	79.50	coding	406	209	1426
PB.6568.1	5	1147	1	full-splice_match	gene: Zm00001d015639	Zm00001d015639_T001	20.17	20.17	coding	211	205	837
PB.2366.1	10	1844	2	full-splice_match	gene: Zm00001d026649	Zm00001d026649_T001	45.59	45.59	coding	527	115	1695
PB.3113.2	2	979	1	genic	gene: Zm00001d005381	novel	49.87	49.87	coding	105	34	348
PB.2157.1	10	1599	9	full-splice_match	gene: Zm00001d025860	Zm00001d025860_T001	13.31	45.68	coding	375	185	1309
PB.5929.1	5	1331	3	novel_in_catalog	gene: Zm00001d012886	novel	272.17	272.17	non_coding	NA	NA	NA
PB.9377.1	8	1638	8	full-splice_match	gene: Zm00001d009813	Zm00001d009813_T001	132.06	132.06	coding	354	199	1260
PB.3881.3	3	1420	1	full-splice_match	gene: Zm00001d040324	Zm00001d040324_T001	0.00	39.42	coding	398	113	1306
PB.1363.1	1	1667	10	full-splice_match	gene: Zm00001d033896	Zm00001d033896_T001	21.89	21.89	coding	462	63	1448
PB.4153.1	3	4348	5	incomplete-splice_match	gene: Zm00001d042028	Zm00001d042028_T013	30.86	31.09	coding	1358	46	4119
PB.5022.1	4	816	3	full-splice_match	gene: Zm00001d049723	Zm00001d049723_T001	287.89	287.89	coding	161	132	614
PB.6775.2	5	1691	2	novel_not_in_catalog	gene: Zm00001d016898	novel	8.58	8.58	coding	460	75	1454
PB.5643.1	4	2813	1	genic	gene: Zm00001d052967	novel	2.62	13.15	non_coding	NA	NA	NA

PB.2705.3	2	1850	14	full-splice_match	gene: Zm00001d003049	Zm00001d003049_T005	27.69	41.57	coding	448	383	1726
PB.2705.2	2	1969	14	full-splice_match	gene: Zm00001d003049	Zm00001d003049_T005	13.88	41.57	coding	448	363	1706
PB.10609.1	9	769	1	genic	gene: Zm00001d047788	novel	83.65	926.68	coding	66	130	327
PB.8254.2	7	2017	4	full-splice_match	gene: Zm00001d019079	Zm00001d019079_T001	32.88	32.88	coding	448	144	1487
PB.2586.3	2	2403	11	novel_in_catalog	gene: Zm00001d002585	novel	1.94	169.75	coding	479	308	1744
PB.4936.2	4	829	2	intergenic	novelGene_73	novel	35.84	38.75	non_coding	NA	NA	NA
PB.8254.1	7	1876	3	incomplete-splice_match	gene: Zm00001d019079	Zm00001d019079_T001	0.00	32.88	coding	383	74	1222
PB.2586.4	2	1966	12	full-splice_match	gene: Zm00001d002585	Zm00001d002585_T006	1.48	169.75	coding	508	345	1868
PB.2474.1	2	2719	9	full-splice_match	gene: Zm00001d002099	Zm00001d002099_T001	11.51	46.36	coding	723	372	2540
PB.8677.1	7	1034	8	incomplete-splice_match	gene: Zm00001d021351	Zm00001d021351_T002	7.18	7.18	coding	53	617	775
PB.1563.1	1	1346	3	novel_in_catalog	gene: Zm00001d034679	novel	7.42	7.42	coding	306	299	1216
PB.2157.2	10	1806	8	novel_in_catalog	gene: Zm00001d025860	novel	8.32	45.68	coding	308	193	1116
PB.7954.2	6	861	6	novel_in_catalog	gene: Zm00001d038533	novel	2.59	30.57	coding	125	296	670
PB.1417.1	1	2584	1	full-splice_match	gene: Zm00001d034112	Zm00001d034112_T001	36.69	36.69	coding	753	93	2351
PB.8924.1	7	1262	5	full-splice_match	gene: Zm00001d022225	Zm00001d022225_T001	17.38	17.38	coding	266	162	959
PB.3894.1	3	1508	2	full-splice_match	gene: Zm00001d040399	Zm00001d040399_T001	200.01	222.24	coding	346	174	1211
PB.5371.2	4	2345	6	novel_not_in_catalog	gene: Zm00001d051879	novel	11.54	48.50	coding	551	274	1926

PB.3894.3	3	1424	2	full-splice_match	gene: Zm00001d040399	Zm00001d040399_T001	0.00	222.24	coding	346	175	1212
PB.3894.2	3	1232	1	novel_in_catalog	gene: Zm00001d040399	novel	22.22	222.24	non_coding	NA	NA	NA
PB.5371.7	4	2427	5	full-splice_match	gene: Zm00001d051879	Zm00001d051879_T001	0.00	48.50	coding	657	277	2247
PB.5371.6	4	2440	5	novel_not_in_catalog	gene: Zm00001d051879	novel	0.00	48.50	coding	396	987	2174
PB.5371.4	4	2588	5	full-splice_match	gene: Zm00001d051879	Zm00001d051879_T001	9.37	48.50	coding	657	332	2302
PB.6260.1	5	1847	15	full-splice_match	gene: Zm00001d014258	Zm00001d014258_T010	61.22	77.62	coding	482	231	1676
PB.8777.1	7	579	1	incomplete-splice_match	gene: Zm00001d021704	Zm00001d021704_T001	34.78	34.78	coding	91	33	305
PB.5915.1	5	806	3	full-splice_match	gene: Zm00001d012832	Zm00001d012832_T004	59.51	59.51	non_coding	NA	NA	NA
PB.8915.5	7	2179	5	full-splice_match	gene: Zm00001d022181	Zm00001d022181_T004	74.30	288.80	coding	538	217	1830
PB.9436.3	8	1522	5	full-splice_match	gene: Zm00001d010159	Zm00001d010159_T002	9.22	80.26	coding	376	100	1227
PB.2157.3	10	1486	9	full-splice_match	gene: Zm00001d025860	Zm00001d025860_T001	24.05	45.68	coding	375	108	1232
PB.6096.1	5	2585	18	full-splice_match	gene: Zm00001d013444	Zm00001d013444_T002	19.36	19.36	coding	700	227	2326
PB.9436.2	8	1749	5	full-splice_match	gene: Zm00001d010159	Zm00001d010159_T002	60.56	80.26	coding	376	217	1344
PB.9075.1	8	1408	3	full-splice_match	gene: Zm00001d008255	Zm00001d008255_T001	13.68	13.68	coding	391	103	1275
PB.7825.1	6	2277	1	full-splice_match	gene: Zm00001d038072	Zm00001d038072_T001	8.53	12.46	coding	472	335	1750
PB.8689.1	7	2455	7	full-splice_match	gene: Zm00001d021392	Zm00001d021392_T002	13.56	174.94	coding	639	79	1995
PB.3913.3	3	1046	2	full-splice_match	gene: Zm00001d040519	Zm00001d040519_T001	99.58	515.93	coding	218	95	748

PB.3913.2	3	1117	1	genic	gene: Zm00001d040519	novel	11.33	515.93	coding	124	530	901
PB.3265.1	2	2077	2	full-splice_match	gene: Zm00001d006193	Zm00001d006193_T001	20.09	20.09	coding	561	94	1776
PB.9226.1	8	802	3	full-splice_match	gene: Zm00001d008956	Zm00001d008956_T001	9.58	9.58	non_coding	NA	NA	NA
PB.4603.1	3	1726	1	genic	gene: Zm00001d043935	novel	9.94	79.60	non_coding	NA	NA	NA
PB.6377.1	5	2172	12	full-splice_match	gene: Zm00001d014756	Zm00001d014756_T004	8.14	8.14	coding	489	326	1792
PB.3913.5	3	815	2	full-splice_match	gene: Zm00001d040519	Zm00001d040519_T001	282.16	515.93	coding	218	95	748
PB.3913.4	3	1157	2	full-splice_match	gene: Zm00001d040519	Zm00001d040519_T001	122.86	515.93	coding	218	314	967
PB.484.2	1	1925	1	novel_in_catalog	gene: Zm00001d029359	novel	7.08	221.73	coding	318	103	1056
PB.2737.1	2	738	1	full-splice_match	gene: Zm00001d003182	Zm00001d003182_T001	38.43	38.43	coding	160	47	526
PB.2089.2	10	1712	6	full-splice_match	gene: Zm00001d025570	Zm00001d025570_T006	7.02	41.02	coding	368	370	1473
PB.484.1	1	1452	2	full-splice_match	gene: Zm00001d029359	Zm00001d029359_T001	214.65	221.73	coding	375	101	1225
PB.10130.1	9	1464	3	novel_in_catalog	gene: Zm00001d045220	novel	137.29	182.87	coding	329	272	1258
PB.8486.3	7	978	3	novel_not_in_catalog	gene: Zm00001d020472	novel	37.51	55.83	non_coding	NA	NA	NA
PB.8486.2	7	951	3	novel_not_in_catalog	gene: Zm00001d020472	novel	18.31	55.83	non_coding	NA	NA	NA
PB.6701.3	5	1513	1	full-splice_match	gene: Zm00001d016582	Zm00001d016582_T001	0.00	371.31	coding	162	666	1151
PB.3033.1	2	1471	2	full-splice_match	gene: Zm00001d004934	Zm00001d004934_T001	12.93	12.93	coding	422	60	1325
PB.6701.1	5	1869	1	full-splice_match	gene: Zm00001d016582	Zm00001d016582_T001	15.13	371.31	coding	162	356	841

PB.798.1	1	659	3	full-splice_match	gene: Zm00001d031220	Zm00001d031220_T001	53.85	53.85	coding	170	10	519
PB.4125.2	3	1159	1	full-splice_match	gene: Zm00001d041927	Zm00001d041927_T001	75.02	158.70	coding	212	246	881
PB.1189.1	1	1112	8	full-splice_match	gene: Zm00001d033058	Zm00001d033058_T002	25.89	25.89	coding	282	106	951
PB.8689.6	7	2420	6	novel_in_catalog	gene: Zm00001d021392	novel	1.73	174.94	coding	370	1093	2202
PB.5075.3	4	1922	6	full-splice_match	gene: Zm00001d050026	Zm00001d050026_T001	0.00	6.63	coding	565	49	1743
PB.1803.2	10	2467	8	full-splice_match	gene: Zm00001d023979	Zm00001d023979_T002	0.00	5.65	coding	697	189	2279
PB.1803.1	10	2633	8	full-splice_match	gene: Zm00001d023979	Zm00001d023979_T002	5.65	5.65	coding	697	182	2272
PB.3272.1	2	1280	7	full-splice_match	gene: Zm00001d006220	Zm00001d006220_T001	184.40	184.40	coding	312	181	1116
PB.8736.1	7	1538	1	antisense	novelGene_gene: Zm00001d021572_A	novel	19.82	19.82	coding	249	657	1403
PB.1064.1	1	2085	7	novel_in_catalog	gene: Zm00001d032516	novel	7.69	7.69	coding	261	1302	2084
PB.9857.2	8	853	5	full-splice_match	gene: Zm00001d012052	Zm00001d012052_T001	0.00	893.24	coding	156	174	641
PB.6746.1	5	2793	13	full-splice_match	gene: Zm00001d016792	Zm00001d016792_T002	27.86	27.86	coding	650	491	2440
PB.1462.1	1	1549	5	novel_in_catalog	gene: Zm00001d034345	novel	89.73	177.64	coding	317	357	1307
PB.9037.3	7	2001	1	full-splice_match	gene: Zm00001d022587	Zm00001d022587_T001	5.73	22.60	coding	506	132	1649
PB.3023.1	2	1363	1	full-splice_match	gene: Zm00001d004882	Zm00001d004882_T001	6.75	6.75	coding	304	154	1065
PB.2095.2	10	3144	3	genic	gene: Zm00001d025625	novel	1.42	1.42	coding	518	66	1619
PB.2727.1	2	1885	3	novel_not_in_catalog	gene: Zm00001d003129	novel	21.14	21.14	coding	440	234	1553

PB.5686.1	4	3068	2	full-splice_match	gene: Zm00001d053208	Zm00001d053208_T001	0.62	5.71	coding	296	386	1273
PB.6175.1	5	1423	4	full-splice_match	gene: Zm00001d013779	Zm00001d013779_T001	2.32	88.91	coding	144	108	539
PB.6175.2	5	974	4	full-splice_match	gene: Zm00001d013779	Zm00001d013779_T001	86.59	88.91	coding	144	109	540
PB.5769.12	4	1463	7	novel_in_catalog	gene: Zm00001d053634	novel	13.23	41.21	coding	305	346	1260
PB.3445.1	2	568	2	antisense	novelGene_gene: Zm00001d007010_A	novel	736.13	736.13	non_coding	NA	NA	NA
PB.5769.11	4	1792	6	novel_in_catalog	gene: Zm00001d053634	novel	3.04	41.21	coding	222	348	1013
PB.2804.1	2	1069	2	full-splice_match	gene: Zm00001d003457	Zm00001d003457_T001	47.08	143.23	coding	204	68	679
PB.9169.7	8	1368	7	novel_in_catalog	gene: Zm00001d008679	novel	1.96	129.95	coding	323	291	1259
PB.9169.4	8	1553	8	full-splice_match	gene: Zm00001d008679	Zm00001d008679_T010	0.00	129.95	coding	357	273	1343
PB.2804.2	2	909	2	full-splice_match	gene: Zm00001d003457	Zm00001d003457_T001	96.15	143.23	coding	204	68	679
PB.9169.2	8	1666	8	full-splice_match	gene: Zm00001d008679	Zm00001d008679_T010	22.34	129.95	coding	357	271	1341
PB.9169.3	8	1628	8	full-splice_match	gene: Zm00001d008679	Zm00001d008679_T005	49.76	129.95	coding	357	339	1409
PB.5890.1	4	1884	1	full-splice_match	gene: Zm00001d054067	Zm00001d054067_T001	45.93	45.93	coding	502	96	1601
PB.9169.1	8	1759	8	full-splice_match	gene: Zm00001d008679	Zm00001d008679_T005	55.89	129.95	coding	357	359	1429
PB.5398.1	4	3004	7	incomplete-splice_match	gene: Zm00001d051980	Zm00001d051980_T006	99.38	99.38	coding	536	980	2587
PB.1433.1	1	2084	9	novel_in_catalog	gene: Zm00001d034206	novel	4.45	4.45	coding	546	173	1810
PB.1419.1	1	718	1	antisense	novelGene_gene: Zm00001d034129_A	novel	99.11	99.11	coding	134	73	474

PB.9502.1	8	2370	4	full-splice_match	gene: Zm00001d010466	Zm00001d010466_T001	20.55	20.55	coding	543	364	1992
PB.5937.5	5	1730	2	full-splice_match	gene: Zm00001d012902	Zm00001d012902_T002	0.00	40.65	coding	115	209	553
PB.5937.4	5	1587	3	novel_in_catalog	gene: Zm00001d012902	novel	11.28	40.65	coding	115	209	553
PB.5937.3	5	1392	2	full-splice_match	gene: Zm00001d012902	Zm00001d012902_T003	22.76	40.65	coding	115	209	553
PB.5937.2	5	2012	1	novel_in_catalog	gene: Zm00001d012902	novel	6.61	40.65	coding	115	167	511
PB.5638.1	4	2458	1	intergenic	novelGene_324	novel	4.14	4.14	coding	252	700	1455
PB.659.1	1	901	1	full-splice_match	gene: Zm00001d030339	Zm00001d030339_T001	69.19	69.19	coding	183	82	630
PB.10362.2	9	2936	19	incomplete-splice_match	gene: Zm00001d046581	Zm00001d046581_T017	2.02	28.82	coding	918	181	2934
PB.6479.2	5	1821	1	intergenic	novelGene_140	novel	9.45	14.51	non_coding	NA	NA	NA
PB.10362.1	9	2672	23	incomplete-splice_match	gene: Zm00001d046581	Zm00001d046581_T011	26.80	28.82	coding	668	542	2545
PB.9929.3	8	1878	10	novel_in_catalog	gene: Zm00001d012380	novel	4.91	27.73	coding	229	387	1073
PB.1256.3	1	1615	6	full-splice_match	gene: Zm00001d033422	Zm00001d033422_T001	27.99	93.70	coding	380	230	1369
PB.1251.2	1	1205	5	novel_not_in_catalog	gene: Zm00001d033393	novel	7.46	11.69	coding	41	273	395
PB.1200.2	1	781	3	intergenic	novelGene_368	novel	42.51	61.74	coding	149	81	527
PB.1311.1	1	2013	11	full-splice_match	gene: Zm00001d033651	Zm00001d033651_T008	25.11	26.12	coding	564	83	1774
PB.5618.1	4	2059	5	full-splice_match	gene: Zm00001d052852	Zm00001d052852_T001	8.02	20.65	coding	523	83	1651
PB.6809.3	5	1004	2	full-splice_match	gene: Zm00001d017095	Zm00001d017095_T001	2229.82	2390.54	coding	203	173	781

PB.1858.1	10	1108	6	full-splice_match	gene: Zm00001d024413	Zm00001d024413_T001	44.39	44.39	coding	277	68	898
PB.4123.1	3	1597	3	novel_in_catalog	gene: Zm00001d041920	novel	8.22	45.02	coding	241	164	886
PB.4250.2	3	2020	3	full-splice_match	gene: Zm00001d042490	Zm00001d042490_T005	10.86	55.01	coding	488	319	1782
PB.4250.1	3	2122	3	full-splice_match	gene: Zm00001d042490	Zm00001d042490_T001	32.95	55.01	coding	492	409	1884
PB.6482.1	5	694	2	full-splice_match	gene: Zm00001d015127	Zm00001d015127_T001	123.36	123.36	coding	119	39	395
PB.4250.5	3	1950	3	full-splice_match	gene: Zm00001d042490	Zm00001d042490_T001	0.00	55.01	coding	492	380	1855
PB.10688.2	9	2390	2	full-splice_match	gene: Zm00001d048054	Zm00001d048054_T002	25.71	78.05	coding	672	112	2127
PB.10688.1	9	2402	2	full-splice_match	gene: Zm00001d048054	Zm00001d048054_T002	32.28	78.05	coding	672	85	2100
PB.133.1	1	3199	19	novel_in_catalog	gene: Zm00001d027803	novel	25.58	25.58	coding	845	244	2778
PB.8937.6	7	2480	3	full-splice_match	gene: Zm00001d022273	Zm00001d022273_T012	0.00	31.12	coding	650	75	2024
PB.133.3	1	3103	19	novel_in_catalog	gene: Zm00001d027803	novel	0.00	25.58	coding	845	234	2768
PB.10688.4	9	1920	1	novel_in_catalog	gene: Zm00001d048054	novel	17.57	78.05	non_coding	NA	NA	NA
PB.3814.1	3	2141	3	full-splice_match	gene: Zm00001d040035	Zm00001d040035_T001	22.14	22.14	coding	598	58	1851
PB.3814.3	3	2018	3	full-splice_match	gene: Zm00001d040035	Zm00001d040035_T001	0.00	22.14	coding	598	58	1851
PB.6032.1	5	792	4	incomplete-splice_match	gene: Zm00001d013252	Zm00001d013252_T001	1356.45	1356.45	coding	206	69	686
PB.9663.6	8	984	8	incomplete-splice_match	gene: Zm00001d011188	Zm00001d011188_T001	2.95	127.52	coding	296	97	984
PB.9044.1	7	995	2	full-splice_match	gene: Zm00001d022613	Zm00001d022613_T001	5.21	5.21	coding	130	211	600

PB.6066.1	5	592	1	full-splice_match	gene: Zm00001d013333	Zm00001d013333_T001	28.35	28.35	coding	104	121	432
PB.8710.2	7	914	6	full-splice_match	gene: Zm00001d021486	Zm00001d021486_T008	0.00	71.29	coding	163	253	741
PB.8710.1	7	1022	6	full-splice_match	gene: Zm00001d021486	Zm00001d021486_T008	71.29	71.29	coding	163	253	741
PB.9722.1	8	2153	15	full-splice_match	gene: Zm00001d011487	Zm00001d011487_T001	8.84	329.73	coding	412	104	1339
PB.10790.2	9	2689	12	full-splice_match	gene: Zm00001d048415	Zm00001d048415_T010	4.51	31.23	coding	396	71	1258
PB.8788.1	7	1221	1	incomplete-splice_match	gene: Zm00001d021731	Zm00001d021731_T001	14.08	14.08	coding	352	29	1084
PB.10513.1	9	1737	10	full-splice_match	gene: Zm00001d047439	Zm00001d047439_T001	31.08	31.08	coding	392	401	1576
PB.9722.5	8	1646	15	full-splice_match	gene: Zm00001d011487	Zm00001d011487_T001	22.48	329.73	coding	412	195	1430
PB.9722.4	8	2004	15	full-splice_match	gene: Zm00001d011487	Zm00001d011487_T001	1.63	329.73	coding	412	105	1340
PB.5927.1	5	2004	7	full-splice_match	gene: Zm00001d012882	Zm00001d012882_T003	23.23	23.23	coding	498	234	1727
PB.6369.1	5	1860	2	full-splice_match	gene: Zm00001d014721	Zm00001d014721_T001	13.45	13.45	coding	488	167	1630
PB.5343.1	4	1663	2	full-splice_match	gene: Zm00001d051693	Zm00001d051693_T001	48.20	53.12	coding	422	160	1425
PB.5343.2	4	1733	1	novel_in_catalog	gene: Zm00001d051693	novel	4.92	53.12	coding	418	269	1522
PB.4596.1	3	1512	1	antisense	novelGene_gene: Zm00001d043891_A	novel	17.88	17.88	non_coding	NA	NA	NA
PB.3267.1	2	674	1	full-splice_match	gene: Zm00001d006196	Zm00001d006196_T001	82.86	82.86	non_coding	NA	NA	NA
PB.4928.1	4	762	1	antisense	novelGene_gene: Zm00001d049233_A	novel	27.71	27.71	coding	38	487	600
PB.6806.5	5	1849	2	full-splice_match	gene: Zm00001d017085	Zm00001d017085_T002	10.36	88.85	coding	388	476	1639

PB.6806.3	5	1827	2	full-splice_match	gene: Zm00001d017085	Zm00001d017085_T002	38.61	88.85	coding	388	310	1473
PB.1850.2	10	767	4	full-splice_match	gene: Zm00001d024347	Zm00001d024347_T001	0.00	69.45	coding	186	33	590
PB.1674.1	10	1519	4	novel_not_in_catalog	gene: Zm00001d023304	novel	14.41	14.41	coding	387	48	1208
PB.6008.1	5	2747	3	genic	gene: Zm00001d013165	novel	2.50	4.76	coding	107	57	377
PB.6008.2	5	2668	2	genic	gene: Zm00001d013165	novel	2.25	4.76	non_coding	NA	NA	NA
PB.7392.1	6	1580	17	full-splice_match	gene: Zm00001d035975	Zm00001d035975_T001	62.08	62.08	coding	429	95	1381
PB.3467.1	2	2070	9	full-splice_match	gene: Zm00001d007090	Zm00001d007090_T002	9.20	9.20	coding	474	121	1542
PB.5506.3	4	1397	9	novel_not_in_catalog	gene: Zm00001d052391	novel	13.43	242.70	coding	318	192	1145
PB.1302.1	1	2580	8	full-splice_match	gene: Zm00001d033624	Zm00001d033624_T014	97.61	105.67	coding	714	220	2361
PB.1302.3	1	2393	6	novel_in_catalog	gene: Zm00001d033624	novel	4.31	105.67	coding	436	866	2173
PB.1302.2	1	2632	7	novel_in_catalog	gene: Zm00001d033624	novel	3.75	105.67	coding	460	1030	2409
PB.3937.1	3	1842	13	novel_not_in_catalog	gene: Zm00001d040686	novel	9.98	9.98	coding	513	10	1548
PB.5848.2	4	2677	10	full-splice_match	gene: Zm00001d053927	Zm00001d053927_T015	7.30	49.24	coding	331	31	1023
PB.5848.3	4	2197	8	incomplete-splice_match	gene: Zm00001d053927	Zm00001d053927_T015	0.00	49.24	coding	331	31	1023
PB.3344.1	2	1571	1	full-splice_match	gene: Zm00001d006525	Zm00001d006525_T001	1.61	1.61	coding	453	39	1397
PB.226.1	1	2308	3	full-splice_match	gene: Zm00001d028230	Zm00001d028230_T001	0.24	40.57	coding	514	134	1675
PB.226.2	1	2063	3	full-splice_match	gene: Zm00001d028230	Zm00001d028230_T001	40.33	40.57	coding	514	134	1675

PB.226.3	1	1940	3	full-splice_match	gene: Zm00001d028230	Zm00001d028230_T001	0.00	40.57	coding	514	126	1667
PB.2192.1	10	939	1	full-splice_match	gene: Zm00001d026029	Zm00001d026029_T001	77.53	210.43	coding	104	136	447
PB.9369.1	8	3482	12	full-splice_match	gene: Zm00001d009765	Zm00001d009765_T008	7.98	7.98	coding	1111	80	3412
PB.2192.2	10	867	1	full-splice_match	gene: Zm00001d026029	Zm00001d026029_T001	132.90	210.43	coding	104	135	446
PB.4462.1	3	1433	3	full-splice_match	gene: Zm00001d043411	Zm00001d043411_T001	8.58	8.58	coding	331	59	1051
PB.1400.1	1	1134	1	genic	gene: Zm00001d034035	novel	14.32	61.04	coding	187	43	603
PB.117.1	1	964	1	genic	gene: Zm00001d027703	novel	28.39	28.39	coding	227	75	755
PB.8329.3	7	1783	13	novel_in_catalog	gene: Zm00001d019534	novel	0.29	84.58	coding	421	98	1360
PB.8329.2	7	1702	14	full-splice_match	gene: Zm00001d019534	Zm00001d019534_T003	0.00	84.58	coding	501	100	1602
PB.8329.1	7	1809	14	full-splice_match	gene: Zm00001d019534	Zm00001d019534_T003	84.29	84.58	coding	501	100	1602
PB.7314.6	6	2427	7	full-splice_match	gene: Zm00001d035514	Zm00001d035514_T016	0.79	19.37	coding	226	1551	2228
PB.7314.7	6	1665	9	full-splice_match	gene: Zm00001d035514	Zm00001d035514_T013	5.40	19.37	coding	226	827	1504
PB.7314.4	6	1561	8	novel_in_catalog	gene: Zm00001d035514	novel	1.60	19.37	coding	226	675	1352
PB.7314.5	6	1813	7	novel_in_catalog	gene: Zm00001d035514	novel	2.75	19.37	coding	310	678	1607
PB.8766.1	7	2453	17	novel_in_catalog	gene: Zm00001d021669	novel	4.27	4.27	coding	665	239	2233
PB.806.1	1	1061	2	full-splice_match	gene: Zm00001d031257	Zm00001d031257_T001	312.16	343.85	coding	178	274	807
PB.4323.7	3	3307	7	full-splice_match	gene: Zm00001d042809	Zm00001d042809_T008	9.59	172.86	coding	457	446	1816

PB.4323.6	3	2286	8	full-splice_match	gene: Zm00001d042809	Zm00001d042809_T003	4.99	172.86	coding	490	496	1965
PB.9021.4	7	2211	4	incomplete-splice_match	gene: Zm00001d022527	Zm00001d022527_T009	4.78	35.63	coding	623	127	1995
PB.5367.6	4	1264	2	full-splice_match	gene: Zm00001d051872	Zm00001d051872_T001	3.41	1415.22	coding	288	204	1067
PB.5367.7	4	1062	2	full-splice_match	gene: Zm00001d051872	Zm00001d051872_T001	0.00	1415.22	coding	288	82	945
PB.5367.4	4	1289	2	full-splice_match	gene: Zm00001d051872	Zm00001d051872_T001	1115.61	1415.22	coding	288	79	942
PB.4272.1	3	1854	1	full-splice_match	gene: Zm00001d042602	Zm00001d042602_T001	0.39	0.39	coding	520	156	1715
PB.5367.2	4	1442	1	genic	gene: Zm00001d051872	novel	296.20	1415.22	coding	319	82	1038
PB.4498.2	3	554	3	full-splice_match	gene: Zm00001d043523	Zm00001d043523_T001	0.00	185.46	coding	132	106	501
PB.1073.1	1	1820	2	full-splice_match	gene: Zm00001d032543	Zm00001d032543_T001	28.49	28.49	coding	517	45	1595
PB.8134.3	6	2301	2	full-splice_match	gene: Zm00001d039188	Zm00001d039188_T001	6.88	10.01	coding	654	134	2095
PB.8134.2	6	2728	1	genic	gene: Zm00001d039188	novel	3.13	10.01	coding	455	134	1498
PB.8134.1	6	1300	1	full-splice_match	gene: Zm00001d039187	Zm00001d039187_T001	0.35	0.35	coding	139	703	1119
PB.7620.6	6	457	2	full-splice_match	gene: Zm00001d037239	Zm00001d037239_T001	24.55	316.09	non_coding	NA	NA	NA
PB.9905.3	8	1248	9	full-splice_match	gene: Zm00001d012263	Zm00001d012263_T003	9.45	10.13	coding	335	58	1062
PB.873.1	1	1056	5	full-splice_match	gene: Zm00001d031637	Zm00001d031637_T001	57.59	71.62	coding	213	133	771
PB.7620.2	6	1748	2	full-splice_match	gene: Zm00001d037239	Zm00001d037239_T001	82.10	316.09	coding	377	90	1220
PB.7620.3	6	1423	2	full-splice_match	gene: Zm00001d037239	Zm00001d037239_T001	146.04	316.09	coding	377	34	1164

PB.7620.1	6	1772	2	full-splice_match	gene: Zm00001d037239	Zm00001d037239_T001	63.39	316.09	coding	377	93	1223
PB.2945.1	2	2235	1	full-splice_match	gene: Zm00001d004375	Zm00001d004375_T001	8.23	15.20	coding	567	365	2065
PB.10654.1	9	520	1	intergenic	novelGene_233	novel	23.87	23.87	coding	99	152	448
PB.10306.3	9	4877	11	incomplete-splice_match	gene: Zm00001d046226	Zm00001d046226_T002	19.11	19.11	coding	1317	472	4422
PB.10449.1	9	1414	1	genic	gene: Zm00001d047060	novel	17.27	17.27	coding	300	213	1112
PB.3199.2	2	2240	4	novel_in_catalog	gene: Zm00001d005848	novel	5.16	19.83	non_coding	NA	NA	NA
PB.814.1	1	1242	1	full-splice_match	gene: Zm00001d031281	Zm00001d031281_T001	23.72	23.72	coding	198	40	633
PB.3199.4	2	1370	9	full-splice_match	gene: Zm00001d005848	Zm00001d005848_T003	13.02	19.83	coding	341	68	1090
PB.587.1	1	864	1	full-splice_match	gene: Zm00001d029859	Zm00001d029859_T001	1096.89	1700.56	coding	133	77	475
PB.254.3	1	2499	4	novel_in_catalog	gene: Zm00001d028355	novel	1.19	9.16	coding	139	41	457
PB.254.1	1	2644	3	novel_in_catalog	gene: Zm00001d028355	novel	7.97	9.16	coding	291	1684	2556
PB.3376.1	2	1703	1	genic	gene: Zm00001d006678	novel	1.38	32.14	coding	268	174	977
PB.587.2	1	648	1	full-splice_match	gene: Zm00001d029859	Zm00001d029859_T001	603.67	1700.56	coding	133	74	472
PB.5909.3	5	1945	5	full-splice_match	gene: Zm00001d012808	Zm00001d012808_T003	10.85	22.04	coding	545	83	1717
PB.2965.1	2	878	1	novel_in_catalog	gene: Zm00001d004523	novel	60.21	60.21	coding	198	68	661
PB.585.2	1	1089	3	incomplete-splice_match	gene: Zm00001d029853	Zm00001d029853_T001	177.36	177.36	coding	172	412	927
PB.5039.3	4	1899	1	intergenic	novelGene_108	novel	0.00	4.13	non_coding	NA	NA	NA

PB.9228.1	8	2087	1	full-splice_match	gene: Zm00001d008960	Zm00001d008960_T001	6.67	26.71	coding	581	72	1814
PB.9228.2	8	1960	1	full-splice_match	gene: Zm00001d008960	Zm00001d008960_T001	20.03	26.71	coding	581	87	1829
PB.10067.1	9	2238	5	novel_not_in_catalog	gene: Zm00001d044917	novel	1.74	17.25	coding	103	192	500
PB.10067.2	9	2202	4	full-splice_match	gene: Zm00001d044917	Zm00001d044917_T002	15.50	17.25	coding	103	187	495
PB.10505.2	9	776	4	full-splice_match	gene: Zm00001d047423	Zm00001d047423_T001	0.00	51.62	coding	217	9	659
PB.2123.11	10	2803	8	novel_in_catalog	gene: Zm00001d025739	novel	5.48	45.61	coding	606	514	2331
PB.2123.10	10	2766	8	novel_not_in_catalog	gene: Zm00001d025739	novel	4.40	45.61	coding	515	277	1821
PB.2123.13	10	2707	7	novel_in_catalog	gene: Zm00001d025739	novel	0.00	45.61	coding	720	280	2439
PB.866.1	1	1095	1	antisense	novelGene_gene: Zm00001d031614_A	novel	10.51	10.51	coding	122	229	594
PB.7962.1	6	1739	3	full-splice_match	gene: Zm00001d038547	Zm00001d038547_T001	49.72	49.72	coding	474	45	1466
PB.3923.2	3	1685	1	novel_in_catalog	gene: Zm00001d040597	novel	5.32	25.26	coding	178	378	911
PB.7291.1	6	570	2	intergenic	novelGene_75	novel	25.61	25.61	non_coding	NA	NA	NA
PB.3923.3	3	1581	2	novel_in_catalog	gene: Zm00001d040597	novel	0.00	25.26	coding	178	286	819
PB.8055.1	6	1255	7	full-splice_match	gene: Zm00001d038896	Zm00001d038896_T001	8.82	94.14	coding	266	84	881
PB.803.1	1	4502	21	full-splice_match	gene: Zm00001d031232	Zm00001d031232_T021	4.67	70.88	coding	846	1770	4307
PB.8055.2	6	1285	7	full-splice_match	gene: Zm00001d038896	Zm00001d038896_T001	85.31	94.14	coding	266	284	1081
PB.7267.1	6	1982	2	genic	gene: Zm00001d035236	novel	9.35	18.56	coding	387	210	1370

PB.7267.3	6	1471	2	genic	gene: Zm00001d035236	novel	9.21	18.56	coding	387	210	1370
PB.7267.2	6	1963	2	genic	gene: Zm00001d035236	novel	0.00	18.56	coding	387	355	1515
PB.7336.2	6	573	2	full-splice_match	gene: Zm00001d035662	Zm00001d035662_T001	1926.32	3104.24	coding	80	87	326
PB.7336.3	6	464	2	full-splice_match	gene: Zm00001d035662	Zm00001d035662_T001	1169.48	3104.24	coding	80	87	326
PB.7336.1	6	1505	1	genic	gene: Zm00001d035662	novel	8.44	3104.24	non_coding	NA	NA	NA
PB.6276.1	5	363	3	intergenic	novelGene_102	novel	152.14	152.14	non_coding	NA	NA	NA
PB.10561.1	9	2725	5	full-splice_match	gene: Zm00001d047633	Zm00001d047633_T002	30.65	30.65	coding	768	184	2487
PB.5475.1	4	2709	4	incomplete-splice_match	gene: Zm00001d052307	Zm00001d052307_T002	12.77	21.79	coding	745	86	2320
PB.3923.4	3	1196	3	full-splice_match	gene: Zm00001d040597	Zm00001d040597_T001	19.94	25.26	coding	356	56	1123
PB.1536.1	1	1425	7	novel_in_catalog	gene: Zm00001d034607	novel	21.06	21.06	coding	269	98	904
PB.649.1	1	791	1	incomplete-splice_match	gene: Zm00001d030239	Zm00001d030239_T005	31.44	44.80	non_coding	NA	NA	NA
PB.2979.1	2	520	1	full-splice_match	gene: Zm00001d000909	Zm00001d000909_T001	21.38	21.38	non_coding	NA	NA	NA
PB.10414.4	9	1582	12	full-splice_match	gene: Zm00001d046909	Zm00001d046909_T001	0.00	39.95	coding	399	194	1390
PB.10414.3	9	1795	12	full-splice_match	gene: Zm00001d046909	Zm00001d046909_T001	0.00	39.95	coding	399	203	1399
PB.10586.4	9	973	5	full-splice_match	gene: Zm00001d047713	Zm00001d047713_T002	0.00	97.32	coding	228	130	813
PB.10414.1	9	1972	12	full-splice_match	gene: Zm00001d046909	Zm00001d046909_T001	39.95	39.95	coding	399	253	1449
PB.10287.4	9	1441	8	full-splice_match	gene: Zm00001d046135	Zm00001d046135_T001	0.00	52.81	coding	395	124	1308

PB.10287.5	9	1800	8	novel_in_catalog	gene: Zm00001d046135	novel	0.00	52.81	coding	206	1072	1689
PB.649.2	1	1775	2	incomplete-splice_match	gene: Zm00001d030239	Zm00001d030239_T017	13.37	44.80	coding	370	63	1172
PB.3746.3	3	1534	2	full-splice_match	gene: Zm00001d039642	Zm00001d039642_T001	0.00	12.21	coding	497	44	1534
PB.3746.2	3	1652	2	full-splice_match	gene: Zm00001d039642	Zm00001d039642_T001	5.92	12.21	coding	506	44	1561
PB.3746.1	3	551	1	incomplete-splice_match	gene: Zm00001d039642	Zm00001d039642_T001	6.29	12.21	non_coding	NA	NA	NA
PB.5860.1	4	2562	1	genic	gene: Zm00001d027041	novel	1.11	9.07	non_coding	NA	NA	NA
PB.10152.1	9	1300	7	full-splice_match	gene: Zm00001d045336	Zm00001d045336_T001	106.84	106.84	coding	303	69	977
PB.10152.3	9	862	7	full-splice_match	gene: Zm00001d045336	Zm00001d045336_T001	0.00	106.84	coding	282	15	860
PB.10152.2	9	1188	7	full-splice_match	gene: Zm00001d045336	Zm00001d045336_T001	0.00	106.84	coding	303	57	965
PB.7908.3	6	1990	5	full-splice_match	gene: Zm00001d038346	Zm00001d038346_T002	42.62	78.74	coding	362	296	1381
PB.2428.2	2	996	6	full-splice_match	gene: Zm00001d001901	Zm00001d001901_T002	38.48	80.21	coding	198	91	684
PB.2428.1	2	1079	6	full-splice_match	gene: Zm00001d001901	Zm00001d001901_T002	41.73	80.21	coding	198	39	632
PB.3285.1	2	975	2	full-splice_match	gene: Zm00001d006268	Zm00001d006268_T001	3.22	3.22	coding	271	9	821
PB.7488.1	6	1485	4	full-splice_match	gene: Zm00001d036542	Zm00001d036542_T001	13.38	13.38	coding	406	61	1278
PB.276.2	1	2024	2	incomplete-splice_match	gene: Zm00001d028429	Zm00001d028429_T006	3.55	23.34	coding	195	1294	1878
PB.276.1	1	1447	7	full-splice_match	gene: Zm00001d028429	Zm00001d028429_T003	19.79	23.34	coding	329	222	1208
PB.1116.3	1	1626	10	full-splice_match	gene: Zm00001d032699	Zm00001d032699_T022	28.50	35.84	coding	285	345	1199

PB.2188.1	10	2002	7	full-splice_match	gene: Zm00001d026018	Zm00001d026018_T005	59.78	59.78	coding	556	193	1860
PB.7203.4	5	1427	2	genic	gene: Zm00001d018567	novel	11.80	52.97	coding	405	74	1288
PB.7203.3	5	1503	2	genic	gene: Zm00001d018567	novel	15.03	52.97	coding	405	48	1262
PB.7203.2	5	2041	1	full-splice_match	gene: Zm00001d018567	Zm00001d018567_T001	13.40	52.97	coding	405	48	1262
PB.7203.1	5	2268	1	full-splice_match	gene: Zm00001d018567	Zm00001d018567_T001	12.74	52.97	coding	405	76	1290
PB.2114.2	10	1242	1	full-splice_match	gene: Zm00001d025704	Zm00001d025704_T001	19.66	25.93	coding	341	85	1107
PB.7474.1	6	1105	1	full-splice_match	gene: Zm00001d036468	Zm00001d036468_T001	66.84	66.84	coding	214	219	860
PB.5598.1	4	1424	2	full-splice_match	gene: Zm00001d052777	Zm00001d052777_T003	22.95	22.95	coding	291	224	1096
PB.9607.1	8	1707	8	full-splice_match	gene: Zm00001d010919	Zm00001d010919_T001	28.79	58.84	coding	356	275	1342
PB.7529.1	6	584	1	novel_in_catalog	gene: Zm00001d036723	novel	7.96	7.96	non_coding	NA	NA	NA
PB.2248.1	10	906	2	full-splice_match	gene: Zm00001d026285	Zm00001d026285_T001	1.31	15.10	coding	60	348	527
PB.9607.2	8	1538	8	full-splice_match	gene: Zm00001d010919	Zm00001d010919_T001	30.05	58.84	coding	310	389	1318
PB.1111.1	1	2773	22	novel_not_in_catalog	gene: Zm00001d032685	novel	28.88	38.67	coding	766	234	2531
PB.4090.1	3	2220	4	full-splice_match	gene: Zm00001d041740	Zm00001d041740_T001	8.70	22.02	coding	494	171	1652
PB.4090.2	3	2000	4	full-splice_match	gene: Zm00001d041740	Zm00001d041740_T001	13.32	22.02	coding	494	51	1532
PB.5266.1	4	1730	2	full-splice_match	gene: Zm00001d051343	Zm00001d051343_T001	25.39	25.39	coding	400	143	1342
PB.4545.2	3	440	1	intergenic	novelGene_325	novel	169.20	379.86	non_coding	NA	NA	NA

PB.840.1	1	1696	7	novel_not_in_catalog	gene: Zm00001d031459	novel	23.68	23.68	coding	335	214	1218
PB.8355.6	7	2001	7	full-splice_match	gene: Zm00001d019664	Zm00001d019664_T010	0.33	30.21	coding	358	615	1688
PB.8355.7	7	1577	8	novel_in_catalog	gene: Zm00001d019664	novel	20.40	30.21	coding	358	222	1295
PB.8355.4	7	2229	4	incomplete-splice_match	gene: Zm00001d019664	Zm00001d019664_T006	2.80	30.21	coding	423	115	1383
PB.8355.5	7	1894	8	full-splice_match	gene: Zm00001d019664	Zm00001d019664_T004	2.41	30.21	coding	358	497	1570
PB.8355.2	7	3345	9	full-splice_match	gene: Zm00001d019664	Zm00001d019664_T001	0.00	30.21	coding	896	222	2909
PB.8701.10	7	3041	6	full-splice_match	gene: Zm00001d021442	Zm00001d021442_T023	0.00	76.11	coding	644	775	2706
PB.2899.5	2	2148	7	full-splice_match	gene: Zm00001d003941	Zm00001d003941_T004	0.00	84.92	coding	668	57	2060
PB.8355.1	7	3671	9	full-splice_match	gene: Zm00001d019664	Zm00001d019664_T001	4.26	30.21	coding	896	313	3000
PB.678.1	1	1453	6	full-splice_match	gene: Zm00001d030507	Zm00001d030507_T001	42.55	52.08	coding	370	162	1271
PB.3716.1	3	1952	6	full-splice_match	gene: Zm00001d039513	Zm00001d039513_T001	2.66	35.66	coding	229	961	1647
PB.678.3	1	1361	6	novel_not_in_catalog	gene: Zm00001d030507	novel	0.00	52.08	coding	353	170	1228
PB.3716.3	3	1726	6	full-splice_match	gene: Zm00001d039513	Zm00001d039513_T001	33.00	35.66	coding	229	781	1467
PB.9296.3	8	1680	3	genic	gene: Zm00001d009401	novel	0.00	64.20	coding	370	284	1393
PB.9296.2	8	1721	3	genic	gene: Zm00001d009401	novel	0.00	64.20	coding	370	238	1347
PB.9296.1	8	1893	3	genic	gene: Zm00001d009401	novel	64.20	64.20	coding	370	270	1379
PB.790.1	1	1095	2	genic	gene: Zm00001d031184	novel	45.12	45.12	coding	241	150	872

PB.6281.2	5	1478	2	genic	gene: Zm00001d014338	novel	40.90	43.73	coding	348	165	1208
PB.6281.3	5	1808	2	genic	gene: Zm00001d014338	novel	2.83	43.73	coding	348	516	1559
PB.9564.4	8	1095	6	novel_in_catalog	gene: Zm00001d010733	novel	20.48	568.49	coding	114	499	840
PB.9945.4	8	2609	8	novel_in_catalog	gene: Zm00001d012452	novel	0.17	15.31	coding	724	299	2470
PB.9248.1	8	594	1	full-splice_match	gene: Zm00001d009032	Zm00001d009032_T001	110.63	110.63	coding	136	93	500
PB.10729.1	9	2421	3	genic	gene: Zm00001d048199	novel	69.19	69.19	coding	545	326	1960
PB.9945.1	8	2725	8	novel_in_catalog	gene: Zm00001d012452	novel	13.98	15.31	coding	724	206	2377
PB.10729.2	9	2184	3	genic	gene: Zm00001d048199	novel	0.00	69.19	coding	545	302	1936
PB.1581.1	1	1338	4	full-splice_match	gene: Zm00001d034740	Zm00001d034740_T001	66.97	74.19	coding	331	57	1049
PB.1581.2	1	1190	4	full-splice_match	gene: Zm00001d034740	Zm00001d034740_T001	7.22	74.19	coding	331	84	1076
PB.4084.1	3	3167	1	antisense	novelGene_gene: Zm00001d041709_A	novel	5.12	5.12	coding	331	577	1569
PB.4053.3	3	1724	1	novel_in_catalog	gene: Zm00001d041568	novel	3.20	973.78	coding	148	79	522
PB.4053.2	3	1046	2	full-splice_match	gene: Zm00001d041568	Zm00001d041568_T001	934.36	973.78	coding	261	79	861
PB.4053.1	3	1137	2	full-splice_match	gene: Zm00001d041568	Zm00001d041568_T001	36.22	973.78	coding	261	77	859
PB.9477.2	8	2780	3	genic	gene: Zm00001d010340	novel	1.93	3.79	coding	494	17	1498
PB.9477.3	8	1646	1	full-splice_match	gene: Zm00001d010340	Zm00001d010340_T001	1.86	3.79	coding	493	21	1499
PB.7781.2	6	1582	13	intergenic	novelGene_227	novel	0.00	40.11	coding	222	197	862

PB.848.1	1	1166	2	full-splice_match	gene: Zm00001d031510	Zm00001d031510_T001	21.39	21.39	coding	252	132	887
PB.646.4	1	1675	2	novel_in_catalog	gene: Zm00001d030232	novel	0.48	82.46	coding	162	339	824
PB.646.5	1	1320	3	novel_not_in_catalog	gene: Zm00001d030232	novel	0.00	82.46	coding	184	13	564
PB.646.2	1	1366	4	novel_not_in_catalog	gene: Zm00001d030232	novel	44.03	82.46	coding	319	31	987
PB.646.3	1	1481	3	novel_not_in_catalog	gene: Zm00001d030232	novel	21.54	82.46	coding	184	29	580
PB.3760.1	3	812	1	full-splice_match	gene: Zm00001d039735	Zm00001d039735_T001	49.60	49.60	coding	179	68	604
PB.646.1	1	1502	3	novel_in_catalog	gene: Zm00001d030232	novel	16.41	82.46	coding	297	230	1120
PB.2162.4	10	1069	2	full-splice_match	gene: Zm00001d025901	Zm00001d025901_T001	19.41	52.05	coding	223	195	863
PB.2162.5	10	1215	1	novel_in_catalog	gene: Zm00001d025901	novel	0.00	52.05	coding	102	710	1015
PB.10735.2	9	1675	3	full-splice_match	gene: Zm00001d048229	Zm00001d048229_T001	7.56	67.55	coding	357	130	1200
PB.7402.1	6	1705	6	full-splice_match	gene: Zm00001d036023	Zm00001d036023_T001	6.75	6.75	coding	522	57	1622
PB.2162.2	10	1395	1	novel_in_catalog	gene: Zm00001d025901	novel	1.45	52.05	coding	102	710	1015
PB.2162.3	10	1225	2	full-splice_match	gene: Zm00001d025901	Zm00001d025901_T001	29.70	52.05	coding	223	195	863
PB.367.1	1	962	5	full-splice_match	gene: Zm00001d028820	Zm00001d028820_T001	94.00	116.10	coding	168	118	621
PB.367.2	1	1201	4	novel_in_catalog	gene: Zm00001d028820	novel	22.10	116.10	coding	144	211	642
PB.2541.1	2	1111	1	full-splice_match	gene: Zm00001d002364	Zm00001d002364_T001	8.77	35.23	coding	222	91	756
PB.1177.1	1	2922	4	incomplete-splice_match	gene: Zm00001d033004	Zm00001d033004_T001	5.13	45.33	coding	153	175	633

PB.7878.2	6	626	3	intergenic	novelGene_250	novel	26.60	99.35	coding	104	46	357
PB.2541.2	2	973	1	full-splice_match	gene: Zm00001d002364	Zm00001d002364_T001	26.46	35.23	coding	222	90	755
PB.74.1	1	4056	5	novel_not_in_catalog	gene: Zm00001d027510	novel	31.71	31.71	coding	902	279	2984
PB.74.2	1	4009	5	novel_not_in_catalog	gene: Zm00001d027510	novel	0.00	31.71	coding	902	311	3016
PB.74.3	1	2203	2	incomplete-splice_match	gene: Zm00001d027510	Zm00001d027510_T002	0.00	31.71	coding	466	348	1745
PB.74.4	1	1913	2	incomplete-splice_match	gene: Zm00001d027510	Zm00001d027510_T002	0.00	31.71	coding	466	187	1584
PB.7016.1	5	1466	5	full-splice_match	gene: Zm00001d017960	Zm00001d017960_T001	46.85	46.85	coding	367	72	1172
PB.1171.1	1	1127	1	full-splice_match	gene: Zm00001d032972	Zm00001d032972_T001	29.94	55.98	coding	253	189	947
PB.1572.1	1	2038	4	full-splice_match	gene: Zm00001d034723	Zm00001d034723_T001	1.39	1.89	coding	480	10	1449
PB.10281.1	9	2254	3	full-splice_match	gene: Zm00001d046029	Zm00001d046029_T001	6.55	6.55	coding	674	52	2073
PB.6014.2	5	2807	1	full-splice_match	gene: Zm00001d013192	Zm00001d013192_T001	11.01	20.41	coding	815	66	2510
PB.6014.3	5	1616	1	full-splice_match	gene: Zm00001d013192	Zm00001d013192_T001	0.00	20.41	coding	451	68	1420
PB.4759.1	3	2476	12	incomplete-splice_match	gene: Zm00001d044584	Zm00001d044584_T020	28.99	28.99	coding	755	11	2275
PB.6014.1	5	2364	1	full-splice_match	gene: Zm00001d013192	Zm00001d013192_T001	9.40	20.41	coding	618	96	1949
PB.2573.2	2	1341	1	full-splice_match	gene: Zm00001d002519	Zm00001d002519_T001	0.00	15.20	coding	397	59	1249
PB.940.1	1	1502	2	full-splice_match	gene: Zm00001d031971	Zm00001d031971_T001	329.15	329.15	coding	377	152	1282
PB.2573.1	2	1504	1	full-splice_match	gene: Zm00001d002519	Zm00001d002519_T001	15.20	15.20	coding	397	60	1250

PB.4980.2	4	2327	1	novel_in_catalog	gene: Zm00001d049541	novel	30.53	2472.19	coding	204	1377	1988
PB.4980.1	4	1624	2	full-splice_match	gene: Zm00001d049541	Zm00001d049541_T001	421.38	2472.19	coding	364	120	1211
PB.4980.6	4	1522	2	full-splice_match	gene: Zm00001d049541	Zm00001d049541_T001	1097.26	2472.19	coding	364	119	1210
PB.1850.1	10	914	4	full-splice_match	gene: Zm00001d024347	Zm00001d024347_T001	69.45	69.45	coding	186	34	591
PB.4980.5	4	1127	2	novel_not_in_catalog	gene: Zm00001d049541	novel	923.03	2472.19	coding	204	203	814
PB.3988.1	3	457	1	genic	gene: Zm00001d041163	novel	5.61	5.61	non_coding	NA	NA	NA
PB.4980.9	4	1418	2	full-splice_match	gene: Zm00001d049541	Zm00001d049541_T001	0.00	2472.19	coding	364	119	1210
PB.1482.1	1	867	3	full-splice_match	gene: Zm00001d034414	Zm00001d034414_T002	506.62	521.97	coding	182	107	652
PB.3768.2	3	712	1	full-splice_match	gene: Zm00001d039790	Zm00001d039790_T001	683.67	716.12	coding	136	88	495
PB.3768.1	3	880	1	full-splice_match	gene: Zm00001d039790	Zm00001d039790_T001	32.45	716.12	coding	136	90	497
PB.4530.1	3	1612	5	full-splice_match	gene: Zm00001d043651	Zm00001d043651_T002	16.58	16.58	coding	486	27	1484
PB.6806.2	5	2800	1	novel_in_catalog	gene: Zm00001d017085	novel	2.66	88.85	coding	358	1351	2424
PB.2111.1	10	1747	4	full-splice_match	gene: Zm00001d025690	Zm00001d025690_T003	9.09	32.52	coding	377	119	1249
PB.1457.1	1	765	1	full-splice_match	gene: Zm00001d034339	Zm00001d034339_T001	39.48	39.48	coding	128	148	531
PB.10426.5	9	1945	8	novel_in_catalog	gene: Zm00001d046952	novel	18.51	426.48	coding	218	1183	1836
PB.10426.4	9	1620	12	novel_not_in_catalog	gene: Zm00001d046952	novel	94.03	426.48	coding	150	1054	1503
PB.10426.7	9	1280	9	novel_not_in_catalog	gene: Zm00001d046952	novel	141.14	426.48	coding	218	519	1172

PB.6898.1	5	1042	4	full-splice_match	gene: Zm00001d017449	Zm00001d017449_T001	60.85	60.85	coding	213	126	764
PB.10426.1	9	706	3	novel_in_catalog	gene: Zm00001d046952	novel	42.58	426.48	coding	150	31	480
PB.10426.3	9	1967	12	full-splice_match	gene: Zm00001d046952	Zm00001d046952_T001	130.23	426.48	coding	505	345	1859
PB.5924.2	5	3548	12	full-splice_match	gene: Zm00001d012863	Zm00001d012863_T009	1.28	18.16	coding	654	80	2041
PB.5599.1	4	2585	13	full-splice_match	gene: Zm00001d052779	Zm00001d052779_T004	3.96	14.33	coding	634	248	2149
PB.7289.1	6	2091	2	intergenic	novelGene_70	novel	5.09	5.09	coding	444	354	1685
PB.961.1	1	1323	3	full-splice_match	gene: Zm00001d032076	Zm00001d032076_T002	5.81	5.81	coding	304	188	1099
PB.2370.2	10	1181	1	full-splice_match	gene: Zm00001d026665	Zm00001d026665_T001	26.38	48.52	coding	265	113	907
PB.2228.1	10	2721	11	full-splice_match	gene: Zm00001d026213	Zm00001d026213_T003	13.55	20.33	coding	640	483	2402
PB.1898.1	10	596	1	genic_intron	novelGene_143	novel	95.06	95.06	non_coding	NA	NA	NA
PB.2228.2	10	2119	10	novel_not_in_catalog	gene: Zm00001d026213	novel	2.67	20.33	coding	438	39	1352
PB.2228.5	10	2622	11	full-splice_match	gene: Zm00001d026213	Zm00001d026213_T003	0.00	20.33	coding	640	467	2386
PB.8414.1	7	3496	6	fusion	gene: Zm00001d020110_g	novel	8.55	8.55	coding	1004	75	3086
PB.8414.2	7	1849	2	incomplete-splice_match	gene: Zm00001d020112	Zm00001d020112_T001	0.00	0.00	coding	548	206	1849
PB.8414.3	7	2172	3	full-splice_match	gene: Zm00001d020112	Zm00001d020112_T001	0.00	0.00	coding	701	69	2171
PB.377.2	1	3612	19	full-splice_match	gene: Zm00001d028896	Zm00001d028896_T007	28.83	28.83	coding	982	363	3308
PB.9930.2	8	1712	6	novel_not_in_catalog	gene: Zm00001d012394	novel	6.14	34.14	coding	144	172	603

PB.4988.1	4	1682	1	antisense	novelGene_gene: Zm00001d049573_A	novel	3.84	3.84	coding	258	394	1167
PB.2028.3	10	2478	8	novel_in_catalog	gene: Zm00001d025180	novel	1.71	38.15	coding	274	1560	2381
PB.4379.1	3	1515	7	full-splice_match	gene: Zm00001d043080	Zm00001d043080_T001	18.72	26.17	coding	289	323	1189
PB.377.4	1	3576	19	full-splice_match	gene: Zm00001d028896	Zm00001d028896_T017	0.00	28.83	coding	982	351	3296
PB.4528.27	3	3111	9	full-splice_match	gene: Zm00001d043649	Zm00001d043649_T058	1.15	96.43	coding	145	1805	2239
PB.7271.1	6	1939	1	full-splice_match	gene: Zm00001d035295	Zm00001d035295_T001	4.82	8.23	coding	513	36	1574
PB.7271.2	6	1778	1	full-splice_match	gene: Zm00001d035295	Zm00001d035295_T001	3.41	8.23	coding	513	35	1573
PB.4528.23	3	2411	10	novel_not_in_catalog	gene: Zm00001d043649	novel	3.17	96.43	non_coding	NA	NA	NA
PB.7855.1	6	1623	1	full-splice_match	gene: Zm00001d038158	Zm00001d038158_T001	28.01	28.01	coding	403	51	1259
PB.10581.1	9	2065	2	genic	gene: Zm00001d047705	novel	57.86	57.86	coding	287	240	1100
PB.8347.2	7	2500	1	novel_in_catalog	gene: Zm00001d019597	novel	4.46	371.96	non_coding	NA	NA	NA
PB.4243.2	3	2038	5	novel_in_catalog	gene: Zm00001d042463	novel	104.82	115.55	coding	584	67	1818
PB.8399.1	7	2020	14	novel_not_in_catalog	gene: Zm00001d020038	novel	6.28	17.43	coding	356	476	1543
PB.4278.1	3	2083	1	intergenic	novelGene_286	novel	0.00	0.00	coding	448	80	1423
PB.4528.28	3	2454	9	novel_in_catalog	gene: Zm00001d043649	novel	1.37	96.43	non_coding	NA	NA	NA
PB.8938.1	7	1298	6	full-splice_match	gene: Zm00001d022274	Zm00001d022274_T001	17.28	17.28	coding	364	88	1179
PB.10652.1	9	1991	1	full-splice_match	gene: Zm00001d047915	Zm00001d047915_T001	2.99	20.07	coding	479	92	1528

PB.10529.13	9	1184	13	novel_in_catalog	gene: Zm00001d047498	novel	0.00	170.79	coding	283	85	933
PB.10578.2	9	1914	12	full-splice_match	gene: Zm00001d047683	Zm00001d047683_T004	26.18	26.18	coding	395	456	1640
PB.10529.10	9	2155	13	novel_in_catalog	gene: Zm00001d047498	novel	3.86	170.79	coding	245	109	843
PB.5147.2	4	1680	1	full-splice_match	gene: Zm00001d050436	Zm00001d050436_T001	0.00	25.84	coding	408	51	1274
PB.5252.3	4	1973	5	novel_in_catalog	gene: Zm00001d051247	novel	1.03	16.18	coding	118	229	582
PB.5147.1	4	2571	1	full-splice_match	gene: Zm00001d050436	Zm00001d050436_T001	1.16	25.84	coding	408	87	1310
PB.4379.2	3	1017	6	novel_in_catalog	gene: Zm00001d043080	novel	7.45	26.17	coding	225	98	772
PB.5147.4	4	1575	1	full-splice_match	gene: Zm00001d050436	Zm00001d050436_T001	24.68	25.84	coding	408	89	1312
PB.7642.2	6	3914	10	novel_in_catalog	gene: Zm00001d037313	novel	6.70	16.07	coding	998	212	3205
PB.7642.3	6	2171	6	novel_in_catalog	gene: Zm00001d037313	novel	7.02	16.07	coding	528	102	1685
PB.1168.1	1	1372	2	full-splice_match	gene: Zm00001d032955	Zm00001d032955_T001	38.23	116.99	coding	300	208	1107
PB.6973.2	5	2869	3	full-splice_match	gene: Zm00001d017793	Zm00001d017793_T001	4.46	16.16	coding	713	544	2682
PB.5252.1	4	1749	7	novel_not_in_catalog	gene: Zm00001d051247	novel	4.30	16.18	coding	148	228	671
PB.7642.4	6	2988	8	incomplete-splice_match	gene: Zm00001d037313	Zm00001d037313_T026	2.35	16.07	coding	892	139	2814
PB.10508.5	9	2838	18	novel_in_catalog	gene: Zm00001d047426	novel	1.52	42.05	coding	412	259	1494
PB.4037.1	3	3034	18	novel_in_catalog	gene: Zm00001d041489	novel	44.08	44.08	coding	854	200	2761
PB.8560.1	7	2755	19	full-splice_match	gene: Zm00001d020827	Zm00001d020827_T001	28.12	28.12	coding	797	159	2549

PB.4037.2	3	2910	18	novel_in_catalog	gene: Zm00001d041489	novel	0.00	44.08	coding	854	195	2756
PB.3102.2	2	700	4	full-splice_match	gene: Zm00001d005334	Zm00001d005334_T001	556.68	603.73	coding	129	93	479
PB.9465.3	8	3795	2	genic	gene: Zm00001d010269	novel	3.31	9.69	coding	1111	125	3457
PB.9465.2	8	3626	1	full-splice_match	gene: Zm00001d010269	Zm00001d010269_T001	6.38	9.69	coding	930	256	3045
PB.4902.1	4	1576	1	full-splice_match	gene: Zm00001d049127	Zm00001d049127_T001	13.85	13.85	coding	422	59	1324
PB.3633.4	2	2241	16	full-splice_match	gene: Zm00001d007966	Zm00001d007966_T001	1.83	97.92	coding	619	131	1987
PB.6404.2	5	2437	9	full-splice_match	gene: Zm00001d014844	Zm00001d014844_T021	17.15	28.87	coding	482	551	1996
PB.1148.1	1	2764	9	full-splice_match	gene: Zm00001d032859	Zm00001d032859_T006	155.63	155.63	coding	810	100	2529
PB.3633.3	2	2331	16	full-splice_match	gene: Zm00001d007966	Zm00001d007966_T001	86.50	97.92	coding	619	115	1971
PB.1148.2	1	2636	9	full-splice_match	gene: Zm00001d032859	Zm00001d032859_T006	0.00	155.63	coding	810	94	2523
PB.5492.1	4	1194	1	genic	gene: Zm00001d052357	novel	4.43	79.45	coding	77	159	389
PB.9913.1	8	1255	3	full-splice_match	gene: Zm00001d012295	Zm00001d012295_T007	32.51	47.92	coding	256	292	1059
PB.1516.1	1	1979	8	novel_in_catalog	gene: Zm00001d034522	novel	18.55	18.55	coding	381	753	1895
PB.7713.2	6	2290	3	genic	gene: Zm00001d037650	novel	16.55	45.14	coding	664	154	2145
PB.3790.2	3	655	1	full-splice_match	gene: Zm00001d039908	Zm00001d039908_T001	209.00	1117.94	coding	157	85	555
PB.8937.1	7	2718	3	full-splice_match	gene: Zm00001d022273	Zm00001d022273_T009	0.00	31.12	coding	650	28	1977
PB.3790.1	3	785	1	full-splice_match	gene: Zm00001d039908	Zm00001d039908_T001	908.94	1117.94	coding	157	89	559

PB.7735.1	6	811	2	incomplete-splice_match	gene: Zm00001d037733	Zm00001d037733_T001	27.20	27.20	coding	173	291	809
PB.3582.1	2	1787	1	antisense	novelGene_gene: Zm00001d007773_A	novel	31.26	31.26	coding	109	241	567
PB.4174.2	3	730	2	full-splice_match	gene: Zm00001d042099	Zm00001d042099_T001	13.03	13.25	coding	127	265	645
PB.4174.1	3	2030	2	full-splice_match	gene: Zm00001d042099	Zm00001d042099_T001	0.22	13.25	coding	127	265	645
PB.9588.7	8	2164	18	novel_not_in_catalog	gene: Zm00001d010835	novel	1.46	25.74	coding	474	139	1560
PB.2369.1	10	1052	1	full-splice_match	gene: Zm00001d026664	Zm00001d026664_T001	54.40	102.06	coding	221	76	738
PB.7766.1	6	2693	4	intergenic	novelGene_224	novel	22.76	22.76	coding	86	106	363
PB.2369.2	10	840	1	full-splice_match	gene: Zm00001d026664	Zm00001d026664_T001	47.66	102.06	coding	221	74	736
PB.5639.1	4	2320	7	full-splice_match	gene: Zm00001d052944	Zm00001d052944_T001	30.66	37.40	coding	528	262	1845
PB.4671.1	3	747	5	incomplete-splice_match	gene: Zm00001d044220	Zm00001d044220_T004	24.22	24.22	non_coding	NA	NA	NA
PB.2569.1	2	1878	8	full-splice_match	gene: Zm00001d002499	Zm00001d002499_T003	27.75	27.75	coding	417	427	1677
PB.6991.3	5	1626	2	incomplete-splice_match	gene: Zm00001d017866	Zm00001d017866_T003	0.00	84.19	coding	335	259	1263
PB.424.1	1	956	4	full-splice_match	gene: Zm00001d029078	Zm00001d029078_T001	31.30	31.30	coding	163	158	646
PB.351.3	1	995	1	novel_in_catalog	gene: Zm00001d028770	novel	24.25	44.64	coding	213	104	742
PB.351.2	1	2047	6	novel_in_catalog	gene: Zm00001d028770	novel	20.39	44.64	coding	511	251	1783
PB.4638.1	3	1297	8	full-splice_match	gene: Zm00001d044090	Zm00001d044090_T001	27.70	32.80	coding	269	183	989
PB.1196.8	1	2333	17	full-splice_match	gene: Zm00001d033108	Zm00001d033108_T006	0.00	82.41	coding	605	121	1935

PB.2082.1	10	1648	1	full-splice_match	gene: Zm00001d025514	Zm00001d025514_T001	14.61	14.61	coding	392	415	1590
PB.9405.1	8	821	4	full-splice_match	gene: Zm00001d009969	Zm00001d009969_T001	32.27	32.27	coding	179	70	606
PB.3090.1	2	434	4	incomplete-splice_match	gene: Zm00001d005270	Zm00001d005270_T001	39.78	39.78	coding	18	280	333
PB.9405.2	8	684	4	full-splice_match	gene: Zm00001d009969	Zm00001d009969_T001	0.00	32.27	coding	179	64	600
PB.1196.1	1	2316	17	full-splice_match	gene: Zm00001d033108	Zm00001d033108_T001	55.59	82.41	coding	605	96	1910
PB.6128.1	5	1073	7	full-splice_match	gene: Zm00001d013583	Zm00001d013583_T001	179.12	179.12	coding	203	197	805
PB.10846.7	B73V4 ctg107	2954	1	full-splice_match	gene: Zm00001d000300	Zm00001d000300_T001	1.35	197.63	coding	451	1386	2738
PB.10846.6	B73V4 ctg107	1812	2	genic	gene: Zm00001d000300	novel	0.00	197.63	coding	451	390	1742
PB.8215.1	7	2792	1	full-splice_match	gene: Zm00001d018871	Zm00001d018871_T001	4.56	4.56	coding	839	100	2616
PB.4765.10	3	1472	2	full-splice_match	gene: Zm00001d044604	Zm00001d044604_T001	0.00	337.61	coding	450	53	1402
PB.10846.3	B73V4 ctg107	1938	2	genic	gene: Zm00001d000300	novel	0.00	197.63	coding	451	393	1745
PB.644.2	1	830	3	full-splice_match	gene: Zm00001d030222	Zm00001d030222_T001	60.39	60.39	coding	189	83	649
PB.10846.1	B73V4 ctg107	2040	2	genic	gene: Zm00001d000300	novel	196.28	197.63	coding	451	393	1745
PB.4445.1	3	3642	8	full-splice_match	gene: Zm00001d043347	Zm00001d043347_T002	10.09	12.29	coding	1144	64	3495
PB.9584.1	8	653	1	full-splice_match	gene: Zm00001d010819	Zm00001d010819_T001	42.17	42.17	non_coding	NA	NA	NA
PB.8168.1	7	460	1	novel_in_catalog	gene: Zm00001d018681	novel	2.75	2.75	coding	133	28	426
PB.5034.1	4	1640	4	full-splice_match	gene: Zm00001d049766	Zm00001d049766_T001	5.96	14.87	coding	400	213	1412

PB.5034.3	4	1524	4	full-splice_match	gene: Zm00001d049766	Zm00001d049766_T001	8.91	14.87	coding	400	210	1409
PB.6380.6	5	2579	10	full-splice_match	gene: Zm00001d014761	Zm00001d014761_T006	1.84	38.61	coding	483	460	1908
PB.2164.2	10	1347	6	full-splice_match	gene: Zm00001d025904	Zm00001d025904_T001	0.94	13.07	coding	415	7	1251
PB.6380.2	5	2641	11	novel_in_catalog	gene: Zm00001d014761	novel	0.00	38.61	coding	645	455	2389
PB.6380.3	5	2534	12	full-splice_match	gene: Zm00001d014761	Zm00001d014761_T001	21.41	38.61	coding	702	177	2282
PB.6380.1	5	1415	7	novel_in_catalog	gene: Zm00001d014761	novel	15.36	38.61	coding	86	370	627
PB.4154.1	3	580	1	full-splice_match	gene: Zm00001d042030	Zm00001d042030_T001	81.24	81.24	non_coding	NA	NA	NA
PB.7133.1	5	815	5	full-splice_match	gene: Zm00001d018377	Zm00001d018377_T010	53.44	53.44	coding	199	22	618
PB.9008.1	7	1109	1	antisense	novelGene_gene: Zm00001d022485_A	novel	69.94	69.94	non_coding	NA	NA	NA
PB.2164.1	10	1409	6	full-splice_match	gene: Zm00001d025904	Zm00001d025904_T001	12.13	13.07	coding	380	40	1179
PB.938.3	1	1228	8	full-splice_match	gene: Zm00001d031967	Zm00001d031967_T002	51.70	94.25	coding	304	126	1037
PB.5780.1	4	1348	12	full-splice_match	gene: Zm00001d053665	Zm00001d053665_T001	37.65	37.65	coding	279	129	965
PB.8533.1	7	1140	1	genic	gene: Zm00001d020697	novel	1.72	38.61	non_coding	NA	NA	NA
PB.8533.2	7	1955	3	full-splice_match	gene: Zm00001d020697	Zm00001d020697_T001	10.90	38.61	coding	406	138	1355
PB.8533.3	7	1615	2	novel_in_catalog	gene: Zm00001d020697	novel	8.77	38.61	coding	45	817	951
PB.8533.4	7	1673	3	full-splice_match	gene: Zm00001d020697	Zm00001d020697_T001	17.23	38.61	coding	406	138	1355
PB.3080.2	2	1142	6	full-splice_match	gene: Zm00001d005228	Zm00001d005228_T001	23.07	55.82	coding	288	64	927

PB.579.2	1	1183	4	novel_not_in_catalog	gene: Zm00001d029811	novel	35.29	35.29	non_coding	NA	NA	NA
PB.9844.2	8	1549	2	genic	gene: Zm00001d011985	novel	66.01	66.55	coding	315	116	1060
PB.9844.3	8	1150	1	full-splice_match	gene: Zm00001d011985	Zm00001d011985_T001	0.00	66.55	coding	315	65	1009
PB.4665.2	3	2776	3	genic	gene: Zm00001d044189	novel	12.90	12.90	coding	644	590	2521
PB.2109.1	10	1928	1	full-splice_match	gene: Zm00001d025675	Zm00001d025675_T001	23.35	43.81	coding	497	125	1615
PB.1543.1	1	1278	5	full-splice_match	gene: Zm00001d034627	Zm00001d034627_T001	50.01	71.31	coding	250	149	898
PB.8517.1	7	1369	5	full-splice_match	gene: Zm00001d020614	Zm00001d020614_T001	6.13	6.13	coding	218	172	825
PB.1931.3	10	1785	11	full-splice_match	gene: Zm00001d024762	Zm00001d024762_T001	23.88	23.88	coding	463	150	1538
PB.2783.1	2	1901	9	novel_not_in_catalog	gene: Zm00001d003349	novel	8.20	8.20	coding	481	145	1587
PB.2236.5	10	2176	14	full-splice_match	gene: Zm00001d026257	Zm00001d026257_T009	4.03	55.22	coding	564	77	1768
PB.5169.1	4	1090	6	full-splice_match	gene: Zm00001d050637	Zm00001d050637_T002	10.18	10.18	coding	270	68	877
PB.8380.1	7	2185	4	full-splice_match	gene: Zm00001d019890	Zm00001d019890_T001	86.99	150.09	coding	534	214	1815
PB.8380.2	7	2111	4	full-splice_match	gene: Zm00001d019890	Zm00001d019890_T001	63.11	150.09	coding	534	213	1814
PB.8380.3	7	2005	4	full-splice_match	gene: Zm00001d019890	Zm00001d019890_T001	0.00	150.09	coding	534	217	1818
PB.4478.1	3	616	1	full-splice_match	gene: Zm00001d043455	Zm00001d043455_T001	390.43	390.43	coding	150	44	493
PB.5611.1	4	1279	1	genic	gene: Zm00001d052820	novel	10.78	100.06	coding	155	5	469
PB.1903.2	10	1747	8	novel_not_in_catalog	gene: Zm00001d024635	novel	0.00	70.13	coding	448	71	1414

PB.8201.1	7	1609	9	novel_not_in_catalog	gene: Zm00001d018810	novel	4.06	4.06	coding	369	194	1300
PB.1903.1	10	1866	8	novel_not_in_catalog	gene: Zm00001d024635	novel	70.13	70.13	coding	448	71	1414
PB.6142.2	5	1207	9	full-splice_match	gene: Zm00001d013631	Zm00001d013631_T002	11.27	31.64	coding	200	230	829
PB.3629.2	2	1363	9	novel_in_catalog	gene: Zm00001d007951	novel	8.06	90.52	coding	350	99	1148
PB.3629.1	2	1599	10	full-splice_match	gene: Zm00001d007951	Zm00001d007951_T001	82.47	90.52	coding	332	317	1312
PB.3186.4	2	1471	1	full-splice_match	gene: Zm00001d005790	Zm00001d005790_T001	2.97	63.26	coding	328	361	1344
PB.9346.1	8	1288	1	full-splice_match	gene: Zm00001d009673	Zm00001d009673_T001	48.50	48.50	coding	121	610	972
PB.2981.1	2	955	1	genic	gene: Zm00001d004614	novel	199.68	279.07	coding	213	72	710
PB.4762.1	3	2854	2	full-splice_match	gene: Zm00001d044596	Zm00001d044596_T001	8.85	8.85	coding	898	102	2795
PB.3908.1	3	1798	5	full-splice_match	gene: Zm00001d040477	Zm00001d040477_T001	61.09	61.09	coding	404	104	1315
PB.6142.1	5	1512	9	full-splice_match	gene: Zm00001d013631	Zm00001d013631_T003	20.37	31.64	coding	200	307	906
PB.1508.3	1	939	5	incomplete-splice_match	gene: Zm00001d034509	Zm00001d034509_T002	26.98	86.87	coding	197	51	641
PB.10522.1	9	767	5	intergenic	novelGene_206	novel	25.83	25.83	coding	124	305	676
PB.2981.2	2	740	1	genic	gene: Zm00001d004614	novel	79.38	279.07	coding	213	72	710
PB.9973.1	8	2003	12	full-splice_match	gene: Zm00001d012571	Zm00001d012571_T001	7.53	7.53	coding	530	12	1601
PB.10896.1	B73V4 ctg23	1057	1	full-splice_match	gene: Zm00001d000339	Zm00001d000339_T001	110.04	110.04	coding	233	103	801
PB.3687.1	3	2156	10	full-splice_match	gene: Zm00001d039403	Zm00001d039403_T001	30.07	30.07	coding	574	100	1821

PB.6545.1	5	2267	2	full-splice_match	gene: Zm00001d015474	Zm00001d015474_T001	11.77	11.77	coding	649	42	1988
PB.5393.2	4	2376	12	novel_not_in_catalog	gene: Zm00001d051945	novel	12.35	19.78	coding	598	189	1982
PB.5393.1	4	3081	13	novel_not_in_catalog	gene: Zm00001d051945	novel	7.43	19.78	coding	579	170	1906
PB.7949.1	6	3353	19	full-splice_match	gene: Zm00001d038522	Zm00001d038522_T002	20.67	66.10	coding	974	338	3259
PB.2246.1	10	908	1	full-splice_match	gene: Zm00001d026282	Zm00001d026282_T001	17.60	17.60	coding	161	64	546
PB.3080.1	2	1306	6	full-splice_match	gene: Zm00001d005228	Zm00001d005228_T001	32.75	55.82	coding	288	12	875
PB.3186.2	2	1537	1	full-splice_match	gene: Zm00001d005790	Zm00001d005790_T001	21.44	63.26	coding	328	212	1195
PB.5752.4	4	1687	1	full-splice_match	gene: Zm00001d053586	Zm00001d053586_T001	0.00	13.88	coding	509	50	1576
PB.7958.1	6	2602	3	novel_not_in_catalog	gene: Zm00001d038541	novel	13.08	30.36	coding	674	202	2223
PB.5611.3	4	1503	6	full-splice_match	gene: Zm00001d052820	Zm00001d052820_T001	0.00	100.06	coding	383	61	1209
PB.3821.1	3	1425	1	full-splice_match	gene: ENSRNA049474264	ENSRNA049474264-T1	14.65	14.65	non_coding	NA	NA	NA
PB.8870.1	7	2425	4	full-splice_match	gene: Zm00001d022016	Zm00001d022016_T001	26.78	43.68	coding	623	205	2073
PB.8870.6	7	2329	4	full-splice_match	gene: Zm00001d022016	Zm00001d022016_T001	0.62	43.68	coding	623	212	2080
PB.7958.2	6	2358	3	full-splice_match	gene: Zm00001d038541	Zm00001d038541_T003	17.28	30.36	coding	652	22	1977
PB.6896.2	5	1533	9	novel_in_catalog	gene: Zm00001d017445	novel	36.07	50.46	coding	266	366	1163
PB.6896.3	5	1465	9	novel_in_catalog	gene: Zm00001d017445	novel	0.00	50.46	coding	266	344	1141
PB.2100.1	10	2516	7	full-splice_match	gene: Zm00001d025651	Zm00001d025651_T001	9.89	9.89	coding	693	76	2154

PB.7460.2	6	703	1	incomplete-splice_match	gene: Zm00001d036420	Zm00001d036420_T004	15.28	23.49	non_coding	NA	NA	NA
PB.7460.3	6	2497	4	incomplete-splice_match	gene: Zm00001d036420	Zm00001d036420_T009	8.20	23.49	non_coding	NA	NA	NA
PB.4784.2	3	959	2	full-splice_match	gene: Zm00001d044691	Zm00001d044691_T001	96.16	108.71	non_coding	NA	NA	NA
PB.4585.1	3	2006	2	full-splice_match	gene: Zm00001d043864	Zm00001d043864_T001	64.65	64.65	coding	512	242	1777
PB.5000.1	4	1489	7	full-splice_match	gene: Zm00001d049630	Zm00001d049630_T002	6.71	6.71	coding	400	53	1252
PB.839.1	1	2519	3	full-splice_match	gene: Zm00001d031454	Zm00001d031454_T001	16.26	16.82	coding	643	255	2183
PB.4338.2	3	823	1	full-splice_match	gene: Zm00001d042875	Zm00001d042875_T001	9.36	9.83	non_coding	NA	NA	NA
PB.6150.1	5	598	5	full-splice_match	gene: Zm00001d013656	Zm00001d013656_T001	38.20	38.20	coding	145	64	498
PB.4092.3	3	962	5	full-splice_match	gene: Zm00001d041750	Zm00001d041750_T002	0.00	233.46	coding	254	77	838
PB.4796.1	4	2494	1	genic	gene: Zm00001d048603	novel	9.90	22.75	coding	686	191	2248
PB.5844.2	4	1242	6	full-splice_match	gene: Zm00001d053923	Zm00001d053923_T002	59.96	73.38	coding	232	257	952
PB.6055.1	5	1160	5	novel_not_in_catalog	gene: Zm00001d013304	novel	30.87	30.87	coding	194	402	983
PB.9873.1	8	1389	3	incomplete-splice_match	gene: Zm00001d012128	Zm00001d012128_T005	94.81	94.81	coding	357	107	1177
PB.4092.1	3	1193	5	full-splice_match	gene: Zm00001d041750	Zm00001d041750_T002	99.07	233.46	coding	254	81	842
PB.9791.1	8	407	2	incomplete-splice_match	gene: Zm00001d011780	Zm00001d011780_T001	72.35	72.35	non_coding	NA	NA	NA
PB.3159.3	2	2303	6	novel_in_catalog	gene: Zm00001d005675	novel	5.16	387.55	coding	545	92	1726
PB.1398.2	1	2444	11	novel_in_catalog	gene: Zm00001d034033	novel	0.00	58.21	coding	263	1271	2059

PB.1398.3	1	1462	11	full-splice_match	gene: Zm00001d034033	Zm00001d034033_T009	0.00	58.21	coding	415	217	1461
PB.6495.2	5	2664	1	intergenic	novelGene_142	novel	15.57	15.57	non_coding	NA	NA	NA
PB.10201.1	9	2208	1	antisense	novelGene_gene: Zm00001d045507_A	novel	8.52	8.52	coding	195	1218	1802
PB.1485.3	1	694	4	novel_in_catalog	gene: Zm00001d034422	novel	978.16	1248.88	coding	152	83	538
PB.9030.4	7	1282	1	full-splice_match	gene: Zm00001d022557	Zm00001d022557_T001	0.00	31.90	coding	363	56	1144
PB.3620.1	2	2849	4	intergenic	novelGene_362	novel	11.00	11.00	coding	137	2031	2441
PB.9030.1	7	1616	1	full-splice_match	gene: Zm00001d022557	Zm00001d022557_T001	7.72	31.90	coding	363	61	1149
PB.2214.1	10	2034	1	antisense	novelGene_gene: Zm00001d026161_A	novel	6.81	12.84	coding	216	19	666
PB.2214.2	10	1219	1	antisense	novelGene_gene: Zm00001d026161_A	novel	6.02	12.84	coding	127	1	381
PB.9030.2	7	1513	1	full-splice_match	gene: Zm00001d022557	Zm00001d022557_T001	9.28	31.90	coding	363	69	1157
PB.3244.1	2	1056	7	full-splice_match	gene: Zm00001d006100	Zm00001d006100_T001	384.09	384.09	coding	258	69	842
PB.7011.1	5	895	6	full-splice_match	gene: Zm00001d017945	Zm00001d017945_T001	32.15	32.15	coding	184	230	781
PB.5383.1	4	3770	11	full-splice_match	gene: Zm00001d051902	Zm00001d051902_T004	28.72	37.10	coding	1002	318	3323
PB.5383.2	4	3856	10	full-splice_match	gene: Zm00001d051902	Zm00001d051902_T005	8.39	37.10	coding	686	320	2377
PB.7707.1	6	896	5	full-splice_match	gene: Zm00001d037620	Zm00001d037620_T002	224.65	224.65	coding	208	106	729
PB.7011.2	5	758	6	full-splice_match	gene: Zm00001d017945	Zm00001d017945_T001	0.00	32.15	coding	184	150	701
PB.6316.3	5	785	1	full-splice_match	gene: Zm00001d014518	Zm00001d014518_T001	192.70	246.12	non_coding	NA	NA	NA

PB.6316.2	5	871	1	full-splice_match	gene: Zm00001d014518	Zm00001d014518_T001	49.80	246.12	coding	92	129	404
PB.6316.1	5	1677	1	full-splice_match	gene: Zm00001d014518	Zm00001d014518_T001	3.62	246.12	coding	92	129	404
PB.9020.2	7	1796	7	full-splice_match	gene: Zm00001d022526	Zm00001d022526_T001	0.00	16.57	coding	367	298	1398
PB.3697.1	3	1843	1	full-splice_match	gene: Zm00001d039444	Zm00001d039444_T001	28.16	28.16	coding	511	83	1615
PB.6316.4	5	649	1	full-splice_match	gene: Zm00001d014518	Zm00001d014518_T001	0.00	246.12	coding	92	129	404
PB.4122.1	3	1721	15	full-splice_match	gene: Zm00001d041918	Zm00001d041918_T001	159.20	159.20	coding	272	742	1557
PB.1029.1	1	3405	6	novel_in_catalog	gene: Zm00001d032376	novel	3.09	18.26	coding	897	131	2821
PB.8146.1	7	1483	1	intergenic	novelGene_11	novel	4.06	4.06	non_coding	NA	NA	NA
PB.8079.4	6	3925	18	novel_in_catalog	gene: Zm00001d038982	novel	0.37	28.25	coding	572	480	2195
PB.2292.1	10	2928	7	full-splice_match	gene: Zm00001d026415	Zm00001d026415_T002	40.38	40.38	coding	773	227	2545
PB.6870.1	5	1234	10	novel_in_catalog	gene: Zm00001d017324	novel	65.50	67.14	coding	272	99	914
PB.6870.2	5	1080	9	novel_in_catalog	gene: Zm00001d017324	novel	1.64	67.14	coding	252	70	825
PB.1413.2	1	2514	2	genic	gene: Zm00001d034090	novel	14.25	23.23	coding	442	383	1708
PB.5540.1	4	702	3	full-splice_match	gene: Zm00001d052493	Zm00001d052493_T002	462.41	1238.52	coding	113	363	701
PB.5904.1	5	1517	4	incomplete-splice_match	gene: Zm00001d012797	Zm00001d012797_T001	64.58	64.58	coding	229	361	1047
PB.6126.2	5	942	1	genic	gene: Zm00001d013568	novel	70.27	3770.33	coding	123	343	711
PB.7798.1	6	2008	2	full-splice_match	gene: Zm00001d037975	Zm00001d037975_T048	10.96	10.96	coding	523	295	1863

PB.6126.6	5	696	2	full-splice_match	gene: Zm00001d013568	Zm00001d013568_T001	0.00	3770.33	coding	159	88	564
PB.10127.5	9	1001	3	full-splice_match	gene: Zm00001d045206	Zm00001d045206_T003	4.02	807.89	coding	299	105	1001
PB.10127.4	9	1193	1	novel_in_catalog	gene: Zm00001d045206	novel	11.09	807.89	coding	138	780	1193
PB.3131.1	2	3871	5	incomplete-splice_match	gene: Zm00001d005507	Zm00001d005507_T005	10.84	29.78	coding	1109	29	3355
PB.9698.2	8	3138	5	full-splice_match	gene: Zm00001d011373	Zm00001d011373_T005	19.25	20.64	coding	797	264	2654
PB.10127.1	9	1176	4	novel_in_catalog	gene: Zm00001d045206	novel	781.94	807.89	coding	263	105	893
PB.4294.12	3	1702	3	novel_not_in_catalog	gene: Zm00001d042684	novel	39.83	417.27	coding	427	236	1516
PB.10127.3	9	974	3	full-splice_match	gene: Zm00001d045206	Zm00001d045206_T005	10.83	807.89	coding	202	367	972
PB.410.1	1	3177	8	novel_in_catalog	gene: Zm00001d029010	novel	10.81	10.81	coding	327	348	1328
PB.2116.1	10	1814	3	full-splice_match	gene: Zm00001d025712	Zm00001d025712_T006	29.19	40.51	coding	459	189	1565
PB.8862.1	7	1555	2	full-splice_match	gene: Zm00001d021974	Zm00001d021974_T002	51.05	51.05	coding	423	123	1391
PB.8088.10	6	2108	1	full-splice_match	gene: Zm00001d039038	Zm00001d039038_T001	42.20	42.20	coding	582	116	1861
PB.2643.1	2	2531	3	novel_in_catalog	gene: Zm00001d002806	novel	6.24	54.26	coding	258	1524	2297
PB.2643.2	2	1013	3	novel_in_catalog	gene: Zm00001d002806	novel	44.93	54.26	coding	258	58	831
PB.2643.3	2	2559	1	novel_in_catalog	gene: Zm00001d002806	novel	3.09	54.26	non_coding	NA	NA	NA
PB.1518.3	1	1143	7	full-splice_match	gene: Zm00001d034532	Zm00001d034532_T002	112.59	125.80	coding	191	77	649
PB.283.2	1	1975	1	full-splice_match	gene: Zm00001d028452	Zm00001d028452_T001	0.00	1.54	coding	580	106	1845

PB.283.1	1	2144	1	full-splice_match	gene: Zm00001d028452	Zm00001d028452_T001	1.54	1.54	coding	580	108	1847
PB.7590.1	6	1922	9	full-splice_match	gene: Zm00001d037098	Zm00001d037098_T001	23.93	24.22	coding	473	210	1628
PB.1818.3	10	1002	1	genic	gene: Zm00001d024102	novel	2.62	18.33	coding	295	87	971
PB.1818.2	10	1446	2	novel_in_catalog	gene: Zm00001d024102	novel	8.18	18.33	coding	387	104	1264
PB.1818.1	10	1370	3	novel_not_in_catalog	gene: Zm00001d024102	novel	7.52	18.33	coding	349	87	1133
PB.815.2	1	758	4	novel_not_in_catalog	gene: Zm00001d022873	novel	2.48	6.24	non_coding	NA	NA	NA
PB.3151.1	2	1170	3	incomplete-splice_match	gene: Zm00001d005636	Zm00001d005636_T003	324.28	324.28	coding	276	46	873
PB.4215.2	3	1829	3	full-splice_match	gene: Zm00001d042319	Zm00001d042319_T001	19.78	24.43	coding	409	427	1653
PB.4215.1	3	1273	2	novel_in_catalog	gene: Zm00001d042319	novel	4.65	24.43	coding	221	323	985
PB.10752.2	9	506	1	full-splice_match	gene: Zm00001d048307	Zm00001d048307_T001	412.75	422.38	non_coding	NA	NA	NA
PB.3196.1	2	2652	6	novel_in_catalog	gene: Zm00001d005826	novel	15.37	18.63	coding	717	351	2501
PB.6188.1	5	1342	8	full-splice_match	gene: Zm00001d013885	Zm00001d013885_T001	10.72	10.72	coding	291	130	1002
PB.6223.3	5	1094	3	novel_in_catalog	gene: Zm00001d014039	novel	7.27	60.24	coding	190	152	721
PB.6223.1	5	1459	3	full-splice_match	gene: Zm00001d014039	Zm00001d014039_T001	42.21	60.24	coding	284	239	1090
PB.6223.4	5	1258	4	full-splice_match	gene: Zm00001d014039	Zm00001d014039_T002	10.76	60.24	coding	284	161	1012
PB.6978.2	5	1869	2	full-splice_match	gene: Zm00001d017820	Zm00001d017820_T001	0.00	6.69	coding	523	109	1677
PB.1413.4	1	2139	2	genic	gene: Zm00001d034090	novel	8.97	23.23	coding	442	382	1707

PB.9533.1	8	914	5	full-splice_match	gene: Zm00001d010618	Zm00001d010618_T002	999.22	1006.66	coding	148	142	585
PB.2938.1	2	1767	4	novel_not_in_catalog	gene: Zm00001d004215	novel	21.13	21.13	non_coding	NA	NA	NA
PB.1680.1	10	1593	1	antisense	novelGene_gene: Zm00001d023336_A	novel	0.79	0.79	non_coding	NA	NA	NA
PB.7703.2	6	1317	12	full-splice_match	gene: Zm00001d037613	Zm00001d037613_T001	28.03	85.26	coding	299	174	1070
PB.7703.3	6	1287	12	full-splice_match	gene: Zm00001d037613	Zm00001d037613_T001	0.00	85.26	coding	299	146	1042
PB.7703.1	6	1513	12	full-splice_match	gene: Zm00001d037613	Zm00001d037613_T001	57.23	85.26	coding	299	146	1042
PB.2312.2	10	1015	1	intergenic	novelGene_239	novel	2.90	13.46	coding	164	33	524
PB.8057.1	6	1956	1	antisense	novelGene_gene: Zm00001d038904_A	novel	5.83	5.83	coding	100	1083	1382
PB.1035.2	1	2083	2	full-splice_match	gene: Zm00001d032407	Zm00001d032407_T001	19.04	24.77	coding	614	58	1899
PB.9868.14	8	2588	8	novel_in_catalog	gene: Zm00001d012100	novel	0.00	79.52	coding	101	443	745
PB.3084.1	2	1692	9	full-splice_match	gene: Zm00001d005244	Zm00001d005244_T003	21.18	21.18	coding	371	153	1265
PB.7973.6	6	2417	13	novel_in_catalog	gene: Zm00001d038589	novel	0.00	16.75	coding	581	575	2317
PB.8149.5	7	679	4	full-splice_match	gene: Zm00001d018613	Zm00001d018613_T009	0.00	208.84	coding	134	68	469
PB.2119.1	10	2617	3	novel_in_catalog	gene: Zm00001d025717	novel	18.13	18.13	coding	473	702	2120
PB.1055.3	1	1079	3	incomplete-splice_match	gene: Zm00001d032480	Zm00001d032480_T001	17.39	129.92	coding	294	40	921
PB.1055.1	1	1495	5	full-splice_match	gene: Zm00001d032480	Zm00001d032480_T002	112.53	129.92	coding	377	89	1219
PB.4275.1	3	2659	13	full-splice_match	gene: Zm00001d042615	Zm00001d042615_T006	16.51	16.51	coding	773	80	2398

PB.6579.1	5	1045	2	novel_not_in_catalog	gene: Zm00001d015741	novel	32.44	32.44	coding	232	37	732
PB.6579.2	5	904	2	novel_not_in_catalog	gene: Zm00001d015741	novel	0.00	32.44	coding	232	6	701
PB.9806.1	8	2258	6	full-splice_match	gene: Zm00001d011831	Zm00001d011831_T001	27.19	27.19	coding	582	240	1985
PB.4886.1	4	2438	10	novel_in_catalog	gene: Zm00001d049080	novel	5.56	28.59	coding	394	905	2086
PB.4886.3	4	2468	10	novel_in_catalog	gene: Zm00001d049080	novel	1.12	28.59	coding	347	100	1140
PB.4886.2	4	2433	11	full-splice_match	gene: Zm00001d049080	Zm00001d049080_T001	21.91	28.59	coding	677	198	2228
PB.5876.4	4	2159	9	novel_in_catalog	gene: Zm00001d054003	novel	16.73	40.22	coding	513	183	1721
PB.8018.1	6	620	2	intergenic	novelGene_269	novel	154.34	154.34	non_coding	NA	NA	NA
PB.3952.2	3	2615	1	full-splice_match	gene: Zm00001d040788	Zm00001d040788_T001	0.22	25.24	coding	810	124	2553
PB.8652.1	7	1437	2	full-splice_match	gene: Zm00001d021268	Zm00001d021268_T001	112.84	112.84	coding	239	395	1111
PB.3952.1	3	2725	1	full-splice_match	gene: Zm00001d040788	Zm00001d040788_T001	25.02	25.24	coding	810	98	2527
PB.800.1	1	1821	1	full-splice_match	gene: Zm00001d031222	Zm00001d031222_T001	83.29	83.29	coding	527	39	1619
PB.9707.1	8	1946	5	full-splice_match	gene: Zm00001d011418	Zm00001d011418_T003	52.66	72.20	coding	528	57	1640
PB.9707.2	8	2542	4	full-splice_match	gene: Zm00001d011418	Zm00001d011418_T001	7.85	72.20	coding	338	1282	2295
PB.3274.4	2	1164	1	novel_in_catalog	gene: Zm00001d006236	novel	9.76	132.37	coding	88	157	420
PB.9767.1	8	1751	2	genic	gene: Zm00001d011692	novel	37.03	37.03	coding	433	94	1392
PB.3274.1	2	1299	2	incomplete-splice_match	gene: Zm00001d006236	Zm00001d006236_T006	72.79	132.37	coding	273	154	972

PB.3906.3	3	1973	17	novel_not_in_catalog	gene: Zm00001d040468	novel	7.07	7.99	coding	488	325	1788
PB.3274.2	2	1383	1	genic	gene: Zm00001d006236	novel	0.00	132.37	coding	88	153	416
PB.3482.1	2	2208	2	full-splice_match	gene: Zm00001d007175	Zm00001d007175_T001	1.67	3.12	coding	615	112	1956
PB.3482.2	2	2371	1	novel_in_catalog	gene: Zm00001d007175	novel	1.45	3.12	coding	607	315	2135
PB.3906.8	3	2271	16	novel_not_in_catalog	gene: Zm00001d040468	novel	0.92	7.99	coding	211	86	718
PB.3829.1	3	1198	2	full-splice_match	gene: Zm00001d040126	Zm00001d040126_T001	77.68	77.68	coding	290	96	965
PB.10898.4	B73V4 ctg24	1125	2	full-splice_match	gene: Zm00001d000380	Zm00001d000380_T001	16.23	29.19	coding	252	92	847
PB.7992.1	6	1346	2	full-splice_match	gene: Zm00001d038703	Zm00001d038703_T001	21.15	21.15	coding	358	40	1113
PB.3689.1	3	2651	16	full-splice_match	gene: Zm00001d039409	Zm00001d039409_T004	26.45	32.10	coding	611	446	2278
PB.10898.3	B73V4 ctg24	923	2	full-splice_match	gene: Zm00001d000380	Zm00001d000380_T001	0.00	29.19	coding	167	36	536
PB.3689.2	3	2598	15	full-splice_match	gene: Zm00001d039409	Zm00001d039409_T009	5.65	32.10	coding	390	361	1530
PB.3166.1	2	2428	10	full-splice_match	gene: Zm00001d005707	Zm00001d005707_T007	14.55	14.55	coding	632	107	2002
PB.5086.1	4	1237	4	incomplete-splice_match	gene: Zm00001d050107	Zm00001d050107_T001	45.60	45.60	coding	359	3	1079
PB.3968.1	3	2639	13	full-splice_match	gene: Zm00001d040960	Zm00001d040960_T005	23.81	23.81	coding	695	170	2254
PB.3968.3	3	2535	13	full-splice_match	gene: Zm00001d040960	Zm00001d040960_T005	0.00	23.81	coding	695	170	2254
PB.10469.1	9	1392	4	full-splice_match	gene: Zm00001d047210	Zm00001d047210_T002	9.72	12.39	coding	222	459	1124
PB.3968.5	3	2396	13	full-splice_match	gene: Zm00001d040960	Zm00001d040960_T005	0.00	23.81	coding	695	167	2251

PB.4872.1	4	1237	1	full-splice_match	gene: Zm00001d049002	Zm00001d049002_T001	6.95	6.95	coding	243	365	1093
PB.5607.1	4	1163	8	full-splice_match	gene: Zm00001d052803	Zm00001d052803_T006	55.20	84.34	coding	256	76	843
PB.6372.1	5	1847	4	incomplete-splice_match	gene: Zm00001d014735	Zm00001d014735_T001	17.91	23.48	coding	369	205	1311
PB.6372.2	5	2270	6	full-splice_match	gene: Zm00001d014735	Zm00001d014735_T001	5.57	23.48	coding	593	81	1859
PB.2495.3	2	2728	6	novel_not_in_catalog	gene: Zm00001d002168	novel	0.00	19.68	coding	668	445	2448
PB.2495.2	2	2950	4	novel_in_catalog	gene: Zm00001d002168	novel	1.36	19.68	coding	528	414	1997
PB.2495.1	2	2848	6	novel_not_in_catalog	gene: Zm00001d002168	novel	4.89	19.68	coding	830	19	2508
PB.667.11	1	1539	10	novel_not_in_catalog	gene: Zm00001d030366	novel	4.36	29.16	coding	423	124	1392
PB.10135.1	9	801	1	full-splice_match	gene: Zm00001d045296	Zm00001d045296_T001	52.51	108.95	coding	117	107	457
PB.2495.5	2	1596	6	full-splice_match	gene: Zm00001d002168	Zm00001d002168_T001	12.61	19.68	coding	420	86	1345
PB.2495.4	2	2894	5	novel_in_catalog	gene: Zm00001d002168	novel	0.83	19.68	coding	690	23	2092
PB.5320.1	4	2193	11	full-splice_match	gene: Zm00001d051609	Zm00001d051609_T004	88.66	88.66	coding	467	397	1797
PB.2090.1	10	1883	2	full-splice_match	gene: Zm00001d025572	Zm00001d025572_T001	13.45	13.45	coding	507	150	1670
PB.2389.1	2	1059	1	full-splice_match	gene: Zm00001d001774	Zm00001d001774_T001	12.64	12.64	coding	297	103	993
PB.3581.1	2	2026	9	full-splice_match	gene: Zm00001d007770	Zm00001d007770_T001	58.70	58.70	coding	523	177	1745
PB.6611.1	5	2042	1	intergenic	novelGene_226	novel	8.00	8.00	coding	110	282	611
PB.299.1	1	1205	7	full-splice_match	gene: Zm00001d028529	Zm00001d028529_T001	108.36	108.36	coding	317	69	1019

PB.7331.1	6	696	3	full-splice_match	gene: Zm00001d035629	Zm00001d035629_T001	128.69	128.69	coding	139	141	557
PB.1051.8	1	1884	14	novel_in_catalog	gene: Zm00001d032472	novel	0.00	37.15	coding	509	121	1647
PB.9484.2	8	951	3	full-splice_match	gene: Zm00001d010401	Zm00001d010401_T001	36.37	76.31	coding	105	121	435
PB.9484.3	8	863	4	novel_not_in_catalog	gene: Zm00001d010401	novel	39.94	76.31	coding	105	90	404
PB.10766.1	9	1222	3	full-splice_match	gene: Zm00001d048347	Zm00001d048347_T001	4.41	4.41	coding	317	134	1084
PB.2489.1	2	1885	18	incomplete-splice_match	gene: Zm00001d002154	Zm00001d002154_T008	7.83	12.87	coding	548	17	1660
PB.2489.2	2	1739	14	novel_in_catalog	gene: Zm00001d002154	novel	5.04	12.87	coding	289	118	984
PB.9149.1	8	724	2	full-splice_match	gene: Zm00001d008601	Zm00001d008601_T001	516.45	516.45	coding	134	41	442
PB.7090.1	5	905	2	full-splice_match	gene: Zm00001d018220	Zm00001d018220_T001	6.01	6.01	coding	238	78	791
PB.4456.1	3	1261	2	full-splice_match	gene: Zm00001d043391	Zm00001d043391_T001	11.43	11.43	coding	7	703	723
PB.8273.1	7	1479	9	novel_in_catalog	gene: Zm00001d019172	novel	41.42	47.72	coding	343	148	1176
PB.5289.3	4	1128	1	full-splice_match	gene: Zm00001d051473	Zm00001d051473_T001	0.00	471.99	coding	335	23	1027
PB.5289.2	4	1258	1	full-splice_match	gene: Zm00001d051473	Zm00001d051473_T001	170.72	471.99	coding	335	18	1022
PB.5289.1	4	1364	1	full-splice_match	gene: Zm00001d051473	Zm00001d051473_T001	301.27	471.99	coding	335	20	1024
PB.8771.1	7	2218	8	full-splice_match	gene: Zm00001d021696	Zm00001d021696_T003	1.92	30.87	coding	596	186	1973
PB.8771.2	7	2189	8	full-splice_match	gene: Zm00001d021696	Zm00001d021696_T003	12.17	30.87	coding	672	76	2091
PB.8771.3	7	2274	7	full-splice_match	gene: Zm00001d021696	Zm00001d021696_T004	13.18	30.87	coding	482	758	2203

PB.8771.4	7	2317	6	novel_in_catalog	gene: Zm00001d021696	novel	3.61	30.87	coding	320	711	1670
PB.8771.5	7	2184	7	novel_in_catalog	gene: Zm00001d021696	novel	0.00	30.87	coding	510	17	1546
PB.1110.3	1	2649	4	full-splice_match	gene: Zm00001d032683	Zm00001d032683_T001	1.81	14.66	coding	681	339	2381
PB.7967.2	6	2095	1	intergenic	novelGene_261	novel	9.18	9.18	non_coding	NA	NA	NA
PB.5562.1	4	1311	3	full-splice_match	gene: Zm00001d052651	Zm00001d052651_T001	53.12	92.26	coding	297	83	973
PB.5863.1	4	814	2	full-splice_match	gene: Zm00001d053979	Zm00001d053979_T001	222.75	222.75	coding	134	94	495
PB.9845.2	8	2166	7	novel_not_in_catalog	gene: Zm00001d011990	novel	14.64	22.74	coding	231	389	1081
PB.9765.1	8	1009	7	incomplete-splice_match	gene: Zm00001d011685	Zm00001d011685_T005	24.65	24.65	coding	143	322	750
PB.4492.1	3	1739	3	full-splice_match	gene: Zm00001d043503	Zm00001d043503_T001	4.61	4.61	coding	383	240	1388
PB.936.1	1	930	6	full-splice_match	gene: Zm00001d031961	Zm00001d031961_T004	79.39	79.39	coding	144	108	539
PB.1780.1	10	1148	1	full-splice_match	gene: Zm00001d023863	Zm00001d023863_T001	40.54	40.54	coding	298	57	950
PB.9083.3	8	1819	6	incomplete-splice_match	gene: Zm00001d008289	Zm00001d008289_T007	0.00	31.90	coding	515	275	1819
PB.9083.1	8	3495	14	novel_not_in_catalog	gene: Zm00001d008289	novel	31.90	31.90	coding	950	291	3140
PB.6286.8	5	1631	4	novel_in_catalog	gene: Zm00001d014347	novel	6.52	26.00	coding	105	445	759
PB.10129.5	9	3449	10	full-splice_match	gene: Zm00001d045217	Zm00001d045217_T011	0.00	5.86	coding	1040	123	3242
PB.6286.5	5	1420	6	full-splice_match	gene: Zm00001d014347	Zm00001d014347_T004	1.61	26.00	non_coding	NA	NA	NA
PB.2481.5	2	2172	16	novel_not_in_catalog	gene: Zm00001d002135	novel	5.35	49.63	coding	296	184	1071

PB.271.2	1	1845	1	full-splice_match	gene: Zm00001d028406	Zm00001d028406_T001	0.00	20.98	coding	547	121	1761
PB.6286.1	5	1881	4	novel_in_catalog	gene: Zm00001d014347	novel	4.28	26.00	non_coding	NA	NA	NA
PB.9566.3	8	1255	1	full-splice_match	gene: Zm00001d010751	Zm00001d010751_T001	136.29	158.47	coding	313	71	1009
PB.2481.2	2	2257	16	novel_not_in_catalog	gene: Zm00001d002135	novel	6.10	49.63	coding	296	139	1026
PB.2481.3	2	2167	17	novel_not_in_catalog	gene: Zm00001d002135	novel	38.18	49.63	coding	573	140	1858
PB.10923.1	B73V4 ctg51	1802	2	genic	gene: Zm00001d000314	novel	23.88	23.88	coding	412	114	1349
PB.9102.2	8	1313	1	full-splice_match	gene: Zm00001d008370	Zm00001d008370_T001	5.69	6.03	coding	363	53	1141
PB.9102.1	8	1427	1	full-splice_match	gene: Zm00001d008370	Zm00001d008370_T001	0.34	6.03	coding	363	52	1140
PB.10923.2	B73V4 ctg51	1673	2	genic	gene: Zm00001d000314	novel	0.00	23.88	coding	412	86	1321
PB.5673.1	4	2679	2	full-splice_match	gene: Zm00001d053119	Zm00001d053119_T001	7.51	48.27	coding	679	176	2212
PB.5730.1	4	2343	2	novel_in_catalog	gene: Zm00001d053438	novel	10.27	22.12	coding	478	459	1892
PB.3076.1	2	1757	3	incomplete-splice_match	gene: Zm00001d005195	Zm00001d005195_T013	25.47	25.47	non_coding	NA	NA	NA
PB.622.1	1	1407	3	full-splice_match	gene: Zm00001d030103	Zm00001d030103_T001	29.26	40.40	coding	347	168	1208
PB.622.2	1	1147	3	full-splice_match	gene: Zm00001d030103	Zm00001d030103_T001	11.14	40.40	coding	347	17	1057
PB.7487.2	6	2766	4	novel_not_in_catalog	gene: Zm00001d036541	novel	4.16	174.28	coding	187	8	568
PB.1337.1	1	1167	8	full-splice_match	gene: Zm00001d033788	Zm00001d033788_T001	80.82	80.82	coding	270	129	938
PB.962.1	1	1804	8	novel_in_catalog	gene: Zm00001d032079	novel	14.64	15.19	coding	493	6	1484

PB.5281.1	4	1288	2	full-splice_match	gene: Zm00001d051420	Zm00001d051420_T001	377.38	377.38	coding	289	75	941
PB.6283.1	5	1340	3	full-splice_match	gene: Zm00001d014341	Zm00001d014341_T001	10.84	10.84	coding	341	77	1099
PB.1774.2	10	3374	8	full-splice_match	gene: Zm00001d023838	Zm00001d023838_T001	34.89	69.36	coding	884	291	2942
PB.1774.3	10	3240	8	full-splice_match	gene: Zm00001d023838	Zm00001d023838_T001	0.00	69.36	coding	884	346	2997
PB.2461.4	2	1365	7	novel_in_catalog	gene: Zm00001d002035	novel	6.23	196.16	coding	159	574	1050
PB.2461.5	2	1998	7	novel_in_catalog	gene: Zm00001d002035	novel	6.14	196.16	coding	186	1203	1760
PB.2461.2	2	1297	8	full-splice_match	gene: Zm00001d002035	Zm00001d002035_T001	59.83	196.16	coding	291	94	966
PB.2461.3	2	2154	6	novel_in_catalog	gene: Zm00001d002035	novel	0.90	196.16	coding	159	1362	1838
PB.7941.1	6	1258	3	full-splice_match	gene: Zm00001d038476	Zm00001d038476_T001	27.97	27.97	coding	245	156	890
PB.9049.1	7	1203	1	full-splice_match	gene: Zm00001d022624	Zm00001d022624_T001	20.03	20.03	coding	111	621	953
PB.1110.1	1	2534	4	full-splice_match	gene: Zm00001d032683	Zm00001d032683_T001	11.43	14.66	coding	681	118	2160
PB.7783.1	6	1914	2	antisense	novelGene_gene: Zm00001d037932_A	novel	5.80	5.80	coding	127	291	671
PB.8424.17	7	1117	2	novel_in_catalog	gene: Zm00001d020185	novel	16.73	341.07	coding	240	16	735
PB.2461.8	2	1228	7	novel_in_catalog	gene: Zm00001d002035	novel	0.00	196.16	coding	159	558	1034
PB.1147.3	1	2913	12	incomplete-splice_match	gene: Zm00001d032855	Zm00001d032855_T001	1.55	25.91	coding	666	319	2316
PB.4931.2	4	2887	9	novel_in_catalog	gene: Zm00001d049251	novel	1.39	18.02	coding	548	1081	2724
PB.4931.3	4	2757	10	incomplete-splice_match	gene: Zm00001d049251	Zm00001d049251_T032	9.60	18.02	coding	507	1061	2581

PB.5461.3	4	2540	20	novel_in_catalog	gene: Zm00001d052248	novel	7.09	19.85	coding	174	641	1162
PB.8898.1	7	3688	17	full-splice_match	gene: Zm00001d022114	Zm00001d022114_T003	15.67	15.67	coding	1015	383	3427
PB.8898.2	7	3612	17	full-splice_match	gene: Zm00001d022114	Zm00001d022114_T003	0.00	15.67	coding	1015	410	3454
PB.9646.1	8	2746	9	full-splice_match	gene: Zm00001d011108	Zm00001d011108_T003	22.30	22.30	coding	759	218	2494
PB.1147.1	1	2419	8	incomplete-splice_match	gene: Zm00001d032855	Zm00001d032855_T001	21.43	25.91	coding	519	88	1644
PB.9646.4	8	2509	9	full-splice_match	gene: Zm00001d011108	Zm00001d011108_T003	0.00	22.30	coding	759	94	2370
PB.9646.5	8	2160	9	full-splice_match	gene: Zm00001d011108	Zm00001d011108_T003	0.00	22.30	coding	644	228	2159
PB.3849.1	3	2591	2	genic	gene: Zm00001d040222	novel	0.35	0.35	coding	784	87	2438
PB.8424.10	7	2141	1	intergenic	novelGene_171	novel	4.47	4.47	non_coding	NA	NA	NA
PB.3064.4	2	2510	10	novel_in_catalog	gene: Zm00001d005144	novel	3.36	38.64	coding	264	69	860
PB.5412.1	4	1750	1	intergenic	novelGene_296	novel	4.08	4.08	coding	113	517	855
PB.5777.2	4	1684	2	genic	gene: Zm00001d053650	novel	0.35	26.54	coding	427	207	1487
PB.5851.2	4	1311	6	full-splice_match	gene: Zm00001d053932	Zm00001d053932_T003	106.51	151.82	coding	311	111	1043
PB.8424.13	7	2947	13	novel_in_catalog	gene: Zm00001d020185	novel	101.67	341.07	coding	896	16	2703
PB.3064.2	2	2298	11	full-splice_match	gene: Zm00001d005144	Zm00001d005144_T005	4.29	38.64	coding	264	92	883
PB.1897.1	10	965	4	fusion	gene: Zm00001d024591_g	novel	85.99	85.99	non_coding	NA	NA	NA
PB.9539.1	8	894	2	genic	gene: Zm00001d010650	novel	752.05	755.88	coding	149	106	552

PB.10024.1	8	764	1	novel_in_catalog	gene: Zm00001d012750	novel	85.41	113.43	non_coding	NA	NA	NA
PB.9539.2	8	2305	1	full-splice_match	gene: Zm00001d010650	Zm00001d010650_T001	3.83	755.88	coding	113	1793	2131
PB.9998.3	8	1361	9	full-splice_match	gene: Zm00001d012659	Zm00001d012659_T007	28.45	33.32	coding	274	259	1080
PB.9998.1	8	2659	9	full-splice_match	gene: Zm00001d012659	Zm00001d012659_T005	4.88	33.32	coding	274	1562	2383
PB.10003.15	8	1794	10	full-splice_match	gene: Zm00001d012667	Zm00001d012667_T029	8.47	43.63	coding	517	14	1564
PB.5793.1	4	898	4	novel_not_in_catalog	gene: Zm00001d053728	novel	214.68	214.68	coding	176	160	687
PB.302.4	1	1202	2	full-splice_match	gene: Zm00001d028540	Zm00001d028540_T001	4.40	26.84	coding	197	144	734
PB.302.3	1	1260	2	full-splice_match	gene: Zm00001d028540	Zm00001d028540_T001	0.00	26.84	coding	197	87	677
PB.9093.1	8	862	1	full-splice_match	gene: Zm00001d008329	Zm00001d008329_T001	98.08	98.08	coding	96	103	390
PB.302.1	1	1391	2	full-splice_match	gene: Zm00001d028540	Zm00001d028540_T001	22.43	26.84	coding	197	87	677
PB.5952.1	5	584	1	full-splice_match	gene: Zm00001d012956	Zm00001d012956_T001	10.24	10.24	coding	95	135	419
PB.10441.2	9	2341	2	full-splice_match	gene: Zm00001d047030	Zm00001d047030_T085	33.52	128.55	coding	668	23	2026
PB.1601.1	1	1809	9	full-splice_match	gene: Zm00001d034814	Zm00001d034814_T001	28.05	28.05	coding	518	202	1755
PB.690.2	1	1552	10	fusion	gene: Zm00001d030588_g	novel	16.00	16.00	coding	166	85	582
PB.8473.1	7	2036	5	full-splice_match	gene: Zm00001d020418	Zm00001d020418_T001	8.68	8.68	coding	527	129	1709
PB.1312.12	1	2480	10	novel_in_catalog	gene: Zm00001d033652	novel	2.46	87.02	coding	540	248	1867
PB.1312.14	1	2217	12	novel_not_in_catalog	gene: Zm00001d033652	novel	0.00	87.02	coding	605	229	2043

PB.4368.9	3	1861	5	full-splice_match	gene: Zm00001d043019	Zm00001d043019_T002	98.95	238.63	coding	572	92	1807
PB.6518.2	5	2207	2	genic	gene: Zm00001d015325	novel	0.00	12.94	coding	470	536	1945
PB.4368.4	3	2413	2	novel_in_catalog	gene: Zm00001d043019	novel	1.28	238.63	coding	520	643	2202
PB.1976.1	10	878	2	full-splice_match	gene: Zm00001d024946	Zm00001d024946_T001	10.98	10.98	coding	201	103	705
PB.638.1	1	1595	5	full-splice_match	gene: Zm00001d030172	Zm00001d030172_T003	7.79	18.58	coding	289	346	1212
PB.6687.1	5	1675	10	full-splice_match	gene: Zm00001d016477	Zm00001d016477_T001	20.25	20.25	coding	491	42	1514
PB.801.1	1	2244	1	full-splice_match	gene: Zm00001d031230	Zm00001d031230_T001	20.00	30.76	coding	674	39	2060
PB.638.2	1	1832	6	full-splice_match	gene: Zm00001d030172	Zm00001d030172_T002	10.79	18.58	coding	495	102	1586
PB.8302.6	7	1996	11	novel_not_in_catalog	gene: Zm00001d019327	novel	1.73	173.25	coding	549	190	1836
PB.8302.7	7	2038	10	novel_not_in_catalog	gene: Zm00001d019327	novel	21.29	173.25	coding	312	946	1881
PB.8302.4	7	1998	11	novel_not_in_catalog	gene: Zm00001d019327	novel	121.92	173.25	coding	549	80	1726
PB.8302.5	7	1822	10	novel_not_in_catalog	gene: Zm00001d019327	novel	4.63	173.25	coding	475	232	1656
PB.5475.4	4	2360	3	incomplete-splice_match	gene: Zm00001d052307	Zm00001d052307_T015	0.00	21.79	coding	712	18	2153
PB.8302.3	7	2377	9	novel_not_in_catalog	gene: Zm00001d019327	novel	0.16	173.25	coding	312	1169	2104
PB.8302.1	7	2507	9	novel_not_in_catalog	gene: Zm00001d019327	novel	5.78	173.25	coding	357	1153	2223
PB.821.1	1	2627	15	full-splice_match	gene: Zm00001d031316	Zm00001d031316_T009	12.43	15.93	coding	608	253	2076
PB.9957.4	8	2119	5	full-splice_match	gene: Zm00001d012515	Zm00001d012515_T002	0.00	24.92	coding	551	281	1933

PB.4308.2	3	2381	5	novel_not_in_catalog	gene: Zm00001d042731	novel	33.63	56.15	coding	376	821	1948
PB.2160.1	10	2034	8	full-splice_match	gene: Zm00001d025893	Zm00001d025893_T001	21.78	21.78	coding	490	168	1637
PB.9957.1	8	2257	5	full-splice_match	gene: Zm00001d012515	Zm00001d012515_T002	24.92	24.92	coding	551	300	1952
PB.8302.9	7	1920	8	novel_not_in_catalog	gene: Zm00001d019327	novel	4.13	173.25	coding	312	830	1765
PB.5088.1	4	1440	6	novel_not_in_catalog	gene: Zm00001d050123	novel	11.27	34.74	coding	309	152	1078
PB.5088.2	4	1504	5	full-splice_match	gene: Zm00001d050123	Zm00001d050123_T001	23.47	34.74	coding	332	153	1148
PB.5635.1	4	2342	2	full-splice_match	gene: Zm00001d052937	Zm00001d052937_T001	41.11	41.11	coding	719	120	2276
PB.7256.1	6	1913	6	full-splice_match	gene: Zm00001d035186	Zm00001d035186_T001	39.52	39.52	coding	389	338	1504
PB.7256.3	6	1775	6	full-splice_match	gene: Zm00001d035186	Zm00001d035186_T001	0.00	39.52	coding	389	338	1504
PB.5767.2	4	912	5	full-splice_match	gene: Zm00001d053632	Zm00001d053632_T001	943.79	1142.28	coding	221	74	736
PB.3458.1	2	744	4	incomplete-splice_match	gene: Zm00001d007067	Zm00001d007067_T002	81.45	81.45	coding	123	127	495
PB.5767.1	4	1068	5	full-splice_match	gene: Zm00001d053632	Zm00001d053632_T001	198.49	1142.28	coding	221	61	723
PB.1944.1	10	2254	6	incomplete-splice_match	gene: Zm00001d024816	Zm00001d024816_T001	30.52	30.52	coding	677	42	2072
PB.2661.1	2	315	2	incomplete-splice_match	gene: Zm00001d002873	Zm00001d002873_T005	107.75	107.75	non_coding	NA	NA	NA
PB.4597.2	3	1209	2	novel_in_catalog	gene: Zm00001d043904	novel	95.46	113.53	coding	148	37	480
PB.1591.3	1	3136	1	full-splice_match	gene: Zm00001d034781	Zm00001d034781_T001	11.79	43.63	coding	852	233	2788
PB.8338.1	7	1269	3	full-splice_match	gene: Zm00001d019565	Zm00001d019565_T001	313.96	324.77	coding	288	95	958

PB.8338.2	7	1335	2	novel_in_catalog	gene: Zm00001d019565	novel	10.81	324.77	coding	269	93	899
PB.10473.7	9	842	1	incomplete-splice_match	gene: Zm00001d047240	Zm00001d047240_T002	8.86	84.90	non_coding	NA	NA	NA
PB.10473.6	9	1086	1	incomplete-splice_match	gene: Zm00001d047240	Zm00001d047240_T003	27.52	84.90	coding	247	345	1085
PB.10473.5	9	2886	3	full-splice_match	gene: Zm00001d047240	Zm00001d047240_T001	13.57	84.90	coding	785	413	2767
PB.10473.4	9	3959	1	novel_in_catalog	gene: Zm00001d047240	novel	11.57	84.90	coding	823	1371	3839
PB.8424.3	7	1922	9	novel_in_catalog	gene: Zm00001d020183	novel	11.76	177.20	coding	397	162	1352
PB.10473.1	9	3127	2	novel_in_catalog	gene: Zm00001d047240	novel	3.55	84.90	coding	823	413	2881
PB.9136.1	8	2543	12	full-splice_match	gene: Zm00001d008512	Zm00001d008512_T003	2.53	2.53	coding	667	153	2153
PB.8936.2	7	1465	5	novel_in_catalog	gene: Zm00001d022254	novel	3.07	22.75	coding	194	127	708
PB.3721.1	3	4505	22	full-splice_match	gene: Zm00001d039522	Zm00001d039522_T002	18.81	18.81	coding	1181	528	4070
PB.8796.1	7	2621	10	novel_in_catalog	gene: Zm00001d021753	novel	10.80	113.38	coding	431	17	1309
PB.4051.1	3	2640	2	full-splice_match	gene: Zm00001d041550	Zm00001d041550_T005	5.17	2134.62	coding	649	86	2032
PB.8796.3	7	1315	2	novel_in_catalog	gene: Zm00001d021753	novel	16.44	113.38	non_coding	NA	NA	NA
PB.8796.2	7	1797	9	novel_not_in_catalog	gene: Zm00001d021753	novel	14.10	113.38	coding	201	24	626
PB.10072.2	9	664	4	full-splice_match	gene: Zm00001d044939	Zm00001d044939_T002	207.33	228.40	coding	122	134	499
PB.8796.4	7	1646	7	novel_in_catalog	gene: Zm00001d021753	novel	51.61	113.38	coding	232	25	720
PB.127.2	1	2309	10	full-splice_match	gene: Zm00001d027749	Zm00001d027749_T001	61.37	68.20	coding	653	172	2130

PB.10072.1	9	730	5	full-splice_match	gene: Zm00001d044939	Zm00001d044939_T001	21.07	228.40	coding	122	78	443
PB.663.1	1	963	2	full-splice_match	gene: Zm00001d030346	Zm00001d030346_T001	98.29	98.29	coding	208	142	765
PB.3701.1	3	1221	7	full-splice_match	gene: Zm00001d039451	Zm00001d039451_T007	35.65	37.85	coding	255	50	814
PB.3701.2	3	1485	6	novel_in_catalog	gene: Zm00001d039451	novel	2.19	37.85	coding	167	51	551
PB.663.2	1	798	2	full-splice_match	gene: Zm00001d030346	Zm00001d030346_T001	0.00	98.29	coding	208	95	718
PB.4051.2	3	2458	2	full-splice_match	gene: Zm00001d041550	Zm00001d041550_T005	61.77	2134.62	coding	649	85	2031
PB.7054.2	5	2255	4	full-splice_match	gene: Zm00001d018090	Zm00001d018090_T001	9.51	433.60	coding	156	55	522
PB.10195.1	9	1562	1	full-splice_match	gene: Zm00001d045496	Zm00001d045496_T001	17.60	17.60	coding	382	86	1231
PB.10341.1	9	1457	7	full-splice_match	gene: Zm00001d046467	Zm00001d046467_T001	119.08	121.40	coding	361	84	1166
PB.8779.7	7	458	1	genic_intron	novelGene_263	novel	0.00	0.00	non_coding	NA	NA	NA
PB.10341.6	9	1231	7	novel_not_in_catalog	gene: Zm00001d046467	novel	2.32	121.40	coding	175	86	610
PB.10341.5	9	1338	7	full-splice_match	gene: Zm00001d046467	Zm00001d046467_T001	0.00	121.40	coding	361	80	1162
PB.4051.4	3	2252	2	full-splice_match	gene: Zm00001d041550	Zm00001d041550_T005	1852.60	2134.62	coding	649	85	2031
PB.2835.15	2	1180	6	full-splice_match	gene: Zm00001d003616	Zm00001d003616_T001	113.61	253.94	coding	269	172	978
PB.2835.14	2	2643	5	novel_in_catalog	gene: Zm00001d003616	novel	4.51	253.94	coding	107	2108	2428
PB.2835.17	2	1158	6	novel_not_in_catalog	gene: Zm00001d003616	novel	13.30	253.94	coding	230	172	861
PB.7888.1	6	2352	1	full-splice_match	gene: Zm00001d038269	Zm00001d038269_T001	9.16	9.16	coding	660	129	2108

PB.8277.1	7	1505	5	full-splice_match	gene: Zm00001d019194	Zm00001d019194_T008	13.66	13.66	coding	251	165	917
PB.2835.13	2	1340	5	novel_in_catalog	gene: Zm00001d003616	novel	6.90	253.94	coding	240	174	893
PB.10683.5	9	2439	6	full-splice_match	gene: Zm00001d048046	Zm00001d048046_T001	0.00	66.01	coding	686	295	2352
PB.2143.3	10	2038	1	genic	gene: Zm00001d025808	novel	15.87	19.68	coding	546	281	1918
PB.2415.2	2	1549	5	full-splice_match	gene: Zm00001d001855	Zm00001d001855_T002	10.16	10.16	coding	354	159	1220
PB.7423.1	6	3525	25	full-splice_match	gene: Zm00001d036161	Zm00001d036161_T001	14.98	34.34	coding	900	196	2895
PB.2835.19	2	1220	6	novel_not_in_catalog	gene: Zm00001d003616	novel	0.24	253.94	coding	251	173	925
PB.2835.18	2	1453	4	novel_in_catalog	gene: Zm00001d003616	novel	0.00	253.94	coding	161	181	663
PB.7423.5	6	3377	25	full-splice_match	gene: Zm00001d036161	Zm00001d036161_T001	6.66	34.34	coding	900	194	2893
PB.9904.3	8	2437	3	intergenic	novelGene_282	novel	27.62	27.62	coding	692	194	2269
PB.2536.1	2	784	3	full-splice_match	gene: Zm00001d002344	Zm00001d002344_T001	73.57	73.57	coding	107	227	547
PB.1059.1	1	1269	9	full-splice_match	gene: Zm00001d032501	Zm00001d032501_T002	33.05	33.05	coding	307	109	1029
PB.9406.1	8	1248	4	incomplete-splice_match	gene: Zm00001d009979	Zm00001d009979_T002	31.09	31.09	coding	243	54	782
PB.4691.5	3	2397	4	full-splice_match	gene: Zm00001d044291	Zm00001d044291_T001	0.00	52.55	coding	701	82	2184
PB.6103.3	5	1666	2	full-splice_match	gene: Zm00001d013468	Zm00001d013468_T002	0.00	21.74	coding	431	245	1537
PB.6103.2	5	1782	2	full-splice_match	gene: Zm00001d013468	Zm00001d013468_T002	0.00	21.74	coding	431	245	1537
PB.6103.1	5	1934	2	full-splice_match	gene: Zm00001d013468	Zm00001d013468_T002	21.74	21.74	coding	431	241	1533

PB.3974.2	3	3038	15	full-splice_match	gene: Zm00001d041056	Zm00001d041056_T014	0.00	15.05	coding	830	261	2750
PB.3974.3	3	2872	11	novel_in_catalog	gene: Zm00001d041056	novel	1.86	15.05	coding	320	431	1390
PB.3974.1	3	3185	15	full-splice_match	gene: Zm00001d041056	Zm00001d041056_T014	13.19	15.05	coding	830	265	2754
PB.1166.1	1	1772	4	full-splice_match	gene: Zm00001d032932	Zm00001d032932_T001	16.70	16.70	coding	428	190	1473
PB.5825.1	4	1297	3	full-splice_match	gene: Zm00001d053856	Zm00001d053856_T001	41.05	41.05	coding	345	89	1123
PB.7917.1	6	1229	9	full-splice_match	gene: Zm00001d038380	Zm00001d038380_T001	198.57	294.87	coding	215	184	828
PB.2888.1	2	1193	4	full-splice_match	gene: Zm00001d003897	Zm00001d003897_T001	5.07	5.07	coding	18	450	503
PB.5487.2	4	1855	2	genic	gene: Zm00001d052340	novel	0.00	2.71	coding	443	378	1706
PB.5487.1	4	2025	2	genic	gene: Zm00001d052340	novel	2.71	2.71	coding	443	455	1783
PB.10441.1	9	2958	3	incomplete-splice_match	gene: Zm00001d047030	Zm00001d047030_T070	95.04	128.55	coding	813	4	2442
PB.7054.4	5	2413	3	novel_in_catalog	gene: Zm00001d018090	novel	3.98	433.60	coding	172	1735	2250
PB.479.1	1	1051	4	full-splice_match	gene: Zm00001d029325	Zm00001d029325_T002	37.81	37.81	coding	154	321	782
PB.10534.1	9	1448	3	full-splice_match	gene: Zm00001d047514	Zm00001d047514_T001	9.89	9.89	coding	321	157	1119
PB.2508.1	2	651	1	full-splice_match	gene: Zm00001d002253	Zm00001d002253_T001	759.18	759.18	coding	137	62	472
PB.7880.1	6	1378	2	incomplete-splice_match	gene: Zm00001d038223	Zm00001d038223_T001	5.42	5.42	coding	264	110	901
PB.9912.1	8	1375	1	novel_in_catalog	gene: Zm00001d012292	novel	2.88	33.83	coding	356	120	1187
PB.2693.2	2	2294	7	novel_not_in_catalog	gene: Zm00001d003012	novel	9.08	19.62	coding	320	174	1133

PB.2693.1	2	2311	7	novel_not_in_catalog	gene: Zm00001d003012	novel	10.54	19.62	coding	320	188	1147
PB.4442.1	3	1594	6	novel_in_catalog	gene: Zm00001d043328	novel	9.23	9.23	coding	204	42	653
PB.3833.1	3	2293	1	full-splice_match	gene: Zm00001d040157	Zm00001d040157_T001	5.76	29.67	coding	640	122	2041
PB.3833.3	3	2216	1	full-splice_match	gene: Zm00001d040157	Zm00001d040157_T001	23.91	29.67	coding	640	195	2114
PB.10329.1	9	2007	4	full-splice_match	gene: Zm00001d046399	Zm00001d046399_T001	45.60	45.60	coding	439	232	1548
PB.4676.5	3	1484	3	incomplete-splice_match	gene: Zm00001d044232	Zm00001d044232_T004	29.29	70.69	coding	260	414	1193
PB.1178.1	1	1656	2	full-splice_match	gene: Zm00001d033005	Zm00001d033005_T001	29.79	32.14	coding	360	152	1231
PB.7516.2	6	1705	7	novel_in_catalog	gene: Zm00001d036654	novel	6.27	102.24	coding	261	577	1359
PB.7516.3	6	1628	8	full-splice_match	gene: Zm00001d036654	Zm00001d036654_T002	38.25	102.24	coding	349	245	1291
PB.1197.1	1	2743	2	novel_in_catalog	gene: Zm00001d033111	novel	8.80	8.80	coding	144	96	527
PB.2740.1	2	1345	3	novel_not_in_catalog	gene: Zm00001d003185	novel	18.44	18.44	coding	375	135	1259
PB.5662.1	4	784	1	full-splice_match	gene: Zm00001d053055	Zm00001d053055_T001	242.10	649.95	coding	171	59	571
PB.881.2	1	1753	7	incomplete-splice_match	gene: Zm00001d031667	Zm00001d031667_T016	15.12	21.70	coding	410	68	1297
PB.7516.4	6	1233	8	full-splice_match	gene: Zm00001d036654	Zm00001d036654_T002	44.27	102.24	coding	325	60	1034
PB.6858.9	5	2473	2	full-splice_match	gene: Zm00001d017274	Zm00001d017274_T002	7.15	2528.25	coding	585	401	2155
PB.8445.2	7	1386	6	full-splice_match	gene: Zm00001d020297	Zm00001d020297_T004	37.05	111.51	coding	335	87	1091
PB.10865.1	B73V4 ctg150	2397	3	full-splice_match	gene: Zm00001d000005	Zm00001d000005_T001	7.99	7.99	coding	664	42	2033

PB.2500.4	2	1621	15	full-splice_match	gene: Zm00001d002185	Zm00001d002185_T002	0.00	11.35	coding	469	65	1471
PB.356.1	1	1532	5	full-splice_match	gene: Zm00001d028782	Zm00001d028782_T002	74.93	74.93	coding	339	137	1153
PB.8826.1	7	922	2	full-splice_match	gene: Zm00001d021850	Zm00001d021850_T001	115.72	115.72	coding	180	211	750
PB.2500.1	2	1841	15	full-splice_match	gene: Zm00001d002185	Zm00001d002185_T002	8.35	11.35	coding	469	113	1519
PB.2500.2	2	2185	14	novel_in_catalog	gene: Zm00001d002185	novel	3.00	11.35	coding	246	83	820
PB.9855.3	8	1583	2	novel_in_catalog	gene: Zm00001d012031	novel	2.86	88.09	coding	102	210	515
PB.773.3	1	2191	5	novel_in_catalog	gene: Zm00001d031092	novel	0.00	81.05	coding	219	245	901
PB.773.2	1	1887	6	full-splice_match	gene: Zm00001d031092	Zm00001d031092_T002	53.25	81.05	coding	330	267	1256
PB.773.1	1	2286	5	novel_in_catalog	gene: Zm00001d031092	novel	19.56	81.05	coding	219	254	910
PB.6858.5	5	3449	1	novel_in_catalog	gene: Zm00001d017274	novel	12.57	2528.25	coding	557	1506	3176
PB.4735.1	3	651	1	intergenic	novelGene_350	novel	955.15	955.15	coding	20	314	373
PB.8538.1	7	1822	2	full-splice_match	gene: Zm00001d020721	Zm00001d020721_T001	4.15	4.15	coding	550	131	1780
PB.773.4	1	2502	5	full-splice_match	gene: Zm00001d031092	Zm00001d031092_T003	0.00	81.05	coding	219	558	1214
PB.6834.2	5	1757	10	full-splice_match	gene: Zm00001d017175	Zm00001d017175_T002	37.46	84.08	coding	467	56	1456
PB.9587.1	8	2409	4	novel_not_in_catalog	gene: Zm00001d010828	novel	0.82	0.82	coding	670	199	2208
PB.5102.3	4	665	2	full-splice_match	gene: Zm00001d050179	Zm00001d050179_T001	0.00	534.87	coding	149	51	497
PB.6113.1	5	946	3	genic	gene: Zm00001d013504	novel	60.99	60.99	coding	224	97	768

PB.5102.1	4	794	2	full-splice_match	gene: Zm00001d050179	Zm00001d050179_T001	532.11	534.87	coding	149	73	519
PB.8308.1	7	1719	9	full-splice_match	gene: Zm00001d019423	Zm00001d019423_T003	21.71	21.71	coding	445	77	1411
PB.2520.4	2	2014	10	full-splice_match	gene: Zm00001d002292	Zm00001d002292_T009	4.30	98.71	coding	321	339	1301
PB.2520.5	2	1331	9	incomplete-splice_match	gene: Zm00001d002292	Zm00001d002292_T006	28.91	98.71	coding	370	70	1179
PB.2520.2	2	2515	8	novel_in_catalog	gene: Zm00001d002292	novel	0.83	98.71	coding	217	1128	1778
PB.2520.3	2	1981	10	full-splice_match	gene: Zm00001d002292	Zm00001d002292_T008	13.96	98.71	coding	290	748	1617
PB.2520.1	2	1976	11	full-splice_match	gene: Zm00001d002292	Zm00001d002292_T006	50.72	98.71	coding	394	348	1529
PB.5166.1	4	1859	7	novel_in_catalog	gene: Zm00001d050610	novel	0.00	57.17	coding	411	3	1235
PB.5166.3	4	2099	9	full-splice_match	gene: Zm00001d050610	Zm00001d050610_T001	48.09	57.17	coding	477	186	1616
PB.5166.2	4	2195	8	novel_in_catalog	gene: Zm00001d050610	novel	8.91	57.17	coding	220	1037	1696
PB.465.1	1	1397	1	intergenic	novelGene_122	novel	30.27	30.27	non_coding	NA	NA	NA
PB.5166.4	4	2228	7	novel_in_catalog	gene: Zm00001d050610	novel	0.17	57.17	coding	180	188	727
PB.4857.1	4	2054	2	genic	gene: Zm00001d048898	novel	57.13	57.13	coding	435	349	1653
PB.465.2	1	842	4	antisense	novelGene_gene: Zm00001d029257_A	novel	33.77	33.77	non_coding	NA	NA	NA
PB.6301.22	5	2855	5	novel_in_catalog	gene: Zm00001d014423	novel	0.89	169.16	coding	491	469	1941
PB.3676.1	3	1359	2	genic	gene: Zm00001d039365	novel	104.73	104.73	coding	305	241	1155
PB.4400.4	3	2166	11	novel_in_catalog	gene: Zm00001d043152	novel	6.09	93.99	coding	309	73	999

PB.1278.2	1	2451	11	novel_not_in_catalog	gene: Zm00001d033525	novel	1.37	5.79	coding	509	744	2270
PB.7744.1	6	460	2	incomplete-splice_match	gene: Zm00001d037775	Zm00001d037775_T003	61.93	61.93	non_coding	NA	NA	NA
PB.9545.1	8	1296	4	full-splice_match	gene: Zm00001d010662	Zm00001d010662_T001	24.00	24.00	coding	285	235	1089
PB.9817.2	8	2230	5	novel_in_catalog	gene: Zm00001d011855	novel	3.27	71.85	coding	485	128	1582
PB.379.2	1	1641	8	full-splice_match	gene: Zm00001d028903	Zm00001d028903_T001	52.29	52.29	coding	368	240	1343
PB.10082.3	9	964	1	full-splice_match	gene: Zm00001d045017	Zm00001d045017_T001	29.09	83.69	coding	119	367	723
PB.10082.2	9	1090	1	full-splice_match	gene: Zm00001d045017	Zm00001d045017_T001	46.31	83.69	coding	119	381	737
PB.10082.1	9	1214	1	full-splice_match	gene: Zm00001d045017	Zm00001d045017_T001	8.30	83.69	coding	119	381	737
PB.1587.1	1	1083	1	full-splice_match	gene: Zm00001d034763	Zm00001d034763_T001	15.28	15.28	coding	216	163	810
PB.6726.5	5	1613	13	full-splice_match	gene: Zm00001d016702	Zm00001d016702_T006	0.00	20.56	coding	427	11	1291
PB.4749.1	3	1901	1	full-splice_match	gene: Zm00001d044537	Zm00001d044537_T001	2.54	2.54	coding	560	47	1726
PB.5265.1	4	3585	1	genic	gene: Zm00001d051334	novel	5.97	5.97	coding	160	115	594
PB.4195.2	3	1397	1	genic	gene: Zm00001d042245	novel	12.33	264.55	coding	162	253	738
PB.4195.3	3	1053	2	full-splice_match	gene: Zm00001d042245	Zm00001d042245_T001	0.00	264.55	coding	144	253	684
PB.6946.2	5	2017	1	full-splice_match	gene: Zm00001d017678	Zm00001d017678_T001	0.00	19.01	coding	405	431	1645
PB.8530.1	7	1418	2	full-splice_match	gene: Zm00001d020686	Zm00001d020686_T001	24.50	24.50	coding	278	44	877
PB.4259.2	3	1296	11	full-splice_match	gene: Zm00001d042530	Zm00001d042530_T003	19.19	21.19	coding	316	80	1027

PB.4028.1	3	3058	11	full-splice_match	gene: Zm00001d041445	Zm00001d041445_T002	60.21	157.23	coding	772	110	2425
PB.4028.3	3	2801	11	full-splice_match	gene: Zm00001d041445	Zm00001d041445_T010	0.00	157.23	coding	772	102	2417
PB.4028.5	3	2633	11	full-splice_match	gene: Zm00001d041445	Zm00001d041445_T010	0.00	157.23	coding	772	109	2424
PB.3648.1	3	2705	5	full-splice_match	gene: Zm00001d039287	Zm00001d039287_T002	23.85	58.02	coding	686	193	2250
PB.3648.2	3	1796	4	incomplete-splice_match	gene: Zm00001d039287	Zm00001d039287_T002	32.94	58.02	coding	364	303	1394
PB.3648.3	3	2537	5	full-splice_match	gene: Zm00001d039287	Zm00001d039287_T002	1.23	58.02	coding	686	217	2274
PB.1204.1	1	1871	3	novel_in_catalog	gene: Zm00001d033160	novel	5.27	74.31	coding	185	1069	1623
PB.4076.1	3	1557	9	full-splice_match	gene: Zm00001d041679	Zm00001d041679_T001	87.23	87.23	coding	394	164	1345
PB.10742.1	9	828	1	full-splice_match	gene: Zm00001d048263	Zm00001d048263_T001	17.20	17.20	coding	170	126	635
PB.3448.1	2	1978	1	full-splice_match	gene: Zm00001d007018	Zm00001d007018_T001	95.81	95.81	coding	125	219	593
PB.3258.1	2	2440	14	full-splice_match	gene: Zm00001d006160	Zm00001d006160_T001	145.69	145.69	coding	704	79	2190
PB.1579.2	1	1665	4	full-splice_match	gene: Zm00001d034738	Zm00001d034738_T001	17.39	19.79	coding	379	103	1239
PB.2167.1	10	898	1	full-splice_match	gene: Zm00001d025913	Zm00001d025913_T001	40.45	962.55	coding	150	82	531
PB.2167.3	10	685	1	full-splice_match	gene: Zm00001d025913	Zm00001d025913_T001	563.30	962.55	coding	150	83	532
PB.2167.2	10	795	1	full-splice_match	gene: Zm00001d025913	Zm00001d025913_T001	358.80	962.55	coding	150	83	532
PB.1978.4	10	3866	19	novel_not_in_catalog	gene: Zm00001d024952	novel	2.44	124.67	coding	882	86	2731
PB.2794.3	2	1597	3	full-splice_match	gene: Zm00001d003414	Zm00001d003414_T002	38.89	96.03	coding	365	190	1284

PB.2794.2	2	1464	4	novel_not_in_catalog	gene: Zm00001d003414	novel	53.08	96.03	coding	365	47	1141
PB.5067.1	4	1116	4	genic	gene: Zm00001d049966	novel	48.44	48.44	coding	232	276	971
PB.2794.4	2	1411	2	novel_in_catalog	gene: Zm00001d003414	novel	4.06	96.03	coding	150	665	1114
PB.1978.2	10	3839	20	novel_not_in_catalog	gene: Zm00001d024952	novel	3.18	124.67	coding	882	89	2734
PB.1978.3	10	3104	19	novel_not_in_catalog	gene: Zm00001d024952	novel	20.37	124.67	coding	888	89	2752
PB.71.1	1	2819	12	incomplete-splice_match	gene: Zm00001d027503	Zm00001d027503_T022	28.45	31.58	coding	833	26	2524
PB.1342.6	1	2574	4	full-splice_match	gene: Zm00001d033799	Zm00001d033799_T035	27.22	73.47	coding	803	3	2411
PB.4157.1	3	2768	6	full-splice_match	gene: Zm00001d042042	Zm00001d042042_T001	26.48	32.48	coding	333	471	1469
PB.1978.8	10	3027	19	novel_not_in_catalog	gene: Zm00001d024952	novel	0.00	124.67	coding	888	89	2752
PB.1978.9	10	2909	19	novel_not_in_catalog	gene: Zm00001d024952	novel	1.01	124.67	coding	854	91	2652
PB.71.7	1	2572	9	full-splice_match	gene: Zm00001d027503	Zm00001d027503_T043	3.13	31.58	coding	758	145	2418
PB.6719.2	5	2695	2	novel_not_in_catalog	gene: Zm00001d016674	novel	3.00	6.38	coding	188	449	1012
PB.3857.1	3	2814	8	full-splice_match	gene: Zm00001d040270	Zm00001d040270_T013	33.04	33.32	coding	693	502	2580
PB.3857.5	3	2354	7	full-splice_match	gene: Zm00001d040270	Zm00001d040270_T018	0.28	33.32	coding	645	374	2308
PB.9833.1	8	896	1	novel_in_catalog	gene: Zm00001d011938	novel	105.76	105.76	non_coding	NA	NA	NA
PB.762.1	1	1355	3	full-splice_match	gene: Zm00001d031071	Zm00001d031071_T001	68.36	68.36	coding	350	124	1173
PB.3640.1	3	3158	5	novel_in_catalog	gene: Zm00001d039266	novel	7.80	7.80	coding	916	35	2782

PB.1640.2	1	1591	9	incomplete-splice_match	gene: Zm00001d034954	Zm00001d034954_T001	21.20	47.91	coding	442	87	1412
PB.3223.1	2	385	1	full-splice_match	gene: Zm00001d006016	Zm00001d006016_T001	590.39	590.39	non_coding	NA	NA	NA
PB.5599.4	4	1414	7	incomplete-splice_match	gene: Zm00001d052779	Zm00001d052779_T002	10.37	14.33	coding	296	179	1066
PB.7685.1	6	2292	18	full-splice_match	gene: Zm00001d037538	Zm00001d037538_T002	53.25	53.25	coding	654	147	2108
PB.7051.2	5	1020	3	novel_not_in_catalog	gene: Zm00001d018072	novel	115.63	349.46	coding	118	230	583
PB.8853.4	7	2390	9	full-splice_match	gene: Zm00001d021956	Zm00001d021956_T006	0.00	13.38	coding	320	1075	2034
PB.8853.3	7	2528	9	full-splice_match	gene: Zm00001d021956	Zm00001d021956_T006	2.63	13.38	coding	320	1080	2039
PB.8853.1	7	2362	10	full-splice_match	gene: Zm00001d021956	Zm00001d021956_T002	10.75	13.38	coding	611	12	1844
PB.550.1	1	2031	4	full-splice_match	gene: Zm00001d029649	Zm00001d029649_T002	0.00	56.91	coding	469	93	1499
PB.7863.1	6	2654	2	full-splice_match	gene: Zm00001d038189	Zm00001d038189_T014	24.13	25.81	coding	654	132	2093
PB.8032.1	6	620	4	full-splice_match	gene: Zm00001d038839	Zm00001d038839_T002	110.17	587.75	coding	80	64	303
PB.8032.2	6	520	4	full-splice_match	gene: Zm00001d038839	Zm00001d038839_T002	477.58	587.75	coding	80	90	329
PB.8141.1	6	1476	3	novel_in_catalog	gene: Zm00001d039222	novel	43.05	43.05	coding	259	529	1305
PB.4012.2	3	945	6	full-splice_match	gene: Zm00001d041352	Zm00001d041352_T001	564.74	604.71	coding	223	38	706
PB.9444.1	8	2314	5	full-splice_match	gene: Zm00001d010200	Zm00001d010200_T001	14.22	14.22	coding	503	175	1683
PB.5991.1	5	833	7	full-splice_match	gene: Zm00001d013079	Zm00001d013079_T001	207.65	207.65	coding	194	17	598
PB.550.3	1	1708	4	full-splice_match	gene: Zm00001d029649	Zm00001d029649_T002	39.07	56.91	coding	469	126	1532

PB.8877.4	7	2472	9	novel_not_in_catalog	gene: Zm00001d022041	novel	21.11	35.21	coding	551	452	2104
PB.10562.1	9	1245	5	full-splice_match	gene: Zm00001d047635	Zm00001d047635_T005	227.26	242.87	coding	309	97	1023
PB.4718.1	3	3215	18	full-splice_match	gene: Zm00001d044433	Zm00001d044433_T010	12.68	12.68	coding	849	271	2817
PB.10712.3	9	2424	4	novel_not_in_catalog	gene: Zm00001d048142	novel	2.96	10.31	coding	510	689	2218
PB.10712.2	9	1923	4	novel_not_in_catalog	gene: Zm00001d048142	novel	7.35	10.31	coding	475	70	1494
PB.8263.1	7	1657	1	intergenic	novelGene_57	novel	0.15	0.15	coding	164	1055	1546
PB.1950.6	10	2704	18	full-splice_match	gene: Zm00001d024855	Zm00001d024855_T014	0.00	127.72	coding	744	166	2397
PB.1950.4	10	2835	18	full-splice_match	gene: Zm00001d024855	Zm00001d024855_T014	76.16	127.72	coding	744	216	2447
PB.10100.3	9	1621	6	full-splice_match	gene: Zm00001d045076	Zm00001d045076_T009	52.40	95.12	coding	184	87	638
PB.1950.2	10	3039	18	full-splice_match	gene: Zm00001d024855	Zm00001d024855_T002	39.87	127.72	coding	744	200	2431
PB.1950.3	10	2655	19	full-splice_match	gene: Zm00001d024855	Zm00001d024855_T010	0.00	127.72	coding	744	167	2398
PB.8552.1	7	1458	1	full-splice_match	gene: Zm00001d020789	Zm00001d020789_T001	6.45	6.45	coding	398	69	1262
PB.1950.1	10	2827	19	full-splice_match	gene: Zm00001d024855	Zm00001d024855_T010	11.68	127.72	coding	744	189	2420
PB.3504.3	2	1507	1	full-splice_match	gene: Zm00001d007274	Zm00001d007274_T001	0.00	338.75	coding	373	86	1204
PB.7119.2	5	2175	11	novel_in_catalog	gene: Zm00001d018336	novel	2.12	9.41	coding	441	182	1504
PB.65.1	1	907	2	full-splice_match	gene: Zm00001d027477	Zm00001d027477_T001	18.83	18.83	coding	153	120	578
PB.3365.1	2	1089	2	full-splice_match	gene: Zm00001d006608	Zm00001d006608_T001	14.25	14.25	coding	170	178	687

PB.477.1	1	2200	1	genic	gene: Zm00001d029321	novel	5.19	14.49	coding	321	135	1097
PB.477.2	1	1855	2	full-splice_match	gene: Zm00001d029321	Zm00001d029321_T001	9.31	14.49	coding	486	185	1642
PB.477.3	1	1703	2	full-splice_match	gene: Zm00001d029321	Zm00001d029321_T001	0.00	14.49	coding	486	134	1591
PB.191.1	1	414	1	novel_in_catalog	gene: Zm00001d028100	novel	26.97	61.76	non_coding	NA	NA	NA
PB.1466.4	1	3263	7	full-splice_match	gene: Zm00001d034350	Zm00001d034350_T010	0.00	23.85	coding	959	92	2968
PB.191.3	1	1323	11	incomplete-splice_match	gene: Zm00001d028100	Zm00001d028100_T002	34.79	61.76	non_coding	NA	NA	NA
PB.4503.2	3	2542	10	incomplete-splice_match	gene: Zm00001d043543	Zm00001d043543_T005	47.36	47.36	coding	533	2	1600
PB.9991.4	8	1979	9	novel_in_catalog	gene: Zm00001d012633	novel	1.84	111.96	coding	462	146	1531
PB.6167.1	5	1742	6	full-splice_match	gene: Zm00001d013745	Zm00001d013745_T001	61.35	69.38	coding	390	104	1273
PB.4936.1	4	1058	2	intergenic	novelGene_73	novel	2.91	38.75	non_coding	NA	NA	NA
PB.6167.3	5	1708	6	full-splice_match	gene: Zm00001d013745	Zm00001d013745_T001	4.63	69.38	coding	390	191	1360
PB.6167.2	5	2188	5	novel_in_catalog	gene: Zm00001d013745	novel	3.40	69.38	coding	271	54	866
PB.778.12	1	1481	10	incomplete-splice_match	gene: Zm00001d031120	Zm00001d031120_T003	0.00	204.87	coding	428	197	1480
PB.4978.3	4	2081	13	novel_in_catalog	gene: Zm00001d049522	novel	0.00	22.64	coding	567	149	1849
PB.1640.1	1	1954	10	full-splice_match	gene: Zm00001d034954	Zm00001d034954_T001	26.71	47.91	coding	502	145	1650
PB.4978.1	4	2245	13	novel_in_catalog	gene: Zm00001d049522	novel	22.64	22.64	coding	567	162	1862
PB.1524.5	1	1867	4	full-splice_match	gene: Zm00001d034568	Zm00001d034568_T005	0.00	20.21	coding	467	177	1577

PB.1524.4	1	1784	5	novel_in_catalog	gene: Zm00001d034568	novel	1.15	20.21	coding	232	786	1481
PB.10627.6	9	1788	3	novel_not_in_catalog	gene: Zm00001d047840	novel	67.33	67.33	coding	450	156	1505
PB.1524.2	1	3453	1	novel_in_catalog	gene: Zm00001d034568	novel	0.69	20.21	coding	177	1451	1981
PB.6204.1	5	3003	8	full-splice_match	gene: Zm00001d013964	Zm00001d013964_T004	30.60	30.60	coding	867	156	2756
PB.4308.7	3	2169	4	novel_in_catalog	gene: Zm00001d042731	novel	1.33	56.15	coding	376	771	1898
PB.5547.9	4	1752	14	full-splice_match	gene: Zm00001d052532	Zm00001d052532_T001	650.10	938.93	coding	452	101	1456
PB.1012.1	1	1625	3	full-splice_match	gene: Zm00001d032316	Zm00001d032316_T001	40.17	82.53	coding	270	486	1295
PB.7411.1	6	1223	5	full-splice_match	gene: Zm00001d036073	Zm00001d036073_T002	101.15	101.15	coding	288	161	1024
PB.2171.2	10	2022	7	novel_not_in_catalog	gene: Zm00001d025931	novel	10.21	25.04	coding	12	304	339
PB.2171.3	10	1898	8	novel_not_in_catalog	gene: Zm00001d025931	novel	0.00	25.04	coding	12	322	357
PB.10776.1	9	1954	14	full-splice_match	gene: Zm00001d048373	Zm00001d048373_T002	59.70	59.70	coding	550	155	1804
PB.2171.5	10	2018	6	novel_not_in_catalog	gene: Zm00001d025931	novel	2.91	25.04	coding	12	335	370
PB.2171.6	10	2024	7	novel_not_in_catalog	gene: Zm00001d025931	novel	4.63	25.04	coding	12	302	337
PB.13.1	1	754	1	full-splice_match	gene: Zm00001d027291	Zm00001d027291_T001	1059.61	1105.07	coding	144	90	521
PB.4489.1	3	2070	7	incomplete-splice_match	gene: Zm00001d043492	Zm00001d043492_T001	5.75	5.75	coding	412	8	1243
PB.6459.2	5	1672	1	novel_in_catalog	gene: Zm00001d015030	novel	3.51	9.59	coding	317	647	1597
PB.6459.1	5	1607	2	full-splice_match	gene: Zm00001d015030	Zm00001d015030_T001	6.08	9.59	coding	484	80	1531

PB.2052.1	10	1175	3	novel_in_catalog	gene: Zm00001d025338	novel	40.14	129.87	coding	110	98	427
PB.7572.2	6	1942	7	novel_in_catalog	gene: Zm00001d037001	novel	13.27	13.27	coding	226	887	1564
PB.2052.3	10	782	4	full-splice_match	gene: Zm00001d025338	Zm00001d025338_T001	89.74	129.87	coding	170	99	608
PB.2263.2	10	1900	2	full-splice_match	gene: Zm00001d026333	Zm00001d026333_T001	10.04	48.08	coding	471	108	1520
PB.9307.1	8	2095	15	full-splice_match	gene: Zm00001d009452	Zm00001d009452_T014	36.03	36.03	coding	564	104	1795
PB.9307.2	8	2026	15	full-splice_match	gene: Zm00001d009452	Zm00001d009452_T014	0.00	36.03	coding	564	106	1797
PB.7335.2	6	741	2	full-splice_match	gene: Zm00001d035659	Zm00001d035659_T001	20.31	373.16	coding	83	68	316
PB.4121.3	3	2157	2	incomplete-splice_match	gene: Zm00001d041914	Zm00001d041914_T004	9.63	9.63	coding	582	76	1821
PB.5151.2	4	2341	10	full-splice_match	gene: Zm00001d050483	Zm00001d050483_T003	13.31	13.31	coding	577	295	2025
PB.1633.1	1	1015	4	incomplete-splice_match	gene: Zm00001d034918	Zm00001d034918_T005	6.03	10.94	coding	204	98	709
PB.6445.1	5	1833	9	full-splice_match	gene: Zm00001d014983	Zm00001d014983_T001	37.54	37.54	coding	516	39	1586
PB.4294.2	3	2181	2	novel_in_catalog	gene: Zm00001d042684	novel	0.00	417.27	coding	241	261	983
PB.10327.2	9	1857	6	novel_not_in_catalog	gene: Zm00001d046388	novel	17.47	17.47	coding	187	107	667
PB.4269.3	3	1944	7	full-splice_match	gene: Zm00001d042590	Zm00001d042590_T001	12.15	119.12	coding	293	445	1323
PB.6656.2	5	1567	11	novel_in_catalog	gene: Zm00001d016308	novel	2.02	29.98	coding	348	111	1154
PB.6656.3	5	3981	10	novel_in_catalog	gene: Zm00001d016308	novel	2.63	29.98	coding	334	2672	3673
PB.9564.3	8	1068	7	full-splice_match	gene: Zm00001d010733	Zm00001d010733_T003	536.84	568.49	coding	206	115	732

PB.5547.1	4	1824	14	full-splice_match	gene: Zm00001d052532	Zm00001d052532_T001	86.17	938.93	coding	452	94	1449
PB.2316.3	10	868	2	incomplete-splice_match	gene: Zm00001d026514	Zm00001d026514_T001	23.13	45.71	coding	72	317	532
PB.2316.2	10	2043	8	novel_not_in_catalog	gene: Zm00001d026514	novel	2.28	45.71	coding	413	230	1468
PB.2316.1	10	2023	8	full-splice_match	gene: Zm00001d026514	Zm00001d026514_T002	20.30	45.71	coding	440	279	1598
PB.8171.1	7	1924	4	novel_not_in_catalog	gene: Zm00001d018696	novel	39.75	39.75	coding	547	85	1725
PB.3935.9	3	2494	3	novel_in_catalog	gene: Zm00001d040667	novel	0.00	252.28	coding	205	67	681
PB.7390.1	6	3178	16	novel_in_catalog	gene: Zm00001d035973	novel	35.72	35.72	coding	355	1815	2879
PB.3935.6	3	2347	4	full-splice_match	gene: Zm00001d040667	Zm00001d040667_T004	3.97	252.28	coding	205	67	681
PB.9885.3	8	2301	6	novel_not_in_catalog	gene: Zm00001d012167	novel	34.10	113.70	coding	560	330	2009
PB.3935.4	3	2253	5	full-splice_match	gene: Zm00001d040667	Zm00001d040667_T003	234.17	252.28	coding	205	74	688
PB.8187.1	7	3649	2	full-splice_match	gene: Zm00001d018752	Zm00001d018752_T001	17.49	17.49	coding	1037	51	3161
PB.5077.3	4	1298	10	novel_in_catalog	gene: Zm00001d050043	novel	4.70	97.16	coding	139	700	1116
PB.8187.3	7	2619	2	full-splice_match	gene: Zm00001d018752	Zm00001d018752_T001	0.00	17.49	coding	796	139	2526
PB.8187.2	7	3344	2	full-splice_match	gene: Zm00001d018752	Zm00001d018752_T001	0.00	17.49	coding	1037	36	3146
PB.97.1	1	1367	2	novel_not_in_catalog	gene: Zm00001d027613	novel	19.40	19.40	coding	342	206	1231
PB.6948.1	5	1828	2	incomplete-splice_match	gene: Zm00001d017685	Zm00001d017685_T002	6.95	17.88	non_coding	NA	NA	NA
PB.5454.1	4	1814	1	full-splice_match	gene: Zm00001d052229	Zm00001d052229_T001	459.68	459.68	coding	263	675	1463

PB.3209.2	2	1069	1	full-splice_match	gene: Zm00001d005940	Zm00001d005940_T001	0.00	20.53	coding	129	479	865
PB.6948.4	5	466	2	incomplete-splice_match	gene: Zm00001d017685	Zm00001d017685_T005	4.59	17.88	non_coding	NA	NA	NA
PB.6948.5	5	1956	8	novel_in_catalog	gene: Zm00001d017685	novel	1.70	17.88	coding	121	142	504
PB.10377.18	9	1770	6	full-splice_match	gene: Zm00001d046661	Zm00001d046661_T007	3.28	391.13	coding	459	320	1696
PB.1687.1	10	2732	23	full-splice_match	gene: Zm00001d023373	Zm00001d023373_T004	8.96	8.96	coding	654	443	2404
PB.10377.17	9	1560	6	full-splice_match	gene: Zm00001d046661	Zm00001d046661_T002	0.00	391.13	coding	459	110	1486
PB.1687.3	10	2534	23	full-splice_match	gene: Zm00001d023373	Zm00001d023373_T004	0.00	8.96	coding	654	439	2400
PB.10377.15	9	1750	6	full-splice_match	gene: Zm00001d046661	Zm00001d046661_T002	274.00	391.13	coding	459	110	1486
PB.10377.12	9	2341	4	novel_in_catalog	gene: Zm00001d046661	novel	0.18	391.13	coding	290	937	1806
PB.10377.13	9	1455	1	novel_in_catalog	gene: Zm00001d046661	novel	0.00	391.13	non_coding	NA	NA	NA
PB.8659.3	7	2527	7	novel_in_catalog	gene: Zm00001d021291	novel	1.51	40.13	coding	602	139	1944
PB.2881.6	2	2903	9	full-splice_match	gene: Zm00001d003861	Zm00001d003861_T002	0.00	98.91	coding	781	368	2710
PB.8659.1	7	2491	8	full-splice_match	gene: Zm00001d021291	Zm00001d021291_T006	38.62	40.13	coding	629	152	2038
PB.3462.7	2	2013	7	novel_not_in_catalog	gene: Zm00001d007073	novel	25.27	70.73	coding	524	209	1780
PB.909.1	1	1774	1	full-splice_match	gene: Zm00001d031833	Zm00001d031833_T001	246.45	246.45	coding	326	546	1523
PB.4823.4	4	1605	2	full-splice_match	gene: Zm00001d048707	Zm00001d048707_T001	91.65	173.30	coding	459	160	1536
PB.4823.5	4	1646	1	genic	gene: Zm00001d048707	novel	0.00	173.30	coding	228	893	1576

PB.4823.2	4	1857	2	full-splice_match	gene: Zm00001d048707	Zm00001d048707_T001	4.06	173.30	coding	459	160	1536
PB.4823.3	4	1665	2	full-splice_match	gene: Zm00001d048707	Zm00001d048707_T001	65.37	173.30	coding	459	81	1457
PB.4823.1	4	1907	1	genic	gene: Zm00001d048707	novel	12.22	173.30	coding	228	883	1566
PB.7184.1	5	2664	10	full-splice_match	gene: Zm00001d018516	Zm00001d018516_T002	7.47	7.47	coding	818	67	2520
PB.3215.1	2	952	1	full-splice_match	gene: Zm00001d005978	Zm00001d005978_T001	78.21	78.21	coding	157	134	604
PB.2881.9	2	2762	9	full-splice_match	gene: Zm00001d003861	Zm00001d003861_T002	0.00	98.91	coding	781	367	2709
PB.9323.2	8	1282	11	full-splice_match	gene: Zm00001d009522	Zm00001d009522_T003	17.89	43.11	coding	242	92	817
PB.10597.1	9	1673	3	full-splice_match	gene: Zm00001d047750	Zm00001d047750_T001	49.90	68.56	coding	352	210	1265
PB.10597.2	9	3711	1	genic	gene: Zm00001d047750	novel	3.67	68.56	coding	216	52	699
PB.9323.1	8	1208	12	full-splice_match	gene: Zm00001d009522	Zm00001d009522_T004	25.22	43.11	coding	242	53	778
PB.6254.1	5	1346	7	fusion	gene: Zm00001d014196_g	novel	37.33	37.33	coding	346	60	1097
PB.9323.4	8	1103	12	full-splice_match	gene: Zm00001d009522	Zm00001d009522_T004	0.00	43.11	coding	242	53	778
PB.1574.3	1	1682	4	full-splice_match	gene: Zm00001d034729	Zm00001d034729_T002	9.33	26.62	coding	491	125	1597
PB.2346.1	10	2202	8	novel_in_catalog	gene: Zm00001d026593	novel	5.63	30.70	coding	164	307	798
PB.2346.2	10	1832	9	full-splice_match	gene: Zm00001d026593	Zm00001d026593_T004	25.07	30.70	coding	313	390	1328
PB.6779.5	5	2073	2	novel_in_catalog	gene: Zm00001d016911	novel	5.71	35.18	coding	388	53	1216
PB.6779.2	5	1656	2	novel_in_catalog	gene: Zm00001d016911	novel	0.00	35.18	coding	359	77	1153

PB.6779.3	5	1436	2	novel_in_catalog	gene: Zm00001d016911	novel	0.00	35.18	coding	359	53	1129
PB.8011.2	6	1688	1	full-splice_match	gene: Zm00001d038765	Zm00001d038765_T001	55.10	63.74	coding	478	87	1520
PB.3110.3	2	2102	8	full-splice_match	gene: Zm00001d005373	Zm00001d005373_T002	9.43	45.50	coding	520	386	1945
PB.7733.1	6	1167	1	antisense	novelGene_gene: Zm00001d037729_A	novel	7.07	7.07	non_coding	NA	NA	NA
PB.1208.1	1	1358	2	antisense	novelGene_gene: Zm00001d033180_A	novel	7.36	7.36	coding	141	862	1284
PB.907.1	1	974	5	full-splice_match	gene: Zm00001d031825	Zm00001d031825_T002	647.45	651.03	coding	170	117	626
PB.4542.2	3	1246	6	full-splice_match	gene: Zm00001d043696	Zm00001d043696_T001	12.46	32.80	coding	288	110	973
PB.4542.3	3	1097	6	full-splice_match	gene: Zm00001d043696	Zm00001d043696_T001	20.33	32.80	coding	288	85	948
PB.8651.3	7	1456	2	full-splice_match	gene: Zm00001d021246	Zm00001d021246_T002	2.24	35.89	coding	248	394	1137
PB.8651.2	7	2622	1	novel_in_catalog	gene: Zm00001d021246	novel	5.18	35.89	coding	183	1648	2196
PB.8651.1	7	1500	2	full-splice_match	gene: Zm00001d021246	Zm00001d021246_T002	28.47	35.89	coding	248	296	1039
PB.9928.1	8	1062	1	full-splice_match	gene: Zm00001d012378	Zm00001d012378_T001	18.82	18.82	coding	225	236	910
PB.6855.1	5	2026	14	full-splice_match	gene: Zm00001d017261	Zm00001d017261_T002	25.94	25.94	coding	465	338	1732
PB.4454.1	3	1643	8	novel_not_in_catalog	gene: Zm00001d043387	novel	32.76	32.76	coding	441	54	1376
PB.1441.2	1	2546	16	full-splice_match	gene: Zm00001d034245	Zm00001d034245_T003	1.20	24.20	coding	422	466	1731
PB.8709.1	7	1117	6	full-splice_match	gene: Zm00001d021485	Zm00001d021485_T003	71.46	83.65	coding	229	145	831
PB.6765.1	5	2017	1	full-splice_match	gene: Zm00001d016865	Zm00001d016865_T001	2.13	2.13	coding	607	82	1902

PB.5422.2	4	2988	19	full-splice_match	gene: Zm00001d052072	Zm00001d052072_T018	9.95	9.95	coding	770	167	2476
PB.3126.2	2	2087	2	full-splice_match	gene: Zm00001d005480	Zm00001d005480_T001	15.99	15.99	coding	558	216	1889
PB.512.1	1	1688	11	incomplete-splice_match	gene: Zm00001d029490	Zm00001d029490_T018	22.86	27.05	coding	422	126	1391
PB.2622.3	2	1930	9	full-splice_match	gene: Zm00001d002716	Zm00001d002716_T002	0.00	57.24	coding	485	296	1750
PB.2330.2	10	838	5	full-splice_match	gene: Zm00001d026562	Zm00001d026562_T001	5.89	32.01	coding	207	87	707
PB.2622.1	2	2042	9	full-splice_match	gene: Zm00001d002716	Zm00001d002716_T002	57.24	57.24	coding	485	296	1750
PB.8709.2	7	1395	5	full-splice_match	gene: Zm00001d021485	Zm00001d021485_T004	12.19	83.65	coding	171	601	1113
PB.8756.1	7	1970	1	full-splice_match	gene: Zm00001d021638	Zm00001d021638_T001	3.69	6.30	coding	521	127	1689
PB.3500.1	2	1205	5	full-splice_match	gene: Zm00001d007267	Zm00001d007267_T002	8.07	8.07	coding	283	40	888
PB.2310.1	10	1595	14	incomplete-splice_match	gene: Zm00001d026501	Zm00001d026501_T001	19.16	19.16	coding	423	82	1350
PB.3783.1	3	542	2	full-splice_match	gene: Zm00001d039873	Zm00001d039873_T003	41.81	41.81	non_coding	NA	NA	NA
PB.1019.1	1	1400	1	full-splice_match	gene: Zm00001d032342	Zm00001d032342_T001	12.01	12.01	coding	381	75	1217
PB.1092.1	1	1788	3	full-splice_match	gene: Zm00001d032600	Zm00001d032600_T001	11.40	55.22	coding	483	38	1486
PB.1092.3	1	1710	3	full-splice_match	gene: Zm00001d032600	Zm00001d032600_T001	41.98	55.22	coding	483	68	1516
PB.1092.2	1	3610	2	novel_in_catalog	gene: Zm00001d032600	novel	1.84	55.22	coding	344	2358	3389
PB.8966.2	7	1792	4	full-splice_match	gene: Zm00001d022366	Zm00001d022366_T001	0.00	36.98	coding	422	233	1498
PB.10252.1	9	1126	6	full-splice_match	gene: Zm00001d045819	Zm00001d045819_T001	48.54	48.54	coding	244	95	826

PB.10611.1	9	2588	3	full-splice_match	gene: Zm00001d047796	Zm00001d047796_T003	44.46	44.46	coding	672	189	2204
PB.7610.2	6	2695	13	novel_in_catalog	gene: Zm00001d037198	novel	7.84	27.52	coding	494	870	2351
PB.10611.2	9	2344	3	full-splice_match	gene: Zm00001d047796	Zm00001d047796_T003	0.00	44.46	coding	672	189	2204
PB.988.1	1	1239	2	intergenic	novelGene_339	novel	5.69	5.69	coding	386	1	1158
PB.7610.4	6	2502	14	novel_not_in_catalog	gene: Zm00001d037198	novel	19.67	27.52	coding	710	34	2163
PB.6532.1	5	2047	7	novel_in_catalog	gene: Zm00001d015401	novel	37.79	37.79	coding	234	1107	1808
PB.6994.4	5	1977	7	incomplete-splice_match	gene: Zm00001d017872	Zm00001d017872_T020	13.73	162.08	coding	234	1102	1803
PB.6994.3	5	1695	10	full-splice_match	gene: Zm00001d017872	Zm00001d017872_T003	3.14	162.08	coding	446	177	1514
PB.6994.2	5	1830	10	full-splice_match	gene: Zm00001d017872	Zm00001d017872_T003	139.13	162.08	coding	446	179	1516
PB.10276.1	9	918	6	incomplete-splice_match	gene: Zm00001d045978	Zm00001d045978_T001	6.89	6.89	coding	223	63	731
PB.2338.7	10	2538	7	full-splice_match	gene: Zm00001d026585	Zm00001d026585_T002	2.07	47.67	coding	581	450	2192
PB.2338.4	10	2738	6	novel_in_catalog	gene: Zm00001d026585	novel	1.59	47.67	coding	289	450	1316
PB.9210.1	8	2394	8	full-splice_match	gene: Zm00001d008874	Zm00001d008874_T007	13.43	13.43	coding	632	70	1965
PB.2338.2	10	2705	8	novel_not_in_catalog	gene: Zm00001d026585	novel	44.01	47.67	coding	581	535	2277
PB.1209.1	1	994	4	full-splice_match	gene: Zm00001d033189	Zm00001d033189_T001	28.78	28.78	coding	188	110	673
PB.9952.1	8	988	1	antisense	novelGene_gene: Zm00001d012480_A	novel	12.34	12.34	non_coding	NA	NA	NA
PB.10160.2	9	3403	25	full-splice_match	gene: Zm00001d045379	Zm00001d045379_T005	21.09	22.29	coding	906	167	2884

PB.10160.1	9	2963	21	novel_not_in_catalog	gene: Zm00001d045379	novel	1.19	22.29	coding	828	6	2489
PB.5737.2	4	1481	1	full-splice_match	gene: Zm00001d053522	Zm00001d053522_T001	39.85	39.85	coding	354	99	1160
PB.2224.1	10	1024	1	full-splice_match	gene: Zm00001d026199	Zm00001d026199_T001	9.71	9.71	coding	270	71	880
PB.2709.1	2	1495	10	full-splice_match	gene: Zm00001d003066	Zm00001d003066_T002	40.39	40.39	coding	439	53	1369
PB.1076.2	1	2541	3	full-splice_match	gene: Zm00001d032547	Zm00001d032547_T010	5.68	33.58	coding	519	593	2149
PB.3886.1	3	626	3	full-splice_match	gene: Zm00001d040361	Zm00001d040361_T001	89.99	89.99	coding	23	387	455
PB.542.1	1	698	3	full-splice_match	gene: Zm00001d029625	Zm00001d029625_T001	323.90	345.12	coding	102	117	422
PB.6179.1	5	1893	1	full-splice_match	gene: Zm00001d013816	Zm00001d013816_T001	3.20	3.20	coding	499	63	1559
PB.542.3	1	625	3	full-splice_match	gene: Zm00001d029625	Zm00001d029625_T001	21.22	345.12	coding	102	66	371
PB.10520.4	9	1045	2	novel_in_catalog	gene: Zm00001d047470	novel	33.89	514.72	coding	103	213	521
PB.9766.1	8	1678	3	full-splice_match	gene: Zm00001d011687	Zm00001d011687_T001	5.18	11.06	coding	443	75	1403
PB.6325.1	5	1650	2	full-splice_match	gene: Zm00001d014554	Zm00001d014554_T001	13.53	13.53	coding	483	95	1543
PB.7405.1	6	1947	1	intergenic	novelGene_125	novel	7.91	7.91	non_coding	NA	NA	NA
PB.4839.1	4	758	1	novel_in_catalog	gene: Zm00001d048831	novel	3.21	3.21	coding	86	41	298
PB.1445.2	1	2301	5	incomplete-splice_match	gene: Zm00001d034282	Zm00001d034282_T009	16.45	21.34	coding	602	9	1814
PB.9065.3	8	1503	2	full-splice_match	gene: Zm00001d008212	Zm00001d008212_T002	11.18	14.55	coding	369	125	1231
PB.9065.2	8	1834	2	full-splice_match	gene: Zm00001d008212	Zm00001d008212_T002	3.36	14.55	coding	369	44	1150

PB.5919.1	5	2855	4	novel_not_in_catalog	gene: Zm00001d012850	novel	5.57	36.94	coding	366	114	1211
PB.3809.4	3	2737	14	incomplete-splice_match	gene: Zm00001d040008	Zm00001d040008_T009	3.69	56.05	coding	822	59	2524
PB.9885.8	8	2061	6	novel_not_in_catalog	gene: Zm00001d012167	novel	0.00	113.70	coding	560	328	2007
PB.5919.4	5	1443	3	full-splice_match	gene: Zm00001d012850	Zm00001d012850_T001	28.16	36.94	coding	366	115	1212
PB.7959.1	6	876	3	full-splice_match	gene: Zm00001d038542	Zm00001d038542_T001	17.53	17.53	coding	169	160	666
PB.9868.9	8	2004	9	novel_in_catalog	gene: Zm00001d012100	novel	16.22	79.52	coding	114	169	510
PB.10228.1	9	597	4	full-splice_match	gene: Zm00001d045650	Zm00001d045650_T001	82.09	82.09	coding	103	122	430
PB.2943.3	2	3649	12	novel_in_catalog	gene: Zm00001d004335	novel	16.24	22.82	coding	599	135	1931
PB.3262.1	2	2031	10	full-splice_match	gene: Zm00001d006176	Zm00001d006176_T001	15.63	17.93	coding	553	106	1764
PB.6092.1	5	871	5	full-splice_match	gene: Zm00001d013431	Zm00001d013431_T001	50.13	50.13	coding	161	216	698
PB.2479.3	2	2621	2	genic	gene: Zm00001d002126	novel	13.02	23.83	coding	658	396	2369
PB.1504.1	1	2371	6	full-splice_match	gene: Zm00001d034498	Zm00001d034498_T007	17.41	17.41	coding	672	95	2110
PB.9679.1	8	1157	3	full-splice_match	gene: Zm00001d011263	Zm00001d011263_T001	50.44	50.44	coding	258	118	891
PB.10248.3	9	1717	1	full-splice_match	gene: Zm00001d045785	Zm00001d045785_T001	0.00	24.42	coding	491	36	1508
PB.5979.4	5	1382	4	novel_not_in_catalog	gene: Zm00001d013043	novel	9.87	91.15	coding	199	577	1173
PB.10248.1	9	1850	1	full-splice_match	gene: Zm00001d045785	Zm00001d045785_T001	24.42	24.42	coding	491	45	1517
PB.3262.3	2	1900	10	full-splice_match	gene: Zm00001d006176	Zm00001d006176_T001	2.30	17.93	coding	553	115	1773

PB.5979.1	5	1226	4	full-splice_match	gene: Zm00001d013043	Zm00001d013043_T001	74.44	91.15	coding	300	128	1027
PB.7037.1	5	2003	1	full-splice_match	gene: Zm00001d018016	Zm00001d018016_T001	2.03	2.03	coding	398	417	1610
PB.5979.3	5	2701	1	novel_in_catalog	gene: Zm00001d013043	novel	2.42	91.15	coding	108	2157	2480
PB.5979.2	5	2535	3	full-splice_match	gene: Zm00001d013043	Zm00001d013043_T004	4.43	91.15	coding	199	1716	2312
PB.10035.1	9	3848	1	full-splice_match	gene: Zm00001d044726	Zm00001d044726_T001	9.64	9.64	coding	1103	253	3561
PB.9868.25	8	2416	10	novel_in_catalog	gene: Zm00001d012100	novel	2.26	79.52	coding	101	435	737
PB.1997.2	10	1192	3	novel_in_catalog	gene: Zm00001d025025	novel	1.47	64.93	non_coding	NA	NA	NA
PB.9278.1	8	3374	15	full-splice_match	gene: Zm00001d009248	Zm00001d009248_T004	10.31	10.31	coding	897	399	3089
PB.3081.6	2	840	7	full-splice_match	gene: Zm00001d005231	Zm00001d005231_T007	1511.97	1988.67	coding	181	116	658
PB.4343.2	3	787	3	intergenic	novelGene_303	novel	36.08	87.77	coding	51	247	399
PB.4615.1	3	472	3	incomplete-splice_match	gene: Zm00001d043982	Zm00001d043982_T073	179.18	179.18	non_coding	NA	NA	NA
PB.9408.2	8	954	4	novel_not_in_catalog	gene: Zm00001d010000	novel	0.00	21.56	coding	221	110	772
PB.9626.3	8	1535	2	full-splice_match	gene: Zm00001d010982	Zm00001d010982_T001	10.48	117.96	coding	294	258	1139
PB.3037.1	2	1789	8	novel_in_catalog	gene: Zm00001d004976	novel	56.08	73.06	coding	350	434	1483
PB.3037.3	2	1669	8	novel_in_catalog	gene: Zm00001d004976	novel	16.98	73.06	coding	350	438	1487
PB.3037.5	2	393	4	incomplete-splice_match	gene: Zm00001d004976	Zm00001d004976_T003	0.00	73.06	coding	96	105	392
PB.8230.1	7	2912	13	full-splice_match	gene: Zm00001d018929	Zm00001d018929_T001	20.37	26.22	coding	578	671	2404

PB.8230.4	7	2544	13	full-splice_match	gene: Zm00001d018929	Zm00001d018929_T001	4.75	26.22	coding	578	434	2167
PB.8230.5	7	391	1	incomplete-splice_match	gene: Zm00001d018929	Zm00001d018929_T010	1.10	26.22	non_coding	NA	NA	NA
PB.526.1	1	636	3	novel_not_in_catalog	gene: Zm00001d029551	novel	65.68	65.68	non_coding	NA	NA	NA
PB.6191.1	5	1650	17	full-splice_match	gene: Zm00001d013892	Zm00001d013892_T004	111.16	111.16	coding	390	176	1345
PB.9645.2	8	1627	13	full-splice_match	gene: Zm00001d011102	Zm00001d011102_T001	4.11	25.76	coding	456	130	1497
PB.9645.1	8	1836	13	full-splice_match	gene: Zm00001d011102	Zm00001d011102_T001	21.66	25.76	coding	456	80	1447
PB.2775.1	2	893	1	full-splice_match	gene: Zm00001d003306	Zm00001d003306_T001	10.69	10.69	coding	179	57	593
PB.5315.1	4	1098	6	full-splice_match	gene: Zm00001d051595	Zm00001d051595_T004	385.33	385.33	coding	233	101	799
PB.8191.1	7	3075	2	genic	gene: Zm00001d018789	novel	3.16	13.90	coding	876	110	2737
PB.179.1	1	594	1	full-splice_match	gene: Zm00001d028043	Zm00001d028043_T001	170.71	170.71	coding	144	42	473
PB.8191.3	7	2987	2	genic	gene: Zm00001d018789	novel	10.75	13.90	coding	876	294	2921
PB.2232.1	10	1859	6	full-splice_match	gene: Zm00001d026244	Zm00001d026244_T001	2.49	10.68	coding	530	55	1644
PB.10939.1	B73V4 ctg74	2243	4	novel_in_catalog	gene: Zm00001d000328	novel	8.52	8.52	coding	501	279	1781
PB.849.12	1	1697	5	novel_not_in_catalog	gene: Zm00001d031529	novel	14.18	121.67	coding	27	284	364
PB.7836.2	6	1286	5	full-splice_match	gene: Zm00001d038114	Zm00001d038114_T001	0.00	35.58	coding	335	138	1142
PB.1034.2	1	1198	4	full-splice_match	gene: Zm00001d032405	Zm00001d032405_T001	22.32	50.38	coding	355	61	1125
PB.8284.1	7	1751	3	full-splice_match	gene: Zm00001d019225	Zm00001d019225_T001	15.34	23.50	coding	428	77	1360

PB.8284.2	7	1586	3	full-splice_match	gene: Zm00001d019225	Zm00001d019225_T001	8.16	23.50	coding	428	104	1387
PB.8017.5	6	1734	9	full-splice_match	gene: Zm00001d038792	Zm00001d038792_T001	0.00	45.08	coding	507	138	1658
PB.8017.4	6	1923	9	full-splice_match	gene: Zm00001d038792	Zm00001d038792_T001	12.86	45.08	coding	507	150	1670
PB.8017.1	6	2043	9	full-splice_match	gene: Zm00001d038792	Zm00001d038792_T001	32.22	45.08	coding	507	170	1690
PB.1306.5	1	1882	4	full-splice_match	gene: Zm00001d033641	Zm00001d033641_T005	0.00	81.41	coding	147	203	643
PB.1874.4	10	2586	3	full-splice_match	gene: Zm00001d024497	Zm00001d024497_T001	23.07	56.38	coding	727	238	2418
PB.1874.7	10	1934	1	novel_in_catalog	gene: Zm00001d024497	novel	7.82	56.38	coding	331	841	1833
PB.2232.2	10	1767	6	full-splice_match	gene: Zm00001d026244	Zm00001d026244_T001	8.19	10.68	coding	530	67	1656
PB.1658.1	10	2604	8	full-splice_match	gene: Zm00001d023260	Zm00001d023260_T001	9.77	15.54	coding	492	713	2188
PB.1658.3	10	1960	9	full-splice_match	gene: Zm00001d023260	Zm00001d023260_T004	5.77	15.54	coding	580	5	1744
PB.10886.2	B73V4 ctg182	2898	12	novel_not_in_catalog	gene: Zm00001d000042	novel	27.58	27.58	coding	597	674	2464
PB.2800.4	2	1296	5	full-splice_match	gene: Zm00001d003434	Zm00001d003434_T001	13.53	55.77	coding	363	104	1192
PB.2800.1	2	1556	5	full-splice_match	gene: Zm00001d003434	Zm00001d003434_T001	31.00	55.77	coding	363	317	1405
PB.10041.1	9	1900	1	full-splice_match	gene: Zm00001d044773	Zm00001d044773_T001	10.82	10.82	coding	508	91	1614
PB.10041.2	9	938	1	full-splice_match	gene: Zm00001d044773	Zm00001d044773_T001	0.00	10.82	coding	238	52	765
PB.9864.3	8	1289	8	novel_in_catalog	gene: Zm00001d012085	novel	6.25	18.09	coding	286	22	879
PB.5619.2	4	2548	3	full-splice_match	gene: Zm00001d052855	Zm00001d052855_T001	157.40	585.42	coding	699	164	2260

PB.9868.26	8	3373	7	novel_in_catalog	gene: Zm00001d012100	novel	1.53	79.52	coding	128	563	946
PB.7026.1	5	2192	13	full-splice_match	gene: Zm00001d017988	Zm00001d017988_T001	2.08	11.21	coding	588	92	1855
PB.4601.4	3	2085	2	incomplete-splice_match	gene: Zm00001d043927	Zm00001d043927_T009	11.36	70.75	coding	374	685	1806
PB.3726.4	3	1891	3	full-splice_match	gene: Zm00001d039532	Zm00001d039532_T001	0.00	12.18	coding	547	103	1743
PB.4785.2	3	2906	3	full-splice_match	gene: Zm00001d044692	Zm00001d044692_T001	15.13	16.58	coding	791	417	2789
PB.3726.1	3	2261	3	full-splice_match	gene: Zm00001d039532	Zm00001d039532_T001	6.74	12.18	coding	547	261	1901
PB.4353.1	3	1295	1	full-splice_match	gene: Zm00001d042949	Zm00001d042949_T001	6.70	6.70	coding	371	54	1166
PB.3726.3	3	1996	3	full-splice_match	gene: Zm00001d039532	Zm00001d039532_T001	4.21	12.18	coding	547	106	1746
PB.3726.2	3	2423	1	genic	gene: Zm00001d039532	novel	1.23	12.18	coding	261	1303	2085
PB.5318.5	4	2240	7	full-splice_match	gene: Zm00001d051600	Zm00001d051600_T031	56.73	171.13	coding	617	389	2239
PB.2860.4	2	1539	7	incomplete-splice_match	gene: Zm00001d003773	Zm00001d003773_T002	0.00	102.00	coding	433	241	1539
PB.2860.2	2	2170	8	full-splice_match	gene: Zm00001d003773	Zm00001d003773_T002	102.00	102.00	coding	536	358	1965
PB.6835.3	5	1806	3	novel_in_catalog	gene: Zm00001d017177	novel	17.78	135.85	coding	141	306	728
PB.6835.2	5	1244	5	full-splice_match	gene: Zm00001d017177	Zm00001d017177_T005	118.06	135.85	non_coding	NA	NA	NA
PB.1088.1	1	3204	13	full-splice_match	gene: Zm00001d032575	Zm00001d032575_T004	17.18	17.18	coding	890	90	2759
PB.1088.2	1	3058	13	full-splice_match	gene: Zm00001d032575	Zm00001d032575_T004	0.00	17.18	coding	838	154	2667
PB.10366.1	9	2741	8	full-splice_match	gene: Zm00001d046591	Zm00001d046591_T001	56.75	56.75	coding	771	117	2429

PB.7591.2	6	1848	9	incomplete-splice_match	gene: Zm00001d037099	Zm00001d037099_T027	35.05	47.76	coding	251	520	1272
PB.5301.1	4	743	1	intergenic	novelGene_264	novel	6.64	6.64	non_coding	NA	NA	NA
PB.5318.6	4	1649	6	incomplete-splice_match	gene: Zm00001d051600	Zm00001d051600_T031	0.00	171.13	coding	538	36	1649
PB.5727.1	4	885	1	full-splice_match	gene: Zm00001d053433	Zm00001d053433_T001	18.14	18.14	coding	181	125	667
PB.6931.5	5	1333	1	full-splice_match	gene: Zm00001d017616	Zm00001d017616_T001	1.92	28.22	coding	353	48	1106
PB.907.3	1	2029	3	novel_in_catalog	gene: Zm00001d031825	novel	3.29	651.03	coding	75	117	341
PB.3586.1	2	433	1	genic	gene: Zm00001d007788	novel	16.35	16.35	coding	101	42	344
PB.5691.1	4	1862	5	full-splice_match	gene: Zm00001d053234	Zm00001d053234_T001	3.65	3.65	coding	506	90	1607
PB.7362.5	6	2064	5	incomplete-splice_match	gene: Zm00001d035789	Zm00001d035789_T002	0.00	23.42	coding	478	630	2063
PB.7362.4	6	2903	6	full-splice_match	gene: Zm00001d035789	Zm00001d035789_T002	0.00	23.42	coding	892	40	2715
PB.8877.1	7	3297	9	full-splice_match	gene: Zm00001d022041	Zm00001d022041_T005	14.10	35.21	coding	715	786	2930
PB.7362.1	6	3031	6	full-splice_match	gene: Zm00001d035789	Zm00001d035789_T002	14.60	23.42	coding	892	95	2770
PB.4736.6	3	1853	13	novel_in_catalog	gene: Zm00001d044494	novel	10.57	63.71	non_coding	NA	NA	NA
PB.7362.2	6	1967	5	novel_in_catalog	gene: Zm00001d035789	novel	8.81	23.42	coding	441	135	1457
PB.9667.4	8	2767	23	novel_not_in_catalog	gene: Zm00001d011213	novel	0.00	122.27	coding	809	153	2579
PB.9667.3	8	2893	23	novel_not_in_catalog	gene: Zm00001d011213	novel	0.43	122.27	coding	809	169	2595
PB.9667.2	8	2978	23	novel_not_in_catalog	gene: Zm00001d011213	novel	121.84	122.27	coding	809	135	2561

PB.44.1	1	857	2	genic	gene: Zm00001d027389	novel	78.61	202.41	coding	132	89	484
PB.2868.2	2	1536	5	full-splice_match	gene: Zm00001d003800	Zm00001d003800_T010	14.17	69.70	coding	372	334	1449
PB.2868.1	2	1245	3	novel_in_catalog	gene: Zm00001d003800	novel	4.91	69.70	coding	115	403	747
PB.44.2	1	774	2	genic	gene: Zm00001d027389	novel	123.80	202.41	coding	132	134	529
PB.7151.1	5	2752	14	incomplete-splice_match	gene: Zm00001d018420	Zm00001d018420_T003	20.03	20.03	coding	335	425	1429
PB.8702.1	7	681	5	full-splice_match	gene: Zm00001d021445	Zm00001d021445_T001	1118.87	1118.87	coding	134	76	477
PB.7346.1	6	1769	15	full-splice_match	gene: Zm00001d035696	Zm00001d035696_T001	24.43	24.43	coding	401	307	1509
PB.2301.17	10	2638	1	novel_in_catalog	gene: Zm00001d026458	novel	5.91	1555.47	coding	111	1792	2124
PB.492.2	1	1667	3	novel_not_in_catalog	gene: Zm00001d029402	novel	27.18	66.43	coding	280	350	1189
PB.492.1	1	2122	3	novel_not_in_catalog	gene: Zm00001d029402	novel	3.03	66.43	coding	280	346	1185
PB.4736.5	3	2934	15	novel_in_catalog	gene: Zm00001d044494	novel	9.00	63.71	coding	201	194	796
PB.492.5	1	1331	3	novel_not_in_catalog	gene: Zm00001d029402	novel	0.00	66.43	coding	280	346	1185
PB.492.4	1	1557	3	novel_not_in_catalog	gene: Zm00001d029402	novel	36.21	66.43	coding	280	348	1187
PB.7670.1	6	1218	6	novel_not_in_catalog	gene: Zm00001d037449	novel	6.94	6.94	coding	330	64	1053
PB.7945.1	6	953	2	incomplete-splice_match	gene: Zm00001d038489	Zm00001d038489_T001	25.25	25.25	coding	145	229	663
PB.10825.2	9	2306	7	novel_in_catalog	gene: Zm00001d048531	novel	1.65	15.52	coding	595	54	1838
PB.10533.1	9	480	1	full-splice_match	gene: Zm00001d047507	Zm00001d047507_T001	0.61	0.61	non_coding	NA	NA	NA

PB.9151.3	8	2421	6	full-splice_match	gene: Zm00001d008611	Zm00001d008611_T006	6.42	49.10	coding	216	359	1006
PB.9151.2	8	1658	7	full-splice_match	gene: Zm00001d008611	Zm00001d008611_T003	9.36	49.10	coding	260	401	1180
PB.5965.4	5	1740	2	novel_in_catalog	gene: Zm00001d013005	novel	7.91	183.42	coding	371	298	1410
PB.10342.7	9	1721	7	novel_not_in_catalog	gene: Zm00001d046468	novel	0.00	17.22	coding	218	893	1546
PB.5965.3	5	1628	3	full-splice_match	gene: Zm00001d013005	Zm00001d013005_T003	83.22	183.42	coding	371	298	1410
PB.5965.1	5	1735	3	full-splice_match	gene: Zm00001d013005	Zm00001d013005_T003	92.30	183.42	coding	371	298	1410
PB.8812.1	7	683	1	full-splice_match	gene: Zm00001d021816	Zm00001d021816_T001	20.73	20.73	coding	166	73	570
PB.5248.4	4	1580	9	novel_in_catalog	gene: Zm00001d051227	novel	2.27	63.45	coding	361	84	1166
PB.5248.5	4	1312	9	incomplete-splice_match	gene: Zm00001d051227	Zm00001d051227_T001	10.39	63.45	coding	355	21	1085
PB.5248.1	4	1584	10	full-splice_match	gene: Zm00001d051227	Zm00001d051227_T001	50.79	63.45	coding	384	170	1321
PB.1486.1	1	1316	5	full-splice_match	gene: Zm00001d034423	Zm00001d034423_T001	61.68	61.68	coding	248	297	1040
PB.222.6	1	2561	17	full-splice_match	gene: Zm00001d028218	Zm00001d028218_T017	0.00	93.04	coding	800	68	2467
PB.3340.1	2	2092	5	incomplete-splice_match	gene: Zm00001d006497	Zm00001d006497_T005	5.58	18.06	coding	225	961	1635
PB.222.1	1	2815	16	novel_in_catalog	gene: Zm00001d028218	novel	1.42	93.04	coding	583	46	1794
PB.222.2	1	2719	16	novel_in_catalog	gene: Zm00001d028218	novel	6.31	93.04	coding	485	1076	2530
PB.222.3	1	2634	17	full-splice_match	gene: Zm00001d028218	Zm00001d028218_T001	85.30	93.04	coding	800	59	2458
PB.113.3	1	1367	1	full-splice_match	gene: Zm00001d027685	Zm00001d027685_T001	0.00	45.89	coding	302	243	1148

PB.113.2	1	1574	1	full-splice_match	gene: Zm00001d027685	Zm00001d027685_T001	35.42	45.89	coding	302	245	1150
PB.113.1	1	763	1	full-splice_match	gene: Zm00001d027685	Zm00001d027685_T001	10.46	45.89	non_coding	NA	NA	NA
PB.2161.1	10	882	3	full-splice_match	gene: Zm00001d025900	Zm00001d025900_T002	21.27	21.27	coding	92	363	638
PB.5602.1	4	2631	4	full-splice_match	gene: Zm00001d052793	Zm00001d052793_T001	17.71	20.67	coding	775	201	2525
PB.5602.2	4	2636	3	novel_in_catalog	gene: Zm00001d052793	novel	2.96	20.67	coding	357	123	1193
PB.2006.2	10	1084	6	full-splice_match	gene: Zm00001d025063	Zm00001d025063_T004	44.47	59.37	coding	240	188	907
PB.2006.1	10	2048	6	full-splice_match	gene: Zm00001d025063	Zm00001d025063_T003	14.90	59.37	coding	138	1386	1799
PB.6498.1	5	1062	2	full-splice_match	gene: Zm00001d015204	Zm00001d015204_T001	51.06	51.06	coding	262	81	866
PB.7557.3	6	1792	1	full-splice_match	gene: Zm00001d036925	Zm00001d036925_T001	10.58	74.09	coding	383	104	1252
PB.2077.2	10	1754	5	novel_not_in_catalog	gene: Zm00001d025475	novel	0.00	3.48	coding	507	20	1540
PB.7557.1	6	1897	1	full-splice_match	gene: Zm00001d036925	Zm00001d036925_T001	1.69	74.09	coding	383	90	1238
PB.32.1	1	1004	1	full-splice_match	gene: Zm00001d027345	Zm00001d027345_T001	83.48	83.48	coding	199	101	697
PB.9677.4	8	2491	11	novel_not_in_catalog	gene: Zm00001d011253	novel	15.02	141.59	coding	210	1002	1631
PB.8585.6	7	2455	3	genic	gene: Zm00001d020939	novel	7.75	33.46	coding	609	380	2206
PB.7557.5	6	1514	1	full-splice_match	gene: Zm00001d036925	Zm00001d036925_T001	61.82	74.09	coding	383	100	1248
PB.206.1	1	1587	2	full-splice_match	gene: Zm00001d028167	Zm00001d028167_T001	130.56	130.56	coding	385	106	1260
PB.9677.8	8	2420	10	novel_not_in_catalog	gene: Zm00001d011253	novel	6.58	141.59	coding	190	1037	1606

PB.8498.1	7	1816	1	novel_in_catalog	gene: Zm00001d020528	novel	3.63	3.63	coding	284	824	1675
PB.8585.8	7	1437	2	genic	gene: Zm00001d020939	novel	0.00	33.46	coding	375	311	1435
PB.5232.1	4	1723	2	genic	gene: Zm00001d051136	novel	21.64	21.64	coding	289	576	1442
PB.5232.2	4	1594	2	genic	gene: Zm00001d051136	novel	0.00	21.64	coding	289	579	1445
PB.7378.1	6	1626	2	genic	gene: Zm00001d035918	novel	14.32	14.32	coding	373	80	1198
PB.442.2	1	1902	10	novel_in_catalog	gene: Zm00001d029144	novel	0.00	138.20	coding	549	256	1902
PB.442.3	1	1761	10	novel_in_catalog	gene: Zm00001d029144	novel	0.00	138.20	coding	502	256	1761
PB.442.4	1	1748	8	novel_in_catalog	gene: Zm00001d029144	novel	2.00	138.20	coding	220	308	967
PB.442.5	1	1638	10	novel_in_catalog	gene: Zm00001d029144	novel	0.00	138.20	coding	459	260	1636
PB.9860.3	8	523	1	full-splice_match	gene: Zm00001d012080	Zm00001d012080_T001	58.91	85.64	coding	107	94	414
PB.5093.1	4	1830	6	full-splice_match	gene: Zm00001d050139	Zm00001d050139_T007	57.29	58.83	coding	458	26	1399
PB.5093.2	4	1648	6	full-splice_match	gene: Zm00001d050139	Zm00001d050139_T007	1.54	58.83	coding	458	35	1408
PB.9895.2	8	2398	5	full-splice_match	gene: Zm00001d012224	Zm00001d012224_T001	7.71	32.16	coding	378	1109	2242
PB.8084.2	6	2978	13	incomplete-splice_match	gene: Zm00001d039005	Zm00001d039005_T009	0.00	23.91	coding	945	28	2862
PB.8724.1	7	1334	3	full-splice_match	gene: Zm00001d021533	Zm00001d021533_T001	14.08	14.08	coding	364	78	1169
PB.595.1	1	1025	1	full-splice_match	gene: Zm00001d029923	Zm00001d029923_T001	487.26	487.26	coding	225	82	756
PB.829.1	1	2615	1	full-splice_match	gene: Zm00001d031398	Zm00001d031398_T001	4.29	4.29	coding	779	64	2400

PB.9204.1	8	2184	4	full-splice_match	gene: Zm00001d008856	Zm00001d008856_T004	26.23	26.23	coding	133	118	516
PB.6351.1	5	1314	1	genic	gene: Zm00001d014652	novel	6.67	6.67	coding	97	16	306
PB.5787.1	4	724	1	full-splice_match	gene: Zm00001d053689	Zm00001d053689_T001	14.93	25.89	coding	114	72	413
PB.1662.6	10	1203	4	novel_not_in_catalog	gene: Zm00001d023267	novel	53.94	155.61	coding	222	67	732
PB.1662.7	10	3464	4	full-splice_match	gene: Zm00001d023267	Zm00001d023267_T001	0.00	155.61	coding	1036	59	3166
PB.1662.4	10	3489	5	novel_not_in_catalog	gene: Zm00001d023267	novel	2.88	155.61	coding	996	63	3050
PB.1662.5	10	3597	4	full-splice_match	gene: Zm00001d023267	Zm00001d023267_T001	98.80	155.61	coding	1036	51	3158
PB.1846.2	10	1590	5	full-splice_match	gene: Zm00001d024325	Zm00001d024325_T002	23.82	37.33	coding	325	112	1086
PB.1846.3	10	1419	5	full-splice_match	gene: Zm00001d024325	Zm00001d024325_T002	0.00	37.33	coding	325	66	1040
PB.1846.1	10	1837	5	full-splice_match	gene: Zm00001d024325	Zm00001d024325_T002	13.51	37.33	coding	325	119	1093
PB.1201.3	1	2160	2	full-splice_match	gene: Zm00001d033153	Zm00001d033153_T001	1.48	15.76	coding	425	732	2006
PB.6921.2	5	458	1	incomplete-splice_match	gene: Zm00001d017547	Zm00001d017547_T003	29.38	75.30	coding	29	241	327
PB.6921.1	5	1335	2	full-splice_match	gene: Zm00001d017547	Zm00001d017547_T001	45.91	75.30	coding	290	119	988
PB.9182.10	8	1539	9	full-splice_match	gene: Zm00001d008744	Zm00001d008744_T001	11.14	74.69	coding	394	199	1380
PB.4582.2	3	1479	1	full-splice_match	gene: Zm00001d043858	Zm00001d043858_T001	0.00	204.46	coding	396	33	1220
PB.2390.1	2	917	3	full-splice_match	gene: Zm00001d001779	Zm00001d001779_T001	59.57	59.57	coding	228	64	747
PB.8442.3	7	2207	1	genic	gene: Zm00001d020282	novel	0.00	29.67	coding	134	969	1370

PB.5822.3	4	2963	9	full-splice_match	gene: Zm00001d053841	Zm00001d053841_T009	4.52	46.13	coding	257	1995	2765
PB.8442.4	7	1989	2	novel_in_catalog	gene: Zm00001d020282	novel	0.00	29.67	coding	300	934	1833
PB.8442.7	7	2002	2	novel_in_catalog	gene: Zm00001d020282	novel	10.17	29.67	coding	358	164	1237
PB.4309.2	3	825	1	full-splice_match	gene: Zm00001d042735	Zm00001d042735_T001	0.00	186.94	coding	224	84	755
PB.4736.8	3	2752	15	full-splice_match	gene: Zm00001d044494	Zm00001d044494_T010	0.00	63.71	coding	185	84	638
PB.258.3	1	979	2	genic	gene: Zm00001d028371	novel	0.00	98.73	coding	219	201	857
PB.258.1	1	1164	2	genic	gene: Zm00001d028371	novel	98.73	98.73	coding	219	281	937
PB.4736.2	3	2694	14	full-splice_match	gene: Zm00001d044494	Zm00001d044494_T013	12.89	63.71	coding	79	69	305
PB.2434.1	2	1245	3	full-splice_match	gene: Zm00001d001933	Zm00001d001933_T001	88.90	111.22	coding	146	577	1014
PB.2434.2	2	1132	2	novel_in_catalog	gene: Zm00001d001933	novel	22.32	111.22	coding	146	517	954
PB.2256.11	10	3401	23	novel_in_catalog	gene: Zm00001d026306	novel	0.00	152.36	coding	809	35	2461
PB.238.1	1	2785	7	full-splice_match	gene: Zm00001d028285	Zm00001d028285_T001	10.04	21.05	coding	607	114	1934
PB.238.3	1	2666	6	novel_in_catalog	gene: Zm00001d028285	novel	0.00	21.05	coding	532	504	2099
PB.581.2	1	1978	3	full-splice_match	gene: Zm00001d029842	Zm00001d029842_T001	81.65	226.49	coding	499	64	1560
PB.238.4	1	2539	6	novel_in_catalog	gene: Zm00001d028285	novel	11.01	21.05	coding	532	573	2168
PB.3356.7	2	1903	6	novel_in_catalog	gene: Zm00001d006578	novel	17.87	51.11	coding	312	314	1249
PB.2984.1	2	1633	8	novel_not_in_catalog	gene: Zm00001d004644	novel	13.70	18.53	coding	408	102	1325

PB.689.1	1	1488	11	full-splice_match	gene: Zm00001d030561	Zm00001d030561_T003	60.91	65.62	coding	323	69	1037
PB.5290.1	4	636	1	full-splice_match	gene: Zm00001d051478	Zm00001d051478_T001	318.16	683.73	coding	103	77	385
PB.7547.1	6	1158	2	full-splice_match	gene: Zm00001d036834	Zm00001d036834_T001	11.03	11.03	coding	288	202	1065
PB.7054.1	5	1260	5	full-splice_match	gene: Zm00001d018090	Zm00001d018090_T002	420.11	433.60	coding	314	157	1098
PB.9446.1	8	1146	1	full-splice_match	gene: Zm00001d010217	Zm00001d010217_T001	2.46	23.27	coding	187	84	644
PB.7131.3	5	1459	14	incomplete-splice_match	gene: Zm00001d018371	Zm00001d018371_T005	3.52	17.94	coding	362	1	1086
PB.4403.1	3	1975	2	full-splice_match	gene: Zm00001d043166	Zm00001d043166_T001	5.53	5.53	coding	470	59	1468
PB.7131.1	5	2371	15	full-splice_match	gene: Zm00001d018371	Zm00001d018371_T005	5.55	17.94	coding	365	552	1646
PB.3878.5	3	2250	5	full-splice_match	gene: Zm00001d040318	Zm00001d040318_T002	6.10	26.48	coding	517	285	1835
PB.8371.1	7	2379	9	full-splice_match	gene: Zm00001d019824	Zm00001d019824_T004	196.75	453.10	coding	656	132	2099
PB.3878.1	3	2323	5	full-splice_match	gene: Zm00001d040318	Zm00001d040318_T002	20.38	26.48	coding	517	255	1805
PB.8371.3	7	2223	9	full-splice_match	gene: Zm00001d019824	Zm00001d019824_T010	1.98	453.10	coding	627	146	2026
PB.2961.3	2	1325	7	novel_not_in_catalog	gene: Zm00001d004498	novel	6.73	30.04	coding	172	179	694
PB.5127.1	4	1028	3	full-splice_match	gene: Zm00001d050347	Zm00001d050347_T004	72.00	75.18	coding	222	88	753
PB.5127.2	4	2641	2	novel_in_catalog	gene: Zm00001d050347	novel	3.18	75.18	coding	187	200	760
PB.7497.1	6	756	6	full-splice_match	gene: Zm00001d036565	Zm00001d036565_T009	803.55	803.55	coding	150	81	530
PB.9814.3	8	1100	5	full-splice_match	gene: Zm00001d011849	Zm00001d011849_T002	6.38	42.13	coding	279	83	919

PB.9814.2	8	1066	5	full-splice_match	gene: Zm00001d011849	Zm00001d011849_T002	17.01	42.13	coding	233	79	777
PB.9814.1	8	2464	1	novel_in_catalog	gene: Zm00001d011849	novel	18.74	42.13	non_coding	NA	NA	NA
PB.2885.1	2	1762	2	novel_not_in_catalog	gene: Zm00001d003884	novel	8.23	8.23	coding	137	912	1322
PB.7782.3	6	806	4	full-splice_match	gene: Zm00001d037929	Zm00001d037929_T002	989.55	1384.77	coding	199	69	665
PB.3580.1	2	1244	5	full-splice_match	gene: Zm00001d007767	Zm00001d007767_T001	187.97	519.93	coding	222	204	869
PB.7834.2	6	2650	4	full-splice_match	gene: Zm00001d038105	Zm00001d038105_T002	0.00	60.46	coding	555	243	1907
PB.769.2	1	1342	5	incomplete-splice_match	gene: Zm00001d031081	Zm00001d031081_T004	17.28	29.83	coding	326	107	1084
PB.769.3	1	2066	7	full-splice_match	gene: Zm00001d031081	Zm00001d031081_T004	1.45	29.83	coding	598	119	1912
PB.10.1	1	1262	4	incomplete-splice_match	gene: Zm00001d027276	Zm00001d027276_T001	58.37	58.37	coding	327	110	1090
PB.7782.1	6	914	4	full-splice_match	gene: Zm00001d037929	Zm00001d037929_T002	395.22	1384.77	coding	199	69	665
PB.7076.3	5	1296	6	full-splice_match	gene: Zm00001d018183	Zm00001d018183_T001	0.00	153.98	coding	366	51	1148
PB.589.1	1	1706	6	full-splice_match	gene: Zm00001d029886	Zm00001d029886_T001	8.05	8.05	coding	396	320	1507
PB.4413.1	3	2005	2	novel_not_in_catalog	gene: Zm00001d043200	novel	1.73	1.73	coding	404	627	1838
PB.5855.1	4	1591	6	full-splice_match	gene: Zm00001d053957	Zm00001d053957_T003	26.66	26.66	coding	369	77	1183
PB.2901.1	2	2624	8	full-splice_match	gene: Zm00001d003947	Zm00001d003947_T022	112.66	112.66	coding	784	50	2401
PB.6070.1	5	1158	7	full-splice_match	gene: Zm00001d013360	Zm00001d013360_T002	41.38	41.38	coding	244	220	951
PB.7834.7	6	1738	4	full-splice_match	gene: Zm00001d038105	Zm00001d038105_T002	0.00	60.46	coding	371	460	1572

PB.4075.2	3	1468	1	genic	gene: Zm00001d041678	novel	9.69	31.06	coding	209	842	1468
PB.4075.1	3	1587	1	genic	gene: Zm00001d041678	novel	21.37	31.06	coding	220	812	1471
PB.4087.1	3	1375	5	full-splice_match	gene: Zm00001d041725	Zm00001d041725_T001	6.08	38.51	coding	196	105	692
PB.4745.11	3	1953	3	novel_in_catalog	gene: Zm00001d044516	novel	0.00	381.23	coding	330	126	1115
PB.4745.10	3	2119	2	novel_in_catalog	gene: Zm00001d044516	novel	0.00	381.23	coding	297	127	1017
PB.4745.13	3	2179	1	novel_in_catalog	gene: Zm00001d044516	novel	0.00	381.23	coding	334	126	1127
PB.4745.12	3	1597	3	novel_in_catalog	gene: Zm00001d044516	novel	0.00	381.23	coding	297	126	1016
PB.8563.1	7	2190	7	novel_in_catalog	gene: Zm00001d020835	novel	6.72	44.58	coding	417	267	1517
PB.8279.1	7	1428	1	genic	gene: Zm00001d019216	novel	69.23	69.23	coding	274	206	1027
PB.8379.5	7	3031	3	novel_in_catalog	gene: Zm00001d019875	novel	0.00	20.40	coding	468	154	1557
PB.8379.6	7	1998	5	novel_not_in_catalog	gene: Zm00001d019875	novel	1.88	20.40	coding	515	169	1713
PB.5269.6	4	738	1	antisense	novelGene_gene: Zm00001d051349_A	novel	33.33	103.06	non_coding	NA	NA	NA
PB.8379.1	7	2927	4	novel_not_in_catalog	gene: Zm00001d019875	novel	3.29	20.40	coding	468	163	1566
PB.8379.3	7	2433	5	novel_not_in_catalog	gene: Zm00001d019875	novel	8.81	20.40	coding	466	204	1601
PB.18.2	1	1685	2	full-splice_match	gene: Zm00001d027305	Zm00001d027305_T002	0.00	132.79	coding	477	117	1547
PB.18.1	1	1819	2	full-splice_match	gene: Zm00001d027305	Zm00001d027305_T002	132.79	132.79	coding	477	119	1549
PB.7969.1	6	1863	6	full-splice_match	gene: Zm00001d038579	Zm00001d038579_T001	20.40	20.40	coding	480	198	1637

PB.3850.1	3	2223	9	novel_not_in_catalog	gene: Zm00001d040233	novel	5.73	34.57	coding	217	598	1248
PB.3528.2	2	1782	2	incomplete-splice_match	gene: Zm00001d007457	Zm00001d007457_T001	18.48	48.71	coding	433	52	1350
PB.3850.2	3	1422	12	novel_not_in_catalog	gene: Zm00001d040233	novel	27.44	34.57	coding	289	361	1227
PB.3850.4	3	1638	11	novel_not_in_catalog	gene: Zm00001d040233	novel	1.39	34.57	coding	217	324	974
PB.874.1	1	1133	7	full-splice_match	gene: Zm00001d031640	Zm00001d031640_T001	115.20	202.20	coding	257	75	845
PB.2184.1	10	620	1	full-splice_match	gene: Zm00001d026002	Zm00001d026002_T001	25.39	25.39	coding	80	61	300
PB.5566.2	4	1645	7	novel_in_catalog	gene: Zm00001d052656	novel	10.32	22.44	non_coding	NA	NA	NA
PB.10410.2	9	2375	7	novel_not_in_catalog	gene: Zm00001d046893	novel	20.11	23.81	coding	625	125	1999
PB.6829.1	5	1880	13	full-splice_match	gene: Zm00001d017153	Zm00001d017153_T005	22.20	26.57	coding	410	230	1459
PB.10410.1	9	2354	7	novel_not_in_catalog	gene: Zm00001d046893	novel	3.70	23.81	coding	503	81	1589
PB.1198.1	1	3623	15	full-splice_match	gene: Zm00001d033112	Zm00001d033112_T001	9.41	9.41	coding	1081	146	3388
PB.7223.1	6	2534	18	novel_in_catalog	gene: Zm00001d035041	novel	18.44	45.85	coding	629	249	2135
PB.606.4	1	1050	5	full-splice_match	gene: Zm00001d030009	Zm00001d030009_T001	49.26	85.64	coding	245	109	843
PB.7223.3	6	2063	15	incomplete-splice_match	gene: Zm00001d035041	Zm00001d035041_T010	27.40	45.85	coding	527	186	1766
PB.317.1	1	1562	6	novel_not_in_catalog	gene: Zm00001d028613	novel	62.26	73.81	coding	374	224	1345
PB.317.3	1	1380	6	novel_not_in_catalog	gene: Zm00001d028613	novel	0.00	73.81	coding	374	98	1219
PB.317.2	1	1555	5	full-splice_match	gene: Zm00001d028613	Zm00001d028613_T001	11.55	73.81	coding	405	95	1309

PB.7052.3	5	2616	6	full-splice_match	gene: Zm00001d018080	Zm00001d018080_T001	0.00	37.21	coding	780	196	2535
PB.7052.2	5	2621	5	full-splice_match	gene: Zm00001d018080	Zm00001d018080_T004	9.16	37.21	coding	452	1139	2494
PB.7052.1	5	2494	6	full-splice_match	gene: Zm00001d018080	Zm00001d018080_T001	28.06	37.21	coding	780	24	2363
PB.9133.7	8	1479	9	full-splice_match	gene: Zm00001d008503	Zm00001d008503_T002	11.53	36.43	coding	271	382	1194
PB.9133.6	8	2271	8	novel_in_catalog	gene: Zm00001d008503	novel	0.94	36.43	coding	175	1460	1984
PB.9133.5	8	1507	9	novel_not_in_catalog	gene: Zm00001d008503	novel	6.30	36.43	coding	235	386	1090
PB.9133.4	8	1818	8	novel_in_catalog	gene: Zm00001d008503	novel	4.58	36.43	coding	257	535	1305
PB.5856.1	4	1155	4	full-splice_match	gene: Zm00001d053963	Zm00001d053963_T002	46.16	50.67	coding	255	184	948
PB.9133.1	8	1781	8	novel_in_catalog	gene: Zm00001d008503	novel	13.08	36.43	coding	257	375	1145
PB.6894.1	5	1192	8	full-splice_match	gene: Zm00001d017435	Zm00001d017435_T001	28.96	28.96	coding	315	12	956
PB.2102.2	10	356	3	full-splice_match	gene: Zm00001d025654	Zm00001d025654_T001	24.60	112.15	coding	77	126	356
PB.5541.1	4	2047	16	full-splice_match	gene: Zm00001d052494	Zm00001d052494_T001	11.97	128.72	coding	527	152	1732
PB.5541.2	4	1756	12	novel_in_catalog	gene: Zm00001d052494	novel	1.75	128.72	coding	161	582	1064
PB.2102.1	10	719	3	full-splice_match	gene: Zm00001d025654	Zm00001d025654_T001	87.54	112.15	coding	150	126	575
PB.10529.2	9	1694	15	full-splice_match	gene: Zm00001d047498	Zm00001d047498_T005	21.74	170.79	coding	257	132	902
PB.7401.1	6	1020	1	full-splice_match	gene: Zm00001d036016	Zm00001d036016_T001	9.60	9.60	coding	229	56	742
PB.6965.3	5	1766	4	full-splice_match	gene: Zm00001d017768	Zm00001d017768_T008	10.62	10.62	coding	449	215	1561

PB.6496.2	5	798	3	full-splice_match	gene: Zm00001d015184	Zm00001d015184_T001	39.50	39.50	coding	151	192	644
PB.2404.1	2	1808	2	full-splice_match	gene: Zm00001d001817	Zm00001d001817_T001	12.64	12.64	coding	538	104	1717
PB.10406.2	9	1523	3	full-splice_match	gene: Zm00001d046852	Zm00001d046852_T001	11.56	16.03	coding	385	220	1374
PB.10406.1	9	1078	1	genic	gene: Zm00001d046852	novel	4.47	16.03	coding	221	19	681
PB.1533.2	1	2341	7	full-splice_match	gene: Zm00001d034597	Zm00001d034597_T003	2.71	27.67	coding	549	387	2033
PB.8836.1	7	1296	12	novel_not_in_catalog	gene: Zm00001d021881	novel	47.72	47.72	coding	342	17	1042
PB.3754.3	3	1828	8	full-splice_match	gene: Zm00001d039706	Zm00001d039706_T001	57.54	57.54	coding	500	37	1536
PB.7779.1	6	2098	6	full-splice_match	gene: Zm00001d037920	Zm00001d037920_T002	8.26	8.26	coding	460	491	1870
PB.2079.4	10	1864	1	full-splice_match	gene: Zm00001d025490	Zm00001d025490_T001	31.54	152.24	coding	146	1228	1665
PB.2079.7	10	607	1	intergenic	novelGene_192	novel	6.55	6.55	non_coding	NA	NA	NA
PB.2079.6	10	1042	2	genic	gene: Zm00001d025490	novel	72.69	152.24	coding	146	493	930
PB.2079.1	10	1576	2	genic	gene: Zm00001d025490	novel	0.00	152.24	coding	146	494	931
PB.1714.2	10	1587	9	full-splice_match	gene: Zm00001d023514	Zm00001d023514_T003	2.11	54.18	coding	355	335	1399
PB.2079.3	10	1382	2	genic	gene: Zm00001d025490	novel	40.09	152.24	coding	146	738	1175
PB.7475.6	6	1300	6	full-splice_match	gene: Zm00001d036481	Zm00001d036481_T006	24.15	373.04	coding	183	487	1035
PB.3220.8	2	2275	7	full-splice_match	gene: Zm00001d006001	Zm00001d006001_T001	0.00	41.26	coding	567	450	2150
PB.1896.1	10	1309	8	full-splice_match	gene: Zm00001d024588	Zm00001d024588_T001	60.75	64.22	coding	241	312	1034

PB.10314.1	9	2533	12	incomplete-splice_match	gene: Zm00001d046277	Zm00001d046277_T023	40.17	40.17	coding	750	45	2294
PB.2244.2	10	1839	16	incomplete-splice_match	gene: Zm00001d026270	Zm00001d026270_T006	15.62	15.62	coding	483	102	1550
PB.2936.1	2	2213	2	full-splice_match	gene: Zm00001d004190	Zm00001d004190_T001	5.05	5.05	coding	351	95	1147
PB.10642.2	9	3109	4	full-splice_match	gene: Zm00001d047881	Zm00001d047881_T001	0.00	30.62	coding	870	126	2735
PB.7458.1	6	1586	5	full-splice_match	gene: Zm00001d036410	Zm00001d036410_T003	62.74	65.13	coding	391	37	1209
PB.5618.2	4	664	1	incomplete-splice_match	gene: Zm00001d052852	Zm00001d052852_T001	12.63	20.65	coding	104	61	372
PB.7458.2	6	2133	4	novel_in_catalog	gene: Zm00001d036410	novel	2.39	65.13	coding	344	79	1110
PB.2789.3	2	867	3	intergenic	novelGene_103	novel	4.30	48.16	coding	176	205	732
PB.4057.1	3	1472	10	full-splice_match	gene: Zm00001d041575	Zm00001d041575_T003	87.71	133.85	coding	353	110	1168
PB.4676.2	3	4231	4	full-splice_match	gene: Zm00001d044232	Zm00001d044232_T002	0.35	70.69	coding	633	1783	3681
PB.8416.1	7	2896	14	incomplete-splice_match	gene: Zm00001d020134	Zm00001d020134_T008	8.50	8.50	coding	892	6	2681
PB.6438.1	5	504	1	full-splice_match	gene: Zm00001d014960	Zm00001d014960_T001	3.46	18.71	coding	79	109	345
PB.6438.2	5	1617	1	full-splice_match	gene: Zm00001d014960	Zm00001d014960_T001	15.26	18.71	coding	427	131	1411
PB.192.1	1	2119	5	full-splice_match	gene: Zm00001d028103	Zm00001d028103_T001	94.94	94.94	coding	572	171	1886
PB.4514.1	3	3598	16	incomplete-splice_match	gene: Zm00001d043598	Zm00001d043598_T005	22.03	26.72	coding	997	216	3206
PB.5502.2	4	2691	8	novel_in_catalog	gene: Zm00001d052385	novel	1.82	18.21	coding	623	14	1882
PB.5050.1	4	566	2	full-splice_match	gene: Zm00001d049858	Zm00001d049858_T001	144.34	144.34	non_coding	NA	NA	NA

PB.4514.2	3	4734	20	full-splice_match	gene: Zm00001d043598	Zm00001d043598_T002	4.69	26.72	coding	1478	121	4554
PB.5502.7	4	2099	9	full-splice_match	gene: Zm00001d052385	Zm00001d052385_T001	0.00	18.21	coding	633	14	1912
PB.916.1	1	3093	7	incomplete-splice_match	gene: Zm00001d031863	Zm00001d031863_T027	71.74	71.74	coding	956	22	2889
PB.9677.13	8	1769	9	novel_not_in_catalog	gene: Zm00001d011253	novel	3.18	141.59	coding	157	475	945
PB.9949.1	8	1715	8	novel_in_catalog	gene: Zm00001d012464	novel	3.53	3.53	coding	501	39	1541
PB.9033.5	7	907	1	full-splice_match	gene: Zm00001d022574	Zm00001d022574_T001	10.81	10.81	coding	228	71	754
PB.363.1	1	1092	1	full-splice_match	gene: Zm00001d028808	Zm00001d028808_T001	31.14	31.14	coding	124	352	723
PB.6010.2	5	581	1	full-splice_match	gene: Zm00001d013175	Zm00001d013175_T001	43.71	98.65	coding	59	282	458
PB.9123.1	8	2334	2	novel_in_catalog	gene: Zm00001d008456	novel	18.10	23.41	coding	66	125	322
PB.6010.1	5	922	1	full-splice_match	gene: Zm00001d013175	Zm00001d013175_T001	54.94	98.65	coding	157	227	697
PB.9123.4	8	2217	2	novel_in_catalog	gene: Zm00001d008456	novel	0.00	23.41	coding	66	124	321
PB.8079.2	6	3859	19	full-splice_match	gene: Zm00001d038982	Zm00001d038982_T024	23.45	28.25	coding	1022	490	3555
PB.8079.1	6	3201	17	incomplete-splice_match	gene: Zm00001d038982	Zm00001d038982_T026	4.43	28.25	coding	604	3	1814
PB.1296.2	1	962	3	full-splice_match	gene: Zm00001d033596	Zm00001d033596_T001	77.16	229.47	coding	211	154	786
PB.1585.4	1	2264	18	full-splice_match	gene: Zm00001d034758	Zm00001d034758_T001	0.00	42.58	coding	659	176	2152
PB.9677.16	8	2136	3	novel_not_in_catalog	gene: Zm00001d011253	novel	10.93	141.59	non_coding	NA	NA	NA
PB.692.1	1	1514	5	full-splice_match	gene: Zm00001d030601	Zm00001d030601_T001	34.70	34.70	coding	356	190	1257

PB.5529.1	4	410	1	intergenic	novelGene_308	novel	0.00	0.00	non_coding	NA	NA	NA
PB.5070.1	4	633	2	full-splice_match	gene: Zm00001d049984	Zm00001d049984_T001	60.86	60.86	non_coding	NA	NA	NA
PB.1585.1	1	2455	18	full-splice_match	gene: Zm00001d034758	Zm00001d034758_T001	42.37	42.58	coding	659	179	2155
PB.1585.2	1	2523	17	novel_in_catalog	gene: Zm00001d034758	novel	0.21	42.58	coding	330	1259	2248
PB.5070.2	4	349	2	full-splice_match	gene: Zm00001d049984	Zm00001d049984_T001	0.00	60.86	non_coding	NA	NA	NA
PB.1126.1	1	2553	21	novel_in_catalog	gene: Zm00001d032748	novel	92.73	92.73	coding	745	131	2365
PB.9516.1	8	1278	9	full-splice_match	gene: Zm00001d010538	Zm00001d010538_T005	154.78	235.70	coding	215	158	802
PB.387.4	1	2761	9	novel_in_catalog	gene: Zm00001d028936	novel	1.80	99.84	coding	225	1795	2469
PB.387.5	1	1769	10	novel_not_in_catalog	gene: Zm00001d028936	novel	4.59	99.84	coding	153	1029	1487
PB.387.2	1	1951	10	full-splice_match	gene: Zm00001d028936	Zm00001d028936_T003	64.33	99.84	coding	335	590	1594
PB.387.3	1	1808	9	full-splice_match	gene: Zm00001d028936	Zm00001d028936_T004	7.44	99.84	coding	335	512	1516
PB.387.1	1	2029	10	full-splice_match	gene: Zm00001d028936	Zm00001d028936_T003	21.68	99.84	coding	335	643	1647
PB.9677.15	8	2376	10	novel_not_in_catalog	gene: Zm00001d011253	novel	0.00	141.59	coding	190	1007	1576
PB.387.8	1	1897	10	full-splice_match	gene: Zm00001d028936	Zm00001d028936_T003	0.00	99.84	coding	335	636	1640
PB.6233.1	5	1407	9	full-splice_match	gene: Zm00001d014094	Zm00001d014094_T002	29.79	29.79	coding	357	155	1225
PB.1513.3	1	2440	13	novel_in_catalog	gene: Zm00001d034519	novel	1.28	31.00	coding	298	65	958
PB.1513.2	1	2627	14	full-splice_match	gene: Zm00001d034519	Zm00001d034519_T001	0.44	31.00	coding	729	200	2386

PB.6937.1	5	877	1	full-splice_match	gene: Zm00001d017645	Zm00001d017645_T001	50.32	50.32	coding	185	90	644
PB.9304.1	8	1252	6	full-splice_match	gene: Zm00001d009440	Zm00001d009440_T001	41.64	41.64	coding	201	335	937
PB.3266.1	2	1203	2	full-splice_match	gene: Zm00001d006195	Zm00001d006195_T001	24.93	24.93	coding	244	142	873
PB.8755.1	7	2647	12	incomplete-splice_match	gene: Zm00001d021636	Zm00001d021636_T005	5.88	5.88	coding	802	4	2409
PB.884.3	1	2433	5	full-splice_match	gene: Zm00001d031700	Zm00001d031700_T002	14.69	26.54	coding	746	69	2306
PB.884.1	1	2939	5	full-splice_match	gene: Zm00001d031700	Zm00001d031700_T002	11.85	26.54	coding	746	476	2713
PB.6156.1	5	2500	16	incomplete-splice_match	gene: Zm00001d013683	Zm00001d013683_T001	35.30	37.88	coding	678	112	2145
PB.8410.1	7	2120	5	full-splice_match	gene: Zm00001d020097	Zm00001d020097_T002	13.67	22.86	coding	414	402	1643
PB.6156.3	5	2311	14	incomplete-splice_match	gene: Zm00001d013683	Zm00001d013683_T001	2.59	37.88	coding	657	1	1971
PB.8410.3	7	2156	1	incomplete-splice_match	gene: Zm00001d020097	Zm00001d020097_T007	2.93	22.86	coding	92	29	304
PB.8410.4	7	2048	4	novel_in_catalog	gene: Zm00001d020097	novel	0.20	22.86	coding	159	1344	1820
PB.1194.3	1	831	6	full-splice_match	gene: Zm00001d033099	Zm00001d033099_T001	207.54	321.05	coding	142	130	555
PB.9990.1	8	544	1	intergenic	novelGene_295	novel	22.73	22.73	non_coding	NA	NA	NA
PB.831.2	1	2585	8	full-splice_match	gene: Zm00001d031430	Zm00001d031430_T004	41.15	58.80	coding	695	259	2343
PB.8091.1	6	3212	15	novel_not_in_catalog	gene: Zm00001d039052	novel	37.41	37.41	coding	401	262	1464
PB.4526.1	3	1458	1	intergenic	novelGene_323	novel	3.27	3.27	coding	114	101	442
PB.8688.1	7	2061	2	genic	gene: Zm00001d021389	novel	29.45	88.17	coding	337	608	1618

PB.1022.1	1	2486	3	full-splice_match	gene: Zm00001d032356	Zm00001d032356_T001	4.60	4.60	coding	768	35	2338
PB.8430.2	7	1644	2	novel_in_catalog	gene: Zm00001d020224	novel	8.91	33.28	coding	101	397	699
PB.8430.3	7	668	3	full-splice_match	gene: Zm00001d020224	Zm00001d020224_T001	16.53	33.28	coding	101	71	373
PB.8430.1	7	1465	3	full-splice_match	gene: Zm00001d020224	Zm00001d020224_T002	1.46	33.28	coding	101	352	654
PB.8430.4	7	1088	3	full-splice_match	gene: Zm00001d020224	Zm00001d020224_T002	6.38	33.28	coding	101	359	661
PB.4984.1	4	669	4	full-splice_match	gene: Zm00001d049557	Zm00001d049557_T002	271.01	271.01	non_coding	NA	NA	NA
PB.7830.3	6	1381	10	full-splice_match	gene: Zm00001d038091	Zm00001d038091_T006	23.50	23.50	coding	397	10	1200
PB.8245.1	7	2017	14	novel_in_catalog	gene: Zm00001d019040	novel	18.07	18.07	coding	203	1117	1725
PB.397.1	1	2671	1	genic	gene: Zm00001d028975	novel	3.12	3.12	coding	703	488	2596
PB.7585.3	6	816	4	full-splice_match	gene: Zm00001d037067	Zm00001d037067_T002	44.95	44.95	coding	161	85	567
PB.3212.1	2	882	2	novel_in_catalog	gene: Zm00001d005952	novel	62.06	62.06	non_coding	NA	NA	NA
PB.1154.1	1	1230	2	full-splice_match	gene: Zm00001d032882	Zm00001d032882_T001	32.58	32.58	coding	257	144	914
PB.8630.1	7	755	1	full-splice_match	gene: Zm00001d021160	Zm00001d021160_T001	2.54	2.54	non_coding	NA	NA	NA
PB.460.2	1	2247	19	novel_not_in_catalog	gene: Zm00001d029231	novel	29.28	29.28	coding	526	409	1986
PB.460.3	1	2206	19	novel_not_in_catalog	gene: Zm00001d029231	novel	0.00	29.28	coding	559	352	2028
PB.8901.3	7	2128	5	novel_in_catalog	gene: Zm00001d022122	novel	8.41	21.12	coding	541	23	1645
PB.8901.1	7	3453	5	novel_in_catalog	gene: Zm00001d022122	novel	12.71	21.12	coding	370	2111	3220

PB.4150.1	3	1269	2	full-splice_match	gene: Zm00001d042022	Zm00001d042022_T001	15.89	15.89	coding	361	71	1153
PB.839.3	1	1652	2	novel_in_catalog	gene: Zm00001d031454	novel	0.57	16.82	coding	343	86	1114
PB.7719.6	6	484	1	full-splice_match	gene: Zm00001d037684	Zm00001d037684_T001	0.00	1550.15	coding	86	93	350
PB.7719.5	6	593	1	full-splice_match	gene: Zm00001d037684	Zm00001d037684_T001	0.00	1550.15	coding	86	94	351
PB.7719.4	6	701	1	full-splice_match	gene: Zm00001d037684	Zm00001d037684_T001	1427.31	1550.15	coding	86	94	351
PB.7719.3	6	802	1	full-splice_match	gene: Zm00001d037684	Zm00001d037684_T001	72.29	1550.15	coding	86	90	347
PB.7719.2	6	926	1	full-splice_match	gene: Zm00001d037684	Zm00001d037684_T001	50.20	1550.15	coding	86	94	351
PB.7719.1	6	1448	1	full-splice_match	gene: Zm00001d037684	Zm00001d037684_T001	0.34	1550.15	coding	86	94	351
PB.2130.1	10	1062	1	full-splice_match	gene: Zm00001d025762	Zm00001d025762_T001	74.93	74.93	coding	191	102	674
PB.8481.1	7	1719	1	full-splice_match	gene: Zm00001d020448	Zm00001d020448_T001	1.94	1.94	coding	450	60	1409
PB.7994.1	6	1346	1	full-splice_match	gene: Zm00001d038717	Zm00001d038717_T001	172.55	172.55	coding	320	111	1070
PB.5353.5	4	2378	6	novel_in_catalog	gene: Zm00001d051804	novel	32.26	848.79	coding	256	1056	1823
PB.6581.3	5	1750	18	full-splice_match	gene: Zm00001d015765	Zm00001d015765_T016	21.34	21.34	coding	440	153	1472
PB.8438.1	7	930	3	novel_not_in_catalog	gene: Zm00001d020274	novel	18.12	18.12	non_coding	NA	NA	NA
PB.4140.2	3	1248	2	full-splice_match	gene: Zm00001d041987	Zm00001d041987_T001	78.04	126.67	coding	341	48	1070
PB.4140.3	3	1338	1	novel_in_catalog	gene: Zm00001d041987	novel	1.90	126.67	coding	110	865	1194
PB.8961.3	7	1833	2	full-splice_match	gene: Zm00001d022351	Zm00001d022351_T001	0.00	20.51	coding	526	160	1737

PB.4140.1	3	1372	2	full-splice_match	gene: Zm00001d041987	Zm00001d041987_T001	46.74	126.67	coding	341	48	1070
PB.9522.1	8	2614	17	novel_in_catalog	gene: Zm00001d010567	novel	8.68	8.68	coding	782	115	2460
PB.10738.1	9	1120	5	full-splice_match	gene: Zm00001d048236	Zm00001d048236_T001	10.63	22.77	coding	40	219	338
PB.6400.1	5	1303	4	full-splice_match	gene: Zm00001d014817	Zm00001d014817_T001	77.95	77.95	coding	378	73	1206
PB.1380.3	1	1217	3	novel_in_catalog	gene: Zm00001d033983	novel	6.24	621.61	coding	112	824	1159
PB.6683.1	5	1438	9	full-splice_match	gene: Zm00001d016465	Zm00001d016465_T002	23.82	23.82	coding	243	410	1138
PB.9511.1	8	1784	3	intergenic	novelGene_191	novel	20.80	20.80	coding	120	283	642
PB.6410.1	5	1521	4	incomplete-splice_match	gene: Zm00001d014865	Zm00001d014865_T001	72.33	72.33	coding	379	134	1270
PB.4714.2	3	1476	9	full-splice_match	gene: Zm00001d044412	Zm00001d044412_T002	49.48	64.89	coding	349	115	1161
PB.10335.1	9	1392	3	novel_in_catalog	gene: Zm00001d046440	novel	15.47	15.47	coding	229	641	1327
PB.4592.1	3	826	1	full-splice_match	gene: Zm00001d043875	Zm00001d043875_T001	11.31	11.31	coding	243	28	756
PB.5279.2	4	1267	1	full-splice_match	gene: Zm00001d051416	Zm00001d051416_T001	13.48	19.71	coding	342	5	1030
PB.1641.2	1	1176	7	fusion	gene: Zm00001d034957_g	novel	46.85	57.51	coding	298	54	947
PB.4675.1	3	1460	2	full-splice_match	gene: Zm00001d044228	Zm00001d044228_T001	317.13	317.13	coding	355	98	1162
PB.6370.1	5	1710	10	novel_in_catalog	gene: Zm00001d014727	novel	11.28	11.28	coding	299	219	1115
PB.5988.1	5	1395	8	novel_not_in_catalog	gene: Zm00001d013072	novel	68.83	68.83	coding	321	142	1104
PB.1928.1	10	1530	7	novel_in_catalog	gene: Zm00001d024755	novel	96.98	96.98	coding	250	602	1351

PB.10616.1	9	2023	8	full-splice_match	gene: Zm00001d047806	Zm00001d047806_T001	13.60	13.60	coding	446	50	1387
PB.1162.1	1	2724	2	novel_not_in_catalog	gene: Zm00001d032909	novel	62.76	62.76	coding	839	114	2630
PB.9692.2	8	1555	2	full-splice_match	gene: Zm00001d011349	Zm00001d011349_T001	0.00	64.46	coding	428	106	1389
PB.9692.1	8	1617	2	full-splice_match	gene: Zm00001d011349	Zm00001d011349_T001	64.46	64.46	coding	428	140	1423
PB.9401.1	8	702	1	full-splice_match	gene: Zm00001d009957	Zm00001d009957_T001	138.53	138.53	coding	138	83	496
PB.5167.2	4	1620	6	full-splice_match	gene: Zm00001d050616	Zm00001d050616_T001	163.23	229.36	coding	420	104	1363
PB.5547.19	4	2288	11	novel_in_catalog	gene: Zm00001d052532	novel	0.00	938.93	coding	102	94	399
PB.3389.4	2	1414	6	full-splice_match	gene: Zm00001d006737	Zm00001d006737_T006	26.31	67.93	coding	335	208	1212
PB.7004.2	5	2704	10	novel_in_catalog	gene: Zm00001d017913	novel	24.49	24.49	coding	419	1041	2297
PB.3389.2	2	1697	5	full-splice_match	gene: Zm00001d006737	Zm00001d006737_T003	40.18	67.93	coding	335	380	1384
PB.3389.3	2	1331	6	novel_not_in_catalog	gene: Zm00001d006737	novel	1.44	67.93	coding	337	193	1203
PB.6384.2	5	1795	3	novel_not_in_catalog	gene: Zm00001d014765	novel	24.12	30.41	coding	308	107	1030
PB.6384.1	5	1969	3	novel_not_in_catalog	gene: Zm00001d014765	novel	6.29	30.41	coding	308	104	1027
PB.2950.1	2	673	3	full-splice_match	gene: Zm00001d004428	Zm00001d004428_T002	50.30	50.30	non_coding	NA	NA	NA
PB.4494.1	3	1916	2	novel_in_catalog	gene: Zm00001d043510	novel	12.16	31.56	coding	255	944	1708
PB.8773.8	7	2119	6	novel_not_in_catalog	gene: Zm00001d021698	novel	0.70	192.02	coding	393	128	1306
PB.2225.1	10	940	1	novel_in_catalog	gene: Zm00001d026204	novel	10.86	10.86	coding	170	220	729

PB.2371.8	10	1397	2	full-splice_match	gene: Zm00001d026668	Zm00001d026668_T001	21.95	72.32	coding	332	242	1237
PB.2371.6	10	2237	2	full-splice_match	gene: Zm00001d026668	Zm00001d026668_T001	30.22	72.32	coding	623	1	1869
PB.2371.7	10	3371	1	novel_in_catalog	gene: Zm00001d026668	novel	20.15	72.32	coding	786	104	2461
PB.2371.4	10	3343	2	intergenic	novelGene_242	novel	3.03	67.98	coding	203	2625	3233
PB.541.1	1	1760	2	full-splice_match	gene: Zm00001d029611	Zm00001d029611_T001	19.41	19.41	coding	426	105	1382
PB.8773.6	7	2414	6	novel_not_in_catalog	gene: Zm00001d021698	novel	0.00	192.02	coding	462	127	1512
PB.2371.3	10	1305	3	intergenic	novelGene_242	novel	62.11	67.98	coding	355	86	1150
PB.8773.4	7	2480	6	novel_not_in_catalog	gene: Zm00001d021698	novel	4.53	192.02	coding	362	130	1215
PB.8773.5	7	2456	7	novel_not_in_catalog	gene: Zm00001d021698	novel	0.00	192.02	coding	677	254	2284
PB.2585.5	2	2105	8	full-splice_match	gene: Zm00001d002580	Zm00001d002580_T004	0.00	31.64	coding	598	42	1835
PB.2585.4	2	2192	8	full-splice_match	gene: Zm00001d002580	Zm00001d002580_T004	0.00	31.64	coding	531	181	1773
PB.8257.1	7	2889	12	full-splice_match	gene: Zm00001d019090	Zm00001d019090_T008	20.51	20.51	coding	760	31	2310
PB.3594.1	2	2558	1	full-splice_match	gene: Zm00001d007823	Zm00001d007823_T001	29.00	55.81	coding	709	119	2245
PB.10785.1	9	1312	1	full-splice_match	gene: Zm00001d048407	Zm00001d048407_T001	24.12	24.12	coding	225	45	719
PB.5259.1	4	3191	13	full-splice_match	gene: Zm00001d051308	Zm00001d051308_T010	8.70	21.70	coding	752	617	2872
PB.2351.1	10	802	4	full-splice_match	gene: Zm00001d026609	Zm00001d026609_T001	56.67	56.67	coding	143	145	573
PB.2585.9	2	2014	8	novel_in_catalog	gene: Zm00001d002580	novel	3.65	31.64	coding	598	42	1835

PB.1467.1	1	1124	1	full-splice_match	gene: Zm00001d034356	Zm00001d034356_T001	882.22	1365.31	coding	224	243	914
PB.5842.2	4	3140	12	incomplete-splice_match	gene: Zm00001d053917	Zm00001d053917_T012	7.17	21.60	coding	275	87	911
PB.5547.25	4	1789	13	novel_not_in_catalog	gene: Zm00001d052532	novel	38.79	938.93	coding	152	88	543
PB.8741.1	7	1203	5	full-splice_match	gene: Zm00001d021582	Zm00001d021582_T001	244.01	244.01	coding	290	146	1015
PB.797.1	1	3146	3	full-splice_match	gene: Zm00001d031217	Zm00001d031217_T002	20.40	24.00	coding	853	275	2833
PB.5259.3	4	2336	10	incomplete-splice_match	gene: Zm00001d051308	Zm00001d051308_T019	7.79	21.70	coding	582	453	2198
PB.5547.20	4	1754	13	novel_in_catalog	gene: Zm00001d052532	novel	30.56	938.93	coding	140	94	513
PB.8608.2	7	1586	8	full-splice_match	gene: Zm00001d021019	Zm00001d021019_T001	28.83	28.83	coding	354	136	1197
PB.8384.1	7	1944	10	novel_in_catalog	gene: Zm00001d019922	novel	6.53	43.32	coding	382	260	1405
PB.8384.2	7	1746	11	full-splice_match	gene: Zm00001d019922	Zm00001d019922_T003	36.79	43.32	coding	452	210	1565
PB.5547.28	4	2174	11	novel_in_catalog	gene: Zm00001d052532	novel	3.64	938.93	coding	102	92	397
PB.859.1	1	1603	2	genic	gene: Zm00001d031572	novel	22.58	22.58	coding	350	282	1331
PB.4649.1	3	1981	6	full-splice_match	gene: Zm00001d044133	Zm00001d044133_T010	7.43	46.80	coding	99	1419	1715
PB.4649.2	3	2482	5	full-splice_match	gene: Zm00001d044133	Zm00001d044133_T009	16.54	46.80	coding	99	1947	2243
PB.4188.1	3	1435	9	full-splice_match	gene: Zm00001d042193	Zm00001d042193_T006	35.66	43.02	coding	286	123	980
PB.4649.4	3	2104	5	novel_in_catalog	gene: Zm00001d044133	novel	22.82	46.80	coding	114	1530	1871
PB.8714.2	7	2201	12	full-splice_match	gene: Zm00001d021498	Zm00001d021498_T003	25.72	25.72	coding	566	251	1948

PB.6091.5	5	2027	18	full-splice_match	gene: Zm00001d013428	Zm00001d013428_T002	0.00	201.54	coding	583	126	1874
PB.6091.4	5	2120	18	full-splice_match	gene: Zm00001d013428	Zm00001d013428_T002	69.54	201.54	coding	583	126	1874
PB.1196.7	1	2011	17	full-splice_match	gene: Zm00001d033108	Zm00001d033108_T001	0.00	82.41	coding	605	98	1912
PB.6091.1	5	2254	18	full-splice_match	gene: Zm00001d013428	Zm00001d013428_T002	132.00	201.54	coding	583	126	1874
PB.721.3	1	1711	1	novel_in_catalog	gene: Zm00001d030845	novel	0.00	20.33	coding	249	211	957
PB.9848.8	8	2249	13	novel_in_catalog	gene: Zm00001d012007	novel	8.98	55.59	coding	406	713	1930
PB.2570.1	2	697	2	genic	gene: Zm00001d002500	novel	119.25	190.18	non_coding	NA	NA	NA
PB.10003.1	8	3040	8	full-splice_match	gene: Zm00001d012667	Zm00001d012667_T004	9.83	43.63	coding	181	19	561
PB.4138.2	3	1210	2	full-splice_match	gene: Zm00001d041984	Zm00001d041984_T001	58.05	593.35	coding	334	55	1056
PB.4138.1	3	1315	2	full-splice_match	gene: Zm00001d041984	Zm00001d041984_T001	535.30	593.35	coding	334	59	1060
PB.9848.2	8	2439	12	novel_in_catalog	gene: Zm00001d012007	novel	14.31	55.59	coding	326	997	1974
PB.10133.1	9	848	8	incomplete-splice_match	gene: Zm00001d045294	Zm00001d045294_T004	219.02	219.02	coding	227	146	826
PB.9848.4	8	2431	13	novel_in_catalog	gene: Zm00001d012007	novel	9.05	55.59	coding	516	258	1805
PB.6517.1	5	1703	8	incomplete-splice_match	gene: Zm00001d015314	Zm00001d015314_T013	25.54	25.54	coding	517	52	1602
PB.9848.6	8	2324	14	full-splice_match	gene: Zm00001d012007	Zm00001d012007_T006	23.25	55.59	coding	545	328	1962
PB.7444.2	6	2088	5	full-splice_match	gene: Zm00001d036337	Zm00001d036337_T001	4.50	62.85	coding	405	661	1875
PB.7444.1	6	1976	6	incomplete-splice_match	gene: Zm00001d036337	Zm00001d036337_T002	8.23	62.85	coding	405	427	1641

PB.3034.6	2	627	2	full-splice_match	gene: Zm00001d004936	Zm00001d004936_T002	402.11	471.76	non_coding	NA	NA	NA
PB.9231.4	8	2035	10	full-splice_match	gene: Zm00001d008975	Zm00001d008975_T002	2.85	61.67	coding	545	161	1795
PB.8714.4	7	2089	12	full-splice_match	gene: Zm00001d021498	Zm00001d021498_T003	0.00	25.72	coding	566	254	1951
PB.9231.3	8	2161	10	full-splice_match	gene: Zm00001d008975	Zm00001d008975_T002	52.55	61.67	coding	545	113	1747
PB.9231.1	8	2606	9	full-splice_match	gene: Zm00001d008975	Zm00001d008975_T006	6.27	61.67	coding	228	114	797
PB.1067.1	1	1749	2	full-splice_match	gene: Zm00001d032527	Zm00001d032527_T001	18.15	18.15	coding	462	72	1457
PB.10696.2	9	2240	12	full-splice_match	gene: Zm00001d048086	Zm00001d048086_T001	0.00	31.05	coding	623	115	1983
PB.8513.4	7	759	6	full-splice_match	gene: Zm00001d020571	Zm00001d020571_T003	18.48	50.36	coding	153	133	591
PB.8513.3	7	997	6	full-splice_match	gene: Zm00001d020571	Zm00001d020571_T003	31.88	50.36	coding	153	216	674
PB.4803.1	4	1660	2	full-splice_match	gene: Zm00001d048620	Zm00001d048620_T001	5.18	5.18	coding	390	312	1481
PB.10670.2	9	2981	1	full-splice_match	gene: Zm00001d047966	Zm00001d047966_T001	4.57	4.57	coding	872	175	2790
PB.9807.1	8	1385	7	full-splice_match	gene: Zm00001d011833	Zm00001d011833_T001	19.75	19.75	coding	369	75	1181
PB.5700.2	4	2405	2	novel_in_catalog	gene: Zm00001d053289	novel	0.60	39.71	coding	252	1479	2234
PB.5700.1	4	1527	3	full-splice_match	gene: Zm00001d053289	Zm00001d053289_T001	39.11	39.71	coding	347	244	1284
PB.10696.1	9	2412	12	full-splice_match	gene: Zm00001d048086	Zm00001d048086_T001	31.05	31.05	coding	623	114	1982
PB.3370.4	2	2251	3	incomplete-splice_match	gene: Zm00001d006628	Zm00001d006628_T008	0.00	71.39	coding	559	231	1907
PB.7763.1	6	1958	2	novel_in_catalog	gene: Zm00001d037849	novel	188.23	188.23	coding	261	170	952

PB.7811.2	6	1822	1	full-splice_match	gene: Zm00001d038030	Zm00001d038030_T001	1.74	12.17	coding	523	61	1629
PB.5593.1	4	1815	5	full-splice_match	gene: Zm00001d052766	Zm00001d052766_T002	38.59	58.65	coding	409	174	1400
PB.4589.1	3	1809	1	novel_in_catalog	gene: Zm00001d043871	novel	1.22	1.22	coding	423	489	1757
PB.1370.3	1	1127	6	novel_not_in_catalog	gene: Zm00001d033925	novel	1179.21	1241.38	coding	205	131	745
PB.10790.5	9	2729	13	novel_in_catalog	gene: Zm00001d048415	novel	5.50	31.23	coding	180	797	1336
PB.10790.4	9	2260	14	full-splice_match	gene: Zm00001d048415	Zm00001d048415_T016	14.36	31.23	coding	406	87	1304
PB.6564.11	5	2388	2	novel_in_catalog	gene: Zm00001d015618	novel	3.65	718.92	coding	229	1491	2177
PB.6564.12	5	1439	4	full-splice_match	gene: Zm00001d015618	Zm00001d015618_T001	206.75	718.92	coding	367	148	1248
PB.7658.5	6	1586	8	novel_in_catalog	gene: Zm00001d037393	novel	0.00	14.11	non_coding	NA	NA	NA
PB.8684.1	7	1612	4	full-splice_match	gene: Zm00001d021379	Zm00001d021379_T003	21.79	21.79	coding	436	74	1381
PB.10582.4	9	825	6	full-splice_match	gene: Zm00001d047707	Zm00001d047707_T002	845.63	1265.54	coding	192	86	661
PB.1370.1	1	2193	5	novel_not_in_catalog	gene: Zm00001d033925	novel	21.25	1241.38	coding	150	190	639
PB.10582.2	9	937	6	full-splice_match	gene: Zm00001d047707	Zm00001d047707_T002	401.93	1265.54	coding	192	84	659
PB.3550.1	2	1603	11	full-splice_match	gene: Zm00001d007577	Zm00001d007577_T001	40.93	40.93	coding	316	465	1412
PB.10582.1	9	1161	6	full-splice_match	gene: Zm00001d047707	Zm00001d047707_T002	17.98	1265.54	coding	192	91	666
PB.3891.1	3	1463	7	novel_in_catalog	gene: Zm00001d040372	novel	8.88	8.88	coding	103	966	1274
PB.5397.1	4	729	2	fusion	gene: Zm00001d051967_g	novel	48.59	48.59	coding	61	179	361

PB.9442.1	8	1901	1	full-splice_match	gene: Zm00001d010197	Zm00001d010197_T001	29.91	29.91	coding	540	95	1714
PB.837.4	1	2483	10	incomplete-splice_match	gene: Zm00001d031447	Zm00001d031447_T001	0.00	69.53	coding	767	6	2306
PB.10205.4	9	1216	8	full-splice_match	gene: Zm00001d045517	Zm00001d045517_T001	51.73	142.56	coding	268	131	934
PB.10205.5	9	962	8	full-splice_match	gene: Zm00001d045517	Zm00001d045517_T001	0.00	142.56	coding	268	72	875
PB.10205.2	9	1259	8	full-splice_match	gene: Zm00001d045517	Zm00001d045517_T001	62.57	142.56	coding	268	116	919
PB.9893.1	8	1396	2	full-splice_match	gene: Zm00001d012221	Zm00001d012221_T001	154.68	154.68	coding	380	68	1207
PB.7858.5	6	2589	7	novel_in_catalog	gene: Zm00001d038167	novel	0.00	24.08	coding	222	295	960
PB.10205.1	9	1333	1	genic	gene: Zm00001d045517	novel	28.27	142.56	coding	256	435	1202
PB.3240.1	2	1377	4	full-splice_match	gene: Zm00001d006082	Zm00001d006082_T001	7.04	7.04	coding	365	102	1196
PB.5534.1	4	2382	2	genic	gene: Zm00001d052474	novel	7.41	7.41	coding	261	74	856
PB.974.1	1	457	1	full-splice_match	gene: Zm00001d032132	Zm00001d032132_T001	30.84	30.84	non_coding	NA	NA	NA
PB.9521.2	8	829	5	intergenic	novelGene_193	novel	23.25	23.25	coding	29	228	314
PB.1077.1	1	842	2	full-splice_match	gene: Zm00001d032556	Zm00001d032556_T001	49.26	49.26	coding	162	52	537
PB.6251.1	5	1205	7	full-splice_match	gene: Zm00001d014183	Zm00001d014183_T008	70.57	98.26	coding	298	42	935
PB.8688.2	7	1988	2	genic	gene: Zm00001d021389	novel	20.66	88.17	coding	337	667	1677
PB.6251.3	5	2530	4	incomplete-splice_match	gene: Zm00001d014183	Zm00001d014183_T007	2.83	98.26	coding	210	1691	2320
PB.6251.2	5	1228	7	full-splice_match	gene: Zm00001d014183	Zm00001d014183_T008	24.86	98.26	coding	298	125	1018

PB.2086.2	10	2234	9	full-splice_match	gene: Zm00001d025548	Zm00001d025548_T001	60.32	60.32	coding	443	404	1732
PB.4663.7	3	1836	8	full-splice_match	gene: Zm00001d044185	Zm00001d044185_T005	0.00	121.37	coding	524	75	1646
PB.6575.1	5	2230	4	novel_not_in_catalog	gene: Zm00001d015702	novel	69.67	69.67	coding	596	239	2026
PB.2663.1	2	543	3	full-splice_match	gene: Zm00001d002878	Zm00001d002878_T001	65.35	65.35	coding	129	24	410
PB.5467.1	4	1330	3	incomplete-splice_match	gene: Zm00001d052264	Zm00001d052264_T001	15.03	15.03	coding	276	78	905
PB.6874.1	5	716	3	full-splice_match	gene: Zm00001d017351	Zm00001d017351_T001	44.35	44.35	coding	169	40	546
PB.1394.3	1	1589	6	incomplete-splice_match	gene: Zm00001d034018	Zm00001d034018_T033	17.29	38.21	coding	300	28	927
PB.4663.1	3	2001	8	full-splice_match	gene: Zm00001d044185	Zm00001d044185_T005	121.37	121.37	coding	524	130	1701
PB.1468.1	1	1431	3	full-splice_match	gene: Zm00001d034360	Zm00001d034360_T001	7.33	7.33	coding	369	48	1154
PB.7389.1	6	602	2	intergenic	novelGene_121	novel	2.49	2.49	non_coding	NA	NA	NA
PB.2179.6	10	2670	7	novel_not_in_catalog	gene: Zm00001d025977	novel	4.63	32.47	coding	397	1284	2474
PB.134.2	1	3211	13	full-splice_match	gene: Zm00001d027807	Zm00001d027807_T001	25.31	25.31	coding	854	34	2595
PB.1289.1	1	2015	5	novel_in_catalog	gene: Zm00001d033572	novel	4.69	4.69	coding	351	692	1744
PB.2718.4	2	1605	7	novel_not_in_catalog	gene: Zm00001d003110	novel	1.03	16.24	coding	383	359	1507
PB.4184.1	3	2992	1	intergenic	novelGene_253	novel	2.93	2.93	coding	753	67	2325
PB.2718.1	2	1746	6	novel_not_in_catalog	gene: Zm00001d003110	novel	2.90	16.24	coding	272	364	1179
PB.2718.2	2	1981	7	novel_not_in_catalog	gene: Zm00001d003110	novel	12.31	16.24	coding	383	270	1418

PB.6712.3	5	3378	17	full-splice_match	gene: Zm00001d016640	Zm00001d016640_T010	19.72	34.20	coding	901	153	2855
PB.1995.2	10	2595	7	full-splice_match	gene: Zm00001d025021	Zm00001d025021_T008	4.13	5.28	coding	757	209	2479
PB.6712.1	5	3073	13	incomplete-splice_match	gene: Zm00001d016640	Zm00001d016640_T012	14.48	34.20	coding	377	395	1525
PB.7598.1	6	1957	5	full-splice_match	gene: Zm00001d037120	Zm00001d037120_T001	4.24	4.24	coding	329	80	1066
PB.6854.4	5	1431	5	incomplete-splice_match	gene: Zm00001d017252	Zm00001d017252_T010	3.65	40.00	coding	125	822	1196
PB.6854.5	5	1637	8	full-splice_match	gene: Zm00001d017252	Zm00001d017252_T008	3.62	40.00	coding	454	47	1408
PB.2179.7	10	2486	8	novel_not_in_catalog	gene: Zm00001d025977	novel	0.00	32.47	coding	672	286	2301
PB.5344.2	4	2185	13	full-splice_match	gene: Zm00001d051740	Zm00001d051740_T002	8.06	23.92	coding	672	98	2113
PB.5344.1	4	382	1	incomplete-splice_match	gene: Zm00001d051740	Zm00001d051740_T002	15.86	23.92	non_coding	NA	NA	NA
PB.5440.1	4	1281	4	full-splice_match	gene: Zm00001d052174	Zm00001d052174_T001	45.29	45.29	coding	263	163	951
PB.4927.3	4	841	2	full-splice_match	gene: Zm00001d049230	Zm00001d049230_T002	687.38	1100.82	coding	176	108	635
PB.4927.2	4	937	2	full-splice_match	gene: Zm00001d049230	Zm00001d049230_T002	413.44	1100.82	coding	176	115	642
PB.10726.1	9	762	5	full-splice_match	gene: Zm00001d048193	Zm00001d048193_T002	21.08	21.08	coding	193	35	613
PB.6520.2	5	2909	3	incomplete-splice_match	gene: Zm00001d015328	Zm00001d015328_T005	27.82	61.20	coding	357	1595	2665
PB.3463.1	2	1520	2	intergenic	novelGene_346	novel	3.52	3.52	coding	10	282	311
PB.1821.2	10	1394	12	full-splice_match	gene: Zm00001d024158	Zm00001d024158_T006	36.35	36.35	coding	343	105	1133
PB.3186.1	2	1668	1	full-splice_match	gene: Zm00001d005790	Zm00001d005790_T001	1.41	63.26	coding	328	103	1086

PB.3186.3	2	1491	1	full-splice_match	gene: Zm00001d005790	Zm00001d005790_T001	37.45	63.26	coding	328	216	1199
PB.2880.4	2	1982	6	full-splice_match	gene: Zm00001d003857	Zm00001d003857_T001	311.89	364.78	coding	534	72	1673
PB.4338.1	3	1208	1	full-splice_match	gene: Zm00001d042875	Zm00001d042875_T001	0.46	9.83	non_coding	NA	NA	NA
PB.2302.1	10	2171	3	full-splice_match	gene: Zm00001d026460	Zm00001d026460_T001	93.63	93.63	coding	452	372	1727
PB.9146.1	8	693	1	full-splice_match	gene: Zm00001d008594	Zm00001d008594_T001	1014.82	1014.82	coding	128	42	425
PB.2880.6	2	2900	1	novel_in_catalog	gene: Zm00001d003857	novel	2.51	364.78	coding	303	1121	2029
PB.6897.3	5	1199	8	novel_in_catalog	gene: Zm00001d017448	novel	30.07	38.54	coding	153	428	886
PB.114.1	1	1114	9	full-splice_match	gene: Zm00001d027688	Zm00001d027688_T002	123.42	123.42	coding	237	209	919
PB.1665.1	10	1365	3	incomplete-splice_match	gene: Zm00001d023272	Zm00001d023272_T007	24.53	24.53	coding	215	290	934
PB.8382.4	7	3446	25	full-splice_match	gene: Zm00001d019907	Zm00001d019907_T001	3.97	23.19	coding	1025	128	3202
PB.7590.2	6	1820	9	full-splice_match	gene: Zm00001d037098	Zm00001d037098_T006	0.29	24.22	coding	462	141	1526
PB.9533.3	8	1840	4	novel_in_catalog	gene: Zm00001d010618	novel	5.66	1006.66	coding	121	135	497
PB.9533.2	8	2132	4	full-splice_match	gene: Zm00001d010618	Zm00001d010618_T001	1.78	1006.66	coding	148	1418	1861
PB.9245.1	8	1157	2	full-splice_match	gene: Zm00001d009022	Zm00001d009022_T001	6.49	6.49	coding	332	48	1043
PB.7973.5	6	2339	14	full-splice_match	gene: Zm00001d038589	Zm00001d038589_T001	13.01	16.75	coding	720	71	2230
PB.9718.1	8	2516	5	full-splice_match	gene: Zm00001d011471	Zm00001d011471_T001	9.91	77.55	coding	634	85	1986
PB.3155.1	2	1739	8	full-splice_match	gene: Zm00001d005662	Zm00001d005662_T001	16.43	22.76	coding	281	15	857

PB.9358.1	8	2137	9	full-splice_match	gene: Zm00001d009719	Zm00001d009719_T002	23.51	36.66	coding	505	196	1710
PB.9358.2	8	2171	8	full-splice_match	gene: Zm00001d009719	Zm00001d009719_T003	13.16	36.66	coding	505	279	1793
PB.3155.2	2	1694	7	novel_in_catalog	gene: Zm00001d005662	novel	6.32	22.76	coding	215	723	1367
PB.1724.1	10	2932	2	incomplete-splice_match	gene: Zm00001d023544	Zm00001d023544_T003	4.22	6.24	coding	819	81	2537
PB.1724.3	10	2502	2	incomplete-splice_match	gene: Zm00001d023544	Zm00001d023544_T011	0.00	6.24	coding	761	70	2352
PB.1724.2	10	2583	2	incomplete-splice_match	gene: Zm00001d023544	Zm00001d023544_T011	2.02	6.24	coding	761	48	2330
PB.1051.2	1	2388	14	novel_in_catalog	gene: Zm00001d032472	novel	37.15	37.15	coding	509	237	1763
PB.9868.1	8	2544	8	full-splice_match	gene: Zm00001d012100	Zm00001d012100_T046	5.19	79.52	coding	138	223	636
PB.8914.2	7	873	2	genic	gene: Zm00001d022177	novel	26.63	36.79	coding	196	53	640
PB.2722.1	2	3049	8	incomplete-splice_match	gene: Zm00001d003119	Zm00001d003119_T006	16.36	16.36	coding	909	81	2807
PB.5364.3	4	1963	4	incomplete-splice_match	gene: Zm00001d051866	Zm00001d051866_T028	6.79	6.79	coding	434	38	1339
PB.7587.4	6	1934	5	novel_in_catalog	gene: Zm00001d037085	novel	0.00	65.08	coding	300	924	1823
PB.10687.1	9	2091	2	full-splice_match	gene: Zm00001d048053	Zm00001d048053_T001	72.30	72.30	coding	461	385	1767
PB.8424.19	7	789	2	novel_in_catalog	gene: Zm00001d020185	novel	141.95	341.07	coding	240	15	734
PB.8424.18	7	905	2	novel_in_catalog	gene: Zm00001d020185	novel	33.95	341.07	coding	240	18	737
PB.3523.1	2	1811	1	genic	gene: Zm00001d001043	novel	13.21	13.21	non_coding	NA	NA	NA
PB.7623.1	6	1737	1	full-splice_match	gene: Zm00001d037245	Zm00001d037245_T001	29.38	29.38	coding	423	177	1445

PB.8688.4	7	2469	1	full-splice_match	gene: Zm00001d021389	Zm00001d021389_T001	5.84	88.17	coding	335	1170	2174
PB.6352.1	5	643	1	genic	gene: Zm00001d014654	novel	9.31	9.31	non_coding	NA	NA	NA
PB.8424.16	7	1394	3	novel_in_catalog	gene: Zm00001d020185	novel	19.25	341.07	coding	395	18	1202
PB.4882.1	4	1716	9	novel_in_catalog	gene: Zm00001d049042	novel	22.34	82.11	coding	367	141	1241
PB.5588.3	4	926	7	full-splice_match	gene: Zm00001d052742	Zm00001d052742_T006	32.32	43.89	coding	189	123	689
PB.4882.3	4	1438	9	incomplete-splice_match	gene: Zm00001d049042	Zm00001d049042_T001	59.77	82.11	coding	367	144	1244
PB.8424.12	7	3395	12	novel_in_catalog	gene: Zm00001d020185	novel	27.54	341.07	coding	765	16	2310
PB.7684.2	6	1923	5	novel_in_catalog	gene: Zm00001d037530	novel	4.38	27.35	coding	279	844	1680
PB.5932.1	5	637	3	incomplete-splice_match	gene: Zm00001d012890	Zm00001d012890_T001	85.72	85.72	coding	100	79	378
PB.9382.1	8	2495	11	full-splice_match	gene: Zm00001d009850	Zm00001d009850_T005	73.79	255.97	coding	529	157	1743
PB.9382.2	8	2378	11	full-splice_match	gene: Zm00001d009850	Zm00001d009850_T005	117.84	255.97	coding	529	157	1743
PB.9382.5	8	2207	11	full-splice_match	gene: Zm00001d009850	Zm00001d009850_T005	61.60	255.97	coding	529	147	1733
PB.7587.5	6	1846	6	full-splice_match	gene: Zm00001d037085	Zm00001d037085_T001	0.00	65.08	coding	535	148	1752
PB.9382.6	8	3148	10	novel_in_catalog	gene: Zm00001d009850	novel	2.74	255.97	coding	360	157	1236
PB.9565.1	8	1392	10	full-splice_match	gene: Zm00001d010744	Zm00001d010744_T001	89.00	89.00	coding	342	107	1132
PB.9727.3	8	2993	10	full-splice_match	gene: Zm00001d011517	Zm00001d011517_T002	5.28	66.94	coding	840	154	2673
PB.9727.1	8	3027	10	full-splice_match	gene: Zm00001d011517	Zm00001d011517_T002	57.22	66.94	coding	840	148	2667

PB.1308.2	1	1535	10	full-splice_match	gene: Zm00001d033646	Zm00001d033646_T013	47.30	99.15	coding	411	69	1301
PB.7201.1	5	816	5	novel_not_in_catalog	gene: Zm00001d018557	novel	65.60	65.60	coding	172	206	721
PB.9727.4	8	2895	5	incomplete-splice_match	gene: Zm00001d011517	Zm00001d011517_T009	4.45	66.94	coding	256	82	849
PB.6025.6	5	936	6	incomplete-splice_match	gene: Zm00001d013232	Zm00001d013232_T001	1.47	4.16	coding	208	32	655
PB.1491.3	1	1845	1	intergenic	novelGene_379	novel	0.00	3.47	non_coding	NA	NA	NA
PB.6025.4	5	1744	2	novel_not_in_catalog	gene: Zm00001d013231	novel	93.01	97.88	coding	513	76	1614
PB.6025.5	5	2039	6	novel_in_catalog	gene: Zm00001d013232	novel	2.69	4.16	coding	338	98	1111
PB.6025.2	5	1886	1	novel_in_catalog	gene: Zm00001d013231	novel	4.87	97.88	coding	339	80	1096
PB.9170.1	8	1282	4	full-splice_match	gene: Zm00001d008693	Zm00001d008693_T002	41.60	41.60	coding	326	75	1052
PB.10504.2	9	1563	6	full-splice_match	gene: Zm00001d047422	Zm00001d047422_T007	30.26	30.26	coding	363	223	1311
PB.1679.2	10	2292	10	incomplete-splice_match	gene: Zm00001d023333	Zm00001d023333_T037	4.70	15.67	coding	566	126	1823
PB.1679.1	10	2051	8	incomplete-splice_match	gene: Zm00001d023333	Zm00001d023333_T037	10.97	15.67	coding	489	9	1475
PB.5908.1	5	1020	6	novel_in_catalog	gene: Zm00001d012806	novel	35.38	47.90	coding	279	53	889
PB.5908.2	5	3098	2	novel_in_catalog	gene: Zm00001d012806	novel	2.46	47.90	coding	112	2676	3011
PB.8356.1	7	4399	25	full-splice_match	gene: Zm00001d019670	Zm00001d019670_T021	6.74	51.39	coding	1032	728	3823
PB.8356.3	7	2136	14	novel_in_catalog	gene: Zm00001d019670	novel	28.92	51.39	coding	537	53	1663
PB.8356.2	7	4256	26	full-splice_match	gene: Zm00001d019670	Zm00001d019670_T001	15.73	51.39	coding	1032	795	3890

PB.8356.5	7	2600	15	novel_in_catalog	gene: Zm00001d019670	novel	0.00	51.39	coding	624	728	2599
PB.8356.4	7	3589	24	incomplete-splice_match	gene: Zm00001d019670	Zm00001d019670_T007	0.00	51.39	coding	952	732	3587
PB.4259.1	3	2268	10	full-splice_match	gene: Zm00001d042530	Zm00001d042530_T002	2.00	21.19	coding	289	122	988
PB.6726.1	5	2387	13	full-splice_match	gene: Zm00001d016702	Zm00001d016702_T006	2.21	20.56	coding	521	182	1744
PB.729.2	1	1908	4	novel_in_catalog	gene: Zm00001d030894	novel	0.63	15.46	coding	285	155	1009
PB.5674.1	4	1702	1	novel_in_catalog	gene: Zm00001d053133	novel	1.82	1.82	coding	271	49	861
PB.5587.1	4	1230	2	full-splice_match	gene: Zm00001d052741	Zm00001d052741_T001	7.73	7.73	coding	284	202	1053
PB.7292.1	6	721	4	full-splice_match	gene: Zm00001d035390	Zm00001d035390_T001	8.76	8.76	coding	119	32	388
PB.8715.1	7	1775	6	full-splice_match	gene: Zm00001d021506	Zm00001d021506_T001	31.38	47.07	coding	414	240	1481
PB.8203.1	7	2054	1	full-splice_match	gene: Zm00001d018813	Zm00001d018813_T001	10.36	41.55	coding	410	312	1541
PB.3486.1	2	502	1	incomplete-splice_match	gene: Zm00001d007195	Zm00001d007195_T018	1.09	21.14	non_coding	NA	NA	NA
PB.7996.4	6	3243	6	novel_in_catalog	gene: Zm00001d038720	novel	6.50	23.97	non_coding	NA	NA	NA
PB.7996.5	6	2307	4	novel_in_catalog	gene: Zm00001d038720	novel	3.31	23.97	non_coding	NA	NA	NA
PB.7335.1	6	878	2	full-splice_match	gene: Zm00001d035659	Zm00001d035659_T001	24.04	373.16	coding	83	68	316
PB.7996.3	6	1808	4	novel_in_catalog	gene: Zm00001d038720	novel	11.39	23.97	coding	111	1453	1785
PB.7335.3	6	624	2	full-splice_match	gene: Zm00001d035659	Zm00001d035659_T001	328.81	373.16	coding	83	69	317
PB.13.2	1	643	1	full-splice_match	gene: Zm00001d027291	Zm00001d027291_T001	45.46	1105.07	coding	144	93	524

PB.825.1	1	1941	4	full-splice_match	gene: Zm00001d031329	Zm00001d031329_T004	24.68	73.11	coding	344	388	1419
PB.3426.2	2	826	1	full-splice_match	gene: Zm00001d006904	Zm00001d006904_T001	41.67	93.07	coding	131	84	476
PB.7512.3	6	2761	11	full-splice_match	gene: Zm00001d036631	Zm00001d036631_T005	22.33	22.33	coding	215	1801	2445
PB.3462.6	2	2146	7	novel_not_in_catalog	gene: Zm00001d007073	novel	0.00	70.73	coding	524	209	1780
PB.4898.1	4	1364	2	full-splice_match	gene: Zm00001d049113	Zm00001d049113_T001	34.28	34.28	coding	363	85	1173
PB.3462.4	2	2722	8	novel_not_in_catalog	gene: Zm00001d007073	novel	1.27	70.73	coding	524	53	1624
PB.3462.5	2	2142	7	novel_not_in_catalog	gene: Zm00001d007073	novel	0.00	70.73	coding	524	53	1624
PB.825.3	1	1641	3	novel_in_catalog	gene: Zm00001d031329	novel	7.56	73.11	coding	162	45	530
PB.3462.1	2	3011	7	novel_not_in_catalog	gene: Zm00001d007073	novel	44.19	70.73	coding	524	209	1780
PB.8709.5	7	1173	5	full-splice_match	gene: Zm00001d021485	Zm00001d021485_T004	0.00	83.65	coding	171	552	1064
PB.9682.1	8	1836	2	full-splice_match	gene: Zm00001d011286	Zm00001d011286_T001	16.78	16.78	coding	107	1298	1618
PB.825.2	1	2334	4	novel_in_catalog	gene: Zm00001d031329	novel	5.44	73.11	coding	333	105	1103
PB.4903.1	4	1514	2	full-splice_match	gene: Zm00001d049129	Zm00001d049129_T001	72.18	333.47	coding	347	128	1168
PB.7543.1	6	1739	1	intergenic	novelGene_175	novel	21.20	21.20	coding	72	142	357
PB.4903.2	4	1378	2	full-splice_match	gene: Zm00001d049129	Zm00001d049129_T001	261.29	333.47	coding	347	128	1168
PB.1710.1	10	1114	1	full-splice_match	gene: Zm00001d023468	Zm00001d023468_T001	321.00	321.00	coding	213	157	795
PB.1710.2	10	983	1	full-splice_match	gene: Zm00001d023468	Zm00001d023468_T001	0.00	321.00	coding	213	146	784

PB.4294.1	3	2096	3	full-splice_match	gene: Zm00001d042684	Zm00001d042684_T002	147.94	417.27	coding	279	54	890
PB.10882.1	B73V4 ctg182	1865	5	novel_in_catalog	gene: Zm00001d000029	novel	90.29	90.29	coding	157	1217	1687
PB.3174.6	2	2172	3	novel_not_in_catalog	gene: Zm00001d005743	novel	2.30	27.10	non_coding	NA	NA	NA
PB.3174.5	2	2563	4	novel_not_in_catalog	gene: Zm00001d005743	novel	5.42	27.10	non_coding	NA	NA	NA
PB.3174.2	2	1697	1	novel_in_catalog	gene: Zm00001d005743	novel	11.49	27.10	non_coding	NA	NA	NA
PB.5566.3	4	1689	7	novel_in_catalog	gene: Zm00001d052656	novel	12.12	22.44	non_coding	NA	NA	NA
PB.3174.1	2	2800	2	novel_not_in_catalog	gene: Zm00001d005743	novel	7.88	27.10	non_coding	NA	NA	NA
PB.2479.1	2	2963	3	genic	gene: Zm00001d002126	novel	10.81	23.83	coding	658	710	2683
PB.10290.1	9	953	1	antisense	novelGene_gene: Zm00001d046148_A	novel	112.81	112.81	non_coding	NA	NA	NA
PB.10151.4	9	3170	1	novel_in_catalog	gene: Zm00001d045335	novel	2.94	19.20	coding	598	1272	3065
PB.2948.1	2	1432	10	full-splice_match	gene: Zm00001d004400	Zm00001d004400_T001	14.10	14.10	coding	357	101	1171
PB.10151.1	9	3262	1	genic	gene: Zm00001d045335	novel	16.26	19.20	coding	598	586	2379
PB.8324.1	7	1905	2	full-splice_match	gene: Zm00001d019510	Zm00001d019510_T001	2.41	2.41	coding	520	138	1697
PB.275.5	1	662	1	novel_in_catalog	gene: Zm00001d028428	novel	12.73	47.44	coding	120	195	554
PB.2230.1	10	802	2	full-splice_match	gene: Zm00001d026223	Zm00001d026223_T001	120.42	120.42	coding	122	254	619
PB.275.1	1	1574	5	novel_not_in_catalog	gene: Zm00001d028428	novel	31.21	47.44	coding	386	122	1279
PB.275.2	1	2025	4	full-splice_match	gene: Zm00001d028428	Zm00001d028428_T001	3.50	47.44	coding	386	586	1743

PB.10823.2	9	3386	18	full-splice_match	gene: Zm00001d048527	Zm00001d048527_T002	7.24	48.39	coding	557	1425	3095
PB.2187.2	10	1866	6	full-splice_match	gene: Zm00001d026017	Zm00001d026017_T001	7.80	11.91	coding	453	192	1550
PB.2187.1	10	2058	6	full-splice_match	gene: Zm00001d026017	Zm00001d026017_T002	4.12	11.91	coding	252	903	1658
PB.3580.2	2	2570	4	novel_in_catalog	gene: Zm00001d007767	novel	3.19	519.93	coding	168	1717	2220
PB.4326.1	3	1988	1	full-splice_match	gene: Zm00001d042826	Zm00001d042826_T001	174.94	174.94	coding	491	131	1603
PB.4326.2	3	1860	1	full-splice_match	gene: Zm00001d042826	Zm00001d042826_T001	0.00	174.94	coding	491	128	1600
PB.4326.3	3	1738	1	full-splice_match	gene: Zm00001d042826	Zm00001d042826_T001	0.00	174.94	coding	491	128	1600
PB.2465.1	2	495	4	incomplete-splice_match	gene: Zm00001d002056	Zm00001d002056_T008	27.08	27.08	coding	28	300	383
PB.1079.1	1	1505	2	full-splice_match	gene: Zm00001d032560	Zm00001d032560_T001	89.24	89.24	coding	375	117	1241
PB.1079.2	1	1394	2	full-splice_match	gene: Zm00001d032560	Zm00001d032560_T001	0.00	89.24	coding	375	121	1245
PB.7526.3	6	2144	7	full-splice_match	gene: Zm00001d036715	Zm00001d036715_T001	0.00	31.15	coding	636	74	1981
PB.7526.2	6	1838	5	incomplete-splice_match	gene: Zm00001d036715	Zm00001d036715_T001	12.58	31.15	coding	520	52	1611
PB.7526.1	6	2552	7	full-splice_match	gene: Zm00001d036715	Zm00001d036715_T001	18.57	31.15	coding	636	132	2039
PB.10425.3	9	1362	5	full-splice_match	gene: Zm00001d046949	Zm00001d046949_T001	6.09	81.12	coding	270	343	1152
PB.5787.2	4	621	1	full-splice_match	gene: Zm00001d053689	Zm00001d053689_T001	10.95	25.89	coding	114	74	415
PB.10425.1	9	1433	5	full-splice_match	gene: Zm00001d046949	Zm00001d046949_T001	75.04	81.12	coding	270	319	1128
PB.3426.1	2	1170	1	full-splice_match	gene: Zm00001d006904	Zm00001d006904_T001	30.70	93.07	coding	131	124	516

PB.8030.1	6	1831	1	full-splice_match	gene: Zm00001d038833	Zm00001d038833_T001	6.32	6.32	coding	482	122	1567
PB.9446.2	8	758	1	full-splice_match	gene: Zm00001d010217	Zm00001d010217_T001	20.80	23.27	coding	187	84	644
PB.10339.2	9	1548	2	full-splice_match	gene: Zm00001d046455	Zm00001d046455_T001	47.43	53.51	coding	425	68	1342
PB.9110.1	8	2348	1	full-splice_match	gene: Zm00001d008412	Zm00001d008412_T001	25.49	25.49	coding	572	64	1779
PB.2940.1	2	687	1	full-splice_match	gene: Zm00001d004231	Zm00001d004231_T001	125.84	125.84	coding	113	92	430
PB.6282.1	5	1989	1	novel_in_catalog	gene: Zm00001d014340	novel	15.60	69.73	coding	446	202	1539
PB.6282.2	5	2960	5	incomplete-splice_match	gene: Zm00001d014340	Zm00001d014340_T010	54.13	69.73	coding	900	29	2728
PB.6282.4	5	2440	4	incomplete-splice_match	gene: Zm00001d014340	Zm00001d014340_T006	0.00	69.73	coding	446	973	2310
PB.4087.2	3	903	5	full-splice_match	gene: Zm00001d041725	Zm00001d041725_T001	32.43	38.51	coding	196	105	692
PB.10058.2	9	1939	1	full-splice_match	gene: Zm00001d044857	Zm00001d044857_T001	0.00	97.98	coding	349	674	1720
PB.10058.1	9	2081	1	full-splice_match	gene: Zm00001d044857	Zm00001d044857_T001	97.98	97.98	coding	349	687	1733
PB.5542.1	4	1357	5	novel_not_in_catalog	gene: Zm00001d052507	novel	13.28	13.28	non_coding	NA	NA	NA
PB.10439.1	9	2670	1	full-splice_match	gene: Zm00001d047017	Zm00001d047017_T001	16.05	16.55	coding	703	123	2231
PB.4432.1	3	671	1	incomplete-splice_match	gene: Zm00001d043286	Zm00001d043286_T007	12.59	12.59	non_coding	NA	NA	NA
PB.10439.2	9	2361	1	full-splice_match	gene: Zm00001d047017	Zm00001d047017_T001	0.50	16.55	coding	703	147	2255
PB.9942.3	8	2963	18	full-splice_match	gene: Zm00001d012446	Zm00001d012446_T002	0.00	32.44	coding	818	145	2598
PB.9942.2	8	1925	7	novel_in_catalog	gene: Zm00001d012446	novel	9.06	32.44	coding	167	1051	1551

PB.9942.1	8	3092	18	full-splice_match	gene: Zm00001d012446	Zm00001d012446_T002	23.38	32.44	coding	818	175	2628
PB.10927.2	B73V4 ctg56	2437	5	full-splice_match	gene: Zm00001d000124	Zm00001d000124_T009	15.11	15.11	coding	521	577	2139
PB.4603.2	3	1253	2	full-splice_match	gene: Zm00001d043935	Zm00001d043935_T001	69.67	79.60	coding	220	337	996
PB.9097.1	8	2186	11	full-splice_match	gene: Zm00001d008345	Zm00001d008345_T001	181.04	181.04	coding	527	138	1718
PB.6910.1	5	1059	4	full-splice_match	gene: Zm00001d017501	Zm00001d017501_T001	67.86	67.86	coding	253	88	846
PB.9097.4	8	2084	11	full-splice_match	gene: Zm00001d008345	Zm00001d008345_T001	0.00	181.04	coding	527	138	1718
PB.5636.2	4	1751	7	full-splice_match	gene: Zm00001d052941	Zm00001d052941_T001	6.61	30.53	coding	515	60	1604
PB.8325.2	7	1611	8	full-splice_match	gene: Zm00001d019515	Zm00001d019515_T001	36.56	63.67	coding	368	227	1330
PB.9474.1	8	819	2	full-splice_match	gene: Zm00001d010322	Zm00001d010322_T001	135.45	135.45	coding	177	35	565
PB.2976.2	2	1150	2	genic	gene: Zm00001d004568	novel	14.49	148.51	coding	198	396	989
PB.2976.1	2	1111	2	genic	gene: Zm00001d004568	novel	134.02	148.51	coding	290	71	940
PB.5797.3	4	2351	4	incomplete-splice_match	gene: Zm00001d053741	Zm00001d053741_T004	6.40	15.97	coding	750	1	2250
PB.3910.1	3	2447	4	full-splice_match	gene: Zm00001d040503	Zm00001d040503_T009	80.08	105.47	coding	636	55	1962
PB.3910.2	3	3321	4	full-splice_match	gene: Zm00001d040503	Zm00001d040503_T009	23.46	105.47	coding	950	111	2960
PB.3910.3	3	3127	4	full-splice_match	gene: Zm00001d040503	Zm00001d040503_T009	1.93	105.47	coding	950	113	2962
PB.3289.1	2	2773	4	full-splice_match	gene: Zm00001d006286	Zm00001d006286_T001	21.03	21.03	coding	509	306	1832
PB.7786.3	6	459	3	incomplete-splice_match	gene: Zm00001d037939	Zm00001d037939_T004	83.08	83.08	non_coding	NA	NA	NA

PB.839.2	1	2341	3	full-splice_match	gene: Zm00001d031454	Zm00001d031454_T001	0.00	16.82	coding	643	230	2158
PB.10831.1	9	1188	6	novel_in_catalog	gene: Zm00001d048550	novel	27.91	27.91	coding	112	782	1117
PB.2791.3	2	1217	6	full-splice_match	gene: Zm00001d003401	Zm00001d003401_T004	1707.00	1842.56	coding	261	93	875
PB.845.1	1	1723	10	full-splice_match	gene: Zm00001d031490	Zm00001d031490_T003	94.29	96.58	coding	390	225	1394
PB.77.1	1	1350	5	full-splice_match	gene: Zm00001d027527	Zm00001d027527_T002	146.79	146.79	coding	308	111	1034
PB.3068.1	2	2813	14	full-splice_match	gene: Zm00001d005157	Zm00001d005157_T002	25.98	26.18	coding	732	170	2365
PB.3068.2	2	2675	13	novel_in_catalog	gene: Zm00001d005157	novel	0.00	26.18	coding	445	170	1504
PB.3068.3	2	2554	13	novel_in_catalog	gene: Zm00001d005157	novel	0.00	26.18	coding	445	157	1491
PB.8477.1	7	1882	3	full-splice_match	gene: Zm00001d020438	Zm00001d020438_T004	17.22	173.17	coding	102	102	407
PB.7262.1	6	3091	5	full-splice_match	gene: Zm00001d035208	Zm00001d035208_T002	9.36	19.00	coding	195	2176	2760
PB.7262.2	6	3044	5	full-splice_match	gene: Zm00001d035208	Zm00001d035208_T002	9.65	19.00	coding	195	2247	2831
PB.5279.1	4	1547	1	full-splice_match	gene: Zm00001d051416	Zm00001d051416_T001	6.23	19.71	coding	342	58	1083
PB.8306.2	7	3029	1	novel_in_catalog	gene: Zm00001d019374	novel	3.67	20.75	coding	496	23	1510
PB.4563.3	3	3093	4	novel_in_catalog	gene: Zm00001d043768	novel	1.32	15.83	coding	326	83	1060
PB.8306.1	7	833	2	full-splice_match	gene: Zm00001d019374	Zm00001d019374_T001	17.08	20.75	coding	207	63	683
PB.1129.1	1	792	2	full-splice_match	gene: Zm00001d032760	Zm00001d032760_T001	740.90	1444.84	coding	164	72	563
PB.3288.2	2	3465	2	full-splice_match	gene: Zm00001d006274	Zm00001d006274_T001	3.46	3.46	coding	149	2873	3319

PB.5879.1	4	1620	6	full-splice_match	gene: Zm00001d054014	Zm00001d054014_T001	39.35	44.48	coding	430	111	1400
PB.6017.1	5	1219	6	full-splice_match	gene: Zm00001d013204	Zm00001d013204_T002	46.97	46.97	coding	328	40	1023
PB.10555.2	9	3250	6	full-splice_match	gene: Zm00001d047601	Zm00001d047601_T010	2.19	46.65	coding	529	79	1665
PB.5259.2	4	2899	13	incomplete-splice_match	gene: Zm00001d051308	Zm00001d051308_T005	4.36	21.70	coding	880	18	2657
PB.10431.2	9	1281	11	full-splice_match	gene: Zm00001d046972	Zm00001d046972_T002	14.54	64.09	coding	215	311	955
PB.5259.4	4	1593	9	incomplete-splice_match	gene: Zm00001d051308	Zm00001d051308_T010	0.85	21.70	coding	271	780	1592
PB.10431.4	9	1080	10	novel_in_catalog	gene: Zm00001d046972	novel	17.46	64.09	coding	149	308	754
PB.1227.1	1	1599	9	full-splice_match	gene: Zm00001d033294	Zm00001d033294_T003	13.08	21.40	coding	272	513	1328
PB.10555.3	9	1961	5	incomplete-splice_match	gene: Zm00001d047601	Zm00001d047601_T019	9.83	46.65	coding	327	37	1017
PB.8262.1	7	1254	9	full-splice_match	gene: Zm00001d019123	Zm00001d019123_T001	35.09	35.09	coding	330	85	1074
PB.4304.6	3	2842	5	full-splice_match	gene: Zm00001d042724	Zm00001d042724_T006	2.77	239.40	coding	480	1152	2591
PB.2570.2	2	550	2	genic	gene: Zm00001d002500	novel	70.92	190.18	non_coding	NA	NA	NA
PB.4304.2	3	2262	6	novel_not_in_catalog	gene: Zm00001d042724	novel	4.40	239.40	coding	480	572	2011
PB.4304.3	3	1976	6	novel_not_in_catalog	gene: Zm00001d042724	novel	210.51	239.40	coding	480	246	1685
PB.2062.1	10	1324	1	incomplete-splice_match	gene: Zm00001d025397	Zm00001d025397_T003	59.69	59.69	coding	253	29	787
PB.10555.1	9	2812	7	full-splice_match	gene: Zm00001d047601	Zm00001d047601_T007	11.63	46.65	coding	722	125	2290
PB.1242.3	1	1656	6	full-splice_match	gene: Zm00001d033366	Zm00001d033366_T002	0.00	65.09	coding	392	186	1361

PB.9602.1	8	374	1	intergenic	novelGene_210	novel	21.22	21.22	non_coding	NA	NA	NA
PB.7214.1	6	1534	5	novel_not_in_catalog	gene: Zm00001d035014	novel	39.77	39.77	coding	355	180	1244
PB.4983.1	4	2235	3	full-splice_match	gene: Zm00001d049554	Zm00001d049554_T001	8.91	14.12	coding	457	402	1772
PB.4983.2	4	2103	3	full-splice_match	gene: Zm00001d049554	Zm00001d049554_T001	5.21	14.12	coding	457	398	1768
PB.7309.2	6	2869	12	incomplete-splice_match	gene: Zm00001d035487	Zm00001d035487_T034	18.76	20.95	coding	636	314	2221
PB.7309.4	6	3290	17	full-splice_match	gene: Zm00001d035487	Zm00001d035487_T058	2.19	20.95	coding	876	196	2823
PB.6914.1	5	1189	2	intergenic	novelGene_314	novel	1.62	1.62	coding	181	4	546
PB.1738.2	10	1949	5	full-splice_match	gene: Zm00001d023606	Zm00001d023606_T012	1.63	23.60	coding	285	168	1022
PB.1738.3	10	1728	6	full-splice_match	gene: Zm00001d023606	Zm00001d023606_T005	0.00	23.60	coding	457	168	1538
PB.3719.1	3	1435	5	full-splice_match	gene: Zm00001d039519	Zm00001d039519_T001	16.68	16.68	coding	287	163	1023
PB.1738.1	10	1874	6	full-splice_match	gene: Zm00001d023606	Zm00001d023606_T005	21.97	23.60	coding	457	168	1538
PB.10325.1	9	1626	5	full-splice_match	gene: Zm00001d046381	Zm00001d046381_T023	15.77	21.97	coding	139	811	1227
PB.10325.2	9	3065	7	full-splice_match	gene: Zm00001d046381	Zm00001d046381_T033	6.20	21.97	coding	163	2320	2808
PB.4382.1	3	978	1	full-splice_match	gene: Zm00001d043087	Zm00001d043087_T001	39.70	39.70	coding	212	175	810
PB.3280.1	2	1305	9	novel_not_in_catalog	gene: Zm00001d006250	novel	49.19	49.19	coding	265	166	960
PB.5072.11	4	1062	5	full-splice_match	gene: Zm00001d050011	Zm00001d050011_T001	57.76	131.70	coding	277	68	898
PB.5072.10	4	1372	4	novel_in_catalog	gene: Zm00001d050011	novel	1.57	131.70	coding	141	68	490

PB.6072.11	5	1664	4	full-splice_match	gene: Zm00001d013367	Zm00001d013367_T001	15.90	3063.25	coding	451	182	1534
PB.2018.5	10	1655	9	full-splice_match	gene: Zm00001d025128	Zm00001d025128_T012	0.00	38.69	coding	491	53	1525
PB.2018.2	10	1837	9	full-splice_match	gene: Zm00001d025128	Zm00001d025128_T012	27.78	38.69	coding	491	53	1525
PB.3510.6	2	1811	6	full-splice_match	gene: Zm00001d007311	Zm00001d007311_T003	2.52	54.58	coding	428	349	1632
PB.2018.1	10	3181	8	full-splice_match	gene: Zm00001d025128	Zm00001d025128_T016	10.91	38.69	coding	292	1925	2800
PB.825.5	1	1135	1	novel_in_catalog	gene: Zm00001d031329	novel	11.39	73.11	non_coding	NA	NA	NA
PB.825.4	1	2033	5	full-splice_match	gene: Zm00001d031329	Zm00001d031329_T005	24.04	73.11	coding	515	101	1645
PB.3510.7	2	1972	3	novel_in_catalog	gene: Zm00001d007311	novel	2.82	54.58	coding	364	200	1291
PB.3779.1	3	1658	11	full-splice_match	gene: Zm00001d039856	Zm00001d039856_T007	31.52	256.04	coding	345	136	1170
PB.1593.3	1	1036	5	novel_not_in_catalog	gene: Zm00001d034790	novel	419.63	1199.41	coding	265	99	893
PB.8413.1	7	1252	10	full-splice_match	gene: Zm00001d020108	Zm00001d020108_T002	33.04	33.04	coding	315	67	1011
PB.3779.2	3	1518	11	full-splice_match	gene: Zm00001d039856	Zm00001d039856_T009	0.71	256.04	coding	345	141	1175
PB.950.1	1	938	1	full-splice_match	gene: Zm00001d032003	Zm00001d032003_T001	4.06	4.06	coding	277	8	838
PB.7192.1	5	2089	3	intergenic	novelGene_347	novel	5.20	6.85	coding	300	1083	1982
PB.4753.2	3	2766	18	full-splice_match	gene: Zm00001d044552	Zm00001d044552_T010	14.10	14.10	coding	737	286	2496
PB.1796.2	10	943	5	full-splice_match	gene: Zm00001d023934	Zm00001d023934_T003	45.37	48.33	coding	228	50	733
PB.10339.3	9	1401	2	full-splice_match	gene: Zm00001d046455	Zm00001d046455_T001	0.00	53.51	coding	425	68	1342

PB.1796.1	10	2143	4	incomplete-splice_match	gene: Zm00001d023934	Zm00001d023934_T002	2.96	48.33	coding	134	1530	1931
PB.249.9	1	1507	7	full-splice_match	gene: Zm00001d028344	Zm00001d028344_T002	0.00	29.38	coding	402	184	1389
PB.6970.1	5	1668	3	full-splice_match	gene: Zm00001d017785	Zm00001d017785_T002	2.91	110.51	coding	328	382	1365
PB.845.5	1	1559	10	full-splice_match	gene: Zm00001d031490	Zm00001d031490_T003	0.00	96.58	coding	390	140	1309
PB.845.2	1	2245	9	full-splice_match	gene: Zm00001d031490	Zm00001d031490_T004	2.29	96.58	coding	178	140	673
PB.7852.1	6	3979	22	novel_not_in_catalog	gene: Zm00001d038150	novel	13.24	13.24	coding	1170	197	3706
PB.3609.1	2	2824	6	full-splice_match	gene: Zm00001d007865	Zm00001d007865_T001	101.69	101.69	coding	732	263	2458
PB.249.1	1	2251	7	full-splice_match	gene: Zm00001d028344	Zm00001d028344_T002	0.00	29.38	coding	402	111	1316
PB.7542.4	6	1560	1	incomplete-splice_match	gene: Zm00001d036798	Zm00001d036798_T001	2.59	35.60	coding	105	164	478
PB.249.2	1	1733	7	full-splice_match	gene: Zm00001d028344	Zm00001d028344_T002	29.38	29.38	coding	402	183	1388
PB.7542.1	6	2219	9	full-splice_match	gene: Zm00001d036798	Zm00001d036798_T002	2.02	35.60	coding	574	33	1754
PB.249.4	1	1638	7	full-splice_match	gene: Zm00001d028344	Zm00001d028344_T002	0.00	29.38	coding	402	186	1391
PB.7542.3	6	2533	8	full-splice_match	gene: Zm00001d036798	Zm00001d036798_T003	2.03	35.60	coding	508	36	1559
PB.7542.2	6	2085	9	full-splice_match	gene: Zm00001d036798	Zm00001d036798_T002	28.95	35.60	coding	574	34	1755
PB.3813.2	3	2224	3	full-splice_match	gene: Zm00001d040034	Zm00001d040034_T001	4.44	7.97	coding	599	180	1976
PB.3813.3	3	1980	3	full-splice_match	gene: Zm00001d040034	Zm00001d040034_T001	0.00	7.97	coding	599	72	1868
PB.3813.1	3	2424	3	full-splice_match	gene: Zm00001d040034	Zm00001d040034_T001	3.54	7.97	coding	599	306	2102

PB.5162.1	4	809	1	full-splice_match	gene: Zm00001d050553	Zm00001d050553_T001	13.55	13.55	coding	193	136	714
PB.7944.1	6	2942	3	full-splice_match	gene: Zm00001d038487	Zm00001d038487_T003	15.12	15.12	coding	182	2072	2617
PB.7240.2	6	3203	11	novel_not_in_catalog	gene: Zm00001d035113	novel	10.04	10.04	coding	811	496	2928
PB.6202.1	5	902	1	full-splice_match	gene: Zm00001d013945	Zm00001d013945_T001	85.82	85.82	coding	172	139	654
PB.2687.1	2	1075	1	full-splice_match	gene: Zm00001d002969	Zm00001d002969_T001	90.23	90.23	coding	259	51	827
PB.6970.3	5	2022	1	novel_in_catalog	gene: Zm00001d017785	novel	17.51	110.51	coding	279	124	960
PB.6202.2	5	796	1	full-splice_match	gene: Zm00001d013945	Zm00001d013945_T001	0.00	85.82	coding	172	136	651
PB.6970.5	5	1346	3	full-splice_match	gene: Zm00001d017785	Zm00001d017785_T002	69.46	110.51	coding	328	124	1107
PB.6970.4	5	1370	2	novel_in_catalog	gene: Zm00001d017785	novel	0.00	110.51	coding	256	1	768
PB.1250.4	1	1878	2	incomplete-splice_match	gene: Zm00001d033388	Zm00001d033388_T002	0.00	34.59	coding	595	94	1878
PB.10453.1	9	588	2	incomplete-splice_match	gene: Zm00001d047105	Zm00001d047105_T001	33.99	61.31	non_coding	NA	NA	NA
PB.6727.3	5	1266	8	novel_in_catalog	gene: Zm00001d016704	novel	0.31	100.81	coding	226	412	1089
PB.1427.1	1	2146	6	novel_in_catalog	gene: Zm00001d034180	novel	6.66	122.73	coding	156	1111	1578
PB.10453.2	9	2411	10	full-splice_match	gene: Zm00001d047105	Zm00001d047105_T001	27.32	61.31	coding	737	2	2212
PB.7641.1	6	1515	7	full-splice_match	gene: Zm00001d037308	Zm00001d037308_T002	3.37	3.37	coding	434	62	1363
PB.667.8	1	2525	20	full-splice_match	gene: Zm00001d030366	Zm00001d030366_T024	0.23	29.16	coding	495	134	1618
PB.667.7	1	2418	21	full-splice_match	gene: Zm00001d030366	Zm00001d030366_T008	9.24	29.16	coding	710	125	2254

PB.667.6	1	1450	12	novel_in_catalog	gene: Zm00001d030366	novel	15.33	29.16	coding	211	653	1285
PB.3705.1	3	1201	1	genic	gene: Zm00001d039467	novel	6.44	25.43	coding	232	52	747
PB.3705.2	3	2471	3	full-splice_match	gene: Zm00001d039467	Zm00001d039467_T001	19.00	25.43	coding	497	664	2154
PB.3705.3	3	708	1	incomplete-splice_match	gene: Zm00001d039467	Zm00001d039467_T001	0.00	25.43	coding	149	56	502
PB.3630.1	2	1777	1	genic	gene: Zm00001d007954	novel	1.67	1.67	non_coding	NA	NA	NA
PB.3191.2	2	1216	5	full-splice_match	gene: Zm00001d005812	Zm00001d005812_T003	29.59	72.85	coding	245	118	852
PB.1401.1	1	2443	4	full-splice_match	gene: Zm00001d034038	Zm00001d034038_T002	24.50	31.79	coding	631	189	2081
PB.812.1	1	1414	1	full-splice_match	gene: Zm00001d031273	Zm00001d031273_T001	2.29	29.53	coding	289	54	920
PB.4710.1	3	1850	1	full-splice_match	gene: Zm00001d044390	Zm00001d044390_T001	60.59	62.28	coding	485	39	1493
PB.3793.1	3	1349	5	full-splice_match	gene: Zm00001d039919	Zm00001d039919_T001	10.45	10.45	coding	328	125	1108
PB.3097.7	2	2503	13	novel_in_catalog	gene: Zm00001d005306	novel	1.28	31.54	coding	225	1744	2418
PB.3097.4	2	1605	9	novel_in_catalog	gene: Zm00001d005306	novel	4.03	31.54	coding	434	27	1328
PB.3970.1	3	662	2	intergenic	novelGene_149	novel	19.24	19.24	coding	57	150	320
PB.3097.2	2	2317	14	novel_in_catalog	gene: Zm00001d005306	novel	17.30	31.54	coding	225	1517	2191
PB.643.2	1	1396	3	full-splice_match	gene: Zm00001d030220	Zm00001d030220_T001	0.00	38.06	coding	355	192	1256
PB.643.1	1	1457	3	full-splice_match	gene: Zm00001d030220	Zm00001d030220_T001	38.06	38.06	coding	355	163	1227
PB.3097.1	2	2348	14	full-splice_match	gene: Zm00001d005306	Zm00001d005306_T003	8.93	31.54	coding	534	652	2253

PB.9501.1	8	2174	2	full-splice_match	gene: Zm00001d010463	Zm00001d010463_T001	40.64	79.92	coding	576	45	1772
PB.9501.2	8	2076	2	full-splice_match	gene: Zm00001d010463	Zm00001d010463_T001	29.59	79.92	coding	576	45	1772
PB.9501.3	8	2645	1	novel_in_catalog	gene: Zm00001d010463	novel	5.05	79.92	non_coding	NA	NA	NA
PB.9501.4	8	1911	2	full-splice_match	gene: Zm00001d010463	Zm00001d010463_T001	0.00	79.92	coding	576	45	1772
PB.2964.2	2	626	1	novel_in_catalog	gene: Zm00001d004521	novel	0.33	0.33	coding	134	40	441
PB.1900.1	10	758	6	incomplete-splice_match	gene: Zm00001d024612	Zm00001d024612_T004	54.54	54.54	non_coding	NA	NA	NA
PB.828.4	1	1045	2	full-splice_match	gene: Zm00001d031344	Zm00001d031344_T001	736.24	1026.24	coding	167	137	637
PB.10855.1	B73V4 ctg129	1881	15	full-splice_match	gene: Zm00001d000205	Zm00001d000205_T002	15.40	15.40	coding	491	146	1618
PB.7948.2	6	1005	3	full-splice_match	gene: Zm00001d038519	Zm00001d038519_T004	8.35	63.23	non_coding	NA	NA	NA
PB.409.7	1	848	1	novel_in_catalog	gene: Zm00001d029009	novel	7.80	7.80	coding	126	360	737
PB.7948.1	6	1343	2	novel_in_catalog	gene: Zm00001d038519	novel	8.43	63.23	non_coding	NA	NA	NA
PB.8206.1	7	2144	10	full-splice_match	gene: Zm00001d018842	Zm00001d018842_T001	20.12	20.12	coding	559	149	1825
PB.2941.2	2	1819	1	genic	gene: Zm00001d004248	novel	7.73	19.51	coding	489	150	1616
PB.4623.3	3	2382	19	full-splice_match	gene: Zm00001d044016	Zm00001d044016_T001	15.38	66.64	coding	655	94	2058
PB.4301.1	3	1207	3	full-splice_match	gene: Zm00001d042719	Zm00001d042719_T001	59.61	59.61	coding	276	90	917
PB.5851.3	4	1118	6	full-splice_match	gene: Zm00001d053932	Zm00001d053932_T003	0.00	151.82	coding	311	85	1017
PB.7128.2	5	1590	10	full-splice_match	gene: Zm00001d018366	Zm00001d018366_T001	11.88	11.88	coding	317	102	1052

PB.2376.1	10	1451	1	intergenic	novelGene_243	novel	6.04	6.04	non_coding	NA	NA	NA
PB.2376.3	10	1479	1	full-splice_match	gene: Zm00001d026678	Zm00001d026678_T001	21.54	30.97	coding	409	36	1262
PB.2376.2	10	1586	1	full-splice_match	gene: Zm00001d026678	Zm00001d026678_T001	9.43	30.97	coding	409	36	1262
PB.9054.3	7	598	3	incomplete-splice_match	gene: Zm00001d022631	Zm00001d022631_T002	0.00	48.42	coding	115	75	419
PB.7948.5	6	875	3	full-splice_match	gene: Zm00001d038519	Zm00001d038519_T005	14.21	63.23	non_coding	NA	NA	NA
PB.352.2	1	2347	3	full-splice_match	gene: Zm00001d028774	Zm00001d028774_T001	17.17	17.17	coding	592	453	2228
PB.4446.1	3	2233	3	full-splice_match	gene: Zm00001d043350	Zm00001d043350_T001	12.17	12.17	coding	604	118	1929
PB.6117.1	5	1220	4	full-splice_match	gene: Zm00001d013526	Zm00001d013526_T005	126.09	284.60	coding	227	128	808
PB.6117.2	5	1127	3	novel_in_catalog	gene: Zm00001d013526	novel	8.18	284.60	coding	159	26	502
PB.6117.3	5	746	3	incomplete-splice_match	gene: Zm00001d013526	Zm00001d013526_T005	150.32	284.60	coding	92	233	508
PB.8051.1	6	779	5	novel_in_catalog	gene: Zm00001d038890	novel	40.69	40.69	coding	190	81	650
PB.1191.1	1	2896	10	novel_not_in_catalog	gene: Zm00001d033071	novel	17.73	19.96	coding	824	142	2613
PB.856.2	1	1448	3	full-splice_match	gene: Zm00001d031555	Zm00001d031555_T001	0.00	27.58	coding	395	123	1307
PB.2524.1	2	2268	2	full-splice_match	gene: Zm00001d002307	Zm00001d002307_T001	8.05	8.05	coding	234	1458	2159
PB.10841.1	B73V4 ctg103	312	1	incomplete-splice_match	gene: Zm00001d000298	Zm00001d000298_T001	22.49	22.49	non_coding	NA	NA	NA
PB.6383.1	5	3594	4	full-splice_match	gene: Zm00001d014764	Zm00001d014764_T005	5.29	6.73	coding	864	313	2904
PB.6848.1	5	3349	16	full-splice_match	gene: Zm00001d017216	Zm00001d017216_T001	11.06	11.06	coding	980	63	3002

PB.8167.1	7	1519	10	full-splice_match	gene: Zm00001d018669	Zm00001d018669_T001	67.01	67.01	coding	332	209	1204
PB.6383.2	5	3301	4	full-splice_match	gene: Zm00001d014764	Zm00001d014764_T005	1.45	6.73	coding	864	271	2862
PB.9788.3	8	1609	9	full-splice_match	gene: Zm00001d011767	Zm00001d011767_T003	0.00	48.45	coding	472	60	1475
PB.9788.1	8	1735	9	full-splice_match	gene: Zm00001d011767	Zm00001d011767_T003	48.45	48.45	coding	472	84	1499
PB.6905.1	5	1104	2	full-splice_match	gene: Zm00001d017485	Zm00001d017485_T002	582.63	674.17	coding	250	70	819
PB.4185.2	3	528	1	novel_in_catalog	gene: Zm00001d042166	novel	7.93	17.59	non_coding	NA	NA	NA
PB.4185.1	3	657	1	novel_in_catalog	gene: Zm00001d042166	novel	9.65	17.59	non_coding	NA	NA	NA
PB.8873.1	7	1234	2	full-splice_match	gene: Zm00001d022027	Zm00001d022027_T001	12.75	12.75	coding	248	18	761
PB.9448.1	8	2029	4	novel_not_in_catalog	gene: Zm00001d010222	novel	2.59	16.51	coding	441	452	1774
PB.9448.2	8	1909	4	novel_not_in_catalog	gene: Zm00001d010222	novel	13.92	16.51	coding	441	344	1666
PB.10086.3	9	3530	12	novel_in_catalog	gene: Zm00001d045026	novel	7.67	20.28	coding	809	695	3121
PB.10086.1	9	4077	13	novel_in_catalog	gene: Zm00001d045026	novel	12.62	20.28	coding	809	1165	3591
PB.7936.1	6	1121	3	incomplete-splice_match	gene: Zm00001d038451	Zm00001d038451_T003	4.52	4.52	coding	248	65	808
PB.6558.1	5	1892	10	full-splice_match	gene: Zm00001d015527	Zm00001d015527_T002	18.04	18.04	coding	506	117	1634
PB.5512.6	4	2435	4	novel_in_catalog	gene: Zm00001d052405	novel	7.27	19.26	coding	253	1363	2121
PB.577.1	1	1083	4	full-splice_match	gene: Zm00001d029806	Zm00001d029806_T001	69.69	69.69	coding	230	139	828
PB.6421.4	5	2343	17	novel_not_in_catalog	gene: Zm00001d014897	novel	3.96	117.26	coding	321	1187	2149

PB.6957.1	5	2096	2	full-splice_match	gene: Zm00001d017728	Zm00001d017728_T001	5.32	5.32	coding	525	224	1798
PB.6421.2	5	2064	17	full-splice_match	gene: Zm00001d014897	Zm00001d014897_T001	98.81	117.26	coding	583	79	1827
PB.9447.1	8	1376	4	full-splice_match	gene: Zm00001d010218	Zm00001d010218_T001	12.28	12.28	coding	330	128	1117
PB.1181.1	1	1336	2	full-splice_match	gene: Zm00001d033018	Zm00001d033018_T001	14.05	14.05	coding	369	68	1174
PB.6421.1	5	2035	17	full-splice_match	gene: Zm00001d014897	Zm00001d014897_T001	14.49	117.26	coding	422	422	1687
PB.4742.7	3	702	7	incomplete-splice_match	gene: Zm00001d044503	Zm00001d044503_T011	11.74	26.50	coding	208	21	644
PB.10758.1	9	1728	8	full-splice_match	gene: Zm00001d048323	Zm00001d048323_T001	7.07	7.07	coding	458	47	1420
PB.8461.1	7	3103	11	fusion	gene: Zm00001d020367_g	novel	5.46	5.46	coding	893	159	2837
PB.9881.2	8	1112	4	full-splice_match	gene: Zm00001d012160	Zm00001d012160_T005	6.11	96.04	coding	133	365	763
PB.4199.1	3	2564	3	full-splice_match	gene: Zm00001d042268	Zm00001d042268_T001	3.42	5.47	coding	414	797	2038
PB.4199.2	3	2367	3	full-splice_match	gene: Zm00001d042268	Zm00001d042268_T001	2.05	5.47	coding	414	799	2040
PB.10849.4	B73V4 ctg107	1766	2	genic	gene: Zm00001d000307	novel	0.87	35.00	coding	319	363	1319
PB.10849.5	B73V4 ctg107	1502	2	genic	gene: Zm00001d000307	novel	7.85	35.00	coding	194	714	1295
PB.1537.1	1	1975	7	novel_in_catalog	gene: Zm00001d034608	novel	50.61	50.61	coding	412	476	1711
PB.7750.2	6	328	1	intergenic	novelGene_221	novel	0.00	0.00	non_coding	NA	NA	NA
PB.10849.1	B73V4 ctg107	1648	3	genic	gene: Zm00001d000307	novel	6.66	35.00	coding	16	272	319
PB.10849.2	B73V4 ctg107	1881	2	genic	gene: Zm00001d000307	novel	4.68	35.00	coding	319	331	1287

PB.10849.3	B73V4 ctg107	1549	3	genic	gene: Zm00001d000307	novel	14.94	35.00	coding	202	714	1319
PB.6782.1	5	1140	5	full-splice_match	gene: Zm00001d016920	Zm00001d016920_T002	63.40	63.40	coding	242	176	901
PB.8514.1	7	1494	1	full-splice_match	gene: Zm00001d020583	Zm00001d020583_T001	5.52	68.45	coding	318	4	957
PB.10064.1	9	1366	2	full-splice_match	gene: Zm00001d044906	Zm00001d044906_T001	11.92	11.92	coding	373	94	1212
PB.8514.2	7	1327	1	full-splice_match	gene: Zm00001d020583	Zm00001d020583_T001	12.17	68.45	coding	318	48	1001
PB.6133.1	5	796	2	full-splice_match	gene: Zm00001d013598	Zm00001d013598_T001	127.92	127.92	coding	210	51	680
PB.2231.3	10	2098	13	full-splice_match	gene: Zm00001d026239	Zm00001d026239_T007	3.51	18.93	coding	468	220	1623
PB.8387.2	7	1958	4	novel_in_catalog	gene: Zm00001d019957	novel	0.57	1.63	coding	514	51	1592
PB.8387.1	7	1912	5	full-splice_match	gene: Zm00001d019957	Zm00001d019957_T003	1.06	1.63	coding	559	49	1725
PB.9023.1	7	522	2	genic	gene: Zm00001d022535	novel	3.45	3.45	non_coding	NA	NA	NA
PB.8052.3	6	2002	12	full-splice_match	gene: Zm00001d038891	Zm00001d038891_T006	0.00	20.71	coding	501	220	1722
PB.8052.1	6	2330	12	full-splice_match	gene: Zm00001d038891	Zm00001d038891_T002	19.25	20.71	coding	501	402	1904
PB.7931.2	6	2355	8	full-splice_match	gene: Zm00001d038432	Zm00001d038432_T001	4.53	22.10	coding	601	311	2113
PB.3690.4	3	985	2	full-splice_match	gene: Zm00001d039411	Zm00001d039411_T009	75.52	112.23	coding	214	39	680
PB.3690.3	3	4202	4	full-splice_match	gene: Zm00001d039411	Zm00001d039411_T003	36.70	112.23	coding	1217	136	3786
PB.7580.1	6	1813	5	incomplete-splice_match	gene: Zm00001d037045	Zm00001d037045_T020	11.21	41.91	coding	211	940	1572
PB.3363.6	2	1649	11	novel_not_in_catalog	gene: Zm00001d006602	novel	1.98	100.06	coding	194	854	1435

PB.5118.1	4	1016	9	full-splice_match	gene: Zm00001d050300	Zm00001d050300_T001	150.91	150.91	coding	215	161	805
PB.429.4	1	455	1	incomplete-splice_match	gene: Zm00001d029102	Zm00001d029102_T001	0.00	335.87	non_coding	NA	NA	NA
PB.5025.1	4	2433	3	novel_not_in_catalog	gene: Zm00001d049727	novel	3.79	6.99	coding	720	56	2215
PB.429.1	1	1031	3	full-splice_match	gene: Zm00001d029102	Zm00001d029102_T001	331.94	335.87	coding	132	97	492
PB.9460.3	8	685	3	novel_not_in_catalog	gene: Zm00001d010244	novel	59.14	100.47	coding	104	157	468
PB.429.2	1	2573	2	novel_in_catalog	gene: Zm00001d029102	novel	3.93	335.87	coding	122	82	447
PB.10750.1	9	3023	14	full-splice_match	gene: Zm00001d048298	Zm00001d048298_T003	5.39	7.53	coding	783	534	2882
PB.9793.3	8	1385	1	novel_in_catalog	gene: Zm00001d011786	novel	5.27	257.23	coding	122	937	1302
PB.6905.2	5	1197	1	novel_in_catalog	gene: Zm00001d017485	novel	6.24	674.17	non_coding	NA	NA	NA
PB.10750.2	9	1879	9	novel_not_in_catalog	gene: Zm00001d048298	novel	2.14	7.53	coding	16	518	565
PB.4867.3	4	3434	3	full-splice_match	gene: Zm00001d048962	Zm00001d048962_T001	0.00	61.26	coding	977	349	3279
PB.4867.1	4	3513	3	full-splice_match	gene: Zm00001d048962	Zm00001d048962_T001	61.26	61.26	coding	977	395	3325
PB.1798.1	10	2131	12	full-splice_match	gene: Zm00001d023943	Zm00001d023943_T002	105.49	111.19	coding	516	210	1757
PB.1798.2	10	2393	11	full-splice_match	gene: Zm00001d023943	Zm00001d023943_T009	1.30	111.19	coding	434	207	1508
PB.1798.3	10	2010	12	full-splice_match	gene: Zm00001d023943	Zm00001d023943_T002	4.40	111.19	coding	516	234	1781
PB.7517.4	6	1715	3	intergenic	novelGene_165	novel	5.08	14.86	non_coding	NA	NA	NA
PB.1798.7	10	1906	12	full-splice_match	gene: Zm00001d023943	Zm00001d023943_T002	0.00	111.19	coding	516	234	1781

PB.1538.4	1	2327	10	novel_in_catalog	gene: Zm00001d034610	novel	0.00	19.55	coding	548	388	2031
PB.4333.2	3	2018	4	novel_in_catalog	gene: Zm00001d042853	novel	2.34	34.14	coding	239	809	1525
PB.7029.1	5	997	3	full-splice_match	gene: Zm00001d017998	Zm00001d017998_T001	94.44	94.44	coding	173	126	644
PB.6218.1	5	1066	6	full-splice_match	gene: Zm00001d014025	Zm00001d014025_T005	234.66	234.66	coding	163	266	754
PB.7029.2	5	923	3	full-splice_match	gene: Zm00001d017998	Zm00001d017998_T001	0.00	94.44	coding	173	123	641
PB.9853.1	8	855	2	genic	gene: Zm00001d012020	novel	11.64	11.64	coding	98	14	307
PB.3664.1	3	1002	1	genic	gene: Zm00001d039327	novel	0.59	0.59	coding	147	561	1001
PB.5237.26	4	2466	1	novel_in_catalog	gene: Zm00001d051161	novel	2.60	110.85	coding	374	1326	2447
PB.1699.4	10	1426	9	novel_in_catalog	gene: Zm00001d023423	novel	8.30	252.64	coding	265	127	921
PB.1699.5	10	1202	10	novel_not_in_catalog	gene: Zm00001d023423	novel	23.79	252.64	coding	135	389	793
PB.10305.1	9	381	1	incomplete-splice_match	gene: Zm00001d046218	Zm00001d046218_T001	73.41	73.41	non_coding	NA	NA	NA
PB.1699.7	10	1275	10	full-splice_match	gene: Zm00001d023423	Zm00001d023423_T008	156.59	252.64	coding	271	82	894
PB.1699.1	10	1412	10	full-splice_match	gene: Zm00001d023423	Zm00001d023423_T006	54.16	252.64	coding	271	196	1008
PB.1699.3	10	1446	9	novel_in_catalog	gene: Zm00001d023423	novel	9.79	252.64	coding	175	518	1042
PB.2987.1	2	2650	22	full-splice_match	gene: Zm00001d004667	Zm00001d004667_T011	52.46	64.73	coding	714	105	2246
PB.6332.1	5	1757	1	intergenic	novelGene_115	novel	5.48	5.48	coding	277	559	1389
PB.5753.1	4	1586	10	full-splice_match	gene: Zm00001d053591	Zm00001d053591_T002	21.03	27.03	coding	439	132	1448

PB.1199.1	1	2112	8	full-splice_match	gene: Zm00001d033130	Zm00001d033130_T008	67.62	67.62	coding	592	192	1967
PB.856.1	1	1665	3	full-splice_match	gene: Zm00001d031555	Zm00001d031555_T001	27.58	27.58	coding	395	125	1309
PB.1284.3	1	2626	2	novel_in_catalog	gene: Zm00001d033544	novel	0.16	1.92	coding	556	794	2461
PB.421.5	1	1706	6	full-splice_match	gene: Zm00001d029050	Zm00001d029050_T001	2.57	2.57	coding	481	135	1577
PB.7191.1	5	1649	1	full-splice_match	gene: Zm00001d018530	Zm00001d018530_T001	3.19	54.38	coding	58	269	442
PB.421.1	1	2168	7	fusion	gene: Zm00001d029050_g	novel	12.87	18.43	coding	481	498	1940
PB.4463.3	3	1587	4	full-splice_match	gene: Zm00001d043414	Zm00001d043414_T001	0.00	40.22	coding	397	237	1427
PB.421.3	1	2169	6	fusion	gene: Zm00001d029050_g	novel	2.81	18.43	coding	292	388	1263
PB.421.2	1	2349	5	fusion	gene: Zm00001d029050_g	novel	2.75	18.43	coding	292	386	1261
PB.9870.1	8	2055	14	full-splice_match	gene: Zm00001d012108	Zm00001d012108_T002	43.65	43.65	coding	517	236	1786
PB.5237.25	4	1813	2	novel_in_catalog	gene: Zm00001d051161	novel	0.00	110.85	coding	520	123	1682
PB.1954.1	10	346	1	antisense	novelGene_gene: Zm00001d024873_A	novel	0.00	0.00	non_coding	NA	NA	NA
PB.8629.2	7	1904	9	intergenic	novelGene_231	novel	6.30	54.18	coding	152	1272	1727
PB.8629.1	7	1897	10	intergenic	novelGene_231	novel	14.23	54.18	coding	287	848	1708
PB.7191.2	5	1296	1	full-splice_match	gene: Zm00001d018530	Zm00001d018530_T001	2.77	54.38	coding	207	31	651
PB.9890.3	8	325	2	novel_in_catalog	gene: Zm00001d012213	novel	9.19	20.94	non_coding	NA	NA	NA
PB.9890.2	8	1056	5	novel_not_in_catalog	gene: Zm00001d012213	novel	11.74	20.94	coding	123	354	722

PB.702.1	1	1200	1	intergenic	novelGene_248	novel	106.48	106.48	non_coding	NA	NA	NA
PB.5031.1	4	3596	10	full-splice_match	gene: Zm00001d049761	Zm00001d049761_T006	19.72	19.72	coding	986	301	3258
PB.9501.5	8	1746	1	novel_in_catalog	gene: Zm00001d010463	novel	4.64	79.92	coding	463	43	1431
PB.2786.1	2	2296	3	incomplete-splice_match	gene: Zm00001d003378	Zm00001d003378_T001	8.50	29.27	coding	525	135	1709
PB.2786.2	2	3588	4	full-splice_match	gene: Zm00001d003378	Zm00001d003378_T002	6.49	29.27	coding	1127	45	3425
PB.2786.3	2	1546	3	incomplete-splice_match	gene: Zm00001d003378	Zm00001d003378_T008	14.27	29.27	coding	271	613	1425
PB.2213.3	10	2550	1	full-splice_match	gene: Zm00001d026158	Zm00001d026158_T001	1.93	19.63	coding	718	194	2347
PB.2213.1	10	2663	1	full-splice_match	gene: Zm00001d026158	Zm00001d026158_T001	17.69	19.63	coding	718	180	2333
PB.7604.1	6	1358	1	intergenic	novelGene_187	novel	4.16	4.16	non_coding	NA	NA	NA
PB.348.1	1	695	1	genic	gene: Zm00001d028746	novel	0.54	0.54	coding	118	194	547
PB.8143.1	7	1572	1	full-splice_match	gene: Zm00001d018599	Zm00001d018599_T001	8.51	8.51	coding	335	331	1335
PB.5192.1	4	1692	1	full-splice_match	gene: Zm00001d050886	Zm00001d050886_T001	3.59	3.59	coding	279	41	877
PB.3243.1	2	1310	3	full-splice_match	gene: Zm00001d006097	Zm00001d006097_T001	13.00	13.00	coding	335	115	1119
PB.1996.1	10	2364	3	full-splice_match	gene: Zm00001d025023	Zm00001d025023_T002	18.82	123.94	coding	194	51	632
PB.1195.2	1	2428	8	incomplete-splice_match	gene: Zm00001d033104	Zm00001d033104_T001	7.56	22.73	coding	690	25	2094
PB.6576.1	5	1294	1	intergenic	novelGene_183	novel	12.49	12.49	coding	127	42	422
PB.9608.10	8	1485	2	full-splice_match	gene: Zm00001d010920	Zm00001d010920_T002	120.14	1709.65	coding	396	153	1340

PB.10623.1	9	3006	7	full-splice_match	gene: Zm00001d047828	Zm00001d047828_T001	15.75	46.02	coding	853	110	2668
PB.10772.1	9	3302	20	full-splice_match	gene: Zm00001d048358	Zm00001d048358_T004	51.67	51.67	coding	989	35	3001
PB.10772.3	9	3193	20	full-splice_match	gene: Zm00001d048358	Zm00001d048358_T004	0.00	51.67	coding	989	35	3001
PB.7728.2	6	1575	1	full-splice_match	gene: Zm00001d037721	Zm00001d037721_T001	0.64	0.64	coding	476	139	1566
PB.7728.1	6	1178	1	full-splice_match	gene: Zm00001d037721	Zm00001d037721_T001	0.00	0.64	coding	329	75	1061
PB.6455.2	5	2227	7	novel_in_catalog	gene: Zm00001d015009	novel	1.63	21.87	coding	344	1024	2055
PB.6455.1	5	1463	8	novel_not_in_catalog	gene: Zm00001d015009	novel	20.23	21.87	coding	374	124	1245
PB.6877.1	5	728	1	genic	gene: Zm00001d017357	novel	35.67	35.67	non_coding	NA	NA	NA
PB.10124.2	9	3064	18	novel_in_catalog	gene: Zm00001d045195	novel	2.63	43.82	coding	727	664	2844
PB.10124.1	9	3102	19	full-splice_match	gene: Zm00001d045195	Zm00001d045195_T002	41.19	43.82	coding	867	181	2781
PB.8605.12	7	4816	18	novel_in_catalog	gene: Zm00001d021000	novel	3.35	202.53	coding	488	3133	4596
PB.10391.1	9	1688	2	novel_in_catalog	gene: Zm00001d046723	novel	6.79	43.04	coding	163	231	719
PB.10391.2	9	1159	3	full-splice_match	gene: Zm00001d046723	Zm00001d046723_T001	36.25	43.04	coding	265	228	1022
PB.4020.1	3	2143	1	full-splice_match	gene: Zm00001d041387	Zm00001d041387_T001	8.00	8.00	coding	414	231	1472
PB.280.3	1	1808	10	intergenic	novelGene_93	novel	0.00	35.46	coding	511	86	1618
PB.4375.2	3	1859	3	full-splice_match	gene: Zm00001d043069	Zm00001d043069_T001	4.00	12.73	coding	446	345	1682
PB.4771.1	3	3075	2	intergenic	novelGene_354	novel	2.20	712.33	coding	130	2470	2859

PB.4771.2	3	636	2	intergenic	novelGene_354	novel	710.12	712.33	non_coding	NA	NA	NA
PB.8526.2	7	540	3	full-splice_match	gene: Zm00001d020658	Zm00001d020658_T001	61.45	64.08	non_coding	NA	NA	NA
PB.8526.3	7	1712	2	novel_in_catalog	gene: Zm00001d020658	novel	0.00	64.08	coding	88	42	305
PB.9510.1	8	992	2	incomplete-splice_match	gene: Zm00001d010512	Zm00001d010512_T003	24.37	24.37	coding	244	18	749
PB.8526.1	7	2525	2	novel_in_catalog	gene: Zm00001d020658	novel	2.62	64.08	non_coding	NA	NA	NA
PB.407.4	1	1537	3	full-splice_match	gene: Zm00001d028999	Zm00001d028999_T001	14.73	27.04	coding	371	248	1360
PB.3231.2	2	1789	6	full-splice_match	gene: Zm00001d006046	Zm00001d006046_T010	15.10	79.51	coding	301	375	1277
PB.7754.1	6	1942	10	novel_in_catalog	gene: Zm00001d037810	novel	18.91	32.81	coding	129	516	902
PB.3231.4	2	1502	5	full-splice_match	gene: Zm00001d006046	Zm00001d006046_T007	15.88	79.51	coding	301	143	1045
PB.3231.5	2	2216	5	full-splice_match	gene: Zm00001d006046	Zm00001d006046_T008	3.50	79.51	coding	301	899	1801
PB.4875.1	4	641	1	incomplete-splice_match	gene: Zm00001d049011	Zm00001d049011_T001	14.06	14.06	coding	108	89	412
PB.407.2	1	1830	1	novel_in_catalog	gene: Zm00001d028999	novel	4.71	27.04	coding	245	738	1472
PB.3231.8	2	2162	5	full-splice_match	gene: Zm00001d006046	Zm00001d006046_T008	0.00	79.51	coding	301	899	1801
PB.3231.9	2	1535	5	full-splice_match	gene: Zm00001d006046	Zm00001d006046_T006	4.29	79.51	coding	301	269	1171
PB.6278.1	5	1716	1	full-splice_match	gene: Zm00001d014328	Zm00001d014328_T001	7.49	7.49	coding	501	135	1637
PB.7700.8	6	2056	11	full-splice_match	gene: Zm00001d037606	Zm00001d037606_T006	0.00	155.33	coding	528	135	1718
PB.5601.2	4	1749	6	novel_not_in_catalog	gene: Zm00001d052785	novel	1.13	1.13	coding	153	746	1204

PB.1500.1	1	2341	1	full-splice_match	gene: Zm00001d034489	Zm00001d034489_T001	35.53	35.53	coding	614	78	1919
PB.8754.1	7	751	1	antisense	novelGene_gene: Zm00001d021634_A	novel	55.24	55.24	coding	149	32	478
PB.4337.3	3	1547	5	novel_in_catalog	gene: Zm00001d042874	novel	114.54	130.44	coding	361	201	1283
PB.7700.1	6	2469	11	full-splice_match	gene: Zm00001d037606	Zm00001d037606_T006	155.33	155.33	coding	528	135	1718
PB.7700.5	6	2333	11	full-splice_match	gene: Zm00001d037606	Zm00001d037606_T006	0.00	155.33	coding	528	133	1716
PB.10399.3	9	2061	8	full-splice_match	gene: Zm00001d046788	Zm00001d046788_T001	13.34	13.34	coding	590	143	1912
PB.5017.4	4	2341	7	full-splice_match	gene: Zm00001d049706	Zm00001d049706_T009	26.14	99.33	coding	706	54	2171
PB.4337.1	3	1750	3	novel_in_catalog	gene: Zm00001d042874	novel	15.90	130.44	coding	302	491	1396
PB.1683.1	10	1287	2	full-splice_match	gene: Zm00001d023366	Zm00001d023366_T001	75.73	109.36	non_coding	NA	NA	NA
PB.5017.1	4	2624	1	incomplete-splice_match	gene: Zm00001d049706	Zm00001d049706_T003	20.87	99.33	coding	430	1016	2305
PB.5017.2	4	2461	7	full-splice_match	gene: Zm00001d049706	Zm00001d049706_T009	43.08	99.33	coding	706	26	2143
PB.1683.2	10	1113	2	full-splice_match	gene: Zm00001d023366	Zm00001d023366_T001	33.63	109.36	non_coding	NA	NA	NA
PB.1020.10	1	2348	7	full-splice_match	gene: Zm00001d032350	Zm00001d032350_T007	4.53	135.32	coding	114	178	519
PB.7739.2	6	1619	4	full-splice_match	gene: Zm00001d037756	Zm00001d037756_T001	49.78	134.17	coding	387	218	1378
PB.1032.2	1	1121	2	full-splice_match	gene: Zm00001d032396	Zm00001d032396_T002	3.40	16.19	coding	334	17	1018
PB.1032.1	1	1443	2	full-splice_match	gene: Zm00001d032396	Zm00001d032396_T002	12.80	16.19	coding	334	44	1045
PB.1056.9	1	908	2	genic	gene: Zm00001d032485	novel	11.70	130.84	coding	143	203	631

PB.10385.2	9	2198	3	full-splice_match	gene: Zm00001d046688	Zm00001d046688_T002	3.63	81.15	coding	243	819	1547
PB.10385.1	9	2050	4	full-splice_match	gene: Zm00001d046688	Zm00001d046688_T003	48.42	81.15	coding	259	521	1297
PB.7114.1	5	2083	1	full-splice_match	gene: Zm00001d018326	Zm00001d018326_T001	57.25	57.25	coding	356	238	1305
PB.10385.5	9	1850	4	full-splice_match	gene: Zm00001d046688	Zm00001d046688_T003	0.00	81.15	coding	259	524	1300
PB.10116.1	9	943	5	full-splice_match	gene: Zm00001d045160	Zm00001d045160_T001	28.87	28.87	coding	154	44	505
PB.3609.2	2	2628	6	full-splice_match	gene: Zm00001d007865	Zm00001d007865_T001	0.00	101.69	coding	732	261	2456
PB.10385.9	9	2443	2	novel_in_catalog	gene: Zm00001d046688	novel	3.16	81.15	coding	194	710	1291
PB.10385.8	9	1368	4	full-splice_match	gene: Zm00001d046688	Zm00001d046688_T003	25.94	81.15	coding	259	144	920
PB.1056.4	1	1879	3	novel_not_in_catalog	gene: Zm00001d032485	novel	53.94	130.84	coding	143	204	632
PB.2999.2	2	1722	10	full-splice_match	gene: Zm00001d004731	Zm00001d004731_T002	90.19	147.54	coding	478	120	1553
PB.7028.1	5	1465	1	genic	gene: Zm00001d017992	novel	8.17	8.17	coding	237	589	1299
PB.4206.1	3	1193	3	full-splice_match	gene: Zm00001d042288	Zm00001d042288_T003	49.05	49.05	coding	246	185	922
PB.2999.1	2	2048	10	full-splice_match	gene: Zm00001d004731	Zm00001d004731_T002	57.35	147.54	coding	478	345	1778
PB.5091.2	4	4438	1	full-splice_match	gene: Zm00001d050132	Zm00001d050132_T001	0.00	5.92	coding	1208	639	4262
PB.10702.12	9	388	2	novel_in_catalog	gene: Zm00001d048101	novel	217.95	217.95	non_coding	NA	NA	NA
PB.3114.2	2	1227	2	full-splice_match	gene: Zm00001d005391	Zm00001d005391_T001	4.27	241.18	coding	374	54	1175
PB.5091.1	4	4162	2	genic	gene: Zm00001d050132	novel	5.92	5.92	coding	1208	351	3974

PB.8111.2	6	1076	1	full-splice_match	gene: Zm00001d039140	Zm00001d039140_T001	0.00	13.15	coding	280	117	956
PB.4793.1	4	2617	1	intergenic	novelGene_13	novel	0.22	0.22	coding	323	534	1502
PB.4869.2	4	1914	7	full-splice_match	gene: Zm00001d048970	Zm00001d048970_T014	6.11	19.86	coding	141	146	568
PB.4869.3	4	1206	9	novel_in_catalog	gene: Zm00001d048970	novel	13.75	19.86	coding	165	552	1046
PB.10702.10	9	1633	7	full-splice_match	gene: Zm00001d048100	Zm00001d048100_T001	93.03	435.97	coding	475	113	1537
PB.1107.2	1	2481	20	full-splice_match	gene: Zm00001d032667	Zm00001d032667_T012	13.10	28.39	coding	698	181	2274
PB.7192.2	5	1433	3	intergenic	novelGene_347	novel	1.65	6.85	non_coding	NA	NA	NA
PB.6318.2	5	2617	8	full-splice_match	gene: Zm00001d014524	Zm00001d014524_T009	1.23	16.33	coding	410	172	1401
PB.2656.2	2	859	5	full-splice_match	gene: Zm00001d002850	Zm00001d002850_T001	48.11	54.42	coding	129	95	481
PB.2656.3	2	755	5	full-splice_match	gene: Zm00001d002850	Zm00001d002850_T001	0.00	54.42	coding	129	95	481
PB.9700.1	8	2817	3	genic	gene: Zm00001d011378	novel	3.49	4.17	coding	683	407	2455
PB.2656.1	2	1092	4	novel_in_catalog	gene: Zm00001d002850	novel	6.31	54.42	coding	87	95	355
PB.508.3	1	1282	2	genic	gene: Zm00001d029475	novel	36.26	99.82	coding	308	140	1063
PB.508.2	1	1370	1	full-splice_match	gene: Zm00001d029475	Zm00001d029475_T001	14.58	99.82	coding	308	115	1038
PB.508.1	1	1499	2	genic	gene: Zm00001d029475	novel	48.99	99.82	coding	308	244	1167
PB.3134.1	2	737	5	full-splice_match	gene: Zm00001d005520	Zm00001d005520_T001	10.11	10.11	coding	173	95	613
PB.4238.2	3	2523	11	full-splice_match	gene: Zm00001d042435	Zm00001d042435_T003	12.28	12.28	coding	492	781	2256

PB.2285.6	10	1477	3	novel_in_catalog	gene: Zm00001d026391	novel	5.90	158.91	coding	197	81	671
PB.3093.1	2	1509	1	genic	gene: Zm00001d005279	novel	2.75	122.07	coding	299	10	906
PB.2285.4	10	2565	1	novel_in_catalog	gene: Zm00001d026391	novel	0.00	158.91	coding	197	74	664
PB.2285.5	10	878	2	full-splice_match	gene: Zm00001d026391	Zm00001d026391_T002	92.40	158.91	coding	197	81	671
PB.2285.2	10	1016	2	full-splice_match	gene: Zm00001d026391	Zm00001d026391_T002	38.40	158.91	coding	197	81	671
PB.2285.3	10	2029	2	full-splice_match	gene: Zm00001d026391	Zm00001d026391_T004	13.79	158.91	coding	197	74	664
PB.2285.1	10	2712	1	genic	gene: Zm00001d026391	novel	8.42	158.91	coding	197	74	664
PB.5085.1	4	1059	2	full-splice_match	gene: Zm00001d050106	Zm00001d050106_T001	57.88	57.88	coding	223	180	848
PB.7015.9	5	1592	9	novel_in_catalog	gene: Zm00001d017958	novel	5.17	1562.37	coding	323	391	1359
PB.7358.1	6	1211	7	full-splice_match	gene: Zm00001d035762	Zm00001d035762_T001	30.61	149.98	coding	228	156	839
PB.7015.1	5	1542	10	full-splice_match	gene: Zm00001d017958	Zm00001d017958_T001	1458.48	1562.37	coding	356	71	1138
PB.7015.2	5	1662	9	novel_in_catalog	gene: Zm00001d017958	novel	15.08	1562.37	coding	163	768	1256
PB.7015.7	5	1483	9	novel_in_catalog	gene: Zm00001d017958	novel	62.75	1562.37	coding	231	77	769
PB.1993.5	10	1448	3	genic	gene: Zm00001d025017	novel	24.73	87.26	coding	197	610	1200
PB.1993.4	10	1645	1	genic	gene: Zm00001d025017	novel	0.00	87.26	coding	166	886	1383
PB.1993.6	10	1591	1	genic	gene: Zm00001d025017	novel	7.34	87.26	coding	166	935	1432
PB.10039.1	9	2323	16	full-splice_match	gene: Zm00001d044754	Zm00001d044754_T002	18.00	139.09	coding	564	76	1767

PB.10039.3	9	2147	16	full-splice_match	gene: Zm00001d044754	Zm00001d044754_T002	1.63	139.09	coding	564	238	1929
PB.10039.2	9	2098	16	full-splice_match	gene: Zm00001d044754	Zm00001d044754_T002	119.46	139.09	coding	564	129	1820
PB.9891.1	8	2185	1	full-splice_match	gene: Zm00001d012216	Zm00001d012216_T001	8.56	8.56	coding	647	87	2027
PB.1993.9	10	1496	2	genic	gene: Zm00001d025017	novel	14.69	87.26	coding	197	761	1351
PB.4380.6	3	2668	10	full-splice_match	gene: Zm00001d043083	Zm00001d043083_T003	0.00	58.95	coding	432	1102	2397
PB.8634.1	7	1108	4	full-splice_match	gene: Zm00001d021189	Zm00001d021189_T001	21.46	21.46	coding	231	33	725
PB.500.3	1	2251	4	incomplete-splice_match	gene: Zm00001d029443	Zm00001d029443_T011	11.76	14.94	coding	714	1	2142
PB.4230.5	3	2344	12	incomplete-splice_match	gene: Zm00001d042394	Zm00001d042394_T013	13.68	20.41	coding	581	101	1843
PB.500.1	1	4914	10	incomplete-splice_match	gene: Zm00001d029443	Zm00001d029443_T004	3.18	14.94	coding	1447	332	4672
PB.3173.1	2	1687	3	full-splice_match	gene: Zm00001d005727	Zm00001d005727_T001	20.60	20.60	coding	435	109	1413
PB.8142.2	7	1987	7	full-splice_match	gene: Zm00001d018598	Zm00001d018598_T001	66.32	72.86	coding	464	455	1846
PB.4230.3	3	3571	12	full-splice_match	gene: Zm00001d042394	Zm00001d042394_T006	6.74	20.41	coding	741	263	2485
PB.2100.2	10	2276	7	full-splice_match	gene: Zm00001d025651	Zm00001d025651_T001	0.00	9.89	coding	693	56	2134
PB.3435.1	2	896	3	full-splice_match	gene: Zm00001d006924	Zm00001d006924_T001	214.45	214.45	coding	193	61	639
PB.9483.7	8	1871	2	full-splice_match	gene: Zm00001d010399	Zm00001d010399_T001	0.00	89.96	coding	498	128	1621
PB.9483.6	8	1625	1	novel_in_catalog	gene: Zm00001d010399	novel	0.00	89.96	coding	376	414	1541
PB.9483.4	8	1969	2	full-splice_match	gene: Zm00001d010399	Zm00001d010399_T001	0.00	89.96	coding	498	26	1519

PB.10025.2	8	2143	10	antisense	novelGene_gene: Zm00001d012761_A	novel	13.02	16.24	coding	139	1447	1863
PB.9483.2	8	2227	1	genic	gene: Zm00001d010399	novel	3.97	89.96	coding	607	121	1941
PB.9483.1	8	2167	2	full-splice_match	gene: Zm00001d010399	Zm00001d010399_T001	85.98	89.96	coding	498	121	1614
PB.10025.1	8	1847	8	antisense	novelGene_gene: Zm00001d012761_A	novel	3.22	16.24	non_coding	NA	NA	NA
PB.3562.2	2	2234	3	full-splice_match	gene: Zm00001d007636	Zm00001d007636_T001	20.80	36.85	coding	617	191	2041
PB.4210.2	3	863	1	full-splice_match	gene: Zm00001d042307	Zm00001d042307_T001	0.00	90.29	coding	192	112	687
PB.4210.1	3	982	1	full-splice_match	gene: Zm00001d042307	Zm00001d042307_T001	90.29	90.29	coding	192	120	695
PB.5442.2	4	2669	3	full-splice_match	gene: Zm00001d052188	Zm00001d052188_T001	24.11	27.63	coding	622	131	1996
PB.5442.3	4	2614	3	full-splice_match	gene: Zm00001d052188	Zm00001d052188_T001	1.23	27.63	coding	666	117	2114
PB.8446.1	7	2193	4	genic	gene: Zm00001d020303	novel	4.58	14.47	coding	370	560	1669
PB.7752.3	6	1812	15	full-splice_match	gene: Zm00001d037808	Zm00001d037808_T004	30.91	63.65	coding	499	38	1534
PB.6072.4	5	2428	3	novel_in_catalog	gene: Zm00001d013367	novel	2.01	3063.25	coding	416	993	2240
PB.3259.1	2	1249	1	full-splice_match	gene: Zm00001d006165	Zm00001d006165_T001	11.79	11.79	coding	330	30	1019
PB.6072.2	5	1816	4	full-splice_match	gene: Zm00001d013367	Zm00001d013367_T001	3037.90	3063.25	coding	451	125	1477
PB.5809.2	4	797	4	full-splice_match	gene: Zm00001d053800	Zm00001d053800_T002	137.57	233.84	coding	160	163	642
PB.6072.1	5	2008	4	full-splice_match	gene: Zm00001d013367	Zm00001d013367_T001	3.31	3063.25	coding	451	125	1477
PB.10553.1	9	2216	12	novel_not_in_catalog	gene: Zm00001d047592	novel	11.84	11.84	coding	179	670	1206

PB.1372.5	1	1948	9	full-splice_match	gene: Zm00001d033931	Zm00001d033931_T006	43.62	1100.09	coding	268	856	1659
PB.3449.1	2	814	1	full-splice_match	gene: Zm00001d007027	Zm00001d007027_T001	14.43	14.43	coding	191	70	642
PB.6072.8	5	1757	3	novel_in_catalog	gene: Zm00001d013367	novel	4.13	3063.25	coding	184	1014	1565
PB.6790.4	5	1697	5	full-splice_match	gene: Zm00001d016993	Zm00001d016993_T012	1.65	45.23	coding	258	571	1344
PB.10272.8	9	1389	6	full-splice_match	gene: Zm00001d045965	Zm00001d045965_T001	131.24	472.17	coding	376	101	1228
PB.10272.9	9	1678	5	novel_in_catalog	gene: Zm00001d045965	novel	10.62	472.17	coding	342	111	1136
PB.9762.2	8	2278	14	full-splice_match	gene: Zm00001d011663	Zm00001d011663_T005	5.08	5.08	coding	589	273	2039
PB.10272.1	9	1594	6	full-splice_match	gene: Zm00001d045965	Zm00001d045965_T001	323.40	472.17	coding	376	103	1230
PB.10272.2	9	1804	5	full-splice_match	gene: Zm00001d045965	Zm00001d045965_T003	5.12	472.17	coding	289	754	1620
PB.5730.4	4	1784	3	full-splice_match	gene: Zm00001d053438	Zm00001d053438_T002	3.57	22.12	coding	490	178	1647
PB.7634.1	6	344	1	incomplete-splice_match	gene: Zm00001d037268	Zm00001d037268_T007	31.53	31.53	non_coding	NA	NA	NA
PB.7081.1	5	1627	5	full-splice_match	gene: Zm00001d018201	Zm00001d018201_T001	8.00	8.00	coding	429	81	1367
PB.4597.1	3	501	1	genic	gene: Zm00001d043904	novel	18.07	113.53	non_coding	NA	NA	NA
PB.7628.2	6	1377	1	incomplete-splice_match	gene: Zm00001d037252	Zm00001d037252_T001	38.99	53.00	coding	366	18	1115
PB.6048.1	5	2892	23	incomplete-splice_match	gene: Zm00001d013276	Zm00001d013276_T013	30.67	30.67	coding	855	57	2621
PB.7073.3	5	2249	3	novel_in_catalog	gene: Zm00001d018161	novel	0.00	110.52	coding	368	64	1167
PB.1243.1	1	894	4	full-splice_match	gene: Zm00001d033371	Zm00001d033371_T008	90.67	125.13	coding	148	274	717

PB.3945.1	3	2624	2	novel_in_catalog	gene: Zm00001d040743	novel	7.03	7.03	coding	806	108	2525
PB.7942.9	6	3665	7	antisense	novelGene_gene: Zm00001d038477_A	novel	7.48	7.48	coding	11	380	412
PB.7942.8	6	1792	5	intergenic	novelGene_260	novel	15.43	54.59	non_coding	NA	NA	NA
PB.2955.4	2	2072	6	incomplete-splice_match	gene: Zm00001d004466	Zm00001d004466_T022	6.15	47.29	coding	301	842	1744
PB.10920.1	B73V4 ctg51	1046	1	full-splice_match	gene: Zm00001d000310	Zm00001d000310_T001	19.66	19.66	coding	244	113	844
PB.7942.1	6	845	2	intergenic	novelGene_260	novel	15.51	54.59	non_coding	NA	NA	NA
PB.9274.2	8	2741	6	full-splice_match	gene: Zm00001d009222	Zm00001d009222_T001	2.86	26.74	coding	799	155	2551
PB.9274.1	8	2666	7	full-splice_match	gene: Zm00001d009222	Zm00001d009222_T002	23.89	26.74	coding	776	140	2467
PB.7628.1	6	3275	1	incomplete-splice_match	gene: Zm00001d037252	Zm00001d037252_T003	14.01	53.00	coding	845	435	2969
PB.10622.1	9	1139	1	full-splice_match	gene: Zm00001d047824	Zm00001d047824_T001	66.64	66.64	coding	278	156	989
PB.6674.2	5	601	2	incomplete-splice_match	gene: Zm00001d016407	Zm00001d016407_T003	11.67	24.95	coding	14	559	600
PB.5292.2	4	429	2	incomplete-splice_match	gene: Zm00001d051499	Zm00001d051499_T001	73.43	124.37	non_coding	NA	NA	NA
PB.5292.1	4	1059	2	novel_in_catalog	gene: Zm00001d051499	novel	50.94	124.37	coding	80	228	467
PB.6674.1	5	3753	5	full-splice_match	gene: Zm00001d016407	Zm00001d016407_T001	10.10	24.95	coding	1082	256	3501
PB.9737.8	8	2151	7	full-splice_match	gene: Zm00001d011565	Zm00001d011565_T004	0.00	970.93	coding	112	1719	2054
PB.2666.1	2	2224	6	novel_not_in_catalog	gene: Zm00001d002891	novel	10.88	10.88	coding	161	1170	1652
PB.9443.2	8	2717	11	novel_in_catalog	gene: Zm00001d010199	novel	4.28	30.45	coding	364	263	1354

PB.315.2	1	2980	3	full-splice_match	gene: Zm00001d028603	Zm00001d028603_T001	11.88	31.14	coding	734	292	2493
PB.315.1	1	2989	3	full-splice_match	gene: Zm00001d028603	Zm00001d028603_T001	19.26	31.14	coding	734	168	2369
PB.161.1	1	1123	1	full-splice_match	gene: Zm00001d027929	Zm00001d027929_T001	483.36	483.36	coding	306	76	993
PB.5747.4	4	1678	2	novel_in_catalog	gene: Zm00001d053566	novel	4.03	115.65	coding	236	197	904
PB.5747.5	4	1785	1	novel_in_catalog	gene: Zm00001d053566	novel	5.31	115.65	coding	161	574	1056
PB.522.1	1	1570	9	full-splice_match	gene: Zm00001d029534	Zm00001d029534_T002	51.08	51.08	coding	371	158	1270
PB.4932.1	4	892	3	incomplete-splice_match	gene: Zm00001d049251	Zm00001d049251_T038	7.03	18.02	non_coding	NA	NA	NA
PB.8470.2	7	1757	7	full-splice_match	gene: Zm00001d020409	Zm00001d020409_T001	38.76	60.04	coding	404	164	1375
PB.5747.2	4	1749	3	novel_in_catalog	gene: Zm00001d053566	novel	0.00	115.65	coding	382	196	1341
PB.6195.5	5	1581	8	incomplete-splice_match	gene: Zm00001d013920	Zm00001d013920_T003	45.61	45.61	coding	415	138	1382
PB.5852.1	4	1671	6	fusion	gene: Zm00001d053939_g	novel	23.27	50.22	coding	421	55	1317
PB.5852.2	4	1571	6	fusion	gene: Zm00001d053939_g	novel	26.95	50.22	coding	421	63	1325
PB.6383.3	5	1869	2	full-splice_match	gene: Zm00001d014764	Zm00001d014764_T006	0.00	6.73	coding	588	49	1812
PB.7089.1	5	2985	9	full-splice_match	gene: Zm00001d018217	Zm00001d018217_T003	8.72	8.72	coding	692	424	2499
PB.9754.2	8	1118	10	incomplete-splice_match	gene: Zm00001d011632	Zm00001d011632_T002	21.29	21.29	coding	289	12	878
PB.1171.2	1	1017	1	full-splice_match	gene: Zm00001d032972	Zm00001d032972_T001	26.04	55.98	coding	253	189	947
PB.4636.2	3	800	1	full-splice_match	gene: Zm00001d044076	Zm00001d044076_T001	121.13	207.04	coding	136	168	575

PB.3063.2	2	2071	11	novel_not_in_catalog	gene: Zm00001d005143	novel	6.61	19.23	coding	431	429	1721
PB.3063.1	2	1945	11	novel_not_in_catalog	gene: Zm00001d005143	novel	12.62	19.23	coding	431	188	1480
PB.141.1	1	1689	7	full-splice_match	gene: Zm00001d027848	Zm00001d027848_T004	39.59	39.59	coding	426	51	1328
PB.5745.1	4	1429	10	full-splice_match	gene: Zm00001d053563	Zm00001d053563_T002	8.61	39.87	coding	350	119	1168
PB.5311.1	4	1378	3	full-splice_match	gene: Zm00001d051577	Zm00001d051577_T001	3.42	3.42	coding	297	264	1154
PB.9445.2	8	1949	2	full-splice_match	gene: Zm00001d010214	Zm00001d010214_T003	5.03	15.42	coding	385	538	1692
PB.7095.1	5	1445	9	full-splice_match	gene: Zm00001d018243	Zm00001d018243_T001	32.01	32.01	coding	362	52	1137
PB.9629.1	8	1481	1	full-splice_match	gene: Zm00001d011002	Zm00001d011002_T001	113.01	113.01	coding	112	119	454
PB.147.1	1	1830	4	full-splice_match	gene: Zm00001d027871	Zm00001d027871_T001	78.76	81.25	coding	484	28	1479
PB.7606.2	6	1507	7	full-splice_match	gene: Zm00001d037174	Zm00001d037174_T003	0.00	53.93	coding	399	151	1347
PB.7606.1	6	1761	7	full-splice_match	gene: Zm00001d037174	Zm00001d037174_T003	53.93	53.93	coding	399	165	1361
PB.147.2	1	1722	4	full-splice_match	gene: Zm00001d027871	Zm00001d027871_T001	2.50	81.25	coding	484	62	1513
PB.9094.1	8	827	3	full-splice_match	gene: Zm00001d008330	Zm00001d008330_T001	96.07	96.07	coding	122	131	496
PB.1084.2	1	1100	5	full-splice_match	gene: Zm00001d032570	Zm00001d032570_T001	65.19	138.86	coding	317	37	987
PB.1372.9	1	1895	9	full-splice_match	gene: Zm00001d033931	Zm00001d033931_T006	0.00	1100.09	coding	268	856	1659
PB.9341.1	8	1017	7	full-splice_match	gene: Zm00001d009645	Zm00001d009645_T001	39.52	39.52	coding	211	33	665
PB.1556.1	1	1179	7	full-splice_match	gene: Zm00001d034665	Zm00001d034665_T001	41.90	41.90	coding	257	116	886

PB.3377.6	2	1257	5	novel_not_in_catalog	gene: Zm00001d006679	novel	32.14	147.77	coding	344	118	1149
PB.1243.2	1	993	5	full-splice_match	gene: Zm00001d033371	Zm00001d033371_T003	25.58	125.13	coding	211	231	863
PB.1614.1	1	932	2	novel_in_catalog	gene: Zm00001d034853	novel	14.40	30.52	coding	214	46	687
PB.1767.1	10	1210	5	full-splice_match	gene: Zm00001d023808	Zm00001d023808_T002	43.14	43.14	coding	225	209	883
PB.8477.3	7	762	4	full-splice_match	gene: Zm00001d020438	Zm00001d020438_T001	155.95	173.17	coding	151	101	553
PB.802.1	1	3815	15	novel_not_in_catalog	gene: Zm00001d031231	novel	20.67	36.66	coding	936	54	2861
PB.802.2	1	1854	11	novel_not_in_catalog	gene: Zm00001d031231	novel	10.70	36.66	coding	182	366	911
PB.802.3	1	1258	7	incomplete-splice_match	gene: Zm00001d031231	Zm00001d031231_T008	2.87	36.66	coding	182	145	690
PB.802.4	1	3214	12	full-splice_match	gene: Zm00001d031231	Zm00001d031231_T056	2.42	36.66	coding	936	104	2911
PB.5941.1	5	678	1	genic	gene: Zm00001d012916	novel	46.65	85.12	coding	71	132	344
PB.5941.2	5	3492	11	full-splice_match	gene: Zm00001d012916	Zm00001d012916_T001	38.47	85.12	coding	961	434	3316
PB.149.9	1	1063	7	full-splice_match	gene: Zm00001d027874	Zm00001d027874_T009	0.00	155.38	coding	124	560	931
PB.149.6	1	1262	7	full-splice_match	gene: Zm00001d027874	Zm00001d027874_T009	38.98	155.38	coding	124	581	952
PB.149.7	1	1674	6	novel_in_catalog	gene: Zm00001d027874	novel	8.10	155.38	coding	131	971	1363
PB.149.4	1	1216	6	full-splice_match	gene: Zm00001d027874	Zm00001d027874_T010	54.77	155.38	coding	124	533	904
PB.149.5	1	2363	5	full-splice_match	gene: Zm00001d027874	Zm00001d027874_T008	2.40	155.38	coding	237	1342	2052
PB.6680.3	5	3229	2	novel_in_catalog	gene: Zm00001d016446	novel	1.20	1001.69	coding	206	41	658

PB.149.3	1	2799	4	full-splice_match	gene: Zm00001d027874	Zm00001d027874_T007	9.36	155.38	coding	131	2095	2487
PB.6680.1	5	1468	4	full-splice_match	gene: Zm00001d016446	Zm00001d016446_T001	995.08	1001.69	coding	371	100	1212
PB.149.1	1	2144	6	full-splice_match	gene: Zm00001d027874	Zm00001d027874_T006	1.59	155.38	coding	244	1085	1816
PB.10754.4	9	1946	2	full-splice_match	gene: Zm00001d048310	Zm00001d048310_T004	7.77	111.41	coding	428	307	1590
PB.6891.1	5	592	3	full-splice_match	gene: Zm00001d017425	Zm00001d017425_T001	155.76	155.76	coding	139	58	474
PB.2759.4	2	1760	1	full-splice_match	gene: Zm00001d003245	Zm00001d003245_T001	0.00	60.24	coding	423	255	1523
PB.1827.1	10	2400	5	incomplete-splice_match	gene: Zm00001d024178	Zm00001d024178_T002	42.08	74.62	coding	654	9	1970
PB.4307.1	3	1540	2	intergenic	novelGene_298	novel	11.70	11.70	coding	169	709	1215
PB.8280.2	7	2053	11	full-splice_match	gene: Zm00001d019217	Zm00001d019217_T004	11.20	11.20	coding	549	179	1825
PB.2759.1	2	2583	1	full-splice_match	gene: Zm00001d003245	Zm00001d003245_T001	1.97	60.24	coding	626	18	1895
PB.2759.2	2	1784	1	full-splice_match	gene: Zm00001d003245	Zm00001d003245_T001	7.24	60.24	coding	315	293	1237
PB.2759.3	2	2307	1	full-splice_match	gene: Zm00001d003245	Zm00001d003245_T001	51.03	60.24	coding	626	18	1895
PB.155.1	1	899	1	full-splice_match	gene: Zm00001d027894	Zm00001d027894_T001	91.49	221.23	coding	129	233	619
PB.3073.1	2	2380	3	incomplete-splice_match	gene: Zm00001d005173	Zm00001d005173_T001	47.67	47.67	non_coding	NA	NA	NA
PB.155.2	1	778	1	full-splice_match	gene: Zm00001d027894	Zm00001d027894_T001	129.74	221.23	coding	129	235	621
PB.10118.1	9	502	2	full-splice_match	gene: Zm00001d045174	Zm00001d045174_T001	772.93	772.93	non_coding	NA	NA	NA
PB.6512.2	5	3550	2	intergenic	novelGene_147	novel	1.48	12.25	non_coding	NA	NA	NA

PB.7480.2	6	1545	3	genic	gene: Zm00001d036529	novel	0.00	43.39	coding	315	238	1182
PB.4286.1	3	1379	3	novel_in_catalog	gene: Zm00001d042653	novel	102.97	114.75	coding	334	166	1167
PB.6810.26	5	1591	11	novel_in_catalog	gene: Zm00001d017096	novel	1.07	118.92	coding	243	106	834
PB.5339.3	4	4196	13	full-splice_match	gene: Zm00001d051678	Zm00001d051678_T024	6.95	30.19	coding	1331	43	4035
PB.7025.3	5	2022	4	full-splice_match	gene: Zm00001d017987	Zm00001d017987_T003	0.60	35.95	coding	612	26	1861
PB.5339.1	4	2670	8	full-splice_match	gene: Zm00001d051678	Zm00001d051678_T013	23.24	30.19	coding	744	112	2343
PB.6661.4	5	2461	1	novel_in_catalog	gene: Zm00001d016332	novel	0.00	386.30	coding	190	1121	1690
PB.3834.2	3	660	5	full-splice_match	gene: Zm00001d040164	Zm00001d040164_T002	118.32	226.88	coding	119	78	434
PB.3834.1	3	745	5	full-splice_match	gene: Zm00001d040164	Zm00001d040164_T002	108.56	226.88	coding	119	74	430
PB.7149.1	5	1080	8	novel_in_catalog	gene: Zm00001d018416	novel	3.57	3.57	coding	280	82	921
PB.7480.1	6	1657	3	genic	gene: Zm00001d036529	novel	43.39	43.39	coding	315	310	1254
PB.1103.2	1	1169	7	full-splice_match	gene: Zm00001d032652	Zm00001d032652_T001	17.88	92.27	coding	222	94	759
PB.5949.3	5	2379	11	novel_not_in_catalog	gene: Zm00001d012938	novel	32.43	32.43	coding	295	1065	1949
PB.3050.1	2	1773	5	full-splice_match	gene: Zm00001d005071	Zm00001d005071_T001	2.01	2.01	coding	473	44	1462
PB.7308.5	6	1321	7	novel_not_in_catalog	gene: Zm00001d035481	novel	31.67	57.76	coding	147	266	706
PB.10474.6	9	3125	14	novel_in_catalog	gene: Zm00001d047253	novel	7.80	821.31	coding	236	1996	2703
PB.10474.7	9	3122	14	novel_in_catalog	gene: Zm00001d047253	novel	7.21	821.31	coding	676	162	2189

PB.8296.1	7	1896	6	full-splice_match	gene: Zm00001d019288	Zm00001d019288_T003	12.31	12.31	coding	431	262	1554
PB.10474.2	9	3410	14	full-splice_match	gene: Zm00001d047253	Zm00001d047253_T003	6.17	821.31	coding	816	576	3023
PB.10474.3	9	2539	10	novel_in_catalog	gene: Zm00001d047253	novel	319.57	821.31	coding	499	614	2110
PB.10390.5	9	2529	11	full-splice_match	gene: Zm00001d046719	Zm00001d046719_T013	0.30	17.88	coding	357	165	1235
PB.2864.1	2	1820	1	novel_in_catalog	gene: Zm00001d003786	novel	9.82	9.82	coding	317	30	980
PB.8027.2	6	2419	9	novel_in_catalog	gene: Zm00001d038819	novel	19.52	28.86	coding	253	665	1423
PB.6299.2	5	2041	1	novel_in_catalog	gene: Zm00001d014419	novel	3.42	25.17	coding	165	243	737
PB.2987.2	2	1467	8	novel_not_in_catalog	gene: Zm00001d004667	novel	12.27	64.73	coding	371	31	1143
PB.10390.6	9	2473	13	novel_not_in_catalog	gene: Zm00001d046719	novel	0.60	17.88	coding	401	166	1368
PB.7155.1	5	3095	2	full-splice_match	gene: Zm00001d018428	Zm00001d018428_T001	56.36	56.36	coding	739	413	2629
PB.8383.1	7	3920	8	full-splice_match	gene: Zm00001d019920	Zm00001d019920_T001	44.29	44.29	coding	1052	99	3254
PB.4373.4	3	1747	2	novel_in_catalog	gene: Zm00001d043047	novel	16.56	92.05	coding	153	73	531
PB.4373.5	3	1084	3	full-splice_match	gene: Zm00001d043047	Zm00001d043047_T001	75.49	92.05	coding	253	73	831
PB.1475.6	1	3094	9	incomplete-splice_match	gene: Zm00001d034388	Zm00001d034388_T002	24.22	212.88	coding	695	925	3009
PB.1475.1	1	4492	10	full-splice_match	gene: Zm00001d034388	Zm00001d034388_T003	52.90	212.88	coding	1357	156	4226
PB.7593.1	6	2418	3	genic	gene: Zm00001d037105	novel	62.41	62.41	coding	669	153	2159
PB.1475.3	1	4688	9	full-splice_match	gene: Zm00001d034388	Zm00001d034388_T012	4.90	212.88	coding	1155	157	3621

PB.6726.3	5	2172	13	full-splice_match	gene: Zm00001d016702	Zm00001d016702_T006	12.70	20.56	coding	521	147	1709
PB.4885.1	4	599	1	full-splice_match	gene: Zm00001d049061	Zm00001d049061_T001	12.21	12.21	coding	92	201	476
PB.9526.1	8	1968	4	full-splice_match	gene: Zm00001d010590	Zm00001d010590_T001	42.79	42.79	coding	527	88	1668
PB.4750.2	3	2792	18	novel_in_catalog	gene: Zm00001d044540	novel	1.41	18.96	coding	754	313	2574
PB.9526.3	8	1835	4	full-splice_match	gene: Zm00001d010590	Zm00001d010590_T001	0.00	42.79	coding	527	73	1653
PB.3083.2	2	2498	6	incomplete-splice_match	gene: Zm00001d005241	Zm00001d005241_T005	26.57	33.55	coding	775	37	2361
PB.1532.1	1	3228	4	full-splice_match	gene: Zm00001d034591	Zm00001d034591_T001	16.37	16.90	coding	807	592	3012
PB.9979.1	8	621	3	full-splice_match	gene: Zm00001d012598	Zm00001d012598_T001	493.97	493.97	coding	108	63	386
PB.6872.1	5	750	5	incomplete-splice_match	gene: Zm00001d017334	Zm00001d017334_T006	379.13	379.13	coding	157	40	510
PB.6723.2	5	1918	1	full-splice_match	gene: Zm00001d016687	Zm00001d016687_T001	5.90	75.42	coding	478	264	1697
PB.6723.1	5	1984	1	full-splice_match	gene: Zm00001d016687	Zm00001d016687_T001	69.52	75.42	coding	478	253	1686
PB.6479.1	5	1212	2	intergenic	novelGene_140	novel	5.06	14.51	non_coding	NA	NA	NA
PB.7243.1	6	2927	1	genic	gene: Zm00001d035125	novel	17.36	17.36	coding	686	680	2737
PB.512.4	1	2223	15	novel_in_catalog	gene: Zm00001d029490	novel	4.20	27.05	coding	599	254	2050
PB.3133.4	2	2141	12	full-splice_match	gene: Zm00001d005516	Zm00001d005516_T004	5.46	14.44	coding	468	294	1697
PB.3518.1	2	629	4	full-splice_match	gene: Zm00001d007394	Zm00001d007394_T001	6.49	6.49	coding	139	16	432
PB.3977.1	3	2040	5	full-splice_match	gene: Zm00001d041072	Zm00001d041072_T001	149.63	149.63	coding	595	89	1873

PB.4728.2	3	1753	2	full-splice_match	gene: Zm00001d044469	Zm00001d044469_T008	25.85	31.20	coding	447	95	1435
PB.2684.5	2	1706	1	novel_in_catalog	gene: Zm00001d002964	novel	0.54	0.54	coding	112	368	703
PB.2684.4	2	2063	16	fusion	gene: Zm00001d002964_g	novel	26.51	51.92	coding	601	132	1934
PB.2684.3	2	3117	15	fusion	gene: Zm00001d002964_g	novel	1.80	51.92	coding	360	1827	2906
PB.2684.2	2	2304	16	fusion	gene: Zm00001d002964_g	novel	0.00	51.92	coding	601	266	2068
PB.2684.1	2	2402	16	fusion	gene: Zm00001d002964_g	novel	23.60	51.92	coding	601	305	2107
PB.8448.1	7	1229	3	intergenic	novelGene_177	novel	19.13	19.13	coding	286	141	998
PB.36.4	1	1473	6	full-splice_match	gene: Zm00001d027353	Zm00001d027353_T001	7.83	89.25	coding	361	230	1312
PB.36.2	1	1561	6	novel_not_in_catalog	gene: Zm00001d027353	novel	5.19	89.25	coding	349	238	1284
PB.36.3	1	1442	6	full-splice_match	gene: Zm00001d027353	Zm00001d027353_T001	68.76	89.25	coding	361	85	1167
PB.36.1	1	1655	6	full-splice_match	gene: Zm00001d027353	Zm00001d027353_T001	7.47	89.25	coding	361	82	1164
PB.10537.1	9	3038	12	full-splice_match	gene: Zm00001d047522	Zm00001d047522_T003	86.95	134.86	coding	629	750	2636
PB.68.3	1	2584	8	novel_in_catalog	gene: Zm00001d027484	novel	1.33	89.29	coding	327	111	1091
PB.68.2	1	2426	9	full-splice_match	gene: Zm00001d027484	Zm00001d027484_T005	1.56	89.29	coding	414	93	1334
PB.68.1	1	2177	10	full-splice_match	gene: Zm00001d027484	Zm00001d027484_T003	86.40	89.29	coding	616	88	1935
PB.1961.1	10	1059	2	full-splice_match	gene: Zm00001d024890	Zm00001d024890_T001	36.39	36.39	coding	285	89	943
PB.1530.1	1	1232	1	full-splice_match	gene: Zm00001d034588	Zm00001d034588_T001	17.96	17.96	coding	291	101	973

PB.2859.1	2	801	4	novel_not_in_catalog	gene: Zm00001d003770	novel	16.37	16.37	coding	135	57	461
PB.9985.1	8	974	4	full-splice_match	gene: Zm00001d012607	Zm00001d012607_T001	233.58	406.73	coding	203	125	733
PB.9635.6	8	1708	6	full-splice_match	gene: Zm00001d011051	Zm00001d011051_T003	0.00	27.71	coding	441	190	1512
PB.6056.1	5	1203	1	full-splice_match	gene: Zm00001d013306	Zm00001d013306_T001	22.08	75.73	coding	273	103	921
PB.6056.2	5	1060	1	full-splice_match	gene: Zm00001d013306	Zm00001d013306_T001	53.65	75.73	coding	273	74	892
PB.9635.5	8	1877	5	full-splice_match	gene: Zm00001d011051	Zm00001d011051_T005	0.00	27.71	coding	468	90	1493
PB.9635.2	8	1994	5	full-splice_match	gene: Zm00001d011051	Zm00001d011051_T005	4.55	27.71	coding	468	90	1493
PB.3864.1	3	963	1	full-splice_match	gene: Zm00001d040292	Zm00001d040292_T001	536.83	3288.33	coding	95	133	417
PB.3864.2	3	865	1	full-splice_match	gene: Zm00001d040292	Zm00001d040292_T001	2238.69	3288.33	coding	95	133	417
PB.3864.3	3	759	1	full-splice_match	gene: Zm00001d040292	Zm00001d040292_T001	512.81	3288.33	coding	95	129	413
PB.9930.1	8	1631	6	full-splice_match	gene: Zm00001d012394	Zm00001d012394_T001	28.00	34.14	coding	402	165	1370
PB.7921.2	6	3566	11	full-splice_match	gene: Zm00001d038389	Zm00001d038389_T002	0.00	16.53	coding	969	299	3205
PB.882.1	1	1958	6	full-splice_match	gene: Zm00001d031669	Zm00001d031669_T001	15.05	15.05	coding	448	94	1437
PB.7893.4	6	811	2	genic	gene: Zm00001d038291	novel	90.09	173.62	coding	154	155	616
PB.7893.2	6	924	2	genic	gene: Zm00001d038291	novel	64.31	173.62	coding	154	150	611
PB.7893.3	6	815	1	full-splice_match	gene: Zm00001d038291	Zm00001d038291_T001	0.00	173.62	coding	154	63	524
PB.1537.3	1	1863	7	novel_in_catalog	gene: Zm00001d034608	novel	0.00	50.61	coding	412	472	1707

PB.7893.1	6	1450	1	full-splice_match	gene: Zm00001d038291	Zm00001d038291_T001	19.22	173.62	coding	154	532	993
PB.10178.1	9	761	1	intergenic	novelGene_65	novel	21.17	21.17	non_coding	NA	NA	NA
PB.8773.14	7	1495	4	full-splice_match	gene: Zm00001d021698	Zm00001d021698_T001	0.00	192.02	coding	463	106	1494
PB.439.5	1	2003	9	novel_in_catalog	gene: Zm00001d029141	novel	7.70	63.05	coding	104	1099	1410
PB.7515.1	6	1203	2	genic	gene: Zm00001d036648	novel	57.68	57.68	coding	255	194	958
PB.9985.6	8	797	4	full-splice_match	gene: Zm00001d012607	Zm00001d012607_T001	65.21	406.73	coding	203	123	731
PB.10815.1	9	710	1	novel_in_catalog	gene: Zm00001d048509	novel	66.90	66.90	coding	26	257	334
PB.2743.2	2	1909	4	full-splice_match	gene: Zm00001d003196	Zm00001d003196_T001	4.68	4.68	coding	485	213	1667
PB.10851.3	B73V4 ctg11	1046	7	full-splice_match	gene: Zm00001d000241	Zm00001d000241_T002	12.18	44.63	coding	206	205	822
PB.9815.3	8	1171	8	full-splice_match	gene: Zm00001d011850	Zm00001d011850_T002	0.00	212.54	coding	300	195	1094
PB.202.1	1	1978	2	full-splice_match	gene: Zm00001d028151	Zm00001d028151_T001	40.79	40.79	coding	524	193	1764
PB.10358.1	9	1890	12	novel_in_catalog	gene: Zm00001d046554	novel	38.72	49.07	coding	445	209	1543
PB.10358.3	9	1748	12	novel_in_catalog	gene: Zm00001d046554	novel	0.00	49.07	coding	445	177	1511
PB.439.3	1	2416	7	novel_in_catalog	gene: Zm00001d029141	novel	1.79	63.05	coding	104	1464	1775
PB.8446.5	7	2114	2	genic	gene: Zm00001d020303	novel	2.12	14.47	coding	370	633	1742
PB.8446.4	7	2065	3	genic	gene: Zm00001d020303	novel	7.78	14.47	coding	370	549	1658
PB.8446.7	7	1769	3	genic	gene: Zm00001d020303	novel	0.00	14.47	coding	370	513	1622

PB.8446.6	7	1840	3	genic	gene: Zm00001d020303	novel	0.00	14.47	coding	370	468	1577
PB.2394.1	2	2602	13	full-splice_match	gene: Zm00001d001786	Zm00001d001786_T004	23.53	23.53	coding	674	417	2438
PB.9843.1	8	4670	6	full-splice_match	gene: Zm00001d011983	Zm00001d011983_T050	30.99	30.99	coding	1458	54	4427
PB.1642.2	1	2172	8	incomplete-splice_match	gene: Zm00001d034962	Zm00001d034962_T005	5.92	8.09	coding	631	19	1911
PB.824.1	1	1791	4	full-splice_match	gene: Zm00001d031328	Zm00001d031328_T001	61.32	61.32	coding	504	141	1652
PB.2430.1	2	1050	1	full-splice_match	gene: Zm00001d001905	Zm00001d001905_T001	32.90	32.90	coding	222	15	680
PB.7910.2	6	1029	6	full-splice_match	gene: Zm00001d038352	Zm00001d038352_T003	53.58	57.62	coding	137	168	578
PB.7910.3	6	698	1	incomplete-splice_match	gene: Zm00001d038352	Zm00001d038352_T004	3.89	57.62	non_coding	NA	NA	NA
PB.10707.1	9	970	1	full-splice_match	gene: Zm00001d048111	Zm00001d048111_T001	15.77	15.77	coding	212	37	672
PB.5842.4	4	2377	8	full-splice_match	gene: Zm00001d053917	Zm00001d053917_T035	5.04	21.60	coding	722	106	2271
PB.2507.1	2	1320	1	incomplete-splice_match	gene: Zm00001d002252	Zm00001d002252_T001	25.13	25.13	coding	333	32	1030
PB.8773.11	7	2271	7	novel_not_in_catalog	gene: Zm00001d021698	novel	0.00	192.02	coding	677	131	2161
PB.52.4	1	1454	7	novel_not_in_catalog	gene: Zm00001d027425	novel	5.87	59.74	coding	194	379	960
PB.52.1	1	1292	7	novel_in_catalog	gene: Zm00001d027425	novel	33.55	59.74	coding	228	340	1023
PB.52.2	1	1488	5	full-splice_match	gene: Zm00001d027425	Zm00001d027425_T002	11.43	59.74	coding	189	217	783
PB.52.3	1	1643	6	novel_in_catalog	gene: Zm00001d027425	novel	8.89	59.74	coding	194	486	1067
PB.2523.1	2	2390	4	full-splice_match	gene: Zm00001d002302	Zm00001d002302_T004	18.08	40.83	coding	89	241	507

PB.5212.2	4	1060	6	full-splice_match	gene: Zm00001d050978	Zm00001d050978_T001	101.17	147.59	coding	229	77	763
PB.5212.1	4	497	2	incomplete-splice_match	gene: Zm00001d050978	Zm00001d050978_T001	46.43	147.59	non_coding	NA	NA	NA
PB.8773.13	7	3125	4	novel_not_in_catalog	gene: Zm00001d021698	novel	1.54	192.02	coding	330	130	1119
PB.2523.5	2	2694	3	full-splice_match	gene: Zm00001d002302	Zm00001d002302_T012	3.64	40.83	coding	89	253	519
PB.2523.4	2	2400	5	full-splice_match	gene: Zm00001d002302	Zm00001d002302_T008	9.30	40.83	coding	89	175	441
PB.6114.1	5	1484	6	full-splice_match	gene: Zm00001d013506	Zm00001d013506_T001	12.09	12.09	coding	386	111	1268
PB.7926.1	6	888	1	full-splice_match	gene: Zm00001d038401	Zm00001d038401_T001	42.45	42.45	coding	80	78	317
PB.5133.1	4	5887	4	novel_in_catalog	gene: Zm00001d050372	novel	8.01	8.01	coding	1413	1231	5469
PB.4277.1	3	1557	6	novel_in_catalog	gene: Zm00001d042621	novel	3.18	28.79	coding	246	22	759
PB.4407.2	3	1872	1	full-splice_match	gene: Zm00001d043183	Zm00001d043183_T001	7.64	45.71	coding	287	680	1540
PB.605.2	1	2228	15	novel_not_in_catalog	gene: Zm00001d030005	novel	0.00	13.85	coding	620	160	2019
PB.4840.2	4	1000	2	incomplete-splice_match	gene: Zm00001d048834	Zm00001d048834_T002	15.38	47.50	coding	184	188	739
PB.4840.1	4	1686	4	full-splice_match	gene: Zm00001d048834	Zm00001d048834_T002	32.11	47.50	coding	399	124	1320
PB.9715.2	8	1567	8	novel_not_in_catalog	gene: Zm00001d011457	novel	1.97	17.99	coding	247	500	1240
PB.9815.1	8	1280	8	full-splice_match	gene: Zm00001d011850	Zm00001d011850_T002	184.64	212.54	coding	300	202	1101
PB.9818.3	8	1693	8	incomplete-splice_match	gene: Zm00001d011855	Zm00001d011855_T009	5.06	71.85	coding	515	21	1565
PB.9818.2	8	2250	9	full-splice_match	gene: Zm00001d011855	Zm00001d011855_T008	4.97	71.85	coding	543	386	2014

PB.9818.1	8	2353	9	full-splice_match	gene: Zm00001d011855	Zm00001d011855_T008	9.43	71.85	coding	543	386	2014
PB.7750.1	6	1287	2	genic	gene: Zm00001d037799	novel	14.87	14.87	coding	238	120	833
PB.10085.1	9	1244	6	full-splice_match	gene: Zm00001d045025	Zm00001d045025_T005	177.22	187.61	coding	246	153	890
PB.10085.2	9	1144	6	full-splice_match	gene: Zm00001d045025	Zm00001d045025_T005	10.39	187.61	coding	246	154	891
PB.6945.1	5	2403	2	novel_in_catalog	gene: Zm00001d017677	novel	4.59	4.59	coding	245	31	765
PB.5565.1	4	1382	2	full-splice_match	gene: Zm00001d052655	Zm00001d052655_T001	66.09	66.09	coding	270	137	946
PB.8567.1	7	977	1	full-splice_match	gene: Zm00001d020861	Zm00001d020861_T001	167.77	167.77	coding	162	158	643
PB.5659.1	4	1603	7	novel_not_in_catalog	gene: Zm00001d053036	novel	38.48	81.90	non_coding	NA	NA	NA
PB.5659.2	4	2073	5	novel_not_in_catalog	gene: Zm00001d053036	novel	43.42	81.90	non_coding	NA	NA	NA
PB.5820.5	4	1952	1	full-splice_match	gene: Zm00001d053834	Zm00001d053834_T002	31.31	1202.82	coding	381	593	1735
PB.7869.1	6	1895	13	full-splice_match	gene: Zm00001d038200	Zm00001d038200_T003	66.10	109.12	coding	445	97	1431
PB.927.2	1	2192	14	full-splice_match	gene: Zm00001d031929	Zm00001d031929_T011	34.35	48.60	coding	591	121	1893
PB.927.1	1	2336	14	full-splice_match	gene: Zm00001d031929	Zm00001d031929_T004	14.24	48.60	coding	591	141	1913
PB.9838.4	8	1388	5	novel_in_catalog	gene: Zm00001d011958	novel	2.55	211.60	coding	287	106	966
PB.5490.1	4	1258	3	full-splice_match	gene: Zm00001d052346	Zm00001d052346_T001	4.72	4.72	non_coding	NA	NA	NA
PB.9838.1	8	585	1	genic	gene: Zm00001d011958	novel	70.45	211.60	non_coding	NA	NA	NA
PB.7608.1	6	1545	2	full-splice_match	gene: Zm00001d037182	Zm00001d037182_T001	26.19	34.89	coding	377	163	1293

PB.7608.2	6	1361	2	full-splice_match	gene: Zm00001d037182	Zm00001d037182_T001	8.70	34.89	coding	377	98	1228
PB.9838.2	8	1387	6	full-splice_match	gene: Zm00001d011958	Zm00001d011958_T001	138.61	211.60	coding	369	31	1137
PB.7938.2	6	3691	7	full-splice_match	gene: Zm00001d038453	Zm00001d038453_T006	18.33	143.82	coding	183	2029	2577
PB.7938.3	6	1915	7	novel_in_catalog	gene: Zm00001d038453	novel	7.78	143.82	coding	414	269	1510
PB.7938.1	6	1947	9	full-splice_match	gene: Zm00001d038453	Zm00001d038453_T003	97.05	143.82	coding	477	70	1500
PB.7938.6	6	3487	8	full-splice_match	gene: Zm00001d038453	Zm00001d038453_T004	0.00	143.82	coding	391	2026	3198
PB.7938.7	6	1826	9	full-splice_match	gene: Zm00001d038453	Zm00001d038453_T003	0.00	143.82	coding	477	81	1511
PB.7938.4	6	1994	8	novel_in_catalog	gene: Zm00001d038453	novel	13.03	143.82	coding	305	705	1619
PB.7938.5	6	1693	7	novel_in_catalog	gene: Zm00001d038453	novel	7.62	143.82	coding	198	784	1377
PB.5327.2	4	1746	2	full-splice_match	gene: Zm00001d051636	Zm00001d051636_T001	3.64	9.69	coding	392	194	1369
PB.5327.3	4	1602	2	full-splice_match	gene: Zm00001d051636	Zm00001d051636_T001	0.00	9.69	coding	392	193	1368
PB.5327.1	4	1857	2	full-splice_match	gene: Zm00001d051636	Zm00001d051636_T001	6.05	9.69	coding	392	194	1369
PB.2268.1	10	2159	5	novel_not_in_catalog	gene: Zm00001d026348	novel	3.60	8.17	coding	305	184	1098
PB.8514.3	7	1260	1	full-splice_match	gene: Zm00001d020583	Zm00001d020583_T001	50.75	68.45	coding	318	189	1142
PB.2268.3	10	2029	5	novel_in_catalog	gene: Zm00001d026348	novel	0.18	8.17	coding	305	175	1089
PB.1797.2	10	959	1	antisense	novelGene_gene: Zm00001d023941_A	novel	20.44	149.54	coding	268	74	877
PB.6785.8	5	1646	3	full-splice_match	gene: Zm00001d016956	Zm00001d016956_T002	0.00	12.39	coding	107	189	509

PB.1161.1	1	1658	3	full-splice_match	gene: Zm00001d032906	Zm00001d032906_T001	6.99	6.99	coding	508	84	1607
PB.10800.1	9	1407	5	novel_in_catalog	gene: Zm00001d048450	novel	17.94	17.94	coding	136	757	1164
PB.1797.1	10	1437	1	antisense	novelGene_gene: Zm00001d023941_A	novel	129.10	149.54	coding	268	235	1038
PB.3075.5	2	2988	5	full-splice_match	gene: Zm00001d005190	Zm00001d005190_T001	0.54	76.54	coding	898	169	2862
PB.3403.1	2	1197	1	full-splice_match	gene: Zm00001d006774	Zm00001d006774_T001	175.51	207.71	coding	262	67	852
PB.3403.3	2	965	1	full-splice_match	gene: Zm00001d006774	Zm00001d006774_T001	0.00	207.71	coding	262	68	853
PB.3403.2	2	1073	1	full-splice_match	gene: Zm00001d006774	Zm00001d006774_T001	32.20	207.71	coding	262	67	852
PB.4423.2	3	910	4	full-splice_match	gene: Zm00001d043249	Zm00001d043249_T001	92.91	171.42	coding	152	287	742
PB.4423.1	3	1178	4	full-splice_match	gene: Zm00001d043249	Zm00001d043249_T001	78.51	171.42	coding	203	272	880
PB.4464.1	3	1358	4	full-splice_match	gene: Zm00001d043420	Zm00001d043420_T001	12.67	13.91	coding	278	129	962
PB.5123.1	4	1197	5	full-splice_match	gene: Zm00001d050319	Zm00001d050319_T001	23.95	23.95	coding	262	168	953
PB.5651.2	4	3011	3	fusion	gene: Zm00001d052993_g	novel	5.75	5.75	coding	887	48	2708
PB.4464.2	3	1389	4	full-splice_match	gene: Zm00001d043420	Zm00001d043420_T001	1.24	13.91	coding	278	267	1100
PB.8823.2	7	1513	2	full-splice_match	gene: Zm00001d021839	Zm00001d021839_T001	115.52	122.88	coding	294	222	1103
PB.9150.10	8	2200	1	novel_in_catalog	gene: Zm00001d008602	novel	12.31	155.50	coding	147	721	1161
PB.8823.1	7	1572	1	genic	gene: Zm00001d021839	novel	7.37	122.88	coding	331	135	1127
PB.3854.1	3	1028	1	antisense	novelGene_gene: Zm00001d040246_A	novel	22.00	22.00	coding	159	291	767

PB.10398.8	9	947	3	full-splice_match	gene: Zm00001d046767	Zm00001d046767_T001	0.00	1894.58	coding	174	121	642
PB.2905.1	2	1184	6	full-splice_match	gene: Zm00001d003975	Zm00001d003975_T002	11.99	11.99	coding	182	90	635
PB.3647.6	3	1812	6	novel_in_catalog	gene: Zm00001d039285	novel	0.00	76.47	coding	384	432	1583
PB.3647.4	3	2493	5	novel_in_catalog	gene: Zm00001d039285	novel	3.46	76.47	coding	384	1124	2275
PB.3647.3	3	1966	5	novel_in_catalog	gene: Zm00001d039285	novel	4.09	76.47	coding	175	439	963
PB.3647.2	3	1925	6	novel_in_catalog	gene: Zm00001d039285	novel	68.92	76.47	coding	384	439	1590
PB.3311.1	2	3602	2	genic	gene: Zm00001d006371	novel	28.69	28.69	coding	1012	316	3351
PB.2231.2	10	2207	12	full-splice_match	gene: Zm00001d026239	Zm00001d026239_T008	4.34	18.93	coding	354	153	1214
PB.7015.12	5	1395	9	novel_in_catalog	gene: Zm00001d017958	novel	20.89	1562.37	coding	379	77	1213
PB.2231.1	10	2202	13	full-splice_match	gene: Zm00001d026239	Zm00001d026239_T007	11.08	18.93	coding	468	223	1626
PB.1503.1	1	1720	5	full-splice_match	gene: Zm00001d034493	Zm00001d034493_T001	9.84	9.84	coding	371	197	1309
PB.3663.1	3	1556	1	full-splice_match	gene: Zm00001d039324	Zm00001d039324_T001	22.80	22.80	coding	351	157	1209
PB.6951.1	5	1747	4	incomplete-splice_match	gene: Zm00001d017698	Zm00001d017698_T006	4.59	12.43	coding	434	128	1429
PB.635.2	1	2022	5	novel_in_catalog	gene: Zm00001d030159	novel	15.94	324.18	coding	566	150	1847
PB.10320.1	9	1675	8	full-splice_match	gene: Zm00001d046344	Zm00001d046344_T001	15.73	15.73	coding	508	85	1608
PB.7761.1	6	985	2	full-splice_match	gene: Zm00001d037842	Zm00001d037842_T001	31.13	31.13	coding	221	46	708
PB.6810.17	5	2348	7	full-splice_match	gene: Zm00001d017096	Zm00001d017096_T002	0.00	118.92	coding	129	171	557

PB.7932.2	6	1039	1	full-splice_match	gene: Zm00001d038442	Zm00001d038442_T001	44.92	74.39	coding	247	190	930
PB.5539.3	4	1401	3	full-splice_match	gene: Zm00001d052489	Zm00001d052489_T001	36.38	36.38	coding	421	139	1401
PB.10398.1	9	1827	3	full-splice_match	gene: Zm00001d046767	Zm00001d046767_T001	1.37	1894.58	coding	174	120	641
PB.631.1	1	882	4	full-splice_match	gene: Zm00001d030146	Zm00001d030146_T001	67.32	69.01	coding	163	44	532
PB.10715.1	9	1039	5	full-splice_match	gene: Zm00001d048153	Zm00001d048153_T001	6.85	6.85	coding	231	72	764
PB.4786.3	3	1039	6	full-splice_match	gene: Zm00001d044694	Zm00001d044694_T002	97.57	277.83	coding	240	110	829
PB.1953.1	10	2352	2	genic	gene: Zm00001d024867	novel	3.46	3.46	coding	683	99	2147
PB.8052.6	6	750	2	novel_in_catalog	gene: Zm00001d038891	novel	1.46	20.71	coding	101	402	704
PB.4005.2	3	1741	6	incomplete-splice_match	gene: Zm00001d041280	Zm00001d041280_T001	16.09	22.48	coding	443	138	1466
PB.4005.1	3	2132	8	incomplete-splice_match	gene: Zm00001d041280	Zm00001d041280_T001	6.39	22.48	coding	571	39	1751
PB.10398.2	9	1182	3	full-splice_match	gene: Zm00001d046767	Zm00001d046767_T001	23.52	1894.58	coding	174	117	638
PB.7227.1	6	1580	5	incomplete-splice_match	gene: Zm00001d035048	Zm00001d035048_T002	6.10	61.02	coding	280	426	1265
PB.7227.3	6	1073	5	incomplete-splice_match	gene: Zm00001d035048	Zm00001d035048_T003	43.83	61.02	non_coding	NA	NA	NA
PB.7227.2	6	1173	4	novel_in_catalog	gene: Zm00001d035048	novel	11.09	61.02	coding	174	80	601
PB.898.1	1	1591	1	genic	gene: Zm00001d031769	novel	13.49	13.49	coding	445	5	1339
PB.8773.12	7	2121	6	novel_not_in_catalog	gene: Zm00001d021698	novel	0.00	192.02	coding	216	338	985
PB.3079.1	2	1288	2	full-splice_match	gene: Zm00001d005208	Zm00001d005208_T001	7.19	57.37	coding	301	49	951

PB.898.2	1	1456	1	genic	gene: Zm00001d031769	novel	0.00	13.49	coding	407	110	1330
PB.631.2	1	734	4	full-splice_match	gene: Zm00001d030146	Zm00001d030146_T001	0.00	69.01	coding	163	36	524
PB.8541.1	7	2079	1	genic	gene: Zm00001d020731	novel	3.94	3.94	coding	95	262	546
PB.4392.3	3	2986	3	novel_not_in_catalog	gene: Zm00001d043135	novel	7.82	12.89	coding	766	251	2548
PB.1600.2	1	1710	13	full-splice_match	gene: Zm00001d034809	Zm00001d034809_T032	54.42	80.83	coding	408	197	1420
PB.10681.1	9	1688	5	incomplete-splice_match	gene: Zm00001d048023	Zm00001d048023_T009	25.43	25.43	coding	439	123	1439
PB.1880.1	10	1239	5	novel_in_catalog	gene: Zm00001d024519	novel	17.30	82.59	coding	186	212	769
PB.7932.1	6	1306	1	full-splice_match	gene: Zm00001d038442	Zm00001d038442_T001	29.47	74.39	coding	247	180	920
PB.4388.1	3	1873	2	genic	gene: Zm00001d043125	novel	4.79	27.68	coding	350	516	1565
PB.6876.1	5	1329	3	full-splice_match	gene: Zm00001d017353	Zm00001d017353_T001	119.52	141.84	coding	243	151	879
PB.9709.3	8	2595	10	full-splice_match	gene: Zm00001d011426	Zm00001d011426_T003	0.00	54.05	coding	613	389	2227
PB.2790.1	2	1358	1	full-splice_match	gene: Zm00001d003398	Zm00001d003398_T001	4.55	4.55	coding	302	204	1109
PB.8606.1	7	1080	2	full-splice_match	gene: Zm00001d021006	Zm00001d021006_T001	161.95	169.98	coding	258	184	957
PB.1573.2	1	1809	7	full-splice_match	gene: Zm00001d034727	Zm00001d034727_T004	11.75	24.82	coding	477	263	1693
PB.6253.1	5	1435	10	full-splice_match	gene: Zm00001d014192	Zm00001d014192_T001	17.51	17.51	coding	277	292	1122
PB.7668.1	6	2168	10	full-splice_match	gene: Zm00001d037438	Zm00001d037438_T002	5.34	14.31	coding	561	62	1744
PB.7668.4	6	1058	4	incomplete-splice_match	gene: Zm00001d037438	Zm00001d037438_T002	8.97	14.31	coding	224	132	803

PB.1407.2	1	1264	6	full-splice_match	gene: Zm00001d034063	Zm00001d034063_T002	0.00	87.06	coding	303	193	1101
PB.10775.1	9	2564	7	full-splice_match	gene: Zm00001d048372	Zm00001d048372_T002	14.61	14.61	coding	715	202	2346
PB.8832.1	7	1621	2	full-splice_match	gene: Zm00001d021867	Zm00001d021867_T001	23.63	23.63	coding	439	87	1403
PB.7048.1	5	2657	4	full-splice_match	gene: Zm00001d018057	Zm00001d018057_T001	9.98	9.98	coding	787	96	2456
PB.10709.2	9	1566	2	full-splice_match	gene: Zm00001d048131	Zm00001d048131_T001	9.48	11.01	coding	391	164	1336
PB.10709.1	9	1468	1	genic	gene: Zm00001d048131	novel	1.54	11.01	coding	194	149	730
PB.8982.1	7	644	4	full-splice_match	gene: Zm00001d022418	Zm00001d022418_T002	230.46	230.46	coding	102	57	362
PB.8682.1	7	1863	6	full-splice_match	gene: Zm00001d021372	Zm00001d021372_T001	7.82	7.82	coding	432	80	1375
PB.4570.1	3	2335	15	incomplete-splice_match	gene: Zm00001d043797	Zm00001d043797_T020	2.28	153.66	coding	546	111	1748
PB.5025.2	4	1820	3	novel_not_in_catalog	gene: Zm00001d049727	novel	3.20	6.99	coding	571	1	1713
PB.6952.1	5	2485	3	novel_not_in_catalog	gene: Zm00001d017704	novel	111.67	111.67	coding	261	181	963
PB.1122.1	1	775	4	full-splice_match	gene: Zm00001d032736	Zm00001d032736_T001	95.59	95.59	coding	166	38	535
PB.9453.1	8	594	1	intergenic	novelGene_176	novel	8.41	8.41	non_coding	NA	NA	NA
PB.6933.2	5	773	1	full-splice_match	gene: Zm00001d017622	Zm00001d017622_T001	63.01	98.02	coding	181	61	603
PB.7986.1	6	2655	1	incomplete-splice_match	gene: Zm00001d038671	Zm00001d038671_T001	20.66	32.27	coding	574	99	1820
PB.6933.1	5	896	1	full-splice_match	gene: Zm00001d017622	Zm00001d017622_T001	35.00	98.02	coding	181	163	705
PB.2895.1	2	655	2	full-splice_match	gene: Zm00001d000842	Zm00001d000842_T001	8.38	8.38	non_coding	NA	NA	NA

PB.4277.2	3	1843	9	full-splice_match	gene: Zm00001d042621	Zm00001d042621_T007	25.61	28.79	coding	359	384	1460
PB.9308.1	8	950	2	incomplete-splice_match	gene: Zm00001d009465	Zm00001d009465_T001	193.49	193.49	coding	206	69	686
PB.5878.2	4	1885	11	full-splice_match	gene: Zm00001d054011	Zm00001d054011_T006	11.47	14.10	coding	429	148	1434
PB.9554.2	8	1794	2	genic	gene: Zm00001d010705	novel	2.86	15.37	coding	225	91	765
PB.9554.3	8	1887	2	genic	gene: Zm00001d010705	novel	12.51	15.37	coding	412	115	1350
PB.9802.1	8	717	2	full-splice_match	gene: Zm00001d011825	Zm00001d011825_T001	134.25	134.25	coding	155	27	491
PB.8982.2	7	477	4	full-splice_match	gene: Zm00001d022418	Zm00001d022418_T002	0.00	230.46	coding	23	229	297
PB.7986.2	6	3237	5	full-splice_match	gene: Zm00001d038671	Zm00001d038671_T003	0.00	32.27	coding	797	136	2526
PB.10572.1	9	707	2	genic	gene: Zm00001d047675	novel	48.78	48.78	coding	112	77	412
PB.7561.1	6	738	4	full-splice_match	gene: Zm00001d036964	Zm00001d036964_T001	75.89	75.89	coding	76	131	358
PB.7393.1	6	1962	1	genic	gene: Zm00001d035989	novel	1.71	1.71	coding	542	44	1669
PB.8918.7	7	1966	3	novel_in_catalog	gene: Zm00001d022190	novel	0.44	98.23	coding	136	954	1361
PB.9460.1	8	791	3	novel_not_in_catalog	gene: Zm00001d010244	novel	41.33	100.47	coding	104	152	463
PB.2252.2	10	2375	18	incomplete-splice_match	gene: Zm00001d026291	Zm00001d026291_T007	29.84	45.74	coding	635	173	2077
PB.2252.3	10	1410	8	incomplete-splice_match	gene: Zm00001d026291	Zm00001d026291_T004	2.11	45.74	coding	428	125	1408
PB.7957.1	6	1570	6	full-splice_match	gene: Zm00001d038540	Zm00001d038540_T001	9.57	9.57	coding	364	236	1327
PB.2924.1	2	734	2	genic	gene: Zm00001d004127	novel	17.46	17.46	coding	97	88	378

PB.1472.1	1	2086	2	full-splice_match	gene: Zm00001d034373	Zm00001d034373_T001	144.72	144.72	coding	465	298	1692
PB.8172.1	7	860	6	full-splice_match	gene: Zm00001d018699	Zm00001d018699_T001	43.71	43.71	coding	189	94	660
PB.3496.1	2	4014	2	novel_in_catalog	gene: Zm00001d007241	novel	8.57	22.24	coding	1165	326	3820
PB.1219.1	1	1725	6	full-splice_match	gene: Zm00001d033248	Zm00001d033248_T002	80.11	80.11	coding	431	81	1373
PB.383.2	1	2346	10	full-splice_match	gene: Zm00001d028920	Zm00001d028920_T003	16.74	17.96	coding	535	147	1751
PB.383.3	1	2525	9	full-splice_match	gene: Zm00001d028920	Zm00001d028920_T001	1.23	17.96	coding	256	137	904
PB.188.4	1	1138	1	novel_in_catalog	gene: Zm00001d028094	novel	10.58	181.73	coding	144	14	445
PB.188.3	1	1764	2	full-splice_match	gene: Zm00001d028094	Zm00001d028094_T001	19.24	181.73	coding	307	617	1537
PB.188.2	1	1428	2	full-splice_match	gene: Zm00001d028094	Zm00001d028094_T001	150.29	181.73	coding	307	29	949
PB.188.1	1	1588	2	full-splice_match	gene: Zm00001d028094	Zm00001d028094_T001	1.62	181.73	coding	307	27	947
PB.6641.1	5	1217	4	full-splice_match	gene: Zm00001d016203	Zm00001d016203_T004	50.90	50.90	coding	174	422	943
PB.4961.1	4	876	2	full-splice_match	gene: Zm00001d049419	Zm00001d049419_T001	61.02	61.02	coding	228	93	776
PB.4496.5	3	1493	5	novel_in_catalog	gene: Zm00001d043515	novel	3.11	78.68	coding	353	93	1151
PB.8639.2	7	1584	6	novel_in_catalog	gene: Zm00001d021203	novel	5.49	20.53	coding	226	786	1463
PB.8241.1	7	693	4	full-splice_match	gene: Zm00001d018979	Zm00001d018979_T001	233.05	233.05	coding	153	39	497
PB.557.2	1	2286	3	novel_in_catalog	gene: Zm00001d029672	novel	32.34	32.34	non_coding	NA	NA	NA
PB.4191.1	3	598	2	incomplete-splice_match	gene: Zm00001d042211	Zm00001d042211_T001	17.30	17.30	coding	30	320	409

PB.4820.1	4	1392	2	novel_not_in_catalog	gene: Zm00001d048691	novel	13.57	47.65	coding	322	130	1095
PB.4506.1	3	1820	14	full-splice_match	gene: Zm00001d043560	Zm00001d043560_T001	28.09	28.09	coding	507	102	1622
PB.4820.2	4	1306	2	novel_not_in_catalog	gene: Zm00001d048691	novel	34.08	47.65	coding	322	197	1162
PB.5518.4	4	2457	16	incomplete-splice_match	gene: Zm00001d052425	Zm00001d052425_T010	10.46	41.50	coding	502	781	2286
PB.10797.1	9	5141	13	full-splice_match	gene: Zm00001d048442	Zm00001d048442_T004	14.83	14.83	coding	1466	75	4472
PB.2122.10	10	1538	6	novel_not_in_catalog	gene: Zm00001d025727	novel	22.00	114.82	coding	353	269	1327
PB.3724.2	3	2213	3	intergenic	novelGene_32	novel	4.95	59.17	non_coding	NA	NA	NA
PB.3328.1	2	1198	2	incomplete-splice_match	gene: Zm00001d006454	Zm00001d006454_T001	20.03	20.03	coding	301	38	940
PB.6474.1	5	2275	1	genic	gene: Zm00001d015098	novel	10.33	10.33	coding	88	118	381
PB.6474.3	5	2164	1	genic	gene: Zm00001d015098	novel	0.00	10.33	coding	88	118	381
PB.3472.1	2	1284	3	full-splice_match	gene: Zm00001d007105	Zm00001d007105_T002	10.95	10.95	coding	277	198	1028
PB.5202.2	4	3262	7	novel_in_catalog	gene: Zm00001d050925	novel	19.14	19.14	coding	338	1921	2934
PB.8660.4	7	3180	4	novel_not_in_catalog	gene: Zm00001d021296	novel	9.70	24.75	coding	275	1935	2759
PB.9181.1	8	2294	2	full-splice_match	gene: Zm00001d008739	Zm00001d008739_T001	28.38	28.38	coding	575	263	1987
PB.9181.2	8	2193	2	full-splice_match	gene: Zm00001d008739	Zm00001d008739_T001	0.00	28.38	coding	575	263	1987
PB.9181.3	8	2086	2	full-splice_match	gene: Zm00001d008739	Zm00001d008739_T001	0.00	28.38	coding	575	263	1987
PB.1017.1	1	1245	2	incomplete-splice_match	gene: Zm00001d032338	Zm00001d032338_T001	27.36	27.36	non_coding	NA	NA	NA

PB.4380.5	3	2123	11	full-splice_match	gene: Zm00001d043083	Zm00001d043083_T002	0.00	58.95	coding	432	380	1675
PB.9312.2	8	1258	3	full-splice_match	gene: Zm00001d009476	Zm00001d009476_T001	22.56	139.08	coding	252	277	1032
PB.3117.2	2	1919	8	full-splice_match	gene: Zm00001d005406	Zm00001d005406_T002	5.95	35.82	coding	352	149	1204
PB.9312.1	8	1132	4	full-splice_match	gene: Zm00001d009476	Zm00001d009476_T002	116.52	139.08	coding	157	413	883
PB.10452.4	9	810	3	incomplete-splice_match	gene: Zm00001d047104	Zm00001d047104_T001	8.11	53.18	coding	236	64	771
PB.8922.2	7	1372	2	full-splice_match	gene: Zm00001d022212	Zm00001d022212_T001	19.88	19.88	coding	220	439	1098
PB.4696.1	3	1865	6	full-splice_match	gene: Zm00001d044315	Zm00001d044315_T002	72.95	275.21	coding	451	251	1603
PB.5046.1	4	2085	17	incomplete-splice_match	gene: Zm00001d049817	Zm00001d049817_T007	8.77	8.77	coding	305	723	1637
PB.2294.1	10	1192	5	full-splice_match	gene: Zm00001d026425	Zm00001d026425_T004	174.84	174.84	coding	244	233	964
PB.7595.9	6	2379	5	novel_in_catalog	gene: Zm00001d037109	novel	0.00	71.30	coding	375	379	1503
PB.2416.1	2	1069	8	full-splice_match	gene: Zm00001d001856	Zm00001d001856_T005	54.71	54.71	coding	263	111	899
PB.7420.2	6	1807	1	novel_in_catalog	gene: Zm00001d036136	novel	19.82	760.62	coding	55	182	346
PB.7420.3	6	399	2	full-splice_match	gene: Zm00001d036136	Zm00001d036136_T002	38.18	760.62	non_coding	NA	NA	NA
PB.4975.2	4	821	1	full-splice_match	gene: Zm00001d049503	Zm00001d049503_T001	87.36	110.19	coding	195	111	695
PB.5425.6	4	3187	6	full-splice_match	gene: Zm00001d052076	Zm00001d052076_T009	1.98	36.14	non_coding	NA	NA	NA
PB.4975.1	4	930	1	full-splice_match	gene: Zm00001d049503	Zm00001d049503_T001	22.83	110.19	coding	195	109	693
PB.5425.2	4	2005	4	full-splice_match	gene: Zm00001d052076	Zm00001d052076_T018	7.32	36.14	non_coding	NA	NA	NA

PB.5425.1	4	1642	7	full-splice_match	gene: Zm00001d052076	Zm00001d052076_T002	26.84	36.14	coding	163	411	899
PB.1205.2	1	3342	13	novel_not_in_catalog	gene: Zm00001d033168	novel	2.88	34.71	coding	593	68	1846
PB.10452.2	9	452	1	genic	gene: Zm00001d047104	novel	8.16	53.18	non_coding	NA	NA	NA
PB.3276.6	2	1730	6	full-splice_match	gene: Zm00001d006242	Zm00001d006242_T009	0.00	142.91	coding	345	63	1097
PB.9229.1	8	2491	2	full-splice_match	gene: Zm00001d008965	Zm00001d008965_T001	30.29	30.29	coding	672	221	2236
PB.3293.3	2	1688	3	full-splice_match	gene: Zm00001d006308	Zm00001d006308_T001	0.00	24.16	coding	424	81	1352
PB.1922.8	10	1871	7	novel_in_catalog	gene: Zm00001d024744	novel	4.95	36.88	coding	216	336	983
PB.3571.1	2	2103	2	novel_not_in_catalog	gene: Zm00001d007706	novel	34.43	34.43	coding	484	131	1582
PB.3780.1	3	4282	17	novel_in_catalog	gene: Zm00001d039860	novel	36.20	53.91	coding	1294	29	3910
PB.3780.3	3	2447	9	incomplete-splice_match	gene: Zm00001d039860	Zm00001d039860_T007	17.71	53.91	coding	455	31	1395
PB.3601.1	2	1267	1	full-splice_match	gene: Zm00001d007836	Zm00001d007836_T001	9.84	93.75	coding	170	196	705
PB.774.1	1	1505	1	full-splice_match	gene: Zm00001d031094	Zm00001d031094_T001	22.06	22.06	coding	341	391	1413
PB.6740.3	5	1946	10	novel_in_catalog	gene: Zm00001d016770	novel	4.26	43.77	coding	363	216	1304
PB.10612.4	9	1285	13	full-splice_match	gene: Zm00001d047797	Zm00001d047797_T006	476.78	476.78	coding	350	108	1157
PB.3295.1	2	3045	3	full-splice_match	gene: Zm00001d006317	Zm00001d006317_T002	37.07	37.07	coding	856	272	2839
PB.1609.3	1	1257	9	full-splice_match	gene: Zm00001d034833	Zm00001d034833_T004	0.00	181.28	coding	310	106	1035
PB.3276.1	2	1755	7	novel_in_catalog	gene: Zm00001d006242	novel	12.70	142.91	coding	363	105	1193

PB.9581.1	8	361	1	intergenic	novelGene_203	novel	260.78	260.78	non_coding	NA	NA	NA
PB.4124.4	3	2903	4	fusion	gene: Zm00001d000620_g	novel	1.79	26.29	coding	772	43	2358
PB.7958.3	6	2430	3	novel_not_in_catalog	gene: Zm00001d038541	novel	0.00	30.36	coding	674	167	2188
PB.6382.1	5	1444	5	intergenic	novelGene_119	novel	23.97	23.97	coding	290	419	1288
PB.4124.1	3	3002	5	fusion	gene: Zm00001d000620_g	novel	17.90	26.29	coding	817	89	2539
PB.4124.2	3	2859	5	fusion	gene: Zm00001d000620_g	novel	6.60	26.29	coding	849	8	2554
PB.8979.2	7	1568	11	full-splice_match	gene: Zm00001d022407	Zm00001d022407_T004	0.00	16.93	coding	422	120	1385
PB.6785.4	5	2192	4	incomplete-splice_match	gene: Zm00001d016956	Zm00001d016956_T012	5.34	12.39	coding	107	194	514
PB.8979.1	7	1677	11	full-splice_match	gene: Zm00001d022407	Zm00001d022407_T004	16.93	16.93	coding	422	121	1386
PB.80.5	1	1863	2	full-splice_match	gene: Zm00001d027537	Zm00001d027537_T001	22.93	96.58	coding	546	49	1686
PB.80.1	1	2098	2	full-splice_match	gene: Zm00001d027537	Zm00001d027537_T001	73.65	96.58	coding	546	49	1686
PB.8654.1	7	3577	3	novel_in_catalog	gene: Zm00001d021278	novel	11.58	11.58	coding	132	2737	3132
PB.5417.2	4	3438	2	novel_in_catalog	gene: Zm00001d052060	novel	5.04	58.94	coding	797	154	2544
PB.695.2	1	2688	1	genic	gene: Zm00001d030618	novel	6.81	852.21	non_coding	NA	NA	NA
PB.4387.2	3	915	7	novel_not_in_catalog	gene: Zm00001d043113	novel	62.84	97.17	coding	181	144	686
PB.9680.2	8	562	2	full-splice_match	gene: Zm00001d011278	Zm00001d011278_T002	0.00	202.67	coding	92	88	363
PB.3091.1	2	1538	6	full-splice_match	gene: Zm00001d005271	Zm00001d005271_T002	79.82	79.82	coding	357	213	1283

PB.9680.1	8	801	3	full-splice_match	gene: Zm00001d011278	Zm00001d011278_T001	202.67	202.67	coding	122	140	505
PB.8182.1	7	1186	8	full-splice_match	gene: Zm00001d018732	Zm00001d018732_T004	34.47	34.47	coding	264	36	827
PB.6756.1	5	2849	15	full-splice_match	gene: Zm00001d016838	Zm00001d016838_T002	21.90	64.46	coding	677	307	2337
PB.7046.3	5	1784	4	incomplete-splice_match	gene: Zm00001d018050	Zm00001d018050_T002	13.81	13.81	coding	153	950	1408
PB.545.2	1	818	1	full-splice_match	gene: Zm00001d029628	Zm00001d029628_T001	406.36	409.71	coding	129	68	454
PB.6756.6	5	2835	14	full-splice_match	gene: Zm00001d016838	Zm00001d016838_T004	1.22	64.46	coding	525	896	2470
PB.6756.5	5	2747	15	full-splice_match	gene: Zm00001d016838	Zm00001d016838_T002	37.38	64.46	coding	677	313	2343
PB.545.1	1	1330	1	full-splice_match	gene: Zm00001d029628	Zm00001d029628_T001	3.35	409.71	coding	129	64	450
PB.2329.5	10	1971	2	genic	gene: Zm00001d026559	novel	0.00	74.04	coding	568	267	1970
PB.7245.1	6	1165	2	full-splice_match	gene: Zm00001d035136	Zm00001d035136_T002	175.16	175.16	coding	281	65	907
PB.6985.2	5	516	2	full-splice_match	gene: Zm00001d017850	Zm00001d017850_T001	871.15	1717.47	coding	123	37	405
PB.2329.1	10	2535	2	genic	gene: Zm00001d026559	novel	71.52	74.04	coding	621	269	2131
PB.10657.1	9	4058	6	full-splice_match	gene: Zm00001d047923	Zm00001d047923_T002	82.51	82.51	coding	1032	607	3702
PB.2329.2	10	2749	1	full-splice_match	gene: Zm00001d026559	Zm00001d026559_T001	2.52	74.04	coding	621	487	2349
PB.6635.3	5	1944	6	full-splice_match	gene: Zm00001d016175	Zm00001d016175_T001	6.11	10.93	coding	532	184	1779
PB.6635.2	5	2104	6	full-splice_match	gene: Zm00001d016175	Zm00001d016175_T001	0.00	10.93	coding	532	175	1770
PB.6635.1	5	2208	6	full-splice_match	gene: Zm00001d016175	Zm00001d016175_T001	4.82	10.93	coding	532	164	1759

PB.5021.4	4	1631	8	full-splice_match	gene: Zm00001d049716	Zm00001d049716_T001	6.07	100.67	coding	417	274	1524
PB.2603.1	2	2152	1	full-splice_match	gene: Zm00001d002623	Zm00001d002623_T001	5.93	5.93	coding	568	258	1961
PB.6148.1	5	1978	1	full-splice_match	gene: Zm00001d013646	Zm00001d013646_T001	9.58	9.58	coding	553	136	1794
PB.8971.1	7	1260	8	novel_in_catalog	gene: Zm00001d022384	novel	29.41	29.41	coding	322	84	1049
PB.576.1	1	1688	14	full-splice_match	gene: Zm00001d029803	Zm00001d029803_T005	18.31	20.58	coding	446	116	1453
PB.8642.3	7	1967	3	novel_in_catalog	gene: Zm00001d021221	novel	5.73	174.12	coding	404	445	1656
PB.8642.2	7	1911	4	full-splice_match	gene: Zm00001d021221	Zm00001d021221_T001	122.54	174.12	coding	334	445	1446
PB.8642.1	7	2251	4	full-splice_match	gene: Zm00001d021221	Zm00001d021221_T001	2.33	174.12	coding	334	447	1448
PB.3160.2	2	1799	7	novel_not_in_catalog	gene: Zm00001d005682	novel	26.59	38.30	coding	351	455	1507
PB.8642.7	7	1788	3	novel_in_catalog	gene: Zm00001d021221	novel	0.98	174.12	coding	257	770	1540
PB.576.2	1	1148	9	incomplete-splice_match	gene: Zm00001d029803	Zm00001d029803_T008	1.50	20.58	coding	127	314	694
PB.8642.5	7	1852	3	novel_in_catalog	gene: Zm00001d021221	novel	0.00	174.12	coding	404	445	1656
PB.8642.4	7	1808	4	full-splice_match	gene: Zm00001d021221	Zm00001d021221_T001	42.54	174.12	coding	334	446	1447
PB.1242.1	1	1699	6	full-splice_match	gene: Zm00001d033366	Zm00001d033366_T002	64.78	65.09	coding	392	180	1355
PB.576.3	1	1827	12	novel_in_catalog	gene: Zm00001d029803	novel	0.77	20.58	coding	360	40	1119
PB.3096.1	2	1562	1	full-splice_match	gene: Zm00001d005302	Zm00001d005302_T001	19.51	19.51	coding	397	126	1316
PB.5197.3	4	1455	4	novel_not_in_catalog	gene: Zm00001d050913	novel	2.20	8.32	coding	340	236	1255

PB.5197.2	4	1930	3	novel_in_catalog	gene: Zm00001d050913	novel	6.13	8.32	coding	340	487	1506
PB.7743.2	6	1874	9	full-splice_match	gene: Zm00001d037771	Zm00001d037771_T001	3.99	18.37	coding	438	238	1551
PB.10743.4	9	3339	6	full-splice_match	gene: Zm00001d048272	Zm00001d048272_T003	3.43	119.34	coding	241	1037	1759
PB.1740.1	10	1314	1	full-splice_match	gene: Zm00001d023620	Zm00001d023620_T001	27.91	27.91	coding	73	698	916
PB.7743.1	6	1952	9	full-splice_match	gene: Zm00001d037771	Zm00001d037771_T001	14.38	18.37	coding	438	177	1490
PB.499.1	1	1610	2	full-splice_match	gene: Zm00001d029442	Zm00001d029442_T001	24.83	24.83	coding	401	216	1418
PB.6513.1	5	1163	6	full-splice_match	gene: Zm00001d015300	Zm00001d015300_T001	429.64	435.90	coding	207	188	808
PB.10789.1	9	1029	4	novel_in_catalog	gene: Zm00001d048414	novel	9.14	26.31	coding	190	98	667
PB.3160.6	2	1592	7	novel_not_in_catalog	gene: Zm00001d005682	novel	0.00	38.30	coding	351	415	1467
PB.6513.5	5	1059	5	novel_in_catalog	gene: Zm00001d015300	novel	6.26	435.90	coding	112	588	923
PB.1606.1	1	1334	1	full-splice_match	gene: Zm00001d034827	Zm00001d034827_T001	1.19	1.19	coding	300	154	1053
PB.186.1	1	1292	2	incomplete-splice_match	gene: Zm00001d028084	Zm00001d028084_T013	21.21	21.21	coding	276	211	1038
PB.9209.1	8	1170	11	full-splice_match	gene: Zm00001d008870	Zm00001d008870_T007	29.91	29.91	coding	271	22	834
PB.7440.1	6	1318	1	full-splice_match	gene: Zm00001d036306	Zm00001d036306_T001	14.81	14.81	coding	357	90	1160
PB.1760.9	10	3627	12	full-splice_match	gene: Zm00001d023784	Zm00001d023784_T001	4.44	32.04	coding	816	863	3310
PB.1760.8	10	2213	8	novel_not_in_catalog	gene: Zm00001d023784	novel	6.25	32.04	coding	308	120	1043
PB.1242.2	1	1648	5	novel_in_catalog	gene: Zm00001d033366	novel	0.31	65.09	coding	355	80	1144

PB.6192.1	5	3815	8	full-splice_match	gene: Zm00001d013908	Zm00001d013908_T002	17.73	17.73	coding	982	609	3554
PB.525.1	1	1415	11	full-splice_match	gene: Zm00001d029548	Zm00001d029548_T008	22.31	22.31	coding	239	635	1351
PB.8672.1	7	1249	8	full-splice_match	gene: Zm00001d021332	Zm00001d021332_T004	81.42	81.42	coding	233	194	892
PB.2355.1	10	1085	2	full-splice_match	gene: Zm00001d026628	Zm00001d026628_T001	140.45	140.45	coding	211	148	780
PB.1760.3	10	3526	11	full-splice_match	gene: Zm00001d023784	Zm00001d023784_T003	21.35	32.04	coding	816	599	3046
PB.2776.1	2	1239	10	full-splice_match	gene: Zm00001d003308	Zm00001d003308_T001	10.06	10.06	coding	302	89	994
PB.6693.2	5	2029	3	full-splice_match	gene: Zm00001d016531	Zm00001d016531_T001	49.65	62.84	coding	521	120	1682
PB.9388.1	8	777	1	antisense	novelGene_gene: Zm00001d009894_A	novel	52.07	52.07	coding	246	33	770
PB.1063.1	1	2982	2	genic	gene: Zm00001d032510	novel	10.32	10.32	coding	941	3	2825
PB.6760.2	5	545	2	full-splice_match	gene: Zm00001d016850	Zm00001d016850_T002	72.64	94.02	coding	106	46	363
PB.6760.3	5	422	2	full-splice_match	gene: Zm00001d016850	Zm00001d016850_T002	21.37	94.02	coding	106	38	355
PB.2985.1	2	1895	7	full-splice_match	gene: Zm00001d004651	Zm00001d004651_T001	28.11	29.88	coding	443	165	1493
PB.3018.7	2	1876	4	novel_in_catalog	gene: Zm00001d004855	novel	6.42	183.36	coding	377	620	1750
PB.3018.6	2	1396	5	novel_in_catalog	gene: Zm00001d004855	novel	0.00	183.36	coding	377	118	1248
PB.3018.1	2	1542	5	novel_in_catalog	gene: Zm00001d004855	novel	176.73	183.36	coding	377	119	1249
PB.3018.3	2	2315	4	novel_in_catalog	gene: Zm00001d004855	novel	0.21	183.36	coding	377	931	2061
PB.2335.1	10	803	4	full-splice_match	gene: Zm00001d026578	Zm00001d026578_T002	150.77	1101.17	coding	111	97	429

PB.6621.4	5	2368	2	novel_in_catalog	gene: Zm00001d016105	novel	0.00	285.45	coding	159	37	513
PB.6621.5	5	1101	3	full-splice_match	gene: Zm00001d016105	Zm00001d016105_T001	191.66	285.45	coding	212	122	757
PB.1103.3	1	1030	7	full-splice_match	gene: Zm00001d032652	Zm00001d032652_T001	0.00	92.27	coding	222	63	728
PB.6621.1	5	2758	2	novel_in_catalog	gene: Zm00001d016105	novel	9.19	285.45	coding	159	248	724
PB.6621.2	5	1347	3	full-splice_match	gene: Zm00001d016105	Zm00001d016105_T001	7.31	285.45	coding	212	105	740
PB.6621.3	5	1283	3	full-splice_match	gene: Zm00001d016105	Zm00001d016105_T001	77.28	285.45	coding	212	179	814
PB.5966.1	5	1008	7	novel_in_catalog	gene: Zm00001d013007	novel	383.21	825.83	coding	181	187	729
PB.5966.3	5	873	7	novel_in_catalog	gene: Zm00001d013007	novel	429.10	825.83	coding	181	130	672
PB.5966.2	5	1048	6	novel_in_catalog	gene: Zm00001d013007	novel	13.52	825.83	coding	183	133	681
PB.3264.2	2	840	1	full-splice_match	gene: Zm00001d006182	Zm00001d006182_T001	0.00	18.73	coding	240	119	838
PB.8612.1	7	1059	1	full-splice_match	gene: Zm00001d021026	Zm00001d021026_T001	6.95	68.08	coding	94	146	427
PB.8612.2	7	1910	1	full-splice_match	gene: Zm00001d021026	Zm00001d021026_T001	61.13	68.08	coding	487	112	1572
PB.3912.1	3	2490	3	full-splice_match	gene: Zm00001d040513	Zm00001d040513_T001	3.69	3.69	coding	613	62	1900
PB.6275.3	5	2510	6	full-splice_match	gene: Zm00001d014321	Zm00001d014321_T003	0.00	34.04	coding	780	49	2388
PB.6275.2	5	2711	4	incomplete-splice_match	gene: Zm00001d014321	Zm00001d014321_T006	5.04	34.04	coding	152	2091	2546
PB.6275.1	5	2606	6	full-splice_match	gene: Zm00001d014321	Zm00001d014321_T003	29.01	34.04	coding	780	42	2381
PB.10257.2	9	427	1	intergenic	novelGene_107	novel	22.13	22.13	non_coding	NA	NA	NA

PB.4114.5	3	3900	2	novel_in_catalog	gene: Zm00001d041857	novel	2.27	32.12	coding	319	43	999
PB.4114.4	3	2222	3	full-splice_match	gene: Zm00001d041857	Zm00001d041857_T001	3.78	32.12	coding	639	21	1937
PB.8000.1	6	1670	14	novel_not_in_catalog	gene: Zm00001d038734	novel	13.65	22.67	coding	486	183	1640
PB.7038.1	5	964	1	full-splice_match	gene: Zm00001d018017	Zm00001d018017_T001	84.64	84.64	coding	179	115	651
PB.4114.3	3	1035	3	novel_not_in_catalog	gene: Zm00001d041857	novel	22.16	32.12	coding	182	190	735
PB.4114.2	3	2431	2	novel_in_catalog	gene: Zm00001d041857	novel	3.91	32.12	coding	182	1593	2138
PB.4463.1	3	1723	4	full-splice_match	gene: Zm00001d043414	Zm00001d043414_T001	40.22	40.22	coding	397	237	1427
PB.6069.2	5	2307	15	full-splice_match	gene: Zm00001d013358	Zm00001d013358_T010	15.28	15.28	coding	634	176	2077
PB.8629.6	7	2190	10	intergenic	novelGene_231	novel	6.08	54.18	coding	287	1161	2021
PB.9060.1	8	1591	1	full-splice_match	gene: Zm00001d008187	Zm00001d008187_T001	26.70	26.70	coding	458	73	1446
PB.1584.2	1	819	1	full-splice_match	gene: Zm00001d034756	Zm00001d034756_T001	297.86	304.16	non_coding	NA	NA	NA
PB.3543.1	2	2230	1	intergenic	novelGene_357	novel	12.69	12.69	non_coding	NA	NA	NA
PB.8445.1	7	1435	6	full-splice_match	gene: Zm00001d020297	Zm00001d020297_T002	74.46	111.51	coding	338	125	1138
PB.6366.2	5	2012	13	full-splice_match	gene: Zm00001d014715	Zm00001d014715_T001	7.05	49.83	coding	426	554	1831
PB.9215.1	8	1875	1	full-splice_match	gene: Zm00001d008901	Zm00001d008901_T001	11.15	11.15	coding	453	202	1560
PB.4354.1	3	2774	1	full-splice_match	gene: Zm00001d042953	Zm00001d042953_T001	11.82	33.34	coding	761	211	2493
PB.6366.1	5	1680	13	full-splice_match	gene: Zm00001d014715	Zm00001d014715_T001	42.78	49.83	coding	426	81	1358

PB.10247.1	9	3015	14	incomplete-splice_match	gene: Zm00001d045783	Zm00001d045783_T048	81.50	81.50	coding	889	6	2672
PB.8824.4	7	1790	7	genic	gene: Zm00001d021845	novel	0.00	43.34	coding	151	136	588
PB.5992.1	5	2380	14	incomplete-splice_match	gene: Zm00001d013083	Zm00001d013083_T014	31.41	36.48	coding	630	11	1900
PB.5992.2	5	5065	21	novel_in_catalog	gene: Zm00001d013083	novel	3.14	36.48	coding	483	129	1577
PB.5992.3	5	3009	20	incomplete-splice_match	gene: Zm00001d013083	Zm00001d013083_T017	1.92	36.48	coding	823	166	2634
PB.10608.1	9	1076	2	full-splice_match	gene: Zm00001d047781	Zm00001d047781_T001	61.29	61.29	coding	306	55	972
PB.8727.1	7	1541	1	full-splice_match	gene: Zm00001d021544	Zm00001d021544_T001	15.56	15.56	coding	339	59	1075
PB.1276.2	1	1068	5	intergenic	novelGene_371	novel	7.89	35.58	coding	145	25	459
PB.5360.1	4	1746	1	genic	gene: Zm00001d051839	novel	14.31	27.80	coding	300	76	975
PB.5360.2	4	3186	1	incomplete-splice_match	gene: Zm00001d051839	Zm00001d051839_T015	13.48	27.80	coding	915	149	2893
PB.2985.2	2	1784	7	full-splice_match	gene: Zm00001d004651	Zm00001d004651_T001	1.76	29.88	coding	443	166	1494
PB.8890.2	7	1731	5	novel_in_catalog	gene: Zm00001d022082	novel	0.39	31.16	coding	223	98	766
PB.5479.1	4	3704	7	full-splice_match	gene: Zm00001d052314	Zm00001d052314_T003	44.24	76.52	coding	1056	241	3408
PB.1523.1	1	2221	13	full-splice_match	gene: Zm00001d034565	Zm00001d034565_T004	32.59	32.59	coding	611	200	2032
PB.6735.1	5	1458	2	full-splice_match	gene: Zm00001d016744	Zm00001d016744_T001	13.34	13.34	coding	118	806	1159
PB.1620.3	1	1175	6	full-splice_match	gene: Zm00001d034871	Zm00001d034871_T001	34.56	117.03	coding	249	134	880
PB.8676.2	7	3090	7	full-splice_match	gene: Zm00001d021347	Zm00001d021347_T003	0.00	36.50	coding	925	195	2969

PB.3363.4	2	1987	11	incomplete-splice_match	gene: Zm00001d006602	Zm00001d006602_T002	84.11	100.06	coding	473	182	1600
PB.8676.1	7	3172	7	full-splice_match	gene: Zm00001d021347	Zm00001d021347_T003	36.50	36.50	coding	925	129	2903
PB.5914.1	5	1614	6	full-splice_match	gene: Zm00001d012825	Zm00001d012825_T001	31.76	31.76	coding	443	53	1381
PB.3895.1	3	1258	6	full-splice_match	gene: Zm00001d040409	Zm00001d040409_T001	19.93	19.93	coding	280	19	858
PB.3895.2	3	1031	6	full-splice_match	gene: Zm00001d040409	Zm00001d040409_T001	0.00	19.93	coding	58	587	760
PB.3436.1	2	1527	6	full-splice_match	gene: Zm00001d006928	Zm00001d006928_T001	24.32	24.32	coding	255	225	989
PB.3436.3	2	1302	6	full-splice_match	gene: Zm00001d006928	Zm00001d006928_T001	0.00	24.32	coding	255	221	985
PB.10460.1	9	2421	9	novel_in_catalog	gene: Zm00001d047162	novel	4.69	4.69	coding	659	72	2048
PB.3961.3	3	3662	25	full-splice_match	gene: Zm00001d040831	Zm00001d040831_T002	11.05	84.83	coding	1106	101	3418
PB.1524.3	1	2125	3	novel_in_catalog	gene: Zm00001d034568	novel	0.95	20.21	coding	288	265	1128
PB.3803.1	3	1122	2	novel_not_in_catalog	gene: Zm00001d039982	novel	10.19	10.19	coding	209	194	820
PB.8227.1	7	1580	2	full-splice_match	gene: Zm00001d018916	Zm00001d018916_T001	8.86	8.86	coding	405	60	1274
PB.6341.2	5	1679	1	genic	gene: Zm00001d014611	novel	5.65	165.59	coding	104	1148	1459
PB.6639.2	5	1905	1	full-splice_match	gene: Zm00001d016199	Zm00001d016199_T001	0.00	17.79	coding	561	26	1708
PB.10235.2	9	557	2	full-splice_match	gene: Zm00001d045685	Zm00001d045685_T001	24.41	142.98	coding	114	86	427
PB.10235.1	9	779	2	full-splice_match	gene: Zm00001d045685	Zm00001d045685_T001	118.57	142.98	coding	114	86	427
PB.10824.1	9	2017	12	novel_in_catalog	gene: Zm00001d048529	novel	49.53	137.46	coding	399	172	1368

PB.3044.2	2	2194	2	full-splice_match	gene: Zm00001d005029	Zm00001d005029_T001	24.50	26.71	coding	607	72	1892
PB.799.1	1	1908	1	full-splice_match	gene: Zm00001d031221	Zm00001d031221_T001	42.01	42.01	coding	529	88	1674
PB.6700.1	5	577	1	full-splice_match	gene: Zm00001d016568	Zm00001d016568_T001	375.31	375.31	non_coding	NA	NA	NA
PB.5625.1	4	2064	2	full-splice_match	gene: Zm00001d052889	Zm00001d052889_T001	7.15	7.15	coding	602	92	1897
PB.485.2	1	403	2	incomplete-splice_match	gene: Zm00001d029361	Zm00001d029361_T001	0.00	138.93	non_coding	NA	NA	NA
PB.485.1	1	561	2	novel_in_catalog	gene: Zm00001d029361	novel	138.93	138.93	non_coding	NA	NA	NA
PB.3044.3	2	3007	1	novel_in_catalog	gene: Zm00001d005029	novel	0.00	26.71	coding	202	691	1296
PB.6758.5	5	1364	5	full-splice_match	gene: Zm00001d016842	Zm00001d016842_T001	3.21	32.55	coding	191	641	1213
PB.10373.1	9	3286	9	full-splice_match	gene: Zm00001d046630	Zm00001d046630_T003	11.44	30.22	coding	969	59	2965
PB.10373.3	9	2021	4	incomplete-splice_match	gene: Zm00001d046630	Zm00001d046630_T015	18.78	30.22	coding	113	346	684
PB.510.1	1	1538	1	intergenic	novelGene_134	novel	7.58	96.53	coding	124	990	1361
PB.10373.5	9	1599	6	full-splice_match	gene: Zm00001d046630	Zm00001d046630_T015	0.00	30.22	coding	457	228	1598
PB.6758.2	5	1501	4	full-splice_match	gene: Zm00001d016842	Zm00001d016842_T002	29.34	32.55	coding	288	417	1280
PB.3044.1	2	3342	1	genic	gene: Zm00001d005029	novel	2.21	26.71	coding	485	73	1527
PB.7894.1	6	2234	4	novel_in_catalog	gene: Zm00001d038292	novel	3.36	17.85	coding	343	148	1176
PB.1124.1	1	2102	3	full-splice_match	gene: Zm00001d032740	Zm00001d032740_T001	6.66	6.66	coding	606	133	1950
PB.7894.2	6	1675	5	novel_in_catalog	gene: Zm00001d038292	novel	14.49	17.85	coding	336	204	1211

PB.10052.1	9	2272	1	genic	gene: Zm00001d044825	novel	5.98	5.98	coding	683	42	2090
PB.110.1	1	960	5	full-splice_match	gene: Zm00001d027680	Zm00001d027680_T002	19.63	788.10	coding	158	69	542
PB.110.2	1	892	5	full-splice_match	gene: Zm00001d027680	Zm00001d027680_T002	768.47	788.10	coding	158	114	587
PB.1802.1	10	2192	2	genic	gene: Zm00001d023974	novel	10.21	10.21	coding	531	332	1924
PB.1524.1	1	2090	4	full-splice_match	gene: Zm00001d034568	Zm00001d034568_T005	17.42	20.21	coding	467	265	1665
PB.4336.3	3	1939	1	incomplete-splice_match	gene: Zm00001d042869	Zm00001d042869_T003	6.78	231.40	non_coding	NA	NA	NA
PB.2457.1	2	1693	3	full-splice_match	gene: Zm00001d002020	Zm00001d002020_T004	9.34	44.58	coding	446	118	1455
PB.2457.2	2	1566	3	full-splice_match	gene: Zm00001d002020	Zm00001d002020_T004	35.24	44.58	coding	446	174	1511
PB.35.1	1	2280	3	full-splice_match	gene: Zm00001d027349	Zm00001d027349_T001	3.80	3.80	coding	485	46	1500
PB.7255.1	6	1916	6	novel_in_catalog	gene: Zm00001d035185	novel	13.04	13.04	coding	411	252	1484
PB.995.1	1	2810	13	full-splice_match	gene: Zm00001d032245	Zm00001d032245_T003	8.05	8.05	coding	862	34	2619
PB.3444.2	2	2538	20	full-splice_match	gene: Zm00001d007009	Zm00001d007009_T001	24.85	24.85	coding	678	88	2121
PB.3444.3	2	2331	20	full-splice_match	gene: Zm00001d007009	Zm00001d007009_T001	0.00	24.85	coding	678	56	2089
PB.100.5	1	1899	12	full-splice_match	gene: Zm00001d027626	Zm00001d027626_T005	2.17	39.01	coding	568	83	1786
PB.100.4	1	2295	11	novel_in_catalog	gene: Zm00001d027626	novel	0.00	39.01	coding	406	937	2154
PB.100.3	1	2842	9	novel_in_catalog	gene: Zm00001d027626	novel	1.85	39.01	coding	406	1440	2657
PB.100.1	1	1980	12	full-splice_match	gene: Zm00001d027626	Zm00001d027626_T005	35.00	39.01	coding	568	63	1766

PB.10528.2	9	2224	9	full-splice_match	gene: Zm00001d047497	Zm00001d047497_T003	0.00	17.62	coding	484	422	1873
PB.8574.1	7	904	1	intergenic	novelGene_216	novel	0.00	0.00	non_coding	NA	NA	NA
PB.10528.1	9	2369	9	full-splice_match	gene: Zm00001d047497	Zm00001d047497_T003	17.62	17.62	coding	484	414	1865
PB.6077.1	5	1003	1	novel_in_catalog	gene: Zm00001d013380	novel	31.78	31.78	coding	269	114	920
PB.1992.1	10	4641	20	full-splice_match	gene: Zm00001d025012	Zm00001d025012_T001	23.19	23.53	coding	1464	55	4446
PB.3279.5	2	753	1	genic	gene: Zm00001d006249	novel	49.62	49.62	coding	147	62	502
PB.10576.1	9	1487	5	incomplete-splice_match	gene: Zm00001d047677	Zm00001d047677_T017	46.69	56.67	coding	393	39	1217
PB.9503.1	8	4216	14	full-splice_match	gene: Zm00001d010470	Zm00001d010470_T016	13.75	13.75	coding	1273	47	3865
PB.5867.1	4	2341	5	novel_in_catalog	gene: Zm00001d053987	novel	17.93	17.93	coding	486	574	2031
PB.2805.1	2	2131	3	full-splice_match	gene: Zm00001d003469	Zm00001d003469_T001	0.78	5.03	coding	513	43	1581
PB.2805.2	2	1932	3	full-splice_match	gene: Zm00001d003469	Zm00001d003469_T001	4.25	5.03	coding	513	146	1684
PB.1845.1	10	1130	1	genic	gene: Zm00001d024317	novel	29.04	29.04	coding	288	100	963
PB.5936.1	5	1679	6	novel_in_catalog	gene: Zm00001d012896	novel	32.48	32.48	coding	423	144	1412
PB.9207.1	8	1242	1	full-splice_match	gene: Zm00001d008866	Zm00001d008866_T001	19.52	19.52	coding	250	148	897
PB.1436.2	1	474	3	full-splice_match	gene: Zm00001d034223	Zm00001d034223_T001	525.07	574.01	non_coding	NA	NA	NA
PB.6356.1	5	2113	11	full-splice_match	gene: Zm00001d014668	Zm00001d014668_T001	40.83	40.83	coding	372	486	1601
PB.9386.4	8	2166	10	novel_in_catalog	gene: Zm00001d009883	novel	12.75	40.14	coding	104	1110	1421

PB.5639.4	4	2078	7	full-splice_match	gene: Zm00001d052944	Zm00001d052944_T001	0.00	37.40	coding	528	262	1845
PB.5639.5	4	1797	7	full-splice_match	gene: Zm00001d052944	Zm00001d052944_T001	0.00	37.40	coding	528	99	1682
PB.9386.1	8	1907	7	full-splice_match	gene: Zm00001d009883	Zm00001d009883_T085	0.00	40.14	coding	104	835	1146
PB.9386.3	8	3502	9	novel_in_catalog	gene: Zm00001d009883	novel	13.10	40.14	coding	104	2444	2755
PB.9386.2	8	1781	8	full-splice_match	gene: Zm00001d009883	Zm00001d009883_T079	14.30	40.14	coding	104	770	1081
PB.3379.1	2	1766	1	genic	gene: Zm00001d006683	novel	3.99	21.87	coding	302	654	1559
PB.9174.1	8	946	1	full-splice_match	gene: Zm00001d008722	Zm00001d008722_T001	10.59	10.59	coding	121	271	633
PB.1313.1	1	2408	9	full-splice_match	gene: Zm00001d033655	Zm00001d033655_T003	24.31	32.74	coding	633	128	2026
PB.3379.2	2	1665	2	full-splice_match	gene: Zm00001d006683	Zm00001d006683_T001	17.87	21.87	coding	474	37	1458
PB.3379.4	2	1500	2	full-splice_match	gene: Zm00001d006683	Zm00001d006683_T001	0.00	21.87	coding	474	37	1458
PB.10363.1	9	773	6	full-splice_match	gene: Zm00001d046583	Zm00001d046583_T002	531.93	531.93	coding	150	141	590
PB.1859.2	10	2259	1	full-splice_match	gene: Zm00001d024416	Zm00001d024416_T001	38.77	38.77	coding	637	140	2050
PB.5619.1	4	2573	3	full-splice_match	gene: Zm00001d052855	Zm00001d052855_T001	252.68	585.42	coding	699	95	2191
PB.2819.1	2	2792	7	novel_in_catalog	gene: Zm00001d003533	novel	16.03	16.03	coding	624	621	2492
PB.435.1	1	1017	1	genic	gene: Zm00001d029130	novel	6.78	6.78	non_coding	NA	NA	NA
PB.5619.5	4	2290	3	full-splice_match	gene: Zm00001d052855	Zm00001d052855_T001	0.00	585.42	coding	699	96	2192
PB.10594.1	9	2347	7	full-splice_match	gene: Zm00001d047746	Zm00001d047746_T001	12.01	12.01	coding	659	201	2177

PB.6483.1	5	1398	10	genic	gene: Zm00001d001580	novel	38.90	38.90	coding	225	369	1043
PB.2433.1	2	1821	2	antisense	novelGene_gene: Zm00001d001924_A	novel	1.41	1.41	non_co ding	NA	NA	NA
PB.6881.1	5	1861	2	genic	gene: Zm00001d017378	novel	9.69	22.37	coding	255	197	961
PB.1258.1	1	2148	8	incomplete-splice_match	gene: Zm00001d033448	Zm00001d033448_T006	9.79	9.79	coding	614	10	1851
PB.5308.1	4	1023	9	novel_not_in_catalog	gene: Zm00001d051564	novel	68.22	68.22	coding	201	129	731
PB.3965.1	3	1042	7	novel_not_in_catalog	gene: Zm00001d040873	novel	5.57	5.57	coding	176	394	921
PB.7927.1	6	2931	18	fusion	gene: Zm00001d038405_g	novel	11.25	11.25	coding	806	134	2551
PB.318.1	1	1586	8	full-splice_match	gene: Zm00001d028616	Zm00001d028616_T001	31.59	31.59	coding	446	94	1431
PB.7367.1	6	2211	11	full-splice_match	gene: Zm00001d035854	Zm00001d035854_T001	58.34	58.34	coding	619	87	1943
PB.7367.2	6	2101	11	full-splice_match	gene: Zm00001d035854	Zm00001d035854_T001	0.00	58.34	coding	619	86	1942
PB.7367.3	6	1697	10	incomplete-splice_match	gene: Zm00001d035854	Zm00001d035854_T001	0.00	58.34	coding	537	87	1697
PB.5227.1	4	1099	8	full-splice_match	gene: Zm00001d051065	Zm00001d051065_T007	52.00	52.00	coding	242	70	795
PB.9662.2	8	981	5	full-splice_match	gene: Zm00001d011180	Zm00001d011180_T003	99.65	176.06	coding	176	117	644
PB.9662.3	8	895	5	full-splice_match	gene: Zm00001d011180	Zm00001d011180_T003	64.34	176.06	coding	176	143	670
PB.7473.1	6	801	1	full-splice_match	gene: Zm00001d036463	Zm00001d036463_T001	8.77	8.77	coding	147	54	494
PB.10512.1	9	1832	2	novel_in_catalog	gene: Zm00001d047437	novel	3.74	66.43	coding	253	765	1523
PB.10512.3	9	1939	1	genic	gene: Zm00001d047437	novel	0.00	66.43	coding	211	1005	1637

PB.10512.5	9	1113	1	novel_in_catalog	gene: Zm00001d047437	novel	8.69	66.43	coding	211	376	1008
PB.10512.4	9	1635	3	full-splice_match	gene: Zm00001d047437	Zm00001d047437_T001	54.00	66.43	coding	440	87	1406
PB.4404.1	3	696	3	full-splice_match	gene: Zm00001d043175	Zm00001d043175_T002	283.42	311.70	coding	135	1	405
PB.10807.1	9	1704	1	full-splice_match	gene: Zm00001d048486	Zm00001d048486_T001	73.32	73.32	coding	492	109	1584
PB.8121.1	6	2836	19	full-splice_match	gene: Zm00001d039165	Zm00001d039165_T005	19.23	72.08	coding	735	271	2475
PB.6368.1	5	1381	6	incomplete-splice_match	gene: Zm00001d014719	Zm00001d014719_T021	146.56	146.56	coding	288	185	1048
PB.8376.2	7	2941	12	full-splice_match	gene: Zm00001d019852	Zm00001d019852_T004	0.00	4.27	coding	962	56	2941
PB.8376.1	7	3219	12	full-splice_match	gene: Zm00001d019852	Zm00001d019852_T004	4.27	4.27	coding	979	56	2992
PB.1675.2	10	1881	3	novel_in_catalog	gene: Zm00001d023309	novel	0.00	55.81	coding	491	214	1686
PB.1851.3	10	1861	2	novel_not_in_catalog	gene: Zm00001d024365	novel	0.00	20.22	coding	468	262	1665
PB.1675.1	10	2075	3	novel_in_catalog	gene: Zm00001d023309	novel	55.81	55.81	coding	491	246	1718
PB.6486.2	5	1097	7	full-splice_match	gene: Zm00001d015134	Zm00001d015134_T001	185.63	391.08	coding	243	92	820
PB.8566.1	7	2260	3	full-splice_match	gene: Zm00001d020858	Zm00001d020858_T002	51.01	51.01	coding	577	279	2009
PB.8060.1	6	3918	14	full-splice_match	gene: Zm00001d038916	Zm00001d038916_T001	15.58	15.58	coding	1205	88	3702
PB.7377.1	6	2898	9	full-splice_match	gene: Zm00001d035913	Zm00001d035913_T004	5.72	5.72	coding	331	44	1036
PB.5353.10	4	1213	2	novel_in_catalog	gene: Zm00001d051804	novel	7.87	848.79	coding	100	401	700
PB.3040.3	2	2551	7	novel_in_catalog	gene: Zm00001d004986	novel	0.96	81.46	coding	279	798	1634

PB.17.1	1	4337	4	incomplete-splice_match	gene: Zm00001d027299	Zm00001d027299_T010	17.28	17.28	coding	1256	170	3937
PB.3936.1	3	2695	6	full-splice_match	gene: Zm00001d040670	Zm00001d040670_T007	12.37	12.37	coding	310	218	1147
PB.10495.1	9	2901	13	novel_in_catalog	gene: Zm00001d047367	novel	45.59	45.59	coding	227	457	1137
PB.9658.1	8	1062	8	full-splice_match	gene: Zm00001d011162	Zm00001d011162_T002	451.99	451.99	coding	203	303	911
PB.6009.1	5	1274	1	full-splice_match	gene: Zm00001d013173	Zm00001d013173_T001	28.03	28.03	coding	373	31	1149
PB.7629.6	6	1933	12	novel_not_in_catalog	gene: Zm00001d037257	novel	0.00	107.46	coding	441	290	1612
PB.7961.1	6	1295	7	full-splice_match	gene: Zm00001d038546	Zm00001d038546_T005	71.11	71.11	coding	257	156	926
PB.3040.1	2	2171	9	full-splice_match	gene: Zm00001d004986	Zm00001d004986_T002	66.46	81.46	coding	506	409	1926
PB.10066.1	9	1443	2	full-splice_match	gene: Zm00001d044908	Zm00001d044908_T001	7.01	22.09	coding	376	106	1233
PB.10066.3	9	1328	2	full-splice_match	gene: Zm00001d044908	Zm00001d044908_T001	15.08	22.09	coding	376	96	1223
PB.227.1	1	816	7	full-splice_match	gene: Zm00001d028232	Zm00001d028232_T004	46.69	46.69	coding	163	26	514
PB.3345.1	2	422	1	full-splice_match	gene: Zm00001d006526	Zm00001d006526_T001	1.76	1.76	non_coding	NA	NA	NA
PB.7811.1	6	2074	1	full-splice_match	gene: Zm00001d038030	Zm00001d038030_T001	10.44	12.17	coding	523	42	1610
PB.6969.1	5	3277	7	full-splice_match	gene: Zm00001d017780	Zm00001d017780_T001	26.77	26.77	coding	773	596	2914
PB.5464.3	4	2330	2	novel_in_catalog	gene: Zm00001d052259	novel	7.37	82.76	coding	115	114	458
PB.3172.1	2	1735	7	novel_not_in_catalog	gene: Zm00001d005726	novel	51.64	53.82	coding	391	201	1373
PB.3172.2	2	1532	7	novel_not_in_catalog	gene: Zm00001d005726	novel	2.19	53.82	coding	391	246	1418

PB.877.1	1	671	3	incomplete-splice_match	gene: Zm00001d031653	Zm00001d031653_T004	31.73	31.73	coding	152	1	456
PB.10564.1	9	1437	2	full-splice_match	gene: Zm00001d047638	Zm00001d047638_T001	86.52	86.52	coding	337	183	1193
PB.3190.1	2	1413	1	full-splice_match	gene: Zm00001d005811	Zm00001d005811_T001	5.77	10.32	coding	291	80	952
PB.3190.2	2	1188	1	full-splice_match	gene: Zm00001d005811	Zm00001d005811_T001	4.54	10.32	coding	291	80	952
PB.8328.1	7	1414	2	intergenic	novelGene_100	novel	126.63	126.63	non_coding	NA	NA	NA
PB.4322.1	3	2487	2	novel_in_catalog	gene: Zm00001d042807	novel	14.91	44.36	coding	358	1053	2126
PB.4322.2	3	2577	2	novel_in_catalog	gene: Zm00001d042807	novel	2.96	44.36	coding	358	1281	2354
PB.4322.3	3	1716	3	full-splice_match	gene: Zm00001d042807	Zm00001d042807_T001	24.13	44.36	coding	478	125	1558
PB.4322.4	3	5061	2	novel_in_catalog	gene: Zm00001d042807	novel	2.36	44.36	coding	358	3846	4919
PB.5237.3	4	2839	2	novel_in_catalog	gene: Zm00001d051161	novel	105.81	110.85	coding	520	123	1682
PB.8348.1	7	2341	3	full-splice_match	gene: Zm00001d019624	Zm00001d019624_T003	9.59	14.57	non_coding	NA	NA	NA
PB.237.1	1	1029	6	incomplete-splice_match	gene: Zm00001d028284	Zm00001d028284_T003	48.47	48.47	coding	253	61	819
PB.1447.3	1	1821	2	novel_not_in_catalog	gene: Zm00001d034301	novel	13.82	19.02	non_coding	NA	NA	NA
PB.8348.5	7	1798	5	novel_not_in_catalog	gene: Zm00001d019624	novel	4.98	14.57	non_coding	NA	NA	NA
PB.8348.6	7	1837	3	full-splice_match	gene: Zm00001d019624	Zm00001d019624_T003	0.00	14.57	non_coding	NA	NA	NA
PB.8348.7	7	959	2	incomplete-splice_match	gene: Zm00001d019624	Zm00001d019624_T001	0.00	14.57	non_coding	NA	NA	NA
PB.7009.16	5	359	2	intergenic	novelGene_327	novel	14.02	14.02	non_coding	NA	NA	NA

PB.8431.12	7	3331	6	full-splice_match	gene: Zm00001d020227	Zm00001d020227_T024	6.90	43.17	coding	201	541	1143
PB.7305.4	6	1219	2	novel_in_catalog	gene: Zm00001d035474	novel	4.35	86.92	coding	289	40	906
PB.1373.1	1	1791	9	full-splice_match	gene: Zm00001d033932	Zm00001d033932_T001	23.45	23.45	coding	489	81	1547
PB.7305.1	6	1242	2	novel_in_catalog	gene: Zm00001d035474	novel	19.49	86.92	coding	288	104	967
PB.10421.8	9	1238	6	full-splice_match	gene: Zm00001d046937	Zm00001d046937_T001	0.00	99.27	coding	282	141	986
PB.7305.3	6	1012	3	full-splice_match	gene: Zm00001d035474	Zm00001d035474_T001	63.08	86.92	coding	287	36	896
PB.10307.1	9	2074	18	full-splice_match	gene: Zm00001d046231	Zm00001d046231_T010	38.18	38.18	coding	519	64	1620
PB.10421.7	9	1371	6	full-splice_match	gene: Zm00001d046937	Zm00001d046937_T001	75.77	99.27	coding	282	140	985
PB.10421.4	9	1913	4	novel_in_catalog	gene: Zm00001d046937	novel	0.00	99.27	coding	156	953	1420
PB.10421.5	9	2005	3	novel_in_catalog	gene: Zm00001d046937	novel	3.43	99.27	coding	143	955	1383
PB.9418.3	8	2368	11	full-splice_match	gene: Zm00001d010050	Zm00001d010050_T002	6.60	32.36	coding	721	73	2235
PB.9418.2	8	2126	9	novel_in_catalog	gene: Zm00001d010050	novel	6.49	32.36	coding	311	54	986
PB.9418.1	8	2473	11	full-splice_match	gene: Zm00001d010050	Zm00001d010050_T002	19.27	32.36	coding	721	46	2208
PB.10421.1	9	2350	5	novel_in_catalog	gene: Zm00001d046937	novel	0.84	99.27	coding	128	71	454
PB.8927.1	7	1319	9	full-splice_match	gene: Zm00001d022231	Zm00001d022231_T001	30.79	30.79	coding	297	157	1047
PB.5464.1	4	1974	4	novel_in_catalog	gene: Zm00001d052259	novel	55.79	82.76	coding	507	237	1757
PB.1414.1	1	1998	2	genic	gene: Zm00001d034104	novel	5.12	5.12	coding	520	94	1653

PB.7063.5	5	1591	6	full-splice_match	gene: Zm00001d018128	Zm00001d018128_T001	0.00	72.50	coding	355	330	1394
PB.7063.4	5	1788	6	full-splice_match	gene: Zm00001d018128	Zm00001d018128_T001	0.00	72.50	coding	355	335	1399
PB.3198.1	2	1765	1	genic	gene: Zm00001d005834	novel	4.08	20.05	coding	244	571	1302
PB.3198.2	2	1481	1	genic	gene: Zm00001d005834	novel	15.97	20.05	coding	244	576	1307
PB.3757.1	3	2955	13	full-splice_match	gene: Zm00001d039729	Zm00001d039729_T005	17.68	18.33	coding	773	394	2712
PB.7122.2	5	1571	7	full-splice_match	gene: Zm00001d018341	Zm00001d018341_T001	8.79	84.75	coding	173	179	697
PB.3757.3	3	2097	12	incomplete-splice_match	gene: Zm00001d039729	Zm00001d039729_T003	0.65	18.33	coding	621	234	2096
PB.3757.2	3	2708	14	full-splice_match	gene: Zm00001d039729	Zm00001d039729_T003	0.00	18.33	coding	773	230	2548
PB.10401.1	9	2111	9	novel_not_in_catalog	gene: Zm00001d046801	novel	7.86	31.05	coding	539	149	1765
PB.10401.2	9	1986	10	novel_not_in_catalog	gene: Zm00001d046801	novel	21.72	31.05	coding	533	159	1757
PB.10401.3	9	1012	6	novel_not_in_catalog	gene: Zm00001d046801	novel	1.47	31.05	coding	204	77	688
PB.8839.3	7	1923	15	full-splice_match	gene: Zm00001d021893	Zm00001d021893_T004	39.34	92.44	coding	570	31	1740
PB.8839.2	7	3751	19	full-splice_match	gene: Zm00001d021893	Zm00001d021893_T002	0.00	92.44	coding	1126	73	3450
PB.8839.1	7	3223	19	full-splice_match	gene: Zm00001d021893	Zm00001d021893_T002	40.77	92.44	coding	891	124	2796
PB.6999.1	5	1346	4	incomplete-splice_match	gene: Zm00001d017883	Zm00001d017883_T031	15.70	91.76	coding	164	167	658
PB.8839.4	7	1200	1	incomplete-splice_match	gene: Zm00001d021893	Zm00001d021893_T002	12.33	92.44	coding	357	129	1199
PB.9281.3	8	2014	6	full-splice_match	gene: Zm00001d009271	Zm00001d009271_T001	12.01	78.80	coding	561	145	1827

PB.9281.2	8	2134	6	full-splice_match	gene: Zm00001d009271	Zm00001d009271_T001	66.80	78.80	coding	561	162	1844
PB.1234.5	1	1934	1	novel_in_catalog	gene: Zm00001d033320	novel	0.00	21.30	coding	163	97	585
PB.10317.1	9	1679	3	genic	gene: Zm00001d046332	novel	9.02	23.89	coding	264	454	1245
PB.5522.1	4	1797	13	full-splice_match	gene: Zm00001d052439	Zm00001d052439_T003	19.88	19.88	coding	475	148	1572
PB.1234.2	1	1740	2	full-splice_match	gene: Zm00001d033320	Zm00001d033320_T002	14.15	21.30	coding	461	99	1481
PB.10317.4	9	1413	3	genic	gene: Zm00001d046332	novel	0.00	23.89	coding	264	451	1242
PB.7953.2	6	951	7	novel_not_in_catalog	gene: Zm00001d038533	novel	21.02	30.57	coding	184	114	665
PB.7800.2	6	3219	20	full-splice_match	gene: Zm00001d037982	Zm00001d037982_T001	4.98	13.32	coding	869	104	2710
PB.1356.1	1	1592	2	incomplete-splice_match	gene: Zm00001d033862	Zm00001d033862_T001	5.85	5.85	coding	347	73	1113
PB.867.1	1	1196	4	full-splice_match	gene: Zm00001d031618	Zm00001d031618_T001	15.59	15.59	coding	250	107	856
PB.3291.1	2	2045	10	novel_not_in_catalog	gene: Zm00001d006295	novel	20.42	20.42	coding	581	43	1785
PB.7481.1	6	2077	8	novel_not_in_catalog	gene: Zm00001d036532	novel	2.76	17.24	coding	567	106	1806
PB.6598.1	5	690	1	full-splice_match	gene: Zm00001d015918	Zm00001d015918_T001	74.31	74.31	coding	127	56	436
PB.2898.7	2	2899	5	novel_in_catalog	gene: Zm00001d003939	novel	2.75	30.14	coding	241	1954	2676
PB.2898.4	2	1827	7	novel_not_in_catalog	gene: Zm00001d003939	novel	1.62	30.14	coding	140	1259	1678
PB.2898.5	2	3358	12	full-splice_match	gene: Zm00001d003939	Zm00001d003939_T005	4.48	30.14	coding	252	2472	3227
PB.245.1	1	1840	8	full-splice_match	gene: Zm00001d028325	Zm00001d028325_T005	8.60	8.60	coding	508	60	1583

PB.2898.3	2	1915	5	novel_in_catalog	gene: Zm00001d003939	novel	2.77	30.14	coding	140	1347	1766
PB.245.3	1	1700	8	full-splice_match	gene: Zm00001d028325	Zm00001d028325_T005	0.00	8.60	coding	508	60	1583
PB.2898.1	2	1664	8	novel_not_in_catalog	gene: Zm00001d003939	novel	10.41	30.14	coding	252	730	1485
PB.5148.1	4	1036	9	full-splice_match	gene: Zm00001d050440	Zm00001d050440_T003	91.51	91.51	coding	220	181	840
PB.7266.1	6	3917	4	full-splice_match	gene: Zm00001d035228	Zm00001d035228_T001	5.32	14.49	coding	1115	52	3396
PB.7266.2	6	2350	4	full-splice_match	gene: Zm00001d035228	Zm00001d035228_T001	9.17	14.49	coding	664	112	2103
PB.5109.2	4	882	8	incomplete-splice_match	gene: Zm00001d050216	Zm00001d050216_T003	26.23	38.71	coding	201	7	609
PB.2898.8	2	2545	7	novel_not_in_catalog	gene: Zm00001d003939	novel	1.48	30.14	coding	368	1262	2365
PB.2898.9	2	1351	3	incomplete-splice_match	gene: Zm00001d003939	Zm00001d003939_T016	1.72	30.14	coding	327	219	1199
PB.1269.4	1	648	2	genic	gene: Zm00001d033483	novel	856.43	876.45	coding	100	95	394
PB.2923.4	2	2091	10	full-splice_match	gene: Zm00001d004119	Zm00001d004119_T003	25.98	78.20	coding	685	31	2085
PB.9946.1	8	1592	4	full-splice_match	gene: Zm00001d012456	Zm00001d012456_T001	3.64	3.64	coding	386	160	1317
PB.7481.4	6	1943	8	novel_not_in_catalog	gene: Zm00001d036532	novel	2.16	17.24	coding	567	197	1897
PB.2923.1	2	2592	10	full-splice_match	gene: Zm00001d004119	Zm00001d004119_T003	52.22	78.20	coding	685	246	2300
PB.10409.1	9	1331	7	novel_not_in_catalog	gene: Zm00001d046890	novel	86.68	86.68	coding	252	336	1091
PB.2255.3	10	1332	10	full-splice_match	gene: Zm00001d026302	Zm00001d026302_T003	0.00	29.94	coding	331	54	1046
PB.2923.2	2	2509	10	full-splice_match	gene: Zm00001d004119	Zm00001d004119_T003	0.00	78.20	coding	685	308	2362

PB.570.2	1	863	1	incomplete-splice_match	gene: Zm00001d029754	Zm00001d029754_T010	22.75	22.75	coding	66	560	757
PB.5255.1	4	1031	8	full-splice_match	gene: Zm00001d051288	Zm00001d051288_T001	22.09	22.09	coding	225	150	824
PB.7073.4	5	2010	4	full-splice_match	gene: Zm00001d018161	Zm00001d018161_T002	86.58	110.52	coding	593	66	1844
PB.7073.5	5	2189	2	novel_in_catalog	gene: Zm00001d018161	novel	8.56	110.52	coding	256	1264	2031
PB.7073.6	5	2106	3	novel_in_catalog	gene: Zm00001d018161	novel	1.88	110.52	coding	368	66	1169
PB.2574.1	2	2420	1	full-splice_match	gene: Zm00001d002536	Zm00001d002536_T001	1.57	2.52	coding	691	101	2173
PB.7073.1	5	2215	4	full-splice_match	gene: Zm00001d018161	Zm00001d018161_T002	13.51	110.52	coding	593	64	1842
PB.8266.3	7	1002	3	genic	gene: Zm00001d019142	novel	44.03	115.31	coding	253	44	802
PB.2574.2	2	2357	1	full-splice_match	gene: Zm00001d002536	Zm00001d002536_T001	0.95	2.52	coding	691	190	2262
PB.7284.5	6	3391	2	intergenic	novelGene_67	novel	2.74	20.78	coding	420	1923	3182
PB.10415.1	9	1902	10	novel_not_in_catalog	gene: Zm00001d046910	novel	2.42	2.42	coding	522	41	1606
PB.10814.1	9	1363	1	full-splice_match	gene: Zm00001d048508	Zm00001d048508_T001	0.00	14.21	coding	171	73	585
PB.1397.1	1	1635	3	novel_not_in_catalog	gene: Zm00001d034032	novel	27.37	27.37	coding	398	167	1360
PB.6420.2	5	1733	11	novel_in_catalog	gene: Zm00001d014896	novel	10.00	24.30	coding	268	203	1006
PB.10286.1	9	1717	4	full-splice_match	gene: Zm00001d046093	Zm00001d046093_T001	6.91	6.91	coding	486	22	1479
PB.3861.4	3	2190	14	incomplete-splice_match	gene: Zm00001d040279	Zm00001d040279_T006	58.74	83.10	coding	651	68	2020
PB.1214.3	1	2106	1	novel_in_catalog	gene: Zm00001d033225	novel	0.68	44.32	coding	310	1014	1943

PB.1214.2	1	1630	4	full-splice_match	gene: Zm00001d033225	Zm00001d033225_T002	22.13	44.32	coding	259	636	1412	
PB.1214.4	1	1370	4	full-splice_match	gene: Zm00001d033225	Zm00001d033225_T002	0.00	44.32	coding	383	125	1273	
PB.3861.2	3	3861	25	full-splice_match	gene: Zm00001d040279	Zm00001d040279_T012	24.37	83.10	coding	1125	181	3555	
PB.2429.2	2	2282	3	novel_not_in_catalog	gene: Zm00001d001904	novel	0.06	5.68	coding	93	129	407	
PB.2429.3	2	1138	2	novel_in_catalog	gene: Zm00001d001904	novel	0.00	5.68	non_coding	NA	NA	NA	
PB.124.13	1	2801	9	novel_in_catalog	gene: Zm00001d027731	novel	55.39	562.10	coding	687	311	2371	
PB.2429.1	2	2642	1	genic	gene: Zm00001d001904	novel	5.62	5.68	non_coding	NA	NA	NA	
PB.1299.6	1	2207	1	novel_in_catalog	gene: Zm00001d033611	novel	1.65	86.53	coding	175	1487	2011	
PB.124.17	1	2671	9	full-splice_match	gene: Zm00001d027731	Zm00001d027731_T007	71.53	562.10	coding	690	311	2380	
PB.7562.2	6	1482	5	novel_not_in_catalog	gene: Zm00001d036965	novel	0.20	13.33	coding	324	70	1041	
PB.7562.1	6	1558	5	full-splice_match	gene: Zm00001d036965	Zm00001d036965_T003	13.13	13.33	coding	408	68	1291	
PB.8529.13	7	1719	7	incomplete-splice_match	gene: Zm00001d020685	Zm00001d020685_T003	50.50	208.26	coding	378	586	1719	
PB.8529.12	7	1993	10	incomplete-splice_match	gene: Zm00001d020685	Zm00001d020685_T003	0.00	208.26	coding	470	583	1992	
PB.7800.1	6	2024	9	incomplete-splice_match	gene: Zm00001d037982	Zm00001d037982_T001	4.37	13.32	coding	227	99	779	
PB.5229.1	4	4456	10	full-splice_match	gene: Zm00001d051075	Zm00001d051075_T002	11.65	11.65	coding	1345	9	4043	
PB.3954.5	3	1610	2	incomplete-splice_match	gene: Zm00001d040795	Zm00001d040795_T001	0.00	0.00	coding	504	98	1609	
PB.459.1	1	2205	14	full-splice_match	gene: Zm00001d029227	Zm00001d029227_T001	13.34	54.78	coding	566	106	1803	

PB.459.2	1	2243	14	full-splice_match	gene: Zm00001d029227	Zm00001d029227_T001	28.35	54.78	coding	566	247	1944
PB.459.3	1	2062	14	full-splice_match	gene: Zm00001d029227	Zm00001d029227_T002	13.09	54.78	coding	564	103	1794
PB.4068.1	3	1740	12	full-splice_match	gene: Zm00001d041627	Zm00001d041627_T001	31.87	32.32	coding	407	277	1497
PB.459.5	1	2122	14	full-splice_match	gene: Zm00001d029227	Zm00001d029227_T001	0.00	54.78	coding	566	243	1940
PB.4068.3	3	1629	11	novel_in_catalog	gene: Zm00001d041627	novel	0.45	32.32	coding	321	233	1195
PB.4068.2	3	1568	12	full-splice_match	gene: Zm00001d041627	Zm00001d041627_T001	0.00	32.32	coding	407	234	1454
PB.10829.3	9	2402	18	incomplete-splice_match	gene: Zm00001d048546	Zm00001d048546_T047	27.83	41.83	coding	693	70	2148
PB.1466.3	1	3342	6	novel_in_catalog	gene: Zm00001d034350	novel	11.03	23.85	coding	616	1184	3031
PB.7202.1	5	2215	10	novel_not_in_catalog	gene: Zm00001d018565	novel	79.22	79.22	coding	638	78	1991
PB.7475.9	6	1709	1	full-splice_match	gene: Zm00001d001390	Zm00001d001390_T001	0.00	0.00	non_coding	NA	NA	NA
PB.4987.1	4	1887	8	full-splice_match	gene: Zm00001d049572	Zm00001d049572_T003	23.55	23.55	coding	470	270	1679
PB.378.1	1	2256	8	novel_not_in_catalog	gene: Zm00001d028901	novel	2.96	2.96	coding	610	83	1912
PB.7475.4	6	3103	6	full-splice_match	gene: Zm00001d036481	Zm00001d036481_T004	3.22	373.04	coding	183	2278	2826
PB.7475.5	6	1004	7	full-splice_match	gene: Zm00001d036481	Zm00001d036481_T002	124.79	373.04	coding	183	209	757
PB.7475.2	6	1092	7	full-splice_match	gene: Zm00001d036481	Zm00001d036481_T002	201.44	373.04	coding	183	174	722
PB.7475.3	6	1484	8	novel_not_in_catalog	gene: Zm00001d036481	novel	7.42	373.04	coding	183	657	1205
PB.7475.1	6	2323	5	novel_in_catalog	gene: Zm00001d036481	novel	12.03	373.04	coding	183	1358	1906

PB.841.3	1	1047	2	incomplete-splice_match	gene: Zm00001d031470	Zm00001d031470_T001	27.81	51.16	coding	95	156	440
PB.841.2	1	2627	2	novel_in_catalog	gene: Zm00001d031470	novel	4.29	51.16	coding	570	60	1769
PB.841.1	1	2523	3	full-splice_match	gene: Zm00001d031470	Zm00001d031470_T001	19.05	51.16	coding	577	55	1785
PB.4520.15	3	3176	11	full-splice_match	gene: Zm00001d043613	Zm00001d043613_T002	0.00	284.34	coding	300	1972	2871
PB.8433.1	7	873	3	novel_not_in_catalog	gene: Zm00001d020236	novel	68.99	68.99	coding	128	159	542
PB.4520.13	3	2902	13	full-splice_match	gene: Zm00001d043613	Zm00001d043613_T008	0.00	284.34	coding	300	1695	2594
PB.4520.10	3	3394	10	novel_in_catalog	gene: Zm00001d043613	novel	0.16	284.34	coding	171	2421	2933
PB.4520.11	3	2886	13	novel_not_in_catalog	gene: Zm00001d043613	novel	0.51	284.34	coding	300	1537	2436
PB.5049.1	4	2091	5	full-splice_match	gene: Zm00001d049857	Zm00001d049857_T002	45.62	58.65	coding	434	298	1599
PB.5049.3	4	1710	5	full-splice_match	gene: Zm00001d049857	Zm00001d049857_T002	0.00	58.65	coding	434	219	1520
PB.5049.2	4	1963	5	full-splice_match	gene: Zm00001d049857	Zm00001d049857_T002	13.03	58.65	coding	434	360	1661
PB.5049.4	4	1663	5	full-splice_match	gene: Zm00001d049857	Zm00001d049857_T002	0.00	58.65	coding	434	358	1659
PB.8242.1	7	2282	5	full-splice_match	gene: Zm00001d018983	Zm00001d018983_T001	23.94	23.94	coding	572	425	2140
PB.9430.1	8	795	4	full-splice_match	gene: Zm00001d010128	Zm00001d010128_T001	425.57	672.93	coding	113	67	405
PB.4950.6	4	633	5	novel_in_catalog	gene: Zm00001d049362	novel	0.43	288.48	non_coding	NA	NA	NA
PB.6387.1	5	904	1	full-splice_match	gene: Zm00001d014774	Zm00001d014774_T001	21.33	21.33	coding	110	103	432
PB.7116.3	5	1928	13	full-splice_match	gene: Zm00001d018333	Zm00001d018333_T001	0.00	42.53	coding	492	136	1611

PB.7116.1	5	2110	13	full-splice_match	gene: Zm00001d018333	Zm00001d018333_T001	42.53	42.53	coding	492	214	1689
PB.1743.4	10	927	3	intergenic	novelGene_47	novel	27.84	39.07	non_coding	NA	NA	NA
PB.10443.1	9	1960	3	full-splice_match	gene: Zm00001d047045	Zm00001d047045_T001	53.32	55.17	coding	534	123	1724
PB.6770.2	5	1633	11	full-splice_match	gene: Zm00001d016891	Zm00001d016891_T001	0.00	46.75	coding	398	265	1458
PB.9297.1	8	2808	4	full-splice_match	gene: Zm00001d009409	Zm00001d009409_T002	25.02	25.02	coding	745	68	2302
PB.1272.2	1	3264	1	full-splice_match	gene: Zm00001d033505	Zm00001d033505_T001	3.72	10.66	coding	1044	7	3138
PB.6180.1	5	1787	5	full-splice_match	gene: Zm00001d013819	Zm00001d013819_T004	3.05	9.97	coding	450	70	1419
PB.2151.2	10	3890	8	novel_in_catalog	gene: Zm00001d025837	novel	0.13	18.57	coding	736	1501	3708
PB.6407.12	5	1047	7	full-splice_match	gene: Zm00001d014858	Zm00001d014858_T001	1775.97	2470.46	coding	157	239	709
PB.849.8	1	2676	4	novel_in_catalog	gene: Zm00001d031529	novel	5.08	121.67	coding	174	1379	1900
PB.1269.3	1	1064	1	full-splice_match	gene: Zm00001d033483	Zm00001d033483_T001	20.02	876.45	coding	100	532	831
PB.280.1	1	1988	10	intergenic	novelGene_93	novel	35.46	35.46	coding	511	172	1704
PB.849.3	1	3085	9	full-splice_match	gene: Zm00001d031529	Zm00001d031529_T010	0.00	121.67	coding	859	184	2760
PB.2549.1	2	2201	5	full-splice_match	gene: Zm00001d002391	Zm00001d002391_T010	2.80	30.53	coding	482	339	1784
PB.849.1	1	1972	7	novel_in_catalog	gene: Zm00001d031529	novel	27.09	121.67	coding	276	146	973
PB.849.7	1	2272	5	novel_not_in_catalog	gene: Zm00001d031529	novel	6.46	121.67	coding	27	237	317
PB.9793.2	8	681	3	full-splice_match	gene: Zm00001d011786	Zm00001d011786_T001	169.10	257.23	coding	135	128	532

PB.3093.2	2	1261	1	genic	gene: Zm00001d005279	novel	119.32	122.07	coding	299	9	905
PB.4052.4	3	3492	3	novel_not_in_catalog	gene: Zm00001d041556	novel	0.00	73.92	coding	872	252	2867
PB.3761.1	3	1413	12	full-splice_match	gene: Zm00001d039740	Zm00001d039740_T002	37.80	37.80	coding	328	75	1058
PB.4052.2	3	3704	3	novel_not_in_catalog	gene: Zm00001d041556	novel	2.03	73.92	coding	872	254	2869
PB.9152.1	8	2377	12	full-splice_match	gene: Zm00001d008614	Zm00001d008614_T001	14.37	14.37	coding	712	84	2219
PB.4052.1	3	3843	3	novel_not_in_catalog	gene: Zm00001d041556	novel	42.71	73.92	coding	872	236	2851
PB.10042.3	9	933	2	genic	gene: Zm00001d044783	novel	12.14	24.29	coding	187	96	656
PB.10042.2	9	1154	2	genic	gene: Zm00001d044783	novel	6.16	24.29	coding	187	97	657
PB.10042.1	9	1659	2	genic	gene: Zm00001d044783	novel	6.00	24.29	coding	187	103	663
PB.8811.1	7	1338	5	incomplete-splice_match	gene: Zm00001d021815	Zm00001d021815_T002	312.08	312.08	coding	349	41	1087
PB.9579.1	8	728	4	full-splice_match	gene: Zm00001d010804	Zm00001d010804_T001	438.31	700.77	coding	140	76	495
PB.9457.1	8	773	3	full-splice_match	gene: Zm00001d010236	Zm00001d010236_T001	106.39	376.64	coding	112	90	425
PB.9579.2	8	628	4	full-splice_match	gene: Zm00001d010804	Zm00001d010804_T001	262.46	700.77	coding	140	78	497
PB.2540.1	2	1560	8	full-splice_match	gene: Zm00001d002358	Zm00001d002358_T001	49.25	87.45	coding	397	183	1373
PB.2540.2	2	1939	7	novel_in_catalog	gene: Zm00001d002358	novel	6.21	87.45	coding	280	25	864
PB.273.1	1	3102	10	novel_in_catalog	gene: Zm00001d028417	novel	23.30	23.30	coding	887	231	2891
PB.4873.1	4	1342	4	novel_not_in_catalog	gene: Zm00001d049007	novel	5.85	5.85	coding	161	122	604

PB.366.4	1	1041	8	incomplete-splice_match	gene: Zm00001d028812	Zm00001d028812_T018	12.82	12.82	coding	136	358	765
PB.4873.3	4	1978	3	fusion	gene: Zm00001d049007_g	novel	1.71	73.19	coding	172	1154	1669
PB.4873.2	4	1242	5	fusion	gene: Zm00001d049007_g	novel	71.47	73.19	coding	254	127	888
PB.4528.5	3	3950	6	full-splice_match	gene: Zm00001d043649	Zm00001d043649_T023	14.53	96.43	coding	125	1843	2217
PB.9482.2	8	1141	6	novel_not_in_catalog	gene: Zm00001d010395	novel	2.67	72.62	coding	213	202	840
PB.2564.1	2	953	2	full-splice_match	gene: Zm00001d002479	Zm00001d002479_T001	6.79	17.76	coding	173	62	580
PB.7481.3	6	1154	5	novel_not_in_catalog	gene: Zm00001d036532	novel	11.12	17.24	coding	310	135	1064
PB.1255.1	1	812	3	full-splice_match	gene: Zm00001d033421	Zm00001d033421_T001	83.28	83.28	coding	171	75	587
PB.1115.1	1	1458	7	full-splice_match	gene: Zm00001d032695	Zm00001d032695_T001	711.12	802.50	coding	332	93	1088
PB.10506.10	9	1390	1	novel_in_catalog	gene: Zm00001d047424	novel	1.95	173.54	non_coding	NA	NA	NA
PB.7384.2	6	2112	14	novel_not_in_catalog	gene: Zm00001d035937	novel	67.57	67.57	coding	579	103	1839
PB.7384.4	6	1345	8	novel_not_in_catalog	gene: Zm00001d035937	novel	0.00	67.57	coding	413	105	1343
PB.1430.1	1	2478	12	full-splice_match	gene: Zm00001d034191	Zm00001d034191_T003	25.26	25.26	coding	203	1697	2305
PB.3769.2	3	571	1	full-splice_match	gene: Zm00001d039791	Zm00001d039791_T001	308.11	342.12	non_coding	NA	NA	NA
PB.897.2	1	2994	10	novel_in_catalog	gene: Zm00001d031764	novel	3.80	35.40	coding	317	118	1068
PB.3769.1	3	1029	1	full-splice_match	gene: Zm00001d039791	Zm00001d039791_T001	34.01	342.12	non_coding	NA	NA	NA
PB.5156.1	4	1318	1	genic	gene: Zm00001d026860	novel	2.52	2.52	non_coding	NA	NA	NA

PB.1115.6	1	1366	6	novel_in_catalog	gene: Zm00001d032695	novel	1.46	802.50	coding	219	91	747
PB.7007.1	5	1014	5	full-splice_match	gene: Zm00001d017927	Zm00001d017927_T001	69.40	69.40	coding	197	172	762
PB.5719.1	4	1209	3	full-splice_match	gene: Zm00001d053396	Zm00001d053396_T001	112.98	153.18	coding	217	255	905
PB.398.1	1	991	8	full-splice_match	gene: Zm00001d028981	Zm00001d028981_T002	81.02	81.02	coding	214	63	704
PB.6608.1	5	1360	1	antisense	novelGene_gene: Zm00001d016019_A	novel	1.55	1.55	coding	114	846	1187
PB.1160.2	1	1389	6	incomplete-splice_match	gene: Zm00001d032905	Zm00001d032905_T013	4.16	44.34	coding	441	53	1375
PB.9784.1	8	418	2	incomplete-splice_match	gene: Zm00001d011753	Zm00001d011753_T001	42.54	42.54	non_coding	NA	NA	NA
PB.960.1	1	2664	1	full-splice_match	gene: Zm00001d032075	Zm00001d032075_T001	18.41	18.41	coding	631	219	2111
PB.960.3	1	2293	1	full-splice_match	gene: Zm00001d032075	Zm00001d032075_T001	0.00	18.41	coding	631	142	2034
PB.9601.1	8	3454	21	full-splice_match	gene: Zm00001d010887	Zm00001d010887_T004	28.40	28.40	coding	895	190	2874
PB.3527.1	2	1335	5	full-splice_match	gene: Zm00001d007446	Zm00001d007446_T002	407.50	490.59	coding	266	140	937
PB.7816.2	6	2827	1	full-splice_match	gene: Zm00001d038048	Zm00001d038048_T001	0.20	9.20	coding	458	1332	2705
PB.7816.1	6	2298	2	genic	gene: Zm00001d038048	novel	9.00	9.20	coding	458	757	2130
PB.1249.2	1	1156	2	genic	gene: Zm00001d033386	novel	0.00	95.47	coding	230	170	859
PB.2996.1	2	2231	7	incomplete-splice_match	gene: Zm00001d004716	Zm00001d004716_T003	19.79	19.79	coding	670	52	2061
PB.2372.1	10	1415	10	novel_not_in_catalog	gene: Zm00001d026670	novel	4.96	4.96	coding	81	105	347
PB.6425.1	5	1906	8	full-splice_match	gene: Zm00001d014911	Zm00001d014911_T010	16.23	20.94	coding	416	60	1307

PB.1899.1	10	2413	8	full-splice_match	gene: Zm00001d024598	Zm00001d024598_T001	5.19	5.19	coding	626	163	2040
PB.6425.9	5	2989	8	full-splice_match	gene: Zm00001d014911	Zm00001d014911_T010	4.71	20.94	coding	778	654	2987
PB.3074.1	2	524	4	intergenic	novelGene_265	novel	138.47	230.11	coding	83	81	329
PB.620.1	1	1459	3	full-splice_match	gene: Zm00001d030101	Zm00001d030101_T001	13.77	13.77	coding	377	83	1213
PB.407.3	1	1842	2	novel_in_catalog	gene: Zm00001d028999	novel	7.60	27.04	coding	242	757	1482
PB.6518.1	5	2302	2	genic	gene: Zm00001d015325	novel	12.94	12.94	coding	470	554	1963
PB.2600.1	2	2558	12	full-splice_match	gene: Zm00001d002614	Zm00001d002614_T006	14.47	14.47	coding	696	261	2348
PB.3231.7	2	1643	6	full-splice_match	gene: Zm00001d006046	Zm00001d006046_T010	0.00	79.51	coding	301	375	1277
PB.1558.2	1	2321	1	full-splice_match	gene: Zm00001d034671	Zm00001d034671_T001	1.68	4.68	coding	682	124	2169
PB.4696.6	3	1682	6	full-splice_match	gene: Zm00001d044315	Zm00001d044315_T002	0.00	275.21	coding	451	265	1617
PB.869.1	1	1113	8	full-splice_match	gene: Zm00001d031621	Zm00001d031621_T006	356.50	356.50	coding	251	96	848
PB.5564.1	4	750	1	genic	gene: Zm00001d052654	novel	4.90	4.90	coding	193	65	643
PB.4696.2	3	1968	5	full-splice_match	gene: Zm00001d044315	Zm00001d044315_T005	8.26	275.21	coding	301	177	1079
PB.4696.3	3	1794	6	full-splice_match	gene: Zm00001d044315	Zm00001d044315_T002	19.94	275.21	coding	451	265	1617
PB.1330.3	1	2345	5	full-splice_match	gene: Zm00001d033753	Zm00001d033753_T001	27.02	103.65	coding	627	291	2171
PB.8939.1	7	748	3	incomplete-splice_match	gene: Zm00001d022276	Zm00001d022276_T006	10.56	10.56	coding	88	62	325
PB.722.1	1	2253	4	full-splice_match	gene: Zm00001d030846	Zm00001d030846_T002	17.69	17.69	coding	544	101	1732

PB.9081.1	8	1232	6	full-splice_match	gene: Zm00001d008284	Zm00001d008284_T006	55.10	125.38	coding	307	54	974
PB.722.3	1	2030	4	full-splice_match	gene: Zm00001d030846	Zm00001d030846_T002	0.00	17.69	coding	544	84	1715
PB.722.2	1	2112	4	full-splice_match	gene: Zm00001d030846	Zm00001d030846_T002	0.00	17.69	coding	544	101	1732
PB.344.1	1	1308	6	full-splice_match	gene: Zm00001d028714	Zm00001d028714_T001	99.79	103.53	coding	297	81	971
PB.6783.3	5	1944	5	novel_not_in_catalog	gene: Zm00001d016922	novel	19.16	19.16	coding	187	1139	1699
PB.344.2	1	2009	4	novel_in_catalog	gene: Zm00001d028714	novel	3.74	103.53	coding	212	72	707
PB.9027.5	7	1991	3	intergenic	novelGene_300	novel	1.23	8.54	coding	101	1160	1462
PB.7481.7	6	2501	7	novel_not_in_catalog	gene: Zm00001d036532	novel	0.00	17.24	coding	195	202	786
PB.6397.4	5	2554	4	novel_in_catalog	gene: Zm00001d014797	novel	1.65	68.14	coding	310	302	1231
PB.6397.5	5	1645	5	full-splice_match	gene: Zm00001d014797	Zm00001d014797_T001	8.38	68.14	coding	394	301	1482
PB.6397.2	5	1846	5	full-splice_match	gene: Zm00001d014797	Zm00001d014797_T007	51.20	68.14	coding	394	221	1402
PB.6397.3	5	2095	5	full-splice_match	gene: Zm00001d014797	Zm00001d014797_T001	0.19	68.14	coding	394	680	1861
PB.6397.1	5	2196	5	full-splice_match	gene: Zm00001d014797	Zm00001d014797_T001	6.72	68.14	coding	394	568	1749
PB.4169.1	3	2174	4	incomplete-splice_match	gene: Zm00001d042074	Zm00001d042074_T005	36.27	36.27	coding	604	48	1859
PB.7828.1	6	1855	9	full-splice_match	gene: Zm00001d038082	Zm00001d038082_T001	22.49	30.96	coding	364	207	1298
PB.6082.1	5	704	2	full-splice_match	gene: Zm00001d013392	Zm00001d013392_T001	314.33	314.33	coding	137	96	506
PB.336.1	1	1602	6	novel_not_in_catalog	gene: Zm00001d028672	novel	20.81	35.78	coding	306	293	1210

PB.7828.4	6	1438	9	novel_not_in_catalog	gene: Zm00001d038082	novel	5.72	30.96	coding	364	239	1330
PB.7162.17	5	2281	12	novel_in_catalog	gene: Zm00001d018445	novel	8.83	433.77	coding	418	85	1338
PB.7447.1	6	1473	3	full-splice_match	gene: Zm00001d036338	Zm00001d036338_T001	14.82	14.82	coding	312	217	1152
PB.8316.1	7	1781	2	incomplete-splice_match	gene: Zm00001d019461	Zm00001d019461_T001	2.20	51.51	coding	504	62	1573
PB.9847.1	8	1405	9	full-splice_match	gene: Zm00001d012003	Zm00001d012003_T001	52.90	52.90	coding	281	271	1113
PB.2127.2	10	1070	2	full-splice_match	gene: Zm00001d025753	Zm00001d025753_T001	43.77	103.28	coding	279	59	895
PB.6510.3	5	2107	2	full-splice_match	gene: Zm00001d015292	Zm00001d015292_T001	0.00	16.57	coding	526	464	2041
PB.6510.2	5	2466	2	full-splice_match	gene: Zm00001d015292	Zm00001d015292_T001	0.72	16.57	coding	526	759	2336
PB.6510.1	5	2335	2	full-splice_match	gene: Zm00001d015292	Zm00001d015292_T001	15.86	16.57	coding	526	463	2040
PB.1772.1	10	2791	7	incomplete-splice_match	gene: Zm00001d023825	Zm00001d023825_T006	22.54	52.78	coding	660	352	2331
PB.6301.3	5	2623	5	incomplete-splice_match	gene: Zm00001d014423	Zm00001d014423_T023	0.00	169.16	coding	491	161	1633
PB.6301.2	5	3122	4	novel_in_catalog	gene: Zm00001d014423	novel	0.23	169.16	coding	451	470	1822
PB.6301.1	5	3489	4	novel_in_catalog	gene: Zm00001d014423	novel	0.00	169.16	coding	410	466	1695
PB.9232.2	8	1376	5	full-splice_match	gene: Zm00001d008976	Zm00001d008976_T002	7.97	10.03	coding	352	111	1166
PB.6301.7	5	2513	5	novel_not_in_catalog	gene: Zm00001d014423	novel	2.06	169.16	coding	369	480	1586
PB.6301.6	5	2627	7	full-splice_match	gene: Zm00001d014423	Zm00001d014423_T021	79.53	169.16	coding	593	469	2247
PB.6301.5	5	2194	1	incomplete-splice_match	gene: Zm00001d014423	Zm00001d014423_T001	5.61	169.16	non_coding	NA	NA	NA

PB.8510.1	7	2459	5	novel_in_catalog	gene: Zm00001d020560	novel	8.44	9.33	coding	675	64	2088
PB.8392.2	7	3092	19	full-splice_match	gene: Zm00001d019988	Zm00001d019988_T001	28.55	28.55	coding	917	118	2868
PB.6301.8	5	2882	6	full-splice_match	gene: Zm00001d014423	Zm00001d014423_T004	47.26	169.16	coding	491	526	1998
PB.10630.1	9	1926	11	novel_not_in_catalog	gene: Zm00001d047849	novel	33.85	34.33	coding	414	456	1697
PB.5840.1	4	2193	8	novel_in_catalog	gene: Zm00001d053909	novel	14.98	14.98	coding	468	82	1485
PB.3583.1	2	2095	1	full-splice_match	gene: Zm00001d007774	Zm00001d007774_T001	5.72	5.72	coding	548	85	1728
PB.1104.1	1	2961	12	full-splice_match	gene: Zm00001d032659	Zm00001d032659_T008	9.90	9.90	coding	701	354	2456
PB.4175.1	3	838	2	full-splice_match	gene: Zm00001d042104	Zm00001d042104_T001	100.83	100.83	coding	227	53	733
PB.10288.3	9	1408	5	novel_in_catalog	gene: Zm00001d046136	novel	21.36	44.56	coding	379	104	1240
PB.3749.1	3	2256	11	full-splice_match	gene: Zm00001d039678	Zm00001d039678_T003	27.74	27.74	coding	590	49	1818
PB.10288.1	9	1526	5	novel_in_catalog	gene: Zm00001d046136	novel	20.72	44.56	coding	379	175	1311
PB.6288.2	5	1813	8	novel_in_catalog	gene: Zm00001d014358	novel	2.59	16.11	coding	353	33	1091
PB.4528.3	3	4060	5	novel_in_catalog	gene: Zm00001d043649	novel	2.51	96.43	coding	125	2024	2398
PB.892.7	1	1669	3	full-splice_match	gene: Zm00001d031732	Zm00001d031732_T001	21.03	218.99	coding	320	445	1404
PB.2568.1	2	1567	1	full-splice_match	gene: Zm00001d002498	Zm00001d002498_T001	16.93	16.93	coding	413	130	1368
PB.4670.4	3	1760	12	novel_in_catalog	gene: Zm00001d044213	novel	0.00	33.65	coding	400	439	1638
PB.8611.2	7	1853	3	full-splice_match	gene: Zm00001d021024	Zm00001d021024_T001	16.09	27.54	coding	479	38	1474

PB.8611.1	7	1959	3	full-splice_match	gene: Zm00001d021024	Zm00001d021024_T001	11.45	27.54	coding	479	43	1479
PB.4670.1	3	2133	13	full-splice_match	gene: Zm00001d044213	Zm00001d044213_T001	7.96	33.65	coding	558	419	2092
PB.4670.2	3	1986	13	novel_not_in_catalog	gene: Zm00001d044213	novel	0.13	33.65	coding	400	439	1638
PB.7635.1	6	2062	1	incomplete-splice_match	gene: Zm00001d037272	Zm00001d037272_T001	26.09	35.67	coding	511	49	1581
PB.10793.2	9	1959	2	incomplete-splice_match	gene: Zm00001d048432	Zm00001d048432_T001	8.88	13.79	coding	548	31	1674
PB.10793.1	9	3625	5	full-splice_match	gene: Zm00001d048432	Zm00001d048432_T002	4.91	13.79	coding	973	290	3208
PB.7635.2	6	2651	1	incomplete-splice_match	gene: Zm00001d037272	Zm00001d037272_T001	9.58	35.67	coding	751	25	2277
PB.4550.1	3	2004	11	full-splice_match	gene: Zm00001d043726	Zm00001d043726_T001	99.05	101.51	coding	429	285	1571
PB.9043.1	7	4447	9	intergenic	novelGene_301	novel	47.70	47.70	coding	102	225	530
PB.10921.3	B73V4 ctg51	1506	10	novel_in_catalog	gene: Zm00001d000311	novel	0.00	6.34	coding	227	635	1315
PB.4605.1	3	689	5	full-splice_match	gene: Zm00001d043946	Zm00001d043946_T002	47.59	47.59	coding	99	124	420
PB.4483.1	3	1014	6	novel_in_catalog	gene: Zm00001d043479	novel	67.02	69.20	coding	133	172	570
PB.4483.2	3	1761	3	novel_in_catalog	gene: Zm00001d043479	novel	2.17	69.20	non_coding	NA	NA	NA
PB.5035.1	4	1177	2	novel_in_catalog	gene: Zm00001d049768	novel	112.47	112.47	coding	260	216	995
PB.3540.1	2	1595	3	full-splice_match	gene: Zm00001d007503	Zm00001d007503_T001	16.80	16.80	coding	456	128	1495
PB.8214.2	7	1831	2	novel_not_in_catalog	gene: Zm00001d018869	novel	1.60	3.97	coding	594	49	1830
PB.9100.3	8	1242	5	full-splice_match	gene: Zm00001d008360	Zm00001d008360_T001	0.00	39.61	coding	318	52	1005

PB.8214.1	7	1929	2	novel_not_in_catalog	gene: Zm00001d018869	novel	2.37	3.97	coding	633	30	1928
PB.4646.3	3	2701	6	full-splice_match	gene: Zm00001d044127	Zm00001d044127_T007	36.25	36.25	coding	327	1348	2328
PB.9461.3	8	1711	13	full-splice_match	gene: Zm00001d010251	Zm00001d010251_T001	178.54	178.54	coding	445	150	1484
PB.9452.2	8	2546	4	full-splice_match	gene: Zm00001d010231	Zm00001d010231_T001	5.46	17.08	coding	478	753	2186
PB.1554.1	1	1413	11	full-splice_match	gene: Zm00001d034659	Zm00001d034659_T001	40.21	40.21	coding	350	99	1148
PB.4787.1	3	1499	5	full-splice_match	gene: Zm00001d044699	Zm00001d044699_T001	54.54	81.61	coding	350	158	1207
PB.9894.1	8	2054	4	novel_not_in_catalog	gene: Zm00001d012223	novel	10.88	10.88	coding	426	453	1730
PB.4421.1	3	1604	4	full-splice_match	gene: Zm00001d043238	Zm00001d043238_T002	65.15	65.15	coding	342	62	1087
PB.9452.5	8	1748	5	novel_not_in_catalog	gene: Zm00001d010231	novel	0.99	17.08	coding	478	219	1652
PB.7132.2	5	2079	10	full-splice_match	gene: Zm00001d018375	Zm00001d018375_T006	2.34	9.82	coding	520	138	1697
PB.9452.4	8	1848	5	novel_not_in_catalog	gene: Zm00001d010231	novel	10.62	17.08	coding	478	208	1641
PB.2660.1	2	1825	6	novel_not_in_catalog	gene: Zm00001d002865	novel	9.19	18.87	coding	273	73	891
PB.2660.3	2	3038	7	full-splice_match	gene: Zm00001d002865	Zm00001d002865_T002	4.15	18.87	coding	785	272	2626
PB.2660.2	2	2947	7	full-splice_match	gene: Zm00001d002865	Zm00001d002865_T002	5.53	18.87	coding	710	190	2319
PB.4550.3	3	1862	11	full-splice_match	gene: Zm00001d043726	Zm00001d043726_T006	0.00	101.51	coding	429	240	1526
PB.6572.3	5	1120	2	genic	gene: Zm00001d015662	novel	13.05	35.96	coding	287	200	1060
PB.6572.2	5	1255	2	genic	gene: Zm00001d015662	novel	22.91	35.96	coding	287	201	1061

PB.482.4	1	2667	6	full-splice_match	gene: Zm00001d029352	Zm00001d029352_T001	21.74	28.11	coding	743	107	2335
PB.482.7	1	2604	7	novel_not_in_catalog	gene: Zm00001d029352	novel	6.37	28.11	coding	714	127	2268
PB.1008.1	1	1505	1	full-splice_match	gene: Zm00001d032295	Zm00001d032295_T001	65.24	65.24	coding	276	442	1269
PB.4240.1	3	548	3	full-splice_match	gene: Zm00001d042454	Zm00001d042454_T001	59.57	59.57	coding	130	56	445
PB.1930.1	10	1712	11	novel_in_catalog	gene: Zm00001d024759	novel	2.41	18.86	coding	368	141	1244
PB.1930.2	10	1611	12	full-splice_match	gene: Zm00001d024759	Zm00001d024759_T001	16.45	18.86	coding	416	141	1388
PB.4832.10	4	1197	6	novel_in_catalog	gene: Zm00001d048785	novel	5.14	169.67	coding	177	136	666
PB.5017.3	4	3188	1	incomplete-splice_match	gene: Zm00001d049706	Zm00001d049706_T035	9.23	99.33	coding	430	1701	2990
PB.10120.2	9	1962	10	full-splice_match	gene: Zm00001d045176	Zm00001d045176_T001	0.73	80.94	coding	453	333	1691
PB.10120.1	9	2035	10	full-splice_match	gene: Zm00001d045176	Zm00001d045176_T001	80.21	80.94	coding	453	326	1684
PB.4550.2	3	2040	10	novel_in_catalog	gene: Zm00001d043726	novel	2.47	101.51	coding	304	201	1112
PB.2380.1	10	4503	10	incomplete-splice_match	gene: Zm00001d026691	Zm00001d026691_T009	8.22	22.99	coding	1328	357	4340
PB.1805.1	10	2603	4	incomplete-splice_match	gene: Zm00001d023998	Zm00001d023998_T004	9.03	9.03	coding	740	4	2223
PB.3233.1	2	1946	3	full-splice_match	gene: Zm00001d006053	Zm00001d006053_T001	21.83	27.30	coding	388	430	1593
PB.564.1	1	2423	7	full-splice_match	gene: Zm00001d029726	Zm00001d029726_T001	31.75	31.75	coding	462	699	2084
PB.564.3	1	2208	7	full-splice_match	gene: Zm00001d029726	Zm00001d029726_T001	0.00	31.75	coding	462	699	2084
PB.564.2	1	2270	7	full-splice_match	gene: Zm00001d029726	Zm00001d029726_T001	0.00	31.75	coding	462	657	2042

PB.2822.1	2	2619	7	full-splice_match	gene: Zm00001d003556	Zm00001d003556_T001	19.69	19.69	coding	712	203	2338
PB.6317.4	5	1710	3	novel_in_catalog	gene: Zm00001d014519	novel	0.00	635.77	non_coding	NA	NA	NA
PB.3628.1	2	3086	16	incomplete-splice_match	gene: Zm00001d007943	Zm00001d007943_T022	21.45	21.45	coding	773	460	2778
PB.6317.2	5	624	3	novel_in_catalog	gene: Zm00001d014519	novel	595.53	635.77	coding	103	105	413
PB.7019.1	5	603	1	full-splice_match	gene: Zm00001d017968	Zm00001d017968_T001	48.86	48.86	coding	80	75	314
PB.6317.1	5	1824	3	novel_in_catalog	gene: Zm00001d014519	novel	40.24	635.77	non_coding	NA	NA	NA
PB.10236.1	9	2271	6	full-splice_match	gene: Zm00001d045696	Zm00001d045696_T001	21.49	21.49	coding	615	139	1983
PB.10236.2	9	2219	6	full-splice_match	gene: Zm00001d045696	Zm00001d045696_T001	0.00	21.49	coding	615	197	2041
PB.10236.3	9	1472	6	full-splice_match	gene: Zm00001d045696	Zm00001d045696_T001	0.00	21.49	coding	461	89	1471
PB.9524.2	8	2700	8	full-splice_match	gene: Zm00001d010578	Zm00001d010578_T005	9.30	10.10	coding	680	399	2438
PB.9256.1	8	2509	16	novel_in_catalog	gene: Zm00001d009127	novel	63.69	63.69	coding	384	1080	2231
PB.1496.2	1	1531	6	fusion	gene: Zm00001d034448_g	novel	84.35	84.35	coding	405	171	1385
PB.813.1	1	2446	1	full-splice_match	gene: Zm00001d031280	Zm00001d031280_T001	10.12	10.12	coding	592	36	1811
PB.10395.1	9	2057	3	novel_in_catalog	gene: Zm00001d046751	novel	70.11	70.11	coding	287	853	1713
PB.8523.3	7	1782	9	full-splice_match	gene: Zm00001d020642	Zm00001d020642_T001	0.00	111.32	coding	525	19	1593
PB.3909.1	3	308	1	genic	gene: Zm00001d040501	novel	40.16	40.16	non_coding	NA	NA	NA
PB.6544.1	5	2249	9	full-splice_match	gene: Zm00001d015464	Zm00001d015464_T003	168.39	187.29	coding	616	105	1952

PB.1497.8	1	2721	3	full-splice_match	gene: Zm00001d034460	Zm00001d034460_T021	0.00	56.18	coding	56	189	356
PB.6544.3	5	2336	8	novel_in_catalog	gene: Zm00001d015464	novel	18.90	187.29	coding	313	1100	2038
PB.6544.4	5	2205	9	full-splice_match	gene: Zm00001d015464	Zm00001d015464_T003	0.00	187.29	coding	616	120	1967
PB.6544.5	5	2105	9	full-splice_match	gene: Zm00001d015464	Zm00001d015464_T003	0.00	187.29	coding	616	120	1967
PB.10897.1	B73V4 ctg24	1470	5	novel_in_catalog	gene: Zm00001d000379	novel	8.86	92.04	coding	92	100	375
PB.3142.7	2	3202	8	novel_in_catalog	gene: Zm00001d005579	novel	0.75	89.25	coding	310	1961	2890
PB.3686.1	3	1679	1	antisense	novelGene_gene: Zm00001d039397_A	novel	4.64	12.53	non_co ding	NA	NA	NA
PB.3222.4	2	2151	2	incomplete-splice_match	gene: Zm00001d006011	Zm00001d006011_T001	0.00	23.08	coding	680	110	2149
PB.1525.1	1	1716	2	genic	gene: Zm00001d034569	novel	4.95	30.52	coding	309	650	1576
PB.3142.6	2	2380	7	full-splice_match	gene: Zm00001d005579	Zm00001d005579_T006	3.60	89.25	coding	310	1138	2067
PB.2721.1	2	1981	3	full-splice_match	gene: Zm00001d003116	Zm00001d003116_T001	17.86	17.86	coding	523	85	1653
PB.3142.1	2	1609	9	full-splice_match	gene: Zm00001d005579	Zm00001d005579_T005	47.62	89.25	coding	310	307	1236
PB.9357.1	8	2464	2	genic	gene: Zm00001d009709	novel	21.43	21.43	coding	572	548	2263
PB.969.3	1	1168	12	novel_in_catalog	gene: Zm00001d032101	novel	11.26	11.26	coding	340	148	1167
PB.5423.4	4	1902	8	novel_in_catalog	gene: Zm00001d052074	novel	12.10	84.05	coding	212	1044	1679
PB.3537.1	2	785	3	novel_not_in_catalog	gene: Zm00001d007498	novel	18.35	18.35	coding	156	83	550
PB.6151.2	5	1816	3	full-splice_match	gene: Zm00001d013658	Zm00001d013658_T001	0.00	49.07	coding	499	65	1561

PB.6151.3	5	1528	3	full-splice_match	gene: Zm00001d013658	Zm00001d013658_T001	4.74	49.07	coding	466	130	1527
PB.6151.1	5	1974	3	full-splice_match	gene: Zm00001d013658	Zm00001d013658_T001	44.32	49.07	coding	499	91	1587
PB.7114.2	5	1972	1	full-splice_match	gene: Zm00001d018326	Zm00001d018326_T001	0.00	57.25	coding	356	234	1301
PB.4424.10	3	2983	6	novel_in_catalog	gene: Zm00001d043256	novel	15.01	34.21	coding	864	179	2770
PB.3832.1	3	2804	21	novel_not_in_catalog	gene: Zm00001d040154	novel	8.68	8.68	coding	755	389	2653
PB.6054.3	5	2589	2	incomplete-splice_match	gene: Zm00001d013301	Zm00001d013301_T001	7.83	18.46	coding	753	39	2297
PB.992.1	1	2698	7	full-splice_match	gene: Zm00001d032226	Zm00001d032226_T011	42.93	42.93	coding	544	631	2262
PB.4811.4	4	2264	11	full-splice_match	gene: Zm00001d048655	Zm00001d048655_T005	21.08	210.28	coding	367	904	2004
PB.4811.6	4	1065	3	incomplete-splice_match	gene: Zm00001d048655	Zm00001d048655_T003	1.72	210.28	coding	31	207	299
PB.4811.1	4	2990	10	full-splice_match	gene: Zm00001d048655	Zm00001d048655_T006	2.40	210.28	coding	367	1625	2725
PB.4811.3	4	1857	11	novel_not_in_catalog	gene: Zm00001d048655	novel	2.99	210.28	coding	367	494	1594
PB.4811.2	4	1912	11	full-splice_match	gene: Zm00001d048655	Zm00001d048655_T012	79.37	210.28	coding	539	34	1650
PB.5931.1	5	1002	1	full-splice_match	gene: Zm00001d012889	Zm00001d012889_T001	6.93	6.93	coding	217	115	765
PB.6219.5	5	1243	6	full-splice_match	gene: Zm00001d014030	Zm00001d014030_T001	36.89	71.87	coding	312	103	1038
PB.4811.8	4	381	3	incomplete-splice_match	gene: Zm00001d048655	Zm00001d048655_T011	102.71	210.28	non_coding	NA	NA	NA
PB.9031.1	7	819	4	novel_not_in_catalog	gene: Zm00001d022558	novel	1.08	38.47	non_coding	NA	NA	NA
PB.9031.2	7	1562	15	full-splice_match	gene: Zm00001d022558	Zm00001d022558_T001	26.37	38.47	coding	389	123	1289

PB.9031.3	7	375	3	incomplete-splice_match	gene: Zm00001d022558	Zm00001d022558_T001	11.02	38.47	non_coding	NA	NA	NA
PB.9965.1	8	1118	11	full-splice_match	gene: Zm00001d012546	Zm00001d012546_T003	194.04	194.04	coding	249	188	934
PB.4742.3	3	2558	15	full-splice_match	gene: Zm00001d044503	Zm00001d044503_T001	9.25	26.50	coding	645	280	2214
PB.4938.2	4	3344	2	incomplete-splice_match	gene: Zm00001d049286	Zm00001d049286_T001	3.17	12.29	coding	1070	51	3260
PB.6026.1	5	2230	4	full-splice_match	gene: Zm00001d013238	Zm00001d013238_T001	1.04	3.08	coding	524	66	1637
PB.4424.19	3	2817	6	novel_in_catalog	gene: Zm00001d043256	novel	0.00	34.21	coding	864	178	2769
PB.6938.1	5	922	2	full-splice_match	gene: Zm00001d017646	Zm00001d017646_T001	13.31	48.93	non_coding	NA	NA	NA
PB.6026.2	5	2158	4	full-splice_match	gene: Zm00001d013238	Zm00001d013238_T001	2.03	3.08	coding	524	103	1674
PB.9724.1	8	1157	5	full-splice_match	gene: Zm00001d011500	Zm00001d011500_T003	59.30	679.31	coding	147	118	558
PB.10698.1	9	2135	12	full-splice_match	gene: Zm00001d048090	Zm00001d048090_T002	16.04	29.50	coding	616	12	1859
PB.10698.2	9	2132	13	novel_not_in_catalog	gene: Zm00001d048090	novel	13.46	29.50	coding	616	111	1958
PB.9724.2	8	1308	4	full-splice_match	gene: Zm00001d011500	Zm00001d011500_T012	16.82	679.31	coding	120	525	884
PB.6808.1	5	1609	3	novel_in_catalog	gene: Zm00001d017091	novel	21.16	21.16	coding	163	956	1444
PB.1400.5	1	1687	3	novel_in_catalog	gene: Zm00001d034035	novel	5.46	61.04	coding	259	104	880
PB.9177.3	8	1970	4	novel_in_catalog	gene: Zm00001d008726	novel	2.10	24.60	coding	152	199	654
PB.6953.2	5	1315	2	novel_in_catalog	gene: Zm00001d017708	novel	10.05	163.14	coding	92	104	379
PB.9177.1	8	1046	5	full-splice_match	gene: Zm00001d008726	Zm00001d008726_T002	22.50	24.60	coding	189	164	730

PB.1299.3	1	1424	3	full-splice_match	gene: Zm00001d033611	Zm00001d033611_T001	49.44	86.53	coding	257	288	1058
PB.3462.10	2	1752	7	novel_not_in_catalog	gene: Zm00001d007073	novel	0.00	70.73	coding	524	53	1624
PB.7498.1	6	605	5	full-splice_match	gene: Zm00001d036567	Zm00001d036567_T005	517.52	517.52	coding	92	126	401
PB.8942.1	7	1410	2	intergenic	novelGene_287	novel	39.99	39.99	coding	352	89	1144
PB.6725.1	5	1142	1	full-splice_match	gene: Zm00001d016697	Zm00001d016697_T001	29.45	33.70	coding	234	163	864
PB.6725.2	5	973	1	full-splice_match	gene: Zm00001d016697	Zm00001d016697_T001	4.25	33.70	coding	234	166	867
PB.1028.1	1	1682	1	intergenic	novelGene_348	novel	18.90	18.90	coding	507	68	1588
PB.9482.1	8	1233	6	full-splice_match	gene: Zm00001d010395	Zm00001d010395_T002	69.95	72.62	coding	224	261	932
PB.3562.1	2	2382	3	full-splice_match	gene: Zm00001d007636	Zm00001d007636_T001	16.05	36.85	coding	617	204	2054
PB.6960.1	5	1002	2	novel_in_catalog	gene: Zm00001d017752	novel	21.96	21.96	coding	149	424	870
PB.1453.2	1	2394	1	genic	gene: Zm00001d034322	novel	3.82	28.13	coding	333	1323	2321
PB.4918.1	4	2421	6	novel_not_in_catalog	gene: Zm00001d049192	novel	2.26	35.18	coding	250	86	835
PB.1048.1	1	1326	2	full-splice_match	gene: Zm00001d032457	Zm00001d032457_T001	46.97	46.97	coding	202	192	797
PB.4918.3	4	2537	5	novel_not_in_catalog	gene: Zm00001d049192	novel	0.16	35.18	coding	133	87	485
PB.4918.2	4	1727	7	novel_not_in_catalog	gene: Zm00001d049192	novel	32.75	35.18	coding	456	87	1454
PB.4918.5	4	2236	6	novel_not_in_catalog	gene: Zm00001d049192	novel	0.00	35.18	coding	250	23	772
PB.10476.1	9	1029	3	full-splice_match	gene: Zm00001d047262	Zm00001d047262_T001	69.82	69.82	coding	129	162	548

PB.4918.6	4	1606	7	novel_not_in_catalog	gene: Zm00001d049192	novel	0.00	35.18	coding	456	86	1453
PB.7009.15	5	497	3	antisense	novelGene_gene: Zm00001d017932_A	novel	0.00	81.66	non_coding	NA	NA	NA
PB.3130.1	2	1690	12	full-splice_match	gene: Zm00001d005498	Zm00001d005498_T004	22.97	22.97	coding	446	157	1494
PB.7995.1	6	816	4	full-splice_match	gene: Zm00001d038718	Zm00001d038718_T001	11.14	11.14	coding	191	54	626
PB.2881.1	2	2978	9	full-splice_match	gene: Zm00001d003861	Zm00001d003861_T002	98.91	98.91	coding	781	368	2710
PB.10690.1	9	1217	7	full-splice_match	gene: Zm00001d048060	Zm00001d048060_T001	25.63	25.63	coding	323	115	1083
PB.3481.1	2	2090	1	genic	gene: Zm00001d007174	novel	28.80	28.80	coding	103	74	382
PB.3633.1	2	2553	16	full-splice_match	gene: Zm00001d007966	Zm00001d007966_T001	9.59	97.92	coding	619	114	1970
PB.4214.1	3	1024	5	genic	gene: Zm00001d042315	novel	27.00	27.00	coding	166	154	651
PB.3114.1	2	1354	2	full-splice_match	gene: Zm00001d005391	Zm00001d005391_T001	236.91	241.18	coding	374	61	1182
PB.3461.1	2	2168	4	full-splice_match	gene: Zm00001d007070	Zm00001d007070_T001	14.56	30.80	coding	537	155	1765
PB.9852.1	8	1020	6	full-splice_match	gene: Zm00001d012017	Zm00001d012017_T002	117.79	117.79	coding	202	148	753
PB.3461.2	2	2112	4	full-splice_match	gene: Zm00001d007070	Zm00001d007070_T001	16.24	30.80	coding	537	258	1868
PB.1819.2	10	1356	4	full-splice_match	gene: Zm00001d024126	Zm00001d024126_T001	23.00	30.09	coding	229	328	1014
PB.6847.3	5	481	3	novel_in_catalog	gene: Zm00001d017215	novel	12.18	12.18	coding	30	214	303
PB.1819.1	10	2064	4	full-splice_match	gene: Zm00001d024126	Zm00001d024126_T001	7.09	30.09	coding	229	245	931
PB.1355.1	1	1793	10	novel_not_in_catalog	gene: Zm00001d033858	novel	15.22	15.22	coding	385	355	1509

PB.929.1	1	683	4	full-splice_match	gene: Zm00001d031939	Zm00001d031939_T001	669.80	669.80	coding	108	76	399
PB.3917.1	3	1720	1	full-splice_match	gene: Zm00001d000526	Zm00001d000526_T001	279.04	279.04	non_coding	NA	NA	NA
PB.2555.1	2	1322	3	full-splice_match	gene: Zm00001d002409	Zm00001d002409_T001	23.38	23.38	coding	297	38	928
PB.6938.2	5	783	2	full-splice_match	gene: Zm00001d017646	Zm00001d017646_T001	35.62	48.93	non_coding	NA	NA	NA
PB.1454.1	1	1909	2	genic	gene: Zm00001d034324	novel	39.93	39.93	coding	458	34	1407
PB.3177.1	2	1366	4	full-splice_match	gene: Zm00001d005762	Zm00001d005762_T001	39.34	39.34	coding	367	82	1182
PB.5769.7	4	2851	7	novel_in_catalog	gene: Zm00001d053634	novel	0.00	41.21	coding	305	324	1238
PB.10885.1	B73V4 ctg182	2805	4	full-splice_match	gene: Zm00001d000039	Zm00001d000039_T001	4.65	4.65	coding	781	122	2464
PB.10557.2	9	1679	6	novel_in_catalog	gene: Zm00001d047612	novel	43.71	84.25	coding	334	420	1421
PB.8685.3	7	1941	12	full-splice_match	gene: Zm00001d021384	Zm00001d021384_T004	6.82	26.30	coding	542	100	1725
PB.1639.2	1	971	7	full-splice_match	gene: Zm00001d034949	Zm00001d034949_T001	0.00	90.81	coding	203	94	702
PB.2254.2	10	2976	7	full-splice_match	gene: Zm00001d026301	Zm00001d026301_T003	0.00	6.96	coding	837	323	2833
PB.7157.2	5	2057	1	novel_in_catalog	gene: Zm00001d018435	novel	6.68	30.49	coding	246	908	1645
PB.3193.1	2	1997	5	full-splice_match	gene: Zm00001d005817	Zm00001d005817_T004	13.79	13.79	coding	515	117	1661
PB.636.4	1	2757	9	novel_in_catalog	gene: Zm00001d030166	novel	20.64	20.64	coding	399	47	1243
PB.7630.1	6	1044	4	novel_in_catalog	gene: Zm00001d037258	novel	9.02	9.02	coding	70	156	365
PB.1721.1	10	1950	11	incomplete-splice_match	gene: Zm00001d023538	Zm00001d023538_T006	36.84	36.84	coding	451	404	1756

PB.4368.5	3	2328	3	novel_in_catalog	gene: Zm00001d043019	novel	1.80	238.63	coding	492	643	2118
PB.10001.1	8	2502	4	incomplete-splice_match	gene: Zm00001d012661	Zm00001d012661_T013	14.01	16.76	coding	603	178	1986
PB.10001.2	8	4060	15	full-splice_match	gene: Zm00001d012661	Zm00001d012661_T005	2.74	16.76	coding	1195	156	3740
PB.10656.1	9	2143	1	full-splice_match	gene: Zm00001d047918	Zm00001d047918_T001	12.03	12.03	coding	646	58	1995
PB.9373.1	8	1007	7	full-splice_match	gene: Zm00001d009792	Zm00001d009792_T001	24.12	24.12	coding	261	60	842
PB.8929.6	7	1657	11	full-splice_match	gene: Zm00001d022239	Zm00001d022239_T001	9.44	122.71	coding	440	184	1503
PB.5211.25	4	2523	3	full-splice_match	gene: Zm00001d050970	Zm00001d050970_T022	5.58	68.24	coding	156	90	557
PB.1020.13	1	2167	7	full-splice_match	gene: Zm00001d032350	Zm00001d032350_T006	0.00	135.32	coding	114	124	465
PB.1020.14	1	1177	6	full-splice_match	gene: Zm00001d032350	Zm00001d032350_T001	0.00	135.32	coding	114	152	493
PB.5211.21	4	1448	8	novel_in_catalog	gene: Zm00001d050970	novel	6.34	68.24	coding	193	90	668
PB.8858.1	7	863	1	full-splice_match	gene: Zm00001d021967	Zm00001d021967_T001	3.33	3.33	coding	183	105	653
PB.8033.1	6	926	1	genic	gene: Zm00001d038841	novel	142.43	142.43	coding	215	64	708
PB.7306.1	6	850	1	genic_intron	novelGene_82	novel	1.14	1.14	non_coding	NA	NA	NA
PB.4887.1	4	1509	5	full-splice_match	gene: Zm00001d049083	Zm00001d049083_T001	64.30	64.30	coding	449	52	1398
PB.817.3	1	887	2	genic	gene: Zm00001d031292	novel	4.94	49.10	coding	125	368	742
PB.3907.1	3	2005	5	full-splice_match	gene: Zm00001d040470	Zm00001d040470_T002	179.75	183.26	coding	436	277	1584
PB.1282.1	1	2825	10	fusion	gene: Zm00001d033532_g	novel	21.99	21.99	coding	722	173	2338

PB.7085.3	5	1401	1	full-splice_match	gene: Zm00001d018208	Zm00001d018208_T001	92.83	96.21	coding	355	67	1131
PB.7085.2	5	1629	1	full-splice_match	gene: Zm00001d018208	Zm00001d018208_T001	2.16	96.21	coding	72	393	608
PB.7085.1	5	1874	1	full-splice_match	gene: Zm00001d018208	Zm00001d018208_T001	1.23	96.21	non_coding	NA	NA	NA
PB.8997.1	7	1500	3	novel_not_in_catalog	gene: Zm00001d022458	novel	13.59	96.94	coding	310	182	1111
PB.2947.1	2	3507	19	full-splice_match	gene: Zm00001d004396	Zm00001d004396_T045	24.77	24.77	coding	988	106	3069
PB.7085.4	5	1290	1	full-splice_match	gene: Zm00001d018208	Zm00001d018208_T001	0.00	96.21	coding	355	60	1124
PB.10899.3	B73V4 ctg24	2154	4	full-splice_match	gene: Zm00001d000381	Zm00001d000381_T001	0.00	14.36	coding	638	32	1945
PB.3688.1	3	538	1	full-splice_match	gene: Zm00001d039408	Zm00001d039408_T001	97.46	97.46	non_coding	NA	NA	NA
PB.10899.1	B73V4 ctg24	2449	4	full-splice_match	gene: Zm00001d000381	Zm00001d000381_T001	14.36	14.36	coding	638	194	2107
PB.9700.4	8	2423	3	genic	gene: Zm00001d011378	novel	0.00	4.17	coding	683	149	2197
PB.4080.3	3	2301	2	genic	gene: Zm00001d041698	novel	0.00	13.84	coding	647	166	2106
PB.4080.2	3	2466	2	genic	gene: Zm00001d041698	novel	4.47	13.84	coding	647	224	2164
PB.4080.1	3	2497	2	genic	gene: Zm00001d041698	novel	9.37	13.84	coding	647	172	2112
PB.5856.2	4	1165	4	full-splice_match	gene: Zm00001d053963	Zm00001d053963_T003	4.50	50.67	coding	255	240	1004
PB.4598.1	3	2107	13	full-splice_match	gene: Zm00001d043907	Zm00001d043907_T003	16.61	18.25	coding	558	108	1781
PB.10924.1	B73V4 ctg52	2166	9	novel_in_catalog	gene: Zm00001d000107	novel	31.78	31.78	coding	400	783	1982
PB.3828.2	3	3479	16	incomplete-splice_match	gene: Zm00001d040123	Zm00001d040123_T010	45.91	45.91	coding	944	234	3065

PB.3828.3	3	2354	10	incomplete-splice_match	gene: Zm00001d040123	Zm00001d040123_T010	0.00	45.91	coding	661	96	2078
PB.4897.1	4	798	2	novel_in_catalog	gene: Zm00001d049109	novel	12.60	12.60	coding	37	246	356
PB.5581.1	4	1895	1	full-splice_match	gene: Zm00001d052731	Zm00001d052731_T001	2.73	9.40	coding	442	58	1383
PB.5581.2	4	2253	1	full-splice_match	gene: Zm00001d052731	Zm00001d052731_T001	6.67	9.40	coding	662	57	2042
PB.9804.1	8	1915	8	novel_in_catalog	gene: Zm00001d011828	novel	5.45	62.09	coding	260	74	853
PB.6373.1	5	934	1	full-splice_match	gene: Zm00001d014745	Zm00001d014745_T001	9.49	9.49	coding	236	22	729
PB.9098.1	8	689	5	full-splice_match	gene: Zm00001d008356	Zm00001d008356_T005	16.04	16.04	coding	60	305	484
PB.2304.1	10	1170	7	full-splice_match	gene: Zm00001d026475	Zm00001d026475_T001	48.48	48.48	coding	243	145	873
PB.7658.3	6	2378	8	full-splice_match	gene: Zm00001d037393	Zm00001d037393_T014	5.70	14.11	non_coding	NA	NA	NA
PB.3969.1	3	830	3	novel_not_in_catalog	gene: Zm00001d000561	novel	15.45	15.45	non_coding	NA	NA	NA
PB.7658.1	6	2153	9	novel_in_catalog	gene: Zm00001d037393	novel	8.41	14.11	non_coding	NA	NA	NA
PB.9716.1	8	441	2	full-splice_match	gene: Zm00001d011461	Zm00001d011461_T001	11.63	11.63	non_coding	NA	NA	NA
PB.10134.1	9	2466	19	novel_in_catalog	gene: Zm00001d045295	novel	17.63	44.24	coding	649	133	2079
PB.4313.1	3	1348	1	full-splice_match	gene: Zm00001d042768	Zm00001d042768_T001	20.26	62.31	coding	301	28	930
PB.246.8	1	3894	13	novel_in_catalog	gene: Zm00001d028330	novel	35.47	86.01	coding	116	1748	2095
PB.10134.2	9	2336	19	novel_in_catalog	gene: Zm00001d045295	novel	26.61	44.24	coding	649	133	2079
PB.10134.4	9	2217	19	novel_in_catalog	gene: Zm00001d045295	novel	0.00	44.24	coding	649	135	2081

PB.7361.5	6	2409	8	incomplete-splice_match	gene: Zm00001d035775	Zm00001d035775_T001	2.11	17.52	coding	725	165	2339
PB.2951.2	2	392	2	intergenic	novelGene_186	novel	37.67	176.52	non_coding	NA	NA	NA
PB.2388.1	2	1376	3	novel_not_in_catalog	gene: Zm00001d001773	novel	1.06	1.06	non_coding	NA	NA	NA
PB.5478.3	4	1774	2	genic	gene: Zm00001d052312	novel	0.33	115.68	coding	422	249	1514
PB.246.5	1	1794	7	incomplete-splice_match	gene: Zm00001d028330	Zm00001d028330_T025	35.78	86.01	coding	141	631	1053
PB.298.2	1	832	6	full-splice_match	gene: Zm00001d028515	Zm00001d028515_T001	0.00	141.45	coding	229	31	717
PB.298.1	1	948	6	full-splice_match	gene: Zm00001d028515	Zm00001d028515_T001	141.45	141.45	coding	229	69	755
PB.5478.2	4	1952	2	genic	gene: Zm00001d052312	novel	102.32	115.68	coding	422	247	1512
PB.8482.1	7	752	4	full-splice_match	gene: Zm00001d020450	Zm00001d020450_T001	459.99	522.38	coding	120	78	437
PB.3067.1	2	2969	5	full-splice_match	gene: Zm00001d005156	Zm00001d005156_T002	8.16	8.16	coding	827	20	2500
PB.145.1	1	668	2	full-splice_match	gene: Zm00001d027855	Zm00001d027855_T001	86.04	86.04	non_coding	NA	NA	NA
PB.4088.2	3	402	3	full-splice_match	gene: Zm00001d041727	Zm00001d041727_T001	1342.56	1395.62	non_coding	NA	NA	NA
PB.7265.1	6	1293	6	novel_not_in_catalog	gene: Zm00001d035224	novel	7.23	7.23	coding	224	197	868
PB.3319.1	2	1327	9	full-splice_match	gene: Zm00001d006410	Zm00001d006410_T011	13.51	58.40	coding	260	110	889
PB.8305.1	7	3295	3	genic	gene: Zm00001d019356	novel	1.89	3.97	coding	987	22	2982
PB.8305.2	7	3276	3	genic	gene: Zm00001d019356	novel	2.08	3.97	coding	987	146	3106
PB.4368.1	3	2192	4	novel_in_catalog	gene: Zm00001d043019	novel	76.79	238.63	coding	544	345	1976

PB.5256.2	4	2521	7	full-splice_match	gene: Zm00001d051291	Zm00001d051291_T002	28.24	28.24	coding	608	250	2073
PB.8599.1	7	1840	10	full-splice_match	gene: Zm00001d020973	Zm00001d020973_T003	32.55	32.55	coding	394	268	1449
PB.5288.2	4	1064	1	full-splice_match	gene: Zm00001d051468	Zm00001d051468_T001	18.38	35.88	coding	159	216	692
PB.2305.1	10	1528	10	incomplete-splice_match	gene: Zm00001d026483	Zm00001d026483_T004	296.34	296.34	coding	247	460	1200
PB.5288.1	4	1141	1	full-splice_match	gene: Zm00001d051468	Zm00001d051468_T001	17.50	35.88	coding	159	221	697
PB.4303.1	3	1712	12	full-splice_match	gene: Zm00001d042722	Zm00001d042722_T014	17.70	17.70	coding	190	71	640
PB.5426.1	4	492	1	full-splice_match	gene: Zm00001d052077	Zm00001d052077_T001	15.48	15.48	non_coding	NA	NA	NA
PB.1045.1	1	2034	14	novel_not_in_catalog	gene: Zm00001d032441	novel	48.54	55.74	coding	487	147	1607
PB.4774.1	3	1002	4	novel_in_catalog	gene: Zm00001d044653	novel	105.46	108.25	coding	187	128	688
PB.5335.1	4	1757	10	full-splice_match	gene: Zm00001d051671	Zm00001d051671_T010	47.70	47.70	coding	450	175	1524
PB.5788.1	4	2320	1	full-splice_match	gene: Zm00001d053695	Zm00001d053695_T001	11.92	11.92	coding	680	117	2156
PB.1955.1	10	1571	1	antisense	novelGene_gene: Zm00001d024875_A	novel	0.64	0.64	coding	166	183	680
PB.1781.1	10	1010	3	full-splice_match	gene: Zm00001d023865	Zm00001d023865_T001	9.90	9.90	coding	254	3	764
PB.3045.1	2	1833	6	novel_in_catalog	gene: Zm00001d005030	novel	22.57	22.57	non_coding	NA	NA	NA
PB.5477.1	4	1065	6	full-splice_match	gene: Zm00001d052310	Zm00001d052310_T001	23.85	29.95	coding	273	77	895
PB.5801.1	4	2864	18	full-splice_match	gene: Zm00001d053765	Zm00001d053765_T006	95.10	198.66	coding	614	784	2625
PB.9611.3	8	2343	11	full-splice_match	gene: Zm00001d010928	Zm00001d010928_T001	11.13	11.13	coding	659	199	2175

PB.6287.1	5	605	1	full-splice_match	gene: Zm00001d014355	Zm00001d014355_T001	664.51	664.51	coding	137	4	414
PB.8881.1	7	1289	1	intergenic	novelGene_277	novel	10.69	10.69	coding	298	84	977
PB.9082.1	8	535	1	full-splice_match	gene: Zm00001d008288	Zm00001d008288_T001	324.12	324.12	coding	91	157	429
PB.6888.1	5	1429	2	full-splice_match	gene: Zm00001d017419	Zm00001d017419_T001	27.29	27.29	coding	357	143	1213
PB.1020.6	1	2360	7	full-splice_match	gene: Zm00001d032350	Zm00001d032350_T006	0.00	135.32	coding	114	227	568
PB.3006.1	2	2682	2	intergenic	novelGene_220	novel	11.97	20.19	non_coding	NA	NA	NA
PB.4783.2	3	3026	9	full-splice_match	gene: Zm00001d044689	Zm00001d044689_T001	4.83	4.83	coding	875	176	2800
PB.2483.10	2	2144	1	incomplete-splice_match	gene: Zm00001d002141	Zm00001d002141_T018	6.25	691.17	coding	521	63	1625
PB.2483.11	2	1960	2	full-splice_match	gene: Zm00001d002141	Zm00001d002141_T021	11.50	691.17	coding	424	281	1552
PB.2483.12	2	1896	2	full-splice_match	gene: Zm00001d002141	Zm00001d002141_T022	0.00	691.17	coding	521	37	1599
PB.5280.1	4	1564	5	full-splice_match	gene: Zm00001d051419	Zm00001d051419_T002	40.82	40.82	coding	371	144	1256
PB.9103.2	8	1468	9	full-splice_match	gene: Zm00001d008376	Zm00001d008376_T003	58.60	88.92	coding	326	252	1229
PB.9103.1	8	679	3	incomplete-splice_match	gene: Zm00001d008376	Zm00001d008376_T002	30.32	88.92	coding	97	60	350
PB.1795.1	10	2075	3	full-splice_match	gene: Zm00001d023929	Zm00001d023929_T001	34.74	34.74	coding	578	44	1777
PB.846.9	1	1795	8	novel_in_catalog	gene: Zm00001d031502	novel	1.14	54.70	coding	184	220	771
PB.1795.3	10	1945	3	full-splice_match	gene: Zm00001d023929	Zm00001d023929_T001	0.00	34.74	coding	578	45	1778
PB.3609.3	2	2600	6	full-splice_match	gene: Zm00001d007865	Zm00001d007865_T001	0.00	101.69	coding	732	253	2448

PB.10538.1	9	3005	8	novel_in_catalog	gene: Zm00001d047527	novel	24.03	25.92	coding	814	25	2466
PB.7991.1	6	1296	2	full-splice_match	gene: Zm00001d038700	Zm00001d038700_T001	16.99	16.99	coding	368	72	1175
PB.846.3	1	3089	8	novel_in_catalog	gene: Zm00001d031502	novel	4.96	54.70	coding	184	147	698
PB.846.4	1	2356	10	full-splice_match	gene: Zm00001d031502	Zm00001d031502_T026	13.23	54.70	coding	283	182	1030
PB.846.5	1	2455	9	full-splice_match	gene: Zm00001d031502	Zm00001d031502_T004	0.00	54.70	coding	353	134	1192
PB.846.6	1	2744	8	novel_in_catalog	gene: Zm00001d031502	novel	1.98	54.70	coding	256	185	952
PB.846.7	1	1377	10	full-splice_match	gene: Zm00001d031502	Zm00001d031502_T023	25.43	54.70	coding	282	192	1037
PB.4998.1	4	733	4	full-splice_match	gene: Zm00001d049625	Zm00001d049625_T002	7.53	7.53	coding	110	301	630
PB.4127.2	3	1624	11	full-splice_match	gene: Zm00001d041945	Zm00001d041945_T005	8.27	124.21	coding	397	219	1409
PB.5460.1	4	1285	10	full-splice_match	gene: Zm00001d052247	Zm00001d052247_T001	73.65	73.65	coding	303	206	1114
PB.9707.4	8	1880	5	full-splice_match	gene: Zm00001d011418	Zm00001d011418_T003	11.70	72.20	coding	528	94	1677
PB.3848.3	3	2767	7	novel_not_in_catalog	gene: Zm00001d040218	novel	4.00	63.01	coding	698	159	2252
PB.3848.1	3	3381	7	novel_not_in_catalog	gene: Zm00001d040218	novel	9.88	63.01	coding	670	657	2666
PB.5840.2	4	2031	8	novel_in_catalog	gene: Zm00001d053909	novel	0.00	14.98	coding	468	72	1475
PB.3848.7	3	2224	2	incomplete-splice_match	gene: Zm00001d040218	Zm00001d040218_T026	38.91	63.01	coding	516	94	1641
PB.3848.4	3	1587	3	incomplete-splice_match	gene: Zm00001d040218	Zm00001d040218_T001	10.23	63.01	non_coding	NA	NA	NA
PB.9996.1	8	3285	10	full-splice_match	gene: Zm00001d012655	Zm00001d012655_T002	6.51	6.51	coding	868	421	3024

PB.4594.1	3	1829	2	full-splice_match	gene: Zm00001d043880	Zm00001d043880_T001	4.24	21.01	coding	370	59	1168
PB.1268.2	1	1919	1	full-splice_match	gene: Zm00001d033482	Zm00001d033482_T001	0.00	18.86	coding	551	118	1770
PB.3810.1	3	1621	5	full-splice_match	gene: Zm00001d040020	Zm00001d040020_T001	30.16	30.16	coding	437	28	1338
PB.7247.2	6	1763	3	full-splice_match	gene: Zm00001d035140	Zm00001d035140_T004	1.29	44.24	coding	506	112	1629
PB.7247.1	6	2409	2	novel_in_catalog	gene: Zm00001d035140	novel	42.95	44.24	coding	506	366	1883
PB.1414.3	1	1884	2	genic	gene: Zm00001d034104	novel	0.00	5.12	coding	520	111	1670
PB.6838.1	5	916	1	intergenic	novelGene_305	novel	24.61	24.61	coding	116	124	471
PB.2688.1	2	1474	7	full-splice_match	gene: Zm00001d002980	Zm00001d002980_T003	37.72	37.72	coding	381	55	1197
PB.5776.1	4	2430	7	novel_in_catalog	gene: Zm00001d053648	novel	4.81	14.22	non_coding	NA	NA	NA
PB.5776.2	4	1454	11	full-splice_match	gene: Zm00001d053648	Zm00001d053648_T002	9.41	14.22	coding	327	229	1209
PB.5683.1	4	1562	9	novel_not_in_catalog	gene: Zm00001d053184	novel	20.97	20.97	coding	91	559	831
PB.1959.2	10	1864	1	full-splice_match	gene: Zm00001d024885	Zm00001d024885_T001	0.00	3.09	coding	527	78	1658
PB.1959.1	10	2175	1	full-splice_match	gene: Zm00001d024885	Zm00001d024885_T001	3.09	3.09	coding	527	266	1846
PB.4266.2	3	940	5	novel_in_catalog	gene: Zm00001d042578	novel	6.48	6.48	coding	121	419	781
PB.3973.1	3	2142	8	novel_not_in_catalog	gene: Zm00001d041036	novel	24.32	50.39	coding	536	75	1682
PB.3973.2	3	2029	8	novel_not_in_catalog	gene: Zm00001d041036	novel	26.07	50.39	coding	536	75	1682
PB.3702.7	3	1712	8	full-splice_match	gene: Zm00001d039453	Zm00001d039453_T002	0.00	61.97	coding	503	106	1614

PB.3702.6	3	1938	8	full-splice_match	gene: Zm00001d039453	Zm00001d039453_T002	0.00	61.97	coding	503	174	1682
PB.3702.4	3	2380	8	novel_not_in_catalog	gene: Zm00001d039453	novel	3.94	61.97	coding	343	179	1207
PB.3702.2	3	2205	8	full-splice_match	gene: Zm00001d039453	Zm00001d039453_T002	58.03	61.97	coding	503	210	1718
PB.660.1	1	1968	3	incomplete-splice_match	gene: Zm00001d030340	Zm00001d030340_T024	18.92	18.92	coding	446	104	1441
PB.1600.8	1	2438	12	full-splice_match	gene: Zm00001d034809	Zm00001d034809_T026	6.79	80.83	coding	340	108	1127
PB.695.3	1	435	2	genic	gene: Zm00001d030618	novel	633.75	852.21	non_coding	NA	NA	NA
PB.4924.1	4	1148	3	full-splice_match	gene: Zm00001d049225	Zm00001d049225_T001	9.76	9.76	coding	300	55	954
PB.5792.1	4	1107	5	full-splice_match	gene: Zm00001d053724	Zm00001d053724_T001	9.87	32.66	coding	178	84	617
PB.3766.1	3	879	2	full-splice_match	gene: Zm00001d039786	Zm00001d039786_T001	196.04	196.04	coding	144	90	521
PB.640.1	1	1708	1	full-splice_match	gene: Zm00001d030181	Zm00001d030181_T001	8.18	18.31	coding	368	138	1241
PB.3766.3	3	776	2	full-splice_match	gene: Zm00001d039786	Zm00001d039786_T001	0.00	196.04	coding	144	90	521
PB.3094.2	2	1337	3	novel_not_in_catalog	gene: Zm00001d005295	novel	91.91	110.79	coding	328	78	1061
PB.4304.13	3	2061	6	novel_not_in_catalog	gene: Zm00001d042724	novel	0.00	239.40	coding	217	1361	2011
PB.8713.2	7	3581	2	genic	gene: Zm00001d021494	novel	2.52	30.53	coding	1024	417	3488
PB.4304.11	3	1983	6	novel_not_in_catalog	gene: Zm00001d042724	novel	21.72	239.40	coding	480	353	1792
PB.2223.1	10	2898	14	full-splice_match	gene: Zm00001d026195	Zm00001d026195_T008	15.02	15.02	coding	886	102	2759
PB.8787.2	7	1267	3	genic	gene: Zm00001d021730	novel	171.93	175.56	coding	269	143	949

PB.8787.1	7	1464	3	genic	gene: Zm00001d021730	novel	3.63	175.56	coding	269	143	949
PB.2414.2	2	1087	7	intergenic	novelGene_15	novel	268.80	307.17	coding	263	78	866
PB.3825.3	3	942	1	full-splice_match	gene: Zm00001d040111	Zm00001d040111_T001	44.39	72.45	coding	172	148	663
PB.10063.2	9	3833	22	incomplete-splice_match	gene: Zm00001d044903	Zm00001d044903_T005	1.33	10.50	coding	991	658	3630
PB.7422.1	6	765	1	full-splice_match	gene: Zm00001d036151	Zm00001d036151_T001	680.71	680.71	coding	144	72	503
PB.3034.8	2	3263	1	novel_in_catalog	gene: Zm00001d004936	novel	4.60	471.76	non_coding	NA	NA	NA
PB.3533.2	2	1694	1	full-splice_match	gene: Zm00001d007486	Zm00001d007486_T001	14.49	66.66	coding	476	71	1498
PB.8472.1	7	1260	2	full-splice_match	gene: Zm00001d020417	Zm00001d020417_T001	41.82	56.85	coding	232	126	821
PB.8472.2	7	1111	2	full-splice_match	gene: Zm00001d020417	Zm00001d020417_T001	15.03	56.85	coding	232	151	846
PB.2033.1	10	3399	12	full-splice_match	gene: Zm00001d025235	Zm00001d025235_T007	16.05	16.05	coding	976	205	3132
PB.9792.1	8	1352	9	full-splice_match	gene: Zm00001d011782	Zm00001d011782_T002	18.61	18.61	coding	282	183	1028
PB.803.4	1	1628	1	novel_in_catalog	gene: Zm00001d031232	novel	7.89	70.88	coding	232	793	1488
PB.1777.1	10	996	6	full-splice_match	gene: Zm00001d023846	Zm00001d023846_T008	33.62	33.62	coding	151	256	708
PB.5130.10	4	1970	6	novel_in_catalog	gene: Zm00001d050357	novel	18.15	42.70	coding	226	1138	1815
PB.9956.4	8	2547	3	novel_not_in_catalog	gene: Zm00001d012514	novel	0.00	15.55	coding	637	484	2394
PB.803.7	1	3021	12	incomplete-splice_match	gene: Zm00001d031232	Zm00001d031232_T056	12.43	70.88	coding	504	239	1750
PB.4746.1	3	1109	3	full-splice_match	gene: Zm00001d044527	Zm00001d044527_T001	70.87	103.91	coding	240	54	773

PB.7257.1	6	758	5	full-splice_match	gene: Zm00001d035195	Zm00001d035195_T001	28.19	28.19	coding	150	120	569
PB.803.6	1	920	2	novel_not_in_catalog	gene: Zm00001d031232	novel	36.08	70.88	coding	172	266	781
PB.1993.3	10	1847	2	genic	gene: Zm00001d025017	novel	0.00	87.26	coding	197	762	1352
PB.7873.2	6	1484	13	incomplete-splice_match	gene: Zm00001d038205	Zm00001d038205_T003	0.00	15.26	coding	430	61	1350
PB.7418.4	6	1213	7	full-splice_match	gene: Zm00001d036123	Zm00001d036123_T002	14.70	42.28	coding	358	56	1129
PB.7418.1	6	1464	7	full-splice_match	gene: Zm00001d036123	Zm00001d036123_T002	20.28	42.28	coding	358	24	1097
PB.7861.4	6	1453	2	incomplete-splice_match	gene: Zm00001d038186	Zm00001d038186_T002	0.00	27.54	coding	480	14	1453
PB.7418.3	6	1399	6	novel_in_catalog	gene: Zm00001d036123	novel	7.30	42.28	coding	129	139	525
PB.671.1	1	1530	9	full-splice_match	gene: Zm00001d030385	Zm00001d030385_T007	23.60	48.91	coding	354	119	1180
PB.10480.1	9	691	3	novel_not_in_catalog	gene: Zm00001d047282	novel	1959.91	1959.91	coding	99	105	401
PB.8404.1	7	1411	11	full-splice_match	gene: Zm00001d020053	Zm00001d020053_T002	18.34	18.34	coding	360	159	1238
PB.803.3	1	3642	22	full-splice_match	gene: Zm00001d031232	Zm00001d031232_T082	9.81	70.88	coding	1136	94	3501
PB.6696.9	5	1921	1	novel_in_catalog	gene: Zm00001d016553	novel	11.91	140.34	coding	202	1040	1645
PB.6118.3	5	2406	4	novel_in_catalog	gene: Zm00001d013527	novel	9.79	74.65	coding	509	197	1723
PB.8339.1	7	2120	17	fusion	gene: Zm00001d019567_g	novel	29.23	39.17	coding	542	143	1768
PB.6118.1	5	2253	5	full-splice_match	gene: Zm00001d013527	Zm00001d013527_T002	15.17	74.65	coding	585	124	1878
PB.8339.3	7	2298	18	fusion	gene: Zm00001d019567_g	novel	9.94	39.17	coding	615	107	1951

PB.6118.7	5	3011	2	novel_in_catalog	gene: Zm00001d013527	novel	1.25	74.65	coding	312	122	1057
PB.6118.6	5	2247	5	full-splice_match	gene: Zm00001d013527	Zm00001d013527_T002	0.29	74.65	coding	585	278	2032
PB.6118.5	5	2274	4	novel_in_catalog	gene: Zm00001d013527	novel	0.00	74.65	coding	509	124	1650
PB.6118.4	5	2287	4	novel_in_catalog	gene: Zm00001d013527	novel	1.57	74.65	coding	660	124	2103
PB.7624.2	6	1132	10	full-splice_match	gene: Zm00001d037246	Zm00001d037246_T003	7.26	182.30	coding	242	163	888
PB.1182.1	1	1050	5	fusion	gene: Zm00001d033021_g	novel	34.55	34.55	non_coding	NA	NA	NA
PB.7624.1	6	1186	11	full-splice_match	gene: Zm00001d037246	Zm00001d037246_T002	175.04	182.30	coding	250	170	919
PB.3667.1	3	2244	2	full-splice_match	gene: Zm00001d039343	Zm00001d039343_T001	7.94	7.94	coding	653	48	2006
PB.709.1	1	2395	4	full-splice_match	gene: Zm00001d030732	Zm00001d030732_T001	43.33	43.33	coding	665	64	2058
PB.10658.1	9	1881	1	full-splice_match	gene: Zm00001d047931	Zm00001d047931_T001	25.27	25.27	coding	399	268	1464
PB.10819.1	9	1735	12	incomplete-splice_match	gene: Zm00001d048514	Zm00001d048514_T009	108.20	108.20	coding	371	50	1162
PB.5165.1	4	858	1	full-splice_match	gene: Zm00001d050609	Zm00001d050609_T001	16.80	16.80	coding	247	34	774
PB.10465.4	9	2537	6	full-splice_match	gene: Zm00001d047191	Zm00001d047191_T004	0.72	44.26	coding	732	83	2278
PB.10538.4	9	4720	9	novel_not_in_catalog	gene: Zm00001d047527	novel	1.88	25.92	coding	1306	467	4384
PB.10073.3	9	2180	3	novel_in_catalog	gene: Zm00001d044940	novel	0.10	15.63	coding	305	115	1029
PB.3605.1	2	3534	3	full-splice_match	gene: Zm00001d007853	Zm00001d007853_T001	7.35	7.35	coding	517	215	1765
PB.10073.1	9	1551	4	full-splice_match	gene: Zm00001d044940	Zm00001d044940_T002	15.53	15.63	coding	351	113	1165

PB.9119.4	8	2192	8	full-splice_match	gene: Zm00001d008439	Zm00001d008439_T001	0.00	101.30	coding	649	145	2091
PB.9119.3	8	2423	7	full-splice_match	gene: Zm00001d008439	Zm00001d008439_T002	1.27	101.30	coding	627	121	2001
PB.9119.2	8	2519	8	full-splice_match	gene: Zm00001d008439	Zm00001d008439_T001	38.60	101.30	coding	649	122	2068
PB.9119.1	8	1719	5	incomplete-splice_match	gene: Zm00001d008439	Zm00001d008439_T001	61.43	101.30	coding	371	71	1183
PB.10089.1	9	2724	16	full-splice_match	gene: Zm00001d045042	Zm00001d045042_T007	526.40	526.40	coding	802	67	2472
PB.6422.2	5	1697	14	full-splice_match	gene: Zm00001d014901	Zm00001d014901_T001	93.90	93.90	coding	536	43	1650
PB.6390.1	5	1596	4	novel_not_in_catalog	gene: Zm00001d014780	novel	14.26	14.26	coding	412	145	1380
PB.8797.1	7	2325	6	full-splice_match	gene: Zm00001d021754	Zm00001d021754_T002	41.73	41.73	coding	640	76	1995
PB.4745.4	3	1702	3	novel_in_catalog	gene: Zm00001d044516	novel	3.68	381.23	coding	297	122	1012
PB.9018.3	7	889	6	full-splice_match	gene: Zm00001d022513	Zm00001d022513_T001	0.00	1516.94	coding	219	93	749
PB.10194.2	9	815	2	novel_in_catalog	gene: Zm00001d045495	novel	11.19	43.59	coding	138	97	510
PB.10194.1	9	1419	5	full-splice_match	gene: Zm00001d045495	Zm00001d045495_T001	32.40	43.59	coding	361	157	1239
PB.7009.14	5	1724	5	antisense	novelGene_gene: Zm00001d017932_A	novel	0.00	81.66	non_coding	NA	NA	NA
PB.7009.12	5	1797	7	antisense	novelGene_gene: Zm00001d017932_A	novel	0.00	81.66	non_coding	NA	NA	NA
PB.4453.1	3	568	2	full-splice_match	gene: Zm00001d043386	Zm00001d043386_T001	2.42	2.42	coding	93	103	381
PB.7009.10	5	1851	5	antisense	novelGene_gene: Zm00001d017932_A	novel	0.00	81.66	non_coding	NA	NA	NA
PB.2112.1	10	2097	1	full-splice_match	gene: Zm00001d025695	Zm00001d025695_T001	1.41	1.41	coding	605	262	2076

PB.10465.1	9	2734	6	full-splice_match	gene: Zm00001d047191	Zm00001d047191_T004	38.06	44.26	coding	732	80	2275
PB.6941.1	5	1389	1	full-splice_match	gene: Zm00001d017657	Zm00001d017657_T001	32.73	32.73	coding	284	149	1000
PB.1622.2	1	983	1	full-splice_match	gene: Zm00001d034874	Zm00001d034874_T001	0.00	61.12	coding	215	66	710
PB.4745.9	3	1487	4	full-splice_match	gene: Zm00001d044516	Zm00001d044516_T001	254.90	381.23	coding	365	127	1221
PB.7928.1	6	2439	8	full-splice_match	gene: Zm00001d038409	Zm00001d038409_T003	5.69	16.41	coding	684	214	2265
PB.2307.3	10	1604	1	full-splice_match	gene: Zm00001d026489	Zm00001d026489_T001	13.07	32.42	coding	392	196	1371
PB.7857.1	6	1623	1	novel_in_catalog	gene: Zm00001d038166	novel	8.33	8.33	coding	532	4	1599
PB.2386.1	2	2879	1	full-splice_match	gene: Zm00001d001766	Zm00001d001766_T001	0.41	52.69	coding	311	197	1129
PB.2386.2	2	1706	1	full-splice_match	gene: Zm00001d001766	Zm00001d001766_T001	22.58	52.69	coding	311	190	1122
PB.2386.3	2	1545	1	full-splice_match	gene: Zm00001d001766	Zm00001d001766_T001	29.71	52.69	coding	311	195	1127
PB.7036.1	5	2020	12	incomplete-splice_match	gene: Zm00001d018014	Zm00001d018014_T013	18.46	18.46	coding	526	181	1758
PB.347.1	1	897	2	novel_in_catalog	gene: Zm00001d028745	novel	13.38	13.38	coding	212	36	671
PB.2537.2	2	798	1	full-splice_match	gene: Zm00001d002347	Zm00001d002347_T001	485.53	488.87	coding	145	54	488
PB.2537.3	2	718	1	full-splice_match	gene: Zm00001d002347	Zm00001d002347_T001	0.00	488.87	coding	145	60	494
PB.5195.1	4	1112	2	full-splice_match	gene: Zm00001d050905	Zm00001d050905_T001	20.94	20.94	coding	194	160	741
PB.2537.1	2	1624	1	full-splice_match	gene: Zm00001d002347	Zm00001d002347_T001	3.34	488.87	coding	145	50	484
PB.2994.2	2	2535	22	incomplete-splice_match	gene: Zm00001d004707	Zm00001d004707_T001	6.17	6.17	coding	746	18	2255

PB.2222.1	10	1224	4	full-splice_match	gene: Zm00001d026194	Zm00001d026194_T001	31.72	31.72	coding	254	158	919
PB.8390.7	7	2550	21	full-splice_match	gene: Zm00001d019978	Zm00001d019978_T010	46.18	46.18	coding	271	167	979
PB.3495.1	2	1089	8	full-splice_match	gene: Zm00001d007234	Zm00001d007234_T003	190.79	419.80	coding	191	359	931
PB.9463.1	8	1395	15	novel_not_in_catalog	gene: Zm00001d010262	novel	41.62	41.62	coding	357	69	1139
PB.2889.1	2	1247	5	full-splice_match	gene: Zm00001d003904	Zm00001d003904_T001	126.86	126.86	coding	173	304	822
PB.9463.2	8	1292	15	novel_not_in_catalog	gene: Zm00001d010262	novel	0.00	41.62	coding	357	69	1139
PB.6102.2	5	2404	1	full-splice_match	gene: Zm00001d013465	Zm00001d013465_T001	93.70	93.70	coding	625	220	2094
PB.6998.1	5	1550	9	full-splice_match	gene: Zm00001d017881	Zm00001d017881_T002	102.51	102.51	coding	332	224	1219
PB.1250.1	1	2956	4	full-splice_match	gene: Zm00001d033388	Zm00001d033388_T002	15.56	34.59	coding	720	19	2178
PB.8921.1	7	1549	9	novel_in_catalog	gene: Zm00001d022202	novel	25.69	25.69	coding	439	55	1371
PB.4864.3	4	1403	5	intergenic	novelGene_43	novel	7.94	18.79	non_coding	NA	NA	NA
PB.4864.2	4	1273	6	intergenic	novelGene_43	novel	10.85	18.79	non_coding	NA	NA	NA
PB.7162.18	5	2757	15	full-splice_match	gene: Zm00001d018445	Zm00001d018445_T004	198.72	433.77	coding	805	52	2466
PB.6951.2	5	2563	6	full-splice_match	gene: Zm00001d017698	Zm00001d017698_T004	7.84	12.43	coding	758	91	2364
PB.5824.1	4	2792	6	full-splice_match	gene: Zm00001d053852	Zm00001d053852_T001	28.76	28.76	coding	685	332	2386
PB.5177.1	4	1869	10	novel_in_catalog	gene: Zm00001d050741	novel	48.69	48.69	coding	466	147	1544
PB.3290.1	2	1050	1	full-splice_match	gene: Zm00001d006294	Zm00001d006294_T001	9.91	9.91	coding	267	64	864

PB.4196.1	3	902	5	full-splice_match	gene: Zm00001d042257	Zm00001d042257_T001	12.77	12.77	coding	229	48	734
PB.3319.2	2	1204	9	full-splice_match	gene: Zm00001d006410	Zm00001d006410_T011	44.88	58.40	coding	260	107	886
PB.4443.1	3	3119	5	full-splice_match	gene: Zm00001d043329	Zm00001d043329_T002	68.58	72.65	coding	807	210	2630
PB.4443.2	3	3072	5	full-splice_match	gene: Zm00001d043329	Zm00001d043329_T002	4.07	72.65	coding	807	332	2752
PB.5396.2	4	2585	2	full-splice_match	gene: Zm00001d051948	Zm00001d051948_T001	5.00	80.23	coding	632	393	2288
PB.4172.1	3	1742	6	novel_in_catalog	gene: Zm00001d042088	novel	24.18	24.18	coding	474	102	1523
PB.2692.2	2	1850	2	genic	gene: Zm00001d003007	novel	0.00	19.50	coding	468	139	1542
PB.6969.2	5	2969	7	full-splice_match	gene: Zm00001d017780	Zm00001d017780_T001	0.00	26.77	coding	765	445	2739
PB.3034.2	2	799	2	full-splice_match	gene: Zm00001d004936	Zm00001d004936_T002	65.05	471.76	non_coding	NA	NA	NA
PB.2692.1	2	2058	2	genic	gene: Zm00001d003007	novel	19.50	19.50	coding	468	245	1648
PB.8525.1	7	1337	1	full-splice_match	gene: Zm00001d020651	Zm00001d020651_T001	7.22	7.22	coding	308	178	1101
PB.3528.1	2	1382	3	incomplete-splice_match	gene: Zm00001d007457	Zm00001d007457_T003	30.23	48.71	coding	219	94	750
PB.1100.2	1	1864	8	incomplete-splice_match	gene: Zm00001d032637	Zm00001d032637_T018	67.84	102.07	coding	503	113	1621
PB.837.1	1	2450	8	incomplete-splice_match	gene: Zm00001d031447	Zm00001d031447_T004	52.83	69.53	coding	581	186	1928
PB.8798.1	7	1941	1	full-splice_match	gene: Zm00001d021757	Zm00001d021757_T001	69.15	69.15	coding	518	142	1695
PB.4776.1	3	1080	1	full-splice_match	gene: Zm00001d044660	Zm00001d044660_T001	24.12	124.96	coding	227	141	821
PB.6659.1	5	1636	1	novel_in_catalog	gene: Zm00001d016326	novel	40.38	83.53	coding	374	128	1249

PB.6659.3	5	2635	1	incomplete-splice_match	gene: Zm00001d016326	Zm00001d016326_T010	13.35	83.53	coding	374	1348	2469
PB.6659.2	5	1989	6	incomplete-splice_match	gene: Zm00001d016326	Zm00001d016326_T001	23.93	83.53	coding	450	363	1712
PB.6659.5	5	1454	3	incomplete-splice_match	gene: Zm00001d016326	Zm00001d016326_T001	0.00	83.53	coding	429	34	1320
PB.6659.4	5	2818	2	incomplete-splice_match	gene: Zm00001d016326	Zm00001d016326_T008	5.87	83.53	coding	374	1514	2635
PB.917.1	1	1986	1	full-splice_match	gene: Zm00001d031866	Zm00001d031866_T001	2.47	2.47	coding	442	494	1819
PB.6799.3	5	1004	4	full-splice_match	gene: Zm00001d017047	Zm00001d017047_T001	214.67	389.64	coding	219	64	720
PB.4460.1	3	2499	12	novel_in_catalog	gene: Zm00001d043404	novel	5.70	8.37	coding	435	977	2281
PB.1341.2	1	2711	2	incomplete-splice_match	gene: Zm00001d033798	Zm00001d033798_T033	3.97	32.54	coding	680	672	2711
PB.7981.1	6	2892	3	full-splice_match	gene: Zm00001d038622	Zm00001d038622_T001	18.82	18.82	coding	751	408	2660
PB.9822.1	8	1460	9	full-splice_match	gene: Zm00001d011889	Zm00001d011889_T001	40.23	40.23	coding	436	51	1358
PB.8158.1	7	2149	10	novel_in_catalog	gene: Zm00001d018639	novel	0.00	75.50	coding	318	518	1471
PB.8158.3	7	2000	11	full-splice_match	gene: Zm00001d018639	Zm00001d018639_T001	17.49	75.50	coding	412	90	1325
PB.8158.2	7	1894	12	incomplete-splice_match	gene: Zm00001d018639	Zm00001d018639_T004	10.16	75.50	coding	478	140	1573
PB.8158.5	7	1672	12	incomplete-splice_match	gene: Zm00001d018639	Zm00001d018639_T004	35.49	75.50	coding	478	85	1518
PB.8158.4	7	1942	10	novel_in_catalog	gene: Zm00001d018639	novel	1.71	75.50	coding	318	500	1453
PB.10864.1	B73V4 ctg150	1817	12	incomplete-splice_match	gene: Zm00001d000002	Zm00001d000002_T011	5.10	5.10	coding	484	48	1499
PB.1957.4	10	2478	6	novel_not_in_catalog	gene: Zm00001d024878	novel	4.00	28.66	coding	274	235	1056

PB.8539.1	7	932	1	full-splice_match	gene: Zm00001d020725	Zm00001d020725_T001	5.75	5.75	coding	252	1	756
PB.1957.1	10	2033	7	novel_not_in_catalog	gene: Zm00001d024878	novel	16.86	28.66	coding	449	420	1766
PB.8827.3	7	2739	3	novel_not_in_catalog	gene: Zm00001d021858	novel	39.53	140.53	coding	776	30	2357
PB.1957.3	10	2636	6	novel_not_in_catalog	gene: Zm00001d024878	novel	0.00	28.66	coding	264	423	1214
PB.1957.2	10	2009	7	novel_not_in_catalog	gene: Zm00001d024878	novel	7.81	28.66	coding	439	426	1742
PB.5103.2	4	878	3	full-splice_match	gene: Zm00001d050182	Zm00001d050182_T001	64.94	85.09	coding	199	65	661
PB.4480.1	3	1854	3	full-splice_match	gene: Zm00001d043469	Zm00001d043469_T005	75.97	75.97	coding	435	150	1454
PB.5103.1	4	1021	3	full-splice_match	gene: Zm00001d050182	Zm00001d050182_T001	20.15	85.09	coding	199	57	653
PB.4760.1	3	3044	11	full-splice_match	gene: Zm00001d044593	Zm00001d044593_T001	65.85	65.85	coding	921	147	2909
PB.10844.4	B73V4 ctg105	1791	1	genic	gene: Zm00001d000424	novel	0.85	58.45	coding	291	473	1345
PB.10844.1	B73V4 ctg105	1493	1	novel_in_catalog	gene: Zm00001d000424	novel	15.03	58.45	coding	291	539	1411
PB.2646.1	2	2172	2	full-splice_match	gene: Zm00001d002811	Zm00001d002811_T001	44.81	120.79	coding	371	392	1504
PB.10844.3	B73V4 ctg105	1199	2	full-splice_match	gene: Zm00001d000424	Zm00001d000424_T001	41.52	58.45	coding	291	248	1120
PB.10844.2	B73V4 ctg105	2432	1	genic	gene: Zm00001d000424	novel	1.06	58.45	coding	291	497	1369
PB.1105.3	1	816	1	full-splice_match	gene: Zm00001d032663	Zm00001d032663_T001	0.00	216.87	coding	180	94	633
PB.1449.1	1	1097	5	full-splice_match	gene: Zm00001d034313	Zm00001d034313_T001	42.23	59.19	coding	248	23	766
PB.4856.1	4	1355	7	full-splice_match	gene: Zm00001d048893	Zm00001d048893_T001	81.49	81.49	coding	316	182	1129

PB.5036.1	4	1087	3	novel_not_in_catalog	gene: Zm00001d049784	novel	3.14	3.14	coding	239	75	791
PB.8794.3	7	1820	7	novel_in_catalog	gene: Zm00001d021748	novel	0.00	36.95	coding	374	300	1421
PB.3576.3	2	1893	8	full-splice_match	gene: Zm00001d007754	Zm00001d007754_T001	6.04	37.31	coding	472	21	1436
PB.7909.1	6	1445	9	full-splice_match	gene: Zm00001d038348	Zm00001d038348_T002	17.09	19.75	non_coding	NA	NA	NA
PB.8794.2	7	1867	8	full-splice_match	gene: Zm00001d021748	Zm00001d021748_T002	5.24	36.95	coding	374	312	1433
PB.715.3	1	1325	1	genic	gene: Zm00001d030772	novel	0.00	14.19	coding	308	61	984
PB.715.2	1	2200	2	genic	gene: Zm00001d030772	novel	0.00	14.19	coding	4	666	677
PB.715.1	1	2759	2	genic	gene: Zm00001d030772	novel	14.19	14.19	coding	308	149	1072
PB.3677.1	3	1562	8	novel_in_catalog	gene: Zm00001d039369	novel	8.57	31.87	coding	364	92	1183
PB.8868.1	7	1177	1	antisense	novelGene_gene: Zm00001d022009_A	novel	31.78	31.78	coding	244	77	808
PB.3254.2	2	2230	16	novel_not_in_catalog	gene: Zm00001d006131	novel	0.51	88.89	coding	335	939	1943
PB.3254.1	2	2286	18	novel_not_in_catalog	gene: Zm00001d006131	novel	85.32	88.89	coding	595	175	1959
PB.3835.1	3	2244	18	incomplete-splice_match	gene: Zm00001d040166	Zm00001d040166_T009	9.97	12.96	coding	527	12	1592
PB.1458.1	1	1656	2	full-splice_match	gene: Zm00001d034340	Zm00001d034340_T001	15.88	15.88	coding	438	118	1431
PB.5108.1	4	1393	9	full-splice_match	gene: Zm00001d050208	Zm00001d050208_T007	50.03	50.03	coding	332	125	1120
PB.8531.1	7	1707	11	full-splice_match	gene: Zm00001d020692	Zm00001d020692_T001	7.25	7.25	coding	398	221	1414
PB.4468.1	3	1444	7	full-splice_match	gene: Zm00001d043432	Zm00001d043432_T001	17.41	18.68	coding	363	108	1196

PB.8794.6	7	1733	7	novel_in_catalog	gene: Zm00001d021748	novel	0.00	36.95	coding	374	297	1418
PB.10771.1	9	3194	2	full-splice_match	gene: Zm00001d048357	Zm00001d048357_T001	0.28	0.28	coding	990	59	3028
PB.9863.1	8	2176	10	novel_in_catalog	gene: Zm00001d012084	novel	19.09	19.09	coding	433	773	2071
PB.2151.1	10	2655	9	full-splice_match	gene: Zm00001d025837	Zm00001d025837_T001	18.44	18.57	coding	736	266	2473
PB.3515.1	2	500	2	full-splice_match	gene: Zm00001d007381	Zm00001d007381_T001	4.95	4.95	coding	81	75	317
PB.6943.2	5	2209	5	novel_not_in_catalog	gene: Zm00001d017666	novel	0.86	14.04	coding	367	135	1235
PB.3557.3	2	1271	9	full-splice_match	gene: Zm00001d007610	Zm00001d007610_T002	40.59	47.46	coding	293	16	894
PB.10743.9	9	1789	8	full-splice_match	gene: Zm00001d048272	Zm00001d048272_T002	2.32	119.34	coding	466	343	1740
PB.10743.8	9	2456	7	novel_in_catalog	gene: Zm00001d048272	novel	2.35	119.34	coding	241	325	1047
PB.10743.5	9	1883	8	novel_not_in_catalog	gene: Zm00001d048272	novel	90.53	119.34	coding	466	192	1589
PB.7406.1	6	1605	2	full-splice_match	gene: Zm00001d036051	Zm00001d036051_T001	5.48	5.48	coding	426	123	1400
PB.10743.7	9	1629	9	novel_in_catalog	gene: Zm00001d048272	novel	3.85	119.34	coding	436	273	1580
PB.10743.6	9	2720	7	full-splice_match	gene: Zm00001d048272	Zm00001d048272_T011	0.00	119.34	coding	466	1263	2660
PB.10743.1	9	526	1	genic	gene: Zm00001d048272	novel	12.08	119.34	non_coding	NA	NA	NA
PB.10743.3	9	3012	7	full-splice_match	gene: Zm00001d048272	Zm00001d048272_T011	4.48	119.34	coding	466	1321	2718
PB.5066.1	4	1330	8	full-splice_match	gene: Zm00001d049959	Zm00001d049959_T003	96.93	101.11	coding	304	132	1043
PB.5066.2	4	1238	8	full-splice_match	gene: Zm00001d049959	Zm00001d049959_T003	4.18	101.11	coding	304	157	1068

PB.4979.3	4	1360	6	full-splice_match	gene: Zm00001d049526	Zm00001d049526_T001	35.55	60.94	coding	275	261	1085
PB.4979.1	4	1451	6	full-splice_match	gene: Zm00001d049526	Zm00001d049526_T001	25.39	60.94	coding	275	261	1085
PB.2795.1	2	1262	3	full-splice_match	gene: Zm00001d003418	Zm00001d003418_T001	0.82	34.37	coding	193	159	737
PB.2795.2	2	1052	3	full-splice_match	gene: Zm00001d003418	Zm00001d003418_T001	33.55	34.37	coding	193	159	737
PB.7144.6	5	1409	9	full-splice_match	gene: Zm00001d018409	Zm00001d018409_T004	0.00	146.45	coding	428	76	1359
PB.7103.1	5	2780	1	full-splice_match	gene: Zm00001d018282	Zm00001d018282_T001	30.72	51.05	coding	529	757	2343
PB.6679.1	5	1566	7	full-splice_match	gene: Zm00001d016441	Zm00001d016441_T006	374.50	374.50	coding	440	148	1467
PB.4036.2	3	3044	9	full-splice_match	gene: Zm00001d041488	Zm00001d041488_T005	7.75	26.75	coding	545	821	2455
PB.400.1	1	3981	7	novel_not_in_catalog	gene: Zm00001d028987	novel	23.52	23.52	coding	1122	374	3739
PB.9875.1	8	2005	6	novel_not_in_catalog	gene: Zm00001d012144	novel	18.52	18.52	coding	555	126	1790
PB.3425.2	2	2049	1	full-splice_match	gene: Zm00001d001023	Zm00001d001023_T001	4.44	4.44	non_coding	NA	NA	NA
PB.6741.1	5	3137	3	genic	gene: Zm00001d016772	novel	14.21	14.21	coding	715	187	2331
PB.6198.2	5	1381	3	novel_in_catalog	gene: Zm00001d013934	novel	12.57	105.36	coding	124	837	1208
PB.6198.1	5	1160	5	full-splice_match	gene: Zm00001d013934	Zm00001d013934_T001	92.79	105.36	coding	315	33	977
PB.7684.3	6	1368	6	full-splice_match	gene: Zm00001d037530	Zm00001d037530_T002	18.49	27.35	coding	363	74	1162
PB.9016.1	7	1264	1	genic	gene: Zm00001d022506	novel	9.37	22.89	coding	187	524	1084
PB.9937.2	8	1851	6	full-splice_match	gene: Zm00001d012425	Zm00001d012425_T002	34.81	34.81	coding	161	1066	1548

PB.9016.2	7	1123	2	novel_not_in_catalog	gene: Zm00001d022506	novel	13.51	22.89	coding	187	358	918
PB.782.1	1	743	3	full-splice_match	gene: Zm00001d031132	Zm00001d031132_T002	80.64	1446.26	coding	84	105	356
PB.7196.2	5	823	2	full-splice_match	gene: Zm00001d018535	Zm00001d018535_T002	7.68	301.71	non_coding	NA	NA	NA
PB.10638.1	9	1098	2	full-splice_match	gene: Zm00001d047868	Zm00001d047868_T001	63.81	63.81	coding	229	184	870
PB.8239.1	7	3392	11	incomplete-splice_match	gene: Zm00001d018976	Zm00001d018976_T055	32.89	32.89	coding	1049	55	3201
PB.3222.1	2	3156	3	full-splice_match	gene: Zm00001d006011	Zm00001d006011_T001	12.00	23.08	coding	880	174	2813
PB.3222.2	2	3032	3	full-splice_match	gene: Zm00001d006011	Zm00001d006011_T001	0.00	23.08	coding	880	124	2763
PB.3222.3	2	1759	3	full-splice_match	gene: Zm00001d006011	Zm00001d006011_T001	11.08	23.08	coding	516	47	1594
PB.9759.1	8	678	3	incomplete-splice_match	gene: Zm00001d011646	Zm00001d011646_T001	77.91	77.91	coding	134	136	537
PB.2789.1	2	995	3	intergenic	novelGene_103	novel	43.85	48.16	coding	176	173	700
PB.7199.1	5	1048	2	full-splice_match	gene: Zm00001d018544	Zm00001d018544_T002	13.94	13.94	coding	293	37	915
PB.5010.1	4	1528	3	full-splice_match	gene: Zm00001d049678	Zm00001d049678_T001	6.49	6.49	coding	379	180	1316
PB.6330.1	5	950	1	full-splice_match	gene: Zm00001d014565	Zm00001d014565_T001	24.40	24.40	coding	193	178	756
PB.1467.2	1	903	1	full-splice_match	gene: Zm00001d034356	Zm00001d034356_T001	483.10	1365.31	coding	224	111	782
PB.4600.1	3	4080	12	fusion	gene: Zm00001d043915_g	novel	11.38	19.80	coding	1052	652	3807
PB.1003.2	1	3133	16	full-splice_match	gene: Zm00001d032279	Zm00001d032279_T006	26.10	26.10	coding	962	97	2982
PB.997.4	1	1799	14	novel_in_catalog	gene: Zm00001d032257	novel	90.28	90.28	coding	393	549	1727

PB.1003.4	1	2989	16	full-splice_match	gene: Zm00001d032279	Zm00001d032279_T006	0.00	26.10	coding	962	102	2987
PB.6470.1	5	1819	5	full-splice_match	gene: Zm00001d015084	Zm00001d015084_T001	58.51	63.09	coding	456	138	1505
PB.6470.2	5	2365	4	novel_in_catalog	gene: Zm00001d015084	novel	4.58	63.09	coding	296	157	1044
PB.8553.2	7	1622	1	full-splice_match	gene: Zm00001d020791	Zm00001d020791_T001	28.98	33.62	coding	361	155	1237
PB.8553.3	7	1419	1	full-splice_match	gene: Zm00001d020791	Zm00001d020791_T001	0.00	33.62	coding	361	149	1231
PB.8553.1	7	1770	1	full-splice_match	gene: Zm00001d020791	Zm00001d020791_T001	4.64	33.62	coding	361	158	1240
PB.1138.1	1	1365	9	full-splice_match	gene: Zm00001d032807	Zm00001d032807_T001	32.23	32.23	coding	359	83	1159
PB.10101.1	9	3000	17	full-splice_match	gene: Zm00001d045080	Zm00001d045080_T002	22.89	22.89	coding	810	92	2521
PB.4794.1	4	609	1	full-splice_match	gene: Zm00001d048598	Zm00001d048598_T001	18.03	18.03	coding	110	114	443
PB.3563.3	2	1637	11	full-splice_match	gene: Zm00001d007638	Zm00001d007638_T001	105.39	111.96	coding	374	240	1361
PB.476.1	1	1748	6	full-splice_match	gene: Zm00001d029303	Zm00001d029303_T001	9.45	23.30	coding	269	132	938
PB.6450.3	5	2395	8	full-splice_match	gene: Zm00001d014993	Zm00001d014993_T003	9.18	190.83	coding	663	130	2118
PB.476.3	1	1457	6	full-splice_match	gene: Zm00001d029303	Zm00001d029303_T001	0.00	23.30	coding	269	132	938
PB.476.2	1	1451	6	full-splice_match	gene: Zm00001d029303	Zm00001d029303_T001	13.85	23.30	coding	269	3	809
PB.6450.6	5	1631	4	novel_in_catalog	gene: Zm00001d014993	novel	20.72	190.83	coding	176	884	1411
PB.6450.4	5	2434	7	novel_in_catalog	gene: Zm00001d014993	novel	4.00	190.83	coding	548	567	2210
PB.9459.4	8	1339	8	novel_in_catalog	gene: Zm00001d010243	novel	2.78	48.05	coding	220	135	794

PB.5018.4	4	2643	4	full-splice_match	gene: Zm00001d049708	Zm00001d049708_T008	3.39	25.50	coding	706	308	2425
PB.5018.3	4	3000	5	full-splice_match	gene: Zm00001d049708	Zm00001d049708_T006	10.66	25.50	coding	830	216	2705
PB.6665.1	5	1598	8	full-splice_match	gene: Zm00001d016358	Zm00001d016358_T001	109.24	120.04	coding	414	148	1389
PB.5018.1	4	2749	4	full-splice_match	gene: Zm00001d049708	Zm00001d049708_T008	11.46	25.50	coding	706	303	2420
PB.2286.1	10	566	5	full-splice_match	gene: Zm00001d026392	Zm00001d026392_T001	173.61	173.61	coding	98	157	450
PB.6860.1	5	2406	1	full-splice_match	gene: Zm00001d017276	Zm00001d017276_T001	38.01	38.01	coding	697	129	2219
PB.2216.2	10	671	1	genic	gene: Zm00001d026163	novel	0.00	4617.38	coding	131	94	486
PB.4549.2	3	546	2	novel_not_in_catalog	gene: Zm00001d043724	novel	19.84	25.55	coding	118	2	355
PB.4549.1	3	2047	5	novel_not_in_catalog	gene: Zm00001d043724	novel	5.71	25.55	coding	375	90	1214
PB.2216.1	10	785	1	genic	gene: Zm00001d026163	novel	4378.30	4617.38	coding	131	94	486
PB.7942.4	6	2426	5	intergenic	novelGene_260	novel	16.25	54.59	non_coding	NA	NA	NA
PB.5429.2	4	2750	1	full-splice_match	gene: Zm00001d052104	Zm00001d052104_T001	56.93	505.95	coding	780	246	2585
PB.5429.1	4	1876	1	full-splice_match	gene: Zm00001d052104	Zm00001d052104_T001	323.84	505.95	coding	502	32	1537
PB.4755.1	3	1629	8	full-splice_match	gene: Zm00001d044554	Zm00001d044554_T003	22.43	22.43	coding	496	35	1522
PB.6939.1	5	835	2	incomplete-splice_match	gene: Zm00001d017649	Zm00001d017649_T001	137.10	137.10	coding	19	538	594
PB.6166.1	5	1634	2	full-splice_match	gene: Zm00001d013737	Zm00001d013737_T002	87.18	87.18	coding	397	122	1312
PB.6166.2	5	1402	2	full-splice_match	gene: Zm00001d013737	Zm00001d013737_T002	0.00	87.18	coding	397	10	1200

PB.8047.1	6	1508	10	full-splice_match	gene: Zm00001d038879	Zm00001d038879_T005	21.41	75.33	coding	338	248	1261
PB.4738.2	3	1135	10	full-splice_match	gene: Zm00001d044497	Zm00001d044497_T001	20.43	44.09	coding	204	318	929
PB.8955.1	7	3216	7	full-splice_match	gene: Zm00001d022335	Zm00001d022335_T003	17.26	17.26	coding	928	261	3044
PB.6205.1	5	1548	2	genic	gene: Zm00001d013971	novel	42.16	42.16	coding	385	104	1258
PB.10596.1	9	1598	6	full-splice_match	gene: Zm00001d047749	Zm00001d047749_T001	42.67	42.67	coding	289	387	1253
PB.9459.5	8	2013	14	novel_in_catalog	gene: Zm00001d010243	novel	28.44	48.05	coding	508	91	1614
PB.4438.4	3	1827	6	full-splice_match	gene: Zm00001d043309	Zm00001d043309_T019	8.87	44.80	coding	247	171	911
PB.1250.3	1	2578	4	full-splice_match	gene: Zm00001d033388	Zm00001d033388_T002	11.85	34.59	coding	720	92	2251
PB.10626.1	9	1496	7	full-splice_match	gene: Zm00001d047838	Zm00001d047838_T001	12.87	12.87	coding	335	276	1280
PB.4033.5	3	1116	7	novel_in_catalog	gene: Zm00001d041474	novel	0.00	137.78	coding	140	304	723
PB.5746.1	4	2609	7	full-splice_match	gene: Zm00001d053564	Zm00001d053564_T002	18.62	79.10	coding	692	68	2143
PB.4033.7	3	925	6	full-splice_match	gene: Zm00001d041474	Zm00001d041474_T002	18.05	137.78	coding	140	170	589
PB.4033.6	3	1523	6	full-splice_match	gene: Zm00001d041474	Zm00001d041474_T001	7.77	137.78	coding	273	336	1154
PB.4033.1	3	1220	7	novel_in_catalog	gene: Zm00001d041474	novel	111.97	137.78	coding	140	312	731
PB.6458.2	5	1592	12	full-splice_match	gene: Zm00001d015026	Zm00001d015026_T004	4.89	34.79	coding	387	177	1337
PB.6458.1	5	1760	12	full-splice_match	gene: Zm00001d015026	Zm00001d015026_T004	29.90	34.79	coding	387	175	1335
PB.4033.8	3	888	7	novel_in_catalog	gene: Zm00001d041474	novel	0.00	137.78	coding	140	289	708

PB.751.1	1	2237	9	incomplete-splice_match	gene: Zm00001d031009	Zm00001d031009_T006	17.52	17.52	coding	588	28	1791
PB.7424.1	6	1626	4	intergenic	novelGene_135	novel	5.16	5.16	coding	393	291	1469
PB.3200.1	2	1605	1	full-splice_match	gene: Zm00001d005860	Zm00001d005860_T001	10.33	10.33	coding	443	83	1411
PB.3200.2	2	1484	1	full-splice_match	gene: Zm00001d005860	Zm00001d005860_T001	0.00	10.33	coding	443	79	1407
PB.1473.1	1	1951	2	full-splice_match	gene: Zm00001d034382	Zm00001d034382_T001	12.03	12.26	coding	529	130	1716
PB.4120.1	3	1548	1	full-splice_match	gene: Zm00001d041913	Zm00001d041913_T001	5.81	5.81	coding	414	125	1366
PB.4541.3	3	1793	1	full-splice_match	gene: Zm00001d043695	Zm00001d043695_T001	1.30	27.30	coding	433	130	1428
PB.4237.2	3	1469	4	novel_in_catalog	gene: Zm00001d042427	novel	43.93	43.93	coding	397	58	1248
PB.4541.1	3	1898	2	genic	gene: Zm00001d043695	novel	26.00	27.30	coding	433	110	1408
PB.9471.5	8	2102	8	novel_not_in_catalog	gene: Zm00001d010292	novel	0.00	49.58	coding	608	170	1993
PB.4708.1	3	1193	1	novel_in_catalog	gene: Zm00001d044376	novel	0.16	1.28	coding	111	125	457
PB.8941.2	7	1273	2	full-splice_match	gene: Zm00001d022284	Zm00001d022284_T001	55.02	110.28	coding	391	4	1176
PB.8941.1	7	1606	2	full-splice_match	gene: Zm00001d022284	Zm00001d022284_T001	55.26	110.28	coding	391	119	1291
PB.7154.1	5	3214	22	incomplete-splice_match	gene: Zm00001d018423	Zm00001d018423_T008	39.08	55.57	coding	843	91	2619
PB.2641.2	2	1294	1	full-splice_match	gene: Zm00001d002798	Zm00001d002798_T001	41.13	217.54	coding	209	211	837
PB.2641.3	2	1124	1	full-splice_match	gene: Zm00001d002798	Zm00001d002798_T001	137.68	217.54	coding	209	167	793
PB.2641.1	2	1324	1	full-splice_match	gene: Zm00001d002798	Zm00001d002798_T001	38.73	217.54	coding	209	103	729

PB.7731.1	6	1903	5	full-splice_match	gene: Zm00001d037728	Zm00001d037728_T001	5.23	5.23	coding	421	119	1381
PB.1382.1	1	2004	2	incomplete-splice_match	gene: Zm00001d033984	Zm00001d033984_T013	2.51	104.75	coding	654	41	2002
PB.9494.1	8	464	2	full-splice_match	gene: Zm00001d010434	Zm00001d010434_T001	229.07	229.07	non_coding	NA	NA	NA
PB.10012.1	8	539	1	full-splice_match	gene: Zm00001d012714	Zm00001d012714_T001	150.78	150.78	non_coding	NA	NA	NA
PB.1042.1	1	1542	1	full-splice_match	gene: Zm00001d032433	Zm00001d032433_T001	5.15	10.38	coding	393	94	1272
PB.6071.1	5	2168	2	genic	gene: Zm00001d013362	novel	7.57	36.81	coding	406	497	1714
PB.8186.1	7	1892	5	novel_not_in_catalog	gene: Zm00001d018751	novel	33.55	33.55	coding	483	119	1567
PB.8186.2	7	1162	3	novel_not_in_catalog	gene: Zm00001d018751	novel	0.00	33.55	coding	336	49	1056
PB.6071.2	5	2056	2	genic	gene: Zm00001d013362	novel	27.88	36.81	coding	406	501	1718
PB.6998.3	5	1431	9	full-splice_match	gene: Zm00001d017881	Zm00001d017881_T002	0.00	102.51	coding	332	223	1218
PB.7989.2	6	1307	10	full-splice_match	gene: Zm00001d038689	Zm00001d038689_T001	79.95	115.37	coding	369	1	1107
PB.96.1	1	2582	7	full-splice_match	gene: Zm00001d027612	Zm00001d027612_T003	49.94	49.94	coding	715	215	2359
PB.1455.1	1	2735	1	intergenic	novelGene_378	novel	9.99	9.99	coding	828	94	2577
PB.965.2	1	940	1	full-splice_match	gene: Zm00001d032088	Zm00001d032088_T001	0.00	69.38	coding	137	134	544
PB.3208.1	2	1120	2	full-splice_match	gene: Zm00001d005934	Zm00001d005934_T002	8.42	8.42	coding	139	539	955
PB.9519.1	8	770	2	novel_not_in_catalog	gene: Zm00001d010560	novel	2.23	2.23	non_coding	NA	NA	NA
PB.8658.2	7	1797	3	full-splice_match	gene: Zm00001d021288	Zm00001d021288_T002	0.00	52.07	coding	276	286	1113

PB.8658.3	7	1741	3	full-splice_match	gene: Zm00001d021288	Zm00001d021288_T002	49.24	52.07	coding	276	396	1223
PB.8658.1	7	2170	3	full-splice_match	gene: Zm00001d021288	Zm00001d021288_T002	2.83	52.07	coding	276	280	1107
PB.5501.3	4	2740	7	full-splice_match	gene: Zm00001d052379	Zm00001d052379_T002	2.79	73.35	coding	190	1995	2564
PB.7525.2	6	1294	4	incomplete-splice_match	gene: Zm00001d036704	Zm00001d036704_T001	9.87	16.94	coding	333	56	1054
PB.8658.4	7	1219	3	full-splice_match	gene: Zm00001d021288	Zm00001d021288_T002	0.00	52.07	coding	276	191	1018
PB.9495.4	8	1116	3	full-splice_match	gene: Zm00001d010445	Zm00001d010445_T001	75.21	239.50	coding	197	178	768
PB.3231.6	2	1385	5	full-splice_match	gene: Zm00001d006046	Zm00001d006046_T007	25.44	79.51	coding	301	117	1019
PB.8949.1	7	1552	9	full-splice_match	gene: Zm00001d022314	Zm00001d022314_T001	16.52	16.52	coding	343	101	1129
PB.9771.2	8	1383	1	full-splice_match	gene: Zm00001d011710	Zm00001d011710_T001	0.00	110.91	coding	312	176	1111
PB.3946.3	3	3211	25	full-splice_match	gene: Zm00001d040748	Zm00001d040748_T072	0.00	31.44	coding	906	145	2862
PB.3946.2	3	3391	25	full-splice_match	gene: Zm00001d040748	Zm00001d040748_T048	31.44	31.44	coding	906	181	2898
PB.8164.1	7	2776	14	incomplete-splice_match	gene: Zm00001d018657	Zm00001d018657_T016	24.65	24.65	coding	781	106	2448
PB.3168.1	2	2019	12	novel_not_in_catalog	gene: Zm00001d005711	novel	3.02	6.71	coding	304	83	994
PB.2997.1	2	824	1	full-splice_match	gene: Zm00001d004721	Zm00001d004721_T001	1519.95	1519.95	coding	193	30	608
PB.5887.1	4	2008	1	full-splice_match	gene: Zm00001d054060	Zm00001d054060_T001	68.47	250.97	coding	524	82	1653
PB.3168.2	2	2010	12	full-splice_match	gene: Zm00001d005711	Zm00001d005711_T003	3.69	6.71	coding	514	99	1640
PB.4110.3	3	3964	8	novel_in_catalog	gene: Zm00001d041847	novel	0.33	7.42	coding	626	99	1976

PB.7185.1	5	592	1	full-splice_match	gene: Zm00001d018521	Zm00001d018521_T001	523.22	523.22	non_coding	NA	NA	NA
PB.9398.3	8	3117	10	novel_in_catalog	gene: Zm00001d009941	novel	6.29	74.78	coding	244	75	806
PB.2708.2	2	1341	3	full-splice_match	gene: Zm00001d003064	Zm00001d003064_T001	54.12	63.81	coding	294	112	993
PB.2708.3	2	1215	3	full-splice_match	gene: Zm00001d003064	Zm00001d003064_T001	0.00	63.81	coding	294	106	987
PB.10604.1	9	1162	1	full-splice_match	gene: Zm00001d047775	Zm00001d047775_T001	51.16	60.58	coding	305	71	985
PB.2708.1	2	1702	3	full-splice_match	gene: Zm00001d003064	Zm00001d003064_T001	9.69	63.81	coding	294	112	993
PB.5746.5	4	3753	5	full-splice_match	gene: Zm00001d053564	Zm00001d053564_T006	10.46	79.10	coding	449	2215	3561
PB.926.2	1	2036	1	novel_in_catalog	gene: Zm00001d031927	novel	8.16	74.67	coding	122	1539	1904
PB.5720.1	4	711	1	antisense	novelGene_gene: Zm00001d053403_A	novel	3.60	3.60	non_coding	NA	NA	NA
PB.8884.2	7	1314	5	full-splice_match	gene: Zm00001d022065	Zm00001d022065_T001	11.47	13.83	coding	308	110	1033
PB.10016.1	8	1085	1	full-splice_match	gene: Zm00001d012720	Zm00001d012720_T001	102.73	102.73	coding	237	182	892
PB.10016.3	8	960	1	full-splice_match	gene: Zm00001d012720	Zm00001d012720_T001	0.00	102.73	coding	237	184	894
PB.6677.1	5	994	1	genic	gene: Zm00001d016414	novel	23.86	23.86	coding	295	24	908
PB.2280.1	10	673	5	novel_in_catalog	gene: Zm00001d026374	novel	9.95	9.95	non_coding	NA	NA	NA
PB.9527.1	8	1024	1	antisense	novelGene_gene: Zm00001d010591_A	novel	0.14	0.14	coding	109	21	347
PB.10631.1	9	2998	1	full-splice_match	gene: Zm00001d047852	Zm00001d047852_T001	7.32	7.32	coding	477	78	1508
PB.8650.1	7	1358	1	full-splice_match	gene: Zm00001d021243	Zm00001d021243_T001	11.62	11.62	coding	371	75	1187

PB.8697.1	7	2632	4	full-splice_match	gene: Zm00001d021425	Zm00001d021425_T001	2.96	2.96	coding	828	50	2533
PB.7522.1	6	1470	7	novel_in_catalog	gene: Zm00001d036679	novel	28.09	28.09	coding	253	346	1104
PB.521.4	1	1213	6	incomplete-splice_match	gene: Zm00001d029530	Zm00001d029530_T001	6.03	33.88	coding	341	190	1212
PB.10175.1	9	1239	4	full-splice_match	gene: Zm00001d045421	Zm00001d045421_T001	19.25	19.25	coding	206	112	729
PB.1250.2	1	2453	4	full-splice_match	gene: Zm00001d033388	Zm00001d033388_T002	7.18	34.59	coding	571	220	1932
PB.4751.1	3	1282	1	genic	gene: Zm00001d044541	novel	14.32	14.32	coding	137	72	482
PB.6196.1	5	2834	4	novel_not_in_catalog	gene: Zm00001d013926	novel	7.68	7.68	coding	840	13	2532
PB.521.1	1	2902	13	full-splice_match	gene: Zm00001d029530	Zm00001d029530_T003	5.03	33.88	coding	853	52	2610
PB.521.2	1	1424	4	incomplete-splice_match	gene: Zm00001d029530	Zm00001d029530_T007	5.01	33.88	coding	230	34	723
PB.521.3	1	2864	13	full-splice_match	gene: Zm00001d029530	Zm00001d029530_T003	17.81	33.88	coding	853	98	2656
PB.4635.1	3	1445	2	intergenic	novelGene_342	novel	34.28	34.28	non_coding	NA	NA	NA
PB.2772.1	2	1993	3	full-splice_match	gene: Zm00001d003288	Zm00001d003288_T002	82.07	82.07	coding	528	70	1653
PB.6685.3	5	1900	5	full-splice_match	gene: Zm00001d016474	Zm00001d016474_T001	0.00	106.01	coding	526	144	1721
PB.2623.2	2	2528	1	full-splice_match	gene: Zm00001d002718	Zm00001d002718_T001	14.22	49.37	coding	721	128	2290
PB.5733.1	4	1678	10	full-splice_match	gene: Zm00001d053452	Zm00001d053452_T001	42.73	42.73	coding	379	201	1337
PB.2623.1	2	914	1	full-splice_match	gene: Zm00001d002718	Zm00001d002718_T001	35.15	49.37	coding	144	128	559
PB.3408.4	2	1758	5	full-splice_match	gene: Zm00001d006823	Zm00001d006823_T001	22.74	38.36	coding	570	47	1756

PB.5888.1	4	523	4	full-splice_match	gene: Zm00001d054065	Zm00001d054065_T001	1237.32	1237.32	non_coding	NA	NA	NA
PB.3501.2	2	783	6	full-splice_match	gene: Zm00001d007270	Zm00001d007270_T001	72.66	101.91	coding	142	142	567
PB.8205.1	7	1310	7	full-splice_match	gene: Zm00001d018825	Zm00001d018825_T003	24.55	34.17	coding	314	61	1002
PB.8205.2	7	1429	5	novel_in_catalog	gene: Zm00001d018825	novel	3.72	34.17	coding	289	443	1309
PB.8205.3	7	1567	5	novel_in_catalog	gene: Zm00001d018825	novel	5.90	34.17	coding	445	112	1446
PB.3127.2	2	927	5	full-splice_match	gene: Zm00001d005485	Zm00001d005485_T001	22.99	198.67	coding	223	157	825
PB.513.2	1	827	1	genic	gene: Zm00001d029495	novel	120.06	167.77	coding	157	146	616
PB.513.1	1	911	1	genic	gene: Zm00001d029495	novel	47.70	167.77	coding	157	125	595
PB.3127.1	2	1316	5	full-splice_match	gene: Zm00001d005485	Zm00001d005485_T001	175.68	198.67	coding	289	149	1015
PB.6722.2	5	1993	1	genic	gene: Zm00001d016682	novel	3.90	39.88	coding	113	218	556
PB.1472.4	1	1829	2	full-splice_match	gene: Zm00001d034373	Zm00001d034373_T001	0.00	144.72	coding	465	268	1662
PB.1472.3	1	1876	2	full-splice_match	gene: Zm00001d034373	Zm00001d034373_T001	0.00	144.72	coding	465	196	1590
PB.6312.2	5	1954	1	full-splice_match	gene: Zm00001d014496	Zm00001d014496_T001	11.04	15.83	coding	580	73	1812
PB.6312.1	5	2075	1	full-splice_match	gene: Zm00001d014496	Zm00001d014496_T001	4.79	15.83	coding	580	73	1812
PB.9743.3	8	707	3	full-splice_match	gene: Zm00001d011581	Zm00001d011581_T001	1048.99	1136.33	coding	162	85	570
PB.10253.2	9	1795	10	novel_not_in_catalog	gene: Zm00001d045837	novel	17.66	24.40	coding	448	163	1506
PB.10253.1	9	2043	10	novel_not_in_catalog	gene: Zm00001d045837	novel	6.74	24.40	coding	448	158	1501

PB.7143.2	5	1634	1	full-splice_match	gene: Zm00001d018407	Zm00001d018407_T001	9.95	9.95	coding	403	63	1271
PB.5962.1	5	547	2	incomplete-splice_match	gene: Zm00001d012998	Zm00001d012998_T001	23.75	23.75	coding	116	3	350
PB.1764.1	10	1375	1	full-splice_match	gene: Zm00001d023799	Zm00001d023799_T001	0.95	0.95	coding	318	91	1044
PB.9342.1	8	1526	6	full-splice_match	gene: Zm00001d009660	Zm00001d009660_T001	81.85	93.43	coding	377	204	1334
PB.1551.2	1	2097	7	full-splice_match	gene: Zm00001d034650	Zm00001d034650_T004	28.87	48.82	coding	474	399	1820
PB.6271.1	5	2056	1	full-splice_match	gene: Zm00001d014314	Zm00001d014314_T001	3.71	3.71	coding	360	52	1131
PB.9342.2	8	1490	5	novel_in_catalog	gene: Zm00001d009660	novel	10.15	93.43	coding	231	68	760
PB.6995.3	5	1053	3	novel_not_in_catalog	gene: Zm00001d017874	novel	53.20	60.54	coding	170	360	869
PB.6995.1	5	1621	3	novel_not_in_catalog	gene: Zm00001d017874	novel	7.34	60.54	coding	170	348	857
PB.5748.2	4	1115	1	full-splice_match	gene: Zm00001d053568	Zm00001d053568_T001	60.24	60.61	coding	199	49	645
PB.5748.1	4	1275	1	full-splice_match	gene: Zm00001d053568	Zm00001d053568_T001	0.37	60.61	coding	15	382	426
PB.7611.1	6	2249	2	full-splice_match	gene: Zm00001d037203	Zm00001d037203_T001	1.62	1.62	coding	562	205	1890
PB.10277.1	9	1790	2	genic	gene: Zm00001d045984	novel	8.82	8.82	coding	381	72	1214
PB.3220.5	2	2015	3	novel_in_catalog	gene: Zm00001d006001	novel	7.37	41.26	coding	195	318	902
PB.5946.2	5	1905	10	full-splice_match	gene: Zm00001d012931	Zm00001d012931_T001	0.00	13.74	coding	491	102	1574
PB.5946.1	5	2120	10	full-splice_match	gene: Zm00001d012931	Zm00001d012931_T001	13.74	13.74	coding	491	206	1678
PB.6533.1	5	1927	6	full-splice_match	gene: Zm00001d015407	Zm00001d015407_T001	8.70	8.70	coding	376	421	1548

PB.2339.2	10	2302	8	novel_in_catalog	gene: Zm00001d026586	novel	2.45	38.46	coding	144	1759	2190
PB.9211.1	8	1080	1	full-splice_match	gene: Zm00001d008878	Zm00001d008878_T001	72.95	72.95	coding	212	172	807
PB.1704.1	10	2611	3	intergenic	novelGene_25	novel	15.63	15.63	coding	455	367	1731
PB.2339.1	10	2073	10	novel_in_catalog	gene: Zm00001d026586	novel	36.01	38.46	coding	144	1523	1954
PB.10020.1	8	2800	3	novel_in_catalog	gene: Zm00001d012745	novel	14.12	14.12	coding	105	1501	1815
PB.3220.3	2	2589	6	novel_in_catalog	gene: Zm00001d006001	novel	2.59	41.26	coding	382	1183	2328
PB.5736.1	4	1027	1	antisense	novelGene_gene: Zm00001d053518_A	novel	7.68	7.68	coding	144	253	684
PB.1083.3	1	2578	9	incomplete-splice_match	gene: Zm00001d032567	Zm00001d032567_T007	28.72	71.27	coding	758	62	2335
PB.1083.1	1	1701	3	incomplete-splice_match	gene: Zm00001d032567	Zm00001d032567_T016	12.74	71.27	coding	276	205	1032
PB.10161.4	9	2888	3	novel_in_catalog	gene: Zm00001d045382	novel	1.57	79.92	coding	211	2122	2754
PB.10161.3	9	1489	5	full-splice_match	gene: Zm00001d045382	Zm00001d045382_T001	50.96	79.92	coding	391	169	1341
PB.10161.2	9	1732	5	full-splice_match	gene: Zm00001d045382	Zm00001d045382_T002	15.86	79.92	coding	159	154	630
PB.10161.1	9	1560	5	full-splice_match	gene: Zm00001d045382	Zm00001d045382_T001	11.52	79.92	coding	391	135	1307
PB.1083.4	1	2355	10	novel_not_in_catalog	gene: Zm00001d032567	novel	29.80	71.27	coding	520	505	2064
PB.9636.1	8	1507	12	full-splice_match	gene: Zm00001d011061	Zm00001d011061_T005	13.77	20.66	coding	434	77	1378
PB.7286.7	6	2055	10	novel_not_in_catalog	gene: Zm00001d035356	novel	2.92	28.23	coding	107	1067	1387
PB.9636.2	8	1942	12	novel_in_catalog	gene: Zm00001d011061	novel	6.89	20.66	coding	212	105	740

PB.7286.2	6	3134	7	novel_not_in_catalog	gene: Zm00001d035356	novel	5.97	28.23	coding	143	1685	2113
PB.7286.3	6	2354	9	novel_not_in_catalog	gene: Zm00001d035356	novel	1.13	28.23	coding	198	111	704
PB.7286.1	6	2271	8	novel_not_in_catalog	gene: Zm00001d035356	novel	10.55	28.23	coding	107	129	449
PB.6059.3	5	2073	19	incomplete-splice_match	gene: Zm00001d013314	Zm00001d013314_T013	0.00	29.09	coding	678	40	2073
PB.6059.2	5	1385	9	incomplete-splice_match	gene: Zm00001d013314	Zm00001d013314_T013	5.70	29.09	coding	358	112	1185
PB.6059.1	5	2988	21	full-splice_match	gene: Zm00001d013314	Zm00001d013314_T005	23.40	29.09	coding	768	90	2393
PB.8723.1	7	1653	6	incomplete-splice_match	gene: Zm00001d021532	Zm00001d021532_T001	3.39	3.39	coding	409	43	1269
PB.10763.1	9	2072	6	full-splice_match	gene: Zm00001d048342	Zm00001d048342_T001	15.21	15.21	coding	408	129	1352
PB.3863.1	3	1390	7	full-splice_match	gene: Zm00001d040286	Zm00001d040286_T001	107.93	107.93	coding	284	236	1087
PB.7286.9	6	2762	7	novel_not_in_catalog	gene: Zm00001d035356	novel	2.42	28.23	coding	100	57	356
PB.6324.1	5	1153	6	novel_not_in_catalog	gene: Zm00001d014549	novel	72.73	72.73	non_coding	NA	NA	NA
PB.1444.2	1	933	2	full-splice_match	gene: Zm00001d034279	Zm00001d034279_T001	125.73	168.32	coding	180	63	602
PB.4838.1	4	2522	6	novel_not_in_catalog	gene: Zm00001d048823	novel	32.44	32.44	coding	652	256	2211
PB.1444.4	1	865	2	full-splice_match	gene: Zm00001d034279	Zm00001d034279_T001	31.84	168.32	coding	180	138	677
PB.3002.1	2	2193	12	full-splice_match	gene: Zm00001d004740	Zm00001d004740_T002	57.08	57.08	coding	586	112	1869
PB.5098.1	4	958	1	full-splice_match	gene: Zm00001d050156	Zm00001d050156_T001	2.05	2.05	coding	242	103	828
PB.7557.4	6	1661	1	full-splice_match	gene: Zm00001d036925	Zm00001d036925_T001	0.00	74.09	coding	383	100	1248

PB.10942.1	B73V4 ctg88	1392	4	full-splice_match	gene: Zm00001d000439	Zm00001d000439_T005	74.87	74.87	coding	254	252	1013		
PB.8024.1	6	876	1	full-splice_match	gene: Zm00001d038810	Zm00001d038810_T001	94.49	94.49	coding	217	50	700		
PB.6265.1	5	1230	1	full-splice_match	gene: Zm00001d014285	Zm00001d014285_T001	55.17	63.52	coding	283	63	911		
PB.6265.2	5	1085	1	full-splice_match	gene: Zm00001d014285	Zm00001d014285_T001	8.35	63.52	coding	283	75	923		
PB.1432.3	1	2369	5	full-splice_match	gene: Zm00001d034205	Zm00001d034205_T001	0.00	22.09	coding	709	208	2334		
PB.9064.2	8	757	3	full-splice_match	gene: Zm00001d008211	Zm00001d008211_T001	35.37	315.97	coding	85	191	445		
PB.9064.3	8	1106	2	novel_in_catalog	gene: Zm00001d008211	novel	5.24	315.97	non_coding	NA	NA	NA		
PB.8291.1	7	1776	1	genic	gene: Zm00001d019254	novel	4.25	4.25	coding	192	55	630		
PB.9064.1	8	702	3	novel_not_in_catalog	gene: Zm00001d008211	novel	275.36	315.97	coding	15	300	344		
PB.1756.2	10	2559	12	full-splice_match	gene: Zm00001d023749	Zm00001d023749_T007	45.26	81.57	coding	731	112	2304		
PB.1756.3	10	3164	1	novel_in_catalog	gene: Zm00001d023749	novel	4.11	81.57	coding	371	139	1251		
PB.1756.1	10	2663	12	full-splice_match	gene: Zm00001d023749	Zm00001d023749_T001	32.21	81.57	coding	731	112	2304		
PB.3432.2	2	1508	9	full-splice_match	gene: Zm00001d006918	Zm00001d006918_T001	22.17	22.17	coding	444	38	1369		
PB.5978.1	5	1821	1	full-splice_match	gene: Zm00001d013042	Zm00001d013042_T001	20.73	21.74	coding	291	705	1577		
PB.5978.2	5	1469	2	genic	gene: Zm00001d013042	novel	1.01	21.74	coding	214	583	1224		
PB.5978.3	5	1710	1	full-splice_match	gene: Zm00001d013042	Zm00001d013042_T001	0.00	21.74	coding	291	700	1572		
PB.3693.1	3	2635	8	full-splice_match	gene: Zm00001d039425	Zm00001d039425_T006	9.99	9.99	coding	694	204	2285		

PB.9279.1	8	1873	1	intergenic	novelGene_110	novel	6.41	6.41	coding	233	950	1648
PB.860.2	1	2273	3	novel_in_catalog	gene: Zm00001d031580	novel	42.28	42.28	coding	222	1296	1961
PB.1335.2	1	1780	2	full-splice_match	gene: Zm00001d033777	Zm00001d033777_T001	12.94	22.30	coding	420	327	1586
PB.1335.1	1	3233	2	full-splice_match	gene: Zm00001d033777	Zm00001d033777_T001	9.36	22.30	coding	962	21	2906
PB.2381.2	10	2433	10	full-splice_match	gene: Zm00001d026698	Zm00001d026698_T026	11.92	32.91	coding	573	126	1844
PB.879.1	1	2042	4	novel_not_in_catalog	gene: Zm00001d031662	novel	17.27	25.59	coding	498	146	1639
PB.4576.2	3	1951	9	full-splice_match	gene: Zm00001d043834	Zm00001d043834_T002	0.00	121.81	coding	554	113	1774
PB.2381.1	10	1151	4	incomplete-splice_match	gene: Zm00001d026698	Zm00001d026698_T035	20.98	32.91	coding	130	6	395
PB.6674.3	5	1724	4	full-splice_match	gene: Zm00001d016407	Zm00001d016407_T004	3.18	24.95	coding	508	199	1722
PB.164.1	1	1809	1	full-splice_match	gene: Zm00001d027947	Zm00001d027947_T001	0.47	0.47	coding	528	168	1751
PB.7758.2	6	2387	16	full-splice_match	gene: Zm00001d037818	Zm00001d037818_T002	0.00	25.81	coding	634	137	2038
PB.4342.1	3	1929	4	full-splice_match	gene: Zm00001d042886	Zm00001d042886_T001	1.37	24.98	coding	408	475	1698
PB.4721.2	3	1372	7	incomplete-splice_match	gene: Zm00001d044443	Zm00001d044443_T001	31.05	41.18	coding	376	60	1187
PB.4881.2	4	1315	1	novel_in_catalog	gene: Zm00001d049032	novel	0.66	6.55	coding	225	598	1272
PB.9644.2	8	1115	2	novel_not_in_catalog	gene: Zm00001d011095	novel	34.10	109.70	coding	219	257	913
PB.807.3	1	1065	1	novel_in_catalog	gene: Zm00001d031258	novel	7.91	62.40	non_coding	NA	NA	NA
PB.9644.1	8	1049	3	novel_not_in_catalog	gene: Zm00001d011095	novel	75.60	109.70	coding	219	192	848

PB.807.4	1	422	1	incomplete-splice_match	gene: Zm00001d031258	Zm00001d031258_T001	0.00	62.40	coding	54	166	327
PB.8299.1	7	795	3	full-splice_match	gene: Zm00001d019305	Zm00001d019305_T001	67.28	67.28	coding	112	247	582
PB.7907.3	6	1781	12	full-splice_match	gene: Zm00001d038343	Zm00001d038343_T001	73.00	97.25	non_coding	NA	NA	NA
PB.1193.1	1	2457	10	full-splice_match	gene: Zm00001d033094	Zm00001d033094_T001	13.86	23.26	coding	615	138	1982
PB.5314.1	4	818	1	full-splice_match	gene: Zm00001d051594	Zm00001d051594_T001	4.23	4.23	coding	180	91	630
PB.7886.1	6	1823	9	novel_in_catalog	gene: Zm00001d038266	novel	33.12	33.12	coding	315	619	1563
PB.8285.1	7	873	3	full-splice_match	gene: Zm00001d019228	Zm00001d019228_T001	22.40	22.40	coding	189	194	760
PB.628.1	1	1442	1	full-splice_match	gene: Zm00001d030129	Zm00001d030129_T001	22.30	22.30	coding	398	42	1235
PB.10938.1	B73V4 ctg73	2420	6	full-splice_match	gene: Zm00001d000295	Zm00001d000295_T001	80.83	85.07	coding	678	119	2152
PB.10938.3	B73V4 ctg73	2332	6	full-splice_match	gene: Zm00001d000295	Zm00001d000295_T001	0.00	85.07	coding	678	116	2149
PB.10938.2	B73V4 ctg73	2463	5	novel_in_catalog	gene: Zm00001d000295	novel	4.24	85.07	coding	291	1369	2241
PB.8016.1	6	2142	13	full-splice_match	gene: Zm00001d038777	Zm00001d038777_T003	16.34	16.34	coding	539	393	2009
PB.2319.8	10	928	8	full-splice_match	gene: Zm00001d026528	Zm00001d026528_T001	102.95	102.95	coding	220	136	795
PB.9150.6	8	1978	2	full-splice_match	gene: Zm00001d008602	Zm00001d008602_T001	9.18	155.50	coding	299	793	1689
PB.1659.1	10	4433	24	full-splice_match	gene: Zm00001d023262	Zm00001d023262_T068	17.22	97.36	coding	1361	192	4274
PB.9150.4	8	1467	3	full-splice_match	gene: Zm00001d008602	Zm00001d008602_T002	86.76	155.50	coding	299	299	1195
PB.9150.2	8	2129	2	full-splice_match	gene: Zm00001d008602	Zm00001d008602_T001	0.00	155.50	coding	299	793	1689

PB.9150.3	8	1621	3	full-splice_match	gene: Zm00001d008602	Zm00001d008602_T002	0.00	155.50	coding	299	299	1195
PB.1875.6	10	1985	2	novel_not_in_catalog	gene: Zm00001d024499	novel	32.72	276.67	coding	246	1057	1794
PB.9150.1	8	1631	3	full-splice_match	gene: Zm00001d008602	Zm00001d008602_T003	42.61	155.50	coding	299	191	1087
PB.10040.1	9	1361	1	full-splice_match	gene: Zm00001d044771	Zm00001d044771_T001	42.13	42.13	coding	370	65	1174
PB.2801.1	2	1430	5	full-splice_match	gene: Zm00001d003436	Zm00001d003436_T001	18.49	18.49	coding	361	102	1184
PB.2801.2	2	1268	5	full-splice_match	gene: Zm00001d003436	Zm00001d003436_T001	0.00	18.49	coding	361	95	1177
PB.8874.1	7	769	2	incomplete-splice_match	gene: Zm00001d022028	Zm00001d022028_T001	19.45	19.45	coding	34	233	334
PB.9150.8	8	1697	3	novel_not_in_catalog	gene: Zm00001d008602	novel	4.63	155.50	coding	299	522	1418
PB.9150.9	8	1783	2	novel_in_catalog	gene: Zm00001d008602	novel	0.00	155.50	coding	147	303	743
PB.3454.2	2	3127	7	full-splice_match	gene: Zm00001d007043	Zm00001d007043_T002	30.70	30.70	coding	927	172	2952
PB.10018.4	8	852	1	novel_in_catalog	gene: Zm00001d012736	novel	45.60	58.42	coding	143	93	521
PB.10018.2	8	1130	1	novel_in_catalog	gene: Zm00001d012736	novel	5.19	58.42	coding	143	97	525
PB.10018.3	8	970	1	novel_in_catalog	gene: Zm00001d012736	novel	7.62	58.42	coding	143	93	521
PB.5601.3	4	1813	10	fusion	gene: Zm00001d052785_g	novel	2.30	56.39	coding	477	144	1574
PB.3727.1	3	2273	5	full-splice_match	gene: Zm00001d039533	Zm00001d039533_T001	2.41	35.08	coding	279	102	938
PB.3727.2	3	1995	5	full-splice_match	gene: Zm00001d039533	Zm00001d039533_T001	18.83	35.08	coding	279	100	936
PB.3727.3	3	1028	2	incomplete-splice_match	gene: Zm00001d039533	Zm00001d039533_T001	13.83	35.08	coding	107	101	421

PB.3727.4	3	1413	5	full-splice_match	gene: Zm00001d039533	Zm00001d039533_T001	0.00	35.08	coding	240	89	808
PB.5601.4	4	1626	9	fusion	gene: Zm00001d052785_g	novel	0.00	56.39	coding	214	864	1505
PB.2861.2	2	1423	10	full-splice_match	gene: Zm00001d003774	Zm00001d003774_T003	6.81	9.75	coding	347	100	1140
PB.7665.1	6	1706	4	novel_in_catalog	gene: Zm00001d037425	novel	10.59	10.59	coding	193	873	1451
PB.2861.1	2	1390	10	full-splice_match	gene: Zm00001d003774	Zm00001d003774_T004	2.94	9.75	coding	347	100	1140
PB.338.2	1	3032	8	full-splice_match	gene: Zm00001d028687	Zm00001d028687_T001	22.42	100.86	coding	872	230	2845
PB.10812.2	9	3169	3	novel_in_catalog	gene: Zm00001d048503	novel	8.46	120.19	coding	345	1953	2987
PB.10812.3	9	3467	2	novel_in_catalog	gene: Zm00001d048503	novel	2.92	120.19	coding	247	2517	3257
PB.4441.1	3	3163	10	full-splice_match	gene: Zm00001d043327	Zm00001d043327_T006	4.78	4.78	coding	859	334	2910
PB.10367.1	9	1069	7	full-splice_match	gene: Zm00001d046592	Zm00001d046592_T003	48.04	48.04	coding	208	96	719
PB.10704.2	9	2324	3	novel_in_catalog	gene: Zm00001d048105	novel	7.53	39.41	coding	534	369	1970
PB.10704.3	9	2593	2	novel_in_catalog	gene: Zm00001d048105	novel	4.37	39.41	coding	442	939	2264
PB.6378.1	5	1066	1	full-splice_match	gene: Zm00001d014757	Zm00001d014757_T001	151.13	151.13	coding	214	140	781
PB.5661.1	4	2435	3	full-splice_match	gene: Zm00001d053049	Zm00001d053049_T001	12.13	12.13	coding	636	173	2080
PB.1645.2	10	1762	4	novel_in_catalog	gene: Zm00001d023220	novel	1.71	16.61	coding	515	86	1630
PB.1645.1	10	2079	4	novel_in_catalog	gene: Zm00001d023220	novel	14.90	16.61	coding	515	30	1574
PB.4106.3	3	2235	15	full-splice_match	gene: Zm00001d041816	Zm00001d041816_T005	8.61	8.61	coding	550	124	1773

PB.7284.9	6	3130	2	intergenic	novelGene_67	novel	0.40	20.78	coding	361	2047	3129
PB.8932.1	7	954	2	full-splice_match	gene: Zm00001d022244	Zm00001d022244_T001	98.88	104.80	coding	128	310	693
PB.2510.3	2	1528	11	novel_in_catalog	gene: Zm00001d002261	novel	8.82	202.64	coding	221	615	1277
PB.2510.2	2	1537	11	novel_in_catalog	gene: Zm00001d002261	novel	21.90	202.64	coding	330	90	1079
PB.2510.1	2	1615	12	full-splice_match	gene: Zm00001d002261	Zm00001d002261_T003	103.24	202.64	coding	367	181	1281
PB.5827.2	4	656	6	full-splice_match	gene: Zm00001d053862	Zm00001d053862_T002	390.02	502.97	coding	125	63	437
PB.2510.7	2	892	8	incomplete-splice_match	gene: Zm00001d002261	Zm00001d002261_T001	58.89	202.64	coding	221	127	789
PB.2510.6	2	1414	12	full-splice_match	gene: Zm00001d002261	Zm00001d002261_T003	0.00	202.64	coding	367	95	1195
PB.2510.5	2	1925	8	novel_in_catalog	gene: Zm00001d002261	novel	3.95	202.64	coding	105	94	408
PB.2510.4	2	1626	10	novel_in_catalog	gene: Zm00001d002261	novel	5.84	202.64	coding	184	616	1167
PB.7363.1	6	4211	1	genic	gene: Zm00001d035812	novel	2.65	60.27	coding	835	162	2666
PB.7363.2	6	2249	1	genic	gene: Zm00001d035812	novel	13.46	60.27	coding	576	47	1774
PB.7363.3	6	2310	1	genic	gene: Zm00001d035812	novel	29.78	60.27	coding	576	361	2088
PB.7363.4	6	2721	1	incomplete-splice_match	gene: Zm00001d035812	Zm00001d035812_T005	14.38	60.27	coding	835	87	2591
PB.9666.2	8	2253	4	full-splice_match	gene: Zm00001d011202	Zm00001d011202_T001	3.37	42.74	coding	438	684	1997
PB.45.1	1	1591	6	full-splice_match	gene: Zm00001d027403	Zm00001d027403_T001	23.42	23.42	coding	424	133	1404
PB.9666.1	8	2294	4	full-splice_match	gene: Zm00001d011202	Zm00001d011202_T001	39.37	42.74	coding	438	609	1922

PB.10471.1	9	1500	5	full-splice_match	gene: Zm00001d047217	Zm00001d047217_T006	11.41	192.52	coding	223	635	1303
PB.10471.3	9	1096	6	full-splice_match	gene: Zm00001d047217	Zm00001d047217_T003	53.90	192.52	coding	307	26	946
PB.262.1	1	932	5	full-splice_match	gene: Zm00001d028375	Zm00001d028375_T002	164.77	164.77	coding	129	233	619
PB.2530.5	2	2190	17	novel_in_catalog	gene: Zm00001d002326	novel	11.82	111.90	coding	500	508	2007
PB.2530.4	2	2215	18	novel_in_catalog	gene: Zm00001d002326	novel	1.24	111.90	coding	451	208	1560
PB.4408.1	3	1628	8	novel_not_in_catalog	gene: Zm00001d043188	novel	19.84	19.84	coding	323	211	1179
PB.2869.1	2	1389	2	incomplete-splice_match	gene: Zm00001d003807	Zm00001d003807_T001	59.34	75.53	coding	361	73	1155
PB.2530.1	2	2145	19	full-splice_match	gene: Zm00001d002326	Zm00001d002326_T012	84.82	111.90	coding	510	265	1794
PB.2530.3	2	2268	17	novel_in_catalog	gene: Zm00001d002326	novel	0.00	111.90	coding	415	170	1414
PB.2530.2	2	2187	18	full-splice_match	gene: Zm00001d002326	Zm00001d002326_T011	14.01	111.90	coding	415	204	1448
PB.7347.2	6	1843	9	full-splice_match	gene: Zm00001d035698	Zm00001d035698_T002	21.25	23.17	coding	490	42	1511
PB.7347.3	6	1916	8	full-splice_match	gene: Zm00001d035698	Zm00001d035698_T003	1.93	23.17	coding	308	34	957
PB.453.1	1	1421	4	full-splice_match	gene: Zm00001d029203	Zm00001d029203_T001	28.50	28.50	coding	301	217	1119
PB.10388.4	9	1658	3	incomplete-splice_match	gene: Zm00001d046717	Zm00001d046717_T011	5.98	18.10	coding	445	150	1484
PB.7150.1	5	982	4	novel_in_catalog	gene: Zm00001d018419	novel	20.64	20.64	coding	122	160	525
PB.1869.1	10	492	3	incomplete-splice_match	gene: Zm00001d024468	Zm00001d024468_T002	83.16	83.16	non_coding	NA	NA	NA
PB.2031.1	10	1141	2	novel_not_in_catalog	gene: Zm00001d025221	novel	34.08	34.08	coding	180	288	827

PB.10532.2	9	1170	7	incomplete-splice_match	gene: Zm00001d047506	Zm00001d047506_T012	10.73	19.54	coding	282	14	859
PB.10532.1	9	2798	22	incomplete-splice_match	gene: Zm00001d047506	Zm00001d047506_T004	8.82	19.54	coding	624	523	2394
PB.1323.1	1	1413	1	genic	gene: Zm00001d033706	novel	35.99	35.99	coding	362	132	1217
PB.10824.7	9	1686	12	novel_in_catalog	gene: Zm00001d048529	novel	2.58	137.46	coding	399	209	1405
PB.10824.6	9	1779	12	novel_not_in_catalog	gene: Zm00001d048529	novel	0.00	137.46	coding	399	217	1413
PB.10824.5	9	1912	12	novel_not_in_catalog	gene: Zm00001d048529	novel	39.93	137.46	coding	399	217	1413
PB.10824.4	9	1855	11	novel_not_in_catalog	gene: Zm00001d048529	novel	0.70	137.46	coding	257	451	1221
PB.1625.1	1	2306	1	full-splice_match	gene: Zm00001d034879	Zm00001d034879_T001	8.31	12.47	coding	603	24	1832
PB.10343.2	9	2152	3	full-splice_match	gene: Zm00001d046472	Zm00001d046472_T002	41.84	41.84	coding	109	117	443
PB.7671.1	6	821	3	full-splice_match	gene: Zm00001d037463	Zm00001d037463_T002	54.73	54.73	coding	190	122	691
PB.9390.4	8	2900	4	full-splice_match	gene: Zm00001d009908	Zm00001d009908_T015	197.40	197.40	coding	225	91	765
PB.10916.1	B73V4 ctg50	1970	1	incomplete-splice_match	gene: Zm00001d000166	Zm00001d000166_T001	21.27	21.27	coding	404	101	1312
PB.749.2	1	1562	2	incomplete-splice_match	gene: Zm00001d031005	Zm00001d031005_T009	22.54	34.82	coding	463	1	1389
PB.789.1	1	1097	7	full-splice_match	gene: Zm00001d031179	Zm00001d031179_T001	89.32	89.32	coding	204	168	779
PB.223.1	1	2270	1	full-splice_match	gene: Zm00001d028221	Zm00001d028221_T001	2.17	2.17	coding	691	25	2097
PB.2385.2	2	2200	12	novel_not_in_catalog	gene: Zm00001d001765	novel	53.87	80.07	coding	631	126	2018
PB.2385.1	2	2736	12	novel_not_in_catalog	gene: Zm00001d001765	novel	20.05	80.07	coding	631	108	2000

PB.8996.1	7	1276	3	full-splice_match	gene: Zm00001d022457	Zm00001d022457_T001	74.24	74.24	coding	320	114	1073
PB.2385.5	2	1596	8	incomplete-splice_match	gene: Zm00001d001765	Zm00001d001765_T001	0.00	80.07	coding	448	251	1594
PB.10803.2	9	2212	11	full-splice_match	gene: Zm00001d048463	Zm00001d048463_T005	2.70	64.75	coding	344	189	1220
PB.10803.3	9	2076	12	full-splice_match	gene: Zm00001d048463	Zm00001d048463_T004	44.60	64.75	coding	520	213	1772
PB.10803.1	9	1522	11	novel_not_in_catalog	gene: Zm00001d048463	novel	17.46	64.75	coding	175	39	563
PB.5324.1	4	1556	9	full-splice_match	gene: Zm00001d051628	Zm00001d051628_T002	19.11	32.01	coding	333	293	1291
PB.5324.3	4	1563	9	full-splice_match	gene: Zm00001d051628	Zm00001d051628_T002	2.23	32.01	coding	437	43	1353
PB.2143.2	10	2182	2	full-splice_match	gene: Zm00001d025808	Zm00001d025808_T001	3.81	19.68	coding	546	319	1956
PB.8800.3	7	2115	3	novel_in_catalog	gene: Zm00001d021764	novel	0.00	31.17	coding	164	1624	2115
PB.8800.2	7	2550	4	full-splice_match	gene: Zm00001d021764	Zm00001d021764_T006	1.02	31.17	coding	194	1623	2204
PB.8372.1	7	765	3	full-splice_match	gene: Zm00001d019836	Zm00001d019836_T006	11.62	49.82	coding	143	122	550
PB.7439.2	6	5386	8	full-splice_match	gene: Zm00001d036301	Zm00001d036301_T014	20.49	163.84	coding	1704	85	5196
PB.7439.3	6	1825	6	incomplete-splice_match	gene: Zm00001d036301	Zm00001d036301_T006	133.74	163.84	coding	468	304	1707
PB.910.1	1	1871	3	full-splice_match	gene: Zm00001d031844	Zm00001d031844_T002	7.07	7.07	coding	440	342	1661
PB.8372.4	7	776	4	full-splice_match	gene: Zm00001d019836	Zm00001d019836_T001	38.20	49.82	coding	175	167	691
PB.3321.2	2	447	2	novel_in_catalog	gene: Zm00001d006421	novel	25.57	249.69	non_coding	NA	NA	NA
PB.1001.1	1	1674	5	full-splice_match	gene: Zm00001d032272	Zm00001d032272_T001	16.35	17.05	coding	440	194	1513

PB.207.1	1	3256	13	incomplete-splice_match	gene: Zm00001d028177	Zm00001d028177_T004	28.43	28.43	coding	744	434	2665
PB.3321.1	2	611	3	full-splice_match	gene: Zm00001d006421	Zm00001d006421_T001	224.12	249.69	coding	80	111	350
PB.6688.1	5	2478	15	full-splice_match	gene: Zm00001d016496	Zm00001d016496_T005	44.75	44.75	coding	603	279	2087
PB.4616.2	3	723	1	genic	gene: Zm00001d043984	novel	15.19	15.19	non_coding	NA	NA	NA
PB.4570.2	3	1805	11	incomplete-splice_match	gene: Zm00001d043797	Zm00001d043797_T020	31.75	153.66	coding	397	134	1324
PB.9670.3	8	2085	6	novel_in_catalog	gene: Zm00001d011226	novel	2.70	45.59	coding	236	1264	1971
PB.8584.4	7	1773	11	full-splice_match	gene: Zm00001d020938	Zm00001d020938_T002	0.00	11.79	coding	486	78	1535
PB.637.1	1	1101	2	full-splice_match	gene: Zm00001d030167	Zm00001d030167_T002	52.14	52.14	coding	258	35	808
PB.8584.2	7	1939	11	full-splice_match	gene: Zm00001d020938	Zm00001d020938_T002	7.85	11.79	coding	486	78	1535
PB.2657.1	2	1671	1	full-splice_match	gene: Zm00001d002851	Zm00001d002851_T001	7.71	21.65	coding	342	73	1098
PB.5124.1	4	2654	1	full-splice_match	gene: Zm00001d050335	Zm00001d050335_T001	8.40	8.40	coding	787	82	2442
PB.7556.1	6	2196	1	antisense	novelGene_gene: Zm00001d036922_A	novel	4.99	4.99	coding	122	503	868
PB.3003.1	2	1217	2	full-splice_match	gene: Zm00001d004744	Zm00001d004744_T001	16.25	16.25	coding	275	213	1037
PB.8820.1	7	1200	3	full-splice_match	gene: Zm00001d021834	Zm00001d021834_T001	61.21	63.48	coding	254	257	1018
PB.6742.1	5	1603	9	full-splice_match	gene: Zm00001d016783	Zm00001d016783_T001	41.46	41.46	coding	364	270	1361
PB.8820.2	7	4049	2	novel_in_catalog	gene: Zm00001d021834	novel	2.27	63.48	coding	124	3418	3789
PB.3399.2	2	865	6	full-splice_match	gene: Zm00001d006760	Zm00001d006760_T001	1313.83	2189.04	coding	160	87	566

PB.443.1	1	1121	2	intergenic	novelGene_118	novel	14.93	14.93	coding	115	326	670
PB.581.3	1	947	1	novel_in_catalog	gene: Zm00001d029842	novel	8.10	226.49	coding	118	232	585
PB.9801.1	8	1906	4	novel_not_in_catalog	gene: Zm00001d011823	novel	10.02	10.02	coding	316	721	1668
PB.3302.2	2	1606	7	full-splice_match	gene: Zm00001d006344	Zm00001d006344_T008	61.55	84.46	coding	354	201	1262
PB.3650.1	3	1489	4	full-splice_match	gene: Zm00001d039295	Zm00001d039295_T001	30.01	30.01	coding	411	202	1434
PB.4528.21	3	3724	7	full-splice_match	gene: Zm00001d043649	Zm00001d043649_T053	0.00	96.43	coding	125	1849	2223
PB.6930.1	5	1047	1	full-splice_match	gene: Zm00001d017612	Zm00001d017612_T001	17.69	17.69	coding	199	77	673
PB.3302.6	2	3598	6	full-splice_match	gene: Zm00001d006344	Zm00001d006344_T002	0.00	84.46	coding	359	124	1200
PB.3302.5	2	1480	7	full-splice_match	gene: Zm00001d006344	Zm00001d006344_T008	0.00	84.46	coding	354	143	1204
PB.3302.4	2	3574	7	novel_in_catalog	gene: Zm00001d006344	novel	1.01	84.46	coding	214	690	1331
PB.7762.1	6	1203	6	novel_in_catalog	gene: Zm00001d037848	novel	6.49	6.49	coding	251	132	884
PB.4564.3	3	2002	11	novel_in_catalog	gene: Zm00001d043771	novel	1.70	15.81	coding	483	195	1643
PB.10740.1	9	3100	16	full-splice_match	gene: Zm00001d048258	Zm00001d048258_T021	32.08	55.35	coding	780	165	2504
PB.712.8	1	1546	1	genic	gene: Zm00001d030760	novel	0.00	53.70	coding	361	346	1428
PB.10015.1	8	1189	4	incomplete-splice_match	gene: Zm00001d012719	Zm00001d012719_T001	10.29	10.29	coding	280	98	937
PB.2218.1	10	1089	4	novel_not_in_catalog	gene: Zm00001d026171	novel	15.16	15.16	coding	285	61	915
PB.712.4	1	2017	2	genic	gene: Zm00001d030760	novel	0.00	53.70	coding	361	346	1428

PB.712.5	1	1862	2	genic	gene: Zm00001d030760	novel	0.00	53.70	coding	361	346	1428
PB.712.6	1	1756	1	genic	gene: Zm00001d030760	novel	9.24	53.70	coding	361	346	1428
PB.712.7	1	1652	1	genic	gene: Zm00001d030760	novel	0.00	53.70	coding	361	346	1428
PB.3670.1	3	992	1	full-splice_match	gene: Zm00001d039354	Zm00001d039354_T001	46.06	46.06	coding	238	67	780
PB.712.1	1	2325	2	genic	gene: Zm00001d030760	novel	10.94	53.70	coding	361	346	1428
PB.3670.2	3	670	1	full-splice_match	gene: Zm00001d039354	Zm00001d039354_T001	0.00	46.06	coding	157	109	579
PB.4006.1	3	1577	2	genic	gene: Zm00001d041299	novel	8.96	8.96	coding	364	226	1317
PB.1609.1	1	1378	9	full-splice_match	gene: Zm00001d034833	Zm00001d034833_T004	177.88	181.28	coding	310	156	1085
PB.7878.3	6	689	2	intergenic	novelGene_250	novel	29.23	99.35	coding	104	78	389
PB.3753.1	3	1838	5	full-splice_match	gene: Zm00001d039703	Zm00001d039703_T001	59.50	59.50	coding	417	260	1510
PB.3085.1	2	764	2	intergenic	novelGene_271	novel	32.34	32.34	coding	183	16	564
PB.580.1	1	3744	25	full-splice_match	gene: Zm00001d029818	Zm00001d029818_T001	9.61	9.61	coding	1087	163	3423
PB.9277.1	8	3519	12	full-splice_match	gene: Zm00001d009243	Zm00001d009243_T001	18.05	64.33	coding	1061	5	3187
PB.9277.2	8	1387	6	incomplete-splice_match	gene: Zm00001d009243	Zm00001d009243_T003	46.29	64.33	coding	349	49	1095
PB.4737.1	3	2875	9	full-splice_match	gene: Zm00001d044495	Zm00001d044495_T001	111.51	807.82	coding	481	1053	2495
PB.9956.1	8	2704	3	novel_not_in_catalog	gene: Zm00001d012514	novel	15.55	15.55	coding	637	487	2397
PB.6594.1	5	740	2	full-splice_match	gene: Zm00001d015865	Zm00001d015865_T001	56.41	56.41	coding	141	151	573

PB.2169.1	10	2309	1	full-splice_match	gene: Zm00001d025920	Zm00001d025920_T001	29.83	29.83	coding	669	66	2072
PB.2854.5	2	1567	1	novel_in_catalog	gene: Zm00001d003707	novel	346.95	346.95	coding	153	99	557
PB.3845.2	3	4132	7	full-splice_match	gene: Zm00001d040203	Zm00001d040203_T001	0.00	7.89	coding	1198	365	3958
PB.3845.1	3	4319	7	full-splice_match	gene: Zm00001d040203	Zm00001d040203_T001	7.89	7.89	coding	1198	359	3952
PB.8880.1	7	1167	1	full-splice_match	gene: Zm00001d022053	Zm00001d022053_T001	96.95	96.95	coding	292	72	947
PB.1561.6	1	940	2	genic	gene: Zm00001d034677	novel	27.35	140.30	coding	123	136	504
PB.10405.8	9	2022	11	full-splice_match	gene: Zm00001d046828	Zm00001d046828_T008	1.39	85.56	coding	193	148	726
PB.10405.9	9	2227	9	full-splice_match	gene: Zm00001d046828	Zm00001d046828_T004	1.29	85.56	coding	193	173	751
PB.3879.1	3	2479	11	incomplete-splice_match	gene: Zm00001d040320	Zm00001d040320_T002	6.57	6.57	coding	568	16	1719
PB.7168.1	5	1002	7	full-splice_match	gene: Zm00001d018471	Zm00001d018471_T001	42.73	42.73	coding	199	44	640
PB.5713.1	4	1073	5	full-splice_match	gene: Zm00001d053375	Zm00001d053375_T001	68.52	70.03	coding	228	108	791
PB.9815.2	8	303	1	novel_in_catalog	gene: Zm00001d011850	novel	27.90	212.54	non_coding	NA	NA	NA
PB.10405.1	9	1766	11	full-splice_match	gene: Zm00001d046828	Zm00001d046828_T014	4.27	85.56	coding	246	175	912
PB.10405.3	9	1590	12	full-splice_match	gene: Zm00001d046828	Zm00001d046828_T005	70.71	85.56	coding	365	198	1292
PB.10405.4	9	2125	10	full-splice_match	gene: Zm00001d046828	Zm00001d046828_T012	6.34	85.56	coding	193	151	729
PB.10405.5	9	1553	11	full-splice_match	gene: Zm00001d046828	Zm00001d046828_T014	0.00	85.56	coding	246	161	898
PB.10405.7	9	1509	12	full-splice_match	gene: Zm00001d046828	Zm00001d046828_T005	0.00	85.56	coding	365	198	1292

PB.2158.1	10	2019	2	novel_in_catalog	gene: Zm00001d025864	novel	5.03	43.22	coding	131	176	568
PB.6118.11	5	1007	4	incomplete-splice_match	gene: Zm00001d013527	Zm00001d013527_T002	43.34	74.65	coding	236	188	895
PB.6118.10	5	2379	3	novel_in_catalog	gene: Zm00001d013527	novel	0.00	74.65	coding	509	148	1674
PB.7514.1	6	905	5	novel_in_catalog	gene: Zm00001d036642	novel	11.08	68.90	coding	140	69	488
PB.5526.1	4	3108	2	genic	gene: Zm00001d052450	novel	2.45	139.39	coding	820	133	2592
PB.588.2	1	1398	14	full-splice_match	gene: Zm00001d029860	Zm00001d029860_T001	1.21	43.82	coding	406	91	1308
PB.588.1	1	1606	14	full-splice_match	gene: Zm00001d029860	Zm00001d029860_T001	42.61	43.82	coding	406	86	1303
PB.2284.4	10	826	1	full-splice_match	gene: Zm00001d026390	Zm00001d026390_T001	62.79	77.39	coding	27	557	637
PB.307.10	1	2611	12	full-splice_match	gene: Zm00001d028570	Zm00001d028570_T007	0.00	67.15	coding	743	184	2412
PB.2900.1	2	1761	5	full-splice_match	gene: Zm00001d003945	Zm00001d003945_T002	88.34	88.34	coding	378	189	1322
PB.10176.1	9	2226	14	incomplete-splice_match	gene: Zm00001d045425	Zm00001d045425_T001	20.68	20.68	coding	617	25	1875
PB.768.1	1	2461	1	antisense	novelGene_gene: Zm00001d031077_A	novel	206.01	206.01	coding	289	1475	2341
PB.775.1	1	1704	7	full-splice_match	gene: Zm00001d031098	Zm00001d031098_T002	25.15	45.68	coding	223	863	1531
PB.4074.1	3	731	1	full-splice_match	gene: Zm00001d041672	Zm00001d041672_T001	617.41	617.41	coding	136	99	506
PB.4412.1	3	1498	1	full-splice_match	gene: Zm00001d043199	Zm00001d043199_T001	1.19	1.19	coding	411	100	1332
PB.9997.1	8	1402	7	full-splice_match	gene: Zm00001d012656	Zm00001d012656_T003	18.59	22.69	coding	361	90	1172
PB.7931.5	6	1789	7	incomplete-splice_match	gene: Zm00001d038432	Zm00001d038432_T001	6.28	22.10	coding	528	78	1661

PB.566.8	1	2833	3	novel_in_catalog	gene: Zm00001d029736	novel	1.22	22.53	coding	393	387	1565
PB.10738.3	9	2871	5	novel_in_catalog	gene: Zm00001d048236	novel	4.02	22.77	coding	605	314	2128
PB.3029.1	2	1703	1	full-splice_match	gene: Zm00001d004918	Zm00001d004918_T001	34.19	34.19	coding	323	321	1289
PB.2411.1	2	830	2	full-splice_match	gene: Zm00001d001843	Zm00001d001843_T001	33.68	33.68	coding	177	73	603
PB.9913.2	8	1002	3	full-splice_match	gene: Zm00001d012295	Zm00001d012295_T006	15.41	47.92	non_coding	NA	NA	NA
PB.566.3	1	2355	5	novel_not_in_catalog	gene: Zm00001d029736	novel	10.92	22.53	coding	612	243	2078
PB.566.2	1	2723	4	novel_not_in_catalog	gene: Zm00001d029736	novel	0.00	22.53	coding	393	223	1401
PB.566.1	1	1541	1	genic	gene: Zm00001d029736	novel	9.32	22.53	coding	214	435	1076
PB.6417.1	5	3580	3	genic	gene: Zm00001d014890	novel	24.67	24.67	coding	918	378	3131
PB.566.6	1	3077	4	novel_not_in_catalog	gene: Zm00001d029736	novel	1.07	22.53	coding	389	1639	2805
PB.2927.1	2	3592	10	incomplete-splice_match	gene: Zm00001d004139	Zm00001d004139_T010	29.26	29.26	coding	1093	81	3359
PB.2046.6	10	1999	5	novel_in_catalog	gene: Zm00001d025305	novel	7.21	267.05	coding	493	63	1541
PB.2251.2	10	3176	8	novel_in_catalog	gene: Zm00001d026289	novel	5.64	11.85	coding	355	1973	3037
PB.7566.2	6	2502	2	full-splice_match	gene: Zm00001d036972	Zm00001d036972_T001	0.00	40.09	coding	619	450	2306
PB.7566.1	6	2797	2	full-splice_match	gene: Zm00001d036972	Zm00001d036972_T001	40.09	40.09	coding	806	6	2423
PB.6942.1	5	1997	4	incomplete-splice_match	gene: Zm00001d017660	Zm00001d017660_T011	11.13	22.57	coding	286	339	1196
PB.5882.1	4	1543	8	full-splice_match	gene: Zm00001d054039	Zm00001d054039_T002	57.18	57.18	coding	407	97	1317

PB.2046.8	10	1873	6	full-splice_match	gene: Zm00001d025305	Zm00001d025305_T001	1.81	267.05	coding	532	127	1722
PB.4529.8	3	1727	5	full-splice_match	gene: Zm00001d043650	Zm00001d043650_T002	32.97	203.44	coding	488	41	1504
PB.8663.1	7	785	1	full-splice_match	gene: Zm00001d021300	Zm00001d021300_T001	923.67	923.67	coding	164	69	560
PB.4560.1	3	2377	4	novel_in_catalog	gene: Zm00001d043764	novel	1.36	6.89	coding	171	1218	1730
PB.2271.3	10	2790	1	genic	gene: Zm00001d026358	novel	5.94	32.03	coding	521	709	2271
PB.2271.2	10	679	1	genic	gene: Zm00001d026358	novel	20.10	32.03	non_coding	NA	NA	NA
PB.2271.1	10	3338	3	novel_in_catalog	gene: Zm00001d026358	novel	5.51	32.03	coding	816	35	2482
PB.4529.5	3	2483	4	novel_in_catalog	gene: Zm00001d043650	novel	1.95	203.44	coding	343	1207	2235
PB.4529.4	3	1816	5	full-splice_match	gene: Zm00001d043650	Zm00001d043650_T002	152.77	203.44	coding	488	104	1567
PB.2271.5	10	3001	3	novel_in_catalog	gene: Zm00001d026358	novel	0.49	32.03	coding	816	90	2537
PB.4529.6	3	2410	3	novel_in_catalog	gene: Zm00001d043650	novel	9.16	203.44	coding	208	1019	1642
PB.7222.1	6	1942	2	full-splice_match	gene: Zm00001d035039	Zm00001d035039_T001	7.60	43.77	coding	355	80	1144
PB.7222.2	6	2174	2	full-splice_match	gene: Zm00001d035039	Zm00001d035039_T001	34.14	43.77	coding	414	496	1737
PB.7222.3	6	2796	1	novel_in_catalog	gene: Zm00001d035039	novel	2.03	43.77	coding	218	363	1016
PB.6810.12	5	1634	9	novel_in_catalog	gene: Zm00001d017096	novel	1.47	118.92	coding	226	711	1388
PB.968.4	1	2619	25	full-splice_match	gene: Zm00001d032099	Zm00001d032099_T004	58.85	58.85	coding	782	77	2422
PB.2590.1	2	534	2	intergenic	novelGene_54	novel	117.89	117.89	non_coding	NA	NA	NA

PB.7053.1	5	1080	1	antisense	novelGene_gene: Zm00001d018089_A	novel	43.11	43.11	coding	160	81	560
PB.1940.1	10	736	1	full-splice_match	gene: Zm00001d024800	Zm00001d024800_T001	43.16	72.67	coding	95	137	421
PB.1940.2	10	538	1	full-splice_match	gene: Zm00001d024800	Zm00001d024800_T001	29.51	72.67	coding	95	91	375
PB.9132.2	8	1236	3	genic	gene: Zm00001d008500	novel	42.42	110.84	coding	275	214	1038
PB.9132.1	8	1214	3	genic	gene: Zm00001d008500	novel	68.42	110.84	coding	275	180	1004
PB.6958.3	5	2557	18	full-splice_match	gene: Zm00001d017729	Zm00001d017729_T014	74.81	110.08	coding	459	623	1999
PB.6958.2	5	2341	19	full-splice_match	gene: Zm00001d017729	Zm00001d017729_T010	21.43	110.08	coding	459	652	2028
PB.6958.1	5	2297	18	full-splice_match	gene: Zm00001d017729	Zm00001d017729_T017	13.84	110.08	coding	459	630	2006
PB.8824.8	7	1183	7	novel_not_in_catalog	gene: Zm00001d021845	novel	6.73	43.34	coding	147	132	572
PB.9434.5	8	1721	3	full-splice_match	gene: Zm00001d010146	Zm00001d010146_T002	0.00	92.18	coding	475	297	1721
PB.9434.4	8	1910	3	full-splice_match	gene: Zm00001d010146	Zm00001d010146_T002	0.00	92.18	coding	512	345	1880
PB.4342.2	3	1619	4	full-splice_match	gene: Zm00001d042886	Zm00001d042886_T001	23.62	24.98	coding	408	227	1450
PB.9434.1	8	2131	3	full-splice_match	gene: Zm00001d010146	Zm00001d010146_T002	92.18	92.18	coding	512	348	1883
PB.8447.2	7	1805	7	novel_not_in_catalog	gene: Zm00001d020309	novel	5.55	42.78	coding	476	227	1654
PB.1598.1	1	1371	5	full-splice_match	gene: Zm00001d034805	Zm00001d034805_T005	78.04	83.94	coding	372	101	1216
PB.9561.2	8	1688	3	full-splice_match	gene: Zm00001d010724	Zm00001d010724_T001	24.88	31.57	coding	427	28	1308
PB.2405.1	2	2015	2	full-splice_match	gene: Zm00001d001818	Zm00001d001818_T001	10.20	10.20	coding	549	176	1822

PB.7437.2	6	1186	3	full-splice_match	gene: Zm00001d036285	Zm00001d036285_T001	0.00	76.84	coding	324	42	1013
PB.9293.1	8	622	1	full-splice_match	gene: Zm00001d009387	Zm00001d009387_T001	37.51	37.51	coding	146	5	442
PB.7437.4	6	1072	3	full-splice_match	gene: Zm00001d036285	Zm00001d036285_T001	0.00	76.84	coding	296	184	1071
PB.9561.4	8	1489	3	full-splice_match	gene: Zm00001d010724	Zm00001d010724_T001	1.03	31.57	coding	427	35	1315
PB.8554.1	7	770	1	intergenic	novelGene_211	novel	818.57	818.57	coding	83	257	505
PB.3325.2	2	1486	5	full-splice_match	gene: Zm00001d006440	Zm00001d006440_T002	12.99	133.58	coding	275	198	1022
PB.5987.4	5	1097	2	full-splice_match	gene: Zm00001d013067	Zm00001d013067_T001	633.61	721.38	coding	261	95	877
PB.5987.2	5	1308	1	novel_in_catalog	gene: Zm00001d013067	novel	87.77	721.38	coding	206	357	974
PB.6473.1	5	2188	1	novel_in_catalog	gene: Zm00001d015097	novel	12.33	12.33	coding	88	118	381
PB.203.2	1	1000	3	full-splice_match	gene: Zm00001d028160	Zm00001d028160_T001	0.00	25.67	coding	206	92	709
PB.6244.3	5	3874	8	full-splice_match	gene: Zm00001d014150	Zm00001d014150_T010	0.00	9.10	coding	427	2107	3387
PB.7558.1	6	2099	12	incomplete-splice_match	gene: Zm00001d036927	Zm00001d036927_T001	14.42	15.39	coding	627	44	1924
PB.6244.1	5	4024	8	full-splice_match	gene: Zm00001d014150	Zm00001d014150_T010	9.10	9.10	coding	427	2164	3444
PB.9311.2	8	1099	2	full-splice_match	gene: Zm00001d009475	Zm00001d009475_T001	23.09	23.09	coding	257	67	837
PB.9186.2	8	1743	1	full-splice_match	gene: Zm00001d008756	Zm00001d008756_T001	65.87	81.88	coding	443	169	1497
PB.9186.1	8	1895	1	full-splice_match	gene: Zm00001d008756	Zm00001d008756_T001	16.01	81.88	coding	443	169	1497
PB.623.4	1	1949	10	full-splice_match	gene: Zm00001d030106	Zm00001d030106_T029	23.40	80.51	coding	54	269	430

PB.8437.1	7	1497	5	novel_not_in_catalog	gene: Zm00001d020272	novel	34.25	34.25	coding	389	90	1256
PB.7014.1	5	2493	1	antisense	novelGene_gene: Zm00001d017957_A	novel	9.58	9.58	non_coding	NA	NA	NA
PB.1018.1	1	1187	1	full-splice_match	gene: Zm00001d032339	Zm00001d032339_T001	2.12	2.12	coding	258	136	909
PB.2937.1	2	1045	3	full-splice_match	gene: Zm00001d004193	Zm00001d004193_T001	56.51	56.51	coding	241	96	818
PB.4147.1	3	1696	1	full-splice_match	gene: Zm00001d042007	Zm00001d042007_T001	21.13	21.13	coding	387	289	1449
PB.7778.1	6	1958	12	full-splice_match	gene: Zm00001d037918	Zm00001d037918_T001	7.49	7.49	coding	532	63	1658
PB.2850.2	2	2482	10	novel_not_in_catalog	gene: Zm00001d003678	novel	0.00	10.39	coding	185	1672	2226
PB.4056.1	3	2029	1	novel_in_catalog	gene: Zm00001d041575	novel	46.14	133.85	coding	127	852	1232
PB.2301.1	10	1944	5	full-splice_match	gene: Zm00001d026458	Zm00001d026458_T001	1435.99	1555.47	coding	469	78	1484
PB.5696.2	4	1368	10	full-splice_match	gene: Zm00001d053275	Zm00001d053275_T001	14.22	26.87	coding	381	62	1204
PB.623.1	1	1841	8	full-splice_match	gene: Zm00001d030106	Zm00001d030106_T007	47.71	80.51	coding	104	843	1154
PB.193.1	1	1462	9	full-splice_match	gene: Zm00001d028104	Zm00001d028104_T003	18.40	18.40	coding	383	72	1220
PB.7459.1	6	2184	11	full-splice_match	gene: Zm00001d036416	Zm00001d036416_T001	44.19	44.19	coding	608	158	1981
PB.10098.1	9	1565	6	full-splice_match	gene: Zm00001d045074	Zm00001d045074_T002	19.95	19.95	coding	196	124	711
PB.9773.1	8	3844	2	full-splice_match	gene: Zm00001d011716	Zm00001d011716_T001	12.13	12.13	coding	1148	20	3463
PB.5269.1	4	1632	1	antisense	novelGene_gene: Zm00001d051349_A	novel	29.53	103.06	non_coding	NA	NA	NA
PB.2850.1	2	2425	10	full-splice_match	gene: Zm00001d003678	Zm00001d003678_T001	6.98	10.39	coding	300	1239	2138

PB.149.8	1	685	1	novel_in_catalog	gene: Zm00001d027874	novel	40.18	155.38	non_coding	NA	NA	NA
PB.362.1	1	503	2	full-splice_match	gene: Zm00001d028807	Zm00001d028807_T002	1793.79	1811.15	non_coding	NA	NA	NA
PB.4002.1	3	3152	6	intergenic	novelGene_182	novel	15.25	61.78	coding	196	2147	2734
PB.362.2	1	1459	1	novel_in_catalog	gene: Zm00001d028807	novel	17.36	1811.15	non_coding	NA	NA	NA
PB.5051.2	4	1436	8	full-splice_match	gene: Zm00001d049873	Zm00001d049873_T001	92.59	97.64	coding	318	174	1127
PB.4515.1	3	1676	7	novel_not_in_catalog	gene: Zm00001d043600	novel	25.95	25.95	coding	414	119	1360
PB.5051.1	4	2593	7	novel_in_catalog	gene: Zm00001d049873	novel	5.05	97.64	coding	252	1509	2264
PB.3991.1	3	1879	5	full-splice_match	gene: Zm00001d041192	Zm00001d041192_T003	103.52	103.52	coding	501	115	1617
PB.9258.1	8	1486	1	full-splice_match	gene: Zm00001d009134	Zm00001d009134_T001	17.93	17.93	coding	410	57	1286
PB.8915.6	7	2683	4	novel_in_catalog	gene: Zm00001d022181	novel	3.33	288.80	coding	463	91	1479
PB.9773.2	8	3794	2	full-splice_match	gene: Zm00001d011716	Zm00001d011716_T001	0.00	12.13	coding	1148	20	3463
PB.5164.5	4	1616	8	incomplete-splice_match	gene: Zm00001d050606	Zm00001d050606_T035	9.73	69.27	coding	502	109	1614
PB.8915.2	7	2417	6	full-splice_match	gene: Zm00001d022181	Zm00001d022181_T005	4.18	288.80	coding	538	91	1704
PB.8915.3	7	2637	5	full-splice_match	gene: Zm00001d022181	Zm00001d022181_T004	0.00	288.80	coding	538	89	1702
PB.5071.1	4	1631	1	genic	gene: Zm00001d049995	novel	276.84	276.84	coding	442	5	1330
PB.4002.2	3	2767	5	intergenic	novelGene_182	novel	18.16	61.78	coding	121	2019	2381
PB.8258.1	7	1157	8	novel_not_in_catalog	gene: Zm00001d019093	novel	126.90	126.90	coding	218	208	861

PB.386.3	1	1549	2	full-splice_match	gene: Zm00001d028930	Zm00001d028930_T001	0.00	26.88	coding	336	192	1199
PB.386.2	1	1728	2	full-splice_match	gene: Zm00001d028930	Zm00001d028930_T001	26.88	26.88	coding	336	192	1199
PB.1571.1	1	1710	6	novel_in_catalog	gene: Zm00001d034719	novel	46.05	46.56	coding	403	220	1428
PB.9258.2	8	1330	1	full-splice_match	gene: Zm00001d009134	Zm00001d009134_T001	0.00	17.93	coding	410	19	1248
PB.7380.4	6	2479	2	full-splice_match	gene: Zm00001d035922	Zm00001d035922_T015	1.34	7.73	coding	634	81	1982
PB.3203.1	2	804	5	full-splice_match	gene: Zm00001d005867	Zm00001d005867_T002	3.94	3.94	coding	134	88	489
PB.8607.2	7	1657	2	full-splice_match	gene: Zm00001d021016	Zm00001d021016_T001	114.03	125.44	coding	403	278	1486
PB.8607.3	7	1698	1	novel_in_catalog	gene: Zm00001d021016	novel	4.65	125.44	coding	243	188	916
PB.7380.2	6	3222	3	full-splice_match	gene: Zm00001d035922	Zm00001d035922_T003	6.38	7.73	coding	401	210	1412
PB.4509.2	3	1758	7	full-splice_match	gene: Zm00001d043573	Zm00001d043573_T001	0.00	57.38	coding	490	164	1633
PB.4509.1	3	1941	7	full-splice_match	gene: Zm00001d043573	Zm00001d043573_T001	57.38	57.38	coding	490	170	1639
PB.4436.3	3	1945	8	novel_in_catalog	gene: Zm00001d043303	novel	2.20	88.18	coding	400	193	1392
PB.3382.3	2	2036	1	novel_in_catalog	gene: Zm00001d006697	novel	0.00	35.13	coding	474	282	1703
PB.3382.2	2	2000	2	full-splice_match	gene: Zm00001d006697	Zm00001d006697_T001	10.84	35.13	coding	520	62	1621
PB.3382.1	2	2056	2	full-splice_match	gene: Zm00001d006697	Zm00001d006697_T001	21.30	35.13	coding	520	66	1625
PB.9305.1	8	765	7	full-splice_match	gene: Zm00001d009445	Zm00001d009445_T001	200.92	200.92	coding	130	121	510
PB.6232.1	5	1743	14	full-splice_match	gene: Zm00001d014093	Zm00001d014093_T002	63.17	63.17	coding	461	234	1616

PB.6232.2	5	1530	14	full-splice_match	gene: Zm00001d014093	Zm00001d014093_T002	0.00	63.17	coding	461	56	1438
PB.3382.4	2	1790	2	full-splice_match	gene: Zm00001d006697	Zm00001d006697_T001	0.00	35.13	coding	520	62	1621
PB.885.2	1	451	3	full-splice_match	gene: Zm00001d031701	Zm00001d031701_T001	549.90	556.53	non_coding	NA	NA	NA
PB.885.1	1	896	1	novel_in_catalog	gene: Zm00001d031701	novel	6.63	556.53	non_coding	NA	NA	NA
PB.5885.2	4	1996	5	full-splice_match	gene: Zm00001d054047	Zm00001d054047_T001	17.89	31.58	coding	582	19	1764
PB.1552.1	1	2226	2	full-splice_match	gene: Zm00001d034651	Zm00001d034651_T001	6.01	6.01	coding	413	496	1734
PB.6626.2	5	1651	1	full-splice_match	gene: Zm00001d016132	Zm00001d016132_T001	4.23	25.28	coding	309	523	1449
PB.6626.1	5	1731	1	full-splice_match	gene: Zm00001d016132	Zm00001d016132_T001	21.05	25.28	coding	309	460	1386
PB.7187.1	5	1705	11	full-splice_match	gene: Zm00001d018523	Zm00001d018523_T003	98.48	145.84	coding	443	165	1493
PB.6115.2	5	1310	4	full-splice_match	gene: Zm00001d013523	Zm00001d013523_T001	12.95	64.64	coding	322	136	1101
PB.8644.3	7	1475	6	full-splice_match	gene: Zm00001d021225	Zm00001d021225_T001	0.00	133.22	coding	175	717	1241
PB.10459.2	9	2134	4	incomplete-splice_match	gene: Zm00001d047126	Zm00001d047126_T007	34.15	41.20	coding	568	32	1735
PB.1386.2	1	1468	2	full-splice_match	gene: Zm00001d033995	Zm00001d033995_T001	167.13	183.77	coding	258	449	1222
PB.10481.3	9	2730	20	full-splice_match	gene: Zm00001d047283	Zm00001d047283_T028	54.84	55.32	coding	700	287	2386
PB.8185.1	7	1785	2	full-splice_match	gene: Zm00001d018749	Zm00001d018749_T001	11.32	11.32	coding	447	176	1516
PB.6115.1	5	1307	4	full-splice_match	gene: Zm00001d013523	Zm00001d013523_T001	51.69	64.64	coding	322	81	1046
PB.3453.2	2	1858	9	novel_in_catalog	gene: Zm00001d007037	novel	21.30	31.18	coding	441	295	1617

PB.1394.1	1	1756	6	incomplete-splice_match	gene: Zm00001d034018	Zm00001d034018_T033	20.92	38.21	coding	300	49	948
PB.3985.1	3	852	4	genic	gene: Zm00001d041135	novel	32.10	32.10	coding	182	49	594
PB.4695.1	3	1860	10	full-splice_match	gene: Zm00001d044304	Zm00001d044304_T004	12.21	12.21	coding	490	246	1715
PB.6975.2	5	3051	8	novel_in_catalog	gene: Zm00001d017810	novel	2.53	41.55	coding	272	1988	2803
PB.5211.4	4	1996	9	full-splice_match	gene: Zm00001d050970	Zm00001d050970_T007	7.67	68.24	coding	171	90	602
PB.5211.5	4	2477	4	full-splice_match	gene: Zm00001d050970	Zm00001d050970_T011	0.00	68.24	coding	123	90	458
PB.5211.6	4	2101	8	full-splice_match	gene: Zm00001d050970	Zm00001d050970_T021	0.00	68.24	coding	220	1241	1900
PB.6975.3	5	2921	9	full-splice_match	gene: Zm00001d017810	Zm00001d017810_T023	10.03	41.55	coding	350	1624	2673
PB.2604.2	2	365	2	incomplete-splice_match	gene: Zm00001d002624	Zm00001d002624_T004	0.00	9.64	non_coding	NA	NA	NA
PB.2604.1	2	1269	8	full-splice_match	gene: Zm00001d002624	Zm00001d002624_T006	9.64	9.64	coding	249	135	881
PB.5211.3	4	2247	7	full-splice_match	gene: Zm00001d050970	Zm00001d050970_T009	4.62	68.24	coding	123	90	458
PB.461.1	1	1233	11	full-splice_match	gene: Zm00001d029234	Zm00001d029234_T003	16.95	16.95	coding	332	31	1026
PB.6147.2	5	2648	1	intergenic	novelGene_52	novel	0.00	0.00	coding	748	404	2647
PB.6147.1	5	4016	3	novel_not_in_catalog	gene: Zm00001d013645	novel	9.93	9.93	coding	1075	369	3593
PB.5211.8	4	2112	8	full-splice_match	gene: Zm00001d050970	Zm00001d050970_T012	0.00	68.24	coding	171	90	602
PB.5211.9	4	1742	11	novel_in_catalog	gene: Zm00001d050970	novel	0.00	68.24	coding	311	611	1543
PB.6975.5	5	3022	8	novel_in_catalog	gene: Zm00001d017810	novel	0.00	41.55	coding	272	1959	2774

PB.8442.1	7	2124	2	novel_in_catalog	gene: Zm00001d020282	novel	19.51	29.67	coding	300	967	1866
PB.760.3	1	347	1	incomplete-splice_match	gene: Zm00001d031064	Zm00001d031064_T008	11.45	21.64	non_coding	NA	NA	NA
PB.1763.1	10	3133	11	incomplete-splice_match	gene: Zm00001d023795	Zm00001d023795_T013	130.48	130.48	coding	854	210	2771
PB.5995.1	5	1648	1	antisense	novelGene_gene: Zm00001d013099_A	novel	6.28	6.28	coding	177	91	621
PB.1078.1	1	540	2	incomplete-splice_match	gene: Zm00001d032557	Zm00001d032557_T002	171.88	171.88	non_coding	NA	NA	NA
PB.10607.1	9	799	3	full-splice_match	gene: Zm00001d047779	Zm00001d047779_T001	19.29	19.29	non_coding	NA	NA	NA
PB.7718.1	6	606	1	full-splice_match	gene: Zm00001d037677	Zm00001d037677_T001	742.67	742.67	coding	101	49	351
PB.9371.3	8	975	7	full-splice_match	gene: Zm00001d009774	Zm00001d009774_T001	45.43	158.07	coding	238	87	800
PB.9371.2	8	1120	7	full-splice_match	gene: Zm00001d009774	Zm00001d009774_T001	51.97	158.07	coding	238	117	830
PB.9371.1	8	1209	7	full-splice_match	gene: Zm00001d009774	Zm00001d009774_T001	60.68	158.07	coding	238	91	804
PB.10935.1	B73V4 ctg72	1051	9	incomplete-splice_match	gene: Zm00001d000191	Zm00001d000191_T039	30.81	30.81	coding	180	196	735
PB.8749.1	7	1975	5	novel_in_catalog	gene: Zm00001d021617	novel	15.80	21.03	coding	181	60	602
PB.7740.1	6	782	1	full-splice_match	gene: Zm00001d037757	Zm00001d037757_T001	182.35	182.35	coding	122	136	501
PB.10786.1	9	2774	8	full-splice_match	gene: Zm00001d048409	Zm00001d048409_T005	2.40	54.04	coding	591	159	1931
PB.10786.2	9	2632	9	full-splice_match	gene: Zm00001d048409	Zm00001d048409_T001	51.64	54.04	coding	804	99	2510
PB.2123.5	10	2860	8	full-splice_match	gene: Zm00001d025739	Zm00001d025739_T006	0.00	45.61	coding	720	278	2437
PB.1497.5	1	2890	7	full-splice_match	gene: Zm00001d034460	Zm00001d034460_T014	1.19	56.18	coding	182	2116	2661

PB.1497.6	1	3352	6	full-splice_match	gene: Zm00001d034460	Zm00001d034460_T001	3.59	56.18	coding	104	2819	3130
PB.2123.6	10	2929	7	novel_in_catalog	gene: Zm00001d025739	novel	24.38	45.61	coding	720	280	2439
PB.7443.1	6	2238	8	full-splice_match	gene: Zm00001d036328	Zm00001d036328_T001	46.51	46.51	coding	628	18	1901
PB.10258.1	9	1624	6	full-splice_match	gene: Zm00001d045872	Zm00001d045872_T002	22.21	22.21	coding	484	30	1481
PB.2123.3	10	3168	9	full-splice_match	gene: Zm00001d025739	Zm00001d025739_T016	11.36	45.61	coding	720	280	2439
PB.9236.5	8	2900	1	incomplete-splice_match	gene: Zm00001d008993	Zm00001d008993_T008	6.62	185.60	coding	155	63	527
PB.9236.4	8	2206	2	novel_in_catalog	gene: Zm00001d008993	novel	17.69	185.60	coding	315	1093	2037
PB.9236.7	8	1120	2	incomplete-splice_match	gene: Zm00001d008993	Zm00001d008993_T003	115.01	185.60	coding	315	65	1009
PB.2352.4	10	1878	10	novel_in_catalog	gene: Zm00001d026612	novel	2.08	23.34	coding	320	691	1650
PB.9236.1	8	1368	2	incomplete-splice_match	gene: Zm00001d008993	Zm00001d008993_T003	46.28	185.60	coding	315	68	1012
PB.2352.1	10	1438	8	incomplete-splice_match	gene: Zm00001d026612	Zm00001d026612_T007	17.94	23.34	coding	320	105	1064
PB.6534.1	5	1048	6	full-splice_match	gene: Zm00001d015415	Zm00001d015415_T004	50.24	50.24	coding	197	273	863
PB.5676.1	4	1893	2	full-splice_match	gene: Zm00001d053144	Zm00001d053144_T001	2.35	2.61	coding	468	24	1427
PB.2624.1	2	1050	1	full-splice_match	gene: Zm00001d002733	Zm00001d002733_T001	14.04	14.04	coding	237	114	824
PB.9338.1	8	2416	20	novel_in_catalog	gene: Zm00001d009631	novel	12.95	12.95	coding	609	202	2028
PB.5349.1	4	2727	17	full-splice_match	gene: Zm00001d051753	Zm00001d051753_T001	23.02	26.14	coding	802	127	2532
PB.6767.1	5	1880	8	novel_in_catalog	gene: Zm00001d016873	novel	2.38	2.38	coding	350	132	1181

PB.5583.6	4	1584	3	full-splice_match	gene: Zm00001d052733	Zm00001d052733_T001	34.74	60.17	coding	309	274	1200
PB.5418.1	4	2394	2	full-splice_match	gene: Zm00001d052061	Zm00001d052061_T001	34.77	34.77	coding	618	273	2126
PB.5583.5	4	1680	2	novel_in_catalog	gene: Zm00001d052733	novel	0.00	60.17	coding	343	266	1294
PB.568.1	1	866	5	full-splice_match	gene: Zm00001d029740	Zm00001d029740_T001	163.73	163.73	coding	160	77	556
PB.5583.3	4	1900	1	genic	gene: Zm00001d052733	novel	2.79	60.17	coding	236	709	1416
PB.568.2	1	732	5	full-splice_match	gene: Zm00001d029740	Zm00001d029740_T001	0.00	163.73	coding	160	69	548
PB.3911.1	3	1749	4	novel_not_in_catalog	gene: Zm00001d040509	novel	1.31	832.21	coding	445	100	1434
PB.9740.2	8	1634	12	novel_not_in_catalog	gene: Zm00001d011572	novel	65.88	65.88	coding	436	123	1430
PB.3911.3	3	1652	4	novel_not_in_catalog	gene: Zm00001d040509	novel	170.80	832.21	coding	445	109	1443
PB.6371.1	5	1374	1	full-splice_match	gene: Zm00001d014734	Zm00001d014734_T001	112.84	112.84	coding	360	79	1158
PB.3911.5	3	1576	4	novel_not_in_catalog	gene: Zm00001d040509	novel	34.99	832.21	coding	445	91	1425
PB.5989.1	5	1903	7	full-splice_match	gene: Zm00001d013073	Zm00001d013073_T002	17.01	41.75	coding	285	125	979
PB.5989.2	5	1243	7	full-splice_match	gene: Zm00001d013073	Zm00001d013073_T002	24.74	41.75	coding	285	132	986
PB.1139.1	1	2642	1	novel_in_catalog	gene: Zm00001d032808	novel	5.37	5.37	coding	277	67	897
PB.8615.2	7	1172	3	novel_not_in_catalog	gene: Zm00001d021048	novel	0.00	9.73	coding	344	54	1085
PB.8615.1	7	1301	3	novel_not_in_catalog	gene: Zm00001d021048	novel	9.73	9.73	coding	344	54	1085
PB.4286.2	3	2285	2	novel_in_catalog	gene: Zm00001d042653	novel	11.78	114.75	coding	201	162	764

PB.1076.17	1	2544	4	full-splice_match	gene: Zm00001d032555	Zm00001d032555_T011	4.98	39.90	coding	517	759	2309
PB.1076.16	1	2696	5	novel_not_in_catalog	gene: Zm00001d032554	novel	2.17	25.20	coding	347	1460	2500
PB.1076.15	1	1637	6	novel_not_in_catalog	gene: Zm00001d032554	novel	5.44	25.20	coding	347	401	1441
PB.5644.1	4	974	3	incomplete-splice_match	gene: Zm00001d052974	Zm00001d052974_T002	227.40	227.40	coding	123	228	596
PB.5438.1	4	2315	2	full-splice_match	gene: Zm00001d052170	Zm00001d052170_T001	67.53	68.30	coding	595	302	2086
PB.1076.12	1	1997	7	novel_not_in_catalog	gene: Zm00001d032552	novel	12.94	48.82	coding	164	1272	1763
PB.1076.11	1	1314	7	novel_not_in_catalog	gene: Zm00001d032552	novel	28.82	48.82	coding	164	576	1067
PB.5438.2	4	3365	1	novel_in_catalog	gene: Zm00001d052170	novel	0.77	68.30	coding	387	2031	3191
PB.9400.1	8	1662	3	full-splice_match	gene: Zm00001d009954	Zm00001d009954_T001	39.37	76.61	coding	313	363	1301
PB.9400.2	8	595	2	incomplete-splice_match	gene: Zm00001d009954	Zm00001d009954_T001	37.24	76.61	coding	45	271	405
PB.9400.3	8	973	3	full-splice_match	gene: Zm00001d009954	Zm00001d009954_T001	0.00	76.61	coding	223	305	973
PB.5332.1	4	1014	1	full-splice_match	gene: Zm00001d051660	Zm00001d051660_T001	38.52	38.52	coding	220	66	725
PB.5671.2	4	2196	6	full-splice_match	gene: Zm00001d053104	Zm00001d053104_T006	2.53	19.94	coding	101	1387	1689
PB.716.2	1	3257	6	full-splice_match	gene: Zm00001d030784	Zm00001d030784_T006	1.03	4.35	coding	792	639	3014
PB.9693.1	8	590	2	incomplete-splice_match	gene: Zm00001d011353	Zm00001d011353_T011	121.86	121.86	non_coding	NA	NA	NA
PB.1405.1	1	2068	6	incomplete-splice_match	gene: Zm00001d034049	Zm00001d034049_T001	183.67	239.03	coding	448	295	1638
PB.9063.4	8	1828	8	full-splice_match	gene: Zm00001d008203	Zm00001d008203_T003	0.00	10.47	coding	266	13	810

PB.1405.3	1	1972	6	incomplete-splice_match	gene: Zm00001d034049	Zm00001d034049_T001	53.86	239.03	coding	448	290	1633
PB.9063.1	8	2012	8	full-splice_match	gene: Zm00001d008203	Zm00001d008203_T003	3.82	10.47	coding	266	125	922
PB.1616.1	1	1829	12	full-splice_match	gene: Zm00001d034856	Zm00001d034856_T003	62.78	128.98	coding	452	44	1399
PB.1405.7	1	1738	6	incomplete-splice_match	gene: Zm00001d034049	Zm00001d034049_T001	0.00	239.03	coding	448	296	1639
PB.9063.2	8	1874	9	novel_in_catalog	gene: Zm00001d008203	novel	6.65	10.47	coding	537	54	1664
PB.1759.1	10	1502	2	genic	gene: Zm00001d023768	novel	16.59	16.59	coding	384	96	1247
PB.4211.1	3	1263	4	full-splice_match	gene: Zm00001d042308	Zm00001d042308_T001	401.54	401.54	coding	208	443	1066
PB.4398.2	3	1768	5	novel_in_catalog	gene: Zm00001d043149	novel	13.89	438.61	coding	194	129	710
PB.3021.1	2	2619	8	full-splice_match	gene: Zm00001d004875	Zm00001d004875_T002	22.33	32.32	coding	695	50	2134
PB.2725.4	2	710	4	full-splice_match	gene: Zm00001d003127	Zm00001d003127_T004	892.49	1102.62	coding	152	79	534
PB.3021.2	2	2429	7	full-splice_match	gene: Zm00001d004875	Zm00001d004875_T006	10.00	32.32	coding	240	62	781
PB.6804.1	5	1907	2	full-splice_match	gene: Zm00001d017077	Zm00001d017077_T001	1.85	17.70	coding	515	53	1597
PB.9469.1	8	436	1	intergenic	novelGene_179	novel	7.21	7.21	non_coding	NA	NA	NA
PB.2725.2	2	823	4	full-splice_match	gene: Zm00001d003127	Zm00001d003127_T004	210.13	1102.62	coding	152	79	534
PB.921.1	1	1682	5	full-splice_match	gene: Zm00001d031902	Zm00001d031902_T002	26.66	26.66	coding	232	180	875
PB.3577.1	2	3066	18	full-splice_match	gene: Zm00001d007758	Zm00001d007758_T003	94.36	125.91	coding	888	162	2825
PB.6779.1	5	1827	2	novel_in_catalog	gene: Zm00001d016911	novel	22.97	35.18	coding	388	22	1185

PB.8467.1	7	1522	4	full-splice_match	gene: Zm00001d020400	Zm00001d020400_T001	2.27	36.97	coding	365	61	1155
PB.2584.1	2	1986	2	full-splice_match	gene: Zm00001d002579	Zm00001d002579_T002	27.20	27.20	coding	514	321	1862
PB.1098.1	1	2299	7	incomplete-splice_match	gene: Zm00001d032615	Zm00001d032615_T006	38.21	38.21	coding	505	420	1934
PB.6524.1	5	558	1	intergenic	novelGene_151	novel	10.08	10.08	non_coding	NA	NA	NA
PB.540.2	1	2642	14	full-splice_match	gene: Zm00001d029608	Zm00001d029608_T008	24.87	24.87	coding	651	370	2322
PB.8675.1	7	1079	2	incomplete-splice_match	gene: Zm00001d021346	Zm00001d021346_T001	8.44	20.01	coding	243	33	761
PB.8675.2	7	1654	6	full-splice_match	gene: Zm00001d021346	Zm00001d021346_T001	11.57	20.01	coding	483	33	1481
PB.4345.1	3	2328	6	full-splice_match	gene: Zm00001d042901	Zm00001d042901_T001	13.29	32.97	coding	582	65	1810
PB.4345.3	3	2233	5	novel_in_catalog	gene: Zm00001d042901	novel	0.87	32.97	coding	555	65	1729
PB.4345.2	3	2133	6	full-splice_match	gene: Zm00001d042901	Zm00001d042901_T001	18.81	32.97	coding	582	65	1810
PB.5373.1	4	2576	2	full-splice_match	gene: Zm00001d051883	Zm00001d051883_T001	14.94	86.49	coding	527	395	1975
PB.8609.1	7	742	6	full-splice_match	gene: Zm00001d021021	Zm00001d021021_T002	154.39	154.39	coding	148	71	514
PB.5373.3	4	2455	1	novel_in_catalog	gene: Zm00001d051883	novel	2.68	86.49	coding	456	341	1708
PB.5373.2	4	2440	2	full-splice_match	gene: Zm00001d051883	Zm00001d051883_T001	65.37	86.49	coding	527	395	1975
PB.5373.5	4	2337	2	full-splice_match	gene: Zm00001d051883	Zm00001d051883_T001	3.50	86.49	coding	527	471	2051
PB.3526.1	2	1593	7	full-splice_match	gene: Zm00001d007442	Zm00001d007442_T001	14.56	14.56	coding	396	118	1305
PB.1436.1	1	377	2	novel_in_catalog	gene: Zm00001d034223	novel	48.93	574.01	non_coding	NA	NA	NA

PB.5373.6	4	2330	1	novel_in_catalog	gene: Zm00001d051883	novel	0.00	86.49	coding	456	349	1716
PB.3563.1	2	1734	11	full-splice_match	gene: Zm00001d007638	Zm00001d007638_T001	6.56	111.96	coding	374	228	1349
PB.3424.1	2	2140	5	full-splice_match	gene: Zm00001d006900	Zm00001d006900_T004	99.31	180.26	coding	537	91	1701
PB.4244.1	3	725	4	full-splice_match	gene: Zm00001d042469	Zm00001d042469_T006	5.45	5.45	coding	138	117	530
PB.3892.2	3	1710	6	full-splice_match	gene: Zm00001d040383	Zm00001d040383_T003	5.94	20.11	coding	171	78	590
PB.3892.1	3	1582	7	full-splice_match	gene: Zm00001d040383	Zm00001d040383_T005	14.18	20.11	coding	420	32	1291
PB.3564.9	2	2102	9	novel_in_catalog	gene: Zm00001d007640	novel	0.00	33.97	coding	623	70	1938
PB.3564.8	2	2293	9	novel_in_catalog	gene: Zm00001d007640	novel	4.10	33.97	coding	628	77	1960
PB.5402.1	4	554	2	full-splice_match	gene: Zm00001d052002	Zm00001d052002_T001	7.26	7.26	coding	141	10	432
PB.6090.1	5	2240	9	full-splice_match	gene: Zm00001d013427	Zm00001d013427_T003	80.40	80.40	coding	603	45	1853
PB.6163.1	5	2334	14	full-splice_match	gene: Zm00001d013729	Zm00001d013729_T002	9.29	9.29	coding	670	178	2187
PB.3564.7	2	2380	8	novel_in_catalog	gene: Zm00001d007640	novel	25.25	33.97	coding	628	68	1951
PB.4228.2	3	1915	7	full-splice_match	gene: Zm00001d042367	Zm00001d042367_T002	58.27	82.90	coding	493	266	1744
PB.4139.1	3	1363	2	full-splice_match	gene: Zm00001d041985	Zm00001d041985_T001	218.45	218.45	coding	348	82	1125
PB.10593.1	9	772	2	genic	gene: Zm00001d047745	novel	6.57	69.88	coding	89	99	365
PB.10593.2	9	911	2	genic	gene: Zm00001d047745	novel	63.31	69.88	coding	93	244	522
PB.3424.2	2	1430	1	genic	gene: Zm00001d006900	novel	13.78	180.26	coding	96	34	321

PB.2826.1	2	1097	1	full-splice_match	gene: Zm00001d003575	Zm00001d003575_T001	7.63	7.63	coding	289	48	914
PB.486.1	1	964	7	full-splice_match	gene: Zm00001d029380	Zm00001d029380_T003	39.28	39.28	coding	216	32	679
PB.918.4	1	2615	2	incomplete-splice_match	gene: Zm00001d031871	Zm00001d031871_T002	6.32	59.27	coding	764	91	2382
PB.4294.15	3	2223	1	novel_in_catalog	gene: Zm00001d042684	novel	0.00	417.27	coding	330	257	1246
PB.3035.2	2	2378	11	full-splice_match	gene: Zm00001d004960	Zm00001d004960_T004	9.53	49.73	coding	336	98	1105
PB.3035.1	2	2286	12	full-splice_match	gene: Zm00001d004960	Zm00001d004960_T006	40.20	49.73	coding	640	100	2019
PB.9054.4	7	657	5	novel_in_catalog	gene: Zm00001d022631	novel	5.44	48.42	coding	154	90	551
PB.3722.1	3	1038	2	intergenic	novelGene_30	novel	7.98	7.98	non_coding	NA	NA	NA
PB.3424.3	2	2012	5	full-splice_match	gene: Zm00001d006900	Zm00001d006900_T004	67.17	180.26	coding	537	89	1699
PB.1801.1	10	2256	7	novel_in_catalog	gene: Zm00001d023968	novel	4.80	38.72	non_coding	NA	NA	NA
PB.9038.1	7	1489	7	full-splice_match	gene: Zm00001d022588	Zm00001d022588_T003	27.89	29.53	coding	411	96	1328
PB.1801.3	10	885	9	full-splice_match	gene: Zm00001d023968	Zm00001d023968_T001	20.58	38.72	coding	213	110	748
PB.1801.2	10	838	10	novel_not_in_catalog	gene: Zm00001d023968	novel	13.34	38.72	coding	206	54	671
PB.1066.2	1	3110	7	incomplete-splice_match	gene: Zm00001d032520	Zm00001d032520_T001	13.05	13.05	coding	912	27	2762
PB.3796.5	3	2134	5	novel_in_catalog	gene: Zm00001d039963	novel	3.42	15.39	coding	294	149	1030
PB.6812.1	5	1085	11	full-splice_match	gene: Zm00001d017103	Zm00001d017103_T005	70.61	70.61	coding	224	137	808
PB.926.3	1	2497	10	novel_in_catalog	gene: Zm00001d031927	novel	12.47	74.67	coding	102	1558	1863

PB.6269.2	5	1019	3	novel_not_in_catalog	gene: Zm00001d014299	novel	77.05	183.89	coding	190	219	788
PB.4359.1	3	1680	9	novel_not_in_catalog	gene: Zm00001d042974	novel	3.47	3.47	coding	430	178	1467
PB.10144.3	9	2707	1	full-splice_match	gene: Zm00001d045319	Zm00001d045319_T001	11.75	11.75	coding	691	50	2122
PB.5701.2	4	3319	5	incomplete-splice_match	gene: Zm00001d053300	Zm00001d053300_T007	5.77	30.94	coding	1001	21	3023
PB.6506.1	5	1765	1	full-splice_match	gene: Zm00001d015247	Zm00001d015247_T001	55.53	55.53	coding	424	215	1486
PB.732.1	1	3105	8	full-splice_match	gene: Zm00001d030916	Zm00001d030916_T001	25.62	31.90	coding	878	112	2745
PB.7394.6	6	2811	10	full-splice_match	gene: Zm00001d035990	Zm00001d035990_T006	10.05	36.35	coding	771	233	2545
PB.7394.5	6	2796	10	full-splice_match	gene: Zm00001d035990	Zm00001d035990_T009	26.30	36.35	coding	771	213	2525
PB.2022.1	10	2554	19	fusion	gene: Zm00001d025153_g	novel	11.12	11.12	coding	804	24	2435
PB.1264.1	1	1468	9	full-splice_match	gene: Zm00001d033472	Zm00001d033472_T001	23.66	23.66	coding	200	213	812
PB.10583.3	9	3140	4	antisense	novelGene_gene: Zm00001d047708_A	novel	2.86	20.54	coding	86	106	363
PB.10583.2	9	2241	4	antisense	novelGene_gene: Zm00001d047708_A	novel	5.67	20.54	coding	140	21	440
PB.10583.1	9	1240	4	antisense	novelGene_gene: Zm00001d047708_A	novel	9.00	20.54	non_coding	NA	NA	NA
PB.3392.3	2	1785	10	novel_not_in_catalog	gene: Zm00001d006751	novel	19.50	58.08	coding	269	612	1418
PB.5892.1	4	2591	1	incomplete-splice_match	gene: Zm00001d054070	Zm00001d054070_T013	11.94	11.94	coding	354	82	1143
PB.1835.1	10	1758	5	novel_in_catalog	gene: Zm00001d024231	novel	3.41	12.83	coding	279	1	837
PB.9689.1	8	2737	1	genic	gene: Zm00001d011336	novel	10.03	60.31	non_coding	NA	NA	NA

PB.1835.3	10	1272	3	novel_in_catalog	gene: Zm00001d024231	novel	9.43	12.83	non_coding	NA	NA	NA
PB.10579.1	9	2014	1	full-splice_match	gene: Zm00001d047685	Zm00001d047685_T001	25.03	32.74	coding	375	605	1729
PB.10579.2	9	1881	1	full-splice_match	gene: Zm00001d047685	Zm00001d047685_T001	7.71	32.74	coding	375	606	1730
PB.9272.1	8	2202	15	full-splice_match	gene: Zm00001d009212	Zm00001d009212_T003	15.28	15.28	coding	562	155	1840
PB.5772.2	4	1975	3	novel_in_catalog	gene: Zm00001d053640	novel	3.48	12.91	coding	466	107	1504
PB.2802.1	2	2709	2	genic	gene: Zm00001d003438	novel	64.18	64.18	coding	625	610	2484
PB.7368.5	6	2434	6	incomplete-splice_match	gene: Zm00001d035869	Zm00001d035869_T024	27.95	96.26	coding	408	147	1370
PB.7368.3	6	3119	13	novel_not_in_catalog	gene: Zm00001d035869	novel	7.35	96.26	coding	794	324	2705
PB.7368.2	6	3154	13	full-splice_match	gene: Zm00001d035869	Zm00001d035869_T023	56.16	96.26	coding	823	269	2737
PB.7368.1	6	3310	8	novel_in_catalog	gene: Zm00001d035869	novel	4.79	96.26	coding	507	602	2122
PB.5396.1	4	2636	2	full-splice_match	gene: Zm00001d051948	Zm00001d051948_T001	75.23	80.23	coding	632	355	2250
PB.6925.4	5	2272	5	full-splice_match	gene: Zm00001d017564	Zm00001d017564_T009	4.09	43.92	coding	166	1610	2107
PB.10519.1	9	1036	4	full-splice_match	gene: Zm00001d047469	Zm00001d047469_T001	78.12	78.12	coding	228	123	806
PB.6250.1	5	1838	1	antisense	novelGene_gene: Zm00001d014181_A	novel	4.17	4.17	coding	195	947	1531
PB.6379.1	5	2267	1	full-splice_match	gene: Zm00001d014759	Zm00001d014759_T001	21.48	24.74	coding	638	45	1958
PB.2713.1	2	524	4	full-splice_match	gene: Zm00001d003084	Zm00001d003084_T001	241.63	241.63	non_coding	NA	NA	NA
PB.4270.1	3	1700	13	full-splice_match	gene: Zm00001d042596	Zm00001d042596_T005	7.94	7.94	coding	400	89	1288

PB.4601.2	3	3969	4	full-splice_match	gene: Zm00001d043927	Zm00001d043927_T013	6.22	70.75	coding	927	764	3544
PB.2030.2	10	3213	5	full-splice_match	gene: Zm00001d025210	Zm00001d025210_T002	12.10	12.10	coding	959	43	2919
PB.2746.3	2	1328	2	full-splice_match	gene: Zm00001d003200	Zm00001d003200_T001	0.00	28.71	coding	124	630	1001
PB.2087.2	10	671	2	novel_in_catalog	gene: Zm00001d025564	novel	94.58	106.44	coding	129	128	514
PB.2087.1	10	418	1	genic	gene: Zm00001d025564	novel	11.86	106.44	non_coding	NA	NA	NA
PB.5359.1	4	2472	3	full-splice_match	gene: Zm00001d051838	Zm00001d051838_T001	45.52	45.52	coding	649	200	2146
PB.2424.2	2	693	3	incomplete-splice_match	gene: Zm00001d001895	Zm00001d001895_T008	64.75	150.50	coding	112	193	528
PB.135.1	1	1531	11	novel_in_catalog	gene: Zm00001d027826	novel	124.91	124.91	coding	313	170	1108
PB.2150.1	10	2077	6	novel_not_in_catalog	gene: Zm00001d025834	novel	3.49	3.49	coding	457	445	1815
PB.5628.1	4	2070	5	full-splice_match	gene: Zm00001d052901	Zm00001d052901_T002	25.40	25.40	coding	369	712	1818
PB.7360.7	6	1825	8	novel_in_catalog	gene: Zm00001d035772	novel	1.97	76.46	coding	185	303	857
PB.7360.5	6	1783	11	novel_in_catalog	gene: Zm00001d035772	novel	40.10	76.46	coding	484	99	1550
PB.4553.1	3	1042	2	full-splice_match	gene: Zm00001d043730	Zm00001d043730_T001	3.34	3.34	coding	236	56	763
PB.7360.3	6	1896	11	full-splice_match	gene: Zm00001d035772	Zm00001d035772_T002	34.39	76.46	coding	485	161	1615
PB.7531.3	6	3107	24	novel_in_catalog	gene: Zm00001d036741	novel	14.29	24.70	coding	753	44	2302
PB.2011.2	10	2077	17	full-splice_match	gene: Zm00001d025100	Zm00001d025100_T008	54.84	88.80	coding	537	164	1774
PB.7531.1	6	3874	27	full-splice_match	gene: Zm00001d036741	Zm00001d036741_T020	10.41	24.70	coding	1172	186	3701

PB.8179.1	7	2703	22	full-splice_match	gene: Zm00001d018724	Zm00001d018724_T002	22.17	37.93	coding	755	106	2370
PB.8712.1	7	2717	1	incomplete-splice_match	gene: Zm00001d021492	Zm00001d021492_T002	1.51	1.51	coding	842	123	2648
PB.3107.1	2	1819	1	full-splice_match	gene: Zm00001d005351	Zm00001d005351_T001	7.86	30.42	coding	422	215	1480
PB.228.1	1	1824	2	full-splice_match	gene: Zm00001d028235	Zm00001d028235_T001	6.02	6.02	coding	506	88	1605
PB.9720.1	8	1081	8	full-splice_match	gene: Zm00001d011474	Zm00001d011474_T001	21.42	93.24	coding	221	128	790
PB.5491.2	4	2377	12	full-splice_match	gene: Zm00001d052354	Zm00001d052354_T005	22.13	26.18	coding	623	280	2148
PB.9720.2	8	965	8	full-splice_match	gene: Zm00001d011474	Zm00001d011474_T001	71.82	93.24	coding	221	130	792
PB.6713.2	5	499	3	intergenic	novelGene_281	novel	109.58	138.85	non_coding	NA	NA	NA
PB.3517.1	2	1830	15	full-splice_match	gene: Zm00001d007391	Zm00001d007391_T005	4.73	4.73	coding	491	38	1510
PB.6713.1	5	579	3	intergenic	novelGene_281	novel	29.27	138.85	non_coding	NA	NA	NA
PB.5345.1	4	925	4	novel_in_catalog	gene: Zm00001d051741	novel	73.24	73.24	coding	149	118	564
PB.3187.1	2	1554	3	intergenic	novelGene_306	novel	6.60	6.60	coding	152	266	721
PB.826.2	1	2267	3	novel_in_catalog	gene: Zm00001d031332	novel	47.81	98.73	coding	351	69	1121
PB.3398.2	2	3283	9	full-splice_match	gene: Zm00001d006759	Zm00001d006759_T003	5.03	36.59	coding	707	737	2857
PB.3450.9	2	2454	11	full-splice_match	gene: Zm00001d007029	Zm00001d007029_T002	0.00	45.54	coding	592	594	2369
PB.9393.1	8	1334	10	novel_not_in_catalog	gene: Zm00001d009919	novel	41.54	41.54	coding	341	5	1027
PB.1820.2	10	2951	1	antisense	novelGene_gene: Zm00001d024134_A	novel	0.00	7.93	coding	161	1561	2043

PB.5620.1	4	1264	2	genic	gene: Zm00001d052864	novel	23.72	89.71	coding	269	181	987
PB.5620.2	4	1097	2	genic	gene: Zm00001d052864	novel	65.99	89.71	coding	269	120	926
PB.1820.1	10	3135	1	antisense	novelGene_gene: Zm00001d024134_A	novel	7.93	7.93	coding	161	1633	2115
PB.810.1	1	1547	10	novel_in_catalog	gene: Zm00001d031269	novel	6.83	51.07	coding	256	104	871
PB.2342.1	10	459	3	full-splice_match	gene: Zm00001d026589	Zm00001d026589_T001	168.87	168.87	non_coding	NA	NA	NA
PB.1096.7	1	1631	12	novel_not_in_catalog	gene: Zm00001d032608	novel	4.64	78.90	coding	224	822	1493
PB.4712.1	3	1477	3	full-splice_match	gene: Zm00001d044395	Zm00001d044395_T007	4.91	35.46	coding	288	119	982
PB.1096.1	1	1750	13	novel_not_in_catalog	gene: Zm00001d032608	novel	5.90	78.90	coding	414	69	1310
PB.9736.1	8	2421	11	full-splice_match	gene: Zm00001d011563	Zm00001d011563_T003	6.36	48.87	coding	285	141	995
PB.9736.2	8	1558	12	full-splice_match	gene: Zm00001d011563	Zm00001d011563_T008	42.51	48.87	coding	398	151	1344
PB.1096.2	1	1641	12	novel_not_in_catalog	gene: Zm00001d032608	novel	11.60	78.90	coding	224	712	1383
PB.1840.1	10	2011	8	full-splice_match	gene: Zm00001d024281	Zm00001d024281_T001	1.60	88.36	coding	500	80	1579
PB.1840.2	10	1902	8	full-splice_match	gene: Zm00001d024281	Zm00001d024281_T001	60.10	88.36	coding	500	80	1579
PB.1840.3	10	1750	8	full-splice_match	gene: Zm00001d024281	Zm00001d024281_T001	26.65	88.36	coding	500	81	1580
PB.220.2	1	3118	4	incomplete-splice_match	gene: Zm00001d028213	Zm00001d028213_T001	0.00	20.74	coding	946	279	3116
PB.220.3	1	2968	4	incomplete-splice_match	gene: Zm00001d028213	Zm00001d028213_T001	0.00	20.74	coding	886	311	2968
PB.1559.4	1	825	2	full-splice_match	gene: Zm00001d034673	Zm00001d034673_T001	29.15	83.84	coding	127	128	508

PB.220.1	1	3643	6	novel_not_in_catalog	gene: Zm00001d028213	novel	20.74	20.74	coding	978	279	3212
PB.4889.1	4	1358	3	intergenic	novelGene_51	novel	18.52	18.52	coding	130	166	555
PB.6684.2	5	902	8	novel_in_catalog	gene: Zm00001d016469	novel	12.07	712.60	coding	141	230	652
PB.2452.4	2	1484	7	novel_in_catalog	gene: Zm00001d001999	novel	4.57	145.70	coding	72	120	335
PB.1725.1	10	1869	3	novel_in_catalog	gene: Zm00001d023557	novel	28.50	28.50	coding	436	126	1433
PB.1725.2	10	1612	3	novel_in_catalog	gene: Zm00001d023557	novel	0.00	28.50	coding	436	111	1418
PB.9244.2	8	3191	5	full-splice_match	gene: Zm00001d009017	Zm00001d009017_T002	33.21	33.21	coding	927	200	2980
PB.2452.1	2	1625	9	full-splice_match	gene: Zm00001d001999	Zm00001d001999_T001	123.61	145.70	coding	336	322	1329
PB.2452.2	2	1517	8	full-splice_match	gene: Zm00001d001999	Zm00001d001999_T007	17.52	145.70	coding	271	122	934
PB.2452.3	2	2474	8	novel_in_catalog	gene: Zm00001d001999	novel	0.00	145.70	coding	178	1657	2190
PB.6897.2	5	1333	7	novel_in_catalog	gene: Zm00001d017448	novel	2.59	38.54	coding	153	561	1019
PB.10619.1	9	2341	2	incomplete-splice_match	gene: Zm00001d047813	Zm00001d047813_T001	22.31	22.31	coding	648	17	1960
PB.5616.1	4	1128	2	genic	gene: Zm00001d052843	novel	5.61	5.61	coding	260	196	975
PB.3197.2	2	2423	6	full-splice_match	gene: Zm00001d005831	Zm00001d005831_T008	0.00	15.48	coding	678	166	2199
PB.3197.1	2	2548	6	full-splice_match	gene: Zm00001d005831	Zm00001d005831_T007	15.48	15.48	coding	678	168	2201
PB.1559.3	1	1020	1	novel_in_catalog	gene: Zm00001d034673	novel	6.01	83.84	non_coding	NA	NA	NA
PB.5230.1	4	502	4	novel_not_in_catalog	gene: Zm00001d051116	novel	127.82	127.82	non_coding	NA	NA	NA

PB.10164.1	9	1883	4	novel_not_in_catalog	gene: Zm00001d045386	novel	13.83	13.83	coding	149	401	847
PB.4715.1	3	2855	7	full-splice_match	gene: Zm00001d044420	Zm00001d044420_T001	7.14	7.14	coding	782	307	2652
PB.1475.2	1	1575	6	novel_not_in_catalog	gene: Zm00001d034388	novel	130.86	212.88	coding	461	20	1402
PB.10485.1	9	1158	2	genic	gene: Zm00001d047303	novel	28.19	28.19	coding	272	229	1044
PB.4883.1	4	1501	5	novel_not_in_catalog	gene: Zm00001d049043	novel	50.61	110.95	coding	412	6	1241
PB.4883.2	4	1420	5	novel_not_in_catalog	gene: Zm00001d049043	novel	60.35	110.95	coding	412	43	1278
PB.8689.3	7	3101	6	novel_in_catalog	gene: Zm00001d021392	novel	0.40	174.94	coding	544	1194	2825
PB.810.3	1	1180	8	full-splice_match	gene: Zm00001d031269	Zm00001d031269_T005	44.24	51.07	coding	256	177	944
PB.8689.5	7	2302	7	full-splice_match	gene: Zm00001d021392	Zm00001d021392_T002	0.00	174.94	coding	639	167	2083
PB.10686.3	9	1966	9	novel_not_in_catalog	gene: Zm00001d048050	novel	44.91	44.91	coding	267	999	1799
PB.8689.7	7	2190	7	full-splice_match	gene: Zm00001d021392	Zm00001d021392_T002	0.00	174.94	coding	639	167	2083
PB.28.1	1	624	1	antisense	novelGene_gene: Zm00001d027337_A	novel	69.58	69.58	coding	26	266	343
PB.10679.3	9	1926	1	full-splice_match	gene: Zm00001d048016	Zm00001d048016_T001	0.00	33.92	coding	637	14	1924
PB.7302.3	6	1736	1	full-splice_match	gene: Zm00001d035455	Zm00001d035455_T001	0.00	174.91	coding	522	29	1594
PB.7302.2	6	1594	1	full-splice_match	gene: Zm00001d035455	Zm00001d035455_T001	37.08	174.91	coding	378	309	1442
PB.7302.1	6	2222	1	full-splice_match	gene: Zm00001d035455	Zm00001d035455_T001	137.82	174.91	coding	600	68	1867
PB.6353.1	5	1749	8	incomplete-splice_match	gene: Zm00001d014655	Zm00001d014655_T001	55.48	55.48	coding	424	235	1506

PB.3459.1	2	1912	4	full-splice_match	gene: Zm00001d007068	Zm00001d007068_T001	71.95	74.37	coding	426	237	1514
PB.3459.2	2	2648	3	novel_in_catalog	gene: Zm00001d007068	novel	2.42	74.37	coding	384	243	1394
PB.3459.3	2	1767	4	full-splice_match	gene: Zm00001d007068	Zm00001d007068_T001	0.00	74.37	coding	426	237	1514
PB.3398.1	2	2690	9	full-splice_match	gene: Zm00001d006759	Zm00001d006759_T015	15.48	36.59	coding	709	153	2279
PB.9254.1	8	2619	2	intergenic	novelGene_94	novel	2.14	2.14	coding	152	274	729
PB.9698.1	8	3345	5	full-splice_match	gene: Zm00001d011373	Zm00001d011373_T005	1.39	20.64	coding	797	273	2663
PB.1978.6	10	2583	5	incomplete-splice_match	gene: Zm00001d024952	Zm00001d024952_T005	20.31	124.67	coding	349	79	1125
PB.4712.2	3	1738	1	novel_in_catalog	gene: Zm00001d044395	novel	3.69	35.46	coding	155	371	835
PB.3409.2	2	3488	5	full-splice_match	gene: Zm00001d006828	Zm00001d006828_T001	11.05	44.28	coding	955	432	3296
PB.5889.5	4	1676	3	full-splice_match	gene: Zm00001d054066	Zm00001d054066_T001	0.00	98.73	coding	497	65	1555
PB.4651.2	3	4026	10	novel_not_in_catalog	gene: Zm00001d044139	novel	14.76	14.76	coding	1118	274	3627
PB.2967.1	2	3343	2	full-splice_match	gene: Zm00001d004526	Zm00001d004526_T001	18.73	20.63	coding	993	52	3030
PB.4750.1	3	2884	18	full-splice_match	gene: Zm00001d044540	Zm00001d044540_T004	17.55	18.96	coding	850	84	2633
PB.2967.2	2	3259	2	full-splice_match	gene: Zm00001d004526	Zm00001d004526_T001	1.89	20.63	coding	993	72	3050
PB.1559.1	1	1466	2	full-splice_match	gene: Zm00001d034673	Zm00001d034673_T001	21.23	83.84	coding	127	128	508
PB.5909.1	5	2139	3	novel_in_catalog	gene: Zm00001d012808	novel	5.95	22.04	coding	422	83	1348
PB.5909.2	5	2107	4	novel_in_catalog	gene: Zm00001d012808	novel	5.23	22.04	coding	453	192	1550

PB.7383.7	6	2416	2	full-splice_match	gene: Zm00001d035934	Zm00001d035934_T002	21.06	156.37	coding	593	354	2132
PB.10505.1	9	1127	5	full-splice_match	gene: Zm00001d047423	Zm00001d047423_T002	51.62	51.62	coding	217	231	881
PB.5909.6	5	1976	3	novel_in_catalog	gene: Zm00001d012808	novel	0.00	22.04	coding	422	84	1349
PB.5909.7	5	1935	4	novel_in_catalog	gene: Zm00001d012808	novel	0.00	22.04	coding	453	83	1441
PB.5264.1	4	894	2	full-splice_match	gene: Zm00001d051333	Zm00001d051333_T001	69.22	383.00	coding	195	63	647
PB.5264.2	4	764	2	full-splice_match	gene: Zm00001d051333	Zm00001d051333_T001	313.77	383.00	coding	195	63	647
PB.4258.1	3	3570	13	full-splice_match	gene: Zm00001d042528	Zm00001d042528_T002	3.93	3.93	coding	968	313	3216
PB.2007.2	10	1271	4	full-splice_match	gene: Zm00001d025075	Zm00001d025075_T003	32.47	33.59	coding	216	417	1064
PB.842.1	1	1766	5	full-splice_match	gene: Zm00001d031473	Zm00001d031473_T001	39.08	39.08	coding	446	75	1412
PB.842.2	1	1607	5	full-splice_match	gene: Zm00001d031473	Zm00001d031473_T001	0.00	39.08	coding	446	74	1411
PB.2007.1	10	3638	3	full-splice_match	gene: Zm00001d025075	Zm00001d025075_T002	1.12	33.59	coding	216	2753	3400
PB.6750.1	5	947	2	novel_in_catalog	gene: Zm00001d016814	novel	42.31	42.31	coding	46	223	360
PB.8357.1	7	2219	10	novel_not_in_catalog	gene: Zm00001d019672	novel	3.12	3.12	coding	604	114	1925
PB.7293.1	6	1367	1	intergenic	novelGene_76	novel	2.59	2.59	coding	321	89	1051
PB.1646.3	10	1896	2	intergenic	novelGene_12	novel	14.05	108.48	coding	422	214	1479
PB.5889.3	4	2435	2	novel_in_catalog	gene: Zm00001d054066	novel	2.96	98.73	coding	393	955	2133
PB.242.1	1	1701	4	full-splice_match	gene: Zm00001d028307	Zm00001d028307_T001	21.55	21.55	coding	431	75	1367

PB.1646.4	10	1796	2	intergenic	novelGene_12	novel	29.87	108.48	coding	422	203	1468
PB.7269.1	6	2219	6	full-splice_match	gene: Zm00001d035256	Zm00001d035256_T002	37.02	37.02	coding	594	101	1882
PB.1261.1	1	1788	3	full-splice_match	gene: Zm00001d033457	Zm00001d033457_T001	455.11	455.11	coding	445	125	1459
PB.7334.1	6	1747	1	full-splice_match	gene: Zm00001d035658	Zm00001d035658_T001	7.14	7.14	coding	424	235	1506
PB.6428.1	5	868	2	full-splice_match	gene: Zm00001d014920	Zm00001d014920_T001	99.99	156.72	coding	163	96	584
PB.700.2	1	2626	5	full-splice_match	gene: Zm00001d030652	Zm00001d030652_T001	4.75	23.57	coding	714	175	2316
PB.7950.1	6	619	3	full-splice_match	gene: Zm00001d038523	Zm00001d038523_T001	130.41	130.41	coding	99	100	396
PB.4351.2	3	562	4	incomplete-splice_match	gene: Zm00001d042923	Zm00001d042923_T001	27.67	27.67	coding	46	195	332
PB.4370.2	3	2079	2	genic	gene: Zm00001d043026	novel	4.40	4.40	coding	540	421	2040
PB.7209.4	6	777	1	full-splice_match	gene: Zm00001d034977	Zm00001d034977_T001	0.00	79.60	coding	214	20	661
PB.8347.1	7	1010	2	full-splice_match	gene: Zm00001d019597	Zm00001d019597_T001	367.50	371.96	coding	182	175	720
PB.2323.4	10	1425	7	full-splice_match	gene: Zm00001d026542	Zm00001d026542_T001	14.84	25.35	coding	365	160	1254
PB.7209.1	6	1324	1	full-splice_match	gene: Zm00001d034977	Zm00001d034977_T001	6.77	79.60	coding	214	184	825
PB.5252.2	4	1687	6	novel_not_in_catalog	gene: Zm00001d051247	novel	0.72	16.18	coding	118	103	456
PB.7209.3	6	1081	1	full-splice_match	gene: Zm00001d034977	Zm00001d034977_T001	48.52	79.60	coding	214	181	822
PB.7209.2	6	1241	1	full-splice_match	gene: Zm00001d034977	Zm00001d034977_T001	24.32	79.60	coding	214	234	875
PB.12.1	1	912	2	full-splice_match	gene: Zm00001d027288	Zm00001d027288_T001	268.32	278.91	coding	211	38	670

PB.12.2	1	1778	1	novel_in_catalog	gene: Zm00001d027288	novel	10.60	278.91	coding	125	40	414
PB.8708.3	7	1944	16	full-splice_match	gene: Zm00001d021473	Zm00001d021473_T006	0.00	13.89	coding	505	101	1615
PB.7074.3	5	1517	1	genic_intron	novelGene_335	novel	3.78	3.78	coding	327	536	1516
PB.7074.2	5	1477	4	full-splice_match	gene: Zm00001d018178	Zm00001d018178_T001	0.00	18.78	coding	351	252	1304
PB.7074.1	5	1701	4	full-splice_match	gene: Zm00001d018178	Zm00001d018178_T001	18.78	18.78	coding	351	250	1302
PB.1251.1	1	1945	4	novel_in_catalog	gene: Zm00001d033393	novel	4.23	11.69	coding	128	118	501
PB.6293.3	5	1863	9	full-splice_match	gene: Zm00001d014385	Zm00001d014385_T001	0.00	27.02	coding	482	30	1475
PB.6293.2	5	1572	8	incomplete-splice_match	gene: Zm00001d014385	Zm00001d014385_T002	0.67	27.02	coding	162	459	944
PB.6293.1	5	2026	9	full-splice_match	gene: Zm00001d014385	Zm00001d014385_T001	24.86	27.02	coding	482	77	1522
PB.3762.1	3	1356	4	full-splice_match	gene: Zm00001d039747	Zm00001d039747_T001	69.09	69.09	coding	187	595	1155
PB.9155.2	8	597	3	full-splice_match	gene: Zm00001d008620	Zm00001d008620_T001	105.19	369.22	non_coding	NA	NA	NA
PB.9155.1	8	713	3	full-splice_match	gene: Zm00001d008620	Zm00001d008620_T001	264.03	369.22	non_coding	NA	NA	NA
PB.6293.4	5	1950	8	novel_in_catalog	gene: Zm00001d014385	novel	1.50	27.02	coding	431	120	1412
PB.7404.2	6	667	2	full-splice_match	gene: Zm00001d036036	Zm00001d036036_T001	2166.96	2369.03	coding	136	97	504
PB.7404.3	6	707	1	novel_in_catalog	gene: Zm00001d036036	novel	147.06	2369.03	non_coding	NA	NA	NA
PB.7404.1	6	906	2	full-splice_match	gene: Zm00001d036036	Zm00001d036036_T001	55.01	2369.03	coding	136	95	502
PB.1444.1	1	1036	2	full-splice_match	gene: Zm00001d034279	Zm00001d034279_T001	8.55	168.32	coding	180	60	599

PB.1595.2	1	1246	5	full-splice_match	gene: Zm00001d034795	Zm00001d034795_T001	125.56	151.38	coding	289	103	969
PB.10150.2	9	1421	8	full-splice_match	gene: Zm00001d045327	Zm00001d045327_T001	28.22	28.42	coding	346	234	1271
PB.10150.1	9	2282	7	novel_in_catalog	gene: Zm00001d045327	novel	0.20	28.42	coding	346	1095	2132
PB.7723.1	6	1631	3	novel_in_catalog	gene: Zm00001d037696	novel	0.84	0.84	coding	394	120	1301
PB.10762.2	9	2118	11	incomplete-splice_match	gene: Zm00001d048338	Zm00001d048338_T039	15.40	33.66	coding	330	890	1879
PB.583.1	1	1944	7	novel_not_in_catalog	gene: Zm00001d029847	novel	0.52	6.14	coding	252	839	1594
PB.10291.1	9	1688	3	full-splice_match	gene: Zm00001d046155	Zm00001d046155_T001	6.84	6.84	coding	156	53	520
PB.2478.1	2	3216	5	full-splice_match	gene: Zm00001d002125	Zm00001d002125_T025	24.71	28.30	coding	902	310	3015
PB.2478.2	2	3860	5	novel_in_catalog	gene: Zm00001d002125	novel	3.59	28.30	coding	829	25	2511
PB.2478.3	2	1394	3	incomplete-splice_match	gene: Zm00001d002125	Zm00001d002125_T017	0.00	28.30	coding	425	118	1392
PB.274.1	1	1583	2	full-splice_match	gene: Zm00001d028419	Zm00001d028419_T001	9.87	9.87	coding	433	141	1439
PB.7825.3	6	1650	1	full-splice_match	gene: Zm00001d038072	Zm00001d038072_T001	0.00	12.46	coding	414	409	1650
PB.7825.2	6	2066	1	full-splice_match	gene: Zm00001d038072	Zm00001d038072_T001	3.93	12.46	coding	472	244	1659
PB.8745.1	7	1908	4	full-splice_match	gene: Zm00001d021596	Zm00001d021596_T001	44.62	44.62	coding	461	97	1479
PB.3546.1	2	1194	2	incomplete-splice_match	gene: Zm00001d007571	Zm00001d007571_T001	5.44	8.51	coding	161	192	674
PB.7476.1	6	3087	11	full-splice_match	gene: Zm00001d036483	Zm00001d036483_T006	16.02	16.02	coding	792	444	2819
PB.2116.3	10	2223	5	full-splice_match	gene: Zm00001d025712	Zm00001d025712_T021	3.13	40.51	coding	712	86	2221

PB.2116.2	10	2361	2	incomplete-splice_match	gene: Zm00001d025712	Zm00001d025712_T019	8.19	40.51	coding	736	154	2361
PB.2186.1	10	556	1	full-splice_match	gene: Zm00001d026015	Zm00001d026015_T001	1108.61	1108.61	coding	103	63	371
PB.583.2	1	1852	8	novel_not_in_catalog	gene: Zm00001d029847	novel	5.61	6.14	coding	479	103	1539
PB.1639.3	1	919	7	full-splice_match	gene: Zm00001d034949	Zm00001d034949_T001	0.00	90.81	coding	203	96	704
PB.8335.4	7	1253	2	full-splice_match	gene: Zm00001d019560	Zm00001d019560_T001	48.63	68.37	coding	308	96	1019
PB.8335.1	7	1579	2	full-splice_match	gene: Zm00001d019560	Zm00001d019560_T001	16.39	68.37	coding	308	104	1027
PB.8335.2	7	1563	1	novel_in_catalog	gene: Zm00001d019560	novel	3.35	68.37	coding	193	570	1148
PB.8335.3	7	1318	2	full-splice_match	gene: Zm00001d019560	Zm00001d019560_T001	0.00	68.37	coding	308	48	971
PB.7115.1	5	2010	7	full-splice_match	gene: Zm00001d018330	Zm00001d018330_T003	12.67	12.67	coding	467	183	1583
PB.7115.2	5	1861	7	full-splice_match	gene: Zm00001d018330	Zm00001d018330_T003	0.00	12.67	coding	467	173	1573
PB.8273.3	7	1238	9	incomplete-splice_match	gene: Zm00001d019172	Zm00001d019172_T001	6.30	47.72	coding	343	73	1101
PB.2565.2	2	2387	2	incomplete-splice_match	gene: Zm00001d002485	Zm00001d002485_T001	6.80	20.65	coding	471	830	2242
PB.2565.1	2	2801	3	full-splice_match	gene: Zm00001d002485	Zm00001d002485_T001	13.85	20.65	coding	754	267	2528
PB.903.4	1	1026	2	incomplete-splice_match	gene: Zm00001d031813	Zm00001d031813_T001	16.61	37.38	coding	178	175	708
PB.3474.2	2	2055	21	full-splice_match	gene: Zm00001d007113	Zm00001d007113_T002	129.75	129.75	coding	482	371	1816
PB.9294.1	8	1382	8	fusion	gene: Zm00001d009396_g	novel	8.42	8.42	coding	397	25	1215
PB.5056.4	4	1528	4	full-splice_match	gene: Zm00001d049916	Zm00001d049916_T001	0.00	62.72	coding	392	166	1341

PB.7504.1	6	2799	8	full-splice_match	gene: Zm00001d036598	Zm00001d036598_T001	9.11	10.39	coding	694	509	2590
PB.9566.2	8	1364	1	full-splice_match	gene: Zm00001d010751	Zm00001d010751_T001	22.18	158.47	coding	313	78	1016
PB.5056.1	4	1828	4	full-splice_match	gene: Zm00001d049916	Zm00001d049916_T001	30.59	62.72	coding	392	166	1341
PB.7342.1	6	1509	1	full-splice_match	gene: Zm00001d035677	Zm00001d035677_T001	14.30	14.30	coding	346	160	1197
PB.5504.3	4	812	2	intergenic	novelGene_307	novel	99.41	113.87	coding	221	27	689
PB.5056.2	4	1656	4	full-splice_match	gene: Zm00001d049916	Zm00001d049916_T001	32.12	62.72	coding	392	158	1333
PB.8985.2	7	2336	8	novel_in_catalog	gene: Zm00001d022425	novel	2.71	67.84	non_coding	NA	NA	NA
PB.8849.2	7	1999	1	novel_in_catalog	gene: Zm00001d021938	novel	2.73	72.90	coding	467	306	1706
PB.10575.1	9	509	5	incomplete-splice_match	gene: Zm00001d047677	Zm00001d047677_T001	9.97	56.67	coding	89	241	507
PB.9943.1	8	551	3	novel_not_in_catalog	gene: Zm00001d012448	novel	66.60	66.60	coding	78	100	333
PB.369.1	1	577	3	incomplete-splice_match	gene: Zm00001d028862	Zm00001d028862_T001	40.50	40.50	non_coding	NA	NA	NA
PB.5687.1	4	1189	2	full-splice_match	gene: Zm00001d053220	Zm00001d053220_T001	101.68	101.68	coding	260	189	968
PB.4051.9	3	2143	2	full-splice_match	gene: Zm00001d041550	Zm00001d041550_T005	0.00	2134.62	coding	649	75	2021
PB.10546.1	9	1483	11	full-splice_match	gene: Zm00001d047566	Zm00001d047566_T014	41.30	41.30	coding	333	113	1111
PB.6911.4	5	1959	11	novel_not_in_catalog	gene: Zm00001d017502	novel	5.12	28.72	coding	369	578	1684
PB.6911.2	5	1639	11	novel_not_in_catalog	gene: Zm00001d017502	novel	23.59	28.72	coding	325	390	1364
PB.1231.1	1	544	2	novel_not_in_catalog	gene: Zm00001d033301	novel	984.82	984.82	non_coding	NA	NA	NA

PB.7674.5	6	2851	6	novel_in_catalog	gene: Zm00001d037480	novel	0.00	36.20	coding	873	66	2684
PB.4051.3	3	2334	2	full-splice_match	gene: Zm00001d041550	Zm00001d041550_T005	215.08	2134.62	coding	649	77	2023
PB.7674.6	6	2614	7	full-splice_match	gene: Zm00001d037480	Zm00001d037480_T001	4.64	36.20	coding	747	260	2500
PB.7674.1	6	2790	6	novel_in_catalog	gene: Zm00001d037480	novel	22.19	36.20	coding	747	314	2554
PB.9475.1	8	3096	16	incomplete-splice_match	gene: Zm00001d010324	Zm00001d010324_T001	25.11	25.11	coding	876	93	2720
PB.7674.3	6	2893	6	novel_in_catalog	gene: Zm00001d037480	novel	9.37	36.20	coding	746	113	2350
PB.2977.1	2	1104	2	novel_not_in_catalog	gene: Zm00001d004573	novel	136.92	136.92	coding	290	63	932
PB.8428.1	7	2164	3	incomplete-splice_match	gene: Zm00001d020204	Zm00001d020204_T011	36.44	42.44	coding	549	8	1654
PB.9924.1	8	2148	1	antisense	novelGene_gene: Zm00001d012337_A	novel	26.35	26.35	coding	116	805	1152
PB.8428.2	7	3336	8	incomplete-splice_match	gene: Zm00001d020204	Zm00001d020204_T007	6.00	42.44	coding	971	141	3053
PB.6667.7	5	2057	17	novel_in_catalog	gene: Zm00001d016365	novel	1.68	79.99	coding	284	140	991
PB.4691.1	3	2590	4	full-splice_match	gene: Zm00001d044291	Zm00001d044291_T001	52.55	52.55	coding	701	91	2193
PB.1387.1	1	2617	8	full-splice_match	gene: Zm00001d034000	Zm00001d034000_T002	18.02	18.02	coding	692	264	2339
PB.8476.1	7	2032	9	novel_not_in_catalog	gene: Zm00001d020432	novel	47.93	47.93	coding	587	69	1829
PB.6756.4	5	2406	9	novel_in_catalog	gene: Zm00001d016838	novel	3.96	64.46	coding	392	793	1968
PB.838.4	1	1193	3	full-splice_match	gene: Zm00001d031453	Zm00001d031453_T001	21.30	21.30	coding	280	78	917
PB.8656.1	7	1770	4	novel_not_in_catalog	gene: Zm00001d021286	novel	11.94	16.76	coding	383	310	1458

PB.3591.1	2	2381	19	full-splice_match	gene: Zm00001d007807	Zm00001d007807_T010	13.52	13.52	coding	628	228	2111
PB.945.5	1	2183	7	full-splice_match	gene: Zm00001d031988	Zm00001d031988_T001	236.60	236.60	coding	639	88	2004
PB.858.1	1	3972	4	novel_in_catalog	gene: Zm00001d031570	novel	1.11	38.14	coding	574	2069	3790
PB.858.3	1	1111	1	incomplete-splice_match	gene: Zm00001d031570	Zm00001d031570_T002	12.42	38.14	coding	345	77	1111
PB.858.2	1	3298	5	full-splice_match	gene: Zm00001d031570	Zm00001d031570_T001	24.61	38.14	coding	1005	102	3116
PB.361.1	1	1154	5	full-splice_match	gene: Zm00001d028804	Zm00001d028804_T001	129.08	129.08	coding	233	90	788
PB.10554.1	9	793	3	full-splice_match	gene: Zm00001d027187	Zm00001d027187_T001	11.68	11.68	non_coding	NA	NA	NA
PB.2571.8	2	2177	10	incomplete-splice_match	gene: Zm00001d002503	Zm00001d002503_T003	0.00	157.95	coding	696	90	2177
PB.2571.9	2	3801	7	incomplete-splice_match	gene: Zm00001d002503	Zm00001d002503_T001	0.00	157.95	coding	1251	47	3799
PB.2571.4	2	4909	11	full-splice_match	gene: Zm00001d002503	Zm00001d002503_T001	19.82	157.95	coding	1540	47	4666
PB.2571.7	2	2207	8	incomplete-splice_match	gene: Zm00001d002503	Zm00001d002503_T003	0.00	157.95	coding	548	400	2043
PB.2571.1	2	2637	9	incomplete-splice_match	gene: Zm00001d002503	Zm00001d002503_T003	24.78	157.95	coding	688	24	2087
PB.2571.2	2	1956	8	incomplete-splice_match	gene: Zm00001d002503	Zm00001d002503_T003	113.36	157.95	coding	548	14	1657
PB.4982.1	4	682	4	full-splice_match	gene: Zm00001d049552	Zm00001d049552_T003	496.38	496.38	coding	119	87	443
PB.523.5	1	1213	4	full-splice_match	gene: Zm00001d029539	Zm00001d029539_T001	7.44	189.73	coding	200	149	748
PB.523.2	1	1736	3	full-splice_match	gene: Zm00001d029539	Zm00001d029539_T003	9.08	189.73	coding	118	146	499
PB.4601.3	3	799	1	incomplete-splice_match	gene: Zm00001d043927	Zm00001d043927_T009	38.72	70.75	coding	122	28	393

PB.446.1	1	899	2	incomplete-splice_match	gene: Zm00001d029180	Zm00001d029180_T021	54.13	54.13	coding	39	180	296
PB.7215.1	6	902	4	novel_not_in_catalog	gene: Zm00001d035015	novel	38.32	38.32	coding	136	7	414
PB.10821.1	9	2264	8	full-splice_match	gene: Zm00001d048516	Zm00001d048516_T001	8.17	8.17	coding	603	259	2067
PB.4176.1	3	1089	1	full-splice_match	gene: Zm00001d042108	Zm00001d042108_T001	71.62	71.62	coding	192	109	684
PB.10537.6	9	2549	12	full-splice_match	gene: Zm00001d047522	Zm00001d047522_T003	47.91	134.86	coding	629	415	2301
PB.676.2	1	2767	10	novel_not_in_catalog	gene: Zm00001d030471	novel	4.11	37.88	coding	858	50	2623
PB.676.1	1	2828	10	novel_not_in_catalog	gene: Zm00001d030471	novel	33.77	37.88	coding	858	46	2619
PB.3718.1	3	2176	5	novel_in_catalog	gene: Zm00001d039518	novel	179.41	179.41	coding	222	116	781
PB.4685.1	3	4002	31	fusion	gene: Zm00001d044273_g	novel	13.29	13.29	coding	1201	108	3710
PB.1978.17	10	2967	17	novel_not_in_catalog	gene: Zm00001d024952	novel	0.00	124.67	coding	882	91	2736
PB.3778.1	3	1483	9	full-splice_match	gene: Zm00001d039856	Zm00001d039856_T001	83.26	256.04	coding	221	484	1146
PB.3778.2	3	1072	9	full-splice_match	gene: Zm00001d039856	Zm00001d039856_T001	140.54	256.04	coding	221	123	785
PB.9480.2	8	1910	1	full-splice_match	gene: Zm00001d010380	Zm00001d010380_T001	0.00	49.89	coding	397	59	1249
PB.10542.1	9	2679	8	full-splice_match	gene: Zm00001d047539	Zm00001d047539_T009	5.95	24.22	coding	416	97	1344
PB.6426.1	5	1734	16	full-splice_match	gene: Zm00001d014912	Zm00001d014912_T008	23.23	25.72	coding	488	107	1570
PB.9169.6	8	1469	8	full-splice_match	gene: Zm00001d008679	Zm00001d008679_T005	0.00	129.95	coding	357	291	1361
PB.6426.2	5	2151	15	full-splice_match	gene: Zm00001d014912	Zm00001d014912_T002	2.49	25.72	coding	319	1032	1988

PB.10338.1	9	2020	6	full-splice_match	gene: Zm00001d046454	Zm00001d046454_T001	53.48	53.48	coding	418	301	1554
PB.2582.1	2	558	1	intergenic	novelGene_50	novel	281.30	281.30	coding	9	339	365
PB.951.1	1	1492	3	full-splice_match	gene: Zm00001d032027	Zm00001d032027_T002	60.28	60.28	coding	319	285	1241
PB.3320.1	2	2426	15	novel_in_catalog	gene: Zm00001d006415	novel	38.87	38.87	coding	291	78	950
PB.6549.1	5	2723	10	full-splice_match	gene: Zm00001d015497	Zm00001d015497_T006	12.63	12.63	coding	129	1306	1692
PB.7853.1	6	1422	9	full-splice_match	gene: Zm00001d038155	Zm00001d038155_T001	17.33	17.33	coding	341	179	1201
PB.10542.4	9	2727	9	full-splice_match	gene: Zm00001d047539	Zm00001d047539_T005	9.72	24.22	coding	488	385	1848
PB.8595.1	7	2260	1	antisense	novelGene_gene: Zm00001d020967_A	novel	5.03	5.03	non_coding	NA	NA	NA
PB.10542.5	9	3136	8	full-splice_match	gene: Zm00001d047539	Zm00001d047539_T009	0.16	24.22	coding	684	114	2165
PB.994.2	1	900	5	full-splice_match	gene: Zm00001d032232	Zm00001d032232_T004	25.92	87.14	coding	157	247	717
PB.1630.2	1	3409	14	incomplete-splice_match	gene: Zm00001d034940	Zm00001d034940_T053	13.41	13.41	coding	1080	34	3273
PB.2426.1	2	2054	13	full-splice_match	gene: Zm00001d001898	Zm00001d001898_T011	6.65	33.36	coding	552	197	1852
PB.1631.1	1	2253	16	incomplete-splice_match	gene: Zm00001d034914	Zm00001d034914_T034	30.95	63.90	coding	526	73	1650
PB.8166.1	7	2061	5	novel_not_in_catalog	gene: Zm00001d018660	novel	4.91	4.91	coding	483	92	1540
PB.6394.1	5	1713	9	full-splice_match	gene: Zm00001d014788	Zm00001d014788_T002	11.98	11.98	coding	346	408	1445
PB.7430.3	6	890	5	incomplete-splice_match	gene: Zm00001d036222	Zm00001d036222_T001	9.45	16.67	coding	148	19	462
PB.7640.2	6	2489	9	full-splice_match	gene: Zm00001d037297	Zm00001d037297_T004	27.78	39.59	coding	589	502	2268

PB.343.2	1	1539	7	full-splice_match	gene: Zm00001d028698	Zm00001d028698_T002	20.99	36.62	coding	318	173	1126
PB.1909.2	10	1043	3	full-splice_match	gene: Zm00001d024700	Zm00001d024700_T001	12.23	12.23	coding	231	48	740
PB.343.1	1	1481	7	full-splice_match	gene: Zm00001d028698	Zm00001d028698_T002	15.63	36.62	coding	318	1	954
PB.4457.3	3	2762	3	full-splice_match	gene: Zm00001d043395	Zm00001d043395_T002	0.00	22.81	coding	674	164	2185
PB.4457.2	3	3039	3	full-splice_match	gene: Zm00001d043395	Zm00001d043395_T002	5.89	22.81	coding	674	178	2199
PB.4457.1	3	2585	3	full-splice_match	gene: Zm00001d043395	Zm00001d043395_T002	12.76	22.81	coding	504	46	1557
PB.2256.10	10	3450	23	novel_in_catalog	gene: Zm00001d026306	novel	0.09	152.36	coding	863	43	2631
PB.7430.1	6	2139	12	incomplete-splice_match	gene: Zm00001d036222	Zm00001d036222_T003	7.22	16.67	coding	426	271	1548
PB.2256.12	10	3306	24	full-splice_match	gene: Zm00001d026306	Zm00001d026306_T009	0.00	152.36	coding	1028	41	3124
PB.2337.1	10	1284	4	full-splice_match	gene: Zm00001d026584	Zm00001d026584_T004	61.94	65.39	coding	288	109	972
PB.2557.1	2	1842	12	full-splice_match	gene: Zm00001d002422	Zm00001d002422_T004	6.78	8.82	coding	397	256	1446
PB.2557.2	2	2039	12	full-splice_match	gene: Zm00001d002422	Zm00001d002422_T004	2.04	8.82	coding	397	567	1757
PB.3631.4	2	1907	9	full-splice_match	gene: Zm00001d007960	Zm00001d007960_T002	0.00	155.16	coding	535	130	1734
PB.1146.1	1	2114	14	novel_in_catalog	gene: Zm00001d032852	novel	9.36	9.36	coding	583	88	1836
PB.4860.1	4	1417	8	full-splice_match	gene: Zm00001d048908	Zm00001d048908_T002	72.24	87.39	coding	286	184	1041
PB.3631.1	2	2134	9	full-splice_match	gene: Zm00001d007960	Zm00001d007960_T002	73.51	155.16	coding	535	130	1734
PB.3631.2	2	2011	9	full-splice_match	gene: Zm00001d007960	Zm00001d007960_T002	81.65	155.16	coding	535	129	1733

PB.4860.2	4	1060	7	incomplete-splice_match	gene: Zm00001d048908	Zm00001d048908_T002	15.15	87.39	coding	145	392	826
PB.5173.5	4	845	1	incomplete-splice_match	gene: Zm00001d050716	Zm00001d050716_T003	1.37	29.88	coding	137	299	709
PB.5173.4	4	2565	3	full-splice_match	gene: Zm00001d050716	Zm00001d050716_T003	1.22	29.88	coding	415	1118	2362
PB.5173.3	4	2372	4	full-splice_match	gene: Zm00001d050716	Zm00001d050716_T008	3.52	29.88	coding	559	410	2086
PB.5173.2	4	2326	2	incomplete-splice_match	gene: Zm00001d050716	Zm00001d050716_T004	6.54	29.88	coding	271	1145	1957
PB.5173.1	4	2484	4	full-splice_match	gene: Zm00001d050716	Zm00001d050716_T008	17.23	29.88	coding	559	456	2132
PB.6213.3	5	2678	3	novel_in_catalog	gene: Zm00001d014013	novel	2.02	24.63	coding	613	392	2230
PB.6213.2	5	2653	4	full-splice_match	gene: Zm00001d014013	Zm00001d014013_T002	22.61	24.63	coding	644	382	2313
PB.1245.1	1	1982	1	full-splice_match	gene: Zm00001d033377	Zm00001d033377_T001	7.93	7.93	coding	282	316	1161
PB.9635.1	8	2107	6	full-splice_match	gene: Zm00001d011051	Zm00001d011051_T003	8.72	27.71	coding	516	68	1615
PB.3792.1	3	2346	2	full-splice_match	gene: Zm00001d039916	Zm00001d039916_T010	129.00	129.00	coding	601	31	1833
PB.5614.1	4	1104	2	genic	gene: Zm00001d052841	novel	512.20	630.36	coding	248	83	826
PB.8207.2	7	590	1	full-splice_match	gene: Zm00001d018854	Zm00001d018854_T001	46.25	72.64	coding	134	4	405
PB.8521.4	7	2774	5	full-splice_match	gene: Zm00001d020639	Zm00001d020639_T004	1.88	38.52	coding	637	318	2228
PB.8207.1	7	1754	1	full-splice_match	gene: Zm00001d018854	Zm00001d018854_T001	26.39	72.64	coding	351	380	1432
PB.8521.1	7	2730	6	full-splice_match	gene: Zm00001d020639	Zm00001d020639_T002	33.34	38.52	coding	722	318	2483
PB.1901.1	10	2364	20	full-splice_match	gene: Zm00001d024625	Zm00001d024625_T002	18.99	18.99	coding	590	134	1903

PB.8521.3	7	2859	4	novel_in_catalog	gene: Zm00001d020639	novel	1.41	38.52	coding	217	1999	2649
PB.8521.2	7	2722	5	full-splice_match	gene: Zm00001d020639	Zm00001d020639_T005	1.88	38.52	coding	217	1918	2568
PB.4501.2	3	2520	4	full-splice_match	gene: Zm00001d043527	Zm00001d043527_T001	3.31	30.27	coding	156	252	719
PB.2377.1	10	1108	9	full-splice_match	gene: Zm00001d026680	Zm00001d026680_T004	152.75	251.56	coding	214	70	711
PB.2377.2	10	1119	9	full-splice_match	gene: Zm00001d026680	Zm00001d026680_T003	98.81	251.56	coding	214	121	762
PB.4677.2	3	1239	10	full-splice_match	gene: Zm00001d044243	Zm00001d044243_T002	6.18	29.32	coding	344	94	1125
PB.4677.1	3	1365	10	full-splice_match	gene: Zm00001d044243	Zm00001d044243_T002	23.13	29.32	coding	344	91	1122
PB.353.1	1	937	5	fusion	gene: Zm00001d028775_g	novel	70.19	70.19	coding	252	52	807
PB.8501.5	7	2248	6	novel_not_in_catalog	gene: Zm00001d020532	novel	1.71	12.22	coding	473	231	1649
PB.977.1	1	1598	1	genic	gene: Zm00001d032158	novel	0.58	56.45	coding	286	239	1096
PB.8501.2	7	2561	7	full-splice_match	gene: Zm00001d020532	Zm00001d020532_T001	10.51	12.22	coding	605	370	2184
PB.8501.1	7	2717	7	full-splice_match	gene: Zm00001d020532	Zm00001d020532_T001	0.00	12.22	coding	605	370	2184
PB.977.2	1	1171	1	genic	gene: Zm00001d032158	novel	5.42	56.45	coding	286	97	954
PB.1190.2	1	1912	6	novel_in_catalog	gene: Zm00001d033063	novel	0.00	111.83	coding	319	125	1081
PB.1190.1	1	1867	7	full-splice_match	gene: Zm00001d033063	Zm00001d033063_T001	111.83	111.83	coding	500	124	1623
PB.8217.1	7	784	4	incomplete-splice_match	gene: Zm00001d018873	Zm00001d018873_T001	18.60	143.91	non_coding	NA	NA	NA
PB.4484.6	3	1721	7	novel_not_in_catalog	gene: Zm00001d043480	novel	68.89	133.65	coding	453	261	1619

PB.4484.5	3	2999	7	novel_not_in_catalog	gene: Zm00001d043480	novel	0.00	133.65	coding	453	1395	2753
PB.8217.2	7	1361	8	full-splice_match	gene: Zm00001d018873	Zm00001d018873_T001	125.32	143.91	coding	326	88	1065
PB.4484.3	3	3105	7	novel_not_in_catalog	gene: Zm00001d043480	novel	20.48	133.65	coding	453	1399	2757
PB.4484.2	3	3191	6	novel_not_in_catalog	gene: Zm00001d043480	novel	1.11	133.65	coding	232	2147	2842
PB.4484.1	3	2697	8	novel_not_in_catalog	gene: Zm00001d043480	novel	43.17	133.65	coding	705	235	2349
PB.9789.1	8	1631	3	novel_not_in_catalog	gene: Zm00001d011769	novel	6.98	6.98	coding	280	108	947
PB.1451.1	1	1158	7	full-splice_match	gene: Zm00001d034319	Zm00001d034319_T001	63.83	63.83	coding	237	259	969
PB.4808.1	4	890	1	genic	gene: Zm00001d048632	novel	44.94	44.94	coding	63	492	680
PB.918.1	1	3233	3	incomplete-splice_match	gene: Zm00001d031871	Zm00001d031871_T002	30.41	59.27	coding	957	9	2879
PB.8872.1	7	628	4	incomplete-splice_match	gene: Zm00001d022021	Zm00001d022021_T001	20.83	20.83	coding	96	117	404
PB.6890.1	5	2167	10	incomplete-splice_match	gene: Zm00001d017424	Zm00001d017424_T008	35.44	35.44	coding	346	636	1673
PB.1370.4	1	2832	3	novel_not_in_catalog	gene: Zm00001d033925	novel	11.00	1241.38	coding	150	792	1241
PB.1370.5	1	2372	2	genic	gene: Zm00001d033925	novel	0.00	1241.38	coding	150	231	680
PB.1370.8	1	1076	5	novel_not_in_catalog	gene: Zm00001d033925	novel	1.60	1241.38	coding	175	41	565
PB.10119.1	9	1923	1	antisense	novelGene_gene: Zm00001d045175_A	novel	5.65	5.65	coding	190	299	868
PB.7697.2	6	1769	6	full-splice_match	gene: Zm00001d037596	Zm00001d037596_T002	53.87	59.20	non_coding	NA	NA	NA
PB.6805.1	5	2265	1	antisense	novelGene_gene: Zm00001d017079_A	novel	45.45	45.45	coding	332	955	1950

PB.3627.1	2	2468	13	full-splice_match	gene: Zm00001d007942	Zm00001d007942_T001	50.88	50.88	coding	652	249	2204
PB.6499.4	5	2694	4	novel_in_catalog	gene: Zm00001d015212	novel	0.00	67.45	coding	205	1881	2495
PB.7920.7	6	1707	3	novel_not_in_catalog	gene: Zm00001d038384	novel	1.18	1.18	coding	254	125	886
PB.1423.1	1	2853	11	full-splice_match	gene: Zm00001d034152	Zm00001d034152_T002	27.33	35.02	coding	755	151	2415
PB.8509.1	7	1376	6	full-splice_match	gene: Zm00001d020556	Zm00001d020556_T001	24.47	24.47	coding	373	70	1188
PB.9842.2	8	392	1	novel_in_catalog	gene: Zm00001d011979	novel	120.85	279.10	non_coding	NA	NA	NA
PB.9842.1	8	1977	11	novel_in_catalog	gene: Zm00001d011979	novel	71.80	279.10	coding	507	58	1578
PB.731.1	1	430	3	novel_not_in_catalog	gene: Zm00001d030910	novel	55.05	55.05	non_coding	NA	NA	NA
PB.10629.2	9	2286	2	novel_in_catalog	gene: Zm00001d047848	novel	1.59	46.93	coding	345	109	1143
PB.3617.2	2	768	1	novel_in_catalog	gene: Zm00001d007901	novel	0.00	63.26	coding	141	345	767
PB.56.1	1	2900	3	novel_in_catalog	gene: Zm00001d027447	novel	3.48	101.03	coding	70	86	295
PB.10848.3	B73V4 ctg107	1402	4	full-splice_match	gene: Zm00001d000305	Zm00001d000305_T002	0.00	68.82	coding	358	99	1172
PB.10848.2	B73V4 ctg107	1521	4	full-splice_match	gene: Zm00001d000305	Zm00001d000305_T002	68.82	68.82	coding	358	112	1185
PB.10848.4	B73V4 ctg107	1245	4	full-splice_match	gene: Zm00001d000305	Zm00001d000305_T002	0.00	68.82	coding	358	56	1129
PB.7789.5	6	1309	1	full-splice_match	gene: Zm00001d037945	Zm00001d037945_T001	191.03	197.91	coding	198	304	897
PB.6561.1	5	1437	5	full-splice_match	gene: Zm00001d015571	Zm00001d015571_T001	78.12	78.12	coding	277	271	1101
PB.2982.9	2	1321	12	full-splice_match	gene: Zm00001d004616	Zm00001d004616_T002	0.43	129.74	coding	331	152	1144

PB.10491.1	9	1433	7	full-splice_match	gene: Zm00001d047345	Zm00001d047345_T001	4.61	4.61	coding	328	105	1088
PB.7789.2	6	2178	1	intergenic	novelGene_229	novel	0.00	0.00	non_coding	NA	NA	NA
PB.7789.3	6	2417	1	full-splice_match	gene: Zm00001d037945	Zm00001d037945_T001	0.00	197.91	coding	198	296	889
PB.8386.2	7	1791	2	full-splice_match	gene: Zm00001d019934	Zm00001d019934_T001	7.94	13.82	coding	492	155	1630
PB.4298.2	3	3977	8	novel_in_catalog	gene: Zm00001d042695	novel	1.92	57.58	coding	212	218	853
PB.8386.1	7	812	1	genic	gene: Zm00001d019934	novel	5.88	13.82	coding	109	195	521
PB.2982.4	2	1435	12	full-splice_match	gene: Zm00001d004616	Zm00001d004616_T002	107.04	129.74	coding	331	148	1140
PB.2982.5	2	2546	10	novel_in_catalog	gene: Zm00001d004616	novel	0.11	129.74	coding	201	1648	2250
PB.1927.4	10	1383	1	full-splice_match	gene: Zm00001d024754	Zm00001d024754_T001	3356.29	3356.29	coding	345	69	1103
PB.1996.5	10	1257	3	full-splice_match	gene: Zm00001d025023	Zm00001d025023_T001	0.00	123.94	coding	361	40	1122
PB.9865.1	8	2552	19	full-splice_match	gene: Zm00001d012086	Zm00001d012086_T004	37.42	41.97	coding	691	144	2216
PB.9865.4	8	2432	18	novel_in_catalog	gene: Zm00001d012086	novel	4.55	41.97	coding	489	49	1515
PB.3691.2	3	1587	6	full-splice_match	gene: Zm00001d039416	Zm00001d039416_T001	37.92	53.81	coding	400	209	1408
PB.2791.1	2	1460	6	full-splice_match	gene: Zm00001d003401	Zm00001d003401_T004	89.41	1842.56	coding	261	91	873
PB.2791.2	2	1371	6	full-splice_match	gene: Zm00001d003401	Zm00001d003401_T004	46.14	1842.56	coding	261	122	904
PB.3691.1	3	1346	6	full-splice_match	gene: Zm00001d039416	Zm00001d039416_T001	15.89	53.81	coding	294	121	1002
PB.9022.1	7	1091	1	full-splice_match	gene: Zm00001d022531	Zm00001d022531_T001	63.30	63.30	coding	214	322	963

PB.1495.4	1	434	1	incomplete-splice_match	gene: Zm00001d034446	Zm00001d034446_T010	79.78	79.78	non_coding	NA	NA	NA
PB.10358.4	9	2743	11	full-splice_match	gene: Zm00001d046554	Zm00001d046554_T011	0.44	49.07	coding	445	1172	2506
PB.10796.1	9	308	1	full-splice_match	gene: Zm00001d048441	Zm00001d048441_T001	29.59	29.59	non_coding	NA	NA	NA
PB.739.1	1	2585	12	novel_in_catalog	gene: Zm00001d030953	novel	31.71	31.71	coding	504	763	2274
PB.1091.3	1	2455	4	full-splice_match	gene: Zm00001d032597	Zm00001d032597_T009	11.69	62.22	coding	171	126	638
PB.3278.1	2	2252	7	full-splice_match	gene: Zm00001d006246	Zm00001d006246_T001	19.04	19.04	coding	500	183	1682
PB.750.1	1	1531	6	full-splice_match	gene: Zm00001d031008	Zm00001d031008_T001	48.50	48.50	coding	394	126	1307
PB.3226.1	2	2289	11	full-splice_match	gene: Zm00001d006027	Zm00001d006027_T002	14.86	14.86	coding	489	127	1593
PB.3685.1	3	1090	2	full-splice_match	gene: Zm00001d039395	Zm00001d039395_T001	48.68	70.07	coding	226	75	752
PB.9054.2	7	1025	7	full-splice_match	gene: Zm00001d022631	Zm00001d022631_T002	27.36	48.42	coding	216	94	741
PB.9054.1	7	704	3	incomplete-splice_match	gene: Zm00001d022631	Zm00001d022631_T002	15.62	48.42	coding	76	78	305
PB.3685.2	3	1039	2	full-splice_match	gene: Zm00001d039395	Zm00001d039395_T001	21.40	70.07	coding	226	77	754
PB.5391.1	4	2057	1	full-splice_match	gene: Zm00001d051939	Zm00001d051939_T001	47.48	51.50	coding	538	68	1681
PB.7113.1	5	939	1	full-splice_match	gene: Zm00001d018324	Zm00001d018324_T001	104.60	104.60	coding	182	83	628
PB.5391.2	4	1937	1	full-splice_match	gene: Zm00001d051939	Zm00001d051939_T001	4.01	51.50	coding	538	103	1716
PB.2588.1	2	2220	1	intergenic	novelGene_53	novel	0.74	0.74	non_coding	NA	NA	NA
PB.10358.2	9	1892	12	full-splice_match	gene: Zm00001d046554	Zm00001d046554_T009	9.91	49.07	coding	445	212	1546

PB.6237.1	5	1135	7	full-splice_match	gene: Zm00001d014122	Zm00001d014122_T002	38.35	38.35	coding	214	230	871
PB.3406.1	2	1676	2	incomplete-splice_match	gene: Zm00001d006798	Zm00001d006798_T001	20.29	28.79	coding	395	35	1219
PB.6844.1	5	2098	3	novel_not_in_catalog	gene: Zm00001d017212	novel	7.07	7.07	coding	162	1403	1888
PB.6553.5	5	1502	4	antisense	novelGene_gene: Zm00001d015513_A	novel	11.71	24.79	non_coding	NA	NA	NA
PB.6553.6	5	1693	3	antisense	novelGene_gene: Zm00001d015513_A	novel	13.08	24.79	non_coding	NA	NA	NA
PB.6553.7	5	1452	1	intergenic	novelGene_163	novel	2.90	2.90	non_coding	NA	NA	NA
PB.4217.1	3	2643	2	genic	gene: Zm00001d042331	novel	19.42	40.47	coding	659	96	2072
PB.3406.2	2	3043	9	incomplete-splice_match	gene: Zm00001d006798	Zm00001d006798_T007	6.83	28.79	coding	917	35	2785
PB.7580.5	6	1374	10	novel_in_catalog	gene: Zm00001d037045	novel	30.70	41.91	coding	236	477	1184
PB.4217.2	3	2528	2	genic	gene: Zm00001d042331	novel	21.05	40.47	coding	659	112	2088
PB.5714.5	4	1360	5	novel_in_catalog	gene: Zm00001d053376	novel	6.80	25.69	coding	393	23	1201
PB.7106.3	5	2132	5	novel_in_catalog	gene: Zm00001d018290	novel	1.81	96.25	coding	110	1417	1746
PB.10139.1	9	1803	7	novel_in_catalog	gene: Zm00001d045309	novel	19.38	57.79	coding	281	632	1474
PB.10139.2	9	1967	7	novel_in_catalog	gene: Zm00001d045309	novel	5.02	57.79	coding	281	802	1644
PB.10139.3	9	1867	8	full-splice_match	gene: Zm00001d045309	Zm00001d045309_T001	33.39	57.79	coding	419	397	1653
PB.10139.4	9	1801	7	novel_in_catalog	gene: Zm00001d045309	novel	0.00	57.79	coding	162	240	725
PB.5656.1	4	2282	14	full-splice_match	gene: Zm00001d053014	Zm00001d053014_T001	11.89	11.89	coding	545	101	1735

PB.4798.3	4	2305	1	full-splice_match	gene: Zm00001d048604	Zm00001d048604_T001	6.46	15.01	coding	621	164	2026
PB.4798.2	4	2033	1	full-splice_match	gene: Zm00001d048604	Zm00001d048604_T001	8.56	15.01	coding	481	84	1526
PB.9871.1	8	508	3	incomplete-splice_match	gene: Zm00001d012126	Zm00001d012126_T005	81.44	105.33	coding	40	177	296
PB.9871.2	8	1085	7	novel_in_catalog	gene: Zm00001d012126	novel	23.90	105.33	coding	203	270	878
PB.2080.2	10	2096	11	novel_in_catalog	gene: Zm00001d025507	novel	22.54	22.54	coding	216	231	878
PB.6661.8	5	1726	2	novel_in_catalog	gene: Zm00001d016332	novel	57.28	386.30	coding	262	188	973
PB.295.1	1	2005	1	full-splice_match	gene: Zm00001d028510	Zm00001d028510_T001	56.37	56.37	coding	381	505	1647
PB.7350.4	6	1610	5	full-splice_match	gene: Zm00001d035720	Zm00001d035720_T002	133.01	791.54	coding	377	212	1342
PB.6661.2	5	1652	3	full-splice_match	gene: Zm00001d016332	Zm00001d016332_T001	313.83	386.30	coding	377	186	1316
PB.1642.3	1	2777	10	full-splice_match	gene: Zm00001d034962	Zm00001d034962_T004	2.17	8.09	coding	875	10	2634
PB.2303.1	10	1508	1	genic	gene: Zm00001d026467	novel	2.68	2.68	coding	172	25	540
PB.6661.6	5	2371	2	novel_in_catalog	gene: Zm00001d016332	novel	15.19	386.30	coding	305	1127	2041
PB.4504.3	3	1712	10	full-splice_match	gene: Zm00001d043548	Zm00001d043548_T001	5.73	18.57	coding	428	88	1371
PB.2212.1	10	2561	1	novel_in_catalog	gene: Zm00001d026148	novel	1.66	1.66	coding	345	1262	2296
PB.7910.1	6	1542	6	full-splice_match	gene: Zm00001d038352	Zm00001d038352_T003	0.15	57.62	coding	137	168	578
PB.3242.2	2	1010	7	full-splice_match	gene: Zm00001d006085	Zm00001d006085_T002	203.13	203.13	coding	221	165	827
PB.8142.1	7	2562	6	full-splice_match	gene: Zm00001d018598	Zm00001d018598_T003	2.77	72.86	coding	432	1126	2421

PB.4941.1	4	2293	12	full-splice_match	gene: Zm00001d049313	Zm00001d049313_T004	5.35	5.35	coding	585	157	1911
PB.8142.3	7	2614	5	novel_in_catalog	gene: Zm00001d018598	novel	3.76	72.86	coding	385	1332	2486
PB.434.2	1	1793	3	full-splice_match	gene: Zm00001d029126	Zm00001d029126_T001	67.93	87.32	coding	462	98	1483
PB.434.1	1	1821	3	full-splice_match	gene: Zm00001d029126	Zm00001d029126_T001	19.39	87.32	coding	462	102	1487
PB.7897.1	6	1579	5	full-splice_match	gene: Zm00001d038304	Zm00001d038304_T001	9.67	9.67	coding	361	240	1322
PB.10903.1	B73V4 ctg28	2582	1	full-splice_match	gene: Zm00001d000403	Zm00001d000403_T001	16.25	33.47	coding	679	81	2117
PB.7910.4	6	887	6	full-splice_match	gene: Zm00001d038352	Zm00001d038352_T003	0.00	57.62	coding	137	166	576
PB.7715.1	6	3222	1	full-splice_match	gene: Zm00001d037666	Zm00001d037666_T001	12.94	12.94	coding	977	144	3074
PB.7823.2	6	1256	4	full-splice_match	gene: Zm00001d038065	Zm00001d038065_T001	61.92	63.52	coding	324	84	1055
PB.1010.1	1	2230	4	novel_in_catalog	gene: Zm00001d032301	novel	4.16	4.16	coding	110	202	531
PB.132.9	1	2538	5	full-splice_match	gene: Zm00001d027802	Zm00001d027802_T003	5.54	63.41	coding	587	389	2149
PB.9160.1	8	1323	1	full-splice_match	gene: Zm00001d008634	Zm00001d008634_T001	51.92	51.92	coding	331	123	1115
PB.7729.1	6	1477	6	full-splice_match	gene: Zm00001d037725	Zm00001d037725_T001	170.61	170.61	coding	308	172	1095
PB.6577.2	5	1221	11	novel_not_in_catalog	gene: Zm00001d015724	novel	0.84	0.84	coding	398	28	1221
PB.2851.1	2	1350	6	incomplete-splice_match	gene: Zm00001d003679	Zm00001d003679_T001	24.22	24.22	coding	304	275	1186
PB.6577.1	5	1889	14	fusion	gene: Zm00001d015724_g	novel	5.93	5.93	coding	363	508	1596
PB.6876.2	5	1432	2	novel_in_catalog	gene: Zm00001d017353	novel	9.12	141.84	coding	129	151	537

PB.6876.3	5	1233	2	novel_in_catalog	gene: Zm00001d017353	novel	13.20	141.84	non_coding	NA	NA	NA
PB.132.2	1	2599	5	novel_in_catalog	gene: Zm00001d027802	novel	23.25	63.41	coding	587	350	2110
PB.132.3	1	2823	3	novel_in_catalog	gene: Zm00001d027802	novel	3.95	63.41	coding	360	389	1468
PB.132.4	1	2777	3	novel_not_in_catalog	gene: Zm00001d027802	novel	1.25	63.41	coding	360	297	1376
PB.132.5	1	2804	4	novel_in_catalog	gene: Zm00001d027802	novel	7.56	63.41	coding	382	426	1571
PB.132.6	1	2719	5	full-splice_match	gene: Zm00001d027802	Zm00001d027802_T001	16.98	63.41	coding	587	476	2236
PB.1827.2	10	2993	6	novel_in_catalog	gene: Zm00001d024178	novel	32.53	74.62	coding	757	544	2814
PB.10390.1	9	2414	12	novel_in_catalog	gene: Zm00001d046719	novel	1.98	17.88	coding	412	166	1401
PB.8569.1	7	1962	2	novel_in_catalog	gene: Zm00001d020879	novel	31.09	31.09	coding	532	195	1790
PB.10390.3	9	2011	13	full-splice_match	gene: Zm00001d046719	Zm00001d046719_T004	14.38	17.88	coding	460	166	1545
PB.10390.2	9	2341	12	novel_in_catalog	gene: Zm00001d046719	novel	0.63	17.88	coding	411	166	1398
PB.6454.2	5	1609	21	full-splice_match	gene: Zm00001d015008	Zm00001d015008_T001	10.36	44.35	coding	467	65	1465
PB.6454.1	5	1717	21	full-splice_match	gene: Zm00001d015008	Zm00001d015008_T001	33.99	44.35	coding	467	58	1458
PB.3133.1	2	1891	12	full-splice_match	gene: Zm00001d005516	Zm00001d005516_T004	5.83	14.44	coding	223	570	1238
PB.1047.2	1	1968	18	full-splice_match	gene: Zm00001d032455	Zm00001d032455_T001	0.00	6.16	coding	584	108	1859
PB.1047.1	1	2131	18	full-splice_match	gene: Zm00001d032455	Zm00001d032455_T001	6.16	6.16	coding	584	93	1844
PB.3133.2	2	1343	5	novel_not_in_catalog	gene: Zm00001d005516	novel	2.03	14.44	coding	196	120	707

PB.3133.5	2	2156	12	novel_not_in_catalog	gene: Zm00001d005516	novel	1.11	14.44	coding	297	299	1189
PB.10811.1	9	1520	7	novel_not_in_catalog	gene: Zm00001d048499	novel	13.16	13.16	coding	344	219	1250
PB.10125.1	9	1670	4	full-splice_match	gene: Zm00001d045202	Zm00001d045202_T002	28.38	28.38	coding	221	797	1459
PB.1572.2	1	1789	4	full-splice_match	gene: Zm00001d034723	Zm00001d034723_T001	0.49	1.89	coding	480	41	1480
PB.1996.3	10	1415	3	full-splice_match	gene: Zm00001d025023	Zm00001d025023_T001	105.11	123.94	coding	361	94	1176
PB.6609.2	5	1106	5	full-splice_match	gene: Zm00001d016041	Zm00001d016041_T001	108.23	126.61	coding	230	103	792
PB.281.4	1	787	2	full-splice_match	gene: Zm00001d028450	Zm00001d028450_T001	113.53	141.69	coding	182	57	602
PB.6609.1	5	2404	3	novel_in_catalog	gene: Zm00001d016041	novel	1.79	126.61	non_coding	NA	NA	NA
PB.2679.3	2	1610	8	full-splice_match	gene: Zm00001d002944	Zm00001d002944_T001	0.00	95.02	coding	481	52	1494
PB.2679.2	2	1721	8	full-splice_match	gene: Zm00001d002944	Zm00001d002944_T001	27.45	95.02	coding	481	126	1568
PB.2679.1	2	1852	8	full-splice_match	gene: Zm00001d002944	Zm00001d002944_T001	67.57	95.02	coding	481	71	1513
PB.6609.5	5	645	4	full-splice_match	gene: Zm00001d016041	Zm00001d016041_T002	0.00	126.61	coding	84	228	479
PB.9715.1	8	1686	8	full-splice_match	gene: Zm00001d011457	Zm00001d011457_T002	16.02	17.99	coding	409	111	1337
PB.533.1	1	885	1	genic	gene: Zm00001d029578	novel	7.82	7.82	coding	224	68	739
PB.3492.1	2	1779	15	full-splice_match	gene: Zm00001d007214	Zm00001d007214_T006	38.10	38.10	coding	474	138	1559
PB.10595.1	9	1848	11	full-splice_match	gene: Zm00001d047747	Zm00001d047747_T002	32.11	32.11	coding	422	264	1529
PB.5586.2	4	1458	2	genic	gene: Zm00001d052738	novel	6.63	6.63	coding	370	168	1277

PB.8209.3	7	1215	1	full-splice_match	gene: Zm00001d018857	Zm00001d018857_T001	579.29	579.29	coding	328	48	1031
PB.7609.1	6	1965	1	full-splice_match	gene: Zm00001d037192	Zm00001d037192_T001	5.44	5.44	coding	421	38	1300
PB.1031.13	1	1279	14	incomplete-splice_match	gene: Zm00001d032386	Zm00001d032386_T007	0.00	154.28	coding	401	76	1278
PB.1031.11	1	2220	19	full-splice_match	gene: Zm00001d032386	Zm00001d032386_T007	0.00	154.28	coding	618	191	2044
PB.1600.4	1	2249	14	full-splice_match	gene: Zm00001d034809	Zm00001d034809_T021	9.02	80.83	coding	321	196	1158
PB.10398.9	9	886	3	full-splice_match	gene: Zm00001d046767	Zm00001d046767_T005	0.00	1894.58	coding	174	149	670
PB.6221.1	5	2449	8	novel_in_catalog	gene: Zm00001d014036	novel	21.04	21.04	coding	616	230	2077
PB.1501.2	1	582	3	full-splice_match	gene: Zm00001d034491	Zm00001d034491_T001	284.43	410.14	coding	133	83	481
PB.5734.1	4	3322	10	novel_in_catalog	gene: Zm00001d053453	novel	62.15	62.15	coding	960	174	3053
PB.3147.1	2	1489	2	novel_not_in_catalog	gene: Zm00001d005612	novel	8.57	11.84	coding	216	230	877
PB.3147.2	2	608	1	genic	gene: Zm00001d005612	novel	3.27	11.84	non_coding	NA	NA	NA
PB.9368.1	8	1843	11	novel_not_in_catalog	gene: Zm00001d009763	novel	83.38	83.38	coding	459	123	1499
PB.9638.3	8	506	2	full-splice_match	gene: Zm00001d011063	Zm00001d011063_T001	974.91	999.97	coding	82	78	323
PB.9368.2	8	1656	11	novel_not_in_catalog	gene: Zm00001d009763	novel	0.00	83.38	coding	459	22	1398
PB.10398.5	9	1653	2	novel_in_catalog	gene: Zm00001d046767	novel	6.87	1894.58	coding	174	763	1284
PB.10398.4	9	1047	3	full-splice_match	gene: Zm00001d046767	Zm00001d046767_T005	52.18	1894.58	coding	174	156	677
PB.10398.6	9	1053	3	full-splice_match	gene: Zm00001d046767	Zm00001d046767_T001	1810.63	1894.58	coding	174	124	645

PB.9709.2	8	2718	10	full-splice_match	gene: Zm00001d011426	Zm00001d011426_T003	50.75	54.05	coding	613	410	2248
PB.1682.1	10	2300	1	full-splice_match	gene: Zm00001d023349	Zm00001d023349_T001	2.39	2.39	coding	433	852	2150
PB.9709.1	8	2754	10	full-splice_match	gene: Zm00001d011426	Zm00001d011426_T003	3.30	54.05	coding	613	339	2177
PB.4888.1	4	1413	11	full-splice_match	gene: Zm00001d049088	Zm00001d049088_T001	14.16	14.16	coding	387	1	1161
PB.1033.2	1	777	3	full-splice_match	gene: Zm00001d032402	Zm00001d032402_T001	0.00	61.46	coding	130	134	523
PB.1033.3	1	2629	2	novel_in_catalog	gene: Zm00001d032402	novel	0.93	61.46	non_coding	NA	NA	NA
PB.1033.1	1	915	3	full-splice_match	gene: Zm00001d032402	Zm00001d032402_T001	60.52	61.46	coding	130	137	526
PB.3511.1	2	830	1	full-splice_match	gene: Zm00001d007316	Zm00001d007316_T001	274.32	274.32	coding	177	53	583
PB.4956.1	4	800	2	full-splice_match	gene: Zm00001d049381	Zm00001d049381_T001	124.63	264.47	non_coding	NA	NA	NA
PB.7570.1	6	720	4	full-splice_match	gene: Zm00001d036985	Zm00001d036985_T001	109.15	109.15	coding	137	88	498
PB.9787.1	8	823	3	full-splice_match	gene: Zm00001d011766	Zm00001d011766_T001	348.21	351.32	coding	137	169	579
PB.9787.2	8	1469	2	novel_in_catalog	gene: Zm00001d011766	novel	3.11	351.32	coding	107	124	444
PB.9787.3	8	676	3	full-splice_match	gene: Zm00001d011766	Zm00001d011766_T001	0.00	351.32	coding	137	168	578
PB.10639.3	9	2122	8	full-splice_match	gene: Zm00001d047869	Zm00001d047869_T007	40.20	44.65	coding	305	902	1816
PB.1726.4	10	1638	2	genic	gene: Zm00001d023558	novel	4.03	63.02	coding	467	157	1557
PB.1726.3	10	1846	2	genic	gene: Zm00001d023558	novel	41.28	63.02	coding	467	260	1660
PB.1726.2	10	2322	1	full-splice_match	gene: Zm00001d023558	Zm00001d023558_T001	12.69	63.02	coding	467	697	2097

PB.1726.1	10	394	1	full-splice_match	gene: Zm00001d023558	Zm00001d023558_T001	5.01	63.02	non_coding	NA	NA	NA
PB.7118.3	5	3154	3	full-splice_match	gene: Zm00001d018335	Zm00001d018335_T001	0.00	20.65	coding	953	48	2906
PB.6740.6	5	1389	9	incomplete-splice_match	gene: Zm00001d016770	Zm00001d016770_T001	0.00	43.77	coding	380	248	1387
PB.6740.7	5	1297	9	incomplete-splice_match	gene: Zm00001d016770	Zm00001d016770_T006	5.84	43.77	coding	376	168	1295
PB.6740.4	5	1675	10	full-splice_match	gene: Zm00001d016770	Zm00001d016770_T001	3.63	43.77	coding	393	273	1451
PB.6740.5	5	2476	9	full-splice_match	gene: Zm00001d016770	Zm00001d016770_T004	0.00	43.77	coding	363	256	1344
PB.6740.2	5	2577	9	full-splice_match	gene: Zm00001d016770	Zm00001d016770_T004	3.79	43.77	coding	363	243	1331
PB.1992.2	10	4585	20	full-splice_match	gene: Zm00001d025012	Zm00001d025012_T001	0.34	23.53	coding	1464	108	4499
PB.6740.1	5	1782	10	full-splice_match	gene: Zm00001d016770	Zm00001d016770_T001	26.25	43.77	coding	393	230	1408
PB.1526.2	1	1972	5	full-splice_match	gene: Zm00001d034575	Zm00001d034575_T001	10.43	70.35	coding	466	235	1632
PB.6985.1	5	628	2	full-splice_match	gene: Zm00001d017850	Zm00001d017850_T001	846.32	1717.47	coding	123	42	410
PB.5645.1	4	1751	4	full-splice_match	gene: Zm00001d052977	Zm00001d052977_T001	3.02	3.02	coding	489	119	1585
PB.6740.8	5	1666	9	novel_in_catalog	gene: Zm00001d016770	novel	0.00	43.77	coding	363	258	1346
PB.4207.1	3	1512	3	full-splice_match	gene: Zm00001d042293	Zm00001d042293_T001	11.15	11.15	coding	310	367	1296
PB.4332.3	3	1832	11	novel_in_catalog	gene: Zm00001d042851	novel	1.10	28.44	coding	229	353	1039
PB.4332.1	3	1799	12	full-splice_match	gene: Zm00001d042851	Zm00001d042851_T009	22.07	28.44	coding	324	353	1324
PB.7505.1	6	1779	6	novel_in_catalog	gene: Zm00001d036602	novel	3.09	33.15	coding	198	991	1584

PB.1298.1	1	2922	2	genic	gene: Zm00001d033610	novel	13.14	13.14	coding	743	289	2517
PB.9865.3	8	2459	19	full-splice_match	gene: Zm00001d012086	Zm00001d012086_T004	0.00	41.97	coding	691	149	2221
PB.4599.1	3	1982	4	novel_in_catalog	gene: Zm00001d043911	novel	6.13	71.56	coding	218	314	967
PB.4599.2	3	1391	5	full-splice_match	gene: Zm00001d043911	Zm00001d043911_T001	65.42	71.56	coding	247	313	1053
PB.4880.2	4	1261	1	full-splice_match	gene: Zm00001d049028	Zm00001d049028_T001	6.87	15.89	coding	286	77	934
PB.4880.1	4	1364	1	full-splice_match	gene: Zm00001d049028	Zm00001d049028_T001	9.02	15.89	coding	286	73	930
PB.4291.1	3	2435	4	incomplete-splice_match	gene: Zm00001d042662	Zm00001d042662_T001	7.42	7.42	coding	638	52	1965
PB.7924.1	6	692	4	intergenic	novelGene_255	novel	525.26	525.26	coding	142	38	463
PB.1285.1	1	4439	6	full-splice_match	gene: Zm00001d033553	Zm00001d033553_T049	3.41	3.41	coding	1143	640	4068
PB.7024.5	5	1680	8	full-splice_match	gene: Zm00001d017985	Zm00001d017985_T001	23.38	214.42	coding	390	100	1269
PB.5479.4	4	2352	4	incomplete-splice_match	gene: Zm00001d052314	Zm00001d052314_T034	32.28	76.52	coding	737	89	2299
PB.10907.1	B73V4 ctg31	3225	2	genic	gene: Zm00001d000093	novel	2.70	2.70	coding	688	435	2498
PB.1535.1	1	1213	1	full-splice_match	gene: Zm00001d034605	Zm00001d034605_T001	8.28	66.56	coding	238	190	903
PB.10621.1	9	1342	9	novel_not_in_catalog	gene: Zm00001d047823	novel	36.59	36.59	coding	280	266	1105
PB.8388.4	7	1631	2	intergenic	novelGene_144	novel	0.00	22.09	non_coding	NA	NA	NA
PB.7990.1	6	1799	2	novel_not_in_catalog	gene: Zm00001d038692	novel	9.45	9.45	coding	513	24	1562
PB.8388.1	7	2717	1	antisense	novelGene_gene: Zm00001d019960_A	novel	2.38	4.56	coding	27	290	370

PB.8388.2	7	2451	1	antisense	novelGene_gene: Zm00001d019960_A	novel	2.18	4.56	coding	18	397	450
PB.4407.1	3	1442	2	genic	gene: Zm00001d043183	novel	38.07	45.71	coding	287	286	1146
PB.594.1	1	1611	6	novel_not_in_catalog	gene: Zm00001d029922	novel	13.17	19.61	coding	211	33	665
PB.1030.2	1	2878	5	novel_in_catalog	gene: Zm00001d032384	novel	0.12	25.25	coding	189	2039	2605
PB.5084.1	4	3062	6	full-splice_match	gene: Zm00001d050104	Zm00001d050104_T002	2.27	2.27	coding	903	117	2825
PB.1595.1	1	1359	5	full-splice_match	gene: Zm00001d034795	Zm00001d034795_T001	25.82	151.38	coding	289	58	924
PB.626.1	1	1896	6	full-splice_match	gene: Zm00001d030126	Zm00001d030126_T005	122.94	122.94	coding	385	260	1414
PB.9275.6	8	2565	1	full-splice_match	gene: Zm00001d009226	Zm00001d009226_T001	0.00	25.18	coding	810	47	2476
PB.6329.1	5	1060	1	full-splice_match	gene: Zm00001d014563	Zm00001d014563_T001	30.17	30.17	coding	249	84	830
PB.1776.2	10	2243	10	full-splice_match	gene: Zm00001d023843	Zm00001d023843_T002	8.98	8.98	coding	650	23	1972
PB.1776.3	10	2068	10	full-splice_match	gene: Zm00001d023843	Zm00001d023843_T002	0.00	8.98	coding	650	23	1972
PB.9275.2	8	2936	3	genic	gene: Zm00001d009226	novel	10.29	25.18	coding	810	49	2478
PB.9275.3	8	2824	3	genic	gene: Zm00001d009226	novel	0.00	25.18	coding	810	43	2472
PB.244.10	1	1734	1	intergenic	novelGene_85	novel	0.00	94.41	non_coding	NA	NA	NA
PB.4571.3	3	1325	3	incomplete-splice_match	gene: Zm00001d043801	Zm00001d043801_T002	0.00	23.74	coding	69	459	665
PB.2497.1	2	2150	7	novel_not_in_catalog	gene: Zm00001d002174	novel	1.75	1.75	coding	246	1145	1882
PB.7629.1	6	2130	12	novel_not_in_catalog	gene: Zm00001d037257	novel	63.38	107.46	coding	441	405	1727

PB.4571.1	3	2227	5	full-splice_match	gene: Zm00001d043801	Zm00001d043801_T002	23.74	23.74	coding	259	118	894
PB.10137.2	9	1484	3	novel_in_catalog	gene: Zm00001d045302	novel	115.13	310.35	coding	364	75	1166
PB.10137.3	9	1438	3	novel_in_catalog	gene: Zm00001d045302	novel	195.22	310.35	coding	364	167	1258
PB.7629.5	6	1986	11	novel_not_in_catalog	gene: Zm00001d037257	novel	0.00	107.46	coding	441	249	1571
PB.6650.8	5	2095	4	full-splice_match	gene: Zm00001d016274	Zm00001d016274_T001	13.24	83.51	coding	671	70	2082
PB.7629.9	6	1779	12	novel_not_in_catalog	gene: Zm00001d037257	novel	0.00	107.46	coding	441	248	1570
PB.7629.8	6	1908	11	novel_not_in_catalog	gene: Zm00001d037257	novel	0.00	107.46	coding	441	281	1603
PB.2301.30	10	930	3	novel_in_catalog	gene: Zm00001d026458	novel	0.00	1555.47	coding	254	10	771
PB.6999.4	5	2780	8	full-splice_match	gene: Zm00001d017883	Zm00001d017883_T031	26.59	91.76	coding	813	85	2523
PB.6999.2	5	1601	6	incomplete-splice_match	gene: Zm00001d017883	Zm00001d017883_T031	49.47	91.76	coding	350	122	1171
PB.2951.1	2	712	4	intergenic	novelGene_186	novel	138.85	176.52	non_coding	NA	NA	NA
PB.9260.1	8	992	7	full-splice_match	gene: Zm00001d009147	Zm00001d009147_T002	14.51	14.51	coding	216	16	663
PB.582.1	1	2195	1	full-splice_match	gene: Zm00001d029846	Zm00001d029846_T001	8.74	8.74	coding	589	24	1790
PB.7481.2	6	3213	6	novel_not_in_catalog	gene: Zm00001d036532	novel	0.00	17.24	coding	310	2019	2948
PB.2473.1	2	1915	11	full-splice_match	gene: Zm00001d002095	Zm00001d002095_T001	17.38	17.38	coding	568	88	1791
PB.9269.6	8	1996	1	genic	gene: Zm00001d009193	novel	3.69	92.88	coding	102	1276	1581
PB.9269.1	8	1112	3	genic	gene: Zm00001d009193	novel	0.00	92.88	coding	136	705	1112

PB.7481.6	6	2885	6	novel_not_in_catalog	gene: Zm00001d036532	novel	1.20	17.24	coding	310	1888	2817
PB.9269.3	8	1016	3	full-splice_match	gene: Zm00001d009193	Zm00001d009193_T001	86.11	92.88	coding	139	169	585
PB.9269.2	8	1920	5	novel_not_in_catalog	gene: Zm00001d009193	novel	3.08	92.88	coding	139	150	566
PB.10199.2	9	2823	15	novel_in_catalog	gene: Zm00001d045501	novel	0.00	29.91	coding	494	1036	2517
PB.1299.1	1	2062	2	full-splice_match	gene: Zm00001d033611	Zm00001d033611_T002	5.11	86.53	coding	257	921	1691
PB.10199.1	9	2631	17	full-splice_match	gene: Zm00001d045501	Zm00001d045501_T003	29.91	29.91	coding	712	172	2307
PB.1299.4	1	1237	3	full-splice_match	gene: Zm00001d033611	Zm00001d033611_T003	30.33	86.53	coding	257	193	963
PB.1700.2	10	1615	6	novel_in_catalog	gene: Zm00001d023424	novel	2.56	9.56	coding	215	110	754
PB.1700.1	10	1205	6	incomplete-splice_match	gene: Zm00001d023424	Zm00001d023424_T001	7.01	9.56	coding	262	25	810
PB.7500.2	6	509	1	full-splice_match	gene: Zm00001d036570	Zm00001d036570_T001	0.00	484.59	coding	57	148	318
PB.2752.1	2	1584	7	full-splice_match	gene: Zm00001d003212	Zm00001d003212_T001	9.55	9.55	coding	374	108	1229
PB.5500.1	4	1824	9	novel_in_catalog	gene: Zm00001d052378	novel	18.65	18.65	coding	422	203	1468
PB.10443.2	9	1695	2	novel_in_catalog	gene: Zm00001d047045	novel	1.84	55.17	coding	280	501	1340
PB.1110.2	1	2764	3	novel_in_catalog	gene: Zm00001d032683	novel	1.42	14.66	coding	653	342	2300
PB.4923.4	4	1204	9	full-splice_match	gene: Zm00001d049222	Zm00001d049222_T002	0.00	73.89	coding	278	134	967
PB.4923.3	4	1217	10	full-splice_match	gene: Zm00001d049222	Zm00001d049222_T001	0.00	73.89	coding	278	143	976
PB.4923.2	4	1395	9	full-splice_match	gene: Zm00001d049222	Zm00001d049222_T002	26.98	73.89	coding	278	205	1038

PB.4923.1	4	1308	10	full-splice_match	gene: Zm00001d049222	Zm00001d049222_T001	46.91	73.89	coding	278	133	966
PB.6180.3	5	1736	5	full-splice_match	gene: Zm00001d013819	Zm00001d013819_T004	6.92	9.97	coding	450	144	1493
PB.1535.2	1	1102	1	full-splice_match	gene: Zm00001d034605	Zm00001d034605_T001	58.28	66.56	coding	238	212	925
PB.5843.1	4	860	7	novel_in_catalog	gene: Zm00001d053919	novel	132.80	132.80	coding	152	166	621
PB.9632.1	8	1452	6	novel_in_catalog	gene: Zm00001d011005	novel	88.12	88.12	coding	324	297	1268
PB.4376.1	3	815	5	full-splice_match	gene: Zm00001d043070	Zm00001d043070_T001	354.87	354.87	coding	148	103	546
PB.9984.2	8	1572	1	full-splice_match	gene: Zm00001d012606	Zm00001d012606_T001	0.00	17.83	coding	453	49	1407
PB.10552.5	9	1897	9	novel_not_in_catalog	gene: Zm00001d047587	novel	9.62	51.20	coding	335	493	1497
PB.8813.3	7	1857	5	full-splice_match	gene: Zm00001d021817	Zm00001d021817_T001	6.46	32.50	coding	431	407	1699
PB.10552.7	9	1731	9	novel_not_in_catalog	gene: Zm00001d047587	novel	0.00	51.20	coding	335	474	1478
PB.158.3	1	1408	3	full-splice_match	gene: Zm00001d027924	Zm00001d027924_T001	159.98	289.31	coding	302	83	988
PB.10552.1	9	2127	9	novel_not_in_catalog	gene: Zm00001d047587	novel	27.87	51.20	coding	335	593	1597
PB.9763.4	8	1198	3	genic	gene: Zm00001d011669	novel	15.61	18.16	coding	301	154	1056
PB.323.1	1	2230	2	full-splice_match	gene: Zm00001d028630	Zm00001d028630_T005	24.54	24.54	coding	649	118	2064
PB.5942.3	5	1593	6	full-splice_match	gene: Zm00001d012921	Zm00001d012921_T002	7.16	52.04	coding	404	200	1411
PB.5942.1	5	1688	6	full-splice_match	gene: Zm00001d012921	Zm00001d012921_T002	44.88	52.04	coding	404	190	1401
PB.8813.1	7	2311	5	full-splice_match	gene: Zm00001d021817	Zm00001d021817_T001	2.45	32.50	coding	431	229	1521

PB.10003.5	8	1479	8	novel_in_catalog	gene: Zm00001d012667	novel	3.27	43.63	non_coding	NA	NA	NA
PB.336.2	1	1523	6	novel_not_in_catalog	gene: Zm00001d028672	novel	14.97	35.78	coding	306	340	1257
PB.8443.1	7	1655	10	full-splice_match	gene: Zm00001d020283	Zm00001d020283_T001	13.63	13.63	coding	423	70	1338
PB.10901.7	B73V4 ctg248	2094	6	novel_not_in_catalog	gene: Zm00001d000401	novel	3.63	21.61	coding	156	6	473
PB.9081.2	8	1140	6	full-splice_match	gene: Zm00001d008284	Zm00001d008284_T006	70.28	125.38	coding	307	85	1005
PB.10273.1	9	1189	6	novel_in_catalog	gene: Zm00001d045973	novel	5.67	5.67	non_coding	NA	NA	NA
PB.2301.23	10	2604	3	novel_in_catalog	gene: Zm00001d026458	novel	7.55	1555.47	coding	298	80	973
PB.10901.3	B73V4 ctg248	2144	5	novel_not_in_catalog	gene: Zm00001d000401	novel	2.08	21.61	coding	156	85	552
PB.10901.2	B73V4 ctg248	2371	7	novel_not_in_catalog	gene: Zm00001d000401	novel	7.87	21.61	coding	245	1200	1934
PB.6288.3	5	2542	9	full-splice_match	gene: Zm00001d014358	Zm00001d014358_T002	6.93	16.11	coding	750	120	2369
PB.6049.1	5	1891	9	incomplete-splice_match	gene: Zm00001d013278	Zm00001d013278_T017	16.96	16.96	coding	392	164	1339
PB.6288.1	5	2663	9	full-splice_match	gene: Zm00001d014358	Zm00001d014358_T002	6.58	16.11	coding	750	108	2357
PB.2487.1	2	2326	2	incomplete-splice_match	gene: Zm00001d002149	Zm00001d002149_T005	5.99	27.58	non_coding	NA	NA	NA
PB.8020.1	6	1509	2	full-splice_match	gene: Zm00001d038801	Zm00001d038801_T001	8.71	8.71	coding	239	416	1132
PB.10901.8	B73V4 ctg248	2235	6	novel_not_in_catalog	gene: Zm00001d000401	novel	8.02	21.61	coding	245	1212	1946
PB.1708.1	10	972	1	full-splice_match	gene: Zm00001d023461	Zm00001d023461_T001	48.36	48.36	coding	176	165	692
PB.9100.1	8	1551	5	full-splice_match	gene: Zm00001d008360	Zm00001d008360_T001	14.22	39.61	coding	318	79	1032

PB.10921.2	B73V4 ctg51	1728	10	novel_in_catalog	gene: Zm00001d000311	novel	0.47	6.34	coding	227	656	1336
PB.10921.1	B73V4 ctg51	1750	11	full-splice_match	gene: Zm00001d000311	Zm00001d000311_T003	5.87	6.34	coding	361	178	1260
PB.9100.2	8	1384	5	full-splice_match	gene: Zm00001d008360	Zm00001d008360_T001	24.31	39.61	coding	318	114	1067
PB.6413.2	5	1591	8	full-splice_match	gene: Zm00001d014879	Zm00001d014879_T004	0.00	16.78	coding	388	134	1297
PB.9100.4	8	1306	4	novel_in_catalog	gene: Zm00001d008360	novel	1.08	39.61	coding	331	50	1042
PB.5779.1	4	1532	2	full-splice_match	gene: Zm00001d053662	Zm00001d053662_T001	2.16	2.16	coding	418	117	1370
PB.3024.1	2	1447	8	full-splice_match	gene: Zm00001d004896	Zm00001d004896_T004	46.74	46.74	coding	366	65	1162
PB.3547.1	2	2849	9	full-splice_match	gene: Zm00001d007572	Zm00001d007572_T001	2.98	2.98	coding	842	15	2540
PB.1339.1	1	2382	20	full-splice_match	gene: Zm00001d033795	Zm00001d033795_T006	27.38	30.20	coding	594	122	1903
PB.8813.4	7	1907	1	novel_in_catalog	gene: Zm00001d021817	novel	0.91	32.50	coding	153	1360	1818
PB.1339.7	1	2122	20	full-splice_match	gene: Zm00001d033795	Zm00001d033795_T006	0.00	30.20	coding	594	122	1903
PB.7172.1	5	1860	14	full-splice_match	gene: Zm00001d018487	Zm00001d018487_T001	138.62	147.71	coding	461	272	1654
PB.6695.1	5	1314	11	full-splice_match	gene: Zm00001d016550	Zm00001d016550_T001	36.04	61.59	coding	312	47	982
PB.5820.2	4	1645	2	full-splice_match	gene: Zm00001d053834	Zm00001d053834_T001	1156.44	1202.82	coding	381	107	1249
PB.6695.3	5	405	5	incomplete-splice_match	gene: Zm00001d016550	Zm00001d016550_T001	0.00	61.59	coding	120	6	365
PB.6695.2	5	581	6	incomplete-splice_match	gene: Zm00001d016550	Zm00001d016550_T001	25.54	61.59	coding	120	72	431
PB.4287.3	3	2416	16	full-splice_match	gene: Zm00001d042654	Zm00001d042654_T009	11.18	11.18	coding	682	99	2144

PB.8984.2	7	1492	2	novel_in_catalog	gene: Zm00001d022421	novel	10.92	510.64	coding	244	426	1157
PB.3268.1	2	1853	2	full-splice_match	gene: Zm00001d006203	Zm00001d006203_T001	303.42	303.42	coding	505	30	1544
PB.160.1	1	1034	1	full-splice_match	gene: Zm00001d027928	Zm00001d027928_T001	129.21	129.21	coding	271	110	922
PB.8336.1	7	2530	13	incomplete-splice_match	gene: Zm00001d019561	Zm00001d019561_T060	25.40	25.40	coding	593	204	1982
PB.3128.2	2	1183	2	full-splice_match	gene: Zm00001d005495	Zm00001d005495_T001	88.81	90.47	coding	223	134	802
PB.5293.2	4	2501	7	full-splice_match	gene: Zm00001d051502	Zm00001d051502_T004	15.16	19.92	coding	562	216	1901
PB.5293.3	4	1894	7	full-splice_match	gene: Zm00001d051502	Zm00001d051502_T004	0.00	19.92	coding	562	21	1706
PB.5293.1	4	2521	8	full-splice_match	gene: Zm00001d051502	Zm00001d051502_T001	4.76	19.92	coding	562	335	2020
PB.4933.1	4	1332	6	full-splice_match	gene: Zm00001d049252	Zm00001d049252_T001	27.12	27.12	coding	293	194	1072
PB.8295.9	7	2808	2	novel_not_in_catalog	gene: Zm00001d019287	novel	4.90	146.35	coding	299	1809	2705
PB.3332.1	2	4614	15	full-splice_match	gene: Zm00001d006459	Zm00001d006459_T004	3.12	22.95	coding	768	380	2683
PB.5463.1	4	1367	2	full-splice_match	gene: Zm00001d052258	Zm00001d052258_T001	34.91	37.18	coding	258	294	1067
PB.5558.3	4	3154	3	full-splice_match	gene: Zm00001d052620	Zm00001d052620_T005	2.22	77.09	coding	158	180	653
PB.5463.3	4	1670	1	novel_in_catalog	gene: Zm00001d052258	novel	2.27	37.18	coding	105	293	607
PB.7088.2	5	1908	3	novel_not_in_catalog	gene: Zm00001d018214	novel	74.11	77.38	coding	481	60	1502
PB.7088.3	5	1664	2	genic	gene: Zm00001d018214	novel	0.00	77.38	coding	344	343	1374
PB.8295.1	7	1510	2	novel_not_in_catalog	gene: Zm00001d019287	novel	124.36	146.35	coding	299	272	1168

PB.7088.1	5	2004	2	genic	gene: Zm00001d018214	novel	3.28	77.38	coding	344	343	1374
PB.8295.7	7	1404	3	novel_not_in_catalog	gene: Zm00001d019287	novel	9.25	146.35	coding	299	295	1191
PB.8295.6	7	1366	2	novel_not_in_catalog	gene: Zm00001d019287	novel	0.00	146.35	coding	299	272	1168
PB.8295.4	7	1525	3	novel_not_in_catalog	gene: Zm00001d019287	novel	7.84	146.35	coding	299	315	1211
PB.6689.2	5	1955	1	novel_in_catalog	gene: Zm00001d016501	novel	10.20	179.93	coding	101	158	460
PB.140.1	1	778	4	full-splice_match	gene: Zm00001d027847	Zm00001d027847_T002	32.23	32.23	coding	188	27	590
PB.6689.1	5	1929	2	full-splice_match	gene: Zm00001d016501	Zm00001d016501_T001	169.73	179.93	coding	465	192	1586
PB.5820.4	4	1458	2	full-splice_match	gene: Zm00001d053834	Zm00001d053834_T001	15.07	1202.82	coding	381	109	1251
PB.4362.2	3	1194	4	full-splice_match	gene: Zm00001d042985	Zm00001d042985_T001	46.92	46.92	coding	259	257	1033
PB.9336.1	8	3812	7	full-splice_match	gene: Zm00001d009617	Zm00001d009617_T003	11.47	11.88	coding	971	124	3036
PB.8997.4	7	2032	2	full-splice_match	gene: Zm00001d022458	Zm00001d022458_T001	1.48	96.94	coding	270	1053	1862
PB.8997.5	7	1248	3	novel_not_in_catalog	gene: Zm00001d022458	novel	20.53	96.94	coding	310	184	1113
PB.8997.3	7	1354	3	novel_not_in_catalog	gene: Zm00001d022458	novel	61.34	96.94	coding	310	182	1111
PB.8449.1	7	1219	2	full-splice_match	gene: Zm00001d020310	Zm00001d020310_T003	46.94	46.94	coding	249	89	835
PB.4454.6	3	1459	8	novel_not_in_catalog	gene: Zm00001d043387	novel	0.00	32.76	coding	441	54	1376
PB.9628.1	8	1876	1	full-splice_match	gene: Zm00001d010997	Zm00001d010997_T001	17.32	17.32	coding	496	123	1610
PB.9812.1	8	705	1	full-splice_match	gene: Zm00001d011841	Zm00001d011841_T001	122.51	122.51	coding	151	110	562

PB.6171.2	5	1858	1	genic	gene: Zm00001d013765	novel	2.05	13.71	coding	268	842	1645
PB.5791.1	4	1477	2	genic	gene: Zm00001d053704	novel	6.95	6.95	coding	386	75	1232
PB.3412.1	2	942	3	full-splice_match	gene: Zm00001d006837	Zm00001d006837_T003	28.26	36.77	non_coding	NA	NA	NA
PB.9095.1	8	1150	2	incomplete-splice_match	gene: Zm00001d008340	Zm00001d008340_T003	2.42	2.42	coding	302	49	954
PB.300.1	1	2238	10	full-splice_match	gene: Zm00001d028536	Zm00001d028536_T001	71.56	81.96	coding	604	117	1928
PB.1639.1	1	1249	7	full-splice_match	gene: Zm00001d034949	Zm00001d034949_T001	90.81	90.81	coding	203	100	708
PB.3412.5	2	828	3	full-splice_match	gene: Zm00001d006837	Zm00001d006837_T003	8.51	36.77	non_coding	NA	NA	NA
PB.300.2	1	2324	10	full-splice_match	gene: Zm00001d028536	Zm00001d028536_T001	10.40	81.96	coding	604	323	2134
PB.611.2	1	1702	5	full-splice_match	gene: Zm00001d030048	Zm00001d030048_T001	7.57	7.57	coding	458	130	1503
PB.3733.1	3	749	2	full-splice_match	gene: Zm00001d039565	Zm00001d039565_T001	11.93	11.93	coding	174	101	622
PB.1621.1	1	1341	4	full-splice_match	gene: Zm00001d034872	Zm00001d034872_T002	9.65	9.65	coding	367	67	1167
PB.8968.1	7	3898	22	full-splice_match	gene: Zm00001d022378	Zm00001d022378_T069	73.46	73.46	coding	1129	125	3511
PB.8984.1	7	1629	3	full-splice_match	gene: Zm00001d022421	Zm00001d022421_T002	499.72	510.64	coding	337	245	1255
PB.5702.7	4	2160	15	novel_in_catalog	gene: Zm00001d053301	novel	2.08	24.99	coding	468	711	2114
PB.5449.2	4	1640	6	novel_not_in_catalog	gene: Zm00001d052212	novel	33.64	33.64	coding	292	520	1395
PB.10768.1	9	759	1	incomplete-splice_match	gene: Zm00001d048353	Zm00001d048353_T001	44.35	44.35	coding	79	205	441
PB.2879.2	2	1257	10	full-splice_match	gene: Zm00001d003855	Zm00001d003855_T005	32.73	32.73	coding	228	267	950

PB.7106.4	5	1292	5	novel_in_catalog	gene: Zm00001d018290	novel	7.67	96.25	coding	196	47	634
PB.9936.1	8	2206	2	full-splice_match	gene: Zm00001d012420	Zm00001d012420_T003	13.97	13.97	coding	648	117	2060
PB.8749.2	7	2417	2	genic	gene: Zm00001d021617	novel	5.22	21.03	coding	675	391	2415
PB.5454.3	4	1700	1	full-splice_match	gene: Zm00001d052229	Zm00001d052229_T001	0.00	459.68	coding	263	663	1451
PB.2332.1	10	979	6	novel_not_in_catalog	gene: Zm00001d026572	novel	14.93	14.93	coding	226	74	751
PB.148.1	1	750	1	full-splice_match	gene: Zm00001d027873	Zm00001d027873_T001	32.92	32.92	non_coding	NA	NA	NA
PB.6681.1	5	1924	3	genic	gene: Zm00001d001673	novel	1.76	1.76	coding	492	431	1906
PB.8475.3	7	1631	6	full-splice_match	gene: Zm00001d020426	Zm00001d020426_T001	0.00	13.56	coding	454	162	1523
PB.8475.2	7	1848	6	full-splice_match	gene: Zm00001d020426	Zm00001d020426_T001	13.56	13.56	coding	454	162	1523
PB.671.2	1	1404	9	full-splice_match	gene: Zm00001d030385	Zm00001d030385_T001	0.26	48.91	coding	155	607	1071
PB.671.3	1	886	6	incomplete-splice_match	gene: Zm00001d030385	Zm00001d030385_T007	25.04	48.91	coding	210	13	642
PB.4745.1	3	2225	2	novel_in_catalog	gene: Zm00001d044516	novel	18.14	381.23	coding	297	123	1013
PB.4745.3	3	2310	1	novel_in_catalog	gene: Zm00001d044516	novel	1.09	381.23	coding	334	125	1126
PB.8064.8	6	2081	9	full-splice_match	gene: Zm00001d038929	Zm00001d038929_T001	3.57	332.63	coding	553	228	1886
PB.4745.5	3	1480	3	novel_in_catalog	gene: Zm00001d044516	novel	9.49	381.23	coding	337	126	1136
PB.3059.1	2	1166	8	full-splice_match	gene: Zm00001d005102	Zm00001d005102_T001	90.77	90.77	coding	258	123	896
PB.7999.1	6	1079	2	full-splice_match	gene: Zm00001d038732	Zm00001d038732_T001	12.84	12.84	coding	257	105	875

PB.4745.6	3	1818	2	novel_in_catalog	gene: Zm00001d044516	novel	10.86	381.23	coding	334	127	1128
PB.10934.1	B73V4 ctg68	1623	5	incomplete-splice_match	gene: Zm00001d000430	Zm00001d000430_T001	19.14	19.14	coding	428	109	1392
PB.9986.3	8	1075	1	full-splice_match	gene: Zm00001d012618	Zm00001d012618_T001	38.09	120.31	coding	213	136	774
PB.3043.1	2	2961	13	full-splice_match	gene: Zm00001d005019	Zm00001d005019_T004	89.88	137.41	coding	726	262	2439
PB.9986.2	8	1651	1	full-splice_match	gene: Zm00001d012618	Zm00001d012618_T001	0.00	120.31	coding	213	87	725
PB.4396.1	3	1275	3	full-splice_match	gene: Zm00001d043145	Zm00001d043145_T001	12.83	20.13	coding	227	141	821
PB.5338.8	4	1229	8	full-splice_match	gene: Zm00001d051676	Zm00001d051676_T003	0.42	76.62	coding	300	193	1092
PB.4396.2	3	1065	3	full-splice_match	gene: Zm00001d043145	Zm00001d043145_T001	7.30	20.13	coding	227	58	738
PB.9354.1	8	866	1	full-splice_match	gene: Zm00001d009701	Zm00001d009701_T001	11.77	11.77	coding	246	60	797
PB.7874.1	6	2372	2	full-splice_match	gene: Zm00001d038206	Zm00001d038206_T002	3.76	3.76	coding	608	17	1840
PB.8919.1	7	851	1	full-splice_match	gene: Zm00001d022197	Zm00001d022197_T001	18.32	54.00	coding	160	7	486
PB.5338.3	4	1569	9	full-splice_match	gene: Zm00001d051676	Zm00001d051676_T002	47.51	76.62	coding	332	285	1280
PB.5338.1	4	1734	9	full-splice_match	gene: Zm00001d051676	Zm00001d051676_T002	11.89	76.62	coding	332	282	1277
PB.5338.6	4	2417	8	novel_not_in_catalog	gene: Zm00001d051676	novel	0.23	76.62	coding	261	1447	2229
PB.8919.2	7	768	1	full-splice_match	gene: Zm00001d022197	Zm00001d022197_T001	35.68	54.00	coding	160	54	533
PB.6665.2	5	1665	7	novel_in_catalog	gene: Zm00001d016358	novel	10.80	120.04	coding	317	483	1433
PB.5338.5	4	1465	9	full-splice_match	gene: Zm00001d051676	Zm00001d051676_T002	0.00	76.62	coding	332	285	1280

PB.3527.2	2	2071	3	novel_in_catalog	gene: Zm00001d007446	novel	5.78	490.59	coding	173	141	659
PB.3835.3	3	2021	20	novel_in_catalog	gene: Zm00001d040166	novel	2.99	12.96	coding	527	231	1811
PB.2691.2	2	2241	1	novel_in_catalog	gene: Zm00001d003006	novel	10.31	644.35	coding	210	74	703
PB.2691.5	2	1167	3	full-splice_match	gene: Zm00001d003006	Zm00001d003006_T001	631.85	644.35	coding	285	79	933
PB.2691.4	2	2153	2	novel_in_catalog	gene: Zm00001d003006	novel	2.20	644.35	coding	286	77	934
PB.3527.5	2	1134	5	full-splice_match	gene: Zm00001d007446	Zm00001d007446_T002	73.93	490.59	coding	266	141	938
PB.1626.1	1	2169	1	full-splice_match	gene: Zm00001d034882	Zm00001d034882_T001	3.92	3.92	coding	630	101	1990
PB.7775.2	6	748	1	genic	gene: Zm00001d037903	novel	12.27	182.38	non_coding	NA	NA	NA
PB.1333.2	1	3501	13	full-splice_match	gene: Zm00001d033776	Zm00001d033776_T002	12.47	22.12	coding	995	107	3091
PB.7627.1	6	1541	2	novel_in_catalog	gene: Zm00001d037250	novel	25.30	29.86	coding	441	69	1391
PB.91.1	1	3079	4	full-splice_match	gene: Zm00001d027570	Zm00001d027570_T002	22.12	22.12	coding	251	434	1186
PB.7627.2	6	1600	1	novel_in_catalog	gene: Zm00001d037250	novel	4.56	29.86	coding	276	656	1483
PB.10816.1	9	2429	3	incomplete-splice_match	gene: Zm00001d048510	Zm00001d048510_T006	37.41	47.52	coding	672	94	2109
PB.10816.2	9	3577	3	incomplete-splice_match	gene: Zm00001d048510	Zm00001d048510_T006	10.11	47.52	coding	1034	157	3258
PB.3051.1	2	2569	8	full-splice_match	gene: Zm00001d005072	Zm00001d005072_T002	6.49	6.49	coding	691	159	2231
PB.3723.2	3	1904	1	full-splice_match	gene: Zm00001d039524	Zm00001d039524_T001	9.71	9.71	coding	472	150	1565
PB.6648.2	5	1206	2	full-splice_match	gene: Zm00001d016255	Zm00001d016255_T001	0.00	33.64	coding	251	224	976

PB.4458.2	3	1737	3	full-splice_match	gene: Zm00001d043400	Zm00001d043400_T001	5.69	14.16	coding	524	109	1680
PB.3601.2	2	774	1	full-splice_match	gene: Zm00001d007836	Zm00001d007836_T001	83.91	93.75	coding	170	165	674
PB.903.3	1	2236	1	genic	gene: Zm00001d031813	novel	0.81	37.38	coding	298	153	1046
PB.1449.2	1	553	3	incomplete-splice_match	gene: Zm00001d034313	Zm00001d034313_T003	16.96	59.19	coding	7	359	379
PB.1333.7	1	3181	12	full-splice_match	gene: Zm00001d033776	Zm00001d033776_T003	0.00	22.12	coding	995	137	3121
PB.2865.1	2	2770	2	genic	gene: Zm00001d003791	novel	6.01	6.01	coding	685	60	2114
PB.5665.1	4	2620	11	full-splice_match	gene: Zm00001d053080	Zm00001d053080_T002	12.90	12.90	coding	739	133	2349
PB.4844.3	4	2186	1	novel_in_catalog	gene: Zm00001d048839	novel	3.64	13.43	coding	66	120	317
PB.7154.3	5	2614	17	novel_in_catalog	gene: Zm00001d018423	novel	16.49	55.57	coding	669	81	2087
PB.8790.2	7	2719	1	full-splice_match	gene: Zm00001d021737	Zm00001d021737_T001	5.12	15.26	coding	826	72	2549
PB.8790.1	7	2223	1	full-splice_match	gene: Zm00001d021737	Zm00001d021737_T001	10.14	15.26	coding	596	184	1971
PB.903.5	1	1607	3	full-splice_match	gene: Zm00001d031813	Zm00001d031813_T001	17.00	37.38	coding	435	153	1457
PB.7689.1	6	732	1	intergenic	novelGene_204	novel	815.37	815.37	coding	155	74	538
PB.4024.6	3	1321	5	full-splice_match	gene: Zm00001d041416	Zm00001d041416_T003	0.00	332.91	coding	271	202	1014
PB.903.6	1	1957	2	novel_in_catalog	gene: Zm00001d031813	novel	2.97	37.38	coding	269	153	959
PB.7854.1	6	3892	9	full-splice_match	gene: Zm00001d038156	Zm00001d038156_T008	14.24	40.49	coding	993	387	3365
PB.9255.1	8	939	3	full-splice_match	gene: Zm00001d009118	Zm00001d009118_T001	292.36	292.36	coding	165	224	718

PB.7854.3	6	1622	4	incomplete-splice_match	gene: Zm00001d038156	Zm00001d038156_T017	18.83	40.49	coding	319	76	1032
PB.7854.2	6	3002	9	full-splice_match	gene: Zm00001d038156	Zm00001d038156_T003	7.42	40.49	coding	749	324	2570
PB.7854.5	6	2198	6	incomplete-splice_match	gene: Zm00001d038156	Zm00001d038156_T017	0.00	40.49	coding	647	59	1999
PB.2197.1	10	2349	1	full-splice_match	gene: Zm00001d026040	Zm00001d026040_T001	0.39	27.22	coding	407	151	1371
PB.7434.1	6	1445	2	full-splice_match	gene: Zm00001d036262	Zm00001d036262_T001	4.56	4.56	coding	431	45	1337
PB.6722.3	5	1954	3	novel_in_catalog	gene: Zm00001d016682	novel	1.25	39.88	coding	108	321	644
PB.5213.4	4	2729	5	full-splice_match	gene: Zm00001d050985	Zm00001d050985_T001	25.27	46.80	coding	531	954	2546
PB.6722.1	5	1257	4	novel_in_catalog	gene: Zm00001d016682	novel	29.92	39.88	coding	238	318	1031
PB.721.1	1	1764	2	full-splice_match	gene: Zm00001d030845	Zm00001d030845_T001	14.10	20.33	coding	525	25	1599
PB.10183.1	9	1724	1	full-splice_match	gene: Zm00001d045465	Zm00001d045465_T001	105.25	105.25	coding	462	93	1478
PB.5213.1	4	2140	5	full-splice_match	gene: Zm00001d050985	Zm00001d050985_T004	21.53	46.80	coding	505	184	1698
PB.7758.1	6	2549	16	full-splice_match	gene: Zm00001d037818	Zm00001d037818_T002	25.81	25.81	coding	634	155	2056
PB.3339.1	2	962	1	full-splice_match	gene: Zm00001d006489	Zm00001d006489_T001	79.05	79.05	coding	192	159	734
PB.5680.1	4	1364	5	incomplete-splice_match	gene: Zm00001d053158	Zm00001d053158_T001	6.45	6.45	coding	317	154	1104
PB.2534.1	2	785	6	full-splice_match	gene: Zm00001d002339	Zm00001d002339_T001	1085.41	1085.41	coding	159	112	588
PB.4579.2	3	1860	7	full-splice_match	gene: Zm00001d043841	Zm00001d043841_T002	11.17	11.17	coding	518	150	1703
PB.7426.2	6	1505	4	full-splice_match	gene: Zm00001d036178	Zm00001d036178_T001	9.98	17.99	coding	439	91	1407

PB.8457.3	7	2062	1	full-splice_match	gene: Zm00001d020358	Zm00001d020358_T001	23.81	42.11	coding	561	236	1918
PB.8457.2	7	2207	1	full-splice_match	gene: Zm00001d020358	Zm00001d020358_T001	12.56	42.11	coding	561	236	1918
PB.8457.1	7	2366	1	full-splice_match	gene: Zm00001d020358	Zm00001d020358_T001	5.74	42.11	coding	561	236	1918
PB.7242.1	6	1985	3	full-splice_match	gene: Zm00001d035119	Zm00001d035119_T001	19.63	19.63	coding	443	342	1670
PB.2685.2	2	2205	15	novel_in_catalog	gene: Zm00001d002967	novel	26.35	26.35	coding	264	1211	2002
PB.69.1	1	3256	24	full-splice_match	gene: Zm00001d027487	Zm00001d027487_T017	24.49	24.49	coding	935	145	2949
PB.338.1	1	2990	8	full-splice_match	gene: Zm00001d028687	Zm00001d028687_T001	78.44	100.86	coding	872	80	2695
PB.69.4	1	3151	24	full-splice_match	gene: Zm00001d027487	Zm00001d027487_T017	0.00	24.49	coding	935	156	2960
PB.7343.1	6	1417	2	full-splice_match	gene: Zm00001d035689	Zm00001d035689_T001	133.49	133.49	coding	369	45	1151
PB.5827.1	4	733	6	full-splice_match	gene: Zm00001d053862	Zm00001d053862_T002	112.95	502.97	coding	125	35	409
PB.2554.2	2	2581	1	antisense	novelGene_gene: Zm00001d002406_A	novel	5.38	5.38	coding	200	1678	2277
PB.2886.1	2	1608	2	full-splice_match	gene: Zm00001d003890	Zm00001d003890_T001	28.26	28.26	coding	438	169	1482
PB.2268.2	10	2250	4	novel_in_catalog	gene: Zm00001d026348	novel	4.38	8.17	coding	305	166	1080
PB.6798.1	5	2242	18	novel_not_in_catalog	gene: Zm00001d017046	novel	3.30	3.30	coding	474	383	1804
PB.7288.1	6	924	1	intergenic	novelGene_69	novel	15.78	15.78	non_coding	NA	NA	NA
PB.2858.1	2	725	2	full-splice_match	gene: Zm00001d003760	Zm00001d003760_T001	27.01	27.01	coding	115	106	450
PB.5211.17	4	1623	12	full-splice_match	gene: Zm00001d050970	Zm00001d050970_T003	22.31	68.24	coding	446	90	1427

PB.10431.1	9	1370	11	full-splice_match	gene: Zm00001d046972	Zm00001d046972_T002	32.10	64.09	coding	215	262	906
PB.624.10	1	3199	5	novel_in_catalog	gene: Zm00001d030110	novel	4.54	158.92	coding	424	470	1741
PB.8189.4	7	2024	1	novel_in_catalog	gene: Zm00001d001080	novel	0.00	333.55	non_coding	NA	NA	NA
PB.8101.5	6	1331	7	full-splice_match	gene: Zm00001d039089	Zm00001d039089_T001	116.38	302.45	coding	340	106	1125
PB.7278.2	6	1188	4	full-splice_match	gene: Zm00001d035318	Zm00001d035318_T001	30.22	30.22	coding	263	11	799
PB.10740.2	9	3329	16	full-splice_match	gene: Zm00001d048258	Zm00001d048258_T011	5.25	55.35	coding	780	549	2888
PB.10740.3	9	1363	3	incomplete-splice_match	gene: Zm00001d048258	Zm00001d048258_T018	16.17	55.35	coding	80	91	330
PB.7807.2	6	3119	3	novel_not_in_catalog	gene: Zm00001d038021	novel	11.40	42.10	coding	870	263	2872
PB.8859.1	7	1945	3	full-splice_match	gene: Zm00001d021968	Zm00001d021968_T001	9.65	9.65	coding	538	169	1782
PB.4078.3	3	915	9	novel_not_in_catalog	gene: Zm00001d041691	novel	20.68	70.19	coding	191	126	698
PB.10740.4	9	3236	16	full-splice_match	gene: Zm00001d048258	Zm00001d048258_T011	1.84	55.35	coding	780	603	2942
PB.4078.1	3	1067	9	full-splice_match	gene: Zm00001d041691	Zm00001d041691_T001	49.52	70.19	coding	192	119	694
PB.2169.2	10	2236	1	full-splice_match	gene: Zm00001d025920	Zm00001d025920_T001	0.00	29.83	coding	669	70	2076
PB.7409.1	6	1860	4	incomplete-splice_match	gene: Zm00001d036055	Zm00001d036055_T001	16.59	16.59	coding	380	333	1472
PB.1994.1	10	855	5	full-splice_match	gene: Zm00001d025020	Zm00001d025020_T002	299.99	299.99	coding	157	133	603
PB.9553.1	8	1322	12	full-splice_match	gene: Zm00001d010703	Zm00001d010703_T007	40.03	40.03	coding	282	120	965
PB.1203.1	1	2451	12	novel_not_in_catalog	gene: Zm00001d033159	novel	130.04	228.23	coding	643	170	2098

PB.10783.1	9	824	5	full-splice_match	gene: Zm00001d048402	Zm00001d048402_T001	177.70	274.26	coding	157	3	473
PB.9553.2	8	1138	12	full-splice_match	gene: Zm00001d010703	Zm00001d010703_T007	0.00	40.03	coding	282	109	954
PB.6208.3	5	1280	8	incomplete-splice_match	gene: Zm00001d013984	Zm00001d013984_T009	0.00	18.50	coding	341	257	1279
PB.6208.2	5	2714	12	full-splice_match	gene: Zm00001d013984	Zm00001d013984_T012	18.50	18.50	coding	699	167	2263
PB.7514.2	6	788	6	full-splice_match	gene: Zm00001d036642	Zm00001d036642_T001	57.82	68.90	coding	179	50	586
PB.8662.1	7	2654	2	novel_in_catalog	gene: Zm00001d021299	novel	27.07	113.27	coding	251	75	827
PB.6810.18	5	1206	7	novel_in_catalog	gene: Zm00001d017096	novel	10.32	118.92	coding	77	148	378
PB.6810.19	5	2158	8	full-splice_match	gene: Zm00001d017096	Zm00001d017096_T024	0.00	118.92	coding	219	1257	1913
PB.1788.1	10	554	1	antisense	novelGene_gene: Zm00001d023888_A	novel	11.10	11.10	non_coding	NA	NA	NA
PB.6810.14	5	1605	9	novel_in_catalog	gene: Zm00001d017096	novel	0.00	118.92	coding	180	106	645
PB.6810.15	5	1357	8	novel_in_catalog	gene: Zm00001d017096	novel	13.66	118.92	coding	99	11	307
PB.6810.16	5	2393	6	full-splice_match	gene: Zm00001d017096	Zm00001d017096_T026	0.00	118.92	coding	129	106	492
PB.10618.3	9	1836	10	full-splice_match	gene: Zm00001d047812	Zm00001d047812_T001	159.82	159.82	coding	466	254	1651
PB.4183.1	3	1963	6	full-splice_match	gene: Zm00001d042152	Zm00001d042152_T005	19.47	28.29	coding	458	394	1767
PB.6810.11	5	1979	9	full-splice_match	gene: Zm00001d017096	Zm00001d017096_T020	0.00	118.92	coding	219	1077	1733
PB.4183.3	3	2348	4	full-splice_match	gene: Zm00001d042152	Zm00001d042152_T008	8.82	28.29	coding	304	43	954
PB.6810.13	5	2242	7	full-splice_match	gene: Zm00001d017096	Zm00001d017096_T015	2.64	118.92	coding	129	106	492

PB.8824.9	7	1770	5	genic	gene: Zm00001d021845	novel	3.36	43.34	coding	151	104	556
PB.8447.1	7	1873	7	novel_not_in_catalog	gene: Zm00001d020309	novel	37.24	42.78	coding	476	119	1546
PB.2395.2	2	1407	11	full-splice_match	gene: Zm00001d001789	Zm00001d001789_T001	62.12	73.51	coding	249	384	1130
PB.203.3	1	2119	2	novel_in_catalog	gene: Zm00001d028160	novel	1.95	25.67	coding	205	122	736
PB.2395.5	2	915	11	full-splice_match	gene: Zm00001d001789	Zm00001d001789_T001	11.40	73.51	coding	249	26	772
PB.203.1	1	1134	3	full-splice_match	gene: Zm00001d028160	Zm00001d028160_T001	23.72	25.67	coding	206	122	739
PB.3325.1	2	1868	4	novel_in_catalog	gene: Zm00001d006440	novel	4.55	133.58	coding	309	198	1124
PB.2506.2	2	1639	6	novel_in_catalog	gene: Zm00001d002244	novel	3.85	217.68	coding	278	68	901
PB.2850.3	2	2467	10	novel_in_catalog	gene: Zm00001d003678	novel	3.42	10.39	coding	364	1133	2224
PB.8824.3	7	1649	8	genic	gene: Zm00001d021845	novel	3.57	43.34	coding	129	55	441
PB.2506.1	2	1785	7	full-splice_match	gene: Zm00001d002244	Zm00001d002244_T004	213.83	217.68	coding	432	158	1453
PB.8824.5	7	1348	8	novel_not_in_catalog	gene: Zm00001d021845	novel	8.24	43.34	coding	151	167	619
PB.10863.1	B73V4 ctg150	2149	2	full-splice_match	gene: Zm00001d000001	Zm00001d000001_T002	37.50	37.50	coding	613	74	1912
PB.8824.7	7	867	7	novel_not_in_catalog	gene: Zm00001d021845	novel	5.28	43.34	non_coding	NA	NA	NA
PB.8824.6	7	1872	6	genic	gene: Zm00001d021845	novel	11.88	43.34	coding	151	203	655
PB.771.1	1	3953	21	full-splice_match	gene: Zm00001d031088	Zm00001d031088_T001	33.76	33.76	coding	996	269	3256
PB.6696.3	5	2172	3	full-splice_match	gene: Zm00001d016553	Zm00001d016553_T001	14.65	140.34	coding	364	627	1718

PB.8029.3	6	1996	7	novel_in_catalog	gene: Zm00001d038832	novel	199.90	199.90	coding	200	1125	1724
PB.1342.5	1	3367	5	novel_not_in_catalog	gene: Zm00001d033799	novel	3.68	73.47	coding	912	327	3062
PB.10002.1	8	1604	3	full-splice_match	gene: Zm00001d012662	Zm00001d012662_T002	51.40	51.40	coding	421	116	1378
PB.10847.2	B73V4 ctg107	1900	6	novel_in_catalog	gene: Zm00001d000302	novel	6.96	358.42	coding	181	246	788
PB.10847.1	B73V4 ctg107	1514	8	full-splice_match	gene: Zm00001d000302	Zm00001d000302_T001	344.12	358.42	coding	290	257	1126
PB.8568.1	7	599	1	full-splice_match	gene: Zm00001d020869	Zm00001d020869_T001	58.53	58.53	coding	68	163	366
PB.10847.4	B73V4 ctg107	1366	7	novel_in_catalog	gene: Zm00001d000302	novel	7.34	358.42	coding	200	23	622
PB.7584.1	6	662	3	intergenic	novelGene_184	novel	100.60	100.60	coding	164	13	504
PB.5459.2	4	1856	8	novel_in_catalog	gene: Zm00001d052237	novel	1.65	26.36	coding	378	679	1812
PB.10369.4	9	2269	1	full-splice_match	gene: Zm00001d046599	Zm00001d046599_T001	0.00	400.31	coding	204	289	900
PB.5993.1	5	1625	2	genic	gene: Zm00001d013087	novel	5.24	5.24	coding	195	106	690
PB.10599.2	9	1471	6	full-splice_match	gene: Zm00001d047758	Zm00001d047758_T004	13.44	168.10	coding	374	151	1272
PB.5132.4	4	1822	4	novel_not_in_catalog	gene: Zm00001d050371	novel	6.78	10.99	coding	532	54	1649
PB.5132.1	4	1938	4	novel_not_in_catalog	gene: Zm00001d050371	novel	4.21	10.99	coding	532	55	1650
PB.3674.1	3	2095	7	full-splice_match	gene: Zm00001d039363	Zm00001d039363_T001	14.42	24.33	coding	428	241	1524
PB.716.1	1	2988	5	full-splice_match	gene: Zm00001d030784	Zm00001d030784_T001	3.31	4.35	coding	792	237	2612
PB.3674.3	3	1868	7	full-splice_match	gene: Zm00001d039363	Zm00001d039363_T001	9.91	24.33	coding	428	116	1399

PB.1342.4	1	3528	4	full-splice_match	gene: Zm00001d033799	Zm00001d033799_T035	4.65	73.47	coding	912	215	2950
PB.3674.5	3	1743	7	full-splice_match	gene: Zm00001d039363	Zm00001d039363_T001	0.00	24.33	coding	428	235	1518
PB.4224.1	3	1062	5	full-splice_match	gene: Zm00001d042350	Zm00001d042350_T003	29.25	46.82	coding	234	118	819
PB.3841.1	3	1952	8	full-splice_match	gene: Zm00001d040191	Zm00001d040191_T001	62.65	62.65	coding	379	345	1481
PB.3674.6	3	1452	7	full-splice_match	gene: Zm00001d039363	Zm00001d039363_T001	0.00	24.33	coding	428	114	1397
PB.10084.1	9	872	1	full-splice_match	gene: Zm00001d045022	Zm00001d045022_T001	0.87	3.32	coding	116	136	483
PB.1863.1	10	1551	5	full-splice_match	gene: Zm00001d024429	Zm00001d024429_T002	8.34	8.34	coding	424	43	1314
PB.10084.2	9	656	1	full-splice_match	gene: Zm00001d045022	Zm00001d045022_T001	2.45	3.32	coding	116	140	487
PB.10702.9	9	1862	7	full-splice_match	gene: Zm00001d048100	Zm00001d048100_T001	48.82	435.97	coding	475	115	1539
PB.5828.1	4	590	5	full-splice_match	gene: Zm00001d053863	Zm00001d053863_T001	2467.27	2468.45	non_coding	NA	NA	NA
PB.1288.1	1	2512	1	antisense	novelGene_gene: Zm00001d033571_A	novel	9.73	9.73	coding	540	26	1645
PB.10625.1	9	1752	4	full-splice_match	gene: Zm00001d047835	Zm00001d047835_T004	114.27	314.58	coding	323	130	1098
PB.732.2	1	3081	8	full-splice_match	gene: Zm00001d030916	Zm00001d030916_T001	6.28	31.90	coding	878	196	2829
PB.6955.2	5	2483	2	incomplete-splice_match	gene: Zm00001d017721	Zm00001d017721_T002	7.53	13.95	coding	613	15	1853
PB.3614.1	2	1460	4	full-splice_match	gene: Zm00001d007888	Zm00001d007888_T003	19.14	19.14	coding	399	69	1265
PB.10369.2	9	2757	1	full-splice_match	gene: Zm00001d046599	Zm00001d046599_T001	10.87	400.31	coding	204	309	920
PB.926.7	1	2671	12	novel_in_catalog	gene: Zm00001d031927	novel	10.49	74.67	coding	102	1617	1922

PB.6944.1	5	1485	1	antisense	novelGene_gene: Zm00001d017671_A	novel	10.53	10.53	coding	341	117	1139
PB.926.4	1	2333	11	novel_in_catalog	gene: Zm00001d031927	novel	0.00	74.67	coding	144	909	1340
PB.9839.1	8	1290	3	full-splice_match	gene: Zm00001d011969	Zm00001d011969_T001	42.08	42.08	coding	259	166	942
PB.3318.1	2	1076	8	full-splice_match	gene: Zm00001d006409	Zm00001d006409_T002	32.00	32.00	coding	271	96	908
PB.8545.1	7	3609	1	genic_intron	novelGene_207	novel	13.86	13.86	coding	732	169	2364
PB.6955.1	5	3533	4	full-splice_match	gene: Zm00001d017721	Zm00001d017721_T001	6.42	13.95	coding	822	389	2854
PB.9470.1	8	954	4	full-splice_match	gene: Zm00001d010290	Zm00001d010290_T001	192.88	192.88	coding	176	137	664
PB.5491.1	4	2486	12	full-splice_match	gene: Zm00001d052354	Zm00001d052354_T005	4.04	26.18	coding	623	277	2145
PB.8823.3	7	1337	1	novel_in_catalog	gene: Zm00001d021839	novel	0.00	122.88	coding	331	137	1129
PB.8226.1	7	2373	3	full-splice_match	gene: Zm00001d018911	Zm00001d018911_T001	186.97	186.97	coding	607	159	1979
PB.1924.1	10	584	1	full-splice_match	gene: Zm00001d024749	Zm00001d024749_T001	63.03	63.03	coding	92	93	368
PB.8226.2	7	2322	3	full-splice_match	gene: Zm00001d018911	Zm00001d018911_T001	0.00	186.97	coding	607	159	1979
PB.538.2	1	1127	2	full-splice_match	gene: Zm00001d029601	Zm00001d029601_T002	31.82	31.82	coding	217	137	787
PB.6161.1	5	498	2	novel_in_catalog	gene: Zm00001d013721	novel	74.96	74.96	coding	103	41	349
PB.538.1	1	1301	1	genic	gene: Zm00001d029602	novel	14.71	14.71	coding	257	409	1179
PB.3402.1	2	1739	7	full-splice_match	gene: Zm00001d006763	Zm00001d006763_T002	19.13	19.13	coding	424	294	1565
PB.9558.1	8	3087	10	incomplete-splice_match	gene: Zm00001d010719	Zm00001d010719_T006	41.48	41.48	coding	743	691	2919

PB.4465.1	3	1020	1	full-splice_match	gene: Zm00001d043422	Zm00001d043422_T001	126.14	126.14	coding	241	85	807
PB.73.1	1	339	4	incomplete-splice_match	gene: Zm00001d027505	Zm00001d027505_T001	29.53	29.53	non_coding	NA	NA	NA
PB.1574.1	1	1727	4	full-splice_match	gene: Zm00001d034729	Zm00001d034729_T002	17.29	26.62	coding	491	59	1531
PB.3309.1	2	1370	5	full-splice_match	gene: Zm00001d006368	Zm00001d006368_T003	63.97	63.97	coding	246	390	1127
PB.10474.14	9	3077	14	novel_in_catalog	gene: Zm00001d047253	novel	18.54	821.31	coding	759	164	2440
PB.3646.2	3	1213	1	full-splice_match	gene: Zm00001d039279	Zm00001d039279_T001	5.74	33.87	coding	304	49	960
PB.3646.3	3	1059	1	full-splice_match	gene: Zm00001d039279	Zm00001d039279_T001	18.09	33.87	coding	304	54	965
PB.764.1	1	3313	3	novel_not_in_catalog	gene: Zm00001d031073	novel	0.00	18.12	coding	735	370	2574
PB.3646.1	3	2054	1	full-splice_match	gene: Zm00001d039279	Zm00001d039279_T001	10.04	33.87	coding	304	54	965
PB.6646.1	5	2073	5	full-splice_match	gene: Zm00001d016234	Zm00001d016234_T001	24.73	24.73	coding	533	267	1865
PB.10474.18	9	3270	14	full-splice_match	gene: Zm00001d047253	Zm00001d047253_T003	2.03	821.31	coding	816	615	3062
PB.4821.1	4	1907	3	novel_in_catalog	gene: Zm00001d048702	novel	8.74	8.74	coding	536	50	1657
PB.5336.1	4	991	3	full-splice_match	gene: Zm00001d051672	Zm00001d051672_T001	39.88	39.88	coding	167	183	683
PB.2275.1	10	1282	6	full-splice_match	gene: Zm00001d026363	Zm00001d026363_T001	64.89	64.89	coding	284	111	962
PB.7582.1	6	2431	4	novel_not_in_catalog	gene: Zm00001d037056	novel	69.04	69.04	coding	568	610	2313
PB.3225.1	2	1527	6	genic	gene: Zm00001d006026	novel	14.99	14.99	coding	410	91	1320
PB.10321.1	9	1660	7	full-splice_match	gene: Zm00001d046352	Zm00001d046352_T001	150.65	783.40	coding	417	167	1417

PB.10321.2	9	1559	7	full-splice_match	gene: Zm00001d046352	Zm00001d046352_T001	632.74	783.40	coding	417	169	1419
PB.7693.1	6	915	2	genic	gene: Zm00001d037574	novel	47.28	47.28	coding	175	124	648
PB.9309.1	8	1459	2	novel_in_catalog	gene: Zm00001d009473	novel	12.80	12.80	coding	164	25	516
PB.994.1	1	701	5	full-splice_match	gene: Zm00001d032232	Zm00001d032232_T006	50.52	87.14	coding	114	162	503
PB.6550.1	5	2590	7	incomplete-splice_match	gene: Zm00001d015504	Zm00001d015504_T014	114.72	114.72	coding	222	1436	2101
PB.994.3	1	964	4	full-splice_match	gene: Zm00001d032232	Zm00001d032232_T009	10.70	87.14	coding	125	284	658
PB.8845.1	7	630	4	full-splice_match	gene: Zm00001d021913	Zm00001d021913_T002	67.92	67.92	coding	121	102	464
PB.10714.2	9	2049	6	novel_not_in_catalog	gene: Zm00001d048152	novel	10.29	10.29	coding	576	168	1895
PB.1374.1	1	1527	11	incomplete-splice_match	gene: Zm00001d033945	Zm00001d033945_T003	52.81	52.81	coding	420	164	1423
PB.5710.1	4	1310	1	incomplete-splice_match	gene: Zm00001d053356	Zm00001d053356_T002	23.76	23.76	coding	322	69	1034
PB.10102.1	9	2301	5	full-splice_match	gene: Zm00001d045082	Zm00001d045082_T002	14.15	14.15	coding	534	454	2055
PB.8550.1	7	920	7	full-splice_match	gene: Zm00001d020783	Zm00001d020783_T008	53.37	133.83	coding	182	109	654
PB.10629.1	9	2314	3	full-splice_match	gene: Zm00001d047848	Zm00001d047848_T001	45.34	46.93	coding	591	153	1925
PB.8550.3	7	873	7	full-splice_match	gene: Zm00001d020783	Zm00001d020783_T008	46.08	133.83	coding	182	109	654
PB.8550.2	7	911	7	full-splice_match	gene: Zm00001d020783	Zm00001d020783_T016	34.38	133.83	coding	182	127	672
PB.9315.4	8	1960	9	full-splice_match	gene: Zm00001d009488	Zm00001d009488_T001	0.00	350.05	coding	553	101	1759
PB.9315.1	8	2207	9	full-splice_match	gene: Zm00001d009488	Zm00001d009488_T001	116.89	350.05	coding	553	101	1759

PB.9315.2	8	2032	9	full-splice_match	gene: Zm00001d009488	Zm00001d009488_T001	233.16	350.05	coding	553	85	1743
PB.6453.1	5	2691	5	novel_in_catalog	gene: Zm00001d015007	novel	4.15	4.15	coding	683	167	2215
PB.3043.3	2	2666	13	full-splice_match	gene: Zm00001d005019	Zm00001d005019_T004	0.00	137.41	coding	726	262	2439
[truncated: 518,018 more chars]
