# Supplementary material for: High-resolution profile of transcriptomes reveals a role of alternative splicing for modulating response to nitrogen in maize
Source: BMC Genomics. 2020 May 11;21:353. doi: 10.1186/s12864-020-6769-8 (PMC7216474; doi:10.1186/s12864-020-6769-8)
Supplement: Supplementary file 16 — Additional file 16: Table S10. The profile of high-confidence isoforms identified in the nitrate-treated sample. [file 12864_2020_6769_MOESM16_ESM.pdf]

Supplemental Table S10: The profile of high-confidence isoforms identified in the nitrate-treated sample.

Isoform	Chr	Length	Exons	Structural Category	Associated Gene	Associated Transcript	Iso Exp	Gene Exp	Coding	ORF Length	CDS Start	CDS End
PB.10565.2	9	1221	2	full-splice_match	gene: Zm00001d047194	Zm00001d047194_T00	0.00	22.63	coding	362	51	1136
PB.10565.1	9	1368	2	full-splice_match	gene: Zm00001d047194	Zm00001d047194_T00	22.63	22.63	coding	362	50	1135
PB.1297.1	1	3378	5	novel_in_catalog	gene: Zm00001d033378	novel	135.54	135.54	coding	358	1984	3057
PB.9776.3	8	1672	5	novel_not_in_catalog	gene: Zm00001d011436	novel	4.21	16.29	non coding	NA	NA	NA
PB.1591.1	1	1777	3	incomplete-splice_match	gene: Zm00001d034636	Zm00001d034636_T00	29.09	61.52	coding	437	60	1370
PB.5575.1	4	2518	18	full-splice_match	gene: Zm00001d052425	Zm00001d052425_T01	35.90	73.52	coding	694	269	2350
PB.7470.1	6	1000	1	full-splice_match	gene: Zm00001d035963	Zm00001d035963_T00	40.40	40.40	coding	246	110	847
PB.3189.1	2	1343	2	full-splice_match	gene: Zm00001d005684	Zm00001d005684_T00	31.17	31.17	coding	172	723	1238
PB.10283.1	9	2760	6	novel_not_in_catalog	gene: Zm00001d045499	novel	6.19	53.54	coding	326	1477	2454
PB.10283.2	9	1810	7	novel_not_in_catalog	gene: Zm00001d045499	novel	45.01	53.54	coding	408	301	1524
PB.10283.3	9	1982	6	novel_not_in_catalog	gene: Zm00001d045499	novel	2.34	53.54	coding	328	410	1393
PB.6488.1	5	1936	16	full-splice_match	gene: Zm00001d014912	Zm00001d014912_T00	6.37	23.07	coding	488	131	1594
PB.6488.2	5	1766	16	full-splice_match	gene: Zm00001d014912	Zm00001d014912_T00	16.70	23.07	coding	488	140	1603
PB.11017.4	B73V4	2245	2	full-splice_match	gene: Zm00001d000408	Zm00001d000408_T00	0.00	59.16	coding	626	33	1910
PB.8351.1	7	1618	1	full-splice_match	gene: Zm00001d019269	Zm00001d019269_T00	3.09	3.09	coding	438	61	1374

PB.11017.2	B73V4	2787	2	full-splice_match	gene: Zm00001d000408	Zm00001d000408_T00	0.00	59.16	coding	626	252	2129
PB.11017.3	B73V4	2510	2	full-splice_match	gene: Zm00001d000408	Zm00001d000408_T00	0.39	59.16	coding	626	253	2130
PB.11017.1	B73V4	2902	2	full-splice_match	gene: Zm00001d000408	Zm00001d000408_T00	58.78	59.16	coding	626	251	2128
PB.3396.16	2	1841	6	novel_in_catalog	gene: Zm00001d006578	novel	6.35	210.21	coding	312	347	1282
PB.7339.1	6	1991	7	incomplete-splice_match	gene: Zm00001d035191	Zm00001d035191_T00	4.91	4.91	coding	507	207	1727
PB.10650.2	9	3101	1	full-splice_match	gene: Zm00001d047533	Zm00001d047533_T00	94.48	99.91	coding	888	216	2879
PB.240.1	1	1726	1	full-splice_match	gene: Zm00001d028275	Zm00001d028275_T00	6.54	6.54	coding	472	110	1525
PB.5555.1	4	1227	1	full-splice_match	gene: Zm00001d052371	Zm00001d052371_T00	0.32	7.97	coding	220	114	773
PB.1280.1	1	2086	16	full-splice_match	gene: Zm00001d033309	Zm00001d033309_T00	44.23	44.23	coding	478	407	1840
PB.4086.1	3	3724	14	full-splice_match	gene: Zm00001d041497	Zm00001d041497_T00	15.70	15.70	coding	888	705	3368
PB.1151.2	1	2126	15	full-splice_match	gene: Zm00001d032715	Zm00001d032715_T00	8.64	10.67	coding	481	410	1852
PB.7847.1	6	1166	14	full-splice_match	gene: Zm00001d037925	Zm00001d037925_T00	39.08	41.20	coding	276	61	888
PB.4689.1	3	1687	1	genic	gene: Zm00001d044106	novel	40.40	48.22	coding	295	603	1487
PB.4689.2	3	1110	1	genic	gene: Zm00001d044106	novel	7.82	48.22	coding	296	140	1027
PB.7847.2	6	1809	12	full-splice_match	gene: Zm00001d037925	Zm00001d037925_T01	2.12	41.20	coding	208	964	1587
PB.2476.3	2	2287	2	novel_in_catalog	gene: Zm00001d001979	novel	10.33	235.86	coding	349	1027	2073
PB.2999.8	2	3220	19	full-splice_match	gene: Zm00001d004564	Zm00001d004564_T00	0.00	65.73	coding	565	1240	2934

PB.2476.1	2	1761	3	novel_in_catalog	gene: Zm00001d001979	novel	162.69	235.86	coding	349	423	1469
PB.2476.5	2	1469	3	novel_not_in_catalog	gene: Zm00001d001979	novel	62.84	235.86	coding	349	304	1350
PB.5267.1	4	1877	12	full-splice_match	gene: Zm00001d051043	Zm00001d051043_T00	7.28	29.99	coding	483	45	1493
PB.4346.1	3	1353	8	incomplete-splice_match	gene: Zm00001d042794	Zm00001d042794_T00	21.12	21.12	coding	306	237	1154
PB.250.4	1	3098	16	novel_in_catalog	gene: Zm00001d028332	novel	4.69	56.74	coding	640	946	2865
PB.9017.1	7	2139	3	novel_in_catalog	gene: Zm00001d022342	novel	7.38	29.33	coding	169	1362	1868
PB.250.2	1	3559	17	novel_in_catalog	gene: Zm00001d028332	novel	14.71	56.74	coding	794	942	3323
PB.250.1	1	3273	17	full-splice_match	gene: Zm00001d028332	Zm00001d028332_T01	37.34	56.74	coding	775	640	2964
PB.3396.5	2	1889	2	novel_in_catalog	gene: Zm00001d006578	novel	40.47	210.21	coding	143	78	506
PB.10418.2	9	1967	1	full-splice_match	gene: Zm00001d046378	Zm00001d046378_T00	12.16	836.48	coding	138	339	752
PB.10418.3	9	1251	2	genic	gene: Zm00001d046378	novel	259.58	836.48	coding	49	153	299
PB.7231.1	5	534	2	full-splice_match	gene: Zm00001d018410	Zm00001d018410_T00	197.67	723.73	non coding	NA	NA	NA
PB.10418.1	9	1917	2	genic	gene: Zm00001d046378	novel	403.33	836.48	coding	138	288	701
PB.10418.6	9	1420	2	genic	gene: Zm00001d046378	novel	0.00	836.48	coding	138	251	664
PB.10418.7	9	1460	1	full-splice_match	gene: Zm00001d046378	Zm00001d046378_T00	0.00	836.48	coding	138	288	701
PB.10418.4	9	1468	3	genic	gene: Zm00001d046378	novel	4.29	836.48	coding	138	200	613
PB.10418.5	9	1522	2	genic	gene: Zm00001d046378	novel	157.12	836.48	coding	138	197	610

PB.9473.2	8	1848	1	novel_in_catalog	gene: Zm00001d010053	novel	6.12	64.01	coding	204	181	792
PB.7267.1	5	902	5	full-splice_match	gene: Zm00001d018511	Zm00001d018511_T00	11.94	44.35	non coding	NA	NA	NA
PB.10418.8	9	1167	2	genic	gene: Zm00001d046378	novel	0.00	836.48	coding	138	200	613
PB.9473.1	8	911	3	full-splice_match	gene: Zm00001d010053	Zm00001d010053_T00	57.90	64.01	coding	154	246	707
PB.7258.3	5	1795	13	full-splice_match	gene: Zm00001d018487	Zm00001d018487_T00	10.19	205.91	coding	328	113	1096
PB.2975.1	2	1477	1	full-splice_match	gene: Zm00001d004439	Zm00001d004439_T00	8.57	8.57	coding	359	166	1242
PB.2078.1	10	2019	5	novel_in_catalog	gene: Zm00001d025305	novel	181.48	367.37	coding	401	558	1760
PB.6291.1	5	2727	9	full-splice_match	gene: Zm00001d014124	Zm00001d014124_T00	175.81	180.46	coding	811	99	2531
PB.5846.3	4	1589	11	full-splice_match	gene: Zm00001d053763	Zm00001d053763_T00	26.42	39.76	coding	388	257	1420
PB.6291.3	5	3948	7	novel_in_catalog	gene: Zm00001d014124	novel	4.65	180.46	coding	627	1086	2966
PB.6291.2	5	2613	9	full-splice_match	gene: Zm00001d014124	Zm00001d014124_T00	0.00	180.46	coding	811	95	2527
PB.9157.1	8	2173	11	full-splice_match	gene: Zm00001d008345	Zm00001d008345_T00	137.02	137.02	coding	527	142	1722
PB.2110.1	10	907	1	incomplete-splice_match	gene: Zm00001d025476	Zm00001d025476_T00	1.69	1.69	coding	68	144	347
PB.6006.1	5	1692	4	novel_in_catalog	gene: Zm00001d012983	novel	28.22	28.22	coding	471	95	1507
PB.10687.4	9	1219	3	novel_not_in_catalog	gene: Zm00001d047676	novel	0.00	125.61	coding	161	399	881
PB.1074.3	1	3180	9	full-splice_match	gene: Zm00001d032449	Zm00001d032449_T00	4.96	57.57	coding	756	243	2510
PB.10739.1	9	1585	7	full-splice_match	gene: Zm00001d047838	Zm00001d047838_T00	10.72	10.72	coding	335	274	1278

PB.3396.14	2	1800	7	novel_in_catalog	gene: Zm00001d006578	novel	4.95	210.21	coding	293	305	1183
PB.9508.5	8	694	2	novel_not_in_catalog	gene: Zm00001d010244	novel	3.04	141.12	coding	104	157	468
PB.9508.4	8	647	3	novel_not_in_catalog	gene: Zm00001d010244	novel	17.71	141.12	coding	104	150	461
PB.1521.1	1	3261	9	full-splice_match	gene: Zm00001d034387	Zm00001d034387_T01	29.80	77.03	coding	784	615	2966
PB.5569.3	4	2211	5	full-splice_match	gene: Zm00001d052405	Zm00001d052405_T00	5.97	38.06	coding	253	1089	1847
PB.9508.3	8	777	3	novel_not_in_catalog	gene: Zm00001d010244	novel	88.42	141.12	coding	104	152	463
PB.9508.2	8	914	3	novel_not_in_catalog	gene: Zm00001d010244	novel	31.95	141.12	coding	104	151	462
PB.3082.4	2	1654	4	full-splice_match	gene: Zm00001d005092	Zm00001d005092_T00	62.33	62.33	coding	472	120	1535
PB.9084.1	7	1810	10	full-splice_match	gene: Zm00001d022542	Zm00001d022542_T00	26.98	154.27	coding	333	259	1257
PB.9084.2	7	1515	9	full-splice_match	gene: Zm00001d022542	Zm00001d022542_T00	10.27	154.27	coding	326	178	1155
PB.9084.3	7	1541	8	full-splice_match	gene: Zm00001d022542	Zm00001d022542_T00	30.23	154.27	coding	311	164	1096
PB.10293.3	9	497	2	novel_not_in_catalog	gene: Zm00001d045520	novel	124.83	205.00	non coding	NA	NA	NA
PB.10293.2	9	622	2	novel_not_in_catalog	gene: Zm00001d045520	novel	69.33	205.00	non coding	NA	NA	NA
PB.10293.1	9	716	2	novel_not_in_catalog	gene: Zm00001d045520	novel	10.84	205.00	non coding	NA	NA	NA
PB.2969.3	2	3064	14	full-splice_match	gene: Zm00001d004396	Zm00001d004396_T01	3.84	49.36	coding	601	805	2607
PB.5549.1	4	1918	6	novel_in_catalog	gene: Zm00001d052346	novel	29.00	38.28	coding	179	16	552
PB.2969.1	2	3522	19	full-splice_match	gene: Zm00001d004396	Zm00001d004396_T04	45.52	49.36	coding	988	75	3038

PB.6882.3	5	3976	17	novel_not_in_catalog	gene: Zm00001d017046	novel	0.37	3.69	coding	326	427	1404
PB.6882.2	5	1982	18	novel_not_in_catalog	gene: Zm00001d017046	novel	3.32	3.69	coding	518	133	1686
PB.1362.1	1	2015	11	full-splice_match	gene: Zm00001d033651	Zm00001d033651_T00	21.39	24.56	coding	564	114	1805
PB.4647.1	3	703	8	full-splice_match	gene: Zm00001d043905	Zm00001d043905_T00	747.00	747.00	coding	153	4	462
PB.7791.3	6	541	3	full-splice_match	gene: Zm00001d037663	Zm00001d037663_T00	231.60	343.20	coding	99	89	385
PB.7791.2	6	628	3	full-splice_match	gene: Zm00001d037663	Zm00001d037663_T00	0.00	343.20	coding	99	147	443
PB.7791.1	6	810	3	full-splice_match	gene: Zm00001d037663	Zm00001d037663_T00	111.60	343.20	coding	99	148	444
PB.1362.4	1	1952	11	full-splice_match	gene: Zm00001d033651	Zm00001d033651_T00	0.00	24.56	coding	564	126	1817
PB.4562.11	3	3094	13	full-splice_match	gene: Zm00001d043613	Zm00001d043613_T00	4.24	178.80	coding	300	1696	2595
PB.8492.1	7	1411	12	full-splice_match	gene: Zm00001d020234	Zm00001d020234_T00	42.91	42.91	coding	381	30	1172
PB.5238.1	4	1084	10	full-splice_match	gene: Zm00001d050874	Zm00001d050874_T00	188.89	188.89	coding	251	125	877
PB.448.2	1	1857	8	full-splice_match	gene: Zm00001d029174	Zm00001d029174_T00	101.97	135.48	coding	545	85	1719
PB.448.3	1	1534	8	full-splice_match	gene: Zm00001d029174	Zm00001d029174_T00	0.00	135.48	coding	483	85	1533
PB.609.3	1	1135	5	full-splice_match	gene: Zm00001d030009	Zm00001d030009_T00	56.63	159.80	coding	302	18	923
PB.448.1	1	771	3	incomplete-splice_match	gene: Zm00001d029174	Zm00001d029174_T00	33.51	135.48	coding	122	151	516
PB.8402.1	7	980	2	full-splice_match	gene: Zm00001d019597	Zm00001d019597_T00	271.73	494.98	coding	182	115	660
PB.609.4	1	1324	5	full-splice_match	gene: Zm00001d030009	Zm00001d030009_T00	9.26	159.80	coding	321	169	1131

PB.448.4	1	1436	8	full-splice_match	gene: Zm00001d029174	Zm00001d029174_T00	0.00	135.48	coding	449	90	1436
PB.8402.2	7	868	2	full-splice_match	gene: Zm00001d019597	Zm00001d019597_T00	223.25	494.98	coding	182	115	660
PB.8333.3	7	1396	9	incomplete-splice_match	gene: Zm00001d019172	Zm00001d019172_T00	0.00	55.19	coding	343	152	1180
PB.8333.1	7	1499	9	novel_in_catalog	gene: Zm00001d019172	novel	38.52	55.19	coding	343	114	1142
PB.8333.4	7	1315	9	incomplete-splice_match	gene: Zm00001d019172	Zm00001d019172_T00	16.68	55.19	coding	343	151	1179
PB.9964.1	8	1919	4	novel_not_in_catalog	gene: Zm00001d012223	novel	15.91	15.91	coding	426	375	1652
PB.4992.2	4	1304	9	full-splice_match	gene: Zm00001d049333	Zm00001d049333_T00	1.29	29.22	coding	262	175	960
PB.4992.1	4	1335	9	full-splice_match	gene: Zm00001d049333	Zm00001d049333_T00	27.93	29.22	coding	262	66	851
PB.10422.4	9	2520	3	novel_in_catalog	gene: Zm00001d046399	novel	3.26	60.23	coding	263	188	976
PB.4534.1	3	1687	2	full-splice_match	gene: Zm00001d043520	Zm00001d043520_T00	5.08	5.08	coding	447	84	1424
PB.8271.1	7	1215	1	full-splice_match	gene: Zm00001d018857	Zm00001d018857_T00	194.19	194.19	coding	328	48	1031
PB.6038.1	5	1336	7	full-splice_match	gene: Zm00001d013072	Zm00001d013072_T00	49.72	49.72	coding	320	142	1101
PB.6038.2	5	889	7	full-splice_match	gene: Zm00001d013072	Zm00001d013072_T00	0.00	49.72	coding	282	44	889
PB.9613.2	8	1010	1	full-splice_match	gene: Zm00001d010676	Zm00001d010676_T00	27.03	57.96	coding	205	230	844
PB.8934.2	7	2392	9	full-splice_match	gene: Zm00001d022038	Zm00001d022038_T01	11.35	11.35	coding	613	281	2119
PB.8426.1	7	1135	3	novel_not_in_catalog	gene: Zm00001d019809	novel	12.52	12.52	coding	217	212	862
PB.9613.1	8	994	1	full-splice_match	gene: Zm00001d010676	Zm00001d010676_T00	30.93	57.96	coding	205	81	695

PB.9976.2	8	2388	3	intergenic	novelGene_349	novel	8.30	34.66	coding	692	221	2296
PB.5058.1	4	1764	5	full-splice_match	gene: Zm00001d049661	Zm00001d049661_T00	58.27	58.27	coding	375	210	1334
PB.6285.1	5	1460	9	full-splice_match	gene: Zm00001d014094	Zm00001d014094_T00	20.98	20.98	coding	357	208	1278
PB.5487.1	4	2249	9	full-splice_match	gene: Zm00001d052147	Zm00001d052147_T00	34.70	52.96	coding	525	271	1845
PB.8622.1	7	2507	5	novel_in_catalog	gene: Zm00001d020881	novel	10.04	74.69	coding	416	403	1650
PB.9941.1	8	1414	2	full-splice_match	gene: Zm00001d012103	Zm00001d012103_T00	545.36	1084.73	coding	355	122	1186
PB.8622.3	7	2415	6	full-splice_match	gene: Zm00001d020881	Zm00001d020881_T00	48.76	74.69	coding	583	407	2155
PB.7704.4	6	2158	11	novel_not_in_catalog	gene: Zm00001d037257	novel	37.74	132.91	coding	441	349	1671
PB.8622.5	7	2232	6	full-splice_match	gene: Zm00001d020881	Zm00001d020881_T00	0.00	74.69	coding	583	404	2152
PB.8622.4	7	2337	5	novel_in_catalog	gene: Zm00001d020881	novel	0.00	74.69	coding	416	363	1610
PB.8622.6	7	2397	4	novel_in_catalog	gene: Zm00001d020881	novel	3.23	74.69	coding	163	1231	1719
PB.713.2	1	870	5	incomplete-splice_match	gene: Zm00001d030657	Zm00001d030657_T00	81.60	91.63	non coding	NA	NA	NA
PB.3806.1	3	1448	12	full-splice_match	gene: Zm00001d039740	Zm00001d039740_T00	48.59	48.59	coding	328	108	1091
PB.5689.1	4	3276	1	incomplete-splice_match	gene: Zm00001d052997	Zm00001d052997_T00	14.39	14.39	coding	1028	3	3086
PB.3806.4	3	1303	12	full-splice_match	gene: Zm00001d039740	Zm00001d039740_T00	0.00	48.59	coding	328	64	1047
PB.7851.2	6	624	1	antisense	novelGene_gene: Zm00001d037932	novel	2.62	2.62	non coding	NA	NA	NA
PB.10302.1	9	1561	5	full-splice_match	gene: Zm00001d045563	Zm00001d045563_T00	13.35	13.35	coding	402	190	1395

PB.398.5	1	2641	14	novel_in_catalog	gene: Zm00001d028949	novel	4.71	453.16	coding	219	1591	2247
PB.7976.3	6	1699	5	full-splice_match	gene: Zm00001d038346	Zm00001d038346_T00	0.00	262.57	coding	362	261	1346
PB.4465.1	3	3225	6	full-splice_match	gene: Zm00001d043295	Zm00001d043295_T00	21.98	61.12	coding	980	154	3093
PB.4693.4	3	2731	4	novel_in_catalog	gene: Zm00001d044127	novel	0.58	51.45	coding	384	114	1265
PB.4693.1	3	2599	7	full-splice_match	gene: Zm00001d044127	Zm00001d044127_T01	31.19	51.45	coding	628	328	2211
PB.4693.3	3	2787	5	full-splice_match	gene: Zm00001d044127	Zm00001d044127_T01	5.79	51.45	coding	229	1731	2417
PB.7550.1	6	1741	5	full-splice_match	gene: Zm00001d036480	Zm00001d036480_T00	294.09	294.09	coding	414	140	1381
PB.7072.5	5	1710	2	genic	gene: Zm00001d017825	novel	18.23	116.32	coding	324	503	1474
PB.9784.1	8	3445	11	full-splice_match	gene: Zm00001d011473	Zm00001d011473_T00	21.84	21.84	coding	887	262	2922
PB.792.1	1	723	1	full-splice_match	gene: Zm00001d031075	Zm00001d031075_T00	2.00	2.00	coding	38	342	455
PB.947.1	1	954	8	full-splice_match	gene: Zm00001d031908	Zm00001d031908_T00	72.74	72.74	coding	206	48	665
PB.1436.1	1	1407	1	genic	gene: Zm00001d033993	novel	1.25	20.22	non coding	NA	NA	NA
PB.4531.5	3	1578	5	novel_in_catalog	gene: Zm00001d043515	novel	0.91	52.60	coding	184	90	641
PB.8478.2	7	1798	6	full-splice_match	gene: Zm00001d020129	Zm00001d020129_T00	3.77	6.58	coding	491	65	1537
PB.993.2	1	953	1	full-splice_match	gene: Zm00001d032088	Zm00001d032088_T00	0.00	130.41	coding	137	145	555
PB.8478.1	7	2076	5	novel_in_catalog	gene: Zm00001d020129	novel	2.81	6.58	coding	377	668	1798
PB.4740.1	3	2014	11	full-splice_match	gene: Zm00001d044297	Zm00001d044297_T00	21.07	32.71	coding	513	132	1670

PB.9902.1	8	1333	1	antisense	novelGene_gene: Zm00001d011964	novel	141.24	141.24	coding	76	219	446
PB.7072.6	5	1978	1	full-splice_match	gene: Zm00001d017825	Zm00001d017825_T00	33.27	116.32	coding	324	786	1757
PB.3479.2	2	1138	3	full-splice_match	gene: Zm00001d006921	Zm00001d006921_T00	4.51	5.70	coding	244	153	884
PB.10053.1	8	703	3	full-splice_match	gene: Zm00001d012598	Zm00001d012598_T00	69.51	363.60	coding	108	59	382
PB.8418.1	7	1001	1	full-splice_match	gene: Zm00001d019734	Zm00001d019734_T00	3.05	3.05	coding	257	60	830
PB.3396.11	2	1912	6	novel_in_catalog	gene: Zm00001d006578	novel	6.49	210.21	coding	293	335	1213
PB.7711.4	6	1988	16	full-splice_match	gene: Zm00001d037278	Zm00001d037278_T00	0.00	114.81	coding	564	104	1795
PB.7551.8	6	4406	5	novel_in_catalog	gene: Zm00001d036481	novel	9.39	416.78	coding	183	3597	4145
PB.856.1	1	841	2	full-splice_match	gene: Zm00001d031427	Zm00001d031427_T00	3.28	3.28	coding	235	9	713
PB.788.1	1	1367	3	full-splice_match	gene: Zm00001d031071	Zm00001d031071_T00	45.91	45.91	coding	350	90	1139
PB.7551.7	6	1022	7	novel_not_in_catalog	gene: Zm00001d036481	novel	304.24	416.78	coding	183	208	756
PB.8265.1	7	947	2	full-splice_match	gene: Zm00001d018811	Zm00001d018811_T00	6.91	6.91	coding	212	92	727
PB.2071.1	10	997	3	novel_not_in_catalog	gene: Zm00001d025252	novel	143.44	143.44	coding	138	177	590
PB.2577.4	2	2160	14	full-splice_match	gene: Zm00001d002388	Zm00001d002388_T00	0.00	86.25	coding	565	156	1850
PB.7551.2	6	1241	6	full-splice_match	gene: Zm00001d036481	Zm00001d036481_T00	89.14	416.78	coding	183	346	894
PB.5579.2	4	1760	12	novel_in_catalog	gene: Zm00001d052439	novel	11.15	39.63	coding	305	150	1064
PB.5171.1	4	960	1	antisense	novelGene_gene: Zm00001d050302	novel	24.69	24.69	non coding	NA	NA	NA

PB.7275.1	5	2073	21	full-splice_match	gene: Zm00001d018525	Zm00001d018525_T00	35.03	35.03	coding	586	116	1873
PB.7275.2	5	1920	21	full-splice_match	gene: Zm00001d018525	Zm00001d018525_T00	0.00	35.03	coding	586	102	1859
PB.10985.1	B73V 4	2673	10	novel_not_in_catalog	gene: Zm00001d000452	novel	2.69	47.26	coding	125	170	544
PB.10985.3	B73V 4	3019	11	novel_not_in_catalog	gene: Zm00001d000452	novel	4.40	47.26	coding	12	365	400
PB.10985.2	B73V 4	2674	9	novel_not_in_catalog	gene: Zm00001d000452	novel	5.87	47.26	coding	125	236	610
PB.10985.5	B73V 4	2107	5	novel_not_in_catalog	gene: Zm00001d000452	novel	13.40	47.26	non coding	NA	NA	NA
PB.10985.4	B73V 4	2471	12	novel_not_in_catalog	gene: Zm00001d000452	novel	9.33	47.26	coding	125	210	584
PB.10985.7	B73V 4	2767	10	novel_not_in_catalog	gene: Zm00001d000452	novel	0.33	47.26	coding	12	373	408
PB.10985.6	B73V 4	2480	9	novel_not_in_catalog	gene: Zm00001d000452	novel	11.24	47.26	non coding	NA	NA	NA
PB.5361.7	4	1431	1	genic_intron	novelGene_327	novel	10.31	10.31	coding	399	103	1299
PB.5143.2	4	1764	6	novel_not_in_catalog	gene: Zm00001d050139	novel	1.10	58.80	coding	441	35	1357
PB.5143.3	4	1646	6	full-splice_match	gene: Zm00001d050139	Zm00001d050139_T00	0.00	58.80	coding	458	35	1408
PB.674.3	1	932	2	full-splice_match	gene: Zm00001d030345	Zm00001d030345_T00	32.80	47.92	coding	221	33	695
PB.5143.1	4	1844	6	full-splice_match	gene: Zm00001d050139	Zm00001d050139_T00	57.69	58.80	coding	458	35	1408
PB.3627.1	2	1616	2	genic	gene: Zm00001d007789	novel	36.40	79.61	coding	455	34	1398
PB.9850.2	8	819	1	full-splice_match	gene: Zm00001d011751	Zm00001d011751_T00	0.72	0.72	coding	141	104	526
PB.7012.1	5	1402	2	full-splice_match	gene: Zm00001d017547	Zm00001d017547_T00	57.14	100.75	coding	290	119	988

PB.3627.2	2	2815	1	full-splice_match	gene: Zm00001d007789	Zm00001d007789_T00	36.21	79.61	coding	866	61	2658
PB.7816.1	6	1412	9	full-splice_match	gene: Zm00001d037775	Zm00001d037775_T00	26.81	26.81	coding	347	40	1080
PB.7012.5	5	1100	2	full-splice_match	gene: Zm00001d017547	Zm00001d017547_T00	0.00	100.75	coding	290	119	988
PB.7012.4	5	1299	2	full-splice_match	gene: Zm00001d017547	Zm00001d017547_T00	38.14	100.75	coding	290	119	988
PB.6948.16	5	2381	2	full-splice_match	gene: Zm00001d017274	Zm00001d017274_T00	0.00	2308.60	coding	703	109	2217
PB.9954.3	8	1745	5	intergenic	novelGene_342	novel	21.22	27.94	non coding	NA	NA	NA
PB.9954.1	8	2652	3	intergenic	novelGene_342	novel	6.71	27.94	non coding	NA	NA	NA
PB.8744.1	7	2087	2	genic	gene: Zm00001d021389	novel	62.06	129.48	coding	337	626	1636
PB.10991.2	B73V4	1860	8	novel_in_catalog	gene: Zm00001d000329	novel	11.96	11.96	coding	105	1273	1587
PB.10819.20	9	3095	8	novel_in_catalog	gene: Zm00001d048171	novel	3.64	25.07	coding	179	2198	2734
PB.7484.1	6	1639	6	full-splice_match	gene: Zm00001d036023	Zm00001d036023_T00	10.84	10.84	coding	522	46	1611
PB.1164.3	1	2027	18	full-splice_match	gene: Zm00001d032763	Zm00001d032763_T00	29.62	76.57	coding	526	181	1758
PB.654.2	1	1289	3	full-splice_match	gene: Zm00001d030220	Zm00001d030220_T00	0.00	26.30	coding	355	90	1154
PB.654.1	1	1482	3	full-splice_match	gene: Zm00001d030220	Zm00001d030220_T00	26.30	26.30	coding	355	163	1227
PB.3776.1	3	2918	10	incomplete-splice_match	gene: Zm00001d039612	Zm00001d039612_T02	13.69	13.69	coding	843	121	2649
PB.4768.3	3	3869	19	incomplete-splice_match	gene: Zm00001d044443	Zm00001d044443_T00	53.17	101.76	coding	1226	9	3686
PB.957.1	1	3002	2	full-splice_match	gene: Zm00001d031940	Zm00001d031940_T00	50.93	50.93	non coding	NA	NA	NA

PB.4768.1	3	4918	20	novel_in_catalog	gene: Zm00001d044443	novel	48.58	101.76	coding	1448	227	4570
PB.4768.4	3	2237	12	incomplete-splice_match	gene: Zm00001d044443	Zm00001d044443_T00	0.00	101.76	coding	676	209	2236
PB.1917.1	10	2582	3	full-splice_match	gene: Zm00001d024497	Zm00001d024497_T00	33.57	33.57	coding	727	238	2418
PB.5385.6	4	1375	9	full-splice_match	gene: Zm00001d051676	Zm00001d051676_T00	0.00	66.22	coding	332	194	1189
PB.10842.1	9	1101	2	novel_in_catalog	gene: Zm00001d048284	novel	2.20	2.20	non coding	NA	NA	NA
PB.4486.1	3	737	1	full-splice_match	gene: Zm00001d043371	Zm00001d043371_T00	27.25	27.25	coding	118	113	466
PB.2109.2	10	2700	7	full-splice_match	gene: Zm00001d025474	Zm00001d025474_T00	26.75	26.75	coding	795	70	2454
PB.5926.4	4	904	9	full-splice_match	gene: Zm00001d054017	Zm00001d054017_T00	33.94	181.85	coding	187	90	650
PB.1367.1	1	2356	8	full-splice_match	gene: Zm00001d033670	Zm00001d033670_T00	36.33	36.33	coding	498	324	1817
PB.10414.1	9	1677	7	full-splice_match	gene: Zm00001d046352	Zm00001d046352_T00	107.28	130.20	coding	417	192	1442
PB.8211.3	7	2043	7	full-splice_match	gene: Zm00001d018598	Zm00001d018598_T00	41.42	80.07	coding	432	628	1923
PB.8211.2	7	2141	7	full-splice_match	gene: Zm00001d018598	Zm00001d018598_T00	3.22	80.07	coding	464	606	1997
PB.8211.1	7	2109	6	full-splice_match	gene: Zm00001d018598	Zm00001d018598_T00	35.43	80.07	coding	432	68	1363
PB.5038.6	4	1219	2	incomplete-splice_match	gene: Zm00001d049558	Zm00001d049558_T00	37.49	37.49	coding	328	236	1219
PB.5385.5	4	2086	7	novel_in_catalog	gene: Zm00001d051676	novel	3.89	66.22	coding	213	194	832
PB.1465.1	1	2031	17	novel_in_catalog	gene: Zm00001d034108	novel	38.97	42.98	coding	498	389	1882
PB.2145.3	10	1476	4	full-splice_match	gene: Zm00001d025690	Zm00001d025690_T00	0.00	47.56	coding	377	80	1210

PB.5385.2	4	1547	9	full-splice_match	gene: Zm00001d051676	Zm00001d051676_T00	46.81	66.22	coding	332	194	1189
PB.2365.1	10	881	5	full-splice_match	gene: Zm00001d026562	Zm00001d026562_T00	34.36	34.36	coding	207	51	671
PB.2227.1	10	1397	1	full-splice_match	gene: Zm00001d026061	Zm00001d026061_T00	7.85	7.85	coding	383	36	1184
PB.688.1	1	2396	3	genic	gene: Zm00001d030500	novel	24.90	24.90	coding	542	347	1972
PB.341.4	1	1362	1	novel_in_catalog	gene: Zm00001d028690	novel	32.63	83.97	coding	325	27	1001
PB.341.5	1	1873	2	incomplete-splice_match	gene: Zm00001d028690	Zm00001d028690_T00	10.50	83.97	coding	494	39	1520
PB.341.1	1	4931	3	full-splice_match	gene: Zm00001d028690	Zm00001d028690_T00	2.91	83.97	coding	1210	37	3666
PB.341.2	1	2844	2	incomplete-splice_match	gene: Zm00001d028690	Zm00001d028690_T00	16.79	83.97	coding	621	142	2004
PB.341.3	1	4163	4	full-splice_match	gene: Zm00001d028690	Zm00001d028690_T00	21.13	83.97	coding	1210	107	3736
PB.8396.5	7	1447	7	full-splice_match	gene: Zm00001d019579	Zm00001d019579_T00	0.00	24.53	coding	352	41	1096
PB.6104.1	5	1748	4	novel_not_in_catalog	gene: Zm00001d013304	novel	8.30	22.56	coding	133	381	779
PB.6392.5	5	1165	12	full-splice_match	gene: Zm00001d014572	Zm00001d014572_T00	18.53	38.20	coding	326	59	1036
PB.6104.3	5	1075	3	novel_in_catalog	gene: Zm00001d013304	novel	0.59	22.56	coding	162	328	813
PB.2992.1	2	3369	2	full-splice_match	gene: Zm00001d004526	Zm00001d004526_T00	20.69	30.45	coding	993	78	3056
PB.8396.3	7	1940	7	full-splice_match	gene: Zm00001d019579	Zm00001d019579_T00	3.12	24.53	coding	352	61	1116
PB.10287.1	9	1904	6	novel_in_catalog	gene: Zm00001d045509	novel	38.72	38.72	coding	129	1188	1574
PB.4241.4	3	1681	4	full-splice_match	gene: Zm00001d042333	Zm00001d042333_T00	0.00	13.24	coding	427	242	1522

PB.2551.1	2	643	1	genic	gene: Zm00001d002302	novel	19.24	50.80	coding	100	118	417
PB.10121.2	9	1105	2	genic	gene: Zm00001d044783	novel	9.52	16.24	coding	187	105	665
PB.3637.2	2	1233	6	incomplete-splice_match	gene: Zm00001d007826	Zm00001d007826_T01	18.16	49.42	coding	306	167	1084
PB.8065.1	6	1228	2	full-splice_match	gene: Zm00001d038699	Zm00001d038699_T00	7.18	7.18	coding	359	20	1096
PB.1144.3	1	3493	7	full-splice_match	gene: Zm00001d032689	Zm00001d032689_T03	2.85	2.85	coding	807	168	2588
PB.373.4	1	568	5	full-splice_match	gene: Zm00001d028820	Zm00001d028820_T00	0.00	113.50	coding	124	114	485
PB.373.3	1	1156	4	novel_in_catalog	gene: Zm00001d028820	novel	9.66	113.50	coding	144	118	549
PB.373.2	1	1260	3	novel_in_catalog	gene: Zm00001d028820	novel	1.34	113.50	coding	144	118	549
PB.373.1	1	1047	5	full-splice_match	gene: Zm00001d028820	Zm00001d028820_T00	102.50	113.50	coding	168	213	716
PB.6563.1	5	2328	1	full-splice_match	gene: Zm00001d015213	Zm00001d015213_T00	23.55	23.55	coding	656	139	2106
PB.836.1	1	1389	1	full-splice_match	gene: Zm00001d031273	Zm00001d031273_T00	2.22	24.47	coding	289	16	882
PB.4178.1	3	1566	3	novel_in_catalog	gene: Zm00001d042017	novel	7.80	58.20	non coding	NA	NA	NA
PB.7839.1	6	1682	8	full-splice_match	gene: Zm00001d037898	Zm00001d037898_T00	4.22	7.48	coding	381	256	1398
PB.1037.1	1	778	5	incomplete-splice_match	gene: Zm00001d032301	Zm00001d032301_T00	6.85	6.85	coding	8	352	375
PB.4023.1	3	849	2	genic	gene: Zm00001d041065	novel	30.10	30.10	coding	79	138	374
PB.677.3	1	1534	10	full-splice_match	gene: Zm00001d030348	Zm00001d030348_T00	46.77	81.97	coding	398	105	1298
PB.9588.4	8	594	5	full-splice_match	gene: Zm00001d010614	Zm00001d010614_T00	900.83	1295.66	coding	135	40	444

PB.7452.1	6	1270	1	antisense	novelGene_gene: Zm00001d035815	novel	9.46	9.46	coding	157	143	613
PB.9825.6	8	1690	1	full-splice_match	gene: Zm00001d011649	Zm00001d011649_T00	273.91	277.42	coding	480	57	1496
PB.9588.1	8	806	5	full-splice_match	gene: Zm00001d010614	Zm00001d010614_T00	394.83	1295.66	coding	135	38	442
PB.5794.2	4	1536	10	full-splice_match	gene: Zm00001d053563	Zm00001d053563_T00	29.51	40.38	coding	350	222	1271
PB.5794.3	4	1459	10	full-splice_match	gene: Zm00001d053563	Zm00001d053563_T00	9.58	40.38	coding	350	258	1307
PB.4158.9	3	1820	6	novel_in_catalog	gene: Zm00001d041945	novel	2.12	133.59	coding	151	1082	1534
PB.2401.1	10	3222	2	full-splice_match	gene: Zm00001d026668	Zm00001d026668_T00	35.76	35.76	coding	958	102	2975
PB.6432.1	5	1261	1	full-splice_match	gene: Zm00001d014752	Zm00001d014752_T00	24.24	24.24	coding	276	97	924
PB.4158.5	3	1823	10	novel_in_catalog	gene: Zm00001d041945	novel	0.00	133.59	coding	211	232	864
PB.569.1	1	2404	4	incomplete-splice_match	gene: Zm00001d029750	Zm00001d029750_T00	36.51	36.51	coding	644	195	2126
PB.4158.7	3	1811	9	novel_in_catalog	gene: Zm00001d041945	novel	0.00	133.59	coding	170	120	629
PB.3918.3	3	913	5	novel_in_catalog	gene: Zm00001d040308	novel	0.00	12.53	coding	175	332	856
PB.4158.1	3	1872	8	novel_in_catalog	gene: Zm00001d041945	novel	5.97	133.59	coding	170	61	570
PB.4158.3	3	1886	11	full-splice_match	gene: Zm00001d041945	Zm00001d041945_T00	101.00	133.59	coding	397	396	1586
PB.4158.2	3	1804	10	novel_in_catalog	gene: Zm00001d041945	novel	1.72	133.59	coding	381	357	1499
PB.1296.3	1	1100	1	full-splice_match	gene: Zm00001d033377	Zm00001d033377_T00	0.00	15.15	non coding	NA	NA	NA
PB.7256.7	5	1669	14	novel_in_catalog	gene: Zm00001d018484	novel	7.37	44.98	coding	211	469	1101

PB.6793.4	5	957	2	genic	gene: Zm00001d016655	novel	37.16	265.24	coding	119	93	449
PB.6793.2	5	1130	2	genic	gene: Zm00001d016655	novel	87.03	265.24	coding	119	97	453
PB.6481.4	5	2407	10	full-splice_match	gene: Zm00001d014895	Zm00001d014895_T00	32.61	95.11	coding	703	219	2327
PB.1804.1	10	2361	2	incomplete-splice_match	gene: Zm00001d023788	Zm00001d023788_T00	4.01	14.45	coding	621	39	1901
PB.2559.2	2	2088	6	full-splice_match	gene: Zm00001d002332	Zm00001d002332_T02	0.00	89.96	coding	285	107	961
PB.2559.3	2	1593	6	full-splice_match	gene: Zm00001d002332	Zm00001d002332_T02	4.96	89.96	coding	285	203	1057
PB.428.2	1	1283	2	incomplete-splice_match	gene: Zm00001d029055	Zm00001d029055_T01	13.90	55.25	coding	157	173	643
PB.2559.1	2	3060	8	incomplete-splice_match	gene: Zm00001d002332	Zm00001d002332_T01	10.60	89.96	coding	256	1901	2668
PB.2559.6	2	1213	4	novel_in_catalog	gene: Zm00001d002332	novel	3.92	89.96	coding	231	108	800
PB.2559.7	2	952	5	full-splice_match	gene: Zm00001d002332	Zm00001d002332_T02	11.98	89.96	coding	256	107	874
PB.2559.4	2	1278	5	novel_in_catalog	gene: Zm00001d002332	novel	4.74	89.96	coding	263	245	1033
PB.2559.5	2	1292	5	full-splice_match	gene: Zm00001d002332	Zm00001d002332_T02	53.75	89.96	coding	297	207	1097
PB.8503.5	7	1689	3	full-splice_match	gene: Zm00001d020282	Zm00001d020282_T00	0.00	38.13	coding	524	60	1631
PB.971.1	1	2169	7	full-splice_match	gene: Zm00001d031986	Zm00001d031986_T00	132.57	132.57	coding	639	71	1987
PB.6856.3	5	1662	2	full-splice_match	gene: Zm00001d016896	Zm00001d016896_T00	15.55	31.67	coding	488	41	1504
PB.8503.1	7	1983	3	full-splice_match	gene: Zm00001d020282	Zm00001d020282_T00	25.38	38.13	coding	524	101	1672
PB.8503.2	7	1867	3	full-splice_match	gene: Zm00001d020282	Zm00001d020282_T00	12.75	38.13	coding	524	99	1670

PB.6856.4	5	1546	1	novel_in_catalog	gene: Zm00001d016896	novel	5.38	31.67	coding	157	954	1424
PB.4803.1	3	2829	20	full-splice_match	gene: Zm00001d044552	Zm00001d044552_T00	15.05	32.32	coding	737	285	2495
PB.6408.1	5	2111	2	incomplete-splice_match	gene: Zm00001d014652	Zm00001d014652_T00	10.39	10.39	coding	440	157	1476
PB.10446.1	9	1393	3	full-splice_match	gene: Zm00001d046500	Zm00001d046500_T00	8.64	8.64	coding	305	229	1143
PB.677.1	1	1670	10	full-splice_match	gene: Zm00001d030348	Zm00001d030348_T00	29.82	81.97	coding	398	105	1298
PB.5159.1	4	1339	5	full-splice_match	gene: Zm00001d050202	Zm00001d050202_T00	36.38	36.38	coding	310	91	1020
PB.6306.5	5	2873	17	novel_in_catalog	gene: Zm00001d014178	novel	30.96	50.79	coding	782	409	2754
PB.1519.2	1	1889	2	full-splice_match	gene: Zm00001d034373	Zm00001d034373_T00	0.00	366.63	coding	465	175	1569
PB.6239.1	5	1273	8	full-splice_match	gene: Zm00001d013885	Zm00001d013885_T00	9.62	9.62	coding	291	5	877
PB.1519.6	1	1613	1	novel_in_catalog	gene: Zm00001d034373	novel	5.66	366.63	coding	492	137	1612
PB.3683.1	3	829	2	full-splice_match	gene: Zm00001d039273	Zm00001d039273_T00	85.88	85.88	coding	227	55	735
PB.8202.2	6	1327	9	full-splice_match	gene: Zm00001d039182	Zm00001d039182_T00	39.04	90.47	coding	347	138	1178
PB.1372.1	1	1360	2	novel_not_in_catalog	gene: Zm00001d033705	novel	7.81	40.41	coding	152	225	680
PB.1372.2	1	1753	2	novel_not_in_catalog	gene: Zm00001d033705	novel	32.59	40.41	coding	322	228	1193
PB.4500.4	3	1119	4	full-splice_match	gene: Zm00001d043420	Zm00001d043420_T00	0.00	8.82	coding	278	123	956
PB.6243.1	5	2304	19	full-splice_match	gene: Zm00001d013911	Zm00001d013911_T00	48.46	57.26	coding	613	180	2018
PB.5527.6	4	766	3	novel_in_catalog	gene: Zm00001d052268	novel	17.36	134.67	coding	126	208	585

PB.7488.1	6	2096	9	intergenic	novelGene_161	novel	14.49	14.49	coding	611	55	1887
PB.4683.3	3	3642	4	novel_in_catalog	gene: Zm00001d044091	novel	3.64	31.00	coding	144	2454	2885
PB.4186.1	3	1959	5	full-splice_match	gene: Zm00001d042043	Zm00001d042043_T00	41.07	41.07	coding	394	423	1604
PB.4878.1	4	1048	2	novel_in_catalog	gene: Zm00001d048831	novel	12.30	12.30	coding	183	36	584
PB.9412.3	8	1186	5	incomplete-splice_match	gene: Zm00001d009702	Zm00001d009702_T00	73.45	181.99	coding	300	58	957
PB.7827.2	6	2368	7	full-splice_match	gene: Zm00001d037832	Zm00001d037832_T00	6.34	15.91	coding	441	64	1386
PB.6954.13	5	1543	9	full-splice_match	gene: Zm00001d017298	Zm00001d017298_T00	19.07	125.29	coding	295	427	1311
PB.10558.3	9	2374	5	novel_in_catalog	gene: Zm00001d047126	novel	0.00	35.72	coding	633	475	2373
PB.6954.11	5	1499	1	novel_in_catalog	gene: Zm00001d017298	novel	4.21	125.29	non coding	NA	NA	NA
PB.9412.2	8	2253	10	incomplete-splice_match	gene: Zm00001d009702	Zm00001d009702_T00	30.55	181.99	coding	561	235	1917
PB.3252.1	2	951	2	novel_in_catalog	gene: Zm00001d005983	novel	7.98	14.40	coding	13	272	310
PB.7795.1	6	631	1	full-splice_match	gene: Zm00001d037677	Zm00001d037677_T00	266.59	266.59	coding	101	49	351
PB.10558.2	9	3578	7	full-splice_match	gene: Zm00001d047126	Zm00001d047126_T00	35.72	35.72	coding	896	492	3179
PB.8072.4	6	1309	1	novel_in_catalog	gene: Zm00001d038720	novel	21.59	48.74	coding	101	1007	1309
PB.9840.1	8	1243	7	novel_not_in_catalog	gene: Zm00001d011717	novel	1.02	69.71	coding	206	107	724
PB.9840.2	8	1083	7	novel_not_in_catalog	gene: Zm00001d011717	novel	34.05	69.71	coding	206	92	709
PB.9840.3	8	986	6	novel_not_in_catalog	gene: Zm00001d011717	novel	3.50	69.71	coding	144	35	466

PB.4162.2	3	1610	3	full-splice_match	gene: Zm00001d041955	Zm00001d041955_T00	13.24	22.55	coding	344	163	1194
PB.10548.1	9	1376	2	full-splice_match	gene: Zm00001d047064	Zm00001d047064_T00	0.95	0.95	coding	331	98	1090
PB.3611.4	2	3539	2	novel_in_catalog	gene: Zm00001d007745	novel	0.00	11.36	coding	375	2239	3363
PB.4162.1	3	756	1	genic	gene: Zm00001d041955	novel	9.31	22.55	non coding	NA	NA	NA
PB.10644.1	9	3606	19	full-splice_match	gene: Zm00001d047518	Zm00001d047518_T00	17.41	17.41	coding	1036	90	3197
PB.9409.1	8	2808	1	full-splice_match	gene: Zm00001d009694	Zm00001d009694_T00	14.25	14.25	coding	881	4	2646
PB.4094.1	3	1463	10	full-splice_match	gene: Zm00001d041575	Zm00001d041575_T00	65.96	65.96	coding	353	60	1118
PB.5800.3	4	1742	1	full-splice_match	gene: Zm00001d053586	Zm00001d053586_T00	0.00	9.84	coding	509	49	1575
PB.2210.2	10	1404	9	novel_not_in_catalog	gene: Zm00001d025991	novel	5.85	9.18	coding	150	586	1035
PB.2210.3	10	1171	9	full-splice_match	gene: Zm00001d025991	Zm00001d025991_T00	3.33	9.18	coding	266	203	1000
PB.1169.1	1	866	4	incomplete-splice_match	gene: Zm00001d032790	Zm00001d032790_T00	7.91	7.91	coding	196	36	623
PB.9157.3	8	2043	11	full-splice_match	gene: Zm00001d008345	Zm00001d008345_T00	0.00	137.02	coding	527	142	1722
PB.4263.1	3	1730	7	novel_in_catalog	gene: Zm00001d042438	novel	8.34	8.34	coding	302	54	959
PB.4943.1	4	1415	4	full-splice_match	gene: Zm00001d049120	Zm00001d049120_T00	31.80	31.80	coding	374	57	1178
PB.7287.1	5	2266	20	fusion	gene: Zm00001d018562	novel	41.16	41.16	coding	714	125	2266
PB.4943.2	4	764	2	incomplete-splice_match	gene: Zm00001d049120	Zm00001d049120_T00	0.00	31.80	coding	139	216	632
PB.1386.1	1	1411	1	intergenic	novelGene_459	novel	8.75	8.75	non coding	NA	NA	NA

PB.763.1	1	1265	1	intergenic	novelGene_348	novel	0.00	0.00	non coding	NA	NA	NA
PB.4474.1	3	3117	10	full-splice_match	gene: Zm00001d043327	Zm00001d043327_T00	5.26	5.26	coding	859	286	2862
PB.9867.1	8	1306	7	full-splice_match	gene: Zm00001d011817	Zm00001d011817_T00	33.83	52.25	coding	295	117	1001
PB.1921.3	10	2421	11	full-splice_match	gene: Zm00001d024512	Zm00001d024512_T00	16.51	19.41	coding	417	1087	2337
PB.4581.1	3	2006	9	novel_in_catalog	gene: Zm00001d043686	novel	2.06	35.10	coding	428	173	1456
PB.9867.2	8	1235	7	full-splice_match	gene: Zm00001d011817	Zm00001d011817_T00	18.42	52.25	coding	295	149	1033
PB.1921.4	10	3677	9	full-splice_match	gene: Zm00001d024512	Zm00001d024512_T00	2.89	19.41	coding	407	2388	3608
PB.2188.1	10	843	3	intergenic	novelGene_255	novel	6.83	6.83	non coding	NA	NA	NA
PB.9024.2	7	1907	7	full-splice_match	gene: Zm00001d022374	Zm00001d022374_T00	31.86	32.53	coding	524	45	1616
PB.7013.1	5	764	1	full-splice_match	gene: Zm00001d017557	Zm00001d017557_T00	301.47	301.47	coding	162	3	488
PB.677.4	1	1415	10	full-splice_match	gene: Zm00001d030348	Zm00001d030348_T00	0.00	81.97	coding	398	105	1298
PB.9825.1	8	1561	2	genic	gene: Zm00001d011648	novel	51.81	88.31	coding	407	75	1295
PB.1814.1	10	1010	7	full-splice_match	gene: Zm00001d023833	Zm00001d023833_T00	30.27	30.27	coding	219	112	768
PB.3619.2	2	2026	4	novel_in_catalog	gene: Zm00001d007767	novel	19.69	412.11	coding	176	188	715
PB.9883.2	8	1738	6	full-splice_match	gene: Zm00001d011855	Zm00001d011855_T00	0.00	70.80	coding	476	80	1507
PB.9883.1	8	1814	6	full-splice_match	gene: Zm00001d011855	Zm00001d011855_T00	42.31	70.80	coding	476	78	1505
PB.1170.1	1	2229	1	antisense	novelGene_gene: Zm00001d032791	novel	4.69	4.69	non coding	NA	NA	NA

PB.5026.1	4	1294	9	full-splice_match	gene: Zm00001d049499	Zm00001d049499_T00	20.04	20.04	coding	328	81	1064
PB.5026.2	4	1079	9	full-splice_match	gene: Zm00001d049499	Zm00001d049499_T00	0.00	20.04	coding	328	15	998
PB.5172.1	4	1663	3	full-splice_match	gene: Zm00001d050310	Zm00001d050310_T00	8.50	106.29	coding	272	490	1305
PB.2823.4	2	710	3	full-splice_match	gene: Zm00001d003502	Zm00001d003502_T00	90.84	138.98	coding	146	122	559
PB.1165.1	1	856	1	full-splice_match	gene: Zm00001d032777	Zm00001d032777_T00	6.72	6.72	coding	152	48	503
PB.10798.1	9	3142	6	full-splice_match	gene: Zm00001d048080	Zm00001d048080_T00	34.80	34.80	coding	915	81	2825
PB.2579.1	2	2060	5	full-splice_match	gene: Zm00001d002391	Zm00001d002391_T01	22.01	24.39	coding	482	339	1784
PB.2579.2	2	985	4	incomplete-splice_match	gene: Zm00001d002391	Zm00001d002391_T00	2.38	24.39	coding	132	326	721
PB.10798.2	9	3316	5	novel_in_catalog	gene: Zm00001d048080	novel	0.00	34.80	coding	529	62	1648
PB.1640.1	1	1298	2	full-splice_match	gene: Zm00001d034803	Zm00001d034803_T00	41.97	41.97	coding	234	276	977
PB.8600.1	7	1484	2	full-splice_match	gene: Zm00001d020781	Zm00001d020781_T00	28.84	28.84	coding	367	56	1156
PB.4661.3	3	1685	2	novel_not_in_catalog	gene: Zm00001d043989	novel	2.25	29.55	coding	402	295	1500
PB.4661.1	3	1833	2	novel_not_in_catalog	gene: Zm00001d043989	novel	19.77	29.55	coding	402	291	1496
PB.1039.1	1	1620	3	full-splice_match	gene: Zm00001d032316	Zm00001d032316_T00	39.69	86.66	coding	270	481	1290
PB.3047.3	2	2913	7	novel_in_catalog	gene: Zm00001d004875	novel	18.81	24.20	coding	458	1102	2475
PB.9525.1	8	1346	9	full-splice_match	gene: Zm00001d010314	Zm00001d010314_T00	7.26	7.26	coding	337	106	1116
PB.9330.2	8	2644	7	full-splice_match	gene: Zm00001d009222	Zm00001d009222_T00	25.37	31.89	coding	776	118	2445

PB.869.3	1	1738	5	novel_not_in_catalog	gene: Zm00001d031481	novel	1.72	42.70	coding	245	235	969
PB.9330.1	8	1546	5	incomplete-splice_match	gene: Zm00001d009222	Zm00001d009222_T00	6.52	31.89	coding	416	15	1262
PB.287.1	1	2028	1	full-splice_match	gene: Zm00001d028452	Zm00001d028452_T00	1.50	1.50	coding	580	106	1845
PB.2114.1	10	1677	1	full-splice_match	gene: Zm00001d025514	Zm00001d025514_T00	11.75	11.75	coding	392	395	1570
PB.7582.1	6	2478	5	novel_not_in_catalog	gene: Zm00001d036630	novel	24.11	24.11	coding	677	255	2285
PB.6555.1	5	1446	6	novel_in_catalog	gene: Zm00001d015175	novel	14.29	14.29	coding	355	32	1096
PB.9257.4	8	2224	10	novel_in_catalog	gene: Zm00001d008816	novel	8.13	174.19	coding	223	1419	2087
PB.5387.2	4	1100	10	novel_not_in_catalog	gene: Zm00001d051682	novel	29.48	43.92	coding	219	162	818
PB.5387.3	4	560	4	novel_not_in_catalog	gene: Zm00001d051682	novel	8.42	43.92	non coding	NA	NA	NA
PB.6842.6	5	1353	4	full-splice_match	gene: Zm00001d016842	Zm00001d016842_T00	0.00	22.28	coding	288	385	1248
PB.6842.5	5	1415	5	full-splice_match	gene: Zm00001d016842	Zm00001d016842_T00	2.17	22.28	coding	191	624	1196
PB.9252.11	8	1641	10	novel_not_in_catalog	gene: Zm00001d008744	novel	0.00	103.79	coding	394	305	1486
PB.6842.1	5	1531	4	full-splice_match	gene: Zm00001d016842	Zm00001d016842_T00	19.00	22.28	coding	288	435	1298
PB.1564.8	1	1232	1	novel_in_catalog	gene: Zm00001d034558	novel	42.93	853.55	coding	309	106	1032
PB.7058.2	5	816	7	incomplete-splice_match	gene: Zm00001d017770	Zm00001d017770_T00	7.68	13.38	non coding	NA	NA	NA
PB.2451.2	2	1205	7	novel_in_catalog	gene: Zm00001d001870	novel	0.33	33.14	coding	238	156	869
PB.2730.3	2	512	4	full-splice_match	gene: Zm00001d003084	Zm00001d003084_T00	127.84	262.90	non coding	NA	NA	NA

PB.9252.17	8	1719	4	novel_in_catalog	gene: Zm00001d008749	novel	7.07	136.63	coding	160	131	610
PB.2730.1	2	584	4	full-splice_match	gene: Zm00001d003084	Zm00001d003084_T00	135.06	262.90	non coding	NA	NA	NA
PB.9877.1	8	829	4	full-splice_match	gene: Zm00001d011840	Zm00001d011840_T00	19.78	19.78	coding	191	41	613
PB.2451.1	2	1232	8	full-splice_match	gene: Zm00001d001870	Zm00001d001870_T00	32.81	33.14	coding	250	222	971
PB.4766.4	3	2289	2	novel_in_catalog	gene: Zm00001d044434	novel	4.30	112.31	coding	685	50	2104
PB.297.1	1	1005	6	full-splice_match	gene: Zm00001d028515	Zm00001d028515_T00	57.60	131.83	coding	229	73	759
PB.297.2	1	896	6	full-splice_match	gene: Zm00001d028515	Zm00001d028515_T00	74.23	131.83	coding	229	69	755
PB.1025.4	1	776	4	novel_in_catalog	gene: Zm00001d032232	novel	4.62	55.59	coding	82	162	407
PB.1025.3	1	1095	4	full-splice_match	gene: Zm00001d032232	Zm00001d032232_T00	5.17	55.59	coding	125	275	649
PB.1025.2	1	1012	5	full-splice_match	gene: Zm00001d032232	Zm00001d032232_T00	29.56	55.59	coding	157	261	731
PB.1025.1	1	767	5	full-splice_match	gene: Zm00001d032232	Zm00001d032232_T00	16.24	55.59	coding	114	145	486
PB.2009.1	10	1186	10	full-splice_match	gene: Zm00001d024939	Zm00001d024939_T00	9.32	9.32	coding	285	175	1029
PB.9971.3	8	2747	3	novel_in_catalog	gene: Zm00001d012247	novel	2.39	44.78	coding	494	283	1764
PB.3047.5	2	2974	6	novel_in_catalog	gene: Zm00001d004875	novel	5.39	24.20	coding	402	1368	2573
PB.6378.2	5	1576	6	incomplete-splice_match	gene: Zm00001d014524	Zm00001d014524_T00	9.98	28.50	coding	321	455	1417
PB.9781.1	8	443	2	full-splice_match	gene: Zm00001d011461	Zm00001d011461_T00	29.65	29.65	non coding	NA	NA	NA
PB.9615.1	8	734	2	genic	gene: Zm00001d010690	novel	21.82	21.82	coding	66	237	434

PB.4800.1	3	1633	1	full-splice_match	gene: Zm00001d044546	Zm00001d044546_T00	45.38	45.38	coding	141	952	1374
PB.3600.1	2	1717	11	full-splice_match	gene: Zm00001d007638	Zm00001d007638_T00	9.38	131.78	coding	374	225	1346
PB.8059.1	6	1352	6	full-splice_match	gene: Zm00001d038660	Zm00001d038660_T00	24.04	24.04	coding	359	52	1128
PB.7183.2	5	2816	10	full-splice_match	gene: Zm00001d018217	Zm00001d018217_T00	5.66	25.03	coding	678	341	2374
PB.4568.3	3	2580	4	novel_in_catalog	gene: Zm00001d043650	novel	96.96	254.84	coding	343	1207	2235
PB.978.1	1	1530	3	novel_not_in_catalog	gene: Zm00001d032027	novel	8.58	39.94	coding	176	755	1282
PB.4568.6	3	2480	4	novel_in_catalog	gene: Zm00001d043650	novel	157.88	254.84	coding	343	1209	2237
PB.9006.2	7	2722	11	full-splice_match	gene: Zm00001d022305	Zm00001d022305_T00	57.30	79.02	coding	592	541	2316
PB.3657.4	2	425	2	full-splice_match	gene: Zm00001d007901	Zm00001d007901_T00	34.75	280.15	coding	72	209	424
PB.9006.1	7	962	2	incomplete-splice_match	gene: Zm00001d022305	Zm00001d022305_T01	9.26	79.02	non coding	NA	NA	NA
PB.10211.1	9	1167	1	antisense	novelGene_gene: Zm00001d045218	novel	11.02	11.02	coding	157	37	507
PB.9084.6	7	1645	10	incomplete-splice_match	gene: Zm00001d022542	Zm00001d022542_T00	17.37	154.27	coding	311	305	1237
PB.9006.5	7	2650	11	full-splice_match	gene: Zm00001d022305	Zm00001d022305_T00	12.47	79.02	coding	592	624	2399
PB.7717.1	6	1969	2	full-splice_match	gene: Zm00001d037318	Zm00001d037318_T00	13.55	13.55	coding	554	171	1832
PB.1592.1	1	1027	1	full-splice_match	gene: Zm00001d034639	Zm00001d034639_T00	0.57	8.79	coding	150	64	513
PB.8787.4	7	1309	5	full-splice_match	gene: Zm00001d021557	Zm00001d021557_T00	94.79	98.16	coding	273	200	1018
PB.9162.1	8	1358	11	novel_in_catalog	gene: Zm00001d008363	novel	45.93	45.93	coding	350	4	1053

PB.6051.1	5	3778	6	genic	gene: Zm00001d013136	novel	1.51	1.51	coding	847	99	2639
PB.9646.8	8	2825	10	full-splice_match	gene: Zm00001d010811	Zm00001d010811_T01	16.99	135.99	coding	540	61	1680
PB.5004.1	4	3497	9	novel_not_in_catalog	gene: Zm00001d049376	novel	32.94	32.94	coding	912	145	2880
PB.5628.1	4	1430	3	full-splice_match	gene: Zm00001d052733	Zm00001d052733_T00	60.09	90.78	coding	309	22	948
PB.5266.3	4	1242	8	full-splice_match	gene: Zm00001d051021	Zm00001d051021_T00	29.81	124.83	coding	189	324	890
PB.3282.1	2	1149	3	full-splice_match	gene: Zm00001d006097	Zm00001d006097_T00	6.67	6.67	coding	335	119	1123
PB.1829.1	10	2111	8	full-splice_match	gene: Zm00001d023910	Zm00001d023910_T00	8.20	8.20	coding	629	60	1946
PB.3369.3	2	2238	4	full-splice_match	gene: Zm00001d006430	Zm00001d006430_T00	9.52	14.50	coding	463	614	2002
PB.5549.2	4	1515	7	novel_not_in_catalog	gene: Zm00001d052346	novel	9.29	38.28	coding	455	16	1380
PB.9672.4	8	2932	1	novel_in_catalog	gene: Zm00001d010950	novel	73.67	73.67	coding	106	417	734
PB.3657.5	2	569	1	novel_in_catalog	gene: Zm00001d007901	novel	0.00	280.15	non coding	NA	NA	NA
PB.3639.5	2	764	1	full-splice_match	gene: Zm00001d007831	Zm00001d007831_T00	586.51	936.69	coding	181	73	615
PB.10392.1	9	555	4	intergenic	novelGene_163	novel	258.17	258.17	coding	76	127	354
PB.3639.1	2	1053	1	full-splice_match	gene: Zm00001d007831	Zm00001d007831_T00	131.01	936.69	coding	181	67	609
PB.3470.8	2	3565	3	full-splice_match	gene: Zm00001d006900	Zm00001d006900_T00	10.80	444.34	coding	271	87	899
PB.10123.1	9	1668	1	full-splice_match	gene: Zm00001d044808	Zm00001d044808_T00	21.23	21.23	coding	411	219	1451
PB.10123.2	9	1509	1	full-splice_match	gene: Zm00001d044808	Zm00001d044808_T00	0.00	21.23	coding	411	165	1397

PB.10986.2	B73V4	1404	2	intergenic	novelGene_2	novel	7.41	53.28	non coding	NA	NA	NA
PB.4801.2	3	1318	1	incomplete-splice_match	gene: Zm00001d044547	Zm00001d044547_T00	0.00	1.29	coding	280	36	875
PB.2238.2	10	3029	1	genic	gene: Zm00001d026148	novel	0.91	2.28	coding	345	1942	2976
PB.2484.14	2	1097	1	novel_in_catalog	gene: Zm00001d002006	novel	111.80	1327.46	non coding	NA	NA	NA
PB.4219.1	3	1289	2	full-splice_match	gene: Zm00001d042245	Zm00001d042245_T00	549.22	578.71	coding	144	257	688
PB.2238.1	10	2462	1	novel_in_catalog	gene: Zm00001d026148	novel	1.37	2.28	coding	345	1163	2197
PB.5266.4	4	1298	7	full-splice_match	gene: Zm00001d051021	Zm00001d051021_T00	12.82	124.83	coding	142	329	754
PB.2484.13	2	3719	10	novel_in_catalog	gene: Zm00001d002006	novel	4.86	1327.46	coding	653	204	2162
PB.2484.12	2	2506	9	novel_in_catalog	gene: Zm00001d002006	novel	134.32	1327.46	coding	365	22	1116
PB.515.1	1	2335	15	incomplete-splice_match	gene: Zm00001d029490	Zm00001d029490_T01	23.14	38.72	coding	599	222	2018
PB.3121.1	2	1391	3	novel_not_in_catalog	gene: Zm00001d005295	novel	39.94	76.03	coding	328	82	1065
PB.515.3	1	989	7	incomplete-splice_match	gene: Zm00001d029490	Zm00001d029490_T01	15.57	38.72	coding	232	127	822
PB.5082.1	4	2808	5	full-splice_match	gene: Zm00001d049762	Zm00001d049762_T00	10.21	10.21	coding	797	236	2626
PB.7256.1	5	1724	15	novel_in_catalog	gene: Zm00001d018484	novel	10.50	44.98	coding	211	353	985
PB.4780.3	3	1439	11	novel_not_in_catalog	gene: Zm00001d044481	novel	0.75	24.61	coding	276	332	1159
PB.3490.1	2	785	1	full-splice_match	gene: Zm00001d006950	Zm00001d006950_T00	31.33	31.33	coding	156	90	557
PB.4876.1	4	933	2	genic	gene: Zm00001d048801	novel	20.37	23.82	coding	129	355	741

PB.4876.2	4	1022	1	full-splice_match	gene: Zm00001d048801	Zm00001d048801_T00	0.94	23.82	coding	129	444	830
PB.4876.3	4	1126	2	genic	gene: Zm00001d048801	novel	2.51	23.82	coding	129	621	1007
PB.5584.1	4	949	5	full-splice_match	gene: Zm00001d052452	Zm00001d052452_T00	35.43	57.24	coding	159	157	633
PB.5584.3	4	777	5	full-splice_match	gene: Zm00001d052452	Zm00001d052452_T00	0.00	57.24	coding	159	157	633
PB.5584.2	4	899	5	full-splice_match	gene: Zm00001d052452	Zm00001d052452_T00	21.81	57.24	coding	159	157	633
PB.9713.6	8	467	4	incomplete-splice_match	gene: Zm00001d011152	Zm00001d011152_T02	8.64	33.09	non coding	NA	NA	NA
PB.1735.1	10	626	5	incomplete-splice_match	gene: Zm00001d023431	Zm00001d023431_T00	8.11	8.11	coding	132	7	402
PB.9027.2	7	1816	5	full-splice_match	gene: Zm00001d022386	Zm00001d022386_T00	24.72	32.03	coding	414	43	1284
PB.4279.1	3	1892	13	full-splice_match	gene: Zm00001d042507	Zm00001d042507_T00	28.01	28.01	coding	480	218	1657
PB.9713.2	8	1587	11	novel_in_catalog	gene: Zm00001d011152	novel	15.98	33.09	coding	104	596	907
PB.9713.1	8	3849	6	novel_in_catalog	gene: Zm00001d011152	novel	8.46	33.09	coding	104	2592	2903
PB.9731.2	8	1940	7	novel_in_catalog	gene: Zm00001d011213	novel	7.14	138.91	coding	43	504	632
PB.3141.2	2	1462	5	full-splice_match	gene: Zm00001d005393	Zm00001d005393_T00	65.31	69.91	coding	354	46	1107
PB.4169.1	3	1387	2	full-splice_match	gene: Zm00001d041984	Zm00001d041984_T00	7.57	528.22	coding	334	55	1056
PB.3141.1	2	3263	1	genic	gene: Zm00001d005393	novel	4.60	69.91	non coding	NA	NA	NA
PB.5698.2	4	2113	5	novel_not_in_catalog	gene: Zm00001d053036	novel	43.50	79.13	non coding	NA	NA	NA
PB.9731.1	8	3006	23	novel_not_in_catalog	gene: Zm00001d011213	novel	131.77	138.91	coding	809	163	2589

PB.9464.3	8	1188	7	full-splice_match	gene: Zm00001d009997	Zm00001d009997_T00	29.35	29.35	coding	207	270	890
PB.7640.4	6	1808	13	full-splice_match	gene: Zm00001d036971	Zm00001d036971_T05	4.15	145.45	coding	243	1078	1806
PB.5453.1	4	539	1	genic	gene: Zm00001d052018	novel	19.11	19.11	non coding	NA	NA	NA
PB.3470.1	2	2151	5	full-splice_match	gene: Zm00001d006900	Zm00001d006900_T00	39.60	444.34	coding	537	87	1697
PB.5859.9	4	2256	6	novel_in_catalog	gene: Zm00001d053818	novel	7.66	105.42	coding	129	1656	2042
PB.2296.2	10	800	3	novel_not_in_catalog	gene: Zm00001d026333	novel	38.79	77.84	coding	138	24	437
PB.7828.2	6	880	2	full-splice_match	gene: Zm00001d037842	Zm00001d037842_T00	0.00	29.99	coding	221	84	746
PB.3470.5	2	2952	4	full-splice_match	gene: Zm00001d006900	Zm00001d006900_T00	0.00	444.34	coding	271	89	901
PB.7823.2	6	2999	1	genic	gene: Zm00001d037810	novel	6.92	8.92	coding	876	169	2796
PB.4169.2	3	1289	2	full-splice_match	gene: Zm00001d041984	Zm00001d041984_T00	520.65	528.22	coding	334	58	1059
PB.8680.1	7	1570	14	novel_not_in_catalog	gene: Zm00001d021132	novel	45.26	54.97	coding	419	69	1325
PB.6122.1	5	1699	1	full-splice_match	gene: Zm00001d013362	Zm00001d013362_T00	44.10	85.92	coding	330	226	1215
PB.6122.2	5	2096	2	genic	gene: Zm00001d013362	novel	38.03	85.92	coding	406	595	1812
PB.6122.3	5	2906	1	full-splice_match	gene: Zm00001d013362	Zm00001d013362_T00	3.79	85.92	coding	406	1362	2579
PB.8248.1	7	1813	2	full-splice_match	gene: Zm00001d018749	Zm00001d018749_T00	9.68	15.65	coding	447	170	1510
PB.9694.7	8	2410	8	incomplete-splice_match	gene: Zm00001d011061	Zm00001d011061_T00	2.88	25.76	coding	179	1789	2325
PB.2899.4	2	1233	4	novel_in_catalog	gene: Zm00001d003904	novel	6.94	109.58	coding	101	287	589

PB.9694.1	8	2465	12	novel_in_catalog	gene: Zm00001d011061	novel	1.87	25.76	coding	212	129	764
PB.9694.3	8	897	8	novel_in_catalog	gene: Zm00001d011061	novel	7.78	25.76	coding	235	76	780
PB.9694.2	8	1535	12	full-splice_match	gene: Zm00001d011061	Zm00001d011061_T00	8.24	25.76	coding	434	105	1406
PB.2750.8	2	1883	3	full-splice_match	gene: Zm00001d003181	Zm00001d003181_T00	11.70	83.26	coding	415	253	1497
PB.3162.1	2	1598	9	full-splice_match	gene: Zm00001d005542	Zm00001d005542_T00	79.83	110.43	coding	390	124	1293
PB.1702.1	10	927	1	full-splice_match	gene: Zm00001d023280	Zm00001d023280_T00	212.99	212.99	coding	212	48	683
PB.2022.1	10	1594	3	novel_not_in_catalog	gene: Zm00001d025008	novel	5.56	27.38	coding	375	127	1251
PB.2022.2	10	1837	2	novel_in_catalog	gene: Zm00001d025008	novel	21.83	27.38	coding	375	214	1338
PB.678.3	1	822	3	incomplete-splice_match	gene: Zm00001d030357	Zm00001d030357_T00	30.21	102.40	coding	152	70	525
PB.2750.6	2	1833	4	full-splice_match	gene: Zm00001d003181	Zm00001d003181_T00	71.56	83.26	coding	378	260	1393
PB.2127.1	10	724	1	antisense	novelGene_gene: Zm00001d025612	novel	173.04	2207.53	non coding	NA	NA	NA
PB.678.2	1	492	1	incomplete-splice_match	gene: Zm00001d030357	Zm00001d030357_T00	38.98	102.40	non coding	NA	NA	NA
PB.9847.2	8	3427	13	novel_in_catalog	gene: Zm00001d011742	novel	6.53	22.85	coding	1048	122	3265
PB.3149.2	2	1730	1	novel_in_catalog	gene: Zm00001d005456	novel	7.73	24.52	coding	308	687	1610
PB.3149.1	2	1573	2	full-splice_match	gene: Zm00001d005456	Zm00001d005456_T00	16.79	24.52	coding	470	40	1449
PB.4205.1	3	1324	10	incomplete-splice_match	gene: Zm00001d042128	Zm00001d042128_T00	8.41	8.41	coding	354	18	1079
PB.4330.1	3	1080	7	full-splice_match	gene: Zm00001d042729	Zm00001d042729_T00	150.59	150.59	coding	210	71	700

PB.7640.6	6	2600	15	full-splice_match	gene: Zm00001d036971	Zm00001d036971_T04	4.52	145.45	coding	773	280	2598
PB.4273.6	3	1550	6	full-splice_match	gene: Zm00001d042482	Zm00001d042482_T00	0.00	130.54	coding	362	164	1249
PB.4752.3	3	2994	22	novel_not_in_catalog	gene: Zm00001d044358	novel	7.93	11.14	coding	877	236	2866
PB.10010.1	8	2208	2	full-splice_match	gene: Zm00001d012420	Zm00001d012420_T00	25.16	25.16	coding	648	117	2060
PB.4265.6	3	767	3	incomplete-splice_match	gene: Zm00001d042443	Zm00001d042443_T00	0.00	364.97	coding	152	175	630
PB.9296.3	8	710	3	full-splice_match	gene: Zm00001d008992	Zm00001d008992_T00	6.81	74.35	coding	215	65	709
PB.4265.4	3	2524	3	novel_in_catalog	gene: Zm00001d042443	novel	23.87	364.97	coding	341	462	1484
PB.4265.5	3	1409	5	full-splice_match	gene: Zm00001d042443	Zm00001d042443_T00	257.90	364.97	coding	344	137	1168
PB.4265.2	3	3852	2	novel_in_catalog	gene: Zm00001d042443	novel	60.89	364.97	coding	103	3054	3362
PB.4642.1	3	1422	3	genic	gene: Zm00001d043881	novel	25.77	25.77	coding	116	649	996
PB.2499.1	2	775	2	incomplete-splice_match	gene: Zm00001d002065	Zm00001d002065_T01	764.88	764.88	coding	140	12	431
PB.6690.1	5	647	1	full-splice_match	gene: Zm00001d016076	Zm00001d016076_T00	15.48	15.48	coding	99	70	366
PB.6734.1	5	1757	16	full-splice_match	gene: Zm00001d016311	Zm00001d016311_T00	69.46	69.46	coding	509	14	1540
PB.9847.4	8	3537	16	novel_not_in_catalog	gene: Zm00001d011742	novel	3.62	22.85	coding	1035	358	3462
PB.5628.2	4	1783	2	novel_in_catalog	gene: Zm00001d052733	novel	30.69	90.78	coding	343	275	1303
PB.9564.1	8	2057	2	full-splice_match	gene: Zm00001d010497	Zm00001d010497_T00	9.72	9.72	coding	242	83	808
PB.3602.3	2	1315	11	full-splice_match	gene: Zm00001d007645	Zm00001d007645_T00	8.34	30.46	coding	172	189	704

PB.6734.4	5	1640	16	full-splice_match	gene: Zm00001d016311	Zm00001d016311_T00	0.00	69.46	coding	509	14	1540
PB.6047.6	5	2615	6	novel_in_catalog	gene: Zm00001d013109	novel	30.32	284.25	coding	314	411	1352
PB.9232.1	8	953	1	intergenic	novelGene_71	novel	36.30	36.30	non coding	NA	NA	NA
PB.6047.4	5	2553	5	novel_in_catalog	gene: Zm00001d013109	novel	0.00	284.25	coding	414	267	1508
PB.6047.5	5	2734	6	novel_in_catalog	gene: Zm00001d013109	novel	26.39	284.25	coding	414	547	1788
PB.6047.2	5	2469	3	novel_in_catalog	gene: Zm00001d013109	novel	7.62	284.25	coding	208	157	780
PB.6047.3	5	1412	6	novel_in_catalog	gene: Zm00001d013109	novel	80.01	284.25	coding	157	722	1192
PB.6047.1	5	2759	7	full-splice_match	gene: Zm00001d013109	Zm00001d013109_T00	139.92	284.25	coding	620	697	2556
PB.609.5	1	996	1	novel_in_catalog	gene: Zm00001d030009	novel	0.00	159.80	non coding	NA	NA	NA
PB.9454.4	8	1923	2	novel_in_catalog	gene: Zm00001d009941	novel	8.03	88.88	non coding	NA	NA	NA
PB.9454.3	8	1338	11	novel_in_catalog	gene: Zm00001d009941	novel	0.00	88.88	coding	262	74	859
PB.9454.2	8	1469	11	novel_in_catalog	gene: Zm00001d009941	novel	7.52	88.88	coding	262	98	883
PB.9454.1	8	1267	12	full-splice_match	gene: Zm00001d009941	Zm00001d009941_T00	73.33	88.88	coding	298	95	988
PB.5545.1	4	1422	4	novel_not_in_catalog	gene: Zm00001d052338	novel	1.84	1.84	coding	139	221	637
PB.3699.2	3	2196	4	novel_in_catalog	gene: Zm00001d039316	novel	5.52	274.74	coding	144	65	496
PB.3699.1	3	1238	5	full-splice_match	gene: Zm00001d039316	Zm00001d039316_T00	269.22	274.74	coding	283	63	911
PB.609.7	1	1149	5	novel_not_in_catalog	gene: Zm00001d030009	novel	6.70	159.80	coding	110	132	461

PB.10265.1	9	706	1	full-splice_match	gene: Zm00001d045460	Zm00001d045460_T00	30.57	958.30	coding	87	177	437
PB.10265.2	9	602	1	full-splice_match	gene: Zm00001d045460	Zm00001d045460_T00	927.73	958.30	coding	87	177	437
PB.609.6	1	1335	4	novel_in_catalog	gene: Zm00001d030009	novel	2.32	159.80	coding	279	30	866
PB.3839.3	3	1439	3	full-splice_match	gene: Zm00001d039952	Zm00001d039952_T00	4.79	4.79	coding	435	59	1363
PB.1491.1	1	2409	17	full-splice_match	gene: Zm00001d034245	Zm00001d034245_T00	31.54	36.73	coding	570	424	2133
PB.7975.4	6	328	5	incomplete-splice_match	gene: Zm00001d038343	Zm00001d038343_T00	0.00	134.01	non coding	NA	NA	NA
PB.3956.8	3	1900	5	full-splice_match	gene: Zm00001d040470	Zm00001d040470_T00	0.00	187.61	coding	436	279	1586
PB.2491.8	2	1139	8	full-splice_match	gene: Zm00001d002035	Zm00001d002035_T00	110.27	177.22	coding	291	99	971
PB.3956.6	3	2081	6	novel_not_in_catalog	gene: Zm00001d040470	novel	12.45	187.61	coding	427	442	1722
PB.7617.1	6	2439	9	novel_in_catalog	gene: Zm00001d036827	novel	0.00	57.72	coding	543	662	2290
PB.2491.5	2	1284	7	novel_in_catalog	gene: Zm00001d002035	novel	4.33	177.22	coding	159	572	1048
PB.3956.5	3	2016	6	novel_not_in_catalog	gene: Zm00001d040470	novel	6.60	187.61	coding	444	297	1628
PB.2491.3	2	1237	8	full-splice_match	gene: Zm00001d002035	Zm00001d002035_T00	46.33	177.22	coding	291	93	965
PB.2491.2	2	1405	7	novel_in_catalog	gene: Zm00001d002035	novel	0.00	177.22	coding	159	569	1045
PB.2491.1	2	1346	8	full-splice_match	gene: Zm00001d002035	Zm00001d002035_T00	16.30	177.22	coding	291	94	966
PB.3956.1	3	2010	5	full-splice_match	gene: Zm00001d040470	Zm00001d040470_T00	159.95	187.61	coding	436	278	1585
PB.6749.1	5	1326	9	full-splice_match	gene: Zm00001d016410	Zm00001d016410_T00	99.49	158.09	coding	329	118	1104

PB.10078.8	8	1886	10	full-splice_match	gene: Zm00001d012667	Zm00001d012667_T02	10.07	49.54	coding	517	39	1589
PB.2225.1	10	2133	2	full-splice_match	gene: Zm00001d026051	Zm00001d026051_T00	38.19	57.74	coding	546	79	1716
PB.2471.1	2	3094	6	novel_not_in_catalog	gene: Zm00001d001953	novel	37.26	37.26	coding	890	188	2857
PB.9267.1	8	1690	11	incomplete-splice_match	gene: Zm00001d008863	Zm00001d008863_T00	33.11	33.11	coding	446	35	1372
PB.7951.2	6	1758	9	novel_in_catalog	gene: Zm00001d038266	novel	31.18	31.18	coding	315	603	1547
PB.3835.1	3	1061	1	intergenic	novelGene_79	novel	3.66	3.66	coding	152	198	653
PB.10078.1	8	3007	8	full-splice_match	gene: Zm00001d012667	Zm00001d012667_T00	11.64	49.54	non coding	NA	NA	NA
PB.10078.2	8	2061	6	novel_in_catalog	gene: Zm00001d012667	novel	3.46	49.54	non coding	NA	NA	NA
PB.10078.3	8	2953	9	incomplete-splice_match	gene: Zm00001d012667	Zm00001d012667_T00	6.71	49.54	coding	105	198	512
PB.6601.2	5	1609	2	full-splice_match	gene: Zm00001d015433	Zm00001d015433_T00	7.92	21.91	coding	440	57	1376
PB.6601.3	5	1501	2	full-splice_match	gene: Zm00001d015433	Zm00001d015433_T00	0.00	21.91	coding	440	47	1366
PB.5285.1	4	2803	2	novel_in_catalog	gene: Zm00001d051161	novel	99.68	167.23	coding	520	117	1676
PB.6601.1	5	1726	2	full-splice_match	gene: Zm00001d015433	Zm00001d015433_T00	13.99	21.91	coding	440	50	1369
PB.2671.3	2	1853	13	full-splice_match	gene: Zm00001d002827	Zm00001d002827_T00	40.97	40.97	coding	420	325	1584
PB.3410.1	2	1739	3	novel_in_catalog	gene: Zm00001d006620	novel	10.97	26.19	coding	254	557	1318
PB.4323.10	3	1473	2	incomplete-splice_match	gene: Zm00001d042695	Zm00001d042695_T00	3.07	95.71	non coding	NA	NA	NA
PB.5285.9	4	2539	1	novel_in_catalog	gene: Zm00001d051161	novel	2.85	167.23	coding	374	1323	2444

PB.3274.1	2	1004	8	full-splice_match	gene: Zm00001d006061	Zm00001d006061_T00	37.53	37.53	coding	179	141	677
PB.3410.5	2	1324	3	novel_in_catalog	gene: Zm00001d006620	novel	0.00	26.19	coding	254	415	1176
PB.3410.4	2	1134	3	full-splice_match	gene: Zm00001d006620	Zm00001d006620_T00	9.26	26.19	coding	254	207	968
PB.6186.2	5	1746	3	full-splice_match	gene: Zm00001d013612	Zm00001d013612_T00	176.46	200.71	coding	447	136	1476
PB.6186.1	5	1909	3	full-splice_match	gene: Zm00001d013612	Zm00001d013612_T00	24.25	200.71	coding	447	131	1471
PB.7321.2	6	931	1	full-splice_match	gene: Zm00001d035095	Zm00001d035095_T00	243.13	492.26	coding	148	90	533
PB.7321.3	6	815	1	full-splice_match	gene: Zm00001d035095	Zm00001d035095_T00	180.50	492.26	coding	148	107	550
PB.8314.1	7	1242	6	novel_in_catalog	gene: Zm00001d019048	novel	115.21	115.21	coding	174	142	663
PB.7321.1	6	954	1	full-splice_match	gene: Zm00001d035095	Zm00001d035095_T00	68.63	492.26	coding	148	95	538
PB.5841.1	4	1280	1	full-splice_match	gene: Zm00001d053736	Zm00001d053736_T00	2.65	2.65	coding	319	109	1065
PB.1576.1	1	776	4	incomplete-splice_match	gene: Zm00001d034597	Zm00001d034597_T00	47.24	70.06	coding	138	40	453
PB.10422.3	9	2015	4	full-splice_match	gene: Zm00001d046399	Zm00001d046399_T00	56.97	60.23	coding	439	240	1556
PB.9761.1	8	1406	7	full-splice_match	gene: Zm00001d011363	Zm00001d011363_T00	128.20	128.20	coding	345	98	1132
PB.9761.2	8	1150	6	incomplete-splice_match	gene: Zm00001d011363	Zm00001d011363_T00	0.00	128.20	coding	317	5	955
PB.602.2	1	713	4	full-splice_match	gene: Zm00001d029968	Zm00001d029968_T00	855.93	865.21	coding	149	64	510
PB.5299.1	4	990	4	incomplete-splice_match	gene: Zm00001d051272	Zm00001d051272_T00	107.37	107.37	coding	236	40	747
PB.602.1	1	824	4	full-splice_match	gene: Zm00001d029968	Zm00001d029968_T00	9.28	865.21	coding	149	67	513

PB.5940.1	4	1176	9	full-splice_match	gene: Zm00001d054078	Zm00001d054078_T00	79.34	79.34	coding	278	95	928
PB.3583.2	2	1148	2	intergenic	novelGene_433	novel	4.47	23.82	coding	165	440	934
PB.10948.5	B73V4	858	3	novel_in_catalog	gene: Zm00001d000302	novel	18.46	281.10	coding	164	65	556
PB.10948.6	B73V4	570	1	novel_in_catalog	gene: Zm00001d000302	novel	0.00	281.10	non coding	NA	NA	NA
PB.4175.1	3	1853	13	full-splice_match	gene: Zm00001d041995	Zm00001d041995_T00	19.44	19.44	coding	488	43	1506
PB.9614.1	8	1661	1	full-splice_match	gene: Zm00001d010689	Zm00001d010689_T00	12.23	12.23	coding	480	58	1497
PB.10948.1	B73V4	1544	8	full-splice_match	gene: Zm00001d000302	Zm00001d000302_T00	260.50	281.10	coding	290	283	1152
PB.10948.2	B73V4	1609	7	novel_in_catalog	gene: Zm00001d000302	novel	2.14	281.10	coding	200	254	853
PB.7082.8	5	1690	2	full-splice_match	gene: Zm00001d017867	Zm00001d017867_T00	22.91	69.14	coding	478	123	1556
PB.7082.9	5	1948	2	full-splice_match	gene: Zm00001d017867	Zm00001d017867_T00	38.50	69.14	coding	478	24	1457
PB.8707.1	7	3698	1	full-splice_match	gene: Zm00001d021243	Zm00001d021243_T00	3.58	12.57	coding	371	42	1154
PB.9087.1	7	1521	2	full-splice_match	gene: Zm00001d022549	Zm00001d022549_T00	5.68	5.68	coding	388	50	1213
PB.3749.1	3	1009	1	incomplete-splice_match	gene: Zm00001d039510	Zm00001d039510_T00	7.74	7.74	coding	217	23	673
PB.9352.2	8	634	1	genic	gene: Zm00001d009415	novel	22.10	622.40	non coding	NA	NA	NA
PB.2567.1	2	1452	2	novel_not_in_catalog	gene: Zm00001d002353	novel	27.48	27.48	coding	357	70	1140
PB.9352.1	8	627	1	genic	gene: Zm00001d009415	novel	144.75	622.40	non coding	NA	NA	NA
PB.7876.1	6	3170	3	novel_not_in_catalog	gene: Zm00001d038021	novel	28.42	34.18	coding	870	215	2824

PB.7876.2	6	3104	3	novel_not_in_catalog	gene: Zm00001d038021	novel	5.76	34.18	coding	870	256	2865
PB.1200.1	1	1393	2	full-splice_match	gene: Zm00001d032911	Zm00001d032911_T00	85.19	85.19	coding	420	23	1282
PB.1778.2	10	1304	3	novel_not_in_catalog	gene: Zm00001d023615	novel	45.61	45.61	coding	150	296	745
PB.6808.1	5	1177	9	full-splice_match	gene: Zm00001d016704	Zm00001d016704_T00	118.80	118.80	coding	300	101	1000
PB.4390.1	3	2292	5	full-splice_match	gene: Zm00001d042993	Zm00001d042993_T00	126.00	126.00	coding	541	340	1962
PB.5633.1	4	954	7	full-splice_match	gene: Zm00001d052742	Zm00001d052742_T00	21.64	32.69	coding	189	124	690
PB.5956.4	5	1556	4	incomplete-splice_match	gene: Zm00001d012808	Zm00001d012808_T00	0.00	43.98	coding	467	50	1450
PB.5956.5	5	1907	3	novel_in_catalog	gene: Zm00001d012808	novel	0.00	43.98	coding	422	79	1344
PB.5956.6	5	1823	4	novel_in_catalog	gene: Zm00001d012808	novel	7.83	43.98	coding	453	83	1441
PB.261.4	1	1654	5	full-splice_match	gene: Zm00001d028366	Zm00001d028366_T00	0.00	62.46	coding	455	169	1533
PB.261.3	1	1720	5	full-splice_match	gene: Zm00001d028366	Zm00001d028366_T00	17.30	62.46	coding	455	172	1536
PB.5956.1	5	2089	3	novel_in_catalog	gene: Zm00001d012808	novel	12.54	43.98	coding	422	83	1348
PB.5956.2	5	1948	5	full-splice_match	gene: Zm00001d012808	Zm00001d012808_T00	23.60	43.98	coding	545	83	1717
PB.5956.3	5	1986	4	novel_in_catalog	gene: Zm00001d012808	novel	0.00	43.98	coding	453	83	1441
PB.3657.3	2	770	1	novel_in_catalog	gene: Zm00001d007901	novel	0.00	280.15	coding	142	345	770
PB.1718.1	10	3109	3	novel_in_catalog	gene: Zm00001d023367	novel	2.87	31.46	coding	795	374	2758
PB.1718.2	10	2950	3	novel_in_catalog	gene: Zm00001d023367	novel	28.60	31.46	coding	795	416	2800

PB.8334.2	7	2464	4	full-splice_match	gene: Zm00001d019173	Zm00001d019173_T00	55.50	94.34	coding	742	7	2232
PB.5291.1	4	873	6	genic_intron	novelGene_295	novel	281.22	281.22	coding	157	177	647
PB.6693.1	5	1059	3	full-splice_match	gene: Zm00001d016098	Zm00001d016098_T00	2.20	2.20	coding	301	54	956
PB.9961.3	8	2581	1	novel_in_catalog	gene: Zm00001d012212	novel	3.64	14.52	coding	218	25	678
PB.1784.3	10	1282	8	full-splice_match	gene: Zm00001d023635	Zm00001d023635_T00	189.20	195.48	coding	275	145	969
PB.1784.2	10	502	1	incomplete-splice_match	gene: Zm00001d023635	Zm00001d023635_T00	6.29	195.48	non coding	NA	NA	NA
PB.2298.2	10	900	6	full-splice_match	gene: Zm00001d026335	Zm00001d026335_T01	269.23	642.33	coding	120	334	693
PB.2298.3	10	749	7	full-splice_match	gene: Zm00001d026335	Zm00001d026335_T00	373.10	642.33	coding	120	179	538
PB.832.3	1	821	2	full-splice_match	gene: Zm00001d031257	Zm00001d031257_T00	404.74	724.27	coding	178	113	646
PB.1784.5	10	1142	8	full-splice_match	gene: Zm00001d023635	Zm00001d023635_T00	0.00	195.48	coding	275	141	965
PB.3000.1	2	1108	2	genic	gene: Zm00001d004568	novel	120.74	120.74	coding	290	71	940
PB.8297.1	7	1263	4	novel_in_catalog	gene: Zm00001d018946	novel	2.51	40.09	non coding	NA	NA	NA
PB.10940.5	9	1679	9	incomplete-splice_match	gene: Zm00001d048575	Zm00001d048575_T01	14.62	38.05	coding	298	190	1083
PB.8297.3	7	866	6	full-splice_match	gene: Zm00001d018946	Zm00001d018946_T00	15.26	40.09	coding	188	63	626
PB.8297.2	7	652	3	novel_in_catalog	gene: Zm00001d018946	novel	22.32	40.09	non coding	NA	NA	NA
PB.4727.1	3	667	1	full-splice_match	gene: Zm00001d044244	Zm00001d044244_T00	32.32	32.32	coding	120	135	494
PB.7518.1	6	3809	8	full-splice_match	gene: Zm00001d036301	Zm00001d036301_T01	96.76	96.76	coding	1197	14	3604

PB.10940.2	9	3037	9	incomplete-splice_match	gene: Zm00001d048575	Zm00001d048575_T02	20.34	38.05	coding	384	1612	2763
PB.2038.3	10	3044	3	full-splice_match	gene: Zm00001d025050	Zm00001d025050_T00	42.14	119.07	coding	767	695	2995
PB.634.1	1	1866	1	full-splice_match	gene: Zm00001d030129	Zm00001d030129_T00	13.86	13.86	coding	398	463	1656
PB.3060.1	2	1652	9	full-splice_match	gene: Zm00001d004976	Zm00001d004976_T00	68.02	78.70	coding	416	145	1392
PB.3060.2	2	1688	8	novel_in_catalog	gene: Zm00001d004976	novel	10.68	78.70	coding	350	462	1511
PB.4364.1	3	1497	7	full-splice_match	gene: Zm00001d042869	Zm00001d042869_T00	455.64	492.67	coding	310	218	1147
PB.5271.2	4	715	4	full-splice_match	gene: Zm00001d051055	Zm00001d051055_T00	199.75	348.82	coding	77	127	357
PB.5271.3	4	621	4	full-splice_match	gene: Zm00001d051055	Zm00001d051055_T00	0.00	348.82	coding	77	131	361
PB.4364.4	3	1435	7	full-splice_match	gene: Zm00001d042869	Zm00001d042869_T00	0.00	492.67	coding	310	224	1153
PB.5271.1	4	839	4	full-splice_match	gene: Zm00001d051055	Zm00001d051055_T00	149.07	348.82	coding	77	145	375
PB.5486.1	4	1040	1	incomplete-splice_match	gene: Zm00001d052144	Zm00001d052144_T00	55.66	55.66	coding	266	31	828
PB.10960.1	B73V4	662	6	full-splice_match	gene: Zm00001d000120	Zm00001d000120_T00	126.53	126.53	coding	151	42	494
PB.6714.1	5	915	4	full-splice_match	gene: Zm00001d016200	Zm00001d016200_T00	264.47	349.37	coding	182	79	624
PB.2399.1	10	1331	1	full-splice_match	gene: Zm00001d026665	Zm00001d026665_T00	64.42	64.42	coding	265	116	910
PB.2399.2	10	1221	1	full-splice_match	gene: Zm00001d026665	Zm00001d026665_T00	0.00	64.42	coding	265	107	901
PB.6714.2	5	1683	2	novel_in_catalog	gene: Zm00001d016200	novel	1.85	349.37	coding	158	212	685
PB.2519.10	2	2113	1	novel_in_catalog	gene: Zm00001d002141	novel	4.83	657.91	coding	134	1479	1880

PB.2519.11	2	1616	3	full-splice_match	gene: Zm00001d002141	Zm00001d002141_T00	3.83	657.91	coding	171	872	1384
PB.2519.13	2	1025	2	full-splice_match	gene: Zm00001d002141	Zm00001d002141_T00	1.77	657.91	non coding	NA	NA	NA
PB.3827.2	3	855	5	full-splice_match	gene: Zm00001d039874	Zm00001d039874_T00	0.00	33.89	coding	191	118	690
PB.3827.1	3	935	5	full-splice_match	gene: Zm00001d039874	Zm00001d039874_T00	33.89	33.89	coding	191	119	691
PB.3266.4	2	1851	6	novel_in_catalog	gene: Zm00001d006034	novel	6.25	40.60	coding	377	458	1588
PB.848.2	1	1683	5	full-splice_match	gene: Zm00001d031329	Zm00001d031329_T00	16.18	39.51	coding	428	10	1293
PB.5763.2	4	1863	7	full-splice_match	gene: Zm00001d053376	Zm00001d053376_T00	9.84	24.27	coding	485	182	1636
PB.5763.3	4	1665	7	full-splice_match	gene: Zm00001d053376	Zm00001d053376_T00	1.42	24.27	coding	485	111	1565
PB.7837.1	6	1697	3	full-splice_match	gene: Zm00001d037873	Zm00001d037873_T00	480.50	480.50	coding	447	83	1423
PB.3731.1	3	1912	1	full-splice_match	gene: Zm00001d039444	Zm00001d039444_T00	59.91	59.91	coding	511	151	1683
PB.613.1	1	2733	1	full-splice_match	gene: Zm00001d030028	Zm00001d030028_T00	114.74	204.20	coding	705	48	2162
PB.613.2	1	2613	1	full-splice_match	gene: Zm00001d030028	Zm00001d030028_T00	11.97	204.20	coding	705	62	2176
PB.613.4	1	2496	1	full-splice_match	gene: Zm00001d030028	Zm00001d030028_T00	0.00	204.20	coding	705	51	2165
PB.613.5	1	780	1	full-splice_match	gene: Zm00001d030028	Zm00001d030028_T00	77.48	204.20	coding	150	105	554
PB.3966.1	3	1814	2	genic	gene: Zm00001d040555	novel	57.55	57.55	coding	470	87	1496
PB.9529.1	8	821	2	full-splice_match	gene: Zm00001d010322	Zm00001d010322_T00	99.37	99.37	coding	177	35	565
PB.1556.1	1	1845	8	novel_in_catalog	gene: Zm00001d034518	novel	76.02	134.53	coding	419	323	1579

PB.2877.1	2	2265	2	full-splice_match	gene: Zm00001d003800	Zm00001d003800_T00	0.28	66.00	coding	347	1057	2097
PB.848.1	1	2103	5	full-splice_match	gene: Zm00001d031329	Zm00001d031329_T00	23.33	39.51	coding	515	140	1684
PB.8038.2	6	1239	2	full-splice_match	gene: Zm00001d038574	Zm00001d038574_T00	15.60	15.60	coding	229	325	1011
PB.5948.2	4	387	2	novel_not_in_catalog	gene: Zm00001d027045	novel	15.12	35.90	non coding	NA	NA	NA
PB.8150.1	6	658	1	full-splice_match	gene: Zm00001d038975	Zm00001d038975_T00	39.47	39.47	coding	104	72	383
PB.1430.2	1	1072	4	full-splice_match	gene: Zm00001d033983	Zm00001d033983_T00	0.00	483.18	coding	255	114	878
PB.3484.1	2	1415	2	full-splice_match	gene: Zm00001d006933	Zm00001d006933_T00	61.97	316.37	coding	362	47	1132
PB.805.3	1	536	3	full-splice_match	gene: Zm00001d031132	Zm00001d031132_T00	943.06	1430.04	coding	84	104	355
PB.1853.4	10	1846	13	fusion	gene: Zm00001d024043	novel	1.39	81.04	coding	361	427	1509
PB.3711.1	3	1292	2	genic	gene: Zm00001d039365	novel	91.08	91.08	coding	305	155	1069
PB.8099.1	6	2073	9	full-splice_match	gene: Zm00001d038830	Zm00001d038830_T00	8.57	26.07	coding	501	299	1801
PB.7160.1	5	886	4	full-splice_match	gene: Zm00001d018135	Zm00001d018135_T00	109.67	109.67	coding	162	122	607
PB.3092.5	2	1679	11	novel_in_catalog	gene: Zm00001d005144	novel	2.18	54.95	coding	288	191	1054
PB.805.4	1	396	3	full-splice_match	gene: Zm00001d031132	Zm00001d031132_T00	0.00	1430.04	coding	84	106	357
PB.4670.4	3	2393	21	full-splice_match	gene: Zm00001d044024	Zm00001d044024_T00	6.25	44.61	coding	619	175	2031
PB.8622.2	7	2235	4	novel_in_catalog	gene: Zm00001d020881	novel	12.66	74.69	coding	345	57	1091
PB.10461.2	9	3414	19	full-splice_match	gene: Zm00001d046587	Zm00001d046587_T02	2.44	13.43	coding	610	1196	3025

PB.5865.1	4	1159	2	full-splice_match	gene: Zm00001d053830	Zm00001d053830_T00	12.32	12.32	coding	275	191	1015
PB.9172.1	8	1655	7	novel_in_catalog	gene: Zm00001d008406	novel	10.92	142.15	coding	213	656	1294
PB.9243.1	8	2825	4	full-splice_match	gene: Zm00001d008727	Zm00001d008727_T00	134.96	134.96	coding	812	107	2542
PB.8611.1	7	2803	19	full-splice_match	gene: Zm00001d020827	Zm00001d020827_T00	28.60	28.60	coding	797	198	2588
PB.9535.3	8	1718	4	novel_in_catalog	gene: Zm00001d010368	novel	10.04	109.71	coding	162	1003	1488
PB.9535.2	8	1193	7	full-splice_match	gene: Zm00001d010368	Zm00001d010368_T00	99.67	109.71	coding	243	174	902
PB.10869.2	9	1060	3	full-splice_match	gene: Zm00001d048360	Zm00001d048360_T00	0.00	9.12	coding	276	58	885
PB.3092.1	2	2285	11	full-splice_match	gene: Zm00001d005144	Zm00001d005144_T00	11.49	54.95	coding	264	92	883
PB.10869.1	9	1185	3	full-splice_match	gene: Zm00001d048360	Zm00001d048360_T00	9.12	9.12	coding	276	73	900
PB.8103.1	6	765	4	full-splice_match	gene: Zm00001d038839	Zm00001d038839_T00	1.65	485.82	coding	80	57	296
PB.8103.3	6	526	4	full-splice_match	gene: Zm00001d038839	Zm00001d038839_T00	407.59	485.82	coding	80	84	323
PB.8103.2	6	650	4	full-splice_match	gene: Zm00001d038839	Zm00001d038839_T00	72.98	485.82	coding	80	79	318
PB.8886.4	7	3569	7	novel_not_in_catalog	gene: Zm00001d021858	novel	13.43	123.94	coding	985	215	3169
PB.10891.1	9	3798	13	full-splice_match	gene: Zm00001d048442	Zm00001d048442_T00	18.75	28.66	coding	864	538	3129
PB.3837.1	3	963	2	incomplete-splice_match	gene: Zm00001d039919	Zm00001d039919_T00	1.83	37.80	coding	62	234	419
PB.3837.2	3	1395	5	full-splice_match	gene: Zm00001d039919	Zm00001d039919_T00	17.65	37.80	coding	328	125	1108
PB.3837.3	3	1468	4	novel_in_catalog	gene: Zm00001d039919	novel	18.32	37.80	coding	194	124	705

PB.1418.1	1	1192	6	novel_not_in_catalog	gene: Zm00001d033925	novel	863.80	979.16	coding	205	134	748
PB.3381.7	2	2317	4	full-splice_match	gene: Zm00001d006480	Zm00001d006480_T00	0.00	92.42	coding	658	114	2087
PB.7355.2	6	774	5	full-splice_match	gene: Zm00001d035304	Zm00001d035304_T00	10.28	56.88	coding	195	45	629
PB.7355.1	6	1082	5	full-splice_match	gene: Zm00001d035304	Zm00001d035304_T00	46.60	56.88	coding	195	290	874
PB.4179.1	3	1259	2	full-splice_match	gene: Zm00001d042022	Zm00001d042022_T00	9.09	9.09	coding	361	71	1153
PB.2697.2	2	1441	5	full-splice_match	gene: Zm00001d002941	Zm00001d002941_T00	77.87	102.35	coding	344	243	1274
PB.2697.1	2	1607	5	full-splice_match	gene: Zm00001d002941	Zm00001d002941_T00	24.48	102.35	coding	344	186	1217
PB.3958.1	3	3338	4	full-splice_match	gene: Zm00001d040503	Zm00001d040503_T00	80.65	324.70	coding	950	106	2955
PB.3958.2	3	2723	4	full-splice_match	gene: Zm00001d040503	Zm00001d040503_T00	30.06	324.70	coding	757	181	2451
PB.3958.3	3	383	2	incomplete-splice_match	gene: Zm00001d040503	Zm00001d040503_T00	213.99	324.70	non coding	NA	NA	NA
PB.43.3	1	1317	1	novel_in_catalog	gene: Zm00001d027405	novel	7.45	74.94	coding	145	760	1194
PB.10477.5	9	2429	2	novel_in_catalog	gene: Zm00001d046688	novel	2.10	42.31	coding	194	710	1291
PB.43.1	1	1007	2	full-splice_match	gene: Zm00001d027405	Zm00001d027405_T00	67.49	74.94	coding	194	282	863
PB.3057.2	2	801	2	full-splice_match	gene: Zm00001d004936	Zm00001d004936_T00	82.02	405.47	non coding	NA	NA	NA
PB.10814.1	9	796	1	incomplete-splice_match	gene: Zm00001d048134	Zm00001d048134_T00	16.78	16.78	coding	169	9	515
PB.1972.2	10	1208	1	intergenic	novelGene_192	novel	6.81	24.22	coding	71	216	428
PB.3057.4	2	3273	1	novel_in_catalog	gene: Zm00001d004936	novel	6.68	405.47	non coding	NA	NA	NA

PB.2162.1	10	971	5	full-splice_match	gene: Zm00001d025763	Zm00001d025763_T00	61.99	68.58	coding	200	98	697
PB.3209.2	2	1485	1	full-splice_match	gene: Zm00001d005790	Zm00001d005790_T00	3.87	94.90	coding	328	363	1346
PB.3260.1	2	3095	3	full-splice_match	gene: Zm00001d006011	Zm00001d006011_T00	19.64	19.64	coding	880	107	2746
PB.8691.4	7	2609	3	full-splice_match	gene: Zm00001d021202	Zm00001d021202_T03	10.60	25.59	coding	315	800	1744
PB.10793.1	9	2474	2	full-splice_match	gene: Zm00001d048054	Zm00001d048054_T00	56.48	68.53	coding	672	112	2127
PB.2162.2	10	852	4	full-splice_match	gene: Zm00001d025763	Zm00001d025763_T00	6.59	68.58	coding	166	116	613
PB.6023.1	5	1141	2	full-splice_match	gene: Zm00001d013034	Zm00001d013034_T00	314.93	317.39	coding	274	87	908
PB.6023.2	5	1789	1	genic	gene: Zm00001d013034	novel	2.46	317.39	coding	248	10	753
PB.10043.1	8	953	4	incomplete-splice_match	gene: Zm00001d012550	Zm00001d012550_T00	12.61	327.31	non coding	NA	NA	NA
PB.9626.2	8	1248	9	full-splice_match	gene: Zm00001d010723	Zm00001d010723_T00	51.19	60.67	coding	280	212	1051
PB.3859.1	3	1959	3	full-splice_match	gene: Zm00001d040034	Zm00001d040034_T00	6.03	11.36	coding	520	16	1575
PB.3859.2	3	1975	3	full-splice_match	gene: Zm00001d040034	Zm00001d040034_T00	5.33	11.36	coding	599	94	1890
PB.2133.3	10	2585	7	full-splice_match	gene: Zm00001d025653	Zm00001d025653_T00	7.55	29.70	coding	220	105	764
PB.10004.1	8	1692	4	full-splice_match	gene: Zm00001d012401	Zm00001d012401_T00	26.89	46.52	coding	413	203	1441
PB.10004.3	8	1565	4	full-splice_match	gene: Zm00001d012401	Zm00001d012401_T00	13.67	46.52	coding	413	191	1429
PB.10004.2	8	1849	2	novel_in_catalog	gene: Zm00001d012401	novel	5.97	46.52	coding	284	230	1081
PB.2058.2	10	2624	16	fusion	gene: Zm00001d025175	novel	4.98	13.50	coding	630	295	2184

PB.891.1	1	863	7	full-splice_match	gene: Zm00001d031611	Zm00001d031611_T00	320.17	320.17	coding	171	73	585
PB.7578.1	6	1567	2	novel_in_catalog	gene: Zm00001d036613	novel	3.14	3.14	coding	246	629	1366
PB.2058.4	10	2586	16	fusion	gene: Zm00001d025175	novel	8.52	13.50	coding	730	94	2283
PB.7884.1	6	791	3	full-splice_match	gene: Zm00001d038038	Zm00001d038038_T00	17.53	17.53	coding	185	154	708
PB.10185.1	9	1424	8	novel_not_in_catalog	gene: Zm00001d045113	novel	2.94	13.00	coding	248	415	1158
PB.6720.1	5	1733	2	intergenic	novelGene_302	novel	6.90	6.90	coding	222	104	769
PB.10185.3	9	1805	7	novel_not_in_catalog	gene: Zm00001d045113	novel	2.24	13.00	coding	180	1089	1628
PB.10185.2	9	1317	9	novel_not_in_catalog	gene: Zm00001d045113	novel	7.82	13.00	coding	321	178	1140
PB.2000.2	10	1438	6	full-splice_match	gene: Zm00001d024908	Zm00001d024908_T00	53.97	53.97	coding	340	253	1272
PB.215.2	1	2571	6	novel_in_catalog	gene: Zm00001d028188	novel	77.29	100.22	coding	560	335	2014
PB.215.1	1	427	1	genic	gene: Zm00001d028188	novel	22.94	100.22	non coding	NA	NA	NA
PB.8780.5	7	788	4	full-splice_match	gene: Zm00001d021526	Zm00001d021526_T00	822.71	822.71	coding	149	127	573
PB.8455.1	7	873	1	incomplete-splice_match	gene: Zm00001d020014	Zm00001d020014_T00	4.10	19.26	non coding	NA	NA	NA
PB.1325.1	1	3640	2	novel_in_catalog	gene: Zm00001d033511	novel	12.03	62.99	coding	259	2316	3092
PB.1325.2	1	2752	1	genic	gene: Zm00001d033511	novel	5.84	62.99	coding	259	1499	2275
PB.8455.2	7	2851	3	incomplete-splice_match	gene: Zm00001d020014	Zm00001d020014_T01	15.16	19.26	coding	877	3	2633
PB.9725.2	8	764	1	full-splice_match	gene: Zm00001d011189	Zm00001d011189_T00	45.99	55.67	coding	119	142	498

PB.9084.4	7	1827	10	full-splice_match	gene: Zm00001d022542	Zm00001d022542_T00	46.45	154.27	coding	333	385	1383
PB.3488.1	2	1819	13	novel_not_in_catalog	gene: Zm00001d006944	novel	40.93	50.84	coding	443	115	1443
PB.6794.1	5	454	1	full-splice_match	gene: Zm00001d016660	Zm00001d016660_T00	5939.33	5939.33	non coding	NA	NA	NA
PB.2884.1	2	3138	12	full-splice_match	gene: Zm00001d003847	Zm00001d003847_T00	13.01	36.16	coding	932	36	2831
PB.2884.3	2	1457	6	incomplete-splice_match	gene: Zm00001d003847	Zm00001d003847_T00	19.88	36.16	coding	365	202	1296
PB.2884.2	2	3477	11	novel_in_catalog	gene: Zm00001d003847	novel	3.27	36.16	coding	670	1162	3171
PB.6107.1	5	1474	7	novel_in_catalog	gene: Zm00001d013310	novel	16.23	16.23	coding	370	52	1161
PB.2556.6	2	1441	4	incomplete-splice_match	gene: Zm00001d002325	Zm00001d002325_T00	144.40	303.54	coding	376	313	1440
PB.308.5	1	1566	11	novel_not_in_catalog	gene: Zm00001d028567	novel	28.31	56.96	coding	261	711	1493
PB.308.4	1	1386	9	novel_not_in_catalog	gene: Zm00001d028567	novel	23.46	56.96	coding	263	524	1312
PB.5106.1	4	1803	10	full-splice_match	gene: Zm00001d049922	Zm00001d049922_T00	5.59	5.59	coding	464	167	1558
PB.8860.3	7	1042	5	full-splice_match	gene: Zm00001d021779	Zm00001d021779_T00	38.42	55.84	coding	110	417	746
PB.308.1	1	1872	9	novel_in_catalog	gene: Zm00001d028567	novel	1.56	56.96	coding	372	614	1729
PB.308.3	1	1594	11	full-splice_match	gene: Zm00001d028567	Zm00001d028567_T00	3.63	56.96	coding	263	727	1515
PB.4414.1	3	2402	5	novel_in_catalog	gene: Zm00001d043108	novel	7.94	7.94	coding	294	1330	2211
PB.6846.1	5	2078	3	incomplete-splice_match	gene: Zm00001d016858	Zm00001d016858_T00	13.23	55.65	coding	482	206	1651
PB.4853.2	4	1240	1	full-splice_match	gene: Zm00001d048671	Zm00001d048671_T00	20.04	20.45	coding	322	118	1083

PB.4853.1	4	1378	1	full-splice_match	gene: Zm00001d048671	Zm00001d048671_T00	0.41	20.45	coding	322	118	1083
PB.3625.1	2	758	3	incomplete-splice_match	gene: Zm00001d007783	Zm00001d007783_T00	18.82	18.82	coding	175	73	597
PB.1359.1	1	2374	6	novel_in_catalog	gene: Zm00001d033642	novel	21.48	85.06	coding	634	134	2035
PB.3535.2	2	1101	3	incomplete-splice_match	gene: Zm00001d007173	Zm00001d007173_T00	0.00	82.88	coding	274	137	958
PB.6899.12	5	1442	9	full-splice_match	gene: Zm00001d017096	Zm00001d017096_T00	41.86	254.51	coding	193	106	684
PB.1185.2	1	1269	4	incomplete-splice_match	gene: Zm00001d032855	Zm00001d032855_T01	31.00	60.27	coding	163	562	1050
PB.1185.4	1	3587	16	novel_not_in_catalog	gene: Zm00001d032855	novel	8.72	60.27	coding	992	82	3057
PB.6027.6	5	2498	3	full-splice_match	gene: Zm00001d013043	Zm00001d013043_T00	7.13	62.01	coding	199	1712	2308
PB.5698.3	4	1604	7	novel_not_in_catalog	gene: Zm00001d053036	novel	35.63	79.13	non coding	NA	NA	NA
PB.4483.1	3	837	2	fusion	gene: Zm00001d000681	novel	1.22	1.22	coding	175	36	560
PB.6978.1	5	639	3	full-splice_match	gene: Zm00001d017425	Zm00001d017425_T00	131.04	131.04	coding	139	71	487
PB.3488.9	2	1770	12	novel_not_in_catalog	gene: Zm00001d006944	novel	3.20	50.84	coding	392	116	1291
PB.5138.1	4	1508	5	full-splice_match	gene: Zm00001d050123	Zm00001d050123_T00	26.91	42.63	coding	332	153	1148
PB.5138.2	4	1322	6	novel_not_in_catalog	gene: Zm00001d050123	novel	15.71	42.63	coding	309	136	1062
PB.5138.3	4	1401	5	full-splice_match	gene: Zm00001d050123	Zm00001d050123_T00	0.00	42.63	coding	332	153	1148
PB.4906.1	4	2083	3	intergenic	novelGene_49	novel	6.34	11.67	non coding	NA	NA	NA
PB.6926.1	5	1411	3	full-splice_match	gene: Zm00001d017187	Zm00001d017187_T00	36.50	36.50	coding	328	107	1090

PB.5726.1	4	884	1	antisense	novelGene_gene: Zm00001d053213	novel	22.68	22.68	coding	22	430	495
PB.726.2	1	1163	1	genic	gene: Zm00001d022836	novel	3.16	11.31	non coding	NA	NA	NA
PB.912.1	1	445	3	full-splice_match	gene: Zm00001d031701	Zm00001d031701_T00	302.56	302.56	non coding	NA	NA	NA
PB.5483.3	4	1169	9	novel_in_catalog	gene: Zm00001d052108	novel	7.78	262.20	coding	176	120	647
PB.5483.1	4	1224	10	full-splice_match	gene: Zm00001d052108	Zm00001d052108_T00	143.13	262.20	coding	212	200	835
PB.9827.1	8	1462	1	full-splice_match	gene: Zm00001d011656	Zm00001d011656_T00	262.60	262.60	coding	450	111	1460
PB.2027.1	10	834	5	full-splice_match	gene: Zm00001d025020	Zm00001d025020_T00	295.58	360.30	coding	157	101	571
PB.9827.2	8	1349	1	full-splice_match	gene: Zm00001d011656	Zm00001d011656_T00	0.00	262.60	coding	413	110	1348
PB.976.1	1	920	1	intergenic	novelGene_405	novel	3.16	3.16	coding	240	82	801
PB.9037.3	7	802	1	full-splice_match	gene: Zm00001d022416	Zm00001d022416_T00	536.50	587.97	coding	113	145	483
PB.3477.1	2	3612	3	full-splice_match	gene: Zm00001d006917	Zm00001d006917_T00	21.54	21.54	coding	1059	251	3427
PB.5711.1	4	1465	8	full-splice_match	gene: Zm00001d053104	Zm00001d053104_T00	13.06	18.41	coding	266	127	924
PB.2277.1	10	1260	2	full-splice_match	gene: Zm00001d026285	Zm00001d026285_T00	11.88	11.88	coding	289	166	1032
PB.5330.7	4	2191	6	full-splice_match	gene: Zm00001d051442	Zm00001d051442_T00	11.85	11.85	coding	647	75	2015
PB.7947.1	6	672	2	incomplete-splice_match	gene: Zm00001d038248	Zm00001d038248_T00	14.31	226.44	non coding	NA	NA	NA
PB.1271.1	1	713	4	novel_not_in_catalog	gene: Zm00001d033284	novel	26.14	26.14	coding	195	94	678
PB.11009.1	B73V 4	985	1	full-splice_match	gene: Zm00001d000310	Zm00001d000310_T00	36.68	36.68	coding	244	113	844

PB.4693.2	3	1347	4	novel_in_catalog	gene: Zm00001d044127	novel	12.83	51.45	coding	118	197	550
PB.7947.2	6	2196	2	novel_in_catalog	gene: Zm00001d038248	novel	7.20	226.44	coding	92	71	346
PB.1395.1	1	2447	5	full-splice_match	gene: Zm00001d033800	Zm00001d033800_T00	32.01	57.51	coding	587	382	2142
PB.8036.1	6	1545	2	full-splice_match	gene: Zm00001d038553	Zm00001d038553_T00	5.57	5.57	coding	457	45	1415
PB.7947.3	6	1712	3	novel_in_catalog	gene: Zm00001d038248	novel	9.14	226.44	coding	130	76	465
PB.320.1	1	1499	1	genic	gene: Zm00001d028611	novel	9.34	9.34	non coding	NA	NA	NA
PB.7947.4	6	930	4	novel_in_catalog	gene: Zm00001d038248	novel	16.15	226.44	coding	176	69	596
PB.4473.1	3	484	4	incomplete-splice_match	gene: Zm00001d043320	Zm00001d043320_T00	9.33	9.33	non coding	NA	NA	NA
PB.5130.1	4	1903	1	genic	gene: Zm00001d026821	novel	6.80	6.80	non coding	NA	NA	NA
PB.8703.3	7	2969	4	novel_in_catalog	gene: Zm00001d021225	novel	2.98	59.68	coding	154	384	845
PB.8794.7	7	1238	5	full-splice_match	gene: Zm00001d021574	Zm00001d021574_T00	16.97	105.83	coding	302	124	1029
PB.3843.1	3	2491	2	genic	gene: Zm00001d039967	novel	3.66	3.66	coding	698	101	2194
PB.710.8	1	1290	9	full-splice_match	gene: Zm00001d030620	Zm00001d030620_T00	9.81	72.87	coding	294	185	1066
PB.710.9	1	1249	9	full-splice_match	gene: Zm00001d030620	Zm00001d030620_T00	10.37	72.87	coding	294	176	1057
PB.710.4	1	1369	9	full-splice_match	gene: Zm00001d030620	Zm00001d030620_T00	0.00	72.87	coding	294	187	1068
PB.710.5	1	1388	9	full-splice_match	gene: Zm00001d030620	Zm00001d030620_T00	19.19	72.87	coding	294	227	1108
PB.710.2	1	1513	9	full-splice_match	gene: Zm00001d030620	Zm00001d030620_T00	0.00	72.87	coding	294	208	1089

PB.4843.1	4	1610	1	full-splice_match	gene: Zm00001d048615	Zm00001d048615_T00	5.43	5.43	coding	265	480	1274
PB.3672.1	2	1833	2	incomplete-splice_match	gene: Zm00001d007975	Zm00001d007975_T00	17.32	17.32	coding	545	15	1649
PB.710.1	1	1495	9	full-splice_match	gene: Zm00001d030620	Zm00001d030620_T00	21.39	72.87	coding	294	190	1071
PB.2798.5	2	1717	2	incomplete-splice_match	gene: Zm00001d003401	Zm00001d003401_T00	7.63	1796.02	non coding	NA	NA	NA
PB.7947.8	6	1525	2	novel_in_catalog	gene: Zm00001d038248	novel	7.59	226.44	coding	92	82	357
PB.2798.6	2	1321	5	novel_in_catalog	gene: Zm00001d003401	novel	6.13	1796.02	coding	170	92	601
PB.2798.1	2	1515	6	full-splice_match	gene: Zm00001d003401	Zm00001d003401_T00	163.61	1796.02	coding	261	106	888
PB.8098.9	6	1740	9	novel_in_catalog	gene: Zm00001d038819	novel	3.30	47.25	coding	261	669	1451
PB.2798.3	2	1788	5	full-splice_match	gene: Zm00001d003401	Zm00001d003401_T01	5.51	1796.02	coding	261	687	1469
PB.2798.2	2	1367	6	full-splice_match	gene: Zm00001d003401	Zm00001d003401_T00	585.48	1796.02	coding	261	93	875
PB.8098.4	6	1742	9	novel_in_catalog	gene: Zm00001d038819	novel	9.33	47.25	coding	252	766	1521
PB.730.1	1	2609	2	genic	gene: Zm00001d030772	novel	7.87	17.39	coding	4	911	922
PB.730.2	1	2547	2	genic	gene: Zm00001d030772	novel	9.53	17.39	coding	308	61	984
PB.2573.1	2	1699	2	full-splice_match	gene: Zm00001d002370	Zm00001d002370_T00	341.10	383.43	coding	460	110	1489
PB.2798.9	2	1164	6	full-splice_match	gene: Zm00001d003401	Zm00001d003401_T00	481.25	1796.02	coding	261	98	880
PB.7772.6	6	1705	6	full-splice_match	gene: Zm00001d037596	Zm00001d037596_T00	4.13	68.64	non coding	NA	NA	NA
PB.7772.7	6	1163	6	full-splice_match	gene: Zm00001d037596	Zm00001d037596_T00	0.00	68.64	non coding	NA	NA	NA

PB.7772.4	6	1625	1	novel_in_catalog	gene: Zm00001d037596	novel	3.27	68.64	coding	139	1012	1428
PB.7772.5	6	2576	5	novel_in_catalog	gene: Zm00001d037596	novel	3.47	68.64	coding	280	1545	2384
PB.7772.2	6	1816	6	full-splice_match	gene: Zm00001d037596	Zm00001d037596_T00	52.69	68.64	non coding	NA	NA	NA
PB.10333.1	9	2151	2	incomplete-splice_match	gene: Zm00001d045739	Zm00001d045739_T00	35.50	73.68	coding	599	24	1820
PB.10333.2	9	3531	4	full-splice_match	gene: Zm00001d045739	Zm00001d045739_T00	27.96	73.68	coding	1029	218	3304
PB.10333.3	9	2608	3	incomplete-splice_match	gene: Zm00001d045739	Zm00001d045739_T00	10.23	73.68	coding	599	686	2482
PB.3484.2	2	1271	2	full-splice_match	gene: Zm00001d006933	Zm00001d006933_T00	163.91	316.37	coding	362	36	1121
PB.1067.1	1	1761	1	genic_intron	novelGene_430	novel	5.50	5.50	non coding	NA	NA	NA
PB.4759.9	3	1344	2	novel_in_catalog	gene: Zm00001d044395	novel	17.04	76.77	coding	273	371	1189
PB.8542.1	7	750	4	full-splice_match	gene: Zm00001d020450	Zm00001d020450_T00	266.74	322.97	coding	120	78	437
PB.8542.2	7	1830	3	novel_in_catalog	gene: Zm00001d020450	novel	3.13	322.97	coding	99	1228	1524
PB.8542.3	7	651	4	full-splice_match	gene: Zm00001d020450	Zm00001d020450_T00	53.11	322.97	coding	120	82	441
PB.10760.1	9	1572	6	novel_in_catalog	gene: Zm00001d047911	novel	30.26	30.26	coding	332	413	1408
PB.1926.1	10	2605	2	novel_in_catalog	gene: Zm00001d024521	novel	6.04	6.04	coding	514	35	1576
PB.5507.15	4	2240	4	novel_in_catalog	gene: Zm00001d052219	novel	2.88	64.47	coding	212	124	759
PB.10695.2	9	1115	2	genic	gene: Zm00001d047709	novel	0.00	53.08	coding	260	25	804
PB.5507.10	4	1551	5	novel_in_catalog	gene: Zm00001d052219	novel	2.29	64.47	coding	165	243	737

PB.5208.1	4	1079	2	intergenic	novelGene_210	novel	99.35	99.35	coding	148	96	539
PB.5507.13	4	1696	7	novel_not_in_catalog	gene: Zm00001d052219	novel	33.24	64.47	coding	231	67	759
PB.8436.2	7	3670	25	full-splice_match	gene: Zm00001d019907	Zm00001d019907_T00	15.03	15.03	coding	1025	97	3171
PB.6588.1	5	906	2	full-splice_match	gene: Zm00001d015347	Zm00001d015347_T00	8.92	8.92	coding	205	45	659
PB.7748.1	6	2744	1	full-splice_match	gene: Zm00001d037471	Zm00001d037471_T00	0.75	0.75	coding	668	68	2071
PB.751.1	1	1707	1	full-splice_match	gene: Zm00001d030901	Zm00001d030901_T00	0.79	0.79	coding	510	26	1555
PB.3852.1	3	590	1	intergenic	novelGene_84	novel	0.65	0.65	non coding	NA	NA	NA
PB.9545.3	8	1318	4	novel_in_catalog	gene: Zm00001d010411	novel	1.80	160.22	coding	127	624	1004
PB.8469.1	7	1151	3	novel_in_catalog	gene: Zm00001d020068	novel	14.39	14.39	coding	108	11	334
PB.181.1	1	1553	4	full-splice_match	gene: Zm00001d028009	Zm00001d028009_T00	62.54	62.54	coding	362	54	1139
PB.1383.1	1	1304	5	full-splice_match	gene: Zm00001d033766	Zm00001d033766_T00	45.68	45.68	coding	283	166	1014
PB.4740.3	3	1895	11	full-splice_match	gene: Zm00001d044297	Zm00001d044297_T00	3.64	32.71	coding	513	133	1671
PB.3205.1	2	1105	1	genic	gene: Zm00001d005762	novel	4.49	46.46	coding	140	30	449
PB.3920.3	3	2076	19	full-splice_match	gene: Zm00001d040311	Zm00001d040311_T02	0.00	25.06	coding	680	35	2074
PB.9898.2	8	1818	11	full-splice_match	gene: Zm00001d011945	Zm00001d011945_T00	2.42	14.44	coding	400	42	1241
PB.3799.2	3	1189	5	full-splice_match	gene: Zm00001d039727	Zm00001d039727_T00	37.14	37.14	coding	185	393	947
PB.8748.1	7	899	3	intergenic	novelGene_300	novel	188.51	188.51	coding	172	165	680

PB.7695.2	6	1058	1	incomplete-splice_match	gene: Zm00001d037240	Zm00001d037240_T00	4.41	13.29	non coding	NA	NA	NA
PB.7695.3	6	1102	7	novel_in_catalog	gene: Zm00001d037240	novel	6.60	13.29	coding	262	69	854
PB.10323.1	9	606	1	full-splice_match	gene: Zm00001d045669	Zm00001d045669_T00	25.34	25.34	non coding	NA	NA	NA
PB.7695.1	6	2445	7	novel_not_in_catalog	gene: Zm00001d037240	novel	2.28	13.29	coding	270	88	897
PB.6552.1	5	2247	6	full-splice_match	gene: Zm00001d015164	Zm00001d015164_T00	38.23	38.23	coding	544	162	1793
PB.9076.1	7	1007	6	full-splice_match	gene: Zm00001d022513	Zm00001d022513_T00	861.10	873.30	coding	219	86	742
PB.4319.17	3	1797	3	full-splice_match	gene: Zm00001d042684	Zm00001d042684_T00	0.00	359.58	coding	279	54	890
PB.4319.10	3	1938	2	novel_in_catalog	gene: Zm00001d042684	novel	0.00	359.58	coding	241	257	979
PB.8847.1	7	1449	5	full-splice_match	gene: Zm00001d021739	Zm00001d021739_T00	19.71	245.14	coding	275	261	1085
PB.854.1	1	2603	4	fusion	gene: Zm00001d031384	novel	1.68	1.68	coding	677	55	2085
PB.8847.3	7	2899	1	novel_in_catalog	gene: Zm00001d021739	novel	46.65	245.14	non coding	NA	NA	NA
PB.9748.2	8	1612	5	intergenic	novelGene_290	novel	9.08	16.96	non coding	NA	NA	NA
PB.10716.1	9	1008	1	full-splice_match	gene: Zm00001d047768	Zm00001d047768_T00	1.97	1.97	coding	186	2	559
PB.4563.2	3	885	2	novel_not_in_catalog	gene: Zm00001d043619	novel	8.57	215.93	coding	202	31	636
PB.4563.1	3	1016	2	novel_not_in_catalog	gene: Zm00001d043619	novel	207.36	215.93	coding	202	76	681
PB.5536.1	4	1800	4	novel_not_in_catalog	gene: Zm00001d052312	novel	20.40	109.40	coding	535	7	1611
PB.5981.1	5	2165	2	full-splice_match	gene: Zm00001d012893	Zm00001d012893_T00	7.12	7.12	coding	561	72	1754

PB.1052.2	1	908	6	full-splice_match	gene: Zm00001d032370	Zm00001d032370_T00	227.09	286.41	coding	205	99	713
PB.1131.1	1	1214	7	full-splice_match	gene: Zm00001d032652	Zm00001d032652_T00	40.79	40.79	coding	222	137	802
PB.1131.2	1	1061	7	full-splice_match	gene: Zm00001d032652	Zm00001d032652_T00	0.00	40.79	coding	222	94	759
PB.9057.4	7	994	3	novel_in_catalog	gene: Zm00001d022469	novel	158.65	384.90	coding	259	107	883
PB.8863.1	7	970	2	genic_intron	novelGene_323	novel	31.26	31.26	coding	77	196	426
PB.1790.1	10	1684	3	novel_in_catalog	gene: Zm00001d023682	novel	36.46	36.46	coding	413	408	1646
PB.9837.2	8	1425	1	full-splice_match	gene: Zm00001d011710	Zm00001d011710_T00	0.00	95.33	coding	312	171	1106
PB.10104.1	8	2760	7	full-splice_match	gene: Zm00001d012774	Zm00001d012774_T00	4.98	22.39	coding	441	466	1788
PB.10104.2	8	2681	8	full-splice_match	gene: Zm00001d012774	Zm00001d012774_T00	17.41	22.39	coding	668	508	2511
PB.9837.1	8	1558	1	full-splice_match	gene: Zm00001d011710	Zm00001d011710_T00	95.33	95.33	coding	312	171	1106
PB.1678.3	1	1095	7	full-splice_match	gene: Zm00001d034949	Zm00001d034949_T00	39.21	143.81	coding	203	137	745
PB.10893.2	9	1376	1	genic	gene: Zm00001d048449	novel	7.86	211.06	coding	201	460	1062
PB.7576.1	6	1679	7	novel_in_catalog	gene: Zm00001d036602	novel	47.77	47.77	coding	381	364	1506
PB.2056.2	10	1361	4	full-splice_match	gene: Zm00001d025166	Zm00001d025166_T00	0.00	92.12	coding	339	145	1161
PB.2056.3	10	1553	3	novel_in_catalog	gene: Zm00001d025166	novel	3.89	92.12	coding	235	649	1353
PB.417.2	1	2071	9	full-splice_match	gene: Zm00001d029009	Zm00001d029009_T00	8.60	13.07	coding	478	383	1816
PB.6292.1	5	1093	1	intergenic	novelGene_120	novel	13.12	13.12	non coding	NA	NA	NA

PB.4740.4	3	538	5	incomplete-splice_match	gene: Zm00001d044297	Zm00001d044297_T00	8.00	32.71	coding	112	203	538
PB.417.1	1	1809	9	full-splice_match	gene: Zm00001d029009	Zm00001d029009_T00	4.47	13.07	coding	449	73	1419
PB.2267.2	10	2226	14	full-splice_match	gene: Zm00001d026257	Zm00001d026257_T00	4.05	67.53	coding	564	121	1812
PB.2267.3	10	2395	6	incomplete-splice_match	gene: Zm00001d026257	Zm00001d026257_T02	29.17	67.53	coding	112	189	524
PB.3106.3	2	1892	6	novel_in_catalog	gene: Zm00001d005231	novel	0.00	1483.63	coding	129	74	460
PB.2267.1	10	3210	13	full-splice_match	gene: Zm00001d026257	Zm00001d026257_T01	34.31	67.53	coding	425	122	1396
PB.2644.2	2	1762	7	full-splice_match	gene: Zm00001d002696	Zm00001d002696_T00	2.71	16.27	coding	462	222	1607
PB.2644.1	2	1759	7	full-splice_match	gene: Zm00001d002696	Zm00001d002696_T00	13.56	16.27	coding	462	147	1532
PB.8161.1	6	2160	5	full-splice_match	gene: Zm00001d039012	Zm00001d039012_T00	7.79	7.79	coding	462	33	1418
PB.6441.1	5	561	2	novel_in_catalog	gene: Zm00001d014770	novel	73.14	73.14	non coding	NA	NA	NA
PB.7117.2	5	2133	5	full-splice_match	gene: Zm00001d017987	Zm00001d017987_T00	0.00	58.40	coding	612	78	1913
PB.9001.1	7	3018	1	genic	gene: Zm00001d022286	novel	50.74	70.42	coding	903	33	2741
PB.9001.2	7	3323	2	incomplete-splice_match	gene: Zm00001d022286	Zm00001d022286_T00	19.68	70.42	coding	1062	19	3204
PB.6652.2	5	1685	9	novel_in_catalog	gene: Zm00001d015788	novel	1.08	160.66	coding	272	523	1338
PB.7180.2	5	1389	1	full-splice_match	gene: Zm00001d018208	Zm00001d018208_T00	65.97	68.31	coding	355	66	1130
PB.7180.1	5	2285	1	full-splice_match	gene: Zm00001d018208	Zm00001d018208_T00	2.34	68.31	coding	355	60	1124
PB.435.1	1	1585	10	full-splice_match	gene: Zm00001d029121	Zm00001d029121_T00	27.78	27.78	coding	339	87	1103

PB.4954.1	4	1326	10	full-splice_match	gene: Zm00001d049163	Zm00001d049163_T00	69.99	85.61	coding	333	116	1114
PB.2577.1	2	2264	14	full-splice_match	gene: Zm00001d002388	Zm00001d002388_T00	86.25	86.25	coding	565	157	1851
PB.9180.1	8	3666	17	incomplete-splice_match	gene: Zm00001d008429	Zm00001d008429_T01	50.43	94.20	coding	1025	97	3171
PB.8583.1	7	1348	4	full-splice_match	gene: Zm00001d020670	Zm00001d020670_T00	6.72	6.72	coding	333	32	1030
PB.5007.1	4	445	5	full-splice_match	gene: Zm00001d049387	Zm00001d049387_T00	11.71	11.71	non coding	NA	NA	NA
PB.6173.5	5	634	2	full-splice_match	gene: Zm00001d013568	Zm00001d013568_T00	365.11	2041.31	coding	159	82	558
PB.8857.1	7	1105	2	full-splice_match	gene: Zm00001d021774	Zm00001d021774_T00	87.82	94.22	coding	266	119	916
PB.9180.4	8	917	5	incomplete-splice_match	gene: Zm00001d008429	Zm00001d008429_T01	39.06	94.20	coding	185	22	576
PB.548.1	1	1470	4	full-splice_match	gene: Zm00001d029632	Zm00001d029632_T00	20.64	28.86	coding	279	198	1034
PB.6173.1	5	804	2	full-splice_match	gene: Zm00001d013568	Zm00001d013568_T00	1657.52	2041.31	coding	159	88	564
PB.4837.1	4	1537	1	full-splice_match	gene: Zm00001d048602	Zm00001d048602_T00	20.64	20.64	coding	434	67	1368
PB.4573.1	3	783	1	full-splice_match	gene: Zm00001d043665	Zm00001d043665_T00	14.71	27.85	coding	168	48	551
PB.3332.3	2	1922	4	novel_not_in_catalog	gene: Zm00001d006308	novel	10.68	94.24	coding	384	76	1227
PB.5186.2	4	1632	6	full-splice_match	gene: Zm00001d050383	Zm00001d050383_T00	0.00	56.99	coding	472	46	1461
PB.5186.1	4	1758	6	full-splice_match	gene: Zm00001d050383	Zm00001d050383_T00	56.99	56.99	coding	472	92	1507
PB.61.1	1	923	1	full-splice_match	gene: Zm00001d027483	Zm00001d027483_T00	171.78	171.78	coding	188	86	649
PB.1119.3	1	1893	5	incomplete-splice_match	gene: Zm00001d032597	Zm00001d032597_T00	0.00	58.45	coding	576	166	1893

PB.5522.2	4	2076	4	novel_in_catalog	gene: Zm00001d052259	novel	0.00	71.54	coding	507	297	1817
PB.1119.1	1	2783	8	full-splice_match	gene: Zm00001d032597	Zm00001d032597_T00	58.45	58.45	coding	770	203	2512
PB.3480.1	2	2613	14	full-splice_match	gene: Zm00001d006922	Zm00001d006922_T00	34.92	34.92	coding	655	193	2157
PB.3384.3	2	1687	3	incomplete-splice_match	gene: Zm00001d006504	Zm00001d006504_T00	0.00	26.41	coding	441	152	1474
PB.4026.1	3	2412	7	fusion	gene: Zm00001d041076	novel	28.90	28.90	coding	715	28	2172
PB.6769.1	5	1878	15	novel_not_in_catalog	gene: Zm00001d016516	novel	31.33	66.76	coding	440	189	1508
PB.6769.2	5	3120	15	novel_not_in_catalog	gene: Zm00001d016516	novel	35.43	66.76	coding	804	339	2750
PB.6449.1	5	1694	13	full-splice_match	gene: Zm00001d014786	Zm00001d014786_T00	23.18	25.23	coding	393	71	1249
PB.6449.2	5	2203	12	novel_in_catalog	gene: Zm00001d014786	novel	2.06	25.23	coding	311	26	958
PB.4222.1	3	2928	14	full-splice_match	gene: Zm00001d042267	Zm00001d042267_T00	161.25	161.91	coding	799	186	2582
PB.4222.3	3	2823	14	novel_not_in_catalog	gene: Zm00001d042267	novel	0.66	161.91	coding	693	142	2220
PB.7978.1	6	1027	6	full-splice_match	gene: Zm00001d038352	Zm00001d038352_T00	23.28	23.28	coding	137	165	575
PB.4222.4	3	2767	14	full-splice_match	gene: Zm00001d042267	Zm00001d042267_T00	0.00	161.91	coding	799	189	2585
PB.8208.1	6	3077	23	full-splice_match	gene: Zm00001d039214	Zm00001d039214_T02	21.92	21.92	coding	910	151	2880
PB.1004.1	1	563	3	full-splice_match	gene: Zm00001d032153	Zm00001d032153_T00	63.54	63.54	coding	98	55	348
PB.9327.4	8	2884	13	full-splice_match	gene: Zm00001d009205	Zm00001d009205_T00	8.52	74.70	coding	287	1873	2733
PB.9327.2	8	1817	14	full-splice_match	gene: Zm00001d009205	Zm00001d009205_T00	33.68	74.70	coding	506	59	1576

PB.9327.3	8	1777	14	novel_not_in_catalog	gene: Zm00001d009205	novel	0.17	74.70	coding	453	103	1461
PB.8561.1	7	837	3	incomplete-splice_match	gene: Zm00001d020555	Zm00001d020555_T00	4.81	32.85	non coding	NA	NA	NA
PB.9327.1	8	1964	14	full-splice_match	gene: Zm00001d009205	Zm00001d009205_T00	32.32	74.70	coding	506	103	1620
PB.886.3	1	2417	3	novel_in_catalog	gene: Zm00001d031580	novel	18.60	228.68	coding	222	1441	2106
PB.8917.1	7	1509	5	full-splice_match	gene: Zm00001d021961	Zm00001d021961_T00	10.33	10.33	coding	396	154	1341
PB.5073.2	4	2155	2	full-splice_match	gene: Zm00001d049724	Zm00001d049724_T00	0.00	3.11	coding	653	78	2036
PB.5073.1	4	2259	2	full-splice_match	gene: Zm00001d049724	Zm00001d049724_T00	3.11	3.11	coding	653	80	2038
PB.5711.3	4	2098	6	full-splice_match	gene: Zm00001d053104	Zm00001d053104_T00	0.54	18.41	coding	125	66	440
PB.5861.1	4	1368	9	full-splice_match	gene: Zm00001d053822	Zm00001d053822_T00	58.85	120.93	coding	208	304	927
PB.6211.1	5	1635	2	full-splice_match	gene: Zm00001d013737	Zm00001d013737_T00	81.39	82.84	coding	397	123	1313
PB.7415.7	6	1923	1	genic	gene: Zm00001d035605	novel	1471.98	1856.08	coding	250	879	1628
PB.7415.1	6	2065	1	genic	gene: Zm00001d035605	novel	270.70	1856.08	coding	250	879	1628
PB.2392.3	10	1309	6	full-splice_match	gene: Zm00001d026639	Zm00001d026639_T00	0.00	94.36	coding	328	78	1061
PB.6211.3	5	1428	1	novel_in_catalog	gene: Zm00001d013737	novel	0.00	82.84	coding	186	662	1219
PB.5450.1	4	2703	16	full-splice_match	gene: Zm00001d052009	Zm00001d052009_T00	12.81	47.74	coding	728	193	2376
PB.2477.2	2	1457	16	full-splice_match	gene: Zm00001d001983	Zm00001d001983_T00	27.79	46.86	coding	378	168	1301
PB.83.2	1	913	2	full-splice_match	gene: Zm00001d027557	Zm00001d027557_T00	25.55	30.31	coding	232	68	763

PB.4455.1	3	724	5	full-splice_match	gene: Zm00001d043259	Zm00001d043259_T00	31.20	31.20	coding	112	265	600
PB.83.1	1	1002	1	novel_in_catalog	gene: Zm00001d027557	novel	4.77	30.31	coding	127	464	844
PB.1553.3	1	1289	7	full-splice_match	gene: Zm00001d034509	Zm00001d034509_T00	18.35	58.85	coding	293	80	958
PB.2477.1	2	1670	16	full-splice_match	gene: Zm00001d001983	Zm00001d001983_T00	19.07	46.86	coding	378	139	1272
PB.7023.1	5	1683	1	full-splice_match	gene: Zm00001d017615	Zm00001d017615_T00	6.55	6.55	coding	154	212	673
PB.2694.4	2	1497	3	novel_not_in_catalog	gene: Zm00001d002934	novel	1.98	6.68	coding	196	567	1154
PB.5861.4	4	972	8	novel_in_catalog	gene: Zm00001d053822	novel	3.37	120.93	coding	120	398	757
PB.2694.5	2	1335	4	novel_not_in_catalog	gene: Zm00001d002934	novel	0.00	6.68	coding	320	130	1089
PB.8958.1	7	1048	8	full-splice_match	gene: Zm00001d022134	Zm00001d022134_T00	785.54	785.54	coding	229	105	791
PB.4018.1	3	2385	9	novel_not_in_catalog	gene: Zm00001d040959	novel	2.15	23.26	coding	612	191	2026
PB.4018.2	3	2281	10	novel_not_in_catalog	gene: Zm00001d040959	novel	21.11	23.26	coding	612	192	2027
PB.5256.10	4	2109	8	full-splice_match	gene: Zm00001d050970	Zm00001d050970_T01	0.00	60.57	coding	171	90	602
PB.8649.1	7	1938	10	full-splice_match	gene: Zm00001d020973	Zm00001d020973_T00	19.80	19.80	coding	394	365	1546
PB.8649.2	7	1657	10	full-splice_match	gene: Zm00001d020973	Zm00001d020973_T00	0.00	19.80	coding	394	191	1372
PB.1693.1	10	3759	6	novel_in_catalog	gene: Zm00001d023258	novel	29.32	29.32	coding	857	153	2723
PB.3239.1	2	778	5	full-splice_match	gene: Zm00001d005926	Zm00001d005926_T00	17.62	17.62	coding	181	120	662
PB.1178.3	1	943	1	intergenic	novelGene_438	novel	0.00	322.84	coding	261	96	878

PB.10860.4	9	1738	1	genic_intron	novelGene_309	novel	5.92	5.92	coding	261	770	1552
PB.1541.4	1	1195	3	full-splice_match	gene: Zm00001d034463	Zm00001d034463_T00	9.51	13.13	coding	202	130	735
PB.10492.1	9	1509	3	novel_not_in_catalog	gene: Zm00001d046803	novel	57.08	57.08	coding	346	135	1172
PB.3931.1	3	1471	1	full-splice_match	gene: Zm00001d040356	Zm00001d040356_T00	11.38	11.38	coding	445	37	1371
PB.590.3	1	1712	13	novel_in_catalog	gene: Zm00001d029860	novel	4.15	49.45	coding	290	69	938
PB.10601.1	9	1751	4	full-splice_match	gene: Zm00001d047388	Zm00001d047388_T00	41.26	41.26	coding	469	79	1485
PB.812.13	1	784	1	novel_in_catalog	gene: Zm00001d031168	novel	0.00	7101.71	non coding	NA	NA	NA
PB.812.12	1	717	2	full-splice_match	gene: Zm00001d031168	Zm00001d031168_T00	1496.69	7101.71	coding	155	100	564
PB.10806.4	9	2114	3	full-splice_match	gene: Zm00001d048105	Zm00001d048105_T00	0.00	56.11	coding	315	902	1846
PB.10867.1	9	1456	6	incomplete-splice_match	gene: Zm00001d048358	Zm00001d048358_T00	70.63	122.58	coding	287	195	1055
PB.5789.3	4	1499	3	novel_in_catalog	gene: Zm00001d053548	novel	90.13	90.13	non coding	NA	NA	NA
PB.4119.1	3	1680	9	novel_in_catalog	gene: Zm00001d041710	novel	62.49	62.49	coding	186	721	1278
PB.2392.1	10	1561	6	full-splice_match	gene: Zm00001d026639	Zm00001d026639_T00	18.29	94.36	coding	328	78	1061
PB.1660.1	1	2040	14	novel_not_in_catalog	gene: Zm00001d034868	novel	12.94	12.94	coding	608	10	1833
PB.7756.1	6	1582	7	full-splice_match	gene: Zm00001d037505	Zm00001d037505_T00	176.97	176.97	coding	354	209	1270
PB.93.1	1	1515	2	novel_not_in_catalog	gene: Zm00001d027613	novel	27.11	27.11	coding	342	202	1227
PB.93.2	1	1370	2	novel_not_in_catalog	gene: Zm00001d027613	novel	0.00	27.11	coding	342	118	1143

PB.5404.1	4	2123	2	full-splice_match	gene: Zm00001d051824	Zm00001d051824_T00	29.23	30.47	coding	628	100	1983
PB.5404.2	4	2160	1	novel_in_catalog	gene: Zm00001d051824	novel	1.24	30.47	coding	612	212	2047
PB.5404.3	4	1891	2	full-splice_match	gene: Zm00001d051824	Zm00001d051824_T00	0.00	30.47	coding	606	72	1889
PB.6747.2	5	915	4	incomplete-splice_match	gene: Zm00001d016406	Zm00001d016406_T00	14.23	37.94	coding	105	73	387
PB.5277.1	4	1091	1	intergenic	novelGene_275	novel	0.35	0.35	non coding	NA	NA	NA
PB.525.3	1	1530	11	full-splice_match	gene: Zm00001d029548	Zm00001d029548_T00	19.69	45.49	coding	239	640	1356
PB.8641.1	7	901	2	full-splice_match	gene: Zm00001d020950	Zm00001d020950_T00	30.72	30.72	coding	117	311	661
PB.5643.1	4	1320	2	full-splice_match	gene: Zm00001d052777	Zm00001d052777_T00	36.33	36.33	coding	291	156	1028
PB.6749.2	5	1302	9	full-splice_match	gene: Zm00001d016410	Zm00001d016410_T00	58.60	158.09	coding	327	127	1107
PB.5198.4	4	2017	10	full-splice_match	gene: Zm00001d050483	Zm00001d050483_T00	6.57	22.58	coding	509	332	1858
PB.8981.7	7	1117	5	full-splice_match	gene: Zm00001d022225	Zm00001d022225_T00	0.00	66.30	coding	266	162	959
PB.5198.2	4	1870	10	novel_not_in_catalog	gene: Zm00001d050483	novel	7.65	22.58	coding	398	105	1298
PB.6169.2	5	1120	3	novel_in_catalog	gene: Zm00001d013532	novel	18.98	20.68	coding	278	122	955
PB.6169.1	5	1622	3	novel_in_catalog	gene: Zm00001d013532	novel	1.71	20.68	coding	278	123	956
PB.5198.1	4	2530	10	full-splice_match	gene: Zm00001d050483	Zm00001d050483_T00	5.67	22.58	coding	577	537	2267
PB.9493.1	8	740	2	genic	gene: Zm00001d010191	novel	31.11	31.11	coding	99	145	441
PB.2610.4	2	2028	8	full-splice_match	gene: Zm00001d002580	Zm00001d002580_T00	0.00	31.40	coding	531	167	1759

PB.2610.5	2	1912	8	novel_in_catalog	gene: Zm00001d002580	novel	6.79	31.40	coding	531	145	1737
PB.2610.6	2	1922	8	full-splice_match	gene: Zm00001d002580	Zm00001d002580_T00	0.00	31.40	coding	531	162	1754
PB.2342.7	10	2270	9	novel_in_catalog	gene: Zm00001d026501	novel	46.83	190.01	coding	116	1665	2012
PB.9746.1	8	839	3	full-splice_match	gene: Zm00001d011286	Zm00001d011286_T00	22.53	78.68	coding	177	102	632
PB.2342.1	10	1736	14	incomplete-splice_match	gene: Zm00001d026501	Zm00001d026501_T00	61.65	190.01	coding	423	131	1399
PB.2610.2	2	2388	8	full-splice_match	gene: Zm00001d002580	Zm00001d002580_T00	21.81	31.40	coding	598	39	1832
PB.2610.3	2	2097	8	full-splice_match	gene: Zm00001d002580	Zm00001d002580_T00	2.80	31.40	coding	531	163	1755
PB.8962.2	7	1237	1	full-splice_match	gene: Zm00001d022154	Zm00001d022154_T00	0.44	36.07	coding	271	294	1106
PB.674.1	1	1047	2	full-splice_match	gene: Zm00001d030345	Zm00001d030345_T00	15.12	47.92	coding	221	33	695
PB.2032.1	10	732	1	full-splice_match	gene: Zm00001d025033	Zm00001d025033_T00	9.99	9.99	coding	176	15	542
PB.3572.1	2	776	2	genic	gene: Zm00001d007448	novel	0.98	0.98	coding	177	39	569
PB.9669.1	8	2000	8	incomplete-splice_match	gene: Zm00001d010928	Zm00001d010928_T00	13.74	13.74	coding	603	42	1850
PB.8655.1	7	1083	2	full-splice_match	gene: Zm00001d021006	Zm00001d021006_T00	50.30	50.30	coding	258	185	958
PB.7802.2	6	2126	3	full-splice_match	gene: Zm00001d037712	Zm00001d037712_T00	6.22	16.28	coding	648	182	2125
PB.953.1	1	771	3	full-splice_match	gene: Zm00001d031928	Zm00001d031928_T00	14.82	17.59	coding	136	125	532
PB.5432.1	4	1481	4	full-splice_match	gene: Zm00001d051912	Zm00001d051912_T00	56.36	88.26	coding	329	173	1159
PB.6060.2	5	1345	1	full-splice_match	gene: Zm00001d013173	Zm00001d013173_T00	7.86	25.12	coding	373	103	1221

PB.6060.1	5	1396	1	full-splice_match	gene: Zm00001d013173	Zm00001d013173_T00	17.26	25.12	coding	373	44	1162
PB.1605.1	1	1352	3	novel_in_catalog	gene: Zm00001d034679	novel	5.55	5.55	coding	306	305	1222
PB.10986.3	B73V4	1033	3	intergenic	novelGene_2	novel	15.45	53.28	non coding	NA	NA	NA
PB.1753.1	10	1346	1	full-splice_match	gene: Zm00001d023524	Zm00001d023524_T00	10.54	10.54	coding	354	67	1128
PB.9335.1	8	763	1	full-splice_match	gene: Zm00001d009247	Zm00001d009247_T00	33.26	33.26	coding	108	100	423
PB.7012.2	5	1462	1	novel_in_catalog	gene: Zm00001d017547	novel	5.47	100.75	coding	194	536	1117
PB.6500.1	5	1830	1	genic	gene: Zm00001d014958	novel	21.13	21.13	coding	408	396	1619
PB.7130.3	5	1747	3	genic	gene: Zm00001d018019	novel	0.54	2.89	coding	97	88	378
PB.4588.1	3	554	1	full-splice_match	gene: Zm00001d043704	Zm00001d043704_T00	19.45	19.45	coding	96	22	309
PB.3627.3	2	2194	1	full-splice_match	gene: Zm00001d007789	Zm00001d007789_T00	6.99	79.61	coding	524	518	2089
PB.7249.1	5	2060	10	incomplete-splice_match	gene: Zm00001d018460	Zm00001d018460_T00	11.24	24.88	coding	530	37	1626
PB.10880.2	9	982	9	novel_in_catalog	gene: Zm00001d048403	novel	113.49	156.39	coding	237	99	809
PB.1202.2	1	2571	6	novel_in_catalog	gene: Zm00001d032931	novel	2.24	15.05	coding	226	1657	2334
PB.1562.4	1	1375	5	novel_not_in_catalog	gene: Zm00001d034534	novel	5.31	47.66	coding	206	409	1026
PB.6181.1	5	1369	4	incomplete-splice_match	gene: Zm00001d013599	Zm00001d013599_T00	17.03	67.07	coding	347	65	1105
PB.6181.3	5	2766	6	novel_in_catalog	gene: Zm00001d013599	novel	3.36	67.07	coding	347	1634	2674
PB.109.1	1	1276	1	full-splice_match	gene: Zm00001d027684	Zm00001d027684_T00	10.45	10.45	coding	313	106	1044

PB.10031.1	8	1226	3	novel_not_in_catalog	gene: Zm00001d012505	novel	9.41	9.41	coding	277	158	988
PB.2765.1	2	1412	14	full-splice_match	gene: Zm00001d003220	Zm00001d003220_T00	36.38	36.38	coding	348	128	1171
PB.10884.1	9	1045	4	novel_in_catalog	gene: Zm00001d048414	novel	10.07	22.51	coding	190	101	670
PB.6520.1	5	1435	8	novel_not_in_catalog	gene: Zm00001d015009	novel	17.21	17.21	coding	374	137	1258
PB.1301.2	1	1207	2	genic	gene: Zm00001d033386	novel	23.41	76.58	coding	230	140	829
PB.6755.1	5	1385	9	full-splice_match	gene: Zm00001d016465	Zm00001d016465_T00	25.00	25.00	coding	243	409	1137
PB.6990.1	5	1370	4	full-splice_match	gene: Zm00001d017468	Zm00001d017468_T00	46.04	46.04	coding	209	152	778
PB.10884.2	9	946	5	full-splice_match	gene: Zm00001d048414	Zm00001d048414_T00	12.45	22.51	coding	238	88	801
PB.169.1	1	1813	1	full-splice_match	gene: Zm00001d027947	Zm00001d027947_T00	3.60	3.60	coding	528	159	1742
PB.4619.1	3	933	1	full-splice_match	gene: Zm00001d043830	Zm00001d043830_T00	60.49	60.49	coding	205	75	689
PB.5379.1	4	864	2	genic	gene: Zm00001d051663	novel	33.59	33.59	coding	163	53	541
PB.1562.2	1	1301	4	novel_not_in_catalog	gene: Zm00001d034534	novel	5.35	47.66	coding	213	178	816
PB.942.2	1	2277	5	novel_not_in_catalog	gene: Zm00001d031863	novel	103.01	103.01	coding	673	47	2065
PB.1937.1	10	839	2	genic	gene: Zm00001d024573	novel	58.85	58.85	coding	146	145	582
PB.5975.1	5	3859	11	full-splice_match	gene: Zm00001d012869	Zm00001d012869_T02	25.38	25.38	coding	199	280	876
PB.7019.4	5	2583	1	genic	gene: Zm00001d017575	novel	9.73	85.92	coding	359	1264	2340
PB.4829.6	3	1753	5	novel_in_catalog	gene: Zm00001d044694	novel	0.00	329.15	coding	138	1150	1563

PB.6335.1	5	2362	1	full-splice_match	gene: Zm00001d014316	Zm00001d014316_T00	19.10	19.10	coding	658	191	2164
PB.2279.4	10	1824	5	full-splice_match	gene: Zm00001d026288	Zm00001d026288_T00	0.00	23.40	coding	432	180	1475
PB.8195.1	6	3522	19	full-splice_match	gene: Zm00001d039165	Zm00001d039165_T04	55.80	78.41	coding	1089	68	3334
PB.3927.1	3	1561	1	full-splice_match	gene: Zm00001d040324	Zm00001d040324_T00	26.21	26.21	coding	398	113	1306
PB.10244.4	9	3113	25	full-splice_match	gene: Zm00001d045379	Zm00001d045379_T00	16.76	46.76	coding	906	63	2780
PB.5826.1	4	3222	12	full-splice_match	gene: Zm00001d053672	Zm00001d053672_T00	37.55	59.75	coding	879	373	3009
PB.10244.2	9	2268	16	incomplete-splice_match	gene: Zm00001d045379	Zm00001d045379_T00	11.15	46.76	coding	595	25	1809
PB.10244.1	9	1752	12	incomplete-splice_match	gene: Zm00001d045379	Zm00001d045379_T00	18.84	46.76	coding	352	199	1254
PB.9329.1	8	2118	15	full-splice_match	gene: Zm00001d009212	Zm00001d009212_T00	20.40	20.40	coding	562	95	1780
PB.8013.1	6	410	1	incomplete-splice_match	gene: Zm00001d038504	Zm00001d038504_T00	47.82	47.82	non coding	NA	NA	NA
PB.3628.1	2	2063	4	full-splice_match	gene: Zm00001d007801	Zm00001d007801_T00	17.48	17.48	coding	429	453	1739
PB.4103.1	3	1313	2	novel_not_in_catalog	gene: Zm00001d041610	novel	7.04	7.04	coding	307	76	996
PB.7922.3	6	1524	1	full-splice_match	gene: Zm00001d038158	Zm00001d038158_T00	0.00	24.98	coding	403	51	1259
PB.4590.1	3	1992	11	full-splice_match	gene: Zm00001d043726	Zm00001d043726_T00	136.48	145.08	coding	429	250	1536
PB.6528.1	5	2182	14	novel_not_in_catalog	gene: Zm00001d015060	novel	9.90	9.90	coding	527	205	1785
PB.5764.1	4	1480	2	incomplete-splice_match	gene: Zm00001d053379	Zm00001d053379_T00	34.37	34.37	coding	268	185	988
PB.1743.2	10	967	1	full-splice_match	gene: Zm00001d023461	Zm00001d023461_T00	47.08	47.08	coding	176	163	690

PB.5969.2	5	1127	7	full-splice_match	gene: Zm00001d012854	Zm00001d012854_T00	7.05	38.86	coding	146	101	538
PB.1533.3	1	1385	1	full-splice_match	gene: Zm00001d034427	Zm00001d034427_T00	161.89	312.51	coding	392	98	1273
PB.5969.1	5	882	8	full-splice_match	gene: Zm00001d012854	Zm00001d012854_T00	31.80	38.86	coding	173	146	664
PB.3882.1	3	2147	1	full-splice_match	gene: Zm00001d040157	Zm00001d040157_T00	39.41	39.41	coding	640	126	2045
PB.1418.11	1	2257	3	novel_not_in_catalog	gene: Zm00001d033925	novel	12.22	979.16	coding	183	126	674
PB.1418.10	1	1170	5	novel_not_in_catalog	gene: Zm00001d033925	novel	0.30	979.16	coding	175	142	666
PB.4611.7	3	1858	10	novel_in_catalog	gene: Zm00001d043797	novel	4.92	166.78	coding	262	39	824
PB.4324.2	3	1000	2	full-splice_match	gene: Zm00001d042699	Zm00001d042699_T00	2.34	16.85	coding	105	18	332
PB.4611.5	3	2807	19	full-splice_match	gene: Zm00001d043797	Zm00001d043797_T02	26.16	166.78	coding	767	134	2434
PB.4262.2	3	2619	11	full-splice_match	gene: Zm00001d042435	Zm00001d042435_T00	11.35	16.89	coding	492	843	2318
PB.4347.3	3	1811	5	full-splice_match	gene: Zm00001d042795	Zm00001d042795_T00	21.68	43.05	non coding	NA	NA	NA
PB.4684.10	3	938	7	full-splice_match	gene: Zm00001d044095	Zm00001d044095_T00	182.12	327.19	coding	200	115	714
PB.4347.1	3	1703	6	full-splice_match	gene: Zm00001d042795	Zm00001d042795_T00	21.37	43.05	coding	138	161	574
PB.8786.1	7	2072	11	incomplete-splice_match	gene: Zm00001d021553	Zm00001d021553_T06	86.19	86.19	coding	428	43	1326
PB.10908.1	9	1394	8	full-splice_match	gene: Zm00001d048497	Zm00001d048497_T00	88.09	88.09	coding	294	317	1198
PB.10819.21	9	1625	4	novel_in_catalog	gene: Zm00001d048171	novel	8.57	25.07	non coding	NA	NA	NA
PB.7922.2	6	1689	1	full-splice_match	gene: Zm00001d038158	Zm00001d038158_T00	23.62	24.98	coding	403	47	1255

PB.9967.2	8	2248	6	full-splice_match	gene: Zm00001d012238	Zm00001d012238_T00	64.41	188.10	coding	256	178	945
PB.1983.1	10	2082	15	full-splice_match	gene: Zm00001d024832	Zm00001d024832_T00	72.60	72.60	coding	546	191	1828
PB.4363.1	3	1589	4	novel_not_in_catalog	gene: Zm00001d042868	novel	5.33	10.06	coding	257	438	1208
PB.4363.3	3	1719	3	novel_not_in_catalog	gene: Zm00001d042868	novel	4.73	10.06	coding	173	822	1340
PB.2771.1	2	2526	1	genic	gene: Zm00001d003251	novel	4.76	4.76	coding	256	1692	2459
PB.1164.2	1	2221	18	full-splice_match	gene: Zm00001d032763	Zm00001d032763_T00	38.62	76.57	coding	526	228	1805
PB.2703.11	2	1191	9	novel_in_catalog	gene: Zm00001d002956	novel	2.43	79.45	coding	325	217	1191
PB.2703.10	2	1697	11	full-splice_match	gene: Zm00001d002956	Zm00001d002956_T00	47.84	79.45	coding	413	270	1508
PB.4306.1	3	3489	3	full-splice_match	gene: Zm00001d042637	Zm00001d042637_T00	14.55	14.55	coding	958	261	3134
PB.10320.1	9	2273	1	intergenic	novelGene_118	novel	1.49	23.84	non coding	NA	NA	NA
PB.744.1	1	2674	4	novel_in_catalog	gene: Zm00001d030864	novel	3.46	7.04	coding	453	454	1812
PB.7943.5	6	2659	5	full-splice_match	gene: Zm00001d038227	Zm00001d038227_T00	16.62	338.21	coding	452	918	2273
PB.3804.2	3	620	1	full-splice_match	gene: Zm00001d039733	Zm00001d039733_T00	789.44	885.19	coding	103	77	385
PB.3804.1	3	745	1	full-splice_match	gene: Zm00001d039733	Zm00001d039733_T00	95.75	885.19	coding	103	77	385
PB.7922.1	6	1636	3	genic	gene: Zm00001d038158	novel	1.37	24.98	coding	375	47	1171
PB.9256.1	8	654	1	intergenic	novelGene_82	novel	23.42	23.42	non coding	NA	NA	NA
PB.5961.1	5	1753	2	novel_not_in_catalog	gene: Zm00001d012823	novel	62.47	62.47	coding	497	226	1716

PB.5.1	1	1730	11	incomplete-splice_match	gene: Zm00001d027259	Zm00001d027259_T06	14.89	20.99	coding	473	30	1448
PB.5305.3	4	1151	7	novel_in_catalog	gene: Zm00001d051320	novel	4.34	8.74	non coding	NA	NA	NA
PB.5.2	1	2634	17	incomplete-splice_match	gene: Zm00001d027259	Zm00001d027259_T05	6.10	20.99	coding	601	235	2037
PB.8728.2	7	1719	2	genic	gene: Zm00001d021321	novel	22.68	36.95	coding	410	371	1600
PB.3425.3	2	3226	4	novel_not_in_catalog	gene: Zm00001d006679	novel	66.47	476.20	coding	243	118	846
PB.8728.1	7	1961	1	full-splice_match	gene: Zm00001d021321	Zm00001d021321_T00	14.27	36.95	coding	410	612	1841
PB.3425.5	2	1517	6	novel_not_in_catalog	gene: Zm00001d006679	novel	80.21	476.20	coding	196	114	701
PB.8003.9	6	1271	1	full-splice_match	gene: Zm00001d038447	Zm00001d038447_T00	0.00	137.96	coding	334	270	1271
PB.3425.9	2	2055	1	genic	gene: Zm00001d006679	novel	64.72	476.20	coding	104	1460	1771
PB.8003.6	6	2242	1	full-splice_match	gene: Zm00001d038447	Zm00001d038447_T00	0.00	137.96	coding	581	300	2042
PB.8003.7	6	1860	1	full-splice_match	gene: Zm00001d038447	Zm00001d038447_T00	13.62	137.96	coding	559	51	1727
PB.981.2	1	1760	5	novel_in_catalog	gene: Zm00001d032031	novel	2.10	144.91	coding	217	4	654
PB.8003.1	6	2610	1	full-splice_match	gene: Zm00001d038447	Zm00001d038447_T00	8.29	137.96	coding	581	264	2006
PB.8003.3	6	2384	1	full-splice_match	gene: Zm00001d038447	Zm00001d038447_T00	116.06	137.96	coding	581	313	2055
PB.3876.1	3	844	4	novel_not_in_catalog	gene: Zm00001d040124	novel	51.84	51.84	coding	137	118	528
PB.9641.1	8	376	1	intergenic	novelGene_246	novel	112.16	112.16	non coding	NA	NA	NA
PB.8234.1	7	1546	10	full-splice_match	gene: Zm00001d018669	Zm00001d018669_T00	64.37	64.37	coding	332	325	1320

PB.6728.1	5	1342	10	incomplete-splice_match	gene: Zm00001d016286	Zm00001d016286_T00	5.93	16.00	coding	288	27	890
PB.3058.1	2	2283	12	full-splice_match	gene: Zm00001d004960	Zm00001d004960_T00	37.04	43.02	coding	640	100	2019
PB.3058.2	2	2378	11	full-splice_match	gene: Zm00001d004960	Zm00001d004960_T00	5.98	43.02	coding	336	100	1107
PB.4598.1	3	522	1	intergenic	novelGene_403	novel	1.72	1.72	non coding	NA	NA	NA
PB.3152.2	2	1814	6	full-splice_match	gene: Zm00001d005462	Zm00001d005462_T00	18.55	18.55	coding	429	254	1540
PB.2458.2	2	361	1	full-splice_match	gene: Zm00001d001905	Zm00001d001905_T00	96.89	171.29	non coding	NA	NA	NA
PB.1426.5	1	1567	11	full-splice_match	gene: Zm00001d033969	Zm00001d033969_T00	25.41	92.30	coding	384	263	1414
PB.2458.1	2	367	1	full-splice_match	gene: Zm00001d001905	Zm00001d001905_T00	74.41	171.29	non coding	NA	NA	NA
PB.5371.2	4	2364	17	novel_in_catalog	gene: Zm00001d051629	novel	3.30	79.62	coding	418	86	1339
PB.4917.2	4	911	2	intergenic	novelGene_54	novel	7.73	20.51	non coding	NA	NA	NA
PB.5610.1	4	1773	2	full-splice_match	gene: Zm00001d052636	Zm00001d052636_T00	4.33	4.33	coding	450	149	1498
PB.6973.4	5	2887	7	novel_in_catalog	gene: Zm00001d017392	novel	0.00	39.92	coding	486	1145	2602
PB.496.1	1	1718	13	full-splice_match	gene: Zm00001d029410	Zm00001d029410_T00	28.93	28.93	coding	458	85	1458
PB.10346.1	9	1776	10	novel_not_in_catalog	gene: Zm00001d045837	novel	30.44	30.44	coding	448	163	1506
PB.10346.2	9	1679	10	novel_not_in_catalog	gene: Zm00001d045837	novel	0.00	30.44	coding	448	163	1506
PB.2812.7	2	1016	1	novel_in_catalog	gene: Zm00001d003457	novel	3.36	95.96	coding	108	479	802
PB.10840.11	9	2622	8	novel_in_catalog	gene: Zm00001d048272	novel	0.00	104.28	coding	436	1268	2575

PB.2812.1	2	1113	2	full-splice_match	gene: Zm00001d003457	Zm00001d003457_T00	32.72	95.96	coding	204	68	679
PB.6747.3	5	2679	14	full-splice_match	gene: Zm00001d016406	Zm00001d016406_T02	20.26	37.94	coding	708	226	2349
PB.2812.3	2	1011	2	full-splice_match	gene: Zm00001d003457	Zm00001d003457_T00	25.51	95.96	coding	204	68	679
PB.7883.4	6	994	3	novel_in_catalog	gene: Zm00001d038035	novel	7.04	16.09	non coding	NA	NA	NA
PB.8720.1	7	3319	17	full-splice_match	gene: Zm00001d021301	Zm00001d021301_T00	72.11	73.13	coding	885	160	2814
PB.2812.8	2	721	2	full-splice_match	gene: Zm00001d003457	Zm00001d003457_T00	0.00	95.96	coding	204	15	626
PB.2447.3	2	1096	8	intergenic	novelGene_13	novel	137.46	209.41	coding	263	66	854
PB.7883.2	6	1116	5	full-splice_match	gene: Zm00001d038035	Zm00001d038035_T00	9.05	16.09	non coding	NA	NA	NA
PB.10733.1	9	1387	7	full-splice_match	gene: Zm00001d047814	Zm00001d047814_T00	3.72	35.66	coding	280	174	1013
PB.10733.2	9	1295	7	full-splice_match	gene: Zm00001d047814	Zm00001d047814_T00	31.94	35.66	coding	280	183	1022
PB.1837.2	10	1996	12	full-splice_match	gene: Zm00001d023943	Zm00001d023943_T00	120.52	215.04	coding	516	234	1781
PB.1837.3	10	1912	12	full-splice_match	gene: Zm00001d023943	Zm00001d023943_T00	3.07	215.04	coding	516	240	1787
PB.5670.1	4	1345	1	full-splice_match	gene: Zm00001d052895	Zm00001d052895_T00	15.68	15.68	coding	372	14	1129
PB.1837.1	10	2057	12	full-splice_match	gene: Zm00001d023943	Zm00001d023943_T00	91.46	215.04	coding	516	190	1737
PB.7902.4	6	2011	4	full-splice_match	gene: Zm00001d038105	Zm00001d038105_T00	0.00	57.90	coding	555	78	1742
PB.10158.1	9	2644	16	full-splice_match	gene: Zm00001d045002	Zm00001d045002_T00	14.92	14.92	coding	721	94	2256
PB.7642.5	6	2172	8	novel_not_in_catalog	gene: Zm00001d036977	novel	35.38	111.08	coding	546	36	1673

PB.2422.1	2	2222	12	full-splice_match	gene: Zm00001d001780	Zm00001d001780_T00	54.83	54.83	coding	490	227	1696
PB.7902.3	6	2446	4	full-splice_match	gene: Zm00001d038105	Zm00001d038105_T00	4.00	57.90	coding	555	349	2013
PB.7902.2	6	2551	4	full-splice_match	gene: Zm00001d038105	Zm00001d038105_T00	0.00	57.90	coding	555	299	1963
PB.2713.2	2	1015	9	incomplete-splice_match	gene: Zm00001d003031	Zm00001d003031_T00	4.57	27.96	coding	264	222	1013
PB.7721.2	6	1259	6	full-splice_match	gene: Zm00001d037336	Zm00001d037336_T00	0.00	93.20	coding	342	60	1085
PB.2713.1	2	3003	15	full-splice_match	gene: Zm00001d003031	Zm00001d003031_T00	23.38	27.96	coding	842	128	2653
PB.3276.2	2	1286	5	full-splice_match	gene: Zm00001d006070	Zm00001d006070_T00	18.98	71.75	coding	198	267	860
PB.4479.5	3	2281	6	novel_not_in_catalog	gene: Zm00001d043354	novel	0.23	31.37	coding	602	188	1993
PB.2283.1	10	659	1	genic	gene: Zm00001d026297	novel	1.31	222.72	non coding	NA	NA	NA
PB.2283.2	10	580	3	full-splice_match	gene: Zm00001d026297	Zm00001d026297_T00	221.41	222.72	coding	80	122	361
PB.175.4	1	1286	10	novel_not_in_catalog	gene: Zm00001d027995	novel	0.00	30.27	coding	382	50	1195
PB.175.3	1	1506	10	novel_not_in_catalog	gene: Zm00001d027995	novel	1.83	30.27	coding	326	332	1309
PB.175.2	1	1441	10	novel_not_in_catalog	gene: Zm00001d027995	novel	5.79	30.27	coding	382	56	1201
PB.175.1	1	1614	10	novel_not_in_catalog	gene: Zm00001d027995	novel	22.65	30.27	coding	382	56	1201
PB.5141.4	4	2313	1	full-splice_match	gene: Zm00001d050132	Zm00001d050132_T00	3.52	11.78	coding	650	86	2035
PB.10829.2	9	1110	3	full-splice_match	gene: Zm00001d048204	Zm00001d048204_T00	2.19	307.47	coding	143	95	523
PB.1815.2	10	1881	2	incomplete-splice_match	gene: Zm00001d023838	Zm00001d023838_T00	2.16	74.52	non coding	NA	NA	NA

PB.7721.1	6	1516	6	full-splice_match	gene: Zm00001d037336	Zm00001d037336_T00	93.20	93.20	coding	377	88	1218
PB.6042.2	5	909	7	full-splice_match	gene: Zm00001d013079	Zm00001d013079_T00	130.55	147.34	coding	194	115	696
PB.6042.1	5	977	7	full-splice_match	gene: Zm00001d013079	Zm00001d013079_T00	16.79	147.34	coding	194	63	644
PB.268.3	1	1318	1	full-splice_match	gene: Zm00001d028389	Zm00001d028389_T00	0.00	36.40	coding	256	409	1176
PB.10503.2	9	2535	7	novel_not_in_catalog	gene: Zm00001d046893	novel	0.00	13.71	coding	625	360	2234
PB.10503.1	9	2700	7	novel_not_in_catalog	gene: Zm00001d046893	novel	13.71	13.71	coding	625	122	1996
PB.4091.2	3	2586	2	incomplete-splice_match	gene: Zm00001d041556	Zm00001d041556_T00	53.33	103.64	coding	615	47	1891
PB.5812.1	4	1335	9	full-splice_match	gene: Zm00001d053628	Zm00001d053628_T00	19.41	22.84	coding	291	208	1080
PB.7704.7	6	2054	12	novel_not_in_catalog	gene: Zm00001d037257	novel	0.00	132.91	coding	441	407	1729
PB.8747.2	7	2308	7	full-splice_match	gene: Zm00001d021392	Zm00001d021392_T00	110.96	240.57	coding	639	157	2073
PB.5903.2	4	1633	3	novel_not_in_catalog	gene: Zm00001d053972	novel	2.96	20.94	coding	338	416	1429
PB.1815.1	10	3459	8	full-splice_match	gene: Zm00001d023838	Zm00001d023838_T00	38.39	74.52	coding	884	285	2936
PB.9272.1	8	1954	12	full-splice_match	gene: Zm00001d008893	Zm00001d008893_T00	29.11	126.70	coding	406	212	1429
PB.6341.2	5	1591	1	full-splice_match	gene: Zm00001d014328	Zm00001d014328_T00	1.04	13.64	coding	472	174	1589
PB.6341.1	5	1726	1	full-splice_match	gene: Zm00001d014328	Zm00001d014328_T00	12.60	13.64	coding	501	145	1647
PB.9272.2	8	1813	12	full-splice_match	gene: Zm00001d008893	Zm00001d008893_T00	97.59	126.70	coding	406	212	1429
PB.2447.1	2	1177	7	intergenic	novelGene_13	novel	58.72	209.41	coding	263	203	991

PB.10182.1	9	2516	1	full-splice_match	gene: Zm00001d045090	Zm00001d045090_T00	1.58	1.58	coding	599	254	2050
PB.5678.1	4	1979	17	incomplete-splice_match	gene: Zm00001d052943	Zm00001d052943_T02	33.21	33.21	coding	409	268	1494
PB.7141.1	5	943	2	incomplete-splice_match	gene: Zm00001d018070	Zm00001d018070_T00	23.32	23.32	non coding	NA	NA	NA
PB.10527.1	9	2730	10	full-splice_match	gene: Zm00001d046973	Zm00001d046973_T00	43.46	43.46	coding	820	72	2531
PB.10835.2	9	3291	9	incomplete-splice_match	gene: Zm00001d048236	Zm00001d048236_T00	18.47	24.13	coding	856	45	2612
PB.9395.1	8	1814	1	full-splice_match	gene: Zm00001d009608	Zm00001d009608_T00	31.79	72.38	coding	388	142	1305
PB.5836.1	4	1049	5	full-splice_match	gene: Zm00001d053724	Zm00001d053724_T00	16.48	28.84	coding	178	146	679
PB.9395.2	8	2203	1	full-splice_match	gene: Zm00001d009608	Zm00001d009608_T00	40.59	72.38	coding	505	301	1815
PB.9141.1	8	1765	9	novel_not_in_catalog	gene: Zm00001d008289	novel	27.94	52.48	coding	362	296	1381
PB.9141.2	8	3379	14	novel_not_in_catalog	gene: Zm00001d008289	novel	24.55	52.48	coding	950	302	3151
PB.10370.1	9	1306	10	incomplete-splice_match	gene: Zm00001d045978	Zm00001d045978_T00	2.67	2.67	coding	223	404	1072
PB.1634.4	1	3198	1	full-splice_match	gene: Zm00001d034781	Zm00001d034781_T00	16.92	60.57	coding	852	363	2918
PB.5606.1	4	1620	2	full-splice_match	gene: Zm00001d052618	Zm00001d052618_T00	21.92	21.92	coding	404	80	1291
PB.6818.1	5	2039	1	full-splice_match	gene: Zm00001d016755	Zm00001d016755_T00	14.86	14.86	coding	405	637	1851
PB.7829.1	6	1158	6	novel_in_catalog	gene: Zm00001d037848	novel	18.71	18.71	coding	251	137	889
PB.10051.2	8	1174	4	full-splice_match	gene: Zm00001d012590	Zm00001d012590_T00	22.68	46.41	coding	299	82	978
PB.10723.1	9	802	3	full-splice_match	gene: Zm00001d047779	Zm00001d047779_T00	21.24	21.24	non coding	NA	NA	NA

PB.9567.1	8	426	2	intergenic	novelGene_236	novel	109.83	109.83	non coding	NA	NA	NA
PB.7704.9	6	1728	12	novel_not_in_catalog	gene: Zm00001d037257	novel	0.00	132.91	coding	441	232	1554
PB.8067.1	6	1344	2	full-splice_match	gene: Zm00001d038703	Zm00001d038703_T00	89.79	89.79	coding	358	38	1111
PB.8341.1	7	856	4	novel_in_catalog	gene: Zm00001d019219	novel	10.90	621.53	coding	140	144	563
PB.10958.1	B73V4	2303	1	intergenic	novelGene_6	novel	75.34	75.34	coding	123	1335	1703
PB.8341.3	7	723	5	full-splice_match	gene: Zm00001d019219	Zm00001d019219_T00	610.63	621.53	coding	140	93	512
PB.14.1	1	970	2	full-splice_match	gene: Zm00001d027296	Zm00001d027296_T00	10.56	10.56	coding	143	351	779
PB.2517.8	2	1112	10	novel_not_in_catalog	gene: Zm00001d002135	novel	0.00	57.34	coding	296	140	1027
PB.6074.1	5	2157	4	full-splice_match	gene: Zm00001d013238	Zm00001d013238_T00	1.89	1.89	coding	524	108	1679
PB.4908.1	4	1188	3	novel_in_catalog	gene: Zm00001d048959	novel	7.30	173.58	coding	243	183	911
PB.8702.6	7	1485	3	full-splice_match	gene: Zm00001d021224	Zm00001d021224_T00	0.00	100.77	coding	296	417	1304
PB.3352.1	2	3549	2	genic	gene: Zm00001d006371	novel	26.93	26.93	coding	1012	278	3313
PB.7937.1	6	1272	6	full-splice_match	gene: Zm00001d038204	Zm00001d038204_T00	22.31	22.31	coding	234	228	929
PB.9657.1	8	2513	1	genic	gene: Zm00001d010869	novel	25.42	43.27	coding	708	60	2183
PB.2845.1	2	1821	1	full-splice_match	gene: Zm00001d003617	Zm00001d003617_T00	4.19	4.19	coding	471	75	1487
PB.4417.5	3	1120	7	novel_not_in_catalog	gene: Zm00001d043113	novel	0.00	109.53	coding	181	348	890
PB.2517.3	2	2221	17	novel_not_in_catalog	gene: Zm00001d002135	novel	27.41	57.34	coding	574	201	1922

PB.2517.4	2	2356	15	novel_not_in_catalog	gene: Zm00001d002135	novel	14.73	57.34	coding	296	304	1191
PB.2517.5	2	2179	16	novel_not_in_catalog	gene: Zm00001d002135	novel	10.56	57.34	coding	546	139	1776
PB.2517.6	2	2616	16	novel_not_in_catalog	gene: Zm00001d002135	novel	4.63	57.34	coding	408	1158	2381
PB.10872.1	9	2019	14	full-splice_match	gene: Zm00001d048373	Zm00001d048373_T00	73.87	73.87	coding	550	171	1820
PB.1504.1	1	1416	1	intergenic	novelGene_465	novel	20.33	20.33	coding	397	38	1228
PB.6355.1	5	2029	9	full-splice_match	gene: Zm00001d014385	Zm00001d014385_T00	20.17	21.43	coding	482	84	1529
PB.6355.2	5	1926	9	full-splice_match	gene: Zm00001d014385	Zm00001d014385_T00	0.00	21.43	coding	482	89	1534
PB.8361.4	7	1876	11	novel_not_in_catalog	gene: Zm00001d019327	novel	68.23	154.99	coding	549	74	1720
PB.9734.2	8	2286	6	novel_in_catalog	gene: Zm00001d011226	novel	6.26	55.35	coding	236	1346	2053
PB.11002.1	B73V 4	1718	5	full-splice_match	gene: Zm00001d000391	Zm00001d000391_T00	24.77	24.77	coding	372	242	1357
PB.8361.1	7	2495	9	novel_not_in_catalog	gene: Zm00001d019327	novel	8.14	154.99	coding	312	1224	2159
PB.9734.1	8	2171	7	full-splice_match	gene: Zm00001d011226	Zm00001d011226_T00	49.09	55.35	coding	340	895	1914
PB.1842.2	10	897	9	full-splice_match	gene: Zm00001d023968	Zm00001d023968_T00	7.56	42.17	coding	213	84	722
PB.1842.1	10	1035	9	full-splice_match	gene: Zm00001d023968	Zm00001d023968_T00	34.61	42.17	coding	213	120	758
PB.8887.3	7	684	1	intergenic	novelGene_329	novel	75.69	154.98	non coding	NA	NA	NA
PB.6810.4	5	1619	7	full-splice_match	gene: Zm00001d016714	Zm00001d016714_T00	7.02	84.19	coding	311	182	1114
PB.6810.5	5	1234	8	full-splice_match	gene: Zm00001d016714	Zm00001d016714_T00	0.00	84.19	coding	314	182	1123

PB.8077.1	6	1106	2	full-splice_match	gene: Zm00001d038741	Zm00001d038741_T00	31.67	61.28	coding	200	113	712
PB.608.4	1	2241	15	novel_not_in_catalog	gene: Zm00001d030005	novel	4.49	19.48	coding	623	138	2006
PB.8077.2	6	905	2	full-splice_match	gene: Zm00001d038741	Zm00001d038741_T00	29.62	61.28	coding	200	110	709
PB.5868.3	4	2365	12	full-splice_match	gene: Zm00001d053841	Zm00001d053841_T01	12.86	59.78	coding	320	1176	2135
PB.3556.1	2	1735	1	full-splice_match	gene: Zm00001d007274	Zm00001d007274_T00	36.84	131.97	coding	373	86	1204
PB.1179.2	1	819	1	full-splice_match	gene: Zm00001d032821	Zm00001d032821_T00	146.53	208.82	coding	196	24	611
PB.6691.4	5	1700	8	novel_in_catalog	gene: Zm00001d016077	novel	14.10	71.60	coding	109	669	995
PB.22.1	1	1775	20	full-splice_match	gene: Zm00001d027326	Zm00001d027326_T00	40.25	55.18	coding	443	82	1410
PB.3943.1	3	1574	8	full-splice_match	gene: Zm00001d040411	Zm00001d040411_T00	19.15	62.24	coding	292	182	1057
PB.22.3	1	1664	20	full-splice_match	gene: Zm00001d027326	Zm00001d027326_T00	14.93	55.18	coding	443	113	1441
PB.3943.3	3	1402	8	full-splice_match	gene: Zm00001d040411	Zm00001d040411_T00	23.01	62.24	coding	292	266	1141
PB.6925.3	5	1734	3	full-splice_match	gene: Zm00001d017186	Zm00001d017186_T00	646.12	841.44	coding	446	147	1484
PB.10929.1	9	1372	2	full-splice_match	gene: Zm00001d048547	Zm00001d048547_T00	28.08	28.08	coding	357	76	1146
PB.10482.5	9	1136	3	full-splice_match	gene: Zm00001d046723	Zm00001d046723_T00	5.96	31.26	coding	265	223	1017
PB.10482.4	9	444	1	novel_in_catalog	gene: Zm00001d046723	novel	13.27	31.26	coding	94	28	309
PB.1086.2	1	1046	6	fusion	gene: Zm00001d032496	novel	25.59	142.32	coding	170	59	568
PB.2180.1	10	1190	11	incomplete-splice_match	gene: Zm00001d025853	Zm00001d025853_T00	11.57	11.57	coding	351	45	1097

PB.1086.1	1	1219	6	fusion	gene: Zm00001d032496	novel	0.87	142.32	coding	170	58	567
PB.5767.1	4	1256	3	full-splice_match	gene: Zm00001d053396	Zm00001d053396_T00	8.57	106.06	coding	217	116	766
PB.1086.4	1	826	6	fusion	gene: Zm00001d032496	novel	77.69	142.32	coding	170	52	561
PB.8098.8	6	1650	10	novel_in_catalog	gene: Zm00001d038819	novel	4.65	47.25	coding	250	692	1441
PB.6691.1	5	1876	9	novel_in_catalog	gene: Zm00001d016077	novel	8.10	71.60	coding	109	586	912
PB.608.1	1	3148	15	novel_not_in_catalog	gene: Zm00001d030005	novel	1.36	19.48	coding	620	164	2023
PB.4071.3	3	3136	15	full-splice_match	gene: Zm00001d041420	Zm00001d041420_T00	10.93	70.50	coding	807	501	2921
PB.2093.1	10	3935	5	novel_in_catalog	gene: Zm00001d025397	novel	29.57	173.03	coding	330	177	1166
PB.4071.1	3	1024	4	incomplete-splice_match	gene: Zm00001d041420	Zm00001d041420_T01	34.59	70.50	coding	144	103	534
PB.4280.1	3	2144	1	full-splice_match	gene: Zm00001d042508	Zm00001d042508_T00	11.26	27.95	coding	598	51	1844
PB.4071.4	3	3544	14	full-splice_match	gene: Zm00001d041420	Zm00001d041420_T01	5.07	70.50	coding	467	1944	3344
PB.5974.5	5	2103	9	incomplete-splice_match	gene: Zm00001d012863	Zm00001d012863_T00	0.00	40.26	coding	262	64	849
PB.5790.1	4	3586	2	genic	gene: Zm00001d053552	novel	24.48	57.09	coding	1038	67	3180
PB.608.3	1	1455	7	incomplete-splice_match	gene: Zm00001d030005	Zm00001d030005_T00	12.00	19.48	coding	340	67	1086
PB.8369.2	7	905	3	full-splice_match	gene: Zm00001d019459	Zm00001d019459_T00	27.91	30.95	coding	166	85	582
PB.8369.1	7	970	3	full-splice_match	gene: Zm00001d019459	Zm00001d019459_T00	3.04	30.95	coding	166	49	546
PB.5541.3	4	749	4	full-splice_match	gene: Zm00001d052325	Zm00001d052325_T00	0.00	32.43	coding	173	82	600

PB.4584.1	3	1858	2	genic	gene: Zm00001d043695	novel	60.90	60.90	coding	433	121	1419
PB.5646.1	4	2050	10	fusion	gene: Zm00001d052785	novel	52.63	52.63	coding	520	167	1726
PB.3606.1	2	4309	28	full-splice_match	gene: Zm00001d007698	Zm00001d007698_T00	39.58	140.33	coding	1203	112	3720
PB.4652.2	3	1719	1	genic	gene: Zm00001d043935	novel	19.49	215.60	non coding	NA	NA	NA
PB.5224.1	4	2429	19	full-splice_match	gene: Zm00001d050737	Zm00001d050737_T00	84.23	84.23	coding	598	237	2030
PB.5224.3	4	2300	19	full-splice_match	gene: Zm00001d050737	Zm00001d050737_T00	0.00	84.23	coding	598	234	2027
PB.11034.3	B73V 4	3002	10	novel_in_catalog	gene: Zm00001d000228	novel	25.71	106.98	coding	313	295	1233
PB.8375.1	7	1638	3	genic	gene: Zm00001d019493	novel	12.66	26.26	coding	423	190	1458
PB.11034.1	B73V 4	2736	13	novel_in_catalog	gene: Zm00001d000228	novel	7.40	106.98	coding	313	388	1326
PB.8375.3	7	3421	1	full-splice_match	gene: Zm00001d019493	Zm00001d019493_T00	4.29	26.26	coding	423	1979	3247
PB.11034.7	B73V 4	2839	11	novel_in_catalog	gene: Zm00001d000228	novel	0.48	106.98	coding	313	266	1204
PB.6948.17	5	2262	2	full-splice_match	gene: Zm00001d017274	Zm00001d017274_T00	0.00	2308.60	coding	703	109	2217
PB.11034.5	B73V 4	2929	11	novel_in_catalog	gene: Zm00001d000228	novel	3.77	106.98	coding	313	286	1224
PB.11034.4	B73V 4	2868	11	full-splice_match	gene: Zm00001d000228	Zm00001d000228_T03	0.00	106.98	coding	313	362	1300
PB.8126.2	6	1115	7	full-splice_match	gene: Zm00001d038896	Zm00001d038896_T00	0.00	69.20	coding	266	171	968
PB.10896.1	9	2200	12	full-splice_match	gene: Zm00001d048463	Zm00001d048463_T00	22.47	79.33	coding	520	213	1772
PB.11034.8	B73V 4	2381	14	novel_in_catalog	gene: Zm00001d000228	novel	8.65	106.98	coding	313	295	1233

PB.8396.7	7	1331	7	full-splice_match	gene: Zm00001d019579	Zm00001d019579_T00	17.94	24.53	coding	352	64	1119
PB.1908.1	10	1382	1	intergenic	novelGene_164	novel	18.68	18.68	non coding	NA	NA	NA
PB.6917.1	5	1207	3	full-splice_match	gene: Zm00001d017147	Zm00001d017147_T00	23.89	216.33	coding	258	301	1074
PB.6917.2	5	1276	2	novel_in_catalog	gene: Zm00001d017147	novel	44.68	216.33	coding	175	228	752
PB.6392.4	5	1192	11	novel_in_catalog	gene: Zm00001d014572	novel	12.88	38.20	coding	167	563	1063
PB.6917.5	5	985	3	full-splice_match	gene: Zm00001d017147	Zm00001d017147_T00	139.08	216.33	coding	258	139	912
PB.10310.1	9	1720	18	novel_not_in_catalog	gene: Zm00001d045604	novel	7.42	7.42	coding	472	126	1541
PB.9143.1	8	2487	6	full-splice_match	gene: Zm00001d008294	Zm00001d008294_T00	6.32	6.32	coding	262	52	837
PB.2971.2	2	695	2	novel_not_in_catalog	gene: Zm00001d004410	novel	23.93	24.84	coding	114	126	467
PB.2971.3	2	1636	1	novel_in_catalog	gene: Zm00001d004410	novel	0.68	24.84	coding	114	1196	1537
PB.9282.1	8	605	6	full-splice_match	gene: Zm00001d008916	Zm00001d008916_T00	255.75	255.75	non coding	NA	NA	NA
PB.11013.2	B73V4	2522	2	novel_in_catalog	gene: Zm00001d000112	novel	85.09	476.95	coding	148	1370	1813
PB.7056.2	5	1557	6	novel_not_in_catalog	gene: Zm00001d017756	novel	16.64	54.96	coding	171	716	1228
PB.7056.1	5	2038	6	novel_not_in_catalog	gene: Zm00001d017756	novel	10.76	54.96	coding	137	335	745
PB.11013.1	B73V4	2027	2	novel_in_catalog	gene: Zm00001d000112	novel	57.71	476.95	coding	232	619	1314
PB.11013.6	B73V4	2268	3	full-splice_match	gene: Zm00001d000112	Zm00001d000112_T00	4.78	476.95	coding	148	1122	1565
PB.6392.1	5	1617	12	full-splice_match	gene: Zm00001d014572	Zm00001d014572_T00	5.97	38.20	coding	326	64	1041

PB.11013.5	B73V4	2623	1	novel_in_catalog	gene: Zm00001d000112	novel	6.74	476.95	coding	148	1475	1918
PB.5551.3	4	991	3	full-splice_match	gene: Zm00001d052357	Zm00001d052357_T00	23.87	63.72	coding	228	84	767
PB.5551.2	4	1144	3	full-splice_match	gene: Zm00001d052357	Zm00001d052357_T00	13.15	63.72	coding	228	74	757
PB.5551.1	4	933	1	incomplete-splice_match	gene: Zm00001d052357	Zm00001d052357_T00	17.39	63.72	coding	77	154	384
PB.11013.9	B73V4	1865	3	full-splice_match	gene: Zm00001d000112	Zm00001d000112_T00	301.83	476.95	coding	359	131	1207
PB.599.1	1	1157	1	full-splice_match	gene: Zm00001d029923	Zm00001d029923_T00	150.77	150.77	coding	225	221	895
PB.9960.1	8	808	7	incomplete-splice_match	gene: Zm00001d012205	Zm00001d012205_T00	24.85	24.85	coding	162	201	686
PB.996.1	1	2745	25	full-splice_match	gene: Zm00001d032099	Zm00001d032099_T00	64.30	64.30	coding	782	116	2461
PB.9706.12	8	3132	8	novel_in_catalog	gene: Zm00001d011119	novel	9.37	78.92	coding	158	2423	2896
PB.7135.2	5	2478	2	full-splice_match	gene: Zm00001d018043	Zm00001d018043_T00	24.21	24.21	coding	736	84	2291
PB.2911.1	2	3647	10	full-splice_match	gene: Zm00001d003947	Zm00001d003947_T00	114.85	114.85	coding	1025	347	3421
PB.3269.14	2	1470	6	full-splice_match	gene: Zm00001d006046	Zm00001d006046_T00	20.30	72.31	coding	301	204	1106
PB.7648.1	6	2559	3	novel_in_catalog	gene: Zm00001d037001	novel	6.36	38.53	coding	254	677	1438
PB.7648.2	6	1600	4	novel_in_catalog	gene: Zm00001d037001	novel	1.73	38.53	coding	226	428	1105
PB.7648.3	6	2826	10	novel_in_catalog	gene: Zm00001d037001	novel	5.78	38.53	coding	277	1807	2637
PB.7648.4	6	2827	11	novel_in_catalog	gene: Zm00001d037001	novel	15.85	38.53	coding	226	1765	2442
PB.7648.5	6	3683	10	novel_in_catalog	gene: Zm00001d037001	novel	8.81	38.53	non coding	NA	NA	NA

PB.7648.7	6	392	1	incomplete-splice_match	gene: Zm00001d037001	Zm00001d037001_T00	0.00	38.53	non coding	NA	NA	NA
PB.10674.2	9	2218	4	novel_in_catalog	gene: Zm00001d047633	novel	6.66	28.42	coding	576	386	2113
PB.6054.2	5	1482	6	full-splice_match	gene: Zm00001d013147	Zm00001d013147_T00	32.20	46.32	coding	382	124	1269
PB.9542.1	8	1492	3	full-splice_match	gene: Zm00001d010403	Zm00001d010403_T00	86.55	86.55	coding	349	216	1262
PB.2883.1	2	1996	7	full-splice_match	gene: Zm00001d003836	Zm00001d003836_T00	16.91	16.91	coding	519	73	1629
PB.6907.3	5	1456	14	incomplete-splice_match	gene: Zm00001d017119	Zm00001d017119_T00	266.21	266.21	coding	358	231	1304
PB.1595.2	1	2173	4	incomplete-splice_match	gene: Zm00001d034655	Zm00001d034655_T00	49.80	77.19	coding	615	35	1879
PB.5531.3	4	3475	10	full-splice_match	gene: Zm00001d052295	Zm00001d052295_T00	15.73	24.82	coding	859	567	3143
PB.4797.1	3	2094	11	full-splice_match	gene: Zm00001d044533	Zm00001d044533_T00	52.19	80.50	coding	462	104	1489
PB.870.2	1	982	5	full-splice_match	gene: Zm00001d031488	Zm00001d031488_T00	3.81	6.53	coding	258	136	909
PB.870.1	1	1325	4	full-splice_match	gene: Zm00001d031488	Zm00001d031488_T00	2.72	6.53	coding	108	907	1230
PB.6854.3	5	584	3	incomplete-splice_match	gene: Zm00001d016892	Zm00001d016892_T00	4.43	168.96	coding	145	65	499
PB.559.1	1	793	1	intergenic	novelGene_185	novel	23.34	23.34	non coding	NA	NA	NA
PB.5859.12	4	1991	8	novel_not_in_catalog	gene: Zm00001d053818	novel	3.54	105.42	coding	405	605	1819
PB.2932.3	2	1376	2	novel_in_catalog	gene: Zm00001d004086	novel	4.40	78.94	coding	248	105	848
PB.2932.2	2	1317	3	full-splice_match	gene: Zm00001d004086	Zm00001d004086_T00	12.26	78.94	coding	331	105	1097
PB.2932.1	2	1261	3	full-splice_match	gene: Zm00001d004086	Zm00001d004086_T00	62.28	78.94	coding	331	47	1039

PB.3520.2	2	1597	5	incomplete-splice_match	gene: Zm00001d007089	Zm00001d007089_T01	0.00	29.14	coding	362	97	1182
PB.8269.1	7	1541	1	full-splice_match	gene: Zm00001d018854	Zm00001d018854_T00	26.08	26.08	coding	351	301	1353
PB.4098.1	3	2267	13	full-splice_match	gene: Zm00001d041599	Zm00001d041599_T00	25.61	38.55	coding	596	92	1879
PB.4098.3	3	3565	13	novel_in_catalog	gene: Zm00001d041599	novel	2.07	38.55	coding	618	1329	3182
PB.4098.4	3	2804	13	novel_not_in_catalog	gene: Zm00001d041599	novel	10.88	38.55	coding	286	339	1196
PB.7836.3	6	2817	15	full-splice_match	gene: Zm00001d037872	Zm00001d037872_T00	11.82	53.51	coding	511	879	2411
PB.8602.1	7	1266	1	full-splice_match	gene: Zm00001d020787	Zm00001d020787_T00	12.76	12.76	coding	341	45	1067
PB.196.2	1	3564	13	full-splice_match	gene: Zm00001d028084	Zm00001d028084_T01	2.34	16.98	coding	1172	48	3563
PB.196.3	1	3739	13	incomplete-splice_match	gene: Zm00001d028084	Zm00001d028084_T00	1.04	16.98	coding	987	779	3739
PB.196.1	1	2723	8	incomplete-splice_match	gene: Zm00001d028084	Zm00001d028084_T00	13.60	16.98	coding	806	141	2558
PB.7692.1	6	892	4	incomplete-splice_match	gene: Zm00001d037228	Zm00001d037228_T00	5.04	5.04	coding	185	34	588
PB.2260.4	10	1103	1	novel_in_catalog	gene: Zm00001d026223	novel	0.00	210.95	coding	76	107	334
PB.4538.1	3	1058	3	full-splice_match	gene: Zm00001d043528	Zm00001d043528_T00	30.78	30.78	coding	256	81	848
PB.2919.1	2	1407	3	full-splice_match	gene: Zm00001d004007	Zm00001d004007_T00	24.92	24.92	coding	358	81	1154
PB.2260.1	10	750	2	full-splice_match	gene: Zm00001d026223	Zm00001d026223_T00	14.10	210.95	coding	122	93	458
PB.2260.3	10	783	2	full-splice_match	gene: Zm00001d026223	Zm00001d026223_T00	192.11	210.95	coding	122	252	617
PB.2260.2	10	1661	1	novel_in_catalog	gene: Zm00001d026223	novel	4.74	210.95	coding	76	110	337

PB.7237.2	5	2324	2	novel_in_catalog	gene: Zm00001d018420	novel	4.06	47.60	coding	215	1341	1985
PB.563.3	1	1423	3	novel_in_catalog	gene: Zm00001d029711	novel	1.30	20.27	coding	306	104	1021
PB.563.1	1	1559	4	full-splice_match	gene: Zm00001d029711	Zm00001d029711_T00	18.97	20.27	coding	397	102	1292
PB.5273.2	4	647	4	full-splice_match	gene: Zm00001d051061	Zm00001d051061_T00	0.00	136.86	coding	103	150	458
PB.4746.4	3	1317	10	full-splice_match	gene: Zm00001d044315	Zm00001d044315_T01	0.00	301.89	coding	351	168	1220
PB.7060.4	5	2973	7	full-splice_match	gene: Zm00001d017780	Zm00001d017780_T00	38.53	172.94	coding	765	449	2743
PB.5343.5	4	2178	3	incomplete-splice_match	gene: Zm00001d051507	Zm00001d051507_T01	0.00	31.79	coding	692	101	2176
PB.8275.1	7	1310	12	full-splice_match	gene: Zm00001d018867	Zm00001d018867_T00	136.46	136.46	coding	302	131	1036
PB.7060.2	5	2240	1	novel_in_catalog	gene: Zm00001d017780	novel	66.06	172.94	coding	253	80	838
PB.7060.3	5	2979	7	full-splice_match	gene: Zm00001d017780	Zm00001d017780_T00	68.36	172.94	coding	765	455	2749
PB.10402.3	9	560	2	novel_in_catalog	gene: Zm00001d046234	novel	20.46	37.55	non coding	NA	NA	NA
PB.1351.5	1	1268	3	full-splice_match	gene: Zm00001d033611	Zm00001d033611_T00	43.60	103.62	coding	257	261	1031
PB.10402.1	9	1335	9	novel_in_catalog	gene: Zm00001d046234	novel	17.09	37.55	coding	302	234	1139
PB.4518.1	3	1456	3	full-splice_match	gene: Zm00001d043474	Zm00001d043474_T00	33.69	71.52	coding	306	285	1202
PB.4518.2	3	1354	3	full-splice_match	gene: Zm00001d043474	Zm00001d043474_T00	37.82	71.52	coding	306	285	1202
PB.5919.1	4	1917	4	full-splice_match	gene: Zm00001d054002	Zm00001d054002_T00	18.86	18.86	coding	565	31	1725
PB.5982.3	5	1922	5	novel_in_catalog	gene: Zm00001d012896	novel	7.17	38.57	coding	172	885	1400

PB.5982.2	5	1532	5	full-splice_match	gene: Zm00001d012896	Zm00001d012896_T00	7.27	38.57	coding	306	89	1006
PB.5982.1	5	1121	5	incomplete-splice_match	gene: Zm00001d012896	Zm00001d012896_T01	24.13	38.57	coding	282	9	854
PB.6466.1	5	469	1	antisense	novelGene_gene: Zm00001d001569	novel	10.70	10.70	non coding	NA	NA	NA
PB.7839.2	6	2164	7	full-splice_match	gene: Zm00001d037898	Zm00001d037898_T00	3.26	7.48	coding	305	297	1211
PB.9938.2	8	2380	1	novel_in_catalog	gene: Zm00001d012098	novel	40.87	41.71	coding	241	1423	2145
PB.9938.3	8	1381	1	novel_in_catalog	gene: Zm00001d012098	novel	0.84	41.71	coding	32	280	375
PB.9512.1	8	1326	15	novel_not_in_catalog	gene: Zm00001d010262	novel	35.11	35.11	coding	357	69	1139
PB.4764.1	3	1751	10	incomplete-splice_match	gene: Zm00001d044432	Zm00001d044432_T01	47.41	47.41	coding	486	44	1501
PB.7575.2	6	2889	8	full-splice_match	gene: Zm00001d036598	Zm00001d036598_T00	13.66	22.52	coding	694	551	2632
PB.7575.1	6	814	1	genic	gene: Zm00001d036598	novel	8.86	22.52	non coding	NA	NA	NA
PB.8563.4	7	2477	5	incomplete-splice_match	gene: Zm00001d020560	Zm00001d020560_T00	6.48	6.48	coding	699	286	2382
PB.6628.1	5	942	3	full-splice_match	gene: Zm00001d015635	Zm00001d015635_T00	31.63	31.63	coding	115	355	699
PB.9300.1	8	703	4	full-splice_match	gene: Zm00001d009000	Zm00001d009000_T00	347.38	347.38	coding	130	94	483
PB.7549.1	6	1102	1	full-splice_match	gene: Zm00001d036468	Zm00001d036468_T00	127.66	127.66	coding	214	219	860
PB.3349.1	2	1265	4	full-splice_match	gene: Zm00001d006365	Zm00001d006365_T00	26.42	26.42	coding	350	79	1128
PB.1615.1	1	703	3	novel_not_in_catalog	gene: Zm00001d034724	novel	3.96	3.96	coding	165	60	554
PB.693.1	1	2115	13	full-splice_match	gene: Zm00001d030533	Zm00001d030533_T00	22.61	32.25	coding	551	46	1698

PB.1801.1	10	1283	1	intergenic	novelGene_75	novel	68.17	68.17	coding	134	283	684
PB.8422.1	7	1587	4	full-splice_match	gene: Zm00001d019759	Zm00001d019759_T00	43.11	43.11	coding	418	185	1438
PB.7084.9	5	981	3	novel_not_in_catalog	gene: Zm00001d017874	novel	25.02	126.53	non coding	NA	NA	NA
PB.7769.2	6	934	2	genic	gene: Zm00001d037574	novel	10.44	26.10	coding	175	145	669
PB.7583.6	6	1460	12	full-splice_match	gene: Zm00001d036631	Zm00001d036631_T01	15.11	46.80	coding	215	499	1143
PB.7769.1	6	942	2	genic	gene: Zm00001d037574	novel	15.67	26.10	coding	175	167	691
PB.2163.1	10	1318	5	novel_in_catalog	gene: Zm00001d025765	novel	0.16	12.76	coding	140	606	1025
PB.10061.2	8	3810	16	novel_in_catalog	gene: Zm00001d012620	novel	4.18	32.26	coding	858	963	3536
PB.693.3	1	781	5	novel_in_catalog	gene: Zm00001d030533	novel	9.64	32.25	non coding	NA	NA	NA
PB.979.1	1	2083	4	full-splice_match	gene: Zm00001d032029	Zm00001d032029_T00	5.69	5.69	coding	576	66	1793
PB.7466.6	6	1760	2	novel_in_catalog	gene: Zm00001d035925	novel	15.59	352.40	coding	482	176	1621
PB.121.4	1	1562	5	incomplete-splice_match	gene: Zm00001d027731	Zm00001d027731_T00	125.64	333.50	coding	390	99	1268
PB.4178.4	3	1361	3	novel_in_catalog	gene: Zm00001d042017	novel	1.43	58.20	non coding	NA	NA	NA
PB.5054.2	4	1553	9	full-splice_match	gene: Zm00001d049636	Zm00001d049636_T00	48.55	82.77	coding	490	84	1553
PB.5054.1	4	1214	4	incomplete-splice_match	gene: Zm00001d049636	Zm00001d049636_T00	34.22	82.77	coding	223	80	748
PB.7466.1	6	2187	2	novel_in_catalog	gene: Zm00001d035925	novel	65.41	352.40	coding	482	138	1583
PB.852.3	1	1062	2	full-splice_match	gene: Zm00001d031344	Zm00001d031344_T00	62.00	103.08	coding	167	139	639

PB.852.2	1	1193	1	novel_in_catalog	gene: Zm00001d031344	novel	0.64	103.08	non coding	NA	NA	NA
PB.852.1	1	1156	2	full-splice_match	gene: Zm00001d031344	Zm00001d031344_T00	5.89	103.08	coding	167	140	640
PB.8910.1	7	2285	6	full-splice_match	gene: Zm00001d021947	Zm00001d021947_T00	88.73	89.10	coding	613	80	1918
PB.852.6	1	982	1	novel_in_catalog	gene: Zm00001d031344	novel	0.00	103.08	non coding	NA	NA	NA
PB.6492.2	5	889	5	incomplete-splice_match	gene: Zm00001d014920	Zm00001d014920_T04	48.95	201.10	coding	36	302	409
PB.6492.3	5	3192	11	incomplete-splice_match	gene: Zm00001d014920	Zm00001d014920_T03	46.94	201.10	coding	901	164	2866
PB.2447.2	2	1257	6	intergenic	novelGene_13	novel	13.23	209.41	coding	278	167	1000
PB.1967.2	10	1460	11	novel_in_catalog	gene: Zm00001d024759	novel	7.06	20.32	coding	368	112	1215
PB.1967.3	10	1600	12	full-splice_match	gene: Zm00001d024759	Zm00001d024759_T00	13.27	20.32	coding	416	159	1406
PB.2132.1	10	2533	7	full-splice_match	gene: Zm00001d025651	Zm00001d025651_T00	7.50	10.55	coding	693	63	2141
PB.573.2	1	2196	14	novel_in_catalog	gene: Zm00001d029768	novel	6.67	6.67	coding	504	444	1955
PB.1140.5	1	2555	22	novel_not_in_catalog	gene: Zm00001d032685	novel	0.00	57.68	coding	766	209	2506
PB.3980.2	3	1830	5	novel_not_in_catalog	gene: Zm00001d040625	novel	3.91	11.00	coding	402	91	1296
PB.3980.1	3	1980	5	novel_not_in_catalog	gene: Zm00001d040625	novel	0.00	11.00	coding	298	67	960
PB.1140.6	1	2624	21	novel_not_in_catalog	gene: Zm00001d032685	novel	0.00	57.68	coding	670	213	2222
PB.1140.1	1	2737	22	novel_not_in_catalog	gene: Zm00001d032685	novel	50.54	57.68	coding	766	197	2494
PB.8069.1	6	970	2	intergenic	novelGene_324	novel	44.25	44.25	coding	220	191	850

PB.1140.3	1	2728	21	novel_not_in_catalog	gene: Zm00001d032685	novel	2.64	57.68	coding	670	137	2146
PB.3980.4	3	1530	6	novel_not_in_catalog	gene: Zm00001d040625	novel	7.08	11.00	coding	370	91	1200
PB.7967.1	6	1293	8	novel_not_in_catalog	gene: Zm00001d038319	novel	34.52	34.52	coding	258	130	903
PB.8619.2	7	1013	1	full-splice_match	gene: Zm00001d020861	Zm00001d020861_T00	259.06	259.06	coding	162	161	646
PB.8485.10	7	630	1	novel_in_catalog	gene: Zm00001d020185	novel	36.28	390.61	coding	41	206	328
PB.8485.11	7	1391	3	novel_in_catalog	gene: Zm00001d020185	novel	0.00	390.61	coding	395	16	1200
PB.8485.12	7	1190	2	novel_in_catalog	gene: Zm00001d020185	novel	15.62	390.61	coding	240	16	735
PB.8485.13	7	900	2	novel_in_catalog	gene: Zm00001d020185	novel	63.57	390.61	coding	240	7	726
PB.393.1	1	756	1	full-splice_match	gene: Zm00001d028922	Zm00001d028922_T00	13.97	13.97	coding	100	167	466
PB.9576.3	8	2236	3	antisense	novelGene_gene: Zm00001d010564	novel	12.97	44.33	non coding	NA	NA	NA
PB.8572.2	7	863	4	incomplete-splice_match	gene: Zm00001d020620	Zm00001d020620_T00	3.56	7.83	coding	120	290	649
PB.9576.1	8	768	4	intergenic	novelGene_237	novel	18.69	29.46	non coding	NA	NA	NA
PB.6414.2	5	1777	5	full-splice_match	gene: Zm00001d014673	Zm00001d014673_T00	236.21	350.21	coding	414	133	1374
PB.3224.1	2	2308	1	intergenic	novelGene_387	novel	0.23	0.23	non coding	NA	NA	NA
PB.6414.1	5	1456	2	incomplete-splice_match	gene: Zm00001d014673	Zm00001d014673_T00	114.00	350.21	coding	286	192	1049
PB.1150.2	1	2686	13	full-splice_match	gene: Zm00001d032708	Zm00001d032708_T00	40.23	45.03	coding	540	262	1881
PB.6912.3	5	1243	1	full-splice_match	gene: Zm00001d017131	Zm00001d017131_T00	5.69	19.00	coding	311	107	1039

PB.1150.1	1	2820	13	full-splice_match	gene: Zm00001d032708	Zm00001d032708_T00	4.80	45.03	coding	540	239	1858
PB.4136.1	3	1747	3	intergenic	novelGene_266	novel	14.05	14.05	coding	362	314	1399
PB.1232.2	1	1465	5	novel_in_catalog	gene: Zm00001d033063	novel	0.17	113.99	coding	209	43	669
PB.10966.8	B73V 4	2607	4	novel_in_catalog	gene: Zm00001d000021	novel	32.34	461.70	coding	313	296	1234
PB.5693.1	4	3214	5	full-splice_match	gene: Zm00001d053011	Zm00001d053011_T00	9.77	9.77	coding	936	263	3070
PB.5654.1	4	2757	19	full-splice_match	gene: Zm00001d052809	Zm00001d052809_T00	21.75	21.75	coding	787	120	2480
PB.650.2	1	2404	8	full-splice_match	gene: Zm00001d030173	Zm00001d030173_T00	51.07	59.17	coding	527	311	1891
PB.650.1	1	2730	7	novel_in_catalog	gene: Zm00001d030173	novel	8.10	59.17	coding	275	315	1139
PB.3772.1	3	719	1	full-splice_match	gene: Zm00001d039581	Zm00001d039581_T00	5.15	5.15	coding	191	25	597
PB.650.7	1	1972	8	full-splice_match	gene: Zm00001d030173	Zm00001d030173_T00	0.00	59.17	coding	527	106	1686
PB.9127.1	8	1576	9	novel_not_in_catalog	gene: Zm00001d008225	novel	14.14	19.24	coding	329	206	1192
PB.9127.2	8	1481	9	novel_not_in_catalog	gene: Zm00001d008225	novel	5.10	19.24	coding	329	247	1233
PB.10701.1	9	1620	5	full-splice_match	gene: Zm00001d047723	Zm00001d047723_T00	16.32	16.32	coding	420	209	1468
PB.3807.2	3	1180	4	full-splice_match	gene: Zm00001d039747	Zm00001d039747_T00	0.00	76.13	coding	187	438	998
PB.5759.3	4	1820	6	full-splice_match	gene: Zm00001d053354	Zm00001d053354_T00	0.00	23.15	coding	537	127	1737
PB.2411.1	10	4166	10	full-splice_match	gene: Zm00001d026698	Zm00001d026698_T02	10.70	21.95	coding	1189	9	3575
PB.6120.2	5	3748	14	full-splice_match	gene: Zm00001d013358	Zm00001d013358_T01	1.53	17.23	coding	402	2303	3508

PB.6120.3	5	2184	15	full-splice_match	gene: Zm00001d013358	Zm00001d013358_T01	0.00	17.23	coding	634	113	2014
PB.6120.1	5	2292	15	full-splice_match	gene: Zm00001d013358	Zm00001d013358_T01	15.70	17.23	coding	634	115	2016
PB.8572.1	7	901	4	incomplete-splice_match	gene: Zm00001d020620	Zm00001d020620_T00	4.27	7.83	coding	120	227	586
PB.7000.3	5	899	1	novel_in_catalog	gene: Zm00001d017503	novel	2.76	8.01	coding	227	33	713
PB.4806.2	3	954	5	full-splice_match	gene: Zm00001d044575	Zm00001d044575_T00	26.14	133.28	coding	245	108	842
PB.1913.1	10	613	3	full-splice_match	gene: Zm00001d024471	Zm00001d024471_T00	318.60	318.60	coding	71	102	314
PB.9696.2	8	494	2	full-splice_match	gene: Zm00001d011063	Zm00001d011063_T00	1612.06	1643.90	coding	82	72	317
PB.9696.1	8	649	2	full-splice_match	gene: Zm00001d011063	Zm00001d011063_T00	31.84	1643.90	coding	82	78	323
PB.3146.4	2	1928	3	genic	gene: Zm00001d005445	novel	6.34	39.10	coding	468	165	1568
PB.7058.1	5	2571	8	novel_in_catalog	gene: Zm00001d017770	novel	5.71	13.38	coding	201	1675	2277
PB.4158.4	3	2870	7	novel_in_catalog	gene: Zm00001d041945	novel	0.00	133.59	coding	84	240	491
PB.1220.2	1	1676	2	full-splice_match	gene: Zm00001d033005	Zm00001d033005_T00	14.46	18.93	coding	360	153	1232
PB.9437.1	8	827	1	full-splice_match	gene: Zm00001d009826	Zm00001d009826_T00	8.69	8.69	coding	147	98	538
PB.1612.1	1	1336	8	incomplete-splice_match	gene: Zm00001d034713	Zm00001d034713_T00	2.95	2.95	coding	297	317	1207
PB.4668.2	3	2399	18	novel_in_catalog	gene: Zm00001d044016	novel	1.88	120.65	coding	431	687	1979
PB.1907.1	10	1631	1	genic	gene: Zm00001d024450	novel	21.65	21.65	non coding	NA	NA	NA
PB.5822.1	4	1781	2	genic	gene: Zm00001d053650	novel	11.76	15.16	coding	427	157	1437

PB.8905.7	7	1915	4	novel_in_catalog	gene: Zm00001d021930	novel	38.80	155.11	coding	243	126	854
PB.658.2	1	1605	2	novel_in_catalog	gene: Zm00001d030232	novel	11.37	82.18	coding	162	223	708
PB.658.1	1	1474	3	novel_in_catalog	gene: Zm00001d030232	novel	23.79	82.18	coding	297	226	1116
PB.7850.2	6	908	4	full-splice_match	gene: Zm00001d037929	Zm00001d037929_T00	675.75	748.79	coding	199	69	665
PB.658.7	1	1162	3	novel_not_in_catalog	gene: Zm00001d030232	novel	0.00	82.18	coding	184	28	579
PB.658.6	1	1110	4	novel_not_in_catalog	gene: Zm00001d030232	novel	10.26	82.18	coding	319	31	987
PB.658.5	1	1259	4	novel_not_in_catalog	gene: Zm00001d030232	novel	26.65	82.18	coding	319	33	989
PB.1196.1	1	1791	1	full-splice_match	gene: Zm00001d032899	Zm00001d032899_T00	5.82	5.82	coding	586	12	1769
PB.7106.1	5	1603	5	full-splice_match	gene: Zm00001d017960	Zm00001d017960_T00	12.19	33.88	coding	367	56	1156
PB.8128.1	6	819	1	full-splice_match	gene: Zm00001d038904	Zm00001d038904_T00	98.68	98.68	coding	175	3	527
PB.6858.2	5	2101	3	intergenic	novelGene_369	novel	5.40	12.21	non coding	NA	NA	NA
PB.9620.1	8	2737	9	full-splice_match	gene: Zm00001d010710	Zm00001d010710_T00	24.87	32.75	coding	658	310	2283
PB.9797.3	8	2036	6	full-splice_match	gene: Zm00001d011541	Zm00001d011541_T00	23.01	54.83	coding	520	282	1841
PB.9797.2	8	2181	7	full-splice_match	gene: Zm00001d011541	Zm00001d011541_T00	10.94	54.83	coding	521	424	1986
PB.9797.1	8	1441	5	incomplete-splice_match	gene: Zm00001d011541	Zm00001d011541_T00	20.88	54.83	coding	348	141	1184
PB.9405.6	8	2043	18	full-splice_match	gene: Zm00001d009669	Zm00001d009669_T00	0.00	65.46	coding	506	318	1835
PB.4522.2	3	3234	4	full-splice_match	gene: Zm00001d043484	Zm00001d043484_T02	11.15	51.49	coding	124	220	591

PB.10588.1	9	1199	4	full-splice_match	gene: Zm00001d047293	Zm00001d047293_T00	23.91	33.88	coding	196	235	822
PB.10588.2	9	993	4	full-splice_match	gene: Zm00001d047293	Zm00001d047293_T00	9.97	33.88	coding	196	172	759
PB.4522.1	3	1796	4	novel_not_in_catalog	gene: Zm00001d043484	novel	9.57	51.49	non coding	NA	NA	NA
PB.6591.2	5	2028	5	novel_in_catalog	gene: Zm00001d015361	novel	47.99	47.99	coding	598	80	1873
PB.10648.2	9	2688	2	novel_in_catalog	gene: Zm00001d047528	novel	32.56	41.79	coding	381	1296	2438
PB.7777.1	6	1012	1	genic	gene: Zm00001d037612	novel	5.75	5.75	coding	203	76	684
PB.8743.2	7	2465	7	full-splice_match	gene: Zm00001d021388	Zm00001d021388_T00	0.00	72.72	coding	715	176	2320
PB.2731.1	2	1114	1	intergenic	novelGene_117	novel	8.50	3539.17	non coding	NA	NA	NA
PB.8764.1	7	1981	1	intergenic	novelGene_306	novel	0.49	0.49	non coding	NA	NA	NA
PB.499.3	1	2669	3	full-splice_match	gene: Zm00001d029420	Zm00001d029420_T00	6.80	49.31	coding	784	235	2586
PB.6436.1	5	2590	11	novel_in_catalog	gene: Zm00001d014761	novel	36.35	36.35	coding	645	411	2345
PB.2136.3	10	1498	7	full-splice_match	gene: Zm00001d025658	Zm00001d025658_T00	0.00	58.55	coding	346	303	1340
PB.198.1	1	1425	2	full-splice_match	gene: Zm00001d028094	Zm00001d028094_T00	44.25	104.68	coding	307	27	947
PB.7456.1	6	976	2	full-splice_match	gene: Zm00001d035872	Zm00001d035872_T00	128.33	128.33	coding	164	212	703
PB.1644.1	1	1153	5	full-splice_match	gene: Zm00001d034824	Zm00001d034824_T00	44.72	44.72	coding	336	50	1057
PB.7161.1	5	1714	8	full-splice_match	gene: Zm00001d018144	Zm00001d018144_T00	21.63	21.63	coding	460	51	1430
PB.10846.2	9	1405	1	full-splice_match	gene: Zm00001d048293	Zm00001d048293_T00	0.00	109.74	coding	330	256	1245

PB.6772.1	5	753	7	incomplete-splice_match	gene: Zm00001d016550	Zm00001d016550_T00	68.66	68.66	coding	149	53	499
PB.2572.3	2	3097	4	full-splice_match	gene: Zm00001d002366	Zm00001d002366_T00	0.00	161.87	coding	279	2038	2874
PB.6029.3	5	1592	14	full-splice_match	gene: Zm00001d013048	Zm00001d013048_T00	0.00	58.70	coding	401	109	1311
PB.4679.1	3	684	1	full-splice_match	gene: Zm00001d044075	Zm00001d044075_T00	12.98	12.98	coding	111	113	445
PB.2341.2	10	1126	1	novel_in_catalog	gene: Zm00001d026495	novel	0.00	23.54	coding	351	74	1126
PB.2613.1	2	465	2	incomplete-splice_match	gene: Zm00001d002589	Zm00001d002589_T00	19.60	19.60	non coding	NA	NA	NA
PB.4780.1	3	1803	12	novel_not_in_catalog	gene: Zm00001d044481	novel	23.86	24.61	coding	462	85	1470
PB.8260.1	7	3250	7	full-splice_match	gene: Zm00001d018798	Zm00001d018798_T00	47.88	114.87	coding	941	40	2862
PB.4793.10	3	1705	2	novel_in_catalog	gene: Zm00001d044516	novel	10.72	891.68	coding	334	146	1147
PB.7725.6	6	1540	7	full-splice_match	gene: Zm00001d037352	Zm00001d037352_T00	8.74	33.69	coding	306	363	1280
PB.684.2	1	1443	6	fusion	gene: Zm00001d030411	novel	6.10	42.29	coding	165	60	554
PB.684.4	1	1026	7	fusion	gene: Zm00001d030411	novel	7.79	42.29	coding	250	70	819
PB.5642.2	4	857	1	full-splice_match	gene: Zm00001d052776	Zm00001d052776_T00	0.00	76.95	coding	213	75	713
PB.428.1	1	913	1	genic	gene: Zm00001d029055	novel	3.29	55.25	non coding	NA	NA	NA
PB.5877.2	4	909	1	full-splice_match	gene: Zm00001d053887	Zm00001d053887_T00	13.18	73.32	coding	189	128	694
PB.7383.1	6	1149	1	intergenic	novelGene_107	novel	8.31	8.31	non coding	NA	NA	NA
PB.1558.1	1	649	5	full-splice_match	gene: Zm00001d034520	Zm00001d034520_T00	268.71	268.71	coding	107	139	459

PB.1520.3	1	1767	2	full-splice_match	gene: Zm00001d034382	Zm00001d034382_T00	0.00	4.18	coding	529	130	1716
PB.10223.2	9	1794	8	full-splice_match	gene: Zm00001d045309	Zm00001d045309_T00	31.11	62.70	coding	419	218	1474
PB.10223.3	9	1539	5	novel_in_catalog	gene: Zm00001d045309	novel	1.42	62.70	coding	281	437	1279
PB.10223.1	9	1429	5	novel_in_catalog	gene: Zm00001d045309	novel	17.21	62.70	coding	281	240	1082
PB.247.1	1	1674	1	intergenic	novelGene_112	novel	11.41	90.35	non coding	NA	NA	NA
PB.10223.4	9	1649	7	novel_in_catalog	gene: Zm00001d045309	novel	6.70	62.70	coding	281	551	1393
PB.10223.5	9	1118	7	novel_in_catalog	gene: Zm00001d045309	novel	6.26	62.70	coding	245	225	959
PB.7725.2	6	1785	6	novel_in_catalog	gene: Zm00001d037352	novel	9.46	33.69	coding	231	399	1091
PB.4556.15	3	2114	18	novel_in_catalog	gene: Zm00001d043601	novel	3.76	284.99	coding	486	77	1534
PB.5877.1	4	1019	1	full-splice_match	gene: Zm00001d053887	Zm00001d053887_T00	60.14	73.32	coding	189	129	695
PB.9052.1	7	1299	3	full-splice_match	gene: Zm00001d022457	Zm00001d022457_T00	14.10	14.10	coding	320	114	1073
PB.3485.1	2	1379	2	full-splice_match	gene: Zm00001d006937	Zm00001d006937_T00	0.26	0.26	coding	373	76	1194
PB.3051.1	2	1426	1	genic	gene: Zm00001d004899	novel	1.15	11.95	coding	226	608	1285
PB.6388.1	5	937	1	full-splice_match	gene: Zm00001d014556	Zm00001d014556_T00	8.34	8.34	coding	103	89	397
PB.9411.2	8	668	1	full-splice_match	gene: Zm00001d009700	Zm00001d009700_T00	23.65	336.83	coding	166	98	595
PB.7767.1	6	1650	6	novel_in_catalog	gene: Zm00001d037553	novel	2.51	55.16	coding	368	65	1168
PB.6095.1	5	1628	9	incomplete-splice_match	gene: Zm00001d013278	Zm00001d013278_T01	32.86	32.86	coding	347	42	1082

PB.9411.1	8	768	1	full-splice_match	gene: Zm00001d009700	Zm00001d009700_T00	313.17	336.83	coding	166	96	593
PB.10660.1	9	2878	9	incomplete-splice_match	gene: Zm00001d047574	Zm00001d047574_T00	31.30	31.30	coding	321	1675	2637
PB.6793.8	5	844	2	genic	gene: Zm00001d016655	novel	141.06	265.24	coding	119	99	455
PB.3667.3	2	1480	10	full-splice_match	gene: Zm00001d007951	Zm00001d007951_T00	6.06	98.41	coding	332	292	1287
PB.5850.1	4	530	1	full-splice_match	gene: Zm00001d053781	Zm00001d053781_T00	24.00	61.38	non coding	NA	NA	NA
PB.7498.1	6	1091	8	full-splice_match	gene: Zm00001d036108	Zm00001d036108_T00	69.61	266.22	coding	244	136	867
PB.7725.1	6	1665	7	full-splice_match	gene: Zm00001d037352	Zm00001d037352_T00	15.49	33.69	coding	306	399	1316
PB.1520.2	1	2009	1	novel_in_catalog	gene: Zm00001d034382	novel	1.42	4.18	coding	408	616	1839
PB.6503.1	5	712	1	genic	gene: Zm00001d014966	novel	42.84	42.84	coding	121	54	416
PB.5591.2	4	1144	1	genic	gene: Zm00001d052482	novel	60.82	144.34	coding	263	167	955
PB.9221.1	8	762	3	full-splice_match	gene: Zm00001d008620	Zm00001d008620_T00	151.09	446.10	non coding	NA	NA	NA
PB.6374.2	5	1342	1	genic	gene: Zm00001d014503	novel	0.00	4.15	non coding	NA	NA	NA
PB.6374.1	5	2546	1	genic	gene: Zm00001d014503	novel	4.15	4.15	non coding	NA	NA	NA
PB.9221.2	8	627	3	full-splice_match	gene: Zm00001d008620	Zm00001d008620_T00	295.01	446.10	non coding	NA	NA	NA
PB.2108.4	10	2363	13	full-splice_match	gene: Zm00001d025461	Zm00001d025461_T00	51.46	51.46	coding	503	298	1806
PB.7468.5	6	1869	2	full-splice_match	gene: Zm00001d035934	Zm00001d035934_T00	0.00	155.42	coding	579	36	1772
PB.7468.6	6	1364	2	full-splice_match	gene: Zm00001d035934	Zm00001d035934_T00	0.00	155.42	coding	399	168	1364

PB.1748.3	10	1107	9	full-splice_match	gene: Zm00001d023506	Zm00001d023506_T00	90.42	90.42	coding	223	177	845
PB.1748.2	10	1058	9	full-splice_match	gene: Zm00001d023504	Zm00001d023504_T00	72.89	72.89	coding	223	192	860
PB.6229.2	5	1941	14	full-splice_match	gene: Zm00001d013838	Zm00001d013838_T00	5.50	45.68	coding	485	173	1627
PB.8507.2	7	2079	3	genic	gene: Zm00001d020303	novel	2.17	24.24	coding	370	445	1554
PB.2375.7	10	2112	9	novel_in_catalog	gene: Zm00001d026586	novel	2.31	56.25	coding	144	1595	2026
PB.2375.4	10	2233	9	novel_in_catalog	gene: Zm00001d026586	novel	4.28	56.25	coding	144	1682	2113
PB.9360.1	8	763	7	full-splice_match	gene: Zm00001d009445	Zm00001d009445_T00	202.04	202.04	coding	130	114	503
PB.3590.1	2	1795	1	genic	gene: Zm00001d007575	novel	3.65	3.65	coding	312	591	1526
PB.10781.1	9	1250	5	full-splice_match	gene: Zm00001d047983	Zm00001d047983_T00	78.68	78.68	coding	226	243	920
PB.5161.1	4	1454	15	novel_in_catalog	gene: Zm00001d050216	novel	7.77	35.71	coding	387	12	1172
PB.4668.5	3	2257	19	full-splice_match	gene: Zm00001d044016	Zm00001d044016_T00	0.00	120.65	coding	655	90	2054
PB.7264.5	5	1640	4	novel_not_in_catalog	gene: Zm00001d018503	novel	111.88	190.25	coding	316	400	1347
PB.6914.1	5	696	4	full-splice_match	gene: Zm00001d017141	Zm00001d017141_T00	107.26	107.26	coding	129	55	441
PB.2595.2	2	1221	4	full-splice_match	gene: Zm00001d002496	Zm00001d002496_T00	8.97	58.12	coding	306	55	972
PB.2595.3	2	1066	4	full-splice_match	gene: Zm00001d002496	Zm00001d002496_T00	40.55	58.12	coding	306	52	969
PB.2595.1	2	1373	3	novel_in_catalog	gene: Zm00001d002496	novel	8.60	58.12	coding	191	524	1096
PB.1486.2	1	1756	1	full-splice_match	gene: Zm00001d034221	Zm00001d034221_T00	89.49	127.95	coding	435	137	1441

PB.432.1	1	1041	3	full-splice_match	gene: Zm00001d029102	Zm00001d029102_T00	150.12	150.12	coding	132	107	502
PB.7999.1	6	2146	7	full-splice_match	gene: Zm00001d038409	Zm00001d038409_T01	22.14	22.14	coding	540	246	1865
PB.8664.1	7	1876	9	full-splice_match	gene: Zm00001d021036	Zm00001d021036_T00	15.23	15.23	coding	374	368	1489
PB.432.2	1	941	3	full-splice_match	gene: Zm00001d029102	Zm00001d029102_T00	0.00	150.12	coding	132	108	503
PB.8196.6	6	1098	5	full-splice_match	gene: Zm00001d039166	Zm00001d039166_T00	0.00	100.76	coding	276	144	971
PB.8196.5	6	1314	5	full-splice_match	gene: Zm00001d039166	Zm00001d039166_T00	10.22	100.76	coding	276	214	1041
PB.1028.3	1	673	1	intergenic	novelGene_417	novel	0.00	15.61	coding	107	101	421
PB.8148.2	6	2337	6	novel_in_catalog	gene: Zm00001d038972	novel	4.61	517.57	coding	261	130	912
PB.8148.1	6	2014	8	full-splice_match	gene: Zm00001d038972	Zm00001d038972_T00	39.74	517.57	coding	507	119	1639
PB.4668.4	3	2481	19	full-splice_match	gene: Zm00001d044016	Zm00001d044016_T00	41.83	120.65	coding	655	121	2085
PB.2042.1	10	1418	4	fusion	gene: Zm00001d025079	novel	9.42	30.44	coding	276	49	876
PB.1028.1	1	884	1	intergenic	novelGene_417	novel	9.14	15.61	coding	107	101	421
PB.8862.2	7	1460	1	novel_in_catalog	gene: Zm00001d021791	novel	1.84	16.15	coding	251	140	892
PB.9028.6	7	925	1	novel_in_catalog	gene: Zm00001d022388	novel	33.49	194.04	non coding	NA	NA	NA
PB.6899.27	5	1611	7	novel_in_catalog	gene: Zm00001d017096	novel	0.41	254.51	coding	129	106	492
PB.9028.4	7	3596	25	incomplete-splice_match	gene: Zm00001d022388	Zm00001d022388_T01	44.51	194.04	coding	1078	9	3242
PB.9028.5	7	1947	10	incomplete-splice_match	gene: Zm00001d022388	Zm00001d022388_T01	20.59	194.04	coding	284	728	1579

PB.7676.1	6	1582	6	full-splice_match	gene: Zm00001d037160	Zm00001d037160_T00	5.99	5.99	coding	383	135	1283
PB.632.1	1	2041	14	full-splice_match	gene: Zm00001d030117	Zm00001d030117_T00	17.07	17.07	coding	447	156	1496
PB.9440.10	8	1684	1	novel_in_catalog	gene: Zm00001d009858	novel	0.00	870.63	coding	262	63	848
PB.9440.11	8	1358	1	novel_in_catalog	gene: Zm00001d009858	novel	0.00	870.63	coding	262	61	846
PB.3553.2	2	3742	2	novel_in_catalog	gene: Zm00001d007241	novel	4.51	26.17	coding	1144	309	3740
PB.3881.1	3	2166	11	novel_not_in_catalog	gene: Zm00001d040155	novel	29.34	29.34	coding	512	45	1580
PB.8777.2	7	1140	7	full-splice_match	gene: Zm00001d021517	Zm00001d021517_T00	0.00	18.05	coding	310	63	992
PB.6081.1	5	2333	7	full-splice_match	gene: Zm00001d013253	Zm00001d013253_T00	31.34	34.18	coding	647	185	2125
PB.6081.2	5	2320	6	novel_in_catalog	gene: Zm00001d013253	novel	2.84	34.18	coding	676	93	2120
PB.8777.1	7	1322	7	full-splice_match	gene: Zm00001d021517	Zm00001d021517_T00	18.05	18.05	coding	310	134	1063
PB.9048.1	7	2034	7	novel_not_in_catalog	gene: Zm00001d022434	novel	26.64	41.47	coding	486	410	1867
PB.9048.2	7	1948	7	novel_not_in_catalog	gene: Zm00001d022434	novel	14.82	41.47	coding	486	413	1870
PB.9048.3	7	1289	6	incomplete-splice_match	gene: Zm00001d022434	Zm00001d022434_T00	0.00	41.47	coding	400	90	1289
PB.5513.1	4	1634	9	full-splice_match	gene: Zm00001d052233	Zm00001d052233_T00	55.99	55.99	coding	331	270	1262
PB.9887.2	8	896	3	full-splice_match	gene: Zm00001d011880	Zm00001d011880_T00	35.03	41.90	coding	143	322	750
PB.9887.1	8	1688	2	full-splice_match	gene: Zm00001d011880	Zm00001d011880_T00	6.87	41.90	coding	183	962	1510
PB.9235.2	8	1338	3	novel_in_catalog	gene: Zm00001d008693	novel	7.14	59.14	coding	266	61	858

PB.6302.5	5	1070	7	full-splice_match	gene: Zm00001d014158	Zm00001d014158_T00	0.00	40.74	coding	257	102	872
PB.3900.1	3	3011	20	full-splice_match	gene: Zm00001d040268	Zm00001d040268_T00	9.50	9.80	coding	699	832	2928
PB.9235.1	8	1215	4	full-splice_match	gene: Zm00001d008693	Zm00001d008693_T00	52.00	59.14	coding	326	62	1039
PB.6302.1	5	1362	7	full-splice_match	gene: Zm00001d014158	Zm00001d014158_T00	10.18	40.74	coding	257	102	872
PB.6302.2	5	1235	7	full-splice_match	gene: Zm00001d014158	Zm00001d014158_T00	24.96	40.74	coding	257	113	883
PB.6302.3	5	2578	5	full-splice_match	gene: Zm00001d014158	Zm00001d014158_T00	5.61	40.74	coding	138	2044	2457
PB.857.1	1	2665	8	full-splice_match	gene: Zm00001d031430	Zm00001d031430_T00	63.73	63.73	coding	695	332	2416
PB.1041.1	1	1000	4	incomplete-splice_match	gene: Zm00001d032319	Zm00001d032319_T00	6.80	18.33	coding	308	77	1000
PB.1041.2	1	1542	5	incomplete-splice_match	gene: Zm00001d032319	Zm00001d032319_T00	11.53	18.33	coding	185	54	608
PB.6716.3	5	2457	1	full-splice_match	gene: Zm00001d016225	Zm00001d016225_T00	17.42	63.99	coding	729	110	2296
PB.6716.2	5	2855	1	full-splice_match	gene: Zm00001d016225	Zm00001d016225_T00	10.88	63.99	coding	729	333	2519
PB.6716.1	5	2749	1	full-splice_match	gene: Zm00001d016225	Zm00001d016225_T00	35.69	63.99	coding	729	130	2316
PB.4459.8	3	1443	10	novel_in_catalog	gene: Zm00001d043272	novel	3.79	143.13	coding	215	166	810
PB.8716.1	7	1826	3	full-splice_match	gene: Zm00001d021288	Zm00001d021288_T00	49.89	77.31	coding	276	279	1106
PB.6892.1	5	2917	1	intergenic	novelGene_378	novel	26.94	26.94	non coding	NA	NA	NA
PB.2.1	1	1678	1	novel_in_catalog	gene: Zm00001d027239	novel	41.11	41.11	non coding	NA	NA	NA
PB.491.4	1	1456	4	novel_in_catalog	gene: Zm00001d029389	novel	1.81	12.98	coding	379	112	1248

PB.3228.2	2	984	1	full-splice_match	gene: Zm00001d005865	Zm00001d005865_T00	8.29	22.33	coding	281	11	853
PB.1810.1	10	1924	6	novel_not_in_catalog	gene: Zm00001d023813	novel	23.65	23.65	coding	449	189	1535
PB.1073.5	1	1890	14	novel_not_in_catalog	gene: Zm00001d032441	novel	17.07	67.31	coding	487	147	1607
PB.491.1	1	1136	5	full-splice_match	gene: Zm00001d029389	Zm00001d029389_T00	2.06	12.98	coding	240	56	775
PB.491.2	1	1596	4	novel_in_catalog	gene: Zm00001d029389	novel	0.00	12.98	coding	379	106	1242
PB.491.3	1	1349	5	full-splice_match	gene: Zm00001d029389	Zm00001d029389_T00	9.11	12.98	coding	378	78	1211
PB.8716.2	7	1729	2	novel_in_catalog	gene: Zm00001d021288	novel	8.72	77.31	coding	149	784	1230
PB.4085.4	3	2884	18	novel_in_catalog	gene: Zm00001d041489	novel	0.00	37.08	coding	854	162	2723
PB.10794.1	9	1916	3	full-splice_match	gene: Zm00001d048055	Zm00001d048055_T00	16.34	16.34	coding	501	233	1735
PB.1399.2	1	2021	1	genic	gene: Zm00001d033839	novel	11.03	36.50	coding	490	552	2021
PB.3228.1	2	1650	1	full-splice_match	gene: Zm00001d005865	Zm00001d005865_T00	14.04	22.33	coding	438	106	1419
PB.1108.2	1	1391	2	full-splice_match	gene: Zm00001d032560	Zm00001d032560_T00	0.00	98.17	coding	375	107	1231
PB.3030.3	2	1338	1	genic	gene: Zm00001d004744	novel	0.00	21.73	coding	155	690	1154
PB.3030.2	2	1272	2	full-splice_match	gene: Zm00001d004744	Zm00001d004744_T00	19.49	21.73	coding	275	219	1043
PB.2734.5	2	1157	7	full-splice_match	gene: Zm00001d003108	Zm00001d003108_T00	21.35	50.93	coding	214	169	810
PB.2734.4	2	2229	5	novel_in_catalog	gene: Zm00001d003108	novel	3.93	50.93	coding	191	163	735
PB.2734.3	2	1470	2	novel_in_catalog	gene: Zm00001d003108	novel	1.12	50.93	non coding	NA	NA	NA

PB.2734.2	2	1300	7	full-splice_match	gene: Zm00001d003108	Zm00001d003108_T00	21.29	50.93	coding	214	223	864
PB.2734.1	2	1768	6	novel_in_catalog	gene: Zm00001d003108	novel	3.24	50.93	coding	141	160	582
PB.6822.2	5	2592	3	novel_in_catalog	gene: Zm00001d016768	novel	52.72	52.72	coding	415	948	2192
PB.551.1	1	3520	1	full-splice_match	gene: Zm00001d029652	Zm00001d029652_T00	18.10	18.10	coding	1063	129	3317
PB.6617.2	5	2538	6	full-splice_match	gene: Zm00001d015521	Zm00001d015521_T00	94.91	161.78	coding	631	269	2161
PB.6617.1	5	2331	5	incomplete-splice_match	gene: Zm00001d015521	Zm00001d015521_T00	5.54	161.78	coding	551	34	1686
PB.8716.6	7	1475	3	full-splice_match	gene: Zm00001d021288	Zm00001d021288_T00	0.00	77.31	coding	276	213	1040
PB.6617.7	5	2432	6	full-splice_match	gene: Zm00001d015521	Zm00001d015521_T00	0.00	161.78	coding	631	267	2159
PB.7298.2	6	1278	3	full-splice_match	gene: Zm00001d035007	Zm00001d035007_T00	36.70	50.47	coding	333	116	1114
PB.7298.1	6	1886	2	novel_in_catalog	gene: Zm00001d035007	novel	13.77	50.47	coding	248	957	1700
PB.6800.5	5	1777	3	incomplete-splice_match	gene: Zm00001d016679	Zm00001d016679_T00	11.82	11.82	coding	176	111	638
PB.3387.1	2	858	1	full-splice_match	gene: Zm00001d006516	Zm00001d006516_T00	64.24	64.24	coding	220	60	719
PB.4302.4	3	983	2	full-splice_match	gene: Zm00001d042627	Zm00001d042627_T01	10.12	159.67	non coding	NA	NA	NA
PB.702.4	1	1093	1	incomplete-splice_match	gene: Zm00001d030593	Zm00001d030593_T00	3.27	44.85	coding	343	63	1091
PB.1728.1	10	1921	1	full-splice_match	gene: Zm00001d023399	Zm00001d023399_T00	7.04	14.83	coding	456	136	1503
PB.10479.1	9	3679	9	full-splice_match	gene: Zm00001d046717	Zm00001d046717_T00	3.26	22.30	coding	1007	115	3135
PB.9241.4	8	2488	12	full-splice_match	gene: Zm00001d008725	Zm00001d008725_T01	45.35	57.62	coding	665	200	2194

PB.9241.1	8	2716	13	novel_not_in_catalog	gene: Zm00001d008725	novel	12.27	57.62	coding	370	1224	2333
PB.9440.4	8	2141	2	novel_in_catalog	gene: Zm00001d009858	novel	10.17	870.63	coding	312	63	998
PB.10892.2	9	1221	8	novel_in_catalog	gene: Zm00001d048445	novel	18.32	18.32	coding	305	128	1042
PB.5924.1	4	3283	7	novel_not_in_catalog	gene: Zm00001d054016	novel	15.68	15.68	coding	926	251	3028
PB.6899.6	5	2562	4	full-splice_match	gene: Zm00001d017096	Zm00001d017096_T01	29.61	254.51	coding	129	106	492
PB.13.20	1	2205	9	novel_in_catalog	gene: Zm00001d027293	novel	1.34	422.53	coding	557	104	1774
PB.13.21	1	2081	10	full-splice_match	gene: Zm00001d027293	Zm00001d027293_T00	0.00	422.53	coding	625	97	1971
PB.2112.11	10	1790	10	novel_in_catalog	gene: Zm00001d025507	novel	6.30	29.40	coding	183	247	795
PB.6899.5	5	1555	8	novel_in_catalog	gene: Zm00001d017096	novel	26.99	254.51	coding	193	106	684
PB.6571.1	5	3043	9	novel_not_in_catalog	gene: Zm00001d015256	novel	21.08	21.08	coding	99	291	587
PB.7713.1	6	2154	11	full-splice_match	gene: Zm00001d037291	Zm00001d037291_T00	37.58	49.71	coding	516	141	1688
PB.7713.3	6	2523	10	novel_in_catalog	gene: Zm00001d037291	novel	9.23	49.71	coding	239	243	959
PB.7713.2	6	2595	10	novel_in_catalog	gene: Zm00001d037291	novel	2.90	49.71	coding	239	186	902
PB.2576.3	2	1202	5	full-splice_match	gene: Zm00001d002386	Zm00001d002386_T00	60.77	70.87	coding	248	184	927
PB.2082.5	10	2289	6	full-splice_match	gene: Zm00001d025323	Zm00001d025323_T00	2.56	158.65	coding	521	544	2106
PB.2082.4	10	1776	2	novel_in_catalog	gene: Zm00001d025323	novel	1.00	158.65	coding	371	42	1154
PB.9166.1	8	1632	9	full-splice_match	gene: Zm00001d008376	Zm00001d008376_T00	63.71	78.87	coding	326	253	1230

PB.2082.2	10	2052	1	novel_in_catalog	gene: Zm00001d025323	novel	14.51	158.65	coding	332	15	1010
PB.2082.1	10	2405	6	full-splice_match	gene: Zm00001d025323	Zm00001d025323_T00	140.58	158.65	coding	521	538	2100
PB.9166.2	8	557	3	incomplete-splice_match	gene: Zm00001d008376	Zm00001d008376_T00	15.16	78.87	coding	97	9	299
PB.7920.1	6	1384	9	full-splice_match	gene: Zm00001d038155	Zm00001d038155_T00	10.93	10.93	coding	341	100	1122
PB.2576.1	2	1698	3	incomplete-splice_match	gene: Zm00001d002386	Zm00001d002386_T00	10.09	70.87	non coding	NA	NA	NA
PB.2714.1	2	1852	5	full-splice_match	gene: Zm00001d003038	Zm00001d003038_T00	2.74	2.74	coding	517	32	1582
PB.7882.1	6	1602	1	intergenic	novelGene_287	novel	70.47	70.47	non coding	NA	NA	NA
PB.10898.3	9	1736	5	novel_in_catalog	gene: Zm00001d048471	novel	7.62	84.97	coding	262	223	1008
PB.2942.1	2	899	2	full-splice_match	gene: Zm00001d004138	Zm00001d004138_T00	202.15	218.55	coding	155	173	637
PB.1531.1	1	2009	9	full-splice_match	gene: Zm00001d034424	Zm00001d034424_T00	9.99	9.99	coding	564	84	1775
PB.453.1	1	3179	15	novel_not_in_catalog	gene: Zm00001d029196	novel	60.93	60.93	coding	895	186	2870
PB.5348.1	4	2037	1	full-splice_match	gene: Zm00001d051534	Zm00001d051534_T00	5.37	5.37	coding	385	39	1193
PB.6142.1	5	2221	18	full-splice_match	gene: Zm00001d013428	Zm00001d013428_T00	144.48	229.68	coding	583	126	1874
PB.5473.2	4	1478	10	incomplete-splice_match	gene: Zm00001d052072	Zm00001d052072_T03	3.56	14.23	coding	340	149	1168
PB.6142.4	5	2112	18	full-splice_match	gene: Zm00001d013428	Zm00001d013428_T00	85.20	229.68	coding	583	126	1874
PB.6142.5	5	2008	18	full-splice_match	gene: Zm00001d013428	Zm00001d013428_T00	0.00	229.68	coding	583	123	1871
PB.3821.1	3	2044	1	full-splice_match	gene: Zm00001d039854	Zm00001d039854_T00	28.47	28.47	coding	537	109	1719

PB.9670.2	8	1605	4	full-splice_match	gene: Zm00001d010944	Zm00001d010944_T00	0.00	41.21	coding	443	62	1390
PB.9670.1	8	1817	4	full-splice_match	gene: Zm00001d010944	Zm00001d010944_T00	41.21	41.21	coding	443	98	1426
PB.6021.1	5	653	4	full-splice_match	gene: Zm00001d013031	Zm00001d013031_T00	96.72	96.72	coding	83	134	382
PB.3159.1	2	1741	8	incomplete-splice_match	gene: Zm00001d005497	Zm00001d005497_T00	10.39	10.39	coding	446	9	1346
PB.4784.5	3	1826	9	full-splice_match	gene: Zm00001d044495	Zm00001d044495_T00	0.00	1158.15	coding	481	79	1521
PB.4784.6	3	2352	2	novel_in_catalog	gene: Zm00001d044495	novel	2.49	1158.15	coding	108	1722	2045
PB.3481.2	2	823	3	full-splice_match	gene: Zm00001d006924	Zm00001d006924_T00	65.60	142.02	coding	193	62	640
PB.8765.1	7	416	1	incomplete-splice_match	gene: Zm00001d021469	Zm00001d021469_T01	13.43	13.43	non coding	NA	NA	NA
PB.5904.1	4	1487	1	genic	gene: Zm00001d027041	novel	4.77	14.87	non coding	NA	NA	NA
PB.5368.4	4	2041	2	incomplete-splice_match	gene: Zm00001d051615	Zm00001d051615_T00	26.65	63.45	coding	459	337	1713
PB.5904.3	4	1309	1	genic	gene: Zm00001d027041	novel	10.11	14.87	non coding	NA	NA	NA
PB.2825.1	2	1196	2	full-splice_match	gene: Zm00001d003511	Zm00001d003511_T00	9.63	9.63	coding	304	2	913
PB.5368.1	4	1274	1	genic	gene: Zm00001d051615	novel	7.27	63.45	non coding	NA	NA	NA
PB.10500.1	9	571	2	genic	gene: Zm00001d046883	novel	56.37	56.37	coding	81	100	342
PB.5368.3	4	1400	1	incomplete-splice_match	gene: Zm00001d051615	Zm00001d051615_T00	16.95	63.45	coding	289	16	882
PB.5368.2	4	1342	1	incomplete-splice_match	gene: Zm00001d051615	Zm00001d051615_T00	12.57	63.45	coding	226	98	775
PB.1333.1	1	2159	7	incomplete-splice_match	gene: Zm00001d033533	Zm00001d033533_T00	7.44	7.44	coding	607	337	2157

PB.9848.3	8	2384	4	full-splice_match	gene: Zm00001d011745	Zm00001d011745_T00	0.10	7.94	coding	731	136	2328
PB.4793.12	3	1201	1	incomplete-splice_match	gene: Zm00001d044516	Zm00001d044516_T00	30.26	891.68	non coding	NA	NA	NA
PB.1825.1	10	914	2	incomplete-splice_match	gene: Zm00001d023880	Zm00001d023880_T00	43.11	43.11	coding	190	108	677
PB.3150.3	2	1113	11	full-splice_match	gene: Zm00001d005460	Zm00001d005460_T00	0.00	121.56	coding	368	9	1112
PB.9733.1	8	656	3	full-splice_match	gene: Zm00001d011224	Zm00001d011224_T00	94.99	94.99	coding	139	69	485
PB.6637.1	5	922	1	full-splice_match	gene: Zm00001d015670	Zm00001d015670_T00	16.63	16.63	coding	143	59	487
PB.3474.1	2	1348	4	full-splice_match	gene: Zm00001d006910	Zm00001d006910_T01	19.97	103.45	coding	208	120	743
PB.1669.1	1	1364	11	full-splice_match	gene: Zm00001d034887	Zm00001d034887_T00	64.15	64.15	coding	306	126	1043
PB.3150.4	2	1144	10	full-splice_match	gene: Zm00001d005460	Zm00001d005460_T00	0.00	121.56	coding	351	91	1143
PB.2695.7	2	1651	4	full-splice_match	gene: Zm00001d002937	Zm00001d002937_T03	0.00	64.00	coding	428	98	1381
PB.9142.1	8	2785	2	full-splice_match	gene: Zm00001d008293	Zm00001d008293_T00	30.39	30.39	coding	806	127	2544
PB.7955.5	6	2039	2	novel_in_catalog	gene: Zm00001d038273	novel	7.14	164.25	coding	358	17	1090
PB.7955.4	6	2140	3	novel_in_catalog	gene: Zm00001d038273	novel	96.53	164.25	coding	599	201	1997
PB.1069.5	1	1662	6	full-splice_match	gene: Zm00001d032428	Zm00001d032428_T00	8.91	97.16	coding	415	142	1386
PB.2475.1	2	1489	7	full-splice_match	gene: Zm00001d001973	Zm00001d001973_T00	13.09	13.09	coding	319	159	1115
PB.3615.1	2	3096	18	full-splice_match	gene: Zm00001d007758	Zm00001d007758_T00	150.84	173.72	coding	888	180	2843
PB.7487.3	6	731	1	novel_in_catalog	gene: Zm00001d036036	novel	25.21	1057.89	non coding	NA	NA	NA

PB.2475.2	2	1317	7	full-splice_match	gene: Zm00001d001973	Zm00001d001973_T00	0.00	13.09	coding	319	89	1045
PB.1330.1	1	1965	2	full-splice_match	gene: Zm00001d033528	Zm00001d033528_T00	344.26	344.26	coding	513	34	1572
PB.5341.1	4	2367	8	full-splice_match	gene: Zm00001d051502	Zm00001d051502_T00	9.45	46.08	coding	562	179	1864
PB.10411.8	9	1803	15	full-splice_match	gene: Zm00001d046336	Zm00001d046336_T00	0.61	483.03	coding	487	131	1591
PB.827.2	1	1961	1	full-splice_match	gene: Zm00001d031230	Zm00001d031230_T00	16.81	16.81	coding	391	623	1795
PB.122.2	1	2669	10	novel_not_in_catalog	gene: Zm00001d027732	novel	14.66	21.28	coding	695	334	2418
PB.122.1	1	1505	4	incomplete-splice_match	gene: Zm00001d027732	Zm00001d027732_T00	6.62	21.28	coding	253	268	1026
PB.8585.4	7	2608	11	novel_in_catalog	gene: Zm00001d020685	novel	101.79	254.34	coding	286	436	1293
PB.1531.2	1	1131	7	incomplete-splice_match	gene: Zm00001d034424	Zm00001d034424_T00	0.00	9.99	coding	340	112	1131
PB.1627.3	1	2291	18	full-splice_match	gene: Zm00001d034758	Zm00001d034758_T00	9.55	108.87	coding	659	178	2154
PB.2125.1	10	2238	11	full-splice_match	gene: Zm00001d025580	Zm00001d025580_T00	11.44	13.70	coding	272	1231	2046
PB.8779.1	7	1307	9	full-splice_match	gene: Zm00001d021522	Zm00001d021522_T00	20.18	49.18	coding	260	84	863
PB.8145.1	6	1210	3	full-splice_match	gene: Zm00001d038960	Zm00001d038960_T00	11.31	16.71	coding	263	82	870
PB.8779.3	7	1497	3	novel_in_catalog	gene: Zm00001d021522	novel	4.98	49.18	non coding	NA	NA	NA
PB.8779.4	7	1146	9	full-splice_match	gene: Zm00001d021522	Zm00001d021522_T00	3.17	49.18	coding	260	104	883
PB.8779.5	7	1168	9	novel_in_catalog	gene: Zm00001d021522	novel	13.99	49.18	coding	246	84	821
PB.8779.6	7	1224	8	novel_in_catalog	gene: Zm00001d021522	novel	5.81	49.18	coding	197	57	647

PB.8779.7	7	1322	8	novel_in_catalog	gene: Zm00001d021522	novel	1.06	49.18	coding	226	60	737
PB.9112.1	7	1320	3	full-splice_match	gene: Zm00001d022643	Zm00001d022643_T00	7.18	7.18	coding	321	82	1044
PB.6847.2	5	986	9	novel_not_in_catalog	gene: Zm00001d016860	novel	17.00	69.05	coding	224	78	749
PB.6963.3	5	725	3	full-splice_match	gene: Zm00001d017351	Zm00001d017351_T00	29.23	31.28	coding	169	42	548
PB.8517.5	7	1516	11	novel_in_catalog	gene: Zm00001d020359	novel	2.19	21.18	coding	249	66	812
PB.4334.1	3	1144	1	full-splice_match	gene: Zm00001d042735	Zm00001d042735_T00	5.58	52.28	coding	224	84	755
PB.10683.1	9	941	1	full-splice_match	gene: Zm00001d047664	Zm00001d047664_T00	53.92	53.92	coding	201	90	692
PB.4334.3	3	830	1	full-splice_match	gene: Zm00001d042735	Zm00001d042735_T00	18.00	52.28	coding	224	81	752
PB.4334.2	3	975	1	full-splice_match	gene: Zm00001d042735	Zm00001d042735_T00	28.70	52.28	coding	224	83	754
PB.4261.2	3	1578	5	full-splice_match	gene: Zm00001d042427	Zm00001d042427_T00	4.35	34.25	coding	397	83	1273
PB.5169.2	4	1239	7	full-splice_match	gene: Zm00001d050258	Zm00001d050258_T00	35.30	39.30	coding	256	155	922
PB.5352.4	4	936	5	full-splice_match	gene: Zm00001d051546	Zm00001d051546_T00	1.22	49.82	coding	172	167	682
PB.4261.1	3	1719	4	novel_in_catalog	gene: Zm00001d042427	novel	16.33	34.25	coding	397	38	1228
PB.5599.11	4	1221	12	novel_not_in_catalog	gene: Zm00001d052532	novel	2.60	583.99	coding	290	56	925
PB.5352.3	4	1097	6	full-splice_match	gene: Zm00001d051546	Zm00001d051546_T00	43.33	49.82	coding	194	130	711
PB.4261.4	3	1406	4	novel_in_catalog	gene: Zm00001d042427	novel	0.00	34.25	coding	397	62	1252
PB.5352.1	4	2049	5	novel_in_catalog	gene: Zm00001d051546	novel	5.27	49.82	coding	156	167	634

PB.2750.11	2	1716	4	full-splice_match	gene: Zm00001d003181	Zm00001d003181_T00	0.00	83.26	coding	378	251	1384
PB.2750.13	2	1713	3	full-splice_match	gene: Zm00001d003181	Zm00001d003181_T00	0.00	83.26	coding	415	254	1498
PB.6624.2	5	1451	5	full-splice_match	gene: Zm00001d015600	Zm00001d015600_T00	0.00	5.79	coding	403	45	1253
PB.6633.1	5	843	2	full-splice_match	gene: Zm00001d015657	Zm00001d015657_T00	18.40	18.40	coding	131	82	474
PB.118.1	1	2132	6	full-splice_match	gene: Zm00001d027711	Zm00001d027711_T00	24.52	24.52	coding	555	46	1710
PB.6858.1	5	1473	4	intergenic	novelGene_369	novel	3.69	12.21	coding	128	854	1237
PB.1637.1	1	745	3	full-splice_match	gene: Zm00001d034793	Zm00001d034793_T00	7.06	205.56	coding	116	51	398
PB.6954.10	5	1888	9	full-splice_match	gene: Zm00001d017298	Zm00001d017298_T00	0.00	125.29	coding	295	559	1443
PB.3922.1	3	1454	1	full-splice_match	gene: Zm00001d040313	Zm00001d040313_T00	5.45	5.45	coding	317	286	1236
PB.1973.1	10	514	1	intergenic	novelGene_193	novel	472.03	657.49	coding	58	136	309
PB.6963.2	5	828	3	full-splice_match	gene: Zm00001d017351	Zm00001d017351_T00	2.05	31.28	coding	169	38	544
PB.1127.2	1	2818	13	novel_in_catalog	gene: Zm00001d032633	novel	5.20	13.48	coding	359	82	1158
PB.2956.1	2	2323	4	genic	gene: Zm00001d004222	novel	6.11	6.11	coding	392	440	1615
PB.6735.4	5	1410	1	incomplete-splice_match	gene: Zm00001d016326	Zm00001d016326_T01	0.00	56.35	coding	374	105	1226
PB.2984.1	2	811	5	incomplete-splice_match	gene: Zm00001d004468	Zm00001d004468_T00	1.83	1.83	coding	97	93	383
PB.5169.4	4	1476	6	full-splice_match	gene: Zm00001d050258	Zm00001d050258_T00	0.00	39.30	coding	256	434	1201
PB.6900.2	5	1970	5	full-splice_match	gene: Zm00001d017100	Zm00001d017100_T00	3.04	57.26	coding	203	1217	1825

PB.6735.2	5	1760	5	incomplete-splice_match	gene: Zm00001d016326	Zm00001d016326_T00	32.03	56.35	coding	450	98	1447
PB.6954.14	5	1367	8	novel_in_catalog	gene: Zm00001d017298	novel	0.00	125.29	coding	298	422	1315
PB.952.8	1	2668	12	novel_in_catalog	gene: Zm00001d031927	novel	0.00	61.88	coding	217	1290	1940
PB.1637.2	1	761	3	full-splice_match	gene: Zm00001d034793	Zm00001d034793_T00	198.49	205.56	coding	116	116	463
PB.2992.2	2	1517	1	incomplete-splice_match	gene: Zm00001d004526	Zm00001d004526_T00	9.76	30.45	coding	413	111	1349
PB.3611.2	2	3651	2	novel_in_catalog	gene: Zm00001d007745	novel	4.26	11.36	coding	375	2239	3363
PB.7827.1	6	3533	8	full-splice_match	gene: Zm00001d037832	Zm00001d037832_T00	9.57	15.91	coding	649	499	2445
PB.3261.1	2	2008	2	full-splice_match	gene: Zm00001d006025	Zm00001d006025_T00	7.67	7.67	coding	588	104	1867
PB.5573.1	4	1928	15	full-splice_match	gene: Zm00001d052416	Zm00001d052416_T00	43.65	60.49	coding	440	330	1649
PB.3611.3	2	1448	2	incomplete-splice_match	gene: Zm00001d007745	Zm00001d007745_T00	4.53	11.36	coding	373	89	1207
PB.4269.2	3	1356	5	full-splice_match	gene: Zm00001d042455	Zm00001d042455_T00	129.92	177.52	coding	249	262	1008
PB.9931.1	8	805	1	full-splice_match	gene: Zm00001d012080	Zm00001d012080_T00	18.72	48.61	coding	107	92	412
PB.592.1	1	1326	1	full-splice_match	gene: Zm00001d029869	Zm00001d029869_T00	2.71	2.71	coding	307	98	1018
PB.1736.1	10	2061	5	full-splice_match	gene: Zm00001d023434	Zm00001d023434_T00	41.51	41.51	coding	501	310	1812
PB.1736.2	10	1624	5	full-splice_match	gene: Zm00001d023434	Zm00001d023434_T00	0.00	41.51	coding	501	28	1530
PB.1736.3	10	1997	4	novel_in_catalog	gene: Zm00001d023434	novel	0.00	41.51	coding	180	1341	1880
PB.3178.1	2	846	4	full-splice_match	gene: Zm00001d005631	Zm00001d005631_T00	58.15	58.15	coding	122	93	458

PB.9931.3	8	526	1	full-splice_match	gene: Zm00001d012080	Zm00001d012080_T00	29.89	48.61	coding	107	94	414
PB.7264.1	5	1786	4	novel_not_in_catalog	gene: Zm00001d018503	novel	38.51	190.25	coding	316	403	1350
PB.10074.1	8	2840	4	full-splice_match	gene: Zm00001d012660	Zm00001d012660_T00	11.32	178.45	coding	621	449	2311
PB.10074.2	8	1260	3	incomplete-splice_match	gene: Zm00001d012660	Zm00001d012660_T00	91.51	178.45	coding	267	87	887
PB.10074.3	8	2634	4	full-splice_match	gene: Zm00001d012660	Zm00001d012660_T00	66.27	178.45	coding	621	559	2421
PB.10074.4	8	2549	3	novel_in_catalog	gene: Zm00001d012660	novel	5.96	178.45	coding	546	327	1964
PB.10074.5	8	3024	3	novel_in_catalog	gene: Zm00001d012660	novel	3.39	178.45	coding	492	460	1935
PB.9840.5	8	983	6	novel_not_in_catalog	gene: Zm00001d011717	novel	12.06	69.71	coding	181	265	807
PB.10964.4	B73V4	1289	2	incomplete-splice_match	gene: Zm00001d000011	Zm00001d000011_T02	0.00	15.34	coding	103	810	1118
PB.10964.2	B73V4	2589	9	novel_in_catalog	gene: Zm00001d000011	novel	6.50	15.34	coding	103	827	1135
PB.10964.3	B73V4	2115	8	full-splice_match	gene: Zm00001d000011	Zm00001d000011_T02	4.59	15.34	coding	103	845	1153
PB.10964.1	B73V4	2093	10	novel_in_catalog	gene: Zm00001d000011	novel	4.25	15.34	coding	103	428	736
PB.8046.1	6	2619	1	genic	gene: Zm00001d038587	novel	1.26	36.15	non coding	NA	NA	NA
PB.3809.2	3	1012	3	full-splice_match	gene: Zm00001d039764	Zm00001d039764_T00	17.45	82.56	coding	256	159	926
PB.3809.1	3	1173	2	incomplete-splice_match	gene: Zm00001d039764	Zm00001d039764_T00	29.20	82.56	coding	204	35	646
PB.9840.7	8	861	7	novel_not_in_catalog	gene: Zm00001d011717	novel	19.08	69.71	coding	206	97	714
PB.358.2	1	2394	3	full-splice_match	gene: Zm00001d028774	Zm00001d028774_T00	16.66	19.48	coding	592	456	2231

PB.3809.6	3	791	1	genic	gene: Zm00001d039764	novel	17.19	82.56	coding	107	374	694
PB.3809.5	3	574	2	novel_in_catalog	gene: Zm00001d039764	novel	0.00	82.56	coding	102	163	468
PB.358.1	1	817	1	genic	gene: Zm00001d028774	novel	2.82	19.48	non coding	NA	NA	NA
PB.7325.2	6	2108	1	incomplete-splice_match	gene: Zm00001d035125	Zm00001d035125_T00	9.81	22.47	coding	428	754	2037
PB.3914.1	3	995	1	full-splice_match	gene: Zm00001d040298	Zm00001d040298_T00	180.40	289.88	coding	108	165	488
PB.3914.2	3	941	1	full-splice_match	gene: Zm00001d040298	Zm00001d040298_T00	2.66	289.88	coding	108	213	536
PB.3914.3	3	616	1	full-splice_match	gene: Zm00001d040298	Zm00001d040298_T00	106.82	289.88	coding	108	165	488
PB.10449.1	9	1975	9	full-splice_match	gene: Zm00001d046531	Zm00001d046531_T00	82.56	82.56	coding	385	498	1652
PB.10449.4	9	1818	9	full-splice_match	gene: Zm00001d046531	Zm00001d046531_T00	0.00	82.56	coding	385	485	1639
PB.1536.1	1	1159	3	full-splice_match	gene: Zm00001d034434	Zm00001d034434_T00	12.58	37.65	coding	99	483	779
PB.2972.1	2	2223	1	intergenic	novelGene_223	novel	13.59	13.59	non coding	NA	NA	NA
PB.3177.1	2	1406	5	novel_in_catalog	gene: Zm00001d005616	novel	10.13	10.13	coding	286	376	1233
PB.10886.1	9	1402	3	full-splice_match	gene: Zm00001d048422	Zm00001d048422_T00	10.33	10.33	coding	355	147	1211
PB.7218.3	5	1960	10	full-splice_match	gene: Zm00001d018370	Zm00001d018370_T00	0.00	161.81	coding	535	182	1786
PB.7218.1	5	2136	10	full-splice_match	gene: Zm00001d018370	Zm00001d018370_T00	161.81	161.81	coding	596	46	1833
PB.10554.4	9	771	1	full-splice_match	gene: Zm00001d047114	Zm00001d047114_T00	267.16	267.16	coding	129	98	484
PB.1604.1	1	2107	14	full-splice_match	gene: Zm00001d034678	Zm00001d034678_T00	6.08	6.08	coding	529	209	1795

PB.3170.1	2	765	1	antisense	novelGene_gene: Zm00001d005583	novel	14.10	14.10	coding	100	137	436
PB.8201.1	6	1374	4	incomplete-splice_match	gene: Zm00001d039181	Zm00001d039181_T00	11.16	11.16	coding	341	180	1202
PB.10429.2	9	1377	2	full-splice_match	gene: Zm00001d046449	Zm00001d046449_T00	811.08	811.08	coding	346	88	1125
PB.5596.1	4	1063	2	full-splice_match	gene: Zm00001d052518	Zm00001d052518_T00	12.55	12.55	coding	285	59	913
PB.7917.1	6	1228	4	full-splice_match	gene: Zm00001d038147	Zm00001d038147_T00	923.71	923.71	coding	264	97	888
PB.6360.1	5	1618	1	novel_in_catalog	gene: Zm00001d014419	novel	20.90	20.90	coding	1	375	377
PB.3085.3	2	1091	8	full-splice_match	gene: Zm00001d005102	Zm00001d005102_T00	43.88	97.60	coding	258	140	913
PB.10294.1	9	1075	4	full-splice_match	gene: Zm00001d045528	Zm00001d045528_T00	39.07	95.95	coding	223	91	759
PB.10294.2	9	926	4	full-splice_match	gene: Zm00001d045528	Zm00001d045528_T00	56.88	95.95	coding	223	45	713
PB.8685.1	7	452	1	full-splice_match	gene: Zm00001d021160	Zm00001d021160_T00	61.66	61.66	non coding	NA	NA	NA
PB.829.1	1	3558	22	full-splice_match	gene: Zm00001d031232	Zm00001d031232_T00	17.85	50.83	coding	1046	227	3364
PB.4300.1	3	2763	13	full-splice_match	gene: Zm00001d042615	Zm00001d042615_T00	21.80	21.80	coding	773	140	2458
PB.829.3	1	1611	1	novel_in_catalog	gene: Zm00001d031232	novel	10.09	50.83	coding	232	777	1472
PB.829.2	1	3437	16	novel_not_in_catalog	gene: Zm00001d031232	novel	12.22	50.83	coding	504	597	2108
PB.3552.1	2	471	1	intergenic	novelGene_429	novel	11.69	11.69	non coding	NA	NA	NA
PB.829.4	1	3888	22	novel_not_in_catalog	gene: Zm00001d031232	novel	10.68	50.83	coding	846	1212	3749
PB.1474.1	1	2761	11	full-splice_match	gene: Zm00001d034152	Zm00001d034152_T00	53.46	56.11	coding	755	80	2344

PB.5758.1	4	1253	2	genic	gene: Zm00001d053352	novel	13.62	13.62	coding	306	43	960
PB.8511.1	7	631	1	full-splice_match	gene: Zm00001d020317	Zm00001d020317_T00	0.86	0.86	coding	93	65	343
PB.7949.5	6	1274	4	full-splice_match	gene: Zm00001d038262	Zm00001d038262_T00	0.00	90.57	coding	310	207	1136
PB.2253.2	10	1656	11	novel_in_catalog	gene: Zm00001d026192	novel	6.67	6.67	coding	436	106	1413
PB.7949.3	6	2355	3	novel_in_catalog	gene: Zm00001d038262	novel	1.75	90.57	coding	152	793	1248
PB.7497.1	6	1560	1	full-splice_match	gene: Zm00001d036103	Zm00001d036103_T00	6.46	6.46	coding	382	199	1344
PB.265.3	1	889	5	novel_not_in_catalog	gene: Zm00001d028375	novel	16.12	180.01	coding	129	120	506
PB.265.2	1	2583	4	full-splice_match	gene: Zm00001d028375	Zm00001d028375_T00	3.12	180.01	coding	88	108	371
PB.265.1	1	818	5	full-splice_match	gene: Zm00001d028375	Zm00001d028375_T00	160.77	180.01	coding	129	118	504
PB.8491.1	7	2658	6	novel_in_catalog	gene: Zm00001d020227	novel	9.03	51.79	coding	201	551	1153
PB.6889.2	5	1719	2	full-splice_match	gene: Zm00001d017077	Zm00001d017077_T00	0.00	37.02	coding	515	52	1596
PB.8491.3	7	3543	5	full-splice_match	gene: Zm00001d020227	Zm00001d020227_T02	40.63	51.79	coding	201	574	1176
PB.2449.1	2	964	1	full-splice_match	gene: Zm00001d001865	Zm00001d001865_T00	11.03	28.43	coding	156	72	539
PB.2449.2	2	791	1	full-splice_match	gene: Zm00001d001865	Zm00001d001865_T00	17.40	28.43	coding	156	69	536
PB.606.1	1	1863	1	full-splice_match	gene: Zm00001d029999	Zm00001d029999_T00	0.77	0.77	coding	529	40	1626
PB.7969.5	6	1431	3	full-splice_match	gene: Zm00001d038325	Zm00001d038325_T00	113.01	162.06	coding	383	41	1189
PB.7969.6	6	1323	3	full-splice_match	gene: Zm00001d038325	Zm00001d038325_T00	0.00	162.06	coding	383	41	1189

PB.8330.5	7	3563	14	full-splice_match	gene: Zm00001d019149	Zm00001d019149_T00	9.30	358.17	coding	803	982	3390
PB.7401.1	6	4413	2	genic	gene: Zm00001d035527	novel	9.15	9.15	coding	1313	305	4243
PB.8330.1	7	3628	14	full-splice_match	gene: Zm00001d019149	Zm00001d019149_T00	348.87	358.17	coding	1077	120	3350
PB.1714.1	10	2536	4	genic	gene: Zm00001d023330	novel	46.05	46.05	coding	537	227	1837
PB.9610.1	8	1755	2	novel_in_catalog	gene: Zm00001d010671	novel	157.86	157.86	coding	211	934	1566
PB.1276.1	1	1586	9	full-splice_match	gene: Zm00001d033294	Zm00001d033294_T00	7.95	20.52	coding	272	491	1306
PB.4490.1	3	741	2	full-splice_match	gene: Zm00001d043386	Zm00001d043386_T00	3.13	8.54	coding	93	98	376
PB.4083.1	3	2845	5	full-splice_match	gene: Zm00001d041484	Zm00001d041484_T00	13.83	13.83	coding	574	521	2242
PB.4083.2	3	2654	5	full-splice_match	gene: Zm00001d041484	Zm00001d041484_T00	0.00	13.83	coding	574	436	2157
PB.835.5	1	1143	8	full-splice_match	gene: Zm00001d031269	Zm00001d031269_T00	32.34	45.70	coding	256	106	873
PB.835.2	1	1227	8	full-splice_match	gene: Zm00001d031269	Zm00001d031269_T00	6.75	45.70	coding	256	84	851
PB.4249.1	3	1156	6	incomplete-splice_match	gene: Zm00001d042355	Zm00001d042355_T00	11.82	11.82	coding	327	87	1067
PB.835.1	1	3250	5	novel_in_catalog	gene: Zm00001d031269	novel	2.02	45.70	coding	107	126	446
PB.10974.1	B73V4	1697	6	full-splice_match	gene: Zm00001d000030	Zm00001d000030_T00	72.04	112.09	coding	441	95	1417
PB.8320.4	7	1416	3	full-splice_match	gene: Zm00001d019086	Zm00001d019086_T00	0.00	41.94	coding	409	188	1414
PB.3491.3	2	779	4	novel_in_catalog	gene: Zm00001d006951	novel	0.00	219.45	coding	172	141	656
PB.8320.2	7	2883	1	incomplete-splice_match	gene: Zm00001d019086	Zm00001d019086_T00	0.73	41.94	coding	119	1706	2062

PB.8320.3	7	2000	4	full-splice_match	gene: Zm00001d019086	Zm00001d019086_T00	27.25	41.94	coding	469	188	1594
PB.8320.1	7	2102	4	full-splice_match	gene: Zm00001d019086	Zm00001d019086_T00	13.96	41.94	coding	469	187	1593
PB.3709.2	3	2087	7	full-splice_match	gene: Zm00001d039363	Zm00001d039363_T00	29.60	30.45	coding	428	239	1522
PB.3709.3	3	1799	7	full-splice_match	gene: Zm00001d039363	Zm00001d039363_T00	0.84	30.45	coding	428	287	1570
PB.3709.4	3	1454	7	full-splice_match	gene: Zm00001d039363	Zm00001d039363_T00	0.00	30.45	coding	428	114	1397
PB.10335.2	9	2980	2	novel_not_in_catalog	gene: Zm00001d045757	novel	0.00	14.47	coding	630	722	2611
PB.10335.3	9	2927	2	novel_not_in_catalog	gene: Zm00001d045757	novel	1.87	14.47	coding	630	771	2660
PB.9511.1	8	487	1	full-splice_match	gene: Zm00001d010256	Zm00001d010256_T00	56.24	56.24	coding	115	20	364
PB.10335.1	9	3124	2	novel_not_in_catalog	gene: Zm00001d045757	novel	7.82	14.47	coding	642	686	2611
PB.5570.1	4	2399	8	full-splice_match	gene: Zm00001d052407	Zm00001d052407_T00	15.00	20.67	coding	577	323	2053
PB.10335.4	9	1805	1	genic	gene: Zm00001d045757	novel	4.78	14.47	coding	541	69	1691
PB.785.1	1	1302	6	novel_not_in_catalog	gene: Zm00001d031063	novel	30.35	30.35	coding	214	292	933
PB.1557.1	1	2025	12	incomplete-splice_match	gene: Zm00001d034519	Zm00001d034519_T00	55.67	55.67	coding	517	171	1721
PB.9606.2	8	1325	4	full-splice_match	gene: Zm00001d010662	Zm00001d010662_T00	2.26	2.26	coding	285	257	1111
PB.8598.1	7	2301	14	full-splice_match	gene: Zm00001d020771	Zm00001d020771_T00	62.47	62.47	coding	617	133	1983
PB.6294.1	5	2291	14	full-splice_match	gene: Zm00001d014138	Zm00001d014138_T00	32.94	71.60	coding	314	970	1911
PB.6294.2	5	2327	13	full-splice_match	gene: Zm00001d014138	Zm00001d014138_T00	13.59	71.60	coding	318	1044	1997

PB.6294.3	5	1979	13	full-splice_match	gene: Zm00001d014138	Zm00001d014138_T01	9.98	71.60	coding	536	43	1650
PB.6294.4	5	2158	14	full-splice_match	gene: Zm00001d014138	Zm00001d014138_T01	15.09	71.60	coding	314	986	1927
PB.4581.3	3	1812	10	incomplete-splice_match	gene: Zm00001d043686	Zm00001d043686_T00	19.99	35.10	coding	404	173	1384
PB.4581.2	3	1881	10	incomplete-splice_match	gene: Zm00001d043686	Zm00001d043686_T00	13.05	35.10	coding	404	136	1347
PB.956.2	1	582	4	full-splice_match	gene: Zm00001d031939	Zm00001d031939_T00	0.00	359.06	coding	108	54	377
PB.1343.2	1	883	2	novel_not_in_catalog	gene: Zm00001d033574	novel	328.51	461.16	coding	170	71	580
PB.7985.1	6	1310	9	full-splice_match	gene: Zm00001d038380	Zm00001d038380_T00	125.35	310.67	coding	215	182	826
PB.1050.3	1	3142	2	novel_in_catalog	gene: Zm00001d032359	novel	127.43	127.43	non coding	NA	NA	NA
PB.7842.1	6	667	1	genic	gene: Zm00001d037903	novel	58.87	125.91	non coding	NA	NA	NA
PB.7842.2	6	615	1	genic	gene: Zm00001d037903	novel	67.04	125.91	non coding	NA	NA	NA
PB.9114.1	8	1389	2	full-splice_match	gene: Zm00001d008173	Zm00001d008173_T00	97.69	97.69	coding	348	98	1141
PB.5546.2	4	1416	1	full-splice_match	gene: Zm00001d052340	Zm00001d052340_T00	0.00	1.40	coding	435	1	1305
PB.5546.1	4	1954	2	genic	gene: Zm00001d052340	novel	1.40	1.40	coding	443	379	1707
PB.3715.1	3	916	1	antisense	novelGene_gene: Zm00001d039375	novel	11.05	11.05	coding	72	207	422
PB.10726.6	9	1489	12	novel_in_catalog	gene: Zm00001d047797	novel	694.15	1301.92	coding	176	102	629
PB.10726.7	9	1598	13	full-splice_match	gene: Zm00001d047797	Zm00001d047797_T00	607.77	1301.92	coding	350	421	1470
PB.7965.1	6	1023	4	full-splice_match	gene: Zm00001d038311	Zm00001d038311_T00	43.99	43.99	coding	248	18	761

PB.2552.4	2	928	4	full-splice_match	gene: Zm00001d002302	Zm00001d002302_T00	8.35	50.80	coding	140	172	591
PB.370.2	1	982	1	full-splice_match	gene: Zm00001d028808	Zm00001d028808_T00	0.00	293.75	coding	124	352	723
PB.370.3	1	315	1	full-splice_match	gene: Zm00001d028808	Zm00001d028808_T00	0.00	293.75	non coding	NA	NA	NA
PB.2552.1	2	2737	2	full-splice_match	gene: Zm00001d002302	Zm00001d002302_T00	2.11	50.80	coding	89	175	441
PB.2552.3	2	2397	4	full-splice_match	gene: Zm00001d002302	Zm00001d002302_T00	8.96	50.80	coding	89	253	519
PB.2552.2	2	2533	5	full-splice_match	gene: Zm00001d002302	Zm00001d002302_T01	12.14	50.80	coding	89	304	570
PB.63.1	1	927	5	incomplete-splice_match	gene: Zm00001d027486	Zm00001d027486_T00	31.06	31.06	coding	229	176	862
PB.9978.1	8	1422	2	full-splice_match	gene: Zm00001d012270	Zm00001d012270_T00	2.90	38.76	coding	205	173	787
PB.9978.2	8	1257	3	full-splice_match	gene: Zm00001d012270	Zm00001d012270_T00	35.86	38.76	coding	276	164	991
PB.4516.2	3	695	2	intergenic	novelGene_393	novel	21.17	21.17	non coding	NA	NA	NA
PB.2890.8	2	1921	6	full-splice_match	gene: Zm00001d003857	Zm00001d003857_T00	84.10	452.88	coding	534	115	1716
PB.6003.1	5	1497	8	incomplete-splice_match	gene: Zm00001d012969	Zm00001d012969_T00	32.62	32.62	coding	204	174	785
PB.1529.2	1	621	4	novel_in_catalog	gene: Zm00001d034422	novel	328.31	798.13	coding	152	74	529
PB.2890.2	2	2087	5	novel_in_catalog	gene: Zm00001d003857	novel	5.89	452.88	coding	403	568	1776
PB.2890.1	2	2142	6	full-splice_match	gene: Zm00001d003857	Zm00001d003857_T00	362.88	452.88	coding	534	115	1716
PB.4997.2	4	2005	3	full-splice_match	gene: Zm00001d049361	Zm00001d049361_T00	43.78	127.88	coding	423	424	1692
PB.4997.3	4	2262	2	novel_in_catalog	gene: Zm00001d049361	novel	1.03	127.88	coding	423	787	2055

PB.4997.1	4	2024	3	full-splice_match	gene: Zm00001d049361	Zm00001d049361_T00	83.07	127.88	coding	423	425	1693
PB.2054.1	10	1156	4	full-splice_match	gene: Zm00001d025141	Zm00001d025141_T00	34.26	34.26	coding	262	72	857
PB.7643.1	6	1275	3	full-splice_match	gene: Zm00001d036978	Zm00001d036978_T00	2.22	98.71	coding	275	25	849
PB.7064.2	5	2839	3	full-splice_match	gene: Zm00001d017793	Zm00001d017793_T00	8.48	25.02	coding	713	484	2622
PB.673.1	1	2068	1	full-splice_match	gene: Zm00001d030343	Zm00001d030343_T00	20.78	20.78	coding	535	70	1674
PB.8165.1	6	2094	1	full-splice_match	gene: Zm00001d039038	Zm00001d039038_T00	25.41	33.09	coding	582	73	1818
PB.7249.2	5	2951	18	full-splice_match	gene: Zm00001d018460	Zm00001d018460_T00	13.64	24.88	coding	767	330	2630
PB.7168.9	5	2141	3	novel_in_catalog	gene: Zm00001d018161	novel	0.00	1797.84	coding	319	971	1927
PB.9024.4	7	1733	7	full-splice_match	gene: Zm00001d022374	Zm00001d022374_T00	0.67	32.53	coding	524	48	1619
PB.3775.1	3	1406	8	full-splice_match	gene: Zm00001d039611	Zm00001d039611_T00	104.60	137.72	coding	330	47	1036
PB.3471.2	2	1869	1	full-splice_match	gene: Zm00001d001023	Zm00001d001023_T00	9.87	9.87	non coding	NA	NA	NA
PB.3775.3	3	1293	8	full-splice_match	gene: Zm00001d039611	Zm00001d039611_T00	0.00	137.72	coding	330	7	996
PB.6966.1	5	737	1	genic	gene: Zm00001d017357	novel	19.31	19.31	non coding	NA	NA	NA
PB.10435.1	9	3207	26	novel_not_in_catalog	gene: Zm00001d046468	novel	24.72	24.72	coding	903	176	2884
PB.3775.4	3	468	5	incomplete-splice_match	gene: Zm00001d039611	Zm00001d039611_T00	33.12	137.72	coding	75	244	468
PB.5879.6	4	2111	6	full-splice_match	gene: Zm00001d053890	Zm00001d053890_T00	0.00	7.14	coding	482	508	1953
PB.8681.5	7	840	1	full-splice_match	gene: Zm00001d021141	Zm00001d021141_T00	115.23	243.60	coding	165	131	625

PB.3401.1	2	2369	11	incomplete-splice_match	gene: Zm00001d006593	Zm00001d006593_T01	56.48	56.48	coding	613	101	1939
PB.8165.2	6	2009	1	full-splice_match	gene: Zm00001d039038	Zm00001d039038_T00	7.68	33.09	coding	582	88	1833
PB.972.1	1	2193	7	full-splice_match	gene: Zm00001d031988	Zm00001d031988_T00	89.60	104.30	coding	639	49	1965
PB.972.2	1	2073	7	full-splice_match	gene: Zm00001d031988	Zm00001d031988_T00	14.69	104.30	coding	639	54	1970
PB.7417.1	6	634	3	full-splice_match	gene: Zm00001d035629	Zm00001d035629_T00	160.46	160.46	coding	139	80	496
PB.8407.1	7	1734	2	antisense	novelGene_gene: Zm00001d019641	novel	3.61	3.61	coding	177	79	609
PB.5573.7	4	1045	11	incomplete-splice_match	gene: Zm00001d052416	Zm00001d052416_T00	0.00	60.49	coding	300	144	1043
PB.6227.14	5	1493	8	novel_in_catalog	gene: Zm00001d013831	novel	0.87	62.18	coding	173	173	691
PB.6227.15	5	1613	9	full-splice_match	gene: Zm00001d013831	Zm00001d013831_T00	0.00	62.18	coding	173	126	644
PB.9923.2	8	1482	3	novel_not_in_catalog	gene: Zm00001d012021	novel	20.51	20.51	coding	383	18	1166
PB.5485.2	4	1530	4	incomplete-splice_match	gene: Zm00001d052133	Zm00001d052133_T00	0.00	3.06	coding	256	133	900
PB.8076.1	6	471	1	incomplete-splice_match	gene: Zm00001d038740	Zm00001d038740_T00	78.16	78.16	non coding	NA	NA	NA
PB.2070.1	10	666	3	full-splice_match	gene: Zm00001d025239	Zm00001d025239_T00	215.54	215.54	non coding	NA	NA	NA
PB.7244.1	5	1775	10	novel_in_catalog	gene: Zm00001d018438	novel	2.04	4.89	coding	409	204	1430
PB.8037.1	6	635	3	full-splice_match	gene: Zm00001d038558	Zm00001d038558_T00	294.53	307.63	coding	110	112	441
PB.10001.3	8	1562	6	full-splice_match	gene: Zm00001d012394	Zm00001d012394_T00	25.22	70.61	coding	402	168	1373
PB.7860.1	6	3446	19	novel_in_catalog	gene: Zm00001d037962	novel	69.59	69.59	coding	524	1693	3264

PB.5826.3	4	1495	4	novel_in_catalog	gene: Zm00001d053672	novel	18.68	59.75	coding	172	790	1305
PB.6545.2	5	1397	5	novel_in_catalog	gene: Zm00001d015134	novel	0.97	330.02	coding	154	671	1132
PB.3632.1	2	736	1	intergenic	novelGene_440	novel	2.00	2.00	non coding	NA	NA	NA
PB.4386.1	3	1720	9	novel_not_in_catalog	gene: Zm00001d042974	novel	4.82	4.82	coding	430	220	1509
PB.8417.3	7	687	4	full-splice_match	gene: Zm00001d019727	Zm00001d019727_T00	3.73	14.61	coding	194	22	603
PB.8417.2	7	854	4	full-splice_match	gene: Zm00001d019727	Zm00001d019727_T00	8.14	14.61	coding	194	95	676
PB.8417.1	7	2665	4	novel_in_catalog	gene: Zm00001d019727	novel	2.74	14.61	coding	423	1251	2519
PB.10560.35	9	1524	9	novel_in_catalog	gene: Zm00001d047166	novel	225.30	415.37	coding	428	92	1375
PB.4113.6	3	556	1	full-splice_match	gene: Zm00001d041685	Zm00001d041685_T00	48.01	150.23	non coding	NA	NA	NA
PB.5684.1	4	2488	3	full-splice_match	gene: Zm00001d052981	Zm00001d052981_T00	11.38	11.38	coding	730	60	2249
PB.4371.1	3	2066	2	intergenic	novelGene_375	novel	43.00	43.00	coding	54	292	453
PB.1195.2	1	1809	4	novel_not_in_catalog	gene: Zm00001d032897	novel	1.69	155.96	coding	262	868	1653
PB.1195.1	1	1928	3	novel_not_in_catalog	gene: Zm00001d032897	novel	9.79	155.96	coding	245	837	1571
PB.7651.1	6	1757	12	full-splice_match	gene: Zm00001d037010	Zm00001d037010_T00	52.07	52.07	coding	403	193	1401
PB.3612.1	2	1259	1	full-splice_match	gene: Zm00001d007753	Zm00001d007753_T00	23.10	23.10	coding	380	18	1157
PB.734.1	1	1865	2	incomplete-splice_match	gene: Zm00001d030795	Zm00001d030795_T00	15.09	25.03	coding	452	204	1559
PB.734.2	1	2363	3	full-splice_match	gene: Zm00001d030795	Zm00001d030795_T00	9.94	25.03	coding	720	57	2216

PB.4020.1	3	2014	8	novel_not_in_catalog	gene: Zm00001d041036	novel	48.57	48.57	coding	536	74	1681
PB.5497.1	4	318	1	full-splice_match	gene: Zm00001d052190	Zm00001d052190_T00	5.29	5.29	non coding	NA	NA	NA
PB.7754.1	6	1437	2	incomplete-splice_match	gene: Zm00001d037495	Zm00001d037495_T00	38.18	38.18	coding	350	61	1110
PB.1689.3	10	1043	9	novel_not_in_catalog	gene: Zm00001d023237	novel	6.41	19.27	coding	185	243	797
PB.1689.2	10	1109	9	novel_not_in_catalog	gene: Zm00001d023237	novel	0.72	19.27	coding	252	95	850
PB.1689.1	10	1402	10	novel_not_in_catalog	gene: Zm00001d023237	novel	12.14	19.27	coding	318	102	1055
PB.1527.4	1	875	4	full-splice_match	gene: Zm00001d034415	Zm00001d034415_T00	81.84	216.73	coding	201	117	719
PB.10060.3	8	946	1	full-splice_match	gene: Zm00001d012618	Zm00001d012618_T00	63.52	97.66	coding	213	148	786
PB.10060.2	8	1097	1	full-splice_match	gene: Zm00001d012618	Zm00001d012618_T00	23.49	97.66	coding	213	137	775
PB.10060.1	8	3360	1	full-splice_match	gene: Zm00001d012618	Zm00001d012618_T00	10.65	97.66	coding	213	186	824
PB.3619.4	2	1415	4	novel_in_catalog	gene: Zm00001d007767	novel	0.00	412.11	coding	176	204	731
PB.6431.1	5	718	5	full-splice_match	gene: Zm00001d014749	Zm00001d014749_T00	33.04	388.40	coding	112	76	411
PB.1437.2	1	1366	3	full-splice_match	gene: Zm00001d033995	Zm00001d033995_T00	11.20	105.44	coding	258	352	1125
PB.6431.2	5	606	5	full-splice_match	gene: Zm00001d014749	Zm00001d014749_T00	355.36	388.40	coding	112	75	410
PB.7868.1	6	422	1	incomplete-splice_match	gene: Zm00001d038002	Zm00001d038002_T00	20.51	56.64	non coding	NA	NA	NA
PB.7868.2	6	873	3	full-splice_match	gene: Zm00001d038002	Zm00001d038002_T00	36.13	56.64	coding	189	16	582
PB.3099.3	2	1931	13	novel_not_in_catalog	gene: Zm00001d005164	novel	2.86	134.75	coding	427	467	1747

PB.6790.1	5	624	3	intergenic	novelGene_347	novel	38.00	116.43	non coding	NA	NA	NA
PB.6790.3	5	1046	4	intergenic	novelGene_347	novel	8.89	116.43	coding	173	184	702
PB.6790.2	5	502	3	intergenic	novelGene_347	novel	69.54	116.43	non coding	NA	NA	NA
PB.5369.3	4	1784	2	genic	gene: Zm00001d051620	novel	0.00	45.97	coding	314	503	1444
PB.7726.1	6	1464	4	full-splice_match	gene: Zm00001d037359	Zm00001d037359_T00	96.18	363.39	coding	345	91	1125
PB.8033.1	6	1231	7	full-splice_match	gene: Zm00001d038546	Zm00001d038546_T00	66.39	66.94	coding	257	92	862
PB.7726.3	6	1450	3	novel_in_catalog	gene: Zm00001d037359	novel	6.46	363.39	coding	213	86	724
PB.7726.2	6	1354	4	full-splice_match	gene: Zm00001d037359	Zm00001d037359_T00	103.57	363.39	coding	345	91	1125
PB.7726.4	6	1427	3	novel_in_catalog	gene: Zm00001d037359	novel	6.59	363.39	coding	142	788	1213
PB.7726.7	6	1337	3	novel_in_catalog	gene: Zm00001d037359	novel	0.00	363.39	coding	142	788	1213
PB.7726.6	6	1257	4	full-splice_match	gene: Zm00001d037359	Zm00001d037359_T00	150.59	363.39	coding	345	93	1127
PB.1423.15	1	797	5	novel_in_catalog	gene: Zm00001d033948	novel	0.00	76.35	non coding	NA	NA	NA
PB.1031.3	1	1869	3	novel_in_catalog	gene: Zm00001d032272	novel	2.95	13.63	coding	125	127	501
PB.8037.2	6	799	2	full-splice_match	gene: Zm00001d038558	Zm00001d038558_T00	13.10	307.63	coding	110	112	441
PB.8033.3	6	1097	7	full-splice_match	gene: Zm00001d038546	Zm00001d038546_T00	0.00	66.94	coding	257	92	862
PB.1423.13	1	1592	8	novel_not_in_catalog	gene: Zm00001d033948	novel	0.56	76.35	coding	120	68	427
PB.6896.1	5	854	1	full-splice_match	gene: Zm00001d017090	Zm00001d017090_T00	36.14	48.45	coding	200	24	623

PB.9101.1	7	655	1	antisense	novelGene_gene: Zm00001d022600	novel	41.93	41.93	non coding	NA	NA	NA
PB.5627.2	4	2536	1	full-splice_match	gene: Zm00001d052731	Zm00001d052731_T00	10.51	36.67	coding	683	264	2312
PB.8465.1	7	1378	11	full-splice_match	gene: Zm00001d020053	Zm00001d020053_T00	16.58	16.58	coding	232	514	1209
PB.7025.1	5	614	2	full-splice_match	gene: Zm00001d017617	Zm00001d017617_T00	74.64	74.64	coding	87	78	338
PB.4697.4	3	2062	5	novel_in_catalog	gene: Zm00001d044133	novel	3.58	60.87	coding	114	1477	1818
PB.8043.1	6	977	1	full-splice_match	gene: Zm00001d038581	Zm00001d038581_T00	2.52	8.95	coding	210	60	689
PB.2528.1	2	3198	4	full-splice_match	gene: Zm00001d002166	Zm00001d002166_T00	145.75	145.75	coding	930	179	2968
PB.2548.8	2	1812	11	full-splice_match	gene: Zm00001d002292	Zm00001d002292_T00	5.34	207.58	coding	394	337	1518
PB.2548.9	2	1849	10	full-splice_match	gene: Zm00001d002292	Zm00001d002292_T00	0.00	207.58	coding	290	742	1611
PB.8383.1	7	992	1	antisense	novelGene_gene: Zm00001d001133	novel	1.21	1.21	non coding	NA	NA	NA
PB.8769.1	7	1024	6	full-splice_match	gene: Zm00001d021486	Zm00001d021486_T00	73.41	92.92	coding	163	252	740
PB.4595.2	3	1040	4	full-splice_match	gene: Zm00001d043737	Zm00001d043737_T00	10.83	101.73	coding	152	149	604
PB.6786.2	5	1368	3	full-splice_match	gene: Zm00001d016604	Zm00001d016604_T00	34.16	34.16	coding	366	5	1102
PB.2987.4	2	926	3	novel_in_catalog	gene: Zm00001d004480	novel	3.19	21.71	coding	260	66	845
PB.9416.1	8	2224	9	full-splice_match	gene: Zm00001d009719	Zm00001d009719_T00	17.54	51.22	coding	505	290	1804
PB.2548.1	2	1957	11	full-splice_match	gene: Zm00001d002292	Zm00001d002292_T00	146.74	207.58	coding	394	330	1511
PB.2548.2	2	2124	9	novel_in_catalog	gene: Zm00001d002292	novel	1.44	207.58	coding	131	740	1132

PB.2548.3	2	2031	10	full-splice_match	gene: Zm00001d002292	Zm00001d002292_T00	14.12	207.58	coding	321	334	1296
PB.2548.4	2	1981	10	full-splice_match	gene: Zm00001d002292	Zm00001d002292_T00	36.55	207.58	coding	290	733	1602
PB.2548.5	2	2103	9	novel_in_catalog	gene: Zm00001d002292	novel	0.00	207.58	coding	217	738	1388
PB.2548.7	2	2406	9	novel_in_catalog	gene: Zm00001d002292	novel	3.39	207.58	coding	336	698	1705
PB.10851.7	9	1843	3	full-splice_match	gene: Zm00001d048310	Zm00001d048310_T00	0.00	282.88	coding	428	374	1657
PB.8750.2	7	2041	1	full-splice_match	gene: Zm00001d021412	Zm00001d021412_T00	0.74	6.44	coding	573	186	1904
PB.8750.1	7	2097	1	full-splice_match	gene: Zm00001d021412	Zm00001d021412_T00	5.70	6.44	coding	573	178	1896
PB.10851.4	9	2266	3	full-splice_match	gene: Zm00001d048310	Zm00001d048310_T00	44.65	282.88	coding	428	755	2038
PB.439.2	1	2725	4	novel_in_catalog	gene: Zm00001d029137	novel	2.39	7.85	coding	125	239	613
PB.439.3	1	934	2	full-splice_match	gene: Zm00001d029137	Zm00001d029137_T00	1.61	7.85	coding	125	242	616
PB.10851.1	9	2221	3	full-splice_match	gene: Zm00001d048310	Zm00001d048310_T00	10.18	282.88	coding	428	375	1658
PB.439.1	1	3484	3	full-splice_match	gene: Zm00001d029137	Zm00001d029137_T00	3.85	7.85	coding	125	241	615
PB.9823.1	8	1968	12	full-splice_match	gene: Zm00001d011642	Zm00001d011642_T00	83.02	83.02	coding	502	236	1741
PB.4661.2	3	2592	1	genic	gene: Zm00001d043989	novel	7.52	29.55	coding	402	1123	2328
PB.9823.3	8	1906	12	full-splice_match	gene: Zm00001d011642	Zm00001d011642_T00	0.00	83.02	coding	502	241	1746
PB.3275.2	2	834	5	incomplete-splice_match	gene: Zm00001d006065	Zm00001d006065_T00	2.17	20.76	coding	130	54	443
PB.4181.1	3	542	1	full-splice_match	gene: Zm00001d042030	Zm00001d042030_T00	8.65	8.65	non coding	NA	NA	NA

PB.4430.9	3	1780	9	novel_in_catalog	gene: Zm00001d043152	novel	2.18	115.37	coding	309	71	997
PB.6885.1	5	1194	5	incomplete-splice_match	gene: Zm00001d017053	Zm00001d017053_T00	4.40	10.57	coding	136	291	698
PB.5114.3	4	1734	8	novel_in_catalog	gene: Zm00001d049954	novel	23.61	379.39	coding	201	104	706
PB.5114.1	4	1968	9	full-splice_match	gene: Zm00001d049954	Zm00001d049954_T00	172.49	379.39	coding	433	406	1704
PB.5114.6	4	1103	5	incomplete-splice_match	gene: Zm00001d049954	Zm00001d049954_T00	173.02	379.39	coding	277	186	1016
PB.5114.5	4	1841	8	novel_in_catalog	gene: Zm00001d049954	novel	10.27	379.39	coding	261	240	1022
PB.4161.1	3	1683	6	full-splice_match	gene: Zm00001d041954	Zm00001d041954_T00	42.04	42.04	coding	362	285	1370
PB.7971.1	6	937	3	novel_in_catalog	gene: Zm00001d038329	novel	21.06	21.06	coding	109	299	625
PB.4459.7	3	2299	9	novel_in_catalog	gene: Zm00001d043272	novel	0.56	143.13	coding	145	122	556
PB.1282.1	1	1739	2	full-splice_match	gene: Zm00001d033320	Zm00001d033320_T00	23.24	23.24	coding	461	99	1481
PB.8518.1	7	2080	9	incomplete-splice_match	gene: Zm00001d020363	Zm00001d020363_T00	23.02	23.02	coding	583	42	1790
PB.6683.1	5	1949	1	intergenic	novelGene_277	novel	8.96	8.96	coding	110	183	512
PB.5401.12	4	1543	9	novel_in_catalog	gene: Zm00001d051804	novel	0.00	1338.89	coding	266	576	1373
PB.4761.3	3	1360	9	full-splice_match	gene: Zm00001d044412	Zm00001d044412_T00	0.00	75.36	coding	349	57	1103
PB.4761.2	3	1518	9	full-splice_match	gene: Zm00001d044412	Zm00001d044412_T00	73.00	75.36	coding	349	104	1150
PB.4761.1	3	1406	8	full-splice_match	gene: Zm00001d044412	Zm00001d044412_T00	2.36	75.36	coding	196	111	698
PB.8546.1	7	1252	5	full-splice_match	gene: Zm00001d020485	Zm00001d020485_T00	88.92	201.89	coding	218	326	979

PB.8546.2	7	1004	5	full-splice_match	gene: Zm00001d020485	Zm00001d020485_T00	112.97	201.89	coding	218	210	863
PB.4641.1	3	1737	2	full-splice_match	gene: Zm00001d043880	Zm00001d043880_T00	2.72	6.08	coding	370	59	1168
PB.9013.1	7	1292	9	novel_not_in_catalog	gene: Zm00001d022332	novel	2.62	2.62	coding	298	131	1024
PB.5682.1	4	1662	3	full-splice_match	gene: Zm00001d052951	Zm00001d052951_T00	37.28	39.00	coding	318	557	1510
PB.4477.1	3	3604	8	full-splice_match	gene: Zm00001d043347	Zm00001d043347_T00	14.49	14.49	coding	1144	67	3498
PB.762.1	1	1854	12	full-splice_match	gene: Zm00001d030967	Zm00001d030967_T00	26.70	72.13	coding	469	163	1569
PB.8892.2	7	852	3	genic	gene: Zm00001d021874	novel	14.14	16.09	non coding	NA	NA	NA
PB.9585.1	8	2446	1	full-splice_match	gene: Zm00001d010602	Zm00001d010602_T00	96.39	96.39	coding	662	214	2199
PB.9585.2	8	2358	1	full-splice_match	gene: Zm00001d010602	Zm00001d010602_T00	0.00	96.39	coding	662	213	2198
PB.9585.3	8	2149	1	full-splice_match	gene: Zm00001d010602	Zm00001d010602_T00	0.00	96.39	coding	662	112	2097
PB.8639.1	7	1439	1	genic	gene: Zm00001d020947	novel	12.60	50.09	coding	308	171	1094
PB.8639.3	7	2589	2	novel_in_catalog	gene: Zm00001d020947	novel	3.39	50.09	coding	284	97	948
PB.8639.2	7	2148	3	full-splice_match	gene: Zm00001d020947	Zm00001d020947_T00	11.15	50.09	coding	574	99	1820
PB.8639.4	7	2047	3	full-splice_match	gene: Zm00001d020947	Zm00001d020947_T00	22.95	50.09	coding	574	135	1856
PB.431.1	1	1019	4	incomplete-splice_match	gene: Zm00001d029091	Zm00001d029091_T01	19.53	19.53	coding	234	30	731
PB.2997.4	2	1338	9	full-splice_match	gene: Zm00001d004552	Zm00001d004552_T00	92.33	114.21	coding	191	126	698
PB.4493.2	3	999	2	full-splice_match	gene: Zm00001d043391	Zm00001d043391_T00	0.00	45.91	coding	7	707	727

PB.4493.1	3	1473	2	full-splice_match	gene: Zm00001d043391	Zm00001d043391_T00	45.91	45.91	coding	270	32	841
PB.8393.1	7	850	3	full-splice_match	gene: Zm00001d019576	Zm00001d019576_T00	33.61	33.61	coding	162	184	669
PB.2221.3	10	1553	4	novel_not_in_catalog	gene: Zm00001d026038	novel	1.71	2.12	coding	100	539	838
PB.11040.1	Mt	2199	1	intergenic	novelGene_8	novel	401.16	629.91	non coding	NA	NA	NA
PB.1039.2	1	1503	3	full-splice_match	gene: Zm00001d032316	Zm00001d032316_T00	46.98	86.66	coding	270	491	1300
PB.5021.3	4	1976	2	novel_in_catalog	gene: Zm00001d049459	novel	6.20	105.17	coding	185	102	656
PB.5021.2	4	1316	3	novel_in_catalog	gene: Zm00001d049459	novel	7.30	105.17	coding	205	102	716
PB.5021.1	4	1219	4	full-splice_match	gene: Zm00001d049459	Zm00001d049459_T00	91.68	105.17	coding	314	102	1043
PB.2882.1	2	1649	1	full-splice_match	gene: Zm00001d003830	Zm00001d003830_T00	164.52	164.52	coding	426	130	1407
PB.10899.2	9	2481	9	novel_in_catalog	gene: Zm00001d048472	novel	0.00	74.45	coding	510	189	1718
PB.9333.1	8	1021	7	incomplete-splice_match	gene: Zm00001d009236	Zm00001d009236_T00	20.89	29.87	coding	88	336	599
PB.4796.1	3	858	2	novel_not_in_catalog	gene: Zm00001d044532	novel	0.17	0.17	non coding	NA	NA	NA
PB.9333.2	8	657	4	novel_in_catalog	gene: Zm00001d009236	novel	8.98	29.87	coding	88	96	359
PB.8147.1	6	1418	4	novel_not_in_catalog	gene: Zm00001d038971	novel	66.54	96.32	coding	350	121	1170
PB.706.1	1	2077	14	full-splice_match	gene: Zm00001d030612	Zm00001d030612_T00	6.23	6.23	coding	545	85	1719
PB.3668.1	2	1259	3	incomplete-splice_match	gene: Zm00001d007954	Zm00001d007954_T00	9.38	9.38	coding	330	27	1016
PB.3247.2	2	848	1	full-splice_match	gene: Zm00001d005962	Zm00001d005962_T00	87.72	89.52	coding	195	106	690

PB.3247.1	2	980	1	full-splice_match	gene: Zm00001d005962	Zm00001d005962_T00	1.81	89.52	coding	195	108	692
PB.4775.3	3	2199	1	full-splice_match	gene: Zm00001d044461	Zm00001d044461_T00	17.36	79.06	coding	311	839	1771
PB.4775.4	3	2098	1	full-splice_match	gene: Zm00001d044461	Zm00001d044461_T00	0.00	79.06	coding	311	839	1771
PB.2782.1	2	3109	3	novel_in_catalog	gene: Zm00001d003302	novel	13.12	13.12	coding	798	230	2623
PB.2016.1	10	1006	3	full-splice_match	gene: Zm00001d024966	Zm00001d024966_T00	31.36	31.36	coding	267	10	810
PB.8522.3	7	1708	8	full-splice_match	gene: Zm00001d020378	Zm00001d020378_T00	17.76	24.20	coding	390	62	1231
PB.3608.2	2	2268	9	novel_in_catalog	gene: Zm00001d007709	novel	25.56	53.13	coding	692	25	2100
PB.8522.1	7	2112	7	full-splice_match	gene: Zm00001d020378	Zm00001d020378_T00	2.92	24.20	coding	256	173	940
PB.8522.6	7	1874	6	novel_in_catalog	gene: Zm00001d020378	novel	3.52	24.20	coding	99	565	861
PB.8522.5	7	1342	6	novel_in_catalog	gene: Zm00001d020378	novel	0.00	24.20	coding	233	316	1014
PB.5387.4	4	928	10	novel_not_in_catalog	gene: Zm00001d051682	novel	0.00	43.92	coding	219	146	802
PB.1592.2	1	751	1	full-splice_match	gene: Zm00001d034639	Zm00001d034639_T00	8.22	8.79	coding	150	62	511
PB.6556.1	5	2236	1	intergenic	novelGene_176	novel	13.73	13.73	non coding	NA	NA	NA
PB.10899.5	9	2380	9	novel_in_catalog	gene: Zm00001d048472	novel	0.60	74.45	coding	502	191	1696
PB.5380.1	4	2678	2	intergenic	novelGene_333	novel	2.05	2.05	coding	718	313	2466
PB.1856.1	10	639	3	intergenic	novelGene_130	novel	43.90	43.90	non coding	NA	NA	NA
PB.3636.3	2	1387	2	full-splice_match	gene: Zm00001d007824	Zm00001d007824_T00	0.00	413.51	coding	404	40	1251

PB.4467.1	3	2536	18	full-splice_match	gene: Zm00001d043301	Zm00001d043301_T00	28.16	28.16	coding	715	111	2255
PB.3636.2	2	1497	2	full-splice_match	gene: Zm00001d007824	Zm00001d007824_T00	375.38	413.51	coding	404	40	1251
PB.6881.4	5	2328	12	novel_in_catalog	gene: Zm00001d017042	novel	0.00	138.01	coding	528	536	2119
PB.1361.5	1	1990	11	full-splice_match	gene: Zm00001d033649	Zm00001d033649_T00	25.87	68.36	coding	502	264	1769
PB.6881.6	5	2245	12	novel_in_catalog	gene: Zm00001d017042	novel	6.62	138.01	coding	528	571	2154
PB.8867.1	7	2153	16	full-splice_match	gene: Zm00001d021810	Zm00001d021810_T00	21.36	84.59	coding	392	646	1821
PB.3301.1	2	1389	7	full-splice_match	gene: Zm00001d006167	Zm00001d006167_T00	50.77	50.77	coding	363	120	1208
PB.6881.1	5	2026	13	full-splice_match	gene: Zm00001d017042	Zm00001d017042_T00	109.97	138.01	coding	533	190	1788
PB.6881.2	5	2100	9	novel_in_catalog	gene: Zm00001d017042	novel	15.49	138.01	coding	365	779	1873
PB.6801.1	5	1952	3	novel_in_catalog	gene: Zm00001d016682	novel	13.95	70.81	coding	108	318	641
PB.10108.1	9	2553	11	full-splice_match	gene: Zm00001d044724	Zm00001d044724_T00	19.52	26.76	coding	606	243	2060
PB.10108.2	9	2437	11	full-splice_match	gene: Zm00001d044724	Zm00001d044724_T00	7.25	26.76	coding	606	243	2060
PB.7473.3	6	1222	15	incomplete-splice_match	gene: Zm00001d035975	Zm00001d035975_T00	0.00	67.27	coding	370	111	1220
PB.10108.4	9	1029	6	incomplete-splice_match	gene: Zm00001d044724	Zm00001d044724_T00	0.00	26.76	coding	267	228	1028
PB.2194.1	10	1300	5	incomplete-splice_match	gene: Zm00001d025911	Zm00001d025911_T00	45.13	45.13	coding	273	63	881
PB.10754.3	9	3473	4	full-splice_match	gene: Zm00001d047882	Zm00001d047882_T00	1.84	51.23	coding	206	517	1134
PB.10754.2	9	2446	7	full-splice_match	gene: Zm00001d047882	Zm00001d047882_T01	49.38	51.23	coding	564	292	1983

PB.5013.1	4	952	2	full-splice_match	gene: Zm00001d049419	Zm00001d049419_T00	88.60	88.60	coding	228	65	748
PB.10924.1	9	937	1	full-splice_match	gene: Zm00001d048533	Zm00001d048533_T00	108.66	108.66	coding	136	221	628
PB.8236.2	7	2079	9	full-splice_match	gene: Zm00001d018694	Zm00001d018694_T00	50.18	69.17	coding	535	92	1696
PB.8236.3	7	1959	9	full-splice_match	gene: Zm00001d018694	Zm00001d018694_T00	17.86	69.17	coding	535	92	1696
PB.6125.1	5	1697	10	full-splice_match	gene: Zm00001d013375	Zm00001d013375_T00	9.79	9.79	coding	407	226	1446
PB.8236.1	7	2185	9	full-splice_match	gene: Zm00001d018694	Zm00001d018694_T00	1.13	69.17	coding	535	90	1694
PB.7602.8	6	1915	4	intergenic	novelGene_209	novel	10.38	145.86	non coding	NA	NA	NA
PB.975.2	1	1628	13	full-splice_match	gene: Zm00001d031996	Zm00001d031996_T00	7.55	15.39	coding	351	89	1141
PB.7110.1	5	528	1	intergenic	novelGene_407	novel	62.66	62.66	coding	56	144	311
PB.4567.9	3	3916	5	full-splice_match	gene: Zm00001d043649	Zm00001d043649_T01	9.02	120.68	coding	125	2005	2379
PB.1325.3	1	1208	2	full-splice_match	gene: Zm00001d033511	Zm00001d033511_T00	45.11	62.99	coding	297	100	990
PB.413.2	1	1167	1	novel_in_catalog	gene: Zm00001d028995	novel	14.71	91.08	coding	110	339	668
PB.413.3	1	1521	3	full-splice_match	gene: Zm00001d028995	Zm00001d028995_T00	22.45	91.08	coding	351	208	1260
PB.413.1	1	1793	3	full-splice_match	gene: Zm00001d028995	Zm00001d028995_T00	53.93	91.08	coding	351	215	1267
PB.4567.3	3	3786	6	full-splice_match	gene: Zm00001d043649	Zm00001d043649_T04	15.55	120.68	coding	125	1763	2137
PB.10212.3	9	1383	4	full-splice_match	gene: Zm00001d045220	Zm00001d045220_T00	238.05	313.04	coding	329	204	1190
PB.9862.2	8	1482	1	full-splice_match	gene: Zm00001d011787	Zm00001d011787_T00	10.83	102.34	coding	381	169	1311

PB.10212.1	9	1543	4	full-splice_match	gene: Zm00001d045220	Zm00001d045220_T00	74.99	313.04	coding	329	257	1243
PB.4567.7	3	3162	8	novel_in_catalog	gene: Zm00001d043649	novel	13.38	120.68	coding	125	998	1372
PB.4567.6	3	1610	5	novel_in_catalog	gene: Zm00001d043649	novel	8.98	120.68	coding	125	13	387
PB.4567.5	3	1257	5	incomplete-splice_match	gene: Zm00001d043649	Zm00001d043649_T03	0.00	120.68	non coding	NA	NA	NA
PB.4567.4	3	3201	8	novel_in_catalog	gene: Zm00001d043649	novel	6.37	120.68	coding	125	1179	1553
PB.2132.2	10	2308	7	full-splice_match	gene: Zm00001d025651	Zm00001d025651_T00	3.04	10.55	coding	693	87	2165
PB.1450.1	1	1853	3	novel_not_in_catalog	gene: Zm00001d034032	novel	20.95	43.53	coding	398	398	1591
PB.4641.2	3	1685	2	full-splice_match	gene: Zm00001d043880	Zm00001d043880_T00	0.00	6.08	coding	370	59	1168
PB.5887.2	4	3083	12	incomplete-splice_match	gene: Zm00001d053917	Zm00001d053917_T01	6.08	30.39	coding	275	87	911
PB.2207.3	10	2319	7	full-splice_match	gene: Zm00001d025977	Zm00001d025977_T00	23.75	23.75	coding	705	1	2115
PB.6899.7	5	2185	8	full-splice_match	gene: Zm00001d017096	Zm00001d017096_T00	5.93	254.51	coding	171	106	618
PB.3257.6	2	1777	5	incomplete-splice_match	gene: Zm00001d006001	Zm00001d006001_T00	0.00	43.84	coding	542	58	1683
PB.8175.1	6	1555	7	full-splice_match	gene: Zm00001d039089	Zm00001d039089_T00	187.50	263.85	coding	340	106	1125
PB.4950.1	4	1400	2	full-splice_match	gene: Zm00001d026757	Zm00001d026757_T00	34.69	34.69	non coding	NA	NA	NA
PB.3257.1	2	2357	7	full-splice_match	gene: Zm00001d006001	Zm00001d006001_T00	30.69	43.84	coding	567	399	2099
PB.3257.2	2	2505	5	novel_in_catalog	gene: Zm00001d006001	novel	6.26	43.84	coding	382	1098	2243
PB.6842.2	5	1567	3	novel_in_catalog	gene: Zm00001d016842	novel	1.11	22.28	coding	288	477	1340

PB.5003.2	4	837	3	full-splice_match	gene: Zm00001d049375	Zm00001d049375_T00	18.37	34.67	coding	91	348	620
PB.5003.1	4	530	1	novel_in_catalog	gene: Zm00001d049375	novel	16.30	34.67	coding	91	34	306
PB.6323.1	5	688	3	incomplete-splice_match	gene: Zm00001d014275	Zm00001d014275_T00	31.16	31.16	coding	150	23	472
PB.4728.2	3	760	1	full-splice_match	gene: Zm00001d044247	Zm00001d044247_T00	733.07	775.40	coding	148	87	530
PB.6470.15	5	777	7	full-splice_match	gene: Zm00001d014858	Zm00001d014858_T00	280.11	2503.17	coding	157	165	635
PB.1036.2	1	1009	2	full-splice_match	gene: Zm00001d032292	Zm00001d032292_T00	135.99	178.27	coding	189	262	828
PB.1036.1	1	1216	2	full-splice_match	gene: Zm00001d032292	Zm00001d032292_T00	42.28	178.27	coding	189	262	828
PB.2281.2	10	3227	20	full-splice_match	gene: Zm00001d026291	Zm00001d026291_T01	39.85	39.85	coding	915	127	2871
PB.7811.3	6	1763	7	full-splice_match	gene: Zm00001d037745	Zm00001d037745_T00	18.49	44.70	coding	522	8	1573
PB.8885.1	7	813	2	full-splice_match	gene: Zm00001d021850	Zm00001d021850_T00	70.15	70.15	coding	180	108	647
PB.6863.1	5	1105	5	full-splice_match	gene: Zm00001d016920	Zm00001d016920_T00	46.03	46.03	coding	242	164	889
PB.1383.2	1	1214	5	full-splice_match	gene: Zm00001d033766	Zm00001d033766_T00	0.00	45.68	coding	283	158	1006
PB.6139.1	5	1293	5	full-splice_match	gene: Zm00001d013419	Zm00001d013419_T00	31.26	31.26	coding	283	139	987
PB.2989.1	2	2792	2	full-splice_match	gene: Zm00001d004506	Zm00001d004506_T00	0.07	2.57	coding	383	432	1580
PB.10381.1	9	723	3	incomplete-splice_match	gene: Zm00001d046097	Zm00001d046097_T00	25.06	25.06	coding	28	383	466
PB.2989.2	2	1747	2	full-splice_match	gene: Zm00001d004506	Zm00001d004506_T00	2.51	2.57	coding	383	420	1568
PB.10739.2	9	1489	7	full-splice_match	gene: Zm00001d047838	Zm00001d047838_T00	0.00	10.72	coding	335	273	1277

PB.7587.1	6	1442	1	full-splice_match	gene: Zm00001d036650	Zm00001d036650_T00	1.73	54.10	coding	129	315	701
PB.7587.2	6	1188	1	full-splice_match	gene: Zm00001d036650	Zm00001d036650_T00	0.00	54.10	coding	129	205	591
PB.7587.3	6	1052	1	full-splice_match	gene: Zm00001d036650	Zm00001d036650_T00	52.37	54.10	coding	240	62	781
PB.10112.1	9	1571	1	full-splice_match	gene: Zm00001d044734	Zm00001d044734_T00	3.49	3.49	coding	438	43	1356
PB.7513.1	6	1667	2	genic	gene: Zm00001d036255	novel	22.75	22.75	coding	507	49	1569
PB.4787.1	3	3507	21	full-splice_match	gene: Zm00001d044498	Zm00001d044498_T00	24.89	24.89	coding	1024	33	3104
PB.2214.1	10	558	1	full-splice_match	gene: Zm00001d026015	Zm00001d026015_T00	841.20	841.20	coding	103	65	373
PB.403.1	1	3122	6	full-splice_match	gene: Zm00001d028960	Zm00001d028960_T00	53.61	53.61	coding	843	330	2858
PB.1237.2	1	845	6	full-splice_match	gene: Zm00001d033099	Zm00001d033099_T00	0.00	259.99	coding	142	66	491
PB.8947.2	7	3395	5	full-splice_match	gene: Zm00001d022072	Zm00001d022072_T00	8.60	8.60	coding	951	436	3288
PB.4607.3	3	1352	8	novel_not_in_catalog	gene: Zm00001d043776	novel	5.75	26.89	coding	211	25	657
PB.10844.1	9	1474	9	full-splice_match	gene: Zm00001d048286	Zm00001d048286_T00	11.67	11.67	coding	304	240	1151
PB.5657.2	4	1620	6	full-splice_match	gene: Zm00001d052820	Zm00001d052820_T00	76.87	85.71	coding	383	100	1248
PB.5870.1	4	2648	6	full-splice_match	gene: Zm00001d053852	Zm00001d053852_T00	37.47	41.26	coding	685	179	2233
PB.4698.9	3	2212	1	genic	gene: Zm00001d044138	novel	134.46	1495.64	non coding	NA	NA	NA
PB.9716.3	8	1039	10	full-splice_match	gene: Zm00001d011159	Zm00001d011159_T00	10.60	116.81	coding	204	178	789
PB.2295.1	10	1690	7	incomplete-splice_match	gene: Zm00001d026331	Zm00001d026331_T03	116.92	116.92	coding	331	214	1206

PB.6873.2	5	1642	6	full-splice_match	gene: Zm00001d016993	Zm00001d016993_T00	49.60	68.80	coding	311	199	1131
PB.1393.1	1	1964	6	full-splice_match	gene: Zm00001d033798	Zm00001d033798_T00	67.24	67.24	coding	446	291	1628
PB.3142.1	2	798	3	full-splice_match	gene: Zm00001d005396	Zm00001d005396_T00	59.16	59.16	coding	162	140	625
PB.7658.1	6	1719	2	full-splice_match	gene: Zm00001d037058	Zm00001d037058_T00	32.95	32.95	coding	481	32	1474
PB.4938.9	4	1189	7	novel_not_in_catalog	gene: Zm00001d049097	novel	1.66	57.34	coding	254	246	1007
PB.4938.8	4	1290	6	novel_in_catalog	gene: Zm00001d049097	novel	21.60	57.34	coding	260	246	1025
PB.4938.7	4	2057	5	novel_in_catalog	gene: Zm00001d049097	novel	0.68	57.34	coding	194	365	946
PB.1938.1	10	1289	8	full-splice_match	gene: Zm00001d024588	Zm00001d024588_T00	68.44	71.06	coding	241	255	977
PB.4938.5	4	1211	6	novel_in_catalog	gene: Zm00001d049097	novel	0.56	57.34	coding	186	246	803
PB.4938.4	4	1056	7	novel_not_in_catalog	gene: Zm00001d049097	novel	1.38	57.34	coding	246	74	811
PB.4938.3	4	1916	6	full-splice_match	gene: Zm00001d049097	Zm00001d049097_T00	10.82	57.34	coding	194	246	827
PB.4938.2	4	2032	5	novel_in_catalog	gene: Zm00001d049097	novel	19.82	57.34	coding	194	249	830
PB.6216.2	5	599	1	incomplete-splice_match	gene: Zm00001d013765	Zm00001d013765_T00	7.99	19.51	coding	17	522	572
PB.8942.1	7	1727	14	full-splice_match	gene: Zm00001d022058	Zm00001d022058_T00	20.79	20.79	coding	434	130	1431
PB.6216.1	5	1996	3	novel_in_catalog	gene: Zm00001d013765	novel	11.53	19.51	coding	417	480	1730
PB.1240.6	1	2908	16	full-splice_match	gene: Zm00001d033108	Zm00001d033108_T00	2.44	77.27	coding	605	103	1917
PB.1240.7	1	2450	17	full-splice_match	gene: Zm00001d033108	Zm00001d033108_T00	0.00	77.27	coding	605	103	1917

PB.6960.4	5	2359	9	novel_in_catalog	gene: Zm00001d017324	novel	5.71	68.26	coding	215	201	845
PB.87.1	1	1064	7	novel_not_in_catalog	gene: Zm00001d027576	novel	92.02	92.02	coding	188	256	819
PB.10029.2	8	2166	4	novel_in_catalog	gene: Zm00001d012477	novel	15.31	70.77	coding	327	1044	2024
PB.1240.8	1	1619	14	incomplete-splice_match	gene: Zm00001d033108	Zm00001d033108_T00	0.00	77.27	coding	464	91	1482
PB.10029.1	8	2390	5	full-splice_match	gene: Zm00001d012477	Zm00001d012477_T00	55.47	70.77	coding	571	529	2241
PB.10389.2	9	2028	5	novel_in_catalog	gene: Zm00001d046174	novel	1.25	12.69	coding	380	719	1858
PB.10389.1	9	1943	6	incomplete-splice_match	gene: Zm00001d046174	Zm00001d046174_T00	11.44	12.69	coding	531	97	1689
PB.1697.2	10	3579	4	novel_in_catalog	gene: Zm00001d023265	novel	56.44	91.16	coding	1063	55	3243
PB.9178.1	8	2669	5	intergenic	novelGene_38	novel	40.62	40.62	coding	133	299	697
PB.1697.1	10	2603	4	incomplete-splice_match	gene: Zm00001d023265	Zm00001d023265_T01	34.72	91.16	coding	724	51	2222
PB.5573.4	4	1332	4	novel_in_catalog	gene: Zm00001d052416	novel	4.48	60.49	non coding	NA	NA	NA
PB.1000.1	1	2188	5	full-splice_match	gene: Zm00001d032114	Zm00001d032114_T00	138.65	138.65	coding	624	70	1941
PB.3235.1	2	2797	1	genic	gene: Zm00001d005899	novel	8.12	8.12	coding	174	2136	2657
PB.4202.3	3	919	2	full-splice_match	gene: Zm00001d042104	Zm00001d042104_T00	53.75	53.75	coding	227	52	732
PB.4155.6	3	2557	3	novel_not_in_catalog	gene: Zm00001d000620	novel	0.23	0.23	coding	712	421	2556
PB.4829.3	3	2100	5	novel_in_catalog	gene: Zm00001d044694	novel	11.14	329.15	coding	138	1192	1605
PB.1020.5	1	1626	3	genic	gene: Zm00001d032221	novel	4.81	4.81	non coding	NA	NA	NA

PB.4537.1	3	2166	6	full-splice_match	gene: Zm00001d043527	Zm00001d043527_T00	1.63	43.35	coding	340	252	1271
PB.9107.3	7	2062	1	intergenic	novelGene_373	novel	0.60	13.58	coding	672	46	2061
PB.4266.1	3	977	2	full-splice_match	gene: Zm00001d042449	Zm00001d042449_T00	42.09	56.36	coding	164	67	558
PB.4266.3	3	1081	1	novel_in_catalog	gene: Zm00001d042449	novel	1.84	56.36	coding	129	67	453
PB.2652.1	2	1020	3	full-splice_match	gene: Zm00001d002738	Zm00001d002738_T00	104.48	104.48	coding	248	42	785
PB.4266.5	3	600	2	full-splice_match	gene: Zm00001d042449	Zm00001d042449_T00	12.43	56.36	coding	164	67	558
PB.4266.4	3	514	2	full-splice_match	gene: Zm00001d042449	Zm00001d042449_T00	0.00	56.36	coding	71	118	330
PB.5817.1	4	1888	4	full-splice_match	gene: Zm00001d053640	Zm00001d053640_T00	8.21	10.82	coding	529	65	1651
PB.5149.1	4	1081	6	novel_not_in_catalog	gene: Zm00001d050165	novel	34.59	41.48	coding	202	127	732
PB.5500.1	4	793	1	incomplete-splice_match	gene: Zm00001d052193	Zm00001d052193_T00	36.50	73.52	non coding	NA	NA	NA
PB.9487.1	8	829	1	genic	gene: Zm00001d010131	novel	29.32	29.32	coding	175	1	525
PB.2388.1	10	3570	18	full-splice_match	gene: Zm00001d026621	Zm00001d026621_T02	28.64	28.64	coding	910	354	3083
PB.5383.6	4	1256	2	incomplete-splice_match	gene: Zm00001d051674	Zm00001d051674_T00	6.19	33.89	non coding	NA	NA	NA
PB.3941.2	3	1930	1	novel_in_catalog	gene: Zm00001d040399	novel	4.47	160.77	coding	193	169	747
PB.3941.3	3	1430	2	full-splice_match	gene: Zm00001d040399	Zm00001d040399_T00	63.04	160.77	coding	346	172	1209
PB.10480.3	9	1009	3	full-splice_match	gene: Zm00001d046718	Zm00001d046718_T00	0.00	144.08	coding	229	186	872
PB.3941.1	3	1651	2	full-splice_match	gene: Zm00001d040399	Zm00001d040399_T00	81.46	160.77	coding	346	315	1352

PB.6044.5	5	918	4	novel_in_catalog	gene: Zm00001d013092	novel	22.55	278.74	coding	105	234	548
PB.9772.1	8	2455	4	full-splice_match	gene: Zm00001d011418	Zm00001d011418_T00	0.00	75.59	coding	338	1137	2150
PB.3941.4	3	1146	1	novel_in_catalog	gene: Zm00001d040399	novel	11.81	160.77	non coding	NA	NA	NA
PB.2953.1	2	1034	3	full-splice_match	gene: Zm00001d004193	Zm00001d004193_T00	54.47	54.47	coding	241	97	819
PB.5461.4	4	810	2	full-splice_match	gene: Zm00001d052053	Zm00001d052053_T00	8.69	62.79	coding	154	191	652
PB.3249.1	2	832	1	antisense	novelGene_gene: Zm00001d005970	novel	42.75	42.75	non coding	NA	NA	NA
PB.9478.1	8	972	6	novel_in_catalog	gene: Zm00001d010068	novel	3.74	3.74	coding	205	80	694
PB.8690.1	7	1806	2	full-splice_match	gene: Zm00001d021197	Zm00001d021197_T00	41.90	41.90	coding	485	47	1501
PB.7281.1	5	496	2	novel_in_catalog	gene: Zm00001d018535	novel	260.74	349.82	non coding	NA	NA	NA
PB.7981.2	6	897	6	full-splice_match	gene: Zm00001d038368	Zm00001d038368_T00	108.83	220.15	coding	186	173	730
PB.8482.5	7	1105	4	incomplete-splice_match	gene: Zm00001d020157	Zm00001d020157_T04	0.00	34.79	coding	311	173	1105
PB.2334.1	10	1166	7	full-splice_match	gene: Zm00001d026475	Zm00001d026475_T00	50.11	50.11	coding	243	84	812
PB.1441.1	1	2023	11	novel_in_catalog	gene: Zm00001d034012	novel	15.16	15.16	coding	471	305	1717
PB.3576.1	2	2864	2	full-splice_match	gene: Zm00001d007474	Zm00001d007474_T00	9.38	9.38	coding	696	677	2764
PB.5095.4	4	1664	5	full-splice_match	gene: Zm00001d049857	Zm00001d049857_T00	4.98	71.63	coding	434	359	1660
PB.5095.2	4	1909	5	full-splice_match	gene: Zm00001d049857	Zm00001d049857_T00	34.98	71.63	coding	434	221	1522
PB.7841.1	6	1463	11	full-splice_match	gene: Zm00001d037900	Zm00001d037900_T00	52.67	52.67	coding	344	195	1226

PB.5095.1	4	2086	5	full-splice_match	gene: Zm00001d049857	Zm00001d049857_T00	31.68	71.63	coding	434	291	1592
PB.3955.1	3	1316	14	novel_not_in_catalog	gene: Zm00001d040468	novel	9.39	9.39	coding	376	4	1131
PB.10748.2	9	1898	5	full-splice_match	gene: Zm00001d047863	Zm00001d047863_T00	0.00	23.10	coding	483	98	1546
PB.9704.1	8	1043	7	full-splice_match	gene: Zm00001d011104	Zm00001d011104_T00	63.19	63.19	coding	248	42	785
PB.10567.6	9	1239	6	novel_in_catalog	gene: Zm00001d047208	novel	21.67	66.33	coding	222	265	930
PB.2179.1	10	2178	11	novel_in_catalog	gene: Zm00001d025846	novel	8.57	46.07	coding	365	779	1873
PB.7618.2	6	1625	9	full-splice_match	gene: Zm00001d036830	Zm00001d036830_T00	11.10	13.77	coding	317	387	1337
PB.7618.1	6	1760	8	novel_in_catalog	gene: Zm00001d036830	novel	2.68	13.77	coding	318	516	1469
PB.7006.1	5	1659	1	full-splice_match	gene: Zm00001d017528	Zm00001d017528_T00	34.61	70.99	coding	388	100	1263
PB.7196.2	5	1349	1	novel_in_catalog	gene: Zm00001d001747	novel	7.20	12.71	coding	139	50	466
PB.7419.2	6	1841	6	full-splice_match	gene: Zm00001d035634	Zm00001d035634_T00	0.00	45.91	coding	443	122	1450
PB.7196.1	5	833	2	full-splice_match	gene: Zm00001d001747	Zm00001d001747_T00	5.51	12.71	non coding	NA	NA	NA
PB.1294.1	1	912	4	full-splice_match	gene: Zm00001d033371	Zm00001d033371_T00	123.79	164.88	coding	148	284	727
PB.2482.2	2	2537	1	full-splice_match	gene: Zm00001d001997	Zm00001d001997_T00	5.29	8.12	coding	411	821	2053
PB.2482.1	2	1143	1	full-splice_match	gene: Zm00001d001997	Zm00001d001997_T00	2.83	8.12	coding	130	129	518
PB.2179.3	10	2055	11	novel_in_catalog	gene: Zm00001d025846	novel	0.00	46.07	coding	365	778	1872
PB.4367.1	3	1915	11	full-splice_match	gene: Zm00001d042880	Zm00001d042880_T00	2.34	2.34	coding	503	114	1622

PB.5468.1	4	947	4	incomplete-splice_match	gene: Zm00001d052062	Zm00001d052062_T00	43.89	43.89	non coding	NA	NA	NA
PB.2179.4	10	1805	12	full-splice_match	gene: Zm00001d025846	Zm00001d025846_T00	21.81	46.07	coding	519	125	1681
PB.8992.3	7	1085	7	full-splice_match	gene: Zm00001d022254	Zm00001d022254_T00	19.02	76.67	coding	264	120	911
PB.8992.2	7	1349	6	novel_in_catalog	gene: Zm00001d022254	novel	4.64	76.67	coding	194	119	700
PB.8071.4	6	1010	3	novel_in_catalog	gene: Zm00001d038718	novel	1.12	139.28	coding	123	67	435
PB.6602.4	5	3056	16	full-splice_match	gene: Zm00001d015435	Zm00001d015435_T00	3.58	29.07	coding	585	872	2626
PB.3660.9	2	2174	7	intergenic	novelGene_443	novel	0.00	53.91	coding	279	1033	1869
PB.6602.1	5	3381	17	full-splice_match	gene: Zm00001d015435	Zm00001d015435_T00	25.49	29.07	coding	787	214	2574
PB.9497.4	8	2030	1	novel_in_catalog	gene: Zm00001d010214	novel	0.00	35.64	coding	385	698	1852
PB.9497.5	8	1756	3	full-splice_match	gene: Zm00001d010214	Zm00001d010214_T00	0.00	35.64	coding	352	619	1674
PB.9497.6	8	1844	2	full-splice_match	gene: Zm00001d010214	Zm00001d010214_T00	10.97	35.64	coding	385	537	1691
PB.7490.1	6	2600	5	full-splice_match	gene: Zm00001d036055	Zm00001d036055_T00	12.35	13.84	coding	646	275	2212
PB.9497.1	8	1899	3	full-splice_match	gene: Zm00001d010214	Zm00001d010214_T00	14.65	35.64	coding	352	582	1637
PB.9497.2	8	2672	1	genic	gene: Zm00001d010214	novel	1.07	35.64	coding	385	1214	2368
PB.7490.2	6	1714	4	incomplete-splice_match	gene: Zm00001d036055	Zm00001d036055_T00	1.49	13.84	coding	380	451	1590
PB.6185.1	5	1462	1	full-splice_match	gene: Zm00001d013611	Zm00001d013611_T00	90.26	90.26	coding	407	167	1387
PB.5753.1	4	1251	4	full-splice_match	gene: Zm00001d053320	Zm00001d053320_T00	3.61	3.61	coding	285	151	1005

PB.10188.7	9	2685	8	full-splice_match	gene: Zm00001d045139	Zm00001d045139_T00	417.45	426.19	coding	762	92	2377
PB.10188.8	9	2599	8	full-splice_match	gene: Zm00001d045139	Zm00001d045139_T00	8.74	426.19	coding	762	104	2389
PB.10188.9	9	2344	8	full-splice_match	gene: Zm00001d045139	Zm00001d045139_T00	0.00	426.19	coding	748	99	2342
PB.2761.1	2	1850	1	full-splice_match	gene: Zm00001d003209	Zm00001d003209_T00	60.42	60.42	coding	498	95	1588
PB.5577.2	4	1799	16	full-splice_match	gene: Zm00001d052434	Zm00001d052434_T00	10.12	12.13	coding	484	119	1570
PB.5692.4	4	723	3	full-splice_match	gene: Zm00001d053010	Zm00001d053010_T00	0.00	825.25	coding	165	128	622
PB.3067.5	2	2571	10	incomplete-splice_match	gene: Zm00001d005019	Zm00001d005019_T00	4.35	190.88	coding	190	347	916
PB.7183.1	5	4089	9	novel_in_catalog	gene: Zm00001d018217	novel	5.19	25.03	coding	674	1566	3587
PB.3512.2	2	1413	8	full-splice_match	gene: Zm00001d007067	Zm00001d007067_T00	31.83	57.30	coding	313	100	1038
PB.8212.1	7	1847	1	novel_in_catalog	gene: Zm00001d018601	novel	66.09	66.09	coding	571	20	1732
PB.8744.2	7	1933	2	genic	gene: Zm00001d021389	novel	13.75	129.48	coding	337	611	1621
PB.4323.9	3	974	9	full-splice_match	gene: Zm00001d042695	Zm00001d042695_T00	0.00	95.71	non coding	NA	NA	NA
PB.8744.4	7	1816	2	genic	gene: Zm00001d021389	novel	28.95	129.48	coding	337	648	1658
PB.8744.5	7	708	1	full-splice_match	gene: Zm00001d021389	Zm00001d021389_T00	24.72	129.48	coding	71	123	335
PB.504.1	1	565	2	genic	gene: Zm00001d029444	novel	65.92	65.92	non coding	NA	NA	NA
PB.3138.1	2	1299	1	genic	gene: Zm00001d005375	novel	14.44	14.44	coding	184	92	643
PB.7183.3	5	2919	9	full-splice_match	gene: Zm00001d018217	Zm00001d018217_T00	14.18	25.03	coding	692	403	2478

PB.9750.1	8	496	1	incomplete-splice_match	gene: Zm00001d011312	Zm00001d011312_T00	20.60	20.60	non coding	NA	NA	NA
PB.4411.1	3	2026	13	incomplete-splice_match	gene: Zm00001d043093	Zm00001d043093_T10	94.85	94.85	coding	552	10	1665
PB.10240.2	9	631	2	full-splice_match	gene: Zm00001d045351	Zm00001d045351_T00	53.69	56.03	coding	119	45	401
PB.10240.1	9	1004	2	full-splice_match	gene: Zm00001d045351	Zm00001d045351_T00	2.34	56.03	coding	119	29	385
PB.6298.1	5	1183	8	incomplete-splice_match	gene: Zm00001d014152	Zm00001d014152_T00	25.53	25.53	coding	308	3	926
PB.3380.4	2	2154	9	full-splice_match	gene: Zm00001d006479	Zm00001d006479_T00	0.00	69.28	coding	531	421	2013
PB.1390.1	1	2172	10	full-splice_match	gene: Zm00001d033794	Zm00001d033794_T00	24.64	32.11	coding	621	141	2003
PB.9090.3	7	2241	14	full-splice_match	gene: Zm00001d022554	Zm00001d022554_T00	12.95	34.22	coding	593	221	1999
PB.9351.4	8	1824	2	genic	gene: Zm00001d009401	novel	0.00	37.57	coding	370	343	1452
PB.9090.1	7	1982	12	incomplete-splice_match	gene: Zm00001d022554	Zm00001d022554_T00	21.27	34.22	coding	526	50	1627
PB.9351.1	8	2032	2	genic	gene: Zm00001d009401	novel	37.57	37.57	coding	370	428	1537
PB.6870.2	5	1157	3	full-splice_match	gene: Zm00001d016974	Zm00001d016974_T00	2.23	3.55	coding	225	250	924
PB.7091.2	5	2361	1	full-splice_match	gene: Zm00001d017894	Zm00001d017894_T00	0.00	4.94	coding	610	142	1971
PB.6972.2	5	1383	7	novel_in_catalog	gene: Zm00001d017390	novel	2.68	32.46	coding	166	710	1207
PB.7602.6	6	1963	4	intergenic	novelGene_209	novel	0.00	145.86	coding	126	309	686
PB.262.1	1	1187	2	genic	gene: Zm00001d028371	novel	40.37	43.18	coding	219	308	964
PB.262.2	1	1226	1	full-splice_match	gene: Zm00001d028371	Zm00001d028371_T00	2.81	43.18	coding	219	441	1097

PB.6986.1	5	860	2	full-splice_match	gene: Zm00001d017455	Zm00001d017455_T00	367.32	367.32	coding	212	47	682
PB.7602.2	6	2037	4	intergenic	novelGene_209	novel	29.92	145.86	coding	126	287	664
PB.1252.1	1	2008	1	antisense	novelGene_gene: Zm00001d033181	novel	0.27	3.32	coding	114	141	482
PB.7602.1	6	2099	4	intergenic	novelGene_209	novel	22.49	145.86	coding	126	291	668
PB.10260.1	9	1199	4	full-splice_match	gene: Zm00001d045421	Zm00001d045421_T00	22.70	22.70	coding	206	97	714
PB.1423.12	1	1026	9	novel_in_catalog	gene: Zm00001d033948	novel	10.59	76.35	non coding	NA	NA	NA
PB.4613.1	3	905	1	genic	gene: Zm00001d043801	novel	55.09	77.07	coding	143	476	904
PB.9109.1	7	1724	8	intergenic	novelGene_374	novel	3.31	3.31	coding	200	265	864
PB.9977.1	8	1471	9	full-splice_match	gene: Zm00001d012263	Zm00001d012263_T00	11.82	13.34	coding	355	209	1273
PB.5951.1	5	3684	10	full-splice_match	gene: Zm00001d012797	Zm00001d012797_T00	38.83	38.83	coding	245	404	1138
PB.3003.1	2	531	1	full-splice_match	gene: Zm00001d000909	Zm00001d000909_T00	25.56	25.56	non coding	NA	NA	NA
PB.1717.2	10	1193	2	full-splice_match	gene: Zm00001d023366	Zm00001d023366_T00	62.98	95.71	non coding	NA	NA	NA
PB.1717.1	10	1266	2	full-splice_match	gene: Zm00001d023366	Zm00001d023366_T00	9.00	95.71	non coding	NA	NA	NA
PB.1717.5	10	1075	2	full-splice_match	gene: Zm00001d023366	Zm00001d023366_T00	23.73	95.71	non coding	NA	NA	NA
PB.1987.2	10	3424	3	novel_in_catalog	gene: Zm00001d024861	novel	0.74	11.64	coding	244	2482	3213
PB.5743.1	4	2506	12	novel_not_in_catalog	gene: Zm00001d053284	novel	9.58	9.58	coding	513	827	2365
PB.1987.1	10	2204	5	full-splice_match	gene: Zm00001d024861	Zm00001d024861_T00	10.90	11.64	coding	289	338	1204

PB.8698.2	7	2315	4	novel_not_in_catalog	gene: Zm00001d021215	novel	13.87	13.87	coding	114	10	351
PB.6691.10	5	1453	7	novel_in_catalog	gene: Zm00001d016077	novel	0.00	71.60	coding	109	419	745
PB.3391.1	2	516	3	novel_not_in_catalog	gene: Zm00001d006555	novel	244.52	244.52	coding	69	93	299
PB.5272.1	4	2098	11	novel_not_in_catalog	gene: Zm00001d051057	novel	13.01	13.01	coding	585	81	1835
PB.6739.1	5	1664	8	full-splice_match	gene: Zm00001d016347	Zm00001d016347_T00	36.05	36.05	coding	377	114	1244
PB.2749.2	2	3898	5	novel_not_in_catalog	gene: Zm00001d003176	novel	25.18	37.08	coding	931	836	3628
PB.10176.1	9	1425	5	full-splice_match	gene: Zm00001d045065	Zm00001d045065_T00	7.57	7.57	coding	346	197	1234
PB.4788.1	3	1607	2	incomplete-splice_match	gene: Zm00001d044500	Zm00001d044500_T00	11.88	11.88	coding	113	731	1069
PB.9625.1	8	2068	1	full-splice_match	gene: Zm00001d010722	Zm00001d010722_T00	11.12	11.12	coding	482	36	1481
PB.10931.2	9	1042	4	full-splice_match	gene: Zm00001d048551	Zm00001d048551_T00	87.96	91.65	coding	239	131	847
PB.4944.1	4	1759	2	full-splice_match	gene: Zm00001d049126	Zm00001d049126_T00	21.34	21.34	coding	184	501	1052
PB.10931.4	9	1143	3	novel_in_catalog	gene: Zm00001d048551	novel	3.70	91.65	coding	80	78	317
PB.7099.9	5	1927	7	antisense	novelGene_gene: Zm00001d017932	novel	14.45	98.88	non coding	NA	NA	NA
PB.5476.1	4	3418	5	full-splice_match	gene: Zm00001d052076	Zm00001d052076_T00	14.41	14.41	non coding	NA	NA	NA
PB.7099.4	5	1923	5	antisense	novelGene_gene: Zm00001d017932	novel	0.00	98.88	non coding	NA	NA	NA
PB.7099.7	5	1813	6	antisense	novelGene_gene: Zm00001d017932	novel	0.00	98.88	non coding	NA	NA	NA
PB.796.4	1	2566	6	novel_in_catalog	gene: Zm00001d031090	novel	2.17	62.61	coding	421	959	2221

PB.7099.1	5	1931	6	antisense	novelGene_gene: Zm00001d017932	novel	25.01	98.88	non coding	NA	NA	NA
PB.7099.2	5	1878	6	antisense	novelGene_gene: Zm00001d017932	novel	0.00	98.88	non coding	NA	NA	NA
PB.7099.3	5	1959	5	antisense	novelGene_gene: Zm00001d017932	novel	18.41	98.88	non coding	NA	NA	NA
PB.9258.9	8	1378	11	full-splice_match	gene: Zm00001d008820	Zm00001d008820_T00	13.74	244.63	coding	329	138	1124
PB.6311.1	5	1406	10	full-splice_match	gene: Zm00001d014192	Zm00001d014192_T00	18.16	18.16	coding	277	282	1112
PB.4680.1	3	1075	1	full-splice_match	gene: Zm00001d044076	Zm00001d044076_T00	21.02	183.25	coding	136	172	579
PB.5760.2	4	2187	3	full-splice_match	gene: Zm00001d053356	Zm00001d053356_T00	0.00	12.93	coding	603	216	2024
PB.151.1	1	1177	4	full-splice_match	gene: Zm00001d027887	Zm00001d027887_T00	52.79	52.79	coding	327	82	1062
PB.686.2	1	2748	9	novel_not_in_catalog	gene: Zm00001d030471	novel	1.78	47.00	coding	727	105	2285
PB.7594.1	6	1448	7	incomplete-splice_match	gene: Zm00001d036673	Zm00001d036673_T00	13.30	13.30	coding	321	21	983
PB.9258.1	8	1618	10	full-splice_match	gene: Zm00001d008820	Zm00001d008820_T00	11.08	244.63	coding	205	137	751
PB.10268.3	9	1790	3	full-splice_match	gene: Zm00001d045468	Zm00001d045468_T00	9.79	41.89	coding	499	80	1576
PB.10268.2	9	1672	1	novel_in_catalog	gene: Zm00001d045468	novel	4.10	41.89	non coding	NA	NA	NA
PB.9258.5	8	1876	8	novel_in_catalog	gene: Zm00001d008820	novel	7.41	244.63	coding	157	566	1036
PB.5959.2	5	1241	1	full-splice_match	gene: Zm00001d012819	Zm00001d012819_T00	12.68	52.76	coding	364	44	1135
PB.5959.1	5	1343	1	full-splice_match	gene: Zm00001d012819	Zm00001d012819_T00	40.08	52.76	coding	364	44	1135
PB.9258.6	8	1501	10	full-splice_match	gene: Zm00001d008820	Zm00001d008820_T00	41.99	244.63	coding	205	137	751

PB.9639.2	8	1652	1	genic	gene: Zm00001d010804	novel	3.18	561.94	coding	134	528	929
PB.9441.1	8	1703	3	full-splice_match	gene: Zm00001d009868	Zm00001d009868_T00	252.11	339.74	coding	447	97	1437
PB.6272.2	5	1506	3	full-splice_match	gene: Zm00001d014039	Zm00001d014039_T00	58.07	58.07	coding	284	238	1089
PB.6272.3	5	1287	3	full-splice_match	gene: Zm00001d014039	Zm00001d014039_T00	0.00	58.07	coding	284	189	1040
PB.1305.3	1	1597	1	novel_in_catalog	gene: Zm00001d033402	novel	48.70	103.87	coding	80	119	358
PB.1763.2	10	1725	3	novel_in_catalog	gene: Zm00001d023557	novel	20.54	27.73	coding	436	145	1452
PB.9698.5	8	329	1	full-splice_match	gene: Zm00001d011076	Zm00001d011076_T00	23.51	104.59	non coding	NA	NA	NA
PB.5875.4	4	3290	24	novel_in_catalog	gene: Zm00001d053881	novel	1.94	120.88	coding	740	165	2384
PB.1763.1	10	1768	4	full-splice_match	gene: Zm00001d023557	Zm00001d023557_T00	7.19	27.73	coding	436	170	1477
PB.3421.1	2	1375	7	full-splice_match	gene: Zm00001d006667	Zm00001d006667_T00	139.54	139.54	coding	331	20	1012
PB.311.2	1	1791	5	full-splice_match	gene: Zm00001d028571	Zm00001d028571_T00	0.00	25.40	coding	464	188	1579
PB.311.1	1	2019	5	full-splice_match	gene: Zm00001d028571	Zm00001d028571_T00	25.40	25.40	coding	464	214	1605
PB.5945.1	4	434	4	full-splice_match	gene: Zm00001d054093	Zm00001d054093_T00	31.43	31.43	coding	115	3	347
PB.1643.3	1	1587	7	novel_in_catalog	gene: Zm00001d034814	novel	1.56	51.40	coding	451	42	1394
PB.10891.3	9	4905	13	full-splice_match	gene: Zm00001d048442	Zm00001d048442_T00	9.91	28.66	coding	1526	21	4598
PB.3680.1	3	891	3	incomplete-splice_match	gene: Zm00001d039267	Zm00001d039267_T00	59.34	169.97	coding	120	4	363
PB.3680.2	3	2740	13	novel_in_catalog	gene: Zm00001d039267	novel	0.00	169.97	coding	610	137	1966

PB.3680.3	3	2681	14	full-splice_match	gene: Zm00001d039267	Zm00001d039267_T00	84.39	169.97	coding	686	220	2277
PB.3680.4	3	2674	13	novel_in_catalog	gene: Zm00001d039267	novel	8.31	169.97	coding	652	117	2072
PB.1340.2	1	1893	13	novel_not_in_catalog	gene: Zm00001d033558	novel	18.03	41.60	coding	473	281	1699
PB.3680.6	3	2715	13	novel_in_catalog	gene: Zm00001d039267	novel	17.94	169.97	coding	610	222	2051
PB.3271.1	2	1846	3	full-splice_match	gene: Zm00001d006053	Zm00001d006053_T00	15.60	15.60	coding	388	292	1455
PB.4291.1	3	1357	5	full-splice_match	gene: Zm00001d042555	Zm00001d042555_T00	37.33	37.33	coding	350	241	1290
PB.4521.11	3	2986	7	novel_not_in_catalog	gene: Zm00001d043480	novel	2.69	197.65	coding	232	2051	2746
PB.6944.1	5	1305	3	antisense	novelGene_gene: Zm00001d017258	novel	5.90	5.90	coding	142	251	676
PB.7905.1	6	1462	5	full-splice_match	gene: Zm00001d038114	Zm00001d038114_T00	35.96	36.27	coding	335	125	1129
PB.5875.5	4	3067	25	full-splice_match	gene: Zm00001d053881	Zm00001d053881_T00	0.00	120.88	coding	892	146	2821
PB.7602.7	6	1604	1	intergenic	novelGene_209	novel	17.72	145.86	non coding	NA	NA	NA
PB.1965.1	10	1671	2	genic	gene: Zm00001d024754	novel	3115.97	3115.97	coding	397	200	1390
PB.159.1	1	1336	7	incomplete-splice_match	gene: Zm00001d027916	Zm00001d027916_T00	31.80	31.80	coding	272	1	816
PB.5312.1	4	1738	2	full-splice_match	gene: Zm00001d051343	Zm00001d051343_T00	26.68	28.39	coding	400	149	1348
PB.4377.1	3	1145	8	fusion	gene: Zm00001d042924	novel	20.75	45.53	coding	196	174	761
PB.5448.1	4	1983	11	full-splice_match	gene: Zm00001d052001	Zm00001d052001_T00	9.82	9.82	coding	567	25	1725
PB.4754.1	3	989	2	full-splice_match	gene: Zm00001d044376	Zm00001d044376_T00	2.91	3.08	coding	197	129	719

PB.8311.1	7	1122	1	full-splice_match	gene: Zm00001d019043	Zm00001d019043_T00	12.76	12.76	coding	233	265	963
PB.9631.2	8	1698	9	novel_in_catalog	gene: Zm00001d010744	novel	4.64	109.33	coding	171	126	638
PB.9631.3	8	1994	8	novel_in_catalog	gene: Zm00001d010744	novel	3.56	109.33	coding	171	16	528
PB.9631.1	8	1390	10	full-splice_match	gene: Zm00001d010744	Zm00001d010744_T00	66.13	109.33	coding	342	57	1082
PB.9631.4	8	1727	9	novel_in_catalog	gene: Zm00001d010744	novel	4.77	109.33	coding	62	491	676
PB.9631.5	8	1264	10	full-splice_match	gene: Zm00001d010744	Zm00001d010744_T00	30.23	109.33	coding	342	31	1056
PB.1305.1	1	816	1	genic	gene: Zm00001d033402	novel	22.84	103.87	coding	65	155	349
PB.3326.3	2	1867	3	novel_in_catalog	gene: Zm00001d006251	novel	3.61	144.23	non coding	NA	NA	NA
PB.1124.2	1	1596	13	novel_not_in_catalog	gene: Zm00001d032608	novel	0.00	59.83	coding	414	70	1311
PB.4049.3	3	3119	6	intergenic	novelGene_219	novel	59.30	79.86	coding	196	2152	2739
PB.4680.2	3	1054	1	full-splice_match	gene: Zm00001d044076	Zm00001d044076_T00	83.50	183.25	coding	136	283	690
PB.5329.1	4	1485	5	full-splice_match	gene: Zm00001d051441	Zm00001d051441_T00	34.15	57.95	coding	375	52	1176
PB.5329.3	4	1361	5	full-splice_match	gene: Zm00001d051441	Zm00001d051441_T00	23.80	57.95	coding	375	64	1188
PB.6684.3	5	1563	3	full-splice_match	gene: Zm00001d016052	Zm00001d016052_T00	0.00	48.76	coding	383	193	1341
PB.8292.1	7	1120	2	novel_in_catalog	gene: Zm00001d018936	novel	100.96	115.61	coding	274	141	962
PB.6684.1	5	1883	3	full-splice_match	gene: Zm00001d016052	Zm00001d016052_T00	25.33	48.76	coding	383	108	1256
PB.2643.1	2	1429	2	antisense	novelGene_gene: Zm00001d002693	novel	6.31	6.31	non coding	NA	NA	NA

PB.2926.1	2	1349	2	intergenic	novelGene_177	novel	19.36	19.36	non coding	NA	NA	NA
PB.1373.1	1	1346	1	genic	gene: Zm00001d033706	novel	25.64	25.64	coding	362	64	1149
PB.8023.1	6	1279	2	genic	gene: Zm00001d038528	novel	6.91	6.91	coding	333	153	1151
PB.3409.1	2	765	1	full-splice_match	gene: Zm00001d006618	Zm00001d006618_T00	4.63	4.63	coding	108	53	376
PB.3551.4	2	1099	8	novel_in_catalog	gene: Zm00001d007234	novel	90.58	532.34	coding	205	82	696
PB.1862.1	10	1501	3	novel_not_in_catalog	gene: Zm00001d024102	novel	10.67	26.72	coding	349	217	1263
PB.1862.2	10	1490	2	novel_not_in_catalog	gene: Zm00001d024102	novel	16.06	26.72	coding	387	122	1282
PB.1314.3	1	1810	2	novel_in_catalog	gene: Zm00001d033457	novel	2.80	420.57	coding	213	922	1560
PB.9125.2	8	1199	5	full-splice_match	gene: Zm00001d008215	Zm00001d008215_T00	180.66	199.23	coding	263	130	918
PB.9125.3	8	1116	5	full-splice_match	gene: Zm00001d008215	Zm00001d008215_T00	18.14	199.23	coding	263	156	944
PB.7182.1	5	2200	3	novel_not_in_catalog	gene: Zm00001d018214	novel	3.50	89.30	coding	481	59	1501
PB.9125.1	8	1363	3	incomplete-splice_match	gene: Zm00001d008215	Zm00001d008215_T00	0.44	199.23	coding	117	77	427
PB.10703.1	9	2509	2	novel_in_catalog	gene: Zm00001d047732	novel	0.19	364.80	coding	187	85	645
PB.10703.3	9	1576	2	novel_in_catalog	gene: Zm00001d047732	novel	7.49	364.80	coding	187	85	645
PB.10703.2	9	1554	3	full-splice_match	gene: Zm00001d047732	Zm00001d047732_T00	357.11	364.80	coding	388	84	1247
PB.7887.1	6	1992	14	full-splice_match	gene: Zm00001d038054	Zm00001d038054_T00	5.95	5.95	coding	588	113	1876
PB.7179.1	5	359	1	incomplete-splice_match	gene: Zm00001d018207	Zm00001d018207_T00	81.03	81.03	non coding	NA	NA	NA

PB.8776.2	7	3163	13	full-splice_match	gene: Zm00001d021516	Zm00001d021516_T00	18.12	55.11	coding	913	103	2841
PB.1484.6	1	1741	5	full-splice_match	gene: Zm00001d034205	Zm00001d034205_T00	16.71	174.54	coding	470	297	1706
PB.10007.1	8	4666	2	full-splice_match	gene: Zm00001d012413	Zm00001d012413_T00	4.57	4.57	coding	68	364	567
PB.616.1	1	1196	1	full-splice_match	gene: Zm00001d030045	Zm00001d030045_T00	24.36	119.59	coding	212	176	811
PB.3734.1	3	1517	11	full-splice_match	gene: Zm00001d039448	Zm00001d039448_T00	35.27	35.27	coding	387	25	1185
PB.3042.2	2	1164	1	full-splice_match	gene: Zm00001d004848	Zm00001d004848_T00	6.15	44.80	coding	148	536	979
PB.616.2	1	1059	1	full-splice_match	gene: Zm00001d030045	Zm00001d030045_T00	95.23	119.59	coding	212	173	808
PB.9821.1	8	713	3	incomplete-splice_match	gene: Zm00001d011636	Zm00001d011636_T00	49.32	49.32	coding	123	1	369
PB.2872.2	2	1319	10	full-splice_match	gene: Zm00001d003774	Zm00001d003774_T00	6.18	13.99	coding	347	52	1092
PB.7698.1	6	1233	11	full-splice_match	gene: Zm00001d037246	Zm00001d037246_T00	137.13	137.13	coding	250	217	966
PB.2872.1	2	1379	10	full-splice_match	gene: Zm00001d003774	Zm00001d003774_T00	7.81	13.99	coding	347	84	1124
PB.10186.2	9	1453	1	full-splice_match	gene: Zm00001d045119	Zm00001d045119_T00	3.28	19.59	coding	351	75	1127
PB.826.4	1	1605	1	full-splice_match	gene: Zm00001d031222	Zm00001d031222_T00	0.00	106.90	coding	522	40	1605
PB.10126.2	9	1658	1	intergenic	novelGene_20	novel	0.00	6.37	non coding	NA	NA	NA
PB.10186.1	9	1554	1	full-splice_match	gene: Zm00001d045119	Zm00001d045119_T00	16.31	19.59	coding	351	61	1113
PB.10374.3	9	2381	9	novel_in_catalog	gene: Zm00001d046020	novel	11.11	25.48	coding	313	112	1050
PB.10374.1	9	2292	10	full-splice_match	gene: Zm00001d046020	Zm00001d046020_T00	14.37	25.48	coding	672	147	2162

PB.9139.1	8	1632	1	full-splice_match	gene: Zm00001d008283	Zm00001d008283_T00	3.61	3.61	coding	476	23	1450
PB.10374.6	9	2171	10	full-splice_match	gene: Zm00001d046020	Zm00001d046020_T00	0.00	25.48	coding	672	106	2121
PB.4722.2	3	1575	5	full-splice_match	gene: Zm00001d044225	Zm00001d044225_T00	20.70	75.86	coding	450	126	1475
PB.8776.1	7	1443	4	incomplete-splice_match	gene: Zm00001d021516	Zm00001d021516_T00	32.73	55.11	coding	292	131	1006
PB.10783.2	9	472	1	intergenic	novelGene_294	novel	0.00	0.00	non coding	NA	NA	NA
PB.1322.1	1	1036	1	antisense	novelGene_gene: Zm00001d033495	novel	0.88	0.88	non coding	NA	NA	NA
PB.3308.1	2	2074	2	full-splice_match	gene: Zm00001d006193	Zm00001d006193_T00	13.89	13.89	coding	561	94	1776
PB.7430.3	6	1169	1	full-splice_match	gene: Zm00001d035694	Zm00001d035694_T00	5.59	8.85	coding	304	104	1015
PB.2150.2	10	1633	2	novel_in_catalog	gene: Zm00001d025717	novel	7.87	39.69	coding	473	105	1523
PB.7430.1	6	1230	1	full-splice_match	gene: Zm00001d035694	Zm00001d035694_T00	3.26	8.85	coding	304	45	956
PB.2150.4	10	693	1	genic	gene: Zm00001d025717	novel	11.91	39.69	non coding	NA	NA	NA
PB.8381.1	7	1780	14	full-splice_match	gene: Zm00001d019534	Zm00001d019534_T00	76.02	78.71	coding	501	84	1586
PB.2503.1	2	334	1	incomplete-splice_match	gene: Zm00001d002089	Zm00001d002089_T03	260.83	260.83	non coding	NA	NA	NA
PB.3151.3	2	1393	2	genic	gene: Zm00001d005461	novel	9.96	61.69	coding	305	224	1138
PB.3151.2	2	1444	2	genic	gene: Zm00001d005461	novel	0.00	61.69	coding	305	165	1079
PB.2511.1	2	1282	2	genic	gene: Zm00001d002105	novel	175.38	192.77	coding	269	123	929
PB.6110.3	5	2222	8	novel_not_in_catalog	gene: Zm00001d013320	novel	0.72	20.40	coding	200	65	664

PB.6110.2	5	1715	8	novel_not_in_catalog	gene: Zm00001d013320	novel	7.44	20.40	coding	202	517	1122
PB.6110.1	5	2278	8	novel_not_in_catalog	gene: Zm00001d013320	novel	4.41	20.40	coding	200	88	687
PB.9653.1	8	1628	7	full-splice_match	gene: Zm00001d010842	Zm00001d010842_T01	12.02	23.19	coding	374	241	1362
PB.10462.1	9	2670	13	full-splice_match	gene: Zm00001d046590	Zm00001d046590_T00	32.93	35.75	coding	639	113	2029
PB.6110.5	5	1406	9	novel_not_in_catalog	gene: Zm00001d013320	novel	7.83	20.40	coding	318	78	1031
PB.6110.4	5	1538	9	novel_not_in_catalog	gene: Zm00001d013320	novel	0.00	20.40	coding	318	57	1010
PB.1101.2	1	3008	2	novel_in_catalog	gene: Zm00001d032536	novel	103.70	116.31	coding	177	99	629
PB.1305.5	1	494	1	novel_in_catalog	gene: Zm00001d033402	novel	32.34	103.87	coding	80	119	358
PB.5799.3	4	2076	18	full-splice_match	gene: Zm00001d053580	Zm00001d053580_T00	19.78	81.19	coding	539	163	1779
PB.5494.2	4	757	7	novel_not_in_catalog	gene: Zm00001d052180	novel	7.59	23.33	coding	167	1	501
PB.5494.1	4	1261	8	novel_not_in_catalog	gene: Zm00001d052180	novel	15.74	23.33	coding	228	167	850
PB.10523.5	9	1338	2	genic	gene: Zm00001d046968	novel	0.00	81.16	coding	364	192	1283
PB.7350.1	6	1060	3	novel_in_catalog	gene: Zm00001d035263	novel	9.42	9.42	coding	87	509	769
PB.10523.1	9	1491	2	genic	gene: Zm00001d046968	novel	70.10	81.16	coding	364	260	1351
PB.2849.1	2	1538	9	full-splice_match	gene: Zm00001d003659	Zm00001d003659_T00	58.59	59.98	coding	356	244	1311
PB.2849.2	2	4082	8	full-splice_match	gene: Zm00001d003659	Zm00001d003659_T00	1.39	59.98	coding	202	200	805
PB.10523.2	9	1693	1	full-splice_match	gene: Zm00001d046968	Zm00001d046968_T00	8.76	81.16	coding	364	477	1568

PB.2988.1	2	1393	7	full-splice_match	gene: Zm00001d004498	Zm00001d004498_T00	13.28	35.29	coding	297	181	1071
PB.7897.6	6	1519	8	full-splice_match	gene: Zm00001d038082	Zm00001d038082_T00	2.91	40.46	coding	364	329	1420
PB.7165.1	5	1158	1	antisense	novelGene_gene: Zm00001d018155	novel	16.23	16.23	non coding	NA	NA	NA
PB.1602.1	1	1251	3	incomplete-splice_match	gene: Zm00001d034675	Zm00001d034675_T00	59.58	65.52	coding	215	203	847
PB.7897.5	6	1782	9	full-splice_match	gene: Zm00001d038082	Zm00001d038082_T00	27.69	40.46	coding	364	242	1333
PB.4437.4	3	1085	10	incomplete-splice_match	gene: Zm00001d043182	Zm00001d043182_T00	0.00	42.01	coding	275	261	1085
PB.10811.1	9	812	3	full-splice_match	gene: Zm00001d048117	Zm00001d048117_T00	18.08	18.08	coding	172	92	607
PB.10955.1	B73V4	2106	4	novel_not_in_catalog	gene: Zm00001d000361	novel	4.97	4.97	coding	517	189	1739
PB.7826.2	6	2218	9	full-splice_match	gene: Zm00001d037829	Zm00001d037829_T00	27.24	102.42	coding	515	440	1984
PB.5649.1	4	1077	4	full-splice_match	gene: Zm00001d052796	Zm00001d052796_T00	219.28	219.28	coding	259	113	889
PB.6940.1	5	802	4	full-splice_match	gene: Zm00001d017242	Zm00001d017242_T00	13.40	232.47	non coding	NA	NA	NA
PB.6929.3	5	1553	1	intergenic	novelGene_382	novel	0.00	15.85	non coding	NA	NA	NA
PB.1209.2	1	996	1	full-splice_match	gene: Zm00001d032972	Zm00001d032972_T00	25.43	79.32	coding	288	80	943
PB.1209.1	1	1130	1	full-splice_match	gene: Zm00001d032972	Zm00001d032972_T00	53.89	79.32	coding	288	87	950
PB.10458.2	9	654	1	full-splice_match	gene: Zm00001d046574	Zm00001d046574_T00	103.62	164.44	coding	108	147	470
PB.7666.1	6	3044	16	full-splice_match	gene: Zm00001d037099	Zm00001d037099_T01	52.73	52.73	coding	765	300	2594
PB.6929.5	5	1060	2	intergenic	novelGene_382	novel	4.87	15.85	non coding	NA	NA	NA

PB.10458.1	9	766	1	full-splice_match	gene: Zm00001d046574	Zm00001d046574_T00	60.82	164.44	coding	108	147	470
PB.2757.2	2	3241	21	novel_in_catalog	gene: Zm00001d003198	novel	4.21	13.79	coding	701	469	2571
PB.6860.6	5	1978	2	novel_in_catalog	gene: Zm00001d016911	novel	5.66	46.96	coding	359	60	1136
PB.2757.1	2	2660	17	incomplete-splice_match	gene: Zm00001d003198	Zm00001d003198_T02	9.58	13.79	coding	610	231	2060
PB.630.5	1	2817	7	full-splice_match	gene: Zm00001d030110	Zm00001d030110_T01	5.48	227.82	coding	489	472	1938
PB.9488.1	8	1496	3	full-splice_match	gene: Zm00001d010137	Zm00001d010137_T00	17.10	17.10	coding	403	144	1352
PB.230.1	1	860	7	full-splice_match	gene: Zm00001d028232	Zm00001d028232_T00	39.70	39.70	coding	163	40	528
PB.978.2	1	1775	2	novel_in_catalog	gene: Zm00001d032027	novel	4.25	39.94	coding	176	1015	1542
PB.4514.3	3	1722	12	full-splice_match	gene: Zm00001d043459	Zm00001d043459_T00	8.56	42.24	coding	471	154	1566
PB.1023.2	1	2542	6	full-splice_match	gene: Zm00001d032226	Zm00001d032226_T01	35.44	35.44	coding	667	137	2137
PB.978.3	1	1447	3	full-splice_match	gene: Zm00001d032027	Zm00001d032027_T00	27.11	39.94	coding	319	278	1234
PB.10444.1	9	1319	1	full-splice_match	gene: Zm00001d046496	Zm00001d046496_T00	22.91	22.91	coding	209	135	761
PB.10668.2	9	2402	5	full-splice_match	gene: Zm00001d047608	Zm00001d047608_T00	6.22	123.54	coding	122	794	1159
PB.5333.1	4	1829	9	full-splice_match	gene: Zm00001d051458	Zm00001d051458_T00	28.15	48.80	coding	284	275	1126
PB.2841.4	2	1023	11	incomplete-splice_match	gene: Zm00001d003590	Zm00001d003590_T00	3.04	33.30	coding	294	140	1021
PB.11006.1	B73V4	3553	4	full-splice_match	gene: Zm00001d000166	Zm00001d000166_T01	21.48	21.48	coding	817	435	2885
PB.323.2	1	2534	2	genic	gene: Zm00001d028620	novel	143.05	170.40	coding	639	379	2295

PB.323.3	1	2974	1	full-splice_match	gene: Zm00001d028620	Zm00001d028620_T00	5.76	170.40	coding	639	835	2751
PB.323.4	1	2460	2	genic	gene: Zm00001d028620	novel	2.23	170.40	coding	639	382	2298
PB.2513.1	2	3358	7	incomplete-splice_match	gene: Zm00001d002125	Zm00001d002125_T00	29.04	29.04	coding	1046	11	3148
PB.2841.2	2	1565	13	full-splice_match	gene: Zm00001d003590	Zm00001d003590_T00	26.52	33.30	coding	345	134	1168
PB.2841.3	2	1256	13	full-splice_match	gene: Zm00001d003590	Zm00001d003590_T00	3.75	33.30	coding	346	135	1172
PB.10857.1	9	1060	3	incomplete-splice_match	gene: Zm00001d048329	Zm00001d048329_T00	33.39	33.39	coding	125	96	470
PB.746.1	1	1899	3	full-splice_match	gene: Zm00001d030877	Zm00001d030877_T00	6.47	6.47	coding	584	69	1820
PB.3332.1	2	1926	3	full-splice_match	gene: Zm00001d006308	Zm00001d006308_T00	62.31	94.24	coding	424	185	1456
PB.1314.1	1	1776	3	full-splice_match	gene: Zm00001d033457	Zm00001d033457_T00	85.96	420.57	coding	445	126	1460
PB.4700.1	3	1674	6	novel_not_in_catalog	gene: Zm00001d044144	novel	60.98	102.99	coding	397	199	1389
PB.4700.2	3	2911	6	full-splice_match	gene: Zm00001d044144	Zm00001d044144_T00	8.28	102.99	coding	460	1247	2626
PB.4479.6	3	2098	5	novel_not_in_catalog	gene: Zm00001d043354	novel	0.91	31.37	coding	599	11	1807
PB.5220.1	4	2370	4	full-splice_match	gene: Zm00001d050716	Zm00001d050716_T00	39.14	49.36	coding	559	297	1973
PB.5220.2	4	2711	3	full-splice_match	gene: Zm00001d050716	Zm00001d050716_T00	2.43	49.36	coding	415	1120	2364
PB.5220.3	4	2392	4	full-splice_match	gene: Zm00001d050716	Zm00001d050716_T00	6.56	49.36	coding	559	430	2106
PB.10852.4	9	1560	9	full-splice_match	gene: Zm00001d048315	Zm00001d048315_T00	22.48	33.62	coding	360	369	1448
PB.5220.5	4	1001	1	incomplete-splice_match	gene: Zm00001d050716	Zm00001d050716_T00	1.23	49.36	coding	137	300	710

PB.5137.1	4	972	3	full-splice_match	gene: Zm00001d050121	Zm00001d050121_T00	38.11	38.11	coding	218	121	774
PB.8579.1	7	2526	6	full-splice_match	gene: Zm00001d020647	Zm00001d020647_T00	44.15	44.15	coding	676	277	2304
PB.961.3	1	837	2	novel_in_catalog	gene: Zm00001d031959	novel	7.86	111.08	coding	80	122	361
PB.4793.6	3	1481	3	novel_in_catalog	gene: Zm00001d044516	novel	17.48	891.68	coding	337	127	1137
PB.4479.4	3	1630	3	novel_in_catalog	gene: Zm00001d043354	novel	6.79	31.37	coding	409	43	1269
PB.6737.1	5	1107	1	full-splice_match	gene: Zm00001d016342	Zm00001d016342_T00	78.03	78.03	coding	199	77	673
PB.10925.1	9	2633	4	incomplete-splice_match	gene: Zm00001d048535	Zm00001d048535_T03	35.49	71.63	coding	536	367	1974
PB.9975.2	8	1974	3	genic	gene: Zm00001d012260	novel	5.56	73.75	coding	488	302	1765
PB.5113.1	4	853	6	full-splice_match	gene: Zm00001d049952	Zm00001d049952_T00	43.39	43.39	coding	144	113	544
PB.4479.3	3	2506	5	novel_not_in_catalog	gene: Zm00001d043354	novel	14.19	31.37	coding	656	212	2179
PB.450.1	1	2139	6	novel_in_catalog	gene: Zm00001d029191	novel	3.00	15.05	coding	459	53	1429
PB.8761.1	7	1408	4	full-splice_match	gene: Zm00001d021447	Zm00001d021447_T00	22.56	22.56	coding	367	53	1153
PB.4844.1	4	1684	2	full-splice_match	gene: Zm00001d048620	Zm00001d048620_T00	5.68	5.68	coding	390	332	1501
PB.450.2	1	2055	7	full-splice_match	gene: Zm00001d029191	Zm00001d029191_T00	12.05	15.05	coding	494	116	1597
PB.9252.31	8	4973	22	full-splice_match	gene: Zm00001d008789	Zm00001d008789_T01	22.98	57.67	coding	1497	17	4507
PB.9252.30	8	1624	9	full-splice_match	gene: Zm00001d008757	Zm00001d008757_T00	63.08	102.00	coding	415	152	1396
PB.9252.33	8	1235	5	incomplete-splice_match	gene: Zm00001d008789	Zm00001d008789_T00	26.14	57.67	coding	214	284	925

PB.9252.32	8	2678	12	novel_in_catalog	gene: Zm00001d008789	novel	8.56	57.67	coding	530	658	2247
PB.2100.1	10	2054	11	full-splice_match	gene: Zm00001d025433	Zm00001d025433_T00	21.26	21.26	coding	553	211	1869
PB.7771.1	6	2467	16	full-splice_match	gene: Zm00001d037593	Zm00001d037593_T00	13.02	13.02	coding	747	68	2308
PB.9286.1	8	1519	9	full-splice_match	gene: Zm00001d008954	Zm00001d008954_T00	12.22	12.22	coding	361	175	1257
PB.2909.1	2	2328	7	full-splice_match	gene: Zm00001d003941	Zm00001d003941_T00	101.26	113.38	coding	668	183	2186
PB.2909.3	2	2435	6	full-splice_match	gene: Zm00001d003941	Zm00001d003941_T00	12.12	113.38	coding	250	21	770
PB.4412.1	3	451	3	incomplete-splice_match	gene: Zm00001d043098	Zm00001d043098_T00	876.75	876.75	non coding	NA	NA	NA
PB.701.4	1	1645	9	fusion	gene: Zm00001d030588	novel	0.16	34.46	coding	166	62	559
PB.701.5	1	1140	12	fusion	gene: Zm00001d030588	novel	19.06	34.46	coding	286	76	933
PB.218.1	1	1187	6	full-splice_match	gene: Zm00001d028197	Zm00001d028197_T00	13.60	13.60	coding	239	179	895
PB.701.7	1	1615	9	fusion	gene: Zm00001d030588	novel	2.17	34.46	coding	166	74	571
PB.933.1	1	965	6	novel_in_catalog	gene: Zm00001d031824	novel	16.39	16.39	coding	208	82	705
PB.4022.1	3	1219	2	incomplete-splice_match	gene: Zm00001d041063	Zm00001d041063_T00	12.11	12.11	coding	305	84	998
PB.701.3	1	1781	8	fusion	gene: Zm00001d030588	novel	8.76	34.46	coding	166	106	603
PB.9558.2	8	2076	2	full-splice_match	gene: Zm00001d010463	Zm00001d010463_T00	12.23	57.43	coding	576	117	1844
PB.9558.3	8	2146	1	novel_in_catalog	gene: Zm00001d010463	novel	3.36	57.43	coding	463	45	1433
PB.4015.2	3	1806	8	novel_not_in_catalog	gene: Zm00001d040883	novel	20.64	20.64	coding	484	17	1468

PB.6806.1	5	1148	1	full-splice_match	gene: Zm00001d016697	Zm00001d016697_T00	37.00	37.00	coding	234	167	868
PB.9558.4	8	1751	1	novel_in_catalog	gene: Zm00001d010463	novel	0.00	57.43	coding	463	45	1433
PB.9941.2	8	1375	2	full-splice_match	gene: Zm00001d012103	Zm00001d012103_T00	539.37	1084.73	coding	355	176	1240
PB.10804.2	9	696	1	incomplete-splice_match	gene: Zm00001d048100	Zm00001d048100_T00	103.62	528.08	non coding	NA	NA	NA
PB.10804.4	9	1540	6	incomplete-splice_match	gene: Zm00001d048100	Zm00001d048100_T00	424.46	528.08	coding	397	21	1211
PB.10645.3	9	2728	10	novel_in_catalog	gene: Zm00001d047522	novel	2.05	184.41	coding	328	315	1298
PB.10081.1	8	1038	5	full-splice_match	gene: Zm00001d012689	Zm00001d012689_T00	29.61	29.61	coding	166	199	696
PB.3490.2	2	751	1	full-splice_match	gene: Zm00001d006950	Zm00001d006950_T00	0.00	31.33	coding	156	90	557
PB.4767.2	3	2671	11	incomplete-splice_match	gene: Zm00001d044442	Zm00001d044442_T01	36.55	87.18	coding	797	8	2398
PB.8848.2	7	1262	6	full-splice_match	gene: Zm00001d021745	Zm00001d021745_T00	0.00	10.81	coding	307	151	1071
PB.8848.1	7	1439	6	full-splice_match	gene: Zm00001d021745	Zm00001d021745_T00	10.81	10.81	coding	307	151	1071
PB.1336.1	1	1672	1	intergenic	novelGene_457	novel	126.08	126.08	non coding	NA	NA	NA
PB.4468.1	3	2057	9	full-splice_match	gene: Zm00001d043303	Zm00001d043303_T00	15.34	118.16	coding	494	168	1649
PB.6587.1	5	2082	16	full-splice_match	gene: Zm00001d015329	Zm00001d015329_T00	17.24	17.24	coding	586	94	1851
PB.4468.2	3	1947	9	full-splice_match	gene: Zm00001d043303	Zm00001d043303_T00	102.82	118.16	coding	494	195	1676
PB.3364.6	2	1143	9	full-splice_match	gene: Zm00001d006410	Zm00001d006410_T00	12.20	41.20	coding	248	54	797
PB.3488.8	2	1503	11	novel_not_in_catalog	gene: Zm00001d006944	novel	5.94	50.84	coding	356	20	1087

PB.3417.1	2	1457	4	incomplete-splice_match	gene: Zm00001d006631	Zm00001d006631_T00	13.98	13.98	coding	332	54	1049
PB.5078.1	4	2050	2	novel_not_in_catalog	gene: Zm00001d049737	novel	6.39	6.39	coding	553	50	1708
PB.4475.2	3	1498	7	full-splice_match	gene: Zm00001d043328	Zm00001d043328_T00	11.04	11.04	coding	367	143	1243
PB.66.1	1	3577	13	full-splice_match	gene: Zm00001d027503	Zm00001d027503_T01	17.84	36.85	coding	1045	145	3279
PB.9103.2	7	1474	6	full-splice_match	gene: Zm00001d022614	Zm00001d022614_T00	5.31	20.55	coding	403	97	1305
PB.66.4	1	1821	11	incomplete-splice_match	gene: Zm00001d027503	Zm00001d027503_T02	19.00	36.85	coding	563	13	1701
PB.8988.3	7	915	2	full-splice_match	gene: Zm00001d022244	Zm00001d022244_T00	0.00	79.64	coding	126	281	658
PB.10865.1	9	1031	3	full-splice_match	gene: Zm00001d048354	Zm00001d048354_T00	20.35	20.35	coding	226	62	739
PB.7177.1	5	1626	5	full-splice_match	gene: Zm00001d018201	Zm00001d018201_T00	3.07	3.07	coding	429	81	1367
PB.7984.2	6	1582	5	novel_in_catalog	gene: Zm00001d038378	novel	2.52	108.28	coding	118	921	1274
PB.9550.2	8	1794	7	novel_not_in_catalog	gene: Zm00001d010431	novel	18.90	106.46	coding	229	770	1456
PB.9103.1	7	1614	5	full-splice_match	gene: Zm00001d022614	Zm00001d022614_T00	15.24	20.55	coding	403	100	1308
PB.9550.6	8	1119	8	novel_not_in_catalog	gene: Zm00001d010431	novel	87.57	106.46	coding	229	274	960
PB.9550.7	8	967	8	novel_not_in_catalog	gene: Zm00001d010431	novel	0.00	106.46	coding	229	230	916
PB.9550.4	8	1718	7	novel_not_in_catalog	gene: Zm00001d010431	novel	0.00	106.46	coding	229	771	1457
PB.9597.2	8	2429	3	genic	gene: Zm00001d010646	novel	0.00	23.62	coding	678	150	2183
PB.4596.1	3	1371	1	full-splice_match	gene: Zm00001d043741	Zm00001d043741_T00	10.95	10.95	coding	427	91	1371

PB.5573.3	4	1954	11	novel_in_catalog	gene: Zm00001d052416	novel	6.83	60.49	coding	212	10	645
PB.2915.2	2	1023	6	full-splice_match	gene: Zm00001d003975	Zm00001d003975_T00	9.07	19.43	coding	182	181	726
PB.2915.1	2	1111	6	full-splice_match	gene: Zm00001d003975	Zm00001d003975_T00	10.36	19.43	coding	182	20	565
PB.5535.1	4	1081	6	full-splice_match	gene: Zm00001d052310	Zm00001d052310_T00	22.16	32.56	coding	273	97	915
PB.7064.1	5	2967	3	full-splice_match	gene: Zm00001d017793	Zm00001d017793_T00	16.54	25.02	coding	713	520	2658
PB.4317.4	3	2301	4	full-splice_match	gene: Zm00001d042664	Zm00001d042664_T00	21.56	152.56	coding	571	241	1953
PB.10046.1	8	1531	2	full-splice_match	gene: Zm00001d012564	Zm00001d012564_T00	94.73	100.15	coding	308	277	1200
PB.6614.6	5	1645	3	antisense	novelGene_gene: Zm00001d015513	novel	1.82	30.48	non coding	NA	NA	NA
PB.8840.1	7	2086	5	full-splice_match	gene: Zm00001d021727	Zm00001d021727_T00	4.13	4.13	coding	589	21	1787
PB.6614.7	5	1619	3	antisense	novelGene_gene: Zm00001d015513	novel	5.84	30.48	non coding	NA	NA	NA
PB.10779.5	9	2019	4	novel_in_catalog	gene: Zm00001d047981	novel	56.88	352.56	coding	301	775	1677
PB.1627.1	1	2397	18	full-splice_match	gene: Zm00001d034758	Zm00001d034758_T00	84.36	108.87	coding	659	178	2154
PB.4001.2	3	1239	1	incomplete-splice_match	gene: Zm00001d040789	Zm00001d040789_T00	3.40	45.23	coding	170	524	1033
PB.9457.1	8	1248	3	full-splice_match	gene: Zm00001d009954	Zm00001d009954_T00	46.26	66.01	coding	45	749	883
PB.9457.2	8	3194	2	novel_in_catalog	gene: Zm00001d009954	novel	0.84	66.01	coding	110	246	575
PB.4001.1	3	1746	6	full-splice_match	gene: Zm00001d040789	Zm00001d040789_T00	41.83	45.23	coding	446	156	1493
PB.8860.5	7	1558	3	novel_in_catalog	gene: Zm00001d021779	novel	7.39	55.84	coding	110	995	1324

PB.984.1	1	1620	6	full-splice_match	gene: Zm00001d032056	Zm00001d032056_T00	5.26	5.26	coding	349	12	1058
PB.4001.4	3	1637	6	full-splice_match	gene: Zm00001d040789	Zm00001d040789_T00	0.00	45.23	non coding	NA	NA	NA
PB.668.3	1	1908	3	full-splice_match	gene: Zm00001d030310	Zm00001d030310_T00	0.00	26.33	coding	544	61	1692
PB.994.1	1	904	1	full-splice_match	gene: Zm00001d032094	Zm00001d032094_T00	40.60	40.60	coding	133	192	590
PB.7263.1	5	577	2	incomplete-splice_match	gene: Zm00001d018499	Zm00001d018499_T00	6.16	44.85	non coding	NA	NA	NA
PB.7412.3	6	1119	1	novel_in_catalog	gene: Zm00001d035603	novel	0.00	4.48	non coding	NA	NA	NA
PB.7412.2	6	1293	1	genic	gene: Zm00001d035603	novel	4.48	4.48	non coding	NA	NA	NA
PB.2172.1	10	2251	1	full-splice_match	gene: Zm00001d025812	Zm00001d025812_T00	23.26	39.92	coding	546	150	1787
PB.553.3	1	2196	10	full-splice_match	gene: Zm00001d029654	Zm00001d029654_T00	5.26	67.98	coding	328	84	1067
PB.553.2	1	2036	11	full-splice_match	gene: Zm00001d029654	Zm00001d029654_T00	42.26	67.98	coding	564	86	1777
PB.553.1	1	1379	9	novel_not_in_catalog	gene: Zm00001d029654	novel	20.46	67.98	coding	242	357	1082
PB.668.1	1	2122	3	full-splice_match	gene: Zm00001d030310	Zm00001d030310_T00	26.33	26.33	coding	544	70	1701
PB.6462.1	5	1322	4	full-splice_match	gene: Zm00001d014817	Zm00001d014817_T00	72.07	72.07	coding	378	114	1247
PB.4193.3	3	1227	4	novel_not_in_catalog	gene: Zm00001d042059	novel	4.65	107.87	coding	107	134	454
PB.2264.1	10	3060	1	genic	gene: Zm00001d026245	novel	2.62	9.59	coding	162	2212	2697
PB.6810.1	5	1265	8	full-splice_match	gene: Zm00001d016714	Zm00001d016714_T00	72.81	84.19	coding	314	124	1065
PB.2264.3	10	2818	1	genic	gene: Zm00001d026245	novel	0.00	9.59	coding	162	2200	2685

PB.2264.2	10	1330	2	novel_not_in_catalog	gene: Zm00001d026245	novel	6.98	9.59	coding	271	250	1062
PB.2051.3	10	2950	16	full-splice_match	gene: Zm00001d025111	Zm00001d025111_T03	9.25	56.41	coding	828	172	2655
PB.7571.2	6	707	1	full-splice_match	gene: Zm00001d036570	Zm00001d036570_T00	504.08	588.19	coding	137	63	473
PB.7571.1	6	826	1	full-splice_match	gene: Zm00001d036570	Zm00001d036570_T00	84.11	588.19	coding	137	63	473
PB.9086.3	7	1970	15	novel_not_in_catalog	gene: Zm00001d022547	novel	31.95	31.95	coding	449	384	1730
PB.5382.1	4	2619	9	full-splice_match	gene: Zm00001d051671	Zm00001d051671_T03	5.73	60.22	coding	345	1398	2432
PB.6446.4	5	631	2	novel_in_catalog	gene: Zm00001d014779	novel	4.59	24.17	non coding	NA	NA	NA
PB.6446.3	5	754	2	novel_in_catalog	gene: Zm00001d014779	novel	14.76	24.17	non coding	NA	NA	NA
PB.6446.2	5	724	3	full-splice_match	gene: Zm00001d014779	Zm00001d014779_T00	2.87	24.17	non coding	NA	NA	NA
PB.6446.1	5	846	3	full-splice_match	gene: Zm00001d014779	Zm00001d014779_T00	1.94	24.17	non coding	NA	NA	NA
PB.7233.1	5	1938	11	full-splice_match	gene: Zm00001d018413	Zm00001d018413_T00	11.16	90.00	coding	488	119	1582
PB.4673.2	3	1269	6	full-splice_match	gene: Zm00001d044036	Zm00001d044036_T00	9.92	32.93	coding	117	387	737
PB.6614.8	5	1548	2	antisense	novelGene_gene: Zm00001d015513	novel	5.90	30.48	non coding	NA	NA	NA
PB.2204.1	10	2245	3	full-splice_match	gene: Zm00001d025950	Zm00001d025950_T00	33.72	33.72	coding	512	233	1768
PB.2928.2	2	2010	9	full-splice_match	gene: Zm00001d004055	Zm00001d004055_T00	3.46	9.68	coding	475	208	1632
PB.3470.3	2	2054	5	full-splice_match	gene: Zm00001d006900	Zm00001d006900_T00	321.34	444.34	coding	537	87	1697
PB.2928.1	2	2004	9	full-splice_match	gene: Zm00001d004055	Zm00001d004055_T00	6.22	9.68	coding	475	200	1624

PB.3508.1	2	562	1	intergenic	novelGene_422	novel	1.09	1.09	non coding	NA	NA	NA
PB.3658.2	2	1755	13	incomplete-splice_match	gene: Zm00001d007905	Zm00001d007905_T00	15.62	15.62	coding	403	153	1361
PB.3470.2	2	1061	1	incomplete-splice_match	gene: Zm00001d006900	Zm00001d006900_T00	66.89	444.34	non coding	NA	NA	NA
PB.8251.3	7	399	2	full-splice_match	gene: Zm00001d001080	Zm00001d001080_T00	164.96	198.40	non coding	NA	NA	NA
PB.2583.2	2	1112	3	full-splice_match	gene: Zm00001d002405	Zm00001d002405_T00	213.59	222.87	coding	298	87	980
PB.8251.1	7	557	2	full-splice_match	gene: Zm00001d001080	Zm00001d001080_T00	33.44	198.40	non coding	NA	NA	NA
PB.9086.5	7	1309	12	novel_not_in_catalog	gene: Zm00001d022547	novel	0.00	31.95	coding	371	197	1309
PB.2583.5	2	1214	2	novel_in_catalog	gene: Zm00001d002405	novel	9.28	222.87	coding	232	90	785
PB.8969.5	7	2999	4	full-splice_match	gene: Zm00001d022181	Zm00001d022181_T00	3.06	327.94	coding	272	87	902
PB.2527.5	2	1652	13	full-splice_match	gene: Zm00001d002163	Zm00001d002163_T00	0.00	26.63	coding	495	74	1558
PB.8969.7	7	1803	5	novel_not_in_catalog	gene: Zm00001d022181	novel	0.37	327.94	coding	405	286	1500
PB.2411.2	10	2796	10	full-splice_match	gene: Zm00001d026698	Zm00001d026698_T02	11.26	21.95	coding	733	84	2282
PB.1920.1	10	1525	5	incomplete-splice_match	gene: Zm00001d024510	Zm00001d024510_T00	9.50	92.14	coding	146	806	1243
PB.8969.3	7	2210	5	full-splice_match	gene: Zm00001d022181	Zm00001d022181_T00	13.58	327.94	coding	538	92	1705
PB.4148.1	3	1294	1	incomplete-splice_match	gene: Zm00001d041873	Zm00001d041873_T00	8.95	14.32	coding	328	153	1136
PB.7759.2	6	2542	11	full-splice_match	gene: Zm00001d037518	Zm00001d037518_T00	4.85	9.61	coding	733	83	2281
PB.7759.1	6	2703	11	full-splice_match	gene: Zm00001d037518	Zm00001d037518_T00	4.76	9.61	coding	733	81	2279

PB.9423.1	8	1990	14	full-splice_match	gene: Zm00001d009756	Zm00001d009756_T00	37.99	51.62	coding	475	157	1581
PB.7877.2	6	2058	2	full-splice_match	gene: Zm00001d038022	Zm00001d038022_T00	30.92	33.74	coding	490	160	1629
PB.9423.3	8	1760	14	full-splice_match	gene: Zm00001d009756	Zm00001d009756_T00	0.00	51.62	coding	475	155	1579
PB.9423.2	8	1861	14	full-splice_match	gene: Zm00001d009756	Zm00001d009756_T00	13.63	51.62	coding	475	155	1579
PB.6338.2	5	1442	5	incomplete-splice_match	gene: Zm00001d014321	Zm00001d014321_T00	14.89	44.92	non coding	NA	NA	NA
PB.6338.1	5	2493	6	full-splice_match	gene: Zm00001d014321	Zm00001d014321_T00	30.03	44.92	coding	780	35	2374
PB.9572.1	8	938	9	full-splice_match	gene: Zm00001d010536	Zm00001d010536_T00	12.70	12.70	coding	218	60	713
PB.10765.1	9	2250	6	full-splice_match	gene: Zm00001d047920	Zm00001d047920_T00	22.75	22.75	coding	239	16	732
PB.2527.1	2	1936	13	full-splice_match	gene: Zm00001d002163	Zm00001d002163_T00	22.35	26.63	coding	495	144	1628
PB.1225.2	1	2764	14	full-splice_match	gene: Zm00001d033028	Zm00001d033028_T00	29.56	46.92	coding	724	428	2599
PB.2296.1	10	1893	2	full-splice_match	gene: Zm00001d026333	Zm00001d026333_T00	39.05	77.84	coding	471	113	1525
PB.3487.1	2	1005	4	novel_in_catalog	gene: Zm00001d006942	novel	1.98	57.82	non coding	NA	NA	NA
PB.5998.1	5	1265	5	incomplete-splice_match	gene: Zm00001d012955	Zm00001d012955_T00	19.69	19.69	coding	248	23	766
PB.9324.1	8	1961	2	full-splice_match	gene: Zm00001d009186	Zm00001d009186_T00	54.63	56.83	coding	520	188	1747
PB.7565.1	6	1769	2	genic	gene: Zm00001d036558	novel	9.31	9.31	coding	452	140	1495
PB.9191.5	8	752	1	incomplete-splice_match	gene: Zm00001d008488	Zm00001d008488_T00	0.53	33.42	coding	176	224	751
PB.9324.2	8	1775	2	full-splice_match	gene: Zm00001d009186	Zm00001d009186_T00	2.20	56.83	coding	501	271	1773

PB.6418.2	5	596	1	full-splice_match	gene: Zm00001d014682	Zm00001d014682_T00	0.00	52.33	non coding	NA	NA	NA
PB.6418.3	5	1018	1	full-splice_match	gene: Zm00001d014682	Zm00001d014682_T00	40.80	52.33	coding	130	310	699
PB.9191.1	8	2400	3	incomplete-splice_match	gene: Zm00001d008488	Zm00001d008488_T00	32.89	33.42	coding	683	184	2232
PB.6418.1	5	1257	1	full-splice_match	gene: Zm00001d014682	Zm00001d014682_T00	11.53	52.33	coding	130	310	699
PB.8914.1	7	888	5	full-splice_match	gene: Zm00001d021958	Zm00001d021958_T00	150.20	214.16	coding	157	214	684
PB.2995.2	2	918	4	full-splice_match	gene: Zm00001d004545	Zm00001d004545_T00	0.00	22.06	coding	208	70	693
PB.3706.4	3	960	7	full-splice_match	gene: Zm00001d039350	Zm00001d039350_T00	167.50	366.27	coding	230	130	819
PB.4132.1	3	1483	2	incomplete-splice_match	gene: Zm00001d041767	Zm00001d041767_T00	5.15	62.77	coding	127	874	1254
PB.6899.1	5	2083	9	full-splice_match	gene: Zm00001d017096	Zm00001d017096_T01	9.08	254.51	coding	171	106	618
PB.6478.1	5	1999	3	full-splice_match	gene: Zm00001d014887	Zm00001d014887_T00	270.26	270.26	coding	562	121	1806
PB.4063.1	3	2783	12	incomplete-splice_match	gene: Zm00001d041378	Zm00001d041378_T00	20.12	20.12	coding	792	110	2485
PB.10352.2	9	2282	5	novel_in_catalog	gene: Zm00001d045872	novel	1.74	30.70	coding	207	1519	2139
PB.10352.1	9	1608	6	full-splice_match	gene: Zm00001d045872	Zm00001d045872_T00	28.96	30.70	coding	484	10	1461
PB.10779.7	9	2021	4	novel_in_catalog	gene: Zm00001d047981	novel	10.06	352.56	coding	388	548	1711
PB.4393.2	3	2173	4	full-splice_match	gene: Zm00001d043019	Zm00001d043019_T00	173.12	374.64	coding	492	474	1949
PB.2635.4	2	601	7	full-splice_match	gene: Zm00001d002659	Zm00001d002659_T00	0.00	163.97	coding	123	84	452
PB.2635.3	2	799	6	full-splice_match	gene: Zm00001d002659	Zm00001d002659_T00	16.36	163.97	non coding	NA	NA	NA

PB.2635.2	2	871	7	full-splice_match	gene: Zm00001d002659	Zm00001d002659_T00	131.70	163.97	coding	123	118	486
PB.2635.1	2	966	6	full-splice_match	gene: Zm00001d002659	Zm00001d002659_T00	15.92	163.97	non coding	NA	NA	NA
PB.10779.1	9	2149	5	full-splice_match	gene: Zm00001d047981	Zm00001d047981_T00	43.70	352.56	coding	490	100	1569
PB.8104.2	6	887	1	incomplete-splice_match	gene: Zm00001d038841	Zm00001d038841_T00	0.00	139.32	coding	215	133	777
PB.7121.1	5	1398	1	genic	gene: Zm00001d017992	novel	67.96	67.96	coding	237	532	1242
PB.8104.1	6	927	1	genic	gene: Zm00001d038841	novel	109.04	139.32	coding	215	61	705
PB.8680.3	7	1916	13	novel_not_in_catalog	gene: Zm00001d021132	novel	4.99	54.97	coding	221	1008	1670
PB.8680.2	7	2550	12	novel_not_in_catalog	gene: Zm00001d021132	novel	4.71	54.97	coding	221	1642	2304
PB.5042.1	4	826	1	full-splice_match	gene: Zm00001d049582	Zm00001d049582_T00	24.69	24.69	coding	207	43	663
PB.84.1	1	3350	20	full-splice_match	gene: Zm00001d027558	Zm00001d027558_T00	119.28	122.86	coding	986	48	3005
PB.8085.3	6	2365	11	novel_in_catalog	gene: Zm00001d038776	novel	0.75	77.48	coding	411	803	2035
PB.5421.1	4	1840	8	full-splice_match	gene: Zm00001d051881	Zm00001d051881_T00	1.29	140.47	coding	344	48	1079
PB.9851.1	8	3131	6	novel_not_in_catalog	gene: Zm00001d011753	novel	9.23	9.23	coding	474	119	1540
PB.84.5	1	3471	20	full-splice_match	gene: Zm00001d027558	Zm00001d027558_T00	3.57	122.86	coding	986	279	3236
PB.9619.2	8	1693	2	genic	gene: Zm00001d010705	novel	0.00	16.45	coding	412	119	1354
PB.8085.4	6	1749	13	novel_in_catalog	gene: Zm00001d038776	novel	0.00	77.48	coding	499	56	1552
PB.7002.1	1	891	5	intergenic	novelGene_297	novel	30.01	30.01	non coding	NA	NA	NA

PB.1690.1	10	1180	5	novel_not_in_catalog	gene: Zm00001d023242	novel	18.18	39.58	coding	235	60	764
PB.7011.1	5	916	1	full-splice_match	gene: Zm00001d017540	Zm00001d017540_T00	12.26	12.26	coding	191	62	634
PB.1690.3	10	640	1	novel_in_catalog	gene: Zm00001d023242	novel	5.43	39.58	non coding	NA	NA	NA
PB.1690.2	10	919	5	novel_not_in_catalog	gene: Zm00001d023242	novel	15.97	39.58	coding	235	60	764
PB.4971.2	4	1106	3	full-splice_match	gene: Zm00001d049228	Zm00001d049228_T00	1.75	7.61	coding	337	80	1090
PB.9199.2	8	1804	8	novel_in_catalog	gene: Zm00001d008503	novel	5.67	65.73	coding	257	533	1303
PB.9199.1	8	1511	9	novel_not_in_catalog	gene: Zm00001d008503	novel	30.08	65.73	coding	235	385	1089
PB.4971.1	4	1293	3	full-splice_match	gene: Zm00001d049228	Zm00001d049228_T00	5.86	7.61	coding	337	26	1036
PB.9694.6	8	1951	12	novel_in_catalog	gene: Zm00001d011061	novel	4.99	25.76	coding	212	134	769
PB.3236.1	2	1159	1	full-splice_match	gene: Zm00001d005910	Zm00001d005910_T00	4.54	4.54	coding	327	83	1063
PB.1134.1	1	402	1	genic	gene: Zm00001d032662	novel	0.00	0.00	non coding	NA	NA	NA
PB.1140.4	1	2792	21	novel_not_in_catalog	gene: Zm00001d032685	novel	0.00	57.68	coding	534	975	2576
PB.8618.1	7	2529	3	full-splice_match	gene: Zm00001d020858	Zm00001d020858_T00	41.98	41.98	coding	577	460	2190
PB.9185.1	8	1529	3	novel_in_catalog	gene: Zm00001d008456	novel	16.99	25.15	coding	62	124	309
PB.9185.2	8	2208	2	novel_in_catalog	gene: Zm00001d008456	novel	8.17	25.15	coding	116	1609	1956
PB.1332.3	1	2462	10	fusion	gene: Zm00001d033532	novel	0.00	28.67	coding	722	168	2333
PB.3487.2	2	697	6	full-splice_match	gene: Zm00001d006942	Zm00001d006942_T00	51.12	57.82	coding	130	91	480

PB.10584.1	9	907	3	novel_not_in_catalog	gene: Zm00001d047282	novel	792.50	792.50	coding	99	300	596
PB.3395.1	2	1713	1	full-splice_match	gene: Zm00001d006574	Zm00001d006574_T00	20.60	25.66	coding	375	169	1293
PB.6247.1	5	1417	6	full-splice_match	gene: Zm00001d013935	Zm00001d013935_T00	22.47	61.56	coding	334	128	1129
PB.1567.2	1	1819	1	genic	gene: Zm00001d034569	novel	9.57	51.59	coding	309	722	1648
PB.1567.3	1	1703	1	novel_in_catalog	gene: Zm00001d034569	novel	0.00	51.59	coding	309	724	1650
PB.896.3	1	2866	1	novel_in_catalog	gene: Zm00001d031635	novel	3.43	217.19	coding	192	1977	2552
PB.4886.1	4	1903	10	full-splice_match	gene: Zm00001d048856	Zm00001d048856_T00	6.01	13.60	coding	512	93	1628
PB.2607.2	2	1202	3	full-splice_match	gene: Zm00001d002548	Zm00001d002548_T00	40.39	49.59	coding	258	139	912
PB.4197.1	3	1424	1	full-splice_match	gene: Zm00001d042067	Zm00001d042067_T00	4.04	4.04	coding	401	9	1211
PB.775.1	1	1106	6	novel_in_catalog	gene: Zm00001d031026	novel	4.03	4.03	coding	273	138	956
PB.896.6	1	1399	3	full-splice_match	gene: Zm00001d031635	Zm00001d031635_T00	0.00	217.19	coding	337	172	1182
PB.3162.2	2	1492	9	full-splice_match	gene: Zm00001d005542	Zm00001d005542_T00	30.60	110.43	coding	390	119	1288
PB.8825.5	7	2335	6	novel_in_catalog	gene: Zm00001d021696	novel	0.00	26.16	coding	320	700	1659
PB.896.8	1	997	1	novel_in_catalog	gene: Zm00001d031635	novel	14.02	217.19	coding	192	349	924
PB.6631.2	5	1525	1	full-splice_match	gene: Zm00001d015651	Zm00001d015651_T00	0.00	13.39	coding	459	63	1439
PB.6631.1	5	1742	1	full-splice_match	gene: Zm00001d015651	Zm00001d015651_T00	13.39	13.39	coding	490	48	1517
PB.2999.7	2	3472	19	full-splice_match	gene: Zm00001d004564	Zm00001d004564_T01	0.00	65.73	coding	565	1489	3183

PB.3098.5	2	1303	9	full-splice_match	gene: Zm00001d005159	Zm00001d005159_T00	9.10	47.24	coding	318	105	1058
PB.5028.1	4	786	3	full-splice_match	gene: Zm00001d049507	Zm00001d049507_T00	16.57	367.45	coding	110	50	379
PB.3355.1	2	1905	15	full-splice_match	gene: Zm00001d006377	Zm00001d006377_T00	26.44	26.44	coding	549	22	1668
PB.4227.1	3	1283	6	full-splice_match	gene: Zm00001d042284	Zm00001d042284_T00	51.49	51.49	coding	225	265	939
PB.4555.2	3	1558	7	novel_not_in_catalog	gene: Zm00001d043600	novel	0.00	35.42	coding	414	119	1360
PB.8646.2	7	2287	4	full-splice_match	gene: Zm00001d020963	Zm00001d020963_T00	109.55	109.55	coding	578	97	1830
PB.1963.1	10	1310	1	full-splice_match	gene: Zm00001d024750	Zm00001d024750_T00	284.36	284.36	coding	237	393	1103
PB.7602.3	6	2802	1	intergenic	novelGene_209	novel	49.39	145.86	non coding	NA	NA	NA
PB.4699.3	3	802	7	novel_not_in_catalog	gene: Zm00001d044139	novel	3.33	50.73	non coding	NA	NA	NA
PB.4402.1	3	827	5	full-splice_match	gene: Zm00001d043070	Zm00001d043070_T00	405.45	553.22	coding	148	107	550
PB.848.3	1	813	1	incomplete-splice_match	gene: Zm00001d031329	Zm00001d031329_T00	0.00	39.51	coding	91	252	524
PB.3996.2	3	3405	25	full-splice_match	gene: Zm00001d040748	Zm00001d040748_T07	42.10	93.71	coding	906	202	2919
PB.3996.3	3	1878	6	incomplete-splice_match	gene: Zm00001d040748	Zm00001d040748_T03	6.12	93.71	coding	248	648	1391
PB.3996.1	3	1755	7	incomplete-splice_match	gene: Zm00001d040748	Zm00001d040748_T04	45.49	93.71	coding	350	99	1148
PB.6776.1	5	1143	2	full-splice_match	gene: Zm00001d016566	Zm00001d016566_T00	49.44	49.44	coding	296	45	932
PB.3996.4	3	1321	6	incomplete-splice_match	gene: Zm00001d040748	Zm00001d040748_T08	0.00	93.71	coding	350	14	1063
PB.5776.1	4	1180	1	full-splice_match	gene: Zm00001d053429	Zm00001d053429_T00	27.57	103.58	coding	188	292	855

PB.3113.1	2	2521	17	fusion	gene: Zm00001d005256	novel	39.87	45.21	coding	695	203	2287
PB.1642.4	1	2198	14	full-splice_match	gene: Zm00001d034809	Zm00001d034809_T02	1.30	87.36	coding	321	143	1105
PB.2345.3	10	857	1	novel_in_catalog	gene: Zm00001d026511	novel	10.03	69.44	non coding	NA	NA	NA
PB.6692.4	5	512	1	full-splice_match	gene: Zm00001d016083	Zm00001d016083_T00	0.00	26.14	coding	82	59	304
PB.2617.1	2	4116	1	genic	gene: Zm00001d002599	novel	9.86	9.86	coding	435	2240	3544
PB.5206.3	4	447	3	genic_intron	novelGene_207	novel	93.60	130.51	coding	43	319	447
PB.7056.4	5	1209	7	novel_not_in_catalog	gene: Zm00001d017756	novel	13.41	54.96	coding	291	125	997
PB.8007.1	6	1131	1	full-splice_match	gene: Zm00001d038466	Zm00001d038466_T00	1.34	1.34	coding	231	176	868
PB.1356.1	1	1210	1	full-splice_match	gene: Zm00001d033637	Zm00001d033637_T00	64.98	64.98	coding	278	203	1036
PB.3575.1	2	2287	3	full-splice_match	gene: Zm00001d007465	Zm00001d007465_T00	3.53	3.53	coding	219	208	864
PB.10966.13	B73V4	1599	6	full-splice_match	gene: Zm00001d000021	Zm00001d000021_T00	0.00	461.70	coding	387	293	1453
PB.4441.2	3	1423	5	full-splice_match	gene: Zm00001d043196	Zm00001d043196_T00	0.00	30.37	coding	377	113	1243
PB.9239.1	8	1001	1	full-splice_match	gene: Zm00001d008722	Zm00001d008722_T00	7.70	7.70	coding	121	326	688
PB.8975.1	7	1861	12	full-splice_match	gene: Zm00001d022199	Zm00001d022199_T00	24.26	24.26	coding	478	91	1524
PB.4491.1	3	1700	8	novel_not_in_catalog	gene: Zm00001d043387	novel	19.51	29.94	coding	441	53	1375
PB.5923.3	4	1839	5	novel_in_catalog	gene: Zm00001d054014	novel	2.13	43.81	coding	164	1187	1678
PB.10664.2	9	2128	12	novel_not_in_catalog	gene: Zm00001d047592	novel	12.36	19.66	coding	179	746	1282

PB.5896.7	4	941	4	intergenic	novelGene_446	novel	61.38	108.08	coding	74	289	510
PB.10664.4	9	2096	9	novel_in_catalog	gene: Zm00001d047592	novel	7.31	19.66	non coding	NA	NA	NA
PB.3229.2	2	2019	8	full-splice_match	gene: Zm00001d005866	Zm00001d005866_T00	57.20	57.20	coding	337	105	1115
PB.5708.1	4	2619	11	full-splice_match	gene: Zm00001d053080	Zm00001d053080_T00	17.05	17.05	coding	739	124	2340
PB.4782.1	3	638	1	intergenic	novelGene_432	novel	671.07	1136.01	coding	20	316	375
PB.10032.2	8	798	5	full-splice_match	gene: Zm00001d012513	Zm00001d012513_T00	275.63	363.47	coding	148	108	551
PB.1484.5	1	1085	1	novel_in_catalog	gene: Zm00001d034205	novel	14.72	174.54	coding	185	467	1021
PB.1404.2	1	1836	10	novel_not_in_catalog	gene: Zm00001d033858	novel	9.64	19.73	coding	385	390	1544
PB.2991.1	2	2022	15	full-splice_match	gene: Zm00001d004525	Zm00001d004525_T00	38.77	92.88	coding	534	163	1764
PB.1484.1	1	2838	5	full-splice_match	gene: Zm00001d034205	Zm00001d034205_T00	21.53	174.54	coding	709	94	2220
PB.1484.2	1	1940	5	full-splice_match	gene: Zm00001d034205	Zm00001d034205_T00	53.98	174.54	coding	470	168	1577
PB.1484.3	1	2733	4	novel_in_catalog	gene: Zm00001d034205	novel	1.35	174.54	coding	455	225	1589
PB.5256.14	4	2282	6	full-splice_match	gene: Zm00001d050970	Zm00001d050970_T01	4.24	60.57	coding	123	90	458
PB.3973.2	3	636	3	intergenic	novelGene_144	novel	6.00	11.05	coding	23	293	361
PB.9225.1	8	1319	1	full-splice_match	gene: Zm00001d008634	Zm00001d008634_T00	37.55	37.55	coding	331	125	1117
PB.4245.1	3	1977	2	novel_in_catalog	gene: Zm00001d042344	novel	107.45	107.45	coding	222	171	836
PB.3522.2	2	1755	10	novel_in_catalog	gene: Zm00001d007095	novel	3.84	25.79	coding	258	107	880

PB.3644.1	2	1441	1	novel_in_catalog	gene: Zm00001d007862	novel	8.60	8.60	coding	163	782	1270
PB.6523.1	5	1516	1	novel_in_catalog	gene: Zm00001d015030	novel	6.16	6.16	coding	317	490	1440
PB.2991.4	2	636	6	incomplete-splice_match	gene: Zm00001d004525	Zm00001d004525_T00	45.90	92.88	coding	156	70	537
PB.5728.3	4	1087	9	novel_not_in_catalog	gene: Zm00001d053223	novel	47.72	131.22	coding	289	113	979
PB.5728.2	4	1309	9	novel_not_in_catalog	gene: Zm00001d053223	novel	40.24	131.22	coding	289	141	1007
PB.2325.1	10	1294	3	full-splice_match	gene: Zm00001d026431	Zm00001d026431_T00	16.13	28.84	coding	320	34	993
PB.3906.1	3	602	2	novel_not_in_catalog	gene: Zm00001d040281	novel	23.61	468.21	non coding	NA	NA	NA
PB.2325.2	10	1140	3	full-splice_match	gene: Zm00001d026431	Zm00001d026431_T00	12.70	28.84	coding	320	34	993
PB.987.1	1	2124	3	full-splice_match	gene: Zm00001d032072	Zm00001d032072_T00	1.06	1.06	coding	247	1281	2021
PB.6752.1	5	1666	6	novel_in_catalog	gene: Zm00001d016441	novel	217.52	217.52	coding	266	140	937
PB.3899.1	3	1018	4	full-splice_match	gene: Zm00001d040244	Zm00001d040244_T00	112.69	117.26	coding	231	171	863
PB.3899.2	3	1054	3	novel_in_catalog	gene: Zm00001d040244	novel	4.57	117.26	coding	140	479	898
PB.8233.3	7	1922	6	novel_not_in_catalog	gene: Zm00001d018660	novel	5.34	6.76	coding	560	100	1779
PB.8233.2	7	1971	5	novel_not_in_catalog	gene: Zm00001d018660	novel	0.00	6.76	coding	483	40	1488
PB.8233.1	7	2065	4	novel_not_in_catalog	gene: Zm00001d018660	novel	1.43	6.76	coding	371	6	1118
PB.9872.2	8	2212	6	full-splice_match	gene: Zm00001d011831	Zm00001d011831_T00	30.37	34.51	coding	582	215	1960
PB.6362.2	5	805	5	full-splice_match	gene: Zm00001d014428	Zm00001d014428_T00	207.28	269.75	coding	159	116	592

PB.9753.2	8	1490	2	genic	gene: Zm00001d011331	novel	50.10	73.83	coding	252	482	1237
PB.9753.1	8	1921	1	full-splice_match	gene: Zm00001d011331	Zm00001d011331_T00	23.73	73.83	coding	252	911	1666
PB.3906.2	3	474	2	novel_not_in_catalog	gene: Zm00001d040281	novel	440.25	468.21	non coding	NA	NA	NA
PB.1402.5	1	1668	4	full-splice_match	gene: Zm00001d033850	Zm00001d033850_T01	602.50	968.13	coding	451	110	1462
PB.6883.2	5	1193	3	novel_in_catalog	gene: Zm00001d017047	novel	2.30	274.64	coding	171	66	578
PB.5996.1	5	2203	4	full-splice_match	gene: Zm00001d012953	Zm00001d012953_T00	27.63	27.63	coding	579	208	1944
PB.1456.7	1	1897	6	incomplete-splice_match	gene: Zm00001d034049	Zm00001d034049_T00	0.00	311.75	coding	448	296	1639
PB.6336.1	5	787	2	full-splice_match	gene: Zm00001d014317	Zm00001d014317_T00	107.38	118.25	non coding	NA	NA	NA
PB.6831.1	5	731	3	full-splice_match	gene: Zm00001d016801	Zm00001d016801_T00	21.11	21.11	non coding	NA	NA	NA
PB.8660.1	7	1759	2	incomplete-splice_match	gene: Zm00001d021023	Zm00001d021023_T00	11.27	11.27	coding	523	63	1631
PB.6336.2	5	968	1	genic	gene: Zm00001d014317	novel	10.87	118.25	coding	104	172	483
PB.9072.1	7	1034	8	incomplete-splice_match	gene: Zm00001d022502	Zm00001d022502_T01	66.63	66.63	coding	172	229	744
PB.10243.1	9	2269	6	novel_not_in_catalog	gene: Zm00001d045375	novel	8.90	8.90	coding	530	432	2021
PB.10243.2	9	2100	6	novel_not_in_catalog	gene: Zm00001d045375	novel	0.00	8.90	coding	530	428	2017
PB.4621.2	3	1366	2	full-splice_match	gene: Zm00001d043837	Zm00001d043837_T00	29.88	89.25	coding	320	188	1147
PB.4385.1	3	1642	8	full-splice_match	gene: Zm00001d042965	Zm00001d042965_T00	52.76	52.76	coding	365	184	1278
PB.4621.1	3	1282	2	full-splice_match	gene: Zm00001d043837	Zm00001d043837_T00	59.37	89.25	coding	201	354	956

PB.6259.1	5	3397	11	full-splice_match	gene: Zm00001d013984	Zm00001d013984_T02	2.50	25.34	coding	619	231	2087
PB.5413.1	4	2452	11	full-splice_match	gene: Zm00001d051856	Zm00001d051856_T01	41.70	103.88	coding	653	145	2103
PB.6259.3	5	2534	11	full-splice_match	gene: Zm00001d013984	Zm00001d013984_T01	3.88	25.34	coding	565	155	1849
PB.6259.2	5	2754	12	full-splice_match	gene: Zm00001d013984	Zm00001d013984_T01	18.96	25.34	coding	699	215	2311
PB.6085.1	5	1706	2	full-splice_match	gene: Zm00001d013261	Zm00001d013261_T00	57.45	57.45	coding	341	123	1145
PB.9684.2	8	1445	2	full-splice_match	gene: Zm00001d010982	Zm00001d010982_T00	41.39	88.95	coding	294	161	1042
PB.1744.2	10	731	3	incomplete-splice_match	gene: Zm00001d023465	Zm00001d023465_T00	0.00	10.11	coding	96	17	304
PB.1744.1	10	1308	9	incomplete-splice_match	gene: Zm00001d023465	Zm00001d023465_T00	10.11	10.11	coding	239	41	757
PB.9684.1	8	1630	2	full-splice_match	gene: Zm00001d010982	Zm00001d010982_T00	47.56	88.95	coding	294	254	1135
PB.7042.4	5	2438	7	full-splice_match	gene: Zm00001d017685	Zm00001d017685_T00	5.35	18.31	coding	121	187	549
PB.7042.5	5	1238	8	full-splice_match	gene: Zm00001d017685	Zm00001d017685_T00	0.00	18.31	coding	121	187	549
PB.7042.6	5	1204	6	full-splice_match	gene: Zm00001d017685	Zm00001d017685_T00	0.00	18.31	coding	121	229	591
PB.7042.1	5	2829	7	full-splice_match	gene: Zm00001d017685	Zm00001d017685_T00	2.13	18.31	coding	121	145	507
PB.3885.1	3	993	3	full-splice_match	gene: Zm00001d040178	Zm00001d040178_T00	230.28	230.28	coding	201	166	768
PB.7042.3	5	1056	9	novel_in_catalog	gene: Zm00001d017685	novel	2.75	18.31	coding	121	142	504
PB.1980.3	10	1001	8	full-splice_match	gene: Zm00001d024815	Zm00001d024815_T00	0.00	91.35	coding	298	106	999
PB.1980.2	10	1491	11	full-splice_match	gene: Zm00001d024815	Zm00001d024815_T00	81.62	91.35	coding	382	91	1236

PB.1980.4	10	944	7	full-splice_match	gene: Zm00001d024815	Zm00001d024815_T00	9.73	91.35	coding	276	116	943
PB.8825.3	7	2370	7	full-splice_match	gene: Zm00001d021696	Zm00001d021696_T00	0.00	26.16	coding	482	712	2157
PB.830.2	1	982	1	genic	gene: Zm00001d031244	novel	1.13	8.32	non coding	NA	NA	NA
PB.3014.1	2	2049	16	incomplete-splice_match	gene: Zm00001d004667	Zm00001d004667_T00	44.65	82.34	coding	525	51	1625
PB.10546.6	9	931	5	novel_in_catalog	gene: Zm00001d047059	novel	2.54	29.99	coding	142	321	746
PB.4602.1	3	1534	5	full-splice_match	gene: Zm00001d043764	Zm00001d043764_T00	6.93	9.71	coding	310	56	985
PB.3014.2	2	3670	23	full-splice_match	gene: Zm00001d004667	Zm00001d004667_T00	37.69	82.34	coding	1086	100	3357
PB.830.1	1	1225	1	genic	gene: Zm00001d031244	novel	7.19	8.32	non coding	NA	NA	NA
PB.2710.3	2	1110	3	full-splice_match	gene: Zm00001d003006	Zm00001d003006_T00	951.46	951.46	coding	285	74	928
PB.7258.2	5	1855	14	full-splice_match	gene: Zm00001d018487	Zm00001d018487_T00	183.61	205.91	coding	461	270	1652
PB.8888.3	7	1849	8	full-splice_match	gene: Zm00001d021861	Zm00001d021861_T00	18.16	44.03	coding	414	184	1425
PB.8888.2	7	1794	9	full-splice_match	gene: Zm00001d021861	Zm00001d021861_T00	16.07	44.03	coding	414	256	1497
PB.8888.1	7	1783	7	incomplete-splice_match	gene: Zm00001d021861	Zm00001d021861_T00	9.80	44.03	coding	349	101	1147
PB.7869.2	6	913	2	full-splice_match	gene: Zm00001d038003	Zm00001d038003_T00	150.59	228.40	coding	158	157	630
PB.9102.1	7	2211	8	intergenic	novelGene_372	novel	22.77	82.50	coding	134	1255	1656
PB.10898.4	9	1443	6	full-splice_match	gene: Zm00001d048471	Zm00001d048471_T00	15.44	84.97	coding	318	223	1176
PB.8524.1	7	1380	4	full-splice_match	gene: Zm00001d020392	Zm00001d020392_T00	6.27	6.27	coding	342	89	1114

PB.2370.2	10	1861	4	novel_in_catalog	gene: Zm00001d026577	novel	2.05	70.13	coding	494	106	1587
PB.8223.1	7	1400	2	genic	gene: Zm00001d018629	novel	20.29	20.29	coding	210	676	1305
PB.535.4	1	1062	2	full-splice_match	gene: Zm00001d029590	Zm00001d029590_T00	113.74	194.15	coding	271	104	916
PB.535.3	1	1170	2	full-splice_match	gene: Zm00001d029590	Zm00001d029590_T00	0.00	194.15	coding	271	71	883
PB.535.2	1	1177	2	full-splice_match	gene: Zm00001d029590	Zm00001d029590_T00	34.06	194.15	coding	219	81	737
PB.535.1	1	1430	2	full-splice_match	gene: Zm00001d029590	Zm00001d029590_T00	46.34	194.15	coding	271	85	897
PB.6715.1	5	1416	4	full-splice_match	gene: Zm00001d016203	Zm00001d016203_T00	47.86	47.86	coding	174	533	1054
PB.10898.2	9	1550	6	full-splice_match	gene: Zm00001d048471	Zm00001d048471_T00	61.92	84.97	coding	318	135	1088
PB.2700.2	2	1660	8	full-splice_match	gene: Zm00001d002944	Zm00001d002944_T00	58.80	95.28	coding	481	68	1510
PB.3433.4	2	2026	2	genic	gene: Zm00001d006725	novel	0.00	251.48	coding	437	155	1465
PB.2700.1	2	1913	8	full-splice_match	gene: Zm00001d002944	Zm00001d002944_T00	36.48	95.28	coding	481	135	1577
PB.5306.1	4	974	3	incomplete-splice_match	gene: Zm00001d051321	Zm00001d051321_T01	68.64	68.64	coding	122	42	407
PB.8616.1	7	2381	7	full-splice_match	gene: Zm00001d020852	Zm00001d020852_T00	7.18	7.18	coding	727	72	2252
PB.1600.1	1	1855	1	genic	gene: Zm00001d034669	novel	2.70	27.63	coding	400	3	1202
PB.1292.2	1	1532	6	full-splice_match	gene: Zm00001d033366	Zm00001d033366_T00	0.00	66.06	coding	392	80	1255
PB.6.1	1	1982	2	full-splice_match	gene: Zm00001d027268	Zm00001d027268_T00	47.03	47.03	coding	525	195	1769
PB.4253.1	3	730	1	full-splice_match	gene: Zm00001d042383	Zm00001d042383_T00	18.98	18.98	coding	106	101	418

PB.7214.6	5	1557	6	full-splice_match	gene: Zm00001d018359	Zm00001d018359_T00	559.70	559.70	coding	413	156	1394
PB.2019.1	10	1685	2	novel_not_in_catalog	gene: Zm00001d024995	novel	12.48	12.48	coding	504	36	1547
PB.182.1	1	2194	12	full-splice_match	gene: Zm00001d028010	Zm00001d028010_T00	9.86	39.83	coding	589	26	1792
PB.1600.4	1	1285	1	genic	gene: Zm00001d034669	novel	6.63	27.63	coding	400	17	1216
PB.182.2	1	2069	12	full-splice_match	gene: Zm00001d028010	Zm00001d028010_T00	29.98	39.83	coding	589	30	1796
PB.529.4	1	2588	16	full-splice_match	gene: Zm00001d029561	Zm00001d029561_T00	0.00	60.49	coding	783	128	2476
PB.7294.1	6	1062	2	full-splice_match	gene: Zm00001d034984	Zm00001d034984_T00	4.15	4.15	coding	273	3	821
PB.4810.2	3	1409	6	incomplete-splice_match	gene: Zm00001d044594	Zm00001d044594_T00	25.21	31.65	coding	421	7	1269
PB.529.1	1	2752	16	full-splice_match	gene: Zm00001d029561	Zm00001d029561_T00	39.65	60.49	coding	783	144	2492
PB.529.2	1	3511	15	novel_in_catalog	gene: Zm00001d029561	novel	14.25	60.49	coding	891	65	2737
PB.4911.3	4	1341	9	novel_in_catalog	gene: Zm00001d048970	novel	16.45	21.57	coding	165	657	1151
PB.4754.2	3	1096	1	novel_in_catalog	gene: Zm00001d044376	novel	0.17	3.08	coding	111	127	459
PB.1849.1	10	3000	5	full-splice_match	gene: Zm00001d023998	Zm00001d023998_T00	13.65	13.65	coding	740	437	2656
PB.1849.2	10	2880	5	full-splice_match	gene: Zm00001d023998	Zm00001d023998_T00	0.00	13.65	coding	740	432	2651
PB.1188.1	1	1578	4	full-splice_match	gene: Zm00001d032873	Zm00001d032873_T00	260.06	341.11	coding	375	119	1243
PB.2835.1	2	1142	3	incomplete-splice_match	gene: Zm00001d003556	Zm00001d003556_T00	28.44	28.44	coding	286	87	944
PB.10724.1	9	938	1	genic	gene: Zm00001d047788	novel	138.38	657.40	coding	148	53	496

PB.2133.2	10	1878	10	full-splice_match	gene: Zm00001d025653	Zm00001d025653_T00	22.15	29.70	coding	481	128	1570
PB.991.1	1	1978	2	novel_in_catalog	gene: Zm00001d032081	novel	6.46	89.17	coding	109	229	555
PB.495.1	1	1664	1	full-splice_match	gene: Zm00001d029409	Zm00001d029409_T00	31.30	47.95	coding	396	114	1301
PB.495.2	1	1451	1	full-splice_match	gene: Zm00001d029409	Zm00001d029409_T00	16.65	47.95	coding	396	92	1279
PB.7934.5	6	1825	13	full-splice_match	gene: Zm00001d038200	Zm00001d038200_T00	71.27	122.54	coding	445	127	1461
PB.9805.1	8	1355	1	full-splice_match	gene: Zm00001d011571	Zm00001d011571_T00	167.50	167.50	coding	315	130	1074
PB.2233.1	10	2678	4	full-splice_match	gene: Zm00001d026113	Zm00001d026113_T00	15.98	15.98	coding	598	341	2134
PB.7835.4	6	1492	9	full-splice_match	gene: Zm00001d037871	Zm00001d037871_T00	0.00	54.28	coding	456	85	1452
PB.4461.5	3	2463	6	full-splice_match	gene: Zm00001d043275	Zm00001d043275_T01	7.02	78.92	coding	345	1131	2165
PB.991.9	1	966	3	full-splice_match	gene: Zm00001d032081	Zm00001d032081_T00	0.00	89.17	coding	131	227	619
PB.2414.2	10	2234	1	incomplete-splice_match	gene: Zm00001d026706	Zm00001d026706_T00	14.63	277.23	coding	653	113	2071
PB.104.1	1	873	6	full-splice_match	gene: Zm00001d027675	Zm00001d027675_T00	84.19	84.19	coding	183	158	706
PB.3026.1	2	2712	4	novel_not_in_catalog	gene: Zm00001d004733	novel	19.34	19.34	non coding	NA	NA	NA
PB.4461.6	3	1862	8	full-splice_match	gene: Zm00001d043275	Zm00001d043275_T00	48.47	78.92	coding	436	263	1570
PB.5812.2	4	1464	8	novel_in_catalog	gene: Zm00001d053628	novel	3.43	22.84	coding	218	253	906
PB.10063.1	8	1558	10	fusion	gene: Zm00001d012628	novel	68.00	68.00	coding	376	210	1337
PB.10469.2	9	980	4	incomplete-splice_match	gene: Zm00001d046630	Zm00001d046630_T00	31.48	59.89	coding	181	117	659

PB.7846.1	6	1942	6	full-splice_match	gene: Zm00001d037920	Zm00001d037920_T00	6.24	6.24	coding	460	353	1732
PB.2815.2	2	2327	5	full-splice_match	gene: Zm00001d003477	Zm00001d003477_T00	0.00	9.94	coding	688	8	2071
PB.2815.1	2	2711	5	full-splice_match	gene: Zm00001d003477	Zm00001d003477_T00	9.94	9.94	coding	688	217	2280
PB.1834.1	10	2961	4	novel_in_catalog	gene: Zm00001d023934	novel	5.87	41.46	coding	134	2277	2678
PB.1834.3	10	910	5	full-splice_match	gene: Zm00001d023934	Zm00001d023934_T00	9.69	41.46	coding	228	46	729
PB.1834.2	10	1068	5	full-splice_match	gene: Zm00001d023934	Zm00001d023934_T00	25.90	41.46	coding	228	175	858
PB.5815.6	4	1453	7	novel_in_catalog	gene: Zm00001d053634	novel	19.89	47.31	coding	305	357	1271
PB.4461.1	3	2444	7	full-splice_match	gene: Zm00001d043275	Zm00001d043275_T01	18.56	78.92	coding	382	901	2046
PB.489.1	1	1272	7	novel_not_in_catalog	gene: Zm00001d029383	novel	12.97	12.97	coding	328	123	1106
PB.10469.1	9	2764	6	full-splice_match	gene: Zm00001d046630	Zm00001d046630_T01	17.40	59.89	coding	325	447	1421
PB.9318.1	8	2198	8	novel_not_in_catalog	gene: Zm00001d009156	novel	9.48	9.48	coding	611	81	1913
PB.10157.1	9	802	1	full-splice_match	gene: Zm00001d045000	Zm00001d045000_T00	53.81	759.36	coding	142	60	485
PB.10157.2	9	722	1	full-splice_match	gene: Zm00001d045000	Zm00001d045000_T00	705.55	759.36	coding	142	101	526
PB.704.2	1	1563	5	full-splice_match	gene: Zm00001d030601	Zm00001d030601_T00	0.61	33.32	coding	356	239	1306
PB.5712.2	4	1538	7	full-splice_match	gene: Zm00001d053107	Zm00001d053107_T00	0.00	66.67	coding	430	100	1389
PB.5712.1	4	1678	7	full-splice_match	gene: Zm00001d053107	Zm00001d053107_T00	66.67	66.67	coding	430	110	1399
PB.9800.1	8	1590	2	genic	gene: Zm00001d011559	novel	3.15	3.15	coding	157	13	483

PB.3450.1	2	797	1	full-splice_match	gene: Zm00001d006815	Zm00001d006815_T00	17.69	17.69	coding	71	151	363
PB.6840.5	5	2851	14	novel_in_catalog	gene: Zm00001d016838	novel	1.39	81.40	coding	392	1363	2538
PB.4265.1	3	1457	5	full-splice_match	gene: Zm00001d042443	Zm00001d042443_T00	22.31	364.97	coding	344	79	1110
PB.7317.1	6	2067	10	full-splice_match	gene: Zm00001d035086	Zm00001d035086_T00	84.66	84.66	coding	328	612	1595
PB.6019.2	5	1048	1	full-splice_match	gene: Zm00001d013028	Zm00001d013028_T00	0.00	42.99	coding	256	128	895
PB.6019.1	5	1093	1	full-splice_match	gene: Zm00001d013028	Zm00001d013028_T00	42.99	42.99	coding	256	123	890
PB.6834.4	5	2744	8	novel_in_catalog	gene: Zm00001d016825	novel	5.80	48.81	coding	183	174	722
PB.10590.2	9	695	3	full-splice_match	gene: Zm00001d047310	Zm00001d047310_T00	0.00	51.99	non coding	NA	NA	NA
PB.10590.1	9	1204	3	full-splice_match	gene: Zm00001d047310	Zm00001d047310_T00	51.99	51.99	coding	177	422	952
PB.3245.2	2	881	2	novel_in_catalog	gene: Zm00001d005952	novel	22.72	51.42	non coding	NA	NA	NA
PB.5881.1	4	1368	7	full-splice_match	gene: Zm00001d053899	Zm00001d053899_T00	108.48	115.84	coding	197	391	981
PB.9674.2	8	1685	1	full-splice_match	gene: Zm00001d010956	Zm00001d010956_T00	31.61	132.74	coding	513	7	1545
PB.9674.1	8	1772	1	full-splice_match	gene: Zm00001d010956	Zm00001d010956_T00	101.14	132.74	coding	372	379	1494
PB.31.1	1	1290	2	incomplete-splice_match	gene: Zm00001d027349	Zm00001d027349_T00	5.34	5.34	coding	123	13	381
PB.3440.1	2	698	1	incomplete-splice_match	gene: Zm00001d006755	Zm00001d006755_T00	14.40	14.40	coding	181	54	596
PB.10231.1	9	1580	3	full-splice_match	gene: Zm00001d045323	Zm00001d045323_T00	32.49	2102.57	coding	375	131	1255
PB.7142.2	5	962	3	novel_not_in_catalog	gene: Zm00001d018072	novel	111.69	440.71	coding	119	169	525

PB.7142.5	5	935	3	novel_not_in_catalog	gene: Zm00001d018072	novel	5.05	440.71	coding	32	442	537
PB.7142.7	5	867	3	novel_not_in_catalog	gene: Zm00001d018072	novel	313.30	440.71	coding	119	194	550
PB.2829.1	2	1104	3	full-splice_match	gene: Zm00001d003524	Zm00001d003524_T00	10.63	10.63	coding	329	19	1005
PB.2133.1	10	2481	8	novel_in_catalog	gene: Zm00001d025653	novel	0.00	29.70	coding	220	84	743
PB.5629.2	4	1596	1	intergenic	novelGene_392	novel	5.94	14.81	coding	320	196	1155
PB.1235.2	1	1730	13	full-splice_match	gene: Zm00001d033090	Zm00001d033090_T00	41.88	76.96	coding	313	322	1260
PB.2809.1	2	2671	2	genic	gene: Zm00001d003438	novel	89.07	89.07	coding	625	560	2434
PB.10466.2	9	2652	1	full-splice_match	gene: Zm00001d046599	Zm00001d046599_T00	21.00	459.37	coding	204	149	760
PB.5185.1	4	1667	12	full-splice_match	gene: Zm00001d050380	Zm00001d050380_T00	29.66	44.17	coding	389	184	1350
PB.5185.2	4	1709	11	full-splice_match	gene: Zm00001d050380	Zm00001d050380_T00	0.49	44.17	coding	389	144	1310
PB.5185.3	4	1565	11	full-splice_match	gene: Zm00001d050380	Zm00001d050380_T00	14.02	44.17	coding	389	114	1280
PB.7898.1	6	795	4	full-splice_match	gene: Zm00001d038088	Zm00001d038088_T00	58.37	80.49	non coding	NA	NA	NA
PB.7652.1	6	1825	1	full-splice_match	gene: Zm00001d037017	Zm00001d037017_T00	7.09	7.09	coding	311	758	1690
PB.7898.2	6	642	4	full-splice_match	gene: Zm00001d038088	Zm00001d038088_T00	22.12	80.49	non coding	NA	NA	NA
PB.5910.1	4	1064	7	full-splice_match	gene: Zm00001d053983	Zm00001d053983_T00	221.21	221.21	coding	243	150	878
PB.8691.3	7	1610	5	novel_not_in_catalog	gene: Zm00001d021202	novel	2.44	25.59	coding	99	1148	1444
PB.2778.1	2	1104	2	incomplete-splice_match	gene: Zm00001d003276	Zm00001d003276_T00	6.24	6.24	coding	278	30	863

PB.1302.3	1	2530	4	full-splice_match	gene: Zm00001d033388	Zm00001d033388_T00	33.66	60.74	coding	720	92	2251
PB.1235.3	1	1693	12	novel_in_catalog	gene: Zm00001d033090	novel	5.35	76.96	coding	228	139	822
PB.3375.1	2	2210	6	full-splice_match	gene: Zm00001d006457	Zm00001d006457_T00	34.06	34.06	coding	610	123	1952
PB.257.1	1	1118	5	full-splice_match	gene: Zm00001d028352	Zm00001d028352_T00	20.67	20.67	coding	245	121	855
PB.8879.2	7	1207	3	full-splice_match	gene: Zm00001d021834	Zm00001d021834_T00	33.20	35.56	coding	254	213	974
PB.2437.1	2	1090	9	full-splice_match	gene: Zm00001d001819	Zm00001d001819_T00	14.11	14.11	coding	263	154	942
PB.2952.2	2	2211	2	full-splice_match	gene: Zm00001d004190	Zm00001d004190_T00	5.55	5.55	coding	351	44	1096
PB.7917.6	6	940	4	full-splice_match	gene: Zm00001d038147	Zm00001d038147_T00	0.00	923.71	coding	264	17	808
PB.6487.8	5	3365	9	full-splice_match	gene: Zm00001d014911	Zm00001d014911_T01	0.29	16.56	coding	803	933	3341
PB.9956.1	8	2296	6	novel_not_in_catalog	gene: Zm00001d012167	novel	145.66	214.85	coding	560	331	2010
PB.6487.6	5	2835	9	full-splice_match	gene: Zm00001d014911	Zm00001d014911_T01	3.73	16.56	coding	803	364	2772
PB.6487.7	5	2701	10	full-splice_match	gene: Zm00001d014911	Zm00001d014911_T01	2.86	16.56	coding	803	265	2673
PB.6487.4	5	3029	10	full-splice_match	gene: Zm00001d014911	Zm00001d014911_T01	7.35	16.56	coding	803	303	2711
PB.9654.2	8	1960	4	novel_not_in_catalog	gene: Zm00001d010851	novel	10.48	15.14	coding	422	176	1441
PB.4158.11	3	2651	8	novel_in_catalog	gene: Zm00001d041945	novel	6.78	133.59	coding	84	194	445
PB.6487.3	5	3937	8	full-splice_match	gene: Zm00001d014911	Zm00001d014911_T01	2.34	16.56	coding	803	1033	3441
PB.7373.1	6	679	2	intergenic	novelGene_95	novel	32.26	32.26	non coding	NA	NA	NA

PB.8701.5	7	1950	3	novel_in_catalog	gene: Zm00001d021221	novel	17.77	356.28	coding	404	438	1649
PB.2064.2	10	2036	4	novel_not_in_catalog	gene: Zm00001d025208	novel	6.46	7.82	coding	583	225	1973
PB.2064.1	10	3171	3	full-splice_match	gene: Zm00001d025208	Zm00001d025208_T00	1.36	7.82	coding	583	1319	3067
PB.3571.3	2	1947	4	full-splice_match	gene: Zm00001d007446	Zm00001d007446_T00	4.98	444.63	coding	230	136	825
PB.8701.1	7	2472	4	full-splice_match	gene: Zm00001d021221	Zm00001d021221_T00	2.08	356.28	coding	334	445	1446
PB.2495.5	2	3105	1	novel_in_catalog	gene: Zm00001d002052	novel	6.10	340.24	coding	182	2336	2881
PB.8701.3	7	1959	4	full-splice_match	gene: Zm00001d021221	Zm00001d021221_T00	15.70	356.28	coding	334	441	1442
PB.8701.2	7	2265	4	full-splice_match	gene: Zm00001d021221	Zm00001d021221_T00	10.42	356.28	coding	334	445	1446
PB.9737.1	8	3080	5	incomplete-splice_match	gene: Zm00001d011231	Zm00001d011231_T00	41.86	74.07	coding	679	906	2942
PB.2518.2	2	2001	7	novel_not_in_catalog	gene: Zm00001d002140	novel	9.65	15.79	coding	526	234	1811
PB.2518.1	2	2137	7	novel_not_in_catalog	gene: Zm00001d002140	novel	6.14	15.79	coding	526	162	1739
PB.9737.2	8	2001	4	novel_in_catalog	gene: Zm00001d011231	novel	32.20	74.07	coding	232	1002	1697
PB.5247.1	4	681	4	full-splice_match	gene: Zm00001d050921	Zm00001d050921_T00	235.98	235.98	coding	131	129	521
PB.5869.1	4	1231	3	full-splice_match	gene: Zm00001d053848	Zm00001d053848_T00	25.19	49.55	coding	291	37	909
PB.3046.1	2	991	1	full-splice_match	gene: Zm00001d004862	Zm00001d004862_T00	0.94	0.94	coding	261	65	847
PB.5356.1	4	1753	11	incomplete-splice_match	gene: Zm00001d051565	Zm00001d051565_T02	7.25	7.25	coding	445	182	1516
PB.4545.2	3	2027	14	full-splice_match	gene: Zm00001d043563	Zm00001d043563_T00	74.51	99.22	coding	564	193	1884

PB.5831.1	4	2278	1	full-splice_match	gene: Zm00001d053695	Zm00001d053695_T00	27.84	27.84	coding	680	54	2093
PB.10566.1	9	1139	5	full-splice_match	gene: Zm00001d047203	Zm00001d047203_T00	42.33	42.33	coding	274	35	856
PB.10921.2	9	1939	12	novel_not_in_catalog	gene: Zm00001d048529	novel	53.54	134.48	coding	399	162	1358
PB.724.3	1	1622	1	genic	gene: Zm00001d030760	novel	14.82	68.18	coding	361	405	1487
PB.8733.5	7	1941	7	full-splice_match	gene: Zm00001d021344	Zm00001d021344_T00	0.00	43.40	coding	532	225	1820
PB.8733.2	7	2076	6	novel_in_catalog	gene: Zm00001d021344	novel	2.80	43.40	coding	396	172	1359
PB.8733.3	7	2073	7	full-splice_match	gene: Zm00001d021344	Zm00001d021344_T00	40.60	43.40	coding	532	230	1825
PB.7607.1	6	1113	7	full-splice_match	gene: Zm00001d036748	Zm00001d036748_T00	129.53	129.53	coding	207	98	718
PB.2113.2	10	514	2	full-splice_match	gene: Zm00001d025509	Zm00001d025509_T00	41.38	89.17	coding	97	100	390
PB.724.1	1	2300	2	genic	gene: Zm00001d030760	novel	16.65	68.18	coding	361	346	1428
PB.5382.2	4	1687	10	full-splice_match	gene: Zm00001d051671	Zm00001d051671_T01	54.49	60.22	coding	450	180	1529
PB.1855.2	10	3316	8	novel_in_catalog	gene: Zm00001d024049	novel	0.93	16.43	coding	768	268	2571
PB.1855.3	10	2677	8	incomplete-splice_match	gene: Zm00001d024049	Zm00001d024049_T00	1.85	16.43	coding	776	237	2564
PB.1855.1	10	3232	9	full-splice_match	gene: Zm00001d024049	Zm00001d024049_T01	13.65	16.43	coding	903	264	2972
PB.9378.2	8	997	5	incomplete-splice_match	gene: Zm00001d009517	Zm00001d009517_T00	0.00	71.77	coding	251	22	774
PB.1085.7	1	1865	3	novel_not_in_catalog	gene: Zm00001d032485	novel	79.04	241.80	coding	143	203	631
PB.9378.1	8	1194	5	novel_in_catalog	gene: Zm00001d009517	novel	44.52	71.77	coding	251	113	865

PB.9106.1	7	910	1	full-splice_match	gene: Zm00001d022624	Zm00001d022624_T00	16.05	16.05	coding	111	309	641
PB.1085.3	1	735	4	novel_not_in_catalog	gene: Zm00001d032485	novel	84.65	241.80	non coding	NA	NA	NA
PB.1085.2	1	2695	2	novel_in_catalog	gene: Zm00001d032485	novel	8.64	241.80	coding	143	1006	1434
PB.1651.1	1	2085	5	full-splice_match	gene: Zm00001d034839	Zm00001d034839_T00	13.18	13.18	coding	545	214	1848
PB.1651.2	1	1946	5	full-splice_match	gene: Zm00001d034839	Zm00001d034839_T00	0.00	13.18	coding	545	213	1847
PB.233.2	1	1821	2	full-splice_match	gene: Zm00001d028241	Zm00001d028241_T00	0.00	33.39	coding	494	207	1688
PB.9158.1	8	2332	5	novel_in_catalog	gene: Zm00001d008354	novel	16.02	16.02	coding	288	195	1058
PB.9892.2	8	880	1	full-splice_match	gene: Zm00001d011902	Zm00001d011902_T00	0.00	40.75	coding	154	73	534
PB.10650.1	9	2075	1	full-splice_match	gene: Zm00001d047533	Zm00001d047533_T00	5.42	99.91	coding	580	1	1740
PB.3369.1	2	1601	3	incomplete-splice_match	gene: Zm00001d006430	Zm00001d006430_T00	4.98	14.50	coding	364	9	1100
PB.2427.4	2	1133	11	full-splice_match	gene: Zm00001d001789	Zm00001d001789_T00	87.60	87.60	coding	249	227	973
PB.1732.1	10	2528	9	novel_in_catalog	gene: Zm00001d023423	novel	1.23	275.94	coding	175	522	1046
PB.1732.2	10	1418	10	full-splice_match	gene: Zm00001d023423	Zm00001d023423_T00	55.36	275.94	coding	271	118	930
PB.1732.3	10	1372	9	novel_in_catalog	gene: Zm00001d023423	novel	5.00	275.94	coding	265	73	867
PB.1732.4	10	1470	9	novel_in_catalog	gene: Zm00001d023423	novel	14.91	275.94	coding	175	543	1067
PB.1732.5	10	1191	9	novel_in_catalog	gene: Zm00001d023423	novel	8.81	275.94	coding	135	384	788
PB.1732.6	10	1306	10	full-splice_match	gene: Zm00001d023423	Zm00001d023423_T00	190.64	275.94	coding	271	115	927

PB.9507.1	8	2243	13	novel_in_catalog	gene: Zm00001d010243	novel	8.40	66.11	coding	324	91	1062
PB.596.7	1	1321	4	full-splice_match	gene: Zm00001d029899	Zm00001d029899_T00	70.03	139.82	coding	282	110	955
PB.596.4	1	1851	3	novel_in_catalog	gene: Zm00001d029899	novel	5.73	139.82	coding	155	913	1377
PB.596.5	1	1776	1	novel_in_catalog	gene: Zm00001d029899	novel	3.36	139.82	non coding	NA	NA	NA
PB.596.2	1	1594	4	full-splice_match	gene: Zm00001d029899	Zm00001d029899_T00	1.01	139.82	coding	282	343	1188
PB.2012.9	10	3149	20	novel_not_in_catalog	gene: Zm00001d024952	novel	0.00	175.10	coding	905	89	2803
PB.596.1	1	1535	4	full-splice_match	gene: Zm00001d029899	Zm00001d029899_T00	30.90	139.82	coding	282	107	952
PB.2012.4	10	3055	19	novel_not_in_catalog	gene: Zm00001d024952	novel	4.35	175.10	coding	888	89	2752
PB.2012.5	10	3889	19	novel_not_in_catalog	gene: Zm00001d024952	novel	14.09	175.10	coding	882	82	2727
PB.4682.1	3	1345	8	full-splice_match	gene: Zm00001d044090	Zm00001d044090_T00	28.64	28.64	coding	269	183	989
PB.2012.7	10	3753	20	novel_not_in_catalog	gene: Zm00001d024952	novel	7.91	175.10	coding	882	86	2731
PB.10173.3	9	1644	7	novel_not_in_catalog	gene: Zm00001d045050	novel	0.37	27.59	coding	331	454	1446
PB.2012.2	10	2393	3	incomplete-splice_match	gene: Zm00001d024952	Zm00001d024952_T00	35.89	175.10	coding	258	80	853
PB.10173.1	9	1937	7	novel_not_in_catalog	gene: Zm00001d045050	novel	27.22	27.59	coding	331	418	1410
PB.4260.2	3	1792	15	full-splice_match	gene: Zm00001d042421	Zm00001d042421_T00	56.45	67.32	coding	401	225	1427
PB.3438.1	2	665	1	incomplete-splice_match	gene: Zm00001d006752	Zm00001d006752_T00	14.23	14.23	coding	87	39	299
PB.4890.1	4	1004	2	novel_not_in_catalog	gene: Zm00001d048883	novel	88.95	88.95	coding	229	49	735

PB.6611.2	5	2278	4	incomplete-splice_match	gene: Zm00001d015493	Zm00001d015493_T00	8.10	17.80	coding	647	180	2120
PB.7126.3	5	2031	10	novel_not_in_catalog	gene: Zm00001d018005	novel	0.19	61.84	coding	421	34	1296
PB.7126.2	5	2143	9	full-splice_match	gene: Zm00001d018005	Zm00001d018005_T00	45.10	61.84	coding	563	60	1748
PB.7126.1	5	2245	9	full-splice_match	gene: Zm00001d018005	Zm00001d018005_T00	16.55	61.84	coding	563	34	1722
PB.1379.2	1	1643	1	incomplete-splice_match	gene: Zm00001d033734	Zm00001d033734_T00	33.94	57.27	coding	498	28	1521
PB.6871.2	5	2753	1	novel_in_catalog	gene: Zm00001d016977	novel	0.84	13.50	non coding	NA	NA	NA
PB.6344.3	5	4095	1	full-splice_match	gene: Zm00001d014338	Zm00001d014338_T00	1.43	31.84	coding	348	2874	3917
PB.11023.1	B73V4	1168	10	incomplete-splice_match	gene: Zm00001d000191	Zm00001d000191_T03	40.08	40.08	coding	258	81	854
PB.6344.1	5	1502	2	genic	gene: Zm00001d014338	novel	30.41	31.84	coding	348	189	1232
PB.8131.6	6	506	1	genic	gene: Zm00001d038908	novel	264.87	264.87	non coding	NA	NA	NA
PB.1391.5	1	2127	20	full-splice_match	gene: Zm00001d033795	Zm00001d033795_T00	0.00	39.36	coding	594	124	1905
PB.4395.1	3	1845	1	full-splice_match	gene: Zm00001d043026	Zm00001d043026_T00	6.82	6.82	coding	540	24	1643
PB.9285.2	8	2408	21	incomplete-splice_match	gene: Zm00001d008941	Zm00001d008941_T00	12.07	12.07	coding	695	17	2101
PB.1236.1	1	2368	10	full-splice_match	gene: Zm00001d033094	Zm00001d033094_T00	18.56	25.33	coding	615	44	1888
PB.1103.3	1	3236	8	novel_not_in_catalog	gene: Zm00001d032540	novel	7.16	32.63	coding	252	2011	2766
PB.10056.1	8	771	4	novel_not_in_catalog	gene: Zm00001d012604	novel	12.06	15.73	coding	141	84	506
PB.9780.1	8	1751	7	novel_in_catalog	gene: Zm00001d011457	novel	7.67	19.85	coding	314	46	987

PB.3593.1	2	1016	9	full-splice_match	gene: Zm00001d007594	Zm00001d007594_T00	62.35	62.35	coding	229	31	717
PB.3819.1	3	800	2	genic	gene: Zm00001d039841	novel	40.21	40.21	coding	168	136	639
PB.1103.1	1	2563	7	novel_not_in_catalog	gene: Zm00001d032540	novel	25.47	32.63	coding	323	1117	2085
PB.1379.1	1	4088	3	novel_in_catalog	gene: Zm00001d033734	novel	23.33	57.27	coding	1218	179	3832
PB.7263.4	5	1911	5	novel_not_in_catalog	gene: Zm00001d018499	novel	38.69	44.85	coding	165	1053	1547
PB.4400.2	3	1203	3	novel_not_in_catalog	gene: Zm00001d043060	novel	10.07	10.07	coding	270	178	987
PB.7647.9	6	2014	12	novel_not_in_catalog	gene: Zm00001d036987	novel	15.85	64.02	coding	611	181	2013
PB.7647.8	6	4067	20	novel_not_in_catalog	gene: Zm00001d036987	novel	0.00	64.02	coding	713	181	2319
PB.2912.1	2	2760	3	genic	gene: Zm00001d003948	novel	7.20	45.19	coding	660	209	2188
PB.6063.1	5	2338	4	full-splice_match	gene: Zm00001d013182	Zm00001d013182_T00	41.57	45.02	coding	682	131	2176
PB.6063.3	5	2266	3	novel_in_catalog	gene: Zm00001d013182	novel	3.45	45.02	coding	506	52	1569
PB.7647.3	6	2338	12	novel_not_in_catalog	gene: Zm00001d036987	novel	21.04	64.02	coding	690	144	2213
PB.10298.1	9	693	6	full-splice_match	gene: Zm00001d045555	Zm00001d045555_T00	200.24	200.24	coding	114	108	449
PB.10298.2	9	557	6	full-splice_match	gene: Zm00001d045555	Zm00001d045555_T00	0.00	200.24	coding	114	93	434
PB.7647.7	6	2894	13	novel_not_in_catalog	gene: Zm00001d036987	novel	5.95	64.02	coding	595	42	1826
PB.4522.5	3	3158	3	full-splice_match	gene: Zm00001d043484	Zm00001d043484_T01	16.52	51.49	coding	123	223	591
PB.7647.5	6	2792	10	novel_not_in_catalog	gene: Zm00001d036987	novel	13.47	64.02	coding	316	3	950

PB.5251.1	4	994	2	full-splice_match	gene: Zm00001d050944	Zm00001d050944_T00	6.31	93.25	non coding	NA	NA	NA
PB.5251.2	4	686	2	full-splice_match	gene: Zm00001d050944	Zm00001d050944_T00	86.95	93.25	coding	96	94	381
PB.5251.3	4	527	2	full-splice_match	gene: Zm00001d050944	Zm00001d050944_T00	0.00	93.25	coding	96	67	354
PB.6495.1	5	740	1	antisense	novelGene_gene: Zm00001d014947	novel	58.17	58.17	non coding	NA	NA	NA
PB.2578.1	2	1177	1	genic	gene: Zm00001d002390	novel	11.38	46.33	coding	147	22	462
PB.4569.1	3	1662	5	full-splice_match	gene: Zm00001d043651	Zm00001d043651_T00	22.54	27.29	coding	486	22	1479
PB.2578.3	2	2483	15	novel_in_catalog	gene: Zm00001d002390	novel	9.72	46.33	coding	567	676	2376
PB.2578.2	2	2341	12	incomplete-splice_match	gene: Zm00001d002390	Zm00001d002390_T01	10.10	46.33	coding	567	435	2135
PB.5831.2	4	2096	1	full-splice_match	gene: Zm00001d053695	Zm00001d053695_T00	0.00	27.84	coding	680	46	2085
PB.2578.4	2	628	1	incomplete-splice_match	gene: Zm00001d002390	Zm00001d002390_T01	15.13	46.33	coding	128	243	626
PB.574.1	1	922	5	incomplete-splice_match	gene: Zm00001d029776	Zm00001d029776_T00	101.98	101.98	coding	155	277	741
PB.7280.1	5	1494	1	full-splice_match	gene: Zm00001d018534	Zm00001d018534_T00	11.93	11.93	coding	419	63	1319
PB.783.5	1	1620	3	incomplete-splice_match	gene: Zm00001d031059	Zm00001d031059_T00	23.81	23.81	coding	320	660	1619
PB.5208.2	4	941	2	intergenic	novelGene_210	novel	0.00	99.35	coding	148	96	539
PB.8897.1	7	1244	9	full-splice_match	gene: Zm00001d021898	Zm00001d021898_T00	6.58	6.58	coding	340	37	1056
PB.874.5	1	1356	5	full-splice_match	gene: Zm00001d031505	Zm00001d031505_T00	0.00	48.34	coding	321	241	1203
PB.896.9	1	1092	3	full-splice_match	gene: Zm00001d031635	Zm00001d031635_T00	0.00	217.19	coding	337	10	1020

PB.4097.2	3	835	6	full-splice_match	gene: Zm00001d041597	Zm00001d041597_T00	83.00	129.00	coding	188	37	600
PB.4097.1	3	969	6	full-splice_match	gene: Zm00001d041597	Zm00001d041597_T00	46.00	129.00	coding	188	39	602
PB.7182.2	5	1917	3	novel_not_in_catalog	gene: Zm00001d018214	novel	85.80	89.30	coding	481	59	1501
PB.863.3	1	1363	4	intergenic	novelGene_385	novel	3.84	3.84	non coding	NA	NA	NA
PB.9534.2	8	2754	19	full-splice_match	gene: Zm00001d010341	Zm00001d010341_T00	21.65	46.58	coding	748	39	2282
PB.7301.1	6	1051	1	full-splice_match	gene: Zm00001d035020	Zm00001d035020_T00	16.34	16.34	coding	240	107	826
PB.9534.3	8	900	6	incomplete-splice_match	gene: Zm00001d010341	Zm00001d010341_T00	24.93	46.58	coding	146	102	539
PB.1602.5	1	1519	1	genic	gene: Zm00001d034676	novel	2.12	2.12	non coding	NA	NA	NA
PB.2445.1	2	3200	6	full-splice_match	gene: Zm00001d001848	Zm00001d001848_T00	55.83	55.83	coding	955	104	2968
PB.2197.2	10	1434	1	full-splice_match	gene: Zm00001d025917	Zm00001d025917_T00	3.82	10.23	coding	343	48	1076
PB.10126.1	9	3233	1	intergenic	novelGene_20	novel	6.37	6.37	non coding	NA	NA	NA
PB.1202.3	1	1097	7	full-splice_match	gene: Zm00001d032931	Zm00001d032931_T00	5.10	15.05	coding	289	11	877
PB.6948.3	5	2676	1	novel_in_catalog	gene: Zm00001d017274	novel	22.79	2308.60	coding	557	701	2371
PB.8808.2	7	1795	1	full-splice_match	gene: Zm00001d021638	Zm00001d021638_T00	4.94	29.45	coding	521	108	1670
PB.8808.1	7	1905	1	full-splice_match	gene: Zm00001d021638	Zm00001d021638_T00	24.51	29.45	coding	521	104	1666
PB.2245.3	10	581	1	novel_in_catalog	gene: Zm00001d026163	novel	49.94	2729.80	coding	122	124	489
PB.7234.1	5	982	5	incomplete-splice_match	gene: Zm00001d018413	Zm00001d018413_T01	51.72	90.00	coding	114	6	347

PB.1176.2	1	2361	1	full-splice_match	gene: Zm00001d032819	Zm00001d032819_T00	50.30	50.30	coding	637	137	2047
PB.4556.8	3	2074	19	full-splice_match	gene: Zm00001d043601	Zm00001d043601_T00	121.76	284.99	coding	593	113	1891
PB.1236.2	1	2601	9	novel_in_catalog	gene: Zm00001d033094	novel	6.77	25.33	coding	384	84	1235
PB.27.1	1	717	3	full-splice_match	gene: Zm00001d027342	Zm00001d027342_T00	201.69	201.69	non coding	NA	NA	NA
PB.7617.2	6	2188	10	full-splice_match	gene: Zm00001d036827	Zm00001d036827_T00	19.05	57.72	coding	582	295	2040
PB.7067.1	5	2041	1	full-splice_match	gene: Zm00001d017811	Zm00001d017811_T00	13.62	13.62	coding	492	84	1559
PB.6918.3	5	1095	10	novel_in_catalog	gene: Zm00001d017153	novel	0.00	21.57	coding	311	162	1094
PB.709.4	1	402	2	genic	gene: Zm00001d030618	novel	711.40	1118.65	non coding	NA	NA	NA
PB.6918.1	5	1888	13	full-splice_match	gene: Zm00001d017153	Zm00001d017153_T00	21.57	21.57	coding	410	272	1501
PB.6883.5	5	886	4	full-splice_match	gene: Zm00001d017047	Zm00001d017047_T00	8.64	274.64	coding	219	64	720
PB.3771.1	3	1541	3	novel_not_in_catalog	gene: Zm00001d039576	novel	30.93	30.93	coding	349	172	1218
PB.9120.2	8	540	2	full-splice_match	gene: Zm00001d008200	Zm00001d008200_T00	523.28	523.28	non coding	NA	NA	NA
PB.5652.2	4	1162	8	full-splice_match	gene: Zm00001d052803	Zm00001d052803_T00	39.21	60.14	coding	256	76	843
PB.6883.1	5	1149	4	full-splice_match	gene: Zm00001d017047	Zm00001d017047_T00	120.86	274.64	coding	219	64	720
PB.2111.2	10	1889	1	full-splice_match	gene: Zm00001d025490	Zm00001d025490_T00	14.49	91.90	coding	146	1267	1704
PB.7617.4	6	1822	3	novel_in_catalog	gene: Zm00001d036827	novel	5.66	57.72	coding	499	66	1562
PB.8905.15	7	2665	4	full-splice_match	gene: Zm00001d021930	Zm00001d021930_T00	6.68	155.11	coding	219	126	782

PB.2111.1	10	1879	1	full-splice_match	gene: Zm00001d025490	Zm00001d025490_T00	12.82	91.90	coding	146	766	1203
PB.2111.6	10	987	2	genic	gene: Zm00001d025490	novel	41.08	91.90	coding	146	449	886
PB.4767.4	3	319	2	incomplete-splice_match	gene: Zm00001d044442	Zm00001d044442_T00	0.00	87.18	coding	98	26	319
PB.7617.5	6	1718	3	novel_in_catalog	gene: Zm00001d036827	novel	33.00	57.72	coding	499	68	1564
PB.8200.1	6	2446	8	novel_not_in_catalog	gene: Zm00001d039180	novel	9.33	55.29	coding	513	400	1938
PB.10411.5	9	1906	15	full-splice_match	gene: Zm00001d046336	Zm00001d046336_T00	445.55	483.03	coding	487	126	1586
PB.7624.1	6	868	1	genic	gene: Zm00001d036895	novel	6.02	101.76	non coding	NA	NA	NA
PB.2439.1	2	970	3	full-splice_match	gene: Zm00001d001826	Zm00001d001826_T00	33.58	49.55	coding	115	319	663
PB.10479.3	9	3692	8	incomplete-splice_match	gene: Zm00001d046717	Zm00001d046717_T01	7.43	22.30	coding	1100	115	3414
PB.10411.1	9	2157	14	full-splice_match	gene: Zm00001d046336	Zm00001d046336_T00	36.87	483.03	coding	498	317	1810
PB.2439.2	2	835	3	full-splice_match	gene: Zm00001d001826	Zm00001d001826_T00	15.97	49.55	coding	115	302	646
PB.11016.3	B73V4	703	2	full-splice_match	gene: Zm00001d000126	Zm00001d000126_T00	0.00	238.48	coding	108	87	410
PB.11016.2	B73V4	984	1	novel_in_catalog	gene: Zm00001d000126	novel	18.08	238.48	coding	108	237	560
PB.11016.1	B73V4	839	2	full-splice_match	gene: Zm00001d000126	Zm00001d000126_T00	158.39	238.48	coding	108	86	409
PB.10479.2	9	1777	4	full-splice_match	gene: Zm00001d046717	Zm00001d046717_T00	11.61	22.30	coding	445	3	1337
PB.10282.1	9	544	3	full-splice_match	gene: Zm00001d045497	Zm00001d045497_T00	344.34	344.34	non coding	NA	NA	NA
PB.3743.3	3	1055	6	novel_not_in_catalog	gene: Zm00001d039483	novel	0.00	208.95	coding	267	96	896

PB.1218.3	1	3262	5	full-splice_match	gene: Zm00001d033004	Zm00001d033004_T00	18.03	68.92	coding	268	167	970
PB.11016.4	B73V4	551	2	full-splice_match	gene: Zm00001d000126	Zm00001d000126_T00	62.01	238.48	coding	108	87	410
PB.241.1	1	1499	3	incomplete-splice_match	gene: Zm00001d028285	Zm00001d028285_T00	3.73	41.13	coding	177	119	649
PB.847.3	1	600	4	full-splice_match	gene: Zm00001d031328	Zm00001d031328_T00	0.00	47.35	coding	169	94	600
PB.241.3	1	1271	3	incomplete-splice_match	gene: Zm00001d028285	Zm00001d028285_T00	7.76	41.13	coding	177	173	703
PB.241.2	1	1272	3	incomplete-splice_match	gene: Zm00001d028285	Zm00001d028285_T00	0.00	41.13	coding	177	44	574
PB.241.5	1	2390	6	novel_in_catalog	gene: Zm00001d028285	novel	7.65	41.13	coding	532	427	2022
PB.241.4	1	2296	7	full-splice_match	gene: Zm00001d028285	Zm00001d028285_T00	22.00	41.13	coding	607	33	1853
PB.1069.1	1	1718	6	full-splice_match	gene: Zm00001d032428	Zm00001d032428_T00	88.25	97.16	coding	415	138	1382
PB.1995.1	10	1081	2	full-splice_match	gene: Zm00001d024890	Zm00001d024890_T00	34.76	34.76	coding	285	103	957
PB.8785.1	7	2577	19	full-splice_match	gene: Zm00001d021546	Zm00001d021546_T00	56.65	56.65	coding	619	363	2219
PB.4614.1	3	1191	2	full-splice_match	gene: Zm00001d043806	Zm00001d043806_T00	12.60	12.60	coding	159	492	968
PB.10303.1	9	2145	10	full-splice_match	gene: Zm00001d045566	Zm00001d045566_T00	21.45	21.45	coding	575	107	1831
PB.4612.1	3	778	1	antisense	novelGene_gene: Zm00001d043800	novel	5.29	5.29	non coding	NA	NA	NA
PB.8257.1	7	1287	2	full-splice_match	gene: Zm00001d018790	Zm00001d018790_T00	44.16	50.42	coding	313	65	1003
PB.10651.2	9	2951	11	full-splice_match	gene: Zm00001d047534	Zm00001d047534_T00	2.11	115.08	coding	568	102	1805
PB.7465.1	6	1483	3	full-splice_match	gene: Zm00001d035921	Zm00001d035921_T00	8.92	8.92	coding	164	542	1033

PB.7465.2	6	744	2	full-splice_match	gene: Zm00001d035922	Zm00001d035922_T00	0.00	0.00	coding	155	25	489
PB.2105.5	10	3301	10	novel_in_catalog	gene: Zm00001d025449	novel	13.76	45.53	coding	497	174	1664
PB.2105.4	10	2715	11	full-splice_match	gene: Zm00001d025449	Zm00001d025449_T00	13.98	45.53	coding	727	174	2354
PB.4044.1	3	3206	1	full-splice_match	gene: Zm00001d041236	Zm00001d041236_T00	39.62	39.62	coding	989	49	3015
PB.7143.2	5	2468	6	full-splice_match	gene: Zm00001d018080	Zm00001d018080_T00	7.92	42.89	coding	780	41	2380
PB.2259.1	10	1347	12	full-splice_match	gene: Zm00001d026214	Zm00001d026214_T00	20.51	104.52	coding	270	150	959
PB.5706.2	4	1401	2	intergenic	novelGene_409	novel	2.36	19.41	non coding	NA	NA	NA
PB.2259.3	10	1202	12	full-splice_match	gene: Zm00001d026214	Zm00001d026214_T00	31.60	104.52	coding	270	115	924
PB.2259.4	10	1180	12	full-splice_match	gene: Zm00001d026214	Zm00001d026214_T00	52.40	104.52	coding	270	226	1035
PB.8749.4	7	2841	4	full-splice_match	gene: Zm00001d021400	Zm00001d021400_T00	50.65	69.85	coding	687	450	2510
PB.1385.3	1	1032	5	incomplete-splice_match	gene: Zm00001d033776	Zm00001d033776_T00	10.51	38.59	coding	239	43	759
PB.4567.13	3	3497	7	novel_in_catalog	gene: Zm00001d043649	novel	0.95	120.68	coding	125	1812	2186
PB.4521.3	3	2712	8	novel_not_in_catalog	gene: Zm00001d043480	novel	45.51	197.65	coding	705	234	2348
PB.4521.5	3	3126	7	novel_not_in_catalog	gene: Zm00001d043480	novel	89.06	197.65	coding	453	1396	2754
PB.4567.16	3	1306	5	incomplete-splice_match	gene: Zm00001d043649	Zm00001d043649_T05	15.63	120.68	coding	98	11	304
PB.4521.6	3	3007	7	novel_not_in_catalog	gene: Zm00001d043480	novel	0.00	197.65	coding	453	1396	2754
PB.3638.1	2	2564	1	genic_intron	novelGene_441	novel	14.45	14.45	coding	660	302	2281

PB.4521.8	3	2594	8	novel_not_in_catalog	gene: Zm00001d043480	novel	60.39	197.65	coding	705	235	2349
PB.4567.19	3	2241	5	full-splice_match	gene: Zm00001d043649	Zm00001d043649_T05	0.00	120.68	coding	125	277	651
PB.4567.18	3	2294	8	full-splice_match	gene: Zm00001d043649	Zm00001d043649_T04	1.95	120.68	coding	125	470	844
PB.10419.1	9	2195	4	full-splice_match	gene: Zm00001d046383	Zm00001d046383_T00	13.44	13.44	coding	592	147	1922
PB.778.1	1	2115	1	antisense	novelGene_gene: Zm00001d031044	novel	0.10	0.10	coding	295	833	1717
PB.8246.3	7	1129	7	full-splice_match	gene: Zm00001d018732	Zm00001d018732_T00	7.57	31.27	coding	238	36	749
PB.8246.2	7	1248	7	full-splice_match	gene: Zm00001d018732	Zm00001d018732_T00	9.89	31.27	coding	238	24	737
PB.8246.1	7	1441	8	full-splice_match	gene: Zm00001d018732	Zm00001d018732_T00	13.82	31.27	coding	264	91	882
PB.6007.1	5	1360	4	full-splice_match	gene: Zm00001d012991	Zm00001d012991_T00	18.87	18.87	coding	345	116	1150
PB.4582.12	3	916	9	novel_not_in_catalog	gene: Zm00001d043693	novel	0.00	284.74	coding	218	261	914
PB.9280.2	8	2360	1	genic_intron	novelGene_100	novel	185.51	185.51	coding	111	1605	1937
PB.4582.10	3	2284	13	novel_not_in_catalog	gene: Zm00001d043693	novel	0.00	284.74	coding	267	1227	2027
PB.8246.4	7	1018	7	full-splice_match	gene: Zm00001d018732	Zm00001d018732_T00	0.00	31.27	coding	238	36	749
PB.5045.5	4	1223	7	novel_not_in_catalog	gene: Zm00001d049598	novel	1.03	329.84	coding	217	223	873
PB.8064.11	6	1274	10	full-splice_match	gene: Zm00001d038689	Zm00001d038689_T00	82.50	137.30	coding	322	140	1105
PB.9575.2	8	2355	11	incomplete-splice_match	gene: Zm00001d010546	Zm00001d010546_T00	34.95	38.77	coding	622	71	1936
PB.5045.1	4	1498	7	full-splice_match	gene: Zm00001d049598	Zm00001d049598_T00	328.81	329.84	coding	228	309	992

PB.6290.1	5	1046	7	full-splice_match	gene: Zm00001d014122	Zm00001d014122_T00	20.94	20.94	coding	214	230	871
PB.5045.3	4	1344	7	full-splice_match	gene: Zm00001d049598	Zm00001d049598_T00	0.00	329.84	coding	228	304	987
PB.1153.1	1	2516	11	incomplete-splice_match	gene: Zm00001d032725	Zm00001d032725_T00	63.70	63.70	coding	725	116	2290
PB.9509.1	8	1900	13	full-splice_match	gene: Zm00001d010251	Zm00001d010251_T00	104.27	229.60	coding	445	94	1428
PB.9509.2	8	1862	13	full-splice_match	gene: Zm00001d010251	Zm00001d010251_T00	123.05	229.60	coding	445	182	1516
PB.9509.4	8	1917	12	novel_in_catalog	gene: Zm00001d010251	novel	2.28	229.60	coding	244	153	884
PB.10976.2	B73V4	2698	4	full-splice_match	gene: Zm00001d000039	Zm00001d000039_T00	11.49	11.49	coding	781	137	2479
PB.8058.1	6	1635	6	full-splice_match	gene: Zm00001d038646	Zm00001d038646_T00	30.25	68.91	coding	309	270	1196
PB.8058.2	6	1440	6	full-splice_match	gene: Zm00001d038646	Zm00001d038646_T00	38.66	68.91	coding	309	295	1221
PB.1487.2	1	331	2	novel_in_catalog	gene: Zm00001d034223	novel	41.22	382.61	non coding	NA	NA	NA
PB.5144.1	4	1635	5	full-splice_match	gene: Zm00001d050140	Zm00001d050140_T00	12.05	12.05	coding	397	112	1302
PB.10721.1	9	306	1	incomplete-splice_match	gene: Zm00001d047776	Zm00001d047776_T00	65.13	65.13	non coding	NA	NA	NA
PB.5144.2	4	1567	5	full-splice_match	gene: Zm00001d050140	Zm00001d050140_T00	0.00	12.05	coding	397	276	1466
PB.1309.2	1	1907	3	full-splice_match	gene: Zm00001d033423	Zm00001d033423_T00	17.52	17.52	coding	461	174	1556
PB.4565.1	3	1551	6	full-splice_match	gene: Zm00001d043625	Zm00001d043625_T00	490.79	509.97	coding	378	208	1341
PB.9985.2	8	2077	1	novel_in_catalog	gene: Zm00001d012292	novel	15.53	83.51	coding	356	829	1896
PB.5918.1	4	1981	10	novel_not_in_catalog	gene: Zm00001d053998	novel	7.58	7.58	coding	530	103	1692

PB.1590.2	1	1162	3	novel_in_catalog	gene: Zm00001d034635	novel	3.06	359.96	coding	137	446	856
PB.2314.4	10	2556	1	novel_in_catalog	gene: Zm00001d026391	novel	5.05	99.60	coding	197	86	676
PB.4064.1	3	1780	13	full-splice_match	gene: Zm00001d041383	Zm00001d041383_T00	24.28	24.28	coding	422	238	1503
PB.7508.1	6	615	2	intergenic	novelGene_170	novel	97.79	97.79	coding	65	193	387
PB.7790.1	6	1535	8	novel_not_in_catalog	gene: Zm00001d037659	novel	2.62	2.62	coding	241	633	1355
PB.7663.1	6	1983	6	full-splice_match	gene: Zm00001d037085	Zm00001d037085_T00	61.70	61.70	coding	535	176	1780
PB.2968.1	2	1000	6	full-splice_match	gene: Zm00001d004392	Zm00001d004392_T00	6.11	6.11	coding	244	12	743
PB.8929.1	7	534	2	incomplete-splice_match	gene: Zm00001d022021	Zm00001d022021_T00	11.80	25.07	coding	42	280	405
PB.9937.2	8	794	2	genic	gene: Zm00001d012097	novel	1.94	5.86	non coding	NA	NA	NA
PB.9937.1	8	1146	3	genic	gene: Zm00001d012097	novel	3.92	5.86	non coding	NA	NA	NA
PB.5239.1	4	1693	1	intergenic	novelGene_247	novel	4.05	4.05	non coding	NA	NA	NA
PB.879.2	1	3388	4	novel_in_catalog	gene: Zm00001d031534	novel	37.84	75.72	coding	778	664	2997
PB.879.1	1	3316	4	novel_in_catalog	gene: Zm00001d031534	novel	37.88	75.72	coding	816	109	2556
PB.6123.1	5	2017	4	full-splice_match	gene: Zm00001d013367	Zm00001d013367_T00	61.01	2327.02	coding	451	125	1477
PB.398.11	1	2956	13	novel_in_catalog	gene: Zm00001d028949	novel	0.00	453.16	coding	219	1921	2577
PB.398.12	1	523	1	novel_in_catalog	gene: Zm00001d028949	novel	208.97	453.16	non coding	NA	NA	NA
PB.8403.3	7	1763	5	novel_not_in_catalog	gene: Zm00001d019624	novel	4.83	18.14	non coding	NA	NA	NA

PB.398.14	1	1314	12	incomplete-splice_match	gene: Zm00001d028949	Zm00001d028949_T00	0.00	453.16	coding	372	198	1313
PB.6123.4	5	1671	4	full-splice_match	gene: Zm00001d013367	Zm00001d013367_T00	2023.29	2327.02	coding	451	123	1475
PB.6123.7	5	1116	1	novel_in_catalog	gene: Zm00001d013367	novel	7.31	2327.02	coding	184	371	922
PB.10316.1	9	1780	1	intergenic	novelGene_109	novel	0.81	0.81	coding	109	551	877
PB.4746.2	3	1490	10	full-splice_match	gene: Zm00001d044315	Zm00001d044315_T01	38.28	301.89	coding	351	244	1296
PB.449.3	1	1736	5	full-splice_match	gene: Zm00001d029189	Zm00001d029189_T00	24.41	49.23	coding	328	230	1213
PB.449.2	1	2937	3	novel_in_catalog	gene: Zm00001d029189	novel	11.19	49.23	coding	113	325	663
PB.449.1	1	1598	3	novel_in_catalog	gene: Zm00001d029189	novel	13.63	49.23	coding	219	260	916
PB.10089.1	8	1131	1	full-splice_match	gene: Zm00001d012720	Zm00001d012720_T00	24.69	24.69	coding	237	181	891
PB.8082.1	6	1856	6	full-splice_match	gene: Zm00001d038769	Zm00001d038769_T00	7.65	10.26	coding	299	168	1064
PB.5285.7	4	976	1	novel_in_catalog	gene: Zm00001d051161	novel	64.71	167.23	coding	218	253	906
PB.2314.2	10	1027	2	full-splice_match	gene: Zm00001d026391	Zm00001d026391_T00	78.72	99.60	coding	197	86	676
PB.8989.1	7	2113	2	genic	gene: Zm00001d022247	novel	5.39	83.51	coding	302	817	1722
PB.8935.2	7	1416	10	novel_not_in_catalog	gene: Zm00001d022040	novel	67.85	67.85	coding	283	19	867
PB.8427.1	7	1094	1	intergenic	novelGene_157	novel	0.12	0.12	non coding	NA	NA	NA
PB.9854.1	8	825	3	full-splice_match	gene: Zm00001d011766	Zm00001d011766_T00	320.10	328.49	coding	137	166	576
PB.6422.5	5	1185	8	incomplete-splice_match	gene: Zm00001d014716	Zm00001d014716_T00	0.55	5.41	non coding	NA	NA	NA

PB.8270.1	7	1337	1	full-splice_match	gene: Zm00001d018855	Zm00001d018855_T00	16.44	16.44	coding	368	63	1166
PB.8797.1	7	1371	5	full-splice_match	gene: Zm00001d021582	Zm00001d021582_T00	227.45	227.45	coding	290	317	1186
PB.9853.1	8	854	2	full-splice_match	gene: Zm00001d011763	Zm00001d011763_T00	9.44	9.44	coding	166	214	711
PB.8430.1	7	2041	4	intergenic	novelGene_162	novel	8.91	8.91	coding	559	78	1754
PB.4820.1	3	2120	3	genic	gene: Zm00001d044635	novel	6.78	6.78	coding	170	109	618
PB.5059.1	4	1540	4	novel_not_in_catalog	gene: Zm00001d049664	novel	7.68	7.68	non coding	NA	NA	NA
PB.3074.2	2	2245	12	full-splice_match	gene: Zm00001d005062	Zm00001d005062_T00	9.46	28.49	coding	624	184	2055
PB.3999.3	3	2524	7	full-splice_match	gene: Zm00001d040784	Zm00001d040784_T00	15.54	36.02	coding	732	136	2331
PB.3999.2	3	3543	5	full-splice_match	gene: Zm00001d040784	Zm00001d040784_T00	10.61	36.02	coding	260	1043	1822
PB.3999.5	3	2564	6	full-splice_match	gene: Zm00001d040784	Zm00001d040784_T01	9.88	36.02	coding	500	866	2365
PB.8623.1	7	1428	4	full-splice_match	gene: Zm00001d020882	Zm00001d020882_T00	15.92	15.92	coding	396	109	1296
PB.8623.2	7	1271	4	full-splice_match	gene: Zm00001d020882	Zm00001d020882_T00	0.00	15.92	coding	396	44	1231
PB.7206.1	5	1890	13	full-splice_match	gene: Zm00001d018333	Zm00001d018333_T00	67.50	73.67	coding	442	144	1469
PB.1511.23	1	1453	5	novel_in_catalog	gene: Zm00001d034345	novel	365.67	1673.99	coding	317	349	1299
PB.3410.2	2	1865	2	novel_in_catalog	gene: Zm00001d006620	novel	5.96	26.19	coding	254	767	1528
PB.4317.3	3	897	1	novel_in_catalog	gene: Zm00001d042664	novel	11.67	152.56	coding	75	87	311
PB.3759.2	3	1350	4	full-splice_match	gene: Zm00001d039529	Zm00001d039529_T00	3.41	3.65	coding	386	96	1253

PB.3759.1	3	2698	3	novel_in_catalog	gene: Zm00001d039529	novel	0.24	3.65	coding	263	1726	2514
PB.9018.2	7	3167	1	genic	gene: Zm00001d022351	novel	0.38	16.62	coding	423	1741	3009
PB.9018.1	7	1900	2	full-splice_match	gene: Zm00001d022351	Zm00001d022351_T00	16.25	16.62	coding	526	159	1736
PB.8894.1	7	1651	5	full-splice_match	gene: Zm00001d021891	Zm00001d021891_T00	452.81	467.58	coding	450	93	1442
PB.964.6	1	1234	8	full-splice_match	gene: Zm00001d031967	Zm00001d031967_T00	49.22	110.89	coding	304	132	1043
PB.7599.1	6	1463	4	incomplete-splice_match	gene: Zm00001d036704	Zm00001d036704_T00	13.05	24.40	coding	333	47	1045
PB.6592.7	5	1088	10	novel_not_in_catalog	gene: Zm00001d015364	novel	13.36	54.32	coding	197	167	757
PB.6592.6	5	1329	9	full-splice_match	gene: Zm00001d015364	Zm00001d015364_T00	14.53	54.32	coding	135	758	1162
PB.6592.1	5	1638	9	novel_in_catalog	gene: Zm00001d015364	novel	10.63	54.32	coding	227	783	1463
PB.10319.1	9	1028	1	full-splice_match	gene: Zm00001d045657	Zm00001d045657_T00	297.94	297.94	coding	170	155	664
PB.1533.2	1	1489	1	full-splice_match	gene: Zm00001d034427	Zm00001d034427_T00	126.67	312.51	coding	392	98	1273
PB.8155.1	6	531	2	incomplete-splice_match	gene: Zm00001d038999	Zm00001d038999_T00	17.98	366.37	non coding	NA	NA	NA
PB.1889.1	10	1804	6	incomplete-splice_match	gene: Zm00001d024339	Zm00001d024339_T00	101.17	176.74	coding	481	6	1448
PB.5666.2	4	963	1	intergenic	novelGene_398	novel	1.69	1.69	coding	125	319	693
PB.9673.1	8	2113	2	genic	gene: Zm00001d010954	novel	48.46	76.76	coding	414	502	1743
PB.1889.2	10	2101	9	full-splice_match	gene: Zm00001d024339	Zm00001d024339_T00	75.58	176.74	coding	592	136	1911
PB.6592.8	5	886	9	novel_in_catalog	gene: Zm00001d015364	novel	15.80	54.32	coding	169	148	654

PB.6781.2	5	1513	6	full-splice_match	gene: Zm00001d016588	Zm00001d016588_T00	0.68	23.47	coding	245	661	1395
PB.9161.1	8	1606	4	novel_in_catalog	gene: Zm00001d008360	novel	4.91	35.92	coding	331	79	1071
PB.10218.1	9	799	1	full-splice_match	gene: Zm00001d045296	Zm00001d045296_T00	19.71	82.03	coding	117	92	442
PB.10218.2	9	655	1	full-splice_match	gene: Zm00001d045296	Zm00001d045296_T00	62.32	82.03	coding	117	93	443
PB.3251.1	2	1910	5	full-splice_match	gene: Zm00001d005981	Zm00001d005981_T00	7.91	7.91	coding	522	31	1596
PB.6435.1	5	2189	1	full-splice_match	gene: Zm00001d014759	Zm00001d014759_T00	24.35	25.97	coding	638	42	1955
PB.6435.2	5	1469	1	full-splice_match	gene: Zm00001d014759	Zm00001d014759_T00	1.61	25.97	coding	465	75	1469
PB.10793.5	9	2214	2	full-splice_match	gene: Zm00001d048054	Zm00001d048054_T00	0.00	68.53	coding	672	112	2127
PB.327.3	1	1485	9	full-splice_match	gene: Zm00001d028631	Zm00001d028631_T00	53.54	58.40	coding	308	239	1162
PB.946.1	1	2107	1	full-splice_match	gene: Zm00001d022897	Zm00001d022897_T00	8.28	8.28	non coding	NA	NA	NA
PB.9083.1	7	976	3	incomplete-splice_match	gene: Zm00001d022535	Zm00001d022535_T00	6.61	6.61	coding	194	57	638
PB.9331.6	8	2666	1	full-splice_match	gene: Zm00001d009226	Zm00001d009226_T00	27.48	63.11	coding	810	42	2471
PB.738.1	1	1996	1	genic	gene: Zm00001d030843	novel	1.27	6.31	coding	76	73	300
PB.4433.1	3	1991	2	full-splice_match	gene: Zm00001d043166	Zm00001d043166_T00	5.95	5.95	coding	470	58	1467
PB.9903.1	8	1206	3	full-splice_match	gene: Zm00001d011969	Zm00001d011969_T00	58.77	58.77	coding	259	172	948
PB.5397.2	4	561	1	novel_in_catalog	gene: Zm00001d051761	novel	4.17	281.69	non coding	NA	NA	NA
PB.4921.1	4	3040	11	novel_in_catalog	gene: Zm00001d049023	novel	14.32	14.32	coding	169	1054	1560

PB.8582.2	7	2544	1	full-splice_match	gene: Zm00001d020659	Zm00001d020659_T00	0.00	83.97	coding	139	1693	2109
PB.8582.3	7	824	2	genic	gene: Zm00001d020659	novel	17.55	83.97	coding	123	160	528
PB.8582.5	7	799	2	genic	gene: Zm00001d020659	novel	57.95	83.97	coding	123	251	619
PB.8582.6	7	2374	1	full-splice_match	gene: Zm00001d020659	Zm00001d020659_T00	4.62	83.97	coding	139	1781	2197
PB.5754.1	4	2342	1	full-splice_match	gene: Zm00001d053322	Zm00001d053322_T00	8.08	8.08	coding	683	127	2175
PB.5170.1	4	1024	9	full-splice_match	gene: Zm00001d050300	Zm00001d050300_T00	99.68	99.68	coding	215	166	810
PB.8672.1	7	855	1	full-splice_match	gene: Zm00001d021062	Zm00001d021062_T00	161.09	348.86	coding	136	64	471
PB.6193.3	5	2613	1	intergenic	novelGene_67	novel	0.00	0.00	coding	748	369	2612
PB.4113.7	3	702	1	full-splice_match	gene: Zm00001d041685	Zm00001d041685_T00	91.93	150.23	coding	124	131	502
PB.5374.1	4	1812	2	full-splice_match	gene: Zm00001d051636	Zm00001d051636_T00	16.80	16.80	coding	392	241	1416
PB.4666.1	3	2199	3	novel_in_catalog	gene: Zm00001d043998	novel	143.09	143.09	coding	118	1636	1989
PB.1381.1	1	1518	12	novel_in_catalog	gene: Zm00001d033747	novel	5.92	5.92	coding	279	498	1334
PB.6305.1	5	1607	1	full-splice_match	gene: Zm00001d014166	Zm00001d014166_T00	1.49	1.49	coding	495	39	1523
PB.1179.1	1	933	1	full-splice_match	gene: Zm00001d032821	Zm00001d032821_T00	62.29	208.82	coding	196	24	611
PB.10663.8	9	1972	7	full-splice_match	gene: Zm00001d047587	Zm00001d047587_T01	2.08	69.93	coding	305	785	1699
PB.10663.9	9	2514	8	novel_not_in_catalog	gene: Zm00001d047587	novel	4.80	69.93	coding	219	1576	2232
PB.10663.2	9	2143	8	novel_not_in_catalog	gene: Zm00001d047587	novel	13.07	69.93	coding	219	939	1595

PB.5428.2	4	447	1	intergenic	novelGene_353	novel	1.29	1.29	non coding	NA	NA	NA
PB.10663.1	9	1614	8	novel_not_in_catalog	gene: Zm00001d047587	novel	27.64	69.93	coding	335	51	1055
PB.10663.6	9	1699	9	novel_not_in_catalog	gene: Zm00001d047587	novel	1.07	69.93	coding	305	253	1167
PB.10663.7	9	1885	10	novel_not_in_catalog	gene: Zm00001d047587	novel	9.43	69.93	coding	305	606	1520
PB.2239.2	10	1099	1	incomplete-splice_match	gene: Zm00001d026149	Zm00001d026149_T00	8.09	14.99	coding	224	209	880
PB.7026.1	5	2646	1	intergenic	novelGene_395	novel	0.24	0.24	non coding	NA	NA	NA
PB.2544.5	2	1864	3	full-splice_match	gene: Zm00001d002284	Zm00001d002284_T00	9.91	44.30	coding	472	179	1594
PB.2544.6	2	1784	3	full-splice_match	gene: Zm00001d002284	Zm00001d002284_T00	0.00	44.30	coding	450	288	1637
PB.761.1	1	1576	8	full-splice_match	gene: Zm00001d030962	Zm00001d030962_T00	1.68	1.68	coding	473	38	1456
PB.9412.1	8	2995	16	full-splice_match	gene: Zm00001d009702	Zm00001d009702_T00	77.99	181.99	coding	843	124	2652
PB.2544.1	2	2236	3	full-splice_match	gene: Zm00001d002284	Zm00001d002284_T00	0.00	44.30	coding	450	297	1646
PB.2544.2	2	2079	3	novel_in_catalog	gene: Zm00001d002284	novel	3.17	44.30	coding	450	148	1497
PB.2544.3	2	1921	3	full-splice_match	gene: Zm00001d002284	Zm00001d002284_T00	31.22	44.30	coding	450	295	1644
PB.7637.2	6	644	4	full-splice_match	gene: Zm00001d036964	Zm00001d036964_T00	32.75	107.56	coding	76	128	355
PB.1487.1	1	500	3	full-splice_match	gene: Zm00001d034223	Zm00001d034223_T00	341.39	382.61	non coding	NA	NA	NA
PB.3626.1	2	440	1	intergenic	novelGene_439	novel	0.00	0.00	non coding	NA	NA	NA
PB.10445.1	9	1183	1	full-splice_match	gene: Zm00001d046499	Zm00001d046499_T00	22.38	22.38	coding	260	81	860

PB.9252.19	8	922	5	full-splice_match	gene: Zm00001d008749	Zm00001d008749_T00	99.02	136.63	coding	186	131	688
PB.9252.18	8	1289	4	novel_in_catalog	gene: Zm00001d008749	novel	4.74	136.63	coding	210	223	852
PB.8989.4	7	2172	1	full-splice_match	gene: Zm00001d022247	Zm00001d022247_T00	48.88	83.51	coding	581	106	1848
PB.8908.1	7	1291	2	full-splice_match	gene: Zm00001d021943	Zm00001d021943_T00	8.52	8.52	coding	109	788	1114
PB.4640.1	3	1311	5	full-splice_match	gene: Zm00001d043878	Zm00001d043878_T00	153.72	153.72	coding	269	105	911
PB.6226.1	5	952	5	full-splice_match	gene: Zm00001d013819	Zm00001d013819_T00	7.86	24.10	coding	280	112	951
PB.10990.3	B73V 4	2272	4	full-splice_match	gene: Zm00001d000381	Zm00001d000381_T00	17.13	17.13	coding	638	150	2063
PB.9252.16	8	1156	5	full-splice_match	gene: Zm00001d008749	Zm00001d008749_T00	25.81	136.63	coding	186	129	686
PB.9252.15	8	1473	9	full-splice_match	gene: Zm00001d008744	Zm00001d008744_T00	37.01	103.79	coding	394	200	1381
PB.4487.1	3	3182	10	incomplete-splice_match	gene: Zm00001d043376	Zm00001d043376_T00	53.52	53.52	coding	266	2217	3014
PB.8989.2	7	2291	1	full-splice_match	gene: Zm00001d022247	Zm00001d022247_T00	29.24	83.51	coding	581	99	1841
PB.3120.1	2	1005	3	novel_in_catalog	gene: Zm00001d005281	novel	16.89	16.89	coding	188	84	647
PB.8746.1	7	679	2	full-splice_match	gene: Zm00001d021391	Zm00001d021391_T00	4.96	4.96	coding	168	53	556
PB.5920.2	4	4746	12	incomplete-splice_match	gene: Zm00001d054003	Zm00001d054003_T04	18.27	75.42	coding	1371	632	4744
PB.1916.1	10	955	1	full-splice_match	gene: Zm00001d024494	Zm00001d024494_T00	17.63	17.63	coding	171	138	650
PB.1477.2	1	423	1	incomplete-splice_match	gene: Zm00001d034175	Zm00001d034175_T00	14.73	67.60	non coding	NA	NA	NA
PB.1162.1	1	844	2	full-splice_match	gene: Zm00001d032760	Zm00001d032760_T00	362.71	497.94	coding	164	80	571

PB.2596.1	2	1879	8	full-splice_match	gene: Zm00001d002499	Zm00001d002499_T00	31.74	31.74	coding	417	426	1676
PB.3396.17	2	2325	4	novel_in_catalog	gene: Zm00001d006578	novel	8.35	210.21	coding	293	306	1184
PB.8248.2	7	1914	1	genic	gene: Zm00001d018749	novel	5.97	15.65	coding	302	173	1078
PB.2364.1	10	2539	2	genic	gene: Zm00001d026559	novel	58.06	69.13	coding	621	288	2150
PB.2364.2	10	2729	1	full-splice_match	gene: Zm00001d026559	Zm00001d026559_T00	11.06	69.13	coding	621	467	2329
PB.2800.2	2	1975	1	intergenic	novelGene_135	novel	8.49	8.49	non coding	NA	NA	NA
PB.2364.4	10	2409	2	genic	gene: Zm00001d026559	novel	0.00	69.13	coding	621	284	2146
PB.2364.5	10	2589	1	full-splice_match	gene: Zm00001d026559	Zm00001d026559_T00	0.00	69.13	coding	621	466	2328
PB.2364.6	10	2153	2	genic	gene: Zm00001d026559	novel	0.00	69.13	coding	621	269	2131
PB.340.1	1	940	1	novel_in_catalog	gene: Zm00001d028687	novel	60.96	206.78	coding	97	284	574
PB.964.4	1	1337	8	full-splice_match	gene: Zm00001d031967	Zm00001d031967_T00	44.28	110.89	coding	304	126	1037
PB.964.7	1	1133	8	full-splice_match	gene: Zm00001d031967	Zm00001d031967_T00	10.69	110.89	coding	304	133	1044
PB.340.2	1	3643	8	full-splice_match	gene: Zm00001d028687	Zm00001d028687_T00	69.24	206.78	coding	1061	193	3375
PB.340.5	1	1112	4	incomplete-splice_match	gene: Zm00001d028687	Zm00001d028687_T00	40.54	206.78	coding	331	21	1013
PB.340.4	1	2129	8	full-splice_match	gene: Zm00001d028687	Zm00001d028687_T00	36.03	206.78	coding	625	4	1878
PB.964.3	1	1922	7	novel_in_catalog	gene: Zm00001d031967	novel	6.70	110.89	coding	263	129	917
PB.6393.2	5	1799	11	incomplete-splice_match	gene: Zm00001d014575	Zm00001d014575_T01	77.86	77.86	coding	492	19	1494

PB.2993.1	2	2864	14	full-splice_match	gene: Zm00001d004541	Zm00001d004541_T00	15.45	15.45	coding	853	31	2589
PB.2225.2	10	1986	2	full-splice_match	gene: Zm00001d026051	Zm00001d026051_T00	18.95	57.74	coding	546	152	1789
PB.2225.3	10	2046	2	full-splice_match	gene: Zm00001d026051	Zm00001d026051_T00	0.61	57.74	coding	606	52	1869
PB.1613.1	1	1703	6	novel_in_catalog	gene: Zm00001d034719	novel	41.33	45.33	coding	403	207	1415
PB.8774.1	7	1710	6	full-splice_match	gene: Zm00001d021506	Zm00001d021506_T00	59.72	59.72	coding	414	195	1436
PB.8774.3	7	1569	6	full-splice_match	gene: Zm00001d021506	Zm00001d021506_T00	0.00	59.72	coding	414	124	1365
PB.4497.4	3	1611	12	full-splice_match	gene: Zm00001d043410	Zm00001d043410_T00	0.00	81.14	coding	383	118	1266
PB.10642.1	9	3335	24	novel_in_catalog	gene: Zm00001d047506	novel	15.23	15.23	coding	883	216	2864
PB.4497.1	3	1743	12	full-splice_match	gene: Zm00001d043410	Zm00001d043410_T00	81.14	81.14	coding	383	119	1267
PB.10696.1	9	1365	3	incomplete-splice_match	gene: Zm00001d047710	Zm00001d047710_T01	13.18	21.87	coding	85	53	307
PB.10938.1	9	1272	2	novel_not_in_catalog	gene: Zm00001d048565	novel	7.18	7.18	coding	251	317	1069
PB.6086.1	5	1128	3	incomplete-splice_match	gene: Zm00001d013262	Zm00001d013262_T00	9.19	9.19	coding	248	135	878
PB.9589.1	8	981	3	incomplete-splice_match	gene: Zm00001d010615	Zm00001d010615_T00	35.58	49.35	coding	162	179	664
PB.9589.2	8	867	4	novel_not_in_catalog	gene: Zm00001d010615	novel	13.77	49.35	coding	183	18	566
PB.6954.3	5	1207	5	novel_in_catalog	gene: Zm00001d017298	novel	2.06	125.29	coding	129	6	392
PB.6562.2	5	2189	4	novel_in_catalog	gene: Zm00001d015212	novel	44.39	44.39	coding	205	1138	1752
PB.6562.3	5	1613	4	novel_in_catalog	gene: Zm00001d015212	novel	0.00	44.39	coding	205	852	1466

PB.2133.4	10	2075	9	full-splice_match	gene: Zm00001d025653	Zm00001d025653_T00	0.00	29.70	coding	220	45	704
PB.10896.2	9	2065	12	full-splice_match	gene: Zm00001d048463	Zm00001d048463_T00	56.86	79.33	coding	520	213	1772
PB.7453.2	6	2187	11	full-splice_match	gene: Zm00001d035854	Zm00001d035854_T00	0.00	69.42	coding	619	80	1936
PB.7453.3	6	1113	5	incomplete-splice_match	gene: Zm00001d035854	Zm00001d035854_T00	30.13	69.42	coding	294	106	987
PB.7453.1	6	2254	11	full-splice_match	gene: Zm00001d035854	Zm00001d035854_T00	39.28	69.42	coding	619	91	1947
PB.7365.10	6	4632	8	novel_in_catalog	gene: Zm00001d035346	novel	6.36	44.35	coding	187	1167	1727
PB.6886.3	5	2404	3	novel_in_catalog	gene: Zm00001d017067	novel	2.99	13.96	coding	555	451	2115
PB.7365.12	6	1036	5	full-splice_match	gene: Zm00001d035346	Zm00001d035346_T17	0.00	44.35	non coding	NA	NA	NA
PB.7365.13	6	985	8	novel_in_catalog	gene: Zm00001d035346	novel	5.04	44.35	non coding	NA	NA	NA
PB.8982.1	7	1181	10	full-splice_match	gene: Zm00001d022226	Zm00001d022226_T00	10.01	10.01	coding	293	59	937
PB.1070.1	1	1604	1	full-splice_match	gene: Zm00001d032433	Zm00001d032433_T00	3.28	24.41	coding	393	75	1253
PB.1070.2	1	1534	1	full-splice_match	gene: Zm00001d032433	Zm00001d032433_T00	21.13	24.41	coding	393	113	1291
PB.726.1	1	1082	2	novel_in_catalog	gene: Zm00001d022836	novel	8.16	11.31	non coding	NA	NA	NA
PB.6886.1	5	2481	4	full-splice_match	gene: Zm00001d017067	Zm00001d017067_T00	9.32	13.96	coding	555	433	2097
PB.1247.1	1	1383	4	full-splice_match	gene: Zm00001d033160	Zm00001d033160_T00	63.62	63.62	coding	335	132	1136
PB.334.1	1	741	3	full-splice_match	gene: Zm00001d028661	Zm00001d028661_T00	30.52	30.52	coding	166	57	554
PB.1117.1	1	867	3	full-splice_match	gene: Zm00001d032582	Zm00001d032582_T00	45.77	49.52	coding	182	170	715

PB.7082.2	5	1719	3	incomplete-splice_match	gene: Zm00001d017866	Zm00001d017866_T00	0.00	83.84	coding	467	91	1491
PB.8502.1	7	1209	1	novel_in_catalog	gene: Zm00001d020281	novel	1.79	7.99	coding	134	226	627
PB.6514.1	5	594	1	genic	gene: Zm00001d014992	novel	20.93	20.93	non coding	NA	NA	NA
PB.1990.1	10	1095	2	full-splice_match	gene: Zm00001d024872	Zm00001d024872_T00	7.71	7.71	coding	277	76	906
PB.5896.9	4	1304	2	intergenic	novelGene_446	novel	10.46	108.08	coding	62	270	455
PB.6409.1	5	2438	12	full-splice_match	gene: Zm00001d014655	Zm00001d014655_T00	32.20	49.96	coding	712	61	2196
PB.6409.2	5	1625	7	incomplete-splice_match	gene: Zm00001d014655	Zm00001d014655_T00	17.76	49.96	coding	424	167	1438
PB.7082.5	5	2207	3	incomplete-splice_match	gene: Zm00001d017866	Zm00001d017866_T00	14.92	83.84	coding	467	90	1490
PB.9898.3	8	1776	11	full-splice_match	gene: Zm00001d011945	Zm00001d011945_T00	8.66	14.44	coding	400	345	1544
PB.2297.3	10	860	2	incomplete-splice_match	gene: Zm00001d026334	Zm00001d026334_T00	8.13	93.80	non coding	NA	NA	NA
PB.9898.1	8	1153	6	novel_in_catalog	gene: Zm00001d011945	novel	3.36	14.44	coding	130	42	431
PB.5158.1	4	956	3	novel_in_catalog	gene: Zm00001d050199	novel	12.35	12.35	coding	113	230	568
PB.7863.1	6	700	3	full-splice_match	gene: Zm00001d037968	Zm00001d037968_T00	17.50	17.50	coding	178	45	578
PB.7082.6	5	1999	3	incomplete-splice_match	gene: Zm00001d017866	Zm00001d017866_T00	0.00	83.84	coding	467	83	1483
PB.1934.2	10	1357	2	incomplete-splice_match	gene: Zm00001d024546	Zm00001d024546_T00	8.43	15.88	coding	360	11	1090
PB.1934.3	10	2131	2	novel_in_catalog	gene: Zm00001d024546	novel	7.45	15.88	coding	490	45	1514
PB.10893.1	9	1282	2	full-splice_match	gene: Zm00001d048449	Zm00001d048449_T00	189.00	211.06	coding	170	459	968

PB.8864.9	7	1849	11	full-splice_match	gene: Zm00001d021802	Zm00001d021802_T00	21.66	166.06	coding	468	216	1619
PB.10893.3	9	1176	2	full-splice_match	gene: Zm00001d048449	Zm00001d048449_T00	14.20	211.06	coding	170	466	975
PB.3682.1	3	1521	1	full-splice_match	gene: Zm00001d039270	Zm00001d039270_T00	4.09	4.09	coding	397	35	1225
PB.1293.1	1	981	3	full-splice_match	gene: Zm00001d033370	Zm00001d033370_T00	46.33	46.33	coding	127	21	401
PB.10893.4	9	845	2	full-splice_match	gene: Zm00001d048449	Zm00001d048449_T00	0.00	211.06	coding	170	240	749
PB.4286.1	3	862	4	novel_not_in_catalog	gene: Zm00001d042535	novel	119.73	119.73	coding	163	136	624
PB.1546.1	1	1722	5	full-splice_match	gene: Zm00001d034493	Zm00001d034493_T00	10.27	10.27	coding	371	100	1212
PB.3384.4	2	2801	6	full-splice_match	gene: Zm00001d006504	Zm00001d006504_T01	8.28	26.41	coding	863	125	2713
PB.4575.5	3	989	10	incomplete-splice_match	gene: Zm00001d043669	Zm00001d043669_T00	20.11	103.17	coding	253	115	873
PB.6238.2	5	857	1	intergenic	novelGene_92	novel	168.99	264.10	non coding	NA	NA	NA
PB.7009.1	5	774	1	full-splice_match	gene: Zm00001d017535	Zm00001d017535_T00	11.85	11.85	coding	126	90	467
PB.3384.2	2	1674	3	incomplete-splice_match	gene: Zm00001d006504	Zm00001d006504_T00	18.12	26.41	coding	441	32	1354
PB.5384.1	4	2543	2	genic	gene: Zm00001d051675	novel	188.18	188.18	coding	656	261	2228
PB.985.1	1	1055	2	full-splice_match	gene: Zm00001d032058	Zm00001d032058_T00	17.85	17.85	coding	221	118	780
PB.3140.2	2	1283	1	genic	gene: Zm00001d005381	novel	12.53	12.53	coding	183	87	635
PB.3919.1	3	1828	3	full-splice_match	gene: Zm00001d040310	Zm00001d040310_T00	16.86	16.86	coding	545	1	1635
PB.7328.2	6	1957	1	incomplete-splice_match	gene: Zm00001d035139	Zm00001d035139_T00	10.87	58.08	coding	535	138	1742

PB.7328.1	6	2592	4	full-splice_match	gene: Zm00001d035139	Zm00001d035139_T00	47.21	58.08	coding	720	168	2327
PB.6199.8	5	2460	5	novel_in_catalog	gene: Zm00001d013669	novel	2.33	1101.77	coding	338	1245	2258
PB.2001.1	10	1831	2	full-splice_match	gene: Zm00001d024916	Zm00001d024916_T00	75.19	75.19	coding	470	152	1561
PB.3253.2	2	2454	2	full-splice_match	gene: Zm00001d005986	Zm00001d005986_T00	14.37	14.37	coding	605	456	2270
PB.2994.1	2	1407	8	full-splice_match	gene: Zm00001d004543	Zm00001d004543_T00	395.21	395.21	coding	321	61	1023
PB.9087.2	7	1311	2	full-splice_match	gene: Zm00001d022549	Zm00001d022549_T00	0.00	5.68	coding	388	53	1216
PB.10903.1	9	1146	1	full-splice_match	gene: Zm00001d048486	Zm00001d048486_T00	57.26	57.26	coding	259	49	825
PB.10559.1	9	3454	14	novel_in_catalog	gene: Zm00001d047162	novel	4.76	4.76	coding	659	1097	3073
PB.4492.2	3	1226	1	full-splice_match	gene: Zm00001d043390	Zm00001d043390_T00	252.51	303.27	coding	297	126	1016
PB.518.1	1	2631	2	full-splice_match	gene: Zm00001d029516	Zm00001d029516_T00	7.89	47.35	coding	543	380	2008
PB.8051.1	6	1503	9	full-splice_match	gene: Zm00001d038600	Zm00001d038600_T00	16.61	16.61	coding	422	65	1330
PB.6242.1	5	3866	8	full-splice_match	gene: Zm00001d013908	Zm00001d013908_T00	29.73	29.73	coding	982	652	3597
PB.518.2	1	2404	2	full-splice_match	gene: Zm00001d029516	Zm00001d029516_T00	39.46	47.35	coding	543	452	2080
PB.1562.3	1	1204	5	novel_not_in_catalog	gene: Zm00001d034534	novel	37.00	47.66	coding	206	239	856
PB.6791.1	5	1685	1	full-splice_match	gene: Zm00001d016653	Zm00001d016653_T00	21.80	26.34	coding	416	82	1329
PB.1809.1	10	849	2	full-splice_match	gene: Zm00001d023811	Zm00001d023811_T00	3.57	3.57	coding	161	52	534
PB.9618.1	8	1142	9	full-splice_match	gene: Zm00001d010703	Zm00001d010703_T01	4.76	47.99	coding	167	123	623

PB.518.3	1	2137	2	full-splice_match	gene: Zm00001d029516	Zm00001d029516_T00	0.00	47.35	coding	543	381	2009
PB.4262.1	3	1930	7	novel_in_catalog	gene: Zm00001d042435	novel	5.54	16.89	coding	297	55	945
PB.9967.1	8	2260	6	full-splice_match	gene: Zm00001d012238	Zm00001d012238_T00	70.76	188.10	coding	284	173	1024
PB.6540.2	5	1825	1	intergenic	novelGene_174	novel	10.67	10.67	non coding	NA	NA	NA
PB.9352.3	8	423	1	genic	gene: Zm00001d009415	novel	455.55	622.40	non coding	NA	NA	NA
PB.5470.1	4	858	1	full-splice_match	gene: Zm00001d052066	Zm00001d052066_T00	52.93	52.93	coding	123	115	483
PB.6867.1	5	1742	3	antisense	novelGene_gene: Zm00001d016957	novel	13.71	16.07	coding	82	91	336
PB.6867.2	5	1051	4	antisense	novelGene_gene: Zm00001d016957	novel	2.36	16.07	non coding	NA	NA	NA
PB.3041.1	2	939	1	intergenic	novelGene_279	novel	0.00	0.00	coding	132	178	573
PB.10894.1	9	1744	6	novel_in_catalog	gene: Zm00001d048450	novel	4.44	41.22	coding	79	128	364
PB.9098.1	7	1481	7	full-splice_match	gene: Zm00001d022588	Zm00001d022588_T00	36.25	36.25	coding	411	73	1305
PB.6141.5	5	622	1	incomplete-splice_match	gene: Zm00001d013427	Zm00001d013427_T00	3.29	134.99	coding	150	26	475
PB.6141.4	5	841	5	incomplete-splice_match	gene: Zm00001d013427	Zm00001d013427_T00	36.44	134.99	coding	156	104	571
PB.1584.3	1	1012	3	novel_in_catalog	gene: Zm00001d034618	novel	1.62	89.40	coding	213	39	677
PB.4931.1	4	1974	10	novel_in_catalog	gene: Zm00001d049080	novel	15.80	40.78	coding	394	475	1656
PB.6141.1	5	2221	9	full-splice_match	gene: Zm00001d013427	Zm00001d013427_T00	95.26	134.99	coding	603	41	1849
PB.9084.13	7	1719	10	full-splice_match	gene: Zm00001d022542	Zm00001d022542_T00	0.00	154.27	coding	333	384	1382

PB.4580.2	3	3268	11	full-splice_match	gene: Zm00001d043684	Zm00001d043684_T00	12.55	22.23	coding	913	186	2924
PB.9866.1	8	865	2	full-splice_match	gene: Zm00001d011813	Zm00001d011813_T00	266.58	350.55	coding	181	52	594
PB.9866.2	8	757	2	full-splice_match	gene: Zm00001d011813	Zm00001d011813_T00	83.98	350.55	coding	181	52	594
PB.5461.2	4	1142	1	novel_in_catalog	gene: Zm00001d052053	novel	7.40	62.79	coding	56	265	432
PB.268.2	1	1574	1	full-splice_match	gene: Zm00001d028389	Zm00001d028389_T00	0.00	36.40	coding	256	411	1178
PB.1815.3	10	3389	8	full-splice_match	gene: Zm00001d023838	Zm00001d023838_T00	33.97	74.52	coding	884	311	2962
PB.268.1	1	1689	1	full-splice_match	gene: Zm00001d028389	Zm00001d028389_T00	36.40	36.40	coding	256	409	1176
PB.1815.4	10	1070	2	full-splice_match	gene: Zm00001d023838	Zm00001d023838_T02	0.00	74.52	coding	297	180	1070
PB.8732.1	7	842	1	full-splice_match	gene: Zm00001d021337	Zm00001d021337_T00	0.00	0.00	coding	114	98	439
PB.3757.8	3	689	4	intergenic	novelGene_36	novel	24.55	79.80	non coding	NA	NA	NA
PB.9646.1	8	2202	10	incomplete-splice_match	gene: Zm00001d010811	Zm00001d010811_T00	0.16	135.99	coding	428	183	1466
PB.4934.2	4	1346	3	intergenic	novelGene_66	novel	18.03	18.03	coding	130	156	545
PB.8601.2	7	980	7	full-splice_match	gene: Zm00001d020783	Zm00001d020783_T00	166.23	166.23	coding	182	155	700
PB.1550.1	1	2677	12	full-splice_match	gene: Zm00001d034502	Zm00001d034502_T00	7.82	12.74	coding	667	225	2225
PB.7589.3	6	1555	8	full-splice_match	gene: Zm00001d036654	Zm00001d036654_T00	50.87	119.84	coding	349	276	1322
PB.5632.1	4	1271	2	full-splice_match	gene: Zm00001d052741	Zm00001d052741_T00	3.63	3.63	coding	284	202	1053
PB.9035.2	7	2077	7	incomplete-splice_match	gene: Zm00001d022405	Zm00001d022405_T03	45.08	45.08	coding	363	534	1622

PB.2382.1	10	1315	1	full-splice_match	gene: Zm00001d026597	Zm00001d026597_T00	119.50	273.37	coding	309	66	992
PB.8986.2	7	458	3	incomplete-splice_match	gene: Zm00001d022240	Zm00001d022240_T00	8.29	19.89	non coding	NA	NA	NA
PB.4390.2	3	2228	5	full-splice_match	gene: Zm00001d042993	Zm00001d042993_T00	0.00	126.00	coding	541	309	1931
PB.9173.1	8	1450	1	full-splice_match	gene: Zm00001d008408	Zm00001d008408_T00	30.74	30.74	coding	345	96	1130
PB.2382.3	10	513	1	full-splice_match	gene: Zm00001d026597	Zm00001d026597_T00	153.88	273.37	coding	11	315	347
PB.10320.3	9	1790	1	intergenic	novelGene_118	novel	4.26	23.84	non coding	NA	NA	NA
PB.7003.4	5	1077	3	full-splice_match	gene: Zm00001d017523	Zm00001d017523_T00	14.87	40.25	coding	224	38	709
PB.7583.4	6	1523	13	full-splice_match	gene: Zm00001d036631	Zm00001d036631_T00	7.44	46.80	coding	215	561	1205
PB.7583.5	6	2731	11	full-splice_match	gene: Zm00001d036631	Zm00001d036631_T00	13.22	46.80	coding	215	1770	2414
PB.121.2	1	2785	9	novel_in_catalog	gene: Zm00001d027731	novel	74.25	333.50	coding	687	311	2371
PB.121.3	1	2783	9	full-splice_match	gene: Zm00001d027731	Zm00001d027731_T00	133.61	333.50	coding	690	304	2373
PB.1852.1	10	654	4	incomplete-splice_match	gene: Zm00001d024036	Zm00001d024036_T00	49.23	49.23	non coding	NA	NA	NA
PB.7583.3	6	1454	12	full-splice_match	gene: Zm00001d036631	Zm00001d036631_T01	0.00	46.80	coding	215	493	1137
PB.9252.2	8	1819	9	full-splice_match	gene: Zm00001d008744	Zm00001d008744_T00	57.41	103.79	coding	394	186	1367
PB.9520.1	8	934	4	full-splice_match	gene: Zm00001d010290	Zm00001d010290_T00	258.93	258.93	coding	176	94	621
PB.9252.6	8	1806	10	novel_not_in_catalog	gene: Zm00001d008744	novel	9.37	103.79	coding	394	306	1487
PB.2665.1	2	1239	1	full-splice_match	gene: Zm00001d002798	Zm00001d002798_T00	43.36	72.12	coding	209	105	731

PB.3146.1	2	2720	3	genic	gene: Zm00001d005445	novel	9.35	39.10	coding	711	61	2193
PB.3146.3	2	2689	2	genic	gene: Zm00001d005445	novel	9.24	39.10	coding	711	84	2216
PB.3146.2	2	2105	2	genic	gene: Zm00001d005445	novel	14.17	39.10	coding	468	175	1578
PB.2665.2	2	1159	1	full-splice_match	gene: Zm00001d002798	Zm00001d002798_T00	28.76	72.12	coding	209	162	788
PB.1024.3	1	1233	6	full-splice_match	gene: Zm00001d032231	Zm00001d032231_T00	395.50	490.73	coding	256	225	992
PB.2731.2	2	644	1	intergenic	novelGene_117	novel	149.45	3539.17	non coding	NA	NA	NA
PB.8893.1	7	2072	11	full-splice_match	gene: Zm00001d021881	Zm00001d021881_T00	37.43	51.42	coding	216	96	743
PB.1024.4	1	715	3	novel_in_catalog	gene: Zm00001d032231	novel	77.79	490.73	coding	94	30	311
PB.1024.5	1	936	7	novel_in_catalog	gene: Zm00001d032231	novel	17.44	490.73	coding	132	300	695
PB.5351.1	4	3372	16	novel_in_catalog	gene: Zm00001d051545	novel	9.03	32.84	coding	780	1	2340
PB.630.1	1	1648	5	full-splice_match	gene: Zm00001d030110	Zm00001d030110_T00	40.73	227.82	coding	214	16	657
PB.5351.3	4	3030	17	full-splice_match	gene: Zm00001d051545	Zm00001d051545_T00	3.71	32.84	coding	877	111	2741
PB.5351.2	4	2441	15	incomplete-splice_match	gene: Zm00001d051545	Zm00001d051545_T00	19.00	32.84	coding	621	110	1972
PB.296.1	1	2276	9	full-splice_match	gene: Zm00001d028514	Zm00001d028514_T00	38.55	38.55	coding	600	115	1914
PB.5351.4	4	3254	16	novel_in_catalog	gene: Zm00001d051545	novel	1.10	32.84	coding	617	1117	2967
PB.8488.1	7	1784	2	incomplete-splice_match	gene: Zm00001d020204	Zm00001d020204_T00	54.48	54.48	coding	494	6	1487
PB.261.2	1	2096	4	novel_in_catalog	gene: Zm00001d028366	novel	13.93	62.46	coding	230	171	860

PB.9728.1	8	2286	4	full-splice_match	gene: Zm00001d011202	Zm00001d011202_T00	40.85	102.93	coding	438	617	1930
PB.10694.1	9	1554	5	antisense	novelGene_gene: Zm00001d047708	novel	14.46	40.46	coding	140	1	420
PB.10694.2	9	2673	3	antisense	novelGene_gene: Zm00001d047708	novel	10.42	40.46	non coding	NA	NA	NA
PB.10694.3	9	1214	4	antisense	novelGene_gene: Zm00001d047708	novel	15.58	40.46	coding	112	37	372
PB.261.1	1	1970	4	novel_in_catalog	gene: Zm00001d028366	novel	31.23	62.46	coding	342	492	1517
PB.5591.1	4	1270	1	genic	gene: Zm00001d052482	novel	83.52	144.34	coding	263	160	948
PB.6700.1	5	3020	5	full-splice_match	gene: Zm00001d016144	Zm00001d016144_T00	4.63	4.63	coding	194	2246	2827
PB.9776.1	8	2817	2	novel_in_catalog	gene: Zm00001d011436	novel	2.20	16.29	non coding	NA	NA	NA
PB.630.2	1	2612	7	full-splice_match	gene: Zm00001d030110	Zm00001d030110_T00	105.10	227.82	coding	592	504	2279
PB.9776.4	8	2056	3	novel_in_catalog	gene: Zm00001d011436	novel	4.51	16.29	coding	83	194	442
PB.9776.5	8	2235	4	full-splice_match	gene: Zm00001d011436	Zm00001d011436_T00	5.38	16.29	non coding	NA	NA	NA
PB.3979.1	3	739	1	full-splice_match	gene: Zm00001d040623	Zm00001d040623_T00	0.43	0.43	coding	72	127	342
PB.4196.1	3	1709	5	full-splice_match	gene: Zm00001d042066	Zm00001d042066_T04	36.32	87.33	coding	242	425	1150
PB.863.6	1	1055	3	full-splice_match	gene: Zm00001d031453	Zm00001d031453_T00	0.00	19.26	coding	280	67	906
PB.5921.1	4	1750	5	full-splice_match	gene: Zm00001d054008	Zm00001d054008_T00	49.76	49.76	coding	405	250	1464
PB.9728.2	8	2028	4	full-splice_match	gene: Zm00001d011202	Zm00001d011202_T00	62.08	102.93	coding	438	464	1777
PB.9973.3	8	2057	4	full-splice_match	gene: Zm00001d012254	Zm00001d012254_T00	3.24	41.83	coding	556	247	1914

PB.8334.3	7	585	1	incomplete-splice_match	gene: Zm00001d019173	Zm00001d019173_T00	38.83	94.34	non coding	NA	NA	NA
PB.7083.5	5	1795	10	full-splice_match	gene: Zm00001d017872	Zm00001d017872_T02	3.22	171.20	coding	313	178	1116
PB.3499.4	2	3874	9	novel_in_catalog	gene: Zm00001d007029	novel	2.64	82.97	coding	262	594	1379
PB.3499.5	2	2647	10	full-splice_match	gene: Zm00001d007029	Zm00001d007029_T00	5.63	82.97	coding	499	594	2090
PB.1114.1	1	788	2	genic	gene: Zm00001d032571	novel	2.82	64.19	coding	89	145	411
PB.3499.7	2	958	6	incomplete-splice_match	gene: Zm00001d007029	Zm00001d007029_T00	18.45	82.97	coding	262	21	806
PB.3499.1	2	744	1	genic	gene: Zm00001d007029	novel	2.71	82.97	non coding	NA	NA	NA
PB.3499.2	2	967	3	incomplete-splice_match	gene: Zm00001d007029	Zm00001d007029_T00	6.83	82.97	coding	78	88	321
PB.6795.1	5	1938	14	novel_in_catalog	gene: Zm00001d016662	novel	21.47	26.52	coding	441	298	1620
PB.3044.11	2	2186	4	novel_in_catalog	gene: Zm00001d004855	novel	0.00	53.34	coding	377	933	2063
PB.3499.8	2	2372	11	full-splice_match	gene: Zm00001d007029	Zm00001d007029_T00	0.00	82.97	coding	592	512	2287
PB.9163.1	8	1905	5	full-splice_match	gene: Zm00001d008367	Zm00001d008367_T00	63.83	80.54	coding	176	667	1194
PB.9163.2	8	2767	4	novel_in_catalog	gene: Zm00001d008367	novel	16.71	80.54	coding	176	431	958
PB.9706.9	8	1799	11	full-splice_match	gene: Zm00001d011119	Zm00001d011119_T01	24.66	78.92	coding	491	82	1554
PB.9794.1	8	1146	3	full-splice_match	gene: Zm00001d011531	Zm00001d011531_T00	22.27	44.49	coding	275	46	870
PB.1828.1	10	403	2	incomplete-splice_match	gene: Zm00001d023908	Zm00001d023908_T00	67.62	67.62	non coding	NA	NA	NA
PB.7083.4	5	1745	10	full-splice_match	gene: Zm00001d017872	Zm00001d017872_T00	140.14	171.20	coding	446	179	1516

PB.1731.1	10	1698	2	genic	gene: Zm00001d023422	novel	16.86	16.86	coding	389	69	1235
PB.8323.1	7	3139	9	incomplete-splice_match	gene: Zm00001d019115	Zm00001d019115_T00	17.21	21.26	coding	899	96	2792
PB.8323.3	7	1545	7	novel_in_catalog	gene: Zm00001d019115	novel	4.04	21.26	coding	328	8	991
PB.6743.2	5	2034	18	full-splice_match	gene: Zm00001d016365	Zm00001d016365_T00	20.87	89.66	coding	542	161	1786
PB.5276.1	4	2926	14	full-splice_match	gene: Zm00001d051067	Zm00001d051067_T01	40.26	40.26	coding	793	231	2609
PB.6735.3	5	1921	1	incomplete-splice_match	gene: Zm00001d016326	Zm00001d016326_T01	0.00	56.35	coding	374	513	1634
PB.5914.2	4	2075	1	intergenic	novelGene_447	novel	0.00	5.99	non coding	NA	NA	NA
PB.5276.2	4	2203	14	full-splice_match	gene: Zm00001d051067	Zm00001d051067_T01	0.00	40.26	coding	678	168	2201
PB.8653.1	7	1560	2	full-splice_match	gene: Zm00001d020984	Zm00001d020984_T00	77.36	77.36	coding	415	152	1396
PB.3296.1	2	2160	4	novel_in_catalog	gene: Zm00001d006133	novel	13.00	13.00	coding	365	225	1319
PB.4218.2	3	2580	13	full-splice_match	gene: Zm00001d042234	Zm00001d042234_T00	10.30	32.08	coding	800	41	2440
PB.4218.1	3	2192	10	novel_in_catalog	gene: Zm00001d042234	novel	21.78	32.08	coding	597	87	1877
PB.4325.1	3	1257	3	full-splice_match	gene: Zm00001d042719	Zm00001d042719_T00	51.82	51.82	coding	276	147	974
PB.9998.1	8	1931	1	full-splice_match	gene: Zm00001d012340	Zm00001d012340_T00	4.56	10.68	coding	503	245	1753
PB.7194.2	5	2702	1	full-splice_match	gene: Zm00001d018282	Zm00001d018282_T00	15.18	56.26	coding	783	13	2361
PB.7799.3	6	2529	5	full-splice_match	gene: Zm00001d037695	Zm00001d037695_T00	0.00	14.94	coding	558	763	2436
PB.1421.1	1	1785	9	full-splice_match	gene: Zm00001d033932	Zm00001d033932_T00	30.30	30.30	coding	489	85	1551

PB.1407.1	1	1500	10	full-splice_match	gene: Zm00001d033870	Zm00001d033870_T00	17.73	17.73	coding	316	107	1054
PB.1002.1	1	704	5	novel_not_in_catalog	gene: Zm00001d032123	novel	76.40	151.22	non coding	NA	NA	NA
PB.4893.2	4	1139	1	full-splice_match	gene: Zm00001d048887	Zm00001d048887_T00	13.91	28.71	coding	330	49	1038
PB.4893.1	4	1217	1	full-splice_match	gene: Zm00001d048887	Zm00001d048887_T00	14.79	28.71	coding	330	19	1008
PB.3169.4	2	1481	8	full-splice_match	gene: Zm00001d005579	Zm00001d005579_T01	20.98	72.57	coding	310	236	1165
PB.7985.6	6	1076	8	novel_in_catalog	gene: Zm00001d038380	novel	2.15	310.67	coding	300	177	1076
PB.7985.4	6	1270	7	novel_in_catalog	gene: Zm00001d038380	novel	0.00	310.67	coding	120	193	552
PB.7985.5	6	1187	8	novel_in_catalog	gene: Zm00001d038380	novel	2.82	310.67	coding	120	184	543
PB.7326.1	6	2282	1	full-splice_match	gene: Zm00001d035133	Zm00001d035133_T00	32.50	42.31	coding	676	7	2034
PB.7985.3	6	1240	9	full-splice_match	gene: Zm00001d038380	Zm00001d038380_T00	5.44	310.67	coding	215	326	970
PB.4520.9	3	650	4	novel_in_catalog	gene: Zm00001d043479	novel	12.50	136.35	coding	106	48	365
PB.7326.2	6	3078	1	full-splice_match	gene: Zm00001d035133	Zm00001d035133_T00	9.82	42.31	coding	982	98	3043
PB.4700.5	3	1915	7	novel_not_in_catalog	gene: Zm00001d044144	novel	27.82	102.99	coding	460	256	1635
PB.6954.7	5	1987	9	full-splice_match	gene: Zm00001d017298	Zm00001d017298_T00	49.72	125.29	coding	295	556	1440
PB.8893.5	7	1138	1	novel_in_catalog	gene: Zm00001d021881	novel	13.99	51.42	non coding	NA	NA	NA
PB.2990.2	2	736	1	genic	gene: Zm00001d004523	novel	13.01	56.95	coding	198	101	694
PB.2407.2	10	1112	9	full-splice_match	gene: Zm00001d026680	Zm00001d026680_T00	204.65	273.34	coding	214	107	748

PB.8681.1	7	1111	1	full-splice_match	gene: Zm00001d021141	Zm00001d021141_T00	17.78	243.60	coding	165	65	559
PB.3471.4	2	587	2	full-splice_match	gene: Zm00001d006903	Zm00001d006903_T00	0.00	181.01	coding	112	203	538
PB.8681.3	7	975	1	full-splice_match	gene: Zm00001d021141	Zm00001d021141_T00	34.53	243.60	coding	165	47	541
PB.8681.4	7	914	1	full-splice_match	gene: Zm00001d021141	Zm00001d021141_T00	76.07	243.60	coding	165	123	617
PB.2226.1	10	1310	6	full-splice_match	gene: Zm00001d026056	Zm00001d026056_T00	18.93	46.80	coding	319	114	1070
PB.3471.1	2	919	2	full-splice_match	gene: Zm00001d006903	Zm00001d006903_T00	181.01	181.01	coding	112	269	604
PB.9695.1	8	1969	3	novel_in_catalog	gene: Zm00001d011062	novel	16.53	16.53	coding	550	65	1714
PB.186.1	1	916	7	full-splice_match	gene: Zm00001d028040	Zm00001d028040_T00	44.55	44.55	coding	140	203	622
PB.4914.2	4	1231	5	fusion	gene: Zm00001d049007	novel	79.49	79.49	coding	254	127	888
PB.831.1	1	2055	4	full-splice_match	gene: Zm00001d031253	Zm00001d031253_T00	23.72	23.72	coding	551	115	1767
PB.7928.1	6	736	1	genic	gene: Zm00001d038186	novel	3.44	27.56	coding	120	18	377
PB.10543.1	9	1665	5	novel_not_in_catalog	gene: Zm00001d047050	novel	4.60	4.60	coding	341	199	1221
PB.9266.1	8	1172	6	full-splice_match	gene: Zm00001d008859	Zm00001d008859_T00	16.49	16.49	coding	217	187	837
PB.10928.3	9	3767	28	incomplete-splice_match	gene: Zm00001d048546	Zm00001d048546_T04	25.28	54.67	coding	1131	36	3428
PB.2038.1	10	3059	3	full-splice_match	gene: Zm00001d025050	Zm00001d025050_T00	76.93	119.07	coding	767	327	2627
PB.7494.1	6	1453	2	genic	gene: Zm00001d036080	novel	44.15	44.15	coding	163	388	876
PB.3339.1	2	1058	4	full-splice_match	gene: Zm00001d006324	Zm00001d006324_T00	16.70	16.70	coding	235	25	729

PB.8041.1	6	646	2	full-splice_match	gene: Zm00001d038578	Zm00001d038578_T00	58.30	58.30	coding	133	35	433
PB.10142.2	9	1436	2	full-splice_match	gene: Zm00001d044908	Zm00001d044908_T00	119.14	134.69	coding	376	103	1230
PB.10142.3	9	1503	1	novel_in_catalog	gene: Zm00001d044908	novel	4.13	134.69	coding	187	759	1319
PB.10142.1	9	748	1	incomplete-splice_match	gene: Zm00001d044908	Zm00001d044908_T00	11.42	134.69	coding	124	54	425
PB.3263.1	2	2932	11	full-splice_match	gene: Zm00001d006027	Zm00001d006027_T00	8.57	17.78	coding	489	127	1593
PB.5781.1	4	3252	14	full-splice_match	gene: Zm00001d053450	Zm00001d053450_T01	26.45	26.45	coding	970	323	3232
PB.832.2	1	1018	1	genic	gene: Zm00001d031257	novel	8.08	724.27	coding	126	370	747
PB.4776.2	3	1203	11	incomplete-splice_match	gene: Zm00001d044468	Zm00001d044468_T00	1.64	6.27	coding	178	481	1014
PB.7084.3	5	1480	3	novel_not_in_catalog	gene: Zm00001d017874	novel	12.48	126.53	coding	168	208	711
PB.768.2	1	1665	1	antisense	novelGene_gene: Zm00001d030998	novel	3.80	7.91	coding	166	195	692
PB.1237.3	1	725	6	full-splice_match	gene: Zm00001d033099	Zm00001d033099_T00	174.23	259.99	coding	142	58	483
PB.10441.1	9	1396	6	full-splice_match	gene: Zm00001d046489	Zm00001d046489_T00	55.59	55.59	coding	346	105	1142
PB.3483.1	2	1697	6	full-splice_match	gene: Zm00001d006931	Zm00001d006931_T00	7.54	7.54	coding	398	25	1218
PB.2446.1	2	770	1	genic	gene: Zm00001d001849	novel	27.49	27.49	coding	49	198	344
PB.4331.1	3	667	1	full-splice_match	gene: Zm00001d042730	Zm00001d042730_T00	428.84	428.84	coding	136	69	476
PB.7099.17	5	2216	3	antisense	novelGene_gene: Zm00001d017932	novel	1.88	98.88	non coding	NA	NA	NA
PB.7099.14	5	1731	6	antisense	novelGene_gene: Zm00001d017932	novel	0.00	98.88	non coding	NA	NA	NA

PB.2498.1	2	474	4	intergenic	novelGene_22	novel	32.30	32.30	non coding	NA	NA	NA
PB.7099.12	5	3511	1	antisense	novelGene_gene: Zm00001d017932	novel	7.37	98.88	non coding	NA	NA	NA
PB.7099.13	5	1825	5	antisense	novelGene_gene: Zm00001d017932	novel	21.50	98.88	non coding	NA	NA	NA
PB.7099.11	5	2013	6	antisense	novelGene_gene: Zm00001d017932	novel	8.25	98.88	non coding	NA	NA	NA
PB.2745.3	2	3010	1	novel_in_catalog	gene: Zm00001d003157	novel	5.28	669.42	coding	400	1522	2721
PB.2745.2	2	2160	2	novel_in_catalog	gene: Zm00001d003157	novel	1.18	669.42	coding	400	674	1873
PB.2745.1	2	2067	3	full-splice_match	gene: Zm00001d003157	Zm00001d003157_T00	662.96	669.42	coding	400	579	1778
PB.10692.1	9	1857	1	intergenic	novelGene_272	novel	21.68	118.74	coding	290	20	889
PB.2745.7	2	898	1	incomplete-splice_match	gene: Zm00001d003157	Zm00001d003157_T00	0.00	669.42	coding	217	171	821
PB.2745.6	2	1988	3	full-splice_match	gene: Zm00001d003157	Zm00001d003157_T00	0.00	669.42	coding	400	579	1778
PB.7099.18	5	497	3	antisense	novelGene_gene: Zm00001d017932	novel	0.00	98.88	non coding	NA	NA	NA
PB.4292.3	3	2484	4	novel_in_catalog	gene: Zm00001d042569	novel	4.75	59.67	coding	526	905	2482
PB.753.1	1	1623	1	novel_in_catalog	gene: Zm00001d030909	novel	0.84	0.84	coding	163	2	490
PB.10436.1	9	1764	4	full-splice_match	gene: Zm00001d046472	Zm00001d046472_T00	93.62	93.62	coding	171	129	641
PB.9107.2	7	2536	1	intergenic	novelGene_373	novel	3.56	13.58	coding	766	237	2534
PB.5876.2	4	2789	2	genic	gene: Zm00001d053882	novel	30.73	65.78	coding	808	7	2430
PB.2407.1	10	1180	9	full-splice_match	gene: Zm00001d026680	Zm00001d026680_T00	68.70	273.34	coding	214	140	781

PB.9455.1	8	799	1	full-splice_match	gene: Zm00001d009945	Zm00001d009945_T00	3.75	3.75	coding	183	40	588
PB.9621.1	8	1636	13	novel_in_catalog	gene: Zm00001d010714	novel	20.48	20.48	coding	303	463	1371
PB.5087.1	4	1003	4	full-splice_match	gene: Zm00001d049798	Zm00001d049798_T00	22.22	27.75	coding	247	40	780
PB.5876.5	4	2962	3	genic	gene: Zm00001d053882	novel	18.82	65.78	coding	839	67	2583
PB.6799.1	5	3128	16	full-splice_match	gene: Zm00001d016675	Zm00001d016675_T00	30.33	30.33	coding	800	434	2833
PB.6028.7	5	1250	11	incomplete-splice_match	gene: Zm00001d013047	Zm00001d013047_T00	51.61	202.57	coding	307	110	1030
PB.6028.6	5	1381	11	incomplete-splice_match	gene: Zm00001d013047	Zm00001d013047_T00	67.88	202.57	coding	307	109	1029
PB.10567.7	9	1350	5	novel_in_catalog	gene: Zm00001d047208	novel	1.41	66.33	coding	328	68	1051
PB.6028.4	5	556	2	incomplete-splice_match	gene: Zm00001d013047	Zm00001d013047_T00	0.00	202.57	coding	127	107	487
PB.6028.3	5	1703	10	novel_in_catalog	gene: Zm00001d013047	novel	3.79	202.57	coding	133	103	501
PB.6028.2	5	1606	11	incomplete-splice_match	gene: Zm00001d013047	Zm00001d013047_T00	59.96	202.57	coding	307	110	1030
PB.6028.1	5	1130	4	incomplete-splice_match	gene: Zm00001d013047	Zm00001d013047_T00	19.32	202.57	coding	115	48	392
PB.5955.2	5	1802	5	novel_in_catalog	gene: Zm00001d012806	novel	1.29	46.16	coding	135	21	425
PB.5955.1	5	1077	6	novel_in_catalog	gene: Zm00001d012806	novel	39.33	46.16	coding	279	53	889
PB.10567.9	9	1459	6	novel_in_catalog	gene: Zm00001d047208	novel	0.00	66.33	coding	316	87	1034
PB.10567.8	9	873	4	novel_in_catalog	gene: Zm00001d047208	novel	6.06	66.33	coding	190	56	625
PB.5577.1	4	1672	16	novel_not_in_catalog	gene: Zm00001d052434	novel	2.00	12.13	coding	421	58	1320

PB.5955.4	5	959	6	novel_in_catalog	gene: Zm00001d012806	novel	0.00	46.16	coding	279	20	856
PB.9913.3	8	832	5	full-splice_match	gene: Zm00001d011992	Zm00001d011992_T00	367.89	367.89	coding	208	81	704
PB.329.1	1	3032	1	full-splice_match	gene: Zm00001d028643	Zm00001d028643_T00	103.24	103.24	coding	533	537	2135
PB.10264.1	9	1473	8	full-splice_match	gene: Zm00001d045450	Zm00001d045450_T00	148.92	148.92	coding	390	77	1246
PB.9977.2	8	2145	7	full-splice_match	gene: Zm00001d012263	Zm00001d012263_T00	1.52	13.34	coding	279	1116	1952
PB.9601.1	8	1066	1	antisense	novelGene_gene: Zm00001d010652	novel	3.03	3.03	coding	69	394	600
PB.705.1	1	981	3	incomplete-splice_match	gene: Zm00001d030609	Zm00001d030609_T00	38.27	38.27	coding	154	121	582
PB.2490.2	2	1145	9	novel_in_catalog	gene: Zm00001d002034	novel	102.55	102.55	coding	214	276	917
PB.1539.2	1	1475	2	full-splice_match	gene: Zm00001d034440	Zm00001d034440_T00	67.04	73.68	coding	404	66	1277
PB.8694.1	7	889	1	full-splice_match	gene: Zm00001d021207	Zm00001d021207_T00	32.78	32.78	coding	238	73	786
PB.3430.1	2	1950	2	full-splice_match	gene: Zm00001d006697	Zm00001d006697_T00	47.08	47.08	coding	520	56	1615
PB.1358.2	1	1222	6	full-splice_match	gene: Zm00001d033641	Zm00001d033641_T00	72.68	88.72	coding	276	202	1029
PB.8995.1	7	841	1	full-splice_match	gene: Zm00001d022270	Zm00001d022270_T00	59.04	59.04	coding	113	92	430
PB.9117.1	8	823	1	genic	gene: Zm00001d008179	novel	28.41	28.41	non coding	NA	NA	NA
PB.5784.6	4	2715	17	full-splice_match	gene: Zm00001d053515	Zm00001d053515_T00	3.73	37.94	coding	787	219	2579
PB.2943.1	2	3500	10	incomplete-splice_match	gene: Zm00001d004139	Zm00001d004139_T01	43.01	43.01	coding	1020	197	3256
PB.1358.3	1	1934	4	full-splice_match	gene: Zm00001d033641	Zm00001d033641_T00	0.74	88.72	coding	147	203	643

PB.3903.1	3	736	3	novel_not_in_catalog	gene: Zm00001d040276	novel	39.79	39.79	coding	106	91	408
PB.3903.2	3	595	3	novel_not_in_catalog	gene: Zm00001d040276	novel	0.00	39.79	coding	106	87	404
PB.10079.1	8	1761	8	incomplete-splice_match	gene: Zm00001d012677	Zm00001d012677_T00	26.11	42.45	coding	391	177	1349
PB.10079.2	8	1544	8	incomplete-splice_match	gene: Zm00001d012677	Zm00001d012677_T00	16.35	42.45	coding	391	229	1401
PB.8996.1	7	3071	3	full-splice_match	gene: Zm00001d022273	Zm00001d022273_T00	30.30	30.30	coding	650	78	2027
PB.7845.1	6	1959	12	full-splice_match	gene: Zm00001d037918	Zm00001d037918_T00	17.25	17.25	coding	532	64	1659
PB.7118.1	5	2202	11	novel_in_catalog	gene: Zm00001d017988	novel	0.63	13.38	coding	297	1125	2015
PB.2670.2	2	1387	2	full-splice_match	gene: Zm00001d002825	Zm00001d002825_T00	6.54	15.20	coding	296	123	1010
PB.2670.3	2	1283	2	full-splice_match	gene: Zm00001d002825	Zm00001d002825_T00	0.00	15.20	coding	296	136	1023
PB.2670.1	2	1297	3	novel_not_in_catalog	gene: Zm00001d002825	novel	8.66	15.20	coding	293	136	1014
PB.60.1	1	1779	14	full-splice_match	gene: Zm00001d027479	Zm00001d027479_T00	13.24	13.24	coding	435	195	1499
PB.10858.1	9	1764	12	incomplete-splice_match	gene: Zm00001d048335	Zm00001d048335_T00	12.81	12.81	coding	457	95	1465
PB.5284.3	4	1244	2	antisense	novelGene_gene: Zm00001d051161	novel	19.87	19.87	coding	198	287	880
PB.2306.1	10	1397	6	full-splice_match	gene: Zm00001d026363	Zm00001d026363_T00	73.65	73.65	coding	284	250	1101
PB.4920.1	4	1315	2	full-splice_match	gene: Zm00001d049020	Zm00001d049020_T00	30.58	30.58	coding	364	43	1134
PB.8315.1	7	1318	1	full-splice_match	gene: Zm00001d019049	Zm00001d019049_T00	16.29	16.29	coding	341	14	1036
PB.5019.4	4	3834	11	full-splice_match	gene: Zm00001d049455	Zm00001d049455_T00	0.00	25.41	coding	1033	408	3506

PB.6000.1	5	635	1	genic	gene: Zm00001d012965	novel	23.65	23.65	coding	32	200	295
PB.5150.1	4	829	2	full-splice_match	gene: Zm00001d050179	Zm00001d050179_T00	158.28	492.06	coding	149	46	492
PB.9609.1	8	3429	1	intergenic	novelGene_240	novel	24.95	24.95	non coding	NA	NA	NA
PB.2893.1	2	2342	3	novel_in_catalog	gene: Zm00001d003878	novel	50.76	50.76	coding	352	1078	2133
PB.603.2	1	1037	1	full-splice_match	gene: Zm00001d029969	Zm00001d029969_T00	60.69	70.85	coding	210	137	766
PB.603.1	1	1739	2	genic	gene: Zm00001d029969	novel	10.15	70.85	coding	210	100	729
PB.4317.7	3	2613	3	novel_in_catalog	gene: Zm00001d042664	novel	3.40	152.56	coding	410	71	1300
PB.818.1	1	2122	4	full-splice_match	gene: Zm00001d031203	Zm00001d031203_T00	8.46	8.46	coding	329	955	1941
PB.4317.1	3	2342	3	novel_in_catalog	gene: Zm00001d042664	novel	3.03	152.56	coding	451	68	1420
PB.8408.1	7	1297	4	full-splice_match	gene: Zm00001d019648	Zm00001d019648_T00	17.10	17.10	coding	303	138	1046
PB.3136.1	2	1315	4	incomplete-splice_match	gene: Zm00001d005373	Zm00001d005373_T00	7.01	50.22	coding	257	237	1007
PB.10975.1	B73V4	1448	9	incomplete-splice_match	gene: Zm00001d000034	Zm00001d000034_T00	8.30	8.30	coding	293	417	1295
PB.932.1	1	1129	3	incomplete-splice_match	gene: Zm00001d031817	Zm00001d031817_T00	105.84	105.84	coding	149	402	848
PB.8988.5	7	841	2	full-splice_match	gene: Zm00001d022244	Zm00001d022244_T00	24.81	79.64	coding	126	317	694
PB.3136.2	2	2091	8	novel_in_catalog	gene: Zm00001d005373	novel	6.81	50.22	coding	585	177	1931
PB.8988.1	7	1096	2	full-splice_match	gene: Zm00001d022244	Zm00001d022244_T00	54.83	79.64	coding	126	340	717
PB.6614.4	5	1526	4	antisense	novelGene_gene: Zm00001d015513	novel	11.13	30.48	non coding	NA	NA	NA

PB.6614.5	5	1070	5	antisense	novelGene_gene: Zm00001d015513	novel	5.78	30.48	non coding	NA	NA	NA
PB.10949.3	B73V 4	1374	4	full-splice_match	gene: Zm00001d000305	Zm00001d000305_T00	32.78	76.68	coding	358	57	1130
PB.10949.2	B73V 4	1524	4	full-splice_match	gene: Zm00001d000305	Zm00001d000305_T00	43.89	76.68	coding	358	119	1192
PB.4994.1	4	1310	1	full-splice_match	gene: Zm00001d049347	Zm00001d049347_T00	7.61	7.61	coding	279	115	951
PB.9967.5	8	2131	6	full-splice_match	gene: Zm00001d012238	Zm00001d012238_T00	52.93	188.10	coding	256	175	942
PB.9648.1	8	2017	7	novel_in_catalog	gene: Zm00001d010823	novel	14.94	14.94	coding	167	155	655
PB.335.5	1	1720	15	novel_in_catalog	gene: Zm00001d028662	novel	1.17	49.70	coding	345	140	1174
PB.335.4	1	1593	16	full-splice_match	gene: Zm00001d028662	Zm00001d028662_T00	3.86	49.70	coding	464	182	1573
PB.3405.1	2	1141	2	full-splice_match	gene: Zm00001d006608	Zm00001d006608_T00	12.03	12.03	coding	170	225	734
PB.335.1	1	2079	16	full-splice_match	gene: Zm00001d028662	Zm00001d028662_T00	39.82	49.70	coding	464	159	1550
PB.6614.9	5	1442	4	antisense	novelGene_gene: Zm00001d015513	novel	0.00	30.48	non coding	NA	NA	NA
PB.335.3	1	1807	16	full-splice_match	gene: Zm00001d028662	Zm00001d028662_T00	0.00	49.70	coding	464	159	1550
PB.335.2	1	2605	15	full-splice_match	gene: Zm00001d028662	Zm00001d028662_T00	4.85	49.70	coding	331	1116	2108
PB.3706.3	3	1059	7	full-splice_match	gene: Zm00001d039350	Zm00001d039350_T00	150.94	366.27	coding	230	144	833
PB.7877.1	6	2225	1	novel_in_catalog	gene: Zm00001d038022	novel	1.98	33.74	coding	257	1023	1793
PB.3706.1	3	1217	7	full-splice_match	gene: Zm00001d039350	Zm00001d039350_T00	47.83	366.27	coding	230	64	753
PB.664.1	1	1048	1	full-splice_match	gene: Zm00001d030267	Zm00001d030267_T00	6.71	6.71	coding	193	143	721

PB.10350.1	9	2221	10	full-splice_match	gene: Zm00001d045864	Zm00001d045864_T00	29.53	29.53	coding	592	184	1959
PB.7955.2	6	1987	3	incomplete-splice_match	gene: Zm00001d038273	Zm00001d038273_T00	23.35	164.25	coding	469	56	1462
PB.10350.2	9	1333	7	incomplete-splice_match	gene: Zm00001d045864	Zm00001d045864_T00	0.00	29.53	coding	362	246	1331
PB.2174.1	10	2988	9	incomplete-splice_match	gene: Zm00001d025817	Zm00001d025817_T03	43.98	66.86	coding	892	24	2699
PB.1410.1	1	1750	1	full-splice_match	gene: Zm00001d033882	Zm00001d033882_T00	0.97	3.75	coding	299	652	1548
PB.3439.2	2	2363	2	full-splice_match	gene: Zm00001d006753	Zm00001d006753_T00	11.62	29.44	coding	614	146	1987
PB.2174.2	10	3337	14	incomplete-splice_match	gene: Zm00001d025817	Zm00001d025817_T00	22.89	66.86	coding	1063	36	3224
PB.9945.1	8	795	1	intergenic	novelGene_339	novel	102.83	102.83	coding	129	316	702
PB.3293.1	2	2938	5	full-splice_match	gene: Zm00001d006130	Zm00001d006130_T00	85.63	94.71	coding	761	324	2606
PB.3293.3	2	2718	5	full-splice_match	gene: Zm00001d006130	Zm00001d006130_T00	9.08	94.71	coding	761	305	2587
PB.8883.4	7	1856	6	genic	gene: Zm00001d021845	novel	3.23	36.30	coding	151	81	533
PB.981.1	1	1209	6	full-splice_match	gene: Zm00001d032031	Zm00001d032031_T00	71.92	144.91	coding	273	93	911
PB.6692.2	5	1755	1	full-splice_match	gene: Zm00001d016083	Zm00001d016083_T00	3.88	26.14	coding	136	166	573
PB.6692.3	5	1653	2	genic	gene: Zm00001d016083	novel	22.26	26.14	coding	136	169	576
PB.876.1	1	1153	1	antisense	novelGene_gene: Zm00001d031520	novel	33.08	33.08	coding	25	340	414
PB.4368.1	3	2522	13	full-splice_match	gene: Zm00001d042884	Zm00001d042884_T00	66.94	66.94	coding	684	209	2260
PB.3098.1	2	1544	9	full-splice_match	gene: Zm00001d005159	Zm00001d005159_T00	3.92	47.24	coding	318	98	1051

PB.8400.1	7	994	2	full-splice_match	gene: Zm00001d019593	Zm00001d019593_T00	18.77	18.77	coding	270	58	867
PB.3098.2	2	1417	9	full-splice_match	gene: Zm00001d005159	Zm00001d005159_T00	34.23	47.24	coding	318	105	1058
PB.4778.1	3	880	1	genic	gene: Zm00001d044475	novel	14.54	81.70	non coding	NA	NA	NA
PB.5868.5	4	1572	11	full-splice_match	gene: Zm00001d053841	Zm00001d053841_T01	0.00	59.78	coding	453	84	1442
PB.4778.2	3	1632	2	full-splice_match	gene: Zm00001d044475	Zm00001d044475_T00	67.15	81.70	coding	428	150	1433
PB.3738.1	3	1542	3	full-splice_match	gene: Zm00001d039456	Zm00001d039456_T00	13.70	13.70	coding	376	85	1212
PB.5864.1	4	794	2	full-splice_match	gene: Zm00001d053829	Zm00001d053829_T00	7.01	7.01	coding	214	31	672
PB.10941.1	9	1415	11	novel_not_in_catalog	gene: Zm00001d048577	novel	112.38	112.38	coding	271	403	1215
PB.4726.1	3	1373	11	novel_not_in_catalog	gene: Zm00001d044243	novel	14.61	20.78	coding	344	89	1120
PB.4726.2	3	1332	10	full-splice_match	gene: Zm00001d044243	Zm00001d044243_T00	6.17	20.78	coding	344	149	1180
PB.1785.2	10	1934	11	novel_not_in_catalog	gene: Zm00001d023636	novel	39.15	59.26	coding	544	67	1698
PB.1785.3	10	2059	10	novel_not_in_catalog	gene: Zm00001d023636	novel	7.47	59.26	coding	596	102	1889
PB.1785.1	10	1942	5	novel_not_in_catalog	gene: Zm00001d023636	novel	12.63	59.26	coding	235	1038	1742
PB.5385.10	4	1275	8	full-splice_match	gene: Zm00001d051676	Zm00001d051676_T00	0.00	66.22	coding	300	194	1093
PB.4729.1	3	2206	4	full-splice_match	gene: Zm00001d044253	Zm00001d044253_T00	96.72	100.04	coding	562	167	1852
PB.3283.1	2	1050	7	full-splice_match	gene: Zm00001d006100	Zm00001d006100_T00	278.37	278.37	coding	258	72	845
PB.4385.2	3	1343	8	full-splice_match	gene: Zm00001d042965	Zm00001d042965_T00	0.00	52.76	coding	229	397	1083

PB.7867.3	6	1285	10	incomplete-splice_match	gene: Zm00001d037982	Zm00001d037982_T00	12.80	25.78	coding	284	98	949
PB.7867.2	6	3213	20	full-splice_match	gene: Zm00001d037982	Zm00001d037982_T00	11.68	25.78	coding	869	98	2704
PB.7867.1	6	3976	20	full-splice_match	gene: Zm00001d037982	Zm00001d037982_T00	1.30	25.78	coding	869	98	2704
PB.913.2	1	2903	16	novel_not_in_catalog	gene: Zm00001d031712	novel	1.74	50.18	coding	475	240	1664
PB.7537.4	6	1750	10	novel_in_catalog	gene: Zm00001d036416	novel	21.97	76.08	coding	326	118	1095
PB.6861.1	5	1592	7	novel_not_in_catalog	gene: Zm00001d016916	novel	19.19	19.19	coding	327	259	1239
PB.7537.1	6	2439	10	novel_in_catalog	gene: Zm00001d036416	novel	13.76	76.08	coding	315	186	1130
PB.3536.1	2	524	2	genic	gene: Zm00001d007174	novel	248.24	260.46	non coding	NA	NA	NA
PB.7537.3	6	2155	11	full-splice_match	gene: Zm00001d036416	Zm00001d036416_T00	35.48	76.08	coding	608	158	1981
PB.7537.2	6	1134	4	novel_in_catalog	gene: Zm00001d036416	novel	4.88	76.08	coding	115	600	944
PB.3826.1	3	1334	1	incomplete-splice_match	gene: Zm00001d039873	Zm00001d039873_T00	3.23	3.23	coding	331	70	1062
PB.5117.1	4	792	3	genic	gene: Zm00001d049966	novel	60.63	90.81	coding	127	255	635
PB.7253.1	5	1661	2	full-splice_match	gene: Zm00001d018469	Zm00001d018469_T00	9.85	9.85	coding	482	12	1457
PB.5117.3	4	1277	2	genic	gene: Zm00001d049966	novel	6.14	90.81	non coding	NA	NA	NA
PB.5117.2	4	1052	4	genic	gene: Zm00001d049966	novel	24.04	90.81	coding	232	215	910
PB.4520.4	3	2059	3	novel_in_catalog	gene: Zm00001d043479	novel	12.35	136.35	non coding	NA	NA	NA
PB.3543.2	2	3333	3	novel_in_catalog	gene: Zm00001d007197	novel	2.48	13.45	coding	616	1154	3001

PB.4747.4	3	2583	5	novel_in_catalog	gene: Zm00001d044324	novel	32.56	32.56	coding	253	208	966
PB.2879.1	2	1405	2	incomplete-splice_match	gene: Zm00001d003807	Zm00001d003807_T00	30.33	50.21	coding	361	73	1155
PB.1259.1	1	815	1	full-splice_match	gene: Zm00001d033216	Zm00001d033216_T00	13.43	13.43	coding	119	300	656
PB.10466.1	9	1105	2	genic	gene: Zm00001d046599	novel	30.32	459.37	coding	204	186	797
PB.4556.1	3	2606	10	novel_not_in_catalog	gene: Zm00001d043601	novel	1.98	284.99	coding	215	235	879
PB.10466.3	9	978	2	genic	gene: Zm00001d046599	novel	10.71	459.37	coding	204	150	761
PB.3967.1	3	1707	1	full-splice_match	gene: Zm00001d000523	Zm00001d000523_T00	215.58	215.58	non coding	NA	NA	NA
PB.10466.5	9	2031	1	full-splice_match	gene: Zm00001d046599	Zm00001d046599_T00	0.00	459.37	coding	204	150	761
PB.10466.4	9	1036	2	genic	gene: Zm00001d046599	novel	397.34	459.37	coding	204	200	811
PB.10466.7	9	896	1	full-splice_match	gene: Zm00001d046599	Zm00001d046599_T00	0.00	459.37	coding	204	150	761
PB.10466.6	9	1083	1	full-splice_match	gene: Zm00001d046599	Zm00001d046599_T00	0.00	459.37	coding	204	150	761
PB.5498.1	4	866	2	full-splice_match	gene: Zm00001d052191	Zm00001d052191_T00	18.80	18.80	coding	205	73	687
PB.612.1	1	755	4	full-splice_match	gene: Zm00001d030018	Zm00001d030018_T00	29.25	29.25	coding	128	41	424
PB.3730.1	3	1567	2	full-splice_match	gene: Zm00001d039439	Zm00001d039439_T00	83.01	90.00	coding	345	234	1268
PB.3730.2	3	1756	1	novel_in_catalog	gene: Zm00001d039439	novel	6.99	90.00	coding	258	685	1458
PB.247.13	1	589	2	intergenic	novelGene_113	novel	0.00	14.11	non coding	NA	NA	NA
PB.247.12	1	863	1	intergenic	novelGene_113	novel	14.11	14.11	non coding	NA	NA	NA

PB.247.11	1	1285	1	intergenic	novelGene_113	novel	0.00	14.11	non coding	NA	NA	NA
PB.9990.1	8	1864	1	antisense	novelGene_gene: Zm00001d012304	novel	10.39	10.39	coding	268	466	1269
PB.2876.3	2	1461	5	novel_not_in_catalog	gene: Zm00001d003800	novel	49.10	66.00	coding	337	305	1315
PB.5683.2	4	1228	2	full-splice_match	gene: Zm00001d052967	Zm00001d052967_T00	5.70	12.07	coding	127	515	895
PB.9826.1	8	1203	1	full-splice_match	gene: Zm00001d011650	Zm00001d011650_T00	53.43	172.52	coding	240	67	786
PB.9957.1	8	1519	11	full-splice_match	gene: Zm00001d012168	Zm00001d012168_T00	10.70	10.70	coding	316	180	1127
PB.5886.1	4	339	1	intergenic	novelGene_445	novel	33.55	33.55	non coding	NA	NA	NA
PB.8783.1	7	594	3	full-splice_match	gene: Zm00001d021536	Zm00001d021536_T00	216.06	216.06	non coding	NA	NA	NA
PB.8920.4	7	3699	25	novel_in_catalog	gene: Zm00001d021972	novel	8.80	21.79	coding	1104	259	3570
PB.4719.5	3	1852	11	novel_in_catalog	gene: Zm00001d044213	novel	4.88	36.19	coding	263	959	1747
PB.1207.2	1	2613	18	full-splice_match	gene: Zm00001d032956	Zm00001d032956_T01	0.00	47.04	coding	774	142	2463
PB.3710.1	3	1382	7	full-splice_match	gene: Zm00001d039364	Zm00001d039364_T00	2.48	2.48	coding	391	50	1222
PB.9242.1	8	957	5	novel_in_catalog	gene: Zm00001d008726	novel	2.53	28.56	coding	148	96	539
PB.9242.2	8	950	5	full-splice_match	gene: Zm00001d008726	Zm00001d008726_T00	23.59	28.56	coding	189	74	640
PB.9242.3	8	1868	4	novel_in_catalog	gene: Zm00001d008726	novel	2.45	28.56	coding	152	58	513
PB.4742.4	3	1758	10	full-splice_match	gene: Zm00001d044304	Zm00001d044304_T00	8.48	20.35	coding	490	218	1687
PB.10868.1	9	1247	4	full-splice_match	gene: Zm00001d048359	Zm00001d048359_T00	80.19	80.19	coding	284	94	945

PB.5204.2	4	2441	6	novel_in_catalog	gene: Zm00001d050518	novel	2.39	78.69	coding	376	115	1242
PB.10190.2	9	2537	8	incomplete-splice_match	gene: Zm00001d045147	Zm00001d045147_T02	16.19	35.24	coding	276	1476	2303
PB.10190.1	9	2477	16	full-splice_match	gene: Zm00001d045147	Zm00001d045147_T01	19.05	35.24	coding	745	2	2236
PB.5204.1	4	2294	7	full-splice_match	gene: Zm00001d050518	Zm00001d050518_T00	76.30	78.69	coding	612	209	2044
PB.4317.9	3	2017	4	full-splice_match	gene: Zm00001d042664	Zm00001d042664_T00	0.00	152.56	coding	571	68	1780
PB.4232.2	3	1797	10	full-splice_match	gene: Zm00001d042303	Zm00001d042303_T00	60.86	67.81	coding	499	159	1655
PB.6577.1	5	2676	2	intergenic	novelGene_188	novel	1.77	13.24	non coding	NA	NA	NA
PB.2798.10	2	1044	6	full-splice_match	gene: Zm00001d003401	Zm00001d003401_T00	546.41	1796.02	coding	261	98	880
PB.4856.2	4	1051	1	intergenic	novelGene_19	novel	0.00	15.73	coding	242	311	1036
PB.868.1	1	1849	5	full-splice_match	gene: Zm00001d031473	Zm00001d031473_T00	11.58	35.92	coding	446	65	1402
PB.10651.1	9	2392	12	full-splice_match	gene: Zm00001d047534	Zm00001d047534_T00	61.49	115.08	coding	604	148	1959
PB.8462.1	7	1803	5	full-splice_match	gene: Zm00001d020043	Zm00001d020043_T00	10.45	10.45	coding	283	793	1641
PB.7277.1	5	1163	3	intergenic	novelGene_425	novel	3.55	3.55	non coding	NA	NA	NA
PB.8679.11	7	2062	8	novel_in_catalog	gene: Zm00001d021119	novel	4.60	157.85	coding	470	119	1528
PB.2696.1	2	1739	6	novel_in_catalog	gene: Zm00001d002938	novel	13.66	13.66	coding	499	16	1512
PB.8679.10	7	1859	9	full-splice_match	gene: Zm00001d021119	Zm00001d021119_T00	9.35	157.85	coding	372	503	1618
PB.327.4	1	1376	9	full-splice_match	gene: Zm00001d028631	Zm00001d028631_T00	0.00	58.40	coding	308	240	1163

PB.6781.5	5	1369	7	full-splice_match	gene: Zm00001d016588	Zm00001d016588_T00	21.90	23.47	coding	376	135	1262
PB.4856.1	4	1348	1	intergenic	novelGene_19	novel	15.73	15.73	coding	242	392	1117
PB.6781.3	5	2506	6	full-splice_match	gene: Zm00001d016588	Zm00001d016588_T00	0.89	23.47	coding	230	1701	2390
PB.327.1	1	1561	8	novel_in_catalog	gene: Zm00001d028631	novel	4.86	58.40	coding	205	593	1207
PB.7354.1	6	1790	3	full-splice_match	gene: Zm00001d035300	Zm00001d035300_T00	4.34	4.34	coding	380	328	1467
PB.5583.2	4	1078	6	full-splice_match	gene: Zm00001d052450	Zm00001d052450_T00	10.87	159.81	coding	246	107	844
PB.2547.1	2	885	1	full-splice_match	gene: Zm00001d002290	Zm00001d002290_T00	28.22	28.22	coding	152	72	527
PB.1186.3	1	713	2	incomplete-splice_match	gene: Zm00001d032859	Zm00001d032859_T00	136.28	254.56	coding	196	16	603
PB.8458.1	7	469	1	intergenic	novelGene_190	novel	139.68	139.68	non coding	NA	NA	NA
PB.1186.1	1	2738	9	full-splice_match	gene: Zm00001d032859	Zm00001d032859_T00	118.29	254.56	coding	810	94	2523
PB.3598.1	2	642	3	novel_not_in_catalog	gene: Zm00001d007633	novel	162.75	356.02	coding	68	114	317
PB.5767.2	4	1172	3	full-splice_match	gene: Zm00001d053396	Zm00001d053396_T00	60.07	106.06	coding	217	255	905
PB.3342.4	2	1481	7	full-splice_match	gene: Zm00001d006344	Zm00001d006344_T00	38.36	96.19	coding	354	136	1197
PB.10856.1	9	1210	9	full-splice_match	gene: Zm00001d048324	Zm00001d048324_T00	74.30	74.30	coding	274	53	874
PB.805.2	1	641	3	full-splice_match	gene: Zm00001d031132	Zm00001d031132_T00	339.50	1430.04	coding	84	162	413
PB.7109.2	5	762	1	full-splice_match	gene: Zm00001d017968	Zm00001d017968_T00	31.42	40.12	coding	114	56	397
PB.4848.1	4	742	1	genic	gene: Zm00001d048632	novel	41.14	41.14	coding	63	404	592

PB.7109.1	5	1080	1	full-splice_match	gene: Zm00001d017968	Zm00001d017968_T00	8.70	40.12	coding	80	72	311
PB.805.1	1	742	3	full-splice_match	gene: Zm00001d031132	Zm00001d031132_T00	147.49	1430.04	coding	84	105	356
PB.8174.4	6	2223	19	novel_in_catalog	gene: Zm00001d039081	novel	15.28	15.28	coding	556	123	1790
PB.8864.4	7	1950	11	full-splice_match	gene: Zm00001d021802	Zm00001d021802_T00	0.00	166.06	coding	468	172	1575
PB.4192.1	3	907	1	genic	gene: Zm00001d042057	novel	68.69	68.69	coding	235	148	852
PB.8864.6	7	2317	8	novel_in_catalog	gene: Zm00001d021802	novel	11.25	166.06	coding	145	1596	2030
PB.8864.1	7	2044	11	full-splice_match	gene: Zm00001d021802	Zm00001d021802_T00	127.94	166.06	coding	468	191	1594
PB.7987.2	6	719	1	full-splice_match	gene: Zm00001d038382	Zm00001d038382_T00	0.00	458.24	coding	172	54	569
PB.8864.3	7	2149	10	full-splice_match	gene: Zm00001d021802	Zm00001d021802_T00	3.57	166.06	coding	233	1074	1772
PB.8864.2	7	2171	10	novel_in_catalog	gene: Zm00001d021802	novel	1.64	166.06	coding	265	157	951
PB.9830.1	8	1145	3	full-splice_match	gene: Zm00001d011680	Zm00001d011680_T00	43.83	43.83	coding	183	108	656
PB.868.3	1	1600	5	full-splice_match	gene: Zm00001d031473	Zm00001d031473_T00	0.00	35.92	coding	446	62	1399
PB.9830.2	8	375	1	incomplete-splice_match	gene: Zm00001d011680	Zm00001d011680_T00	0.00	43.83	non coding	NA	NA	NA
PB.7521.1	6	1043	1	full-splice_match	gene: Zm00001d036319	Zm00001d036319_T00	132.30	317.25	coding	250	79	828
PB.7521.2	6	938	1	full-splice_match	gene: Zm00001d036319	Zm00001d036319_T00	184.95	317.25	coding	250	79	828
PB.3790.1	3	467	4	incomplete-splice_match	gene: Zm00001d039664	Zm00001d039664_T00	495.69	495.69	non coding	NA	NA	NA
PB.4447.1	3	1137	1	incomplete-splice_match	gene: Zm00001d043218	Zm00001d043218_T00	17.75	17.75	coding	279	67	903

PB.2390.1	10	1178	1	full-splice_match	gene: Zm00001d026632	Zm00001d026632_T00	54.11	54.11	coding	265	92	886
PB.8549.2	7	899	3	incomplete-splice_match	gene: Zm00001d020505	Zm00001d020505_T00	7.55	22.25	coding	223	44	712
PB.8549.1	7	4696	4	full-splice_match	gene: Zm00001d020505	Zm00001d020505_T00	14.71	22.25	coding	268	3663	4466
PB.6789.1	5	1172	1	genic	gene: Zm00001d016648	novel	4.36	4.36	non coding	NA	NA	NA
PB.1560.4	1	1739	8	novel_in_catalog	gene: Zm00001d034522	novel	6.30	41.43	coding	323	693	1661
PB.8454.1	7	2067	3	full-splice_match	gene: Zm00001d020013	Zm00001d020013_T00	14.47	14.47	coding	526	195	1772
PB.8454.2	7	1963	3	full-splice_match	gene: Zm00001d020013	Zm00001d020013_T00	0.00	14.47	coding	526	191	1768
PB.214.4	1	866	5	incomplete-splice_match	gene: Zm00001d028185	Zm00001d028185_T00	0.00	23.94	coding	213	227	865
PB.10259.1	9	1173	2	intergenic	novelGene_83	novel	2.76	2.76	non coding	NA	NA	NA
PB.214.2	1	1488	6	full-splice_match	gene: Zm00001d028185	Zm00001d028185_T00	5.07	23.94	coding	283	472	1320
PB.214.1	1	1272	6	novel_not_in_catalog	gene: Zm00001d028185	novel	18.87	23.94	coding	283	215	1063
PB.3336.1	2	3039	3	full-splice_match	gene: Zm00001d006317	Zm00001d006317_T00	33.20	33.20	coding	856	264	2831
PB.4011.5	3	831	4	novel_not_in_catalog	gene: Zm00001d040834	novel	15.91	92.35	coding	110	90	419
PB.4011.4	3	857	4	novel_not_in_catalog	gene: Zm00001d040834	novel	53.82	92.35	coding	113	137	475
PB.4011.6	3	973	5	novel_not_in_catalog	gene: Zm00001d040834	novel	22.62	92.35	coding	129	90	476
PB.4562.12	3	3309	11	full-splice_match	gene: Zm00001d043613	Zm00001d043613_T00	18.38	178.80	coding	300	1942	2841
PB.6697.1	5	1722	5	novel_in_catalog	gene: Zm00001d016130	novel	335.33	341.80	coding	378	251	1384

PB.8235.1	7	1175	1	genic	gene: Zm00001d018693	novel	12.73	20.17	coding	230	23	712
PB.8529.7	7	1338	4	novel_not_in_catalog	gene: Zm00001d020402	novel	31.69	53.45	coding	414	16	1257
PB.6795.3	5	1476	11	novel_in_catalog	gene: Zm00001d016662	novel	5.06	26.52	coding	298	49	942
PB.1027.4	1	1648	14	novel_in_catalog	gene: Zm00001d032257	novel	0.00	110.69	coding	393	416	1594
PB.8529.3	7	1639	2	novel_in_catalog	gene: Zm00001d020402	novel	13.49	53.45	coding	114	1121	1462
PB.8529.2	7	1509	3	full-splice_match	gene: Zm00001d020402	Zm00001d020402_T00	8.27	53.45	coding	187	784	1344
PB.1898.1	10	1455	9	full-splice_match	gene: Zm00001d024408	Zm00001d024408_T00	55.97	55.97	coding	332	124	1119
PB.6106.1	5	1749	4	novel_in_catalog	gene: Zm00001d013307	novel	53.11	53.11	coding	395	117	1301
PB.8235.3	7	2085	2	full-splice_match	gene: Zm00001d018693	Zm00001d018693_T00	7.44	20.17	coding	571	277	1989
PB.2990.1	2	864	1	novel_in_catalog	gene: Zm00001d004523	novel	43.93	56.95	coding	198	81	674
PB.4969.2	4	1298	9	full-splice_match	gene: Zm00001d049222	Zm00001d049222_T00	23.34	85.66	coding	278	199	1032
PB.7040.1	5	1731	1	full-splice_match	gene: Zm00001d017678	Zm00001d017678_T00	20.27	20.27	coding	405	40	1254
PB.715.3	1	1298	2	novel_in_catalog	gene: Zm00001d030678	novel	118.18	337.18	coding	177	536	1066
PB.3056.2	2	1481	2	full-splice_match	gene: Zm00001d004934	Zm00001d004934_T00	3.98	10.01	coding	422	7	1272
PB.5107.1	4	1537	3	full-splice_match	gene: Zm00001d049926	Zm00001d049926_T00	9.57	9.57	coding	396	188	1375
PB.5032.3	4	1144	2	novel_not_in_catalog	gene: Zm00001d049541	novel	1341.31	3282.06	coding	204	206	817
PB.5032.2	4	2304	1	novel_in_catalog	gene: Zm00001d049541	novel	27.39	3282.06	coding	204	1364	1975

PB.5032.1	4	1619	2	full-splice_match	gene: Zm00001d049541	Zm00001d049541_T00	1883.14	3282.06	coding	364	113	1204
PB.6823.6	5	1624	9	novel_in_catalog	gene: Zm00001d016770	novel	2.56	44.81	coding	363	216	1304
PB.5032.7	4	1407	2	full-splice_match	gene: Zm00001d049541	Zm00001d049541_T00	30.21	3282.06	coding	364	120	1211
PB.8795.1	7	1831	1	full-splice_match	gene: Zm00001d021576	Zm00001d021576_T00	3.80	3.80	coding	493	86	1564
PB.10747.1	9	840	4	full-splice_match	gene: Zm00001d047857	Zm00001d047857_T00	117.14	224.89	coding	111	228	560
PB.5699.1	4	1123	3	incomplete-splice_match	gene: Zm00001d053038	Zm00001d053038_T00	28.55	28.55	coding	154	361	822
PB.10747.2	9	624	4	full-splice_match	gene: Zm00001d047857	Zm00001d047857_T00	107.75	224.89	coding	111	91	423
PB.4852.2	4	1111	1	incomplete-splice_match	gene: Zm00001d048667	Zm00001d048667_T01	0.00	5.88	coding	283	120	968
PB.9858.2	8	617	3	incomplete-splice_match	gene: Zm00001d011776	Zm00001d011776_T00	23.31	27.64	non coding	NA	NA	NA
PB.9858.1	8	4230	3	incomplete-splice_match	gene: Zm00001d011776	Zm00001d011776_T00	4.33	27.64	coding	74	76	297
PB.4852.1	4	4276	8	incomplete-splice_match	gene: Zm00001d048667	Zm00001d048667_T01	5.88	5.88	coding	788	30	2393
PB.5191.1	4	1170	5	full-splice_match	gene: Zm00001d050412	Zm00001d050412_T00	11.01	11.01	coding	284	107	958
PB.1607.1	1	550	2	full-splice_match	gene: Zm00001d034688	Zm00001d034688_T00	67.31	67.31	non coding	NA	NA	NA
PB.3607.3	2	1873	2	novel_not_in_catalog	gene: Zm00001d007706	novel	8.41	36.81	coding	484	131	1582
PB.725.3	1	1012	8	incomplete-splice_match	gene: Zm00001d030760	Zm00001d030760_T01	12.71	68.18	coding	139	27	443
PB.913.1	1	2963	16	full-splice_match	gene: Zm00001d031712	Zm00001d031712_T00	48.45	50.18	coding	810	268	2697
PB.6979.7	5	2297	7	full-splice_match	gene: Zm00001d017429	Zm00001d017429_T00	0.00	471.48	coding	682	39	2084

PB.4766.1	3	2499	2	novel_in_catalog	gene: Zm00001d044434	novel	75.78	112.31	coding	685	198	2252
PB.6979.1	5	2402	7	full-splice_match	gene: Zm00001d017429	Zm00001d017429_T00	232.41	471.48	coding	682	38	2083
PB.725.4	1	1238	10	incomplete-splice_match	gene: Zm00001d030760	Zm00001d030760_T01	17.37	68.18	coding	323	90	1058
PB.6979.3	5	1343	1	genic	gene: Zm00001d017429	novel	212.24	471.48	coding	304	127	1038
PB.719.2	1	2420	4	full-splice_match	gene: Zm00001d030732	Zm00001d030732_T00	37.63	37.63	coding	665	63	2057
PB.9826.2	8	1229	1	full-splice_match	gene: Zm00001d011650	Zm00001d011650_T00	55.94	172.52	coding	240	260	979
PB.4748.3	3	1979	5	novel_in_catalog	gene: Zm00001d044327	novel	1.77	192.78	coding	130	1111	1500
PB.9826.4	8	967	1	full-splice_match	gene: Zm00001d011650	Zm00001d011650_T00	63.16	172.52	coding	240	102	821
PB.9088.1	7	2122	3	intergenic	novelGene_370	novel	5.52	5.52	coding	101	1157	1459
PB.8157.1	6	2099	4	incomplete-splice_match	gene: Zm00001d039002	Zm00001d039002_T00	67.16	67.16	coding	587	16	1776
PB.8712.1	7	1350	1	antisense	novelGene_gene: Zm00001d021283	novel	7.93	7.93	coding	109	167	493
PB.3786.1	3	1676	2	full-splice_match	gene: Zm00001d039642	Zm00001d039642_T00	6.52	6.52	coding	506	44	1561
PB.4039.1	3	2370	4	full-splice_match	gene: Zm00001d041201	Zm00001d041201_T00	8.15	8.15	coding	697	93	2183
PB.5482.1	4	2846	12	novel_in_catalog	gene: Zm00001d052107	novel	18.75	18.75	coding	136	169	576
PB.2276.1	10	1243	7	full-splice_match	gene: Zm00001d026283	Zm00001d026283_T00	276.91	279.00	coding	287	104	964
PB.2276.2	10	1260	6	novel_in_catalog	gene: Zm00001d026283	novel	2.09	279.00	coding	231	103	795
PB.4540.1	3	998	1	antisense	novelGene_gene: Zm00001d043543	novel	33.65	33.65	coding	234	100	801

PB.180.1	1	1632	4	incomplete-splice_match	gene: Zm00001d028007	Zm00001d028007_T00	14.23	14.23	coding	453	35	1393
PB.1321.1	1	864	6	full-splice_match	gene: Zm00001d033484	Zm00001d033484_T00	21.35	21.35	coding	165	32	526
PB.4969.1	4	1308	10	full-splice_match	gene: Zm00001d049222	Zm00001d049222_T00	62.32	85.66	coding	278	230	1063
PB.7616.1	6	1336	2	intergenic	novelGene_212	novel	14.12	20.33	coding	72	170	385
PB.4969.3	4	1246	10	full-splice_match	gene: Zm00001d049222	Zm00001d049222_T00	0.00	85.66	coding	278	197	1030
PB.7415.15	6	1615	2	novel_not_in_catalog	gene: Zm00001d035605	novel	3.24	1856.08	non coding	NA	NA	NA
PB.7415.13	6	2109	1	genic	gene: Zm00001d035605	novel	27.36	1856.08	coding	250	1167	1916
PB.7415.10	6	1414	2	novel_not_in_catalog	gene: Zm00001d035605	novel	57.44	1856.08	non coding	NA	NA	NA
PB.7415.11	6	1626	2	full-splice_match	gene: Zm00001d035605	Zm00001d035605_T00	25.35	1856.08	coding	316	482	1429
PB.9593.1	8	1386	7	full-splice_match	gene: Zm00001d010622	Zm00001d010622_T00	10.53	16.25	coding	290	107	976
PB.4748.8	3	1783	6	novel_in_catalog	gene: Zm00001d044327	novel	2.54	192.78	coding	130	939	1328
PB.1616.1	1	1231	1	genic_intron	novelGene_466	novel	2.89	2.89	coding	255	467	1231
PB.9593.2	8	1245	6	full-splice_match	gene: Zm00001d010622	Zm00001d010622_T00	5.72	16.25	coding	170	353	862
PB.8880.3	7	1947	8	full-splice_match	gene: Zm00001d021835	Zm00001d021835_T00	0.00	125.51	coding	539	153	1769
PB.4472.1	3	953	2	full-splice_match	gene: Zm00001d043314	Zm00001d043314_T00	81.15	81.15	coding	187	108	668
PB.7616.2	6	1464	2	intergenic	novelGene_212	novel	6.21	20.33	coding	72	128	343
PB.9715.1	8	1518	8	novel_not_in_catalog	gene: Zm00001d011156	novel	7.29	7.29	coding	427	46	1326

PB.1854.2	10	1253	5	intergenic	novelGene_128	novel	5.95	29.39	non coding	NA	NA	NA
PB.3842.1	3	887	1	antisense	novelGene_gene: Zm00001d039966	novel	8.58	8.58	non coding	NA	NA	NA
PB.7625.1	6	774	1	full-splice_match	gene: Zm00001d036902	Zm00001d036902_T00	41.08	41.08	coding	150	122	571
PB.3491.1	2	989	4	novel_in_catalog	gene: Zm00001d006951	novel	219.45	219.45	coding	229	114	800
PB.3673.2	3	2028	4	novel_in_catalog	gene: Zm00001d039245	novel	1.77	22.23	coding	274	1053	1874
PB.7199.3	5	1498	5	novel_in_catalog	gene: Zm00001d018297	novel	18.18	118.62	coding	114	206	547
PB.711.1	1	1491	10	incomplete-splice_match	gene: Zm00001d030623	Zm00001d030623_T00	11.03	11.03	coding	389	1	1167
PB.3673.1	3	1982	5	full-splice_match	gene: Zm00001d039245	Zm00001d039245_T00	20.47	22.23	coding	541	121	1743
PB.6655.1	5	1430	10	novel_not_in_catalog	gene: Zm00001d015804	novel	4.66	32.62	coding	322	234	1199
PB.1511.7	1	1864	5	novel_in_catalog	gene: Zm00001d034345	novel	258.91	1673.99	coding	160	1132	1611
PB.6655.2	5	1370	9	full-splice_match	gene: Zm00001d015804	Zm00001d015804_T00	27.96	32.62	coding	322	179	1144
PB.422.2	1	3425	1	novel_in_catalog	gene: Zm00001d029033	novel	6.51	31.35	coding	196	1522	2109
PB.2799.1	2	1598	8	intergenic	novelGene_134	novel	7.85	7.85	coding	162	227	712
PB.422.1	1	2079	6	full-splice_match	gene: Zm00001d029033	Zm00001d029033_T00	15.48	31.35	coding	216	731	1378
PB.2200.3	10	3260	3	novel_not_in_catalog	gene: Zm00001d025931	novel	7.39	24.51	non coding	NA	NA	NA
PB.2200.2	10	2027	7	novel_not_in_catalog	gene: Zm00001d025931	novel	7.85	24.51	coding	12	311	346
PB.422.4	1	1698	5	novel_in_catalog	gene: Zm00001d029033	novel	5.77	31.35	coding	216	829	1476

PB.422.5	1	2133	3	full-splice_match	gene: Zm00001d029033	Zm00001d029033_T00	3.59	31.35	coding	216	1419	2066
PB.6238.1	5	1297	2	intergenic	novelGene_92	novel	95.11	264.10	non coding	NA	NA	NA
PB.8626.1	7	1864	6	full-splice_match	gene: Zm00001d020901	Zm00001d020901_T00	19.01	19.01	coding	535	150	1754
PB.7276.1	5	1060	1	full-splice_match	gene: Zm00001d018530	Zm00001d018530_T00	42.31	42.31	coding	207	31	651
PB.8154.3	6	1943	6	novel_not_in_catalog	gene: Zm00001d038991	novel	3.56	57.46	coding	427	178	1458
PB.8154.2	6	1877	7	full-splice_match	gene: Zm00001d038991	Zm00001d038991_T00	30.67	57.46	coding	473	221	1639
PB.10332.1	9	880	1	full-splice_match	gene: Zm00001d045738	Zm00001d045738_T00	15.57	24.14	coding	207	51	671
PB.5853.2	4	1622	14	novel_in_catalog	gene: Zm00001d053798	novel	4.11	24.83	coding	412	206	1441
PB.10332.2	9	772	1	full-splice_match	gene: Zm00001d045738	Zm00001d045738_T00	8.57	24.14	coding	207	51	671
PB.7680.1	6	1293	1	full-splice_match	gene: Zm00001d037180	Zm00001d037180_T00	68.74	175.03	coding	325	59	1033
PB.7680.2	6	1182	1	full-splice_match	gene: Zm00001d037180	Zm00001d037180_T00	106.29	175.03	coding	325	75	1049
PB.10634.6	9	758	7	full-splice_match	gene: Zm00001d047479	Zm00001d047479_T01	236.67	667.71	coding	118	132	485
PB.10818.2	9	1339	2	full-splice_match	gene: Zm00001d048146	Zm00001d048146_T00	9.46	17.30	coding	292	272	1147
PB.6543.1	5	809	2	full-splice_match	gene: Zm00001d015127	Zm00001d015127_T00	64.84	64.84	coding	119	149	505
PB.10634.5	9	958	7	full-splice_match	gene: Zm00001d047479	Zm00001d047479_T00	404.95	667.71	non coding	NA	NA	NA
PB.7371.1	6	922	4	intergenic	novelGene_94	novel	18.13	18.13	coding	123	148	516
PB.5653.1	4	786	1	full-splice_match	gene: Zm00001d052807	Zm00001d052807_T00	8.66	8.66	coding	172	48	563

PB.10634.1	9	1381	6	full-splice_match	gene: Zm00001d047479	Zm00001d047479_T01	26.09	667.71	non coding	NA	NA	NA
PB.10761.1	9	1354	6	full-splice_match	gene: Zm00001d047912	Zm00001d047912_T00	0.90	68.75	coding	295	48	932
PB.10761.2	9	1212	6	full-splice_match	gene: Zm00001d047912	Zm00001d047912_T00	67.85	68.75	coding	295	105	989
PB.8784.1	7	1492	1	full-splice_match	gene: Zm00001d021544	Zm00001d021544_T00	10.32	10.32	coding	339	31	1047
PB.7730.1	6	1784	1	full-splice_match	gene: Zm00001d037383	Zm00001d037383_T00	15.13	15.13	coding	482	111	1556
PB.6442.3	5	566	5	full-splice_match	gene: Zm00001d014772	Zm00001d014772_T00	136.18	508.61	coding	114	73	414
PB.6442.2	5	665	5	full-splice_match	gene: Zm00001d014772	Zm00001d014772_T00	361.71	508.61	coding	114	61	402
PB.6442.1	5	740	5	full-splice_match	gene: Zm00001d014772	Zm00001d014772_T00	10.72	508.61	coding	114	35	376
PB.3053.1	2	1059	3	full-splice_match	gene: Zm00001d004913	Zm00001d004913_T00	16.16	16.16	coding	238	199	912
PB.9491.10	8	598	1	novel_in_catalog	gene: Zm00001d010172	novel	40.01	93.09	non coding	NA	NA	NA
PB.1384.2	1	2366	2	incomplete-splice_match	gene: Zm00001d033775	Zm00001d033775_T00	27.31	47.67	coding	693	117	2195
PB.2880.1	2	1094	8	novel_not_in_catalog	gene: Zm00001d003807	novel	11.82	50.21	coding	179	219	755
PB.8684.12	7	1508	4	intergenic	novelGene_282	novel	0.00	53.03	coding	111	797	1129
PB.10808.1	9	1701	1	intergenic	novelGene_299	novel	14.41	14.41	coding	48	478	621
PB.4791.5	3	1666	2	full-splice_match	gene: Zm00001d044504	Zm00001d044504_T00	0.00	283.31	coding	482	124	1569
PB.10386.1	9	735	1	full-splice_match	gene: Zm00001d046157	Zm00001d046157_T00	29.77	29.77	coding	175	102	626
PB.3525.1	2	1177	3	full-splice_match	gene: Zm00001d007105	Zm00001d007105_T00	12.48	12.48	coding	277	93	923

PB.7749.1	6	2726	6	novel_in_catalog	gene: Zm00001d037480	novel	13.33	42.34	coding	650	173	2122
PB.7749.2	6	2680	7	full-splice_match	gene: Zm00001d037480	Zm00001d037480_T00	18.43	42.34	coding	747	269	2509
PB.7749.3	6	1043	4	novel_not_in_catalog	gene: Zm00001d037480	novel	2.46	42.34	coding	191	31	603
PB.9320.1	8	1908	2	full-splice_match	gene: Zm00001d009163	Zm00001d009163_T00	83.03	83.03	coding	499	139	1635
PB.7749.5	6	2747	5	novel_in_catalog	gene: Zm00001d037480	novel	0.00	42.34	coding	650	315	2264
PB.9320.2	8	1764	2	full-splice_match	gene: Zm00001d009163	Zm00001d009163_T00	0.00	83.03	coding	499	122	1618
PB.6655.5	5	1236	9	full-splice_match	gene: Zm00001d015804	Zm00001d015804_T00	0.00	32.62	coding	322	157	1122
PB.1047.8	1	2452	7	full-splice_match	gene: Zm00001d032350	Zm00001d032350_T00	6.47	141.86	coding	114	255	596
PB.801.12	1	332	2	incomplete-splice_match	gene: Zm00001d031120	Zm00001d031120_T00	0.00	247.40	non coding	NA	NA	NA
PB.2639.3	2	3699	4	full-splice_match	gene: Zm00001d002678	Zm00001d002678_T01	7.95	65.85	non coding	NA	NA	NA
PB.2639.2	2	2098	6	novel_in_catalog	gene: Zm00001d002678	novel	0.00	65.85	coding	17	352	402
PB.2639.1	2	2037	6	novel_in_catalog	gene: Zm00001d002678	novel	22.14	65.85	non coding	NA	NA	NA
PB.4791.1	3	1969	2	full-splice_match	gene: Zm00001d044504	Zm00001d044504_T00	26.40	283.31	coding	482	124	1569
PB.8535.2	7	1642	6	full-splice_match	gene: Zm00001d020426	Zm00001d020426_T00	0.00	15.14	coding	454	176	1537
PB.2639.6	2	1798	7	novel_in_catalog	gene: Zm00001d002678	novel	3.60	65.85	non coding	NA	NA	NA
PB.2639.5	2	1042	8	novel_in_catalog	gene: Zm00001d002678	novel	6.04	65.85	coding	46	353	490
PB.2639.4	2	1150	8	novel_in_catalog	gene: Zm00001d002678	novel	26.13	65.85	non coding	NA	NA	NA

PB.3204.1	2	1578	1	full-splice_match	gene: Zm00001d005752	Zm00001d005752_T00	24.38	24.38	coding	421	15	1277
PB.5187.4	4	1390	2	full-splice_match	gene: Zm00001d050394	Zm00001d050394_T00	0.00	49.77	coding	370	136	1245
PB.472.1	1	1260	2	full-splice_match	gene: Zm00001d029279	Zm00001d029279_T00	82.08	82.08	coding	336	62	1069
PB.5187.3	4	2746	1	genic	gene: Zm00001d050394	novel	5.57	49.77	coding	206	1893	2510
PB.472.3	1	1246	2	full-splice_match	gene: Zm00001d029280	Zm00001d029280_T00	66.37	66.37	coding	337	54	1064
PB.5187.1	4	1137	1	genic	gene: Zm00001d050394	novel	11.86	49.77	coding	206	84	701
PB.9036.1	7	1767	11	full-splice_match	gene: Zm00001d022407	Zm00001d022407_T00	24.18	24.18	coding	422	290	1555
PB.1769.2	10	508	1	intergenic	novelGene_48	novel	137.29	168.40	non coding	NA	NA	NA
PB.4949.1	4	1054	1	intergenic	novelGene_73	novel	0.37	0.37	coding	30	314	403
PB.3581.3	2	875	2	novel_not_in_catalog	gene: Zm00001d007498	novel	13.16	22.63	coding	128	104	487
PB.10989.1	B73V4	1273	2	full-splice_match	gene: Zm00001d000380	Zm00001d000380_T00	14.67	15.83	coding	252	103	858
PB.3798.3	3	1401	6	full-splice_match	gene: Zm00001d039719	Zm00001d039719_T00	0.00	40.90	coding	179	529	1065
PB.10989.3	B73V4	1261	1	novel_in_catalog	gene: Zm00001d000380	novel	1.16	15.83	coding	291	103	975
PB.3798.1	3	1270	6	full-splice_match	gene: Zm00001d039719	Zm00001d039719_T00	40.90	40.90	coding	179	281	817
PB.1053.2	1	2924	8	full-splice_match	gene: Zm00001d032373	Zm00001d032373_T01	2.42	8.66	coding	441	10	1332
PB.9195.2	8	2402	6	novel_not_in_catalog	gene: Zm00001d008495	novel	42.95	42.95	coding	629	342	2228
PB.5667.1	4	1790	3	novel_not_in_catalog	gene: Zm00001d052878	novel	13.08	13.08	coding	191	946	1518

PB.4562.2	3	1858	12	full-splice_match	gene: Zm00001d043613	Zm00001d043613_T00	29.85	178.80	coding	300	209	1108
PB.4562.3	3	3449	12	full-splice_match	gene: Zm00001d043613	Zm00001d043613_T00	48.34	178.80	coding	300	1865	2764
PB.4562.1	3	3613	11	full-splice_match	gene: Zm00001d043613	Zm00001d043613_T00	0.00	178.80	coding	300	2029	2928
PB.1694.1	10	2079	9	full-splice_match	gene: Zm00001d023260	Zm00001d023260_T00	11.70	15.60	coding	492	188	1663
PB.5062.1	4	2055	2	full-splice_match	gene: Zm00001d049683	Zm00001d049683_T00	131.49	131.49	coding	593	66	1844
PB.1694.3	10	1874	9	full-splice_match	gene: Zm00001d023260	Zm00001d023260_T00	3.90	15.60	coding	492	201	1676
PB.4789.2	3	1695	2	genic	gene: Zm00001d044501	novel	0.00	30.27	coding	188	639	1202
PB.1985.7	10	2834	18	full-splice_match	gene: Zm00001d024855	Zm00001d024855_T01	112.07	198.71	coding	744	192	2423
PB.9749.2	8	3388	18	novel_in_catalog	gene: Zm00001d011309	novel	46.65	55.36	coding	898	358	3051
PB.9749.3	8	3652	17	novel_in_catalog	gene: Zm00001d011309	novel	0.12	55.36	coding	616	335	2182
PB.1007.1	1	1427	1	genic	gene: Zm00001d032162	novel	0.20	130.62	coding	246	87	824
PB.9749.1	8	1896	7	incomplete-splice_match	gene: Zm00001d011309	Zm00001d011309_T02	8.58	55.36	coding	232	792	1487
PB.2200.4	10	3542	5	novel_not_in_catalog	gene: Zm00001d025931	novel	4.19	24.51	coding	12	315	350
PB.3232.2	2	671	1	full-splice_match	gene: Zm00001d005879	Zm00001d005879_T00	48.29	111.87	coding	91	176	448
PB.3232.1	2	775	1	full-splice_match	gene: Zm00001d005879	Zm00001d005879_T00	63.58	111.87	coding	91	176	448
PB.10105.1	8	1118	5	full-splice_match	gene: Zm00001d012776	Zm00001d012776_T00	279.83	515.03	coding	253	57	815
PB.4112.1	3	1631	1	full-splice_match	gene: Zm00001d041684	Zm00001d041684_T00	12.36	12.36	coding	489	101	1567

PB.10105.2	8	1036	5	full-splice_match	gene: Zm00001d012776	Zm00001d012776_T00	235.20	515.03	coding	253	77	835
PB.10995.2	B73V4	2481	1	full-splice_match	gene: Zm00001d000403	Zm00001d000403_T00	11.49	30.80	coding	679	236	2272
PB.2378.2	10	403	3	full-splice_match	gene: Zm00001d026589	Zm00001d026589_T00	99.31	137.34	non coding	NA	NA	NA
PB.9348.1	8	1165	1	full-splice_match	gene: Zm00001d009387	Zm00001d009387_T00	17.59	17.59	coding	320	49	1008
PB.10995.1	B73V4	2559	1	full-splice_match	gene: Zm00001d000403	Zm00001d000403_T00	19.31	30.80	coding	679	81	2117
PB.4233.3	3	801	1	full-splice_match	gene: Zm00001d042305	Zm00001d042305_T00	0.00	32.90	coding	133	184	582
PB.507.1	1	2605	13	full-splice_match	gene: Zm00001d029462	Zm00001d029462_T00	49.47	49.47	coding	756	128	2395
PB.4545.1	3	2526	15	novel_in_catalog	gene: Zm00001d043563	novel	11.00	99.22	coding	691	187	2259
PB.2321.1	10	619	1	antisense	novelGene_gene: Zm00001d026421	novel	4.15	4.15	non coding	NA	NA	NA
PB.2200.1	10	3189	7	novel_not_in_catalog	gene: Zm00001d025931	novel	5.08	24.51	non coding	NA	NA	NA
PB.10477.2	9	1971	4	full-splice_match	gene: Zm00001d046688	Zm00001d046688_T00	34.89	42.31	coding	259	521	1297
PB.6928.1	5	1642	8	full-splice_match	gene: Zm00001d017193	Zm00001d017193_T00	0.57	28.58	coding	243	79	807
PB.3107.2	2	2153	13	full-splice_match	gene: Zm00001d005240	Zm00001d005240_T00	29.38	29.38	coding	581	109	1851
PB.10477.1	9	2051	4	full-splice_match	gene: Zm00001d046688	Zm00001d046688_T00	5.32	42.31	coding	259	521	1297
PB.8739.3	7	1947	7	novel_not_in_catalog	gene: Zm00001d021371	novel	11.09	43.60	coding	267	844	1644
PB.3311.2	2	1924	2	full-splice_match	gene: Zm00001d006203	Zm00001d006203_T00	24.80	106.00	coding	505	30	1544
PB.416.1	1	1416	4	incomplete-splice_match	gene: Zm00001d029008	Zm00001d029008_T00	7.55	7.55	coding	251	53	805

PB.9495.1	8	2507	12	full-splice_match	gene: Zm00001d010199	Zm00001d010199_T00	38.17	44.11	coding	649	295	2241
PB.2645.2	2	2456	9	novel_in_catalog	gene: Zm00001d002703	novel	21.74	55.77	coding	377	1033	2163
PB.9775.1	8	1310	1	antisense	novelGene_gene: Zm00001d011434	novel	8.72	8.72	non coding	NA	NA	NA
PB.2645.1	2	2406	9	novel_in_catalog	gene: Zm00001d002703	novel	34.03	55.77	coding	377	800	1930
PB.5445.1	4	1131	4	full-splice_match	gene: Zm00001d051995	Zm00001d051995_T00	130.45	130.45	coding	263	103	891
PB.7915.1	6	416	2	incomplete-splice_match	gene: Zm00001d038143	Zm00001d038143_T00	157.97	157.97	coding	55	150	314
PB.4559.1	3	699	1	full-splice_match	gene: Zm00001d043610	Zm00001d043610_T00	7.43	24.08	coding	108	40	363
PB.4559.2	3	520	1	full-splice_match	gene: Zm00001d043610	Zm00001d043610_T00	16.65	24.08	coding	108	40	363
PB.8154.1	6	1827	7	novel_not_in_catalog	gene: Zm00001d038991	novel	23.24	57.46	coding	473	185	1603
PB.10477.4	9	1751	4	full-splice_match	gene: Zm00001d046688	Zm00001d046688_T00	0.00	42.31	coding	259	520	1296
PB.4955.1	4	675	1	full-splice_match	gene: Zm00001d049166	Zm00001d049166_T00	138.51	138.51	coding	69	160	366
PB.4955.2	4	632	1	full-splice_match	gene: Zm00001d049166	Zm00001d049166_T00	0.00	138.51	coding	69	158	364
PB.5096.1	4	603	2	full-splice_match	gene: Zm00001d049858	Zm00001d049858_T00	97.72	97.72	non coding	NA	NA	NA
PB.4790.2	3	1687	15	novel_in_catalog	gene: Zm00001d044503	novel	1.52	19.57	coding	302	437	1342
PB.4790.1	3	2711	14	novel_in_catalog	gene: Zm00001d044503	novel	2.96	19.57	coding	414	332	1573
PB.5006.2	4	787	1	novel_in_catalog	gene: Zm00001d049381	novel	54.17	686.85	non coding	NA	NA	NA
PB.5006.3	4	578	2	full-splice_match	gene: Zm00001d049381	Zm00001d049381_T00	230.03	686.85	non coding	NA	NA	NA

PB.6108.2	5	2967	20	novel_in_catalog	gene: Zm00001d013314	novel	0.36	32.59	coding	765	104	2398
PB.5006.1	4	800	2	full-splice_match	gene: Zm00001d049381	Zm00001d049381_T00	402.65	686.85	non coding	NA	NA	NA
PB.3579.3	2	1583	1	full-splice_match	gene: Zm00001d007486	Zm00001d007486_T00	0.00	62.51	coding	476	71	1498
PB.3579.2	2	1698	1	full-splice_match	gene: Zm00001d007486	Zm00001d007486_T00	29.25	62.51	coding	476	70	1497
PB.4790.9	3	2616	13	novel_in_catalog	gene: Zm00001d044503	novel	4.71	19.57	coding	484	159	1610
PB.1118.1	1	980	3	novel_in_catalog	gene: Zm00001d032588	novel	71.77	266.58	coding	118	418	771
PB.4396.3	3	1746	2	intergenic	novelGene_379	novel	0.00	10.38	non coding	NA	NA	NA
PB.4836.1	4	2235	1	full-splice_match	gene: Zm00001d048601	Zm00001d048601_T00	72.03	72.03	coding	642	96	2021
PB.4836.2	4	2123	1	full-splice_match	gene: Zm00001d048601	Zm00001d048601_T00	0.00	72.03	coding	642	61	1986
PB.1768.5	10	2025	12	full-splice_match	gene: Zm00001d023576	Zm00001d023576_T00	0.00	221.57	coding	596	128	1915
PB.1768.4	10	2101	12	full-splice_match	gene: Zm00001d023576	Zm00001d023576_T00	156.88	221.57	coding	596	111	1898
PB.1984.7	10	875	3	full-splice_match	gene: Zm00001d024839	Zm00001d024839_T00	518.77	1330.84	coding	223	108	776
PB.5807.1	4	1342	5	novel_in_catalog	gene: Zm00001d053613	novel	11.17	26.54	non coding	NA	NA	NA
PB.1768.1	10	2373	12	full-splice_match	gene: Zm00001d023576	Zm00001d023576_T00	28.65	221.57	coding	596	102	1889
PB.1768.3	10	2770	11	novel_in_catalog	gene: Zm00001d023576	novel	1.98	221.57	coding	343	44	1072
PB.1768.2	10	2382	12	full-splice_match	gene: Zm00001d023576	Zm00001d023576_T00	34.07	221.57	coding	596	131	1918
PB.10171.4	9	1614	3	fusion	gene: Zm00001d027055	novel	10.98	10.98	coding	363	189	1277

PB.10171.1	9	1810	2	full-splice_match	gene: Zm00001d045044	Zm00001d045044_T00	17.10	176.80	coding	363	179	1267
PB.10171.2	9	1702	2	full-splice_match	gene: Zm00001d045044	Zm00001d045044_T00	159.70	176.80	coding	363	192	1280
PB.9716.4	8	1070	11	full-splice_match	gene: Zm00001d011159	Zm00001d011159_T00	0.00	116.81	coding	246	136	873
PB.1329.3	1	1190	8	novel_not_in_catalog	gene: Zm00001d033523	novel	0.00	51.91	coding	299	44	940
PB.4223.4	3	503	3	novel_not_in_catalog	gene: Zm00001d042272	novel	3.22	13.49	non coding	NA	NA	NA
PB.4223.5	3	1838	8	novel_in_catalog	gene: Zm00001d042272	novel	0.26	13.49	coding	263	103	891
PB.517.1	1	1499	4	full-splice_match	gene: Zm00001d029497	Zm00001d029497_T00	81.29	81.29	coding	362	146	1231
PB.3057.5	2	626	2	full-splice_match	gene: Zm00001d004936	Zm00001d004936_T00	316.77	405.47	non coding	NA	NA	NA
PB.3164.1	2	775	1	genic	gene: Zm00001d005558	novel	36.53	92.13	coding	97	113	403
PB.5731.1	4	897	2	genic	gene: Zm00001d053233	novel	7.50	7.50	coding	126	227	604
PB.4223.2	3	2489	16	full-splice_match	gene: Zm00001d042272	Zm00001d042272_T00	4.93	13.49	coding	662	127	2112
PB.4223.3	3	1771	8	full-splice_match	gene: Zm00001d042272	Zm00001d042272_T00	5.07	13.49	coding	315	451	1395
PB.10883.1	9	1373	4	full-splice_match	gene: Zm00001d048411	Zm00001d048411_T00	33.40	33.40	coding	350	92	1141
PB.8951.2	7	1523	5	full-splice_match	gene: Zm00001d022104	Zm00001d022104_T00	25.87	25.87	coding	366	177	1274
PB.5583.1	4	1281	5	full-splice_match	gene: Zm00001d052450	Zm00001d052450_T00	25.16	159.81	coding	144	484	915
PB.4979.1	4	1899	7	full-splice_match	gene: Zm00001d049251	Zm00001d049251_T03	5.60	16.24	coding	308	581	1504
PB.9490.2	8	1579	5	full-splice_match	gene: Zm00001d010159	Zm00001d010159_T00	98.64	117.95	coding	376	100	1227

PB.9490.1	8	1308	2	incomplete-splice_match	gene: Zm00001d010159	Zm00001d010159_T00	19.31	117.95	coding	186	69	626
PB.747.9	1	1768	4	novel_not_in_catalog	gene: Zm00001d030891	novel	8.81	622.65	coding	186	904	1461
PB.7094.3	5	2158	9	novel_in_catalog	gene: Zm00001d017908	novel	0.70	15.70	coding	204	135	746
PB.10635.1	9	1109	1	intergenic	novelGene_253	novel	3.10	3.10	non coding	NA	NA	NA
PB.739.2	1	1772	1	novel_in_catalog	gene: Zm00001d030845	novel	2.49	20.30	coding	249	202	948
PB.2252.1	10	1525	1	full-splice_match	gene: Zm00001d026191	Zm00001d026191_T00	49.01	49.01	coding	221	63	725
PB.4246.10	3	1030	4	novel_in_catalog	gene: Zm00001d042347	novel	17.36	869.05	non coding	NA	NA	NA
PB.4713.1	3	817	1	full-splice_match	gene: Zm00001d044190	Zm00001d044190_T00	11.75	11.75	coding	137	255	665
PB.7022.2	5	1041	1	full-splice_match	gene: Zm00001d017612	Zm00001d017612_T00	83.16	96.90	coding	199	81	677
PB.7022.1	5	1042	1	full-splice_match	gene: Zm00001d017612	Zm00001d017612_T00	13.74	96.90	coding	138	55	468
PB.6061.1	5	1059	1	full-splice_match	gene: Zm00001d013178	Zm00001d013178_T00	81.50	81.50	coding	186	150	707
PB.4323.8	3	1258	9	full-splice_match	gene: Zm00001d042695	Zm00001d042695_T00	9.55	95.71	coding	319	302	1258
PB.10540.1	9	1587	4	incomplete-splice_match	gene: Zm00001d047044	Zm00001d047044_T01	68.68	68.68	coding	401	101	1303
PB.3629.1	2	1862	7	full-splice_match	gene: Zm00001d007802	Zm00001d007802_T00	41.18	41.18	coding	6	469	486
PB.4790.4	3	2635	14	novel_in_catalog	gene: Zm00001d044503	novel	4.32	19.57	coding	502	291	1796
PB.4639.1	3	879	1	full-splice_match	gene: Zm00001d043875	Zm00001d043875_T00	10.47	10.47	coding	243	33	761
PB.4134.2	3	1058	1	full-splice_match	gene: Zm00001d041774	Zm00001d041774_T00	3.01	23.64	coding	117	657	1007

PB.2795.1	2	1029	3	intergenic	novelGene_133	novel	38.18	38.18	coding	176	177	704
PB.9757.1	8	1643	2	full-splice_match	gene: Zm00001d011349	Zm00001d011349_T00	62.94	62.94	coding	428	141	1424
PB.7935.2	6	586	1	full-splice_match	gene: Zm00001d038203	Zm00001d038203_T00	21.67	67.68	coding	124	22	393
PB.1361.6	1	2224	10	novel_in_catalog	gene: Zm00001d033649	novel	8.65	68.36	coding	194	1385	1966
PB.1511.5	1	3207	2	novel_in_catalog	gene: Zm00001d034345	novel	12.33	1673.99	coding	167	435	935
PB.1630.1	1	2097	14	fusion	gene: Zm00001d034766	novel	9.35	9.35	coding	583	92	1840
PB.5705.1	4	654	4	incomplete-splice_match	gene: Zm00001d053070	Zm00001d053070_T00	21.56	21.56	non coding	NA	NA	NA
PB.2143.1	10	1917	1	full-splice_match	gene: Zm00001d025675	Zm00001d025675_T00	39.11	39.11	coding	497	90	1580
PB.8959.2	7	619	4	full-splice_match	gene: Zm00001d022141	Zm00001d022141_T00	53.97	56.30	coding	114	57	398
PB.3505.1	2	842	2	full-splice_match	gene: Zm00001d007045	Zm00001d007045_T00	209.45	209.45	coding	197	86	676
PB.7601.1	6	1664	1	genic	gene: Zm00001d036715	novel	13.27	40.28	coding	347	111	1151
PB.10600.2	9	1185	7	novel_not_in_catalog	gene: Zm00001d047366	novel	4.69	15.66	coding	133	545	943
PB.7601.3	6	2291	7	full-splice_match	gene: Zm00001d036715	Zm00001d036715_T00	15.70	40.28	coding	636	77	1984
PB.7601.2	6	2518	7	full-splice_match	gene: Zm00001d036715	Zm00001d036715_T00	10.23	40.28	coding	636	101	2008
PB.4830.3	3	1291	5	full-splice_match	gene: Zm00001d044699	Zm00001d044699_T00	0.00	79.62	coding	350	135	1184
PB.7096.2	5	704	4	full-splice_match	gene: Zm00001d017914	Zm00001d017914_T00	90.56	133.23	coding	150	65	514
PB.7601.7	6	2147	6	novel_in_catalog	gene: Zm00001d036715	novel	0.39	40.28	coding	517	501	2051

PB.7601.6	6	2151	6	novel_in_catalog	gene: Zm00001d036715	novel	0.70	40.28	coding	659	67	2043
PB.6527.1	5	937	6	full-splice_match	gene: Zm00001d015058	Zm00001d015058_T00	65.37	65.37	coding	152	200	655
PB.6527.3	5	777	6	full-splice_match	gene: Zm00001d015058	Zm00001d015058_T00	0.00	65.37	coding	152	171	626
PB.7103.2	5	3239	14	incomplete-splice_match	gene: Zm00001d017946	Zm00001d017946_T01	24.06	69.55	coding	912	1	2736
PB.10493.3	9	1243	3	full-splice_match	gene: Zm00001d046805	Zm00001d046805_T00	40.61	40.61	coding	351	54	1106
PB.3930.1	3	1725	10	full-splice_match	gene: Zm00001d040351	Zm00001d040351_T00	33.52	33.52	coding	396	162	1349
PB.92.1	1	2557	7	full-splice_match	gene: Zm00001d027612	Zm00001d027612_T00	52.56	52.56	coding	715	211	2355
PB.7108.1	5	1673	7	full-splice_match	gene: Zm00001d017966	Zm00001d017966_T00	142.53	143.60	coding	415	85	1329
PB.10263.1	9	2283	4	full-splice_match	gene: Zm00001d045445	Zm00001d045445_T00	4.33	4.33	coding	682	14	2059
PB.7103.4	5	913	4	incomplete-splice_match	gene: Zm00001d017946	Zm00001d017946_T01	34.38	69.55	coding	128	258	641
PB.9201.1	8	1710	2	full-splice_match	gene: Zm00001d008528	Zm00001d008528_T00	2.88	2.88	coding	355	244	1308
PB.9626.1	8	2436	6	novel_in_catalog	gene: Zm00001d010723	novel	6.09	60.67	coding	105	1862	2176
PB.10012.1	8	1828	1	antisense	novelGene_gene: Zm00001d012426	novel	4.92	9.88	coding	202	341	946
PB.9618.2	8	1324	12	full-splice_match	gene: Zm00001d010703	Zm00001d010703_T00	43.23	47.99	coding	282	103	948
PB.3471.3	2	705	2	full-splice_match	gene: Zm00001d006903	Zm00001d006903_T00	0.00	181.01	coding	112	200	535
PB.8948.1	7	1753	6	full-splice_match	gene: Zm00001d022082	Zm00001d022082_T00	19.48	84.71	coding	447	98	1438
PB.2309.3	10	749	3	full-splice_match	gene: Zm00001d026368	Zm00001d026368_T00	47.58	54.35	coding	129	141	527

PB.2309.1	10	1327	2	novel_in_catalog	gene: Zm00001d026368	novel	6.77	54.35	coding	100	140	439
PB.5378.1	4	983	1	full-splice_match	gene: Zm00001d051660	Zm00001d051660_T00	36.25	36.25	coding	220	34	693
PB.1095.1	1	657	1	full-splice_match	gene: Zm00001d032522	Zm00001d032522_T00	5.01	5.01	non coding	NA	NA	NA
PB.3010.1	2	917	6	novel_not_in_catalog	gene: Zm00001d004655	novel	67.52	67.52	coding	153	135	593
PB.172.1	1	987	6	novel_not_in_catalog	gene: Zm00001d027966	novel	96.18	96.18	coding	189	31	597
PB.172.2	1	855	6	novel_not_in_catalog	gene: Zm00001d027966	novel	0.00	96.18	coding	113	227	565
PB.3514.1	2	716	5	full-splice_match	gene: Zm00001d007069	Zm00001d007069_T00	78.07	78.07	coding	127	84	464
PB.6168.2	5	1551	4	full-splice_match	gene: Zm00001d013528	Zm00001d013528_T00	0.00	53.77	coding	457	58	1428
PB.531.1	1	360	2	incomplete-splice_match	gene: Zm00001d029567	Zm00001d029567_T00	232.54	232.54	non coding	NA	NA	NA
PB.6168.1	5	1666	4	full-splice_match	gene: Zm00001d013528	Zm00001d013528_T00	53.77	53.77	coding	457	65	1435
PB.2288.2	10	1046	4	full-splice_match	gene: Zm00001d026307	Zm00001d026307_T00	14.53	29.23	coding	246	118	855
PB.2288.1	10	1186	4	full-splice_match	gene: Zm00001d026307	Zm00001d026307_T00	14.71	29.23	coding	246	105	842
PB.10783.1	9	785	1	full-splice_match	gene: Zm00001d048008	Zm00001d048008_T00	27.64	27.64	non coding	NA	NA	NA
PB.9491.5	8	932	5	novel_in_catalog	gene: Zm00001d010172	novel	53.07	93.09	non coding	NA	NA	NA
PB.5283.1	4	1323	13	full-splice_match	gene: Zm00001d051157	Zm00001d051157_T00	88.65	88.65	coding	342	105	1130
PB.6748.1	5	1298	1	novel_in_catalog	gene: Zm00001d016407	novel	27.02	27.02	coding	147	532	972
PB.9747.1	8	1273	1	full-splice_match	gene: Zm00001d011302	Zm00001d011302_T00	22.86	22.86	coding	329	51	1037

PB.156.3	1	938	8	full-splice_match	gene: Zm00001d027896	Zm00001d027896_T00	0.00	339.94	coding	246	64	801
PB.3070.1	2	1759	6	novel_in_catalog	gene: Zm00001d005030	novel	18.83	24.99	non coding	NA	NA	NA
PB.156.1	1	1115	8	full-splice_match	gene: Zm00001d027896	Zm00001d027896_T00	339.94	339.94	coding	246	132	869
PB.5927.1	4	1535	8	full-splice_match	gene: Zm00001d054039	Zm00001d054039_T00	53.47	53.47	coding	407	101	1321
PB.6848.1	5	1800	7	full-splice_match	gene: Zm00001d016866	Zm00001d016866_T00	28.46	42.40	coding	393	267	1445
PB.312.1	1	1842	3	full-splice_match	gene: Zm00001d028572	Zm00001d028572_T00	7.52	192.86	coding	361	494	1576
PB.312.2	1	1721	4	full-splice_match	gene: Zm00001d028572	Zm00001d028572_T00	69.99	192.86	coding	361	367	1449
PB.5433.1	4	1626	5	full-splice_match	gene: Zm00001d051916	Zm00001d051916_T00	23.26	32.05	non coding	NA	NA	NA
PB.5433.6	4	1068	4	full-splice_match	gene: Zm00001d051916	Zm00001d051916_T00	4.47	32.05	coding	83	572	820
PB.312.5	1	1792	4	full-splice_match	gene: Zm00001d028572	Zm00001d028572_T00	11.50	192.86	coding	361	439	1521
PB.9963.1	8	1401	2	full-splice_match	gene: Zm00001d012221	Zm00001d012221_T00	17.41	17.41	coding	380	80	1219
PB.312.8	1	1695	4	full-splice_match	gene: Zm00001d028572	Zm00001d028572_T00	103.85	192.86	coding	361	415	1497
PB.7615.3	6	800	5	incomplete-splice_match	gene: Zm00001d036798	Zm00001d036798_T00	0.00	42.64	coding	250	51	800
PB.8654.4	7	3585	19	novel_in_catalog	gene: Zm00001d021000	novel	21.48	167.68	coding	488	1795	3258
PB.7615.1	6	2117	9	full-splice_match	gene: Zm00001d036798	Zm00001d036798_T00	34.39	42.64	coding	574	34	1755
PB.9668.1	8	1541	2	full-splice_match	gene: Zm00001d010924	Zm00001d010924_T00	19.38	19.38	coding	336	204	1211
PB.7615.4	6	566	1	incomplete-splice_match	gene: Zm00001d036798	Zm00001d036798_T00	4.64	42.64	coding	102	15	320

PB.4036.1	3	1313	6	full-splice_match	gene: Zm00001d041179	Zm00001d041179_T00	5.58	5.58	coding	334	15	1016
PB.9334.2	8	2233	8	incomplete-splice_match	gene: Zm00001d009243	Zm00001d009243_T00	55.28	95.10	coding	569	197	1903
PB.9334.3	8	3094	10	novel_in_catalog	gene: Zm00001d009243	novel	39.81	95.10	coding	592	120	1895
PB.2058.1	10	1476	7	novel_not_in_catalog	gene: Zm00001d025174	novel	8.04	8.04	coding	189	264	830
PB.6261.1	5	1469	1	full-splice_match	gene: Zm00001d013995	Zm00001d013995_T00	20.98	20.98	coding	251	587	1339
PB.5838.1	4	2369	2	full-splice_match	gene: Zm00001d053732	Zm00001d053732_T00	11.08	12.08	coding	655	93	2057
PB.7684.1	6	2544	14	novel_not_in_catalog	gene: Zm00001d037198	novel	34.49	34.49	coding	710	81	2210
PB.6493.1	5	2075	7	full-splice_match	gene: Zm00001d014924	Zm00001d014924_T00	3.43	29.38	coding	378	73	1206
PB.6493.2	5	1990	8	novel_in_catalog	gene: Zm00001d014924	novel	25.95	29.38	coding	511	72	1604
PB.1218.2	1	2443	5	novel_in_catalog	gene: Zm00001d033004	novel	3.76	68.92	coding	423	1050	2318
PB.1167.1	1	1558	3	full-splice_match	gene: Zm00001d032788	Zm00001d032788_T00	5.29	5.29	coding	390	158	1327
PB.4374.1	3	775	5	full-splice_match	gene: Zm00001d042909	Zm00001d042909_T00	289.17	289.17	coding	157	97	567
PB.1331.1	1	1805	9	novel_not_in_catalog	gene: Zm00001d033529	novel	3.56	3.56	coding	439	150	1466
PB.10111.1	9	1279	2	intergenic	novelGene_9	novel	7.54	7.54	non coding	NA	NA	NA
PB.8312.1	7	1749	1	genic	gene: Zm00001d019044	novel	13.25	24.78	coding	335	624	1628
PB.9016.1	7	1501	6	full-splice_match	gene: Zm00001d022340	Zm00001d022340_T00	33.59	33.59	coding	89	98	364
PB.8312.2	7	1553	1	genic	gene: Zm00001d019044	novel	11.53	24.78	coding	335	502	1506

PB.4701.3	3	1956	4	novel_not_in_catalog	gene: Zm00001d044146	novel	15.67	90.95	coding	350	683	1732
PB.1248.1	1	2296	8	incomplete-splice_match	gene: Zm00001d033168	Zm00001d033168_T00	41.75	41.75	coding	598	54	1847
PB.5295.9	4	1394	10	full-splice_match	gene: Zm00001d051227	Zm00001d051227_T00	0.00	100.94	coding	384	18	1169
PB.1270.1	1	918	1	full-splice_match	gene: Zm00001d033279	Zm00001d033279_T00	388.10	388.10	coding	141	120	542
PB.902.3	1	2115	5	full-splice_match	gene: Zm00001d031657	Zm00001d031657_T00	12.18	46.70	coding	626	68	1945
PB.108.1	1	1515	6	full-splice_match	gene: Zm00001d027682	Zm00001d027682_T00	20.79	20.79	coding	377	96	1226
PB.2764.4	2	2767	15	full-splice_match	gene: Zm00001d003214	Zm00001d003214_T00	5.28	41.09	coding	266	1699	2496
PB.2764.6	2	2009	17	full-splice_match	gene: Zm00001d003214	Zm00001d003214_T00	16.87	41.09	coding	511	209	1741
PB.2764.7	2	1826	16	novel_in_catalog	gene: Zm00001d003214	novel	2.00	41.09	coding	203	954	1562
PB.2764.1	2	2370	14	incomplete-splice_match	gene: Zm00001d003214	Zm00001d003214_T01	9.62	41.09	coding	266	1235	2032
PB.2764.2	2	1867	12	novel_in_catalog	gene: Zm00001d003214	novel	4.48	41.09	coding	203	926	1534
PB.2764.3	2	2875	15	novel_in_catalog	gene: Zm00001d003214	novel	2.83	41.09	coding	266	1792	2589
PB.168.1	1	1592	2	full-splice_match	gene: Zm00001d027946	Zm00001d027946_T00	9.02	9.02	coding	440	112	1431
PB.8852.2	7	1819	1	full-splice_match	gene: Zm00001d021757	Zm00001d021757_T00	0.00	47.38	coding	518	142	1695
PB.3006.2	2	1440	12	full-splice_match	gene: Zm00001d004616	Zm00001d004616_T00	130.69	156.83	coding	331	154	1146
PB.4618.1	3	1233	1	full-splice_match	gene: Zm00001d043829	Zm00001d043829_T00	0.00	2.35	coding	318	22	975
PB.1420.8	1	1892	9	full-splice_match	gene: Zm00001d033931	Zm00001d033931_T00	0.90	439.36	coding	268	856	1659

PB.10486.1	9	1080	1	full-splice_match	gene: Zm00001d046746	Zm00001d046746_T00	12.27	12.27	coding	312	44	979
PB.1222.1	1	1433	1	intergenic	novelGene_449	novel	17.32	17.32	non coding	NA	NA	NA
PB.3006.7	2	1507	11	full-splice_match	gene: Zm00001d004616	Zm00001d004616_T00	10.09	156.83	coding	201	631	1233
PB.3006.8	2	2339	11	novel_in_catalog	gene: Zm00001d004616	novel	16.06	156.83	coding	271	1256	2068
PB.10457.1	9	4294	9	full-splice_match	gene: Zm00001d046569	Zm00001d046569_T00	48.48	57.45	coding	1114	346	3687
PB.1451.2	1	1497	11	full-splice_match	gene: Zm00001d034033	Zm00001d034033_T00	0.00	55.24	coding	415	252	1496
PB.6991.2	5	1020	7	incomplete-splice_match	gene: Zm00001d017470	Zm00001d017470_T00	41.21	59.18	coding	138	318	731
PB.293.1	1	1779	3	novel_not_in_catalog	gene: Zm00001d028493	novel	10.42	22.92	coding	467	116	1516
PB.293.2	1	1295	3	novel_not_in_catalog	gene: Zm00001d028493	novel	12.50	22.92	coding	389	42	1208
PB.5796.11	4	2460	2	novel_in_catalog	gene: Zm00001d053566	novel	23.84	614.41	coding	307	1311	2231
PB.8852.1	7	1933	1	full-splice_match	gene: Zm00001d021757	Zm00001d021757_T00	47.38	47.38	coding	518	143	1696
PB.740.1	1	2095	4	full-splice_match	gene: Zm00001d030846	Zm00001d030846_T00	22.60	22.60	coding	544	42	1673
PB.9126.1	8	2233	3	full-splice_match	gene: Zm00001d008224	Zm00001d008224_T00	46.04	46.04	coding	622	177	2042
PB.8239.1	7	1807	1	full-splice_match	gene: Zm00001d018708	Zm00001d018708_T00	1.96	1.96	coding	583	52	1800
PB.9126.2	8	2150	3	full-splice_match	gene: Zm00001d008224	Zm00001d008224_T00	0.00	46.04	coding	622	112	1977
PB.5974.1	5	1651	6	incomplete-splice_match	gene: Zm00001d012863	Zm00001d012863_T00	18.03	40.26	coding	260	123	902
PB.5974.2	5	3593	12	full-splice_match	gene: Zm00001d012863	Zm00001d012863_T00	18.21	40.26	coding	654	78	2039

PB.5974.3	5	2292	10	incomplete-splice_match	gene: Zm00001d012863	Zm00001d012863_T00	3.24	40.26	coding	501	163	1665
PB.4102.1	3	1938	12	full-splice_match	gene: Zm00001d041608	Zm00001d041608_T00	41.77	41.77	coding	313	650	1588
PB.8281.1	7	2051	4	full-splice_match	gene: Zm00001d018887	Zm00001d018887_T00	23.64	34.73	coding	432	79	1374
PB.3948.1	3	1818	1	full-splice_match	gene: Zm00001d040442	Zm00001d040442_T00	23.49	23.49	coding	478	56	1489
PB.8281.3	7	1568	4	full-splice_match	gene: Zm00001d018887	Zm00001d018887_T00	0.00	34.73	coding	432	68	1363
PB.8009.2	6	1248	3	full-splice_match	gene: Zm00001d038476	Zm00001d038476_T00	0.00	10.81	coding	245	156	890
PB.10638.5	9	2985	13	full-splice_match	gene: Zm00001d047491	Zm00001d047491_T00	5.35	17.21	coding	895	120	2804
PB.10489.9	9	1079	2	novel_in_catalog	gene: Zm00001d046767	novel	11.88	778.69	coding	174	275	796
PB.1621.1	1	1991	9	full-splice_match	gene: Zm00001d034736	Zm00001d034736_T00	3.77	3.77	coding	564	67	1758
PB.8012.1	6	1991	3	novel_in_catalog	gene: Zm00001d038489	novel	15.34	15.34	coding	489	222	1688
PB.5838.2	4	1218	1	novel_in_catalog	gene: Zm00001d053732	novel	1.00	12.08	coding	109	583	909
PB.5419.3	4	2905	6	novel_in_catalog	gene: Zm00001d051875	novel	5.73	21.42	coding	498	1232	2725
PB.743.1	1	2191	6	full-splice_match	gene: Zm00001d030863	Zm00001d030863_T00	16.59	41.23	coding	239	1115	1831
PB.524.3	1	536	2	full-splice_match	gene: Zm00001d029546	Zm00001d029546_T00	2963.12	7497.45	coding	79	82	318
PB.4610.2	3	1445	10	full-splice_match	gene: Zm00001d043797	Zm00001d043797_T00	89.96	166.78	coding	303	281	1189
PB.8009.1	6	1362	3	full-splice_match	gene: Zm00001d038476	Zm00001d038476_T00	10.81	10.81	coding	245	159	893
PB.7146.1	5	1070	1	antisense	novelGene_gene: Zm00001d018089	novel	36.45	36.45	coding	160	81	560

PB.1442.1	1	1682	8	incomplete-splice_match	gene: Zm00001d034013	Zm00001d034013_T01	8.29	8.29	coding	440	179	1498
PB.4610.4	3	1887	9	full-splice_match	gene: Zm00001d043797	Zm00001d043797_T00	0.00	166.78	coding	123	231	599
PB.4610.5	3	2503	3	incomplete-splice_match	gene: Zm00001d043797	Zm00001d043797_T00	5.52	166.78	coding	102	1110	1415
PB.2871.5	2	1037	7	novel_in_catalog	gene: Zm00001d003773	novel	102.67	206.03	coding	309	14	940
PB.4284.1	3	1759	8	incomplete-splice_match	gene: Zm00001d042528	Zm00001d042528_T00	6.14	6.14	coding	413	112	1350
PB.7199.6	5	830	1	genic_intron	novelGene_416	novel	2.68	2.68	non coding	NA	NA	NA
PB.2871.1	2	2325	8	full-splice_match	gene: Zm00001d003773	Zm00001d003773_T00	103.37	206.03	coding	536	399	2006
PB.6320.1	5	1722	15	full-splice_match	gene: Zm00001d014258	Zm00001d014258_T01	64.98	77.15	coding	482	106	1551
PB.5850.2	4	771	1	full-splice_match	gene: Zm00001d053781	Zm00001d053781_T00	37.38	61.38	coding	120	145	504
PB.1545.1	1	480	1	antisense	novelGene_gene: Zm00001d023019	novel	10.59	10.59	non coding	NA	NA	NA
PB.6856.2	5	1772	2	full-splice_match	gene: Zm00001d016896	Zm00001d016896_T00	4.32	31.67	coding	488	37	1500
PB.4725.5	3	1064	1	incomplete-splice_match	gene: Zm00001d044232	Zm00001d044232_T00	20.48	62.54	coding	247	37	777
PB.4725.4	3	3532	3	full-splice_match	gene: Zm00001d044232	Zm00001d044232_T00	4.07	62.54	coding	384	333	1484
PB.10909.1	9	1127	5	novel_not_in_catalog	gene: Zm00001d048499	novel	17.18	17.18	coding	249	16	762
PB.4638.2	3	1901	3	novel_in_catalog	gene: Zm00001d043874	novel	16.46	131.42	coding	107	128	448
PB.4725.1	3	3176	4	full-splice_match	gene: Zm00001d044232	Zm00001d044232_T00	32.59	62.54	coding	756	239	2506
PB.1240.2	1	2623	17	full-splice_match	gene: Zm00001d033108	Zm00001d033108_T00	18.82	77.27	coding	605	108	1922

PB.4638.3	3	1205	4	novel_in_catalog	gene: Zm00001d043874	novel	2.15	131.42	coding	107	128	448
PB.6730.2	5	1306	7	full-splice_match	gene: Zm00001d016299	Zm00001d016299_T00	0.00	26.88	coding	335	97	1101
PB.6730.1	5	1501	7	full-splice_match	gene: Zm00001d016299	Zm00001d016299_T00	26.88	26.88	coding	335	97	1101
PB.8129.1	6	1590	4	novel_in_catalog	gene: Zm00001d038905	novel	279.98	279.98	coding	415	66	1310
PB.6838.1	5	1160	5	novel_not_in_catalog	gene: Zm00001d016834	novel	9.71	9.71	coding	246	51	788
PB.2548.10	2	1668	11	full-splice_match	gene: Zm00001d002292	Zm00001d002292_T00	0.00	207.58	coding	394	335	1516
PB.2548.11	2	1709	10	full-splice_match	gene: Zm00001d002292	Zm00001d002292_T00	0.00	207.58	coding	290	744	1613
PB.8289.1	7	1611	2	full-splice_match	gene: Zm00001d018916	Zm00001d018916_T00	12.13	12.13	coding	405	60	1274
PB.457.1	1	785	1	incomplete-splice_match	gene: Zm00001d029205	Zm00001d029205_T00	9.65	9.65	non coding	NA	NA	NA
PB.1102.3	1	1813	7	novel_in_catalog	gene: Zm00001d032539	novel	3.11	50.15	coding	291	771	1643
PB.1102.2	1	940	4	incomplete-splice_match	gene: Zm00001d032539	Zm00001d032539_T00	18.39	50.15	coding	181	59	601
PB.3960.1	3	1843	2	novel_not_in_catalog	gene: Zm00001d040509	novel	7.00	791.03	coding	299	672	1568
PB.2383.1	10	2664	6	full-splice_match	gene: Zm00001d026607	Zm00001d026607_T00	80.88	80.88	coding	720	148	2307
PB.3960.3	3	1701	4	novel_not_in_catalog	gene: Zm00001d040509	novel	229.09	791.03	coding	445	100	1434
PB.10461.1	9	3278	19	full-splice_match	gene: Zm00001d046587	Zm00001d046587_T02	10.99	13.43	coding	904	175	2886
PB.1102.1	1	2986	8	incomplete-splice_match	gene: Zm00001d032539	Zm00001d032539_T00	15.98	50.15	coding	727	29	2209
PB.3960.7	3	1587	4	novel_not_in_catalog	gene: Zm00001d040509	novel	0.00	791.03	coding	445	100	1434

PB.3960.6	3	1620	4	novel_not_in_catalog	gene: Zm00001d040509	novel	554.95	791.03	coding	445	90	1424
PB.9650.4	8	2142	18	full-splice_match	gene: Zm00001d010835	Zm00001d010835_T00	24.79	24.79	coding	604	142	1953
PB.10520.9	9	802	2	novel_in_catalog	gene: Zm00001d046952	novel	8.84	244.88	coding	77	77	307
PB.7028.1	5	872	1	full-splice_match	gene: Zm00001d017622	Zm00001d017622_T00	20.72	20.72	coding	181	144	686
PB.6676.1	5	1340	4	full-splice_match	gene: Zm00001d015977	Zm00001d015977_T00	6.77	6.77	coding	300	132	1031
PB.10520.2	9	1693	12	full-splice_match	gene: Zm00001d046952	Zm00001d046952_T00	192.59	244.88	coding	505	65	1579
PB.2383.2	10	2538	6	full-splice_match	gene: Zm00001d026607	Zm00001d026607_T00	0.00	80.88	coding	720	105	2264
PB.10520.6	9	2367	10	novel_in_catalog	gene: Zm00001d046952	novel	15.72	244.88	coding	218	1605	2258
PB.10520.7	9	1550	12	novel_not_in_catalog	gene: Zm00001d046952	novel	4.99	244.88	coding	218	791	1444
PB.55.1	1	3075	13	full-splice_match	gene: Zm00001d027462	Zm00001d027462_T00	6.01	6.01	coding	878	231	2864
PB.10520.5	9	1486	12	novel_not_in_catalog	gene: Zm00001d046952	novel	22.75	244.88	coding	150	929	1378
PB.7353.4	6	809	4	full-splice_match	gene: Zm00001d035298	Zm00001d035298_T00	0.00	28.06	coding	172	84	599
PB.9756.1	8	1579	2	full-splice_match	gene: Zm00001d011348	Zm00001d011348_T00	113.10	122.22	coding	428	133	1416
PB.7353.1	6	1200	1	incomplete-splice_match	gene: Zm00001d035298	Zm00001d035298_T00	2.92	28.06	non coding	NA	NA	NA
PB.7353.2	6	1210	5	full-splice_match	gene: Zm00001d035298	Zm00001d035298_T00	25.14	28.06	coding	172	172	687
PB.9640.4	8	945	1	genic	gene: Zm00001d010805	novel	13.32	81.10	coding	258	36	809
PB.8535.1	7	1783	6	full-splice_match	gene: Zm00001d020426	Zm00001d020426_T00	15.14	15.14	coding	454	176	1537

PB.9640.2	8	1930	1	genic	gene: Zm00001d010805	novel	7.41	81.10	coding	258	34	807
PB.3877.1	3	1205	2	full-splice_match	gene: Zm00001d040126	Zm00001d040126_T00	168.58	168.58	coding	290	96	965
PB.7162.1	5	2035	7	incomplete-splice_match	gene: Zm00001d018146	Zm00001d018146_T00	2.37	67.45	coding	330	259	1248
PB.9640.1	8	1846	2	full-splice_match	gene: Zm00001d010805	Zm00001d010805_T00	8.21	81.10	coding	229	32	718
PB.4717.1	3	1884	1	genic	gene: Zm00001d044204	novel	5.06	5.06	coding	244	466	1197
PB.2168.1	10	1689	1	full-splice_match	gene: Zm00001d025788	Zm00001d025788_T00	10.95	22.46	coding	446	71	1408
PB.10266.1	9	1721	1	genic	gene: Zm00001d045463	novel	8.05	30.61	coding	199	85	681
PB.9263.1	8	1545	1	full-splice_match	gene: Zm00001d008850	Zm00001d008850_T00	104.23	104.23	coding	282	229	1074
PB.7552.2	6	1445	10	full-splice_match	gene: Zm00001d036496	Zm00001d036496_T00	18.07	31.17	coding	408	71	1294
PB.10193.1	9	1400	3	full-splice_match	gene: Zm00001d045159	Zm00001d045159_T00	11.39	32.04	coding	303	262	1170
PB.10193.2	9	1308	3	novel_not_in_catalog	gene: Zm00001d045159	novel	20.65	32.04	coding	303	170	1078
PB.2072.1	10	1947	13	full-splice_match	gene: Zm00001d025258	Zm00001d025258_T00	91.01	91.01	coding	446	212	1549
PB.1568.1	1	443	1	full-splice_match	gene: Zm00001d034572	Zm00001d034572_T00	36.90	36.90	non coding	NA	NA	NA
PB.3204.2	2	1391	1	full-splice_match	gene: Zm00001d005752	Zm00001d005752_T00	0.00	24.38	coding	263	458	1246
PB.7552.4	6	1584	9	novel_in_catalog	gene: Zm00001d036496	novel	10.85	31.17	coding	113	68	406
PB.2026.5	10	1679	1	genic	gene: Zm00001d025017	novel	0.00	100.34	coding	166	934	1431
PB.2026.4	10	1448	3	genic	gene: Zm00001d025017	novel	11.00	100.34	coding	197	608	1198

PB.2026.7	10	1554	1	genic	gene: Zm00001d025017	novel	0.00	100.34	coding	166	934	1431
PB.2026.6	10	1338	3	genic	gene: Zm00001d025017	novel	30.42	100.34	coding	197	605	1195
PB.2754.1	2	1586	3	full-splice_match	gene: Zm00001d003186	Zm00001d003186_T00	11.65	11.65	coding	379	248	1384
PB.2026.3	10	1972	1	genic	gene: Zm00001d025017	novel	22.10	100.34	coding	166	933	1430
PB.2754.2	2	1408	3	full-splice_match	gene: Zm00001d003186	Zm00001d003186_T00	0.00	11.65	coding	379	103	1239
PB.5187.2	4	1508	2	full-splice_match	gene: Zm00001d050394	Zm00001d050394_T00	32.34	49.77	coding	370	141	1250
PB.3351.1	2	571	2	novel_not_in_catalog	gene: Zm00001d006369	novel	12.60	12.60	non coding	NA	NA	NA
PB.4079.3	3	1076	6	full-splice_match	gene: Zm00001d041474	Zm00001d041474_T00	15.52	117.69	coding	140	170	589
PB.2026.9	10	1190	1	genic	gene: Zm00001d025017	novel	0.00	100.34	non coding	NA	NA	NA
PB.2026.8	10	1476	2	genic	gene: Zm00001d025017	novel	28.91	100.34	coding	197	763	1353
PB.8892.1	7	987	2	genic	gene: Zm00001d021874	novel	1.95	16.09	non coding	NA	NA	NA
PB.7429.1	6	768	3	full-splice_match	gene: Zm00001d035693	Zm00001d035693_T00	21.70	71.69	non coding	NA	NA	NA
PB.1871.6	10	1863	11	novel_in_catalog	gene: Zm00001d024212	novel	0.00	55.15	coding	255	851	1615
PB.7429.2	6	659	3	full-splice_match	gene: Zm00001d035693	Zm00001d035693_T00	49.99	71.69	non coding	NA	NA	NA
PB.2149.5	10	2470	2	full-splice_match	gene: Zm00001d025714	Zm00001d025714_T00	87.28	87.28	coding	510	674	2203
PB.1871.1	10	2151	12	novel_in_catalog	gene: Zm00001d024212	novel	2.86	55.15	coding	255	1062	1826
PB.2514.9	2	2939	2	genic	gene: Zm00001d002126	novel	12.66	202.56	coding	658	796	2769

PB.1871.3	10	2038	13	full-splice_match	gene: Zm00001d024212	Zm00001d024212_T00	40.54	55.15	coding	500	220	1719
PB.2514.7	2	2863	3	genic	gene: Zm00001d002126	novel	6.06	202.56	coding	658	609	2582
PB.2423.1	2	2343	1	full-splice_match	gene: Zm00001d001781	Zm00001d001781_T00	5.39	8.30	coding	380	904	2043
PB.7903.2	6	5286	15	full-splice_match	gene: Zm00001d038106	Zm00001d038106_T01	19.16	19.16	coding	1627	75	4955
PB.2514.4	2	2800	2	genic	gene: Zm00001d002126	novel	2.86	202.56	coding	658	548	2521
PB.2514.3	2	3494	2	genic	gene: Zm00001d002126	novel	21.05	202.56	coding	658	1333	3306
PB.2514.2	2	2760	2	genic	gene: Zm00001d002126	novel	77.45	202.56	coding	658	402	2375
PB.2514.1	2	2585	1	full-splice_match	gene: Zm00001d002126	Zm00001d002126_T00	18.84	202.56	coding	658	75	2048
PB.10044.2	8	1566	4	full-splice_match	gene: Zm00001d012556	Zm00001d012556_T00	0.00	74.47	coding	448	84	1427
PB.7568.1	6	2211	4	novel_not_in_catalog	gene: Zm00001d036564	novel	31.38	31.38	coding	454	496	1857
PB.2048.3	10	2079	4	novel_not_in_catalog	gene: Zm00001d025106	novel	3.82	27.26	coding	100	423	722
PB.2048.2	10	1223	5	full-splice_match	gene: Zm00001d025106	Zm00001d025106_T00	12.64	27.26	coding	146	111	548
PB.213.1	1	2233	14	full-splice_match	gene: Zm00001d028183	Zm00001d028183_T00	45.04	58.75	coding	558	244	1917
PB.213.2	1	2135	14	full-splice_match	gene: Zm00001d028183	Zm00001d028183_T00	13.71	58.75	coding	558	251	1924
PB.745.3	1	1219	3	novel_not_in_catalog	gene: Zm00001d030867	novel	3.04	6.63	coding	100	218	517
PB.5436.1	4	2096	1	full-splice_match	gene: Zm00001d051939	Zm00001d051939_T00	40.18	40.18	coding	538	168	1781
PB.10017.2	8	809	1	full-splice_match	gene: Zm00001d012438	Zm00001d012438_T00	2.42	11.10	coding	148	33	476

PB.4297.1	3	1675	13	full-splice_match	gene: Zm00001d042596	Zm00001d042596_T00	7.53	7.53	coding	400	84	1283
PB.3521.1	2	2134	8	novel_in_catalog	gene: Zm00001d007090	novel	9.36	9.36	coding	281	99	941
PB.4424.1	3	803	1	full-splice_match	gene: Zm00001d043136	Zm00001d043136_T00	76.75	76.75	coding	129	80	466
PB.6043.3	5	3577	22	full-splice_match	gene: Zm00001d013083	Zm00001d013083_T00	6.90	32.77	coding	990	129	3098
PB.5120.1	4	442	2	full-splice_match	gene: Zm00001d049984	Zm00001d049984_T00	96.84	96.84	non coding	NA	NA	NA
PB.6043.1	5	3793	22	full-splice_match	gene: Zm00001d013083	Zm00001d013083_T00	24.94	32.77	coding	990	208	3177
PB.6043.6	5	2786	19	novel_not_in_catalog	gene: Zm00001d013083	novel	0.93	32.77	coding	571	791	2503
PB.5365.1	4	702	1	genic	gene: Zm00001d051608	novel	6.08	62.79	coding	104	177	488
PB.1522.3	1	1370	5	incomplete-splice_match	gene: Zm00001d034388	Zm00001d034388_T02	212.88	388.07	coding	367	110	1210
PB.5813.1	4	1091	5	full-splice_match	gene: Zm00001d053632	Zm00001d053632_T00	264.43	264.43	coding	221	72	734
PB.9458.1	8	703	1	full-splice_match	gene: Zm00001d009957	Zm00001d009957_T00	133.55	133.55	coding	138	82	495
PB.5813.4	4	582	3	incomplete-splice_match	gene: Zm00001d053632	Zm00001d053632_T00	0.00	264.43	coding	14	452	493
PB.10828.2	9	2054	9	novel_not_in_catalog	gene: Zm00001d048203	novel	8.43	67.94	coding	120	1661	2020
PB.10828.1	9	2298	9	novel_not_in_catalog	gene: Zm00001d048203	novel	59.51	67.94	coding	596	285	2072
PB.8109.3	6	2272	2	novel_in_catalog	gene: Zm00001d038852	novel	2.48	55.39	coding	233	1374	2072
PB.9273.1	8	1864	1	full-splice_match	gene: Zm00001d008901	Zm00001d008901_T00	9.62	9.62	coding	453	202	1560
PB.8109.1	6	1480	3	full-splice_match	gene: Zm00001d038852	Zm00001d038852_T00	22.64	55.39	coding	233	214	912

PB.7647.10	6	1407	10	novel_not_in_catalog	gene: Zm00001d036987	novel	0.00	64.02	coding	430	118	1407
PB.1420.6	1	1564	11	full-splice_match	gene: Zm00001d033931	Zm00001d033931_T00	143.04	439.36	coding	379	139	1275
PB.1105.1	1	1875	3	full-splice_match	gene: Zm00001d032546	Zm00001d032546_T00	58.92	145.01	coding	499	96	1592
PB.1420.5	1	1755	10	novel_in_catalog	gene: Zm00001d033931	novel	6.65	439.36	coding	379	311	1447
PB.1879.4	10	1865	8	full-splice_match	gene: Zm00001d024281	Zm00001d024281_T00	15.12	33.52	coding	500	82	1581
PB.9289.2	8	2012	1	full-splice_match	gene: Zm00001d008960	Zm00001d008960_T00	9.00	14.58	coding	581	100	1842
PB.9289.1	8	1463	1	full-splice_match	gene: Zm00001d008960	Zm00001d008960_T00	5.58	14.58	coding	409	9	1235
PB.5679.1	4	2176	7	full-splice_match	gene: Zm00001d052944	Zm00001d052944_T00	38.34	54.99	coding	528	83	1666
PB.5100.1	4	1076	6	full-splice_match	gene: Zm00001d049881	Zm00001d049881_T00	159.98	159.98	coding	181	200	742
PB.1879.1	10	2006	8	full-splice_match	gene: Zm00001d024281	Zm00001d024281_T00	0.00	33.52	coding	500	83	1582
PB.1879.2	10	1963	8	full-splice_match	gene: Zm00001d024281	Zm00001d024281_T00	6.45	33.52	coding	500	79	1578
PB.5100.2	4	945	6	full-splice_match	gene: Zm00001d049881	Zm00001d049881_T00	0.00	159.98	coding	187	174	734
PB.6956.1	5	506	3	incomplete-splice_match	gene: Zm00001d017307	Zm00001d017307_T00	1.85	1.85	non coding	NA	NA	NA
PB.9394.1	8	900	1	intergenic	novelGene_168	novel	13.68	13.68	coding	63	118	306
PB.8088.1	6	2049	9	full-splice_match	gene: Zm00001d038792	Zm00001d038792_T00	112.91	112.91	coding	507	174	1694
PB.5837.1	4	917	4	novel_not_in_catalog	gene: Zm00001d053728	novel	192.43	192.43	coding	176	165	692
PB.934.6	1	870	5	full-splice_match	gene: Zm00001d031825	Zm00001d031825_T00	100.22	940.49	coding	170	117	626

PB.934.7	1	510	4	full-splice_match	gene: Zm00001d031825	Zm00001d031825_T00	215.77	940.49	coding	131	117	509
PB.934.4	1	1796	4	full-splice_match	gene: Zm00001d031825	Zm00001d031825_T00	1.52	940.49	coding	75	95	319
PB.934.5	1	2918	2	novel_in_catalog	gene: Zm00001d031825	novel	4.18	940.49	coding	75	94	318
PB.5295.2	4	1568	10	full-splice_match	gene: Zm00001d051227	Zm00001d051227_T00	95.04	100.94	coding	384	84	1235
PB.934.3	1	1098	4	novel_in_catalog	gene: Zm00001d031825	novel	2.16	940.49	coding	119	117	473
PB.934.1	1	961	5	full-splice_match	gene: Zm00001d031825	Zm00001d031825_T00	616.64	940.49	coding	170	95	604
PB.7421.2	6	738	2	full-splice_match	gene: Zm00001d035659	Zm00001d035659_T00	45.33	895.56	coding	83	62	310
PB.1635.4	1	1053	2	novel_in_catalog	gene: Zm00001d034784	novel	0.00	97.45	coding	202	211	816
PB.4592.2	3	1162	1	novel_in_catalog	gene: Zm00001d043728	novel	18.30	18.30	coding	65	166	360
PB.1635.2	1	1560	3	novel_in_catalog	gene: Zm00001d034784	novel	40.14	97.45	coding	214	667	1308
PB.2556.7	2	1952	2	incomplete-splice_match	gene: Zm00001d002325	Zm00001d002325_T00	63.03	303.54	coding	586	194	1951
PB.1327.1	1	304	1	genic	gene: Zm00001d033517	novel	22.15	22.15	non coding	NA	NA	NA
PB.6215.1	5	1198	8	full-splice_match	gene: Zm00001d013755	Zm00001d013755_T00	73.30	100.28	coding	217	176	826
PB.5270.1	4	834	5	full-splice_match	gene: Zm00001d051053	Zm00001d051053_T00	4.37	4.37	coding	152	113	568
PB.6798.1	5	2262	2	full-splice_match	gene: Zm00001d016674	Zm00001d016674_T00	4.46	6.36	coding	508	469	1992
PB.1329.2	1	2232	7	novel_not_in_catalog	gene: Zm00001d033523	novel	3.74	51.91	coding	136	314	721
PB.6215.3	5	1124	7	full-splice_match	gene: Zm00001d013755	Zm00001d013755_T00	0.00	100.28	coding	197	346	936

PB.8340.1	7	1576	8	full-splice_match	gene: Zm00001d019218	Zm00001d019218_T00	2.17	2.17	coding	427	30	1310
PB.10989.2	B73V 4	1087	2	full-splice_match	gene: Zm00001d000380	Zm00001d000380_T00	0.00	15.83	coding	252	103	858
PB.7516.1	6	1523	3	full-splice_match	gene: Zm00001d036285	Zm00001d036285_T00	41.85	76.26	coding	324	202	1173
PB.2230.1	10	938	3	incomplete-splice_match	gene: Zm00001d026079	Zm00001d026079_T00	115.06	115.06	coding	180	58	597
PB.4609.1	3	3724	8	full-splice_match	gene: Zm00001d043785	Zm00001d043785_T00	5.74	14.58	coding	372	21	1136
PB.3414.2	2	661	3	incomplete-splice_match	gene: Zm00001d006628	Zm00001d006628_T00	46.24	104.94	coding	56	130	297
PB.10873.1	9	3351	2	full-splice_match	gene: Zm00001d048390	Zm00001d048390_T00	2.02	2.02	coding	1047	109	3249
PB.8015.2	6	2769	2	novel_in_catalog	gene: Zm00001d038508	novel	57.81	57.81	coding	64	119	310
PB.11003.1	B73V 4	1769	1	full-splice_match	gene: Zm00001d000237	Zm00001d000237_T00	9.71	30.95	coding	462	68	1453
PB.8816.3	7	1009	5	full-splice_match	gene: Zm00001d021668	Zm00001d021668_T00	1381.02	1881.70	coding	115	269	613
PB.11003.3	B73V 4	1494	1	full-splice_match	gene: Zm00001d000237	Zm00001d000237_T00	0.00	30.95	coding	467	1	1401
PB.8360.1	7	1016	8	full-splice_match	gene: Zm00001d019326	Zm00001d019326_T00	3.38	3.38	coding	213	49	687
PB.3869.1	3	764	1	incomplete-splice_match	gene: Zm00001d040082	Zm00001d040082_T00	16.05	47.34	coding	63	129	317
PB.8816.7	7	862	4	full-splice_match	gene: Zm00001d021668	Zm00001d021668_T00	30.52	1881.70	non coding	NA	NA	NA
PB.3869.3	3	2576	13	incomplete-splice_match	gene: Zm00001d040082	Zm00001d040082_T01	15.19	47.34	coding	773	116	2434
PB.3869.2	3	1549	6	incomplete-splice_match	gene: Zm00001d040082	Zm00001d040082_T01	16.10	47.34	coding	418	4	1257
PB.2916.1	2	778	1	full-splice_match	gene: Zm00001d003981	Zm00001d003981_T00	1219.88	1697.93	coding	126	102	479

PB.8816.8	7	1575	1	novel_in_catalog	gene: Zm00001d021668	novel	19.04	1881.70	non coding	NA	NA	NA
PB.2916.2	2	661	1	full-splice_match	gene: Zm00001d003981	Zm00001d003981_T00	478.06	1697.93	coding	126	105	482
PB.8115.1	6	1813	10	novel_in_catalog	gene: Zm00001d038866	novel	7.02	225.75	coding	295	252	1136
PB.8115.2	6	1551	10	incomplete-splice_match	gene: Zm00001d038866	Zm00001d038866_T00	0.00	225.75	coding	295	197	1081
PB.7929.1	6	2600	2	full-splice_match	gene: Zm00001d038189	Zm00001d038189_T01	15.08	25.38	coding	654	134	2095
PB.4365.4	3	1698	4	novel_in_catalog	gene: Zm00001d042874	novel	0.91	120.14	coding	361	515	1597
PB.958.1	1	733	1	full-splice_match	gene: Zm00001d031944	Zm00001d031944_T00	26.54	26.54	coding	190	88	657
PB.4365.3	3	1935	3	novel_in_catalog	gene: Zm00001d042874	novel	3.77	120.14	coding	212	1059	1694
PB.6924.1	5	1381	4	full-splice_match	gene: Zm00001d017177	Zm00001d017177_T00	42.95	87.19	non coding	NA	NA	NA
PB.330.1	1	1126	2	full-splice_match	gene: Zm00001d028651	Zm00001d028651_T00	9.40	25.19	coding	268	52	855
PB.330.2	1	1009	2	full-splice_match	gene: Zm00001d028651	Zm00001d028651_T00	15.79	25.19	coding	268	52	855
PB.4365.2	3	1623	5	novel_in_catalog	gene: Zm00001d042874	novel	87.50	120.14	coding	361	198	1280
PB.4126.5	3	1633	1	novel_in_catalog	gene: Zm00001d041741	novel	0.00	13.46	coding	212	154	789
PB.6450.2	5	1764	13	full-splice_match	gene: Zm00001d014787	Zm00001d014787_T00	4.62	15.88	coding	455	161	1525
PB.8831.1	7	1110	2	full-splice_match	gene: Zm00001d021704	Zm00001d021704_T00	2.94	29.99	coding	210	85	714
PB.6903.1	5	1379	3	full-splice_match	gene: Zm00001d017111	Zm00001d017111_T00	47.45	127.91	coding	326	87	1064
PB.10355.1	9	1176	2	genic	gene: Zm00001d045896	novel	6.88	6.88	coding	151	545	997

PB.1340.1	1	1244	11	novel_not_in_catalog	gene: Zm00001d033558	novel	23.56	41.60	coding	333	12	1010
PB.899.1	1	1261	7	full-splice_match	gene: Zm00001d031640	Zm00001d031640_T00	21.24	140.89	coding	257	70	840
PB.7449.4	6	3141	8	incomplete-splice_match	gene: Zm00001d035775	Zm00001d035775_T00	44.90	74.66	coding	959	42	2918
PB.2129.3	10	1770	15	full-splice_match	gene: Zm00001d025626	Zm00001d025626_T00	0.00	40.89	coding	506	122	1639
PB.7449.2	6	2474	8	incomplete-splice_match	gene: Zm00001d035775	Zm00001d035775_T00	18.25	74.66	coding	710	29	2158
PB.7449.1	6	1980	6	incomplete-splice_match	gene: Zm00001d035775	Zm00001d035775_T00	11.51	74.66	coding	473	76	1494
PB.5493.1	4	1285	4	full-splice_match	gene: Zm00001d052174	Zm00001d052174_T00	49.39	49.39	coding	263	161	949
PB.10013.1	8	853	1	full-splice_match	gene: Zm00001d012427	Zm00001d012427_T00	9.29	9.29	coding	169	63	569
PB.4281.2	3	894	5	full-splice_match	gene: Zm00001d042510	Zm00001d042510_T00	43.33	43.33	coding	199	124	720
PB.6450.1	5	1816	13	full-splice_match	gene: Zm00001d014787	Zm00001d014787_T00	11.26	15.88	coding	455	54	1418
PB.8851.8	7	706	4	incomplete-splice_match	gene: Zm00001d021753	Zm00001d021753_T00	49.68	164.99	coding	155	151	615
PB.8851.5	7	1609	8	novel_in_catalog	gene: Zm00001d021753	novel	42.36	164.99	coding	215	40	684
PB.8851.4	7	1607	8	novel_in_catalog	gene: Zm00001d021753	novel	13.79	164.99	coding	189	900	1466
PB.8851.7	7	1674	7	novel_in_catalog	gene: Zm00001d021753	novel	13.79	164.99	coding	215	6	650
PB.8368.1	7	2723	6	full-splice_match	gene: Zm00001d019450	Zm00001d019450_T00	30.08	30.08	coding	815	72	2516
PB.8851.1	7	1659	11	novel_not_in_catalog	gene: Zm00001d021753	novel	21.87	164.99	coding	431	18	1310
PB.4070.1	3	1282	3	full-splice_match	gene: Zm00001d041418	Zm00001d041418_T00	96.54	96.54	coding	230	135	824

PB.10669.1	9	1833	7	full-splice_match	gene: Zm00001d047612	Zm00001d047612_T00	10.82	48.10	coding	401	95	1297
PB.10669.2	9	1652	7	full-splice_match	gene: Zm00001d047612	Zm00001d047612_T00	35.70	48.10	coding	401	112	1314
PB.10669.3	9	1529	7	full-splice_match	gene: Zm00001d047612	Zm00001d047612_T00	0.00	48.10	coding	401	93	1295
PB.10669.4	9	1602	6	novel_in_catalog	gene: Zm00001d047612	novel	1.59	48.10	coding	334	422	1423
PB.4562.4	3	3414	11	full-splice_match	gene: Zm00001d043613	Zm00001d043613_T00	40.42	178.80	coding	300	1942	2841
PB.10768.3	9	1280	6	novel_in_catalog	gene: Zm00001d047940	novel	5.96	5.96	coding	110	813	1142
PB.7116.4	5	1795	8	full-splice_match	gene: Zm00001d017985	Zm00001d017985_T00	0.00	272.89	coding	390	218	1387
PB.161.4	1	1138	3	full-splice_match	gene: Zm00001d027924	Zm00001d027924_T00	0.00	50.97	coding	302	77	982
PB.2397.1	10	675	2	incomplete-splice_match	gene: Zm00001d026652	Zm00001d026652_T00	4.29	31.75	coding	137	4	414
PB.1352.3	1	3582	21	novel_not_in_catalog	gene: Zm00001d033616	novel	19.45	34.71	coding	397	2247	3437
PB.1016.9	1	1718	9	full-splice_match	gene: Zm00001d032198	Zm00001d032198_T00	0.00	168.87	coding	140	1084	1503
PB.8991.2	7	1503	4	full-splice_match	gene: Zm00001d022252	Zm00001d022252_T00	8.60	14.34	coding	309	197	1123
PB.8991.4	7	1977	3	novel_in_catalog	gene: Zm00001d022252	novel	2.01	14.34	coding	232	69	764
PB.1893.1	10	1295	2	full-splice_match	gene: Zm00001d024382	Zm00001d024382_T00	11.32	50.31	coding	281	83	925
PB.1893.2	10	1182	2	full-splice_match	gene: Zm00001d024382	Zm00001d024382_T00	37.88	50.31	coding	281	83	925
PB.1893.3	10	922	1	novel_in_catalog	gene: Zm00001d024382	novel	1.12	50.31	coding	190	125	694
PB.2308.2	10	593	1	full-splice_match	gene: Zm00001d026367	Zm00001d026367_T00	3.59	4.58	coding	121	104	466

PB.800.3	1	1955	1	novel_in_catalog	gene: Zm00001d031115	novel	7.08	20.85	coding	383	647	1795
PB.1007.3	1	884	2	full-splice_match	gene: Zm00001d032162	Zm00001d032162_T00	119.44	130.62	coding	181	101	643
PB.6240.5	5	842	2	genic	gene: Zm00001d013886	novel	0.00	154.72	coding	143	148	576
PB.6240.4	5	1768	3	novel_not_in_catalog	gene: Zm00001d013886	novel	33.83	154.72	coding	143	199	627
PB.4525.1	3	1569	15	full-splice_match	gene: Zm00001d043490	Zm00001d043490_T00	42.27	42.27	coding	384	230	1381
PB.6240.2	5	2649	2	novel_in_catalog	gene: Zm00001d013886	novel	17.88	154.72	coding	143	1041	1469
PB.6240.1	5	1986	3	novel_not_in_catalog	gene: Zm00001d013886	novel	103.02	154.72	coding	143	197	625
PB.6665.1	5	2391	21	novel_not_in_catalog	gene: Zm00001d015890	novel	24.37	62.56	coding	661	249	2231
PB.9939.20	8	2155	10	full-splice_match	gene: Zm00001d012100	Zm00001d012100_T03	23.55	77.85	coding	101	448	750
PB.10349.1	9	1308	2	full-splice_match	gene: Zm00001d045845	Zm00001d045845_T00	57.93	57.93	coding	320	152	1111
PB.2308.1	10	762	1	full-splice_match	gene: Zm00001d026367	Zm00001d026367_T00	0.99	4.58	coding	121	102	464
PB.3458.5	2	624	1	incomplete-splice_match	gene: Zm00001d006844	Zm00001d006844_T00	192.79	605.08	coding	145	69	503
PB.8826.2	7	2210	4	novel_not_in_catalog	gene: Zm00001d021697	novel	26.02	46.46	coding	660	89	2068
PB.10766.2	9	3221	4	full-splice_match	gene: Zm00001d047923	Zm00001d047923_T02	118.91	182.37	coding	897	258	2948
PB.10766.3	9	797	3	incomplete-splice_match	gene: Zm00001d047923	Zm00001d047923_T03	18.00	182.37	non coding	NA	NA	NA
PB.10766.1	9	4031	6	full-splice_match	gene: Zm00001d047923	Zm00001d047923_T00	45.46	182.37	coding	1032	584	3679
PB.10094.6	8	2101	12	novel_in_catalog	gene: Zm00001d012747	novel	0.00	38.83	coding	470	170	1579

PB.11012.2	B73V4	1660	2	genic	gene: Zm00001d000314	novel	0.00	25.05	coding	412	76	1311
PB.11012.1	B73V4	1775	2	genic	gene: Zm00001d000314	novel	25.05	25.05	coding	412	86	1321
PB.9144.1	8	1803	1	full-splice_match	gene: Zm00001d008296	Zm00001d008296_T00	38.20	38.20	coding	509	68	1594
PB.8882.1	7	1489	2	full-splice_match	gene: Zm00001d021839	Zm00001d021839_T00	165.29	645.47	coding	294	226	1107
PB.899.4	1	1043	7	full-splice_match	gene: Zm00001d031640	Zm00001d031640_T00	77.80	140.89	coding	257	74	844
PB.5550.2	4	2286	12	full-splice_match	gene: Zm00001d052354	Zm00001d052354_T00	11.24	23.11	coding	623	227	2095
PB.4505.2	3	4258	10	incomplete-splice_match	gene: Zm00001d043434	Zm00001d043434_T00	25.13	64.97	coding	965	78	2972
PB.4505.1	3	2118	3	incomplete-splice_match	gene: Zm00001d043434	Zm00001d043434_T03	39.84	64.97	coding	586	116	1873
PB.5550.1	4	2434	12	full-splice_match	gene: Zm00001d052354	Zm00001d052354_T00	11.87	23.11	coding	623	224	2092
PB.1157.1	1	2157	3	full-splice_match	gene: Zm00001d032744	Zm00001d032744_T00	10.10	14.24	coding	596	92	1879
PB.6860.1	5	2046	3	full-splice_match	gene: Zm00001d016911	Zm00001d016911_T00	16.76	46.96	coding	359	78	1154
PB.10746.1	9	1269	4	full-splice_match	gene: Zm00001d047856	Zm00001d047856_T00	122.80	122.80	coding	329	108	1094
PB.1594.2	1	1986	7	full-splice_match	gene: Zm00001d034650	Zm00001d034650_T01	14.50	63.38	coding	474	362	1783
PB.173.1	1	2408	3	full-splice_match	gene: Zm00001d027975	Zm00001d027975_T00	11.42	20.60	coding	598	8	1801
PB.5530.2	4	3785	3	novel_in_catalog	gene: Zm00001d052294	novel	21.82	21.82	coding	774	289	2610
PB.6906.1	5	476	4	novel_not_in_catalog	gene: Zm00001d017118	novel	22.20	22.20	non coding	NA	NA	NA
PB.3011.1	2	1637	2	novel_in_catalog	gene: Zm00001d000915	novel	27.99	27.99	non coding	NA	NA	NA

PB.3137.1	2	2784	9	full-splice_match	gene: Zm00001d005373	Zm00001d005373_T00	28.54	50.22	coding	709	114	2240
PB.10675.5	9	1464	1	novel_in_catalog	gene: Zm00001d047635	novel	8.00	286.92	coding	160	760	1239
PB.10675.4	9	1227	5	full-splice_match	gene: Zm00001d047635	Zm00001d047635_T00	215.03	286.92	coding	309	76	1002
PB.10675.3	9	2876	4	full-splice_match	gene: Zm00001d047635	Zm00001d047635_T00	41.42	286.92	coding	188	2086	2649
PB.173.3	1	2277	3	full-splice_match	gene: Zm00001d027975	Zm00001d027975_T00	9.18	20.60	coding	598	232	2025
PB.9543.1	8	1499	10	incomplete-splice_match	gene: Zm00001d010406	Zm00001d010406_T00	22.96	22.96	coding	379	104	1240
PB.7906.1	6	1496	1	full-splice_match	gene: Zm00001d038122	Zm00001d038122_T00	8.29	8.29	coding	388	164	1327
PB.1437.3	1	1265	1	novel_in_catalog	gene: Zm00001d033995	novel	10.50	105.44	coding	258	254	1027
PB.5201.1	4	508	1	full-splice_match	gene: Zm00001d050496	Zm00001d050496_T00	69.24	69.24	coding	106	11	328
PB.3137.2	2	1357	1	incomplete-splice_match	gene: Zm00001d005373	Zm00001d005373_T01	7.86	50.22	non coding	NA	NA	NA
PB.3106.8	2	2098	5	novel_in_catalog	gene: Zm00001d005231	novel	6.12	1483.63	coding	129	362	748
PB.471.1	1	1269	2	full-splice_match	gene: Zm00001d029275	Zm00001d029275_T00	82.72	82.72	coding	295	85	969
PB.2241.2	10	2555	1	full-splice_match	gene: Zm00001d026158	Zm00001d026158_T00	0.00	18.32	coding	718	142	2295
PB.2933.1	2	1101	6	full-splice_match	gene: Zm00001d004090	Zm00001d004090_T00	14.26	14.26	coding	297	20	910
PB.5041.1	4	1940	8	full-splice_match	gene: Zm00001d049572	Zm00001d049572_T00	13.68	18.95	coding	470	229	1638
PB.5429.3	4	1208	2	full-splice_match	gene: Zm00001d051896	Zm00001d051896_T00	44.50	130.26	coding	350	53	1102
PB.5517.3	4	1218	10	full-splice_match	gene: Zm00001d052247	Zm00001d052247_T00	0.00	77.03	coding	303	156	1064

PB.5041.2	4	1911	8	full-splice_match	gene: Zm00001d049572	Zm00001d049572_T00	5.27	18.95	coding	326	633	1610
PB.5517.4	4	1391	9	novel_in_catalog	gene: Zm00001d052247	novel	3.05	77.03	coding	249	203	949
PB.928.1	1	1663	3	full-splice_match	gene: Zm00001d031813	Zm00001d031813_T00	55.75	55.75	coding	435	153	1457
PB.390.1	1	1133	1	full-splice_match	gene: Zm00001d022725	Zm00001d022725_T00	4.12	4.12	non coding	NA	NA	NA
PB.8207.1	6	3205	7	full-splice_match	gene: Zm00001d039196	Zm00001d039196_T00	14.52	14.87	coding	862	221	2806
PB.197.2	1	2839	5	incomplete-splice_match	gene: Zm00001d028093	Zm00001d028093_T00	0.00	38.79	coding	847	171	2711
PB.197.1	1	3421	7	novel_in_catalog	gene: Zm00001d028093	novel	38.79	38.79	coding	1020	47	3106
PB.4735.1	3	3680	18	incomplete-splice_match	gene: Zm00001d044281	Zm00001d044281_T03	15.41	15.41	coding	1218	27	3680
PB.1550.2	1	1993	11	incomplete-splice_match	gene: Zm00001d034502	Zm00001d034502_T00	4.92	12.74	coding	527	62	1642
PB.4972.3	4	840	2	full-splice_match	gene: Zm00001d049230	Zm00001d049230_T00	513.24	1055.67	coding	176	105	632
PB.5061.8	4	1719	9	full-splice_match	gene: Zm00001d049680	Zm00001d049680_T00	0.00	135.97	coding	467	97	1497
PB.8086.1	6	2126	13	full-splice_match	gene: Zm00001d038777	Zm00001d038777_T00	19.20	19.20	coding	539	337	1953
PB.9343.1	8	1163	5	full-splice_match	gene: Zm00001d009339	Zm00001d009339_T00	46.56	46.56	coding	310	112	1041
PB.9438.1	8	647	3	full-splice_match	gene: Zm00001d009847	Zm00001d009847_T00	39.66	39.66	coding	145	25	459
PB.2562.1	2	1925	3	full-splice_match	gene: Zm00001d002342	Zm00001d002342_T00	56.25	56.25	coding	496	239	1726
PB.5061.1	4	1731	9	full-splice_match	gene: Zm00001d049680	Zm00001d049680_T00	87.83	135.97	coding	467	97	1497
PB.4789.1	3	1561	3	genic	gene: Zm00001d044501	novel	13.11	30.27	coding	188	485	1048

PB.5061.7	4	1934	8	novel_in_catalog	gene: Zm00001d049680	novel	3.83	135.97	coding	432	193	1488
PB.1194.1	1	1002	2	full-splice_match	gene: Zm00001d032893	Zm00001d032893_T00	29.29	29.29	coding	200	146	745
PB.5061.4	4	1780	10	novel_in_catalog	gene: Zm00001d049680	novel	11.57	135.97	coding	467	115	1515
PB.1164.1	1	1946	19	full-splice_match	gene: Zm00001d032763	Zm00001d032763_T00	8.33	76.57	coding	526	184	1761
PB.4519.1	3	1550	1	full-splice_match	gene: Zm00001d043478	Zm00001d043478_T00	2.29	2.29	coding	414	84	1325
PB.5983.8	5	761	2	novel_in_catalog	gene: Zm00001d012902	novel	19.48	38.87	non coding	NA	NA	NA
PB.10933.1	9	753	1	full-splice_match	gene: Zm00001d048558	Zm00001d048558_T00	22.96	22.96	coding	227	36	716
PB.1206.1	1	1372	2	full-splice_match	gene: Zm00001d032955	Zm00001d032955_T00	33.34	33.34	coding	300	207	1106
PB.7744.2	6	1335	2	full-splice_match	gene: Zm00001d037429	Zm00001d037429_T00	26.40	50.84	coding	370	100	1209
PB.8860.2	7	1079	4	novel_in_catalog	gene: Zm00001d021779	novel	10.03	55.84	coding	110	439	768
PB.5983.1	5	1512	2	full-splice_match	gene: Zm00001d012902	Zm00001d012902_T00	19.40	38.87	coding	115	209	553
PB.7084.1	5	1730	3	novel_not_in_catalog	gene: Zm00001d017874	novel	19.17	126.53	coding	168	375	878
PB.1133.2	1	707	4	incomplete-splice_match	gene: Zm00001d032659	Zm00001d032659_T00	21.36	38.09	non coding	NA	NA	NA
PB.1133.1	1	2753	12	full-splice_match	gene: Zm00001d032659	Zm00001d032659_T00	14.28	38.09	coding	701	96	2198
PB.1437.1	1	1654	2	full-splice_match	gene: Zm00001d033995	Zm00001d033995_T00	83.74	105.44	coding	258	640	1413
PB.4545.3	3	1910	14	novel_not_in_catalog	gene: Zm00001d043563	novel	13.71	99.22	coding	320	101	1060
PB.3114.3	2	931	4	intergenic	novelGene_334	novel	5.36	17.84	coding	123	23	391

PB.4233.2	3	1576	2	genic	gene: Zm00001d042305	novel	32.90	32.90	coding	324	147	1118
PB.9706.8	8	1877	11	full-splice_match	gene: Zm00001d011119	Zm00001d011119_T00	20.41	78.92	coding	494	82	1563
PB.8039.4	6	1698	1	intergenic	novelGene_320	novel	8.78	8.78	coding	109	282	608
PB.3639.3	2	885	1	full-splice_match	gene: Zm00001d007831	Zm00001d007831_T00	219.18	936.69	coding	181	74	616
PB.5102.2	4	2025	6	full-splice_match	gene: Zm00001d049888	Zm00001d049888_T00	5.84	39.14	coding	570	125	1834
PB.5215.1	4	1083	2	intergenic	novelGene_226	novel	7.59	7.59	coding	109	45	371
PB.6977.1	5	521	1	incomplete-splice_match	gene: Zm00001d017424	Zm00001d017424_T01	43.12	43.12	non coding	NA	NA	NA
PB.8562.2	7	1525	5	novel_in_catalog	gene: Zm00001d020556	novel	1.19	17.05	coding	320	418	1377
PB.5102.1	4	2379	6	full-splice_match	gene: Zm00001d049888	Zm00001d049888_T00	33.30	39.14	coding	570	266	1975
PB.895.2	1	758	2	full-splice_match	gene: Zm00001d031627	Zm00001d031627_T00	322.72	577.64	coding	124	166	537
PB.7574.1	6	2211	7	full-splice_match	gene: Zm00001d036594	Zm00001d036594_T00	10.42	10.42	coding	601	113	1915
PB.9301.2	8	943	5	full-splice_match	gene: Zm00001d009010	Zm00001d009010_T00	610.32	610.32	coding	194	94	675
PB.3104.2	2	1344	1	novel_in_catalog	gene: Zm00001d005208	novel	15.25	206.90	coding	184	100	651
PB.3104.1	2	1363	2	full-splice_match	gene: Zm00001d005208	Zm00001d005208_T00	124.71	206.90	coding	301	109	1011
PB.1137.5	1	1610	11	incomplete-splice_match	gene: Zm00001d032667	Zm00001d032667_T00	0.66	70.46	coding	419	148	1404
PB.5908.1	4	1880	6	full-splice_match	gene: Zm00001d053981	Zm00001d053981_T00	6.93	55.85	coding	187	210	770
PB.5507.9	4	1731	6	full-splice_match	gene: Zm00001d052219	Zm00001d052219_T00	0.40	64.47	coding	212	73	708

PB.4827.2	3	874	2	full-splice_match	gene: Zm00001d044691	Zm00001d044691_T00	0.00	118.61	non coding	NA	NA	NA
PB.10810.1	9	1337	2	genic	gene: Zm00001d048112	novel	31.89	31.89	coding	261	296	1078
PB.8931.1	7	852	1	full-splice_match	gene: Zm00001d022023	Zm00001d022023_T00	2.20	2.20	non coding	NA	NA	NA
PB.5055.3	4	1363	10	full-splice_match	gene: Zm00001d049641	Zm00001d049641_T00	2.92	607.71	coding	295	275	1159
PB.5055.1	4	1368	11	incomplete-splice_match	gene: Zm00001d049641	Zm00001d049641_T00	598.02	607.71	coding	337	73	1083
PB.5055.7	4	1190	11	incomplete-splice_match	gene: Zm00001d049641	Zm00001d049641_T00	0.00	607.71	coding	337	78	1088
PB.5055.5	4	1404	10	novel_in_catalog	gene: Zm00001d049641	novel	6.77	607.71	coding	295	322	1206
PB.6477.3	5	1221	3	full-splice_match	gene: Zm00001d014885	Zm00001d014885_T00	8.63	12.05	coding	312	37	972
PB.6477.2	5	1275	2	novel_in_catalog	gene: Zm00001d014885	novel	1.43	12.05	coding	66	516	713
PB.6477.1	5	1334	3	full-splice_match	gene: Zm00001d014885	Zm00001d014885_T00	1.99	12.05	coding	312	37	972
PB.2153.7	10	1539	6	novel_not_in_catalog	gene: Zm00001d025727	novel	5.20	97.72	coding	353	273	1331
PB.7433.1	6	1901	9	full-splice_match	gene: Zm00001d035698	Zm00001d035698_T00	19.84	19.84	coding	490	64	1533
PB.2401.2	10	3057	2	full-splice_match	gene: Zm00001d026668	Zm00001d026668_T00	0.00	35.76	coding	846	278	2815
PB.2153.3	10	4730	11	novel_not_in_catalog	gene: Zm00001d025727	novel	27.11	97.72	coding	432	3309	4604
PB.2029.1	10	1398	3	full-splice_match	gene: Zm00001d025023	Zm00001d025023_T00	85.00	103.68	coding	361	79	1161
PB.8558.1	7	3059	13	full-splice_match	gene: Zm00001d020541	Zm00001d020541_T01	9.11	9.11	coding	217	1605	2255
PB.3620.2	2	1918	9	full-splice_match	gene: Zm00001d007770	Zm00001d007770_T00	0.00	50.07	coding	523	130	1698

PB.9057.1	7	1350	3	novel_in_catalog	gene: Zm00001d022469	novel	226.25	384.90	coding	259	367	1143
PB.6724.1	5	1263	1	full-splice_match	gene: Zm00001d016271	Zm00001d016271_T00	14.15	14.15	coding	315	85	1029
PB.3275.1	2	1670	8	full-splice_match	gene: Zm00001d006065	Zm00001d006065_T00	18.60	20.76	coding	350	134	1183
PB.1346.1	1	865	1	full-splice_match	gene: Zm00001d033586	Zm00001d033586_T00	13.21	13.21	coding	191	163	735
PB.1493.2	1	1008	2	full-splice_match	gene: Zm00001d034279	Zm00001d034279_T00	91.64	132.58	coding	180	138	677
PB.1493.3	1	793	2	full-splice_match	gene: Zm00001d034279	Zm00001d034279_T00	33.75	132.58	coding	180	64	603
PB.10876.1	9	905	3	full-splice_match	gene: Zm00001d048396	Zm00001d048396_T00	3.08	3.08	coding	190	96	665
PB.7045.2	5	1255	1	full-splice_match	gene: Zm00001d017696	Zm00001d017696_T00	0.00	366.69	coding	337	92	1102
PB.7045.1	5	1454	1	full-splice_match	gene: Zm00001d017696	Zm00001d017696_T00	278.93	366.69	coding	337	189	1199
PB.9796.1	8	938	4	full-splice_match	gene: Zm00001d011536	Zm00001d011536_T00	79.15	79.15	coding	183	145	693
PB.7988.6	6	951	3	novel_not_in_catalog	gene: Zm00001d038383	novel	0.59	0.59	coding	98	332	625
PB.9804.2	8	2054	8	novel_not_in_catalog	gene: Zm00001d011570	novel	15.15	15.15	coding	280	371	1210
PB.5483.4	4	1074	10	full-splice_match	gene: Zm00001d052108	Zm00001d052108_T00	111.29	262.20	coding	212	115	750
PB.625.1	1	1354	1	intergenic	novelGene_214	novel	6.24	6.24	non coding	NA	NA	NA
PB.1263.1	1	1166	4	incomplete-splice_match	gene: Zm00001d033228	Zm00001d033228_T00	4.42	42.12	coding	165	48	542
PB.8976.1	7	3465	3	full-splice_match	gene: Zm00001d022201	Zm00001d022201_T00	16.70	16.70	coding	1058	97	3270
PB.5947.1	4	1918	15	full-splice_match	gene: Zm00001d054105	Zm00001d054105_T00	24.29	24.40	coding	480	150	1589

PB.4792.1	3	2314	5	full-splice_match	gene: Zm00001d044511	Zm00001d044511_T00	11.36	11.36	coding	687	56	2116
PB.5633.4	4	1709	5	full-splice_match	gene: Zm00001d052742	Zm00001d052742_T01	2.73	32.69	coding	37	284	394
PB.3063.3	2	2115	9	full-splice_match	gene: Zm00001d004986	Zm00001d004986_T00	64.27	77.06	coding	506	449	1966
PB.10667.1	9	1886	10	full-splice_match	gene: Zm00001d047603	Zm00001d047603_T00	22.12	22.12	coding	543	67	1695
PB.8952.3	7	848	6	full-splice_match	gene: Zm00001d022111	Zm00001d022111_T00	51.41	61.82	coding	232	37	732
PB.8952.2	7	2238	6	full-splice_match	gene: Zm00001d022111	Zm00001d022111_T00	0.00	61.82	coding	232	38	733
PB.8952.1	7	2419	6	full-splice_match	gene: Zm00001d022111	Zm00001d022111_T00	10.41	61.82	coding	232	37	732
PB.4137.1	3	939	1	full-splice_match	gene: Zm00001d041790	Zm00001d041790_T00	42.80	119.26	coding	163	96	584
PB.4137.2	3	824	1	full-splice_match	gene: Zm00001d041790	Zm00001d041790_T00	76.46	119.26	coding	163	89	577
PB.7056.8	5	972	4	novel_in_catalog	gene: Zm00001d017756	novel	4.99	54.96	coding	167	166	666
PB.4827.1	3	992	2	full-splice_match	gene: Zm00001d044691	Zm00001d044691_T00	118.61	118.61	non coding	NA	NA	NA
PB.2608.2	2	1952	1	full-splice_match	gene: Zm00001d002550	Zm00001d002550_T00	1.22	4.25	coding	603	46	1854
PB.2608.1	2	1010	1	full-splice_match	gene: Zm00001d002550	Zm00001d002550_T00	3.03	4.25	coding	181	93	635
PB.6405.1	5	1381	3	novel_not_in_catalog	gene: Zm00001d014641	novel	64.98	69.76	coding	324	87	1058
PB.6405.2	5	2392	2	full-splice_match	gene: Zm00001d014641	Zm00001d014641_T00	4.78	69.76	coding	89	370	636
PB.6405.3	5	1195	2	full-splice_match	gene: Zm00001d014641	Zm00001d014641_T00	0.00	69.76	coding	325	88	1062
PB.5597.1	4	2312	8	full-splice_match	gene: Zm00001d052525	Zm00001d052525_T00	18.50	55.88	coding	426	681	1958

PB.1912.1	10	1820	3	incomplete-splice_match	gene: Zm00001d024470	Zm00001d024470_T00	2.92	2.92	coding	462	207	1592
PB.5597.3	4	1858	8	novel_in_catalog	gene: Zm00001d052525	novel	37.38	55.88	coding	183	141	689
PB.9697.1	8	1607	7	novel_in_catalog	gene: Zm00001d011067	novel	17.40	17.40	coding	273	270	1088
PB.8718.3	7	3477	1	novel_in_catalog	gene: Zm00001d021296	novel	4.96	24.15	coding	139	300	716
PB.4916.2	4	1248	3	full-splice_match	gene: Zm00001d049011	Zm00001d049011_T00	2.66	5.58	coding	300	159	1058
PB.4916.1	4	1090	3	full-splice_match	gene: Zm00001d049011	Zm00001d049011_T00	2.92	5.58	coding	263	70	858
PB.5597.6	4	1848	8	novel_in_catalog	gene: Zm00001d052525	novel	0.00	55.88	coding	183	141	689
PB.7059.1	5	1073	5	full-splice_match	gene: Zm00001d017771	Zm00001d017771_T00	17.91	17.91	coding	310	78	1007
PB.7228.1	5	1198	10	full-splice_match	gene: Zm00001d018405	Zm00001d018405_T00	9.78	13.02	coding	202	102	707
PB.1349.1	1	1136	3	full-splice_match	gene: Zm00001d033596	Zm00001d033596_T00	74.80	190.80	coding	211	161	793
PB.5766.1	4	1855	5	full-splice_match	gene: Zm00001d053395	Zm00001d053395_T00	6.18	7.53	coding	451	407	1759
PB.5842.1	4	4192	5	full-splice_match	gene: Zm00001d053741	Zm00001d053741_T00	16.10	33.12	coding	1139	566	3982
PB.6804.1	5	1645	8	novel_in_catalog	gene: Zm00001d016694	novel	4.19	4.19	coding	205	138	752
PB.4288.2	3	2578	4	full-splice_match	gene: Zm00001d042543	Zm00001d042543_T00	0.00	10.34	coding	733	87	2285
PB.2628.1	2	2163	1	full-splice_match	gene: Zm00001d002623	Zm00001d002623_T00	53.67	53.67	coding	568	259	1962
PB.6233.1	5	1030	1	intergenic	novelGene_85	novel	9.17	9.17	coding	209	319	945
PB.699.2	1	2136	10	novel_in_catalog	gene: Zm00001d030561	novel	6.40	80.49	coding	323	967	1935

PB.699.3	1	650	6	incomplete-splice_match	gene: Zm00001d030561	Zm00001d030561_T00	0.00	80.49	coding	193	70	648
PB.6622.1	5	2714	8	full-splice_match	gene: Zm00001d015569	Zm00001d015569_T00	121.52	121.52	coding	766	108	2405
PB.699.1	1	1287	11	full-splice_match	gene: Zm00001d030561	Zm00001d030561_T00	74.09	80.49	coding	323	77	1045
PB.5805.1	4	2687	14	full-splice_match	gene: Zm00001d053610	Zm00001d053610_T00	40.09	40.09	coding	692	184	2259
PB.2368.1	10	1205	6	novel_not_in_catalog	gene: Zm00001d026572	novel	20.20	25.18	coding	295	64	948
PB.2368.3	10	1270	5	novel_not_in_catalog	gene: Zm00001d026572	novel	4.98	25.18	coding	237	30	740
PB.8904.2	7	1403	2	full-splice_match	gene: Zm00001d021929	Zm00001d021929_T00	0.00	27.78	coding	363	214	1302
PB.9555.2	8	1162	2	novel_in_catalog	gene: Zm00001d010456	novel	7.57	53.48	non coding	NA	NA	NA
PB.9687.1	8	1462	1	full-splice_match	gene: Zm00001d011002	Zm00001d011002_T00	71.93	71.93	coding	112	99	434
PB.2405.10	10	2638	16	novel_in_catalog	gene: Zm00001d026677	novel	7.02	621.14	coding	372	188	1303
PB.4622.1	3	1323	1	genic	gene: Zm00001d043839	novel	6.91	22.56	coding	201	374	976
PB.5414.1	4	777	1	full-splice_match	gene: Zm00001d051859	Zm00001d051859_T00	10.60	10.60	non coding	NA	NA	NA
PB.9817.1	8	2229	8	full-splice_match	gene: Zm00001d011622	Zm00001d011622_T01	106.22	106.22	coding	451	658	2010
PB.10589.1	9	1175	2	genic	gene: Zm00001d047303	novel	21.21	21.21	coding	272	244	1059
PB.4817.1	3	1057	8	full-splice_match	gene: Zm00001d044615	Zm00001d044615_T00	81.83	81.83	coding	231	89	781
PB.2313.1	10	1606	1	full-splice_match	gene: Zm00001d026390	Zm00001d026390_T00	3.47	3.47	coding	27	558	638
PB.1495.2	1	1104	1	genic_intron	novelGene_463	novel	6.71	23.57	non coding	NA	NA	NA

PB.4829.5	3	1093	6	full-splice_match	gene: Zm00001d044694	Zm00001d044694_T00	131.02	329.15	coding	240	185	904
PB.6538.1	5	1889	12	full-splice_match	gene: Zm00001d015120	Zm00001d015120_T00	23.12	28.53	coding	455	104	1468
PB.7800.3	6	1439	6	incomplete-splice_match	gene: Zm00001d037696	Zm00001d037696_T03	18.50	27.46	coding	339	49	1065
PB.6538.3	5	963	8	incomplete-splice_match	gene: Zm00001d015120	Zm00001d015120_T00	4.32	28.53	coding	196	99	686
PB.6538.2	5	1936	11	novel_in_catalog	gene: Zm00001d015120	novel	1.10	28.53	coding	375	93	1217
PB.9404.1	8	1760	6	novel_not_in_catalog	gene: Zm00001d009667	novel	18.50	19.32	coding	496	51	1538
PB.9404.2	8	1942	5	novel_not_in_catalog	gene: Zm00001d009667	novel	0.83	19.32	coding	390	574	1743
PB.9404.3	8	1269	6	novel_not_in_catalog	gene: Zm00001d009667	novel	0.00	19.32	coding	276	373	1200
PB.4248.1	3	476	2	incomplete-splice_match	gene: Zm00001d042350	Zm00001d042350_T00	59.02	59.02	non coding	NA	NA	NA
PB.6566.1	5	2939	17	full-splice_match	gene: Zm00001d015234	Zm00001d015234_T00	17.24	62.67	coding	588	989	2752
PB.6566.2	5	2678	17	novel_in_catalog	gene: Zm00001d015234	novel	45.43	62.67	coding	588	731	2494
PB.199.1	1	1942	8	incomplete-splice_match	gene: Zm00001d028096	Zm00001d028096_T00	4.77	13.67	coding	465	160	1554
PB.9216.1	8	1156	3	full-splice_match	gene: Zm00001d008610	Zm00001d008610_T00	12.03	12.03	coding	274	48	869
PB.199.2	1	1446	7	incomplete-splice_match	gene: Zm00001d028096	Zm00001d028096_T01	8.90	13.67	coding	286	97	954
PB.7457.4	6	1140	11	full-splice_match	gene: Zm00001d035874	Zm00001d035874_T00	13.92	62.74	coding	196	346	933
PB.5599.18	4	1821	13	novel_in_catalog	gene: Zm00001d052532	novel	21.20	583.99	coding	330	92	1081
PB.1755.5	10	644	2	full-splice_match	gene: Zm00001d023529	Zm00001d023529_T00	0.00	1922.47	coding	138	93	506

PB.1755.4	10	859	1	novel_in_catalog	gene: Zm00001d023529	novel	17.95	1922.47	non coding	NA	NA	NA
PB.536.2	1	1121	2	full-splice_match	gene: Zm00001d029594	Zm00001d029594_T00	55.70	271.73	coding	238	57	770
PB.536.3	1	1043	2	full-splice_match	gene: Zm00001d029594	Zm00001d029594_T00	208.98	271.73	coding	238	85	798
PB.1755.1	10	853	2	full-splice_match	gene: Zm00001d023529	Zm00001d023529_T00	229.47	1922.47	coding	138	92	505
PB.2360.1	10	1231	5	full-splice_match	gene: Zm00001d026549	Zm00001d026549_T00	12.95	17.53	coding	294	88	969
PB.1755.3	10	754	2	full-splice_match	gene: Zm00001d023529	Zm00001d023529_T00	1664.04	1922.47	coding	138	95	508
PB.1755.2	10	961	1	novel_in_catalog	gene: Zm00001d023529	novel	11.01	1922.47	non coding	NA	NA	NA
PB.2703.1	2	1997	11	full-splice_match	gene: Zm00001d002956	Zm00001d002956_T00	11.78	79.45	coding	413	414	1652
PB.4155.5	3	3050	6	fusion	gene: Zm00001d000620	novel	4.02	29.71	coding	817	388	2838
PB.9375.1	8	1762	2	full-splice_match	gene: Zm00001d009510	Zm00001d009510_T00	24.39	24.39	coding	469	240	1646
PB.2703.2	2	1825	11	novel_not_in_catalog	gene: Zm00001d002956	novel	16.74	79.45	coding	266	672	1469
PB.4155.1	3	817	3	novel_not_in_catalog	gene: Zm00001d041923	novel	26.65	26.65	non coding	NA	NA	NA
PB.4155.2	3	2881	5	fusion	gene: Zm00001d000620	novel	7.93	29.71	coding	849	79	2625
PB.2703.6	2	2346	11	full-splice_match	gene: Zm00001d002956	Zm00001d002956_T00	0.65	79.45	coding	413	872	2110
PB.6773.4	5	1407	1	novel_in_catalog	gene: Zm00001d016553	novel	2.82	153.95	coding	202	527	1132
PB.6773.5	5	1915	3	full-splice_match	gene: Zm00001d016553	Zm00001d016553_T00	6.16	153.95	coding	364	565	1656
PB.6773.6	5	1851	4	full-splice_match	gene: Zm00001d016553	Zm00001d016553_T00	96.24	153.95	coding	364	537	1628

PB.3200.3	2	1879	6	novel_not_in_catalog	gene: Zm00001d005726	novel	2.26	66.59	coding	320	260	1219
PB.1074.1	1	3307	9	full-splice_match	gene: Zm00001d032449	Zm00001d032449_T00	39.11	57.57	coding	756	243	2510
PB.6773.1	5	2080	4	full-splice_match	gene: Zm00001d016553	Zm00001d016553_T00	8.82	153.95	coding	364	522	1613
PB.6773.2	5	1975	4	full-splice_match	gene: Zm00001d016553	Zm00001d016553_T00	39.92	153.95	coding	364	538	1629
PB.1074.2	1	3301	8	novel_in_catalog	gene: Zm00001d032449	novel	2.15	57.57	coding	440	1264	2583
PB.747.14	1	1451	5	novel_not_in_catalog	gene: Zm00001d030891	novel	0.00	622.65	coding	361	162	1244
PB.2612.2	2	1928	12	full-splice_match	gene: Zm00001d002585	Zm00001d002585_T00	11.07	149.98	coding	508	309	1832
PB.6584.1	5	2312	2	genic	gene: Zm00001d015325	novel	22.52	26.47	coding	470	478	1887
PB.6584.2	5	2116	2	genic	gene: Zm00001d015325	novel	3.95	26.47	coding	470	473	1882
PB.2612.1	2	1985	12	full-splice_match	gene: Zm00001d002585	Zm00001d002585_T00	138.91	149.98	coding	508	304	1827
PB.5434.1	4	3131	6	novel_not_in_catalog	gene: Zm00001d051917	novel	19.78	19.78	coding	926	274	3051
PB.6849.1	5	1728	9	full-splice_match	gene: Zm00001d016873	Zm00001d016873_T00	11.32	11.32	coding	489	95	1561
PB.685.1	1	1466	9	full-splice_match	gene: Zm00001d030421	Zm00001d030421_T00	39.10	39.10	coding	360	113	1192
PB.5805.2	4	2465	14	full-splice_match	gene: Zm00001d053610	Zm00001d053610_T00	0.00	40.09	coding	692	123	2198
PB.3896.2	3	1430	12	novel_not_in_catalog	gene: Zm00001d040233	novel	28.86	42.38	coding	289	385	1251
PB.3896.1	3	797	2	incomplete-splice_match	gene: Zm00001d040233	Zm00001d040233_T00	13.52	42.38	non coding	NA	NA	NA
PB.1129.1	1	1359	1	full-splice_match	gene: Zm00001d032643	Zm00001d032643_T00	10.76	10.76	coding	320	72	1031

PB.3901.2	3	2883	8	full-splice_match	gene: Zm00001d040270	Zm00001d040270_T01	40.77	40.77	coding	754	369	2630
PB.10222.9	9	827	3	incomplete-splice_match	gene: Zm00001d045305	Zm00001d045305_T00	71.38	336.50	coding	212	123	758
PB.2190.1	10	1007	3	full-splice_match	gene: Zm00001d025900	Zm00001d025900_T00	8.95	8.95	coding	234	53	754
PB.10222.6	9	1666	4	novel_in_catalog	gene: Zm00001d045305	novel	0.00	336.50	coding	155	935	1399
PB.6262.1	5	1527	8	full-splice_match	gene: Zm00001d013996	Zm00001d013996_T00	24.43	24.43	coding	320	52	1011
PB.10222.4	9	1749	5	novel_in_catalog	gene: Zm00001d045305	novel	175.19	336.50	coding	335	487	1491
PB.9053.4	7	1072	1	incomplete-splice_match	gene: Zm00001d022458	Zm00001d022458_T00	0.00	66.73	non coding	NA	NA	NA
PB.10222.2	9	2131	5	novel_in_catalog	gene: Zm00001d045305	novel	60.25	336.50	coding	335	754	1758
PB.10222.1	9	1541	4	novel_in_catalog	gene: Zm00001d045305	novel	29.69	336.50	coding	155	682	1146
PB.1606.1	1	662	1	full-splice_match	gene: Zm00001d034680	Zm00001d034680_T00	28.35	28.35	coding	96	29	316
PB.1320.6	1	311	1	full-splice_match	gene: Zm00001d033483	Zm00001d033483_T00	494.85	1516.71	coding	76	84	311
PB.4129.1	3	2189	3	novel_in_catalog	gene: Zm00001d041753	novel	7.62	7.62	coding	106	1546	1863
PB.4541.1	3	2614	11	incomplete-splice_match	gene: Zm00001d043543	Zm00001d043543_T00	135.54	135.54	coding	656	2	1969
PB.6502.3	5	3052	2	novel_in_catalog	gene: Zm00001d014961	novel	7.06	109.00	coding	569	78	1784
PB.6094.5	5	4278	34	incomplete-splice_match	gene: Zm00001d013276	Zm00001d013276_T01	27.76	27.76	coding	1299	117	4013
PB.3568.1	2	2386	4	novel_not_in_catalog	gene: Zm00001d007395	novel	9.99	9.99	coding	660	112	2091
PB.6946.4	5	2183	11	novel_in_catalog	gene: Zm00001d017271	novel	2.64	445.51	coding	189	869	1435

PB.6502.4	5	2227	3	full-splice_match	gene: Zm00001d014961	Zm00001d014961_T00	0.00	109.00	coding	612	173	2008
PB.6502.5	5	1117	2	incomplete-splice_match	gene: Zm00001d014961	Zm00001d014961_T00	26.16	109.00	coding	337	50	1060
PB.6946.5	5	1414	12	novel_in_catalog	gene: Zm00001d017271	novel	1.43	445.51	coding	238	109	822
PB.3114.2	2	1579	4	intergenic	novelGene_334	novel	12.49	17.84	coding	123	69	437
PB.9031.3	7	1249	5	novel_in_catalog	gene: Zm00001d022393	novel	2.56	67.81	non coding	NA	NA	NA
PB.1320.2	1	710	2	genic	gene: Zm00001d033483	novel	422.57	1516.71	coding	100	84	383
PB.9220.1	8	1255	9	full-splice_match	gene: Zm00001d008619	Zm00001d008619_T00	128.55	628.71	coding	250	115	864
PB.9220.2	8	1136	9	full-splice_match	gene: Zm00001d008619	Zm00001d008619_T00	500.16	628.71	coding	250	82	831
PB.6375.1	5	3144	15	novel_in_catalog	gene: Zm00001d014516	novel	6.32	6.32	coding	266	163	960
PB.1749.6	10	1404	9	full-splice_match	gene: Zm00001d023514	Zm00001d023514_T00	4.66	68.47	coding	355	280	1344
PB.1749.7	10	1805	6	novel_in_catalog	gene: Zm00001d023514	novel	0.27	68.47	coding	150	1294	1743
PB.1749.4	10	1452	8	novel_in_catalog	gene: Zm00001d023514	novel	4.48	68.47	coding	332	229	1224
PB.1749.5	10	1533	9	full-splice_match	gene: Zm00001d023514	Zm00001d023514_T00	0.00	68.47	coding	355	278	1342
PB.1749.2	10	2311	6	novel_in_catalog	gene: Zm00001d023514	novel	6.06	68.47	coding	208	256	879
PB.1749.3	10	2341	7	full-splice_match	gene: Zm00001d023514	Zm00001d023514_T00	0.00	68.47	coding	231	242	934
PB.3524.2	2	1306	5	novel_not_in_catalog	gene: Zm00001d007102	novel	26.70	64.37	coding	242	319	1044
PB.1749.1	10	1589	9	full-splice_match	gene: Zm00001d023514	Zm00001d023514_T00	50.92	68.47	coding	355	274	1338

PB.4349.1	3	2540	7	novel_in_catalog	gene: Zm00001d042809	novel	16.75	125.79	coding	490	645	2114
PB.7982.1	6	1701	1	novel_in_catalog	gene: Zm00001d038371	novel	7.12	52.95	coding	357	320	1390
PB.4349.3	3	2255	8	full-splice_match	gene: Zm00001d042809	Zm00001d042809_T00	101.19	125.79	coding	490	447	1916
PB.6946.1	5	1575	13	full-splice_match	gene: Zm00001d017271	Zm00001d017271_T00	8.62	445.51	coding	342	109	1134
PB.4349.5	3	1159	1	incomplete-splice_match	gene: Zm00001d042809	Zm00001d042809_T00	5.98	125.79	non coding	NA	NA	NA
PB.9361.1	8	482	4	incomplete-splice_match	gene: Zm00001d009450	Zm00001d009450_T00	7.54	55.60	non coding	NA	NA	NA
PB.1749.8	10	2139	7	novel_in_catalog	gene: Zm00001d023514	novel	2.07	68.47	coding	140	208	627
PB.1749.9	10	1246	8	novel_in_catalog	gene: Zm00001d023514	novel	0.00	68.47	coding	332	191	1186
PB.8730.1	7	1275	8	full-splice_match	gene: Zm00001d021332	Zm00001d021332_T00	85.13	85.13	coding	233	220	918
PB.10935.1	9	842	1	intergenic	novelGene_317	novel	1.38	1.38	coding	61	280	462
PB.8959.1	7	2149	2	full-splice_match	gene: Zm00001d022141	Zm00001d022141_T00	2.33	56.30	non coding	NA	NA	NA
PB.8722.3	7	2224	6	full-splice_match	gene: Zm00001d021304	Zm00001d021304_T00	140.35	169.93	coding	526	219	1796
PB.6616.2	5	1091	2	incomplete-splice_match	gene: Zm00001d015520	Zm00001d015520_T00	6.12	19.43	coding	284	136	987
PB.9498.1	8	813	1	full-splice_match	gene: Zm00001d010217	Zm00001d010217_T00	15.85	15.85	coding	187	84	644
PB.6616.1	5	2309	7	incomplete-splice_match	gene: Zm00001d015520	Zm00001d015520_T00	13.31	19.43	coding	657	158	2128
PB.3532.1	2	1361	10	full-splice_match	gene: Zm00001d007146	Zm00001d007146_T00	29.94	29.94	coding	306	119	1036
PB.7982.2	6	2109	5	incomplete-splice_match	gene: Zm00001d038371	Zm00001d038371_T00	45.83	52.95	coding	587	26	1786

PB.9626.3	8	1570	8	novel_in_catalog	gene: Zm00001d010723	novel	3.39	60.67	coding	157	128	598
PB.9981.2	8	1158	2	incomplete-splice_match	gene: Zm00001d012277	Zm00001d012277_T00	55.53	549.67	non coding	NA	NA	NA
PB.8665.1	7	792	1	full-splice_match	gene: Zm00001d021038	Zm00001d021038_T00	18.06	18.06	coding	184	61	612
PB.9981.3	8	2022	5	full-splice_match	gene: Zm00001d012277	Zm00001d012277_T00	49.56	549.67	coding	376	129	1256
PB.3579.1	2	1815	1	full-splice_match	gene: Zm00001d007486	Zm00001d007486_T00	33.26	62.51	coding	476	61	1488
PB.2002.1	10	1089	9	novel_not_in_catalog	gene: Zm00001d024919	novel	47.94	47.94	coding	244	64	795
PB.9772.4	8	2404	4	full-splice_match	gene: Zm00001d011418	Zm00001d011418_T00	8.59	75.59	coding	338	1188	2201
PB.3443.3	2	2321	4	incomplete-splice_match	gene: Zm00001d006762	Zm00001d006762_T00	13.41	50.70	coding	611	489	2321
PB.2317.1	10	2381	12	full-splice_match	gene: Zm00001d026396	Zm00001d026396_T00	43.60	51.75	coding	623	229	2097
PB.6833.1	5	986	4	incomplete-splice_match	gene: Zm00001d016824	Zm00001d016824_T00	17.23	17.23	coding	231	84	776
PB.2580.4	2	1073	7	full-splice_match	gene: Zm00001d002395	Zm00001d002395_T00	315.14	437.57	coding	244	93	824
PB.2580.5	2	963	6	novel_in_catalog	gene: Zm00001d002395	novel	5.16	437.57	coding	244	95	826
PB.9886.1	8	1949	3	full-splice_match	gene: Zm00001d011874	Zm00001d011874_T00	4.21	45.06	coding	416	260	1507
PB.8679.8	7	2148	7	novel_in_catalog	gene: Zm00001d021119	novel	3.86	157.85	coding	508	64	1587
PB.2580.2	2	1155	7	full-splice_match	gene: Zm00001d002395	Zm00001d002395_T00	117.26	437.57	coding	244	62	793
PB.3880.1	3	2832	21	novel_not_in_catalog	gene: Zm00001d040154	novel	19.61	19.61	coding	755	378	2642
PB.4833.5	4	1312	9	full-splice_match	gene: Zm00001d048583	Zm00001d048583_T00	52.08	103.55	coding	310	68	997

PB.8679.4	7	2041	8	novel_in_catalog	gene: Zm00001d021119	novel	5.28	157.85	coding	235	1058	1762
PB.8679.7	7	1995	8	novel_in_catalog	gene: Zm00001d021119	novel	4.93	157.85	coding	430	71	1360
PB.8679.6	7	2112	7	full-splice_match	gene: Zm00001d021119	Zm00001d021119_T00	14.88	157.85	coding	468	67	1470
PB.4833.1	4	1546	9	full-splice_match	gene: Zm00001d048583	Zm00001d048583_T00	51.48	103.55	coding	310	205	1134
PB.8679.3	7	1950	9	full-splice_match	gene: Zm00001d021119	Zm00001d021119_T00	88.22	157.85	coding	533	71	1669
PB.8679.2	7	2060	8	full-splice_match	gene: Zm00001d021119	Zm00001d021119_T00	14.25	157.85	coding	571	67	1779
PB.1040.1	1	695	1	full-splice_match	gene: Zm00001d032318	Zm00001d032318_T00	144.47	144.47	non coding	NA	NA	NA
PB.10200.4	9	2528	4	novel_not_in_catalog	gene: Zm00001d045185	novel	19.59	36.02	coding	322	1364	2329
PB.10200.3	9	2743	2	genic	gene: Zm00001d045185	novel	4.90	36.02	coding	322	1578	2543
PB.10200.2	9	2640	3	novel_not_in_catalog	gene: Zm00001d045185	novel	0.00	36.02	coding	322	1475	2440
PB.10200.1	9	2807	3	novel_not_in_catalog	gene: Zm00001d045185	novel	11.52	36.02	coding	322	1475	2440
PB.6717.2	5	1655	7	full-splice_match	gene: Zm00001d016228	Zm00001d016228_T00	7.85	23.41	coding	501	18	1520
PB.3836.2	3	570	2	full-splice_match	gene: Zm00001d039914	Zm00001d039914_T00	0.00	43.70	non coding	NA	NA	NA
PB.5636.1	4	508	2	full-splice_match	gene: Zm00001d052760	Zm00001d052760_T00	94.92	94.92	coding	115	71	415
PB.7535.1	6	1139	2	full-splice_match	gene: Zm00001d036409	Zm00001d036409_T00	8.30	8.30	coding	131	391	783
PB.710.3	1	1504	9	full-splice_match	gene: Zm00001d030620	Zm00001d030620_T00	12.11	72.87	coding	294	205	1086
PB.2729.1	2	1775	15	full-splice_match	gene: Zm00001d003083	Zm00001d003083_T00	25.54	29.58	coding	489	118	1584

PB.490.8	1	1955	5	full-splice_match	gene: Zm00001d029387	Zm00001d029387_T00	0.00	1055.64	coding	354	91	1152
PB.2729.2	2	1861	14	novel_in_catalog	gene: Zm00001d003083	novel	4.03	29.58	coding	297	212	1102
PB.10360.4	9	2615	6	full-splice_match	gene: Zm00001d045944	Zm00001d045944_T00	38.42	38.42	coding	654	375	2336
PB.2838.1	2	2544	9	full-splice_match	gene: Zm00001d003572	Zm00001d003572_T00	30.60	30.60	coding	652	280	2235
PB.4578.1	3	1137	3	full-splice_match	gene: Zm00001d043675	Zm00001d043675_T00	25.40	25.40	coding	233	265	963
PB.2838.3	2	1777	5	incomplete-splice_match	gene: Zm00001d003572	Zm00001d003572_T00	0.00	30.60	coding	416	430	1677
PB.490.1	1	2346	5	full-splice_match	gene: Zm00001d029387	Zm00001d029387_T00	0.00	1055.64	coding	354	91	1152
PB.1984.1	10	1264	1	novel_in_catalog	gene: Zm00001d024839	novel	15.99	1330.84	coding	128	664	1047
PB.490.3	1	2331	4	full-splice_match	gene: Zm00001d029387	Zm00001d029387_T00	0.00	1055.64	coding	244	501	1232
PB.490.4	1	2228	5	full-splice_match	gene: Zm00001d029387	Zm00001d029387_T00	57.96	1055.64	coding	354	91	1152
PB.490.7	1	2124	5	full-splice_match	gene: Zm00001d029387	Zm00001d029387_T00	0.00	1055.64	coding	354	91	1152
PB.490.6	1	2456	3	novel_in_catalog	gene: Zm00001d029387	novel	0.00	1055.64	coding	422	86	1351
PB.1588.1	1	1516	7	incomplete-splice_match	gene: Zm00001d034633	Zm00001d034633_T03	24.95	24.95	coding	422	75	1340
PB.5872.5	4	2086	14	full-splice_match	gene: Zm00001d053857	Zm00001d053857_T00	70.33	112.35	coding	560	142	1821
PB.7963.1	6	1582	12	incomplete-splice_match	gene: Zm00001d038302	Zm00001d038302_T07	15.65	15.65	coding	372	153	1268
PB.125.1	1	1428	5	novel_in_catalog	gene: Zm00001d027749	novel	38.90	76.92	coding	335	68	1072
PB.125.2	1	2308	10	full-splice_match	gene: Zm00001d027749	Zm00001d027749_T00	38.02	76.92	coding	653	175	2133

PB.1811.1	10	2157	10	full-splice_match	gene: Zm00001d023817	Zm00001d023817_T00	13.96	13.96	coding	559	210	1886
PB.4256.4	3	2873	17	novel_in_catalog	gene: Zm00001d042398	novel	71.41	71.41	coding	369	69	1175
PB.4495.1	3	1736	2	novel_in_catalog	gene: Zm00001d043400	novel	9.65	9.65	coding	364	62	1153
PB.1984.2	10	1089	3	full-splice_match	gene: Zm00001d024839	Zm00001d024839_T00	788.84	1330.84	coding	223	205	873
PB.4375.1	3	2393	8	novel_in_catalog	gene: Zm00001d042910	novel	4.16	34.15	coding	560	353	2032
PB.2735.2	2	1297	1	genic	gene: Zm00001d003110	novel	11.43	60.58	coding	117	407	757
PB.1831.1	10	2144	1	full-splice_match	gene: Zm00001d023919	Zm00001d023919_T00	38.12	38.12	coding	669	7	2013
PB.6823.2	5	1937	11	full-splice_match	gene: Zm00001d016770	Zm00001d016770_T00	17.19	44.81	coding	446	273	1610
PB.2735.1	2	2512	6	novel_not_in_catalog	gene: Zm00001d003110	novel	21.37	60.58	coding	153	1514	1972
PB.6823.4	5	2345	8	incomplete-splice_match	gene: Zm00001d016770	Zm00001d016770_T00	4.35	44.81	coding	363	15	1103
PB.6823.5	5	1389	9	incomplete-splice_match	gene: Zm00001d016770	Zm00001d016770_T00	0.00	44.81	coding	380	248	1387
PB.2735.4	2	2849	6	novel_not_in_catalog	gene: Zm00001d003110	novel	14.80	60.58	coding	383	1166	2314
PB.2735.5	2	2383	4	novel_not_in_catalog	gene: Zm00001d003110	novel	1.26	60.58	coding	153	1827	2285
PB.5110.2	4	612	4	novel_in_catalog	gene: Zm00001d049933	novel	168.43	235.30	coding	108	67	390
PB.8484.1	7	2210	4	incomplete-splice_match	gene: Zm00001d020178	Zm00001d020178_T00	44.05	44.05	coding	593	27	1805
PB.7299.1	6	2303	6	full-splice_match	gene: Zm00001d035008	Zm00001d035008_T00	16.39	16.39	coding	555	236	1900
PB.3031.2	2	804	1	full-splice_match	gene: Zm00001d004753	Zm00001d004753_T00	0.00	253.07	coding	188	35	598

PB.3031.1	2	914	1	full-splice_match	gene: Zm00001d004753	Zm00001d004753_T00	253.07	253.07	coding	188	35	598
PB.10515.2	9	1343	6	full-splice_match	gene: Zm00001d046937	Zm00001d046937_T00	13.13	38.48	coding	282	156	1001
PB.10515.3	9	1175	6	full-splice_match	gene: Zm00001d046937	Zm00001d046937_T00	10.46	38.48	coding	282	76	921
PB.6071.2	5	1039	4	incomplete-splice_match	gene: Zm00001d013218	Zm00001d013218_T00	4.51	10.51	coding	233	150	848
PB.6064.2	5	1674	1	full-splice_match	gene: Zm00001d013192	Zm00001d013192_T00	1.23	18.29	coding	451	26	1378
PB.6064.1	5	3575	1	full-splice_match	gene: Zm00001d013192	Zm00001d013192_T00	17.06	18.29	coding	940	357	3176
PB.9699.3	8	1869	2	novel_in_catalog	gene: Zm00001d011081	novel	3.29	342.12	coding	46	180	317
PB.2830.4	2	1939	5	novel_in_catalog	gene: Zm00001d003525	novel	1.40	6.56	coding	360	685	1764
PB.9536.3	8	1373	1	full-splice_match	gene: Zm00001d010380	Zm00001d010380_T00	4.36	20.12	coding	397	54	1244
PB.5110.1	4	744	4	novel_in_catalog	gene: Zm00001d049933	novel	66.87	235.30	coding	108	67	390
PB.2830.1	2	1599	2	incomplete-splice_match	gene: Zm00001d003525	Zm00001d003525_T00	1.07	6.56	non coding	NA	NA	NA
PB.2830.3	2	1165	5	novel_in_catalog	gene: Zm00001d003525	novel	4.09	6.56	coding	117	230	580
PB.5925.2	4	1471	12	full-splice_match	gene: Zm00001d054017	Zm00001d054017_T00	79.49	181.85	coding	346	225	1262
PB.3616.1	2	2781	7	full-splice_match	gene: Zm00001d007760	Zm00001d007760_T00	29.15	29.15	coding	778	177	2510
PB.3640.3	2	861	3	full-splice_match	gene: Zm00001d007832	Zm00001d007832_T00	93.94	241.96	coding	191	113	685
PB.4756.2	3	1338	1	genic	gene: Zm00001d044388	novel	5.00	14.31	coding	379	111	1247
PB.9218.1	8	2844	12	novel_not_in_catalog	gene: Zm00001d008614	novel	14.02	14.02	coding	579	957	2693

PB.697.2	1	1133	10	incomplete-splice_match	gene: Zm00001d030551	Zm00001d030551_T00	1.77	57.05	coding	314	191	1132
PB.8098.5	6	2621	6	full-splice_match	gene: Zm00001d038819	Zm00001d038819_T01	13.46	47.25	coding	253	882	1640
PB.3561.6	2	862	1	novel_in_catalog	gene: Zm00001d007311	novel	11.41	74.66	coding	105	143	457
PB.8098.6	6	2701	5	incomplete-splice_match	gene: Zm00001d038819	Zm00001d038819_T04	11.13	47.25	coding	242	991	1716
PB.3599.1	2	2342	3	full-splice_match	gene: Zm00001d007636	Zm00001d007636_T00	22.23	31.48	coding	617	137	1987
PB.5351.5	4	1778	9	novel_in_catalog	gene: Zm00001d051545	novel	0.00	32.84	coding	325	164	1138
PB.10509.1	9	1101	1	antisense	novelGene_gene: Zm00001d046914	novel	1.16	1.16	coding	87	241	501
PB.7712.1	6	2817	11	full-splice_match	gene: Zm00001d037289	Zm00001d037289_T00	45.16	46.87	coding	842	116	2641
PB.7712.2	6	2897	10	novel_in_catalog	gene: Zm00001d037289	novel	1.71	46.87	coding	541	1100	2722
PB.8098.7	6	1455	8	novel_in_catalog	gene: Zm00001d038819	novel	5.38	47.25	coding	243	542	1270
PB.5534.1	4	1110	3	incomplete-splice_match	gene: Zm00001d052307	Zm00001d052307_T00	39.90	39.90	coding	149	237	683
PB.5818.2	4	1002	5	full-splice_match	gene: Zm00001d053641	Zm00001d053641_T00	77.54	77.54	coding	109	455	781
PB.1300.2	1	1319	1	full-splice_match	gene: Zm00001d033385	Zm00001d033385_T00	33.24	34.58	coding	351	29	1081
PB.1872.1	10	1293	3	novel_not_in_catalog	gene: Zm00001d024213	novel	9.24	9.24	coding	262	140	925
PB.10476.1	9	900	1	full-splice_match	gene: Zm00001d046687	Zm00001d046687_T00	6.06	6.06	coding	209	51	677
PB.9504.1	8	2182	1	full-splice_match	gene: Zm00001d010234	Zm00001d010234_T00	30.15	30.15	coding	650	74	2023
PB.2806.2	2	1384	4	novel_in_catalog	gene: Zm00001d003432	novel	6.34	53.20	coding	413	28	1266

PB.2806.1	2	1425	5	full-splice_match	gene: Zm00001d003432	Zm00001d003432_T00	46.86	53.20	coding	361	217	1299
PB.3396.7	2	2485	4	novel_in_catalog	gene: Zm00001d006578	novel	4.74	210.21	coding	158	300	773
PB.3396.1	2	2761	2	novel_in_catalog	gene: Zm00001d006578	novel	0.00	210.21	coding	158	297	770
PB.10587.1	9	1573	9	full-splice_match	gene: Zm00001d047292	Zm00001d047292_T00	34.10	34.10	coding	371	65	1177
PB.3396.3	2	1948	7	full-splice_match	gene: Zm00001d006578	Zm00001d006578_T00	84.53	210.21	coding	367	370	1470
PB.5773.2	4	1231	6	novel_not_in_catalog	gene: Zm00001d027011	novel	5.70	20.70	non coding	NA	NA	NA
PB.7960.1	6	1509	2	genic	gene: Zm00001d038291	novel	1.58	1563.14	coding	154	155	616
PB.2420.4	2	1081	1	full-splice_match	gene: Zm00001d001774	Zm00001d001774_T00	49.24	57.65	coding	297	103	993
PB.2420.3	2	1354	2	genic	gene: Zm00001d001774	novel	1.53	57.65	coding	297	103	993
PB.2420.2	2	1612	1	full-splice_match	gene: Zm00001d001774	Zm00001d001774_T00	6.89	57.65	coding	297	93	983
PB.7900.1	6	2164	9	full-splice_match	gene: Zm00001d038100	Zm00001d038100_T00	17.29	17.29	coding	533	206	1804
PB.4759.2	3	1731	2	novel_in_catalog	gene: Zm00001d044395	novel	0.00	76.77	coding	234	374	1075
PB.2379.1	10	1706	5	incomplete-splice_match	gene: Zm00001d026590	Zm00001d026590_T02	24.38	24.38	coding	417	57	1307
PB.8857.2	7	1399	1	novel_in_catalog	gene: Zm00001d021774	novel	6.39	94.22	coding	186	114	671
PB.6215.2	5	1296	7	full-splice_match	gene: Zm00001d013755	Zm00001d013755_T00	26.98	100.28	coding	197	359	949
PB.3158.1	2	1181	2	full-splice_match	gene: Zm00001d005495	Zm00001d005495_T00	69.15	70.91	coding	223	134	802
PB.3158.2	2	1093	2	full-splice_match	gene: Zm00001d005495	Zm00001d005495_T00	0.00	70.91	coding	223	134	802

PB.3158.3	2	1008	2	full-splice_match	gene: Zm00001d005495	Zm00001d005495_T00	1.76	70.91	coding	223	172	840
PB.8327.1	7	1272	3	genic	gene: Zm00001d019142	novel	48.38	96.71	coding	253	48	806
PB.7312.1	6	3411	7	novel_in_catalog	gene: Zm00001d035050	novel	6.82	51.73	coding	550	387	2036
PB.7312.2	6	3137	8	full-splice_match	gene: Zm00001d035050	Zm00001d035050_T00	44.91	51.73	coding	753	513	2771
PB.8327.2	7	1159	3	genic	gene: Zm00001d019142	novel	48.34	96.71	coding	253	66	824
PB.5234.3	4	1357	7	full-splice_match	gene: Zm00001d050851	Zm00001d050851_T00	2.38	17.00	coding	141	563	985
PB.5234.2	4	1451	7	full-splice_match	gene: Zm00001d050851	Zm00001d050851_T00	14.62	17.00	coding	327	85	1065
PB.3469.2	2	2276	11	novel_in_catalog	gene: Zm00001d006885	novel	14.55	41.73	coding	598	128	1921
PB.3056.1	2	1592	2	full-splice_match	gene: Zm00001d004934	Zm00001d004934_T00	6.02	10.01	coding	422	7	1272
PB.9671.1	8	1715	4	full-splice_match	gene: Zm00001d010945	Zm00001d010945_T00	15.35	15.35	coding	385	411	1565
PB.6347.1	5	1947	5	novel_not_in_catalog	gene: Zm00001d014344	novel	7.81	78.54	coding	372	39	1154
PB.7804.2	6	582	2	incomplete-splice_match	gene: Zm00001d037717	Zm00001d037717_T00	0.00	26.27	coding	66	110	307
PB.6347.3	5	2661	2	full-splice_match	gene: Zm00001d014344	Zm00001d014344_T03	18.63	78.54	coding	636	84	1991
PB.6347.2	5	2200	5	incomplete-splice_match	gene: Zm00001d014344	Zm00001d014344_T02	11.00	78.54	coding	429	120	1406
PB.6347.5	5	864	1	novel_in_catalog	gene: Zm00001d014344	novel	3.98	78.54	non coding	NA	NA	NA
PB.6347.4	5	5244	8	full-splice_match	gene: Zm00001d014344	Zm00001d014344_T03	37.11	78.54	coding	1399	433	4629
PB.5810.1	4	2253	2	antisense	novelGene_gene: Zm00001d053623	novel	11.13	73.94	non coding	NA	NA	NA

PB.4759.7	3	1288	3	full-splice_match	gene: Zm00001d044395	Zm00001d044395_T00	18.00	76.77	coding	230	376	1065
PB.8132.1	6	1080	5	full-splice_match	gene: Zm00001d038910	Zm00001d038910_T00	32.04	32.04	coding	227	37	717
PB.9995.3	8	1186	1	full-splice_match	gene: Zm00001d012336	Zm00001d012336_T00	13.70	115.03	coding	327	62	1042
PB.235.11	1	1497	10	full-splice_match	gene: Zm00001d028260	Zm00001d028260_T00	92.30	1792.39	coding	389	83	1249
PB.247.10	1	1219	2	intergenic	novelGene_113	novel	0.00	14.11	non coding	NA	NA	NA
PB.11024.1	B73V4	1701	2	full-splice_match	gene: Zm00001d000294	Zm00001d000294_T00	4.10	4.10	coding	473	72	1490
PB.235.14	1	1530	10	novel_in_catalog	gene: Zm00001d028260	novel	18.15	1792.39	coding	401	84	1286
PB.235.15	1	1632	10	novel_in_catalog	gene: Zm00001d028260	novel	8.97	1792.39	coding	164	899	1390
PB.1824.3	10	1344	12	full-splice_match	gene: Zm00001d023874	Zm00001d023874_T00	68.96	123.88	coding	293	99	977
PB.235.18	1	1669	9	novel_in_catalog	gene: Zm00001d028260	novel	0.00	1792.39	coding	131	1033	1425
PB.235.19	1	1940	8	novel_in_catalog	gene: Zm00001d028260	novel	9.85	1792.39	coding	164	1205	1696
PB.9995.1	8	1431	1	full-splice_match	gene: Zm00001d012336	Zm00001d012336_T00	58.34	115.03	coding	327	58	1038
PB.1824.4	10	1268	12	full-splice_match	gene: Zm00001d023874	Zm00001d023874_T00	0.00	123.88	coding	293	94	972
PB.3475.2	2	787	1	full-splice_match	gene: Zm00001d006911	Zm00001d006911_T00	34.48	52.44	coding	213	61	699
PB.9732.1	8	2680	7	full-splice_match	gene: Zm00001d011220	Zm00001d011220_T00	65.83	65.83	coding	704	222	2333
PB.7364.1	6	3399	2	intergenic	novelGene_88	novel	4.77	17.85	coding	420	1930	3189
PB.3475.1	2	951	1	full-splice_match	gene: Zm00001d006911	Zm00001d006911_T00	17.96	52.44	coding	213	61	699

PB.6034.9	5	2826	22	full-splice_match	gene: Zm00001d013059	Zm00001d013059_T00	0.00	107.28	coding	803	127	2535
PB.7960.3	6	1164	2	genic	gene: Zm00001d038291	novel	142.61	1563.14	coding	154	150	611
PB.1341.2	1	2265	1	genic_intron	novelGene_458	novel	4.78	18.88	coding	694	183	2264
PB.3816.3	3	564	2	full-splice_match	gene: Zm00001d039826	Zm00001d039826_T00	0.00	89.21	coding	10	534	563
PB.3816.2	3	891	2	full-splice_match	gene: Zm00001d039826	Zm00001d039826_T00	74.87	89.21	coding	10	582	611
PB.3816.1	3	1139	1	novel_in_catalog	gene: Zm00001d039826	novel	14.33	89.21	non coding	NA	NA	NA
PB.6034.7	5	2906	21	novel_in_catalog	gene: Zm00001d013059	novel	6.29	107.28	coding	454	1206	2567
PB.6034.1	5	3006	21	novel_in_catalog	gene: Zm00001d013059	novel	3.97	107.28	coding	599	127	1923
PB.6034.3	5	2931	22	full-splice_match	gene: Zm00001d013059	Zm00001d013059_T00	62.55	107.28	coding	803	126	2534
PB.1068.2	1	2001	10	full-splice_match	gene: Zm00001d032427	Zm00001d032427_T00	13.98	26.36	coding	467	124	1524
PB.2474.1	2	642	3	full-splice_match	gene: Zm00001d001972	Zm00001d001972_T00	28.78	46.33	coding	67	232	432
PB.2474.2	2	563	3	full-splice_match	gene: Zm00001d001972	Zm00001d001972_T00	17.55	46.33	coding	67	263	463
PB.1068.1	1	920	3	incomplete-splice_match	gene: Zm00001d032427	Zm00001d032427_T00	12.38	26.36	coding	54	133	294
PB.7931.1	6	1131	2	full-splice_match	gene: Zm00001d038192	Zm00001d038192_T00	10.73	10.73	coding	267	37	837
PB.3370.4	2	1696	2	novel_in_catalog	gene: Zm00001d006440	novel	3.16	53.60	coding	167	605	1105
PB.252.4	1	1170	7	incomplete-splice_match	gene: Zm00001d028339	Zm00001d028339_T04	0.00	86.23	coding	366	72	1169
PB.252.1	1	454	1	genic	gene: Zm00001d028339	novel	4.11	86.23	non coding	NA	NA	NA

PB.3370.1	2	1487	5	full-splice_match	gene: Zm00001d006440	Zm00001d006440_T00	49.58	53.60	coding	275	197	1021
PB.252.3	1	1829	7	full-splice_match	gene: Zm00001d028339	Zm00001d028339_T04	0.86	86.23	coding	499	182	1678
PB.252.2	1	3927	8	full-splice_match	gene: Zm00001d028339	Zm00001d028339_T00	81.26	86.23	coding	1168	261	3764
PB.3515.2	2	2550	2	novel_in_catalog	gene: Zm00001d007070	novel	1.02	26.16	coding	414	1079	2320
PB.7960.2	6	1637	1	full-splice_match	gene: Zm00001d038291	Zm00001d038291_T00	0.00	1563.14	coding	154	532	993
PB.4829.7	3	1004	6	novel_not_in_catalog	gene: Zm00001d044694	novel	16.82	329.15	coding	240	113	832
PB.1334.3	1	1208	2	novel_in_catalog	gene: Zm00001d033539	novel	7.85	90.40	coding	254	148	909
PB.4590.4	3	1815	11	full-splice_match	gene: Zm00001d043726	Zm00001d043726_T00	8.60	145.08	coding	429	242	1528
PB.161.5	1	1215	2	novel_in_catalog	gene: Zm00001d027924	novel	4.79	50.97	coding	338	85	1098
PB.1914.1	10	935	2	genic	gene: Zm00001d023122	novel	11.05	15.86	non coding	NA	NA	NA
PB.860.1	1	1247	6	full-splice_match	gene: Zm00001d031439	Zm00001d031439_T00	0.86	4.91	coding	288	11	874
PB.860.2	1	1041	7	novel_not_in_catalog	gene: Zm00001d031439	novel	4.05	4.91	coding	254	12	773
PB.3183.1	2	1498	8	full-splice_match	gene: Zm00001d005662	Zm00001d005662_T00	13.94	26.81	coding	281	148	990
PB.3183.2	2	1311	8	full-splice_match	gene: Zm00001d005662	Zm00001d005662_T00	11.22	26.81	coding	281	122	964
PB.3183.3	2	1092	7	full-splice_match	gene: Zm00001d005662	Zm00001d005662_T00	1.66	26.81	coding	281	117	959
PB.10682.1	9	2074	3	full-splice_match	gene: Zm00001d047663	Zm00001d047663_T00	6.55	6.55	coding	582	128	1873
PB.4335.1	3	1293	3	full-splice_match	gene: Zm00001d042758	Zm00001d042758_T00	4.57	4.57	coding	292	199	1074

PB.5242.1	4	1117	2	full-splice_match	gene: Zm00001d050905	Zm00001d050905_T00	16.82	16.82	coding	194	158	739
PB.5242.2	4	939	2	full-splice_match	gene: Zm00001d050905	Zm00001d050905_T00	0.00	16.82	coding	194	158	739
PB.224.2	1	1723	2	full-splice_match	gene: Zm00001d028214	Zm00001d028214_T00	19.43	198.62	coding	371	265	1377
PB.224.3	1	1593	2	full-splice_match	gene: Zm00001d028214	Zm00001d028214_T00	136.69	198.62	coding	371	162	1274
PB.3346.1	2	2903	5	full-splice_match	gene: Zm00001d006355	Zm00001d006355_T01	27.87	38.31	coding	734	216	2417
PB.224.1	1	944	1	genic	gene: Zm00001d028214	novel	13.24	198.62	non coding	NA	NA	NA
PB.224.6	1	854	2	full-splice_match	gene: Zm00001d028214	Zm00001d028214_T00	0.00	198.62	coding	232	157	852
PB.2442.2	2	1435	1	full-splice_match	gene: Zm00001d001839	Zm00001d001839_T00	22.66	33.02	coding	268	107	910
PB.224.4	1	1946	3	novel_not_in_catalog	gene: Zm00001d028214	novel	23.13	198.62	coding	361	581	1663
PB.224.5	1	1604	2	novel_not_in_catalog	gene: Zm00001d028214	novel	6.13	198.62	coding	361	241	1323
PB.119.1	1	1408	2	full-splice_match	gene: Zm00001d027721	Zm00001d027721_T00	44.43	44.43	coding	356	152	1219
PB.6632.1	5	1637	6	novel_in_catalog	gene: Zm00001d015656	novel	23.24	30.76	coding	415	168	1412
PB.6632.2	5	943	2	novel_in_catalog	gene: Zm00001d015656	novel	7.52	30.76	coding	97	237	527
PB.10092.1	8	490	2	full-splice_match	gene: Zm00001d012741	Zm00001d012741_T00	711.93	711.93	non coding	NA	NA	NA
PB.3923.3	3	2446	6	novel_not_in_catalog	gene: Zm00001d040318	novel	32.56	91.95	coding	231	1303	1995
PB.3458.4	2	738	1	incomplete-splice_match	gene: Zm00001d006844	Zm00001d006844_T00	405.30	605.08	coding	145	78	512
PB.3923.6	3	2023	5	full-splice_match	gene: Zm00001d040318	Zm00001d040318_T00	0.00	91.95	coding	517	285	1835

PB.7330.1	6	1702	5	novel_not_in_catalog	gene: Zm00001d035149	novel	5.47	5.47	coding	504	147	1658
PB.3923.5	3	2234	5	full-splice_match	gene: Zm00001d040318	Zm00001d040318_T00	59.39	91.95	coding	517	278	1828
PB.6880.1	5	807	1	full-splice_match	gene: Zm00001d017041	Zm00001d017041_T00	30.61	30.61	coding	109	309	635
PB.2957.1	2	679	1	full-splice_match	gene: Zm00001d004231	Zm00001d004231_T00	134.78	134.78	coding	113	89	427
PB.10432.2	9	1488	2	full-splice_match	gene: Zm00001d046455	Zm00001d046455_T00	64.72	74.53	coding	425	68	1342
PB.10432.1	9	1633	2	full-splice_match	gene: Zm00001d046455	Zm00001d046455_T00	9.81	74.53	coding	425	42	1316
PB.7070.3	5	1824	2	full-splice_match	gene: Zm00001d017821	Zm00001d017821_T00	0.00	15.54	coding	513	44	1582
PB.5507.14	4	1943	6	novel_in_catalog	gene: Zm00001d052219	novel	5.75	64.47	coding	165	224	718
PB.4959.7	4	1670	1	full-splice_match	gene: Zm00001d049187	Zm00001d049187_T00	0.00	662.52	coding	507	112	1632
PB.10563.2	9	976	6	full-splice_match	gene: Zm00001d047187	Zm00001d047187_T00	56.32	56.32	coding	176	171	698
PB.5872.2	4	825	5	incomplete-splice_match	gene: Zm00001d053857	Zm00001d053857_T00	37.79	112.35	coding	144	1	432
PB.4730.1	3	1195	5	full-splice_match	gene: Zm00001d044254	Zm00001d044254_T00	15.77	57.89	coding	279	117	953
PB.3129.5	2	2523	4	novel_in_catalog	gene: Zm00001d005338	novel	14.09	72.24	coding	463	171	1559
PB.10036.1	8	1678	1	full-splice_match	gene: Zm00001d012528	Zm00001d012528_T00	3.76	3.76	coding	378	339	1472
PB.3740.1	3	836	1	full-splice_match	gene: Zm00001d039468	Zm00001d039468_T00	54.81	127.18	coding	120	96	455
PB.6736.19	5	2216	2	novel_in_catalog	gene: Zm00001d016332	novel	58.13	221.47	coding	305	1127	2041
PB.3740.3	3	719	1	full-splice_match	gene: Zm00001d039468	Zm00001d039468_T00	72.37	127.18	coding	120	97	456

PB.10281.1	9	1563	1	full-splice_match	gene: Zm00001d045496	Zm00001d045496_T00	12.69	12.69	coding	382	79	1224
PB.4490.2	3	568	2	full-splice_match	gene: Zm00001d043386	Zm00001d043386_T00	5.41	8.54	coding	93	99	377
PB.10416.1	9	2598	3	incomplete-splice_match	gene: Zm00001d046366	Zm00001d046366_T00	18.93	18.93	coding	738	108	2321
PB.11019.1	B73V 4	883	3	novel_not_in_catalog	gene: Zm00001d000135	novel	333.77	333.77	coding	147	178	618
PB.2198.3	10	962	1	full-splice_match	gene: Zm00001d025920	Zm00001d025920_T00	7.61	27.75	coding	283	70	918
PB.2198.2	10	2213	1	full-splice_match	gene: Zm00001d025920	Zm00001d025920_T00	0.00	27.75	coding	669	54	2060
PB.2198.1	10	2342	1	full-splice_match	gene: Zm00001d025920	Zm00001d025920_T00	20.14	27.75	coding	669	55	2061
PB.3640.1	2	1184	3	full-splice_match	gene: Zm00001d007832	Zm00001d007832_T00	148.03	241.96	coding	191	181	753
PB.7660.2	6	801	4	full-splice_match	gene: Zm00001d037067	Zm00001d037067_T00	48.59	48.59	coding	161	70	552
PB.5557.1	4	1808	6	novel_not_in_catalog	gene: Zm00001d052376	novel	56.66	56.66	coding	224	997	1668
PB.3640.2	2	1052	3	full-splice_match	gene: Zm00001d007832	Zm00001d007832_T00	0.00	241.96	coding	191	110	682
PB.593.1	1	1632	6	full-splice_match	gene: Zm00001d029886	Zm00001d029886_T00	28.04	28.04	coding	396	317	1504
PB.10456.1	9	2063	9	full-splice_match	gene: Zm00001d046561	Zm00001d046561_T00	8.49	36.53	coding	347	230	1270
PB.762.2	1	1735	12	full-splice_match	gene: Zm00001d030967	Zm00001d030967_T00	45.43	72.13	coding	469	160	1566
PB.10456.2	9	1922	10	full-splice_match	gene: Zm00001d046561	Zm00001d046561_T00	25.02	36.53	coding	465	230	1624
PB.8164.1	6	2353	10	novel_not_in_catalog	gene: Zm00001d039037	novel	85.30	85.30	coding	589	144	1910
PB.270.5	1	348	1	incomplete-splice_match	gene: Zm00001d028392	Zm00001d028392_T00	0.00	224.58	non coding	NA	NA	NA

PB.270.4	1	2418	2	novel_in_catalog	gene: Zm00001d028392	novel	12.99	224.58	non coding	NA	NA	NA
PB.7205.3	5	916	1	full-splice_match	gene: Zm00001d018326	Zm00001d018326_T00	120.67	286.71	non coding	NA	NA	NA
PB.7205.2	5	2083	1	full-splice_match	gene: Zm00001d018326	Zm00001d018326_T00	123.83	286.71	coding	356	231	1298
PB.270.1	1	1047	3	full-splice_match	gene: Zm00001d028392	Zm00001d028392_T00	79.54	224.58	coding	143	148	576
PB.6719.1	5	2091	5	full-splice_match	gene: Zm00001d016234	Zm00001d016234_T00	26.82	26.82	coding	533	281	1879
PB.270.3	1	946	3	full-splice_match	gene: Zm00001d028392	Zm00001d028392_T00	122.85	224.58	coding	143	151	579
PB.270.2	1	2577	1	novel_in_catalog	gene: Zm00001d028392	novel	9.20	224.58	coding	130	145	534
PB.359.2	1	916	5	fusion	gene: Zm00001d028775	novel	5.82	5.82	coding	252	35	790
PB.359.1	1	912	5	novel_not_in_catalog	gene: Zm00001d028775	novel	52.47	52.47	coding	247	17	757
PB.8624.2	7	1160	2	antisense	novelGene_gene: Zm00001d020883	novel	33.55	33.55	coding	217	202	852
PB.8156.1	6	1556	3	full-splice_match	gene: Zm00001d039000	Zm00001d039000_T00	16.67	16.67	coding	362	251	1336
PB.5887.3	4	2150	5	novel_in_catalog	gene: Zm00001d053917	novel	1.96	30.39	coding	200	942	1541
PB.3006.10	2	1289	12	full-splice_match	gene: Zm00001d004616	Zm00001d004616_T00	0.00	156.83	coding	331	118	1110
PB.3808.2	3	1239	6	full-splice_match	gene: Zm00001d039750	Zm00001d039750_T00	14.22	20.40	coding	286	263	1120
PB.2059.1	10	1187	6	full-splice_match	gene: Zm00001d025178	Zm00001d025178_T00	21.10	81.34	coding	212	106	741
PB.3808.1	3	1424	5	full-splice_match	gene: Zm00001d039750	Zm00001d039750_T00	6.18	20.40	coding	231	591	1283
PB.2973.2	2	647	3	full-splice_match	gene: Zm00001d004428	Zm00001d004428_T00	5.62	32.62	coding	2	295	300

PB.2973.3	2	619	3	full-splice_match	gene: Zm00001d004428	Zm00001d004428_T00	16.09	32.62	non coding	NA	NA	NA
PB.9605.1	8	1747	9	full-splice_match	gene: Zm00001d010656	Zm00001d010656_T00	9.71	9.71	coding	446	176	1513
PB.2973.1	2	898	3	full-splice_match	gene: Zm00001d004428	Zm00001d004428_T00	10.91	32.62	non coding	NA	NA	NA
PB.2934.1	2	1774	13	full-splice_match	gene: Zm00001d004091	Zm00001d004091_T00	27.98	40.25	coding	474	103	1524
PB.2934.2	2	904	9	incomplete-splice_match	gene: Zm00001d004091	Zm00001d004091_T00	12.27	40.25	coding	208	153	776
PB.4092.2	3	1056	2	full-splice_match	gene: Zm00001d041568	Zm00001d041568_T00	596.30	614.92	coding	261	76	858
PB.10488.1	9	1815	2	novel_not_in_catalog	gene: Zm00001d046758	novel	11.89	11.89	coding	539	28	1644
PB.1049.1	1	1425	2	incomplete-splice_match	gene: Zm00001d032356	Zm00001d032356_T00	3.29	3.29	coding	389	149	1315
PB.4092.1	3	1202	2	full-splice_match	gene: Zm00001d041568	Zm00001d041568_T00	18.62	614.92	coding	261	81	863
PB.10448.1	9	930	2	full-splice_match	gene: Zm00001d046510	Zm00001d046510_T00	31.24	54.03	coding	147	125	565
PB.5260.1	4	1327	3	full-splice_match	gene: Zm00001d050992	Zm00001d050992_T00	74.62	102.92	coding	326	32	1009
PB.10448.3	9	795	2	full-splice_match	gene: Zm00001d046510	Zm00001d046510_T00	16.57	54.03	coding	147	130	570
PB.10448.2	9	1048	1	genic	gene: Zm00001d046510	novel	6.23	54.03	coding	70	130	339
PB.6297.3	5	1689	4	incomplete-splice_match	gene: Zm00001d014150	Zm00001d014150_T00	6.44	24.40	coding	386	44	1201
PB.6297.2	5	3478	6	incomplete-splice_match	gene: Zm00001d014150	Zm00001d014150_T01	8.17	24.40	coding	427	1598	2878
PB.6297.1	5	2467	7	incomplete-splice_match	gene: Zm00001d014150	Zm00001d014150_T00	9.79	24.40	coding	524	295	1866
PB.2897.2	2	1341	2	full-splice_match	gene: Zm00001d003896	Zm00001d003896_T00	171.14	242.41	coding	166	593	1090

PB.5839.1	4	1122	1	full-splice_match	gene: Zm00001d053733	Zm00001d053733_T00	4.86	4.86	coding	341	69	1091
PB.10428.2	9	1698	7	full-splice_match	gene: Zm00001d046445	Zm00001d046445_T00	0.00	137.66	coding	340	337	1356
PB.10428.1	9	1814	7	full-splice_match	gene: Zm00001d046445	Zm00001d046445_T00	137.66	137.66	coding	340	340	1359
PB.10971.1	B73V 4	1367	1	full-splice_match	gene: Zm00001d000179	Zm00001d000179_T00	16.34	111.82	coding	281	95	937
PB.10971.2	B73V 4	1295	1	full-splice_match	gene: Zm00001d000179	Zm00001d000179_T00	95.48	111.82	coding	281	177	1019
PB.10971.3	B73V 4	1128	1	full-splice_match	gene: Zm00001d000179	Zm00001d000179_T00	0.00	111.82	coding	281	91	933
PB.6901.1	5	1040	11	full-splice_match	gene: Zm00001d017103	Zm00001d017103_T00	66.71	66.71	coding	224	120	791
PB.7973.2	6	2085	4	novel_not_in_catalog	gene: Zm00001d038334	novel	71.12	71.12	coding	565	271	1965
PB.8057.1	6	2349	8	full-splice_match	gene: Zm00001d038625	Zm00001d038625_T00	104.32	104.32	coding	635	107	2011
PB.3084.1	2	2977	3	incomplete-splice_match	gene: Zm00001d005100	Zm00001d005100_T03	28.21	84.39	coding	619	127	1983
PB.3872.1	3	1151	1	full-splice_match	gene: Zm00001d040111	Zm00001d040111_T00	28.73	107.46	coding	172	229	744
PB.3872.3	3	935	1	full-splice_match	gene: Zm00001d040111	Zm00001d040111_T00	78.73	107.46	coding	172	157	672
PB.3872.2	3	1142	1	full-splice_match	gene: Zm00001d040111	Zm00001d040111_T00	0.00	107.46	coding	172	250	765
PB.7836.10	6	925	9	incomplete-splice_match	gene: Zm00001d037872	Zm00001d037872_T00	0.00	53.51	coding	298	31	924
PB.5777.1	4	869	1	full-splice_match	gene: Zm00001d053433	Zm00001d053433_T00	7.71	7.71	coding	181	109	651
PB.2874.2	2	1826	2	genic	gene: Zm00001d003791	novel	16.95	16.95	coding	387	9	1169
PB.603.3	1	755	1	full-splice_match	gene: Zm00001d029969	Zm00001d029969_T00	0.00	70.85	coding	210	21	650

PB.1267.1	1	1628	6	full-splice_match	gene: Zm00001d033248	Zm00001d033248_T00	83.56	83.56	coding	431	53	1345
PB.1137.4	1	2340	20	full-splice_match	gene: Zm00001d032667	Zm00001d032667_T01	0.00	70.46	coding	698	147	2240
PB.3433.1	2	1990	3	genic	gene: Zm00001d006725	novel	246.03	251.48	coding	437	155	1465
PB.8490.1	7	1148	3	full-splice_match	gene: Zm00001d020224	Zm00001d020224_T00	9.02	25.72	coding	101	435	737
PB.8490.2	7	1518	2	novel_in_catalog	gene: Zm00001d020224	novel	6.66	25.72	coding	101	402	704
PB.8490.3	7	1275	2	novel_in_catalog	gene: Zm00001d020224	novel	0.00	25.72	coding	101	395	697
PB.8490.4	7	1159	3	full-splice_match	gene: Zm00001d020224	Zm00001d020224_T00	10.03	25.72	coding	101	434	736
PB.7783.8	6	1731	5	full-splice_match	gene: Zm00001d037625	Zm00001d037625_T00	0.00	56.78	coding	422	290	1555
PB.7783.9	6	1719	4	novel_in_catalog	gene: Zm00001d037625	novel	4.76	56.78	coding	287	686	1546
PB.7783.6	6	2012	5	full-splice_match	gene: Zm00001d037625	Zm00001d037625_T00	0.00	56.78	coding	310	905	1834
PB.7783.7	6	2155	4	full-splice_match	gene: Zm00001d037625	Zm00001d037625_T00	0.00	56.78	coding	335	975	1979
PB.7783.4	6	1857	6	full-splice_match	gene: Zm00001d037625	Zm00001d037625_T00	7.97	56.78	coding	310	679	1608
PB.7783.5	6	2242	4	full-splice_match	gene: Zm00001d037625	Zm00001d037625_T00	9.93	56.78	coding	335	974	1978
PB.7783.2	6	2176	5	full-splice_match	gene: Zm00001d037625	Zm00001d037625_T00	12.50	56.78	coding	310	963	1892
PB.7783.3	6	1828	5	full-splice_match	gene: Zm00001d037625	Zm00001d037625_T00	21.62	56.78	coding	422	292	1557
PB.5268.2	4	1717	1	full-splice_match	gene: Zm00001d051044	Zm00001d051044_T00	27.43	81.31	coding	429	1	1287
PB.5268.3	4	1994	2	genic	gene: Zm00001d051044	novel	48.63	81.31	coding	451	328	1680

PB.8331.1	7	2043	13	full-splice_match	gene: Zm00001d019164	Zm00001d019164_T00	17.39	139.53	coding	440	66	1385
PB.8331.2	7	1704	13	full-splice_match	gene: Zm00001d019164	Zm00001d019164_T00	122.14	139.53	coding	440	62	1381
PB.8331.3	7	1562	13	full-splice_match	gene: Zm00001d019164	Zm00001d019164_T00	0.00	139.53	coding	440	45	1364
PB.607.1	1	1288	1	full-splice_match	gene: Zm00001d030004	Zm00001d030004_T00	30.95	30.95	coding	298	161	1054
PB.4990.1	4	1133	1	intergenic	novelGene_104	novel	27.01	27.01	coding	161	161	643
PB.3518.1	2	913	1	intergenic	novelGene_424	novel	1.82	1.82	non coding	NA	NA	NA
PB.2160.4	10	1072	2	novel_in_catalog	gene: Zm00001d025761	novel	9.41	134.48	coding	161	410	892
PB.2160.5	10	2109	2	novel_in_catalog	gene: Zm00001d025761	novel	3.87	134.48	coding	102	100	405
PB.2160.2	10	2672	2	novel_in_catalog	gene: Zm00001d025761	novel	9.81	134.48	coding	161	1889	2371
PB.2160.3	10	2523	3	novel_in_catalog	gene: Zm00001d025761	novel	23.46	134.48	coding	161	1738	2220
PB.7400.1	6	990	4	full-splice_match	gene: Zm00001d035519	Zm00001d035519_T00	415.21	415.21	coding	196	132	719
PB.8424.5	7	981	2	full-splice_match	gene: Zm00001d019766	Zm00001d019766_T00	4.29	4.29	coding	200	121	720
PB.8424.4	7	2379	7	fusion	gene: Zm00001d019766	novel	0.00	23.35	coding	678	39	2072
PB.3660.5	2	2141	8	intergenic	novelGene_443	novel	6.51	53.91	coding	279	921	1757
PB.9928.3	8	747	5	full-splice_match	gene: Zm00001d012052	Zm00001d012052_T00	104.92	523.95	coding	156	174	641
PB.9928.2	8	832	5	full-splice_match	gene: Zm00001d012052	Zm00001d012052_T00	375.76	523.95	coding	156	120	587
PB.10154.1	9	721	2	novel_in_catalog	gene: Zm00001d044972	novel	6.69	6.69	coding	145	104	538

PB.8424.2	7	2739	7	fusion	gene: Zm00001d019766	novel	12.27	23.35	coding	701	121	2223
PB.5508.1	4	1009	1	novel_in_catalog	gene: Zm00001d052224	novel	15.50	148.46	coding	142	278	703
PB.5508.3	4	797	2	full-splice_match	gene: Zm00001d052224	Zm00001d052224_T00	0.00	148.46	coding	187	44	604
PB.5508.2	4	951	2	full-splice_match	gene: Zm00001d052224	Zm00001d052224_T00	132.96	148.46	coding	187	72	632
PB.8277.2	7	2860	1	full-splice_match	gene: Zm00001d018871	Zm00001d018871_T00	8.34	13.09	coding	839	173	2689
PB.8277.1	7	1265	1	full-splice_match	gene: Zm00001d018871	Zm00001d018871_T00	4.75	13.09	coding	303	58	966
PB.1262.2	1	926	1	incomplete-splice_match	gene: Zm00001d033225	Zm00001d033225_T00	0.00	44.86	coding	253	9	767
PB.4344.2	3	1727	1	full-splice_match	gene: Zm00001d042786	Zm00001d042786_T00	33.64	57.68	coding	391	216	1388
PB.6398.1	5	2183	6	full-splice_match	gene: Zm00001d014609	Zm00001d014609_T00	36.64	54.25	coding	585	176	1930
PB.3321.2	2	1641	8	full-splice_match	gene: Zm00001d006242	Zm00001d006242_T00	108.90	173.20	coding	440	63	1382
PB.6398.3	5	887	5	incomplete-splice_match	gene: Zm00001d014609	Zm00001d014609_T00	16.11	54.25	coding	251	37	789
PB.6398.2	5	2611	5	novel_in_catalog	gene: Zm00001d014609	novel	1.50	54.25	coding	577	176	1906
PB.1361.14	1	1880	10	novel_in_catalog	gene: Zm00001d033650	novel	0.84	13.18	coding	414	484	1725
PB.3660.1	2	4360	17	intergenic	novelGene_443	novel	14.42	53.91	coding	279	914	1750
PB.1361.12	1	1337	10	incomplete-splice_match	gene: Zm00001d033649	Zm00001d033649_T00	0.00	68.36	coding	400	138	1337
PB.10997.1	B73V4	1505	4	full-splice_match	gene: Zm00001d000074	Zm00001d000074_T00	55.65	73.27	coding	445	36	1370
PB.10997.2	B73V4	1415	5	novel_not_in_catalog	gene: Zm00001d000074	novel	17.62	73.27	coding	397	73	1263

PB.7105.9	5	1478	9	novel_in_catalog	gene: Zm00001d017958	novel	188.87	2019.88	coding	379	77	1213
PB.648.1	1	1757	1	antisense	novelGene_gene: Zm00001d030171	novel	10.65	10.65	coding	148	67	510
PB.379.1	1	667	7	full-splice_match	gene: Zm00001d028867	Zm00001d028867_T00	56.87	59.45	non coding	NA	NA	NA
PB.4986.1	4	3366	2	full-splice_match	gene: Zm00001d049293	Zm00001d049293_T00	3.09	3.09	coding	1043	28	3156
PB.379.2	1	887	5	novel_in_catalog	gene: Zm00001d028867	novel	2.59	59.45	non coding	NA	NA	NA
PB.5930.2	4	1954	5	full-splice_match	gene: Zm00001d054047	Zm00001d054047_T00	25.97	28.11	coding	582	19	1764
PB.3986.1	3	922	1	genic	gene: Zm00001d040660	novel	14.70	105.25	non coding	NA	NA	NA
PB.1262.3	1	1225	4	full-splice_match	gene: Zm00001d033225	Zm00001d033225_T00	0.00	44.86	coding	383	38	1186
PB.7250.1	5	1252	3	full-splice_match	gene: Zm00001d018461	Zm00001d018461_T00	216.89	219.73	coding	264	244	1035
PB.3986.2	3	617	2	full-splice_match	gene: Zm00001d040660	Zm00001d040660_T00	90.55	105.25	coding	102	142	447
PB.2012.16	10	3967	17	novel_not_in_catalog	gene: Zm00001d024952	novel	0.32	175.10	coding	882	89	2734
PB.2012.17	10	2495	13	incomplete-splice_match	gene: Zm00001d024952	Zm00001d024952_T00	0.00	175.10	coding	708	141	2264
PB.9444.3	8	3555	7	novel_in_catalog	gene: Zm00001d009883	novel	5.74	30.35	coding	104	2598	2909
PB.1638.4	1	1130	5	full-splice_match	gene: Zm00001d034795	Zm00001d034795_T00	57.20	121.02	coding	289	106	972
PB.2012.13	10	3236	19	novel_not_in_catalog	gene: Zm00001d024952	novel	0.00	175.10	coding	372	1777	2892
PB.2012.10	10	4159	18	novel_not_in_catalog	gene: Zm00001d024952	novel	0.00	175.10	coding	882	86	2731
PB.2012.11	10	2291	14	novel_in_catalog	gene: Zm00001d024952	novel	26.03	175.10	coding	671	5	2017

PB.4438.2	3	1761	1	full-splice_match	gene: Zm00001d043183	Zm00001d043183_T00	5.49	41.28	coding	287	592	1452
PB.5888.2	4	884	4	incomplete-splice_match	gene: Zm00001d053919	Zm00001d053919_T00	6.98	74.41	coding	116	297	644
PB.9920.3	8	851	6	full-splice_match	gene: Zm00001d012017	Zm00001d012017_T00	0.00	82.35	coding	202	7	612
PB.9920.2	8	1483	5	novel_in_catalog	gene: Zm00001d012017	novel	1.47	82.35	coding	165	106	600
PB.9920.1	8	1063	6	full-splice_match	gene: Zm00001d012017	Zm00001d012017_T00	80.88	82.35	coding	202	111	716
PB.4438.3	3	1220	2	genic	gene: Zm00001d043183	novel	0.00	41.28	coding	325	125	1099
PB.3714.1	3	2059	5	full-splice_match	gene: Zm00001d039369	Zm00001d039369_T00	23.92	30.43	coding	490	133	1602
PB.4041.2	3	2794	6	full-splice_match	gene: Zm00001d041204	Zm00001d041204_T00	14.92	16.35	coding	850	81	2630
PB.4041.1	3	2907	5	novel_in_catalog	gene: Zm00001d041204	novel	1.42	16.35	coding	535	1128	2732
PB.10727.3	9	1276	1	full-splice_match	gene: Zm00001d047800	Zm00001d047800_T00	22.67	69.20	coding	189	12	578
PB.9900.3	8	2289	15	incomplete-splice_match	gene: Zm00001d011956	Zm00001d011956_T00	0.00	15.89	coding	687	22	2082
PB.9900.2	8	1383	8	full-splice_match	gene: Zm00001d011956	Zm00001d011956_T00	10.16	15.89	coding	122	730	1095
PB.10727.6	9	2446	2	genic	gene: Zm00001d047800	novel	0.00	69.20	coding	534	493	2094
PB.10727.5	9	2420	2	genic	gene: Zm00001d047800	novel	9.86	69.20	coding	534	432	2033
PB.10727.4	9	2748	3	genic	gene: Zm00001d047800	novel	36.67	69.20	coding	534	538	2139
PB.2553.1	2	2158	2	full-splice_match	gene: Zm00001d002316	Zm00001d002316_T00	28.07	28.07	coding	613	38	1876
PB.2881.2	2	1663	14	novel_not_in_catalog	gene: Zm00001d003813	novel	19.60	23.47	coding	430	144	1433

PB.2881.3	2	1695	13	novel_not_in_catalog	gene: Zm00001d003813	novel	2.60	23.47	coding	198	877	1470
PB.847.1	1	1777	4	full-splice_match	gene: Zm00001d031328	Zm00001d031328_T00	47.35	47.35	coding	504	141	1652
PB.371.1	1	2236	2	genic	gene: Zm00001d028809	novel	30.37	46.72	coding	564	365	2056
PB.371.2	1	2510	1	full-splice_match	gene: Zm00001d028809	Zm00001d028809_T00	16.36	46.72	coding	564	661	2352
PB.7273.2	5	2560	10	full-splice_match	gene: Zm00001d018523	Zm00001d018523_T01	5.86	177.48	coding	231	80	772
PB.7273.3	5	1654	11	full-splice_match	gene: Zm00001d018523	Zm00001d018523_T00	24.28	177.48	coding	443	161	1489
PB.7273.1	5	1657	11	full-splice_match	gene: Zm00001d018523	Zm00001d018523_T00	40.69	177.48	coding	443	111	1439
PB.7273.6	5	1736	10	novel_in_catalog	gene: Zm00001d018523	novel	2.54	177.48	coding	296	686	1573
PB.7273.7	5	1510	11	full-splice_match	gene: Zm00001d018523	Zm00001d018523_T00	93.89	177.48	coding	443	111	1439
PB.7273.4	5	2665	9	novel_in_catalog	gene: Zm00001d018523	novel	3.00	177.48	coding	231	113	805
PB.7273.5	5	1706	10	full-splice_match	gene: Zm00001d018523	Zm00001d018523_T01	7.22	177.48	coding	322	113	1078
PB.1262.1	1	1572	4	full-splice_match	gene: Zm00001d033225	Zm00001d033225_T00	44.86	44.86	coding	383	198	1346
PB.5367.2	4	1430	1	novel_in_catalog	gene: Zm00001d051611	novel	1.13	37.75	coding	224	489	1160
PB.1508.1	1	2100	15	full-splice_match	gene: Zm00001d034343	Zm00001d034343_T00	25.40	44.63	coding	435	478	1782
PB.1919.7	10	1323	1	genic_intron	novelGene_169	novel	8.82	8.82	non coding	NA	NA	NA
PB.1919.4	10	1937	2	novel_in_catalog	gene: Zm00001d024499	novel	5.87	139.41	coding	246	1056	1793
PB.1919.3	10	2891	2	novel_in_catalog	gene: Zm00001d024499	novel	7.21	139.41	coding	438	1459	2772

PB.1919.2	10	2012	3	full-splice_match	gene: Zm00001d024499	Zm00001d024499_T00	126.33	139.41	coding	438	535	1848
PB.4479.2	3	2486	5	novel_not_in_catalog	gene: Zm00001d043354	novel	9.26	31.37	coding	656	206	2173
PB.6130.5	5	2962	9	full-splice_match	gene: Zm00001d013391	Zm00001d013391_T02	13.63	32.35	coding	879	122	2758
PB.6130.4	5	1381	5	novel_in_catalog	gene: Zm00001d013391	novel	2.43	32.35	coding	301	277	1179
PB.672.1	1	3723	12	incomplete-splice_match	gene: Zm00001d030340	Zm00001d030340_T01	12.24	12.24	coding	1067	19	3219
PB.3205.2	2	1320	4	full-splice_match	gene: Zm00001d005762	Zm00001d005762_T00	41.97	46.46	coding	367	84	1184
PB.6130.1	5	2494	9	novel_not_in_catalog	gene: Zm00001d013391	novel	13.27	32.35	coding	636	385	2292
PB.5141.2	4	1593	2	genic	gene: Zm00001d050132	novel	2.10	11.78	non coding	NA	NA	NA
PB.6130.2	5	4045	7	full-splice_match	gene: Zm00001d013391	Zm00001d013391_T00	3.03	32.35	coding	775	146	2470
PB.365.2	1	1035	1	full-splice_match	gene: Zm00001d028793	Zm00001d028793_T00	149.20	558.90	coding	220	54	713
PB.365.3	1	948	1	full-splice_match	gene: Zm00001d028793	Zm00001d028793_T00	190.51	558.90	coding	220	68	727
PB.2543.4	2	771	1	genic	gene: Zm00001d002278	novel	8.70	102.20	coding	144	66	497
PB.2543.3	2	1049	2	genic	gene: Zm00001d002278	novel	11.36	102.20	coding	144	343	774
PB.2543.2	2	1047	2	genic	gene: Zm00001d002278	novel	57.25	102.20	coding	144	341	772
PB.2543.1	2	828	1	genic	gene: Zm00001d002278	novel	24.88	102.20	coding	144	46	477
PB.10988.1	B73V4	888	6	full-splice_match	gene: Zm00001d000379	Zm00001d000379_T00	77.43	77.43	coding	184	97	648
PB.8073.5	6	1302	8	full-splice_match	gene: Zm00001d038730	Zm00001d038730_T00	0.00	38.73	coding	211	208	840

PB.8073.4	6	1543	7	full-splice_match	gene: Zm00001d038730	Zm00001d038730_T00	2.76	38.73	coding	197	210	800
PB.8073.6	6	870	4	full-splice_match	gene: Zm00001d038730	Zm00001d038730_T00	0.00	38.73	coding	148	210	653
PB.8073.1	6	2392	5	novel_in_catalog	gene: Zm00001d038730	novel	2.91	38.73	coding	148	210	653
PB.8073.3	6	1704	6	novel_in_catalog	gene: Zm00001d038730	novel	3.42	38.73	coding	148	168	611
PB.8073.2	6	1452	8	full-splice_match	gene: Zm00001d038730	Zm00001d038730_T00	29.63	38.73	coding	211	212	844
PB.7583.14	6	693	3	novel_in_catalog	gene: Zm00001d036631	novel	0.00	46.80	non coding	NA	NA	NA
PB.7583.12	6	1504	11	novel_in_catalog	gene: Zm00001d036631	novel	2.73	46.80	coding	200	624	1223
PB.7583.13	6	2081	1	incomplete-splice_match	gene: Zm00001d036631	Zm00001d036631_T01	0.00	46.80	non coding	NA	NA	NA
PB.7583.10	6	1414	11	full-splice_match	gene: Zm00001d036631	Zm00001d036631_T01	1.29	46.80	coding	215	456	1100
PB.521.5	1	2019	10	incomplete-splice_match	gene: Zm00001d029530	Zm00001d029530_T00	2.29	30.10	coding	620	14	1873
PB.6406.2	5	948	4	full-splice_match	gene: Zm00001d014643	Zm00001d014643_T00	3.31	10.59	non coding	NA	NA	NA
PB.6406.1	5	1229	4	novel_not_in_catalog	gene: Zm00001d014643	novel	7.29	10.59	non coding	NA	NA	NA
PB.10753.2	9	2581	4	full-splice_match	gene: Zm00001d047881	Zm00001d047881_T00	48.38	64.07	coding	658	88	2061
PB.10600.1	9	1114	6	novel_not_in_catalog	gene: Zm00001d047366	novel	9.34	15.66	coding	133	458	856
PB.4583.5	3	1688	9	full-splice_match	gene: Zm00001d043694	Zm00001d043694_T00	18.76	47.02	coding	343	382	1410
PB.9635.4	8	1859	2	novel_in_catalog	gene: Zm00001d010783	novel	0.00	58.04	coding	232	1	696
PB.4267.2	3	848	1	genic	gene: Zm00001d042453	novel	2014.69	2014.69	coding	128	145	528

PB.2118.1	10	2108	2	full-splice_match	gene: Zm00001d025547	Zm00001d025547_T00	26.23	26.23	coding	580	28	1767
PB.6785.2	5	2523	9	full-splice_match	gene: Zm00001d016602	Zm00001d016602_T00	0.00	46.52	coding	743	74	2302
PB.6785.1	5	2636	9	full-splice_match	gene: Zm00001d016602	Zm00001d016602_T00	46.52	46.52	coding	743	72	2300
PB.3321.3	2	1728	7	novel_in_catalog	gene: Zm00001d006242	novel	4.43	173.20	coding	363	63	1151
PB.521.1	1	2932	13	full-splice_match	gene: Zm00001d029530	Zm00001d029530_T00	11.09	30.10	coding	853	109	2667
PB.7084.5	5	2429	1	novel_in_catalog	gene: Zm00001d017874	novel	12.41	126.53	non coding	NA	NA	NA
PB.7737.1	6	1345	2	full-splice_match	gene: Zm00001d037410	Zm00001d037410_T00	36.14	36.14	coding	338	151	1164
PB.7096.1	5	1001	4	full-splice_match	gene: Zm00001d017914	Zm00001d017914_T00	42.68	133.23	coding	150	65	514
PB.3580.1	2	1638	5	full-splice_match	gene: Zm00001d007489	Zm00001d007489_T00	13.40	13.40	coding	410	52	1281
PB.521.3	1	1780	8	incomplete-splice_match	gene: Zm00001d029530	Zm00001d029530_T00	7.75	30.10	coding	507	90	1610
PB.6559.2	5	1355	6	full-splice_match	gene: Zm00001d015195	Zm00001d015195_T00	31.58	59.52	coding	334	73	1074
PB.4583.4	3	1515	7	novel_in_catalog	gene: Zm00001d043694	novel	5.81	47.02	coding	227	221	901
PB.6559.1	5	1319	6	full-splice_match	gene: Zm00001d015195	Zm00001d015195_T00	27.94	59.52	coding	334	14	1015
PB.4681.4	3	1582	12	full-splice_match	gene: Zm00001d044088	Zm00001d044088_T00	3.15	42.81	coding	404	90	1301
PB.4681.5	3	798	6	incomplete-splice_match	gene: Zm00001d044088	Zm00001d044088_T00	3.87	42.81	coding	223	14	682
PB.963.2	1	1118	6	full-splice_match	gene: Zm00001d031963	Zm00001d031963_T00	69.13	69.13	coding	274	35	856
PB.5367.1	4	1231	2	full-splice_match	gene: Zm00001d051611	Zm00001d051611_T00	29.95	37.75	coding	271	104	916

PB.4681.2	3	3341	10	novel_in_catalog	gene: Zm00001d044088	novel	7.82	42.81	coding	352	237	1292
PB.4681.3	3	1633	12	novel_in_catalog	gene: Zm00001d044088	novel	0.76	42.81	coding	404	51	1262
PB.10100.4	8	2420	9	antisense	novelGene_gene: Zm00001d012761	novel	0.61	19.16	coding	139	1698	2114
PB.8416.2	7	1382	9	full-splice_match	gene: Zm00001d019721	Zm00001d019721_T00	1.17	20.05	coding	412	31	1266
PB.6764.2	5	2463	15	full-splice_match	gene: Zm00001d016496	Zm00001d016496_T00	17.79	37.45	coding	603	274	2082
PB.6158.1	5	1283	3	incomplete-splice_match	gene: Zm00001d013493	Zm00001d013493_T00	14.14	14.14	coding	284	228	1079
PB.8416.1	7	1541	9	full-splice_match	gene: Zm00001d019721	Zm00001d019721_T00	18.88	20.05	coding	412	26	1261
PB.735.1	1	1172	4	full-splice_match	gene: Zm00001d030796	Zm00001d030796_T00	18.23	29.24	coding	289	50	916
PB.7836.2	6	2353	16	full-splice_match	gene: Zm00001d037872	Zm00001d037872_T00	14.75	53.51	coding	589	188	1954
PB.3613.2	2	916	5	incomplete-splice_match	gene: Zm00001d007754	Zm00001d007754_T00	9.47	35.14	coding	170	15	524
PB.735.2	1	433	1	incomplete-splice_match	gene: Zm00001d030796	Zm00001d030796_T00	11.01	29.24	coding	96	21	308
PB.357.1	1	1931	6	novel_in_catalog	gene: Zm00001d028770	novel	43.18	67.71	coding	510	155	1684
PB.10100.6	8	2551	8	antisense	novelGene_gene: Zm00001d012761	novel	5.88	19.16	coding	139	1833	2249
PB.357.3	1	1986	5	novel_in_catalog	gene: Zm00001d028770	novel	1.25	67.71	coding	211	1105	1737
PB.357.2	1	1967	6	novel_in_catalog	gene: Zm00001d028770	novel	23.28	67.71	coding	513	181	1719
PB.9407.1	8	1233	1	full-splice_match	gene: Zm00001d009687	Zm00001d009687_T00	3.38	3.38	coding	291	78	950
PB.1688.1	10	1929	9	incomplete-splice_match	gene: Zm00001d023230	Zm00001d023230_T01	43.19	43.19	coding	452	29	1384

PB.7050.2	5	1402	4	full-splice_match	gene: Zm00001d017727	Zm00001d017727_T00	7.89	37.81	coding	347	90	1130
PB.8847.4	7	1200	5	full-splice_match	gene: Zm00001d021739	Zm00001d021739_T00	178.78	245.14	coding	275	40	864
PB.884.10	1	1528	2	genic	gene: Zm00001d031572	novel	9.43	20.52	coding	350	281	1330
PB.7450.1	6	3065	6	full-splice_match	gene: Zm00001d035789	Zm00001d035789_T00	23.61	23.61	coding	892	50	2725
PB.6565.6	5	2341	22	fusion	gene: Zm00001d015228	novel	0.00	30.63	coding	653	152	2110
PB.6565.5	5	2468	22	fusion	gene: Zm00001d015228	novel	30.63	30.63	coding	653	159	2117
PB.6565.4	5	694	6	full-splice_match	gene: Zm00001d015228	Zm00001d015228_T00	0.00	39.82	coding	185	140	694
PB.6565.3	5	2069	5	novel_in_catalog	gene: Zm00001d015227	novel	3.55	3.55	coding	82	135	380
PB.6565.2	5	1003	6	full-splice_match	gene: Zm00001d015228	Zm00001d015228_T00	39.82	39.82	coding	197	227	817
PB.5492.4	4	696	4	full-splice_match	gene: Zm00001d052171	Zm00001d052171_T00	35.63	55.20	coding	189	41	607
PB.5167.4	4	1554	4	novel_not_in_catalog	gene: Zm00001d050250	novel	1.91	23.12	non coding	NA	NA	NA
PB.5167.5	4	1937	4	novel_not_in_catalog	gene: Zm00001d050250	novel	4.24	23.12	non coding	NA	NA	NA
PB.5167.6	4	1476	4	novel_not_in_catalog	gene: Zm00001d050250	novel	6.44	23.12	non coding	NA	NA	NA
PB.5167.1	4	791	6	novel_not_in_catalog	gene: Zm00001d050250	novel	7.95	23.12	non coding	NA	NA	NA
PB.5167.2	4	1516	5	novel_not_in_catalog	gene: Zm00001d050250	novel	2.58	23.12	non coding	NA	NA	NA
PB.5167.3	4	1094	5	novel_not_in_catalog	gene: Zm00001d050250	novel	0.00	23.12	non coding	NA	NA	NA
PB.1595.1	1	2114	4	novel_in_catalog	gene: Zm00001d034655	novel	27.39	77.19	coding	462	350	1735

PB.10516.1	9	657	1	genic	gene: Zm00001d046938	novel	31.13	31.13	coding	149	16	462
PB.2331.1	10	2180	3	full-splice_match	gene: Zm00001d026460	Zm00001d026460_T00	215.47	236.03	coding	452	373	1728
PB.1143.1	1	1253	6	novel_in_catalog	gene: Zm00001d032688	novel	4.53	4.53	coding	196	171	758
PB.7644.1	6	1916	16	full-splice_match	gene: Zm00001d036982	Zm00001d036982_T00	26.58	36.79	coding	494	87	1568
PB.7644.2	6	2096	15	full-splice_match	gene: Zm00001d036982	Zm00001d036982_T00	10.22	36.79	coding	360	129	1208
PB.721.1	1	1179	8	novel_not_in_catalog	gene: Zm00001d030742	novel	11.61	11.61	coding	243	77	805
PB.9667.2	8	1389	5	intergenic	novelGene_257	novel	0.00	26.72	non coding	NA	NA	NA
PB.609.10	1	1454	4	novel_in_catalog	gene: Zm00001d030009	novel	0.00	159.80	coding	281	161	1003
PB.609.13	1	1009	5	full-splice_match	gene: Zm00001d030009	Zm00001d030009_T00	67.79	159.80	coding	245	113	847
PB.609.12	1	1367	3	novel_in_catalog	gene: Zm00001d030009	novel	9.35	159.80	coding	132	810	1205
PB.609.15	1	999	5	full-splice_match	gene: Zm00001d030009	Zm00001d030009_T00	0.00	159.80	coding	243	109	837
PB.609.14	1	1270	4	novel_in_catalog	gene: Zm00001d030009	novel	0.00	159.80	coding	95	109	393
PB.10670.1	9	2940	1	full-splice_match	gene: Zm00001d047617	Zm00001d047617_T00	7.37	16.94	coding	828	72	2555
PB.4488.1	3	639	5	novel_in_catalog	gene: Zm00001d043382	novel	261.69	261.69	coding	96	350	637
PB.1457.1	1	1342	6	full-splice_match	gene: Zm00001d034063	Zm00001d034063_T00	111.56	111.56	coding	303	228	1136
PB.1633.1	1	2932	3	full-splice_match	gene: Zm00001d034773	Zm00001d034773_T00	278.12	278.12	coding	843	150	2678
PB.10915.1	9	3766	26	incomplete-splice_match	gene: Zm00001d048511	Zm00001d048511_T00	61.24	61.24	coding	1045	188	3322

PB.8631.1	7	3081	8	full-splice_match	gene: Zm00001d020929	Zm00001d020929_T00	2.12	2.12	coding	792	404	2779
PB.4663.2	3	1106	6	full-splice_match	gene: Zm00001d043991	Zm00001d043991_T00	86.61	378.43	coding	230	189	878
PB.4663.3	3	868	5	novel_in_catalog	gene: Zm00001d043991	novel	0.61	378.43	coding	135	149	553
PB.1933.3	10	1125	1	full-splice_match	gene: Zm00001d024545	Zm00001d024545_T00	0.00	44.94	coding	249	241	987
PB.1933.2	10	1253	1	full-splice_match	gene: Zm00001d024545	Zm00001d024545_T00	36.51	44.94	coding	249	257	1003
PB.1933.1	10	1123	1	full-splice_match	gene: Zm00001d024545	Zm00001d024545_T00	8.42	44.94	coding	249	126	872
PB.8205.1	6	2299	2	full-splice_match	gene: Zm00001d039188	Zm00001d039188_T00	8.94	12.03	coding	654	134	2095
PB.2781.3	2	890	2	incomplete-splice_match	gene: Zm00001d003288	Zm00001d003288_T00	0.00	91.55	coding	296	1	888
PB.2781.2	2	2358	2	novel_in_catalog	gene: Zm00001d003288	novel	2.55	91.55	coding	523	106	1674
PB.2781.1	2	2000	3	full-splice_match	gene: Zm00001d003288	Zm00001d003288_T00	89.00	91.55	coding	528	74	1657
PB.8382.5	7	346	1	incomplete-splice_match	gene: Zm00001d019542	Zm00001d019542_T01	0.00	107.73	coding	83	96	344
PB.5936.1	4	1885	1	full-splice_match	gene: Zm00001d054067	Zm00001d054067_T00	75.29	77.11	coding	502	95	1600
PB.8382.3	7	3450	8	novel_in_catalog	gene: Zm00001d019542	novel	17.76	107.73	coding	797	744	3134
PB.9050.1	7	1588	1	full-splice_match	gene: Zm00001d022451	Zm00001d022451_T00	355.65	355.65	coding	437	103	1413
PB.8382.1	7	3243	10	full-splice_match	gene: Zm00001d019542	Zm00001d019542_T00	89.96	107.73	coding	884	280	2931
PB.5588.1	4	1825	7	full-splice_match	gene: Zm00001d052475	Zm00001d052475_T00	10.00	10.00	coding	503	71	1579
PB.2501.1	2	503	1	genic	gene: Zm00001d002075	novel	11.08	54.75	non coding	NA	NA	NA

PB.438.1	1	2683	2	incomplete-splice_match	gene: Zm00001d029130	Zm00001d029130_T00	5.49	5.49	coding	331	882	1874
PB.5588.2	4	1754	6	full-splice_match	gene: Zm00001d052475	Zm00001d052475_T00	0.00	10.00	coding	303	381	1289
PB.4917.1	4	1936	2	intergenic	novelGene_54	novel	5.30	20.51	non coding	NA	NA	NA
PB.7188.1	5	1410	9	full-splice_match	gene: Zm00001d018243	Zm00001d018243_T00	25.52	25.52	coding	362	42	1127
PB.7234.4	5	433	3	incomplete-splice_match	gene: Zm00001d018413	Zm00001d018413_T01	0.00	90.00	non coding	NA	NA	NA
PB.4180.2	3	3955	5	incomplete-splice_match	gene: Zm00001d042028	Zm00001d042028_T01	24.47	82.07	coding	1305	19	3933
PB.5340.2	4	1068	2	novel_in_catalog	gene: Zm00001d051499	novel	13.66	82.04	coding	80	237	476
PB.4180.1	3	2419	5	incomplete-splice_match	gene: Zm00001d042028	Zm00001d042028_T01	57.60	82.07	coding	689	99	2165
PB.2003.3	10	1143	3	novel_not_in_catalog	gene: Zm00001d024925	novel	53.69	102.06	coding	321	70	1032
PB.729.1	1	2507	8	full-splice_match	gene: Zm00001d030770	Zm00001d030770_T00	26.77	30.79	coding	714	114	2255
PB.4503.1	3	1175	1	full-splice_match	gene: Zm00001d043430	Zm00001d043430_T00	16.76	16.76	coding	279	52	888
PB.4673.1	3	1713	5	full-splice_match	gene: Zm00001d044036	Zm00001d044036_T00	1.59	32.93	coding	117	800	1150
PB.3653.1	2	1515	10	full-splice_match	gene: Zm00001d007886	Zm00001d007886_T01	8.76	25.96	coding	463	127	1515
PB.9869.2	8	1979	1	novel_in_catalog	gene: Zm00001d011825	novel	1.37	101.77	coding	120	30	389
PB.9869.1	8	729	2	full-splice_match	gene: Zm00001d011825	Zm00001d011825_T00	100.40	101.77	coding	155	30	494
PB.4673.4	3	1524	5	full-splice_match	gene: Zm00001d044036	Zm00001d044036_T00	0.00	32.93	coding	117	786	1136
PB.7125.1	5	1252	8	full-splice_match	gene: Zm00001d018004	Zm00001d018004_T00	126.36	134.02	coding	214	211	852

PB.7125.2	5	1201	8	full-splice_match	gene: Zm00001d018004	Zm00001d018004_T00	0.00	134.02	coding	214	295	936
PB.7125.3	5	589	8	full-splice_match	gene: Zm00001d018004	Zm00001d018004_T00	0.00	134.02	coding	182	43	588
PB.7125.4	5	891	7	full-splice_match	gene: Zm00001d018004	Zm00001d018004_T01	7.66	134.02	coding	184	340	891
PB.2217.1	10	1174	5	full-splice_match	gene: Zm00001d026025	Zm00001d026025_T00	32.18	32.18	coding	250	103	852
PB.9012.1	7	1369	4	novel_not_in_catalog	gene: Zm00001d022330	novel	42.11	42.11	coding	318	14	967
PB.7956.1	6	884	1	full-splice_match	gene: Zm00001d038284	Zm00001d038284_T00	7.51	7.51	coding	218	40	693
PB.4760.1	3	936	1	full-splice_match	gene: Zm00001d044411	Zm00001d044411_T00	9.52	9.52	coding	193	71	649
PB.10109.1	9	1953	1	full-splice_match	gene: Zm00001d044726	Zm00001d044726_T00	16.92	16.92	coding	499	182	1678
PB.9653.3	8	1721	6	full-splice_match	gene: Zm00001d010842	Zm00001d010842_T01	5.83	23.19	coding	175	982	1506
PB.291.4	1	2810	6	full-splice_match	gene: Zm00001d028472	Zm00001d028472_T00	7.09	48.92	coding	666	493	2490
PB.291.2	1	3509	4	novel_in_catalog	gene: Zm00001d028472	novel	4.16	48.92	coding	575	601	2325
PB.8519.1	7	2937	11	fusion	gene: Zm00001d020367	novel	10.93	10.93	coding	879	34	2670
PB.291.1	1	2886	5	full-splice_match	gene: Zm00001d028472	Zm00001d028472_T00	37.66	48.92	coding	666	466	2463
PB.1638.2	1	1256	5	full-splice_match	gene: Zm00001d034795	Zm00001d034795_T00	35.79	121.02	coding	289	106	972
PB.1585.1	1	3508	5	incomplete-splice_match	gene: Zm00001d034624	Zm00001d034624_T00	28.81	53.35	coding	605	18	1832
PB.6635.1	5	1136	2	genic	gene: Zm00001d015662	novel	31.76	31.76	coding	287	172	1032
PB.6547.1	5	3858	1	incomplete-splice_match	gene: Zm00001d015138	Zm00001d015138_T00	21.60	311.43	coding	1249	111	3857

PB.4476.4	3	2002	1	incomplete-splice_match	gene: Zm00001d043329	Zm00001d043329_T00	28.44	108.90	coding	558	26	1699
PB.1363.1	1	2385	12	full-splice_match	gene: Zm00001d033652	Zm00001d033652_T00	22.85	57.54	coding	605	243	2057
PB.430.1	1	1090	4	full-splice_match	gene: Zm00001d029078	Zm00001d029078_T00	22.36	22.36	coding	164	145	636
PB.7815.2	6	1121	8	incomplete-splice_match	gene: Zm00001d037771	Zm00001d037771_T00	9.23	24.41	coding	247	54	794
PB.4476.3	3	3401	5	full-splice_match	gene: Zm00001d043329	Zm00001d043329_T00	10.94	108.90	coding	807	606	3026
PB.7989.5	6	1283	9	incomplete-splice_match	gene: Zm00001d038389	Zm00001d038389_T00	23.24	47.31	coding	311	143	1075
PB.1923.1	10	2109	2	full-splice_match	gene: Zm00001d024516	Zm00001d024516_T00	49.19	49.19	coding	476	228	1655
PB.1923.2	10	1843	2	full-splice_match	gene: Zm00001d024516	Zm00001d024516_T00	0.00	49.19	coding	476	147	1574
PB.2999.10	2	1380	5	incomplete-splice_match	gene: Zm00001d004564	Zm00001d004564_T01	8.46	65.73	coding	280	345	1184
PB.2999.11	2	965	5	incomplete-splice_match	gene: Zm00001d004564	Zm00001d004564_T01	0.00	65.73	coding	311	32	964
PB.4583.7	3	1436	9	full-splice_match	gene: Zm00001d043694	Zm00001d043694_T00	13.83	47.02	coding	343	221	1249
PB.4583.6	3	1323	8	novel_in_catalog	gene: Zm00001d043694	novel	2.20	47.02	coding	202	177	782
PB.4583.8	3	2754	5	novel_in_catalog	gene: Zm00001d043694	novel	6.42	47.02	coding	123	66	434
PB.5702.2	4	681	1	full-splice_match	gene: Zm00001d053055	Zm00001d053055_T00	436.08	608.28	coding	171	16	528
PB.1564.10	1	1083	1	novel_in_catalog	gene: Zm00001d034558	novel	0.00	853.55	coding	309	115	1041
PB.9598.4	8	869	1	full-splice_match	gene: Zm00001d010649	Zm00001d010649_T00	8.82	72.86	coding	142	198	623
PB.9861.1	8	755	3	full-splice_match	gene: Zm00001d011786	Zm00001d011786_T00	229.70	233.61	coding	135	92	496

PB.6848.4	5	1658	6	novel_in_catalog	gene: Zm00001d016866	novel	0.00	42.40	coding	293	114	992
PB.9598.5	8	747	2	genic	gene: Zm00001d010649	novel	56.47	72.86	coding	152	47	502
PB.1816.1	10	2257	10	full-splice_match	gene: Zm00001d023843	Zm00001d023843_T00	41.19	41.19	coding	650	23	1972
PB.5791.1	4	2075	14	full-splice_match	gene: Zm00001d053553	Zm00001d053553_T00	38.21	38.21	coding	558	163	1836
PB.1816.3	10	2179	10	full-splice_match	gene: Zm00001d023843	Zm00001d023843_T00	0.00	41.19	coding	650	80	2029
PB.6848.2	5	1907	6	novel_in_catalog	gene: Zm00001d016866	novel	6.59	42.40	coding	293	242	1120
PB.577.1	1	1686	14	full-splice_match	gene: Zm00001d029803	Zm00001d029803_T00	41.01	41.01	coding	446	116	1453
PB.9598.2	8	1151	1	full-splice_match	gene: Zm00001d010649	Zm00001d010649_T00	0.00	72.86	coding	142	204	629
PB.6671.1	5	1958	15	full-splice_match	gene: Zm00001d015948	Zm00001d015948_T01	37.82	47.70	coding	559	56	1732
PB.5020.1	4	2598	14	full-splice_match	gene: Zm00001d049457	Zm00001d049457_T04	92.65	92.65	coding	727	77	2257
PB.4706.1	3	1907	6	novel_in_catalog	gene: Zm00001d044170	novel	2.80	21.01	coding	221	78	740
PB.6671.6	5	634	5	novel_in_catalog	gene: Zm00001d015948	novel	2.53	47.70	coding	120	35	394
PB.6671.5	5	612	5	incomplete-splice_match	gene: Zm00001d015948	Zm00001d015948_T00	6.25	47.70	coding	121	150	512
PB.6671.4	5	1731	14	novel_in_catalog	gene: Zm00001d015948	novel	0.00	47.70	coding	506	28	1545
PB.54.2	1	2048	9	novel_not_in_catalog	gene: Zm00001d027460	novel	7.28	20.30	coding	440	284	1603
PB.5527.1	4	1129	5	full-splice_match	gene: Zm00001d052268	Zm00001d052268_T00	61.92	134.67	coding	230	294	983
PB.4498.1	3	628	1	full-splice_match	gene: Zm00001d043413	Zm00001d043413_T00	134.11	134.11	coding	74	156	377

PB.9793.3	8	1384	1	genic	gene: Zm00001d011531	novel	2.78	44.49	coding	328	238	1221
PB.9793.2	8	1381	2	genic	gene: Zm00001d011531	novel	14.89	44.49	coding	378	55	1188
PB.9793.1	8	1492	2	genic	gene: Zm00001d011531	novel	4.55	44.49	coding	378	11	1144
PB.9651.1	8	1426	2	full-splice_match	gene: Zm00001d010838	Zm00001d010838_T00	8.88	8.88	coding	396	98	1285
PB.3561.7	2	1758	5	novel_in_catalog	gene: Zm00001d007311	novel	7.95	74.66	coding	225	199	873
PB.10203.1	9	2477	17	full-splice_match	gene: Zm00001d045195	Zm00001d045195_T00	51.53	69.10	coding	685	102	2156
PB.10203.3	9	2417	17	full-splice_match	gene: Zm00001d045195	Zm00001d045195_T00	0.00	69.10	coding	727	74	2254
PB.10203.4	9	3093	17	novel_in_catalog	gene: Zm00001d045195	novel	17.56	69.10	coding	447	655	1995
PB.4435.5	3	516	3	full-splice_match	gene: Zm00001d043175	Zm00001d043175_T00	81.08	331.13	coding	135	1	405
PB.7191.2	5	1157	5	novel_not_in_catalog	gene: Zm00001d018255	novel	83.96	111.57	coding	271	99	911
PB.9032.1	7	1329	8	incomplete-splice_match	gene: Zm00001d022396	Zm00001d022396_T00	9.90	9.90	coding	301	2	904
PB.7955.1	6	2356	3	incomplete-splice_match	gene: Zm00001d038273	Zm00001d038273_T00	26.38	164.25	coding	571	34	1746
PB.10382.1	9	1926	8	novel_in_catalog	gene: Zm00001d046135	novel	12.47	53.16	coding	206	1129	1746
PB.10382.2	9	1524	8	full-splice_match	gene: Zm00001d046135	Zm00001d046135_T00	40.68	53.16	coding	395	160	1344
PB.10382.3	9	1815	8	novel_in_catalog	gene: Zm00001d046135	novel	0.00	53.16	coding	206	1070	1687
PB.3246.2	2	2431	2	incomplete-splice_match	gene: Zm00001d005958	Zm00001d005958_T00	0.00	28.54	coding	744	133	2364
PB.4978.1	4	2389	10	incomplete-splice_match	gene: Zm00001d049251	Zm00001d049251_T03	10.64	16.24	coding	730	17	2206

PB.4435.2	3	636	4	full-splice_match	gene: Zm00001d043175	Zm00001d043175_T00	18.33	331.13	coding	97	38	328
PB.5381.2	4	1438	7	full-splice_match	gene: Zm00001d051664	Zm00001d051664_T00	84.54	84.54	coding	356	128	1195
PB.1988.1	10	2206	1	full-splice_match	gene: Zm00001d024867	Zm00001d024867_T00	11.62	11.62	coding	683	77	2125
PB.7739.1	6	2310	10	full-splice_match	gene: Zm00001d037413	Zm00001d037413_T00	24.77	24.77	coding	584	204	1955
PB.7154.8	5	1252	7	novel_in_catalog	gene: Zm00001d018112	novel	29.48	135.59	coding	93	47	325
PB.7907.1	6	1838	1	full-splice_match	gene: Zm00001d038125	Zm00001d038125_T00	12.56	37.20	coding	180	342	881
PB.3695.2	3	724	3	novel_not_in_catalog	gene: Zm00001d039303	novel	8.48	24.33	coding	149	54	500
PB.3695.3	3	523	3	full-splice_match	gene: Zm00001d039303	Zm00001d039303_T00	0.00	24.33	coding	41	322	444
PB.7584.1	6	1416	9	full-splice_match	gene: Zm00001d036632	Zm00001d036632_T00	27.04	27.04	coding	333	72	1070
PB.3695.1	3	1042	3	full-splice_match	gene: Zm00001d039303	Zm00001d039303_T00	15.84	24.33	coding	163	107	595
PB.5830.1	4	3755	9	incomplete-splice_match	gene: Zm00001d053688	Zm00001d053688_T00	17.50	17.50	coding	1082	34	3279
PB.2732.1	2	1051	1	full-splice_match	gene: Zm00001d003091	Zm00001d003091_T00	7.80	7.80	coding	259	164	940
PB.9342.1	8	3819	20	fusion	gene: Zm00001d009337	novel	24.51	24.51	coding	1181	139	3681
PB.9344.1	8	2314	8	full-splice_match	gene: Zm00001d009352	Zm00001d009352_T00	36.91	36.91	coding	638	97	2010
PB.3665.4	2	795	1	novel_in_catalog	gene: Zm00001d007942	novel	50.05	99.43	coding	81	318	560
PB.7664.4	6	1120	9	full-splice_match	gene: Zm00001d037096	Zm00001d037096_T00	0.00	155.67	coding	139	652	1068
PB.5527.3	4	1267	4	novel_in_catalog	gene: Zm00001d052268	novel	17.63	134.67	coding	153	30	488

PB.5732.1	4	1798	5	full-splice_match	gene: Zm00001d053234	Zm00001d053234_T00	4.32	4.32	coding	506	27	1544
PB.6124.2	5	1926	12	novel_in_catalog	gene: Zm00001d013371	novel	100.63	107.44	coding	515	173	1717
PB.7782.1	6	1994	5	full-splice_match	gene: Zm00001d037624	Zm00001d037624_T00	25.77	25.77	coding	412	498	1733
PB.6124.1	5	2047	10	novel_in_catalog	gene: Zm00001d013371	novel	6.81	107.44	coding	395	652	1836
PB.1551.1	1	1118	6	full-splice_match	gene: Zm00001d034503	Zm00001d034503_T00	23.27	23.27	coding	255	136	900
PB.4793.5	3	2328	1	genic	gene: Zm00001d044516	novel	1.02	891.68	coding	334	145	1146
PB.1207.1	1	2729	18	full-splice_match	gene: Zm00001d032956	Zm00001d032956_T01	47.04	47.04	coding	774	153	2474
PB.7925.3	6	2404	8	full-splice_match	gene: Zm00001d038170	Zm00001d038170_T00	0.00	9.69	coding	719	199	2355
PB.503.1	1	3328	7	incomplete-splice_match	gene: Zm00001d029443	Zm00001d029443_T00	22.85	37.30	coding	1027	1	3081
PB.503.2	1	1367	3	incomplete-splice_match	gene: Zm00001d029443	Zm00001d029443_T01	14.45	37.30	coding	375	165	1289
PB.8586.1	7	1611	2	full-splice_match	gene: Zm00001d020686	Zm00001d020686_T00	20.09	20.09	coding	326	93	1070
PB.9783.1	8	2236	5	full-splice_match	gene: Zm00001d011471	Zm00001d011471_T00	61.20	71.99	coding	634	130	2031
PB.9783.2	8	745	2	incomplete-splice_match	gene: Zm00001d011471	Zm00001d011471_T00	10.79	71.99	coding	214	38	679
PB.10627.1	9	926	4	full-splice_match	gene: Zm00001d047469	Zm00001d047469_T00	51.41	58.67	coding	228	127	810
PB.8219.1	7	2798	16	full-splice_match	gene: Zm00001d018624	Zm00001d018624_T00	12.51	12.51	coding	760	94	2373
PB.5922.1	4	1888	11	full-splice_match	gene: Zm00001d054011	Zm00001d054011_T00	25.82	25.82	coding	429	142	1428
PB.8954.1	7	2727	6	novel_not_in_catalog	gene: Zm00001d022122	novel	6.75	19.63	coding	370	1374	2483

PB.8718.1	7	3118	4	novel_not_in_catalog	gene: Zm00001d021296	novel	19.19	24.15	coding	275	1858	2682
PB.7234.2	5	1917	7	novel_in_catalog	gene: Zm00001d018413	novel	27.12	90.00	coding	228	760	1443
PB.9653.4	8	2315	5	incomplete-splice_match	gene: Zm00001d010842	Zm00001d010842_T01	5.34	23.19	coding	256	1383	2150
PB.9599.2	8	813	2	genic	gene: Zm00001d010650	novel	428.38	788.18	coding	149	104	550
PB.2056.1	10	1499	4	full-splice_match	gene: Zm00001d025166	Zm00001d025166_T00	88.22	92.12	coding	339	145	1161
PB.8318.2	7	1870	2	full-splice_match	gene: Zm00001d019078	Zm00001d019078_T00	21.83	36.44	coding	512	171	1706
PB.8318.1	7	1788	2	full-splice_match	gene: Zm00001d019078	Zm00001d019078_T00	14.60	36.44	coding	507	100	1620
PB.1524.1	1	2152	10	full-splice_match	gene: Zm00001d034401	Zm00001d034401_T00	21.27	21.27	coding	561	203	1885
PB.2172.2	10	1367	1	full-splice_match	gene: Zm00001d025812	Zm00001d025812_T00	16.67	39.92	coding	187	703	1263
PB.464.1	1	2084	19	novel_not_in_catalog	gene: Zm00001d029231	novel	36.93	36.93	coding	559	236	1912
PB.952.10	1	2420	10	novel_in_catalog	gene: Zm00001d031927	novel	0.00	61.88	coding	102	1560	1865
PB.4951.1	4	1527	10	full-splice_match	gene: Zm00001d049152	Zm00001d049152_T00	18.09	18.09	coding	294	219	1100
PB.952.12	1	2430	10	novel_in_catalog	gene: Zm00001d031927	novel	1.72	61.88	coding	265	873	1667
PB.8560.3	7	968	1	full-splice_match	gene: Zm00001d020550	Zm00001d020550_T00	0.00	25.30	coding	234	178	879
PB.952.15	1	2572	11	novel_in_catalog	gene: Zm00001d031927	novel	0.00	61.88	coding	102	1560	1865
PB.6053.1	5	1846	3	novel_not_in_catalog	gene: Zm00001d013139	novel	93.24	93.77	coding	445	239	1573
PB.10578.10	9	2814	15	full-splice_match	gene: Zm00001d047253	Zm00001d047253_T00	0.00	1036.48	coding	816	164	2611

PB.6053.3	5	2778	2	genic	gene: Zm00001d013139	novel	0.53	93.77	coding	299	1617	2513
PB.10917.1	9	1165	7	novel_not_in_catalog	gene: Zm00001d048515	novel	2.59	5.90	coding	316	113	1060
PB.9561.10	8	2392	3	novel_in_catalog	gene: Zm00001d010478	novel	12.82	122.36	coding	163	1575	2063
PB.9719.3	8	1340	6	novel_not_in_catalog	gene: Zm00001d011166	novel	1.45	24.27	coding	128	96	479
PB.9719.2	8	1389	6	novel_in_catalog	gene: Zm00001d011166	novel	0.00	24.27	coding	174	520	1041
PB.9164.1	8	2060	10	full-splice_match	gene: Zm00001d008369	Zm00001d008369_T00	125.60	125.60	coding	525	201	1775
PB.5660.5	4	1096	1	novel_in_catalog	gene: Zm00001d052842	novel	1.86	178.76	coding	202	367	972
PB.10905.1	9	715	5	full-splice_match	gene: Zm00001d048492	Zm00001d048492_T00	14.18	14.18	coding	141	153	575
PB.8669.1	7	1607	1	full-splice_match	gene: Zm00001d021053	Zm00001d021053_T00	1.06	1.06	coding	456	197	1564
PB.7961.1	6	1915	5	novel_in_catalog	gene: Zm00001d038292	novel	0.65	23.23	coding	336	166	1173
PB.1686.1	10	1724	4	novel_in_catalog	gene: Zm00001d023225	novel	12.30	111.77	coding	196	16	603
PB.1686.2	10	1671	5	full-splice_match	gene: Zm00001d023225	Zm00001d023225_T00	64.36	111.77	coding	439	78	1394
PB.1686.3	10	1562	5	full-splice_match	gene: Zm00001d023225	Zm00001d023225_T00	35.10	111.77	coding	439	73	1389
PB.6736.1	5	1874	2	novel_in_catalog	gene: Zm00001d016332	novel	34.16	221.47	coding	262	188	973
PB.3106.1	2	989	7	full-splice_match	gene: Zm00001d005231	Zm00001d005231_T00	1477.52	1483.63	coding	181	74	616
PB.4295.4	3	960	1	novel_in_catalog	gene: Zm00001d042590	novel	36.07	105.26	non coding	NA	NA	NA
PB.4295.3	3	1573	6	novel_in_catalog	gene: Zm00001d042590	novel	0.00	105.26	coding	221	668	1330

PB.4295.2	3	1961	6	novel_in_catalog	gene: Zm00001d042590	novel	69.20	105.26	coding	221	818	1480
PB.6736.6	5	1721	2	novel_in_catalog	gene: Zm00001d016332	novel	129.19	221.47	coding	262	183	968
PB.1208.2	1	1133	4	intergenic	novelGene_448	novel	37.50	81.86	coding	188	243	806
PB.1722.2	10	1185	5	full-splice_match	gene: Zm00001d023376	Zm00001d023376_T00	597.94	672.76	coding	238	108	821
PB.1722.1	10	1292	5	full-splice_match	gene: Zm00001d023376	Zm00001d023376_T00	69.06	672.76	coding	238	108	821
PB.2988.3	2	1329	7	novel_not_in_catalog	gene: Zm00001d004498	novel	8.51	35.29	coding	172	179	694
PB.1722.7	10	1065	5	full-splice_match	gene: Zm00001d023376	Zm00001d023376_T00	0.00	672.76	coding	238	108	821
PB.2988.5	2	1508	6	novel_in_catalog	gene: Zm00001d004498	novel	0.00	35.29	coding	159	182	658
PB.1722.4	10	1928	4	novel_in_catalog	gene: Zm00001d023376	novel	5.76	672.76	coding	114	1297	1638
PB.8763.1	7	601	2	genic	gene: Zm00001d001256	novel	15.40	15.40	non coding	NA	NA	NA
PB.4786.3	3	1072	10	novel_not_in_catalog	gene: Zm00001d044497	novel	16.69	47.06	coding	204	241	852
PB.1722.8	10	956	5	full-splice_match	gene: Zm00001d023376	Zm00001d023376_T00	0.00	672.76	coding	238	108	821
PB.4628.2	3	1527	3	full-splice_match	gene: Zm00001d043850	Zm00001d043850_T00	4.34	150.66	coding	447	78	1418
PB.3070.2	2	943	1	incomplete-splice_match	gene: Zm00001d005030	Zm00001d005030_T00	6.16	24.99	non coding	NA	NA	NA
PB.5873.1	4	1025	7	novel_not_in_catalog	gene: Zm00001d053862	novel	9.42	560.87	coding	125	60	434
PB.8581.1	7	497	3	full-splice_match	gene: Zm00001d020658	Zm00001d020658_T00	64.09	64.09	coding	88	41	304
PB.7986.3	6	983	1	novel_in_catalog	gene: Zm00001d038381	novel	7.08	566.39	non coding	NA	NA	NA

PB.624.1	1	1315	7	full-splice_match	gene: Zm00001d030091	Zm00001d030091_T00	29.85	29.85	coding	328	70	1053
PB.2669.1	2	2016	7	full-splice_match	gene: Zm00001d002823	Zm00001d002823_T00	24.65	24.65	coding	580	95	1834
PB.3496.1	2	896	1	full-splice_match	gene: Zm00001d007015	Zm00001d007015_T00	17.05	17.05	coding	201	57	659
PB.10917.3	9	1242	6	novel_not_in_catalog	gene: Zm00001d048515	novel	3.10	5.90	coding	252	387	1142
PB.4748.12	3	1401	7	full-splice_match	gene: Zm00001d044327	Zm00001d044327_T00	0.00	192.78	coding	257	256	1026
PB.3143.1	2	3110	21	full-splice_match	gene: Zm00001d005403	Zm00001d005403_T00	15.11	15.11	coding	891	86	2758
PB.7267.5	5	1783	5	novel_in_catalog	gene: Zm00001d018511	novel	0.00	44.35	coding	174	1023	1544
PB.777.2	1	2387	14	full-splice_match	gene: Zm00001d031040	Zm00001d031040_T01	12.31	12.31	coding	573	298	2016
PB.4939.2	4	1919	10	full-splice_match	gene: Zm00001d049099	Zm00001d049099_T00	268.88	279.99	coding	514	55	1596
PB.1012.5	1	1612	2	genic	gene: Zm00001d032187	novel	142.81	534.29	coding	394	90	1271
PB.3472.3	2	692	1	full-splice_match	gene: Zm00001d006904	Zm00001d006904_T00	0.00	67.19	coding	131	73	465
PB.4939.1	4	2039	10	full-splice_match	gene: Zm00001d049099	Zm00001d049099_T00	11.11	279.99	coding	514	55	1596
PB.1392.2	1	2016	7	full-splice_match	gene: Zm00001d033797	Zm00001d033797_T00	50.70	50.70	coding	394	393	1574
PB.680.1	1	1463	14	incomplete-splice_match	gene: Zm00001d030366	Zm00001d030366_T00	24.83	24.83	coding	364	85	1176
PB.2294.2	10	2189	2	genic	gene: Zm00001d026326	novel	27.37	58.91	coding	564	123	1814
PB.2294.3	10	2042	2	genic	gene: Zm00001d026326	novel	0.00	58.91	coding	564	123	1814
PB.6961.1	5	1945	1	genic	gene: Zm00001d017329	novel	10.96	24.87	coding	175	44	568

PB.5873.6	4	690	6	full-splice_match	gene: Zm00001d053862	Zm00001d053862_T00	351.65	560.87	coding	125	107	481
PB.3276.4	2	1086	5	full-splice_match	gene: Zm00001d006070	Zm00001d006070_T00	25.13	71.75	coding	198	226	819
PB.3904.3	3	922	3	full-splice_match	gene: Zm00001d040278	Zm00001d040278_T00	11.87	228.26	coding	193	141	719
PB.3904.2	3	1047	3	full-splice_match	gene: Zm00001d040278	Zm00001d040278_T00	93.03	228.26	coding	193	135	713
PB.3904.1	3	1157	3	full-splice_match	gene: Zm00001d040278	Zm00001d040278_T00	123.36	228.26	coding	193	137	715
PB.6585.8	5	1813	2	full-splice_match	gene: Zm00001d015327	Zm00001d015327_T00	0.00	996.94	coding	533	73	1671
PB.9265.1	8	793	2	novel_in_catalog	gene: Zm00001d008856	novel	25.62	25.62	non coding	NA	NA	NA
PB.7853.1	6	907	1	full-splice_match	gene: Zm00001d037937	Zm00001d037937_T00	72.03	72.03	coding	173	52	570
PB.1012.6	1	2148	1	full-splice_match	gene: Zm00001d032187	Zm00001d032187_T00	57.93	534.29	coding	394	734	1915
PB.2361.2	10	1045	9	fusion	gene: Zm00001d026553	novel	9.73	9.73	coding	245	86	820
PB.10020.2	8	539	3	novel_not_in_catalog	gene: Zm00001d012448	novel	75.61	100.47	coding	78	98	331
PB.3160.1	2	6043	9	full-splice_match	gene: Zm00001d005507	Zm00001d005507_T00	16.43	16.43	coding	1721	365	5527
PB.10083.1	8	1376	8	incomplete-splice_match	gene: Zm00001d012694	Zm00001d012694_T00	26.51	26.51	coding	308	325	1248
PB.10544.1	9	2221	12	full-splice_match	gene: Zm00001d047054	Zm00001d047054_T00	29.27	85.19	coding	561	246	1928
PB.10544.2	9	2149	12	full-splice_match	gene: Zm00001d047054	Zm00001d047054_T00	55.92	85.19	coding	561	248	1930
PB.7994.3	6	901	2	novel_not_in_catalog	gene: Zm00001d038400	novel	0.00	36.41	coding	107	403	723
PB.819.1	1	890	6	full-splice_match	gene: Zm00001d031205	Zm00001d031205_T00	195.49	259.84	coding	161	84	566

PB.8609.1	7	634	2	intergenic	novelGene_259	novel	26.62	26.62	non coding	NA	NA	NA
PB.1348.1	1	1080	3	incomplete-splice_match	gene: Zm00001d033591	Zm00001d033591_T00	19.30	19.30	coding	290	32	901
PB.5433.3	4	1181	6	full-splice_match	gene: Zm00001d051916	Zm00001d051916_T00	4.32	32.05	non coding	NA	NA	NA
PB.4203.2	3	899	1	full-splice_match	gene: Zm00001d042108	Zm00001d042108_T00	79.03	140.70	coding	192	106	681
PB.3180.2	2	2671	4	incomplete-splice_match	gene: Zm00001d005638	Zm00001d005638_T01	0.00	28.52	coding	725	195	2369
PB.3180.1	2	3515	5	full-splice_match	gene: Zm00001d005638	Zm00001d005638_T00	28.52	28.52	coding	982	123	3068
PB.4203.1	3	1081	1	full-splice_match	gene: Zm00001d042108	Zm00001d042108_T00	61.67	140.70	coding	192	103	678
PB.7605.1	6	3362	27	full-splice_match	gene: Zm00001d036741	Zm00001d036741_T01	35.47	56.00	coding	1038	30	3143
PB.7605.3	6	1560	8	full-splice_match	gene: Zm00001d036741	Zm00001d036741_T04	16.78	56.00	coding	465	165	1559
PB.7605.2	6	3324	25	novel_in_catalog	gene: Zm00001d036741	novel	3.75	56.00	coding	503	994	2502
PB.6225.1	5	327	1	incomplete-splice_match	gene: Zm00001d013819	Zm00001d013819_T00	16.24	24.10	non coding	NA	NA	NA
PB.4066.1	3	1632	2	intergenic	novelGene_232	novel	14.96	14.96	coding	100	158	457
PB.6652.1	5	1597	10	full-splice_match	gene: Zm00001d015788	Zm00001d015788_T00	159.58	160.66	coding	400	40	1239
PB.9364.1	8	1167	2	full-splice_match	gene: Zm00001d009475	Zm00001d009475_T00	10.17	10.17	coding	257	126	896
PB.4336.1	3	1065	1	full-splice_match	gene: Zm00001d042760	Zm00001d042760_T00	14.46	14.46	coding	211	163	795
PB.1386.2	1	1239	1	intergenic	novelGene_459	novel	0.00	8.75	coding	188	453	1016
PB.9961.2	8	1506	3	full-splice_match	gene: Zm00001d012212	Zm00001d012212_T00	10.88	14.52	coding	417	24	1274

PB.6613.3	5	919	5	full-splice_match	gene: Zm00001d015509	Zm00001d015509_T00	0.00	113.71	coding	253	38	796
PB.6613.2	5	1135	5	full-splice_match	gene: Zm00001d015509	Zm00001d015509_T00	102.68	113.71	coding	253	138	896
PB.6613.1	5	2292	4	novel_in_catalog	gene: Zm00001d015509	novel	11.03	113.71	coding	140	1570	1989
PB.2052.1	10	1431	12	novel_in_catalog	gene: Zm00001d025125	novel	22.02	22.02	coding	378	1	1134
PB.7333.2	6	849	1	full-splice_match	gene: Zm00001d035171	Zm00001d035171_T00	24.85	29.47	coding	218	110	763
PB.7333.1	6	982	1	full-splice_match	gene: Zm00001d035171	Zm00001d035171_T00	4.62	29.47	coding	218	113	766
PB.332.5	1	949	5	incomplete-splice_match	gene: Zm00001d028655	Zm00001d028655_T00	84.30	209.54	coding	253	24	782
PB.332.2	1	1531	6	full-splice_match	gene: Zm00001d028655	Zm00001d028655_T00	91.64	209.54	coding	253	54	812
PB.332.3	1	1276	4	novel_in_catalog	gene: Zm00001d028655	novel	0.00	209.54	coding	86	59	316
PB.5804.1	4	531	1	full-splice_match	gene: Zm00001d053605	Zm00001d053605_T00	14.84	14.84	coding	139	73	489
PB.332.1	1	2006	5	novel_in_catalog	gene: Zm00001d028655	novel	33.60	209.54	coding	205	361	975
PB.7267.6	5	1571	4	novel_in_catalog	gene: Zm00001d018511	novel	2.22	44.35	non coding	NA	NA	NA
PB.7615.2	6	2543	8	full-splice_match	gene: Zm00001d036798	Zm00001d036798_T00	3.61	42.64	coding	508	46	1569
PB.3248.2	2	2083	1	full-splice_match	gene: Zm00001d005966	Zm00001d005966_T00	18.30	90.24	coding	505	232	1746
PB.4954.2	4	1188	9	full-splice_match	gene: Zm00001d049163	Zm00001d049163_T00	15.62	85.61	coding	294	96	977
PB.10564.2	9	1178	5	full-splice_match	gene: Zm00001d047191	Zm00001d047191_T00	15.63	52.31	coding	164	333	824
PB.10564.3	9	1070	2	incomplete-splice_match	gene: Zm00001d047191	Zm00001d047191_T01	0.00	52.31	coding	310	141	1070

PB.2843.1	2	3801	6	full-splice_match	gene: Zm00001d003615	Zm00001d003615_T00	42.04	70.99	coding	1124	121	3492
PB.7482.1	6	1275	1	full-splice_match	gene: Zm00001d036016	Zm00001d036016_T00	19.87	30.85	coding	229	170	856
PB.9560.1	8	2259	3	incomplete-splice_match	gene: Zm00001d010470	Zm00001d010470_T00	25.33	33.92	coding	625	21	1895
PB.1375.1	1	1834	14	full-splice_match	gene: Zm00001d033708	Zm00001d033708_T00	21.53	36.87	coding	367	291	1391
PB.5760.1	4	2319	3	full-splice_match	gene: Zm00001d053356	Zm00001d053356_T00	12.93	12.93	coding	676	94	2121
PB.8654.5	7	1004	6	incomplete-splice_match	gene: Zm00001d021000	Zm00001d021000_T04	65.13	167.68	coding	256	31	798
PB.3512.1	2	1680	8	full-splice_match	gene: Zm00001d007067	Zm00001d007067_T00	25.47	57.30	coding	313	173	1111
PB.2755.1	2	1753	4	full-splice_match	gene: Zm00001d003196	Zm00001d003196_T00	4.64	4.64	coding	485	229	1683
PB.10267.1	9	1714	1	full-splice_match	gene: Zm00001d045465	Zm00001d045465_T00	113.30	113.30	coding	462	90	1475
PB.3529.1	2	897	3	incomplete-splice_match	gene: Zm00001d007121	Zm00001d007121_T06	52.95	70.33	non coding	NA	NA	NA
PB.10911.4	9	1840	7	full-splice_match	gene: Zm00001d048507	Zm00001d048507_T00	113.50	157.79	coding	418	71	1324
PB.8654.3	7	2644	14	novel_in_catalog	gene: Zm00001d021000	novel	9.75	167.68	coding	417	1065	2315
PB.4884.1	4	401	1	intergenic	novelGene_30	novel	7.93	7.93	non coding	NA	NA	NA
PB.9668.2	8	763	1	incomplete-splice_match	gene: Zm00001d010924	Zm00001d010924_T00	0.00	19.38	coding	108	198	521
PB.3341.1	2	1164	3	full-splice_match	gene: Zm00001d006337	Zm00001d006337_T00	51.24	51.24	coding	194	262	843
PB.9307.1	8	499	1	intergenic	novelGene_111	novel	8.15	8.15	non coding	NA	NA	NA
PB.6306.1	5	1565	11	novel_in_catalog	gene: Zm00001d014178	novel	18.66	50.79	coding	289	279	1145

PB.1147.4	1	2369	8	full-splice_match	gene: Zm00001d032703	Zm00001d032703_T00	51.06	85.19	coding	609	313	2139
PB.8654.1	7	4060	18	novel_in_catalog	gene: Zm00001d021000	novel	35.22	167.68	coding	466	2339	3736
PB.4591.1	3	1453	11	full-splice_match	gene: Zm00001d043727	Zm00001d043727_T00	26.41	26.41	coding	355	194	1258
PB.9180.3	8	2011	7	novel_in_catalog	gene: Zm00001d008429	novel	4.71	94.20	coding	94	419	700
PB.6306.6	5	3213	1	novel_in_catalog	gene: Zm00001d014178	novel	1.17	50.79	non coding	NA	NA	NA
PB.9705.1	8	2652	9	full-splice_match	gene: Zm00001d011108	Zm00001d011108_T00	19.68	19.68	coding	759	126	2402
PB.9980.1	8	643	1	full-splice_match	gene: Zm00001d012276	Zm00001d012276_T00	868.51	868.51	coding	136	63	470
PB.7566.1	6	2457	1	intergenic	novelGene_196	novel	5.68	5.68	non coding	NA	NA	NA
PB.2263.1	10	1784	6	full-splice_match	gene: Zm00001d026244	Zm00001d026244_T00	13.08	13.08	coding	530	71	1660
PB.5826.2	4	2998	11	novel_in_catalog	gene: Zm00001d053672	novel	3.53	59.75	coding	433	77	1375
PB.2483.6	2	2414	8	novel_in_catalog	gene: Zm00001d001999	novel	2.50	116.60	coding	178	1600	2133
PB.2483.7	2	1541	7	novel_in_catalog	gene: Zm00001d001999	novel	7.11	116.60	coding	113	120	458
PB.273.1	1	1821	3	full-splice_match	gene: Zm00001d028399	Zm00001d028399_T00	26.96	69.53	coding	335	97	1101
PB.2483.2	2	1597	9	full-splice_match	gene: Zm00001d001999	Zm00001d001999_T00	86.85	116.60	coding	336	294	1301
PB.2483.3	2	1644	8	full-splice_match	gene: Zm00001d001999	Zm00001d001999_T00	4.83	116.60	coding	271	251	1063
PB.1295.2	1	2761	13	incomplete-splice_match	gene: Zm00001d033375	Zm00001d033375_T01	36.28	86.31	coding	674	422	2443
PB.2483.1	2	1666	9	full-splice_match	gene: Zm00001d001999	Zm00001d001999_T00	15.31	116.60	coding	336	120	1127

PB.5287.1	4	2982	8	novel_in_catalog	gene: Zm00001d051180	novel	14.00	75.05	coding	295	1573	2457
PB.5287.3	4	1781	9	novel_in_catalog	gene: Zm00001d051180	novel	35.49	75.05	coding	295	516	1400
PB.5287.2	4	1810	9	novel_in_catalog	gene: Zm00001d051180	novel	22.44	75.05	coding	295	523	1407
PB.2673.1	2	2330	13	full-splice_match	gene: Zm00001d002835	Zm00001d002835_T00	40.78	51.18	coding	653	170	2128
PB.5287.4	4	2971	8	novel_in_catalog	gene: Zm00001d051180	novel	3.12	75.05	coding	295	1694	2578
PB.5287.7	4	2711	8	novel_in_catalog	gene: Zm00001d051180	novel	0.00	75.05	coding	295	1573	2457
PB.2673.2	2	2498	12	full-splice_match	gene: Zm00001d002835	Zm00001d002835_T00	10.40	51.18	coding	351	1273	2325
PB.152.2	1	985	4	full-splice_match	gene: Zm00001d027888	Zm00001d027888_T00	27.58	27.58	coding	201	20	622
PB.7491.1	6	1125	4	full-splice_match	gene: Zm00001d036057	Zm00001d036057_T00	68.54	68.54	coding	250	100	849
PB.3074.1	2	2483	12	full-splice_match	gene: Zm00001d005062	Zm00001d005062_T00	19.03	28.49	coding	624	257	2128
PB.10008.1	8	1650	1	full-splice_match	gene: Zm00001d012415	Zm00001d012415_T00	50.60	50.60	coding	435	87	1391
PB.4212.5	3	1245	9	full-splice_match	gene: Zm00001d042193	Zm00001d042193_T00	0.00	33.54	coding	286	123	980
PB.1541.1	1	1496	2	novel_in_catalog	gene: Zm00001d034463	novel	0.00	13.13	coding	144	465	896
PB.5752.2	4	2173	15	full-splice_match	gene: Zm00001d053316	Zm00001d053316_T00	13.03	44.66	coding	414	178	1419
PB.6184.1	5	2399	9	novel_in_catalog	gene: Zm00001d013606	novel	15.95	15.95	coding	12	329	364
PB.9449.2	8	447	2	full-splice_match	gene: Zm00001d009910	Zm00001d009910_T00	0.00	57.61	non coding	NA	NA	NA
PB.6860.4	5	1422	2	novel_in_catalog	gene: Zm00001d016911	novel	0.00	46.96	coding	359	55	1131

PB.1772.1	10	2575	17	full-splice_match	gene: Zm00001d023583	Zm00001d023583_T00	3.69	3.69	coding	758	140	2413
PB.5752.4	4	3268	13	novel_in_catalog	gene: Zm00001d053316	novel	0.72	44.66	coding	190	161	730
PB.1996.3	10	1471	11	incomplete-splice_match	gene: Zm00001d024891	Zm00001d024891_T00	0.00	101.63	coding	469	64	1470
PB.9988.1	8	3427	2	novel_in_catalog	gene: Zm00001d012297	novel	11.35	21.82	non coding	NA	NA	NA
PB.2760.1	2	2560	14	full-splice_match	gene: Zm00001d003207	Zm00001d003207_T01	6.16	121.31	coding	511	256	1788
PB.2760.2	2	2449	15	full-splice_match	gene: Zm00001d003207	Zm00001d003207_T00	51.68	121.31	coding	589	313	2079
PB.10189.6	9	2028	11	full-splice_match	gene: Zm00001d045146	Zm00001d045146_T00	3.39	62.82	coding	309	34	960
PB.4417.2	3	1270	7	novel_not_in_catalog	gene: Zm00001d043113	novel	31.41	109.53	coding	181	354	896
PB.2760.5	2	1095	8	incomplete-splice_match	gene: Zm00001d003207	Zm00001d003207_T01	50.64	121.31	coding	230	141	830
PB.10189.3	9	1430	11	novel_in_catalog	gene: Zm00001d045146	novel	1.74	62.82	coding	206	36	653
PB.10189.2	9	1358	12	full-splice_match	gene: Zm00001d045146	Zm00001d045146_T00	57.69	62.82	coding	353	119	1177
PB.8459.1	7	1669	12	incomplete-splice_match	gene: Zm00001d020036	Zm00001d020036_T00	32.95	32.95	coding	213	818	1456
PB.7288.1	5	2122	1	full-splice_match	gene: Zm00001d018567	Zm00001d018567_T00	12.46	45.31	coding	405	48	1262
PB.10917.2	9	1327	5	novel_not_in_catalog	gene: Zm00001d048515	novel	0.21	5.90	coding	271	388	1200
PB.6699.1	5	1557	1	full-splice_match	gene: Zm00001d016132	Zm00001d016132_T00	35.25	47.19	coding	309	379	1305
PB.600.1	1	958	2	incomplete-splice_match	gene: Zm00001d029946	Zm00001d029946_T00	12.45	12.45	coding	263	110	898
PB.4310.1	3	761	3	novel_in_catalog	gene: Zm00001d042642	novel	9.38	10.62	non coding	NA	NA	NA

PB.6699.2	5	1504	1	full-splice_match	gene: Zm00001d016132	Zm00001d016132_T00	11.94	47.19	coding	309	422	1348
PB.5322.3	4	333	4	incomplete-splice_match	gene: Zm00001d051405	Zm00001d051405_T00	0.00	119.73	coding	101	30	332
PB.8363.2	7	1983	10	novel_in_catalog	gene: Zm00001d019349	novel	6.03	6.03	coding	582	237	1982
PB.5322.1	4	859	5	full-splice_match	gene: Zm00001d051405	Zm00001d051405_T00	119.73	119.73	coding	174	81	602
PB.5775.3	4	2635	5	full-splice_match	gene: Zm00001d053427	Zm00001d053427_T01	9.91	25.80	coding	665	293	2287
PB.5775.2	4	2148	5	full-splice_match	gene: Zm00001d053427	Zm00001d053427_T00	13.05	25.80	coding	467	272	1672
PB.5775.1	4	876	1	genic	gene: Zm00001d053427	novel	2.84	25.80	non coding	NA	NA	NA
PB.1481.2	1	2468	12	full-splice_match	gene: Zm00001d034191	Zm00001d034191_T00	17.23	27.52	coding	203	1706	2314
PB.998.2	1	969	1	full-splice_match	gene: Zm00001d032102	Zm00001d032102_T00	33.49	59.33	coding	196	131	718
PB.5970.1	5	2567	6	full-splice_match	gene: Zm00001d012855	Zm00001d012855_T00	8.13	8.13	coding	725	134	2308
PB.10353.1	9	2044	7	novel_not_in_catalog	gene: Zm00001d045887	novel	24.51	24.51	coding	369	164	1270
PB.7288.2	5	1462	2	genic	gene: Zm00001d018567	novel	11.83	45.31	coding	405	24	1238
PB.996.2	1	2640	25	full-splice_match	gene: Zm00001d032099	Zm00001d032099_T00	0.00	64.30	coding	782	116	2461
PB.7213.1	5	2073	11	full-splice_match	gene: Zm00001d018355	Zm00001d018355_T00	10.46	10.46	coding	371	255	1367
PB.9091.2	7	1508	1	full-splice_match	gene: Zm00001d022557	Zm00001d022557_T00	47.69	54.81	coding	363	67	1155
PB.9091.3	7	1383	1	full-splice_match	gene: Zm00001d022557	Zm00001d022557_T00	0.00	54.81	coding	363	56	1144
PB.9091.1	7	1603	1	full-splice_match	gene: Zm00001d022557	Zm00001d022557_T00	7.11	54.81	coding	363	56	1144

PB.1355.1	1	1205	7	incomplete-splice_match	gene: Zm00001d033635	Zm00001d033635_T00	17.63	17.63	coding	183	302	850
PB.4328.1	3	339	1	incomplete-splice_match	gene: Zm00001d042723	Zm00001d042723_T00	2069.65	2069.65	non coding	NA	NA	NA
PB.5950.1	5	2569	5	full-splice_match	gene: Zm00001d012794	Zm00001d012794_T00	11.09	11.09	coding	108	1499	1822
PB.5769.1	4	619	1	antisense	novelGene_gene: Zm00001d053406	novel	15.10	15.10	non coding	NA	NA	NA
PB.1112.1	1	1724	5	incomplete-splice_match	gene: Zm00001d032567	Zm00001d032567_T00	40.41	79.93	coding	423	1	1269
PB.5297.2	4	1079	5	full-splice_match	gene: Zm00001d051234	Zm00001d051234_T00	1.21	9.78	coding	319	61	1017
PB.5297.1	4	1507	5	full-splice_match	gene: Zm00001d051234	Zm00001d051234_T00	8.57	9.78	coding	319	57	1013
PB.10480.1	9	1273	3	full-splice_match	gene: Zm00001d046718	Zm00001d046718_T00	144.08	144.08	coding	300	189	1088
PB.4318.2	3	1140	2	full-splice_match	gene: Zm00001d042665	Zm00001d042665_T00	32.75	141.28	coding	262	88	873
PB.4318.1	3	1461	1	genic	gene: Zm00001d042665	novel	3.37	141.28	coding	180	470	1009
PB.817.1	1	1587	1	genic	gene: Zm00001d031201	novel	26.87	26.87	coding	108	388	711
PB.1786.1	10	3169	23	incomplete-splice_match	gene: Zm00001d023640	Zm00001d023640_T01	25.29	128.70	coding	388	1505	2668
PB.9548.1	8	1051	7	full-splice_match	gene: Zm00001d010424	Zm00001d010424_T00	9.28	9.28	coding	273	73	891
PB.1786.3	10	2451	21	incomplete-splice_match	gene: Zm00001d023640	Zm00001d023640_T01	64.12	128.70	coding	601	188	1990
PB.1786.2	10	1224	12	novel_in_catalog	gene: Zm00001d023640	novel	12.74	128.70	coding	243	276	1004
PB.1716.2	10	2482	7	novel_not_in_catalog	gene: Zm00001d023342	novel	8.41	20.74	coding	182	216	761
PB.1786.4	10	877	6	incomplete-splice_match	gene: Zm00001d023640	Zm00001d023640_T02	26.55	128.70	coding	114	142	483

PB.3049.1	2	1498	8	full-splice_match	gene: Zm00001d004896	Zm00001d004896_T00	35.22	35.22	coding	366	92	1189
PB.1716.1	10	1568	9	novel_not_in_catalog	gene: Zm00001d023342	novel	2.46	20.74	coding	182	222	767
PB.2748.2	2	1097	2	full-splice_match	gene: Zm00001d003175	Zm00001d003175_T00	16.00	18.23	coding	257	167	937
PB.1112.4	1	2657	8	full-splice_match	gene: Zm00001d032567	Zm00001d032567_T01	4.25	79.93	coding	704	38	2149
PB.833.2	1	1192	1	genic	gene: Zm00001d031258	novel	45.38	157.24	non coding	NA	NA	NA
PB.2748.1	2	1038	1	novel_in_catalog	gene: Zm00001d003175	novel	2.23	18.23	coding	239	162	878
PB.833.4	1	1016	1	novel_in_catalog	gene: Zm00001d031258	novel	0.00	157.24	non coding	NA	NA	NA
PB.8124.2	6	2213	15	full-splice_match	gene: Zm00001d038892	Zm00001d038892_T00	21.86	81.42	coding	563	246	1934
PB.833.6	1	930	1	novel_in_catalog	gene: Zm00001d031258	novel	0.00	157.24	non coding	NA	NA	NA
PB.9767.3	8	501	3	novel_not_in_catalog	gene: Zm00001d011384	novel	67.05	172.95	non coding	NA	NA	NA
PB.4983.1	4	800	2	intergenic	novelGene_93	novel	38.53	38.53	non coding	NA	NA	NA
PB.2538.7	2	2804	1	novel_in_catalog	gene: Zm00001d002258	novel	0.00	346.26	coding	211	63	695
PB.2538.6	2	1393	3	full-splice_match	gene: Zm00001d002258	Zm00001d002258_T00	243.37	346.26	coding	409	65	1291
PB.2538.3	2	1562	2	novel_in_catalog	gene: Zm00001d002258	novel	97.73	346.26	coding	432	63	1358
PB.2538.2	2	2870	1	novel_in_catalog	gene: Zm00001d002258	novel	5.16	346.26	coding	211	42	674
PB.4077.1	3	1716	2	intergenic	novelGene_238	novel	22.83	22.83	non coding	NA	NA	NA
PB.10930.1	9	1079	8	full-splice_match	gene: Zm00001d048550	Zm00001d048550_T00	36.18	59.69	coding	303	15	923

PB.9996.1	8	1380	1	antisense	novelGene_gene: Zm00001d012337	novel	61.75	61.75	coding	116	34	381
PB.9624.2	8	2886	12	novel_in_catalog	gene: Zm00001d010720	novel	103.47	103.47	coding	342	253	1278
PB.10930.2	9	1101	7	novel_in_catalog	gene: Zm00001d048550	novel	19.39	59.69	coding	112	663	998
PB.8171.1	6	1655	8	novel_not_in_catalog	gene: Zm00001d039059	novel	6.84	8.56	coding	430	36	1325
PB.3825.1	3	2123	11	full-splice_match	gene: Zm00001d039871	Zm00001d039871_T00	68.69	177.28	coding	510	128	1657
PB.3825.3	3	512	5	incomplete-splice_match	gene: Zm00001d039871	Zm00001d039871_T00	57.78	177.28	coding	121	149	511
PB.3825.2	3	1084	8	incomplete-splice_match	gene: Zm00001d039871	Zm00001d039871_T00	50.81	177.28	coding	196	115	702
PB.5958.2	5	2202	13	full-splice_match	gene: Zm00001d012817	Zm00001d012817_T00	45.89	103.47	coding	496	500	1987
PB.10950.2	B73V 4	1480	3	genic	gene: Zm00001d000307	novel	17.64	34.34	coding	16	277	324
PB.10950.1	B73V 4	1781	2	genic	gene: Zm00001d000307	novel	10.41	34.34	coding	319	339	1295
PB.5958.1	5	1689	12	full-splice_match	gene: Zm00001d012817	Zm00001d012817_T00	42.38	103.47	coding	445	22	1356
PB.5958.6	5	1583	11	novel_in_catalog	gene: Zm00001d012817	novel	0.33	103.47	coding	132	1002	1397
PB.5761.1	4	1127	3	full-splice_match	gene: Zm00001d053371	Zm00001d053371_T00	88.35	249.18	coding	188	248	811
PB.5958.4	5	2637	12	full-splice_match	gene: Zm00001d012817	Zm00001d012817_T00	10.28	103.47	coding	445	1110	2444
PB.5958.5	5	1377	11	incomplete-splice_match	gene: Zm00001d012817	Zm00001d012817_T00	4.59	103.47	coding	132	792	1187
PB.51.2	1	584	4	full-splice_match	gene: Zm00001d027447	Zm00001d027447_T00	62.52	81.18	coding	105	85	399
PB.51.1	1	743	4	full-splice_match	gene: Zm00001d027447	Zm00001d027447_T00	15.78	81.18	coding	105	41	355

PB.10641.1	9	1329	14	full-splice_match	gene: Zm00001d047498	Zm00001d047498_T00	0.00	128.79	coding	285	85	939
PB.3964.1	3	1911	3	novel_not_in_catalog	gene: Zm00001d040527	novel	23.60	38.80	coding	427	186	1466
PB.10465.3	9	1006	2	genic	gene: Zm00001d046596	novel	0.00	83.21	coding	198	118	711
PB.3964.3	3	1130	1	genic	gene: Zm00001d040527	novel	0.00	38.80	coding	197	358	948
PB.51.4	1	1747	1	incomplete-splice_match	gene: Zm00001d027447	Zm00001d027447_T01	2.88	81.18	non coding	NA	NA	NA
PB.3420.1	2	1293	2	genic	gene: Zm00001d006659	novel	22.96	22.96	coding	351	223	1275
PB.7390.1	6	2065	3	novel_not_in_catalog	gene: Zm00001d035479	novel	7.60	7.60	coding	614	117	1958
PB.5879.2	4	2416	5	full-splice_match	gene: Zm00001d053890	Zm00001d053890_T00	4.90	7.14	coding	482	627	2072
PB.3737.1	3	1694	1	full-splice_match	gene: Zm00001d039454	Zm00001d039454_T00	21.48	21.48	coding	468	112	1515
PB.615.1	1	978	1	full-splice_match	gene: Zm00001d030038	Zm00001d030038_T00	8.37	8.37	coding	232	7	702
PB.9968.3	8	1134	5	full-splice_match	gene: Zm00001d012239	Zm00001d012239_T00	4.45	27.09	coding	114	88	429
PB.9968.2	8	1027	1	incomplete-splice_match	gene: Zm00001d012239	Zm00001d012239_T00	22.64	27.09	coding	239	225	941
PB.4429.3	3	1785	5	novel_in_catalog	gene: Zm00001d043149	novel	18.97	368.18	coding	194	150	731
PB.4937.1	4	1415	1	full-splice_match	gene: Zm00001d049096	Zm00001d049096_T00	6.73	6.73	coding	346	117	1154
PB.9968.4	8	839	5	full-splice_match	gene: Zm00001d012239	Zm00001d012239_T00	0.00	27.09	coding	114	106	447
PB.10840.1	9	1312	6	novel_in_catalog	gene: Zm00001d048272	novel	20.44	104.28	coding	131	224	616
PB.9633.2	8	1654	2	intergenic	novelGene_243	novel	27.99	27.99	coding	169	546	1052

PB.3713.3	3	1903	8	novel_in_catalog	gene: Zm00001d039369	novel	6.51	30.43	coding	364	92	1183
PB.1617.1	1	1952	7	full-splice_match	gene: Zm00001d034727	Zm00001d034727_T00	32.66	40.18	coding	477	309	1739
PB.6031.1	5	1117	6	novel_not_in_catalog	gene: Zm00001d013055	novel	192.78	192.78	coding	220	148	807
PB.5896.3	4	1418	1	intergenic	novelGene_446	novel	15.29	108.08	coding	43	285	413
PB.7216.2	5	1137	4	full-splice_match	gene: Zm00001d018364	Zm00001d018364_T00	23.06	31.42	coding	211	225	857
PB.7878.3	6	2110	2	novel_in_catalog	gene: Zm00001d038023	novel	34.89	97.71	coding	332	110	1105
PB.7878.2	6	2058	2	novel_in_catalog	gene: Zm00001d038023	novel	23.30	97.71	coding	254	901	1662
PB.8794.1	7	1545	5	full-splice_match	gene: Zm00001d021574	Zm00001d021574_T00	77.59	105.83	coding	302	124	1029
PB.2023.1	10	1325	3	novel_in_catalog	gene: Zm00001d025010	novel	12.17	62.83	non coding	NA	NA	NA
PB.7431.2	6	3930	8	full-splice_match	gene: Zm00001d035695	Zm00001d035695_T00	7.34	24.76	coding	453	2083	3441
PB.918.1	1	1429	2	full-splice_match	gene: Zm00001d031737	Zm00001d031737_T00	9.26	9.26	coding	290	299	1168
PB.2413.1	10	826	3	full-splice_match	gene: Zm00001d026704	Zm00001d026704_T00	101.23	101.23	coding	166	55	552
PB.7431.1	6	2597	9	novel_in_catalog	gene: Zm00001d035695	novel	17.42	24.76	coding	646	181	2118
PB.5207.1	4	657	1	full-splice_match	gene: Zm00001d050553	Zm00001d050553_T00	11.87	11.87	coding	159	61	537
PB.8794.8	7	1107	5	full-splice_match	gene: Zm00001d021574	Zm00001d021574_T00	11.26	105.83	coding	302	188	1093
PB.5669.1	4	2084	2	full-splice_match	gene: Zm00001d052889	Zm00001d052889_T00	11.12	11.12	coding	602	157	1962
PB.10496.1	9	2141	10	novel_in_catalog	gene: Zm00001d046828	novel	0.61	114.57	coding	193	132	710

PB.1423.8	1	2843	10	novel_not_in_catalog	gene: Zm00001d033948	novel	5.89	76.35	coding	115	906	1250
PB.10197.1	9	2126	10	full-splice_match	gene: Zm00001d045176	Zm00001d045176_T00	31.20	73.53	coding	453	264	1622
PB.10197.2	9	2095	9	novel_in_catalog	gene: Zm00001d045176	novel	3.15	73.53	coding	319	253	1209
PB.10197.3	9	2015	10	full-splice_match	gene: Zm00001d045176	Zm00001d045176_T00	39.19	73.53	coding	453	329	1687
PB.5863.5	4	1729	10	novel_in_catalog	gene: Zm00001d053825	novel	0.00	114.09	coding	200	838	1437
PB.9912.3	8	2492	7	novel_not_in_catalog	gene: Zm00001d011990	novel	16.64	25.63	coding	130	317	706
PB.8045.1	6	1044	1	full-splice_match	gene: Zm00001d038585	Zm00001d038585_T00	163.94	163.94	coding	211	177	809
PB.1578.1	1	1261	1	full-splice_match	gene: Zm00001d034605	Zm00001d034605_T00	9.80	60.25	coding	238	164	877
PB.7858.1	6	3092	21	incomplete-splice_match	gene: Zm00001d037950	Zm00001d037950_T01	24.15	44.37	coding	788	337	2700
PB.4732.1	3	1253	1	full-splice_match	gene: Zm00001d044270	Zm00001d044270_T00	27.24	27.24	coding	320	141	1100
PB.7858.2	6	1749	13	incomplete-splice_match	gene: Zm00001d037950	Zm00001d037950_T01	20.22	44.37	coding	373	348	1466
PB.920.2	1	2162	6	novel_in_catalog	gene: Zm00001d031749	novel	6.52	52.73	coding	521	229	1791
PB.2023.2	10	1793	7	incomplete-splice_match	gene: Zm00001d025010	Zm00001d025010_T02	50.66	62.83	coding	417	93	1343
PB.880.1	1	1466	4	full-splice_match	gene: Zm00001d031536	Zm00001d031536_T00	19.64	23.92	coding	394	184	1365
PB.6948.1	5	2532	2	full-splice_match	gene: Zm00001d017274	Zm00001d017274_T00	2285.81	2308.60	coding	703	109	2217
PB.7357.1	6	709	4	full-splice_match	gene: Zm00001d035309	Zm00001d035309_T00	37.88	64.19	non coding	NA	NA	NA
PB.1263.3	1	1879	10	full-splice_match	gene: Zm00001d033228	Zm00001d033228_T00	37.71	42.12	coding	472	180	1595

PB.7357.2	6	679	4	full-splice_match	gene: Zm00001d035309	Zm00001d035309_T00	26.31	64.19	non coding	NA	NA	NA
PB.7065.2	5	1784	9	full-splice_match	gene: Zm00001d017802	Zm00001d017802_T00	54.29	57.19	coding	417	86	1336
PB.2049.1	10	1651	2	genic	gene: Zm00001d025107	novel	44.60	45.08	coding	375	284	1408
PB.79.1	1	1821	8	full-splice_match	gene: Zm00001d027544	Zm00001d027544_T00	5.59	5.59	coding	431	69	1361
PB.8471.1	7	1833	2	incomplete-splice_match	gene: Zm00001d020088	Zm00001d020088_T01	27.71	27.71	non coding	NA	NA	NA
PB.5504.1	4	1197	1	full-splice_match	gene: Zm00001d052205	Zm00001d052205_T00	121.00	121.00	coding	315	109	1053
PB.7794.2	6	1761	4	intergenic	novelGene_258	novel	0.00	2.89	non coding	NA	NA	NA
PB.2049.2	10	1492	2	genic	gene: Zm00001d025107	novel	0.49	45.08	coding	375	292	1416
PB.5863.4	4	1735	11	full-splice_match	gene: Zm00001d053825	Zm00001d053825_T00	60.71	114.09	coding	402	179	1384
PB.747.1	1	1485	4	novel_not_in_catalog	gene: Zm00001d030891	novel	34.97	622.65	coding	154	183	644
PB.3408.1	2	1008	4	incomplete-splice_match	gene: Zm00001d006614	Zm00001d006614_T00	27.33	27.33	coding	265	56	850
PB.6685.2	5	2397	1	novel_in_catalog	gene: Zm00001d016059	novel	0.94	124.68	coding	75	123	347
PB.4314.1	3	947	2	intergenic	novelGene_358	novel	9.21	9.21	coding	91	96	368
PB.6685.1	5	627	2	full-splice_match	gene: Zm00001d016059	Zm00001d016059_T00	123.74	124.68	non coding	NA	NA	NA
PB.5481.1	4	2789	1	full-splice_match	gene: Zm00001d052104	Zm00001d052104_T00	92.72	165.96	coding	780	79	2418
PB.1208.7	1	926	5	intergenic	novelGene_448	novel	9.03	81.86	coding	103	186	494
PB.5481.2	4	2730	1	full-splice_match	gene: Zm00001d052104	Zm00001d052104_T00	28.43	165.96	coding	780	242	2581

PB.3678.1	3	1727	2	incomplete-splice_match	gene: Zm00001d039266	Zm00001d039266_T00	9.08	40.68	coding	464	82	1473
PB.6948.6	5	2475	2	full-splice_match	gene: Zm00001d017274	Zm00001d017274_T00	0.00	2308.60	coding	703	109	2217
PB.3678.2	3	3073	6	novel_in_catalog	gene: Zm00001d039266	novel	7.10	40.68	coding	635	156	2060
PB.4420.1	3	1308	2	genic	gene: Zm00001d043128	novel	6.95	8.31	coding	287	232	1092
PB.4420.2	3	1479	1	full-splice_match	gene: Zm00001d043128	Zm00001d043128_T00	1.37	8.31	coding	296	436	1323
PB.10637.1	9	1196	5	incomplete-splice_match	gene: Zm00001d047482	Zm00001d047482_T00	14.72	14.72	coding	249	76	822
PB.10006.1	8	1450	3	incomplete-splice_match	gene: Zm00001d012408	Zm00001d012408_T00	33.80	33.80	coding	357	118	1188
PB.7178.8	5	3016	3	novel_in_catalog	gene: Zm00001d018206	novel	0.00	677.05	coding	484	79	1530
PB.7886.4	6	2439	1	novel_in_catalog	gene: Zm00001d038049	novel	7.67	115.55	coding	300	1224	2123
PB.7886.5	6	1489	2	full-splice_match	gene: Zm00001d038049	Zm00001d038049_T00	0.00	115.55	coding	336	222	1229
PB.7886.6	6	991	1	novel_in_catalog	gene: Zm00001d038049	novel	6.82	115.55	non coding	NA	NA	NA
PB.7178.5	5	3246	3	novel_in_catalog	gene: Zm00001d018206	novel	23.81	677.05	coding	457	1631	3001
PB.7178.2	5	3101	4	novel_in_catalog	gene: Zm00001d018206	novel	545.16	677.05	coding	890	188	2857
PB.7886.1	6	1737	2	full-splice_match	gene: Zm00001d038049	Zm00001d038049_T00	101.06	115.55	coding	336	222	1229
PB.7886.2	6	2215	1	novel_in_catalog	gene: Zm00001d038049	novel	0.00	115.55	coding	300	843	1742
PB.7886.3	6	1593	2	full-splice_match	gene: Zm00001d038049	Zm00001d038049_T00	0.00	115.55	coding	336	225	1232
PB.9772.2	8	1894	5	full-splice_match	gene: Zm00001d011418	Zm00001d011418_T00	65.74	75.59	coding	528	53	1636

PB.10187.2	9	1083	5	novel_in_catalog	gene: Zm00001d045133	novel	2.15	84.30	coding	113	449	787
PB.10187.1	9	1060	6	full-splice_match	gene: Zm00001d045133	Zm00001d045133_T00	82.15	84.30	coding	153	307	765
PB.4440.4	3	2622	1	novel_in_catalog	gene: Zm00001d043195	novel	0.00	201.86	coding	186	1938	2495
PB.4440.3	3	1654	2	full-splice_match	gene: Zm00001d043195	Zm00001d043195_T00	145.65	201.86	coding	421	228	1490
PB.4440.2	3	2808	1	novel_in_catalog	gene: Zm00001d043195	novel	19.23	201.86	coding	186	2037	2594
PB.4440.1	3	1836	2	full-splice_match	gene: Zm00001d043195	Zm00001d043195_T00	36.99	201.86	coding	421	368	1630
PB.3335.1	2	1461	6	novel_not_in_catalog	gene: Zm00001d006315	novel	3.35	3.35	coding	318	136	1089
PB.217.1	1	2156	9	full-splice_match	gene: Zm00001d028194	Zm00001d028194_T00	81.98	101.09	coding	530	182	1771
PB.217.3	1	1407	9	full-splice_match	gene: Zm00001d028194	Zm00001d028194_T00	19.11	101.09	coding	366	42	1139
PB.6952.1	5	980	1	full-splice_match	gene: Zm00001d017287	Zm00001d017287_T00	1.10	1.10	coding	170	177	686
PB.1272.2	1	1184	2	intergenic	novelGene_452	novel	2.71	9.78	coding	123	17	385
PB.1323.2	1	895	3	full-splice_match	gene: Zm00001d033504	Zm00001d033504_T00	5.98	20.50	coding	212	33	668
PB.5863.6	4	1657	11	full-splice_match	gene: Zm00001d053825	Zm00001d053825_T00	38.22	114.09	coding	402	212	1417
PB.1323.1	1	1946	3	full-splice_match	gene: Zm00001d033504	Zm00001d033504_T00	14.52	20.50	coding	497	145	1635
PB.3122.1	2	671	1	intergenic	novelGene_338	novel	7.87	7.87	non coding	NA	NA	NA
PB.7862.1	6	1358	1	full-splice_match	gene: Zm00001d037969	Zm00001d037969_T00	22.98	22.98	coding	120	633	992
PB.5925.1	4	1344	10	novel_in_catalog	gene: Zm00001d054017	novel	17.16	181.85	coding	293	128	1006

PB.516.1	1	932	1	genic	gene: Zm00001d029495	novel	8.41	68.39	coding	157	51	521
PB.9772.3	8	1710	3	novel_not_in_catalog	gene: Zm00001d011418	novel	1.26	75.59	coding	354	526	1587
PB.2391.4	10	1079	6	full-splice_match	gene: Zm00001d026634	Zm00001d026634_T00	14.97	39.13	coding	217	126	776
PB.7519.1	6	1253	1	full-splice_match	gene: Zm00001d036306	Zm00001d036306_T00	20.72	20.72	coding	357	79	1149
PB.10867.2	9	3468	20	full-splice_match	gene: Zm00001d048358	Zm00001d048358_T00	49.87	122.58	coding	989	253	3219
PB.8443.1	7	2354	8	novel_not_in_catalog	gene: Zm00001d019949	novel	59.82	59.82	coding	604	201	2012
PB.2391.2	10	2047	5	novel_in_catalog	gene: Zm00001d026634	novel	2.95	39.13	coding	114	1403	1744
PB.10489.14	9	883	2	novel_in_catalog	gene: Zm00001d046767	novel	0.00	778.69	coding	174	200	721
PB.354.2	1	2292	3	full-splice_match	gene: Zm00001d028755	Zm00001d028755_T00	11.35	305.74	coding	163	83	571
PB.354.1	1	1153	4	full-splice_match	gene: Zm00001d028755	Zm00001d028755_T00	294.39	305.74	coding	209	186	812
PB.2854.2	2	797	5	full-splice_match	gene: Zm00001d003672	Zm00001d003672_T00	494.51	538.09	coding	181	86	628
PB.2502.3	2	1628	2	incomplete-splice_match	gene: Zm00001d002080	Zm00001d002080_T00	0.00	94.99	coding	520	69	1628
PB.354.4	1	1003	4	full-splice_match	gene: Zm00001d028755	Zm00001d028755_T00	0.00	305.74	coding	209	135	761
PB.2502.1	2	2862	7	full-splice_match	gene: Zm00001d002080	Zm00001d002080_T00	94.99	94.99	coding	663	588	2576
PB.5104.1	4	1889	5	full-splice_match	gene: Zm00001d049905	Zm00001d049905_T00	12.44	12.44	coding	471	299	1711
PB.3649.1	2	475	1	full-splice_match	gene: Zm00001d007872	Zm00001d007872_T00	169.88	169.88	coding	76	73	300
PB.8042.1	6	1844	6	full-splice_match	gene: Zm00001d038579	Zm00001d038579_T00	9.34	9.34	coding	480	262	1701

PB.9279.3	8	1616	10	full-splice_match	gene: Zm00001d008907	Zm00001d008907_T00	7.15	93.40	coding	302	424	1329
PB.5495.1	4	1395	1	full-splice_match	gene: Zm00001d052182	Zm00001d052182_T00	7.34	7.34	coding	246	345	1082
PB.4448.1	3	720	3	novel_not_in_catalog	gene: Zm00001d043223	novel	303.26	303.26	coding	108	184	507
PB.1384.1	1	3606	2	incomplete-splice_match	gene: Zm00001d033775	Zm00001d033775_T00	20.36	47.67	coding	1134	32	3433
PB.4851.1	4	3147	9	novel_in_catalog	gene: Zm00001d048655	novel	4.09	113.35	coding	364	1792	2883
PB.3317.1	2	1362	9	full-splice_match	gene: Zm00001d006221	Zm00001d006221_T00	53.02	53.02	coding	298	68	961
PB.4851.3	4	1937	11	full-splice_match	gene: Zm00001d048655	Zm00001d048655_T01	93.95	113.35	coding	539	59	1675
PB.4851.2	4	2226	11	full-splice_match	gene: Zm00001d048655	Zm00001d048655_T00	15.31	113.35	coding	367	861	1961
PB.4851.5	4	1557	10	incomplete-splice_match	gene: Zm00001d048655	Zm00001d048655_T01	0.00	113.35	coding	391	276	1448
PB.6859.1	5	2353	7	novel_in_catalog	gene: Zm00001d016902	novel	21.88	84.05	coding	387	792	1952
PB.5325.1	4	1567	5	full-splice_match	gene: Zm00001d051419	Zm00001d051419_T00	36.20	36.20	coding	371	146	1258
PB.9809.1	8	1064	3	full-splice_match	gene: Zm00001d011581	Zm00001d011581_T00	52.04	1267.35	coding	162	85	570
PB.8111.1	6	2052	16	full-splice_match	gene: Zm00001d038857	Zm00001d038857_T00	28.07	28.07	coding	580	36	1775
PB.9809.3	8	706	3	full-splice_match	gene: Zm00001d011581	Zm00001d011581_T00	0.00	1267.35	coding	162	85	570
PB.9809.2	8	804	3	full-splice_match	gene: Zm00001d011581	Zm00001d011581_T00	1215.31	1267.35	coding	162	85	570
PB.1511.8	1	1957	5	novel_in_catalog	gene: Zm00001d034345	novel	0.00	1673.99	coding	317	702	1652
PB.5325.2	4	1037	4	full-splice_match	gene: Zm00001d051419	Zm00001d051419_T00	0.00	36.20	coding	108	515	838

PB.467.1	1	1424	1	antisense	novelGene_gene: Zm00001d029247	novel	2.96	2.96	coding	262	26	811
PB.6991.3	5	1173	7	novel_in_catalog	gene: Zm00001d017470	novel	17.98	59.18	coding	50	391	540
PB.5648.2	4	1496	1	full-splice_match	gene: Zm00001d052795	Zm00001d052795_T00	6.40	52.22	coding	368	393	1496
PB.5648.1	4	1560	2	genic	gene: Zm00001d052795	novel	45.82	52.22	coding	376	191	1318
PB.1535.1	1	1166	4	full-splice_match	gene: Zm00001d034432	Zm00001d034432_T00	129.37	196.53	coding	243	139	867
PB.1014.1	1	2223	4	novel_not_in_catalog	gene: Zm00001d032190	novel	15.84	64.90	coding	336	217	1224
PB.10459.3	9	1972	15	incomplete-splice_match	gene: Zm00001d046581	Zm00001d046581_T01	5.42	33.96	coding	281	974	1816
PB.10459.1	9	2420	19	incomplete-splice_match	gene: Zm00001d046581	Zm00001d046581_T00	28.55	33.96	coding	668	26	2029
PB.6252.1	5	2228	1	intergenic	novelGene_105	novel	0.91	0.91	non coding	NA	NA	NA
PB.2681.1	2	1293	2	full-splice_match	gene: Zm00001d002859	Zm00001d002859_T00	45.77	45.77	coding	325	169	1143
PB.8536.1	7	2962	14	novel_in_catalog	gene: Zm00001d020431	novel	5.22	5.67	coding	359	91	1167
PB.6586.4	5	6160	2	novel_in_catalog	gene: Zm00001d015328	novel	17.12	99.81	coding	161	5494	5976
PB.6586.5	5	1769	4	full-splice_match	gene: Zm00001d015328	Zm00001d015328_T01	38.54	99.81	coding	357	420	1490
PB.986.1	1	2767	6	full-splice_match	gene: Zm00001d032060	Zm00001d032060_T00	8.78	19.11	coding	713	355	2493
PB.6586.1	5	4785	3	incomplete-splice_match	gene: Zm00001d015328	Zm00001d015328_T00	20.22	99.81	coding	357	3445	4515
PB.183.1	1	1571	5	full-splice_match	gene: Zm00001d028018	Zm00001d028018_T00	14.77	14.77	coding	416	77	1324
PB.2384.1	10	799	4	full-splice_match	gene: Zm00001d026609	Zm00001d026609_T00	45.36	45.36	coding	143	130	558

PB.922.2	1	3213	3	novel_in_catalog	gene: Zm00001d031769	novel	14.67	222.47	coding	469	117	1523
PB.6586.8	5	1805	5	novel_not_in_catalog	gene: Zm00001d015328	novel	3.67	99.81	coding	357	490	1560
PB.6586.9	5	2928	3	incomplete-splice_match	gene: Zm00001d015328	Zm00001d015328_T00	18.89	99.81	coding	357	1670	2740
PB.4398.7	3	956	3	full-splice_match	gene: Zm00001d043047	Zm00001d043047_T00	12.40	141.58	coding	253	73	831
PB.5332.1	4	905	3	full-splice_match	gene: Zm00001d051458	Zm00001d051458_T00	20.65	48.80	coding	226	75	752
PB.8467.4	7	2011	7	novel_in_catalog	gene: Zm00001d020063	novel	4.32	23.77	coding	145	66	500
PB.8467.2	7	1612	8	full-splice_match	gene: Zm00001d020063	Zm00001d020063_T00	5.12	23.77	coding	264	64	855
PB.8467.3	7	1611	8	full-splice_match	gene: Zm00001d020063	Zm00001d020063_T00	6.41	23.77	coding	468	88	1491
PB.4398.1	3	1271	3	full-splice_match	gene: Zm00001d043047	Zm00001d043047_T00	107.20	141.58	coding	253	73	831
PB.8467.1	7	2005	8	full-splice_match	gene: Zm00001d020063	Zm00001d020063_T00	7.92	23.77	coding	468	40	1443
PB.7172.14	5	1461	6	full-splice_match	gene: Zm00001d018187	Zm00001d018187_T00	22.85	145.24	coding	366	118	1215
PB.4823.1	3	976	4	incomplete-splice_match	gene: Zm00001d044653	Zm00001d044653_T00	13.29	119.30	coding	187	101	661
PB.10877.3	9	1485	1	full-splice_match	gene: Zm00001d048398	Zm00001d048398_T00	0.00	19.76	coding	429	118	1404
PB.10877.2	9	1607	1	full-splice_match	gene: Zm00001d048398	Zm00001d048398_T00	0.00	19.76	coding	429	118	1404
PB.10877.1	9	1717	1	full-splice_match	gene: Zm00001d048398	Zm00001d048398_T00	19.76	19.76	coding	429	118	1404
PB.4398.8	3	916	2	novel_in_catalog	gene: Zm00001d043047	novel	0.00	141.58	coding	153	73	531
PB.7184.3	5	548	4	incomplete-splice_match	gene: Zm00001d018230	Zm00001d018230_T00	0.00	88.41	non coding	NA	NA	NA

PB.8812.4	7	1610	5	full-splice_match	gene: Zm00001d021653	Zm00001d021653_T00	0.00	69.38	coding	391	156	1328
PB.8812.5	7	2260	3	novel_in_catalog	gene: Zm00001d021653	novel	0.00	69.38	coding	244	156	887
PB.1948.1	10	1321	2	novel_in_catalog	gene: Zm00001d024700	novel	9.59	15.37	coding	205	50	664
PB.8812.3	7	1981	4	full-splice_match	gene: Zm00001d021653	Zm00001d021653_T00	9.63	69.38	coding	317	156	1106
PB.7415.5	6	1740	2	novel_not_in_catalog	gene: Zm00001d035605	novel	0.00	1856.08	non coding	NA	NA	NA
PB.6964.2	5	2640	5	full-splice_match	gene: Zm00001d017352	Zm00001d017352_T00	8.79	72.75	coding	193	1904	2482
PB.5860.3	4	1033	1	novel_in_catalog	gene: Zm00001d053819	novel	0.36	11.39	non coding	NA	NA	NA
PB.9832.2	8	2483	15	novel_in_catalog	gene: Zm00001d011685	novel	6.04	28.93	coding	447	827	2167
PB.5974.4	5	3504	12	full-splice_match	gene: Zm00001d012863	Zm00001d012863_T00	0.78	40.26	coding	654	98	2059
PB.7598.1	6	868	1	intergenic	novelGene_208	novel	3.29	3.29	coding	19	588	644
PB.10853.1	9	501	2	incomplete-splice_match	gene: Zm00001d048317	Zm00001d048317_T00	404.37	404.37	non coding	NA	NA	NA
PB.2746.1	2	1209	2	full-splice_match	gene: Zm00001d003162	Zm00001d003162_T00	11.34	20.90	coding	320	35	994
PB.2746.2	2	1306	1	novel_in_catalog	gene: Zm00001d003162	novel	0.96	20.90	coding	209	6	632
PB.2746.3	2	1147	2	full-splice_match	gene: Zm00001d003162	Zm00001d003162_T00	8.59	20.90	coding	320	67	1026
PB.4845.2	4	1257	1	full-splice_match	gene: Zm00001d048625	Zm00001d048625_T00	23.17	60.51	coding	354	46	1107
PB.4845.1	4	1361	1	full-splice_match	gene: Zm00001d048625	Zm00001d048625_T00	37.34	60.51	coding	354	48	1109
PB.2175.1	10	1935	1	genic	gene: Zm00001d025823	novel	8.02	8.02	coding	68	196	399

PB.1597.1	1	1295	3	full-splice_match	gene: Zm00001d034663	Zm00001d034663_T00	23.56	23.56	coding	268	153	956
PB.3851.1	3	1005	1	full-splice_match	gene: Zm00001d039991	Zm00001d039991_T00	44.00	187.43	coding	185	185	739
PB.3851.2	3	894	1	full-splice_match	gene: Zm00001d039991	Zm00001d039991_T00	143.43	187.43	coding	185	189	743
PB.9319.1	8	502	1	full-splice_match	gene: Zm00001d009160	Zm00001d009160_T00	5.81	18.72	non coding	NA	NA	NA
PB.2908.1	2	2905	6	novel_in_catalog	gene: Zm00001d003939	novel	11.06	20.54	coding	252	1996	2751
PB.2908.2	2	2279	7	full-splice_match	gene: Zm00001d003939	Zm00001d003939_T01	9.48	20.54	coding	679	62	2098
PB.9319.2	8	1857	1	full-splice_match	gene: Zm00001d009160	Zm00001d009160_T00	12.91	18.72	coding	533	52	1650
PB.3314.2	2	720	1	full-splice_match	gene: Zm00001d006213	Zm00001d006213_T00	832.09	1108.46	coding	157	87	557
PB.9835.1	8	3478	13	full-splice_match	gene: Zm00001d011700	Zm00001d011700_T00	31.37	31.37	coding	987	240	3200
PB.760.1	1	2199	2	novel_not_in_catalog	gene: Zm00001d022855	novel	11.23	11.23	non coding	NA	NA	NA
PB.3314.1	2	827	1	full-splice_match	gene: Zm00001d006213	Zm00001d006213_T00	276.36	1108.46	coding	157	87	557
PB.8855.1	7	1727	4	novel_in_catalog	gene: Zm00001d021764	novel	1.06	30.20	coding	254	129	890
PB.8855.3	7	2613	4	full-splice_match	gene: Zm00001d021764	Zm00001d021764_T00	1.55	30.20	coding	194	1613	2194
PB.8801.5	7	1485	5	novel_not_in_catalog	gene: Zm00001d021615	novel	4.17	834.08	coding	119	302	658
PB.8855.5	7	923	4	full-splice_match	gene: Zm00001d021764	Zm00001d021764_T00	0.00	30.20	coding	194	110	691
PB.7661.1	6	561	1	full-splice_match	gene: Zm00001d037072	Zm00001d037072_T00	13.46	13.46	coding	121	41	403
PB.7687.1	6	1418	6	full-splice_match	gene: Zm00001d037209	Zm00001d037209_T00	3.73	3.73	coding	331	198	1190

PB.10331.1	9	311	1	incomplete-splice_match	gene: Zm00001d045725	Zm00001d045725_T00	33.92	33.92	non coding	NA	NA	NA
PB.8544.2	7	1598	1	full-splice_match	gene: Zm00001d020470	Zm00001d020470_T00	79.47	83.78	coding	421	170	1432
PB.8801.2	7	1558	10	novel_not_in_catalog	gene: Zm00001d021615	novel	20.78	834.08	coding	192	804	1379
PB.8544.1	7	1810	2	genic	gene: Zm00001d020470	novel	4.31	83.78	coding	266	311	1108
PB.1477.1	1	1010	3	full-splice_match	gene: Zm00001d034175	Zm00001d034175_T00	52.86	67.60	coding	209	83	709
PB.8544.4	7	1389	1	full-splice_match	gene: Zm00001d020470	Zm00001d020470_T00	0.00	83.78	coding	421	24	1286
PB.10090.4	8	718	3	full-splice_match	gene: Zm00001d012729	Zm00001d012729_T01	15.97	36.18	non coding	NA	NA	NA
PB.10597.3	9	1125	2	genic	gene: Zm00001d047350	novel	4.30	12.28	coding	258	61	834
PB.10762.2	9	1593	1	full-splice_match	gene: Zm00001d047915	Zm00001d047915_T00	6.17	20.20	coding	479	33	1469
PB.10762.1	9	1983	1	full-splice_match	gene: Zm00001d047915	Zm00001d047915_T00	14.03	20.20	coding	479	163	1599
PB.7985.2	6	1196	9	full-splice_match	gene: Zm00001d038380	Zm00001d038380_T00	174.91	310.67	coding	215	229	873
PB.2140.2	10	2196	2	genic	gene: Zm00001d025667	novel	1.79	15.93	coding	182	1385	1930
PB.8875.1	7	1456	1	incomplete-splice_match	gene: Zm00001d021824	Zm00001d021824_T00	27.14	27.14	coding	116	13	360
PB.8000.10	6	2609	7	full-splice_match	gene: Zm00001d038432	Zm00001d038432_T00	0.00	103.47	coding	234	1780	2481
PB.4014.1	3	1145	7	full-splice_match	gene: Zm00001d040873	Zm00001d040873_T00	7.69	7.69	coding	282	107	952
PB.10597.5	9	1009	3	novel_not_in_catalog	gene: Zm00001d047350	novel	7.98	12.28	coding	212	60	695
PB.8849.3	7	2134	6	novel_in_catalog	gene: Zm00001d021748	novel	11.10	45.01	coding	374	443	1564

PB.4109.1	3	790	1	full-splice_match	gene: Zm00001d041672	Zm00001d041672_T00	405.32	405.32	coding	136	99	506
PB.8849.4	7	1375	7	incomplete-splice_match	gene: Zm00001d021748	Zm00001d021748_T00	10.13	45.01	coding	277	193	1023
PB.8849.5	7	1810	7	novel_in_catalog	gene: Zm00001d021748	novel	23.78	45.01	coding	374	291	1412
PB.7746.1	6	814	3	full-splice_match	gene: Zm00001d037463	Zm00001d037463_T00	56.52	56.52	coding	190	115	684
PB.10080.1	8	1534	8	full-splice_match	gene: Zm00001d012681	Zm00001d012681_T01	27.83	28.75	coding	328	133	1116
PB.3661.1	2	1136	10	full-splice_match	gene: Zm00001d007930	Zm00001d007930_T00	79.95	79.95	coding	293	51	929
PB.2315.1	10	865	5	full-splice_match	gene: Zm00001d026392	Zm00001d026392_T00	76.97	76.97	coding	98	148	441
PB.10489.1	9	1462	3	full-splice_match	gene: Zm00001d046767	Zm00001d046767_T00	265.86	778.69	coding	174	120	641
PB.2279.3	10	2687	4	novel_in_catalog	gene: Zm00001d026288	novel	8.66	23.40	coding	132	292	687
PB.5079.1	4	596	1	novel_in_catalog	gene: Zm00001d049743	novel	19.19	19.19	coding	152	4	459
PB.2279.1	10	1951	5	full-splice_match	gene: Zm00001d026288	Zm00001d026288_T00	14.74	23.40	coding	432	203	1498
PB.872.2	1	2326	9	full-splice_match	gene: Zm00001d031490	Zm00001d031490_T00	8.33	103.09	coding	178	219	752
PB.5474.6	4	1762	9	full-splice_match	gene: Zm00001d052074	Zm00001d052074_T00	28.21	129.01	coding	212	802	1437
PB.8281.2	7	1860	4	full-splice_match	gene: Zm00001d018887	Zm00001d018887_T00	11.09	34.73	coding	432	142	1437
PB.4324.1	3	1286	3	full-splice_match	gene: Zm00001d042699	Zm00001d042699_T00	14.51	16.85	coding	278	12	845
PB.2582.1	2	810	2	novel_not_in_catalog	gene: Zm00001d002403	novel	14.66	14.66	coding	185	11	565
PB.5184.1	4	1468	6	full-splice_match	gene: Zm00001d050375	Zm00001d050375_T00	10.33	544.99	coding	256	89	856

PB.8089.2	6	604	2	intergenic	novelGene_328	novel	107.71	137.41	non coding	NA	NA	NA
PB.5184.2	4	1281	6	full-splice_match	gene: Zm00001d050375	Zm00001d050375_T00	395.34	544.99	coding	256	83	850
PB.5184.5	4	959	6	full-splice_match	gene: Zm00001d050375	Zm00001d050375_T00	139.33	544.99	coding	256	91	858
PB.67.1	1	3156	6	full-splice_match	gene: Zm00001d027504	Zm00001d027504_T00	21.35	21.35	coding	919	217	2973
PB.4831.1	3	904	4	incomplete-splice_match	gene: Zm00001d044705	Zm00001d044705_T00	19.14	19.14	coding	173	91	609
PB.9596.8	8	1773	2	genic	gene: Zm00001d010640	novel	60.23	364.13	coding	354	534	1595
PB.9596.9	8	1943	1	full-splice_match	gene: Zm00001d010640	Zm00001d010640_T00	17.41	364.13	coding	354	704	1765
PB.6666.1	5	1344	5	full-splice_match	gene: Zm00001d015905	Zm00001d015905_T00	167.36	186.15	coding	252	160	915
PB.7176.3	5	646	1	full-splice_match	gene: Zm00001d018198	Zm00001d018198_T00	0.00	20.05	coding	154	91	552
PB.475.1	1	1454	1	full-splice_match	gene: Zm00001d029287	Zm00001d029287_T00	21.33	21.33	coding	383	91	1239
PB.9596.1	8	1410	1	full-splice_match	gene: Zm00001d010640	Zm00001d010640_T00	72.68	364.13	coding	260	107	886
PB.9540.6	8	571	1	incomplete-splice_match	gene: Zm00001d010399	Zm00001d010399_T00	0.00	53.39	coding	53	172	330
PB.9596.3	8	2141	1	full-splice_match	gene: Zm00001d010640	Zm00001d010640_T00	40.76	364.13	coding	354	705	1766
PB.2219.1	10	1023	1	full-splice_match	gene: Zm00001d026028	Zm00001d026028_T00	158.70	220.78	non coding	NA	NA	NA
PB.9596.5	8	2053	1	full-splice_match	gene: Zm00001d010640	Zm00001d010640_T00	0.00	364.13	coding	354	704	1765
PB.9596.6	8	1874	2	genic	gene: Zm00001d010640	novel	170.68	364.13	coding	354	525	1586
PB.2219.2	10	916	1	full-splice_match	gene: Zm00001d026028	Zm00001d026028_T00	37.98	220.78	non coding	NA	NA	NA

PB.7697.1	6	1668	1	full-splice_match	gene: Zm00001d037245	Zm00001d037245_T00	21.52	21.52	coding	423	182	1450
PB.6165.1	5	808	2	incomplete-splice_match	gene: Zm00001d013524	Zm00001d013524_T00	73.07	73.07	coding	115	213	557
PB.8841.2	7	3092	6	novel_in_catalog	gene: Zm00001d021729	novel	0.59	66.04	coding	563	39	1727
PB.8841.3	7	2889	8	full-splice_match	gene: Zm00001d021729	Zm00001d021729_T00	6.92	66.04	coding	654	29	1990
PB.8788.1	7	1323	13	novel_in_catalog	gene: Zm00001d021558	novel	72.78	94.47	coding	311	29	961
PB.8841.1	7	2926	9	full-splice_match	gene: Zm00001d021729	Zm00001d021729_T00	52.57	66.04	coding	863	121	2709
PB.4976.1	4	1846	2	novel_not_in_catalog	gene: Zm00001d049241	novel	7.84	7.84	coding	181	151	693
PB.1586.2	1	1145	5	full-splice_match	gene: Zm00001d034627	Zm00001d034627_T00	21.38	54.00	coding	248	207	950
PB.10904.2	9	691	1	full-splice_match	gene: Zm00001d048491	Zm00001d048491_T00	327.45	712.51	coding	149	87	533
PB.5065.3	4	1086	6	novel_in_catalog	gene: Zm00001d049692	novel	17.49	17.49	coding	257	219	989
PB.3406.1	2	1337	1	full-splice_match	gene: Zm00001d006611	Zm00001d006611_T00	16.33	21.32	coding	334	49	1050
PB.3406.2	2	1302	1	full-splice_match	gene: Zm00001d006611	Zm00001d006611_T00	5.00	21.32	coding	334	165	1166
PB.1628.2	1	1790	5	full-splice_match	gene: Zm00001d034761	Zm00001d034761_T00	31.94	176.84	coding	377	214	1344
PB.1208.3	1	1810	2	intergenic	novelGene_448	novel	2.09	81.86	coding	188	832	1395
PB.7503.1	6	593	2	incomplete-splice_match	gene: Zm00001d036136	Zm00001d036136_T00	851.61	1003.33	coding	55	164	328
PB.7413.2	6	1239	2	genic	gene: Zm00001d035604	novel	99.74	208.68	coding	145	580	1014
PB.1010.2	1	1777	8	full-splice_match	gene: Zm00001d032183	Zm00001d032183_T00	2.88	6.03	coding	191	22	594

PB.1010.1	1	2059	9	novel_in_catalog	gene: Zm00001d032183	novel	3.15	6.03	coding	191	90	662
PB.7413.1	6	1447	1	full-splice_match	gene: Zm00001d035604	Zm00001d035604_T00	108.94	208.68	coding	368	139	1242
PB.8596.1	7	1592	8	novel_not_in_catalog	gene: Zm00001d020751	novel	25.91	25.91	coding	487	50	1510
PB.7503.2	6	1820	1	novel_in_catalog	gene: Zm00001d036136	novel	9.98	1003.33	coding	55	182	346
PB.3415.3	2	545	2	intergenic	novelGene_410	novel	0.00	27.26	non coding	NA	NA	NA
PB.6463.1	5	1359	4	full-splice_match	gene: Zm00001d014818	Zm00001d014818_T00	13.48	13.48	coding	361	12	1094
PB.7503.3	6	466	2	full-splice_match	gene: Zm00001d036136	Zm00001d036136_T00	141.74	1003.33	non coding	NA	NA	NA
PB.4610.8	3	1981	10	novel_not_in_catalog	gene: Zm00001d043797	novel	3.36	166.78	coding	124	141	512
PB.9339.3	8	495	4	novel_not_in_catalog	gene: Zm00001d009317	novel	0.00	0.00	coding	44	183	314
PB.9339.2	8	1419	12	fusion	gene: Zm00001d009317	novel	4.75	26.39	coding	390	26	1195
PB.9339.1	8	1614	13	fusion	gene: Zm00001d009317	novel	21.64	26.39	coding	436	71	1378
PB.8566.1	7	1518	1	full-splice_match	gene: Zm00001d020583	Zm00001d020583_T00	14.12	93.25	coding	318	32	985
PB.6825.1	5	1659	5	full-splice_match	gene: Zm00001d016786	Zm00001d016786_T00	18.00	18.00	coding	418	89	1342
PB.8580.2	7	1177	1	full-splice_match	gene: Zm00001d020651	Zm00001d020651_T00	9.39	11.97	coding	308	93	1016
PB.1314.2	1	1713	3	full-splice_match	gene: Zm00001d033457	Zm00001d033457_T00	331.81	420.57	coding	445	125	1459
PB.415.1	1	1734	3	full-splice_match	gene: Zm00001d028999	Zm00001d028999_T00	19.65	19.65	coding	371	246	1358
PB.415.2	1	1366	3	full-splice_match	gene: Zm00001d028999	Zm00001d028999_T00	0.00	19.65	coding	371	71	1183

PB.3219.1	2	2680	6	novel_in_catalog	gene: Zm00001d005826	novel	29.16	29.16	coding	717	375	2525
PB.1030.1	1	868	6	full-splice_match	gene: Zm00001d032266	Zm00001d032266_T00	40.58	40.58	coding	118	210	563
PB.6940.2	5	554	4	full-splice_match	gene: Zm00001d017242	Zm00001d017242_T00	94.69	232.47	non coding	NA	NA	NA
PB.2646.1	2	1023	6	full-splice_match	gene: Zm00001d002704	Zm00001d002704_T00	405.94	460.24	coding	168	162	665
PB.2310.3	10	1214	6	full-splice_match	gene: Zm00001d026370	Zm00001d026370_T00	0.00	93.07	coding	343	57	1085
PB.6447.1	5	1612	4	novel_not_in_catalog	gene: Zm00001d014780	novel	19.24	19.24	coding	412	188	1423
PB.2646.5	2	987	5	novel_in_catalog	gene: Zm00001d002704	novel	6.06	460.24	coding	101	167	469
PB.2646.4	2	1080	6	full-splice_match	gene: Zm00001d002704	Zm00001d002704_T00	48.24	460.24	coding	168	441	944
PB.5256.12	4	1898	10	novel_in_catalog	gene: Zm00001d050970	novel	0.92	60.57	coding	211	90	722
PB.6650.4	5	1622	8	full-splice_match	gene: Zm00001d015780	Zm00001d015780_T00	10.00	31.02	coding	388	227	1390
PB.6650.5	5	1665	7	full-splice_match	gene: Zm00001d015780	Zm00001d015780_T00	2.01	31.02	coding	388	348	1511
PB.6650.1	5	1714	8	full-splice_match	gene: Zm00001d015780	Zm00001d015780_T00	0.00	31.02	coding	388	166	1329
PB.6650.2	5	1825	7	full-splice_match	gene: Zm00001d015780	Zm00001d015780_T00	1.85	31.02	coding	388	287	1450
PB.6650.3	5	1758	7	full-splice_match	gene: Zm00001d015780	Zm00001d015780_T00	17.15	31.02	coding	388	329	1492
PB.2205.7	10	1901	8	novel_in_catalog	gene: Zm00001d025951	novel	2.19	76.16	coding	243	592	1320
PB.4392.1	3	2231	1	genic	gene: Zm00001d043015	novel	10.75	10.75	coding	647	94	2034
PB.2205.2	10	2913	11	incomplete-splice_match	gene: Zm00001d025951	Zm00001d025951_T03	21.14	76.16	coding	605	93	1907

PB.2205.3	10	3659	13	full-splice_match	gene: Zm00001d025951	Zm00001d025951_T05	52.83	76.16	coding	939	687	3503
PB.3128.2	2	655	4	full-splice_match	gene: Zm00001d005334	Zm00001d005334_T00	356.34	435.26	coding	129	91	477
PB.6173.3	5	899	1	novel_in_catalog	gene: Zm00001d013568	novel	18.68	2041.31	coding	123	344	712
PB.4952.2	4	1013	1	genic	gene: Zm00001d049157	novel	0.61	8.55	coding	99	98	394
PB.4952.1	4	914	2	full-splice_match	gene: Zm00001d049157	Zm00001d049157_T00	7.94	8.55	coding	228	92	775
PB.1061.2	1	1305	4	full-splice_match	gene: Zm00001d032405	Zm00001d032405_T00	5.54	31.54	coding	355	61	1125
PB.8968.1	7	1065	6	full-splice_match	gene: Zm00001d022180	Zm00001d022180_T00	1086.98	1086.98	coding	251	94	846
PB.10585.6	9	1239	1	incomplete-splice_match	gene: Zm00001d047283	Zm00001d047283_T03	5.13	70.97	non coding	NA	NA	NA
PB.10585.1	9	2952	19	full-splice_match	gene: Zm00001d047283	Zm00001d047283_T01	16.24	70.97	coding	700	569	2668
PB.8090.1	6	2037	10	full-splice_match	gene: Zm00001d038796	Zm00001d038796_T00	17.22	17.22	coding	564	85	1776
PB.10585.3	9	2730	20	full-splice_match	gene: Zm00001d047283	Zm00001d047283_T03	49.60	70.97	coding	700	349	2448
PB.6931.1	5	2980	14	full-splice_match	gene: Zm00001d017197	Zm00001d017197_T00	55.84	58.79	coding	797	287	2677
PB.9422.1	8	3121	7	full-splice_match	gene: Zm00001d009753	Zm00001d009753_T00	19.88	19.88	coding	939	135	2951
PB.1685.1	10	1032	8	full-splice_match	gene: Zm00001d023223	Zm00001d023223_T00	22.23	22.23	coding	252	94	849
PB.9422.3	8	2093	7	full-splice_match	gene: Zm00001d009753	Zm00001d009753_T01	0.00	19.88	coding	656	126	2093
PB.10631.2	9	1388	8	full-splice_match	gene: Zm00001d047474	Zm00001d047474_T00	14.47	14.47	coding	398	63	1256
PB.2173.1	10	402	1	genic	gene: Zm00001d025816	novel	6.99	6.99	non coding	NA	NA	NA

PB.7175.4	5	1537	5	full-splice_match	gene: Zm00001d018195	Zm00001d018195_T00	69.42	311.25	coding	394	112	1293
PB.4024.1	3	1635	3	incomplete-splice_match	gene: Zm00001d041072	Zm00001d041072_T00	108.29	108.29	coding	471	49	1461
PB.7758.1	6	979	9	full-splice_match	gene: Zm00001d037509	Zm00001d037509_T00	66.76	66.76	coding	227	66	746
PB.7169.2	5	2747	3	novel_in_catalog	gene: Zm00001d018178	novel	0.00	49.27	coding	374	200	1321
PB.6778.1	5	602	1	full-splice_match	gene: Zm00001d016568	Zm00001d016568_T00	277.80	277.80	non coding	NA	NA	NA
PB.4793.11	3	2061	2	novel_in_catalog	gene: Zm00001d044516	novel	0.00	891.68	coding	297	121	1011
PB.4801.1	3	3590	1	incomplete-splice_match	gene: Zm00001d044547	Zm00001d044547_T01	1.29	1.29	coding	741	218	2440
PB.628.2	1	1200	3	full-splice_match	gene: Zm00001d030103	Zm00001d030103_T00	22.71	39.63	coding	347	22	1062
PB.3213.1	2	1188	1	full-splice_match	gene: Zm00001d005811	Zm00001d005811_T00	12.22	12.22	coding	291	80	952
PB.6015.12	5	1037	2	full-splice_match	gene: Zm00001d013015	Zm00001d013015_T00	22.84	206.41	coding	283	76	924
PB.4133.1	3	2087	12	novel_in_catalog	gene: Zm00001d041769	novel	2.58	63.55	coding	480	128	1567
PB.4133.2	3	1931	13	full-splice_match	gene: Zm00001d041769	Zm00001d041769_T00	57.78	63.55	coding	517	177	1727
PB.4133.3	3	2182	11	novel_in_catalog	gene: Zm00001d041769	novel	3.19	63.55	coding	390	128	1297
PB.6256.2	5	1350	2	genic	gene: Zm00001d013971	novel	0.00	42.28	coding	385	57	1211
PB.9325.1	8	1425	1	antisense	novelGene_gene: Zm00001d009187	novel	61.23	61.23	coding	131	474	866
PB.4224.1	3	1192	6	full-splice_match	gene: Zm00001d042274	Zm00001d042274_T00	31.27	43.54	coding	251	214	966
PB.6256.1	5	1510	2	genic	gene: Zm00001d013971	novel	42.28	42.28	coding	385	58	1212

PB.8956.7	7	795	1	full-splice_match	gene: Zm00001d022130	Zm00001d022130_T00	498.94	776.57	coding	155	149	613
PB.2581.1	2	1336	3	full-splice_match	gene: Zm00001d002402	Zm00001d002402_T00	28.79	28.79	coding	325	75	1049
PB.5586.2	4	1480	4	novel_in_catalog	gene: Zm00001d052458	novel	14.49	174.28	coding	256	157	924
PB.628.1	1	1481	3	full-splice_match	gene: Zm00001d030103	Zm00001d030103_T00	16.92	39.63	coding	347	244	1284
PB.8956.2	7	906	1	full-splice_match	gene: Zm00001d022130	Zm00001d022130_T00	273.36	776.57	coding	155	150	614
PB.8956.1	7	2075	1	full-splice_match	gene: Zm00001d022130	Zm00001d022130_T00	4.26	776.57	coding	155	149	613
PB.2634.2	2	794	5	novel_in_catalog	gene: Zm00001d002656	novel	4.40	16.85	coding	100	355	654
PB.10778.2	9	839	2	incomplete-splice_match	gene: Zm00001d047977	Zm00001d047977_T00	185.07	384.22	coding	140	224	643
PB.10778.1	9	2279	7	full-splice_match	gene: Zm00001d047977	Zm00001d047977_T00	118.89	384.22	coding	593	281	2059
PB.2634.1	2	3581	11	full-splice_match	gene: Zm00001d002656	Zm00001d002656_T00	12.45	16.85	coding	568	169	1872
PB.7722.1	6	891	1	genic	gene: Zm00001d037340	novel	29.04	29.04	coding	244	63	794
PB.7865.1	6	4530	4	novel_not_in_catalog	gene: Zm00001d037975	novel	6.91	19.15	coding	634	1	1902
PB.2319.1	10	2014	3	novel_in_catalog	gene: Zm00001d026405	novel	67.91	67.91	coding	531	241	1833
PB.8657.1	7	1820	8	full-splice_match	gene: Zm00001d021019	Zm00001d021019_T00	21.78	55.30	coding	354	379	1440
PB.8657.5	7	1557	8	full-splice_match	gene: Zm00001d021019	Zm00001d021019_T00	33.52	55.30	coding	354	133	1194
PB.1962.7	10	1451	6	novel_in_catalog	gene: Zm00001d024744	novel	8.15	42.47	coding	58	135	308
PB.5430.3	4	1740	8	incomplete-splice_match	gene: Zm00001d051902	Zm00001d051902_T00	6.36	82.21	coding	488	35	1498

PB.5430.2	4	3057	9	full-splice_match	gene: Zm00001d051902	Zm00001d051902_T00	33.13	82.21	coding	426	260	1537
PB.6062.1	5	983	4	full-splice_match	gene: Zm00001d013179	Zm00001d013179_T00	205.36	205.36	coding	214	39	680
PB.4824.1	3	1076	1	full-splice_match	gene: Zm00001d044660	Zm00001d044660_T00	38.46	96.21	coding	227	137	817
PB.39.2	1	740	2	genic	gene: Zm00001d027389	novel	131.17	280.61	coding	132	100	495
PB.9556.1	8	1998	7	novel_not_in_catalog	gene: Zm00001d010456	novel	11.62	53.48	coding	439	67	1383
PB.85.4	1	2199	5	full-splice_match	gene: Zm00001d027571	Zm00001d027571_T00	91.19	146.43	coding	399	607	1803
PB.1628.3	1	1691	5	full-splice_match	gene: Zm00001d034761	Zm00001d034761_T00	134.88	176.84	coding	377	234	1364
PB.85.7	1	1214	4	incomplete-splice_match	gene: Zm00001d027571	Zm00001d027571_T00	0.00	146.43	coding	269	407	1213
PB.7021.1	5	1513	7	incomplete-splice_match	gene: Zm00001d017609	Zm00001d017609_T00	8.08	8.08	non coding	NA	NA	NA
PB.85.2	1	2449	5	full-splice_match	gene: Zm00001d027571	Zm00001d027571_T00	51.12	146.43	coding	399	468	1664
PB.85.3	1	3172	4	novel_in_catalog	gene: Zm00001d027571	novel	4.12	146.43	coding	241	607	1329
PB.9198.2	8	1333	3	genic	gene: Zm00001d008500	novel	17.90	66.77	coding	275	213	1037
PB.9198.3	8	1383	1	full-splice_match	gene: Zm00001d008500	Zm00001d008500_T00	3.17	66.77	coding	275	273	1097
PB.5420.4	4	2276	6	novel_not_in_catalog	gene: Zm00001d051879	novel	3.23	67.38	coding	551	330	1982
PB.9198.1	8	1334	3	genic	gene: Zm00001d008500	novel	0.00	66.77	coding	275	174	998
PB.5420.2	4	2564	5	full-splice_match	gene: Zm00001d051879	Zm00001d051879_T00	21.50	67.38	coding	657	301	2271
PB.9198.4	8	1233	3	genic	gene: Zm00001d008500	novel	17.06	66.77	coding	275	213	1037

PB.9198.5	8	1196	3	genic	gene: Zm00001d008500	novel	28.64	66.77	coding	275	187	1011
PB.9.1	1	940	3	incomplete-splice_match	gene: Zm00001d027276	Zm00001d027276_T00	8.13	48.14	coding	200	10	609
PB.9.2	1	1303	4	incomplete-splice_match	gene: Zm00001d027276	Zm00001d027276_T00	40.01	48.14	coding	372	12	1127
PB.6710.10	5	725	1	novel_in_catalog	gene: Zm00001d016185	novel	18.63	123.88	coding	212	46	681
PB.4725.3	3	3096	4	full-splice_match	gene: Zm00001d044232	Zm00001d044232_T00	5.39	62.54	coding	756	291	2558
PB.9561.3	8	1400	4	novel_in_catalog	gene: Zm00001d010478	novel	1.00	122.36	coding	162	8	493
PB.7567.2	6	873	1	intergenic	novelGene_197	novel	13.46	22.61	non coding	NA	NA	NA
PB.10490.1	9	939	4	incomplete-splice_match	gene: Zm00001d046788	Zm00001d046788_T00	19.70	19.70	coding	187	199	759
PB.8177.1	6	1051	1	genic	gene: Zm00001d039097	novel	5.32	5.32	non coding	NA	NA	NA
PB.5422.5	4	2052	2	full-splice_match	gene: Zm00001d051883	Zm00001d051883_T00	0.00	95.68	coding	527	344	1924
PB.5560.2	4	2409	9	full-splice_match	gene: Zm00001d052385	Zm00001d052385_T00	15.39	18.11	coding	633	120	2018
PB.560.1	1	3620	10	full-splice_match	gene: Zm00001d029684	Zm00001d029684_T00	15.80	15.80	coding	1048	125	3268
PB.5894.1	4	1378	6	full-splice_match	gene: Zm00001d053932	Zm00001d053932_T00	113.91	118.57	coding	311	92	1024
PB.1204.2	1	1442	2	full-splice_match	gene: Zm00001d032937	Zm00001d032937_T00	0.44	0.99	coding	278	203	1036
PB.10599.3	9	1819	1	full-splice_match	gene: Zm00001d047364	Zm00001d047364_T00	6.09	23.30	coding	357	468	1538
PB.5894.4	4	1168	6	full-splice_match	gene: Zm00001d053932	Zm00001d053932_T00	4.67	118.57	coding	311	105	1037
PB.6992.3	5	1816	2	full-splice_match	gene: Zm00001d017473	Zm00001d017473_T00	16.27	16.27	coding	409	349	1575

PB.812.10	1	821	2	full-splice_match	gene: Zm00001d031168	Zm00001d031168_T00	2147.57	7101.71	coding	155	104	568
PB.3237.1	2	1591	9	full-splice_match	gene: Zm00001d005920	Zm00001d005920_T00	17.56	17.56	coding	304	97	1008
PB.754.1	1	790	4	novel_not_in_catalog	gene: Zm00001d030910	novel	51.21	51.21	coding	87	208	468
PB.1307.1	1	829	3	full-splice_match	gene: Zm00001d033421	Zm00001d033421_T00	45.21	45.21	coding	171	70	582
PB.5715.4	4	2612	5	novel_not_in_catalog	gene: Zm00001d053151	novel	29.88	66.45	coding	671	277	2289
PB.6630.1	5	1070	1	full-splice_match	gene: Zm00001d015639	Zm00001d015639_T00	14.72	14.72	coding	211	115	747
PB.1084.5	1	1762	4	novel_in_catalog	gene: Zm00001d032480	novel	8.20	158.15	coding	254	726	1487
PB.6108.1	5	3000	21	full-splice_match	gene: Zm00001d013314	Zm00001d013314_T00	32.22	32.59	coding	768	127	2430
PB.7567.3	6	2074	1	intergenic	novelGene_197	novel	4.96	22.61	non coding	NA	NA	NA
PB.5537.3	4	1825	2	genic	gene: Zm00001d052312	novel	1.32	109.40	coding	422	276	1541
PB.10023.2	8	1408	3	full-splice_match	gene: Zm00001d012456	Zm00001d012456_T00	5.41	10.54	coding	386	4	1161
PB.10023.3	8	1458	4	full-splice_match	gene: Zm00001d012456	Zm00001d012456_T00	5.13	10.54	coding	386	160	1317
PB.6668.1	5	373	1	full-splice_match	gene: Zm00001d015918	Zm00001d015918_T00	86.45	86.45	non coding	NA	NA	NA
PB.3671.1	2	2552	16	full-splice_match	gene: Zm00001d007966	Zm00001d007966_T00	18.76	151.61	coding	619	114	1970
PB.8647.2	7	1300	5	novel_in_catalog	gene: Zm00001d020970	novel	3.10	6.55	coding	103	804	1112
PB.10827.1	9	2410	3	genic	gene: Zm00001d048199	novel	116.42	120.79	coding	545	315	1949
PB.2484.7	2	3509	12	novel_in_catalog	gene: Zm00001d002006	novel	3.02	1327.46	coding	660	184	2163

PB.2484.6	2	3620	11	novel_in_catalog	gene: Zm00001d002006	novel	10.61	1327.46	coding	653	185	2143
PB.2484.1	2	3503	13	full-splice_match	gene: Zm00001d002006	Zm00001d002006_T00	1058.22	1327.46	coding	744	204	2435
PB.7225.1	5	2651	5	novel_in_catalog	gene: Zm00001d018391	novel	2.35	48.23	coding	472	830	2245
PB.10162.1	9	1420	3	full-splice_match	gene: Zm00001d045021	Zm00001d045021_T00	23.01	62.71	coding	220	329	988
PB.10162.2	9	2531	3	incomplete-splice_match	gene: Zm00001d045021	Zm00001d045021_T01	12.64	62.71	coding	220	1474	2133
PB.10162.3	9	1319	3	full-splice_match	gene: Zm00001d045021	Zm00001d045021_T00	20.32	62.71	coding	220	343	1002
PB.10162.5	9	2448	3	incomplete-splice_match	gene: Zm00001d045021	Zm00001d045021_T01	2.57	62.71	coding	78	108	341
PB.10162.6	9	1350	4	novel_not_in_catalog	gene: Zm00001d045021	novel	4.17	62.71	coding	78	128	361
PB.6777.1	5	723	1	full-splice_match	gene: Zm00001d016567	Zm00001d016567_T00	394.20	394.20	coding	69	150	356
PB.6777.2	5	334	1	full-splice_match	gene: Zm00001d016567	Zm00001d016567_T00	0.00	394.20	non coding	NA	NA	NA
PB.2707.1	2	793	2	incomplete-splice_match	gene: Zm00001d002980	Zm00001d002980_T00	11.14	47.33	non coding	NA	NA	NA
PB.2707.2	2	1384	7	full-splice_match	gene: Zm00001d002980	Zm00001d002980_T00	36.20	47.33	coding	381	30	1172
PB.10040.1	8	992	1	full-splice_match	gene: Zm00001d012545	Zm00001d012545_T00	73.93	73.93	coding	131	286	678
PB.2616.1	2	1815	9	novel_not_in_catalog	gene: Zm00001d002598	novel	33.72	33.72	coding	321	676	1638
PB.10827.2	9	2250	1	full-splice_match	gene: Zm00001d048199	Zm00001d048199_T00	4.37	120.79	coding	408	54	1277
PB.3574.1	2	939	3	incomplete-splice_match	gene: Zm00001d007457	Zm00001d007457_T00	39.22	39.22	coding	219	11	667
PB.3112.2	2	1679	3	novel_in_catalog	gene: Zm00001d005253	novel	4.54	337.88	non coding	NA	NA	NA

PB.3112.3	2	643	4	full-splice_match	gene: Zm00001d005253	Zm00001d005253_T00	321.76	337.88	coding	110	111	440
PB.3112.1	2	752	4	full-splice_match	gene: Zm00001d005253	Zm00001d005253_T00	11.58	337.88	coding	110	114	443
PB.10278.2	9	3192	9	novel_in_catalog	gene: Zm00001d045491	novel	10.03	69.96	coding	348	1782	2825
PB.9092.1	7	1562	15	full-splice_match	gene: Zm00001d022558	Zm00001d022558_T00	13.63	31.77	coding	389	91	1257
PB.1111.5	1	2306	11	full-splice_match	gene: Zm00001d032566	Zm00001d032566_T00	5.26	54.09	coding	239	33	749
PB.10278.1	9	2089	8	incomplete-splice_match	gene: Zm00001d045491	Zm00001d045491_T00	41.42	69.96	coding	496	311	1798
PB.6327.3	5	2390	4	incomplete-splice_match	gene: Zm00001d014286	Zm00001d014286_T04	10.76	110.00	coding	616	128	1975
PB.6327.2	5	3470	13	full-splice_match	gene: Zm00001d014286	Zm00001d014286_T01	99.23	110.00	coding	1020	111	3170
PB.10278.4	9	2497	10	full-splice_match	gene: Zm00001d045491	Zm00001d045491_T00	18.51	69.96	coding	749	65	2311
PB.10226.2	9	2204	11	novel_in_catalog	gene: Zm00001d045316	novel	1.01	16.81	coding	203	220	828
PB.10226.1	9	2180	12	full-splice_match	gene: Zm00001d045316	Zm00001d045316_T00	15.80	16.81	coding	431	293	1585
PB.5917.6	4	2084	10	novel_not_in_catalog	gene: Zm00001d053997	novel	6.19	70.44	coding	234	1078	1779
PB.5917.1	4	1161	4	novel_in_catalog	gene: Zm00001d053997	novel	10.08	70.44	non coding	NA	NA	NA
PB.7147.5	5	2268	4	full-splice_match	gene: Zm00001d018090	Zm00001d018090_T00	16.66	297.01	coding	156	68	535
PB.5917.3	4	1969	11	novel_not_in_catalog	gene: Zm00001d053997	novel	13.27	70.44	coding	491	191	1663
PB.5917.2	4	2102	12	full-splice_match	gene: Zm00001d053997	Zm00001d053997_T01	13.52	70.44	coding	491	324	1796
PB.1751.1	10	1607	1	incomplete-splice_match	gene: Zm00001d023523	Zm00001d023523_T01	8.98	140.26	coding	457	94	1464

PB.9337.1	8	1879	1	intergenic	novelGene_142	novel	2.46	2.46	coding	181	105	647
PB.6266.2	5	2298	2	incomplete-splice_match	gene: Zm00001d014017	Zm00001d014017_T00	2.38	69.56	coding	9	431	457
PB.6266.1	5	862	3	full-splice_match	gene: Zm00001d014017	Zm00001d014017_T00	67.18	69.56	non coding	NA	NA	NA
PB.288.2	1	723	3	full-splice_match	gene: Zm00001d028453	Zm00001d028453_T00	75.86	75.86	coding	92	142	417
PB.1542.1	1	1036	1	full-splice_match	gene: Zm00001d034469	Zm00001d034469_T00	22.11	22.11	coding	227	118	798
PB.3029.1	2	2184	12	full-splice_match	gene: Zm00001d004740	Zm00001d004740_T00	60.35	60.35	coding	586	122	1879
PB.9224.1	8	2465	2	full-splice_match	gene: Zm00001d008633	Zm00001d008633_T00	1.25	3.11	coding	756	1	2268
PB.1771.1	10	816	1	antisense	novelGene_gene: Zm00001d023581	novel	0.00	0.00	non coding	NA	NA	NA
PB.10033.2	8	2582	3	novel_not_in_catalog	gene: Zm00001d012514	novel	0.00	22.15	coding	637	482	2392
PB.10033.1	8	2696	3	novel_not_in_catalog	gene: Zm00001d012514	novel	22.15	22.15	coding	637	487	2397
PB.3500.2	2	1685	5	novel_in_catalog	gene: Zm00001d007032	novel	7.83	49.67	non coding	NA	NA	NA
PB.9399.1	8	2113	10	full-splice_match	gene: Zm00001d009638	Zm00001d009638_T00	149.00	187.01	coding	583	116	1864
PB.6522.2	5	1763	12	full-splice_match	gene: Zm00001d015026	Zm00001d015026_T00	26.61	47.32	coding	387	177	1337
PB.6522.3	5	1641	12	full-splice_match	gene: Zm00001d015026	Zm00001d015026_T00	14.44	47.32	coding	387	177	1337
PB.6522.4	5	1542	12	full-splice_match	gene: Zm00001d015026	Zm00001d015026_T00	6.28	47.32	coding	387	203	1363
PB.3785.1	3	1744	3	full-splice_match	gene: Zm00001d039639	Zm00001d039639_T00	5.50	5.50	coding	464	59	1450
PB.9399.7	8	2084	9	novel_in_catalog	gene: Zm00001d009638	novel	15.93	187.01	coding	474	49	1470

PB.9399.6	8	2060	9	novel_in_catalog	gene: Zm00001d009638	novel	2.88	187.01	coding	316	47	994
PB.4513.1	3	784	1	full-splice_match	gene: Zm00001d043456	Zm00001d043456_T00	3.87	3.87	coding	157	4	474
PB.9399.8	8	2297	9	novel_in_catalog	gene: Zm00001d009638	novel	7.46	187.01	coding	239	113	829
PB.1090.1	1	1285	2	incomplete-splice_match	gene: Zm00001d032504	Zm00001d032504_T00	2.35	2.35	coding	266	156	953
PB.5298.2	4	1257	7	full-splice_match	gene: Zm00001d051247	Zm00001d051247_T00	21.31	21.31	coding	267	123	923
PB.2763.1	2	1597	1	genic	gene: Zm00001d003213	novel	7.77	7.77	coding	395	139	1323
PB.10018.3	8	611	1	full-splice_match	gene: Zm00001d012439	Zm00001d012439_T00	1346.76	1346.76	coding	103	65	373
PB.9643.1	8	1082	6	full-splice_match	gene: Zm00001d010808	Zm00001d010808_T00	60.84	60.84	coding	215	166	810
PB.3655.1	2	2764	17	full-splice_match	gene: Zm00001d007889	Zm00001d007889_T00	98.20	98.20	coding	704	210	2321
PB.5458.1	4	1137	1	full-splice_match	gene: Zm00001d052041	Zm00001d052041_T00	5.55	5.55	coding	271	36	848
PB.7295.1	6	1182	5	full-splice_match	gene: Zm00001d034990	Zm00001d034990_T00	25.56	25.56	coding	284	199	1050
PB.8734.1	7	1501	6	full-splice_match	gene: Zm00001d021346	Zm00001d021346_T00	18.41	18.41	coding	483	25	1473
PB.3870.1	3	1498	5	novel_in_catalog	gene: Zm00001d040084	novel	96.68	96.68	coding	252	533	1288
PB.4007.1	3	1514	7	incomplete-splice_match	gene: Zm00001d040805	Zm00001d040805_T00	29.18	29.18	coding	404	65	1276
PB.8661.2	7	1801	3	full-splice_match	gene: Zm00001d021024	Zm00001d021024_T00	0.00	13.26	coding	479	28	1464
PB.6337.1	5	757	1	full-splice_match	gene: Zm00001d014319	Zm00001d014319_T00	53.58	53.58	coding	131	139	531
PB.5317.1	4	1049	4	fusion	gene: Zm00001d051374	novel	32.27	32.27	coding	252	69	824

PB.8661.1	7	1950	3	full-splice_match	gene: Zm00001d021024	Zm00001d021024_T00	13.26	13.26	coding	479	42	1478
PB.9752.2	8	1507	12	full-splice_match	gene: Zm00001d011329	Zm00001d011329_T00	6.28	13.80	coding	357	73	1143
PB.9752.3	8	1486	12	novel_not_in_catalog	gene: Zm00001d011329	novel	0.00	13.80	coding	247	403	1143
PB.3396.4	2	2671	3	novel_in_catalog	gene: Zm00001d006578	novel	0.00	210.21	coding	158	309	782
PB.9752.1	8	1605	12	novel_not_in_catalog	gene: Zm00001d011329	novel	7.51	13.80	coding	247	386	1126
PB.10242.1	9	915	6	full-splice_match	gene: Zm00001d045366	Zm00001d045366_T00	39.34	40.11	coding	14	346	387
PB.10242.2	9	953	5	novel_in_catalog	gene: Zm00001d045366	novel	0.77	40.11	coding	146	233	670
PB.5601.1	4	1398	4	incomplete-splice_match	gene: Zm00001d052561	Zm00001d052561_T00	5.83	49.72	non coding	NA	NA	NA
PB.5973.1	5	4682	1	intergenic	novelGene_12	novel	13.32	13.32	coding	763	2204	4492
PB.8011.1	6	2936	3	full-splice_match	gene: Zm00001d038487	Zm00001d038487_T00	15.94	18.05	coding	182	2069	2614
PB.6258.1	5	1760	3	full-splice_match	gene: Zm00001d013983	Zm00001d013983_T00	17.84	17.84	coding	359	250	1326
PB.8011.3	6	2765	3	full-splice_match	gene: Zm00001d038487	Zm00001d038487_T00	0.00	18.05	coding	182	2059	2604
PB.3727.3	3	1062	3	novel_in_catalog	gene: Zm00001d039427	novel	13.78	44.03	coding	129	460	846
PB.2404.1	10	1347	8	novel_not_in_catalog	gene: Zm00001d026676	novel	4.96	10.81	coding	127	21	401
PB.3591.2	2	1245	11	full-splice_match	gene: Zm00001d007577	Zm00001d007577_T00	0.00	37.49	coding	316	188	1135
PB.9073.1	7	2435	14	full-splice_match	gene: Zm00001d022504	Zm00001d022504_T00	2.94	2.94	coding	539	489	2105
PB.1745.2	10	1191	1	full-splice_match	gene: Zm00001d023468	Zm00001d023468_T00	7.92	307.80	non coding	NA	NA	NA

PB.1745.3	10	1151	1	full-splice_match	gene: Zm00001d023468	Zm00001d023468_T00	297.40	307.80	coding	213	196	834
PB.1745.1	10	1957	1	full-splice_match	gene: Zm00001d023468	Zm00001d023468_T00	2.48	307.80	coding	213	139	777
PB.7289.1	6	3093	11	incomplete-splice_match	gene: Zm00001d034964	Zm00001d034964_T00	29.35	34.50	coding	789	35	2401
PB.9836.1	8	1536	8	novel_not_in_catalog	gene: Zm00001d011709	novel	6.51	14.60	coding	277	106	936
PB.1745.4	10	1036	1	full-splice_match	gene: Zm00001d023468	Zm00001d023468_T00	0.00	307.80	coding	213	199	837
PB.7289.2	6	2680	9	incomplete-splice_match	gene: Zm00001d034964	Zm00001d034964_T01	5.15	34.50	coding	464	609	2000
PB.8637.2	7	2512	3	genic	gene: Zm00001d020939	novel	63.48	70.58	coding	609	295	2121
PB.3556.3	2	1507	1	full-splice_match	gene: Zm00001d007274	Zm00001d007274_T00	0.00	131.97	coding	373	87	1205
PB.3884.1	3	2879	26	incomplete-splice_match	gene: Zm00001d040166	Zm00001d040166_T03	9.32	9.32	coding	860	19	2598
PB.6359.5	5	1038	1	incomplete-splice_match	gene: Zm00001d014414	Zm00001d014414_T00	18.29	25.06	coding	211	225	857
PB.3156.2	2	1184	5	full-splice_match	gene: Zm00001d005485	Zm00001d005485_T00	58.85	131.44	coding	289	100	966
PB.1304.2	1	2450	10	full-splice_match	gene: Zm00001d033397	Zm00001d033397_T00	5.86	15.60	coding	261	111	893
PB.6359.2	5	3200	1	genic	gene: Zm00001d014414	novel	4.34	25.06	coding	211	84	716
PB.6359.3	5	2359	3	novel_not_in_catalog	gene: Zm00001d014414	novel	2.42	25.06	coding	211	80	712
PB.7659.1	6	686	2	intergenic	novelGene_224	novel	39.70	39.70	non coding	NA	NA	NA
PB.2711.1	2	2020	2	genic	gene: Zm00001d003007	novel	20.13	20.13	coding	468	245	1648
PB.10156.1	9	2449	5	full-splice_match	gene: Zm00001d044976	Zm00001d044976_T00	12.83	14.76	coding	724	74	2245

PB.7084.7	5	948	3	novel_not_in_catalog	gene: Zm00001d017874	novel	57.46	126.53	coding	170	202	711
PB.1433.1	1	975	1	genic	gene: Zm00001d033987	novel	0.69	0.69	coding	156	40	507
PB.10156.2	9	1879	5	full-splice_match	gene: Zm00001d044976	Zm00001d044976_T00	1.93	14.76	coding	537	207	1817
PB.5650.1	4	1896	5	full-splice_match	gene: Zm00001d052797	Zm00001d052797_T00	1.53	12.31	coding	511	22	1554
PB.177.4	1	1967	1	intergenic	novelGene_81	novel	8.20	8.20	non coding	NA	NA	NA
PB.7076.2	5	1868	4	full-splice_match	gene: Zm00001d017849	Zm00001d017849_T00	63.24	63.24	coding	494	184	1665
PB.4351.2	3	1994	1	full-splice_match	gene: Zm00001d042814	Zm00001d042814_T00	85.77	158.53	coding	547	110	1750
PB.4351.3	3	1807	1	full-splice_match	gene: Zm00001d042814	Zm00001d042814_T00	0.00	158.53	coding	547	110	1750
PB.177.2	1	1002	2	full-splice_match	gene: Zm00001d028002	Zm00001d028002_T00	103.24	103.24	coding	217	100	750
PB.11034.13	B73V4	2315	15	full-splice_match	gene: Zm00001d000228	Zm00001d000228_T07	10.90	106.98	coding	332	402	1397
PB.11034.12	B73V4	2791	12	full-splice_match	gene: Zm00001d000228	Zm00001d000228_T05	11.02	106.98	coding	313	372	1310
PB.11034.11	B73V4	2227	15	full-splice_match	gene: Zm00001d000228	Zm00001d000228_T06	3.18	106.98	coding	332	349	1344
PB.11034.10	B73V4	2257	15	novel_in_catalog	gene: Zm00001d000228	novel	1.66	106.98	coding	332	389	1384
PB.8889.1	7	1832	2	full-splice_match	gene: Zm00001d021864	Zm00001d021864_T00	57.98	57.98	coding	440	316	1635
PB.6837.2	5	1270	6	full-splice_match	gene: Zm00001d016832	Zm00001d016832_T00	68.13	101.52	coding	329	158	1144
PB.6837.1	5	1395	6	full-splice_match	gene: Zm00001d016832	Zm00001d016832_T00	33.39	101.52	coding	329	161	1147
PB.8966.1	7	2234	2	incomplete-splice_match	gene: Zm00001d022176	Zm00001d022176_T00	12.35	12.35	coding	508	650	2173

PB.7805.1	6	748	2	intergenic	novelGene_262	novel	19.66	19.66	coding	55	146	310
PB.10812.1	9	579	1	full-splice_match	gene: Zm00001d048123	Zm00001d048123_T00	2.01	32.29	non coding	NA	NA	NA
PB.2404.3	10	1315	4	novel_in_catalog	gene: Zm00001d026676	novel	0.93	10.81	coding	135	7	411
PB.10812.2	9	655	1	full-splice_match	gene: Zm00001d048123	Zm00001d048123_T00	30.27	32.29	coding	142	68	493
PB.7033.1	5	879	2	full-splice_match	gene: Zm00001d017646	Zm00001d017646_T00	8.64	43.03	non coding	NA	NA	NA
PB.6480.1	5	2677	7	full-splice_match	gene: Zm00001d014895	Zm00001d014895_T00	10.24	95.11	coding	762	261	2546
PB.7.2	1	1978	4	full-splice_match	gene: Zm00001d027271	Zm00001d027271_T00	16.10	23.26	non coding	NA	NA	NA
PB.7.1	1	2109	7	full-splice_match	gene: Zm00001d027271	Zm00001d027271_T00	7.16	23.26	non coding	NA	NA	NA
PB.6480.2	5	2712	6	novel_in_catalog	gene: Zm00001d014895	novel	2.14	95.11	coding	394	1440	2621
PB.3009.2	2	1761	7	full-splice_match	gene: Zm00001d004651	Zm00001d004651_T00	21.26	47.68	coding	443	143	1471
PB.3009.1	2	1563	6	incomplete-splice_match	gene: Zm00001d004651	Zm00001d004651_T00	26.42	47.68	coding	380	19	1158
PB.167.1	1	1816	1	incomplete-splice_match	gene: Zm00001d027937	Zm00001d027937_T01	3.78	3.78	coding	482	157	1602
PB.528.1	1	1558	3	genic	gene: Zm00001d029559	novel	2.97	28.71	coding	250	222	971
PB.3423.5	2	1153	2	full-splice_match	gene: Zm00001d006677	Zm00001d006677_T00	0.00	40.92	coding	324	18	989
PB.528.2	1	1317	3	genic	gene: Zm00001d029559	novel	25.74	28.71	coding	250	230	979
PB.8895.1	7	1201	1	full-splice_match	gene: Zm00001d021892	Zm00001d021892_T00	18.53	18.53	coding	308	90	1013
PB.7870.1	6	860	3	full-splice_match	gene: Zm00001d038004	Zm00001d038004_T00	49.84	49.84	coding	189	112	678

PB.2404.2	10	1353	6	novel_in_catalog	gene: Zm00001d026676	novel	1.87	10.81	coding	127	22	402
PB.5198.3	4	1969	10	novel_not_in_catalog	gene: Zm00001d050483	novel	2.69	22.58	coding	319	381	1337
PB.1848.1	10	792	7	novel_in_catalog	gene: Zm00001d023995	novel	6.89	31.32	coding	141	102	524
PB.1848.2	10	683	5	novel_in_catalog	gene: Zm00001d023995	novel	22.16	31.32	coding	41	295	417
PB.1848.3	10	565	5	novel_not_in_catalog	gene: Zm00001d023995	novel	2.28	31.32	non coding	NA	NA	NA
PB.8377.10	7	1363	3	incomplete-splice_match	gene: Zm00001d019507	Zm00001d019507_T04	261.39	655.78	coding	294	101	982
PB.8377.11	7	2343	12	incomplete-splice_match	gene: Zm00001d019507	Zm00001d019507_T03	18.46	655.78	coding	718	188	2341
PB.8377.12	7	2224	11	full-splice_match	gene: Zm00001d019507	Zm00001d019507_T06	0.00	655.78	coding	679	188	2224
PB.1626.2	1	812	1	full-splice_match	gene: Zm00001d034756	Zm00001d034756_T00	320.41	498.31	non coding	NA	NA	NA
PB.10725.1	9	1553	1	genic	gene: Zm00001d047796	novel	27.99	134.07	coding	153	581	1039
PB.2834.1	2	1563	1	full-splice_match	gene: Zm00001d003549	Zm00001d003549_T00	9.15	9.15	coding	385	338	1492
PB.10725.3	9	1750	1	novel_in_catalog	gene: Zm00001d047796	novel	0.00	134.07	coding	215	240	884
PB.10725.2	9	2579	3	full-splice_match	gene: Zm00001d047796	Zm00001d047796_T00	106.08	134.07	coding	672	190	2205
PB.10511.2	9	1064	3	incomplete-splice_match	gene: Zm00001d046928	Zm00001d046928_T00	4.86	11.97	coding	250	22	771
PB.9568.2	8	1759	8	full-splice_match	gene: Zm00001d010523	Zm00001d010523_T00	5.81	58.50	coding	348	496	1539
PB.9568.1	8	1663	9	full-splice_match	gene: Zm00001d010523	Zm00001d010523_T00	52.70	58.50	coding	423	149	1417
PB.10511.1	9	3747	13	novel_in_catalog	gene: Zm00001d046928	novel	7.10	11.97	coding	963	433	3321

PB.2412.1	10	1367	3	novel_not_in_catalog	gene: Zm00001d026701	novel	15.45	15.45	coding	328	356	1339
PB.7153.2	5	1012	1	antisense	novelGene_gene: Zm00001d018111	novel	69.33	115.23	coding	247	167	907
PB.4333.6	3	876	2	incomplete-splice_match	gene: Zm00001d042731	Zm00001d042731_T00	36.85	100.59	coding	186	48	605
PB.3424.2	2	1434	2	full-splice_match	gene: Zm00001d006678	Zm00001d006678_T00	15.41	45.68	coding	367	144	1244
PB.4624.2	3	1807	7	full-splice_match	gene: Zm00001d043841	Zm00001d043841_T00	49.34	49.34	coding	518	101	1654
PB.8944.1	7	1392	3	full-splice_match	gene: Zm00001d022067	Zm00001d022067_T00	2.88	2.88	coding	372	64	1179
PB.494.1	1	1648	3	novel_not_in_catalog	gene: Zm00001d029402	novel	43.07	50.95	coding	280	346	1185
PB.494.3	1	1509	3	novel_not_in_catalog	gene: Zm00001d029402	novel	7.87	50.95	coding	280	348	1187
PB.7084.2	5	2382	1	genic	gene: Zm00001d017874	novel	0.00	126.53	non coding	NA	NA	NA
PB.10827.4	9	2259	3	genic	gene: Zm00001d048199	novel	0.00	120.79	coding	545	286	1920
PB.10704.2	9	1502	5	novel_in_catalog	gene: Zm00001d047737	novel	3.63	7.06	coding	383	104	1252
PB.5650.2	4	1728	5	full-splice_match	gene: Zm00001d052797	Zm00001d052797_T00	10.78	12.31	coding	511	25	1557
PB.10344.1	9	1029	5	novel_not_in_catalog	gene: Zm00001d045812	novel	25.30	25.30	coding	210	56	685
PB.4730.4	3	1209	5	full-splice_match	gene: Zm00001d044254	Zm00001d044254_T00	0.00	57.89	coding	279	223	1059
PB.10531.1	9	1548	2	genic	gene: Zm00001d046998	novel	73.54	93.57	coding	277	392	1222
PB.10531.2	9	1907	1	full-splice_match	gene: Zm00001d046998	Zm00001d046998_T00	18.46	93.57	coding	277	748	1578
PB.10531.3	9	1481	2	genic	gene: Zm00001d046998	novel	1.57	93.57	coding	277	346	1176

PB.8816.2	7	1124	5	full-splice_match	gene: Zm00001d021668	Zm00001d021668_T00	62.94	1881.70	coding	115	259	603
PB.10047.2	8	583	5	full-splice_match	gene: Zm00001d012571	Zm00001d012571_T00	0.00	5.14	coding	191	11	583
PB.2128.2	10	1088	1	full-splice_match	gene: Zm00001d025619	Zm00001d025619_T00	51.00	159.79	coding	284	128	979
PB.1263.2	1	2017	10	full-splice_match	gene: Zm00001d033228	Zm00001d033228_T00	0.00	42.12	coding	472	185	1600
PB.10047.1	8	1969	12	full-splice_match	gene: Zm00001d012571	Zm00001d012571_T00	5.14	5.14	coding	530	11	1600
PB.10731.6	9	1695	10	full-splice_match	gene: Zm00001d047812	Zm00001d047812_T00	75.35	128.95	coding	466	254	1651
PB.4111.1	3	1660	9	full-splice_match	gene: Zm00001d041679	Zm00001d041679_T00	82.85	82.85	coding	394	140	1321
PB.1876.1	10	1202	2	full-splice_match	gene: Zm00001d024265	Zm00001d024265_T00	9.77	9.77	coding	300	213	1112
PB.1317.3	1	1492	10	incomplete-splice_match	gene: Zm00001d033480	Zm00001d033480_T00	0.00	268.39	coding	467	91	1491
PB.8830.4	7	737	1	antisense	novelGene_gene: Zm00001d021702	novel	3.79	48.64	non coding	NA	NA	NA
PB.8190.2	6	1793	10	full-splice_match	gene: Zm00001d039157	Zm00001d039157_T00	5.42	108.89	coding	163	149	637
PB.3451.2	2	1875	5	full-splice_match	gene: Zm00001d006823	Zm00001d006823_T00	16.79	27.82	coding	570	37	1746
PB.115.1	1	964	1	genic	gene: Zm00001d027703	novel	24.55	24.55	coding	227	75	755
PB.1317.1	1	2606	14	full-splice_match	gene: Zm00001d033480	Zm00001d033480_T00	268.39	268.39	coding	765	91	2385
PB.7316.1	6	2950	16	full-splice_match	gene: Zm00001d035065	Zm00001d035065_T00	33.15	63.66	coding	837	47	2557
PB.20.1	1	1174	2	full-splice_match	gene: Zm00001d027313	Zm00001d027313_T00	48.63	48.63	coding	301	68	970
PB.6018.1	5	1418	5	full-splice_match	gene: Zm00001d013026	Zm00001d013026_T00	10.60	10.60	non coding	NA	NA	NA

PB.8071.3	6	1220	1	novel_in_catalog	gene: Zm00001d038718	novel	13.98	139.28	non coding	NA	NA	NA
PB.2342.6	10	1502	1	novel_in_catalog	gene: Zm00001d026501	novel	81.53	190.01	coding	136	439	846
PB.8071.1	6	1116	2	novel_in_catalog	gene: Zm00001d038718	novel	51.44	139.28	non coding	NA	NA	NA
PB.4826.2	3	2801	1	incomplete-splice_match	gene: Zm00001d044673	Zm00001d044673_T00	1.07	66.59	coding	280	1695	2534
PB.30.1	1	813	2	incomplete-splice_match	gene: Zm00001d027347	Zm00001d027347_T00	34.84	68.66	coding	92	233	508
PB.9675.1	8	1266	3	full-splice_match	gene: Zm00001d010958	Zm00001d010958_T00	20.34	20.34	coding	326	69	1046
PB.30.4	1	1323	1	novel_in_catalog	gene: Zm00001d027347	novel	33.83	68.66	coding	86	264	521
PB.4826.1	3	2017	3	full-splice_match	gene: Zm00001d044673	Zm00001d044673_T00	65.52	66.59	coding	489	202	1668
PB.2505.1	2	3200	7	novel_not_in_catalog	gene: Zm00001d002091	novel	10.61	10.61	coding	812	362	2797
PB.1378.1	1	773	5	full-splice_match	gene: Zm00001d033727	Zm00001d033727_T00	29.50	29.50	coding	115	116	460
PB.2853.3	2	2474	9	full-splice_match	gene: Zm00001d003670	Zm00001d003670_T00	6.28	13.08	coding	397	852	2042
PB.2853.2	2	2672	9	full-splice_match	gene: Zm00001d003670	Zm00001d003670_T00	6.79	13.08	coding	699	162	2258
PB.6343.5	5	3758	7	full-splice_match	gene: Zm00001d014330	Zm00001d014330_T05	0.00	90.41	coding	69	334	540
PB.6343.4	5	3573	8	full-splice_match	gene: Zm00001d014330	Zm00001d014330_T01	0.00	90.41	coding	69	290	496
PB.5901.2	4	1883	12	full-splice_match	gene: Zm00001d053968	Zm00001d053968_T00	27.58	29.90	coding	455	145	1509
PB.5901.3	4	1975	11	full-splice_match	gene: Zm00001d053968	Zm00001d053968_T00	2.32	29.90	coding	300	146	1045
PB.6343.1	5	3909	7	full-splice_match	gene: Zm00001d014330	Zm00001d014330_T03	14.11	90.41	coding	69	316	522

PB.9746.3	8	650	1	novel_in_catalog	gene: Zm00001d011286	novel	4.04	78.68	non coding	NA	NA	NA
PB.6343.3	5	2708	8	full-splice_match	gene: Zm00001d014330	Zm00001d014330_T02	7.90	90.41	coding	153	1211	1669
PB.6343.2	5	3803	8	full-splice_match	gene: Zm00001d014330	Zm00001d014330_T01	14.70	90.41	non coding	NA	NA	NA
PB.1652.5	1	923	2	full-splice_match	gene: Zm00001d034841	Zm00001d034841_T00	25.88	25.88	coding	217	139	789
PB.9746.2	8	532	2	full-splice_match	gene: Zm00001d011286	Zm00001d011286_T00	52.11	78.68	coding	107	45	365
PB.1423.9	1	1802	10	novel_not_in_catalog	gene: Zm00001d033948	novel	6.33	76.35	coding	115	654	998
PB.7143.1	5	2514	6	full-splice_match	gene: Zm00001d018080	Zm00001d018080_T00	34.97	42.89	coding	780	40	2379
PB.3386.2	2	752	1	full-splice_match	gene: Zm00001d006515	Zm00001d006515_T00	9.19	12.48	coding	194	50	631
PB.9640.3	8	877	2	full-splice_match	gene: Zm00001d010805	Zm00001d010805_T00	52.17	81.10	coding	229	35	721
PB.10525.2	9	2625	5	novel_not_in_catalog	gene: Zm00001d046971	novel	17.10	17.10	coding	677	415	2445
PB.10837.1	9	1386	5	full-splice_match	gene: Zm00001d048253	Zm00001d048253_T00	171.98	171.98	coding	308	126	1049
PB.5383.5	4	2624	13	novel_in_catalog	gene: Zm00001d051674	novel	27.71	33.89	coding	313	175	1113
PB.10837.6	9	1322	5	full-splice_match	gene: Zm00001d048256	Zm00001d048256_T00	119.54	119.54	coding	308	110	1033
PB.5834.2	4	1245	4	full-splice_match	gene: Zm00001d053716	Zm00001d053716_T00	50.87	67.85	coding	274	51	872
PB.10376.1	9	1160	3	incomplete-splice_match	gene: Zm00001d046039	Zm00001d046039_T01	9.90	34.48	coding	279	4	840
PB.2170.1	10	2548	9	full-splice_match	gene: Zm00001d025801	Zm00001d025801_T00	228.67	366.57	coding	652	197	2152
PB.1324.3	1	3332	1	full-splice_match	gene: Zm00001d033505	Zm00001d033505_T00	0.00	10.18	coding	1044	74	3205

PB.10376.2	9	3373	17	incomplete-splice_match	gene: Zm00001d046039	Zm00001d046039_T01	24.58	34.48	coding	1016	115	3162
PB.3951.1	3	4043	7	full-splice_match	gene: Zm00001d040456	Zm00001d040456_T00	7.33	18.56	coding	1178	169	3702
PB.3951.2	3	1803	5	incomplete-splice_match	gene: Zm00001d040456	Zm00001d040456_T00	11.23	18.56	coding	500	133	1632
PB.2510.3	2	647	2	incomplete-splice_match	gene: Zm00001d002103	Zm00001d002103_T00	0.00	42.38	coding	112	8	343
PB.2779.1	2	2040	3	novel_not_in_catalog	gene: Zm00001d003281	novel	1.64	1.64	coding	576	108	1835
PB.4683.2	3	1631	6	full-splice_match	gene: Zm00001d044091	Zm00001d044091_T00	27.36	31.00	coding	382	370	1515
PB.9383.1	8	978	7	incomplete-splice_match	gene: Zm00001d009541	Zm00001d009541_T00	40.24	40.24	coding	128	260	643
PB.2510.2	2	1806	8	full-splice_match	gene: Zm00001d002103	Zm00001d002103_T00	42.38	42.38	coding	401	178	1380
PB.256.1	1	1504	4	full-splice_match	gene: Zm00001d028349	Zm00001d028349_T00	199.61	257.20	coding	334	150	1151
PB.8440.2	7	1561	13	full-splice_match	gene: Zm00001d019926	Zm00001d019926_T00	0.00	27.70	coding	374	240	1361
PB.256.3	1	1093	4	full-splice_match	gene: Zm00001d028349	Zm00001d028349_T00	0.00	257.20	coding	334	29	1030
PB.256.2	1	1233	4	full-splice_match	gene: Zm00001d028349	Zm00001d028349_T00	57.60	257.20	coding	334	56	1057
PB.4729.3	3	1791	3	novel_in_catalog	gene: Zm00001d044253	novel	3.32	100.04	coding	266	31	828
PB.5319.1	4	1447	1	genic	gene: Zm00001d051389	novel	47.66	47.66	coding	290	485	1354
PB.7916.8	6	1670	4	novel_in_catalog	gene: Zm00001d038146	novel	19.43	442.81	coding	198	581	1174
PB.7916.6	6	2603	6	novel_in_catalog	gene: Zm00001d038146	novel	24.24	442.81	coding	253	193	951
PB.309.1	1	1920	15	full-splice_match	gene: Zm00001d028569	Zm00001d028569_T00	66.29	70.92	coding	441	249	1571

PB.309.3	1	1986	14	novel_in_catalog	gene: Zm00001d028569	novel	1.79	70.92	coding	392	249	1424
PB.2436.2	2	1868	2	full-splice_match	gene: Zm00001d001818	Zm00001d001818_T00	0.00	16.44	coding	549	104	1750
PB.309.5	1	1788	15	full-splice_match	gene: Zm00001d028569	Zm00001d028569_T00	0.00	70.92	coding	441	249	1571
PB.925.2	1	1008	4	full-splice_match	gene: Zm00001d031799	Zm00001d031799_T00	2.93	4.53	coding	283	88	936
PB.2436.1	2	2174	2	full-splice_match	gene: Zm00001d001818	Zm00001d001818_T00	16.44	16.44	coding	549	174	1820
PB.5218.1	4	896	5	full-splice_match	gene: Zm00001d050696	Zm00001d050696_T00	18.34	157.46	non coding	NA	NA	NA
PB.8700.1	7	1765	1	genic	gene: Zm00001d021219	novel	20.87	20.87	coding	497	188	1678
PB.5218.3	4	809	5	full-splice_match	gene: Zm00001d050696	Zm00001d050696_T00	48.98	157.46	non coding	NA	NA	NA
PB.5218.2	4	867	5	full-splice_match	gene: Zm00001d050696	Zm00001d050696_T00	48.53	157.46	non coding	NA	NA	NA
PB.5218.5	4	717	5	full-splice_match	gene: Zm00001d050696	Zm00001d050696_T00	19.20	157.46	non coding	NA	NA	NA
PB.5218.4	4	725	5	full-splice_match	gene: Zm00001d050696	Zm00001d050696_T00	22.40	157.46	non coding	NA	NA	NA
PB.3131.1	2	1057	6	full-splice_match	gene: Zm00001d005344	Zm00001d005344_T00	205.80	205.80	coding	145	299	733
PB.5246.1	4	986	4	full-splice_match	gene: Zm00001d050920	Zm00001d050920_T00	267.94	267.94	coding	219	22	678
PB.5246.2	4	662	4	full-splice_match	gene: Zm00001d050920	Zm00001d050920_T00	0.00	267.94	coding	131	121	513
PB.8139.1	6	2031	12	full-splice_match	gene: Zm00001d038927	Zm00001d038927_T00	12.40	63.06	coding	515	139	1683
PB.2065.2	10	1646	2	incomplete-splice_match	gene: Zm00001d025210	Zm00001d025210_T00	19.94	19.94	coding	387	199	1359
PB.864.1	1	1506	2	incomplete-splice_match	gene: Zm00001d031454	Zm00001d031454_T00	13.89	54.12	coding	247	346	1086

PB.864.2	1	2385	3	full-splice_match	gene: Zm00001d031454	Zm00001d031454_T00	27.88	54.12	coding	643	187	2115
PB.7545.1	6	736	1	full-splice_match	gene: Zm00001d036452	Zm00001d036452_T00	0.22	0.22	coding	94	113	394
PB.2519.6	2	1047	5	novel_not_in_catalog	gene: Zm00001d002141	novel	0.52	657.91	coding	171	271	783
PB.2519.7	2	1016	3	full-splice_match	gene: Zm00001d002141	Zm00001d002141_T00	418.04	657.91	coding	171	256	768
PB.11000.4	B73V4 ctg4	1478	1	genic	gene: Zm00001d000400	novel	3.29	32.63	coding	105	732	1046
PB.2519.5	2	1410	2	full-splice_match	gene: Zm00001d002141	Zm00001d002141_T00	0.00	657.91	coding	171	686	1198
PB.2519.2	2	1296	3	novel_not_in_catalog	gene: Zm00001d002141	novel	5.56	657.91	coding	171	447	959
PB.11000.3	B73V4 ctg4	860	3	full-splice_match	gene: Zm00001d000400	Zm00001d000400_T00	23.96	32.63	coding	168	100	603
PB.11000.1	B73V4 ctg4	1169	2	novel_in_catalog	gene: Zm00001d000400	novel	5.38	32.63	coding	159	100	576
PB.5139.1	4	3710	14	full-splice_match	gene: Zm00001d050125	Zm00001d050125_T01	52.07	52.07	coding	1059	163	3339
PB.5432.3	4	1346	4	full-splice_match	gene: Zm00001d051912	Zm00001d051912_T00	31.90	88.26	coding	329	133	1119
PB.7227.3	5	1195	1	intergenic	novelGene_418	novel	23.62	56.56	non coding	NA	NA	NA
PB.8112.2	6	1054	1	genic	gene: Zm00001d038860	novel	3.57	23.90	coding	56	820	987
PB.8112.1	6	1533	1	genic	gene: Zm00001d038860	novel	20.33	23.90	coding	421	103	1365
PB.2519.9	2	783	2	novel_in_catalog	gene: Zm00001d002141	novel	152.40	657.91	coding	134	132	533
PB.9540.1	8	2175	2	full-splice_match	gene: Zm00001d010399	Zm00001d010399_T00	53.39	53.39	coding	498	128	1621
PB.9812.3	8	2896	23	novel_in_catalog	gene: Zm00001d011595	novel	17.32	17.32	coding	749	416	2662

PB.8580.1	7	1209	1	full-splice_match	gene: Zm00001d020651	Zm00001d020651_T00	2.58	11.97	coding	308	20	943
PB.10299.1	9	1069	8	novel_in_catalog	gene: Zm00001d045556	novel	25.63	25.63	coding	278	62	895
PB.9540.4	8	2079	2	full-splice_match	gene: Zm00001d010399	Zm00001d010399_T00	0.00	53.39	coding	498	128	1621
PB.6905.3	5	1916	10	full-splice_match	gene: Zm00001d017114	Zm00001d017114_T01	0.00	16.03	coding	628	32	1915
PB.7646.1	6	565	4	full-splice_match	gene: Zm00001d036985	Zm00001d036985_T00	89.92	89.92	coding	120	3	362
PB.5517.1	4	742	2	incomplete-splice_match	gene: Zm00001d052247	Zm00001d052247_T00	0.76	77.03	non coding	NA	NA	NA
PB.1084.2	1	1606	5	full-splice_match	gene: Zm00001d032480	Zm00001d032480_T00	77.90	158.15	coding	377	203	1333
PB.549.1	1	1840	4	full-splice_match	gene: Zm00001d029649	Zm00001d029649_T00	31.06	52.48	coding	469	128	1534
PB.1084.4	1	2504	4	full-splice_match	gene: Zm00001d032480	Zm00001d032480_T00	1.84	158.15	coding	377	1099	2229
PB.8139.8	6	1655	11	full-splice_match	gene: Zm00001d038927	Zm00001d038927_T00	0.00	63.06	coding	515	90	1634
PB.1084.6	1	1425	5	full-splice_match	gene: Zm00001d032480	Zm00001d032480_T00	70.21	158.15	coding	377	89	1219
PB.2971.1	2	1765	1	novel_in_catalog	gene: Zm00001d004410	novel	0.22	24.84	coding	114	1191	1532
PB.2053.1	10	1539	8	novel_in_catalog	gene: Zm00001d025128	novel	19.63	57.83	coding	322	8	973
PB.232.1	1	587	1	incomplete-splice_match	gene: Zm00001d028238	Zm00001d028238_T01	1.98	28.05	non coding	NA	NA	NA
PB.3313.1	2	2390	8	full-splice_match	gene: Zm00001d006212	Zm00001d006212_T00	9.35	9.35	coding	631	43	1935
PB.767.1	1	1083	2	full-splice_match	gene: Zm00001d030997	Zm00001d030997_T00	1.19	1.19	coding	312	76	1011
PB.5923.2	4	1564	6	full-splice_match	gene: Zm00001d054014	Zm00001d054014_T00	5.95	43.81	coding	430	110	1399

PB.1215.1	1	2364	9	full-splice_match	gene: Zm00001d032994	Zm00001d032994_T00	11.21	13.01	coding	618	1	1854
PB.4073.1	3	2026	2	incomplete-splice_match	gene: Zm00001d041443	Zm00001d041443_T01	11.50	11.50	coding	528	142	1725
PB.6973.1	5	3065	7	novel_in_catalog	gene: Zm00001d017392	novel	15.07	39.92	coding	486	1170	2627
PB.8565.3	7	634	6	full-splice_match	gene: Zm00001d020571	Zm00001d020571_T00	0.00	30.88	coding	153	40	498
PB.2903.1	2	2418	1	intergenic	novelGene_160	novel	26.62	26.62	coding	68	108	311
PB.9666.3	8	2149	1	novel_in_catalog	gene: Zm00001d010920	novel	9.43	1651.48	coding	396	739	1926
PB.3428.1	2	1616	2	novel_in_catalog	gene: Zm00001d006684	novel	11.45	11.45	coding	470	49	1458
PB.10022.4	8	2394	8	novel_in_catalog	gene: Zm00001d012452	novel	0.45	23.78	coding	724	111	2282
PB.10285.2	9	1945	11	novel_in_catalog	gene: Zm00001d045501	novel	16.90	20.22	coding	500	122	1621
PB.3744.2	3	1590	4	full-splice_match	gene: Zm00001d039489	Zm00001d039489_T00	7.38	7.38	coding	365	199	1293
PB.597.2	1	2014	5	novel_not_in_catalog	gene: Zm00001d029920	novel	30.17	58.01	coding	486	288	1745
PB.597.1	1	1258	3	incomplete-splice_match	gene: Zm00001d029920	Zm00001d029920_T00	27.84	58.01	coding	258	91	864
PB.4702.2	3	1992	5	novel_not_in_catalog	gene: Zm00001d044153	novel	30.43	34.32	coding	543	151	1779
PB.9202.1	8	867	6	novel_not_in_catalog	gene: Zm00001d008530	novel	43.29	43.29	coding	235	56	760
PB.939.1	1	689	2	full-splice_match	gene: Zm00001d031850	Zm00001d031850_T00	22.73	26.33	coding	125	155	529
PB.4702.1	3	645	1	genic	gene: Zm00001d044153	novel	3.89	34.32	non coding	NA	NA	NA
PB.5222.2	4	2681	6	fusion	gene: Zm00001d050726	novel	0.00	17.67	coding	809	96	2522

PB.7506.1	6	786	1	full-splice_match	gene: Zm00001d036151	Zm00001d036151_T00	199.37	717.42	coding	144	67	498
PB.10172.1	9	833	8	incomplete-splice_match	gene: Zm00001d045049	Zm00001d045049_T00	86.93	86.93	coding	167	124	624
PB.5222.1	4	2845	6	fusion	gene: Zm00001d050726	novel	17.67	17.67	coding	809	96	2522
PB.5608.2	4	1973	8	full-splice_match	gene: Zm00001d052622	Zm00001d052622_T00	8.95	8.95	coding	546	162	1799
PB.10000.4	8	1335	12	full-splice_match	gene: Zm00001d012380	Zm00001d012380_T00	1.28	34.77	coding	199	498	1094
PB.3439.3	2	2302	1	novel_in_catalog	gene: Zm00001d006753	novel	0.00	29.44	coding	454	757	2118
PB.3439.1	2	2563	1	novel_in_catalog	gene: Zm00001d006753	novel	3.46	29.44	coding	454	757	2118
PB.10107.1	8	1711	1	genic	gene: Zm00001d012789	novel	5.44	5.44	coding	210	81	710
PB.3439.4	2	2125	2	full-splice_match	gene: Zm00001d006753	Zm00001d006753_T00	14.36	29.44	coding	614	146	1987
PB.48.1	1	1343	1	intergenic	novelGene_23	novel	5.95	5.95	non coding	NA	NA	NA
PB.10668.1	9	2410	6	full-splice_match	gene: Zm00001d047608	Zm00001d047608_T00	117.32	123.54	coding	149	684	1130
PB.1596.1	1	1443	11	full-splice_match	gene: Zm00001d034659	Zm00001d034659_T00	41.53	44.31	coding	350	132	1181
PB.7506.2	6	680	1	full-splice_match	gene: Zm00001d036151	Zm00001d036151_T00	518.05	717.42	coding	144	71	502
PB.7201.1	5	1660	10	full-splice_match	gene: Zm00001d018309	Zm00001d018309_T00	139.13	172.12	coding	426	109	1386
PB.7201.3	5	1575	10	full-splice_match	gene: Zm00001d018309	Zm00001d018309_T00	27.72	172.12	coding	426	147	1424
PB.7201.2	5	2308	9	full-splice_match	gene: Zm00001d018309	Zm00001d018309_T01	5.27	172.12	coding	271	1318	2130
PB.1308.4	1	4520	4	full-splice_match	gene: Zm00001d033422	Zm00001d033422_T00	2.88	85.23	coding	287	2754	3614

PB.4408.2	3	971	1	full-splice_match	gene: Zm00001d043087	Zm00001d043087_T00	11.64	19.36	coding	212	175	810
PB.10412.1	9	1715	8	full-splice_match	gene: Zm00001d046344	Zm00001d046344_T00	28.34	28.34	coding	508	85	1608
PB.9284.1	8	1239	3	full-splice_match	gene: Zm00001d008934	Zm00001d008934_T00	4.84	4.84	coding	322	110	1075
PB.7054.1	5	3739	18	full-splice_match	gene: Zm00001d017754	Zm00001d017754_T01	39.81	85.14	coding	940	484	3303
PB.8927.1	7	2346	4	full-splice_match	gene: Zm00001d022016	Zm00001d022016_T00	29.98	40.60	coding	623	172	2040
PB.7054.3	5	2107	10	incomplete-splice_match	gene: Zm00001d017754	Zm00001d017754_T03	33.45	85.14	coding	599	27	1823
PB.7054.2	5	3892	17	novel_in_catalog	gene: Zm00001d017754	novel	10.85	85.14	coding	479	481	1917
PB.7054.4	5	3318	18	full-splice_match	gene: Zm00001d017754	Zm00001d017754_T02	0.00	85.14	coding	940	321	3140
PB.7054.6	5	3598	16	full-splice_match	gene: Zm00001d017754	Zm00001d017754_T04	1.03	85.14	coding	836	483	2990
PB.11015.4	B73V4	2281	6	full-splice_match	gene: Zm00001d000124	Zm00001d000124_T00	0.00	47.03	coding	521	542	2104
PB.11015.5	B73V4	550	3	incomplete-splice_match	gene: Zm00001d000124	Zm00001d000124_T00	7.30	47.03	non coding	NA	NA	NA
PB.5517.2	4	1264	10	full-splice_match	gene: Zm00001d052247	Zm00001d052247_T00	73.22	77.03	coding	303	156	1064
PB.11015.1	B73V4	2618	4	novel_in_catalog	gene: Zm00001d000124	novel	4.44	47.03	coding	486	537	1994
PB.11015.2	B73V4	2406	6	full-splice_match	gene: Zm00001d000124	Zm00001d000124_T00	35.29	47.03	coding	521	544	2106
PB.11015.3	B73V4	2490	5	novel_in_catalog	gene: Zm00001d000124	novel	0.00	47.03	coding	486	544	2001
PB.7137.1	5	1224	2	full-splice_match	gene: Zm00001d018056	Zm00001d018056_T00	13.25	13.25	non coding	NA	NA	NA
PB.2913.1	2	2382	2	novel_in_catalog	gene: Zm00001d003950	novel	21.35	51.54	coding	481	598	2040

PB.2913.3	2	2102	3	full-splice_match	gene: Zm00001d003950	Zm00001d003950_T00	30.19	51.54	coding	595	120	1904
PB.5707.1	4	1989	3	incomplete-splice_match	gene: Zm00001d053079	Zm00001d053079_T00	17.16	17.16	coding	536	285	1892
PB.7567.1	6	1318	1	intergenic	novelGene_197	novel	4.19	22.61	non coding	NA	NA	NA
PB.3131.4	2	916	6	full-splice_match	gene: Zm00001d005344	Zm00001d005344_T00	0.00	205.80	coding	145	299	733
PB.4401.3	3	1803	3	full-splice_match	gene: Zm00001d043069	Zm00001d043069_T00	0.00	21.29	coding	446	298	1635
PB.4401.1	3	1991	3	full-splice_match	gene: Zm00001d043069	Zm00001d043069_T00	21.29	21.29	coding	446	397	1734
PB.1871.4	10	2403	11	full-splice_match	gene: Zm00001d024212	Zm00001d024212_T00	4.41	55.15	coding	255	1369	2133
PB.6494.1	5	788	4	full-splice_match	gene: Zm00001d014926	Zm00001d014926_T00	67.75	67.75	coding	132	140	535
PB.2406.3	10	1450	1	full-splice_match	gene: Zm00001d026678	Zm00001d026678_T00	14.15	17.93	coding	409	36	1262
PB.2406.2	10	1791	1	full-splice_match	gene: Zm00001d026678	Zm00001d026678_T00	0.00	17.93	coding	409	14	1240
PB.7434.1	6	1544	6	full-splice_match	gene: Zm00001d035701	Zm00001d035701_T00	23.57	23.57	coding	382	147	1292
PB.9904.2	8	1164	2	full-splice_match	gene: Zm00001d011971	Zm00001d011971_T00	21.72	49.53	coding	288	193	1056
PB.7693.1	6	960	5	full-splice_match	gene: Zm00001d037237	Zm00001d037237_T00	28.90	34.18	non coding	NA	NA	NA
PB.575.2	1	2312	7	novel_in_catalog	gene: Zm00001d029782	novel	2.70	118.69	coding	489	106	1572
PB.575.1	1	2212	8	full-splice_match	gene: Zm00001d029782	Zm00001d029782_T00	109.28	118.69	coding	578	139	1872
PB.7172.15	5	1329	6	full-splice_match	gene: Zm00001d018187	Zm00001d018187_T00	0.00	145.24	coding	366	95	1192
PB.7172.13	5	1550	6	full-splice_match	gene: Zm00001d018187	Zm00001d018187_T00	122.39	145.24	coding	366	137	1234

PB.575.5	1	3047	6	full-splice_match	gene: Zm00001d029782	Zm00001d029782_T01	6.72	118.69	coding	489	140	1606
PB.7172.11	5	2848	1	full-splice_match	gene: Zm00001d018185	Zm00001d018185_T00	0.00	37.92	coding	882	93	2738
PB.2930.1	2	1608	2	novel_not_in_catalog	gene: Zm00001d004072	novel	6.79	6.79	coding	371	125	1237
PB.782.2	1	2240	8	full-splice_match	gene: Zm00001d031050	Zm00001d031050_T00	5.96	65.50	coding	611	108	1940
PB.8597.3	7	1632	8	novel_not_in_catalog	gene: Zm00001d020764	novel	1.11	57.76	coding	324	137	1108
PB.1871.7	10	2119	13	full-splice_match	gene: Zm00001d024212	Zm00001d024212_T00	7.33	55.15	coding	500	404	1903
PB.8589.3	7	1215	3	full-splice_match	gene: Zm00001d020697	Zm00001d020697_T00	69.47	108.04	coding	89	631	897
PB.8589.2	7	1620	2	novel_in_catalog	gene: Zm00001d020697	novel	38.56	108.04	coding	45	664	798
PB.5527.2	4	1290	4	novel_in_catalog	gene: Zm00001d052268	novel	12.50	134.67	coding	153	31	489
PB.1969.1	10	994	2	incomplete-splice_match	gene: Zm00001d024768	Zm00001d024768_T00	9.39	9.39	coding	249	71	817
PB.10000.1	8	1819	12	full-splice_match	gene: Zm00001d012380	Zm00001d012380_T00	22.22	34.77	coding	324	442	1413
PB.5527.8	4	1110	3	novel_in_catalog	gene: Zm00001d052268	novel	0.00	134.67	coding	153	30	488
PB.7229.2	5	1579	1	full-splice_match	gene: Zm00001d018407	Zm00001d018407_T00	9.48	9.91	coding	403	8	1216
PB.7229.1	5	2649	1	full-splice_match	gene: Zm00001d018407	Zm00001d018407_T00	0.43	9.91	coding	403	57	1265
PB.1303.1	1	1935	3	novel_in_catalog	gene: Zm00001d033393	novel	9.03	9.03	coding	41	191	313
PB.8914.2	7	756	5	full-splice_match	gene: Zm00001d021958	Zm00001d021958_T00	63.97	214.16	coding	157	107	577
PB.194.1	1	2948	5	full-splice_match	gene: Zm00001d028075	Zm00001d028075_T00	32.56	62.03	coding	613	845	2683

PB.2149.7	10	2348	2	full-splice_match	gene: Zm00001d025714	Zm00001d025714_T00	0.00	87.28	coding	510	624	2153
PB.6468.1	5	1591	6	full-splice_match	gene: Zm00001d014850	Zm00001d014850_T00	8.52	8.52	coding	413	107	1345
PB.4096.1	3	1862	1	antisense	novelGene_gene: Zm00001d041584	novel	0.83	0.83	coding	101	1438	1740
PB.4548.5	3	1692	7	full-splice_match	gene: Zm00001d043573	Zm00001d043573_T00	0.00	48.90	coding	490	98	1567
PB.561.1	1	2640	1	full-splice_match	gene: Zm00001d029686	Zm00001d029686_T00	7.84	7.84	coding	753	113	2371
PB.4619.2	3	839	1	full-splice_match	gene: Zm00001d043830	Zm00001d043830_T00	0.00	60.49	coding	205	70	684
PB.4381.3	3	2246	6	full-splice_match	gene: Zm00001d042943	Zm00001d042943_T00	160.11	160.11	coding	231	50	742
PB.4674.1	3	2352	6	full-splice_match	gene: Zm00001d044038	Zm00001d044038_T00	18.38	18.38	coding	639	198	2114
PB.7947.5	6	973	4	full-splice_match	gene: Zm00001d038248	Zm00001d038248_T00	45.85	226.44	coding	137	68	478
PB.7066.3	5	2974	9	novel_in_catalog	gene: Zm00001d017810	novel	0.00	42.14	coding	435	1421	2725
PB.7066.6	5	3971	6	novel_in_catalog	gene: Zm00001d017810	novel	10.89	42.14	coding	237	2167	2877
PB.7066.7	5	3217	7	novel_in_catalog	gene: Zm00001d017810	novel	13.38	42.14	coding	272	2167	2982
PB.1975.3	10	1352	7	full-splice_match	gene: Zm00001d024788	Zm00001d024788_T00	5.97	14.88	coding	344	139	1170
PB.1975.2	10	1455	7	full-splice_match	gene: Zm00001d024788	Zm00001d024788_T00	8.91	14.88	coding	344	82	1113
PB.652.1	1	1273	3	full-splice_match	gene: Zm00001d030191	Zm00001d030191_T00	8.59	8.59	coding	262	64	849
PB.3770.1	3	1536	9	full-splice_match	gene: Zm00001d039568	Zm00001d039568_T00	6.37	30.58	coding	390	31	1200
PB.3770.2	3	1466	9	full-splice_match	gene: Zm00001d039568	Zm00001d039568_T00	24.21	30.58	coding	390	72	1241

PB.9121.1	8	1502	3	novel_not_in_catalog	gene: Zm00001d008201	novel	5.54	5.54	non coding	NA	NA	NA
PB.6016.1	5	696	2	incomplete-splice_match	gene: Zm00001d013019	Zm00001d013019_T02	133.85	133.85	non coding	NA	NA	NA
PB.10732.1	9	2977	3	full-splice_match	gene: Zm00001d047813	Zm00001d047813_T00	24.78	24.78	coding	734	239	2440
PB.8597.1	7	2785	10	full-splice_match	gene: Zm00001d020764	Zm00001d020764_T00	4.21	57.76	coding	304	1558	2469
PB.7150.1	5	2715	1	full-splice_match	gene: Zm00001d018102	Zm00001d018102_T00	6.26	6.26	coding	849	44	2590
PB.4059.1	3	1426	10	incomplete-splice_match	gene: Zm00001d041353	Zm00001d041353_T00	6.49	6.49	coding	342	121	1146
PB.2438.1	2	3199	7	full-splice_match	gene: Zm00001d001825	Zm00001d001825_T00	16.83	25.82	coding	787	580	2940
PB.2438.2	2	2789	7	full-splice_match	gene: Zm00001d001825	Zm00001d001825_T00	8.99	25.82	coding	787	340	2700
PB.2146.1	10	1378	1	full-splice_match	gene: Zm00001d025704	Zm00001d025704_T00	18.13	28.93	coding	341	62	1084
PB.1124.7	1	1568	12	novel_not_in_catalog	gene: Zm00001d032608	novel	2.82	59.83	coding	224	762	1433
PB.4528.1	3	2092	4	full-splice_match	gene: Zm00001d043500	Zm00001d043500_T00	14.83	14.83	coding	543	135	1763
PB.1124.5	1	1646	12	novel_not_in_catalog	gene: Zm00001d032608	novel	0.00	59.83	coding	224	842	1513
PB.1124.4	1	1454	13	novel_not_in_catalog	gene: Zm00001d032608	novel	43.65	59.83	coding	414	70	1311
PB.7577.1	6	987	3	incomplete-splice_match	gene: Zm00001d036611	Zm00001d036611_T00	12.60	12.60	coding	227	132	812
PB.6939.1	5	716	2	incomplete-splice_match	gene: Zm00001d017216	Zm00001d017216_T00	18.28	18.28	coding	66	168	365
PB.8561.3	7	1610	5	novel_in_catalog	gene: Zm00001d020555	novel	0.00	32.85	coding	127	323	703
PB.2057.2	10	2069	4	full-splice_match	gene: Zm00001d025171	Zm00001d025171_T00	13.85	61.22	coding	232	1133	1828

PB.6460.2	5	998	4	full-splice_match	gene: Zm00001d014811	Zm00001d014811_T00	43.17	52.33	coding	208	75	698
PB.6460.1	5	1042	4	full-splice_match	gene: Zm00001d014811	Zm00001d014811_T00	9.17	52.33	coding	208	70	693
PB.9302.1	8	2542	1	full-splice_match	gene: Zm00001d009013	Zm00001d009013_T00	29.00	29.00	coding	611	350	2182
PB.2048.1	10	1590	5	novel_in_catalog	gene: Zm00001d025106	novel	9.93	27.26	coding	105	169	483
PB.6181.2	5	2559	12	full-splice_match	gene: Zm00001d013599	Zm00001d013599_T00	19.60	67.07	coding	750	218	2467
PB.9429.3	8	1009	7	full-splice_match	gene: Zm00001d009774	Zm00001d009774_T00	119.51	182.71	coding	238	86	799
PB.9429.2	8	1205	7	full-splice_match	gene: Zm00001d009774	Zm00001d009774_T00	54.85	182.71	coding	238	163	876
PB.9429.1	8	1308	7	full-splice_match	gene: Zm00001d009774	Zm00001d009774_T00	8.35	182.71	coding	238	86	799
PB.1511.4	1	2166	5	full-splice_match	gene: Zm00001d034345	Zm00001d034345_T00	315.87	1673.99	coding	169	1357	1863
PB.8420.1	7	1366	8	full-splice_match	gene: Zm00001d019754	Zm00001d019754_T00	72.94	106.12	coding	316	39	986
PB.3121.2	2	1295	3	novel_not_in_catalog	gene: Zm00001d005295	novel	36.09	76.03	coding	328	129	1112
PB.8420.3	7	2329	3	incomplete-splice_match	gene: Zm00001d019754	Zm00001d019754_T00	1.31	106.12	non coding	NA	NA	NA
PB.8420.2	7	1363	8	full-splice_match	gene: Zm00001d019754	Zm00001d019754_T00	31.88	106.12	coding	316	176	1123
PB.10647.1	9	3435	8	novel_in_catalog	gene: Zm00001d047527	novel	26.23	40.68	coding	984	13	2964
PB.7819.1	6	514	4	full-splice_match	gene: Zm00001d037785	Zm00001d037785_T00	49.84	52.93	non coding	NA	NA	NA
PB.7819.2	6	3255	1	novel_in_catalog	gene: Zm00001d037785	novel	3.09	52.93	coding	200	117	716
PB.4045.2	3	1238	4	intergenic	novelGene_216	novel	33.05	113.17	non coding	NA	NA	NA

PB.3397.1	2	1419	2	full-splice_match	gene: Zm00001d006581	Zm00001d006581_T00	32.17	41.01	coding	345	58	1092
PB.4045.1	3	1083	5	intergenic	novelGene_216	novel	80.12	113.17	non coding	NA	NA	NA
PB.10000.2	8	1900	10	novel_in_catalog	gene: Zm00001d012380	novel	11.27	34.77	coding	229	360	1046
PB.10378.2	9	1344	8	full-splice_match	gene: Zm00001d046060	Zm00001d046060_T00	25.20	25.20	coding	322	149	1114
PB.10586.3	9	1444	2	genic	gene: Zm00001d027176	novel	7.02	69.51	non coding	NA	NA	NA
PB.7464.1	6	1258	5	full-splice_match	gene: Zm00001d035920	Zm00001d035920_T00	120.10	164.36	coding	270	110	919
PB.2104.2	10	1743	10	novel_in_catalog	gene: Zm00001d025446	novel	0.00	19.82	coding	517	23	1573
PB.7464.3	6	778	3	incomplete-splice_match	gene: Zm00001d035920	Zm00001d035920_T00	44.26	164.36	coding	170	150	659
PB.5587.2	4	1087	4	full-splice_match	gene: Zm00001d052473	Zm00001d052473_T00	28.72	66.04	coding	204	334	945
PB.389.1	1	926	1	full-splice_match	gene: Zm00001d028912	Zm00001d028912_T00	10.27	10.27	coding	142	362	787
PB.10586.1	9	884	4	novel_not_in_catalog	gene: Zm00001d027176	novel	62.49	69.51	non coding	NA	NA	NA
PB.4520.8	3	1379	1	novel_in_catalog	gene: Zm00001d043479	novel	6.23	136.35	non coding	NA	NA	NA
PB.745.2	1	1237	4	novel_not_in_catalog	gene: Zm00001d030867	novel	1.93	6.63	coding	100	283	582
PB.10228.4	9	457	1	intergenic	novelGene_77	novel	6.97	6.97	non coding	NA	NA	NA
PB.375.1	1	1154	3	novel_in_catalog	gene: Zm00001d028862	novel	9.85	55.77	coding	186	148	705
PB.4520.5	3	1027	6	novel_in_catalog	gene: Zm00001d043479	novel	0.00	136.35	coding	216	216	863
PB.375.3	1	869	4	full-splice_match	gene: Zm00001d028862	Zm00001d028862_T00	37.09	55.77	coding	178	179	712

PB.375.2	1	957	4	full-splice_match	gene: Zm00001d028862	Zm00001d028862_T00	8.83	55.77	coding	178	147	680
PB.7949.4	6	2150	4	full-splice_match	gene: Zm00001d038262	Zm00001d038262_T00	0.00	90.57	coding	620	93	1952
PB.4520.2	3	959	5	novel_in_catalog	gene: Zm00001d043479	novel	22.48	136.35	non coding	NA	NA	NA
PB.4520.3	3	1224	6	full-splice_match	gene: Zm00001d043479	Zm00001d043479_T00	5.90	136.35	coding	208	299	922
PB.3982.1	3	2167	1	genic	gene: Zm00001d040644	novel	21.69	21.69	coding	475	486	1910
PB.1625.1	1	2264	17	full-splice_match	gene: Zm00001d034750	Zm00001d034750_T00	28.37	28.37	coding	664	59	2050
PB.546.2	1	734	1	full-splice_match	gene: Zm00001d029628	Zm00001d029628_T00	82.35	183.64	coding	129	65	451
PB.8920.1	7	1914	12	incomplete-splice_match	gene: Zm00001d021972	Zm00001d021972_T00	12.98	21.79	coding	472	173	1588
PB.7342.3	6	1197	4	full-splice_match	gene: Zm00001d035201	Zm00001d035201_T00	314.41	508.78	coding	319	132	1088
PB.6264.1	5	1003	2	incomplete-splice_match	gene: Zm00001d014013	Zm00001d014013_T00	17.85	30.58	coding	175	83	607
PB.9010.4	7	1211	5	full-splice_match	gene: Zm00001d022324	Zm00001d022324_T00	0.00	156.21	coding	273	205	1023
PB.5652.1	4	1360	9	novel_in_catalog	gene: Zm00001d052803	novel	11.81	60.14	coding	264	79	870
PB.2599.3	2	2031	8	novel_in_catalog	gene: Zm00001d002504	novel	4.69	38.68	coding	158	1230	1703
PB.8247.1	7	3227	3	novel_in_catalog	gene: Zm00001d018746	novel	3.49	3.49	coding	123	5	373
PB.395.2	1	1528	2	full-splice_match	gene: Zm00001d028930	Zm00001d028930_T00	4.21	55.77	coding	336	192	1199
PB.395.1	1	1745	2	full-splice_match	gene: Zm00001d028930	Zm00001d028930_T00	51.56	55.77	coding	336	193	1200
PB.1544.3	1	585	3	full-splice_match	gene: Zm00001d034491	Zm00001d034491_T00	192.54	426.33	coding	133	86	484

PB.950.2	1	1511	4	full-splice_match	gene: Zm00001d031913	Zm00001d031913_T00	0.00	37.65	coding	393	131	1309
PB.5145.1	4	995	4	incomplete-splice_match	gene: Zm00001d050153	Zm00001d050153_T00	45.51	45.51	coding	175	77	601
PB.3210.1	2	1564	3	intergenic	novelGene_383	novel	4.83	4.83	coding	152	268	723
PB.6264.2	5	2604	4	full-splice_match	gene: Zm00001d014013	Zm00001d014013_T00	12.73	30.58	coding	644	406	2337
PB.445.6	1	1901	10	novel_in_catalog	gene: Zm00001d029144	novel	0.00	100.94	coding	549	255	1901
PB.1152.1	1	908	1	novel_in_catalog	gene: Zm00001d032724	novel	7.67	7.67	coding	114	86	427
PB.7671.1	6	750	1	novel_in_catalog	gene: Zm00001d037114	novel	1.25	1.25	coding	119	155	511
PB.1606.2	1	438	1	full-splice_match	gene: Zm00001d034680	Zm00001d034680_T00	0.00	28.35	non coding	NA	NA	NA
PB.10462.3	9	1640	9	incomplete-splice_match	gene: Zm00001d046590	Zm00001d046590_T00	0.00	35.75	coding	403	72	1280
PB.4516.1	3	3723	1	antisense	novelGene_gene: Zm00001d043467	novel	0.89	0.89	non coding	NA	NA	NA
PB.8575.1	7	3356	7	full-splice_match	gene: Zm00001d020636	Zm00001d020636_T00	268.51	268.51	coding	171	72	584
PB.10834.1	9	2830	20	full-splice_match	gene: Zm00001d048235	Zm00001d048235_T00	39.04	39.04	coding	746	121	2358
PB.7662.1	6	1591	1	full-splice_match	gene: Zm00001d037073	Zm00001d037073_T00	9.60	9.60	coding	438	55	1368
PB.7256.4	5	1757	14	novel_in_catalog	gene: Zm00001d018484	novel	5.41	44.98	coding	211	361	993
PB.4130.2	3	1263	5	full-splice_match	gene: Zm00001d041758	Zm00001d041758_T00	0.00	34.04	coding	363	80	1168
PB.4130.1	3	1415	5	full-splice_match	gene: Zm00001d041758	Zm00001d041758_T00	34.04	34.04	coding	363	81	1169
PB.1552.2	1	2143	7	novel_not_in_catalog	gene: Zm00001d034508	novel	15.66	27.43	coding	633	25	1923

PB.5652.3	4	1111	8	full-splice_match	gene: Zm00001d052803	Zm00001d052803_T00	9.12	60.14	coding	264	67	858
PB.878.1	1	2099	5	full-splice_match	gene: Zm00001d031531	Zm00001d031531_T00	3.39	3.39	coding	446	516	1853
PB.10577.3	9	3831	2	full-splice_match	gene: Zm00001d047240	Zm00001d047240_T00	25.05	89.69	coding	785	1373	3727
PB.950.3	1	1400	4	full-splice_match	gene: Zm00001d031913	Zm00001d031913_T00	7.99	37.65	coding	393	133	1311
PB.6583.1	5	1846	11	incomplete-splice_match	gene: Zm00001d015314	Zm00001d015314_T01	7.47	27.84	coding	587	86	1846
PB.749.1	1	1565	5	incomplete-splice_match	gene: Zm00001d030894	Zm00001d030894_T00	16.52	16.52	coding	213	333	971
PB.682.2	1	1935	8	fusion	gene: Zm00001d030379	novel	2.71	16.22	coding	376	130	1257
PB.8928.1	7	2383	2	full-splice_match	gene: Zm00001d022017	Zm00001d022017_T00	4.07	14.03	coding	614	152	1993
PB.8928.2	7	2246	2	full-splice_match	gene: Zm00001d022017	Zm00001d022017_T00	9.96	14.03	coding	614	150	1991
PB.682.1	1	1886	9	fusion	gene: Zm00001d030379	novel	13.51	16.22	coding	493	164	1642
PB.7342.1	6	1326	4	full-splice_match	gene: Zm00001d035201	Zm00001d035201_T00	194.37	508.78	coding	319	171	1127
PB.6402.3	5	1737	1	full-splice_match	gene: Zm00001d014625	Zm00001d014625_T00	7.63	34.69	coding	506	109	1626
PB.6402.2	5	2125	1	full-splice_match	gene: Zm00001d014625	Zm00001d014625_T00	0.31	34.69	coding	506	328	1845
PB.6402.1	5	2029	1	full-splice_match	gene: Zm00001d014625	Zm00001d014625_T00	26.76	34.69	coding	506	105	1622
PB.8891.1	7	2043	7	novel_not_in_catalog	gene: Zm00001d021871	novel	5.35	5.35	coding	571	56	1768
PB.4402.4	3	721	5	full-splice_match	gene: Zm00001d043070	Zm00001d043070_T00	0.00	553.22	coding	148	103	546
PB.8981.2	7	1570	4	novel_in_catalog	gene: Zm00001d022225	novel	0.29	66.30	coding	135	879	1283

PB.454.1	1	1302	6	novel_in_catalog	gene: Zm00001d029198	novel	21.18	21.18	coding	273	216	1034
PB.6154.1	5	532	1	full-splice_match	gene: Zm00001d013481	Zm00001d013481_T00	62.38	62.38	non coding	NA	NA	NA
PB.454.2	1	1130	6	novel_in_catalog	gene: Zm00001d029198	novel	0.00	21.18	coding	273	216	1034
PB.1225.1	1	2757	14	full-splice_match	gene: Zm00001d033028	Zm00001d033028_T00	17.36	46.92	coding	724	406	2577
PB.7037.1	5	2677	7	incomplete-splice_match	gene: Zm00001d017660	Zm00001d017660_T00	19.66	19.66	coding	526	225	1802
PB.2844.15	2	1031	2	novel_in_catalog	gene: Zm00001d003616	novel	4.57	198.34	coding	155	103	567
PB.3758.3	3	2052	13	full-splice_match	gene: Zm00001d039526	Zm00001d039526_T00	111.45	111.45	coding	484	52	1503
PB.6227.4	5	1828	9	full-splice_match	gene: Zm00001d013831	Zm00001d013831_T00	0.00	62.18	coding	173	214	732
PB.6227.5	5	2258	7	novel_in_catalog	gene: Zm00001d013831	novel	10.18	62.18	coding	179	363	899
PB.2844.11	2	1443	4	novel_in_catalog	gene: Zm00001d003616	novel	6.88	198.34	coding	107	888	1208
PB.6227.3	5	2112	7	full-splice_match	gene: Zm00001d013831	Zm00001d013831_T01	19.95	62.18	coding	179	264	800
PB.6227.1	5	1965	8	full-splice_match	gene: Zm00001d013831	Zm00001d013831_T01	7.73	62.18	coding	179	367	903
PB.10614.1	9	2817	19	full-splice_match	gene: Zm00001d047426	Zm00001d047426_T00	6.40	60.48	coding	412	306	1541
PB.10614.2	9	2348	20	full-splice_match	gene: Zm00001d047426	Zm00001d047426_T00	43.95	60.48	coding	631	257	2149
PB.10248.4	9	1396	1	novel_in_catalog	gene: Zm00001d045386	novel	66.87	89.45	coding	206	677	1294
PB.2844.18	2	1370	9	full-splice_match	gene: Zm00001d003616	Zm00001d003616_T02	31.81	198.34	coding	334	11	1012
PB.6227.8	5	1992	7	novel_in_catalog	gene: Zm00001d013831	novel	8.07	62.18	coding	173	214	732

PB.8981.6	7	2020	3	novel_in_catalog	gene: Zm00001d022225	novel	8.95	66.30	coding	135	1423	1827
PB.10318.1	9	963	2	genic	gene: Zm00001d045651	novel	23.45	23.45	coding	200	142	741
PB.6593.1	5	1606	7	full-splice_match	gene: Zm00001d015374	Zm00001d015374_T00	7.58	7.58	coding	388	46	1209
PB.7775.6	6	1906	11	full-splice_match	gene: Zm00001d037606	Zm00001d037606_T00	0.00	169.87	coding	518	1	1554
PB.7775.1	6	1294	2	incomplete-splice_match	gene: Zm00001d037606	Zm00001d037606_T00	12.68	169.87	non coding	NA	NA	NA
PB.8981.5	7	1648	1	novel_in_catalog	gene: Zm00001d022225	novel	6.41	66.30	coding	135	1051	1455
PB.9403.1	8	1683	14	novel_in_catalog	gene: Zm00001d009661	novel	9.22	9.22	coding	266	613	1410
PB.7775.2	6	2533	11	full-splice_match	gene: Zm00001d037606	Zm00001d037606_T00	98.50	169.87	coding	528	135	1718
PB.8973.1	7	1357	5	full-splice_match	gene: Zm00001d022190	Zm00001d022190_T00	88.05	105.00	coding	289	211	1077
PB.8973.2	7	1389	4	full-splice_match	gene: Zm00001d022190	Zm00001d022190_T00	16.95	105.00	coding	213	134	772
PB.4582.11	3	1974	14	novel_not_in_catalog	gene: Zm00001d043693	novel	0.00	284.74	coding	470	308	1717
PB.4065.1	3	2022	1	full-splice_match	gene: Zm00001d041387	Zm00001d041387_T00	22.99	22.99	coding	414	127	1368
PB.6434.1	5	1059	1	full-splice_match	gene: Zm00001d014757	Zm00001d014757_T00	107.63	107.63	coding	214	138	779
PB.6434.2	5	969	1	full-splice_match	gene: Zm00001d014757	Zm00001d014757_T00	0.00	107.63	coding	214	143	784
PB.7454.1	6	1260	9	incomplete-splice_match	gene: Zm00001d035862	Zm00001d035862_T00	25.78	25.78	coding	290	18	887
PB.6382.1	5	1435	2	full-splice_match	gene: Zm00001d014532	Zm00001d014532_T00	58.38	62.29	coding	321	312	1274
PB.6382.2	5	1395	2	full-splice_match	gene: Zm00001d014532	Zm00001d014532_T00	3.92	62.29	coding	321	330	1292

PB.8109.2	6	1124	3	full-splice_match	gene: Zm00001d038852	Zm00001d038852_T00	30.26	55.39	coding	233	249	947
PB.9278.3	8	1228	1	full-splice_match	gene: Zm00001d008905	Zm00001d008905_T00	10.80	27.04	coding	313	88	1026
PB.10219.1	9	1476	3	full-splice_match	gene: Zm00001d045298	Zm00001d045298_T00	146.38	155.16	coding	366	208	1305
PB.2367.1	10	1554	3	novel_not_in_catalog	gene: Zm00001d026568	novel	2.43	2.43	coding	179	451	987
PB.10219.2	9	1690	2	novel_in_catalog	gene: Zm00001d045298	novel	8.78	155.16	coding	253	766	1524
PB.7916.18	6	990	1	novel_in_catalog	gene: Zm00001d038146	novel	102.81	442.81	coding	161	97	579
PB.7916.19	6	2171	6	novel_in_catalog	gene: Zm00001d038146	novel	0.00	442.81	coding	189	189	755
PB.1420.1	1	1702	10	novel_in_catalog	gene: Zm00001d033931	novel	189.36	439.36	coding	379	209	1345
PB.2415.1	10	932	6	full-splice_match	gene: Zm00001d026706	Zm00001d026706_T00	203.49	277.23	coding	215	110	754
PB.6509.3	5	2623	4	incomplete-splice_match	gene: Zm00001d014977	Zm00001d014977_T00	50.35	84.86	coding	797	60	2450
PB.6509.2	5	5253	13	full-splice_match	gene: Zm00001d014977	Zm00001d014977_T02	34.52	84.86	coding	1674	56	5077
PB.2611.1	2	759	3	novel_not_in_catalog	gene: Zm00001d002584	novel	0.20	0.20	coding	90	196	465
PB.7916.11	6	2472	6	novel_in_catalog	gene: Zm00001d038146	novel	0.00	442.81	coding	253	189	947
PB.7916.14	6	3309	4	novel_in_catalog	gene: Zm00001d038146	novel	7.87	442.81	coding	189	193	759
PB.6774.1	5	1144	1	intergenic	novelGene_336	novel	27.50	27.50	non coding	NA	NA	NA
PB.7916.17	6	2377	7	novel_not_in_catalog	gene: Zm00001d038146	novel	86.48	442.81	coding	253	189	947
PB.4394.1	3	2259	5	full-splice_match	gene: Zm00001d043025	Zm00001d043025_T00	25.05	25.05	coding	555	221	1885

PB.1156.3	1	2707	3	full-splice_match	gene: Zm00001d032739	Zm00001d032739_T00	7.38	110.46	coding	443	80	1408
PB.2587.2	2	1021	4	incomplete-splice_match	gene: Zm00001d002426	Zm00001d002426_T00	36.63	36.63	coding	215	115	759
PB.8255.1	7	1642	3	full-splice_match	gene: Zm00001d018788	Zm00001d018788_T00	10.54	10.54	coding	466	83	1480
PB.4343.2	3	1075	4	novel_not_in_catalog	gene: Zm00001d042778	novel	1.35	26.10	coding	134	191	592
PB.3902.1	3	1211	3	novel_not_in_catalog	gene: Zm00001d040275	novel	20.28	20.28	coding	297	69	959
PB.4667.1	3	1995	1	antisense	novelGene_gene: Zm00001d044004	novel	9.69	9.69	non coding	NA	NA	NA
PB.6304.1	5	1022	2	genic	gene: Zm00001d014162	novel	8.51	8.51	coding	185	440	994
PB.4343.1	3	1169	3	full-splice_match	gene: Zm00001d042778	Zm00001d042778_T00	24.75	26.10	coding	256	189	956
PB.5387.1	4	1606	9	novel_not_in_catalog	gene: Zm00001d051682	novel	6.02	43.92	coding	219	662	1318
PB.6643.1	5	1952	14	fusion	gene: Zm00001d015724	novel	6.65	6.65	coding	548	35	1678
PB.4708.3	3	1565	10	full-splice_match	gene: Zm00001d044172	Zm00001d044172_T00	321.45	349.37	coding	361	206	1288
PB.5679.2	4	1321	3	novel_in_catalog	gene: Zm00001d052944	novel	5.00	54.99	coding	224	49	720
PB.1107.1	1	2329	5	full-splice_match	gene: Zm00001d032547	Zm00001d032547_T00	37.49	42.63	coding	517	563	2113
PB.4807.1	3	1889	7	full-splice_match	gene: Zm00001d044584	Zm00001d044584_T01	35.64	35.64	coding	499	164	1660
PB.8372.2	7	1373	9	full-splice_match	gene: Zm00001d019467	Zm00001d019467_T00	28.06	50.06	coding	333	107	1105
PB.4714.1	3	2117	11	novel_in_catalog	gene: Zm00001d044195	novel	44.93	44.93	coding	314	1021	1962
PB.10225.1	9	3324	1	full-splice_match	gene: Zm00001d045313	Zm00001d045313_T00	20.65	76.72	coding	705	115	2229

PB.10225.2	9	2598	1	full-splice_match	gene: Zm00001d045313	Zm00001d045313_T00	3.44	76.72	coding	705	188	2302
PB.10225.3	9	2349	1	full-splice_match	gene: Zm00001d045313	Zm00001d045313_T00	52.64	76.72	coding	385	921	2075
PB.6097.1	5	1129	2	full-splice_match	gene: Zm00001d013289	Zm00001d013289_T00	6.38	6.38	coding	250	193	942
PB.9413.1	8	2200	8	full-splice_match	gene: Zm00001d009708	Zm00001d009708_T00	120.08	149.99	coding	539	240	1856
PB.9413.2	8	1406	7	novel_in_catalog	gene: Zm00001d009708	novel	29.91	149.99	coding	154	333	794
PB.2159.5	10	1412	5	novel_in_catalog	gene: Zm00001d025752	novel	9.32	98.80	coding	124	466	837
PB.9413.4	8	1284	5	incomplete-splice_match	gene: Zm00001d009708	Zm00001d009708_T00	0.00	149.99	coding	422	19	1284
PB.7439.2	6	3016	6	novel_in_catalog	gene: Zm00001d035736	novel	1.09	55.73	coding	233	2115	2813
PB.8825.1	7	2520	6	novel_in_catalog	gene: Zm00001d021696	novel	3.71	26.16	coding	320	758	1717
PB.4706.2	3	2574	12	full-splice_match	gene: Zm00001d044170	Zm00001d044170_T00	18.21	21.01	coding	702	86	2191
PB.6718.3	5	1454	3	novel_not_in_catalog	gene: Zm00001d016230	novel	2.44	9.50	coding	328	93	1076
PB.2159.3	10	1369	6	novel_not_in_catalog	gene: Zm00001d025752	novel	5.73	98.80	coding	134	467	868
PB.6376.1	5	1438	1	full-splice_match	gene: Zm00001d014518	Zm00001d014518_T00	0.43	181.76	non coding	NA	NA	NA
PB.9074.4	7	1252	1	genic	gene: Zm00001d022506	novel	23.75	48.71	coding	187	481	1041
PB.6376.3	5	865	1	full-splice_match	gene: Zm00001d014518	Zm00001d014518_T00	68.05	181.76	non coding	NA	NA	NA
PB.6376.2	5	1611	1	full-splice_match	gene: Zm00001d014518	Zm00001d014518_T00	3.91	181.76	non coding	NA	NA	NA
PB.6376.5	5	570	1	full-splice_match	gene: Zm00001d014518	Zm00001d014518_T00	31.93	181.76	coding	92	129	404

PB.6376.4	5	776	1	full-splice_match	gene: Zm00001d014518	Zm00001d014518_T00	77.44	181.76	non coding	NA	NA	NA
PB.9074.3	7	1160	2	novel_not_in_catalog	gene: Zm00001d022506	novel	2.32	48.71	coding	187	423	983
PB.9074.2	7	1390	1	genic	gene: Zm00001d022506	novel	0.00	48.71	coding	187	477	1037
PB.3284.1	2	1099	1	full-splice_match	gene: Zm00001d006101	Zm00001d006101_T00	69.98	369.14	coding	151	86	538
PB.1879.3	10	2004	7	novel_in_catalog	gene: Zm00001d024281	novel	0.00	33.52	coding	331	691	1683
PB.5752.1	4	2253	14	full-splice_match	gene: Zm00001d053316	Zm00001d053316_T01	10.58	44.66	coding	308	527	1450
PB.9227.1	8	1057	3	full-splice_match	gene: Zm00001d008645	Zm00001d008645_T00	65.44	65.44	coding	267	51	851
PB.2736.5	2	1357	8	full-splice_match	gene: Zm00001d003114	Zm00001d003114_T01	3.27	38.10	coding	255	353	1117
PB.2736.4	2	3195	7	novel_in_catalog	gene: Zm00001d003114	novel	3.50	38.10	coding	217	116	766
PB.2736.6	2	1730	9	novel_in_catalog	gene: Zm00001d003114	novel	25.66	38.10	coding	458	121	1494
PB.2088.1	10	2974	3	full-splice_match	gene: Zm00001d025342	Zm00001d025342_T00	39.81	39.81	coding	791	398	2770
PB.2736.3	2	1916	9	novel_in_catalog	gene: Zm00001d003114	novel	2.46	38.10	coding	217	118	768
PB.2736.2	2	1292	2	novel_in_catalog	gene: Zm00001d003114	novel	3.22	38.10	non coding	NA	NA	NA
PB.6615.1	5	1713	1	novel_in_catalog	gene: Zm00001d015515	novel	212.74	212.74	coding	282	459	1304
PB.755.1	1	2320	8	full-splice_match	gene: Zm00001d030916	Zm00001d030916_T00	17.07	42.36	coding	597	93	1883
PB.1184.1	1	2170	13	novel_in_catalog	gene: Zm00001d032852	novel	10.90	10.90	coding	423	644	1912
PB.8249.1	7	1565	3	novel_in_catalog	gene: Zm00001d018751	novel	15.80	47.68	coding	203	615	1223

PB.8249.2	7	1746	5	novel_not_in_catalog	gene: Zm00001d018751	novel	31.88	47.68	coding	483	120	1568
PB.1276.2	1	1510	9	full-splice_match	gene: Zm00001d033294	Zm00001d033294_T00	12.57	20.52	coding	272	541	1356
PB.8666.1	7	1253	3	novel_not_in_catalog	gene: Zm00001d021048	novel	9.24	9.24	coding	344	77	1108
PB.5752.6	4	828	1	novel_in_catalog	gene: Zm00001d053316	novel	17.06	44.66	non coding	NA	NA	NA
PB.4356.1	3	1360	2	full-splice_match	gene: Zm00001d042843	Zm00001d042843_T00	9.36	12.13	coding	262	237	1022
PB.4356.2	3	1331	1	novel_in_catalog	gene: Zm00001d042843	novel	2.77	12.13	coding	262	55	840
PB.8876.2	7	2244	4	intergenic	novelGene_325	novel	13.52	31.00	coding	120	81	440
PB.5663.5	4	823	3	full-splice_match	gene: Zm00001d052855	Zm00001d052855_T00	0.00	384.89	coding	243	94	822
PB.1941.3	10	1285	3	full-splice_match	gene: Zm00001d024630	Zm00001d024630_T00	0.00	13.72	coding	351	76	1128
PB.3551.1	2	1122	8	full-splice_match	gene: Zm00001d007234	Zm00001d007234_T00	87.88	532.34	coding	191	357	929
PB.4587.1	3	700	1	intergenic	novelGene_400	novel	11.66	628.03	non coding	NA	NA	NA
PB.2405.1	10	2183	16	full-splice_match	gene: Zm00001d026677	Zm00001d026677_T00	549.42	621.14	coding	562	188	1873
PB.4587.2	3	574	1	intergenic	novelGene_400	novel	611.95	628.03	non coding	NA	NA	NA
PB.1640.2	1	945	2	full-splice_match	gene: Zm00001d034803	Zm00001d034803_T00	0.00	41.97	coding	234	55	756
PB.4496.1	3	1603	4	novel_in_catalog	gene: Zm00001d043404	novel	9.01	9.01	non coding	NA	NA	NA
PB.9080.2	7	2120	2	incomplete-splice_match	gene: Zm00001d022527	Zm00001d022527_T02	26.28	41.03	coding	622	54	1919
PB.5411.1	4	2275	6	incomplete-splice_match	gene: Zm00001d051854	Zm00001d051854_T00	47.96	47.96	coding	660	59	2038

PB.3883.1	3	783	5	full-splice_match	gene: Zm00001d040164	Zm00001d040164_T00	49.08	188.13	coding	119	78	434
PB.6087.1	5	1067	3	full-splice_match	gene: Zm00001d013263	Zm00001d013263_T00	125.40	125.40	coding	217	113	763
PB.6174.1	5	1108	1	antisense	novelGene_gene: Zm00001d013572	novel	1.84	1.84	non coding	NA	NA	NA
PB.2076.4	10	2054	11	novel_not_in_catalog	gene: Zm00001d025300	novel	9.42	57.17	coding	186	1415	1972
PB.3551.7	2	944	9	full-splice_match	gene: Zm00001d007234	Zm00001d007234_T00	347.83	532.34	coding	250	82	831
PB.5741.1	4	2737	4	full-splice_match	gene: Zm00001d053283	Zm00001d053283_T00	9.45	31.68	coding	183	1981	2529
PB.10580.1	9	1075	3	full-splice_match	gene: Zm00001d047262	Zm00001d047262_T00	31.77	62.71	coding	129	167	553
PB.10580.2	9	962	3	full-splice_match	gene: Zm00001d047262	Zm00001d047262_T00	30.94	62.71	coding	129	167	553
PB.10580.3	9	824	3	full-splice_match	gene: Zm00001d047262	Zm00001d047262_T00	0.00	62.71	coding	129	165	551
PB.2076.2	10	1644	13	novel_not_in_catalog	gene: Zm00001d025300	novel	1.77	57.17	coding	266	757	1554
PB.2405.2	10	2520	15	novel_in_catalog	gene: Zm00001d026677	novel	24.71	621.14	coding	444	501	1832
PB.873.1	1	2973	9	full-splice_match	gene: Zm00001d031502	Zm00001d031502_T00	8.50	59.06	coding	353	185	1243
PB.5200.1	4	867	5	novel_not_in_catalog	gene: Zm00001d050495	novel	21.68	21.68	coding	100	377	676
PB.10207.1	9	871	3	novel_not_in_catalog	gene: Zm00001d045210	novel	26.26	133.02	coding	154	71	532
PB.10207.2	9	729	3	novel_not_in_catalog	gene: Zm00001d045210	novel	101.06	133.02	coding	154	67	528
PB.10207.3	9	2482	2	full-splice_match	gene: Zm00001d045210	Zm00001d045210_T00	5.70	133.02	coding	154	67	528
PB.8584.1	7	1698	2	full-splice_match	gene: Zm00001d020673	Zm00001d020673_T00	2.84	2.84	coding	432	137	1432

PB.5879.3	4	2564	5	novel_in_catalog	gene: Zm00001d053890	novel	0.65	7.14	coding	138	1811	2224
PB.1812.8	10	470	3	incomplete-splice_match	gene: Zm00001d023824	Zm00001d023824_T00	15.08	81.60	coding	126	93	470
PB.8726.1	7	1721	1	novel_in_catalog	gene: Zm00001d021318	novel	173.33	182.56	coding	333	454	1452
PB.8878.1	7	3331	2	incomplete-splice_match	gene: Zm00001d021830	Zm00001d021830_T00	9.87	9.87	coding	887	78	2738
PB.1576.2	1	1628	5	incomplete-splice_match	gene: Zm00001d034597	Zm00001d034597_T00	22.82	70.06	coding	420	369	1628
PB.1972.1	10	1044	2	intergenic	novelGene_192	novel	17.41	24.22	non coding	NA	NA	NA
PB.8115.3	6	1365	10	incomplete-splice_match	gene: Zm00001d038866	Zm00001d038866_T00	29.74	225.75	coding	295	228	1112
PB.1812.3	10	1182	5	full-splice_match	gene: Zm00001d023824	Zm00001d023824_T00	27.64	81.60	coding	145	77	511
PB.2347.2	10	1957	8	full-splice_match	gene: Zm00001d026514	Zm00001d026514_T00	42.45	52.71	coding	440	225	1544
PB.1812.5	10	875	6	full-splice_match	gene: Zm00001d023824	Zm00001d023824_T00	0.00	81.60	coding	195	83	667
PB.1812.4	10	978	6	full-splice_match	gene: Zm00001d023824	Zm00001d023824_T00	34.40	81.60	coding	195	83	667
PB.1812.7	10	722	3	incomplete-splice_match	gene: Zm00001d023824	Zm00001d023824_T00	4.48	81.60	coding	146	83	520
PB.1635.5	1	1209	2	incomplete-splice_match	gene: Zm00001d034784	Zm00001d034784_T00	34.70	97.45	coding	214	440	1081
PB.5752.5	4	2326	14	novel_in_catalog	gene: Zm00001d053316	novel	3.27	44.66	coding	190	169	738
PB.6515.6	5	2297	8	full-splice_match	gene: Zm00001d014993	Zm00001d014993_T00	45.00	264.21	coding	663	120	2108
PB.10131.1	9	1083	3	full-splice_match	gene: Zm00001d044844	Zm00001d044844_T00	32.87	32.87	coding	118	267	620
PB.6515.2	5	2316	8	full-splice_match	gene: Zm00001d014993	Zm00001d014993_T00	60.06	264.21	coding	663	128	2116

PB.9435.2	8	1901	7	novel_not_in_catalog	gene: Zm00001d009808	novel	9.69	9.69	coding	192	683	1258
PB.4251.1	3	1914	7	full-splice_match	gene: Zm00001d042367	Zm00001d042367_T00	38.10	38.10	coding	493	263	1741
PB.6515.1	5	2524	8	full-splice_match	gene: Zm00001d014993	Zm00001d014993_T00	159.15	264.21	coding	663	128	2116
PB.4609.4	3	2851	9	novel_in_catalog	gene: Zm00001d043785	novel	5.03	14.58	coding	482	70	1515
PB.10689.2	9	1779	1	full-splice_match	gene: Zm00001d047685	Zm00001d047685_T00	10.03	48.44	coding	375	506	1630
PB.4935.1	4	1803	9	full-splice_match	gene: Zm00001d049092	Zm00001d049092_T00	70.80	70.80	coding	375	171	1295
PB.1044.1	1	413	1	antisense	novelGene_gene: Zm00001d032332	novel	0.00	0.00	non coding	NA	NA	NA
PB.9899.2	8	1246	4	incomplete-splice_match	gene: Zm00001d011953	Zm00001d011953_T00	61.10	149.53	coding	314	28	969
PB.8191.2	6	787	2	full-splice_match	gene: Zm00001d039158	Zm00001d039158_T00	96.12	145.49	coding	154	85	546
PB.9899.1	8	3139	15	full-splice_match	gene: Zm00001d011953	Zm00001d011953_T00	88.44	149.53	coding	801	208	2610
PB.7008.3	5	659	1	full-splice_match	gene: Zm00001d017534	Zm00001d017534_T00	0.00	41.70	coding	131	156	548
PB.7008.1	5	875	1	full-splice_match	gene: Zm00001d017534	Zm00001d017534_T00	41.70	41.70	coding	131	156	548
PB.1800.1	10	882	1	full-splice_match	gene: Zm00001d023753	Zm00001d023753_T00	5.29	5.29	coding	155	143	607
PB.6852.2	5	1473	11	full-splice_match	gene: Zm00001d016891	Zm00001d016891_T00	17.34	56.25	coding	369	21	1127
PB.8747.4	7	2191	7	full-splice_match	gene: Zm00001d021392	Zm00001d021392_T00	0.00	240.57	coding	639	167	2083
PB.5578.1	4	601	3	novel_not_in_catalog	gene: Zm00001d052436	novel	202.13	419.23	non coding	NA	NA	NA
PB.2837.1	2	909	1	incomplete-splice_match	gene: Zm00001d003567	Zm00001d003567_T00	85.65	85.65	coding	170	228	737

PB.5315.4	4	1092	2	antisense	novelGene_gene: Zm00001d051349	novel	5.60	36.67	non coding	NA	NA	NA
PB.10217.3	9	2408	18	full-splice_match	gene: Zm00001d045295	Zm00001d045295_T00	8.43	85.91	coding	675	126	2150
PB.10217.2	9	2635	18	full-splice_match	gene: Zm00001d045295	Zm00001d045295_T00	0.00	85.91	coding	451	917	2269
PB.10217.1	9	2489	19	novel_in_catalog	gene: Zm00001d045295	novel	41.67	85.91	coding	649	135	2081
PB.8805.1	7	1591	8	incomplete-splice_match	gene: Zm00001d021623	Zm00001d021623_T00	124.98	124.98	coding	397	158	1348
PB.10217.7	9	2436	18	full-splice_match	gene: Zm00001d045295	Zm00001d045295_T00	7.50	85.91	coding	451	919	2271
PB.10217.5	9	2243	19	novel_in_catalog	gene: Zm00001d045295	novel	28.30	85.91	coding	649	133	2079
PB.8695.2	7	892	1	full-splice_match	gene: Zm00001d021208	Zm00001d021208_T00	693.31	799.63	coding	231	98	790
PB.5833.1	4	1317	1	antisense	novelGene_gene: Zm00001d053715	novel	20.42	20.42	coding	95	125	409
PB.4929.1	4	462	1	full-splice_match	gene: Zm00001d049061	Zm00001d049061_T00	112.62	112.62	coding	92	70	345
PB.3918.1	3	700	4	incomplete-splice_match	gene: Zm00001d040308	Zm00001d040308_T00	5.27	12.53	coding	95	166	450
PB.3918.2	3	961	7	novel_not_in_catalog	gene: Zm00001d040308	novel	7.26	12.53	coding	259	74	850
PB.7329.1	6	2617	2	novel_in_catalog	gene: Zm00001d035140	novel	61.79	61.79	coding	506	574	2091
PB.5849.1	4	639	1	intergenic	novelGene_442	novel	5.29	5.29	non coding	NA	NA	NA
PB.1468.2	1	1641	10	full-splice_match	gene: Zm00001d034125	Zm00001d034125_T00	119.80	228.74	coding	383	153	1301
PB.10939.1	9	2294	10	full-splice_match	gene: Zm00001d048574	Zm00001d048574_T00	30.80	30.80	coding	597	249	2039
PB.9168.2	8	1063	1	antisense	novelGene_gene: Zm00001d008377	novel	43.33	143.94	non coding	NA	NA	NA

PB.9168.1	8	1378	1	antisense	novelGene_gene: Zm00001d008377	novel	100.60	143.94	non coding	NA	NA	NA
PB.6243.3	5	2673	16	novel_in_catalog	gene: Zm00001d013911	novel	7.12	57.26	coding	301	80	982
PB.6243.4	5	2172	18	novel_in_catalog	gene: Zm00001d013911	novel	1.68	57.26	coding	311	44	976
PB.6243.5	5	2054	19	full-splice_match	gene: Zm00001d013911	Zm00001d013911_T00	0.00	57.26	coding	618	80	1933
PB.9046.1	7	963	1	full-splice_match	gene: Zm00001d022430	Zm00001d022430_T00	10.25	10.25	coding	200	130	729
PB.9801.1	8	1761	4	incomplete-splice_match	gene: Zm00001d011560	Zm00001d011560_T02	16.31	16.31	coding	301	300	1202
PB.7701.1	6	1842	1	novel_in_catalog	gene: Zm00001d037250	novel	4.86	14.96	coding	276	703	1530
PB.7701.2	6	1550	1	novel_in_catalog	gene: Zm00001d037250	novel	10.10	14.96	coding	276	644	1471
PB.116.3	1	2437	8	incomplete-splice_match	gene: Zm00001d027707	Zm00001d027707_T00	14.36	100.41	coding	756	36	2303
PB.116.1	1	2783	9	full-splice_match	gene: Zm00001d027707	Zm00001d027707_T00	86.04	100.41	coding	807	119	2539
PB.1808.1	10	1243	5	full-splice_match	gene: Zm00001d023808	Zm00001d023808_T00	43.50	43.50	coding	225	241	915
PB.6140.1	5	2325	16	full-splice_match	gene: Zm00001d013426	Zm00001d013426_T00	59.43	80.17	coding	611	127	1959
PB.6140.2	5	2279	16	full-splice_match	gene: Zm00001d013426	Zm00001d013426_T00	20.73	80.17	coding	611	192	2024
PB.4611.1	3	2557	16	incomplete-splice_match	gene: Zm00001d043797	Zm00001d043797_T02	10.41	166.78	coding	546	309	1946
PB.1210.1	1	1171	7	full-splice_match	gene: Zm00001d032974	Zm00001d032974_T00	59.98	59.98	coding	299	94	990
PB.7315.2	6	1251	1	full-splice_match	gene: Zm00001d035055	Zm00001d035055_T00	0.00	824.02	coding	318	123	1076
PB.7315.1	6	1352	1	full-splice_match	gene: Zm00001d035055	Zm00001d035055_T00	824.02	824.02	coding	318	123	1076

PB.8767.5	7	767	8	novel_not_in_catalog	gene: Zm00001d021480	novel	7.56	33.61	coding	134	56	457
PB.2405.7	10	2133	16	full-splice_match	gene: Zm00001d026677	Zm00001d026677_T00	0.00	621.14	coding	562	188	1873
PB.3238.6	2	2350	12	novel_in_catalog	gene: Zm00001d005925	novel	0.00	190.45	coding	350	108	1157
PB.6704.1	5	1222	12	fusion	gene: Zm00001d016155	novel	12.47	12.47	coding	400	21	1220
PB.8767.3	7	1106	9	novel_in_catalog	gene: Zm00001d021480	novel	12.48	33.61	non coding	NA	NA	NA
PB.8767.2	7	1621	7	novel_in_catalog	gene: Zm00001d021480	novel	13.57	33.61	non coding	NA	NA	NA
PB.4543.1	3	1734	13	full-splice_match	gene: Zm00001d043555	Zm00001d043555_T00	31.12	35.77	coding	452	141	1496
PB.5366.1	4	2224	11	full-splice_match	gene: Zm00001d051609	Zm00001d051609_T00	129.94	129.94	coding	467	445	1845
PB.269.4	1	2736	1	full-splice_match	gene: Zm00001d028391	Zm00001d028391_T00	0.00	103.79	coding	794	94	2475
PB.269.3	1	2847	1	full-splice_match	gene: Zm00001d028391	Zm00001d028391_T00	0.00	103.79	coding	794	94	2475
PB.269.2	1	2908	1	full-splice_match	gene: Zm00001d028391	Zm00001d028391_T00	74.20	103.79	coding	794	94	2475
PB.269.1	1	1862	1	full-splice_match	gene: Zm00001d028391	Zm00001d028391_T00	29.58	103.79	coding	387	58	1218
PB.4273.7	3	1372	1	genic	gene: Zm00001d042482	novel	0.00	130.54	coding	165	164	658
PB.7810.1	6	693	1	novel_in_catalog	gene: Zm00001d037736	novel	33.96	33.96	coding	147	16	456
PB.4273.4	3	3164	1	genic	gene: Zm00001d042482	novel	12.58	130.54	coding	153	1549	2007
PB.8747.1	7	2456	7	full-splice_match	gene: Zm00001d021392	Zm00001d021392_T00	129.61	240.57	coding	639	167	2083
PB.4273.2	3	1787	5	novel_in_catalog	gene: Zm00001d042482	novel	21.23	130.54	coding	314	203	1144

PB.4273.1	3	1721	6	full-splice_match	gene: Zm00001d042482	Zm00001d042482_T00	96.73	130.54	coding	362	209	1294
PB.8057.2	6	2275	8	full-splice_match	gene: Zm00001d038625	Zm00001d038625_T00	0.00	104.32	coding	635	107	2011
PB.5465.1	4	2440	11	full-splice_match	gene: Zm00001d052057	Zm00001d052057_T00	29.08	36.17	coding	497	698	2188
PB.4543.2	3	1776	12	full-splice_match	gene: Zm00001d043555	Zm00001d043555_T00	4.65	35.77	coding	247	110	850
PB.5465.5	4	2462	11	full-splice_match	gene: Zm00001d052057	Zm00001d052057_T00	7.10	36.17	coding	497	728	2218
PB.843.4	1	1905	7	novel_not_in_catalog	gene: Zm00001d031315	novel	4.14	14.23	coding	179	584	1120
PB.1106.1	1	843	2	full-splice_match	gene: Zm00001d032556	Zm00001d032556_T00	34.95	34.95	coding	162	47	532
PB.7924.2	6	1839	8	full-splice_match	gene: Zm00001d038167	Zm00001d038167_T00	49.12	74.79	coding	476	295	1722
PB.4650.1	3	3744	9	fusion	gene: Zm00001d043915	novel	27.18	27.18	coding	1052	300	3455
PB.9916.5	8	2237	14	full-splice_match	gene: Zm00001d012007	Zm00001d012007_T00	14.46	40.89	coding	545	243	1877
PB.9380.3	8	1140	12	full-splice_match	gene: Zm00001d009522	Zm00001d009522_T00	9.33	33.74	coding	242	101	826
PB.9380.2	8	1284	11	full-splice_match	gene: Zm00001d009522	Zm00001d009522_T00	14.61	33.74	coding	242	101	826
PB.9380.1	8	1157	12	full-splice_match	gene: Zm00001d009522	Zm00001d009522_T00	9.80	33.74	coding	242	53	778
PB.6658.3	5	1117	1	novel_in_catalog	gene: Zm00001d015829	novel	12.25	85.07	coding	133	555	953
PB.5423.3	4	1953	4	novel_in_catalog	gene: Zm00001d051884	novel	0.00	41.91	coding	125	1250	1624
PB.5633.2	4	1038	7	full-splice_match	gene: Zm00001d052742	Zm00001d052742_T00	4.34	32.69	coding	189	224	790
PB.5633.3	4	1419	6	full-splice_match	gene: Zm00001d052742	Zm00001d052742_T00	3.99	32.69	non coding	NA	NA	NA

PB.5423.2	4	2041	4	full-splice_match	gene: Zm00001d051884	Zm00001d051884_T00	22.50	41.91	coding	424	382	1653
PB.1879.5	10	1766	8	full-splice_match	gene: Zm00001d024281	Zm00001d024281_T00	11.94	33.52	coding	500	86	1585
PB.255.6	1	708	4	intergenic	novelGene_116	novel	270.82	468.24	coding	129	147	533
PB.1853.1	10	2789	15	fusion	gene: Zm00001d024043	novel	9.88	81.04	coding	376	187	1314
PB.255.4	1	721	5	intergenic	novelGene_115	novel	5.13	392.10	coding	159	70	546
PB.1853.3	10	1691	13	fusion	gene: Zm00001d024043	novel	66.40	81.04	coding	376	194	1321
PB.9172.2	8	1499	8	full-splice_match	gene: Zm00001d008406	Zm00001d008406_T00	114.23	142.15	coding	372	95	1210
PB.9172.3	8	1383	8	full-splice_match	gene: Zm00001d008406	Zm00001d008406_T00	17.01	142.15	coding	372	96	1211
PB.3377.1	2	1623	10	full-splice_match	gene: Zm00001d006461	Zm00001d006461_T00	43.23	43.23	coding	348	200	1243
PB.1853.7	10	2260	11	fusion	gene: Zm00001d024043	novel	3.37	81.04	coding	345	1225	2259
PB.1577.1	1	2289	12	full-splice_match	gene: Zm00001d034602	Zm00001d034602_T00	271.63	369.66	coding	593	50	1828
PB.5768.2	4	1164	2	full-splice_match	gene: Zm00001d053397	Zm00001d053397_T00	7.32	24.48	coding	331	48	1040
PB.9916.2	8	2316	14	novel_not_in_catalog	gene: Zm00001d012007	novel	4.71	40.89	coding	265	244	1038
PB.7033.2	5	780	2	full-splice_match	gene: Zm00001d017646	Zm00001d017646_T00	34.39	43.03	non coding	NA	NA	NA
PB.9521.1	8	2216	8	novel_not_in_catalog	gene: Zm00001d010292	novel	53.66	53.66	coding	608	173	1996
PB.5623.1	4	511	3	full-splice_match	gene: Zm00001d052718	Zm00001d052718_T00	763.54	763.54	non coding	NA	NA	NA
PB.4906.2	4	1547	4	intergenic	novelGene_49	novel	5.33	11.67	non coding	NA	NA	NA

PB.3184.1	2	5131	2	novel_in_catalog	gene: Zm00001d005672	novel	21.13	21.13	coding	162	3512	3997
PB.8138.1	6	1327	8	incomplete-splice_match	gene: Zm00001d038922	Zm00001d038922_T00	42.40	42.40	coding	347	171	1211
PB.3414.1	2	3457	7	incomplete-splice_match	gene: Zm00001d006628	Zm00001d006628_T00	58.70	104.94	coding	969	63	2969
PB.3551.2	2	1180	7	novel_in_catalog	gene: Zm00001d007234	novel	6.06	532.34	coding	168	354	857
PB.8489.1	7	808	2	full-splice_match	gene: Zm00001d020214	Zm00001d020214_T00	16.20	16.20	non coding	NA	NA	NA
PB.5245.2	4	903	1	full-splice_match	gene: Zm00001d050918	Zm00001d050918_T00	37.18	52.51	coding	106	323	640
PB.5245.1	4	1151	1	full-splice_match	gene: Zm00001d050918	Zm00001d050918_T00	15.33	52.51	coding	106	455	772
PB.5590.2	4	1604	1	genic	gene: Zm00001d052480	novel	0.00	12.61	coding	351	473	1525
PB.5590.1	4	1649	1	genic	gene: Zm00001d052480	novel	12.61	12.61	coding	351	472	1524
PB.10928.1	9	2220	15	incomplete-splice_match	gene: Zm00001d048546	Zm00001d048546_T01	29.39	54.67	coding	608	21	1844
PB.7290.1	6	1796	5	antisense	novelGene_gene: Zm00001d034967	novel	3.09	3.09	non coding	NA	NA	NA
PB.9089.1	7	1438	3	full-splice_match	gene: Zm00001d022550	Zm00001d022550_T00	8.05	8.05	coding	280	486	1325
PB.7998.1	6	745	2	full-splice_match	gene: Zm00001d038408	Zm00001d038408_T00	5.48	5.48	coding	144	109	540
PB.3484.4	2	1204	2	full-splice_match	gene: Zm00001d006933	Zm00001d006933_T00	90.49	316.37	coding	362	48	1133
PB.3978.1	3	476	3	full-splice_match	gene: Zm00001d040617	Zm00001d040617_T00	865.63	865.63	non coding	NA	NA	NA
PB.10695.1	9	1464	2	genic	gene: Zm00001d047709	novel	53.08	53.08	coding	333	55	1053
PB.9729.1	8	1279	3	full-splice_match	gene: Zm00001d011208	Zm00001d011208_T00	47.15	47.15	coding	313	92	1030

PB.6245.3	5	1895	3	full-splice_match	gene: Zm00001d013927	Zm00001d013927_T00	8.37	35.42	coding	499	225	1721
PB.10653.4	9	2291	9	full-splice_match	gene: Zm00001d047539	Zm00001d047539_T00	0.00	36.82	coding	631	33	1925
PB.7337.1	6	1809	6	novel_in_catalog	gene: Zm00001d035185	novel	8.58	8.58	coding	411	250	1482
PB.3920.2	3	3785	23	full-splice_match	gene: Zm00001d040311	Zm00001d040311_T00	25.06	25.06	coding	1066	80	3277
PB.1306.1	1	875	3	novel_not_in_catalog	gene: Zm00001d033407	novel	3.08	3.08	coding	229	34	720
PB.2958.1	2	975	1	intergenic	novelGene_199	novel	0.28	0.28	coding	162	341	826
PB.1565.1	1	2127	13	full-splice_match	gene: Zm00001d034565	Zm00001d034565_T00	32.92	32.92	coding	611	180	2012
PB.920.1	1	1792	7	incomplete-splice_match	gene: Zm00001d031749	Zm00001d031749_T00	46.22	52.73	coding	497	102	1592
PB.10560.5	9	2668	11	novel_not_in_catalog	gene: Zm00001d047166	novel	172.66	415.37	coding	363	91	1179
PB.10365.15	9	678	3	incomplete-splice_match	gene: Zm00001d045965	Zm00001d045965_T00	2.28	432.40	coding	188	113	676
PB.10365.14	9	2108	4	novel_in_catalog	gene: Zm00001d045965	novel	5.47	432.40	coding	255	761	1525
PB.10365.11	9	1337	6	novel_not_in_catalog	gene: Zm00001d045965	novel	0.27	432.40	coding	357	103	1173
PB.10365.10	9	1408	6	full-splice_match	gene: Zm00001d045965	Zm00001d045965_T00	384.51	432.40	coding	376	109	1236
PB.10365.12	9	1889	4	novel_in_catalog	gene: Zm00001d045965	novel	0.96	432.40	coding	184	759	1310
PB.5861.3	4	1125	9	full-splice_match	gene: Zm00001d053822	Zm00001d053822_T00	0.00	120.93	coding	208	280	903
PB.4814.5	3	1647	2	full-splice_match	gene: Zm00001d044604	Zm00001d044604_T00	99.59	217.43	coding	450	53	1402
PB.4814.6	3	1623	1	novel_in_catalog	gene: Zm00001d044604	novel	0.00	217.43	coding	221	819	1481

PB.4814.7	3	1520	2	full-splice_match	gene: Zm00001d044604	Zm00001d044604_T00	0.00	217.43	coding	450	49	1398
PB.2978.1	2	1204	1	full-splice_match	gene: Zm00001d004451	Zm00001d004451_T00	37.45	37.45	coding	337	68	1078
PB.6089.1	5	3042	15	novel_in_catalog	gene: Zm00001d013267	novel	22.47	22.47	coding	350	421	1470
PB.4814.2	3	1750	2	full-splice_match	gene: Zm00001d044604	Zm00001d044604_T00	95.10	217.43	coding	450	55	1404
PB.4814.3	3	1799	1	novel_in_catalog	gene: Zm00001d044604	novel	19.37	217.43	coding	221	819	1481
PB.590.1	1	1431	14	full-splice_match	gene: Zm00001d029860	Zm00001d029860_T00	45.30	49.45	coding	406	69	1286
PB.7621.1	6	925	2	full-splice_match	gene: Zm00001d036839	Zm00001d036839_T00	212.65	212.65	coding	112	262	597
PB.1730.1	10	1679	1	genic	gene: Zm00001d023418	novel	3.22	3.22	coding	309	644	1570
PB.8564.3	7	1405	3	full-splice_match	gene: Zm00001d020569	Zm00001d020569_T00	0.00	28.23	coding	369	164	1270
PB.3660.10	2	3207	5	intergenic	novelGene_443	novel	10.85	53.91	coding	279	2070	2906
PB.7489.2	6	874	6	full-splice_match	gene: Zm00001d036054	Zm00001d036054_T00	99.01	180.24	coding	152	119	574
PB.7489.1	6	985	6	full-splice_match	gene: Zm00001d036054	Zm00001d036054_T00	81.23	180.24	coding	152	207	662
PB.10153.1	9	1526	2	genic	gene: Zm00001d044953	novel	18.86	18.86	coding	310	265	1194
PB.7065.3	5	2141	7	novel_in_catalog	gene: Zm00001d017802	novel	2.90	57.19	coding	288	86	949
PB.7512.1	6	2124	12	incomplete-splice_match	gene: Zm00001d036222	Zm00001d036222_T00	26.81	26.81	coding	426	256	1533
PB.8322.4	7	1125	8	novel_not_in_catalog	gene: Zm00001d019093	novel	91.48	97.39	coding	218	208	861
PB.1291.1	1	1656	7	full-splice_match	gene: Zm00001d033365	Zm00001d033365_T00	13.22	13.22	coding	372	123	1238

PB.2155.1	10	1777	2	full-splice_match	gene: Zm00001d025735	Zm00001d025735_T00	15.70	15.70	coding	467	160	1560
PB.8322.1	7	1304	5	novel_in_catalog	gene: Zm00001d019093	novel	4.39	97.39	coding	130	617	1006
PB.8322.3	7	1190	7	novel_not_in_catalog	gene: Zm00001d019093	novel	1.53	97.39	coding	192	172	747
PB.3234.1	2	1475	2	full-splice_match	gene: Zm00001d005897	Zm00001d005897_T00	13.19	22.67	coding	146	327	764
PB.11003.2	B73V 4	1713	1	full-splice_match	gene: Zm00001d000237	Zm00001d000237_T00	21.24	30.95	coding	462	177	1562
PB.277.2	1	1366	5	incomplete-splice_match	gene: Zm00001d028417	Zm00001d028417_T00	39.14	71.72	coding	378	94	1227
PB.7149.1	5	1882	3	novel_in_catalog	gene: Zm00001d018099	novel	16.63	164.45	coding	183	168	716
PB.277.1	1	3094	10	novel_in_catalog	gene: Zm00001d028417	novel	32.59	71.72	coding	887	250	2910
PB.4892.2	4	749	1	full-splice_match	gene: Zm00001d048886	Zm00001d048886_T00	61.17	79.49	coding	203	120	728
PB.8403.2	7	2288	3	full-splice_match	gene: Zm00001d019624	Zm00001d019624_T00	8.98	18.14	non coding	NA	NA	NA
PB.4892.1	4	830	1	full-splice_match	gene: Zm00001d048886	Zm00001d048886_T00	18.32	79.49	coding	220	44	703
PB.2189.1	10	1970	8	full-splice_match	gene: Zm00001d025893	Zm00001d025893_T00	25.32	25.32	coding	490	168	1637
PB.4239.3	3	977	6	full-splice_match	gene: Zm00001d042329	Zm00001d042329_T00	56.39	121.86	coding	203	142	750
PB.2189.2	10	1786	8	full-splice_match	gene: Zm00001d025893	Zm00001d025893_T00	0.00	25.32	coding	490	127	1596
PB.7327.1	6	1175	2	full-splice_match	gene: Zm00001d035136	Zm00001d035136_T00	189.06	221.45	coding	281	62	904
PB.7327.2	6	1368	2	novel_in_catalog	gene: Zm00001d035136	novel	32.39	221.45	coding	350	65	1114
PB.7757.6	6	1582	6	novel_in_catalog	gene: Zm00001d037507	novel	25.49	153.37	non coding	NA	NA	NA

PB.7943.1	6	2772	5	full-splice_match	gene: Zm00001d038227	Zm00001d038227_T00	321.60	338.21	coding	452	918	2273
PB.5555.2	4	1027	1	full-splice_match	gene: Zm00001d052371	Zm00001d052371_T00	7.64	7.97	coding	220	111	770
PB.7809.1	6	1210	2	novel_in_catalog	gene: Zm00001d037735	novel	4.38	4.38	coding	118	448	801
PB.2008.4	10	1308	2	novel_in_catalog	gene: Zm00001d024935	novel	4.35	11.42	coding	311	170	1102
PB.6728.2	5	2053	12	full-splice_match	gene: Zm00001d016286	Zm00001d016286_T01	10.07	16.00	coding	536	102	1709
PB.1077.2	1	2104	2	genic	gene: Zm00001d032460	novel	43.43	43.43	coding	439	196	1512
PB.7528.1	6	2547	4	incomplete-splice_match	gene: Zm00001d036343	Zm00001d036343_T03	27.16	27.16	coding	760	48	2327
PB.1590.1	1	1077	4	full-splice_match	gene: Zm00001d034635	Zm00001d034635_T00	356.90	359.96	coding	231	70	762
PB.2008.2	10	1420	2	novel_in_catalog	gene: Zm00001d024935	novel	7.07	11.42	coding	311	113	1045
PB.8354.3	7	1993	5	novel_in_catalog	gene: Zm00001d019288	novel	1.49	20.72	coding	472	225	1640
PB.8354.1	7	1976	6	full-splice_match	gene: Zm00001d019288	Zm00001d019288_T00	11.76	20.72	coding	431	333	1625
PB.4091.1	3	1799	2	incomplete-splice_match	gene: Zm00001d041556	Zm00001d041556_T00	50.31	103.64	coding	264	32	823
PB.5267.2	4	1751	12	full-splice_match	gene: Zm00001d051043	Zm00001d051043_T00	22.72	29.99	coding	483	63	1511
PB.4091.3	3	1285	2	incomplete-splice_match	gene: Zm00001d041556	Zm00001d041556_T00	0.00	103.64	coding	217	13	663
PB.8354.4	7	1383	6	full-splice_match	gene: Zm00001d019288	Zm00001d019288_T00	7.47	20.72	coding	289	202	1068
PB.4915.1	4	1867	9	full-splice_match	gene: Zm00001d049008	Zm00001d049008_T00	3.12	3.12	coding	464	103	1494
PB.609.1	1	1964	1	genic	gene: Zm00001d030009	novel	5.00	159.80	non coding	NA	NA	NA

PB.1019.9	1	1902	13	novel_in_catalog	gene: Zm00001d032220	novel	7.89	44.15	non coding	NA	NA	NA
PB.5544.1	4	749	2	full-splice_match	gene: Zm00001d052333	Zm00001d052333_T00	59.27	59.27	coding	109	82	408
PB.4618.2	3	1208	1	full-splice_match	gene: Zm00001d043829	Zm00001d043829_T00	2.35	2.35	coding	333	141	1139
PB.6280.1	5	577	2	full-splice_match	gene: Zm00001d014082	Zm00001d014082_T00	588.09	588.09	coding	113	62	400
PB.8816.9	7	734	5	full-splice_match	gene: Zm00001d021668	Zm00001d021668_T00	388.18	1881.70	coding	115	257	601
PB.3717.1	3	2373	24	novel_not_in_catalog	gene: Zm00001d039380	novel	10.72	10.72	coding	699	47	2143
PB.5853.1	4	1746	14	novel_in_catalog	gene: Zm00001d053798	novel	20.72	24.83	coding	412	186	1421
PB.679.2	1	814	1	full-splice_match	gene: Zm00001d030363	Zm00001d030363_T00	80.02	80.02	coding	166	56	553
PB.1961.1	10	1364	2	full-splice_match	gene: Zm00001d024738	Zm00001d024738_T00	8.18	8.18	coding	338	189	1202
PB.10728.2	9	2105	2	incomplete-splice_match	gene: Zm00001d047805	Zm00001d047805_T01	11.81	11.81	coding	180	767	1306
PB.8167.3	6	400	2	full-splice_match	gene: Zm00001d001482	Zm00001d001482_T00	10.55	23.73	non coding	NA	NA	NA
PB.3013.2	2	1992	1	full-splice_match	gene: Zm00001d004664	Zm00001d004664_T00	0.00	27.41	coding	515	209	1753
PB.10143.1	9	1289	3	novel_in_catalog	gene: Zm00001d044915	novel	52.47	52.47	non coding	NA	NA	NA
PB.7710.4	6	565	3	full-splice_match	gene: Zm00001d037275	Zm00001d037275_T00	1058.84	2715.69	coding	87	105	365
PB.4501.1	3	965	1	full-splice_match	gene: Zm00001d043422	Zm00001d043422_T00	58.38	58.38	coding	241	85	807
PB.8403.4	7	2170	4	novel_not_in_catalog	gene: Zm00001d019624	novel	4.33	18.14	coding	129	1289	1675
PB.8773.5	7	2052	12	full-splice_match	gene: Zm00001d021498	Zm00001d021498_T00	18.57	66.97	coding	566	219	1916

PB.5405.1	4	1446	9	novel_in_catalog	gene: Zm00001d051830	novel	15.67	15.67	coding	215	69	713
PB.3298.1	2	1497	1	antisense	novelGene_gene: Zm00001d006149	novel	133.41	133.41	non coding	NA	NA	NA
PB.7938.2	6	1735	14	full-splice_match	gene: Zm00001d038205	Zm00001d038205_T00	0.00	22.73	coding	492	119	1594
PB.1580.1	1	2052	6	novel_in_catalog	gene: Zm00001d034608	novel	14.18	86.52	coding	412	560	1795
PB.1580.3	1	1344	1	incomplete-splice_match	gene: Zm00001d034608	Zm00001d034608_T00	4.89	86.52	non coding	NA	NA	NA
PB.7780.1	6	1217	3	full-splice_match	gene: Zm00001d037617	Zm00001d037617_T00	8.88	24.91	coding	193	196	774
PB.9476.1	8	982	6	full-splice_match	gene: Zm00001d010061	Zm00001d010061_T00	18.56	18.56	coding	136	176	583
PB.7780.2	6	1096	3	full-splice_match	gene: Zm00001d037617	Zm00001d037617_T00	16.03	24.91	coding	193	194	772
PB.7836.5	6	2593	15	full-splice_match	gene: Zm00001d037872	Zm00001d037872_T00	0.00	53.51	coding	511	795	2327
PB.7836.4	6	2888	15	novel_in_catalog	gene: Zm00001d037872	novel	2.38	53.51	coding	451	1213	2565
PB.2224.5	10	1489	4	full-splice_match	gene: Zm00001d026047	Zm00001d026047_T00	127.58	201.68	coding	253	138	896
PB.7836.6	6	2248	16	full-splice_match	gene: Zm00001d037872	Zm00001d037872_T00	15.15	53.51	coding	589	199	1965
PB.7836.9	6	2125	15	novel_in_catalog	gene: Zm00001d037872	novel	8.95	53.51	coding	406	643	1860
PB.7836.8	6	2752	14	novel_in_catalog	gene: Zm00001d037872	novel	0.45	53.51	coding	406	1270	2487
PB.604.2	1	1501	9	full-splice_match	gene: Zm00001d029975	Zm00001d029975_T00	68.87	75.65	coding	333	281	1279
PB.604.4	1	1365	8	novel_in_catalog	gene: Zm00001d029975	novel	6.78	75.65	coding	140	772	1191
PB.1579.2	1	1211	7	novel_in_catalog	gene: Zm00001d034607	novel	8.04	25.01	coding	45	251	385

PB.1603.3	1	740	2	genic	gene: Zm00001d034677	novel	66.92	85.79	coding	123	127	495
PB.3558.1	2	1860	12	full-splice_match	gene: Zm00001d007288	Zm00001d007288_T00	30.92	30.92	coding	476	124	1551
PB.7403.1	6	1296	3	full-splice_match	gene: Zm00001d035543	Zm00001d035543_T00	871.58	908.96	coding	199	111	707
PB.2447.7	2	427	2	intergenic	novelGene_14	novel	1.46	1.46	non coding	NA	NA	NA
PB.7403.3	6	1673	1	novel_in_catalog	gene: Zm00001d035543	novel	19.45	908.96	coding	109	1105	1431
PB.7403.2	6	1272	2	novel_in_catalog	gene: Zm00001d035543	novel	17.93	908.96	coding	199	433	1029
PB.2163.5	10	1001	6	full-splice_match	gene: Zm00001d025765	Zm00001d025765_T00	0.00	12.76	coding	215	316	960
PB.2163.4	10	1180	6	full-splice_match	gene: Zm00001d025765	Zm00001d025765_T00	11.61	12.76	coding	215	322	966
PB.2195.1	10	899	1	full-splice_match	gene: Zm00001d025913	Zm00001d025913_T00	43.68	789.31	coding	150	78	527
PB.2447.8	2	725	1	genic	gene: Zm00001d001855	novel	9.71	9.71	non coding	NA	NA	NA
PB.2447.9	2	1259	8	full-splice_match	gene: Zm00001d001856	Zm00001d001856_T00	39.15	39.15	coding	263	229	1017
PB.6744.1	5	2927	19	full-splice_match	gene: Zm00001d016373	Zm00001d016373_T00	82.44	83.25	coding	879	89	2725
PB.3425.1	2	1591	4	novel_not_in_catalog	gene: Zm00001d006679	novel	19.65	476.20	coding	253	549	1307
PB.8828.8	7	1477	3	novel_not_in_catalog	gene: Zm00001d021699	novel	1.33	63.08	coding	370	22	1131
PB.989.1	1	2524	1	full-splice_match	gene: Zm00001d032075	Zm00001d032075_T00	17.28	17.28	coding	631	142	2034
PB.3404.2	2	1373	12	novel_in_catalog	gene: Zm00001d006605	novel	0.00	17.97	coding	281	134	976
PB.8300.1	7	1258	7	full-splice_match	gene: Zm00001d018967	Zm00001d018967_T00	79.83	79.83	coding	285	158	1012

PB.3404.1	2	1372	13	full-splice_match	gene: Zm00001d006605	Zm00001d006605_T00	17.97	17.97	coding	305	134	1048
PB.6015.3	5	1587	1	novel_in_catalog	gene: Zm00001d013015	novel	32.74	206.41	coding	244	716	1447
PB.6015.2	5	1178	2	full-splice_match	gene: Zm00001d013015	Zm00001d013015_T00	144.04	206.41	coding	283	113	961
PB.9620.2	8	2605	9	full-splice_match	gene: Zm00001d010710	Zm00001d010710_T00	0.00	32.75	coding	658	296	2269
PB.7106.2	5	1526	5	full-splice_match	gene: Zm00001d017960	Zm00001d017960_T00	0.00	33.88	coding	367	70	1170
PB.9620.5	8	2498	9	full-splice_match	gene: Zm00001d010710	Zm00001d010710_T00	3.92	32.75	coding	658	333	2306
PB.10437.1	9	505	2	full-splice_match	gene: Zm00001d046474	Zm00001d046474_T00	149.84	149.84	non coding	NA	NA	NA
PB.6029.2	5	1807	14	full-splice_match	gene: Zm00001d013048	Zm00001d013048_T00	25.91	58.70	coding	401	134	1336
PB.8260.2	7	1076	3	incomplete-splice_match	gene: Zm00001d018798	Zm00001d018798_T00	67.00	114.87	coding	241	86	808
PB.2572.1	2	1290	5	full-splice_match	gene: Zm00001d002366	Zm00001d002366_T00	53.40	161.87	coding	279	117	953
PB.6029.1	5	1976	14	full-splice_match	gene: Zm00001d013048	Zm00001d013048_T00	32.79	58.70	coding	401	214	1416
PB.609.2	1	1314	5	full-splice_match	gene: Zm00001d030009	Zm00001d030009_T00	2.76	159.80	coding	243	109	837
PB.5799.4	4	2240	17	novel_in_catalog	gene: Zm00001d053580	novel	2.29	81.19	coding	455	220	1584
PB.5799.7	4	1025	10	incomplete-splice_match	gene: Zm00001d053580	Zm00001d053580_T00	33.24	81.19	coding	150	360	809
PB.2572.4	2	1180	5	full-splice_match	gene: Zm00001d002366	Zm00001d002366_T00	94.96	161.87	coding	279	128	964
PB.9513.1	8	1752	3	novel_not_in_catalog	gene: Zm00001d010265	novel	83.33	83.33	coding	498	135	1628
PB.5576.1	4	1586	2	full-splice_match	gene: Zm00001d052433	Zm00001d052433_T00	84.88	84.88	coding	349	368	1414

PB.7006.2	5	2756	1	full-splice_match	gene: Zm00001d017528	Zm00001d017528_T00	36.38	70.99	coding	534	981	2582
PB.9600.2	8	707	1	full-splice_match	gene: Zm00001d010651	Zm00001d010651_T00	0.00	48.60	coding	165	104	598
PB.9600.1	8	898	1	full-splice_match	gene: Zm00001d010651	Zm00001d010651_T00	48.60	48.60	coding	165	143	637
PB.5954.2	5	1511	2	novel_not_in_catalog	gene: Zm00001d012806	novel	0.24	46.16	coding	176	799	1326
PB.4981.1	4	1009	1	full-splice_match	gene: Zm00001d049258	Zm00001d049258_T00	53.01	53.01	coding	179	199	735
PB.5954.1	5	1166	4	novel_not_in_catalog	gene: Zm00001d012806	novel	5.29	46.16	coding	176	408	935
PB.811.1	1	1474	11	full-splice_match	gene: Zm00001d031163	Zm00001d031163_T00	8.04	8.04	coding	366	118	1215
PB.328.1	1	581	3	full-splice_match	gene: Zm00001d028641	Zm00001d028641_T00	74.21	74.21	non coding	NA	NA	NA
PB.23.1	1	3386	3	full-splice_match	gene: Zm00001d027335	Zm00001d027335_T00	7.49	7.49	coding	809	542	2968
PB.5785.1	4	1474	1	full-splice_match	gene: Zm00001d053522	Zm00001d053522_T00	46.90	46.90	coding	354	91	1152
PB.9440.9	8	1134	2	novel_in_catalog	gene: Zm00001d009858	novel	0.00	870.63	coding	312	63	998
PB.6899.8	5	1708	6	novel_in_catalog	gene: Zm00001d017096	novel	0.00	254.51	coding	193	106	684
PB.9116.4	8	1481	9	full-splice_match	gene: Zm00001d008177	Zm00001d008177_T00	0.00	44.51	coding	357	168	1238
PB.9116.5	8	1413	9	full-splice_match	gene: Zm00001d008177	Zm00001d008177_T00	5.69	44.51	coding	357	218	1288
PB.8124.1	6	2145	15	full-splice_match	gene: Zm00001d038892	Zm00001d038892_T00	58.34	81.42	coding	563	64	1752
PB.9440.7	8	2607	2	novel_in_catalog	gene: Zm00001d009858	novel	9.23	870.63	coding	262	63	848
PB.2942.2	2	980	1	novel_in_catalog	gene: Zm00001d004138	novel	16.40	218.55	coding	182	174	719

PB.9116.1	8	1840	9	full-splice_match	gene: Zm00001d008177	Zm00001d008177_T00	24.41	44.51	coding	357	164	1234
PB.9116.2	8	1734	9	full-splice_match	gene: Zm00001d008177	Zm00001d008177_T00	14.41	44.51	coding	357	178	1248
PB.9440.3	8	1809	3	full-splice_match	gene: Zm00001d009858	Zm00001d009858_T00	158.96	870.63	coding	501	144	1646
PB.8415.1	7	1592	10	full-splice_match	gene: Zm00001d019719	Zm00001d019719_T00	2.86	2.86	coding	466	8	1405
PB.8831.3	7	429	2	full-splice_match	gene: Zm00001d021704	Zm00001d021704_T00	6.50	29.99	non coding	NA	NA	NA
PB.9951.1	8	2047	12	full-splice_match	gene: Zm00001d012159	Zm00001d012159_T00	15.77	15.77	coding	203	1178	1786
PB.3446.1	2	777	1	full-splice_match	gene: Zm00001d006775	Zm00001d006775_T00	30.87	30.87	coding	164	71	562
PB.10835.1	9	945	2	novel_in_catalog	gene: Zm00001d048236	novel	5.66	24.13	non coding	NA	NA	NA
PB.4686.1	3	1340	1	full-splice_match	gene: Zm00001d044100	Zm00001d044100_T00	18.35	18.35	coding	261	83	865
PB.8831.2	7	990	2	full-splice_match	gene: Zm00001d021704	Zm00001d021704_T00	20.54	29.99	coding	210	81	710
PB.6109.1	5	1134	2	incomplete-splice_match	gene: Zm00001d013319	Zm00001d013319_T00	196.55	529.46	coding	124	207	578
PB.6109.2	5	2464	6	full-splice_match	gene: Zm00001d013319	Zm00001d013319_T00	268.26	529.46	coding	621	257	2119
PB.6109.4	5	2450	6	full-splice_match	gene: Zm00001d013319	Zm00001d013319_T00	64.65	529.46	coding	621	286	2148
PB.1088.1	1	1360	9	full-splice_match	gene: Zm00001d032501	Zm00001d032501_T00	71.38	71.38	coding	307	105	1025
PB.952.2	1	2716	12	novel_in_catalog	gene: Zm00001d031927	novel	10.10	61.88	coding	102	1631	1936
PB.952.3	1	2336	12	novel_in_catalog	gene: Zm00001d031927	novel	1.32	61.88	coding	227	1049	1729
PB.8684.6	7	2229	10	intergenic	novelGene_282	novel	11.65	53.03	coding	287	1199	2059

PB.952.6	1	2524	13	novel_in_catalog	gene: Zm00001d031927	novel	6.89	61.88	coding	247	1046	1786
PB.952.7	1	2348	10	novel_in_catalog	gene: Zm00001d031927	novel	3.80	61.88	coding	202	929	1534
PB.952.4	1	1259	9	novel_in_catalog	gene: Zm00001d031927	novel	15.76	61.88	coding	102	347	652
PB.952.5	1	2416	10	full-splice_match	gene: Zm00001d031927	Zm00001d031927_T03	0.00	61.88	coding	217	1227	1877
PB.8068.1	6	479	1	full-splice_match	gene: Zm00001d038706	Zm00001d038706_T00	18.11	18.11	non coding	NA	NA	NA
PB.3250.1	2	967	1	full-splice_match	gene: Zm00001d005978	Zm00001d005978_T00	61.56	61.56	coding	157	134	604
PB.9608.1	8	515	1	intergenic	novelGene_239	novel	135.70	135.70	non coding	NA	NA	NA
PB.8828.5	7	1981	7	novel_not_in_catalog	gene: Zm00001d021699	novel	0.00	63.08	coding	582	127	1872
PB.4989.1	4	920	5	incomplete-splice_match	gene: Zm00001d049313	Zm00001d049313_T00	9.62	9.62	coding	168	35	538
PB.9931.2	8	673	1	full-splice_match	gene: Zm00001d012080	Zm00001d012080_T00	0.00	48.61	coding	107	94	414
PB.7877.3	6	4094	1	novel_in_catalog	gene: Zm00001d038022	novel	0.83	33.74	coding	259	148	924
PB.8409.1	7	1987	2	genic	gene: Zm00001d019656	novel	13.73	13.73	coding	318	996	1949
PB.7270.1	5	2700	10	full-splice_match	gene: Zm00001d018516	Zm00001d018516_T00	4.72	4.72	coding	818	105	2558
PB.2699.1	2	1723	2	full-splice_match	gene: Zm00001d002943	Zm00001d002943_T01	191.22	191.22	coding	450	245	1594
PB.4623.2	3	2490	16	novel_in_catalog	gene: Zm00001d043840	novel	4.18	20.90	coding	267	374	1174
PB.828.2	1	3383	14	novel_not_in_catalog	gene: Zm00001d031231	novel	18.30	29.39	coding	619	577	2433
PB.2892.1	2	1230	4	novel_in_catalog	gene: Zm00001d003866	novel	8.38	8.38	coding	288	115	978

PB.4995.1	4	1122	8	full-splice_match	gene: Zm00001d049348	Zm00001d049348_T00	28.64	207.58	coding	182	84	629
PB.4995.2	4	1015	8	full-splice_match	gene: Zm00001d049348	Zm00001d049348_T00	173.59	207.58	coding	182	168	713
PB.4995.3	4	2719	6	novel_in_catalog	gene: Zm00001d049348	novel	5.35	207.58	coding	86	248	505
PB.75.1	1	1283	2	full-splice_match	gene: Zm00001d027539	Zm00001d027539_T00	69.10	69.10	coding	305	63	977
PB.4995.5	4	888	8	full-splice_match	gene: Zm00001d049348	Zm00001d049348_T00	0.00	207.58	coding	182	89	634
PB.8487.2	7	598	4	full-splice_match	gene: Zm00001d020201	Zm00001d020201_T00	581.08	662.56	coding	112	65	400
PB.10974.2	B73V 4	1581	6	full-splice_match	gene: Zm00001d000030	Zm00001d000030_T00	40.04	112.09	coding	441	109	1431
PB.7260.2	5	1174	9	full-splice_match	gene: Zm00001d018489	Zm00001d018489_T00	14.56	17.90	coding	298	115	1008
PB.5146.2	4	743	4	full-splice_match	gene: Zm00001d050155	Zm00001d050155_T00	10.93	32.09	coding	197	82	672
PB.5146.1	4	866	4	full-splice_match	gene: Zm00001d050155	Zm00001d050155_T00	21.16	32.09	coding	197	80	670
PB.7260.1	5	1432	10	full-splice_match	gene: Zm00001d018489	Zm00001d018489_T00	3.34	17.90	coding	298	124	1017
PB.7877.4	6	1849	2	full-splice_match	gene: Zm00001d038022	Zm00001d038022_T00	0.00	33.74	coding	490	135	1604
PB.4562.15	3	3007	12	full-splice_match	gene: Zm00001d043613	Zm00001d043613_T00	23.42	178.80	coding	300	1785	2684
PB.4562.16	3	3150	11	full-splice_match	gene: Zm00001d043613	Zm00001d043613_T00	0.00	178.80	coding	300	1942	2841
PB.7130.1	5	2898	1	full-splice_match	gene: Zm00001d018019	Zm00001d018019_T00	0.07	2.89	coding	312	6	941
PB.7643.3	6	691	3	full-splice_match	gene: Zm00001d036978	Zm00001d036978_T00	0.00	98.71	coding	39	421	537
PB.7643.2	6	1189	3	full-splice_match	gene: Zm00001d036978	Zm00001d036978_T00	96.49	98.71	coding	275	115	939

PB.10455.1	9	925	3	full-splice_match	gene: Zm00001d046555	Zm00001d046555_T00	11.93	11.93	coding	143	64	492
PB.5485.1	4	2138	7	incomplete-splice_match	gene: Zm00001d052133	Zm00001d052133_T00	3.06	3.06	coding	426	117	1394
PB.7720.1	6	4488	42	full-splice_match	gene: Zm00001d037333	Zm00001d037333_T02	60.36	60.36	coding	1379	98	4234
PB.3707.1	3	1045	1	full-splice_match	gene: Zm00001d039354	Zm00001d039354_T00	23.71	23.71	coding	238	68	781
PB.4423.2	3	3091	3	novel_not_in_catalog	gene: Zm00001d043135	novel	5.26	24.38	coding	766	153	2450
PB.3099.9	2	4073	9	novel_in_catalog	gene: Zm00001d005164	novel	4.68	134.75	coding	235	2065	2769
PB.9802.2	8	1605	12	full-splice_match	gene: Zm00001d011563	Zm00001d011563_T00	49.23	60.42	coding	398	250	1443
PB.2129.1	10	2040	15	full-splice_match	gene: Zm00001d025626	Zm00001d025626_T00	40.89	40.89	coding	506	233	1750
PB.1438.3	1	2466	8	full-splice_match	gene: Zm00001d034000	Zm00001d034000_T00	0.00	24.42	coding	692	278	2353
PB.3099.1	2	3946	9	novel_in_catalog	gene: Zm00001d005164	novel	5.14	134.75	coding	235	1881	2585
PB.3099.2	2	1992	12	full-splice_match	gene: Zm00001d005164	Zm00001d005164_T00	92.88	134.75	coding	512	273	1808
PB.8401.1	7	2308	6	full-splice_match	gene: Zm00001d019595	Zm00001d019595_T00	102.70	102.70	coding	483	599	2047
PB.3099.5	2	2619	11	novel_in_catalog	gene: Zm00001d005164	novel	18.51	134.75	coding	396	124	1311
PB.3099.7	2	1818	11	novel_in_catalog	gene: Zm00001d005164	novel	1.82	134.75	coding	182	1143	1688
PB.10980.1	B73V4	3096	6	full-splice_match	gene: Zm00001d000435	Zm00001d000435_T01	25.75	37.64	coding	466	883	2280
PB.10980.3	B73V4	1253	1	incomplete-splice_match	gene: Zm00001d000435	Zm00001d000435_T01	8.42	37.64	non coding	NA	NA	NA
PB.10980.4	B73V4	1067	2	novel_in_catalog	gene: Zm00001d000435	novel	3.47	37.64	non coding	NA	NA	NA

PB.7168.4	5	2299	3	novel_in_catalog	gene: Zm00001d018161	novel	89.08	1797.84	coding	319	977	1933
PB.10095.2	8	1355	1	incomplete-splice_match	gene: Zm00001d012749	Zm00001d012749_T00	0.00	28.68	coding	279	243	1079
PB.5829.1	4	1830	6	full-splice_match	gene: Zm00001d053687	Zm00001d053687_T00	13.25	27.83	coding	488	67	1530
PB.1085.10	1	890	2	genic	gene: Zm00001d032485	novel	61.17	241.80	coding	143	206	634
PB.1309.3	1	1689	3	full-splice_match	gene: Zm00001d033423	Zm00001d033423_T00	0.00	17.52	coding	461	14	1396
PB.7866.1	6	2273	15	fusion	gene: Zm00001d037981	novel	10.61	10.61	coding	625	50	1924
PB.3324.2	2	821	1	genic	gene: Zm00001d006249	novel	31.78	31.78	coding	147	61	501
PB.7536.1	6	1781	5	full-splice_match	gene: Zm00001d036410	Zm00001d036410_T00	57.37	61.13	coding	467	3	1403
PB.7536.3	6	755	3	incomplete-splice_match	gene: Zm00001d036410	Zm00001d036410_T00	3.76	61.13	non coding	NA	NA	NA
PB.4384.1	3	1523	10	incomplete-splice_match	gene: Zm00001d042961	Zm00001d042961_T00	26.11	48.10	coding	337	145	1155
PB.4384.2	3	2025	10	incomplete-splice_match	gene: Zm00001d042961	Zm00001d042961_T00	22.00	48.10	coding	598	33	1826
PB.4384.3	3	905	8	incomplete-splice_match	gene: Zm00001d042961	Zm00001d042961_T00	0.00	48.10	coding	283	7	855
PB.10912.1	9	769	1	full-splice_match	gene: Zm00001d048508	Zm00001d048508_T00	7.59	7.59	coding	171	140	652
PB.8175.4	6	1201	7	full-splice_match	gene: Zm00001d039089	Zm00001d039089_T00	0.00	263.85	coding	340	40	1059
PB.5116.3	4	1217	8	full-splice_match	gene: Zm00001d049959	Zm00001d049959_T00	99.20	99.20	coding	304	128	1039
PB.4849.2	4	727	2	incomplete-splice_match	gene: Zm00001d048644	Zm00001d048644_T00	0.00	91.38	coding	71	313	525
PB.8175.3	6	1375	7	full-splice_match	gene: Zm00001d039089	Zm00001d039089_T00	0.00	263.85	coding	340	106	1125

PB.4849.1	4	1322	5	incomplete-splice_match	gene: Zm00001d048644	Zm00001d048644_T00	91.38	91.38	coding	337	7	1017
PB.1102.4	1	2423	8	incomplete-splice_match	gene: Zm00001d032539	Zm00001d032539_T00	12.67	50.15	coding	727	128	2308
PB.8882.4	7	1230	2	full-splice_match	gene: Zm00001d021839	Zm00001d021839_T00	107.34	645.47	coding	294	140	1021
PB.1197.1	1	2016	1	full-splice_match	gene: Zm00001d032900	Zm00001d032900_T00	13.61	13.61	coding	583	35	1783
PB.7653.1	6	1684	5	full-splice_match	gene: Zm00001d037034	Zm00001d037034_T00	52.99	52.99	coding	412	183	1418
PB.6129.1	5	1644	3	full-splice_match	gene: Zm00001d013390	Zm00001d013390_T00	28.20	28.20	coding	442	112	1437
PB.8828.1	7	2433	6	novel_not_in_catalog	gene: Zm00001d021699	novel	9.51	63.08	coding	559	18	1694
PB.350.1	1	1309	6	full-splice_match	gene: Zm00001d028714	Zm00001d028714_T00	56.51	56.51	coding	297	80	970
PB.4444.2	3	988	1	full-splice_match	gene: Zm00001d043205	Zm00001d043205_T00	199.90	303.35	coding	237	83	793
PB.4444.1	3	1109	1	full-splice_match	gene: Zm00001d043205	Zm00001d043205_T00	103.45	303.35	coding	237	83	793
PB.10649.1	9	1214	7	novel_in_catalog	gene: Zm00001d047528	novel	9.23	41.79	coding	330	30	1019
PB.8204.1	6	790	2	intergenic	novelGene_351	novel	36.68	67.56	non coding	NA	NA	NA
PB.3105.1	2	1360	6	full-splice_match	gene: Zm00001d005228	Zm00001d005228_T00	36.51	41.58	coding	288	59	922
PB.4348.4	3	2449	3	incomplete-splice_match	gene: Zm00001d042807	Zm00001d042807_T00	8.24	40.70	coding	367	1196	2296
PB.9815.1	8	1709	1	full-splice_match	gene: Zm00001d011618	Zm00001d011618_T00	15.28	15.28	coding	363	16	1104
PB.4855.9	4	1410	3	full-splice_match	gene: Zm00001d048680	Zm00001d048680_T00	154.60	232.95	coding	194	63	644
PB.5499.1	4	1423	3	full-splice_match	gene: Zm00001d052192	Zm00001d052192_T00	29.53	29.53	coding	345	174	1208

PB.4855.5	4	1523	3	full-splice_match	gene: Zm00001d048680	Zm00001d048680_T00	14.18	232.95	coding	194	58	639
PB.4855.4	4	1729	2	novel_in_catalog	gene: Zm00001d048680	novel	31.17	232.95	coding	194	63	644
PB.6381.1	5	1769	1	antisense	novelGene_gene: Zm00001d014530	novel	10.31	10.31	coding	153	568	1026
PB.4855.6	4	1054	4	novel_not_in_catalog	gene: Zm00001d048680	novel	32.61	232.95	coding	191	63	635
PB.8169.1	6	2442	14	novel_not_in_catalog	gene: Zm00001d039052	novel	50.07	50.07	coding	305	8	922
PB.3583.3	2	1162	4	intergenic	novelGene_433	novel	1.84	23.82	coding	213	332	970
PB.9009.1	7	1624	9	full-splice_match	gene: Zm00001d022314	Zm00001d022314_T00	28.98	28.98	coding	343	153	1181
PB.10067.1	8	1297	3	novel_not_in_catalog	gene: Zm00001d012642	novel	5.25	7.05	coding	297	64	954
PB.5160.3	4	887	6	incomplete-splice_match	gene: Zm00001d050208	Zm00001d050208_T00	0.00	54.35	coding	128	326	709
PB.5160.2	4	1359	9	full-splice_match	gene: Zm00001d050208	Zm00001d050208_T00	54.35	54.35	coding	332	102	1097
PB.5692.1	4	993	3	full-splice_match	gene: Zm00001d053010	Zm00001d053010_T00	578.37	825.25	coding	165	155	649
PB.6972.3	5	1227	8	full-splice_match	gene: Zm00001d017390	Zm00001d017390_T00	2.13	32.46	coding	225	382	1056
PB.4229.2	3	1292	2	novel_in_catalog	gene: Zm00001d042288	novel	10.02	68.03	coding	107	699	1019
PB.6972.1	5	1124	8	full-splice_match	gene: Zm00001d017390	Zm00001d017390_T00	27.65	32.46	coding	225	169	843
PB.8851.2	7	2763	8	novel_in_catalog	gene: Zm00001d021753	novel	16.20	164.99	coding	232	42	737
PB.10394.4	9	1510	6	full-splice_match	gene: Zm00001d046190	Zm00001d046190_T00	6.08	61.71	coding	401	251	1453
PB.4229.3	3	1279	2	novel_in_catalog	gene: Zm00001d042288	novel	6.10	68.03	coding	195	161	745

PB.3868.12	3	3643	11	novel_in_catalog	gene: Zm00001d040076	novel	3.06	33.65	coding	308	2587	3510
PB.3868.13	3	2465	12	full-splice_match	gene: Zm00001d040076	Zm00001d040076_T01	0.00	33.65	coding	179	1176	1712
PB.3868.14	3	2573	12	full-splice_match	gene: Zm00001d040076	Zm00001d040076_T00	0.00	33.65	coding	423	1294	2562
PB.3868.15	3	1561	13	full-splice_match	gene: Zm00001d040076	Zm00001d040076_T00	0.00	33.65	coding	423	282	1550
PB.779.1	1	894	3	full-splice_match	gene: Zm00001d031047	Zm00001d031047_T00	13.50	13.50	coding	205	64	678
PB.578.1	1	1081	4	full-splice_match	gene: Zm00001d029806	Zm00001d029806_T00	52.19	52.19	coding	230	139	828
PB.8532.1	7	1279	2	full-splice_match	gene: Zm00001d020417	Zm00001d020417_T00	30.79	31.11	coding	232	149	844
PB.7940.3	6	630	3	intergenic	novelGene_301	novel	16.47	111.88	coding	104	58	369
PB.8532.2	7	1112	2	full-splice_match	gene: Zm00001d020417	Zm00001d020417_T00	0.31	31.11	coding	232	151	846
PB.8463.3	7	1384	7	full-splice_match	gene: Zm00001d020046	Zm00001d020046_T00	12.95	32.03	coding	382	128	1273
PB.8463.1	7	1569	7	full-splice_match	gene: Zm00001d020046	Zm00001d020046_T00	19.08	32.03	coding	382	189	1334
PB.2073.1	10	2670	5	full-splice_match	gene: Zm00001d025268	Zm00001d025268_T00	87.03	87.03	coding	573	595	2313
PB.8559.1	7	1993	3	full-splice_match	gene: Zm00001d020544	Zm00001d020544_T00	268.41	268.41	coding	392	516	1691
PB.4748.13	3	1300	6	novel_in_catalog	gene: Zm00001d044327	novel	2.98	192.78	coding	148	485	928
PB.8559.2	7	1622	2	incomplete-splice_match	gene: Zm00001d020544	Zm00001d020544_T00	0.00	268.41	coding	392	282	1457
PB.4664.1	3	1741	8	full-splice_match	gene: Zm00001d043993	Zm00001d043993_T00	12.45	12.45	coding	314	629	1570
PB.4698.26	3	1470	6	full-splice_match	gene: Zm00001d044138	Zm00001d044138_T00	687.93	1495.64	coding	379	25	1161

PB.326.1	1	1729	2	full-splice_match	gene: Zm00001d028625	Zm00001d028625_T00	8.86	8.86	coding	386	211	1368
PB.8381.3	7	1865	13	novel_in_catalog	gene: Zm00001d019534	novel	2.69	78.71	coding	164	1274	1765
PB.4122.1	3	816	2	incomplete-splice_match	gene: Zm00001d041726	Zm00001d041726_T00	3.23	13.31	coding	169	34	540
PB.902.1	1	2220	5	full-splice_match	gene: Zm00001d031657	Zm00001d031657_T00	34.53	46.70	coding	619	18	1874
PB.8381.4	7	1685	14	full-splice_match	gene: Zm00001d019534	Zm00001d019534_T00	0.00	78.71	coding	501	84	1586
PB.8516.1	7	1318	1	full-splice_match	gene: Zm00001d020358	Zm00001d020358_T00	17.84	17.84	coding	343	1	1029
PB.2812.5	2	939	2	full-splice_match	gene: Zm00001d003457	Zm00001d003457_T00	34.37	95.96	coding	204	115	726
PB.10044.1	8	1678	4	full-splice_match	gene: Zm00001d012556	Zm00001d012556_T00	74.47	74.47	coding	448	89	1432
PB.1187.1	1	1630	1	full-splice_match	gene: Zm00001d032869	Zm00001d032869_T00	22.12	22.12	coding	300	259	1158
PB.6135.1	5	681	2	novel_not_in_catalog	gene: Zm00001d013399	novel	15.06	15.06	coding	131	2	394
PB.5168.1	4	1301	11	incomplete-splice_match	gene: Zm00001d050251	Zm00001d050251_T00	50.65	50.65	coding	256	99	866
PB.4699.4	3	687	6	novel_not_in_catalog	gene: Zm00001d044139	novel	47.40	50.73	coding	135	17	421
PB.1902.3	10	2052	11	full-splice_match	gene: Zm00001d024420	Zm00001d024420_T00	8.63	13.87	coding	548	62	1705
PB.1902.2	10	2089	11	novel_not_in_catalog	gene: Zm00001d024420	novel	3.61	13.87	coding	539	110	1726
PB.8752.3	7	2027	4	novel_in_catalog	gene: Zm00001d021413	novel	52.66	65.96	non coding	NA	NA	NA
PB.8200.2	6	2329	8	novel_not_in_catalog	gene: Zm00001d039180	novel	37.14	55.29	coding	513	400	1938
PB.8752.5	7	2316	7	novel_in_catalog	gene: Zm00001d021413	novel	13.30	65.96	non coding	NA	NA	NA

PB.8200.4	6	2243	8	full-splice_match	gene: Zm00001d039180	Zm00001d039180_T00	8.82	55.29	coding	513	350	1888
PB.1902.5	10	2127	10	novel_in_catalog	gene: Zm00001d024420	novel	1.62	13.87	coding	364	62	1153
PB.2546.1	2	1293	1	full-splice_match	gene: Zm00001d002286	Zm00001d002286_T00	3.73	3.73	coding	344	93	1124
PB.6747.1	5	2307	1	incomplete-splice_match	gene: Zm00001d016406	Zm00001d016406_T00	3.45	37.94	non coding	NA	NA	NA
PB.3033.1	2	2606	2	intergenic	novelGene_267	novel	17.32	21.68	non coding	NA	NA	NA
PB.1501.1	1	1971	4	full-splice_match	gene: Zm00001d034320	Zm00001d034320_T00	12.37	22.47	coding	501	111	1613
PB.7077.2	5	1992	1	novel_in_catalog	gene: Zm00001d017850	novel	9.99	1123.79	non coding	NA	NA	NA
PB.10840.10	9	1783	9	novel_not_in_catalog	gene: Zm00001d048272	novel	0.30	104.28	coding	251	297	1049
PB.6887.2	5	566	2	full-splice_match	gene: Zm00001d017070	Zm00001d017070_T00	97.15	109.87	coding	96	52	339
PB.8865.1	7	1569	12	novel_in_catalog	gene: Zm00001d021805	novel	33.60	77.47	coding	261	421	1203
PB.6887.1	5	856	2	full-splice_match	gene: Zm00001d017070	Zm00001d017070_T00	12.72	109.87	coding	96	36	323
PB.8865.4	7	1302	13	full-splice_match	gene: Zm00001d021805	Zm00001d021805_T00	43.87	77.47	coding	305	146	1060
PB.5326.6	4	1065	2	full-splice_match	gene: Zm00001d051420	Zm00001d051420_T00	0.00	277.20	coding	289	77	943
PB.3787.1	3	1113	9	full-splice_match	gene: Zm00001d039653	Zm00001d039653_T00	156.84	156.84	coding	255	90	854
PB.4193.6	3	1277	4	novel_not_in_catalog	gene: Zm00001d042059	novel	39.68	107.87	coding	283	132	980
PB.4193.7	3	1092	4	novel_not_in_catalog	gene: Zm00001d042059	novel	0.00	107.87	coding	107	86	406
PB.4193.4	3	1478	3	novel_not_in_catalog	gene: Zm00001d042059	novel	22.41	107.87	coding	107	154	474

PB.10840.12	9	1254	3	novel_not_in_catalog	gene: Zm00001d048272	novel	9.74	104.28	coding	241	234	956
PB.2160.1	10	1301	4	full-splice_match	gene: Zm00001d025761	Zm00001d025761_T00	87.93	134.48	coding	292	101	976
PB.10756.1	9	465	1	full-splice_match	gene: Zm00001d047888	Zm00001d047888_T00	88.76	88.76	coding	91	63	335
PB.4193.1	3	1363	4	novel_not_in_catalog	gene: Zm00001d042059	novel	41.12	107.87	coding	283	134	982
PB.828.3	1	3410	14	full-splice_match	gene: Zm00001d031231	Zm00001d031231_T01	4.48	29.39	coding	936	110	2917
PB.8720.3	7	3000	17	full-splice_match	gene: Zm00001d021301	Zm00001d021301_T00	1.03	73.13	coding	723	454	2622
PB.3555.1	2	1454	3	novel_in_catalog	gene: Zm00001d007270	novel	35.28	35.28	non coding	NA	NA	NA
PB.1185.1	1	3738	15	novel_not_in_catalog	gene: Zm00001d032855	novel	6.99	60.27	coding	841	87	2609
PB.6788.1	5	3488	17	full-splice_match	gene: Zm00001d016640	Zm00001d016640_T01	53.98	53.98	coding	901	237	2939
PB.9851.3	8	3176	2	incomplete-splice_match	gene: Zm00001d011754	Zm00001d011754_T00	1.77	1.77	coding	858	151	2724
PB.9583.1	8	1011	1	full-splice_match	gene: Zm00001d010594	Zm00001d010594_T00	16.75	16.75	coding	249	109	855
PB.5326.5	4	1172	2	full-splice_match	gene: Zm00001d051420	Zm00001d051420_T00	0.00	277.20	coding	289	77	943
PB.3662.1	2	2719	4	full-splice_match	gene: Zm00001d007932	Zm00001d007932_T00	6.97	6.97	coding	523	1074	2642
PB.8124.3	6	1716	11	novel_in_catalog	gene: Zm00001d038892	novel	1.22	81.42	coding	315	551	1495
PB.8548.1	7	467	2	genic	gene: Zm00001d020503	novel	182.30	182.30	non coding	NA	NA	NA
PB.4010.1	3	711	3	incomplete-splice_match	gene: Zm00001d040831	Zm00001d040831_T01	21.78	89.12	non coding	NA	NA	NA
PB.4010.2	3	3699	25	full-splice_match	gene: Zm00001d040831	Zm00001d040831_T00	53.12	89.12	coding	1106	99	3416

PB.4010.3	3	3614	25	full-splice_match	gene: Zm00001d040831	Zm00001d040831_T00	14.22	89.12	coding	1106	135	3452
PB.4471.3	3	1315	3	incomplete-splice_match	gene: Zm00001d043309	Zm00001d043309_T02	1.75	49.60	coding	218	379	1032
PB.4471.2	3	1919	6	full-splice_match	gene: Zm00001d043309	Zm00001d043309_T01	25.20	49.60	coding	472	199	1614
PB.4471.1	3	2123	6	full-splice_match	gene: Zm00001d043309	Zm00001d043309_T01	0.18	49.60	coding	472	177	1592
PB.8528.1	7	1436	2	novel_in_catalog	gene: Zm00001d020401	novel	25.25	145.69	coding	186	685	1242
PB.8528.3	7	1155	1	genic	gene: Zm00001d020401	novel	48.45	145.69	coding	186	407	964
PB.8528.4	7	1340	3	novel_in_catalog	gene: Zm00001d020401	novel	71.99	145.69	coding	358	123	1196
PB.8871.2	7	682	1	full-splice_match	gene: Zm00001d021816	Zm00001d021816_T00	195.53	304.93	coding	166	75	572
PB.8871.1	7	862	1	full-splice_match	gene: Zm00001d021816	Zm00001d021816_T00	109.40	304.93	coding	166	179	676
PB.2991.2	2	2377	13	novel_in_catalog	gene: Zm00001d004525	novel	5.33	92.88	coding	430	166	1455
PB.2991.3	2	2449	12	novel_in_catalog	gene: Zm00001d004525	novel	2.88	92.88	coding	411	165	1397
PB.4716.1	3	2149	10	full-splice_match	gene: Zm00001d044203	Zm00001d044203_T00	28.78	28.78	coding	560	67	1746
PB.8395.1	7	499	4	novel_in_catalog	gene: Zm00001d019577	novel	89.17	89.17	non coding	NA	NA	NA
PB.7461.1	6	1721	2	full-splice_match	gene: Zm00001d035899	Zm00001d035899_T00	347.27	350.73	coding	486	69	1526
PB.4491.4	3	1523	8	novel_not_in_catalog	gene: Zm00001d043387	novel	10.43	29.94	coding	441	7	1329
PB.7461.3	6	1610	2	full-splice_match	gene: Zm00001d035899	Zm00001d035899_T00	0.00	350.73	coding	486	64	1521
PB.7461.2	6	2001	1	novel_in_catalog	gene: Zm00001d035899	novel	3.46	350.73	coding	439	542	1858

PB.9819.1	8	2481	5	novel_not_in_catalog	gene: Zm00001d011625	novel	93.95	119.76	coding	717	74	2224
PB.9872.1	8	4411	5	full-splice_match	gene: Zm00001d011831	Zm00001d011831_T00	4.15	34.51	coding	345	3123	4157
PB.5001.1	4	3308	3	novel_not_in_catalog	gene: Zm00001d049370	novel	8.91	8.91	coding	193	2278	2856
PB.10045.1	8	881	3	full-splice_match	gene: Zm00001d012561	Zm00001d012561_T00	194.49	194.49	coding	134	120	521
PB.6321.1	5	1039	7	full-splice_match	gene: Zm00001d014272	Zm00001d014272_T00	18.49	18.49	coding	226	49	726
PB.5706.1	4	858	3	intergenic	novelGene_409	novel	17.06	19.41	coding	29	234	320
PB.1117.2	1	2486	1	incomplete-splice_match	gene: Zm00001d032582	Zm00001d032582_T00	3.75	49.52	non coding	NA	NA	NA
PB.3394.3	2	1072	4	novel_in_catalog	gene: Zm00001d006570	novel	1.59	31.20	coding	109	517	843
PB.4722.1	3	1626	5	full-splice_match	gene: Zm00001d044225	Zm00001d044225_T00	55.16	75.86	coding	450	50	1399
PB.7019.5	5	1496	2	full-splice_match	gene: Zm00001d017575	Zm00001d017575_T00	0.00	85.92	coding	374	215	1336
PB.986.2	1	2149	6	full-splice_match	gene: Zm00001d032060	Zm00001d032060_T00	10.33	19.11	coding	438	618	1931
PB.11040.2	Mt	978	1	intergenic	novelGene_8	novel	0.00	629.91	non coding	NA	NA	NA
PB.11040.3	Mt	811	1	intergenic	novelGene_8	novel	228.75	629.91	non coding	NA	NA	NA
PB.1450.3	1	1074	1	novel_in_catalog	gene: Zm00001d034032	novel	6.61	43.53	coding	232	170	865
PB.9859.1	8	1029	2	full-splice_match	gene: Zm00001d011778	Zm00001d011778_T00	51.92	51.92	coding	254	47	808
PB.704.1	1	1561	5	full-splice_match	gene: Zm00001d030601	Zm00001d030601_T00	32.71	33.32	coding	356	190	1257
PB.5033.1	4	2456	4	novel_in_catalog	gene: Zm00001d049543	novel	10.92	10.92	coding	644	80	2011

PB.2784.1	2	775	4	full-splice_match	gene: Zm00001d003310	Zm00001d003310_T00	63.28	63.28	coding	122	155	520
PB.3245.7	2	676	2	novel_in_catalog	gene: Zm00001d005952	novel	23.06	51.42	non coding	NA	NA	NA
PB.3245.6	2	884	4	full-splice_match	gene: Zm00001d005952	Zm00001d005952_T00	5.65	51.42	non coding	NA	NA	NA
PB.4074.6	3	2841	10	novel_in_catalog	gene: Zm00001d041445	novel	3.47	127.03	coding	772	81	2396
PB.10940.1	9	708	1	genic	gene: Zm00001d048575	novel	3.09	38.05	non coding	NA	NA	NA
PB.8879.3	7	4169	1	novel_in_catalog	gene: Zm00001d021834	novel	2.36	35.56	coding	124	3540	3911
PB.5190.3	4	709	4	full-splice_match	gene: Zm00001d050411	Zm00001d050411_T00	159.32	159.32	coding	119	122	478
PB.8991.5	7	1065	1	novel_in_catalog	gene: Zm00001d022252	novel	3.73	14.34	coding	157	94	564
PB.3696.1	3	2657	10	full-splice_match	gene: Zm00001d039304	Zm00001d039304_T00	43.05	88.83	coding	673	375	2393
PB.3606.4	2	3515	24	incomplete-splice_match	gene: Zm00001d007698	Zm00001d007698_T02	16.31	140.33	coding	958	240	3113
PB.3696.2	3	3368	8	novel_in_catalog	gene: Zm00001d039304	novel	2.81	88.83	coding	449	1190	2536
PB.3696.5	3	3210	9	novel_in_catalog	gene: Zm00001d039304	novel	0.00	88.83	coding	588	1275	3038
PB.724.2	1	1126	2	genic	gene: Zm00001d030760	novel	6.64	68.18	coding	156	72	539
PB.3696.7	3	1716	9	novel_in_catalog	gene: Zm00001d039304	novel	11.69	88.83	coding	511	182	1714
PB.3696.6	3	2228	10	full-splice_match	gene: Zm00001d039304	Zm00001d039304_T00	31.29	88.83	coding	673	116	2134
PB.4038.2	3	2131	1	incomplete-splice_match	gene: Zm00001d041192	Zm00001d041192_T00	4.62	122.97	coding	129	1724	2110
PB.4038.1	3	1966	5	full-splice_match	gene: Zm00001d041192	Zm00001d041192_T00	118.34	122.97	coding	501	202	1704

PB.492.1	1	1622	3	novel_not_in_catalog	gene: Zm00001d029390	novel	11.38	17.43	coding	78	135	368
PB.7940.4	6	703	3	intergenic	novelGene_301	novel	57.40	111.88	coding	104	123	434
PB.6871.1	5	1090	2	novel_in_catalog	gene: Zm00001d016977	novel	12.66	13.50	coding	197	196	786
PB.6871.3	5	850	2	novel_in_catalog	gene: Zm00001d016977	novel	0.00	13.50	coding	197	103	693
PB.2209.1	10	1954	8	novel_not_in_catalog	gene: Zm00001d025988	novel	13.18	23.86	coding	543	5	1633
PB.4208.1	3	827	2	full-splice_match	gene: Zm00001d042178	Zm00001d042178_T00	6.03	6.03	coding	179	97	633
PB.1391.4	1	3021	19	full-splice_match	gene: Zm00001d033795	Zm00001d033795_T00	2.74	39.36	coding	316	1850	2797
PB.2638.1	2	1220	4	novel_not_in_catalog	gene: Zm00001d002677	novel	55.99	55.99	coding	236	182	889
PB.3394.1	2	941	2	novel_in_catalog	gene: Zm00001d006570	novel	6.99	31.20	coding	99	314	610
PB.7902.1	6	2699	4	full-splice_match	gene: Zm00001d038105	Zm00001d038105_T00	53.90	57.90	coding	555	310	1974
PB.2293.1	10	1980	7	full-splice_match	gene: Zm00001d026322	Zm00001d026322_T00	48.60	48.60	coding	374	660	1781
PB.2864.1	2	1599	2	intergenic	novelGene_147	novel	136.03	136.03	coding	371	74	1186
PB.7178.10	5	3125	3	novel_in_catalog	gene: Zm00001d018206	novel	0.00	677.05	coding	457	1631	3001
PB.1185.5	1	2248	8	incomplete-splice_match	gene: Zm00001d032855	Zm00001d032855_T00	13.57	60.27	coding	555	49	1713
PB.2147.4	10	1459	11	novel_not_in_catalog	gene: Zm00001d025705	novel	0.55	23.54	coding	363	32	1120
PB.6127.1	5	1746	2	full-splice_match	gene: Zm00001d013383	Zm00001d013383_T00	18.29	18.29	coding	478	56	1489
PB.1936.1	10	2124	3	full-splice_match	gene: Zm00001d024572	Zm00001d024572_T00	118.85	118.85	coding	464	411	1802

PB.9592.5	8	1793	4	novel_in_catalog	gene: Zm00001d010618	novel	6.80	814.05	coding	121	59	421
PB.9592.6	8	729	5	full-splice_match	gene: Zm00001d010618	Zm00001d010618_T00	0.00	814.05	coding	148	143	586
PB.9592.1	8	897	5	full-splice_match	gene: Zm00001d010618	Zm00001d010618_T00	804.43	814.05	coding	148	138	581
PB.9592.2	8	2168	4	full-splice_match	gene: Zm00001d010618	Zm00001d010618_T00	2.81	814.05	coding	148	1410	1853
PB.2147.2	10	2008	14	novel_in_catalog	gene: Zm00001d025705	novel	3.84	23.54	coding	436	334	1641
PB.282.3	1	1853	13	full-splice_match	gene: Zm00001d028443	Zm00001d028443_T00	46.05	73.79	coding	485	263	1717
PB.2147.1	10	2181	15	full-splice_match	gene: Zm00001d025705	Zm00001d025705_T00	18.18	23.54	coding	476	330	1757
PB.282.1	1	2206	13	full-splice_match	gene: Zm00001d028443	Zm00001d028443_T00	27.74	73.79	coding	485	420	1874
PB.2642.9	2	1108	4	full-splice_match	gene: Zm00001d002690	Zm00001d002690_T00	1159.81	1838.05	coding	288	78	941
PB.411.1	1	1269	4	full-splice_match	gene: Zm00001d028992	Zm00001d028992_T00	27.45	27.45	coding	284	73	924
PB.2314.5	10	1483	3	novel_in_catalog	gene: Zm00001d026391	novel	10.06	99.60	coding	197	86	676
PB.6654.1	5	1861	1	antisense	novelGene_gene: Zm00001d015791	novel	0.00	0.00	non coding	NA	NA	NA
PB.4565.3	3	902	3	incomplete-splice_match	gene: Zm00001d043625	Zm00001d043625_T00	0.00	509.97	coding	232	205	900
PB.4565.2	3	1620	5	novel_in_catalog	gene: Zm00001d043625	novel	19.19	509.97	coding	307	207	1127
PB.2642.1	2	1144	1	genic	gene: Zm00001d002690	novel	4.18	1838.05	non coding	NA	NA	NA
PB.2294.4	10	2080	2	genic	gene: Zm00001d026326	novel	22.49	58.91	coding	564	176	1867
PB.2642.3	2	1523	3	novel_in_catalog	gene: Zm00001d002690	novel	98.91	1838.05	coding	286	80	937

PB.2642.2	2	1343	4	full-splice_match	gene: Zm00001d002690	Zm00001d002690_T00	575.15	1838.05	coding	288	273	1136
PB.6189.1	5	1420	7	novel_not_in_catalog	gene: Zm00001d013632	novel	9.34	9.34	coding	284	71	922
PB.2201.3	10	1231	9	full-splice_match	gene: Zm00001d025939	Zm00001d025939_T00	55.17	92.35	coding	250	216	965
PB.2201.1	10	979	6	incomplete-splice_match	gene: Zm00001d025939	Zm00001d025939_T00	17.42	92.35	coding	166	28	525
PB.2201.6	10	1148	9	full-splice_match	gene: Zm00001d025939	Zm00001d025939_T00	2.09	92.35	coding	248	281	1024
PB.2201.4	10	1187	9	full-splice_match	gene: Zm00001d025939	Zm00001d025939_T00	16.21	92.35	coding	248	173	916
PB.2201.5	10	1196	8	novel_in_catalog	gene: Zm00001d025939	novel	1.46	92.35	coding	128	233	616
PB.8627.1	7	868	1	full-splice_match	gene: Zm00001d001230	Zm00001d001230_T00	12.49	12.49	non coding	NA	NA	NA
PB.8627.2	7	619	1	full-splice_match	gene: Zm00001d001230	Zm00001d001230_T00	0.00	12.49	non coding	NA	NA	NA
PB.8155.3	6	1296	4	novel_in_catalog	gene: Zm00001d038999	novel	0.77	366.37	non coding	NA	NA	NA
PB.423.3	1	1041	8	novel_not_in_catalog	gene: Zm00001d029036	novel	3.95	121.70	coding	268	82	885
PB.423.2	1	1167	7	full-splice_match	gene: Zm00001d029036	Zm00001d029036_T00	117.74	121.70	coding	258	108	881
PB.7670.4	6	1232	3	novel_in_catalog	gene: Zm00001d037112	novel	59.68	249.69	coding	306	225	1142
PB.95.3	1	2728	11	full-splice_match	gene: Zm00001d027626	Zm00001d027626_T00	3.91	29.39	coding	394	1353	2534
PB.95.2	1	1932	12	full-splice_match	gene: Zm00001d027626	Zm00001d027626_T00	2.54	29.39	coding	361	634	1716
PB.95.1	1	1980	12	full-splice_match	gene: Zm00001d027626	Zm00001d027626_T00	22.94	29.39	coding	568	61	1764
PB.4113.5	3	1065	1	full-splice_match	gene: Zm00001d041685	Zm00001d041685_T00	5.01	150.23	coding	124	122	493

PB.6222.3	5	1692	5	novel_not_in_catalog	gene: Zm00001d013797	novel	16.83	41.16	coding	283	205	1053
PB.6222.2	5	1154	3	incomplete-splice_match	gene: Zm00001d013797	Zm00001d013797_T00	24.33	41.16	coding	310	64	993
PB.1502.1	1	2413	1	genic	gene: Zm00001d034322	novel	10.82	10.82	coding	333	1323	2321
PB.4113.1	3	1142	1	full-splice_match	gene: Zm00001d041685	Zm00001d041685_T00	5.28	150.23	coding	124	95	466
PB.10497.1	9	1398	8	full-splice_match	gene: Zm00001d046837	Zm00001d046837_T00	5.57	5.57	coding	268	452	1255
PB.7702.1	6	601	2	intergenic	novelGene_234	novel	173.44	173.44	non coding	NA	NA	NA
PB.10533.1	9	1386	1	intergenic	novelGene_228	novel	1.79	1.79	non coding	NA	NA	NA
PB.10666.1	9	2813	7	full-splice_match	gene: Zm00001d047601	Zm00001d047601_T00	12.03	44.94	coding	722	122	2287
PB.1560.2	1	2814	7	novel_in_catalog	gene: Zm00001d034522	novel	5.24	41.43	coding	325	205	1179
PB.1034.1	1	2160	5	full-splice_match	gene: Zm00001d032284	Zm00001d032284_T00	1.98	1.98	coding	579	144	1880
PB.2816.8	2	1749	8	novel_in_catalog	gene: Zm00001d003491	novel	10.67	13.80	coding	98	190	483
PB.3647.2	2	1597	1	full-splice_match	gene: Zm00001d007869	Zm00001d007869_T00	8.98	13.47	coding	479	68	1504
PB.6965.2	5	1468	1	genic	gene: Zm00001d017353	novel	13.03	204.80	non coding	NA	NA	NA
PB.1560.1	1	2030	8	novel_in_catalog	gene: Zm00001d034522	novel	29.89	41.43	coding	381	798	1940
PB.1681.1	10	932	4	incomplete-splice_match	gene: Zm00001d023218	Zm00001d023218_T17	276.53	276.53	coding	161	77	559
PB.6542.1	5	893	1	full-splice_match	gene: Zm00001d015126	Zm00001d015126_T00	10.85	10.85	coding	106	183	500
PB.1385.2	1	3307	13	full-splice_match	gene: Zm00001d033776	Zm00001d033776_T00	9.53	38.59	coding	911	200	2932

PB.5900.1	4	1142	2	full-splice_match	gene: Zm00001d053964	Zm00001d053964_T00	62.73	62.73	coding	194	198	779
PB.6865.1	5	2437	11	full-splice_match	gene: Zm00001d016928	Zm00001d016928_T01	95.30	95.30	coding	626	270	2147
PB.8887.2	7	806	1	intergenic	novelGene_329	novel	51.74	154.98	non coding	NA	NA	NA
PB.6865.4	5	2150	11	full-splice_match	gene: Zm00001d016928	Zm00001d016928_T01	0.00	95.30	coding	626	131	2008
PB.547.1	1	722	1	intergenic	novelGene_182	novel	6.89	6.89	non coding	NA	NA	NA
PB.2935.2	2	3134	6	intergenic	novelGene_187	novel	37.76	45.37	coding	453	1050	2408
PB.2927.1	2	435	1	intergenic	novelGene_178	novel	0.00	0.00	non coding	NA	NA	NA
PB.8039.3	6	2691	1	intergenic	novelGene_319	novel	2.51	30.36	non coding	NA	NA	NA
PB.10387.2	9	791	3	full-splice_match	gene: Zm00001d046169	Zm00001d046169_T00	60.21	60.21	coding	181	96	638
PB.6443.2	5	1188	1	full-splice_match	gene: Zm00001d014774	Zm00001d014774_T00	14.94	14.94	coding	145	290	724
PB.4633.1	3	1995	2	full-splice_match	gene: Zm00001d043864	Zm00001d043864_T00	82.01	103.64	coding	512	238	1773
PB.2209.2	10	2080	8	novel_not_in_catalog	gene: Zm00001d025988	novel	10.68	23.86	coding	543	308	1936
PB.9321.1	8	1638	5	novel_in_catalog	gene: Zm00001d009171	novel	8.90	15.88	coding	357	83	1153
PB.9321.2	8	865	4	incomplete-splice_match	gene: Zm00001d009171	Zm00001d009171_T00	6.98	15.88	coding	132	51	446
PB.9321.3	8	1478	5	novel_in_catalog	gene: Zm00001d009171	novel	0.00	15.88	coding	357	78	1148
PB.9939.22	8	1557	7	novel_in_catalog	gene: Zm00001d012100	novel	0.55	77.85	coding	151	796	1248
PB.10110.1	9	1373	3	full-splice_match	gene: Zm00001d044729	Zm00001d044729_T00	17.16	17.16	coding	317	99	1049

PB.4972.2	4	921	2	full-splice_match	gene: Zm00001d049230	Zm00001d049230_T00	542.43	1055.67	coding	176	111	638
PB.7589.1	6	1633	8	full-splice_match	gene: Zm00001d036654	Zm00001d036654_T00	68.96	119.84	coding	349	247	1293
PB.3434.2	2	1664	5	full-splice_match	gene: Zm00001d006737	Zm00001d006737_T00	58.48	58.48	coding	335	430	1434
PB.3017.1	2	2046	1	genic	gene: Zm00001d000919	novel	60.91	60.91	non coding	NA	NA	NA
PB.6578.1	5	1162	6	full-splice_match	gene: Zm00001d015300	Zm00001d015300_T00	1.42	363.03	coding	207	188	808
PB.6578.3	5	927	6	full-splice_match	gene: Zm00001d015300	Zm00001d015300_T00	0.00	363.03	coding	207	189	809
PB.6578.2	5	1035	6	full-splice_match	gene: Zm00001d015300	Zm00001d015300_T00	361.62	363.03	coding	207	191	811
PB.8039.2	6	6392	1	intergenic	novelGene_319	novel	23.37	30.36	coding	109	1535	1861
PB.5882.2	4	1632	5	full-splice_match	gene: Zm00001d053900	Zm00001d053900_T00	18.72	24.48	coding	369	149	1255
PB.9706.3	8	1866	11	full-splice_match	gene: Zm00001d011119	Zm00001d011119_T02	22.32	78.92	coding	483	82	1530
PB.4653.2	3	1666	9	full-splice_match	gene: Zm00001d043948	Zm00001d043948_T00	24.24	29.62	coding	409	229	1455
PB.8574.1	7	1594	4	full-splice_match	gene: Zm00001d020631	Zm00001d020631_T00	29.64	34.26	coding	409	59	1285
PB.5029.1	4	1203	1	full-splice_match	gene: Zm00001d049521	Zm00001d049521_T00	56.29	56.29	coding	299	3	899
PB.3578.1	2	1543	3	full-splice_match	gene: Zm00001d007479	Zm00001d007479_T00	19.05	19.05	coding	446	91	1428
PB.7637.3	6	439	4	full-splice_match	gene: Zm00001d036964	Zm00001d036964_T00	41.76	107.56	coding	76	99	326
PB.6309.2	5	1235	5	full-splice_match	gene: Zm00001d014186	Zm00001d014186_T01	44.47	44.47	non coding	NA	NA	NA
PB.1469.1	1	1290	4	novel_not_in_catalog	gene: Zm00001d034128	novel	191.30	191.30	coding	332	76	1071

PB.3953.3	3	550	4	incomplete-splice_match	gene: Zm00001d040462	Zm00001d040462_T00	18.62	85.15	coding	138	137	550
PB.7194.1	5	2806	1	full-splice_match	gene: Zm00001d018282	Zm00001d018282_T00	41.08	56.26	coding	783	13	2361
PB.3953.1	3	1565	12	full-splice_match	gene: Zm00001d040462	Zm00001d040462_T00	66.53	85.15	coding	438	73	1386
PB.6214.1	5	1517	4	full-splice_match	gene: Zm00001d013751	Zm00001d013751_T00	424.25	424.25	coding	364	122	1213
PB.6962.2	5	665	2	novel_not_in_catalog	gene: Zm00001d017347	novel	95.40	99.80	coding	102	137	442
PB.8502.2	7	1619	3	full-splice_match	gene: Zm00001d020281	Zm00001d020281_T00	6.20	7.99	coding	509	69	1595
PB.9037.2	7	922	1	full-splice_match	gene: Zm00001d022416	Zm00001d022416_T00	38.99	587.97	coding	113	145	483
PB.3132.1	2	969	5	novel_in_catalog	gene: Zm00001d005346	novel	3.16	8.67	coding	133	328	726
PB.3132.2	2	1348	9	full-splice_match	gene: Zm00001d005346	Zm00001d005346_T00	5.50	8.67	coding	382	14	1159
PB.9037.1	7	1076	1	full-splice_match	gene: Zm00001d022416	Zm00001d022416_T00	12.48	587.97	coding	113	145	483
PB.6799.7	5	2962	16	full-splice_match	gene: Zm00001d016675	Zm00001d016675_T00	0.00	30.33	coding	800	416	2815
PB.4948.2	4	1538	3	novel_not_in_catalog	gene: Zm00001d049136	novel	5.98	81.87	coding	106	1041	1358
PB.1485.1	1	1196	3	full-splice_match	gene: Zm00001d034207	Zm00001d034207_T00	15.61	15.61	coding	314	127	1068
PB.4948.1	4	587	4	novel_not_in_catalog	gene: Zm00001d049136	novel	75.89	81.87	coding	106	89	406
PB.5087.3	4	757	4	full-splice_match	gene: Zm00001d049798	Zm00001d049798_T00	0.00	27.75	coding	211	19	651
PB.5087.2	4	2388	3	novel_in_catalog	gene: Zm00001d049798	novel	5.52	27.75	coding	146	1729	2166
PB.1695.2	10	4526	24	full-splice_match	gene: Zm00001d023262	Zm00001d023262_T00	57.50	72.23	coding	1361	198	4280

PB.1695.3	10	1085	2	incomplete-splice_match	gene: Zm00001d023262	Zm00001d023262_T01	14.73	72.23	coding	191	329	901
PB.9673.2	8	2106	2	genic	gene: Zm00001d010954	novel	28.31	76.76	coding	414	445	1686
PB.7993.1	6	787	4	intergenic	novelGene_310	novel	341.22	341.22	coding	142	114	539
PB.89.1	1	2182	3	full-splice_match	gene: Zm00001d027593	Zm00001d027593_T00	50.76	50.76	coding	612	87	1922
PB.89.3	1	2032	3	full-splice_match	gene: Zm00001d027593	Zm00001d027593_T00	0.00	50.76	coding	612	81	1916
PB.3502.1	2	635	2	full-splice_match	gene: Zm00001d007035	Zm00001d007035_T00	17.46	17.46	coding	102	89	394
PB.3081.1	2	935	1	intergenic	novelGene_314	novel	2.05	2.05	coding	127	412	792
PB.6637.2	5	767	1	full-splice_match	gene: Zm00001d015670	Zm00001d015670_T00	0.00	16.63	coding	143	17	445
PB.10994.1	B73V4	2237	3	novel_not_in_catalog	gene: Zm00001d000402	novel	4.76	4.76	coding	549	433	2079
PB.9349.1	8	838	3	novel_in_catalog	gene: Zm00001d009394	novel	13.37	13.37	non coding	NA	NA	NA
PB.2320.1	10	2837	7	full-splice_match	gene: Zm00001d026415	Zm00001d026415_T00	32.76	39.38	coding	773	223	2541
PB.2320.2	10	2792	7	full-splice_match	gene: Zm00001d026415	Zm00001d026415_T00	6.61	39.38	coding	773	195	2513
PB.8890.1	7	1740	2	full-splice_match	gene: Zm00001d021866	Zm00001d021866_T00	40.78	40.78	coding	328	485	1468
PB.890.3	1	3229	1	genic	gene: Zm00001d022889	novel	8.01	8.01	non coding	NA	NA	NA
PB.4232.6	3	1634	10	full-splice_match	gene: Zm00001d042303	Zm00001d042303_T00	0.00	67.81	coding	499	118	1614
PB.7913.2	6	1955	16	incomplete-splice_match	gene: Zm00001d038134	Zm00001d038134_T00	13.86	29.90	coding	466	151	1548
PB.4544.4	3	2020	13	novel_in_catalog	gene: Zm00001d043560	novel	0.64	26.48	coding	408	81	1304

PB.506.1	1	2149	4	full-splice_match	gene: Zm00001d029457	Zm00001d029457_T00	75.62	75.62	coding	569	240	1946
PB.4232.3	3	1940	8	full-splice_match	gene: Zm00001d042303	Zm00001d042303_T00	3.46	67.81	coding	355	120	1184
PB.1389.2	1	1255	6	novel_not_in_catalog	gene: Zm00001d033792	novel	9.26	14.95	coding	233	57	755
PB.4232.1	3	1947	9	novel_in_catalog	gene: Zm00001d042303	novel	3.50	67.81	coding	373	159	1277
PB.9485.1	8	638	5	full-splice_match	gene: Zm00001d010120	Zm00001d010120_T00	93.16	93.16	non coding	NA	NA	NA
PB.9774.2	8	924	1	novel_in_catalog	gene: Zm00001d011428	novel	18.09	152.42	coding	211	102	734
PB.3340.2	2	514	2	full-splice_match	gene: Zm00001d006336	Zm00001d006336_T00	321.21	409.74	coding	81	124	366
PB.9774.6	8	2480	3	novel_in_catalog	gene: Zm00001d011428	novel	31.18	152.42	coding	211	22	654
PB.9774.7	8	2306	4	novel_in_catalog	gene: Zm00001d011428	novel	103.16	152.42	coding	265	22	816
PB.1654.1	1	1063	2	genic	gene: Zm00001d034849	novel	19.90	20.47	coding	246	99	836
PB.7913.1	6	2835	19	full-splice_match	gene: Zm00001d038134	Zm00001d038134_T00	9.15	29.90	coding	784	34	2385
PB.4603.3	3	1134	5	full-splice_match	gene: Zm00001d043766	Zm00001d043766_T01	7.24	7.24	coding	332	76	1071
PB.5444.6	4	1367	1	incomplete-splice_match	gene: Zm00001d051981	Zm00001d051981_T00	53.73	197.79	coding	152	300	755
PB.5444.7	4	1970	3	full-splice_match	gene: Zm00001d051981	Zm00001d051981_T00	0.00	197.79	coding	405	495	1709
PB.2158.1	10	2236	6	novel_not_in_catalog	gene: Zm00001d025749	novel	24.78	24.78	coding	509	381	1907
PB.5444.2	4	1963	1	novel_in_catalog	gene: Zm00001d051981	novel	58.76	197.79	coding	301	196	1098
PB.4707.1	3	481	1	intergenic	novelGene_423	novel	12.71	12.71	non coding	NA	NA	NA

PB.7983.1	6	1568	1	genic	gene: Zm00001d038373	novel	8.15	8.15	coding	430	13	1302
PB.9499.1	8	1377	4	full-splice_match	gene: Zm00001d010218	Zm00001d010218_T00	26.58	26.58	coding	330	128	1117
PB.2229.1	10	1805	4	novel_not_in_catalog	gene: Zm00001d026067	novel	3.74	3.74	coding	512	163	1698
PB.4791.6	3	2789	1	genic	gene: Zm00001d044504	novel	0.00	283.31	coding	393	123	1301
PB.4791.7	3	1467	1	genic	gene: Zm00001d044504	novel	0.00	283.31	coding	393	126	1304
PB.10034.3	8	2724	4	novel_in_catalog	gene: Zm00001d012515	novel	0.91	16.84	coding	501	992	2494
PB.4791.2	3	3557	1	genic	gene: Zm00001d044504	novel	93.32	283.31	coding	393	127	1305
PB.4791.3	3	1881	2	full-splice_match	gene: Zm00001d044504	Zm00001d044504_T00	163.60	283.31	coding	482	128	1573
PB.8770.2	7	1755	7	full-splice_match	gene: Zm00001d021489	Zm00001d021489_T00	3.17	3.17	coding	464	172	1563
PB.5994.2	5	2897	12	novel_not_in_catalog	gene: Zm00001d012938	novel	13.64	41.25	coding	358	1395	2468
PB.1985.2	10	2677	19	full-splice_match	gene: Zm00001d024855	Zm00001d024855_T01	20.69	198.71	coding	744	166	2397
PB.1985.4	10	3009	18	full-splice_match	gene: Zm00001d024855	Zm00001d024855_T00	33.37	198.71	coding	744	192	2423
PB.1985.5	10	3109	17	novel_in_catalog	gene: Zm00001d024855	novel	0.00	198.71	coding	440	163	1482
PB.9200.1	8	1495	1	antisense	novelGene_gene: Zm00001d008515	novel	15.64	15.64	coding	172	695	1210
PB.1985.8	10	2853	17	novel_in_catalog	gene: Zm00001d024855	novel	2.07	198.71	coding	374	160	1281
PB.1985.9	10	2923	17	novel_in_catalog	gene: Zm00001d024855	novel	6.17	198.71	coding	440	187	1506
PB.1368.1	1	2314	13	novel_in_catalog	gene: Zm00001d033674	novel	5.71	66.10	coding	456	115	1482

PB.2480.1	2	819	2	novel_not_in_catalog	gene: Zm00001d001989	novel	31.94	113.12	coding	124	21	392
PB.2480.3	2	530	2	novel_not_in_catalog	gene: Zm00001d001989	novel	75.76	113.12	coding	124	27	398
PB.2480.2	2	948	2	novel_not_in_catalog	gene: Zm00001d001989	novel	5.41	113.12	coding	124	443	814
PB.10128.1	9	2835	8	full-splice_match	gene: Zm00001d044823	Zm00001d044823_T00	29.19	29.19	coding	637	493	2403
PB.6897.1	5	1749	3	novel_in_catalog	gene: Zm00001d017091	novel	2.71	41.54	coding	289	96	962
PB.3165.1	2	1134	7	full-splice_match	gene: Zm00001d005570	Zm00001d005570_T00	82.09	82.82	coding	205	118	732
PB.3165.2	2	1223	7	full-splice_match	gene: Zm00001d005570	Zm00001d005570_T00	0.73	82.82	coding	205	334	948
PB.7134.1	5	1735	10	full-splice_match	gene: Zm00001d018036	Zm00001d018036_T00	31.62	31.62	coding	394	51	1232
PB.2048.4	10	3038	3	full-splice_match	gene: Zm00001d025106	Zm00001d025106_T00	0.87	27.26	non coding	NA	NA	NA
PB.1292.1	1	1718	6	full-splice_match	gene: Zm00001d033366	Zm00001d033366_T00	66.06	66.06	coding	392	168	1343
PB.2126.1	10	2147	3	incomplete-splice_match	gene: Zm00001d025590	Zm00001d025590_T01	42.54	42.80	coding	545	144	1778
PB.6965.3	5	1192	3	full-splice_match	gene: Zm00001d017353	Zm00001d017353_T00	133.70	204.80	coding	243	151	879
PB.10922.1	9	1138	5	incomplete-splice_match	gene: Zm00001d048531	Zm00001d048531_T00	3.15	16.32	coding	189	265	831
PB.10922.2	9	2216	8	full-splice_match	gene: Zm00001d048531	Zm00001d048531_T00	11.05	16.32	coding	655	54	2018
PB.10922.3	9	2261	7	novel_in_catalog	gene: Zm00001d048531	novel	2.12	16.32	coding	595	49	1833
PB.2460.3	2	1888	12	novel_not_in_catalog	gene: Zm00001d001914	novel	0.29	20.55	coding	115	43	387
PB.2460.2	2	2088	11	full-splice_match	gene: Zm00001d001914	Zm00001d001914_T00	14.76	20.55	coding	115	100	444

PB.10037.1	8	830	1	incomplete-splice_match	gene: Zm00001d012529	Zm00001d012529_T00	17.28	17.28	coding	78	349	582
PB.7940.6	6	602	2	intergenic	novelGene_301	novel	13.50	111.88	coding	104	64	375
PB.2460.5	2	1800	12	novel_in_catalog	gene: Zm00001d001914	novel	3.27	20.55	coding	292	529	1404
PB.2460.4	2	3133	9	novel_in_catalog	gene: Zm00001d001914	novel	2.24	20.55	coding	115	98	442
PB.7296.1	6	2002	3	incomplete-splice_match	gene: Zm00001d035004	Zm00001d035004_T03	89.16	89.16	coding	508	83	1606
PB.10906.1	9	1348	4	incomplete-splice_match	gene: Zm00001d048493	Zm00001d048493_T00	3.45	3.45	coding	441	26	1348
PB.1775.3	10	1861	3	full-splice_match	gene: Zm00001d023593	Zm00001d023593_T00	0.00	79.08	coding	477	168	1598
PB.1775.2	10	2139	2	novel_in_catalog	gene: Zm00001d023593	novel	2.85	79.08	coding	281	165	1007
PB.1775.1	10	2019	3	full-splice_match	gene: Zm00001d023593	Zm00001d023593_T00	76.22	79.08	coding	477	168	1598
PB.4917.3	4	1051	2	intergenic	novelGene_54	novel	7.48	20.51	non coding	NA	NA	NA
PB.2694.2	2	1468	4	novel_not_in_catalog	gene: Zm00001d002934	novel	4.71	6.68	coding	320	131	1090
PB.1578.2	1	1098	1	full-splice_match	gene: Zm00001d034605	Zm00001d034605_T00	50.45	60.25	coding	238	208	921
PB.8274.1	7	2577	16	novel_in_catalog	gene: Zm00001d018864	novel	17.49	17.49	coding	618	408	2261
PB.4638.8	3	1926	2	novel_in_catalog	gene: Zm00001d043874	novel	1.62	131.42	coding	107	127	447
PB.524.4	1	426	2	full-splice_match	gene: Zm00001d029546	Zm00001d029546_T00	3193.71	7497.45	coding	79	79	315
PB.8719.1	7	787	1	full-splice_match	gene: Zm00001d021300	Zm00001d021300_T00	800.21	800.21	coding	164	69	560
PB.524.1	1	842	2	full-splice_match	gene: Zm00001d029546	Zm00001d029546_T00	1328.78	7497.45	non coding	NA	NA	NA

PB.6193.1	5	4069	3	novel_not_in_catalog	gene: Zm00001d013645	novel	9.09	15.22	coding	1075	369	3593
PB.6193.2	5	2625	4	novel_not_in_catalog	gene: Zm00001d013645	novel	6.13	15.22	coding	428	156	1439
PB.524.2	1	719	1	novel_in_catalog	gene: Zm00001d029546	novel	11.85	7497.45	non coding	NA	NA	NA
PB.4638.1	3	1044	4	novel_in_catalog	gene: Zm00001d043874	novel	40.63	131.42	coding	160	128	607
PB.7916.36	6	1635	1	novel_in_catalog	gene: Zm00001d038146	novel	35.86	442.81	coding	161	1023	1505
PB.10675.8	9	2605	5	novel_in_catalog	gene: Zm00001d047635	novel	5.21	286.92	coding	188	1827	2390
PB.9647.1	8	644	1	full-splice_match	gene: Zm00001d010819	Zm00001d010819_T00	23.57	23.57	coding	88	192	455
PB.8585.1	7	1366	1	genic	gene: Zm00001d020685	novel	8.54	254.34	non coding	NA	NA	NA
PB.4638.6	3	924	4	novel_in_catalog	gene: Zm00001d043874	novel	0.00	131.42	coding	160	116	595
PB.3374.1	2	2179	16	novel_in_catalog	gene: Zm00001d006456	novel	4.91	4.91	coding	653	221	2179
PB.9677.3	8	2357	1	full-splice_match	gene: Zm00001d010961	Zm00001d010961_T00	9.67	41.66	coding	490	510	1979
PB.10541.1	9	1619	3	full-splice_match	gene: Zm00001d047045	Zm00001d047045_T00	119.37	119.37	coding	292	507	1382
PB.9756.4	8	1527	2	full-splice_match	gene: Zm00001d011348	Zm00001d011348_T00	9.12	122.22	coding	428	152	1435
PB.3794.1	3	2195	2	intergenic	novelGene_52	novel	9.42	9.42	coding	189	163	729
PB.9677.4	8	2706	1	full-splice_match	gene: Zm00001d010961	Zm00001d010961_T00	5.36	41.66	coding	814	75	2516
PB.2357.1	10	1123	7	full-splice_match	gene: Zm00001d026544	Zm00001d026544_T00	17.32	17.32	coding	124	32	403
PB.6965.1	5	1304	3	full-splice_match	gene: Zm00001d017353	Zm00001d017353_T00	36.14	204.80	coding	243	155	883

PB.4787.2	3	1125	10	incomplete-splice_match	gene: Zm00001d044498	Zm00001d044498_T01	0.00	24.89	coding	353	66	1124
PB.303.2	1	1356	2	full-splice_match	gene: Zm00001d028540	Zm00001d028540_T00	14.01	26.71	coding	197	85	675
PB.303.3	1	1205	2	full-splice_match	gene: Zm00001d028540	Zm00001d028540_T00	8.04	26.71	coding	197	87	677
PB.303.1	1	1077	3	novel_not_in_catalog	gene: Zm00001d028540	novel	4.65	26.71	coding	268	80	883
PB.9999.1	8	1014	1	full-splice_match	gene: Zm00001d012378	Zm00001d012378_T00	36.74	36.74	coding	225	88	762
PB.7741.1	6	413	1	intergenic	novelGene_242	novel	2527.97	2527.97	coding	65	101	295
PB.10818.1	9	2452	2	full-splice_match	gene: Zm00001d048146	Zm00001d048146_T00	7.83	17.30	coding	292	272	1147
PB.206.1	1	1938	2	full-splice_match	gene: Zm00001d028151	Zm00001d028151_T00	56.05	56.05	coding	524	189	1760
PB.7600.1	6	1144	1	antisense	novelGene_gene: Zm00001d036706	novel	7.26	7.26	non coding	NA	NA	NA
PB.1999.1	10	1193	2	full-splice_match	gene: Zm00001d024906	Zm00001d024906_T00	37.81	37.81	coding	329	99	1085
PB.10675.2	9	1325	6	full-splice_match	gene: Zm00001d047635	Zm00001d047635_T00	13.15	286.92	coding	188	535	1098
PB.10914.3	9	1277	3	incomplete-splice_match	gene: Zm00001d048510	Zm00001d048510_T00	37.02	100.80	coding	266	312	1109
PB.10914.2	9	985	3	incomplete-splice_match	gene: Zm00001d048510	Zm00001d048510_T00	5.16	100.80	coding	205	72	686
PB.10914.1	9	3606	3	incomplete-splice_match	gene: Zm00001d048510	Zm00001d048510_T00	58.61	100.80	coding	1034	184	3285
PB.5953.1	5	1210	3	incomplete-splice_match	gene: Zm00001d012801	Zm00001d012801_T00	12.64	12.64	coding	97	576	866
PB.4638.9	3	1039	1	genic	gene: Zm00001d043874	novel	0.00	131.42	coding	107	128	448
PB.1715.1	10	3309	13	incomplete-splice_match	gene: Zm00001d023333	Zm00001d023333_T03	16.71	22.61	coding	899	38	2734

PB.10606.1	9	1235	4	incomplete-splice_match	gene: Zm00001d047403	Zm00001d047403_T00	30.38	30.38	coding	237	54	764
PB.1715.2	10	1461	3	novel_in_catalog	gene: Zm00001d023333	novel	5.90	22.61	coding	231	437	1129
PB.1326.1	1	1242	1	genic	gene: Zm00001d033514	novel	7.58	7.58	coding	157	593	1063
PB.5718.1	4	748	1	intergenic	novelGene_413	novel	0.00	0.00	non coding	NA	NA	NA
PB.6686.1	5	1265	2	genic	gene: Zm00001d016062	novel	33.33	33.33	coding	301	115	1017
PB.639.1	1	903	4	full-splice_match	gene: Zm00001d030146	Zm00001d030146_T00	50.98	52.56	coding	163	50	538
PB.639.2	1	967	3	novel_in_catalog	gene: Zm00001d030146	novel	1.58	52.56	coding	106	44	361
PB.639.3	1	758	4	full-splice_match	gene: Zm00001d030146	Zm00001d030146_T00	0.00	52.56	coding	163	44	532
PB.6531.2	5	1759	5	full-splice_match	gene: Zm00001d015084	Zm00001d015084_T00	24.42	55.78	coding	456	79	1446
PB.4369.5	3	2540	8	full-splice_match	gene: Zm00001d042885	Zm00001d042885_T01	8.37	50.26	coding	234	210	911
PB.4369.4	3	2235	8	novel_in_catalog	gene: Zm00001d042885	novel	0.00	50.26	coding	334	210	1211
PB.3956.10	3	1928	6	novel_not_in_catalog	gene: Zm00001d040470	novel	4.85	187.61	coding	439	328	1644
PB.3956.11	3	1853	6	novel_not_in_catalog	gene: Zm00001d040470	novel	3.76	187.61	coding	427	290	1570
PB.8303.1	7	1548	2	incomplete-splice_match	gene: Zm00001d018976	Zm00001d018976_T08	18.16	40.63	coding	486	91	1548
PB.173.2	1	889	1	incomplete-splice_match	gene: Zm00001d027975	Zm00001d027975_T00	0.00	20.60	coding	73	221	439
PB.1977.1	10	1475	4	full-splice_match	gene: Zm00001d024796	Zm00001d024796_T00	70.80	70.80	coding	323	344	1312
PB.10655.5	9	1609	1	full-splice_match	gene: Zm00001d047560	Zm00001d047560_T00	9.19	39.22	coding	309	397	1323

PB.4946.1	4	1478	2	full-splice_match	gene: Zm00001d049129	Zm00001d049129_T00	71.31	237.01	coding	347	123	1163
PB.4946.2	4	1375	2	full-splice_match	gene: Zm00001d049129	Zm00001d049129_T00	161.41	237.01	coding	347	125	1165
PB.2289.1	10	1279	9	full-splice_match	gene: Zm00001d026312	Zm00001d026312_T00	263.83	284.49	coding	295	111	995
PB.4946.4	4	1433	1	novel_in_catalog	gene: Zm00001d049129	novel	4.29	237.01	coding	241	542	1264
PB.1823.2	10	720	1	intergenic	novelGene_103	novel	0.00	0.00	non coding	NA	NA	NA
PB.5282.1	4	1388	7	novel_not_in_catalog	gene: Zm00001d051139	novel	3.00	6.15	coding	149	173	619
PB.5282.2	4	1118	6	full-splice_match	gene: Zm00001d051139	Zm00001d051139_T00	3.15	6.15	coding	231	127	819
PB.2029.5	10	1100	2	incomplete-splice_match	gene: Zm00001d025023	Zm00001d025023_T00	0.00	103.68	coding	194	69	650
PB.2029.2	10	2383	3	full-splice_match	gene: Zm00001d025023	Zm00001d025023_T00	18.68	103.68	coding	194	79	660
PB.2029.3	10	2196	3	full-splice_match	gene: Zm00001d025023	Zm00001d025023_T00	0.00	103.68	coding	99	280	576
PB.9623.1	8	1284	1	incomplete-splice_match	gene: Zm00001d010719	Zm00001d010719_T00	51.55	51.55	coding	337	35	1045
PB.7509.1	6	1750	4	full-splice_match	gene: Zm00001d036178	Zm00001d036178_T00	12.17	12.17	coding	439	264	1580
PB.5303.8	4	1505	10	incomplete-splice_match	gene: Zm00001d051308	Zm00001d051308_T00	0.00	36.69	coding	399	308	1504
PB.157.2	1	838	1	full-splice_match	gene: Zm00001d027899	Zm00001d027899_T00	12.36	22.26	coding	164	111	602
PB.157.1	1	1012	1	full-splice_match	gene: Zm00001d027899	Zm00001d027899_T00	9.90	22.26	coding	164	107	598
PB.5089.1	4	1760	2	genic	gene: Zm00001d049809	novel	5.44	5.44	coding	175	177	701
PB.4870.1	4	1899	1	intergenic	novelGene_24	novel	0.46	0.46	coding	259	83	859

PB.5303.1	4	2119	3	full-splice_match	gene: Zm00001d051307	Zm00001d051307_T00	63.16	72.41	coding	536	234	1841
PB.5303.3	4	2213	3	full-splice_match	gene: Zm00001d051307	Zm00001d051307_T00	9.25	72.41	coding	536	432	2039
PB.4288.1	3	2734	4	full-splice_match	gene: Zm00001d042543	Zm00001d042543_T00	10.34	10.34	coding	771	19	2331
PB.5766.2	4	1394	5	full-splice_match	gene: Zm00001d053395	Zm00001d053395_T00	1.35	7.53	coding	429	28	1314
PB.5303.6	4	3347	13	full-splice_match	gene: Zm00001d051308	Zm00001d051308_T01	22.94	36.69	coding	752	773	3028
PB.5303.7	4	3076	14	full-splice_match	gene: Zm00001d051308	Zm00001d051308_T00	13.75	36.69	coding	880	310	2949
PB.5363.1	4	1108	8	full-splice_match	gene: Zm00001d051605	Zm00001d051605_T00	20.92	20.92	coding	176	300	827
PB.6672.1	5	1942	1	full-splice_match	gene: Zm00001d015965	Zm00001d015965_T00	20.08	53.93	coding	555	42	1706
PB.6672.2	5	770	1	full-splice_match	gene: Zm00001d015965	Zm00001d015965_T00	33.85	53.93	coding	201	98	700
PB.5782.1	4	3345	10	novel_in_catalog	gene: Zm00001d053453	novel	43.52	86.60	coding	960	209	3088
PB.1409.1	1	1998	3	full-splice_match	gene: Zm00001d033879	Zm00001d033879_T00	18.05	18.05	coding	500	88	1587
PB.9025.3	7	909	1	intergenic	novelGene_360	novel	8.64	28.61	non coding	NA	NA	NA
PB.5696.3	4	331	5	incomplete-splice_match	gene: Zm00001d053022	Zm00001d053022_T00	23.42	96.59	coding	41	208	330
PB.313.1	1	1077	2	incomplete-splice_match	gene: Zm00001d028574	Zm00001d028574_T00	12.84	24.04	coding	152	219	674
PB.5734.3	4	2943	11	novel_in_catalog	gene: Zm00001d053247	novel	2.89	30.72	coding	485	1235	2689
PB.313.2	1	1536	3	full-splice_match	gene: Zm00001d028574	Zm00001d028574_T00	11.20	24.04	coding	396	162	1349
PB.1320.5	1	501	2	genic	gene: Zm00001d033483	novel	0.00	1516.71	coding	100	82	381

PB.3720.2	3	2128	10	full-splice_match	gene: Zm00001d039403	Zm00001d039403_T00	31.55	31.55	coding	574	89	1810
PB.3052.1	2	771	1	antisense	novelGene_gene: Zm00001d004910	novel	27.45	27.45	coding	127	145	525
PB.8603.1	7	1464	1	full-splice_match	gene: Zm00001d020789	Zm00001d020789_T00	7.00	7.00	coding	398	70	1263
PB.1632.1	1	2958	3	full-splice_match	gene: Zm00001d034771	Zm00001d034771_T00	46.85	46.85	coding	843	187	2715
PB.1320.3	1	605	2	genic	gene: Zm00001d033483	novel	519.55	1516.71	coding	100	84	383
PB.2122.1	10	1885	6	full-splice_match	gene: Zm00001d025570	Zm00001d025570_T00	15.80	54.67	coding	368	334	1437
PB.7432.1	6	2020	15	full-splice_match	gene: Zm00001d035696	Zm00001d035696_T00	36.01	36.01	coding	401	558	1760
PB.7200.5	5	1401	3	novel_not_in_catalog	gene: Zm00001d018305	novel	0.00	204.57	coding	363	86	1174
PB.2400.3	10	1231	3	intergenic	novelGene_298	novel	36.78	60.81	coding	355	58	1122
PB.2400.2	10	1689	3	intergenic	novelGene_298	novel	4.20	60.81	coding	154	1035	1496
PB.7178.1	5	3382	2	novel_in_catalog	gene: Zm00001d018206	novel	32.28	677.05	coding	443	1782	3110
PB.9969.2	8	1514	1	genic	gene: Zm00001d012242	novel	1.90	51.26	coding	122	868	1233
PB.6965.5	5	512	2	novel_in_catalog	gene: Zm00001d017353	novel	11.54	204.80	coding	120	151	510
PB.442.9	1	2251	7	novel_in_catalog	gene: Zm00001d029141	novel	9.02	78.59	coding	77	117	347
PB.4375.4	3	2249	9	full-splice_match	gene: Zm00001d042910	Zm00001d042910_T00	20.09	34.15	coding	560	247	1926
PB.8190.1	6	1640	11	full-splice_match	gene: Zm00001d039157	Zm00001d039157_T00	103.47	108.89	coding	377	171	1301
PB.4375.6	3	491	4	incomplete-splice_match	gene: Zm00001d042910	Zm00001d042910_T00	0.00	34.15	coding	85	235	489

PB.2981.1	2	929	3	full-splice_match	gene: Zm00001d004465	Zm00001d004465_T00	10.78	49.09	coding	198	39	632
PB.662.1	1	1929	8	novel_in_catalog	gene: Zm00001d030256	novel	38.49	38.49	coding	302	805	1710
PB.4375.3	3	2322	9	full-splice_match	gene: Zm00001d042910	Zm00001d042910_T00	9.90	34.15	coding	560	251	1930
PB.4756.1	3	2342	1	genic	gene: Zm00001d044388	novel	9.30	14.31	coding	665	94	2088
PB.9561.2	8	1546	5	full-splice_match	gene: Zm00001d010478	Zm00001d010478_T00	92.14	122.36	coding	335	158	1162
PB.4611.6	3	2762	18	novel_in_catalog	gene: Zm00001d043797	novel	1.03	166.78	coding	556	74	1741
PB.5701.2	4	1913	3	full-splice_match	gene: Zm00001d053049	Zm00001d053049_T00	13.59	30.01	coding	474	387	1808
PB.7437.1	6	1589	5	full-splice_match	gene: Zm00001d035722	Zm00001d035722_T00	333.93	522.84	coding	377	149	1279
PB.1417.4	1	2073	4	novel_in_catalog	gene: Zm00001d033917	novel	3.43	186.86	coding	297	191	1081
PB.10655.6	9	1445	2	genic	gene: Zm00001d047560	novel	7.52	39.22	coding	309	236	1162
PB.178.1	1	1567	6	full-splice_match	gene: Zm00001d028005	Zm00001d028005_T00	45.01	45.01	coding	366	112	1209
PB.10340.2	9	609	1	incomplete-splice_match	gene: Zm00001d045783	Zm00001d045783_T05	78.18	212.40	coding	202	3	608
PB.10340.3	9	964	9	incomplete-splice_match	gene: Zm00001d045783	Zm00001d045783_T08	23.12	212.40	coding	34	863	964
PB.10340.1	9	1959	10	incomplete-splice_match	gene: Zm00001d045783	Zm00001d045783_T05	96.41	212.40	coding	336	612	1619
PB.9561.1	8	1616	4	novel_in_catalog	gene: Zm00001d010478	novel	0.99	122.36	coding	359	156	1232
PB.4315.2	3	2197	5	novel_not_in_catalog	gene: Zm00001d042661	novel	2.56	19.03	coding	446	635	1972
PB.9347.1	8	404	2	genic	gene: Zm00001d008035	novel	115.40	115.40	non coding	NA	NA	NA

PB.7889.1	6	683	2	full-splice_match	gene: Zm00001d038057	Zm00001d038057_T00	233.49	233.49	coding	125	94	468
PB.4315.1	3	2360	4	full-splice_match	gene: Zm00001d042661	Zm00001d042661_T00	16.47	19.03	coding	588	354	2117
PB.292.1	1	1614	2	full-splice_match	gene: Zm00001d028482	Zm00001d028482_T00	18.79	27.03	coding	460	23	1402
PB.2405.4	10	2230	17	novel_in_catalog	gene: Zm00001d026677	novel	9.32	621.14	coding	370	180	1289
PB.292.2	1	2001	1	genic	gene: Zm00001d028482	novel	8.24	27.03	coding	493	335	1813
PB.2405.5	10	1175	4	novel_in_catalog	gene: Zm00001d026677	novel	20.04	621.14	coding	128	9	392
PB.2682.1	2	1285	2	genic	gene: Zm00001d002865	novel	1.14	44.29	coding	52	203	358
PB.595.1	1	666	2	intergenic	novelGene_198	novel	16.98	16.98	non coding	NA	NA	NA
PB.2682.3	2	3051	7	full-splice_match	gene: Zm00001d002865	Zm00001d002865_T00	28.00	44.29	coding	785	271	2625
PB.2682.2	2	1198	2	incomplete-splice_match	gene: Zm00001d002865	Zm00001d002865_T00	15.16	44.29	coding	52	475	630
PB.9561.4	8	1613	4	novel_in_catalog	gene: Zm00001d010478	novel	6.11	122.36	coding	241	157	879
PB.3975.3	3	2275	15	novel_not_in_catalog	gene: Zm00001d040603	novel	0.34	154.33	coding	475	33	1457
PB.3975.2	3	1450	8	novel_in_catalog	gene: Zm00001d040603	novel	11.74	154.33	coding	129	362	748
PB.3975.1	3	2231	17	novel_not_in_catalog	gene: Zm00001d040603	novel	142.25	154.33	coding	608	100	1923
PB.10456.3	9	2028	9	novel_in_catalog	gene: Zm00001d046561	novel	0.00	36.53	coding	355	230	1294
PB.10456.4	9	1243	2	novel_in_catalog	gene: Zm00001d046561	novel	2.29	36.53	non coding	NA	NA	NA
PB.10456.5	9	1542	3	novel_in_catalog	gene: Zm00001d046561	novel	0.41	36.53	coding	112	36	371

PB.10456.6	9	1951	9	full-splice_match	gene: Zm00001d046561	Zm00001d046561_T01	0.00	36.53	coding	347	230	1270
PB.10456.7	9	1637	10	full-splice_match	gene: Zm00001d046561	Zm00001d046561_T00	0.00	36.53	coding	465	53	1447
PB.3949.2	3	2293	2	novel_in_catalog	gene: Zm00001d040446	novel	4.06	222.57	coding	388	898	2061
PB.2405.8	10	2320	15	novel_in_catalog	gene: Zm00001d026677	novel	10.63	621.14	coding	431	768	2060
PB.3949.1	3	2031	3	full-splice_match	gene: Zm00001d040446	Zm00001d040446_T00	193.21	222.57	coding	509	158	1684
PB.7638.1	6	1566	5	full-splice_match	gene: Zm00001d036965	Zm00001d036965_T00	20.77	20.77	coding	408	73	1296
PB.3949.4	3	1778	3	full-splice_match	gene: Zm00001d040446	Zm00001d040446_T00	25.30	222.57	coding	509	136	1662
PB.2261.1	10	1221	6	intergenic	novelGene_276	novel	41.38	41.38	coding	206	102	719
PB.10535.1	9	1448	1	full-splice_match	gene: Zm00001d047017	Zm00001d047017_T00	27.07	64.40	coding	269	150	956
PB.10535.2	9	2509	1	full-splice_match	gene: Zm00001d047017	Zm00001d047017_T00	37.33	64.40	coding	692	2	2077
PB.10535.3	9	998	1	full-splice_match	gene: Zm00001d047017	Zm00001d047017_T00	0.00	64.40	coding	152	241	696
PB.1294.2	1	802	4	full-splice_match	gene: Zm00001d033371	Zm00001d033371_T00	12.45	164.88	coding	128	231	614
PB.5605.5	4	1536	3	full-splice_match	gene: Zm00001d052591	Zm00001d052591_T00	19.27	29.18	coding	307	88	1008
PB.1431.1	1	2693	5	incomplete-splice_match	gene: Zm00001d033984	Zm00001d033984_T00	56.67	109.76	coding	650	45	1994
PB.2810.7	2	969	6	novel_not_in_catalog	gene: Zm00001d003445	novel	26.82	26.82	non coding	NA	NA	NA
PB.2810.4	2	2192	1	intergenic	novelGene_138	novel	14.14	14.14	coding	86	68	325
PB.8554.2	7	1180	2	full-splice_match	gene: Zm00001d020530	Zm00001d020530_T00	0.00	36.15	coding	317	99	1049

PB.1435.1	1	1577	12	incomplete-splice_match	gene: Zm00001d033993	Zm00001d033993_T01	18.97	20.22	coding	295	201	1085
PB.5676.1	4	1857	2	novel_in_catalog	gene: Zm00001d052933	novel	8.42	8.42	coding	509	74	1600
PB.7147.1	5	1853	4	novel_in_catalog	gene: Zm00001d018090	novel	10.81	297.01	coding	230	61	750
PB.7147.2	5	1278	5	full-splice_match	gene: Zm00001d018090	Zm00001d018090_T00	226.48	297.01	coding	314	66	1007
PB.7147.4	5	1336	4	novel_in_catalog	gene: Zm00001d018090	novel	43.06	297.01	coding	56	154	321
PB.7881.2	6	1905	1	full-splice_match	gene: Zm00001d038030	Zm00001d038030_T00	0.00	7.59	coding	523	62	1630
PB.7881.1	6	2088	1	full-splice_match	gene: Zm00001d038030	Zm00001d038030_T00	7.59	7.59	coding	523	64	1632
PB.3866.1	3	1691	4	full-splice_match	gene: Zm00001d040060	Zm00001d040060_T00	7.11	7.11	coding	465	97	1491
PB.2870.1	2	792	4	novel_not_in_catalog	gene: Zm00001d003770	novel	11.98	11.98	coding	135	63	467
PB.5415.1	4	1446	3	incomplete-splice_match	gene: Zm00001d051866	Zm00001d051866_T00	6.54	16.94	coding	105	843	1157
PB.7861.1	6	1270	8	full-splice_match	gene: Zm00001d037967	Zm00001d037967_T00	12.33	12.33	coding	313	125	1063
PB.3561.11	2	1751	5	novel_in_catalog	gene: Zm00001d007311	novel	0.00	74.66	coding	457	200	1570
PB.9137.1	8	1891	8	full-splice_match	gene: Zm00001d008271	Zm00001d008271_T00	20.48	20.48	coding	434	153	1454
PB.4724.1	3	1460	2	full-splice_match	gene: Zm00001d044228	Zm00001d044228_T00	457.41	457.41	coding	355	100	1164
PB.10328.1	9	2312	6	full-splice_match	gene: Zm00001d045696	Zm00001d045696_T00	19.34	19.34	coding	615	197	2041
PB.10328.2	9	2133	6	full-splice_match	gene: Zm00001d045696	Zm00001d045696_T00	0.00	19.34	coding	615	127	1971
PB.6244.3	5	1937	8	incomplete-splice_match	gene: Zm00001d013920	Zm00001d013920_T00	36.42	68.18	coding	464	71	1462

PB.6731.1	5	917	3	novel_not_in_catalog	gene: Zm00001d016301	novel	3.17	3.17	coding	236	2	709
PB.2741.1	2	1878	3	novel_not_in_catalog	gene: Zm00001d003129	novel	33.60	33.60	coding	440	232	1551
PB.7558.1	6	1999	1	full-splice_match	gene: Zm00001d036536	Zm00001d036536_T00	6.86	30.83	coding	197	50	640
PB.7558.2	6	1766	2	genic	gene: Zm00001d036536	novel	2.46	30.83	coding	197	111	701
PB.7374.1	6	1132	1	intergenic	novelGene_96	novel	1.67	1.67	non coding	NA	NA	NA
PB.2857.1	2	997	3	incomplete-splice_match	gene: Zm00001d003679	Zm00001d003679_T00	27.63	27.63	coding	240	124	843
PB.2501.2	2	1870	10	novel_not_in_catalog	gene: Zm00001d002075	novel	43.67	54.75	coding	308	301	1224
PB.8444.3	7	1386	2	intergenic	novelGene_180	novel	11.43	11.43	non coding	NA	NA	NA
PB.2396.2	10	1847	2	full-splice_match	gene: Zm00001d026649	Zm00001d026649_T00	43.17	72.24	coding	527	115	1695
PB.2396.1	10	1343	1	genic	gene: Zm00001d026649	novel	29.07	72.24	coding	267	221	1021
PB.5501.1	4	1036	2	full-splice_match	gene: Zm00001d052194	Zm00001d052194_T00	2.40	2.40	coding	218	93	746
PB.1564.7	1	1133	2	full-splice_match	gene: Zm00001d034558	Zm00001d034558_T00	64.75	853.55	coding	260	114	893
PB.6814.1	5	1175	7	novel_in_catalog	gene: Zm00001d016736	novel	88.02	88.02	coding	260	97	876
PB.7153.1	5	1132	1	antisense	novelGene_gene: Zm00001d018111	novel	45.90	115.23	coding	247	148	888
PB.6112.1	5	3019	11	novel_in_catalog	gene: Zm00001d013330	novel	28.46	28.46	coding	815	204	2648
PB.4908.2	4	1093	3	novel_in_catalog	gene: Zm00001d048959	novel	166.27	173.58	coding	243	204	932
PB.54.1	1	1853	9	novel_not_in_catalog	gene: Zm00001d027460	novel	13.03	20.30	coding	440	295	1614

PB.772.2	1	1478	6	full-splice_match	gene: Zm00001d031008	Zm00001d031008_T00	24.55	35.82	coding	394	126	1307
PB.9186.1	8	1849	4	novel_not_in_catalog	gene: Zm00001d008462	novel	15.30	15.30	coding	547	81	1721
PB.772.1	1	1608	6	full-splice_match	gene: Zm00001d031008	Zm00001d031008_T00	11.27	35.82	coding	394	126	1307
PB.4419.1	3	2849	2	incomplete-splice_match	gene: Zm00001d043126	Zm00001d043126_T00	14.42	23.67	coding	747	58	2298
PB.4419.2	3	1039	1	incomplete-splice_match	gene: Zm00001d043126	Zm00001d043126_T00	9.25	23.67	coding	238	129	842
PB.8180.3	6	580	5	full-splice_match	gene: Zm00001d039106	Zm00001d039106_T00	0.00	164.01	coding	140	73	492
PB.7352.1	6	706	1	full-splice_match	gene: Zm00001d035295	Zm00001d035295_T00	0.35	12.05	coding	52	213	368
PB.7352.2	6	831	1	full-splice_match	gene: Zm00001d035295	Zm00001d035295_T00	11.70	12.05	coding	52	459	614
PB.8702.1	7	1817	3	full-splice_match	gene: Zm00001d021224	Zm00001d021224_T00	90.24	100.77	coding	296	662	1549
PB.10878.1	9	2037	3	full-splice_match	gene: Zm00001d048400	Zm00001d048400_T00	7.97	7.97	coding	551	107	1759
PB.5061.2	4	1947	9	novel_in_catalog	gene: Zm00001d049680	novel	32.74	135.97	coding	467	256	1656
PB.7749.4	6	2778	6	novel_in_catalog	gene: Zm00001d037480	novel	8.12	42.34	coding	747	413	2653
PB.1188.2	1	1277	1	genic	gene: Zm00001d032873	novel	10.50	341.11	coding	155	529	993
PB.1564.9	1	974	2	full-splice_match	gene: Zm00001d034558	Zm00001d034558_T00	0.00	853.55	coding	260	115	894
PB.9262.2	8	2332	16	full-splice_match	gene: Zm00001d008847	Zm00001d008847_T00	0.00	62.70	coding	639	161	2077
PB.9262.3	8	2291	16	full-splice_match	gene: Zm00001d008847	Zm00001d008847_T00	34.17	62.70	coding	639	142	2058
PB.9262.1	8	2541	16	full-splice_match	gene: Zm00001d008847	Zm00001d008847_T00	28.52	62.70	coding	639	148	2064

PB.3602.4	2	1207	11	full-splice_match	gene: Zm00001d007645	Zm00001d007645_T00	9.32	30.46	coding	262	75	860
PB.9262.4	8	1364	12	incomplete-splice_match	gene: Zm00001d008847	Zm00001d008847_T00	0.00	62.70	coding	430	73	1362
PB.6585.1	5	1916	2	full-splice_match	gene: Zm00001d015327	Zm00001d015327_T00	978.83	996.94	coding	533	78	1676
PB.6585.2	5	2904	1	novel_in_catalog	gene: Zm00001d015327	novel	18.10	996.94	coding	533	1087	2685
PB.9544.1	8	1274	3	full-splice_match	gene: Zm00001d010410	Zm00001d010410_T00	39.41	39.41	coding	257	163	933
PB.1648.1	1	1391	9	full-splice_match	gene: Zm00001d034833	Zm00001d034833_T00	173.38	173.38	coding	310	156	1085
PB.5826.4	4	3164	12	full-splice_match	gene: Zm00001d053672	Zm00001d053672_T00	0.00	59.75	coding	879	376	3012
PB.10192.4	9	968	8	incomplete-splice_match	gene: Zm00001d045153	Zm00001d045153_T00	30.68	63.31	coding	208	224	847
PB.2027.2	10	755	5	full-splice_match	gene: Zm00001d025020	Zm00001d025020_T00	64.72	360.30	coding	157	118	588
PB.7507.3	6	3333	25	full-splice_match	gene: Zm00001d036161	Zm00001d036161_T00	0.00	39.06	coding	900	178	2877
PB.10192.1	9	1864	12	full-splice_match	gene: Zm00001d045153	Zm00001d045153_T00	17.60	63.31	coding	470	58	1467
PB.921.5	1	1740	11	full-splice_match	gene: Zm00001d031764	Zm00001d031764_T00	0.00	35.67	coding	483	120	1568
PB.10192.3	9	1753	12	full-splice_match	gene: Zm00001d045153	Zm00001d045153_T00	12.85	63.31	coding	137	1074	1484
PB.10192.2	9	1977	11	novel_in_catalog	gene: Zm00001d045153	novel	2.19	63.31	coding	137	1241	1651
PB.1628.1	1	1893	5	full-splice_match	gene: Zm00001d034761	Zm00001d034761_T00	9.38	176.84	coding	377	202	1332
PB.8464.3	7	2105	1	novel_in_catalog	gene: Zm00001d020052	novel	1.67	44.10	coding	334	945	1946
PB.8464.2	7	1378	2	novel_in_catalog	gene: Zm00001d020052	novel	26.18	44.10	coding	334	127	1128

PB.1628.4	1	1594	5	full-splice_match	gene: Zm00001d034761	Zm00001d034761_T00	0.64	176.84	coding	377	242	1372
PB.8464.4	7	1260	2	novel_not_in_catalog	gene: Zm00001d020052	novel	16.25	44.10	coding	334	123	1124
PB.7557.2	6	642	2	intergenic	novelGene_194	novel	31.38	45.73	non coding	NA	NA	NA
PB.6928.2	5	1190	8	full-splice_match	gene: Zm00001d017193	Zm00001d017193_T00	28.01	28.58	coding	243	79	807
PB.4417.4	3	1026	7	novel_not_in_catalog	gene: Zm00001d043113	novel	20.85	109.53	coding	181	255	797
PB.5671.2	4	908	1	full-splice_match	gene: Zm00001d052898	Zm00001d052898_T00	17.74	25.83	coding	158	139	612
PB.8367.1	7	1070	6	full-splice_match	gene: Zm00001d019439	Zm00001d019439_T00	121.15	121.15	coding	253	75	833
PB.4417.3	3	1093	7	novel_not_in_catalog	gene: Zm00001d043113	novel	56.91	109.53	coding	181	253	795
PB.2847.1	2	2069	11	full-splice_match	gene: Zm00001d003632	Zm00001d003632_T00	40.48	40.48	coding	443	405	1733
PB.1390.2	1	1673	8	novel_not_in_catalog	gene: Zm00001d033794	novel	7.47	32.11	coding	351	450	1502
PB.2148.1	10	644	3	full-splice_match	gene: Zm00001d025712	Zm00001d025712_T00	31.24	31.24	coding	83	169	417
PB.10870.2	9	3650	10	novel_not_in_catalog	gene: Zm00001d048364	novel	16.62	16.62	coding	970	418	3327
PB.7428.3	6	1263	2	full-splice_match	gene: Zm00001d035689	Zm00001d035689_T00	266.91	266.91	coding	369	49	1155
PB.212.1	1	1926	2	novel_in_catalog	gene: Zm00001d028181	novel	21.35	21.35	coding	72	103	318
PB.744.2	1	2408	5	full-splice_match	gene: Zm00001d030864	Zm00001d030864_T00	3.58	7.04	coding	641	445	2367
PB.6954.1	5	3898	8	novel_in_catalog	gene: Zm00001d017298	novel	16.23	125.29	coding	298	434	1327
PB.4417.1	3	1576	7	novel_not_in_catalog	gene: Zm00001d043113	novel	0.37	109.53	coding	181	253	795

PB.8887.1	7	922	1	intergenic	novelGene_329	novel	27.56	154.98	non coding	NA	NA	NA
PB.880.2	1	1521	3	novel_in_catalog	gene: Zm00001d031536	novel	4.28	23.92	coding	318	184	1137
PB.4296.1	3	811	1	full-splice_match	gene: Zm00001d042593	Zm00001d042593_T00	24.88	24.88	coding	178	165	698
PB.1585.2	1	2128	5	incomplete-splice_match	gene: Zm00001d034624	Zm00001d034624_T01	0.00	53.35	coding	515	38	1582
PB.10850.1	9	3520	6	full-splice_match	gene: Zm00001d048308	Zm00001d048308_T00	16.82	16.82	coding	1006	307	3324
PB.7569.1	6	2144	5	full-splice_match	gene: Zm00001d036565	Zm00001d036565_T02	5.35	743.17	coding	153	1303	1761
PB.7569.2	6	905	6	full-splice_match	gene: Zm00001d036565	Zm00001d036565_T00	141.52	743.17	coding	150	85	534
PB.7569.3	6	777	6	full-splice_match	gene: Zm00001d036565	Zm00001d036565_T00	596.30	743.17	coding	150	85	534
PB.6970.2	5	728	2	full-splice_match	gene: Zm00001d017379	Zm00001d017379_T00	51.47	74.23	coding	173	71	589
PB.5412.1	4	1398	7	novel_not_in_catalog	gene: Zm00001d051856	novel	2.07	103.88	coding	144	88	519
PB.5121.8	4	1214	4	novel_in_catalog	gene: Zm00001d050011	novel	7.58	194.14	coding	151	557	1009
PB.5121.9	4	1379	4	full-splice_match	gene: Zm00001d050011	Zm00001d050011_T00	16.24	194.14	coding	141	68	490
PB.5121.7	4	1512	3	novel_in_catalog	gene: Zm00001d050011	novel	0.00	194.14	coding	134	819	1220
PB.5121.4	4	1491	4	full-splice_match	gene: Zm00001d050011	Zm00001d050011_T00	0.00	194.14	coding	141	68	490
PB.5121.2	4	1645	3	novel_in_catalog	gene: Zm00001d050011	novel	0.00	194.14	coding	134	819	1220
PB.5121.3	4	1369	4	novel_in_catalog	gene: Zm00001d050011	novel	0.00	194.14	coding	151	557	1009
PB.5121.1	4	1397	5	full-splice_match	gene: Zm00001d050011	Zm00001d050011_T00	150.54	194.14	coding	277	68	898

PB.8825.4	7	2299	7	full-splice_match	gene: Zm00001d021696	Zm00001d021696_T00	22.45	26.16	coding	482	755	2200
PB.3856.6	3	1769	5	novel_not_in_catalog	gene: Zm00001d040023	novel	8.69	48.31	coding	294	264	1145
PB.3856.5	3	2021	4	novel_not_in_catalog	gene: Zm00001d040023	novel	14.08	48.31	coding	333	799	1797
PB.6355.3	5	1582	8	incomplete-splice_match	gene: Zm00001d014385	Zm00001d014385_T00	1.26	21.43	coding	482	101	1546
PB.3856.3	3	1390	4	novel_not_in_catalog	gene: Zm00001d040023	novel	7.01	48.31	coding	183	610	1158
PB.3856.2	3	1714	6	novel_not_in_catalog	gene: Zm00001d040023	novel	16.81	48.31	coding	224	277	948
PB.3856.1	3	1554	4	novel_not_in_catalog	gene: Zm00001d040023	novel	1.72	48.31	coding	147	221	661
PB.9459.1	8	472	1	full-splice_match	gene: Zm00001d009961	Zm00001d009961_T00	59.80	59.80	non coding	NA	NA	NA
PB.5868.2	4	1729	11	full-splice_match	gene: Zm00001d053841	Zm00001d053841_T01	34.63	59.78	coding	453	89	1447
PB.2907.1	2	1348	7	full-splice_match	gene: Zm00001d003935	Zm00001d003935_T01	47.55	47.55	coding	212	433	1068
PB.8825.8	7	512	1	incomplete-splice_match	gene: Zm00001d021696	Zm00001d021696_T00	0.00	26.16	coding	143	15	443
PB.1985.13	10	2763	18	full-splice_match	gene: Zm00001d024855	Zm00001d024855_T01	6.20	198.71	coding	744	227	2458
PB.9832.1	8	2387	16	full-splice_match	gene: Zm00001d011685	Zm00001d011685_T00	22.88	28.93	coding	657	98	2068
PB.8361.2	7	2003	11	novel_not_in_catalog	gene: Zm00001d019327	novel	78.62	154.99	coding	549	87	1733
PB.4352.1	3	1976	1	full-splice_match	gene: Zm00001d042826	Zm00001d042826_T00	32.30	32.30	coding	491	128	1600
PB.8817.1	7	2449	17	full-splice_match	gene: Zm00001d021669	Zm00001d021669_T00	3.99	3.99	coding	665	256	2250
PB.9288.1	8	1047	2	incomplete-splice_match	gene: Zm00001d008957	Zm00001d008957_T00	83.76	83.76	coding	286	1	858

PB.935.2	1	2255	6	novel_not_in_catalog	gene: Zm00001d031832	novel	30.12	102.21	coding	452	715	2070
PB.935.1	1	2746	11	full-splice_match	gene: Zm00001d031832	Zm00001d031832_T00	43.99	102.21	coding	786	188	2545
PB.5868.1	4	663	3	incomplete-splice_match	gene: Zm00001d053841	Zm00001d053841_T01	10.05	59.78	non coding	NA	NA	NA
PB.7050.1	5	1491	4	full-splice_match	gene: Zm00001d017727	Zm00001d017727_T00	29.91	37.81	coding	347	140	1180
PB.1277.1	1	1400	8	full-splice_match	gene: Zm00001d033295	Zm00001d033295_T00	93.14	93.14	coding	225	307	981
PB.7050.3	5	1156	4	full-splice_match	gene: Zm00001d017727	Zm00001d017727_T00	0.00	37.81	coding	347	42	1082
PB.2139.1	10	3630	6	full-splice_match	gene: Zm00001d025665	Zm00001d025665_T00	8.18	44.73	coding	302	2429	3334
PB.6810.2	5	1636	6	novel_in_catalog	gene: Zm00001d016714	novel	4.37	84.19	coding	170	52	561
PB.4352.2	3	1842	1	full-splice_match	gene: Zm00001d042826	Zm00001d042826_T00	0.00	32.30	coding	491	128	1600
PB.2695.10	2	1521	3	incomplete-splice_match	gene: Zm00001d002937	Zm00001d002937_T02	16.39	64.00	coding	309	15	941
PB.2012.1	10	3269	20	novel_not_in_catalog	gene: Zm00001d024952	novel	86.50	175.10	coding	905	180	2894
PB.1600.3	1	1537	1	genic	gene: Zm00001d034669	novel	9.54	27.63	coding	400	141	1340
PB.7041.1	5	1305	4	full-splice_match	gene: Zm00001d017682	Zm00001d017682_T00	64.76	69.17	coding	252	160	915
PB.9445.1	8	1983	2	full-splice_match	gene: Zm00001d009891	Zm00001d009891_T00	3.68	14.51	coding	195	360	944
PB.4013.1	3	1508	6	incomplete-splice_match	gene: Zm00001d040864	Zm00001d040864_T00	51.16	51.16	coding	370	150	1259
PB.9445.2	8	961	2	full-splice_match	gene: Zm00001d009891	Zm00001d009891_T00	10.82	14.51	coding	195	127	711
PB.5671.1	4	1417	1	full-splice_match	gene: Zm00001d052898	Zm00001d052898_T00	8.09	25.83	coding	158	139	612

PB.2330.14	10	1741	5	full-splice_match	gene: Zm00001d026458	Zm00001d026458_T00	7.75	1064.10	coding	469	82	1488
PB.1365.1	1	2421	9	full-splice_match	gene: Zm00001d033655	Zm00001d033655_T00	45.39	45.39	coding	633	150	2048
PB.2330.11	10	2637	3	novel_in_catalog	gene: Zm00001d026458	novel	4.37	1064.10	coding	298	74	967
PB.8253.1	7	856	1	novel_in_catalog	gene: Zm00001d018787	novel	4.03	24.50	non coding	NA	NA	NA
PB.1951.1	10	1599	9	full-splice_match	gene: Zm00001d024704	Zm00001d024704_T00	12.88	12.88	coding	373	127	1245
PB.2581.2	2	747	1	incomplete-splice_match	gene: Zm00001d002402	Zm00001d002402_T00	0.00	28.79	coding	186	6	563
PB.7602.4	6	2076	3	intergenic	novelGene_209	novel	15.95	145.86	coding	126	289	666
PB.6176.2	5	1077	6	novel_in_catalog	gene: Zm00001d013583	novel	11.61	248.69	coding	199	98	694
PB.6176.1	5	1079	7	full-splice_match	gene: Zm00001d013583	Zm00001d013583_T00	237.08	248.69	coding	203	232	840
PB.9518.3	8	1315	4	novel_not_in_catalog	gene: Zm00001d010282	novel	74.59	172.07	coding	349	160	1206
PB.1239.1	1	853	1	genic	gene: Zm00001d033107	novel	3.04	3.04	non coding	NA	NA	NA
PB.10251.1	9	713	3	full-splice_match	gene: Zm00001d045391	Zm00001d045391_T00	13.41	13.41	coding	115	123	467
PB.3415.2	2	1077	1	intergenic	novelGene_410	novel	15.97	27.26	non coding	NA	NA	NA
PB.4698.1	3	2417	1	genic	gene: Zm00001d044138	novel	10.09	1495.64	non coding	NA	NA	NA
PB.4630.2	3	1536	1	full-splice_match	gene: Zm00001d043858	Zm00001d043858_T00	8.87	119.15	coding	396	88	1275
PB.64.3	1	2468	19	novel_in_catalog	gene: Zm00001d027487	novel	14.49	38.83	coding	732	81	2276
PB.7225.2	5	1942	6	novel_in_catalog	gene: Zm00001d018391	novel	45.89	48.23	coding	510	45	1574

PB.64.1	1	3202	24	full-splice_match	gene: Zm00001d027487	Zm00001d027487_T01	22.49	38.83	coding	935	65	2869
PB.9518.1	8	1619	4	novel_not_in_catalog	gene: Zm00001d010282	novel	97.48	172.07	coding	349	354	1400
PB.64.5	1	4201	23	full-splice_match	gene: Zm00001d027487	Zm00001d027487_T00	1.85	38.83	coding	536	116	1723
PB.3868.1	3	1748	13	full-splice_match	gene: Zm00001d040076	Zm00001d040076_T00	15.93	33.65	coding	423	283	1551
PB.3868.5	3	1707	13	full-splice_match	gene: Zm00001d040076	Zm00001d040076_T01	8.30	33.65	coding	179	283	819
PB.3868.6	3	2609	12	novel_not_in_catalog	gene: Zm00001d040076	novel	2.40	33.65	coding	168	1211	1714
PB.3868.7	3	1697	13	full-splice_match	gene: Zm00001d040076	Zm00001d040076_T00	0.00	33.65	coding	423	283	1551
PB.3868.8	3	1650	13	full-splice_match	gene: Zm00001d040076	Zm00001d040076_T00	2.94	33.65	coding	423	237	1505
PB.3868.9	3	2720	12	full-splice_match	gene: Zm00001d040076	Zm00001d040076_T01	1.02	33.65	coding	179	1297	1833
PB.9599.1	8	916	2	genic	gene: Zm00001d010650	novel	359.79	788.18	coding	149	101	547
PB.3307.1	2	1464	1	full-splice_match	gene: Zm00001d006182	Zm00001d006182_T00	12.88	12.88	coding	350	148	1197
PB.1882.1	10	2496	3	genic	gene: Zm00001d024306	novel	36.10	36.10	coding	601	261	2063
PB.4698.6	3	2862	4	novel_in_catalog	gene: Zm00001d044138	novel	63.08	1495.64	non coding	NA	NA	NA
PB.3291.4	2	1717	4	novel_not_in_catalog	gene: Zm00001d006127	novel	0.86	43.35	coding	232	234	929
PB.3453.1	2	3466	5	full-splice_match	gene: Zm00001d006828	Zm00001d006828_T00	11.97	11.97	coding	955	432	3296
PB.5321.1	4	3899	13	full-splice_match	gene: Zm00001d051404	Zm00001d051404_T01	15.47	15.47	coding	895	633	3317
PB.6822.4	5	2416	3	novel_in_catalog	gene: Zm00001d016768	novel	0.00	52.72	coding	415	880	2124

PB.7557.1	6	1343	2	intergenic	novelGene_194	novel	14.35	45.73	non coding	NA	NA	NA
PB.331.1	1	1461	2	incomplete-splice_match	gene: Zm00001d028654	Zm00001d028654_T00	47.43	47.43	coding	334	241	1242
PB.7038.1	5	2419	5	full-splice_match	gene: Zm00001d017666	Zm00001d017666_T00	61.41	61.41	coding	613	134	1972
PB.10354.1	9	841	2	novel_in_catalog	gene: Zm00001d045893	novel	91.62	320.25	coding	138	104	517
PB.4061.1	3	2290	3	novel_in_catalog	gene: Zm00001d041362	novel	13.66	14.86	coding	535	165	1769
PB.4061.2	3	1784	2	novel_in_catalog	gene: Zm00001d041362	novel	1.19	14.86	coding	364	43	1134
PB.10354.2	9	684	2	novel_in_catalog	gene: Zm00001d045893	novel	228.63	320.25	coding	138	95	508
PB.7448.2	6	1827	11	full-splice_match	gene: Zm00001d035772	Zm00001d035772_T00	81.29	81.29	coding	485	98	1552
PB.10777.1	9	2556	2	full-splice_match	gene: Zm00001d047969	Zm00001d047969_T00	129.67	132.58	coding	735	92	2296
PB.10777.2	9	3378	1	novel_in_catalog	gene: Zm00001d047969	novel	2.91	132.58	coding	603	39	1847
PB.2128.1	10	1185	1	full-splice_match	gene: Zm00001d025619	Zm00001d025619_T00	108.79	159.79	coding	284	97	948
PB.8830.2	7	534	2	antisense	novelGene_gene: Zm00001d021702	novel	17.28	48.64	non coding	NA	NA	NA
PB.8830.3	7	1779	2	antisense	novelGene_gene: Zm00001d021702	novel	9.30	48.64	coding	179	1197	1733
PB.8830.1	7	352	1	antisense	novelGene_gene: Zm00001d021702	novel	18.28	48.64	non coding	NA	NA	NA
PB.2170.5	10	2248	9	full-splice_match	gene: Zm00001d025801	Zm00001d025801_T00	0.00	366.57	coding	652	195	2150
PB.10482.3	9	1160	3	full-splice_match	gene: Zm00001d046723	Zm00001d046723_T00	0.00	31.26	coding	265	112	906
PB.2170.6	10	775	1	novel_in_catalog	gene: Zm00001d025801	novel	2.03	366.57	coding	104	195	506

PB.7410.1	6	1681	2	novel_not_in_catalog	gene: Zm00001d035599	novel	38.76	38.76	coding	90	957	1226
PB.1029.1	1	999	5	novel_not_in_catalog	gene: Zm00001d032263	novel	82.13	82.13	coding	209	35	661
PB.10482.2	9	1396	3	full-splice_match	gene: Zm00001d046723	Zm00001d046723_T00	5.32	31.26	coding	265	232	1026
PB.8850.1	7	1691	9	novel_not_in_catalog	gene: Zm00001d021752	novel	84.71	84.71	coding	417	167	1417
PB.3943.2	3	1449	8	full-splice_match	gene: Zm00001d040411	Zm00001d040411_T00	20.09	62.24	coding	292	191	1066
PB.9667.4	8	3550	4	intergenic	novelGene_257	novel	11.08	26.72	non coding	NA	NA	NA
PB.7077.1	5	566	2	full-splice_match	gene: Zm00001d017850	Zm00001d017850_T00	772.64	1123.79	coding	123	45	413
PB.2053.5	10	1929	9	full-splice_match	gene: Zm00001d025128	Zm00001d025128_T01	14.34	57.83	coding	491	75	1547
PB.2053.6	10	3011	8	full-splice_match	gene: Zm00001d025128	Zm00001d025128_T01	0.00	57.83	coding	292	1877	2752
PB.7573.1	6	739	1	full-splice_match	gene: Zm00001d036574	Zm00001d036574_T00	2.14	2.14	coding	127	167	547
PB.6935.1	5	1781	2	full-splice_match	gene: Zm00001d017210	Zm00001d017210_T00	8.42	8.42	coding	499	9	1505
PB.2053.3	10	1923	4	incomplete-splice_match	gene: Zm00001d025128	Zm00001d025128_T00	4.84	57.83	coding	202	48	653
PB.8565.2	7	931	6	full-splice_match	gene: Zm00001d020571	Zm00001d020571_T00	28.44	30.88	coding	153	153	611
PB.10668.5	9	1507	4	incomplete-splice_match	gene: Zm00001d047608	Zm00001d047608_T00	0.00	123.54	coding	122	834	1199
PB.10668.4	9	1435	5	full-splice_match	gene: Zm00001d047608	Zm00001d047608_T00	0.00	123.54	coding	149	683	1129
PB.2053.9	10	2925	8	full-splice_match	gene: Zm00001d025128	Zm00001d025128_T01	19.02	57.83	coding	292	1922	2797
PB.4698.4	3	1730	6	full-splice_match	gene: Zm00001d044138	Zm00001d044138_T00	271.42	1495.64	coding	379	25	1161

PB.10668.3	9	2297	6	full-splice_match	gene: Zm00001d047608	Zm00001d047608_T00	0.00	123.54	coding	149	614	1060
PB.2067.1	10	1053	2	novel_not_in_catalog	gene: Zm00001d025221	novel	24.09	24.09	coding	180	287	826
PB.6444.1	5	1123	1	antisense	novelGene_gene: Zm00001d014775	novel	36.92	36.92	coding	224	111	782
PB.6925.1	5	1822	3	full-splice_match	gene: Zm00001d017186	Zm00001d017186_T00	93.01	841.44	coding	446	233	1570
PB.6444.3	5	789	1	antisense	novelGene_gene: Zm00001d014775	novel	0.00	36.92	coding	224	27	698
PB.6444.2	5	986	1	antisense	novelGene_gene: Zm00001d014775	novel	0.00	36.92	coding	224	106	777
PB.1075.2	1	2176	18	full-splice_match	gene: Zm00001d032455	Zm00001d032455_T00	7.23	7.23	coding	584	95	1846
PB.6657.1	5	2442	7	novel_not_in_catalog	gene: Zm00001d015818	novel	27.65	27.65	coding	215	446	1090
PB.895.1	1	929	2	full-splice_match	gene: Zm00001d031627	Zm00001d031627_T00	75.74	577.64	coding	124	128	499
PB.458.1	1	3232	22	full-splice_match	gene: Zm00001d029212	Zm00001d029212_T00	62.06	62.06	coding	871	305	2917
PB.8170.2	6	534	1	intergenic	novelGene_343	novel	24.86	24.86	non coding	NA	NA	NA
PB.8170.1	6	2416	1	full-splice_match	gene: Zm00001d039053	Zm00001d039053_T00	27.59	27.59	coding	514	272	1813
PB.458.2	1	3176	22	full-splice_match	gene: Zm00001d029212	Zm00001d029212_T00	0.00	62.06	coding	871	296	2908
PB.10586.2	9	735	4	novel_not_in_catalog	gene: Zm00001d027176	novel	0.00	69.51	non coding	NA	NA	NA
PB.6241.3	5	1577	17	full-splice_match	gene: Zm00001d013892	Zm00001d013892_T00	98.63	98.63	coding	390	166	1335
PB.101.12	1	2733	4	novel_in_catalog	gene: Zm00001d027667	novel	2.21	198.17	coding	535	823	2427
PB.101.13	1	2498	4	full-splice_match	gene: Zm00001d027667	Zm00001d027667_T00	14.23	198.17	coding	535	595	2199

PB.101.14	1	2526	4	full-splice_match	gene: Zm00001d027667	Zm00001d027667_T00	16.48	198.17	coding	535	683	2287
PB.101.15	1	2399	5	full-splice_match	gene: Zm00001d027667	Zm00001d027667_T01	7.14	198.17	coding	535	578	2182
PB.6241.5	5	1424	17	full-splice_match	gene: Zm00001d013892	Zm00001d013892_T01	0.00	98.63	coding	390	104	1273
PB.4963.3	4	725	6	novel_not_in_catalog	gene: Zm00001d049193	novel	0.00	76.78	non coding	NA	NA	NA
PB.5935.2	4	2266	3	full-splice_match	gene: Zm00001d054066	Zm00001d054066_T00	13.25	143.46	coding	571	238	1950
PB.4963.1	4	809	6	novel_not_in_catalog	gene: Zm00001d049193	novel	76.78	76.78	non coding	NA	NA	NA
PB.1269.1	1	548	3	novel_not_in_catalog	gene: Zm00001d033267	novel	3.83	3.83	non coding	NA	NA	NA
PB.8097.2	6	553	4	incomplete-splice_match	gene: Zm00001d038815	Zm00001d038815_T00	3.95	21.74	non coding	NA	NA	NA
PB.8097.1	6	1421	8	incomplete-splice_match	gene: Zm00001d038815	Zm00001d038815_T00	17.79	21.74	coding	263	27	815
PB.7682.1	6	1580	1	full-splice_match	gene: Zm00001d037188	Zm00001d037188_T00	11.49	11.49	coding	416	120	1367
PB.10051.1	8	1218	4	full-splice_match	gene: Zm00001d012590	Zm00001d012590_T00	23.73	46.41	coding	282	30	875
PB.4913.1	4	856	1	full-splice_match	gene: Zm00001d048985	Zm00001d048985_T00	11.34	61.74	coding	168	21	524
PB.6949.9	5	1692	1	incomplete-splice_match	gene: Zm00001d017275	Zm00001d017275_T00	126.38	271.71	coding	459	22	1398
PB.5520.1	4	1698	1	full-splice_match	gene: Zm00001d052256	Zm00001d052256_T00	8.79	8.79	coding	389	110	1276
PB.3350.1	2	1363	5	full-splice_match	gene: Zm00001d006368	Zm00001d006368_T00	73.16	73.16	coding	246	383	1120
PB.4504.2	3	1448	4	novel_in_catalog	gene: Zm00001d043432	novel	2.68	21.23	coding	240	125	844
PB.9570.1	8	1991	2	full-splice_match	gene: Zm00001d010529	Zm00001d010529_T00	27.68	27.68	coding	587	57	1817

PB.4504.1	3	1382	7	full-splice_match	gene: Zm00001d043432	Zm00001d043432_T00	18.55	21.23	coding	363	108	1196
PB.5048.1	4	1713	5	full-splice_match	gene: Zm00001d049610	Zm00001d049610_T00	23.94	23.94	coding	474	63	1484
PB.9062.1	7	1459	1	full-splice_match	gene: Zm00001d022478	Zm00001d022478_T00	3.30	30.65	coding	314	36	977
PB.3104.3	2	1213	2	full-splice_match	gene: Zm00001d005208	Zm00001d005208_T00	66.94	206.90	coding	301	109	1011
PB.1156.2	1	2037	4	full-splice_match	gene: Zm00001d032739	Zm00001d032739_T00	0.00	110.46	coding	443	80	1408
PB.1086.5	1	710	6	fusion	gene: Zm00001d032496	novel	38.17	142.32	coding	170	65	574
PB.9092.2	7	1448	15	full-splice_match	gene: Zm00001d022558	Zm00001d022558_T00	18.14	31.77	coding	389	89	1255
PB.1156.6	1	1411	2	novel_not_in_catalog	gene: Zm00001d032739	novel	2.19	110.46	non coding	NA	NA	NA
PB.1156.7	1	1890	2	incomplete-splice_match	gene: Zm00001d032739	Zm00001d032739_T00	96.64	110.46	coding	443	80	1408
PB.1575.1	1	3385	4	full-splice_match	gene: Zm00001d034591	Zm00001d034591_T00	23.78	23.78	coding	807	768	3188
PB.9193.1	8	1505	6	full-splice_match	gene: Zm00001d008491	Zm00001d008491_T00	112.69	112.69	coding	387	101	1261
PB.3214.2	2	1011	5	full-splice_match	gene: Zm00001d005812	Zm00001d005812_T00	23.42	48.55	coding	245	118	852
PB.3214.1	2	1164	5	full-splice_match	gene: Zm00001d005812	Zm00001d005812_T00	25.14	48.55	coding	245	97	831
PB.1009.1	1	4586	10	novel_in_catalog	gene: Zm00001d032166	novel	31.06	54.86	coding	412	3107	4342
PB.1312.1	1	3132	13	full-splice_match	gene: Zm00001d033451	Zm00001d033451_T00	8.23	8.23	coding	559	857	2533
PB.1009.3	1	2118	19	fusion	gene: Zm00001d032166	novel	1.71	44.05	coding	620	24	1883
PB.1009.2	1	2590	22	fusion	gene: Zm00001d032166	novel	35.11	44.05	coding	766	49	2346

PB.1009.4	1	2354	21	fusion	gene: Zm00001d032166	novel	7.24	44.05	coding	519	28	1584
PB.1009.6	1	2056	1	genic	gene: Zm00001d032166	novel	23.80	54.86	coding	507	87	1607
PB.2615.5	2	1972	4	novel_in_catalog	gene: Zm00001d002597	novel	9.04	180.40	coding	290	115	984
PB.2615.4	2	1880	5	full-splice_match	gene: Zm00001d002597	Zm00001d002597_T00	53.69	180.40	coding	475	115	1539
PB.2615.7	2	2712	4	novel_in_catalog	gene: Zm00001d002597	novel	9.18	180.40	coding	131	117	509
PB.2615.6	2	1736	5	full-splice_match	gene: Zm00001d002597	Zm00001d002597_T00	0.00	180.40	coding	475	113	1537
PB.2615.1	2	1979	5	full-splice_match	gene: Zm00001d002597	Zm00001d002597_T00	16.32	180.40	coding	475	115	1539
PB.2347.3	10	1989	8	full-splice_match	gene: Zm00001d026514	Zm00001d026514_T00	9.59	52.71	coding	216	936	1583
PB.2615.2	2	1696	5	full-splice_match	gene: Zm00001d002597	Zm00001d002597_T00	57.66	180.40	coding	411	51	1283
PB.2615.9	2	1619	5	full-splice_match	gene: Zm00001d002597	Zm00001d002597_T00	34.51	180.40	coding	475	120	1544
PB.7131.2	5	2450	6	full-splice_match	gene: Zm00001d018024	Zm00001d018024_T00	0.00	42.14	coding	595	221	2005
PB.7105.10	5	1331	10	full-splice_match	gene: Zm00001d017958	Zm00001d017958_T00	1831.01	2019.88	coding	356	77	1144
PB.8106.1	6	1889	4	full-splice_match	gene: Zm00001d038846	Zm00001d038846_T00	25.14	27.76	coding	370	417	1526
PB.8106.2	6	3088	3	novel_in_catalog	gene: Zm00001d038846	novel	2.62	27.76	coding	268	350	1153
PB.470.1	1	601	3	full-splice_match	gene: Zm00001d029263	Zm00001d029263_T00	45.89	45.89	coding	97	97	387
PB.6965.4	5	1248	2	novel_in_catalog	gene: Zm00001d017353	novel	10.38	204.80	non coding	NA	NA	NA
PB.4235.1	3	964	5	full-splice_match	gene: Zm00001d042308	Zm00001d042308_T00	420.07	420.07	coding	208	86	709

PB.5516.1	4	431	2	incomplete-splice_match	gene: Zm00001d052237	Zm00001d052237_T00	59.85	59.85	non coding	NA	NA	NA
PB.5040.2	4	2033	1	intergenic	novelGene_124	novel	41.54	41.54	non coding	NA	NA	NA
PB.4235.2	3	845	5	full-splice_match	gene: Zm00001d042308	Zm00001d042308_T00	0.00	420.07	coding	208	85	708
PB.5423.1	4	2024	4	novel_in_catalog	gene: Zm00001d051884	novel	19.41	41.91	coding	125	1283	1657
PB.8087.1	6	1026	2	full-splice_match	gene: Zm00001d038780	Zm00001d038780_T00	135.37	150.37	coding	171	152	664
PB.9916.6	8	2329	13	novel_in_catalog	gene: Zm00001d012007	novel	1.27	40.89	coding	406	750	1967
PB.8087.3	6	917	2	full-splice_match	gene: Zm00001d038780	Zm00001d038780_T00	0.00	150.37	coding	171	152	664
PB.8087.4	6	1039	1	novel_in_catalog	gene: Zm00001d038780	novel	15.00	150.37	coding	85	152	406
PB.9916.1	8	2426	13	novel_in_catalog	gene: Zm00001d012007	novel	20.44	40.89	coding	406	746	1963
PB.391.1	1	1852	12	novel_in_catalog	gene: Zm00001d028915	novel	9.64	9.64	coding	273	367	1185
PB.4071.2	3	2430	12	incomplete-splice_match	gene: Zm00001d041420	Zm00001d041420_T01	19.90	70.50	coding	679	62	2098
PB.1883.1	10	872	5	novel_in_catalog	gene: Zm00001d024311	novel	24.96	24.96	coding	188	308	871
PB.9916.8	8	1935	14	full-splice_match	gene: Zm00001d012007	Zm00001d012007_T00	0.00	40.89	coding	545	121	1755
PB.4973.1	4	1184	1	antisense	novelGene_gene: Zm00001d049233	novel	5.35	5.35	coding	38	466	579
PB.3264.1	2	1107	7	incomplete-splice_match	gene: Zm00001d006030	Zm00001d006030_T00	38.67	38.67	coding	231	31	723
PB.1127.4	1	2406	14	novel_not_in_catalog	gene: Zm00001d032633	novel	0.22	13.48	coding	359	83	1159
PB.5060.4	4	604	4	full-splice_match	gene: Zm00001d049674	Zm00001d049674_T00	977.50	1071.99	coding	109	102	428

PB.7391.8	6	2768	4	novel_not_in_catalog	gene: Zm00001d035481	novel	2.50	65.74	coding	92	267	542
PB.5060.2	4	786	3	novel_in_catalog	gene: Zm00001d049674	novel	13.23	1071.99	non coding	NA	NA	NA
PB.5060.3	4	707	4	full-splice_match	gene: Zm00001d049674	Zm00001d049674_T00	81.27	1071.99	coding	109	102	428
PB.5152.1	4	1516	5	full-splice_match	gene: Zm00001d050187	Zm00001d050187_T00	14.00	14.00	coding	369	171	1277
PB.7391.3	6	1182	7	novel_not_in_catalog	gene: Zm00001d035481	novel	3.92	65.74	coding	83	214	462
PB.7391.2	6	1222	7	novel_not_in_catalog	gene: Zm00001d035481	novel	30.86	65.74	coding	83	267	515
PB.7391.1	6	1715	6	novel_not_in_catalog	gene: Zm00001d035481	novel	0.00	65.74	coding	132	1121	1516
PB.3234.2	2	1205	2	full-splice_match	gene: Zm00001d005897	Zm00001d005897_T00	9.47	22.67	coding	146	330	767
PB.7391.7	6	1403	6	novel_not_in_catalog	gene: Zm00001d035481	novel	11.12	65.74	coding	147	267	707
PB.7391.4	6	1253	9	novel_not_in_catalog	gene: Zm00001d035481	novel	2.50	65.74	coding	155	571	1035
PB.8967.2	7	956	2	genic	gene: Zm00001d022177	novel	18.78	30.16	coding	196	114	701
PB.8967.1	7	1332	4	genic	gene: Zm00001d022177	novel	11.37	30.16	coding	196	117	704
PB.5507.1	4	1912	5	novel_in_catalog	gene: Zm00001d052219	novel	12.87	64.47	coding	212	188	823
PB.3782.1	3	1371	5	novel_not_in_catalog	gene: Zm00001d039626	novel	7.70	7.70	coding	294	337	1218
PB.497.3	1	507	4	full-splice_match	gene: Zm00001d029418	Zm00001d029418_T00	489.53	578.04	non coding	NA	NA	NA
PB.989.3	1	2205	1	full-splice_match	gene: Zm00001d032075	Zm00001d032075_T00	0.00	17.28	coding	631	141	2033
PB.4280.2	3	2042	1	full-splice_match	gene: Zm00001d042508	Zm00001d042508_T00	16.69	27.95	coding	598	53	1846

PB.5507.7	4	2201	3	novel_in_catalog	gene: Zm00001d052219	novel	7.05	64.47	coding	212	158	793
PB.894.1	1	1501	8	full-splice_match	gene: Zm00001d031621	Zm00001d031621_T00	157.31	497.36	coding	251	107	859
PB.894.2	1	867	6	incomplete-splice_match	gene: Zm00001d031621	Zm00001d031621_T00	340.05	497.36	coding	118	248	601
PB.10610.1	9	724	4	incomplete-splice_match	gene: Zm00001d047422	Zm00001d047422_T00	43.30	73.93	coding	128	39	422
PB.10610.2	9	1500	6	full-splice_match	gene: Zm00001d047422	Zm00001d047422_T00	30.63	73.93	coding	363	221	1309
PB.3252.2	2	746	3	full-splice_match	gene: Zm00001d005983	Zm00001d005983_T00	6.42	14.40	coding	13	269	307
PB.3105.2	2	1191	6	full-splice_match	gene: Zm00001d005228	Zm00001d005228_T00	5.07	41.58	coding	288	60	923
PB.4126.4	3	1596	2	novel_not_in_catalog	gene: Zm00001d041741	novel	0.00	13.46	coding	212	196	831
PB.8607.1	7	1399	3	full-splice_match	gene: Zm00001d020808	Zm00001d020808_T00	49.24	49.24	coding	347	159	1199
PB.4126.2	3	1739	2	novel_not_in_catalog	gene: Zm00001d041741	novel	10.29	13.46	coding	286	1	858
PB.5655.2	4	1660	6	full-splice_match	gene: Zm00001d052810	Zm00001d052810_T00	12.40	22.24	coding	398	310	1503
PB.4126.1	3	1923	1	novel_in_catalog	gene: Zm00001d041741	novel	3.17	13.46	coding	212	178	813
PB.10745.2	9	2737	1	full-splice_match	gene: Zm00001d047855	Zm00001d047855_T00	0.47	29.48	coding	843	67	2595
PB.2153.4	10	2673	5	full-splice_match	gene: Zm00001d025727	Zm00001d025727_T01	6.60	97.72	coding	432	1284	2579
PB.9814.4	8	2644	7	incomplete-splice_match	gene: Zm00001d011610	Zm00001d011610_T03	93.38	158.78	coding	874	21	2642
PB.8189.2	6	1388	3	incomplete-splice_match	gene: Zm00001d039156	Zm00001d039156_T00	17.63	48.66	coding	299	215	1111
PB.2153.5	10	1843	2	incomplete-splice_match	gene: Zm00001d025727	Zm00001d025727_T01	21.76	97.72	coding	378	710	1843

PB.9814.3	8	4294	10	full-splice_match	gene: Zm00001d011610	Zm00001d011610_T01	29.83	158.78	coding	1332	188	4183
PB.9803.2	8	988	8	full-splice_match	gene: Zm00001d011565	Zm00001d011565_T00	55.81	608.45	coding	153	148	606
PB.1414.2	1	1155	4	full-splice_match	gene: Zm00001d033905	Zm00001d033905_T00	1.66	3.46	coding	227	255	935
PB.2153.6	10	1597	6	novel_not_in_catalog	gene: Zm00001d025727	novel	0.00	97.72	coding	355	266	1330
PB.11013.10	B73V4	1650	3	novel_not_in_catalog	gene: Zm00001d000112	novel	6.17	476.95	coding	232	326	1021
PB.7912.1	6	1078	3	full-splice_match	gene: Zm00001d038133	Zm00001d038133_T00	17.49	30.22	coding	234	168	869
PB.11013.12	B73V4	2424	2	novel_in_catalog	gene: Zm00001d000112	novel	0.00	476.95	coding	148	1364	1807
PB.9907.1	8	1568	3	novel_not_in_catalog	gene: Zm00001d011979	novel	91.43	206.91	coding	437	72	1382
PB.11013.14	B73V4	2218	3	novel_in_catalog	gene: Zm00001d000112	novel	14.63	476.95	coding	148	1076	1519
PB.11013.15	B73V4	1548	3	full-splice_match	gene: Zm00001d000112	Zm00001d000112_T00	0.00	476.95	coding	359	131	1207
PB.11013.16	B73V4	843	1	novel_in_catalog	gene: Zm00001d000112	novel	0.00	476.95	non coding	NA	NA	NA
PB.4229.1	3	1187	3	full-splice_match	gene: Zm00001d042288	Zm00001d042288_T00	51.90	68.03	coding	246	185	922
PB.3997.2	3	1533	2	full-splice_match	gene: Zm00001d040777	Zm00001d040777_T00	39.84	70.08	coding	399	171	1367
PB.6476.4	5	841	2	genic	gene: Zm00001d014883	novel	213.20	213.20	coding	168	194	697
PB.6476.2	5	1090	8	full-splice_match	gene: Zm00001d014880	Zm00001d014880_T00	0.00	68.72	coding	287	221	1081
PB.10745.1	9	2902	1	full-splice_match	gene: Zm00001d047855	Zm00001d047855_T00	29.01	29.48	coding	843	107	2635
PB.6476.1	5	1282	8	full-splice_match	gene: Zm00001d014880	Zm00001d014880_T00	68.72	68.72	coding	287	248	1108

PB.7044.2	5	1314	5	novel_not_in_catalog	gene: Zm00001d017691	novel	8.97	34.86	coding	243	341	1069
PB.3887.4	3	382	2	incomplete-splice_match	gene: Zm00001d040191	Zm00001d040191_T00	66.62	145.18	non coding	NA	NA	NA
PB.7044.1	5	1355	5	full-splice_match	gene: Zm00001d017691	Zm00001d017691_T00	17.83	34.86	coding	254	341	1102
PB.3887.1	3	2012	8	full-splice_match	gene: Zm00001d040191	Zm00001d040191_T00	13.23	145.18	coding	379	317	1453
PB.7921.2	6	3391	7	incomplete-splice_match	gene: Zm00001d038156	Zm00001d038156_T01	5.84	48.74	coding	887	157	2817
PB.3887.3	3	1903	8	full-splice_match	gene: Zm00001d040191	Zm00001d040191_T00	65.33	145.18	coding	379	367	1503
PB.4816.1	3	1240	6	incomplete-splice_match	gene: Zm00001d044614	Zm00001d044614_T17	190.85	190.85	coding	308	25	948
PB.2401.3	10	3079	2	full-splice_match	gene: Zm00001d026668	Zm00001d026668_T00	0.00	35.76	coding	958	77	2950
PB.1594.1	1	2039	7	full-splice_match	gene: Zm00001d034650	Zm00001d034650_T00	48.88	63.38	coding	474	417	1838
PB.5496.1	4	2665	3	full-splice_match	gene: Zm00001d052188	Zm00001d052188_T00	40.72	40.72	coding	622	128	1993
PB.10229.3	9	2125	9	intergenic	novelGene_78	novel	32.71	32.71	coding	533	296	1894
PB.8977.1	7	1557	9	novel_in_catalog	gene: Zm00001d022202	novel	26.49	26.49	coding	436	73	1380
PB.9678.3	8	1488	3	novel_not_in_catalog	gene: Zm00001d010969	novel	7.85	26.56	coding	279	220	1056
PB.9678.2	8	1547	2	full-splice_match	gene: Zm00001d010969	Zm00001d010969_T00	3.92	26.56	coding	318	44	997
PB.9678.1	8	1684	3	novel_not_in_catalog	gene: Zm00001d010969	novel	6.93	26.56	coding	279	48	884
PB.4633.2	3	2406	1	genic	gene: Zm00001d043864	novel	21.63	103.64	coding	163	1686	2174
PB.10666.5	9	3202	6	full-splice_match	gene: Zm00001d047601	Zm00001d047601_T01	0.00	44.94	coding	529	114	1700

PB.9678.4	8	1005	3	novel_not_in_catalog	gene: Zm00001d010969	novel	7.87	26.56	coding	279	51	887
PB.2153.8	10	1881	5	novel_not_in_catalog	gene: Zm00001d025727	novel	2.86	97.72	coding	224	1027	1698
PB.7129.1	5	962	1	full-splice_match	gene: Zm00001d018017	Zm00001d018017_T00	203.01	203.01	coding	179	93	629
PB.1589.1	1	1267	3	full-splice_match	gene: Zm00001d034634	Zm00001d034634_T00	18.98	18.98	coding	210	96	725
PB.1541.2	1	1636	1	novel_in_catalog	gene: Zm00001d034463	novel	3.62	13.13	coding	126	468	845
PB.6093.3	5	1361	11	novel_in_catalog	gene: Zm00001d013274	novel	4.37	20.57	coding	393	181	1359
PB.6093.2	5	2941	21	novel_in_catalog	gene: Zm00001d013274	novel	16.20	20.57	coding	479	1199	2635
PB.1482.1	1	851	2	full-splice_match	gene: Zm00001d034192	Zm00001d034192_T00	9.66	9.66	coding	224	44	715
PB.8953.2	7	3594	17	full-splice_match	gene: Zm00001d022114	Zm00001d022114_T00	24.72	24.72	coding	1015	343	3387
PB.10249.1	9	1540	9	incomplete-splice_match	gene: Zm00001d045389	Zm00001d045389_T00	35.80	35.80	coding	343	258	1286
PB.5870.3	4	1187	5	novel_in_catalog	gene: Zm00001d053852	novel	2.19	41.26	coding	216	540	1187
PB.10034.2	8	2231	5	full-splice_match	gene: Zm00001d012515	Zm00001d012515_T00	15.93	16.84	coding	551	274	1926
PB.8739.1	7	1773	8	novel_not_in_catalog	gene: Zm00001d021371	novel	28.69	43.60	coding	359	165	1241
PB.184.1	1	921	3	full-splice_match	gene: Zm00001d028025	Zm00001d028025_T00	18.16	18.16	coding	172	120	635
PB.769.1	1	2515	15	full-splice_match	gene: Zm00001d030999	Zm00001d030999_T00	11.03	11.03	coding	370	1146	2255
PB.5759.2	4	2266	5	novel_in_catalog	gene: Zm00001d053354	novel	2.53	23.15	coding	358	1086	2159
PB.7446.1	6	2818	9	full-splice_match	gene: Zm00001d035764	Zm00001d035764_T00	12.85	12.85	coding	759	77	2353

PB.2126.2	10	1563	2	incomplete-splice_match	gene: Zm00001d025590	Zm00001d025590_T01	0.00	42.80	coding	438	49	1362
PB.2126.3	10	2172	1	novel_in_catalog	gene: Zm00001d025590	novel	0.26	42.80	coding	399	817	2013
PB.6190.3	5	1109	1	novel_in_catalog	gene: Zm00001d013635	novel	0.00	34.81	coding	298	166	1059
PB.6190.2	5	1866	2	novel_not_in_catalog	gene: Zm00001d013635	novel	30.97	34.81	coding	492	194	1669
PB.6190.1	5	1867	2	novel_in_catalog	gene: Zm00001d013635	novel	3.84	34.81	coding	298	716	1609
PB.527.1	1	826	1	full-splice_match	gene: Zm00001d029558	Zm00001d029558_T00	200.76	200.76	coding	160	121	600
PB.2609.1	2	1664	1	full-splice_match	gene: Zm00001d002573	Zm00001d002573_T00	209.98	209.98	coding	440	46	1365
PB.2774.1	2	1480	6	novel_in_catalog	gene: Zm00001d003257	novel	79.06	79.06	coding	252	520	1275
PB.6531.1	5	1934	5	full-splice_match	gene: Zm00001d015084	Zm00001d015084_T00	16.75	55.78	coding	456	130	1497
PB.8231.3	7	4856	24	incomplete-splice_match	gene: Zm00001d018657	Zm00001d018657_T03	32.68	32.68	coding	1470	144	4553
PB.6531.3	5	830	3	incomplete-splice_match	gene: Zm00001d015084	Zm00001d015084_T00	14.61	55.78	coding	137	204	614
PB.9751.1	8	1222	5	full-splice_match	gene: Zm00001d011325	Zm00001d011325_T00	82.88	82.88	coding	291	142	1014
PB.2629.1	2	1778	4	full-splice_match	gene: Zm00001d002630	Zm00001d002630_T00	71.63	71.63	coding	457	174	1544
PB.9751.3	8	1089	5	full-splice_match	gene: Zm00001d011325	Zm00001d011325_T00	0.00	82.88	coding	291	93	965
PB.8063.1	6	2253	5	full-splice_match	gene: Zm00001d038687	Zm00001d038687_T00	6.88	7.84	coding	701	19	2121
PB.8721.5	7	1984	3	novel_in_catalog	gene: Zm00001d021303	novel	0.00	177.34	coding	150	1332	1781
PB.6762.1	5	1400	7	incomplete-splice_match	gene: Zm00001d016486	Zm00001d016486_T00	23.99	23.99	coding	385	34	1188

PB.1892.1	10	1370	1	full-splice_match	gene: Zm00001d024379	Zm00001d024379_T00	119.20	402.10	coding	286	125	982
PB.4344.3	3	1610	1	full-splice_match	gene: Zm00001d042786	Zm00001d042786_T00	0.00	57.68	coding	391	190	1362
PB.8662.1	7	1862	1	full-splice_match	gene: Zm00001d021026	Zm00001d021026_T00	45.58	45.58	coding	487	115	1575
PB.178.3	1	767	6	full-splice_match	gene: Zm00001d028005	Zm00001d028005_T00	0.00	45.01	coding	238	53	766
PB.8046.6	6	3167	8	full-splice_match	gene: Zm00001d038587	Zm00001d038587_T02	0.00	36.15	coding	593	188	1966
PB.11034.2	B73V 4	2637	6	novel_in_catalog	gene: Zm00001d000228	novel	34.22	106.98	non coding	NA	NA	NA
PB.9070.1	7	1845	10	full-splice_match	gene: Zm00001d022493	Zm00001d022493_T00	16.68	16.68	coding	298	657	1550
PB.1099.2	1	683	1	full-splice_match	gene: Zm00001d032533	Zm00001d032533_T00	50.06	67.88	non coding	NA	NA	NA
PB.1315.1	1	706	1	full-splice_match	gene: Zm00001d033469	Zm00001d033469_T00	15.04	15.04	coding	150	7	456
PB.6623.2	5	1672	4	full-splice_match	gene: Zm00001d015581	Zm00001d015581_T00	17.31	17.31	coding	370	221	1330
PB.8375.2	7	1594	4	genic	gene: Zm00001d019493	novel	9.32	26.26	coding	324	481	1452
PB.698.1	1	2878	11	full-splice_match	gene: Zm00001d030554	Zm00001d030554_T00	14.33	14.33	coding	838	200	2713
PB.4907.1	4	624	4	full-splice_match	gene: Zm00001d048957	Zm00001d048957_T00	459.24	475.40	non coding	NA	NA	NA
PB.4623.1	3	1955	17	full-splice_match	gene: Zm00001d043840	Zm00001d043840_T00	16.72	20.90	coding	422	360	1625
PB.4907.3	4	700	3	full-splice_match	gene: Zm00001d048957	Zm00001d048957_T00	6.36	475.40	coding	67	119	319
PB.4907.2	4	836	2	novel_in_catalog	gene: Zm00001d048957	novel	9.81	475.40	coding	97	119	409
PB.6626.6	5	1682	4	novel_not_in_catalog	gene: Zm00001d015618	novel	3.01	992.44	coding	229	794	1480

PB.8375.4	7	2588	2	genic	gene: Zm00001d019493	novel	0.00	26.26	coding	423	1164	2432
PB.5899.1	4	1122	4	full-splice_match	gene: Zm00001d053963	Zm00001d053963_T00	36.94	36.94	coding	255	188	952
PB.8180.2	6	712	5	full-splice_match	gene: Zm00001d039106	Zm00001d039106_T00	148.64	164.01	coding	140	77	496
PB.9686.1	8	2898	1	full-splice_match	gene: Zm00001d010997	Zm00001d010997_T00	16.42	16.42	coding	854	102	2663
PB.8180.1	6	803	4	full-splice_match	gene: Zm00001d039106	Zm00001d039106_T00	15.38	164.01	coding	140	169	588
PB.4116.2	3	1287	6	incomplete-splice_match	gene: Zm00001d041700	Zm00001d041700_T00	4.41	14.44	coding	23	263	331
PB.4116.1	3	1975	9	full-splice_match	gene: Zm00001d041700	Zm00001d041700_T00	10.03	14.44	coding	284	287	1138
PB.10155.1	9	946	1	intergenic	novelGene_43	novel	3.75	3.75	non coding	NA	NA	NA
PB.6539.1	5	1445	4	full-splice_match	gene: Zm00001d015123	Zm00001d015123_T00	43.44	48.18	coding	244	421	1152
PB.6539.2	5	1347	4	novel_not_in_catalog	gene: Zm00001d015123	novel	4.74	48.18	coding	171	421	933
PB.9066.4	7	656	4	novel_in_catalog	gene: Zm00001d022489	novel	251.15	487.65	coding	130	133	522
PB.4600.3	3	1503	6	incomplete-splice_match	gene: Zm00001d043762	Zm00001d043762_T00	4.00	34.99	coding	480	64	1503
PB.5203.4	4	1850	11	novel_in_catalog	gene: Zm00001d050501	novel	4.83	30.27	coding	160	1006	1485
PB.7837.12	6	1632	3	full-splice_match	gene: Zm00001d037877	Zm00001d037877_T00	188.91	647.16	coding	447	76	1416
PB.7837.13	6	1525	3	full-splice_match	gene: Zm00001d037877	Zm00001d037877_T00	153.46	647.16	coding	447	77	1417
PB.7837.11	6	1751	3	full-splice_match	gene: Zm00001d037877	Zm00001d037877_T00	304.79	647.16	coding	447	76	1416
PB.1754.1	10	1621	1	intergenic	novelGene_41	novel	0.49	0.49	coding	242	99	824

PB.9217.2	8	1428	7	full-splice_match	gene: Zm00001d008611	Zm00001d008611_T00	50.18	64.80	coding	260	359	1138
PB.3012.2	2	1273	3	incomplete-splice_match	gene: Zm00001d004659	Zm00001d004659_T01	22.57	39.36	coding	188	120	683
PB.3012.1	2	3668	13	full-splice_match	gene: Zm00001d004659	Zm00001d004659_T00	16.79	39.36	coding	907	249	2969
PB.9217.1	8	2342	6	full-splice_match	gene: Zm00001d008611	Zm00001d008611_T00	14.62	64.80	coding	216	329	976
PB.1089.1	1	2002	1	full-splice_match	gene: Zm00001d032503	Zm00001d032503_T00	45.93	45.93	coding	470	196	1605
PB.8126.1	6	1328	7	full-splice_match	gene: Zm00001d038896	Zm00001d038896_T00	69.20	69.20	coding	266	275	1072
PB.8139.6	6	4016	10	novel_in_catalog	gene: Zm00001d038927	novel	1.86	63.06	coding	409	2372	3598
PB.7763.3	6	2250	17	novel_in_catalog	gene: Zm00001d037538	novel	28.34	53.04	coding	635	139	2043
PB.1075.3	1	2079	18	full-splice_match	gene: Zm00001d032455	Zm00001d032455_T00	0.00	7.23	coding	584	50	1801
PB.2702.1	2	527	1	genic	gene: Zm00001d002954	novel	8.93	33.21	non coding	NA	NA	NA
PB.2702.2	2	845	4	full-splice_match	gene: Zm00001d002954	Zm00001d002954_T00	24.28	33.21	coding	149	70	516
PB.9374.1	8	2337	16	novel_not_in_catalog	gene: Zm00001d009504	novel	60.35	60.35	coding	609	227	2053
PB.7745.4	6	3523	8	novel_in_catalog	gene: Zm00001d037448	novel	1.27	27.81	coding	100	3024	3323
PB.7745.6	6	2645	9	novel_in_catalog	gene: Zm00001d037448	novel	0.17	27.81	coding	100	2172	2471
PB.7745.7	6	3464	7	full-splice_match	gene: Zm00001d037448	Zm00001d037448_T01	9.46	27.81	coding	100	3007	3306
PB.7745.1	6	2826	9	full-splice_match	gene: Zm00001d037448	Zm00001d037448_T00	11.90	27.81	coding	100	2299	2598
PB.7745.3	6	2366	7	incomplete-splice_match	gene: Zm00001d037448	Zm00001d037448_T00	5.01	27.81	coding	100	1864	2163

PB.5300.4	4	3922	6	full-splice_match	gene: Zm00001d051291	Zm00001d051291_T00	3.42	32.00	coding	529	1951	3537
PB.5300.3	4	3649	7	full-splice_match	gene: Zm00001d051291	Zm00001d051291_T00	3.18	32.00	coding	489	1798	3264
PB.5300.2	4	2510	7	full-splice_match	gene: Zm00001d051291	Zm00001d051291_T00	25.40	32.00	coding	608	235	2058
PB.6967.1	5	1511	4	full-splice_match	gene: Zm00001d017363	Zm00001d017363_T00	9.85	9.85	coding	368	299	1402
PB.9788.7	8	2414	2	full-splice_match	gene: Zm00001d011500	Zm00001d011500_T01	4.07	501.01	non coding	NA	NA	NA
PB.1589.2	1	1085	3	full-splice_match	gene: Zm00001d034634	Zm00001d034634_T00	0.00	18.98	coding	180	64	603
PB.5183.1	4	3293	2	incomplete-splice_match	gene: Zm00001d050372	Zm00001d050372_T00	19.71	19.71	coding	913	135	2873
PB.8139.7	6	2079	10	full-splice_match	gene: Zm00001d038927	Zm00001d038927_T00	2.63	63.06	coding	224	90	761
PB.1237.1	1	1025	6	full-splice_match	gene: Zm00001d033099	Zm00001d033099_T00	85.76	259.99	coding	142	80	505
PB.5889.2	4	983	6	full-splice_match	gene: Zm00001d053923	Zm00001d053923_T00	0.00	91.26	coding	232	132	827
PB.3897.4	3	2030	7	incomplete-splice_match	gene: Zm00001d040234	Zm00001d040234_T00	10.19	10.19	coding	513	492	2030
PB.9994.1	8	460	1	full-splice_match	gene: Zm00001d012332	Zm00001d012332_T00	2.37	2.37	coding	9	283	309
PB.3549.2	2	1806	4	novel_in_catalog	gene: Zm00001d007215	novel	4.70	306.75	coding	256	103	870
PB.5912.1	4	1026	1	full-splice_match	gene: Zm00001d053988	Zm00001d053988_T00	21.70	21.70	coding	162	265	750
PB.3549.1	2	1673	5	full-splice_match	gene: Zm00001d007215	Zm00001d007215_T00	302.06	306.75	coding	447	112	1452
PB.1250.1	1	1533	4	full-splice_match	gene: Zm00001d033174	Zm00001d033174_T00	638.84	779.80	coding	364	120	1211
PB.4128.1	3	1134	5	full-splice_match	gene: Zm00001d041750	Zm00001d041750_T00	151.17	151.17	coding	254	69	830

PB.4128.2	3	739	2	incomplete-splice_match	gene: Zm00001d041750	Zm00001d041750_T00	0.00	151.17	coding	18	469	522
PB.8396.2	7	1904	9	full-splice_match	gene: Zm00001d019579	Zm00001d019579_T00	3.47	24.53	coding	352	79	1134
PB.6263.2	5	1712	3	novel_not_in_catalog	gene: Zm00001d013997	novel	13.43	34.61	coding	286	174	1031
PB.6263.3	5	603	2	novel_not_in_catalog	gene: Zm00001d013997	novel	21.00	34.61	coding	93	155	433
PB.8739.2	7	2371	5	novel_not_in_catalog	gene: Zm00001d021371	novel	3.82	43.60	coding	240	1144	1863
PB.6263.1	5	2151	4	novel_not_in_catalog	gene: Zm00001d013997	novel	0.17	34.61	coding	203	197	805
PB.3569.1	2	4372	15	full-splice_match	gene: Zm00001d007407	Zm00001d007407_T02	11.19	36.87	coding	866	128	2725
PB.10655.3	9	1602	2	genic	gene: Zm00001d047560	novel	1.81	39.22	coding	309	273	1199
PB.7209.1	5	2125	12	full-splice_match	gene: Zm00001d018336	Zm00001d018336_T00	5.06	11.41	coding	543	143	1771
PB.3569.5	2	2588	9	incomplete-splice_match	gene: Zm00001d007407	Zm00001d007407_T04	6.92	36.87	coding	453	378	1736
PB.3569.6	2	2984	15	full-splice_match	gene: Zm00001d007407	Zm00001d007407_T02	14.29	36.87	coding	866	74	2671
PB.4790.6	3	2441	15	novel_in_catalog	gene: Zm00001d044503	novel	2.98	19.57	coding	414	221	1462
PB.1408.2	1	2578	4	full-splice_match	gene: Zm00001d033873	Zm00001d033873_T00	1.70	20.98	coding	202	20	625
PB.3801.1	3	366	1	incomplete-splice_match	gene: Zm00001d039730	Zm00001d039730_T00	266.60	266.60	non coding	NA	NA	NA
PB.2980.1	2	1410	10	incomplete-splice_match	gene: Zm00001d004463	Zm00001d004463_T01	81.23	81.23	coding	364	16	1107
PB.10301.11	9	1073	4	novel_in_catalog	gene: Zm00001d045558	novel	0.00	105.73	coding	219	48	704
PB.10301.10	9	1442	3	novel_in_catalog	gene: Zm00001d045558	novel	0.00	105.73	coding	151	41	493

PB.493.2	1	2500	3	novel_not_in_catalog	gene: Zm00001d029397	novel	17.77	17.77	coding	754	108	2369
PB.3494.2	2	422	2	antisense	novelGene_gene: Zm00001d007010	novel	547.84	1098.91	non coding	NA	NA	NA
PB.567.6	1	1497	1	novel_in_catalog	gene: Zm00001d029738	novel	5.52	134.73	coding	161	53	535
PB.2817.1	2	836	5	full-splice_match	gene: Zm00001d003493	Zm00001d003493_T00	172.24	172.24	coding	136	124	531
PB.8185.1	6	2414	10	novel_not_in_catalog	gene: Zm00001d039141	novel	52.52	52.52	coding	601	209	2011
PB.2817.2	2	735	5	full-splice_match	gene: Zm00001d003493	Zm00001d003493_T00	0.00	172.24	coding	136	124	531
PB.9803.9	8	676	8	full-splice_match	gene: Zm00001d011565	Zm00001d011565_T00	34.60	608.45	coding	153	148	606
PB.10255.1	9	815	3	full-splice_match	gene: Zm00001d045404	Zm00001d045404_T00	16.33	16.33	coding	39	611	727
PB.567.4	1	1015	2	novel_in_catalog	gene: Zm00001d029738	novel	11.89	134.73	coding	161	60	542
PB.8485.6	7	2942	13	novel_in_catalog	gene: Zm00001d020185	novel	180.40	390.61	coding	896	18	2705
PB.8485.7	7	4036	9	novel_in_catalog	gene: Zm00001d020185	novel	18.09	390.61	coding	395	16	1200
PB.8485.4	7	2232	12	novel_not_in_catalog	gene: Zm00001d020183	novel	32.96	86.02	coding	612	251	2086
PB.8485.5	7	782	2	genic	gene: Zm00001d020184	novel	210.19	210.19	coding	252	12	767
PB.8485.2	7	3343	11	novel_not_in_catalog	gene: Zm00001d020183	novel	9.98	86.02	coding	323	2218	3186
PB.8485.1	7	1891	4	novel_in_catalog	gene: Zm00001d020183	novel	43.08	86.02	coding	138	113	526
PB.8731.1	7	941	7	full-splice_match	gene: Zm00001d021335	Zm00001d021335_T00	18.89	18.89	coding	202	197	802
PB.8485.8	7	2414	9	novel_in_catalog	gene: Zm00001d020185	novel	7.99	390.61	coding	325	521	1495

PB.567.3	1	1032	3	full-splice_match	gene: Zm00001d029738	Zm00001d029738_T00	94.06	134.73	coding	216	60	707
PB.10807.1	9	1066	2	genic	gene: Zm00001d048110	novel	88.32	88.32	coding	144	351	782
PB.6499.7	5	1341	4	full-splice_match	gene: Zm00001d014957	Zm00001d014957_T01	8.15	2924.75	coding	129	75	461
PB.6499.6	5	715	5	full-splice_match	gene: Zm00001d014957	Zm00001d014957_T00	2916.60	2924.75	coding	167	75	575
PB.6639.1	5	2698	13	fusion	gene: Zm00001d015693	novel	42.60	49.74	coding	711	143	2275
PB.112.1	1	657	1	full-splice_match	gene: Zm00001d027695	Zm00001d027695_T00	0.00	0.00	coding	104	102	413
PB.7319.2	6	1417	11	full-splice_match	gene: Zm00001d035090	Zm00001d035090_T00	8.48	17.40	coding	394	121	1302
PB.7319.1	6	1561	11	full-splice_match	gene: Zm00001d035090	Zm00001d035090_T00	8.92	17.40	coding	394	95	1276
PB.3476.1	2	751	2	genic	gene: Zm00001d006915	novel	21.61	21.61	coding	160	91	570
PB.6832.2	5	2062	9	full-splice_match	gene: Zm00001d016811	Zm00001d016811_T00	14.30	26.58	coding	460	471	1850
PB.1047.6	1	2764	6	full-splice_match	gene: Zm00001d032350	Zm00001d032350_T00	9.89	141.86	coding	114	178	519
PB.6832.1	5	1923	8	novel_in_catalog	gene: Zm00001d016811	novel	12.29	26.58	coding	460	304	1683
PB.2720.1	2	1908	14	full-splice_match	gene: Zm00001d003049	Zm00001d003049_T00	60.24	60.24	coding	448	404	1747
PB.2720.2	2	1870	14	full-splice_match	gene: Zm00001d003049	Zm00001d003049_T00	0.00	60.24	coding	448	404	1747
PB.9676.2	8	1846	12	intergenic	novelGene_261	novel	19.49	19.49	coding	93	49	327
PB.8886.1	7	4917	12	novel_not_in_catalog	gene: Zm00001d021858	novel	58.43	123.94	coding	1342	174	4199
PB.8499.1	7	845	3	novel_not_in_catalog	gene: Zm00001d020274	novel	21.59	21.59	non coding	NA	NA	NA

PB.33.2	1	1605	6	full-splice_match	gene: Zm00001d027359	Zm00001d027359_T00	54.49	55.09	coding	445	84	1418
PB.33.1	1	1334	6	novel_not_in_catalog	gene: Zm00001d027359	novel	0.59	55.09	coding	242	58	783
PB.6846.5	5	1799	3	incomplete-splice_match	gene: Zm00001d016858	Zm00001d016858_T01	0.00	55.65	coding	482	275	1720
PB.5268.1	4	339	2	intergenic	novelGene_270	novel	2.92	2.92	non coding	NA	NA	NA
PB.8725.2	7	1254	5	full-splice_match	gene: Zm00001d021313	Zm00001d021313_T00	0.00	29.24	coding	276	155	982
PB.8725.1	7	1358	5	full-splice_match	gene: Zm00001d021313	Zm00001d021313_T00	29.24	29.24	coding	276	155	982
PB.5362.4	4	2239	7	full-splice_match	gene: Zm00001d051600	Zm00001d051600_T03	10.50	151.77	coding	617	389	2239
PB.7379.1	6	1297	1	full-splice_match	gene: Zm00001d035440	Zm00001d035440_T00	141.43	230.21	coding	135	564	968
PB.5362.2	4	1856	3	incomplete-splice_match	gene: Zm00001d051600	Zm00001d051600_T02	0.00	151.77	coding	499	97	1593
PB.5362.1	4	3407	6	incomplete-splice_match	gene: Zm00001d051600	Zm00001d051600_T02	73.64	151.77	coding	802	673	3078
PB.7379.2	6	1188	1	full-splice_match	gene: Zm00001d035440	Zm00001d035440_T00	88.78	230.21	coding	135	560	964
PB.7056.7	5	1068	7	novel_not_in_catalog	gene: Zm00001d017756	novel	9.15	54.96	coding	269	168	974
PB.4832.2	3	1939	8	novel_in_catalog	gene: Zm00001d044713	novel	112.43	112.43	coding	102	485	790
PB.5092.1	4	786	2	full-splice_match	gene: Zm00001d049826	Zm00001d049826_T00	384.37	636.01	coding	124	89	460
PB.7144.2	5	1269	2	genic	gene: Zm00001d018087	novel	1.38	37.47	coding	331	13	1005
PB.7144.1	5	1296	1	full-splice_match	gene: Zm00001d018087	Zm00001d018087_T00	36.09	37.47	coding	331	15	1007
PB.5092.2	4	663	2	full-splice_match	gene: Zm00001d049826	Zm00001d049826_T00	251.65	636.01	coding	124	92	463

PB.594.4	1	1138	3	novel_not_in_catalog	gene: Zm00001d029887	novel	7.81	19.08	coding	173	482	1000
PB.6815.1	5	842	1	full-splice_match	gene: Zm00001d016740	Zm00001d016740_T00	3.96	3.96	coding	181	136	678
PB.5930.1	4	676	2	incomplete-splice_match	gene: Zm00001d054047	Zm00001d054047_T00	2.14	28.11	coding	115	332	676
PB.594.2	1	1651	3	novel_in_catalog	gene: Zm00001d029887	novel	11.27	19.08	coding	187	950	1510
PB.7534.2	6	1161	8	full-splice_match	gene: Zm00001d036403	Zm00001d036403_T00	236.86	292.17	coding	258	175	948
PB.7534.3	6	1999	7	novel_in_catalog	gene: Zm00001d036403	novel	2.02	292.17	coding	142	1365	1790
PB.4680.3	3	816	1	full-splice_match	gene: Zm00001d044076	Zm00001d044076_T00	78.73	183.25	coding	136	171	578
PB.7534.1	6	1192	8	full-splice_match	gene: Zm00001d036403	Zm00001d036403_T00	53.29	292.17	coding	258	87	860
PB.2014.4	10	871	1	novel_in_catalog	gene: Zm00001d024963	novel	0.00	717.24	coding	109	456	782
PB.6156.2	5	1039	1	intergenic	novelGene_55	novel	25.23	38.69	non coding	NA	NA	NA
PB.9384.1	8	2224	8	fusion	gene: Zm00001d009542	novel	23.05	23.05	coding	578	490	2223
PB.3657.1	2	1187	2	full-splice_match	gene: Zm00001d007901	Zm00001d007901_T00	190.47	280.15	coding	224	280	951
PB.259.3	1	1242	2	incomplete-splice_match	gene: Zm00001d028355	Zm00001d028355_T00	7.09	13.68	coding	237	441	1151
PB.259.1	1	1897	5	full-splice_match	gene: Zm00001d028355	Zm00001d028355_T00	6.59	13.68	coding	586	22	1779
PB.10361.1	9	1420	1	genic	gene: Zm00001d045946	novel	4.69	29.47	coding	249	112	858
PB.2728.1	2	718	4	full-splice_match	gene: Zm00001d003081	Zm00001d003081_T00	261.00	758.42	coding	131	95	487
PB.2728.2	2	576	4	full-splice_match	gene: Zm00001d003081	Zm00001d003081_T00	497.42	758.42	coding	113	53	391

PB.10361.2	9	1147	2	full-splice_match	gene: Zm00001d045946	Zm00001d045946_T00	17.11	29.47	coding	316	14	961
PB.2435.1	2	1785	2	full-splice_match	gene: Zm00001d001817	Zm00001d001817_T00	9.58	9.58	coding	538	79	1692
PB.1281.1	1	1206	2	full-splice_match	gene: Zm00001d033319	Zm00001d033319_T00	38.39	38.39	coding	226	148	825
PB.9519.1	8	313	1	full-splice_match	gene: Zm00001d010284	Zm00001d010284_T00	16.65	16.65	non coding	NA	NA	NA
PB.874.4	1	2332	4	full-splice_match	gene: Zm00001d031505	Zm00001d031505_T00	3.62	48.34	coding	321	1065	2027
PB.1665.1	1	1256	2	novel_not_in_catalog	gene: Zm00001d034878	novel	14.16	14.16	coding	140	37	456
PB.7159.4	5	1660	1	novel_in_catalog	gene: Zm00001d018134	novel	1.52	549.21	non coding	NA	NA	NA
PB.3305.4	2	1621	1	incomplete-splice_match	gene: Zm00001d006177	Zm00001d006177_T02	11.14	19.31	non coding	NA	NA	NA
PB.6485.1	5	1604	7	full-splice_match	gene: Zm00001d014903	Zm00001d014903_T00	42.47	98.22	coding	408	5	1228
PB.6485.2	5	2098	9	full-splice_match	gene: Zm00001d014903	Zm00001d014903_T00	51.64	98.22	coding	586	162	1919
PB.6485.3	5	2193	8	novel_in_catalog	gene: Zm00001d014903	novel	4.11	98.22	coding	474	131	1552
PB.7546.1	6	3112	1	full-splice_match	gene: Zm00001d036455	Zm00001d036455_T00	2.74	5.59	coding	801	107	2509
PB.7546.2	6	2707	1	full-splice_match	gene: Zm00001d036455	Zm00001d036455_T00	2.84	5.59	coding	801	105	2507
PB.5459.5	4	2735	21	full-splice_match	gene: Zm00001d052050	Zm00001d052050_T00	5.83	75.60	coding	775	79	2403
PB.239.1	1	941	1	full-splice_match	gene: Zm00001d028273	Zm00001d028273_T00	6.45	115.35	coding	239	6	722
PB.239.2	1	1168	1	full-splice_match	gene: Zm00001d028273	Zm00001d028273_T00	108.90	115.35	coding	330	63	1052
PB.1617.2	1	1707	4	novel_in_catalog	gene: Zm00001d034727	novel	7.53	40.18	coding	183	947	1495

PB.2848.1	2	1467	11	full-splice_match	gene: Zm00001d003652	Zm00001d003652_T00	13.76	47.25	coding	318	67	1020
PB.5249.2	4	1298	4	full-splice_match	gene: Zm00001d050942	Zm00001d050942_T00	11.68	32.51	coding	295	143	1027
PB.2848.3	2	1310	10	novel_in_catalog	gene: Zm00001d003652	novel	1.31	47.25	coding	163	60	548
PB.2848.2	2	1282	11	full-splice_match	gene: Zm00001d003652	Zm00001d003652_T00	28.01	47.25	coding	318	25	978
PB.2848.4	2	1353	11	novel_in_catalog	gene: Zm00001d003652	novel	4.17	47.25	coding	121	759	1121
PB.6065.2	5	2087	1	novel_in_catalog	gene: Zm00001d013196	novel	6.18	58.95	coding	255	1135	1899
PB.4402.3	3	1492	4	novel_in_catalog	gene: Zm00001d043070	novel	3.97	553.22	coding	132	887	1282
PB.6065.1	5	1558	2	full-splice_match	gene: Zm00001d013196	Zm00001d013196_T00	52.77	58.95	coding	432	48	1343
PB.4402.5	3	329	4	incomplete-splice_match	gene: Zm00001d043070	Zm00001d043070_T00	143.80	553.22	coding	75	103	327
PB.5211.2	4	2071	8	incomplete-splice_match	gene: Zm00001d050610	Zm00001d050610_T00	11.66	65.05	coding	220	956	1615
PB.4919.2	4	742	1	genic	gene: Zm00001d049019	novel	125.78	125.78	non coding	NA	NA	NA
PB.10514.2	9	2456	10	novel_in_catalog	gene: Zm00001d046936	novel	4.81	75.54	coding	272	362	1177
PB.10514.1	9	1943	11	full-splice_match	gene: Zm00001d046936	Zm00001d046936_T00	70.72	75.54	coding	378	425	1558
PB.8123.3	6	2039	12	full-splice_match	gene: Zm00001d038891	Zm00001d038891_T00	0.00	56.47	coding	501	398	1900
PB.9219.1	8	2578	7	full-splice_match	gene: Zm00001d008618	Zm00001d008618_T00	26.31	32.33	coding	691	249	2321
PB.9219.2	8	2656	6	full-splice_match	gene: Zm00001d008618	Zm00001d008618_T00	6.02	32.33	coding	452	197	1552
PB.5613.2	4	1304	2	full-splice_match	gene: Zm00001d052655	Zm00001d052655_T00	0.00	54.77	coding	270	173	982

PB.8970.1	7	1301	5	full-splice_match	gene: Zm00001d022182	Zm00001d022182_T00	13.61	13.61	coding	330	60	1049
PB.1555.1	1	4222	18	full-splice_match	gene: Zm00001d034515	Zm00001d034515_T00	6.85	6.85	coding	1146	183	3620
PB.8485.9	7	2075	1	novel_in_catalog	gene: Zm00001d020185	novel	41.12	390.61	non coding	NA	NA	NA
PB.5126.1	4	1311	11	full-splice_match	gene: Zm00001d050043	Zm00001d050043_T00	85.70	85.70	coding	318	178	1131
PB.1857.1	10	2028	7	full-splice_match	gene: Zm00001d024059	Zm00001d024059_T00	18.91	18.91	coding	430	512	1801
PB.1857.2	10	1591	7	full-splice_match	gene: Zm00001d024059	Zm00001d024059_T00	0.00	18.91	coding	430	132	1421
PB.6801.3	5	1055	4	novel_in_catalog	gene: Zm00001d016682	novel	43.24	70.81	coding	238	123	836
PB.8062.1	6	878	7	full-splice_match	gene: Zm00001d038681	Zm00001d038681_T00	13.77	21.90	coding	116	186	533
PB.5819.1	4	2976	2	full-splice_match	gene: Zm00001d053642	Zm00001d053642_T00	196.94	196.94	coding	651	242	2194
PB.3460.1	2	1574	2	intergenic	novelGene_415	novel	45.30	58.56	non coding	NA	NA	NA
PB.3460.2	2	2443	1	intergenic	novelGene_415	novel	13.26	58.56	coding	176	165	692
PB.8062.4	6	746	7	full-splice_match	gene: Zm00001d038681	Zm00001d038681_T00	8.13	21.90	coding	116	236	583
PB.1230.4	1	2332	4	novel_in_catalog	gene: Zm00001d033056	novel	0.00	179.46	coding	294	58	939
PB.10645.4	9	2523	12	full-splice_match	gene: Zm00001d047522	Zm00001d047522_T00	0.00	184.41	coding	629	396	2282
PB.4701.9	3	1798	4	novel_not_in_catalog	gene: Zm00001d044146	novel	0.00	90.95	coding	350	683	1732
PB.10838.1	9	3340	16	full-splice_match	gene: Zm00001d048258	Zm00001d048258_T01	48.76	63.38	coding	780	449	2788
PB.10838.2	9	3210	16	full-splice_match	gene: Zm00001d048258	Zm00001d048258_T02	8.80	63.38	coding	780	429	2768

PB.1873.2	10	1200	6	full-splice_match	gene: Zm00001d024231	Zm00001d024231_T00	0.00	26.09	coding	322	63	1028
PB.7184.2	5	1557	9	novel_in_catalog	gene: Zm00001d018230	novel	3.87	88.41	coding	305	33	947
PB.5897.2	4	868	9	incomplete-splice_match	gene: Zm00001d053954	Zm00001d053954_T00	5.71	37.13	coding	128	183	566
PB.1873.1	10	1326	6	full-splice_match	gene: Zm00001d024231	Zm00001d024231_T00	26.09	26.09	coding	322	81	1046
PB.9505.4	8	1912	6	incomplete-splice_match	gene: Zm00001d010235	Zm00001d010235_T01	12.26	12.26	coding	537	159	1769
PB.2807.4	2	1351	5	full-splice_match	gene: Zm00001d003434	Zm00001d003434_T00	31.45	41.84	coding	363	133	1221
PB.2421.1	2	1002	2	novel_in_catalog	gene: Zm00001d001779	novel	60.57	60.57	coding	111	501	833
PB.4701.5	3	2022	3	novel_not_in_catalog	gene: Zm00001d044146	novel	2.72	90.95	coding	275	995	1819
PB.9754.2	8	1762	2	full-splice_match	gene: Zm00001d011336	Zm00001d011336_T00	38.57	46.99	coding	448	60	1403
PB.2807.1	2	1481	4	novel_in_catalog	gene: Zm00001d003434	novel	10.39	41.84	coding	187	736	1296
PB.8378.1	7	1666	8	full-splice_match	gene: Zm00001d019515	Zm00001d019515_T00	58.75	78.00	coding	368	210	1313
PB.1301.4	1	1025	2	genic	gene: Zm00001d033386	novel	0.00	76.58	coding	230	137	826
PB.4345.1	3	1649	5	full-splice_match	gene: Zm00001d042787	Zm00001d042787_T00	20.46	26.34	coding	398	14	1207
PB.8378.2	7	1531	8	full-splice_match	gene: Zm00001d019515	Zm00001d019515_T00	19.25	78.00	coding	368	199	1302
PB.2356.2	10	1982	7	novel_not_in_catalog	gene: Zm00001d026542	novel	14.69	44.70	coding	297	108	998
PB.8326.1	7	1596	14	full-splice_match	gene: Zm00001d019139	Zm00001d019139_T00	180.01	180.01	coding	344	200	1231
PB.8326.5	7	1485	14	full-splice_match	gene: Zm00001d019139	Zm00001d019139_T00	0.00	180.01	coding	344	200	1231

PB.875.2	1	1108	2	full-splice_match	gene: Zm00001d031510	Zm00001d031510_T00	80.48	87.58	coding	252	130	885
PB.875.3	1	1073	2	full-splice_match	gene: Zm00001d031510	Zm00001d031510_T00	0.00	87.58	coding	326	60	1037
PB.875.1	1	1446	1	genic	gene: Zm00001d031510	novel	7.10	87.58	coding	178	638	1171
PB.5926.1	4	993	9	full-splice_match	gene: Zm00001d054017	Zm00001d054017_T00	40.40	181.85	coding	187	72	632
PB.19.6	1	1584	15	novel_not_in_catalog	gene: Zm00001d027309	novel	20.64	44.73	coding	373	126	1244
PB.19.5	1	1951	13	novel_in_catalog	gene: Zm00001d027309	novel	8.71	44.73	coding	299	132	1028
PB.19.4	1	2270	14	novel_in_catalog	gene: Zm00001d027309	novel	8.15	44.73	coding	169	1431	1937
PB.19.3	1	1767	13	novel_in_catalog	gene: Zm00001d027309	novel	7.24	44.73	coding	339	119	1135
PB.3468.1	2	2800	7	full-splice_match	gene: Zm00001d006884	Zm00001d006884_T00	3.53	21.39	coding	163	337	825
PB.3468.2	2	2078	8	full-splice_match	gene: Zm00001d006884	Zm00001d006884_T00	9.60	21.39	coding	529	50	1636
PB.5235.1	4	589	2	full-splice_match	gene: Zm00001d050860	Zm00001d050860_T00	44.24	44.24	coding	116	241	588
PB.11025.1	B73V4	2435	6	full-splice_match	gene: Zm00001d000295	Zm00001d000295_T00	57.16	101.48	coding	678	116	2149
PB.11025.2	B73V4	1433	3	incomplete-splice_match	gene: Zm00001d000295	Zm00001d000295_T00	44.32	101.48	coding	291	435	1307
PB.1246.3	1	1303	8	novel_not_in_catalog	gene: Zm00001d033159	novel	0.00	343.80	coding	408	79	1302
PB.2356.5	10	884	5	novel_in_catalog	gene: Zm00001d026542	novel	0.00	44.70	coding	156	250	717
PB.6034.10	5	1725	15	novel_in_catalog	gene: Zm00001d013059	novel	23.12	107.28	coding	534	44	1645
PB.9542.2	8	1259	3	full-splice_match	gene: Zm00001d010403	Zm00001d010403_T00	0.00	86.55	coding	349	195	1241

PB.10076.1	8	1747	3	full-splice_match	gene: Zm00001d012662	Zm00001d012662_T00	62.64	62.64	coding	421	252	1514
PB.2356.4	10	975	1	novel_in_catalog	gene: Zm00001d026542	novel	18.25	44.70	non coding	NA	NA	NA
PB.9810.1	8	789	1	full-splice_match	gene: Zm00001d011589	Zm00001d011589_T00	16.07	16.07	coding	215	33	677
PB.10674.1	9	2640	5	full-splice_match	gene: Zm00001d047633	Zm00001d047633_T00	21.76	28.42	coding	768	99	2402
PB.10607.1	9	3666	4	novel_in_catalog	gene: Zm00001d047412	novel	26.50	26.50	coding	964	566	3457
PB.7365.6	6	2991	10	novel_in_catalog	gene: Zm00001d035346	novel	7.39	44.35	coding	187	931	1491
PB.7365.7	6	1400	6	full-splice_match	gene: Zm00001d035346	Zm00001d035346_T15	11.50	44.35	coding	99	63	359
PB.7365.4	6	2209	8	novel_in_catalog	gene: Zm00001d035346	novel	3.42	44.35	coding	187	279	839
PB.316.1	1	2211	5	novel_in_catalog	gene: Zm00001d028599	novel	6.17	18.42	coding	283	1059	1907
PB.316.2	1	2042	6	full-splice_match	gene: Zm00001d028599	Zm00001d028599_T00	12.25	18.42	coding	576	100	1827
PB.7365.1	6	1950	8	novel_in_catalog	gene: Zm00001d035346	novel	10.64	44.35	coding	116	360	707
PB.4046.5	3	676	2	full-splice_match	gene: Zm00001d041246	Zm00001d041246_T00	131.23	779.74	coding	145	107	541
PB.4046.4	3	825	1	novel_in_catalog	gene: Zm00001d041246	novel	0.00	779.74	coding	100	71	370
PB.4046.1	3	868	2	full-splice_match	gene: Zm00001d041246	Zm00001d041246_T00	40.32	779.74	coding	145	81	515
PB.8962.1	7	1277	1	full-splice_match	gene: Zm00001d022154	Zm00001d022154_T00	35.63	36.07	coding	271	223	1035
PB.4046.3	3	771	2	full-splice_match	gene: Zm00001d041246	Zm00001d041246_T00	595.44	779.74	coding	145	81	515
PB.4046.2	3	940	1	genic	gene: Zm00001d041246	novel	12.74	779.74	coding	100	83	382

PB.4697.3	3	1938	6	full-splice_match	gene: Zm00001d044133	Zm00001d044133_T01	13.03	60.87	coding	99	1405	1701
PB.2103.3	10	747	3	full-splice_match	gene: Zm00001d025445	Zm00001d025445_T00	31.61	105.64	coding	154	100	561
PB.2103.2	10	868	3	full-splice_match	gene: Zm00001d025445	Zm00001d025445_T00	70.84	105.64	coding	154	124	585
PB.7463.1	6	1478	2	genic	gene: Zm00001d035918	novel	12.02	12.02	coding	373	81	1199
PB.5597.4	4	2152	8	full-splice_match	gene: Zm00001d052525	Zm00001d052525_T00	0.00	55.88	coding	426	623	1900
PB.5926.3	4	1271	2	full-splice_match	gene: Zm00001d054017	Zm00001d054017_T00	4.82	181.85	non coding	NA	NA	NA
PB.3755.1	3	1299	2	intergenic	novelGene_34	novel	19.02	19.02	coding	90	59	328
PB.10526.7	9	1077	10	novel_in_catalog	gene: Zm00001d046972	novel	0.00	66.39	coding	149	303	749
PB.6497.1	5	1560	2	full-splice_match	gene: Zm00001d014952	Zm00001d014952_T00	44.55	44.55	coding	382	99	1244
PB.2962.1	2	395	1	genic	gene: Zm00001d004293	novel	22.11	22.11	non coding	NA	NA	NA
PB.8342.1	7	1371	3	novel_in_catalog	gene: Zm00001d019220	novel	3.11	3.11	coding	377	81	1211
PB.8837.1	7	1346	9	full-splice_match	gene: Zm00001d021719	Zm00001d021719_T00	56.80	56.80	coding	301	155	1057
PB.770.2	1	1357	8	full-splice_match	gene: Zm00001d031002	Zm00001d031002_T00	20.53	42.89	coding	324	79	1050
PB.8837.3	7	1153	9	full-splice_match	gene: Zm00001d021719	Zm00001d021719_T00	0.00	56.80	coding	301	66	968
PB.2092.1	10	966	2	full-splice_match	gene: Zm00001d025371	Zm00001d025371_T00	17.04	304.72	coding	224	86	757
PB.10643.1	9	1349	3	full-splice_match	gene: Zm00001d047514	Zm00001d047514_T00	17.31	17.31	coding	321	156	1118
PB.781.6	1	1860	5	novel_in_catalog	gene: Zm00001d031049	novel	6.55	124.30	coding	211	503	1135

PB.6925.2	5	2760	1	incomplete-splice_match	gene: Zm00001d017186	Zm00001d017186_T00	74.42	841.44	coding	286	1667	2524
PB.2092.2	10	831	2	full-splice_match	gene: Zm00001d025371	Zm00001d025371_T00	287.68	304.72	coding	224	52	723
PB.781.2	1	1591	7	full-splice_match	gene: Zm00001d031049	Zm00001d031049_T00	84.89	124.30	coding	377	308	1438
PB.7975.3	6	1743	12	full-splice_match	gene: Zm00001d038343	Zm00001d038343_T00	93.07	134.01	non coding	NA	NA	NA
PB.4095.1	3	3008	9	incomplete-splice_match	gene: Zm00001d041582	Zm00001d041582_T00	18.82	29.14	coding	780	201	2540
PB.4095.2	3	4031	14	novel_in_catalog	gene: Zm00001d041582	novel	10.31	29.14	coding	1203	150	3758
PB.4095.5	3	814	5	novel_in_catalog	gene: Zm00001d041582	novel	0.00	29.14	coding	221	150	812
PB.4095.4	3	2479	9	incomplete-splice_match	gene: Zm00001d041582	Zm00001d041582_T00	0.00	29.14	coding	826	1	2478
PB.861.3	1	1494	1	antisense	novelGene_gene: Zm00001d031443	novel	0.00	16.47	non coding	NA	NA	NA
PB.7784.2	6	529	4	novel_not_in_catalog	gene: Zm00001d037633	novel	114.70	119.13	coding	80	75	314
PB.6439.1	5	1745	4	full-splice_match	gene: Zm00001d014764	Zm00001d014764_T00	21.62	21.62	coding	405	90	1304
PB.2191.3	10	1095	2	full-splice_match	gene: Zm00001d025901	Zm00001d025901_T00	12.58	40.40	coding	223	193	861
PB.3273.2	2	1314	1	genic	gene: Zm00001d006059	novel	5.82	32.67	coding	113	193	531
PB.2443.1	2	828	2	full-splice_match	gene: Zm00001d001843	Zm00001d001843_T00	24.24	24.24	coding	177	71	601
PB.1093.1	1	1890	1	full-splice_match	gene: Zm00001d032518	Zm00001d032518_T00	2.32	2.32	coding	504	107	1618
PB.2191.4	10	1205	1	novel_in_catalog	gene: Zm00001d025901	novel	0.00	40.40	coding	102	712	1017
PB.2191.5	10	985	2	full-splice_match	gene: Zm00001d025901	Zm00001d025901_T00	0.00	40.40	coding	223	193	861

PB.1250.10	1	2209	3	novel_in_catalog	gene: Zm00001d033174	novel	0.00	779.80	coding	161	1593	2075
PB.1447.1	1	1714	3	novel_not_in_catalog	gene: Zm00001d034022	novel	34.58	34.58	coding	445	88	1422
PB.770.3	1	1240	8	full-splice_match	gene: Zm00001d031002	Zm00001d031002_T00	0.00	42.89	coding	324	90	1061
PB.3347.2	2	1129	4	full-splice_match	gene: Zm00001d006360	Zm00001d006360_T00	0.00	42.87	coding	272	72	887
PB.225.1	1	2671	17	full-splice_match	gene: Zm00001d028218	Zm00001d028218_T00	93.00	93.00	coding	800	60	2459
PB.3347.1	2	1289	4	full-splice_match	gene: Zm00001d006360	Zm00001d006360_T00	42.87	42.87	coding	272	106	921
PB.8056.6	6	1084	12	full-splice_match	gene: Zm00001d038623	Zm00001d038623_T00	0.00	154.61	coding	282	69	914
PB.6917.3	5	1443	1	genic	gene: Zm00001d017147	novel	8.69	216.33	coding	140	530	949
PB.8056.4	6	1250	12	full-splice_match	gene: Zm00001d038623	Zm00001d038623_T00	42.79	154.61	coding	282	113	958
PB.8056.5	6	1580	11	novel_in_catalog	gene: Zm00001d038623	novel	1.45	154.61	coding	167	893	1393
PB.3238.11	2	2093	13	novel_in_catalog	gene: Zm00001d005925	novel	1.41	190.45	coding	433	74	1372
PB.7975.2	6	1862	11	novel_in_catalog	gene: Zm00001d038343	novel	18.34	134.01	non coding	NA	NA	NA
PB.8056.1	6	1373	12	full-splice_match	gene: Zm00001d038623	Zm00001d038623_T00	110.36	154.61	coding	282	113	958
PB.10433.3	9	1390	10	full-splice_match	gene: Zm00001d046456	Zm00001d046456_T00	36.94	73.19	coding	380	138	1277
PB.10433.2	9	1274	9	novel_not_in_catalog	gene: Zm00001d046456	novel	1.11	73.19	coding	323	137	1105
PB.10433.1	9	1657	10	full-splice_match	gene: Zm00001d046456	Zm00001d046456_T00	29.27	73.19	coding	380	112	1251
PB.9948.1	8	1990	6	novel_not_in_catalog	gene: Zm00001d012144	novel	14.21	26.51	coding	555	29	1693

PB.10433.4	9	1292	9	full-splice_match	gene: Zm00001d046456	Zm00001d046456_T00	5.88	73.19	coding	267	386	1186
PB.6011.6	5	1881	1	novel_in_catalog	gene: Zm00001d013005	novel	1.65	145.27	coding	371	438	1550
PB.6011.5	5	1761	2	novel_in_catalog	gene: Zm00001d013005	novel	7.37	145.27	coding	371	317	1429
PB.6011.4	5	1644	3	full-splice_match	gene: Zm00001d013005	Zm00001d013005_T00	60.91	145.27	coding	371	307	1419
PB.6011.3	5	1564	4	novel_not_in_catalog	gene: Zm00001d013005	novel	0.29	145.27	coding	349	293	1339
PB.6011.2	5	1772	3	full-splice_match	gene: Zm00001d013005	Zm00001d013005_T00	75.05	145.27	coding	371	307	1419
PB.5873.4	4	804	6	full-splice_match	gene: Zm00001d053862	Zm00001d053862_T00	199.81	560.87	coding	125	101	475
PB.8960.1	7	1819	12	incomplete-splice_match	gene: Zm00001d022144	Zm00001d022144_T00	21.59	21.59	coding	433	4	1302
PB.5903.1	4	1660	4	novel_not_in_catalog	gene: Zm00001d053972	novel	17.98	20.94	coding	338	246	1259
PB.6847.1	5	1062	9	novel_not_in_catalog	gene: Zm00001d016860	novel	6.28	69.05	coding	224	33	704
PB.7043.1	5	1369	4	full-splice_match	gene: Zm00001d017689	Zm00001d017689_T00	23.57	23.57	coding	254	268	1029
PB.5528.2	4	2432	9	full-splice_match	gene: Zm00001d052270	Zm00001d052270_T01	4.18	36.92	coding	167	1704	2204
PB.5528.1	4	1118	11	full-splice_match	gene: Zm00001d052270	Zm00001d052270_T00	32.75	36.92	coding	246	144	881
PB.7071.1	5	1463	2	full-splice_match	gene: Zm00001d017823	Zm00001d017823_T00	10.29	64.23	coding	331	86	1078
PB.6780.2	5	1197	6	incomplete-splice_match	gene: Zm00001d016587	Zm00001d016587_T00	6.27	10.35	coding	328	15	998
PB.9701.4	8	947	3	novel_not_in_catalog	gene: Zm00001d011095	novel	0.00	99.86	coding	219	193	849
PB.9517.1	8	655	1	genic	gene: Zm00001d010275	novel	9.70	18.00	coding	146	7	444

PB.3038.1	2	2751	7	novel_not_in_catalog	gene: Zm00001d004785	novel	47.68	47.68	coding	833	30	2528
PB.9517.2	8	1595	4	novel_not_in_catalog	gene: Zm00001d010275	novel	8.30	18.00	coding	444	100	1431
PB.2199.2	10	1374	4	novel_in_catalog	gene: Zm00001d025922	novel	2.29	45.44	coding	188	598	1161
PB.6936.1	5	919	4	novel_not_in_catalog	gene: Zm00001d017212	novel	14.00	14.00	non coding	NA	NA	NA
PB.2932.4	2	1130	3	full-splice_match	gene: Zm00001d004086	Zm00001d004086_T00	0.00	78.94	coding	331	54	1046
PB.3741.1	3	3222	16	full-splice_match	gene: Zm00001d039475	Zm00001d039475_T01	5.77	5.77	coding	879	267	2903
PB.10280.1	9	1250	5	full-splice_match	gene: Zm00001d045495	Zm00001d045495_T00	21.66	21.66	coding	226	293	970
PB.10805.1	9	2112	9	full-splice_match	gene: Zm00001d048102	Zm00001d048102_T00	143.75	148.22	coding	530	139	1728
PB.5556.1	4	675	2	full-splice_match	gene: Zm00001d052375	Zm00001d052375_T00	117.94	124.60	coding	135	48	452
PB.5556.2	4	857	2	novel_not_in_catalog	gene: Zm00001d052375	novel	6.66	124.60	coding	112	299	634
PB.10050.1	8	1092	1	full-splice_match	gene: Zm00001d012585	Zm00001d012585_T00	60.33	60.33	coding	241	78	800
PB.11018.1	B73V4	627	2	novel_not_in_catalog	gene: Zm00001d000345	novel	135.40	135.40	coding	80	113	352
PB.10301.9	9	1279	5	novel_not_in_catalog	gene: Zm00001d045558	novel	0.76	105.73	coding	371	47	1159
PB.10301.8	9	1407	4	novel_in_catalog	gene: Zm00001d045558	novel	0.00	105.73	coding	219	40	696
PB.10301.7	9	1373	4	novel_in_catalog	gene: Zm00001d045558	novel	14.65	105.73	coding	151	40	492
PB.10301.6	9	1302	5	full-splice_match	gene: Zm00001d045558	Zm00001d045558_T00	0.00	105.73	coding	219	48	704
PB.10301.5	9	1193	6	novel_not_in_catalog	gene: Zm00001d045558	novel	0.00	105.73	coding	333	48	1046

PB.10301.4	9	1384	3	novel_in_catalog	gene: Zm00001d045558	novel	0.00	105.73	coding	105	75	389
PB.10301.3	9	1328	6	novel_not_in_catalog	gene: Zm00001d045558	novel	66.16	105.73	coding	333	41	1039
PB.10301.2	9	1408	5	full-splice_match	gene: Zm00001d045558	Zm00001d045558_T00	0.00	105.73	coding	219	11	667
PB.10301.1	9	1627	4	novel_in_catalog	gene: Zm00001d045558	novel	24.16	105.73	coding	219	132	788
PB.2368.4	10	950	6	novel_not_in_catalog	gene: Zm00001d026572	novel	0.00	25.18	coding	226	123	800
PB.10711.3	9	1584	12	full-splice_match	gene: Zm00001d047755	Zm00001d047755_T00	23.73	55.03	coding	343	110	1138
PB.5607.3	4	3526	3	full-splice_match	gene: Zm00001d052620	Zm00001d052620_T00	14.40	65.70	coding	158	157	630
PB.8625.1	7	1362	8	full-splice_match	gene: Zm00001d020900	Zm00001d020900_T00	1.57	1.57	coding	357	108	1178
PB.8411.1	7	2699	18	novel_in_catalog	gene: Zm00001d019670	novel	56.40	96.02	coding	650	122	2071
PB.7204.1	5	940	1	full-splice_match	gene: Zm00001d018324	Zm00001d018324_T00	57.64	57.64	coding	182	80	625
PB.8411.3	7	4364	26	full-splice_match	gene: Zm00001d019670	Zm00001d019670_T00	39.62	96.02	coding	1032	906	4001
PB.10711.5	9	627	2	full-splice_match	gene: Zm00001d047755	Zm00001d047755_T00	0.00	55.03	non coding	NA	NA	NA
PB.5287.6	4	1536	9	novel_in_catalog	gene: Zm00001d051180	novel	0.00	75.05	coding	295	382	1266
PB.4771.1	3	1446	6	novel_in_catalog	gene: Zm00001d044451	novel	57.36	57.36	coding	310	128	1057
PB.2123.1	10	3335	1	genic	gene: Zm00001d025572	novel	7.74	32.53	coding	185	2480	3034
PB.4985.1	4	508	2	incomplete-splice_match	gene: Zm00001d049286	Zm00001d049286_T00	11.01	11.01	coding	126	6	383
PB.2935.3	2	3032	6	intergenic	novelGene_187	novel	7.61	45.37	coding	453	1111	2469

PB.4527.2	3	742	8	full-splice_match	gene: Zm00001d043497	Zm00001d043497_T00	0.00	302.11	coding	163	90	578
PB.4527.1	3	886	8	full-splice_match	gene: Zm00001d043497	Zm00001d043497_T00	302.11	302.11	coding	163	142	630
PB.8244.1	7	2696	22	full-splice_match	gene: Zm00001d018724	Zm00001d018724_T00	33.37	36.35	coding	755	111	2375
PB.6005.1	5	1207	5	full-splice_match	gene: Zm00001d012980	Zm00001d012980_T00	10.74	10.74	coding	331	71	1063
PB.8244.3	7	3037	22	novel_not_in_catalog	gene: Zm00001d018724	novel	2.98	36.35	coding	501	70	1572
PB.8927.3	7	1729	4	full-splice_match	gene: Zm00001d022016	Zm00001d022016_T00	10.62	40.60	coding	171	212	724
PB.8431.1	7	1934	6	full-splice_match	gene: Zm00001d019850	Zm00001d019850_T00	82.69	89.46	coding	496	102	1589
PB.9604.1	8	1522	5	novel_not_in_catalog	gene: Zm00001d010655	novel	51.78	51.78	coding	392	141	1316
PB.6296.2	5	1927	3	novel_in_catalog	gene: Zm00001d014141	novel	5.07	15.37	coding	117	295	645
PB.2896.1	2	960	4	full-splice_match	gene: Zm00001d003888	Zm00001d003888_T00	4.94	4.94	coding	191	46	618
PB.6296.1	5	1099	4	novel_in_catalog	gene: Zm00001d014141	novel	10.30	15.37	coding	265	78	872
PB.7436.7	6	1658	5	full-splice_match	gene: Zm00001d035720	Zm00001d035720_T00	610.83	655.78	coding	377	206	1336
PB.10970.1	B73V4	1345	10	novel_not_in_catalog	gene: Zm00001d000178	novel	166.69	166.69	coding	311	170	1102
PB.8147.3	6	1199	3	incomplete-splice_match	gene: Zm00001d038971	Zm00001d038971_T00	0.00	96.32	coding	350	4	1053
PB.396.7	1	1912	10	novel_not_in_catalog	gene: Zm00001d028936	novel	0.00	113.38	coding	153	1167	1625
PB.396.1	1	2124	10	full-splice_match	gene: Zm00001d028936	Zm00001d028936_T00	66.35	113.38	coding	335	642	1646
PB.396.2	1	2051	10	full-splice_match	gene: Zm00001d028936	Zm00001d028936_T00	31.32	113.38	coding	335	755	1759

PB.396.3	1	2003	9	novel_in_catalog	gene: Zm00001d028936	novel	10.30	113.38	coding	225	1005	1679
PB.3873.1	3	1540	4	full-splice_match	gene: Zm00001d040117	Zm00001d040117_T00	27.45	27.45	coding	182	577	1122
PB.3873.2	3	1440	4	full-splice_match	gene: Zm00001d040117	Zm00001d040117_T00	0.00	27.45	coding	182	564	1109
PB.5362.8	4	1410	7	full-splice_match	gene: Zm00001d051600	Zm00001d051600_T03	5.54	151.77	coding	418	155	1408
PB.396.8	1	1723	11	novel_not_in_catalog	gene: Zm00001d028936	novel	5.41	113.38	coding	166	581	1078
PB.1155.1	1	852	4	full-splice_match	gene: Zm00001d032736	Zm00001d032736_T00	106.89	106.89	coding	166	103	600
PB.9560.2	8	1313	1	incomplete-splice_match	gene: Zm00001d010470	Zm00001d010470_T00	8.59	33.92	coding	52	832	987
PB.8904.1	7	1509	2	full-splice_match	gene: Zm00001d021929	Zm00001d021929_T00	27.78	27.78	coding	363	218	1306
PB.7436.3	6	2275	4	full-splice_match	gene: Zm00001d035720	Zm00001d035720_T00	44.95	655.78	coding	377	776	1906
PB.2156.2	10	2584	7	full-splice_match	gene: Zm00001d025739	Zm00001d025739_T03	7.61	63.34	coding	402	89	1294
PB.1663.1	1	1016	1	full-splice_match	gene: Zm00001d034874	Zm00001d034874_T00	69.08	69.08	coding	215	64	708
PB.2170.4	10	2437	9	full-splice_match	gene: Zm00001d025801	Zm00001d025801_T00	135.87	366.57	coding	652	195	2150
PB.9935.1	8	2572	19	full-splice_match	gene: Zm00001d012086	Zm00001d012086_T00	31.79	55.58	coding	691	139	2211
PB.2260.5	10	467	1	novel_in_catalog	gene: Zm00001d026223	novel	0.00	210.95	coding	76	110	337
PB.8722.1	7	2365	6	full-splice_match	gene: Zm00001d021304	Zm00001d021304_T00	29.58	169.93	coding	526	220	1797
PB.9935.2	8	2655	18	novel_in_catalog	gene: Zm00001d012086	novel	4.74	55.58	coding	489	148	1614
PB.9935.5	8	2492	18	novel_in_catalog	gene: Zm00001d012086	novel	0.00	55.58	coding	489	115	1581

PB.9935.4	8	1755	12	novel_in_catalog	gene: Zm00001d012086	novel	0.00	55.58	coding	468	114	1517
PB.8439.2	7	1809	11	full-splice_match	gene: Zm00001d019922	Zm00001d019922_T00	19.78	27.81	coding	452	221	1576
PB.8439.1	7	1873	10	novel_in_catalog	gene: Zm00001d019922	novel	8.03	27.81	coding	382	258	1403
PB.5342.5	4	2183	1	full-splice_match	gene: Zm00001d051505	Zm00001d051505_T00	35.52	63.49	coding	291	346	1218
PB.641.1	1	2161	7	novel_not_in_catalog	gene: Zm00001d030154	novel	12.00	12.00	coding	270	64	873
PB.3767.1	3	1785	1	full-splice_match	gene: Zm00001d039548	Zm00001d039548_T00	2.80	2.80	coding	454	324	1685
PB.2404.9	10	1097	6	novel_in_catalog	gene: Zm00001d026676	novel	0.00	10.81	coding	127	1	381
PB.9915.1	8	1352	9	full-splice_match	gene: Zm00001d012003	Zm00001d012003_T00	49.37	49.37	coding	281	220	1062
PB.6629.3	5	2108	1	novel_in_catalog	gene: Zm00001d015638	novel	2.08	24.20	coding	194	1192	1773
PB.5810.4	4	1468	3	antisense	novelGene_gene: Zm00001d053623	novel	8.54	73.94	non coding	NA	NA	NA
PB.8181.1	6	1280	1	full-splice_match	gene: Zm00001d039108	Zm00001d039108_T00	3.40	3.40	coding	305	212	1126
PB.3754.3	3	4548	22	full-splice_match	gene: Zm00001d039522	Zm00001d039522_T00	0.95	30.56	coding	1429	161	4447
PB.6411.2	5	863	1	full-splice_match	gene: Zm00001d014667	Zm00001d014667_T00	0.00	55.89	coding	205	126	740
PB.6411.1	5	940	1	full-splice_match	gene: Zm00001d014667	Zm00001d014667_T00	55.89	55.89	coding	205	126	740
PB.9929.1	8	2254	14	full-splice_match	gene: Zm00001d012060	Zm00001d012060_T00	22.99	22.99	coding	492	221	1696
PB.4270.6	3	2104	1	novel_in_catalog	gene: Zm00001d042461	novel	8.65	70.70	coding	335	895	1899
PB.8937.6	7	759	5	full-splice_match	gene: Zm00001d022042	Zm00001d022042_T00	37.66	1497.07	coding	141	87	509

PB.2014.3	10	865	2	full-splice_match	gene: Zm00001d024963	Zm00001d024963_T00	688.24	717.24	coding	232	10	705
PB.8937.8	7	612	6	full-splice_match	gene: Zm00001d022042	Zm00001d022042_T01	0.00	1497.07	coding	160	90	569
PB.4301.1	3	1794	9	full-splice_match	gene: Zm00001d042621	Zm00001d042621_T00	61.69	61.69	coding	359	365	1441
PB.8276.1	7	1857	2	novel_not_in_catalog	gene: Zm00001d018869	novel	8.84	8.84	coding	594	74	1855
PB.9656.1	8	2345	3	full-splice_match	gene: Zm00001d010863	Zm00001d010863_T00	28.77	28.77	coding	590	186	1955
PB.6399.1	5	1232	4	novel_not_in_catalog	gene: Zm00001d014611	novel	90.13	122.40	coding	262	158	943
PB.6399.2	5	1234	3	novel_not_in_catalog	gene: Zm00001d014611	novel	3.20	122.40	coding	348	121	1164
PB.6399.3	5	1061	4	novel_not_in_catalog	gene: Zm00001d014611	novel	29.08	122.40	coding	262	94	879
PB.3319.3	2	1375	1	genic	gene: Zm00001d006236	novel	3.84	62.99	coding	88	154	417
PB.1671.1	1	1681	1	genic	gene: Zm00001d034898	novel	6.09	6.09	coding	371	374	1486
PB.3396.18	2	2178	3	novel_in_catalog	gene: Zm00001d006578	novel	6.74	210.21	coding	158	294	767
PB.6224.1	5	1638	1	full-splice_match	gene: Zm00001d013814	Zm00001d013814_T00	6.01	6.01	coding	509	85	1611
PB.5607.11	4	1527	3	incomplete-splice_match	gene: Zm00001d052620	Zm00001d052620_T00	0.00	65.70	non coding	NA	NA	NA
PB.10996.3	B73V4 ctg3	2774	1	full-splice_match	gene: Zm00001d000434	Zm00001d000434_T00	1.59	10.91	coding	573	26	1744
PB.10996.2	B73V4 ctg3	2096	1	full-splice_match	gene: Zm00001d000434	Zm00001d000434_T00	9.32	10.91	coding	573	25	1743
PB.3396.12	2	2195	6	novel_in_catalog	gene: Zm00001d006578	novel	11.31	210.21	coding	365	306	1400
PB.3396.13	2	2652	2	novel_in_catalog	gene: Zm00001d006578	novel	0.00	210.21	coding	158	298	771

PB.5607.10	4	857	4	novel_in_catalog	gene: Zm00001d052620	novel	0.00	65.70	non coding	NA	NA	NA
PB.2336.1	10	1520	10	novel_in_catalog	gene: Zm00001d026483	novel	307.62	307.62	coding	247	463	1203
PB.7386.1	6	2088	1	novel_in_catalog	gene: Zm00001d035468	novel	3.15	3.15	non coding	NA	NA	NA
PB.7237.1	5	1248	5	incomplete-splice_match	gene: Zm00001d018420	Zm00001d018420_T00	43.54	47.60	coding	215	264	908
PB.5154.2	4	1154	1	full-splice_match	gene: Zm00001d050195	Zm00001d050195_T00	114.50	114.50	coding	306	94	1011
PB.1233.1	1	1937	7	novel_not_in_catalog	gene: Zm00001d033068	novel	4.46	4.46	coding	465	404	1798
PB.3388.1	2	852	4	incomplete-splice_match	gene: Zm00001d006536	Zm00001d006536_T00	9.47	9.47	coding	163	173	661
PB.7005.1	5	2386	11	incomplete-splice_match	gene: Zm00001d017525	Zm00001d017525_T00	16.00	16.00	coding	595	78	1862
PB.3398.1	2	2675	6	full-splice_match	gene: Zm00001d006582	Zm00001d006582_T00	13.27	13.27	coding	624	314	2185
PB.6954.2	5	1673	9	full-splice_match	gene: Zm00001d017298	Zm00001d017298_T00	20.08	125.29	coding	352	556	1611
PB.1493.1	1	1046	2	full-splice_match	gene: Zm00001d034279	Zm00001d034279_T00	7.19	132.58	coding	180	65	604
PB.5810.2	4	2395	2	antisense	novelGene_gene: Zm00001d053623	novel	18.42	73.94	non coding	NA	NA	NA
PB.6767.3	5	3595	28	novel_in_catalog	gene: Zm00001d016506	novel	7.19	33.13	coding	692	174	2249
PB.7118.2	5	2030	13	full-splice_match	gene: Zm00001d017988	Zm00001d017988_T00	12.75	13.38	coding	588	90	1853
PB.9921.1	8	991	1	antisense	novelGene_gene: Zm00001d012019	novel	1.75	1.75	coding	189	59	625
PB.6860.7	5	1526	2	incomplete-splice_match	gene: Zm00001d016911	Zm00001d016911_T00	7.01	46.96	coding	388	16	1179
PB.7148.3	5	1140	3	novel_not_in_catalog	gene: Zm00001d018097	novel	0.00	49.75	coding	322	106	1071

PB.5898.1	4	1601	6	full-splice_match	gene: Zm00001d053957	Zm00001d053957_T00	29.99	29.99	coding	369	83	1189
PB.6385.1	5	963	2	full-splice_match	gene: Zm00001d014554	Zm00001d014554_T00	25.14	25.14	coding	213	192	830
PB.7785.1	6	1129	2	incomplete-splice_match	gene: Zm00001d037636	Zm00001d037636_T00	684.04	684.04	coding	189	244	810
PB.9299.1	8	1835	7	full-splice_match	gene: Zm00001d008996	Zm00001d008996_T00	45.26	45.26	coding	521	77	1639
PB.940.1	1	742	5	incomplete-splice_match	gene: Zm00001d031851	Zm00001d031851_T00	8.87	18.33	coding	34	215	316
PB.8703.2	7	1438	6	full-splice_match	gene: Zm00001d021225	Zm00001d021225_T00	0.00	59.68	coding	175	672	1196
PB.2370.3	10	1779	4	novel_in_catalog	gene: Zm00001d026577	novel	0.52	70.13	coding	502	106	1611
PB.7776.2	6	1650	7	full-splice_match	gene: Zm00001d037609	Zm00001d037609_T00	22.99	34.84	coding	32	775	870
PB.2370.1	10	1741	5	full-splice_match	gene: Zm00001d026577	Zm00001d026577_T00	67.57	70.13	coding	470	106	1515
PB.8399.2	7	2300	13	full-splice_match	gene: Zm00001d019591	Zm00001d019591_T00	5.49	19.29	coding	717	96	2246
PB.8399.3	7	2493	4	novel_in_catalog	gene: Zm00001d019591	novel	7.83	19.29	coding	156	117	584
PB.8399.1	7	2242	13	novel_in_catalog	gene: Zm00001d019591	novel	5.97	19.29	coding	469	747	2153
PB.2563.1	2	787	3	full-splice_match	gene: Zm00001d002344	Zm00001d002344_T00	89.40	89.40	coding	107	262	582
PB.8772.4	7	549	2	intergenic	novelGene_308	novel	7.45	7.45	coding	30	216	305
PB.5583.4	4	1160	5	full-splice_match	gene: Zm00001d052450	Zm00001d052450_T00	11.40	159.81	coding	144	488	919
PB.8772.1	7	3545	2	genic	gene: Zm00001d021494	novel	47.93	47.93	coding	1024	269	3340
PB.2244.2	10	711	1	genic	gene: Zm00001d026163	novel	0.00	2729.80	coding	131	94	486

PB.5739.2	4	1308	10	full-splice_match	gene: Zm00001d053275	Zm00001d053275_T00	0.00	25.95	coding	381	2	1144
PB.7776.1	6	1714	6	novel_in_catalog	gene: Zm00001d037609	novel	11.85	34.84	non coding	NA	NA	NA
PB.4301.2	3	1432	9	full-splice_match	gene: Zm00001d042621	Zm00001d042621_T00	0.00	61.69	coding	359	113	1189
PB.2936.1	2	2736	5	full-splice_match	gene: Zm00001d004099	Zm00001d004099_T00	7.59	12.19	coding	527	58	1638
PB.5869.2	4	1693	3	full-splice_match	gene: Zm00001d053848	Zm00001d053848_T00	24.36	49.55	coding	497	89	1579
PB.6873.3	5	1456	6	full-splice_match	gene: Zm00001d016993	Zm00001d016993_T00	19.20	68.80	coding	311	194	1126
PB.3088.1	2	450	1	full-splice_match	gene: Zm00001d005109	Zm00001d005109_T00	34.05	34.05	non coding	NA	NA	NA
PB.2350.1	10	674	4	full-splice_match	gene: Zm00001d026521	Zm00001d026521_T00	65.30	65.30	coding	94	59	340
PB.9735.1	8	1881	2	incomplete-splice_match	gene: Zm00001d011228	Zm00001d011228_T00	18.69	18.69	coding	290	14	883
PB.5593.5	4	917	2	incomplete-splice_match	gene: Zm00001d052489	Zm00001d052489_T00	0.00	35.08	coding	265	121	915
PB.6538.4	5	1783	11	novel_in_catalog	gene: Zm00001d015120	novel	0.00	28.53	coding	375	93	1217
PB.2138.1	10	884	6	full-splice_match	gene: Zm00001d025663	Zm00001d025663_T00	22.15	98.07	coding	219	51	707
PB.2803.1	2	1168	3	full-splice_match	gene: Zm00001d003418	Zm00001d003418_T00	15.87	43.54	coding	193	233	811
PB.6131.1	5	704	2	full-splice_match	gene: Zm00001d013392	Zm00001d013392_T00	216.67	216.67	coding	137	96	506
PB.5418.4	4	2239	13	incomplete-splice_match	gene: Zm00001d051873	Zm00001d051873_T00	30.95	52.66	coding	507	364	1884
PB.8385.1	7	552	3	full-splice_match	gene: Zm00001d019550	Zm00001d019550_T00	133.90	133.90	coding	78	106	339
PB.2981.2	2	717	3	full-splice_match	gene: Zm00001d004465	Zm00001d004465_T00	38.31	49.09	coding	198	41	634

PB.3267.1	2	2353	5	full-splice_match	gene: Zm00001d006036	Zm00001d006036_T00	57.40	57.40	coding	681	121	2163
PB.2542.1	2	1304	4	full-splice_match	gene: Zm00001d002277	Zm00001d002277_T00	8.58	8.58	coding	268	176	979
PB.4648.1	3	2170	13	full-splice_match	gene: Zm00001d043907	Zm00001d043907_T00	20.24	20.24	coding	597	56	1846
PB.10361.3	9	1268	1	novel_in_catalog	gene: Zm00001d045946	novel	7.68	29.47	coding	249	84	830
PB.4648.2	3	2005	13	full-splice_match	gene: Zm00001d043907	Zm00001d043907_T00	0.00	20.24	coding	558	125	1798
PB.4871.3	4	1332	6	novel_in_catalog	gene: Zm00001d048785	novel	0.00	66.19	coding	115	136	480
PB.4871.2	4	1582	7	full-splice_match	gene: Zm00001d048785	Zm00001d048785_T00	8.61	66.19	coding	275	133	957
PB.4871.1	4	1809	6	novel_in_catalog	gene: Zm00001d048785	novel	4.05	66.19	coding	115	136	480
PB.3620.1	2	2004	9	full-splice_match	gene: Zm00001d007770	Zm00001d007770_T00	50.07	50.07	coding	523	130	1698
PB.364.3	1	1645	8	novel_in_catalog	gene: Zm00001d028787	novel	0.91	72.57	coding	130	618	1007
PB.364.2	1	1698	8	novel_not_in_catalog	gene: Zm00001d028787	novel	2.03	72.57	coding	110	1169	1498
PB.364.1	1	1583	10	full-splice_match	gene: Zm00001d028787	Zm00001d028787_T00	69.62	72.57	coding	368	257	1360
PB.4871.4	4	1216	7	full-splice_match	gene: Zm00001d048785	Zm00001d048785_T00	19.43	66.19	coding	275	133	957
PB.6516.1	5	1130	5	full-splice_match	gene: Zm00001d014995	Zm00001d014995_T00	54.81	54.81	coding	235	128	832
PB.124.1	1	674	1	full-splice_match	gene: Zm00001d027742	Zm00001d027742_T00	27.17	27.17	coding	162	33	518
PB.3911.2	3	1460	1	full-splice_match	gene: Zm00001d040293	Zm00001d040293_T00	11.38	48.83	coding	276	631	1458
PB.9462.1	8	1764	4	novel_in_catalog	gene: Zm00001d009979	novel	23.16	23.16	coding	242	11	736

PB.9462.2	8	1649	4	novel_in_catalog	gene: Zm00001d009979	novel	0.00	23.16	coding	242	3	728
PB.5934.1	4	531	4	full-splice_match	gene: Zm00001d054065	Zm00001d054065_T00	862.88	862.88	non coding	NA	NA	NA
PB.1854.1	10	1322	5	intergenic	novelGene_128	novel	22.10	29.39	non coding	NA	NA	NA
PB.2125.2	10	2828	9	novel_in_catalog	gene: Zm00001d025580	novel	2.25	13.70	coding	175	1673	2197
PB.7445.1	6	1132	7	full-splice_match	gene: Zm00001d035762	Zm00001d035762_T00	219.05	219.05	coding	228	168	851
PB.9849.1	8	961	2	full-splice_match	gene: Zm00001d011747	Zm00001d011747_T00	21.55	21.55	coding	174	305	826
PB.9230.3	8	2050	7	full-splice_match	gene: Zm00001d008649	Zm00001d008649_T00	36.92	36.92	coding	514	276	1817
PB.7479.3	6	1949	15	full-splice_match	gene: Zm00001d036004	Zm00001d036004_T01	12.12	21.91	coding	571	36	1748
PB.7479.1	6	2946	15	novel_in_catalog	gene: Zm00001d036004	novel	5.06	21.91	coding	268	1695	2498
PB.3534.1	2	1594	4	full-splice_match	gene: Zm00001d007172	Zm00001d007172_T00	22.22	22.22	coding	362	114	1199
PB.2119.7	10	2401	8	novel_in_catalog	gene: Zm00001d025548	novel	8.49	137.33	coding	261	121	903
PB.7479.5	6	1963	15	full-splice_match	gene: Zm00001d036004	Zm00001d036004_T02	0.00	21.91	coding	555	40	1704
PB.1910.2	10	6095	6	full-splice_match	gene: Zm00001d024463	Zm00001d024463_T00	5.38	27.93	coding	1927	313	6093
PB.8512.2	7	2911	9	full-splice_match	gene: Zm00001d020325	Zm00001d020325_T00	1.12	44.54	coding	852	104	2659
PB.8512.1	7	2999	9	full-splice_match	gene: Zm00001d020325	Zm00001d020325_T00	43.43	44.54	coding	852	103	2658
PB.1910.1	10	867	1	incomplete-splice_match	gene: Zm00001d024463	Zm00001d024463_T00	22.55	27.93	coding	136	360	767
PB.6970.1	5	1040	2	full-splice_match	gene: Zm00001d017379	Zm00001d017379_T00	22.76	74.23	coding	173	177	695

PB.4759.12	3	1438	1	novel_in_catalog	gene: Zm00001d044395	novel	3.60	76.77	coding	155	347	811
PB.2137.2	10	596	4	incomplete-splice_match	gene: Zm00001d025663	Zm00001d025663_T00	27.46	98.07	coding	116	75	422
PB.3581.1	2	958	3	novel_not_in_catalog	gene: Zm00001d007498	novel	9.47	22.63	coding	156	106	573
PB.10100.7	8	1187	3	antisense	novelGene_gene: Zm00001d012761	novel	1.70	19.16	non coding	NA	NA	NA
PB.4173.1	3	2063	7	novel_not_in_catalog	gene: Zm00001d041991	novel	36.36	60.72	coding	590	73	1842
PB.4173.2	3	1905	7	novel_not_in_catalog	gene: Zm00001d041991	novel	24.36	60.72	coding	590	21	1790
PB.7736.4	6	1629	6	incomplete-splice_match	gene: Zm00001d037408	Zm00001d037408_T01	5.39	41.48	coding	299	288	1184
PB.7736.2	6	2330	6	incomplete-splice_match	gene: Zm00001d037408	Zm00001d037408_T02	19.83	41.48	coding	602	12	1817
PB.7736.1	6	789	6	incomplete-splice_match	gene: Zm00001d037408	Zm00001d037408_T00	16.26	41.48	non coding	NA	NA	NA
PB.962.1	1	932	6	full-splice_match	gene: Zm00001d031961	Zm00001d031961_T00	68.85	68.85	coding	144	112	543
PB.7457.1	6	1378	11	full-splice_match	gene: Zm00001d035874	Zm00001d035874_T00	48.82	62.74	coding	334	40	1041
PB.2244.3	10	598	1	genic	gene: Zm00001d026163	novel	471.70	2729.80	coding	131	94	486
PB.6159.2	5	2980	2	genic	gene: Zm00001d013497	novel	5.71	30.90	coding	747	299	2539
PB.1897.1	10	1514	10	full-splice_match	gene: Zm00001d024393	Zm00001d024393_T00	9.21	57.90	coding	374	76	1197
PB.1897.2	10	1437	10	full-splice_match	gene: Zm00001d024393	Zm00001d024393_T00	48.69	57.90	coding	383	100	1248
PB.6159.1	5	3126	2	genic	gene: Zm00001d013497	novel	25.20	30.90	coding	747	286	2526
PB.6954.5	5	1575	1	genic	gene: Zm00001d017298	novel	0.00	125.29	non coding	NA	NA	NA

PB.10921.1	9	1086	3	incomplete-splice_match	gene: Zm00001d048529	Zm00001d048529_T00	25.83	134.48	non coding	NA	NA	NA
PB.9045.1	7	4344	12	full-splice_match	gene: Zm00001d022429	Zm00001d022429_T03	24.18	52.55	coding	1304	102	4013
PB.10791.1	9	1190	4	incomplete-splice_match	gene: Zm00001d048047	Zm00001d048047_T01	16.39	52.44	coding	212	42	677
PB.10791.2	9	1752	6	incomplete-splice_match	gene: Zm00001d048047	Zm00001d048047_T01	36.05	52.44	coding	353	351	1409
PB.9045.2	7	993	1	incomplete-splice_match	gene: Zm00001d022429	Zm00001d022429_T03	28.37	52.55	coding	236	158	865
PB.5599.19	4	1611	14	full-splice_match	gene: Zm00001d052532	Zm00001d052532_T00	5.39	583.99	coding	452	95	1450
PB.1202.1	1	1209	6	novel_in_catalog	gene: Zm00001d032931	novel	4.44	15.05	coding	163	478	966
PB.1391.6	1	1752	11	novel_in_catalog	gene: Zm00001d033795	novel	3.98	39.36	coding	273	715	1533
PB.5502.1	4	2582	10	full-splice_match	gene: Zm00001d052197	Zm00001d052197_T01	27.55	27.55	coding	727	186	2366
PB.10578.8	9	3084	14	novel_in_catalog	gene: Zm00001d047253	novel	5.95	1036.48	coding	499	1199	2695
PB.8828.2	7	2281	7	novel_not_in_catalog	gene: Zm00001d021699	novel	52.24	63.08	coding	662	102	2087
PB.2360.4	10	1998	4	novel_in_catalog	gene: Zm00001d026549	novel	1.08	17.53	coding	127	48	428
PB.9406.2	8	1139	4	full-splice_match	gene: Zm00001d009686	Zm00001d009686_T00	30.10	30.10	coding	245	104	838
PB.2726.2	2	759	3	intergenic	novelGene_114	novel	40.37	53.62	coding	121	134	496
PB.536.1	1	1248	1	genic	gene: Zm00001d029594	novel	7.06	271.73	coding	117	32	382
PB.3589.2	2	1999	7	full-splice_match	gene: Zm00001d007571	Zm00001d007571_T00	0.00	9.09	coding	539	156	1772
PB.3424.3	2	1668	1	genic	gene: Zm00001d006678	novel	1.05	45.68	coding	268	172	975

PB.3589.1	2	2294	7	full-splice_match	gene: Zm00001d007571	Zm00001d007571_T00	6.85	9.09	coding	539	156	1772
PB.3890.1	3	2632	1	full-splice_match	gene: Zm00001d040201	Zm00001d040201_T00	14.15	14.15	coding	661	433	2415
PB.3589.4	2	2271	6	full-splice_match	gene: Zm00001d007571	Zm00001d007571_T00	2.24	9.09	coding	276	166	993
PB.6858.4	5	925	4	intergenic	novelGene_369	novel	3.12	12.21	non coding	NA	NA	NA
PB.2066.1	10	1136	3	full-splice_match	gene: Zm00001d025218	Zm00001d025218_T00	13.44	13.44	coding	324	114	1085
PB.2241.1	10	2659	1	full-splice_match	gene: Zm00001d026158	Zm00001d026158_T00	18.32	18.32	coding	718	144	2297
PB.9059.1	7	608	1	full-splice_match	gene: Zm00001d022472	Zm00001d022472_T00	4.30	4.30	coding	128	36	419
PB.9587.2	8	2148	2	genic	gene: Zm00001d010607	novel	0.00	56.26	coding	565	303	1997
PB.10351.5	9	577	1	intergenic	novelGene_139	novel	12.32	12.32	non coding	NA	NA	NA
PB.1028.2	1	695	2	intergenic	novelGene_417	novel	6.46	15.61	non coding	NA	NA	NA
PB.9587.1	8	2270	2	genic	gene: Zm00001d010607	novel	56.26	56.26	coding	565	313	2007
PB.5599.12	4	1835	13	novel_not_in_catalog	gene: Zm00001d052532	novel	20.04	583.99	coding	152	89	544
PB.3602.2	2	1440	11	full-splice_match	gene: Zm00001d007645	Zm00001d007645_T00	0.00	30.46	coding	172	193	708
PB.2244.1	10	815	1	genic	gene: Zm00001d026163	novel	1909.77	2729.80	coding	131	88	480
PB.720.1	1	1259	8	full-splice_match	gene: Zm00001d030739	Zm00001d030739_T00	126.75	133.72	non coding	NA	NA	NA
PB.3602.1	2	1430	11	full-splice_match	gene: Zm00001d007645	Zm00001d007645_T00	9.43	30.46	coding	262	170	955
PB.4157.2	3	1257	1	full-splice_match	gene: Zm00001d041927	Zm00001d041927_T00	45.73	173.64	coding	212	246	881

PB.4157.3	3	1134	1	full-splice_match	gene: Zm00001d041927	Zm00001d041927_T00	44.68	173.64	coding	212	246	881
PB.7640.7	6	1986	12	novel_in_catalog	gene: Zm00001d036971	novel	0.14	145.45	coding	230	1295	1984
PB.4157.1	3	1317	1	full-splice_match	gene: Zm00001d041927	Zm00001d041927_T00	83.23	173.64	coding	212	180	815
PB.1666.1	1	3448	1	full-splice_match	gene: Zm00001d034879	Zm00001d034879_T00	8.86	8.86	coding	959	245	3121
PB.10671.1	9	883	1	full-splice_match	gene: Zm00001d047618	Zm00001d047618_T00	226.90	226.90	coding	173	138	656
PB.2360.3	10	1290	4	full-splice_match	gene: Zm00001d026549	Zm00001d026549_T00	0.88	17.53	coding	183	14	562
PB.2055.1	10	2746	15	full-splice_match	gene: Zm00001d025153	Zm00001d025153_T01	11.41	11.41	coding	642	702	2627
PB.1076.1	1	1875	1	full-splice_match	gene: Zm00001d032458	Zm00001d032458_T00	2.19	10.05	coding	514	75	1616
PB.8905.5	7	3452	2	novel_in_catalog	gene: Zm00001d021930	novel	0.19	155.11	coding	104	2851	3162
PB.6670.2	5	1191	1	incomplete-splice_match	gene: Zm00001d015927	Zm00001d015927_T00	0.00	16.92	coding	264	283	1074
PB.6670.1	5	2089	2	full-splice_match	gene: Zm00001d015927	Zm00001d015927_T00	16.92	16.92	coding	583	101	1849
PB.4936.1	4	2038	4	novel_in_catalog	gene: Zm00001d049094	novel	15.29	25.34	coding	411	102	1334
PB.4936.2	4	2388	4	novel_in_catalog	gene: Zm00001d049094	novel	6.80	25.34	coding	386	996	2153
PB.4936.5	4	1307	2	novel_in_catalog	gene: Zm00001d049094	novel	3.25	25.34	coding	336	79	1086
PB.4936.4	4	1855	4	novel_in_catalog	gene: Zm00001d049094	novel	0.00	25.34	coding	411	102	1334
PB.4489.1	3	2029	4	novel_in_catalog	gene: Zm00001d043383	novel	1.49	1.49	coding	114	1577	1918
PB.1932.2	10	1626	9	full-splice_match	gene: Zm00001d024540	Zm00001d024540_T00	28.35	28.35	coding	394	136	1317

PB.10365.1	9	1928	5	full-splice_match	gene: Zm00001d045965	Zm00001d045965_T00	36.30	432.40	coding	289	753	1619
PB.4242.1	3	3056	22	full-splice_match	gene: Zm00001d042335	Zm00001d042335_T00	28.72	28.72	coding	869	86	2692
PB.4654.1	3	2608	10	full-splice_match	gene: Zm00001d043954	Zm00001d043954_T00	82.24	85.09	coding	588	359	2122
PB.7388.1	6	1480	13	novel_in_catalog	gene: Zm00001d035475	novel	12.70	12.70	coding	407	116	1336
PB.7487.1	6	897	2	full-splice_match	gene: Zm00001d036036	Zm00001d036036_T00	16.58	1057.89	coding	136	86	493
PB.9051.3	7	1295	3	novel_in_catalog	gene: Zm00001d022456	novel	0.00	67.58	coding	321	106	1068
PB.9051.1	7	1383	3	novel_in_catalog	gene: Zm00001d022456	novel	66.18	67.58	coding	321	101	1063
PB.5249.1	4	1399	4	full-splice_match	gene: Zm00001d050942	Zm00001d050942_T00	20.82	32.51	coding	295	70	954
PB.9051.4	7	1181	3	novel_in_catalog	gene: Zm00001d022456	novel	0.00	67.58	coding	321	98	1060
PB.7832.1	6	4469	11	full-splice_match	gene: Zm00001d037864	Zm00001d037864_T01	12.01	21.42	coding	695	78	2162
PB.1807.1	10	538	1	full-splice_match	gene: Zm00001d023804	Zm00001d023804_T00	49.58	49.58	non coding	NA	NA	NA
PB.6010.3	5	1412	2	full-splice_match	gene: Zm00001d013003	Zm00001d013003_T00	52.73	203.08	coding	302	197	1102
PB.5386.1	4	1714	7	full-splice_match	gene: Zm00001d051678	Zm00001d051678_T01	37.35	64.88	coding	418	123	1376
PB.4155.3	3	2853	5	fusion	gene: Zm00001d000620	novel	17.76	29.71	coding	849	75	2621
PB.5589.1	4	1643	1	full-splice_match	gene: Zm00001d052479	Zm00001d052479_T00	7.36	7.36	coding	499	61	1557
PB.6369.10	5	2747	10	novel_not_in_catalog	gene: Zm00001d014472	novel	1.37	73.79	coding	214	1810	2451
PB.1074.5	1	445	2	incomplete-splice_match	gene: Zm00001d032449	Zm00001d032449_T00	11.36	57.57	non coding	NA	NA	NA

PB.6010.1	5	1411	2	full-splice_match	gene: Zm00001d013003	Zm00001d013003_T00	17.48	203.08	coding	302	67	972
PB.6954.9	5	1584	7	novel_in_catalog	gene: Zm00001d017298	novel	8.18	125.29	coding	189	93	659
PB.8278.1	7	2227	4	incomplete-splice_match	gene: Zm00001d018872	Zm00001d018872_T03	30.54	48.47	coding	578	72	1805
PB.8278.3	7	4252	12	full-splice_match	gene: Zm00001d018872	Zm00001d018872_T01	17.92	48.47	coding	1078	750	3983
PB.3269.5	2	1862	6	novel_not_in_catalog	gene: Zm00001d006046	novel	4.25	72.31	coding	301	454	1356
PB.10483.1	9	587	3	novel_not_in_catalog	gene: Zm00001d046729	novel	18.15	18.15	non coding	NA	NA	NA
PB.3269.3	2	2650	4	full-splice_match	gene: Zm00001d006046	Zm00001d006046_T00	8.31	72.31	coding	301	1228	2130
PB.3269.2	2	1653	6	full-splice_match	gene: Zm00001d006046	Zm00001d006046_T00	0.00	72.31	coding	301	216	1118
PB.3269.1	2	1611	5	full-splice_match	gene: Zm00001d006046	Zm00001d006046_T00	2.57	72.31	coding	301	98	1000
PB.6796.2	5	1056	6	full-splice_match	gene: Zm00001d016665	Zm00001d016665_T00	10.83	56.96	coding	271	40	852
PB.10998.1	B73V4	1282	12	incomplete-splice_match	gene: Zm00001d000080	Zm00001d000080_T00	36.96	36.96	coding	95	744	1028
PB.9345.1	8	1840	10	novel_not_in_catalog	gene: Zm00001d009364	novel	18.05	18.05	coding	522	17	1582
PB.290.1	1	967	1	full-splice_match	gene: Zm00001d028469	Zm00001d028469_T00	33.08	33.08	coding	211	94	726
PB.2074.1	10	3411	10	full-splice_match	gene: Zm00001d025273	Zm00001d025273_T01	112.76	112.76	coding	921	198	2960
PB.6546.3	5	1550	1	incomplete-splice_match	gene: Zm00001d015138	Zm00001d015138_T00	149.69	311.43	coding	420	149	1408
PB.6954.8	5	2178	7	novel_in_catalog	gene: Zm00001d017298	novel	5.75	125.29	coding	238	430	1143
PB.6546.1	5	5335	6	full-splice_match	gene: Zm00001d015138	Zm00001d015138_T00	91.44	311.43	coding	1584	284	5035

PB.6546.6	5	2933	6	full-splice_match	gene: Zm00001d015138	Zm00001d015138_T00	0.00	311.43	coding	761	650	2932
PB.6546.7	5	1738	5	novel_in_catalog	gene: Zm00001d015138	novel	1.52	311.43	coding	537	127	1737
PB.6546.4	5	2698	5	incomplete-splice_match	gene: Zm00001d015138	Zm00001d015138_T00	31.00	311.43	coding	750	448	2697
PB.6546.5	5	2179	5	incomplete-splice_match	gene: Zm00001d015138	Zm00001d015138_T00	0.00	311.43	coding	711	45	2177
PB.6844.1	5	716	1	genic	gene: Zm00001d016850	novel	1.13	71.10	non coding	NA	NA	NA
PB.8905.2	7	2409	5	full-splice_match	gene: Zm00001d021930	Zm00001d021930_T00	26.82	155.11	coding	219	126	782
PB.6546.8	5	1624	5	incomplete-splice_match	gene: Zm00001d015138	Zm00001d015138_T00	0.00	311.43	coding	541	1	1623
PB.6546.9	5	2598	5	incomplete-splice_match	gene: Zm00001d015138	Zm00001d015138_T00	0.00	311.43	coding	832	102	2597
PB.8930.1	7	738	4	incomplete-splice_match	gene: Zm00001d022021	Zm00001d022021_T00	13.27	25.07	coding	136	36	443
PB.8717.1	7	2466	8	full-splice_match	gene: Zm00001d021291	Zm00001d021291_T00	24.47	24.47	coding	629	149	2035
PB.6869.4	5	2142	6	full-splice_match	gene: Zm00001d016972	Zm00001d016972_T00	20.92	69.89	coding	525	116	1690
PB.6869.5	5	1176	5	incomplete-splice_match	gene: Zm00001d016972	Zm00001d016972_T00	19.62	69.89	coding	287	2	862
PB.6869.2	5	1975	6	full-splice_match	gene: Zm00001d016972	Zm00001d016972_T00	10.69	69.89	coding	370	132	1241
PB.1389.3	1	996	6	novel_not_in_catalog	gene: Zm00001d033792	novel	3.78	14.95	coding	233	58	756
PB.5304.1	4	1355	1	intergenic	novelGene_305	novel	13.26	13.26	non coding	NA	NA	NA
PB.6869.1	5	2789	5	novel_in_catalog	gene: Zm00001d016972	novel	18.66	69.89	coding	390	109	1278
PB.2723.3	2	1222	3	full-splice_match	gene: Zm00001d003064	Zm00001d003064_T00	0.00	27.44	coding	294	112	993

PB.2723.2	2	1396	3	full-splice_match	gene: Zm00001d003064	Zm00001d003064_T00	20.03	27.44	coding	294	113	994
PB.2723.1	2	1699	3	full-splice_match	gene: Zm00001d003064	Zm00001d003064_T00	7.40	27.44	coding	294	112	993
PB.9860.1	8	1310	9	full-splice_match	gene: Zm00001d011782	Zm00001d011782_T00	10.09	10.09	coding	282	161	1006
PB.9359.1	8	1337	6	full-splice_match	gene: Zm00001d009440	Zm00001d009440_T00	6.77	47.32	coding	201	291	893
PB.4789.4	3	1306	3	genic	gene: Zm00001d044501	novel	14.13	30.27	coding	188	454	1017
PB.4582.2	3	2256	13	novel_not_in_catalog	gene: Zm00001d043693	novel	5.24	284.74	coding	504	247	1758
PB.2723.4	2	1047	3	full-splice_match	gene: Zm00001d003064	Zm00001d003064_T00	0.00	27.44	coding	294	112	993
PB.1817.1	10	1154	5	novel_in_catalog	gene: Zm00001d023846	novel	26.06	26.06	coding	151	414	866
PB.7487.2	6	698	2	full-splice_match	gene: Zm00001d036036	Zm00001d036036_T00	1016.10	1057.89	coding	136	97	504
PB.1405.2	1	2160	3	full-splice_match	gene: Zm00001d033862	Zm00001d033862_T00	2.30	9.59	coding	485	224	1678
PB.8352.11	7	922	7	incomplete-splice_match	gene: Zm00001d019283	Zm00001d019283_T01	0.00	603.21	coding	283	74	922
PB.8352.10	7	1151	10	full-splice_match	gene: Zm00001d019283	Zm00001d019283_T01	0.00	603.21	coding	360	71	1150
PB.3367.1	2	618	3	full-splice_match	gene: Zm00001d006421	Zm00001d006421_T00	281.99	307.00	coding	80	104	343
PB.2662.2	2	1951	5	novel_not_in_catalog	gene: Zm00001d002790	novel	11.94	12.98	coding	416	388	1635
PB.2662.1	2	2197	5	novel_not_in_catalog	gene: Zm00001d002790	novel	1.04	12.98	coding	416	329	1576
PB.1585.3	1	2303	5	incomplete-splice_match	gene: Zm00001d034624	Zm00001d034624_T03	24.54	53.35	coding	654	45	2006
PB.1783.1	10	1520	1	intergenic	novelGene_58	novel	19.72	34.02	coding	126	17	394

PB.10573.2	9	913	5	full-splice_match	gene: Zm00001d047218	Zm00001d047218_T00	49.41	188.40	coding	166	55	552
PB.10573.3	9	668	4	incomplete-splice_match	gene: Zm00001d047218	Zm00001d047218_T00	38.83	188.40	non coding	NA	NA	NA
PB.10573.1	9	1050	5	full-splice_match	gene: Zm00001d047218	Zm00001d047218_T00	96.81	188.40	coding	166	89	586
PB.747.12	1	1865	2	novel_in_catalog	gene: Zm00001d030891	novel	0.00	622.65	coding	151	123	575
PB.10573.4	9	1381	5	novel_not_in_catalog	gene: Zm00001d047218	novel	3.35	188.40	coding	47	447	587
PB.8606.2	7	2824	25	novel_in_catalog	gene: Zm00001d020799	novel	8.50	12.63	coding	777	85	2415
PB.5309.2	4	1404	2	full-splice_match	gene: Zm00001d051334	Zm00001d051334_T00	7.26	31.85	coding	291	231	1103
PB.7190.1	5	3548	23	full-splice_match	gene: Zm00001d018246	Zm00001d018246_T00	30.88	31.93	coding	994	170	3151
PB.3934.2	3	653	3	full-splice_match	gene: Zm00001d040361	Zm00001d040361_T00	79.44	92.08	coding	23	387	455
PB.2340.1	10	2349	1	full-splice_match	gene: Zm00001d026492	Zm00001d026492_T00	6.38	6.38	coding	421	644	1906
PB.7159.2	5	804	5	full-splice_match	gene: Zm00001d018134	Zm00001d018134_T00	289.88	549.21	coding	112	122	457
PB.7145.1	5	2318	6	novel_not_in_catalog	gene: Zm00001d018088	novel	13.35	23.22	coding	328	1	984
PB.10202.1	9	1335	6	full-splice_match	gene: Zm00001d045193	Zm00001d045193_T00	31.85	31.85	coding	333	54	1052
PB.10202.2	9	1227	6	full-splice_match	gene: Zm00001d045193	Zm00001d045193_T00	0.00	31.85	coding	333	42	1040
PB.1246.1	1	2120	11	novel_not_in_catalog	gene: Zm00001d033159	novel	213.24	343.80	coding	583	36	1784
PB.5634.1	4	1703	11	full-splice_match	gene: Zm00001d052747	Zm00001d052747_T00	23.78	23.78	coding	481	65	1507
PB.9171.1	8	890	4	full-splice_match	gene: Zm00001d008405	Zm00001d008405_T00	69.55	69.55	coding	130	249	638

PB.10383.4	9	1402	5	novel_in_catalog	gene: Zm00001d046136	novel	0.00	42.36	coding	379	86	1222
PB.6024.1	5	865	2	full-splice_match	gene: Zm00001d013038	Zm00001d013038_T00	183.68	183.68	coding	213	137	775
PB.9726.1	8	1569	8	full-splice_match	gene: Zm00001d011193	Zm00001d011193_T00	84.75	84.75	coding	362	221	1306
PB.10383.2	9	1514	5	novel_in_catalog	gene: Zm00001d046136	novel	41.19	42.36	coding	379	102	1238
PB.920.3	1	1730	7	incomplete-splice_match	gene: Zm00001d031749	Zm00001d031749_T00	0.00	52.73	coding	497	49	1539
PB.6319.1	5	1705	15	full-splice_match	gene: Zm00001d014258	Zm00001d014258_T00	12.17	77.15	coding	486	131	1588
PB.1678.1	1	932	5	incomplete-splice_match	gene: Zm00001d034949	Zm00001d034949_T00	52.06	143.81	coding	125	61	435
PB.7585.1	6	813	6	full-splice_match	gene: Zm00001d036642	Zm00001d036642_T00	37.54	37.54	coding	179	44	580
PB.8123.1	6	2319	12	full-splice_match	gene: Zm00001d038891	Zm00001d038891_T00	56.47	56.47	coding	501	402	1904
PB.1058.1	1	2313	19	full-splice_match	gene: Zm00001d032386	Zm00001d032386_T00	102.08	130.95	coding	618	245	2098
PB.1678.4	1	995	7	full-splice_match	gene: Zm00001d034949	Zm00001d034949_T00	7.23	143.81	coding	203	174	782
PB.9250.1	8	1372	5	full-splice_match	gene: Zm00001d008758	Zm00001d008758_T00	12.71	30.35	coding	344	133	1164
PB.1257.1	1	2999	6	novel_in_catalog	gene: Zm00001d033211	novel	11.02	11.02	coding	226	2072	2749
PB.2820.1	2	1386	1	full-splice_match	gene: Zm00001d003499	Zm00001d003499_T00	27.62	27.62	coding	388	78	1241
PB.9250.2	8	1479	5	full-splice_match	gene: Zm00001d008758	Zm00001d008758_T00	17.63	30.35	coding	344	118	1149
PB.7466.7	6	2346	1	novel_in_catalog	gene: Zm00001d035925	novel	4.26	352.40	coding	482	761	2206
PB.1783.3	10	933	4	intergenic	novelGene_58	novel	14.30	34.02	non coding	NA	NA	NA

PB.10238.2	9	1001	2	intergenic	novelGene_80	novel	6.25	32.56	coding	271	22	834
PB.10238.3	9	924	2	intergenic	novelGene_80	novel	9.13	32.56	coding	271	59	871
PB.1450.2	1	1532	3	novel_not_in_catalog	gene: Zm00001d034032	novel	15.96	43.53	coding	398	170	1363
PB.1254.1	1	848	3	full-splice_match	gene: Zm00001d033190	Zm00001d033190_T00	11.76	11.76	coding	183	194	742
PB.502.1	1	1609	2	full-splice_match	gene: Zm00001d029442	Zm00001d029442_T00	6.64	6.64	coding	401	216	1418
PB.8714.2	7	1760	4	novel_not_in_catalog	gene: Zm00001d021286	novel	29.61	29.61	coding	383	313	1461
PB.6780.1	5	2095	8	novel_in_catalog	gene: Zm00001d016587	novel	4.08	10.35	coding	272	145	960
PB.3548.1	2	1778	15	full-splice_match	gene: Zm00001d007214	Zm00001d007214_T00	33.44	33.44	coding	474	138	1559
PB.7466.3	6	1888	2	novel_in_catalog	gene: Zm00001d035925	novel	267.15	352.40	coding	482	175	1620
PB.8218.2	7	1221	7	full-splice_match	gene: Zm00001d018616	Zm00001d018616_T00	54.83	62.16	coding	307	97	1017
PB.10696.3	9	3163	5	novel_in_catalog	gene: Zm00001d047710	novel	5.68	21.87	coding	188	198	761
PB.4803.2	3	1005	8	incomplete-splice_match	gene: Zm00001d044552	Zm00001d044552_T01	17.26	32.32	coding	238	8	721
PB.8218.1	7	1593	8	novel_in_catalog	gene: Zm00001d018616	novel	7.33	62.16	coding	307	96	1016
PB.5506.1	4	802	2	full-splice_match	gene: Zm00001d052216	Zm00001d052216_T00	16.02	16.02	coding	161	35	517
PB.5593.3	4	1557	3	full-splice_match	gene: Zm00001d052489	Zm00001d052489_T00	8.73	35.08	coding	430	121	1410
PB.5358.5	4	3476	2	novel_in_catalog	gene: Zm00001d051589	novel	0.33	109.09	coding	643	515	2443
PB.2097.1	10	1174	4	novel_not_in_catalog	gene: Zm00001d025425	novel	5.86	64.66	coding	150	335	784

PB.7997.1	6	2745	2	full-splice_match	gene: Zm00001d038407	Zm00001d038407_T00	2.37	2.37	coding	860	94	2673
PB.2106.1	10	1892	15	full-splice_match	gene: Zm00001d025453	Zm00001d025453_T00	29.58	29.58	coding	450	164	1513
PB.8235.2	7	2277	2	full-splice_match	gene: Zm00001d018693	Zm00001d018693_T00	0.00	20.17	coding	571	260	1972
PB.5358.6	4	2207	2	incomplete-splice_match	gene: Zm00001d051589	Zm00001d051589_T00	0.00	109.09	coding	571	493	2205
PB.4212.4	3	1306	9	novel_in_catalog	gene: Zm00001d042193	novel	2.23	33.54	coding	268	174	977
PB.2013.1	10	2491	13	incomplete-splice_match	gene: Zm00001d024953	Zm00001d024953_T00	12.73	12.73	coding	477	40	1470
PB.7070.1	5	2835	3	novel_not_in_catalog	gene: Zm00001d017821	novel	1.80	15.54	coding	749	75	2321
PB.5613.1	4	1362	2	full-splice_match	gene: Zm00001d052655	Zm00001d052655_T00	54.77	54.77	coding	270	140	949
PB.5182.1	4	912	2	full-splice_match	gene: Zm00001d050368	Zm00001d050368_T00	104.79	104.79	coding	183	47	595
PB.4212.2	3	1453	9	full-splice_match	gene: Zm00001d042193	Zm00001d042193_T00	27.19	33.54	coding	286	175	1032
PB.9778.1	8	1277	7	incomplete-splice_match	gene: Zm00001d011445	Zm00001d011445_T00	7.40	7.40	coding	264	138	929
PB.7905.2	6	1339	5	full-splice_match	gene: Zm00001d038114	Zm00001d038114_T00	0.31	36.27	coding	162	124	609
PB.5394.3	4	1882	9	full-splice_match	gene: Zm00001d051754	Zm00001d051754_T00	71.22	148.08	coding	550	125	1774
PB.3273.1	2	1122	2	full-splice_match	gene: Zm00001d006059	Zm00001d006059_T00	26.85	32.67	coding	262	150	935
PB.4369.2	3	2370	8	novel_in_catalog	gene: Zm00001d042885	novel	11.05	50.26	coding	334	210	1211
PB.9988.2	8	2783	1	genic	gene: Zm00001d012297	novel	10.47	21.82	non coding	NA	NA	NA
PB.8319.1	7	1994	4	full-splice_match	gene: Zm00001d019079	Zm00001d019079_T00	50.84	50.84	coding	448	119	1462

PB.9718.1	8	1038	8	full-splice_match	gene: Zm00001d011162	Zm00001d011162_T00	524.34	531.74	coding	203	222	830
PB.9718.2	8	1190	5	novel_in_catalog	gene: Zm00001d011162	novel	7.39	531.74	non coding	NA	NA	NA
PB.4899.1	4	1590	5	novel_not_in_catalog	gene: Zm00001d048907	novel	18.71	18.71	coding	367	314	1414
PB.6801.2	5	1998	1	genic	gene: Zm00001d016682	novel	13.63	70.81	coding	113	223	561
PB.9802.3	8	2369	11	full-splice_match	gene: Zm00001d011563	Zm00001d011563_T00	11.20	60.42	coding	285	151	1005
PB.3830.3	3	529	6	full-splice_match	gene: Zm00001d039899	Zm00001d039899_T00	0.00	79.56	coding	150	79	528
PB.3830.2	3	896	6	full-splice_match	gene: Zm00001d039899	Zm00001d039899_T00	64.14	79.56	coding	172	91	606
PB.3830.1	3	486	1	genic	gene: Zm00001d039899	novel	15.42	79.56	non coding	NA	NA	NA
PB.9165.1	8	1449	1	full-splice_match	gene: Zm00001d008370	Zm00001d008370_T00	21.03	21.03	coding	363	51	1139
PB.9683.1	8	1896	3	full-splice_match	gene: Zm00001d010976	Zm00001d010976_T00	167.30	173.74	coding	365	425	1519
PB.6052.1	5	1272	2	genic	gene: Zm00001d013138	novel	26.80	26.80	coding	257	103	873
PB.2026.1	10	1819	2	novel_not_in_catalog	gene: Zm00001d025017	novel	7.91	100.34	non coding	NA	NA	NA
PB.9683.2	8	1466	1	novel_in_catalog	gene: Zm00001d010976	novel	6.44	173.74	non coding	NA	NA	NA
PB.5225.1	4	2031	11	novel_in_catalog	gene: Zm00001d050741	novel	27.81	47.93	coding	466	64	1461
PB.7159.6	5	486	5	full-splice_match	gene: Zm00001d018134	Zm00001d018134_T00	0.00	549.21	coding	112	74	409
PB.852.5	1	886	2	full-splice_match	gene: Zm00001d031344	Zm00001d031344_T00	34.55	103.08	coding	167	139	639
PB.4294.2	3	661	1	genic	gene: Zm00001d042585	novel	0.00	130.03	non coding	NA	NA	NA

PB.4294.3	3	761	3	incomplete-splice_match	gene: Zm00001d042585	Zm00001d042585_T00	46.74	130.03	non coding	NA	NA	NA
PB.7511.3	6	2597	3	intergenic	novelGene_171	novel	5.96	9.99	non coding	NA	NA	NA
PB.2031.2	10	2699	9	incomplete-splice_match	gene: Zm00001d025031	Zm00001d025031_T00	11.99	11.99	coding	730	369	2558
PB.4217.3	3	1260	5	novel_not_in_catalog	gene: Zm00001d042233	novel	9.38	35.14	coding	247	355	1095
PB.5225.3	4	1903	10	novel_in_catalog	gene: Zm00001d050741	novel	18.36	47.93	coding	466	147	1544
PB.4294.4	3	706	3	incomplete-splice_match	gene: Zm00001d042585	Zm00001d042585_T00	29.82	130.03	coding	50	184	333
PB.4294.5	3	1855	13	full-splice_match	gene: Zm00001d042585	Zm00001d042585_T00	38.99	130.03	coding	503	159	1667
PB.3126.3	2	1959	9	full-splice_match	gene: Zm00001d005324	Zm00001d005324_T00	7.65	15.59	coding	554	66	1727
PB.8325.3	7	2491	13	full-splice_match	gene: Zm00001d019130	Zm00001d019130_T00	15.69	29.36	coding	714	80	2221
PB.1723.3	10	2105	16	full-splice_match	gene: Zm00001d023379	Zm00001d023379_T00	140.23	174.70	coding	499	325	1821
PB.8325.1	7	560	1	genic	gene: Zm00001d019130	novel	6.80	29.36	non coding	NA	NA	NA
PB.1723.1	10	2141	16	full-splice_match	gene: Zm00001d023379	Zm00001d023379_T00	34.46	174.70	coding	527	174	1754
PB.4327.1	3	2068	11	full-splice_match	gene: Zm00001d042722	Zm00001d042722_T00	3.73	18.07	coding	132	1410	1805
PB.3169.5	2	3345	7	full-splice_match	gene: Zm00001d005579	Zm00001d005579_T00	3.28	72.57	coding	310	2101	3030
PB.4327.3	3	1389	13	novel_in_catalog	gene: Zm00001d042722	novel	14.33	18.07	coding	162	79	564
PB.3499.3	2	2622	11	full-splice_match	gene: Zm00001d007029	Zm00001d007029_T00	46.72	82.97	coding	592	645	2420
PB.4815.1	3	1030	2	full-splice_match	gene: Zm00001d044606	Zm00001d044606_T00	25.09	25.09	coding	289	17	883

PB.3169.1	2	1563	9	full-splice_match	gene: Zm00001d005579	Zm00001d005579_T00	48.30	72.57	coding	310	307	1236
PB.8256.1	7	3087	2	genic	gene: Zm00001d018789	novel	13.03	18.75	coding	876	295	2922
PB.3802.1	3	1210	3	genic_intron	novelGene_60	novel	12.74	12.74	coding	151	34	486
PB.3602.5	2	2049	9	full-splice_match	gene: Zm00001d007645	Zm00001d007645_T00	3.36	30.46	coding	110	187	516
PB.5554.2	4	1643	1	incomplete-splice_match	gene: Zm00001d052362	Zm00001d052362_T00	31.11	31.11	coding	119	553	909
PB.8256.2	7	2974	2	genic	gene: Zm00001d018789	novel	5.72	18.75	coding	876	285	2912
PB.2153.12	10	932	2	intergenic	novelGene_244	novel	9.55	9.55	coding	145	268	702
PB.8108.3	6	1440	11	full-splice_match	gene: Zm00001d038851	Zm00001d038851_T00	153.52	198.75	coding	370	156	1265
PB.4276.2	3	1026	2	genic	gene: Zm00001d042503	novel	16.03	47.63	coding	162	10	495
PB.4276.3	3	919	2	genic	gene: Zm00001d042503	novel	31.60	47.63	coding	162	20	505
PB.2464.1	2	1037	1	antisense	novelGene_gene: Zm00001d001924	novel	1.89	1.89	non coding	NA	NA	NA
PB.4276.1	3	1129	2	genic	gene: Zm00001d042503	novel	0.00	47.63	coding	162	10	495
PB.681.1	1	1733	17	full-splice_match	gene: Zm00001d030373	Zm00001d030373_T00	32.70	573.60	coding	446	51	1388
PB.6727.1	5	3207	2	full-splice_match	gene: Zm00001d016285	Zm00001d016285_T00	7.35	7.35	coding	1032	36	3131
PB.681.2	1	1608	17	full-splice_match	gene: Zm00001d030373	Zm00001d030373_T00	540.90	573.60	coding	446	51	1388
PB.3792.10	3	788	7	incomplete-splice_match	gene: Zm00001d039674	Zm00001d039674_T00	8.79	100.53	coding	227	13	693
PB.5927.2	4	1397	8	full-splice_match	gene: Zm00001d054039	Zm00001d054039_T00	0.00	53.47	coding	407	68	1288

PB.1296.2	1	2048	1	full-splice_match	gene: Zm00001d033377	Zm00001d033377_T00	15.15	15.15	coding	282	314	1159
PB.3277.2	2	3622	3	full-splice_match	gene: Zm00001d006079	Zm00001d006079_T00	6.69	17.57	coding	1061	262	3444
PB.3905.3	3	2334	16	incomplete-splice_match	gene: Zm00001d040279	Zm00001d040279_T00	71.65	128.90	coding	713	28	2166
PB.3905.1	3	3900	25	full-splice_match	gene: Zm00001d040279	Zm00001d040279_T01	57.26	128.90	coding	1125	217	3591
PB.5786.1	4	1609	2	incomplete-splice_match	gene: Zm00001d053524	Zm00001d053524_T00	76.41	76.41	coding	400	112	1311
PB.884.7	1	873	2	novel_not_in_catalog	gene: Zm00001d031562	novel	34.09	73.26	coding	216	93	740
PB.1618.1	1	1793	4	full-splice_match	gene: Zm00001d034729	Zm00001d034729_T00	14.29	14.29	coding	491	128	1600
PB.5938.1	4	1819	7	full-splice_match	gene: Zm00001d054072	Zm00001d054072_T00	11.47	12.49	coding	413	169	1407
PB.4698.42	3	2356	3	novel_in_catalog	gene: Zm00001d044138	novel	0.00	1495.64	coding	250	16	765
PB.1140.7	1	2630	21	novel_not_in_catalog	gene: Zm00001d032685	novel	4.51	57.68	coding	367	1489	2589
PB.9447.1	8	1331	2	novel_not_in_catalog	gene: Zm00001d009904	novel	5.30	5.30	coding	391	76	1248
PB.10545.1	9	1106	6	full-splice_match	gene: Zm00001d047057	Zm00001d047057_T00	56.46	56.46	coding	174	89	610
PB.4730.6	3	803	3	full-splice_match	gene: Zm00001d044254	Zm00001d044254_T00	11.26	57.89	coding	169	150	656
PB.6946.3	5	2434	12	full-splice_match	gene: Zm00001d017271	Zm00001d017271_T00	3.70	445.51	coding	133	114	512
PB.5854.2	4	779	4	full-splice_match	gene: Zm00001d053800	Zm00001d053800_T00	97.45	274.30	coding	160	185	664
PB.5854.1	4	926	4	full-splice_match	gene: Zm00001d053800	Zm00001d053800_T00	176.85	274.30	coding	160	153	632
PB.2026.2	10	2112	2	genic	gene: Zm00001d025017	novel	0.00	100.34	coding	197	739	1329

PB.10926.1	9	1334	8	incomplete-splice_match	gene: Zm00001d048536	Zm00001d048536_T00	17.30	17.30	coding	225	64	738
PB.2010.1	10	1785	2	full-splice_match	gene: Zm00001d024943	Zm00001d024943_T00	80.06	80.06	coding	532	9	1604
PB.3161.1	2	1272	6	full-splice_match	gene: Zm00001d005513	Zm00001d005513_T00	16.45	20.37	coding	303	64	972
PB.3161.2	2	1165	6	full-splice_match	gene: Zm00001d005513	Zm00001d005513_T00	3.92	20.37	coding	303	75	983
PB.5897.4	4	1335	11	full-splice_match	gene: Zm00001d053954	Zm00001d053954_T00	31.42	37.13	coding	291	269	1141
PB.5403.1	4	2389	16	full-splice_match	gene: Zm00001d051809	Zm00001d051809_T00	30.86	30.86	coding	671	48	2060
PB.6502.1	5	2330	3	full-splice_match	gene: Zm00001d014961	Zm00001d014961_T00	75.78	109.00	coding	612	165	2000
PB.10438.1	9	1732	8	full-splice_match	gene: Zm00001d046476	Zm00001d046476_T00	12.02	22.81	coding	300	434	1333
PB.10438.2	9	1667	7	novel_in_catalog	gene: Zm00001d046476	novel	10.79	22.81	coding	300	467	1366
PB.2012.15	10	3185	15	incomplete-splice_match	gene: Zm00001d024952	Zm00001d024952_T00	0.00	175.10	coding	708	72	2195
PB.7604.1	6	778	1	full-splice_match	gene: Zm00001d036737	Zm00001d036737_T00	10.94	158.16	coding	106	97	414
PB.7604.2	6	742	1	full-splice_match	gene: Zm00001d036737	Zm00001d036737_T00	147.22	158.16	coding	106	177	494
PB.5755.2	4	3103	14	novel_not_in_catalog	gene: Zm00001d053334	novel	5.73	58.57	coding	335	1700	2704
PB.9096.1	7	1180	8	novel_in_catalog	gene: Zm00001d022585	novel	39.96	39.96	coding	211	327	959
PB.5755.4	4	2220	15	novel_not_in_catalog	gene: Zm00001d053334	novel	34.38	58.57	coding	485	375	1829
PB.8108.1	6	1568	11	full-splice_match	gene: Zm00001d038851	Zm00001d038851_T00	45.23	198.75	coding	370	157	1266
PB.3181.2	2	669	4	full-splice_match	gene: Zm00001d005642	Zm00001d005642_T00	55.12	522.42	coding	112	40	375

PB.3181.3	2	571	4	full-splice_match	gene: Zm00001d005642	Zm00001d005642_T00	467.29	522.42	coding	112	43	378
PB.4580.1	3	3291	11	novel_not_in_catalog	gene: Zm00001d043684	novel	9.68	22.23	coding	910	186	2915
PB.2444.1	2	2732	2	novel_in_catalog	gene: Zm00001d001847	novel	6.63	6.63	coding	296	1437	2324
PB.10838.4	9	3260	16	full-splice_match	gene: Zm00001d048258	Zm00001d048258_T01	5.82	63.38	coding	780	598	2937
PB.9365.2	8	1199	3	full-splice_match	gene: Zm00001d009476	Zm00001d009476_T00	94.21	94.21	coding	252	219	974
PB.4613.2	3	911	1	incomplete-splice_match	gene: Zm00001d043801	Zm00001d043801_T00	21.98	77.07	non coding	NA	NA	NA
PB.6156.3	5	437	1	intergenic	novelGene_55	novel	0.00	38.69	non coding	NA	NA	NA
PB.1138.1	1	747	1	full-splice_match	gene: Zm00001d032672	Zm00001d032672_T00	53.13	53.13	coding	166	11	508
PB.5278.3	4	1677	5	incomplete-splice_match	gene: Zm00001d051075	Zm00001d051075_T01	0.15	24.91	coding	310	747	1676
PB.5278.2	4	1366	4	incomplete-splice_match	gene: Zm00001d051075	Zm00001d051075_T01	0.00	24.91	coding	373	247	1365
PB.5278.1	4	2645	7	incomplete-splice_match	gene: Zm00001d051075	Zm00001d051075_T00	24.75	24.91	coding	520	726	2285
PB.6612.1	5	2765	10	full-splice_match	gene: Zm00001d015497	Zm00001d015497_T00	19.08	19.08	coding	129	1330	1716
PB.7263.5	5	1782	5	novel_not_in_catalog	gene: Zm00001d018499	novel	0.00	44.85	coding	165	1053	1547
PB.8480.2	7	1945	2	genic	gene: Zm00001d020139	novel	26.09	57.62	coding	451	235	1587
PB.8480.1	7	1230	2	genic	gene: Zm00001d020139	novel	31.53	57.62	coding	255	81	845
PB.7263.6	5	919	2	full-splice_match	gene: Zm00001d018501	Zm00001d018501_T00	18.04	18.16	coding	187	76	636
PB.5914.1	4	2179	1	intergenic	novelGene_447	novel	5.99	5.99	non coding	NA	NA	NA

PB.333.1	1	1693	3	full-splice_match	gene: Zm00001d028656	Zm00001d028656_T00	220.94	220.94	coding	454	97	1458
PB.1301.1	1	1235	2	genic	gene: Zm00001d033386	novel	53.16	76.58	coding	230	147	836
PB.6682.1	5	2496	6	full-splice_match	gene: Zm00001d016044	Zm00001d016044_T00	23.73	41.22	coding	677	242	2272
PB.8000.4	6	2158	4	incomplete-splice_match	gene: Zm00001d038432	Zm00001d038432_T00	37.06	103.47	coding	234	1131	1832
PB.6682.2	5	2021	5	incomplete-splice_match	gene: Zm00001d016044	Zm00001d016044_T00	17.49	41.22	coding	574	95	1816
PB.7901.2	6	2701	3	full-splice_match	gene: Zm00001d038101	Zm00001d038101_T00	0.00	84.96	coding	778	31	2364
PB.7332.2	6	1345	10	full-splice_match	gene: Zm00001d035169	Zm00001d035169_T00	12.88	20.13	coding	291	143	1015
PB.884.8	1	3339	5	full-splice_match	gene: Zm00001d031570	Zm00001d031570_T00	23.12	23.12	coding	1005	144	3158
PB.8307.1	7	987	2	genic	gene: Zm00001d018984	novel	34.11	34.11	coding	173	92	610
PB.7332.1	6	1433	10	full-splice_match	gene: Zm00001d035169	Zm00001d035169_T00	7.24	20.13	coding	291	107	979
PB.5874.1	4	591	5	full-splice_match	gene: Zm00001d053863	Zm00001d053863_T00	1481.55	1627.71	non coding	NA	NA	NA
PB.2955.1	2	1376	2	full-splice_match	gene: Zm00001d004209	Zm00001d004209_T00	0.17	0.17	coding	242	336	1061
PB.5258.1	4	1199	6	full-splice_match	gene: Zm00001d050978	Zm00001d050978_T00	41.16	99.06	coding	229	75	761
PB.3437.10	2	2129	12	full-splice_match	gene: Zm00001d006751	Zm00001d006751_T00	0.00	70.92	coding	631	52	1944
PB.10430.2	9	1886	2	novel_in_catalog	gene: Zm00001d046450	novel	2.93	20.69	coding	200	935	1534
PB.10430.3	9	1522	4	full-splice_match	gene: Zm00001d046450	Zm00001d046450_T00	17.76	20.69	coding	360	91	1170
PB.9576.6	8	2793	1	antisense	novelGene_gene: Zm00001d010564	novel	22.16	44.33	non coding	NA	NA	NA

PB.7901.1	6	2781	3	full-splice_match	gene: Zm00001d038101	Zm00001d038101_T00	84.96	84.96	coding	778	31	2364
PB.6847.3	5	862	9	novel_not_in_catalog	gene: Zm00001d016860	novel	37.45	69.05	coding	224	68	739
PB.7694.3	6	1572	2	full-splice_match	gene: Zm00001d037239	Zm00001d037239_T00	63.30	296.70	coding	377	190	1320
PB.7093.1	5	1312	1	full-splice_match	gene: Zm00001d017900	Zm00001d017900_T00	13.55	13.55	coding	300	130	1029
PB.5957.1	5	1526	1	antisense	novelGene_gene: Zm00001d012813	novel	13.96	13.96	coding	300	625	1524
PB.9576.4	8	2365	2	antisense	novelGene_gene: Zm00001d010564	novel	9.21	44.33	non coding	NA	NA	NA
PB.4701.4	3	1930	4	novel_not_in_catalog	gene: Zm00001d044146	novel	7.25	90.95	coding	275	887	1711
PB.3368.3	2	2459	15	novel_in_catalog	gene: Zm00001d006422	novel	1.79	49.80	coding	406	1068	2285
PB.9975.4	8	1560	2	genic	gene: Zm00001d012260	novel	20.03	73.75	coding	473	141	1559
PB.9576.5	8	2138	3	antisense	novelGene_gene: Zm00001d010564	novel	0.00	44.33	non coding	NA	NA	NA
PB.9603.1	8	1666	2	genic	gene: Zm00001d010654	novel	141.67	315.30	coding	357	232	1302
PB.1230.3	1	2467	3	full-splice_match	gene: Zm00001d033056	Zm00001d033056_T00	4.62	179.46	coding	294	14	895
PB.9975.3	8	1389	1	full-splice_match	gene: Zm00001d012260	Zm00001d012260_T00	12.45	73.75	coding	363	127	1215
PB.9603.2	8	1507	2	genic	gene: Zm00001d010654	novel	173.63	315.30	coding	357	195	1265
PB.133.1	1	3767	13	full-splice_match	gene: Zm00001d027807	Zm00001d027807_T00	30.25	48.13	coding	1045	3	3137
PB.8485.14	7	793	2	novel_in_catalog	gene: Zm00001d020185	novel	27.54	390.61	coding	240	19	738
PB.4885.4	4	919	3	full-splice_match	gene: Zm00001d048839	Zm00001d048839_T00	4.24	12.76	non coding	NA	NA	NA

PB.4701.6	3	1807	5	novel_not_in_catalog	gene: Zm00001d044146	novel	51.41	90.95	coding	521	50	1612
PB.7308.1	6	2680	12	novel_in_catalog	gene: Zm00001d035046	novel	11.76	24.45	coding	521	1043	2605
PB.4885.3	4	1052	2	novel_in_catalog	gene: Zm00001d048839	novel	2.13	12.76	non coding	NA	NA	NA
PB.7308.2	6	2303	10	incomplete-splice_match	gene: Zm00001d035046	Zm00001d035046_T00	12.68	24.45	coding	698	151	2244
PB.3281.1	2	1025	7	full-splice_match	gene: Zm00001d006085	Zm00001d006085_T00	167.05	167.05	coding	221	180	842
PB.7865.4	6	2013	1	incomplete-splice_match	gene: Zm00001d037975	Zm00001d037975_T00	8.39	19.15	coding	634	1	1902
PB.1739.3	10	1805	4	novel_in_catalog	gene: Zm00001d023450	novel	20.73	46.66	coding	225	932	1606
PB.1739.2	10	1509	4	incomplete-splice_match	gene: Zm00001d023450	Zm00001d023450_T00	21.37	46.66	coding	354	242	1303
PB.1739.1	10	1295	2	novel_in_catalog	gene: Zm00001d023450	novel	4.57	46.66	coding	187	576	1136
PB.4728.1	3	874	1	full-splice_match	gene: Zm00001d044247	Zm00001d044247_T00	42.33	775.40	coding	148	87	530
PB.9281.2	8	2008	2	genic_intron	novelGene_101	novel	0.00	15.62	coding	594	161	1942
PB.7539.1	6	2110	13	full-splice_match	gene: Zm00001d036429	Zm00001d036429_T00	59.80	59.80	coding	549	123	1769
PB.4383.1	3	685	1	full-splice_match	gene: Zm00001d042954	Zm00001d042954_T00	229.23	229.23	coding	103	81	389
PB.824.1	1	1899	1	full-splice_match	gene: Zm00001d031221	Zm00001d031221_T00	45.13	62.08	coding	529	88	1674
PB.2672.2	2	1353	5	incomplete-splice_match	gene: Zm00001d002830	Zm00001d002830_T00	40.55	40.55	coding	359	46	1122
PB.824.3	1	873	1	full-splice_match	gene: Zm00001d031221	Zm00001d031221_T00	16.95	62.08	coding	146	352	789
PB.272.1	1	1883	2	novel_in_catalog	gene: Zm00001d028397	novel	24.08	24.08	non coding	NA	NA	NA

PB.142.2	1	1621	1	intergenic	novelGene_65	novel	0.21	0.21	non coding	NA	NA	NA
PB.6916.1	5	1152	5	full-splice_match	gene: Zm00001d017145	Zm00001d017145_T00	96.58	96.58	coding	245	117	851
PB.1415.1	1	1735	10	full-splice_match	gene: Zm00001d033906	Zm00001d033906_T00	27.11	27.11	coding	473	123	1541
PB.3523.4	2	776	1	full-splice_match	gene: Zm00001d007097	Zm00001d007097_T00	35.02	191.58	coding	159	101	577
PB.5286.1	4	2060	1	incomplete-splice_match	gene: Zm00001d051163	Zm00001d051163_T00	191.44	191.44	coding	557	67	1737
PB.6735.1	5	2437	1	novel_in_catalog	gene: Zm00001d016326	novel	24.32	56.35	coding	374	926	2047
PB.1797.2	10	1300	11	full-splice_match	gene: Zm00001d023738	Zm00001d023738_T00	27.13	78.78	coding	304	117	1028
PB.1797.1	10	1424	11	full-splice_match	gene: Zm00001d023738	Zm00001d023738_T00	49.97	78.78	coding	304	137	1048
PB.621.3	1	1434	1	full-splice_match	gene: Zm00001d030083	Zm00001d030083_T00	39.59	51.23	coding	342	105	1130
PB.621.2	1	1722	1	full-splice_match	gene: Zm00001d030083	Zm00001d030083_T00	10.15	51.23	coding	342	113	1138
PB.153.1	1	1140	3	novel_in_catalog	gene: Zm00001d027892	novel	156.49	156.49	coding	171	249	761
PB.1797.4	10	2089	8	novel_in_catalog	gene: Zm00001d023738	novel	1.67	78.78	coding	186	117	674
PB.844.2	1	1336	9	full-splice_match	gene: Zm00001d031323	Zm00001d031323_T00	9.73	30.67	coding	332	85	1080
PB.844.1	1	1472	9	full-splice_match	gene: Zm00001d031323	Zm00001d031323_T00	20.94	30.67	coding	332	166	1161
PB.7772.1	6	2280	1	genic	gene: Zm00001d037596	novel	5.08	68.64	coding	128	27	410
PB.10953.1	B73V4	1673	3	incomplete-splice_match	gene: Zm00001d000358	Zm00001d000358_T00	27.50	28.69	coding	387	299	1459
PB.10953.2	B73V4	1806	2	novel_in_catalog	gene: Zm00001d000358	novel	1.19	28.69	coding	379	171	1307

PB.601.1	1	1114	1	genic	gene: Zm00001d029948	novel	4.47	8.19	coding	236	20	727
PB.6698.1	5	2339	5	full-splice_match	gene: Zm00001d016131	Zm00001d016131_T00	12.04	28.24	coding	464	94	1485
PB.10132.5	9	1956	8	novel_in_catalog	gene: Zm00001d044854	novel	4.38	20.43	coding	209	160	786
PB.10132.4	9	1633	9	novel_in_catalog	gene: Zm00001d044854	novel	0.26	20.43	coding	275	1	825
PB.10132.3	9	1917	9	novel_in_catalog	gene: Zm00001d044854	novel	2.05	20.43	coding	209	160	786
PB.10132.2	9	1789	9	novel_in_catalog	gene: Zm00001d044854	novel	0.42	20.43	coding	163	81	569
PB.10132.1	9	1750	10	full-splice_match	gene: Zm00001d044854	Zm00001d044854_T00	13.32	20.43	coding	384	149	1300
PB.5262.1	4	1356	12	full-splice_match	gene: Zm00001d051001	Zm00001d051001_T00	687.91	687.91	coding	337	92	1102
PB.6688.3	5	1187	1	full-splice_match	gene: Zm00001d016066	Zm00001d016066_T00	0.00	15.28	coding	319	6	962
PB.10977.1	B73V4	3413	12	novel_not_in_catalog	gene: Zm00001d000042	novel	33.63	33.63	coding	922	197	2962
PB.2895.3	2	1971	9	full-splice_match	gene: Zm00001d003879	Zm00001d003879_T01	0.00	375.02	coding	564	142	1833
PB.248.1	1	1820	8	full-splice_match	gene: Zm00001d028325	Zm00001d028325_T00	5.77	5.77	coding	506	58	1575
PB.2895.1	2	2271	9	full-splice_match	gene: Zm00001d003879	Zm00001d003879_T01	54.99	375.02	coding	679	18	2054
PB.6703.2	5	1109	4	full-splice_match	gene: Zm00001d016154	Zm00001d016154_T00	0.00	27.58	coding	252	154	909
PB.3812.1	3	880	2	full-splice_match	gene: Zm00001d039786	Zm00001d039786_T00	306.44	306.44	coding	144	91	522
PB.1540.6	1	1925	6	full-splice_match	gene: Zm00001d034452	Zm00001d034452_T00	1.26	20.68	coding	183	233	781
PB.4584.2	3	1700	2	genic	gene: Zm00001d043695	novel	0.00	60.90	coding	433	109	1407

PB.2686.1	2	2096	1	full-splice_match	gene: Zm00001d002882	Zm00001d002882_T00	3.63	36.01	non coding	NA	NA	NA
PB.1376.1	1	902	2	full-splice_match	gene: Zm00001d033711	Zm00001d033711_T00	138.00	138.00	coding	130	184	573
PB.2686.3	2	1426	1	full-splice_match	gene: Zm00001d002882	Zm00001d002882_T00	10.28	36.01	coding	229	493	1179
PB.2686.2	2	1619	1	full-splice_match	gene: Zm00001d002882	Zm00001d002882_T00	22.10	36.01	coding	409	41	1267
PB.3704.1	3	1033	1	full-splice_match	gene: Zm00001d039324	Zm00001d039324_T00	45.67	45.67	non coding	NA	NA	NA
PB.5681.2	4	2036	13	full-splice_match	gene: Zm00001d052948	Zm00001d052948_T00	20.20	97.00	coding	542	239	1864
PB.666.2	1	1411	2	full-splice_match	gene: Zm00001d030300	Zm00001d030300_T00	14.35	14.35	coding	176	884	1411
PB.5770.1	4	803	2	genic	gene: Zm00001d053410	novel	106.89	106.89	coding	117	107	457
PB.3971.1	3	1329	2	full-splice_match	gene: Zm00001d040581	Zm00001d040581_T00	198.86	198.86	coding	348	71	1114
PB.6688.2	5	1885	3	genic	gene: Zm00001d016066	novel	12.67	15.28	coding	415	310	1554
PB.3523.2	2	892	1	full-splice_match	gene: Zm00001d007097	Zm00001d007097_T00	135.88	191.58	coding	159	98	574
PB.3295.1	2	919	2	incomplete-splice_match	gene: Zm00001d006132	Zm00001d006132_T00	7.98	7.98	coding	245	111	845
PB.1514.3	1	3389	6	full-splice_match	gene: Zm00001d034350	Zm00001d034350_T00	3.39	43.80	coding	615	1237	3081
PB.3564.2	2	1874	10	full-splice_match	gene: Zm00001d007369	Zm00001d007369_T02	32.35	36.05	coding	458	136	1509
PB.2116.3	10	1983	5	novel_in_catalog	gene: Zm00001d025539	novel	14.90	40.33	coding	194	197	778
PB.1097.1	1	2616	3	novel_in_catalog	gene: Zm00001d032528	novel	2.85	40.15	coding	244	1447	2178
PB.3764.3	3	2018	7	incomplete-splice_match	gene: Zm00001d039537	Zm00001d039537_T03	20.36	20.36	coding	226	10	687

PB.642.3	1	1218	6	full-splice_match	gene: Zm00001d030157	Zm00001d030157_T00	78.00	88.96	coding	194	201	782
PB.3096.1	2	2813	14	full-splice_match	gene: Zm00001d005157	Zm00001d005157_T00	25.64	27.86	coding	732	169	2364
PB.642.1	1	1180	5	incomplete-splice_match	gene: Zm00001d030157	Zm00001d030157_T00	6.70	88.96	coding	79	276	512
PB.4319.7	3	1939	3	novel_in_catalog	gene: Zm00001d042684	novel	53.40	359.58	coding	504	261	1772
PB.642.4	1	887	6	full-splice_match	gene: Zm00001d030157	Zm00001d030157_T00	0.00	88.96	coding	194	201	782
PB.3096.4	2	2705	14	full-splice_match	gene: Zm00001d005157	Zm00001d005157_T00	0.00	27.86	coding	732	168	2363
PB.4312.2	3	597	2	incomplete-splice_match	gene: Zm00001d042654	Zm00001d042654_T01	6.32	18.30	coding	90	176	445
PB.9361.2	8	731	5	full-splice_match	gene: Zm00001d009450	Zm00001d009450_T00	48.06	55.60	coding	190	68	637
PB.10324.1	9	1690	12	full-splice_match	gene: Zm00001d045673	Zm00001d045673_T00	12.30	12.30	coding	406	38	1255
PB.9500.1	8	726	4	full-splice_match	gene: Zm00001d010220	Zm00001d010220_T00	62.76	62.76	coding	140	147	566
PB.1787.1	10	1651	2	full-splice_match	gene: Zm00001d023654	Zm00001d023654_T00	14.44	14.44	coding	412	218	1453
PB.5561.1	4	482	3	incomplete-splice_match	gene: Zm00001d052386	Zm00001d052386_T00	5.76	5.76	coding	80	146	385
PB.10059.1	8	969	4	full-splice_match	gene: Zm00001d012607	Zm00001d012607_T00	425.95	540.20	coding	203	120	728
PB.2539.1	2	799	4	full-splice_match	gene: Zm00001d002259	Zm00001d002259_T00	73.26	95.61	coding	131	128	520
PB.2539.2	2	704	4	full-splice_match	gene: Zm00001d002259	Zm00001d002259_T00	22.35	95.61	coding	131	141	533
PB.5844.2	4	2602	14	novel_not_in_catalog	gene: Zm00001d053751	novel	6.91	9.03	coding	480	604	2043
PB.8176.1	6	902	4	incomplete-splice_match	gene: Zm00001d039090	Zm00001d039090_T00	288.00	288.00	coding	28	304	387

PB.3623.1	2	2031	3	full-splice_match	gene: Zm00001d007778	Zm00001d007778_T00	14.45	14.45	coding	568	115	1818
PB.4607.1	3	2637	5	novel_in_catalog	gene: Zm00001d043776	novel	7.41	26.89	coding	249	517	1263
PB.8176.4	6	1113	8	full-splice_match	gene: Zm00001d039091	Zm00001d039091_T00	620.37	620.37	coding	221	93	755
PB.7686.1	6	1062	1	incomplete-splice_match	gene: Zm00001d037203	Zm00001d037203_T00	1.46	1.46	coding	220	44	703
PB.408.1	1	724	2	full-splice_match	gene: Zm00001d028986	Zm00001d028986_T00	5.97	5.97	non coding	NA	NA	NA
PB.3824.1	3	361	2	incomplete-splice_match	gene: Zm00001d039870	Zm00001d039870_T00	91.76	91.76	non coding	NA	NA	NA
PB.3532.2	2	1243	10	full-splice_match	gene: Zm00001d007146	Zm00001d007146_T00	0.00	29.94	coding	306	119	1036
PB.7255.1	5	2660	1	antisense	novelGene_gene: Zm00001d018475	novel	6.09	6.09	coding	125	42	416
PB.4554.2	3	4653	20	full-splice_match	gene: Zm00001d043598	Zm00001d043598_T00	100.73	158.68	coding	1478	123	4556
PB.3984.1	3	1368	5	novel_in_catalog	gene: Zm00001d040652	novel	7.11	7.11	coding	370	99	1208
PB.5355.1	4	1052	9	novel_not_in_catalog	gene: Zm00001d051564	novel	30.53	30.53	coding	201	112	714
PB.9981.9	8	1733	5	full-splice_match	gene: Zm00001d012277	Zm00001d012277_T00	393.49	549.67	coding	376	162	1289
PB.1168.1	1	2222	17	full-splice_match	gene: Zm00001d032789	Zm00001d032789_T00	68.15	68.15	coding	576	175	1902
PB.10464.2	9	918	6	novel_in_catalog	gene: Zm00001d046592	novel	0.27	65.36	coding	148	63	506
PB.3965.2	3	2355	6	full-splice_match	gene: Zm00001d040536	Zm00001d040536_T00	9.88	13.09	coding	567	161	1861
PB.3965.3	3	2990	3	novel_in_catalog	gene: Zm00001d040536	novel	3.21	13.09	coding	398	214	1407
PB.1371.1	1	1287	1	incomplete-splice_match	gene: Zm00001d033704	Zm00001d033704_T00	30.70	30.70	coding	340	1	1020

PB.4542.1	3	1644	10	novel_not_in_catalog	gene: Zm00001d043548	novel	0.66	17.49	coding	193	16	594
PB.50.1	1	883	3	full-splice_match	gene: Zm00001d027442	Zm00001d027442_T00	17.28	17.28	coding	155	127	591
PB.3040.2	2	1303	10	full-splice_match	gene: Zm00001d004837	Zm00001d004837_T00	28.00	115.63	coding	276	127	954
PB.3040.3	2	1328	9	full-splice_match	gene: Zm00001d004837	Zm00001d004837_T00	74.95	115.63	coding	124	673	1044
PB.614.1	1	1135	1	antisense	novelGene_gene: Zm00001d030032	novel	87.39	87.39	coding	268	296	1099
PB.3736.1	3	2154	8	full-splice_match	gene: Zm00001d039453	Zm00001d039453_T00	48.77	66.86	coding	503	106	1614
PB.6116.1	5	1825	9	novel_in_catalog	gene: Zm00001d013335	novel	12.88	12.88	coding	479	64	1500
PB.4067.4	3	2808	2	novel_in_catalog	gene: Zm00001d041397	novel	3.46	14.14	coding	343	473	1501
PB.3736.4	3	1874	8	full-splice_match	gene: Zm00001d039453	Zm00001d039453_T00	18.08	66.86	coding	503	109	1617
PB.1947.1	10	341	1	novel_in_catalog	gene: Zm00001d024698	novel	26.27	46.03	non coding	NA	NA	NA
PB.5466.4	4	400	1	incomplete-splice_match	gene: Zm00001d052060	Zm00001d052060_T00	107.56	193.91	coding	100	43	342
PB.3523.1	2	1359	1	full-splice_match	gene: Zm00001d007097	Zm00001d007097_T00	20.68	191.58	coding	159	93	569
PB.5466.1	4	3045	3	full-splice_match	gene: Zm00001d052060	Zm00001d052060_T00	67.46	193.91	coding	855	200	2764
PB.5466.2	4	2927	3	full-splice_match	gene: Zm00001d052060	Zm00001d052060_T00	13.87	193.91	coding	855	131	2695
PB.5466.3	4	3437	2	novel_in_catalog	gene: Zm00001d052060	novel	5.02	193.91	coding	797	190	2580
PB.5810.6	4	806	5	antisense	novelGene_gene: Zm00001d053623	novel	24.22	73.94	non coding	NA	NA	NA
PB.6030.1	5	985	4	incomplete-splice_match	gene: Zm00001d013049	Zm00001d013049_T00	18.49	37.97	coding	213	74	712

PB.3468.3	2	1986	8	full-splice_match	gene: Zm00001d006884	Zm00001d006884_T00	8.26	21.39	coding	529	186	1772
PB.6030.4	5	2660	7	full-splice_match	gene: Zm00001d013049	Zm00001d013049_T00	19.48	37.97	coding	775	225	2549
PB.9969.3	8	821	1	novel_in_catalog	gene: Zm00001d012242	novel	30.58	51.26	coding	122	281	646
PB.650.6	1	559	1	novel_in_catalog	gene: Zm00001d030173	novel	0.00	59.17	non coding	NA	NA	NA
PB.10071.1	8	3237	10	full-splice_match	gene: Zm00001d012655	Zm00001d012655_T00	6.90	6.90	coding	868	354	2957
PB.8910.2	7	2160	6	full-splice_match	gene: Zm00001d021947	Zm00001d021947_T00	0.37	89.10	coding	613	72	1910
PB.919.2	1	1378	3	full-splice_match	gene: Zm00001d031740	Zm00001d031740_T00	30.32	67.76	coding	348	101	1144
PB.6420.2	5	3113	3	novel_in_catalog	gene: Zm00001d014698	novel	3.03	18.81	coding	206	1113	1730
PB.6420.1	5	1931	5	full-splice_match	gene: Zm00001d014698	Zm00001d014698_T00	7.09	18.81	coding	439	448	1764
PB.7879.2	6	505	3	full-splice_match	gene: Zm00001d038027	Zm00001d038027_T00	18.15	18.15	non coding	NA	NA	NA
PB.6420.6	5	1736	5	full-splice_match	gene: Zm00001d014698	Zm00001d014698_T00	2.74	18.81	coding	439	257	1573
PB.6420.4	5	2311	4	novel_in_catalog	gene: Zm00001d014698	novel	5.95	18.81	coding	206	316	933
PB.10196.1	9	620	2	full-splice_match	gene: Zm00001d045174	Zm00001d045174_T00	286.33	286.33	coding	62	220	405
PB.5488.1	4	823	1	full-splice_match	gene: Zm00001d052148	Zm00001d052148_T00	175.64	175.64	coding	117	184	534
PB.10867.8	9	3472	18	novel_in_catalog	gene: Zm00001d048358	novel	2.08	122.58	coding	510	1774	3303
PB.7859.1	6	2236	20	full-splice_match	gene: Zm00001d037961	Zm00001d037961_T01	195.12	195.12	coding	644	78	2009
PB.776.1	1	1439	8	novel_in_catalog	gene: Zm00001d031028	novel	28.44	28.44	coding	105	94	408

PB.10759.4	9	3236	17	novel_in_catalog	gene: Zm00001d047910	novel	11.26	148.90	coding	749	678	2924
PB.7751.2	6	2227	15	novel_in_catalog	gene: Zm00001d037484	novel	29.42	139.64	coding	371	138	1250
PB.7751.1	6	2269	16	full-splice_match	gene: Zm00001d037484	Zm00001d037484_T00	23.83	139.64	coding	551	273	1925
PB.8460.1	7	735	5	novel_not_in_catalog	gene: Zm00001d020038	novel	30.50	30.50	non coding	NA	NA	NA
PB.10759.1	9	2930	18	full-splice_match	gene: Zm00001d047910	Zm00001d047910_T00	137.64	148.90	coding	820	133	2592
PB.7751.5	6	2036	16	full-splice_match	gene: Zm00001d037484	Zm00001d037484_T00	86.39	139.64	coding	551	143	1795
PB.486.1	1	407	3	intergenic	novelGene_159	novel	20.22	20.22	non coding	NA	NA	NA
PB.1021.1	1	1793	1	genic	gene: Zm00001d032222	novel	10.33	10.33	coding	496	91	1578
PB.7751.8	6	1987	16	full-splice_match	gene: Zm00001d037484	Zm00001d037484_T00	0.00	139.64	coding	551	238	1890
PB.8630.1	7	902	1	antisense	novelGene_gene: Zm00001d020925	novel	20.75	20.75	non coding	NA	NA	NA
PB.4339.4	3	2450	1	genic	gene: Zm00001d042767	novel	0.00	255.64	coding	349	1345	2391
PB.6783.1	5	1000	1	full-splice_match	gene: Zm00001d016593	Zm00001d016593_T00	3.59	3.59	coding	130	187	576
PB.4339.1	3	1544	2	genic	gene: Zm00001d042767	novel	128.91	255.64	coding	403	143	1351
PB.78.2	1	950	2	full-splice_match	gene: Zm00001d027542	Zm00001d027542_T00	22.06	22.06	coding	224	93	764
PB.4339.3	3	1437	2	genic	gene: Zm00001d042767	novel	105.34	255.64	coding	403	98	1306
PB.2545.1	2	1082	2	incomplete-splice_match	gene: Zm00001d002285	Zm00001d002285_T00	12.45	23.34	coding	196	50	637
PB.6171.2	5	359	2	intergenic	novelGene_63	novel	20.40	87.53	non coding	NA	NA	NA

PB.8470.4	7	1925	13	novel_in_catalog	gene: Zm00001d020069	novel	6.46	205.57	coding	587	131	1891
PB.2545.2	2	1398	2	novel_in_catalog	gene: Zm00001d002285	novel	10.89	23.34	coding	196	579	1166
PB.6132.2	5	861	2	antisense	novelGene_gene: Zm00001d013393	novel	7.55	20.50	coding	145	158	592
PB.6132.1	5	987	2	antisense	novelGene_gene: Zm00001d013393	novel	12.95	20.50	coding	145	68	502
PB.8470.1	7	1984	13	novel_in_catalog	gene: Zm00001d020069	novel	142.58	205.57	coding	587	129	1889
PB.5119.3	4	1250	10	full-splice_match	gene: Zm00001d049982	Zm00001d049982_T00	4.25	220.75	coding	289	190	1056
PB.5119.2	4	1367	9	full-splice_match	gene: Zm00001d049982	Zm00001d049982_T00	174.34	220.75	coding	289	142	1008
PB.5119.1	4	1353	10	full-splice_match	gene: Zm00001d049982	Zm00001d049982_T00	4.11	220.75	coding	289	142	1008
PB.5119.6	4	1163	9	full-splice_match	gene: Zm00001d049982	Zm00001d049982_T00	0.00	220.75	coding	289	141	1007
PB.5119.5	4	1313	9	full-splice_match	gene: Zm00001d049982	Zm00001d049982_T00	38.06	220.75	coding	289	223	1089
PB.3784.1	3	1619	4	novel_not_in_catalog	gene: Zm00001d039637	novel	7.50	7.50	coding	499	81	1577
PB.4421.1	3	3125	2	incomplete-splice_match	gene: Zm00001d043129	Zm00001d043129_T00	31.80	31.80	coding	844	160	2691
PB.9461.1	8	821	4	full-splice_match	gene: Zm00001d009969	Zm00001d009969_T00	56.44	56.44	coding	179	74	610
PB.8197.1	6	1180	2	novel_not_in_catalog	gene: Zm00001d039169	novel	14.44	14.44	coding	304	83	994
PB.5480.1	4	2385	15	full-splice_match	gene: Zm00001d052098	Zm00001d052098_T00	29.41	29.41	coding	616	286	2133
PB.8133.1	6	739	1	full-splice_match	gene: Zm00001d038911	Zm00001d038911_T00	427.61	427.61	coding	101	117	419
PB.4190.1	3	2319	10	novel_in_catalog	gene: Zm00001d042052	novel	4.25	32.36	coding	368	192	1295

PB.6884.4	5	1288	8	full-splice_match	gene: Zm00001d017049	Zm00001d017049_T00	0.00	24.38	coding	322	168	1133
PB.4866.1	4	1761	1	full-splice_match	gene: Zm00001d048737	Zm00001d048737_T00	4.87	4.87	coding	515	68	1612
PB.4190.2	3	2116	11	full-splice_match	gene: Zm00001d042052	Zm00001d042052_T00	28.11	32.36	coding	565	193	1887
PB.6884.1	5	1530	8	full-splice_match	gene: Zm00001d017049	Zm00001d017049_T00	19.80	24.38	coding	322	168	1133
PB.6884.3	5	1392	8	full-splice_match	gene: Zm00001d017049	Zm00001d017049_T00	0.00	24.38	coding	322	156	1121
PB.6884.2	5	1459	8	full-splice_match	gene: Zm00001d017049	Zm00001d017049_T00	4.58	24.38	coding	266	131	928
PB.1192.1	1	1255	2	full-splice_match	gene: Zm00001d032883	Zm00001d032883_T00	9.99	63.83	coding	250	79	828
PB.1192.2	1	1121	2	full-splice_match	gene: Zm00001d032883	Zm00001d032883_T00	53.84	63.83	coding	250	79	828
PB.1192.3	1	1009	2	full-splice_match	gene: Zm00001d032883	Zm00001d032883_T00	0.00	63.83	coding	250	81	830
PB.5179.1	4	763	3	intergenic	novelGene_186	novel	29.37	29.37	non coding	NA	NA	NA
PB.10751.1	9	1156	4	full-splice_match	gene: Zm00001d047876	Zm00001d047876_T00	39.14	39.14	coding	225	42	716
PB.5920.1	4	2719	11	incomplete-splice_match	gene: Zm00001d054003	Zm00001d054003_T02	57.15	75.42	coding	513	607	2145
PB.6219.1	5	2159	3	full-splice_match	gene: Zm00001d013779	Zm00001d013779_T00	1.46	77.38	coding	144	108	539
PB.2245.2	10	723	1	novel_in_catalog	gene: Zm00001d026163	novel	0.00	2729.80	coding	122	124	489
PB.6219.2	5	981	4	full-splice_match	gene: Zm00001d013779	Zm00001d013779_T00	75.91	77.38	coding	144	108	539
PB.1799.1	10	2621	12	full-splice_match	gene: Zm00001d023749	Zm00001d023749_T00	97.88	101.33	coding	771	31	2343
PB.1799.3	10	3104	11	full-splice_match	gene: Zm00001d023749	Zm00001d023749_T00	2.13	101.33	coding	649	9	1955

PB.1799.2	10	2867	11	novel_in_catalog	gene: Zm00001d023749	novel	1.33	101.33	coding	625	710	2584
PB.4170.2	3	1265	2	full-splice_match	gene: Zm00001d041985	Zm00001d041985_T00	0.00	281.49	coding	348	82	1125
PB.4170.1	3	1372	2	full-splice_match	gene: Zm00001d041985	Zm00001d041985_T00	281.49	281.49	coding	348	82	1125
PB.8103.4	6	1144	3	novel_in_catalog	gene: Zm00001d038839	novel	3.61	485.82	coding	85	57	311
PB.7000.2	5	996	2	full-splice_match	gene: Zm00001d017503	Zm00001d017503_T00	5.25	8.01	coding	227	118	798
PB.1698.2	10	2224	3	incomplete-splice_match	gene: Zm00001d023267	Zm00001d023267_T00	169.53	169.53	coding	599	36	1832
PB.9580.1	8	2654	8	full-splice_match	gene: Zm00001d010578	Zm00001d010578_T00	18.56	18.56	coding	680	345	2384
PB.1698.4	10	2059	3	incomplete-splice_match	gene: Zm00001d023267	Zm00001d023267_T00	0.00	169.53	coding	567	28	1728
PB.3665.1	2	2459	13	full-splice_match	gene: Zm00001d007942	Zm00001d007942_T00	49.39	99.43	coding	652	246	2201
PB.965.1	1	1494	11	novel_not_in_catalog	gene: Zm00001d031970	novel	3.83	11.26	coding	179	251	787
PB.965.2	1	1833	10	novel_not_in_catalog	gene: Zm00001d031970	novel	5.27	11.26	coding	120	1400	1759
PB.965.3	1	1334	12	novel_not_in_catalog	gene: Zm00001d031970	novel	2.15	11.26	coding	179	252	788
PB.10304.2	9	1788	4	novel_not_in_catalog	gene: Zm00001d045576	novel	1.84	17.52	coding	305	405	1319
PB.10304.3	9	1692	5	novel_not_in_catalog	gene: Zm00001d045576	novel	0.00	17.52	coding	366	409	1506
PB.10304.1	9	1939	5	novel_not_in_catalog	gene: Zm00001d045576	novel	15.68	17.52	coding	366	526	1623
PB.4462.2	3	1444	6	full-splice_match	gene: Zm00001d043278	Zm00001d043278_T00	18.74	34.02	coding	352	153	1208
PB.420.2	1	2302	1	full-splice_match	gene: Zm00001d029028	Zm00001d029028_T00	38.16	826.32	coding	188	90	653

PB.2202.1	10	1326	2	genic	gene: Zm00001d025940	novel	9.39	9.39	coding	181	98	640
PB.420.5	1	2204	1	full-splice_match	gene: Zm00001d029028	Zm00001d029028_T00	0.00	826.32	coding	188	93	656
PB.420.6	1	1205	1	full-splice_match	gene: Zm00001d029028	Zm00001d029028_T00	43.47	826.32	coding	188	90	653
PB.420.7	1	1106	1	full-splice_match	gene: Zm00001d029028	Zm00001d029028_T00	0.00	826.32	coding	188	93	656
PB.420.8	1	956	1	full-splice_match	gene: Zm00001d029028	Zm00001d029028_T00	413.91	826.32	coding	188	93	656
PB.8628.2	7	1164	2	genic	gene: Zm00001d020903	novel	190.03	190.03	coding	265	127	921
PB.8829.2	7	1559	4	novel_in_catalog	gene: Zm00001d021701	novel	10.99	57.88	coding	179	84	620
PB.10866.2	9	1294	1	incomplete-splice_match	gene: Zm00001d048355	Zm00001d048355_T00	24.80	24.80	coding	323	61	1029
PB.5231.2	4	924	6	full-splice_match	gene: Zm00001d050823	Zm00001d050823_T00	20.35	51.36	coding	174	222	743
PB.8043.2	6	876	1	full-splice_match	gene: Zm00001d038581	Zm00001d038581_T00	6.42	8.95	coding	210	60	689
PB.3443.2	2	1346	5	incomplete-splice_match	gene: Zm00001d006762	Zm00001d006762_T00	12.63	50.70	coding	413	22	1260
PB.355.3	1	2100	6	full-splice_match	gene: Zm00001d028759	Zm00001d028759_T00	0.00	247.84	coding	606	147	1964
PB.355.2	1	2186	6	full-splice_match	gene: Zm00001d028759	Zm00001d028759_T00	208.84	247.84	coding	606	157	1974
PB.355.1	1	2375	6	full-splice_match	gene: Zm00001d028759	Zm00001d028759_T00	39.00	247.84	coding	606	157	1974
PB.5105.4	4	1528	4	full-splice_match	gene: Zm00001d049916	Zm00001d049916_T00	0.00	28.85	coding	392	166	1341
PB.4449.1	3	1626	2	genic	gene: Zm00001d043225	novel	25.38	25.38	coding	393	35	1213
PB.5105.1	4	1826	4	full-splice_match	gene: Zm00001d049916	Zm00001d049916_T00	6.38	28.85	coding	392	166	1341

PB.5105.2	4	1657	4	full-splice_match	gene: Zm00001d049916	Zm00001d049916_T00	22.47	28.85	coding	392	166	1341
PB.5030.1	4	2157	13	novel_in_catalog	gene: Zm00001d049522	novel	32.40	32.40	coding	567	162	1862
PB.5030.2	4	2124	13	novel_in_catalog	gene: Zm00001d049522	novel	0.00	32.40	coding	567	146	1846
PB.6392.6	5	1069	10	novel_in_catalog	gene: Zm00001d014572	novel	0.83	38.20	coding	186	410	967
PB.4850.1	4	1075	1	genic	gene: Zm00001d048651	novel	11.27	11.27	coding	309	50	976
PB.5193.1	4	1269	1	full-splice_match	gene: Zm00001d050433	Zm00001d050433_T00	19.90	19.90	coding	280	393	1232
PB.1149.1	1	2476	2	full-splice_match	gene: Zm00001d032707	Zm00001d032707_T00	4.19	4.19	coding	582	330	2075
PB.2787.1	2	3124	1	novel_in_catalog	gene: Zm00001d003334	novel	9.95	9.95	coding	179	2388	2924
PB.727.1	1	1761	9	novel_in_catalog	gene: Zm00001d030765	novel	100.36	100.36	coding	318	555	1508
PB.7034.1	5	1085	2	incomplete-splice_match	gene: Zm00001d017649	Zm00001d017649_T00	187.36	187.36	coding	262	1	786
PB.2245.1	10	840	1	novel_in_catalog	gene: Zm00001d026163	novel	298.39	2729.80	coding	122	124	489
PB.349.1	1	1444	5	full-splice_match	gene: Zm00001d028711	Zm00001d028711_T00	20.65	20.65	coding	376	79	1206
PB.5851.1	4	1466	11	novel_in_catalog	gene: Zm00001d053782	novel	12.94	12.94	coding	422	8	1273
PB.6853.2	5	834	4	incomplete-splice_match	gene: Zm00001d016892	Zm00001d016892_T00	58.71	168.96	coding	142	140	565
PB.1172.1	1	4471	25	full-splice_match	gene: Zm00001d032801	Zm00001d032801_T01	13.74	13.74	coding	1319	294	4250
PB.7747.1	6	2402	10	incomplete-splice_match	gene: Zm00001d037465	Zm00001d037465_T01	84.24	84.24	coding	614	183	2024
PB.8081.1	6	1647	1	full-splice_match	gene: Zm00001d038765	Zm00001d038765_T00	47.42	47.42	coding	478	48	1481

PB.8537.1	7	2084	10	novel_not_in_catalog	gene: Zm00001d020432	novel	36.13	55.70	coding	587	132	1892
PB.8537.2	7	3634	24	novel_not_in_catalog	gene: Zm00001d020432	novel	19.58	55.70	coding	1204	23	3634
PB.6797.2	5	1990	1	full-splice_match	gene: Zm00001d016669	Zm00001d016669_T00	33.81	50.22	coding	468	399	1802
PB.10773.5	9	2452	13	novel_in_catalog	gene: Zm00001d047960	novel	30.02	40.23	coding	737	16	2226
PB.1645.4	1	2775	20	full-splice_match	gene: Zm00001d034826	Zm00001d034826_T00	41.48	41.48	coding	753	104	2362
PB.4399.1	3	1143	2	incomplete-splice_match	gene: Zm00001d043052	Zm00001d043052_T00	32.27	32.27	non coding	NA	NA	NA
PB.10773.2	9	2804	13	full-splice_match	gene: Zm00001d047960	Zm00001d047960_T00	10.21	40.23	coding	451	1215	2567
PB.1935.1	10	692	4	full-splice_match	gene: Zm00001d024571	Zm00001d024571_T00	250.09	250.09	coding	119	179	535
PB.1949.1	10	737	3	incomplete-splice_match	gene: Zm00001d024702	Zm00001d024702_T00	37.12	37.12	coding	155	52	516
PB.2060.2	10	1564	9	full-splice_match	gene: Zm00001d025180	Zm00001d025180_T00	22.64	29.43	coding	440	114	1433
PB.9886.3	8	1771	3	full-splice_match	gene: Zm00001d011874	Zm00001d011874_T00	28.58	45.06	coding	416	219	1466
PB.5605.2	4	1842	2	novel_in_catalog	gene: Zm00001d052591	novel	1.65	29.18	coding	268	90	893
PB.8008.1	6	1600	11	incomplete-splice_match	gene: Zm00001d038467	Zm00001d038467_T00	34.85	34.85	coding	453	89	1447
PB.9595.2	8	1245	2	genic	gene: Zm00001d010635	novel	0.00	35.52	coding	312	78	1013
PB.9595.1	8	1351	2	genic	gene: Zm00001d010635	novel	35.52	35.52	coding	312	96	1031
PB.9934.1	8	1455	12	novel_not_in_catalog	gene: Zm00001d012085	novel	1.93	42.88	coding	294	144	1025
PB.8527.2	7	1317	4	full-splice_match	gene: Zm00001d020400	Zm00001d020400_T00	0.00	103.85	coding	365	65	1159

PB.8527.1	7	1440	4	full-splice_match	gene: Zm00001d020400	Zm00001d020400_T00	103.85	103.85	coding	365	62	1156
PB.8961.3	7	1925	15	full-splice_match	gene: Zm00001d022152	Zm00001d022152_T00	0.00	59.60	coding	541	104	1726
PB.9934.3	8	2618	13	incomplete-splice_match	gene: Zm00001d012085	Zm00001d012085_T00	16.50	42.88	coding	752	182	2437
PB.7101.3	5	1159	12	full-splice_match	gene: Zm00001d017937	Zm00001d017937_T00	13.68	24.03	coding	287	141	1001
PB.7101.1	5	1324	11	novel_in_catalog	gene: Zm00001d017937	novel	10.35	24.03	coding	287	205	1065
PB.3230.1	2	802	5	full-splice_match	gene: Zm00001d005867	Zm00001d005867_T00	9.69	9.69	coding	134	91	492
PB.9886.4	8	2025	1	genic	gene: Zm00001d011874	novel	5.41	45.06	coding	450	270	1619
PB.4110.1	3	694	1	incomplete-splice_match	gene: Zm00001d041678	Zm00001d041678_T00	8.59	8.59	coding	218	41	694
PB.5540.1	4	1140	6	full-splice_match	gene: Zm00001d052317	Zm00001d052317_T00	2.43	2.43	coding	253	86	844
PB.9886.5	8	1776	2	novel_in_catalog	gene: Zm00001d011874	novel	0.00	45.06	coding	416	123	1370
PB.1559.2	1	593	4	novel_not_in_catalog	gene: Zm00001d034521	novel	71.41	71.41	coding	110	89	418
PB.1677.1	1	946	6	full-splice_match	gene: Zm00001d034937	Zm00001d034937_T00	46.09	46.09	coding	249	128	874
PB.10330.3	9	1072	1	genic	gene: Zm00001d045724	novel	6.27	61.25	non coding	NA	NA	NA
PB.10330.2	9	564	5	novel_not_in_catalog	gene: Zm00001d045724	novel	13.92	61.25	coding	95	21	305
PB.10330.1	9	716	5	novel_not_in_catalog	gene: Zm00001d045724	novel	41.06	61.25	coding	95	72	356
PB.658.8	1	1022	4	novel_not_in_catalog	gene: Zm00001d030232	novel	10.12	82.18	coding	316	75	1022
PB.897.1	1	900	3	full-splice_match	gene: Zm00001d031636	Zm00001d031636_T00	2.90	14.53	coding	181	72	614

PB.2103.1	10	2769	2	novel_in_catalog	gene: Zm00001d025445	novel	3.18	105.64	coding	125	118	492
PB.9014.1	7	3146	7	full-splice_match	gene: Zm00001d022335	Zm00001d022335_T00	21.14	21.14	coding	928	240	3023
PB.6541.1	5	811	3	incomplete-splice_match	gene: Zm00001d015125	Zm00001d015125_T00	3.48	3.48	non coding	NA	NA	NA
PB.2374.3	10	2690	8	full-splice_match	gene: Zm00001d026585	Zm00001d026585_T00	21.17	79.99	coding	581	525	2267
PB.3220.2	2	1252	3	incomplete-splice_match	gene: Zm00001d005831	Zm00001d005831_T00	10.32	31.32	coding	328	45	1028
PB.8854.1	7	1667	8	novel_in_catalog	gene: Zm00001d021762	novel	11.02	11.02	coding	322	415	1380
PB.3220.1	2	2569	6	full-splice_match	gene: Zm00001d005831	Zm00001d005831_T00	21.00	31.32	coding	678	205	2238
PB.10091.2	8	804	1	novel_in_catalog	gene: Zm00001d012736	novel	10.65	21.22	coding	143	43	471
PB.10091.1	8	1140	1	novel_in_catalog	gene: Zm00001d012736	novel	10.56	21.22	coding	143	93	521
PB.1264.1	1	917	2	full-splice_match	gene: Zm00001d033232	Zm00001d033232_T00	30.53	30.53	coding	205	63	677
PB.967.1	1	516	2	full-splice_match	gene: Zm00001d031975	Zm00001d031975_T00	372.05	372.05	non coding	NA	NA	NA
PB.6440.1	5	1821	3	novel_not_in_catalog	gene: Zm00001d014765	novel	23.82	27.32	coding	308	72	995
PB.8679.1	7	2313	8	full-splice_match	gene: Zm00001d021119	Zm00001d021119_T00	3.03	157.85	coding	571	74	1786
PB.6440.2	5	1195	3	novel_not_in_catalog	gene: Zm00001d014765	novel	3.50	27.32	coding	308	10	933
PB.7145.3	5	614	3	novel_not_in_catalog	gene: Zm00001d018088	novel	9.86	23.22	non coding	NA	NA	NA
PB.8874.2	7	806	5	full-splice_match	gene: Zm00001d021825	Zm00001d021825_T00	6.80	6.80	coding	185	73	627
PB.10384.1	9	1268	1	antisense	novelGene_gene: Zm00001d046148	novel	62.43	62.43	non coding	NA	NA	NA

PB.7099.8	5	3588	5	antisense	novelGene_gene: Zm00001d017932	novel	2.00	98.88	non coding	NA	NA	NA
PB.5701.1	4	1235	1	incomplete-splice_match	gene: Zm00001d053049	Zm00001d053049_T00	16.43	30.01	coding	226	227	904
PB.10117.1	9	767	1	full-splice_match	gene: Zm00001d044769	Zm00001d044769_T00	118.10	118.10	non coding	NA	NA	NA
PB.9828.1	8	1448	9	full-splice_match	gene: Zm00001d011668	Zm00001d011668_T00	26.85	26.85	coding	336	116	1123
PB.8555.4	7	1551	2	incomplete-splice_match	gene: Zm00001d020532	Zm00001d020532_T00	0.00	13.42	coding	397	361	1551
PB.2998.2	2	1355	9	full-splice_match	gene: Zm00001d004554	Zm00001d004554_T00	3.58	3.95	coding	235	504	1208
PB.2998.1	2	1358	9	full-splice_match	gene: Zm00001d004554	Zm00001d004554_T00	0.37	3.95	coding	235	368	1072
PB.4830.2	3	1427	5	full-splice_match	gene: Zm00001d044699	Zm00001d044699_T00	37.82	79.62	coding	350	158	1207
PB.8905.3	7	1605	4	novel_in_catalog	gene: Zm00001d021930	novel	82.62	155.11	coding	304	400	1311
PB.4830.1	3	1536	5	full-splice_match	gene: Zm00001d044699	Zm00001d044699_T00	41.80	79.62	coding	350	196	1245
PB.5090.3	4	852	7	full-splice_match	gene: Zm00001d049815	Zm00001d049815_T00	45.77	274.60	coding	171	130	642
PB.2278.3	10	1693	15	full-splice_match	gene: Zm00001d026287	Zm00001d026287_T00	0.00	83.17	coding	493	51	1529
PB.5090.1	4	919	7	full-splice_match	gene: Zm00001d049815	Zm00001d049815_T00	228.84	274.60	coding	171	171	683
PB.2278.1	10	1970	15	full-splice_match	gene: Zm00001d026287	Zm00001d026287_T00	83.17	83.17	coding	493	279	1757
PB.2657.2	2	1502	9	full-splice_match	gene: Zm00001d002759	Zm00001d002759_T00	24.69	38.12	coding	371	114	1226
PB.2657.1	2	1138	8	incomplete-splice_match	gene: Zm00001d002759	Zm00001d002759_T00	13.43	38.12	coding	192	173	748
PB.1324.1	1	3789	1	full-splice_match	gene: Zm00001d033505	Zm00001d033505_T00	9.01	10.18	coding	1044	27	3158

PB.4868.1	4	989	1	full-splice_match	gene: Zm00001d048760	Zm00001d048760_T00	25.14	25.14	coding	238	42	755
PB.8905.1	7	3048	3	novel_in_catalog	gene: Zm00001d021930	novel	0.00	155.11	coding	104	2397	2708
PB.6955.4	5	1127	7	full-splice_match	gene: Zm00001d017303	Zm00001d017303_T04	9.85	39.33	coding	370	18	1127
PB.3465.1	2	1208	5	full-splice_match	gene: Zm00001d006871	Zm00001d006871_T00	170.36	170.36	coding	307	12	932
PB.9034.3	7	1138	1	full-splice_match	gene: Zm00001d022403	Zm00001d022403_T00	55.36	62.43	coding	203	148	756
PB.9034.5	7	754	1	full-splice_match	gene: Zm00001d022403	Zm00001d022403_T00	0.00	62.43	coding	203	68	676
PB.9034.4	7	998	1	full-splice_match	gene: Zm00001d022403	Zm00001d022403_T00	7.07	62.43	coding	203	142	750
PB.6667.1	5	809	1	full-splice_match	gene: Zm00001d015917	Zm00001d015917_T00	247.23	247.23	coding	142	82	507
PB.8592.1	7	1259	1	full-splice_match	gene: Zm00001d020707	Zm00001d020707_T00	28.33	28.33	coding	315	84	1028
PB.8880.2	7	2242	7	novel_in_catalog	gene: Zm00001d021835	novel	2.77	125.51	coding	460	153	1532
PB.2284.1	10	1785	5	incomplete-splice_match	gene: Zm00001d026301	Zm00001d026301_T00	14.71	18.86	coding	443	60	1388
PB.4500.3	3	1266	4	full-splice_match	gene: Zm00001d043420	Zm00001d043420_T00	7.24	8.82	coding	278	133	966
PB.3135.1	2	1270	4	full-splice_match	gene: Zm00001d005359	Zm00001d005359_T00	9.17	9.17	coding	322	47	1012
PB.4500.1	3	1445	3	novel_in_catalog	gene: Zm00001d043420	novel	1.58	8.82	coding	160	129	608
PB.9197.1	8	1544	6	novel_not_in_catalog	gene: Zm00001d008498	novel	7.27	33.31	coding	49	156	302
PB.4701.10	3	1767	4	novel_not_in_catalog	gene: Zm00001d044146	novel	0.00	90.95	coding	350	673	1722
PB.4926.1	4	1519	5	novel_not_in_catalog	gene: Zm00001d049043	novel	25.63	92.77	coding	315	281	1225

PB.5427.1	4	2664	2	full-splice_match	gene: Zm00001d051889	Zm00001d051889_T00	7.31	11.25	coding	646	3	1940
PB.5427.3	4	2745	2	full-splice_match	gene: Zm00001d051889	Zm00001d051889_T00	0.00	11.25	coding	754	91	2352
PB.6164.1	5	1215	4	full-splice_match	gene: Zm00001d013523	Zm00001d013523_T00	59.71	59.71	coding	322	56	1021
PB.4560.1	3	1865	1	genic	gene: Zm00001d043611	novel	5.05	31.25	coding	132	1241	1636
PB.4560.2	3	1691	2	novel_not_in_catalog	gene: Zm00001d043611	novel	3.34	31.25	coding	318	512	1465
PB.5064.1	4	1674	2	full-splice_match	gene: Zm00001d049690	Zm00001d049690_T00	38.41	38.41	coding	516	23	1570
PB.4560.4	3	1570	2	novel_in_catalog	gene: Zm00001d043611	novel	2.08	31.25	coding	132	949	1344
PB.76.1	1	1143	3	full-splice_match	gene: Zm00001d027540	Zm00001d027540_T00	90.04	90.04	coding	225	127	801
PB.7014.1	5	1904	7	full-splice_match	gene: Zm00001d017559	Zm00001d017559_T00	34.13	34.13	coding	500	71	1570
PB.3553.1	2	3905	2	novel_in_catalog	gene: Zm00001d007241	novel	21.66	26.17	coding	1165	220	3714
PB.2797.1	2	2227	1	full-splice_match	gene: Zm00001d003397	Zm00001d003397_T00	2.48	2.48	coding	612	10	1845
PB.4806.1	3	1080	5	full-splice_match	gene: Zm00001d044575	Zm00001d044575_T00	107.14	133.28	coding	245	112	846
PB.6717.1	5	1931	7	full-splice_match	gene: Zm00001d016228	Zm00001d016228_T00	15.56	23.41	coding	501	108	1610
PB.3928.1	3	1365	6	full-splice_match	gene: Zm00001d040334	Zm00001d040334_T00	73.39	187.42	coding	307	70	990
PB.4031.1	3	1239	5	novel_not_in_catalog	gene: Zm00001d041127	novel	20.54	20.54	coding	157	387	857
PB.781.5	1	1831	1	novel_in_catalog	gene: Zm00001d031049	novel	22.06	124.30	coding	99	1382	1678
PB.4522.9	3	812	2	incomplete-splice_match	gene: Zm00001d043484	Zm00001d043484_T02	14.25	51.49	non coding	NA	NA	NA

PB.1011.1	1	1121	6	incomplete-splice_match	gene: Zm00001d032184	Zm00001d032184_T00	35.97	35.97	coding	248	20	763
PB.4547.1	3	1750	1	antisense	novelGene_gene: Zm00001d043569	novel	48.51	48.51	coding	148	69	512
PB.9811.1	8	914	1	full-splice_match	gene: Zm00001d011594	Zm00001d011594_T00	2.58	2.58	coding	167	246	746
PB.1031.1	1	1658	5	full-splice_match	gene: Zm00001d032272	Zm00001d032272_T00	10.68	13.63	coding	440	132	1451
PB.9338.4	8	2181	5	novel_in_catalog	gene: Zm00001d009271	novel	0.00	83.06	coding	482	140	1585
PB.98.1	1	951	5	full-splice_match	gene: Zm00001d027648	Zm00001d027648_T00	69.38	69.38	coding	208	112	735
PB.9338.2	8	2227	5	novel_in_catalog	gene: Zm00001d009271	novel	3.03	83.06	coding	482	85	1530
PB.9338.3	8	2058	6	full-splice_match	gene: Zm00001d009271	Zm00001d009271_T00	58.29	83.06	coding	561	140	1822
PB.9338.1	8	2114	6	full-splice_match	gene: Zm00001d009271	Zm00001d009271_T00	21.74	83.06	coding	561	142	1824
PB.3109.6	2	1714	8	novel_in_catalog	gene: Zm00001d005244	novel	0.57	28.08	coding	405	166	1380
PB.490.9	1	1830	5	full-splice_match	gene: Zm00001d029387	Zm00001d029387_T00	934.93	1055.64	coding	354	91	1152
PB.4144.1	3	656	4	incomplete-splice_match	gene: Zm00001d041855	Zm00001d041855_T00	36.52	36.52	coding	125	39	413
PB.3109.2	2	1609	9	full-splice_match	gene: Zm00001d005244	Zm00001d005244_T00	18.50	28.08	coding	371	161	1273
PB.3109.3	2	1596	9	full-splice_match	gene: Zm00001d005244	Zm00001d005244_T00	9.02	28.08	coding	368	156	1259
PB.3392.1	2	410	2	incomplete-splice_match	gene: Zm00001d006562	Zm00001d006562_T00	48.65	48.65	non coding	NA	NA	NA
PB.8648.1	7	2555	3	incomplete-splice_match	gene: Zm00001d020972	Zm00001d020972_T00	4.60	43.24	coding	638	347	2260
PB.8648.3	7	2359	4	incomplete-splice_match	gene: Zm00001d020972	Zm00001d020972_T00	7.51	43.24	coding	709	42	2168

PB.8648.2	7	2459	4	incomplete-splice_match	gene: Zm00001d020972	Zm00001d020972_T00	31.13	43.24	coding	709	42	2168
PB.2647.1	2	1328	4	incomplete-splice_match	gene: Zm00001d002705	Zm00001d002705_T00	27.59	27.59	coding	273	222	1040
PB.3129.1	2	2528	5	novel_in_catalog	gene: Zm00001d005338	novel	49.44	72.24	coding	463	194	1582
PB.10819.19	9	2141	10	full-splice_match	gene: Zm00001d048171	Zm00001d048171_T00	12.85	25.07	coding	426	418	1695
PB.3129.3	2	3148	4	novel_in_catalog	gene: Zm00001d005338	novel	8.71	72.24	coding	360	168	1247
PB.9400.2	8	1522	7	novel_in_catalog	gene: Zm00001d009640	novel	26.67	94.64	coding	340	251	1270
PB.3129.6	2	2392	5	novel_in_catalog	gene: Zm00001d005338	novel	0.00	72.24	coding	463	173	1561
PB.10819.13	9	1116	6	full-splice_match	gene: Zm00001d048158	Zm00001d048158_T00	158.37	158.37	coding	260	67	846
PB.10819.12	9	1086	6	full-splice_match	gene: Zm00001d048157	Zm00001d048157_T00	223.77	234.17	coding	260	63	842
PB.10819.11	9	337	1	genic	gene: Zm00001d048157	novel	10.40	234.17	non coding	NA	NA	NA
PB.4953.1	4	1364	1	full-splice_match	gene: Zm00001d049162	Zm00001d049162_T00	3.39	3.39	coding	327	143	1123
PB.10819.17	9	665	2	full-splice_match	gene: Zm00001d048163	Zm00001d048163_T00	67.01	113.26	coding	131	83	475
PB.10819.16	9	778	2	full-splice_match	gene: Zm00001d048163	Zm00001d048163_T00	46.25	113.26	coding	131	83	475
PB.10819.15	9	1004	6	full-splice_match	gene: Zm00001d048159	Zm00001d048159_T00	157.29	206.65	coding	260	79	858
PB.10819.14	9	1106	6	full-splice_match	gene: Zm00001d048159	Zm00001d048159_T00	49.35	206.65	coding	260	76	855
PB.90.2	1	1821	2	full-splice_match	gene: Zm00001d027596	Zm00001d027596_T00	53.82	129.99	coding	497	12	1502
PB.90.3	1	2893	2	full-splice_match	gene: Zm00001d027596	Zm00001d027596_T00	49.91	129.99	coding	552	730	2385

PB.90.1	1	3169	3	full-splice_match	gene: Zm00001d027596	Zm00001d027596_T00	2.22	129.99	coding	537	1101	2711
PB.90.6	1	3599	3	full-splice_match	gene: Zm00001d027596	Zm00001d027596_T00	0.78	129.99	coding	580	1663	3402
PB.5892.3	4	1253	11	full-splice_match	gene: Zm00001d053927	Zm00001d053927_T01	4.74	46.47	coding	211	374	1006
PB.90.4	1	4687	1	genic	gene: Zm00001d027596	novel	7.85	129.99	coding	552	2548	4203
PB.90.5	1	3305	3	full-splice_match	gene: Zm00001d027596	Zm00001d027596_T00	15.42	129.99	coding	537	1356	2966
PB.9203.1	8	2230	10	full-splice_match	gene: Zm00001d008531	Zm00001d008531_T00	44.63	89.44	coding	580	73	1812
PB.7784.1	6	728	4	novel_not_in_catalog	gene: Zm00001d037633	novel	4.43	119.13	non coding	NA	NA	NA
PB.9203.3	8	2912	9	full-splice_match	gene: Zm00001d008531	Zm00001d008531_T00	4.30	89.44	coding	349	1469	2515
PB.9203.2	8	2600	9	full-splice_match	gene: Zm00001d008531	Zm00001d008531_T00	17.38	89.44	coding	421	942	2204
PB.9203.7	8	2177	10	full-splice_match	gene: Zm00001d008531	Zm00001d008531_T00	23.13	89.44	coding	580	87	1826
PB.3517.3	2	2045	7	novel_not_in_catalog	gene: Zm00001d007073	novel	35.61	108.09	coding	524	36	1607
PB.3517.2	2	2787	8	novel_not_in_catalog	gene: Zm00001d007073	novel	6.52	108.09	coding	524	103	1674
PB.1684.2	10	2813	4	incomplete-splice_match	gene: Zm00001d023222	Zm00001d023222_T01	28.31	44.14	coding	800	191	2590
PB.3517.5	2	2084	7	novel_not_in_catalog	gene: Zm00001d007073	novel	30.94	108.09	coding	524	209	1780
PB.1684.1	10	3602	7	full-splice_match	gene: Zm00001d023222	Zm00001d023222_T01	15.83	44.14	coding	1051	201	3353
PB.8696.2	7	625	1	intergenic	novelGene_289	novel	87.21	91.34	coding	2	404	409
PB.4548.3	3	1942	7	full-splice_match	gene: Zm00001d043573	Zm00001d043573_T00	24.32	48.90	coding	490	177	1646

PB.7832.2	6	1623	5	novel_in_catalog	gene: Zm00001d037864	novel	9.41	21.42	coding	215	182	826
PB.7801.2	6	1665	1	full-splice_match	gene: Zm00001d037711	Zm00001d037711_T00	0.00	18.80	coding	412	245	1480
PB.10485.1	9	1170	1	full-splice_match	gene: Zm00001d046745	Zm00001d046745_T00	37.33	37.33	coding	316	103	1050
PB.3944.1	3	2608	21	full-splice_match	gene: Zm00001d040416	Zm00001d040416_T00	13.86	13.86	coding	726	99	2276
PB.7801.1	6	1993	1	full-splice_match	gene: Zm00001d037711	Zm00001d037711_T00	18.80	18.80	coding	412	249	1484
PB.7669.4	6	2344	5	novel_in_catalog	gene: Zm00001d037109	novel	0.00	112.60	coding	375	344	1468
PB.4425.1	3	1175	11	full-splice_match	gene: Zm00001d043137	Zm00001d043137_T00	183.10	183.10	coding	249	250	996
PB.10598.3	9	933	5	full-splice_match	gene: Zm00001d047354	Zm00001d047354_T00	66.06	123.74	coding	160	73	552
PB.4225.3	3	1225	5	novel_in_catalog	gene: Zm00001d042275	novel	5.09	47.92	coding	164	210	701
PB.10598.1	9	1270	1	genic	gene: Zm00001d047354	novel	0.81	123.74	coding	100	971	1270
PB.4225.1	3	1446	5	novel_in_catalog	gene: Zm00001d042275	novel	8.32	47.92	coding	148	248	691
PB.5889.1	4	1278	6	full-splice_match	gene: Zm00001d053923	Zm00001d053923_T00	91.26	91.26	coding	232	261	956
PB.4225.4	3	526	3	novel_in_catalog	gene: Zm00001d042275	novel	25.03	47.92	coding	109	39	365
PB.10598.4	9	788	5	full-splice_match	gene: Zm00001d047354	Zm00001d047354_T00	56.86	123.74	coding	160	64	543
PB.1523.5	1	1567	4	full-splice_match	gene: Zm00001d034400	Zm00001d034400_T00	57.27	99.36	coding	428	152	1435
PB.1523.6	1	1293	4	full-splice_match	gene: Zm00001d034400	Zm00001d034400_T00	0.00	99.36	coding	380	152	1291
PB.2191.1	10	1241	2	full-splice_match	gene: Zm00001d025901	Zm00001d025901_T00	26.55	40.40	coding	223	193	861

PB.6203.1	5	1346	5	novel_in_catalog	gene: Zm00001d013705	novel	5.31	5.31	coding	255	256	1020
PB.1523.2	1	2098	4	full-splice_match	gene: Zm00001d034400	Zm00001d034400_T00	13.15	99.36	coding	428	144	1427
PB.1523.3	1	1873	5	novel_not_in_catalog	gene: Zm00001d034400	novel	20.69	99.36	coding	427	168	1448
PB.7723.1	6	1335	3	novel_not_in_catalog	gene: Zm00001d037342	novel	17.90	17.90	coding	324	20	991
PB.7713.4	6	2324	11	full-splice_match	gene: Zm00001d037291	Zm00001d037291_T00	0.00	49.71	coding	606	186	2003
PB.10038.4	8	1189	6	full-splice_match	gene: Zm00001d012537	Zm00001d012537_T00	9.82	505.76	coding	181	376	918
PB.4364.6	3	2884	3	incomplete-splice_match	gene: Zm00001d042869	Zm00001d042869_T00	10.53	492.67	coding	149	1360	1806
PB.10038.6	8	859	7	full-splice_match	gene: Zm00001d012537	Zm00001d012537_T00	103.85	505.76	coding	181	151	693
PB.2575.2	2	2841	3	intergenic	novelGene_51	novel	29.32	64.33	non coding	NA	NA	NA
PB.1881.3	10	2051	1	novel_in_catalog	gene: Zm00001d024294	novel	5.87	261.74	coding	205	1262	1876
PB.2156.4	10	2779	9	full-splice_match	gene: Zm00001d025739	Zm00001d025739_T01	5.74	63.34	coding	606	548	2365
PB.314.2	1	902	5	full-splice_match	gene: Zm00001d028575	Zm00001d028575_T00	0.00	68.28	coding	273	17	835
PB.8656.1	7	1587	2	full-splice_match	gene: Zm00001d021016	Zm00001d021016_T00	144.40	144.40	coding	403	183	1391
PB.8654.6	7	1963	3	full-splice_match	gene: Zm00001d021000	Zm00001d021000_T06	36.10	167.68	coding	35	379	483
PB.314.1	1	1108	5	full-splice_match	gene: Zm00001d028575	Zm00001d028575_T00	68.28	68.28	coding	273	94	912
PB.523.6	1	1217	4	full-splice_match	gene: Zm00001d029539	Zm00001d029539_T00	207.98	293.59	coding	190	149	718
PB.523.7	1	1318	3	full-splice_match	gene: Zm00001d029539	Zm00001d029539_T00	46.01	293.59	coding	118	152	505

PB.523.4	1	764	1	genic	gene: Zm00001d029539	novel	7.12	293.59	non coding	NA	NA	NA
PB.523.5	1	869	2	incomplete-splice_match	gene: Zm00001d029539	Zm00001d029539_T00	19.72	293.59	non coding	NA	NA	NA
PB.523.2	1	1633	4	full-splice_match	gene: Zm00001d029539	Zm00001d029539_T00	4.69	293.59	coding	190	150	719
PB.523.3	1	1706	3	full-splice_match	gene: Zm00001d029539	Zm00001d029539_T00	0.00	293.59	coding	118	138	491
PB.3474.3	2	1452	3	full-splice_match	gene: Zm00001d006910	Zm00001d006910_T00	2.78	103.45	coding	243	120	848
PB.523.1	1	1217	3	novel_not_in_catalog	gene: Zm00001d029539	novel	3.27	293.59	non coding	NA	NA	NA
PB.9480.1	8	1656	1	full-splice_match	gene: Zm00001d010079	Zm00001d010079_T00	8.18	8.18	coding	498	44	1537
PB.4637.1	3	1530	9	full-splice_match	gene: Zm00001d043872	Zm00001d043872_T00	35.87	38.76	coding	342	152	1177
PB.523.8	1	1475	1	novel_in_catalog	gene: Zm00001d029539	novel	4.80	293.59	coding	118	655	1008
PB.697.1	1	2511	16	full-splice_match	gene: Zm00001d030551	Zm00001d030551_T00	55.28	57.05	coding	720	168	2327
PB.10011.1	8	886	6	full-splice_match	gene: Zm00001d012425	Zm00001d012425_T00	82.61	82.61	coding	161	98	580
PB.5815.5	4	1563	3	incomplete-splice_match	gene: Zm00001d053634	Zm00001d053634_T00	6.67	47.31	coding	88	32	295
PB.9755.1	8	2601	15	incomplete-splice_match	gene: Zm00001d011347	Zm00001d011347_T00	40.42	55.52	coding	705	192	2306
PB.2625.1	2	1878	10	full-splice_match	gene: Zm00001d002615	Zm00001d002615_T00	25.74	25.74	coding	469	221	1627
PB.2337.2	10	2263	11	full-splice_match	gene: Zm00001d026487	Zm00001d026487_T00	25.61	34.14	coding	579	101	1837
PB.2337.3	10	386	1	incomplete-splice_match	gene: Zm00001d026487	Zm00001d026487_T00	0.00	34.14	non coding	NA	NA	NA
PB.3507.4	2	1735	5	novel_not_in_catalog	gene: Zm00001d007050	novel	0.00	84.89	coding	451	83	1435

PB.3507.5	2	2016	3	novel_not_in_catalog	gene: Zm00001d007050	novel	0.00	84.89	coding	451	75	1427
PB.3113.5	2	2534	17	fusion	gene: Zm00001d005256	novel	5.34	45.21	coding	695	248	2332
PB.3507.1	2	2162	5	novel_not_in_catalog	gene: Zm00001d007050	novel	49.76	84.89	coding	340	98	1117
PB.3507.2	2	1873	5	novel_not_in_catalog	gene: Zm00001d007050	novel	0.00	84.89	coding	340	83	1102
PB.941.1	1	756	5	full-splice_match	gene: Zm00001d031856	Zm00001d031856_T00	51.50	51.50	coding	167	69	569
PB.7094.4	5	1701	11	full-splice_match	gene: Zm00001d017908	Zm00001d017908_T00	4.00	15.70	coding	429	137	1423
PB.6073.8	5	1099	2	novel_not_in_catalog	gene: Zm00001d013231	novel	0.32	70.85	coding	340	80	1099
PB.7094.1	5	1212	8	incomplete-splice_match	gene: Zm00001d017908	Zm00001d017908_T00	11.00	15.70	coding	188	261	824
PB.5767.3	4	1621	2	novel_in_catalog	gene: Zm00001d053396	novel	8.02	106.06	coding	191	150	722
PB.10491.9	9	1519	9	novel_not_in_catalog	gene: Zm00001d046801	novel	0.00	68.20	coding	497	29	1519
PB.10491.8	9	1713	10	novel_not_in_catalog	gene: Zm00001d046801	novel	0.00	68.20	coding	533	47	1645
PB.6073.3	5	1838	2	genic	gene: Zm00001d013230	novel	17.98	19.18	coding	521	74	1636
PB.10491.6	9	2171	7	novel_in_catalog	gene: Zm00001d046801	novel	2.74	68.20	coding	444	26	1357
PB.6073.1	5	2185	2	genic	gene: Zm00001d013230	novel	1.20	19.18	coding	521	72	1634
PB.10491.4	9	2022	9	novel_not_in_catalog	gene: Zm00001d046801	novel	7.71	68.20	coding	539	60	1676
PB.6073.7	5	1740	2	novel_not_in_catalog	gene: Zm00001d013231	novel	14.65	70.85	coding	513	74	1612
PB.6073.6	5	1941	1	novel_in_catalog	gene: Zm00001d013231	novel	4.01	70.85	coding	339	76	1092

PB.3932.2	3	1192	7	full-splice_match	gene: Zm00001d040357	Zm00001d040357_T00	39.73	39.73	coding	254	120	881
PB.6073.4	5	1719	2	genic	gene: Zm00001d013230	novel	0.00	19.18	coding	521	68	1630
PB.1766.3	10	1684	1	full-splice_match	gene: Zm00001d023565	Zm00001d023565_T00	14.30	39.69	coding	405	268	1482
PB.1766.2	10	1310	1	full-splice_match	gene: Zm00001d023565	Zm00001d023565_T00	0.00	39.69	coding	284	86	937
PB.1766.1	10	986	1	full-splice_match	gene: Zm00001d023565	Zm00001d023565_T00	25.39	39.69	coding	126	90	467
PB.1091.2	1	1168	3	full-splice_match	gene: Zm00001d032508	Zm00001d032508_T00	20.97	56.26	coding	249	136	882
PB.198.2	1	1356	2	full-splice_match	gene: Zm00001d028094	Zm00001d028094_T00	60.43	104.68	coding	307	205	1125
PB.9340.1	8	1940	14	full-splice_match	gene: Zm00001d009324	Zm00001d009324_T00	25.39	25.39	coding	500	188	1687
PB.1091.1	1	1195	3	full-splice_match	gene: Zm00001d032508	Zm00001d032508_T00	35.30	56.26	coding	249	67	813
PB.6993.4	5	1136	4	novel_in_catalog	gene: Zm00001d017482	novel	9.93	85.85	coding	294	53	934
PB.1332.1	1	3371	12	fusion	gene: Zm00001d033532	novel	28.67	28.67	coding	722	713	2878
PB.6993.1	5	1345	5	full-splice_match	gene: Zm00001d017482	Zm00001d017482_T00	49.61	85.85	coding	303	82	990
PB.6993.2	5	1298	5	full-splice_match	gene: Zm00001d017482	Zm00001d017482_T00	14.01	85.85	coding	303	124	1032
PB.6993.3	5	1243	4	novel_in_catalog	gene: Zm00001d017482	novel	12.30	85.85	coding	294	5	886
PB.6669.1	5	1310	4	full-splice_match	gene: Zm00001d015921	Zm00001d015921_T00	3.87	45.00	coding	239	110	826
PB.6669.2	5	1183	4	full-splice_match	gene: Zm00001d015921	Zm00001d015921_T00	6.72	45.00	coding	239	198	914
PB.6669.3	5	1069	4	full-splice_match	gene: Zm00001d015921	Zm00001d015921_T00	34.41	45.00	coding	239	109	825

PB.2659.1	2	3008	2	genic	gene: Zm00001d002787	novel	8.98	8.98	coding	886	188	2845
PB.9113.1	7	2414	1	full-splice_match	gene: Zm00001d022647	Zm00001d022647_T00	22.51	22.51	coding	642	159	2084
PB.10271.1	9	1047	1	incomplete-splice_match	gene: Zm00001d045477	Zm00001d045477_T00	11.57	11.57	coding	115	331	675
PB.4364.5	3	1577	1	incomplete-splice_match	gene: Zm00001d042869	Zm00001d042869_T00	26.49	492.67	non coding	NA	NA	NA
PB.2485.1	2	2771	10	novel_in_catalog	gene: Zm00001d002012	novel	2.94	19.30	coding	534	679	2280
PB.2485.2	2	2168	11	full-splice_match	gene: Zm00001d002012	Zm00001d002012_T00	16.36	19.30	coding	578	75	1808
PB.10022.1	8	2481	7	novel_in_catalog	gene: Zm00001d012452	novel	2.15	23.78	coding	272	349	1164
PB.10022.3	8	2381	8	novel_in_catalog	gene: Zm00001d012452	novel	0.00	23.78	coding	388	111	1274
PB.10022.2	8	2675	8	novel_in_catalog	gene: Zm00001d012452	novel	21.18	23.78	coding	724	163	2334
PB.2758.1	2	1578	8	full-splice_match	gene: Zm00001d003200	Zm00001d003200_T00	90.18	90.18	coding	346	219	1256
PB.6707.1	5	878	2	incomplete-splice_match	gene: Zm00001d016175	Zm00001d016175_T00	15.15	15.15	coding	255	29	793
PB.8696.1	7	727	1	intergenic	novelGene_289	novel	4.13	91.34	coding	2	361	366
PB.2758.2	2	1419	8	full-splice_match	gene: Zm00001d003200	Zm00001d003200_T00	0.00	90.18	coding	346	219	1256
PB.1758.1	10	1858	11	incomplete-splice_match	gene: Zm00001d023538	Zm00001d023538_T00	25.20	37.09	coding	451	348	1700
PB.1758.2	10	1977	11	incomplete-splice_match	gene: Zm00001d023538	Zm00001d023538_T00	11.89	37.09	coding	451	339	1691
PB.2735.3	2	2582	5	novel_not_in_catalog	gene: Zm00001d003110	novel	11.72	60.58	coding	153	1585	2043
PB.1366.4	1	4534	15	full-splice_match	gene: Zm00001d033657	Zm00001d033657_T00	16.13	43.19	coding	135	3563	3967

PB.2706.1	2	1172	1	full-splice_match	gene: Zm00001d002969	Zm00001d002969_T00	22.39	55.65	coding	259	51	827
PB.2706.2	2	1059	1	full-splice_match	gene: Zm00001d002969	Zm00001d002969_T00	33.25	55.65	coding	259	75	851
PB.2760.3	2	738	3	novel_not_in_catalog	gene: Zm00001d003207	novel	12.83	121.31	non coding	NA	NA	NA
PB.10163.3	9	509	1	full-splice_match	gene: Zm00001d045022	Zm00001d045022_T00	20.73	40.19	coding	116	12	359
PB.10163.2	9	859	1	full-splice_match	gene: Zm00001d045022	Zm00001d045022_T00	19.47	40.19	coding	116	136	483
PB.6983.3	5	2515	6	novel_in_catalog	gene: Zm00001d017445	novel	3.03	73.20	coding	202	353	958
PB.6983.2	5	1592	9	novel_in_catalog	gene: Zm00001d017445	novel	49.51	73.20	coding	266	367	1164
PB.6983.1	5	1647	9	novel_in_catalog	gene: Zm00001d017445	novel	20.66	73.20	coding	266	226	1023
PB.6823.3	5	1768	10	full-splice_match	gene: Zm00001d016770	Zm00001d016770_T00	20.70	44.81	coding	393	266	1444
PB.3072.1	2	763	1	full-splice_match	gene: Zm00001d005055	Zm00001d005055_T00	0.40	0.40	coding	166	36	533
PB.154.1	1	895	1	full-splice_match	gene: Zm00001d027894	Zm00001d027894_T00	75.49	120.09	coding	129	235	621
PB.154.2	1	778	1	full-splice_match	gene: Zm00001d027894	Zm00001d027894_T00	44.60	120.09	coding	129	235	621
PB.626.3	1	1287	1	full-splice_match	gene: Zm00001d030098	Zm00001d030098_T00	19.73	41.27	coding	308	91	1014
PB.790.2	1	1571	2	genic	gene: Zm00001d031073	novel	0.00	15.54	coding	405	357	1571
PB.6326.2	5	1244	1	full-splice_match	gene: Zm00001d014285	Zm00001d014285_T00	42.37	42.76	coding	283	75	923
PB.6326.1	5	1379	1	full-splice_match	gene: Zm00001d014285	Zm00001d014285_T00	0.40	42.76	coding	283	75	923
PB.8674.1	7	2013	5	full-splice_match	gene: Zm00001d021065	Zm00001d021065_T00	42.13	42.13	coding	443	359	1687

PB.6326.4	5	1142	1	full-splice_match	gene: Zm00001d014285	Zm00001d014285_T00	0.00	42.76	coding	283	75	923
PB.5916.1	4	317	1	incomplete-splice_match	gene: Zm00001d053996	Zm00001d053996_T00	162.03	162.03	non coding	NA	NA	NA
PB.6823.7	5	2187	8	incomplete-splice_match	gene: Zm00001d016770	Zm00001d016770_T00	0.00	44.81	coding	363	221	1309
PB.10279.2	9	2155	3	full-splice_match	gene: Zm00001d045492	Zm00001d045492_T00	14.34	24.53	coding	536	126	1733
PB.10279.1	9	2199	2	full-splice_match	gene: Zm00001d045492	Zm00001d045492_T00	10.19	24.53	coding	536	16	1623
PB.6267.2	5	931	5	novel_in_catalog	gene: Zm00001d014025	novel	13.22	48.21	coding	161	263	745
PB.9336.1	8	2392	10	incomplete-splice_match	gene: Zm00001d009248	Zm00001d009248_T01	7.71	22.04	coding	623	105	1973
PB.9336.2	8	3328	15	full-splice_match	gene: Zm00001d009248	Zm00001d009248_T00	14.32	22.04	coding	897	335	3025
PB.1547.1	1	778	1	full-splice_match	gene: Zm00001d034495	Zm00001d034495_T00	21.93	21.93	coding	162	17	502
PB.1570.1	1	2821	12	full-splice_match	gene: Zm00001d034577	Zm00001d034577_T00	4.79	4.79	coding	793	83	2461
PB.9948.2	8	1935	6	novel_not_in_catalog	gene: Zm00001d012144	novel	12.30	26.51	coding	555	117	1781
PB.5773.1	4	1451	7	novel_not_in_catalog	gene: Zm00001d027011	novel	5.10	20.70	non coding	NA	NA	NA
PB.1750.1	10	1972	5	full-splice_match	gene: Zm00001d023523	Zm00001d023523_T00	131.28	140.26	coding	436	234	1541
PB.5773.3	4	1446	7	novel_not_in_catalog	gene: Zm00001d027011	novel	9.91	20.70	non coding	NA	NA	NA
PB.1750.3	10	1759	5	full-splice_match	gene: Zm00001d023523	Zm00001d023523_T00	0.00	140.26	coding	436	125	1432
PB.2156.3	10	2576	7	incomplete-splice_match	gene: Zm00001d025739	Zm00001d025739_T02	25.28	63.34	coding	606	262	2079
PB.7768.3	6	3345	27	full-splice_match	gene: Zm00001d037557	Zm00001d037557_T06	0.00	60.48	coding	1027	144	3224

PB.1366.2	1	2675	10	incomplete-splice_match	gene: Zm00001d033657	Zm00001d033657_T03	7.31	43.19	coding	135	1670	2074
PB.6689.2	5	2562	6	incomplete-splice_match	gene: Zm00001d016070	Zm00001d016070_T00	15.78	56.48	coding	678	345	2378
PB.7768.2	6	3277	24	incomplete-splice_match	gene: Zm00001d037557	Zm00001d037557_T01	5.77	60.48	coding	390	509	1678
PB.1627.4	1	1089	1	genic	gene: Zm00001d034758	novel	1.14	108.87	non coding	NA	NA	NA
PB.4372.4	3	2059	6	full-splice_match	gene: Zm00001d042901	Zm00001d042901_T00	0.00	60.63	coding	582	65	1810
PB.4372.3	3	2136	5	novel_in_catalog	gene: Zm00001d042901	novel	1.13	60.63	coding	555	65	1729
PB.7768.1	6	3626	27	full-splice_match	gene: Zm00001d037557	Zm00001d037557_T01	51.88	60.48	coding	1027	170	3250
PB.4372.1	3	2267	6	full-splice_match	gene: Zm00001d042901	Zm00001d042901_T00	14.77	60.63	coding	582	65	1810
PB.3586.1	2	1044	3	incomplete-splice_match	gene: Zm00001d007522	Zm00001d007522_T02	254.33	254.33	coding	123	208	576
PB.5696.1	4	2678	10	full-splice_match	gene: Zm00001d053022	Zm00001d053022_T00	52.60	96.59	coding	612	379	2214
PB.1770.1	10	1550	6	novel_in_catalog	gene: Zm00001d023579	novel	72.12	72.12	non coding	NA	NA	NA
PB.2762.2	2	1445	4	novel_in_catalog	gene: Zm00001d003212	novel	6.05	25.08	coding	314	109	1050
PB.2762.3	2	1543	3	novel_in_catalog	gene: Zm00001d003212	novel	8.04	25.08	coding	206	108	725
PB.819.2	1	3939	5	full-splice_match	gene: Zm00001d031205	Zm00001d031205_T00	3.27	259.84	coding	105	99	413
PB.2762.1	2	1738	7	full-splice_match	gene: Zm00001d003212	Zm00001d003212_T00	11.00	25.08	coding	374	268	1389
PB.2341.1	10	1881	1	novel_in_catalog	gene: Zm00001d026495	novel	23.54	23.54	coding	561	79	1761
PB.9398.2	8	2440	20	novel_in_catalog	gene: Zm00001d009631	novel	37.27	58.67	coding	609	205	2031

PB.9398.3	8	2467	19	novel_in_catalog	gene: Zm00001d009631	novel	7.95	58.67	coding	469	133	1539
PB.5459.2	4	2862	21	full-splice_match	gene: Zm00001d052050	Zm00001d052050_T00	64.21	75.60	coding	775	67	2391
PB.1366.1	1	710	1	incomplete-splice_match	gene: Zm00001d033657	Zm00001d033657_T00	6.70	43.19	non coding	NA	NA	NA
PB.5459.1	4	2765	20	novel_in_catalog	gene: Zm00001d052050	novel	5.56	75.60	coding	442	943	2268
PB.5552.2	4	1848	1	incomplete-splice_match	gene: Zm00001d052361	Zm00001d052361_T00	0.00	11.77	coding	598	55	1848
PB.5459.4	4	2617	20	novel_in_catalog	gene: Zm00001d052050	novel	0.00	75.60	coding	442	949	2274
PB.4270.1	3	2947	2	full-splice_match	gene: Zm00001d042461	Zm00001d042461_T00	5.26	70.70	coding	582	58	1803
PB.7916.35	6	1891	6	novel_in_catalog	gene: Zm00001d038146	novel	0.00	442.81	coding	189	189	755
PB.8735.1	7	2988	7	full-splice_match	gene: Zm00001d021347	Zm00001d021347_T00	43.18	43.18	coding	925	90	2864
PB.3871.1	3	2916	9	full-splice_match	gene: Zm00001d040090	Zm00001d040090_T00	15.04	15.04	coding	803	202	2610
PB.9642.1	8	1190	7	full-splice_match	gene: Zm00001d010806	Zm00001d010806_T00	121.82	121.82	coding	273	156	974
PB.2144.1	10	1750	4	full-splice_match	gene: Zm00001d025689	Zm00001d025689_T00	35.77	35.77	coding	458	165	1538
PB.10515.1	9	1416	6	full-splice_match	gene: Zm00001d046937	Zm00001d046937_T00	14.89	38.48	coding	282	78	923
PB.3519.1	2	1328	8	novel_in_catalog	gene: Zm00001d007080	novel	35.15	35.15	coding	322	199	1164
PB.5316.9	4	967	2	full-splice_match	gene: Zm00001d051362	Zm00001d051362_T00	3339.72	3339.72	coding	249	99	845
PB.10936.2	9	1183	9	novel_in_catalog	gene: Zm00001d048562	novel	4.71	21.96	coding	225	40	714
PB.10936.1	9	1513	10	novel_in_catalog	gene: Zm00001d048562	novel	17.25	21.96	coding	336	54	1061

PB.5972.1	5	656	2	incomplete-splice_match	gene: Zm00001d012856	Zm00001d012856_T00	29.37	77.54	coding	23	280	348
PB.9699.1	8	776	3	full-splice_match	gene: Zm00001d011081	Zm00001d011081_T00	15.88	342.12	coding	133	39	437
PB.3418.1	2	2537	6	novel_in_catalog	gene: Zm00001d006644	novel	77.18	77.18	coding	510	827	2356
PB.37.2	1	970	4	full-splice_match	gene: Zm00001d027385	Zm00001d027385_T00	20.85	39.99	coding	238	85	798
PB.37.1	1	1062	4	full-splice_match	gene: Zm00001d027385	Zm00001d027385_T00	19.14	39.99	coding	238	85	798
PB.8010.8	6	1231	3	intergenic	novelGene_316	novel	10.28	39.85	non coding	NA	NA	NA
PB.9699.2	8	666	3	full-splice_match	gene: Zm00001d011081	Zm00001d011081_T00	322.95	342.12	coding	133	39	437
PB.684.1	1	1356	7	fusion	gene: Zm00001d030411	novel	28.40	42.29	coding	250	206	955
PB.2813.1	2	2457	4	novel_in_catalog	gene: Zm00001d003464	novel	38.47	38.47	coding	113	1840	2178
PB.8010.5	6	3096	7	antisense	novelGene_gene: Zm00001d038477	novel	5.84	46.22	coding	11	406	438
PB.4698.23	3	1194	5	novel_in_catalog	gene: Zm00001d044138	novel	12.57	1495.64	coding	43	579	707
PB.4698.22	3	1991	6	novel_not_in_catalog	gene: Zm00001d044138	novel	2.30	1495.64	coding	250	25	774
PB.7288.4	5	1394	1	full-splice_match	gene: Zm00001d018567	Zm00001d018567_T00	0.00	45.31	coding	405	26	1240
PB.4698.24	3	3310	3	novel_in_catalog	gene: Zm00001d044138	novel	0.00	1495.64	coding	250	21	770
PB.8010.2	6	2311	8	antisense	novelGene_gene: Zm00001d038477	novel	14.05	46.22	coding	113	1136	1474
PB.8010.3	6	3181	10	antisense	novelGene_gene: Zm00001d038477	novel	9.74	46.22	non coding	NA	NA	NA
PB.3865.1	3	876	1	full-splice_match	gene: Zm00001d040057	Zm00001d040057_T00	11.34	11.34	coding	202	2	607

PB.8911.1	7	1557	1	antisense	novelGene_gene: Zm00001d021949	novel	28.40	28.40	non coding	NA	NA	NA
PB.9634.1	8	1138	5	incomplete-splice_match	gene: Zm00001d010782	Zm00001d010782_T01	15.51	15.51	coding	291	6	878
PB.3739.1	3	2297	3	full-splice_match	gene: Zm00001d039467	Zm00001d039467_T00	11.08	11.08	coding	497	517	2007
PB.7148.2	5	1532	2	novel_in_catalog	gene: Zm00001d018097	novel	2.22	49.75	coding	204	673	1284
PB.9769.2	8	2605	3	novel_in_catalog	gene: Zm00001d011393	novel	60.24	164.93	coding	634	359	2260
PB.3739.2	3	1507	3	full-splice_match	gene: Zm00001d039467	Zm00001d039467_T00	0.00	11.08	coding	424	139	1410
PB.6057.1	5	1812	1	full-splice_match	gene: Zm00001d013163	Zm00001d013163_T00	11.68	59.43	coding	437	136	1446
PB.5857.5	4	1360	10	novel_in_catalog	gene: Zm00001d053816	novel	16.45	31.66	coding	241	530	1252
PB.6057.3	5	1593	1	full-splice_match	gene: Zm00001d013163	Zm00001d013163_T00	0.00	59.43	coding	437	132	1442
PB.6057.2	5	1713	1	full-splice_match	gene: Zm00001d013163	Zm00001d013163_T00	47.75	59.43	coding	437	136	1446
PB.7892.3	6	1367	3	full-splice_match	gene: Zm00001d038065	Zm00001d038065_T00	9.71	61.97	coding	178	82	615
PB.7892.2	6	1292	4	full-splice_match	gene: Zm00001d038065	Zm00001d038065_T00	50.03	61.97	coding	324	84	1055
PB.7892.1	6	2566	4	full-splice_match	gene: Zm00001d038065	Zm00001d038065_T00	2.22	61.97	coding	324	84	1055
PB.9240.1	8	2685	11	full-splice_match	gene: Zm00001d008724	Zm00001d008724_T00	22.30	22.30	coding	251	1601	2353
PB.4705.1	3	3056	11	full-splice_match	gene: Zm00001d044164	Zm00001d044164_T00	32.95	63.26	coding	845	83	2617
PB.7892.4	6	1153	4	full-splice_match	gene: Zm00001d038065	Zm00001d038065_T00	0.00	61.97	coding	324	84	1055
PB.4299.2	3	1487	2	genic	gene: Zm00001d042613	novel	1.05	9.53	coding	173	588	1106

PB.2330.1	10	1840	5	full-splice_match	gene: Zm00001d026458	Zm00001d026458_T00	1048.43	1064.10	coding	469	75	1481
PB.2107.1	10	1567	4	full-splice_match	gene: Zm00001d025460	Zm00001d025460_T00	29.71	29.71	coding	314	270	1211
PB.1647.1	1	2658	18	full-splice_match	gene: Zm00001d034832	Zm00001d034832_T00	7.95	7.95	coding	746	45	2282
PB.10179.4	9	1416	8	full-splice_match	gene: Zm00001d045076	Zm00001d045076_T00	18.51	107.23	coding	184	136	687
PB.1510.7	1	1454	1	incomplete-splice_match	gene: Zm00001d034344	Zm00001d034344_T00	0.00	59.69	coding	450	105	1454
PB.10359.2	9	2019	10	full-splice_match	gene: Zm00001d045938	Zm00001d045938_T00	22.34	22.34	coding	560	87	1766
PB.10179.7	9	997	6	full-splice_match	gene: Zm00001d045076	Zm00001d045076_T00	51.07	107.23	coding	184	136	687
PB.6732.1	5	1597	1	full-splice_match	gene: Zm00001d016306	Zm00001d016306_T00	7.63	7.63	coding	444	141	1472
PB.2867.1	2	856	3	full-splice_match	gene: Zm00001d003756	Zm00001d003756_T00	11.08	11.08	coding	194	90	671
PB.7252.1	5	1238	8	incomplete-splice_match	gene: Zm00001d018468	Zm00001d018468_T00	166.17	166.17	coding	255	227	991
PB.2087.1	10	1273	4	full-splice_match	gene: Zm00001d025340	Zm00001d025340_T00	13.24	13.24	coding	316	62	1009
PB.8792.1	7	1818	5	full-splice_match	gene: Zm00001d021569	Zm00001d021569_T00	11.20	11.20	coding	483	193	1641
PB.3257.5	2	2291	6	novel_in_catalog	gene: Zm00001d006001	novel	6.90	43.84	coding	332	282	1277
PB.8792.3	7	1706	5	full-splice_match	gene: Zm00001d021569	Zm00001d021569_T00	0.00	11.20	coding	483	193	1641
PB.8374.4	7	1218	1	intergenic	novelGene_126	novel	24.91	24.91	coding	132	370	765
PB.10706.1	9	1259	2	genic	gene: Zm00001d047745	novel	32.72	60.38	coding	258	91	864
PB.10706.2	9	1061	2	genic	gene: Zm00001d047745	novel	27.66	60.38	coding	258	13	786

PB.5663.4	4	2286	3	full-splice_match	gene: Zm00001d052855	Zm00001d052855_T00	0.00	384.89	coding	699	98	2194
PB.6836.2	5	2155	6	novel_not_in_catalog	gene: Zm00001d016827	novel	12.30	30.41	coding	246	52	789
PB.6836.3	5	3712	5	full-splice_match	gene: Zm00001d016827	Zm00001d016827_T00	14.64	30.41	coding	213	2507	3145
PB.1258.1	1	2162	5	novel_in_catalog	gene: Zm00001d033215	novel	22.06	22.06	coding	167	1161	1661
PB.6836.1	5	975	2	novel_not_in_catalog	gene: Zm00001d016827	novel	3.47	30.41	non coding	NA	NA	NA
PB.6481.1	5	1742	9	incomplete-splice_match	gene: Zm00001d014895	Zm00001d014895_T00	35.35	95.11	coding	433	155	1453
PB.6481.2	5	2479	10	full-splice_match	gene: Zm00001d014895	Zm00001d014895_T00	14.77	95.11	coding	703	212	2320
PB.2431.1	2	732	1	incomplete-splice_match	gene: Zm00001d001806	Zm00001d001806_T00	3.04	3.04	non coding	NA	NA	NA
PB.3447.5	2	935	2	full-splice_match	gene: Zm00001d006789	Zm00001d006789_T04	27.44	27.44	coding	244	204	935
PB.53.1	1	1874	14	full-splice_match	gene: Zm00001d027455	Zm00001d027455_T00	23.03	46.99	coding	445	148	1482
PB.745.1	1	1384	5	novel_not_in_catalog	gene: Zm00001d030867	novel	1.65	6.63	coding	100	277	576
PB.7071.2	5	1347	2	full-splice_match	gene: Zm00001d017823	Zm00001d017823_T00	53.94	64.23	coding	331	108	1100
PB.53.2	1	1934	13	novel_in_catalog	gene: Zm00001d027455	novel	5.94	46.99	coding	424	148	1419
PB.53.5	1	1705	14	full-splice_match	gene: Zm00001d027455	Zm00001d027455_T00	17.60	46.99	coding	445	149	1483
PB.53.4	1	1785	13	novel_in_catalog	gene: Zm00001d027455	novel	0.00	46.99	coding	424	147	1418
PB.53.7	1	1549	14	novel_not_in_catalog	gene: Zm00001d027455	novel	0.19	46.99	coding	307	146	1066
PB.53.6	1	1892	13	novel_in_catalog	gene: Zm00001d027455	novel	0.24	46.99	coding	415	146	1390

PB.3422.2	2	911	4	full-splice_match	gene: Zm00001d006670	Zm00001d006670_T00	13.32	92.90	coding	121	214	576
PB.53.8	1	1658	13	novel_in_catalog	gene: Zm00001d027455	novel	0.00	46.99	coding	424	148	1419
PB.8894.2	7	1449	4	novel_in_catalog	gene: Zm00001d021891	novel	14.77	467.58	coding	183	692	1240
PB.3422.1	2	987	4	full-splice_match	gene: Zm00001d006670	Zm00001d006670_T00	14.84	92.90	coding	121	162	524
PB.3008.2	2	1306	8	novel_not_in_catalog	gene: Zm00001d004644	novel	0.00	20.49	coding	348	151	1194
PB.3008.3	2	516	4	incomplete-splice_match	gene: Zm00001d004644	Zm00001d004644_T00	7.32	20.49	coding	136	107	514
PB.166.1	1	2624	14	novel_in_catalog	gene: Zm00001d027936	novel	45.91	45.91	coding	388	1238	2401
PB.3008.1	2	1539	8	novel_not_in_catalog	gene: Zm00001d004644	novel	13.17	20.49	coding	408	72	1295
PB.1656.1	1	1829	12	full-splice_match	gene: Zm00001d034856	Zm00001d034856_T00	79.72	106.99	coding	452	44	1399
PB.3188.2	2	1815	6	full-splice_match	gene: Zm00001d005682	Zm00001d005682_T00	9.28	65.78	coding	377	462	1592
PB.2199.1	10	1193	5	full-splice_match	gene: Zm00001d025922	Zm00001d025922_T00	43.14	45.44	coding	311	37	969
PB.9660.1	8	1057	1	genic	gene: Zm00001d010884	novel	2.21	2.21	coding	106	217	534
PB.1229.2	1	671	3	full-splice_match	gene: Zm00001d033054	Zm00001d033054_T00	32.40	152.11	coding	119	139	495
PB.1161.2	1	2228	4	full-splice_match	gene: Zm00001d032754	Zm00001d032754_T00	9.76	9.76	coding	528	368	1951
PB.10510.1	9	805	1	full-splice_match	gene: Zm00001d046927	Zm00001d046927_T00	35.36	35.36	coding	184	123	674
PB.9569.1	8	1890	1	antisense	novelGene_gene: Zm00001d010527	novel	13.53	13.53	coding	121	1005	1367
PB.5801.1	4	1593	10	full-splice_match	gene: Zm00001d053591	Zm00001d053591_T00	17.31	17.31	coding	439	141	1457

PB.6817.4	5	1159	1	full-splice_match	gene: Zm00001d016748	Zm00001d016748_T00	6.40	17.52	coding	287	239	1099
PB.4406.3	3	2208	11	full-splice_match	gene: Zm00001d043083	Zm00001d043083_T00	77.74	100.25	coding	432	364	1659
PB.4406.2	3	3109	10	full-splice_match	gene: Zm00001d043083	Zm00001d043083_T00	14.24	100.25	coding	432	1253	2548
PB.1319.1	1	2171	1	full-splice_match	gene: Zm00001d033482	Zm00001d033482_T00	16.27	16.27	coding	551	215	1867
PB.1319.2	1	1955	1	full-splice_match	gene: Zm00001d033482	Zm00001d033482_T00	0.00	16.27	coding	551	121	1773
PB.7341.1	6	2610	5	full-splice_match	gene: Zm00001d035199	Zm00001d035199_T00	17.91	17.91	coding	657	228	2198
PB.9261.2	8	1794	3	full-splice_match	gene: Zm00001d008842	Zm00001d008842_T00	7.99	7.99	coding	467	212	1612
PB.10191.1	9	575	2	full-splice_match	gene: Zm00001d045152	Zm00001d045152_T00	544.87	544.87	non coding	NA	NA	NA
PB.5664.1	4	651	1	intergenic	novelGene_397	novel	1.61	1.61	non coding	NA	NA	NA
PB.7152.1	5	1220	1	genic	gene: Zm00001d018106	novel	48.08	48.08	coding	330	93	1082
PB.4604.1	3	1906	11	full-splice_match	gene: Zm00001d043768	Zm00001d043768_T00	22.94	22.94	coding	391	220	1392
PB.10530.3	9	1346	6	novel_not_in_catalog	gene: Zm00001d046997	novel	13.99	13.99	coding	304	43	954
PB.5821.3	4	2649	9	novel_in_catalog	gene: Zm00001d053648	novel	1.55	21.53	coding	298	1521	2414
PB.1128.1	1	4239	23	full-splice_match	gene: Zm00001d032637	Zm00001d032637_T01	64.21	64.21	coding	1191	389	3961
PB.5821.1	4	1338	11	full-splice_match	gene: Zm00001d053648	Zm00001d053648_T00	15.69	21.53	coding	327	90	1070
PB.7208.1	5	3198	3	full-splice_match	gene: Zm00001d018335	Zm00001d018335_T00	9.69	9.69	coding	953	52	2910
PB.3353.1	2	2094	12	full-splice_match	gene: Zm00001d006373	Zm00001d006373_T00	39.76	44.31	coding	565	204	1898

PB.1627.2	1	1748	12	novel_in_catalog	gene: Zm00001d034758	novel	13.82	108.87	coding	330	514	1503
PB.3353.3	2	1983	13	novel_not_in_catalog	gene: Zm00001d006373	novel	2.16	44.31	coding	476	200	1627
PB.3353.2	2	3260	11	novel_in_catalog	gene: Zm00001d006373	novel	2.39	44.31	coding	363	224	1312
PB.864.3	1	1010	1	incomplete-splice_match	gene: Zm00001d031454	Zm00001d031454_T00	12.36	54.12	coding	247	76	816
PB.9000.1	7	1422	2	intergenic	novelGene_356	novel	38.99	38.99	coding	352	91	1146
PB.10345.1	9	1104	6	full-splice_match	gene: Zm00001d045819	Zm00001d045819_T00	34.66	34.66	coding	244	73	804
PB.5612.2	4	1250	5	incomplete-splice_match	gene: Zm00001d052653	Zm00001d052653_T00	453.63	453.63	coding	339	40	1056
PB.1305.2	1	1720	1	genic	gene: Zm00001d033402	novel	0.00	103.87	coding	80	119	358
PB.8773.2	7	1183	6	incomplete-splice_match	gene: Zm00001d021498	Zm00001d021498_T02	28.43	66.97	coding	294	46	927
PB.5673.2	4	1429	7	novel_not_in_catalog	gene: Zm00001d052911	novel	11.57	11.57	coding	356	57	1124
PB.1877.1	10	3334	7	full-splice_match	gene: Zm00001d024270	Zm00001d024270_T00	23.05	23.05	coding	441	284	1606
PB.1305.4	1	639	1	novel_in_catalog	gene: Zm00001d033402	novel	0.00	103.87	coding	80	119	358
PB.4556.16	3	2162	18	novel_in_catalog	gene: Zm00001d043601	novel	9.62	284.99	coding	326	1006	1983
PB.263.2	1	1976	15	full-splice_match	gene: Zm00001d028372	Zm00001d028372_T01	13.02	25.19	coding	381	395	1537
PB.7939.5	6	1963	14	full-splice_match	gene: Zm00001d038209	Zm00001d038209_T00	0.00	79.55	coding	507	92	1612
PB.10730.4	9	1687	8	incomplete-splice_match	gene: Zm00001d047808	Zm00001d047808_T01	8.82	97.88	coding	498	88	1581
PB.10730.3	9	2569	10	full-splice_match	gene: Zm00001d047808	Zm00001d047808_T00	16.07	97.88	coding	680	169	2208

PB.2419.2	2	1144	1	novel_in_catalog	gene: Zm00001d001769	novel	11.11	11.11	coding	130	486	875
PB.10730.1	9	3239	10	full-splice_match	gene: Zm00001d047808	Zm00001d047808_T00	70.85	97.88	coding	680	361	2400
PB.10801.1	9	1500	9	full-splice_match	gene: Zm00001d048091	Zm00001d048091_T00	179.80	481.19	coding	330	92	1081
PB.10801.2	9	2032	8	novel_in_catalog	gene: Zm00001d048091	novel	6.22	481.19	coding	190	90	659
PB.8773.1	7	460	2	incomplete-splice_match	gene: Zm00001d021498	Zm00001d021498_T00	16.64	66.97	non coding	NA	NA	NA
PB.10801.4	9	1386	9	full-splice_match	gene: Zm00001d048091	Zm00001d048091_T00	295.17	481.19	coding	330	83	1072
PB.5326.2	4	1405	1	genic	gene: Zm00001d051420	novel	12.73	277.20	coding	198	465	1058
PB.5326.1	4	1385	2	full-splice_match	gene: Zm00001d051420	Zm00001d051420_T00	7.10	277.20	coding	289	77	943
PB.8070.2	6	1423	1	full-splice_match	gene: Zm00001d038717	Zm00001d038717_T00	62.83	76.71	coding	320	119	1078
PB.8070.3	6	614	1	full-splice_match	gene: Zm00001d038717	Zm00001d038717_T00	13.41	76.71	coding	105	123	437
PB.11018.2	B73V4	386	2	novel_not_in_catalog	gene: Zm00001d000345	novel	0.00	135.40	non coding	NA	NA	NA
PB.21.2	1	962	1	novel_in_catalog	gene: Zm00001d027318	novel	1.78	294.54	non coding	NA	NA	NA
PB.7349.1	6	2210	6	full-splice_match	gene: Zm00001d035256	Zm00001d035256_T00	25.07	25.07	coding	594	94	1875
PB.21.1	1	898	6	full-splice_match	gene: Zm00001d027318	Zm00001d027318_T00	109.58	294.54	coding	169	104	610
PB.5122.1	4	2103	4	full-splice_match	gene: Zm00001d050023	Zm00001d050023_T00	36.95	36.95	coding	566	83	1780
PB.6041.1	5	2492	17	full-splice_match	gene: Zm00001d013075	Zm00001d013075_T00	45.84	45.84	coding	612	262	2097
PB.6041.2	5	2353	17	full-splice_match	gene: Zm00001d013075	Zm00001d013075_T00	0.00	45.84	coding	612	233	2068

PB.5122.2	4	494	1	incomplete-splice_match	gene: Zm00001d050023	Zm00001d050023_T00	0.00	36.95	coding	18	328	381
PB.201.1	1	1908	3	full-splice_match	gene: Zm00001d028111	Zm00001d028111_T00	27.73	27.73	coding	467	271	1671
PB.3327.1	2	1883	2	full-splice_match	gene: Zm00001d006254	Zm00001d006254_T00	12.18	12.18	coding	463	313	1701
PB.10538.1	9	2000	1	full-splice_match	gene: Zm00001d047039	Zm00001d047039_T00	42.60	42.60	coding	525	180	1754
PB.2504.1	2	2299	13	full-splice_match	gene: Zm00001d002090	Zm00001d002090_T00	5.86	5.86	coding	532	376	1971
PB.2504.2	2	2034	13	full-splice_match	gene: Zm00001d002090	Zm00001d002090_T00	0.00	5.86	coding	576	162	1889
PB.2852.3	2	1374	2	full-splice_match	gene: Zm00001d003669	Zm00001d003669_T00	61.98	109.10	coding	336	105	1112
PB.5829.3	4	1788	5	novel_in_catalog	gene: Zm00001d053687	novel	1.51	27.83	coding	439	100	1416
PB.10861.3	9	2871	6	novel_in_catalog	gene: Zm00001d048343	novel	5.99	51.96	coding	448	191	1534
PB.4422.1	3	1257	2	incomplete-splice_match	gene: Zm00001d043131	Zm00001d043131_T00	10.56	10.56	coding	315	22	966
PB.10861.1	9	2176	8	full-splice_match	gene: Zm00001d048343	Zm00001d048343_T00	2.18	51.96	coding	533	4	1602
PB.6342.1	5	1307	8	novel_not_in_catalog	gene: Zm00001d014330	novel	4.20	90.41	coding	339	165	1181
PB.6342.3	5	1457	7	full-splice_match	gene: Zm00001d014330	Zm00001d014330_T00	40.97	90.41	coding	339	200	1216
PB.6342.4	5	1667	6	novel_in_catalog	gene: Zm00001d014330	novel	8.53	90.41	coding	234	295	996
PB.5086.1	4	2418	1	intergenic	novelGene_140	novel	1.79	1.79	non coding	NA	NA	NA
PB.8822.1	7	1641	6	full-splice_match	gene: Zm00001d021682	Zm00001d021682_T00	6.15	6.15	coding	420	1	1260
PB.7815.1	6	1854	9	full-splice_match	gene: Zm00001d037771	Zm00001d037771_T00	15.18	24.41	coding	438	79	1392

PB.10653.3	9	2823	9	full-splice_match	gene: Zm00001d047539	Zm00001d047539_T00	18.07	36.82	coding	684	299	2350
PB.10574.1	9	691	1	full-splice_match	gene: Zm00001d047231	Zm00001d047231_T00	60.49	60.49	coding	117	123	473
PB.7767.2	6	1485	7	full-splice_match	gene: Zm00001d037553	Zm00001d037553_T00	52.65	55.16	coding	404	102	1313
PB.441.1	1	490	2	incomplete-splice_match	gene: Zm00001d029140	Zm00001d029140_T00	4.52	4.52	non coding	NA	NA	NA
PB.10653.2	9	3136	8	full-splice_match	gene: Zm00001d047539	Zm00001d047539_T01	7.46	36.82	coding	399	115	1311
PB.832.1	1	1021	2	full-splice_match	gene: Zm00001d031257	Zm00001d031257_T00	311.45	724.27	coding	178	212	745
PB.5835.1	4	1678	5	full-splice_match	gene: Zm00001d053719	Zm00001d053719_T00	5.10	5.10	coding	403	54	1262
PB.5835.2	4	1529	5	full-splice_match	gene: Zm00001d053719	Zm00001d053719_T00	0.00	5.10	coding	403	54	1262
PB.896.7	1	2543	2	novel_in_catalog	gene: Zm00001d031635	novel	7.92	217.19	coding	159	1967	2443
PB.10653.1	9	556	1	incomplete-splice_match	gene: Zm00001d047539	Zm00001d047539_T00	11.29	36.82	non coding	NA	NA	NA
PB.9167.1	8	816	1	intergenic	novelGene_33	novel	1.27	1.27	non coding	NA	NA	NA
PB.5615.1	4	1611	1	full-splice_match	gene: Zm00001d052662	Zm00001d052662_T00	9.45	9.45	coding	402	195	1400
PB.3950.1	3	2056	16	fusion	gene: Zm00001d040448	novel	60.35	60.35	coding	431	466	1758
PB.1966.2	10	473	2	intergenic	novelGene_189	novel	22.94	44.37	non coding	NA	NA	NA
PB.1252.2	1	1483	2	antisense	novelGene_gene: Zm00001d033181	novel	3.05	3.32	non coding	NA	NA	NA
PB.1300.1	1	1398	1	full-splice_match	gene: Zm00001d033385	Zm00001d033385_T00	1.34	34.58	coding	290	93	962
PB.6950.1	5	2504	1	full-splice_match	gene: Zm00001d017276	Zm00001d017276_T00	37.87	37.87	coding	697	230	2320

PB.645.1	1	2276	5	novel_in_catalog	gene: Zm00001d030159	novel	113.66	229.11	coding	566	391	2088
PB.2083.1	10	976	3	full-splice_match	gene: Zm00001d025325	Zm00001d025325_T00	11.29	11.29	coding	99	475	771
PB.10785.1	9	555	1	antisense	novelGene_gene: Zm00001d048019	novel	12.30	12.30	non coding	NA	NA	NA
PB.9154.3	8	935	1	full-splice_match	gene: Zm00001d008329	Zm00001d008329_T00	643.53	1295.28	coding	96	103	390
PB.4719.6	3	1662	12	novel_in_catalog	gene: Zm00001d044213	novel	0.00	36.19	coding	400	377	1576
PB.1895.3	10	1278	2	full-splice_match	gene: Zm00001d024386	Zm00001d024386_T00	57.93	316.06	coding	280	113	952
PB.4719.4	3	1824	12	novel_in_catalog	gene: Zm00001d044213	novel	9.84	36.19	coding	400	438	1637
PB.1895.1	10	1442	2	full-splice_match	gene: Zm00001d024386	Zm00001d024386_T00	26.14	316.06	coding	280	235	1074
PB.209.1	1	1632	2	full-splice_match	gene: Zm00001d028167	Zm00001d028167_T00	304.81	309.53	coding	385	108	1262
PB.3763.2	3	2180	1	novel_in_catalog	gene: Zm00001d039535	novel	10.10	489.98	coding	265	1133	1927
PB.209.3	1	1510	2	full-splice_match	gene: Zm00001d028167	Zm00001d028167_T00	0.00	309.53	coding	385	108	1262
PB.209.2	1	1160	1	novel_in_catalog	gene: Zm00001d028167	novel	4.72	309.53	coding	190	301	870
PB.5318.1	4	2237	1	full-splice_match	gene: Zm00001d051387	Zm00001d051387_T00	25.43	25.43	coding	708	25	2148
PB.4004.1	3	1225	7	novel_not_in_catalog	gene: Zm00001d040798	novel	21.55	21.55	coding	259	154	930
PB.3763.3	3	1472	2	full-splice_match	gene: Zm00001d039535	Zm00001d039535_T00	0.00	489.98	coding	417	82	1332
PB.2393.2	10	1491	6	novel_in_catalog	gene: Zm00001d026641	novel	12.11	60.75	coding	289	400	1266
PB.9549.1	8	1171	6	full-splice_match	gene: Zm00001d010425	Zm00001d010425_T00	166.78	167.84	coding	215	211	855

PB.9549.3	8	1048	5	novel_in_catalog	gene: Zm00001d010425	novel	1.07	167.84	coding	146	208	645
PB.924.1	1	1151	4	incomplete-splice_match	gene: Zm00001d031779	Zm00001d031779_T00	26.61	26.61	coding	278	47	880
PB.4739.1	3	2601	4	full-splice_match	gene: Zm00001d044291	Zm00001d044291_T00	45.16	69.82	coding	701	90	2192
PB.4739.2	3	1376	2	incomplete-splice_match	gene: Zm00001d044291	Zm00001d044291_T00	24.66	69.82	coding	331	82	1074
PB.4224.2	3	985	6	full-splice_match	gene: Zm00001d042274	Zm00001d042274_T00	12.26	43.54	coding	251	100	852
PB.4739.4	3	2389	4	full-splice_match	gene: Zm00001d044291	Zm00001d044291_T00	0.00	69.82	coding	701	94	2196
PB.645.4	1	1817	5	novel_in_catalog	gene: Zm00001d030159	novel	115.45	229.11	coding	215	1116	1760
PB.6924.4	5	1805	3	novel_in_catalog	gene: Zm00001d017177	novel	10.04	87.19	coding	141	306	728
PB.899.2	1	1150	7	full-splice_match	gene: Zm00001d031640	Zm00001d031640_T00	41.85	140.89	coding	257	75	845
PB.11021.1	B73V4	2327	7	full-splice_match	gene: Zm00001d000428	Zm00001d000428_T00	59.29	62.43	coding	640	117	2036
PB.6001.2	5	859	3	full-splice_match	gene: Zm00001d012966	Zm00001d012966_T00	2827.97	2898.46	coding	139	182	598
PB.11021.2	B73V4	2415	6	novel_in_catalog	gene: Zm00001d000428	novel	3.14	62.43	coding	390	1022	2191
PB.3422.3	2	726	4	full-splice_match	gene: Zm00001d006670	Zm00001d006670_T00	64.74	92.90	coding	121	217	579
PB.1324.2	1	3645	1	full-splice_match	gene: Zm00001d033505	Zm00001d033505_T00	1.17	10.18	coding	1044	67	3198
PB.1881.1	10	1535	2	full-splice_match	gene: Zm00001d024294	Zm00001d024294_T00	255.87	261.74	coding	419	78	1334
PB.8814.1	7	1959	11	full-splice_match	gene: Zm00001d021659	Zm00001d021659_T01	126.40	126.40	coding	452	309	1664
PB.8362.2	7	3199	17	full-splice_match	gene: Zm00001d019335	Zm00001d019335_T01	22.97	22.97	coding	297	913	1803

PB.1157.2	1	1988	3	full-splice_match	gene: Zm00001d032744	Zm00001d032744_T00	4.14	14.24	coding	596	84	1871
PB.1881.4	10	1377	2	full-splice_match	gene: Zm00001d024294	Zm00001d024294_T00	0.00	261.74	coding	419	42	1298
PB.8113.2	6	857	4	full-splice_match	gene: Zm00001d038865	Zm00001d038865_T00	119.84	904.85	coding	143	92	520
PB.8113.3	6	963	3	full-splice_match	gene: Zm00001d038865	Zm00001d038865_T00	4.26	904.85	coding	143	375	803
PB.8113.1	6	852	4	full-splice_match	gene: Zm00001d038865	Zm00001d038865_T00	148.72	904.85	coding	143	74	502
PB.8113.6	6	658	4	full-splice_match	gene: Zm00001d038865	Zm00001d038865_T01	632.04	904.85	coding	143	73	501
PB.8113.4	6	707	4	full-splice_match	gene: Zm00001d038865	Zm00001d038865_T00	0.00	904.85	coding	143	89	517
PB.6904.1	5	3729	8	novel_not_in_catalog	gene: Zm00001d017112	novel	19.20	20.94	coding	972	394	3309
PB.10560.41	9	1715	8	novel_not_in_catalog	gene: Zm00001d047166	novel	0.00	415.37	coding	176	549	1076
PB.10824.1	9	700	5	full-splice_match	gene: Zm00001d048193	Zm00001d048193_T00	18.55	18.55	coding	193	20	598
PB.3396.2	2	1913	6	novel_in_catalog	gene: Zm00001d006578	novel	26.03	210.21	coding	158	140	613
PB.815.1	1	1124	2	genic	gene: Zm00001d031184	novel	42.22	42.22	coding	241	107	829
PB.7468.1	6	2589	2	full-splice_match	gene: Zm00001d035934	Zm00001d035934_T00	155.42	155.42	coding	593	360	2138
PB.9541.4	8	661	3	full-splice_match	gene: Zm00001d010401	Zm00001d010401_T00	0.00	64.29	coding	105	121	435
PB.7468.2	6	2482	2	full-splice_match	gene: Zm00001d035934	Zm00001d035934_T00	0.00	155.42	coding	593	360	2138
PB.9541.2	8	955	3	full-splice_match	gene: Zm00001d010401	Zm00001d010401_T00	25.51	64.29	coding	105	124	438
PB.9541.3	8	627	4	novel_not_in_catalog	gene: Zm00001d010401	novel	13.06	64.29	coding	105	142	456

PB.7669.7	6	2054	6	full-splice_match	gene: Zm00001d037109	Zm00001d037109_T00	4.40	112.60	coding	528	430	2013
PB.7669.6	6	2347	6	novel_in_catalog	gene: Zm00001d037109	novel	0.00	112.60	coding	375	349	1473
PB.7669.5	6	2515	5	novel_in_catalog	gene: Zm00001d037109	novel	0.75	112.60	coding	375	435	1559
PB.7468.3	6	2399	2	full-splice_match	gene: Zm00001d035934	Zm00001d035934_T00	0.00	155.42	coding	593	362	2140
PB.7669.2	6	2233	7	full-splice_match	gene: Zm00001d037109	Zm00001d037109_T00	52.03	112.60	coding	528	432	2015
PB.7669.1	6	2136	6	full-splice_match	gene: Zm00001d037109	Zm00001d037109_T00	41.23	112.60	coding	528	355	1938
PB.10357.1	9	493	1	full-splice_match	gene: Zm00001d045913	Zm00001d045913_T00	48.94	48.94	non coding	NA	NA	NA
PB.766.1	1	3179	15	full-splice_match	gene: Zm00001d030996	Zm00001d030996_T00	18.66	57.29	coding	798	413	2806
PB.9076.3	7	887	6	full-splice_match	gene: Zm00001d022513	Zm00001d022513_T00	1.13	873.30	coding	219	93	749
PB.5524.1	4	1548	5	novel_not_in_catalog	gene: Zm00001d052261	novel	177.77	572.74	coding	303	158	1066
PB.766.2	1	3206	15	full-splice_match	gene: Zm00001d030996	Zm00001d030996_T00	38.62	57.29	coding	798	480	2873
PB.5524.6	4	1342	5	novel_not_in_catalog	gene: Zm00001d052261	novel	275.05	572.74	coding	303	158	1066
PB.7669.9	6	1594	4	incomplete-splice_match	gene: Zm00001d037109	Zm00001d037109_T00	14.20	112.60	coding	228	200	883
PB.7669.8	6	2042	7	full-splice_match	gene: Zm00001d037109	Zm00001d037109_T00	0.00	112.60	coding	528	416	1999
PB.5750.1	4	854	3	incomplete-splice_match	gene: Zm00001d053313	Zm00001d053313_T00	22.41	22.41	coding	124	57	428
PB.2902.1	2	1755	13	full-splice_match	gene: Zm00001d003923	Zm00001d003923_T00	96.46	96.46	coding	446	130	1467
PB.10708.2	9	1994	10	novel_in_catalog	gene: Zm00001d047747	novel	4.16	37.88	coding	239	911	1627

PB.10708.3	9	1827	11	full-splice_match	gene: Zm00001d047747	Zm00001d047747_T00	18.62	37.88	coding	422	266	1531
PB.10708.1	9	1944	11	full-splice_match	gene: Zm00001d047747	Zm00001d047747_T00	15.09	37.88	coding	422	264	1529
PB.3396.8	2	2378	4	novel_in_catalog	gene: Zm00001d006578	novel	3.11	210.21	coding	293	301	1179
PB.10284.1	9	610	3	full-splice_match	gene: Zm00001d045500	Zm00001d045500_T00	75.78	75.78	coding	59	221	397
PB.3745.4	3	1799	2	intergenic	novelGene_27	novel	12.14	53.07	coding	183	1224	1772
PB.1432.2	1	1067	4	full-splice_match	gene: Zm00001d033986	Zm00001d033986_T00	100.96	106.02	coding	213	211	849
PB.9803.3	8	2399	7	full-splice_match	gene: Zm00001d011565	Zm00001d011565_T00	0.00	608.45	coding	112	1680	2015
PB.8507.1	7	2147	4	genic	gene: Zm00001d020303	novel	5.34	24.24	coding	370	500	1609
PB.9803.1	8	1139	8	full-splice_match	gene: Zm00001d011565	Zm00001d011565_T00	13.03	608.45	coding	153	148	606
PB.9555.7	8	1062	1	novel_in_catalog	gene: Zm00001d010456	novel	11.78	53.48	non coding	NA	NA	NA
PB.9555.6	8	935	4	novel_not_in_catalog	gene: Zm00001d010456	novel	9.31	53.48	non coding	NA	NA	NA
PB.9803.5	8	2878	6	full-splice_match	gene: Zm00001d011565	Zm00001d011565_T00	2.25	608.45	coding	109	965	1291
PB.6229.1	5	1996	14	full-splice_match	gene: Zm00001d013838	Zm00001d013838_T00	40.18	45.68	coding	485	87	1541
PB.7812.2	6	1618	4	full-splice_match	gene: Zm00001d037756	Zm00001d037756_T00	30.90	77.81	coding	387	218	1378
PB.7760.1	6	751	3	full-splice_match	gene: Zm00001d037522	Zm00001d037522_T00	42.80	42.80	coding	142	76	501
PB.9803.8	8	2515	6	novel_in_catalog	gene: Zm00001d011565	novel	4.25	608.45	coding	109	966	1292
PB.1782.1	10	3286	4	novel_not_in_catalog	gene: Zm00001d023634	novel	33.00	33.00	coding	810	535	2964

PB.8507.3	7	2034	3	genic	gene: Zm00001d020303	novel	16.72	24.24	coding	370	553	1662
PB.1782.3	10	1709	1	intergenic	novelGene_57	novel	38.48	38.48	coding	439	111	1427
PB.5890.1	4	873	1	full-splice_match	gene: Zm00001d053926	Zm00001d053926_T00	55.20	55.20	coding	167	198	698
PB.2925.3	2	1570	2	genic	gene: Zm00001d004033	novel	0.00	48.06	coding	466	173	1570
PB.2925.2	2	2438	2	genic	gene: Zm00001d004033	novel	48.06	48.06	coding	653	170	2128
PB.2375.1	10	2121	10	novel_in_catalog	gene: Zm00001d026586	novel	48.66	56.25	coding	144	1519	1950
PB.2925.5	2	1219	2	genic	gene: Zm00001d004033	novel	0.00	48.06	coding	350	170	1219
PB.2925.4	2	1483	2	genic	gene: Zm00001d004033	novel	0.00	48.06	coding	384	330	1481
PB.1783.4	10	862	1	intergenic	novelGene_59	novel	9.90	9.90	non coding	NA	NA	NA
PB.7200.2	5	1382	4	novel_not_in_catalog	gene: Zm00001d018305	novel	12.98	204.57	coding	310	57	986
PB.7200.3	5	1516	3	novel_not_in_catalog	gene: Zm00001d018305	novel	191.59	204.57	coding	363	96	1184
PB.1432.1	1	1305	3	novel_in_catalog	gene: Zm00001d033986	novel	5.06	106.02	coding	165	183	677
PB.49.1	1	527	1	antisense	novelGene_gene: Zm00001d027435	novel	20.56	20.56	non coding	NA	NA	NA
PB.2040.1	10	993	6	full-splice_match	gene: Zm00001d025063	Zm00001d025063_T00	32.53	52.71	coding	240	124	843
PB.189.1	1	2289	2	genic	gene: Zm00001d028051	novel	11.31	11.31	coding	660	80	2059
PB.4669.8	3	3005	1	genic_intron	novelGene_412	novel	0.00	0.00	coding	185	1819	2373
PB.11004.1	B73V4	939	2	intergenic	novelGene_3	novel	2.62	2.62	non coding	NA	NA	NA

PB.2247.1	10	1089	4	novel_not_in_catalog	gene: Zm00001d026171	novel	10.60	10.60	coding	285	61	915
PB.7681.1	6	1524	2	full-splice_match	gene: Zm00001d037182	Zm00001d037182_T00	41.61	69.40	coding	377	94	1224
PB.7681.3	6	1418	2	full-splice_match	gene: Zm00001d037182	Zm00001d037182_T00	25.78	69.40	coding	377	97	1227
PB.7681.2	6	1546	1	novel_in_catalog	gene: Zm00001d037182	novel	2.01	69.40	coding	141	99	521
PB.7681.4	6	1273	2	full-splice_match	gene: Zm00001d037182	Zm00001d037182_T00	0.00	69.40	coding	377	89	1219
PB.556.2	1	523	6	incomplete-splice_match	gene: Zm00001d029664	Zm00001d029664_T01	44.73	88.06	coding	105	186	500
PB.10413.4	9	1996	12	full-splice_match	gene: Zm00001d046348	Zm00001d046348_T01	0.80	9.03	coding	240	152	871
PB.556.1	1	2056	13	full-splice_match	gene: Zm00001d029664	Zm00001d029664_T00	43.33	88.06	coding	473	328	1746
PB.10413.1	9	1428	12	full-splice_match	gene: Zm00001d046348	Zm00001d046348_T01	2.00	9.03	coding	218	154	807
PB.2939.1	2	1071	2	genic	gene: Zm00001d004127	novel	26.03	26.03	coding	176	198	725
PB.10413.3	9	1406	13	novel_not_in_catalog	gene: Zm00001d046348	novel	0.24	9.03	coding	160	184	663
PB.10413.2	9	1460	13	full-splice_match	gene: Zm00001d046348	Zm00001d046348_T00	5.99	9.03	coding	351	149	1201
PB.7055.4	5	2229	9	full-splice_match	gene: Zm00001d017755	Zm00001d017755_T02	0.00	21.44	coding	331	963	1955
PB.7055.6	5	1664	6	full-splice_match	gene: Zm00001d017755	Zm00001d017755_T01	4.13	21.44	coding	441	182	1504
PB.7055.7	5	2231	9	full-splice_match	gene: Zm00001d017755	Zm00001d017755_T02	4.19	21.44	coding	331	1090	2082
PB.8240.1	7	701	1	full-splice_match	gene: Zm00001d018710	Zm00001d018710_T00	30.69	30.69	coding	128	91	474
PB.7055.1	5	4028	8	full-splice_match	gene: Zm00001d017755	Zm00001d017755_T00	1.46	21.44	coding	441	2426	3748

PB.7055.2	5	2169	9	full-splice_match	gene: Zm00001d017755	Zm00001d017755_T00	11.65	21.44	coding	541	270	1892
PB.9093.2	7	2686	5	full-splice_match	gene: Zm00001d022573	Zm00001d022573_T00	10.60	155.12	coding	228	1	684
PB.4507.1	3	2019	5	full-splice_match	gene: Zm00001d043443	Zm00001d043443_T00	23.65	29.78	coding	453	139	1497
PB.5519.1	4	1930	1	antisense	novelGene_gene: Zm00001d052252	novel	67.65	67.65	non coding	NA	NA	NA
PB.4507.2	3	1921	5	full-splice_match	gene: Zm00001d043443	Zm00001d043443_T00	0.00	29.78	coding	453	167	1525
PB.1159.1	1	1896	17	incomplete-splice_match	gene: Zm00001d032748	Zm00001d032748_T00	82.48	82.48	coding	196	1115	1702
PB.7136.2	5	2733	6	novel_in_catalog	gene: Zm00001d018050	novel	10.91	10.91	non coding	NA	NA	NA
PB.7435.2	6	834	3	novel_not_in_catalog	gene: Zm00001d035702	novel	97.38	253.12	coding	166	122	619
PB.7435.3	6	725	3	novel_not_in_catalog	gene: Zm00001d035702	novel	0.00	253.12	coding	166	122	619
PB.6475.3	5	1596	8	full-splice_match	gene: Zm00001d014879	Zm00001d014879_T00	0.39	16.24	coding	388	173	1336
PB.7435.1	6	954	3	novel_not_in_catalog	gene: Zm00001d035702	novel	155.73	253.12	coding	166	125	622
PB.9295.1	8	1330	2	full-splice_match	gene: Zm00001d008987	Zm00001d008987_T00	17.36	17.36	coding	413	31	1269
PB.1363.3	1	2322	11	novel_in_catalog	gene: Zm00001d033652	novel	5.66	57.54	coding	631	247	2139
PB.8431.6	7	1683	6	full-splice_match	gene: Zm00001d019850	Zm00001d019850_T00	0.00	89.46	coding	496	101	1588
PB.5349.2	4	779	2	genic	gene: Zm00001d051543	novel	316.18	416.29	coding	166	58	555
PB.8556.1	7	1420	1	antisense	novelGene_gene: Zm00001d020536	novel	9.42	9.42	non coding	NA	NA	NA
PB.8431.5	7	1783	5	novel_in_catalog	gene: Zm00001d019850	novel	6.78	89.46	coding	378	541	1674

PB.1116.4	1	1483	5	full-splice_match	gene: Zm00001d032575	Zm00001d032575_T00	3.24	25.13	coding	250	11	760
PB.8588.2	7	926	5	novel_in_catalog	gene: Zm00001d020694	novel	12.01	206.30	coding	89	105	371
PB.9313.2	8	527	3	full-splice_match	gene: Zm00001d009132	Zm00001d009132_T00	58.76	89.52	coding	103	82	390
PB.9313.1	8	703	3	full-splice_match	gene: Zm00001d009132	Zm00001d009132_T00	30.76	89.52	coding	103	166	474
PB.8588.1	7	1777	6	full-splice_match	gene: Zm00001d020694	Zm00001d020694_T00	187.45	206.30	coding	396	275	1462
PB.5203.1	4	1496	14	full-splice_match	gene: Zm00001d050501	Zm00001d050501_T00	25.44	30.27	coding	351	49	1101
PB.1016.8	1	2622	7	full-splice_match	gene: Zm00001d032198	Zm00001d032198_T01	9.63	168.87	coding	120	651	1010
PB.8588.4	7	1552	6	full-splice_match	gene: Zm00001d020694	Zm00001d020694_T00	0.00	206.30	coding	396	250	1437
PB.5203.2	4	1340	12	novel_in_catalog	gene: Zm00001d050501	novel	0.00	30.27	coding	312	10	945
PB.8435.3	7	2626	5	novel_not_in_catalog	gene: Zm00001d019890	novel	7.85	138.41	coding	428	214	1497
PB.473.1	1	1446	10	full-splice_match	gene: Zm00001d029281	Zm00001d029281_T00	62.04	100.16	coding	275	253	1077
PB.473.2	1	1627	7	novel_in_catalog	gene: Zm00001d029281	novel	2.13	100.16	coding	115	157	501
PB.473.3	1	1299	9	novel_in_catalog	gene: Zm00001d029281	novel	2.17	100.16	coding	183	154	702
PB.473.4	1	1385	10	full-splice_match	gene: Zm00001d029281	Zm00001d029281_T00	31.95	100.16	coding	275	313	1137
PB.473.5	1	1329	8	novel_in_catalog	gene: Zm00001d029281	novel	1.87	100.16	coding	115	126	470
PB.2243.2	10	698	1	full-splice_match	gene: Zm00001d026162	Zm00001d026162_T00	555.01	904.74	coding	134	116	517
PB.6913.3	5	2600	20	full-splice_match	gene: Zm00001d017139	Zm00001d017139_T00	21.60	83.11	coding	746	203	2440

PB.6469.2	5	1048	2	full-splice_match	gene: Zm00001d014853	Zm00001d014853_T00	41.51	41.51	coding	309	57	983
PB.3469.1	2	2529	10	novel_in_catalog	gene: Zm00001d006885	novel	27.18	41.73	coding	434	228	1529
PB.4050.1	3	1167	5	full-splice_match	gene: Zm00001d041267	Zm00001d041267_T00	109.01	112.52	coding	257	194	964
PB.4050.2	3	1193	4	novel_in_catalog	gene: Zm00001d041267	novel	3.52	112.52	coding	131	159	551
PB.392.1	1	2420	10	full-splice_match	gene: Zm00001d028920	Zm00001d028920_T00	19.41	19.41	coding	535	223	1827
PB.9972.1	8	2248	2	intergenic	novelGene_346	novel	5.92	5.92	non coding	NA	NA	NA
PB.195.1	1	1522	6	novel_in_catalog	gene: Zm00001d028075	novel	17.56	62.03	non coding	NA	NA	NA
PB.5511.2	4	1750	1	full-splice_match	gene: Zm00001d052229	Zm00001d052229_T00	307.23	383.47	coding	263	675	1463
PB.5047.1	4	666	1	incomplete-splice_match	gene: Zm00001d049606	Zm00001d049606_T00	3.34	49.88	coding	196	77	664
PB.195.2	1	942	7	novel_in_catalog	gene: Zm00001d028075	novel	11.91	62.03	coding	200	254	853
PB.1511.17	1	2518	4	novel_in_catalog	gene: Zm00001d034345	novel	0.00	1673.99	coding	169	1774	2280
PB.4770.2	3	1919	15	full-splice_match	gene: Zm00001d044447	Zm00001d044447_T00	25.89	25.89	coding	538	39	1652
PB.9911.2	8	1586	2	genic	gene: Zm00001d011985	novel	41.72	43.41	coding	315	161	1105
PB.9911.1	8	2840	2	genic	gene: Zm00001d011985	novel	1.69	43.41	coding	315	5	949
PB.5063.1	4	1875	4	full-splice_match	gene: Zm00001d049684	Zm00001d049684_T00	9.97	9.97	coding	544	100	1731
PB.1974.1	10	2462	3	novel_not_in_catalog	gene: Zm00001d024784	novel	5.56	21.07	coding	430	297	1586
PB.1974.2	10	2124	3	novel_not_in_catalog	gene: Zm00001d024784	novel	15.51	21.07	coding	430	297	1586

PB.4974.4	4	422	2	incomplete-splice_match	gene: Zm00001d049234	Zm00001d049234_T00	0.00	815.22	coding	77	70	300
PB.4974.3	4	2911	3	novel_in_catalog	gene: Zm00001d049234	novel	10.66	815.22	coding	391	96	1268
PB.4974.2	4	1858	4	full-splice_match	gene: Zm00001d049234	Zm00001d049234_T00	581.85	815.22	coding	526	3	1580
PB.158.1	1	2479	4	novel_not_in_catalog	gene: Zm00001d027907	novel	27.60	27.60	coding	363	1245	2333
PB.3378.1	2	1254	1	full-splice_match	gene: Zm00001d006466	Zm00001d006466_T00	3.89	3.89	coding	289	1	867
PB.9188.1	8	2379	2	novel_in_catalog	gene: Zm00001d008477	novel	5.40	21.24	coding	428	913	2196
PB.9188.3	8	2241	3	full-splice_match	gene: Zm00001d008477	Zm00001d008477_T00	15.83	21.24	coding	674	62	2083
PB.5715.6	4	2589	5	novel_not_in_catalog	gene: Zm00001d053151	novel	24.30	66.45	coding	671	202	2214
PB.1133.3	1	2195	1	incomplete-splice_match	gene: Zm00001d032659	Zm00001d032659_T01	2.45	38.09	non coding	NA	NA	NA
PB.1481.3	1	2449	12	full-splice_match	gene: Zm00001d034191	Zm00001d034191_T00	10.29	27.52	coding	203	1687	2295
PB.3993.4	3	550	1	incomplete-splice_match	gene: Zm00001d040721	Zm00001d040721_T00	7.66	125.61	coding	148	27	470
PB.1119.2	1	2666	8	full-splice_match	gene: Zm00001d032597	Zm00001d032597_T00	0.00	58.45	coding	770	176	2485
PB.2220.1	10	938	1	full-splice_match	gene: Zm00001d026029	Zm00001d026029_T00	108.49	108.49	coding	104	135	446
PB.9736.3	8	2025	1	antisense	novelGene_gene: Zm00001d011228	novel	19.78	24.05	coding	130	1552	1941
PB.6920.1	5	2734	14	full-splice_match	gene: Zm00001d017166	Zm00001d017166_T00	8.15	21.80	coding	341	1379	2401
PB.4587.3	3	769	1	intergenic	novelGene_400	novel	4.42	628.03	non coding	NA	NA	NA
PB.7796.1	6	1559	1	full-splice_match	gene: Zm00001d037682	Zm00001d037682_T00	1.47	1.47	coding	441	57	1379

PB.380.5	1	1938	3	full-splice_match	gene: Zm00001d028868	Zm00001d028868_T00	0.00	100.13	coding	567	85	1785
PB.380.4	1	2782	1	novel_in_catalog	gene: Zm00001d028868	novel	6.95	100.13	coding	238	1796	2509
PB.1634.2	1	3147	1	full-splice_match	gene: Zm00001d034781	Zm00001d034781_T00	27.35	60.57	coding	852	213	2768
PB.380.1	1	2062	3	full-splice_match	gene: Zm00001d028868	Zm00001d028868_T00	93.17	100.13	coding	567	88	1788
PB.5369.1	4	2202	1	full-splice_match	gene: Zm00001d051620	Zm00001d051620_T00	8.62	45.97	coding	314	804	1745
PB.4058.5	3	738	5	full-splice_match	gene: Zm00001d041352	Zm00001d041352_T00	14.27	510.45	coding	143	98	526
PB.4058.6	3	846	6	full-splice_match	gene: Zm00001d041352	Zm00001d041352_T00	170.43	510.45	coding	223	39	707
PB.6461.1	5	2894	7	incomplete-splice_match	gene: Zm00001d014813	Zm00001d014813_T00	3.79	3.79	coding	724	17	2188
PB.1125.2	1	2365	3	novel_not_in_catalog	gene: Zm00001d032613	novel	24.60	30.92	coding	647	161	2101
PB.10501.3	9	1216	6	incomplete-splice_match	gene: Zm00001d046889	Zm00001d046889_T01	15.04	36.78	coding	135	525	929
PB.1125.1	1	1807	1	novel_in_catalog	gene: Zm00001d032613	novel	6.32	30.92	coding	181	942	1484
PB.4125.1	3	2066	4	full-splice_match	gene: Zm00001d041740	Zm00001d041740_T00	26.14	26.14	coding	494	145	1626
PB.9428.3	8	574	1	antisense	novelGene_gene: Zm00001d009771	novel	0.00	19.15	non coding	NA	NA	NA
PB.9428.1	8	1530	3	antisense	novelGene_gene: Zm00001d009771	novel	18.18	19.15	coding	131	680	1072
PB.1892.3	10	1135	1	full-splice_match	gene: Zm00001d024379	Zm00001d024379_T00	0.00	402.10	coding	286	129	986
PB.9428.4	8	1346	2	antisense	novelGene_gene: Zm00001d009771	novel	0.30	19.15	non coding	NA	NA	NA
PB.9428.5	8	1522	1	antisense	novelGene_gene: Zm00001d009771	novel	0.68	19.15	non coding	NA	NA	NA

PB.5369.4	4	2055	1	full-splice_match	gene: Zm00001d051620	Zm00001d051620_T00	3.52	45.97	coding	314	845	1786
PB.6230.4	5	1868	5	full-splice_match	gene: Zm00001d013840	Zm00001d013840_T00	0.00	37.15	coding	559	69	1745
PB.8936.5	7	1429	1	novel_in_catalog	gene: Zm00001d022041	novel	0.00	86.37	coding	146	790	1227
PB.1510.2	1	3500	4	novel_in_catalog	gene: Zm00001d034344	novel	15.54	59.69	coding	694	1008	3089
PB.9303.1	8	3217	5	full-splice_match	gene: Zm00001d009017	Zm00001d009017_T00	21.44	21.44	coding	927	200	2980
PB.9919.1	8	1180	1	genic	gene: Zm00001d012016	novel	300.50	300.50	coding	102	417	722
PB.6230.1	5	2256	2	novel_in_catalog	gene: Zm00001d013840	novel	12.03	37.15	coding	460	449	1828
PB.8987.1	7	1080	5	full-splice_match	gene: Zm00001d022242	Zm00001d022242_T00	71.37	71.37	coding	210	180	809
PB.1634.1	1	1092	1	full-splice_match	gene: Zm00001d034781	Zm00001d034781_T00	16.30	60.57	non coding	NA	NA	NA
PB.8147.2	6	1593	5	novel_not_in_catalog	gene: Zm00001d038971	novel	29.78	96.32	coding	245	613	1347
PB.3342.1	2	1616	7	full-splice_match	gene: Zm00001d006344	Zm00001d006344_T00	34.23	96.19	coding	354	206	1267
PB.9134.2	8	890	8	novel_in_catalog	gene: Zm00001d008262	novel	5.06	15.21	coding	177	93	623
PB.9134.3	8	915	8	full-splice_match	gene: Zm00001d008262	Zm00001d008262_T00	10.15	15.21	coding	244	68	799
PB.8196.1	6	1567	5	full-splice_match	gene: Zm00001d039166	Zm00001d039166_T00	13.44	100.76	coding	276	144	971
PB.8183.1	6	989	2	full-splice_match	gene: Zm00001d039118	Zm00001d039118_T00	23.64	23.64	non coding	NA	NA	NA
PB.1892.2	10	1256	1	full-splice_match	gene: Zm00001d024379	Zm00001d024379_T00	282.90	402.10	coding	286	151	1008
PB.5416.1	4	2432	7	full-splice_match	gene: Zm00001d051871	Zm00001d051871_T00	8.46	17.54	coding	542	186	1811

PB.6155.1	5	1736	10	full-splice_match	gene: Zm00001d013485	Zm00001d013485_T00	39.96	39.96	coding	267	70	870
PB.6088.1	5	2374	12	full-splice_match	gene: Zm00001d013265	Zm00001d013265_T00	25.66	25.66	coding	565	278	1972
PB.6706.1	5	1189	1	full-splice_match	gene: Zm00001d016170	Zm00001d016170_T00	32.04	32.04	coding	319	77	1033
PB.5474.9	4	1196	9	full-splice_match	gene: Zm00001d052074	Zm00001d052074_T00	56.13	129.01	coding	212	338	973
PB.6088.2	5	1236	9	novel_in_catalog	gene: Zm00001d013265	novel	0.00	25.66	coding	328	252	1235
PB.9681.2	8	3366	17	full-splice_match	gene: Zm00001d010974	Zm00001d010974_T00	42.89	89.78	coding	876	293	2920
PB.9681.1	8	2392	6	incomplete-splice_match	gene: Zm00001d010974	Zm00001d010974_T02	46.88	89.78	coding	504	157	1668
PB.4645.1	3	637	2	full-splice_match	gene: Zm00001d043898	Zm00001d043898_T00	21.49	21.49	coding	99	116	412
PB.1230.1	1	1751	5	full-splice_match	gene: Zm00001d033056	Zm00001d033056_T00	174.84	179.46	coding	460	74	1453
PB.8132.2	6	771	5	full-splice_match	gene: Zm00001d038910	Zm00001d038910_T00	0.00	32.04	coding	227	36	716
PB.7047.1	5	865	3	full-splice_match	gene: Zm00001d017708	Zm00001d017708_T00	65.79	214.32	coding	157	60	530
PB.7227.4	5	778	1	intergenic	novelGene_418	novel	22.49	56.56	non coding	NA	NA	NA
PB.10077.1	8	2024	5	full-splice_match	gene: Zm00001d012666	Zm00001d012666_T00	23.82	23.82	coding	458	274	1647
PB.8092.1	6	1395	7	full-splice_match	gene: Zm00001d038805	Zm00001d038805_T00	5.75	5.75	coding	306	119	1036
PB.3983.1	3	2009	2	full-splice_match	gene: Zm00001d040649	Zm00001d040649_T00	19.22	58.72	coding	551	77	1729
PB.3983.2	3	1776	2	full-splice_match	gene: Zm00001d040649	Zm00001d040649_T00	39.50	58.72	coding	551	74	1726
PB.940.2	1	3134	7	novel_in_catalog	gene: Zm00001d031851	novel	0.23	18.33	coding	567	264	1964

PB.6149.1	5	602	3	incomplete-splice_match	gene: Zm00001d013456 Zm00001d013456_T00	20.38	44.74	non coding	NA	NA	NA
PB.6149.3	5	1282	9	full-splice_match	gene: Zm00001d013456 Zm00001d013456_T00	24.36	44.74	coding	291	217	1089
PB.9104.1	7	1511	9	full-splice_match	gene: Zm00001d022619 Zm00001d022619_T00	14.79	15.84	coding	414	52	1293
PB.3211.1	2	3133	16	full-splice_match	gene: Zm00001d005794 Zm00001d005794_T01	60.30	60.30	coding	768	623	2926
PB.4131.2	3	860	3	full-splice_match	gene: Zm00001d041760 Zm00001d041760_T00	497.90	927.93	coding	194	91	672
PB.4517.4	3	3134	3	full-splice_match	gene: Zm00001d043469 Zm00001d043469_T01	52.80	176.41	coding	911	74	2806
PB.4131.1	3	963	3	full-splice_match	gene: Zm00001d041760 Zm00001d041760_T00	430.02	927.93	coding	194	85	666
PB.4517.2	3	975	1	incomplete-splice_match	gene: Zm00001d043469 Zm00001d043469_T00	11.68	176.41	coding	89	64	330
PB.4517.3	3	2997	3	full-splice_match	gene: Zm00001d043469 Zm00001d043469_T00	93.23	176.41	coding	793	130	2508
PB.4517.1	3	3709	3	full-splice_match	gene: Zm00001d043469 Zm00001d043469_T00	18.70	176.41	coding	911	231	2963
PB.873.6	1	1346	10	full-splice_match	gene: Zm00001d031502 Zm00001d031502_T02	40.12	59.06	coding	282	176	1021
PB.1824.1	10	1482	12	full-splice_match	gene: Zm00001d023874 Zm00001d023874_T00	54.92	123.88	coding	293	94	972
PB.7742.2	6	1747	6	full-splice_match	gene: Zm00001d037425 Zm00001d037425_T00	8.01	10.51	coding	354	439	1500
PB.7742.1	6	1985	6	full-splice_match	gene: Zm00001d037425 Zm00001d037425_T00	2.49	10.51	coding	354	471	1532
PB.683.3	1	2994	8	full-splice_match	gene: Zm00001d030385 Zm00001d030385_T00	5.07	35.29	coding	287	1838	2698
PB.9532.2	8	1276	1	full-splice_match	gene: Zm00001d010329 Zm00001d010329_T00	0.00	13.64	coding	384	1	1152
PB.683.1	1	1557	9	full-splice_match	gene: Zm00001d030385 Zm00001d030385_T00	30.22	35.29	coding	354	65	1126

PB.6512.1	5	1895	3	full-splice_match	gene: Zm00001d014986	Zm00001d014986_T00	28.54	28.54	coding	203	1006	1614
PB.9244.3	8	703	4	novel_in_catalog	gene: Zm00001d008731	novel	1.95	110.07	coding	18	446	499
PB.6403.2	5	1227	9	full-splice_match	gene: Zm00001d014626	Zm00001d014626_T00	50.04	56.93	coding	293	164	1042
PB.6403.1	5	1319	8	novel_in_catalog	gene: Zm00001d014626	novel	6.89	56.93	coding	293	248	1126
PB.187.1	1	637	1	full-splice_match	gene: Zm00001d028043	Zm00001d028043_T00	105.25	105.25	coding	144	85	516
PB.7441.1	6	1122	1	full-splice_match	gene: Zm00001d035753	Zm00001d035753_T00	21.23	21.23	coding	341	30	1052
PB.2452.3	2	633	4	full-splice_match	gene: Zm00001d001882	Zm00001d001882_T00	57.23	363.91	coding	146	100	537
PB.6761.1	5	1728	10	full-splice_match	gene: Zm00001d016477	Zm00001d016477_T00	30.42	30.42	coding	491	44	1516
PB.7364.3	6	1941	3	intergenic	novelGene_88	novel	13.08	17.85	coding	352	725	1780
PB.1016.7	1	1802	10	full-splice_match	gene: Zm00001d032198	Zm00001d032198_T00	61.80	168.87	coding	464	172	1563
PB.6761.2	5	1535	10	full-splice_match	gene: Zm00001d016477	Zm00001d016477_T00	0.00	30.42	coding	491	43	1515
PB.558.1	1	2402	5	novel_not_in_catalog	gene: Zm00001d029672	novel	31.98	31.98	coding	105	66	380
PB.5616.2	4	1261	6	novel_not_in_catalog	gene: Zm00001d052667	novel	0.00	4.62	coding	241	333	1055
PB.2773.4	2	1511	7	full-splice_match	gene: Zm00001d003254	Zm00001d003254_T00	5.85	60.39	coding	178	122	655
PB.2626.1	2	1079	1	full-splice_match	gene: Zm00001d002618	Zm00001d002618_T00	66.44	66.44	coding	222	82	747
PB.5859.10	4	1992	7	novel_in_catalog	gene: Zm00001d053818	novel	3.22	105.42	coding	405	607	1821
PB.6620.1	5	1581	9	novel_in_catalog	gene: Zm00001d015527	novel	24.27	37.05	coding	324	237	1208

PB.4430.2	3	2080	13	full-splice_match	gene: Zm00001d043152	Zm00001d043152_T00	73.13	115.37	coding	538	176	1789
PB.6620.2	5	1895	10	full-splice_match	gene: Zm00001d015527	Zm00001d015527_T00	12.78	37.05	coding	506	120	1637
PB.1362.2	1	2095	10	full-splice_match	gene: Zm00001d033651	Zm00001d033651_T01	3.17	24.56	coding	392	83	1258
PB.8202.1	6	1470	9	full-splice_match	gene: Zm00001d039182	Zm00001d039182_T00	51.42	90.47	coding	347	137	1177
PB.1188.10	1	1413	4	full-splice_match	gene: Zm00001d032873	Zm00001d032873_T00	0.00	341.11	coding	375	120	1244
PB.8080.1	6	915	4	full-splice_match	gene: Zm00001d038753	Zm00001d038753_T00	105.52	105.52	coding	113	177	515
PB.4829.4	3	1239	6	full-splice_match	gene: Zm00001d044694	Zm00001d044694_T00	155.87	329.15	coding	240	178	897
PB.1420.11	1	1318	10	incomplete-splice_match	gene: Zm00001d033931	Zm00001d033931_T00	99.40	439.36	coding	379	98	1234
PB.6899.26	5	2390	6	full-splice_match	gene: Zm00001d017096	Zm00001d017096_T02	0.48	254.51	coding	129	103	489
PB.10256.1	9	1157	2	full-splice_match	gene: Zm00001d045405	Zm00001d045405_T00	9.32	9.32	coding	319	36	992
PB.2859.11	2	1313	6	full-splice_match	gene: Zm00001d003683	Zm00001d003683_T00	0.00	357.68	coding	389	56	1222
PB.2859.10	2	1529	5	incomplete-splice_match	gene: Zm00001d003683	Zm00001d003683_T00	0.00	357.68	coding	389	221	1387
PB.9367.2	8	1226	7	full-splice_match	gene: Zm00001d009482	Zm00001d009482_T00	28.72	35.20	coding	243	310	1038
PB.5859.11	4	2828	5	novel_in_catalog	gene: Zm00001d053818	novel	6.86	105.42	coding	129	2245	2631
PB.9367.1	8	1828	6	novel_in_catalog	gene: Zm00001d009482	novel	6.48	35.20	coding	169	252	758
PB.9857.1	8	2096	1	genic	gene: Zm00001d011774	novel	0.46	0.46	coding	624	83	1954
PB.6899.24	5	1760	8	novel_in_catalog	gene: Zm00001d017096	novel	0.29	254.51	coding	129	106	492

PB.9041.2	7	921	1	full-splice_match	gene: Zm00001d022420	Zm00001d022420_T00	334.41	1152.02	coding	205	98	712
PB.9402.2	8	1651	6	full-splice_match	gene: Zm00001d009660	Zm00001d009660_T00	27.61	44.28	coding	377	330	1460
PB.6034.4	5	3287	20	novel_in_catalog	gene: Zm00001d013059	novel	3.81	107.28	coding	618	139	1992
PB.6068.1	5	1392	2	full-splice_match	gene: Zm00001d013210	Zm00001d013210_T00	26.28	26.28	coding	94	261	542
PB.7774.3	6	504	1	incomplete-splice_match	gene: Zm00001d037605	Zm00001d037605_T00	30.78	85.55	coding	44	191	322
PB.9402.6	8	706	2	novel_in_catalog	gene: Zm00001d009660	novel	0.00	44.28	coding	143	176	604
PB.4652.1	3	1408	2	full-splice_match	gene: Zm00001d043935	Zm00001d043935_T00	79.39	215.60	coding	220	356	1015
PB.6248.1	5	2303	12	incomplete-splice_match	gene: Zm00001d013935	Zm00001d013935_T00	39.09	61.56	coding	566	151	1848
PB.9402.5	8	1082	4	novel_in_catalog	gene: Zm00001d009660	novel	7.26	44.28	coding	167	82	582
PB.9028.2	7	2505	16	incomplete-splice_match	gene: Zm00001d022388	Zm00001d022388_T01	64.58	194.04	coding	649	120	2066
PB.9855.1	8	1718	9	full-splice_match	gene: Zm00001d011767	Zm00001d011767_T00	58.13	58.13	coding	472	67	1482
PB.4329.9	3	2124	5	full-splice_match	gene: Zm00001d042724	Zm00001d042724_T00	0.00	343.97	coding	480	554	1993
PB.6034.6	5	2981	21	novel_in_catalog	gene: Zm00001d013059	novel	7.54	107.28	coding	560	936	2615
PB.5717.1	4	1421	2	full-splice_match	gene: Zm00001d053156	Zm00001d053156_T00	11.07	11.07	coding	365	72	1166
PB.8936.3	7	3174	8	full-splice_match	gene: Zm00001d022041	Zm00001d022041_T00	13.82	86.37	coding	551	1173	2825
PB.9214.1	8	1832	3	full-splice_match	gene: Zm00001d008602	Zm00001d008602_T00	11.35	150.46	coding	299	208	1104
PB.7455.1	6	3194	13	full-splice_match	gene: Zm00001d035869	Zm00001d035869_T02	54.52	107.19	coding	823	270	2738

PB.6367.1	5	1246	6	full-splice_match	gene: Zm00001d014455	Zm00001d014455_T00	10.97	10.97	coding	347	71	1111
PB.7455.3	6	3085	13	full-splice_match	gene: Zm00001d035869	Zm00001d035869_T02	30.06	107.19	coding	823	270	2738
PB.9214.5	8	1620	3	full-splice_match	gene: Zm00001d008602	Zm00001d008602_T00	0.00	150.46	coding	299	286	1182
PB.9214.4	8	2317	2	full-splice_match	gene: Zm00001d008602	Zm00001d008602_T00	0.00	150.46	coding	299	793	1689
PB.9214.7	8	1644	3	full-splice_match	gene: Zm00001d008602	Zm00001d008602_T00	46.24	150.46	coding	299	303	1199
PB.9214.6	8	2186	1	novel_in_catalog	gene: Zm00001d008602	novel	3.54	150.46	coding	147	477	917
PB.9214.9	8	1900	2	full-splice_match	gene: Zm00001d008602	Zm00001d008602_T00	18.92	150.46	coding	299	792	1688
PB.9214.8	8	2150	2	full-splice_match	gene: Zm00001d008602	Zm00001d008602_T00	0.00	150.46	coding	299	828	1724
PB.5763.1	4	1936	6	full-splice_match	gene: Zm00001d053376	Zm00001d053376_T00	13.00	24.27	coding	327	702	1682
PB.2366.1	10	1038	1	full-splice_match	gene: Zm00001d026563	Zm00001d026563_T00	157.32	173.65	coding	178	231	764
PB.5906.2	4	1687	5	full-splice_match	gene: Zm00001d053977	Zm00001d053977_T00	9.28	41.79	coding	219	888	1544
PB.9935.3	8	2465	19	full-splice_match	gene: Zm00001d012086	Zm00001d012086_T00	19.05	55.58	coding	691	135	2207
PB.2157.1	10	1153	8	full-splice_match	gene: Zm00001d025745	Zm00001d025745_T00	27.26	28.74	coding	259	132	908
PB.5929.1	4	850	1	full-splice_match	gene: Zm00001d054046	Zm00001d054046_T00	11.54	11.54	coding	175	98	622
PB.9377.1	8	2055	8	novel_not_in_catalog	gene: Zm00001d009513	novel	11.97	24.52	coding	504	121	1632
PB.6508.1	5	2410	2	novel_in_catalog	gene: Zm00001d014972	novel	26.68	26.68	coding	135	1905	2309
PB.9377.3	8	2513	1	novel_in_catalog	gene: Zm00001d009513	novel	2.49	24.52	non coding	NA	NA	NA

PB.9377.2	8	2104	9	novel_not_in_catalog	gene: Zm00001d009513	novel	10.05	24.52	coding	520	125	1684
PB.4358.2	3	2408	5	novel_in_catalog	gene: Zm00001d042848	novel	4.85	102.93	coding	362	1065	2150
PB.2705.5	2	1559	14	incomplete-splice_match	gene: Zm00001d002967	Zm00001d002967_T01	52.00	77.43	coding	440	82	1401
PB.6775.1	5	1037	1	full-splice_match	gene: Zm00001d016561	Zm00001d016561_T00	117.33	117.33	coding	107	207	527
PB.2705.3	2	2152	15	full-splice_match	gene: Zm00001d002967	Zm00001d002967_T00	14.82	77.43	coding	504	266	1777
PB.2705.2	2	2380	16	full-splice_match	gene: Zm00001d002967	Zm00001d002967_T00	10.62	77.43	coding	504	446	1957
PB.10609.1	9	957	1	full-splice_match	gene: Zm00001d047420	Zm00001d047420_T00	0.47	53.99	coding	188	43	606
PB.2586.2	2	1006	9	incomplete-splice_match	gene: Zm00001d002422	Zm00001d002422_T00	0.00	9.73	coding	230	151	840
PB.8254.3	7	2187	7	novel_not_in_catalog	gene: Zm00001d018787	novel	6.12	24.50	coding	301	62	964
PB.9884.2	8	2256	9	full-splice_match	gene: Zm00001d011855	Zm00001d011855_T00	24.12	70.80	coding	547	380	2020
PB.2586.1	2	1592	12	full-splice_match	gene: Zm00001d002422	Zm00001d002422_T00	9.73	9.73	coding	397	125	1315
PB.7954.1	6	2016	8	full-splice_match	gene: Zm00001d038272	Zm00001d038272_T00	46.93	46.93	coding	619	60	1916
PB.8677.1	7	3189	19	full-splice_match	gene: Zm00001d021087	Zm00001d021087_T00	14.76	14.76	coding	837	326	2836
PB.1563.1	1	3612	14	full-splice_match	gene: Zm00001d034553	Zm00001d034553_T00	51.98	51.98	coding	1076	214	3441
PB.2157.2	10	1574	6	novel_in_catalog	gene: Zm00001d025745	novel	1.49	28.74	coding	180	165	704
PB.8924.1	7	1236	1	full-splice_match	gene: Zm00001d022001	Zm00001d022001_T00	7.63	7.63	coding	285	65	919
PB.5180.5	4	2605	5	novel_in_catalog	gene: Zm00001d050357	novel	1.98	39.84	coding	154	1138	1599

PB.5180.4	4	2241	7	novel_in_catalog	gene: Zm00001d050357	novel	10.62	39.84	coding	226	1326	2003
PB.5371.1	4	2239	18	full-splice_match	gene: Zm00001d051629	Zm00001d051629_T00	63.66	79.62	coding	616	95	1942
PB.940.4	1	2878	8	full-splice_match	gene: Zm00001d031851	Zm00001d031851_T00	9.23	18.33	coding	677	288	2318
PB.5371.5	4	1285	12	incomplete-splice_match	gene: Zm00001d051629	Zm00001d051629_T00	12.65	79.62	coding	352	42	1097
PB.5180.2	4	2200	6	novel_in_catalog	gene: Zm00001d050357	novel	27.25	39.84	coding	223	1208	1876
PB.6260.1	5	1060	1	full-splice_match	gene: Zm00001d013993	Zm00001d013993_T00	146.05	242.10	coding	186	193	750
PB.10075.1	8	4095	15	full-splice_match	gene: Zm00001d012661	Zm00001d012661_T00	10.57	10.57	coding	1195	176	3760
PB.6260.2	5	811	1	full-splice_match	gene: Zm00001d013993	Zm00001d013993_T00	96.04	242.10	coding	186	54	611
PB.2414.1	10	2269	1	incomplete-splice_match	gene: Zm00001d026706	Zm00001d026706_T00	43.87	277.23	coding	653	33	1991
PB.10224.4	9	1731	11	novel_not_in_catalog	gene: Zm00001d045311	novel	3.19	9.07	coding	299	589	1485
PB.9069.1	7	922	1	incomplete-splice_match	gene: Zm00001d022492	Zm00001d022492_T00	95.80	95.80	coding	117	40	390
PB.10224.6	9	2374	8	novel_in_catalog	gene: Zm00001d045311	novel	4.08	9.07	coding	170	1688	2197
PB.7811.4	6	1539	7	novel_not_in_catalog	gene: Zm00001d037745	novel	5.77	44.70	coding	400	237	1436
PB.7811.5	6	1411	1	novel_in_catalog	gene: Zm00001d037745	novel	1.49	44.70	coding	100	631	930
PB.8768.4	7	1176	5	full-splice_match	gene: Zm00001d021485	Zm00001d021485_T00	0.00	88.97	coding	171	554	1066
PB.8768.2	7	1418	5	full-splice_match	gene: Zm00001d021485	Zm00001d021485_T00	15.88	88.97	coding	171	615	1127
PB.6096.1	5	622	4	novel_in_catalog	gene: Zm00001d013282	novel	514.01	514.01	non coding	NA	NA	NA

PB.8768.1	7	1475	5	full-splice_match	gene: Zm00001d021484	Zm00001d021484_T00	3.35	3.35	coding	171	564	1076
PB.9075.1	7	1668	12	incomplete-splice_match	gene: Zm00001d022510	Zm00001d022510_T01	14.32	14.32	coding	444	127	1458
PB.7825.1	6	2481	16	full-splice_match	gene: Zm00001d037818	Zm00001d037818_T00	24.09	24.09	coding	634	113	2014
PB.8689.1	7	1403	8	full-splice_match	gene: Zm00001d021195	Zm00001d021195_T00	48.63	48.63	coding	409	28	1254
PB.9226.2	8	1396	8	incomplete-splice_match	gene: Zm00001d008640	Zm00001d008640_T00	32.18	32.18	coding	299	170	1066
PB.3913.2	3	693	1	full-splice_match	gene: Zm00001d040297	Zm00001d040297_T00	1034.82	1956.93	coding	138	122	535
PB.3913.1	3	806	1	full-splice_match	gene: Zm00001d040297	Zm00001d040297_T00	922.11	1956.93	coding	138	120	533
PB.6377.1	5	561	4	full-splice_match	gene: Zm00001d014519	Zm00001d014519_T00	1132.26	1433.32	non coding	NA	NA	NA
PB.6377.2	5	456	4	full-splice_match	gene: Zm00001d014519	Zm00001d014519_T00	301.06	1433.32	coding	62	116	301
PB.1417.3	1	2083	6	novel_in_catalog	gene: Zm00001d033917	novel	140.03	186.86	coding	621	170	2032
PB.484.2	1	2355	1	novel_in_catalog	gene: Zm00001d029356	novel	7.54	184.41	coding	325	75	1049
PB.2737.1	2	1471	2	incomplete-splice_match	gene: Zm00001d003116	Zm00001d003116_T00	14.00	22.11	coding	365	56	1150
PB.2737.2	2	2278	3	full-splice_match	gene: Zm00001d003116	Zm00001d003116_T00	8.11	22.11	coding	582	302	2047
PB.484.1	1	1559	2	full-splice_match	gene: Zm00001d029356	Zm00001d029356_T00	106.10	184.41	coding	375	75	1199
PB.10130.1	9	2260	1	genic	gene: Zm00001d044825	novel	4.24	5.14	coding	683	38	2086
PB.7811.2	6	2000	6	novel_in_catalog	gene: Zm00001d037745	novel	10.91	44.70	coding	386	641	1798
PB.11001.1	B73V4	737	1	novel_in_catalog	gene: Zm00001d000293	novel	3.43	3.43	coding	181	76	618

PB.1486.3	1	1568	1	full-splice_match	gene: Zm00001d034221	Zm00001d034221_T00	0.00	127.95	coding	435	134	1438
PB.4042.1	3	3318	21	full-splice_match	gene: Zm00001d041214	Zm00001d041214_T00	49.67	49.67	coding	956	205	3072
PB.484.8	1	1470	2	full-splice_match	gene: Zm00001d029359	Zm00001d029359_T00	178.21	318.34	coding	375	104	1228
PB.484.9	1	1366	2	full-splice_match	gene: Zm00001d029359	Zm00001d029359_T00	140.13	318.34	coding	375	103	1227
PB.798.2	1	1715	7	full-splice_match	gene: Zm00001d031098	Zm00001d031098_T00	23.66	31.90	coding	223	863	1531
PB.798.3	1	1654	7	novel_in_catalog	gene: Zm00001d031098	novel	8.24	31.90	coding	223	837	1505
PB.3033.2	2	1883	1	intergenic	novelGene_267	novel	4.35	21.68	non coding	NA	NA	NA
PB.4829.1	3	1552	5	novel_in_catalog	gene: Zm00001d044694	novel	14.30	329.15	coding	240	355	1074
PB.3370.2	2	1556	3	novel_in_catalog	gene: Zm00001d006440	novel	0.86	53.60	coding	133	837	1235
PB.3900.2	3	3191	19	novel_in_catalog	gene: Zm00001d040268	novel	0.30	9.80	coding	548	1466	3109
PB.1803.3	10	3782	12	full-splice_match	gene: Zm00001d023784	Zm00001d023784_T00	23.03	62.18	coding	816	864	3311
PB.4357.1	3	2113	3	full-splice_match	gene: Zm00001d042847	Zm00001d042847_T00	3.05	3.05	coding	521	36	1598
PB.1803.1	10	3546	11	full-splice_match	gene: Zm00001d023784	Zm00001d023784_T00	18.72	62.18	coding	816	598	3045
PB.5657.3	4	1538	5	novel_in_catalog	gene: Zm00001d052820	novel	0.34	85.71	coding	327	318	1298
PB.8736.3	7	1439	11	novel_not_in_catalog	gene: Zm00001d021351	novel	0.00	7.63	coding	261	84	866
PB.3272.1	2	1698	6	full-splice_match	gene: Zm00001d006057	Zm00001d006057_T00	53.28	53.28	coding	421	216	1478
PB.8736.1	7	1548	11	novel_not_in_catalog	gene: Zm00001d021351	novel	7.63	7.63	coding	261	89	871

PB.1803.4	10	714	3	incomplete-splice_match	gene: Zm00001d023784	Zm00001d023784_T02	20.44	62.18	coding	76	205	432
PB.6746.1	5	1801	15	full-splice_match	gene: Zm00001d016397	Zm00001d016397_T00	6.28	6.28	coding	532	49	1644
PB.4586.1	3	1250	5	full-splice_match	gene: Zm00001d043700	Zm00001d043700_T00	120.42	214.22	coding	221	211	873
PB.4586.2	3	1175	5	full-splice_match	gene: Zm00001d043700	Zm00001d043700_T00	93.81	214.22	coding	221	293	955
PB.1462.1	1	1032	2	full-splice_match	gene: Zm00001d034077	Zm00001d034077_T00	25.82	55.18	coding	169	221	727
PB.101.2	1	2368	4	full-splice_match	gene: Zm00001d027667	Zm00001d027667_T01	86.60	198.17	coding	535	445	2049
PB.3023.2	2	834	1	full-splice_match	gene: Zm00001d004721	Zm00001d004721_T00	1161.28	1281.88	coding	193	31	609
PB.3023.1	2	938	1	full-splice_match	gene: Zm00001d004721	Zm00001d004721_T00	120.60	1281.88	coding	193	34	612
PB.101.1	1	2701	4	full-splice_match	gene: Zm00001d027667	Zm00001d027667_T00	35.46	198.17	coding	535	790	2394
PB.101.6	1	2347	6	novel_not_in_catalog	gene: Zm00001d027667	novel	0.00	198.17	coding	206	504	1121
PB.2095.2	10	774	5	full-splice_match	gene: Zm00001d025407	Zm00001d025407_T00	43.79	76.17	coding	196	35	622
PB.2727.1	2	1717	4	full-splice_match	gene: Zm00001d003076	Zm00001d003076_T00	17.61	17.61	coding	317	169	1119
PB.101.5	1	2514	5	full-splice_match	gene: Zm00001d027667	Zm00001d027667_T01	29.29	198.17	coding	535	593	2197
PB.6175.2	5	1815	3	full-splice_match	gene: Zm00001d013573	Zm00001d013573_T00	26.35	26.35	coding	463	106	1494
PB.3445.3	2	964	1	full-splice_match	gene: Zm00001d006774	Zm00001d006774_T00	0.00	294.42	coding	262	67	852
PB.3445.2	2	1086	1	full-splice_match	gene: Zm00001d006774	Zm00001d006774_T00	27.48	294.42	coding	262	64	849
PB.3445.1	2	1209	1	full-splice_match	gene: Zm00001d006774	Zm00001d006774_T00	266.94	294.42	coding	262	67	852

PB.2804.1	2	1388	11	novel_not_in_catalog	gene: Zm00001d003423	novel	18.38	18.38	coding	374	6	1127
PB.3390.1	2	4901	3	incomplete-splice_match	gene: Zm00001d006552	Zm00001d006552_T01	46.52	46.52	coding	827	528	3008
PB.5890.2	4	821	1	full-splice_match	gene: Zm00001d053926	Zm00001d053926_T00	0.00	55.20	coding	167	198	698
PB.10577.8	9	388	1	incomplete-splice_match	gene: Zm00001d047240	Zm00001d047240_T00	0.00	89.69	non coding	NA	NA	NA
PB.9169.1	8	978	4	full-splice_match	gene: Zm00001d008395	Zm00001d008395_T00	115.36	115.36	coding	221	194	856
PB.10577.6	9	1703	3	full-splice_match	gene: Zm00001d047240	Zm00001d047240_T00	0.00	89.69	coding	430	413	1702
PB.10577.7	9	690	1	incomplete-splice_match	gene: Zm00001d047240	Zm00001d047240_T00	0.00	89.69	non coding	NA	NA	NA
PB.10577.4	9	3198	2	full-splice_match	gene: Zm00001d047240	Zm00001d047240_T00	0.00	89.69	coding	670	1189	3198
PB.10577.5	9	2779	1	novel_in_catalog	gene: Zm00001d047240	novel	0.13	89.69	coding	469	1372	2778
PB.9502.3	8	1260	1	novel_in_catalog	gene: Zm00001d010227	novel	49.66	667.99	coding	91	50	322
PB.9502.2	8	862	1	novel_in_catalog	gene: Zm00001d010227	novel	618.33	667.99	coding	135	21	425
PB.10577.1	9	3082	3	full-splice_match	gene: Zm00001d047240	Zm00001d047240_T00	38.27	89.69	coding	785	538	2892
PB.10206.1	9	1257	1	full-splice_match	gene: Zm00001d045208	Zm00001d045208_T00	13.66	13.66	coding	127	723	1103
PB.1251.1	1	788	1	antisense	novelGene_gene: Zm00001d033180	novel	6.90	6.90	coding	141	294	716
PB.7914.1	6	946	6	full-splice_match	gene: Zm00001d038139	Zm00001d038139_T00	28.46	92.46	coding	177	70	600
PB.5657.1	4	1939	6	full-splice_match	gene: Zm00001d052820	Zm00001d052820_T00	8.49	85.71	coding	327	116	1096
PB.5638.3	4	1768	5	full-splice_match	gene: Zm00001d052766	Zm00001d052766_T00	31.08	41.31	coding	409	234	1460

PB.5638.2	4	3021	4	novel_in_catalog	gene: Zm00001d052766	novel	4.30	41.31	coding	285	1822	2676
PB.5638.1	4	1889	5	full-splice_match	gene: Zm00001d052766	Zm00001d052766_T00	5.94	41.31	coding	409	238	1464
PB.8621.1	7	1770	1	intergenic	novelGene_263	novel	5.84	5.84	coding	334	33	1034
PB.10362.1	9	404	2	incomplete-splice_match	gene: Zm00001d045953	Zm00001d045953_T00	55.12	62.07	coding	44	195	326
PB.3346.3	2	2681	5	full-splice_match	gene: Zm00001d006355	Zm00001d006355_T01	0.00	38.31	coding	734	118	2319
PB.2159.7	10	1722	3	novel_in_catalog	gene: Zm00001d025752	novel	12.34	98.80	coding	173	467	985
PB.9175.1	8	3722	3	genic	gene: Zm00001d008413	novel	22.65	22.65	coding	1018	356	3409
PB.4523.1	3	1115	1	intergenic	novelGene_394	novel	5.70	5.70	non coding	NA	NA	NA
PB.1311.1	1	1238	3	incomplete-splice_match	gene: Zm00001d033448	Zm00001d033448_T01	19.47	19.47	coding	319	63	1019
PB.5618.1	4	874	4	full-splice_match	gene: Zm00001d052678	Zm00001d052678_T00	153.29	153.29	coding	180	73	612
PB.1858.2	10	910	3	full-splice_match	gene: Zm00001d024066	Zm00001d024066_T00	0.00	36.58	coding	199	182	778
PB.1858.1	10	1160	3	full-splice_match	gene: Zm00001d024066	Zm00001d024066_T00	36.58	36.58	coding	199	208	804
PB.8937.4	7	983	4	novel_in_catalog	gene: Zm00001d022042	novel	44.60	1497.07	coding	85	84	338
PB.6482.5	5	1118	6	novel_in_catalog	gene: Zm00001d014896	novel	3.00	27.46	non coding	NA	NA	NA
PB.4250.1	3	2034	2	full-splice_match	gene: Zm00001d042366	Zm00001d042366_T00	5.33	5.33	coding	551	209	1861
PB.6482.3	5	1792	12	full-splice_match	gene: Zm00001d014896	Zm00001d014896_T00	0.00	27.46	coding	372	203	1318
PB.6482.1	5	1895	12	full-splice_match	gene: Zm00001d014896	Zm00001d014896_T00	24.46	27.46	coding	372	203	1318

PB.10688.2	9	1131	8	incomplete-splice_match	gene: Zm00001d047683	Zm00001d047683_T00	78.89	78.89	coding	309	35	961
PB.7366.2	6	2178	9	novel_not_in_catalog	gene: Zm00001d035356	novel	11.52	45.01	coding	107	1203	1523
PB.7366.5	6	821	1	genic	gene: Zm00001d035356	novel	16.58	45.01	coding	100	57	356
PB.7366.4	6	1173	8	novel_not_in_catalog	gene: Zm00001d035356	novel	16.91	45.01	coding	107	334	654
PB.133.3	1	841	1	incomplete-splice_match	gene: Zm00001d027807	Zm00001d027807_T02	13.91	48.13	coding	256	73	840
PB.133.2	1	1919	8	novel_in_catalog	gene: Zm00001d027807	novel	3.96	48.13	coding	357	138	1208
PB.3814.1	3	777	1	full-splice_match	gene: Zm00001d039790	Zm00001d039790_T00	367.15	367.15	coding	136	87	494
PB.9663.2	8	1832	8	full-splice_match	gene: Zm00001d010915	Zm00001d010915_T00	17.80	31.41	coding	421	54	1316
PB.9663.1	8	756	2	incomplete-splice_match	gene: Zm00001d010915	Zm00001d010915_T00	2.79	31.41	non coding	NA	NA	NA
PB.6032.1	5	864	3	full-splice_match	gene: Zm00001d013057	Zm00001d013057_T00	92.50	92.50	coding	22	564	629
PB.1204.3	1	1151	2	full-splice_match	gene: Zm00001d032937	Zm00001d032937_T00	0.00	0.99	coding	278	286	1119
PB.6066.4	5	2130	1	genic	gene: Zm00001d013202	novel	19.60	336.86	coding	71	161	373
PB.6066.7	5	1599	4	novel_in_catalog	gene: Zm00001d013202	novel	0.00	336.86	coding	132	64	459
PB.6066.6	5	1207	3	novel_in_catalog	gene: Zm00001d013202	novel	11.53	336.86	coding	113	547	885
PB.6066.1	5	1669	5	full-splice_match	gene: Zm00001d013202	Zm00001d013202_T00	135.50	336.86	coding	362	150	1235
PB.6066.3	5	1864	3	novel_in_catalog	gene: Zm00001d013202	novel	11.88	336.86	coding	132	149	544
PB.6066.2	5	1938	5	full-splice_match	gene: Zm00001d013202	Zm00001d013202_T00	7.04	336.86	coding	362	532	1617

PB.9722.3	8	969	4	novel_in_catalog	gene: Zm00001d011180	novel	9.97	162.62	coding	188	117	680
PB.9722.2	8	923	5	full-splice_match	gene: Zm00001d011180	Zm00001d011180_T00	125.02	162.62	coding	176	114	641
PB.9722.1	8	1221	5	full-splice_match	gene: Zm00001d011180	Zm00001d011180_T00	27.63	162.62	coding	176	109	636
PB.2836.1	2	1175	1	incomplete-splice_match	gene: Zm00001d003566	Zm00001d003566_T00	115.76	115.76	coding	310	72	1001
PB.6066.9	5	1963	4	novel_in_catalog	gene: Zm00001d013202	novel	0.22	336.86	coding	287	536	1396
PB.6066.8	5	1465	5	full-splice_match	gene: Zm00001d013202	Zm00001d013202_T00	151.09	336.86	coding	362	154	1239
PB.5449.4	4	2084	16	full-splice_match	gene: Zm00001d052008	Zm00001d052008_T00	40.44	74.57	coding	553	278	1936
PB.6369.5	5	1582	10	novel_not_in_catalog	gene: Zm00001d014472	novel	47.74	73.79	coding	393	102	1280
PB.6369.4	5	2069	8	novel_not_in_catalog	gene: Zm00001d014472	novel	10.60	73.79	coding	214	1128	1769
PB.5343.6	4	813	1	incomplete-splice_match	gene: Zm00001d051507	Zm00001d051507_T08	0.00	31.79	coding	254	50	811
PB.2560.1	2	1623	14	novel_in_catalog	gene: Zm00001d002338	novel	82.85	82.85	coding	352	8	1063
PB.6369.1	5	2109	9	novel_not_in_catalog	gene: Zm00001d014472	novel	4.10	73.79	coding	214	945	1586
PB.5343.1	4	3021	6	incomplete-splice_match	gene: Zm00001d051507	Zm00001d051507_T05	1.50	31.79	coding	630	7	1896
PB.6369.3	5	2942	10	novel_not_in_catalog	gene: Zm00001d014472	novel	2.98	73.79	coding	328	1648	2631
PB.6369.2	5	3220	8	novel_not_in_catalog	gene: Zm00001d014472	novel	6.99	73.79	coding	214	2195	2836
PB.4928.1	4	598	1	full-splice_match	gene: Zm00001d049060	Zm00001d049060_T00	69.55	69.55	non coding	NA	NA	NA
PB.3279.1	2	2377	4	full-splice_match	gene: Zm00001d006082	Zm00001d006082_T00	0.85	11.89	coding	365	114	1208

PB.1674.3	1	913	3	incomplete-splice_match	gene: Zm00001d034919	Zm00001d034919_T00	39.26	126.09	coding	57	644	814
PB.1850.2	10	1004	2	incomplete-splice_match	gene: Zm00001d024004	Zm00001d024004_T00	0.00	27.89	coding	63	400	588
PB.1850.1	10	1185	2	novel_in_catalog	gene: Zm00001d024004	novel	27.89	27.89	coding	198	48	641
PB.7392.4	6	3502	17	full-splice_match	gene: Zm00001d035487	Zm00001d035487_T05	16.98	34.97	coding	876	209	2836
PB.7392.5	6	2512	11	incomplete-splice_match	gene: Zm00001d035487	Zm00001d035487_T04	8.50	34.97	coding	636	168	2075
PB.7392.1	6	2672	3	incomplete-splice_match	gene: Zm00001d035487	Zm00001d035487_T03	0.60	34.97	coding	358	9	1082
PB.7392.2	6	1447	3	incomplete-splice_match	gene: Zm00001d035487	Zm00001d035487_T03	5.26	34.97	coding	42	219	344
PB.7392.3	6	1320	3	incomplete-splice_match	gene: Zm00001d035487	Zm00001d035487_T03	3.63	34.97	coding	42	318	443
PB.1870.1	10	1478	1	full-splice_match	gene: Zm00001d024200	Zm00001d024200_T00	11.80	37.86	coding	278	98	931
PB.3393.1	2	767	4	genic	gene: Zm00001d006568	novel	6.62	6.62	non coding	NA	NA	NA
PB.1870.3	10	1233	1	full-splice_match	gene: Zm00001d024200	Zm00001d024200_T00	0.00	37.86	coding	278	91	924
PB.1870.2	10	1344	1	full-splice_match	gene: Zm00001d024200	Zm00001d024200_T00	26.06	37.86	coding	278	98	931
PB.1870.4	10	1139	1	full-splice_match	gene: Zm00001d024200	Zm00001d024200_T00	0.00	37.86	coding	278	98	931
PB.3937.3	3	2986	8	full-splice_match	gene: Zm00001d040368	Zm00001d040368_T01	19.61	49.07	coding	223	2200	2868
PB.7960.5	6	1325	2	genic	gene: Zm00001d038291	novel	19.81	1563.14	coding	154	408	869
PB.7960.4	6	1529	1	full-splice_match	gene: Zm00001d038291	Zm00001d038291_T00	0.00	1563.14	coding	154	532	993
PB.226.2	1	1726	6	full-splice_match	gene: Zm00001d028220	Zm00001d028220_T00	20.67	20.67	coding	454	26	1387

PB.7960.6	6	1052	2	genic	gene: Zm00001d038291	novel	124.70	1563.14	coding	154	140	601
PB.2440.1	2	1006	1	incomplete-splice_match	gene: Zm00001d001831	Zm00001d001831_T00	120.23	120.23	coding	229	24	710
PB.9369.1	8	2182	9	full-splice_match	gene: Zm00001d009488	Zm00001d009488_T00	120.37	372.68	coding	553	76	1734
PB.9369.2	8	2053	9	full-splice_match	gene: Zm00001d009488	Zm00001d009488_T00	249.87	372.68	coding	553	87	1745
PB.9369.3	8	1947	9	full-splice_match	gene: Zm00001d009488	Zm00001d009488_T00	2.43	372.68	coding	553	84	1742
PB.4605.2	3	1975	12	full-splice_match	gene: Zm00001d043771	Zm00001d043771_T00	13.80	32.17	coding	501	200	1702
PB.7960.9	6	1169	2	genic	gene: Zm00001d038291	novel	0.00	1563.14	coding	154	403	864
PB.7960.8	6	1299	1	full-splice_match	gene: Zm00001d038291	Zm00001d038291_T00	55.78	1563.14	coding	154	528	989
PB.10069.3	8	1398	2	genic	gene: Zm00001d012644	novel	30.11	30.11	coding	104	837	1148
PB.117.5	1	1118	3	full-splice_match	gene: Zm00001d027708	Zm00001d027708_T00	0.00	34.40	coding	182	372	917
PB.117.4	1	1733	3	full-splice_match	gene: Zm00001d027708	Zm00001d027708_T00	1.62	34.40	coding	398	128	1321
PB.117.3	1	1654	4	novel_in_catalog	gene: Zm00001d027708	novel	14.58	34.40	coding	372	125	1240
PB.793.5	1	2679	6	novel_in_catalog	gene: Zm00001d031081	novel	2.83	23.18	coding	522	1013	2578
PB.117.1	1	1881	4	novel_in_catalog	gene: Zm00001d027708	novel	18.21	34.40	coding	372	129	1244
PB.2333.1	10	1557	1	genic	gene: Zm00001d026467	novel	0.88	0.88	coding	184	88	639
PB.7314.2	6	780	4	novel_not_in_catalog	gene: Zm00001d035054	novel	198.18	285.67	coding	141	115	537
PB.6590.3	5	1545	2	full-splice_match	gene: Zm00001d015356	Zm00001d015356_T00	0.00	22.37	coding	444	123	1454

PB.8329.1	7	1060	2	full-splice_match	gene: Zm00001d019147	Zm00001d019147_T00	519.92	519.92	coding	279	89	925
PB.7314.1	6	909	4	novel_not_in_catalog	gene: Zm00001d035054	novel	87.50	285.67	coding	141	129	551
PB.2450.2	2	1475	5	full-splice_match	gene: Zm00001d001868	Zm00001d001868_T00	0.00	56.42	coding	404	128	1339
PB.5397.1	4	2156	9	full-splice_match	gene: Zm00001d051761	Zm00001d051761_T00	196.53	281.69	coding	557	151	1821
PB.2450.1	2	1650	5	full-splice_match	gene: Zm00001d001868	Zm00001d001868_T00	56.42	56.42	coding	404	159	1370
PB.5236.1	4	1681	4	novel_in_catalog	gene: Zm00001d050868	novel	32.50	132.49	coding	152	160	615
PB.8766.1	7	2371	16	full-splice_match	gene: Zm00001d021473	Zm00001d021473_T00	30.72	32.55	coding	594	58	1839
PB.5236.3	4	2401	4	novel_in_catalog	gene: Zm00001d050868	novel	3.39	132.49	coding	152	491	946
PB.5236.2	4	2487	11	full-splice_match	gene: Zm00001d050868	Zm00001d050868_T00	30.71	132.49	coding	623	212	2080
PB.5236.5	4	2853	10	full-splice_match	gene: Zm00001d050868	Zm00001d050868_T00	15.10	132.49	coding	364	1454	2545
PB.5236.4	4	2395	11	full-splice_match	gene: Zm00001d050868	Zm00001d050868_T00	50.80	132.49	coding	623	219	2087
PB.1147.5	1	3862	7	full-splice_match	gene: Zm00001d032703	Zm00001d032703_T01	4.18	85.19	coding	535	260	1864
PB.5236.6	4	2491	10	novel_in_catalog	gene: Zm00001d050868	novel	0.00	132.49	coding	411	212	1444
PB.4323.5	3	1808	7	novel_in_catalog	gene: Zm00001d042695	novel	3.83	95.71	coding	111	1285	1617
PB.5236.8	4	2220	11	full-splice_match	gene: Zm00001d050868	Zm00001d050868_T00	0.00	132.49	coding	623	216	2084
PB.4323.7	3	1467	9	full-splice_match	gene: Zm00001d042695	Zm00001d042695_T00	42.97	95.71	coding	361	215	1297
PB.4323.6	3	1574	8	novel_in_catalog	gene: Zm00001d042695	novel	5.83	95.71	coding	261	218	1000

PB.4323.1	3	4604	4	novel_in_catalog	gene: Zm00001d042695	novel	2.06	95.71	coding	111	214	546
PB.3535.1	2	1663	5	incomplete-splice_match	gene: Zm00001d007173	Zm00001d007173_T00	82.88	82.88	coding	437	128	1438
PB.4323.3	3	3723	8	novel_in_catalog	gene: Zm00001d042695	novel	5.03	95.71	coding	192	15	590
PB.4323.2	3	1575	9	full-splice_match	gene: Zm00001d042695	Zm00001d042695_T00	23.38	95.71	coding	361	218	1300
PB.10996.1	B73V4 ctg3	1876	1	full-splice_match	gene: Zm00001d000434	Zm00001d000434_T00	0.00	10.91	coding	573	28	1746
PB.1887.4	10	420	2	novel_not_in_catalog	gene: Zm00001d024325	novel	7.81	45.06	non coding	NA	NA	NA
PB.8349.1	7	585	3	full-splice_match	gene: Zm00001d019260	Zm00001d019260_T00	46.15	308.88	non coding	NA	NA	NA
PB.5367.3	4	1103	2	full-splice_match	gene: Zm00001d051611	Zm00001d051611_T00	6.67	37.75	coding	271	93	905
PB.8349.2	7	463	3	full-splice_match	gene: Zm00001d019260	Zm00001d019260_T00	262.73	308.88	non coding	NA	NA	NA
PB.2442.3	2	681	1	full-splice_match	gene: Zm00001d001839	Zm00001d001839_T00	0.00	33.02	coding	60	162	341
PB.1073.1	1	1969	14	novel_not_in_catalog	gene: Zm00001d032441	novel	50.24	67.31	coding	487	125	1585
PB.9925.4	8	1062	3	full-splice_match	gene: Zm00001d012031	Zm00001d012031_T00	36.37	50.23	coding	228	266	949
PB.9010.1	7	1348	5	full-splice_match	gene: Zm00001d022324	Zm00001d022324_T00	48.85	156.21	coding	273	148	966
PB.3396.15	2	1792	7	full-splice_match	gene: Zm00001d006578	Zm00001d006578_T00	7.15	210.21	coding	367	380	1480
PB.2442.1	2	1246	1	full-splice_match	gene: Zm00001d001839	Zm00001d001839_T00	10.35	33.02	coding	60	148	327
PB.7620.1	6	1337	3	novel_in_catalog	gene: Zm00001d036835	novel	19.22	148.78	coding	254	424	1185
PB.8084.3	6	2100	14	full-splice_match	gene: Zm00001d038775	Zm00001d038775_T00	0.00	52.66	coding	575	117	1841

PB.9925.2	8	2153	1	genic	gene: Zm00001d012031	novel	8.10	50.23	coding	118	1583	1936
PB.3346.4	2	2618	5	full-splice_match	gene: Zm00001d006355	Zm00001d006355_T01	0.00	38.31	coding	734	216	2417
PB.2945.1	2	1209	5	full-splice_match	gene: Zm00001d004143	Zm00001d004143_T00	9.69	9.69	coding	274	36	857
PB.10654.1	9	3133	10	incomplete-splice_match	gene: Zm00001d047540	Zm00001d047540_T00	42.42	66.90	coding	860	179	2758
PB.10654.2	9	2482	6	full-splice_match	gene: Zm00001d047540	Zm00001d047540_T01	24.49	66.90	coding	652	241	2196
PB.3199.1	2	675	2	full-splice_match	gene: Zm00001d005716	Zm00001d005716_T00	27.29	35.35	coding	142	65	490
PB.3199.2	2	813	1	full-splice_match	gene: Zm00001d005716	Zm00001d005716_T00	8.06	35.35	coding	142	65	490
PB.1240.1	1	1268	8	incomplete-splice_match	gene: Zm00001d033108	Zm00001d033108_T00	19.67	77.27	coding	284	16	867
PB.8429.2	7	878	2	full-splice_match	gene: Zm00001d019836	Zm00001d019836_T00	17.69	83.80	coding	143	234	662
PB.9381.1	8	1091	5	full-splice_match	gene: Zm00001d009525	Zm00001d009525_T00	7.75	7.75	coding	201	137	739
PB.254.3	1	1598	7	full-splice_match	gene: Zm00001d028344	Zm00001d028344_T00	0.00	27.37	coding	402	184	1389
PB.3346.5	2	1387	1	novel_in_catalog	gene: Zm00001d006355	novel	10.43	38.31	coding	240	330	1049
PB.254.1	1	1641	7	full-splice_match	gene: Zm00001d028344	Zm00001d028344_T00	27.37	27.37	coding	402	186	1391
PB.8429.3	7	1682	1	genic	gene: Zm00001d019836	novel	11.13	83.80	coding	128	1090	1473
PB.9893.1	8	2737	10	full-splice_match	gene: Zm00001d011913	Zm00001d011913_T00	57.76	58.51	coding	782	69	2414
PB.1054.1	1	1713	1	intergenic	novelGene_427	novel	13.15	13.15	coding	507	71	1591
PB.1586.1	1	1273	5	full-splice_match	gene: Zm00001d034627	Zm00001d034627_T00	32.61	54.00	coding	250	145	894

PB.5362.6	4	2169	7	incomplete-splice_match	gene: Zm00001d051600	Zm00001d051600_T01	6.39	151.77	coding	616	321	2168
PB.585.1	1	3468	2	genic	gene: Zm00001d029846	novel	6.77	6.77	coding	997	74	3064
PB.2014.1	10	984	2	full-splice_match	gene: Zm00001d024963	Zm00001d024963_T00	10.19	717.24	coding	232	12	707
PB.9228.5	8	1151	5	incomplete-splice_match	gene: Zm00001d008646	Zm00001d008646_T00	32.07	61.00	coding	241	60	782
PB.3185.1	2	1788	7	full-splice_match	gene: Zm00001d005674	Zm00001d005674_T00	18.13	18.13	coding	476	258	1685
PB.9228.1	8	1112	2	novel_in_catalog	gene: Zm00001d008646	novel	28.94	61.00	coding	296	109	996
PB.5244.3	4	604	4	full-splice_match	gene: Zm00001d050916	Zm00001d050916_T00	110.89	147.44	coding	114	58	399
PB.10067.2	8	1100	3	novel_not_in_catalog	gene: Zm00001d012642	novel	1.81	7.05	coding	297	61	951
PB.5244.1	4	710	4	full-splice_match	gene: Zm00001d050916	Zm00001d050916_T00	36.55	147.44	coding	114	59	400
PB.866.2	1	2267	2	novel_in_catalog	gene: Zm00001d031470	novel	24.91	102.58	coding	407	250	1470
PB.866.3	1	2325	3	full-splice_match	gene: Zm00001d031470	Zm00001d031470_T00	0.00	102.58	coding	577	43	1773
PB.4699.1	3	1903	1	genic	gene: Zm00001d000707	novel	3.34	3.34	non coding	NA	NA	NA
PB.866.1	1	2561	3	full-splice_match	gene: Zm00001d031470	Zm00001d031470_T00	55.90	102.58	coding	577	171	1901
PB.7291.2	6	750	1	incomplete-splice_match	gene: Zm00001d034968	Zm00001d034968_T00	9.16	13.19	coding	204	102	713
PB.1216.1	1	1371	1	antisense	novelGene_gene: Zm00001d032999	novel	16.43	16.43	coding	155	527	991
PB.7291.1	6	1289	2	incomplete-splice_match	gene: Zm00001d034968	Zm00001d034968_T00	4.03	13.19	non coding	NA	NA	NA
PB.4462.5	3	2119	4	novel_in_catalog	gene: Zm00001d043278	novel	6.39	34.02	coding	157	1516	1986

PB.7267.8	5	1409	6	novel_not_in_catalog	gene: Zm00001d018511	novel	0.44	44.35	coding	174	780	1301
PB.10973.1	B73V4	987	6	full-splice_match	gene: Zm00001d000029	Zm00001d000029_T00	185.23	185.23	coding	157	342	812
PB.8055.1	6	1451	1	incomplete-splice_match	gene: Zm00001d038622	Zm00001d038622_T00	20.42	20.42	coding	370	135	1244
PB.7267.4	5	1884	5	novel_in_catalog	gene: Zm00001d018511	novel	12.56	44.35	coding	174	1023	1544
PB.7267.7	5	938	6	full-splice_match	gene: Zm00001d018511	Zm00001d018511_T00	17.19	44.35	coding	174	267	788
PB.8055.2	6	1315	1	incomplete-splice_match	gene: Zm00001d038622	Zm00001d038622_T00	0.00	20.42	coding	370	39	1148
PB.2987.1	2	1300	2	novel_in_catalog	gene: Zm00001d004480	novel	5.76	21.71	coding	148	312	755
PB.3561.10	2	1745	5	novel_in_catalog	gene: Zm00001d007311	novel	0.00	74.66	coding	427	200	1480
PB.2153.10	10	1035	6	novel_not_in_catalog	gene: Zm00001d025727	novel	34.20	97.72	coding	219	199	855
PB.2153.11	10	1505	6	novel_not_in_catalog	gene: Zm00001d025727	novel	0.00	97.72	coding	355	272	1336
PB.7336.2	6	1865	9	novel_in_catalog	gene: Zm00001d035183	novel	11.57	51.00	coding	404	274	1485
PB.3921.1	3	2075	5	incomplete-splice_match	gene: Zm00001d040312	Zm00001d040312_T01	10.96	12.85	coding	506	54	1571
PB.3921.2	3	1435	2	incomplete-splice_match	gene: Zm00001d040312	Zm00001d040312_T01	1.89	12.85	coding	364	49	1140
PB.7336.1	6	1953	10	full-splice_match	gene: Zm00001d035183	Zm00001d035183_T00	24.18	51.00	coding	435	322	1626
PB.1116.1	1	3236	13	full-splice_match	gene: Zm00001d032575	Zm00001d032575_T00	21.89	25.13	coding	890	83	2752
PB.7230.1	5	1790	9	full-splice_match	gene: Zm00001d018409	Zm00001d018409_T00	55.70	143.21	coding	428	92	1375
PB.5510.1	4	1923	9	full-splice_match	gene: Zm00001d052227	Zm00001d052227_T00	6.94	6.94	coding	356	177	1244

PB.10561.1	9	1514	1	antisense	novelGene_gene: Zm00001d047182	novel	4.57	4.57	coding	129	368	754
PB.7072.2	5	2187	1	full-splice_match	gene: Zm00001d017825	Zm00001d017825_T00	60.04	116.32	coding	324	829	1800
PB.2959.2	2	1693	1	genic	gene: Zm00001d004248	novel	0.00	15.52	coding	489	149	1615
PB.7072.7	5	1096	1	full-splice_match	gene: Zm00001d017825	Zm00001d017825_T00	4.78	116.32	coding	324	54	1025
PB.2959.1	2	1852	1	genic	gene: Zm00001d004248	novel	15.52	15.52	coding	489	145	1611
PB.5579.1	4	1276	10	incomplete-splice_match	gene: Zm00001d052439	Zm00001d052439_T00	28.48	39.63	coding	315	43	987
PB.649.1	1	1789	6	full-splice_match	gene: Zm00001d030172	Zm00001d030172_T00	14.70	14.70	coding	495	95	1579
PB.842.3	1	2495	13	full-splice_match	gene: Zm00001d031300	Zm00001d031300_T00	0.00	11.87	coding	747	118	2358
PB.6617.9	5	3086	5	novel_in_catalog	gene: Zm00001d015521	novel	13.22	161.78	coding	595	1076	2860
PB.1274.1	1	2452	6	full-splice_match	gene: Zm00001d033292	Zm00001d033292_T00	6.47	112.19	coding	559	178	1854
PB.2979.1	2	1423	14	full-splice_match	gene: Zm00001d004460	Zm00001d004460_T00	2.18	4.94	coding	399	82	1278
PB.2979.2	2	1490	13	full-splice_match	gene: Zm00001d004460	Zm00001d004460_T00	2.77	4.94	coding	399	53	1249
PB.6617.8	5	2236	5	novel_in_catalog	gene: Zm00001d015521	novel	4.29	161.78	coding	580	269	2008
PB.10414.2	9	1582	6	novel_in_catalog	gene: Zm00001d046352	novel	0.26	130.20	coding	352	388	1443
PB.10414.3	9	1495	7	full-splice_match	gene: Zm00001d046352	Zm00001d046352_T00	22.66	130.20	coding	417	119	1369
PB.3746.8	3	1168	7	incomplete-splice_match	gene: Zm00001d039499	Zm00001d039499_T00	0.00	118.28	coding	306	249	1166
PB.3746.6	3	2140	9	novel_in_catalog	gene: Zm00001d039499	novel	2.25	118.28	coding	362	168	1253

PB.1214.1	1	1256	8	novel_not_in_catalog	gene: Zm00001d032990	novel	11.25	11.25	coding	303	63	971
PB.5860.2	4	3691	14	full-splice_match	gene: Zm00001d053819	Zm00001d053819_T00	0.00	11.39	coding	914	705	3446
PB.3746.2	3	2064	10	novel_in_catalog	gene: Zm00001d039499	novel	3.11	118.28	coding	362	160	1245
PB.3746.1	3	2047	11	full-splice_match	gene: Zm00001d039499	Zm00001d039499_T00	112.92	118.28	coding	528	285	1868
PB.5860.1	4	3792	14	full-splice_match	gene: Zm00001d053819	Zm00001d053819_T00	11.04	11.39	coding	914	731	3472
PB.10152.1	9	1029	4	incomplete-splice_match	gene: Zm00001d044951	Zm00001d044951_T00	5.87	5.87	coding	299	4	900
PB.836.3	1	1144	1	full-splice_match	gene: Zm00001d031273	Zm00001d031273_T00	0.00	24.47	coding	289	32	898
PB.836.2	1	1291	1	full-splice_match	gene: Zm00001d031273	Zm00001d031273_T00	22.25	24.47	coding	289	48	914
PB.2428.2	2	1247	7	full-splice_match	gene: Zm00001d001799	Zm00001d001799_T00	22.81	22.81	coding	197	233	823
PB.7908.1	6	1129	2	full-splice_match	gene: Zm00001d038126	Zm00001d038126_T00	75.44	93.75	coding	146	167	604
PB.1360.5	1	1528	10	full-splice_match	gene: Zm00001d033646	Zm00001d033646_T01	22.22	78.89	coding	411	68	1300
PB.3285.1	2	1953	5	full-splice_match	gene: Zm00001d006102	Zm00001d006102_T00	60.28	71.28	coding	351	557	1609
PB.3285.3	2	1751	5	full-splice_match	gene: Zm00001d006102	Zm00001d006102_T00	7.10	71.28	coding	351	560	1612
PB.3285.2	2	2083	4	full-splice_match	gene: Zm00001d006102	Zm00001d006102_T00	3.90	71.28	coding	179	1232	1768
PB.6617.3	5	3149	5	novel_in_catalog	gene: Zm00001d015521	novel	38.05	161.78	coding	595	1012	2796
PB.276.1	1	1243	2	full-splice_match	gene: Zm00001d028413	Zm00001d028413_T00	9.64	9.64	coding	260	93	872
PB.8695.1	7	988	1	full-splice_match	gene: Zm00001d021208	Zm00001d021208_T00	106.33	799.63	coding	231	93	785

PB.6691.5	5	1578	9	full-splice_match	gene: Zm00001d016077	Zm00001d016077_T00	49.40	71.60	coding	279	628	1464
PB.1077.3	1	1868	2	genic	gene: Zm00001d032460	novel	0.00	43.43	coding	439	156	1472
PB.7230.3	5	1720	9	full-splice_match	gene: Zm00001d018409	Zm00001d018409_T00	87.51	143.21	coding	428	158	1441
PB.5672.1	4	1924	5	full-splice_match	gene: Zm00001d052901	Zm00001d052901_T00	25.58	25.58	coding	369	570	1676
PB.3125.1	2	1527	3	incomplete-splice_match	gene: Zm00001d005323	Zm00001d005323_T00	45.59	75.29	coding	303	89	997
PB.7474.1	6	850	1	full-splice_match	gene: Zm00001d035978	Zm00001d035978_T00	2.72	2.72	coding	203	59	667
PB.5598.1	4	1779	1	genic	gene: Zm00001d052530	novel	116.74	116.74	coding	507	98	1618
PB.7070.2	5	2738	2	full-splice_match	gene: Zm00001d017821	Zm00001d017821_T00	13.73	15.54	coding	749	65	2311
PB.9971.1	8	1729	4	full-splice_match	gene: Zm00001d012247	Zm00001d012247_T00	13.55	44.78	coding	344	138	1169
PB.7529.1	6	1975	8	full-splice_match	gene: Zm00001d036359	Zm00001d036359_T00	15.38	15.38	coding	458	469	1842
PB.2248.1	10	666	1	antisense	novelGene_gene: Zm00001d026176	novel	21.60	21.60	coding	136	182	589
PB.9971.2	8	2379	4	full-splice_match	gene: Zm00001d012247	Zm00001d012247_T00	17.98	44.78	coding	585	209	1963
PB.9971.4	8	2286	4	full-splice_match	gene: Zm00001d012247	Zm00001d012247_T00	10.86	44.78	coding	585	313	2067
PB.4566.1	3	724	1	intergenic	novelGene_399	novel	0.44	0.44	non coding	NA	NA	NA
PB.6617.6	5	1947	3	novel_in_catalog	gene: Zm00001d015521	novel	5.78	161.78	coding	641	22	1944
PB.5266.2	4	1214	8	full-splice_match	gene: Zm00001d051021	Zm00001d051021_T00	82.21	124.83	coding	213	324	962
PB.4090.1	3	2418	2	full-splice_match	gene: Zm00001d041550	Zm00001d041550_T00	128.99	1251.30	coding	649	85	2031

PB.4090.2	3	2246	2	full-splice_match	gene: Zm00001d041550	Zm00001d041550_T00	1086.73	1251.30	coding	649	81	2027
PB.5872.4	4	2236	14	full-splice_match	gene: Zm00001d053857	Zm00001d053857_T00	4.22	112.35	coding	452	63	1418
PB.4090.6	3	2236	2	full-splice_match	gene: Zm00001d041550	Zm00001d041550_T00	35.58	1251.30	coding	649	86	2032
PB.840.1	1	856	1	full-splice_match	gene: Zm00001d031290	Zm00001d031290_T00	43.99	43.99	coding	146	170	607
PB.2899.2	2	1241	5	full-splice_match	gene: Zm00001d003904	Zm00001d003904_T00	102.65	109.58	coding	173	341	859
PB.8701.10	7	1645	4	full-splice_match	gene: Zm00001d021221	Zm00001d021221_T00	0.00	356.28	coding	334	445	1446
PB.2899.5	2	1109	5	full-splice_match	gene: Zm00001d003904	Zm00001d003904_T00	0.00	109.58	coding	173	283	801
PB.8355.1	7	2958	1	full-splice_match	gene: Zm00001d019297	Zm00001d019297_T00	3.78	3.78	coding	879	39	2675
PB.678.1	1	3468	13	full-splice_match	gene: Zm00001d030357	Zm00001d030357_T00	33.22	102.40	coding	935	126	2930
PB.6575.4	5	2181	2	full-splice_match	gene: Zm00001d015292	Zm00001d015292_T00	0.00	89.68	coding	526	458	2035
PB.3716.2	3	1760	7	genic	gene: Zm00001d039379	novel	5.14	141.45	coding	266	707	1504
PB.3716.3	3	2162	6	genic	gene: Zm00001d039379	novel	17.14	141.45	coding	266	1116	1913
PB.6709.1	5	1296	3	full-splice_match	gene: Zm00001d016182	Zm00001d016182_T00	6.12	13.60	coding	322	243	1208
PB.3716.8	3	1961	7	genic	gene: Zm00001d039379	novel	119.17	141.45	coding	575	75	1799
PB.9296.2	8	951	3	full-splice_match	gene: Zm00001d008992	Zm00001d008992_T00	65.98	74.35	coding	225	61	735
PB.9296.1	8	2413	2	novel_in_catalog	gene: Zm00001d008992	novel	1.56	74.35	coding	108	111	434
PB.790.1	1	3057	3	novel_not_in_catalog	gene: Zm00001d031073	novel	15.54	15.54	coding	735	510	2714

PB.2086.1	10	2304	1	novel_in_catalog	gene: Zm00001d025339	novel	11.63	360.35	coding	124	276	647
PB.10913.1	9	2392	13	novel_in_catalog	gene: Zm00001d048509	novel	27.56	27.56	coding	398	22	1215
PB.6281.1	5	1113	2	full-splice_match	gene: Zm00001d014089	Zm00001d014089_T00	3.88	3.88	coding	222	102	767
PB.9248.2	8	2152	2	full-splice_match	gene: Zm00001d008739	Zm00001d008739_T00	0.00	26.25	coding	575	259	1983
PB.7217.1	5	1675	2	full-splice_match	gene: Zm00001d018367	Zm00001d018367_T00	11.23	11.23	coding	442	138	1463
PB.8282.1	7	1001	1	intergenic	novelGene_40	novel	20.97	20.97	coding	268	95	898
PB.9248.1	8	2299	2	full-splice_match	gene: Zm00001d008739	Zm00001d008739_T00	26.25	26.25	coding	575	268	1992
PB.10729.1	9	2097	8	full-splice_match	gene: Zm00001d047806	Zm00001d047806_T00	15.46	15.46	coding	446	79	1416
PB.1670.1	1	856	5	full-splice_match	gene: Zm00001d034893	Zm00001d034893_T00	69.51	168.92	coding	147	122	562
PB.1178.2	1	1073	1	intergenic	novelGene_438	novel	264.13	322.84	coding	261	92	874
PB.1581.1	1	2379	10	novel_in_catalog	gene: Zm00001d034610	novel	20.31	20.31	coding	548	325	1968
PB.5926.2	4	1146	8	novel_in_catalog	gene: Zm00001d054017	novel	6.04	181.85	coding	107	43	363
PB.7269.1	5	1955	5	full-splice_match	gene: Zm00001d018515	Zm00001d018515_T00	45.30	48.97	coding	388	540	1703
PB.6575.1	5	2272	2	full-splice_match	gene: Zm00001d015292	Zm00001d015292_T00	89.68	89.68	coding	526	464	2041
PB.4762.2	3	2764	7	full-splice_match	gene: Zm00001d044420	Zm00001d044420_T00	6.34	9.53	coding	782	215	2560
PB.7837.9	6	1557	3	full-splice_match	gene: Zm00001d037875	Zm00001d037875_T00	169.43	497.66	coding	447	78	1418
PB.5790.3	4	1395	2	genic	gene: Zm00001d053552	novel	32.61	57.09	coding	362	54	1139

PB.7837.4	6	1665	3	full-splice_match	gene: Zm00001d037875	Zm00001d037875_T00	328.23	497.66	coding	447	83	1423
PB.1350.1	1	2841	23	novel_in_catalog	gene: Zm00001d033597	novel	28.23	39.02	coding	798	99	2492
PB.10379.1	9	573	1	intergenic	novelGene_149	novel	0.00	0.00	non coding	NA	NA	NA
PB.1350.3	1	2101	11	novel_in_catalog	gene: Zm00001d033597	novel	7.58	39.02	coding	304	287	1198
PB.9477.2	8	853	1	full-splice_match	gene: Zm00001d010067	Zm00001d010067_T00	52.28	73.03	coding	166	124	621
PB.7781.1	6	914	5	full-splice_match	gene: Zm00001d037620	Zm00001d037620_T00	122.92	122.92	coding	208	113	736
PB.9477.1	8	948	1	full-splice_match	gene: Zm00001d010067	Zm00001d010067_T00	20.74	73.03	coding	166	80	577
PB.5559.2	4	1225	8	full-splice_match	gene: Zm00001d052379	Zm00001d052379_T00	40.46	103.90	coding	223	289	957
PB.646.4	1	1553	2	incomplete-splice_match	gene: Zm00001d030166	Zm00001d030166_T00	9.92	46.00	coding	144	1098	1529
PB.646.2	1	1705	10	full-splice_match	gene: Zm00001d030166	Zm00001d030166_T00	36.08	46.00	coding	521	91	1653
PB.3092.2	2	1355	12	full-splice_match	gene: Zm00001d005144	Zm00001d005144_T00	41.28	54.95	coding	364	97	1188
PB.3760.1	3	2066	3	full-splice_match	gene: Zm00001d039532	Zm00001d039532_T00	13.53	13.53	coding	547	106	1746
PB.5559.4	4	3011	3	novel_in_catalog	gene: Zm00001d052379	novel	2.92	103.90	non coding	NA	NA	NA
PB.10043.6	8	990	6	full-splice_match	gene: Zm00001d012550	Zm00001d012550_T00	0.00	327.31	coding	213	153	791
PB.10043.4	8	1363	5	novel_in_catalog	gene: Zm00001d012550	novel	2.12	327.31	coding	143	199	627
PB.10043.2	8	1148	6	full-splice_match	gene: Zm00001d012550	Zm00001d012550_T00	312.58	327.31	coding	213	278	916
PB.7402.1	6	1288	3	full-splice_match	gene: Zm00001d035542	Zm00001d035542_T00	125.20	137.49	coding	175	383	907

PB.7402.2	6	1293	3	full-splice_match	gene: Zm00001d035542	Zm00001d035542_T00	12.30	137.49	coding	175	424	948
PB.10735.1	9	1316	9	novel_not_in_catalog	gene: Zm00001d047823	novel	34.38	34.38	coding	280	261	1100
PB.10899.11	9	1953	6	novel_in_catalog	gene: Zm00001d048472	novel	2.96	74.45	coding	440	186	1505
PB.1728.2	10	1622	1	full-splice_match	gene: Zm00001d023399	Zm00001d023399_T00	7.79	14.83	coding	456	83	1450
PB.2541.1	2	3030	20	full-splice_match	gene: Zm00001d002272	Zm00001d002272_T00	95.82	136.44	coding	736	386	2593
PB.1177.1	1	1367	10	intergenic	novelGene_437	novel	23.88	38.15	coding	374	19	1140
PB.2541.3	2	1562	8	novel_in_catalog	gene: Zm00001d002272	novel	20.13	136.44	coding	308	279	1202
PB.2541.2	2	1621	3	novel_in_catalog	gene: Zm00001d002272	novel	20.49	136.44	coding	232	472	1167
PB.74.1	1	1930	2	full-splice_match	gene: Zm00001d027537	Zm00001d027537_T00	265.05	265.05	coding	546	46	1683
PB.74.3	1	1567	2	full-splice_match	gene: Zm00001d027537	Zm00001d027537_T00	0.00	265.05	coding	456	61	1428
PB.7016.1	5	2437	7	full-splice_match	gene: Zm00001d017568	Zm00001d017568_T00	65.55	65.55	coding	610	551	2380
PB.1171.1	1	1505	10	full-splice_match	gene: Zm00001d032798	Zm00001d032798_T00	7.39	7.39	coding	292	232	1107
PB.1657.2	1	1608	3	full-splice_match	gene: Zm00001d034863	Zm00001d034863_T00	3.03	19.60	coding	520	13	1572
PB.5150.2	4	757	2	full-splice_match	gene: Zm00001d050179	Zm00001d050179_T00	333.78	492.06	coding	149	92	538
PB.9537.1	8	1225	3	full-splice_match	gene: Zm00001d010381	Zm00001d010381_T00	49.53	49.53	coding	15	323	367
PB.4759.1	3	1641	3	full-splice_match	gene: Zm00001d044395	Zm00001d044395_T00	8.23	76.77	coding	288	119	982
PB.5150.3	4	592	2	full-splice_match	gene: Zm00001d050179	Zm00001d050179_T00	0.00	492.06	coding	149	37	483

PB.2573.6	2	640	1	genic	gene: Zm00001d002370	novel	18.04	383.43	coding	160	109	588
PB.2573.4	2	1109	1	genic	gene: Zm00001d002370	novel	11.81	383.43	coding	160	109	588
PB.2573.5	2	872	1	genic	gene: Zm00001d002370	novel	0.00	383.43	coding	160	110	589
PB.2573.2	2	3709	1	genic	gene: Zm00001d002370	novel	12.48	383.43	coding	294	2627	3508
PB.2573.3	2	1590	2	full-splice_match	gene: Zm00001d002370	Zm00001d002370_T00	0.00	383.43	coding	460	110	1489
PB.5798.2	4	1485	8	full-splice_match	gene: Zm00001d053576	Zm00001d053576_T00	44.01	82.20	coding	414	89	1330
PB.8261.1	7	1892	14	novel_in_catalog	gene: Zm00001d018799	novel	101.82	101.82	coding	449	343	1689
PB.5157.1	4	1080	7	full-splice_match	gene: Zm00001d050198	Zm00001d050198_T00	267.16	267.16	coding	264	65	856
PB.4980.1	4	1358	6	full-splice_match	gene: Zm00001d049252	Zm00001d049252_T00	50.07	50.07	coding	293	194	1072
PB.2159.2	10	1765	3	novel_in_catalog	gene: Zm00001d025752	novel	6.49	98.80	coding	156	470	937
PB.447.5	1	2409	5	novel_in_catalog	gene: Zm00001d029165	novel	0.00	103.12	coding	191	723	1295
PB.3768.1	3	690	2	full-splice_match	gene: Zm00001d039565	Zm00001d039565_T00	7.71	7.71	coding	174	65	586
PB.873.3	1	2516	10	novel_in_catalog	gene: Zm00001d031502	novel	2.24	59.06	coding	353	126	1184
PB.4530.1	3	1871	4	novel_not_in_catalog	gene: Zm00001d043510	novel	9.64	21.10	coding	414	289	1530
PB.4530.7	3	1858	4	novel_not_in_catalog	gene: Zm00001d043510	novel	2.11	21.10	coding	414	373	1614
PB.447.7	1	2272	6	novel_in_catalog	gene: Zm00001d029165	novel	10.57	103.12	coding	191	593	1165
PB.4530.5	3	1865	4	full-splice_match	gene: Zm00001d043510	Zm00001d043510_T00	4.69	21.10	coding	410	390	1619

PB.4530.4	3	2138	2	novel_in_catalog	gene: Zm00001d043510	novel	4.66	21.10	coding	255	1103	1867
PB.6898.2	5	1271	1	genic	gene: Zm00001d017095	novel	0.00	3802.15	coding	158	493	966
PB.6898.3	5	988	2	full-splice_match	gene: Zm00001d017095	Zm00001d017095_T00	2391.21	3802.15	coding	203	175	783
PB.7117.1	5	2526	5	full-splice_match	gene: Zm00001d017987	Zm00001d017987_T00	58.40	58.40	coding	612	186	2021
PB.6898.1	5	1103	2	full-splice_match	gene: Zm00001d017095	Zm00001d017095_T00	116.91	3802.15	coding	203	174	782
PB.10426.1	9	1365	10	novel_in_catalog	gene: Zm00001d046434	novel	41.65	41.65	coding	127	320	700
PB.6898.7	5	866	2	full-splice_match	gene: Zm00001d017095	Zm00001d017095_T00	965.97	3802.15	coding	203	163	771
PB.6898.4	5	1155	1	novel_in_catalog	gene: Zm00001d017095	novel	328.06	3802.15	coding	158	493	966
PB.4687.2	3	2115	13	full-splice_match	gene: Zm00001d044101	Zm00001d044101_T00	8.87	21.54	coding	383	420	1568
PB.4687.3	3	1943	13	full-splice_match	gene: Zm00001d044101	Zm00001d044101_T00	12.67	21.54	coding	383	412	1560
PB.961.1	1	1964	6	full-splice_match	gene: Zm00001d031959	Zm00001d031959_T00	80.29	111.08	coding	516	169	1716
PB.961.2	1	2005	5	novel_in_catalog	gene: Zm00001d031959	novel	22.94	111.08	coding	373	671	1789
PB.4177.1	3	1720	1	full-splice_match	gene: Zm00001d042007	Zm00001d042007_T00	16.91	16.91	coding	387	322	1482
PB.2228.1	10	1482	6	full-splice_match	gene: Zm00001d026064	Zm00001d026064_T00	11.98	11.98	coding	359	144	1220
PB.1898.2	10	1138	8	novel_in_catalog	gene: Zm00001d024408	novel	0.00	55.97	coding	128	478	861
PB.490.13	1	1533	4	full-splice_match	gene: Zm00001d029387	Zm00001d029387_T00	30.48	1055.64	coding	293	89	967
PB.9950.2	8	1636	6	novel_in_catalog	gene: Zm00001d012155	novel	24.82	80.70	coding	210	405	1034

PB.490.14	1	1378	4	full-splice_match	gene: Zm00001d029387	Zm00001d029387_T00	32.27	1055.64	coding	244	495	1226
PB.8414.2	7	1484	1	full-splice_match	gene: Zm00001d019718	Zm00001d019718_T00	56.99	56.99	coding	371	131	1243
PB.9950.1	8	3960	20	full-splice_match	gene: Zm00001d012155	Zm00001d012155_T00	38.36	80.70	coding	983	743	3691
PB.6424.1	5	567	1	genic	gene: Zm00001d014721	novel	20.62	20.62	coding	99	25	321
PB.4988.2	4	1018	6	novel_not_in_catalog	gene: Zm00001d049305	novel	83.95	266.13	coding	192	85	660
PB.9930.1	8	2172	3	full-splice_match	gene: Zm00001d012074	Zm00001d012074_T00	8.94	42.01	coding	500	255	1754
PB.9930.2	8	2079	3	full-splice_match	gene: Zm00001d012074	Zm00001d012074_T00	33.07	42.01	coding	500	262	1761
PB.9930.4	8	1946	3	full-splice_match	gene: Zm00001d012074	Zm00001d012074_T00	0.00	42.01	coding	500	252	1751
PB.953.2	1	878	2	novel_in_catalog	gene: Zm00001d031928	novel	2.77	17.59	coding	59	178	354
PB.4988.4	4	940	6	novel_not_in_catalog	gene: Zm00001d049305	novel	182.18	266.13	coding	192	144	719
PB.6212.3	5	1637	6	full-splice_match	gene: Zm00001d013745	Zm00001d013745_T00	28.68	60.62	coding	390	105	1274
PB.7271.1	5	618	1	full-splice_match	gene: Zm00001d018521	Zm00001d018521_T00	366.59	366.59	non coding	NA	NA	NA
PB.7855.1	6	1216	1	full-splice_match	gene: Zm00001d037943	Zm00001d037943_T00	72.36	72.36	coding	197	126	716
PB.8347.2	7	2089	1	novel_in_catalog	gene: Zm00001d019241	novel	4.11	62.93	non coding	NA	NA	NA
PB.4243.2	3	1451	3	full-splice_match	gene: Zm00001d042337	Zm00001d042337_T00	73.53	91.62	coding	357	200	1270
PB.6964.1	5	1743	6	full-splice_match	gene: Zm00001d017352	Zm00001d017352_T01	63.95	72.75	coding	351	535	1587
PB.1464.2	1	2372	2	genic	gene: Zm00001d034090	novel	11.91	40.64	coding	442	383	1708

PB.8938.2	7	1275	1	incomplete-splice_match	gene: Zm00001d022044	Zm00001d022044_T00	2.17	13.89	coding	389	32	1198
PB.8938.1	7	3686	2	full-splice_match	gene: Zm00001d022044	Zm00001d022044_T00	11.72	13.89	coding	1101	174	3476
PB.3030.1	2	1440	1	novel_in_catalog	gene: Zm00001d004744	novel	2.24	21.73	coding	155	684	1148
PB.5147.2	4	1476	4	novel_not_in_catalog	gene: Zm00001d050163	novel	0.47	24.48	coding	223	546	1214
PB.5147.3	4	2350	3	full-splice_match	gene: Zm00001d050163	Zm00001d050163_T00	3.99	24.48	coding	151	1722	2174
PB.5147.1	4	1711	4	novel_not_in_catalog	gene: Zm00001d050163	novel	15.31	24.48	coding	243	630	1358
PB.8193.3	6	1870	14	full-splice_match	gene: Zm00001d039162	Zm00001d039162_T00	0.00	105.52	coding	474	211	1632
PB.7705.1	6	852	3	novel_in_catalog	gene: Zm00001d037258	novel	4.06	4.06	coding	70	392	601
PB.5147.4	4	1294	4	novel_not_in_catalog	gene: Zm00001d050163	novel	4.71	24.48	coding	243	396	1124
PB.5252.2	4	1899	2	full-splice_match	gene: Zm00001d050961	Zm00001d050961_T00	93.20	168.34	coding	388	373	1536
PB.4037.8	3	963	2	incomplete-splice_match	gene: Zm00001d041183	Zm00001d041183_T06	3.91	49.39	coding	30	873	962
PB.6396.1	5	1608	4	novel_not_in_catalog	gene: Zm00001d014596	novel	1.70	1.70	coding	386	311	1468
PB.7642.1	6	3047	10	novel_not_in_catalog	gene: Zm00001d036977	novel	41.83	111.08	coding	342	1372	2397
PB.7642.6	6	1413	1	genic	gene: Zm00001d036977	novel	18.28	111.08	coding	236	7	714
PB.723.1	1	2358	1	full-splice_match	gene: Zm00001d030759	Zm00001d030759_T00	10.44	10.44	coding	690	54	2123
PB.7642.4	6	2843	6	novel_not_in_catalog	gene: Zm00001d036977	novel	15.59	111.08	coding	539	608	2224
PB.6201.1	5	2069	12	incomplete-splice_match	gene: Zm00001d013683	Zm00001d013683_T00	63.76	63.76	coding	513	136	1674

PB.4037.1	3	3411	15	full-splice_match	gene: Zm00001d041183	Zm00001d041183_T00	16.64	49.39	coding	953	91	2949
PB.5484.1	4	2670	20	full-splice_match	gene: Zm00001d052110	Zm00001d052110_T00	18.16	18.16	coding	746	89	2326
PB.8560.1	7	1104	1	full-splice_match	gene: Zm00001d020550	Zm00001d020550_T00	25.30	25.30	coding	234	178	879
PB.4037.2	3	1494	1	incomplete-splice_match	gene: Zm00001d041183	Zm00001d041183_T00	11.96	49.39	non coding	NA	NA	NA
PB.4459.1	3	1335	12	full-splice_match	gene: Zm00001d043272	Zm00001d043272_T00	121.61	143.13	coding	287	126	986
PB.4037.7	3	2240	11	incomplete-splice_match	gene: Zm00001d041183	Zm00001d041183_T06	9.44	49.39	coding	522	375	1940
PB.4037.6	3	1470	7	incomplete-splice_match	gene: Zm00001d041183	Zm00001d041183_T06	7.43	49.39	coding	278	216	1049
PB.6854.1	5	853	3	incomplete-splice_match	gene: Zm00001d016892	Zm00001d016892_T00	7.43	168.96	coding	145	24	458
PB.9465.3	8	1186	4	novel_not_in_catalog	gene: Zm00001d010000	novel	31.91	31.91	coding	221	243	905
PB.4902.1	4	1981	13	full-splice_match	gene: Zm00001d048944	Zm00001d048944_T00	6.75	6.75	coding	552	24	1679
PB.6404.1	5	3067	3	full-splice_match	gene: Zm00001d014632	Zm00001d014632_T00	5.83	5.83	coding	120	173	532
PB.3745.2	3	1925	2	intergenic	novelGene_27	novel	34.53	53.07	coding	183	1196	1744
PB.9105.2	7	2817	12	full-splice_match	gene: Zm00001d022621	Zm00001d022621_T01	16.31	23.96	coding	561	804	2486
PB.1148.1	1	2428	17	incomplete-splice_match	gene: Zm00001d032704	Zm00001d032704_T03	30.09	34.81	coding	544	253	1884
PB.3265.2	2	1622	1	full-splice_match	gene: Zm00001d006031	Zm00001d006031_T00	12.38	12.38	coding	366	191	1288
PB.1148.3	1	2069	11	incomplete-splice_match	gene: Zm00001d032704	Zm00001d032704_T05	4.72	34.81	coding	336	123	1130
PB.3633.1	2	1287	1	novel_in_catalog	gene: Zm00001d007819	novel	170.60	170.60	coding	133	746	1144

PB.4670.1	3	2495	21	full-splice_match	gene: Zm00001d044024	Zm00001d044024_T00	30.40	44.61	coding	603	225	2033
PB.10981.8	B73V4	1508	6	novel_in_catalog	gene: Zm00001d000436	novel	0.00	38.73	coding	303	452	1360
PB.6787.1	5	729	1	full-splice_match	gene: Zm00001d016625	Zm00001d016625_T00	121.34	3297.01	non coding	NA	NA	NA
PB.10981.7	B73V4	2254	7	novel_in_catalog	gene: Zm00001d000436	novel	5.67	38.73	coding	303	822	1730
PB.6787.2	5	636	1	full-splice_match	gene: Zm00001d016625	Zm00001d016625_T00	3175.67	3297.01	non coding	NA	NA	NA
PB.10981.1	B73V4	2301	8	full-splice_match	gene: Zm00001d000436	Zm00001d000436_T00	32.20	38.73	coding	552	158	1813
PB.10981.3	B73V4	1970	8	full-splice_match	gene: Zm00001d000436	Zm00001d000436_T00	0.00	38.73	coding	552	152	1807
PB.10981.2	B73V4	2155	7	novel_in_catalog	gene: Zm00001d000436	novel	0.86	38.73	coding	150	152	601
PB.3582.1	2	1202	2	novel_not_in_catalog	gene: Zm00001d007501	novel	7.40	7.40	coding	230	204	893
PB.6123.11	5	1614	4	full-splice_match	gene: Zm00001d013367	Zm00001d013367_T00	0.00	2327.02	coding	451	125	1477
PB.9105.3	7	2326	11	novel_in_catalog	gene: Zm00001d022621	novel	2.11	23.96	coding	356	283	1350
PB.2403.1	10	613	2	full-splice_match	gene: Zm00001d026671	Zm00001d026671_T00	51.94	51.94	non coding	NA	NA	NA
PB.2369.2	10	1524	1	novel_in_catalog	gene: Zm00001d026573	novel	3.76	290.81	coding	105	259	573
PB.1417.2	1	2746	5	novel_in_catalog	gene: Zm00001d033917	novel	6.32	186.86	coding	420	180	1439
PB.4671.1	3	1687	1	full-splice_match	gene: Zm00001d044030	Zm00001d044030_T00	15.73	15.73	coding	501	93	1595
PB.7205.1	5	1498	1	full-splice_match	gene: Zm00001d018326	Zm00001d018326_T00	40.54	286.71	coding	125	144	518
PB.9410.1	8	1077	1	intergenic	novelGene_172	novel	5.38	5.38	coding	93	32	310

PB.2569.1	2	1549	8	full-splice_match	gene: Zm00001d002358	Zm00001d002358_T00	51.34	51.34	coding	397	148	1338
PB.1516.2	1	1113	2	genic	gene: Zm00001d034359	novel	10.39	10.39	coding	244	130	861
PB.424.4	1	1320	6	novel_in_catalog	gene: Zm00001d029039	novel	0.00	51.94	coding	303	60	968
PB.424.5	1	1102	7	full-splice_match	gene: Zm00001d029039	Zm00001d029039_T00	0.00	51.94	coding	303	53	961
PB.1231.1	1	1127	8	novel_in_catalog	gene: Zm00001d033058	novel	17.40	17.40	coding	282	119	964
PB.424.1	1	1060	3	novel_in_catalog	gene: Zm00001d029039	novel	8.55	51.94	coding	115	8	352
PB.424.2	1	1467	7	full-splice_match	gene: Zm00001d029039	Zm00001d029039_T00	43.40	51.94	coding	303	111	1019
PB.424.3	1	1575	6	novel_in_catalog	gene: Zm00001d029039	novel	0.00	51.94	coding	303	83	991
PB.351.2	1	808	4	full-splice_match	gene: Zm00001d028726	Zm00001d028726_T00	186.51	298.40	coding	150	75	524
PB.351.1	1	889	4	full-splice_match	gene: Zm00001d028726	Zm00001d028726_T00	111.89	298.40	coding	150	25	474
PB.3501.1	2	1508	9	incomplete-splice_match	gene: Zm00001d007034	Zm00001d007034_T02	58.70	91.96	coding	422	15	1280
PB.9105.4	7	2762	10	novel_in_catalog	gene: Zm00001d022621	novel	1.59	23.96	coding	301	882	1784
PB.3090.1	2	2729	10	incomplete-splice_match	gene: Zm00001d005137	Zm00001d005137_T02	31.96	57.43	coding	695	255	2339
PB.9405.3	8	2173	18	full-splice_match	gene: Zm00001d009669	Zm00001d009669_T00	65.46	65.46	coding	506	340	1857
PB.10888.2	9	1934	4	novel_in_catalog	gene: Zm00001d048437	novel	14.58	14.58	coding	396	346	1533
PB.4668.1	3	2567	19	full-splice_match	gene: Zm00001d044016	Zm00001d044016_T00	74.68	120.65	coding	655	92	2056
PB.1196.2	1	1787	1	full-splice_match	gene: Zm00001d032899	Zm00001d032899_T00	0.00	5.82	coding	586	13	1770

PB.6128.1	5	1911	1	full-splice_match	gene: Zm00001d013385	Zm00001d013385_T00	17.65	17.65	coding	569	108	1814
PB.9479.1	8	2238	2	genic	gene: Zm00001d010075	novel	17.94	17.94	coding	498	146	1639
PB.8743.1	7	2591	7	full-splice_match	gene: Zm00001d021388	Zm00001d021388_T00	72.72	72.72	coding	715	225	2369
PB.644.2	1	2155	3	full-splice_match	gene: Zm00001d030158	Zm00001d030158_T00	9.21	21.98	coding	596	106	1893
PB.10846.1	9	1639	1	full-splice_match	gene: Zm00001d048293	Zm00001d048293_T00	109.74	109.74	coding	330	328	1317
PB.4445.1	3	650	1	full-splice_match	gene: Zm00001d043209	Zm00001d043209_T00	16.16	16.16	non coding	NA	NA	NA
PB.9856.1	8	651	5	incomplete-splice_match	gene: Zm00001d011773	Zm00001d011773_T00	15.54	15.54	coding	118	107	460
PB.4854.1	4	831	1	intergenic	novelGene_17	novel	7.21	7.21	coding	121	64	426
PB.5034.1	4	805	4	full-splice_match	gene: Zm00001d049552	Zm00001d049552_T00	244.96	244.96	coding	119	230	586
PB.4668.6	3	2428	18	novel_in_catalog	gene: Zm00001d044016	novel	2.26	120.65	coding	569	121	1827
PB.5161.2	4	1597	15	full-splice_match	gene: Zm00001d050216	Zm00001d050216_T00	22.77	35.71	coding	389	153	1319
PB.5161.3	4	1627	14	full-splice_match	gene: Zm00001d050216	Zm00001d050216_T00	5.17	35.71	coding	372	135	1250
PB.6380.4	5	2455	15	novel_in_catalog	gene: Zm00001d014526	novel	6.13	81.73	coding	287	1344	2204
PB.6380.5	5	2171	14	novel_in_catalog	gene: Zm00001d014526	novel	2.51	81.73	coding	441	605	1927
PB.6380.2	5	2014	16	full-splice_match	gene: Zm00001d014526	Zm00001d014526_T00	24.87	81.73	coding	544	106	1737
PB.6380.3	5	2114	15	novel_in_catalog	gene: Zm00001d014526	novel	2.06	81.73	coding	417	613	1863
PB.6380.1	5	2093	16	full-splice_match	gene: Zm00001d014526	Zm00001d014526_T00	46.16	81.73	coding	544	93	1724

PB.4154.1	3	1004	3	novel_in_catalog	gene: Zm00001d041920	novel	9.66	70.62	non coding	NA	NA	NA
PB.7190.2	5	3535	22	novel_in_catalog	gene: Zm00001d018246	novel	1.04	31.93	coding	977	216	3146
PB.4154.2	3	1407	4	full-splice_match	gene: Zm00001d041920	Zm00001d041920_T00	60.96	70.62	coding	321	164	1126
PB.7133.1	5	2085	10	full-splice_match	gene: Zm00001d018032	Zm00001d018032_T00	8.43	11.03	coding	537	118	1728
PB.9008.1	7	680	6	full-splice_match	gene: Zm00001d022308	Zm00001d022308_T00	68.78	68.78	coding	107	34	354
PB.7133.2	5	1718	10	full-splice_match	gene: Zm00001d018032	Zm00001d018032_T00	2.60	11.03	coding	537	54	1664
PB.1519.1	1	1957	2	full-splice_match	gene: Zm00001d034373	Zm00001d034373_T00	360.97	366.63	coding	465	177	1571
PB.5399.1	4	1079	1	intergenic	novelGene_344	novel	20.51	20.51	non coding	NA	NA	NA
PB.2164.1	10	1590	10	full-splice_match	gene: Zm00001d025767	Zm00001d025767_T00	36.13	36.13	coding	448	35	1378
PB.6673.1	5	691	5	full-splice_match	gene: Zm00001d015970	Zm00001d015970_T00	37.74	58.60	coding	70	171	380
PB.4309.1	3	1153	1	novel_in_catalog	gene: Zm00001d042641	novel	90.75	90.75	non coding	NA	NA	NA
PB.6673.2	5	692	4	full-splice_match	gene: Zm00001d015970	Zm00001d015970_T00	20.86	58.60	coding	70	150	359
PB.6864.1	5	1236	3	incomplete-splice_match	gene: Zm00001d016922	Zm00001d016922_T00	13.57	13.57	coding	187	432	992
PB.8156.2	6	1317	3	full-splice_match	gene: Zm00001d039000	Zm00001d039000_T00	0.00	16.67	coding	362	120	1205
PB.4247.2	3	1613	5	novel_not_in_catalog	gene: Zm00001d042349	novel	23.68	25.15	coding	404	198	1409
PB.1918.1	10	1157	1	full-splice_match	gene: Zm00001d024500	Zm00001d024500_T00	4.77	4.77	coding	255	239	1003
PB.9844.1	8	1089	2	genic	gene: Zm00001d011730	novel	14.95	14.95	coding	186	273	830

PB.579.1	1	2050	4	full-splice_match	gene: Zm00001d029808	Zm00001d029808_T00	19.25	19.25	coding	476	135	1562
PB.3634.1	2	790	2	novel_not_in_catalog	gene: Zm00001d007822	novel	11.52	75.29	coding	76	242	469
PB.8517.2	7	1497	12	full-splice_match	gene: Zm00001d020359	Zm00001d020359_T00	6.25	21.18	coding	409	113	1339
PB.1543.1	1	1261	1	full-splice_match	gene: Zm00001d034488	Zm00001d034488_T00	111.03	111.03	coding	264	49	840
PB.8517.1	7	1692	12	full-splice_match	gene: Zm00001d020359	Zm00001d020359_T00	10.39	21.18	coding	409	47	1273
PB.1931.1	10	945	2	genic	gene: Zm00001d024538	novel	0.32	19.37	coding	137	116	526
PB.8517.4	7	1553	11	novel_in_catalog	gene: Zm00001d020359	novel	2.35	21.18	coding	308	113	1036
PB.1931.2	10	1320	2	genic	gene: Zm00001d024538	novel	19.06	19.37	coding	317	61	1011
PB.2783.1	2	890	1	full-splice_match	gene: Zm00001d003306	Zm00001d003306_T00	1.58	1.58	coding	179	57	593
PB.5169.1	4	1535	6	full-splice_match	gene: Zm00001d050258	Zm00001d050258_T00	3.99	39.30	coding	256	342	1109
PB.5169.6	4	1105	7	full-splice_match	gene: Zm00001d050258	Zm00001d050258_T00	0.00	39.30	coding	256	130	897
PB.8380.1	7	1990	3	full-splice_match	gene: Zm00001d019527	Zm00001d019527_T00	7.06	7.61	coding	411	205	1437
PB.8380.2	7	2421	2	full-splice_match	gene: Zm00001d019527	Zm00001d019527_T00	0.55	7.61	coding	377	205	1335
PB.2423.3	2	1527	1	full-splice_match	gene: Zm00001d001781	Zm00001d001781_T00	2.91	8.30	coding	380	252	1391
PB.4478.1	3	2314	3	full-splice_match	gene: Zm00001d043350	Zm00001d043350_T00	22.07	22.07	coding	604	118	1929
PB.5611.1	4	1319	3	full-splice_match	gene: Zm00001d052651	Zm00001d052651_T00	5.99	27.97	coding	297	81	971
PB.7591.4	6	1659	3	intergenic	novelGene_205	novel	9.31	15.00	non coding	NA	NA	NA

PB.8753.5	7	1610	13	novel_in_catalog	gene: Zm00001d021421	novel	197.24	197.24	coding	225	250	924
PB.743.2	1	1092	6	incomplete-splice_match	gene: Zm00001d030863	Zm00001d030863_T00	24.64	41.23	coding	239	137	853
PB.565.1	1	1129	5	full-splice_match	gene: Zm00001d029733	Zm00001d029733_T00	30.35	30.35	coding	167	409	909
PB.7272.1	5	2367	18	full-splice_match	gene: Zm00001d018522	Zm00001d018522_T00	72.27	72.27	coding	720	63	2222
PB.1903.1	10	1255	9	novel_not_in_catalog	gene: Zm00001d024421	novel	16.60	16.60	coding	317	39	989
PB.1467.1	1	2607	1	full-splice_match	gene: Zm00001d034112	Zm00001d034112_T00	57.89	57.89	coding	753	95	2353
PB.4567.11	3	3768	6	novel_in_catalog	gene: Zm00001d043649	novel	0.85	120.68	coding	125	1974	2348
PB.9346.1	8	1296	7	novel_not_in_catalog	gene: Zm00001d009365	novel	34.52	34.52	coding	243	238	966
PB.10992.1	B73V4	1916	8	novel_not_in_catalog	gene: Zm00001d000401	novel	12.66	22.77	coding	442	85	1410
PB.9002.1	7	1377	2	full-splice_match	gene: Zm00001d022289	Zm00001d022289_T00	114.58	114.58	coding	361	81	1163
PB.10816.5	9	1189	1	incomplete-splice_match	gene: Zm00001d048140	Zm00001d048140_T00	5.43	5.43	non coding	NA	NA	NA
PB.3908.3	3	926	7	full-splice_match	gene: Zm00001d040286	Zm00001d040286_T00	113.63	188.02	coding	57	562	732
PB.3908.2	3	1335	7	full-splice_match	gene: Zm00001d040286	Zm00001d040286_T00	35.09	188.02	coding	284	235	1086
PB.3908.1	3	1714	7	full-splice_match	gene: Zm00001d040286	Zm00001d040286_T00	39.29	188.02	coding	284	237	1088
PB.2374.1	10	1281	2	incomplete-splice_match	gene: Zm00001d026585	Zm00001d026585_T00	17.39	79.99	coding	57	442	612
PB.9973.2	8	2099	4	full-splice_match	gene: Zm00001d012254	Zm00001d012254_T00	0.00	41.83	coding	556	100	1767
PB.5879.1	4	2363	5	novel_in_catalog	gene: Zm00001d053890	novel	1.59	7.14	coding	212	1379	2014

PB.1508.2	1	2060	15	novel_in_catalog	gene: Zm00001d034343	novel	19.24	44.63	coding	476	384	1811
PB.7049.1	5	1684	1	incomplete-splice_match	gene: Zm00001d017721	Zm00001d017721_T00	19.63	19.63	coding	339	23	1039
PB.9973.1	8	2161	4	full-splice_match	gene: Zm00001d012254	Zm00001d012254_T00	38.59	41.83	coding	556	104	1771
PB.3687.3	3	1644	7	full-splice_match	gene: Zm00001d039285	Zm00001d039285_T00	48.01	100.37	coding	450	77	1426
PB.6545.4	5	1031	7	full-splice_match	gene: Zm00001d015134	Zm00001d015134_T00	54.96	330.02	coding	243	96	824
PB.3687.1	3	1943	6	novel_in_catalog	gene: Zm00001d039285	novel	47.26	100.37	coding	384	427	1578
PB.7678.2	6	665	1	full-splice_match	gene: Zm00001d037173	Zm00001d037173_T00	61.27	79.51	coding	98	155	448
PB.6545.1	5	1251	7	full-splice_match	gene: Zm00001d015134	Zm00001d015134_T00	274.09	330.02	coding	243	100	828
PB.3687.4	3	1750	6	novel_in_catalog	gene: Zm00001d039285	novel	5.10	100.37	coding	241	76	798
PB.7949.1	6	2045	4	full-splice_match	gene: Zm00001d038262	Zm00001d038262_T00	21.37	90.57	coding	310	663	1592
PB.8803.1	7	1377	1	full-splice_match	gene: Zm00001d021620	Zm00001d021620_T00	9.29	9.29	coding	359	100	1176
PB.5473.1	4	3016	19	full-splice_match	gene: Zm00001d052072	Zm00001d052072_T01	10.68	14.23	coding	770	179	2488
PB.7958.1	6	1354	2	novel_not_in_catalog	gene: Zm00001d038289	novel	0.44	0.44	coding	184	112	663
PB.5728.1	4	1385	9	novel_not_in_catalog	gene: Zm00001d053223	novel	43.26	131.22	coding	289	80	946
PB.8870.1	7	1458	9	novel_not_in_catalog	gene: Zm00001d021815	novel	241.67	241.67	coding	464	30	1421
PB.5876.3	4	1673	1	intergenic	novelGene_444	novel	3.05	3.05	non coding	NA	NA	NA
PB.9841.3	8	801	2	full-splice_match	gene: Zm00001d011718	Zm00001d011718_T00	59.83	100.93	coding	207	35	655

PB.6896.2	5	753	1	full-splice_match	gene: Zm00001d017090	Zm00001d017090_T00	12.32	48.45	coding	200	24	623
PB.4784.1	3	1968	9	full-splice_match	gene: Zm00001d044495	Zm00001d044495_T00	1155.67	1158.15	coding	481	90	1532
PB.7460.1	6	5871	13	full-splice_match	gene: Zm00001d035889	Zm00001d035889_T00	17.48	43.36	coding	1822	119	5584
PB.7460.2	6	4579	13	full-splice_match	gene: Zm00001d035889	Zm00001d035889_T01	25.88	43.36	coding	1288	429	4292
PB.3080.1	2	2744	8	incomplete-splice_match	gene: Zm00001d005090	Zm00001d005090_T03	193.91	224.83	coding	746	61	2298
PB.4585.3	3	1109	6	full-splice_match	gene: Zm00001d043696	Zm00001d043696_T00	32.58	38.68	coding	288	82	945
PB.4585.2	3	1289	6	full-splice_match	gene: Zm00001d043696	Zm00001d043696_T00	0.00	38.68	coding	288	100	963
PB.4585.1	3	1427	6	full-splice_match	gene: Zm00001d043696	Zm00001d043696_T00	6.09	38.68	coding	288	110	973
PB.7797.1	6	853	1	full-splice_match	gene: Zm00001d037684	Zm00001d037684_T00	42.17	981.00	coding	86	94	351
PB.3622.1	2	5790	9	full-splice_match	gene: Zm00001d007776	Zm00001d007776_T00	2.96	2.96	coding	1569	172	4878
PB.6150.3	5	2343	1	full-splice_match	gene: Zm00001d013465	Zm00001d013465_T00	0.00	120.54	coding	625	228	2102
PB.6150.1	5	2450	1	full-splice_match	gene: Zm00001d013465	Zm00001d013465_T00	120.54	120.54	coding	625	228	2102
PB.10780.1	9	1111	1	full-splice_match	gene: Zm00001d047982	Zm00001d047982_T00	3.78	3.78	coding	279	274	1110
PB.6055.3	5	1908	2	incomplete-splice_match	gene: Zm00001d013154	Zm00001d013154_T00	0.00	75.68	coding	594	126	1907
PB.6055.2	5	3277	4	novel_not_in_catalog	gene: Zm00001d013154	novel	2.31	75.68	coding	821	686	3148
PB.6055.1	5	3333	5	novel_not_in_catalog	gene: Zm00001d013154	novel	73.37	75.68	coding	949	332	3178
PB.9873.1	8	1635	1	full-splice_match	gene: Zm00001d011832	Zm00001d011832_T00	1.73	1.73	coding	467	71	1471

PB.9791.4	8	3031	10	full-splice_match	gene: Zm00001d011517	Zm00001d011517_T00	29.59	64.27	coding	840	221	2740
PB.7219.1	5	1749	15	full-splice_match	gene: Zm00001d018371	Zm00001d018371_T00	26.78	26.78	coding	365	225	1319
PB.9791.1	8	3086	10	full-splice_match	gene: Zm00001d011517	Zm00001d011517_T00	30.71	64.27	coding	840	141	2660
PB.6878.1	5	1029	1	incomplete-splice_match	gene: Zm00001d017029	Zm00001d017029_T00	67.45	67.45	coding	221	73	735
PB.9791.3	8	3939	9	full-splice_match	gene: Zm00001d011517	Zm00001d011517_T00	3.96	64.27	coding	545	218	1852
PB.8072.1	6	1840	4	novel_in_catalog	gene: Zm00001d038720	novel	17.69	48.74	coding	111	1468	1800
PB.6004.1	5	1079	9	intergenic	novelGene_15	novel	219.23	219.23	coding	214	300	941
PB.10201.1	9	1704	1	full-splice_match	gene: Zm00001d045188	Zm00001d045188_T00	45.62	45.62	coding	525	34	1608
PB.7239.5	5	1337	10	incomplete-splice_match	gene: Zm00001d018423	Zm00001d018423_T01	24.78	95.51	coding	284	201	1052
PB.5844.5	4	2231	15	novel_not_in_catalog	gene: Zm00001d053751	novel	2.12	9.03	coding	480	446	1885
PB.4698.29	3	2260	5	novel_in_catalog	gene: Zm00001d044138	novel	0.00	1495.64	coding	257	25	795
PB.3557.1	2	1204	3	full-splice_match	gene: Zm00001d007287	Zm00001d007287_T00	8.62	8.62	non coding	NA	NA	NA
PB.3777.1	3	1539	1	genic_intron	novelGene_46	novel	41.81	41.81	coding	450	86	1435
PB.5260.2	4	1286	3	full-splice_match	gene: Zm00001d050992	Zm00001d050992_T00	28.30	102.92	coding	326	98	1075
PB.9030.2	7	2014	8	full-splice_match	gene: Zm00001d022391	Zm00001d022391_T00	9.75	9.75	coding	400	338	1537
PB.10380.2	9	1645	4	full-splice_match	gene: Zm00001d046093	Zm00001d046093_T00	0.00	8.82	coding	486	22	1479
PB.4871.7	4	1091	7	novel_not_in_catalog	gene: Zm00001d048785	novel	0.00	66.19	non coding	NA	NA	NA

PB.10380.1	9	1766	4	full-splice_match	gene: Zm00001d046093	Zm00001d046093_T00	8.82	8.82	coding	486	22	1479
PB.3244.1	2	815	1	full-splice_match	gene: Zm00001d005945	Zm00001d005945_T00	479.38	479.38	coding	146	71	508
PB.8072.5	6	1726	6	novel_not_in_catalog	gene: Zm00001d038720	novel	5.09	48.74	non coding	NA	NA	NA
PB.5876.1	4	2161	2	genic	gene: Zm00001d053882	novel	13.08	65.78	coding	439	90	1406
PB.1413.1	1	856	1	genic_intron	novelGene_460	novel	33.92	33.92	coding	177	33	563
PB.5383.2	4	891	3	full-splice_match	gene: Zm00001d051672	Zm00001d051672_T00	18.48	48.66	coding	169	78	584
PB.5383.3	4	875	3	full-splice_match	gene: Zm00001d051672	Zm00001d051672_T00	30.18	48.66	coding	167	187	687
PB.2243.1	10	815	1	full-splice_match	gene: Zm00001d026162	Zm00001d026162_T00	349.73	904.74	coding	134	121	522
PB.4871.5	4	1245	6	novel_in_catalog	gene: Zm00001d048785	novel	34.11	66.19	coding	115	133	477
PB.5149.2	4	765	5	novel_not_in_catalog	gene: Zm00001d050165	novel	6.89	41.48	coding	202	33	638
PB.6316.3	5	2471	13	incomplete-splice_match	gene: Zm00001d014232	Zm00001d014232_T02	46.72	46.72	coding	704	22	2133
PB.6646.2	5	2753	4	intergenic	novelGene_230	novel	3.95	3.95	coding	124	929	1300
PB.3697.1	3	1462	4	incomplete-splice_match	gene: Zm00001d039305	Zm00001d039305_T00	35.03	35.03	non coding	NA	NA	NA
PB.4209.1	3	1872	2	genic	gene: Zm00001d042184	novel	268.62	356.46	coding	480	156	1595
PB.4209.2	3	2957	1	full-splice_match	gene: Zm00001d042184	Zm00001d042184_T00	7.17	356.46	coding	480	1279	2718
PB.4209.3	3	1869	2	genic	gene: Zm00001d042184	novel	80.67	356.46	coding	480	196	1635
PB.4209.5	3	1323	1	full-splice_match	gene: Zm00001d042184	Zm00001d042184_T00	0.00	356.46	coding	363	109	1197

PB.8146.1	6	792	1	genic	gene: Zm00001d038969	novel	27.51	27.51	coding	157	201	671
PB.2292.2	10	1303	8	novel_in_catalog	gene: Zm00001d026319	novel	9.59	31.90	non coding	NA	NA	NA
PB.8896.3	7	2997	19	full-splice_match	gene: Zm00001d021893	Zm00001d021893_T00	16.86	105.38	coding	891	29	2701
PB.1453.1	1	3940	4	full-splice_match	gene: Zm00001d034038	Zm00001d034038_T00	22.55	22.55	coding	1135	202	3606
PB.8896.1	7	2014	15	full-splice_match	gene: Zm00001d021893	Zm00001d021893_T00	65.04	105.38	coding	553	21	1679
PB.8896.6	7	3660	19	full-splice_match	gene: Zm00001d021893	Zm00001d021893_T00	23.48	105.38	coding	1126	124	3501
PB.6870.1	5	1452	2	novel_in_catalog	gene: Zm00001d016974	novel	1.32	3.55	coding	178	261	794
PB.2292.4	10	1059	8	novel_in_catalog	gene: Zm00001d026319	novel	22.31	31.90	non coding	NA	NA	NA
PB.6126.1	5	1876	4	full-splice_match	gene: Zm00001d013376	Zm00001d013376_T00	17.81	17.81	coding	245	591	1325
PB.7798.1	6	1773	3	full-splice_match	gene: Zm00001d037688	Zm00001d037688_T00	20.82	20.82	coding	462	128	1513
PB.2897.3	2	932	1	novel_in_catalog	gene: Zm00001d003896	novel	71.27	242.41	coding	166	75	572
PB.10396.1	9	1309	7	full-splice_match	gene: Zm00001d046200	Zm00001d046200_T00	7.94	7.94	coding	81	65	307
PB.4701.8	3	1678	5	novel_not_in_catalog	gene: Zm00001d044146	novel	0.00	90.95	coding	521	50	1612
PB.10127.4	9	1224	2	full-splice_match	gene: Zm00001d044821	Zm00001d044821_T00	0.00	136.04	coding	355	67	1131
PB.9698.3	8	1665	1	full-splice_match	gene: Zm00001d011076	Zm00001d011076_T00	63.21	104.59	coding	387	177	1337
PB.9698.2	8	1507	1	full-splice_match	gene: Zm00001d011076	Zm00001d011076_T00	17.87	104.59	coding	365	25	1119
PB.10127.1	9	1358	2	full-splice_match	gene: Zm00001d044821	Zm00001d044821_T00	130.88	136.04	coding	355	67	1131

PB.10127.2	9	1434	1	genic	gene: Zm00001d044821	novel	5.16	136.04	coding	213	615	1253
PB.410.1	1	2153	2	incomplete-splice_match	gene: Zm00001d028989	Zm00001d028989_T00	34.06	34.06	coding	300	1001	1900
PB.2116.1	10	2273	4	novel_in_catalog	gene: Zm00001d025539	novel	2.30	40.33	coding	194	493	1074
PB.5340.3	4	865	3	full-splice_match	gene: Zm00001d051499	Zm00001d051499_T00	51.80	82.04	coding	152	240	695
PB.114.4	1	2066	14	full-splice_match	gene: Zm00001d027701	Zm00001d027701_T00	0.00	64.47	coding	579	126	1862
PB.5340.1	4	1290	1	genic	gene: Zm00001d051499	novel	9.19	82.04	non coding	NA	NA	NA
PB.9896.2	8	1052	1	novel_in_catalog	gene: Zm00001d011938	novel	26.97	217.92	coding	170	112	621
PB.283.2	1	822	2	full-splice_match	gene: Zm00001d028445	Zm00001d028445_T00	231.56	248.68	non coding	NA	NA	NA
PB.283.1	1	961	2	full-splice_match	gene: Zm00001d028445	Zm00001d028445_T00	17.12	248.68	non coding	NA	NA	NA
PB.5340.4	4	858	3	novel_not_in_catalog	gene: Zm00001d051499	novel	7.38	82.04	coding	80	113	352
PB.6846.3	5	2302	3	novel_in_catalog	gene: Zm00001d016858	novel	42.42	55.65	coding	589	307	2073
PB.3151.4	2	1546	1	full-splice_match	gene: Zm00001d005461	Zm00001d005461_T00	2.28	61.69	coding	314	352	1293
PB.1818.1	10	1356	4	novel_not_in_catalog	gene: Zm00001d023853	novel	28.59	28.59	coding	303	64	972
PB.3151.1	2	1612	2	genic	gene: Zm00001d005461	novel	49.45	61.69	coding	305	229	1143
PB.4215.2	3	851	1	incomplete-splice_match	gene: Zm00001d042212	Zm00001d042212_T01	0.00	46.67	non coding	NA	NA	NA
PB.4215.1	3	1539	2	incomplete-splice_match	gene: Zm00001d042212	Zm00001d042212_T01	29.64	46.67	coding	225	520	1194
PB.10752.2	9	970	1	novel_in_catalog	gene: Zm00001d047878	novel	13.32	30.78	coding	184	152	703

PB.6813.2	5	2724	6	novel_in_catalog	gene: Zm00001d016731	novel	1.67	49.01	coding	430	83	1372
PB.6188.1	5	1451	9	full-splice_match	gene: Zm00001d013631	Zm00001d013631_T00	26.52	37.11	coding	200	254	853
PB.6188.2	5	1204	9	full-splice_match	gene: Zm00001d013631	Zm00001d013631_T00	10.58	37.11	coding	200	256	855
PB.7297.1	6	1046	1	full-splice_match	gene: Zm00001d035005	Zm00001d035005_T00	1.12	1.12	coding	268	136	939
PB.5607.5	4	1838	3	full-splice_match	gene: Zm00001d052620	Zm00001d052620_T01	23.89	65.70	non coding	NA	NA	NA
PB.94.4	1	2628	4	incomplete-splice_match	gene: Zm00001d027625	Zm00001d027625_T00	6.14	69.37	coding	730	438	2627
PB.1503.1	1	1237	2	genic	gene: Zm00001d034324	novel	8.42	8.42	coding	160	255	734
PB.94.2	1	4391	11	incomplete-splice_match	gene: Zm00001d027625	Zm00001d027625_T00	34.78	69.37	coding	1334	24	4025
PB.94.3	1	2038	8	novel_not_in_catalog	gene: Zm00001d027625	novel	2.40	69.37	coding	293	791	1669
PB.94.1	1	1861	6	incomplete-splice_match	gene: Zm00001d027625	Zm00001d027625_T00	26.04	69.37	coding	335	82	1086
PB.9533.1	8	1648	1	full-splice_match	gene: Zm00001d010340	Zm00001d010340_T00	6.38	6.38	coding	493	21	1499
PB.1593.1	1	1297	4	full-splice_match	gene: Zm00001d034649	Zm00001d034649_T00	25.80	35.53	non coding	NA	NA	NA
PB.1680.1	10	1637	1	full-splice_match	gene: Zm00001d023216	Zm00001d023216_T00	5.05	5.05	coding	458	77	1450
PB.7703.1	6	4829	5	full-splice_match	gene: Zm00001d037252	Zm00001d037252_T00	23.12	23.12	coding	1453	230	4588
PB.1035.1	1	1040	1	full-splice_match	gene: Zm00001d032285	Zm00001d032285_T00	3.89	3.89	coding	257	98	868
PB.1720.1	10	1243	6	full-splice_match	gene: Zm00001d023371	Zm00001d023371_T00	187.90	289.34	coding	239	313	1029
PB.7479.2	6	2233	16	novel_in_catalog	gene: Zm00001d036004	novel	4.74	21.91	coding	555	40	1704

PB.1720.3	10	1535	5	novel_in_catalog	gene: Zm00001d023371	novel	10.89	289.34	coding	156	855	1322
PB.1720.2	10	1257	6	novel_not_in_catalog	gene: Zm00001d023371	novel	90.55	289.34	coding	241	325	1047
PB.1720.4	10	1111	6	full-splice_match	gene: Zm00001d023371	Zm00001d023371_T00	0.00	289.34	coding	239	231	947
PB.2119.3	10	1892	9	full-splice_match	gene: Zm00001d025548	Zm00001d025548_T00	3.68	137.33	coding	440	173	1492
PB.2291.1	10	519	2	intergenic	novelGene_281	novel	138.52	143.48	non coding	NA	NA	NA
PB.8149.5	6	4263	11	full-splice_match	gene: Zm00001d038972	Zm00001d038972_T01	0.00	517.57	coding	871	1331	3943
PB.938.1	1	1569	11	full-splice_match	gene: Zm00001d031849	Zm00001d031849_T00	242.44	258.47	coding	365	140	1234
PB.7239.2	5	1818	9	novel_in_catalog	gene: Zm00001d018423	novel	3.79	95.51	coding	343	68	1096
PB.2119.1	10	2541	7	novel_in_catalog	gene: Zm00001d025548	novel	8.33	137.33	coding	261	150	932
PB.1055.2	1	3088	4	novel_in_catalog	gene: Zm00001d032376	novel	8.27	19.38	coding	828	25	2508
PB.3084.4	2	2293	3	incomplete-splice_match	gene: Zm00001d005100	Zm00001d005100_T02	12.92	84.39	coding	541	46	1668
PB.1055.1	1	3294	7	novel_in_catalog	gene: Zm00001d032376	novel	11.11	19.38	coding	861	127	2709
PB.4275.1	3	2069	3	full-splice_match	gene: Zm00001d042490	Zm00001d042490_T00	33.84	49.58	coding	492	410	1885
PB.6579.2	5	2045	2	full-splice_match	gene: Zm00001d015307	Zm00001d015307_T00	62.63	62.63	coding	323	806	1774
PB.3084.5	2	3702	11	incomplete-splice_match	gene: Zm00001d005100	Zm00001d005100_T08	42.64	84.39	coding	952	425	3280
PB.5028.2	4	684	3	full-splice_match	gene: Zm00001d049507	Zm00001d049507_T00	350.88	367.45	coding	110	50	379
PB.9578.1	8	559	1	genic	gene: Zm00001d010577	novel	22.74	33.02	coding	22	282	347

PB.4886.2	4	1781	10	full-splice_match	gene: Zm00001d048856	Zm00001d048856_T00	7.59	13.60	coding	512	91	1626
PB.8149.1	6	4410	11	full-splice_match	gene: Zm00001d038972	Zm00001d038972_T01	241.55	517.57	coding	871	1382	3994
PB.5876.4	4	2729	2	genic	gene: Zm00001d053882	novel	3.14	65.78	coding	697	47	2137
PB.8652.1	7	2008	3	full-splice_match	gene: Zm00001d020982	Zm00001d020982_T00	25.42	28.32	coding	376	352	1479
PB.8652.2	7	1724	3	full-splice_match	gene: Zm00001d020982	Zm00001d020982_T00	2.90	28.32	coding	376	352	1479
PB.3952.1	3	1115	1	full-splice_match	gene: Zm00001d040461	Zm00001d040461_T00	28.26	28.26	coding	249	119	865
PB.800.1	1	1936	5	full-splice_match	gene: Zm00001d031115	Zm00001d031115_T00	13.77	20.85	coding	28	629	712
PB.9707.1	8	614	1	novel_in_catalog	gene: Zm00001d011122	novel	15.71	43.55	non coding	NA	NA	NA
PB.9707.2	8	988	6	novel_in_catalog	gene: Zm00001d011122	novel	27.84	43.55	coding	238	126	839
PB.1065.1	1	1026	1	full-splice_match	gene: Zm00001d032410	Zm00001d032410_T00	3.07	3.07	coding	224	34	705
PB.4329.10	3	1877	7	novel_not_in_catalog	gene: Zm00001d042724	novel	0.00	343.97	coding	377	321	1451
PB.936.5	1	1432	1	full-splice_match	gene: Zm00001d031833	Zm00001d031833_T00	1276.55	1772.06	coding	326	334	1311
PB.6308.2	5	3008	3	novel_in_catalog	gene: Zm00001d014183	novel	3.33	89.14	coding	141	2377	2799
PB.6308.3	5	1096	7	full-splice_match	gene: Zm00001d014183	Zm00001d014183_T00	0.00	89.14	coding	298	32	925
PB.3906.3	3	2743	1	genic	gene: Zm00001d040281	novel	4.35	468.21	coding	111	1669	2001
PB.6308.1	5	1251	7	full-splice_match	gene: Zm00001d014183	Zm00001d014183_T00	85.80	89.14	coding	298	125	1018
PB.10693.2	9	932	6	full-splice_match	gene: Zm00001d047707	Zm00001d047707_T00	513.86	748.77	coding	192	176	751

PB.10693.1	9	1022	6	full-splice_match	gene: Zm00001d047707	Zm00001d047707_T00	234.91	748.77	coding	192	91	666
PB.5607.6	4	1185	3	full-splice_match	gene: Zm00001d052620	Zm00001d052620_T01	7.44	65.70	non coding	NA	NA	NA
PB.2119.8	10	1163	6	incomplete-splice_match	gene: Zm00001d025548	Zm00001d025548_T00	3.12	137.33	coding	183	113	661
PB.9538.1	8	654	1	full-splice_match	gene: Zm00001d010388	Zm00001d010388_T00	36.19	36.19	non coding	NA	NA	NA
PB.3829.1	3	1501	2	novel_not_in_catalog	gene: Zm00001d039880	novel	14.64	14.64	coding	437	20	1330
PB.10546.7	9	1567	5	novel_in_catalog	gene: Zm00001d047059	novel	0.00	29.99	coding	129	1008	1394
PB.10546.4	9	1139	7	incomplete-splice_match	gene: Zm00001d047059	Zm00001d047059_T00	17.08	29.99	coding	279	93	929
PB.3829.2	3	1466	2	novel_not_in_catalog	gene: Zm00001d039880	novel	0.00	14.64	coding	437	12	1322
PB.3689.1	3	860	2	full-splice_match	gene: Zm00001d039293	Zm00001d039293_T00	6.67	6.67	coding	213	115	753
PB.10546.3	9	2045	3	novel_in_catalog	gene: Zm00001d047059	novel	7.10	29.99	coding	129	1447	1833
PB.10546.1	9	1719	6	novel_in_catalog	gene: Zm00001d047059	novel	3.27	29.99	coding	129	926	1312
PB.3503.1	2	1208	6	novel_in_catalog	gene: Zm00001d007037	novel	25.70	119.55	coding	190	377	946
PB.3503.2	2	2027	9	novel_in_catalog	gene: Zm00001d007037	novel	60.63	119.55	coding	441	457	1779
PB.3503.4	2	1364	1	novel_in_catalog	gene: Zm00001d007037	novel	21.16	119.55	coding	103	333	641
PB.3503.5	2	1652	8	novel_in_catalog	gene: Zm00001d007037	novel	12.06	119.55	coding	251	127	879
PB.3166.1	2	3066	15	full-splice_match	gene: Zm00001d005573	Zm00001d005573_T01	26.28	26.28	coding	885	122	2776
PB.715.2	1	1140	3	full-splice_match	gene: Zm00001d030678	Zm00001d030678_T00	55.26	337.18	coding	211	246	878

PB.3968.1	3	1772	1	full-splice_match	gene: Zm00001d000526	Zm00001d000526_T00	145.68	145.68	non coding	NA	NA	NA
PB.10469.3	9	3416	4	incomplete-splice_match	gene: Zm00001d046630	Zm00001d046630_T01	11.01	59.89	coding	1039	299	3415
PB.9717.1	8	2354	3	full-splice_match	gene: Zm00001d011160	Zm00001d011160_T00	12.62	12.62	coding	738	1	2214
PB.3968.2	3	1650	1	full-splice_match	gene: Zm00001d000526	Zm00001d000526_T00	0.00	145.68	non coding	NA	NA	NA
PB.4872.1	4	380	3	novel_not_in_catalog	gene: Zm00001d048787	novel	1094.09	1100.51	non coding	NA	NA	NA
PB.4872.2	4	1336	2	genic	gene: Zm00001d048787	novel	6.41	1100.51	non coding	NA	NA	NA
PB.3182.1	2	918	2	antisense	novelGene_gene: Zm00001d005648	novel	9.05	9.05	coding	100	328	627
PB.5607.1	4	2007	4	full-splice_match	gene: Zm00001d052620	Zm00001d052620_T00	2.02	65.70	non coding	NA	NA	NA
PB.6372.1	5	1989	1	full-splice_match	gene: Zm00001d014496	Zm00001d014496_T00	8.74	8.74	coding	580	112	1851
PB.3474.2	2	1257	5	full-splice_match	gene: Zm00001d006910	Zm00001d006910_T01	80.70	103.45	coding	288	133	996
PB.2495.3	2	1409	3	full-splice_match	gene: Zm00001d002052	Zm00001d002052_T00	324.12	340.24	coding	337	142	1152
PB.8691.2	7	2758	5	full-splice_match	gene: Zm00001d021202	Zm00001d021202_T03	12.55	25.59	coding	99	2296	2592
PB.2495.1	2	1577	3	full-splice_match	gene: Zm00001d002052	Zm00001d002052_T00	8.53	340.24	coding	337	142	1152
PB.4312.1	3	1490	8	incomplete-splice_match	gene: Zm00001d042654	Zm00001d042654_T00	11.99	18.30	coding	298	325	1218
PB.10135.1	9	940	6	full-splice_match	gene: Zm00001d044869	Zm00001d044869_T00	134.71	154.35	coding	199	72	668
PB.10135.2	9	1195	7	full-splice_match	gene: Zm00001d044869	Zm00001d044869_T00	19.64	154.35	coding	127	108	488
PB.2495.4	2	1251	2	novel_in_catalog	gene: Zm00001d002052	novel	1.49	340.24	coding	299	128	1024

PB.5320.1	4	1249	5	antisense	novelGene_gene: Zm00001d051399	novel	11.30	14.68	non coding	NA	NA	NA
PB.6997.1	5	1387	4	full-splice_match	gene: Zm00001d017495	Zm00001d017495_T00	35.86	35.86	coding	294	106	987
PB.5320.2	4	1215	4	antisense	novelGene_gene: Zm00001d051399	novel	3.38	14.68	non coding	NA	NA	NA
PB.9466.2	8	1043	1	full-splice_match	gene: Zm00001d010001	Zm00001d010001_T00	0.00	17.39	coding	231	186	878
PB.2389.1	10	1090	2	full-splice_match	gene: Zm00001d026628	Zm00001d026628_T00	143.58	143.58	coding	211	153	785
PB.6611.1	5	3713	5	incomplete-splice_match	gene: Zm00001d015493	Zm00001d015493_T00	9.69	17.80	coding	1143	63	3491
PB.299.1	1	779	3	full-splice_match	gene: Zm00001d028530	Zm00001d028530_T00	25.21	25.21	coding	85	204	458
PB.1385.1	1	3486	13	full-splice_match	gene: Zm00001d033776	Zm00001d033776_T00	18.56	38.59	coding	995	134	3118
PB.7331.3	6	1820	6	novel_in_catalog	gene: Zm00001d035156	novel	47.14	1760.44	coding	122	127	492
PB.7331.2	6	1238	10	incomplete-splice_match	gene: Zm00001d035156	Zm00001d035156_T00	1713.30	1760.44	coding	295	115	999
PB.9484.1	8	2325	1	full-splice_match	gene: Zm00001d010116	Zm00001d010116_T00	8.02	8.02	coding	665	108	2102
PB.1110.2	1	761	1	full-splice_match	gene: Zm00001d032563	Zm00001d032563_T00	206.71	213.97	coding	194	38	619
PB.5364.2	4	1373	10	incomplete-splice_match	gene: Zm00001d051608	Zm00001d051608_T01	41.98	62.79	coding	262	271	1056
PB.2489.1	2	1145	5	full-splice_match	gene: Zm00001d002029	Zm00001d002029_T00	230.40	379.68	coding	216	106	753
PB.2489.2	2	815	5	full-splice_match	gene: Zm00001d002029	Zm00001d002029_T00	145.66	379.68	coding	157	146	616
PB.2489.3	2	790	4	novel_in_catalog	gene: Zm00001d002029	novel	3.62	379.68	coding	189	42	608
PB.7090.1	5	1814	5	full-splice_match	gene: Zm00001d017890	Zm00001d017890_T00	69.21	69.21	coding	446	93	1430

PB.10900.1	9	889	6	full-splice_match	gene: Zm00001d048473	Zm00001d048473_T00	34.01	199.31	coding	160	107	586
PB.1221.2	1	2005	2	genic	gene: Zm00001d033012	novel	58.41	58.41	coding	446	297	1634
PB.8771.1	7	2622	1	incomplete-splice_match	gene: Zm00001d021492	Zm00001d021492_T00	2.86	2.86	coding	840	41	2560
PB.5562.1	4	1678	1	full-splice_match	gene: Zm00001d052387	Zm00001d052387_T00	5.50	5.50	coding	501	50	1552
PB.3371.1	2	1487	2	full-splice_match	gene: Zm00001d006446	Zm00001d006446_T00	2.96	2.96	coding	427	8	1288
PB.9765.1	8	2594	3	genic	gene: Zm00001d011378	novel	4.54	4.54	coding	683	320	2368
PB.3478.2	2	1574	9	full-splice_match	gene: Zm00001d006918	Zm00001d006918_T00	21.19	21.71	coding	444	50	1381
PB.936.1	1	1901	1	full-splice_match	gene: Zm00001d031833	Zm00001d031833_T00	495.51	1772.06	coding	326	670	1647
PB.10687.2	9	3251	4	full-splice_match	gene: Zm00001d047676	Zm00001d047676_T00	62.19	125.61	coding	868	287	2890
PB.1780.1	10	1679	2	novel_not_in_catalog	gene: Zm00001d023625	novel	4.83	4.83	coding	517	61	1611
PB.9845.4	8	790	2	full-splice_match	gene: Zm00001d011732	Zm00001d011732_T00	0.00	305.60	coding	200	77	676
PB.5268.4	4	2078	1	full-splice_match	gene: Zm00001d051044	Zm00001d051044_T00	5.25	81.31	coding	451	504	1856
PB.10129.1	9	920	2	novel_in_catalog	gene: Zm00001d044825	novel	0.90	5.14	coding	178	182	715
PB.5863.2	4	1833	10	novel_in_catalog	gene: Zm00001d053825	novel	15.16	114.09	coding	200	841	1440
PB.7086.1	5	2471	5	novel_in_catalog	gene: Zm00001d017880	novel	82.25	82.25	coding	193	1672	2250
PB.7211.3	5	1593	7	novel_in_catalog	gene: Zm00001d018341	novel	13.71	47.69	coding	173	179	697
PB.271.1	1	1624	11	full-splice_match	gene: Zm00001d028396	Zm00001d028396_T00	148.76	242.34	coding	400	111	1310

PB.271.2	1	1504	11	full-splice_match	gene: Zm00001d028396	Zm00001d028396_T00	93.58	242.34	coding	400	106	1305
PB.6718.2	5	2188	4	novel_not_in_catalog	gene: Zm00001d016230	novel	7.06	9.50	coding	328	22	1005
PB.6286.1	5	2222	3	incomplete-splice_match	gene: Zm00001d014098	Zm00001d014098_T00	1.78	1.78	coding	608	141	1964
PB.2481.1	2	1870	5	novel_in_catalog	gene: Zm00001d001993	novel	7.78	19.56	coding	305	181	1095
PB.2481.2	2	743	3	incomplete-splice_match	gene: Zm00001d001993	Zm00001d001993_T00	11.78	19.56	coding	122	160	525
PB.7637.1	6	739	4	full-splice_match	gene: Zm00001d036964	Zm00001d036964_T00	33.04	107.56	coding	76	100	327
PB.10923.1	9	1906	3	full-splice_match	gene: Zm00001d048532	Zm00001d048532_T00	10.35	17.13	coding	428	209	1492
PB.5172.2	4	1622	3	full-splice_match	gene: Zm00001d050310	Zm00001d050310_T00	29.42	106.29	coding	272	505	1320
PB.9450.1	8	1525	8	full-splice_match	gene: Zm00001d009930	Zm00001d009930_T00	9.44	9.44	coding	394	37	1218
PB.10923.2	9	1378	3	novel_not_in_catalog	gene: Zm00001d048532	novel	6.78	17.13	coding	171	452	964
PB.7955.3	6	2427	2	novel_in_catalog	gene: Zm00001d038273	novel	10.86	164.25	coding	358	165	1238
PB.9049.4	7	1737	15	full-splice_match	gene: Zm00001d022445	Zm00001d022445_T00	51.54	51.54	coding	425	160	1434
PB.622.1	1	1315	2	full-splice_match	gene: Zm00001d030086	Zm00001d030086_T00	16.89	16.89	coding	249	71	817
PB.150.2	1	1695	14	full-splice_match	gene: Zm00001d027886	Zm00001d027886_T00	34.12	34.49	coding	380	262	1401
PB.150.3	1	1800	13	novel_in_catalog	gene: Zm00001d027886	novel	0.37	34.49	coding	296	290	1177
PB.1337.1	1	3658	4	incomplete-splice_match	gene: Zm00001d033553	Zm00001d033553_T07	2.80	2.80	coding	1182	112	3657
PB.2196.1	10	1395	2	genic	gene: Zm00001d025916	novel	19.28	19.28	coding	229	407	1093

PB.5281.1	4	1664	2	genic	gene: Zm00001d051136	novel	25.89	25.89	coding	289	564	1430
PB.7835.3	6	1828	8	novel_in_catalog	gene: Zm00001d037871	novel	1.46	54.28	coding	378	65	1198
PB.1774.3	10	2423	6	full-splice_match	gene: Zm00001d023590	Zm00001d023590_T00	0.00	78.86	coding	208	1650	2273
PB.1774.1	10	2443	6	full-splice_match	gene: Zm00001d023590	Zm00001d023590_T00	73.16	78.86	coding	208	1581	2204
PB.2461.2	2	977	3	full-splice_match	gene: Zm00001d001915	Zm00001d001915_T00	61.25	209.78	coding	137	107	517
PB.2461.3	2	905	3	full-splice_match	gene: Zm00001d001915	Zm00001d001915_T00	148.52	209.78	coding	137	108	518
PB.1774.4	10	2860	5	full-splice_match	gene: Zm00001d023590	Zm00001d023590_T00	5.71	78.86	coding	318	1756	2709
PB.7941.1	6	850	1	antisense	novelGene_gene: Zm00001d038221	novel	20.03	20.03	non coding	NA	NA	NA
PB.5261.1	4	1348	3	full-splice_match	gene: Zm00001d050993	Zm00001d050993_T00	44.96	46.25	coding	348	47	1090
PB.1147.2	1	2468	9	full-splice_match	gene: Zm00001d032703	Zm00001d032703_T01	24.31	85.19	coding	609	379	2205
PB.1110.1	1	1058	1	full-splice_match	gene: Zm00001d032563	Zm00001d032563_T00	7.26	213.97	coding	194	46	627
PB.5261.2	4	1251	3	full-splice_match	gene: Zm00001d050993	Zm00001d050993_T00	1.30	46.25	coding	348	58	1101
PB.7623.3	6	2408	2	full-splice_match	gene: Zm00001d036880	Zm00001d036880_T00	0.00	16.35	coding	626	200	2077
PB.1147.3	1	2359	8	full-splice_match	gene: Zm00001d032703	Zm00001d032703_T00	5.64	85.19	coding	609	270	2096
PB.4931.2	4	2286	11	full-splice_match	gene: Zm00001d049080	Zm00001d049080_T00	22.91	40.78	coding	677	51	2081
PB.4931.3	4	1959	9	novel_in_catalog	gene: Zm00001d049080	novel	2.08	40.78	coding	285	345	1199
PB.10944.2	B73V4	1232	4	full-splice_match	gene: Zm00001d000288	Zm00001d000288_T00	3.23	7.87	coding	194	211	792

PB.10944.3	B73V 4	1092	4	full-splice_match	gene: Zm00001d000288	Zm00001d000288_T00	4.64	7.87	coding	194	204	785
PB.5461.3	4	2213	1	novel_in_catalog	gene: Zm00001d052053	novel	42.69	62.79	coding	440	626	1945
PB.8898.1	7	900	1	full-splice_match	gene: Zm00001d021901	Zm00001d021901_T00	7.15	7.15	coding	227	33	713
PB.5461.1	4	1434	1	genic	gene: Zm00001d052053	novel	4.01	62.79	coding	154	82	543
PB.10572.3	9	1099	6	full-splice_match	gene: Zm00001d047217	Zm00001d047217_T00	48.22	160.92	coding	307	29	949
PB.9646.2	8	1940	9	incomplete-splice_match	gene: Zm00001d010811	Zm00001d010811_T00	9.56	135.99	coding	372	93	1208
PB.9646.3	8	2076	11	full-splice_match	gene: Zm00001d010811	Zm00001d010811_T00	0.00	135.99	coding	567	1	1701
PB.9646.4	8	4979	1	novel_in_catalog	gene: Zm00001d010811	novel	20.03	135.99	coding	615	358	2202
PB.9646.5	8	1952	11	full-splice_match	gene: Zm00001d010811	Zm00001d010811_T00	80.38	135.99	coding	503	186	1694
PB.9646.6	8	715	5	incomplete-splice_match	gene: Zm00001d010811	Zm00001d010811_T00	8.88	135.99	coding	130	80	469
PB.146.5	1	1136	6	full-splice_match	gene: Zm00001d027874	Zm00001d027874_T01	51.65	72.33	coding	249	79	825
PB.146.4	1	2454	5	novel_in_catalog	gene: Zm00001d027874	novel	5.57	72.33	coding	131	1751	2143
PB.9943.1	8	1392	9	full-splice_match	gene: Zm00001d012126	Zm00001d012126_T00	27.41	35.71	coding	367	84	1184
PB.146.1	1	1968	6	full-splice_match	gene: Zm00001d027874	Zm00001d027874_T00	3.77	72.33	coding	244	926	1657
PB.3064.1	2	470	3	novel_not_in_catalog	gene: Zm00001d004997	novel	283.94	283.94	non coding	NA	NA	NA
PB.146.2	1	1593	6	novel_in_catalog	gene: Zm00001d027874	novel	11.34	72.33	coding	131	890	1282
PB.9539.1	8	1238	6	full-splice_match	gene: Zm00001d010395	Zm00001d010395_T00	75.23	75.23	coding	224	264	935

PB.10024.1	8	2058	7	full-splice_match	gene: Zm00001d012457	Zm00001d012457_T00	36.64	48.39	coding	536	122	1729
PB.9539.2	8	1105	6	full-splice_match	gene: Zm00001d010395	Zm00001d010395_T00	0.00	75.23	coding	224	237	908
PB.630.9	1	1344	5	novel_in_catalog	gene: Zm00001d030110	novel	14.55	227.82	coding	183	39	587
PB.630.8	1	1118	1	incomplete-splice_match	gene: Zm00001d030110	Zm00001d030110_T00	14.02	227.82	coding	112	35	370
PB.7216.5	5	726	3	novel_in_catalog	gene: Zm00001d018364	novel	3.86	31.42	non coding	NA	NA	NA
PB.9998.2	8	2265	1	full-splice_match	gene: Zm00001d012340	Zm00001d012340_T00	6.12	10.68	coding	702	96	2201
PB.5412.2	4	1475	7	incomplete-splice_match	gene: Zm00001d051856	Zm00001d051856_T00	60.11	103.88	coding	308	110	1033
PB.5522.4	4	3380	3	novel_in_catalog	gene: Zm00001d052259	novel	6.74	71.54	coding	413	273	1511
PB.3524.1	2	766	2	incomplete-splice_match	gene: Zm00001d007102	Zm00001d007102_T00	20.10	64.37	coding	93	63	341
PB.8014.1	6	1253	7	full-splice_match	gene: Zm00001d038506	Zm00001d038506_T00	19.94	19.94	coding	284	122	973
PB.1998.1	10	2621	1	antisense	novelGene_gene: Zm00001d024904	novel	1.75	1.75	coding	107	323	643
PB.9093.1	7	1632	5	full-splice_match	gene: Zm00001d022573	Zm00001d022573_T00	144.52	155.12	coding	447	70	1410
PB.302.1	1	1947	1	full-splice_match	gene: Zm00001d028537	Zm00001d028537_T00	2.13	2.13	coding	462	197	1582
PB.5952.1	5	1475	7	full-splice_match	gene: Zm00001d012800	Zm00001d012800_T00	30.23	48.61	coding	339	120	1136
PB.5952.2	5	1383	7	full-splice_match	gene: Zm00001d012800	Zm00001d012800_T00	14.93	48.61	coding	315	56	1000
PB.5952.4	5	1286	7	full-splice_match	gene: Zm00001d012800	Zm00001d012800_T00	0.00	48.61	coding	339	94	1110
PB.5952.5	5	1237	7	full-splice_match	gene: Zm00001d012800	Zm00001d012800_T00	0.00	48.61	coding	315	80	1024

PB.5952.6	5	1437	6	novel_in_catalog	gene: Zm00001d012800	novel	3.45	48.61	coding	326	47	1024
PB.3454.1	2	1440	7	full-splice_match	gene: Zm00001d006835	Zm00001d006835_T00	21.08	27.60	coding	293	204	1082
PB.1601.5	1	825	2	full-splice_match	gene: Zm00001d034673	Zm00001d034673_T00	10.93	18.39	coding	127	128	508
PB.1601.4	1	918	1	novel_in_catalog	gene: Zm00001d034673	novel	0.00	18.39	non coding	NA	NA	NA
PB.1601.3	1	929	2	full-splice_match	gene: Zm00001d034673	Zm00001d034673_T00	0.00	18.39	coding	127	136	516
PB.1601.2	1	1065	1	novel_in_catalog	gene: Zm00001d034673	novel	2.63	18.39	non coding	NA	NA	NA
PB.1353.1	1	912	6	novel_not_in_catalog	gene: Zm00001d033620	novel	59.26	59.26	coding	174	119	640
PB.690.2	1	2332	6	incomplete-splice_match	gene: Zm00001d030518	Zm00001d030518_T05	15.15	54.36	coding	630	243	2132
PB.8473.1	7	3607	3	novel_in_catalog	gene: Zm00001d020097	novel	15.31	15.31	coding	268	403	1206
PB.8473.3	7	1907	1	incomplete-splice_match	gene: Zm00001d020097	Zm00001d020097_T00	0.00	15.31	non coding	NA	NA	NA
PB.690.1	1	2293	5	incomplete-splice_match	gene: Zm00001d030518	Zm00001d030518_T05	39.20	54.36	coding	630	148	2037
PB.801.9	1	2804	14	novel_not_in_catalog	gene: Zm00001d031120	novel	4.41	247.40	coding	426	1257	2534
PB.2976.3	2	480	1	incomplete-splice_match	gene: Zm00001d004443	Zm00001d004443_T00	25.21	58.50	coding	121	20	382
PB.1662.1	1	1331	4	full-splice_match	gene: Zm00001d034872	Zm00001d034872_T00	6.65	6.65	coding	367	76	1176
PB.1976.1	10	1687	1	full-splice_match	gene: Zm00001d024789	Zm00001d024789_T00	3.20	3.20	coding	466	174	1571
PB.801.3	1	2356	14	full-splice_match	gene: Zm00001d031120	Zm00001d031120_T00	239.46	247.40	coding	624	123	1994
PB.6687.1	5	1846	4	incomplete-splice_match	gene: Zm00001d016063	Zm00001d016063_T00	11.73	38.27	coding	380	95	1234

PB.6687.2	5	1954	6	novel_not_in_catalog	gene: Zm00001d016063	novel	0.68	38.27	coding	525	190	1764
PB.6687.3	5	1990	6	full-splice_match	gene: Zm00001d016063	Zm00001d016063_T00	25.86	38.27	coding	539	189	1805
PB.5475.1	4	672	4	full-splice_match	gene: Zm00001d052075	Zm00001d052075_T00	227.23	227.23	coding	112	87	422
PB.8302.2	7	2883	10	incomplete-splice_match	gene: Zm00001d018976	Zm00001d018976_T05	15.03	40.63	coding	898	10	2703
PB.8302.1	7	2786	10	incomplete-splice_match	gene: Zm00001d018976	Zm00001d018976_T05	7.44	40.63	coding	777	231	2561
PB.1622.2	1	1580	4	full-splice_match	gene: Zm00001d034738	Zm00001d034738_T00	15.90	26.96	coding	379	163	1299
PB.4308.1	3	1783	12	incomplete-splice_match	gene: Zm00001d042639	Zm00001d042639_T00	25.66	45.96	coding	449	267	1613
PB.9622.1	8	3103	6	incomplete-splice_match	gene: Zm00001d010716	Zm00001d010716_T00	65.06	65.06	coding	962	24	2909
PB.10932.3	9	1978	11	incomplete-splice_match	gene: Zm00001d048552	Zm00001d048552_T00	4.85	17.19	coding	647	38	1978
PB.10932.2	9	2637	9	novel_in_catalog	gene: Zm00001d048552	novel	12.34	17.19	coding	492	5	1480
PB.513.4	1	888	2	intergenic	novelGene_166	novel	20.46	90.46	coding	124	341	712
PB.7256.5	5	1803	15	novel_in_catalog	gene: Zm00001d018484	novel	7.55	44.98	coding	258	306	1079
PB.5088.1	4	1693	2	genic	gene: Zm00001d049800	novel	16.72	23.58	coding	348	289	1332
PB.5088.2	4	1487	2	genic	gene: Zm00001d049800	novel	6.85	23.58	coding	348	214	1257
PB.8283.2	7	2646	7	novel_in_catalog	gene: Zm00001d018896	novel	0.18	85.48	coding	522	696	2261
PB.8283.3	7	2341	8	full-splice_match	gene: Zm00001d018896	Zm00001d018896_T00	0.00	85.48	coding	637	149	2059
PB.7256.3	5	1839	15	novel_in_catalog	gene: Zm00001d018484	novel	14.15	44.98	coding	258	263	1036

PB.8283.1	7	2494	8	full-splice_match	gene: Zm00001d018896	Zm00001d018896_T00	85.30	85.48	coding	637	157	2067
PB.8573.1	7	723	1	full-splice_match	gene: Zm00001d020626	Zm00001d020626_T00	10.65	10.65	coding	127	103	483
PB.4289.1	3	1610	6	full-splice_match	gene: Zm00001d042549	Zm00001d042549_T00	20.40	20.40	coding	260	678	1457
PB.5734.2	4	3088	5	novel_in_catalog	gene: Zm00001d053247	novel	1.31	30.72	coding	152	2384	2839
PB.4317.2	3	2238	4	full-splice_match	gene: Zm00001d042664	Zm00001d042664_T00	112.90	152.56	coding	571	68	1780
PB.6911.4	5	1039	1	novel_in_catalog	gene: Zm00001d017129	novel	4.06	24.18	coding	247	159	899
PB.5767.4	4	879	3	full-splice_match	gene: Zm00001d053396	Zm00001d053396_T00	29.40	106.06	coding	217	96	746
PB.5767.5	4	1452	2	novel_in_catalog	gene: Zm00001d053396	novel	0.00	106.06	coding	191	126	698
PB.1944.1	10	1458	2	novel_in_catalog	gene: Zm00001d024646	novel	2.21	29.62	coding	418	56	1309
PB.6697.2	5	2210	3	incomplete-splice_match	gene: Zm00001d016130	Zm00001d016130_T00	6.47	341.80	coding	119	113	469
PB.1944.3	10	2724	3	novel_in_catalog	gene: Zm00001d024646	novel	2.90	29.62	coding	234	546	1247
PB.1944.2	10	2126	4	full-splice_match	gene: Zm00001d024646	Zm00001d024646_T00	24.52	29.62	coding	482	536	1981
PB.4597.2	3	1401	1	genic	gene: Zm00001d043742	novel	354.66	354.66	coding	434	100	1401
PB.7216.6	5	766	3	novel_in_catalog	gene: Zm00001d018364	novel	0.00	31.42	coding	142	179	604
PB.8936.1	7	3305	9	full-splice_match	gene: Zm00001d022041	Zm00001d022041_T00	66.30	86.37	coding	715	790	2934
PB.3246.1	2	3210	3	incomplete-splice_match	gene: Zm00001d005958	Zm00001d005958_T00	28.54	28.54	coding	814	216	2657
PB.8338.1	7	1404	1	genic	gene: Zm00001d019216	novel	59.88	59.88	coding	274	205	1026

PB.5796.7	4	1642	3	novel_in_catalog	gene: Zm00001d053566	novel	364.06	614.41	coding	382	197	1342
PB.5243.1	4	1630	4	novel_not_in_catalog	gene: Zm00001d050913	novel	10.36	10.36	coding	340	236	1255
PB.8520.3	7	2227	3	novel_in_catalog	gene: Zm00001d020371	novel	2.27	37.61	coding	243	1160	1888
PB.9136.2	8	568	1	incomplete-splice_match	gene: Zm00001d008266	Zm00001d008266_T00	0.00	29.20	coding	98	256	549
PB.8424.3	7	2729	7	fusion	gene: Zm00001d019766	novel	11.08	23.35	coding	695	198	2282
PB.10473.1	9	907	5	incomplete-splice_match	gene: Zm00001d046681	Zm00001d046681_T00	4.15	4.15	coding	240	43	762
PB.9136.1	8	1369	2	full-splice_match	gene: Zm00001d008266	Zm00001d008266_T00	29.20	29.20	coding	343	204	1232
PB.3721.4	3	2501	15	full-splice_match	gene: Zm00001d039409	Zm00001d039409_T00	0.72	38.48	coding	390	445	1614
PB.8936.2	7	3430	8	novel_in_catalog	gene: Zm00001d022041	novel	6.25	86.37	coding	747	831	3071
PB.3721.2	3	2612	16	full-splice_match	gene: Zm00001d039409	Zm00001d039409_T00	20.22	38.48	coding	611	402	2234
PB.3721.3	3	2686	15	full-splice_match	gene: Zm00001d039409	Zm00001d039409_T00	9.40	38.48	coding	390	402	1571
PB.3721.1	3	2681	16	full-splice_match	gene: Zm00001d039409	Zm00001d039409_T00	8.14	38.48	coding	611	400	2232
PB.8796.1	7	1758	12	full-splice_match	gene: Zm00001d021580	Zm00001d021580_T00	16.86	16.86	coding	475	114	1538
PB.7787.2	6	1861	4	full-splice_match	gene: Zm00001d037640	Zm00001d037640_T00	2.15	15.10	coding	406	370	1587
PB.8796.2	7	694	7	incomplete-splice_match	gene: Zm00001d021580	Zm00001d021580_T00	0.00	16.86	coding	140	275	694
PB.10072.2	8	1524	6	novel_in_catalog	gene: Zm00001d012656	novel	4.29	31.27	coding	395	120	1304
PB.10072.1	8	1569	7	full-splice_match	gene: Zm00001d012656	Zm00001d012656_T00	26.99	31.27	coding	361	219	1301

PB.3701.1	3	583	1	incomplete-splice_match	gene: Zm00001d039319	Zm00001d039319_T00	4.46	9.07	non coding	NA	NA	NA
PB.3701.2	3	2232	9	full-splice_match	gene: Zm00001d039319	Zm00001d039319_T00	4.62	9.07	coding	616	16	1863
PB.550.3	1	1792	2	incomplete-splice_match	gene: Zm00001d029650	Zm00001d029650_T00	9.20	9.20	coding	304	644	1555
PB.4615.2	3	1448	7	incomplete-splice_match	gene: Zm00001d043807	Zm00001d043807_T00	23.90	46.97	coding	408	41	1264
PB.1072.3	1	569	1	novel_in_catalog	gene: Zm00001d032440	novel	30.86	62.10	non coding	NA	NA	NA
PB.7831.1	6	1595	5	incomplete-splice_match	gene: Zm00001d037858	Zm00001d037858_T00	47.05	47.05	coding	376	203	1330
PB.3910.1	3	443	1	intergenic	novelGene_125	novel	7.91	7.91	non coding	NA	NA	NA
PB.4616.1	3	1435	11	full-splice_match	gene: Zm00001d043815	Zm00001d043815_T00	6.03	6.03	coding	360	38	1117
PB.4757.4	3	1472	1	full-splice_match	gene: Zm00001d044390	Zm00001d044390_T00	0.00	113.06	coding	342	327	1352
PB.2977.2	2	717	3	intergenic	novelGene_227	novel	26.08	151.94	non coding	NA	NA	NA
PB.10341.1	9	3074	21	incomplete-splice_match	gene: Zm00001d045783	Zm00001d045783_T08	14.70	212.40	coding	1004	63	3074
PB.3145.1	2	587	1	genic	gene: Zm00001d005437	novel	40.93	40.93	non coding	NA	NA	NA
PB.4757.1	3	1852	1	full-splice_match	gene: Zm00001d044390	Zm00001d044390_T00	109.64	113.06	coding	485	40	1494
PB.4757.3	3	1782	1	full-splice_match	gene: Zm00001d044390	Zm00001d044390_T00	3.42	113.06	coding	485	192	1646
PB.7888.1	6	849	4	full-splice_match	gene: Zm00001d038056	Zm00001d038056_T00	27.04	27.04	coding	145	82	516
PB.10823.1	9	1484	2	full-splice_match	gene: Zm00001d048191	Zm00001d048191_T00	94.97	96.83	coding	386	54	1211
PB.2561.1	2	1491	5	novel_in_catalog	gene: Zm00001d002339	novel	281.91	281.91	non coding	NA	NA	NA

PB.1623.1	1	1326	4	full-splice_match	gene: Zm00001d034740	Zm00001d034740_T00	40.86	63.81	coding	331	57	1049
PB.2415.3	10	1491	6	novel_not_in_catalog	gene: Zm00001d026706	novel	4.15	277.23	coding	75	110	334
PB.2415.2	10	1024	5	full-splice_match	gene: Zm00001d026706	Zm00001d026706_T00	11.07	277.23	coding	173	110	628
PB.7423.1	6	521	2	full-splice_match	gene: Zm00001d035663	Zm00001d035663_T00	159.56	159.56	non coding	NA	NA	NA
PB.2977.1	2	672	4	intergenic	novelGene_227	novel	125.86	151.94	non coding	NA	NA	NA
PB.5522.3	4	1933	5	novel_not_in_catalog	gene: Zm00001d052259	novel	64.80	71.54	coding	507	150	1670
PB.9904.3	8	716	1	incomplete-splice_match	gene: Zm00001d011971	Zm00001d011971_T00	0.00	49.53	coding	224	45	716
PB.2536.1	2	2521	3	full-splice_match	gene: Zm00001d002252	Zm00001d002252_T00	36.57	36.57	coding	686	127	2184
PB.1059.1	1	1204	2	full-splice_match	gene: Zm00001d032396	Zm00001d032396_T00	14.47	14.47	coding	334	104	1105
PB.2683.4	2	717	3	full-splice_match	gene: Zm00001d002873	Zm00001d002873_T00	19.36	19.36	coding	124	45	416
PB.2683.5	2	471	3	full-splice_match	gene: Zm00001d002873	Zm00001d002873_T00	0.00	19.36	coding	124	9	380
PB.9011.2	7	1356	1	full-splice_match	gene: Zm00001d022327	Zm00001d022327_T00	113.67	131.61	coding	357	139	1209
PB.8684.4	7	1644	8	intergenic	novelGene_282	novel	5.93	53.03	coding	216	669	1316
PB.7639.1	6	2118	8	incomplete-splice_match	gene: Zm00001d036968	Zm00001d036968_T01	56.16	56.16	coding	567	35	1735
PB.10823.2	9	1574	2	full-splice_match	gene: Zm00001d048191	Zm00001d048191_T00	1.86	96.83	coding	450	39	1388
PB.4691.4	3	1664	1	full-splice_match	gene: Zm00001d044120	Zm00001d044120_T00	0.00	31.82	coding	494	122	1603
PB.3974.2	3	1730	1	novel_in_catalog	gene: Zm00001d040597	novel	5.08	23.96	coding	178	434	967

PB.3974.3	3	1196	2	novel_in_catalog	gene: Zm00001d040597	novel	1.22	23.96	coding	257	366	1136
PB.3974.1	3	1098	3	full-splice_match	gene: Zm00001d040597	Zm00001d040597_T00	17.66	23.96	coding	257	254	1024
PB.2888.5	2	1597	12	full-splice_match	gene: Zm00001d003851	Zm00001d003851_T00	33.13	62.51	coding	415	193	1437
PB.1166.1	1	1296	2	full-splice_match	gene: Zm00001d032787	Zm00001d032787_T00	53.78	53.78	coding	294	267	1148
PB.5825.1	4	1403	12	full-splice_match	gene: Zm00001d053665	Zm00001d053665_T00	62.98	62.98	coding	279	159	995
PB.2888.6	2	2284	11	full-splice_match	gene: Zm00001d003851	Zm00001d003851_T00	5.87	62.51	coding	248	513	1256
PB.5487.3	4	2011	9	full-splice_match	gene: Zm00001d052147	Zm00001d052147_T00	0.00	52.96	coding	525	108	1682
PB.5487.2	4	2173	5	novel_in_catalog	gene: Zm00001d052147	novel	18.26	52.96	coding	194	1188	1769
PB.2888.3	2	2543	11	novel_not_in_catalog	gene: Zm00001d003851	novel	23.51	62.51	coding	241	612	1334
PB.4691.2	3	1999	1	full-splice_match	gene: Zm00001d044120	Zm00001d044120_T00	4.85	31.82	coding	494	125	1606
PB.4676.1	3	2445	7	full-splice_match	gene: Zm00001d044059	Zm00001d044059_T00	15.77	15.77	coding	652	281	2236
PB.10534.1	9	806	1	full-splice_match	gene: Zm00001d047013	Zm00001d047013_T00	2.59	2.59	coding	130	48	437
PB.2508.1	2	2209	1	intergenic	novelGene_29	novel	7.80	7.80	non coding	NA	NA	NA
PB.4261.3	3	1494	4	novel_in_catalog	gene: Zm00001d042427	novel	13.57	34.25	coding	397	112	1302
PB.7367.1	6	437	2	intergenic	novelGene_89	novel	8.90	8.90	non coding	NA	NA	NA
PB.7880.1	6	806	1	full-splice_match	gene: Zm00001d001437	Zm00001d001437_T00	135.01	135.01	non coding	NA	NA	NA
PB.1622.3	1	2919	3	novel_in_catalog	gene: Zm00001d034738	novel	2.22	26.96	coding	315	120	1064

PB.4593.1	3	1044	6	full-splice_match	gene: Zm00001d043735	Zm00001d043735_T00	6.25	6.25	coding	230	152	841
PB.4442.1	3	1877	2	novel_not_in_catalog	gene: Zm00001d043200	novel	4.45	4.45	coding	404	547	1758
PB.3833.1	3	1696	1	full-splice_match	gene: Zm00001d039907	Zm00001d039907_T00	0.00	18.39	coding	378	88	1221
PB.3833.2	3	1717	1	full-splice_match	gene: Zm00001d039907	Zm00001d039907_T00	18.39	18.39	coding	378	285	1418
PB.3833.3	3	1533	1	full-splice_match	gene: Zm00001d039907	Zm00001d039907_T00	0.00	18.39	coding	378	285	1418
PB.10329.1	9	805	5	intergenic	novelGene_122	novel	9.07	9.07	non coding	NA	NA	NA
PB.1679.2	1	1155	7	fusion	gene: Zm00001d034957	novel	38.89	68.49	coding	298	59	952
PB.1178.1	1	1214	1	intergenic	novelGene_438	novel	58.71	322.84	coding	261	96	878
PB.7516.2	6	1349	3	full-splice_match	gene: Zm00001d036285	Zm00001d036285_T00	34.41	76.26	coding	324	160	1131
PB.2740.3	2	760	4	full-splice_match	gene: Zm00001d003127	Zm00001d003127_T00	579.75	637.40	coding	152	79	534
PB.2740.1	2	869	4	full-splice_match	gene: Zm00001d003127	Zm00001d003127_T00	57.65	637.40	coding	152	74	529
PB.5662.1	4	2308	4	full-splice_match	gene: Zm00001d052850	Zm00001d052850_T00	7.16	7.16	coding	604	51	1862
PB.881.2	1	1229	2	full-splice_match	gene: Zm00001d031545	Zm00001d031545_T00	74.55	74.55	coding	272	211	1026
PB.2740.4	2	646	4	full-splice_match	gene: Zm00001d003127	Zm00001d003127_T00	0.00	637.40	coding	152	74	529
PB.1278.4	1	2313	14	full-splice_match	gene: Zm00001d033297	Zm00001d033297_T01	34.51	48.14	coding	503	487	1995
PB.1361.13	1	1817	11	full-splice_match	gene: Zm00001d033650	Zm00001d033650_T00	12.33	13.18	coding	506	104	1621
PB.2397.2	10	1610	5	full-splice_match	gene: Zm00001d026652	Zm00001d026652_T00	23.74	31.75	coding	397	315	1505

PB.2397.3	10	1063	4	novel_in_catalog	gene: Zm00001d026652	novel	3.71	31.75	coding	291	55	927
PB.5624.1	4	1054	4	full-splice_match	gene: Zm00001d052719	Zm00001d052719_T00	48.20	48.20	coding	262	43	828
PB.8445.1	7	1919	5	full-splice_match	gene: Zm00001d019957	Zm00001d019957_T00	2.68	2.68	coding	559	51	1727
PB.356.1	1	1577	19	incomplete-splice_match	gene: Zm00001d028762	Zm00001d028762_T00	29.98	29.98	coding	447	13	1353
PB.8826.1	7	2354	3	novel_not_in_catalog	gene: Zm00001d021697	novel	20.45	46.46	coding	568	89	1792
PB.1361.11	1	1804	11	full-splice_match	gene: Zm00001d033649	Zm00001d033649_T00	33.84	68.36	coding	502	136	1641
PB.10209.1	9	355	1	intergenic	novelGene_69	novel	24.13	24.13	non coding	NA	NA	NA
PB.2856.1	2	2473	10	full-splice_match	gene: Zm00001d003678	Zm00001d003678_T00	5.29	7.38	coding	300	1288	2187
PB.2500.2	2	1520	1	full-splice_match	gene: Zm00001d002066	Zm00001d002066_T00	110.02	110.02	coding	335	291	1295
PB.2856.3	2	2389	10	novel_in_catalog	gene: Zm00001d003678	novel	2.09	7.38	coding	300	1257	2156
PB.773.1	1	2799	11	full-splice_match	gene: Zm00001d031009	Zm00001d031009_T00	11.91	11.91	coding	770	115	2424
PB.3454.2	2	1258	7	full-splice_match	gene: Zm00001d006835	Zm00001d006835_T00	6.52	27.60	coding	293	129	1007
PB.826.3	1	1691	1	full-splice_match	gene: Zm00001d031222	Zm00001d031222_T00	0.00	106.90	coding	527	41	1621
PB.8538.2	7	787	4	full-splice_match	gene: Zm00001d020438	Zm00001d020438_T00	130.37	139.50	coding	151	104	556
PB.8538.1	7	1884	3	full-splice_match	gene: Zm00001d020438	Zm00001d020438_T00	9.13	139.50	coding	102	102	407
PB.928.2	1	1546	3	full-splice_match	gene: Zm00001d031813	Zm00001d031813_T00	0.00	55.75	coding	435	153	1457
PB.10845.1	9	1523	10	full-splice_match	gene: Zm00001d048292	Zm00001d048292_T00	24.25	50.90	coding	311	38	970

PB.10845.3	9	1394	10	full-splice_match	gene: Zm00001d048292	Zm00001d048292_T00	18.80	50.90	coding	311	33	965
PB.10845.4	9	1811	8	novel_in_catalog	gene: Zm00001d048292	novel	7.85	50.90	coding	226	125	802
PB.6113.1	5	1247	8	full-splice_match	gene: Zm00001d013331	Zm00001d013331_T00	10.34	10.97	coding	278	75	908
PB.6113.2	5	1089	8	full-splice_match	gene: Zm00001d013331	Zm00001d013331_T00	0.64	10.97	coding	278	85	918
PB.6113.3	5	555	1	genic_intron	novelGene_42	novel	5.59	5.59	non coding	NA	NA	NA
PB.2520.4	2	629	1	incomplete-splice_match	gene: Zm00001d002141	Zm00001d002141_T01	4.62	657.91	coding	101	230	532
PB.2520.2	2	1450	2	full-splice_match	gene: Zm00001d002141	Zm00001d002141_T02	8.41	657.91	coding	207	482	1102
PB.2520.3	2	1912	2	full-splice_match	gene: Zm00001d002141	Zm00001d002141_T02	0.00	657.91	coding	521	64	1626
PB.2520.1	2	2050	2	full-splice_match	gene: Zm00001d002141	Zm00001d002141_T02	21.07	657.91	coding	521	63	1625
PB.5694.1	4	2376	14	full-splice_match	gene: Zm00001d053014	Zm00001d053014_T00	14.74	14.74	coding	545	95	1729
PB.465.4	1	1246	11	full-splice_match	gene: Zm00001d029234	Zm00001d029234_T00	9.77	31.52	coding	213	35	673
PB.5694.3	4	2135	14	full-splice_match	gene: Zm00001d053014	Zm00001d053014_T00	0.00	14.74	coding	545	27	1661
PB.5908.2	4	1185	7	full-splice_match	gene: Zm00001d053981	Zm00001d053981_T00	48.92	55.85	coding	255	198	962
PB.465.1	1	2613	9	novel_in_catalog	gene: Zm00001d029234	novel	5.86	31.52	coding	230	36	725
PB.4857.1	4	1938	1	full-splice_match	gene: Zm00001d048681	Zm00001d048681_T00	0.62	0.62	coding	442	29	1354
PB.465.2	1	1420	11	full-splice_match	gene: Zm00001d029234	Zm00001d029234_T00	15.88	31.52	coding	332	111	1106
PB.714.2	1	1765	10	full-splice_match	gene: Zm00001d030675	Zm00001d030675_T00	14.28	14.28	coding	429	112	1398

PB.714.3	1	1694	9	fusion	gene: Zm00001d030675	novel	1.79	62.75	coding	429	112	1398
PB.3676.1	3	573	1	incomplete-splice_match	gene: Zm00001d039259	Zm00001d039259_T00	31.27	31.27	non coding	NA	NA	NA
PB.714.1	1	1867	10	fusion	gene: Zm00001d030675	novel	60.96	62.75	coding	444	112	1443
PB.11039.1	Mt	991	1	intergenic	novelGene_7	novel	66.80	66.80	non coding	NA	NA	NA
PB.7744.1	6	1549	2	full-splice_match	gene: Zm00001d037429	Zm00001d037429_T00	24.45	50.84	coding	370	138	1247
PB.9545.1	8	1490	5	full-splice_match	gene: Zm00001d010411	Zm00001d010411_T00	131.87	160.22	coding	246	77	814
PB.9545.2	8	2301	2	novel_in_catalog	gene: Zm00001d010411	novel	4.62	160.22	coding	174	60	581
PB.9510.1	8	1810	13	novel_not_in_catalog	gene: Zm00001d010255	novel	29.91	29.91	coding	445	166	1500
PB.9545.4	8	1632	4	novel_in_catalog	gene: Zm00001d010411	novel	21.93	160.22	coding	214	154	795
PB.10082.1	8	2272	13	novel_in_catalog	gene: Zm00001d012693	novel	6.97	6.97	coding	466	688	2085
PB.1587.1	1	816	4	full-splice_match	gene: Zm00001d034628	Zm00001d034628_T00	262.51	262.51	coding	142	202	627
PB.4749.1	3	1578	8	novel_not_in_catalog	gene: Zm00001d044328	novel	9.91	9.91	coding	146	736	1173
PB.10056.2	8	1169	4	novel_not_in_catalog	gene: Zm00001d012604	novel	3.66	15.73	coding	141	526	948
PB.6946.6	5	1290	13	full-splice_match	gene: Zm00001d017271	Zm00001d017271_T00	0.00	445.51	coding	342	114	1139
PB.4195.1	3	3938	17	full-splice_match	gene: Zm00001d042066	Zm00001d042066_T00	9.71	87.33	coding	552	2049	3704
PB.4195.2	3	3830	18	full-splice_match	gene: Zm00001d042066	Zm00001d042066_T01	31.13	87.33	coding	1113	304	3642
PB.4195.3	3	3848	17	full-splice_match	gene: Zm00001d042066	Zm00001d042066_T00	5.72	87.33	coding	552	2071	3726

PB.6946.2	5	1402	13	full-splice_match	gene: Zm00001d017271	Zm00001d017271_T00	429.13	445.51	coding	342	109	1134
PB.8530.2	7	1753	6	novel_in_catalog	gene: Zm00001d020409	novel	0.57	75.47	coding	184	157	708
PB.8530.1	7	1757	7	full-splice_match	gene: Zm00001d020409	Zm00001d020409_T00	74.90	75.47	coding	404	166	1377
PB.4259.2	3	2251	10	full-splice_match	gene: Zm00001d042416	Zm00001d042416_T00	35.37	123.25	coding	571	167	1879
PB.4028.1	3	1424	8	incomplete-splice_match	gene: Zm00001d041092	Zm00001d041092_T00	51.33	51.33	coding	391	94	1266
PB.3542.1	2	1475	6	novel_not_in_catalog	gene: Zm00001d007195	novel	9.13	22.60	coding	133	81	479
PB.1670.2	1	706	5	full-splice_match	gene: Zm00001d034893	Zm00001d034893_T00	99.42	168.92	coding	147	73	513
PB.3648.1	2	1964	4	novel_in_catalog	gene: Zm00001d007870	novel	81.45	81.45	coding	186	128	685
PB.720.2	1	1204	8	full-splice_match	gene: Zm00001d030739	Zm00001d030739_T00	6.97	133.72	non coding	NA	NA	NA
PB.1204.1	1	1574	1	genic	gene: Zm00001d032937	novel	0.55	0.99	coding	253	194	952
PB.4076.1	3	1670	2	genic	gene: Zm00001d041456	novel	115.18	119.86	coding	426	123	1400
PB.4076.2	3	1580	2	genic	gene: Zm00001d041456	novel	4.69	119.86	coding	426	129	1406
PB.10871.2	9	1582	3	incomplete-splice_match	gene: Zm00001d048372	Zm00001d048372_T00	16.73	16.73	coding	429	81	1367
PB.9969.1	8	1358	3	full-splice_match	gene: Zm00001d012242	Zm00001d012242_T00	18.79	51.26	coding	316	77	1024
PB.10742.1	9	2270	3	full-splice_match	gene: Zm00001d047848	Zm00001d047848_T00	32.91	32.91	coding	591	42	1814
PB.4485.1	3	2680	1	intergenic	novelGene_391	novel	7.74	7.74	coding	746	190	2427
PB.900.3	1	1827	5	full-splice_match	gene: Zm00001d031648	Zm00001d031648_T00	0.00	217.09	coding	435	306	1610

PB.900.2	1	318	1	incomplete-splice_match	gene: Zm00001d031648	Zm00001d031648_T00	111.68	217.09	non coding	NA	NA	NA
PB.900.1	1	2036	5	full-splice_match	gene: Zm00001d031648	Zm00001d031648_T00	105.41	217.09	coding	435	299	1603
PB.2167.1	10	2669	6	novel_in_catalog	gene: Zm00001d025777	novel	3.23	85.42	coding	556	575	2242
PB.2167.2	10	2922	9	novel_in_catalog	gene: Zm00001d025777	novel	82.19	85.42	coding	785	142	2496
PB.2794.1	2	1257	4	full-splice_match	gene: Zm00001d003393	Zm00001d003393_T00	123.65	123.65	coding	312	106	1041
PB.3400.1	2	2206	2	full-splice_match	gene: Zm00001d006590	Zm00001d006590_T00	9.05	9.05	coding	603	189	1997
PB.5067.1	4	2667	4	full-splice_match	gene: Zm00001d049708	Zm00001d049708_T00	23.43	43.55	coding	743	138	2366
PB.1978.1	10	681	1	full-splice_match	gene: Zm00001d024800	Zm00001d024800_T00	71.35	124.65	coding	95	84	368
PB.1978.2	10	530	1	full-splice_match	gene: Zm00001d024800	Zm00001d024800_T00	53.30	124.65	coding	95	85	369
PB.5067.2	4	1328	2	incomplete-splice_match	gene: Zm00001d049708	Zm00001d049708_T00	20.12	43.55	coding	321	195	1157
PB.71.1	1	1380	5	full-splice_match	gene: Zm00001d027527	Zm00001d027527_T00	73.27	94.69	coding	308	149	1072
PB.71.2	1	1301	5	full-splice_match	gene: Zm00001d027527	Zm00001d027527_T00	21.43	94.69	coding	308	148	1071
PB.3203.2	2	2949	3	novel_not_in_catalog	gene: Zm00001d005743	novel	17.80	30.73	non coding	NA	NA	NA
PB.5512.1	4	1385	5	incomplete-splice_match	gene: Zm00001d052232	Zm00001d052232_T00	4.61	4.61	coding	333	216	1214
PB.3857.1	3	510	2	full-splice_match	gene: Zm00001d040028	Zm00001d040028_T00	91.49	91.49	non coding	NA	NA	NA
PB.7102.1	5	1792	6	full-splice_match	gene: Zm00001d017945	Zm00001d017945_T00	2.72	37.97	coding	184	287	838
PB.7102.2	5	910	6	full-splice_match	gene: Zm00001d017945	Zm00001d017945_T00	35.26	37.97	coding	184	142	693

PB.3640.5	2	824	3	full-splice_match	gene: Zm00001d007832	Zm00001d007832_T00	0.00	241.96	coding	191	93	665
PB.9833.1	8	1610	3	full-splice_match	gene: Zm00001d011687	Zm00001d011687_T00	39.88	39.88	coding	443	105	1433
PB.9874.1	8	1555	1	genic	gene: Zm00001d011834	novel	3.30	13.59	coding	323	23	991
PB.3316.1	2	1533	4	full-splice_match	gene: Zm00001d006217	Zm00001d006217_T00	24.61	24.61	coding	422	44	1309
PB.6883.3	5	1026	4	full-splice_match	gene: Zm00001d017047	Zm00001d017047_T00	142.84	274.64	coding	219	97	753
PB.9874.2	8	2059	2	full-splice_match	gene: Zm00001d011834	Zm00001d011834_T00	10.30	13.59	coding	446	473	1810
PB.3223.1	2	629	1	full-splice_match	gene: Zm00001d005841	Zm00001d005841_T00	24.10	24.10	non coding	NA	NA	NA
PB.5599.4	4	1676	13	novel_not_in_catalog	gene: Zm00001d052532	novel	7.13	583.99	coding	95	15	299
PB.2906.2	2	885	1	incomplete-splice_match	gene: Zm00001d003931	Zm00001d003931_T00	0.00	21.49	coding	229	57	743
PB.2906.1	2	1715	4	full-splice_match	gene: Zm00001d003931	Zm00001d003931_T00	21.49	21.49	coding	451	169	1521
PB.7051.1	5	1885	2	full-splice_match	gene: Zm00001d017728	Zm00001d017728_T00	7.40	7.40	coding	525	122	1696
PB.1466.2	1	1719	4	full-splice_match	gene: Zm00001d034109	Zm00001d034109_T00	23.47	127.30	coding	376	285	1412
PB.8853.1	7	2352	1	novel_in_catalog	gene: Zm00001d021759	novel	37.72	37.72	coding	241	1414	2136
PB.8740.1	7	1850	6	novel_not_in_catalog	gene: Zm00001d021372	novel	9.77	9.77	coding	175	30	554
PB.2484.28	2	2406	11	incomplete-splice_match	gene: Zm00001d002006	Zm00001d002006_T01	0.00	1327.46	coding	739	189	2405
PB.2484.29	2	1942	6	novel_in_catalog	gene: Zm00001d002006	novel	0.00	1327.46	coding	589	33	1799
PB.3660.4	2	2275	7	intergenic	novelGene_443	novel	20.02	53.91	coding	279	1019	1855

PB.1830.1	10	1704	2	full-splice_match	gene: Zm00001d023915	Zm00001d023915_T00	6.25	6.25	coding	501	56	1558
PB.8800.1	7	1869	4	full-splice_match	gene: Zm00001d021596	Zm00001d021596_T00	36.81	36.81	coding	461	94	1476
PB.8032.1	6	893	2	incomplete-splice_match	gene: Zm00001d038543	Zm00001d038543_T00	277.82	428.03	coding	149	119	565
PB.8032.2	6	788	2	incomplete-splice_match	gene: Zm00001d038543	Zm00001d038543_T00	150.20	428.03	coding	149	119	565
PB.7551.6	6	1302	6	full-splice_match	gene: Zm00001d036481	Zm00001d036481_T00	14.02	416.78	coding	183	445	993
PB.8877.1	7	2568	7	full-splice_match	gene: Zm00001d021828	Zm00001d021828_T00	20.57	20.57	coding	776	101	2428
PB.5991.2	5	1893	10	full-splice_match	gene: Zm00001d012931	Zm00001d012931_T00	13.80	17.46	coding	491	86	1558
PB.5991.1	5	2106	10	full-splice_match	gene: Zm00001d012931	Zm00001d012931_T00	3.66	17.46	coding	491	280	1752
PB.4012.1	3	520	2	antisense	novelGene_gene: Zm00001d000551	novel	58.49	58.49	non coding	NA	NA	NA
PB.9444.4	8	2092	10	novel_in_catalog	gene: Zm00001d009883	novel	15.83	30.35	coding	135	1549	1953
PB.2484.25	2	3312	13	full-splice_match	gene: Zm00001d002006	Zm00001d002006_T00	4.64	1327.46	coding	744	183	2414
PB.3660.8	2	2975	6	intergenic	novelGene_443	novel	0.00	53.91	coding	279	1806	2642
PB.4438.1	3	1459	2	genic	gene: Zm00001d043183	novel	35.79	41.28	coding	287	265	1125
PB.10712.7	9	641	2	full-splice_match	gene: Zm00001d047758	Zm00001d047758_T00	15.80	193.91	coding	115	82	426
PB.10712.4	9	1589	4	novel_in_catalog	gene: Zm00001d047758	novel	178.11	193.91	coding	404	152	1363
PB.4793.7	3	1483	4	full-splice_match	gene: Zm00001d044516	Zm00001d044516_T00	0.00	891.68	coding	365	127	1221
PB.3442.2	2	851	6	full-splice_match	gene: Zm00001d006760	Zm00001d006760_T00	1201.97	1201.97	coding	160	79	558

PB.7119.1	5	738	1	incomplete-splice_match	gene: Zm00001d017989	Zm00001d017989_T00	9.85	9.85	coding	15	466	510
PB.7020.2	5	1816	2	novel_in_catalog	gene: Zm00001d017608	novel	2.77	50.54	coding	154	29	490
PB.8715.1	7	2647	9	full-splice_match	gene: Zm00001d021287	Zm00001d021287_T00	26.62	26.62	coding	683	136	2184
PB.10100.1	8	2014	9	antisense	novelGene_gene: Zm00001d012761	novel	6.55	19.16	coding	139	1166	1582
PB.1950.4	10	823	3	full-splice_match	gene: Zm00001d024703	Zm00001d024703_T01	17.27	41.31	non coding	NA	NA	NA
PB.10100.3	8	1908	8	antisense	novelGene_gene: Zm00001d012761	novel	4.42	19.16	non coding	NA	NA	NA
PB.1950.2	10	3697	6	full-splice_match	gene: Zm00001d024703	Zm00001d024703_T00	9.55	41.31	coding	962	506	3391
PB.8552.1	7	1650	3	novel_not_in_catalog	gene: Zm00001d020524	novel	31.70	31.70	coding	538	36	1649
PB.1950.1	10	2989	3	incomplete-splice_match	gene: Zm00001d024703	Zm00001d024703_T01	14.48	41.31	coding	705	447	2561
PB.10100.8	8	1120	4	intergenic	novelGene_371	novel	0.00	0.00	non coding	NA	NA	NA
PB.65.2	1	712	2	genic	gene: Zm00001d027493	novel	238.70	336.91	coding	108	121	444
PB.65.1	1	804	2	genic	gene: Zm00001d027493	novel	98.21	336.91	coding	108	107	430
PB.6664.1	5	723	2	full-splice_match	gene: Zm00001d015865	Zm00001d015865_T00	41.71	41.71	coding	141	141	563
PB.3365.1	2	1067	5	incomplete-splice_match	gene: Zm00001d006415	Zm00001d006415_T00	84.66	84.66	coding	144	282	713
PB.1506.1	1	404	1	full-splice_match	gene: Zm00001d034339	Zm00001d034339_T00	48.23	48.23	non coding	NA	NA	NA
PB.477.1	1	1539	6	full-splice_match	gene: Zm00001d029303	Zm00001d029303_T00	28.16	28.16	coding	269	111	917
PB.6451.1	5	1592	9	full-splice_match	gene: Zm00001d014788	Zm00001d014788_T00	26.27	26.27	coding	346	260	1297

PB.7224.1	5	2603	1	full-splice_match	gene: Zm00001d018389	Zm00001d018389_T00	108.52	108.52	coding	661	306	2288
PB.191.1	1	1789	3	novel_not_in_catalog	gene: Zm00001d028055	novel	3.18	181.34	coding	238	79	792
PB.191.3	1	1824	1	novel_in_catalog	gene: Zm00001d028055	novel	21.32	181.34	coding	169	1037	1543
PB.191.2	1	1205	2	full-splice_match	gene: Zm00001d028055	Zm00001d028055_T00	38.83	181.34	coding	272	55	870
PB.191.5	1	1007	2	full-splice_match	gene: Zm00001d028055	Zm00001d028055_T00	79.69	181.34	coding	272	80	895
PB.191.4	1	1371	2	full-splice_match	gene: Zm00001d028055	Zm00001d028055_T00	38.32	181.34	coding	272	276	1091
PB.6167.1	5	2388	5	full-splice_match	gene: Zm00001d013527	Zm00001d013527_T00	27.25	108.40	coding	585	258	2012
PB.6167.3	5	1935	4	novel_in_catalog	gene: Zm00001d013527	novel	16.48	108.40	coding	346	178	1215
PB.6167.2	5	3182	2	novel_in_catalog	gene: Zm00001d013527	novel	1.85	108.40	coding	312	124	1059
PB.6167.5	5	2086	5	full-splice_match	gene: Zm00001d013527	Zm00001d013527_T00	13.72	108.40	coding	585	119	1873
PB.6167.4	5	2421	3	novel_in_catalog	gene: Zm00001d013527	novel	8.58	108.40	coding	509	121	1647
PB.6167.7	5	2234	4	novel_in_catalog	gene: Zm00001d013527	novel	0.00	108.40	coding	509	124	1650
PB.9598.1	8	1060	2	genic	gene: Zm00001d010649	novel	7.57	72.86	coding	152	51	506
PB.6167.9	5	1118	5	full-splice_match	gene: Zm00001d013527	Zm00001d013527_T00	40.52	108.40	coding	306	101	1018
PB.8224.2	7	1052	1	full-splice_match	gene: Zm00001d018631	Zm00001d018631_T00	260.28	280.61	coding	250	105	854
PB.8224.1	7	1125	1	full-splice_match	gene: Zm00001d018631	Zm00001d018631_T00	20.33	280.61	coding	250	63	812
PB.7039.1	5	1656	1	antisense	novelGene_gene: Zm00001d017671	novel	4.50	4.50	coding	341	153	1175

PB.3542.2	2	2190	17	full-splice_match	gene: Zm00001d007195	Zm00001d007195_T01	13.48	22.60	coding	663	26	2014
PB.4544.1	3	1788	14	full-splice_match	gene: Zm00001d043560	Zm00001d043560_T00	20.24	26.48	coding	507	107	1627
PB.10627.2	9	899	4	novel_not_in_catalog	gene: Zm00001d047469	novel	7.26	58.67	coding	217	128	778
PB.6204.4	5	1423	1	novel_in_catalog	gene: Zm00001d013707	novel	0.00	32.34	coding	69	114	320
PB.6204.3	5	1265	2	novel_in_catalog	gene: Zm00001d013707	novel	5.19	32.34	coding	224	114	785
PB.6204.2	5	1295	2	novel_in_catalog	gene: Zm00001d013707	novel	3.92	32.34	coding	69	112	318
PB.6204.1	5	1285	3	full-splice_match	gene: Zm00001d013707	Zm00001d013707_T00	23.23	32.34	coding	224	114	785
PB.9755.3	8	972	7	incomplete-splice_match	gene: Zm00001d011347	Zm00001d011347_T00	15.10	55.52	coding	303	62	970
PB.5575.2	4	2125	13	incomplete-splice_match	gene: Zm00001d052425	Zm00001d052425_T01	37.62	73.52	coding	430	666	1955
PB.6875.1	5	1946	4	full-splice_match	gene: Zm00001d016999	Zm00001d016999_T00	19.31	19.31	coding	340	371	1390
PB.1012.1	1	2477	1	full-splice_match	gene: Zm00001d032187	Zm00001d032187_T00	3.73	534.29	coding	394	731	1912
PB.2171.1	10	1887	1	full-splice_match	gene: Zm00001d025807	Zm00001d025807_T00	16.55	16.55	coding	542	64	1689
PB.7411.2	6	1363	10	novel_not_in_catalog	gene: Zm00001d035601	novel	6.36	6.36	coding	154	46	507
PB.1012.2	1	2325	1	full-splice_match	gene: Zm00001d032187	Zm00001d032187_T00	9.98	534.29	coding	394	736	1917
PB.10776.1	9	1852	1	full-splice_match	gene: Zm00001d047966	Zm00001d047966_T00	4.27	7.80	coding	505	150	1664
PB.4577.1	3	985	1	full-splice_match	gene: Zm00001d043674	Zm00001d043674_T00	34.65	34.65	coding	193	170	748
PB.1012.7	1	1498	2	genic	gene: Zm00001d032187	novel	287.86	534.29	coding	394	86	1267

PB.10776.2	9	2657	1	full-splice_match	gene: Zm00001d047966	Zm00001d047966_T00	3.53	7.80	coding	872	25	2640
PB.5541.1	4	953	4	full-splice_match	gene: Zm00001d052325	Zm00001d052325_T00	30.15	32.43	coding	173	141	659
PB.1395.2	1	2079	5	full-splice_match	gene: Zm00001d033800	Zm00001d033800_T00	25.50	57.51	coding	587	128	1888
PB.6459.3	5	1652	7	full-splice_match	gene: Zm00001d014809	Zm00001d014809_T00	0.53	31.76	coding	474	137	1558
PB.10055.1	8	1007	3	full-splice_match	gene: Zm00001d012603	Zm00001d012603_T00	55.84	55.84	coding	245	84	818
PB.6459.1	5	1747	7	full-splice_match	gene: Zm00001d014809	Zm00001d014809_T00	31.23	31.76	coding	474	135	1556
PB.7572.1	6	2797	9	full-splice_match	gene: Zm00001d036571	Zm00001d036571_T00	11.77	25.09	coding	753	206	2464
PB.7572.2	6	1467	9	full-splice_match	gene: Zm00001d036571	Zm00001d036571_T00	6.09	25.09	coding	371	13	1125
PB.7572.3	6	650	4	incomplete-splice_match	gene: Zm00001d036571	Zm00001d036571_T01	7.24	25.09	coding	99	129	425
PB.8564.4	7	1329	4	novel_not_in_catalog	gene: Zm00001d020569	novel	6.71	28.23	coding	344	168	1199
PB.4242.2	3	1174	8	incomplete-splice_match	gene: Zm00001d042335	Zm00001d042335_T02	0.00	28.72	coding	373	55	1173
PB.1664.1	1	2345	5	novel_in_catalog	gene: Zm00001d034876	novel	18.94	18.94	coding	697	19	2109
PB.13.2	1	2623	10	full-splice_match	gene: Zm00001d027293	Zm00001d027293_T00	5.32	422.53	coding	625	105	1979
PB.4121.1	3	958	5	full-splice_match	gene: Zm00001d041725	Zm00001d041725_T00	53.88	58.95	coding	196	105	692
PB.8612.3	7	2812	2	genic	gene: Zm00001d020828	novel	18.60	37.46	coding	635	432	2336
PB.4121.2	3	912	4	novel_in_catalog	gene: Zm00001d041725	novel	5.07	58.95	coding	126	105	482
PB.6445.1	5	881	1	full-splice_match	gene: Zm00001d014778	Zm00001d014778_T00	26.78	26.78	coding	187	18	578

PB.10327.3	9	560	2	full-splice_match	gene: Zm00001d045685	Zm00001d045685_T00	0.00	53.41	coding	114	83	424
PB.10327.2	9	701	2	full-splice_match	gene: Zm00001d045685	Zm00001d045685_T00	25.69	53.41	coding	114	86	427
PB.10327.1	9	834	2	full-splice_match	gene: Zm00001d045685	Zm00001d045685_T00	27.72	53.41	coding	114	86	427
PB.4269.3	3	2753	3	novel_in_catalog	gene: Zm00001d042455	novel	21.23	177.52	coding	114	257	598
PB.5541.2	4	1662	3	novel_in_catalog	gene: Zm00001d052325	novel	2.28	32.43	coding	100	1188	1487
PB.8577.1	7	1887	9	full-splice_match	gene: Zm00001d020642	Zm00001d020642_T00	152.10	152.10	coding	525	60	1634
PB.5547.1	4	2897	3	novel_in_catalog	gene: Zm00001d052343	novel	38.19	42.57	coding	667	483	2483
PB.6899.15	5	2169	8	full-splice_match	gene: Zm00001d017096	Zm00001d017096_T02	0.00	254.51	coding	129	106	492
PB.2316.2	10	1036	2	full-splice_match	gene: Zm00001d026395	Zm00001d026395_T00	0.00	25.59	coding	337	26	1036
PB.2316.1	10	1737	2	full-splice_match	gene: Zm00001d026395	Zm00001d026395_T00	25.59	25.59	coding	455	86	1450
PB.3413.5	2	1098	2	full-splice_match	gene: Zm00001d006626	Zm00001d006626_T00	106.24	281.14	coding	261	67	849
PB.8171.2	6	1578	8	novel_not_in_catalog	gene: Zm00001d039059	novel	1.71	8.56	coding	430	139	1428
PB.5896.8	4	1232	3	intergenic	novelGene_446	novel	20.96	108.08	coding	75	281	505
PB.4269.1	3	1484	4	novel_in_catalog	gene: Zm00001d042455	novel	26.38	177.52	coding	130	748	1137
PB.3935.9	3	1375	3	novel_in_catalog	gene: Zm00001d040364	novel	0.00	354.56	coding	115	861	1205
PB.3935.8	3	1241	4	novel_in_catalog	gene: Zm00001d040364	novel	300.19	354.56	coding	333	75	1073
PB.10496.4	9	2148	10	full-splice_match	gene: Zm00001d046828	Zm00001d046828_T01	14.93	114.57	coding	193	193	771

PB.10496.5	9	1546	12	full-splice_match	gene: Zm00001d046828	Zm00001d046828_T00	99.03	114.57	coding	365	154	1248
PB.10496.6	9	1135	9	incomplete-splice_match	gene: Zm00001d046831	Zm00001d046831_T00	39.07	57.24	coding	269	45	851
PB.10496.7	9	1514	11	novel_in_catalog	gene: Zm00001d046831	novel	18.16	57.24	coding	246	93	830
PB.8187.1	6	1573	11	incomplete-splice_match	gene: Zm00001d039144	Zm00001d039144_T00	232.32	232.32	coding	361	72	1154
PB.3935.2	3	1889	3	novel_in_catalog	gene: Zm00001d040364	novel	4.85	354.56	coding	115	1361	1705
PB.3935.1	3	1466	3	novel_in_catalog	gene: Zm00001d040364	novel	49.52	354.56	coding	115	861	1205
PB.97.1	1	3430	2	full-splice_match	gene: Zm00001d027645	Zm00001d027645_T00	4.78	5.87	coding	1040	94	3213
PB.9051.2	7	1493	2	full-splice_match	gene: Zm00001d022456	Zm00001d022456_T00	1.40	67.58	coding	254	484	1245
PB.5454.1	4	1002	3	intergenic	novelGene_363	novel	20.05	20.05	non coding	NA	NA	NA
PB.97.2	1	3517	1	novel_in_catalog	gene: Zm00001d027645	novel	0.24	5.87	coding	912	94	2829
PB.8149.7	6	3408	10	full-splice_match	gene: Zm00001d038972	Zm00001d038972_T02	225.95	517.57	coding	871	622	3234
PB.97.4	1	2053	2	full-splice_match	gene: Zm00001d027645	Zm00001d027645_T00	0.85	5.87	coding	485	513	1967
PB.3118.2	2	1955	9	full-splice_match	gene: Zm00001d005276	Zm00001d005276_T01	338.05	338.05	coding	546	121	1758
PB.9011.1	7	1425	1	full-splice_match	gene: Zm00001d022327	Zm00001d022327_T00	17.94	131.61	coding	357	40	1110
PB.1687.1	10	1761	1	full-splice_match	gene: Zm00001d023228	Zm00001d023228_T00	4.36	4.36	coding	485	160	1614
PB.4962.2	4	1586	7	novel_not_in_catalog	gene: Zm00001d049192	novel	0.00	48.80	coding	456	82	1449
PB.10014.2	8	1321	1	novel_in_catalog	gene: Zm00001d012428	novel	5.54	168.66	coding	218	369	1022

PB.4962.1	4	1756	7	novel_not_in_catalog	gene: Zm00001d049192	novel	48.80	48.80	coding	456	87	1454
PB.1397.2	1	1723	10	full-splice_match	gene: Zm00001d033830	Zm00001d033830_T00	0.00	61.91	coding	488	69	1532
PB.8659.2	7	570	6	full-splice_match	gene: Zm00001d021021	Zm00001d021021_T00	34.19	149.32	coding	148	36	479
PB.8659.1	7	777	6	full-splice_match	gene: Zm00001d021021	Zm00001d021021_T00	115.13	149.32	coding	148	117	560
PB.909.1	1	1688	9	incomplete-splice_match	gene: Zm00001d031678	Zm00001d031678_T01	9.52	9.52	coding	522	16	1581
PB.1109.6	1	1449	4	novel_in_catalog	gene: Zm00001d032562	novel	17.96	36.00	coding	141	707	1129
PB.1109.7	1	866	1	genic	gene: Zm00001d032562	novel	0.00	36.00	coding	114	367	708
PB.3520.1	2	2751	11	incomplete-splice_match	gene: Zm00001d007089	Zm00001d007089_T04	29.14	29.14	coding	677	88	2118
PB.1109.1	1	1916	3	novel_in_catalog	gene: Zm00001d032562	novel	7.86	36.00	non coding	NA	NA	NA
PB.1109.3	1	3265	3	novel_in_catalog	gene: Zm00001d032562	novel	10.18	36.00	coding	114	1097	1438
PB.3215.2	2	2111	5	full-splice_match	gene: Zm00001d005817	Zm00001d005817_T00	16.17	21.51	coding	515	137	1681
PB.9192.1	8	2307	2	novel_in_catalog	gene: Zm00001d008489	novel	19.39	19.39	coding	352	997	2052
PB.7184.1	5	1413	10	full-splice_match	gene: Zm00001d018230	Zm00001d018230_T00	84.53	88.41	coding	395	64	1248
PB.3215.1	2	2429	3	full-splice_match	gene: Zm00001d005817	Zm00001d005817_T00	4.95	21.51	coding	388	39	1202
PB.1057.1	1	1349	6	full-splice_match	gene: Zm00001d032384	Zm00001d032384_T00	20.48	20.48	coding	267	212	1012
PB.3215.4	2	1765	4	novel_in_catalog	gene: Zm00001d005817	novel	0.39	21.51	coding	483	37	1485
PB.9323.2	8	1608	6	incomplete-splice_match	gene: Zm00001d009183	Zm00001d009183_T00	0.79	5.00	coding	480	168	1607

PB.9323.1	8	2989	10	full-splice_match	gene: Zm00001d009183	Zm00001d009183_T00	4.21	5.00	coding	866	88	2685
PB.6254.1	5	2806	8	full-splice_match	gene: Zm00001d013964	Zm00001d013964_T00	58.29	63.30	coding	674	500	2521
PB.6254.2	5	2555	6	novel_in_catalog	gene: Zm00001d013964	novel	5.02	63.30	coding	316	115	1062
PB.2614.1	2	620	1	full-splice_match	gene: Zm00001d002596	Zm00001d002596_T00	10.33	10.33	coding	108	47	370
PB.6779.2	5	1519	1	full-splice_match	gene: Zm00001d016582	Zm00001d016582_T00	2.44	295.38	coding	162	671	1156
PB.6779.3	5	1350	1	full-splice_match	gene: Zm00001d016582	Zm00001d016582_T00	0.00	295.38	coding	162	655	1140
PB.8011.2	6	3952	2	novel_in_catalog	gene: Zm00001d038487	novel	2.11	18.05	coding	450	715	2064
PB.3110.1	2	1329	4	intergenic	novelGene_331	novel	4.66	13.49	coding	45	311	445
PB.3110.2	2	1195	5	intergenic	novelGene_331	novel	8.82	13.49	coding	45	310	444
PB.676.2	1	1020	6	incomplete-splice_match	gene: Zm00001d030347	Zm00001d030347_T00	7.14	19.40	coding	261	31	813
PB.676.1	1	1371	8	full-splice_match	gene: Zm00001d030347	Zm00001d030347_T00	12.25	19.40	coding	261	269	1051
PB.9682.1	8	2091	6	full-splice_match	gene: Zm00001d010975	Zm00001d010975_T00	103.76	108.82	coding	467	401	1801
PB.4234.1	3	843	1	full-splice_match	gene: Zm00001d042307	Zm00001d042307_T00	19.78	19.78	non coding	NA	NA	NA
PB.4542.2	3	1809	9	novel_in_catalog	gene: Zm00001d043548	novel	1.85	17.49	coding	229	736	1422
PB.4542.3	3	1738	10	full-splice_match	gene: Zm00001d043548	Zm00001d043548_T00	14.98	17.49	coding	428	87	1370
PB.1107.3	1	2467	3	novel_in_catalog	gene: Zm00001d032547	novel	5.14	42.63	coding	509	683	2209
PB.9825.4	8	2285	1	full-splice_match	gene: Zm00001d011649	Zm00001d011649_T00	0.00	277.42	coding	480	57	1496

PB.8651.1	7	4060	8	incomplete-splice_match	gene: Zm00001d020978	Zm00001d020978_T00	6.02	6.02	coding	1172	114	3629
PB.9928.1	8	918	5	full-splice_match	gene: Zm00001d012052	Zm00001d012052_T00	43.27	523.95	coding	156	125	592
PB.6812.5	5	644	5	full-splice_match	gene: Zm00001d016722	Zm00001d016722_T00	0.00	300.61	coding	69	210	416
PB.5357.5	4	819	3	novel_in_catalog	gene: Zm00001d051584	novel	7.02	70.81	coding	142	26	451
PB.6855.1	5	656	2	incomplete-splice_match	gene: Zm00001d016894	Zm00001d016894_T00	49.47	49.47	non coding	NA	NA	NA
PB.5422.4	4	1048	2	full-splice_match	gene: Zm00001d051883	Zm00001d051883_T00	28.97	95.68	coding	197	171	761
PB.9682.4	8	1993	6	full-splice_match	gene: Zm00001d010975	Zm00001d010975_T00	0.00	108.82	coding	467	358	1758
PB.5422.6	4	1725	1	novel_in_catalog	gene: Zm00001d051883	novel	2.28	95.68	coding	456	340	1707
PB.2780.1	2	2158	7	full-splice_match	gene: Zm00001d003284	Zm00001d003284_T00	11.92	11.92	coding	557	185	1855
PB.6765.1	5	934	1	genic	gene: Zm00001d016501	novel	45.20	193.67	coding	168	85	588
PB.5422.1	4	2578	2	full-splice_match	gene: Zm00001d051883	Zm00001d051883_T00	25.13	95.68	coding	527	342	1922
PB.5422.2	4	2542	2	full-splice_match	gene: Zm00001d051883	Zm00001d051883_T00	26.70	95.68	coding	527	487	2067
PB.6765.2	5	1781	2	full-splice_match	gene: Zm00001d016501	Zm00001d016501_T00	148.47	193.67	coding	465	163	1557
PB.512.2	1	1165	7	full-splice_match	gene: Zm00001d029478	Zm00001d029478_T00	0.00	18.78	coding	216	149	796
PB.512.3	1	1147	6	novel_in_catalog	gene: Zm00001d029478	novel	4.78	18.78	coding	129	539	925
PB.3126.1	2	1241	2	incomplete-splice_match	gene: Zm00001d005324	Zm00001d005324_T00	7.94	15.59	coding	291	6	878
PB.512.1	1	1302	7	full-splice_match	gene: Zm00001d029478	Zm00001d029478_T00	8.19	18.78	coding	216	159	806

PB.7082.4	5	1853	3	incomplete-splice_match	gene: Zm00001d017866	Zm00001d017866_T00	25.91	83.84	coding	467	91	1491
PB.2091.1	10	1114	1	intergenic	novelGene_231	novel	1.94	1.94	non coding	NA	NA	NA
PB.2622.1	2	1598	1	full-splice_match	gene: Zm00001d002609	Zm00001d002609_T00	1.07	1.07	coding	476	9	1436
PB.8709.2	7	1518	2	full-splice_match	gene: Zm00001d021268	Zm00001d021268_T00	29.42	77.76	coding	239	475	1191
PB.8756.1	7	782	8	full-splice_match	gene: Zm00001d021433	Zm00001d021433_T00	255.41	255.41	coding	126	87	464
PB.2224.7	10	942	4	full-splice_match	gene: Zm00001d026047	Zm00001d026047_T00	68.39	201.68	coding	253	34	792
PB.3500.1	2	622	6	full-splice_match	gene: Zm00001d007032	Zm00001d007032_T00	41.84	49.67	coding	106	122	439
PB.2310.1	10	1507	6	full-splice_match	gene: Zm00001d026370	Zm00001d026370_T00	89.53	93.07	coding	343	262	1290
PB.8684.2	7	3546	9	intergenic	novelGene_282	novel	20.85	53.03	coding	287	2530	3390
PB.3783.1	3	3049	1	full-splice_match	gene: Zm00001d039636	Zm00001d039636_T00	12.93	12.93	coding	940	77	2896
PB.1092.1	1	1155	1	genic	gene: Zm00001d032510	novel	8.25	8.25	coding	323	32	1000
PB.8175.2	6	1554	7	full-splice_match	gene: Zm00001d039089	Zm00001d039089_T00	76.35	263.85	coding	340	164	1183
PB.10252.1	9	1993	1	antisense	novelGene_gene: Zm00001d045395	novel	2.67	2.67	coding	174	70	591
PB.10611.1	9	1214	5	full-splice_match	gene: Zm00001d047423	Zm00001d047423_T00	39.37	39.37	coding	217	304	954
PB.5932.1	4	2045	1	full-splice_match	gene: Zm00001d054060	Zm00001d054060_T00	309.57	1485.90	coding	524	96	1667
PB.988.2	1	836	3	novel_not_in_catalog	gene: Zm00001d032073	novel	0.00	102.24	coding	194	180	761
PB.988.1	1	1042	3	novel_not_in_catalog	gene: Zm00001d032073	novel	102.24	102.24	coding	194	260	841

PB.6532.3	5	2029	14	full-splice_match	gene: Zm00001d015088	Zm00001d015088_T00	4.81	53.29	coding	505	310	1824
PB.6532.1	5	1949	14	full-splice_match	gene: Zm00001d015088	Zm00001d015088_T00	48.48	53.29	coding	505	110	1624
PB.728.4	1	879	3	novel_not_in_catalog	gene: Zm00001d030767	novel	14.98	14.98	non coding	NA	NA	NA
PB.6994.3	5	1193	1	novel_in_catalog	gene: Zm00001d017485	novel	180.21	1045.18	non coding	NA	NA	NA
PB.10882.2	9	1408	7	full-splice_match	gene: Zm00001d048410	Zm00001d048410_T00	2.53	107.65	coding	157	152	622
PB.10021.2	8	968	2	full-splice_match	gene: Zm00001d012450	Zm00001d012450_T00	11.43	14.48	coding	163	130	618
PB.3778.3	3	2571	2	novel_in_catalog	gene: Zm00001d039613	novel	2.65	108.14	coding	233	1630	2328
PB.1427.2	1	1331	4	novel_in_catalog	gene: Zm00001d033976	novel	2.12	378.11	coding	226	212	889
PB.10276.1	9	566	2	incomplete-splice_match	gene: Zm00001d045487	Zm00001d045487_T00	0.74	18.92	non coding	NA	NA	NA
PB.10276.2	9	1026	5	full-splice_match	gene: Zm00001d045487	Zm00001d045487_T00	18.18	18.92	coding	231	91	783
PB.8907.1	7	1632	1	genic	gene: Zm00001d021942	novel	20.90	20.90	coding	447	48	1388
PB.9210.1	8	1122	4	incomplete-splice_match	gene: Zm00001d008595	Zm00001d008595_T00	76.14	76.14	coding	218	195	848
PB.2338.1	10	1974	1	full-splice_match	gene: Zm00001d026489	Zm00001d026489_T00	25.53	25.53	coding	392	499	1674
PB.9952.1	8	1059	5	full-splice_match	gene: Zm00001d012160	Zm00001d012160_T00	48.36	66.86	coding	196	123	710
PB.9868.1	8	3116	4	novel_not_in_catalog	gene: Zm00001d011823	novel	18.48	18.48	coding	581	172	1914
PB.9952.2	8	935	5	full-splice_match	gene: Zm00001d012160	Zm00001d012160_T00	18.50	66.86	coding	196	119	706
PB.7952.1	6	1716	8	full-splice_match	gene: Zm00001d038268	Zm00001d038268_T00	10.96	10.96	coding	522	54	1619

PB.2709.1	2	746	2	genic	gene: Zm00001d003005	novel	46.86	46.86	coding	105	89	403
PB.3427.2	2	1031	2	full-splice_match	gene: Zm00001d006683	Zm00001d006683_T00	0.00	20.29	coding	302	81	986
PB.1076.2	1	1718	1	full-splice_match	gene: Zm00001d032458	Zm00001d032458_T00	7.86	10.05	coding	514	75	1616
PB.3554.1	2	443	1	incomplete-splice_match	gene: Zm00001d007267	Zm00001d007267_T00	7.25	7.25	non coding	NA	NA	NA
PB.8046.2	6	3310	8	full-splice_match	gene: Zm00001d038587	Zm00001d038587_T00	21.88	36.15	coding	593	189	1967
PB.542.1	1	1030	1	full-splice_match	gene: Zm00001d029617	Zm00001d029617_T00	0.89	0.89	coding	266	58	855
PB.6179.1	5	953	2	full-splice_match	gene: Zm00001d013596	Zm00001d013596_T00	4.75	48.91	coding	191	43	615
PB.6179.2	5	890	2	full-splice_match	gene: Zm00001d013596	Zm00001d013596_T00	0.00	48.91	coding	191	112	684
PB.6179.3	5	795	2	full-splice_match	gene: Zm00001d013596	Zm00001d013596_T00	44.15	48.91	coding	191	119	691
PB.8046.5	6	3262	1	incomplete-splice_match	gene: Zm00001d038587	Zm00001d038587_T01	5.17	36.15	non coding	NA	NA	NA
PB.1057.2	1	1383	5	novel_in_catalog	gene: Zm00001d032384	novel	0.00	20.48	coding	148	716	1159
PB.8046.4	6	3105	7	full-splice_match	gene: Zm00001d038587	Zm00001d038587_T02	6.05	36.15	coding	593	189	1967
PB.1113.5	1	1160	5	full-splice_match	gene: Zm00001d032570	Zm00001d032570_T00	0.00	196.91	coding	317	34	984
PB.10014.4	8	1455	4	full-splice_match	gene: Zm00001d012428	Zm00001d012428_T00	0.00	168.66	coding	370	64	1173
PB.1113.7	1	353	1	novel_in_catalog	gene: Zm00001d032570	novel	47.98	196.91	non coding	NA	NA	NA
PB.1113.6	1	1203	4	novel_in_catalog	gene: Zm00001d032570	novel	9.55	196.91	coding	193	36	614
PB.1113.1	1	1285	5	full-splice_match	gene: Zm00001d032570	Zm00001d032570_T00	139.39	196.91	coding	317	36	986

PB.2165.1	10	606	3	full-splice_match	gene: Zm00001d025772	Zm00001d025772_T00	45.69	897.54	non coding	NA	NA	NA
PB.4839.1	4	1930	1	novel_in_catalog	gene: Zm00001d048603	novel	19.65	34.26	coding	540	26	1645
PB.8883.12	7	1849	5	genic	gene: Zm00001d021845	novel	0.00	36.30	coding	151	99	551
PB.9065.1	7	1352	1	antisense	novelGene_gene: Zm00001d022485	novel	78.71	78.71	non coding	NA	NA	NA
PB.3809.4	3	758	2	novel_in_catalog	gene: Zm00001d039764	novel	18.73	82.56	coding	107	224	544
PB.3255.3	2	2975	1	novel_in_catalog	gene: Zm00001d005997	novel	1.89	46.59	coding	390	155	1324
PB.10228.5	9	824	1	full-splice_match	gene: Zm00001d045319	Zm00001d045319_T00	7.76	18.49	coding	175	76	600
PB.7959.1	6	2667	14	full-splice_match	gene: Zm00001d038290	Zm00001d038290_T00	38.77	41.44	coding	747	98	2338
PB.3269.4	2	1512	5	full-splice_match	gene: Zm00001d006046	Zm00001d006046_T00	26.29	72.31	coding	301	104	1006
PB.10228.1	9	1146	3	antisense	novelGene_gene: Zm00001d045318	novel	3.71	3.71	coding	114	205	546
PB.5400.1	4	1283	1	full-splice_match	gene: Zm00001d051802	Zm00001d051802_T00	0.19	0.19	coding	115	171	515
PB.10228.3	9	2794	1	full-splice_match	gene: Zm00001d045319	Zm00001d045319_T00	8.86	18.49	coding	691	125	2197
PB.10228.2	9	4206	3	genic	gene: Zm00001d045319	novel	1.87	18.49	coding	551	55	1707
PB.6092.1	5	2128	4	full-splice_match	gene: Zm00001d013273	Zm00001d013273_T00	24.53	24.53	coding	564	153	1844
PB.3570.10	2	1122	1	incomplete-splice_match	gene: Zm00001d007434	Zm00001d007434_T01	0.00	72.29	non coding	NA	NA	NA
PB.8883.11	7	1773	6	genic	gene: Zm00001d021845	novel	20.33	36.30	coding	151	101	553
PB.7959.3	6	2704	12	novel_in_catalog	gene: Zm00001d038290	novel	2.66	41.44	coding	338	76	1089

PB.1757.1	10	2235	17	full-splice_match	gene: Zm00001d023537	Zm00001d023537_T00	32.32	32.32	coding	176	24	551
PB.9679.1	8	1109	12	incomplete-splice_match	gene: Zm00001d010970	Zm00001d010970_T00	22.08	22.08	coding	309	16	942
PB.10248.2	9	1664	4	novel_not_in_catalog	gene: Zm00001d045386	novel	11.58	89.45	coding	149	358	804
PB.10248.1	9	1549	3	novel_not_in_catalog	gene: Zm00001d045386	novel	11.00	89.45	coding	149	306	752
PB.7325.1	6	1802	1	genic	gene: Zm00001d035125	novel	12.66	22.47	coding	428	343	1626
PB.5979.1	5	1624	5	full-splice_match	gene: Zm00001d012887	Zm00001d012887_T00	48.30	59.69	coding	408	162	1385
PB.2224.3	10	953	1	genic	gene: Zm00001d026047	novel	5.70	201.68	coding	122	566	931
PB.3562.1	2	916	1	full-splice_match	gene: Zm00001d007316	Zm00001d007316_T00	170.66	170.66	coding	177	92	622
PB.5979.2	5	1324	4	full-splice_match	gene: Zm00001d012887	Zm00001d012887_T00	11.39	59.69	coding	146	635	1072
PB.7263.7	5	1935	1	novel_in_catalog	gene: Zm00001d018501	novel	0.12	18.16	coding	18	275	328
PB.10151.2	9	1165	1	full-splice_match	gene: Zm00001d044950	Zm00001d044950_T00	0.00	11.69	coding	261	216	998
PB.3692.1	3	1900	15	novel_in_catalog	gene: Zm00001d039296	novel	31.72	31.72	coding	432	225	1520
PB.9278.2	8	2170	1	full-splice_match	gene: Zm00001d008905	Zm00001d008905_T00	15.51	27.04	coding	587	54	1814
PB.9278.1	8	2274	1	full-splice_match	gene: Zm00001d008905	Zm00001d008905_T00	0.72	27.04	coding	587	51	1811
PB.2948.1	2	1335	1	genic	gene: Zm00001d004167	novel	3.13	7.66	coding	339	85	1101
PB.165.1	1	1072	1	genic	gene: Zm00001d027932	novel	8.77	48.45	coding	111	155	487
PB.165.2	1	901	2	full-splice_match	gene: Zm00001d027932	Zm00001d027932_T00	37.92	48.45	coding	146	229	666

PB.165.3	1	1025	1	genic	gene: Zm00001d027932	novel	1.76	48.45	coding	111	273	605
PB.1026.1	1	2738	13	full-splice_match	gene: Zm00001d032245	Zm00001d032245_T00	5.90	5.90	coding	862	29	2614
PB.3037.1	2	1447	6	incomplete-splice_match	gene: Zm00001d004782	Zm00001d004782_T00	37.28	37.28	coding	192	284	859
PB.4321.5	3	1903	1	full-splice_match	gene: Zm00001d042686	Zm00001d042686_T00	0.31	83.67	non coding	NA	NA	NA
PB.526.3	1	672	2	novel_in_catalog	gene: Zm00001d029551	novel	35.41	35.41	non coding	NA	NA	NA
PB.6191.1	5	3407	1	antisense	novelGene_gene: Zm00001d013639	novel	8.16	8.16	coding	68	136	339
PB.1769.1	10	662	1	intergenic	novelGene_48	novel	31.11	168.40	non coding	NA	NA	NA
PB.5141.3	4	2930	5	genic	gene: Zm00001d050132	novel	6.15	11.78	coding	723	82	2250
PB.5408.1	4	2142	1	incomplete-splice_match	gene: Zm00001d051839	Zm00001d051839_T00	27.25	27.25	coding	576	115	1842
PB.9645.1	8	1075	1	antisense	novelGene_gene: Zm00001d010812	novel	47.23	47.23	coding	169	399	905
PB.2775.1	2	2067	18	incomplete-splice_match	gene: Zm00001d003259	Zm00001d003259_T01	22.35	22.35	coding	520	177	1736
PB.5315.1	4	985	1	antisense	novelGene_gene: Zm00001d051349	novel	5.85	36.67	non coding	NA	NA	NA
PB.8663.1	7	1086	6	novel_not_in_catalog	gene: Zm00001d021032	novel	41.03	41.03	coding	197	118	708
PB.179.1	1	1864	8	full-splice_match	gene: Zm00001d028006	Zm00001d028006_T00	53.20	53.20	coding	466	191	1588
PB.8191.3	6	688	2	full-splice_match	gene: Zm00001d039158	Zm00001d039158_T00	0.00	145.49	coding	154	72	533
PB.2232.1	10	3209	10	full-splice_match	gene: Zm00001d026097	Zm00001d026097_T00	11.27	24.76	coding	844	138	2669
PB.4609.2	3	2193	10	full-splice_match	gene: Zm00001d043785	Zm00001d043785_T00	3.81	14.58	coding	592	107	1882

PB.5315.9	4	814	1	antisense	novelGene_gene: Zm00001d051349	novel	22.11	36.67	non coding	NA	NA	NA
PB.5315.8	4	888	1	antisense	novelGene_gene: Zm00001d051349	novel	3.10	36.67	non coding	NA	NA	NA
PB.10939.2	9	2078	10	full-splice_match	gene: Zm00001d048574	Zm00001d048574_T00	0.00	30.80	coding	597	138	1928
PB.4321.4	3	1669	2	genic	gene: Zm00001d042686	novel	0.00	83.67	coding	485	215	1669
PB.4557.1	3	1886	9	full-splice_match	gene: Zm00001d043607	Zm00001d043607_T00	74.13	74.13	coding	504	106	1617
PB.3372.3	2	2208	2	novel_in_catalog	gene: Zm00001d006453	novel	3.96	3.96	coding	107	82	402
PB.6658.4	5	2953	8	incomplete-splice_match	gene: Zm00001d015829	Zm00001d015829_T00	24.31	85.07	coding	858	248	2821
PB.365.1	1	551	1	full-splice_match	gene: Zm00001d028793	Zm00001d028793_T00	219.19	558.90	non coding	NA	NA	NA
PB.8284.1	7	1457	3	full-splice_match	gene: Zm00001d018902	Zm00001d018902_T00	32.73	32.73	coding	354	105	1166
PB.6658.1	5	3373	9	full-splice_match	gene: Zm00001d015829	Zm00001d015829_T00	48.50	85.07	coding	974	233	3154
PB.8284.2	7	1286	3	full-splice_match	gene: Zm00001d018902	Zm00001d018902_T00	0.00	32.73	coding	354	104	1165
PB.5768.1	4	1325	2	full-splice_match	gene: Zm00001d053397	Zm00001d053397_T00	17.16	24.48	coding	331	48	1040
PB.7287.2	5	2311	10	novel_not_in_catalog	gene: Zm00001d018565	novel	101.69	101.69	coding	638	179	2092
PB.8017.5	6	535	3	full-splice_match	gene: Zm00001d038519	Zm00001d038519_T00	38.45	72.46	non coding	NA	NA	NA
PB.8017.4	6	834	3	full-splice_match	gene: Zm00001d038519	Zm00001d038519_T00	8.17	72.46	non coding	NA	NA	NA
PB.8017.3	6	996	3	full-splice_match	gene: Zm00001d038519	Zm00001d038519_T00	11.25	72.46	coding	28	227	310
PB.8017.2	6	2440	3	full-splice_match	gene: Zm00001d038519	Zm00001d038519_T00	2.56	72.46	coding	28	227	310

PB.8017.1	6	887	5	novel_not_in_catalog	gene: Zm00001d038519	novel	12.03	72.46	non coding	NA	NA	NA
PB.4117.2	3	795	4	full-splice_match	gene: Zm00001d041701	Zm00001d041701_T00	60.69	85.66	coding	140	126	545
PB.3203.4	2	2187	2	novel_not_in_catalog	gene: Zm00001d005743	novel	7.55	30.73	non coding	NA	NA	NA
PB.4117.1	3	901	4	full-splice_match	gene: Zm00001d041701	Zm00001d041701_T00	24.97	85.66	coding	140	126	545
PB.1874.1	10	1348	9	novel_not_in_catalog	gene: Zm00001d024245	novel	303.43	339.31	coding	290	161	1030
PB.1874.3	10	1175	9	novel_not_in_catalog	gene: Zm00001d024245	novel	35.87	339.31	coding	310	5	934
PB.10737.1	9	316	1	incomplete-splice_match	gene: Zm00001d047828	Zm00001d047828_T00	43.44	76.02	non coding	NA	NA	NA
PB.10737.2	9	2285	7	full-splice_match	gene: Zm00001d047828	Zm00001d047828_T00	32.58	76.02	coding	666	55	2052
PB.10041.3	8	1098	11	full-splice_match	gene: Zm00001d012546	Zm00001d012546_T00	0.00	199.25	coding	249	186	932
PB.3470.10	2	2616	4	full-splice_match	gene: Zm00001d006900	Zm00001d006900_T00	5.71	444.34	coding	490	85	1554
PB.8595.1	7	1185	1	full-splice_match	gene: Zm00001d020740	Zm00001d020740_T00	15.10	15.10	coding	330	150	1139
PB.6211.2	5	2760	1	genic	gene: Zm00001d013737	novel	1.45	82.84	coding	158	47	520
PB.3013.3	2	1124	1	full-splice_match	gene: Zm00001d004664	Zm00001d004664_T00	5.31	27.41	coding	346	6	1043
PB.3013.1	2	2041	1	full-splice_match	gene: Zm00001d004664	Zm00001d004664_T00	22.09	27.41	coding	515	213	1757
PB.3726.1	3	1667	2	antisense	novelGene_gene: Zm00001d039425	novel	16.23	16.23	non coding	NA	NA	NA
PB.4353.1	3	1135	1	novel_in_catalog	gene: Zm00001d042827	novel	30.02	30.02	non coding	NA	NA	NA
PB.2860.6	2	992	3	full-splice_match	gene: Zm00001d003707	Zm00001d003707_T00	308.88	486.33	coding	275	49	873

PB.2860.4	2	1567	1	novel_in_catalog	gene: Zm00001d003707	novel	177.44	486.33	coding	153	99	557
PB.6835.1	5	1356	1	novel_in_catalog	gene: Zm00001d016826	novel	23.04	54.99	coding	110	792	1121
PB.6835.3	5	1012	3	full-splice_match	gene: Zm00001d016826	Zm00001d016826_T00	23.09	54.99	coding	231	94	786
PB.6835.2	5	1165	2	novel_in_catalog	gene: Zm00001d016826	novel	8.86	54.99	coding	110	608	937
PB.6835.4	5	1197	1	novel_in_catalog	gene: Zm00001d016826	novel	0.00	54.99	coding	110	771	1100
PB.1088.3	1	1223	9	full-splice_match	gene: Zm00001d032501	Zm00001d032501_T00	0.00	71.38	coding	307	71	991
PB.10830.2	9	2996	6	intergenic	novelGene_304	novel	19.65	19.65	coding	441	1089	2411
PB.8487.1	7	715	4	full-splice_match	gene: Zm00001d020201	Zm00001d020201_T00	81.48	662.56	coding	112	63	398
PB.3621.1	2	1883	1	full-splice_match	gene: Zm00001d007774	Zm00001d007774_T00	3.79	3.79	coding	393	402	1580
PB.1580.4	1	1878	7	novel_in_catalog	gene: Zm00001d034608	novel	67.45	86.52	coding	412	468	1703
PB.1094.3	1	2793	6	incomplete-splice_match	gene: Zm00001d032520	Zm00001d032520_T00	6.39	30.82	coding	702	686	2791
PB.5727.2	4	1060	2	full-splice_match	gene: Zm00001d053220	Zm00001d053220_T00	86.66	91.34	coding	260	54	833
PB.5308.3	4	762	2	full-splice_match	gene: Zm00001d051333	Zm00001d051333_T00	226.25	306.93	coding	195	64	648
PB.5394.1	4	2260	9	full-splice_match	gene: Zm00001d051754	Zm00001d051754_T00	40.43	148.08	coding	550	1	1650
PB.5394.2	4	1894	9	full-splice_match	gene: Zm00001d051754	Zm00001d051754_T00	36.44	148.08	coding	550	125	1774
PB.5691.1	4	1869	6	full-splice_match	gene: Zm00001d053004	Zm00001d053004_T00	9.60	9.60	coding	485	89	1543
PB.10470.1	9	1297	1	full-splice_match	gene: Zm00001d046634	Zm00001d046634_T00	1.77	1.77	coding	108	684	1007

PB.1813.3	10	2726	7	novel_in_catalog	gene: Zm00001d023825	novel	0.00	51.51	coding	660	426	2405
PB.7362.1	6	1053	2	full-splice_match	gene: Zm00001d035343	Zm00001d035343_T00	7.04	7.04	coding	264	10	801
PB.973.3	1	1894	4	novel_not_in_catalog	gene: Zm00001d031991	novel	12.64	22.94	non coding	NA	NA	NA
PB.6340.2	5	731	4	intergenic	novelGene_131	novel	5.77	28.86	coding	123	88	456
PB.974.2	1	2504	4	full-splice_match	gene: Zm00001d031992	Zm00001d031992_T00	67.78	67.78	coding	509	695	2221
PB.6211.4	5	1483	2	full-splice_match	gene: Zm00001d013737	Zm00001d013737_T00	0.00	82.84	coding	397	106	1296
PB.7190.4	5	3303	23	full-splice_match	gene: Zm00001d018246	Zm00001d018246_T00	0.00	31.93	coding	994	174	3155
PB.9667.1	8	1008	5	intergenic	novelGene_257	novel	10.91	26.72	non coding	NA	NA	NA
PB.44.4	1	1380	3	full-splice_match	gene: Zm00001d027411	Zm00001d027411_T00	74.03	90.40	coding	329	188	1174
PB.1544.1	1	909	3	full-splice_match	gene: Zm00001d034491	Zm00001d034491_T00	16.58	426.33	coding	133	83	481
PB.44.1	1	1681	2	novel_in_catalog	gene: Zm00001d027411	novel	16.37	90.40	coding	274	554	1375
PB.2868.1	2	723	2	full-splice_match	gene: Zm00001d003760	Zm00001d003760_T00	21.74	21.74	coding	115	105	449
PB.7799.2	6	3913	3	novel_in_catalog	gene: Zm00001d037695	novel	6.29	14.94	coding	493	2202	3680
PB.7151.1	5	1202	1	full-splice_match	gene: Zm00001d018105	Zm00001d018105_T00	23.14	23.14	coding	315	85	1029
PB.813.2	1	324	2	incomplete-splice_match	gene: Zm00001d031179	Zm00001d031179_T00	13.76	129.33	non coding	NA	NA	NA
PB.7799.1	6	2669	5	full-splice_match	gene: Zm00001d037695	Zm00001d037695_T00	8.64	14.94	coding	558	763	2436
PB.7346.1	6	2352	2	intergenic	novelGene_61	novel	16.31	16.31	non coding	NA	NA	NA

PB.8404.1	7	3655	1	genic_intron	novelGene_141	novel	10.39	13.83	coding	151	609	1061
PB.492.2	1	1518	3	novel_not_in_catalog	gene: Zm00001d029390	novel	6.04	17.43	coding	78	141	374
PB.3800.4	3	2087	12	incomplete-splice_match	gene: Zm00001d039729	Zm00001d039729_T00	2.81	34.47	coding	619	230	2086
PB.7583.11	6	3273	11	full-splice_match	gene: Zm00001d036631	Zm00001d036631_T01	7.01	46.80	coding	215	2348	2992
PB.5744.1	4	1295	10	incomplete-splice_match	gene: Zm00001d053286	Zm00001d053286_T00	74.69	74.69	coding	285	58	912
PB.3800.3	3	859	5	incomplete-splice_match	gene: Zm00001d039729	Zm00001d039729_T00	14.00	34.47	coding	186	125	682
PB.5734.4	4	3397	11	novel_in_catalog	gene: Zm00001d053247	novel	10.67	30.72	coding	387	1096	2256
PB.3800.1	3	2953	13	full-splice_match	gene: Zm00001d039729	Zm00001d039729_T00	17.67	34.47	coding	773	353	2671
PB.7670.1	6	1753	4	full-splice_match	gene: Zm00001d037112	Zm00001d037112_T00	166.73	249.69	coding	444	167	1498
PB.7670.3	6	2051	2	novel_in_catalog	gene: Zm00001d037112	novel	23.29	249.69	coding	195	1324	1908
PB.7945.1	6	1359	1	intergenic	novelGene_303	novel	4.11	4.11	coding	71	94	306
PB.2816.5	2	1969	5	novel_in_catalog	gene: Zm00001d003491	novel	3.14	13.80	coding	90	98	367
PB.1094.1	1	2377	3	incomplete-splice_match	gene: Zm00001d032520	Zm00001d032520_T00	24.43	30.82	coding	630	68	1957
PB.10825.1	9	1138	6	full-splice_match	gene: Zm00001d048194	Zm00001d048194_T00	24.93	24.93	coding	214	256	897
PB.9151.3	8	692	3	full-splice_match	gene: Zm00001d008312	Zm00001d008312_T00	275.55	546.19	coding	100	181	480
PB.9151.2	8	2155	2	novel_in_catalog	gene: Zm00001d008312	novel	4.57	546.19	non coding	NA	NA	NA
PB.9151.1	8	746	3	full-splice_match	gene: Zm00001d008312	Zm00001d008312_T00	266.07	546.19	coding	100	147	446

PB.10342.1	9	1801	1	full-splice_match	gene: Zm00001d045785	Zm00001d045785_T00	27.26	27.26	coding	491	44	1516
PB.10342.2	9	1726	1	full-splice_match	gene: Zm00001d045785	Zm00001d045785_T00	0.00	27.26	coding	491	44	1516
PB.5965.1	5	1217	2	novel_in_catalog	gene: Zm00001d012839	novel	26.09	26.09	coding	205	68	682
PB.8812.1	7	1710	5	full-splice_match	gene: Zm00001d021653	Zm00001d021653_T00	54.26	69.38	coding	391	150	1322
PB.5248.2	4	1314	8	full-splice_match	gene: Zm00001d050923	Zm00001d050923_T00	33.40	45.17	coding	322	134	1099
PB.5248.3	4	1196	8	full-splice_match	gene: Zm00001d050923	Zm00001d050923_T00	0.00	45.17	coding	322	117	1082
PB.5796.3	4	1534	1	genic	gene: Zm00001d053566	novel	0.00	614.41	coding	161	80	562
PB.5248.1	4	1416	8	full-splice_match	gene: Zm00001d050923	Zm00001d050923_T00	11.77	45.17	coding	322	132	1097
PB.1486.1	1	1905	1	full-splice_match	gene: Zm00001d034221	Zm00001d034221_T00	38.46	127.95	coding	435	131	1435
PB.3340.1	2	582	2	full-splice_match	gene: Zm00001d006336	Zm00001d006336_T00	88.53	409.74	coding	81	53	295
PB.222.1	1	2114	5	full-splice_match	gene: Zm00001d028211	Zm00001d028211_T00	10.77	19.29	coding	475	267	1691
PB.222.2	1	1411	3	incomplete-splice_match	gene: Zm00001d028211	Zm00001d028211_T00	8.52	19.29	coding	239	388	1104
PB.10753.3	9	700	2	incomplete-splice_match	gene: Zm00001d047881	Zm00001d047881_T00	15.69	64.07	coding	100	12	311
PB.5602.4	4	2120	8	fusion	gene: Zm00001d052565	novel	30.69	53.24	coding	504	243	1754
PB.113.2	1	1623	1	full-splice_match	gene: Zm00001d027700	Zm00001d027700_T00	0.00	281.96	coding	417	229	1479
PB.113.1	1	2026	1	full-splice_match	gene: Zm00001d027700	Zm00001d027700_T00	281.96	281.96	coding	524	208	1779
PB.6638.1	5	851	1	intergenic	novelGene_217	novel	14.40	14.40	coding	78	83	316

PB.3425.13	2	1231	5	novel_not_in_catalog	gene: Zm00001d006679	novel	245.14	476.20	coding	344	115	1146
PB.5602.1	4	2189	7	fusion	gene: Zm00001d052565	novel	1.07	53.24	coding	373	165	1283
PB.5602.2	4	2040	8	fusion	gene: Zm00001d052565	novel	21.48	53.24	coding	502	165	1670
PB.1400.1	1	679	3	full-splice_match	gene: Zm00001d033840	Zm00001d033840_T00	68.74	68.74	non coding	NA	NA	NA
PB.4582.8	3	2075	14	novel_not_in_catalog	gene: Zm00001d043693	novel	131.71	284.74	coding	470	250	1659
PB.1966.1	10	606	3	intergenic	novelGene_189	novel	21.43	44.37	non coding	NA	NA	NA
PB.2006.1	10	1516	8	full-splice_match	gene: Zm00001d024933	Zm00001d024933_T00	11.62	337.93	coding	319	61	1017
PB.6498.1	5	1818	9	full-splice_match	gene: Zm00001d014953	Zm00001d014953_T00	84.36	84.36	coding	484	146	1597
PB.9677.1	8	3471	1	full-splice_match	gene: Zm00001d010961	Zm00001d010961_T00	26.64	41.66	coding	814	94	2535
PB.2077.1	10	1896	9	full-splice_match	gene: Zm00001d025304	Zm00001d025304_T00	4.40	4.40	coding	493	8	1486
PB.32.1	1	1468	6	full-splice_match	gene: Zm00001d027353	Zm00001d027353_T00	45.19	45.19	coding	361	108	1190
PB.8585.7	7	2789	8	novel_in_catalog	gene: Zm00001d020685	novel	6.32	254.34	coding	298	337	1230
PB.8585.6	7	2695	12	full-splice_match	gene: Zm00001d020685	Zm00001d020685_T01	35.09	254.34	coding	472	586	2001
PB.7318.1	6	1794	8	incomplete-splice_match	gene: Zm00001d035088	Zm00001d035088_T00	27.39	27.39	coding	396	189	1376
PB.8498.1	7	1573	5	novel_not_in_catalog	gene: Zm00001d020272	novel	39.74	39.74	coding	389	114	1280
PB.8585.9	7	2596	10	novel_in_catalog	gene: Zm00001d020685	novel	2.51	254.34	coding	484	330	1781
PB.4311.1	3	1797	1	novel_in_catalog	gene: Zm00001d042653	novel	11.10	87.81	coding	121	1225	1587

PB.5232.1	4	1528	1	full-splice_match	gene: Zm00001d050840	Zm00001d050840_T00	25.39	25.39	coding	331	182	1174
PB.7168.11	5	1997	4	full-splice_match	gene: Zm00001d018161	Zm00001d018161_T00	1382.67	1797.84	coding	593	62	1840
PB.7168.13	5	2140	2	novel_in_catalog	gene: Zm00001d018161	novel	109.95	1797.84	coding	256	1269	2036
PB.5232.2	4	1387	1	full-splice_match	gene: Zm00001d050840	Zm00001d050840_T00	0.00	25.39	coding	331	153	1145
PB.5363.2	4	928	8	full-splice_match	gene: Zm00001d051605	Zm00001d051605_T00	0.00	20.92	coding	176	300	827
PB.7378.1	6	2069	3	novel_not_in_catalog	gene: Zm00001d035434	novel	20.50	20.50	coding	530	82	1671
PB.442.2	1	1829	9	novel_in_catalog	gene: Zm00001d029141	novel	10.56	78.59	coding	139	114	530
PB.442.3	1	1455	10	novel_in_catalog	gene: Zm00001d029141	novel	1.82	78.59	coding	138	403	816
PB.442.4	1	1576	10	novel_in_catalog	gene: Zm00001d029141	novel	1.86	78.59	coding	114	901	1242
PB.442.5	1	1218	12	full-splice_match	gene: Zm00001d029141	Zm00001d029141_T00	53.48	78.59	coding	256	117	884
PB.442.7	1	1443	11	novel_in_catalog	gene: Zm00001d029141	novel	1.43	78.59	coding	246	117	854
PB.442.8	1	1377	11	novel_in_catalog	gene: Zm00001d029141	novel	0.42	78.59	coding	114	765	1106
PB.6394.5	5	3122	4	novel_in_catalog	gene: Zm00001d014580	novel	2.31	26.61	coding	108	2397	2720
PB.5093.2	4	1026	5	full-splice_match	gene: Zm00001d049835	Zm00001d049835_T00	129.94	183.34	coding	213	193	831
PB.5093.3	4	1021	5	novel_not_in_catalog	gene: Zm00001d049835	novel	53.40	183.34	coding	213	196	834
PB.4582.6	3	2221	14	novel_not_in_catalog	gene: Zm00001d043693	novel	140.51	284.74	coding	470	310	1719
PB.813.1	1	1084	7	full-splice_match	gene: Zm00001d031179	Zm00001d031179_T00	115.57	129.33	coding	204	183	794

PB.8724.1	7	1319	9	full-splice_match	gene: Zm00001d021310	Zm00001d021310_T00	59.10	59.10	coding	304	117	1028
PB.1431.4	1	5153	5	incomplete-splice_match	gene: Zm00001d033984	Zm00001d033984_T00	21.56	109.76	coding	1520	136	4695
PB.9204.1	8	557	2	full-splice_match	gene: Zm00001d008548	Zm00001d008548_T00	2.59	2.59	coding	102	61	366
PB.6351.1	5	1301	1	antisense	novelGene_gene: Zm00001d014367	novel	0.19	0.19	non coding	NA	NA	NA
PB.1431.3	1	5497	5	incomplete-splice_match	gene: Zm00001d033984	Zm00001d033984_T00	31.53	109.76	coding	1643	1	4929
PB.1846.5	10	902	7	novel_in_catalog	gene: Zm00001d023987	novel	4.09	189.58	coding	106	265	582
PB.1846.2	10	1812	10	full-splice_match	gene: Zm00001d023987	Zm00001d023987_T00	4.98	189.58	coding	264	163	954
PB.1846.1	10	1863	10	full-splice_match	gene: Zm00001d023987	Zm00001d023987_T00	180.51	189.58	coding	490	212	1681
PB.1201.3	1	2425	3	novel_in_catalog	gene: Zm00001d032923	novel	10.70	35.58	coding	227	985	1665
PB.7640.8	6	2395	15	full-splice_match	gene: Zm00001d036971	Zm00001d036971_T04	0.00	145.45	coding	720	236	2395
PB.1201.1	1	2096	4	novel_in_catalog	gene: Zm00001d032923	novel	0.00	35.58	coding	227	924	1604
PB.9385.1	8	2167	7	incomplete-splice_match	gene: Zm00001d009549	Zm00001d009549_T00	2.32	2.32	coding	549	235	1881
PB.2995.1	2	1537	4	full-splice_match	gene: Zm00001d004545	Zm00001d004545_T00	22.06	22.06	coding	387	18	1178
PB.991.6	1	1663	1	novel_in_catalog	gene: Zm00001d032081	novel	4.76	89.17	coding	109	217	543
PB.1201.5	1	1192	3	incomplete-splice_match	gene: Zm00001d032923	Zm00001d032923_T00	0.00	35.58	coding	132	382	777
PB.1201.4	1	2300	4	full-splice_match	gene: Zm00001d032923	Zm00001d032923_T00	8.80	35.58	coding	227	861	1541
PB.8442.1	7	1771	2	full-splice_match	gene: Zm00001d019934	Zm00001d019934_T00	11.05	11.05	coding	492	154	1629

PB.9359.2	8	1256	6	full-splice_match	gene: Zm00001d009440	Zm00001d009440_T00	40.56	47.32	coding	201	339	941
PB.258.3	1	2803	4	novel_in_catalog	gene: Zm00001d028354	novel	2.19	20.35	coding	319	212	1168
PB.258.2	1	1905	5	full-splice_match	gene: Zm00001d028354	Zm00001d028354_T00	3.57	20.35	coding	592	75	1850
PB.258.1	1	2067	5	full-splice_match	gene: Zm00001d028354	Zm00001d028354_T00	14.59	20.35	coding	592	126	1901
PB.5390.1	4	1796	2	full-splice_match	gene: Zm00001d051693	Zm00001d051693_T00	44.83	44.83	coding	422	331	1596
PB.2434.4	2	352	1	novel_in_catalog	gene: Zm00001d001814	novel	7.89	191.59	non coding	NA	NA	NA
PB.4736.1	3	1826	7	incomplete-splice_match	gene: Zm00001d044283	Zm00001d044283_T00	9.74	9.74	coding	604	15	1826
PB.11022.1	B73V 4	1619	5	incomplete-splice_match	gene: Zm00001d000430	Zm00001d000430_T00	28.53	28.53	coding	428	103	1386
PB.6933.1	5	2667	15	full-splice_match	gene: Zm00001d017203	Zm00001d017203_T01	15.36	72.13	coding	293	299	1177
PB.2434.1	2	1336	3	full-splice_match	gene: Zm00001d001814	Zm00001d001814_T00	154.45	191.59	coding	299	102	998
PB.7914.2	6	851	6	full-splice_match	gene: Zm00001d038139	Zm00001d038139_T00	41.63	92.46	coding	177	78	608
PB.2434.3	2	1197	3	full-splice_match	gene: Zm00001d001814	Zm00001d001814_T00	29.24	191.59	coding	299	102	998
PB.238.1	1	3491	3	full-splice_match	gene: Zm00001d028267	Zm00001d028267_T01	40.92	69.05	coding	863	645	3233
PB.238.2	1	3005	3	full-splice_match	gene: Zm00001d028267	Zm00001d028267_T01	28.13	69.05	coding	863	245	2833
PB.6973.2	5	2321	7	full-splice_match	gene: Zm00001d017392	Zm00001d017392_T00	24.85	39.92	coding	605	244	2058
PB.4695.1	3	1505	13	incomplete-splice_match	gene: Zm00001d044129	Zm00001d044129_T01	18.43	18.43	coding	395	9	1193
PB.1574.1	1	2436	14	full-splice_match	gene: Zm00001d034590	Zm00001d034590_T00	69.83	102.49	coding	647	115	2055

PB.8046.7	6	2255	5	full-splice_match	gene: Zm00001d038587	Zm00001d038587_T03	1.79	36.15	coding	593	190	1968
PB.7547.1	6	824	1	full-splice_match	gene: Zm00001d036463	Zm00001d036463_T00	3.57	3.57	coding	147	60	500
PB.8591.2	7	2851	1	full-splice_match	gene: Zm00001d020703	Zm00001d020703_T00	0.00	34.48	coding	430	1400	2689
PB.787.1	1	1305	1	genic	gene: Zm00001d031066	novel	8.76	8.76	coding	216	74	721
PB.7949.2	6	2442	4	full-splice_match	gene: Zm00001d038262	Zm00001d038262_T00	67.44	90.57	coding	620	255	2114
PB.4403.1	3	2259	5	novel_in_catalog	gene: Zm00001d043074	novel	13.42	13.42	coding	553	361	2019
PB.7131.1	5	2553	6	full-splice_match	gene: Zm00001d018024	Zm00001d018024_T00	42.14	42.14	coding	595	220	2004
PB.4403.3	3	1939	5	incomplete-splice_match	gene: Zm00001d043074	Zm00001d043074_T00	0.00	13.42	coding	553	213	1871
PB.4772.1	3	1303	6	full-splice_match	gene: Zm00001d044452	Zm00001d044452_T00	8.22	8.22	coding	262	195	980
PB.8371.1	7	3920	4	incomplete-splice_match	gene: Zm00001d019461	Zm00001d019461_T00	1.60	23.46	coding	1116	284	3631
PB.11030.1	B73V4	1338	4	full-splice_match	gene: Zm00001d000439	Zm00001d000439_T00	58.99	58.99	coding	254	198	959
PB.3413.1	2	1675	1	genic	gene: Zm00001d006626	novel	0.00	281.14	coding	295	71	955
PB.5655.1	4	1294	6	full-splice_match	gene: Zm00001d052810	Zm00001d052810_T00	9.85	22.24	coding	316	80	1027
PB.2961.1	2	1619	5	novel_not_in_catalog	gene: Zm00001d004287	novel	12.06	12.06	coding	508	28	1551
PB.9546.4	8	3378	12	full-splice_match	gene: Zm00001d010413	Zm00001d010413_T00	51.47	123.06	coding	989	84	3050
PB.9546.3	8	2311	5	novel_in_catalog	gene: Zm00001d010413	novel	1.65	123.06	coding	333	19	1017
PB.9546.2	8	3499	12	full-splice_match	gene: Zm00001d010413	Zm00001d010413_T00	25.36	123.06	coding	989	89	3055

PB.9546.1	8	2068	6	incomplete-splice_match	gene: Zm00001d010413	Zm00001d010413_T00	44.58	123.06	coding	506	3	1520
PB.8122.1	6	668	5	novel_in_catalog	gene: Zm00001d038890	novel	38.62	38.62	coding	103	233	541
PB.8491.5	7	3493	5	full-splice_match	gene: Zm00001d020227	Zm00001d020227_T02	2.14	51.79	coding	201	592	1194
PB.9467.3	8	1018	7	full-splice_match	gene: Zm00001d010009	Zm00001d010009_T00	114.56	188.20	coding	171	119	631
PB.7834.2	6	2641	4	intergenic	novelGene_274	novel	21.96	21.96	coding	86	45	302
PB.1180.3	1	1201	3	incomplete-splice_match	gene: Zm00001d032831	Zm00001d032831_T00	11.78	11.78	non coding	NA	NA	NA
PB.10.1	1	938	2	full-splice_match	gene: Zm00001d027281	Zm00001d027281_T00	21.48	21.48	coding	273	68	886
PB.589.2	1	769	1	full-splice_match	gene: Zm00001d029859	Zm00001d029859_T00	787.91	1141.39	coding	133	76	474
PB.589.3	1	656	1	full-splice_match	gene: Zm00001d029859	Zm00001d029859_T00	0.00	1141.39	coding	133	77	475
PB.1279.1	1	577	2	novel_not_in_catalog	gene: Zm00001d033301	novel	516.81	516.81	non coding	NA	NA	NA
PB.589.1	1	870	1	full-splice_match	gene: Zm00001d029859	Zm00001d029859_T00	353.49	1141.39	coding	133	77	475
PB.4413.1	3	968	2	full-splice_match	gene: Zm00001d043107	Zm00001d043107_T00	7.39	7.39	coding	280	36	875
PB.2901.1	2	1242	11	novel_in_catalog	gene: Zm00001d003917	novel	49.27	49.27	coding	300	191	1090
PB.6070.1	5	1331	3	full-splice_match	gene: Zm00001d013212	Zm00001d013212_T00	23.51	36.87	coding	328	37	1020
PB.6070.2	5	1191	3	full-splice_match	gene: Zm00001d013212	Zm00001d013212_T00	13.37	36.87	coding	328	39	1022
PB.1016.12	1	1050	9	incomplete-splice_match	gene: Zm00001d032198	Zm00001d032198_T00	26.60	168.87	coding	271	238	1050
PB.10839.1	9	908	1	full-splice_match	gene: Zm00001d048263	Zm00001d048263_T00	499.12	499.12	coding	170	127	636

PB.1494.1	1	4802	7	novel_in_catalog	gene: Zm00001d034282	novel	3.21	15.68	coding	878	1815	4448
PB.4075.1	3	1306	4	novel_in_catalog	gene: Zm00001d041450	novel	43.29	43.29	coding	100	144	443
PB.4087.1	3	2454	10	full-splice_match	gene: Zm00001d041511	Zm00001d041511_T00	27.90	56.87	coding	540	584	2203
PB.7246.4	5	1506	9	novel_in_catalog	gene: Zm00001d018440	novel	5.56	110.38	coding	281	9	851
PB.9792.2	8	992	2	novel_in_catalog	gene: Zm00001d011527	novel	50.93	78.99	coding	225	164	838
PB.5578.2	4	378	3	novel_not_in_catalog	gene: Zm00001d052436	novel	217.10	419.23	non coding	NA	NA	NA
PB.8279.1	7	1425	8	full-splice_match	gene: Zm00001d018873	Zm00001d018873_T00	47.92	95.54	coding	326	91	1068
PB.1056.1	1	517	1	intergenic	novelGene_428	novel	5.27	5.27	non coding	NA	NA	NA
PB.8279.3	7	1299	8	full-splice_match	gene: Zm00001d018873	Zm00001d018873_T00	47.18	95.54	coding	326	109	1086
PB.8279.2	7	1280	7	novel_in_catalog	gene: Zm00001d018873	novel	0.44	95.54	coding	217	11	661
PB.567.2	1	1137	3	full-splice_match	gene: Zm00001d029738	Zm00001d029738_T00	23.26	134.73	coding	216	60	707
PB.8379.1	7	2387	3	full-splice_match	gene: Zm00001d019522	Zm00001d019522_T00	41.31	41.31	coding	654	215	2176
PB.11038.1	Mt	1486	1	full-splice_match	gene: GRMZM5G8517	GRMZM5G851769_T0	9.36	9.36	coding	174	110	631
PB.3631.1	2	2245	19	full-splice_match	gene: Zm00001d007807	Zm00001d007807_T01	17.68	17.68	coding	628	92	1975
PB.18.2	1	1658	2	full-splice_match	gene: Zm00001d027305	Zm00001d027305_T00	0.71	177.04	coding	477	114	1544
PB.3289.2	2	818	1	full-splice_match	gene: Zm00001d006119	Zm00001d006119_T00	122.24	132.28	coding	159	102	578
PB.18.1	1	1798	2	full-splice_match	gene: Zm00001d027305	Zm00001d027305_T00	176.33	177.04	coding	477	119	1549

PB.7969.1	6	2065	3	full-splice_match	gene: Zm00001d038325	Zm00001d038325_T00	27.85	162.06	coding	383	78	1226
PB.3850.1	3	1792	2	full-splice_match	gene: Zm00001d039989	Zm00001d039989_T00	260.22	278.45	coding	329	456	1442
PB.3850.3	3	1605	2	full-splice_match	gene: Zm00001d039989	Zm00001d039989_T00	0.00	278.45	coding	329	365	1351
PB.3850.2	3	1969	1	genic	gene: Zm00001d039989	novel	18.24	278.45	coding	329	636	1622
PB.874.3	1	1542	5	full-splice_match	gene: Zm00001d031505	Zm00001d031505_T00	6.17	48.34	coding	321	275	1237
PB.874.2	1	1355	5	novel_not_in_catalog	gene: Zm00001d031505	novel	0.00	48.34	coding	319	229	1185
PB.874.1	1	1523	5	novel_not_in_catalog	gene: Zm00001d031505	novel	38.55	48.34	coding	319	296	1252
PB.2184.4	10	965	2	full-splice_match	gene: Zm00001d025864	Zm00001d025864_T00	9.08	55.07	non coding	NA	NA	NA
PB.2184.5	10	1286	3	full-splice_match	gene: Zm00001d025864	Zm00001d025864_T00	16.42	55.07	coding	277	175	1005
PB.2184.6	10	1901	2	novel_in_catalog	gene: Zm00001d025864	novel	0.00	55.07	coding	131	176	568
PB.2184.7	10	1197	3	full-splice_match	gene: Zm00001d025864	Zm00001d025864_T00	0.00	55.07	coding	277	175	1005
PB.2184.1	10	1399	3	full-splice_match	gene: Zm00001d025864	Zm00001d025864_T00	25.58	55.07	coding	277	176	1006
PB.5566.2	4	1518	9	novel_not_in_catalog	gene: Zm00001d052391	novel	120.40	247.20	coding	318	132	1085
PB.2184.3	10	2018	2	novel_in_catalog	gene: Zm00001d025864	novel	4.00	55.07	coding	131	176	568
PB.10410.2	9	1923	3	genic	gene: Zm00001d046332	novel	5.04	11.68	coding	264	699	1490
PB.10410.3	9	1527	3	genic	gene: Zm00001d046332	novel	3.56	11.68	coding	264	450	1241
PB.10410.1	9	1804	3	genic	gene: Zm00001d046332	novel	0.00	11.68	coding	264	450	1241

PB.9896.1	8	1002	2	full-splice_match	gene: Zm00001d011938	Zm00001d011938_T00	190.95	217.92	coding	183	152	700
PB.10410.4	9	939	1	full-splice_match	gene: Zm00001d046332	Zm00001d046332_T00	3.07	11.68	coding	264	29	820
PB.7223.1	5	4643	12	full-splice_match	gene: Zm00001d018382	Zm00001d018382_T01	42.51	42.51	coding	1480	84	4523
PB.7210.1	5	801	1	full-splice_match	gene: Zm00001d018337	Zm00001d018337_T00	81.01	81.01	coding	188	85	648
PB.317.1	1	1139	6	incomplete-splice_match	gene: Zm00001d028601	Zm00001d028601_T00	706.13	706.13	coding	236	113	820
PB.1198.6	1	2164	10	full-splice_match	gene: Zm00001d032905	Zm00001d032905_T01	0.00	74.90	coding	471	751	2163
PB.7052.3	5	2314	19	full-splice_match	gene: Zm00001d017729	Zm00001d017729_T01	61.76	177.47	coding	459	648	2024
PB.7052.2	5	2530	18	full-splice_match	gene: Zm00001d017729	Zm00001d017729_T01	115.71	177.47	coding	459	627	2003
PB.4681.1	3	1842	12	full-splice_match	gene: Zm00001d044088	Zm00001d044088_T00	27.21	42.81	coding	404	232	1443
PB.4047.1	3	1917	7	full-splice_match	gene: Zm00001d041255	Zm00001d041255_T00	23.01	23.01	coding	485	25	1479
PB.1599.1	1	1151	7	full-splice_match	gene: Zm00001d034665	Zm00001d034665_T00	49.34	49.34	coding	257	116	886
PB.6894.2	5	1141	2	genic	gene: Zm00001d017088	novel	2.19	13.29	coding	116	122	469
PB.9133.1	8	575	3	full-splice_match	gene: Zm00001d008259	Zm00001d008259_T00	481.20	481.20	coding	75	105	329
PB.6894.1	5	1549	3	genic	gene: Zm00001d017088	novel	11.10	13.29	coding	116	219	566
PB.10711.1	9	1745	1	genic	gene: Zm00001d047755	novel	1.32	55.03	non coding	NA	NA	NA
PB.10711.2	9	844	2	full-splice_match	gene: Zm00001d047755	Zm00001d047755_T00	21.25	55.03	non coding	NA	NA	NA
PB.7462.1	6	825	3	full-splice_match	gene: Zm00001d035902	Zm00001d035902_T00	764.41	764.41	coding	190	95	664

PB.10711.4	9	1703	12	full-splice_match	gene: Zm00001d047755	Zm00001d047755_T00	8.73	55.03	coding	392	68	1243
PB.2161.1	10	927	1	full-splice_match	gene: Zm00001d025762	Zm00001d025762_T00	76.69	76.69	coding	191	141	713
PB.5986.3	5	2605	10	full-splice_match	gene: Zm00001d012913	Zm00001d012913_T00	0.00	45.72	coding	721	285	2447
PB.6496.1	5	797	1	full-splice_match	gene: Zm00001d014949	Zm00001d014949_T00	42.97	42.97	coding	203	35	643
PB.5986.1	5	2718	10	full-splice_match	gene: Zm00001d012913	Zm00001d012913_T00	45.72	45.72	coding	721	268	2430
PB.2404.6	10	1168	6	novel_in_catalog	gene: Zm00001d026676	novel	2.40	10.81	coding	135	2	406
PB.2156.1	10	3244	10	full-splice_match	gene: Zm00001d025739	Zm00001d025739_T00	24.71	63.34	coding	720	277	2436
PB.10406.2	9	1027	4	full-splice_match	gene: Zm00001d046274	Zm00001d046274_T00	12.04	12.04	coding	54	322	483
PB.9292.1	8	1142	2	full-splice_match	gene: Zm00001d008974	Zm00001d008974_T00	4.19	4.19	coding	240	98	817
PB.5986.4	5	2387	10	full-splice_match	gene: Zm00001d012913	Zm00001d012913_T00	0.00	45.72	coding	712	252	2387
PB.3754.2	3	4756	22	full-splice_match	gene: Zm00001d039522	Zm00001d039522_T00	29.61	30.56	coding	1429	158	4444
PB.6213.1	5	1541	1	full-splice_match	gene: Zm00001d013747	Zm00001d013747_T00	14.80	14.80	coding	449	35	1381
PB.7779.1	6	1312	4	full-splice_match	gene: Zm00001d037616	Zm00001d037616_T00	14.65	14.65	coding	222	428	1093
PB.5521.1	4	1428	2	full-splice_match	gene: Zm00001d052258	Zm00001d052258_T00	33.97	33.97	coding	258	328	1101
PB.2079.1	10	1202	10	full-splice_match	gene: Zm00001d025307	Zm00001d025307_T00	27.11	27.89	coding	228	194	877
PB.6913.1	5	2627	20	full-splice_match	gene: Zm00001d017139	Zm00001d017139_T00	61.52	83.11	coding	746	203	2440
PB.2079.2	10	1313	9	novel_in_catalog	gene: Zm00001d025307	novel	0.78	27.89	coding	156	571	1038

PB.10314.4	9	302	1	full-splice_match	gene: Zm00001d045625	Zm00001d045625_T00	0.00	27.22	non coding	NA	NA	NA
PB.10314.1	9	1337	2	genic	gene: Zm00001d045625	novel	14.16	27.22	coding	310	116	1045
PB.2936.2	2	2265	5	full-splice_match	gene: Zm00001d004099	Zm00001d004099_T00	4.59	12.19	coding	527	69	1649
PB.10314.3	9	1437	1	full-splice_match	gene: Zm00001d045625	Zm00001d045625_T00	7.93	27.22	coding	310	124	1053
PB.10314.2	9	1547	1	full-splice_match	gene: Zm00001d045625	Zm00001d045625_T00	5.13	27.22	coding	141	469	891
PB.7458.1	6	2257	6	full-splice_match	gene: Zm00001d035876	Zm00001d035876_T00	1.93	10.49	coding	620	145	2004
PB.9962.1	8	2188	1	full-splice_match	gene: Zm00001d012216	Zm00001d012216_T00	6.25	6.25	coding	647	84	2024
PB.7458.2	6	2322	7	full-splice_match	gene: Zm00001d035876	Zm00001d035876_T00	8.55	10.49	coding	620	210	2069
PB.3792.2	3	682	1	genic	gene: Zm00001d039674	novel	6.75	100.53	non coding	NA	NA	NA
PB.6910.2	5	1994	8	novel_in_catalog	gene: Zm00001d017126	novel	31.39	98.45	coding	235	40	744
PB.822.3	1	2871	3	full-splice_match	gene: Zm00001d031217	Zm00001d031217_T00	0.00	39.46	coding	853	240	2798
PB.8688.1	7	2668	8	incomplete-splice_match	gene: Zm00001d021170	Zm00001d021170_T00	10.75	13.67	coding	350	1500	2549
PB.192.2	1	1009	3	full-splice_match	gene: Zm00001d028062	Zm00001d028062_T00	0.00	11.73	coding	181	312	854
PB.6910.1	5	3116	10	full-splice_match	gene: Zm00001d017126	Zm00001d017126_T00	24.39	98.45	coding	696	350	2437
PB.192.1	1	1280	3	full-splice_match	gene: Zm00001d028062	Zm00001d028062_T00	11.73	11.73	coding	181	427	969
PB.5050.1	4	1679	4	full-splice_match	gene: Zm00001d049624	Zm00001d049624_T00	14.48	14.48	coding	474	8	1429
PB.4514.2	3	1796	12	full-splice_match	gene: Zm00001d043459	Zm00001d043459_T00	33.67	42.24	coding	471	119	1531

PB.916.1	1	2600	1	full-splice_match	gene: Zm00001d031726	Zm00001d031726_T00	22.41	22.41	coding	692	320	2395
PB.3613.1	2	1986	8	full-splice_match	gene: Zm00001d007754	Zm00001d007754_T00	25.68	35.14	coding	472	65	1480
PB.363.4	1	581	2	full-splice_match	gene: Zm00001d028785	Zm00001d028785_T00	30.17	113.56	coding	121	94	456
PB.9949.1	8	1354	12	incomplete-splice_match	gene: Zm00001d012153	Zm00001d012153_T00	14.59	14.59	coding	264	27	818
PB.1678.2	1	1208	7	full-splice_match	gene: Zm00001d034949	Zm00001d034949_T00	45.32	143.81	coding	203	142	750
PB.363.1	1	1010	2	full-splice_match	gene: Zm00001d028785	Zm00001d028785_T00	66.12	113.56	coding	170	202	711
PB.363.2	1	1351	1	genic	gene: Zm00001d028785	novel	8.63	113.56	coding	143	625	1053
PB.363.3	1	1191	1	novel_in_catalog	gene: Zm00001d028785	novel	8.64	113.56	coding	143	572	1000
PB.6010.2	5	1527	1	novel_in_catalog	gene: Zm00001d013003	novel	33.21	203.08	coding	243	2	730
PB.9123.1	8	673	3	novel_not_in_catalog	gene: Zm00001d008211	novel	51.22	251.70	coding	15	264	308
PB.8259.1	7	1451	6	full-splice_match	gene: Zm00001d018794	Zm00001d018794_T00	38.84	38.84	coding	381	144	1286
PB.9123.3	8	575	3	novel_not_in_catalog	gene: Zm00001d008211	novel	200.48	251.70	coding	15	268	312
PB.6010.6	5	1275	2	full-splice_match	gene: Zm00001d013003	Zm00001d013003_T00	99.66	203.08	coding	302	183	1088
PB.7246.9	5	1983	14	full-splice_match	gene: Zm00001d018440	Zm00001d018440_T00	0.00	110.38	coding	468	417	1820
PB.6010.5	5	1405	1	novel_in_catalog	gene: Zm00001d013003	novel	0.00	203.08	coding	178	191	724
PB.1585.4	1	1949	5	incomplete-splice_match	gene: Zm00001d034624	Zm00001d034624_T03	0.00	53.35	coding	605	12	1826
PB.5529.1	4	1170	3	full-splice_match	gene: Zm00001d052286	Zm00001d052286_T00	33.81	33.81	coding	115	485	829

PB.5070.1	4	993	3	full-splice_match	gene: Zm00001d049715	Zm00001d049715_T00	12.89	12.89	coding	233	91	789
PB.3144.1	2	1841	8	full-splice_match	gene: Zm00001d005406	Zm00001d005406_T00	18.16	18.16	coding	352	66	1121
PB.4698.41	3	1204	6	full-splice_match	gene: Zm00001d044138	Zm00001d044138_T00	0.00	1495.64	coding	379	25	1161
PB.4698.40	3	1325	6	novel_not_in_catalog	gene: Zm00001d044138	novel	10.47	1495.64	coding	254	25	786
PB.1126.1	1	886	2	full-splice_match	gene: Zm00001d032618	Zm00001d032618_T00	19.90	19.90	coding	228	57	740
PB.9516.1	8	1143	6	full-splice_match	gene: Zm00001d010274	Zm00001d010274_T00	71.69	71.69	coding	296	32	919
PB.8606.1	7	710	2	incomplete-splice_match	gene: Zm00001d020799	Zm00001d020799_T01	4.13	12.63	non coding	NA	NA	NA
PB.5153.1	4	648	1	intergenic	novelGene_167	novel	22.28	22.28	coding	3	287	295
PB.835.4	1	1254	7	novel_in_catalog	gene: Zm00001d031269	novel	2.63	45.70	coding	169	463	969
PB.7368.1	6	1160	1	intergenic	novelGene_90	novel	3.07	13.38	coding	9	289	315
PB.5391.1	4	903	4	novel_in_catalog	gene: Zm00001d051741	novel	59.06	59.06	coding	149	96	542
PB.1513.1	1	906	1	incomplete-splice_match	gene: Zm00001d034350	Zm00001d034350_T00	5.08	43.80	coding	199	16	612
PB.8207.2	6	3493	6	novel_in_catalog	gene: Zm00001d039196	novel	0.34	14.87	coding	663	1217	3205
PB.5275.1	4	1353	1	intergenic	novelGene_273	novel	5.80	5.80	non coding	NA	NA	NA
PB.6937.1	5	880	1	incomplete-splice_match	gene: Zm00001d017213	Zm00001d017213_T00	5.79	5.79	coding	169	108	614
PB.835.3	1	1247	7	novel_in_catalog	gene: Zm00001d031269	novel	1.96	45.70	coding	283	109	957
PB.884.6	1	1124	2	novel_not_in_catalog	gene: Zm00001d031562	novel	39.18	73.26	coding	216	326	973

PB.10383.5	9	1515	4	novel_in_catalog	gene: Zm00001d046136	novel	1.17	42.36	coding	338	101	1114
PB.884.4	1	1445	3	full-splice_match	gene: Zm00001d031555	Zm00001d031555_T00	0.00	43.71	coding	395	125	1309
PB.8755.1	7	542	2	full-splice_match	gene: Zm00001d001250	Zm00001d001250_T00	2.73	6.72	non coding	NA	NA	NA
PB.884.2	1	1678	3	full-splice_match	gene: Zm00001d031555	Zm00001d031555_T00	38.90	43.71	coding	395	123	1307
PB.884.3	1	1569	3	full-splice_match	gene: Zm00001d031555	Zm00001d031555_T00	4.81	43.71	coding	395	125	1309
PB.7738.1	6	1372	5	full-splice_match	gene: Zm00001d037412	Zm00001d037412_T00	30.86	30.86	coding	275	236	1060
PB.6156.1	5	1186	1	intergenic	novelGene_55	novel	13.46	38.69	non coding	NA	NA	NA
PB.8410.1	7	1707	1	genic	gene: Zm00001d019664	novel	2.09	33.27	coding	350	47	1096
PB.8410.2	7	3425	9	full-splice_match	gene: Zm00001d019664	Zm00001d019664_T00	6.00	33.27	coding	896	246	2933
PB.8410.3	7	2402	4	incomplete-splice_match	gene: Zm00001d019664	Zm00001d019664_T00	0.00	33.27	coding	678	8	2041
PB.8410.4	7	1639	8	novel_in_catalog	gene: Zm00001d019664	novel	19.66	33.27	coding	358	219	1292
PB.8410.5	7	1595	8	novel_in_catalog	gene: Zm00001d019664	novel	5.53	33.27	coding	358	310	1383
PB.8410.6	7	1392	8	novel_in_catalog	gene: Zm00001d019664	novel	0.00	33.27	coding	358	227	1300
PB.1901.1	10	1957	5	full-splice_match	gene: Zm00001d024418	Zm00001d024418_T00	33.18	36.10	coding	357	416	1486
PB.6340.1	5	844	4	intergenic	novelGene_131	novel	23.09	28.86	coding	123	88	456
PB.10021.1	8	942	3	novel_not_in_catalog	gene: Zm00001d012450	novel	3.04	14.48	coding	163	130	618
PB.8091.1	6	1704	2	full-splice_match	gene: Zm00001d038801	Zm00001d038801_T00	6.91	6.91	coding	382	183	1328

PB.9372.1	8	716	1	intergenic	novelGene_158	novel	12.08	12.08	non coding	NA	NA	NA
PB.4526.1	3	2441	8	full-splice_match	gene: Zm00001d043492	Zm00001d043492_T00	7.79	7.79	coding	509	75	1601
PB.1544.2	1	759	3	full-splice_match	gene: Zm00001d034491	Zm00001d034491_T00	217.21	426.33	coding	133	88	486
PB.8926.2	7	2266	12	full-splice_match	gene: Zm00001d022010	Zm00001d022010_T00	42.29	50.09	coding	632	12	1907
PB.8926.3	7	1874	12	full-splice_match	gene: Zm00001d022010	Zm00001d022010_T00	7.80	50.09	coding	606	57	1874
PB.10822.1	9	2343	9	full-splice_match	gene: Zm00001d048175	Zm00001d048175_T00	5.46	5.46	coding	596	191	1978
PB.10738.3	9	1442	4	full-splice_match	gene: Zm00001d047835	Zm00001d047835_T00	104.36	264.03	coding	323	147	1115
PB.4984.1	4	1403	8	incomplete-splice_match	gene: Zm00001d049276	Zm00001d049276_T00	27.36	27.36	coding	318	32	985
PB.8245.1	7	903	1	full-splice_match	gene: Zm00001d018725	Zm00001d018725_T00	35.20	50.25	coding	136	110	517
PB.8245.2	7	671	1	full-splice_match	gene: Zm00001d018725	Zm00001d018725_T00	15.06	50.25	coding	136	102	509
PB.1058.7	1	1538	17	incomplete-splice_match	gene: Zm00001d032386	Zm00001d032386_T00	0.00	130.95	coding	494	57	1538
PB.2302.1	10	1069	1	genic	gene: Zm00001d026353	novel	18.97	18.97	coding	104	682	993
PB.3212.1	2	1252	5	full-splice_match	gene: Zm00001d005795	Zm00001d005795_T00	11.98	11.98	coding	271	331	1143
PB.6899.2	5	3245	2	novel_in_catalog	gene: Zm00001d017096	novel	13.48	254.51	non coding	NA	NA	NA
PB.1154.1	1	1552	5	novel_in_catalog	gene: Zm00001d032735	novel	20.04	20.04	coding	360	76	1155
PB.9263.2	8	1280	1	full-splice_match	gene: Zm00001d008850	Zm00001d008850_T00	0.00	104.23	coding	282	112	957
PB.460.1	1	1587	2	genic	gene: Zm00001d029217	novel	7.90	7.90	coding	265	656	1450

PB.8901.2	7	737	4	full-splice_match	gene: Zm00001d021913	Zm00001d021913_T00	43.09	55.36	coding	121	171	533
PB.8901.1	7	687	4	full-splice_match	gene: Zm00001d021913	Zm00001d021913_T00	12.27	55.36	coding	117	11	361
PB.4150.1	3	1203	6	incomplete-splice_match	gene: Zm00001d041880	Zm00001d041880_T01	260.85	260.85	coding	245	202	936
PB.2375.2	10	3491	8	novel_in_catalog	gene: Zm00001d026586	novel	1.00	56.25	coding	295	2485	3369
PB.5796.6	4	1499	2	novel_in_catalog	gene: Zm00001d053566	novel	113.79	614.41	coding	161	88	570
PB.7719.4	6	1607	11	novel_in_catalog	gene: Zm00001d037326	novel	6.57	192.16	coding	335	141	1145
PB.7719.3	6	1594	11	novel_in_catalog	gene: Zm00001d037326	novel	0.81	192.16	coding	323	111	1079
PB.7719.2	6	1616	12	full-splice_match	gene: Zm00001d037326	Zm00001d037326_T00	131.83	192.16	coding	402	173	1378
PB.7719.1	6	1695	12	full-splice_match	gene: Zm00001d037326	Zm00001d037326_T00	52.96	192.16	coding	402	114	1319
PB.3188.1	2	1677	7	novel_not_in_catalog	gene: Zm00001d005682	novel	13.47	65.78	coding	351	300	1352
PB.839.1	1	1525	2	genic	gene: Zm00001d022873	novel	3.10	3.10	non coding	NA	NA	NA
PB.6581.1	5	1374	7	full-splice_match	gene: Zm00001d015313	Zm00001d015313_T00	18.62	18.62	coding	307	157	1077
PB.9854.2	8	684	3	full-splice_match	gene: Zm00001d011766	Zm00001d011766_T00	8.39	328.49	coding	137	169	579
PB.8961.4	7	979	10	incomplete-splice_match	gene: Zm00001d022152	Zm00001d022152_T00	0.00	59.60	coding	299	83	979
PB.8438.1	7	4051	8	full-splice_match	gene: Zm00001d019920	Zm00001d019920_T00	66.35	66.35	coding	1052	207	3362
PB.8961.1	7	2104	15	full-splice_match	gene: Zm00001d022152	Zm00001d022152_T00	59.01	59.60	coding	541	128	1750
PB.9586.1	8	2093	5	novel_in_catalog	gene: Zm00001d010604	novel	17.51	17.51	coding	306	171	1088

PB.9934.2	8	1184	9	incomplete-splice_match	gene: Zm00001d012085	Zm00001d012085_T00	24.45	42.88	coding	131	466	858
PB.4140.1	3	2262	14	novel_in_catalog	gene: Zm00001d041816	novel	10.57	10.57	coding	354	49	1110
PB.9522.1	8	1781	10	full-splice_match	gene: Zm00001d010305	Zm00001d010305_T00	26.07	26.07	coding	460	174	1553
PB.10738.1	9	1690	4	full-splice_match	gene: Zm00001d047835	Zm00001d047835_T00	159.67	264.03	coding	323	74	1042
PB.6400.1	5	2036	1	full-splice_match	gene: Zm00001d014612	Zm00001d014612_T00	4.73	4.73	coding	397	133	1323
PB.6859.2	5	2152	8	full-splice_match	gene: Zm00001d016902	Zm00001d016902_T00	62.16	84.05	coding	554	208	1869
PB.4260.5	3	1823	14	novel_in_catalog	gene: Zm00001d042421	novel	5.00	67.32	coding	268	219	1022
PB.4677.2	3	1563	2	full-splice_match	gene: Zm00001d044061	Zm00001d044061_T00	0.00	23.42	coding	183	730	1278
PB.1792.2	10	1596	8	full-splice_match	gene: Zm00001d023698	Zm00001d023698_T00	26.25	26.25	coding	414	28	1269
PB.7994.2	6	2807	3	novel_not_in_catalog	gene: Zm00001d038400	novel	15.35	36.41	coding	701	422	2524
PB.9914.1	8	2918	2	novel_in_catalog	gene: Zm00001d011997	novel	2.15	2.15	coding	806	274	2691
PB.6410.4	5	1222	7	full-splice_match	gene: Zm00001d014656	Zm00001d014656_T00	0.00	63.69	coding	275	221	1045
PB.6410.2	5	2286	6	novel_in_catalog	gene: Zm00001d014656	novel	8.05	63.69	coding	354	97	1158
PB.6410.3	5	1367	7	full-splice_match	gene: Zm00001d014656	Zm00001d014656_T00	44.64	63.69	coding	357	18	1088
PB.6410.1	5	1623	7	full-splice_match	gene: Zm00001d014656	Zm00001d014656_T00	11.00	63.69	coding	357	12	1082
PB.4669.7	3	4613	1	genic	gene: Zm00001d044017	novel	10.88	10.88	coding	638	268	2181
PB.4870.2	4	1613	1	intergenic	novelGene_24	novel	0.00	0.46	coding	227	19	699

PB.4247.1	3	1602	5	novel_not_in_catalog	gene: Zm00001d042349	novel	1.47	25.15	coding	395	198	1382
PB.1641.2	1	2183	4	full-splice_match	gene: Zm00001d034805	Zm00001d034805_T00	9.97	80.10	coding	228	59	742
PB.4675.1	3	738	4	incomplete-splice_match	gene: Zm00001d044054	Zm00001d044054_T00	7.01	130.36	coding	130	1	390
PB.1410.2	1	1656	2	genic	gene: Zm00001d033882	novel	2.78	3.75	coding	340	448	1467
PB.6370.1	5	855	4	full-splice_match	gene: Zm00001d014488	Zm00001d014488_T00	22.20	22.20	coding	192	88	663
PB.4675.2	3	998	8	full-splice_match	gene: Zm00001d044054	Zm00001d044054_T00	120.42	130.36	coding	221	105	767
PB.5988.1	5	1719	6	full-splice_match	gene: Zm00001d012921	Zm00001d012921_T00	49.09	58.01	coding	404	210	1421
PB.5570.2	4	2362	8	full-splice_match	gene: Zm00001d052407	Zm00001d052407_T00	5.68	20.67	coding	577	340	2070
PB.5988.3	5	1563	6	full-splice_match	gene: Zm00001d012921	Zm00001d012921_T00	8.92	58.01	coding	404	196	1407
PB.8123.2	6	2180	12	full-splice_match	gene: Zm00001d038891	Zm00001d038891_T00	0.00	56.47	coding	501	402	1904
PB.10616.2	9	2038	8	full-splice_match	gene: Zm00001d047434	Zm00001d047434_T00	2.85	10.70	coding	273	401	1219
PB.10616.1	9	2032	8	novel_in_catalog	gene: Zm00001d047434	novel	7.85	10.70	coding	279	499	1335
PB.7387.2	6	1371	1	genic	gene: Zm00001d035474	novel	2.73	49.93	coding	288	36	899
PB.7387.3	6	1347	2	novel_in_catalog	gene: Zm00001d035474	novel	20.39	49.93	coding	289	107	973
PB.7387.1	6	1196	2	novel_in_catalog	gene: Zm00001d035474	novel	26.80	49.93	coding	288	160	1023
PB.9692.2	8	764	3	full-splice_match	gene: Zm00001d011049	Zm00001d011049_T00	165.20	204.29	coding	127	28	408
PB.9692.1	8	914	3	full-splice_match	gene: Zm00001d011049	Zm00001d011049_T00	39.08	204.29	coding	127	72	452

PB.4655.4	3	1336	1	full-splice_match	gene: Zm00001d043955	Zm00001d043955_T00	6.49	78.55	coding	321	232	1194
PB.9401.1	8	446	1	genic	gene: Zm00001d009645	novel	22.72	49.42	non coding	NA	NA	NA
PB.4655.2	3	2092	1	full-splice_match	gene: Zm00001d043955	Zm00001d043955_T00	12.97	78.55	coding	580	140	1879
PB.4655.1	3	2186	1	full-splice_match	gene: Zm00001d043955	Zm00001d043955_T00	59.10	78.55	coding	580	89	1828
PB.9401.2	8	1000	7	full-splice_match	gene: Zm00001d009645	Zm00001d009645_T00	26.70	49.42	coding	211	68	700
PB.7552.3	6	2057	7	novel_in_catalog	gene: Zm00001d036496	novel	2.25	31.17	coding	112	57	392
PB.3389.1	2	3035	2	novel_in_catalog	gene: Zm00001d006541	novel	5.88	5.88	coding	155	2261	2725
PB.7004.1	5	679	3	incomplete-splice_match	gene: Zm00001d017526	Zm00001d017526_T00	2544.77	2544.77	coding	128	56	439
PB.6384.2	5	976	7	novel_not_in_catalog	gene: Zm00001d014549	novel	21.84	55.61	non coding	NA	NA	NA
PB.1190.2	1	1964	13	full-splice_match	gene: Zm00001d032875	Zm00001d032875_T00	6.75	54.12	coding	537	35	1645
PB.6384.1	5	1062	7	novel_not_in_catalog	gene: Zm00001d014549	novel	33.77	55.61	non coding	NA	NA	NA
PB.4615.1	3	1474	7	incomplete-splice_match	gene: Zm00001d043807	Zm00001d043807_T00	23.07	46.97	coding	375	93	1217
PB.7418.1	6	1715	1	intergenic	novelGene_127	novel	15.51	15.51	non coding	NA	NA	NA
PB.10238.1	9	1070	2	intergenic	novelGene_80	novel	17.18	32.56	coding	163	300	788
PB.9433.1	8	1176	3	novel_not_in_catalog	gene: Zm00001d009797	novel	32.84	32.84	coding	221	256	918
PB.4494.1	3	945	1	genic	gene: Zm00001d043395	novel	2.00	34.52	non coding	NA	NA	NA
PB.4494.2	3	1737	2	incomplete-splice_match	gene: Zm00001d043395	Zm00001d043395_T00	13.06	34.52	coding	251	105	857

PB.4494.3	3	2735	3	full-splice_match	gene: Zm00001d043395	Zm00001d043395_T00	19.46	34.52	coding	674	172	2193
PB.4494.4	3	2535	3	full-splice_match	gene: Zm00001d043395	Zm00001d043395_T00	0.00	34.52	coding	674	113	2134
PB.541.2	1	1151	1	genic	gene: Zm00001d029613	novel	0.00	4.98	coding	355	13	1077
PB.8773.3	7	2102	11	novel_in_catalog	gene: Zm00001d021498	novel	1.47	66.97	coding	440	646	1965
PB.541.1	1	1876	2	full-splice_match	gene: Zm00001d029613	Zm00001d029613_T00	4.98	4.98	coding	530	66	1655
PB.2371.2	10	703	4	full-splice_match	gene: Zm00001d026578	Zm00001d026578_T00	305.10	904.82	coding	111	99	431
PB.2371.3	10	582	4	full-splice_match	gene: Zm00001d026578	Zm00001d026578_T00	444.31	904.82	coding	111	100	432
PB.8773.4	7	2453	11	full-splice_match	gene: Zm00001d021498	Zm00001d021498_T02	1.87	66.97	coding	399	1120	2316
PB.2371.1	10	730	4	full-splice_match	gene: Zm00001d026578	Zm00001d026578_T00	155.41	904.82	coding	111	96	428
PB.2351.8	10	922	8	full-splice_match	gene: Zm00001d026528	Zm00001d026528_T00	45.63	102.25	coding	220	133	792
PB.10431.1	9	2308	6	full-splice_match	gene: Zm00001d046454	Zm00001d046454_T00	100.12	100.26	coding	418	571	1824
PB.3594.6	2	2131	10	novel_in_catalog	gene: Zm00001d007595	novel	2.92	19.77	coding	166	1068	1565
PB.2585.1	2	1496	3	full-splice_match	gene: Zm00001d002419	Zm00001d002419_T00	11.30	11.30	coding	328	206	1189
PB.3594.1	2	3517	9	novel_in_catalog	gene: Zm00001d007595	novel	16.85	19.77	coding	172	1104	1619
PB.8257.3	7	1150	2	full-splice_match	gene: Zm00001d018790	Zm00001d018790_T00	0.00	50.42	coding	313	65	1003
PB.8257.2	7	1360	1	novel_in_catalog	gene: Zm00001d018790	novel	6.27	50.42	coding	139	57	473
PB.1016.11	1	1666	8	full-splice_match	gene: Zm00001d032198	Zm00001d032198_T01	18.85	168.87	coding	276	654	1481

PB.2351.5	10	1225	12	novel_not_in_catalog	gene: Zm00001d026528	novel	9.07	102.25	coding	321	129	1091
PB.5842.2	4	832	3	incomplete-splice_match	gene: Zm00001d053741	Zm00001d053741_T00	17.01	33.12	coding	149	278	724
PB.2351.7	10	1022	8	full-splice_match	gene: Zm00001d026528	Zm00001d026528_T00	47.55	102.25	coding	220	132	791
PB.8741.1	7	1616	4	full-splice_match	gene: Zm00001d021379	Zm00001d021379_T00	17.49	17.49	coding	436	78	1385
PB.797.1	1	2255	5	novel_in_catalog	gene: Zm00001d031092	novel	19.75	36.47	coding	219	264	920
PB.8608.1	7	2843	15	full-splice_match	gene: Zm00001d020810	Zm00001d020810_T00	21.39	21.39	coding	802	167	2572
PB.8384.1	7	1409	3	incomplete-splice_match	gene: Zm00001d019547	Zm00001d019547_T00	25.83	99.94	coding	270	165	974
PB.8384.2	7	2146	9	full-splice_match	gene: Zm00001d019547	Zm00001d019547_T00	55.00	99.94	coding	517	279	1829
PB.8384.3	7	2186	8	novel_in_catalog	gene: Zm00001d019547	novel	19.11	99.94	coding	379	819	1955
PB.10840.3	9	2882	7	full-splice_match	gene: Zm00001d048272	Zm00001d048272_T01	13.04	104.28	coding	466	1268	2665
PB.8384.6	7	2018	9	full-splice_match	gene: Zm00001d019547	Zm00001d019547_T00	0.00	99.94	coding	517	280	1830
PB.4669.9	3	1023	6	novel_in_catalog	gene: Zm00001d044018	novel	25.60	25.60	coding	266	74	871
PB.4649.1	3	1430	5	full-splice_match	gene: Zm00001d043911	Zm00001d043911_T00	107.51	157.86	coding	247	350	1090
PB.4188.1	3	1605	8	full-splice_match	gene: Zm00001d042050	Zm00001d042050_T00	5.95	5.95	coding	415	53	1297
PB.4649.4	3	1360	5	full-splice_match	gene: Zm00001d043911	Zm00001d043911_T00	34.37	157.86	coding	247	363	1103
PB.4649.5	3	1886	4	novel_in_catalog	gene: Zm00001d043911	novel	11.59	157.86	coding	218	312	965
PB.4649.6	3	3009	2	novel_in_catalog	gene: Zm00001d043911	novel	4.39	157.86	coding	106	135	452

PB.9536.1	8	1949	1	full-splice_match	gene: Zm00001d010380	Zm00001d010380_T00	15.76	20.12	coding	395	1	1185
PB.5582.1	4	1910	10	novel_in_catalog	gene: Zm00001d052445	novel	41.42	41.42	coding	527	3	1583
PB.6091.2	5	1351	8	full-splice_match	gene: Zm00001d013271	Zm00001d013271_T00	49.66	49.66	coding	276	221	1048
PB.1380.2	1	2490	2	full-splice_match	gene: Zm00001d033745	Zm00001d033745_T00	0.00	50.03	coding	694	73	2154
PB.8262.1	7	773	1	full-splice_match	gene: Zm00001d018801	Zm00001d018801_T00	128.65	128.65	coding	93	70	348
PB.10003.1	8	1652	10	novel_not_in_catalog	gene: Zm00001d012398	novel	2.61	10.73	coding	297	346	1236
PB.4138.2	3	820	3	full-splice_match	gene: Zm00001d041793	Zm00001d041793_T00	11.86	11.86	non coding	NA	NA	NA
PB.10133.2	9	1949	1	full-splice_match	gene: Zm00001d044857	Zm00001d044857_T00	2.84	48.13	coding	349	673	1719
PB.9848.1	8	3657	4	full-splice_match	gene: Zm00001d011745	Zm00001d011745_T00	4.44	7.94	coding	731	46	2238
PB.9848.2	8	3546	4	full-splice_match	gene: Zm00001d011745	Zm00001d011745_T00	3.40	7.94	coding	731	91	2283
PB.10133.1	9	2086	1	full-splice_match	gene: Zm00001d044857	Zm00001d044857_T00	45.29	48.13	coding	349	689	1735
PB.6517.1	5	1499	6	novel_in_catalog	gene: Zm00001d014998	novel	2.01	58.32	coding	185	574	1128
PB.3410.3	2	1711	2	novel_in_catalog	gene: Zm00001d006620	novel	0.00	26.19	coding	254	724	1485
PB.4719.2	3	1937	12	novel_in_catalog	gene: Zm00001d044213	novel	21.47	36.19	coding	400	434	1633
PB.7444.1	6	1126	7	full-splice_match	gene: Zm00001d035761	Zm00001d035761_T00	53.51	53.51	coding	251	75	827
PB.2179.2	10	1949	12	full-splice_match	gene: Zm00001d025846	Zm00001d025846_T00	15.68	46.07	coding	519	106	1662
PB.4035.1	3	546	3	incomplete-splice_match	gene: Zm00001d041173	Zm00001d041173_T00	448.67	448.67	coding	103	19	327

PB.9231.1	8	1489	5	incomplete-splice_match	gene: Zm00001d008651	Zm00001d008651_T00	9.10	9.10	coding	371	116	1228
PB.1641.1	1	1399	5	full-splice_match	gene: Zm00001d034805	Zm00001d034805_T00	70.13	80.10	coding	372	104	1219
PB.10696.2	9	2867	1	incomplete-splice_match	gene: Zm00001d047710	Zm00001d047710_T00	3.01	21.87	non coding	NA	NA	NA
PB.4370.1	3	1673	4	full-splice_match	gene: Zm00001d042886	Zm00001d042886_T00	21.00	22.14	coding	408	224	1447
PB.5593.4	4	1375	3	full-splice_match	gene: Zm00001d052489	Zm00001d052489_T00	10.85	35.08	coding	417	123	1373
PB.3167.1	2	819	2	genic	gene: Zm00001d000978	novel	66.94	66.94	coding	68	159	362
PB.10670.2	9	2685	1	full-splice_match	gene: Zm00001d047617	Zm00001d047617_T00	9.57	16.94	coding	828	39	2522
PB.4358.1	3	2309	6	full-splice_match	gene: Zm00001d042848	Zm00001d042848_T00	20.63	102.93	coding	641	125	2047
PB.1495.3	1	1173	1	genic_intron	novelGene_463	novel	16.86	23.57	non coding	NA	NA	NA
PB.5308.2	4	877	2	full-splice_match	gene: Zm00001d051333	Zm00001d051333_T00	80.68	306.93	coding	195	65	649
PB.9807.1	8	1429	4	full-splice_match	gene: Zm00001d011578	Zm00001d011578_T00	107.13	121.95	coding	358	66	1139
PB.7727.1	6	1059	1	antisense	novelGene_gene: Zm00001d037367	novel	16.16	16.16	coding	24	336	407
PB.6507.3	5	1708	2	full-splice_match	gene: Zm00001d014971	Zm00001d014971_T00	3.90	17.50	coding	485	187	1641
PB.6507.2	5	1610	1	novel_in_catalog	gene: Zm00001d014971	novel	3.91	17.50	coding	247	146	886
PB.6507.1	5	4686	2	full-splice_match	gene: Zm00001d014971	Zm00001d014971_T00	9.69	17.50	coding	1033	1414	4512
PB.7714.5	6	2164	10	full-splice_match	gene: Zm00001d037297	Zm00001d037297_T00	11.83	40.38	coding	636	58	1965
PB.7811.1	6	2137	7	full-splice_match	gene: Zm00001d037745	Zm00001d037745_T00	8.04	44.70	coding	522	83	1648

PB.7763.2	6	2256	18	full-splice_match	gene: Zm00001d037538	Zm00001d037538_T00	24.69	53.04	coding	653	147	2105
PB.6507.4	5	939	1	full-splice_match	gene: Zm00001d001575	Zm00001d001575_T00	0.00	0.00	non coding	NA	NA	NA
PB.4589.3	3	1551	5	novel_not_in_catalog	gene: Zm00001d043724	novel	0.00	12.49	coding	375	236	1360
PB.4589.2	3	1702	5	novel_not_in_catalog	gene: Zm00001d043724	novel	5.16	12.49	coding	375	259	1383
PB.4589.1	3	2001	5	novel_not_in_catalog	gene: Zm00001d043724	novel	7.33	12.49	coding	375	44	1168
PB.797.2	1	1989	4	incomplete-splice_match	gene: Zm00001d031092	Zm00001d031092_T00	0.00	36.47	coding	219	87	743
PB.4589.4	3	1411	5	novel_not_in_catalog	gene: Zm00001d043724	novel	0.00	12.49	coding	375	197	1321
PB.1370.2	1	1572	8	novel_in_catalog	gene: Zm00001d033684	novel	6.17	81.63	coding	243	517	1245
PB.10099.1	8	1388	9	incomplete-splice_match	gene: Zm00001d012761	Zm00001d012761_T00	13.23	13.23	coding	335	219	1223
PB.7395.2	6	1437	2	full-splice_match	gene: Zm00001d035501	Zm00001d035501_T00	44.90	53.45	coding	294	199	1080
PB.7395.1	6	1280	2	full-splice_match	gene: Zm00001d035501	Zm00001d035501_T00	8.56	53.45	coding	240	65	784
PB.1343.4	1	733	2	novel_not_in_catalog	gene: Zm00001d033574	novel	98.05	461.16	coding	170	71	580
PB.5593.2	4	1644	3	full-splice_match	gene: Zm00001d052489	Zm00001d052489_T00	15.50	35.08	coding	430	120	1409
PB.10790.1	9	2561	6	full-splice_match	gene: Zm00001d048046	Zm00001d048046_T00	51.12	80.26	coding	686	194	2251
PB.9044.1	7	1049	3	incomplete-splice_match	gene: Zm00001d022428	Zm00001d022428_T00	51.78	71.16	coding	269	31	837
PB.9044.2	7	962	3	incomplete-splice_match	gene: Zm00001d022428	Zm00001d022428_T00	19.38	71.16	coding	269	35	841
PB.10790.2	9	2398	6	full-splice_match	gene: Zm00001d048046	Zm00001d048046_T00	29.14	80.26	coding	686	163	2220

PB.1370.1	1	1328	9	full-splice_match	gene: Zm00001d033684	Zm00001d033684_T00	75.46	81.63	coding	282	86	931
PB.10582.2	9	2386	2	full-splice_match	gene: Zm00001d047273	Zm00001d047273_T00	0.00	33.72	coding	452	628	1983
PB.10582.3	9	2282	2	full-splice_match	gene: Zm00001d047273	Zm00001d047273_T00	0.00	33.72	coding	452	630	1985
PB.10582.1	9	2485	2	full-splice_match	gene: Zm00001d047273	Zm00001d047273_T00	33.72	33.72	coding	452	652	2007
PB.5397.3	4	2003	9	full-splice_match	gene: Zm00001d051761	Zm00001d051761_T00	81.00	281.69	coding	557	100	1770
PB.3891.1	3	2497	3	incomplete-splice_match	gene: Zm00001d040203	Zm00001d040203_T00	5.61	13.09	coding	629	1	1887
PB.3891.2	3	4310	7	full-splice_match	gene: Zm00001d040203	Zm00001d040203_T00	0.00	13.09	coding	1198	358	3951
PB.3891.3	3	4180	7	full-splice_match	gene: Zm00001d040203	Zm00001d040203_T00	7.48	13.09	coding	1198	353	3946
PB.5397.5	4	792	3	incomplete-splice_match	gene: Zm00001d051761	Zm00001d051761_T00	0.00	281.69	coding	232	95	790
PB.2314.1	10	2672	1	novel_in_catalog	gene: Zm00001d026391	novel	5.78	99.60	coding	197	86	676
PB.10205.6	9	1001	3	full-splice_match	gene: Zm00001d045206	Zm00001d045206_T00	7.57	1099.39	coding	299	105	1001
PB.10205.4	9	899	4	incomplete-splice_match	gene: Zm00001d045206	Zm00001d045206_T00	0.00	1099.39	coding	263	109	897
PB.10205.5	9	1082	2	incomplete-splice_match	gene: Zm00001d045206	Zm00001d045206_T00	12.21	1099.39	coding	238	367	1080
PB.10205.2	9	1190	4	novel_not_in_catalog	gene: Zm00001d045206	novel	29.22	1099.39	coding	267	105	905
PB.10205.3	9	1103	4	novel_in_catalog	gene: Zm00001d045206	novel	504.40	1099.39	coding	263	105	893
PB.9893.2	8	1590	9	full-splice_match	gene: Zm00001d011913	Zm00001d011913_T01	0.75	58.51	coding	464	75	1466
PB.10205.1	9	1193	4	novel_in_catalog	gene: Zm00001d045206	novel	545.99	1099.39	coding	263	89	877

PB.6251.5	5	1335	1	full-splice_match	gene: Zm00001d013947	Zm00001d013947_T00	151.59	262.47	coding	265	164	958
PB.6251.4	5	1442	1	full-splice_match	gene: Zm00001d013947	Zm00001d013947_T00	0.00	262.47	coding	265	164	958
PB.6251.6	5	1442	1	full-splice_match	gene: Zm00001d013947	Zm00001d013947_T00	21.05	262.47	coding	265	410	1204
PB.6251.1	5	1694	1	full-splice_match	gene: Zm00001d013947	Zm00001d013947_T00	42.42	262.47	coding	265	166	960
PB.8688.2	7	1331	3	novel_in_catalog	gene: Zm00001d021170	novel	2.92	13.67	coding	251	429	1181
PB.4370.2	3	1677	3	novel_in_catalog	gene: Zm00001d042886	novel	1.15	22.14	coding	278	199	1032
PB.6251.2	5	1547	1	full-splice_match	gene: Zm00001d013947	Zm00001d013947_T00	47.42	262.47	coding	265	164	958
PB.2738.3	2	958	2	incomplete-splice_match	gene: Zm00001d003119	Zm00001d003119_T00	10.56	29.37	coding	158	485	958
PB.2086.2	10	604	2	full-splice_match	gene: Zm00001d025339	Zm00001d025339_T00	348.72	360.35	non coding	NA	NA	NA
PB.2738.1	2	3743	10	full-splice_match	gene: Zm00001d003119	Zm00001d003119_T00	18.80	29.37	coding	1178	13	3546
PB.4271.1	3	1732	4	novel_not_in_catalog	gene: Zm00001d042473	novel	36.59	36.59	coding	482	195	1640
PB.2663.3	2	1874	19	full-splice_match	gene: Zm00001d002791	Zm00001d002791_T00	4.89	17.34	coding	498	36	1529
PB.2663.1	2	2180	18	full-splice_match	gene: Zm00001d002791	Zm00001d002791_T01	12.45	17.34	coding	262	1005	1790
PB.5467.1	4	2390	2	full-splice_match	gene: Zm00001d052061	Zm00001d052061_T00	59.35	59.35	coding	618	267	2120
PB.6874.1	5	1584	3	novel_in_catalog	gene: Zm00001d016998	novel	7.56	7.56	coding	396	107	1294
PB.1394.2	1	3944	6	full-splice_match	gene: Zm00001d033799	Zm00001d033799_T00	30.17	74.68	coding	1131	281	3673
PB.1394.3	1	2130	4	full-splice_match	gene: Zm00001d033799	Zm00001d033799_T03	18.40	74.68	coding	625	102	1976

PB.8684.3	7	1872	10	intergenic	novelGene_282	novel	10.21	53.03	coding	287	823	1683
PB.4663.1	3	1150	6	full-splice_match	gene: Zm00001d043991	Zm00001d043991_T00	291.22	378.43	coding	230	125	814
PB.1468.1	1	1712	10	full-splice_match	gene: Zm00001d034125	Zm00001d034125_T00	108.94	228.74	coding	383	161	1309
PB.7389.1	6	2525	1	genic	gene: Zm00001d035476	novel	11.31	11.31	coding	771	57	2369
PB.134.2	1	1911	10	novel_in_catalog	gene: Zm00001d027826	novel	2.89	121.54	coding	140	129	548
PB.1380.1	1	2693	2	full-splice_match	gene: Zm00001d033745	Zm00001d033745_T00	50.03	50.03	coding	694	124	2205
PB.134.1	1	1516	11	novel_in_catalog	gene: Zm00001d027826	novel	118.65	121.54	coding	313	171	1109
PB.4243.6	3	1281	4	full-splice_match	gene: Zm00001d042337	Zm00001d042337_T00	18.09	91.62	coding	357	82	1152
PB.2410.1	10	1904	2	incomplete-splice_match	gene: Zm00001d026691	Zm00001d026691_T01	32.88	32.88	coding	509	170	1696
PB.2718.1	2	3324	1	full-splice_match	gene: Zm00001d003047	Zm00001d003047_T00	3.14	3.14	coding	1021	14	3076
PB.6854.2	5	687	3	incomplete-splice_match	gene: Zm00001d016892	Zm00001d016892_T00	17.21	168.96	coding	145	15	449
PB.6712.2	5	3112	9	full-splice_match	gene: Zm00001d016198	Zm00001d016198_T00	5.78	36.32	coding	394	122	1303
PB.6712.1	5	1782	10	full-splice_match	gene: Zm00001d016198	Zm00001d016198_T00	30.54	36.32	coding	430	228	1517
PB.1806.1	10	1656	13	incomplete-splice_match	gene: Zm00001d023801	Zm00001d023801_T00	8.86	8.86	coding	412	84	1319
PB.1006.1	1	820	3	incomplete-splice_match	gene: Zm00001d032160	Zm00001d032160_T00	124.55	124.55	coding	127	279	659
PB.6854.4	5	648	2	novel_not_in_catalog	gene: Zm00001d016892	novel	27.26	168.96	non coding	NA	NA	NA
PB.6854.5	5	596	2	novel_not_in_catalog	gene: Zm00001d016892	novel	24.04	168.96	non coding	NA	NA	NA

PB.4212.3	3	1448	8	novel_in_catalog	gene: Zm00001d042193	novel	4.11	33.54	coding	219	174	830
PB.5809.5	4	834	9	full-splice_match	gene: Zm00001d053620	Zm00001d053620_T04	0.55	56.84	coding	218	179	832
PB.1522.2	1	2122	5	incomplete-splice_match	gene: Zm00001d034388	Zm00001d034388_T01	31.06	388.07	coding	332	120	1115
PB.5344.5	4	1138	4	full-splice_match	gene: Zm00001d051511	Zm00001d051511_T00	6.93	41.76	coding	251	148	900
PB.5344.2	4	1270	4	full-splice_match	gene: Zm00001d051511	Zm00001d051511_T00	25.76	41.76	coding	251	122	874
PB.5344.1	4	1341	3	novel_in_catalog	gene: Zm00001d051511	novel	9.06	41.76	coding	191	89	661
PB.10039.1	8	448	1	incomplete-splice_match	gene: Zm00001d012544	Zm00001d012544_T00	18.52	18.52	non coding	NA	NA	NA
PB.4927.2	4	1974	16	full-splice_match	gene: Zm00001d049049	Zm00001d049049_T00	38.29	49.57	coding	527	147	1727
PB.4927.1	4	2594	15	novel_in_catalog	gene: Zm00001d049049	novel	10.04	49.57	coding	221	146	808
PB.10054.1	8	953	2	full-splice_match	gene: Zm00001d012602	Zm00001d012602_T00	41.07	41.07	coding	208	155	778
PB.3463.1	2	2278	13	full-splice_match	gene: Zm00001d006866	Zm00001d006866_T00	48.21	48.21	coding	505	344	1858
PB.4927.4	4	2661	13	novel_in_catalog	gene: Zm00001d049049	novel	1.24	49.57	coding	221	101	763
PB.5621.1	4	575	1	incomplete-splice_match	gene: Zm00001d052696	Zm00001d052696_T00	16.51	29.66	non coding	NA	NA	NA
PB.3186.4	2	1396	4	novel_in_catalog	gene: Zm00001d005675	novel	5.46	373.03	non coding	NA	NA	NA
PB.1821.1	10	978	3	full-splice_match	gene: Zm00001d023865	Zm00001d023865_T00	11.94	11.94	coding	190	160	729
PB.5621.2	4	1848	5	full-splice_match	gene: Zm00001d052696	Zm00001d052696_T00	13.14	29.66	coding	420	363	1622
PB.3186.1	2	742	5	full-splice_match	gene: Zm00001d005675	Zm00001d005675_T00	44.23	373.03	coding	78	78	311

PB.3186.3	2	472	5	full-splice_match	gene: Zm00001d005675	Zm00001d005675_T00	76.44	373.03	coding	78	74	307
PB.370.1	1	1104	1	full-splice_match	gene: Zm00001d028808	Zm00001d028808_T00	293.75	293.75	coding	124	352	723
PB.4338.1	3	1510	3	full-splice_match	gene: Zm00001d042764	Zm00001d042764_T00	26.32	26.32	coding	401	105	1307
PB.8684.5	7	2198	7	intergenic	novelGene_282	novel	4.39	53.03	coding	152	1570	2025
PB.8788.2	7	1145	13	novel_in_catalog	gene: Zm00001d021558	novel	21.69	94.47	coding	311	97	1029
PB.9146.1	8	1289	4	full-splice_match	gene: Zm00001d008299	Zm00001d008299_T00	54.92	54.92	coding	341	72	1094
PB.7707.1	6	1372	1	full-splice_match	gene: Zm00001d037263	Zm00001d037263_T00	39.74	39.74	coding	301	202	1104
PB.6897.3	5	1402	4	full-splice_match	gene: Zm00001d017091	Zm00001d017091_T00	1.53	41.54	coding	375	168	1292
PB.2402.3	10	1435	10	novel_not_in_catalog	gene: Zm00001d026670	novel	0.00	4.65	coding	75	180	404
PB.114.1	1	2196	14	full-splice_match	gene: Zm00001d027701	Zm00001d027701_T00	64.47	64.47	coding	579	137	1873
PB.3163.1	2	1754	2	novel_in_catalog	gene: Zm00001d005554	novel	7.50	7.50	coding	455	147	1511
PB.1841.1	10	1308	5	full-splice_match	gene: Zm00001d023962	Zm00001d023962_T00	60.26	61.13	coding	256	223	990
PB.114.5	1	1597	11	incomplete-splice_match	gene: Zm00001d027701	Zm00001d027701_T00	0.00	64.47	coding	491	125	1597
PB.1841.3	10	1058	5	full-splice_match	gene: Zm00001d023962	Zm00001d023962_T00	0.00	61.13	coding	256	104	871
PB.1841.2	10	1307	5	full-splice_match	gene: Zm00001d023962	Zm00001d023962_T00	0.86	61.13	coding	256	263	1030
PB.2453.1	2	1830	10	full-splice_match	gene: Zm00001d001895	Zm00001d001895_T00	96.36	96.36	coding	461	117	1499
PB.9245.1	8	4891	10	full-splice_match	gene: Zm00001d008733	Zm00001d008733_T00	20.94	20.94	coding	1512	28	4563

PB.2453.2	2	1615	10	full-splice_match	gene: Zm00001d001895	Zm00001d001895_T00	0.00	96.36	coding	461	68	1450
PB.2880.3	2	992	7	novel_not_in_catalog	gene: Zm00001d003807	novel	8.06	50.21	coding	179	221	757
PB.9358.1	8	1671	9	novel_in_catalog	gene: Zm00001d009436	novel	0.65	37.44	coding	150	259	708
PB.9358.2	8	1581	10	full-splice_match	gene: Zm00001d009436	Zm00001d009436_T00	36.79	37.44	coding	338	281	1294
PB.9358.3	8	1350	10	full-splice_match	gene: Zm00001d009436	Zm00001d009436_T00	0.00	37.44	coding	338	215	1228
PB.1724.1	10	1011	2	full-splice_match	gene: Zm00001d023384	Zm00001d023384_T00	35.85	35.85	coding	131	244	636
PB.9439.10	8	1260	9	incomplete-splice_match	gene: Zm00001d009850	Zm00001d009850_T00	0.00	207.80	coding	369	152	1258
PB.2986.3	2	1619	3	full-splice_match	gene: Zm00001d004473	Zm00001d004473_T00	0.00	157.64	coding	428	193	1476
PB.1051.1	1	1750	8	full-splice_match	gene: Zm00001d032368	Zm00001d032368_T00	1.85	1.85	coding	486	95	1552
PB.2722.2	2	1336	9	full-splice_match	gene: Zm00001d003058	Zm00001d003058_T00	13.50	28.40	coding	370	91	1200
PB.8509.1	7	1117	2	intergenic	novelGene_213	novel	19.55	19.55	coding	286	12	869
PB.1764.5	10	2174	1	full-splice_match	gene: Zm00001d023558	Zm00001d023558_T00	7.72	51.52	coding	467	585	1985
PB.2722.1	2	1505	9	full-splice_match	gene: Zm00001d003058	Zm00001d003058_T00	14.90	28.40	coding	370	162	1271
PB.5364.1	4	1377	10	incomplete-splice_match	gene: Zm00001d051608	Zm00001d051608_T02	14.74	62.79	coding	81	336	578
PB.4974.1	4	1472	4	full-splice_match	gene: Zm00001d049234	Zm00001d049234_T00	222.71	815.22	coding	256	271	1038
PB.4556.5	3	1560	15	incomplete-splice_match	gene: Zm00001d043601	Zm00001d043601_T00	127.65	284.99	coding	388	91	1254
PB.2986.1	2	1709	3	full-splice_match	gene: Zm00001d004473	Zm00001d004473_T00	157.64	157.64	coding	428	251	1534

PB.4307.6	3	1534	4	novel_in_catalog	gene: Zm00001d042639	novel	4.83	45.96	coding	211	165	797
PB.8121.2	6	681	7	full-splice_match	gene: Zm00001d038885	Zm00001d038885_T00	0.00	115.22	coding	126	136	513
PB.4812.2	3	2797	7	full-splice_match	gene: Zm00001d044601	Zm00001d044601_T00	0.00	14.15	coding	724	292	2463
PB.10687.1	9	2348	3	incomplete-splice_match	gene: Zm00001d047676	Zm00001d047676_T00	52.80	125.61	coding	303	810	1718
PB.3831.4	3	2499	4	novel_in_catalog	gene: Zm00001d039901	novel	5.16	21.78	coding	634	354	2255
PB.4812.1	3	2882	7	full-splice_match	gene: Zm00001d044601	Zm00001d044601_T00	9.05	14.15	coding	724	295	2466
PB.3831.2	3	2274	5	full-splice_match	gene: Zm00001d039901	Zm00001d039901_T00	10.70	21.78	coding	600	63	1862
PB.3831.3	3	2168	5	full-splice_match	gene: Zm00001d039901	Zm00001d039901_T00	5.69	21.78	coding	594	112	1893
PB.10687.6	9	1318	2	full-splice_match	gene: Zm00001d047676	Zm00001d047676_T00	10.61	125.61	coding	341	294	1316
PB.3831.1	3	2399	4	novel_in_catalog	gene: Zm00001d039901	novel	0.24	21.78	coding	640	66	1985
PB.7623.1	6	2892	2	full-splice_match	gene: Zm00001d036880	Zm00001d036880_T00	16.35	16.35	coding	626	193	2070
PB.9703.1	8	1819	13	full-splice_match	gene: Zm00001d011102	Zm00001d011102_T00	17.32	25.11	coding	456	80	1447
PB.6352.1	5	2163	7	full-splice_match	gene: Zm00001d014378	Zm00001d014378_T00	17.38	17.38	coding	640	100	2019
PB.7623.2	6	2600	2	full-splice_match	gene: Zm00001d036880	Zm00001d036880_T00	0.00	16.35	coding	626	195	2072
PB.4882.1	4	926	2	incomplete-splice_match	gene: Zm00001d048836	Zm00001d048836_T00	6.85	12.57	coding	88	394	657
PB.10572.2	9	1406	5	full-splice_match	gene: Zm00001d047217	Zm00001d047217_T00	0.61	160.92	coding	223	643	1311
PB.10572.1	9	1164	6	full-splice_match	gene: Zm00001d047217	Zm00001d047217_T00	107.66	160.92	coding	332	16	1011

PB.4882.2	4	1224	7	novel_not_in_catalog	gene: Zm00001d048836	novel	5.72	12.57	coding	350	67	1116
PB.9165.2	8	1227	1	full-splice_match	gene: Zm00001d008370	Zm00001d008370_T00	0.00	21.03	coding	363	14	1102
PB.5932.4	4	1795	1	full-splice_match	gene: Zm00001d054060	Zm00001d054060_T00	1068.14	1485.90	coding	524	96	1667
PB.2402.1	10	1611	10	novel_not_in_catalog	gene: Zm00001d026670	novel	4.65	4.65	coding	75	179	403
PB.5932.2	4	1907	1	full-splice_match	gene: Zm00001d054060	Zm00001d054060_T00	108.19	1485.90	coding	524	97	1668
PB.9683.4	8	1301	3	full-splice_match	gene: Zm00001d010976	Zm00001d010976_T00	0.00	173.74	coding	365	86	1180
PB.9382.1	8	1242	5	full-splice_match	gene: Zm00001d009532	Zm00001d009532_T00	52.76	52.76	coding	281	120	962
PB.731.1	1	968	1	incomplete-splice_match	gene: Zm00001d030784	Zm00001d030784_T00	0.88	6.97	coding	56	425	592
PB.5635.1	4	2035	14	full-splice_match	gene: Zm00001d052758	Zm00001d052758_T00	60.70	60.70	coding	552	144	1799
PB.3522.1	2	1796	11	novel_in_catalog	gene: Zm00001d007095	novel	21.96	25.79	coding	422	219	1484
PB.3458.2	2	1885	5	novel_in_catalog	gene: Zm00001d006844	novel	6.99	605.08	coding	151	800	1252
PB.9727.2	8	880	4	incomplete-splice_match	gene: Zm00001d011199	Zm00001d011199_T00	5.97	20.85	coding	122	349	714
PB.9727.1	8	1413	6	full-splice_match	gene: Zm00001d011199	Zm00001d011199_T00	14.88	20.85	coding	298	143	1036
PB.10015.1	8	919	1	full-splice_match	gene: Zm00001d012432	Zm00001d012432_T00	8.28	8.28	coding	212	31	666
PB.235.20	1	1526	10	novel_in_catalog	gene: Zm00001d028260	novel	19.88	1792.39	coding	131	890	1282
PB.6025.3	5	763	4	full-splice_match	gene: Zm00001d013041	Zm00001d013041_T00	262.39	302.66	coding	126	128	505
PB.6025.1	5	869	4	full-splice_match	gene: Zm00001d013041	Zm00001d013041_T00	40.27	302.66	coding	126	132	509

PB.10504.1	9	1424	3	full-splice_match	gene: Zm00001d046898	Zm00001d046898_T00	17.79	17.79	coding	380	97	1236
PB.5674.2	4	1677	3	novel_not_in_catalog	gene: Zm00001d052916	novel	12.79	17.73	coding	427	287	1567
PB.9683.3	8	1796	3	full-splice_match	gene: Zm00001d010976	Zm00001d010976_T00	0.00	173.74	coding	365	425	1519
PB.1679.1	1	1434	7	fusion	gene: Zm00001d034957	novel	29.60	68.49	coding	298	49	942
PB.4684.1	3	1388	6	novel_in_catalog	gene: Zm00001d044095	novel	9.07	327.19	coding	200	454	1053
PB.2821.2	2	2394	1	novel_in_catalog	gene: Zm00001d003500	novel	0.14	17.15	coding	312	1354	2289
PB.2821.3	2	1409	2	full-splice_match	gene: Zm00001d003500	Zm00001d003500_T00	10.90	17.15	coding	423	39	1307
PB.2821.1	2	1501	2	full-splice_match	gene: Zm00001d003500	Zm00001d003500_T00	6.11	17.15	coding	423	49	1317
PB.4259.6	3	3413	9	full-splice_match	gene: Zm00001d042416	Zm00001d042416_T00	4.81	123.25	coding	473	169	1587
PB.4259.5	3	3535	8	full-splice_match	gene: Zm00001d042416	Zm00001d042416_T01	9.42	123.25	coding	394	99	1280
PB.4259.4	3	1992	9	novel_in_catalog	gene: Zm00001d042416	novel	14.32	123.25	coding	397	105	1295
PB.5265.1	4	970	3	novel_in_catalog	gene: Zm00001d051018	novel	26.87	27.09	coding	253	76	834
PB.6726.3	5	1694	1	genic	gene: Zm00001d016278	novel	4.63	61.62	coding	51	194	346
PB.4259.1	3	3411	9	full-splice_match	gene: Zm00001d042416	Zm00001d042416_T00	59.32	123.25	coding	473	102	1520
PB.5265.2	4	972	2	novel_in_catalog	gene: Zm00001d051018	novel	0.23	27.09	coding	170	1	510
PB.729.2	1	2433	8	full-splice_match	gene: Zm00001d030770	Zm00001d030770_T00	4.02	30.79	coding	714	129	2270
PB.2018.1	10	853	3	full-splice_match	gene: Zm00001d024982	Zm00001d024982_T00	17.05	17.05	coding	90	64	333

PB.8801.9	7	1555	10	novel_not_in_catalog	gene: Zm00001d021615	novel	1.46	834.08	coding	245	85	819
PB.5592.1	4	1044	8	incomplete-splice_match	gene: Zm00001d052485	Zm00001d052485_T01	49.73	49.73	coding	241	8	730
PB.5674.1	4	1846	2	genic	gene: Zm00001d052916	novel	4.94	17.73	coding	336	726	1733
PB.5587.1	4	1201	4	full-splice_match	gene: Zm00001d052473	Zm00001d052473_T00	37.33	66.04	coding	272	244	1059
PB.7292.1	6	2126	14	full-splice_match	gene: Zm00001d034970	Zm00001d034970_T00	16.15	24.93	coding	550	87	1736
PB.8715.3	7	2429	9	full-splice_match	gene: Zm00001d021287	Zm00001d021287_T00	0.00	26.62	coding	683	114	2162
PB.7292.3	6	2091	14	full-splice_match	gene: Zm00001d034970	Zm00001d034970_T00	5.30	24.93	coding	550	158	1807
PB.7292.2	6	1272	8	incomplete-splice_match	gene: Zm00001d034970	Zm00001d034970_T00	3.47	24.93	coding	218	169	822
PB.3486.1	2	619	5	incomplete-splice_match	gene: Zm00001d006939	Zm00001d006939_T00	92.98	92.98	coding	108	10	333
PB.10697.1	9	1768	2	full-splice_match	gene: Zm00001d047712	Zm00001d047712_T00	1.07	1.07	coding	484	107	1558
PB.13.5	1	2307	10	full-splice_match	gene: Zm00001d027293	Zm00001d027293_T00	211.60	422.53	coding	625	105	1979
PB.13.6	1	2151	10	full-splice_match	gene: Zm00001d027293	Zm00001d027293_T00	160.40	422.53	coding	625	105	1979
PB.13.3	1	2469	10	full-splice_match	gene: Zm00001d027293	Zm00001d027293_T00	15.67	422.53	coding	625	105	1979
PB.7335.2	6	3349	3	full-splice_match	gene: Zm00001d035183	Zm00001d035183_T00	15.25	51.00	coding	905	197	2911
PB.4684.8	3	2925	2	novel_in_catalog	gene: Zm00001d044095	novel	2.95	327.19	coding	14	389	430
PB.4898.1	4	2119	8	full-splice_match	gene: Zm00001d048902	Zm00001d048902_T00	12.38	70.35	coding	433	214	1512
PB.4898.2	4	2034	8	full-splice_match	gene: Zm00001d048902	Zm00001d048902_T00	57.98	70.35	coding	433	230	1528

PB.4898.3	4	1914	8	full-splice_match	gene: Zm00001d048902	Zm00001d048902_T00	0.00	70.35	coding	433	230	1528
PB.1804.2	10	2643	2	incomplete-splice_match	gene: Zm00001d023788	Zm00001d023788_T00	10.44	14.45	coding	702	180	2285
PB.9682.2	8	2265	5	novel_in_catalog	gene: Zm00001d010975	novel	5.06	108.82	coding	297	1061	1951
PB.4903.1	4	1937	6	full-splice_match	gene: Zm00001d048945	Zm00001d048945_T00	6.99	6.99	coding	361	376	1458
PB.8709.3	7	1336	2	full-splice_match	gene: Zm00001d021268	Zm00001d021268_T00	31.24	77.76	coding	239	391	1107
PB.7789.1	6	1831	3	novel_not_in_catalog	gene: Zm00001d037650	novel	11.86	101.86	coding	523	261	1829
PB.5140.1	4	1312	7	novel_not_in_catalog	gene: Zm00001d050131	novel	123.93	123.93	coding	303	58	966
PB.10882.4	9	1257	7	full-splice_match	gene: Zm00001d048410	Zm00001d048410_T00	0.00	107.65	coding	157	140	610
PB.10882.3	9	988	8	full-splice_match	gene: Zm00001d048410	Zm00001d048410_T00	47.27	107.65	coding	200	194	793
PB.10550.2	9	1484	5	full-splice_match	gene: Zm00001d047078	Zm00001d047078_T00	0.77	68.89	coding	351	177	1229
PB.10550.1	9	1569	5	full-splice_match	gene: Zm00001d047078	Zm00001d047078_T00	68.12	68.89	coding	351	136	1188
PB.10675.16	9	2875	3	novel_in_catalog	gene: Zm00001d047635	novel	4.11	286.92	coding	188	2170	2733
PB.5566.7	4	1303	9	novel_not_in_catalog	gene: Zm00001d052391	novel	105.19	247.20	coding	318	124	1077
PB.5566.1	4	1625	9	novel_not_in_catalog	gene: Zm00001d052391	novel	0.00	247.20	coding	318	134	1087
PB.2165.2	10	457	3	full-splice_match	gene: Zm00001d025772	Zm00001d025772_T00	851.85	897.54	non coding	NA	NA	NA
PB.7405.1	6	749	5	novel_not_in_catalog	gene: Zm00001d035569	novel	5.22	5.22	coding	179	9	545
PB.3174.1	2	1660	1	full-splice_match	gene: Zm00001d005600	Zm00001d005600_T00	2.92	2.92	coding	473	71	1489

PB.2479.1	2	1467	8	novel_in_catalog	gene: Zm00001d001987	novel	4.52	4.52	coding	281	55	897
PB.10290.1	9	1801	3	novel_in_catalog	gene: Zm00001d045517	novel	45.65	155.38	coding	256	852	1619
PB.10290.2	9	1249	8	full-splice_match	gene: Zm00001d045517	Zm00001d045517_T00	81.00	155.38	coding	268	121	924
PB.7959.2	6	2505	14	full-splice_match	gene: Zm00001d038290	Zm00001d038290_T00	0.00	41.44	coding	747	62	2302
PB.10290.4	9	1354	7	novel_in_catalog	gene: Zm00001d045517	novel	3.25	155.38	coding	199	554	1150
PB.10290.5	9	1094	8	full-splice_match	gene: Zm00001d045517	Zm00001d045517_T00	25.47	155.38	coding	268	131	934
PB.10151.1	9	1299	1	full-splice_match	gene: Zm00001d044950	Zm00001d044950_T00	11.69	11.69	coding	261	218	1000
PB.8324.3	7	1138	9	full-splice_match	gene: Zm00001d019123	Zm00001d019123_T00	3.55	49.10	coding	330	84	1073
PB.8324.1	7	1204	9	full-splice_match	gene: Zm00001d019123	Zm00001d019123_T00	45.55	49.10	coding	330	58	1047
PB.5796.13	4	1540	3	novel_in_catalog	gene: Zm00001d053566	novel	0.00	614.41	coding	382	197	1342
PB.5742.1	4	633	1	intergenic	novelGene_426	novel	13.32	13.32	coding	105	202	516
PB.2982.1	2	3721	12	full-splice_match	gene: Zm00001d004466	Zm00001d004466_T02	8.80	57.39	coding	760	167	2446
PB.275.1	1	2180	1	full-splice_match	gene: Zm00001d028406	Zm00001d028406_T00	119.58	131.21	coding	547	121	1761
PB.275.2	1	1974	1	full-splice_match	gene: Zm00001d028406	Zm00001d028406_T00	11.62	131.21	coding	547	218	1858
PB.2187.1	10	1338	1	full-splice_match	gene: Zm00001d025875	Zm00001d025875_T00	14.64	14.64	coding	289	409	1275
PB.4326.1	3	2319	14	full-splice_match	gene: Zm00001d042720	Zm00001d042720_T00	13.53	18.04	coding	620	169	2028
PB.4326.2	3	2277	14	full-splice_match	gene: Zm00001d042720	Zm00001d042720_T00	0.00	18.04	coding	620	272	2131

PB.4326.3	3	2258	14	full-splice_match	gene: Zm00001d042720	Zm00001d042720_T00	4.51	18.04	coding	620	308	2167
PB.2465.1	2	3053	2	novel_in_catalog	gene: Zm00001d001929	novel	27.81	27.81	non coding	NA	NA	NA
PB.10491.1	9	1058	6	novel_not_in_catalog	gene: Zm00001d046801	novel	30.51	68.20	coding	215	40	684
PB.9897.1	8	2033	15	full-splice_match	gene: Zm00001d011941	Zm00001d011941_T00	19.12	19.12	coding	487	241	1701
PB.2006.2	10	1396	8	full-splice_match	gene: Zm00001d024933	Zm00001d024933_T00	326.31	337.93	coding	319	61	1017
PB.7526.1	6	1715	2	novel_in_catalog	gene: Zm00001d036338	novel	40.88	40.88	coding	228	711	1394
PB.7180.3	5	1272	1	full-splice_match	gene: Zm00001d018208	Zm00001d018208_T00	0.00	68.31	coding	355	58	1122
PB.3361.1	2	1668	1	full-splice_match	gene: Zm00001d006397	Zm00001d006397_T00	3.18	3.18	coding	480	93	1532
PB.5787.1	4	468	1	full-splice_match	gene: Zm00001d053534	Zm00001d053534_T00	61.18	61.18	coding	84	51	302
PB.8030.1	6	2573	3	novel_not_in_catalog	gene: Zm00001d038541	novel	40.83	40.83	coding	674	174	2195
PB.9110.2	7	1086	3	full-splice_match	gene: Zm00001d022629	Zm00001d022629_T00	246.58	258.64	coding	249	131	877
PB.9110.3	7	926	3	full-splice_match	gene: Zm00001d022629	Zm00001d022629_T00	0.00	258.64	coding	249	92	838
PB.9446.1	8	1742	9	full-splice_match	gene: Zm00001d009903	Zm00001d009903_T00	18.40	18.40	coding	418	101	1354
PB.2940.1	2	1230	5	full-splice_match	gene: Zm00001d004133	Zm00001d004133_T00	7.18	7.18	coding	323	70	1038
PB.6282.1	5	2237	17	full-splice_match	gene: Zm00001d014090	Zm00001d014090_T00	58.87	58.87	coding	577	197	1927
PB.6282.3	5	2198	16	novel_in_catalog	gene: Zm00001d014090	novel	0.00	58.87	coding	318	126	1079
PB.5855.1	4	800	4	full-splice_match	gene: Zm00001d053805	Zm00001d053805_T00	14.78	14.78	coding	209	11	637

PB.9148.1	8	4242	3	novel_in_catalog	gene: Zm00001d008306	novel	32.52	32.52	coding	262	3042	3827
PB.6282.4	5	2071	17	full-splice_match	gene: Zm00001d014090	Zm00001d014090_T00	0.00	58.87	coding	577	124	1854
PB.4087.2	3	1901	10	full-splice_match	gene: Zm00001d041511	Zm00001d041511_T00	28.97	56.87	coding	540	214	1833
PB.10058.2	8	1629	1	full-splice_match	gene: Zm00001d012606	Zm00001d012606_T00	7.03	15.38	coding	453	52	1410
PB.1027.1	1	1812	14	novel_in_catalog	gene: Zm00001d032257	novel	110.69	110.69	coding	393	493	1671
PB.1796.1	10	1040	3	full-splice_match	gene: Zm00001d023733	Zm00001d023733_T00	79.40	79.40	coding	187	265	825
PB.10680.1	9	1315	1	full-splice_match	gene: Zm00001d047651	Zm00001d047651_T00	780.20	780.20	coding	327	109	1089
PB.4430.3	3	2088	12	novel_in_catalog	gene: Zm00001d043152	novel	1.38	115.37	coding	309	71	997
PB.10439.1	9	1251	10	full-splice_match	gene: Zm00001d046483	Zm00001d046483_T00	3.31	3.31	coding	324	226	1197
PB.4432.1	3	1694	2	full-splice_match	gene: Zm00001d043165	Zm00001d043165_T00	0.80	0.80	coding	474	59	1480
PB.6910.8	5	1897	3	novel_in_catalog	gene: Zm00001d017126	novel	9.47	98.45	coding	494	350	1831
PB.9942.2	8	2461	13	full-splice_match	gene: Zm00001d012108	Zm00001d012108_T01	2.25	40.88	coding	359	1119	2195
PB.9942.1	8	2071	14	full-splice_match	gene: Zm00001d012108	Zm00001d012108_T00	38.62	40.88	coding	517	234	1784
PB.6910.3	5	2984	10	full-splice_match	gene: Zm00001d017126	Zm00001d017126_T00	33.19	98.45	coding	696	354	2441
PB.9097.1	7	1942	2	genic	gene: Zm00001d022587	novel	15.35	21.92	coding	506	81	1598
PB.9097.2	7	2238	1	full-splice_match	gene: Zm00001d022587	Zm00001d022587_T00	6.57	21.92	coding	552	173	1828
PB.7493.1	6	1217	5	full-splice_match	gene: Zm00001d036073	Zm00001d036073_T00	88.93	88.93	coding	288	162	1025

PB.6910.7	5	864	7	incomplete-splice_match	gene: Zm00001d017126	Zm00001d017126_T00	0.00	98.45	coding	274	42	863
PB.6910.6	5	2439	10	full-splice_match	gene: Zm00001d017126	Zm00001d017126_T00	0.00	98.45	coding	696	351	2438
PB.1230.5	1	1783	4	novel_in_catalog	gene: Zm00001d033056	novel	0.00	179.46	coding	357	26	1096
PB.8325.2	7	2988	12	novel_in_catalog	gene: Zm00001d019130	novel	6.87	29.36	coding	387	1505	2665
PB.9474.1	8	1274	8	full-splice_match	gene: Zm00001d010055	Zm00001d010055_T00	38.26	124.90	coding	209	190	816
PB.2976.2	2	1356	2	full-splice_match	gene: Zm00001d004443	Zm00001d004443_T00	27.00	58.50	coding	361	68	1150
PB.2976.1	2	1199	1	novel_in_catalog	gene: Zm00001d004443	novel	6.28	58.50	coding	314	50	991
PB.9474.2	8	1169	8	full-splice_match	gene: Zm00001d010055	Zm00001d010055_T00	86.64	124.90	coding	209	186	812
PB.3266.1	2	1873	7	full-splice_match	gene: Zm00001d006034	Zm00001d006034_T00	32.42	40.60	coding	377	353	1483
PB.3266.2	2	1485	4	novel_in_catalog	gene: Zm00001d006034	novel	1.93	40.60	coding	188	153	716
PB.4531.1	3	1547	7	novel_not_in_catalog	gene: Zm00001d043515	novel	5.51	52.60	coding	357	93	1163
PB.3289.1	2	835	1	full-splice_match	gene: Zm00001d006119	Zm00001d006119_T00	10.04	132.28	coding	159	38	514
PB.7786.2	6	1153	3	novel_not_in_catalog	gene: Zm00001d037637	novel	7.09	8.43	coding	222	230	895
PB.7786.1	6	1802	1	novel_in_catalog	gene: Zm00001d037637	novel	1.34	8.43	coding	124	1148	1519
PB.845.2	1	832	5	incomplete-splice_match	gene: Zm00001d031326	Zm00001d031326_T00	20.04	42.48	coding	190	1	570
PB.10555.6	9	703	1	full-splice_match	gene: Zm00001d047117	Zm00001d047117_T00	0.00	696.41	coding	129	225	611
PB.8481.1	7	1955	5	full-splice_match	gene: Zm00001d020140	Zm00001d020140_T00	7.22	24.86	coding	133	728	1126

PB.8481.6	7	1723	4	full-splice_match	gene: Zm00001d020140	Zm00001d020140_T01	0.00	24.86	coding	156	757	1224
PB.8481.7	7	1983	4	novel_in_catalog	gene: Zm00001d020140	novel	0.74	24.86	coding	133	712	1110
PB.77.1	1	944	3	full-splice_match	gene: Zm00001d027541	Zm00001d027541_T00	163.30	163.30	coding	226	59	736
PB.8481.5	7	1844	4	full-splice_match	gene: Zm00001d020140	Zm00001d020140_T01	16.90	24.86	coding	156	760	1227
PB.3068.1	2	1382	3	full-splice_match	gene: Zm00001d005028	Zm00001d005028_T00	16.26	16.26	coding	322	136	1101
PB.7262.6	5	1409	12	full-splice_match	gene: Zm00001d018498	Zm00001d018498_T00	19.43	68.81	coding	280	114	953
PB.7262.7	5	1498	11	novel_in_catalog	gene: Zm00001d018498	novel	2.66	68.81	coding	280	111	950
PB.8477.1	7	3507	6	fusion	gene: Zm00001d020110	novel	8.35	8.35	coding	1004	77	3088
PB.1928.2	10	2301	8	full-splice_match	gene: Zm00001d024525	Zm00001d024525_T00	0.00	93.66	coding	589	352	2118
PB.1928.1	10	2460	8	full-splice_match	gene: Zm00001d024525	Zm00001d024525_T00	93.66	93.66	coding	589	354	2120
PB.5279.1	4	488	4	novel_not_in_catalog	gene: Zm00001d051116	novel	70.53	70.53	non coding	NA	NA	NA
PB.10968.2	B73V4	2407	4	novel_not_in_catalog	gene: Zm00001d000396	novel	22.13	65.38	coding	602	447	2252
PB.2894.2	2	1115	2	full-splice_match	gene: Zm00001d003879	Zm00001d003879_T00	0.00	375.02	coding	273	96	914
PB.8306.1	7	2226	5	full-splice_match	gene: Zm00001d018983	Zm00001d018983_T00	14.15	14.15	coding	572	351	2066
PB.10968.6	B73V4	3520	3	novel_not_in_catalog	gene: Zm00001d000396	novel	5.67	65.38	coding	357	308	1378
PB.10968.4	B73V4	2405	4	novel_not_in_catalog	gene: Zm00001d000396	novel	37.58	65.38	coding	602	447	2252
PB.6702.1	5	2083	3	full-splice_match	gene: Zm00001d016149	Zm00001d016149_T00	22.11	22.39	coding	471	174	1586

PB.6017.1	5	1159	5	full-splice_match	gene: Zm00001d013022	Zm00001d013022_T00	22.09	30.50	coding	320	48	1007
PB.5259.1	4	2095	5	full-splice_match	gene: Zm00001d050985	Zm00001d050985_T00	30.86	68.48	coding	509	162	1688
PB.6017.3	5	686	1	novel_in_catalog	gene: Zm00001d013022	novel	8.41	30.50	non coding	NA	NA	NA
PB.5259.4	4	2729	5	full-splice_match	gene: Zm00001d050985	Zm00001d050985_T00	37.62	68.48	coding	531	954	2546
PB.859.1	1	1445	1	full-splice_match	gene: Zm00001d031434	Zm00001d031434_T00	8.41	8.41	coding	381	100	1242
PB.10431.7	9	2079	6	full-splice_match	gene: Zm00001d046454	Zm00001d046454_T00	0.14	100.26	coding	418	635	1888
PB.2570.1	2	862	2	antisense	novelGene_gene: Zm00001d002364	novel	12.01	12.01	non coding	NA	NA	NA
PB.2894.1	2	1220	2	full-splice_match	gene: Zm00001d003879	Zm00001d003879_T00	314.01	375.02	coding	273	100	918
PB.10779.10	9	1792	5	full-splice_match	gene: Zm00001d047981	Zm00001d047981_T00	191.25	352.56	coding	490	100	1569
PB.10779.11	9	1676	5	full-splice_match	gene: Zm00001d047981	Zm00001d047981_T00	9.08	352.56	coding	490	108	1577
PB.2062.1	10	1162	11	full-splice_match	gene: Zm00001d025185	Zm00001d025185_T00	180.76	180.76	coding	311	59	991
PB.10887.1	9	2918	9	novel_in_catalog	gene: Zm00001d048424	novel	310.42	310.42	coding	701	476	2578
PB.5879.5	4	2456	5	full-splice_match	gene: Zm00001d053890	Zm00001d053890_T00	0.00	7.14	coding	482	853	2298
PB.9602.1	8	1283	2	novel_not_in_catalog	gene: Zm00001d010653	novel	9.24	18.81	non coding	NA	NA	NA
PB.9602.2	8	1105	3	novel_not_in_catalog	gene: Zm00001d010653	novel	9.57	18.81	non coding	NA	NA	NA
PB.9602.4	8	946	2	novel_not_in_catalog	gene: Zm00001d010653	novel	0.00	18.81	non coding	NA	NA	NA
PB.7542.3	6	1135	6	novel_in_catalog	gene: Zm00001d036442	novel	4.52	479.54	coding	170	218	727

PB.7309.1	6	2792	9	full-splice_match	gene: Zm00001d035047	Zm00001d035047_T00	21.18	34.79	coding	771	216	2528
PB.7309.3	6	2779	9	full-splice_match	gene: Zm00001d035047	Zm00001d035047_T00	13.61	34.79	coding	771	268	2580
PB.10831.3	9	889	1	full-splice_match	gene: Zm00001d048214	Zm00001d048214_T00	0.00	2.32	coding	178	142	675
PB.677.2	1	829	3	novel_in_catalog	gene: Zm00001d030348	novel	5.38	81.97	non coding	NA	NA	NA
PB.10325.4	9	2015	14	full-splice_match	gene: Zm00001d045675	Zm00001d045675_T00	26.87	152.97	coding	591	23	1795
PB.3719.1	3	1199	2	full-splice_match	gene: Zm00001d039395	Zm00001d039395_T00	44.99	44.99	coding	226	210	887
PB.1738.1	10	1914	2	genic	gene: Zm00001d023447	novel	2.06	2.06	coding	501	252	1754
PB.10325.1	9	2312	14	full-splice_match	gene: Zm00001d045675	Zm00001d045675_T00	118.18	152.97	coding	591	259	2031
PB.10325.3	9	2219	13	full-splice_match	gene: Zm00001d045675	Zm00001d045675_T00	7.92	152.97	coding	316	156	1103
PB.3280.4	2	1788	14	full-splice_match	gene: Zm00001d006084	Zm00001d006084_T00	0.00	55.49	coding	490	58	1527
PB.3171.3	2	1069	5	novel_in_catalog	gene: Zm00001d005584	novel	2.43	21.19	coding	215	135	779
PB.4382.1	3	2061	1	full-splice_match	gene: Zm00001d042953	Zm00001d042953_T00	40.50	40.50	coding	565	87	1781
PB.1861.1	10	1677	2	intergenic	novelGene_137	novel	4.53	18.74	non coding	NA	NA	NA
PB.3280.1	2	1885	14	full-splice_match	gene: Zm00001d006084	Zm00001d006084_T00	45.72	55.49	coding	490	54	1523
PB.3280.3	2	1759	13	novel_in_catalog	gene: Zm00001d006084	novel	9.77	55.49	coding	393	75	1253
PB.4684.5	3	2152	6	novel_in_catalog	gene: Zm00001d044095	novel	14.82	327.19	coding	167	1344	1844
PB.4684.4	3	2684	4	novel_in_catalog	gene: Zm00001d044095	novel	5.45	327.19	coding	14	385	426

PB.4684.7	3	3006	1	novel_in_catalog	gene: Zm00001d044095	novel	5.93	327.19	coding	14	368	409
PB.7538.5	6	2967	4	novel_in_catalog	gene: Zm00001d036420	novel	10.14	21.47	non coding	NA	NA	NA
PB.9953.5	8	1173	9	full-splice_match	gene: Zm00001d012161	Zm00001d012161_T00	479.61	528.84	coding	302	77	982
PB.9953.4	8	1301	8	novel_in_catalog	gene: Zm00001d012161	novel	49.22	528.84	coding	278	55	888
PB.4684.3	3	1205	6	novel_in_catalog	gene: Zm00001d044095	novel	106.85	327.19	coding	121	113	475
PB.7538.1	6	2250	1	incomplete-splice_match	gene: Zm00001d036420	Zm00001d036420_T00	11.33	21.47	non coding	NA	NA	NA
PB.5690.1	4	669	2	incomplete-splice_match	gene: Zm00001d053003	Zm00001d053003_T00	340.53	340.53	non coding	NA	NA	NA
PB.3779.1	3	1387	5	full-splice_match	gene: Zm00001d039614	Zm00001d039614_T00	55.20	55.20	coding	343	105	1133
PB.6868.1	5	994	3	incomplete-splice_match	gene: Zm00001d016963	Zm00001d016963_T00	41.65	41.65	coding	204	93	704
PB.8413.1	7	1130	2	intergenic	novelGene_143	novel	9.17	9.17	coding	191	279	851
PB.6899.35	5	1343	8	novel_in_catalog	gene: Zm00001d017096	novel	14.81	254.51	coding	226	425	1102
PB.950.1	1	1693	4	full-splice_match	gene: Zm00001d031913	Zm00001d031913_T00	29.66	37.65	coding	393	131	1309
PB.3287.2	2	940	1	full-splice_match	gene: Zm00001d006111	Zm00001d006111_T00	408.52	510.20	coding	220	78	737
PB.6899.36	5	2274	7	novel_in_catalog	gene: Zm00001d017096	novel	0.00	254.51	coding	171	106	618
PB.3292.1	2	1935	10	full-splice_match	gene: Zm00001d006128	Zm00001d006128_T00	27.62	27.62	coding	572	22	1737
PB.6899.30	5	2200	8	novel_in_catalog	gene: Zm00001d017096	novel	0.81	254.51	coding	129	108	494
PB.6899.33	5	2629	3	novel_in_catalog	gene: Zm00001d017096	novel	1.75	254.51	coding	130	106	495

PB.6899.32	5	1979	9	full-splice_match	gene: Zm00001d017096	Zm00001d017096_T02	3.93	254.51	coding	129	108	494
PB.6984.8	5	963	9	full-splice_match	gene: Zm00001d017448	Zm00001d017448_T00	0.00	50.18	coding	254	17	778
PB.10952.3	B73V4	1153	7	full-splice_match	gene: Zm00001d000241	Zm00001d000241_T00	26.60	29.48	coding	206	205	822
PB.2177.1	10	2232	7	incomplete-splice_match	gene: Zm00001d025830	Zm00001d025830_T00	42.45	42.45	coding	436	400	1707
PB.6899.39	5	1562	12	full-splice_match	gene: Zm00001d017096	Zm00001d017096_T00	14.99	254.51	coding	445	102	1436
PB.10952.4	B73V4	1153	6	novel_in_catalog	gene: Zm00001d000241	novel	2.88	29.48	coding	151	204	656
PB.10339.1	9	966	2	full-splice_match	gene: Zm00001d045778	Zm00001d045778_T00	6.71	6.71	coding	275	52	876
PB.249.8	1	5257	11	full-splice_match	gene: Zm00001d028330	Zm00001d028330_T10	16.25	102.09	coding	123	364	732
PB.845.4	1	1990	16	incomplete-splice_match	gene: Zm00001d031326	Zm00001d031326_T00	5.22	42.48	coding	533	392	1990
PB.845.3	1	2305	17	full-splice_match	gene: Zm00001d031326	Zm00001d031326_T00	17.22	42.48	coding	588	388	2151
PB.7852.2	6	1532	1	novel_in_catalog	gene: Zm00001d037935	novel	1.87	9.58	coding	126	1155	1532
PB.7852.1	6	1585	2	full-splice_match	gene: Zm00001d037935	Zm00001d037935_T00	7.70	9.58	coding	451	79	1431
PB.249.1	1	6135	11	novel_in_catalog	gene: Zm00001d028330	novel	14.96	102.09	coding	123	365	733
PB.7542.4	6	1675	6	novel_in_catalog	gene: Zm00001d036442	novel	2.26	479.54	coding	176	990	1517
PB.6702.2	5	1895	3	full-splice_match	gene: Zm00001d016149	Zm00001d016149_T00	0.28	22.39	coding	471	207	1619
PB.249.2	1	2464	11	incomplete-splice_match	gene: Zm00001d028330	Zm00001d028330_T02	14.30	102.09	coding	209	1091	1717
PB.7542.1	6	1095	7	full-splice_match	gene: Zm00001d036442	Zm00001d036442_T00	138.76	479.54	coding	215	92	736

PB.1250.3	1	1591	3	novel_in_catalog	gene: Zm00001d033174	novel	16.06	779.80	coding	272	123	938
PB.249.7	1	5113	13	novel_in_catalog	gene: Zm00001d028330	novel	30.66	102.09	coding	123	411	779
PB.7542.2	6	1038	7	full-splice_match	gene: Zm00001d036442	Zm00001d036442_T00	333.99	479.54	coding	215	222	866
PB.1250.8	1	1432	4	full-splice_match	gene: Zm00001d033174	Zm00001d033174_T00	110.54	779.80	coding	364	120	1211
PB.3813.1	3	1434	1	full-splice_match	gene: Zm00001d039788	Zm00001d039788_T00	50.85	50.85	coding	362	106	1191
PB.5162.1	4	1426	1	incomplete-splice_match	gene: Zm00001d050227	Zm00001d050227_T00	27.70	27.70	coding	264	380	1171
PB.7240.1	5	2717	2	full-splice_match	gene: Zm00001d018428	Zm00001d018428_T00	46.29	46.29	coding	739	86	2302
PB.966.1	1	1505	2	full-splice_match	gene: Zm00001d031971	Zm00001d031971_T00	537.41	537.41	coding	377	154	1284
PB.6202.1	5	858	1	antisense	novelGene_gene: Zm00001d013703	novel	4.52	4.52	coding	196	27	614
PB.2687.2	2	1755	7	novel_not_in_catalog	gene: Zm00001d002891	novel	14.33	14.90	coding	286	326	1183
PB.2687.3	2	1978	3	novel_not_in_catalog	gene: Zm00001d002891	novel	0.57	14.90	non coding	NA	NA	NA
PB.1250.5	1	2520	1	novel_in_catalog	gene: Zm00001d033174	novel	7.78	779.80	coding	173	113	631
PB.10453.1	9	3363	10	novel_in_catalog	gene: Zm00001d046549	novel	31.82	31.82	coding	995	81	3065
PB.2890.10	2	1763	6	full-splice_match	gene: Zm00001d003857	Zm00001d003857_T00	0.00	452.88	coding	534	69	1670
PB.1427.1	1	1270	5	full-splice_match	gene: Zm00001d033976	Zm00001d033976_T00	375.99	378.11	coding	237	214	924
PB.3970.1	3	2943	3	novel_in_catalog	gene: Zm00001d040563	novel	8.46	8.46	non coding	NA	NA	NA
PB.7641.1	6	849	1	novel_in_catalog	gene: Zm00001d036972	novel	37.05	37.05	coding	130	84	473

PB.1727.3	10	1296	2	full-splice_match	gene: Zm00001d023396	Zm00001d023396_T00	4.21	4.21	coding	279	101	937
PB.3705.4	3	4324	7	full-splice_match	gene: Zm00001d039341	Zm00001d039341_T01	59.49	79.17	coding	1273	127	3945
PB.3705.5	3	1677	1	incomplete-splice_match	gene: Zm00001d039341	Zm00001d039341_T01	0.00	79.17	coding	543	48	1676
PB.667.1	1	1767	3	full-splice_match	gene: Zm00001d030303	Zm00001d030303_T00	15.87	15.87	coding	384	251	1402
PB.3705.3	3	5308	7	full-splice_match	gene: Zm00001d039341	Zm00001d039341_T01	19.68	79.17	coding	1193	1348	4926
PB.3630.3	2	1624	11	full-splice_match	gene: Zm00001d007806	Zm00001d007806_T00	32.07	44.89	coding	323	350	1318
PB.1727.1	10	751	2	full-splice_match	gene: Zm00001d023395	Zm00001d023395_T00	63.88	63.88	coding	107	71	391
PB.3630.4	2	1913	10	full-splice_match	gene: Zm00001d007806	Zm00001d007806_T00	3.51	44.89	coding	323	795	1763
PB.3619.1	2	1233	5	full-splice_match	gene: Zm00001d007767	Zm00001d007767_T00	392.41	412.11	coding	222	251	916
PB.1401.1	1	1360	2	full-splice_match	gene: Zm00001d033849	Zm00001d033849_T00	9.70	9.70	coding	319	65	1021
PB.812.1	1	922	2	full-splice_match	gene: Zm00001d031168	Zm00001d031168_T00	3426.12	7101.71	coding	155	102	566
PB.4710.1	3	3327	8	full-splice_match	gene: Zm00001d044185	Zm00001d044185_T00	23.51	178.87	coding	302	2146	3051
PB.1642.1	1	1701	13	full-splice_match	gene: Zm00001d034809	Zm00001d034809_T03	61.70	87.36	coding	408	190	1413
PB.10982.1	B73V4	3249	7	full-splice_match	gene: Zm00001d000147	Zm00001d000147_T00	2.78	2.78	coding	737	760	2970
PB.3793.5	3	1050	7	full-splice_match	gene: Zm00001d039683	Zm00001d039683_T00	12.77	12.77	coding	276	35	862
PB.1199.1	1	2333	1	genic	gene: Zm00001d032909	novel	29.30	29.30	coding	746	7	2244
PB.3097.1	2	841	3	full-splice_match	gene: Zm00001d005158	Zm00001d005158_T00	33.49	33.49	coding	203	23	631

PB.9501.1	8	1994	4	novel_not_in_catalog	gene: Zm00001d010222	novel	16.14	17.56	coding	441	320	1642
PB.9501.2	8	1911	4	novel_not_in_catalog	gene: Zm00001d010222	novel	1.42	17.56	coding	441	345	1667
PB.1900.1	10	2313	1	full-splice_match	gene: Zm00001d024416	Zm00001d024416_T00	20.74	32.85	coding	637	149	2059
PB.10855.2	9	1764	1	incomplete-splice_match	gene: Zm00001d048320	Zm00001d048320_T00	5.49	44.80	coding	119	1272	1628
PB.10855.1	9	1199	2	full-splice_match	gene: Zm00001d048320	Zm00001d048320_T00	39.31	44.80	coding	329	46	1032
PB.1900.2	10	2206	1	full-splice_match	gene: Zm00001d024416	Zm00001d024416_T00	12.12	32.85	coding	637	155	2065
PB.7948.2	6	1136	5	novel_in_catalog	gene: Zm00001d038258	novel	6.06	36.26	coding	210	128	757
PB.409.2	1	2188	6	novel_not_in_catalog	gene: Zm00001d028987	novel	60.13	60.13	coding	619	107	1963
PB.7948.1	6	1095	6	novel_not_in_catalog	gene: Zm00001d038258	novel	30.20	36.26	coding	215	199	843
PB.8754.1	7	2632	4	full-splice_match	gene: Zm00001d021425	Zm00001d021425_T00	7.90	7.90	coding	828	52	2535
PB.9861.2	8	1240	2	novel_in_catalog	gene: Zm00001d011786	novel	3.91	233.61	non coding	NA	NA	NA
PB.4178.3	3	635	4	full-splice_match	gene: Zm00001d042017	Zm00001d042017_T00	48.98	58.20	non coding	NA	NA	NA
PB.3277.1	2	2879	2	incomplete-splice_match	gene: Zm00001d006079	Zm00001d006079_T00	10.88	17.57	coding	763	24	2312
PB.7501.2	6	1278	7	full-splice_match	gene: Zm00001d036123	Zm00001d036123_T00	16.57	34.97	coding	358	63	1136
PB.1285.5	1	1967	8	intergenic	novelGene_453	novel	10.47	28.16	coding	73	97	315
PB.2376.1	10	2464	3	full-splice_match	gene: Zm00001d026587	Zm00001d026587_T00	20.84	20.84	coding	679	223	2259
PB.828.1	1	1627	2	novel_not_in_catalog	gene: Zm00001d031231	novel	3.05	29.39	non coding	NA	NA	NA

PB.10894.2	9	1022	8	full-splice_match	gene: Zm00001d048450	Zm00001d048450_T00	28.09	41.22	coding	218	128	781
PB.352.1	1	1190	3	full-splice_match	gene: Zm00001d028731	Zm00001d028731_T00	22.43	55.87	coding	227	126	806
PB.352.2	1	1223	3	full-splice_match	gene: Zm00001d028731	Zm00001d028731_T00	33.44	55.87	coding	227	286	966
PB.4446.1	3	1694	8	novel_not_in_catalog	gene: Zm00001d043211	novel	7.71	19.06	coding	290	345	1214
PB.6117.1	5	510	1	intergenic	novelGene_44	novel	119.44	119.44	non coding	NA	NA	NA
PB.1012.4	1	1713	2	genic	gene: Zm00001d032187	novel	31.98	534.29	coding	394	198	1379
PB.7501.1	6	1623	7	full-splice_match	gene: Zm00001d036123	Zm00001d036123_T00	18.40	34.97	coding	358	207	1280
PB.5806.1	4	3151	14	incomplete-splice_match	gene: Zm00001d053612	Zm00001d053612_T00	14.50	14.50	coding	940	66	2885
PB.4446.4	3	2374	10	novel_not_in_catalog	gene: Zm00001d043211	novel	0.00	19.06	coding	588	187	1950
PB.1191.1	1	1238	2	full-splice_match	gene: Zm00001d032882	Zm00001d032882_T00	26.15	26.15	coding	257	149	919
PB.2524.2	2	2227	2	incomplete-splice_match	gene: Zm00001d002149	Zm00001d002149_T00	25.45	48.76	coding	555	199	1863
PB.2524.3	2	1276	1	incomplete-splice_match	gene: Zm00001d002149	Zm00001d002149_T00	12.70	48.76	coding	334	81	1082
PB.2524.1	2	2466	2	incomplete-splice_match	gene: Zm00001d002149	Zm00001d002149_T00	10.61	48.76	coding	555	308	1972
PB.1397.1	1	1847	10	full-splice_match	gene: Zm00001d033830	Zm00001d033830_T00	61.91	61.91	coding	488	69	1532
PB.6383.1	5	1363	10	novel_in_catalog	gene: Zm00001d014533	novel	35.55	35.55	coding	306	63	980
PB.9788.6	8	848	5	full-splice_match	gene: Zm00001d011500	Zm00001d011500_T00	403.74	501.01	coding	147	168	608
PB.9788.5	8	1342	4	full-splice_match	gene: Zm00001d011500	Zm00001d011500_T01	0.00	501.01	coding	120	566	925

PB.667.2	1	1621	3	full-splice_match	gene: Zm00001d030303	Zm00001d030303_T00	0.00	15.87	coding	384	251	1402
PB.9788.3	8	1532	4	full-splice_match	gene: Zm00001d011500	Zm00001d011500_T01	7.59	501.01	coding	120	571	930
PB.9788.2	8	1427	1	novel_in_catalog	gene: Zm00001d011500	novel	2.10	501.01	non coding	NA	NA	NA
PB.9788.1	8	1305	5	full-splice_match	gene: Zm00001d011500	Zm00001d011500_T00	21.83	501.01	coding	147	265	705
PB.3238.10	2	2164	14	full-splice_match	gene: Zm00001d005925	Zm00001d005925_T00	0.00	190.45	coding	616	129	1976
PB.4185.2	3	2510	6	novel_not_in_catalog	gene: Zm00001d042042	novel	11.17	35.71	coding	324	258	1229
PB.4185.1	3	2588	6	full-splice_match	gene: Zm00001d042042	Zm00001d042042_T00	24.55	35.71	coding	333	347	1345
PB.6891.4	5	2629	1	novel_in_catalog	gene: Zm00001d017085	novel	6.53	291.06	coding	358	1346	2419
PB.8873.1	7	2331	5	full-splice_match	gene: Zm00001d021823	Zm00001d021823_T00	55.61	78.93	coding	614	11	1852
PB.6848.3	5	1598	7	full-splice_match	gene: Zm00001d016866	Zm00001d016866_T00	7.34	42.40	coding	393	107	1285
PB.6891.1	5	1927	2	full-splice_match	gene: Zm00001d017085	Zm00001d017085_T00	93.07	291.06	coding	388	307	1470
PB.8873.2	7	1439	1	novel_in_catalog	gene: Zm00001d021823	novel	2.99	78.93	non coding	NA	NA	NA
PB.5759.1	4	1976	6	full-splice_match	gene: Zm00001d053354	Zm00001d053354_T00	20.61	23.15	coding	537	168	1778
PB.10086.1	8	593	1	full-splice_match	gene: Zm00001d012714	Zm00001d012714_T00	85.95	85.95	non coding	NA	NA	NA
PB.4492.1	3	1406	1	full-splice_match	gene: Zm00001d043390	Zm00001d043390_T00	50.76	303.27	coding	297	35	925
PB.9814.1	8	6973	23	full-splice_match	gene: Zm00001d011610	Zm00001d011610_T00	35.57	158.78	coding	2163	267	6755
PB.7244.2	5	1807	11	full-splice_match	gene: Zm00001d018438	Zm00001d018438_T00	2.85	4.89	coding	429	357	1643

PB.6859.3	5	2081	8	full-splice_match	gene: Zm00001d016902	Zm00001d016902_T00	0.00	84.05	coding	554	208	1869
PB.4454.4	3	3052	5	novel_in_catalog	gene: Zm00001d043256	novel	4.07	28.15	coding	566	174	1871
PB.6671.3	5	2320	13	novel_in_catalog	gene: Zm00001d015948	novel	1.11	47.70	coding	360	1030	2109
PB.4454.6	3	3136	4	novel_in_catalog	gene: Zm00001d043256	novel	0.00	28.15	coding	566	170	1867
PB.4454.7	3	2598	6	novel_in_catalog	gene: Zm00001d043256	novel	0.00	28.15	coding	704	395	2506
PB.9111.1	7	1046	7	full-splice_match	gene: Zm00001d022631	Zm00001d022631_T00	47.48	47.48	coding	216	151	798
PB.4454.2	3	2999	6	novel_in_catalog	gene: Zm00001d043256	novel	20.87	28.15	coding	864	173	2764
PB.6984.7	5	1127	8	full-splice_match	gene: Zm00001d017448	Zm00001d017448_T00	0.54	50.18	coding	153	483	941
PB.10758.1	9	3524	1	intergenic	novelGene_283	novel	6.60	6.60	coding	975	313	3237
PB.8461.1	7	1737	9	full-splice_match	gene: Zm00001d020040	Zm00001d020040_T00	5.90	22.73	coding	309	287	1213
PB.8461.2	7	1669	9	full-splice_match	gene: Zm00001d020040	Zm00001d020040_T00	16.83	22.73	coding	309	447	1373
PB.9374.3	8	1364	12	novel_not_in_catalog	gene: Zm00001d009504	novel	0.00	60.35	coding	382	218	1363
PB.777.3	1	2305	13	novel_in_catalog	gene: Zm00001d031040	novel	0.00	12.31	coding	356	153	1220
PB.4199.1	3	1740	6	novel_in_catalog	gene: Zm00001d042088	novel	12.19	12.19	coding	474	98	1519
PB.9843.1	8	1770	1	full-splice_match	gene: Zm00001d011721	Zm00001d011721_T00	45.69	45.69	coding	399	211	1407
PB.1427.4	1	668	3	incomplete-splice_match	gene: Zm00001d033976	Zm00001d033976_T00	0.00	378.11	coding	173	148	666
PB.1517.1	1	1551	3	full-splice_match	gene: Zm00001d034360	Zm00001d034360_T00	6.51	6.51	coding	369	104	1210

PB.1537.1	1	2425	1	genic	gene: Zm00001d034434	novel	14.03	37.65	coding	75	93	317
PB.7750.3	6	1070	4	full-splice_match	gene: Zm00001d037483	Zm00001d037483_T00	67.75	181.99	coding	221	180	842
PB.7750.1	6	1172	4	full-splice_match	gene: Zm00001d037483	Zm00001d037483_T00	114.24	181.99	coding	221	146	808
PB.904.3	1	1988	4	novel_not_in_catalog	gene: Zm00001d031662	novel	13.92	23.58	coding	498	146	1639
PB.6782.1	5	2043	5	full-splice_match	gene: Zm00001d016591	Zm00001d016591_T00	71.88	71.88	coding	509	305	1831
PB.904.1	1	2091	3	novel_in_catalog	gene: Zm00001d031662	novel	9.66	23.58	coding	465	145	1539
PB.8514.1	7	2120	6	full-splice_match	gene: Zm00001d020353	Zm00001d020353_T00	8.47	8.47	coding	325	121	1095
PB.10064.1	8	646	3	full-splice_match	gene: Zm00001d012632	Zm00001d012632_T00	10.08	10.08	non coding	NA	NA	NA
PB.4763.1	3	677	1	full-splice_match	gene: Zm00001d044425	Zm00001d044425_T00	14.97	14.97	coding	55	175	339
PB.1602.3	1	1776	1	novel_in_catalog	gene: Zm00001d034675	novel	1.85	65.52	non coding	NA	NA	NA
PB.8387.1	7	500	3	novel_not_in_catalog	gene: Zm00001d019553	novel	756.83	756.83	non coding	NA	NA	NA
PB.8052.2	6	1704	1	genic	gene: Zm00001d038605	novel	20.84	36.15	coding	493	38	1516
PB.8052.3	6	1634	1	genic	gene: Zm00001d038605	novel	0.00	36.15	coding	493	68	1546
PB.8052.1	6	1805	1	genic	gene: Zm00001d038605	novel	15.31	36.15	coding	493	11	1489
PB.7035.2	5	1764	14	full-splice_match	gene: Zm00001d017654	Zm00001d017654_T00	25.30	31.85	coding	452	51	1406
PB.7035.3	5	1626	14	full-splice_match	gene: Zm00001d017654	Zm00001d017654_T00	0.00	31.85	coding	452	52	1407
PB.1642.2	1	2359	13	full-splice_match	gene: Zm00001d034809	Zm00001d034809_T00	15.83	87.36	coding	321	201	1163

PB.7035.1	5	2145	13	full-splice_match	gene: Zm00001d017654	Zm00001d017654_T00	6.56	31.85	coding	286	66	923
PB.3441.9	2	1339	4	incomplete-splice_match	gene: Zm00001d006759	Zm00001d006759_T00	0.00	47.31	coding	244	332	1063
PB.3690.3	3	1611	10	novel_in_catalog	gene: Zm00001d039294	novel	6.32	13.36	coding	381	195	1337
PB.3690.2	3	1709	11	full-splice_match	gene: Zm00001d039294	Zm00001d039294_T00	7.04	13.36	coding	380	222	1361
PB.5118.1	4	1588	13	full-splice_match	gene: Zm00001d049975	Zm00001d049975_T00	80.18	84.38	coding	369	188	1294
PB.5025.3	4	1090	7	full-splice_match	gene: Zm00001d049496	Zm00001d049496_T00	71.18	178.51	coding	215	200	844
PB.5025.2	4	1173	7	full-splice_match	gene: Zm00001d049496	Zm00001d049496_T00	86.12	178.51	coding	215	168	812
PB.5025.1	4	1264	7	full-splice_match	gene: Zm00001d049496	Zm00001d049496_T00	21.21	178.51	coding	215	131	775
PB.10641.13	9	1175	14	full-splice_match	gene: Zm00001d047498	Zm00001d047498_T00	10.96	128.79	coding	285	162	1016
PB.429.1	1	1624	9	incomplete-splice_match	gene: Zm00001d029055	Zm00001d029055_T02	10.68	55.25	coding	507	102	1622
PB.901.1	1	607	2	full-splice_match	gene: Zm00001d031651	Zm00001d031651_T00	46.57	46.57	non coding	NA	NA	NA
PB.5025.5	4	732	6	incomplete-splice_match	gene: Zm00001d049496	Zm00001d049496_T00	0.00	178.51	coding	183	184	732
PB.9460.1	8	1471	2	incomplete-splice_match	gene: Zm00001d009967	Zm00001d009967_T00	29.00	29.00	coding	390	99	1268
PB.10750.1	9	2169	8	full-splice_match	gene: Zm00001d047869	Zm00001d047869_T00	25.05	43.80	coding	305	960	1874
PB.5178.1	4	1275	7	incomplete-splice_match	gene: Zm00001d050336	Zm00001d050336_T00	2.02	12.73	coding	156	476	943
PB.5178.2	4	1901	10	full-splice_match	gene: Zm00001d050336	Zm00001d050336_T00	10.72	12.73	coding	578	15	1748
PB.10750.4	9	1408	8	novel_in_catalog	gene: Zm00001d047869	novel	11.72	43.80	coding	236	370	1077

PB.4191.2	3	1933	11	novel_not_in_catalog	gene: Zm00001d042055	novel	2.32	13.25	coding	436	62	1369
PB.10797.1	9	4374	2	full-splice_match	gene: Zm00001d048074	Zm00001d048074_T00	28.26	28.26	coding	1364	34	4125
PB.3570.3	2	471	2	full-splice_match	gene: Zm00001d007434	Zm00001d007434_T00	29.17	72.29	coding	59	130	306
PB.1798.1	10	3286	22	full-splice_match	gene: Zm00001d023741	Zm00001d023741_T00	92.22	107.70	coding	954	122	2983
PB.1798.2	10	3204	22	full-splice_match	gene: Zm00001d023741	Zm00001d023741_T00	15.48	107.70	coding	954	127	2988
PB.6218.8	5	816	4	novel_not_in_catalog	gene: Zm00001d013777	novel	1349.95	1538.01	coding	220	62	721
PB.7580.5	6	1580	3	novel_not_in_catalog	gene: Zm00001d036626	novel	0.00	232.37	coding	445	73	1407
PB.7968.1	6	1790	5	full-splice_match	gene: Zm00001d038321	Zm00001d038321_T00	14.47	20.58	coding	432	195	1490
PB.6218.7	5	1461	1	novel_in_catalog	gene: Zm00001d013777	novel	11.66	1538.01	coding	183	305	853
PB.7029.1	5	1715	2	antisense	novelGene_gene: Zm00001d017641	novel	2.09	2.09	non coding	NA	NA	NA
PB.6218.1	5	1301	4	novel_not_in_catalog	gene: Zm00001d013777	novel	172.88	1538.01	coding	220	62	721
PB.5469.1	4	3116	3	novel_not_in_catalog	gene: Zm00001d052064	novel	8.86	8.86	coding	420	625	1884
PB.6218.3	5	947	5	novel_not_in_catalog	gene: Zm00001d013777	novel	3.51	1538.01	coding	220	67	726
PB.9581.1	8	2189	7	novel_in_catalog	gene: Zm00001d010579	novel	43.10	44.87	coding	541	169	1791
PB.9581.2	8	2079	7	novel_in_catalog	gene: Zm00001d010579	novel	1.77	44.87	coding	541	181	1803
PB.3664.1	2	834	6	full-splice_match	gene: Zm00001d007940	Zm00001d007940_T00	41.98	41.98	coding	160	116	595
PB.4171.2	3	1247	2	full-splice_match	gene: Zm00001d041987	Zm00001d041987_T00	60.14	97.44	coding	341	48	1070

PB.4171.1	3	1355	2	full-splice_match	gene: Zm00001d041987	Zm00001d041987_T00	37.30	97.44	coding	341	48	1070
PB.10657.3	9	1800	10	full-splice_match	gene: Zm00001d047566	Zm00001d047566_T01	0.00	37.66	coding	237	709	1419
PB.10657.2	9	1604	11	full-splice_match	gene: Zm00001d047566	Zm00001d047566_T00	22.79	37.66	coding	333	147	1145
PB.10305.1	9	1472	4	full-splice_match	gene: Zm00001d045579	Zm00001d045579_T00	10.66	10.66	coding	443	28	1356
PB.4333.1	3	2367	4	novel_in_catalog	gene: Zm00001d042731	novel	6.71	100.59	coding	376	793	1920
PB.1699.1	10	1607	6	full-splice_match	gene: Zm00001d023271	Zm00001d023271_T00	5.31	5.31	coding	337	166	1176
PB.10657.4	9	1453	11	full-splice_match	gene: Zm00001d047566	Zm00001d047566_T01	7.38	37.66	coding	333	173	1171
PB.2203.1	10	2270	3	novel_in_catalog	gene: Zm00001d025941	novel	2.58	13.11	non coding	NA	NA	NA
PB.2203.2	10	1982	4	novel_in_catalog	gene: Zm00001d025941	novel	10.53	13.11	non coding	NA	NA	NA
PB.2680.2	2	419	1	intergenic	novelGene_98	novel	204.90	396.97	non coding	NA	NA	NA
PB.7191.1	5	1304	5	novel_not_in_catalog	gene: Zm00001d018255	novel	27.61	111.57	coding	271	57	869
PB.421.1	1	1857	5	full-splice_match	gene: Zm00001d029031	Zm00001d029031_T00	19.64	19.64	coding	524	136	1707
PB.4463.1	3	2233	6	incomplete-splice_match	gene: Zm00001d043286	Zm00001d043286_T00	19.71	19.71	coding	132	255	650
PB.9870.1	8	920	4	full-splice_match	gene: Zm00001d011826	Zm00001d011826_T00	53.65	53.65	coding	150	162	611
PB.1954.3	10	1738	1	full-splice_match	gene: Zm00001d024717	Zm00001d024717_T00	6.55	6.55	coding	501	25	1527
PB.8629.2	7	908	2	full-splice_match	gene: Zm00001d020910	Zm00001d020910_T00	1669.02	1669.02	coding	171	9	521
PB.917.2	1	2055	2	novel_in_catalog	gene: Zm00001d031732	novel	61.99	316.35	coding	180	441	980

PB.4053.2	3	1718	3	intergenic	novelGene_225	novel	3.99	24.54	coding	141	987	1409
PB.3078.1	2	5767	3	intergenic	novelGene_312	novel	34.67	34.67	coding	50	245	394
PB.2680.1	2	655	1	intergenic	novelGene_98	novel	192.07	396.97	coding	20	514	573
PB.6152.4	5	1714	2	full-splice_match	gene: Zm00001d013468	Zm00001d013468_T00	0.00	92.12	coding	431	245	1537
PB.6660.4	5	1873	10	fusion	gene: Zm00001d015846	novel	18.04	37.67	coding	495	248	1732
PB.702.1	1	2425	6	full-splice_match	gene: Zm00001d030593	Zm00001d030593_T00	41.58	44.85	coding	733	56	2254
PB.6660.1	5	2770	8	fusion	gene: Zm00001d015846	novel	5.95	37.67	coding	264	1711	2502
PB.5031.1	4	1377	6	full-splice_match	gene: Zm00001d049526	Zm00001d049526_T00	54.66	59.33	coding	275	258	1082
PB.5031.2	4	1325	6	full-splice_match	gene: Zm00001d049526	Zm00001d049526_T00	0.00	59.33	coding	275	258	1082
PB.5031.3	4	1670	5	novel_in_catalog	gene: Zm00001d049526	novel	4.67	59.33	coding	126	258	635
PB.435.2	1	1475	10	full-splice_match	gene: Zm00001d029121	Zm00001d029121_T00	0.00	27.78	coding	339	87	1103
PB.2786.2	2	1480	5	incomplete-splice_match	gene: Zm00001d003329	Zm00001d003329_T01	5.94	26.42	coding	132	175	570
PB.2786.3	2	2820	9	full-splice_match	gene: Zm00001d003329	Zm00001d003329_T00	20.49	26.42	coding	765	198	2492
PB.5870.2	4	2519	6	full-splice_match	gene: Zm00001d053852	Zm00001d053852_T00	1.59	41.26	coding	685	182	2236
PB.2213.1	10	828	2	full-splice_match	gene: Zm00001d026012	Zm00001d026012_T00	21.77	21.77	coding	164	118	609
PB.4640.2	3	1124	5	full-splice_match	gene: Zm00001d043878	Zm00001d043878_T00	0.00	153.72	coding	269	51	857
PB.8143.1	6	932	6	full-splice_match	gene: Zm00001d038946	Zm00001d038946_T00	15.04	15.04	coding	184	5	556

PB.5755.3	4	2045	15	novel_not_in_catalog	gene: Zm00001d053334	novel	4.54	58.57	coding	306	235	1152
PB.3243.1	2	1521	1	full-splice_match	gene: Zm00001d005940	Zm00001d005940_T00	19.42	19.42	coding	357	129	1199
PB.5884.3	4	1160	1	full-splice_match	gene: Zm00001d053908	Zm00001d053908_T00	27.00	508.09	coding	249	86	832
PB.3660.11	2	2346	6	intergenic	novelGene_443	novel	2.11	53.91	coding	279	1209	2045
PB.1577.5	1	2044	12	full-splice_match	gene: Zm00001d034602	Zm00001d034602_T00	5.89	369.66	coding	593	69	1847
PB.6576.2	5	1071	1	incomplete-splice_match	gene: Zm00001d015293	Zm00001d015293_T00	10.57	85.91	coding	316	8	955
PB.6576.1	5	2916	4	full-splice_match	gene: Zm00001d015293	Zm00001d015293_T00	75.34	85.91	coding	753	114	2372
PB.4567.21	3	2579	8	novel_in_catalog	gene: Zm00001d043649	novel	6.58	120.68	coding	125	803	1177
PB.1625.4	1	1045	9	incomplete-splice_match	gene: Zm00001d034750	Zm00001d034750_T00	0.00	28.37	coding	328	60	1043
PB.10772.1	9	869	5	full-splice_match	gene: Zm00001d047958	Zm00001d047958_T00	104.83	453.42	coding	127	160	540
PB.10772.3	9	751	5	full-splice_match	gene: Zm00001d047958	Zm00001d047958_T00	348.59	453.42	coding	127	151	531
PB.7728.4	6	4632	29	incomplete-splice_match	gene: Zm00001d037377	Zm00001d037377_T02	6.50	59.63	coding	1423	43	4311
PB.7728.3	6	891	9	incomplete-splice_match	gene: Zm00001d037377	Zm00001d037377_T00	0.00	59.63	coding	286	34	891
PB.7728.2	6	3094	22	incomplete-splice_match	gene: Zm00001d037377	Zm00001d037377_T03	5.26	59.63	coding	992	119	3094
PB.7728.1	6	1882	10	incomplete-splice_match	gene: Zm00001d037377	Zm00001d037377_T00	47.87	59.63	coding	413	195	1433
PB.4798.2	3	1915	15	incomplete-splice_match	gene: Zm00001d044540	Zm00001d044540_T00	30.47	30.47	coding	540	56	1675
PB.1195.7	1	1912	3	novel_not_in_catalog	gene: Zm00001d032897	novel	12.84	155.96	coding	245	1063	1797

PB.6877.2	5	694	2	full-splice_match	gene: Zm00001d017019	Zm00001d017019_T00	111.48	130.81	coding	122	179	544
PB.6455.5	5	1535	11	incomplete-splice_match	gene: Zm00001d014792	Zm00001d014792_T00	177.37	550.89	coding	490	66	1535
PB.6455.4	5	1315	5	novel_in_catalog	gene: Zm00001d014792	novel	373.52	550.89	coding	353	47	1105
PB.10124.2	9	1763	6	full-splice_match	gene: Zm00001d044812	Zm00001d044812_T00	14.75	61.46	coding	486	47	1504
PB.10124.1	9	2350	6	full-splice_match	gene: Zm00001d044812	Zm00001d044812_T00	46.71	61.46	coding	601	160	1962
PB.10391.1	9	1351	3	full-splice_match	gene: Zm00001d046186	Zm00001d046186_T00	26.30	26.66	coding	323	113	1081
PB.10391.2	9	1367	3	full-splice_match	gene: Zm00001d046186	Zm00001d046186_T00	0.36	26.66	coding	323	235	1203
PB.3291.1	2	1614	4	full-splice_match	gene: Zm00001d006127	Zm00001d006127_T00	33.85	43.35	coding	364	228	1319
PB.4771.4	3	1339	6	novel_in_catalog	gene: Zm00001d044451	novel	0.00	57.36	coding	310	105	1034
PB.280.1	1	2303	5	novel_in_catalog	gene: Zm00001d028429	novel	2.61	24.93	coding	195	1423	2007
PB.280.2	1	1528	7	full-splice_match	gene: Zm00001d028429	Zm00001d028429_T00	22.32	24.93	coding	329	290	1276
PB.280.3	1	1392	7	full-splice_match	gene: Zm00001d028429	Zm00001d028429_T00	0.00	24.93	coding	329	258	1244
PB.9871.2	8	1750	12	full-splice_match	gene: Zm00001d011828	Zm00001d011828_T00	41.80	41.80	coding	422	96	1361
PB.8526.4	7	2045	5	full-splice_match	gene: Zm00001d020399	Zm00001d020399_T00	12.16	26.66	coding	426	369	1646
PB.9871.3	8	1646	12	full-splice_match	gene: Zm00001d011828	Zm00001d011828_T00	0.00	41.80	non coding	NA	NA	NA
PB.8526.2	7	2403	5	full-splice_match	gene: Zm00001d020399	Zm00001d020399_T00	14.49	26.66	coding	426	307	1584
PB.6608.2	5	2121	9	full-splice_match	gene: Zm00001d015464	Zm00001d015464_T00	44.75	82.61	coding	616	63	1910

PB.6608.1	5	2338	8	novel_in_catalog	gene: Zm00001d015464	novel	37.86	82.61	coding	313	1104	2042
PB.5936.2	4	1800	1	full-splice_match	gene: Zm00001d054067	Zm00001d054067_T00	1.82	77.11	coding	502	102	1607
PB.1351.1	1	2051	2	full-splice_match	gene: Zm00001d033611	Zm00001d033611_T00	10.93	103.62	coding	257	939	1709
PB.407.1	1	998	8	full-splice_match	gene: Zm00001d028981	Zm00001d028981_T00	82.09	82.09	coding	214	78	719
PB.407.3	1	882	8	full-splice_match	gene: Zm00001d028981	Zm00001d028981_T00	0.00	82.09	coding	214	64	705
PB.8876.1	7	3995	3	intergenic	novelGene_325	novel	17.49	31.00	coding	120	1633	1992
PB.6278.3	5	1048	5	full-splice_match	gene: Zm00001d014065	Zm00001d014065_T00	93.01	188.30	coding	216	151	798
PB.6278.1	5	1265	5	full-splice_match	gene: Zm00001d014065	Zm00001d014065_T00	70.68	188.30	coding	216	174	821
PB.6278.4	5	909	5	full-splice_match	gene: Zm00001d014065	Zm00001d014065_T00	24.60	188.30	coding	216	79	726
PB.6220.5	5	1225	13	novel_not_in_catalog	gene: Zm00001d013787	novel	46.96	67.10	coding	315	119	1063
PB.6220.4	5	1392	11	novel_not_in_catalog	gene: Zm00001d013787	novel	5.37	67.10	coding	111	254	586
PB.5601.2	4	1580	7	novel_in_catalog	gene: Zm00001d052561	novel	6.02	49.72	coding	254	72	833
PB.9015.1	7	1434	6	incomplete-splice_match	gene: Zm00001d022338	Zm00001d022338_T00	58.61	108.21	coding	328	236	1219
PB.6220.1	5	1378	12	novel_not_in_catalog	gene: Zm00001d013787	novel	11.45	67.10	coding	284	307	1158
PB.1500.3	1	1096	7	full-splice_match	gene: Zm00001d034319	Zm00001d034319_T00	32.83	44.75	coding	237	232	942
PB.6220.2	5	1747	10	novel_not_in_catalog	gene: Zm00001d013787	novel	3.31	67.10	coding	161	291	773
PB.7700.1	6	1530	2	full-splice_match	gene: Zm00001d037249	Zm00001d037249_T00	15.04	15.04	coding	454	53	1414

PB.1610.1	1	1903	1	full-splice_match	gene: Zm00001d034700	Zm00001d034700_T00	16.13	16.13	coding	472	132	1547
PB.10399.1	9	4281	11	incomplete-splice_match	gene: Zm00001d046226	Zm00001d046226_T00	27.20	27.20	coding	1172	309	3824
PB.1683.5	10	1699	2	intergenic	novelGene_10	novel	0.00	110.80	coding	422	186	1451
PB.1683.4	10	1866	2	intergenic	novelGene_10	novel	60.57	110.80	coding	422	238	1503
PB.3538.1	2	1213	9	full-splice_match	gene: Zm00001d007179	Zm00001d007179_T00	95.53	115.96	coding	267	130	930
PB.1451.1	1	2379	12	full-splice_match	gene: Zm00001d034033	Zm00001d034033_T00	55.24	55.24	coding	565	288	1982
PB.9708.2	8	2181	7	full-splice_match	gene: Zm00001d011138	Zm00001d011138_T00	0.30	39.06	coding	578	249	1982
PB.9708.1	8	2334	7	full-splice_match	gene: Zm00001d011138	Zm00001d011138_T00	38.76	39.06	coding	578	231	1964
PB.1683.2	10	1913	2	intergenic	novelGene_10	novel	27.44	110.80	coding	422	215	1480
PB.1032.2	1	3315	15	novel_in_catalog	gene: Zm00001d032279	novel	10.76	72.98	coding	618	102	1955
PB.1032.1	1	3212	16	full-splice_match	gene: Zm00001d032279	Zm00001d032279_T00	62.22	72.98	coding	962	102	2987
PB.10385.2	9	1504	3	full-splice_match	gene: Zm00001d046155	Zm00001d046155_T00	6.92	6.92	coding	140	1084	1503
PB.7114.1	5	1969	2	full-splice_match	gene: Zm00001d017983	Zm00001d017983_T00	47.34	79.54	coding	520	252	1811
PB.7114.3	5	2934	2	full-splice_match	gene: Zm00001d017983	Zm00001d017983_T00	32.20	79.54	coding	921	72	2834
PB.10116.1	9	2321	16	full-splice_match	gene: Zm00001d044754	Zm00001d044754_T00	26.15	144.53	coding	564	76	1767
PB.4105.2	3	2007	9	novel_in_catalog	gene: Zm00001d041634	novel	0.76	2.49	coding	347	628	1668
PB.4105.1	3	1686	10	full-splice_match	gene: Zm00001d041634	Zm00001d041634_T00	1.72	2.49	coding	347	291	1331

PB.10116.2	9	2259	16	full-splice_match	gene: Zm00001d044754	Zm00001d044754_T00	26.42	144.53	coding	564	244	1935
PB.10116.4	9	1977	16	full-splice_match	gene: Zm00001d044754	Zm00001d044754_T00	91.97	144.53	coding	564	73	1764
PB.4206.2	3	2102	5	novel_in_catalog	gene: Zm00001d042152	novel	3.33	46.94	coding	458	571	1944
PB.9252.20	8	2533	3	novel_in_catalog	gene: Zm00001d008752	novel	25.97	25.97	coding	236	144	851
PB.2999.1	2	3231	20	full-splice_match	gene: Zm00001d004564	Zm00001d004564_T00	33.15	65.73	coding	905	106	2820
PB.9252.26	8	701	1	full-splice_match	gene: Zm00001d008753	Zm00001d008753_T00	111.24	111.24	coding	24	247	318
PB.9252.27	8	1885	1	full-splice_match	gene: Zm00001d008756	Zm00001d008756_T00	31.08	363.20	coding	443	158	1486
PB.2999.4	2	3593	19	full-splice_match	gene: Zm00001d004564	Zm00001d004564_T01	24.12	65.73	coding	565	1497	3191
PB.2999.5	2	3334	19	full-splice_match	gene: Zm00001d004564	Zm00001d004564_T00	0.00	65.73	coding	565	1240	2934
PB.4793.4	3	2196	2	novel_in_catalog	gene: Zm00001d044516	novel	6.16	891.68	coding	297	122	1012
PB.6318.1	5	1223	5	incomplete-splice_match	gene: Zm00001d014255	Zm00001d014255_T01	92.99	92.99	coding	255	39	803
PB.9252.28	8	1733	1	full-splice_match	gene: Zm00001d008756	Zm00001d008756_T00	332.12	363.20	coding	443	158	1486
PB.9252.29	8	1726	9	full-splice_match	gene: Zm00001d008757	Zm00001d008757_T00	38.92	102.00	coding	415	115	1359
PB.1363.2	1	2268	12	full-splice_match	gene: Zm00001d033652	Zm00001d033652_T00	29.02	57.54	coding	605	243	2057
PB.4793.1	3	1584	4	full-splice_match	gene: Zm00001d044516	Zm00001d044516_T00	796.92	891.68	coding	365	127	1221
PB.4793.2	3	1037	4	novel_not_in_catalog	gene: Zm00001d044516	novel	10.77	891.68	coding	266	16	813
PB.4793.3	3	1709	3	novel_in_catalog	gene: Zm00001d044516	novel	18.35	891.68	coding	297	129	1019

PB.6586.10	5	2897	1	incomplete-splice_match	gene: Zm00001d015328	Zm00001d015328_T00	1.39	99.81	non coding	NA	NA	NA
PB.4869.1	4	1436	1	genic	gene: Zm00001d048773	novel	5.11	5.11	coding	150	902	1351
PB.7108.4	5	1561	7	full-splice_match	gene: Zm00001d017966	Zm00001d017966_T00	1.07	143.60	coding	415	88	1332
PB.7192.2	5	777	6	full-splice_match	gene: Zm00001d018259	Zm00001d018259_T00	23.31	31.38	non coding	NA	NA	NA
PB.6754.1	5	1595	1	novel_in_catalog	gene: Zm00001d016456	novel	3.93	3.93	coding	160	120	599
PB.7192.1	5	2188	5	novel_in_catalog	gene: Zm00001d018259	novel	8.07	31.38	non coding	NA	NA	NA
PB.2656.2	2	1288	1	full-splice_match	gene: Zm00001d002748	Zm00001d002748_T00	17.13	24.29	coding	150	510	959
PB.2656.3	2	1134	1	full-splice_match	gene: Zm00001d002748	Zm00001d002748_T00	6.94	24.29	coding	150	501	950
PB.9700.1	8	5514	24	full-splice_match	gene: Zm00001d011091	Zm00001d011091_T01	9.39	42.20	coding	770	2778	5087
PB.2656.1	2	1550	1	full-splice_match	gene: Zm00001d002748	Zm00001d002748_T00	0.23	24.29	coding	150	507	956
PB.3134.2	2	1508	2	genic	gene: Zm00001d005351	novel	0.24	29.28	coding	394	68	1249
PB.508.2	1	1713	3	full-splice_match	gene: Zm00001d029468	Zm00001d029468_T00	5.50	20.93	coding	472	150	1565
PB.508.1	1	1819	3	full-splice_match	gene: Zm00001d029468	Zm00001d029468_T00	15.43	20.93	coding	472	154	1569
PB.3134.1	2	1654	1	full-splice_match	gene: Zm00001d005351	Zm00001d005351_T00	17.44	29.28	coding	422	108	1373
PB.4238.1	3	1053	5	genic	gene: Zm00001d042315	novel	27.16	27.16	coding	166	190	687
PB.5426.1	4	555	1	intergenic	novelGene_352	novel	13.41	13.41	non coding	NA	NA	NA
PB.1338.1	1	462	2	incomplete-splice_match	gene: Zm00001d033554	Zm00001d033554_T00	517.76	517.76	non coding	NA	NA	NA

PB.2285.1	10	1449	10	full-splice_match	gene: Zm00001d026302	Zm00001d026302_T00	43.34	43.34	coding	331	79	1071
PB.5085.1	4	364	1	genic	gene: Zm00001d049786	novel	109.53	109.53	non coding	NA	NA	NA
PB.4783.2	3	2681	14	full-splice_match	gene: Zm00001d044494	Zm00001d044494_T01	0.00	46.54	coding	239	103	819
PB.4783.1	3	2979	15	full-splice_match	gene: Zm00001d044494	Zm00001d044494_T01	13.73	46.54	coding	239	116	832
PB.7991.1	6	1790	5	full-splice_match	gene: Zm00001d038394	Zm00001d038394_T00	2.36	2.36	coding	426	273	1550
PB.4783.6	3	2691	16	novel_in_catalog	gene: Zm00001d044494	novel	6.45	46.54	coding	79	82	318
PB.4783.4	3	2640	16	novel_in_catalog	gene: Zm00001d044494	novel	1.87	46.54	coding	79	74	310
PB.7015.1	5	1391	7	full-splice_match	gene: Zm00001d017564	Zm00001d017564_T00	36.41	36.41	coding	353	30	1088
PB.4219.2	3	1346	1	novel_in_catalog	gene: Zm00001d042245	novel	29.50	578.71	coding	162	254	739
PB.4783.8	3	2790	14	full-splice_match	gene: Zm00001d044494	Zm00001d044494_T00	8.65	46.54	coding	79	67	303
PB.4030.2	3	1116	6	full-splice_match	gene: Zm00001d041112	Zm00001d041112_T00	11.27	167.94	coding	241	50	772
PB.4030.3	3	1052	6	full-splice_match	gene: Zm00001d041112	Zm00001d041112_T00	78.41	167.94	coding	241	93	815
PB.4030.1	3	1257	6	full-splice_match	gene: Zm00001d041112	Zm00001d041112_T00	78.27	167.94	coding	241	84	806
PB.1777.1	10	3637	2	full-splice_match	gene: Zm00001d023608	Zm00001d023608_T00	5.29	14.55	coding	1046	29	3166
PB.1777.2	10	3381	2	full-splice_match	gene: Zm00001d023608	Zm00001d023608_T00	9.26	14.55	coding	1046	28	3165
PB.3069.1	2	2883	1	novel_in_catalog	gene: Zm00001d005029	novel	14.77	14.77	coding	202	573	1178
PB.9891.1	8	897	1	novel_in_catalog	gene: Zm00001d011901	novel	6.87	6.87	coding	115	405	749

PB.8142.1	6	823	4	intergenic	novelGene_335	novel	211.63	211.63	coding	111	186	518
PB.500.1	1	632	3	novel_not_in_catalog	gene: Zm00001d029424	novel	27.57	27.57	non coding	NA	NA	NA
PB.3173.1	2	884	1	antisense	novelGene_gene: Zm00001d005593	novel	1.12	1.12	coding	45	675	809
PB.8634.2	7	1720	2	novel_in_catalog	gene: Zm00001d020931	novel	13.28	13.28	coding	204	996	1607
PB.3537.1	2	2236	2	full-splice_match	gene: Zm00001d007175	Zm00001d007175_T00	30.35	30.35	coding	615	161	2005
PB.3435.1	2	1082	2	intergenic	novelGene_411	novel	72.00	72.00	coding	231	72	764
PB.8946.1	7	2175	10	full-splice_match	gene: Zm00001d022071	Zm00001d022071_T00	17.22	17.22	coding	586	124	1881
PB.8946.2	7	2061	10	full-splice_match	gene: Zm00001d022071	Zm00001d022071_T00	0.00	17.22	coding	586	113	1870
PB.9483.2	8	1957	7	novel_in_catalog	gene: Zm00001d010115	novel	3.56	41.56	coding	353	202	1260
PB.9483.1	8	1938	8	full-splice_match	gene: Zm00001d010115	Zm00001d010115_T00	38.01	41.56	coding	516	263	1810
PB.10025.1	8	1240	4	full-splice_match	gene: Zm00001d012460	Zm00001d012460_T00	21.80	21.80	coding	241	343	1065
PB.4210.6	3	518	1	incomplete-splice_match	gene: Zm00001d042185	Zm00001d042185_T01	6.96	114.80	coding	55	183	347
PB.4210.4	3	2737	4	full-splice_match	gene: Zm00001d042185	Zm00001d042185_T01	0.00	114.80	coding	309	1401	2327
PB.4210.5	3	1487	3	incomplete-splice_match	gene: Zm00001d042185	Zm00001d042185_T00	36.34	114.80	coding	334	77	1078
PB.4210.2	3	2428	4	novel_in_catalog	gene: Zm00001d042185	novel	13.49	114.80	coding	363	791	1879
PB.4210.3	3	2251	5	full-splice_match	gene: Zm00001d042185	Zm00001d042185_T01	42.52	114.80	coding	491	230	1702
PB.9102.3	7	3026	8	intergenic	novelGene_372	novel	34.02	82.50	coding	134	2060	2461

PB.4210.1	3	2917	4	full-splice_match	gene: Zm00001d042185	Zm00001d042185_T01	15.49	114.80	coding	309	1415	2341
PB.5442.2	4	1797	1	novel_in_catalog	gene: Zm00001d026942	novel	1.23	1.23	non coding	NA	NA	NA
PB.5442.1	4	668	2	fusion	gene: Zm00001d051967	novel	44.25	44.25	coding	61	122	304
PB.1654.2	1	1055	1	full-splice_match	gene: Zm00001d034849	Zm00001d034849_T00	0.56	20.47	coding	246	67	804
PB.3259.2	2	2020	2	incomplete-splice_match	gene: Zm00001d006008	Zm00001d006008_T00	443.73	443.73	coding	592	50	1825
PB.4446.2	3	2571	11	novel_not_in_catalog	gene: Zm00001d043211	novel	11.35	19.06	coding	593	186	1964
PB.6072.2	5	1425	8	full-splice_match	gene: Zm00001d013223	Zm00001d013223_T00	17.56	34.46	coding	377	86	1216
PB.5601.4	4	1435	7	novel_in_catalog	gene: Zm00001d052561	novel	0.69	49.72	coding	307	315	1235
PB.6072.1	5	1774	8	full-splice_match	gene: Zm00001d013223	Zm00001d013223_T00	16.89	34.46	coding	377	136	1266
PB.10881.1	9	1087	3	novel_in_catalog	gene: Zm00001d048409	novel	62.86	104.17	coding	292	51	926
PB.10881.2	9	2630	9	full-splice_match	gene: Zm00001d048409	Zm00001d048409_T00	41.31	104.17	coding	804	98	2509
PB.3449.1	2	3775	12	full-splice_match	gene: Zm00001d006798	Zm00001d006798_T00	19.72	19.72	coding	1116	168	3515
PB.9986.1	8	934	1	incomplete-splice_match	gene: Zm00001d012295	Zm00001d012295_T00	35.42	75.77	coding	190	320	889
PB.2378.1	10	528	3	full-splice_match	gene: Zm00001d026589	Zm00001d026589_T00	38.03	137.34	non coding	NA	NA	NA
PB.9762.1	8	1607	11	incomplete-splice_match	gene: Zm00001d011366	Zm00001d011366_T00	33.74	33.74	coding	370	86	1195
PB.10272.1	9	1049	2	genic	gene: Zm00001d045478	novel	282.08	282.08	coding	235	80	784
PB.10900.2	9	2225	5	incomplete-splice_match	gene: Zm00001d048473	Zm00001d048473_T00	7.31	199.31	coding	160	110	589

PB.10900.3	9	808	5	incomplete-splice_match	gene: Zm00001d048473	Zm00001d048473_T00	157.99	199.31	coding	160	111	590
PB.796.2	1	2281	7	full-splice_match	gene: Zm00001d031090	Zm00001d031090_T00	57.18	62.61	coding	596	123	1910
PB.3516.1	2	2524	1	genic	gene: Zm00001d007073	novel	18.45	108.09	coding	238	1774	2487
PB.7634.1	6	1060	2	full-splice_match	gene: Zm00001d036956	Zm00001d036956_T00	11.98	11.98	coding	196	220	807
PB.5874.2	4	549	5	full-splice_match	gene: Zm00001d053863	Zm00001d053863_T00	146.16	1627.71	non coding	NA	NA	NA
PB.149.1	1	2093	13	incomplete-splice_match	gene: Zm00001d027884	Zm00001d027884_T00	19.04	33.01	coding	584	67	1818
PB.7081.1	5	624	1	intergenic	novelGene_404	novel	0.00	0.00	non coding	NA	NA	NA
PB.7628.2	6	1379	3	novel_in_catalog	gene: Zm00001d036918	novel	13.02	59.91	coding	72	705	920
PB.5733.3	4	2636	15	full-splice_match	gene: Zm00001d053245	Zm00001d053245_T00	16.22	16.22	coding	603	303	2111
PB.4260.10	3	1764	13	novel_in_catalog	gene: Zm00001d042421	novel	3.32	67.32	coding	268	219	1022
PB.6048.1	5	2825	17	full-splice_match	gene: Zm00001d013116	Zm00001d013116_T00	61.10	61.10	coding	831	172	2664
PB.2486.1	2	1441	1	genic	gene: Zm00001d002013	novel	6.65	6.65	coding	344	138	1169
PB.3945.1	3	2066	17	full-splice_match	gene: Zm00001d040422	Zm00001d040422_T00	304.35	304.35	coding	490	195	1664
PB.10920.4	9	3307	18	full-splice_match	gene: Zm00001d048527	Zm00001d048527_T00	8.70	66.90	coding	557	1425	3095
PB.5258.3	4	895	6	full-splice_match	gene: Zm00001d050978	Zm00001d050978_T00	0.00	99.06	coding	229	75	761
PB.10920.1	9	1936	10	incomplete-splice_match	gene: Zm00001d048527	Zm00001d048527_T01	17.23	66.90	coding	344	119	1150
PB.2462.1	2	1696	3	full-splice_match	gene: Zm00001d001916	Zm00001d001916_T00	6.42	6.42	coding	389	32	1198

PB.4479.7	3	2269	6	novel_not_in_catalog	gene: Zm00001d043354	novel	0.00	31.37	coding	602	180	1985
PB.9274.1	8	2002	6	full-splice_match	gene: Zm00001d008902	Zm00001d008902_T00	22.65	22.65	coding	507	184	1704
PB.5258.2	4	1088	6	full-splice_match	gene: Zm00001d050978	Zm00001d050978_T00	57.89	99.06	coding	229	105	791
PB.3090.3	2	1771	10	incomplete-splice_match	gene: Zm00001d005137	Zm00001d005137_T01	25.47	57.43	coding	511	237	1769
PB.10622.1	9	1126	6	full-splice_match	gene: Zm00001d047448	Zm00001d047448_T00	4.02	137.53	coding	170	117	626
PB.5794.4	4	1128	7	novel_in_catalog	gene: Zm00001d053563	novel	1.29	40.38	coding	123	619	987
PB.5292.2	4	1711	2	full-splice_match	gene: Zm00001d051219	Zm00001d051219_T00	0.00	28.54	coding	417	103	1353
PB.5292.1	4	1851	2	full-splice_match	gene: Zm00001d051219	Zm00001d051219_T00	28.54	28.54	coding	417	105	1355
PB.315.8	1	2388	12	full-splice_match	gene: Zm00001d028579	Zm00001d028579_T01	0.00	74.65	coding	743	160	2388
PB.2666.3	2	2734	3	novel_in_catalog	gene: Zm00001d002806	novel	0.47	20.07	coding	258	1779	2552
PB.4189.1	3	1934	2	full-splice_match	gene: Zm00001d042051	Zm00001d042051_T00	10.97	10.97	coding	437	317	1627
PB.2666.1	2	1433	3	novel_in_catalog	gene: Zm00001d002806	novel	17.35	20.07	coding	258	101	874
PB.315.3	1	2314	7	incomplete-splice_match	gene: Zm00001d028579	Zm00001d028579_T00	22.53	74.65	coding	191	1316	1888
PB.315.2	1	4445	12	novel_in_catalog	gene: Zm00001d028579	novel	4.04	74.65	coding	809	1510	3936
PB.315.1	1	439	3	incomplete-splice_match	gene: Zm00001d028579	Zm00001d028579_T00	5.41	74.65	non coding	NA	NA	NA
PB.315.7	1	2924	12	full-splice_match	gene: Zm00001d028579	Zm00001d028579_T01	0.00	74.65	coding	901	118	2820
PB.161.1	1	1602	3	full-splice_match	gene: Zm00001d027924	Zm00001d027924_T00	16.48	50.97	coding	302	74	979

PB.315.5	1	3154	12	full-splice_match	gene: Zm00001d028579	Zm00001d028579_T01	42.67	74.65	coding	901	160	2862
PB.315.4	1	3132	13	novel_in_catalog	gene: Zm00001d028579	novel	0.00	74.65	coding	901	159	2861
PB.8673.1	7	1006	2	genic	gene: Zm00001d021063	novel	3.43	3.43	coding	247	15	755
PB.522.2	1	1807	8	novel_in_catalog	gene: Zm00001d029534	novel	4.35	56.23	coding	260	779	1558
PB.522.1	1	1526	9	full-splice_match	gene: Zm00001d029534	Zm00001d029534_T00	51.88	56.23	coding	371	175	1287
PB.6195.1	5	701	5	full-splice_match	gene: Zm00001d013656	Zm00001d013656_T00	33.74	33.74	coding	145	48	482
PB.4932.1	4	1559	5	full-splice_match	gene: Zm00001d049083	Zm00001d049083_T00	64.64	64.64	coding	449	53	1399
PB.5747.1	4	2502	5	incomplete-splice_match	gene: Zm00001d053300	Zm00001d053300_T00	25.81	25.81	coding	669	116	2122
PB.1423.14	1	1620	6	novel_not_in_catalog	gene: Zm00001d033948	novel	0.00	76.35	coding	115	1066	1410
PB.9649.3	8	1654	5	full-splice_match	gene: Zm00001d010825	Zm00001d010825_T00	0.00	110.83	coding	440	86	1405
PB.9649.1	8	1777	5	full-splice_match	gene: Zm00001d010825	Zm00001d010825_T00	110.83	110.83	coding	440	86	1405
PB.10622.4	9	2003	4	full-splice_match	gene: Zm00001d047448	Zm00001d047448_T00	4.86	137.53	coding	69	122	328
PB.8167.1	6	1557	2	full-splice_match	gene: Zm00001d001482	Zm00001d001482_T00	13.18	23.73	non coding	NA	NA	NA
PB.7089.1	5	2194	5	full-splice_match	gene: Zm00001d017885	Zm00001d017885_T00	74.05	74.05	coding	394	331	1512
PB.1341.1	1	1743	1	genic_intron	novelGene_458	novel	14.10	18.88	coding	504	39	1550
PB.8996.3	7	2697	3	full-splice_match	gene: Zm00001d022273	Zm00001d022273_T00	0.00	30.30	coding	650	8	1957
PB.9754.1	8	2682	1	genic	gene: Zm00001d011336	novel	8.42	46.99	non coding	NA	NA	NA

PB.637.4	1	2895	10	novel_in_catalog	gene: Zm00001d030133	novel	7.42	47.43	coding	612	120	1955
PB.9788.4	8	1004	5	full-splice_match	gene: Zm00001d011500	Zm00001d011500_T00	61.68	501.01	coding	147	72	512
PB.141.2	1	689	2	full-splice_match	gene: Zm00001d027855	Zm00001d027855_T00	30.77	32.75	non coding	NA	NA	NA
PB.637.3	1	2811	11	full-splice_match	gene: Zm00001d030133	Zm00001d030133_T00	30.04	47.43	coding	810	146	2575
PB.6688.1	5	2123	2	genic	gene: Zm00001d016066	novel	2.61	15.28	coding	415	404	1648
PB.141.1	1	803	2	full-splice_match	gene: Zm00001d027855	Zm00001d027855_T00	1.98	32.75	coding	92	154	429
PB.975.3	1	1344	14	novel_not_in_catalog	gene: Zm00001d031996	novel	0.00	15.39	coding	351	88	1140
PB.5745.1	4	1598	3	full-splice_match	gene: Zm00001d053289	Zm00001d053289_T00	35.83	35.83	coding	347	245	1285
PB.5311.1	4	1874	3	novel_in_catalog	gene: Zm00001d051338	novel	14.14	14.14	coding	597	82	1872
PB.6577.4	5	2399	1	intergenic	novelGene_188	novel	0.00	13.24	non coding	NA	NA	NA
PB.1423.10	1	2337	11	novel_not_in_catalog	gene: Zm00001d033948	novel	15.09	76.35	coding	115	192	536
PB.801.4	1	1861	10	novel_in_catalog	gene: Zm00001d031120	novel	3.53	247.40	coding	318	556	1509
PB.301.1	1	2253	10	full-splice_match	gene: Zm00001d028536	Zm00001d028536_T00	37.73	61.09	coding	604	119	1930
PB.301.2	1	2460	10	full-splice_match	gene: Zm00001d028536	Zm00001d028536_T00	0.00	61.09	coding	259	72	848
PB.301.3	1	2252	10	full-splice_match	gene: Zm00001d028536	Zm00001d028536_T00	14.95	61.09	coding	604	251	2062
PB.301.4	1	2348	10	full-splice_match	gene: Zm00001d028536	Zm00001d028536_T00	8.41	61.09	coding	259	120	896
PB.7095.5	5	2401	11	full-splice_match	gene: Zm00001d017913	Zm00001d017913_T00	31.49	31.49	coding	626	235	2112

PB.9629.3	8	1456	10	full-splice_match	gene: Zm00001d010731	Zm00001d010731_T00	0.00	12.25	coding	378	96	1229
PB.631.1	1	1201	6	full-splice_match	gene: Zm00001d030116	Zm00001d030116_T00	18.01	18.81	coding	291	179	1051
PB.7606.2	6	1806	7	incomplete-splice_match	gene: Zm00001d036742	Zm00001d036742_T00	10.13	33.03	coding	133	635	1033
PB.7606.3	6	2397	10	novel_in_catalog	gene: Zm00001d036742	novel	3.98	33.03	coding	211	1068	1700
PB.7606.1	6	2251	11	incomplete-splice_match	gene: Zm00001d036742	Zm00001d036742_T00	18.92	33.03	coding	402	228	1433
PB.631.2	1	1247	5	novel_in_catalog	gene: Zm00001d030116	novel	0.80	18.81	coding	198	543	1136
PB.9341.2	8	1547	5	novel_not_in_catalog	gene: Zm00001d009330	novel	1.92	26.95	coding	433	247	1545
PB.4662.1	3	1009	1	full-splice_match	gene: Zm00001d043990	Zm00001d043990_T00	4.99	4.99	coding	278	39	872
PB.9341.1	8	2235	8	novel_not_in_catalog	gene: Zm00001d009330	novel	25.03	26.95	coding	571	174	1886
PB.6276.1	5	1990	3	full-splice_match	gene: Zm00001d014060	Zm00001d014060_T00	15.89	15.89	coding	610	83	1912
PB.8873.4	7	2059	5	full-splice_match	gene: Zm00001d021823	Zm00001d021823_T00	20.33	78.93	coding	614	77	1918
PB.1342.1	1	1528	3	incomplete-splice_match	gene: Zm00001d033572	Zm00001d033572_T00	9.38	9.38	coding	351	86	1138
PB.6915.3	5	2085	15	novel_in_catalog	gene: Zm00001d017144	novel	1.81	81.71	coding	246	358	1095
PB.6614.10	5	1735	1	intergenic	novelGene_200	novel	0.64	0.64	non coding	NA	NA	NA
PB.1767.1	10	477	2	incomplete-splice_match	gene: Zm00001d023566	Zm00001d023566_T00	13.46	13.46	non coding	NA	NA	NA
PB.2658.1	2	1498	5	novel_not_in_catalog	gene: Zm00001d002778	novel	14.26	73.37	coding	276	264	1091
PB.1344.1	1	1834	8	incomplete-splice_match	gene: Zm00001d033575	Zm00001d033575_T00	47.20	47.20	coding	430	25	1314

PB.2658.3	2	1388	4	novel_not_in_catalog	gene: Zm00001d002778	novel	4.38	73.37	coding	193	191	769
PB.2658.5	2	2142	4	full-splice_match	gene: Zm00001d002778	Zm00001d002778_T00	7.93	73.37	coding	240	266	985
PB.2658.6	2	1350	5	novel_not_in_catalog	gene: Zm00001d002778	novel	46.81	73.37	coding	276	266	1093
PB.8301.1	7	1174	2	novel_in_catalog	gene: Zm00001d018973	novel	26.76	197.03	coding	182	104	649
PB.6891.3	5	1679	2	full-splice_match	gene: Zm00001d017085	Zm00001d017085_T00	120.40	291.06	coding	388	307	1470
PB.8301.2	7	1078	3	full-splice_match	gene: Zm00001d018973	Zm00001d018973_T00	160.33	197.03	coding	202	170	775
PB.8301.5	7	1234	1	novel_in_catalog	gene: Zm00001d018973	novel	9.95	197.03	coding	73	112	330
PB.1388.2	1	1234	7	novel_in_catalog	gene: Zm00001d033788	novel	11.97	119.83	coding	125	656	1030
PB.6680.1	5	995	2	novel_not_in_catalog	gene: Zm00001d016035	novel	3.88	3.88	coding	132	398	793
PB.8301.6	7	1076	2	novel_in_catalog	gene: Zm00001d018973	novel	0.00	197.03	coding	182	114	659
PB.5727.1	4	1109	2	full-splice_match	gene: Zm00001d053220	Zm00001d053220_T00	4.68	91.34	coding	2	751	756
PB.1396.1	1	1192	2	full-splice_match	gene: Zm00001d033818	Zm00001d033818_T00	23.58	71.29	coding	199	135	731
PB.975.1	1	1503	14	novel_not_in_catalog	gene: Zm00001d031996	novel	6.22	15.39	coding	351	81	1133
PB.1188.11	1	419	2	incomplete-splice_match	gene: Zm00001d032873	Zm00001d032873_T00	70.55	341.11	coding	100	120	419
PB.4307.2	3	1652	5	novel_in_catalog	gene: Zm00001d042639	novel	4.30	45.96	coding	211	108	740
PB.4307.1	3	1743	4	novel_in_catalog	gene: Zm00001d042639	novel	1.33	45.96	coding	211	144	776
PB.4307.7	3	1753	6	novel_in_catalog	gene: Zm00001d042639	novel	5.36	45.96	coding	331	8	1000

PB.8280.4	7	833	5	novel_in_catalog	gene: Zm00001d018884	novel	626.18	1750.35	non coding	NA	NA	NA
PB.6161.11	5	1196	1	novel_in_catalog	gene: Zm00001d013501	novel	8.52	79.68	non coding	NA	NA	NA
PB.8280.6	7	712	5	full-splice_match	gene: Zm00001d018884	Zm00001d018884_T00	1124.17	1750.35	coding	152	205	660
PB.1430.1	1	1188	4	full-splice_match	gene: Zm00001d033983	Zm00001d033983_T00	483.18	483.18	coding	255	114	878
PB.3073.1	2	1525	8	full-splice_match	gene: Zm00001d005059	Zm00001d005059_T00	73.57	73.57	coding	162	135	620
PB.5730.1	4	1874	8	full-splice_match	gene: Zm00001d053232	Zm00001d053232_T00	134.49	134.49	coding	491	142	1614
PB.10118.1	9	1381	1	full-splice_match	gene: Zm00001d044771	Zm00001d044771_T00	103.20	103.20	coding	357	152	1222
PB.9975.1	8	1156	1	full-splice_match	gene: Zm00001d012260	Zm00001d012260_T00	35.71	73.75	coding	280	22	861
PB.8110.1	6	1456	17	full-splice_match	gene: Zm00001d038855	Zm00001d038855_T00	29.47	29.47	coding	379	87	1223
PB.6844.3	5	550	2	full-splice_match	gene: Zm00001d016850	Zm00001d016850_T00	69.97	71.10	coding	106	46	363
PB.6982.1	5	1017	2	full-splice_match	gene: Zm00001d017441	Zm00001d017441_T00	118.51	118.51	coding	209	80	706
PB.7149.4	5	1275	4	full-splice_match	gene: Zm00001d018099	Zm00001d018099_T00	102.42	164.45	coding	321	155	1117
PB.7149.5	5	1498	3	novel_in_catalog	gene: Zm00001d018099	novel	0.00	164.45	coding	183	168	716
PB.7149.2	5	1419	4	full-splice_match	gene: Zm00001d018099	Zm00001d018099_T00	45.39	164.45	coding	321	171	1133
PB.7149.3	5	1617	3	novel_in_catalog	gene: Zm00001d018099	novel	0.00	164.45	coding	183	142	690
PB.3834.1	3	787	1	full-splice_match	gene: Zm00001d039908	Zm00001d039908_T00	1108.95	1108.95	coding	157	75	545
PB.9004.1	7	1301	1	genic	gene: Zm00001d022296	novel	23.41	23.41	coding	163	813	1301

PB.9135.1	8	1064	5	full-splice_match	gene: Zm00001d008264	Zm00001d008264_T00	26.16	26.16	coding	229	167	853
PB.7480.1	6	1444	2	incomplete-splice_match	gene: Zm00001d036008	Zm00001d036008_T00	37.44	87.18	coding	298	3	896
PB.7745.12	6	2136	7	full-splice_match	gene: Zm00001d037448	Zm00001d037448_T00	0.00	27.81	non coding	NA	NA	NA
PB.7745.11	6	2155	9	full-splice_match	gene: Zm00001d037448	Zm00001d037448_T00	0.00	27.81	non coding	NA	NA	NA
PB.3722.1	3	3618	3	incomplete-splice_match	gene: Zm00001d039411	Zm00001d039411_T00	70.29	95.29	coding	1034	84	3185
PB.3722.2	3	986	3	full-splice_match	gene: Zm00001d039411	Zm00001d039411_T01	25.01	95.29	coding	113	290	628
PB.3050.5	2	2113	8	full-splice_match	gene: Zm00001d004898	Zm00001d004898_T00	16.38	29.56	coding	505	218	1732
PB.10474.1	9	1350	1	full-splice_match	gene: Zm00001d046682	Zm00001d046682_T00	4.84	145.11	coding	233	108	806
PB.10474.2	9	761	1	full-splice_match	gene: Zm00001d046682	Zm00001d046682_T00	0.00	145.11	coding	80	70	309
PB.10474.3	9	1106	1	full-splice_match	gene: Zm00001d046682	Zm00001d046682_T00	65.13	145.11	coding	233	68	766
PB.5772.1	4	1881	3	novel_in_catalog	gene: Zm00001d053416	novel	3.69	10.82	coding	214	296	937
PB.6726.2	5	1514	2	novel_not_in_catalog	gene: Zm00001d016278	novel	0.00	61.62	coding	51	193	345
PB.5772.3	4	1155	4	novel_in_catalog	gene: Zm00001d053416	novel	5.63	10.82	coding	214	220	861
PB.8031.1	6	804	3	full-splice_match	gene: Zm00001d038542	Zm00001d038542_T00	11.69	11.69	coding	169	88	594
PB.1629.1	1	1146	1	full-splice_match	gene: Zm00001d034763	Zm00001d034763_T00	12.18	12.18	coding	216	279	926
PB.8027.1	6	1781	2	genic	gene: Zm00001d038537	novel	15.42	15.42	coding	354	543	1604
PB.2987.3	2	823	4	full-splice_match	gene: Zm00001d004480	Zm00001d004480_T00	12.76	21.71	coding	230	53	742

PB.1460.1	1	1413	4	full-splice_match	gene: Zm00001d034073	Zm00001d034073_T00	28.21	28.21	coding	331	216	1208
PB.1454.1	1	1470	3	full-splice_match	gene: Zm00001d034039	Zm00001d034039_T00	14.79	29.36	coding	347	142	1182
PB.3153.1	2	1578	8	full-splice_match	gene: Zm00001d005473	Zm00001d005473_T00	25.47	25.47	coding	384	173	1324
PB.4454.5	3	2728	7	full-splice_match	gene: Zm00001d043256	Zm00001d043256_T00	0.00	28.15	coding	874	54	2675
PB.4373.2	3	2252	1	full-splice_match	gene: Zm00001d042907	Zm00001d042907_T00	1.17	13.65	coding	693	8	2086
PB.4885.2	4	2061	2	full-splice_match	gene: Zm00001d048839	Zm00001d048839_T00	0.80	12.76	coding	66	118	315
PB.5121.12	4	1770	1	novel_in_catalog	gene: Zm00001d050011	novel	3.61	194.14	coding	133	68	466
PB.5121.13	4	1499	3	novel_in_catalog	gene: Zm00001d050011	novel	0.00	194.14	coding	141	68	490
PB.7155.1	5	1625	1	full-splice_match	gene: Zm00001d018123	Zm00001d018123_T00	11.30	11.30	coding	371	144	1256
PB.5121.15	4	1406	3	novel_in_catalog	gene: Zm00001d050011	novel	6.03	194.14	coding	134	819	1220
PB.5121.16	4	1139	5	full-splice_match	gene: Zm00001d050011	Zm00001d050011_T00	9.00	194.14	coding	315	67	1011
PB.7155.2	5	1302	1	full-splice_match	gene: Zm00001d018124	Zm00001d018124_T00	0.00	0.00	coding	371	29	1141
PB.6899.3	5	1760	6	novel_in_catalog	gene: Zm00001d017096	novel	38.09	254.51	coding	129	106	492
PB.6315.1	5	1540	2	full-splice_match	gene: Zm00001d014204	Zm00001d014204_T00	6.19	6.19	coding	370	271	1380
PB.952.11	1	2582	11	full-splice_match	gene: Zm00001d031927	Zm00001d031927_T02	19.88	61.88	coding	217	1224	1874
PB.9254.4	8	1858	3	full-splice_match	gene: Zm00001d008794	Zm00001d008794_T00	65.40	127.11	coding	540	82	1701
PB.4885.1	4	2183	1	novel_in_catalog	gene: Zm00001d048839	novel	5.58	12.76	coding	66	119	316

PB.9254.1	8	2166	2	novel_in_catalog	gene: Zm00001d008794	novel	60.48	127.11	coding	471	437	1849
PB.4750.2	3	847	3	full-splice_match	gene: Zm00001d044332	Zm00001d044332_T00	0.00	80.22	coding	201	70	672
PB.9254.3	8	2285	1	genic	gene: Zm00001d008794	novel	1.23	127.11	coding	263	1180	1968
PB.4454.1	3	2972	7	full-splice_match	gene: Zm00001d043256	Zm00001d043256_T00	3.22	28.15	coding	874	173	2794
PB.1532.1	1	1042	2	novel_not_in_catalog	gene: Zm00001d034425	novel	10.78	10.78	coding	138	266	679
PB.952.13	1	2355	10	novel_in_catalog	gene: Zm00001d031927	novel	2.40	61.88	coding	198	1285	1878
PB.6723.2	5	1205	2	full-splice_match	gene: Zm00001d016255	Zm00001d016255_T00	42.07	73.58	coding	251	224	976
PB.6723.1	5	1331	2	full-splice_match	gene: Zm00001d016255	Zm00001d016255_T00	31.51	73.58	coding	251	181	933
PB.4067.3	3	2718	3	full-splice_match	gene: Zm00001d041397	Zm00001d041397_T00	10.68	14.14	coding	343	381	1409
PB.1047.7	1	1062	7	full-splice_match	gene: Zm00001d032350	Zm00001d032350_T00	7.73	141.86	coding	196	245	832
PB.7243.1	5	752	1	incomplete-splice_match	gene: Zm00001d018435	Zm00001d018435_T00	19.07	19.07	coding	72	147	362
PB.462.2	1	3262	10	full-splice_match	gene: Zm00001d029223	Zm00001d029223_T01	23.04	37.43	coding	947	23	2863
PB.512.4	1	1148	1	novel_in_catalog	gene: Zm00001d029478	novel	5.81	18.78	non coding	NA	NA	NA
PB.462.1	1	2548	9	incomplete-splice_match	gene: Zm00001d029223	Zm00001d029223_T01	14.39	37.43	coding	439	573	1889
PB.1181.4	1	1028	2	novel_in_catalog	gene: Zm00001d032838	novel	9.68	51.34	coding	93	140	418
PB.9979.2	8	1107	7	full-splice_match	gene: Zm00001d012275	Zm00001d012275_T00	60.54	68.95	coding	210	73	702
PB.1047.5	1	2525	7	full-splice_match	gene: Zm00001d032350	Zm00001d032350_T00	0.00	141.86	coding	114	170	511

PB.9105.1	7	2850	11	novel_in_catalog	gene: Zm00001d022621	novel	0.46	23.96	coding	356	655	1722
PB.3977.1	3	1467	6	full-splice_match	gene: Zm00001d040614	Zm00001d040614_T00	7.21	62.55	coding	114	119	460
PB.10454.3	9	1717	12	full-splice_match	gene: Zm00001d046554	Zm00001d046554_T00	74.16	148.69	coding	445	128	1462
PB.3977.3	3	1063	7	full-splice_match	gene: Zm00001d040614	Zm00001d040614_T00	50.70	62.55	coding	229	124	810
PB.3977.2	3	1215	6	novel_in_catalog	gene: Zm00001d040614	novel	4.65	62.55	coding	173	124	642
PB.3977.5	3	848	7	full-splice_match	gene: Zm00001d040614	Zm00001d040614_T00	0.00	62.55	coding	229	84	770
PB.2684.2	2	3811	9	novel_not_in_catalog	gene: Zm00001d002876	novel	15.68	15.68	coding	1056	243	3410
PB.10454.4	9	1624	12	novel_in_catalog	gene: Zm00001d046554	novel	33.28	148.69	coding	445	126	1460
PB.3413.3	2	1230	2	full-splice_match	gene: Zm00001d006626	Zm00001d006626_T00	131.55	281.14	coding	261	42	824
PB.420.10	1	856	1	full-splice_match	gene: Zm00001d029028	Zm00001d029028_T00	330.78	826.32	coding	188	93	656
PB.36.2	1	2110	8	novel_not_in_catalog	gene: Zm00001d027376	novel	24.51	42.03	coding	490	280	1749
PB.7865.3	6	1173	2	full-splice_match	gene: Zm00001d037975	Zm00001d037975_T04	3.85	19.15	coding	247	46	786
PB.36.1	1	1647	4	incomplete-splice_match	gene: Zm00001d027376	Zm00001d027376_T00	17.52	42.03	coding	376	2	1129
PB.10537.2	9	2270	2	genic	gene: Zm00001d047028	novel	168.95	168.95	coding	382	642	1787
PB.6137.2	5	1692	3	full-splice_match	gene: Zm00001d013411	Zm00001d013411_T00	27.63	34.13	coding	416	135	1382
PB.10821.1	9	2052	11	full-splice_match	gene: Zm00001d048174	Zm00001d048174_T00	18.28	44.35	coding	458	69	1442
PB.68.2	1	4062	5	novel_not_in_catalog	gene: Zm00001d027510	novel	4.85	48.29	coding	902	385	3090

PB.68.1	1	3673	5	novel_not_in_catalog	gene: Zm00001d027510	novel	43.44	48.29	coding	734	399	2600
PB.6137.3	5	1706	3	full-splice_match	gene: Zm00001d013411	Zm00001d013411_T00	6.49	34.13	coding	416	260	1507
PB.2859.7	2	1845	4	novel_in_catalog	gene: Zm00001d003683	novel	2.13	357.68	coding	219	1039	1695
PB.6922.1	5	1388	6	full-splice_match	gene: Zm00001d017171	Zm00001d017171_T00	21.65	21.65	coding	373	97	1215
PB.2859.5	2	1625	5	novel_in_catalog	gene: Zm00001d003683	novel	17.51	357.68	coding	389	215	1381
PB.2859.4	2	1667	5	novel_in_catalog	gene: Zm00001d003683	novel	0.00	357.68	coding	389	136	1302
PB.6726.1	5	812	3	novel_not_in_catalog	gene: Zm00001d016278	novel	56.99	61.62	coding	100	312	611
PB.2859.2	2	1696	6	full-splice_match	gene: Zm00001d003683	Zm00001d003683_T00	19.77	357.68	coding	389	56	1222
PB.2859.1	2	1823	6	full-splice_match	gene: Zm00001d003683	Zm00001d003683_T00	1.98	357.68	coding	389	63	1229
PB.9635.6	8	1038	3	novel_not_in_catalog	gene: Zm00001d010783	novel	0.00	58.04	coding	322	47	1012
PB.6056.1	5	1851	4	full-splice_match	gene: Zm00001d013159	Zm00001d013159_T00	392.19	515.67	coding	450	158	1507
PB.6056.2	5	2007	4	full-splice_match	gene: Zm00001d013159	Zm00001d013159_T00	1.17	515.67	coding	450	515	1864
PB.9635.5	8	1010	3	novel_not_in_catalog	gene: Zm00001d010783	novel	0.50	58.04	coding	297	69	959
PB.9635.2	8	2074	2	novel_in_catalog	gene: Zm00001d010783	novel	24.85	58.04	coding	232	74	769
PB.3864.1	3	1392	1	full-splice_match	gene: ENSRNA049474	ENSRNA049474264-T1	17.78	17.78	non coding	NA	NA	NA
PB.6056.6	5	1632	4	full-splice_match	gene: Zm00001d013159	Zm00001d013159_T00	122.30	515.67	coding	450	155	1504
PB.2859.8	2	1423	6	full-splice_match	gene: Zm00001d003683	Zm00001d003683_T00	316.29	357.68	coding	389	62	1228

PB.4430.6	3	1874	13	full-splice_match	gene: Zm00001d043152	Zm00001d043152_T00	0.00	115.37	coding	538	51	1664
PB.9985.3	8	1388	2	full-splice_match	gene: Zm00001d012292	Zm00001d012292_T00	43.10	83.51	coding	410	71	1300
PB.10851.6	9	1752	2	novel_not_in_catalog	gene: Zm00001d048310	novel	15.73	282.88	coding	428	241	1524
PB.7893.2	6	2201	1	full-splice_match	gene: Zm00001d038068	Zm00001d038068_T00	12.20	58.88	coding	665	28	2022
PB.10851.5	9	2194	2	full-splice_match	gene: Zm00001d048310	Zm00001d048310_T00	13.31	282.88	coding	428	683	1966
PB.1537.3	1	2197	3	genic	gene: Zm00001d034434	novel	3.82	37.65	coding	75	96	320
PB.7893.1	6	2433	1	full-splice_match	gene: Zm00001d038068	Zm00001d038068_T00	46.68	58.88	coding	694	117	2198
PB.10178.1	9	1828	6	incomplete-splice_match	gene: Zm00001d045075	Zm00001d045075_T00	26.96	26.96	coding	478	107	1540
PB.6733.1	5	1524	11	novel_in_catalog	gene: Zm00001d016308	novel	26.79	30.88	coding	348	111	1154
PB.6733.2	5	2695	10	novel_in_catalog	gene: Zm00001d016308	novel	4.09	30.88	coding	334	1411	2412
PB.2035.1	10	1488	3	full-splice_match	gene: Zm00001d025037	Zm00001d025037_T00	11.92	11.92	coding	242	659	1384
PB.1636.1	1	1141	5	novel_not_in_catalog	gene: Zm00001d034790	novel	432.03	762.63	coding	265	94	888
PB.4298.1	3	2853	4	full-splice_match	gene: Zm00001d042608	Zm00001d042608_T00	7.05	13.19	coding	711	460	2592
PB.750.2	1	1158	5	novel_in_catalog	gene: Zm00001d030895	novel	4.46	695.94	coding	154	497	958
PB.10851.3	9	1983	2	full-splice_match	gene: Zm00001d048310	Zm00001d048310_T00	29.73	282.88	coding	428	452	1735
PB.8793.2	7	2060	9	full-splice_match	gene: Zm00001d021570	Zm00001d021570_T00	0.00	10.90	coding	446	234	1571
PB.202.1	1	1070	6	full-splice_match	gene: Zm00001d028117	Zm00001d028117_T00	467.03	467.03	coding	260	70	849

PB.10358.1	9	1050	1	full-splice_match	gene: Zm00001d045931	Zm00001d045931_T00	29.63	55.05	coding	162	173	658
PB.750.4	1	816	6	incomplete-splice_match	gene: Zm00001d030895	Zm00001d030895_T00	0.00	695.94	coding	180	138	677
PB.10358.2	9	941	1	full-splice_match	gene: Zm00001d045931	Zm00001d045931_T00	25.42	55.05	coding	162	168	653
PB.2394.5	10	1081	7	full-splice_match	gene: Zm00001d026647	Zm00001d026647_T00	92.69	119.94	coding	202	140	745
PB.8446.4	7	1774	2	intergenic	novelGene_181	novel	20.65	20.65	non coding	NA	NA	NA
PB.5342.2	4	2156	1	full-splice_match	gene: Zm00001d051505	Zm00001d051505_T00	0.00	63.49	coding	291	210	1082
PB.8998.1	7	3141	13	incomplete-splice_match	gene: Zm00001d022276	Zm00001d022276_T00	6.55	6.55	coding	936	32	2839
PB.1866.1	10	2240	4	novel_in_catalog	gene: Zm00001d024160	novel	5.69	78.38	coding	122	1292	1657
PB.2394.3	10	1218	7	full-splice_match	gene: Zm00001d026647	Zm00001d026647_T00	27.25	119.94	coding	202	142	747
PB.1866.3	10	2006	8	full-splice_match	gene: Zm00001d024160	Zm00001d024160_T00	46.67	78.38	coding	424	167	1438
PB.2430.1	2	2593	15	full-splice_match	gene: Zm00001d001804	Zm00001d001804_T00	5.47	5.47	coding	594	689	2470
PB.4430.1	3	2079	13	full-splice_match	gene: Zm00001d043152	Zm00001d043152_T00	31.86	115.37	coding	538	51	1664
PB.1866.8	10	1918	8	full-splice_match	gene: Zm00001d024160	Zm00001d024160_T00	11.26	78.38	coding	422	165	1430
PB.1866.9	10	2011	7	novel_in_catalog	gene: Zm00001d024160	novel	0.87	78.38	coding	453	165	1523
PB.2851.1	2	2712	1	full-splice_match	gene: Zm00001d003668	Zm00001d003668_T00	81.31	81.31	coding	771	195	2507
PB.5342.4	4	1889	2	genic	gene: Zm00001d051505	novel	2.18	63.49	coding	291	83	955
PB.8569.1	7	1060	1	genic	gene: Zm00001d020606	novel	0.54	11.69	coding	179	128	664

PB.8569.3	7	956	1	genic	gene: Zm00001d020606	novel	11.15	11.69	coding	179	128	664
PB.52.1	1	2315	7	full-splice_match	gene: Zm00001d027450	Zm00001d027450_T00	8.87	31.57	coding	643	70	1998
PB.52.2	1	2182	7	full-splice_match	gene: Zm00001d027450	Zm00001d027450_T00	22.70	31.57	coding	643	70	1998
PB.52.3	1	2033	7	full-splice_match	gene: Zm00001d027450	Zm00001d027450_T00	0.00	31.57	coding	643	52	1980
PB.922.4	1	3039	4	novel_in_catalog	gene: Zm00001d031769	novel	121.87	222.47	coding	906	118	2835
PB.8190.4	6	1471	11	full-splice_match	gene: Zm00001d039157	Zm00001d039157_T00	0.00	108.89	coding	377	101	1231
PB.5212.1	4	2005	11	full-splice_match	gene: Zm00001d050615	Zm00001d050615_T00	20.59	20.59	coding	562	59	1744
PB.5342.6	4	1868	1	full-splice_match	gene: Zm00001d051505	Zm00001d051505_T00	0.00	63.49	coding	291	210	1082
PB.6114.2	5	623	1	full-splice_match	gene: Zm00001d013332	Zm00001d013332_T00	0.00	140.90	coding	70	282	491
PB.6114.1	5	835	1	full-splice_match	gene: Zm00001d013332	Zm00001d013332_T00	140.90	140.90	coding	167	89	589
PB.5133.3	4	2403	4	full-splice_match	gene: Zm00001d050103	Zm00001d050103_T00	3.38	27.27	coding	481	581	2023
PB.5133.2	4	1798	5	full-splice_match	gene: Zm00001d050103	Zm00001d050103_T00	9.03	27.27	coding	455	53	1417
PB.5133.1	4	2021	5	full-splice_match	gene: Zm00001d050103	Zm00001d050103_T00	7.45	27.27	coding	455	156	1520
PB.8035.1	6	2475	2	genic	gene: Zm00001d038548	novel	1.70	1.70	coding	690	17	2086
PB.5133.4	4	1331	3	incomplete-splice_match	gene: Zm00001d050103	Zm00001d050103_T00	7.41	27.27	coding	186	532	1089
PB.4277.1	3	1719	2	incomplete-splice_match	gene: Zm00001d042504	Zm00001d042504_T00	4.69	4.69	coding	191	995	1567
PB.5800.2	4	1905	1	full-splice_match	gene: Zm00001d053586	Zm00001d053586_T00	5.40	9.84	coding	509	41	1567

PB.605.2	1	892	2	incomplete-splice_match	gene: Zm00001d029988	Zm00001d029988_T00	21.58	21.58	coding	190	80	649
PB.4344.1	3	1858	1	full-splice_match	gene: Zm00001d042786	Zm00001d042786_T00	24.03	57.68	coding	391	220	1392
PB.10849.1	9	589	1	full-splice_match	gene: Zm00001d048307	Zm00001d048307_T00	36.71	36.71	non coding	NA	NA	NA
PB.4840.3	4	1332	1	full-splice_match	gene: Zm00001d048604	Zm00001d048604_T00	0.00	19.07	coding	318	45	998
PB.4840.2	4	1410	1	full-splice_match	gene: Zm00001d048604	Zm00001d048604_T00	8.20	19.07	coding	301	1	903
PB.4840.1	4	2461	1	full-splice_match	gene: Zm00001d048604	Zm00001d048604_T00	10.87	19.07	coding	546	98	1735
PB.7916.40	6	937	1	incomplete-splice_match	gene: Zm00001d038146	Zm00001d038146_T00	0.00	442.81	coding	198	188	781
PB.4430.8	3	1422	11	incomplete-splice_match	gene: Zm00001d043152	Zm00001d043152_T00	1.59	115.37	coding	449	72	1418
PB.9818.1	8	727	5	novel_not_in_catalog	gene: Zm00001d011623	novel	58.44	58.44	coding	140	22	441
PB.10085.1	8	1756	3	full-splice_match	gene: Zm00001d012712	Zm00001d012712_T00	8.00	8.00	coding	338	128	1141
PB.1318.2	1	1595	1	intergenic	novelGene_455	novel	0.64	0.64	coding	479	159	1595
PB.8209.1	6	1646	1	full-splice_match	gene: Zm00001d039216	Zm00001d039216_T00	41.13	41.13	coding	391	236	1408
PB.6945.1	5	1213	2	full-splice_match	gene: Zm00001d017268	Zm00001d017268_T00	2.32	15.13	coding	244	101	832
PB.6945.2	5	1027	2	full-splice_match	gene: Zm00001d017268	Zm00001d017268_T00	12.81	15.13	coding	244	101	832
PB.5586.1	4	1235	5	full-splice_match	gene: Zm00001d052458	Zm00001d052458_T00	159.79	174.28	coding	296	157	1044
PB.4450.1	3	990	2	full-splice_match	gene: Zm00001d043232	Zm00001d043232_T00	56.17	56.17	coding	214	179	820
PB.7869.1	6	1005	1	genic	gene: Zm00001d038003	novel	15.44	228.40	coding	116	23	370

PB.927.2	1	1219	6	full-splice_match	gene: Zm00001d031809	Zm00001d031809_T00	20.52	29.36	coding	234	204	905
PB.927.3	1	1054	6	full-splice_match	gene: Zm00001d031809	Zm00001d031809_T00	0.00	29.36	coding	234	177	878
PB.927.1	1	1375	6	full-splice_match	gene: Zm00001d031809	Zm00001d031809_T00	8.85	29.36	coding	306	10	927
PB.9838.4	8	1460	9	incomplete-splice_match	gene: Zm00001d011713	Zm00001d011713_T00	20.50	112.91	coding	403	251	1459
PB.5490.1	4	2136	1	genic	gene: Zm00001d052168	novel	0.00	4.30	coding	586	78	1835
PB.9838.1	8	2827	18	incomplete-splice_match	gene: Zm00001d011713	Zm00001d011713_T00	64.79	112.91	coding	655	449	2413
PB.3319.2	2	1300	2	incomplete-splice_match	gene: Zm00001d006236	Zm00001d006236_T00	57.21	62.99	coding	273	154	972
PB.9838.3	8	1247	8	incomplete-splice_match	gene: Zm00001d011713	Zm00001d011713_T01	27.63	112.91	coding	272	431	1246
PB.1626.1	1	920	1	full-splice_match	gene: Zm00001d034756	Zm00001d034756_T00	33.60	498.31	non coding	NA	NA	NA
PB.2418.2	2	1651	1	full-splice_match	gene: Zm00001d001766	Zm00001d001766_T00	21.49	76.58	coding	311	224	1156
PB.7938.3	6	1381	13	incomplete-splice_match	gene: Zm00001d038205	Zm00001d038205_T00	0.00	22.73	coding	415	136	1380
PB.4079.1	3	1282	7	novel_in_catalog	gene: Zm00001d041474	novel	64.63	117.69	coding	178	260	793
PB.2418.1	2	1684	1	full-splice_match	gene: Zm00001d001766	Zm00001d001766_T00	12.71	76.58	coding	311	140	1072
PB.4079.6	3	922	7	novel_in_catalog	gene: Zm00001d041474	novel	0.00	117.69	coding	178	209	742
PB.2418.4	2	1515	1	full-splice_match	gene: Zm00001d001766	Zm00001d001766_T00	42.38	76.58	coding	311	199	1131
PB.4079.4	3	1099	7	novel_in_catalog	gene: Zm00001d041474	novel	37.54	117.69	coding	178	171	704
PB.5327.3	4	2073	7	full-splice_match	gene: Zm00001d051431	Zm00001d051431_T00	4.73	40.22	coding	546	188	1825

PB.7632.1	6	3311	17	novel_in_catalog	gene: Zm00001d036927	novel	9.82	9.82	coding	345	2157	3191
PB.2268.1	10	2124	5	full-splice_match	gene: Zm00001d026258	Zm00001d026258_T00	25.86	25.86	coding	482	405	1850
PB.8317.1	7	3316	21	novel_in_catalog	gene: Zm00001d019062	novel	125.62	125.62	coding	956	114	2981
PB.4783.11	3	2171	15	novel_in_catalog	gene: Zm00001d044494	novel	5.37	46.54	non coding	NA	NA	NA
PB.4783.10	3	2713	14	novel_in_catalog	gene: Zm00001d044494	novel	4.83	46.54	coding	79	68	304
PB.2372.1	10	1685	3	full-splice_match	gene: Zm00001d026580	Zm00001d026580_T00	15.75	15.75	coding	458	115	1488
PB.8029.1	6	1505	6	full-splice_match	gene: Zm00001d038540	Zm00001d038540_T00	11.52	11.52	coding	364	164	1255
PB.922.1	1	3131	4	novel_in_catalog	gene: Zm00001d031769	novel	85.93	222.47	coding	906	101	2818
PB.9965.1	8	2501	5	full-splice_match	gene: Zm00001d012224	Zm00001d012224_T00	34.84	34.84	coding	378	1107	2240
PB.3403.1	2	1772	9	incomplete-splice_match	gene: Zm00001d006602	Zm00001d006602_T00	47.27	113.44	coding	422	44	1309
PB.3403.3	2	1664	3	novel_in_catalog	gene: Zm00001d006602	novel	9.38	113.44	coding	100	542	841
PB.3403.2	2	1626	9	incomplete-splice_match	gene: Zm00001d006602	Zm00001d006602_T00	56.79	113.44	coding	422	7	1272
PB.10860.3	9	3342	13	full-splice_match	gene: Zm00001d048338	Zm00001d048338_T02	0.00	34.93	coding	1054	70	3231
PB.3075.2	2	2570	8	full-splice_match	gene: Zm00001d005072	Zm00001d005072_T00	19.61	19.61	coding	691	147	2219
PB.10860.1	9	3568	13	full-splice_match	gene: Zm00001d048338	Zm00001d048338_T02	34.93	34.93	coding	1054	120	3281
PB.4423.1	3	1455	2	incomplete-splice_match	gene: Zm00001d043135	Zm00001d043135_T00	11.98	24.38	coding	300	8	907
PB.4464.1	3	863	1	incomplete-splice_match	gene: Zm00001d043288	Zm00001d043288_T00	28.46	56.97	non coding	NA	NA	NA

PB.5651.1	4	1169	1	full-splice_match	gene: Zm00001d052799	Zm00001d052799_T00	8.44	8.44	coding	307	48	968
PB.10919.2	9	502	1	genic	gene: Zm00001d048522	novel	0.00	5.18	non coding	NA	NA	NA
PB.4464.2	3	2474	6	full-splice_match	gene: Zm00001d043288	Zm00001d043288_T00	28.51	56.97	coding	376	741	1868
PB.6133.1	5	1591	1	intergenic	novelGene_47	novel	29.94	29.94	coding	105	791	1105
PB.3854.1	3	3449	15	full-splice_match	gene: Zm00001d040008	Zm00001d040008_T00	67.33	80.99	coding	822	676	3141
PB.621.1	1	1953	1	full-splice_match	gene: Zm00001d030083	Zm00001d030083_T00	1.50	51.23	coding	342	113	1138
PB.3854.3	3	3955	10	novel_in_catalog	gene: Zm00001d040008	novel	13.65	80.99	coding	666	1745	3742
PB.3854.2	3	2850	14	incomplete-splice_match	gene: Zm00001d040008	Zm00001d040008_T00	0.00	80.99	coding	822	170	2635
PB.9258.4	8	1373	11	full-splice_match	gene: Zm00001d008820	Zm00001d008820_T00	68.36	244.63	coding	334	137	1138
PB.765.7	1	606	1	full-splice_match	gene: Zm00001d022856	Zm00001d022856_T00	39.25	45.59	non coding	NA	NA	NA
PB.765.4	1	2129	1	full-splice_match	gene: Zm00001d022856	Zm00001d022856_T00	6.34	45.59	coding	226	605	1282
PB.2905.2	2	1839	5	full-splice_match	gene: Zm00001d003925	Zm00001d003925_T00	0.00	23.75	coding	486	198	1655
PB.7930.1	6	944	1	full-splice_match	gene: Zm00001d038191	Zm00001d038191_T00	31.42	31.42	non coding	NA	NA	NA
PB.2988.2	2	1648	6	novel_in_catalog	gene: Zm00001d004498	novel	9.95	35.29	coding	159	199	675
PB.3311.1	2	1653	2	full-splice_match	gene: Zm00001d006203	Zm00001d006203_T00	81.21	106.00	coding	505	43	1557
PB.765.1	1	1675	3	full-splice_match	gene: Zm00001d030989	Zm00001d030989_T00	141.52	141.52	coding	159	251	727
PB.4752.5	3	1199	7	incomplete-splice_match	gene: Zm00001d044358	Zm00001d044358_T01	3.21	11.14	coding	350	150	1199

PB.2983.1	2	1800	2	full-splice_match	gene: Zm00001d004467	Zm00001d004467_T00	19.66	19.66	coding	421	188	1450
PB.8021.1	6	757	3	full-splice_match	gene: Zm00001d038526	Zm00001d038526_T00	44.71	44.71	coding	100	8	307
PB.3663.1	2	967	1	full-splice_match	gene: Zm00001d007935	Zm00001d007935_T00	39.36	39.36	coding	220	51	710
PB.2988.4	2	1203	3	full-splice_match	gene: Zm00001d004498	Zm00001d004498_T00	3.55	35.29	coding	122	326	691
PB.6951.1	5	2175	1	full-splice_match	gene: Zm00001d017279	Zm00001d017279_T00	150.19	150.19	coding	669	16	2022
PB.10320.4	9	426	2	intergenic	novelGene_119	novel	13.29	13.29	non coding	NA	NA	NA
PB.9023.1	7	1922	4	full-splice_match	gene: Zm00001d022366	Zm00001d022366_T00	43.06	43.06	coding	422	230	1495
PB.7761.2	6	1046	3	novel_in_catalog	gene: Zm00001d037530	novel	4.56	31.37	coding	161	316	798
PB.7761.3	6	1404	6	full-splice_match	gene: Zm00001d037530	Zm00001d037530_T00	21.52	31.37	coding	363	74	1162
PB.10320.2	9	1001	3	intergenic	novelGene_118	novel	18.08	23.84	non coding	NA	NA	NA
PB.7761.1	6	1919	5	novel_in_catalog	gene: Zm00001d037530	novel	5.29	31.37	coding	279	832	1668
PB.7932.2	6	1892	4	full-splice_match	gene: Zm00001d038194	Zm00001d038194_T00	0.00	21.16	coding	424	370	1641
PB.5539.1	4	1136	8	novel_in_catalog	gene: Zm00001d052316	novel	64.63	64.63	coding	254	102	863
PB.3604.1	2	1749	9	novel_in_catalog	gene: Zm00001d007677	novel	51.67	51.67	coding	449	98	1444
PB.9080.1	7	1104	2	incomplete-splice_match	gene: Zm00001d022527	Zm00001d022527_T02	9.79	41.03	coding	3	654	662
PB.10398.1	9	2460	14	incomplete-splice_match	gene: Zm00001d046221	Zm00001d046221_T02	38.67	38.67	coding	741	238	2460
PB.9023.2	7	1711	4	full-splice_match	gene: Zm00001d022366	Zm00001d022366_T00	0.00	43.06	coding	422	231	1496

PB.1347.1	1	531	2	novel_not_in_catalog	gene: Zm00001d033589	novel	46.17	46.17	non coding	NA	NA	NA
PB.10715.1	9	1055	6	full-splice_match	gene: Zm00001d047766	Zm00001d047766_T00	63.54	64.47	coding	234	93	794
PB.10715.2	9	2462	5	full-splice_match	gene: Zm00001d047766	Zm00001d047766_T00	0.94	64.47	coding	112	1865	2200
PB.6899.18	5	2241	7	full-splice_match	gene: Zm00001d017096	Zm00001d017096_T01	1.27	254.51	coding	129	107	493
PB.1953.1	10	1614	6	full-splice_match	gene: Zm00001d024715	Zm00001d024715_T00	25.15	39.61	coding	303	292	1200
PB.4005.4	3	638	1	full-splice_match	gene: Zm00001d040803	Zm00001d040803_T00	139.48	421.74	coding	113	158	496
PB.1953.2	10	1500	6	full-splice_match	gene: Zm00001d024715	Zm00001d024715_T00	14.46	39.61	coding	303	293	1201
PB.4005.2	3	1021	1	full-splice_match	gene: Zm00001d040803	Zm00001d040803_T00	28.04	421.74	coding	113	327	665
PB.4005.3	3	734	1	full-splice_match	gene: Zm00001d040803	Zm00001d040803_T00	175.98	421.74	coding	113	152	490
PB.6899.11	5	1819	5	novel_in_catalog	gene: Zm00001d017096	novel	29.06	254.51	coding	129	106	492
PB.4005.1	3	964	1	full-splice_match	gene: Zm00001d040803	Zm00001d040803_T00	78.24	421.74	coding	113	160	498
PB.4311.2	3	1361	3	novel_in_catalog	gene: Zm00001d042653	novel	76.71	87.81	coding	334	162	1163
PB.4786.1	3	1095	10	full-splice_match	gene: Zm00001d044497	Zm00001d044497_T00	21.93	47.06	coding	204	263	874
PB.877.5	1	1661	5	novel_in_catalog	gene: Zm00001d031529	novel	15.18	90.73	coding	174	363	884
PB.7227.1	5	1451	1	intergenic	novelGene_418	novel	10.45	56.56	non coding	NA	NA	NA
PB.10572.4	9	1337	5	full-splice_match	gene: Zm00001d047217	Zm00001d047217_T00	4.43	160.92	coding	198	612	1205
PB.7227.2	5	1314	1	intergenic	novelGene_418	novel	0.00	56.56	non coding	NA	NA	NA

PB.898.1	1	1210	5	full-splice_match	gene: Zm00001d031637	Zm00001d031637_T00	56.16	73.48	coding	213	179	817
PB.6698.2	5	2718	5	full-splice_match	gene: Zm00001d016131	Zm00001d016131_T00	16.21	28.24	coding	667	241	2241
PB.3079.1	2	3612	6	novel_in_catalog	gene: Zm00001d005085	novel	34.46	34.46	coding	1051	213	3365
PB.898.2	1	1028	5	full-splice_match	gene: Zm00001d031637	Zm00001d031637_T00	17.32	73.48	coding	213	107	745
PB.3186.2	2	597	5	full-splice_match	gene: Zm00001d005675	Zm00001d005675_T00	246.89	373.03	coding	78	74	307
PB.7806.1	6	1398	6	full-splice_match	gene: Zm00001d037725	Zm00001d037725_T00	112.29	112.29	coding	308	94	1017
PB.8541.1	7	1473	4	full-splice_match	gene: Zm00001d020444	Zm00001d020444_T00	16.96	16.96	coding	414	60	1301
PB.601.2	1	1217	1	novel_in_catalog	gene: Zm00001d029948	novel	3.73	8.19	coding	297	125	1015
PB.5799.1	4	1989	16	novel_in_catalog	gene: Zm00001d053580	novel	25.89	81.19	coding	419	232	1488
PB.4786.4	3	1045	10	full-splice_match	gene: Zm00001d044497	Zm00001d044497_T00	8.43	47.06	coding	204	233	844
PB.3293.4	2	1191	2	incomplete-splice_match	gene: Zm00001d006130	Zm00001d006130_T00	0.00	94.71	coding	309	265	1191
PB.1600.2	1	1739	1	genic	gene: Zm00001d034669	novel	8.76	27.63	coding	400	60	1259
PB.2021.2	10	1782	2	full-splice_match	gene: Zm00001d025005	Zm00001d025005_T00	0.00	15.52	coding	422	176	1441
PB.1488.1	1	1319	4	incomplete-splice_match	gene: Zm00001d034237	Zm00001d034237_T00	36.10	36.10	coding	365	36	1130
PB.1880.1	10	1085	6	full-splice_match	gene: Zm00001d024292	Zm00001d024292_T00	68.05	68.05	coding	264	113	904
PB.7869.4	6	665	2	full-splice_match	gene: Zm00001d038003	Zm00001d038003_T00	61.01	228.40	coding	158	108	581
PB.1019.11	1	964	9	novel_in_catalog	gene: Zm00001d032220	novel	5.72	44.15	non coding	NA	NA	NA

PB.3194.1	2	776	1	full-splice_match	gene: Zm00001d005698	Zm00001d005698_T00	181.42	181.42	coding	180	62	601
PB.3507.10	2	1727	3	novel_not_in_catalog	gene: Zm00001d007050	novel	0.00	84.89	coding	340	77	1096
PB.3216.1	2	1958	14	full-splice_match	gene: Zm00001d005818	Zm00001d005818_T00	80.59	80.59	coding	509	166	1692
PB.3216.3	2	1782	14	full-splice_match	gene: Zm00001d005818	Zm00001d005818_T00	0.00	80.59	coding	509	60	1586
PB.1158.1	1	2420	2	full-splice_match	gene: Zm00001d032745	Zm00001d032745_T00	8.44	8.44	coding	701	86	2188
PB.4510.1	3	3907	12	incomplete-splice_match	gene: Zm00001d043452	Zm00001d043452_T01	61.31	61.31	coding	1182	105	3650
PB.6253.2	5	1456	5	novel_in_catalog	gene: Zm00001d013957	novel	28.97	79.79	coding	109	184	510
PB.6253.1	5	928	6	full-splice_match	gene: Zm00001d013957	Zm00001d013957_T00	50.81	79.79	coding	243	31	759
PB.5118.3	4	2256	12	full-splice_match	gene: Zm00001d049975	Zm00001d049975_T00	4.20	84.38	coding	178	128	661
PB.10356.2	9	2156	8	novel_in_catalog	gene: Zm00001d045897	novel	48.68	48.68	coding	630	70	1959
PB.7668.1	6	1826	7	full-splice_match	gene: Zm00001d037108	Zm00001d037108_T00	97.14	97.14	coding	461	135	1517
PB.908.1	1	1671	2	full-splice_match	gene: Zm00001d031677	Zm00001d031677_T00	66.54	66.54	coding	418	86	1339
PB.1407.2	1	1347	10	full-splice_match	gene: Zm00001d033870	Zm00001d033870_T00	0.00	17.73	coding	316	107	1054
PB.10775.1	9	431	4	full-splice_match	gene: Zm00001d047962	Zm00001d047962_T00	181.56	181.56	non coding	NA	NA	NA
PB.8832.1	7	1437	2	novel_in_catalog	gene: Zm00001d021705	novel	0.90	0.90	coding	217	126	776
PB.1359.2	1	2291	6	incomplete-splice_match	gene: Zm00001d033642	Zm00001d033642_T00	63.58	85.06	coding	634	182	2083
PB.7048.1	5	1034	2	incomplete-splice_match	gene: Zm00001d017712	Zm00001d017712_T00	13.43	13.43	coding	118	489	842

PB.10709.2	9	1629	3	full-splice_match	gene: Zm00001d047750	Zm00001d047750_T00	109.12	109.12	coding	352	211	1266
PB.4570.1	3	1041	4	incomplete-splice_match	gene: Zm00001d043653	Zm00001d043653_T00	28.98	28.98	coding	173	19	537
PB.10641.11	9	1592	15	full-splice_match	gene: Zm00001d047498	Zm00001d047498_T00	0.00	128.79	coding	257	85	855
PB.6703.1	5	1264	4	full-splice_match	gene: Zm00001d016154	Zm00001d016154_T00	25.44	27.58	coding	252	223	978
PB.10882.1	9	1119	8	full-splice_match	gene: Zm00001d048410	Zm00001d048410_T00	57.85	107.65	coding	200	194	793
PB.1122.1	1	2351	11	novel_in_catalog	gene: Zm00001d032605	novel	89.86	89.86	coding	136	102	509
PB.738.2	1	1741	2	novel_in_catalog	gene: Zm00001d030843	novel	5.05	6.31	coding	206	933	1550
PB.3745.1	3	2787	1	intergenic	novelGene_27	novel	6.40	53.07	coding	439	552	1868
PB.2895.2	2	2500	8	incomplete-splice_match	gene: Zm00001d003879	Zm00001d003879_T00	6.02	375.02	coding	356	1244	2311
PB.6933.2	5	2392	15	full-splice_match	gene: Zm00001d017203	Zm00001d017203_T01	0.00	72.13	coding	293	277	1155
PB.6933.3	5	2282	16	full-splice_match	gene: Zm00001d017203	Zm00001d017203_T00	47.73	72.13	coding	582	342	2087
PB.6885.3	5	1684	9	full-splice_match	gene: Zm00001d017053	Zm00001d017053_T00	6.17	10.57	coding	446	141	1478
PB.6703.3	5	1399	3	novel_in_catalog	gene: Zm00001d016154	novel	2.14	27.58	coding	135	154	558
PB.6933.4	5	2846	14	novel_in_catalog	gene: Zm00001d017203	novel	2.85	72.13	coding	403	329	1537
PB.6933.5	5	1696	10	incomplete-splice_match	gene: Zm00001d017203	Zm00001d017203_T00	6.18	72.13	non coding	NA	NA	NA
PB.9308.3	8	1783	4	novel_not_in_catalog	gene: Zm00001d009054	novel	6.14	53.97	non coding	NA	NA	NA
PB.9308.2	8	1033	6	novel_not_in_catalog	gene: Zm00001d009054	novel	15.90	53.97	non coding	NA	NA	NA

PB.9308.1	8	1689	3	novel_in_catalog	gene: Zm00001d009054	novel	7.64	53.97	non coding	NA	NA	NA
PB.5878.2	4	1260	6	novel_in_catalog	gene: Zm00001d053888	novel	29.14	49.82	non coding	NA	NA	NA
PB.9554.2	8	1074	4	full-splice_match	gene: Zm00001d010452	Zm00001d010452_T00	18.82	28.37	coding	264	11	802
PB.9308.6	8	2210	3	novel_in_catalog	gene: Zm00001d009054	novel	8.59	53.97	non coding	NA	NA	NA
PB.9308.5	8	1132	5	novel_not_in_catalog	gene: Zm00001d009054	novel	15.70	53.97	non coding	NA	NA	NA
PB.9554.1	8	1178	3	novel_in_catalog	gene: Zm00001d010452	novel	8.89	28.37	coding	180	18	557
PB.1661.1	1	1541	6	full-splice_match	gene: Zm00001d034871	Zm00001d034871_T00	44.70	109.09	coding	249	181	927
PB.7986.2	6	1049	2	full-splice_match	gene: Zm00001d038381	Zm00001d038381_T00	559.31	566.39	coding	160	254	733
PB.7561.1	6	1921	5	novel_not_in_catalog	gene: Zm00001d036541	novel	66.39	146.57	coding	484	81	1532
PB.7561.3	6	1809	5	novel_not_in_catalog	gene: Zm00001d036541	novel	80.18	146.57	coding	484	81	1532
PB.404.2	1	931	1	full-splice_match	gene: Zm00001d028968	Zm00001d028968_T00	95.72	109.50	coding	165	141	635
PB.404.3	1	791	1	full-splice_match	gene: Zm00001d028968	Zm00001d028968_T00	12.61	109.50	coding	165	137	631
PB.7393.2	6	2877	5	novel_not_in_catalog	gene: Zm00001d035492	novel	87.37	87.37	coding	304	1687	2598
PB.8918.1	7	1433	2	incomplete-splice_match	gene: Zm00001d021968	Zm00001d021968_T00	13.56	13.56	coding	387	112	1272
PB.7957.1	6	1165	1	genic	gene: Zm00001d038288	novel	4.94	4.94	coding	139	92	508
PB.2924.1	2	1036	6	full-splice_match	gene: Zm00001d004023	Zm00001d004023_T00	89.27	89.27	coding	213	69	707
PB.968.1	1	1250	1	incomplete-splice_match	gene: Zm00001d031977	Zm00001d031977_T00	42.45	42.45	coding	261	41	823

PB.2631.1	2	1471	7	full-splice_match	gene: Zm00001d002641	Zm00001d002641_T00	7.74	7.74	coding	217	430	1080
PB.13.12	1	2287	10	full-splice_match	gene: Zm00001d027293	Zm00001d027293_T00	25.46	422.53	coding	625	209	2083
PB.2021.1	10	1936	2	full-splice_match	gene: Zm00001d025005	Zm00001d025005_T00	15.52	15.52	coding	422	176	1441
PB.8172.3	6	2070	7	incomplete-splice_match	gene: Zm00001d039072	Zm00001d039072_T00	1.28	18.98	coding	475	242	1666
PB.4191.5	3	723	1	novel_in_catalog	gene: Zm00001d042055	novel	0.63	13.25	coding	152	65	520
PB.383.2	1	904	6	incomplete-splice_match	gene: Zm00001d028890	Zm00001d028890_T01	38.64	47.85	coding	157	193	663
PB.13.18	1	2037	10	novel_not_in_catalog	gene: Zm00001d027293	novel	2.74	422.53	coding	450	102	1451
PB.4634.2	3	836	1	intergenic	novelGene_408	novel	4.18	4.18	non coding	NA	NA	NA
PB.383.1	1	1054	6	incomplete-splice_match	gene: Zm00001d028890	Zm00001d028890_T01	9.20	47.85	coding	157	169	639
PB.4578.3	3	927	3	full-splice_match	gene: Zm00001d043675	Zm00001d043675_T00	0.00	25.40	coding	206	309	926
PB.10750.3	9	3320	8	full-splice_match	gene: Zm00001d047869	Zm00001d047869_T00	7.02	43.80	coding	305	2251	3165
PB.188.1	1	1547	1	novel_in_catalog	gene: Zm00001d028046	novel	4.30	4.30	coding	461	109	1491
PB.6641.1	5	668	1	intergenic	novelGene_221	novel	0.26	0.26	non coding	NA	NA	NA
PB.4961.1	4	2457	14	incomplete-splice_match	gene: Zm00001d049190	Zm00001d049190_T01	25.69	25.69	coding	689	125	2191
PB.1708.1	10	1714	1	intergenic	novelGene_16	novel	21.44	21.44	coding	118	160	513
PB.7946.1	6	2483	5	full-splice_match	gene: Zm00001d038229	Zm00001d038229_T00	17.57	17.57	coding	632	284	2179
PB.8095.2	6	964	1	full-splice_match	gene: Zm00001d038810	Zm00001d038810_T00	188.72	210.98	coding	217	74	724

PB.8241.1	7	1300	1	full-splice_match	gene: Zm00001d018712	Zm00001d018712_T00	0.56	7.49	coding	232	325	1020
PB.5014.1	4	902	2	full-splice_match	gene: Zm00001d049423	Zm00001d049423_T00	271.86	271.86	coding	229	92	778
PB.557.3	1	733	1	full-splice_match	gene: Zm00001d029669	Zm00001d029669_T00	59.80	77.47	coding	129	119	505
PB.557.2	1	841	1	full-splice_match	gene: Zm00001d029669	Zm00001d029669_T00	9.51	77.47	coding	129	119	505
PB.557.1	1	1106	1	full-splice_match	gene: Zm00001d029669	Zm00001d029669_T00	8.16	77.47	coding	129	119	505
PB.4191.1	3	2354	10	novel_not_in_catalog	gene: Zm00001d042055	novel	10.30	13.25	coding	436	65	1372
PB.5518.2	4	3506	24	novel_in_catalog	gene: Zm00001d052248	novel	6.35	29.80	coding	516	145	1692
PB.5518.3	4	3459	24	full-splice_match	gene: Zm00001d052248	Zm00001d052248_T00	3.30	29.80	coding	983	197	3145
PB.5510.2	4	784	8	incomplete-splice_match	gene: Zm00001d052227	Zm00001d052227_T00	0.00	6.94	coding	232	27	722
PB.5518.1	4	2302	17	incomplete-splice_match	gene: Zm00001d052248	Zm00001d052248_T01	15.67	29.80	coding	505	301	1815
PB.5518.4	4	2908	24	full-splice_match	gene: Zm00001d052248	Zm00001d052248_T01	4.48	29.80	coding	737	567	2777
PB.1661.3	1	1235	6	full-splice_match	gene: Zm00001d034871	Zm00001d034871_T00	64.39	109.09	coding	249	134	880
PB.4748.14	3	1666	6	novel_in_catalog	gene: Zm00001d044327	novel	0.00	192.78	coding	130	939	1328
PB.6474.4	5	3974	2	full-splice_match	gene: Zm00001d014876	Zm00001d014876_T04	0.00	96.45	coding	1073	531	3749
PB.5774.2	4	1299	1	novel_in_catalog	gene: Zm00001d053425	novel	3.64	96.28	non coding	NA	NA	NA
PB.6474.1	5	4176	3	full-splice_match	gene: Zm00001d014876	Zm00001d014876_T03	32.65	96.45	coding	1073	624	3842
PB.6474.2	5	3959	2	full-splice_match	gene: Zm00001d014876	Zm00001d014876_T04	13.71	96.45	coding	1073	273	3491

PB.6474.3	5	1469	2	incomplete-splice_match	gene: Zm00001d014876	Zm00001d014876_T04	50.09	96.45	coding	260	434	1213
PB.5202.1	4	803	2	full-splice_match	gene: Zm00001d026860	Zm00001d026860_T00	7.44	7.44	non coding	NA	NA	NA
PB.9181.1	8	926	5	full-splice_match	gene: Zm00001d008430	Zm00001d008430_T00	13.32	35.95	coding	126	230	607
PB.9181.2	8	859	4	full-splice_match	gene: Zm00001d008430	Zm00001d008430_T00	22.63	35.95	coding	126	163	540
PB.8557.1	7	1863	8	full-splice_match	gene: Zm00001d020537	Zm00001d020537_T00	19.79	19.79	coding	529	106	1692
PB.1017.1	1	1445	4	full-splice_match	gene: Zm00001d032208	Zm00001d032208_T00	4.69	4.69	coding	341	85	1107
PB.5774.1	4	1604	5	full-splice_match	gene: Zm00001d053425	Zm00001d053425_T00	92.64	96.28	coding	382	178	1323
PB.1661.2	1	1409	6	full-splice_match	gene: Zm00001d034871	Zm00001d034871_T00	0.00	109.09	coding	249	178	924
PB.3117.1	2	2263	2	genic	gene: Zm00001d005275	novel	11.32	11.32	coding	578	143	1876
PB.8434.7	7	1850	3	novel_in_catalog	gene: Zm00001d019875	novel	9.60	36.56	coding	269	140	946
PB.8434.4	7	2245	5	novel_not_in_catalog	gene: Zm00001d019875	novel	5.89	36.56	coding	468	127	1530
PB.8434.5	7	2392	5	novel_not_in_catalog	gene: Zm00001d019875	novel	14.60	36.56	coding	466	169	1566
PB.8434.3	7	2943	4	novel_not_in_catalog	gene: Zm00001d019875	novel	4.81	36.56	coding	468	169	1572
PB.8922.1	7	637	1	full-splice_match	gene: Zm00001d021977	Zm00001d021977_T00	7.84	7.84	coding	99	109	405
PB.5046.2	4	692	3	full-splice_match	gene: Zm00001d049606	Zm00001d049606_T00	0.00	49.88	coding	164	83	574
PB.5046.1	4	830	3	full-splice_match	gene: Zm00001d049606	Zm00001d049606_T00	46.54	49.88	coding	164	114	605
PB.5681.1	4	2149	13	full-splice_match	gene: Zm00001d052948	Zm00001d052948_T00	76.80	97.00	coding	542	218	1843

PB.8434.8	7	1663	3	novel_in_catalog	gene: Zm00001d019875	novel	1.65	36.56	coding	468	192	1595
PB.2294.1	10	2225	1	full-splice_match	gene: Zm00001d026326	Zm00001d026326_T00	9.05	58.91	coding	564	48	1739
PB.9910.2	8	1219	4	incomplete-splice_match	gene: Zm00001d011984	Zm00001d011984_T00	1.38	11.44	coding	128	345	728
PB.4493.3	3	518	2	full-splice_match	gene: Zm00001d043391	Zm00001d043391_T00	0.00	45.91	coding	7	352	372
PB.9910.1	8	2202	10	full-splice_match	gene: Zm00001d011984	Zm00001d011984_T00	10.06	11.44	coding	500	191	1690
PB.7420.1	6	975	1	full-splice_match	gene: Zm00001d035658	Zm00001d035658_T00	2.24	6.62	coding	226	16	693
PB.7420.2	6	1542	1	full-splice_match	gene: Zm00001d035658	Zm00001d035658_T00	4.37	6.62	coding	424	200	1471
PB.8229.2	7	2315	2	full-splice_match	gene: Zm00001d018653	Zm00001d018653_T00	0.00	42.00	coding	654	20	1981
PB.4975.1	4	1859	3	full-splice_match	gene: Zm00001d049239	Zm00001d049239_T00	1283.22	1283.22	coding	485	79	1533
PB.4418.2	3	1732	2	genic	gene: Zm00001d043125	novel	14.94	20.31	coding	350	515	1564
PB.5425.1	4	1211	7	novel_in_catalog	gene: Zm00001d051885	novel	216.22	295.46	coding	308	64	987
PB.1480.1	1	1777	1	antisense	novelGene_gene: Zm00001d034189	novel	19.97	19.97	coding	323	101	1069
PB.10897.1	9	1834	7	full-splice_match	gene: Zm00001d048467	Zm00001d048467_T00	55.63	55.63	coding	526	123	1700
PB.3992.8	3	1439	2	genic	gene: Zm00001d040697	novel	58.61	2382.29	coding	394	90	1271
PB.3992.9	3	1956	1	full-splice_match	gene: Zm00001d040697	Zm00001d040697_T00	0.00	2382.29	coding	394	621	1802
PB.3992.6	3	1566	2	genic	gene: Zm00001d040697	novel	415.73	2382.29	coding	394	87	1268
PB.3992.7	3	2075	1	full-splice_match	gene: Zm00001d040697	Zm00001d040697_T00	0.00	2382.29	coding	394	619	1800

PB.3992.4	3	2184	1	full-splice_match	gene: Zm00001d040697	Zm00001d040697_T00	32.91	2382.29	coding	394	624	1805
PB.9229.2	8	3019	13	full-splice_match	gene: Zm00001d008648	Zm00001d008648_T00	15.35	15.35	coding	908	66	2789
PB.3992.2	3	2281	1	full-splice_match	gene: Zm00001d040697	Zm00001d040697_T00	0.00	2382.29	coding	394	619	1800
PB.3992.3	3	1643	2	genic	gene: Zm00001d040697	novel	1492.38	2382.29	coding	394	83	1264
PB.3992.1	3	1784	2	genic	gene: Zm00001d040697	novel	382.66	2382.29	coding	394	89	1270
PB.8965.1	7	1315	7	novel_not_in_catalog	gene: Zm00001d022172	novel	20.23	57.27	coding	203	238	846
PB.3571.2	2	1364	5	full-splice_match	gene: Zm00001d007446	Zm00001d007446_T00	267.29	444.63	coding	266	136	933
PB.3571.1	2	2144	3	novel_in_catalog	gene: Zm00001d007446	novel	1.84	444.63	coding	173	136	654
PB.8965.2	7	1190	7	novel_not_in_catalog	gene: Zm00001d022172	novel	37.04	57.27	coding	203	243	851
PB.3780.1	3	919	4	incomplete-splice_match	gene: Zm00001d039617	Zm00001d039617_T01	7.98	7.98	coding	186	93	650
PB.3571.5	2	1133	5	full-splice_match	gene: Zm00001d007446	Zm00001d007446_T00	170.51	444.63	coding	266	140	937
PB.3571.4	2	1244	5	full-splice_match	gene: Zm00001d007446	Zm00001d007446_T00	0.00	444.63	coding	266	140	937
PB.1661.4	1	1224	6	full-splice_match	gene: Zm00001d034871	Zm00001d034871_T00	0.00	109.09	coding	249	231	977
PB.3276.3	2	1036	5	full-splice_match	gene: Zm00001d006070	Zm00001d006070_T00	14.22	71.75	coding	198	174	767
PB.428.3	1	2613	7	incomplete-splice_match	gene: Zm00001d029055	Zm00001d029055_T01	27.37	55.25	coding	724	34	2205
PB.6740.3	5	1470	8	full-splice_match	gene: Zm00001d016358	Zm00001d016358_T00	0.00	105.07	coding	414	142	1383
PB.5611.2	4	1201	3	full-splice_match	gene: Zm00001d052651	Zm00001d052651_T00	21.98	27.97	coding	297	83	973

PB.3276.1	2	1526	4	novel_in_catalog	gene: Zm00001d006070	novel	13.43	71.75	non coding	NA	NA	NA
PB.10612.1	9	1788	3	full-splice_match	gene: Zm00001d047424	Zm00001d047424_T00	121.30	121.30	coding	527	39	1619
PB.9918.2	8	2825	1	novel_in_catalog	gene: Zm00001d012015	novel	9.23	56.52	coding	208	2064	2687
PB.8945.2	7	2385	1	genic	gene: Zm00001d022069	novel	3.63	13.03	coding	192	487	1062
PB.8945.1	7	1607	2	full-splice_match	gene: Zm00001d022069	Zm00001d022069_T00	9.40	13.03	coding	307	488	1408
PB.7619.1	6	1328	1	incomplete-splice_match	gene: Zm00001d036833	Zm00001d036833_T00	4.06	4.06	coding	421	64	1326
PB.4124.1	3	625	4	incomplete-splice_match	gene: Zm00001d041734	Zm00001d041734_T00	114.77	114.77	coding	98	150	443
PB.8979.1	7	1317	2	full-splice_match	gene: Zm00001d022212	Zm00001d022212_T00	13.39	13.39	coding	220	439	1098
PB.80.1	1	1813	4	full-splice_match	gene: Zm00001d027546	Zm00001d027546_T00	98.17	98.17	coding	368	332	1435
PB.80.2	1	1608	4	full-splice_match	gene: Zm00001d027546	Zm00001d027546_T00	0.00	98.17	coding	368	332	1435
PB.324.1	1	611	1	intergenic	novelGene_132	novel	1.22	1.22	non coding	NA	NA	NA
PB.5417.1	4	1458	2	full-splice_match	gene: Zm00001d051872	Zm00001d051872_T00	184.91	1787.53	coding	288	82	945
PB.2349.1	10	370	3	incomplete-splice_match	gene: Zm00001d026516	Zm00001d026516_T00	232.11	232.11	non coding	NA	NA	NA
PB.5257.1	4	1068	2	intergenic	novelGene_260	novel	3.27	3.27	coding	143	304	732
PB.5417.6	4	1225	2	full-splice_match	gene: Zm00001d051872	Zm00001d051872_T00	0.00	1787.53	coding	288	82	945
PB.5417.7	4	1124	2	full-splice_match	gene: Zm00001d051872	Zm00001d051872_T00	0.00	1787.53	coding	288	82	945
PB.9680.2	8	2240	1	novel_in_catalog	gene: Zm00001d010971	novel	26.63	26.63	coding	613	154	1992

PB.808.1	1	761	1	full-splice_match	gene: Zm00001d031152	Zm00001d031152_T00	257.08	257.08	coding	171	29	541
PB.3091.1	2	1969	11	novel_not_in_catalog	gene: Zm00001d005143	novel	25.06	25.06	coding	431	345	1637
PB.4901.2	4	696	3	full-splice_match	gene: Zm00001d048909	Zm00001d048909_T00	8.13	13.34	coding	112	223	558
PB.4901.1	4	868	3	full-splice_match	gene: Zm00001d048909	Zm00001d048909_T00	5.22	13.34	coding	112	203	538
PB.6756.3	5	958	9	full-splice_match	gene: Zm00001d016469	Zm00001d016469_T00	0.00	259.43	coding	218	226	879
PB.7046.1	5	2613	6	full-splice_match	gene: Zm00001d017698	Zm00001d017698_T00	23.63	23.63	coding	758	93	2366
PB.6756.1	5	1057	9	full-splice_match	gene: Zm00001d016469	Zm00001d016469_T00	259.43	259.43	coding	218	226	879
PB.545.1	1	2201	9	incomplete-splice_match	gene: Zm00001d029626	Zm00001d029626_T00	46.68	46.68	coding	589	186	1952
PB.4651.3	3	2392	3	incomplete-splice_match	gene: Zm00001d043927	Zm00001d043927_T01	17.15	59.83	coding	734	23	2224
PB.4651.2	3	2340	2	incomplete-splice_match	gene: Zm00001d043927	Zm00001d043927_T00	21.42	59.83	coding	604	79	1890
PB.4651.1	3	1061	1	incomplete-splice_match	gene: Zm00001d043927	Zm00001d043927_T00	21.26	59.83	coding	143	1	429
PB.2329.1	10	1078	1	full-splice_match	gene: Zm00001d026457	Zm00001d026457_T00	2.32	476.12	coding	84	124	375
PB.10657.1	9	1850	10	full-splice_match	gene: Zm00001d047566	Zm00001d047566_T01	7.49	37.66	coding	237	649	1359
PB.2329.3	10	737	1	full-splice_match	gene: Zm00001d026457	Zm00001d026457_T00	219.04	476.12	non coding	NA	NA	NA
PB.9444.1	8	2750	7	full-splice_match	gene: Zm00001d009883	Zm00001d009883_T04	8.77	30.35	coding	104	1763	2074
PB.2603.4	2	2247	7	full-splice_match	gene: Zm00001d002540	Zm00001d002540_T00	17.84	618.35	coding	547	426	2066
PB.2603.5	2	363	2	incomplete-splice_match	gene: Zm00001d002540	Zm00001d002540_T00	338.68	618.35	non coding	NA	NA	NA

PB.2603.6	2	1087	1	novel_in_catalog	gene: Zm00001d002540	novel	7.66	618.35	coding	260	127	906
PB.2603.1	2	2121	7	full-splice_match	gene: Zm00001d002540	Zm00001d002540_T00	221.47	618.35	coding	490	125	1594
PB.2603.2	2	2571	7	full-splice_match	gene: Zm00001d002540	Zm00001d002540_T00	32.70	618.35	coding	547	574	2214
PB.8971.2	7	764	2	novel_in_catalog	gene: Zm00001d022184	novel	94.61	146.72	coding	112	228	563
PB.6148.1	5	2077	3	genic	gene: Zm00001d013453	novel	26.27	26.27	coding	535	184	1788
PB.8971.1	7	894	2	novel_in_catalog	gene: Zm00001d022184	novel	52.11	146.72	coding	112	237	572
PB.642.2	1	1859	5	novel_in_catalog	gene: Zm00001d030157	novel	4.26	88.96	coding	116	230	577
PB.8642.1	7	1744	11	full-splice_match	gene: Zm00001d020953	Zm00001d020953_T00	9.22	9.22	coding	418	290	1543
PB.576.2	1	954	3	full-splice_match	gene: Zm00001d029801	Zm00001d029801_T00	58.24	58.24	coding	246	113	850
PB.5407.1	4	2443	3	full-splice_match	gene: Zm00001d051838	Zm00001d051838_T00	25.76	38.57	coding	649	144	2090
PB.3096.2	2	2318	7	incomplete-splice_match	gene: Zm00001d005157	Zm00001d005157_T01	2.22	27.86	coding	284	1018	1869
PB.1242.1	1	2029	8	full-splice_match	gene: Zm00001d033130	Zm00001d033130_T00	57.79	57.79	coding	592	90	1865
PB.816.1	1	882	4	full-splice_match	gene: Zm00001d031186	Zm00001d031186_T00	36.40	36.40	coding	186	138	695
PB.9742.11	8	1917	10	novel_not_in_catalog	gene: Zm00001d011253	novel	19.31	138.23	coding	190	575	1144
PB.7994.1	6	1928	2	novel_not_in_catalog	gene: Zm00001d038400	novel	21.06	36.41	coding	463	26	1414
PB.1740.3	10	1587	8	full-splice_match	gene: Zm00001d023452	Zm00001d023452_T00	21.97	52.01	coding	425	120	1394
PB.1740.1	10	1814	8	full-splice_match	gene: Zm00001d023452	Zm00001d023452_T00	29.41	52.01	coding	425	120	1394

PB.7743.1	6	683	1	full-splice_match	gene: Zm00001d037426	Zm00001d037426_T00	25.79	25.79	coding	148	50	493
PB.1740.6	10	1478	8	full-splice_match	gene: Zm00001d023452	Zm00001d023452_T00	0.00	52.01	coding	425	114	1388
PB.1740.4	10	1494	8	novel_not_in_catalog	gene: Zm00001d023452	novel	0.64	52.01	coding	400	100	1299
PB.1540.8	1	1264	8	full-splice_match	gene: Zm00001d034452	Zm00001d034452_T00	10.60	20.68	coding	297	182	1072
PB.6513.1	5	1236	3	full-splice_match	gene: Zm00001d014989	Zm00001d014989_T00	7.63	7.63	coding	308	115	1038
PB.3598.3	2	533	3	novel_not_in_catalog	gene: Zm00001d007633	novel	193.27	356.02	coding	68	116	319
PB.1354.1	1	2592	8	full-splice_match	gene: Zm00001d033624	Zm00001d033624_T01	114.06	114.06	coding	714	241	2382
PB.6192.9	5	1717	10	incomplete-splice_match	gene: Zm00001d013644	Zm00001d013644_T00	410.58	802.37	coding	435	283	1587
PB.9209.1	8	2571	2	full-splice_match	gene: Zm00001d008591	Zm00001d008591_T00	81.01	81.01	coding	665	37	2031
PB.2120.1	10	675	2	novel_in_catalog	gene: Zm00001d025564	novel	208.84	208.84	coding	129	128	514
PB.10910.2	9	1454	3	novel_in_catalog	gene: Zm00001d048503	novel	200.17	200.17	coding	362	163	1248
PB.4319.4	3	2107	3	full-splice_match	gene: Zm00001d042684	Zm00001d042684_T00	95.53	359.58	coding	279	140	976
PB.4599.1	3	2746	3	novel_not_in_catalog	gene: Zm00001d043743	novel	8.18	8.18	coding	700	157	2256
PB.6192.1	5	2730	14	full-splice_match	gene: Zm00001d013644	Zm00001d013644_T00	70.06	802.37	coding	765	117	2411
PB.1760.4	10	2261	14	novel_not_in_catalog	gene: Zm00001d023543	novel	9.62	86.99	coding	534	119	1720
PB.525.2	1	1391	10	full-splice_match	gene: Zm00001d029548	Zm00001d029548_T00	19.57	45.49	coding	312	369	1304
PB.6192.2	5	2648	14	full-splice_match	gene: Zm00001d013644	Zm00001d013644_T00	313.82	802.37	coding	765	118	2412

PB.525.4	1	2072	10	full-splice_match	gene: Zm00001d029548	Zm00001d029548_T01	6.22	45.49	coding	171	638	1150
PB.6192.4	5	2773	13	novel_in_catalog	gene: Zm00001d013644	novel	7.91	802.37	coding	617	116	1966
PB.1760.3	10	2362	15	novel_not_in_catalog	gene: Zm00001d023543	novel	70.31	86.99	coding	610	314	2143
PB.2776.1	2	2727	10	full-splice_match	gene: Zm00001d003262	Zm00001d003262_T00	65.85	65.85	coding	591	784	2556
PB.9388.1	8	3306	14	full-splice_match	gene: Zm00001d009564	Zm00001d009564_T00	12.77	12.77	coding	1049	68	3214
PB.6537.1	5	1462	10	full-splice_match	gene: Zm00001d015115	Zm00001d015115_T00	24.75	41.66	coding	399	98	1294
PB.3123.2	2	2247	15	full-splice_match	gene: Zm00001d005306	Zm00001d005306_T00	33.14	49.72	coding	625	246	2120
PB.7923.1	6	3446	1	novel_in_catalog	gene: Zm00001d038166	novel	4.27	4.27	non coding	NA	NA	NA
PB.629.2	1	1895	9	full-splice_match	gene: Zm00001d030106	Zm00001d030106_T07	24.93	62.33	coding	54	313	474
PB.6760.2	5	3815	2	novel_in_catalog	gene: Zm00001d016475	novel	4.87	4.87	coding	370	82	1191
PB.9118.14	8	588	2	full-splice_match	gene: Zm00001d008181	Zm00001d008181_T00	0.00	148.26	non coding	NA	NA	NA
PB.9118.15	8	642	1	genic	gene: Zm00001d008181	novel	24.09	148.26	non coding	NA	NA	NA
PB.9118.10	8	1201	2	full-splice_match	gene: Zm00001d008181	Zm00001d008181_T00	0.00	148.26	non coding	NA	NA	NA
PB.9118.11	8	976	1	novel_in_catalog	gene: Zm00001d008181	novel	0.00	148.26	non coding	NA	NA	NA
PB.2627.1	2	1258	3	full-splice_match	gene: Zm00001d002621	Zm00001d002621_T00	15.10	15.10	coding	198	353	946
PB.10963.1	B73V4	2324	3	full-splice_match	gene: Zm00001d000005	Zm00001d000005_T00	6.65	6.65	coding	664	86	2077
PB.1113.4	1	1316	4	novel_in_catalog	gene: Zm00001d032570	novel	0.00	196.91	coding	193	35	613

PB.128.1	1	1191	5	incomplete-splice_match	gene: Zm00001d027752	Zm00001d027752_T02	5.31	5.31	coding	198	222	815
PB.6621.1	5	562	1	full-splice_match	gene: Zm00001d015550	Zm00001d015550_T00	53.28	53.28	coding	101	37	339
PB.4369.1	3	2274	9	full-splice_match	gene: Zm00001d042885	Zm00001d042885_T00	30.85	50.26	coding	574	210	1931
PB.5966.1	5	776	2	full-splice_match	gene: Zm00001d012845	Zm00001d012845_T00	60.17	60.17	coding	172	75	590
PB.3889.1	3	321	1	incomplete-splice_match	gene: Zm00001d040197	Zm00001d040197_T00	5.15	5.15	non coding	NA	NA	NA
PB.3912.2	3	876	7	full-splice_match	gene: Zm00001d040294	Zm00001d040294_T00	99.97	132.03	coding	146	197	634
PB.3912.3	3	951	6	full-splice_match	gene: Zm00001d040294	Zm00001d040294_T00	1.69	132.03	coding	178	180	713
PB.8612.2	7	5433	2	genic	gene: Zm00001d020828	novel	4.13	37.46	coding	647	3014	4954
PB.3912.1	3	945	7	full-splice_match	gene: Zm00001d040294	Zm00001d040294_T00	30.38	132.03	coding	146	136	573
PB.8819.1	7	1575	12	full-splice_match	gene: Zm00001d021675	Zm00001d021675_T00	44.27	49.49	coding	371	118	1230
PB.9040.1	7	982	4	full-splice_match	gene: Zm00001d022418	Zm00001d022418_T00	2.02	202.45	coding	102	57	362
PB.9040.2	7	638	4	full-splice_match	gene: Zm00001d022418	Zm00001d022418_T00	200.43	202.45	coding	102	57	362
PB.8612.8	7	360	1	full-splice_match	gene: Zm00001d020828	Zm00001d020828_T00	11.45	37.46	non coding	NA	NA	NA
PB.10257.2	9	1881	6	full-splice_match	gene: Zm00001d045417	Zm00001d045417_T00	19.34	42.31	coding	488	237	1700
PB.10257.1	9	1601	5	incomplete-splice_match	gene: Zm00001d045417	Zm00001d045417_T00	22.97	42.31	coding	369	168	1274
PB.5574.1	4	1766	3	full-splice_match	gene: Zm00001d052423	Zm00001d052423_T00	4.35	4.35	coding	446	81	1418
PB.1569.2	1	1728	4	novel_in_catalog	gene: Zm00001d034575	novel	0.00	54.73	coding	466	48	1445

PB.8000.2	6	2825	7	full-splice_match	gene: Zm00001d038432	Zm00001d038432_T00	1.08	103.47	coding	234	1828	2529
PB.8000.1	6	2479	8	full-splice_match	gene: Zm00001d038432	Zm00001d038432_T00	41.41	103.47	coding	601	355	2157
PB.6249.1	5	841	4	full-splice_match	gene: Zm00001d013943	Zm00001d013943_T00	6.73	6.73	coding	214	84	725
PB.7964.1	6	548	3	incomplete-splice_match	gene: Zm00001d038306	Zm00001d038306_T00	20.70	20.70	coding	101	9	311
PB.8000.6	6	2354	8	full-splice_match	gene: Zm00001d038432	Zm00001d038432_T00	0.00	103.47	coding	601	309	2111
PB.8000.5	6	3555	7	full-splice_match	gene: Zm00001d038432	Zm00001d038432_T00	14.35	103.47	coding	530	1649	3238
PB.4114.2	3	952	9	full-splice_match	gene: Zm00001d041691	Zm00001d041691_T00	68.42	68.42	coding	192	126	701
PB.1285.3	1	3045	5	intergenic	novelGene_453	novel	6.70	28.16	coding	73	97	315
PB.8000.8	6	2343	8	full-splice_match	gene: Zm00001d038432	Zm00001d038432_T00	9.57	103.47	coding	603	311	2119
PB.6069.1	5	4213	12	novel_in_catalog	gene: Zm00001d013211	novel	26.29	26.29	coding	65	197	391
PB.9060.1	7	1355	3	full-splice_match	gene: Zm00001d022474	Zm00001d022474_T00	15.98	15.98	coding	329	124	1110
PB.1584.2	1	1248	4	full-splice_match	gene: Zm00001d034618	Zm00001d034618_T00	87.78	89.40	coding	318	104	1057
PB.2404.4	10	1584	3	novel_in_catalog	gene: Zm00001d026676	novel	0.65	10.81	coding	135	9	413
PB.2572.2	2	3563	4	full-splice_match	gene: Zm00001d002366	Zm00001d002366_T00	13.51	161.87	coding	279	2391	3227
PB.4775.1	3	1774	2	genic	gene: Zm00001d044461	novel	1.27	79.06	coding	311	231	1163
PB.3928.4	3	1076	6	full-splice_match	gene: Zm00001d040334	Zm00001d040334_T00	68.00	187.42	coding	307	73	993
PB.4606.4	3	1963	15	full-splice_match	gene: Zm00001d043773	Zm00001d043773_T00	0.00	28.64	coding	560	129	1808

PB.6366.2	5	1574	5	full-splice_match	gene: Zm00001d014449	Zm00001d014449_T00	33.18	40.79	coding	380	163	1302
PB.9215.1	8	2276	3	novel_in_catalog	gene: Zm00001d008604	novel	104.93	104.93	coding	465	669	2063
PB.4354.1	3	3014	2	novel_in_catalog	gene: Zm00001d042828	novel	17.31	17.31	coding	155	43	507
PB.3928.2	3	1212	6	full-splice_match	gene: Zm00001d040334	Zm00001d040334_T00	46.03	187.42	coding	307	102	1022
PB.5992.4	5	1928	1	novel_in_catalog	gene: Zm00001d012932	novel	1.20	52.63	coding	271	848	1660
PB.10247.1	9	577	2	full-splice_match	gene: Zm00001d045385	Zm00001d045385_T00	59.56	59.56	non coding	NA	NA	NA
PB.5992.2	5	1476	2	full-splice_match	gene: Zm00001d012932	Zm00001d012932_T00	51.43	52.63	coding	278	137	970
PB.5992.3	5	1157	2	full-splice_match	gene: Zm00001d012932	Zm00001d012932_T00	0.00	52.63	coding	278	69	902
PB.2704.2	2	2340	16	fusion	gene: Zm00001d002964	novel	59.50	92.89	coding	601	302	2104
PB.10608.1	9	1797	6	novel_not_in_catalog	gene: Zm00001d047418	novel	2.28	2.28	coding	527	71	1651
PB.2704.1	2	2440	16	fusion	gene: Zm00001d002964	novel	33.39	92.89	coding	601	309	2111
PB.9376.1	8	1257	1	full-splice_match	gene: Zm00001d009511	Zm00001d009511_T00	12.74	27.72	coding	302	127	1032
PB.9376.2	8	1821	1	full-splice_match	gene: Zm00001d009511	Zm00001d009511_T00	14.98	27.72	coding	575	11	1735
PB.8727.1	7	2778	11	full-splice_match	gene: Zm00001d021318	Zm00001d021318_T01	9.22	182.56	coding	621	215	2077
PB.9102.2	7	3807	8	intergenic	novelGene_372	novel	25.71	82.50	coding	165	2786	3280
PB.8727.2	7	2628	11	full-splice_match	gene: Zm00001d021318	Zm00001d021318_T01	0.00	182.56	coding	621	214	2076
PB.5360.1	4	1141	6	full-splice_match	gene: Zm00001d051595	Zm00001d051595_T00	427.25	427.25	coding	233	155	853

PB.5360.3	4	1041	6	full-splice_match	gene: Zm00001d051595	Zm00001d051595_T00	0.00	427.25	coding	233	130	828
PB.1838.1	10	1444	10	full-splice_match	gene: Zm00001d023946	Zm00001d023946_T00	12.30	12.30	coding	343	25	1053
PB.10854.4	9	1138	7	incomplete-splice_match	gene: Zm00001d048319	Zm00001d048319_T00	7.75	7.75	coding	296	82	969
PB.5479.1	4	1290	8	incomplete-splice_match	gene: Zm00001d052079	Zm00001d052079_T02	20.11	40.24	coding	211	308	940
PB.5181.1	4	2026	7	novel_in_catalog	gene: Zm00001d050365	novel	5.21	38.35	coding	228	169	852
PB.5181.2	4	1830	7	full-splice_match	gene: Zm00001d050365	Zm00001d050365_T00	20.55	38.35	coding	426	181	1458
PB.5181.3	4	1758	7	novel_in_catalog	gene: Zm00001d050365	novel	0.00	38.35	coding	228	179	862
PB.5181.4	4	572	1	incomplete-splice_match	gene: Zm00001d050365	Zm00001d050365_T00	12.59	38.35	non coding	NA	NA	NA
PB.3608.1	2	3557	9	novel_in_catalog	gene: Zm00001d007709	novel	27.57	53.13	coding	1052	115	3270
PB.4369.6	3	2118	9	full-splice_match	gene: Zm00001d042885	Zm00001d042885_T00	0.00	50.26	coding	574	206	1927
PB.5914.3	4	1960	1	intergenic	novelGene_447	novel	0.00	5.99	non coding	NA	NA	NA
PB.9068.1	7	2974	10	incomplete-splice_match	gene: Zm00001d022491	Zm00001d022491_T01	21.06	21.06	coding	930	26	2815
PB.5370.2	4	1244	9	full-splice_match	gene: Zm00001d051628	Zm00001d051628_T00	0.00	45.41	coding	333	77	1075
PB.3895.1	3	1331	9	full-splice_match	gene: Zm00001d040221	Zm00001d040221_T00	13.11	18.68	coding	335	93	1097
PB.3895.2	3	1462	8	full-splice_match	gene: Zm00001d040221	Zm00001d040221_T00	4.02	18.68	coding	335	94	1098
PB.3895.3	3	1533	7	novel_in_catalog	gene: Zm00001d040221	novel	1.55	18.68	coding	300	59	958
PB.3436.1	2	1979	3	full-splice_match	gene: Zm00001d006749	Zm00001d006749_T00	13.03	13.03	coding	505	252	1766

PB.3643.1	2	3532	3	full-splice_match	gene: Zm00001d007853	Zm00001d007853_T00	16.44	16.44	coding	517	207	1757
PB.2839.1	2	1077	1	full-splice_match	gene: Zm00001d003575	Zm00001d003575_T00	4.30	4.30	coding	289	48	914
PB.3803.1	3	1556	9	novel_in_catalog	gene: Zm00001d039732	novel	65.62	65.62	coding	335	293	1297
PB.8227.5	7	1737	12	incomplete-splice_match	gene: Zm00001d018639	Zm00001d018639_T00	14.70	47.47	coding	478	150	1583
PB.1643.1	1	1704	9	full-splice_match	gene: Zm00001d034814	Zm00001d034814_T00	45.34	51.40	coding	518	57	1610
PB.8227.1	7	1823	12	incomplete-splice_match	gene: Zm00001d018639	Zm00001d018639_T00	19.56	47.47	coding	478	82	1515
PB.8703.1	7	1544	6	full-splice_match	gene: Zm00001d021225	Zm00001d021225_T00	56.69	59.68	coding	175	717	1241
PB.8227.3	7	1959	11	full-splice_match	gene: Zm00001d018639	Zm00001d018639_T00	6.08	47.47	coding	412	91	1326
PB.1100.1	1	436	1	incomplete-splice_match	gene: Zm00001d032535	Zm00001d032535_T00	21.56	21.56	non coding	NA	NA	NA
PB.5888.1	4	1181	7	novel_in_catalog	gene: Zm00001d053919	novel	67.43	74.41	coding	152	202	657
PB.6639.2	5	2615	13	fusion	gene: Zm00001d015693	novel	7.14	49.74	coding	711	151	2283
PB.3044.4	2	2317	4	novel_in_catalog	gene: Zm00001d004855	novel	39.24	53.34	coding	377	935	2065
PB.1505.1	1	896	8	full-splice_match	gene: Zm00001d034337	Zm00001d034337_T00	214.98	214.98	coding	161	167	649
PB.8819.5	7	1306	12	full-splice_match	gene: Zm00001d021675	Zm00001d021675_T00	5.22	49.49	coding	371	115	1227
PB.10235.2	9	3062	1	genic	gene: Zm00001d045335	novel	23.62	38.39	coding	598	1119	2912
PB.10235.1	9	1485	1	genic	gene: Zm00001d045335	novel	14.77	38.39	coding	170	94	603
PB.4388.1	3	2738	8	novel_in_catalog	gene: Zm00001d042979	novel	15.35	15.35	coding	389	186	1352

PB.799.3	1	1657	3	full-splice_match	gene: Zm00001d031101	Zm00001d031101_T00	34.06	171.35	coding	428	119	1402
PB.799.2	1	1425	1	novel_in_catalog	gene: Zm00001d031101	novel	30.60	171.35	coding	300	218	1117
PB.799.1	1	1846	3	full-splice_match	gene: Zm00001d031101	Zm00001d031101_T00	106.69	171.35	coding	428	256	1539
PB.485.4	1	429	2	incomplete-splice_match	gene: Zm00001d029361	Zm00001d029361_T00	0.00	89.25	non coding	NA	NA	NA
PB.3743.1	3	1203	6	novel_not_in_catalog	gene: Zm00001d039483	novel	208.95	208.95	coding	267	168	968
PB.485.2	1	567	2	novel_in_catalog	gene: Zm00001d029361	novel	89.25	89.25	coding	84	52	303
PB.7540.1	6	1495	5	novel_in_catalog	gene: Zm00001d036432	novel	34.03	87.07	coding	353	29	1087
PB.10373.1	9	2499	5	full-splice_match	gene: Zm00001d046016	Zm00001d046016_T00	51.27	61.27	coding	568	606	2309
PB.10373.2	9	2604	5	novel_not_in_catalog	gene: Zm00001d046016	novel	10.01	61.27	coding	568	711	2414
PB.6758.1	5	1351	1	antisense	novelGene_gene: Zm00001d016470	novel	25.20	25.20	non coding	NA	NA	NA
PB.610.1	1	2431	11	full-splice_match	gene: Zm00001d030011	Zm00001d030011_T00	113.62	113.62	coding	720	107	2266
PB.4652.4	3	1062	2	full-splice_match	gene: Zm00001d043935	Zm00001d043935_T00	19.86	215.60	coding	220	219	878
PB.3044.1	2	2396	4	novel_in_catalog	gene: Zm00001d004855	novel	14.10	53.34	coding	377	930	2060
PB.7894.1	6	1777	1	full-splice_match	gene: Zm00001d038072	Zm00001d038072_T00	23.83	23.83	coding	348	242	1285
PB.5605.7	4	1632	2	novel_in_catalog	gene: Zm00001d052591	novel	4.01	29.18	coding	308	88	1011
PB.10720.1	9	1150	1	full-splice_match	gene: Zm00001d047775	Zm00001d047775_T00	66.42	66.42	coding	305	71	985
PB.10720.2	9	1027	1	full-splice_match	gene: Zm00001d047775	Zm00001d047775_T00	0.00	66.42	coding	305	69	983

PB.1802.2	10	1967	8	full-splice_match	gene: Zm00001d023762	Zm00001d023762_T00	31.62	65.00	coding	483	239	1687
PB.110.1	1	1567	1	full-splice_match	gene: Zm00001d027685	Zm00001d027685_T00	50.78	50.78	coding	302	240	1145
PB.110.2	1	1423	1	full-splice_match	gene: Zm00001d027685	Zm00001d027685_T00	0.00	50.78	coding	302	243	1148
PB.110.3	1	1264	1	full-splice_match	gene: Zm00001d027685	Zm00001d027685_T00	0.00	50.78	coding	302	243	1148
PB.7977.5	6	1684	8	novel_in_catalog	gene: Zm00001d038348	novel	2.64	50.47	coding	99	267	563
PB.7580.4	6	1262	1	intergenic	novelGene_202	novel	2.71	2.71	coding	235	309	1013
PB.7977.1	6	1701	9	full-splice_match	gene: Zm00001d038348	Zm00001d038348_T00	29.46	50.47	non coding	NA	NA	NA
PB.7977.3	6	3106	3	novel_in_catalog	gene: Zm00001d038348	novel	10.84	50.47	coding	99	256	552
PB.7977.2	6	1901	8	novel_in_catalog	gene: Zm00001d038348	novel	7.53	50.47	coding	211	385	1017
PB.35.1	1	1625	5	novel_not_in_catalog	gene: Zm00001d027374	novel	95.70	95.70	coding	349	306	1352
PB.2094.3	10	645	2	full-splice_match	gene: Zm00001d025401	Zm00001d025401_T00	17.53	582.41	coding	104	136	447
PB.6830.2	5	2309	2	full-splice_match	gene: Zm00001d016800	Zm00001d016800_T00	3.85	154.47	coding	112	158	493
PB.2094.1	10	854	1	genic	gene: Zm00001d025401	novel	46.69	582.41	coding	181	139	681
PB.995.1	1	938	2	full-splice_match	gene: Zm00001d032098	Zm00001d032098_T00	37.38	37.38	coding	186	150	707
PB.4652.5	3	1058	2	full-splice_match	gene: Zm00001d043935	Zm00001d043935_T00	0.00	215.60	coding	220	337	996
PB.9890.2	8	1860	9	full-splice_match	gene: Zm00001d011900	Zm00001d011900_T00	22.40	23.59	coding	455	145	1509
PB.8497.3	7	1283	1	novel_in_catalog	gene: Zm00001d020260	novel	7.80	58.15	coding	316	229	1176

PB.8497.2	7	1993	2	full-splice_match	gene: Zm00001d020260	Zm00001d020260_T00	48.92	58.15	coding	569	128	1834
PB.8497.1	7	2553	2	full-splice_match	gene: Zm00001d020260	Zm00001d020260_T00	1.43	58.15	coding	569	114	1820
PB.3444.1	2	1703	7	full-splice_match	gene: Zm00001d006763	Zm00001d006763_T00	21.66	21.66	coding	424	285	1556
PB.3123.5	2	2477	13	novel_in_catalog	gene: Zm00001d005306	novel	3.76	49.72	coding	225	1719	2393
PB.9890.1	8	1982	9	full-splice_match	gene: Zm00001d011900	Zm00001d011900_T00	1.18	23.59	coding	455	145	1509
PB.100.1	1	2013	17	incomplete-splice_match	gene: Zm00001d027665	Zm00001d027665_T03	26.19	26.19	coding	609	4	1830
PB.3022.1	2	3305	6	full-splice_match	gene: Zm00001d004716	Zm00001d004716_T01	19.06	19.06	coding	730	131	2320
PB.5891.2	4	1388	6	full-splice_match	gene: Zm00001d053927	Zm00001d053927_T01	13.00	46.47	coding	287	177	1037
PB.5891.3	4	1956	6	full-splice_match	gene: Zm00001d053927	Zm00001d053927_T00	16.89	46.47	coding	270	806	1615
PB.10528.1	9	1395	9	novel_in_catalog	gene: Zm00001d046974	novel	27.82	27.82	coding	273	100	918
PB.6077.3	5	2231	4	novel_not_in_catalog	gene: Zm00001d013242	novel	2.34	20.50	coding	163	357	845
PB.6077.2	5	1233	5	novel_not_in_catalog	gene: Zm00001d013242	novel	4.31	20.50	coding	244	328	1059
PB.6077.1	5	1122	5	novel_not_in_catalog	gene: Zm00001d013242	novel	13.85	20.50	coding	240	49	768
PB.1992.1	10	2027	7	novel_not_in_catalog	gene: Zm00001d024878	novel	29.04	36.94	coding	449	415	1761
PB.9503.2	8	2593	4	full-splice_match	gene: Zm00001d010231	Zm00001d010231_T00	24.80	108.41	coding	478	759	2192
PB.9503.3	8	1831	5	novel_not_in_catalog	gene: Zm00001d010231	novel	83.61	108.41	coding	478	215	1648
PB.10576.1	9	1219	4	full-splice_match	gene: Zm00001d047238	Zm00001d047238_T00	2.32	2.32	coding	316	40	987

PB.10061.1	8	2982	17	full-splice_match	gene: Zm00001d012620	Zm00001d012620_T02	28.08	32.26	coding	834	153	2654
PB.5867.1	4	4188	1	novel_in_catalog	gene: Zm00001d053838	novel	506.39	506.39	coding	533	2475	4073
PB.2805.1	2	1749	12	full-splice_match	gene: Zm00001d003426	Zm00001d003426_T00	7.46	7.46	coding	127	3	383
PB.1845.1	10	1427	6	incomplete-splice_match	gene: Zm00001d023979	Zm00001d023979_T00	10.85	21.36	coding	276	92	919
PB.9207.4	8	2274	4	full-splice_match	gene: Zm00001d008581	Zm00001d008581_T00	0.00	14.83	coding	654	73	2034
PB.6817.3	5	1327	1	full-splice_match	gene: Zm00001d016748	Zm00001d016748_T00	5.53	17.52	coding	287	240	1100
PB.1845.2	10	2763	8	full-splice_match	gene: Zm00001d023979	Zm00001d023979_T00	10.51	21.36	coding	697	284	2374
PB.8566.2	7	1303	1	full-splice_match	gene: Zm00001d020583	Zm00001d020583_T00	47.23	93.25	coding	318	30	983
PB.6660.3	5	1508	6	fusion	gene: Zm00001d015846	novel	0.00	37.67	coding	264	555	1346
PB.9207.3	8	2384	4	fusion	gene: Zm00001d008581	novel	22.43	22.43	coding	644	96	2027
PB.9207.2	8	2563	4	full-splice_match	gene: Zm00001d008581	Zm00001d008581_T00	14.83	14.83	coding	654	121	2082
PB.973.4	1	1003	4	novel_not_in_catalog	gene: Zm00001d031991	novel	10.30	22.94	coding	111	670	1002
PB.6660.2	5	2190	9	fusion	gene: Zm00001d015846	novel	13.68	37.67	coding	264	1132	1923
PB.1552.1	1	810	1	genic	gene: Zm00001d034508	novel	11.77	27.43	non coding	NA	NA	NA
PB.9386.5	8	1166	8	full-splice_match	gene: Zm00001d009554	Zm00001d009554_T00	0.00	125.02	coding	298	72	965
PB.9386.4	8	1332	8	full-splice_match	gene: Zm00001d009554	Zm00001d009554_T00	2.95	125.02	coding	298	92	985
PB.1423.5	1	2378	11	novel_not_in_catalog	gene: Zm00001d033948	novel	13.26	76.35	coding	115	158	502

PB.8474.1	7	1033	1	intergenic	novelGene_195	novel	1.90	1.90	coding	273	34	852
PB.9386.1	8	1447	8	full-splice_match	gene: Zm00001d009554	Zm00001d009554_T00	118.83	125.02	coding	298	82	975
PB.5639.3	4	1543	6	full-splice_match	gene: Zm00001d052767	Zm00001d052767_T00	13.81	18.75	coding	415	101	1345
PB.9386.3	8	1247	7	novel_in_catalog	gene: Zm00001d009554	novel	2.22	125.02	coding	181	355	897
PB.9386.2	8	1533	7	novel_in_catalog	gene: Zm00001d009554	novel	1.03	125.02	coding	287	37	897
PB.1641.3	1	1191	5	full-splice_match	gene: Zm00001d034805	Zm00001d034805_T00	0.00	80.10	coding	308	190	1113
PB.9174.1	8	1515	1	full-splice_match	gene: Zm00001d008412	Zm00001d008412_T00	35.58	35.58	coding	272	123	938
PB.1865.1	10	673	3	incomplete-splice_match	gene: Zm00001d024159	Zm00001d024159_T00	11.89	11.89	coding	125	19	393
PB.3379.2	2	808	5	novel_in_catalog	gene: Zm00001d006474	novel	327.44	345.94	coding	119	246	602
PB.5704.1	4	929	1	full-splice_match	gene: Zm00001d053068	Zm00001d053068_T00	36.93	36.93	coding	211	89	721
PB.3379.4	2	1765	4	novel_in_catalog	gene: Zm00001d006474	novel	18.50	345.94	non coding	NA	NA	NA
PB.10363.1	9	3176	1	incomplete-splice_match	gene: Zm00001d045953	Zm00001d045953_T01	6.95	62.07	coding	1028	91	3174
PB.1859.2	10	1280	2	full-splice_match	gene: Zm00001d024071	Zm00001d024071_T00	13.76	36.56	coding	287	92	952
PB.1859.3	10	1233	1	novel_in_catalog	gene: Zm00001d024071	novel	4.68	36.56	coding	181	447	989
PB.6483.8	5	881	5	novel_not_in_catalog	gene: Zm00001d014897	novel	0.00	100.90	coding	217	56	706
PB.1859.1	10	1780	2	full-splice_match	gene: Zm00001d024071	Zm00001d024071_T00	0.31	36.56	coding	287	92	952
PB.10594.3	9	1096	2	full-splice_match	gene: Zm00001d047340	Zm00001d047340_T00	208.51	304.25	coding	238	74	787

PB.10594.2	9	1205	1	genic	gene: Zm00001d047340	novel	86.36	304.25	coding	123	74	442
PB.1859.4	10	1162	2	full-splice_match	gene: Zm00001d024071	Zm00001d024071_T00	17.82	36.56	coding	287	92	952
PB.1859.5	10	437	1	novel_in_catalog	gene: Zm00001d024071	novel	0.00	36.56	coding	105	27	341
PB.6483.2	5	2118	17	novel_not_in_catalog	gene: Zm00001d014897	novel	3.95	100.90	coding	321	962	1924
PB.6483.3	5	2530	16	novel_in_catalog	gene: Zm00001d014897	novel	0.00	100.90	coding	232	60	755
PB.7913.5	6	2281	18	incomplete-splice_match	gene: Zm00001d038134	Zm00001d038134_T01	0.00	29.90	coding	627	15	1895
PB.6483.1	5	2056	17	full-splice_match	gene: Zm00001d014897	Zm00001d014897_T00	96.70	100.90	coding	583	78	1826
PB.6483.6	5	1707	16	incomplete-splice_match	gene: Zm00001d014897	Zm00001d014897_T00	0.00	100.90	coding	550	57	1706
PB.2433.2	2	3137	17	novel_in_catalog	gene: Zm00001d001809	novel	12.01	38.14	coding	451	1575	2927
PB.2433.1	2	3319	17	novel_in_catalog	gene: Zm00001d001809	novel	26.14	38.14	coding	451	1578	2930
PB.4158.15	3	1556	10	novel_in_catalog	gene: Zm00001d041945	novel	14.03	133.59	coding	211	83	715
PB.4158.14	3	1411	11	novel_not_in_catalog	gene: Zm00001d041945	novel	0.83	133.59	coding	173	65	583
PB.4158.16	3	1884	8	novel_in_catalog	gene: Zm00001d041945	novel	1.14	133.59	coding	151	1271	1723
PB.8352.9	7	1181	11	full-splice_match	gene: Zm00001d019283	Zm00001d019283_T00	0.00	603.21	coding	369	75	1181
PB.8352.8	7	1516	14	full-splice_match	gene: Zm00001d019283	Zm00001d019283_T00	288.45	603.21	coding	421	75	1337
PB.4158.12	3	1742	11	full-splice_match	gene: Zm00001d041945	Zm00001d041945_T00	0.00	133.59	coding	397	354	1544
PB.318.2	1	1459	2	incomplete-splice_match	gene: Zm00001d028603	Zm00001d028603_T00	21.09	38.19	coding	303	111	1019

PB.318.3	1	1443	2	incomplete-splice_match	gene: Zm00001d028603	Zm00001d028603_T00	0.00	38.19	coding	303	214	1122
PB.7927.1	6	2184	3	full-splice_match	gene: Zm00001d038181	Zm00001d038181_T00	5.83	5.83	coding	595	152	1936
PB.8352.6	7	1970	12	novel_in_catalog	gene: Zm00001d019283	novel	0.93	603.21	coding	366	74	1171
PB.8352.1	7	1779	14	full-splice_match	gene: Zm00001d019283	Zm00001d019283_T00	312.91	603.21	coding	421	74	1336
PB.7391.11	6	3119	2	genic	gene: Zm00001d035481	novel	6.29	65.74	coding	92	80	355
PB.1655.1	1	1239	3	novel_in_catalog	gene: Zm00001d034852	novel	17.78	17.78	coding	250	45	794
PB.9486.3	8	590	4	full-splice_match	gene: Zm00001d010128	Zm00001d010128_T00	66.52	771.87	coding	113	72	410
PB.2235.3	10	733	3	full-splice_match	gene: Zm00001d026137	Zm00001d026137_T00	80.36	192.33	coding	147	150	590
PB.7633.3	6	1812	6	intergenic	novelGene_215	novel	20.65	20.65	coding	127	1274	1654
PB.6067.2	5	1213	6	full-splice_match	gene: Zm00001d013204	Zm00001d013204_T00	37.28	37.28	coding	328	35	1018
PB.7391.10	6	3022	3	novel_not_in_catalog	gene: Zm00001d035481	novel	8.56	65.74	coding	92	77	352
PB.9662.2	8	614	1	incomplete-splice_match	gene: Zm00001d010914	Zm00001d010914_T00	4.90	16.50	coding	125	25	399
PB.2113.1	10	632	2	full-splice_match	gene: Zm00001d025509	Zm00001d025509_T00	47.78	89.17	coding	97	97	387
PB.10512.1	9	656	3	full-splice_match	gene: Zm00001d046929	Zm00001d046929_T00	431.84	431.84	non coding	NA	NA	NA
PB.1286.1	1	758	2	incomplete-splice_match	gene: Zm00001d033334	Zm00001d033334_T00	19.43	19.43	coding	139	4	420
PB.9486.1	8	773	4	full-splice_match	gene: Zm00001d010128	Zm00001d010128_T00	167.14	771.87	coding	113	46	384
PB.4404.3	3	2393	1	novel_in_catalog	gene: Zm00001d043076	novel	7.14	22.98	coding	128	491	874

PB.7473.2	6	1562	17	full-splice_match	gene: Zm00001d035975	Zm00001d035975_T00	67.27	67.27	coding	429	114	1400
PB.4404.1	3	1758	2	novel_in_catalog	gene: Zm00001d043076	novel	15.84	22.98	coding	220	823	1482
PB.527.2	1	706	1	full-splice_match	gene: Zm00001d029558	Zm00001d029558_T00	0.00	200.76	coding	160	102	581
PB.8121.1	6	833	7	full-splice_match	gene: Zm00001d038885	Zm00001d038885_T00	115.22	115.22	coding	126	139	516
PB.8693.1	7	961	1	full-splice_match	gene: Zm00001d021205	Zm00001d021205_T00	213.64	213.64	coding	246	102	839
PB.8376.5	7	1115	6	full-splice_match	gene: Zm00001d019505	Zm00001d019505_T00	0.00	241.40	coding	284	175	1026
PB.8376.4	7	1557	5	novel_in_catalog	gene: Zm00001d019505	novel	0.85	241.40	coding	295	215	1099
PB.8376.3	7	1461	5	novel_in_catalog	gene: Zm00001d019505	novel	3.00	241.40	coding	221	215	877
PB.8376.2	7	1404	6	full-splice_match	gene: Zm00001d019505	Zm00001d019505_T00	104.06	241.40	coding	284	271	1122
PB.8376.1	7	1488	6	full-splice_match	gene: Zm00001d019505	Zm00001d019505_T00	133.49	241.40	coding	284	253	1104
PB.9723.1	8	925	2	full-splice_match	gene: Zm00001d011183	Zm00001d011183_T00	18.49	18.49	coding	274	2	823
PB.1851.2	10	1408	1	full-splice_match	gene: Zm00001d024027	Zm00001d024027_T00	0.00	57.80	coding	378	111	1244
PB.982.1	1	1675	3	incomplete-splice_match	gene: Zm00001d032044	Zm00001d032044_T00	63.96	63.96	coding	379	169	1305
PB.5128.1	4	785	5	novel_not_in_catalog	gene: Zm00001d050056	novel	59.32	59.32	non coding	NA	NA	NA
PB.1851.1	10	1491	1	full-splice_match	gene: Zm00001d024027	Zm00001d024027_T00	57.80	57.80	coding	378	111	1244
PB.5192.1	4	2851	3	intergenic	novelGene_191	novel	9.43	9.43	non coding	NA	NA	NA
PB.8060.1	6	3380	5	full-splice_match	gene: Zm00001d038671	Zm00001d038671_T00	18.15	37.16	coding	797	148	2538

PB.7377.2	6	1320	5	novel_in_catalog	gene: Zm00001d035420	novel	25.71	25.71	coding	149	286	732
PB.8060.3	6	3167	5	full-splice_match	gene: Zm00001d038671	Zm00001d038671_T00	4.85	37.16	coding	797	201	2591
PB.3466.1	2	1102	5	incomplete-splice_match	gene: Zm00001d006875	Zm00001d006875_T00	7.30	7.30	coding	172	201	716
PB.8060.5	6	2753	5	full-splice_match	gene: Zm00001d038671	Zm00001d038671_T00	0.00	37.16	coding	797	126	2516
PB.8060.4	6	2825	5	full-splice_match	gene: Zm00001d038671	Zm00001d038671_T00	14.16	37.16	coding	797	110	2500
PB.5464.2	4	2219	16	novel_in_catalog	gene: Zm00001d052056	novel	1.64	152.23	coding	429	116	1402
PB.10495.1	9	1566	1	antisense	novelGene_gene: Zm00001d046823	novel	3.29	3.29	coding	181	492	1034
PB.3936.3	3	709	6	novel_not_in_catalog	gene: Zm00001d040366	novel	14.46	14.46	coding	139	124	540
PB.6009.1	5	974	3	full-splice_match	gene: Zm00001d012998	Zm00001d012998_T00	12.98	12.98	non coding	NA	NA	NA
PB.9658.2	8	947	2	full-splice_match	gene: Zm00001d010869	Zm00001d010869_T00	17.85	43.27	coding	301	44	946
PB.2441.1	2	2565	9	incomplete-splice_match	gene: Zm00001d001834	Zm00001d001834_T03	61.50	61.50	coding	667	123	2123
PB.7961.2	6	2341	4	novel_in_catalog	gene: Zm00001d038292	novel	4.87	23.23	coding	343	252	1280
PB.3040.1	2	1357	9	full-splice_match	gene: Zm00001d004837	Zm00001d004837_T00	4.75	115.63	coding	276	122	949
PB.10066.1	8	1452	4	incomplete-splice_match	gene: Zm00001d012634	Zm00001d012634_T01	13.95	47.94	coding	220	23	682
PB.7961.5	6	1568	5	novel_in_catalog	gene: Zm00001d038292	novel	0.00	23.23	coding	336	181	1188
PB.10066.3	8	3938	6	full-splice_match	gene: Zm00001d012634	Zm00001d012634_T00	33.99	47.94	coding	1068	90	3293
PB.1947.2	10	1725	13	full-splice_match	gene: Zm00001d024698	Zm00001d024698_T00	19.76	46.03	coding	379	421	1557

PB.227.1	1	1378	2	novel_not_in_catalog	gene: Zm00001d028226	novel	16.84	16.84	coding	345	127	1161
PB.3345.1	2	915	4	incomplete-splice_match	gene: Zm00001d006354	Zm00001d006354_T00	1.21	1.21	coding	189	31	597
PB.4734.2	3	1430	8	full-splice_match	gene: Zm00001d044278	Zm00001d044278_T00	12.94	12.94	coding	294	306	1187
PB.992.1	1	905	6	novel_not_in_catalog	gene: Zm00001d032084	novel	69.11	76.62	coding	157	82	552
PB.3040.4	2	2073	8	novel_in_catalog	gene: Zm00001d004837	novel	6.37	115.63	coding	224	1076	1747
PB.3040.5	2	1700	9	novel_in_catalog	gene: Zm00001d004837	novel	1.55	115.63	coding	276	550	1377
PB.8191.1	6	941	2	full-splice_match	gene: Zm00001d039158	Zm00001d039158_T00	49.37	145.49	coding	154	85	546
PB.877.1	1	1418	6	novel_in_catalog	gene: Zm00001d031529	novel	34.41	90.73	coding	233	372	1070
PB.877.2	1	4099	9	novel_in_catalog	gene: Zm00001d031529	novel	16.27	90.73	coding	935	532	3336
PB.10564.1	9	2798	6	full-splice_match	gene: Zm00001d047191	Zm00001d047191_T00	36.68	52.31	coding	732	146	2341
PB.8328.2	7	2987	2	full-splice_match	gene: Zm00001d019145	Zm00001d019145_T00	10.02	13.37	coding	904	24	2735
PB.3190.1	2	3435	4	full-splice_match	gene: Zm00001d005687	Zm00001d005687_T00	21.61	27.10	coding	864	243	2834
PB.3190.2	2	2873	3	full-splice_match	gene: Zm00001d005687	Zm00001d005687_T00	5.48	27.10	coding	704	221	2332
PB.8328.1	7	2077	2	full-splice_match	gene: Zm00001d019145	Zm00001d019145_T00	3.35	13.37	coding	427	262	1542
PB.4322.1	3	2182	14	full-splice_match	gene: Zm00001d042690	Zm00001d042690_T00	68.19	68.19	coding	571	255	1967
PB.4652.3	3	1260	2	full-splice_match	gene: Zm00001d043935	Zm00001d043935_T00	96.85	215.60	coding	220	343	1002
PB.5237.1	4	2611	1	intergenic	novelGene_245	novel	41.99	41.99	coding	111	318	650

PB.8348.1	7	1765	2	full-splice_match	gene: Zm00001d019254	Zm00001d019254_T00	12.04	12.04	coding	472	122	1537
PB.8348.2	7	1518	2	full-splice_match	gene: Zm00001d019254	Zm00001d019254_T00	0.00	12.04	coding	472	103	1518
PB.237.1	1	1291	2	novel_in_catalog	gene: Zm00001d028266	novel	49.19	49.19	coding	379	4	1140
PB.2183.2	10	1532	9	full-splice_match	gene: Zm00001d025860	Zm00001d025860_T00	7.31	21.64	coding	375	197	1321
PB.2183.1	10	1518	9	full-splice_match	gene: Zm00001d025860	Zm00001d025860_T00	14.33	21.64	coding	375	40	1164
PB.10526.1 1	9	946	11	full-splice_match	gene: Zm00001d046972	Zm00001d046972_T00	0.00	66.39	coding	215	260	904
PB.9108.1	7	2283	4	novel_not_in_catalog	gene: Zm00001d022626	novel	3.81	3.81	coding	642	293	2218
PB.1511.9	1	2797	3	novel_in_catalog	gene: Zm00001d034345	novel	85.94	1673.99	coding	168	690	1193
PB.4567.20	3	2949	6	novel_in_catalog	gene: Zm00001d043649	novel	0.00	120.68	coding	125	1044	1418
PB.7305.4	6	1461	3	full-splice_match	gene: Zm00001d035037	Zm00001d035037_T00	133.70	637.36	coding	335	121	1125
PB.7305.5	6	1358	3	full-splice_match	gene: Zm00001d035037	Zm00001d035037_T00	0.00	637.36	coding	335	122	1126
PB.7305.6	6	1430	2	novel_in_catalog	gene: Zm00001d035037	novel	0.00	637.36	coding	305	118	1032
PB.7305.1	6	1562	3	full-splice_match	gene: Zm00001d035037	Zm00001d035037_T00	456.80	637.36	coding	335	122	1126
PB.7305.2	6	2362	2	novel_in_catalog	gene: Zm00001d035037	novel	31.35	637.36	coding	256	1181	1948
PB.7305.3	6	1607	2	novel_in_catalog	gene: Zm00001d035037	novel	15.51	637.36	coding	305	116	1030
PB.10307.1	9	1729	1	full-splice_match	gene: Zm00001d045589	Zm00001d045589_T00	4.46	4.46	coding	507	21	1541
PB.2944.2	2	3471	9	full-splice_match	gene: Zm00001d004140	Zm00001d004140_T00	19.09	19.09	coding	967	98	2998

PB.10655.2	9	1550	2	genic	gene: Zm00001d047560	novel	5.75	39.22	coding	309	220	1146
PB.7305.8	6	1103	3	full-splice_match	gene: Zm00001d035037	Zm00001d035037_T00	0.00	637.36	coding	328	119	1102
PB.10655.4	9	1580	2	genic	gene: Zm00001d047560	novel	7.74	39.22	coding	309	368	1294
PB.1358.1	1	1824	5	novel_in_catalog	gene: Zm00001d033641	novel	6.61	88.72	coding	232	203	898
PB.10421.1	9	1150	5	novel_not_in_catalog	gene: Zm00001d046397	novel	1.71	9.61	coding	142	330	755
PB.7063.3	5	1096	1	novel_in_catalog	gene: Zm00001d017787	novel	15.18	49.61	coding	225	162	836
PB.4397.4	3	2385	2	genic	gene: Zm00001d043038	novel	35.44	65.83	coding	513	623	2161
PB.7063.1	5	1973	7	full-splice_match	gene: Zm00001d017787	Zm00001d017787_T00	5.89	49.61	coding	478	79	1512
PB.1414.1	1	1266	4	full-splice_match	gene: Zm00001d033905	Zm00001d033905_T00	1.79	3.46	coding	227	253	933
PB.1304.1	1	623	2	incomplete-splice_match	gene: Zm00001d033397	Zm00001d033397_T00	9.74	15.60	non coding	NA	NA	NA
PB.3198.5	2	1311	12	full-splice_match	gene: Zm00001d005711	Zm00001d005711_T00	7.96	24.98	coding	245	327	1061
PB.1162.2	1	697	2	full-splice_match	gene: Zm00001d032760	Zm00001d032760_T00	135.23	497.94	coding	164	55	546
PB.3198.1	2	520	2	incomplete-splice_match	gene: Zm00001d005711	Zm00001d005711_T00	11.49	24.98	non coding	NA	NA	NA
PB.3198.3	2	1964	13	novel_in_catalog	gene: Zm00001d005711	novel	5.53	24.98	coding	515	107	1651
PB.7122.3	5	868	3	full-splice_match	gene: Zm00001d017998	Zm00001d017998_T00	28.59	46.86	coding	173	117	635
PB.7122.2	5	1047	3	full-splice_match	gene: Zm00001d017998	Zm00001d017998_T00	0.00	46.86	coding	173	183	701
PB.7122.1	5	1274	3	full-splice_match	gene: Zm00001d017998	Zm00001d017998_T00	18.27	46.86	coding	173	334	852

PB.2964.1	2	3553	13	full-splice_match	gene: Zm00001d004335	Zm00001d004335_T00	97.76	97.76	coding	1094	125	3406
PB.6994.4	5	1022	2	full-splice_match	gene: Zm00001d017485	Zm00001d017485_T00	864.97	1045.18	coding	250	76	825
PB.10401.1	9	1786	18	full-splice_match	gene: Zm00001d046231	Zm00001d046231_T01	69.44	69.44	coding	377	311	1441
PB.3757.6	3	763	5	intergenic	novelGene_36	novel	19.89	79.80	non coding	NA	NA	NA
PB.8839.2	7	581	1	incomplete-splice_match	gene: Zm00001d021726	Zm00001d021726_T00	0.00	6.88	non coding	NA	NA	NA
PB.8839.1	7	2204	6	full-splice_match	gene: Zm00001d021726	Zm00001d021726_T00	6.88	6.88	coding	328	675	1658
PB.6999.1	5	1756	12	novel_not_in_catalog	gene: Zm00001d017502	novel	85.61	85.61	coding	369	368	1474
PB.10078.6	8	2121	9	novel_in_catalog	gene: Zm00001d012667	novel	11.81	49.54	coding	379	595	1731
PB.10317.2	9	1427	6	novel_not_in_catalog	gene: Zm00001d045647	novel	39.90	46.40	coding	383	49	1197
PB.9482.2	8	2856	15	full-splice_match	gene: Zm00001d010109	Zm00001d010109_T00	25.15	42.54	coding	763	236	2524
PB.9281.1	8	2361	2	genic_intron	novelGene_101	novel	15.62	15.62	coding	594	355	2136
PB.10317.1	9	1586	6	novel_not_in_catalog	gene: Zm00001d045647	novel	6.50	46.40	coding	383	17	1165
PB.584.3	1	1815	3	full-splice_match	gene: Zm00001d029842	Zm00001d029842_T00	163.24	262.63	coding	499	64	1560
PB.584.2	1	1930	3	full-splice_match	gene: Zm00001d029842	Zm00001d029842_T00	59.46	262.63	coding	499	64	1560
PB.584.1	1	2050	3	full-splice_match	gene: Zm00001d029842	Zm00001d029842_T00	39.93	262.63	coding	499	61	1557
PB.10024.2	8	3396	6	full-splice_match	gene: Zm00001d012457	Zm00001d012457_T00	11.76	48.39	coding	430	141	1430
PB.7800.2	6	2146	6	incomplete-splice_match	gene: Zm00001d037696	Zm00001d037696_T00	8.96	27.46	coding	316	257	1204

PB.10748.1	9	2054	5	full-splice_match	gene: Zm00001d047863	Zm00001d047863_T00	23.10	23.10	coding	483	101	1549
PB.10641.8	9	1235	14	full-splice_match	gene: Zm00001d047498	Zm00001d047498_T01	0.00	128.79	coding	285	153	1007
PB.10641.9	9	1799	14	novel_in_catalog	gene: Zm00001d047498	novel	1.44	128.79	coding	168	66	569
PB.4476.1	3	3116	5	full-splice_match	gene: Zm00001d043329	Zm00001d043329_T00	69.52	108.90	coding	807	210	2630
PB.7953.1	6	1660	1	antisense	novelGene_gene: Zm00001d038270	novel	234.12	234.12	coding	113	316	654
PB.6598.1	5	1936	6	full-splice_match	gene: Zm00001d015407	Zm00001d015407_T00	8.46	8.46	coding	376	430	1557
PB.4698.5	3	2560	5	novel_in_catalog	gene: Zm00001d044138	novel	73.11	1495.64	coding	257	25	795
PB.7238.1	5	3314	3	full-splice_match	gene: Zm00001d018422	Zm00001d018422_T00	8.81	8.81	coding	932	280	3075
PB.919.1	1	1506	3	full-splice_match	gene: Zm00001d031740	Zm00001d031740_T00	37.45	67.76	coding	348	100	1143
PB.10972.1	B73V4	1075	1	full-splice_match	gene: Zm00001d000028	Zm00001d000028_T00	17.98	17.98	coding	181	377	919
PB.10911.1	9	2152	7	full-splice_match	gene: Zm00001d048507	Zm00001d048507_T00	44.29	157.79	coding	418	328	1581
PB.245.1	1	1751	4	full-splice_match	gene: Zm00001d028307	Zm00001d028307_T00	150.42	150.42	coding	431	162	1454
PB.797.3	1	1393	5	incomplete-splice_match	gene: Zm00001d031092	Zm00001d031092_T00	16.72	36.47	coding	330	1	990
PB.245.2	1	1726	4	full-splice_match	gene: Zm00001d028307	Zm00001d028307_T00	0.00	150.42	coding	431	237	1529
PB.5148.1	4	2206	2	novel_in_catalog	gene: Zm00001d050164	novel	20.33	20.33	coding	266	34	831
PB.7266.1	5	1217	7	full-splice_match	gene: Zm00001d018506	Zm00001d018506_T00	33.78	45.43	coding	312	59	994
PB.7266.2	5	1105	7	full-splice_match	gene: Zm00001d018506	Zm00001d018506_T00	11.65	45.43	coding	312	69	1004

PB.7162.2	5	1529	7	incomplete-splice_match	gene: Zm00001d018146	Zm00001d018146_T00	51.12	67.45	coding	330	312	1301
PB.8637.3	7	2261	3	genic	gene: Zm00001d020939	novel	0.00	70.58	coding	609	188	2014
PB.2529.1	2	2809	6	full-splice_match	gene: Zm00001d002168	Zm00001d002168_T00	13.42	27.39	coding	671	418	2430
PB.8637.1	7	2823	1	full-splice_match	gene: Zm00001d020939	Zm00001d020939_T00	7.10	70.58	coding	162	2200	2685
PB.10489.3	9	1075	3	full-splice_match	gene: Zm00001d046767	Zm00001d046767_T00	500.95	778.69	coding	174	147	668
PB.9482.4	8	2199	13	incomplete-splice_match	gene: Zm00001d010109	Zm00001d010109_T00	0.00	42.54	coding	676	170	2197
PB.9862.1	8	1537	1	full-splice_match	gene: Zm00001d011787	Zm00001d011787_T00	91.50	102.34	coding	381	84	1226
PB.9946.1	8	1448	1	intergenic	novelGene_340	novel	17.83	17.83	coding	269	512	1318
PB.2923.1	2	1889	4	full-splice_match	gene: Zm00001d004022	Zm00001d004022_T00	95.00	170.30	coding	232	937	1632
PB.10409.1	9	1566	2	full-splice_match	gene: Zm00001d046305	Zm00001d046305_T00	29.93	30.76	coding	355	316	1380
PB.2923.2	2	1062	5	full-splice_match	gene: Zm00001d004022	Zm00001d004022_T00	75.29	170.30	coding	232	109	804
PB.1473.3	1	1265	1	full-splice_match	gene: Zm00001d034145	Zm00001d034145_T00	53.19	218.33	coding	278	113	946
PB.5871.1	4	1311	3	full-splice_match	gene: Zm00001d053856	Zm00001d053856_T00	40.74	40.74	coding	345	93	1127
PB.570.1	1	1682	1	full-splice_match	gene: Zm00001d029757	Zm00001d029757_T00	15.05	15.05	coding	335	387	1391
PB.6013.1	5	915	1	full-splice_match	gene: Zm00001d013009	Zm00001d013009_T00	72.67	173.02	coding	130	106	495
PB.5255.1	4	1535	4	fusion	gene: Zm00001d050967	novel	77.17	77.17	coding	373	150	1268
PB.6013.3	5	649	1	full-splice_match	gene: Zm00001d013009	Zm00001d013009_T00	45.85	173.02	coding	130	106	495

PB.6013.2	5	727	1	full-splice_match	gene: Zm00001d013009	Zm00001d013009_T00	54.50	173.02	coding	130	83	472
PB.7073.5	5	2268	17	full-splice_match	gene: Zm00001d017830	Zm00001d017830_T01	0.00	50.77	coding	480	109	1548
PB.7073.6	5	2147	17	novel_not_in_catalog	gene: Zm00001d017830	novel	0.19	50.77	coding	447	109	1449
PB.2574.1	2	827	1	full-splice_match	gene: Zm00001d002374	Zm00001d002374_T00	4.61	4.61	coding	167	100	600
PB.7073.1	5	2373	17	full-splice_match	gene: Zm00001d017830	Zm00001d017830_T01	50.59	50.77	coding	480	109	1548
PB.1582.1	1	1145	3	novel_not_in_catalog	gene: Zm00001d034615	novel	3.98	32.35	coding	200	121	720
PB.8266.2	7	2010	1	full-splice_match	gene: Zm00001d018813	Zm00001d018813_T00	23.97	23.97	coding	410	470	1699
PB.10415.3	9	2797	8	novel_not_in_catalog	gene: Zm00001d046364	novel	9.65	71.08	coding	800	60	2459
PB.10415.2	9	2440	8	novel_not_in_catalog	gene: Zm00001d046364	novel	28.41	71.08	coding	690	19	2088
PB.10415.1	9	2742	9	novel_not_in_catalog	gene: Zm00001d046364	novel	33.02	71.08	coding	768	26	2329
PB.1299.3	1	2285	6	novel_not_in_catalog	gene: Zm00001d033383	novel	14.67	22.91	coding	643	72	2000
PB.4351.1	3	1147	1	full-splice_match	gene: Zm00001d042814	Zm00001d042814_T00	72.76	158.53	coding	219	118	774
PB.6877.1	5	806	2	full-splice_match	gene: Zm00001d017019	Zm00001d017019_T00	19.33	130.81	coding	122	179	544
PB.7284.2	5	2010	6	novel_in_catalog	gene: Zm00001d018545	novel	0.37	23.11	coding	390	148	1317
PB.5668.1	4	1640	3	full-splice_match	gene: Zm00001d052885	Zm00001d052885_T00	19.92	572.70	coding	130	1059	1448
PB.6934.1	5	566	2	full-splice_match	gene: Zm00001d017206	Zm00001d017206_T00	38.88	68.21	non coding	NA	NA	NA
PB.3747.3	3	2002	13	full-splice_match	gene: Zm00001d039505	Zm00001d039505_T00	155.81	155.81	coding	505	143	1657

PB.6934.3	5	1585	1	novel_in_catalog	gene: Zm00001d017206	novel	1.12	68.21	coding	166	118	615
PB.6934.2	5	763	2	full-splice_match	gene: Zm00001d017206	Zm00001d017206_T00	28.21	68.21	coding	141	148	570
PB.5668.2	4	658	4	full-splice_match	gene: Zm00001d052885	Zm00001d052885_T00	552.77	572.70	coding	130	87	476
PB.3861.3	3	2106	6	full-splice_match	gene: Zm00001d040038	Zm00001d040038_T00	14.11	58.55	coding	610	25	1854
PB.3861.2	3	2191	6	full-splice_match	gene: Zm00001d040038	Zm00001d040038_T00	44.44	58.55	coding	610	25	1854
PB.6413.1	5	3588	9	full-splice_match	gene: Zm00001d014669	Zm00001d014669_T00	95.36	182.96	coding	946	319	3156
PB.2429.3	2	1268	6	fusion	gene: Zm00001d001802	novel	11.56	35.28	coding	255	344	1108
PB.4048.1	3	1716	3	genic	gene: Zm00001d041258	novel	8.94	8.94	coding	424	91	1362
PB.6413.2	5	4025	9	full-splice_match	gene: Zm00001d014669	Zm00001d014669_T00	8.42	182.96	coding	1099	344	3640
PB.6413.5	5	3661	10	full-splice_match	gene: Zm00001d014669	Zm00001d014669_T00	0.00	182.96	coding	1099	198	3494
PB.6413.4	5	3862	10	full-splice_match	gene: Zm00001d014669	Zm00001d014669_T00	79.18	182.96	coding	1099	198	3494
PB.1358.5	1	1194	6	novel_in_catalog	gene: Zm00001d033641	novel	8.70	88.72	coding	147	129	569
PB.7562.2	6	2016	3	full-splice_match	gene: Zm00001d036543	Zm00001d036543_T00	37.55	226.81	coding	531	174	1766
PB.7562.1	6	2090	3	full-splice_match	gene: Zm00001d036543	Zm00001d036543_T00	189.26	226.81	coding	531	176	1768
PB.3284.3	2	830	1	full-splice_match	gene: Zm00001d006101	Zm00001d006101_T00	0.00	369.14	coding	151	89	541
PB.3284.4	2	737	1	full-splice_match	gene: Zm00001d006101	Zm00001d006101_T00	209.69	369.14	coding	151	77	529
PB.2020.2	10	3649	2	full-splice_match	gene: Zm00001d025002	Zm00001d025002_T00	4.78	4.78	coding	1147	42	3482

PB.459.1	1	1202	5	full-splice_match	gene: Zm00001d029214	Zm00001d029214_T00	71.28	71.28	coding	210	194	823
PB.459.3	1	1066	5	full-splice_match	gene: Zm00001d029214	Zm00001d029214_T00	0.00	71.28	coding	210	159	788
PB.4068.1	3	1709	2	novel_in_catalog	gene: Zm00001d041410	novel	80.90	200.03	coding	165	432	926
PB.7500.1	6	1950	3	full-splice_match	gene: Zm00001d036122	Zm00001d036122_T00	8.85	159.16	coding	496	30	1517
PB.7826.1	6	2748	9	full-splice_match	gene: Zm00001d037829	Zm00001d037829_T01	75.19	102.42	coding	515	789	2333
PB.5489.2	4	2133	10	incomplete-splice_match	gene: Zm00001d052149	Zm00001d052149_T05	26.95	48.43	coding	399	347	1543
PB.9017.2	7	1091	4	full-splice_match	gene: Zm00001d022342	Zm00001d022342_T00	21.94	29.33	coding	249	109	855
PB.9816.1	8	1385	8	full-splice_match	gene: Zm00001d011619	Zm00001d011619_T00	37.89	37.89	coding	383	44	1192
PB.4987.1	4	2877	14	novel_in_catalog	gene: Zm00001d049295	novel	8.27	95.09	coding	497	1063	2553
PB.378.1	1	2450	3	full-splice_match	gene: Zm00001d028866	Zm00001d028866_T00	23.16	83.57	coding	628	145	2028
PB.4987.3	4	2859	15	novel_in_catalog	gene: Zm00001d049295	novel	0.00	95.09	coding	218	2109	2762
PB.378.3	1	2271	3	full-splice_match	gene: Zm00001d028866	Zm00001d028866_T00	60.41	83.57	coding	628	145	2028
PB.954.1	1	1659	15	full-splice_match	gene: Zm00001d031929	Zm00001d031929_T00	4.73	42.16	coding	432	36	1331
PB.954.2	1	2192	14	full-splice_match	gene: Zm00001d031929	Zm00001d031929_T01	37.43	42.16	coding	591	121	1893
PB.3954.1	3	2681	9	full-splice_match	gene: Zm00001d040466	Zm00001d040466_T00	19.44	19.44	coding	782	79	2424
PB.841.1	1	422	1	full-splice_match	gene: Zm00001d031292	Zm00001d031292_T00	50.75	50.75	coding	94	74	355
PB.8433.1	7	576	1	incomplete-splice_match	gene: Zm00001d019856	Zm00001d019856_T00	35.73	35.73	non coding	NA	NA	NA

PB.1672.4	1	604	3	incomplete-splice_match	gene: Zm00001d034914	Zm00001d034914_T03	24.61	104.93	non coding	NA	NA	NA
PB.2249.1	10	1220	2	novel_in_catalog	gene: Zm00001d026177	novel	462.11	462.11	coding	105	682	996
PB.5049.1	4	2108	7	full-splice_match	gene: Zm00001d049617	Zm00001d049617_T00	13.78	13.78	coding	455	250	1614
PB.5049.2	4	1906	7	full-splice_match	gene: Zm00001d049617	Zm00001d049617_T00	0.00	13.78	coding	455	173	1537
PB.8242.1	7	1420	2	full-splice_match	gene: Zm00001d018718	Zm00001d018718_T00	13.14	13.14	coding	322	144	1109
PB.9430.1	8	1541	10	full-splice_match	gene: Zm00001d009783	Zm00001d009783_T00	86.65	86.65	coding	350	224	1273
PB.2232.2	10	2840	10	full-splice_match	gene: Zm00001d026097	Zm00001d026097_T00	13.49	24.76	coding	844	87	2618
PB.7116.5	5	1628	8	full-splice_match	gene: Zm00001d017985	Zm00001d017985_T00	95.74	272.89	coding	390	141	1310
PB.7116.2	5	1914	8	full-splice_match	gene: Zm00001d017985	Zm00001d017985_T00	177.15	272.89	coding	390	218	1387
PB.1490.2	1	970	8	full-splice_match	gene: Zm00001d034244	Zm00001d034244_T00	11.82	334.45	coding	223	174	842
PB.4366.1	3	852	1	full-splice_match	gene: Zm00001d042875	Zm00001d042875_T00	7.44	7.44	non coding	NA	NA	NA
PB.6136.4	5	1499	5	full-splice_match	gene: Zm00001d013410	Zm00001d013410_T00	194.45	216.44	coding	377	103	1233
PB.1195.5	1	1898	3	novel_not_in_catalog	gene: Zm00001d032897	novel	131.64	155.96	coding	245	954	1688
PB.9297.7	8	2774	1	incomplete-splice_match	gene: Zm00001d008993	Zm00001d008993_T00	0.00	152.33	coding	155	61	525
PB.9297.4	8	2895	1	incomplete-splice_match	gene: Zm00001d008993	Zm00001d008993_T00	0.00	152.33	coding	155	61	525
PB.9297.5	8	1171	2	incomplete-splice_match	gene: Zm00001d008993	Zm00001d008993_T00	74.58	152.33	coding	315	104	1048
PB.9297.2	8	1304	2	incomplete-splice_match	gene: Zm00001d008993	Zm00001d008993_T00	3.66	152.33	coding	315	10	954

PB.9297.3	8	1307	2	incomplete-splice_match	gene: Zm00001d008993	Zm00001d008993_T00	64.97	152.33	coding	315	104	1048
PB.9297.1	8	3112	1	genic	gene: Zm00001d008993	novel	6.79	152.33	coding	155	53	517
PB.3995.1	3	1386	3	full-splice_match	gene: Zm00001d040737	Zm00001d040737_T00	6.74	6.74	coding	349	109	1155
PB.937.2	1	1674	2	novel_in_catalog	gene: Zm00001d031844	novel	4.37	4.37	coding	158	1087	1560
PB.10829.1	9	842	3	full-splice_match	gene: Zm00001d048204	Zm00001d048204_T00	305.27	307.47	coding	143	107	535
PB.9297.9	8	1158	2	full-splice_match	gene: Zm00001d008993	Zm00001d008993_T02	2.34	152.33	coding	313	63	1001
PB.4143.2	3	2503	9	full-splice_match	gene: Zm00001d041847	Zm00001d041847_T00	7.95	7.95	coding	773	90	2408
PB.10443.4	9	1356	3	novel_in_catalog	gene: Zm00001d046492	novel	5.69	217.73	coding	164	188	679
PB.849.3	1	2347	3	novel_in_catalog	gene: Zm00001d031332	novel	12.49	22.17	coding	351	126	1178
PB.849.2	1	2088	1	novel_in_catalog	gene: Zm00001d031332	novel	2.83	22.17	coding	243	1250	1978
PB.849.1	1	2440	3	novel_in_catalog	gene: Zm00001d031332	novel	6.85	22.17	coding	351	126	1178
PB.10443.5	9	1204	4	incomplete-splice_match	gene: Zm00001d046492	Zm00001d046492_T00	0.00	217.73	coding	285	250	1104
PB.9933.3	8	1886	10	novel_in_catalog	gene: Zm00001d012084	novel	11.59	20.17	coding	433	491	1789
PB.9933.2	8	1809	11	novel_in_catalog	gene: Zm00001d012084	novel	7.34	20.17	coding	569	6	1712
PB.9933.1	8	1713	6	novel_in_catalog	gene: Zm00001d012084	novel	1.25	20.17	coding	311	5	937
PB.849.4	1	838	3	incomplete-splice_match	gene: Zm00001d031332	Zm00001d031332_T00	0.00	22.17	coding	239	121	837
PB.3761.2	3	1567	5	full-splice_match	gene: Zm00001d039533	Zm00001d039533_T00	0.00	10.28	coding	279	98	934

PB.273.2	1	1843	3	full-splice_match	gene: Zm00001d028399	Zm00001d028399_T00	21.74	69.53	coding	335	269	1273
PB.647.1	1	1094	2	full-splice_match	gene: Zm00001d030167	Zm00001d030167_T00	33.74	42.59	coding	258	29	802
PB.3761.1	3	1803	5	full-splice_match	gene: Zm00001d039533	Zm00001d039533_T00	8.17	10.28	coding	279	100	936
PB.9152.1	8	1392	5	full-splice_match	gene: Zm00001d008314	Zm00001d008314_T00	108.15	115.69	coding	306	130	1047
PB.9152.2	8	1058	3	incomplete-splice_match	gene: Zm00001d008314	Zm00001d008314_T00	7.54	115.69	coding	129	445	831
PB.273.3	1	1706	3	full-splice_match	gene: Zm00001d028399	Zm00001d028399_T00	20.83	69.53	coding	335	271	1275
PB.9579.5	8	555	3	incomplete-splice_match	gene: Zm00001d010577	Zm00001d010577_T00	0.00	33.02	coding	85	112	366
PB.2025.1	10	4688	20	full-splice_match	gene: Zm00001d025012	Zm00001d025012_T00	39.72	152.42	coding	1464	58	4449
PB.10734.1	9	1450	1	novel_in_catalog	gene: Zm00001d047821	novel	4.27	4.27	non coding	NA	NA	NA
PB.9917.2	8	818	1	full-splice_match	gene: Zm00001d012010	Zm00001d012010_T00	0.00	35.17	coding	119	123	479
PB.9579.1	8	1987	4	novel_not_in_catalog	gene: Zm00001d010577	novel	5.24	33.02	coding	142	275	700
PB.5821.2	4	2532	7	novel_in_catalog	gene: Zm00001d053648	novel	4.29	21.53	coding	170	1756	2265
PB.9579.3	8	2497	2	genic	gene: Zm00001d010577	novel	0.00	33.02	coding	183	548	1096
PB.5558.1	4	1853	9	novel_in_catalog	gene: Zm00001d052378	novel	18.14	18.14	coding	422	225	1490
PB.2540.1	2	1568	12	full-splice_match	gene: Zm00001d002261	Zm00001d002261_T00	102.16	122.78	coding	367	98	1198
PB.2540.2	2	1408	12	full-splice_match	gene: Zm00001d002261	Zm00001d002261_T00	11.18	122.78	coding	367	90	1190
PB.8993.1	7	1767	1	full-splice_match	gene: Zm00001d022265	Zm00001d022265_T00	6.42	6.42	coding	566	70	1767

PB.2540.5	2	1375	12	full-splice_match	gene: Zm00001d002261	Zm00001d002261_T00	9.45	122.78	coding	399	70	1266
PB.4709.1	3	900	3	novel_in_catalog	gene: Zm00001d044179	novel	136.76	136.76	coding	165	134	628
PB.9632.1	8	1234	1	full-splice_match	gene: Zm00001d010751	Zm00001d010751_T00	18.56	18.56	coding	313	65	1003
PB.7017.1	5	1879	3	full-splice_match	gene: Zm00001d017570	Zm00001d017570_T00	77.37	77.37	coding	489	248	1714
PB.366.1	1	992	4	full-splice_match	gene: Zm00001d028802	Zm00001d028802_T00	4.68	42.78	coding	170	9	518
PB.366.2	1	845	4	full-splice_match	gene: Zm00001d028802	Zm00001d028802_T00	38.11	42.78	coding	170	17	526
PB.1255.1	1	1463	2	incomplete-splice_match	gene: Zm00001d033193	Zm00001d033193_T00	7.96	7.96	coding	104	816	1127
PB.9742.9	8	2316	10	novel_not_in_catalog	gene: Zm00001d011253	novel	0.00	138.23	coding	187	954	1514
PB.1295.3	1	1597	7	novel_not_in_catalog	gene: Zm00001d033375	novel	13.60	86.31	coding	259	498	1274
PB.1115.1	1	1259	8	novel_not_in_catalog	gene: Zm00001d032573	novel	62.66	62.66	coding	279	161	997
PB.1564.2	1	1450	1	genic	gene: Zm00001d034558	novel	17.89	853.55	coding	309	252	1178
PB.1624.1	1	1134	2	full-splice_match	gene: Zm00001d034745	Zm00001d034745_T00	395.18	395.18	coding	180	411	950
PB.4819.4	3	647	1	intergenic	novelGene_434	novel	0.00	791.98	non coding	NA	NA	NA
PB.5156.4	4	1236	6	novel_in_catalog	gene: Zm00001d050196	novel	7.23	220.69	coding	109	78	404
PB.5156.5	4	1180	6	novel_in_catalog	gene: Zm00001d050196	novel	10.53	220.69	coding	200	81	680
PB.897.2	1	809	3	full-splice_match	gene: Zm00001d031636	Zm00001d031636_T00	11.64	14.53	coding	181	137	679
PB.3769.1	3	3045	1	full-splice_match	gene: Zm00001d039567	Zm00001d039567_T00	6.88	6.88	coding	997	30	3020

PB.5156.1	4	1230	6	novel_in_catalog	gene: Zm00001d050196	novel	2.79	220.69	coding	156	81	548
PB.5156.2	4	1099	7	full-splice_match	gene: Zm00001d050196	Zm00001d050196_T00	195.02	220.69	coding	264	81	872
PB.5156.3	4	2415	1	novel_in_catalog	gene: Zm00001d050196	novel	5.13	220.69	coding	113	79	417
PB.398.2	1	2022	14	novel_in_catalog	gene: Zm00001d028949	novel	7.45	453.16	coding	309	183	1109
PB.398.3	1	2756	14	novel_in_catalog	gene: Zm00001d028949	novel	15.36	453.16	coding	387	1203	2363
PB.398.1	1	1992	15	full-splice_match	gene: Zm00001d028949	Zm00001d028949_T00	185.08	453.16	coding	449	198	1544
PB.398.6	1	2112	16	novel_not_in_catalog	gene: Zm00001d028949	novel	8.04	453.16	coding	363	632	1720
PB.398.7	1	2028	14	novel_in_catalog	gene: Zm00001d028949	novel	11.86	453.16	coding	432	198	1493
PB.9742.8	8	1418	8	novel_not_in_catalog	gene: Zm00001d011253	novel	18.37	138.23	coding	157	122	592
PB.3989.1	3	3525	20	novel_not_in_catalog	gene: Zm00001d040686	novel	4.97	4.97	coding	1006	111	3128
PB.10880.1	9	1134	9	novel_in_catalog	gene: Zm00001d048403	novel	42.90	156.39	coding	237	119	829
PB.398.8	1	2110	13	novel_in_catalog	gene: Zm00001d028949	novel	0.00	453.16	coding	309	197	1123
PB.398.9	1	2143	14	novel_in_catalog	gene: Zm00001d028949	novel	8.52	453.16	coding	266	198	995
PB.4531.2	3	1889	5	novel_in_catalog	gene: Zm00001d043515	novel	2.94	52.60	coding	317	91	1041
PB.4531.3	3	1438	6	full-splice_match	gene: Zm00001d043515	Zm00001d043515_T00	24.82	52.60	coding	357	90	1160
PB.4376.2	3	1459	3	full-splice_match	gene: Zm00001d042918	Zm00001d042918_T00	58.00	66.79	coding	269	323	1129
PB.10925.2	9	2133	4	incomplete-splice_match	gene: Zm00001d048535	Zm00001d048535_T03	36.14	71.63	coding	536	136	1743

PB.7764.4	6	614	2	intergenic	novelGene_249	novel	0.00	0.00	non coding	NA	NA	NA
PB.7764.2	6	3572	1	full-splice_match	gene: Zm00001d037544	Zm00001d037544_T00	9.39	77.96	coding	836	920	3427
PB.7764.1	6	3694	2	genic	gene: Zm00001d037544	novel	68.56	77.96	coding	836	1005	3512
PB.3231.1	2	585	1	intergenic	novelGene_388	novel	40.56	40.56	non coding	NA	NA	NA
PB.8791.6	7	2194	15	novel_not_in_catalog	gene: Zm00001d021567	novel	0.00	96.58	coding	569	321	2027
PB.10552.2	9	1986	5	full-splice_match	gene: Zm00001d047104	Zm00001d047104_T00	96.44	146.62	coding	488	259	1722
PB.6425.2	5	1569	11	novel_in_catalog	gene: Zm00001d014727	novel	8.21	10.35	coding	113	193	531
PB.620.2	1	527	3	full-splice_match	gene: Zm00001d030079	Zm00001d030079_T00	81.83	81.83	coding	91	73	345
PB.2372.3	10	1650	3	full-splice_match	gene: Zm00001d026580	Zm00001d026580_T00	0.00	15.75	coding	458	124	1497
PB.6425.1	5	1695	10	novel_in_catalog	gene: Zm00001d014727	novel	2.14	10.35	coding	134	245	646
PB.1899.2	10	1084	6	full-splice_match	gene: Zm00001d024413	Zm00001d024413_T00	57.74	61.86	coding	277	13	843
PB.1899.1	10	1158	6	full-splice_match	gene: Zm00001d024413	Zm00001d024413_T00	4.11	61.86	coding	241	39	761
PB.8791.5	7	2239	15	novel_not_in_catalog	gene: Zm00001d021567	novel	0.00	96.58	coding	569	296	2002
PB.4453.2	3	2848	6	full-splice_match	gene: Zm00001d043253	Zm00001d043253_T00	3.83	19.43	coding	852	131	2686
PB.10782.1	9	2074	8	full-splice_match	gene: Zm00001d047998	Zm00001d047998_T00	77.10	77.10	coding	534	176	1777
PB.6518.1	5	3781	2	genic	gene: Zm00001d014998	novel	56.31	58.32	coding	1055	241	3405
PB.255.3	1	657	4	intergenic	novelGene_115	novel	154.25	392.10	coding	129	70	456

PB.2600.1	2	1541	1	full-splice_match	gene: Zm00001d002519	Zm00001d002519_T00	12.67	12.67	coding	397	59	1249
PB.2026.10	10	1112	2	genic	gene: Zm00001d025017	novel	0.00	100.34	coding	116	763	1110
PB.869.4	1	1504	6	novel_not_in_catalog	gene: Zm00001d031481	novel	10.52	42.70	coding	376	208	1335
PB.2726.1	2	1461	2	intergenic	novelGene_114	novel	13.24	53.62	coding	182	138	683
PB.869.1	1	1550	6	novel_not_in_catalog	gene: Zm00001d031481	novel	30.46	42.70	coding	376	204	1331
PB.4696.1	3	1309	4	incomplete-splice_match	gene: Zm00001d044133	Zm00001d044133_T00	14.33	60.87	coding	297	98	988
PB.9981.5	8	1724	6	full-splice_match	gene: Zm00001d012277	Zm00001d012277_T00	2.31	549.67	coding	376	131	1258
PB.1558.3	1	356	3	incomplete-splice_match	gene: Zm00001d034520	Zm00001d034520_T00	0.00	268.71	non coding	NA	NA	NA
PB.10002.7	8	1503	5	full-splice_match	gene: Zm00001d012395	Zm00001d012395_T00	17.37	54.28	coding	361	229	1311
PB.1885.1	10	1658	2	incomplete-splice_match	gene: Zm00001d024318	Zm00001d024318_T01	18.54	18.54	coding	385	70	1224
PB.8939.1	7	1210	1	full-splice_match	gene: Zm00001d022053	Zm00001d022053_T00	124.68	124.68	coding	292	72	947
PB.722.1	1	4420	1	intergenic	novelGene_315	novel	0.16	0.16	coding	114	1000	1341
PB.9081.1	7	957	2	novel_in_catalog	gene: Zm00001d022527	novel	4.96	41.03	coding	161	230	712
PB.3600.2	2	1571	11	full-splice_match	gene: Zm00001d007638	Zm00001d007638_T00	35.30	131.78	coding	374	175	1296
PB.3600.3	2	1476	11	full-splice_match	gene: Zm00001d007638	Zm00001d007638_T00	87.11	131.78	coding	374	240	1361
PB.344.1	1	1122	9	full-splice_match	gene: Zm00001d028693	Zm00001d028693_T00	17.59	207.10	coding	243	59	787
PB.4339.2	3	2583	1	genic	gene: Zm00001d042767	novel	21.39	255.64	coding	349	1345	2391

PB.344.3	1	894	9	full-splice_match	gene: Zm00001d028693	Zm00001d028693_T00	15.06	207.10	coding	243	59	787
PB.344.2	1	1000	9	full-splice_match	gene: Zm00001d028693	Zm00001d028693_T00	174.45	207.10	coding	243	56	784
PB.9027.4	7	1550	5	full-splice_match	gene: Zm00001d022386	Zm00001d022386_T00	0.00	32.03	coding	414	23	1264
PB.905.1	1	1348	5	full-splice_match	gene: Zm00001d031666	Zm00001d031666_T00	42.16	42.16	coding	346	54	1091
PB.9027.1	7	1908	5	full-splice_match	gene: Zm00001d022386	Zm00001d022386_T00	7.32	32.03	coding	209	407	1033
PB.9027.3	7	1677	5	full-splice_match	gene: Zm00001d022386	Zm00001d022386_T00	0.00	32.03	coding	414	38	1279
PB.6397.1	5	863	2	full-splice_match	gene: Zm00001d014600	Zm00001d014600_T00	1.16	1.16	coding	190	117	686
PB.6082.3	5	2438	16	novel_in_catalog	gene: Zm00001d013254	novel	3.41	64.80	coding	433	161	1459
PB.8470.6	7	1046	8	novel_not_in_catalog	gene: Zm00001d020069	novel	26.86	205.57	coding	189	446	1012
PB.6082.1	5	2371	17	full-splice_match	gene: Zm00001d013254	Zm00001d013254_T00	55.39	64.80	coding	648	169	2112
PB.336.1	1	1093	1	full-splice_match	gene: Zm00001d028665	Zm00001d028665_T00	39.71	39.71	coding	267	65	865
PB.6082.5	5	2311	17	full-splice_match	gene: Zm00001d013254	Zm00001d013254_T00	6.01	64.80	coding	648	235	2178
PB.7447.1	6	1283	2	full-splice_match	gene: Zm00001d035766	Zm00001d035766_T00	22.53	22.53	coding	308	197	1120
PB.10901.1	9	1283	1	full-splice_match	gene: Zm00001d048476	Zm00001d048476_T00	24.14	24.14	coding	347	25	1065
PB.2127.3	10	439	1	antisense	novelGene_gene: Zm00001d025612	novel	1386.02	2207.53	non coding	NA	NA	NA
PB.2127.2	10	609	1	antisense	novelGene_gene: Zm00001d025612	novel	648.47	2207.53	non coding	NA	NA	NA
PB.9154.1	8	1375	1	intergenic	novelGene_25	novel	3.31	3.31	non coding	NA	NA	NA

PB.9847.6	8	1013	2	incomplete-splice_match	gene: Zm00001d011742	Zm00001d011742_T05	9.64	22.85	coding	265	16	810
PB.6510.1	5	1529	9	full-splice_match	gene: Zm00001d014983	Zm00001d014983_T00	98.55	98.55	coding	400	82	1281
PB.1996.1	10	1908	12	full-splice_match	gene: Zm00001d024891	Zm00001d024891_T00	101.63	101.63	coding	546	142	1779
PB.3086.1	2	1856	9	novel_in_catalog	gene: Zm00001d005106	novel	29.50	29.50	coding	259	930	1706
PB.9015.2	7	3490	10	incomplete-splice_match	gene: Zm00001d022338	Zm00001d022338_T00	49.60	108.21	coding	1073	84	3302
PB.1461.1	1	1297	7	incomplete-splice_match	gene: Zm00001d034074	Zm00001d034074_T02	15.24	15.24	coding	296	81	968
PB.8470.5	7	2014	12	novel_in_catalog	gene: Zm00001d020069	novel	15.70	205.57	coding	618	125	1978
PB.5174.1	4	1548	5	incomplete-splice_match	gene: Zm00001d050316	Zm00001d050316_T03	23.41	112.12	coding	498	53	1546
PB.8510.1	7	1355	2	full-splice_match	gene: Zm00001d020310	Zm00001d020310_T00	23.97	30.26	coding	249	95	841
PB.8510.2	7	1228	2	full-splice_match	gene: Zm00001d020310	Zm00001d020310_T00	6.29	30.26	coding	249	151	897
PB.8470.3	7	831	6	novel_not_in_catalog	gene: Zm00001d020069	novel	13.97	205.57	non coding	NA	NA	NA
PB.4175.2	3	1714	13	full-splice_match	gene: Zm00001d041995	Zm00001d041995_T00	0.00	19.44	coding	488	25	1488
PB.3583.1	2	1138	3	intergenic	novelGene_433	novel	17.51	23.82	coding	213	294	932
PB.10288.5	9	1401	4	full-splice_match	gene: Zm00001d045513	Zm00001d045513_T00	0.00	158.46	coding	322	62	1027
PB.10288.4	9	1402	4	novel_not_in_catalog	gene: Zm00001d045513	novel	0.00	158.46	coding	154	103	564
PB.10288.3	9	1643	4	full-splice_match	gene: Zm00001d045513	Zm00001d045513_T00	69.10	158.46	coding	322	255	1220
PB.10288.2	9	1388	4	full-splice_match	gene: Zm00001d045513	Zm00001d045513_T00	57.45	158.46	coding	118	369	722

PB.10288.1	9	1601	4	novel_not_in_catalog	gene: Zm00001d045513	novel	31.91	158.46	coding	154	97	558
PB.1500.1	1	1431	7	full-splice_match	gene: Zm00001d034319	Zm00001d034319_T00	11.92	44.75	non coding	NA	NA	NA
PB.7734.1	6	405	2	antisense	novelGene_gene: Zm00001d037401	novel	283.81	283.81	non coding	NA	NA	NA
PB.2568.2	2	2520	15	full-splice_match	gene: Zm00001d002357	Zm00001d002357_T00	23.11	43.12	coding	572	554	2269
PB.2568.1	2	1148	9	incomplete-splice_match	gene: Zm00001d002357	Zm00001d002357_T00	20.00	43.12	coding	217	120	770
PB.3536.2	2	2057	1	genic	gene: Zm00001d007174	novel	1.65	260.46	coding	103	40	348
PB.3679.1	3	1130	8	full-splice_match	gene: Zm00001d039266	Zm00001d039266_T01	24.50	40.68	coding	314	109	1050
PB.425.1	1	2267	9	novel_in_catalog	gene: Zm00001d029041	novel	11.73	28.54	coding	446	532	1869
PB.425.3	1	1832	10	full-splice_match	gene: Zm00001d029041	Zm00001d029041_T00	16.80	28.54	coding	446	284	1621
PB.4766.3	3	924	1	novel_in_catalog	gene: Zm00001d044434	novel	32.23	112.31	coding	205	123	737
PB.425.4	1	1575	10	full-splice_match	gene: Zm00001d029041	Zm00001d029041_T00	0.00	28.54	coding	446	181	1518
PB.4670.2	3	2059	15	novel_in_catalog	gene: Zm00001d044024	novel	4.20	44.61	coding	331	216	1208
PB.4670.3	3	2587	20	novel_in_catalog	gene: Zm00001d044024	novel	3.76	44.61	coding	447	845	2185
PB.8506.4	7	1149	6	full-splice_match	gene: Zm00001d020297	Zm00001d020297_T00	0.00	107.68	coding	335	86	1090
PB.10793.3	9	917	2	full-splice_match	gene: Zm00001d048054	Zm00001d048054_T00	12.05	68.53	coding	53	504	662
PB.2003.1	10	1432	3	novel_not_in_catalog	gene: Zm00001d024925	novel	48.37	102.06	coding	321	270	1232
PB.8710.1	7	651	1	novel_in_catalog	gene: Zm00001d021269	novel	90.47	90.47	non coding	NA	NA	NA

PB.8506.1	7	1529	4	novel_in_catalog	gene: Zm00001d020297	novel	13.34	107.68	coding	197	694	1284
PB.8506.3	7	1358	6	full-splice_match	gene: Zm00001d020297	Zm00001d020297_T00	94.33	107.68	coding	335	110	1114
PB.9028.1	7	2874	19	incomplete-splice_match	gene: Zm00001d022388	Zm00001d022388_T01	30.87	194.04	coding	713	250	2388
PB.4094.2	3	1334	10	full-splice_match	gene: Zm00001d041575	Zm00001d041575_T00	0.00	65.96	coding	353	38	1096
PB.9043.2	7	2314	9	full-splice_match	gene: Zm00001d022425	Zm00001d022425_T00	99.55	99.55	coding	467	584	1984
PB.7908.2	6	495	2	full-splice_match	gene: Zm00001d038126	Zm00001d038126_T00	18.31	93.75	coding	146	16	453
PB.10921.3	9	1871	12	novel_in_catalog	gene: Zm00001d048529	novel	0.00	134.48	coding	399	145	1341
PB.8053.1	6	514	1	full-splice_match	gene: Zm00001d038607	Zm00001d038607_T00	50.38	50.38	coding	101	73	375
PB.8702.2	7	2076	2	novel_in_catalog	gene: Zm00001d021224	novel	3.44	100.77	coding	169	593	1099
PB.2950.2	2	947	4	novel_not_in_catalog	gene: Zm00001d004176	novel	15.30	89.67	coding	210	113	742
PB.5035.1	4	2215	3	full-splice_match	gene: Zm00001d049554	Zm00001d049554_T00	29.65	29.91	coding	457	399	1769
PB.5035.2	4	2256	3	full-splice_match	gene: Zm00001d049554	Zm00001d049554_T00	0.26	29.91	coding	457	581	1951
PB.5035.3	4	1879	3	full-splice_match	gene: Zm00001d049554	Zm00001d049554_T00	0.00	29.91	coding	457	396	1766
PB.5035.4	4	880	2	incomplete-splice_match	gene: Zm00001d049554	Zm00001d049554_T00	0.00	29.91	coding	160	400	879
PB.8742.3	7	1928	11	novel_in_catalog	gene: Zm00001d021384	novel	0.00	18.81	coding	395	51	1235
PB.8742.1	7	2188	11	novel_in_catalog	gene: Zm00001d021384	novel	18.81	18.81	coding	395	100	1284
PB.10292.2	9	508	3	intergenic	novelGene_102	novel	35.61	43.88	non coding	NA	NA	NA

PB.4646.2	3	354	1	novel_in_catalog	gene: Zm00001d043904	novel	32.46	106.73	non coding	NA	NA	NA
PB.4646.1	3	1341	2	novel_in_catalog	gene: Zm00001d043904	novel	74.27	106.73	coding	148	162	605
PB.9894.4	8	1396	8	novel_not_in_catalog	gene: Zm00001d011918	novel	0.00	46.25	coding	336	81	1088
PB.9894.3	8	2612	6	novel_in_catalog	gene: Zm00001d011918	novel	4.39	46.25	coding	175	1401	1925
PB.9894.2	8	1891	7	novel_in_catalog	gene: Zm00001d011918	novel	3.94	46.25	coding	340	153	1172
PB.9894.1	8	1523	8	novel_not_in_catalog	gene: Zm00001d011918	novel	37.92	46.25	coding	336	79	1086
PB.7132.1	5	921	3	full-splice_match	gene: Zm00001d018029	Zm00001d018029_T00	38.53	38.53	coding	202	46	651
PB.4310.2	3	1039	3	novel_in_catalog	gene: Zm00001d042642	novel	1.24	10.62	coding	169	115	621
PB.2668.1	2	1027	1	novel_in_catalog	gene: Zm00001d002815	novel	2.30	2.30	coding	172	73	588
PB.948.1	1	2054	4	full-splice_match	gene: Zm00001d031909	Zm00001d031909_T00	358.82	1000.22	coding	589	65	1831
PB.1052.1	1	1074	6	full-splice_match	gene: Zm00001d032370	Zm00001d032370_T00	59.32	286.41	coding	205	94	708
PB.5779.1	4	465	2	incomplete-splice_match	gene: Zm00001d053442	Zm00001d053442_T00	28.18	55.52	non coding	NA	NA	NA
PB.948.5	1	2115	3	novel_in_catalog	gene: Zm00001d031909	novel	8.82	1000.22	coding	263	1213	2001
PB.948.4	1	1956	4	full-splice_match	gene: Zm00001d031909	Zm00001d031909_T00	451.11	1000.22	coding	589	69	1835
PB.2660.1	2	1036	2	full-splice_match	gene: Zm00001d002788	Zm00001d002788_T00	42.43	42.43	coding	207	90	710
PB.10921.4	9	1833	12	novel_not_in_catalog	gene: Zm00001d048529	novel	32.92	134.48	coding	399	161	1357
PB.938.6	1	1475	11	full-splice_match	gene: Zm00001d031849	Zm00001d031849_T00	4.39	258.47	coding	365	226	1320

PB.1665.2	1	948	1	genic	gene: Zm00001d034878	novel	0.00	14.16	non coding	NA	NA	NA
PB.6572.3	5	1544	3	full-splice_match	gene: Zm00001d015269	Zm00001d015269_T00	122.03	122.03	coding	370	210	1319
PB.5322.2	4	715	5	full-splice_match	gene: Zm00001d051405	Zm00001d051405_T00	0.00	119.73	coding	174	71	592
PB.1683.1	10	1946	2	intergenic	novelGene_10	novel	21.22	110.80	coding	422	257	1522
PB.482.1	1	767	2	full-splice_match	gene: Zm00001d029349	Zm00001d029349_T00	0.80	0.80	coding	181	85	627
PB.4240.1	3	2701	2	genic	gene: Zm00001d042331	novel	15.11	46.93	coding	659	111	2087
PB.4240.2	3	2513	2	genic	gene: Zm00001d042331	novel	31.83	46.93	coding	659	111	2087
PB.1683.3	10	2376	1	intergenic	novelGene_10	novel	1.56	110.80	coding	422	693	1958
PB.10120.1	9	1097	3	full-splice_match	gene: Zm00001d044781	Zm00001d044781_T00	7.69	7.69	coding	211	81	713
PB.992.2	1	1783	1	genic	gene: Zm00001d032084	novel	7.51	76.62	non coding	NA	NA	NA
PB.2380.1	10	1056	6	full-splice_match	gene: Zm00001d026591	Zm00001d026591_T00	7.87	7.87	coding	100	435	734
PB.1805.1	10	3835	12	incomplete-splice_match	gene: Zm00001d023795	Zm00001d023795_T05	164.85	164.85	coding	1112	148	3483
PB.3233.1	2	2344	7	full-splice_match	gene: Zm00001d005881	Zm00001d005881_T00	28.96	28.96	coding	374	475	1596
PB.564.1	1	2598	7	full-splice_match	gene: Zm00001d029726	Zm00001d029726_T00	0.00	29.31	coding	462	637	2022
PB.6604.2	5	1961	15	incomplete-splice_match	gene: Zm00001d015446	Zm00001d015446_T01	6.94	18.64	coding	459	158	1534
PB.6604.1	5	2059	15	incomplete-splice_match	gene: Zm00001d015446	Zm00001d015446_T01	11.71	18.64	coding	459	155	1531
PB.564.2	1	2353	7	full-splice_match	gene: Zm00001d029726	Zm00001d029726_T00	29.31	29.31	coding	462	657	2042

PB.2822.1	2	1052	5	full-splice_match	gene: Zm00001d003501	Zm00001d003501_T00	11.87	11.87	coding	300	58	957
PB.8816.1	7	1404	1	novel_in_catalog	gene: Zm00001d021668	novel	0.00	1881.70	non coding	NA	NA	NA
PB.7205.4	5	1342	1	full-splice_match	gene: Zm00001d018326	Zm00001d018326_T00	1.66	286.71	coding	356	234	1301
PB.9110.1	7	1456	3	full-splice_match	gene: Zm00001d022629	Zm00001d022629_T00	12.06	258.64	coding	249	112	858
PB.7019.2	5	1885	2	full-splice_match	gene: Zm00001d017575	Zm00001d017575_T00	39.27	85.92	coding	374	343	1464
PB.7019.3	5	1634	2	full-splice_match	gene: Zm00001d017575	Zm00001d017575_T00	36.92	85.92	coding	374	214	1335
PB.10236.1	9	1325	7	full-splice_match	gene: Zm00001d045336	Zm00001d045336_T00	98.97	111.78	coding	303	58	966
PB.10236.3	9	1212	7	full-splice_match	gene: Zm00001d045336	Zm00001d045336_T00	12.81	111.78	coding	303	84	992
PB.9524.1	8	2008	2	full-splice_match	gene: Zm00001d010308	Zm00001d010308_T00	164.50	164.50	coding	350	400	1449
PB.6695.2	5	1470	8	full-splice_match	gene: Zm00001d016106	Zm00001d016106_T00	74.19	74.19	coding	365	161	1255
PB.5559.6	4	880	6	full-splice_match	gene: Zm00001d052379	Zm00001d052379_T00	55.68	103.90	coding	223	126	794
PB.10395.1	9	1608	6	incomplete-splice_match	gene: Zm00001d046194	Zm00001d046194_T02	18.87	18.87	coding	412	101	1336
PB.1112.3	1	2742	8	novel_in_catalog	gene: Zm00001d032567	novel	2.51	79.93	coding	467	1080	2480
PB.509.1	1	525	1	intergenic	novelGene_165	novel	0.42	0.42	non coding	NA	NA	NA
PB.3909.3	3	758	1	full-splice_match	gene: Zm00001d040292	Zm00001d040292_T00	1398.63	2798.64	coding	95	129	413
PB.3909.1	3	989	1	full-splice_match	gene: Zm00001d040292	Zm00001d040292_T00	1400.01	2798.64	coding	95	129	413
PB.7704.5	6	2086	11	novel_not_in_catalog	gene: Zm00001d037257	novel	1.22	132.91	coding	355	290	1354

PB.6544.1	5	1590	5	full-splice_match	gene: Zm00001d015130	Zm00001d015130_T00	24.26	37.09	coding	356	193	1260
PB.6544.2	5	1087	2	incomplete-splice_match	gene: Zm00001d015130	Zm00001d015130_T00	12.83	37.09	coding	200	250	849
PB.5392.1	4	427	1	genic	gene: Zm00001d051742	novel	14.42	102.59	non coding	NA	NA	NA
PB.7704.1	6	2055	12	novel_not_in_catalog	gene: Zm00001d037257	novel	90.56	132.91	coding	441	331	1653
PB.10549.1	9	1246	1	full-splice_match	gene: Zm00001d047068	Zm00001d047068_T00	4.31	4.31	coding	32	689	784
PB.7704.8	6	2060	11	novel_not_in_catalog	gene: Zm00001d037257	novel	3.38	132.91	coding	441	443	1765
PB.7597.1	6	1790	5	novel_not_in_catalog	gene: Zm00001d036688	novel	2.79	2.79	coding	428	28	1311
PB.4575.7	3	1053	10	incomplete-splice_match	gene: Zm00001d043669	Zm00001d043669_T00	0.00	103.17	coding	310	124	1053
PB.1525.1	1	685	2	full-splice_match	gene: Zm00001d034407	Zm00001d034407_T00	47.27	47.27	coding	104	138	449
PB.2721.1	2	2054	1	full-splice_match	gene: Zm00001d003050	Zm00001d003050_T00	3.00	3.00	coding	643	60	1988
PB.2093.3	10	2237	5	novel_in_catalog	gene: Zm00001d025397	novel	34.00	173.03	coding	253	1273	2031
PB.2093.2	10	929	1	incomplete-splice_match	gene: Zm00001d025397	Zm00001d025397_T00	64.66	173.03	coding	114	54	395
PB.2093.5	10	739	1	novel_in_catalog	gene: Zm00001d025397	novel	44.80	173.03	coding	154	174	635
PB.10148.1	9	769	5	full-splice_match	gene: Zm00001d044939	Zm00001d044939_T00	31.93	234.90	coding	122	133	498
PB.10148.2	9	466	2	novel_in_catalog	gene: Zm00001d044939	novel	34.94	234.90	non coding	NA	NA	NA
PB.10148.3	9	743	4	full-splice_match	gene: Zm00001d044939	Zm00001d044939_T00	168.03	234.90	coding	122	240	605
PB.8392.1	7	2263	18	fusion	gene: Zm00001d019567	novel	45.48	45.48	coding	615	109	1953

PB.6151.1	5	1182	6	incomplete-splice_match	gene: Zm00001d013466	Zm00001d013466_T00	46.29	46.29	coding	88	293	556
PB.7044.3	5	1240	2	novel_not_in_catalog	gene: Zm00001d017691	novel	8.06	34.86	non coding	NA	NA	NA
PB.3832.1	3	1767	15	novel_not_in_catalog	gene: Zm00001d039903	novel	2.83	2.83	coding	295	623	1507
PB.5293.2	4	1064	2	full-splice_match	gene: Zm00001d051223	Zm00001d051223_T00	4.99	4.99	coding	229	100	786
PB.4797.2	3	2007	11	full-splice_match	gene: Zm00001d044533	Zm00001d044533_T00	25.47	80.50	coding	462	371	1756
PB.6054.1	5	1529	6	full-splice_match	gene: Zm00001d013147	Zm00001d013147_T00	14.12	46.32	coding	382	74	1219
PB.7083.11	5	1640	10	full-splice_match	gene: Zm00001d017872	Zm00001d017872_T00	0.00	171.20	coding	446	179	1516
PB.7083.10	5	2832	7	novel_in_catalog	gene: Zm00001d017872	novel	7.31	171.20	coding	216	12	659
PB.8178.1	6	1231	3	full-splice_match	gene: Zm00001d039102	Zm00001d039102_T00	74.17	75.20	coding	318	68	1021
PB.9790.1	8	999	3	full-splice_match	gene: Zm00001d011514	Zm00001d011514_T00	77.50	306.18	coding	180	83	622
PB.9790.2	8	835	3	full-splice_match	gene: Zm00001d011514	Zm00001d011514_T00	228.69	306.18	coding	180	89	628
PB.6797.1	5	2094	1	full-splice_match	gene: Zm00001d016669	Zm00001d016669_T00	16.41	50.22	coding	468	325	1728
PB.4811.1	3	1903	1	genic	gene: Zm00001d044596	novel	23.45	23.45	coding	588	80	1843
PB.3489.2	2	1809	1	full-splice_match	gene: Zm00001d006945	Zm00001d006945_T00	3.68	20.62	coding	369	100	1206
PB.10741.2	9	2372	14	full-splice_match	gene: Zm00001d047842	Zm00001d047842_T00	45.53	45.53	coding	594	376	2157
PB.1938.2	10	1878	6	novel_in_catalog	gene: Zm00001d024588	novel	2.62	71.06	coding	167	230	730
PB.1316.3	1	1050	9	full-splice_match	gene: Zm00001d033472	Zm00001d033472_T00	0.00	25.59	coding	200	252	851

PB.9031.1	7	970	6	full-splice_match	gene: Zm00001d022393	Zm00001d022393_T00	37.54	67.81	coding	153	67	525
PB.9031.2	7	890	6	full-splice_match	gene: Zm00001d022393	Zm00001d022393_T00	0.00	67.81	coding	153	66	524
PB.1938.3	10	1161	8	full-splice_match	gene: Zm00001d024588	Zm00001d024588_T00	0.00	71.06	coding	241	230	952
PB.9031.4	7	765	6	full-splice_match	gene: Zm00001d022393	Zm00001d022393_T00	27.71	67.81	coding	153	68	526
PB.9031.5	7	1108	5	novel_in_catalog	gene: Zm00001d022393	novel	0.00	67.81	non coding	NA	NA	NA
PB.9350.1	8	460	2	intergenic	novelGene_152	novel	13.06	13.06	non coding	NA	NA	NA
PB.2222.1	10	1342	1	full-splice_match	gene: Zm00001d026040	Zm00001d026040_T00	9.23	9.23	coding	407	66	1286
PB.6918.2	5	1276	13	full-splice_match	gene: Zm00001d017153	Zm00001d017153_T00	0.00	21.57	coding	399	16	1212
PB.6026.1	5	1975	1	full-splice_match	gene: Zm00001d013042	Zm00001d013042_T00	5.23	9.70	coding	291	705	1577
PB.6026.2	5	1768	1	full-splice_match	gene: Zm00001d013042	Zm00001d013042_T00	4.48	9.70	coding	291	705	1577
PB.9724.1	8	1326	10	full-splice_match	gene: Zm00001d011188	Zm00001d011188_T00	110.31	110.98	coding	339	104	1120
PB.10698.1	9	1408	5	full-splice_match	gene: Zm00001d047713	Zm00001d047713_T00	151.89	156.51	coding	339	121	1137
PB.10698.2	9	2900	4	full-splice_match	gene: Zm00001d047713	Zm00001d047713_T00	4.62	156.51	coding	208	118	741
PB.6953.1	5	1717	1	genic	gene: Zm00001d017291	novel	21.96	50.24	coding	449	49	1395
PB.8702.5	7	2462	1	genic	gene: Zm00001d021224	novel	7.09	100.77	coding	147	646	1086
PB.1211.1	1	1069	1	full-splice_match	gene: Zm00001d032976	Zm00001d032976_T00	2.05	2.05	coding	54	566	727
PB.6953.2	5	3276	1	genic	gene: Zm00001d017291	novel	28.28	50.24	coding	1046	5	3142

PB.9177.1	8	2181	1	full-splice_match	gene: Zm00001d008424	Zm00001d008424_T00	16.97	16.97	coding	643	164	2092
PB.3362.1	2	1870	2	novel_not_in_catalog	gene: Zm00001d006401	novel	9.25	9.25	non coding	NA	NA	NA
PB.6960.2	5	1143	9	novel_in_catalog	gene: Zm00001d017324	novel	15.58	68.26	coding	252	69	824
PB.3362.3	2	489	3	novel_not_in_catalog	gene: Zm00001d006401	novel	0.00	9.25	non coding	NA	NA	NA
PB.3362.2	2	1234	7	fusion	gene: Zm00001d006402	novel	7.73	7.73	coding	229	55	741
PB.2466.1	2	1132	7	full-splice_match	gene: Zm00001d001932	Zm00001d001932_T00	23.99	23.99	coding	299	45	941
PB.3362.4	2	984	4	full-splice_match	gene: Zm00001d006402	Zm00001d006402_T00	4.88	4.88	coding	244	46	777
PB.7498.2	6	1034	8	full-splice_match	gene: Zm00001d036108	Zm00001d036108_T00	196.61	266.22	coding	244	148	879
PB.6725.2	5	3050	7	full-splice_match	gene: Zm00001d016274	Zm00001d016274_T00	73.61	101.80	coding	745	480	2714
PB.6725.5	5	2774	7	full-splice_match	gene: Zm00001d016274	Zm00001d016274_T00	28.19	101.80	coding	745	360	2594
PB.4206.1	3	1978	6	full-splice_match	gene: Zm00001d042152	Zm00001d042152_T00	43.61	46.94	coding	458	388	1761
PB.7942.1	6	1940	9	full-splice_match	gene: Zm00001d038222	Zm00001d038222_T00	39.12	39.12	coding	361	466	1548
PB.131.7	1	2522	4	novel_in_catalog	gene: Zm00001d027802	novel	0.00	94.04	coding	382	307	1452
PB.131.6	1	3694	1	novel_in_catalog	gene: Zm00001d027802	novel	4.51	94.04	coding	230	2562	3251
PB.131.5	1	3332	2	novel_in_catalog	gene: Zm00001d027802	novel	0.00	94.04	coding	230	2197	2886
PB.131.4	1	2984	5	full-splice_match	gene: Zm00001d027802	Zm00001d027802_T00	15.14	94.04	coding	587	807	2567
PB.131.3	1	2698	4	novel_in_catalog	gene: Zm00001d027802	novel	27.42	94.04	coding	382	320	1465

PB.131.2	1	2593	5	full-splice_match	gene: Zm00001d027802	Zm00001d027802_T00	46.98	94.04	coding	587	391	2151
PB.4918.1	4	2291	1	full-splice_match	gene: Zm00001d049016	Zm00001d049016_T00	9.93	109.24	coding	372	104	1219
PB.1048.1	1	972	8	novel_in_catalog	gene: Zm00001d032353	novel	6.79	6.79	coding	111	354	686
PB.4918.3	4	1585	1	full-splice_match	gene: Zm00001d049016	Zm00001d049016_T00	0.00	109.24	coding	372	101	1216
PB.4918.2	4	1849	1	full-splice_match	gene: Zm00001d049016	Zm00001d049016_T00	99.32	109.24	coding	372	92	1207
PB.4918.4	4	1368	1	full-splice_match	gene: Zm00001d049016	Zm00001d049016_T00	0.00	109.24	coding	372	101	1216
PB.8716.7	7	892	2	incomplete-splice_match	gene: Zm00001d021288	Zm00001d021288_T00	11.16	77.31	coding	149	237	683
PB.131.8	1	2457	5	full-splice_match	gene: Zm00001d027802	Zm00001d027802_T00	0.00	94.04	coding	587	390	2150
PB.3130.1	2	1358	9	full-splice_match	gene: Zm00001d005343	Zm00001d005343_T00	117.78	117.78	coding	315	255	1199
PB.7995.1	6	895	1	full-splice_match	gene: Zm00001d038401	Zm00001d038401_T00	36.20	36.20	coding	80	84	323
PB.3294.3	2	2394	17	novel_not_in_catalog	gene: Zm00001d006131	novel	19.88	139.42	coding	335	1061	2065
PB.2881.1	2	1520	13	novel_not_in_catalog	gene: Zm00001d003813	novel	1.26	23.47	coding	241	518	1240
PB.10690.1	9	1063	1	full-splice_match	gene: Zm00001d047694	Zm00001d047694_T00	66.03	66.03	coding	258	9	782
PB.3481.1	2	929	3	full-splice_match	gene: Zm00001d006924	Zm00001d006924_T00	76.42	142.02	coding	193	63	641
PB.3294.2	2	2349	18	novel_not_in_catalog	gene: Zm00001d006131	novel	119.55	139.42	coding	595	242	2026
PB.1454.2	1	2177	2	full-splice_match	gene: Zm00001d034039	Zm00001d034039_T00	14.57	29.36	coding	347	919	1959
PB.3150.1	2	1649	14	full-splice_match	gene: Zm00001d005460	Zm00001d005460_T00	121.17	121.56	coding	418	90	1343

PB.3150.2	2	2028	12	novel_in_catalog	gene: Zm00001d005460	novel	0.40	121.56	coding	368	192	1295
PB.4214.1	3	1225	3	full-splice_match	gene: Zm00001d042211	Zm00001d042211_T00	25.87	25.87	coding	283	206	1054
PB.5341.2	4	1533	6	incomplete-splice_match	gene: Zm00001d051502	Zm00001d051502_T00	23.87	46.08	coding	227	234	914
PB.5341.3	4	2244	7	full-splice_match	gene: Zm00001d051502	Zm00001d051502_T00	12.76	46.08	coding	562	335	2020
PB.10821.2	9	2114	10	full-splice_match	gene: Zm00001d048174	Zm00001d048174_T00	0.00	44.35	coding	458	71	1444
PB.3461.1	2	1327	5	full-splice_match	gene: Zm00001d006860	Zm00001d006860_T00	309.53	309.53	coding	237	184	894
PB.9852.1	8	444	2	intergenic	novelGene_322	novel	1.96	1.96	non coding	NA	NA	NA
PB.6847.4	5	891	8	novel_in_catalog	gene: Zm00001d016860	novel	8.32	69.05	coding	142	47	472
PB.6624.1	5	1705	5	full-splice_match	gene: Zm00001d015600	Zm00001d015600_T00	5.79	5.79	coding	403	56	1264
PB.1819.3	10	2077	4	novel_in_catalog	gene: Zm00001d023859	novel	7.21	22.01	coding	617	78	1928
PB.1819.1	10	3158	8	novel_not_in_catalog	gene: Zm00001d023859	novel	14.80	22.01	coding	891	195	2867
PB.636.3	1	2351	6	full-splice_match	gene: Zm00001d030131	Zm00001d030131_T00	27.75	219.51	coding	121	1727	2089
PB.2999.9	2	3346	20	full-splice_match	gene: Zm00001d004564	Zm00001d004564_T01	0.00	65.73	coding	975	136	3060
PB.5933.1	4	620	5	novel_not_in_catalog	gene: Zm00001d027043	novel	42.57	42.57	non coding	NA	NA	NA
PB.3917.1	3	446	1	antisense	novelGene_gene: Zm00001d040304	novel	32.33	32.33	non coding	NA	NA	NA
PB.6689.3	5	2103	6	full-splice_match	gene: Zm00001d016070	Zm00001d016070_T00	0.00	56.48	coding	674	81	2102
PB.3342.3	2	3570	7	full-splice_match	gene: Zm00001d006344	Zm00001d006344_T00	2.75	96.19	coding	359	130	1206

PB.2887.1	2	2716	2	intergenic	novelGene_151	novel	38.80	38.80	non coding	NA	NA	NA
PB.636.1	1	1341	8	full-splice_match	gene: Zm00001d030131	Zm00001d030131_T00	142.25	219.51	coding	290	92	961
PB.1294.3	1	1047	5	full-splice_match	gene: Zm00001d033371	Zm00001d033371_T00	28.64	164.88	coding	211	284	916
PB.10885.4	9	2241	14	full-splice_match	gene: Zm00001d048415	Zm00001d048415_T01	7.11	30.49	coding	406	84	1301
PB.10885.5	9	2309	15	full-splice_match	gene: Zm00001d048415	Zm00001d048415_T02	0.00	30.49	coding	190	802	1371
PB.10885.6	9	2652	12	full-splice_match	gene: Zm00001d048415	Zm00001d048415_T01	0.00	30.49	coding	396	84	1271
PB.3062.1	2	1272	3	full-splice_match	gene: Zm00001d004983	Zm00001d004983_T00	6.48	6.48	coding	209	46	672
PB.10557.1	9	635	3	incomplete-splice_match	gene: Zm00001d047124	Zm00001d047124_T00	333.16	333.16	coding	71	287	499
PB.10885.2	9	2726	13	full-splice_match	gene: Zm00001d048415	Zm00001d048415_T02	9.82	30.49	coding	224	76	747
PB.7279.1	5	1820	12	full-splice_match	gene: Zm00001d018533	Zm00001d018533_T00	8.57	8.57	coding	497	118	1608
PB.4698.31	3	1419	4	novel_in_catalog	gene: Zm00001d044138	novel	0.96	1495.64	non coding	NA	NA	NA
PB.9900.1	8	2624	16	full-splice_match	gene: Zm00001d011956	Zm00001d011956_T00	5.73	15.89	coding	696	202	2289
PB.10885.8	9	1969	13	full-splice_match	gene: Zm00001d048415	Zm00001d048415_T00	3.76	30.49	coding	534	84	1685
PB.10885.9	9	2867	12	novel_in_catalog	gene: Zm00001d048415	novel	2.85	30.49	coding	224	69	740
PB.1085.6	1	967	3	novel_not_in_catalog	gene: Zm00001d032485	novel	2.36	241.80	non coding	NA	NA	NA
PB.1446.2	1	3100	16	full-splice_match	gene: Zm00001d034018	Zm00001d034018_T00	21.43	21.43	coding	675	408	2432
PB.1389.1	1	1429	5	full-splice_match	gene: Zm00001d033792	Zm00001d033792_T00	1.91	14.95	coding	203	51	659

PB.3342.2	2	3707	6	full-splice_match	gene: Zm00001d006344	Zm00001d006344_T00	20.84	96.19	coding	359	134	1210
PB.3193.2	2	529	3	full-splice_match	gene: Zm00001d005695	Zm00001d005695_T00	163.71	544.44	coding	90	42	311
PB.3193.1	2	690	3	full-splice_match	gene: Zm00001d005695	Zm00001d005695_T00	380.73	544.44	coding	90	97	366
PB.636.4	1	316	2	incomplete-splice_match	gene: Zm00001d030131	Zm00001d030131_T00	0.00	219.51	non coding	NA	NA	NA
PB.7630.1	6	1587	2	full-splice_match	gene: Zm00001d036922	Zm00001d036922_T00	19.47	19.47	coding	411	354	1586
PB.1721.1	10	2644	23	full-splice_match	gene: Zm00001d023373	Zm00001d023373_T00	7.89	12.08	coding	654	439	2400
PB.1721.2	10	2113	23	full-splice_match	gene: Zm00001d023373	Zm00001d023373_T00	4.19	12.08	coding	654	15	1976
PB.10656.3	9	2392	2	genic	gene: Zm00001d047563	novel	108.07	247.81	coding	641	239	2161
PB.4220.1	3	2030	10	novel_not_in_catalog	gene: Zm00001d042259	novel	5.28	5.28	coding	305	291	1205
PB.1593.2	1	1493	3	novel_in_catalog	gene: Zm00001d034649	novel	9.72	35.53	coding	254	490	1251
PB.7921.1	6	3223	8	incomplete-splice_match	gene: Zm00001d038156	Zm00001d038156_T00	42.90	48.74	coding	881	48	2690
PB.5310.1	4	805	6	incomplete-splice_match	gene: Zm00001d051336	Zm00001d051336_T00	643.41	643.41	coding	61	325	507
PB.10001.1	8	1908	5	novel_in_catalog	gene: Zm00001d012394	novel	4.36	70.61	coding	289	172	1038
PB.10001.2	8	1698	6	novel_not_in_catalog	gene: Zm00001d012394	novel	41.04	70.61	coding	144	170	601
PB.10656.1	9	2441	2	genic	gene: Zm00001d047563	novel	139.74	247.81	coding	641	239	2161
PB.9373.1	8	544	2	novel_in_catalog	gene: Zm00001d009502	novel	101.74	101.74	non coding	NA	NA	NA
PB.4321.2	3	3374	1	full-splice_match	gene: Zm00001d042686	Zm00001d042686_T00	8.19	83.67	coding	514	1655	3196

PB.4321.1	3	1952	2	genic	gene: Zm00001d042686	novel	75.17	83.67	coding	514	235	1776
PB.1452.1	1	1821	4	incomplete-splice_match	gene: Zm00001d034035	Zm00001d034035_T00	54.20	54.20	coding	526	52	1629
PB.4318.4	3	1280	1	genic	gene: Zm00001d042665	novel	10.40	141.28	coding	180	622	1161
PB.3669.2	2	1978	9	full-splice_match	gene: Zm00001d007960	Zm00001d007960_T00	80.59	125.99	coding	535	129	1733
PB.7306.3	6	1774	2	full-splice_match	gene: Zm00001d035039	Zm00001d035039_T00	0.00	106.13	coding	414	364	1605
PB.7306.2	6	2870	1	novel_in_catalog	gene: Zm00001d035039	novel	3.08	106.13	coding	218	445	1098
PB.7306.1	6	2141	2	full-splice_match	gene: Zm00001d035039	Zm00001d035039_T00	103.04	106.13	coding	414	461	1702
PB.8033.2	6	1369	7	full-splice_match	gene: Zm00001d038546	Zm00001d038546_T00	0.55	66.94	coding	257	240	1010
PB.4887.1	4	1701	7	novel_not_in_catalog	gene: Zm00001d048868	novel	23.87	24.57	coding	380	144	1283
PB.4887.2	4	1107	7	novel_not_in_catalog	gene: Zm00001d048868	novel	0.70	24.57	coding	220	95	754
PB.5771.2	4	1792	7	full-splice_match	gene: Zm00001d053412	Zm00001d053412_T00	2.10	6.21	coding	439	317	1633
PB.3907.1	3	1095	1	full-splice_match	gene: Zm00001d040285	Zm00001d040285_T00	0.00	8.58	coding	194	58	639
PB.3907.2	3	973	1	full-splice_match	gene: Zm00001d040285	Zm00001d040285_T00	8.58	8.58	coding	194	57	638
PB.1608.1	1	1333	7	incomplete-splice_match	gene: Zm00001d034689	Zm00001d034689_T00	7.08	7.08	coding	339	22	1038
PB.5771.1	4	1719	6	incomplete-splice_match	gene: Zm00001d053412	Zm00001d053412_T00	4.11	6.21	coding	251	501	1253
PB.7163.2	5	1269	10	full-splice_match	gene: Zm00001d018146	Zm00001d018146_T00	13.97	67.45	coding	348	55	1098
PB.5598.2	4	1657	1	genic	gene: Zm00001d052530	novel	0.00	116.74	coding	507	87	1607

PB.8997.1	7	1574	6	full-splice_match	gene: Zm00001d022274	Zm00001d022274_T00	16.76	16.76	coding	364	89	1180
PB.2947.1	2	2490	21	novel_in_catalog	gene: Zm00001d004159	novel	14.48	14.48	coding	667	274	2274
PB.10899.3	9	2272	10	full-splice_match	gene: Zm00001d048472	Zm00001d048472_T00	45.96	74.45	coding	513	187	1725
PB.3688.1	3	2344	5	full-splice_match	gene: Zm00001d039287	Zm00001d039287_T00	45.59	55.22	coding	598	128	1921
PB.10899.1	9	1155	1	genic	gene: Zm00001d048472	novel	12.02	74.45	non coding	NA	NA	NA
PB.3688.3	3	1629	4	incomplete-splice_match	gene: Zm00001d039287	Zm00001d039287_T00	9.63	55.22	coding	364	272	1363
PB.5856.1	4	1361	5	novel_in_catalog	gene: Zm00001d053808	novel	10.04	10.04	coding	221	462	1124
PB.10899.6	9	2281	9	novel_in_catalog	gene: Zm00001d048472	novel	8.02	74.45	coding	510	103	1632
PB.4080.1	3	1519	7	novel_not_in_catalog	gene: Zm00001d041476	novel	6.98	6.98	coding	378	212	1345
PB.10899.4	9	2400	9	novel_in_catalog	gene: Zm00001d048472	novel	4.48	74.45	coding	452	219	1574
PB.1298.1	1	774	2	intergenic	novelGene_454	novel	4.94	4.94	coding	95	259	543
PB.9700.3	8	3297	21	incomplete-splice_match	gene: Zm00001d011091	Zm00001d011091_T03	32.80	42.20	coding	947	86	2926
PB.10899.8	9	2291	9	novel_in_catalog	gene: Zm00001d048472	novel	0.41	74.45	coding	213	79	717
PB.3828.1	3	1095	8	full-splice_match	gene: Zm00001d039879	Zm00001d039879_T00	110.53	116.14	coding	218	113	766
PB.3828.2	3	964	8	full-splice_match	gene: Zm00001d039879	Zm00001d039879_T00	5.62	116.14	coding	218	134	787
PB.8628.3	7	562	1	intergenic	novelGene_264	novel	14.86	14.86	non coding	NA	NA	NA
PB.4897.1	4	1549	6	novel_not_in_catalog	gene: Zm00001d048899	novel	26.14	26.14	coding	311	285	1217

PB.5859.3	4	1321	1	genic	gene: Zm00001d053818	novel	17.76	105.42	coding	129	612	998
PB.8829.1	7	1150	5	full-splice_match	gene: Zm00001d021701	Zm00001d021701_T00	46.88	57.88	coding	252	168	923
PB.6373.1	5	1326	13	incomplete-splice_match	gene: Zm00001d014500	Zm00001d014500_T00	7.60	24.74	coding	328	55	1038
PB.6373.2	5	1441	15	incomplete-splice_match	gene: Zm00001d014500	Zm00001d014500_T00	17.14	24.74	coding	412	2	1237
PB.9716.5	8	1198	10	novel_in_catalog	gene: Zm00001d011159	novel	2.51	116.81	coding	174	98	619
PB.3969.1	3	1913	5	incomplete-splice_match	gene: Zm00001d040562	Zm00001d040562_T02	270.09	270.09	coding	522	190	1755
PB.10468.1	9	1063	7	novel_in_catalog	gene: Zm00001d046628	novel	20.34	20.34	coding	203	138	746
PB.1716.3	10	2915	8	novel_not_in_catalog	gene: Zm00001d023342	novel	9.87	20.74	coding	182	174	719
PB.4313.1	3	1123	3	full-splice_match	gene: Zm00001d042658	Zm00001d042658_T00	15.91	15.91	coding	231	156	848
PB.5688.3	4	3247	3	fusion	gene: Zm00001d052993	novel	9.12	9.12	coding	916	208	2955
PB.3134.3	2	1484	1	full-splice_match	gene: Zm00001d005351	Zm00001d005351_T00	11.60	29.28	coding	422	114	1379
PB.794.3	1	1030	5	intergenic	novelGene_361	novel	13.50	13.50	non coding	NA	NA	NA
PB.2307.1	10	2974	11	incomplete-splice_match	gene: Zm00001d026366	Zm00001d026366_T00	9.25	10.54	coding	868	87	2690
PB.246.1	1	579	6	novel_not_in_catalog	gene: Zm00001d028309	novel	39.25	39.25	coding	127	30	410
PB.1156.8	1	1774	2	incomplete-splice_match	gene: Zm00001d032739	Zm00001d032739_T00	0.00	110.46	coding	443	73	1401
PB.2494.1	2	2159	5	full-splice_match	gene: Zm00001d002051	Zm00001d002051_T00	121.49	121.49	coding	626	71	1948
PB.298.3	1	1158	7	full-splice_match	gene: Zm00001d028529	Zm00001d028529_T00	40.86	79.74	coding	317	71	1021

PB.6610.1	5	848	1	full-splice_match	gene: Zm00001d015475	Zm00001d015475_T00	8.01	8.01	coding	180	53	592
PB.298.1	1	1271	7	full-splice_match	gene: Zm00001d028529	Zm00001d028529_T00	38.88	79.74	coding	317	66	1016
PB.8482.1	7	3751	14	full-splice_match	gene: Zm00001d020157	Zm00001d020157_T01	21.56	34.79	coding	1075	173	3397
PB.8482.2	7	1886	7	incomplete-splice_match	gene: Zm00001d020157	Zm00001d020157_T02	13.23	34.79	coding	67	1061	1261
PB.544.1	1	717	3	full-splice_match	gene: Zm00001d029625	Zm00001d029625_T00	354.94	354.94	coding	102	117	422
PB.3067.1	2	2975	13	full-splice_match	gene: Zm00001d005019	Zm00001d005019_T00	61.80	190.88	coding	726	285	2462
PB.145.1	1	867	1	full-splice_match	gene: Zm00001d027873	Zm00001d027873_T00	43.27	43.27	non coding	NA	NA	NA
PB.3067.7	2	2643	13	full-splice_match	gene: Zm00001d005019	Zm00001d005019_T00	39.37	190.88	coding	726	241	2418
PB.4088.2	3	1516	1	full-splice_match	gene: Zm00001d041518	Zm00001d041518_T00	13.05	28.78	coding	341	285	1307
PB.4088.1	3	1648	1	full-splice_match	gene: Zm00001d041518	Zm00001d041518_T00	15.74	28.78	coding	341	105	1127
PB.5256.9	4	1801	10	full-splice_match	gene: Zm00001d050970	Zm00001d050970_T02	1.49	60.57	coding	171	90	602
PB.5256.8	4	1451	8	novel_in_catalog	gene: Zm00001d050970	novel	0.00	60.57	coding	193	90	668
PB.8305.1	7	894	4	full-splice_match	gene: Zm00001d018979	Zm00001d018979_T00	3.99	395.16	coding	153	116	574
PB.1707.1	10	1543	6	novel_not_in_catalog	gene: Zm00001d023296	novel	4.91	4.91	coding	102	366	671
PB.8305.2	7	768	4	full-splice_match	gene: Zm00001d018979	Zm00001d018979_T00	391.17	395.16	coding	153	110	568
PB.5256.2	4	1477	10	full-splice_match	gene: Zm00001d050970	Zm00001d050970_T01	8.93	60.57	coding	173	90	608
PB.5256.1	4	1670	12	full-splice_match	gene: Zm00001d050970	Zm00001d050970_T00	20.04	60.57	coding	446	90	1427

PB.5755.5	4	1967	15	novel_not_in_catalog	gene: Zm00001d053334	novel	13.92	58.57	coding	485	235	1689
PB.5256.7	4	1370	9	full-splice_match	gene: Zm00001d050970	Zm00001d050970_T00	13.58	60.57	coding	193	90	668
PB.5256.6	4	1543	9	novel_in_catalog	gene: Zm00001d050970	novel	0.00	60.57	coding	173	90	608
PB.5256.5	4	3304	3	novel_in_catalog	gene: Zm00001d050970	novel	5.30	60.57	coding	110	2774	3103
PB.5256.4	4	1986	9	full-splice_match	gene: Zm00001d050970	Zm00001d050970_T00	4.64	60.57	coding	171	90	602
PB.8818.1	7	1097	5	full-splice_match	gene: Zm00001d021673	Zm00001d021673_T00	58.33	60.15	coding	194	194	775
PB.5288.1	4	4174	17	full-splice_match	gene: Zm00001d051183	Zm00001d051183_T01	20.19	20.19	coding	1193	191	3769
PB.7969.3	6	1637	2	novel_in_catalog	gene: Zm00001d038325	novel	21.20	162.06	coding	349	297	1343
PB.752.2	1	1208	3	full-splice_match	gene: Zm00001d030902	Zm00001d030902_T00	22.37	22.37	non coding	NA	NA	NA
PB.2168.2	10	1996	1	full-splice_match	gene: Zm00001d025788	Zm00001d025788_T00	11.51	22.46	coding	446	527	1864
PB.5335.1	4	2286	1	full-splice_match	gene: Zm00001d051461	Zm00001d051461_T00	5.38	43.48	coding	607	73	1893
PB.5688.1	4	1358	1	incomplete-splice_match	gene: Zm00001d052993	Zm00001d052993_T00	8.77	8.77	coding	258	24	797
PB.1955.2	10	1988	7	novel_in_catalog	gene: Zm00001d024718	novel	6.74	13.06	coding	182	1305	1850
PB.5788.2	4	1093	3	full-splice_match	gene: Zm00001d027016	Zm00001d027016_T00	6.36	18.78	non coding	NA	NA	NA
PB.5788.1	4	1216	3	full-splice_match	gene: Zm00001d027016	Zm00001d027016_T00	12.43	18.78	non coding	NA	NA	NA
PB.1955.1	10	1032	8	full-splice_match	gene: Zm00001d024718	Zm00001d024718_T00	6.32	13.06	coding	277	22	852
PB.3733.2	3	793	5	intergenic	novelGene_21	novel	0.00	107.94	coding	182	51	596

PB.9486.2	8	681	4	full-splice_match	gene: Zm00001d010128	Zm00001d010128_T00	538.21	771.87	coding	113	61	399
PB.10326.1	9	786	2	full-splice_match	gene: Zm00001d045680	Zm00001d045680_T00	36.20	36.20	coding	142	112	537
PB.1316.1	1	1396	9	full-splice_match	gene: Zm00001d033472	Zm00001d033472_T00	25.59	25.59	coding	200	252	851
PB.3733.1	3	957	5	intergenic	novelGene_21	novel	107.94	107.94	coding	182	113	658
PB.5563.2	4	841	2	intergenic	novelGene_384	novel	96.31	96.31	coding	221	28	690
PB.1019.10	1	2339	10	novel_in_catalog	gene: Zm00001d032220	novel	5.31	44.15	coding	116	113	460
PB.5477.1	4	1261	1	full-splice_match	gene: Zm00001d052077	Zm00001d052077_T00	8.68	8.68	coding	343	86	1114
PB.9611.1	8	1727	6	novel_not_in_catalog	gene: Zm00001d010672	novel	15.15	15.15	coding	477	46	1476
PB.8802.1	7	4038	6	novel_not_in_catalog	gene: Zm00001d021617	novel	12.43	12.70	coding	779	377	2713
PB.6287.2	5	1022	5	novel_in_catalog	gene: Zm00001d014108	novel	189.86	268.70	coding	171	225	737
PB.8881.1	7	1389	1	full-splice_match	gene: Zm00001d021836	Zm00001d021836_T00	30.35	30.35	coding	332	53	1048
PB.9082.2	7	1058	1	full-splice_match	gene: Zm00001d022531	Zm00001d022531_T00	54.92	57.27	coding	214	298	939
PB.6287.5	5	866	5	novel_in_catalog	gene: Zm00001d014108	novel	78.84	268.70	coding	171	198	710
PB.3287.1	2	1042	1	full-splice_match	gene: Zm00001d006111	Zm00001d006111_T00	101.68	510.20	coding	220	79	738
PB.9082.1	7	1248	1	full-splice_match	gene: Zm00001d022531	Zm00001d022531_T00	2.35	57.27	coding	214	272	913
PB.6888.1	5	658	2	novel_not_in_catalog	gene: Zm00001d017072	novel	6.76	222.10	non coding	NA	NA	NA
PB.1496.1	1	707	1	intergenic	novelGene_464	novel	1928.04	1928.04	coding	144	47	478

PB.6888.3	5	3053	7	full-splice_match	gene: Zm00001d017072	Zm00001d017072_T00	113.24	222.10	coding	842	194	2719
PB.1368.2	1	2225	14	full-splice_match	gene: Zm00001d033674	Zm00001d033674_T00	60.40	66.10	coding	473	383	1801
PB.6888.5	5	2158	6	full-splice_match	gene: Zm00001d017072	Zm00001d017072_T00	88.17	222.10	coding	582	193	1938
PB.6888.4	5	2981	7	full-splice_match	gene: Zm00001d017072	Zm00001d017072_T00	13.93	222.10	coding	809	198	2624
PB.731.2	1	1646	2	incomplete-splice_match	gene: Zm00001d030784	Zm00001d030784_T00	6.09	6.97	coding	471	50	1462
PB.3609.1	2	1198	1	incomplete-splice_match	gene: Zm00001d007719	Zm00001d007719_T04	31.62	31.62	coding	252	132	887
PB.5280.1	4	1206	1	full-splice_match	gene: Zm00001d051128	Zm00001d051128_T00	21.91	21.91	coding	156	284	751
PB.9451.2	8	713	3	full-splice_match	gene: Zm00001d009932	Zm00001d009932_T00	19.29	93.67	coding	169	81	587
PB.9103.3	7	1460	5	full-splice_match	gene: Zm00001d022614	Zm00001d022614_T00	0.00	20.55	coding	403	98	1306
PB.9451.1	8	843	3	full-splice_match	gene: Zm00001d009932	Zm00001d009932_T00	74.39	93.67	coding	169	82	588
PB.1795.1	10	2243	3	incomplete-splice_match	gene: Zm00001d023724	Zm00001d023724_T00	9.71	57.50	coding	141	938	1360
PB.7421.1	6	879	2	full-splice_match	gene: Zm00001d035659	Zm00001d035659_T00	48.96	895.56	coding	83	68	316
PB.1795.3	10	1800	6	full-splice_match	gene: Zm00001d023724	Zm00001d023724_T01	20.76	57.50	coding	124	1228	1599
PB.1795.2	10	1012	7	novel_in_catalog	gene: Zm00001d023724	novel	3.91	57.50	coding	157	336	806
PB.1795.5	10	1759	8	novel_in_catalog	gene: Zm00001d023724	novel	5.73	57.50	coding	147	1114	1554
PB.1795.4	10	1182	8	novel_in_catalog	gene: Zm00001d023724	novel	17.38	57.50	coding	151	542	994
PB.5573.5	4	2120	14	novel_in_catalog	gene: Zm00001d052416	novel	5.52	60.49	coding	263	141	929

PB.1795.6	10	1686	7	novel_in_catalog	gene: Zm00001d023724	novel	0.00	57.50	coding	124	1129	1500
PB.5334.1	4	1211	1	full-splice_match	gene: Zm00001d051460	Zm00001d051460_T00	40.64	40.64	coding	220	116	775
PB.1529.1	1	750	4	novel_in_catalog	gene: Zm00001d034422	novel	469.82	798.13	coding	152	87	542
PB.7421.3	6	597	2	full-splice_match	gene: Zm00001d035659	Zm00001d035659_T00	801.27	895.56	coding	83	68	316
PB.4998.1	4	793	6	full-splice_match	gene: Zm00001d049362	Zm00001d049362_T00	330.30	339.97	coding	110	225	554
PB.9824.2	8	674	3	incomplete-splice_match	gene: Zm00001d011646	Zm00001d011646_T00	59.81	85.93	coding	134	136	537
PB.4998.3	4	2669	4	full-splice_match	gene: Zm00001d049362	Zm00001d049362_T00	4.53	339.97	coding	115	947	1291
PB.4998.2	4	702	5	novel_in_catalog	gene: Zm00001d049362	novel	5.15	339.97	non coding	NA	NA	NA
PB.4998.5	4	714	6	full-splice_match	gene: Zm00001d049362	Zm00001d049362_T00	0.00	339.97	coding	110	223	552
PB.2527.3	2	1901	12	novel_in_catalog	gene: Zm00001d002163	novel	4.29	26.63	coding	304	730	1641
PB.10945.5	B73V 4	1572	2	full-splice_match	gene: Zm00001d000424	Zm00001d000424_T00	2.21	83.44	coding	291	250	1122
PB.10945.4	B73V 4	1805	1	genic	gene: Zm00001d000424	novel	0.00	83.44	coding	291	493	1365
PB.3848.1	3	1064	1	full-splice_match	gene: Zm00001d039977	Zm00001d039977_T00	85.50	85.50	coding	138	304	717
PB.10945.1	B73V 4	1252	2	full-splice_match	gene: Zm00001d000424	Zm00001d000424_T00	62.61	83.44	coding	291	298	1170
PB.9619.1	8	1866	2	genic	gene: Zm00001d010705	novel	16.45	16.45	coding	412	134	1369
PB.10945.3	B73V 4	2197	2	full-splice_match	gene: Zm00001d000424	Zm00001d000424_T00	2.10	83.44	coding	291	296	1168
PB.10945.2	B73V 4	1481	1	novel_in_catalog	gene: Zm00001d000424	novel	16.52	83.44	coding	291	527	1399

PB.4594.1	3	1431	4	full-splice_match	gene: Zm00001d043736	Zm00001d043736_T00	30.12	30.12	coding	286	99	956
PB.4698.39	3	1403	5	novel_in_catalog	gene: Zm00001d044138	novel	0.00	1495.64	coding	354	12	1073
PB.10930.3	9	921	8	novel_not_in_catalog	gene: Zm00001d048550	novel	4.12	59.69	coding	112	484	819
PB.7247.1	5	3068	6	full-splice_match	gene: Zm00001d018441	Zm00001d018441_T00	9.15	9.15	coding	895	170	2854
PB.2688.3	2	1333	3	full-splice_match	gene: Zm00001d002897	Zm00001d002897_T00	0.00	367.69	coding	357	139	1209
PB.2688.2	2	1431	3	full-splice_match	gene: Zm00001d002897	Zm00001d002897_T00	304.36	367.69	coding	357	150	1220
PB.2688.1	2	1556	3	full-splice_match	gene: Zm00001d002897	Zm00001d002897_T00	63.33	367.69	coding	357	147	1217
PB.2879.2	2	1304	2	incomplete-splice_match	gene: Zm00001d003807	Zm00001d003807_T00	0.00	50.21	coding	361	74	1156
PB.4783.9	3	2476	15	novel_in_catalog	gene: Zm00001d044494	novel	5.63	46.54	coding	79	68	304
PB.5776.2	4	1634	1	full-splice_match	gene: Zm00001d053429	Zm00001d053429_T00	76.01	103.58	coding	320	546	1505
PB.5683.1	4	1044	2	full-splice_match	gene: Zm00001d052967	Zm00001d052967_T00	6.37	12.07	coding	127	214	594
PB.1959.1	10	642	1	incomplete-splice_match	gene: Zm00001d024734	Zm00001d024734_T00	237.90	237.90	coding	19	396	452
PB.2300.1	10	1177	4	genic	gene: Zm00001d023190	novel	2.12	2.12	coding	110	339	668
PB.3973.1	3	2268	2	intergenic	novelGene_144	novel	2.92	11.05	coding	53	465	623
PB.3973.3	3	834	4	intergenic	novelGene_144	novel	2.13	11.05	coding	33	376	474
PB.10450.1	9	1807	4	incomplete-splice_match	gene: Zm00001d046537	Zm00001d046537_T00	1.78	1.78	coding	489	150	1616
PB.3702.6	3	2050	11	novel_in_catalog	gene: Zm00001d039321	novel	0.00	42.72	coding	595	36	1820

PB.3702.4	3	2325	10	novel_in_catalog	gene: Zm00001d039321	novel	4.06	42.72	coding	554	120	1781
PB.3702.3	3	2126	11	novel_not_in_catalog	gene: Zm00001d039321	novel	9.50	42.72	coding	418	83	1336
PB.3702.1	3	2192	11	novel_in_catalog	gene: Zm00001d039321	novel	29.16	42.72	coding	595	125	1909
PB.660.1	1	2068	4	full-splice_match	gene: Zm00001d030235	Zm00001d030235_T00	11.85	11.85	coding	583	141	1889
PB.4698.32	3	2333	4	novel_in_catalog	gene: Zm00001d044138	novel	79.93	1495.64	coding	257	25	795
PB.1205.1	1	2351	4	novel_in_catalog	gene: Zm00001d032939	novel	28.87	35.03	coding	190	463	1032
PB.1245.2	1	2790	14	full-splice_match	gene: Zm00001d033155	Zm00001d033155_T00	14.82	37.12	coding	675	177	2201
PB.4924.1	4	877	2	incomplete-splice_match	gene: Zm00001d049032	Zm00001d049032_T00	11.32	11.32	coding	211	90	722
PB.5792.1	4	1564	6	full-splice_match	gene: Zm00001d053555	Zm00001d053555_T00	14.41	14.41	coding	352	53	1108
PB.3094.1	2	1001	1	full-splice_match	gene: Zm00001d005146	Zm00001d005146_T00	239.57	2092.10	coding	115	54	398
PB.640.1	1	1220	2	novel_in_catalog	gene: Zm00001d030149	novel	41.83	41.83	coding	289	96	962
PB.3094.3	2	869	1	full-splice_match	gene: Zm00001d005146	Zm00001d005146_T00	562.64	2092.10	coding	115	156	500
PB.3094.2	2	903	1	full-splice_match	gene: Zm00001d005146	Zm00001d005146_T00	67.12	2092.10	coding	115	149	493
PB.10440.1	9	2778	7	full-splice_match	gene: Zm00001d046488	Zm00001d046488_T00	38.03	38.03	coding	765	212	2506
PB.3094.4	2	679	1	full-splice_match	gene: Zm00001d005146	Zm00001d005146_T00	1222.77	2092.10	coding	115	57	401
PB.8713.2	7	2980	1	full-splice_match	gene: Zm00001d021284	Zm00001d021284_T00	5.77	68.37	coding	87	97	357
PB.2223.1	10	1121	1	genic	gene: Zm00001d026042	novel	0.23	0.23	coding	312	107	1042

PB.7422.4	6	429	1	novel_in_catalog	gene: Zm00001d035662	novel	0.00	4830.23	non coding	NA	NA	NA
PB.7422.5	6	508	2	novel_not_in_catalog	gene: Zm00001d035662	novel	5.90	4830.23	non coding	NA	NA	NA
PB.7422.2	6	813	2	full-splice_match	gene: Zm00001d035662	Zm00001d035662_T00	3.23	4830.23	coding	80	87	326
PB.7422.3	6	571	2	full-splice_match	gene: Zm00001d035662	Zm00001d035662_T00	4806.24	4830.23	coding	80	87	326
PB.8787.5	7	1745	4	novel_in_catalog	gene: Zm00001d021557	novel	3.37	98.16	coding	252	202	957
PB.7422.1	6	1109	2	full-splice_match	gene: Zm00001d035662	Zm00001d035662_T00	14.86	4830.23	coding	80	87	326
PB.8472.1	7	1452	9	novel_not_in_catalog	gene: Zm00001d020093	novel	13.98	13.98	coding	241	235	957
PB.2033.1	10	2917	9	full-splice_match	gene: Zm00001d025034	Zm00001d025034_T00	6.86	6.86	coding	776	194	2521
PB.10950.3	B73V4	1539	2	genic	gene: Zm00001d000307	novel	6.28	34.34	coding	194	743	1324
PB.4698.34	3	1654	5	novel_in_catalog	gene: Zm00001d044138	novel	0.00	1495.64	coding	203	25	633
PB.9792.1	8	474	1	incomplete-splice_match	gene: Zm00001d011527	Zm00001d011527_T00	28.05	78.99	non coding	NA	NA	NA
PB.235.31	1	1293	11	novel_in_catalog	gene: Zm00001d028260	novel	583.08	1792.39	coding	357	84	1154
PB.1993.1	10	1709	3	full-splice_match	gene: Zm00001d024883	Zm00001d024883_T00	23.35	23.35	coding	499	23	1519
PB.9956.2	8	2181	6	novel_not_in_catalog	gene: Zm00001d012167	novel	69.20	214.85	coding	560	318	1997
PB.9956.4	8	2088	6	novel_not_in_catalog	gene: Zm00001d012167	novel	0.00	214.85	coding	560	331	2010
PB.4746.3	3	1299	10	novel_not_in_catalog	gene: Zm00001d044315	novel	0.49	301.89	coding	273	148	966
PB.4746.1	3	1535	10	full-splice_match	gene: Zm00001d044315	Zm00001d044315_T01	176.47	301.89	coding	351	170	1222

PB.2234.4	10	1114	4	novel_in_catalog	gene: Zm00001d026124	novel	28.29	28.29	coding	271	32	844
PB.7257.1	5	1245	4	full-splice_match	gene: Zm00001d018485	Zm00001d018485_T00	24.60	24.60	coding	266	58	855
PB.7873.1	6	1058	3	full-splice_match	gene: Zm00001d038012	Zm00001d038012_T00	104.47	104.47	coding	170	239	748
PB.983.1	1	1627	5	full-splice_match	gene: Zm00001d032049	Zm00001d032049_T00	48.33	65.32	coding	399	225	1421
PB.1613.2	1	1584	6	novel_in_catalog	gene: Zm00001d034719	novel	4.01	45.33	coding	403	220	1428
PB.2178.1	10	2678	9	full-splice_match	gene: Zm00001d025837	Zm00001d025837_T00	12.24	18.98	coding	736	289	2496
PB.975.5	1	1317	13	full-splice_match	gene: Zm00001d031996	Zm00001d031996_T00	1.62	15.39	coding	351	97	1149
PB.2178.2	10	1292	8	incomplete-splice_match	gene: Zm00001d025837	Zm00001d025837_T00	6.74	18.98	coding	328	174	1157
PB.8404.3	7	1619	1	genic_intron	novelGene_141	novel	3.44	13.83	coding	239	91	807
PB.6044.1	5	985	5	full-splice_match	gene: Zm00001d013092	Zm00001d013092_T00	241.55	278.74	coding	152	134	589
PB.1244.1	1	2092	3	full-splice_match	gene: Zm00001d033153	Zm00001d033153_T00	22.10	22.10	coding	425	597	1871
PB.975.4	1	1457	13	full-splice_match	gene: Zm00001d031996	Zm00001d031996_T00	0.00	15.39	coding	351	90	1142
PB.896.2	1	2752	2	novel_in_catalog	gene: Zm00001d031635	novel	27.62	217.19	coding	159	1963	2439
PB.671.2	1	742	1	full-splice_match	gene: Zm00001d030339	Zm00001d030339_T00	0.00	122.55	coding	183	87	635
PB.9772.5	8	1733	5	full-splice_match	gene: Zm00001d011418	Zm00001d011418_T00	0.00	75.59	coding	528	2	1585
PB.1250.11	1	1329	4	full-splice_match	gene: Zm00001d033174	Zm00001d033174_T00	0.00	779.80	coding	364	120	1211
PB.8339.1	7	1963	11	full-splice_match	gene: Zm00001d019217	Zm00001d019217_T00	12.49	12.49	coding	549	80	1726

PB.5761.3	4	1028	3	full-splice_match	gene: Zm00001d053371	Zm00001d053371_T00	160.83	249.18	coding	188	249	812
PB.1945.1	10	1309	7	full-splice_match	gene: Zm00001d024661	Zm00001d024661_T00	30.31	30.31	coding	229	299	985
PB.6696.1	5	1532	2	genic	gene: Zm00001d016129	novel	20.50	20.50	coding	380	107	1246
PB.7624.2	6	2093	4	full-splice_match	gene: Zm00001d036895	Zm00001d036895_T00	95.74	101.76	coding	441	227	1549
PB.709.5	1	2297	1	genic	gene: Zm00001d030618	novel	5.03	1118.65	non coding	NA	NA	NA
PB.709.6	1	414	2	genic	gene: Zm00001d030618	novel	124.05	1118.65	non coding	NA	NA	NA
PB.6044.2	5	1285	3	incomplete-splice_match	gene: Zm00001d013092	Zm00001d013092_T00	14.64	278.74	non coding	NA	NA	NA
PB.3667.1	2	1364	10	full-splice_match	gene: Zm00001d007951	Zm00001d007951_T00	92.35	98.41	coding	332	105	1100
PB.709.1	1	509	2	genic	gene: Zm00001d030618	novel	187.85	1118.65	non coding	NA	NA	NA
PB.233.1	1	2011	2	full-splice_match	gene: Zm00001d028241	Zm00001d028241_T00	33.39	33.39	coding	494	207	1688
PB.709.3	1	1269	1	genic	gene: Zm00001d030618	novel	0.00	1118.65	non coding	NA	NA	NA
PB.10658.1	9	1053	1	full-splice_match	gene: Zm00001d047568	Zm00001d047568_T00	37.17	37.17	coding	206	105	722
PB.10819.7	9	1693	3	full-splice_match	gene: Zm00001d048154	Zm00001d048154_T00	14.68	15.24	coding	467	83	1483
PB.10819.6	9	2077	2	full-splice_match	gene: Zm00001d048154	Zm00001d048154_T00	0.56	15.24	coding	467	478	1878
PB.10819.5	9	1108	4	novel_in_catalog	gene: Zm00001d048153	novel	0.71	7.38	coding	234	36	737
PB.10819.4	9	1064	5	full-splice_match	gene: Zm00001d048153	Zm00001d048153_T00	6.66	7.38	coding	231	36	728
PB.10073.3	8	2505	10	novel_in_catalog	gene: Zm00001d012659	novel	1.32	40.43	coding	274	1403	2224

PB.10073.2	8	1314	9	full-splice_match	gene: Zm00001d012659	Zm00001d012659_T00	28.27	40.43	coding	274	211	1032
PB.10073.1	8	2679	9	full-splice_match	gene: Zm00001d012659	Zm00001d012659_T00	7.01	40.43	coding	274	1498	2319
PB.9119.2	8	1541	1	full-splice_match	gene: Zm00001d008187	Zm00001d008187_T00	2.96	24.32	coding	458	78	1451
PB.9119.1	8	1600	1	full-splice_match	gene: Zm00001d008187	Zm00001d008187_T00	21.36	24.32	coding	458	15	1388
PB.10073.4	8	2587	9	full-splice_match	gene: Zm00001d012659	Zm00001d012659_T00	3.83	40.43	coding	274	1569	2390
PB.6422.1	5	1963	13	full-splice_match	gene: Zm00001d014715	Zm00001d014715_T00	2.47	49.99	coding	426	81	1358
PB.10465.2	9	1371	2	genic	gene: Zm00001d046596	novel	80.92	83.21	coding	304	46	957
PB.10089.2	8	905	1	full-splice_match	gene: Zm00001d012720	Zm00001d012720_T00	0.00	24.69	coding	237	174	884
PB.6422.2	5	1812	13	full-splice_match	gene: Zm00001d014715	Zm00001d014715_T00	19.72	49.99	coding	426	155	1432
PB.6390.1	5	920	1	full-splice_match	gene: Zm00001d014563	Zm00001d014563_T00	15.63	15.63	coding	249	84	830
PB.6422.4	5	1525	13	full-splice_match	gene: Zm00001d014715	Zm00001d014715_T00	27.80	49.99	coding	426	76	1353
PB.6422.7	5	2138	13	full-splice_match	gene: Zm00001d014716	Zm00001d014716_T00	4.86	5.41	coding	633	56	1954
PB.6422.6	5	1266	11	incomplete-splice_match	gene: Zm00001d014716	Zm00001d014716_T00	0.00	5.41	coding	206	341	958
PB.10194.3	9	682	5	full-splice_match	gene: Zm00001d045160	Zm00001d045160_T00	10.55	25.36	non coding	NA	NA	NA
PB.10194.2	9	844	5	full-splice_match	gene: Zm00001d045160	Zm00001d045160_T00	4.09	25.36	coding	154	37	498
PB.10194.1	9	1060	5	full-splice_match	gene: Zm00001d045160	Zm00001d045160_T00	10.72	25.36	coding	154	33	494
PB.4453.4	3	1563	1	novel_in_catalog	gene: Zm00001d043253	novel	0.13	19.43	coding	441	131	1453

PB.4453.3	3	2962	5	novel_in_catalog	gene: Zm00001d043253	novel	2.05	19.43	coding	701	142	2244
PB.4282.4	3	1662	3	incomplete-splice_match	gene: Zm00001d042520	Zm00001d042520_T00	0.00	17.23	coding	397	472	1662
PB.4453.1	3	3177	6	full-splice_match	gene: Zm00001d043253	Zm00001d043253_T00	13.42	19.43	coding	852	131	2686
PB.1672.2	1	1483	10	incomplete-splice_match	gene: Zm00001d034914	Zm00001d034914_T03	23.24	104.93	coding	354	54	1115
PB.3964.2	3	1914	3	novel_not_in_catalog	gene: Zm00001d040527	novel	15.21	38.80	coding	427	367	1647
PB.1261.1	1	1787	1	full-splice_match	gene: Zm00001d033222	Zm00001d033222_T00	3.54	3.54	coding	429	196	1482
PB.1071.1	1	1977	8	full-splice_match	gene: Zm00001d032435	Zm00001d032435_T00	27.08	27.08	coding	456	220	1587
PB.1507.6	1	493	4	incomplete-splice_match	gene: Zm00001d034341	Zm00001d034341_T00	0.00	20.99	non coding	NA	NA	NA
PB.7857.4	6	1307	1	full-splice_match	gene: Zm00001d037945	Zm00001d037945_T00	127.37	138.96	coding	198	275	868
PB.7857.5	6	1161	1	full-splice_match	gene: Zm00001d037945	Zm00001d037945_T00	0.00	138.96	coding	198	230	823
PB.7857.2	6	952	1	intergenic	novelGene_280	novel	0.00	0.00	non coding	NA	NA	NA
PB.7857.3	6	1259	1	intergenic	novelGene_280	novel	0.00	0.00	non coding	NA	NA	NA
PB.7857.1	6	3597	1	full-splice_match	gene: Zm00001d037945	Zm00001d037945_T00	11.59	138.96	coding	198	289	882
PB.2028.1	10	2629	7	full-splice_match	gene: Zm00001d025021	Zm00001d025021_T00	4.83	4.83	coding	757	228	2498
PB.3596.1	2	1570	10	full-splice_match	gene: Zm00001d007610	Zm00001d007610_T00	49.94	49.94	coding	393	95	1273
PB.8450.1	7	3060	19	full-splice_match	gene: Zm00001d019988	Zm00001d019988_T00	34.12	34.12	coding	917	69	2819
PB.2386.3	10	2294	10	full-splice_match	gene: Zm00001d026618	Zm00001d026618_T00	4.58	4.58	coding	727	70	2250

PB.7036.1	5	1391	1	full-splice_match	gene: Zm00001d017657	Zm00001d017657_T00	15.71	15.71	coding	284	148	999
PB.347.1	1	848	1	full-splice_match	gene: Zm00001d028702	Zm00001d028702_T00	13.97	13.97	coding	222	43	708
PB.2537.2	2	313	1	full-splice_match	gene: Zm00001d002253	Zm00001d002253_T00	190.33	600.07	non coding	NA	NA	NA
PB.5195.2	4	1060	10	full-splice_match	gene: Zm00001d050440	Zm00001d050440_T00	26.63	117.71	coding	247	185	925
PB.5195.1	4	997	9	full-splice_match	gene: Zm00001d050440	Zm00001d050440_T00	91.07	117.71	coding	220	184	843
PB.2537.1	2	656	1	full-splice_match	gene: Zm00001d002253	Zm00001d002253_T00	409.74	600.07	coding	137	62	472
PB.8390.3	7	1299	3	full-splice_match	gene: Zm00001d019563	Zm00001d019563_T00	31.59	2063.77	coding	290	178	1047
PB.8390.1	7	1389	3	full-splice_match	gene: Zm00001d019563	Zm00001d019563_T00	772.33	2063.77	coding	290	89	958
PB.8390.7	7	1180	3	full-splice_match	gene: Zm00001d019563	Zm00001d019563_T00	1254.81	2063.77	coding	290	89	958
PB.8390.6	7	3037	1	novel_in_catalog	gene: Zm00001d019563	novel	2.77	2063.77	coding	247	91	831
PB.6791.2	5	1636	1	full-splice_match	gene: Zm00001d016653	Zm00001d016653_T00	4.54	26.34	coding	416	150	1397
PB.8390.4	7	3033	2	novel_in_catalog	gene: Zm00001d019563	novel	2.27	2063.77	coding	247	88	828
PB.9463.1	8	1553	7	full-splice_match	gene: Zm00001d009983	Zm00001d009983_T00	9.30	9.30	coding	214	830	1471
PB.2889.1	2	1231	10	full-splice_match	gene: Zm00001d003855	Zm00001d003855_T00	53.56	60.70	coding	228	212	895
PB.9084.10	7	1356	9	novel_in_catalog	gene: Zm00001d022542	novel	3.42	154.27	coding	305	98	1012
PB.8390.8	7	2344	1	novel_in_catalog	gene: Zm00001d019563	novel	0.00	2063.77	coding	247	91	831
PB.6998.1	5	1178	4	full-splice_match	gene: Zm00001d017501	Zm00001d017501_T00	48.08	48.08	coding	253	181	939

PB.3588.5	2	593	2	novel_in_catalog	gene: Zm00001d007560	novel	526.09	526.09	coding	159	84	560
PB.6102.1	5	2120	2	incomplete-splice_match	gene: Zm00001d013301	Zm00001d013301_T00	25.55	25.55	coding	537	220	1830
PB.4864.1	4	1378	10	full-splice_match	gene: Zm00001d048721	Zm00001d048721_T00	45.44	45.44	coding	359	45	1121
PB.8921.1	7	1565	2	full-splice_match	gene: Zm00001d021974	Zm00001d021974_T00	47.89	47.89	coding	423	128	1396
PB.5824.1	4	1553	16	full-splice_match	gene: Zm00001d053658	Zm00001d053658_T00	111.48	111.48	coding	408	64	1287
PB.4196.6	3	1291	5	full-splice_match	gene: Zm00001d042066	Zm00001d042066_T04	4.45	87.33	coding	242	131	856
PB.5177.1	4	1607	1	full-splice_match	gene: Zm00001d050335	Zm00001d050335_T00	14.86	14.86	coding	440	82	1401
PB.478.3	1	1705	2	full-splice_match	gene: Zm00001d029321	Zm00001d029321_T00	9.71	15.96	coding	486	137	1594
PB.478.2	1	2180	1	genic	gene: Zm00001d029321	novel	0.00	15.96	coding	321	137	1099
PB.478.1	1	2296	1	genic	gene: Zm00001d029321	novel	6.25	15.96	coding	321	137	1099
PB.4443.1	3	1851	7	novel_in_catalog	gene: Zm00001d043202	novel	5.80	17.32	coding	434	231	1532
PB.4443.2	3	1708	7	novel_in_catalog	gene: Zm00001d043202	novel	11.53	17.32	coding	434	231	1532
PB.4172.1	3	1783	9	novel_not_in_catalog	gene: Zm00001d041989	novel	7.35	7.35	coding	524	79	1650
PB.6969.5	5	1358	2	genic	gene: Zm00001d017378	novel	2.14	14.03	coding	255	358	1122
PB.6969.4	5	1565	2	genic	gene: Zm00001d017378	novel	0.00	14.03	coding	255	335	1099
PB.2692.2	2	860	2	incomplete-splice_match	gene: Zm00001d002905	Zm00001d002905_T00	19.39	101.64	coding	215	94	738
PB.6969.2	5	1993	2	genic	gene: Zm00001d017378	novel	11.89	14.03	coding	255	337	1101

PB.1249.1	1	1089	3	full-splice_match	gene: Zm00001d033169	Zm00001d033169_T00	69.26	69.26	coding	253	149	907
PB.2692.1	2	1301	2	incomplete-splice_match	gene: Zm00001d002905	Zm00001d002905_T00	82.25	101.64	coding	334	73	1074
PB.8525.1	7	3450	3	full-splice_match	gene: Zm00001d020396	Zm00001d020396_T00	137.80	137.80	coding	888	645	3308
PB.3528.1	2	1659	5	incomplete-splice_match	gene: Zm00001d007121	Zm00001d007121_T03	17.38	70.33	coding	422	394	1659
PB.4776.3	3	1517	13	full-splice_match	gene: Zm00001d044468	Zm00001d044468_T00	0.92	6.27	coding	435	132	1436
PB.7874.1	6	1232	1	full-splice_match	gene: Zm00001d038016	Zm00001d038016_T00	9.85	9.85	coding	301	147	1049
PB.4776.1	3	1717	13	full-splice_match	gene: Zm00001d044468	Zm00001d044468_T00	3.71	6.27	coding	480	89	1528
PB.7981.3	6	650	6	full-splice_match	gene: Zm00001d038368	Zm00001d038368_T00	62.12	220.15	coding	151	198	650
PB.6659.1	5	1662	8	full-splice_match	gene: Zm00001d015839	Zm00001d015839_T00	7.94	43.23	coding	284	60	911
PB.917.6	1	1636	3	full-splice_match	gene: Zm00001d031732	Zm00001d031732_T00	127.80	316.35	coding	320	449	1408
PB.6659.3	5	1540	8	full-splice_match	gene: Zm00001d015839	Zm00001d015839_T00	13.61	43.23	coding	284	150	1001
PB.6659.2	5	1502	8	full-splice_match	gene: Zm00001d015839	Zm00001d015839_T00	0.00	43.23	coding	284	11	862
PB.10409.3	9	2868	2	novel_not_in_catalog	gene: Zm00001d046305	novel	0.84	30.76	coding	280	1844	2683
PB.6659.4	5	1222	8	full-splice_match	gene: Zm00001d015839	Zm00001d015839_T00	21.69	43.23	coding	284	60	911
PB.917.1	1	2153	3	full-splice_match	gene: Zm00001d031732	Zm00001d031732_T00	106.98	316.35	coding	320	646	1605
PB.10046.2	8	1568	1	novel_in_catalog	gene: Zm00001d012564	novel	5.42	100.15	coding	308	314	1237
PB.7981.1	6	537	2	incomplete-splice_match	gene: Zm00001d038368	Zm00001d038368_T00	49.19	220.15	non coding	NA	NA	NA

PB.9822.1	8	1555	1	full-splice_match	gene: Zm00001d011639	Zm00001d011639_T00	25.70	25.70	coding	405	98	1312
PB.9822.2	8	1442	1	full-splice_match	gene: Zm00001d011639	Zm00001d011639_T00	0.00	25.70	coding	405	98	1312
PB.8158.1	6	1714	3	incomplete-splice_match	gene: Zm00001d039005	Zm00001d039005_T00	27.33	53.47	coding	347	219	1259
PB.1105.2	1	3270	1	genic	gene: Zm00001d032546	novel	23.99	145.01	coding	363	1880	2968
PB.8158.2	6	3539	14	full-splice_match	gene: Zm00001d039005	Zm00001d039005_T00	26.15	53.47	coding	1046	101	3238
PB.10864.1	9	1067	3	full-splice_match	gene: Zm00001d048353	Zm00001d048353_T00	9.33	9.33	coding	225	65	739
PB.426.1	1	1360	1	full-splice_match	gene: Zm00001d029047	Zm00001d029047_T00	328.76	328.76	coding	305	213	1127
PB.8827.6	7	2271	7	novel_not_in_catalog	gene: Zm00001d021698	novel	0.00	152.16	coding	677	130	2160
PB.8827.7	7	1511	6	novel_not_in_catalog	gene: Zm00001d021698	novel	0.63	152.16	coding	247	17	757
PB.8827.4	7	2512	5	novel_not_in_catalog	gene: Zm00001d021698	novel	2.94	152.16	coding	462	126	1511
PB.8539.1	7	1897	10	full-splice_match	gene: Zm00001d020441	Zm00001d020441_T00	39.18	39.18	coding	511	34	1566
PB.8827.2	7	2480	6	novel_not_in_catalog	gene: Zm00001d021698	novel	4.22	152.16	coding	535	134	1738
PB.8827.3	7	2331	7	novel_not_in_catalog	gene: Zm00001d021698	novel	84.88	152.16	coding	677	134	2164
PB.8827.1	7	2465	7	novel_not_in_catalog	gene: Zm00001d021698	novel	59.49	152.16	coding	685	88	2142
PB.5103.2	4	1433	9	novel_not_in_catalog	gene: Zm00001d049892	novel	24.06	25.25	coding	336	102	1109
PB.5103.3	4	1504	8	novel_not_in_catalog	gene: Zm00001d049892	novel	1.19	25.25	coding	258	99	872
PB.4480.1	3	1841	7	full-splice_match	gene: Zm00001d043358	Zm00001d043358_T00	6.17	6.17	coding	379	52	1188

PB.8588.3	7	2044	5	novel_in_catalog	gene: Zm00001d020694	novel	5.14	206.30	coding	335	248	1252
PB.7525.6	6	1849	11	full-splice_match	gene: Zm00001d036337	Zm00001d036337_T00	53.04	98.48	coding	394	134	1315
PB.7525.7	6	2012	10	full-splice_match	gene: Zm00001d036337	Zm00001d036337_T00	3.84	98.48	coding	260	196	975
PB.9721.2	8	1244	6	full-splice_match	gene: Zm00001d011178	Zm00001d011178_T00	17.79	17.79	coding	279	163	999
PB.2005.1	10	1996	6	full-splice_match	gene: Zm00001d024927	Zm00001d024927_T00	24.27	24.27	coding	517	240	1790
PB.2400.1	10	1418	3	intergenic	novelGene_298	novel	19.83	60.81	coding	355	125	1189
PB.7525.3	6	1722	6	incomplete-splice_match	gene: Zm00001d036337	Zm00001d036337_T00	39.44	98.48	coding	405	275	1489
PB.1105.3	1	1856	3	full-splice_match	gene: Zm00001d032546	Zm00001d032546_T00	62.11	145.01	coding	499	131	1627
PB.7111.1	5	840	1	full-splice_match	gene: Zm00001d017976	Zm00001d017976_T00	6.40	6.40	coding	138	139	552
PB.4282.3	3	2520	4	full-splice_match	gene: Zm00001d042520	Zm00001d042520_T00	3.19	17.23	coding	496	460	1947
PB.7525.8	6	2023	10	novel_in_catalog	gene: Zm00001d036337	novel	2.16	98.48	coding	347	283	1323
PB.7525.9	6	1541	11	full-splice_match	gene: Zm00001d036337	Zm00001d036337_T00	0.00	98.48	coding	288	279	1142
PB.3846.5	3	1739	12	full-splice_match	gene: Zm00001d039971	Zm00001d039971_T00	33.40	43.54	coding	484	48	1499
PB.7113.3	5	1187	8	incomplete-splice_match	gene: Zm00001d017979	Zm00001d017979_T00	0.00	53.65	non coding	NA	NA	NA
PB.7113.2	5	2740	19	incomplete-splice_match	gene: Zm00001d017979	Zm00001d017979_T00	27.14	53.65	coding	760	81	2360
PB.3846.2	3	1322	11	incomplete-splice_match	gene: Zm00001d039971	Zm00001d039971_T00	10.14	43.54	coding	378	86	1219
PB.5990.1	5	2159	3	full-splice_match	gene: Zm00001d012924	Zm00001d012924_T00	236.19	236.19	coding	542	247	1872

PB.715.5	1	987	3	full-splice_match	gene: Zm00001d030678	Zm00001d030678_T00	78.91	337.18	coding	211	235	867
PB.715.4	1	991	3	novel_not_in_catalog	gene: Zm00001d030678	novel	25.95	337.18	coding	177	239	769
PB.3677.2	3	1395	4	novel_in_catalog	gene: Zm00001d039264	novel	0.00	78.06	coding	340	143	1162
PB.3677.3	3	1348	4	novel_in_catalog	gene: Zm00001d039264	novel	78.06	78.06	coding	340	222	1241
PB.715.1	1	1268	3	full-splice_match	gene: Zm00001d030678	Zm00001d030678_T00	58.87	337.18	coding	242	211	936
PB.3677.1	3	2243	5	fusion	gene: Zm00001d039263	novel	91.94	91.94	coding	340	143	1162
PB.8868.1	7	2148	5	full-splice_match	gene: Zm00001d021810	Zm00001d021810_T01	11.58	84.59	coding	590	92	1861
PB.4748.1	3	1850	7	full-splice_match	gene: Zm00001d044327	Zm00001d044327_T00	13.07	192.78	coding	257	256	1026
PB.884.9	1	1632	2	genic	gene: Zm00001d031572	novel	11.09	20.52	coding	350	235	1284
PB.3527.1	2	2003	21	full-splice_match	gene: Zm00001d007113	Zm00001d007113_T00	73.85	73.85	coding	482	310	1755
PB.4748.4	3	1507	7	full-splice_match	gene: Zm00001d044327	Zm00001d044327_T00	128.11	192.78	coding	257	259	1029
PB.4748.5	3	2299	5	novel_in_catalog	gene: Zm00001d044327	novel	9.06	192.78	coding	130	1432	1821
PB.4748.6	3	2205	6	novel_in_catalog	gene: Zm00001d044327	novel	3.89	192.78	coding	198	250	843
PB.4748.7	3	2299	5	novel_in_catalog	gene: Zm00001d044327	novel	13.97	192.78	coding	198	263	856
PB.8531.2	7	1631	3	full-splice_match	gene: Zm00001d020411	Zm00001d020411_T00	2.27	20.47	coding	441	190	1512
PB.2403.2	10	463	2	full-splice_match	gene: Zm00001d026671	Zm00001d026671_T00	0.00	51.94	non coding	NA	NA	NA
PB.8531.1	7	1733	3	full-splice_match	gene: Zm00001d020411	Zm00001d020411_T00	18.20	20.47	coding	441	161	1483

PB.10771.6	9	1943	2	novel_in_catalog	gene: Zm00001d047955	novel	0.00	164.32	coding	131	1550	1942
PB.10771.4	9	2893	2	novel_in_catalog	gene: Zm00001d047955	novel	0.00	164.32	coding	268	1879	2682
PB.10771.5	9	2426	2	novel_in_catalog	gene: Zm00001d047955	novel	0.00	164.32	coding	182	1880	2425
PB.10771.2	9	1786	3	full-splice_match	gene: Zm00001d047955	Zm00001d047955_T00	53.29	164.32	coding	448	197	1540
PB.10771.3	9	3001	1	genic	gene: Zm00001d047955	novel	3.41	164.32	coding	285	1933	2787
PB.10771.1	9	2946	2	novel_in_catalog	gene: Zm00001d047955	novel	107.61	164.32	coding	268	1881	2684
PB.1673.2	1	2207	6	full-splice_match	gene: Zm00001d034918	Zm00001d034918_T00	15.29	15.29	coding	443	316	1644
PB.9863.1	8	690	1	novel_in_catalog	gene: Zm00001d011789	novel	65.63	532.32	non coding	NA	NA	NA
PB.7878.4	6	1089	2	novel_in_catalog	gene: Zm00001d038023	novel	39.53	97.71	coding	254	189	950
PB.5875.2	4	3219	25	full-splice_match	gene: Zm00001d053881	Zm00001d053881_T00	118.94	120.88	coding	892	147	2822
PB.9863.3	8	435	2	novel_not_in_catalog	gene: Zm00001d011789	novel	91.87	532.32	non coding	NA	NA	NA
PB.5858.1	4	1718	1	full-splice_match	gene: Zm00001d053817	Zm00001d053817_T00	6.84	6.84	coding	441	120	1442
PB.2166.1	10	816	2	novel_in_catalog	gene: Zm00001d025774	novel	1.99	1.99	coding	204	9	620
PB.7406.2	6	2942	20	full-splice_match	gene: Zm00001d035574	Zm00001d035574_T00	28.94	28.94	coding	818	219	2672
PB.10743.1	9	1845	11	novel_not_in_catalog	gene: Zm00001d047849	novel	39.09	39.09	coding	414	393	1634
PB.10743.3	9	1677	11	novel_not_in_catalog	gene: Zm00001d047849	novel	0.00	39.09	coding	414	302	1543
PB.5066.1	4	2494	7	full-splice_match	gene: Zm00001d049706	Zm00001d049706_T00	54.35	74.87	coding	706	50	2167

PB.5066.2	4	2861	1	incomplete-splice_match	gene: Zm00001d049706	Zm00001d049706_T03	20.52	74.87	coding	430	1403	2692
PB.8225.2	7	808	5	novel_in_catalog	gene: Zm00001d018634	novel	0.00	27.22	coding	241	86	808
PB.8225.1	7	1274	8	full-splice_match	gene: Zm00001d018634	Zm00001d018634_T00	27.22	27.22	coding	322	91	1056
PB.8588.6	7	1534	6	full-splice_match	gene: Zm00001d020694	Zm00001d020694_T00	0.00	206.30	coding	396	246	1433
PB.70.1	1	2080	8	full-splice_match	gene: Zm00001d027514	Zm00001d027514_T00	42.42	42.42	coding	536	233	1840
PB.400.4	1	2490	18	full-splice_match	gene: Zm00001d028952	Zm00001d028952_T00	3.43	95.15	coding	670	281	2290
PB.7103.3	5	3672	14	incomplete-splice_match	gene: Zm00001d017946	Zm00001d017946_T01	11.11	69.55	coding	574	179	1900
PB.400.2	1	2659	18	full-splice_match	gene: Zm00001d028952	Zm00001d028952_T00	91.72	95.15	coding	670	299	2308
PB.4166.2	3	804	4	novel_not_in_catalog	gene: Zm00001d041969	novel	5.94	5.94	coding	109	317	643
PB.9039.1	7	1380	2	incomplete-splice_match	gene: Zm00001d022417	Zm00001d022417_T02	6.60	48.28	coding	388	215	1378
PB.10999.2	B73V4	1810	8	fusion	gene: Zm00001d000350	novel	0.00	19.54	coding	455	264	1628
PB.9875.1	8	2207	11	full-splice_match	gene: Zm00001d011835	Zm00001d011835_T00	29.45	36.26	coding	569	238	1944
PB.9875.2	8	2295	10	full-splice_match	gene: Zm00001d011835	Zm00001d011835_T00	6.80	36.26	coding	371	245	1357
PB.10999.1	B73V4	1991	8	fusion	gene: Zm00001d000350	novel	19.54	19.54	coding	553	47	1705
PB.4708.4	3	958	8	incomplete-splice_match	gene: Zm00001d044172	Zm00001d044172_T00	0.00	349.37	coding	288	95	958
PB.6198.2	5	2517	8	full-splice_match	gene: Zm00001d013664	Zm00001d013664_T00	5.82	33.64	coding	180	78	617
PB.6198.1	5	758	6	incomplete-splice_match	gene: Zm00001d013664	Zm00001d013664_T00	27.82	33.64	coding	120	136	495

PB.5810.3	4	1524	5	antisense	novelGene_gene: Zm00001d053623	novel	11.64	73.94	non coding	NA	NA	NA
PB.7684.4	6	2502	14	novel_not_in_catalog	gene: Zm00001d037198	novel	0.00	34.49	coding	710	97	2226
PB.2272.1	10	1892	3	full-splice_match	gene: Zm00001d026267	Zm00001d026267_T00	148.50	148.50	coding	414	311	1552
PB.2272.2	10	1817	3	full-splice_match	gene: Zm00001d026267	Zm00001d026267_T00	0.00	148.50	coding	414	309	1550
PB.10638.2	9	3326	12	full-splice_match	gene: Zm00001d047491	Zm00001d047491_T01	2.61	17.21	coding	757	290	2560
PB.10638.3	9	2138	11	novel_in_catalog	gene: Zm00001d047491	novel	3.19	17.21	coding	531	221	1813
PB.10638.1	9	3501	13	full-splice_match	gene: Zm00001d047491	Zm00001d047491_T00	6.06	17.21	coding	895	494	3178
PB.4965.1	4	1944	1	full-splice_match	gene: Zm00001d049201	Zm00001d049201_T00	21.00	56.61	coding	518	89	1642
PB.3222.1	2	1475	1	genic	gene: Zm00001d005834	novel	14.73	14.73	coding	244	554	1285
PB.4965.3	4	1747	1	full-splice_match	gene: Zm00001d049201	Zm00001d049201_T00	27.77	56.61	coding	518	95	1648
PB.4965.2	4	1835	1	full-splice_match	gene: Zm00001d049201	Zm00001d049201_T00	7.84	56.61	coding	518	91	1644
PB.7199.5	5	1153	6	full-splice_match	gene: Zm00001d018297	Zm00001d018297_T00	57.09	118.62	coding	260	156	935
PB.7199.4	5	1572	4	novel_in_catalog	gene: Zm00001d018297	novel	4.47	118.62	coding	114	212	553
PB.2789.1	2	1892	9	novel_not_in_catalog	gene: Zm00001d003349	novel	13.51	13.51	coding	481	142	1584
PB.9759.2	8	1658	12	full-splice_match	gene: Zm00001d011355	Zm00001d011355_T00	28.83	28.83	coding	444	96	1427
PB.7199.1	5	1354	6	full-splice_match	gene: Zm00001d018297	Zm00001d018297_T00	34.97	118.62	coding	260	215	994
PB.5010.1	4	2030	2	full-splice_match	gene: Zm00001d049407	Zm00001d049407_T00	4.91	4.91	coding	645	47	1981

PB.7199.2	5	3684	2	novel_in_catalog	gene: Zm00001d018297	novel	3.91	118.62	coding	119	2063	2419
PB.6330.3	5	1072	1	incomplete-splice_match	gene: Zm00001d014294	Zm00001d014294_T02	0.00	136.69	coding	313	134	1072
PB.6330.2	5	3044	5	incomplete-splice_match	gene: Zm00001d014294	Zm00001d014294_T02	76.76	136.69	coding	952	28	2883
PB.6330.1	5	3858	8	incomplete-splice_match	gene: Zm00001d014294	Zm00001d014294_T02	59.93	136.69	coding	1174	83	3604
PB.4600.1	3	3876	12	novel_in_catalog	gene: Zm00001d043762	novel	13.77	34.99	coding	1207	32	3652
PB.4259.12	3	3233	9	full-splice_match	gene: Zm00001d042416	Zm00001d042416_T00	0.00	123.25	coding	473	104	1522
PB.6470.8	5	1118	6	novel_in_catalog	gene: Zm00001d014858	novel	7.20	2503.17	coding	106	443	760
PB.1003.2	1	607	1	full-splice_match	gene: Zm00001d032132	Zm00001d032132_T00	20.75	25.73	coding	80	92	331
PB.1003.1	1	726	1	full-splice_match	gene: Zm00001d032132	Zm00001d032132_T00	4.99	25.73	coding	80	90	329
PB.8726.3	7	1567	1	incomplete-splice_match	gene: Zm00001d021318	Zm00001d021318_T00	0.00	182.56	coding	333	407	1405
PB.997.2	1	760	10	incomplete-splice_match	gene: Zm00001d032101	Zm00001d032101_T00	5.34	27.13	coding	248	16	759
PB.997.1	1	2052	15	full-splice_match	gene: Zm00001d032101	Zm00001d032101_T00	21.80	27.13	coding	466	185	1582
PB.6470.1	5	1237	7	full-splice_match	gene: Zm00001d014858	Zm00001d014858_T00	91.03	2503.17	coding	157	167	637
PB.6470.3	5	1293	6	novel_in_catalog	gene: Zm00001d014858	novel	5.13	2503.17	non coding	NA	NA	NA
PB.6470.4	5	1118	7	full-splice_match	gene: Zm00001d014858	Zm00001d014858_T00	2115.87	2503.17	coding	157	167	637
PB.6470.5	5	1289	5	novel_in_catalog	gene: Zm00001d014858	novel	3.84	2503.17	non coding	NA	NA	NA
PB.8553.2	7	1407	2	full-splice_match	gene: Zm00001d020528	Zm00001d020528_T00	2.45	6.55	coding	444	36	1367

PB.8553.1	7	1511	2	full-splice_match	gene: Zm00001d020528	Zm00001d020528_T00	4.10	6.55	coding	444	31	1362
PB.1227.1	1	2560	2	full-splice_match	gene: Zm00001d033039	Zm00001d033039_T00	21.37	21.37	coding	773	26	2344
PB.4794.2	3	1065	3	full-splice_match	gene: Zm00001d044527	Zm00001d044527_T00	35.76	136.03	coding	240	105	824
PB.4794.1	3	1125	3	full-splice_match	gene: Zm00001d044527	Zm00001d044527_T00	100.28	136.03	coding	240	51	770
PB.10101.2	8	962	6	novel_not_in_catalog	gene: Zm00001d012763	novel	17.16	17.16	coding	229	275	961
PB.2655.1	2	1213	1	full-splice_match	gene: Zm00001d002744	Zm00001d002744_T00	8.53	8.53	coding	220	87	746
PB.3615.6	2	2998	18	full-splice_match	gene: Zm00001d007758	Zm00001d007758_T00	6.35	173.72	coding	888	202	2865
PB.2307.3	10	3186	8	novel_in_catalog	gene: Zm00001d026366	novel	1.29	10.54	coding	402	54	1259
PB.2655.2	2	893	1	full-splice_match	gene: Zm00001d002744	Zm00001d002744_T00	0.00	8.53	coding	220	88	747
PB.3615.7	2	3050	17	novel_in_catalog	gene: Zm00001d007758	novel	0.00	173.72	coding	619	1060	2916
PB.6665.5	5	1030	1	genic_intron	novelGene_252	novel	11.08	11.08	non coding	NA	NA	NA
PB.5018.4	4	2205	5	novel_not_in_catalog	gene: Zm00001d049452	novel	2.44	5.53	coding	137	114	524
PB.5018.2	4	2226	6	novel_not_in_catalog	gene: Zm00001d049452	novel	3.09	5.53	coding	209	35	661
PB.6665.3	5	1906	17	novel_not_in_catalog	gene: Zm00001d015890	novel	38.19	62.56	coding	539	57	1673
PB.2286.1	10	390	1	incomplete-splice_match	gene: Zm00001d026303	Zm00001d026303_T00	36.37	36.37	non coding	NA	NA	NA
PB.6860.2	5	1677	2	novel_in_catalog	gene: Zm00001d016911	novel	17.53	46.96	coding	359	79	1155
PB.8882.3	7	1331	2	full-splice_match	gene: Zm00001d021839	Zm00001d021839_T00	224.32	645.47	coding	294	140	1021

PB.8882.2	7	1542	1	genic	gene: Zm00001d021839	novel	148.51	645.47	coding	331	140	1132
PB.2216.2	10	2147	14	novel_in_catalog	gene: Zm00001d026022	novel	1.27	21.15	coding	355	844	1908
PB.2216.3	10	2389	14	novel_in_catalog	gene: Zm00001d026022	novel	0.10	21.15	coding	467	75	1475
PB.3137.3	2	342	1	incomplete-splice_match	gene: Zm00001d005373	Zm00001d005373_T01	0.00	50.22	non coding	NA	NA	NA
PB.2216.1	10	2149	15	full-splice_match	gene: Zm00001d026022	Zm00001d026022_T00	19.78	21.15	coding	593	71	1849
PB.6166.4	5	1064	4	full-splice_match	gene: Zm00001d013526	Zm00001d013526_T00	67.19	144.86	coding	227	125	805
PB.5429.2	4	1321	2	full-splice_match	gene: Zm00001d051896	Zm00001d051896_T00	56.02	130.26	coding	350	28	1077
PB.5429.1	4	1422	2	full-splice_match	gene: Zm00001d051896	Zm00001d051896_T00	29.73	130.26	coding	350	24	1073
PB.4755.1	3	963	2	full-splice_match	gene: Zm00001d044378	Zm00001d044378_T00	0.70	0.70	coding	164	231	722
PB.6166.1	5	1180	4	full-splice_match	gene: Zm00001d013526	Zm00001d013526_T00	77.68	144.86	coding	227	125	805
PB.1274.2	1	2803	5	full-splice_match	gene: Zm00001d033292	Zm00001d033292_T00	14.38	112.19	coding	487	764	2224
PB.6205.2	5	1858	7	full-splice_match	gene: Zm00001d013712	Zm00001d013712_T00	12.00	17.20	coding	527	122	1702
PB.8955.1	7	762	1	full-splice_match	gene: Zm00001d022124	Zm00001d022124_T00	452.50	942.02	coding	145	67	501
PB.8955.2	7	652	1	full-splice_match	gene: Zm00001d022124	Zm00001d022124_T00	489.53	942.02	coding	145	61	495
PB.6205.1	5	2155	7	novel_in_catalog	gene: Zm00001d013712	novel	5.20	17.20	coding	228	1292	1975
PB.10596.1	9	1757	6	novel_in_catalog	gene: Zm00001d047349	novel	77.34	77.34	coding	280	583	1422
PB.10851.2	9	2042	3	full-splice_match	gene: Zm00001d048310	Zm00001d048310_T00	169.28	282.88	coding	428	365	1648

PB.3099.10	2	3606	9	novel_in_catalog	gene: Zm00001d005164	novel	8.87	134.75	coding	309	1225	2151
PB.3099.11	2	1070	7	incomplete-splice_match	gene: Zm00001d005164	Zm00001d005164_T00	0.00	134.75	coding	288	205	1068
PB.10626.1	9	1096	2	incomplete-splice_match	gene: Zm00001d047467	Zm00001d047467_T00	7.15	7.15	coding	267	5	805
PB.5746.1	4	884	6	novel_not_in_catalog	gene: Zm00001d053293	novel	201.11	201.11	coding	82	272	517
PB.4033.1	3	485	1	genic	gene: Zm00001d041163	novel	18.73	31.61	coding	11	263	295
PB.8992.1	7	1192	7	full-splice_match	gene: Zm00001d022254	Zm00001d022254_T00	53.02	76.67	coding	264	119	910
PB.4600.2	3	2124	5	novel_in_catalog	gene: Zm00001d043762	novel	17.22	34.99	coding	629	72	1958
PB.4033.2	3	1398	3	incomplete-splice_match	gene: Zm00001d041163	Zm00001d041163_T00	12.87	31.61	coding	345	325	1359
PB.6458.2	5	1824	5	full-splice_match	gene: Zm00001d014797	Zm00001d014797_T00	62.07	134.39	coding	394	210	1391
PB.6458.3	5	1807	5	full-splice_match	gene: Zm00001d014797	Zm00001d014797_T00	0.00	134.39	coding	394	297	1478
PB.6458.1	5	1924	5	full-splice_match	gene: Zm00001d014797	Zm00001d014797_T00	42.94	134.39	coding	394	298	1479
PB.6458.6	5	1768	4	novel_in_catalog	gene: Zm00001d014797	novel	6.28	134.39	coding	280	775	1614
PB.1013.1	1	1175	1	incomplete-splice_match	gene: Zm00001d032190	Zm00001d032190_T00	18.88	64.90	non coding	NA	NA	NA
PB.6458.4	5	1697	5	full-splice_match	gene: Zm00001d014797	Zm00001d014797_T00	0.00	134.39	coding	394	187	1368
PB.6458.5	5	1684	5	full-splice_match	gene: Zm00001d014797	Zm00001d014797_T00	23.11	134.39	coding	394	301	1482
PB.3200.4	2	1770	6	novel_not_in_catalog	gene: Zm00001d005726	novel	1.46	66.59	coding	160	201	680
PB.3200.5	2	1642	7	novel_not_in_catalog	gene: Zm00001d005726	novel	0.00	66.59	coding	391	201	1373

PB.3200.1	2	1777	7	novel_not_in_catalog	gene: Zm00001d005726	novel	62.87	66.59	coding	391	264	1436
PB.10009.1	8	1068	3	full-splice_match	gene: Zm00001d012418	Zm00001d012418_T00	14.76	14.76	coding	240	142	861
PB.1473.1	1	1376	1	full-splice_match	gene: Zm00001d034145	Zm00001d034145_T00	165.14	218.33	coding	278	99	932
PB.4120.2	3	2443	4	full-splice_match	gene: Zm00001d041715	Zm00001d041715_T00	29.08	29.08	coding	697	215	2305
PB.4237.3	3	1431	12	incomplete-splice_match	gene: Zm00001d042314	Zm00001d042314_T01	8.20	52.81	coding	430	23	1312
PB.4237.1	3	2246	14	full-splice_match	gene: Zm00001d042314	Zm00001d042314_T01	44.61	52.81	coding	550	267	1916
PB.10832.1	9	985	1	full-splice_match	gene: Zm00001d048226	Zm00001d048226_T00	31.25	31.25	coding	185	80	634
PB.4708.1	3	1593	9	novel_in_catalog	gene: Zm00001d044172	novel	27.92	349.37	coding	314	316	1257
PB.5756.1	4	1235	2	intergenic	novelGene_431	novel	25.11	25.11	non coding	NA	NA	NA
PB.8941.1	7	454	1	full-splice_match	gene: Zm00001d022057	Zm00001d022057_T00	236.89	236.89	non coding	NA	NA	NA
PB.2317.2	10	2303	12	full-splice_match	gene: Zm00001d026396	Zm00001d026396_T00	8.15	51.75	coding	623	231	2099
PB.2641.1	2	890	3	full-splice_match	gene: Zm00001d002685	Zm00001d002685_T00	37.41	37.41	non coding	NA	NA	NA
PB.7731.1	6	2653	3	novel_not_in_catalog	gene: Zm00001d037386	novel	20.41	20.41	coding	816	56	2503
PB.7154.2	5	1373	6	novel_in_catalog	gene: Zm00001d018112	novel	0.00	135.59	non coding	NA	NA	NA
PB.9494.1	8	1283	1	full-splice_match	gene: Zm00001d010197	Zm00001d010197_T00	34.98	34.98	coding	279	261	1097
PB.5665.2	4	2278	1	full-splice_match	gene: Zm00001d052864	Zm00001d052864_T00	2.46	84.75	coding	269	1210	2016
PB.1377.1	1	1224	4	full-splice_match	gene: Zm00001d033718	Zm00001d033718_T00	18.89	19.97	coding	235	255	959

PB.3963.1	3	357	2	full-splice_match	gene: Zm00001d040526	Zm00001d040526_T00	28.72	28.72	non coding	NA	NA	NA
PB.6071.1	5	1306	3	novel_in_catalog	gene: Zm00001d013218	novel	6.00	10.51	coding	191	228	800
PB.8186.1	6	1588	6	full-splice_match	gene: Zm00001d039142	Zm00001d039142_T00	21.25	21.25	coding	407	82	1302
PB.3934.1	3	784	3	full-splice_match	gene: Zm00001d040361	Zm00001d040361_T00	12.64	92.08	coding	23	356	424
PB.7989.2	6	3648	11	full-splice_match	gene: Zm00001d038389	Zm00001d038389_T00	24.07	47.31	coding	969	300	3206
PB.4329.14	3	1101	5	incomplete-splice_match	gene: Zm00001d042724	Zm00001d042724_T00	0.00	343.97	non coding	NA	NA	NA
PB.4855.11	4	1394	3	full-splice_match	gene: Zm00001d048680	Zm00001d048680_T00	0.00	232.95	coding	194	63	644
PB.4855.10	4	1727	1	novel_in_catalog	gene: Zm00001d048680	novel	0.38	232.95	coding	162	51	536
PB.4329.11	3	2090	5	novel_in_catalog	gene: Zm00001d042724	novel	16.54	343.97	coding	350	846	1895
PB.4855.12	4	1223	2	novel_in_catalog	gene: Zm00001d048680	novel	0.00	232.95	coding	194	51	632
PB.7154.7	5	2273	3	incomplete-splice_match	gene: Zm00001d018112	Zm00001d018112_T00	11.58	135.59	non coding	NA	NA	NA
PB.5455.2	4	1620	1	full-splice_match	gene: Zm00001d052020	Zm00001d052020_T00	5.58	22.74	coding	446	168	1505
PB.3208.2	2	2557	5	full-splice_match	gene: Zm00001d005769	Zm00001d005769_T00	12.99	12.99	coding	625	512	2386
PB.5455.1	4	1723	1	full-splice_match	gene: Zm00001d052020	Zm00001d052020_T00	17.16	22.74	coding	446	147	1484
PB.3515.1	2	2128	4	full-splice_match	gene: Zm00001d007070	Zm00001d007070_T00	25.14	26.16	coding	537	100	1710
PB.10012.2	8	746	1	antisense	novelGene_gene: Zm00001d012426	novel	4.96	9.88	coding	202	41	646
PB.6856.1	5	1935	2	full-splice_match	gene: Zm00001d016896	Zm00001d016896_T00	6.42	31.67	coding	488	37	1500

PB.8658.1	7	714	4	full-splice_match	gene: Zm00001d021020	Zm00001d021020_T00	269.82	269.82	coding	133	140	538
PB.10095.1	8	1485	1	incomplete-splice_match	gene: Zm00001d012749	Zm00001d012749_T00	28.68	28.68	coding	279	243	1079
PB.5826.6	4	2777	12	full-splice_match	gene: Zm00001d053672	Zm00001d053672_T00	0.00	59.75	coding	879	72	2708
PB.7265.2	5	1550	5	full-splice_match	gene: Zm00001d018504	Zm00001d018504_T00	0.00	48.08	coding	430	32	1321
PB.8949.1	7	1431	2	novel_not_in_catalog	gene: Zm00001d022083	novel	62.28	62.28	coding	220	425	1084
PB.3946.1	3	3181	24	full-splice_match	gene: Zm00001d040429	Zm00001d040429_T00	27.26	28.69	coding	901	169	2871
PB.6736.18	5	1598	2	novel_in_catalog	gene: Zm00001d016332	novel	0.00	221.47	coding	262	183	968
PB.9771.1	8	590	1	full-splice_match	gene: Zm00001d011412	Zm00001d011412_T00	290.29	290.29	coding	79	93	329
PB.3946.2	3	4203	23	full-splice_match	gene: Zm00001d040429	Zm00001d040429_T01	1.43	28.69	coding	901	1198	3900
PB.4823.2	3	876	4	incomplete-splice_match	gene: Zm00001d044653	Zm00001d044653_T00	106.02	119.30	coding	187	114	674
PB.4858.1	4	1389	3	novel_not_in_catalog	gene: Zm00001d048686	novel	9.48	13.42	coding	60	284	463
PB.4858.2	4	1533	2	novel_not_in_catalog	gene: Zm00001d048686	novel	3.93	13.42	coding	60	278	457
PB.3168.1	2	1266	2	full-splice_match	gene: Zm00001d005578	Zm00001d005578_T00	23.13	23.13	coding	170	433	942
PB.2997.1	2	1484	9	full-splice_match	gene: Zm00001d004552	Zm00001d004552_T00	21.88	114.21	coding	191	128	700
PB.5887.1	4	2839	9	novel_in_catalog	gene: Zm00001d053917	novel	6.36	30.39	coding	167	93	593
PB.9497.3	8	1965	2	full-splice_match	gene: Zm00001d010214	Zm00001d010214_T00	8.94	35.64	coding	385	538	1692
PB.4531.4	3	1323	7	novel_not_in_catalog	gene: Zm00001d043515	novel	18.42	52.60	coding	357	93	1163

PB.6736.17	5	2324	1	novel_in_catalog	gene: Zm00001d016332	novel	0.00	221.47	coding	190	1125	1694
PB.7185.1	5	861	3	full-splice_match	gene: Zm00001d018235	Zm00001d018235_T00	6.32	6.32	coding	228	125	808
PB.6981.1	5	1223	8	full-splice_match	gene: Zm00001d017435	Zm00001d017435_T00	19.39	19.39	coding	321	1	963
PB.4361.4	3	2095	3	novel_in_catalog	gene: Zm00001d042853	novel	8.29	84.08	coding	223	807	1475
PB.8884.4	7	3167	26	full-splice_match	gene: Zm00001d021846	Zm00001d021846_T00	12.24	112.48	coding	992	124	3099
PB.4378.1	3	714	3	intergenic	novelGene_377	novel	19.27	19.27	coding	108	32	355
PB.10604.1	9	703	3	full-splice_match	gene: Zm00001d047396	Zm00001d047396_T00	130.28	130.28	coding	131	57	449
PB.2708.1	2	1109	5	novel_not_in_catalog	gene: Zm00001d003002	novel	4.13	4.13	coding	305	114	1028
PB.7265.1	5	1666	5	full-splice_match	gene: Zm00001d018504	Zm00001d018504_T00	48.08	48.08	coding	430	37	1326
PB.5720.1	4	525	4	full-splice_match	gene: Zm00001d053175	Zm00001d053175_T00	5.40	33.55	non coding	NA	NA	NA
PB.5720.2	4	398	4	full-splice_match	gene: Zm00001d053175	Zm00001d053175_T00	28.15	33.55	non coding	NA	NA	NA
PB.5852.1	4	1455	6	full-splice_match	gene: Zm00001d053786	Zm00001d053786_T00	13.54	65.72	coding	175	194	718
PB.8937.7	7	814	6	full-splice_match	gene: Zm00001d022042	Zm00001d022042_T01	1414.80	1497.07	coding	160	90	569
PB.6677.1	5	1571	4	full-splice_match	gene: Zm00001d015986	Zm00001d015986_T00	30.05	30.05	coding	137	122	532
PB.8884.1	7	2113	13	incomplete-splice_match	gene: Zm00001d021846	Zm00001d021846_T01	55.46	112.48	coding	463	474	1862
PB.3570.9	2	1359	2	novel_in_catalog	gene: Zm00001d007434	novel	0.00	72.29	coding	160	879	1358
PB.9527.1	8	646	3	incomplete-splice_match	gene: Zm00001d010315	Zm00001d010315_T00	16.70	56.94	non coding	NA	NA	NA

PB.7211.2	5	1581	7	full-splice_match	gene: Zm00001d018341	Zm00001d018341_T00	2.22	47.69	coding	173	179	697
PB.8650.1	7	1634	5	novel_not_in_catalog	gene: Zm00001d020975	novel	46.15	46.15	coding	297	283	1173
PB.8697.1	7	2230	2	full-splice_match	gene: Zm00001d001240	Zm00001d001240_T00	4.83	4.83	non coding	NA	NA	NA
PB.9527.2	8	3899	9	full-splice_match	gene: Zm00001d010315	Zm00001d010315_T00	40.24	56.94	coding	1005	331	3345
PB.7522.1	6	3052	17	full-splice_match	gene: Zm00001d036322	Zm00001d036322_T00	29.99	29.99	coding	815	119	2563
PB.521.4	1	664	3	incomplete-splice_match	gene: Zm00001d029530	Zm00001d029530_T00	6.23	30.10	coding	211	31	663
PB.10175.1	9	1599	1	full-splice_match	gene: Zm00001d045064	Zm00001d045064_T00	45.78	45.78	coding	347	96	1136
PB.6196.1	5	1874	3	full-splice_match	gene: Zm00001d013658	Zm00001d013658_T00	38.68	38.68	coding	499	129	1625
PB.2772.8	2	2608	15	novel_not_in_catalog	gene: Zm00001d003252	novel	0.48	57.15	coding	233	1770	2468
PB.521.2	1	3041	12	full-splice_match	gene: Zm00001d029530	Zm00001d029530_T00	2.72	30.10	coding	718	106	2259
PB.6196.2	5	1778	3	full-splice_match	gene: Zm00001d013658	Zm00001d013658_T00	0.00	38.68	coding	499	129	1625
PB.4635.1	3	2973	1	full-splice_match	gene: Zm00001d043870	Zm00001d043870_T00	9.34	9.34	coding	819	202	2658
PB.6764.1	5	2778	15	full-splice_match	gene: Zm00001d016496	Zm00001d016496_T00	19.66	37.45	coding	603	275	2083
PB.2772.7	2	2039	15	full-splice_match	gene: Zm00001d003252	Zm00001d003252_T00	3.87	57.15	coding	278	1064	1897
PB.2772.2	2	1821	16	full-splice_match	gene: Zm00001d003252	Zm00001d003252_T00	52.80	57.15	coding	507	161	1681
PB.2331.3	10	2651	2	novel_in_catalog	gene: Zm00001d026460	novel	20.56	236.03	coding	369	103	1209
PB.2623.1	2	1181	6	full-splice_match	gene: Zm00001d002611	Zm00001d002611_T00	35.81	35.81	coding	308	107	1030

PB.6751.1	5	846	1	genic	gene: Zm00001d016414	novel	11.12	11.12	coding	242	57	782
PB.3501.2	2	1650	8	incomplete-splice_match	gene: Zm00001d007034	Zm00001d007034_T00	33.25	91.96	coding	110	216	545
PB.1925.2	10	2326	5	incomplete-splice_match	gene: Zm00001d024519	Zm00001d024519_T00	1.22	78.60	coding	132	110	505
PB.8205.2	6	2574	1	genic	gene: Zm00001d039188	novel	3.09	12.03	coding	455	19	1383
PB.3127.5	2	1581	2	genic	gene: Zm00001d005329	novel	11.03	36.05	coding	386	213	1370
PB.513.3	1	1522	2	intergenic	novelGene_166	novel	6.67	90.46	coding	124	958	1329
PB.3127.3	2	1675	1	full-splice_match	gene: Zm00001d005329	Zm00001d005329_T00	25.01	36.05	coding	386	303	1460
PB.2346.1	10	568	2	full-splice_match	gene: Zm00001d026513	Zm00001d026513_T00	42.85	42.85	coding	142	85	510
PB.10919.1	9	929	1	genic	gene: Zm00001d048522	novel	5.18	5.18	coding	119	192	548
PB.9743.1	8	1168	3	full-splice_match	gene: Zm00001d011263	Zm00001d011263_T00	34.00	34.00	coding	258	118	891
PB.6312.1	5	1366	7	fusion	gene: Zm00001d014196	novel	30.06	30.06	coding	346	144	1181
PB.10484.1	9	1125	1	full-splice_match	gene: Zm00001d046743	Zm00001d046743_T00	10.80	10.80	coding	327	21	1001
PB.10253.1	9	1555	2	full-splice_match	gene: Zm00001d045398	Zm00001d045398_T00	20.04	20.04	coding	293	183	1061
PB.1925.1	10	2143	13	novel_in_catalog	gene: Zm00001d024519	novel	9.20	78.60	coding	169	256	762
PB.5962.1	5	1634	6	full-splice_match	gene: Zm00001d012825	Zm00001d012825_T00	22.73	22.73	coding	443	49	1377
PB.3651.1	2	1203	3	novel_in_catalog	gene: Zm00001d007884	novel	283.48	283.48	coding	139	62	478
PB.1764.1	10	1963	2	genic	gene: Zm00001d023558	novel	4.30	51.52	coding	467	163	1563

PB.1764.3	10	1771	2	genic	gene: Zm00001d023558	novel	20.30	51.52	coding	467	179	1579
PB.1764.2	10	1788	2	genic	gene: Zm00001d023558	novel	16.21	51.52	coding	467	131	1531
PB.6271.3	5	2288	13	novel_in_catalog	gene: Zm00001d014037	novel	0.00	41.89	coding	540	256	1875
PB.6271.2	5	2531	13	novel_in_catalog	gene: Zm00001d014037	novel	0.00	41.89	coding	540	291	1910
PB.6271.1	5	2553	13	novel_in_catalog	gene: Zm00001d014037	novel	41.89	41.89	coding	540	297	1916
PB.1764.6	10	2040	2	genic	gene: Zm00001d023558	novel	2.99	51.52	coding	467	495	1895
PB.5481.5	4	2539	1	full-splice_match	gene: Zm00001d052104	Zm00001d052104_T00	44.81	165.96	coding	780	79	2418
PB.5748.5	4	2221	16	novel_not_in_catalog	gene: Zm00001d053301	novel	0.00	40.50	coding	496	634	2121
PB.6995.1	5	2014	1	novel_in_catalog	gene: Zm00001d017487	novel	8.13	8.13	non coding	NA	NA	NA
PB.4371.3	3	2040	2	intergenic	novelGene_375	novel	0.00	43.00	coding	54	277	438
PB.5748.2	4	2428	15	novel_not_in_catalog	gene: Zm00001d053301	novel	9.95	40.50	coding	349	727	1773
PB.5748.1	4	2397	16	novel_not_in_catalog	gene: Zm00001d053301	novel	30.55	40.50	coding	496	731	2218
PB.7611.1	6	728	1	full-splice_match	gene: Zm00001d036780	Zm00001d036780_T00	116.38	116.38	coding	124	113	484
PB.10277.1	9	2458	1	novel_in_catalog	gene: Zm00001d045490	novel	18.47	52.15	coding	604	304	2115
PB.10277.3	9	2240	2	full-splice_match	gene: Zm00001d045490	Zm00001d045490_T00	21.71	52.15	coding	669	1	2007
PB.10277.2	9	2406	2	full-splice_match	gene: Zm00001d045490	Zm00001d045490_T00	6.57	52.15	coding	698	1	2094
PB.10277.5	9	2298	1	novel_in_catalog	gene: Zm00001d045490	novel	5.39	52.15	coding	604	372	2183

PB.10277.4	9	2350	1	novel_in_catalog	gene: Zm00001d045490	novel	0.00	52.15	coding	604	312	2123
PB.5946.1	4	1319	2	novel_in_catalog	gene: Zm00001d054095	novel	2.36	2.36	coding	245	47	781
PB.9211.1	8	1820	8	full-splice_match	gene: Zm00001d008596	Zm00001d008596_T00	201.32	287.54	coding	435	219	1523
PB.1704.1	10	2350	1	full-splice_match	gene: Zm00001d023283	Zm00001d023283_T00	5.80	5.80	coding	608	75	1898
PB.2339.1	10	3130	13	full-splice_match	gene: Zm00001d026490	Zm00001d026490_T00	1014.75	1014.75	coding	951	192	3044
PB.9211.4	8	1936	7	novel_in_catalog	gene: Zm00001d008596	novel	12.97	287.54	coding	358	237	1310
PB.9211.5	8	1097	8	full-splice_match	gene: Zm00001d008596	Zm00001d008596_T00	73.25	287.54	coding	230	248	937
PB.10020.1	8	645	3	novel_not_in_catalog	gene: Zm00001d012448	novel	24.86	100.47	coding	78	98	331
PB.5736.1	4	2798	1	full-splice_match	gene: Zm00001d053254	Zm00001d053254_T00	3.84	3.84	coding	801	297	2699
PB.2322.1	10	543	1	intergenic	novelGene_286	novel	24.66	24.66	non coding	NA	NA	NA
PB.7211.1	5	1843	7	novel_in_catalog	gene: Zm00001d018341	novel	2.08	47.69	coding	173	179	697
PB.1083.2	1	925	1	full-splice_match	gene: Zm00001d032479	Zm00001d032479_T00	3.69	4.79	coding	275	48	872
PB.1083.1	1	1501	1	full-splice_match	gene: Zm00001d032479	Zm00001d032479_T00	1.10	4.79	coding	341	365	1387
PB.10161.4	9	851	1	full-splice_match	gene: Zm00001d045017	Zm00001d045017_T00	79.37	189.79	coding	119	382	738
PB.10161.3	9	1009	1	full-splice_match	gene: Zm00001d045017	Zm00001d045017_T00	40.78	189.79	coding	119	382	738
PB.10161.2	9	1149	1	full-splice_match	gene: Zm00001d045017	Zm00001d045017_T00	50.04	189.79	coding	119	440	796
PB.10161.1	9	1252	1	full-splice_match	gene: Zm00001d045017	Zm00001d045017_T00	19.60	189.79	coding	119	380	736

PB.10920.3	9	3215	19	full-splice_match	gene: Zm00001d048527	Zm00001d048527_T03	40.97	66.90	coding	842	478	3003
PB.9636.1	8	335	1	novel_in_catalog	gene: Zm00001d010784	novel	21.16	21.16	non coding	NA	NA	NA
PB.7262.5	5	1419	12	full-splice_match	gene: Zm00001d018498	Zm00001d018498_T00	46.73	68.81	coding	300	170	1069
PB.3448.4	2	1978	19	novel_in_catalog	gene: Zm00001d006797	novel	0.00	13.91	coding	227	1102	1782
PB.3448.2	2	2098	19	novel_in_catalog	gene: Zm00001d006797	novel	13.91	13.91	coding	227	1114	1794
PB.7286.1	5	863	5	novel_not_in_catalog	gene: Zm00001d018557	novel	50.40	50.40	coding	172	252	767
PB.6059.2	5	2817	2	genic	gene: Zm00001d013165	novel	4.18	5.67	coding	107	34	354
PB.6059.1	5	2704	3	genic	gene: Zm00001d013165	novel	1.49	5.67	coding	107	14	334
PB.8723.1	7	1587	2	full-splice_match	gene: Zm00001d021305	Zm00001d021305_T00	21.70	21.70	coding	462	127	1512
PB.3863.1	3	1931	4	novel_not_in_catalog	gene: Zm00001d040047	novel	45.79	45.79	coding	402	259	1464
PB.6255.1	5	324	1	intergenic	novelGene_106	novel	0.00	0.00	non coding	NA	NA	NA
PB.8672.2	7	716	1	full-splice_match	gene: Zm00001d021062	Zm00001d021062_T00	187.77	348.86	coding	136	64	471
PB.6324.1	5	1028	7	incomplete-splice_match	gene: Zm00001d014280	Zm00001d014280_T00	91.35	91.35	coding	202	143	748
PB.1112.2	1	3417	12	full-splice_match	gene: Zm00001d032567	Zm00001d032567_T00	32.76	79.93	coding	954	294	3155
PB.4838.1	4	1233	1	genic	gene: Zm00001d048603	novel	14.61	34.26	coding	327	62	1042
PB.9767.2	8	1451	3	novel_not_in_catalog	gene: Zm00001d011384	novel	14.71	172.95	non coding	NA	NA	NA
PB.3002.1	2	1650	1	incomplete-splice_match	gene: Zm00001d004591	Zm00001d004591_T00	14.72	14.72	non coding	NA	NA	NA

PB.5098.1	4	2019	8	fusion	gene: Zm00001d049872	novel	10.68	10.68	coding	502	83	1588
PB.9767.1	8	630	3	novel_not_in_catalog	gene: Zm00001d011384	novel	91.19	172.95	non coding	NA	NA	NA
PB.1208.9	1	1010	4	intergenic	novelGene_448	novel	33.25	81.86	coding	188	190	753
PB.10942.1	B73V4	954	7	full-splice_match	gene: Zm00001d000298	Zm00001d000298_T00	2.99	2.99	coding	201	36	638
PB.8024.1	6	1097	6	incomplete-splice_match	gene: Zm00001d038533	Zm00001d038533_T00	22.61	30.80	coding	184	206	757
PB.6265.1	5	1189	6	full-splice_match	gene: Zm00001d014016	Zm00001d014016_T00	3.59	3.59	coding	207	51	671
PB.3757.3	3	869	5	intergenic	novelGene_36	novel	16.08	79.80	non coding	NA	NA	NA
PB.8024.2	6	593	5	incomplete-splice_match	gene: Zm00001d038533	Zm00001d038533_T00	0.00	30.80	coding	75	162	386
PB.9064.2	7	2340	5	incomplete-splice_match	gene: Zm00001d022481	Zm00001d022481_T05	0.34	77.76	coding	684	237	2288
PB.8291.2	7	1594	7	incomplete-splice_match	gene: Zm00001d018929	Zm00001d018929_T01	4.84	25.11	coding	353	304	1362
PB.8291.1	7	2734	13	full-splice_match	gene: Zm00001d018929	Zm00001d018929_T00	20.26	25.11	coding	578	513	2246
PB.9064.1	7	2522	6	full-splice_match	gene: Zm00001d022481	Zm00001d022481_T06	19.15	77.76	coding	739	305	2521
PB.1528.12	1	809	1	novel_in_catalog	gene: Zm00001d034420	novel	22.68	276.86	non coding	NA	NA	NA
PB.1223.1	1	1316	2	full-splice_match	gene: Zm00001d033018	Zm00001d033018_T00	10.67	10.67	coding	369	68	1174
PB.1756.1	10	1393	3	full-splice_match	gene: Zm00001d023536	Zm00001d023536_T00	3.90	3.90	coding	413	42	1280
PB.3432.1	2	874	5	full-splice_match	gene: Zm00001d006714	Zm00001d006714_T00	126.95	126.95	coding	157	71	541
PB.8985.5	7	2284	10	novel_in_catalog	gene: Zm00001d022239	novel	5.18	128.04	coding	344	1071	2102

PB.5978.1	5	480	4	incomplete-splice_match	gene: Zm00001d012886	Zm00001d012886_T00	1477.79	1477.79	coding	81	107	349
PB.3563.1	2	594	2	full-splice_match	gene: Zm00001d007347	Zm00001d007347_T00	2.58	2.58	coding	135	88	492
PB.9279.2	8	1641	10	full-splice_match	gene: Zm00001d008907	Zm00001d008907_T00	48.41	93.40	coding	302	348	1253
PB.3693.1	3	822	1	full-splice_match	gene: Zm00001d039297	Zm00001d039297_T00	17.54	17.54	coding	185	113	667
PB.9279.1	8	1857	10	full-splice_match	gene: Zm00001d008907	Zm00001d008907_T00	37.84	93.40	coding	302	382	1287
PB.1335.1	1	2932	1	genic	gene: Zm00001d033544	novel	20.65	20.65	coding	382	356	1501
PB.9716.1	8	1225	11	full-splice_match	gene: Zm00001d011159	Zm00001d011159_T00	103.71	116.81	coding	246	209	946
PB.2381.1	10	1846	9	full-splice_match	gene: Zm00001d026593	Zm00001d026593_T00	43.15	43.15	coding	313	388	1326
PB.6960.3	5	2899	7	novel_in_catalog	gene: Zm00001d017324	novel	5.22	68.26	coding	130	102	491
PB.164.1	1	1144	1	full-splice_match	gene: Zm00001d027929	Zm00001d027929_T00	1167.98	1167.98	coding	306	76	993
PB.4342.3	3	855	6	full-splice_match	gene: Zm00001d042772	Zm00001d042772_T00	0.00	236.19	coding	216	102	749
PB.7758.2	6	851	9	full-splice_match	gene: Zm00001d037509	Zm00001d037509_T00	0.00	66.76	coding	227	49	729
PB.4342.1	3	1262	6	full-splice_match	gene: Zm00001d042772	Zm00001d042772_T00	4.70	236.19	coding	216	102	749
PB.7178.6	5	2920	4	novel_in_catalog	gene: Zm00001d018206	novel	22.73	677.05	coding	890	88	2757
PB.4721.1	3	1287	6	novel_not_in_catalog	gene: Zm00001d044224	novel	18.87	18.87	coding	277	169	999
PB.2666.4	2	1781	3	novel_in_catalog	gene: Zm00001d002806	novel	2.26	20.07	coding	258	857	1630
PB.7082.10	5	2065	2	full-splice_match	gene: Zm00001d017867	Zm00001d017867_T00	7.73	69.14	coding	478	24	1457

PB.7178.7	5	3216	2	novel_in_catalog	gene: Zm00001d018206	novel	0.00	677.05	coding	443	1727	3055
PB.807.1	1	652	1	full-splice_match	gene: Zm00001d031146	Zm00001d031146_T00	3.21	3.21	coding	111	29	361
PB.9644.1	8	965	1	genic_intron	novelGene_248	novel	2.10	2.10	coding	58	165	338
PB.5386.2	4	393	1	incomplete-splice_match	gene: Zm00001d051678	Zm00001d051678_T00	27.53	64.88	non coding	NA	NA	NA
PB.8299.1	7	1169	1	full-splice_match	gene: Zm00001d018965	Zm00001d018965_T00	4.27	4.27	coding	307	55	975
PB.1220.5	1	1438	2	full-splice_match	gene: Zm00001d033005	Zm00001d033005_T00	0.00	18.93	coding	363	10	1098
PB.1156.1	1	2886	4	full-splice_match	gene: Zm00001d032739	Zm00001d032739_T00	4.26	110.46	coding	443	82	1410
PB.6813.1	5	2787	7	full-splice_match	gene: Zm00001d016731	Zm00001d016731_T00	47.34	49.01	coding	792	189	2564
PB.1193.1	1	855	1	genic	gene: Zm00001d032884	novel	41.21	41.21	coding	126	255	632
PB.914.1	1	996	1	full-splice_match	gene: Zm00001d031717	Zm00001d031717_T00	2.67	2.67	coding	135	205	609
PB.9907.4	8	1557	2	novel_not_in_catalog	gene: Zm00001d011979	novel	0.00	206.91	coding	298	583	1476
PB.5314.1	4	390	1	genic	gene: Zm00001d051345	novel	49.72	75.87	non coding	NA	NA	NA
PB.5314.2	4	1764	11	incomplete-splice_match	gene: Zm00001d051345	Zm00001d051345_T00	26.15	75.87	coding	466	163	1560
PB.7178.3	5	3121	3	novel_in_catalog	gene: Zm00001d018206	novel	53.08	677.05	coding	484	85	1536
PB.1962.1	10	3310	8	full-splice_match	gene: Zm00001d024744	Zm00001d024744_T00	26.93	42.47	coding	966	193	3090
PB.8285.1	7	914	4	full-splice_match	gene: Zm00001d018903	Zm00001d018903_T00	835.53	835.53	coding	204	57	668
PB.1962.3	10	3154	9	full-splice_match	gene: Zm00001d024744	Zm00001d024744_T00	4.08	42.47	coding	818	159	2612

PB.1962.2	10	3186	9	full-splice_match	gene: Zm00001d024744	Zm00001d024744_T00	3.30	42.47	coding	831	153	2645
PB.39.1	1	1013	2	genic	gene: Zm00001d027389	novel	56.67	280.61	coding	132	188	583
PB.6015.16	5	1355	2	novel_in_catalog	gene: Zm00001d013015	novel	6.78	206.41	coding	244	516	1247
PB.39.3	1	464	2	genic	gene: Zm00001d027389	novel	92.77	280.61	coding	123	96	464
PB.5971.4	5	1043	1	incomplete-splice_match	gene: Zm00001d012856	Zm00001d012856_T00	48.17	77.54	coding	267	243	1043
PB.2195.4	10	692	1	full-splice_match	gene: Zm00001d025913	Zm00001d025913_T00	574.64	789.31	coding	150	84	533
PB.1287.1	1	1762	3	genic	gene: Zm00001d033335	novel	12.76	12.76	coding	281	201	1043
PB.1875.1	10	648	1	genic	gene: Zm00001d024252	novel	3.25	3.25	coding	65	264	458
PB.1659.3	1	1527	6	full-splice_match	gene: Zm00001d034867	Zm00001d034867_T00	16.06	155.07	coding	322	156	1121
PB.9150.1	8	1210	4	novel_in_catalog	gene: Zm00001d008311	novel	23.07	23.07	non coding	NA	NA	NA
PB.10736.1	9	955	1	full-splice_match	gene: Zm00001d047824	Zm00001d047824_T00	58.72	58.72	coding	225	127	801
PB.2801.1	2	1545	3	full-splice_match	gene: Zm00001d003414	Zm00001d003414_T00	34.36	111.67	coding	365	189	1283
PB.2801.3	2	1448	4	novel_not_in_catalog	gene: Zm00001d003414	novel	77.31	111.67	coding	365	51	1145
PB.4440.5	3	854	1	novel_in_catalog	gene: Zm00001d043195	novel	0.00	201.86	coding	142	143	568
PB.7106.3	5	1414	5	full-splice_match	gene: Zm00001d017960	Zm00001d017960_T00	21.69	33.88	coding	367	82	1182
PB.7890.1	6	2767	3	full-splice_match	gene: Zm00001d038059	Zm00001d038059_T00	20.02	20.02	coding	849	2	2548
PB.7166.1	5	1908	2	novel_in_catalog	gene: Zm00001d018156	novel	1.03	1.03	coding	573	57	1775

PB.161.2	1	1374	3	full-splice_match	gene: Zm00001d027924	Zm00001d027924_T00	29.70	50.97	coding	302	85	990
PB.5601.3	4	1381	8	full-splice_match	gene: Zm00001d052561	Zm00001d052561_T00	37.18	49.72	coding	367	77	1177
PB.3727.1	3	2679	4	full-splice_match	gene: Zm00001d039427	Zm00001d039427_T00	19.74	44.03	coding	574	742	2463
PB.3727.2	3	989	1	novel_in_catalog	gene: Zm00001d039427	novel	10.51	44.03	coding	107	256	576
PB.161.3	1	1312	3	full-splice_match	gene: Zm00001d027924	Zm00001d027924_T00	0.00	50.97	coding	302	83	988
PB.3055.5	2	1871	4	novel_not_in_catalog	gene: Zm00001d004931	novel	46.02	46.02	coding	532	129	1724
PB.7343.3	6	1829	6	full-splice_match	gene: Zm00001d035207	Zm00001d035207_T00	2.91	13.28	coding	294	757	1638
PB.1451.3	1	900	8	novel_in_catalog	gene: Zm00001d034033	novel	0.00	55.24	coding	230	210	899
PB.7665.2	6	793	2	novel_in_catalog	gene: Zm00001d037097	novel	25.69	25.69	coding	107	68	388
PB.7665.1	6	3741	6	fusion	gene: Zm00001d037097	novel	17.77	17.77	coding	171	3019	3531
PB.2861.1	2	636	1	full-splice_match	gene: Zm00001d003725	Zm00001d003725_T00	598.67	598.67	coding	136	89	496
PB.7665.6	6	1694	8	incomplete-splice_match	gene: Zm00001d037098	Zm00001d037098_T00	38.54	119.35	coding	473	67	1485
PB.7665.5	6	2721	8	novel_in_catalog	gene: Zm00001d037098	novel	0.00	119.35	coding	400	187	1386
PB.7665.4	6	1977	9	full-splice_match	gene: Zm00001d037098	Zm00001d037098_T00	80.81	119.35	coding	473	330	1748
PB.4441.1	3	1443	5	full-splice_match	gene: Zm00001d043196	Zm00001d043196_T00	30.37	30.37	coding	377	117	1247
PB.10367.1	9	1265	4	novel_in_catalog	gene: Zm00001d045973	novel	3.65	3.65	non coding	NA	NA	NA
PB.1345.1	1	948	3	full-splice_match	gene: Zm00001d033579	Zm00001d033579_T00	55.23	55.23	coding	190	38	607

PB.4106.4	3	2733	7	full-splice_match	gene: Zm00001d041635	Zm00001d041635_T00	34.70	128.11	coding	663	416	2404
PB.10704.3	9	1508	6	full-splice_match	gene: Zm00001d047737	Zm00001d047737_T00	0.58	7.06	coding	428	116	1399
PB.10704.1	9	2605	5	novel_in_catalog	gene: Zm00001d047737	novel	2.85	7.06	coding	262	1568	2353
PB.5661.1	4	1086	2	genic	gene: Zm00001d052843	novel	4.49	4.49	coding	260	154	933
PB.4106.1	3	1742	4	incomplete-splice_match	gene: Zm00001d041635	Zm00001d041635_T00	58.53	128.11	coding	272	270	1085
PB.4106.2	3	2874	7	full-splice_match	gene: Zm00001d041635	Zm00001d041635_T00	34.88	128.11	coding	663	359	2347
PB.4106.3	3	2752	7	full-splice_match	gene: Zm00001d041635	Zm00001d041635_T00	0.00	128.11	coding	663	329	2317
PB.6123.2	5	1820	4	full-splice_match	gene: Zm00001d013367	Zm00001d013367_T00	232.53	2327.02	coding	451	125	1477
PB.2886.1	2	2454	1	intergenic	novelGene_150	novel	12.94	12.94	non coding	NA	NA	NA
PB.8932.1	7	1270	2	full-splice_match	gene: Zm00001d022027	Zm00001d022027_T00	9.70	15.65	coding	248	9	752
PB.2842.3	2	2357	10	novel_in_catalog	gene: Zm00001d003601	novel	25.94	122.13	coding	502	292	1797
PB.2842.2	2	3219	13	novel_in_catalog	gene: Zm00001d003601	novel	12.95	122.13	coding	411	499	1731
PB.2842.1	2	3079	14	full-splice_match	gene: Zm00001d003601	Zm00001d003601_T00	45.95	122.13	coding	661	496	2478
PB.8139.5	6	2062	11	full-splice_match	gene: Zm00001d038927	Zm00001d038927_T00	4.03	63.06	coding	515	99	1643
PB.8139.2	6	1950	12	full-splice_match	gene: Zm00001d038927	Zm00001d038927_T00	42.14	63.06	coding	511	106	1638
PB.2842.6	2	2478	14	full-splice_match	gene: Zm00001d003601	Zm00001d003601_T00	30.71	122.13	coding	657	8	1978
PB.2842.5	2	3202	13	novel_in_catalog	gene: Zm00001d003601	novel	3.46	122.13	coding	397	1452	2642

PB.2842.4	2	2952	13	full-splice_match	gene: Zm00001d003601	Zm00001d003601_T00	3.11	122.13	coding	575	326	2050
PB.7363.1	6	1609	1	intergenic	novelGene_87	novel	25.78	25.78	coding	152	840	1295
PB.1108.1	1	1503	2	full-splice_match	gene: Zm00001d032560	Zm00001d032560_T00	98.17	98.17	coding	375	116	1240
PB.4062.3	3	2364	3	incomplete-splice_match	gene: Zm00001d041363	Zm00001d041363_T00	22.79	100.65	coding	647	114	2054
PB.45.1	1	2767	6	incomplete-splice_match	gene: Zm00001d027412	Zm00001d027412_T06	28.96	28.96	coding	708	258	2381
PB.6666.3	5	1194	5	full-splice_match	gene: Zm00001d015905	Zm00001d015905_T00	18.79	186.15	coding	252	165	920
PB.9666.1	8	1628	2	full-splice_match	gene: Zm00001d010920	Zm00001d010920_T00	1642.04	1651.48	coding	396	156	1343
PB.10471.1	9	1664	6	novel_not_in_catalog	gene: Zm00001d046660	novel	13.35	13.35	coding	384	229	1380
PB.9666.4	8	1514	2	full-splice_match	gene: Zm00001d010920	Zm00001d010920_T00	0.00	1651.48	coding	396	157	1344
PB.1398.1	1	1068	1	full-splice_match	gene: Zm00001d033835	Zm00001d033835_T00	7.38	7.38	coding	195	222	806
PB.2869.2	2	1156	2	full-splice_match	gene: Zm00001d003762	Zm00001d003762_T00	0.00	34.66	coding	325	47	1021
PB.8943.1	7	1538	4	novel_in_catalog	gene: Zm00001d022065	novel	5.52	5.52	coding	308	363	1286
PB.4408.1	3	1143	1	full-splice_match	gene: Zm00001d043087	Zm00001d043087_T00	7.72	19.36	coding	212	175	810
PB.2869.1	2	1255	2	full-splice_match	gene: Zm00001d003762	Zm00001d003762_T00	34.66	34.66	coding	325	47	1021
PB.2530.1	2	2320	1	full-splice_match	gene: Zm00001d002175	Zm00001d002175_T00	59.97	59.97	coding	605	289	2103
PB.5852.3	4	1200	6	full-splice_match	gene: Zm00001d053786	Zm00001d053786_T00	36.78	65.72	non coding	NA	NA	NA
PB.1537.2	1	2283	2	genic	gene: Zm00001d034434	novel	4.00	37.65	coding	75	96	320

PB.7347.2	6	1861	2	genic	gene: Zm00001d035236	novel	0.00	13.42	coding	399	218	1414
PB.7347.3	6	1472	2	genic	gene: Zm00001d035236	novel	3.28	13.42	coding	399	174	1370
PB.7347.1	6	1952	2	genic	gene: Zm00001d035236	novel	10.14	13.42	coding	399	163	1359
PB.453.6	1	2517	13	incomplete-splice_match	gene: Zm00001d029196	Zm00001d029196_T00	0.00	60.93	coding	784	12	2363
PB.8555.2	7	2546	7	full-splice_match	gene: Zm00001d020532	Zm00001d020532_T00	13.42	13.42	coding	605	380	2194
PB.453.4	1	3113	15	novel_not_in_catalog	gene: Zm00001d029196	novel	0.00	60.93	coding	895	167	2851
PB.9878.1	8	673	1	full-splice_match	gene: Zm00001d011841	Zm00001d011841_T00	100.97	100.97	coding	151	107	559
PB.1473.4	1	1129	1	full-splice_match	gene: Zm00001d034145	Zm00001d034145_T00	0.00	218.33	coding	278	114	947
PB.8125.1	6	1859	10	full-splice_match	gene: Zm00001d038894	Zm00001d038894_T00	22.51	22.51	coding	497	193	1683
PB.1869.2	10	1599	4	incomplete-splice_match	gene: Zm00001d024178	Zm00001d024178_T00	36.11	75.33	coding	443	43	1371
PB.1869.1	10	3168	6	novel_in_catalog	gene: Zm00001d024178	novel	39.21	75.33	coding	757	539	2809
PB.981.3	1	1120	6	full-splice_match	gene: Zm00001d032031	Zm00001d032031_T00	70.89	144.91	coding	273	123	941
PB.10532.3	9	3356	1	novel_in_catalog	gene: Zm00001d046999	novel	3.10	22.90	non coding	NA	NA	NA
PB.10532.1	9	2827	3	novel_not_in_catalog	gene: Zm00001d046999	novel	13.55	22.90	coding	105	2022	2336
PB.4428.1	3	1547	5	full-splice_match	gene: Zm00001d043147	Zm00001d043147_T00	12.33	15.56	coding	321	80	1042
PB.10532.6	9	1813	2	novel_not_in_catalog	gene: Zm00001d046999	novel	3.45	22.90	non coding	NA	NA	NA
PB.10532.5	9	1703	3	novel_not_in_catalog	gene: Zm00001d046999	novel	0.00	22.90	coding	99	59	355

PB.10532.4	9	3164	2	novel_not_in_catalog	gene: Zm00001d046999	novel	2.80	22.90	non coding	NA	NA	NA
PB.10343.1	9	2257	5	full-splice_match	gene: Zm00001d045804	Zm00001d045804_T01	28.04	49.91	coding	407	323	1543
PB.10343.3	9	2104	5	full-splice_match	gene: Zm00001d045804	Zm00001d045804_T01	0.00	49.91	coding	407	331	1551
PB.10343.5	9	1649	5	full-splice_match	gene: Zm00001d045804	Zm00001d045804_T01	0.00	49.91	coding	407	321	1541
PB.10343.4	9	1345	4	incomplete-splice_match	gene: Zm00001d045804	Zm00001d045804_T00	17.47	49.91	coding	281	61	903
PB.10343.6	9	2407	3	incomplete-splice_match	gene: Zm00001d045804	Zm00001d045804_T01	4.40	49.91	coding	305	379	1293
PB.10916.1	9	1882	7	incomplete-splice_match	gene: Zm00001d048512	Zm00001d048512_T01	8.29	8.29	coding	409	431	1657
PB.930.1	1	483	1	incomplete-splice_match	gene: Zm00001d031815	Zm00001d031815_T00	4.69	4.69	non coding	NA	NA	NA
PB.5738.1	4	970	3	incomplete-splice_match	gene: Zm00001d053259	Zm00001d053259_T00	72.67	72.67	coding	141	199	621
PB.9390.2	8	1930	6	full-splice_match	gene: Zm00001d009580	Zm00001d009580_T00	30.07	30.07	coding	219	42	698
PB.4694.1	3	1172	1	incomplete-splice_match	gene: Zm00001d044127	Zm00001d044127_T01	1.06	51.45	non coding	NA	NA	NA
PB.223.1	1	1833	3	novel_not_in_catalog	gene: Zm00001d028213	novel	20.83	37.09	coding	439	36	1352
PB.3115.1	2	840	5	full-splice_match	gene: Zm00001d005270	Zm00001d005270_T00	27.15	27.15	coding	235	95	799
PB.8981.1	7	1404	5	full-splice_match	gene: Zm00001d022225	Zm00001d022225_T00	18.19	66.30	coding	266	226	1023
PB.223.2	1	1807	3	novel_not_in_catalog	gene: Zm00001d028213	novel	16.26	37.09	coding	439	111	1427
PB.1887.3	10	1417	5	full-splice_match	gene: Zm00001d024325	Zm00001d024325_T00	0.00	45.06	coding	325	69	1043
PB.1887.2	10	1577	5	full-splice_match	gene: Zm00001d024325	Zm00001d024325_T00	24.12	45.06	coding	325	107	1081

PB.1887.1	10	1801	5	full-splice_match	gene: Zm00001d024325	Zm00001d024325_T00	13.13	45.06	coding	325	82	1056
PB.8981.4	7	1212	5	full-splice_match	gene: Zm00001d022225	Zm00001d022225_T00	32.46	66.30	coding	266	140	937
PB.2159.8	10	1469	5	novel_in_catalog	gene: Zm00001d025752	novel	17.59	98.80	coding	135	467	871
PB.2159.9	10	1564	4	novel_in_catalog	gene: Zm00001d025752	novel	0.00	98.80	coding	124	467	838
PB.2159.6	10	1456	5	novel_in_catalog	gene: Zm00001d025752	novel	12.90	98.80	coding	210	469	1098
PB.11033.1	B73V 4	1751	3	genic	gene: Zm00001d000227	novel	33.70	174.98	coding	307	521	1441
PB.8372.1	7	1220	8	full-splice_match	gene: Zm00001d019467	Zm00001d019467_T00	22.00	50.06	coding	258	18	791
PB.11033.3	B73V 4	1644	4	genic	gene: Zm00001d000227	novel	140.29	174.98	coding	396	230	1417
PB.11033.4	B73V 4	1702	3	genic	gene: Zm00001d000227	novel	0.98	174.98	coding	307	573	1493
PB.7439.3	6	1891	7	full-splice_match	gene: Zm00001d035736	Zm00001d035736_T00	21.89	55.73	coding	521	129	1691
PB.910.1	1	1546	8	full-splice_match	gene: Zm00001d031689	Zm00001d031689_T00	45.13	45.13	coding	363	256	1344
PB.7439.1	6	2598	7	full-splice_match	gene: Zm00001d035736	Zm00001d035736_T00	32.76	55.73	coding	610	517	2346
PB.755.3	1	776	1	incomplete-splice_match	gene: Zm00001d030916	Zm00001d030916_T00	10.56	42.36	coding	182	47	592
PB.207.2	1	821	3	full-splice_match	gene: Zm00001d028160	Zm00001d028160_T00	0.00	56.10	coding	165	37	531
[truncated: 407,742 more chars]
